# The unintended consequences of inconsistent closure policies and mobility restrictions during epidemics

**DOI:** 10.1186/s44263-023-00028-z

**Published:** 2023-12-04

**Authors:** Benjamin M. Althouse, Brendan Wallace, B. K. M. Case, Samuel V. Scarpino, Antoine Allard, Andrew M. Berdahl, Easton R. White, Laurent Hébert-Dufresne

**Affiliations:** 1 University of Washington, Seattle, 98105 WA USA; 2 New Mexico State University, Las Cruces, 88003 NM USA; 3 Department of Applied Mathematics, University of Washington, Seattle, 98195 WA USA; 4 Present Address: Quantitative Ecology and Resource Management, University of Washington, Seattle, WA 98195 USA; 5 School of Aquatic & Fishery Sciences,, University of Washington, Seattle, WA 98195 USA; 6 Department of Computer Science, University of Vermont, Burlington, 05405 VT USA; 7 Vermont Complex Systems Center, University of Vermont, Burlington, 05405 VT USA; 8 Institute for Experiential AI, Northeastern University, Boston, Massachusetts USA; 9 Department of Health Sciences, Northeastern University, Boston, MA USA; 10 Khoury College of Computer Sciences, Northeastern University, Boston, MA USA; 11 Santa Fe Institute, Santa Fe, NM USA; 12 Département de physique, de génie physique et d’optique, Université Laval, Québec (Québec), G1V 0A6 Canada; 13 Centre interdisciplinaire en modélisation mathématique, Université Laval, Québec (Québec), G1V 0A6 Canada; 14 School of Aquatic & Fishery Sciences, University of Washington, Seattle, 98195 WA USA; 15 Department of Biological Sciences, University of New Hampshire, Durham, 03824 NH USA; 16 Gund Institute for Environment, University of Vermont, Burlington, 05405 VT USA

**Keywords:** Disease modeling, Metapopulation models, Adaptive behavior, Mobility data, COVID-19

## Abstract

**Background:**

Controlling the spread of infectious diseases―even when safe, transmission-blocking vaccines are available―may require the effective use of non-pharmaceutical interventions (NPIs), e.g., mask wearing, testing, limits on group sizes, venue closure. During the SARS-CoV-2 pandemic, many countries implemented NPIs inconsistently in space and time. This inconsistency was especially pronounced for policies in the United States of America (US) related to venue closure.

**Methods:**

Here, we investigate the impact of inconsistent policies associated with venue closure using mathematical modeling and high-resolution human mobility, Google search, and county-level SARS-CoV-2 incidence data from the USA. Specifically, we look at high-resolution location data and perform a US-county-level analysis of nearly 8 million SARS-CoV-2 cases and 150 million location visits, including 120 million church visitors across 184,677 churches, 14 million grocery visitors across 7662 grocery stores, and 13.5 million gym visitors across 5483 gyms.

**Results:**

Analyzing the interaction between venue closure and changing mobility using a mathematical model shows that, across a broad range of model parameters, inconsistent or partial closure can be worse in terms of disease transmission as compared to scenarios with no closures at all. Importantly, changes in mobility patterns due to epidemic control measures can lead to increase in the future number of cases. In the most severe cases, individuals traveling to neighboring jurisdictions with different closure policies can result in an outbreak that would otherwise have been contained. To motivate our mathematical models, we turn to mobility data and find that while stay-at-home orders and closures decreased contacts in most areas of the USA, some specific activities and venues saw an increase in attendance and an increase in the distance visitors traveled to attend. We support this finding using search query data, which clearly shows a shift in information seeking behavior concurrent with the changing mobility patterns.

**Conclusions:**

While coarse-grained observations are not sufficient to validate our models, taken together, they highlight the potential unintended consequences of inconsistent epidemic control policies related to venue closure and stress the importance of balancing the societal needs of a population with the risk of an outbreak growing into a large epidemic.

**Supplementary Information:**

The online version contains supplementary material available at 10.1186/s44263-023-00028-z.

## Background

Severe acute respiratory syndrome coronavirus 2 (SARS-CoV-2, the virus that causes COVID-19) has swept the globe, revealing the strengths and weaknesses of our international, national, state, and local public health systems [1]. Evidence from countries such as Vietnam [2], Thailand [3], Singapore [4], South Korea [5], New Zealand [6], China [7], and others [8] have shown that coordinated, national-level policies can control SARS-CoV-2 transmission. However, in many locations—in particular the USA—initial efforts to stem the spread of SARS-CoV-2 using non-pharmaceutical interventions (NPIs) were implemented as a patchwork of self-isolation, school closures, and business restrictions [9, 10]. For example, throughout the months of March and April 2020, US states, counties, and cities often independently implemented stay-at-home orders, mask mandates, limits on gathering sizes, etc. [11]. May and June 2020 saw nearly all states begin to reopen leading to increased cases through July, August, and September of that year and in turn leading again to restrictions/venue closure in half a dozen states including New York, California, and Texas [12]. Over the course of the next 2.5 years, NPI policies in the USA continued to be recommended, imposed, and lifted inconsistently [10, 13].

Even in countries with more uniform policies, some activities were the subject of much debate as the local risks associated with the activity [14–16] clashed with protections of the activity as an essential service to individuals and the community [17]. For example, religious activities, including choirs and large services, in particular have led to many super-spreading events [18], with attack rates well-above 50% in some cases [19]. However, in certain cases, individuals have defied church closures and attended mass gatherings, leading to legal prosecution [20, 21]. Other essential services have seen similar patterns, with public spaces such as urban and suburban parks and trails also being the subject of inconsistent visitation patterns and closures. Data shows that when some, but not all, parks and trails close, individuals may travel further to areas remaining open [22]. Although it is now clear that transmission risk in outdoor settings is typically low [23, 24].

Numerous studies have demonstrated that even inconsistently applied NPIs had pronounced local effects on case rates, hospitalizations, and mortality during COVID-19 pandemic [25–27]. For example, testing at colleges and universities reduced case burden both on campus and in surrounding counties [28], eviction moratoria reduced urban transmission [29], NPIs meant to control SARS-CoV-2 transmission also reduced influenza burden by as much as 60% [30–32], shelter-in-place orders led to reduced mortality [13, 33], and variability in mask-wearing was associated with variability in SARS-CoV-2 case rates [34, 35]. What remains less clear is whether variability in NPI policies/adoption related to venue closure effects population-level aspects of an epidemic.

Here, we develop a simple mathematical model for studying the effect of non-uniform public health policies related to venue closure on the spread of an epidemic in a single population. With this model, we show how―for certain real-world parameter ranges―no closures can be better for epidemic control than inconsistent closure policies. Next, we study a model of epidemics with partial gathering restrictions― and partial adoption of said restrictions―over a mobility network of interconnected populations. To motivate future work on the mechanisms behind our models, we then examine online information seeking and physical foot traffic data to see how gathering-specific behavior has varied across the USA during the COVID-19 pandemic. With these data, we investigate how movement patterns changed during local business closure. We discuss the implications of these results especially as they relate to current discussions around how global societies should plan for and respond to resurgent COVID-19 waves driven by novel SARS-CoV-2 variants.

## Methods

### Simple mathematical framework: the cloSIR model

To explore the consequences of inconsistent epidemic control policies related to venue closure, we formulate a simple, mathematical model which we call *cloSIR* to couple disease dynamics with closure policies. As a first approximation, we take a mean-field perspective and ignore any spatial features or contact structure, which allows us to focus on the average dynamics of gatherings within a population.

We model an epidemic in a population of *N* individuals uniformly distributed across *M* gatherings of size *N*/*M*, and no mixing between gatherings occurs other than a single redistribution event after some gatherings are closed. We assume that a fraction *X* of gatherings are closed at time $t_c$ to help contain an outbreak and that a fraction *Y* of members in closed gatherings then decide to defy the closure by traveling to one of the remaining open gatherings at random, thereby increasing the number of interacting individuals in gatherings which remain open. These open gatherings could be under a different set of rules in a different location or the venue/location itself may be defying government restrictions. Closures therefore protect the local community in compliance with the closures, but can potentially increase opportunities for transmission in any open venues.

We track susceptible-infectious-recovered (SIR) dynamics within a typical open/closed gathering by assuming that the natural normalized transmission rate of the disease is $\lambda$ (with a recovery rate equal to 1 for time units set to the recovery period). We use $S_{o/c}$, $I_{o/c}$, and $R_{o/c}$ to denote the number of susceptible, infectious, and recovered individual in a typical open/closed gathering, respectively. Applying standard SIR dynamics in open gatherings but removing transmission events in closed gatherings, ﻿we write

1 $$\begin{aligned}{} & {} \frac{dS_o}{dt} = -\lambda S_o I_o \quad \frac{dI_o}{dt} = \lambda S_o I_o - I_o \quad \dfrac{dR_o}{dt} = I_o \end{aligned}$$

2 $$\begin{aligned}{} & {} \frac{dS_c}{dt} = 0 \quad \qquad \;\;\; \frac{dI_c}{dt} = -I_c \qquad \quad \;\;\; \frac{dR_c}{dt} = I_c \; . \end{aligned}$$

The critical part of the cloSIR model is the implementation of closure policies at time $t_c$. At time $t<t_c$, all gatherings are open, and we have $S_o+I_o+R_o = N/M$ and $S_c=I_c=R_c=0$ such that all derivatives are equal to zero in closed gatherings for $t<t_c$. Once the intervention is implemented at time $t=t_c$, we uniformly redistribute non-compliant individuals, regardless of their epidemiological state, from closed to open gatherings.

Since *XM* gatherings are closed, we have $XM\times (YN/M)=XYN$ non-compliant individuals to redistribute evenly across $(1-X)M$ open gatherings. This mechanism adds $\frac{XYN}{(1 - X)M}$ individuals to a typical open gathering so that the population of each open gathering is increased to $\frac{N}{M}\times [1+XY/(1-X)]$. Similarly, the population of each gathering which now has become closed is decreased to $\frac{N}{M}(1-Y)$.

After closures are implemented, the dynamics of the cloSIR model are still governed by the same set of ordinary differential equations. As the outbreak progresses, the state of typical open/closed gatherings may be used to quantify two key observables for the population as a whole: first, the total number of infectious individuals

3 $$\begin{aligned} I(t) = \left\{ \begin{array}{ll} MI_o(t), &{} \text {for } t<t_c \\ (1-X)MI_o(t) + XMI_c(t), &{} \text {for } t\ge t_c \end{array}\right. \end{aligned}$$

and, second, the total fraction of recovered individuals

4 $$\begin{aligned} R(t) = \left\{ \begin{array}{ll} MR_o(t), &{} \text {for } t<t_c \\ (1-X)MR_o(t) + XMR_c(t), &{} \text {for } t\ge t_c. \end{array}\right. \end{aligned}$$

Finally, note that the population size *N* and number of gatherings *M* only act as scale factors in our results. Therefore, in all subsequent analyses, we set to $N=M=1$ for simplicity and without loss of generality.

Ultimately, although the dynamics are governed by the standard SIR differential equations for all time, the cloSIR model offers an interesting trade-off between controlling transmission by closing venues and *intensifying* transmission by aggregating contacts in a smaller number of still open venues. The question then becomes whether the redistribution of participants among gathering locations, e.g., churches or parks, will have a positive or negative impact on the epidemic. Assuming one cannot ensure the closure of all venues, is closing a certain percentage of venues worth the increase in visitors to those that remain open?

### A cloSIR model on networks of interconnected populations

The cloSIR model is a general adaptive mechanism where populations adapt to an epidemic through top-down interventions and agents adapt to such interventions. It therefore combines different scales of population adaptation [36], resulting in a balloon effect where adaptation to restrict gatherings squeezes the balloon and pushes the pressure in other unrestricted areas. This phenomenon is reminiscent of more mechanistic adaptive behaviors, such as coupling of epidemics and fear [37] or network rewiring around infectious essential works [38]. Indeed, two important assumptions of the cloSIR model as implemented above is the homogeneous and fixed behavior of agents. Future modeling efforts should couple mobility changes to mechanistic adaptive models. In this section, we focus on a first attempt at extending the cloSIR model towards network metapopulation models with variable behaviors.

There is a long history of using metapopulation models to encode the spatial and coupled structure between populations in epidemiology [39] and also specifically for COVID-19 [40–42]. This literature includes models accounting for how individuals might adapt their mobility in response to an epidemic [43–45]. Importantly, this adaptive behavior change is always a bottom-up response to the epidemic itself (i.e., one individual choosing to avoid infectious contacts or move due to the local prevalence of a disease). The cloSIR mechanism introduced above is different as it considers a top-down intervention (i.e., closure of certain venues) and bottom-up adaptive response to the intervention rather than to the epidemic itself.

We are interested not only in what happens in a population on average but also in how changes in mobility can change the coupling of interconnected populations. We can adapt the cloSIR model to gather insight on the role of coupling of communities as follows.

Let us assume a set of populations―called counties―where every individual county *i* has its own infectious state $\lbrace S_i, I_i, R_i \rbrace$. Counties are not independent and are coupled through a directed and weighted network of human mobility where $\rho _{i,j}$ specifies the rate of visits of individuals *from* county *i* *to* county *j*. The epidemic dynamics within a given county *i* is therefore specified by:

5 $$\begin{aligned} \dot{S}_i{} & {} = -\lambda \rho _{i,i} S_i I_i - \lambda S_i \sum \limits _{j\ne i} (\rho _{i,j}+\rho _{j,i})I_j \end{aligned}$$

6 $$\begin{aligned} \dot{I}_i{} & {} = \lambda \rho _{i,i} S_i I_i + \lambda S_i \sum \limits _{j\ne i} (\rho _{i,j}+\rho _{j,i})I_j - I_i\end{aligned}$$

7 $$\begin{aligned} \dot{R}_i{} & {} = I_i \end{aligned}$$

where the first term in $\dot{S}_i$ accounts for transmission within county *i* and where the sum over other counties account for coupling through mobility. Importantly, individuals from *i* do not permanently move to a different county *j*, they are simply coupled to it through commuting or visit patterns but always return to in *i*. We also assume that both infectious and susceptible individuals continue moving at essentially the same rate given the high rate of asymptomatic individuals.

How is the cloSIR mechanism implemented? In the previous section, we focused on individual gatherings that could eventually close at time $t_c$ with probability *X* where *X* quantified the scale of restrictions. Here, counties themselves do not fully close, but the contact patterns are shifted by the closure of a fraction $X_i$ of gatherings in county *i*. For time $t>t_c$, we change the contact patterns from $\rho$ to $\rho '$ as follows:

8 $$\begin{aligned} \rho '_{i,i}{} & {} = (1-X)\rho _{i,i} \end{aligned}$$

9 $$\begin{aligned} \rho '_{i,j}{} & {} = (1-X_j)\rho _{i,j} + X_i\rho _{i,i}\frac{Y_i}{\mathcal {N}_i}\rho _{i,j}(1-X_j) \; . \end{aligned}$$

Our logic is that a fraction $X_i$ of contacts within county *i* are stopped by closures, and therefore a fraction $(1-X_i)$ of contacts remain after the intervention. Likewise, a fraction $X_j$ of visits from *i* to *j* would target gatherings closed by the intervention in *j* such that only a fraction $(1-X_j)$ remain. Importantly, we model the impact of non-compliance by redirecting a fraction of activities that would have occurred within county *i* but were stopped by the intervention. We therefore send a fraction $X_iY_i/N_i$ individuals to county *j*, where $\mathcal {N}_i = \sum _{j\ne i} \rho _{i,j}$ is the total coupling of county *i* with neighboring counties. These visits then create contacts if those gatherings are still open in county *j*, which occurs with probability $(1-X_j)$.

### Mobility patterns and information-seeking activity

The mechanisms behind our modeling results have clear signatures as some individuals fail to reduce their mobility, visit new places, or even travel further while the rest of the local populations reduce their activity. In practice, however, the unreliable and sensitive nature of available geolocation data [46] means that we should not track individuals but instead coarse-grain and normalize mobility data as much as possible. These issues are detailed in our the “Discussion” section, and we now simply note that mobility data can not be used to validate our models or make any causal claims. Instead, we aim to find general trends coherent with the mechanisms found in our models.

To assess the degree to which the mechanisms included in our mathematical models may have occurred during the COVID-19 pandemic, we use publicly available data from SafeGraph for a fixed time period in the first year of the pandemic. SafeGraph is a data company that aggregates anonymized location data from numerous applications in order to provide insights about physical places [47, 48]. These data allow us quantify human mobility to specific establishments after the adoption of NPIs, e.g., business closure, group-size limits. To enhance privacy, SafeGraph excludes census block group information if fewer than five devices visited a given location in a month (two devices in a week) from a given census block group. Using these data, we use counts of visits and unique visitors to businesses across the USA as well as the distance traveled from “home” (defined as the common nighttime location for the device over a 6 week period where nighttime is 6pm to 7am).

To complement the mobility data, we also use Google search trends (as downloaded from the Google API for Trends), where queries for “churches” increased beginning in March 2020. We compare search volumes for church between March 13 and April 13, 2020, to Sundays in the previous 10 years of searches occurring on the same date across all US states. By normalizing to previous years, we are able to capture deviations during 2020 above and beyond typical searching patterns over this period, which encompasses Lent where individuals may have increased interest in attending church. Comparison to previous years should alleviate potential biases as the previous years act as counterfactuals to 2020.

Finally, SARS-CoV-2 case data and county populations were downloaded from the COVID-19 Data Repository by the Center for Systems Science and Engineering at Johns Hopkins University at the county level beginning in February, 2020 [49, 50], and for the entire time period for which we have mobility data. These are useful to investigate potential correlations between mobility patterns and case data. All data sources are further described in Additional file 1.

## Results

### The cloSIR model

**Fig. 1:**
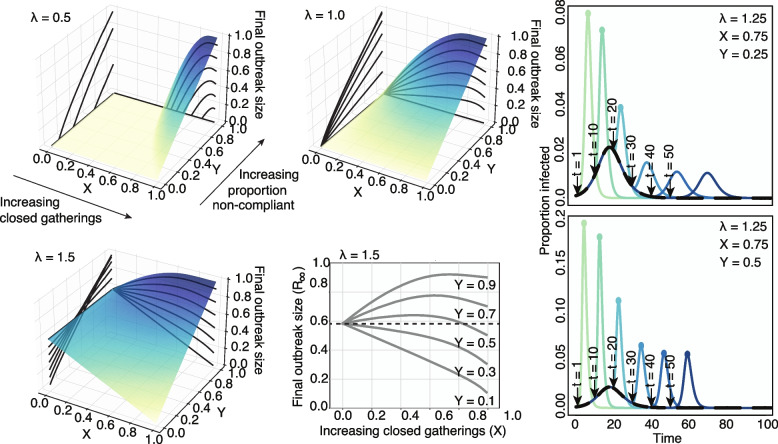
Final state of the SIR dynamics with variable disease infectiousness and intervention scale. Surfaces and contours show the final sizes of outbreaks across a range of intervention effects (*X*, proportion of closed gatherings) and proportion of non-compliant individuals who travel to open gatherings (*Y*). Three levels of infectiousness are illustrated ($\lambda = 0.5, 1.0, 1.5$). We see that the worst-case scenario is a function of $\lambda$, *X* and *Y*, as there is no consistent ranking in outbreak sizes. Small interventions appear beneficial against very transmissible pathogens but risk lowering the epidemic threshold at high frequency of non-compliant individuals, and larger interventions accentuate this effect. The bottom middle panel highlights this effect: we see that the impact of the fraction of non-compliant individuals is non-linear close to the epidemic threshold. At low values of *Y*, i.e., in a population with high compliance to recommendations, closing more gatherings is always beneficial. At the opposite end, for high values of *Y*, keeping all gatherings open is the optimal intervention. However, at medium values of *Y*, while closing all gatherings is still the optimal intervention, keeping all gatherings open is better than partial closures. The right-hand panels show the effects of changing intervention time across ranges of Y. The black curve depicts the course of the outbreak without any intervention. The various colored curves peeling off from the black curve show the course of the outbreak given differently timed interventions. Colored dots indicate epidemic peaks larger than the no intervention baseline scenario. Intuitively, we find that earlier interventions are always better and that delayed and imperfect interventions can cause second epidemic waves

Strikingly, we find that in many scenarios the optimal strategy to minimize the size of the outbreak is often no intervention at all. Figure 1 shows that depending on the proportion of the population that chooses to go to another open gathering (*Y*) the final outbreak size is often minimized when $X=0$ (no closures). In fact, below the epidemic threshold $\lambda < 1$, interventions can only worsen the final size of the outbreak since no closures leads to isolated communities each with a subcritical outbreak. However, when $X>0$ open communities otherwise not at risk may become supercritical by increasing the concentration of susceptible individuals. For stronger epidemics ($\lambda > 1$), although a complete closure of gatherings $X=1$ might be the optimal strategy, the expected outbreak size often follows a non-monotonous function of *X* such that the optimal outcome at $X=1$ is next to a worst-case scenario at large values of *X* just below 1. What this implies is that the outcome is highly dependent on the amount of non-compliance that one can expect in a population (i.e., larger values of non-compliance *Y*).

Similarly, poorly-timed interventions can actually lead to additional waves of infection. Figure 1 shows that secondary peaks of infection occur if intervention is initiated too late. Interestingly, stronger interventions tend to dramatically heighten the epidemic peak under many closure scenarios (colored curves) compared to the no-closure baseline (black curve).

The cloSIR model therefore provides a simple, yet telling, illustration of the potential impact of the collective behavior observed in the empirical mobility and search data from the USA around essential services. Although future research should layer in additional complexity into models of policy interventions, within the idealized scenario considered by the model, one can solve for specific features, e.g., the final outbreak size, the optimal closure percentage *X*, and the critical value of non-compliance *Y* such that weak interventions increase outbreak size.

### Final state of the cloSIR model

The mathematical simplicity of the classic SIR model, on which the cloSIR model is based, allows for a formal analysis of the role of *X* and *Y* on the final outbreak size. In general, the final outbreak size for a given $\lambda$, *X* and *Y* in our model is given by

10 $$\begin{aligned} R(\infty ) = (1 - X) R_o(\infty ) + X R_c(\infty ) \end{aligned}$$

We assume here that $t_c = 0$, and that $S_o(0) \approx 1$, $I_o(0) \ll 1$, and $R_o(0)=0$. These assumptions serve as a natural motivating example while allowing for a less cumbersome mathematical analysis. In this case, $R_o(t_c)$ becomes 0 and so Eq. 10 simplifies to $(1 - X) R_o(\infty )$. Therefore, for convenience, we simply write *R* and *S* to denote the open compartments, since closed compartments will always be empty.

After redistribution, the population sizes are no longer normalized to 1, and we let $P = 1 + \frac{XY}{1 - X}$ be defined as the size of a typical open compartment. Since the equations in (1) are then multiplied through by *P* for each compartment, the infectious dynamics of open compartments will be altered; in particular, the reproductive number $R_0$ is increased to $\lambda P$, leading to a greater force of infection within open compartments. We also let *r*, *s* be the proportion of recovered/susceptible individuals, so that after redistribution, we have $s(t) = S(t) / P$ and $r(t) = R(t) / P$. The outbreak size as a proportion is then

11 $$\begin{aligned} r(\infty ){} & {} = 1 - s(\infty ) \nonumber \\{} & {} = 1 - s(0) \exp (-R_0(r(\infty ) - r(0))\nonumber \\{} & {} = 1 - \exp (-R_0r(\infty ))). \end{aligned}$$

This transcendental equation can then be solved for $r(\infty )$ with respect to a particular set of parameters though numerical means or using the Lambert *W* function. Following Appendix A of Ma & Earn (2006) [51] and elsewhere, $s(\infty ) = -\frac{1}{R_0} W(-R_0 e^{-R_0})$, where *W* is the principal branch of the Lambert *W*-function. Therefore we may write (11) in closed-form, which in turn gives

12 $$\begin{aligned} R(\infty ) = (1 - X + XY)\left( 1 + \frac{W(-R_0 e^{-R_0})}{R_0}\right) . \end{aligned}$$

### Finding an optimal closing intervention

A notable aspect of the cloSIR model is that in many cases, the impact of *X* on the final outbreak size is such that intermediate values of *X* lead to a far worse outbreak, even if increasing *X* far enough towards 1 will be beneficial. In fact, we now show that the optimal value of *X* minimizing Eq. (10) for a given *Y* and $\lambda$ is always 0 (no closures) or 1 (complete closure).

Assuming $\lambda \ge 1$ (when $\lambda < 1$, $X=0$ is clearly as optimal as anything else given that there is no epidemic), one can see from Fig. 1 that $R(\infty )$ as a function of *X* has either a single intermediate peak for higher values of *Y*, or is monotone decreasing for lower values of *Y*. This pervasive downward parabolic shape arises from the fact that $R(\infty )$ is the product of the linearly decreasing, positive function $f(X) = 1 - X + XY$, and the sigmoidal, positive function $g(X) = 1 + (W(-R_0 e^{-R_0})) / R_0$, where $\frac{dg}{dX}$ approaches 0 as *X* approaches 1. This guarantees that $R(\infty )$ is maximized at one of the extreme values $X=0$ or $X=1$.

While Eq. (12) is not defined at $X=1$, we can obtain a right-hand limit. Using that $\lim _{X\rightarrow 1+} -R_0e^{-R_0} = 0$ and $W(x) \approx x$ for small values of *x*, we have as $X\rightarrow 1$ that

$$\begin{aligned} R(\infty ){} & {} = (1 + e^{-R_0})P(1 - X)\\{} & {} = 1 - X + XY + e^{-R_0}(1 - X + XY)\\{} & {} \rightarrow Y. \end{aligned}$$

This makes sense, since we would expect that $r(\infty )$ be equal to 1 when $R_0\rightarrow \infty$, so plugging this into Eq. (11) and simplifying gives $R(\infty ) = Y$ for $X=1$.

**Fig. 2:**
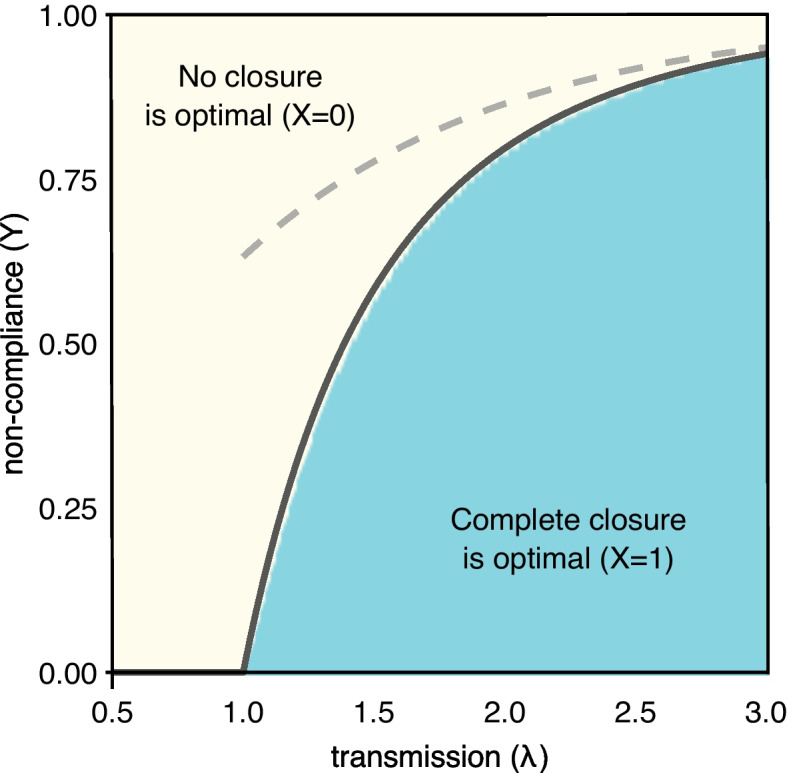
Value of *X* giving the minimum value of Eq. (12), as a function of *Y* and $\lambda$, based on numerical simulation of the cloSIR model. A clear transition from $X = 0$ (yellow) to $X=1$ (blue) is seen, with no intermediary values. The solid black line corresponds to the theoretical closed-form solution from Eq. (13), while the dashed grey line corresponds to the rough approximation $Y > 1 - e^{-\lambda }$ past which it is best to not have any closures

This leads to the section’s main result, which is summarized in Fig. 2:

13 $$\begin{aligned} \underset{X}{\mathrm {arg\,min}} R(\infty ) = \left\{ \begin{array}{ll} 0 &{} \text {if } Y > \left( 1 + \frac{W(-\lambda e^{-\lambda })}{\lambda }\right) \\ 1 &{} \text {otherwise.} \end{array}\right. \end{aligned}$$

While this is in closed form, *W* cannot be expressed with elementary functions and hence poses similar interpretability issues to practitioners as implicit solutions or numerical approximations. Thankfully, a number of useful approximations for *W* exist. For example, here we can use the crude estimate $W(x) < x$ for $-1/e \le x < 0$ to obtain the bound $Y > 1 - e^{-\lambda }$, which serves as sufficient criteria to be certain that no closure is the best option.

### Using the cloSIR model on a network to explore the impact of mobility shifts

The general model of cloSIR on networks is more complicated: we specify the epidemic dynamics with the normalized transmission rate $\lambda$ (equal to the ratio of transmission rate to recovery rate). The intervention and compliance of the population are specified independently for each county *i* through $X_i$, $Y_i$ and potentially through its timing $t_{c,i}$. The size of populations $N_i$ are implicitly specified by $S_i(0)+I_i(0)+R_i(0)$. And finally, the contact patterns both within and across populations are specified by the coupling terms $\rho _{i,j}$.

The goal of this general model however is to explain how uneven changes in mobility patterns across multiple populations can contribute to spreading an epidemic even if the total contact rate goes down by shifting the direction of contacts to lower incidence communities. To do so, we focus on the simplest possible network of two communities, $i \in {1,2}$, of equal size and density $\rho _{1,1} = \rho _{2,2} = 1$, with an initially symmetric coupling $\rho _{1,2} = \rho _{2,1} \equiv \rho$ but where community 1 has no intervention and community 2 has a variable intervention specified by $t_{c,2} = 1$, $X_2 \equiv X$ and $Y_2 \equiv Y$. With this set-up, the total frequency of contacts always goes down following an intervention with $X>0$, but as we will see the final outcome does not always improve.

**Fig. 3:**
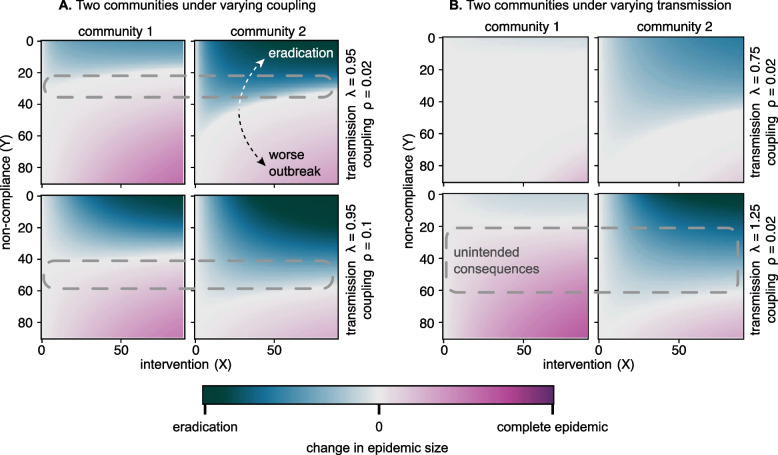
**A** Fixing $\lambda = 0.95$, we show the change in final epidemic size per community (community 1 on the left and 2 on the right) for different interventions (axes) and coupling strength (rows). **B** Fixing $\rho = 0.02$, we repeat the experiment now varying the epidemic transmission rate (rows). Color scale goes from best outcome (eradication of epidemic) in dark blue, to no change in grey, to worst possible outcome ($R(\infty ) = 1$) in dark magenta. In both panels, we use a dashed area to highlight the regime of unintended consequences where a useful intervention in community 2 worsen the epidemic in community 1

In Fig. 3A, we first look at the outcome of this scenario against epidemics close to their epidemic threshold. We fix $\lambda = 0.95$ and explore the impact of different interventions (*X*, *Y*) in community 2 for a series of different coupling strengths $\rho$. The key outcome of these results is that, in every considered case, we find a range of parameters such that community 2 can implement an intervention to improve its own situation while worsening the epidemic in community 1.

We then look at the impact of epidemic strength by changing the transmission rate $\lambda$ and keeping a fixed low value of coupling across communities $\rho =0.02$, shown in Fig. 3B. We find that at low coupling and against strong epidemics, it is extremely hard for an intervention in community 2 to not worsen the outbreak in the neighboring community with weaker intervention (and in this case, no intervention at all).

Together, these results show that the risk of unintended consequences from an intervention here barely depend on the scale of the intervention *X* (the fraction of gatherings closed). This result is consistent with what we found in the previous section. For community 2, the optimal intervention is to either close nothing ($X=0$) or everything ($X=1$). Unfortunately, the latter can lead to unintended consequences and an increased incidence in community 1. We find that the risk of these unintended consequences is determined by three factors outside of the control of community 2:

1. The compliance of the population *Y*; there exists a critical range of *Y* values for which an intervention in community 2 will cause unintended consequences in community 1.
2. The coupling $\rho$; surprisingly, a weaker coupling between populations increases the risk of unintended consequences.
3. The transmission rate of the disease $\lambda$, where strong epidemics mean that an intervention in community 2 almost always leads to unintended consequences for community 1.

These results might represent what we would expect if one county sees a much higher incidence than surrounding populations and therefore implements closures without coordinating with its neighbors. The higher incidence drives closures, which shifts mobility patterns towards counties with fewer closures; and this shift in turn can increase the total spread of the disease. This unintended consequence will be seen in community 1 if non-compliance *Y* is larger than some threshold $Y_1$ (determined by the coupling $\rho$ and transmission rate $\lambda$) and will be seen in both communities if *Y* is larger than some other threshold $Y_2 > Y_1$. These results therefore suggest, much like previous results on a single population, that large enough shifts in mobility patterns can precede increases in incidence.

Altogether, results from the cloSIR model on networks are in line with those from the simpler cloSIR on individual gatherings. Just like partial closures can worsen an epidemic by concentrating individuals in a fraction of open gatherings, a strong intervention in one location can worsen an epidemic since shifts in the mobility patterns can increase contacts across communities even if the total contact rate is decreased. Interventions therefore need to be uniform across gatherings of a certain type at a local level, and coordinated with neighboring populations at the global level.

### Adaptive behavior during the COVID-19 pandemic

**Fig. 4:**
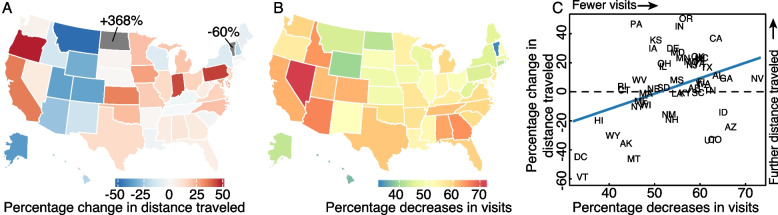
Changes in distances traveled and number of visits to churches. **A** The percent change in average distance traveled to churches by state between the first and last week of March 2020. **B** The percent decreases in numbers of unique visitors to churches by state between the first and last week of March 2020. **C** The relationship between percent changes in distance traveled by percent change in number of visits between the first and last week of March 2020. A decreasing number of visits is weakly correlated with an increase in distance traveled

Turning our attention to mobility data, we find that attendees were on average traveling further to attend certain gatherings, in particular church services, during early periods of NPI adoption in the USA. We find that despite seeing an overall 56% (95% CI: 40–76) decrease in visits to churches, comparing the first to the last week of March, individuals that do visit a church travel on average 13% (95% CI: 4–26) further across most states in the country (Fig. 4). Many of these changes could be do to exogenous factors (such as the creation of food banks at many churches) or selection biases (e.g., people traveling further for church might be more committed and therefore less likely to stop going). These are common issues with these mobility data, which we will discuss later, but can be partially tested by looking at other data sets.

**Fig. 5:**
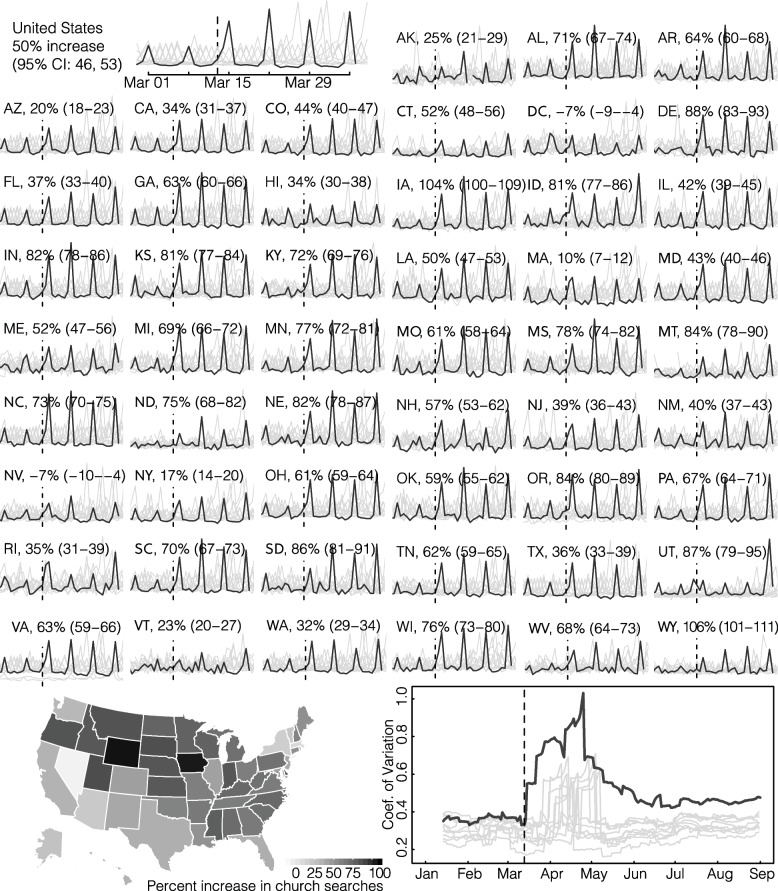
Changes in information-seeking for churches. Sparklines show Google searches for “church + churches” (obtained using the Google Trends API for search) for all states in the USA. Dark line indicates searches in 2020 and lighter lines 2010–2019. Percent increases are comparing Sunday search volume in 2020 to Sunday volumes in 2010–2019. Map on bottom left shows the percent increases as displayed in the sparklines. Bottom right plot shows a 14-day running coefficient of variation of searches for “church + churches.” 2020 saw significant increases in the coefficient of variation that have remained elevated since the US national emergency declaration (March 13th, dashed vertical line)

That individuals are looking and traveling further for churches is also seen in Google search trends, where queries for “churches” increased beginning in March 2020. We show the full data in Fig. 5, with expected weekly spikes on Sundays and we therefore focus our qualitative comparisons on the Sunday values. Overall, we see 49.6% (95% CI: 46.4–52.9) higher search volumes for churches in 2020 as compared to 2010–2019, with similarly higher volumes across states. Indeed, only Nevada and D.C. failed to show statistically significantly higher search volumes in 2020, with the others ranging from 10% (95% CI: 7.0–11.8) in MA to 106% (95% CI: 100.5–111.3) in WY (Fig. 5). Some of these searches may be for individuals looking for online or virtual services which have also increased in recent years, see Additional file 1: Figs. S1 and S2 for a comparison of searches for “church online + church remote + church virtual” to searches for “church” without the terms “online”, “virtual”, or “remote.” While we do indeed find a large increase for individuals searching for online church services, we find even larger differences in search volume between the two with online services being 21- up to 79-fold lower across states than churches without online. Additionally, we compare a 14-day running coefficient of variation of church searches in 2020 to 2010–2019. We see an immediate spike in the coefficient of variation on the day the US president declared a national emergency, March 13th, which peaks in early May and remains elevated through August 2020. These patterns of searches are consistent with our findings using the SafeGraph data, namely increased information-seeking for churches, potentially because an individual’s normal church is closed and they are looking for an open venue. Together, the mobility and search data also support our findings that individuals were physically traveling further to attend church services.

Turning our attention to average church attendance, we find a positive association between distance traveled for church and decrease in average church attendance. More specifically, using a linear regression at the state-level, we estimate that an increase of about 5 additional km traveled is related to a 25% decrease in visitors (95% CI, 15–34, $p=0.002$). While these results do not provide additional insight into the mechanism, they are consistent with individuals traveling further to seek an open church when their local church closes. In addition, we observe that early on in the epidemic (March and April 2020), we see reductions in the mean number of visitors to churches in counties across the USA while concurrently seeing increases in the maximum number of visitors to those churches. This implies that the density of individuals attending church services increased early on.

**Fig. 6:**
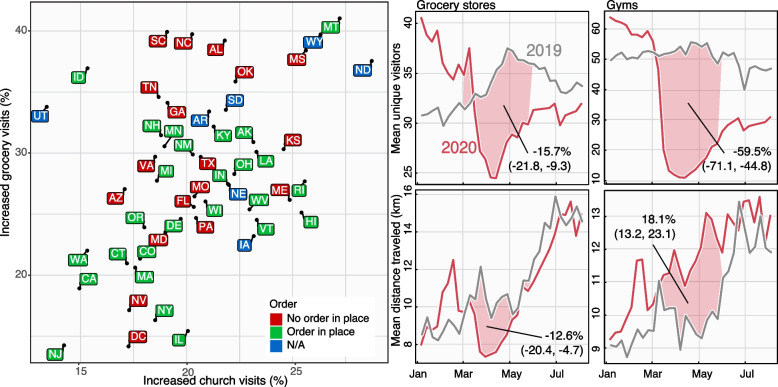
Prevalence of churches and grocery stores with increased numbers of visitors and visitation patterns in 2019 and 2020. Scatter plot of different states based on their increase in visits to essential services as well as whether the state had a stay-at-home order in place before March 29th [52]. These are specific grocery stores or churches who had an increase in visits comparing the first and last week of March. The horizontal axis shows the fraction of churches with an increase in number of visits, and the vertical axis shows the fraction of grocery stores with an increase in number of visits. There is a positive correlation between both but not clear distinction with local policy, suggesting that the phenomenon is related to the local distribution of services and behavior of the local population. Right hand panels show the mean numbers of visitors for grocery stores and gyms (top panels) and the mean distance traveled (bottom panels). While visits and distance traveled both decreased for grocery stores―as would be expected from movement restrictions in place―most states saw increases in visits in 20% to 40% of grocery stores with the remainder driving the decrease. Visits to gyms also dropped drastically, but must like churches, with an associated and sizeable increase in the distance traveled

This phenomenon of increased travel during periods of venue closure is not limited to religious services. We compared differences in numbers of visits to grocery stores that had increases in visits to churches who had increases in visits. We find a correlation between increases to visits to churches with increases in visits to grocery stores at the state level (Pearson’s $r = 0.44$) with increases to grocery stores being higher than to churches (slope = 0.9, 95% CI: 0.37–1.44; Fig. 6). Additionally, we find increases to churches and grocery stores to be largely independent of whether the state had a stay-at-home order in place, suggesting that the phenomenon is closely related to the local distribution of services, individual burden such as food insecurity and behavior of the local population. Comparing mean numbers of visitors and distance traveled for all grocery stores revealed decreases in both―as would be expected from movement restrictions in place―on the other hand, number of gym visits dropped drastically, but saw a sizeable increase in the distance traveled for those visits, which increased throughout the summer.

These mobility and search patterns suggest that individuals may indeed travel further during periods of venue closure and therefore may also increase the epidemiological coupling between distinct communities. Given that the expected number of contacts is expected to increase non-linearly with the number *n* of participants in a gathering (i.e., potential contacts are proportional to $n(n-1)/2 \sim n^2$), it is unclear whether or not closing some venues (e.g., churches, gyms) might be worth the increased risk in the remaining open venues.

### Potential correlations with SARS-CoV-2 incidence

**Fig. 7:**
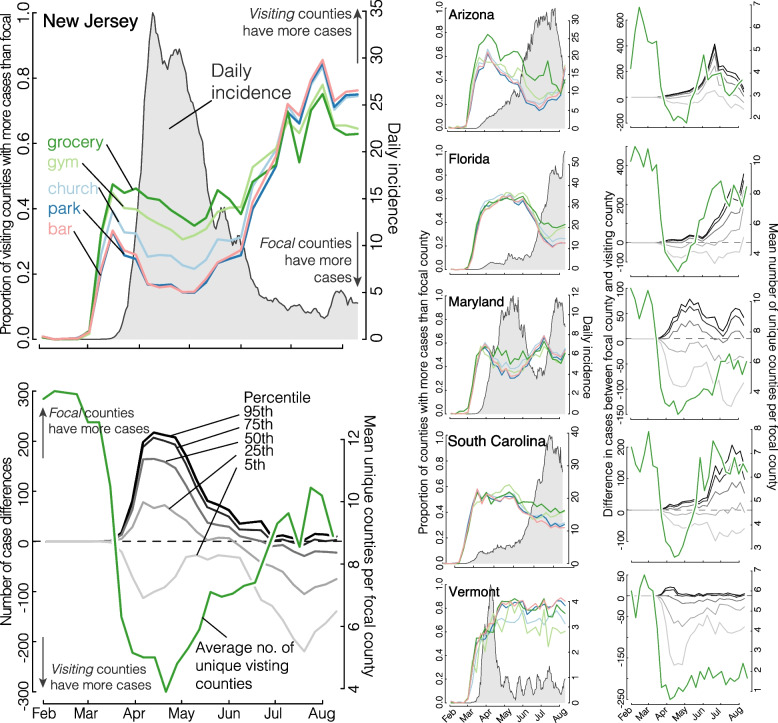
Population movement is influenced by COVID incidence and is highly heterogeneous across states. The large left-hand column shows summaries of cases, movement, and county opening and closing in New Jersey and serves as a guide to interpreting the other panels. New Jersey saw a surge in incidence in early April (cases per 100,000, 7-day running mean, grey shade, top panel). We distinguish between the *focal county*, which is the county receiving visitors from listed *visiting counties*, which are recorded in the SafeGraph data set. We can then calculate the proportion of visiting counties which have more cases than the focal county being visited for churches, gyms, grocery stores, parks, and bars (colored lines in the top left panel). The frequency of trips from higher-incidence to lower-incidence counties appear to lead daily incidence. Finally, we look at the magnitude of population flux in response to incidence in the bottom panel. Grey lines are the 95th, 75th, 50th, 25th, and 5th quantiles of the difference in the numbers of cases between the focal county and the visiting county. There is substantial variation in the numbers of population movement across states. The green line indicates the average number of unique visiting counties per focal county for that state. While most states saw a rebound in the number of unique visiting counties, Vermont maintained a low number of influx of population

Finally, the natural question suggested by the mobility data and the cloSIR model is *Does differential mobility from non-uniform policy implementations lead to unintended consequences in incidence?* Figure 7 summarizes the results. We first distinguish between a *focal county*, which is the county receiving visitors from other, listed *visiting counties*, which are recorded in the SafeGraph data set. We can then calculate the proportion of visiting counties which have more cases than the focal county being visited for churches, gyms, grocery stores, parks, and bars. Nearly uniformly we see that when the focal county has more cases than the visiting counties, COVID incidence goes up, and it goes down when the visiting counties have more cases. That being said, we cannot be more quantitative or definitive about this correlation given the limited number of waves over of COVID-19 over the few months for which we have mobility data. Future studies of these results using separate, better understood, mobility data will be crucial.

We also find large heterogeneity in the magnitude of population movement and cases across states (see Additional file 2) as well as in the average number and population of unique counties visiting a focal county across states. For example, while all states saw a large decline in unique visiting counties, states such as Florida and South Carolina saw rapid rebounds to pre-closure levels (around 2 months) leading to an increase in incidence, contrasted to Vermont which has kept unique visiting counties low and subsequently had not seen a rise in cases over the studied period. Importantly, while we find that movement from dense cities to smaller communities might have been a driving factor in the early epidemic peak, the population size of visiting counties with higher incidence typically returned to a steady-state value fairly quickly (e.g., by mid-April 2020 in Maryland) such that second epidemic peaks are more likely driven by the direction of mobility from high incidence to lower incidence counties rather than by population size alone. To further explore the results, the analysis of Fig. 7 is reproduced for all 50 states and D.C. are presented as additional figures (see Additional file 3).

## Discussion

Through a mathematical model―termed cloSIR―we found that, under certain conditions, interventions meant to stem disease transmission can lead to increased case burden, either locally or in neighboring populations, relative to scenarios with no intervention at all. Then, using real-time mobility and search data in the USA, we found that while overall visits to various types of venues decreased in response to state-level COVID-19 policies implemented during the first 2020 wave, the average distance individuals traveled to visit certain locations increased significantly. This observed increase in travel was corroborated by Google search queries in March, 2020, indicating an increase in information seeking for venues like churches. Our findings are in line with other research showing heterogeneity in mobility and internet search responses across different institutions and locations throughout the pandemic [33, 53–55] and an increase in activities associated with outdoor recreation [23, 56, 57]. Finally, using county-level COVID case data, we found that local SARS-CoV-2 incidence influenced individual movement and vice versa. These dynamics were further modified by the average number of unique visiting counties where states that saw a rapid return to normal unique visiting counties saw large increases in incidence (such as in South Carolina and Florida) and states that maintained a limited number of unique visiting counties saw no increases in cases (Vermont). Taken together, these results indicate the sensitivity and complexity of epidemic dynamics to the distribution across space and time of closure policies.

Balancing the mental, economic, and social health of populations with the serious risks of COVID-19 means that the decision to implement movement restrictions (e.g., *cordons sanitaires*) should be carefully considered. This is the same for other NPIs (e.g., hand washing, facial covering, social distancing, etc.) [58]. Indeed, evidence from China suggests that while the cordon sanitaire of Wuhan delayed the outbreak, it was local measures that slowed transmission and ultimately controlled it [7, 59, 60]. Additionally, studies have shown that outdoor transmission risk of SARS-CoV-2 remains quite low [24] and variability in movement to “blue and green” spaces was not associated with increased risk of COVID-19 cases [23]. Despite evidence of the efficacy of consistent NPIs, many countries (especially the USA) continue to implement control measures in a scattered, patchwork manner [10, 13].

While distance traveled and the number of visits to essential services did not correlate strongly with any demographic variables (e.g., population density, average age), both of these responses did correlate with community tightness, with tight communities being those “with strong norms and little tolerance for deviance” (Fig. S1). Gelfand et al. (2020) found that countries with both efficient governments and those with tight cultures were the most effective in limiting COVID-19 cases and deaths [61]. However, White and Hébert-Dufresne (2020) found the opposite for the USA, with tighter states having faster COVID-19 growth rates early in the pandemic [11]. In the context of our cloSIR model, if a government expects compliance issues and complete lockdown is not possible, it could be best to have no lockdown at all (Figs. 1 and 2) or at the very least coordination with neighboring populations (Fig. 3). This is an extreme example and we do not advocate foregoing NPIs but instead an acknowledgment that one community’s ability to control a pandemic like COVID-19 is dependent both on its own policies and the policies of neighboring communities. This also stresses how local interventions aimed at reducing transmission/disease severity, e.g., mask wearing, testing, vaccination, etc., may be even more important in scenarios where the heterogeneity in or failure of regional/global policies leads to increased case numbers. Finally, additional work should focus on assessing practical values of the rate of non-compliance. Periodic surveys of Facebook users reveal a wide range of mask use and percentage of those experiencing COVID-like symptoms across counties in the USA (see Supplementary Information). In addition, future work could examine how these effects vary at different spatial scales of human mobility and NPI implementation.

There are several important caveats to our study. First, we developed a simple model that was able to illustrate the potential unintended consequences of individuals adapting their behavior to seek essential services under inconsistent physical distancing policies. While the simplicity of this model is a strength when trying to isolate the effects of inconsistent control policies on COVID-19 transmission, future work will be needed before such models could be used to actively inform specific policy decisions. Additionally, models with heterogeneous closure policies can result in both reduced case numbers and increased social activity if interventions are targeted at reducing super-spreading [62, 63]. Second, because the SafeGraph data do not track individual users over long periods of time, those observed in late March are not necessarily the same individuals observed earlier in the month. Moreover, we may expect biases in the diversity and behaviors of individuals tracked by the system since different types of gatherings attract different individuals. Third, the sample-based nature of the SafeGraph data as well as our method for selecting churches, bars, groceries, gyms, and parks mean we fail to capture all of these venues in the USA. However, we do not expect geographical biases with these two limitations and that our lists are representative of the USA. These limitations mean that small geographic regions should not be directly compared to one another, or even to themselves at a different time, and different locations should not be directly compared. This is why we coarse-grained our results over states, why we mostly compared relative changes and not absolute differences, and why we attempted to correlate our findings with a secondary data source like online searches. Future work is therefore warranted, on both data collection and analysis (comparing changing movement patterns for various other business types) and mathematical modeling.

## Conclusions

Our results suggest second-order interactions between disease transmission and population movement: High local incidence could drive local closures which decrease global connectivity but, as in our cloSIR model, could increase coupling across gatherings and populations. Both our models and data stress that interventions need to be uniform across gatherings of a certain type at a local level, and coordinated with neighboring populations at the global level. We also note that while we cannot assess causality, the association between movement and cases provides a starting point for future research.

Altogether, it appears of key importance that NPIs―specifically related to business and venue closure―be implemented in a coordinated way across space in time. Similarly, relaxation of such interventions must be done methodically and over time, with a strong emphasis on equity across incomes and geographies to avoid endangering individuals with lower socioeconomic status [55, 64]. Our findings also have indirect implications for the roll-out of vaccination booster programs. Vaccination acceptance already varies across the USA as it did with past vaccination efforts [65–68]. This allows for the possibility of unvaccinated individuals being clustered geographically and where concentration of contacts among unvaccinated individuals can cause or worsen outbreaks. Human behavior is a strong driver of the transmission dynamics of SARS-CoV-2 and care must be taken to reduce the heavy burden imposed by COVID-19 and avoid unintended, negative consequences from inconsistent policies around implementing and relaxing mobility restrictions and/or venue closure policies [36].

## Supplementary information

**Additional file 1.** Additional details on materials and analyses.

**Additional file 2.** Statistics on mobility patterns for every county.

**Additional file 3.** Mobility and incidence for all 50 states and Washington, D.C.

## Data Availability

All data were freely available from their respective sources during the project. As of this writing, the COVID-19 Data Repository (https://github.com/CSSEGISandData/COVID-19) [50] remains openly available. However, SafeGraph data (https://www.safegraph.com/academics) [47] are now available only through an institutional subscription model. Code for the cloSIR model is also available online (https://github.com/LaurentHebert/cloSIR) [69].
