# Supplementary material for: The unintended consequences of inconsistent closure policies and mobility restrictions during epidemics
Source: BMC Glob Public Health. 2023 Dec 4;1:28. doi: 10.1186/s44263-023-00028-z (PMC11116187; doi:10.1186/s44263-023-00028-z)
Supplement: Supplementary file 1 — Additional file 1. Additional details on materials and analyses. [file 44263_2023_28_MOESM1_ESM.pdf]

Additional File 1: The unintended consequences of inconsistent closure policies and mobility restrictions during epidemics

Benjamin M. Althouse[1,2], Brendan Wallace[3], B. K. M. Case[4,5], Samuel V. Scarpino[5,6,7,8,9], Antoine Allard[5,10,11], Andrew M. Berdahl[12], Easton R. White[13,14], and Laurent Hébert-Dufresne[4,5,10,*]

[1]University of Washington, Seattle, WA 98105
[2]New Mexico State University, Las Cruces, NM 88003
[3]Department of Applied Mathematics, University of Washington, Seattle, WA 98195, USA
[4]Department of Computer Science, University of Vermont, Burlington, VT 05405, USA
[5]Vermont Complex Systems Center, University of Vermont, Burlington, VT 05405, USA
[6]Institute for Experiential AI, Northeastern University, Boston, Massachusetts, USA
[7]Department of Health Sciences, Northeastern University, Boston, MA, USA
[8]Khoury College of Computer Sciences, Northeastern University, Boston, MA, USA
[9]Santa Fe Institute, Santa Fe, NM, USA
[10]Département de physique, de génie physique et d'optique, Université Laval, Québec (Québec), Canada G1V 0A6
[11]Centre interdisciplinaire en modélisation mathématique, Université Laval, Québec (Québec), Canada G1V 0A6
[12]School of Aquatic & Fishery Sciences, University of Washington, Seattle, WA 98195, USA
[13]Department of Biological Sciences, University of New Hampshire, Durham, NH, 03824, USA
[14]Gund Institute for Environment, University of Vermont, Burlington, VT 05405, USA
[*]laurent.hebert-dufresne@uvm.edu

Data

SafeGraph movement data

SafeGraph is a data company that aggregates anonymized location data from numerous applications in order to provide insights about physical places. To enhance privacy, SafeGraph excludes census block group information if fewer than five devices visited an establishment in a month (two devices in a week) from a given census block group. SafeGraph has made public data on foot traffic and visits to places free for COVID-19 researchers. This data is built on a panel of 50 million devices that collect anonymous location data across the US and includes movements by census block group and weekly numbers of visitors for over 6 million places. Using these data we use counts of visits and unique visitors to these places as well as the distance traveled from 'home' (defined as the common nighttime location for the device over a 6 week period where nighttime is 6 pm to 7 am). We calculate the changes in visits over time by location and the distance traveled from each unique county. To get movement patterns for specific venues, we search the business name for 'church' not 'chicken' (to remove visits to the fast-food chain Church's Chicken), 'bar' or 'tavern', 'park', 'grocery', and 'gym'.

Google search data

Google searches for 'church + churches', 'bar + bars', 'park + parks', 'grocery', and 'gym + gyms' were monitored using Google Trends for all 50 US states, D.C., and nationally from January 1, 2010 to October 1, 2020 and were normalized by the number of searches per 10,000,000 searches over the time period. Data were downloaded using the Trends Application Programming Interface for health. To examine increases in searches for churches in the month following the declaration of emergency on March 13, 2020 we compare search volumes for Sundays from March 13 to April 13 to Sundays in March 13 to April 13 from 2010–2019. This approximates a counterfactual scenario to account for the Catholic observance of Lent that occurs around this period. To assess potential differences in search behaviors for the first six months of the epidemic, we calculate the coefficient of variation (search volume standard deviation divided by mean search volume) for January to September for all years.

SARS-CoV-2 case data

SARS-CoV-2 incidence data at the county level were downloaded from the COVID-19 Data Repository by the Center for Systems Science and Engineering at Johns Hopkins University at the county level beginning in February, 2020. We join SafeGraph movement data with case counts at the county level and to assess movement in response to cases, we distinguish a *focal county* to *visiting counties*. That is, for each venue type (church, bar, park, grocery, gym), and each unique county represented by that venue type (this is the focal county) we calculate the total number of cases in that county for that week. We keep this as well as total cases divided by the population of that county. Next we calculate the total cases for each represented visiting counties to venues represented. Finally, we calculate the mean number of unique visiting counties by week and county.

With these data we calculate two metrics, one is the proportion of visiting counties which have more cases than the focal county being visited, and the other is the raw difference in cases between the focal and visiting counties. For the latter we calculate the 5th, 25th, 50th, 75th, and 95th quantiles of numbers of cases. We can then compare these two metrics by state with observed incidence in that state. To quantify any associations between the proportion of visiting counties larger than focal counties, we take the cross-wavelet of the two time series and see where the power is significant and the relative phase angle between the two.

Included as supplementary materials are plots of proportions of visiting counties with more cases than the focal county and differences in cases between visiting and focal counties for all 50 US states and D.C., and cross-wavelet plots for churches, parks, gyms, groceries, and bars and cases for all 50 US states and D.C..

Alternative motives for searching for churches

It is not possible to understand the purpose of an individual search query, nor who is the searcher. For example a search containing the word “church” may be for individuals searching for remote, or online broadcast of church services. Naturally this is for retaining individual privacy. We can, however, try to disentangle aggregate general search queries for “churches” from seeking “virutal” church services. We compare searches for “church online + church remote + church virtual” to searches for “church” without the terms “online”, “virtual”, or “remote”. Searches for online services all saw large increases in the month of March 2020 (top panel Fig. S1). The middle panel of Fig. S1 shows searches for only “church” without the terms “online”, “virtual”, or “remote”; we see similar patterns to those presented in the main text. However, when we compare the volume of searches between those seeking online services versus not online services we see 21- to 79- fold differences across states between the two (Fig. S2).

Mobility data correlates

The results presented in the main text on variations in mobility are affected by many factors. First, in Fig. S3, we split the signal of mobility to churches (in number of participants and distance travelled) across time and across smaller and larger venues. This additional analysis shows that overall visits to churches remained low from March through August, the distance traveled to larger churches (those with more than a mean of 50 visitors per week) saw an increase in average distance traveled.

Second, we used the social, demographic, and economical variables compiled by White & Hébert-Dufresne[1] to examine potential correlates for the mobility data. We found that none of these variables strongly correlated with either percentage decreases in visits or percent change in distance traveled (results not shown). However, increases in state-level “tightness" was correlated with larger decreases in church visits and farther distanced traveled (Fig. S4). Tight cultures are typically defined as those with strong social norms and little tolerance for deviance[2].

Statistics of county-to-county mobility patterns

See Additional file 2 for a collection of figures showing some of the studied statistics on county-to-county mobility patterns. We look for instance at the average population size of the origin counties of visitors (called visiting counties) to counties of interest (called focal counties) conditioned on the visiting county having a higher incidence than the focal county. This statistic is especially useful for states that had two clear epidemic peaks over the studied period and we here focus our discussion on Maryland (MD). Looking at individual counties allows us to determine that the epidemic peaks were not simply single epidemic peaks in different regions, but also to parse out the different mobility patterns driving case data at different times. To see that, we can focus on specific counties such as the Somerset, St. Mary’s, Prince George’s, Howard, Harford, Charles, Baltimore County, and Anne Arundel counties. There, the average size of visiting counties with higher incidence tends to start very high as the first peak in case data might have been driven by mobility from high-incidence dense-population centers to smaller counties. However, the average size of these high incidence visiting counties falls back to a relatively steady value by early to mid-April, over 60 days before the start of the second peak. This second epidemic peak is then not simply driven by mobility from large cities to smaller communities but by the directionality of mobility among these smaller counties.

Heterogeneity of COVID-related behaviors

One could look at data from surveys of random Facebook users to attempt to quantify the rates of non-compliance by county, but these tend to be incredibly heterogeneous. Data were gathered by the Carnegie Mellon Delphi Group (accessible at delphi.cmu.edu/covidcast)[3] and report the percentage of people who report wearing a mask most or all of the time while in public as well as the percentage of people with COVID-like symptoms. Figure S5 shows wide variation in mask wearing across counties in the US.

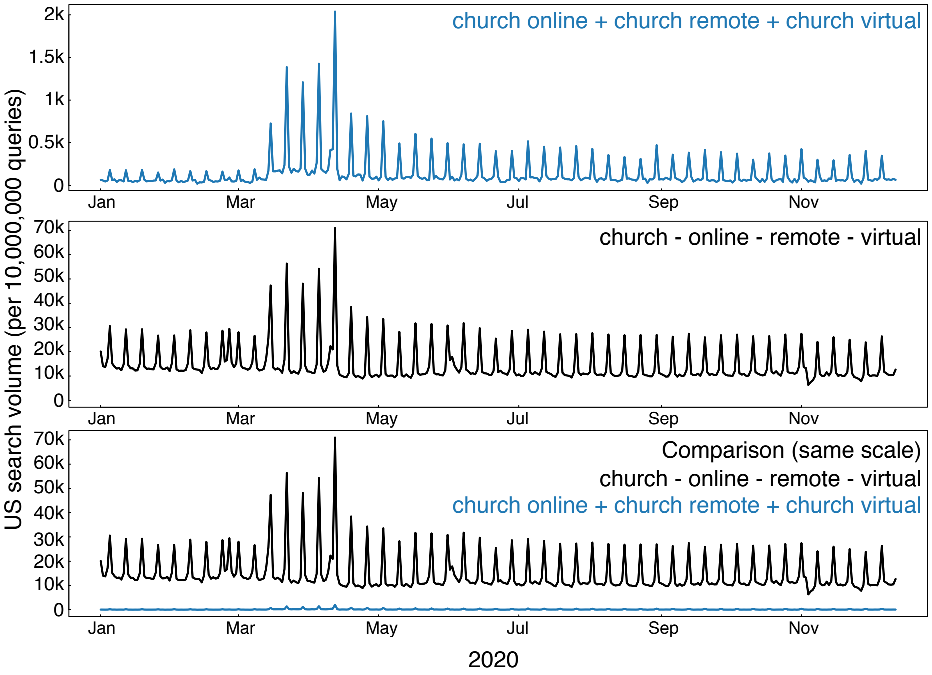

Figure S1. Comparing searches for online church services versus no online services. Figure shows searches for "church online + church remote + church virtual" possibly indicating individuals seeking information on remote services from their local church (top panel). The middle panel shows searches for "church" without the terms "online", "virtual", or "remote". The bottom panel compares the relative search volume comparing the two differing queries.

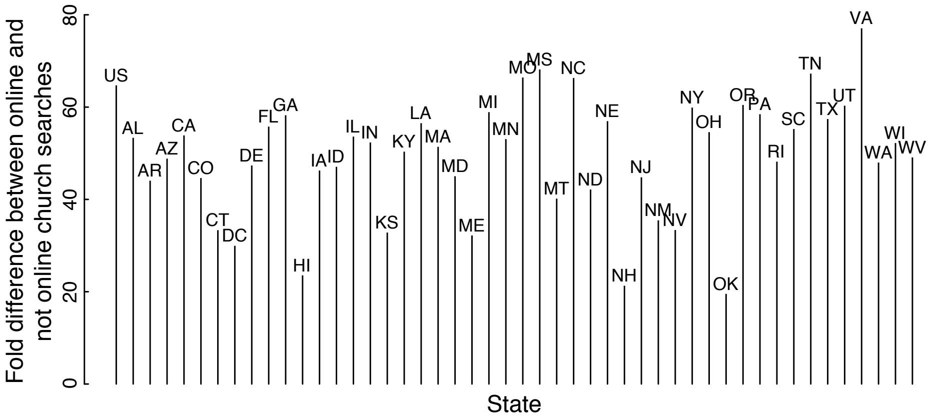

Figure S2. Fold difference between online and not online church services. AK, SD, VT, and WY are omitted due to having zero search volume for online church searches.

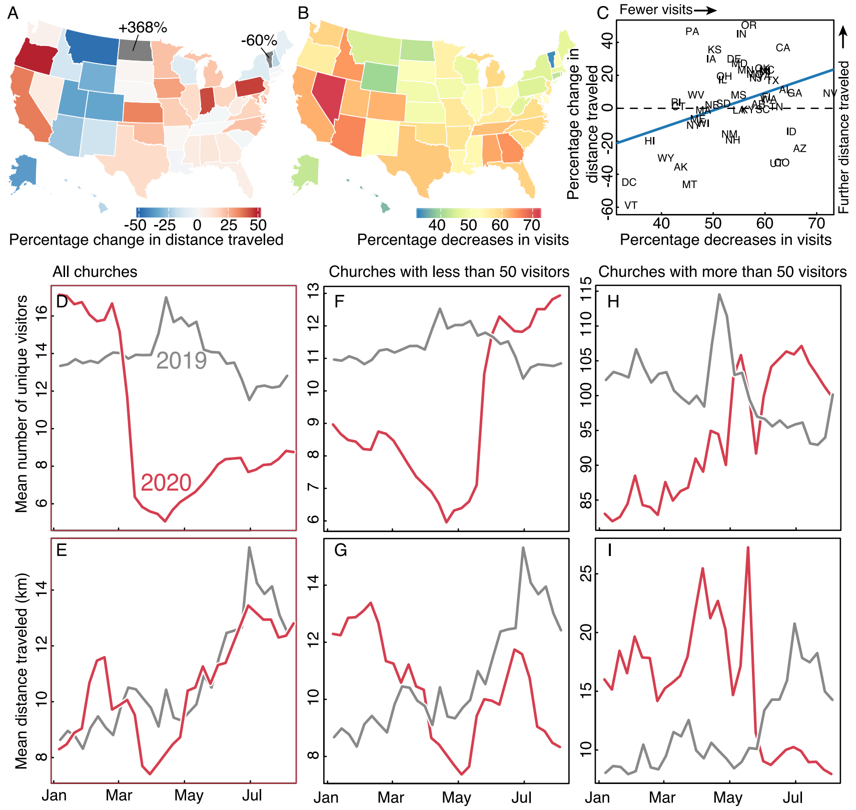

Figure S3. Panels A, B and C are reproductions of Figure 4 in the main text. Panels D & E illustrate the expected variations through time in mobility to churches and show the mean unique visitors and distance traveled (respectively) for all churches in the US from January through August comparing 2019 and 2020. Panels F & G show visitors and distance in churches with less than 50 visitors, and panels H & I show visitors and distance in churches with more than 50 visitors. While social distancing measures limited overall church visits, the distance traveled to large churches increased.

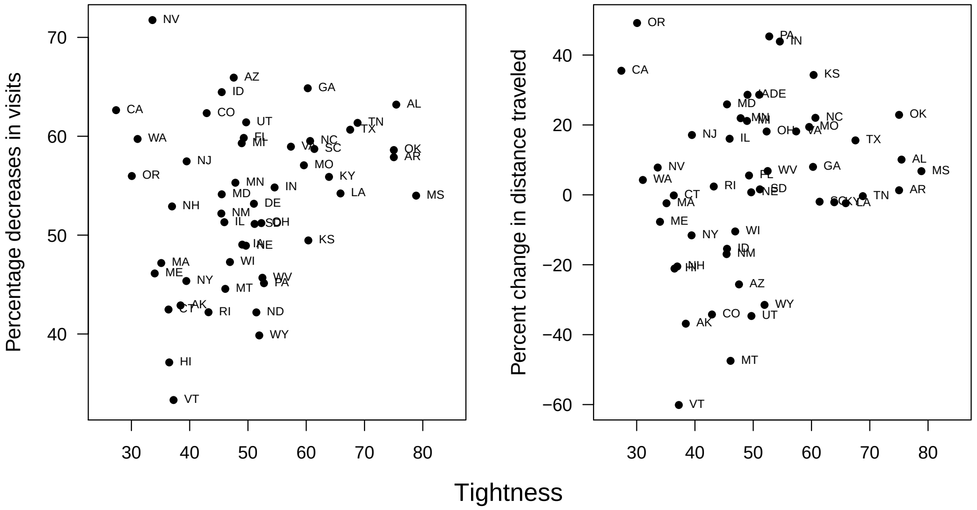

Figure S4. (a) Percent decreases in numbers of unique visitors to churches versus tightness by state. (b) Percent change in distances traveled to churches versus tightness by state.

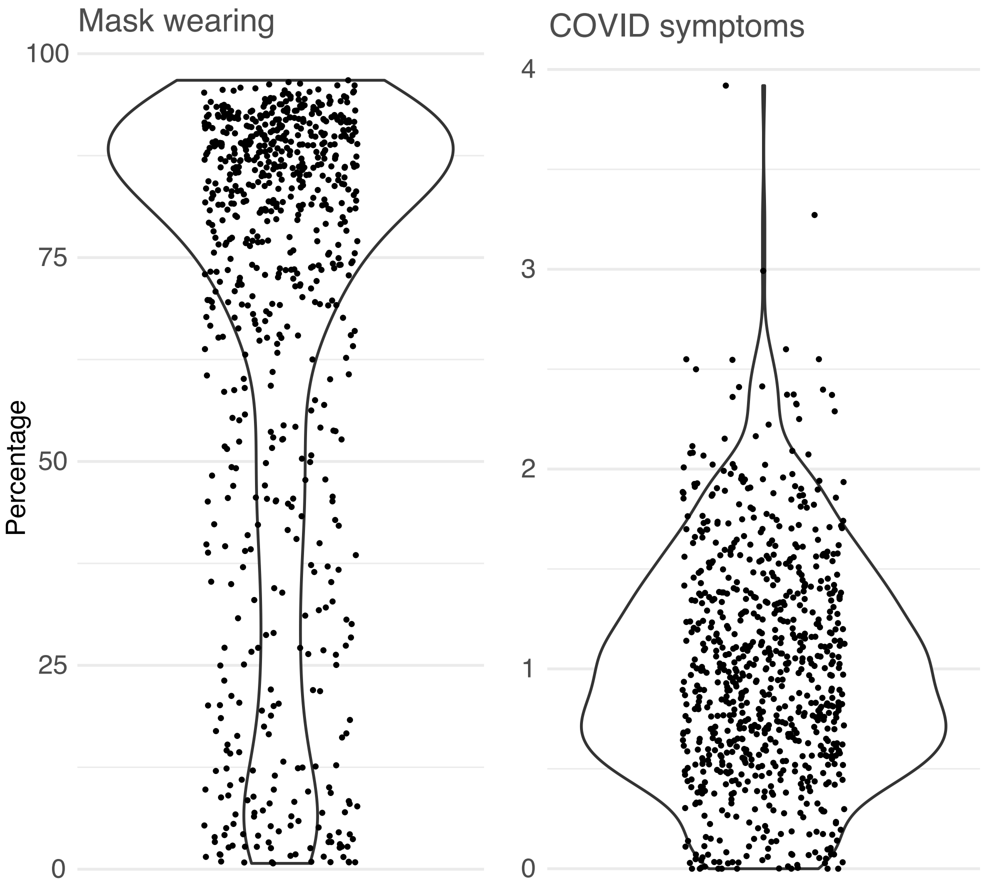

Figure S5. Behavioral metrics. Figure shows the average percentages of those who report wearing a mask most or all of the time while in public (left) and those reporting COVID-like symptoms. Averages are across county from September 8, 2020 for mask usage, and across county from November 18, 2020, both until December 18, 2020.

References

1. White, E. R. & Hébert-Dufresne, L. State-level variation of initial covid-19 dynamics in the united states. *PLOS ONE* **15**, e0240648, DOI: 10.1371/journal.pone.0240648 (2020).

2. Gelfand, M. J. *et al.* The importance of cultural tightness and government efficiency for understanding covid-19 growth and death rates. *Preprint]. PsyArXiv. https://doi. org/10.31234/osf. io/m7f8a* (2020).

3. Nguyen, Q. C., Yardi, I., Gutierrez, F. X. M., Mane, H. & Yue, X. Leveraging 13 million responses to the us covid-19 trends and impact survey to examine vaccine hesitancy, vaccination, and mask wearing, january 2021-february 2022. *BMC Public Health* **22**, 1–15 (2022).
