# Supplementary material for: The unintended consequences of inconsistent closure policies and mobility restrictions during epidemics
Source: BMC Glob Public Health. 2023 Dec 4;1:28. doi: 10.1186/s44263-023-00028-z (PMC11116187; doi:10.1186/s44263-023-00028-z)
Supplement: Supplementary file 3 — Additional file 3. Mobility and incidence for all 50 states and Washington, D.C. [file 44263_2023_28_MOESM3_ESM.pdf]

Additional File 3: The unintended consequences of inconsistent closure policies and mobility restrictions during epidemics

Benjamin M. Althouse[1,2], Brendan Wallace[3], B. K. M. Case[4,5], Samuel V. Scarpino[5,6,7,8,9], Antoine Allard[5,10,11], Andrew M. Berdahl[12], Easton R. White[13,14], and Laurent Hébert-Dufresne[4,5,10,*]

[1]University of Washington, Seattle, WA 98105
[2]New Mexico State University, Las Cruces, NM 88003
[3]Department of Applied Mathematics, University of Washington, Seattle, WA 98195, USA
[4]Department of Computer Science, University of Vermont, Burlington, VT 05405, USA
[5]Vermont Complex Systems Center, University of Vermont, Burlington, VT 05405, USA
[6]Institute for Experiential AI, Northeastern University, Boston, Massachusetts, USA
[7]Department of Health Sciences, Northeastern University, Boston, MA, USA
[8]Khoury College of Computer Sciences, Northeastern University, Boston, MA, USA
[9]Santa Fe Institute, Santa Fe, NM, USA
[10]Département de physique, de génie physique et d'optique, Université Laval, Québec (Québec), Canada G1V 0A6
[11]Centre interdisciplinaire en modélisation mathématique, Université Laval, Québec (Québec), Canada G1V 0A6
[12]School of Aquatic & Fishery Sciences, University of Washington, Seattle, WA 98195, USA
[13]Department of Biological Sciences, University of New Hampshire, Durham, NH, 03824, USA
[14]Gund Institute for Environment, University of Vermont, Burlington, VT 05405, USA
[*]laurent.hebert-dufresne@uvm.edu

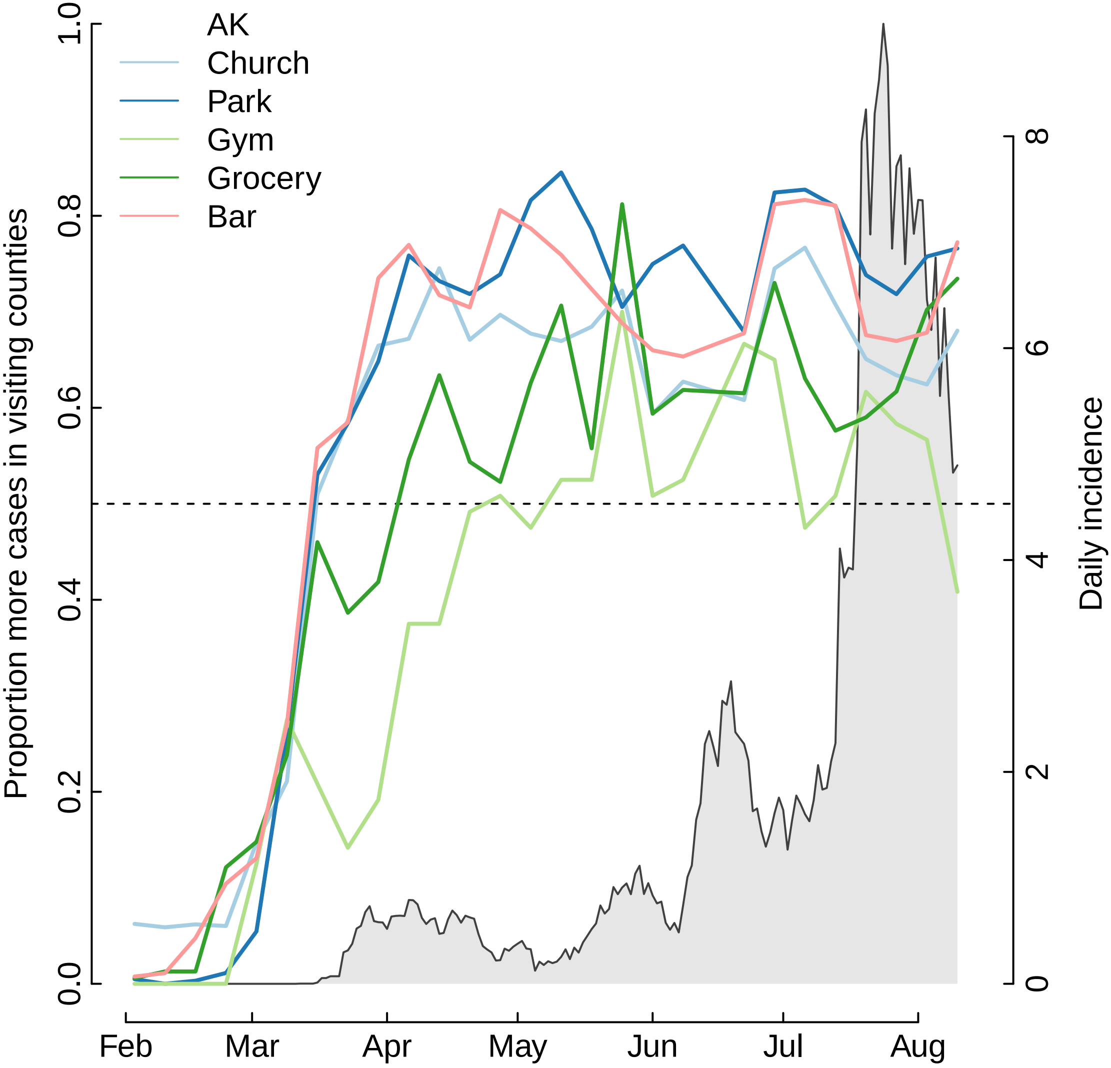

AK
Church
Park
Gym
Grocery
Bar
Proportion more cases in visiting counties
Daily incidence
0.0
0.2
0.4
0.6
0.8
1.0
0
2
4
6
8
Feb
Mar
Apr
May
Jun
Jul
Aug

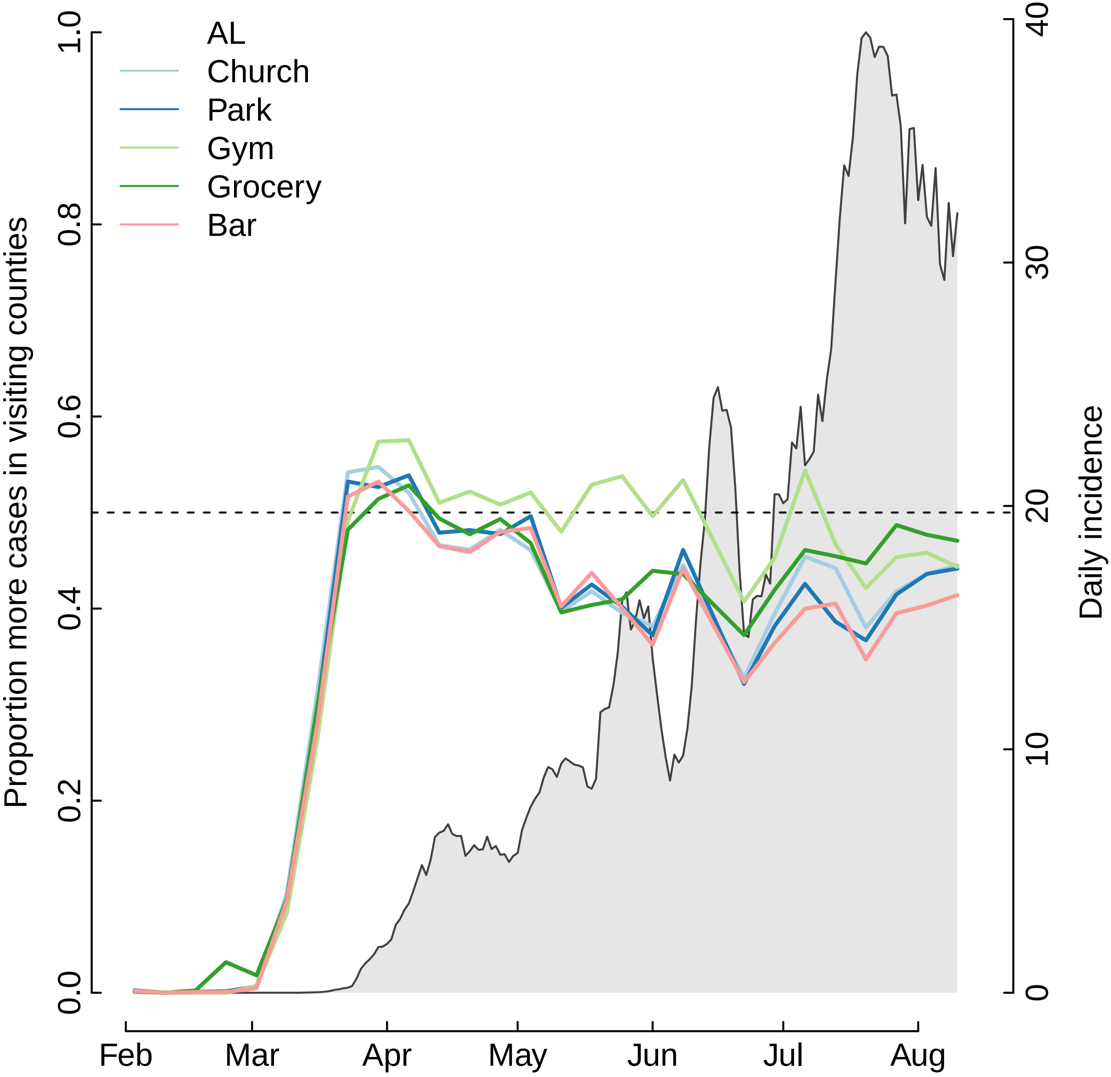
AL
Church
Park
Gym
Grocery
Bar
Proportion more cases in visiting counties
Daily incidence
0.0
0.2
0.4
0.6
0.8
1.0
0
10
20
30
40
Feb
Mar
Apr
May
Jun
Jul
Aug

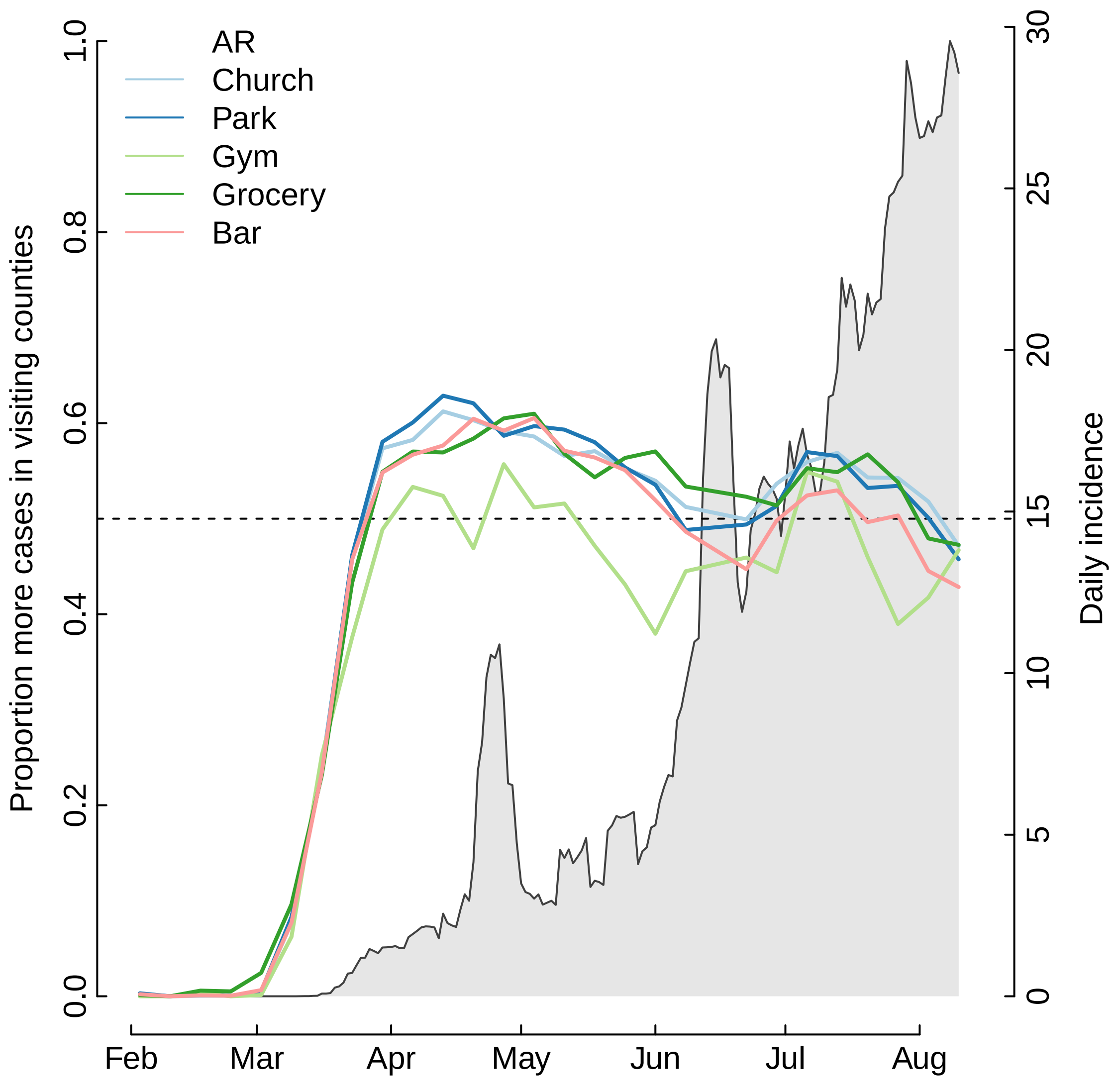

AR
Church
Park
Gym
Grocery
Bar
Proportion more cases in visiting counties
Daily incidence
0.0
0.2
0.4
0.6
0.8
1.0
0
5
10
15
20
25
30
Feb
Mar
Apr
May
Jun
Jul
Aug

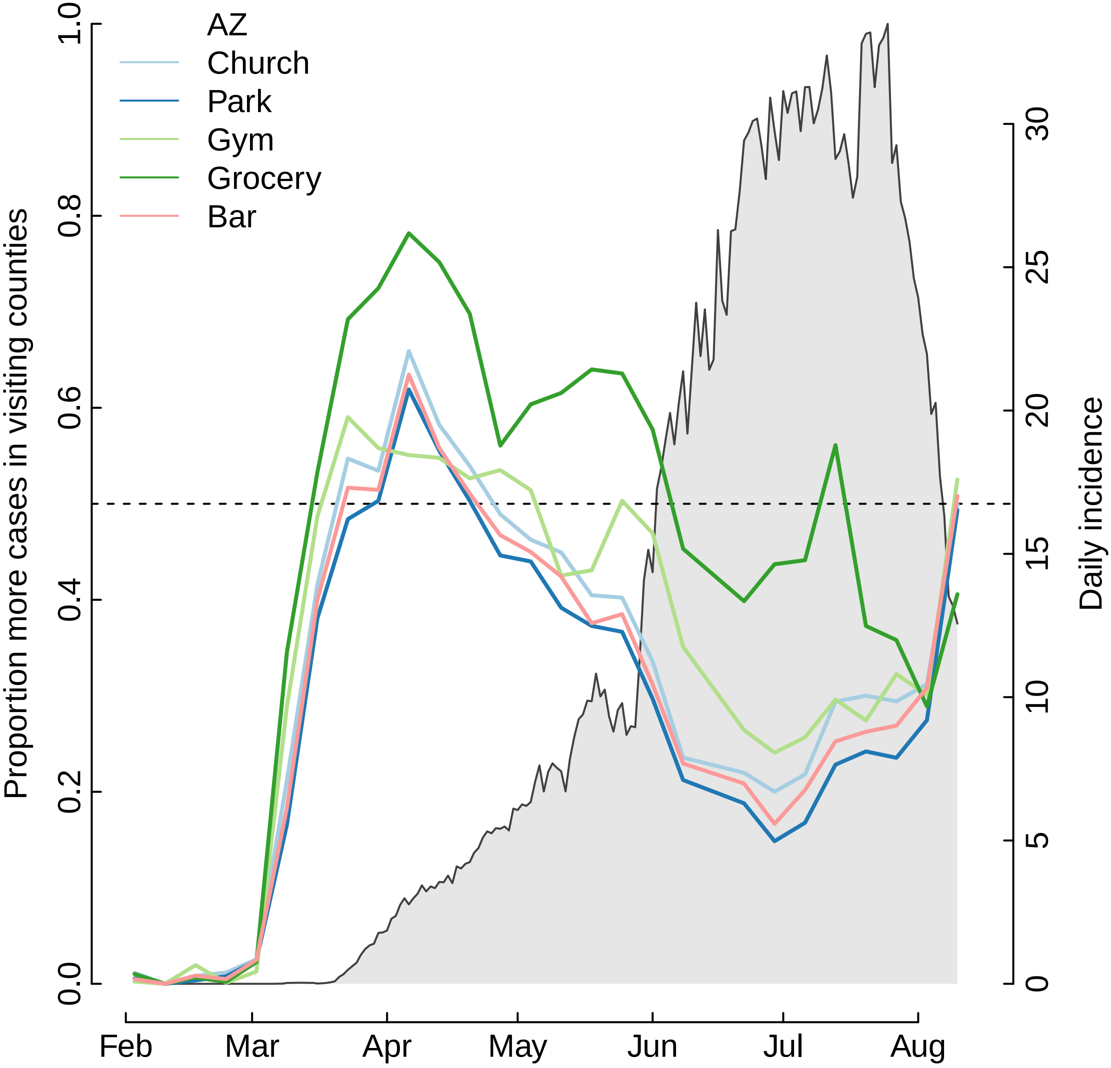

AZ
Church
Park
Gym
Grocery
Bar
Proportion more cases in visiting counties
Daily incidence
0.0
0.2
0.4
0.6
0.8
1.0
0
5
10
15
20
25
30
Feb
Mar
Apr
May
Jun
Jul
Aug

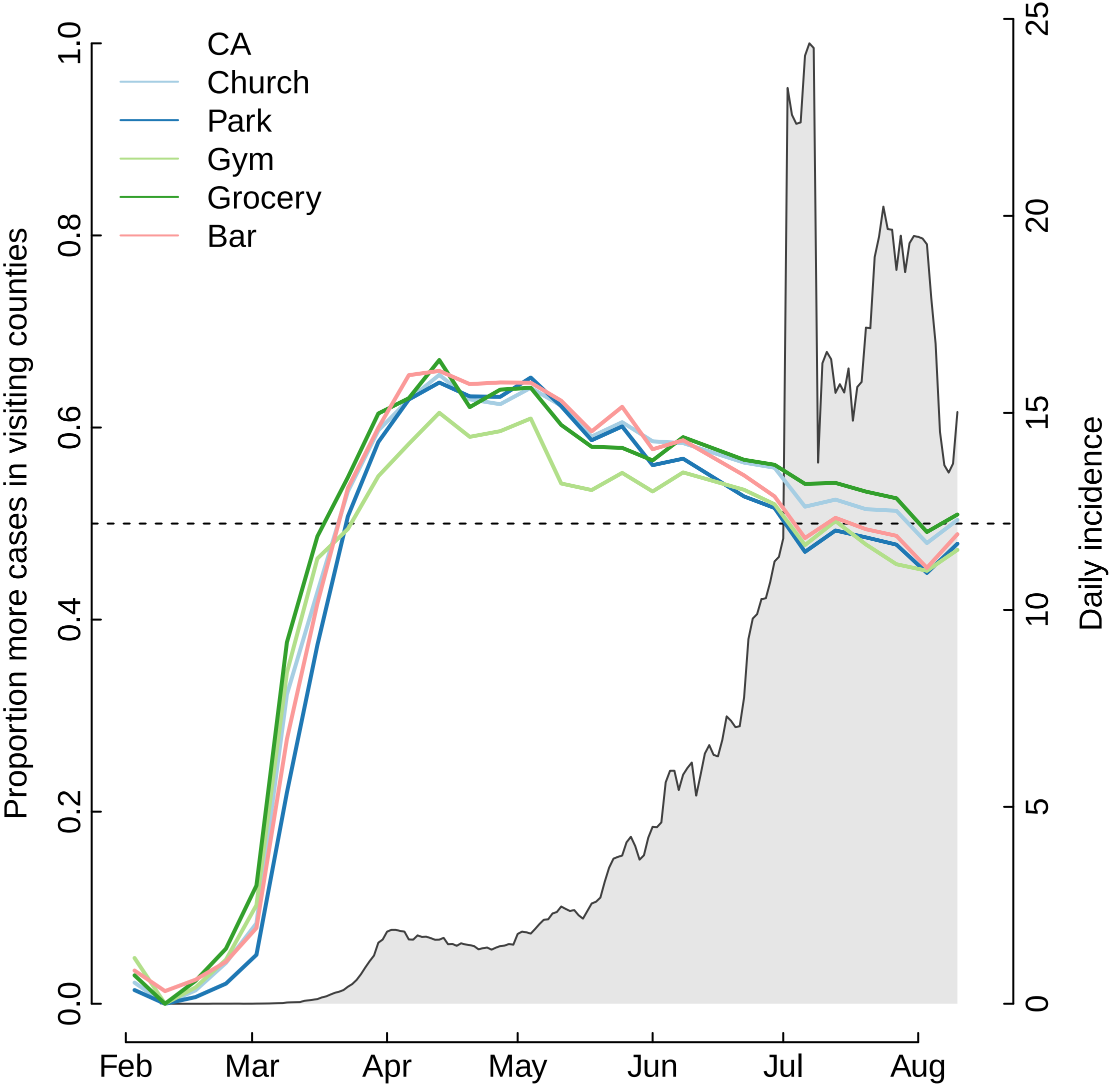

CA
Church
Park
Gym
Grocery
Bar
Proportion more cases in visiting counties
Daily incidence
0.0
0.2
0.4
0.6
0.8
1.0
0
5
10
15
20
25
Feb
Mar
Apr
May
Jun
Jul
Aug

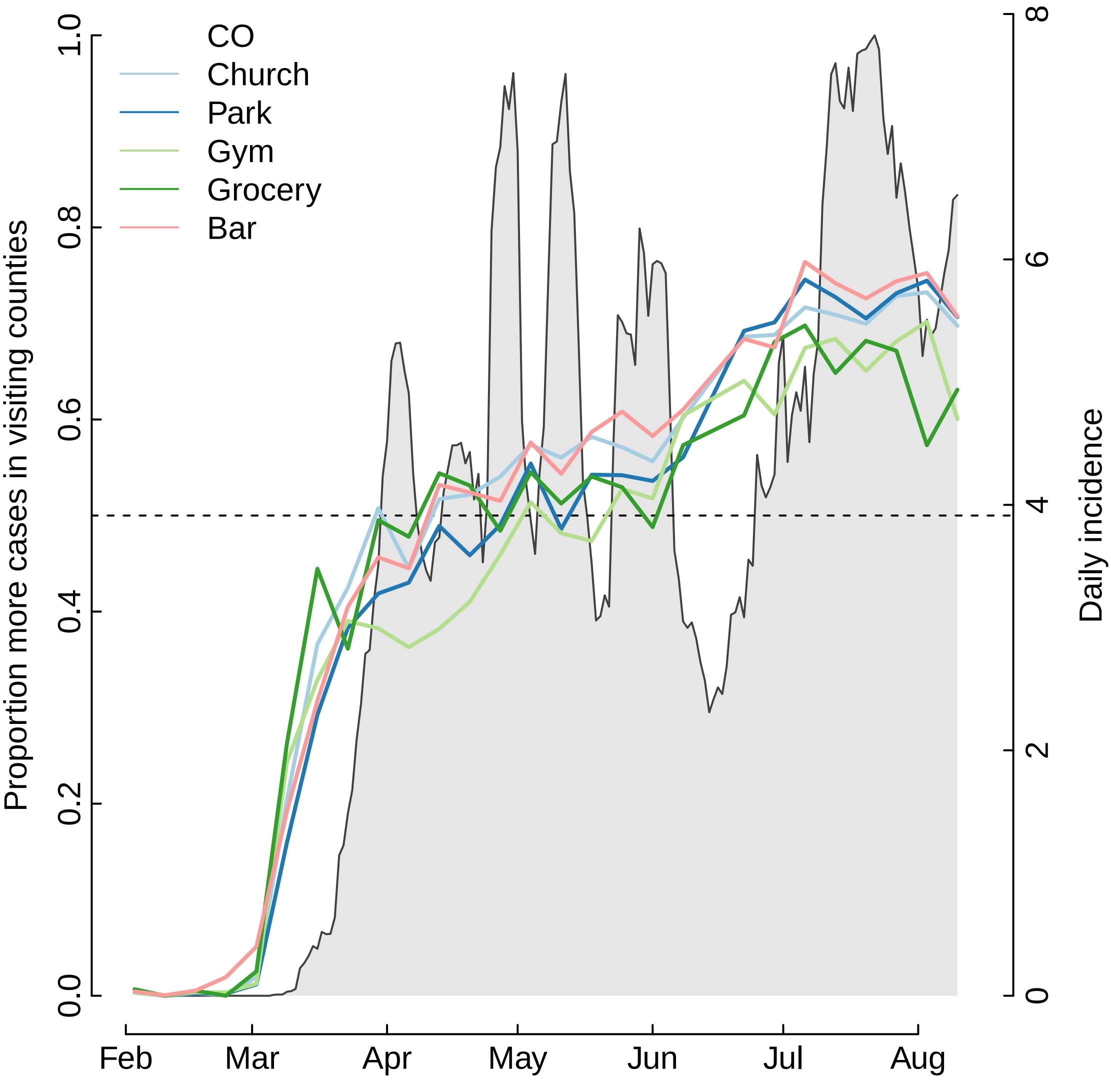
CO
Church
Park
Gym
Grocery
Bar
Proportion more cases in visiting counties
0.0
0.2
0.4
0.6
0.8
1.0
Daily incidence
0
2
4
6
8
Feb
Mar
Apr
May
Jun
Jul
Aug

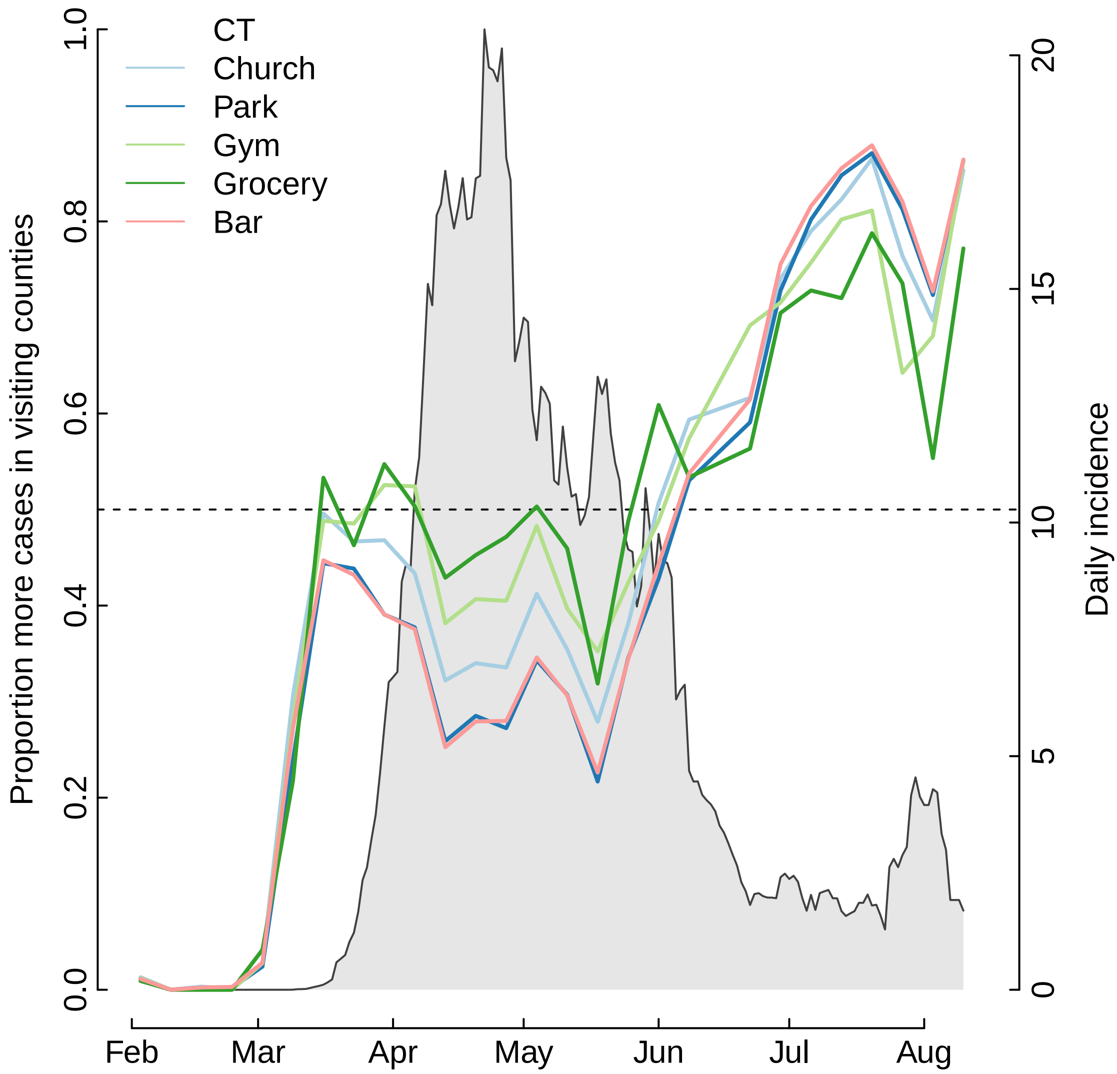

CT
Church
Park
Gym
Grocery
Bar
Proportion more cases in visiting counties
0.0
0.2
0.4
0.6
0.8
1.0
Daily incidence
0
5
10
15
20
Feb
Mar
Apr
May
Jun
Jul
Aug

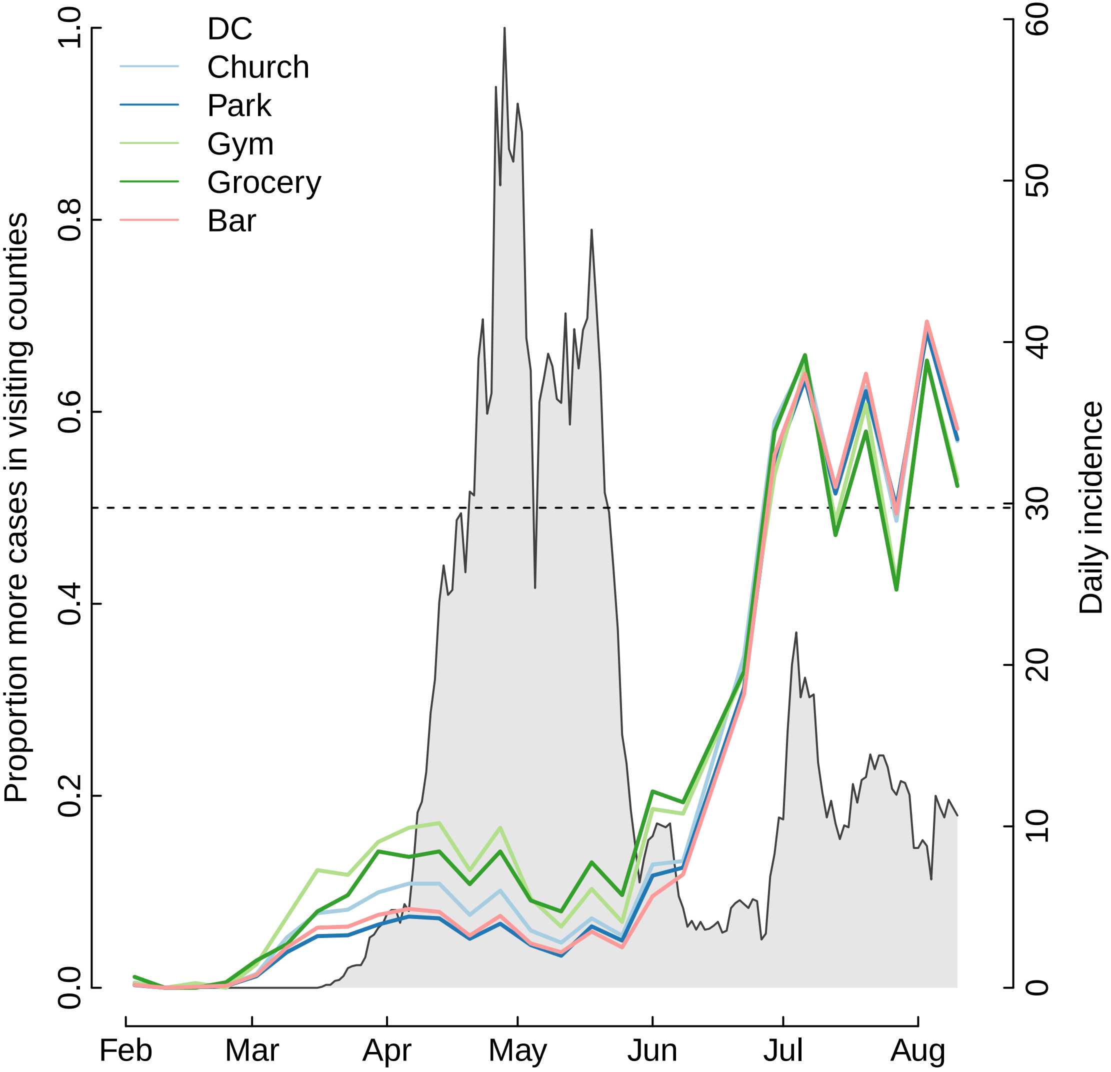

DC
Church
Park
Gym
Grocery
Bar
Proportion more cases in visiting counties
Daily incidence
0.0
0.2
0.4
0.6
0.8
1.0
0
10
20
30
40
50
60
Feb
Mar
Apr
May
Jun
Jul
Aug

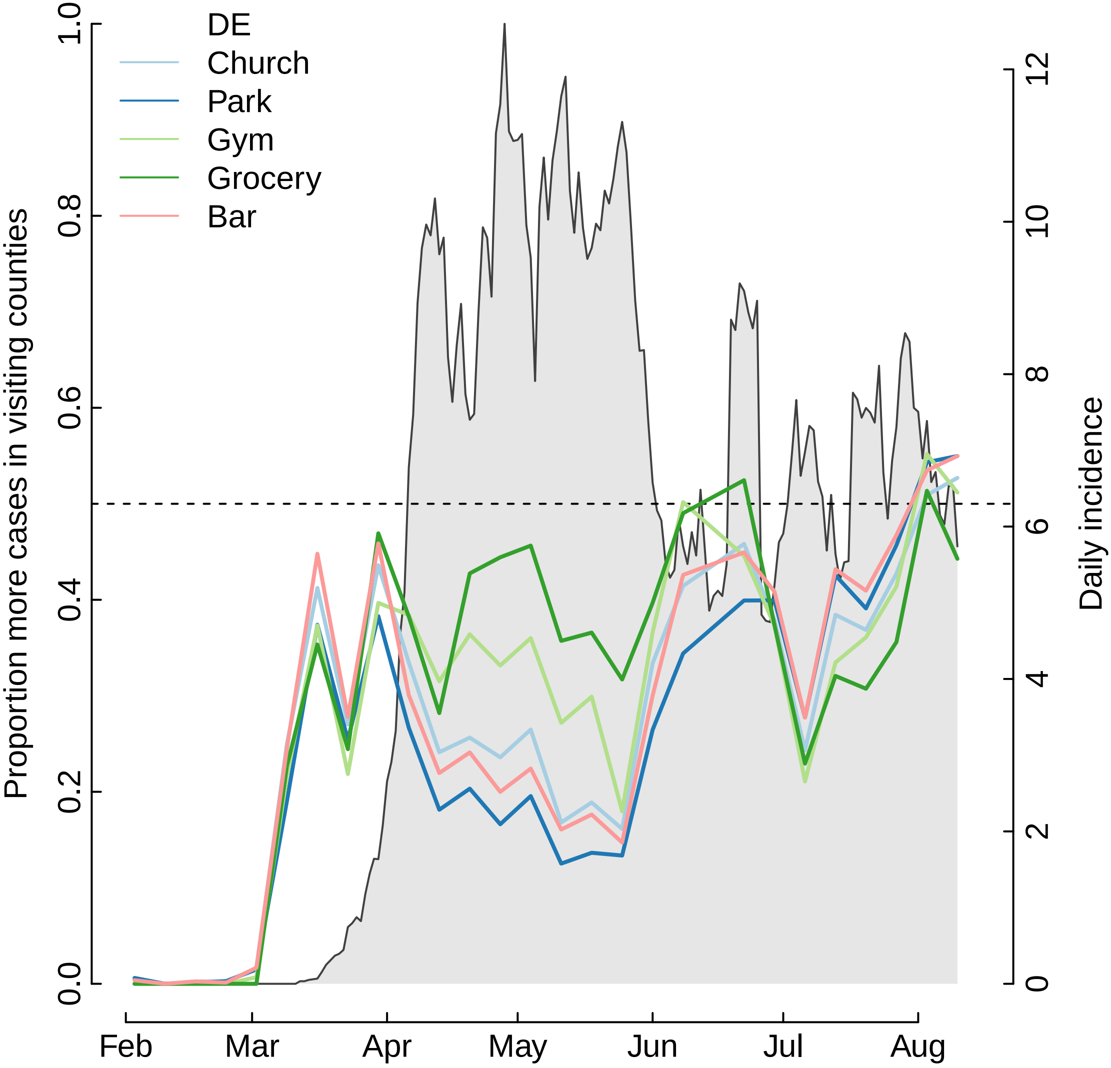
DE
Church
Park
Gym
Grocery
Bar
Proportion more cases in visiting counties
Daily incidence
0.0
0.2
0.4
0.6
0.8
1.0
0
2
4
6
8
10
12
Feb
Mar
Apr
May
Jun
Jul
Aug

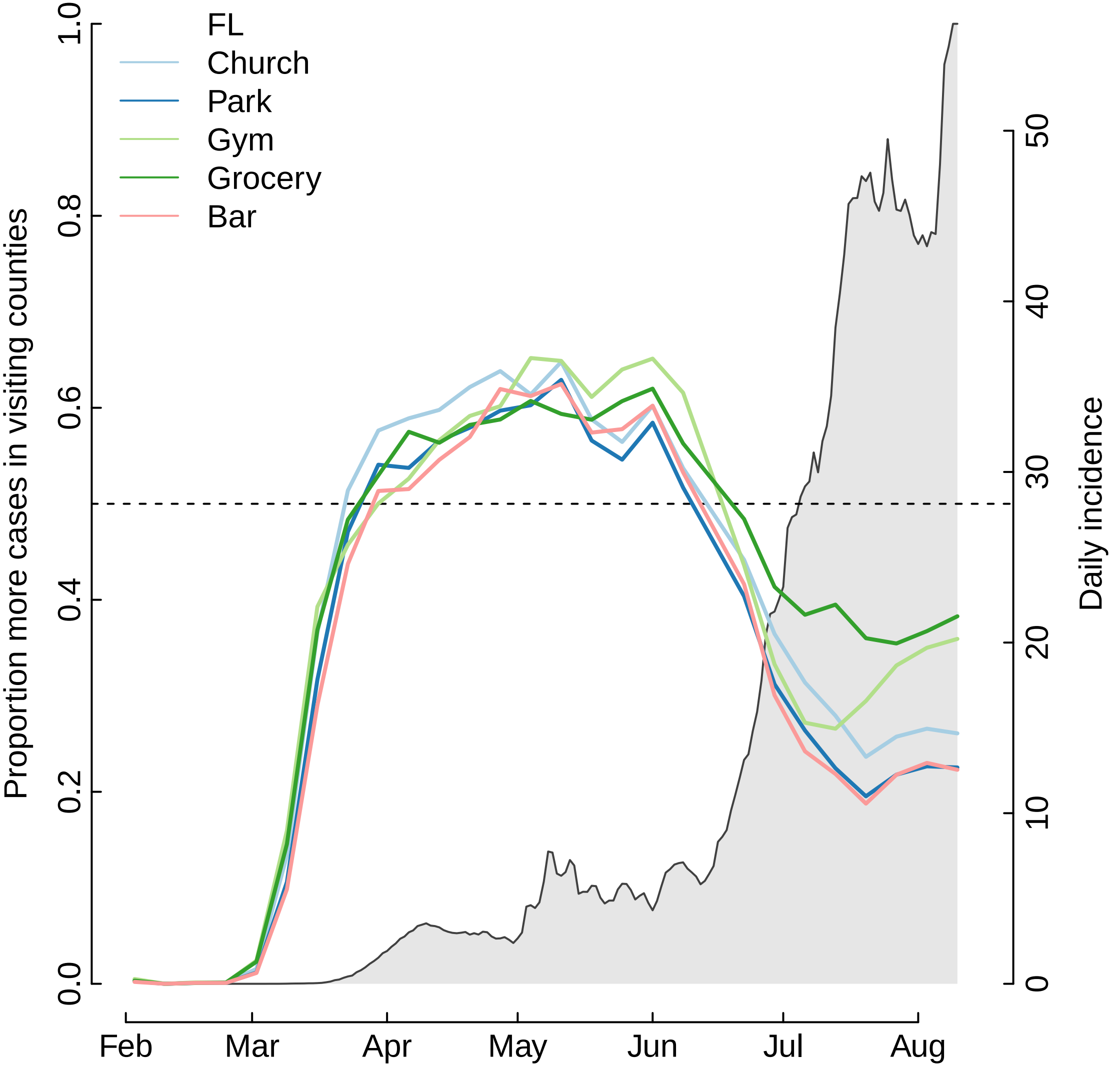
FL
Church
Park
Gym
Grocery
Bar
Proportion more cases in visiting counties
Daily incidence
0.0
0.2
0.4
0.6
0.8
1.0
0
10
20
30
40
50
Feb
Mar
Apr
May
Jun
Jul
Aug

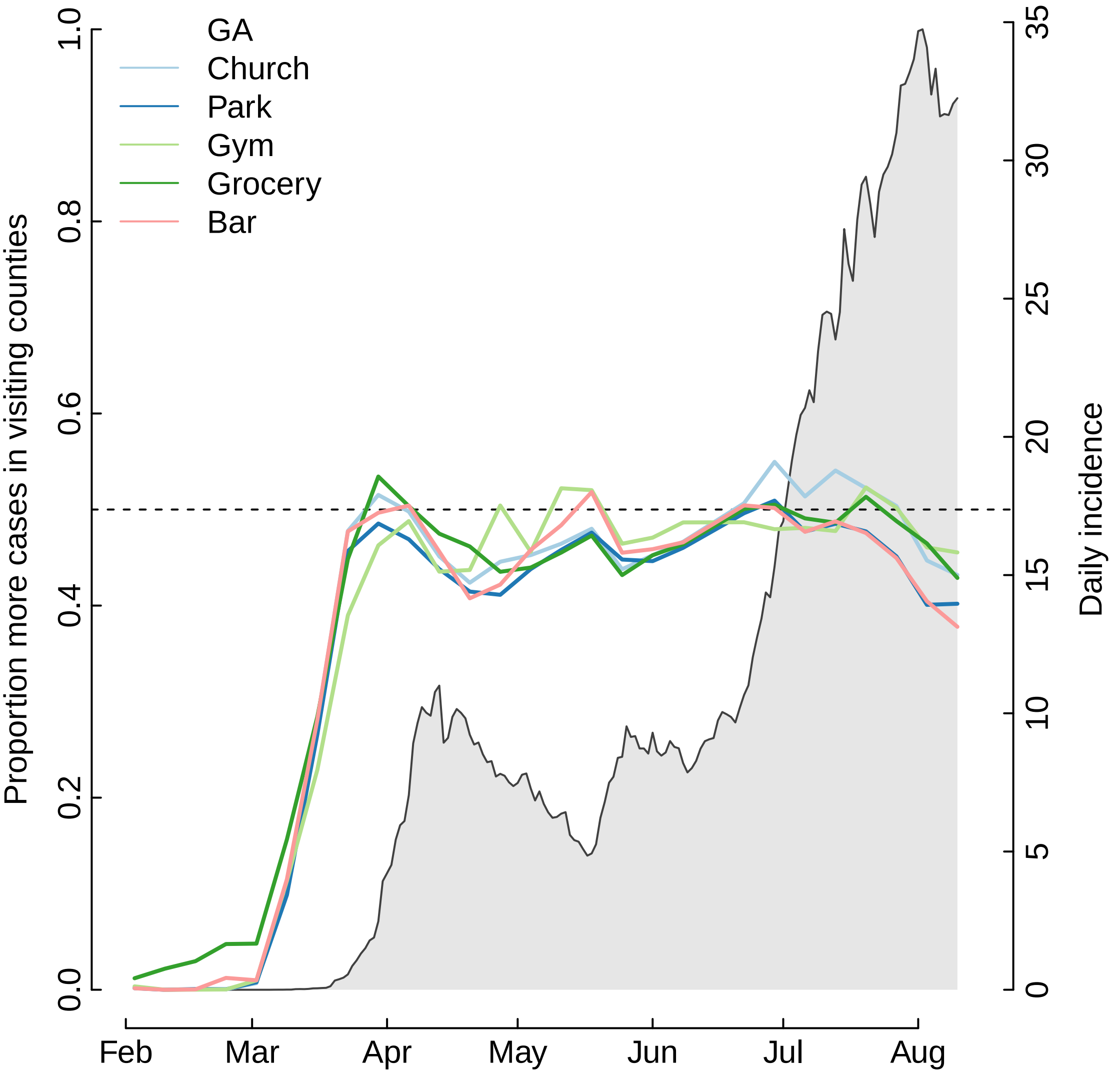

GA
Church
Park
Gym
Grocery
Bar
Proportion more cases in visiting counties
Daily incidence
0.0
0.2
0.4
0.6
0.8
1.0
0
5
10
15
20
25
30
35
Feb
Mar
Apr
May
Jun
Jul
Aug

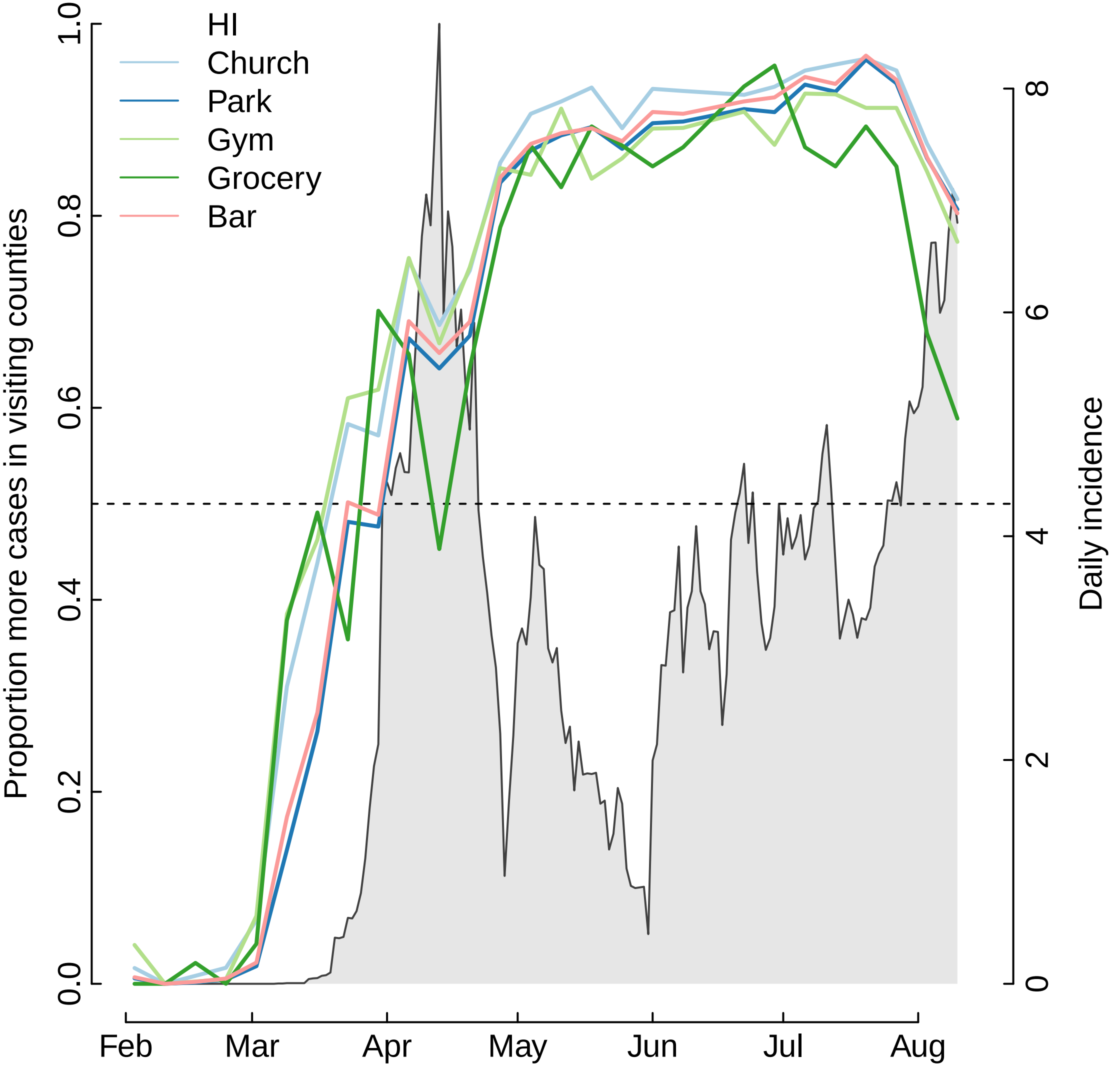

HI
Church
Park
Gym
Grocery
Bar
Proportion more cases in visiting counties
Daily incidence
0.0
0.2
0.4
0.6
0.8
1.0
0
2
4
6
8
Feb
Mar
Apr
May
Jun
Jul
Aug

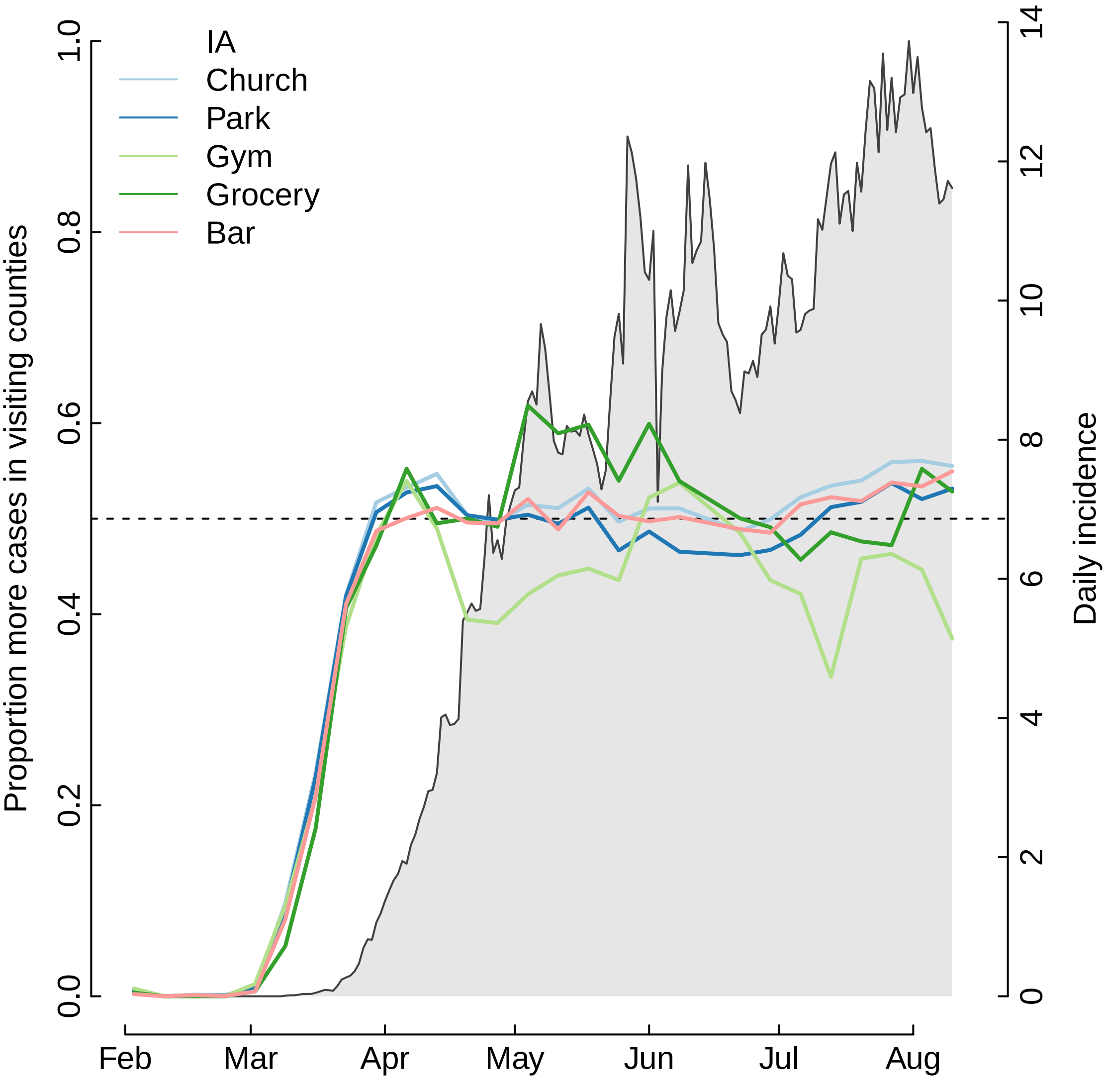

IA
Church
Park
Gym
Grocery
Bar
Proportion more cases in visiting counties
Daily incidence
0.0
0.2
0.4
0.6
0.8
1.0
0
2
4
6
8
10
12
14
Feb
Mar
Apr
May
Jun
Jul
Aug

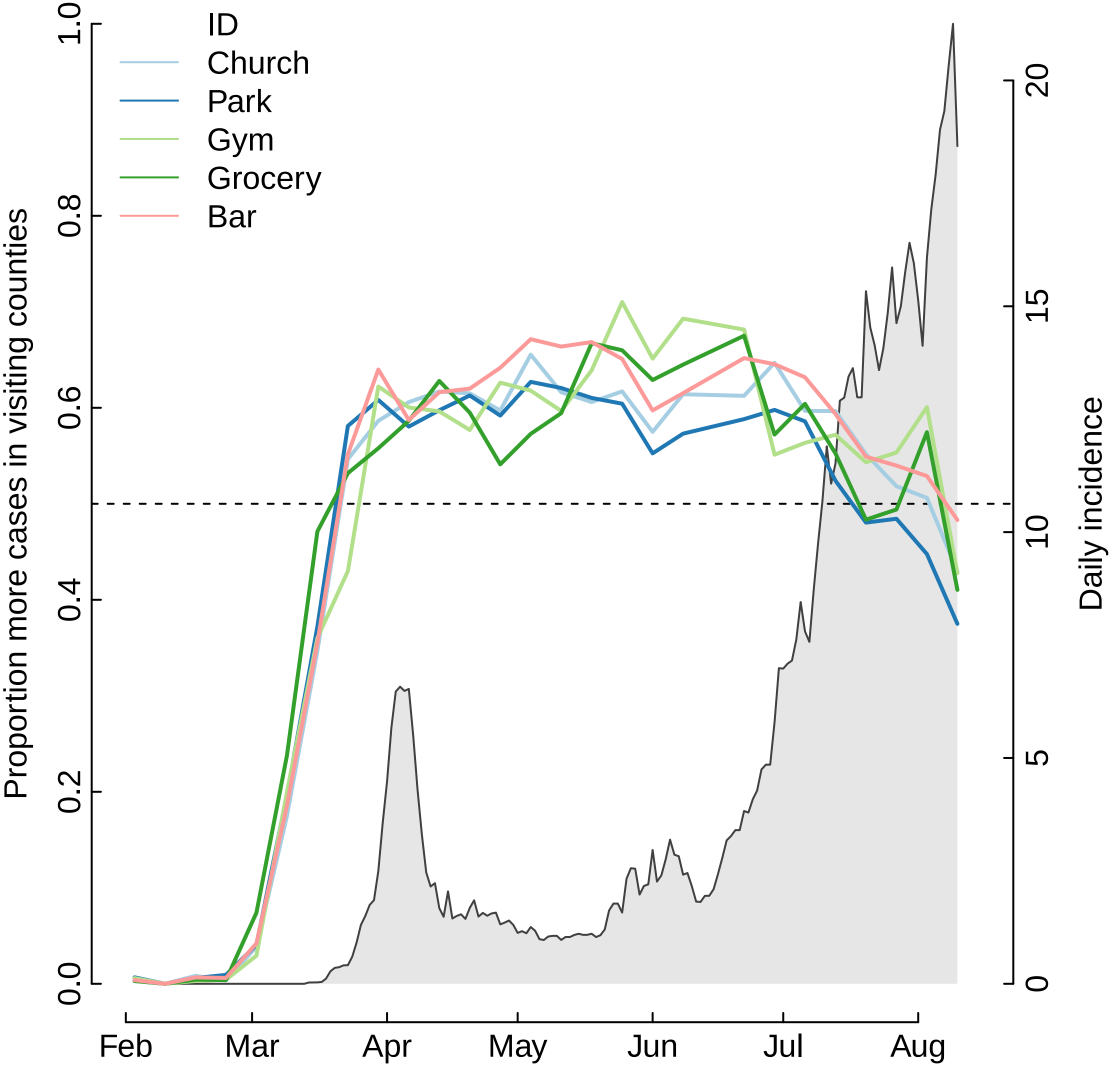

ID
Church
Park
Gym
Grocery
Bar
Proportion more cases in visiting counties
Daily incidence
0.0
0.2
0.4
0.6
0.8
1.0
0
5
10
15
20
Feb
Mar
Apr
May
Jun
Jul
Aug

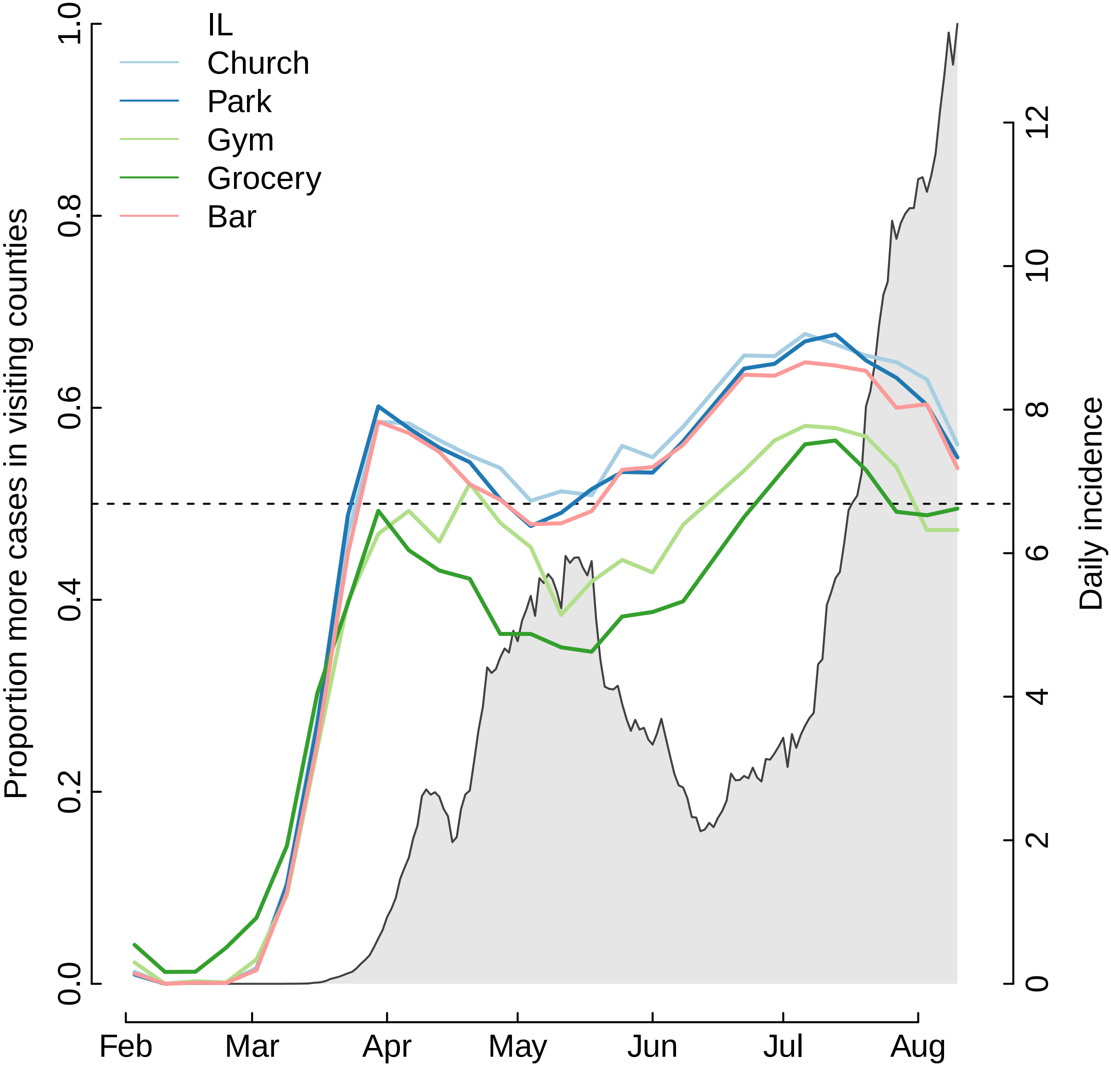

IL
Church
Park
Gym
Grocery
Bar
Proportion more cases in visiting counties
Daily incidence
0.0
0.2
0.4
0.6
0.8
1.0
0
2
4
6
8
10
12
Feb
Mar
Apr
May
Jun
Jul
Aug

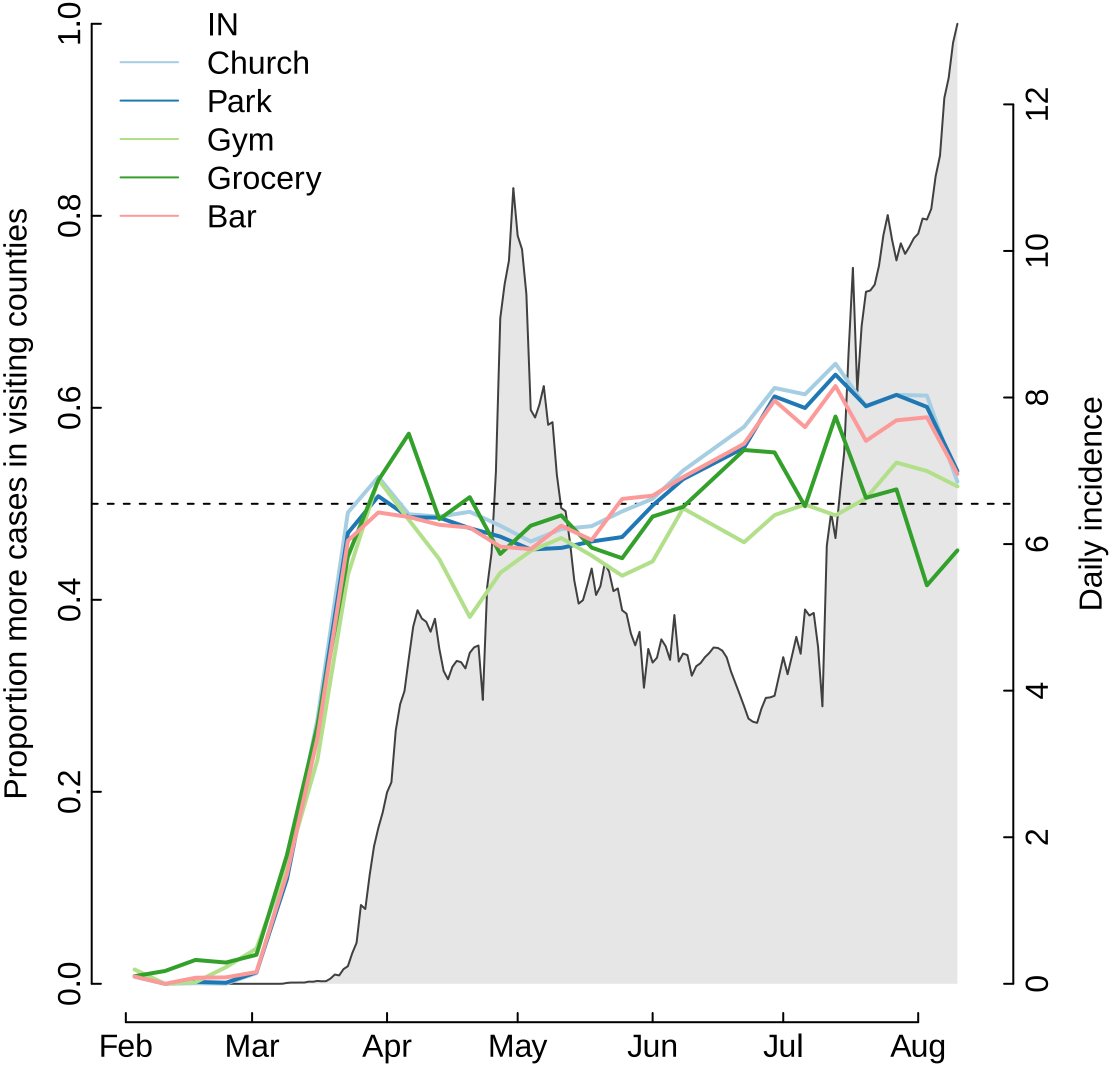

IN
Church
Park
Gym
Grocery
Bar
Proportion more cases in visiting counties
Daily incidence
0.0
0.2
0.4
0.6
0.8
1.0
0
2
4
6
8
10
12
Feb
Mar
Apr
May
Jun
Jul
Aug

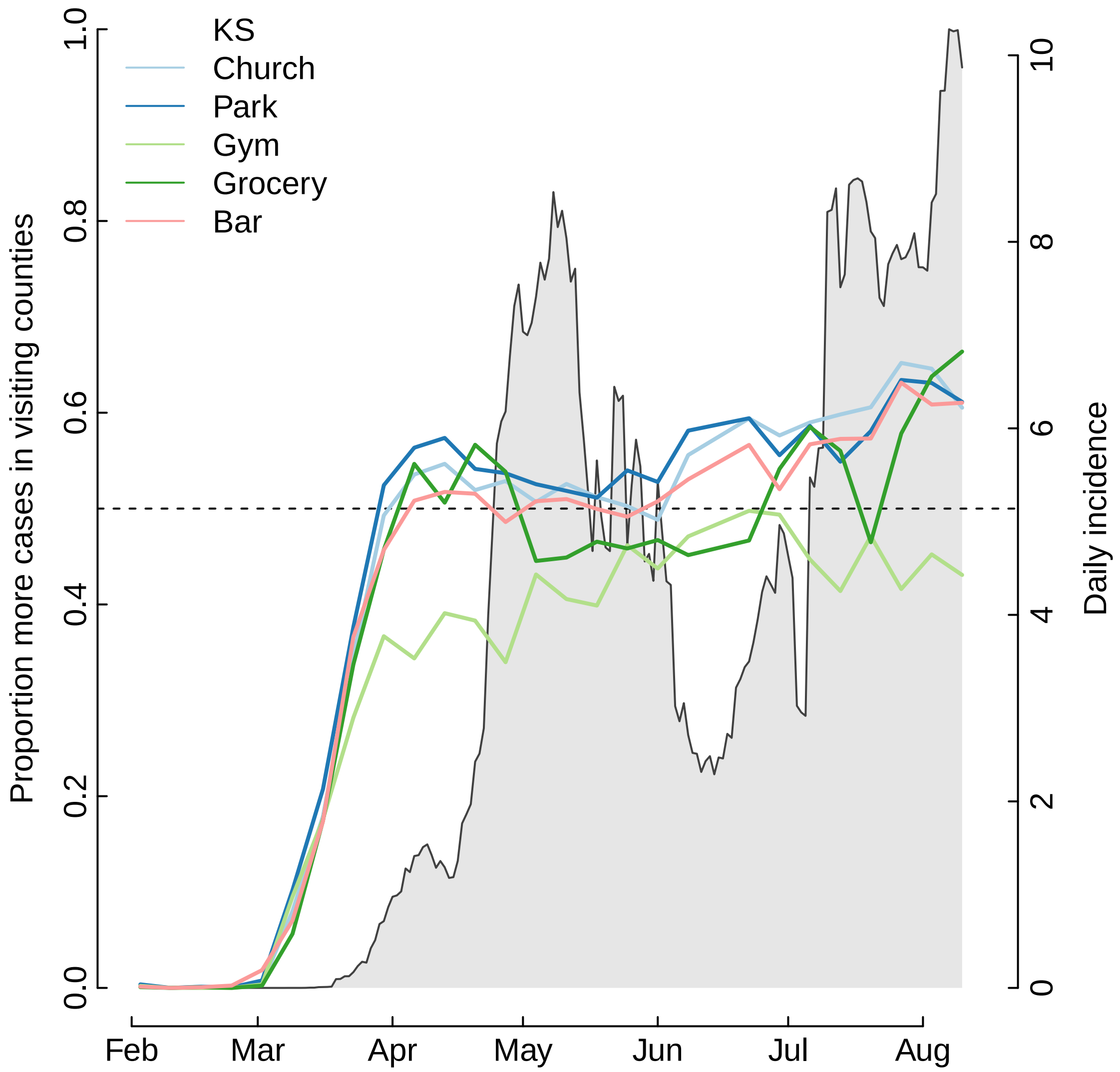

KS
Church
Park
Gym
Grocery
Bar
Proportion more cases in visiting counties
Daily incidence
0.0
0.2
0.4
0.6
0.8
1.0
0
2
4
6
8
10
Feb
Mar
Apr
May
Jun
Jul
Aug

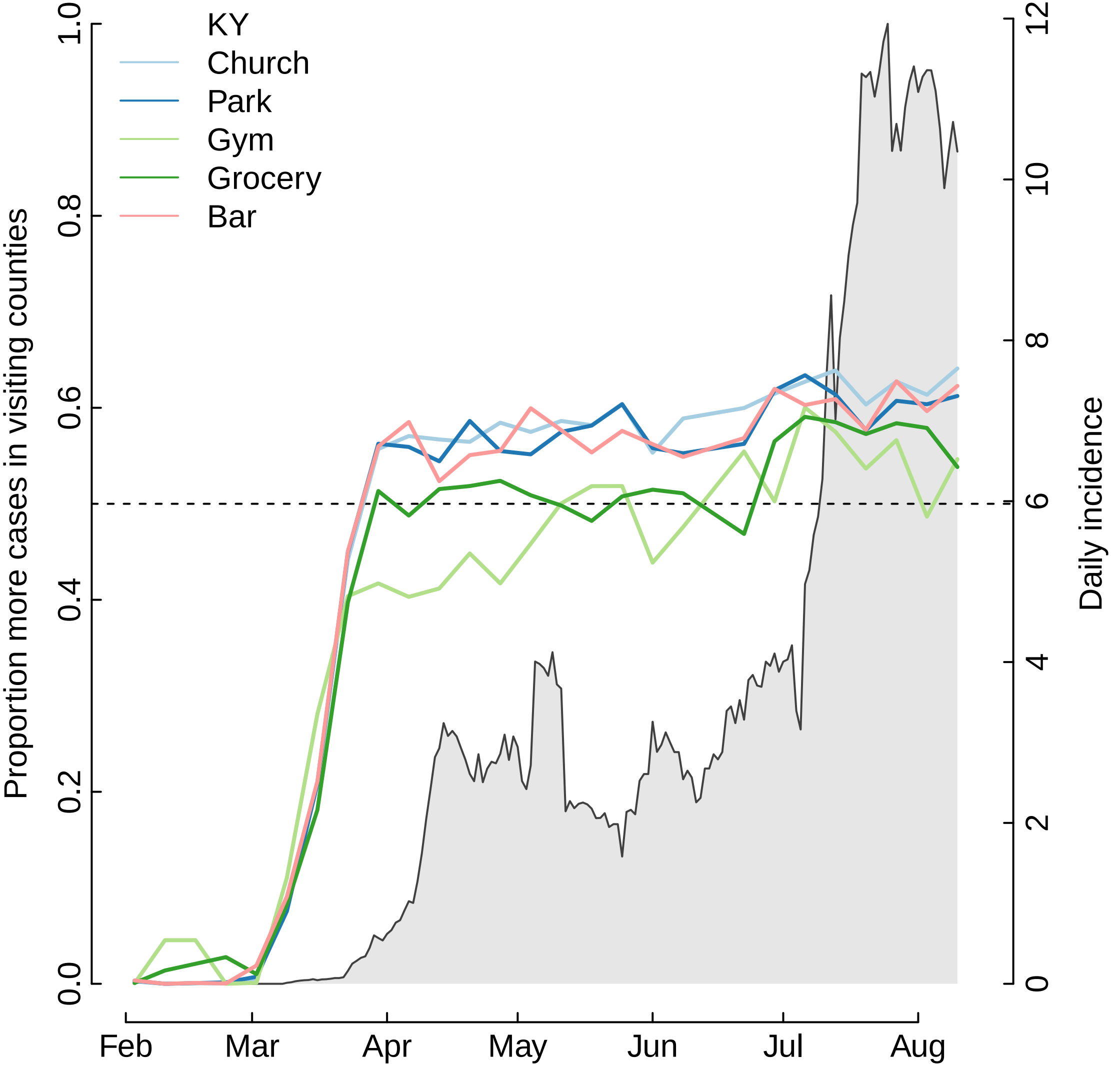

KY
Church
Park
Gym
Grocery
Bar
Proportion more cases in visiting counties
Daily incidence
0.0
0.2
0.4
0.6
0.8
1.0
0
2
4
6
8
10
12
Feb
Mar
Apr
May
Jun
Jul
Aug

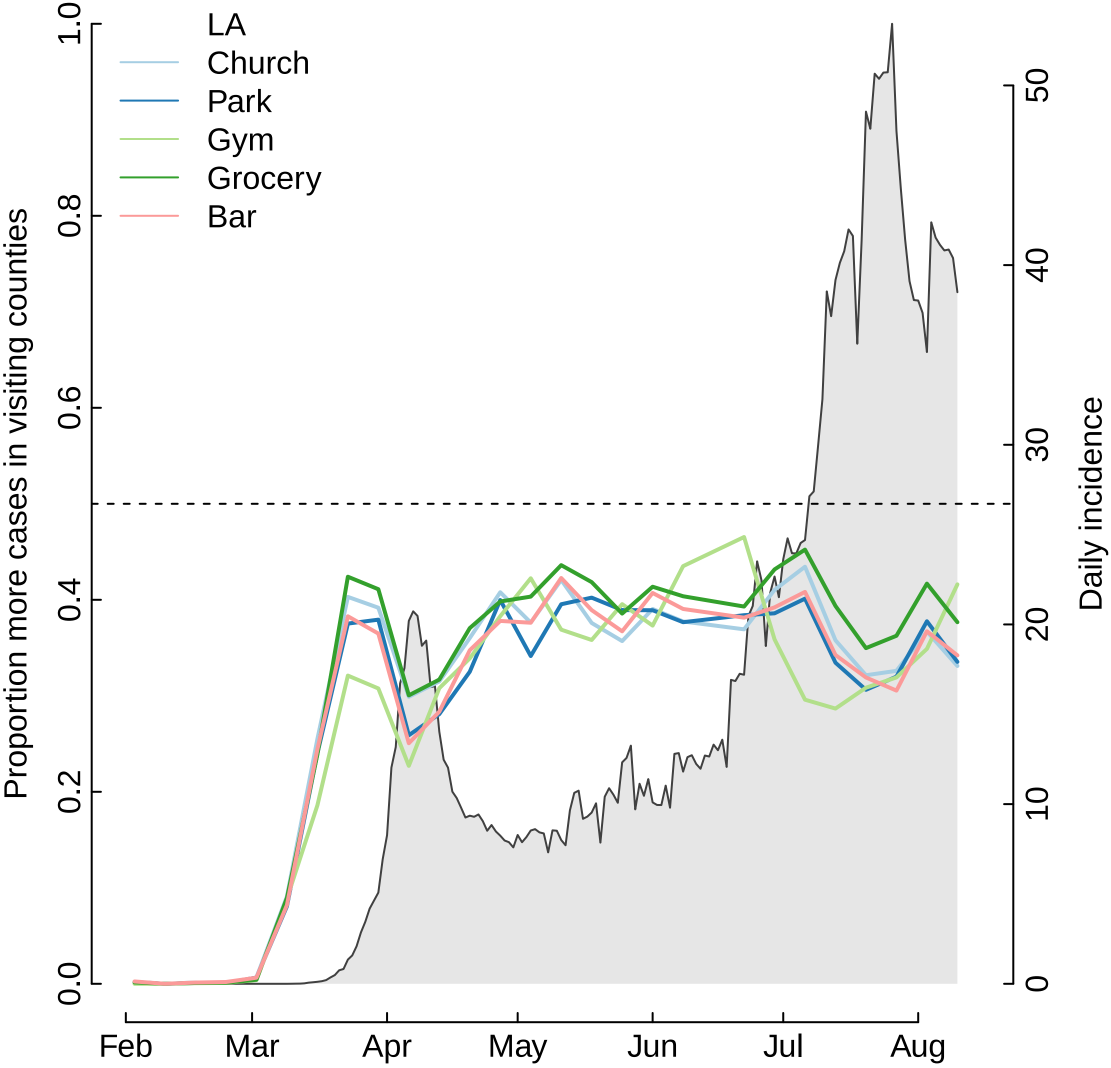
LA
Church
Park
Gym
Grocery
Bar
Proportion more cases in visiting counties
Daily incidence
0.0
0.2
0.4
0.6
0.8
1.0
0
10
20
30
40
50
Feb
Mar
Apr
May
Jun
Jul
Aug

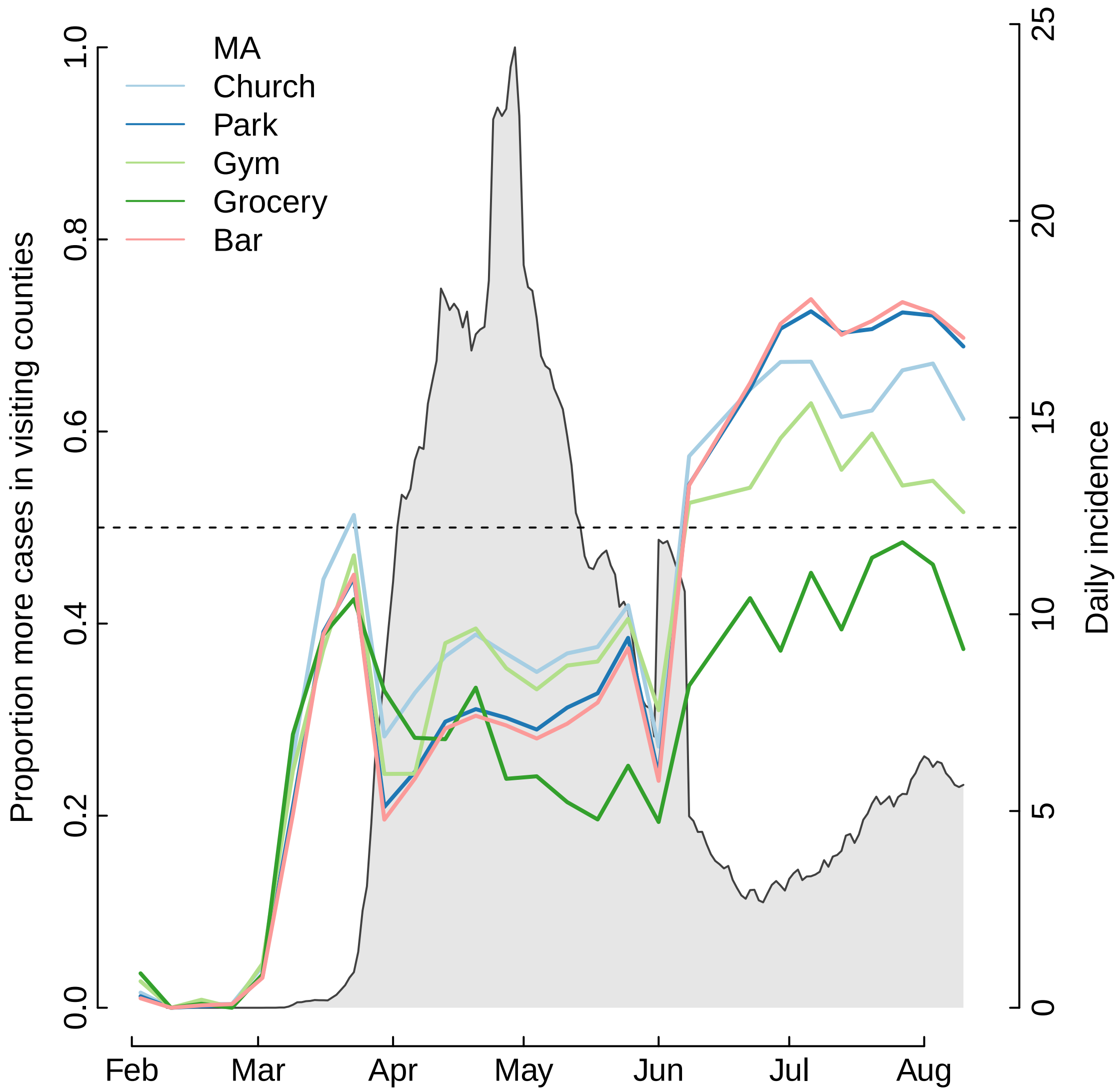

MA
Church
Park
Gym
Grocery
Bar
Proportion more cases in visiting counties
Daily incidence
0.0
0.2
0.4
0.6
0.8
1.0
0
5
10
15
20
25
Feb
Mar
Apr
May
Jun
Jul
Aug

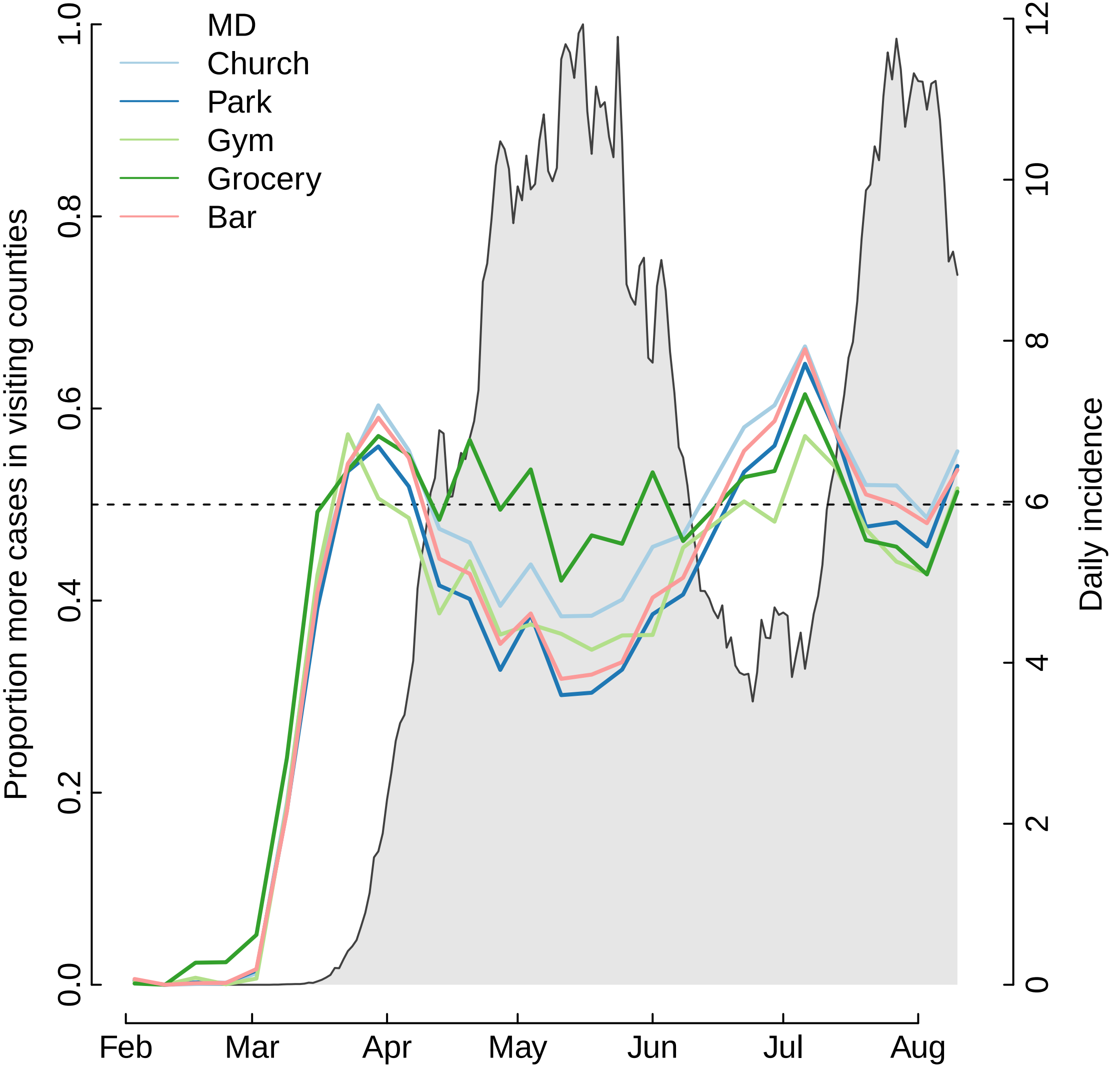

MD
Church
Park
Gym
Grocery
Bar
Proportion more cases in visiting counties
Daily incidence
0.0
0.2
0.4
0.6
0.8
1.0
0
2
4
6
8
10
12
Feb
Mar
Apr
May
Jun
Jul
Aug

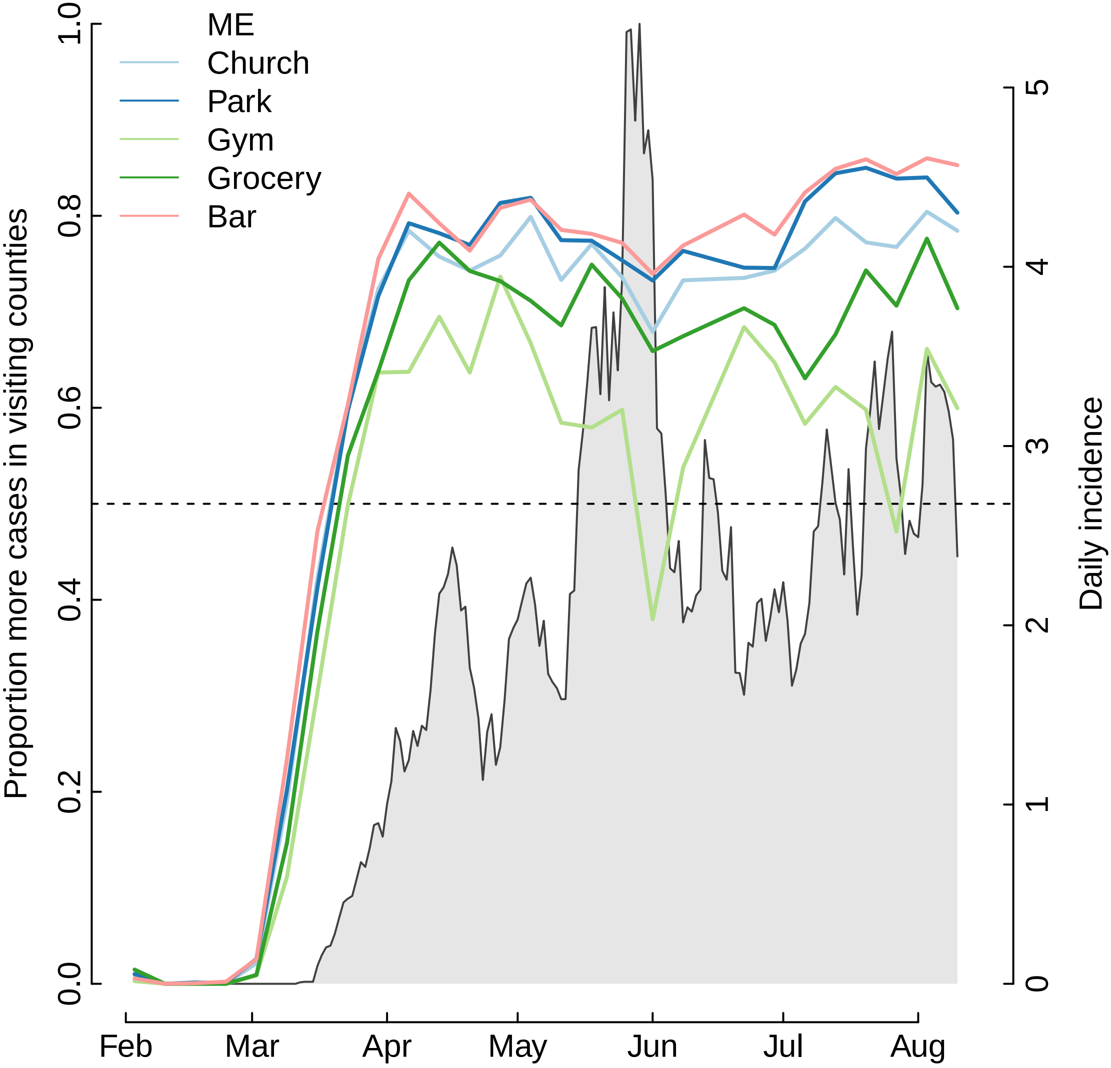

ME
Church
Park
Gym
Grocery
Bar
Proportion more cases in visiting counties
Daily incidence
0.0
0.2
0.4
0.6
0.8
1.0
0
1
2
3
4
5
Feb
Mar
Apr
May
Jun
Jul
Aug

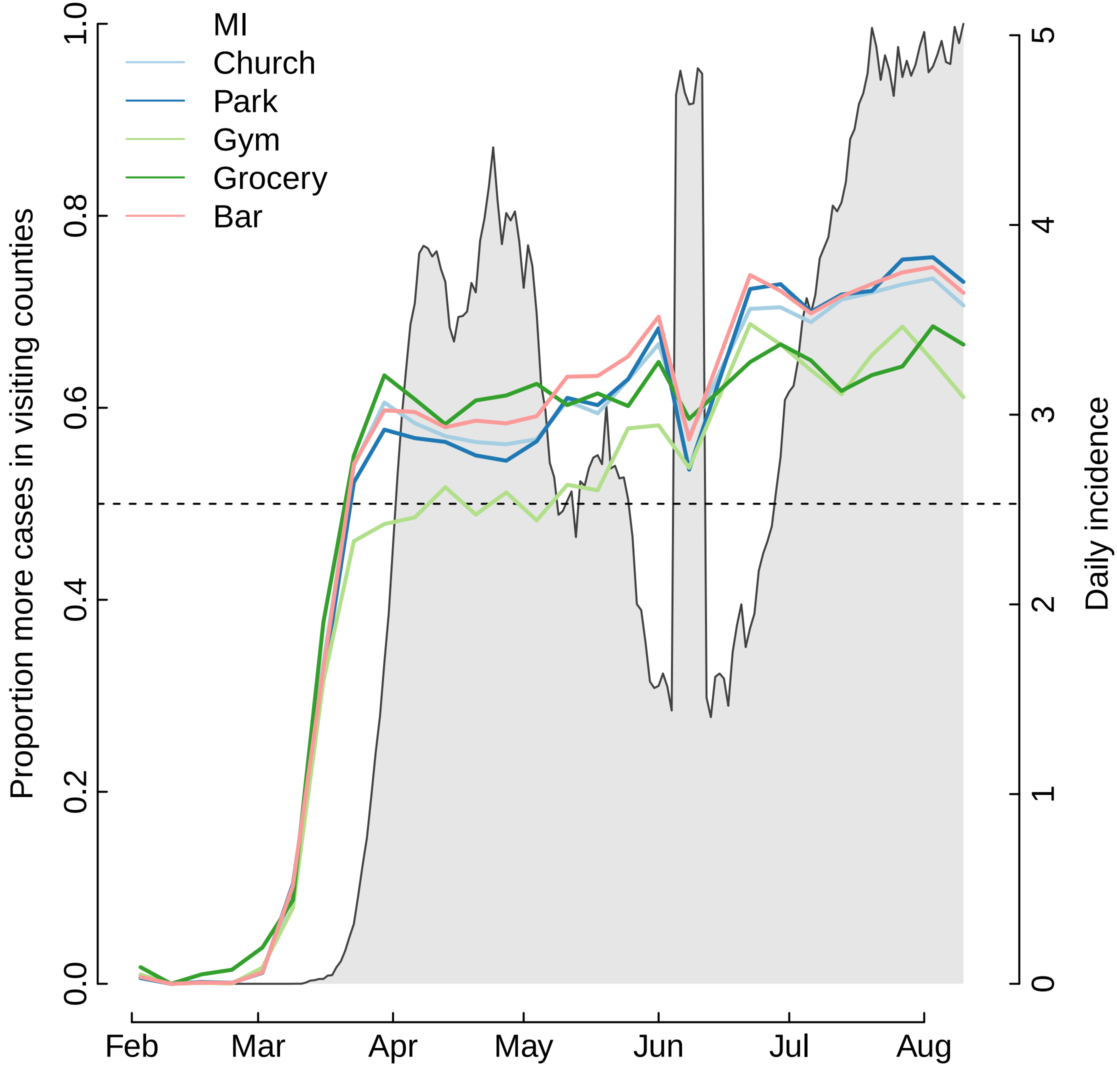

MI
Church
Park
Gym
Grocery
Bar
Proportion more cases in visiting counties
Daily incidence
0.0
0.2
0.4
0.6
0.8
1.0
0
1
2
3
4
5
Feb
Mar
Apr
May
Jun
Jul
Aug

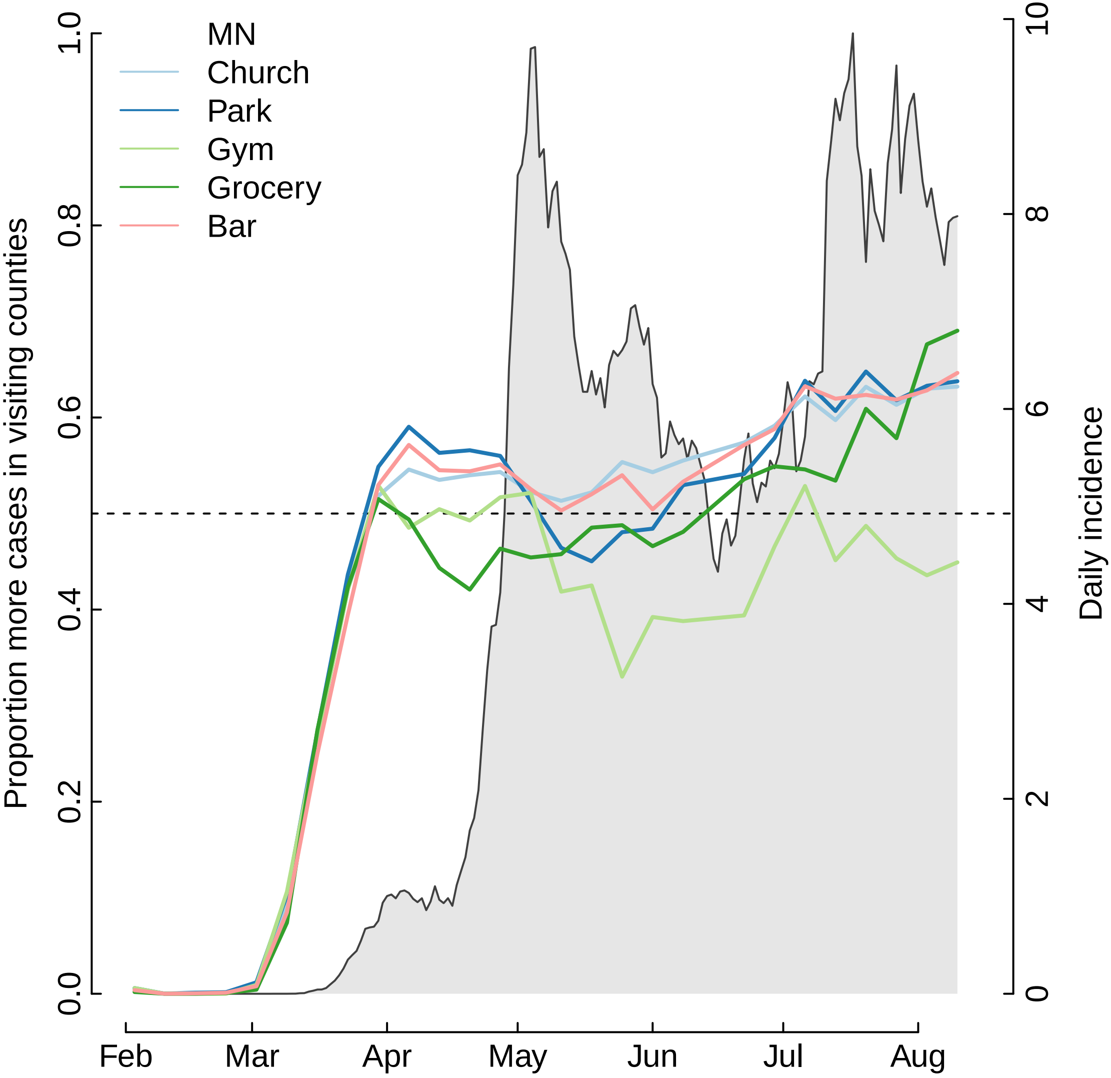

MN
Church
Park
Gym
Grocery
Bar
Proportion more cases in visiting counties
Daily incidence
0.0
0.2
0.4
0.6
0.8
1.0
0
2
4
6
8
10
Feb
Mar
Apr
May
Jun
Jul
Aug

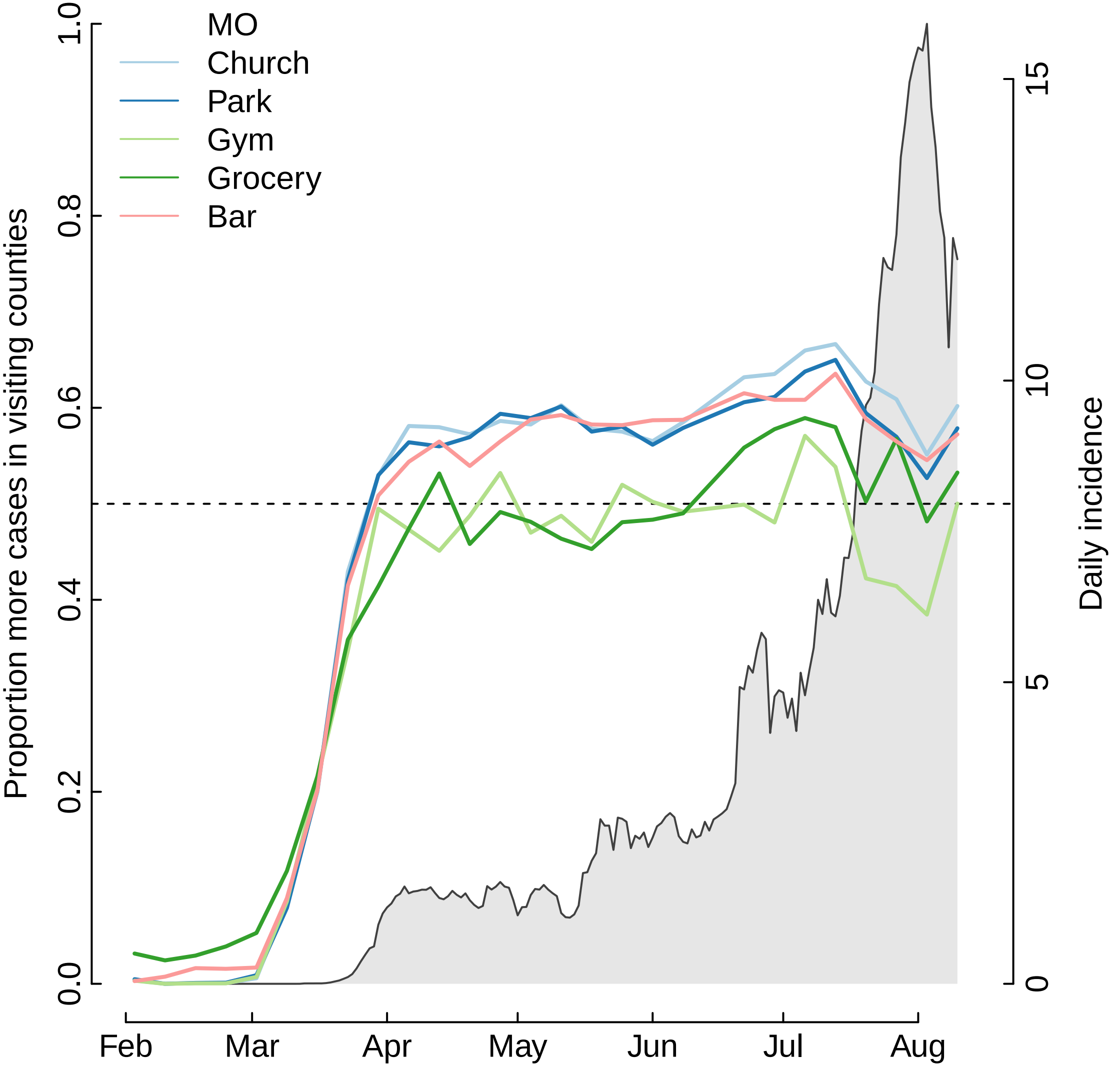

MO
Church
Park
Gym
Grocery
Bar
Proportion more cases in visiting counties
Daily incidence
0.0
0.2
0.4
0.6
0.8
1.0
0
5
10
15
Feb
Mar
Apr
May
Jun
Jul
Aug

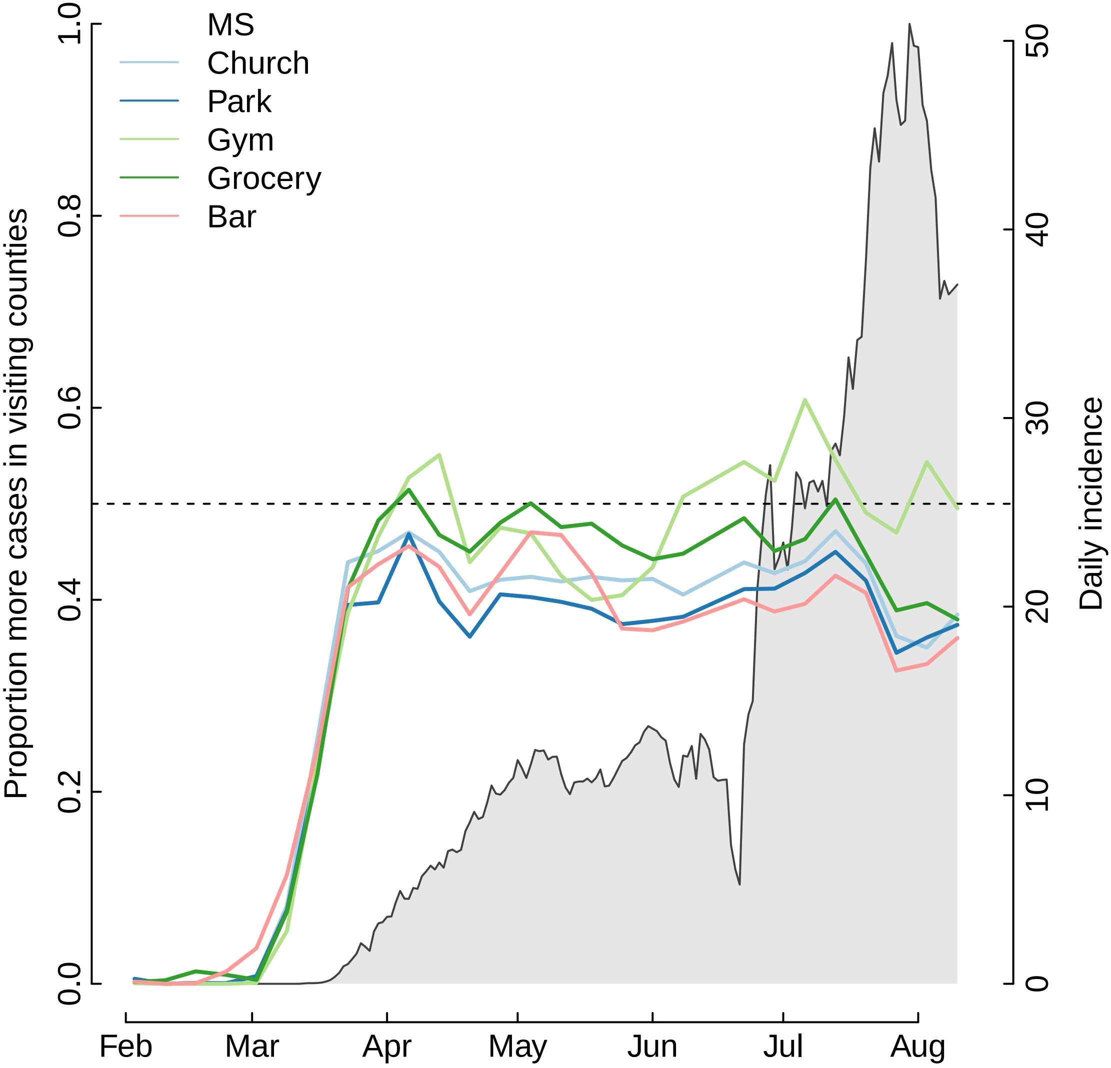

MS
Church
Park
Gym
Grocery
Bar
Proportion more cases in visiting counties
Daily incidence
0.0
0.2
0.4
0.6
0.8
1.0
0
10
20
30
40
50
Feb
Mar
Apr
May
Jun
Jul
Aug

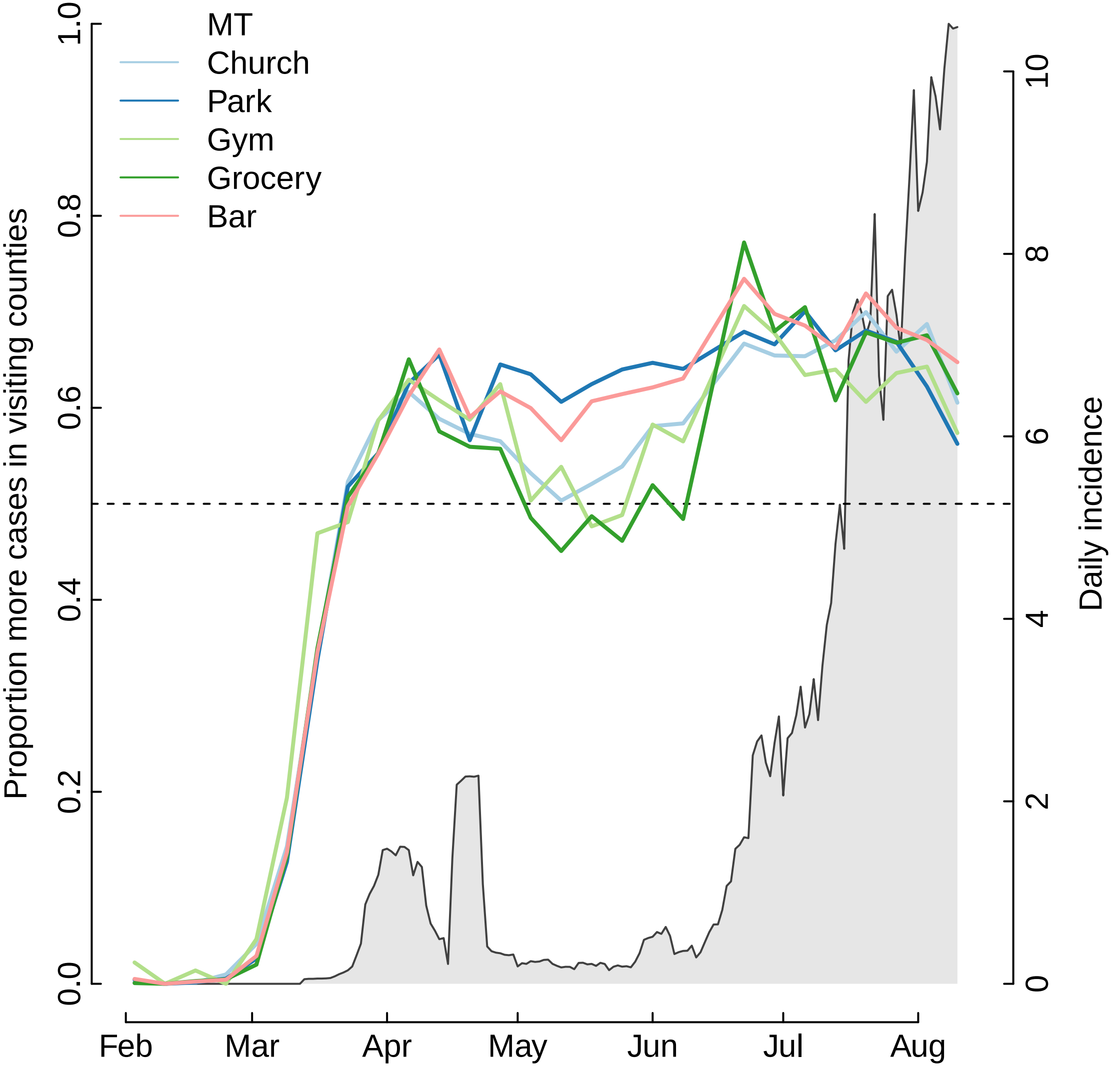

MT
Church
Park
Gym
Grocery
Bar
Proportion more cases in visiting counties
Daily incidence
0.0
0.2
0.4
0.6
0.8
1.0
0
2
4
6
8
10
Feb
Mar
Apr
May
Jun
Jul
Aug

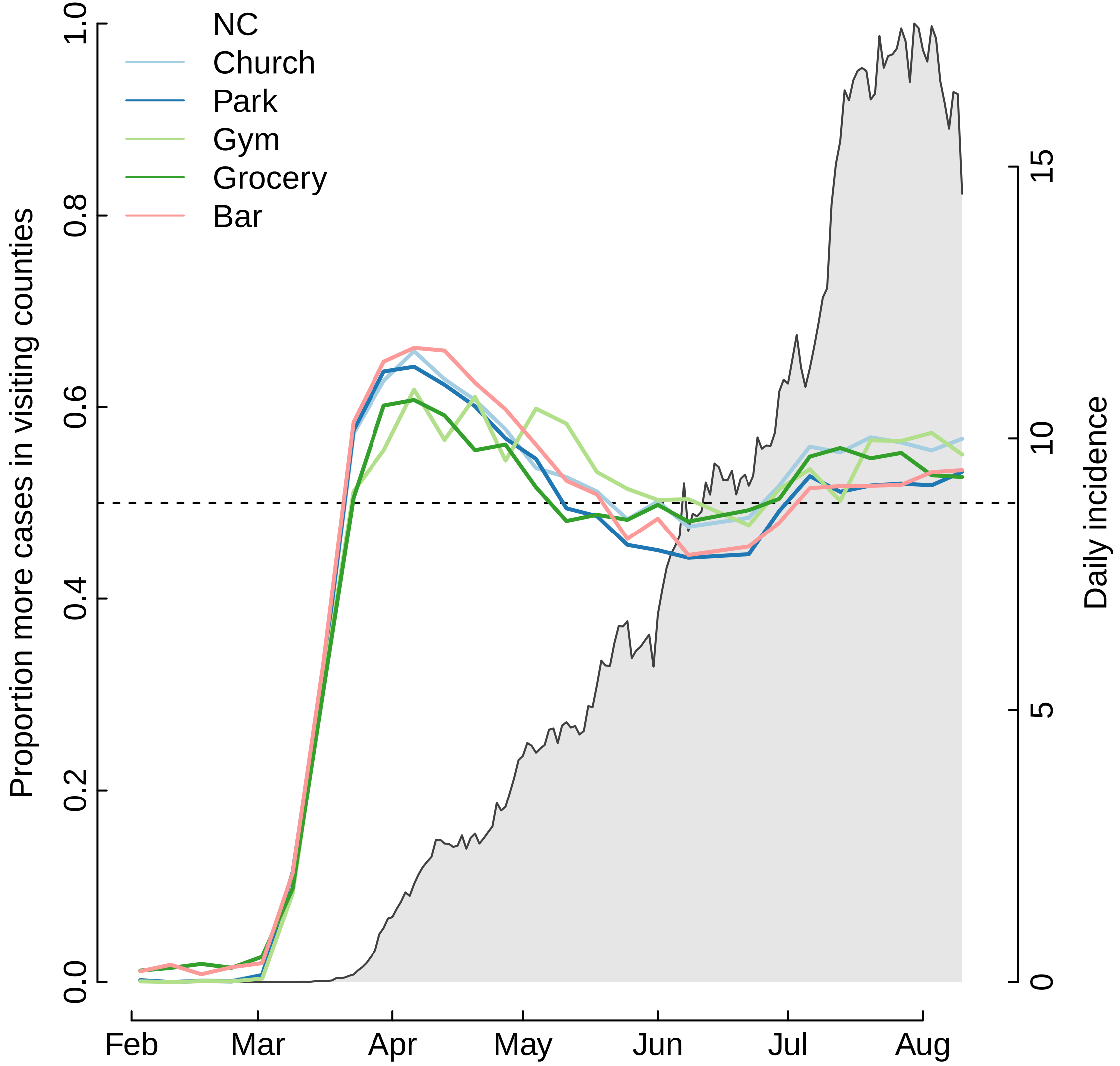

NC
Church
Park
Gym
Grocery
Bar
Proportion more cases in visiting counties
Daily incidence
0.0
0.2
0.4
0.6
0.8
1.0
0
5
10
15
Feb
Mar
Apr
May
Jun
Jul
Aug

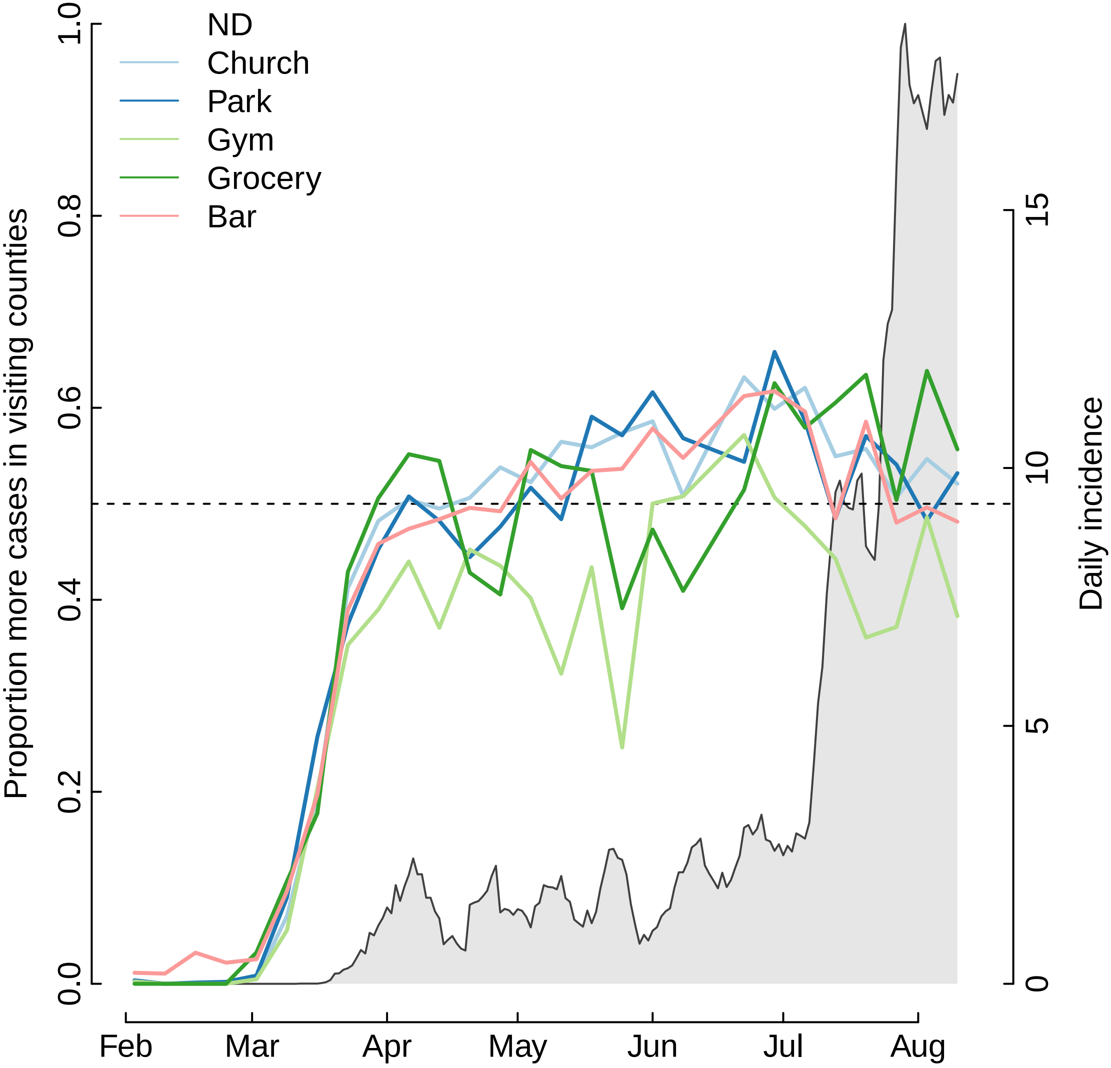

ND
Church
Park
Gym
Grocery
Bar
Proportion more cases in visiting counties
Daily incidence
0.0
0.2
0.4
0.6
0.8
1.0
0
5
10
15
Feb
Mar
Apr
May
Jun
Jul
Aug

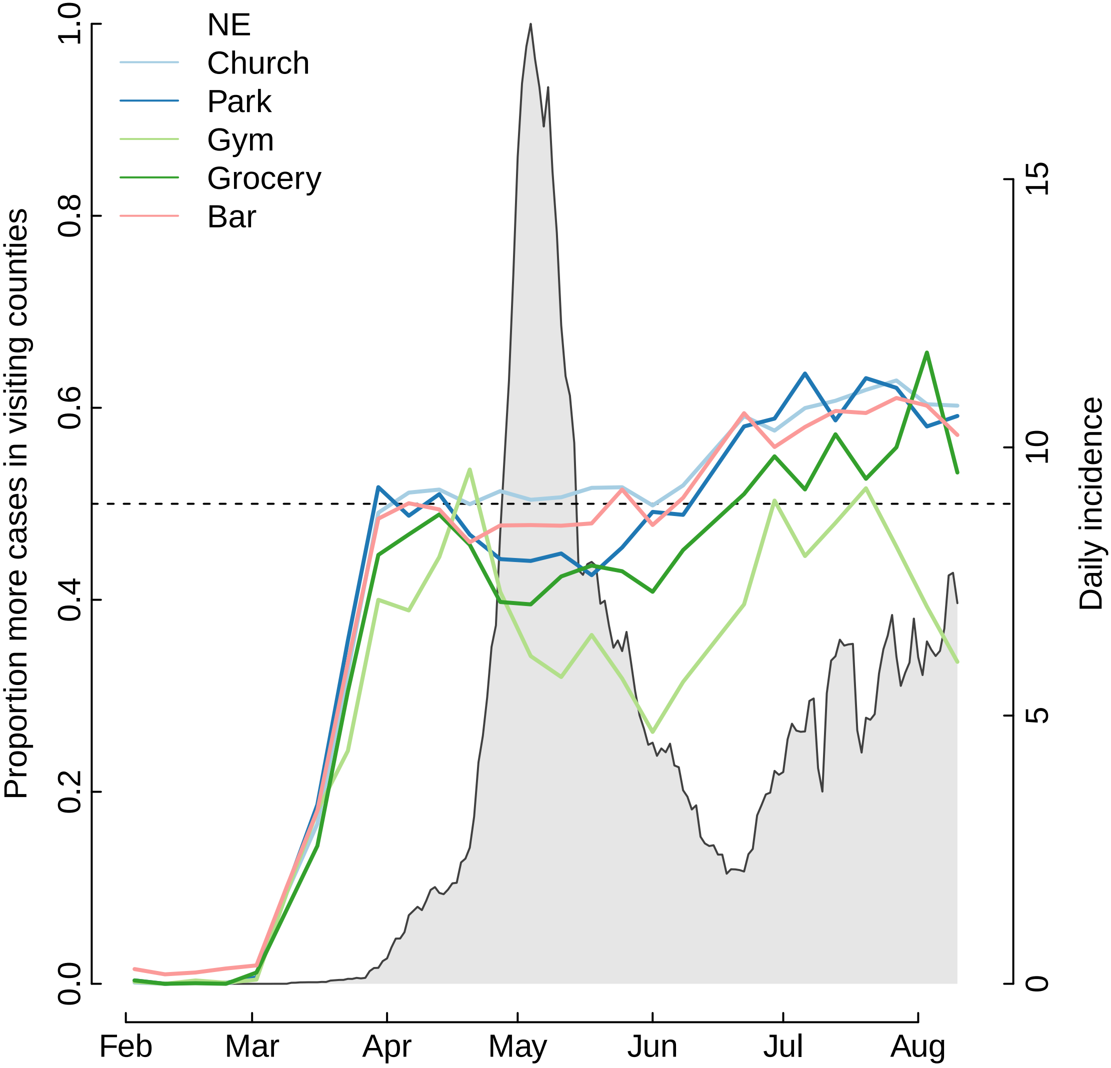

NE
Church
Park
Gym
Grocery
Bar
Proportion more cases in visiting counties
Daily incidence
0.0
0.2
0.4
0.6
0.8
1.0
0
5
10
15
Feb
Mar
Apr
May
Jun
Jul
Aug

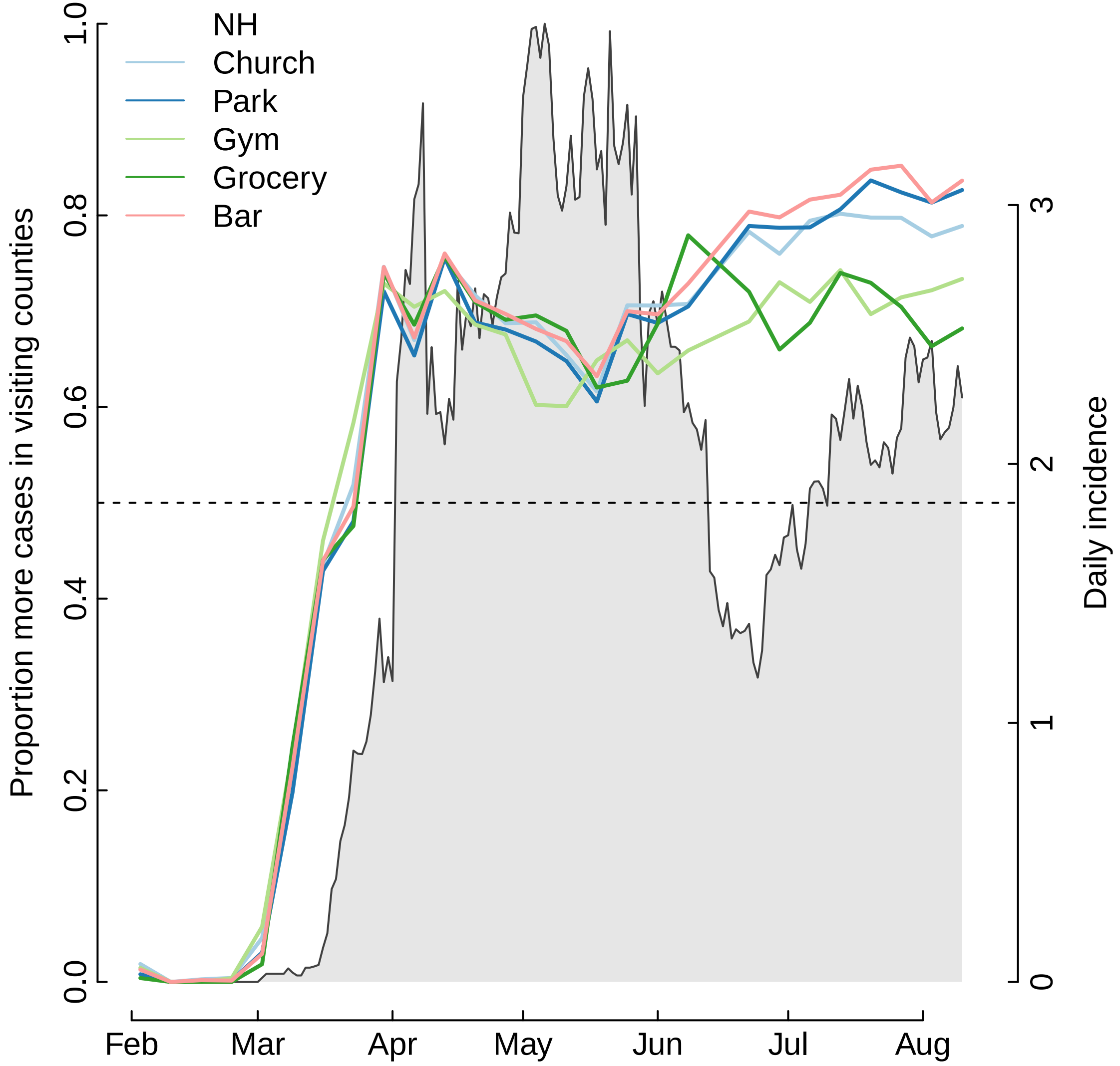

NH
Church
Park
Gym
Grocery
Bar
Proportion more cases in visiting counties
Daily incidence
0.0
0.2
0.4
0.6
0.8
1.0
0
1
2
3
Feb
Mar
Apr
May
Jun
Jul
Aug

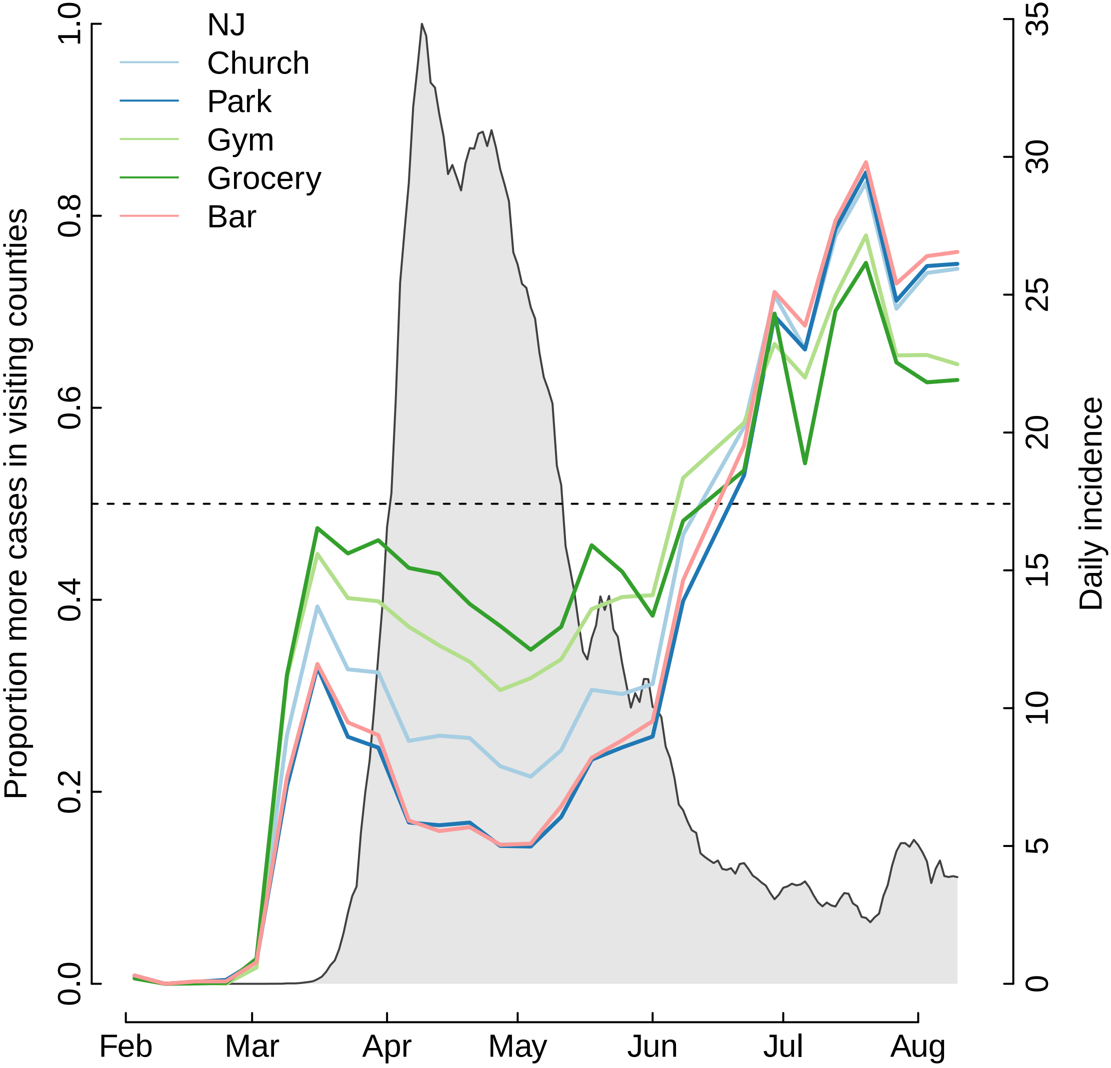
NJ
Church
Park
Gym
Grocery
Bar
Proportion more cases in visiting counties
Daily incidence
0.0
0.2
0.4
0.6
0.8
1.0
0
5
10
15
20
25
30
35
Feb
Mar
Apr
May
Jun
Jul
Aug

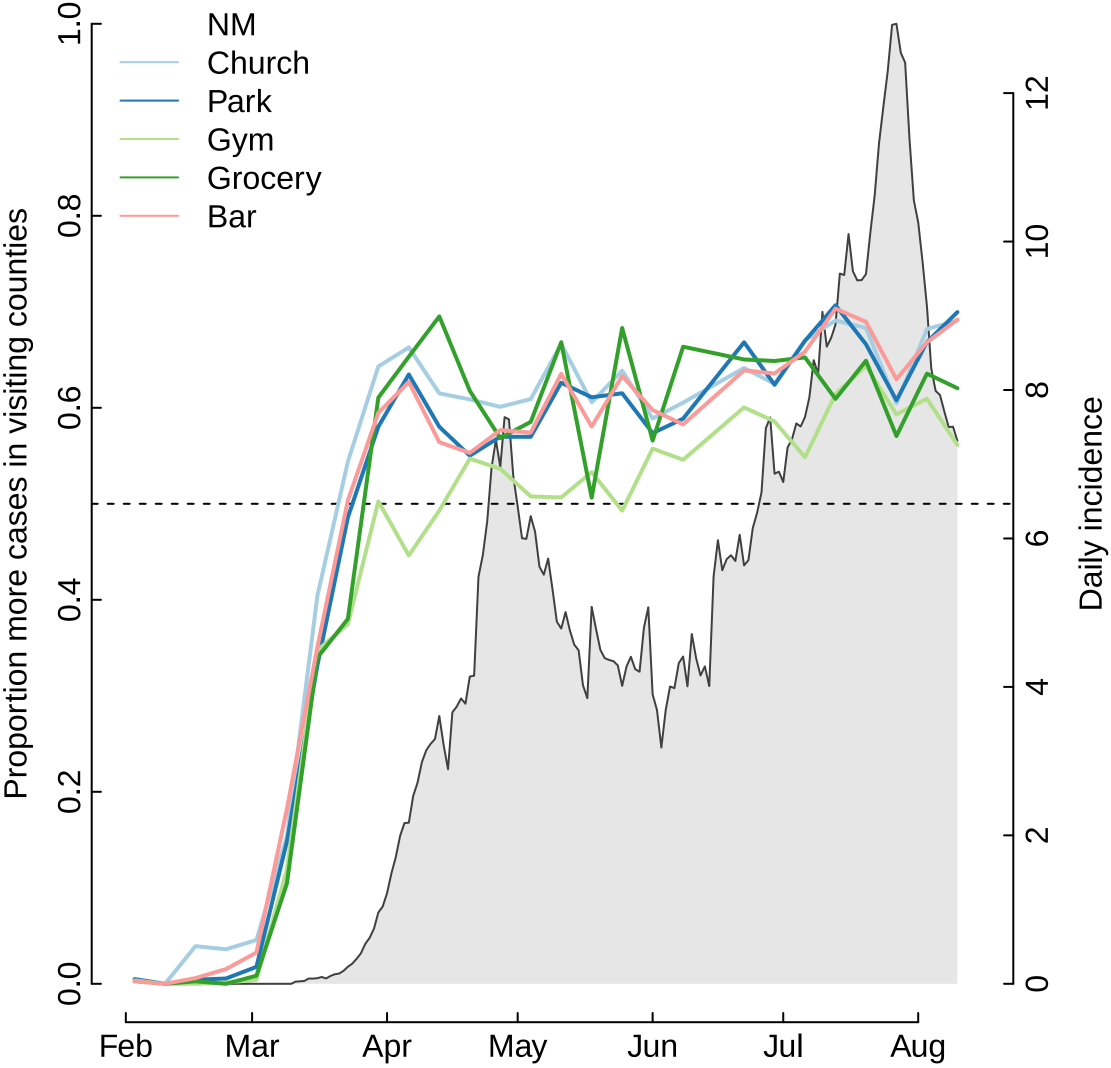

NM
Church
Park
Gym
Grocery
Bar
Proportion more cases in visiting counties
0.0
0.2
0.4
0.6
0.8
1.0
Daily incidence
0
2
4
6
8
10
12
Feb
Mar
Apr
May
Jun
Jul
Aug

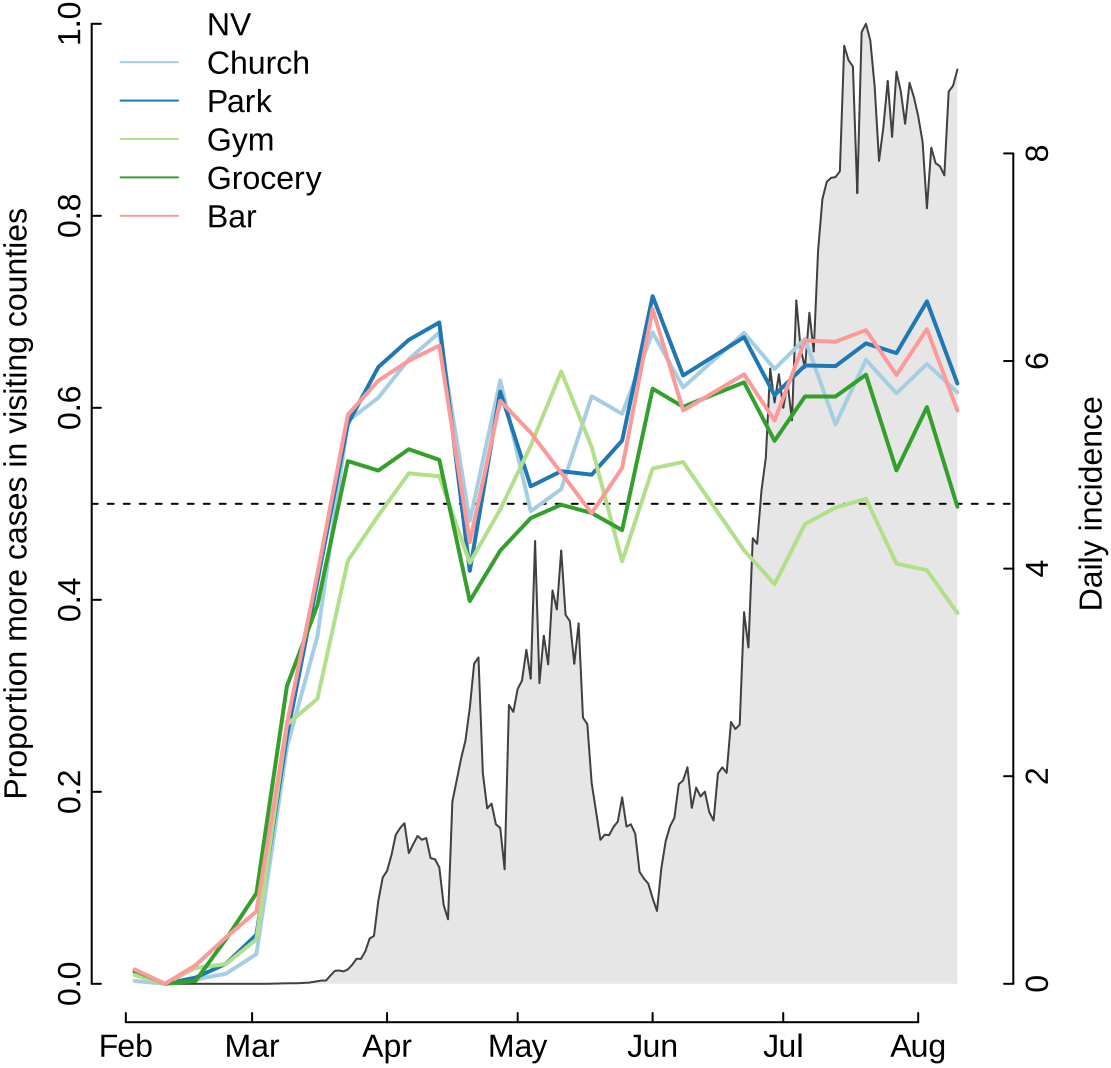

NV
Church
Park
Gym
Grocery
Bar
Proportion more cases in visiting counties
Daily incidence
0.0
0.2
0.4
0.6
0.8
1.0
0
2
4
6
8
Feb
Mar
Apr
May
Jun
Jul
Aug

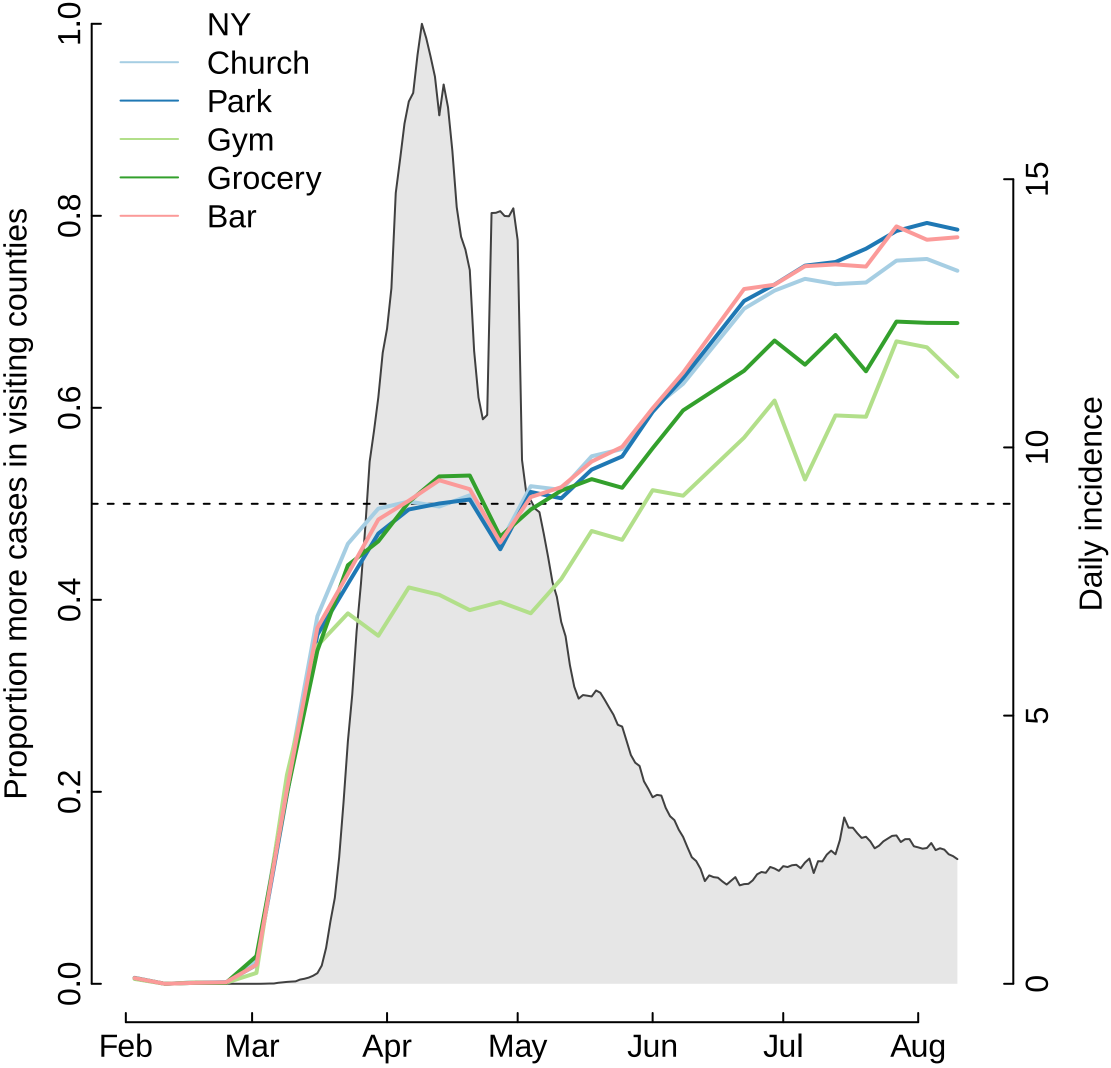

NY
Church
Park
Gym
Grocery
Bar
Proportion more cases in visiting counties
Daily incidence
0.0
0.2
0.4
0.6
0.8
1.0
0
5
10
15
Feb
Mar
Apr
May
Jun
Jul
Aug

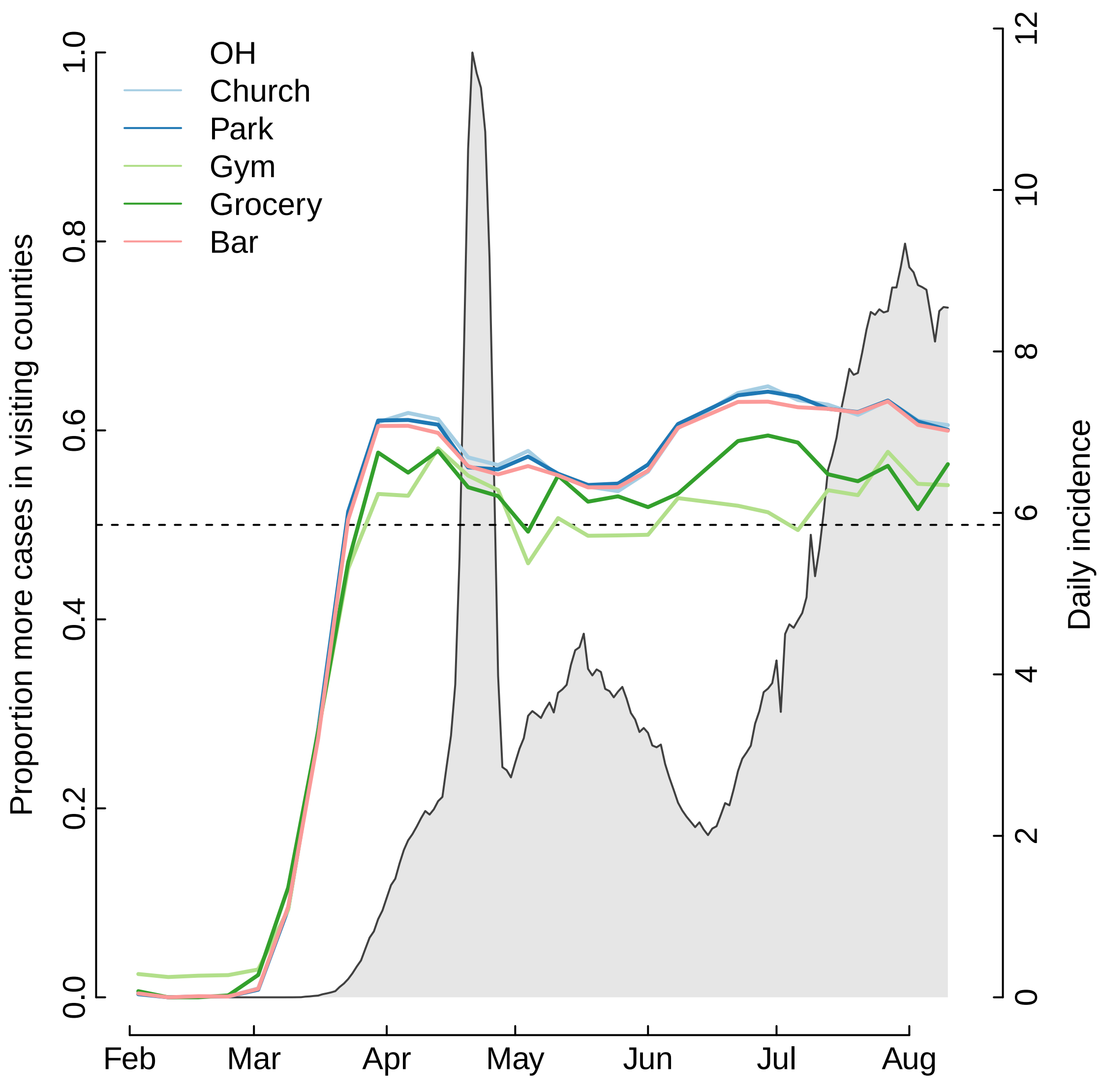

OH
Church
Park
Gym
Grocery
Bar
Proportion more cases in visiting counties
Daily incidence
0.0
0.2
0.4
0.6
0.8
1.0
0
2
4
6
8
10
12
Feb
Mar
Apr
May
Jun
Jul
Aug

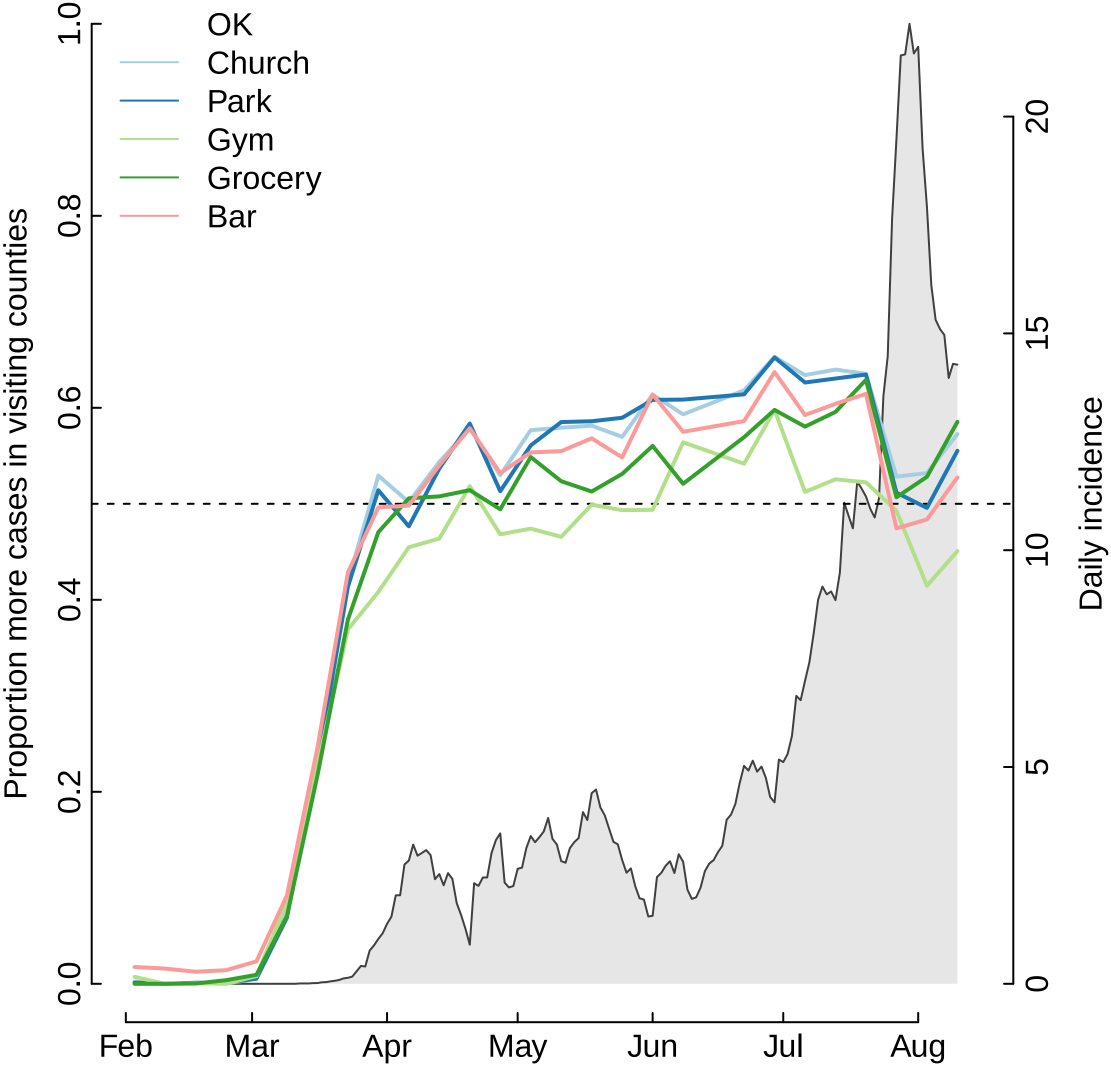

OK
Church
Park
Gym
Grocery
Bar
Proportion more cases in visiting counties
Daily incidence
0.0
0.2
0.4
0.6
0.8
1.0
0
5
10
15
20
Feb
Mar
Apr
May
Jun
Jul
Aug

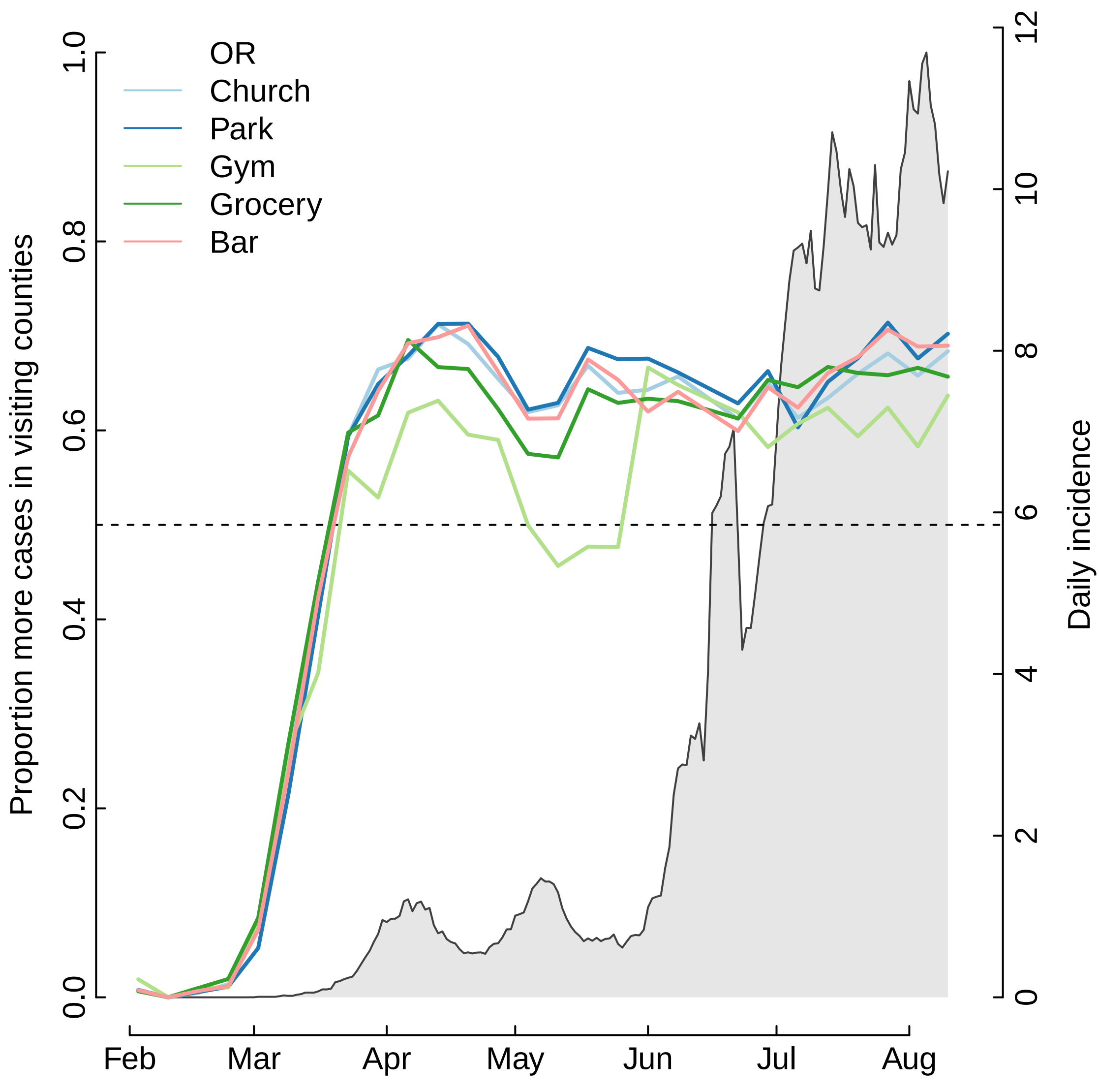

OR
Church
Park
Gym
Grocery
Bar
Proportion more cases in visiting counties
Daily incidence
0.0
0.2
0.4
0.6
0.8
1.0
0
2
4
6
8
10
12
Feb
Mar
Apr
May
Jun
Jul
Aug

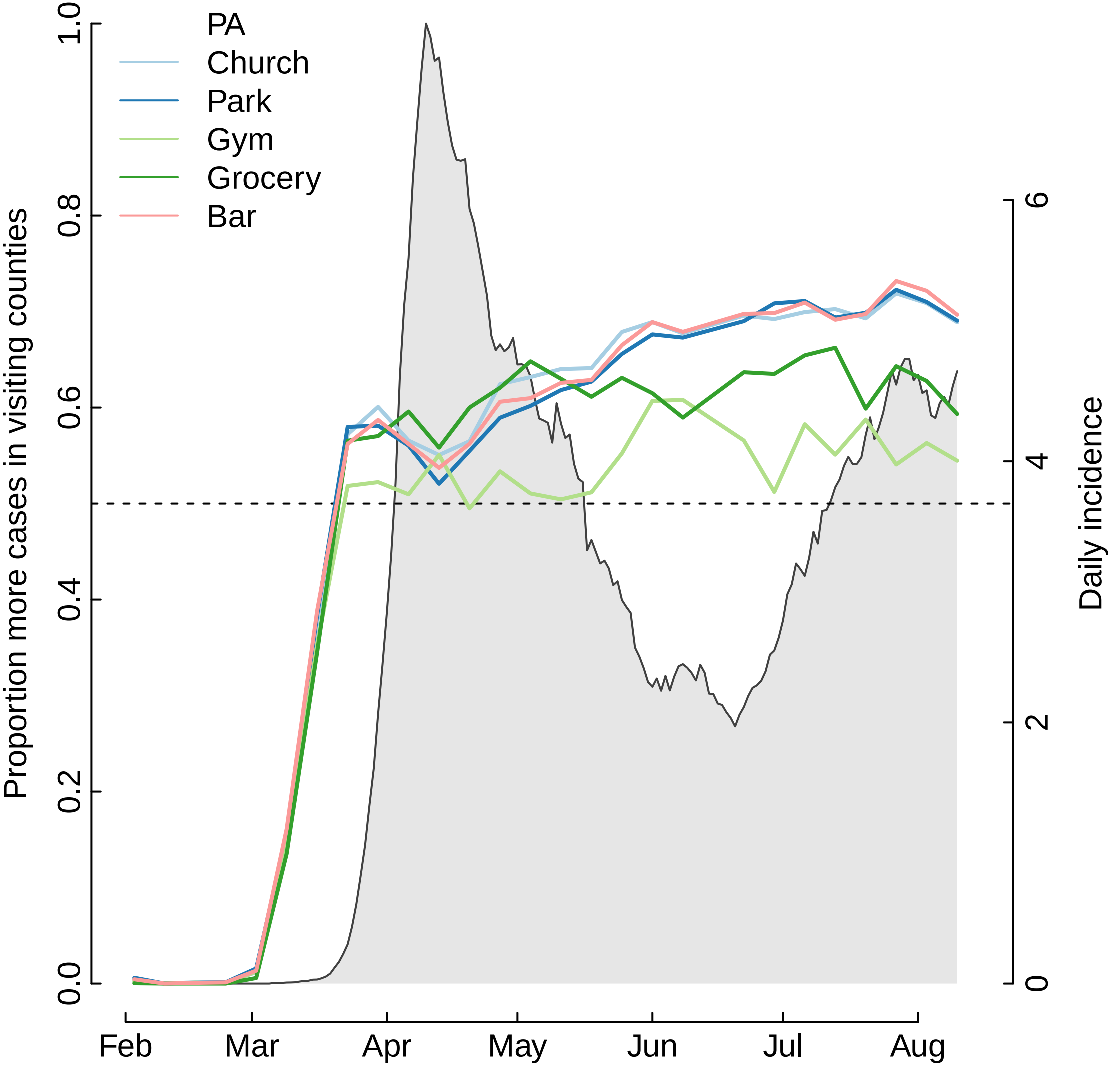

PA
Church
Park
Gym
Grocery
Bar
Proportion more cases in visiting counties
Daily incidence
0.0
0.2
0.4
0.6
0.8
1.0
0
2
4
6
Feb
Mar
Apr
May
Jun
Jul
Aug

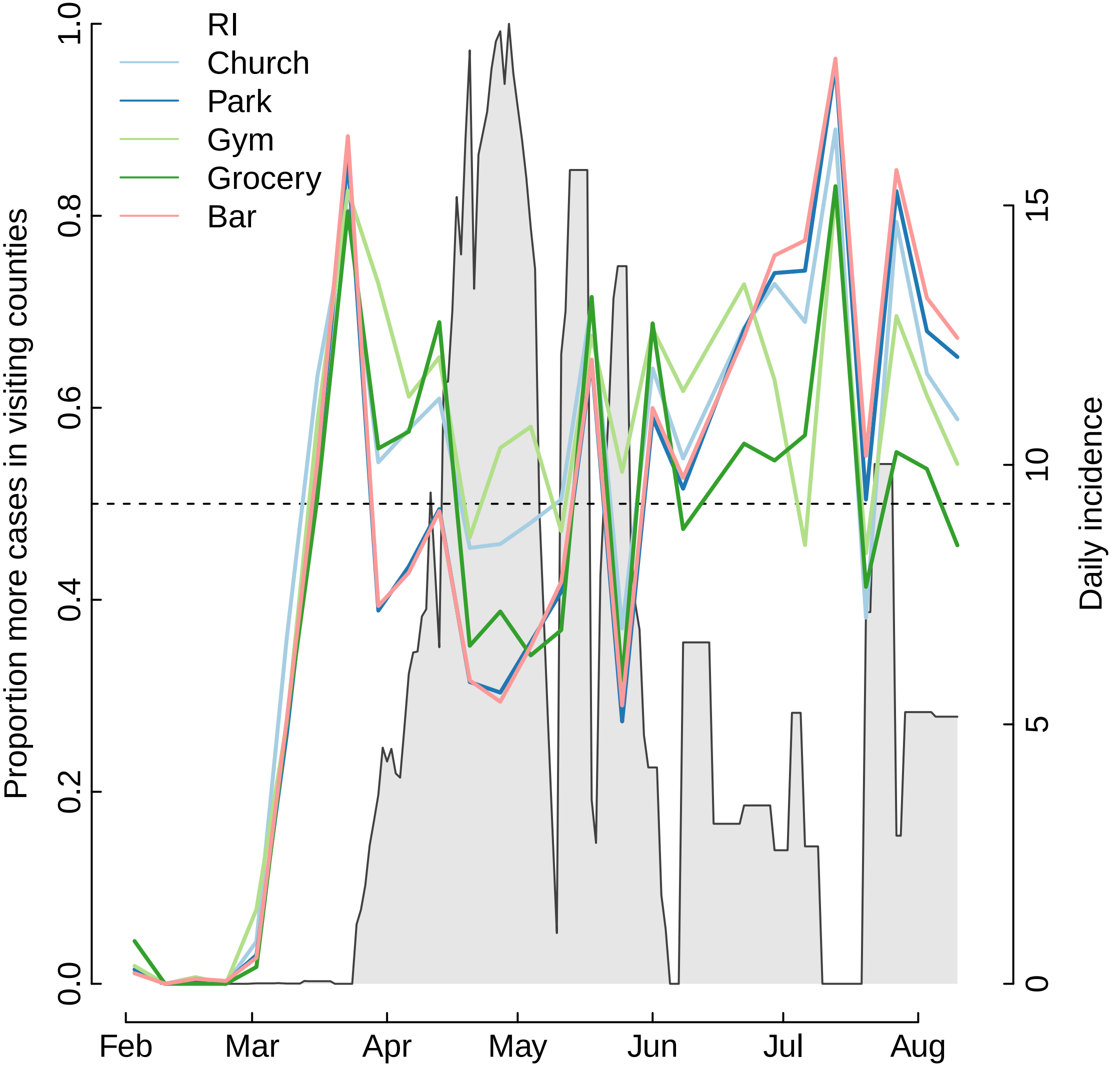

RI
Church
Park
Gym
Grocery
Bar
Proportion more cases in visiting counties
0.0
0.2
0.4
0.6
0.8
1.0
Daily incidence
0
5
10
15
Feb
Mar
Apr
May
Jun
Jul
Aug

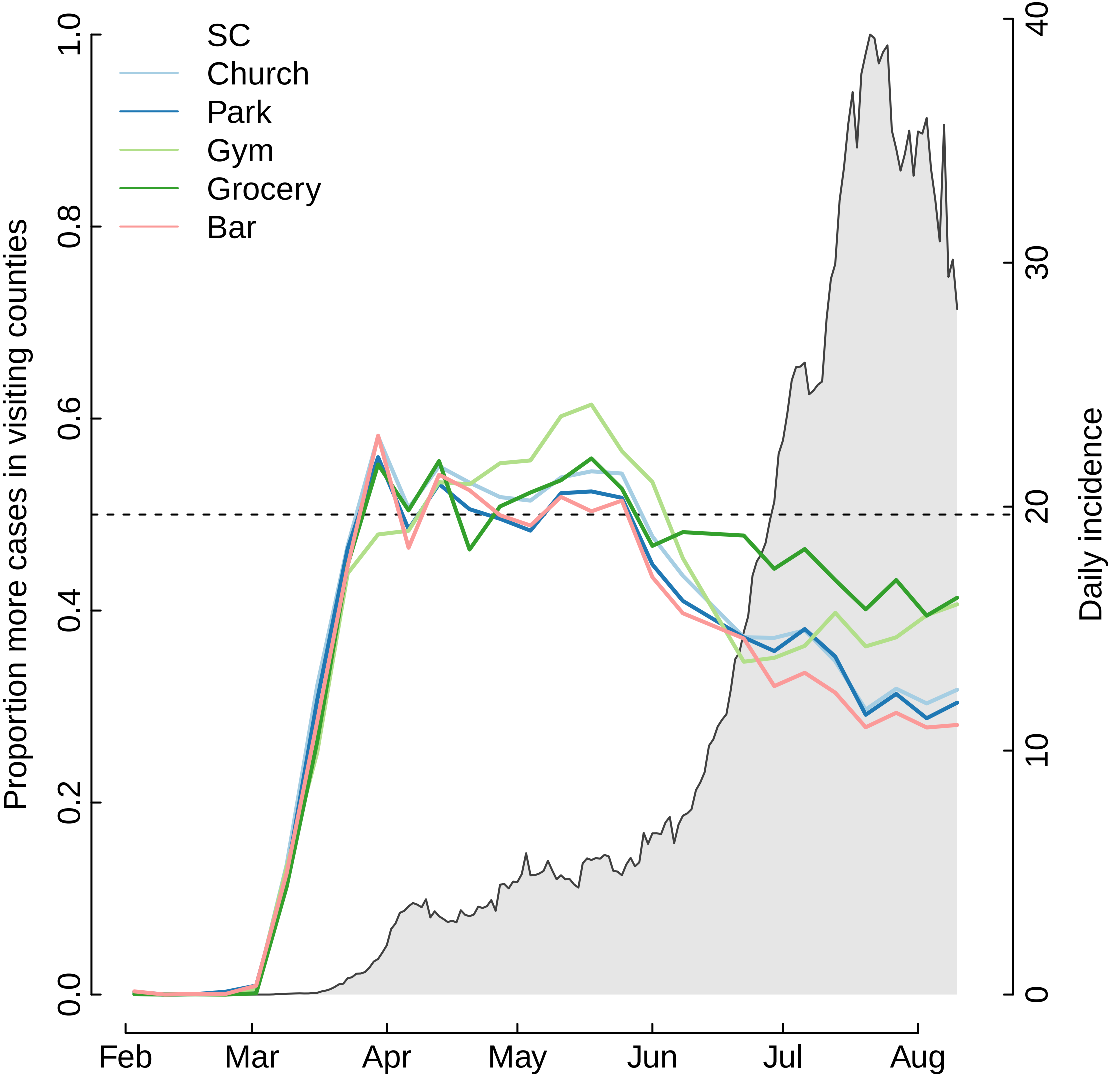
SC
Church
Park
Gym
Grocery
Bar
Proportion more cases in visiting counties
Daily incidence
0.0
0.2
0.4
0.6
0.8
1.0
0
10
20
30
40
Feb
Mar
Apr
May
Jun
Jul
Aug

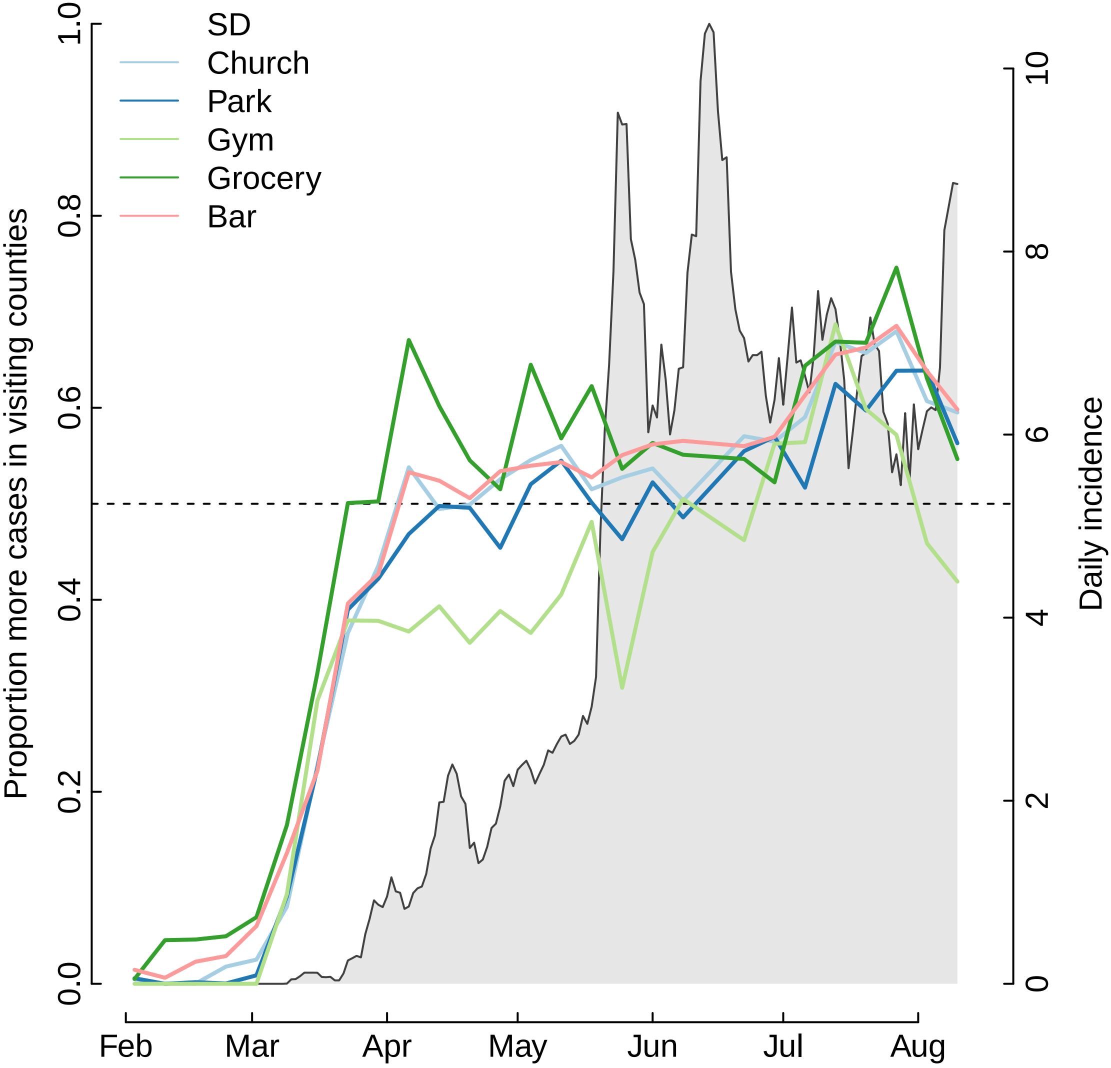

SD
Church
Park
Gym
Grocery
Bar
Proportion more cases in visiting counties
Daily incidence
0.0
0.2
0.4
0.6
0.8
1.0
0
2
4
6
8
10
Feb
Mar
Apr
May
Jun
Jul
Aug

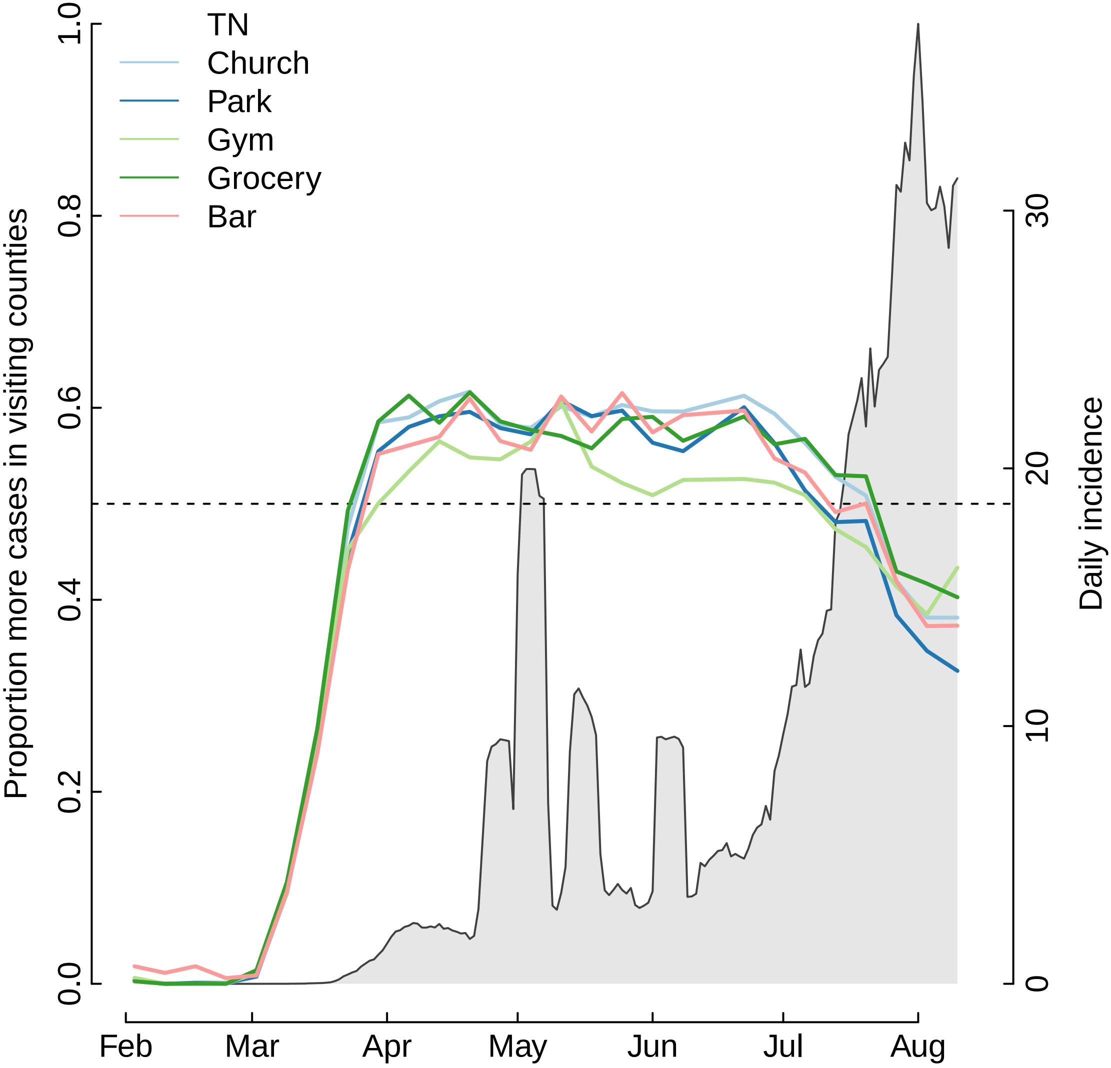

TN
Church
Park
Gym
Grocery
Bar
Proportion more cases in visiting counties
Daily incidence
0.0
0.2
0.4
0.6
0.8
1.0
0
10
20
30
Feb
Mar
Apr
May
Jun
Jul
Aug

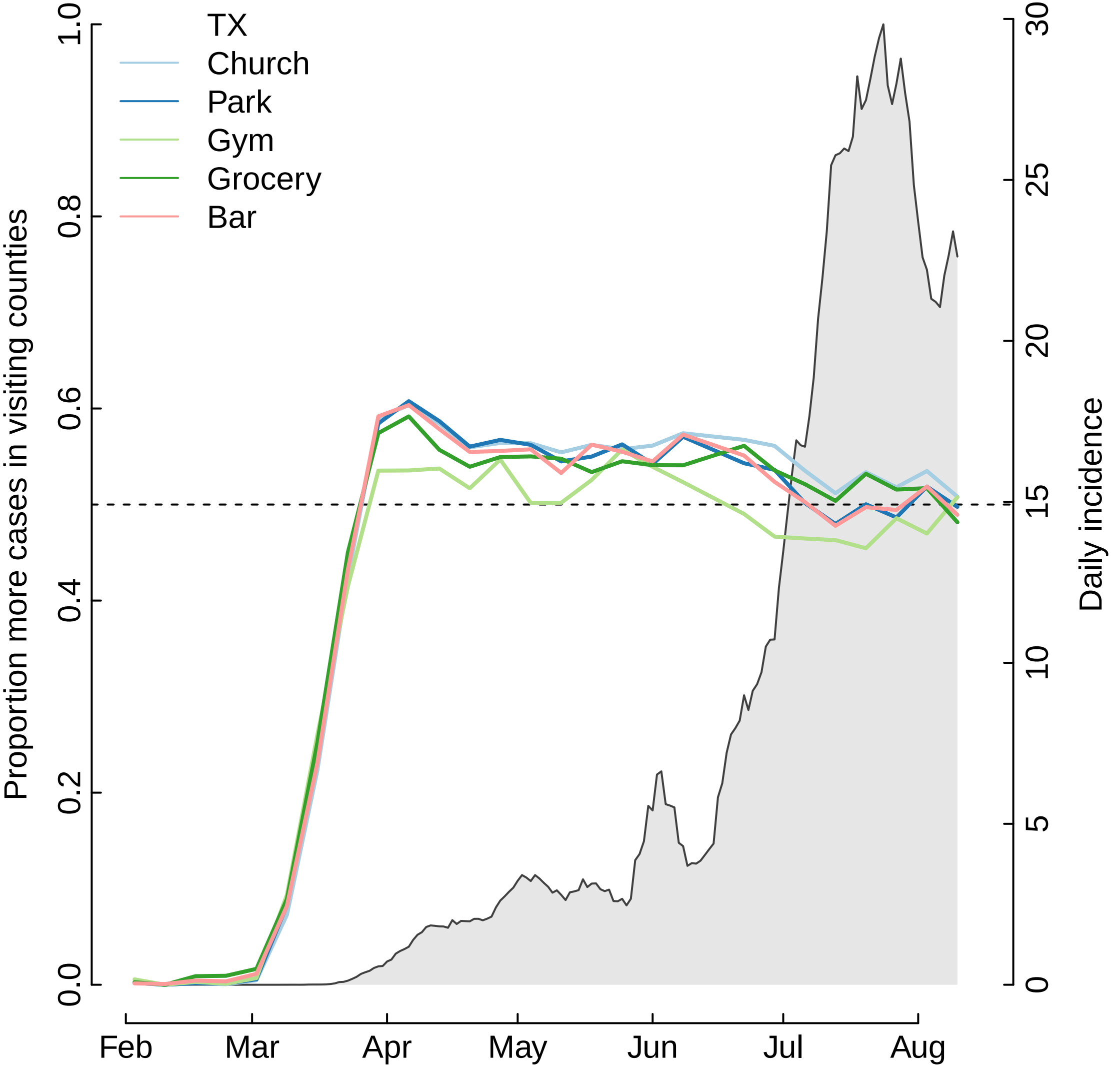

TX
Church
Park
Gym
Grocery
Bar
Proportion more cases in visiting counties
Daily incidence
0.0
0.2
0.4
0.6
0.8
1.0
0
5
10
15
20
25
30
Feb
Mar
Apr
May
Jun
Jul
Aug

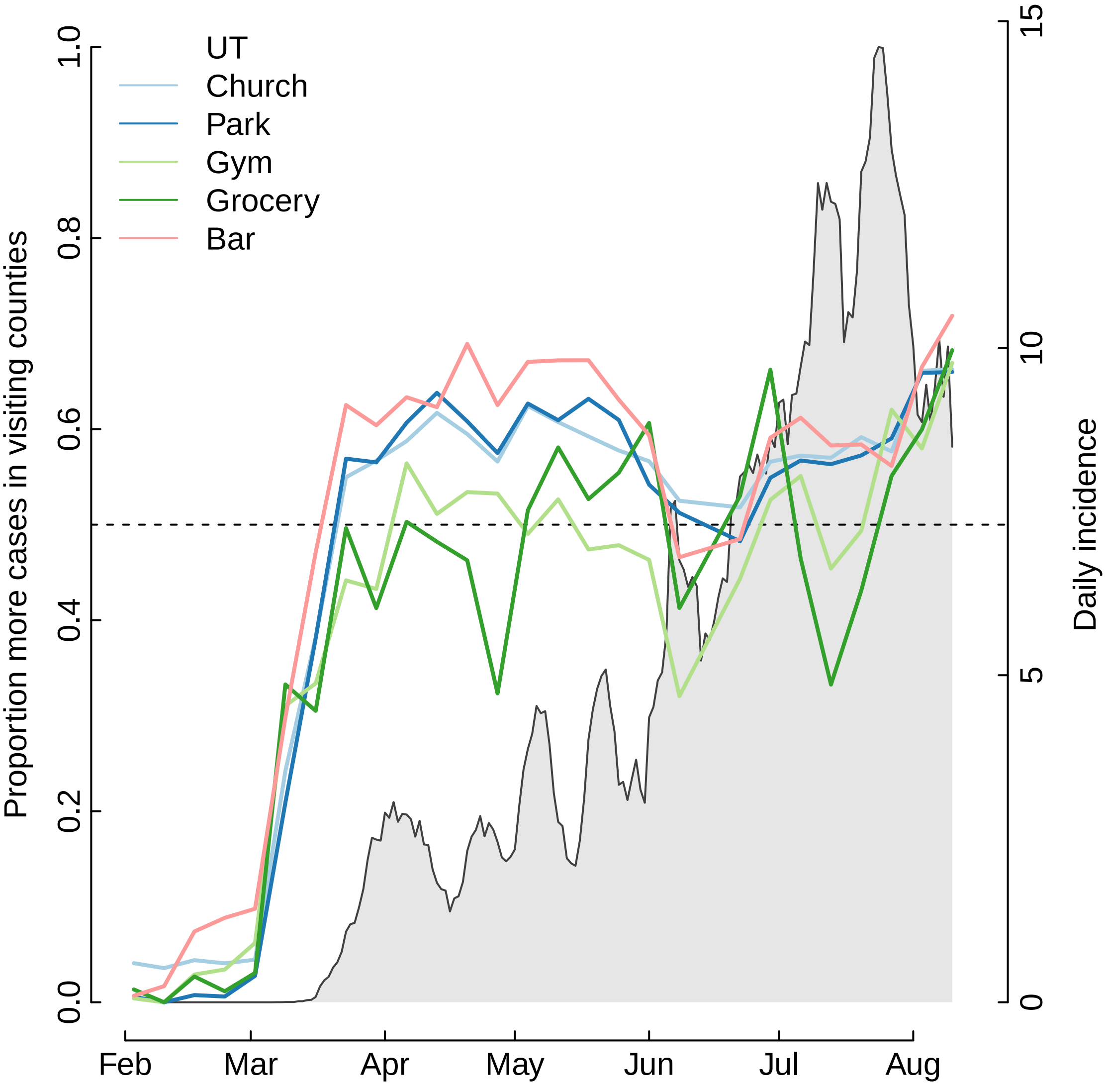

UT
Church
Park
Gym
Grocery
Bar
Proportion more cases in visiting counties
Daily incidence
0.0
0.2
0.4
0.6
0.8
1.0
0
5
10
15
Feb
Mar
Apr
May
Jun
Jul
Aug

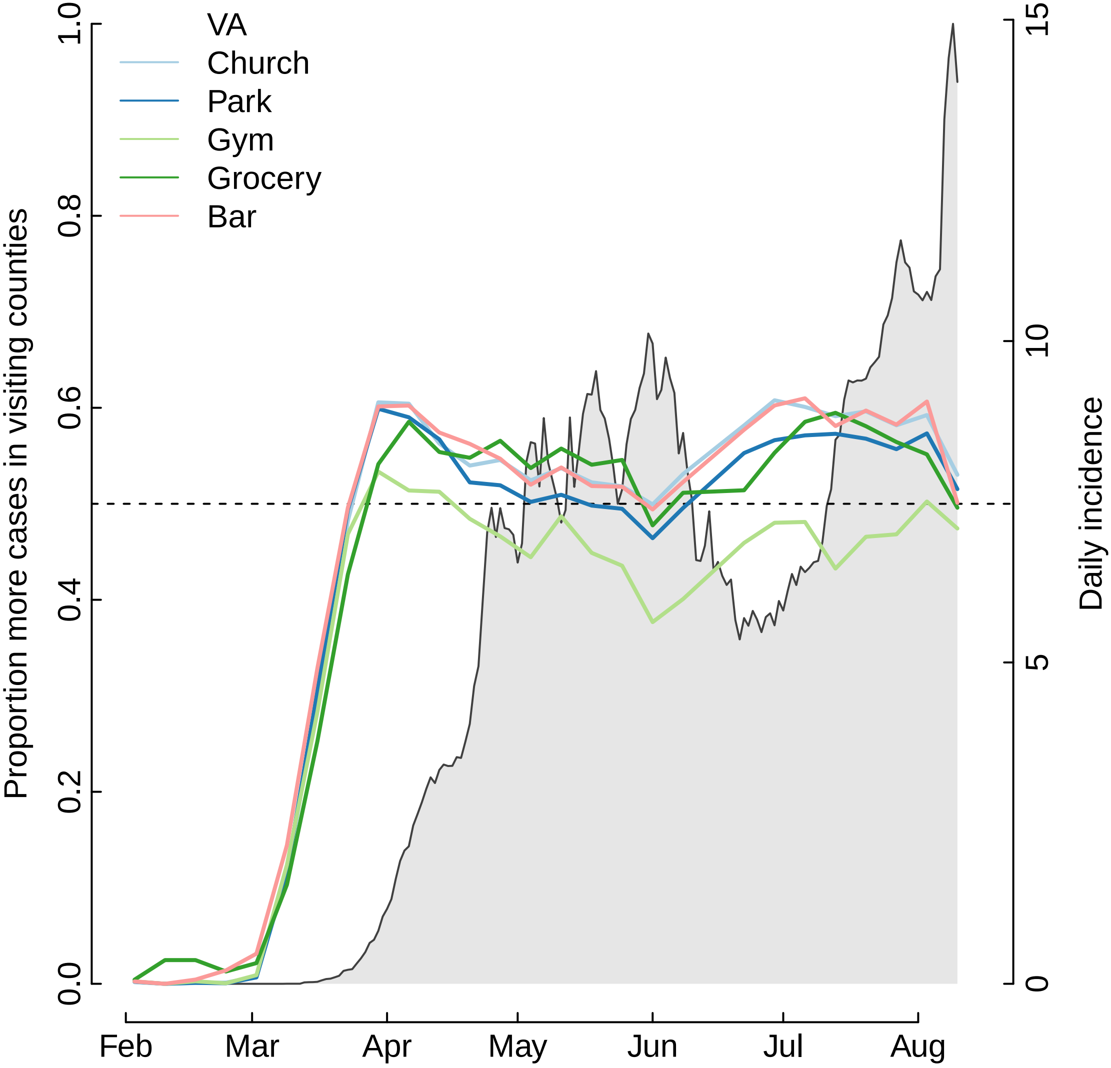

VA
Church
Park
Gym
Grocery
Bar
Proportion more cases in visiting counties
Daily incidence
0.0
0.2
0.4
0.6
0.8
1.0
0
5
10
15
Feb
Mar
Apr
May
Jun
Jul
Aug

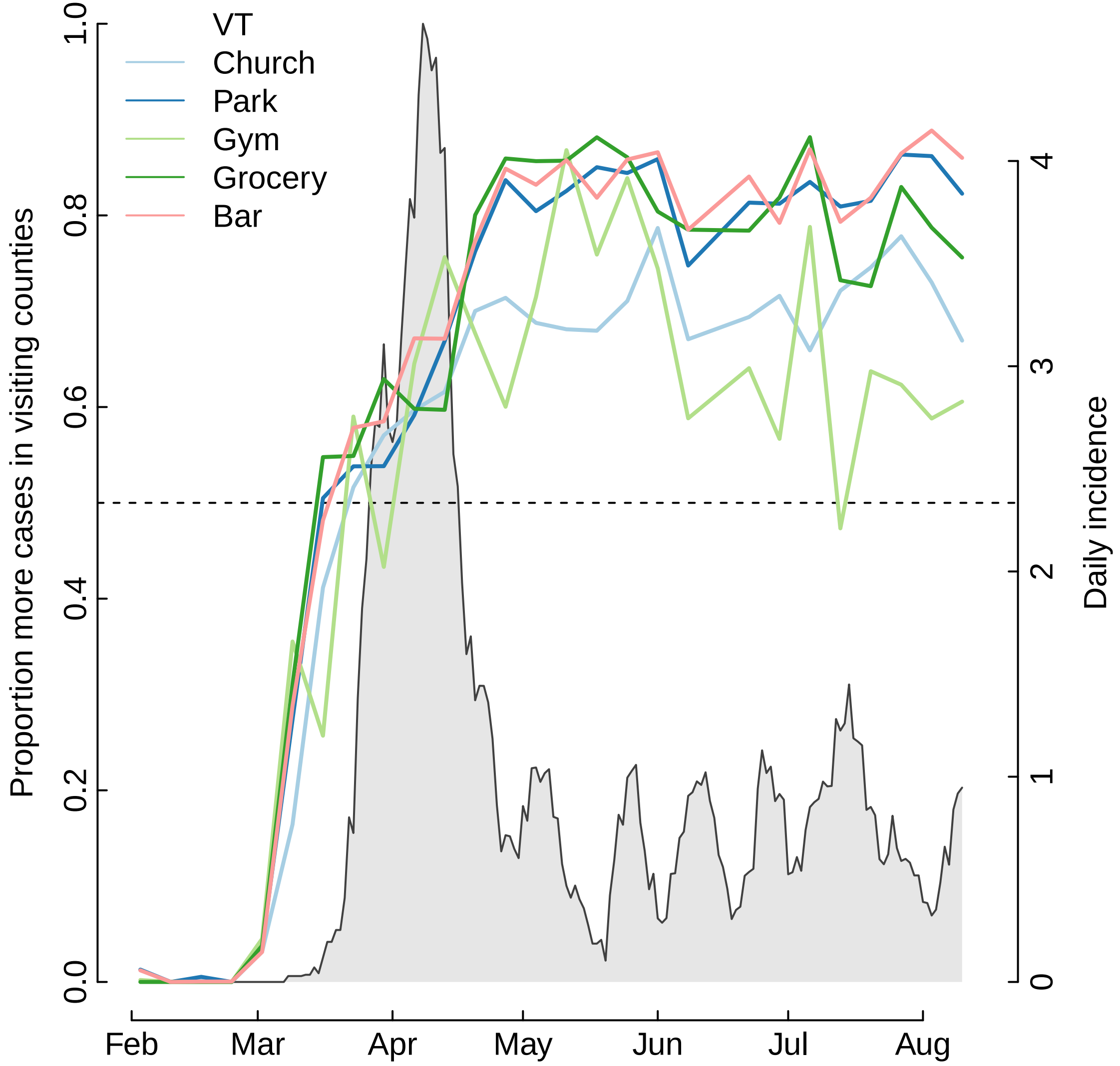

VT
Church
Park
Gym
Grocery
Bar
Proportion more cases in visiting counties
Daily incidence
0.0
0.2
0.4
0.6
0.8
1.0
0
1
2
3
4
Feb
Mar
Apr
May
Jun
Jul
Aug

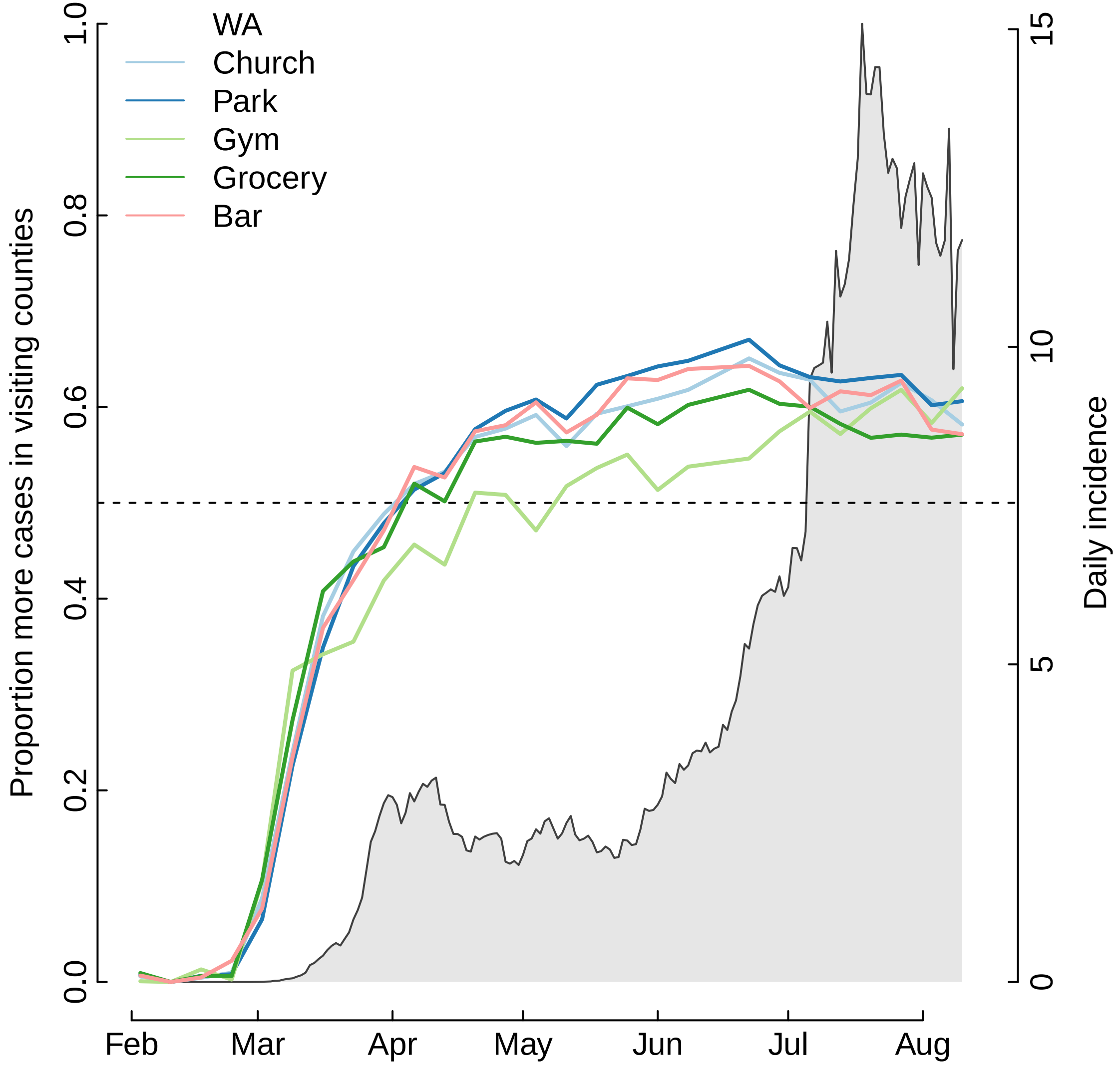

WA
Church
Park
Gym
Grocery
Bar
Proportion more cases in visiting counties
Daily incidence
0.0
0.2
0.4
0.6
0.8
1.0
0
5
10
15
Feb
Mar
Apr
May
Jun
Jul
Aug

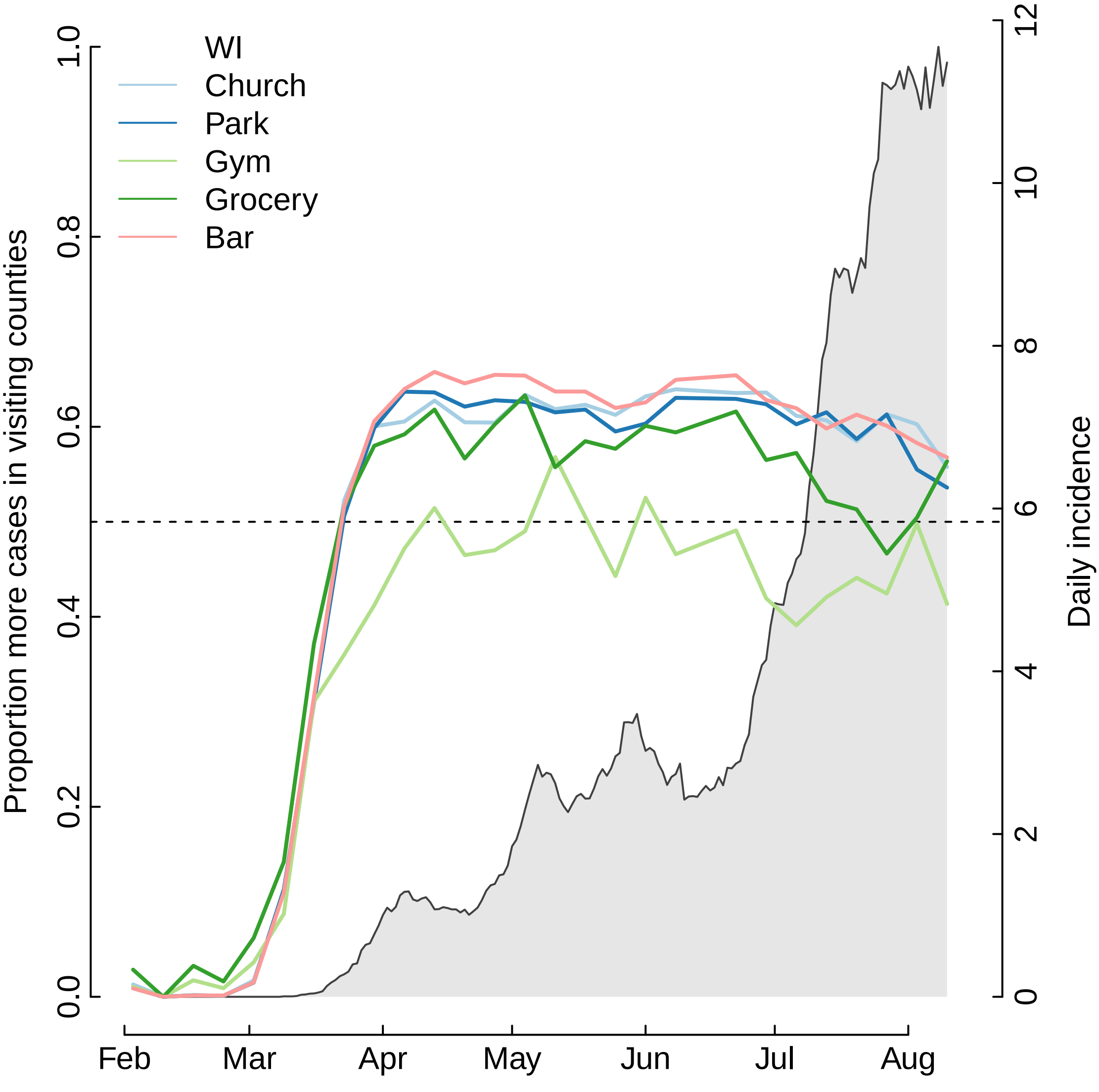

WI
Church
Park
Gym
Grocery
Bar
Proportion more cases in visiting counties
Daily incidence
0.0
0.2
0.4
0.6
0.8
1.0
0
2
4
6
8
10
12
Feb
Mar
Apr
May
Jun
Jul
Aug

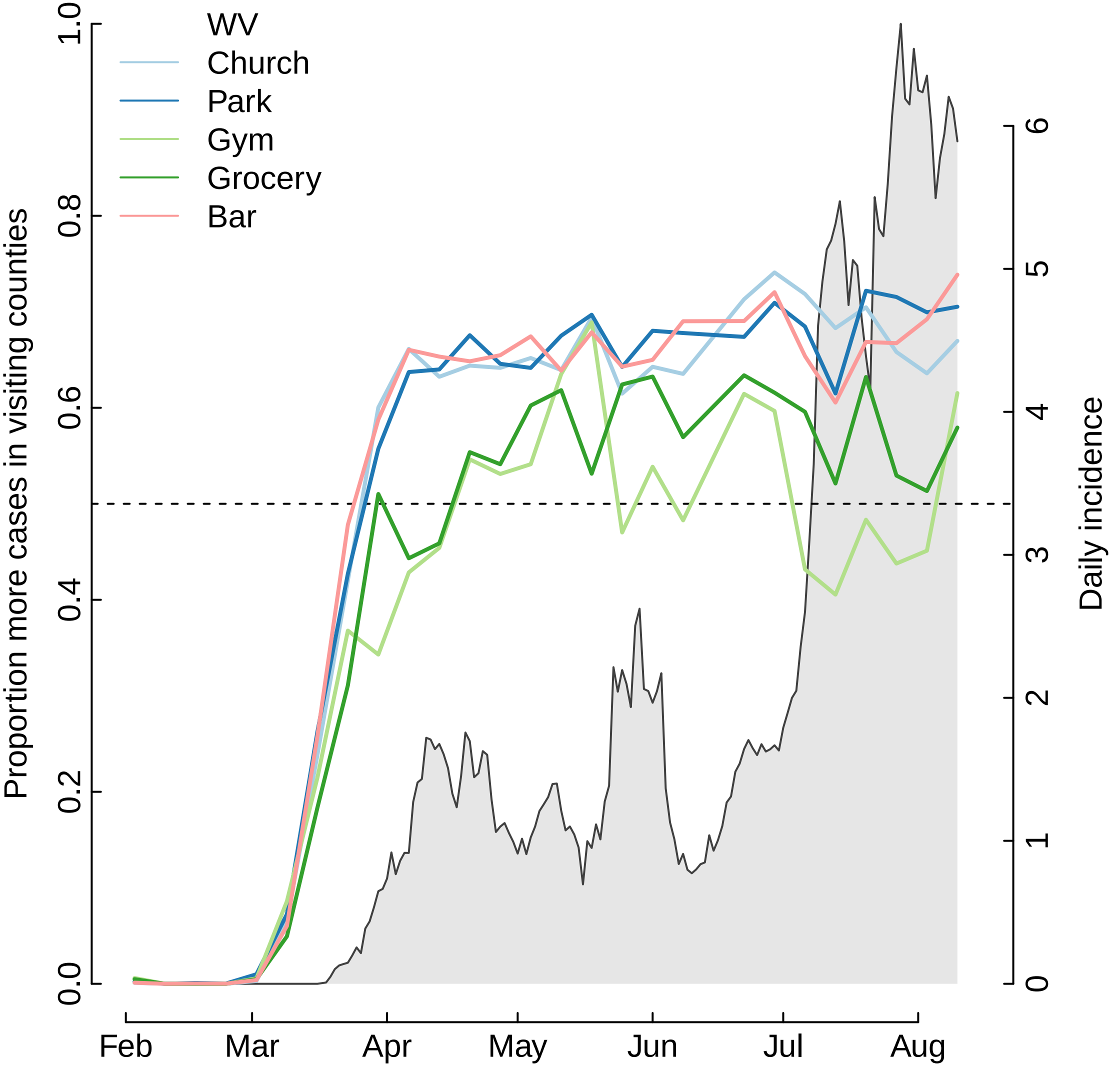

WV
Church
Park
Gym
Grocery
Bar
Proportion more cases in visiting counties
Daily incidence
0.0
0.2
0.4
0.6
0.8
1.0
0
1
2
3
4
5
6
Feb
Mar
Apr
May
Jun
Jul
Aug

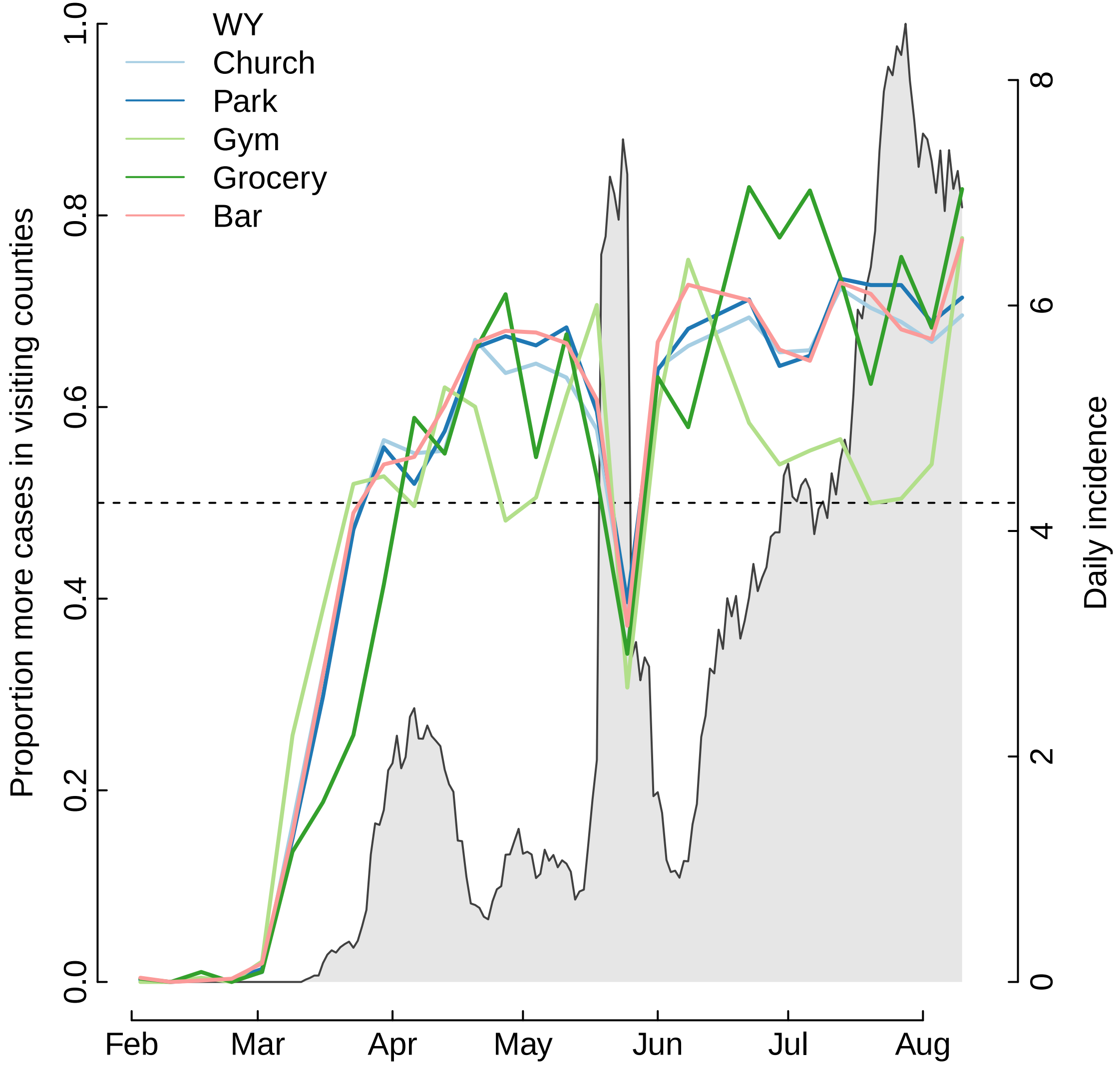

WY
Church
Park
Gym
Grocery
Bar
Proportion more cases in visiting counties
Daily incidence
0.0
0.2
0.4
0.6
0.8
1.0
0
2
4
6
8
Feb
Mar
Apr
May
Jun
Jul
Aug
