# Supplementary material for: The unintended consequences of inconsistent closure policies and mobility restrictions during epidemics
Source: BMC Glob Public Health. 2023 Dec 4;1:28. doi: 10.1186/s44263-023-00028-z (PMC11116187; doi:10.1186/s44263-023-00028-z)
Supplement: Supplementary file 2 — Additional file 2. Statistics on mobility patterns for every county. [file 44263_2023_28_MOESM2_ESM.pdf]

Additional File 2:
The unintended consequences of inconsistent closure policies and mobility restrictions during epidemics

Benjamin M. Althouse[1,2], **Brendan Wallace**[3], **B. K. M. Case**[4,5], **Samuel V. Scarpino**[5,6,7,8,9], **Antoine Allard**[5,10,11], **Andrew M. Berdahl**[12], **Easton R. White**[13,14], **and Laurent Hébert-Dufresne**[4,5,10,*]

[1]University of Washington, Seattle, WA 98105
[2]New Mexico State University, Las Cruces, NM 88003
[3]Department of Applied Mathematics, University of Washington, Seattle, WA 98195, USA
[4]Department of Computer Science, University of Vermont, Burlington, VT 05405, USA
[5]Vermont Complex Systems Center, University of Vermont, Burlington, VT 05405, USA
[6]Institute for Experiential AI, Northeastern University, Boston, Massachusetts, USA
[7]Department of Health Sciences, Northeastern University, Boston, MA, USA
[8]Khoury College of Computer Sciences, Northeastern University, Boston, MA, USA
[9]Santa Fe Institute, Santa Fe, NM, USA
[10]Département de physique, de génie physique et d'optique, Université Laval, Québec (Québec), Canada G1V 0A6
[11]Centre interdisciplinaire en modélisation mathématique, Université Laval, Québec (Québec), Canada G1V 0A6
[12]School of Aquatic & Fishery Sciences, University of Washington, Seattle, WA 98195, USA
[13]Department of Biological Sciences, University of New Hampshire, Durham, NH, 03824, USA
[14]Gund Institute for Environment, University of Vermont, Burlington, VT 05405, USA
[*]laurent.hebert-dufresne@uvm.edu

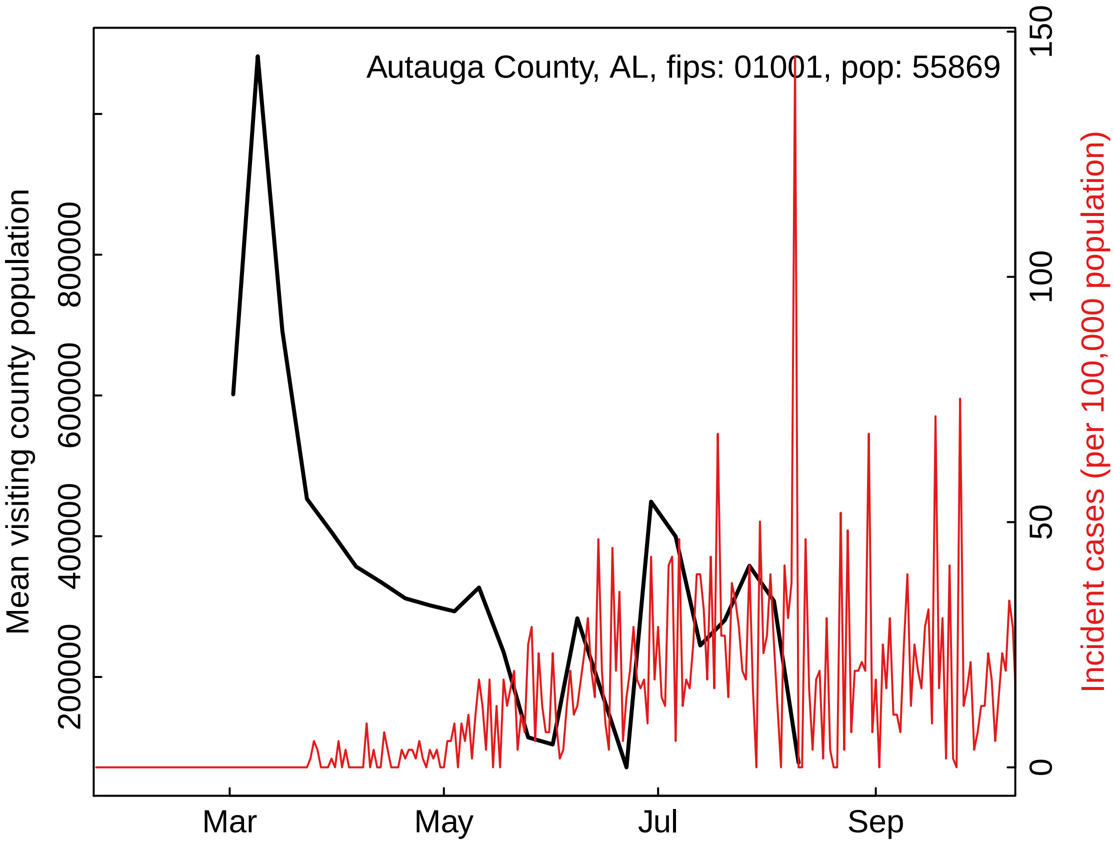

Autauga County, AL, fips: 01001, pop: 55869
Mean visiting county population
200000
400000
600000
800000
Incident cases (per 100,000 population)
0
50
100
150
Mar
May
Jul
Sep

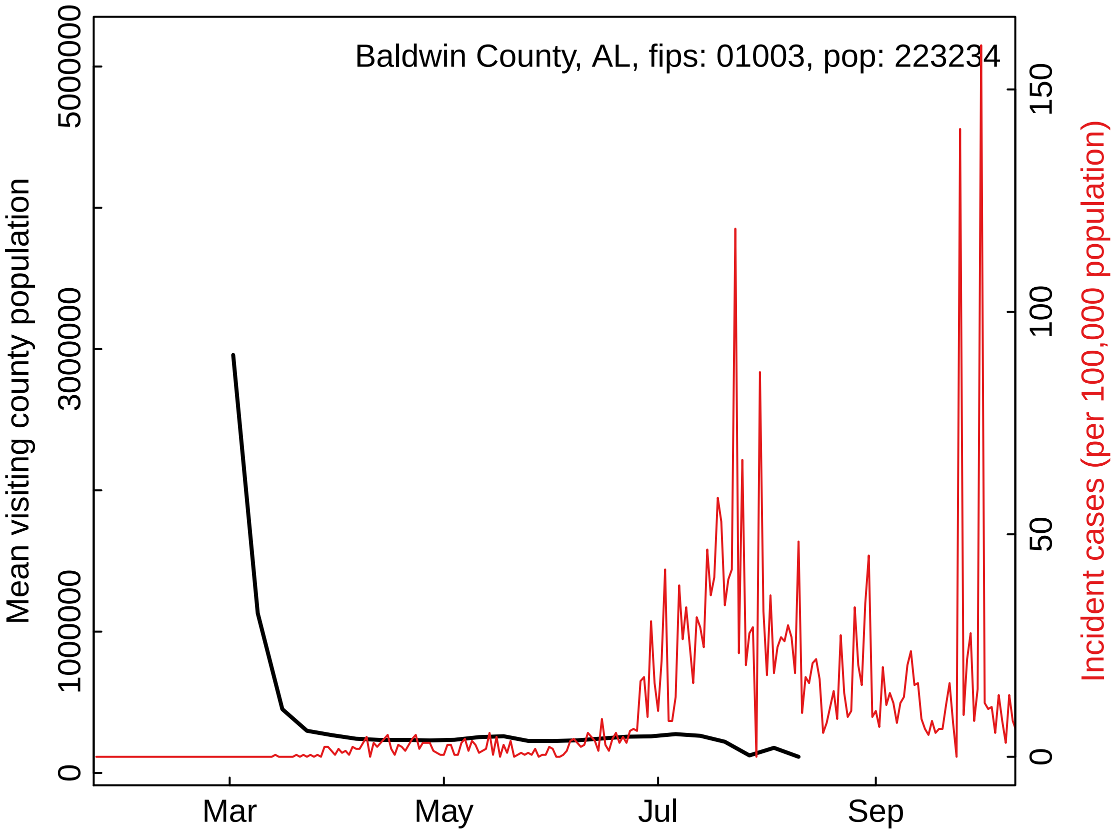
Baldwin County, AL, fips: 01003, pop: 223234
Mean visiting county population
0
1000000
3000000
5000000
Incident cases (per 100,000 population)
0
50
100
150
Mar
May
Jul
Sep

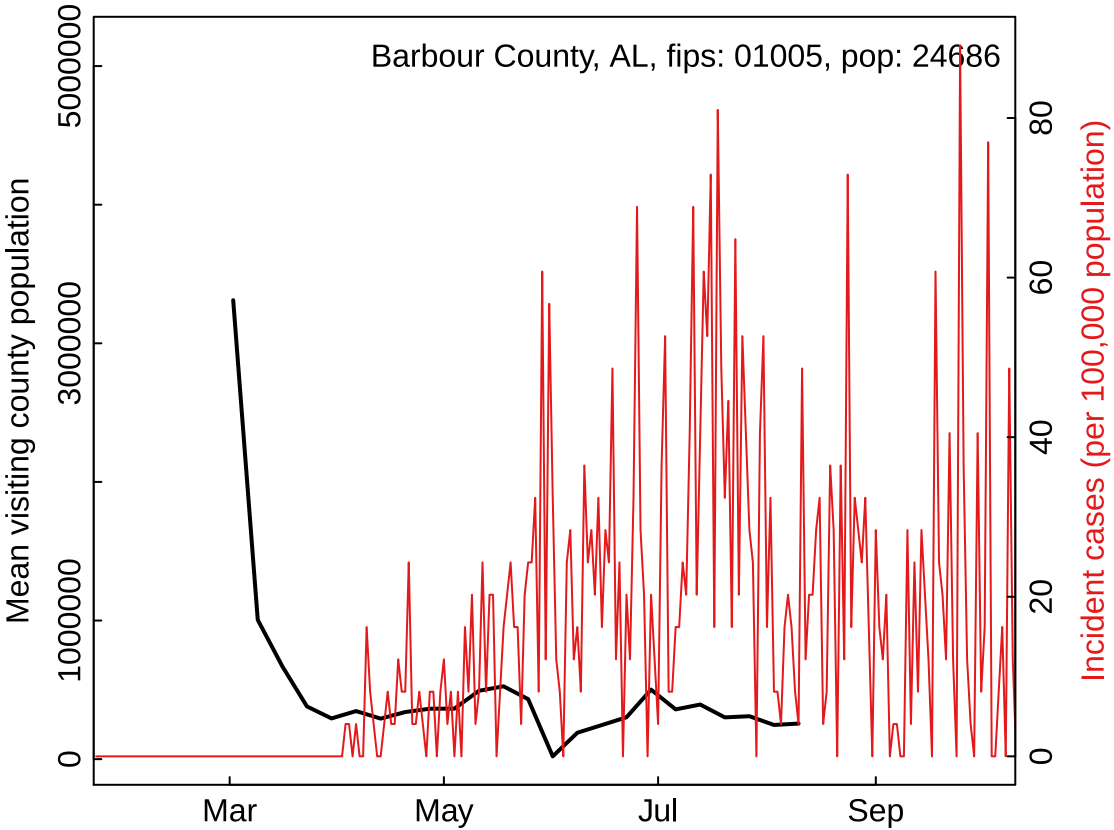

Barbour County, AL, fips: 01005, pop: 24686
Mean visiting county population
Incident cases (per 100,000 population)
0
1000000
3000000
5000000
0
20
40
60
80
Mar
May
Jul
Sep

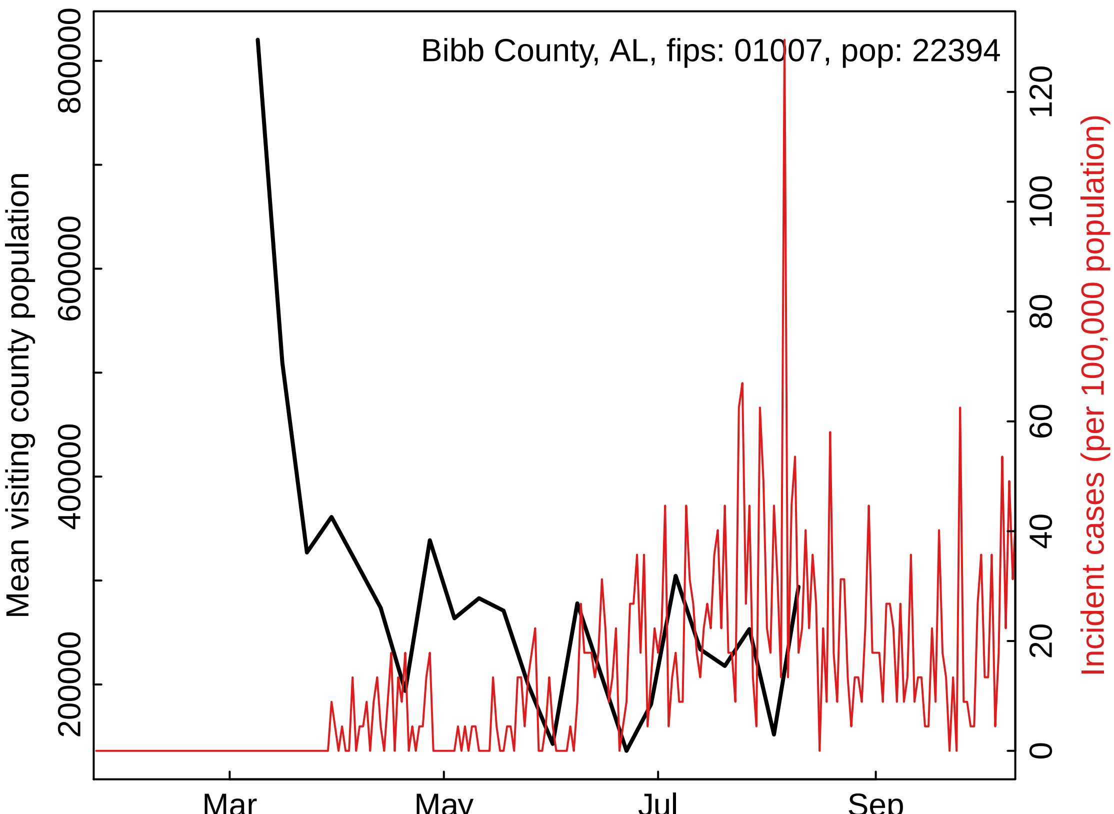

Bibb County, AL, fips: 01007, pop: 22394
Mean visiting county population
200000
400000
600000
800000
Incident cases (per 100,000 population)
0
20
40
60
80
100
120
Mar
May
Jul
Sep

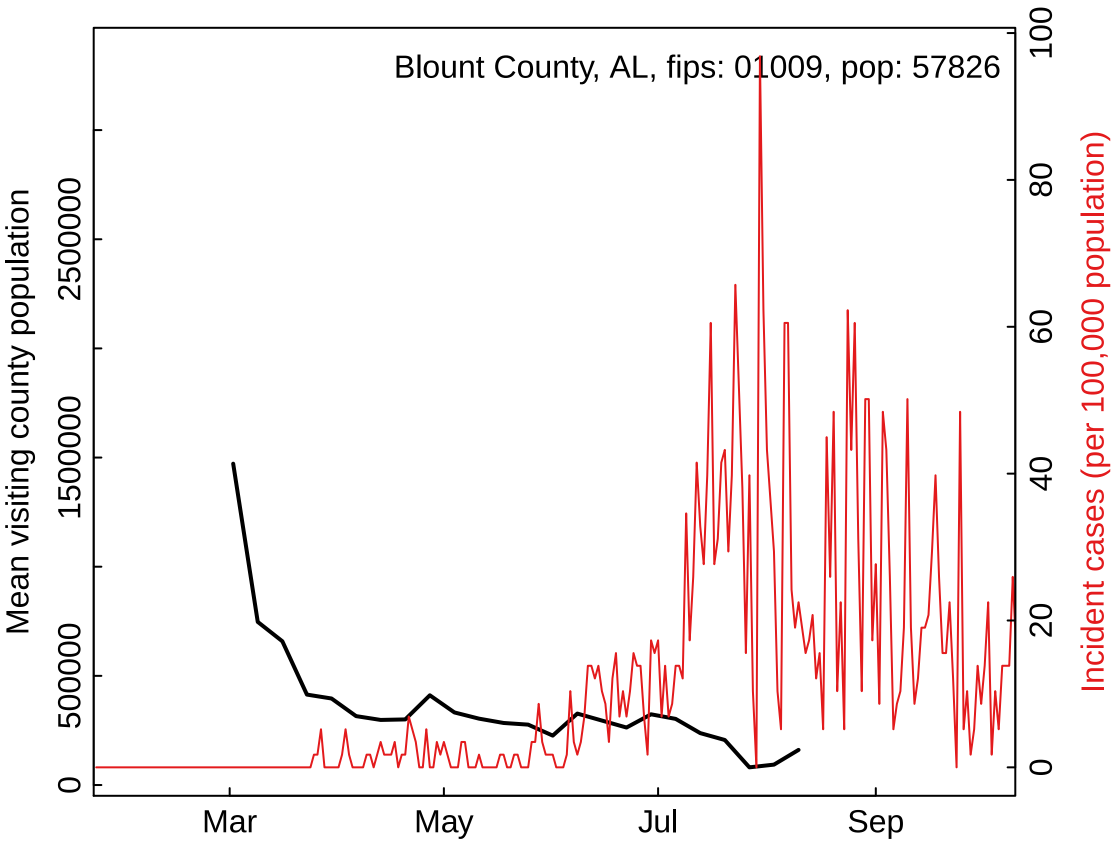

Blount County, AL, fips: 01009, pop: 57826
Mean visiting county population
0
500000
1500000
2500000
Incident cases (per 100,000 population)
0
20
40
60
80
100
Mar
May
Jul
Sep

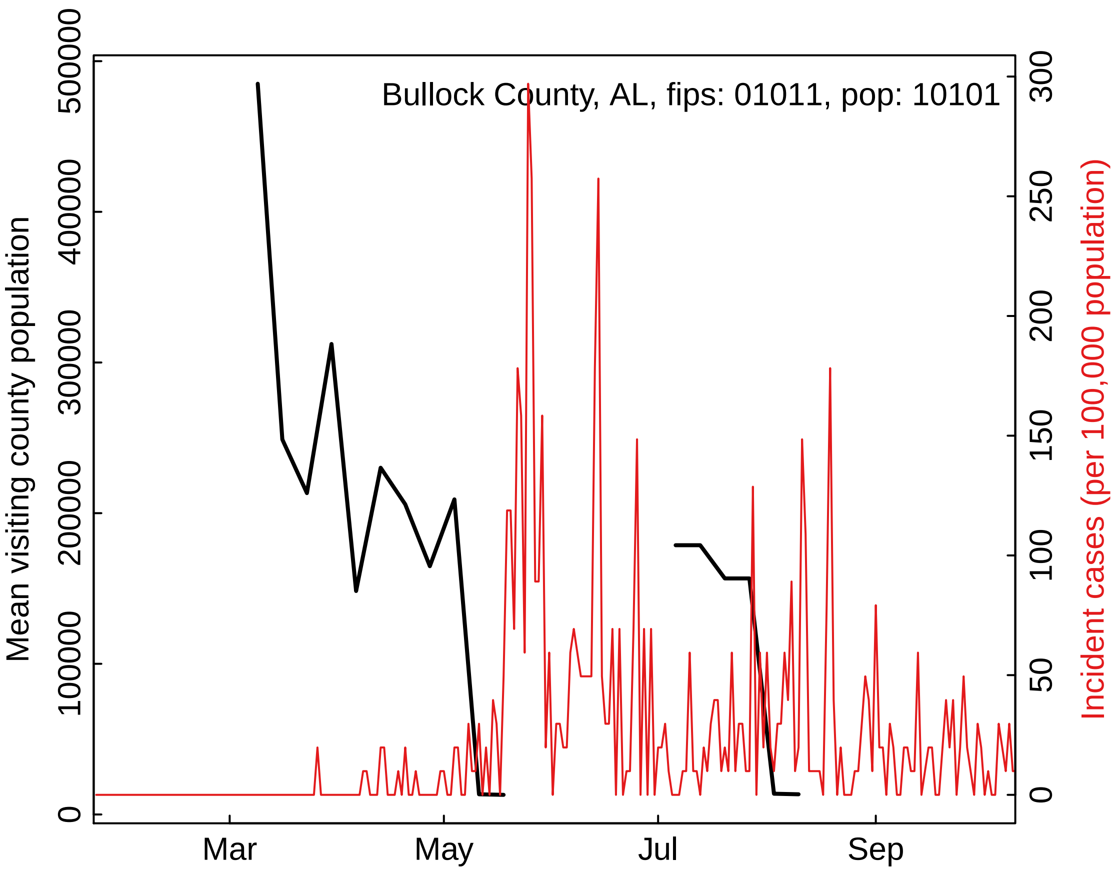

Bullock County, AL, fips: 01011, pop: 10101
Mean visiting county population
Incident cases (per 100,000 population)
0
100000
200000
300000
400000
500000
0
50
100
150
200
250
300
Mar
May
Jul
Sep

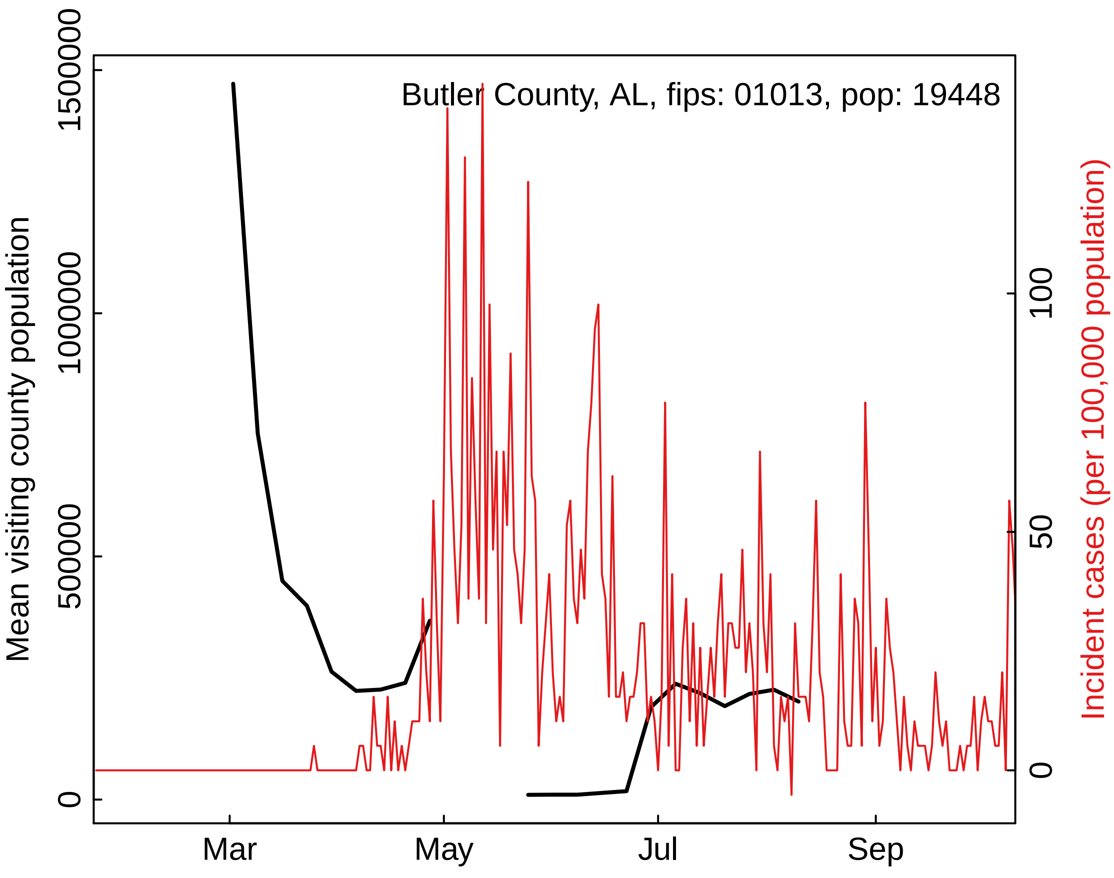

Butler County, AL, fips: 01013, pop: 19448
Mean visiting county population
0
500000
1000000
1500000
Incident cases (per 100,000 population)
0
50
100
Mar
May
Jul
Sep

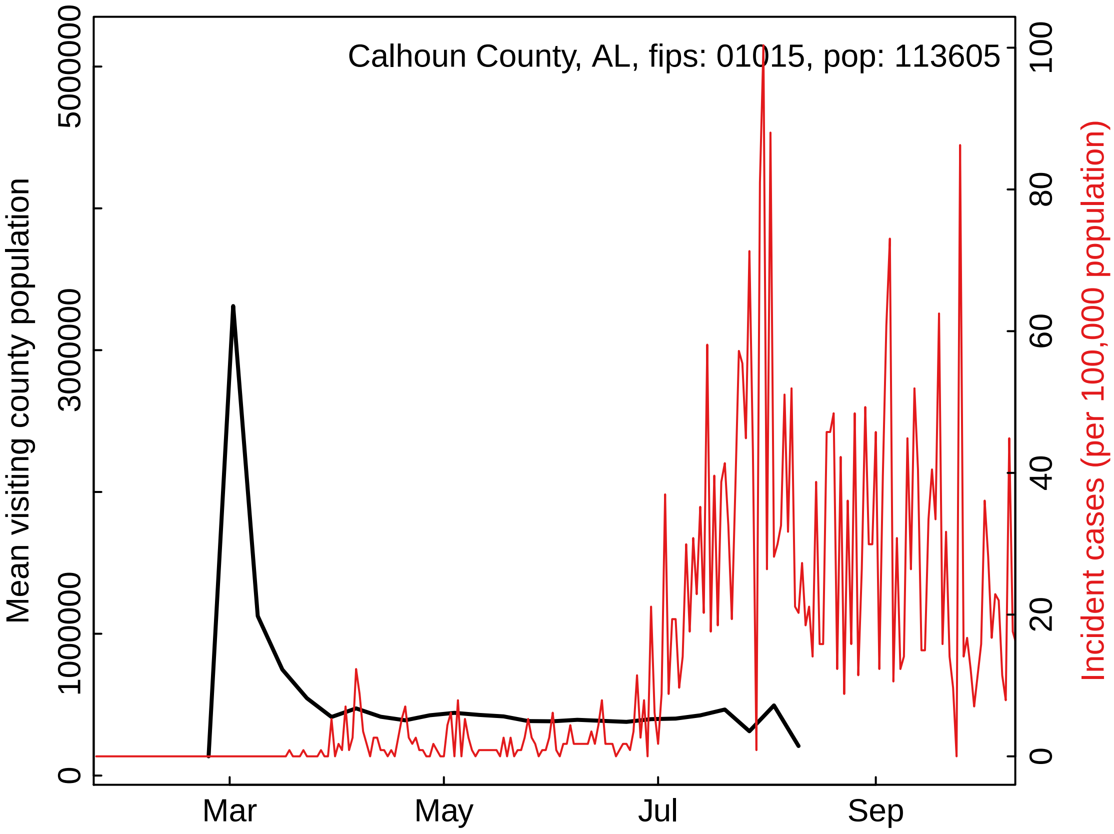

Calhoun County, AL, fips: 01015, pop: 113605
Mean visiting county population
Incident cases (per 100,000 population)
0
1000000
3000000
5000000
0
20
40
60
80
100
Mar
May
Jul
Sep

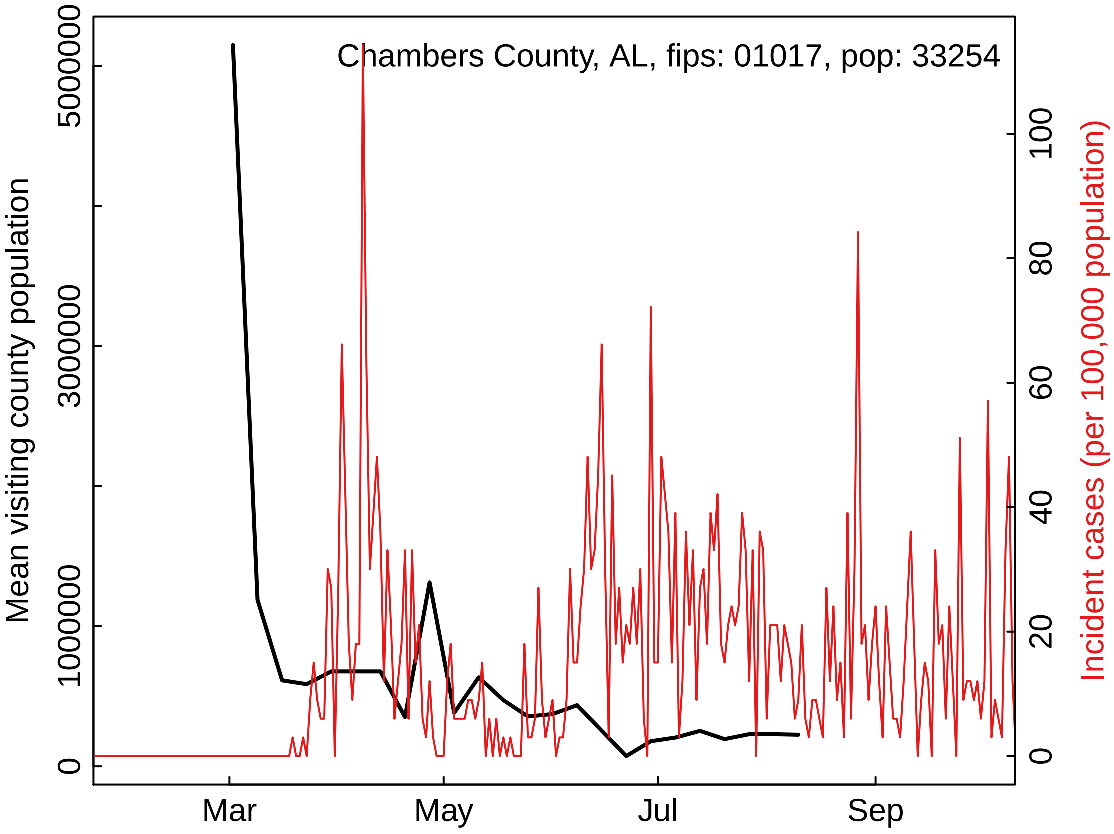
Chambers County, AL, fips: 01017, pop: 33254
Mean visiting county population
0
1000000
3000000
5000000
Incident cases (per 100,000 population)
0
20
40
60
80
100
Mar
May
Jul
Sep

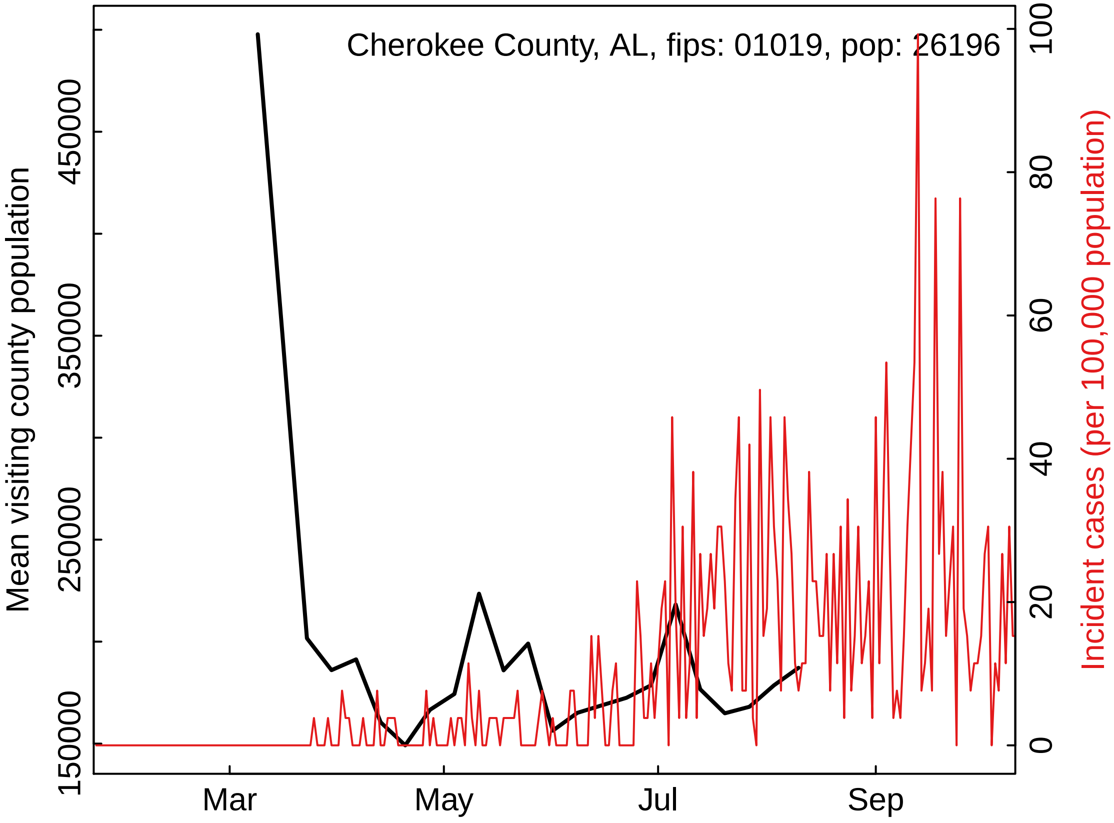

Cherokee County, AL, fips: 01019, pop: 26196
Mean visiting county population
150000
250000
350000
450000
Incident cases (per 100,000 population)
0
20
40
60
80
100
Mar
May
Jul
Sep

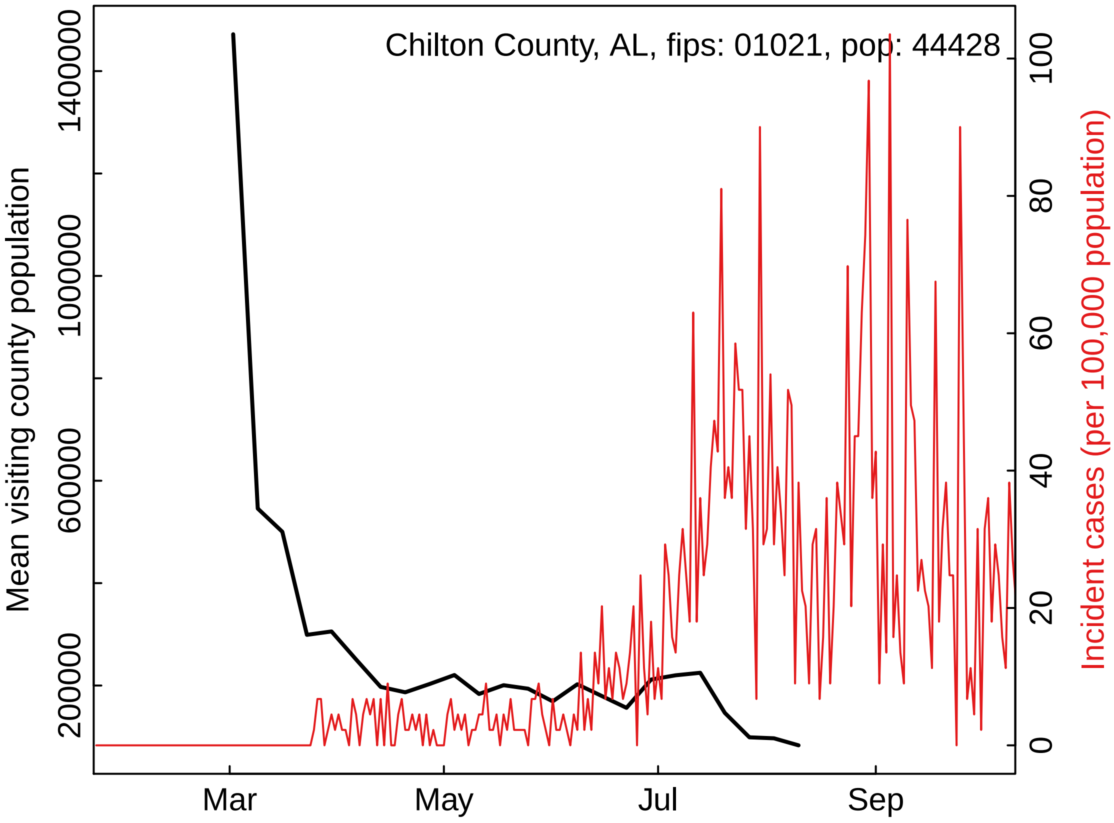

Chilton County, AL, fips: 01021, pop: 44428
Mean visiting county population
200000
600000
1000000
1400000
Mar
May
Jul
Sep
0
20
40
60
80
100
Incident cases (per 100,000 population)

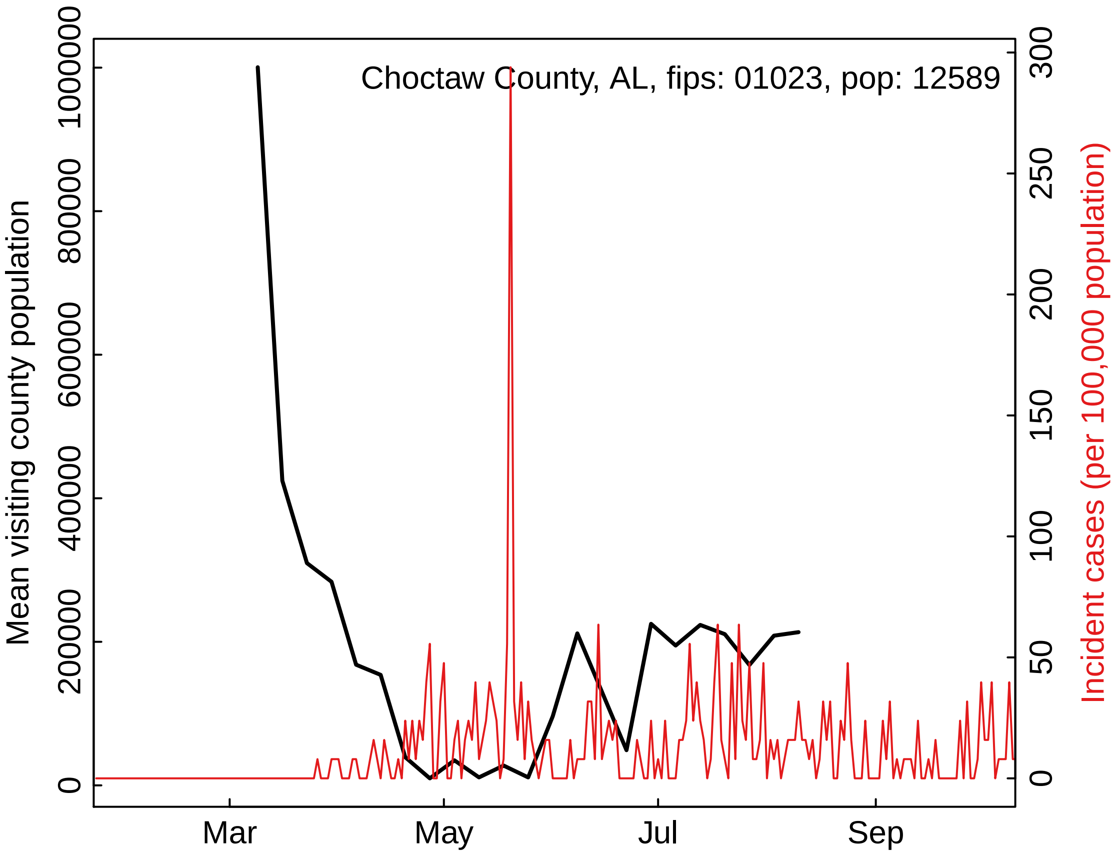

Choctaw County, AL, fips: 01023, pop: 12589
Mean visiting county population
0
200000
400000
600000
800000
1000000
Incident cases (per 100,000 population)
0
50
100
150
200
250
300
Mar
May
Jul
Sep

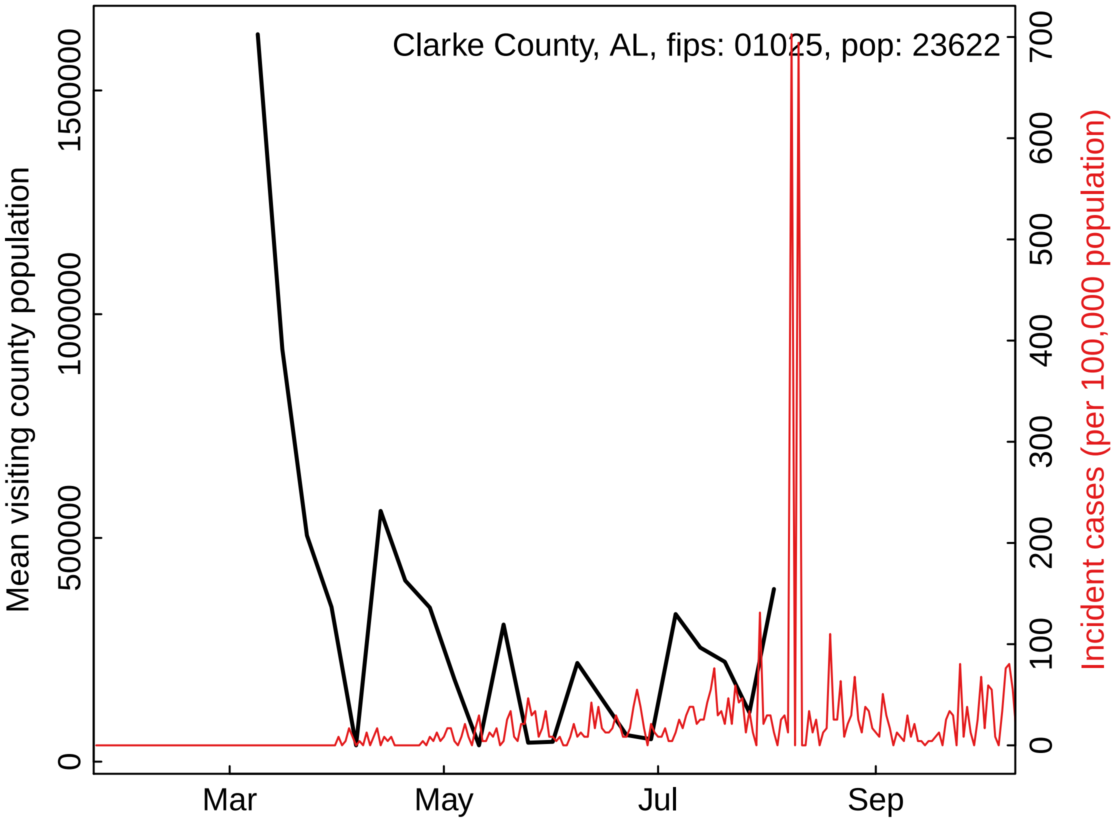
Clarke County, AL, fips: 01025, pop: 23622
Mean visiting county population
Incident cases (per 100,000 population)
0
500000
1000000
1500000
0
100
200
300
400
500
600
700
Mar
May
Jul
Sep

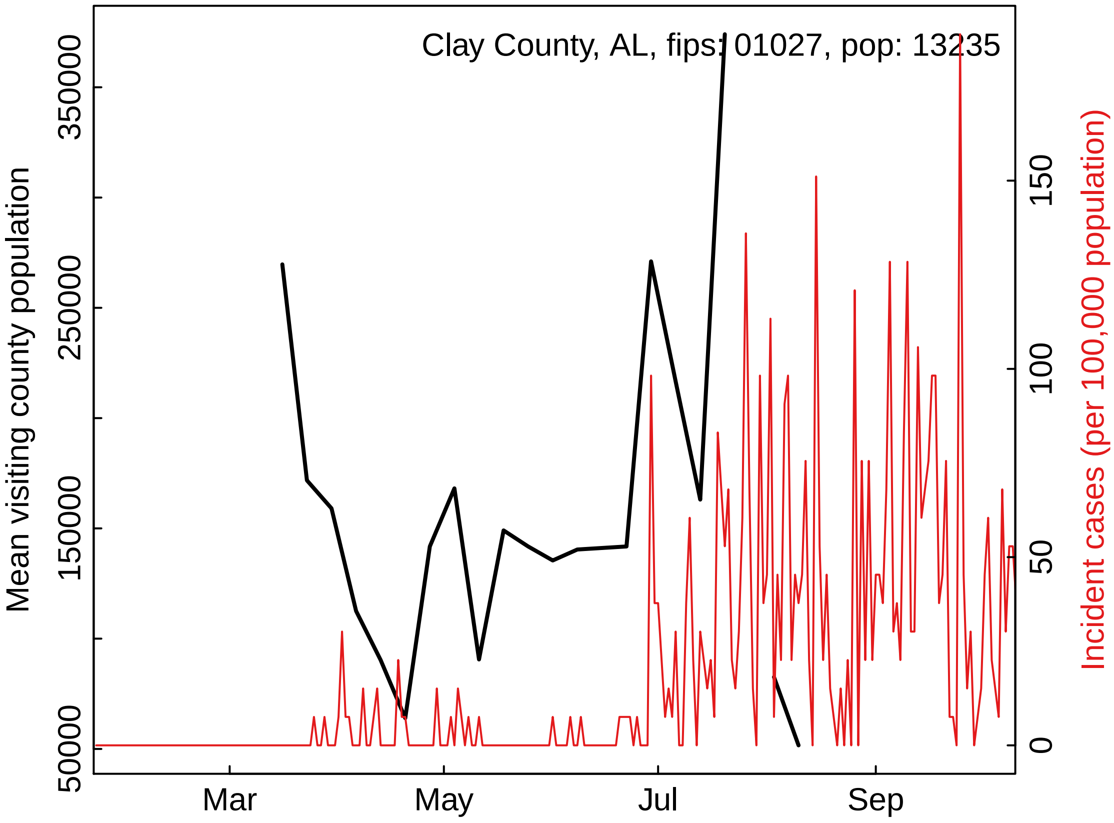
Clay County, AL, fips: 01027, pop: 13235
Mean visiting county population
50000
150000
250000
350000
Incident cases (per 100,000 population)
0
50
100
150
Mar
May
Jul
Sep

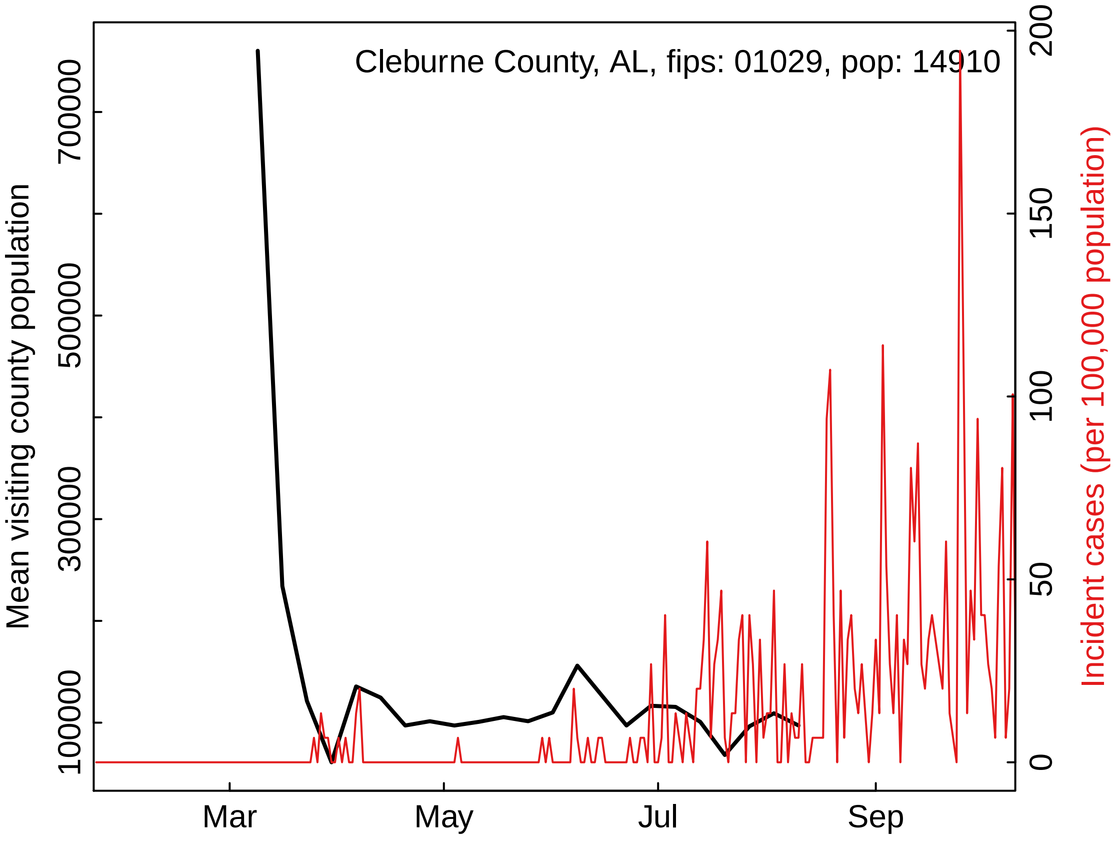

Cleburne County, AL, fips: 01029, pop: 14910
Mean visiting county population
100000
300000
500000
700000
Incident cases (per 100,000 population)
0
50
100
150
200
Mar
May
Jul
Sep

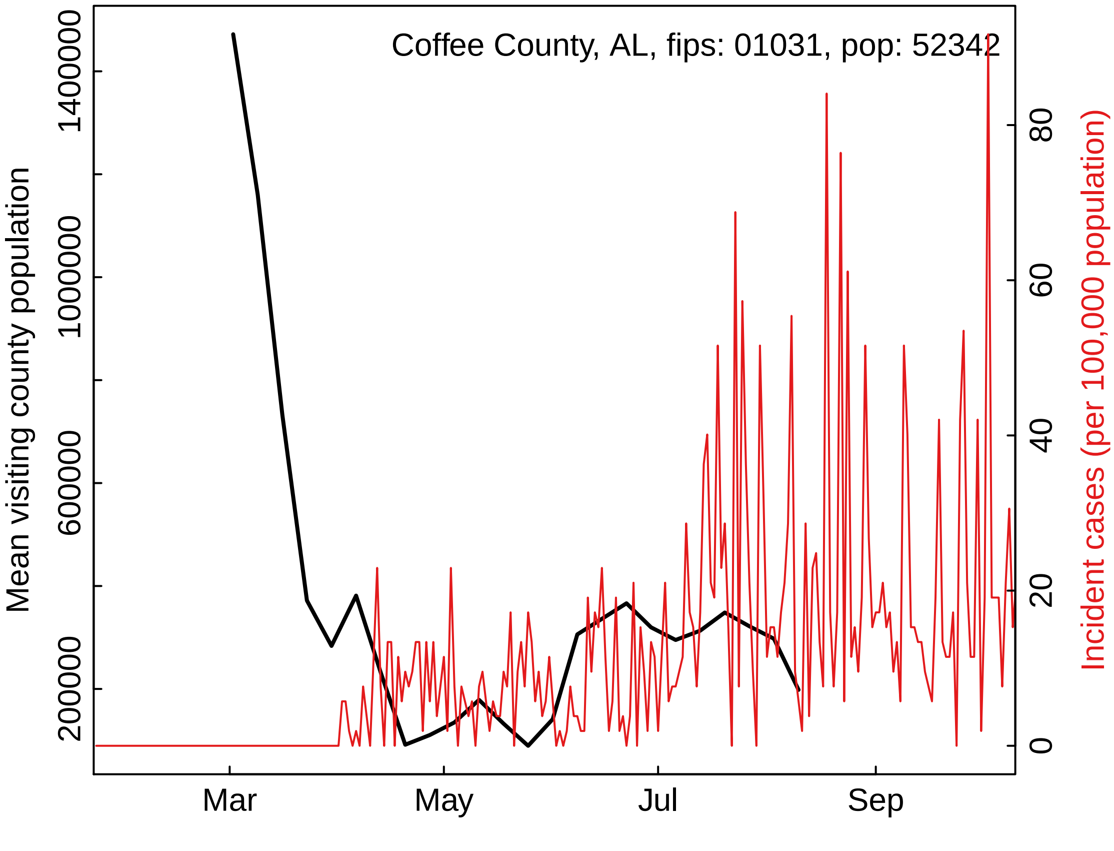

Coffee County, AL, fips: 01031, pop: 52342
Mean visiting county population
200000
600000
1000000
1400000
Mar
May
Jul
Sep
0
20
40
60
80
Incident cases (per 100,000 population)

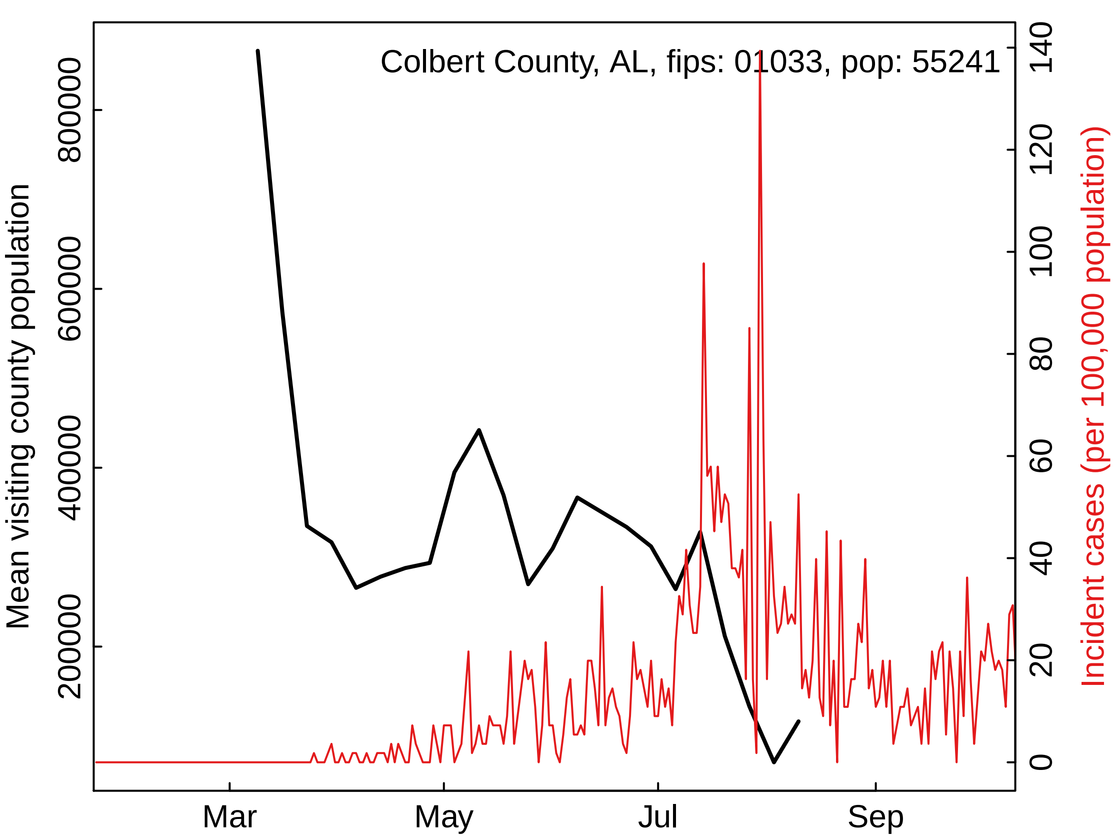

Colbert County, AL, fips: 01033, pop: 55241
Mean visiting county population
200000
400000
600000
800000
Incident cases (per 100,000 population)
0
20
40
60
80
100
120
140
Mar
May
Jul
Sep

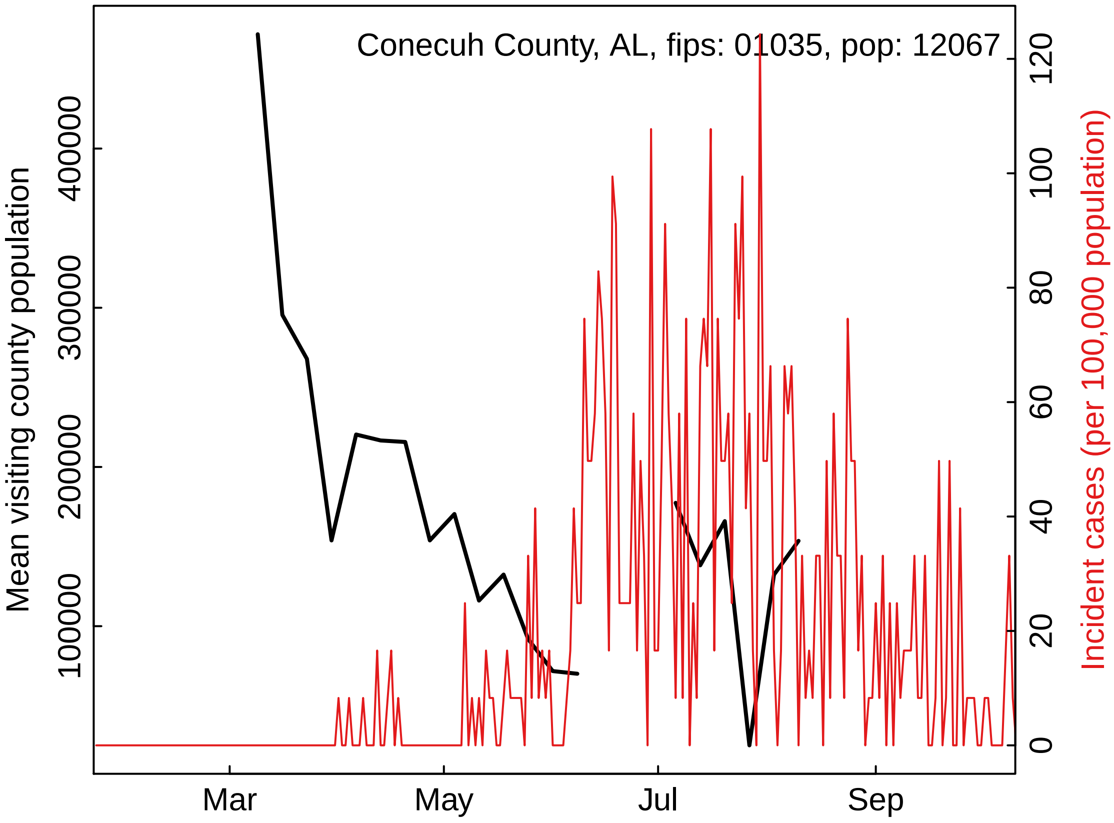
Conecuh County, AL, fips: 01035, pop: 12067
Mean visiting county population
100000
200000
300000
400000
Incident cases (per 100,000 population)
0
20
40
60
80
100
120
Mar
May
Jul
Sep

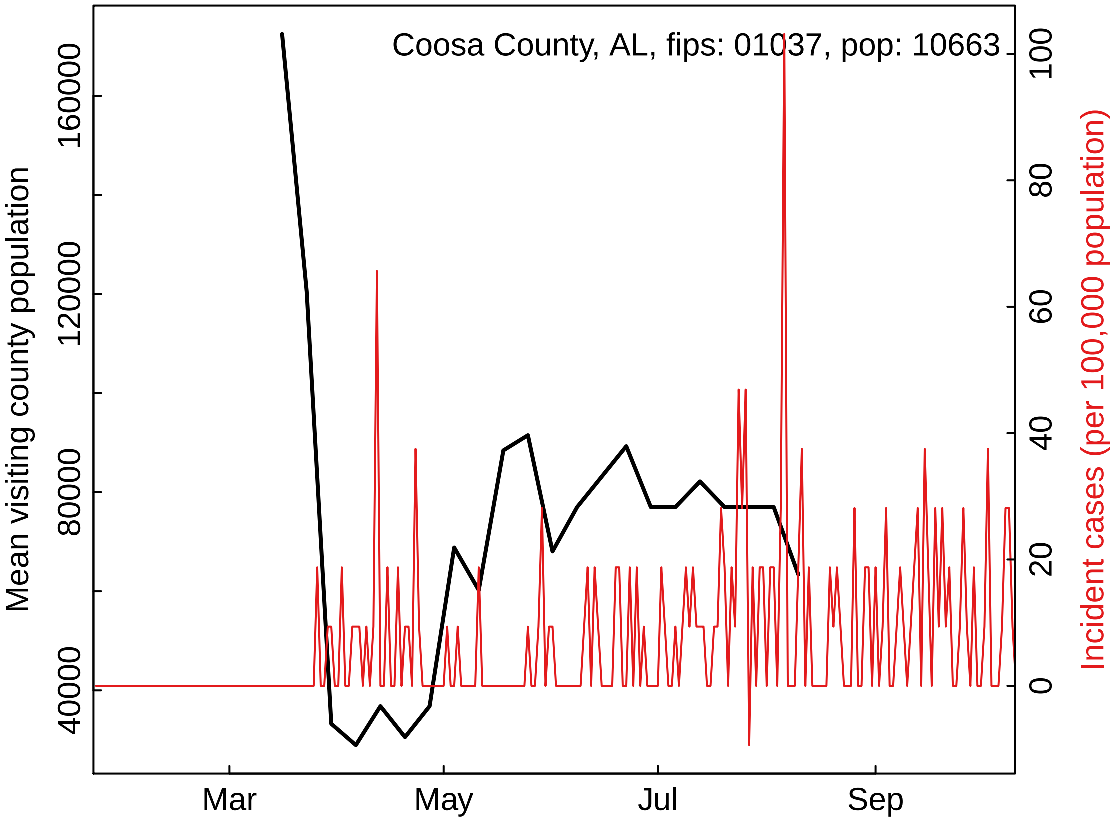
Coosa County, AL, fips: 01037, pop: 10663
Mean visiting county population
40000
80000
120000
160000
Incident cases (per 100,000 population)
0
20
40
60
80
100
Mar
May
Jul
Sep

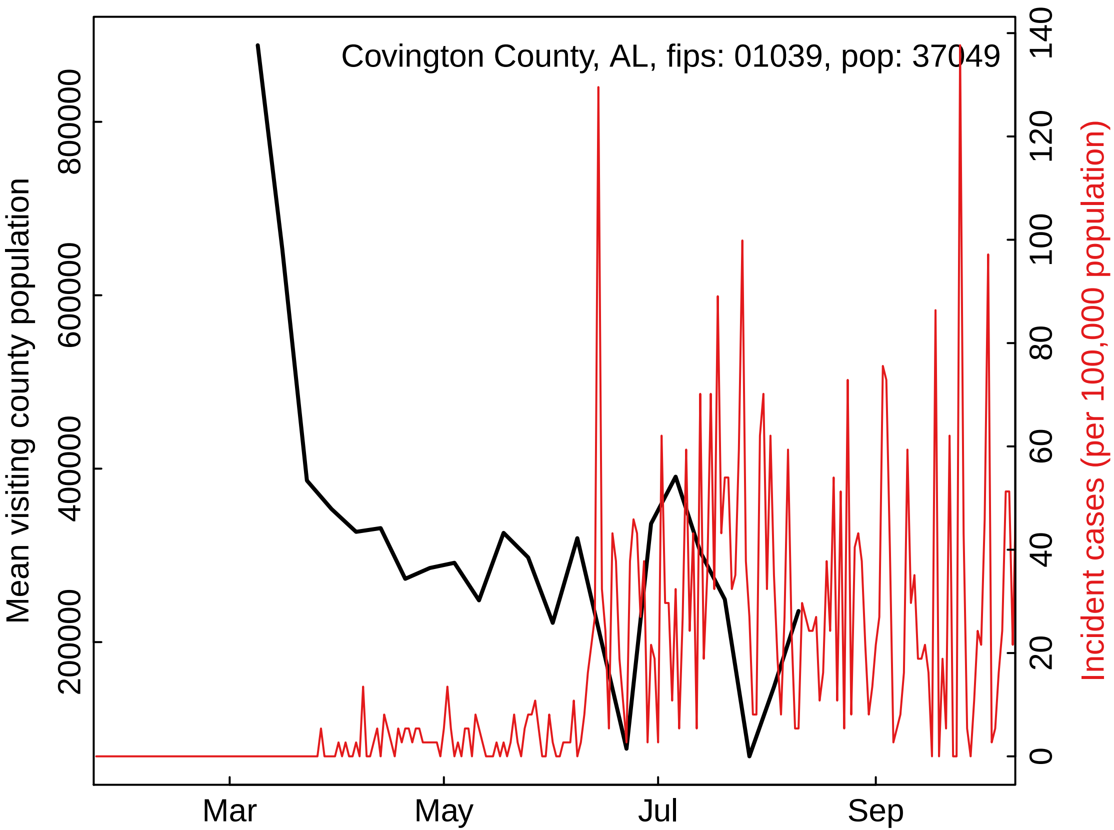

Covington County, AL, fips: 01039, pop: 37049
Mean visiting county population
200000
400000
600000
800000
Incident cases (per 100,000 population)
0
20
40
60
80
100
120
140
Mar
May
Jul
Sep

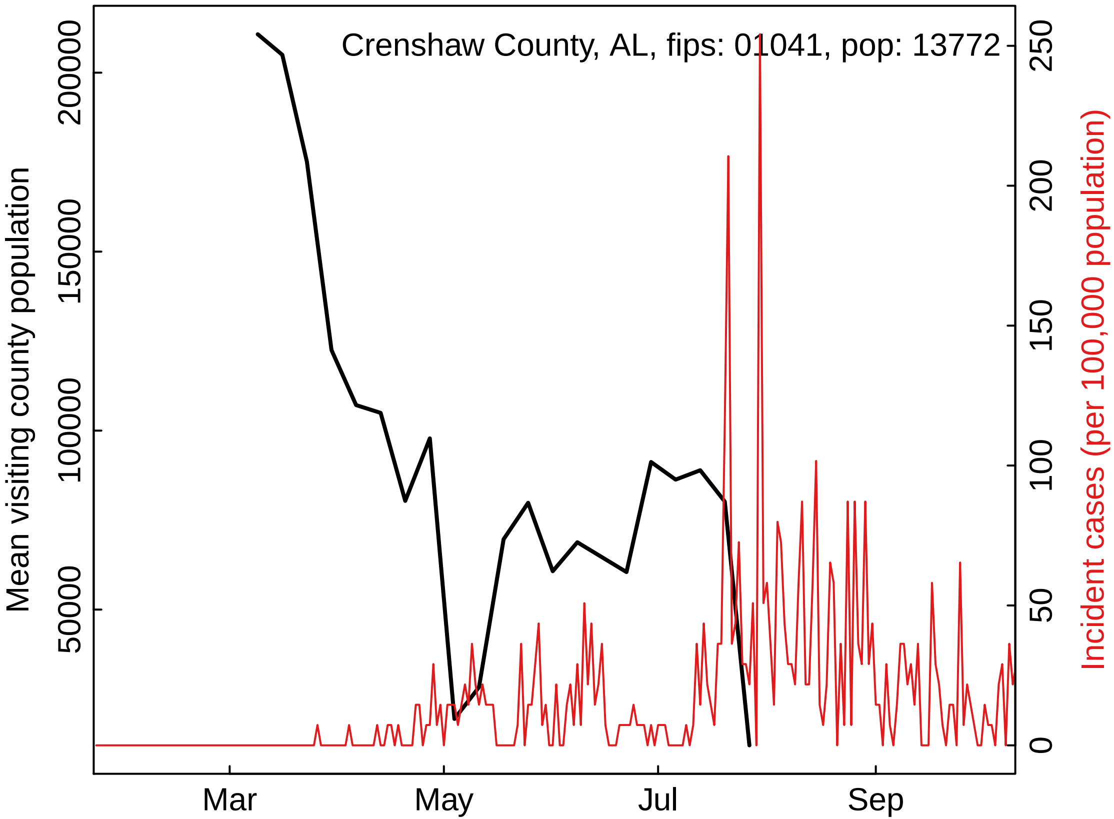

Crenshaw County, AL, fips: 01041, pop: 13772
Mean visiting county population
Incident cases (per 100,000 population)
50000
100000
150000
200000
0
50
100
150
200
250
Mar
May
Jul
Sep

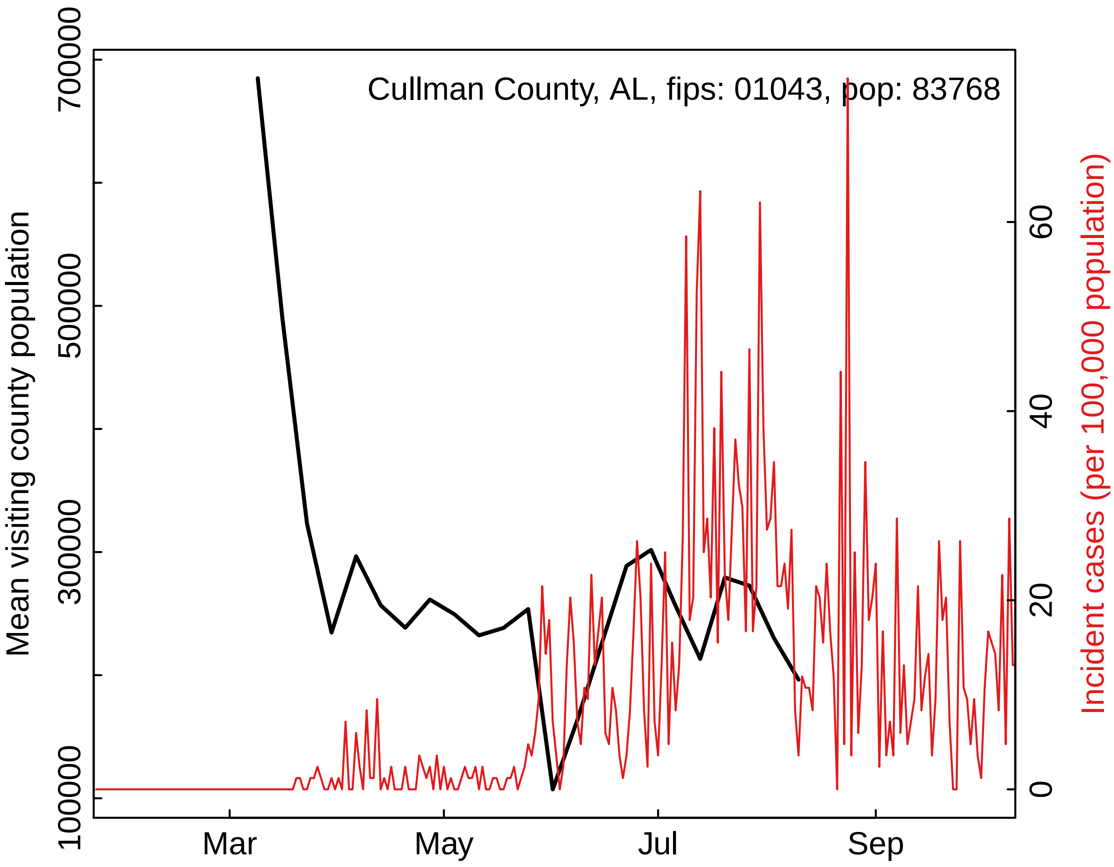

Cullman County, AL, fips: 01043, pop: 83768
Mean visiting county population
Incident cases (per 100,000 population)
100000
300000
500000
700000
0
20
40
60
Mar
May
Jul
Sep

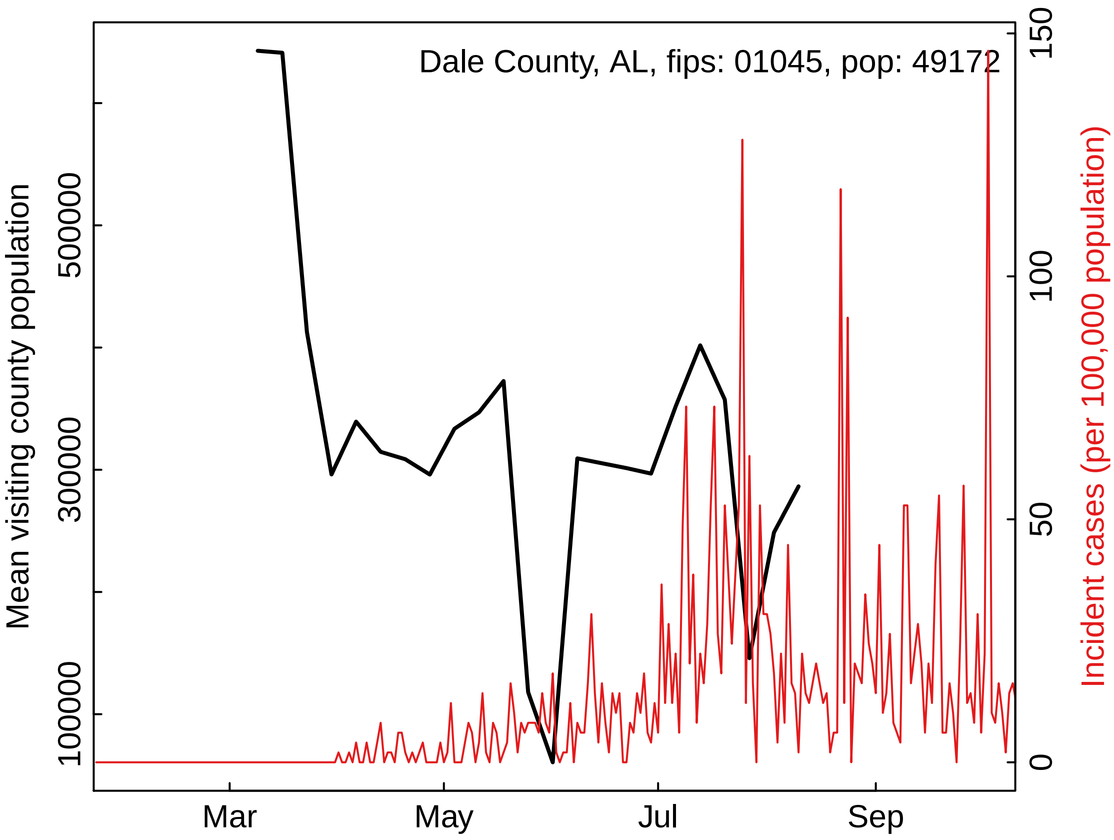

Dale County, AL, fips: 01045, pop: 49172
Mean visiting county population
100000
300000
500000
Mar
May
Jul
Sep
Incident cases (per 100,000 population)
0
50
100
150

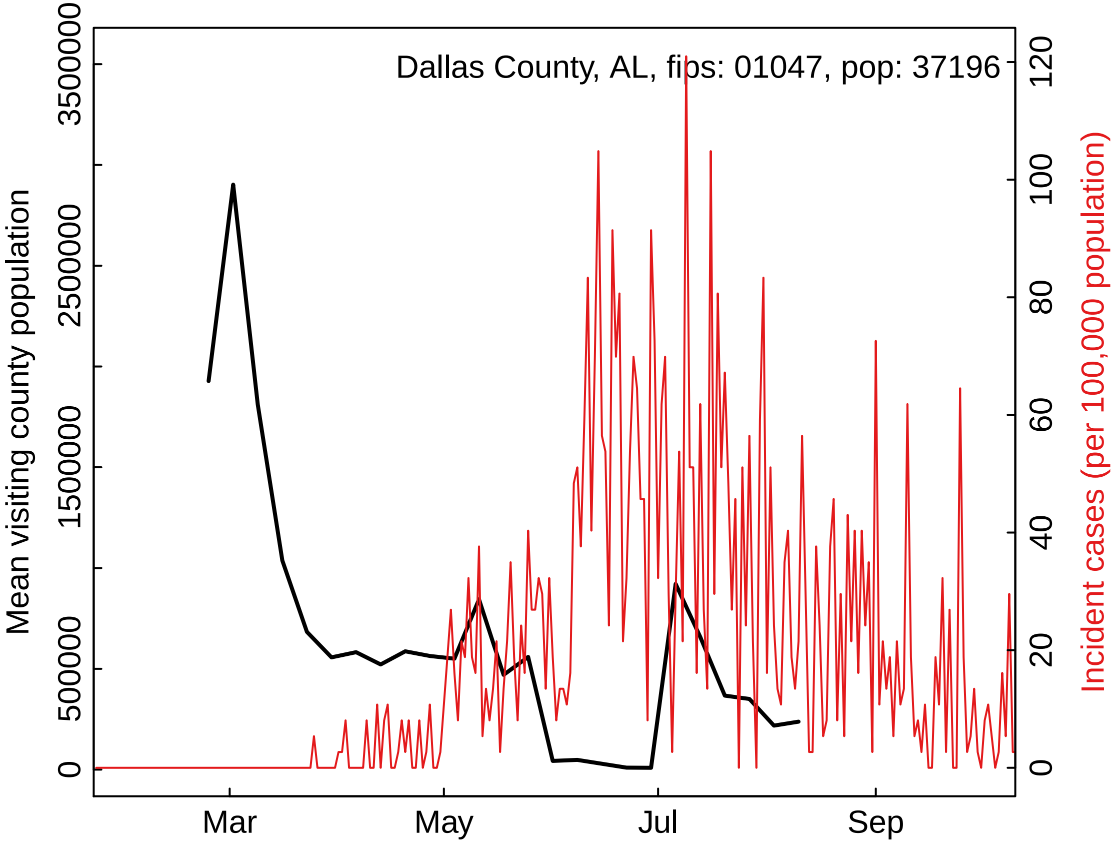

Dallas County, AL, fips: 01047, pop: 37196
Mean visiting county population
0
500000
1500000
2500000
3500000
Incident cases (per 100,000 population)
0
20
40
60
80
100
120
Mar
May
Jul
Sep

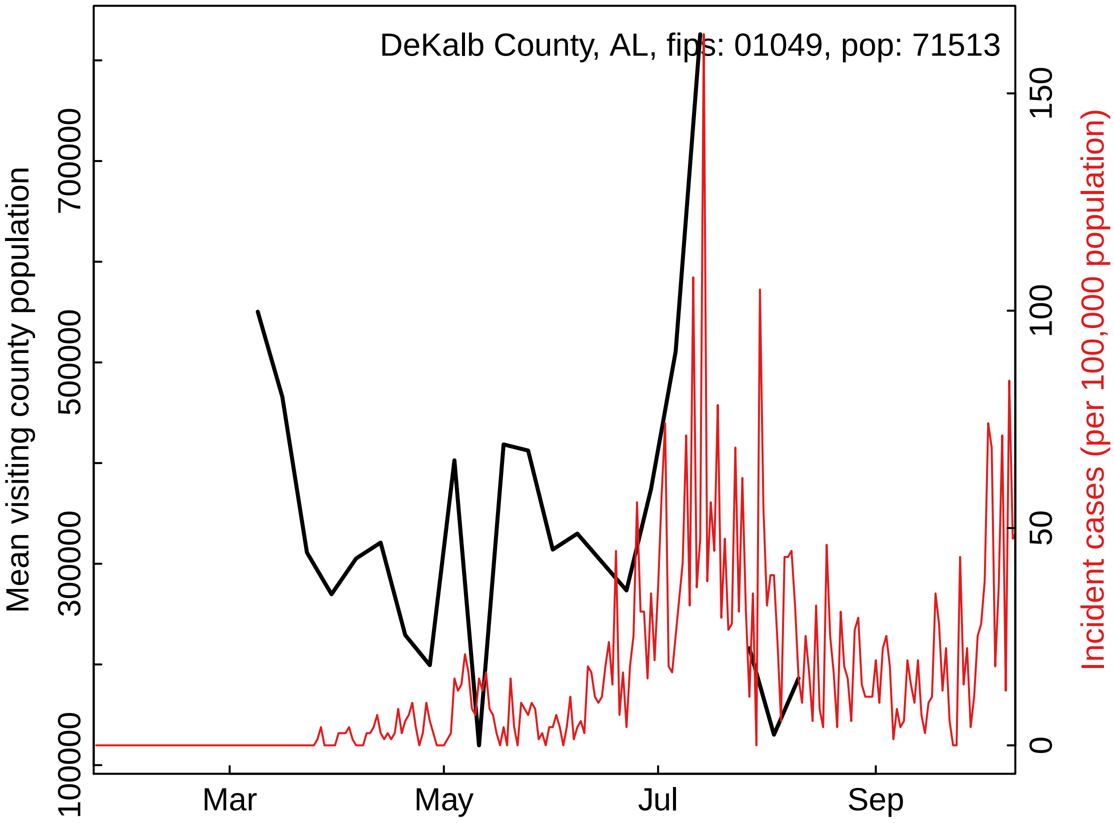

DeKalb County, AL, fips: 01049, pop: 71513
Mean visiting county population
100000
300000
500000
700000
Mar
May
Jul
Sep
0
50
100
150
Incident cases (per 100,000 population)

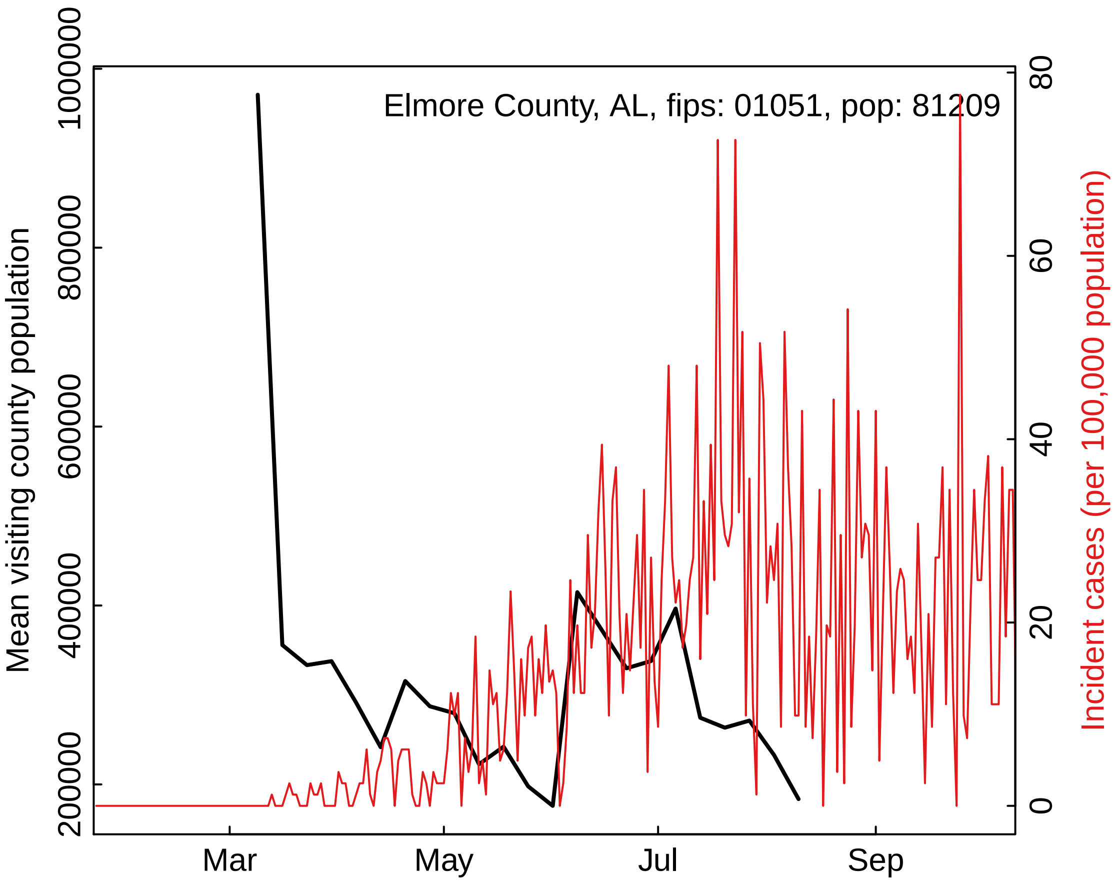

Elmore County, AL, fips: 01051, pop: 81209
Mean visiting county population
Incident cases (per 100,000 population)
200000
400000
600000
800000
1000000
0
20
40
60
80
Mar
May
Jul
Sep

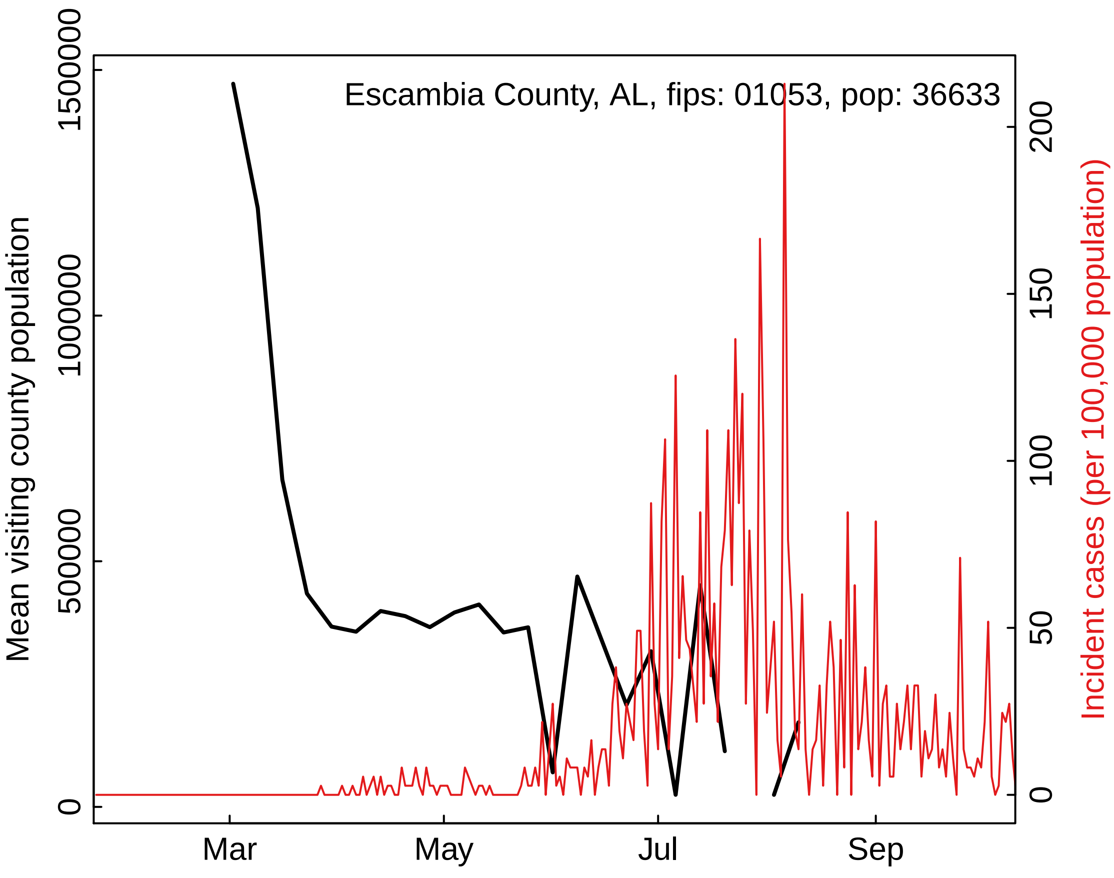
Escambia County, AL, fips: 01053, pop: 36633
Mean visiting county population
Incident cases (per 100,000 population)
0
500000
1000000
1500000
0
50
100
150
200
Mar
May
Jul
Sep

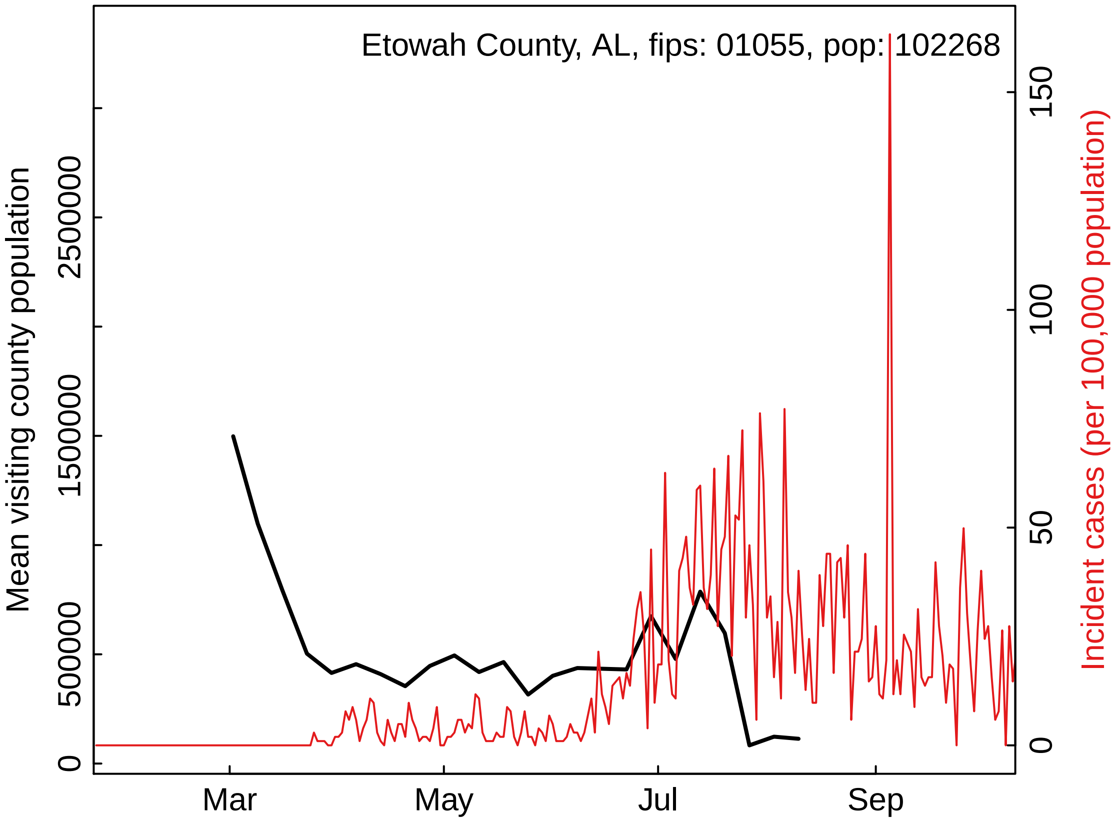

Etowah County, AL, fips: 01055, pop: 102268
Mean visiting county population
0
500000
1500000
2500000
Mar
May
Jul
Sep
0
50
100
150
Incident cases (per 100,000 population)

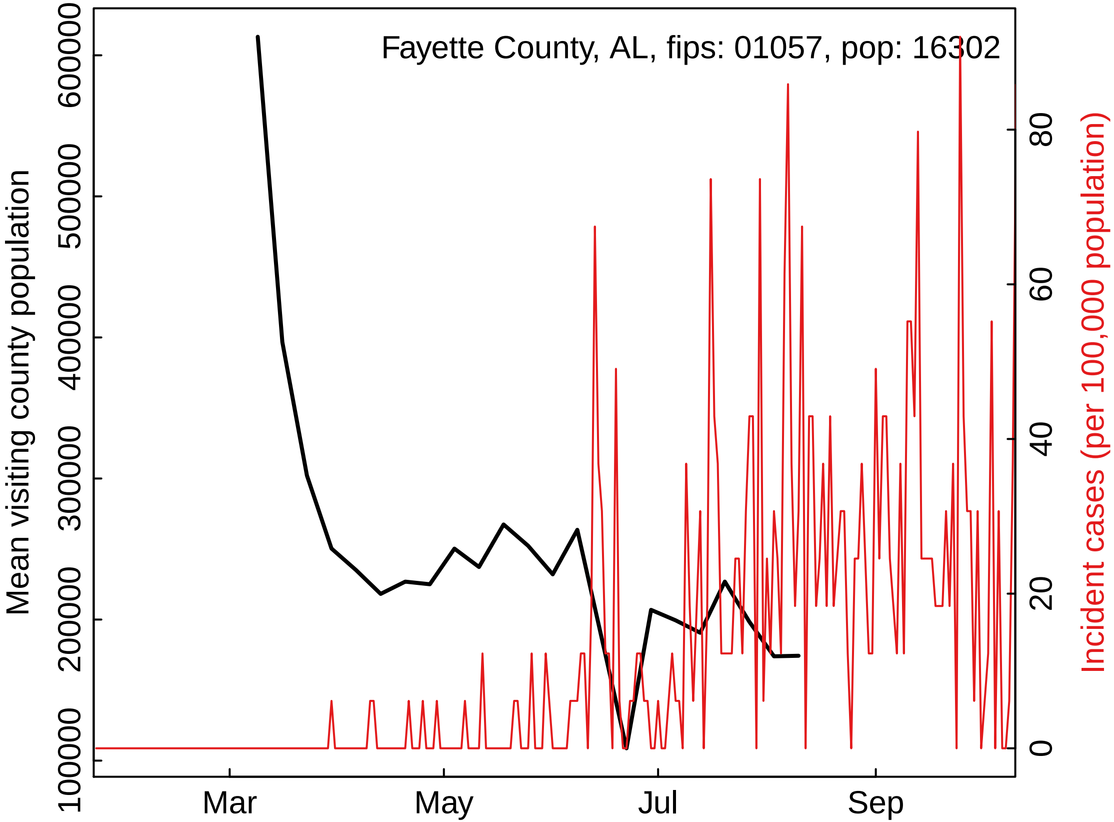
Fayette County, AL, fips: 01057, pop: 16302
Mean visiting county population
Incident cases (per 100,000 population)
100000
200000
300000
400000
500000
600000
0
20
40
60
80
Mar
May
Jul
Sep

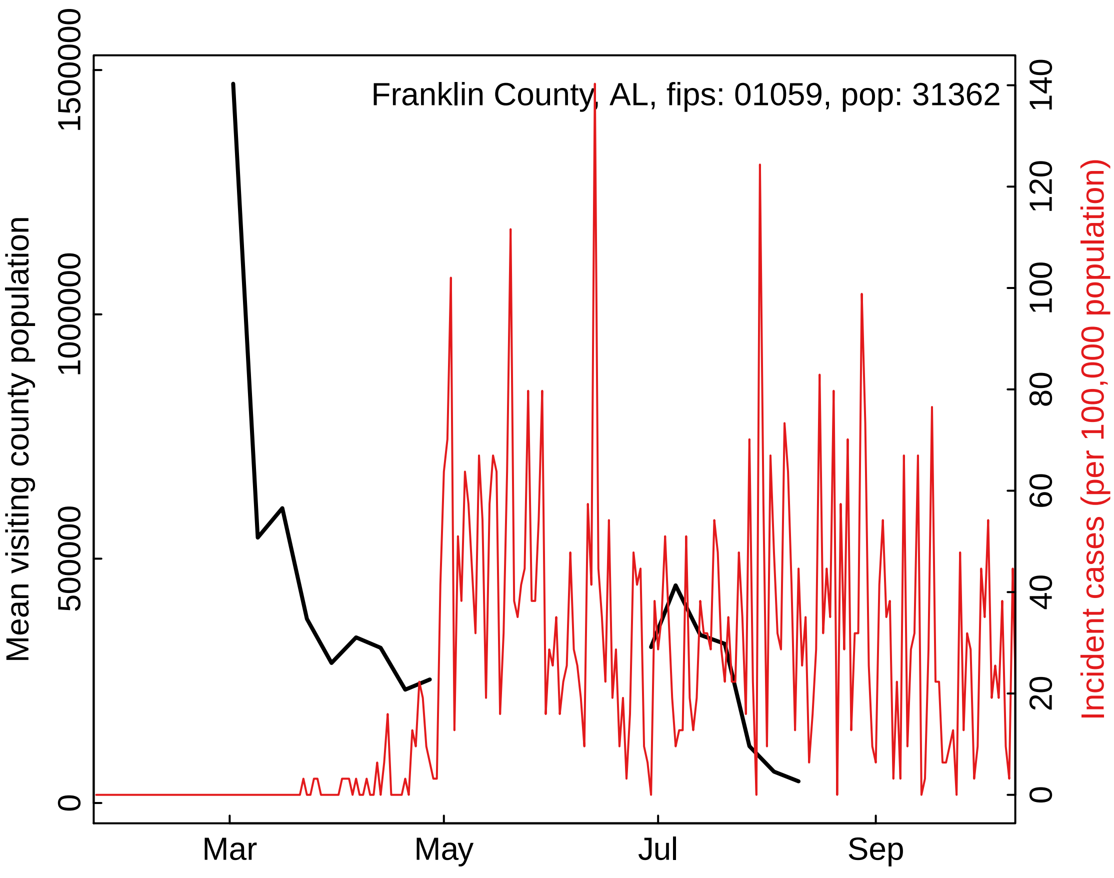

Franklin County, AL, fips: 01059, pop: 31362
Mean visiting county population
0
500000
1000000
1500000
Incident cases (per 100,000 population)
0
20
40
60
80
100
120
140
Mar
May
Jul
Sep

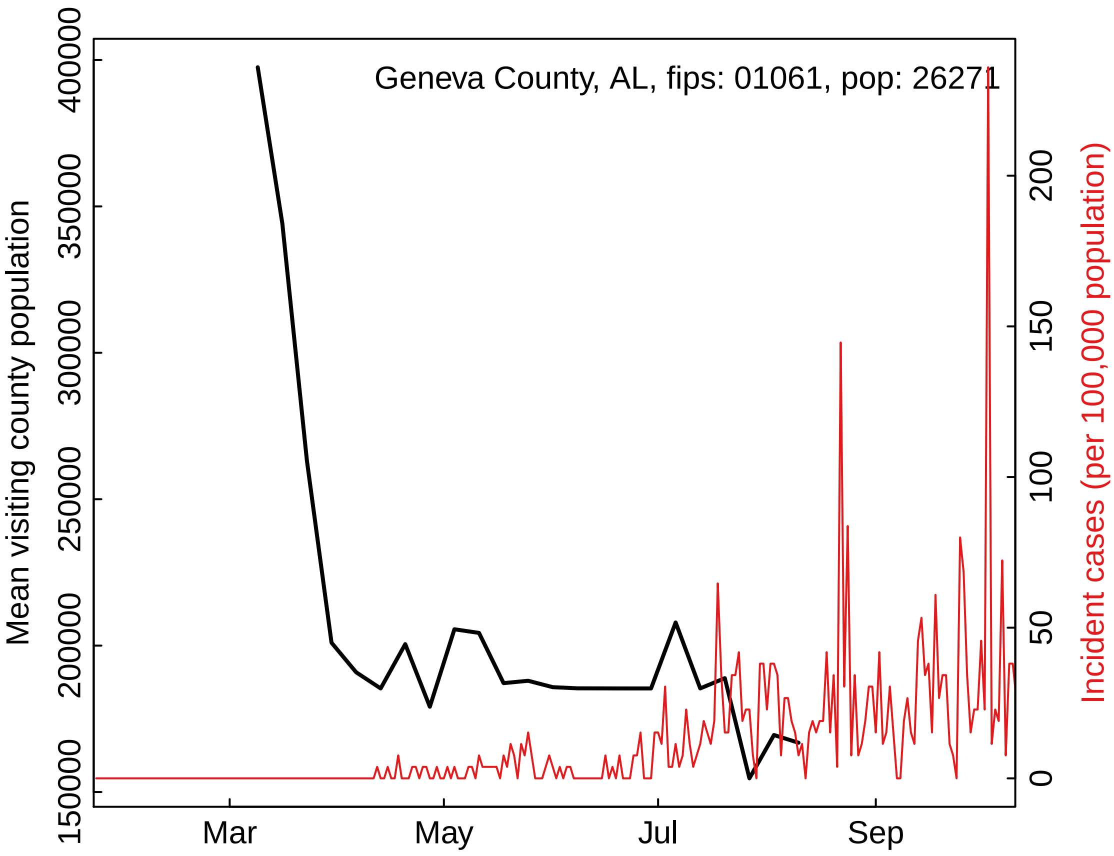

Geneva County, AL, fips: 01061, pop: 26271
Mean visiting county population
150000
200000
250000
300000
350000
400000
Mar
May
Jul
Sep
0
50
100
150
200
Incident cases (per 100,000 population)

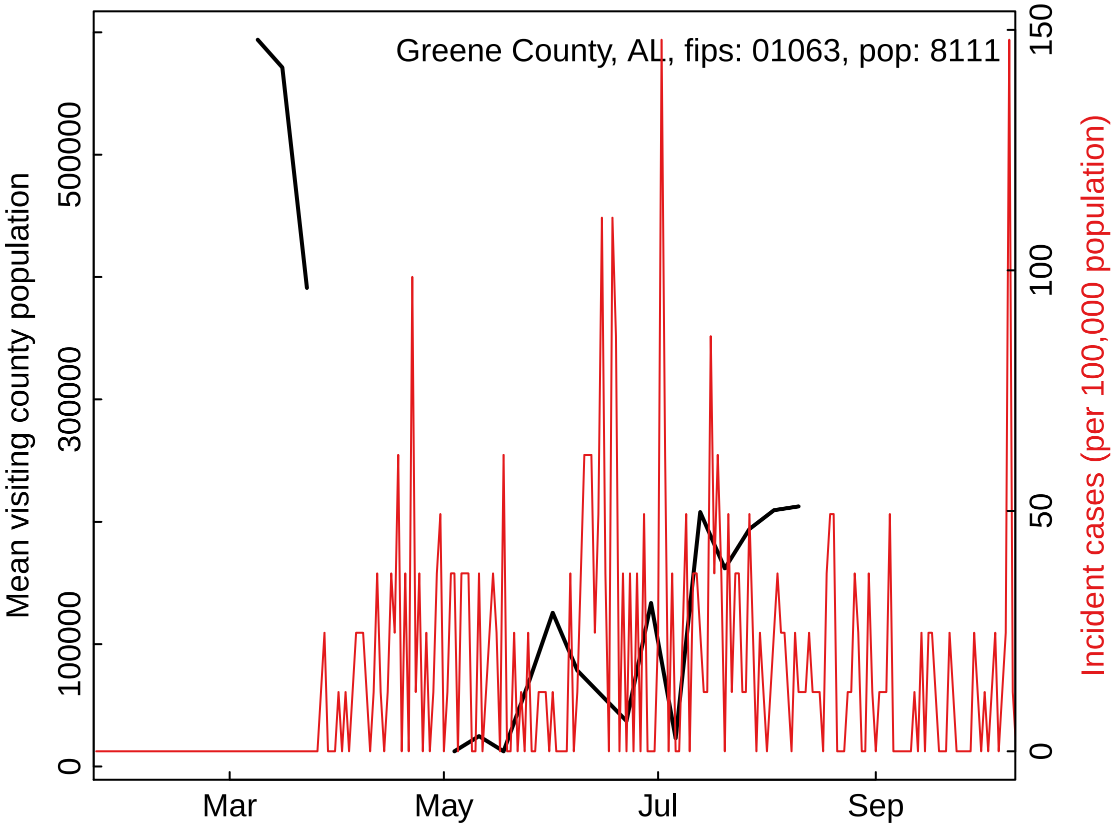

Greene County, AL, fips: 01063, pop: 8111
Mean visiting county population
Incident cases (per 100,000 population)
0
100000
300000
500000
0
50
100
150
Mar
May
Jul
Sep

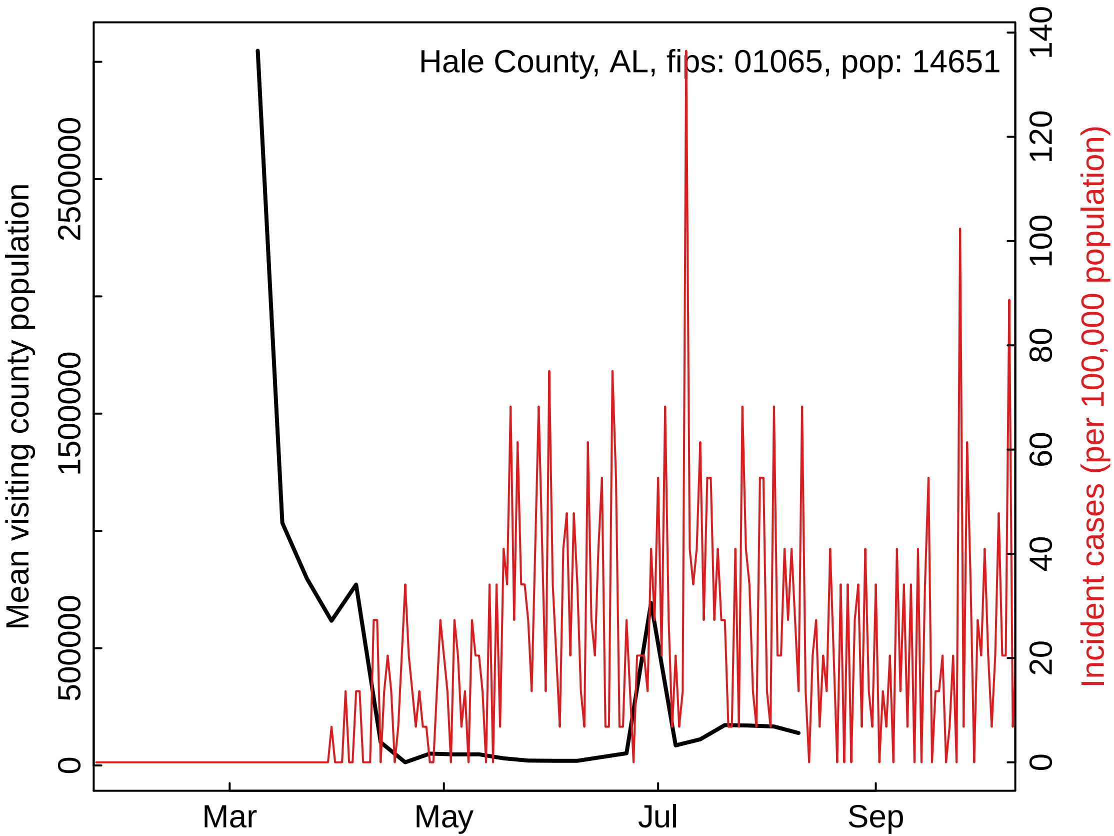

Hale County, AL, fips: 01065, pop: 14651
Mean visiting county population
Incident cases (per 100,000 population)
0
500000
1500000
2500000
0
20
40
60
80
100
120
140
Mar
May
Jul
Sep

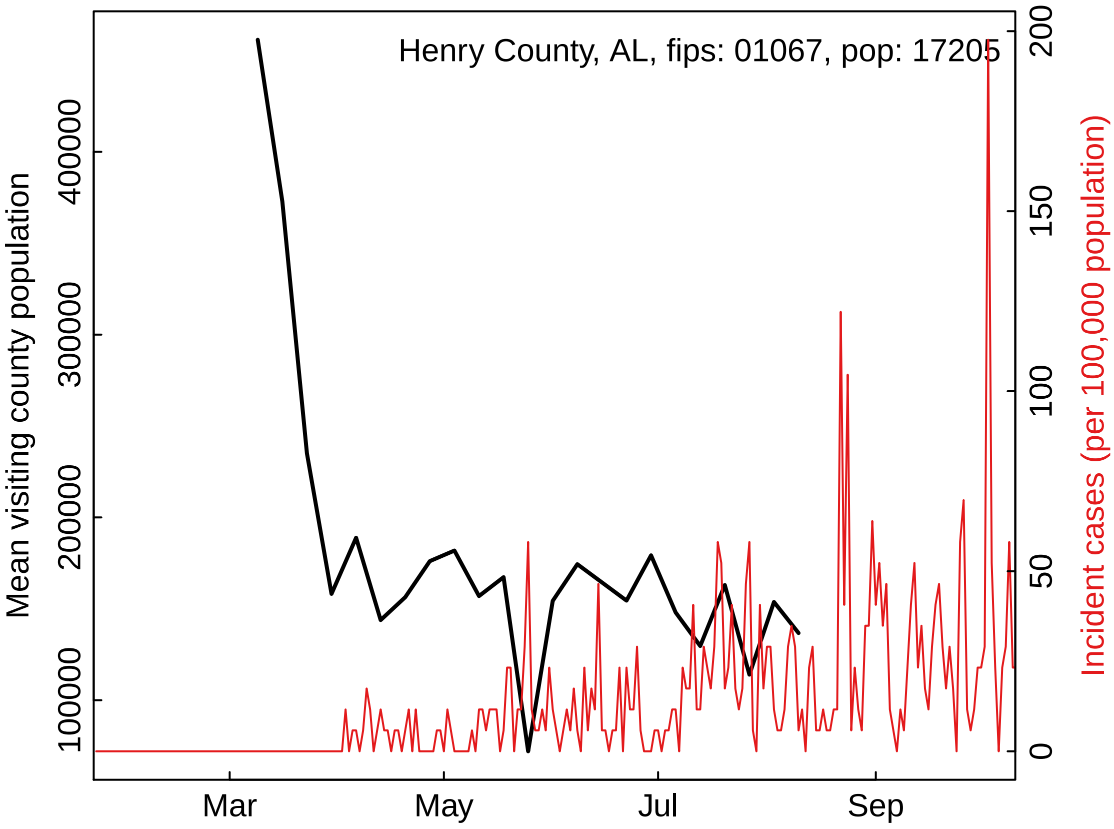

Henry County, AL, fips: 01067, pop: 17205
Mean visiting county population
100000
200000
300000
400000
Incident cases (per 100,000 population)
0
50
100
150
200
Mar
May
Jul
Sep

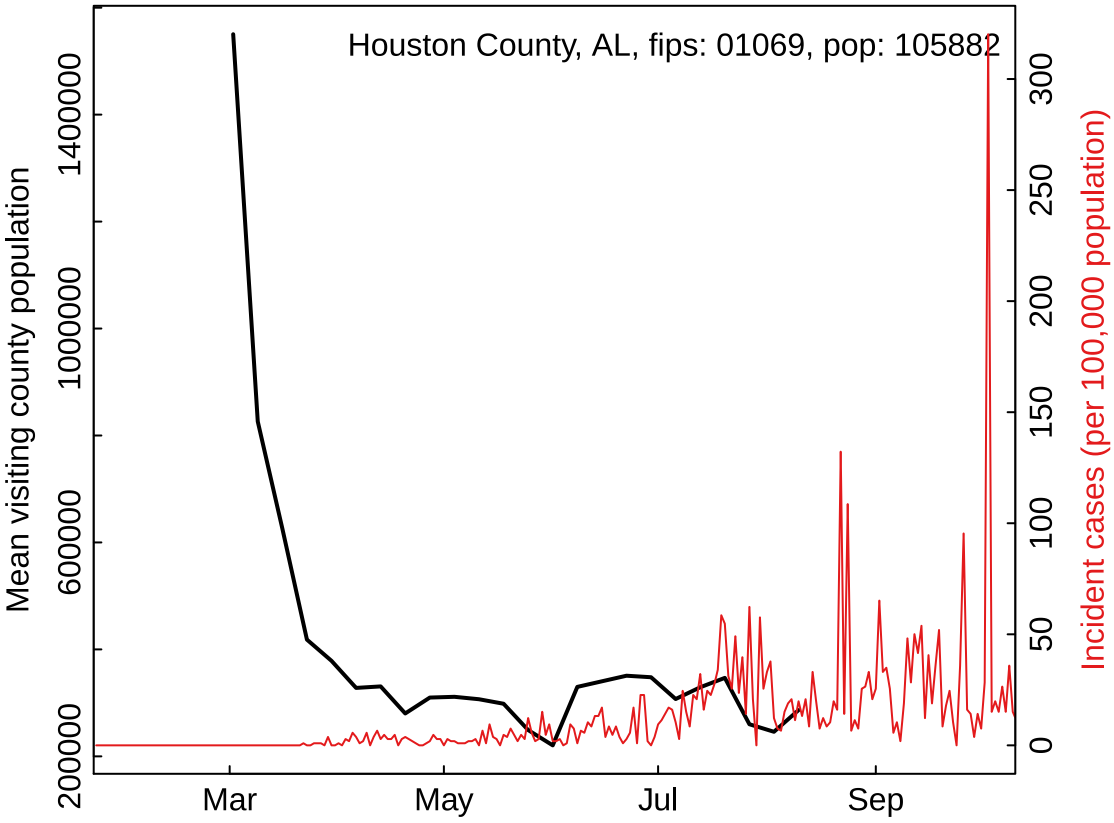

Houston County, AL, fips: 01069, pop: 105882
Mean visiting county population
200000
600000
1000000
1400000
Incident cases (per 100,000 population)
0
50
100
150
200
250
300
Mar
May
Jul
Sep

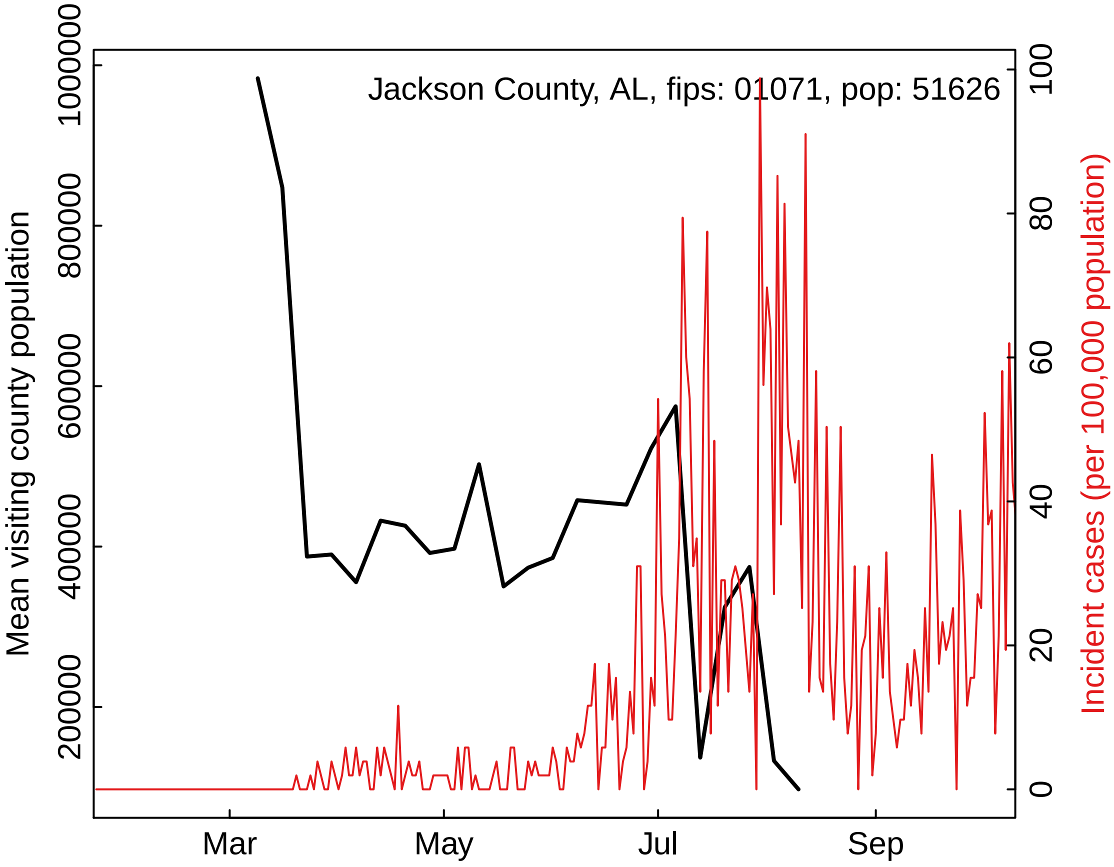

Jackson County, AL, fips: 01071, pop: 51626
Mean visiting county population
Incident cases (per 100,000 population)
200000
400000
600000
800000
1000000
0
20
40
60
80
100
Mar
May
Jul
Sep

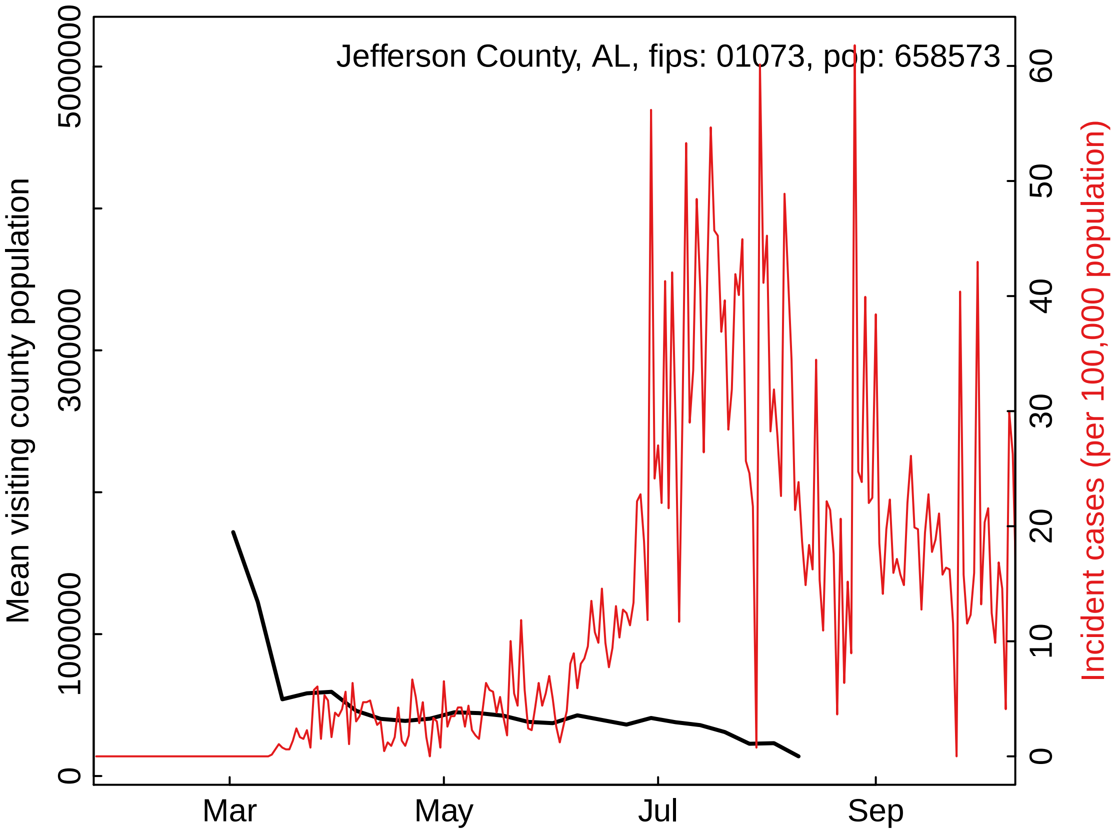

Jefferson County, AL, fips: 01073, pop: 658573
Mean visiting county population
0
1000000
3000000
5000000
Incident cases (per 100,000 population)
0
10
20
30
40
50
60
Mar
May
Jul
Sep

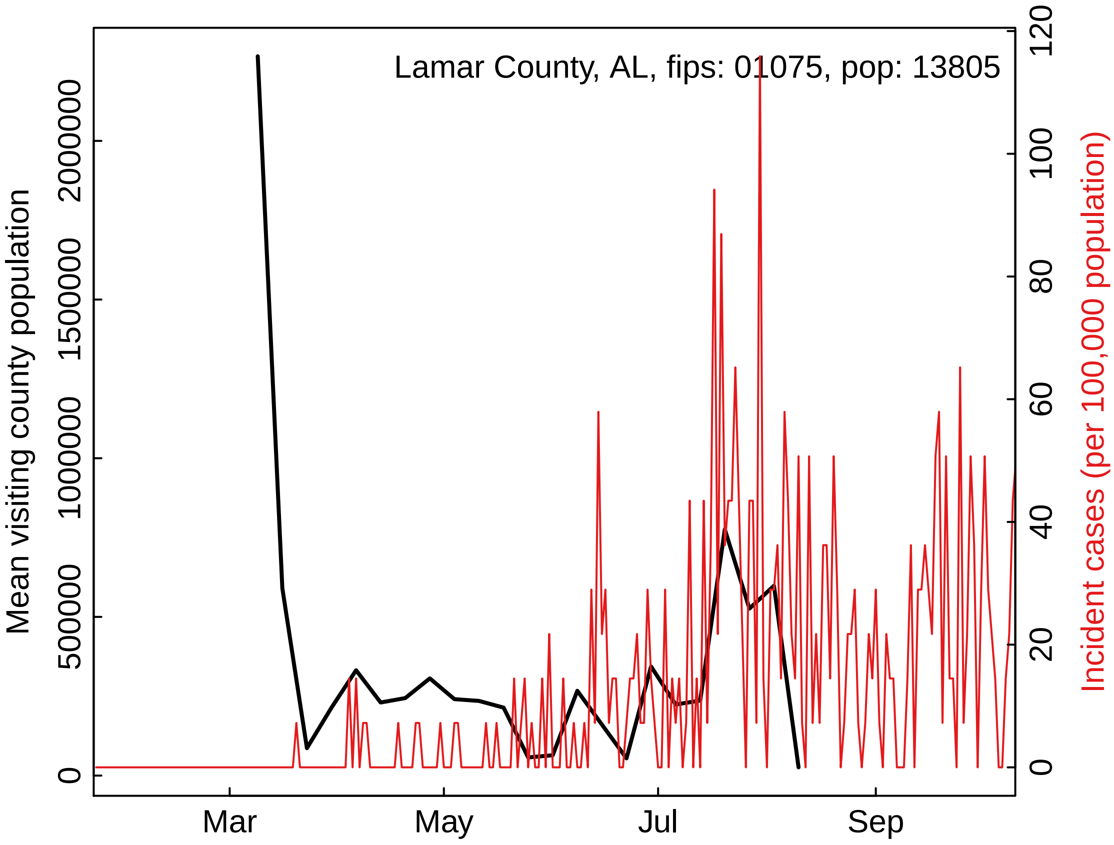

Lamar County, AL, fips: 01075, pop: 13805
Mean visiting county population
0
500000
1000000
1500000
2000000
Incident cases (per 100,000 population)
0
20
40
60
80
100
120
Mar
May
Jul
Sep

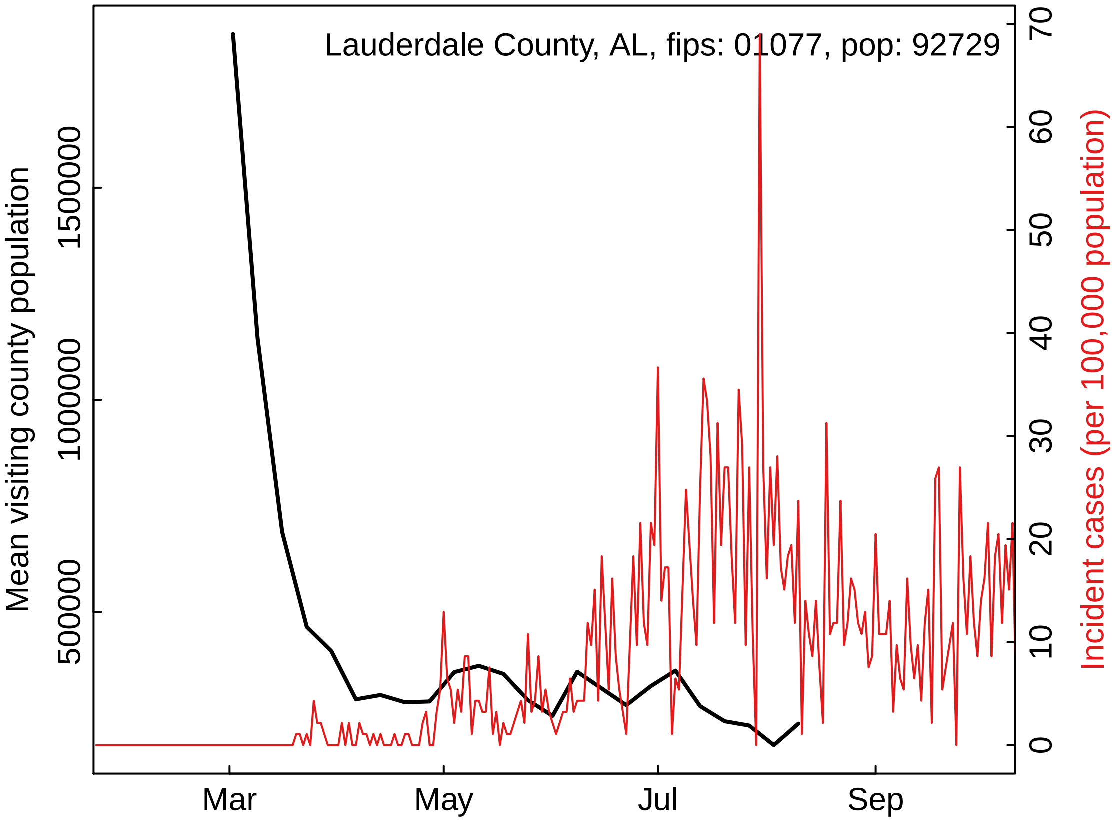

Lauderdale County, AL, fips: 01077, pop: 92729
Mean visiting county population
500000
1000000
1500000
0
10
20
30
40
50
60
70
Incident cases (per 100,000 population)
Mar
May
Jul
Sep

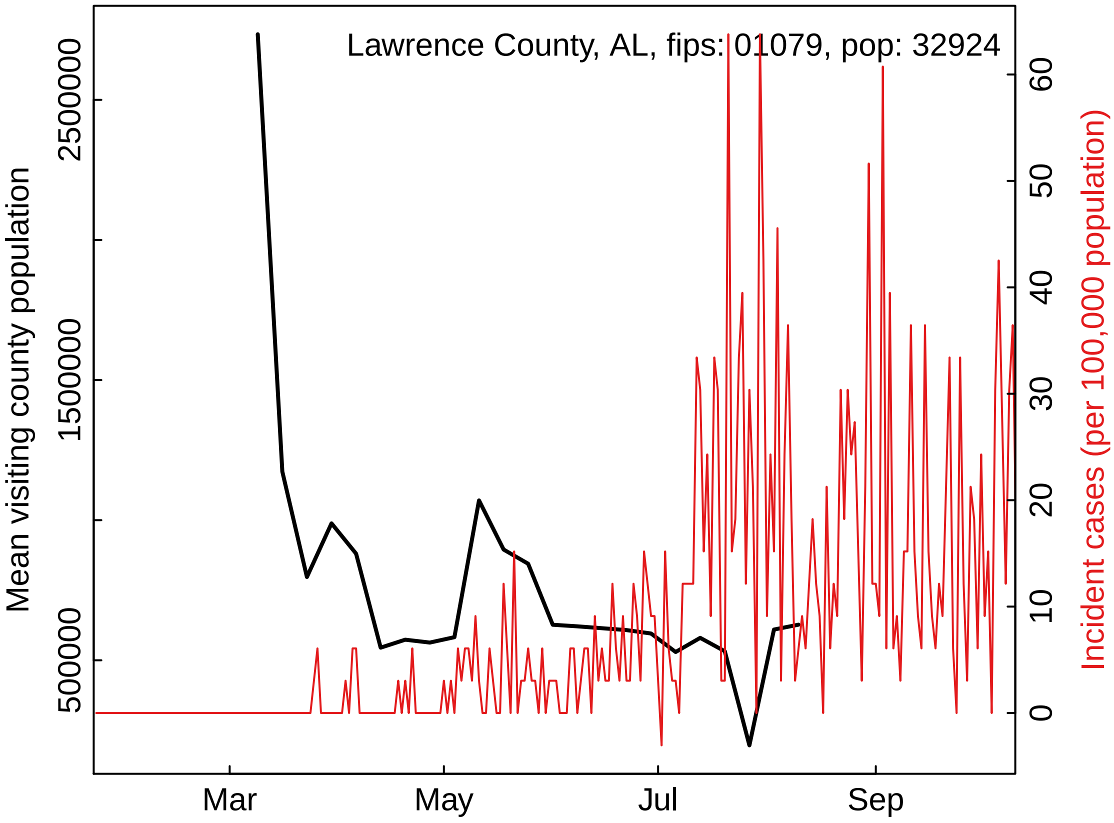

Lawrence County, AL, fips: 01079, pop: 32924
Mean visiting county population
500000
1500000
2500000
Incident cases (per 100,000 population)
0
10
20
30
40
50
60
Mar
May
Jul
Sep

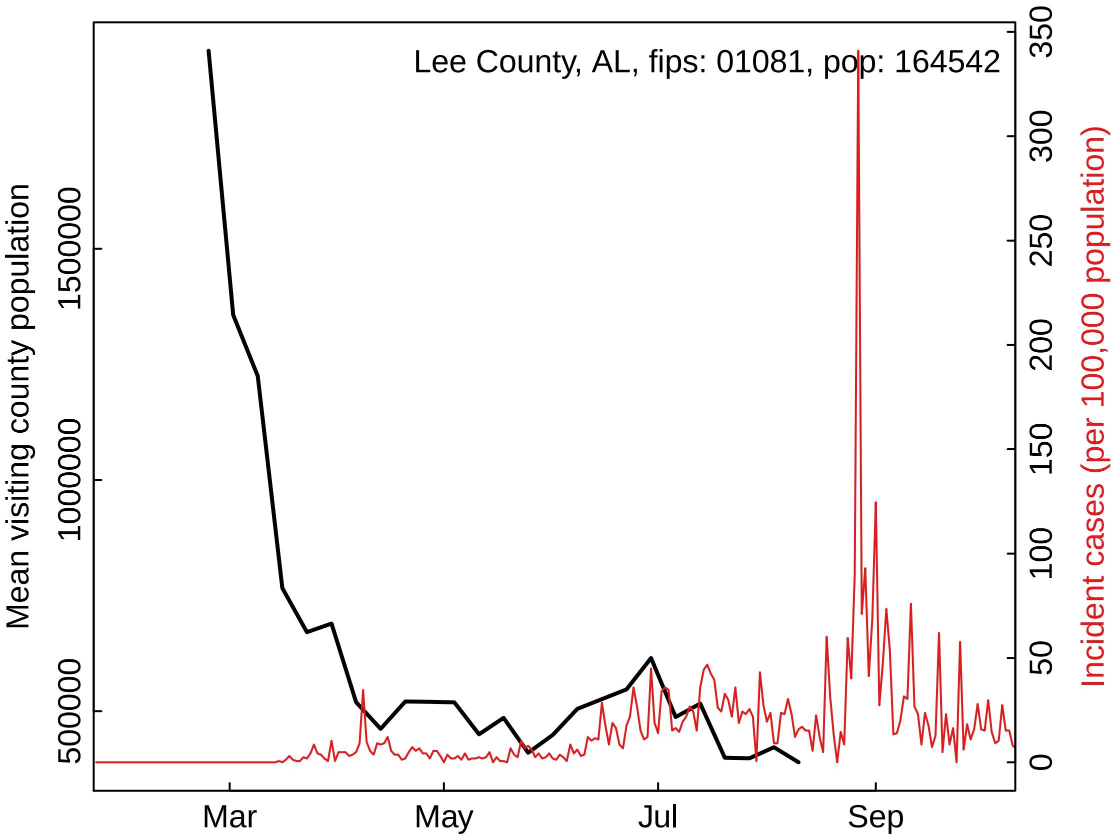

Lee County, AL, fips: 01081, pop: 164542
Mean visiting county population
500000
1000000
1500000
Mar
May
Jul
Sep
0
50
100
150
200
250
300
350
Incident cases (per 100,000 population)

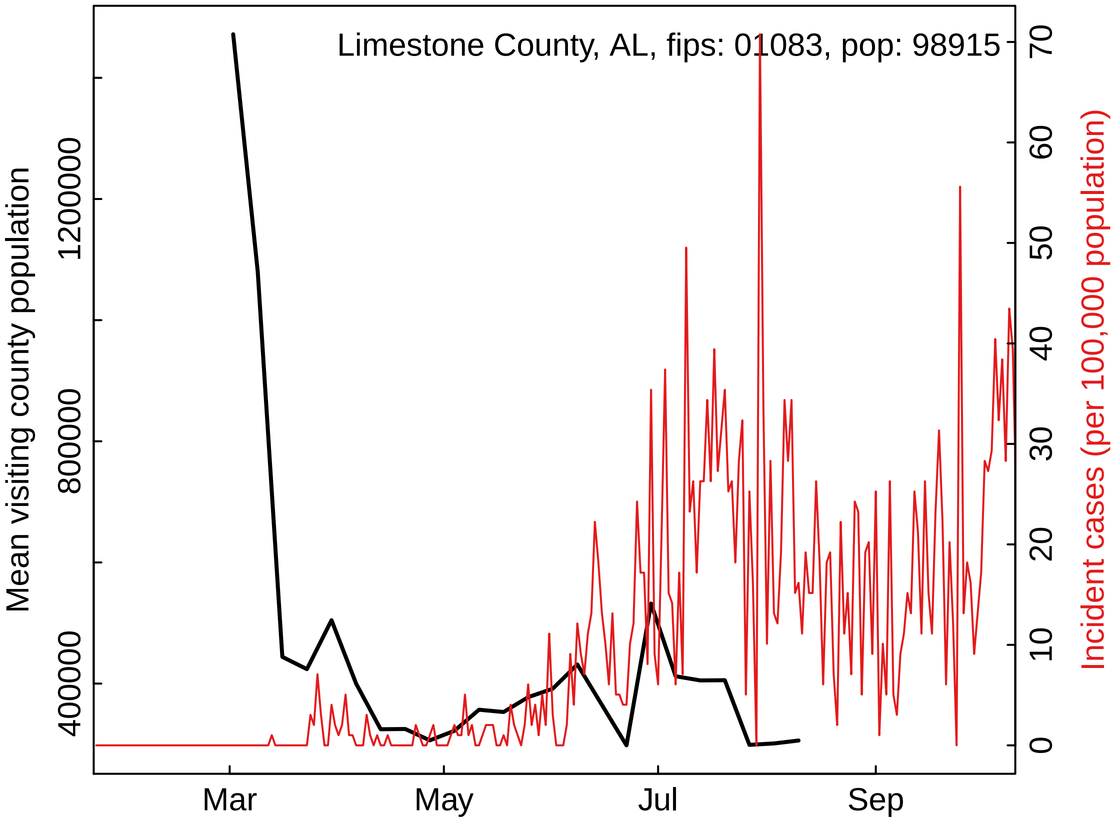

Limestone County, AL, fips: 01083, pop: 98915
Mean visiting county population
400000
800000
1200000
Mar
May
Jul
Sep
0
10
20
30
40
50
60
70
Incident cases (per 100,000 population)

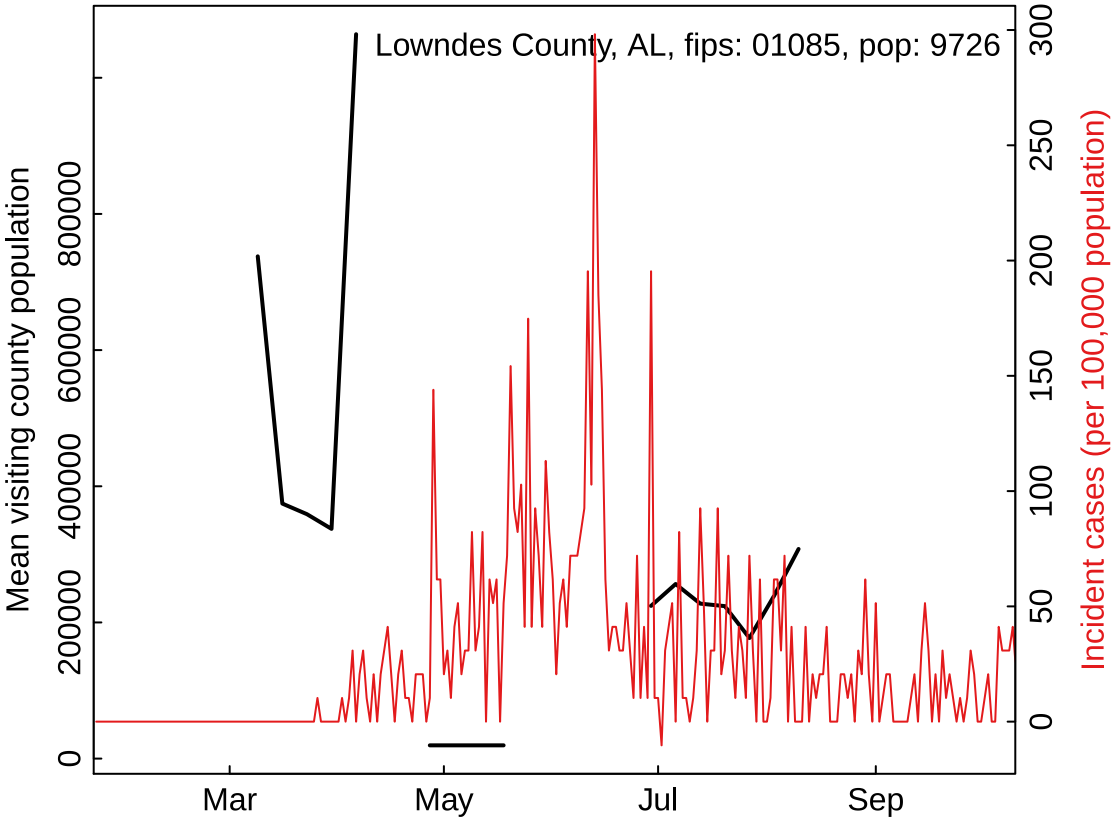

Lowndes County, AL, fips: 01085, pop: 9726
Mean visiting county population
0
200000
400000
600000
800000
Incident cases (per 100,000 population)
0
50
100
150
200
250
300
Mar
May
Jul
Sep

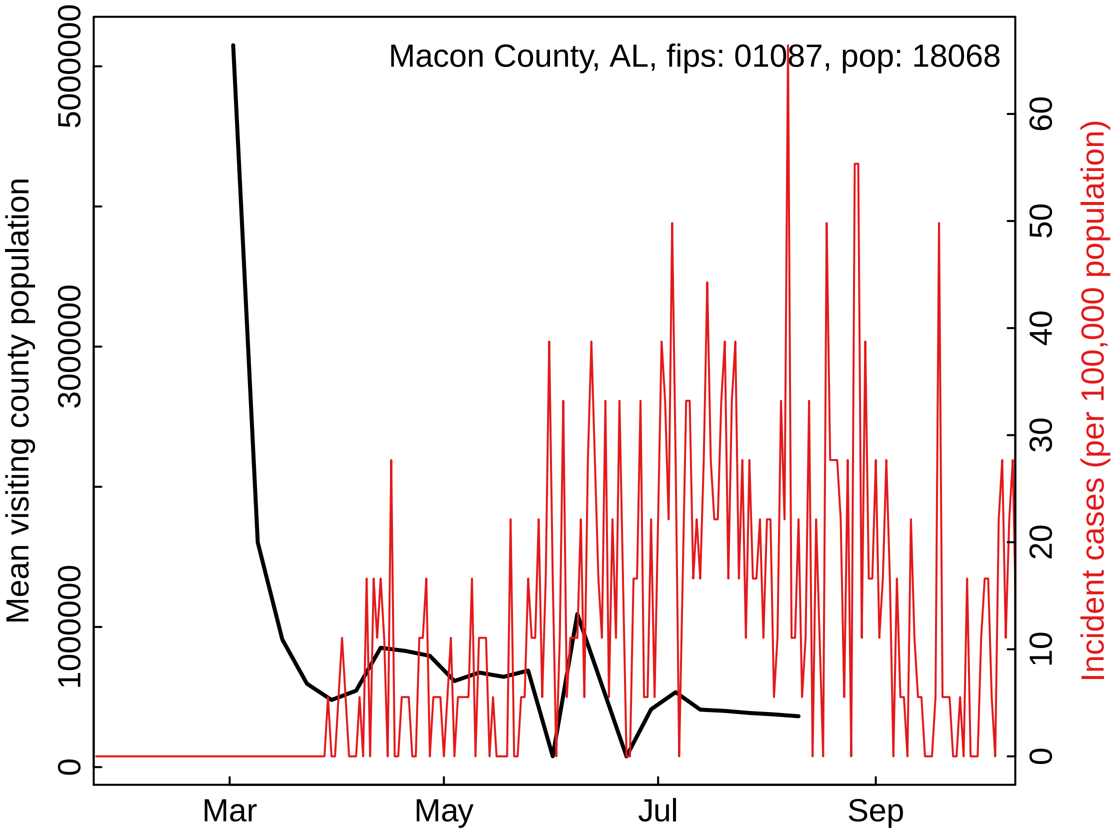

Macon County, AL, fips: 01087, pop: 18068
Mean visiting county population
Incident cases (per 100,000 population)
0
1000000
3000000
5000000
0
10
20
30
40
50
60
Mar
May
Jul
Sep

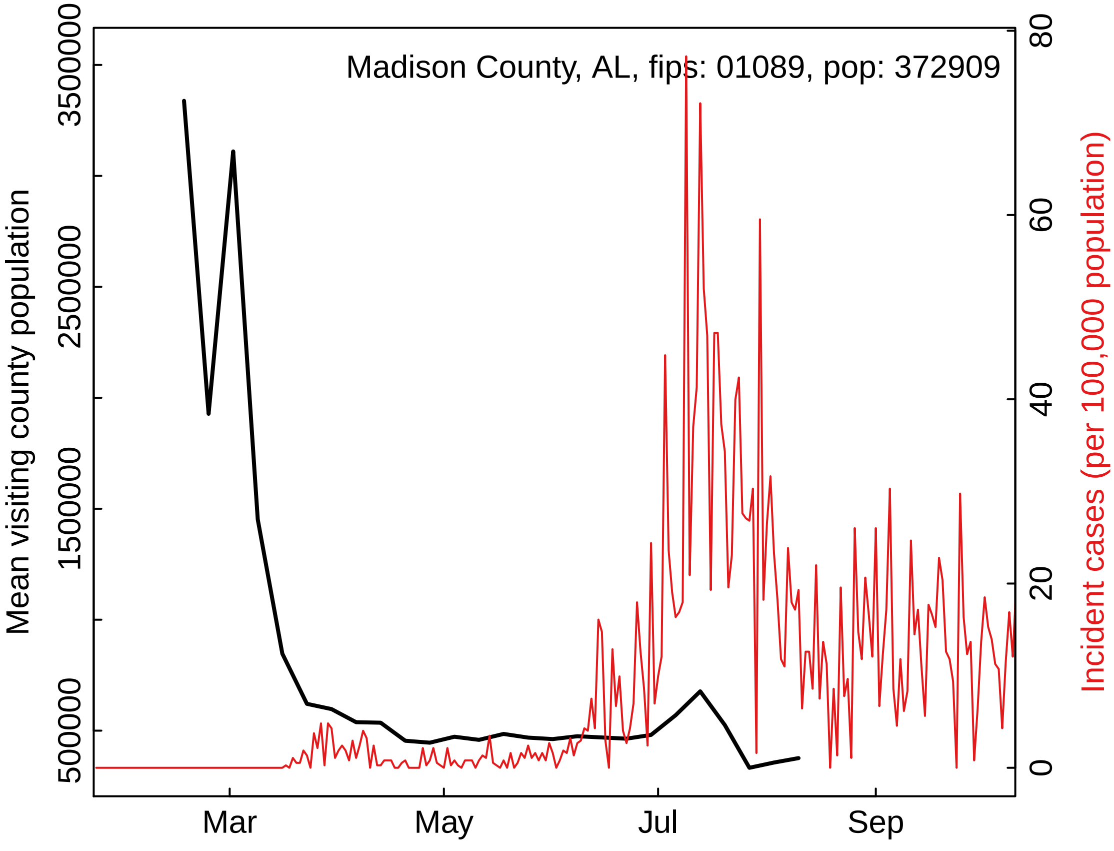

Madison County, AL, fips: 01089, pop: 372909
Mean visiting county population
500000
1500000
2500000
3500000
Mar
May
Jul
Sep
0
20
40
60
80
Incident cases (per 100,000 population)

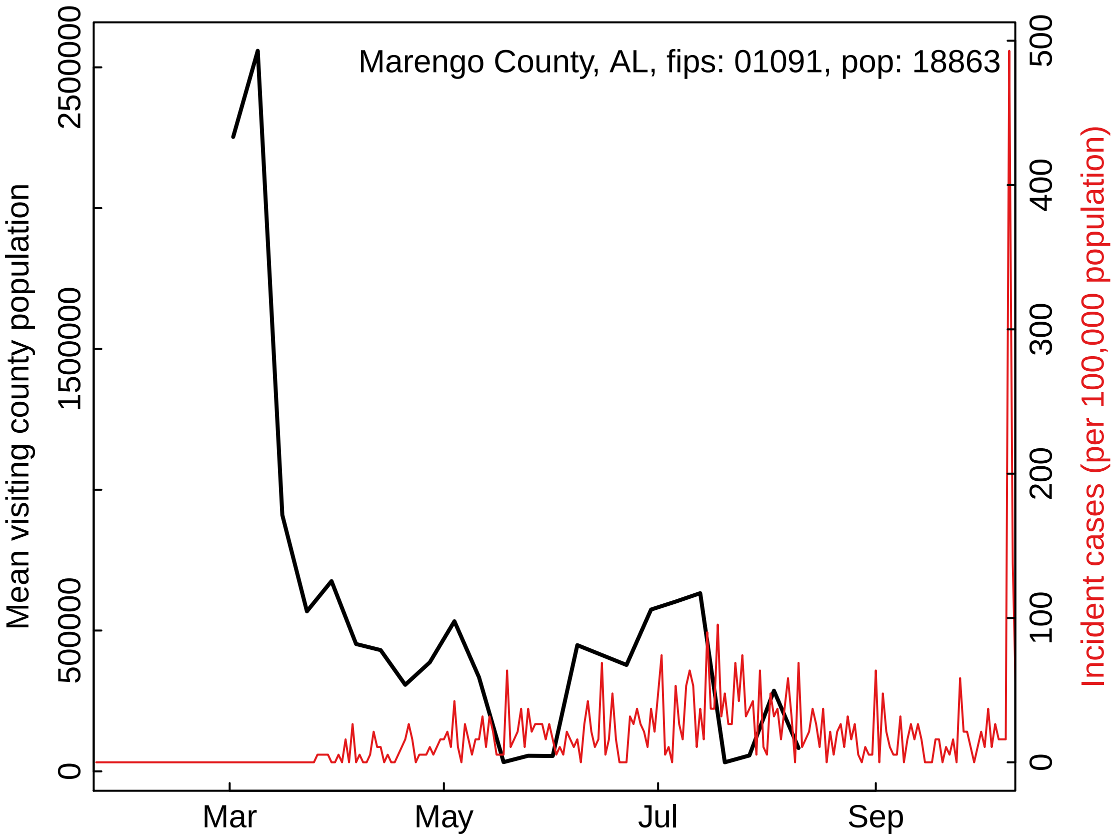

Marengo County, AL, fips: 01091, pop: 18863
Mean visiting county population
Incident cases (per 100,000 population)
0
500000
1500000
2500000
0
100
200
300
400
500
Mar
May
Jul
Sep

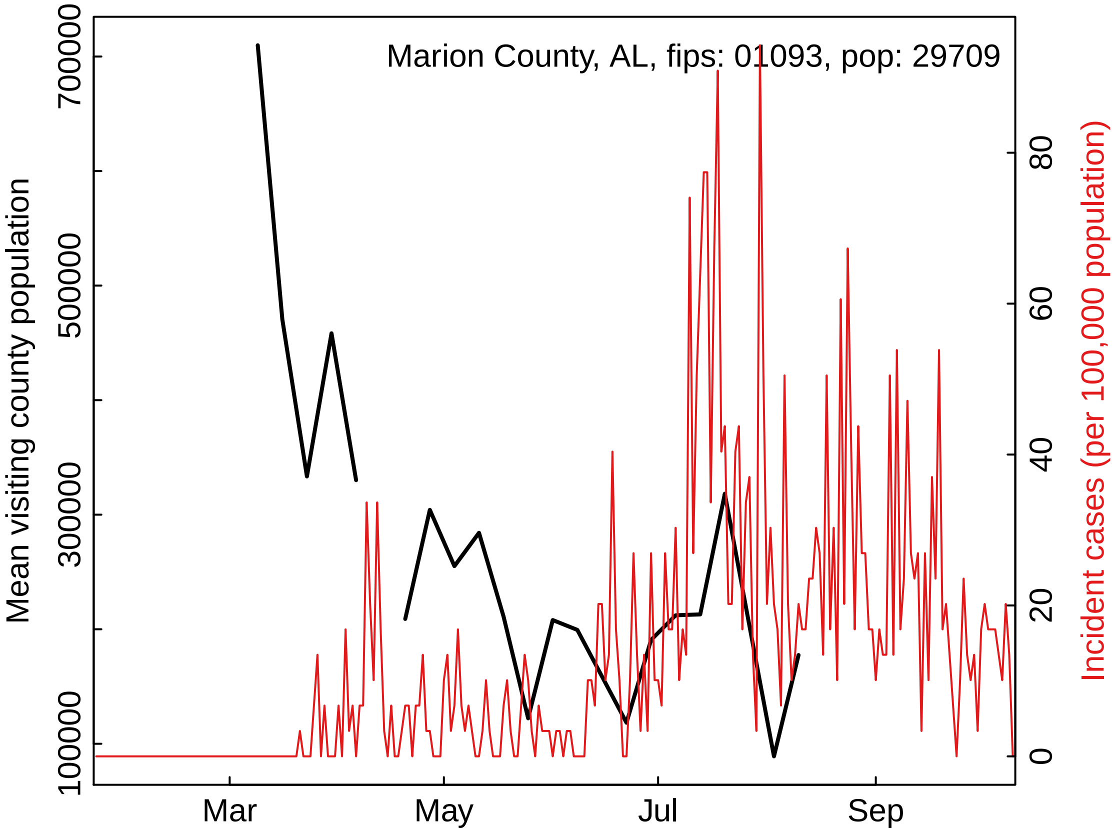

Marion County, AL, fips: 01093, pop: 29709
Mean visiting county population
100000
300000
500000
700000
Incident cases (per 100,000 population)
0
20
40
60
80
Mar
May
Jul
Sep

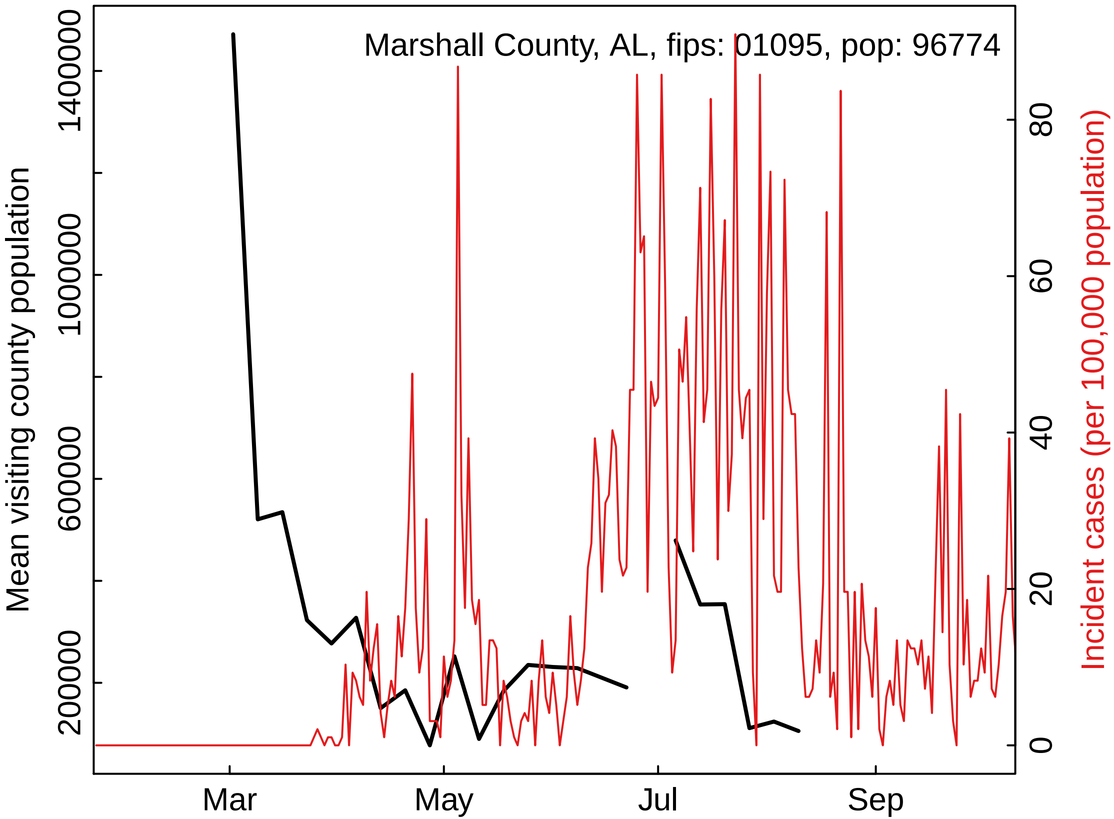
Marshall County, AL, fips: 01095, pop: 96774
Mean visiting county population
200000
600000
1000000
1400000
Incident cases (per 100,000 population)
0
20
40
60
80
Mar
May
Jul
Sep

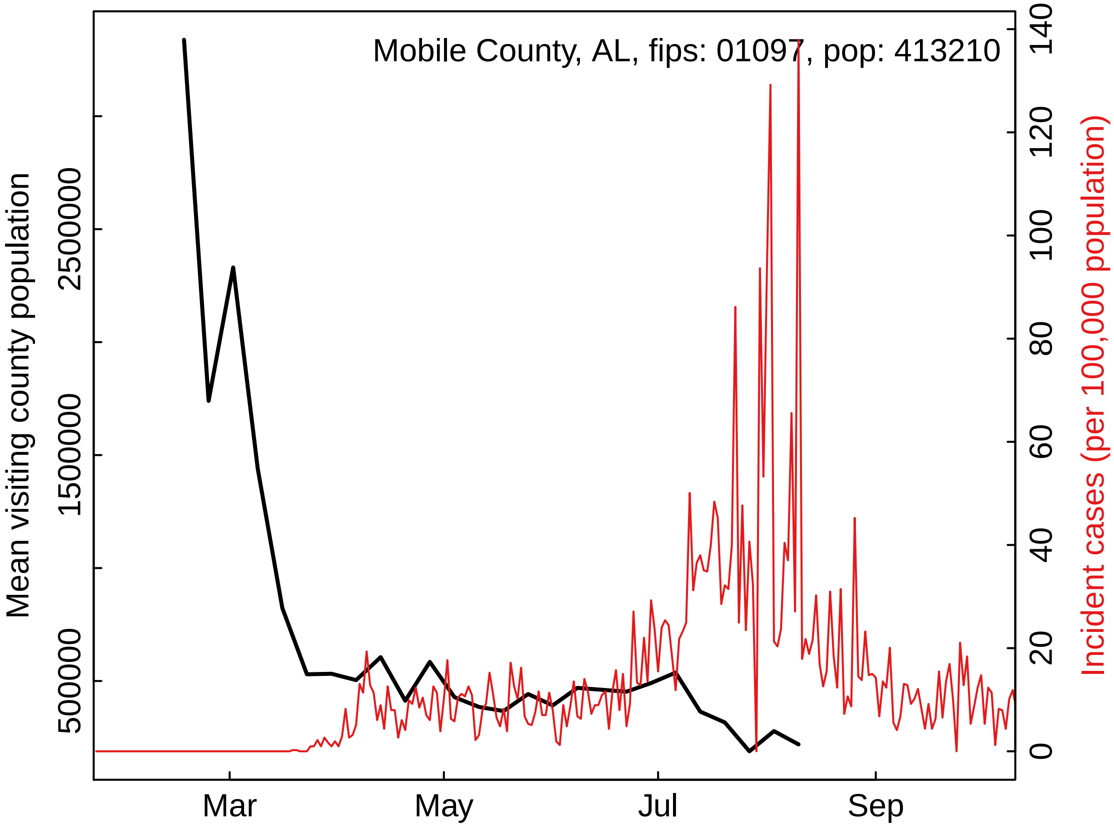

Mobile County, AL, fips: 01097, pop: 413210
Mean visiting county population
500000
1500000
2500000
Incident cases (per 100,000 population)
0
20
40
60
80
100
120
140
Mar
May
Jul
Sep

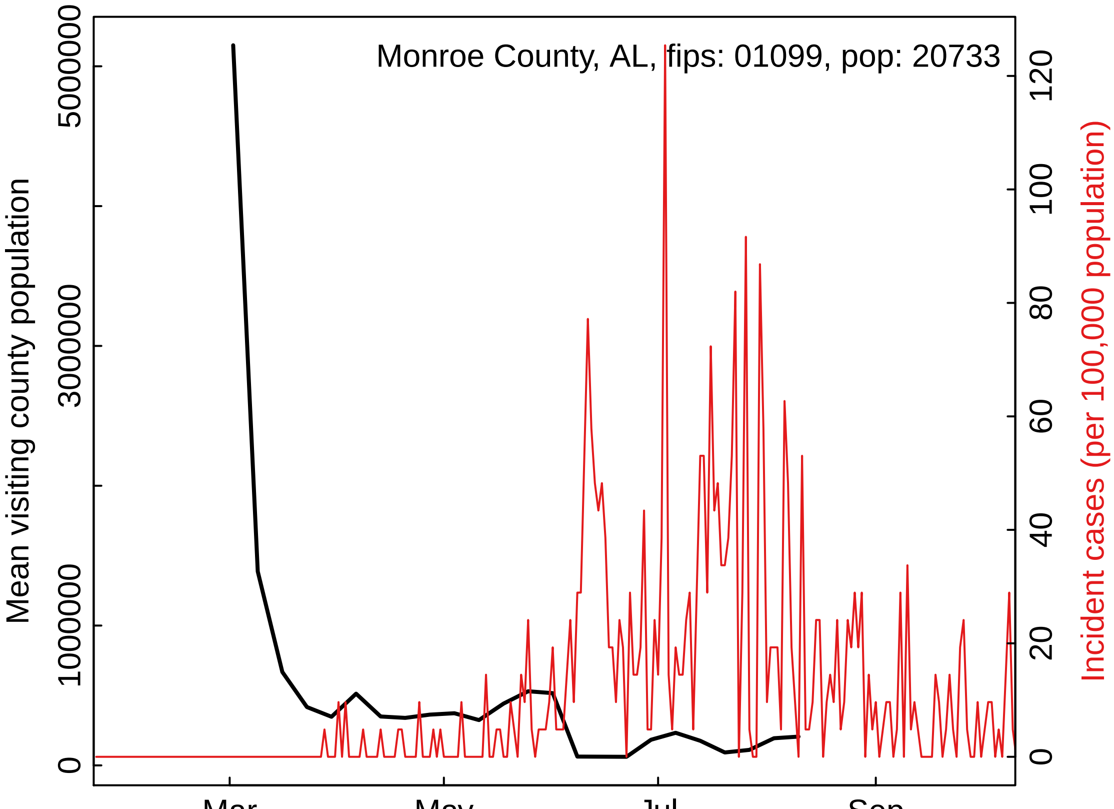

Monroe County, AL, fips: 01099, pop: 20733
Mean visiting county population
0
1000000
3000000
5000000
Incident cases (per 100,000 population)
0
20
40
60
80
100
120
Mar
May
Jul
Sep

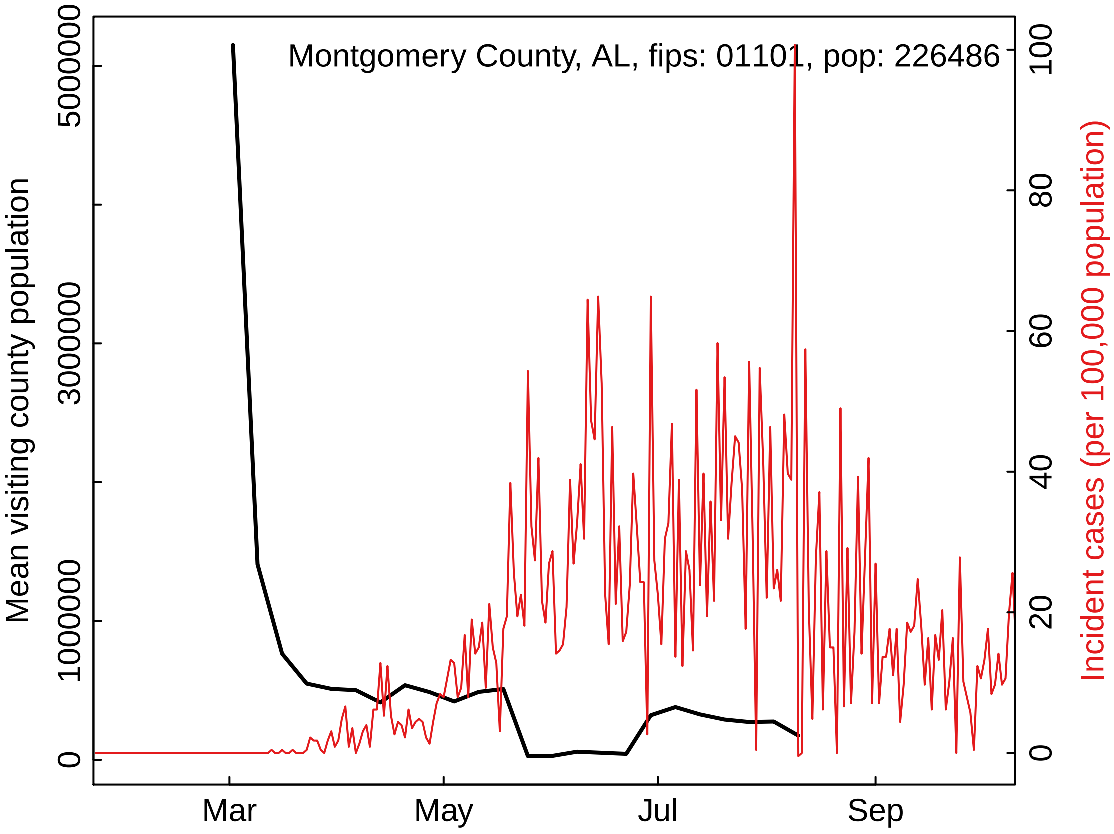

Montgomery County, AL, fips: 01101, pop: 226486
Mean visiting county population
0
1000000
3000000
5000000
Incident cases (per 100,000 population)
0
20
40
60
80
100
Mar
May
Jul
Sep

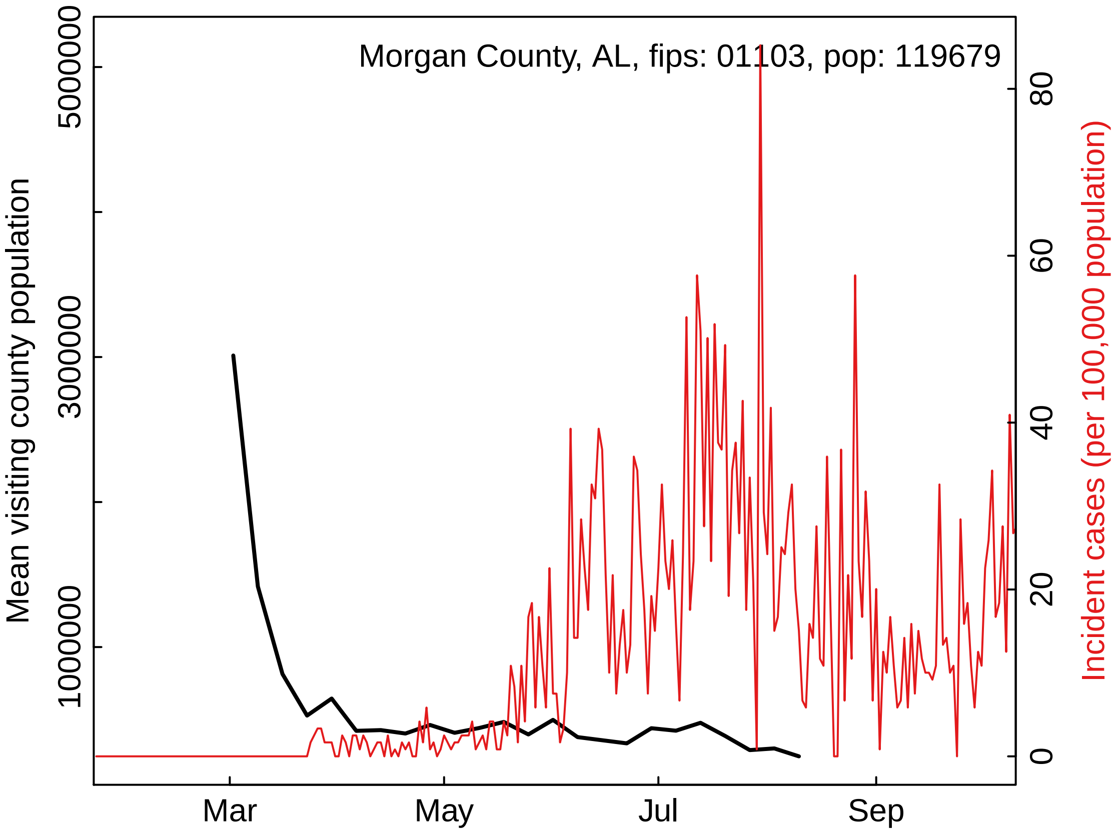

Morgan County, AL, fips: 01103, pop: 119679
Mean visiting county population
1000000
3000000
5000000
Mar
May
Jul
Sep
0
20
40
60
80
Incident cases (per 100,000 population)

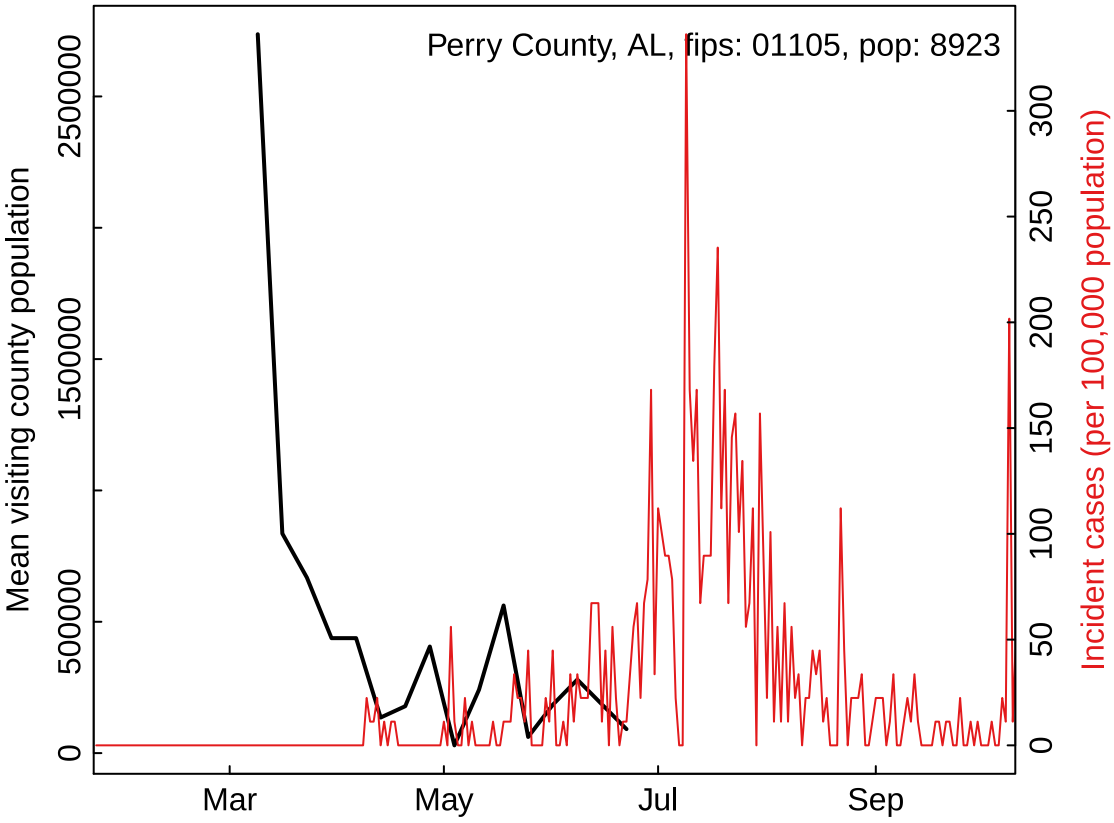
Perry County, AL, fips: 01105, pop: 8923
Mean visiting county population
0
500000
1500000
2500000
Incident cases (per 100,000 population)
0
50
100
150
200
250
300
Mar
May
Jul
Sep

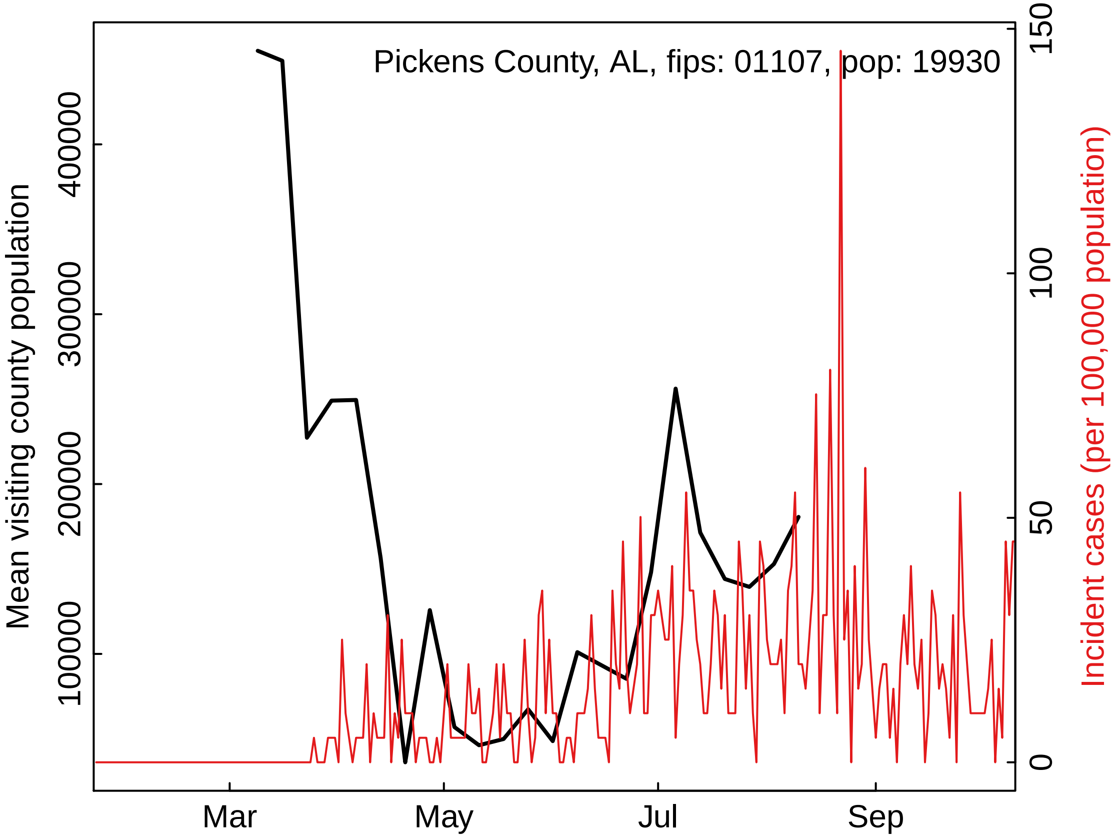
Pickens County, AL, fips: 01107, pop: 19930
Mean visiting county population
100000
200000
300000
400000
Incident cases (per 100,000 population)
0
50
100
150
Mar
May
Jul
Sep

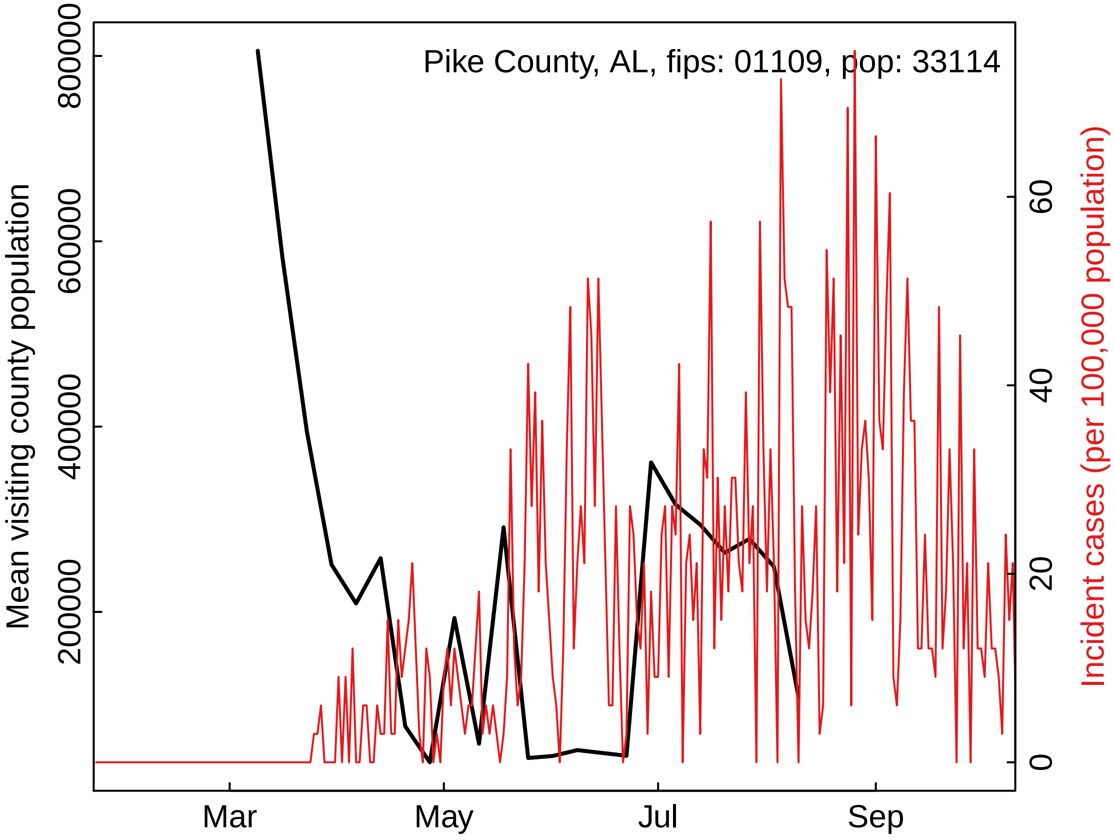
Pike County, AL, fips: 01109, pop: 33114
Mean visiting county population
200000
400000
600000
800000
Incident cases (per 100,000 population)
0
20
40
60
Mar
May
Jul
Sep

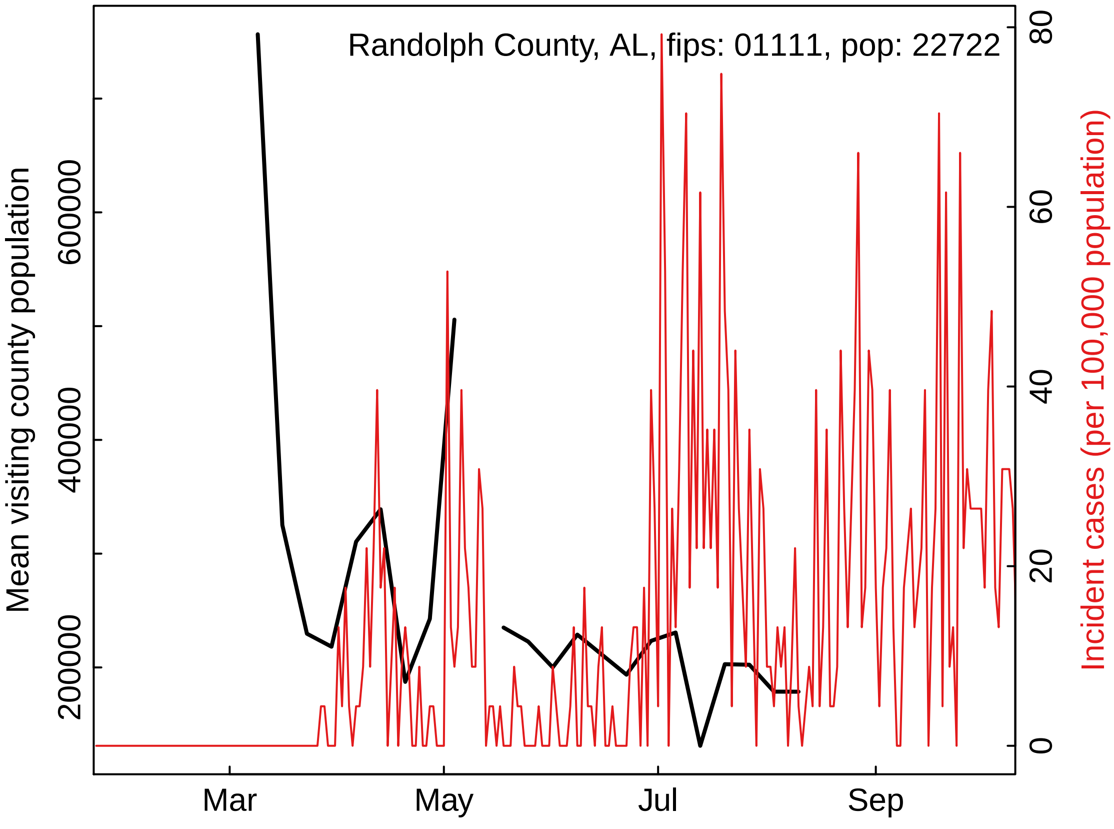

Randolph County, AL, fips: 01111, pop: 22722
Mean visiting county population
200000
400000
600000
Incident cases (per 100,000 population)
0
20
40
60
80
Mar
May
Jul
Sep

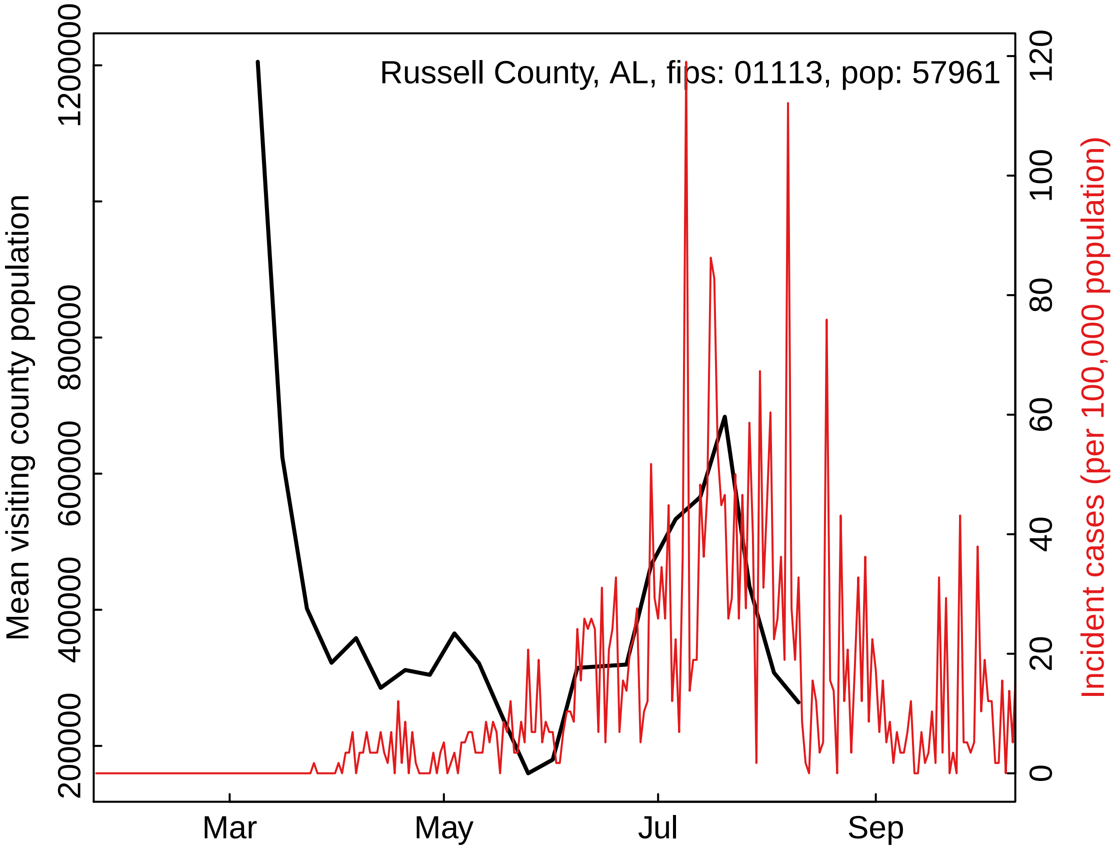

Russell County, AL, fips: 01113, pop: 57961
Mean visiting county population
200000
400000
600000
800000
1200000
Incident cases (per 100,000 population)
0
20
40
60
80
100
120
Mar
May
Jul
Sep

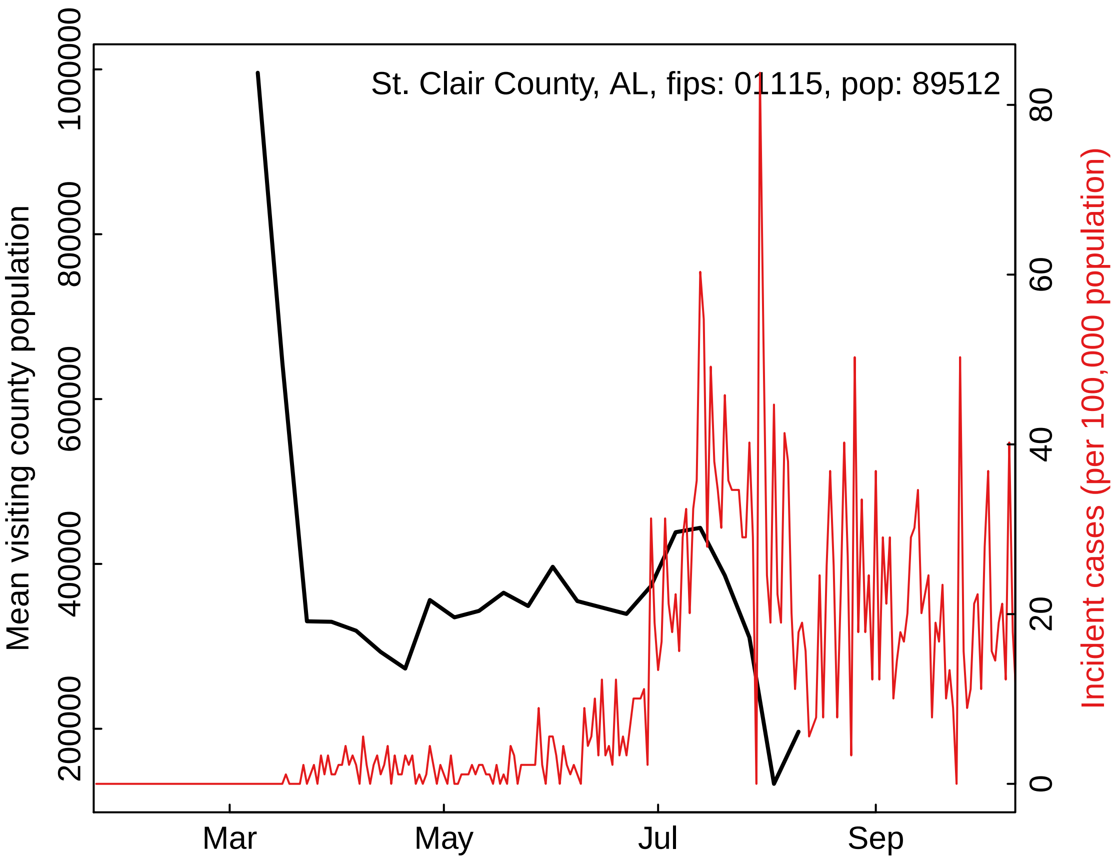

St. Clair County, AL, fips: 01115, pop: 89512
Mean visiting county population
200000
400000
600000
800000
1000000
Incident cases (per 100,000 population)
0
20
40
60
80
Mar
May
Jul
Sep

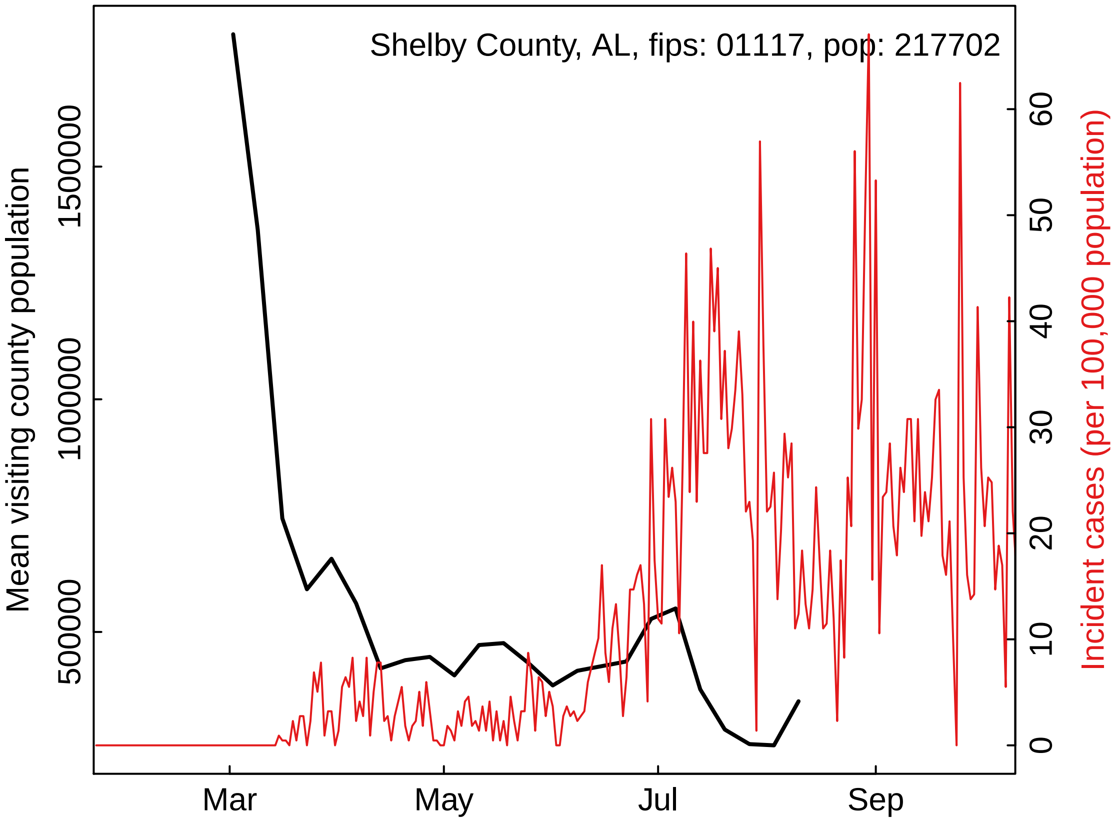

Shelby County, AL, fips: 01117, pop: 217702
Mean visiting county population
500000
1000000
1500000
Mar
May
Jul
Sep
0
10
20
30
40
50
60
Incident cases (per 100,000 population)

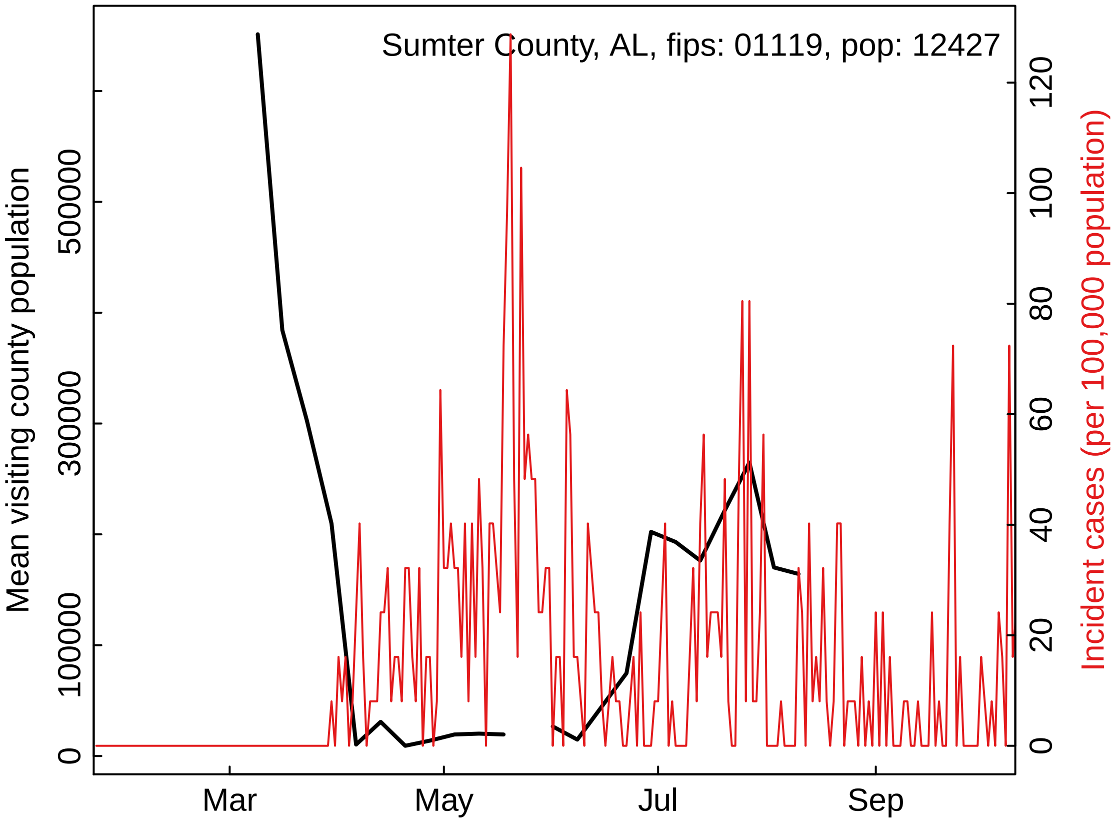

Sumter County, AL, fips: 01119, pop: 12427
Mean visiting county population
0
100000
300000
500000
Incident cases (per 100,000 population)
0
20
40
60
80
100
120
Mar
May
Jul
Sep

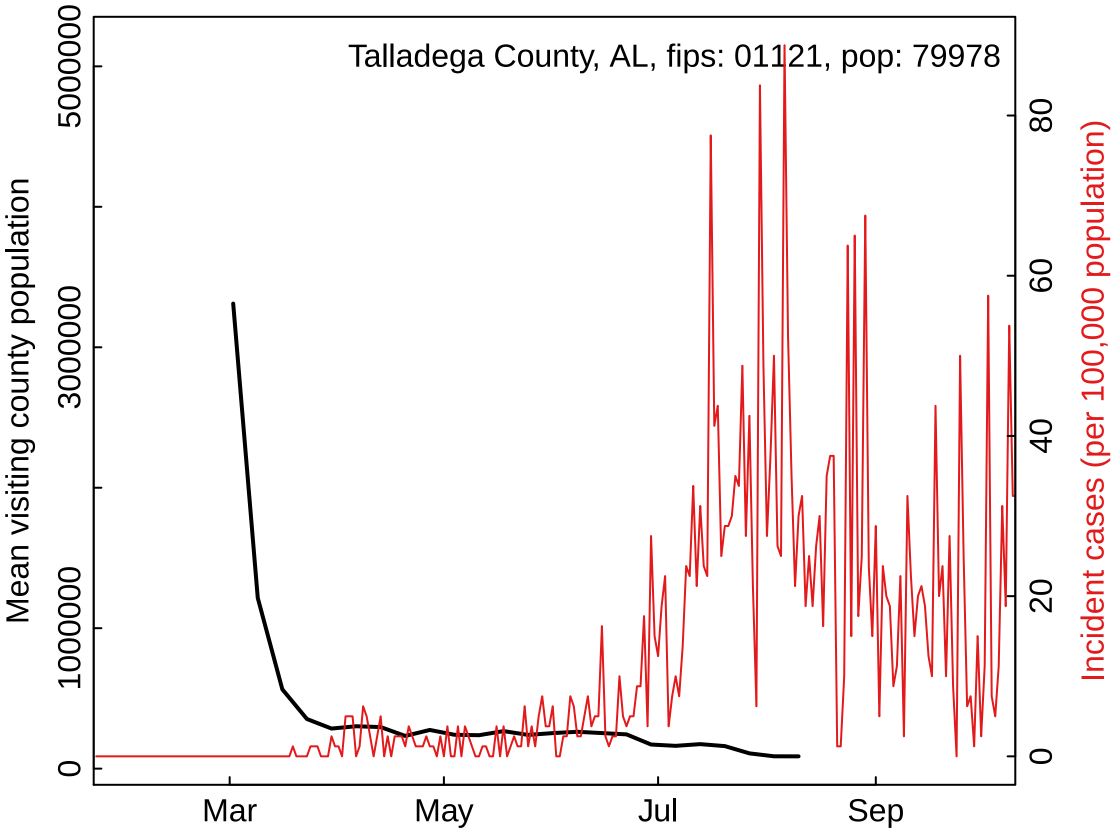

Talladega County, AL, fips: 01121, pop: 79978
Mean visiting county population
0
1000000
3000000
5000000
Mar
May
Jul
Sep
0
20
40
60
80
Incident cases (per 100,000 population)

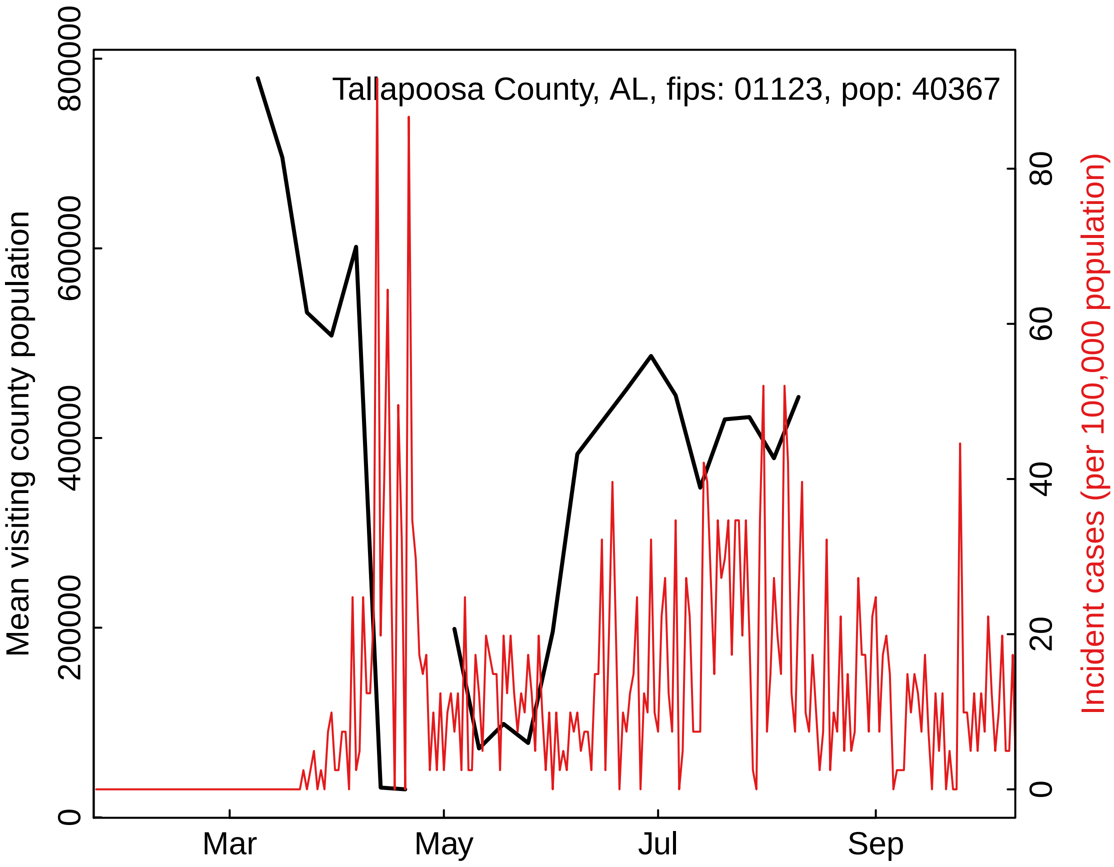
Tallapoosa County, AL, fips: 01123, pop: 40367
Mean visiting county population
Incident cases (per 100,000 population)
0
200000
400000
600000
800000
0
20
40
60
80
Mar
May
Jul
Sep

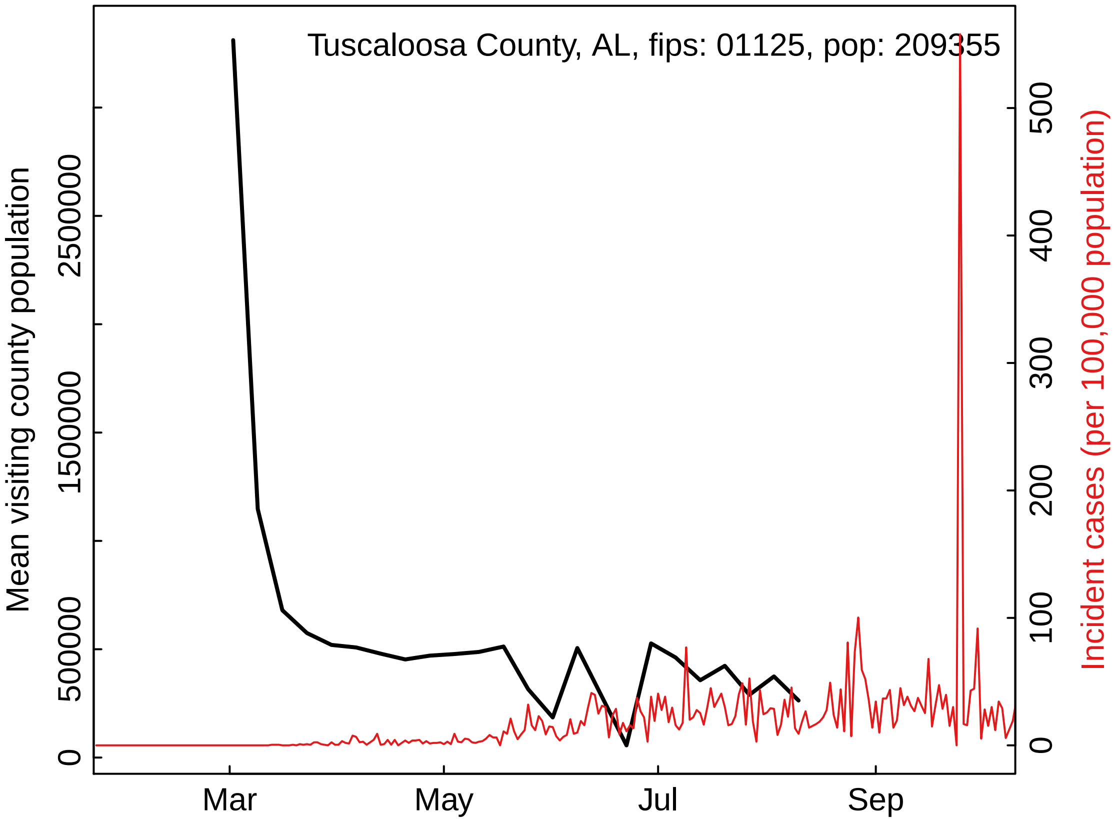

Tuscaloosa County, AL, fips: 01125, pop: 209355
Mean visiting county population
0
500000
1500000
2500000
Incident cases (per 100,000 population)
0
100
200
300
400
500
Mar
May
Jul
Sep

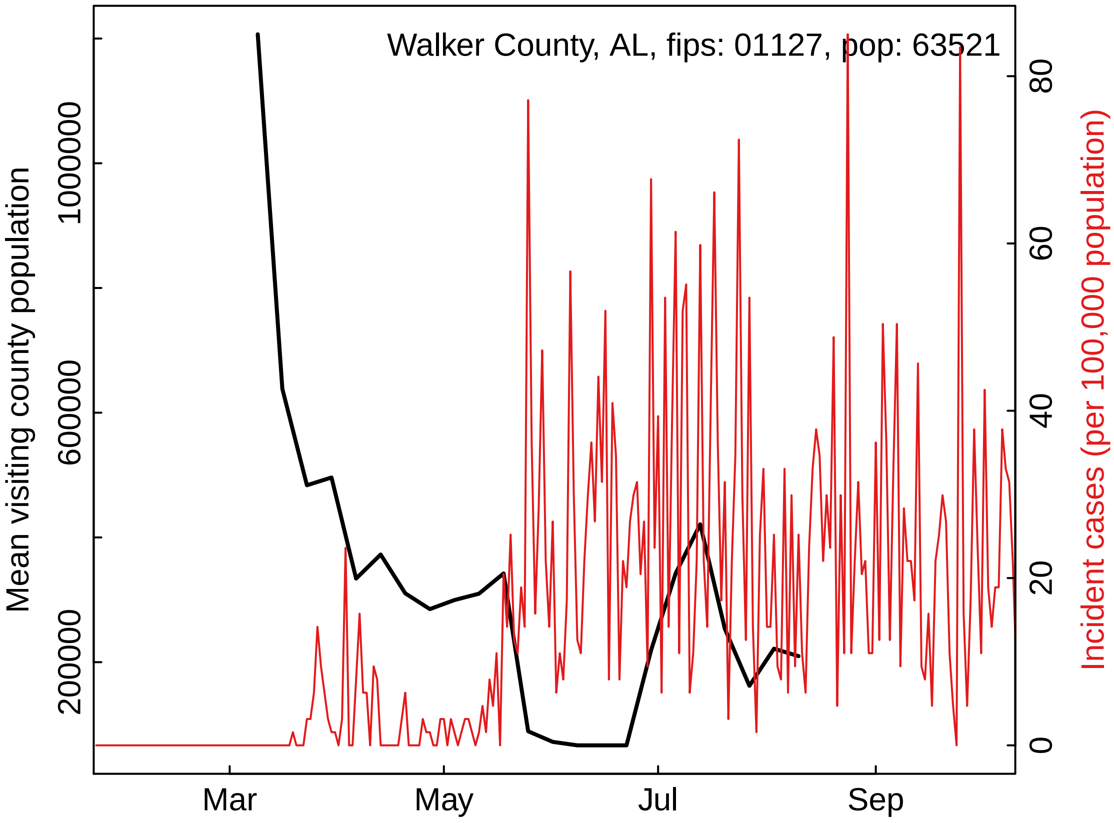

Walker County, AL, fips: 01127, pop: 63521
Mean visiting county population
200000
600000
1000000
Incident cases (per 100,000 population)
0
20
40
60
80
Mar
May
Jul
Sep

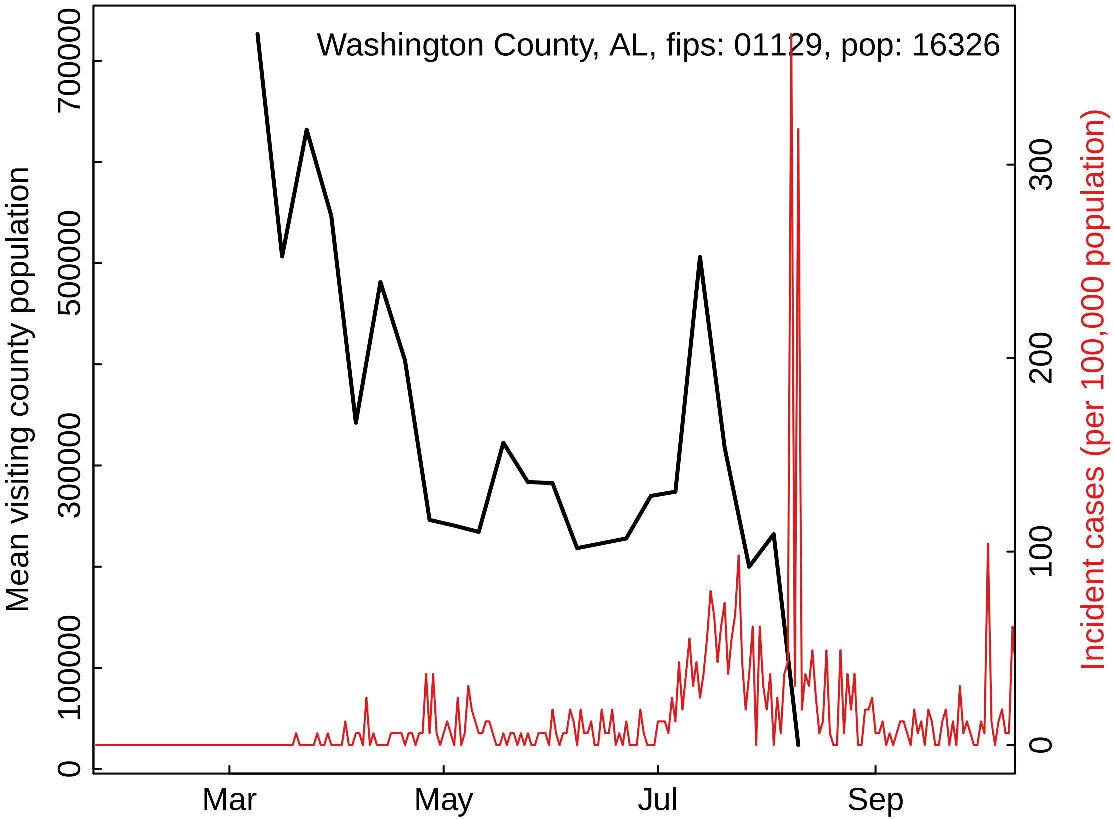

Washington County, AL, fips: 01129, pop: 16326
Mean visiting county population
0
100000
300000
500000
700000
Incident cases (per 100,000 population)
0
100
200
300
Mar
May
Jul
Sep

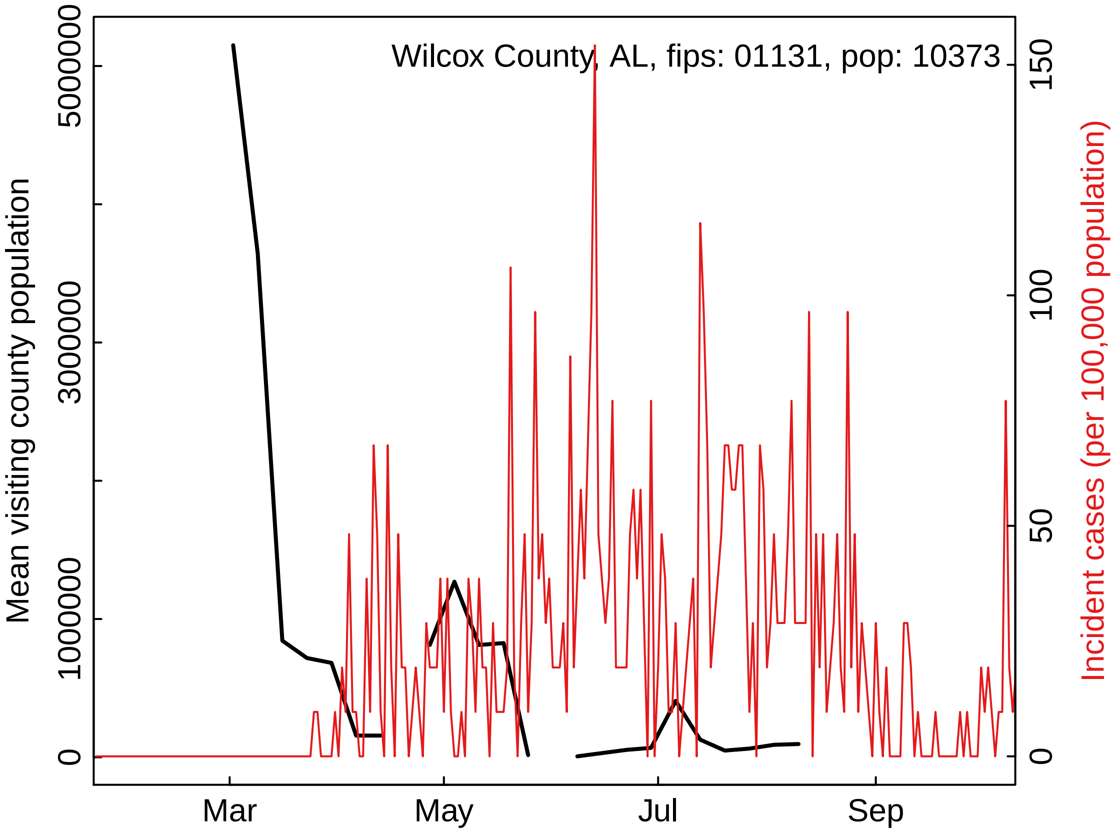

Wilcox County, AL, fips: 01131, pop: 10373
Mean visiting county population
0
1000000
3000000
5000000
Incident cases (per 100,000 population)
0
50
100
150
Mar
May
Jul
Sep

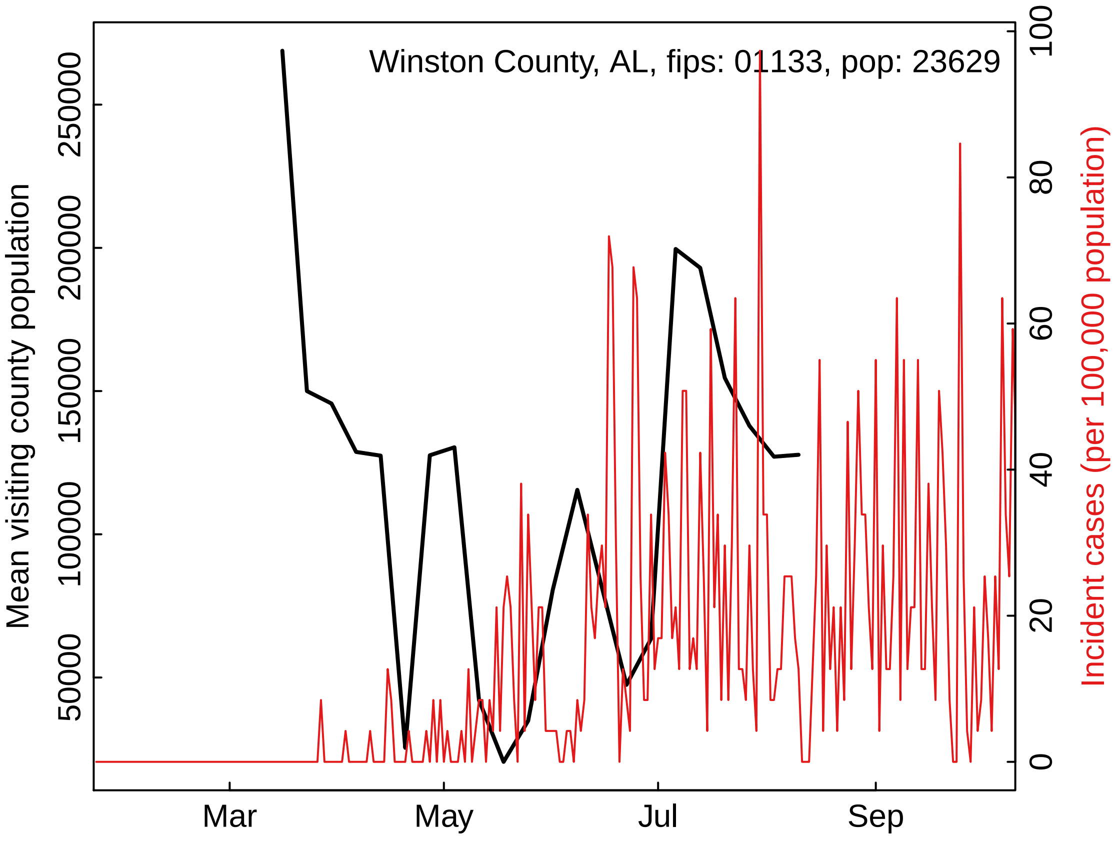

Winston County, AL, fips: 01133, pop: 23629
Mean visiting county population
50000
100000
150000
200000
250000
Incident cases (per 100,000 population)
0
20
40
60
80
100
Mar
May
Jul
Sep

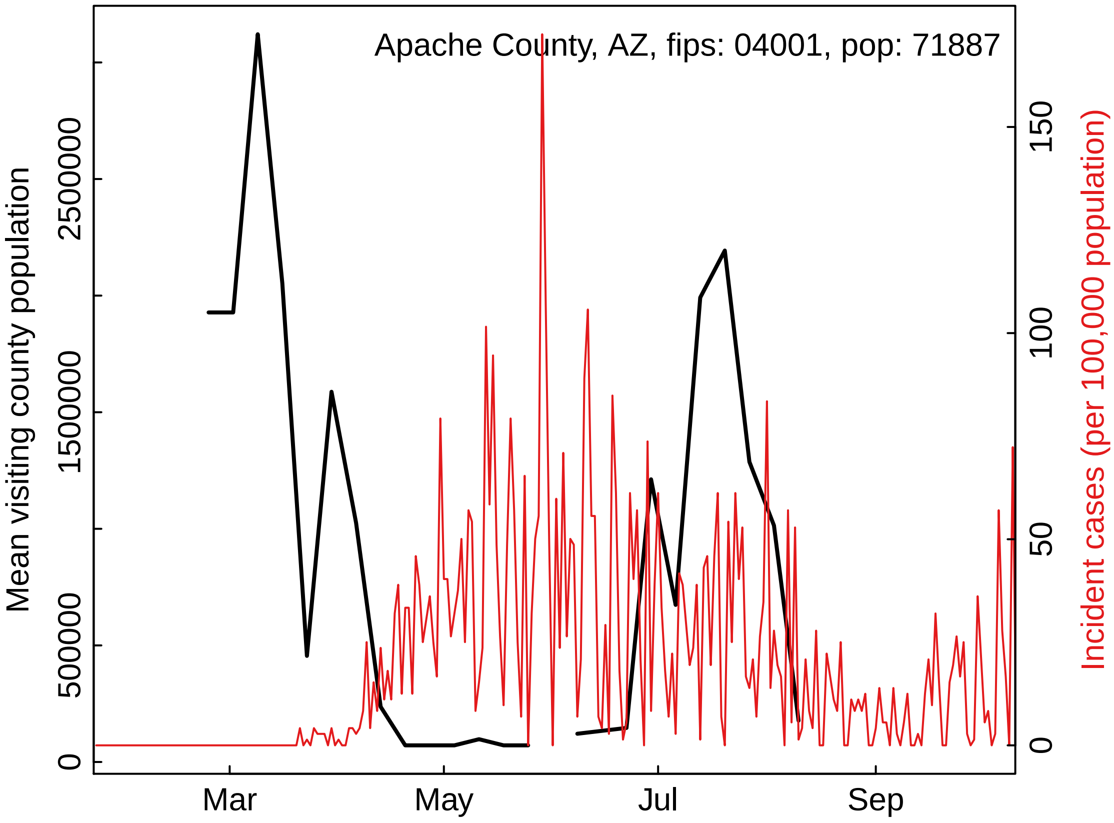

Apache County, AZ, fips: 04001, pop: 71887
Mean visiting county population
0
500000
1500000
2500000
Incident cases (per 100,000 population)
0
50
100
150
Mar
May
Jul
Sep

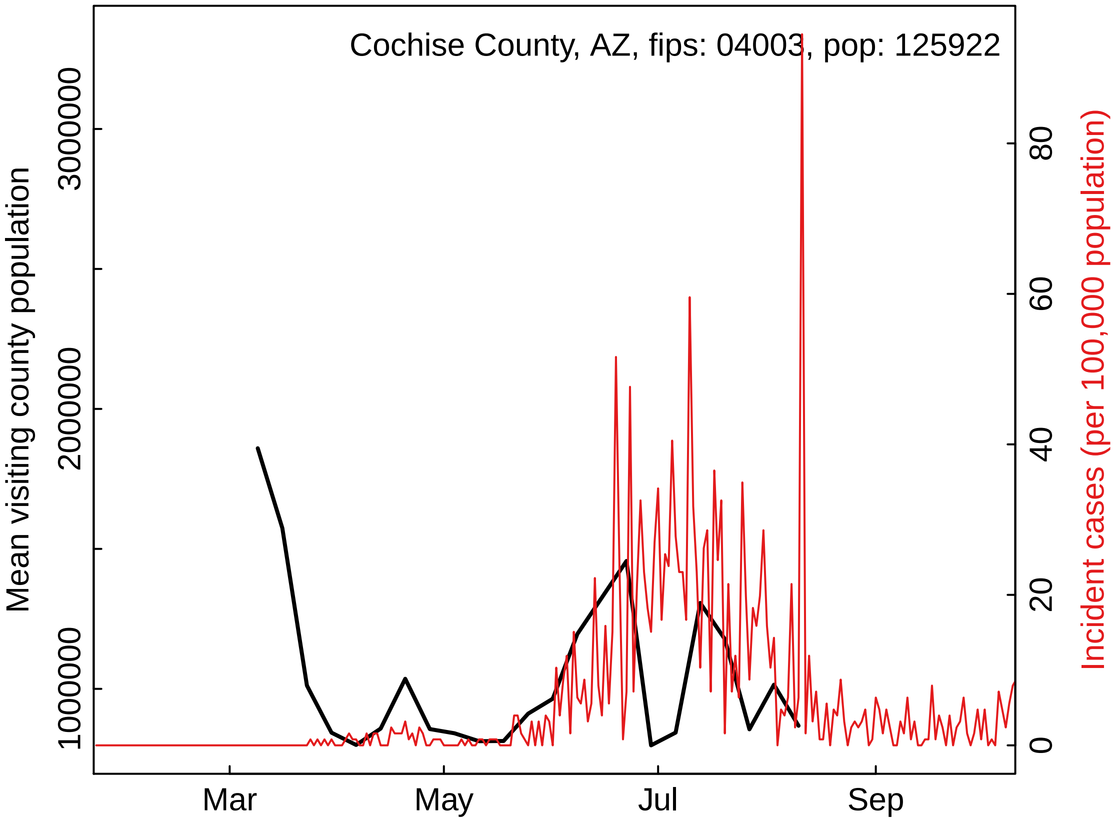
Cochise County, AZ, fips: 04003, pop: 125922
Mean visiting county population
1000000
2000000
3000000
Incident cases (per 100,000 population)
0
20
40
60
80
Mar
May
Jul
Sep

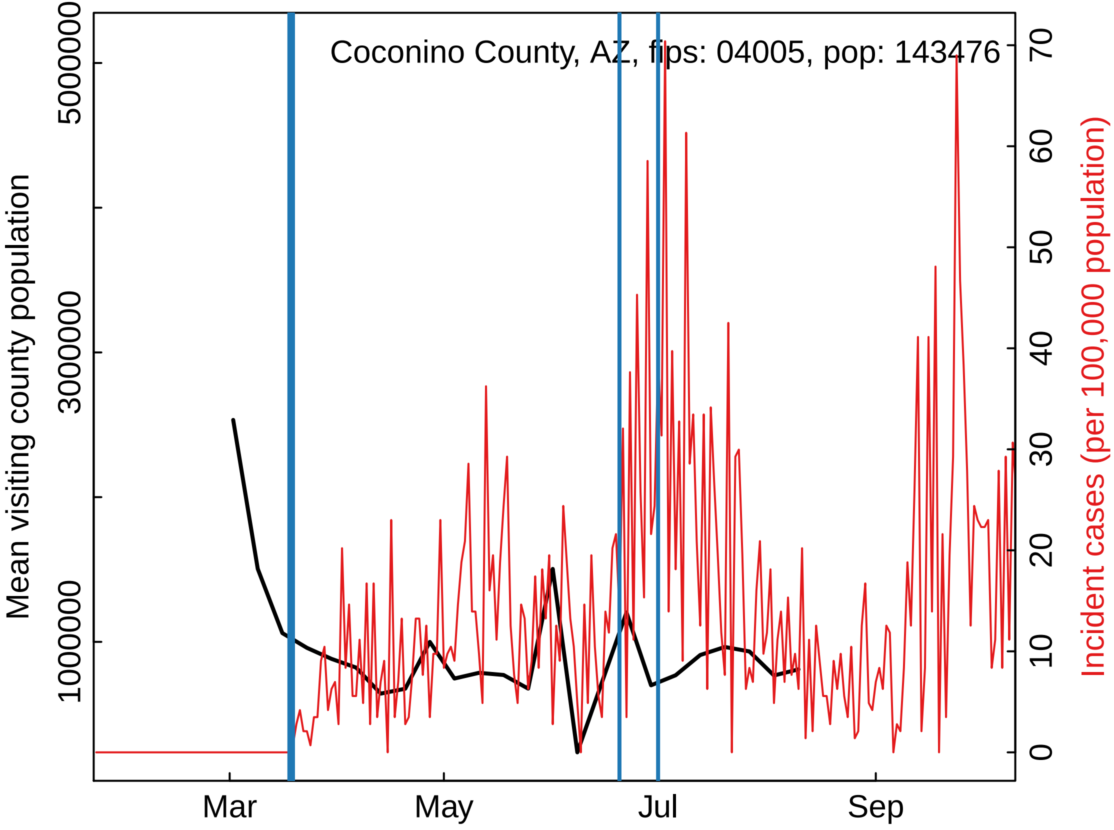

Coconino County, AZ, fips: 04005, pop: 143476
Mean visiting county population
1000000
3000000
5000000
Incident cases (per 100,000 population)
0
10
20
30
40
50
60
70
Mar
May
Jul
Sep

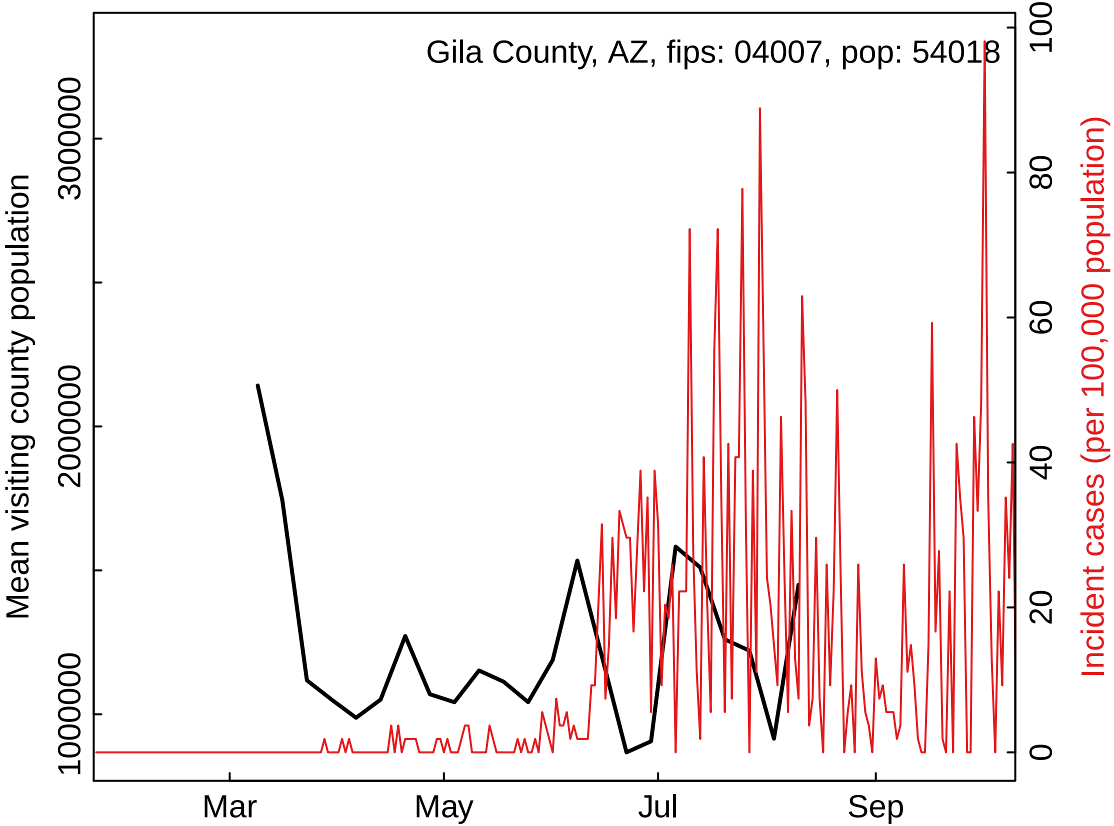

Gila County, AZ, fips: 04007, pop: 54018
Mean visiting county population
1000000
2000000
3000000
Mar
May
Jul
Sep
0
20
40
60
80
100
Incident cases (per 100,000 population)

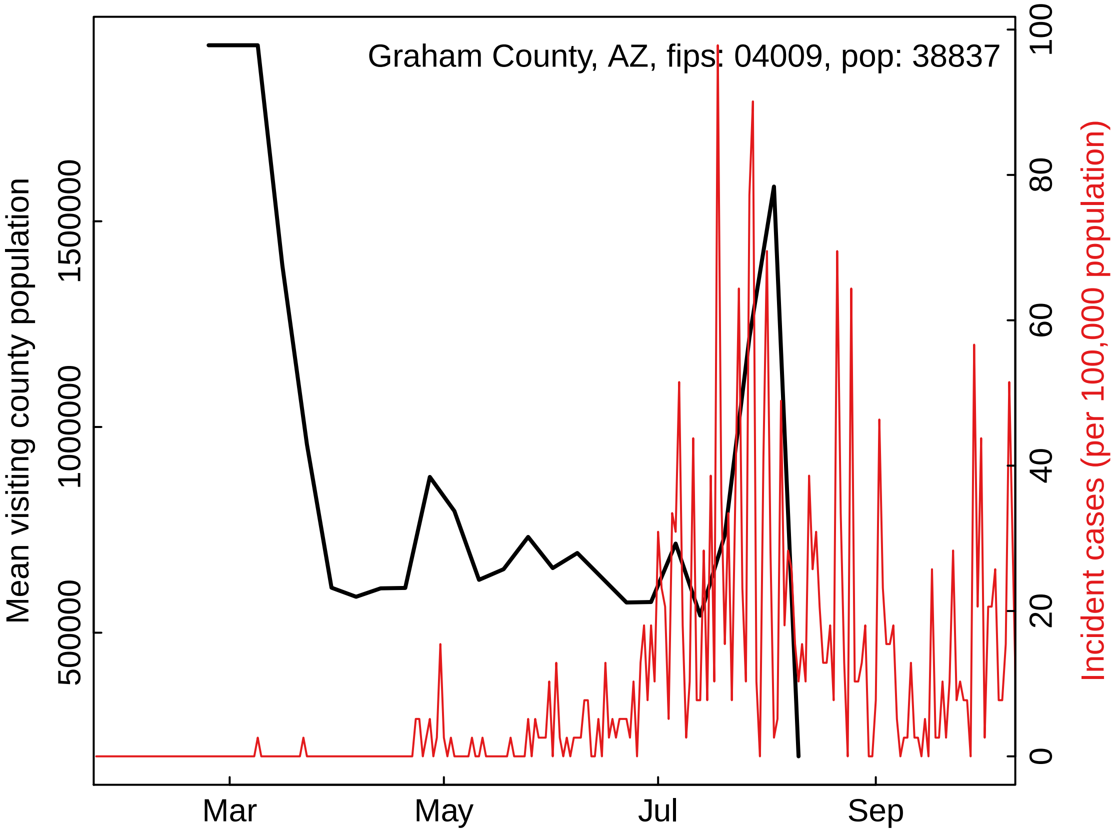
Graham County, AZ, fips: 04009, pop: 38837
Mean visiting county population
500000
1000000
1500000
Incident cases (per 100,000 population)
0
20
40
60
80
100
Mar
May
Jul
Sep

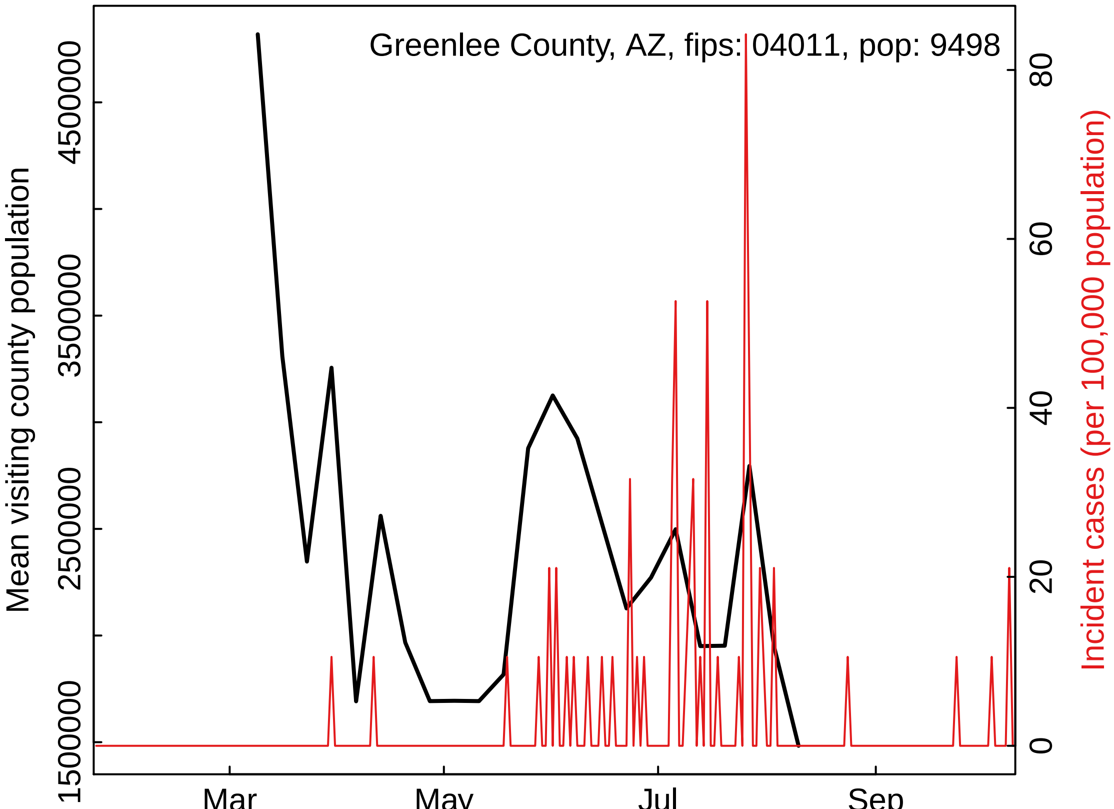
Greenlee County, AZ, fips: 04011, pop: 9498
Mean visiting county population
1500000
2500000
3500000
4500000
Incident cases (per 100,000 population)
0
20
40
60
80
Mar
May
Jul
Sep

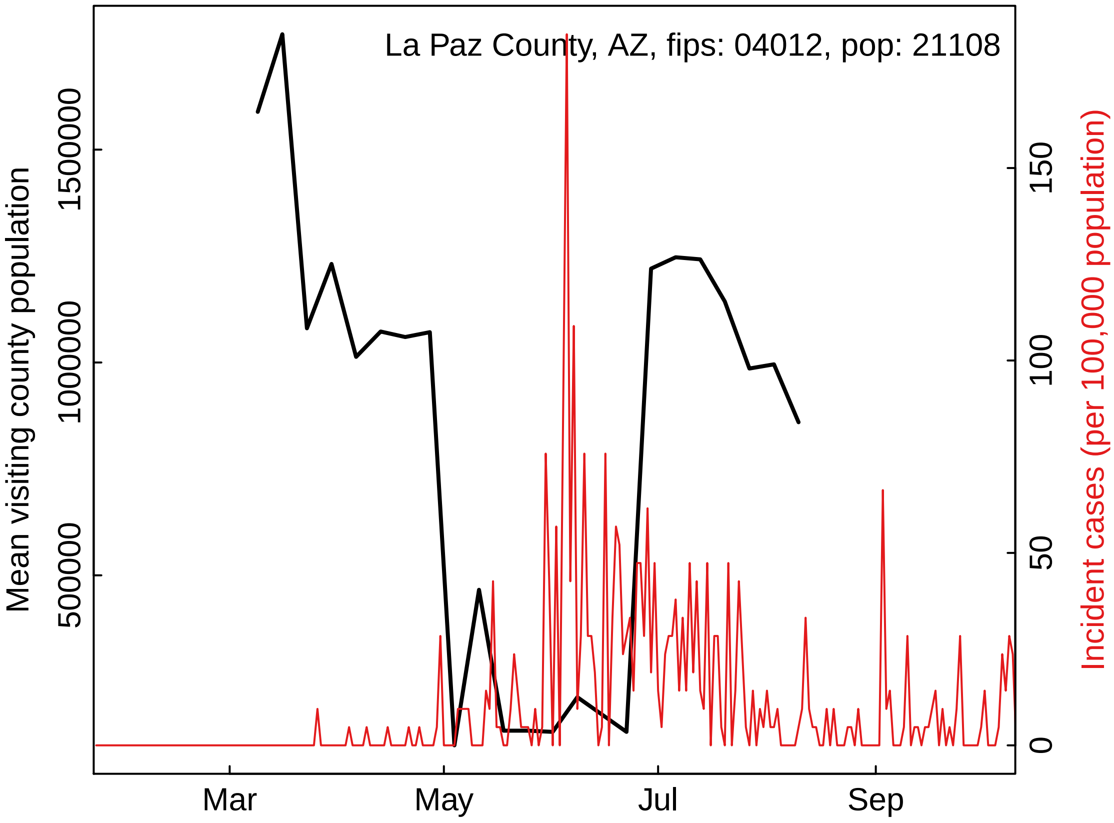
La Paz County, AZ, fips: 04012, pop: 21108
Mean visiting county population
500000
1000000
1500000
Incident cases (per 100,000 population)
0
50
100
150
Mar
May
Jul
Sep

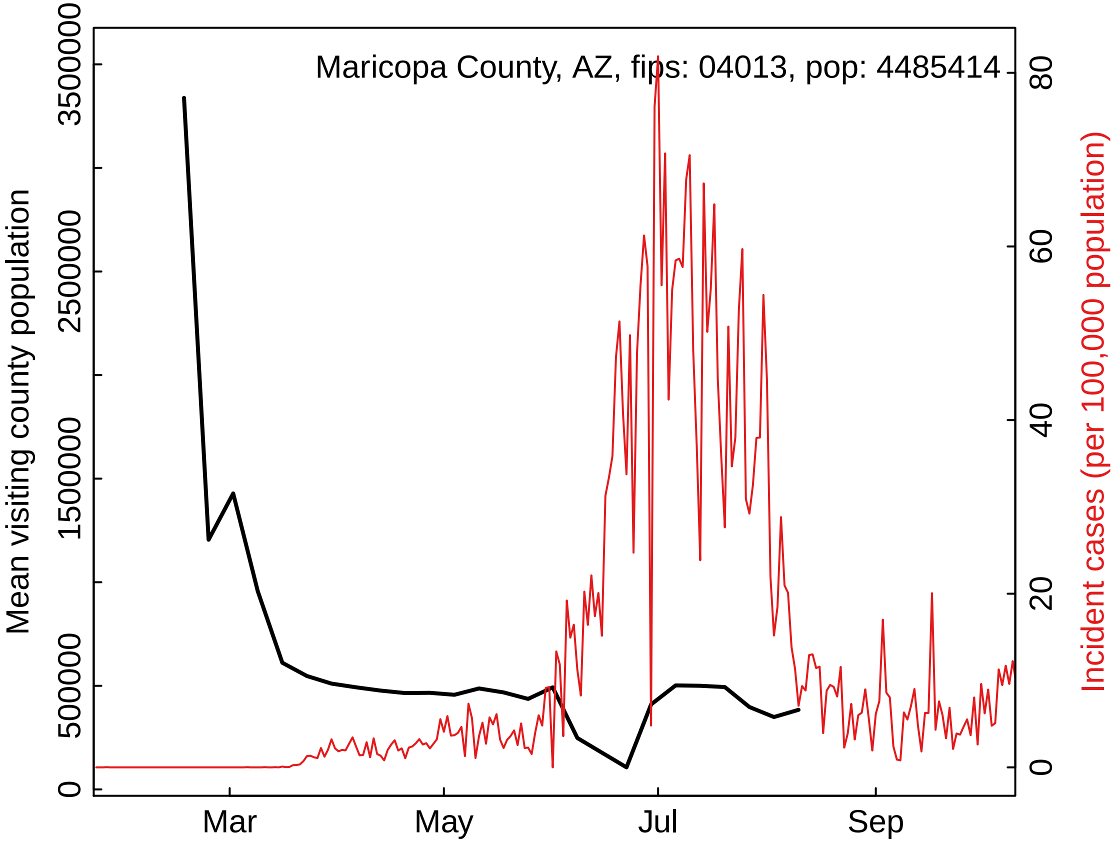

Maricopa County, AZ, fips: 04013, pop: 4485414
Mean visiting county population
Incident cases (per 100,000 population)
0
500000
1500000
2500000
3500000
0
20
40
60
80
Mar
May
Jul
Sep

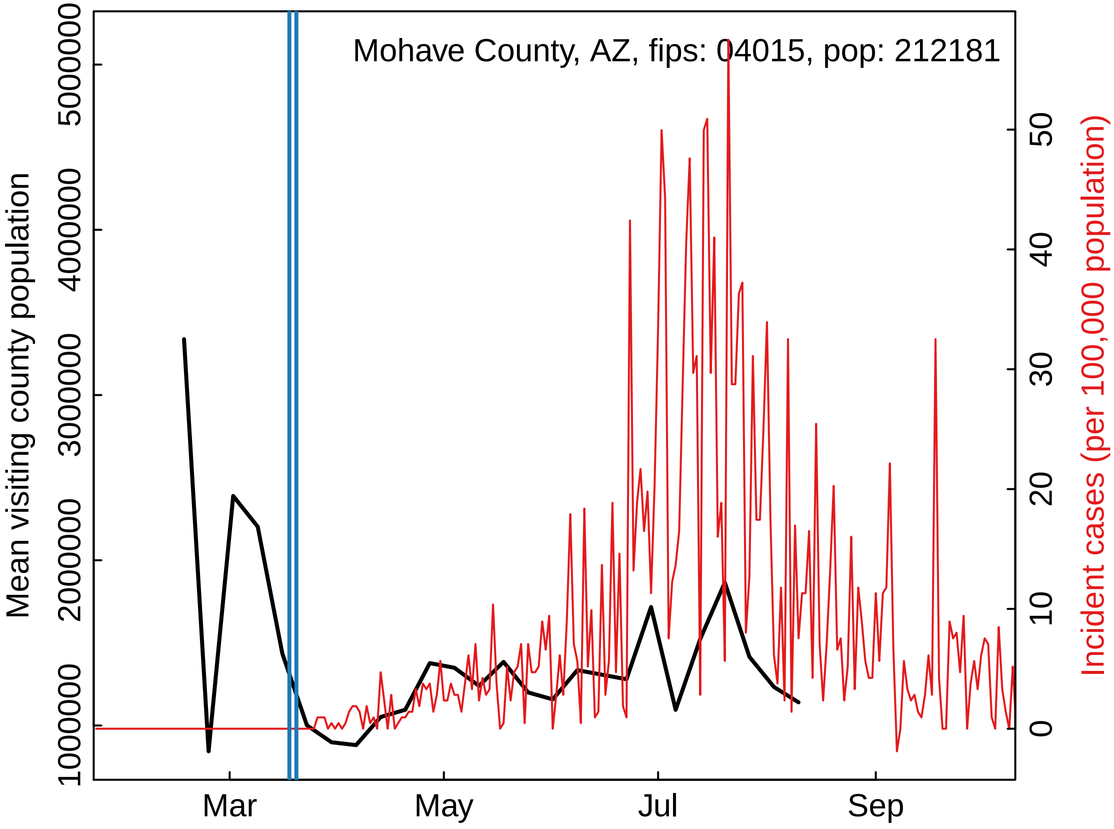

Mohave County, AZ, fips: 04015, pop: 212181
Mean visiting county population
10000000
20000000
30000000
40000000
50000000
Incident cases (per 100,000 population)
0
10
20
30
40
50
Mar
May
Jul
Sep

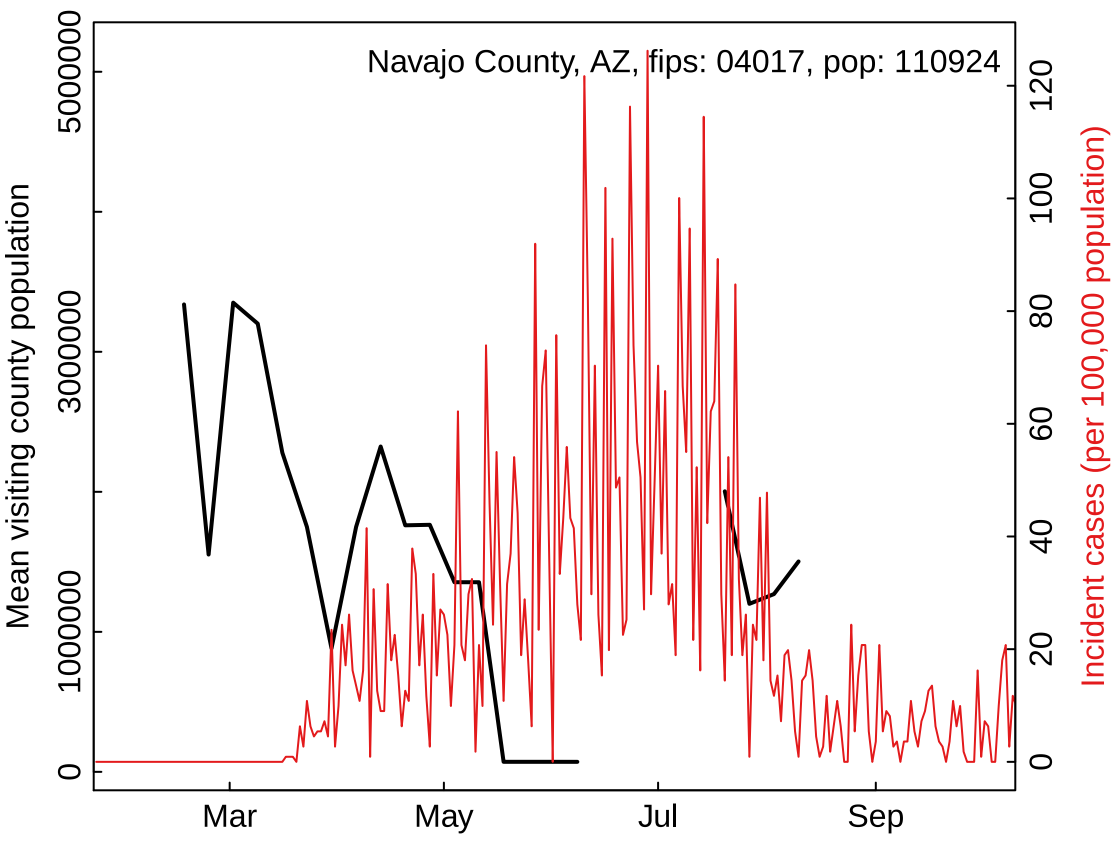

Navajo County, AZ, fips: 04017, pop: 110924
Mean visiting county population
0
1000000
3000000
5000000
Incident cases (per 100,000 population)
0
20
40
60
80
100
120
Mar
May
Jul
Sep

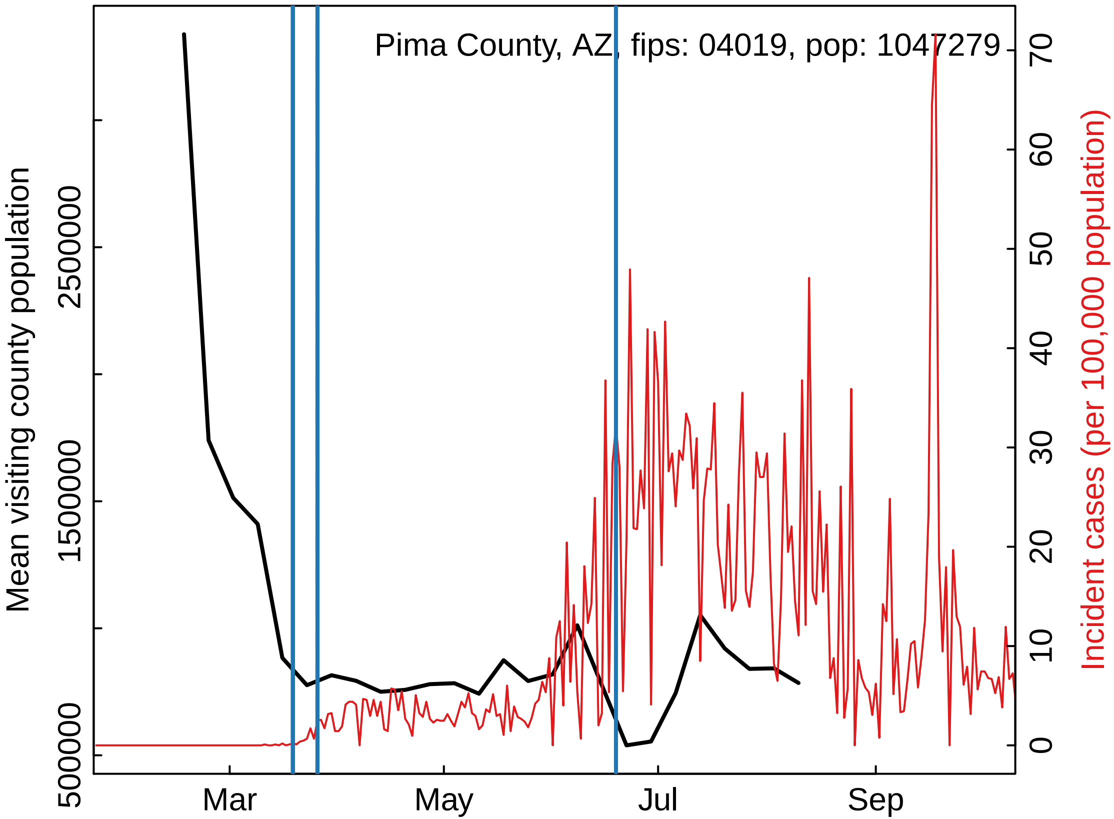

Pima County, AZ, fips: 04019, pop: 1047279
Mean visiting county population
500000
1500000
2500000
Mar
May
Jul
Sep
0
10
20
30
40
50
60
70
Incident cases (per 100,000 population)

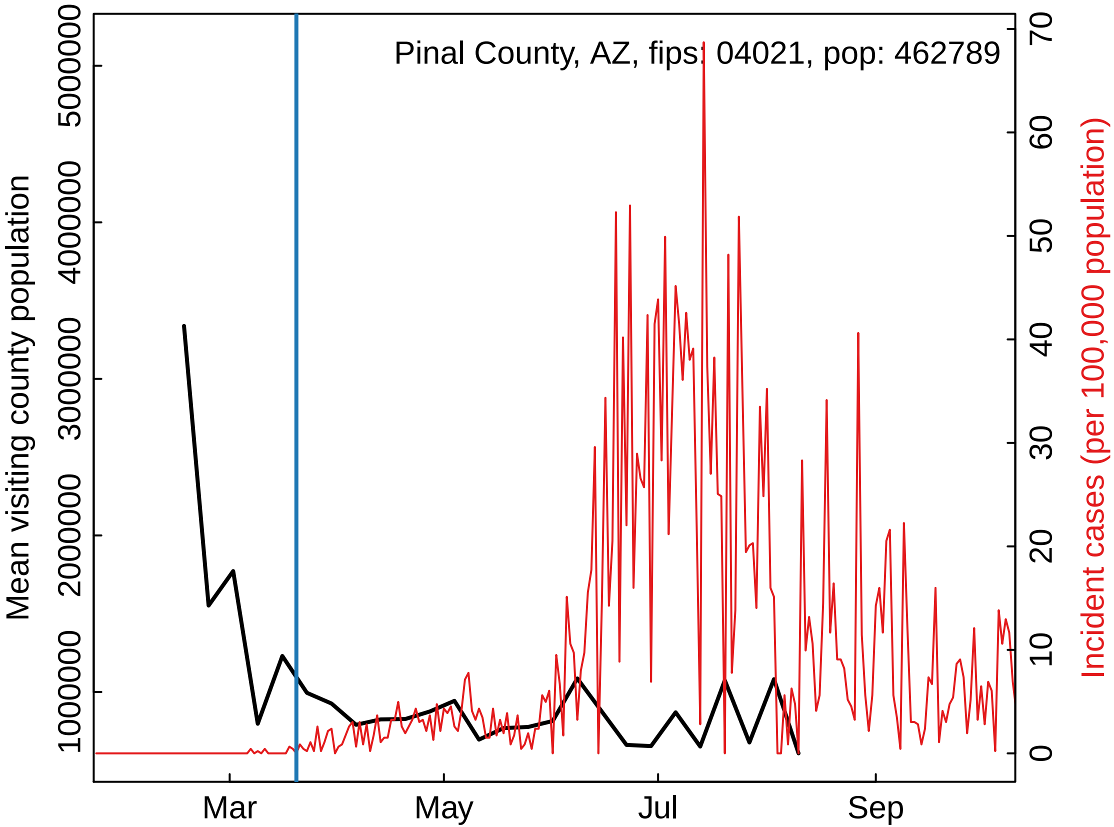

Pinal County, AZ, fips: 04021, pop: 462789
Mean visiting county population
Incident cases (per 100,000 population)
10000000
20000000
30000000
40000000
50000000
0
10
20
30
40
50
60
70
Mar
May
Jul
Sep

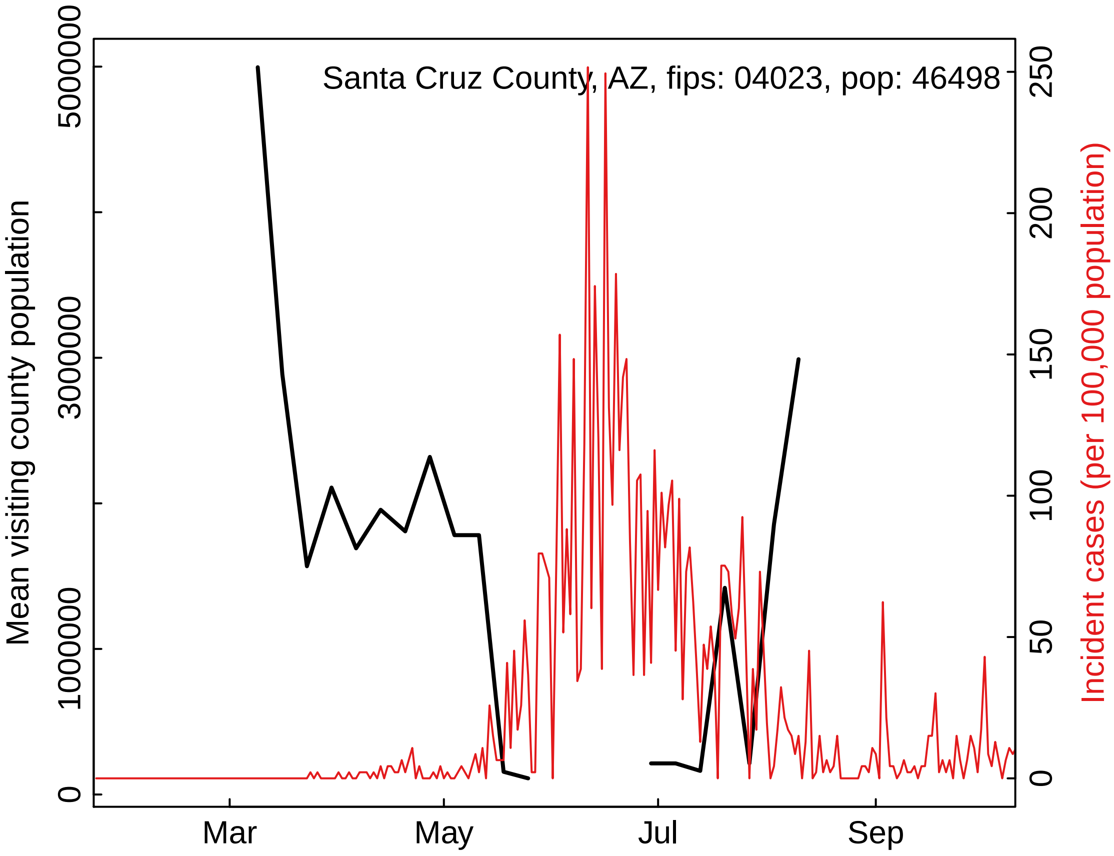

Santa Cruz County, AZ, fips: 04023, pop: 46498
Mean visiting county population
Incident cases (per 100,000 population)
0
1000000
3000000
5000000
0
50
100
150
200
250
Mar
May
Jul
Sep

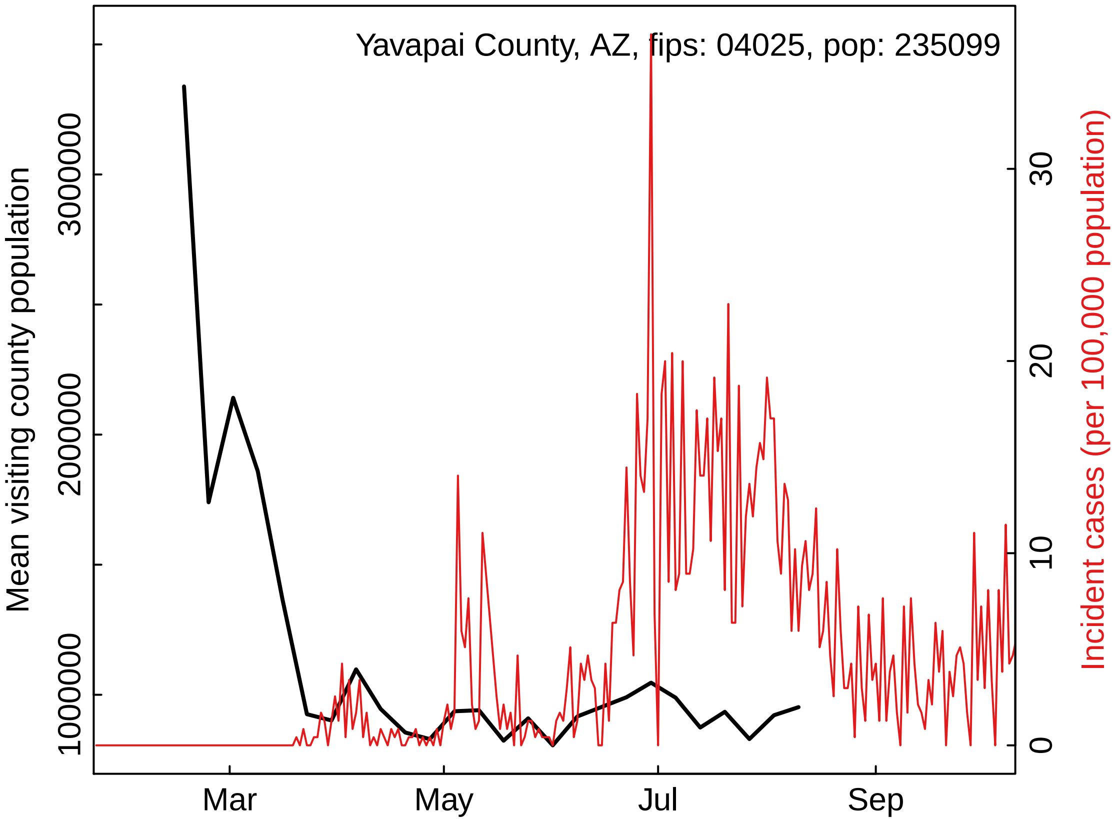

Yavapai County, AZ, fips: 04025, pop: 235099
Mean visiting county population
10000000
20000000
30000000
Incident cases (per 100,000 population)
0
10
20
30
Mar
May
Jul
Sep

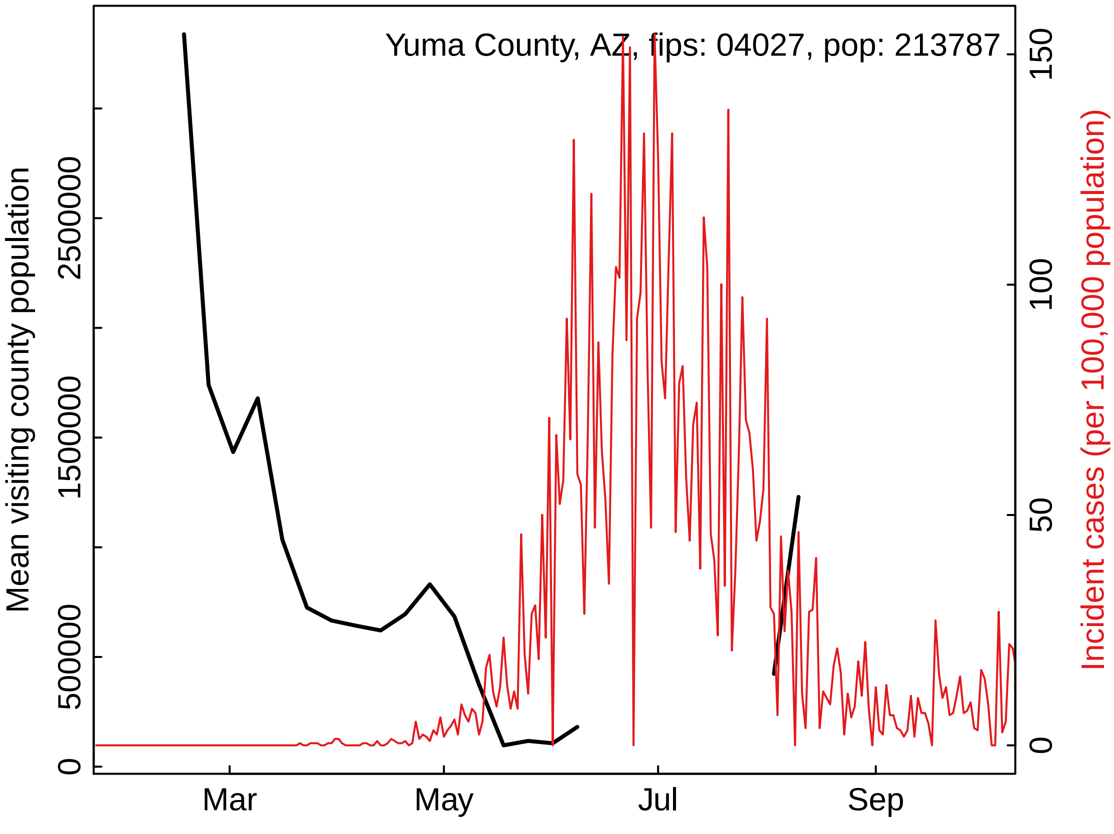

Yuma County, AZ, fips: 04027, pop: 213787
Mean visiting county population
0
500000
1500000
2500000
Incident cases (per 100,000 population)
0
50
100
150
Mar
May
Jul
Sep

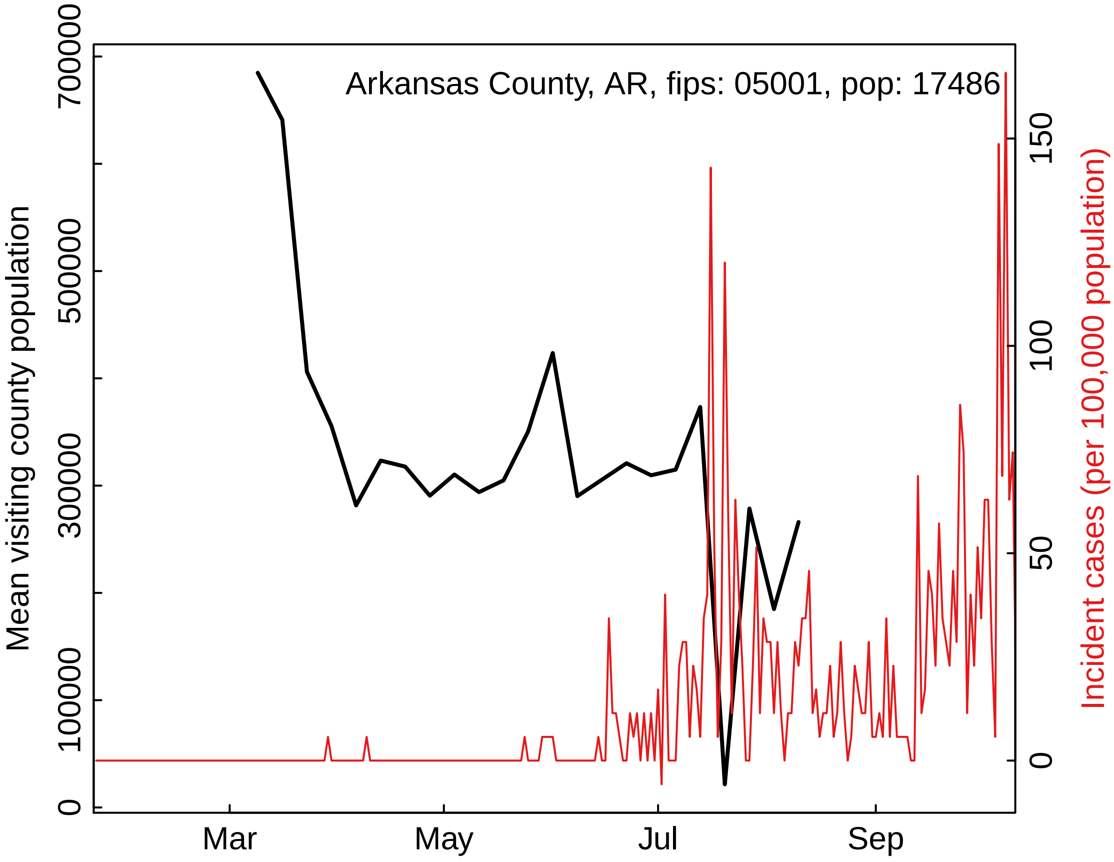

Arkansas County, AR, fips: 05001, pop: 17486
Mean visiting county population
0
100000
300000
500000
700000
Incident cases (per 100,000 population)
0
50
100
150
Mar
May
Jul
Sep

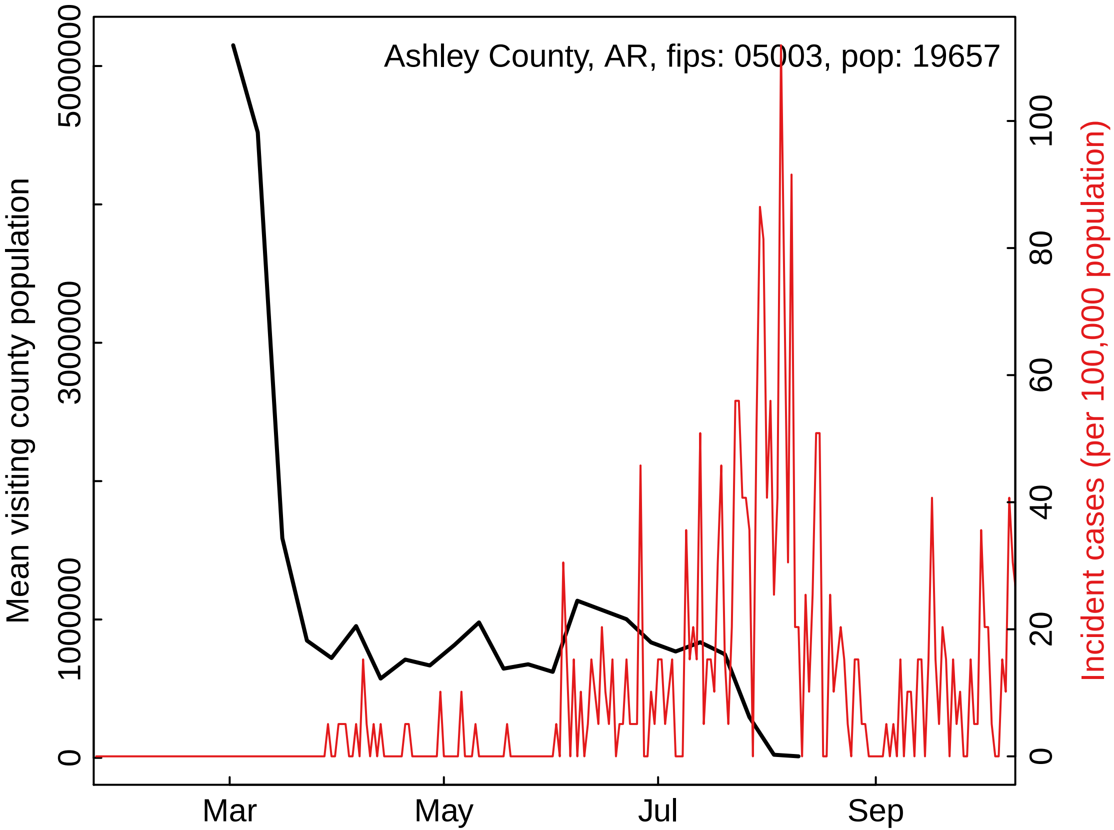

Ashley County, AR, fips: 05003, pop: 19657
Mean visiting county population
Incident cases (per 100,000 population)
0
1000000
3000000
5000000
0
20
40
60
80
100
Mar
May
Jul
Sep

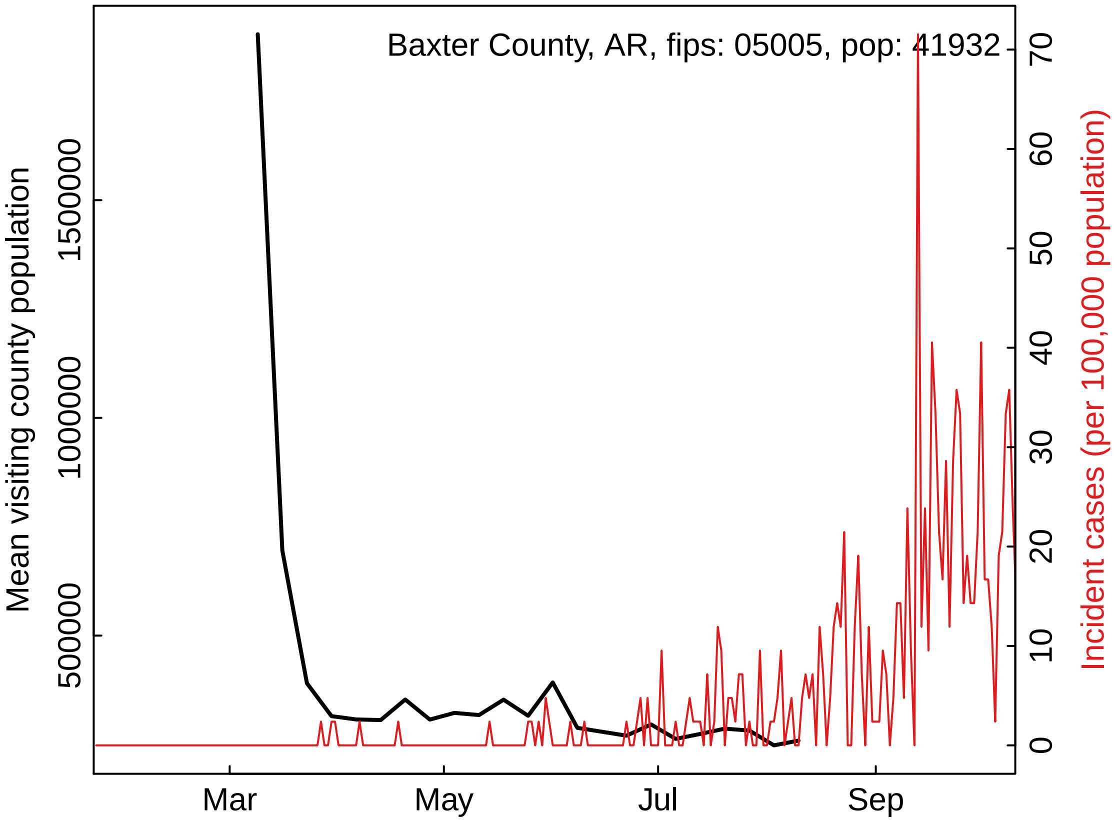

Baxter County, AR, fips: 05005, pop: 41932
Mean visiting county population
500000
1000000
1500000
Mar
May
Jul
Sep
0
10
20
30
40
50
60
70
Incident cases (per 100,000 population)

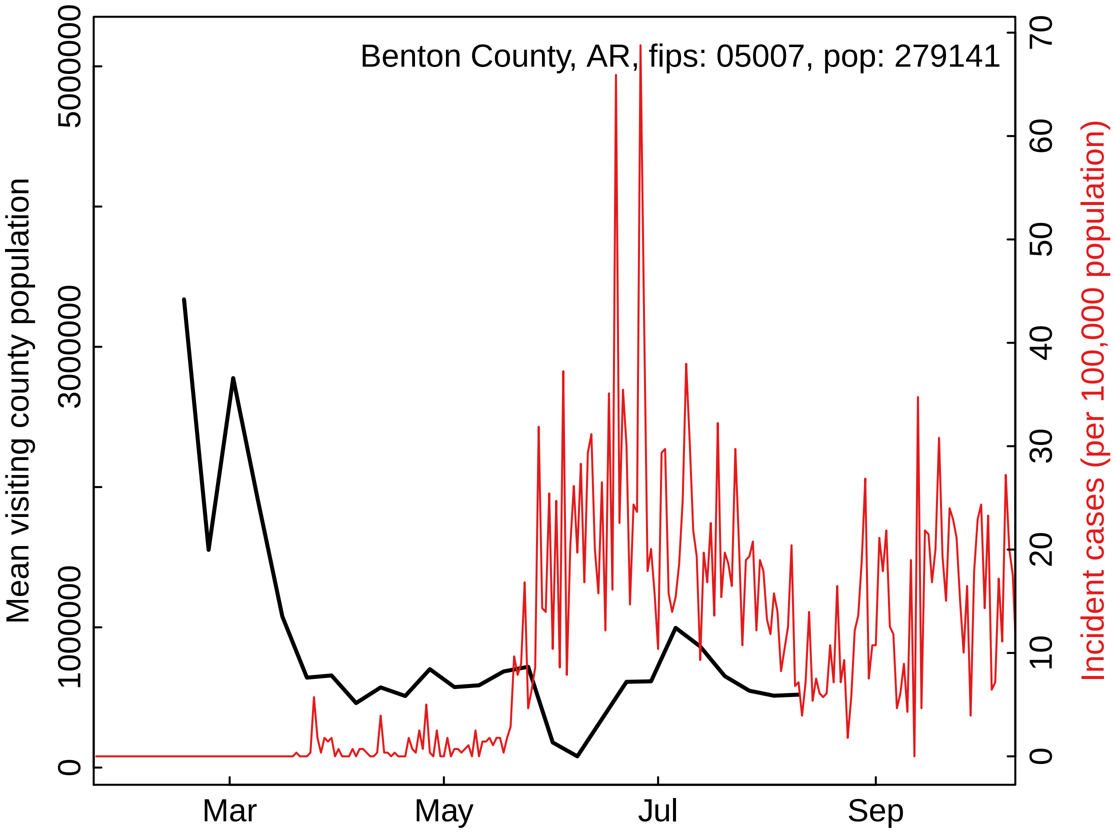

Benton County, AR, fips: 05007, pop: 279141
Mean visiting county population
0
1000000
3000000
5000000
Incident cases (per 100,000 population)
0
10
20
30
40
50
60
70
Mar
May
Jul
Sep

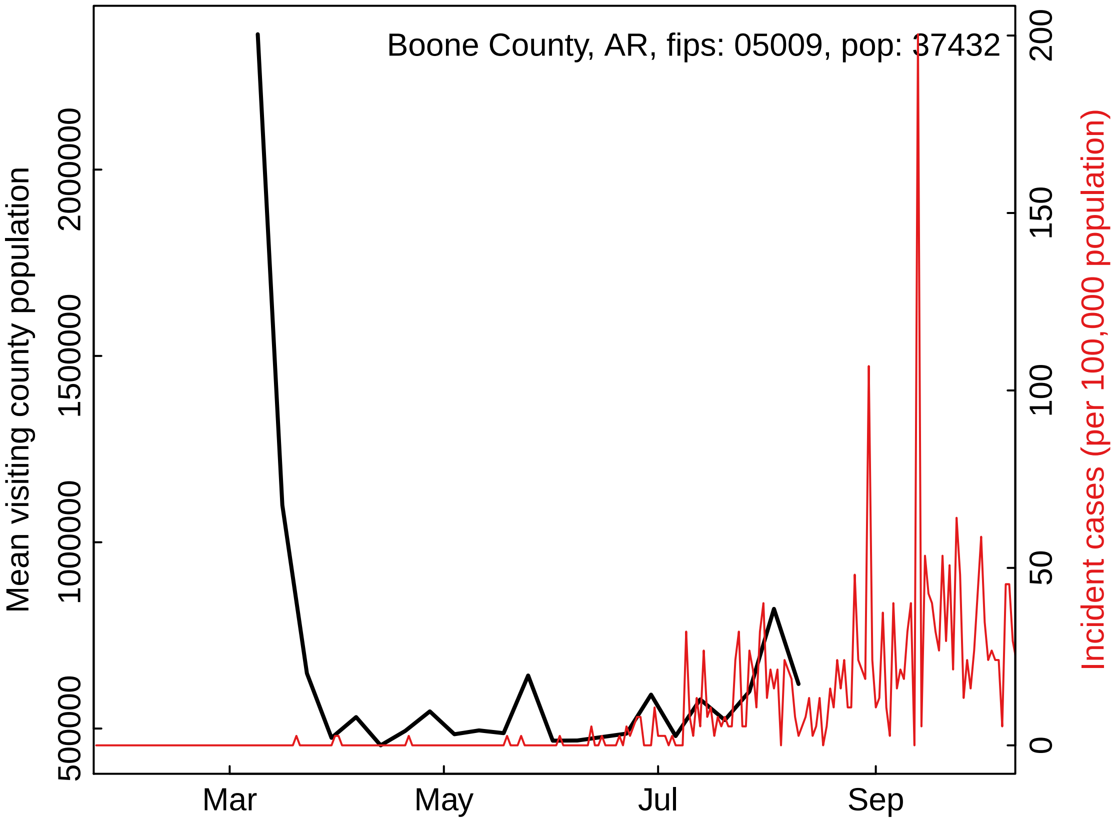

Boone County, AR, fips: 05009, pop: 37432
Mean visiting county population
500000
1000000
1500000
2000000
Incident cases (per 100,000 population)
0
50
100
150
200
Mar
May
Jul
Sep

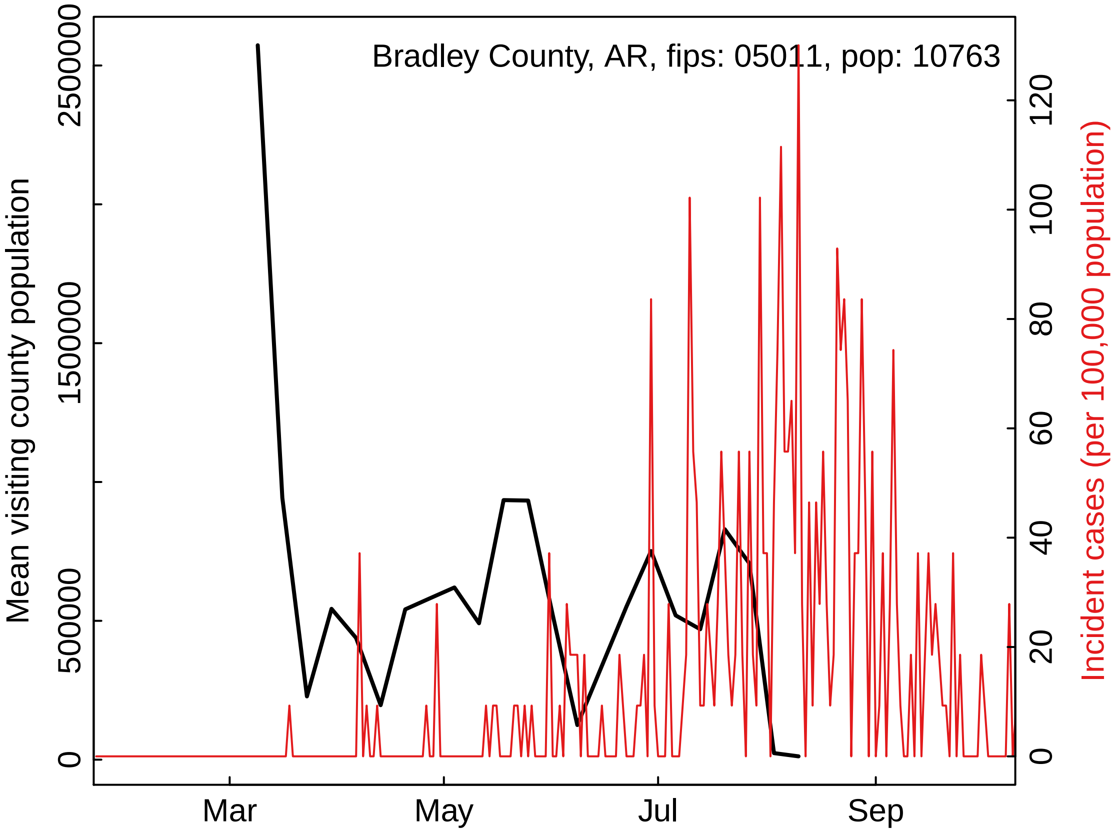

Bradley County, AR, fips: 05011, pop: 10763
Mean visiting county population
0
500000
1500000
2500000
Incident cases (per 100,000 population)
0
20
40
60
80
100
120
Mar
May
Jul
Sep

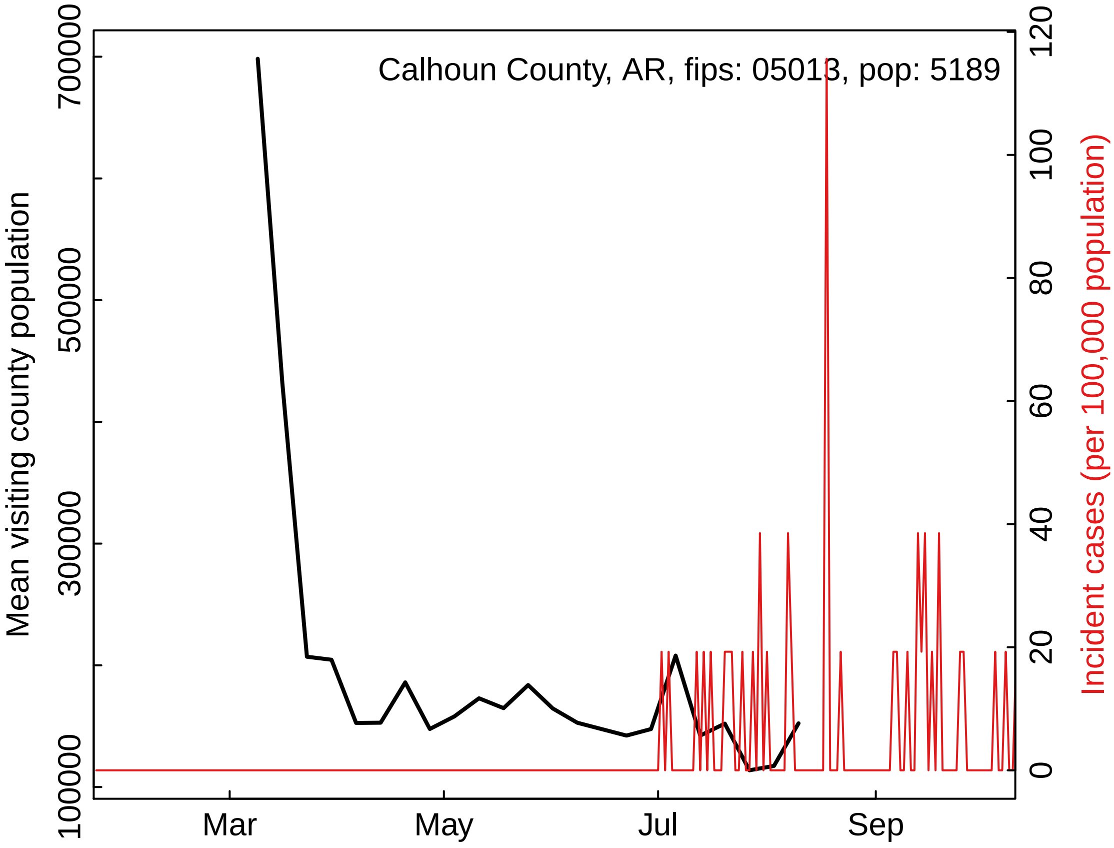
Calhoun County, AR, fips: 05013, pop: 5189
Mean visiting county population
Incident cases (per 100,000 population)
100000
300000
500000
700000
0
20
40
60
80
100
120
Mar
May
Jul
Sep

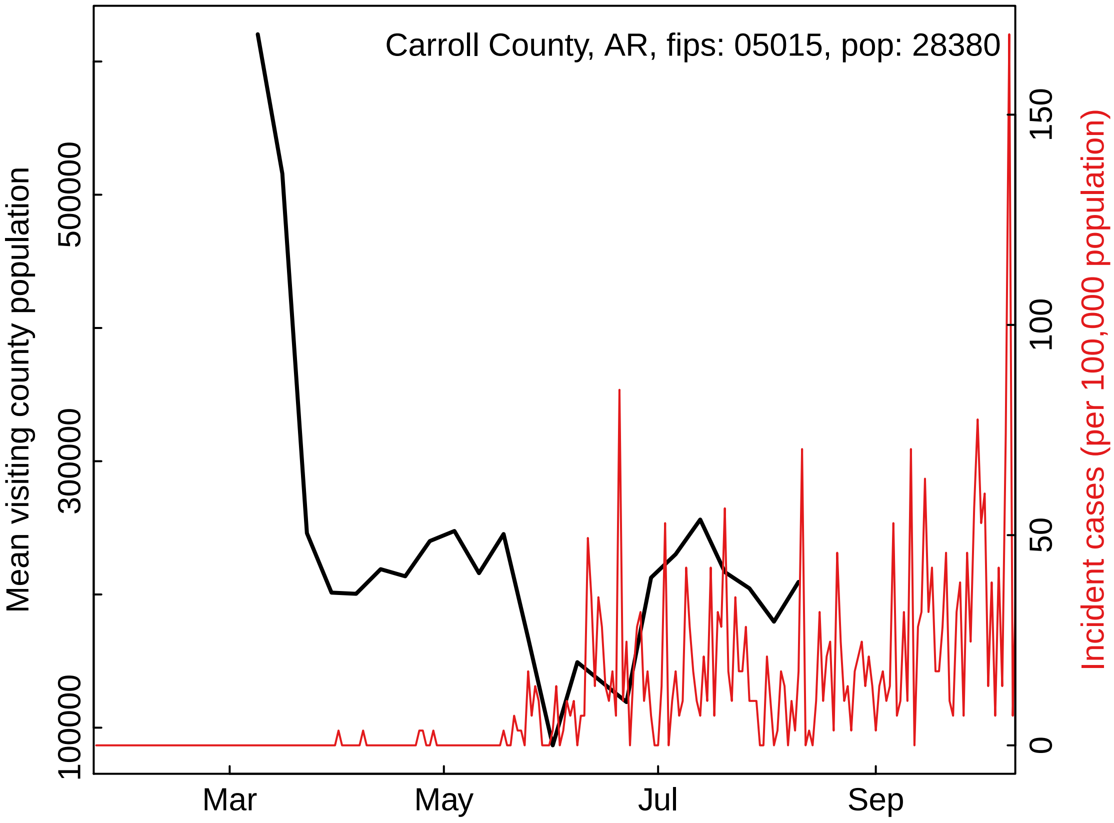

Carroll County, AR, fips: 05015, pop: 28380
Mean visiting county population
100000
300000
500000
Incident cases (per 100,000 population)
0
50
100
150
Mar
May
Jul
Sep

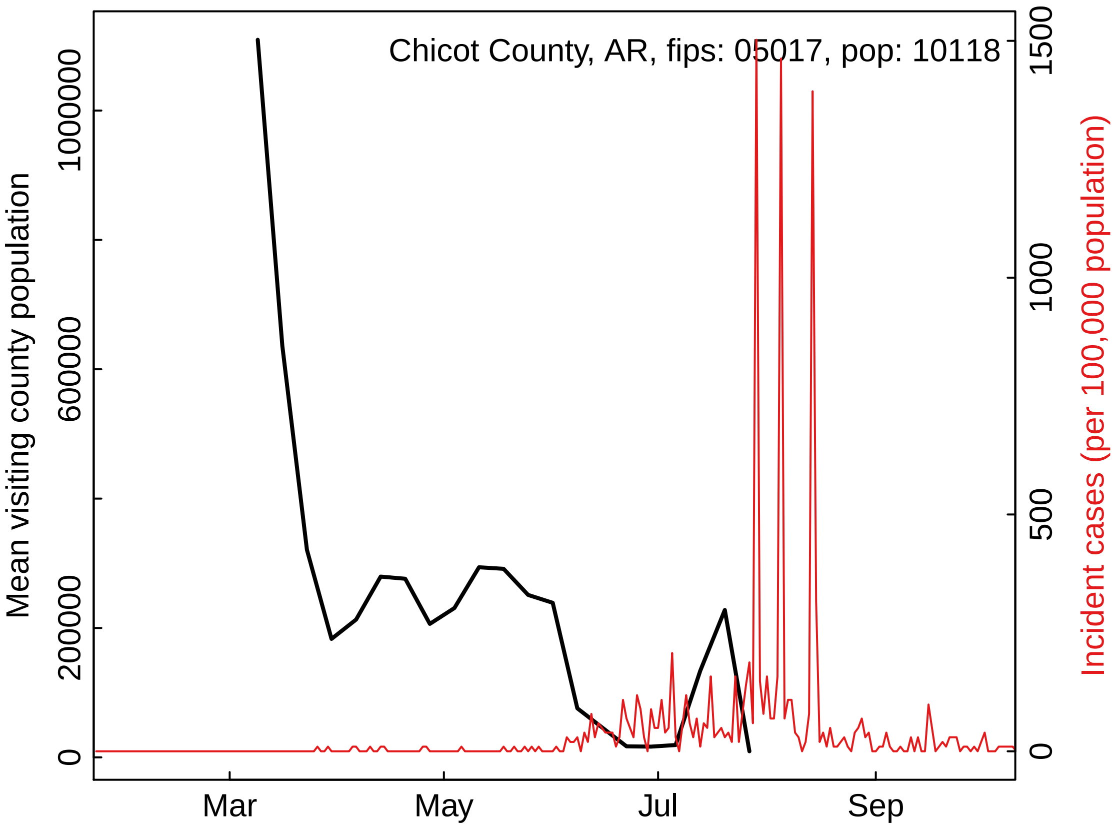

Chicot County, AR, fips: 05017, pop: 10118
Mean visiting county population
0
200000
600000
1000000
Incident cases (per 100,000 population)
0
500
1000
1500
Mar
May
Jul
Sep

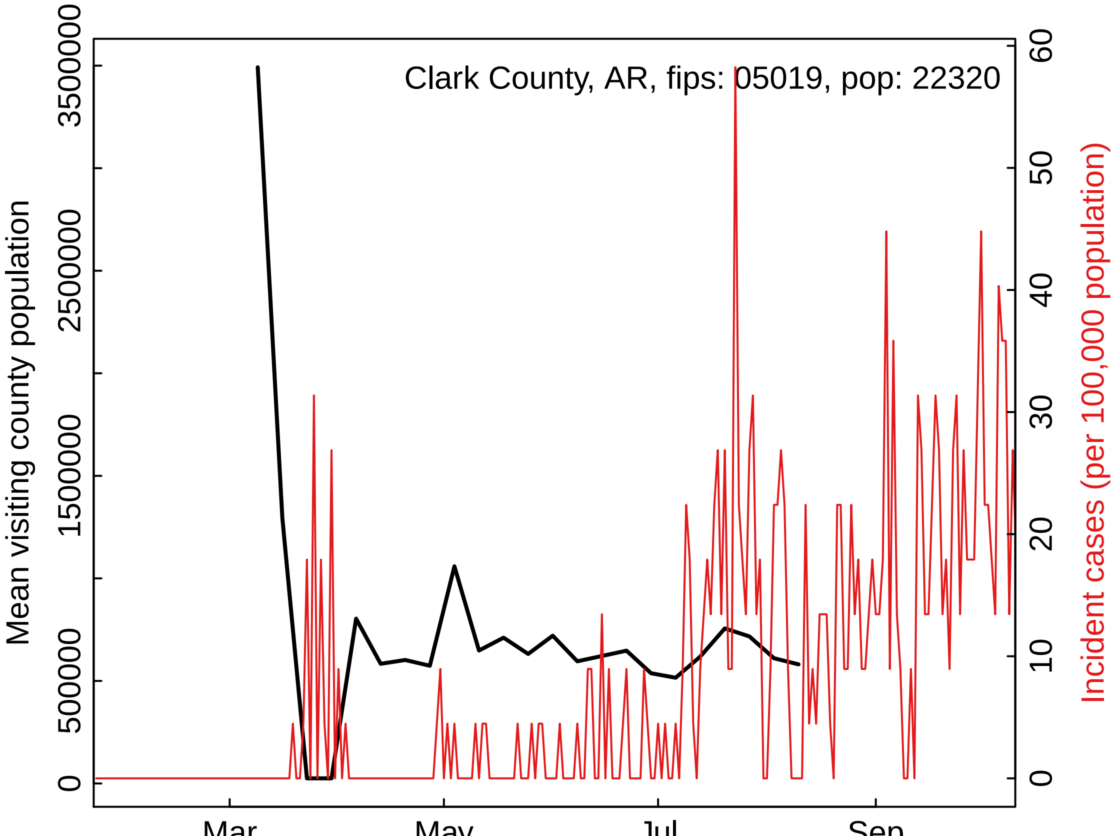

Clark County, AR, fips: 05019, pop: 22320
Mean visiting county population
Incident cases (per 100,000 population)
0
500000
1500000
2500000
3500000
0
10
20
30
40
50
60
Mar
May
Jul
Sep

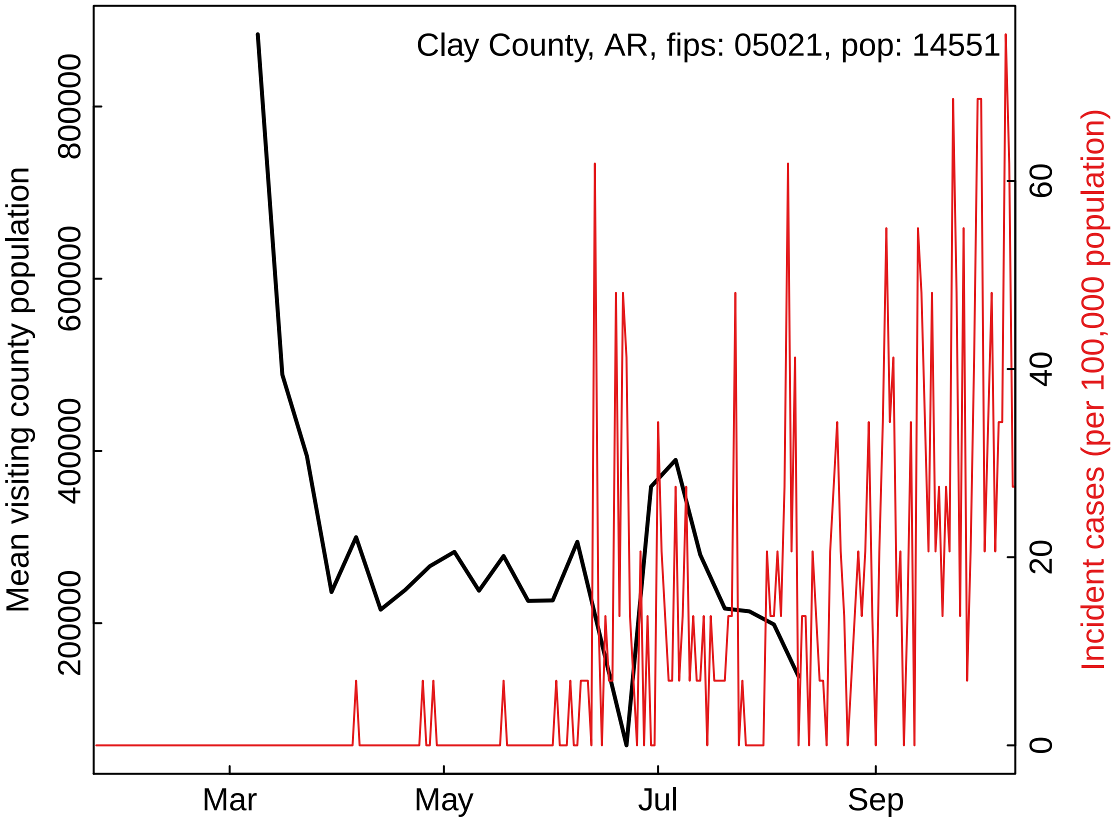

Clay County, AR, fips: 05021, pop: 14551
Mean visiting county population
200000
400000
600000
800000
Incident cases (per 100,000 population)
0
20
40
60
Mar
May
Jul
Sep

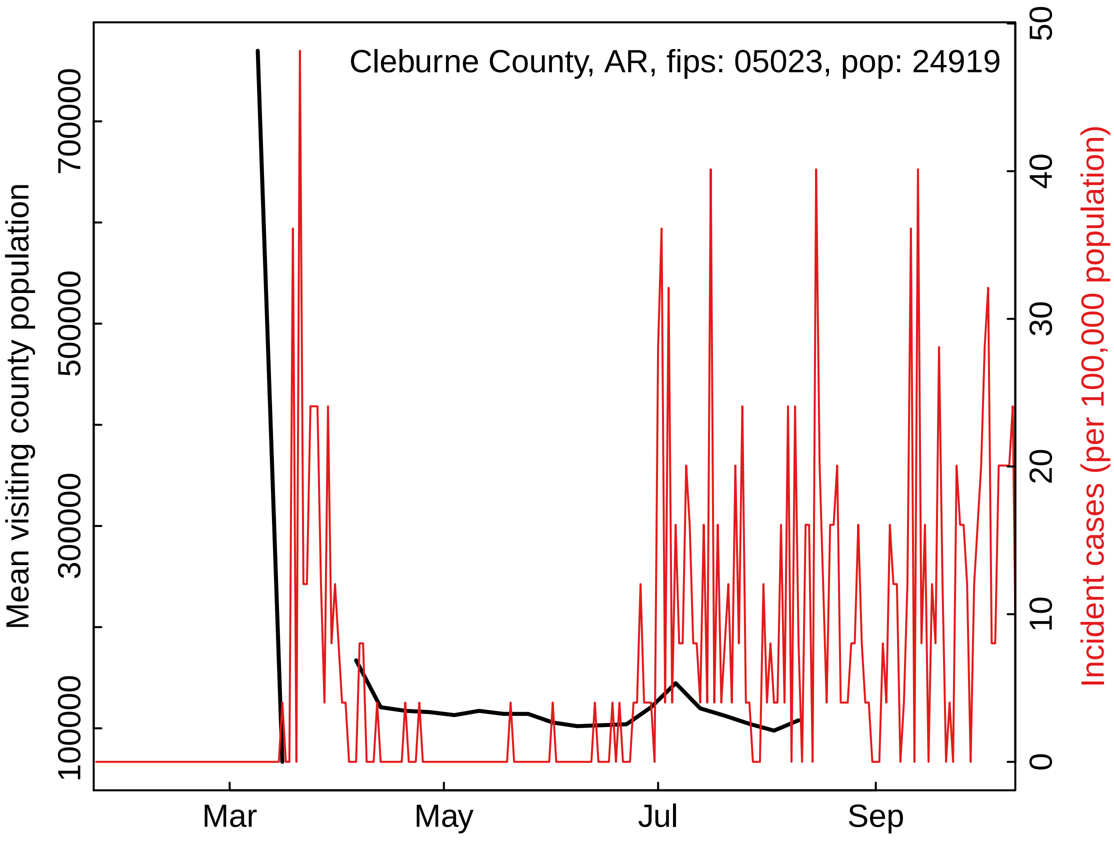

Cleburne County, AR, fips: 05023, pop: 24919
Mean visiting county population
100000
300000
500000
700000
Incident cases (per 100,000 population)
0
10
20
30
40
50
Mar
May
Jul
Sep

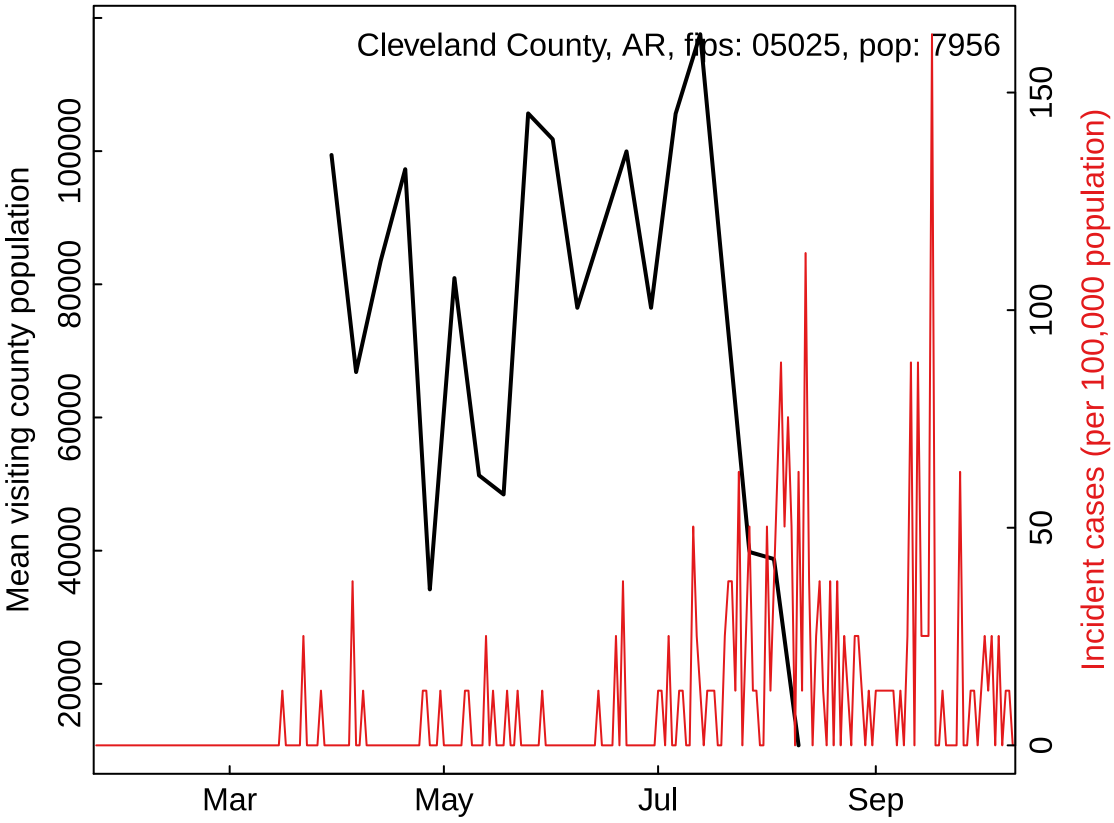

Cleveland County, AR, fips: 05025, pop: 7956
Mean visiting county population
20000
40000
60000
80000
100000
Incident cases (per 100,000 population)
0
50
100
150
Mar
May
Jul
Sep

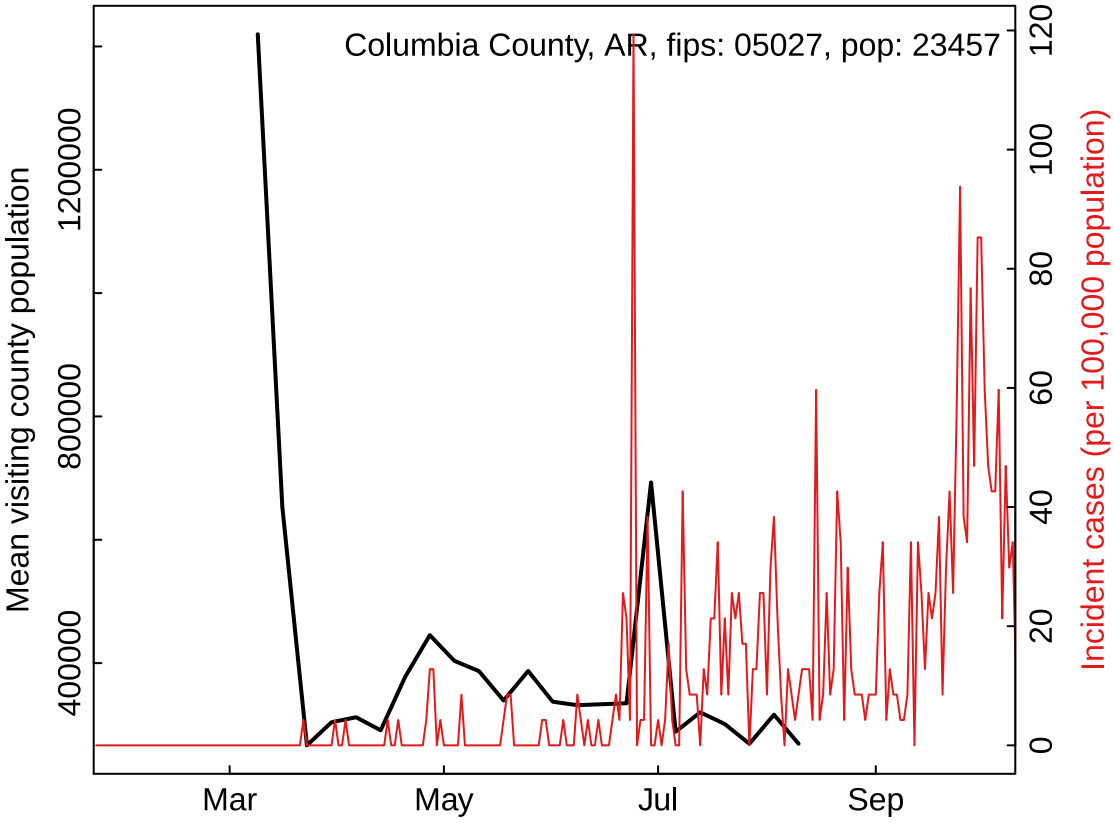

Columbia County, AR, fips: 05027, pop: 23457
Mean visiting county population
400000
800000
1200000
Mar
May
Jul
Sep
0
20
40
60
80
100
120
Incident cases (per 100,000 population)

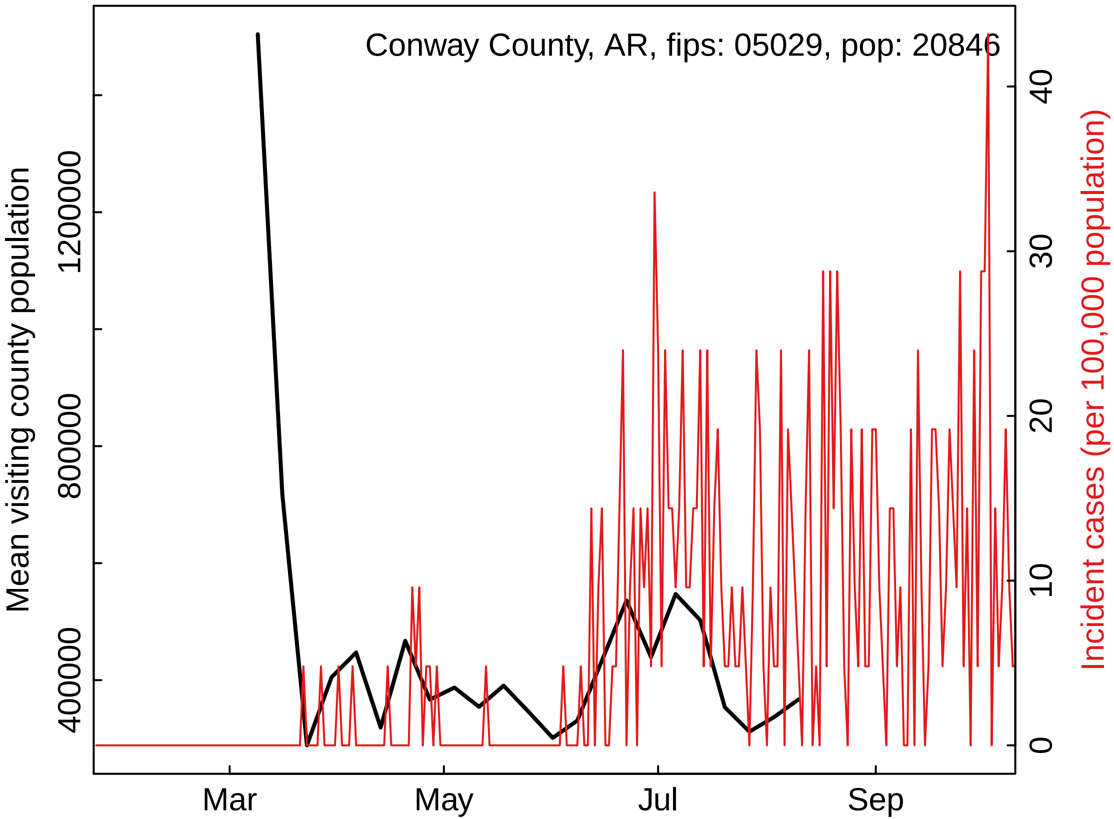

Conway County, AR, fips: 05029, pop: 20846
Mean visiting county population
400000
800000
1200000
Incident cases (per 100,000 population)
0
10
20
30
40
Mar
May
Jul
Sep

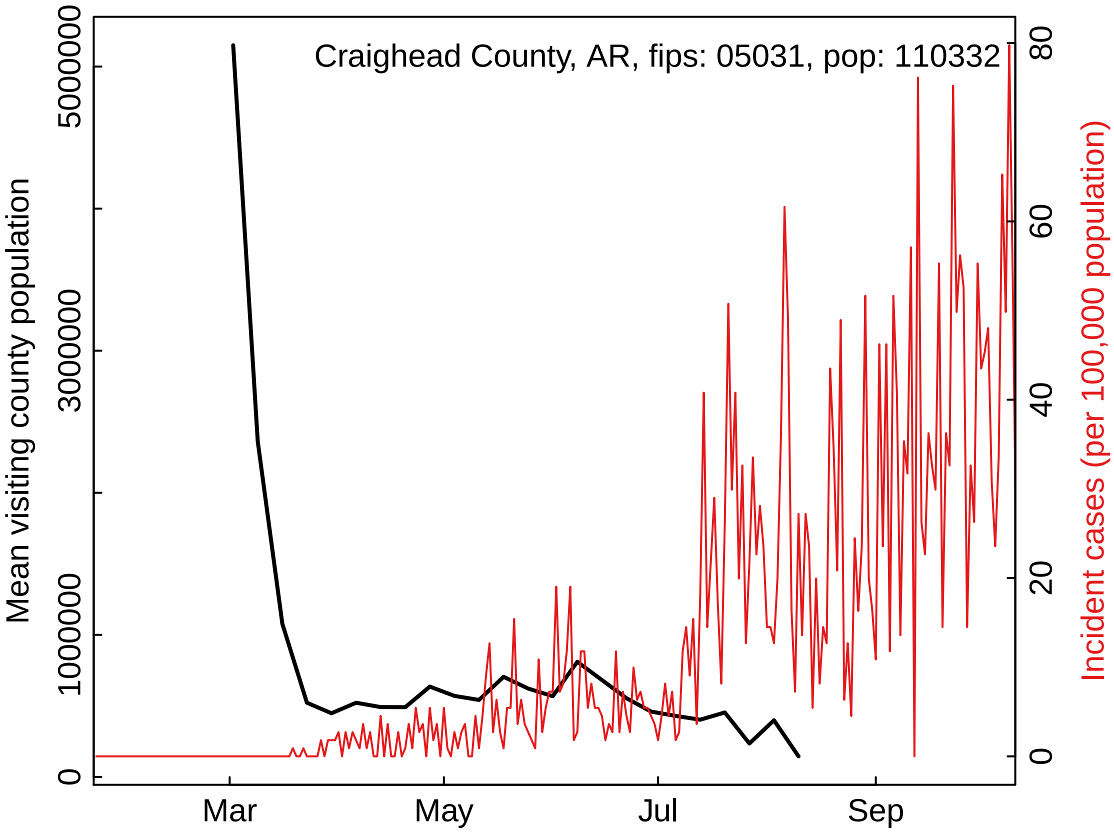
Craighead County, AR, fips: 05031, pop: 110332
Mean visiting county population
0
1000000
3000000
5000000
Incident cases (per 100,000 population)
0
20
40
60
80
Mar
May
Jul
Sep

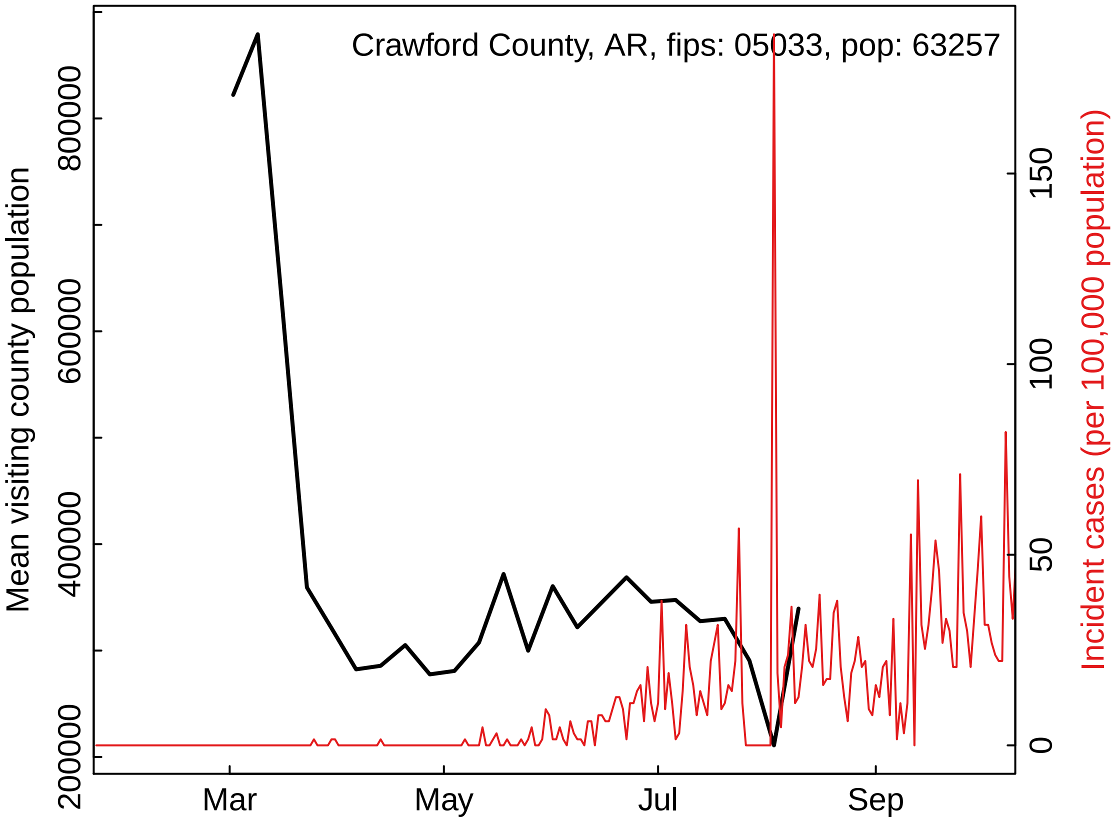

Crawford County, AR, fips: 05033, pop: 63257
Mean visiting county population
200000
400000
600000
800000
Incident cases (per 100,000 population)
0
50
100
150
Mar
May
Jul
Sep

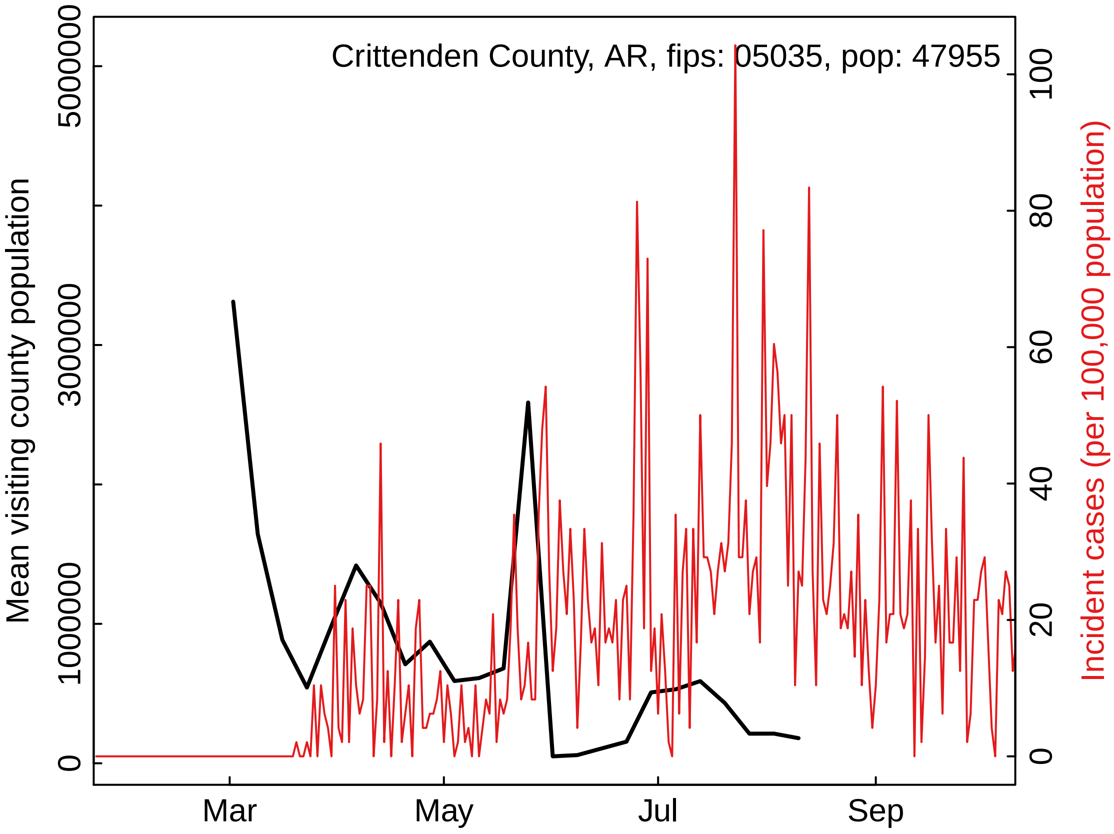

Crittenden County, AR, fips: 05035, pop: 47955
Mean visiting county population
0
1000000
3000000
5000000
Incident cases (per 100,000 population)
0
20
40
60
80
100
Mar
May
Jul
Sep

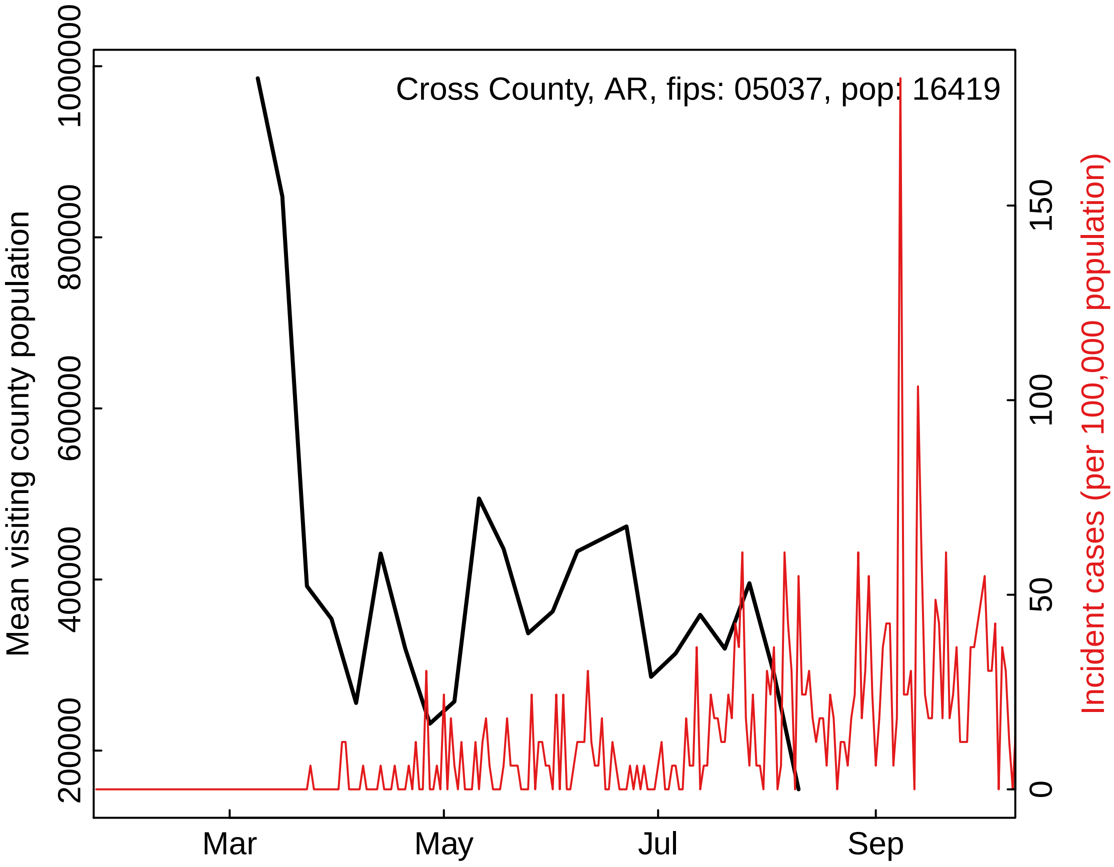

Cross County, AR, fips: 05037, pop: 16419
Mean visiting county population
Incident cases (per 100,000 population)
200000
400000
600000
800000
1000000
0
50
100
150
Mar
May
Jul
Sep

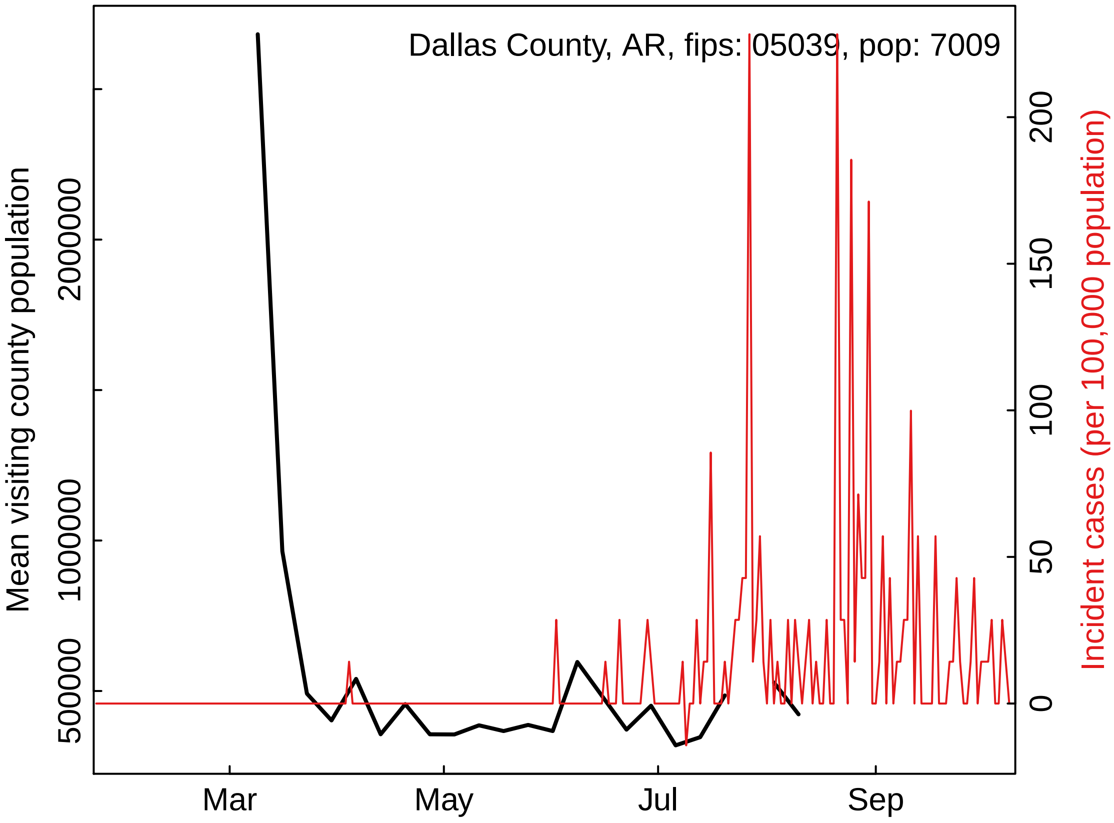

Dallas County, AR, fips: 05039, pop: 7009
Mean visiting county population
500000
1000000
2000000
Incident cases (per 100,000 population)
0
50
100
150
200
Mar
May
Jul
Sep

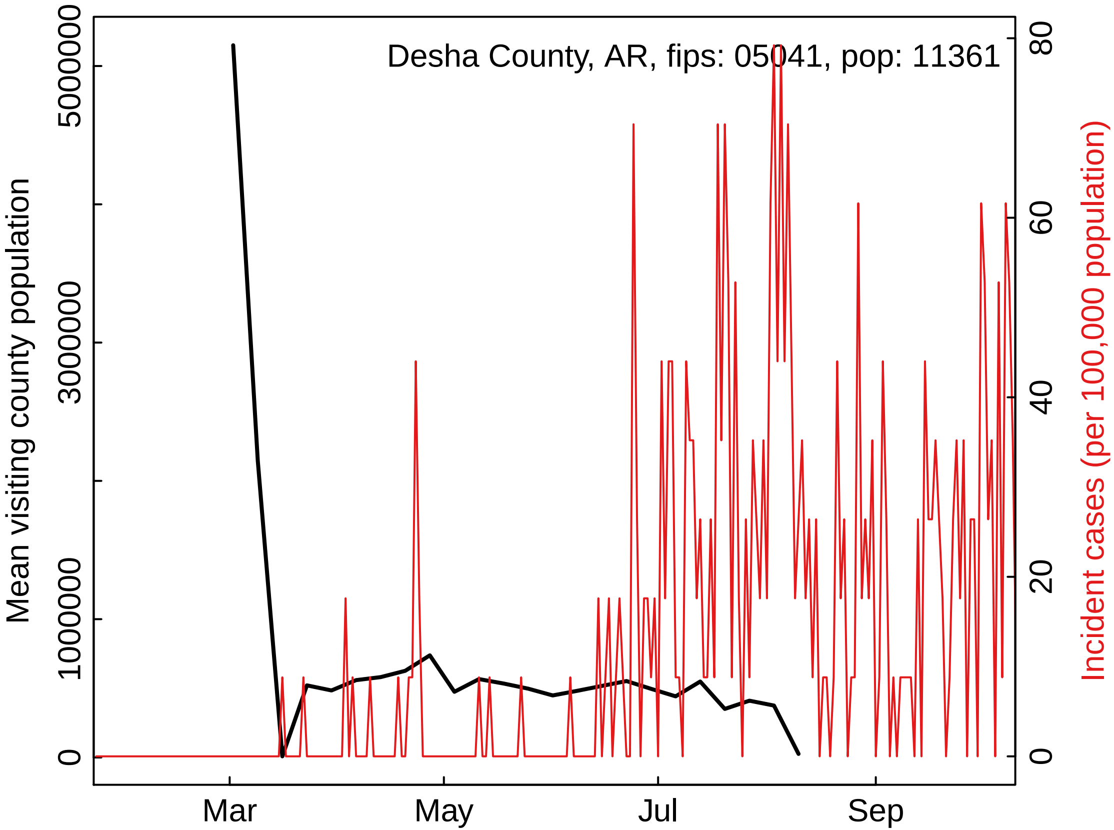
Desha County, AR, fips: 05041, pop: 11361
Mean visiting county population
0
1000000
3000000
5000000
Incident cases (per 100,000 population)
0
20
40
60
80
Mar
May
Jul
Sep

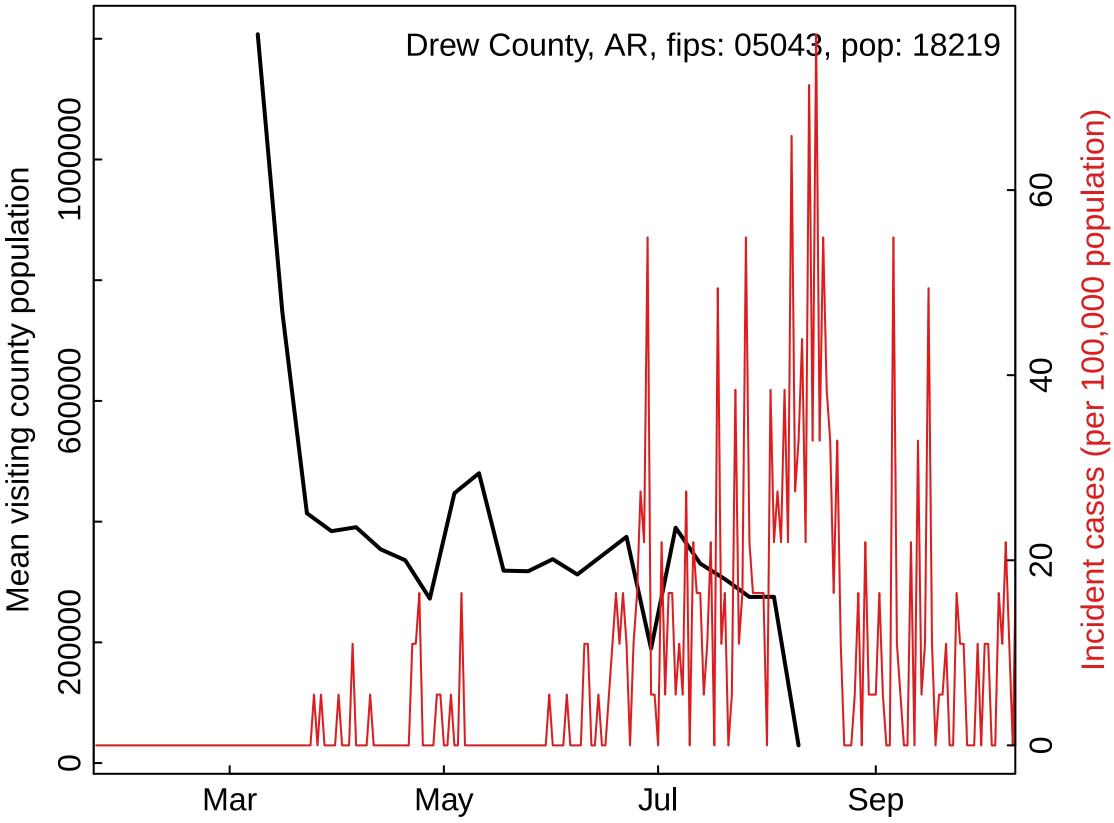

Drew County, AR, fips: 05043, pop: 18219
Mean visiting county population
0
200000
600000
1000000
Incident cases (per 100,000 population)
0
20
40
60
Mar
May
Jul
Sep

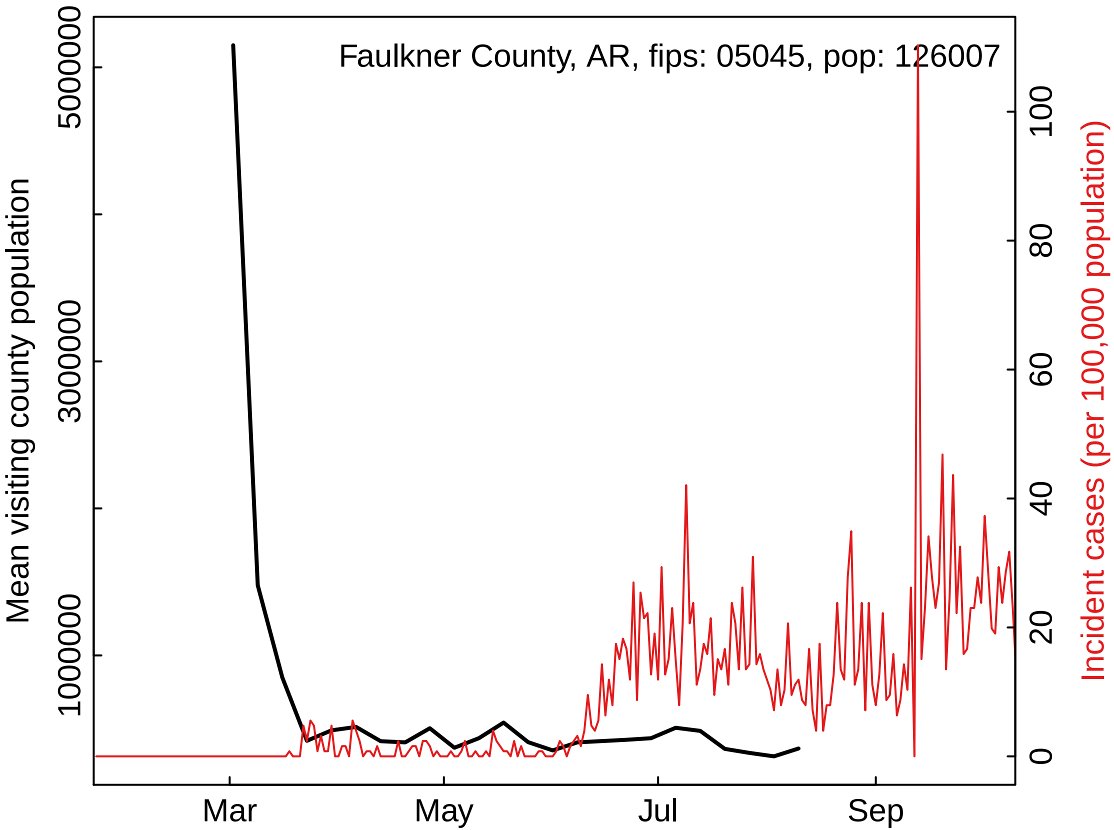

Faulkner County, AR, fips: 05045, pop: 126007
Mean visiting county population
5000000
3000000
1000000
Incident cases (per 100,000 population)
0
20
40
60
80
100
Mar
May
Jul
Sep

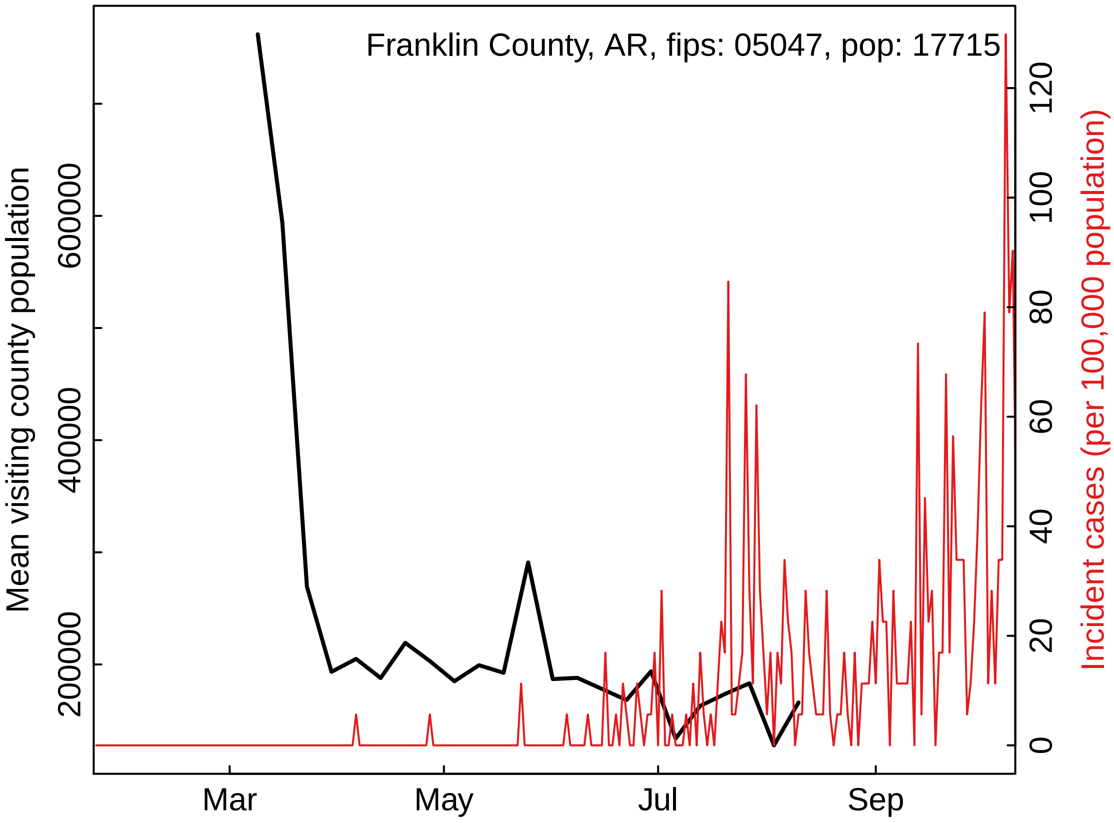

Franklin County, AR, fips: 05047, pop: 17715
Mean visiting county population
200000
400000
600000
Mar
May
Jul
Sep
0
20
40
60
80
100
120
Incident cases (per 100,000 population)

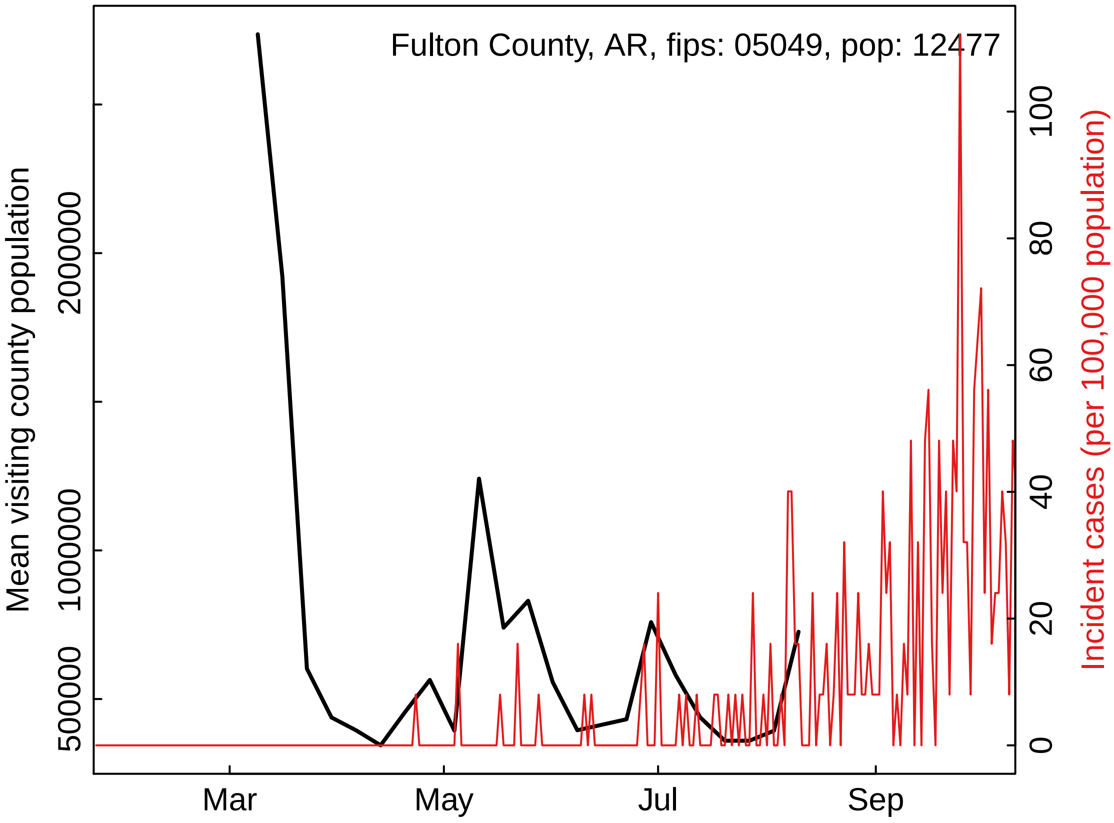
Fulton County, AR, fips: 05049, pop: 12477
Mean visiting county population
500000
1000000
2000000
Incident cases (per 100,000 population)
0
20
40
60
80
100
Mar
May
Jul
Sep

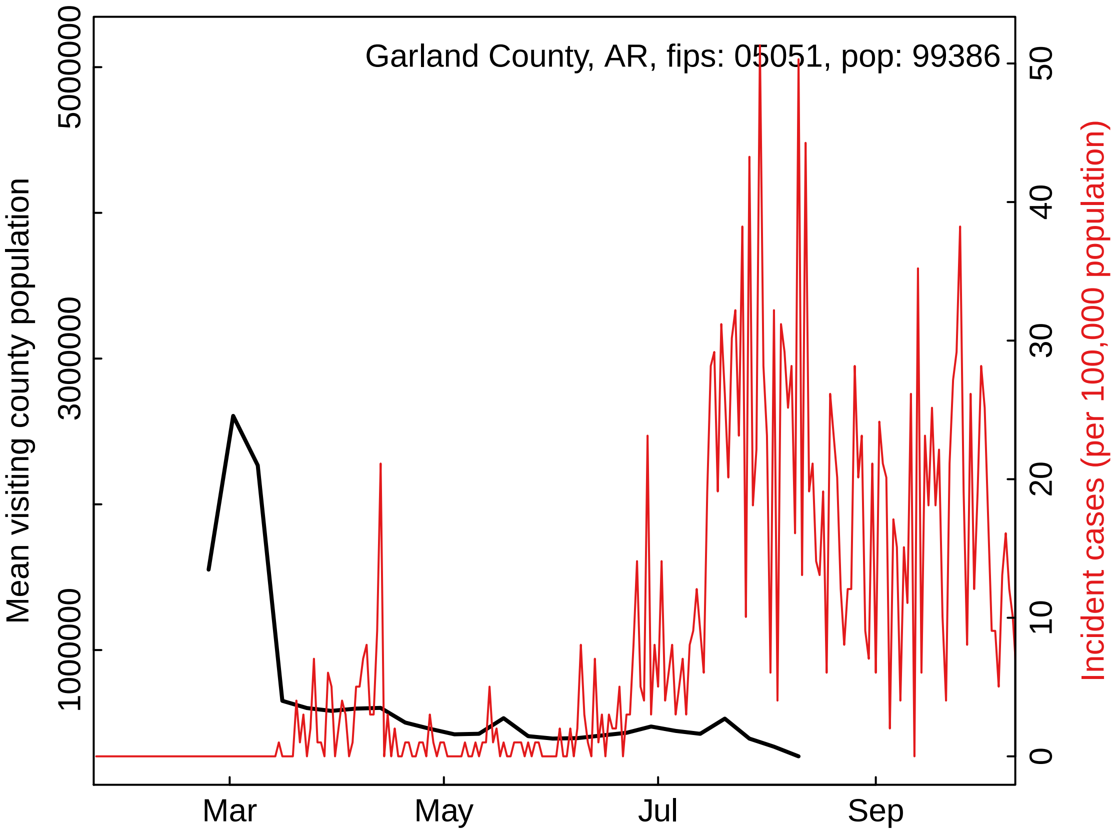

Garland County, AR, fips: 05051, pop: 99386
Mean visiting county population
1000000
3000000
5000000
Incident cases (per 100,000 population)
0
10
20
30
40
50
Mar
May
Jul
Sep

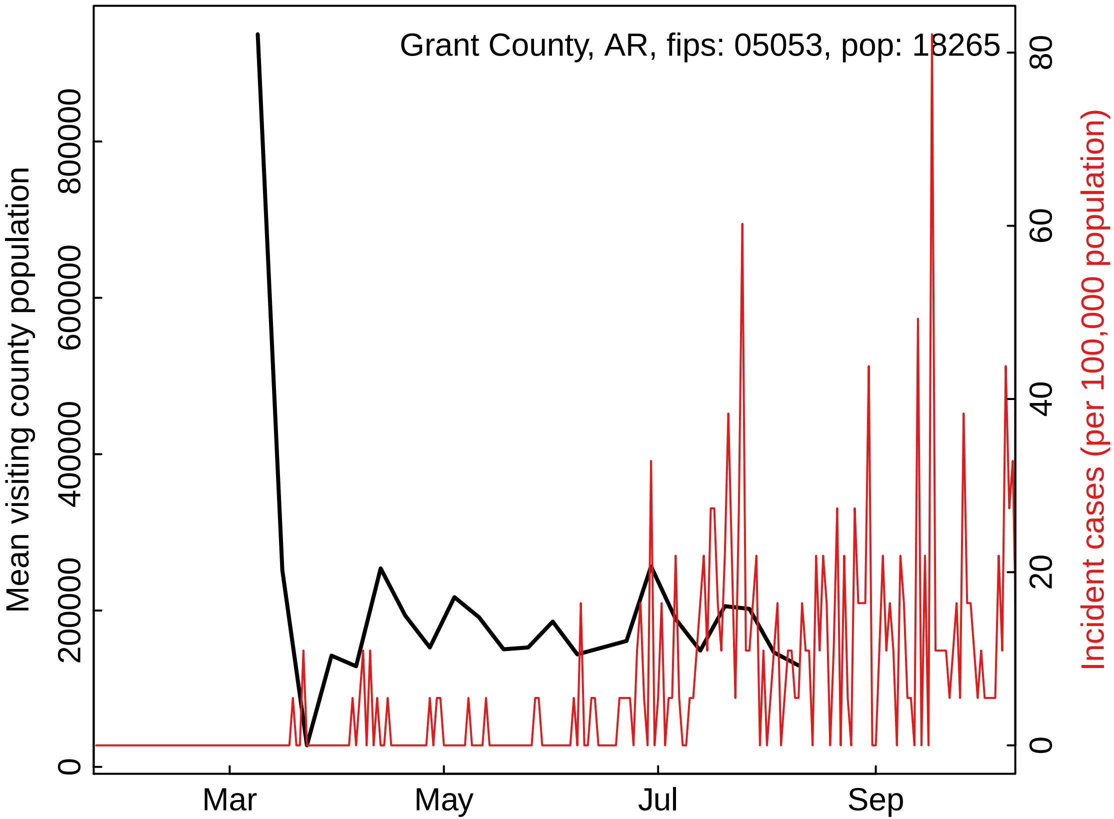
Grant County, AR, fips: 05053, pop: 18265
Mean visiting county population
0
200000
400000
600000
800000
Mar
May
Jul
Sep
Incident cases (per 100,000 population)
0
20
40
60
80

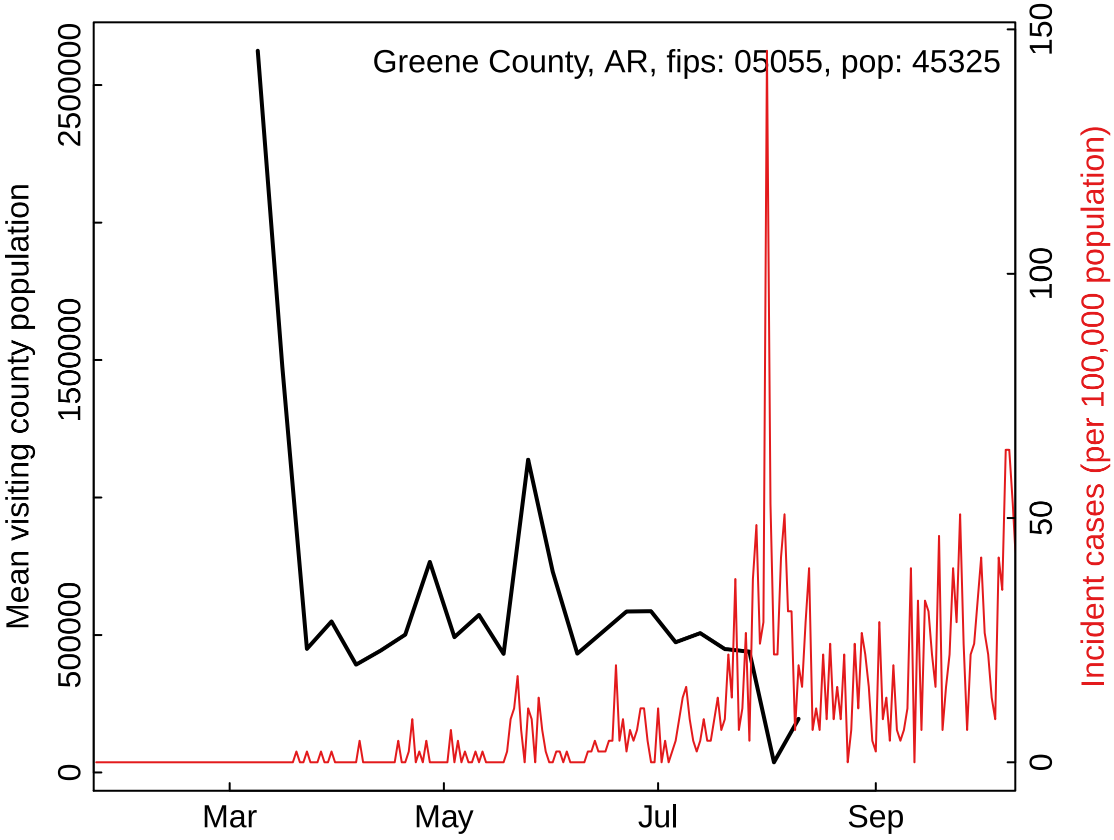
Greene County, AR, fips: 05055, pop: 45325
Mean visiting county population
0
500000
1500000
2500000
Incident cases (per 100,000 population)
0
50
100
150
Mar
May
Jul
Sep

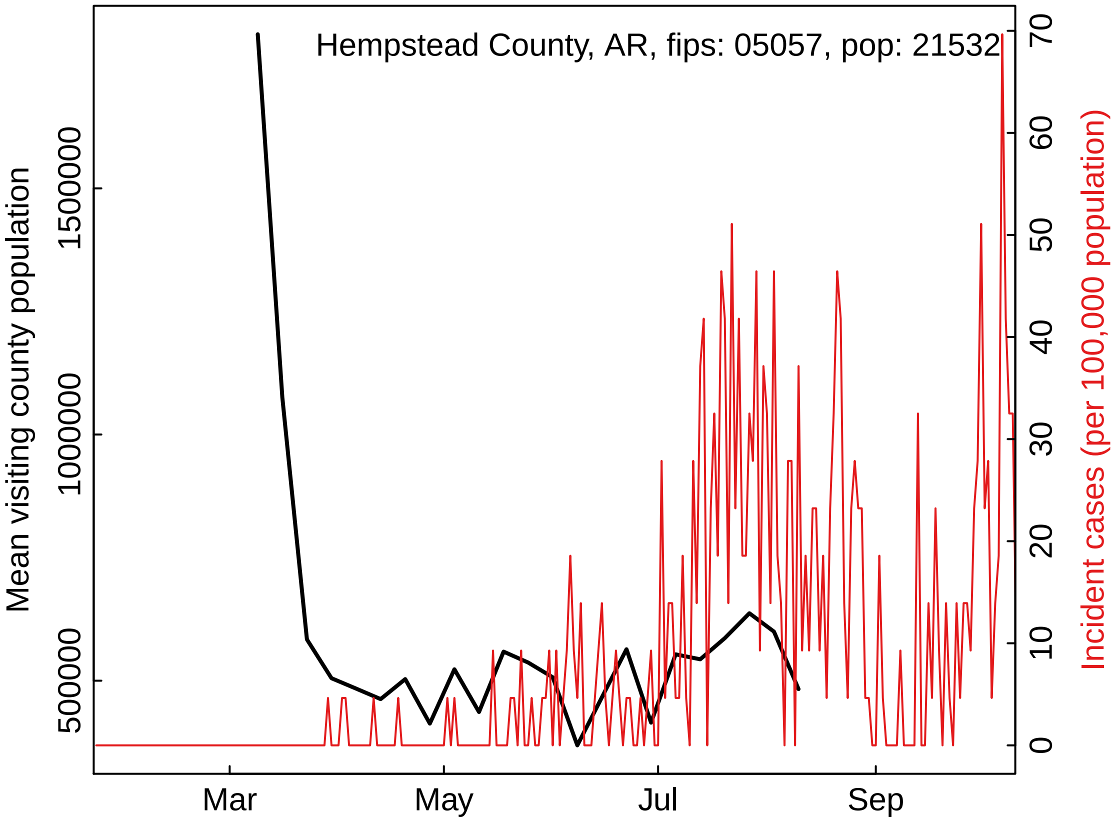
Hempstead County, AR, fips: 05057, pop: 21532
Mean visiting county population
500000
1000000
1500000
Incident cases (per 100,000 population)
0
10
20
30
40
50
60
70
Mar
May
Jul
Sep

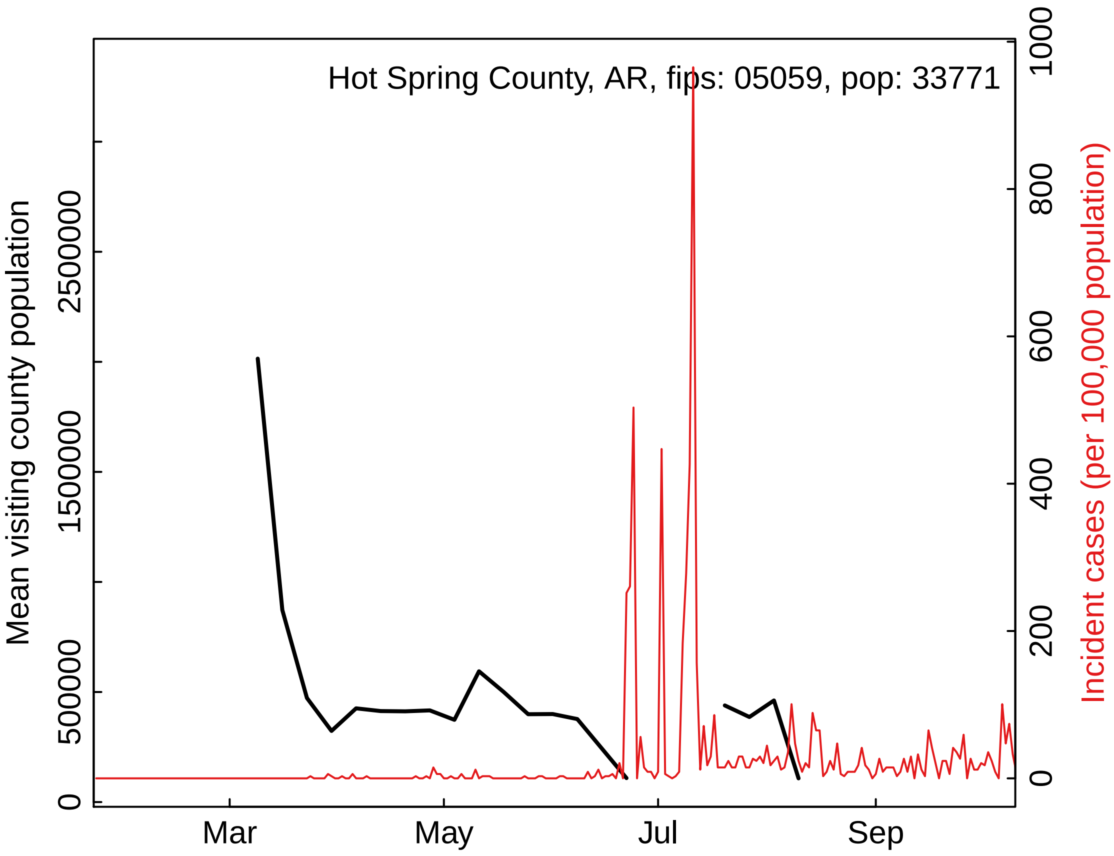
Hot Spring County, AR, fips: 05059, pop: 33771
Mean visiting county population
0
500000
1500000
2500000
Mar
May
Jul
Sep
Incident cases (per 100,000 population)
0
200
400
600
800
1000

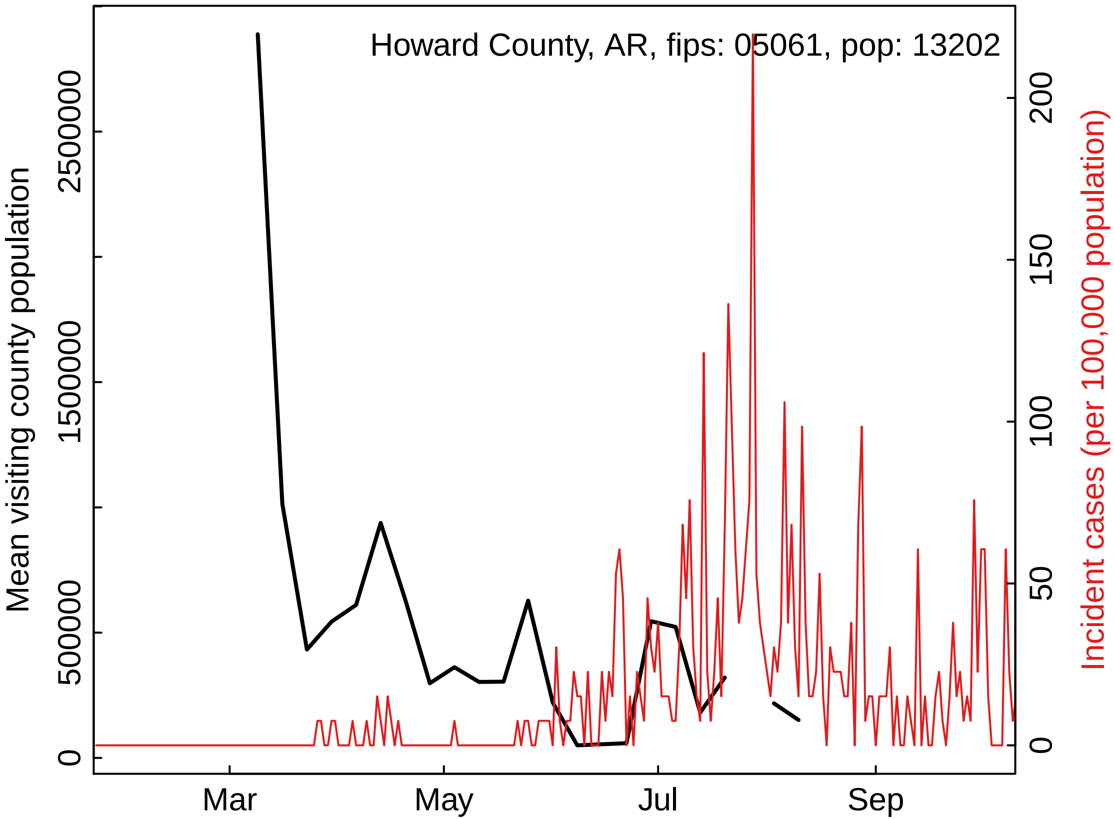
Howard County, AR, fips: 05061, pop: 13202
Mean visiting county population
0
500000
1500000
2500000
Incident cases (per 100,000 population)
0
50
100
150
200
Mar
May
Jul
Sep

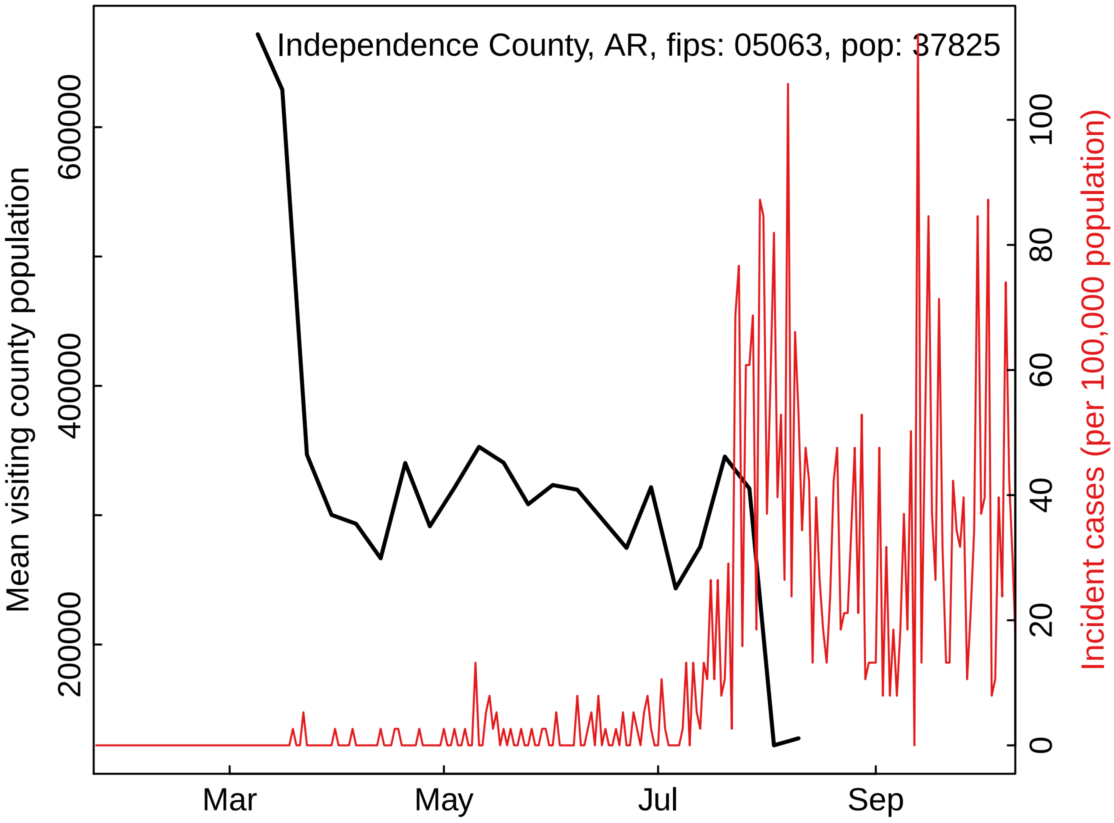

Independence County, AR, fips: 05063, pop: 37825
Mean visiting county population
200000
400000
600000
Mar
May
Jul
Sep
0
20
40
60
80
100
Incident cases (per 100,000 population)

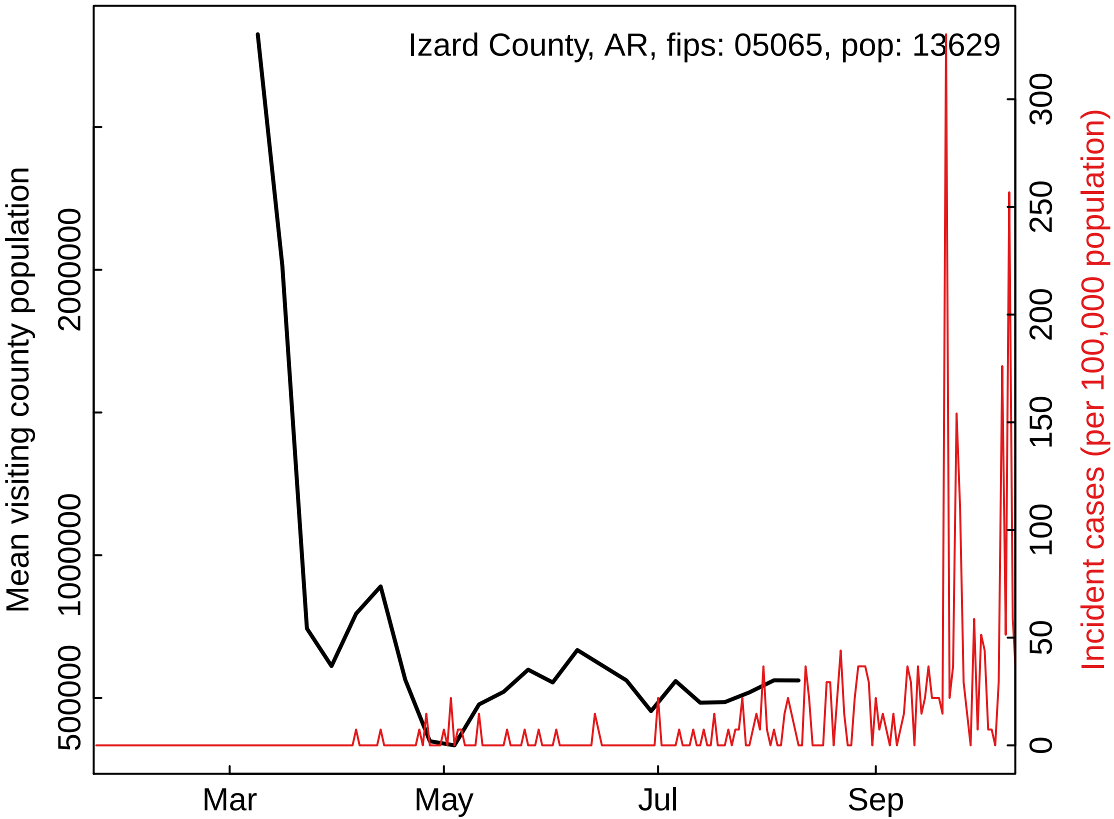

Izard County, AR, fips: 05065, pop: 13629
Mean visiting county population
500000
1000000
2000000
Mar
May
Jul
Sep
0
50
100
150
200
250
300
Incident cases (per 100,000 population)

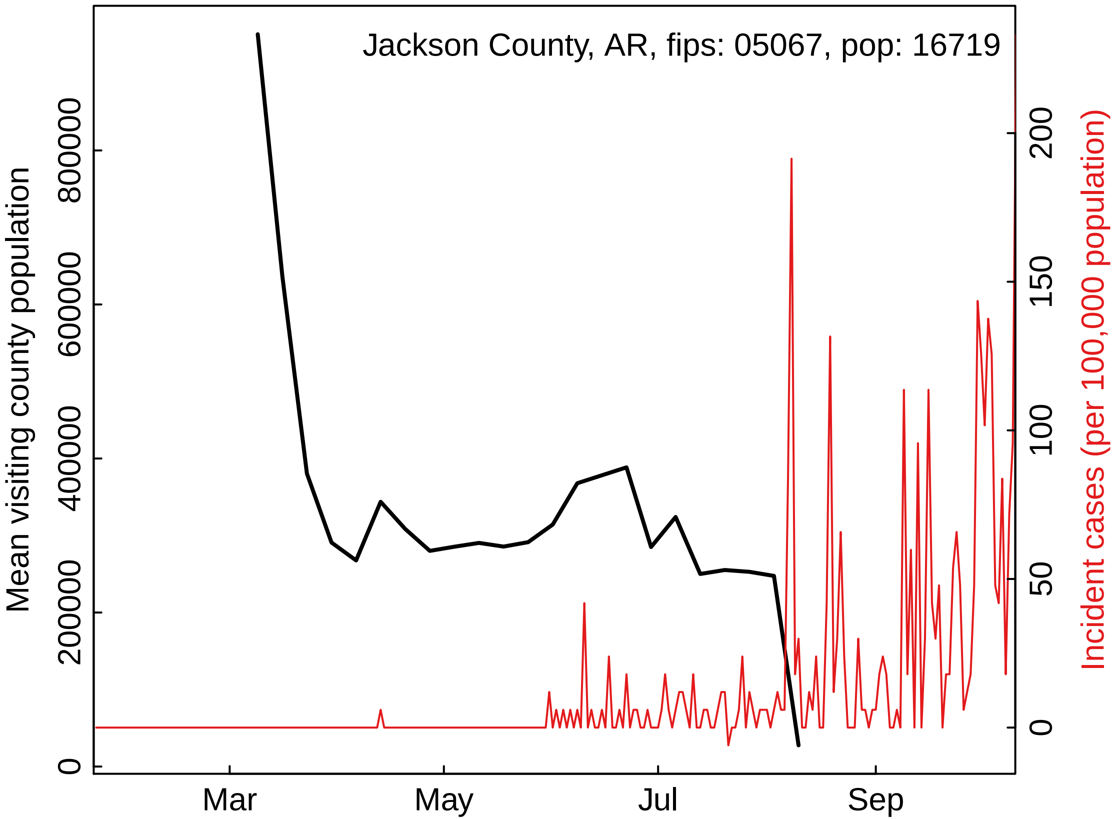

Jackson County, AR, fips: 05067, pop: 16719
Mean visiting county population
0
200000
400000
600000
800000
Mar
May
Jul
Sep
0
50
100
150
200
Incident cases (per 100,000 population)

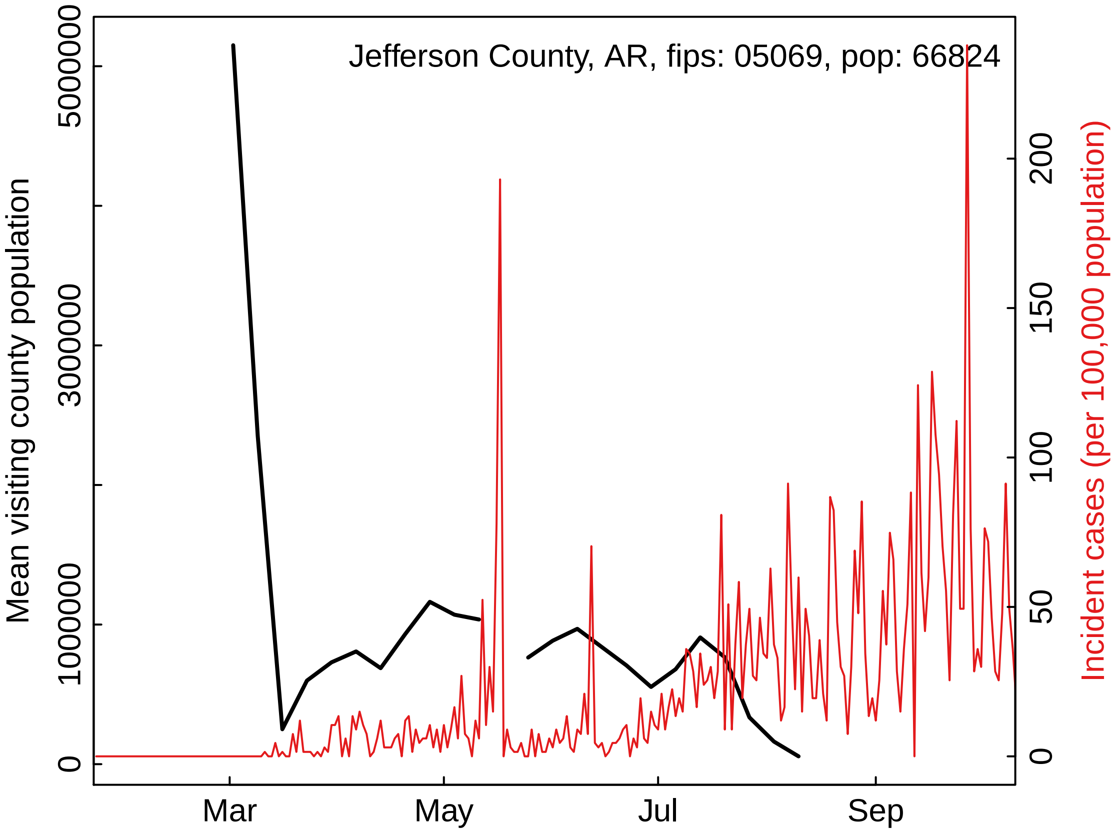

Jefferson County, AR, fips: 05069, pop: 66824
Mean visiting county population
Incident cases (per 100,000 population)
0
1000000
3000000
5000000
0
50
100
150
200
Mar
May
Jul
Sep

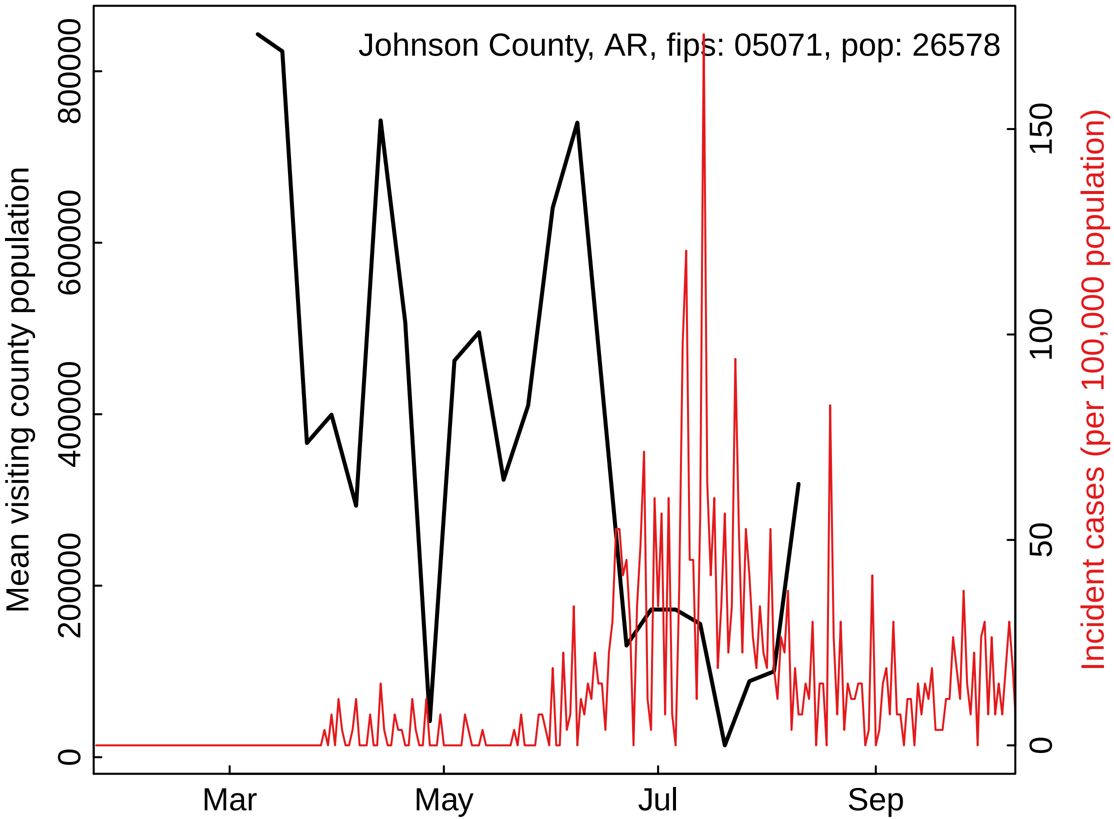

Johnson County, AR, fips: 05071, pop: 26578
Mean visiting county population
0
200000
400000
600000
800000
Incident cases (per 100,000 population)
0
50
100
150
Mar
May
Jul
Sep

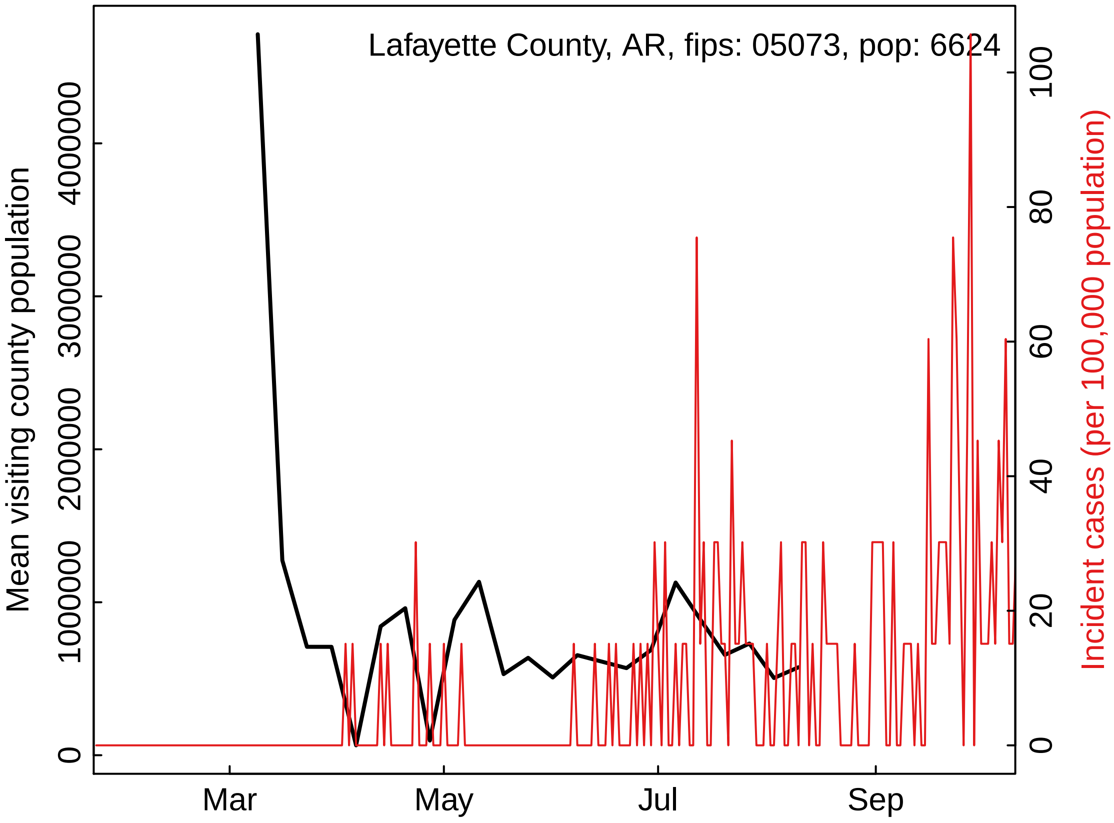

Lafayette County, AR, fips: 05073, pop: 6624
Mean visiting county population
0
1000000
2000000
3000000
4000000
Mar
May
Jul
Sep
0
20
40
60
80
100
Incident cases (per 100,000 population)

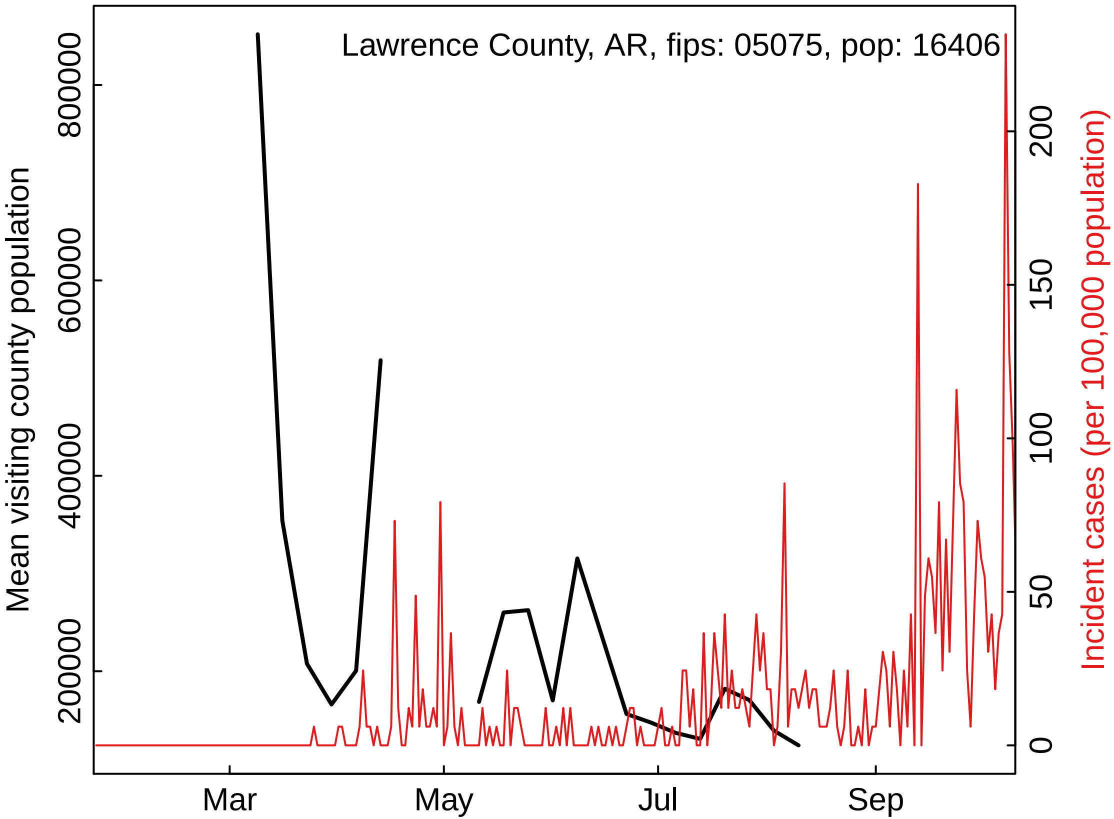

Lawrence County, AR, fips: 05075, pop: 16406
Mean visiting county population
200000
400000
600000
800000
Mar
May
Jul
Sep
0
50
100
150
200
Incident cases (per 100,000 population)

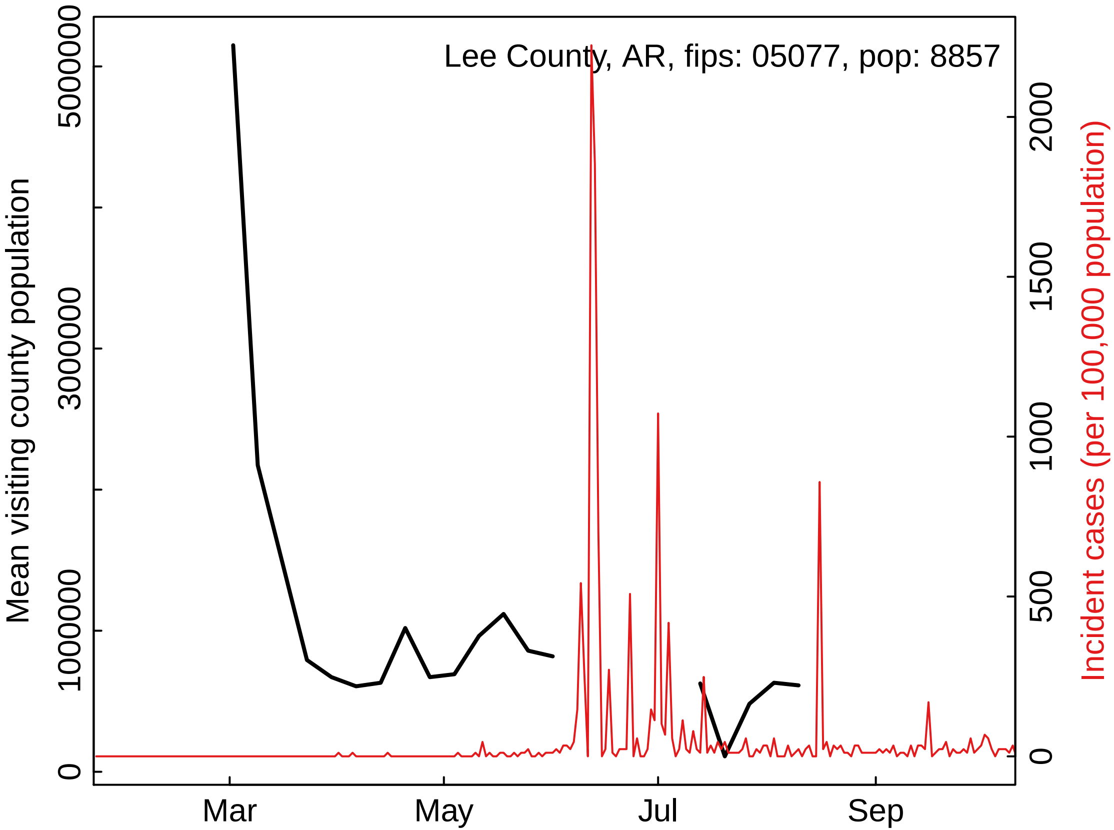

Lee County, AR, fips: 05077, pop: 8857
Mean visiting county population
0
10000000
30000000
50000000
Incident cases (per 100,000 population)
0
500
1000
1500
2000
Mar
May
Jul
Sep

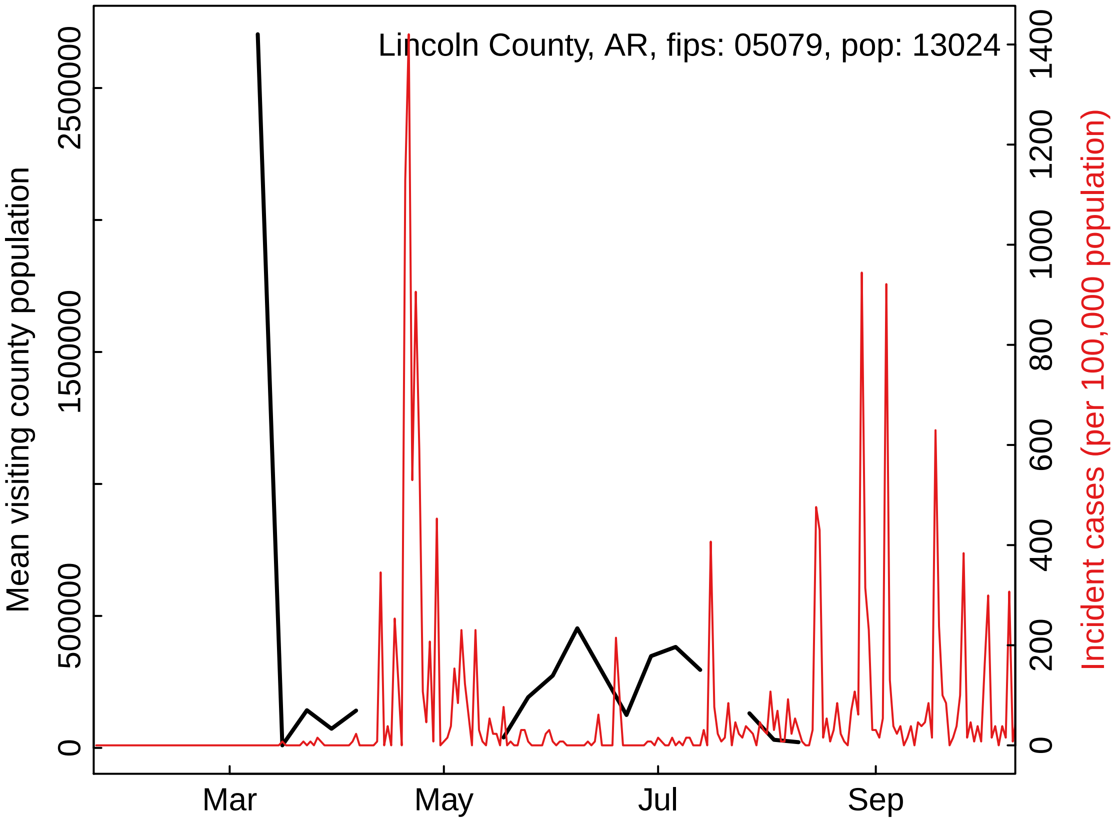

Lincoln County, AR, fips: 05079, pop: 13024
Mean visiting county population
0
500000
1500000
2500000
Incident cases (per 100,000 population)
0
200
400
600
800
1000
1200
1400
Mar
May
Jul
Sep

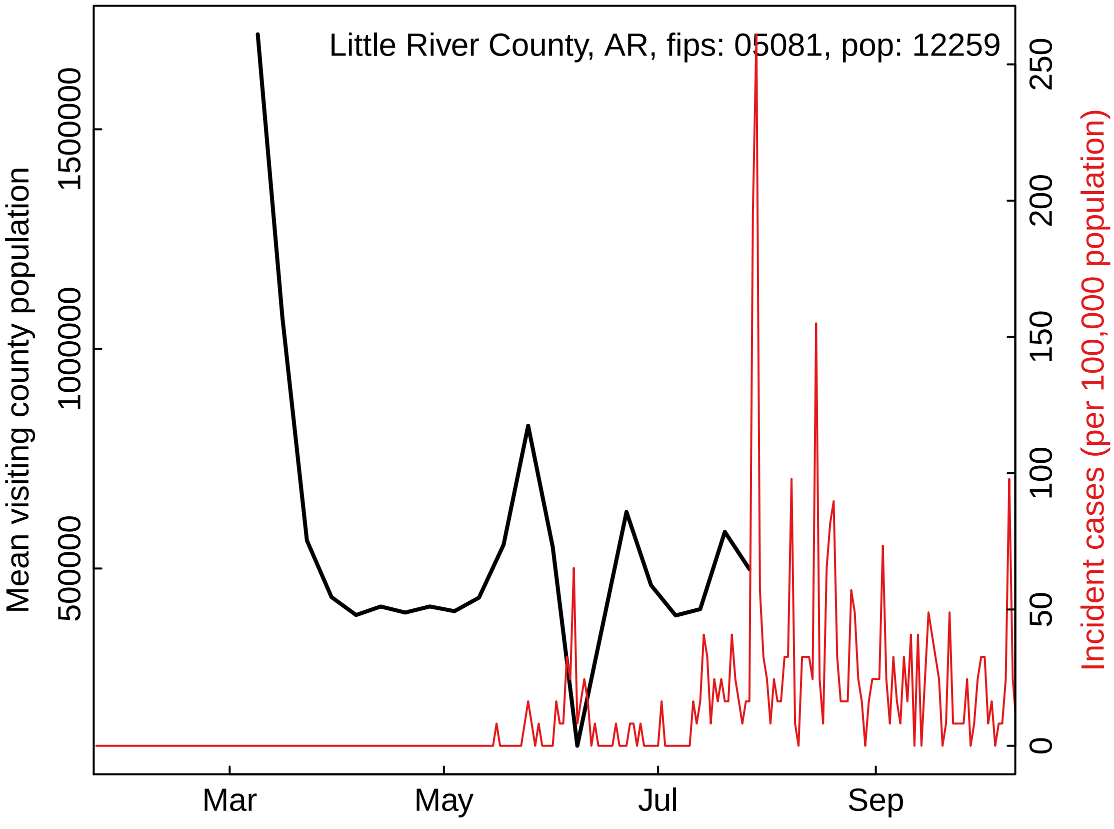

Little River County, AR, fips: 05081, pop: 12259
Mean visiting county population
500000
1000000
1500000
Incident cases (per 100,000 population)
0
50
100
150
200
250
Mar
May
Jul
Sep

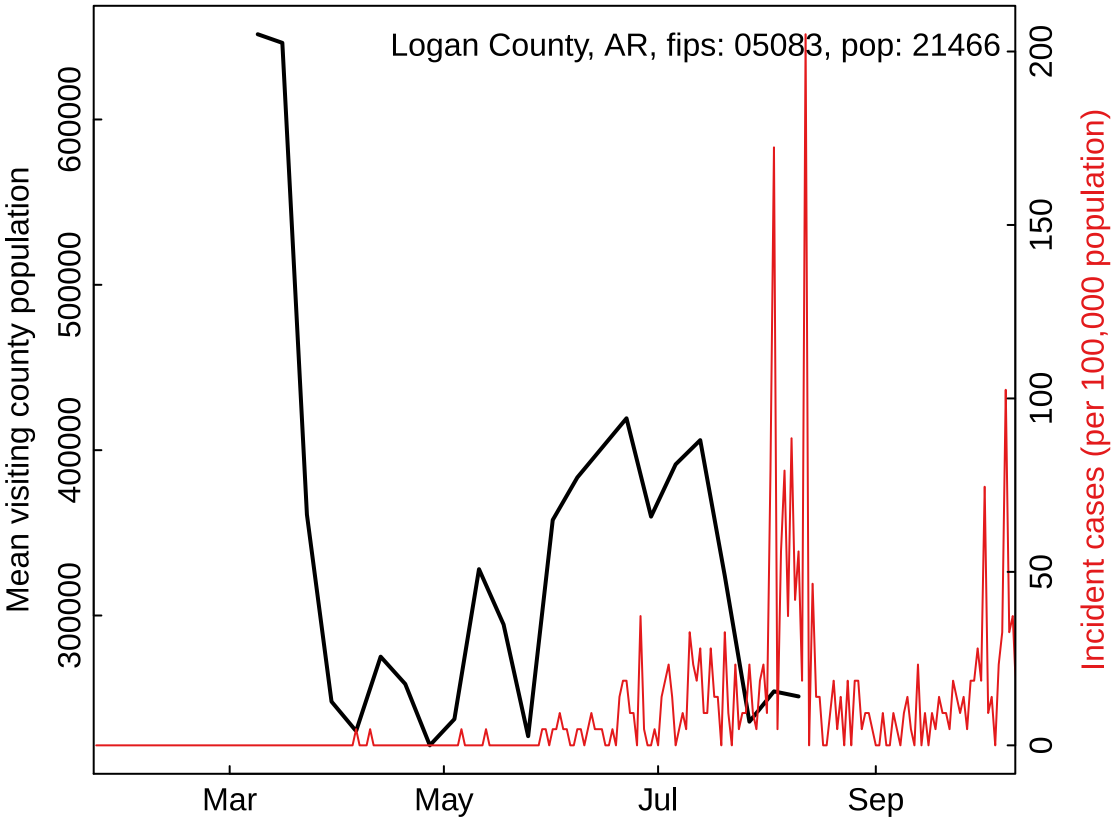
Logan County, AR, fips: 05083, pop: 21466
Mean visiting county population
300000
400000
500000
600000
Incident cases (per 100,000 population)
0
50
100
150
200
Mar
May
Jul
Sep

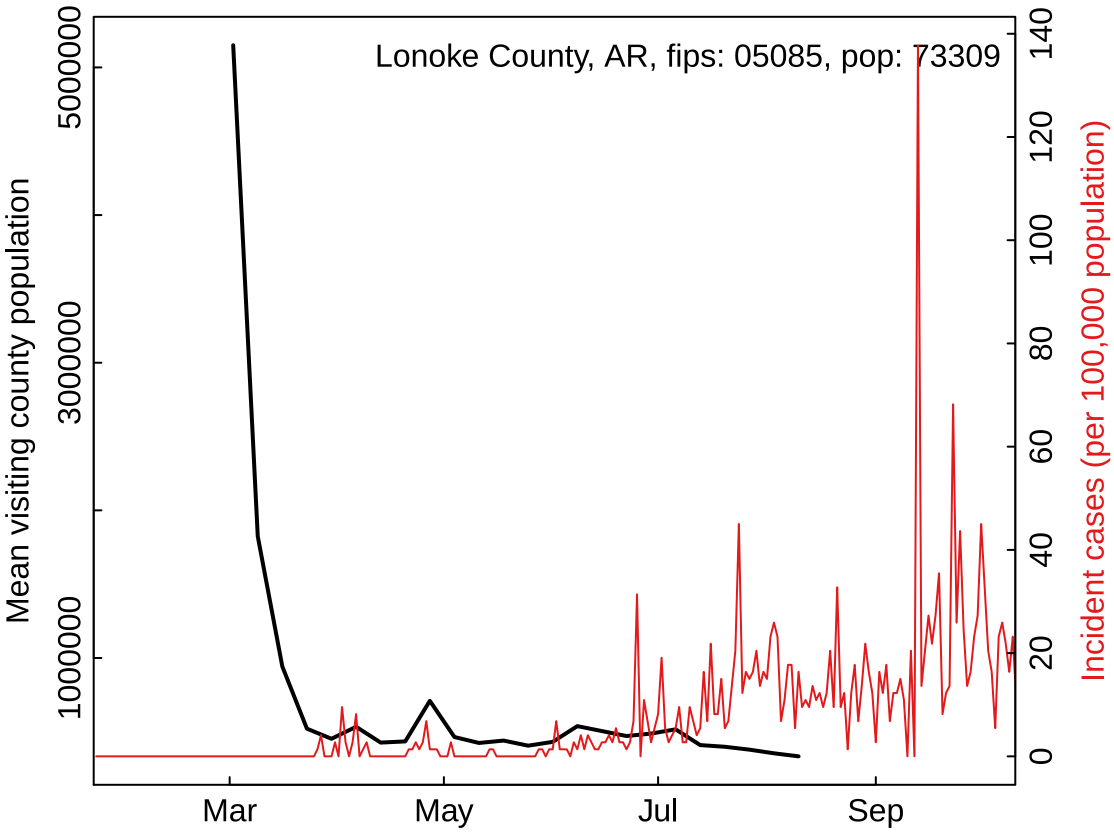

Lonoke County, AR, fips: 05085, pop: 73309
Mean visiting county population
Incident cases (per 100,000 population)
1000000
3000000
5000000
0
20
40
60
80
100
120
140
Mar
May
Jul
Sep

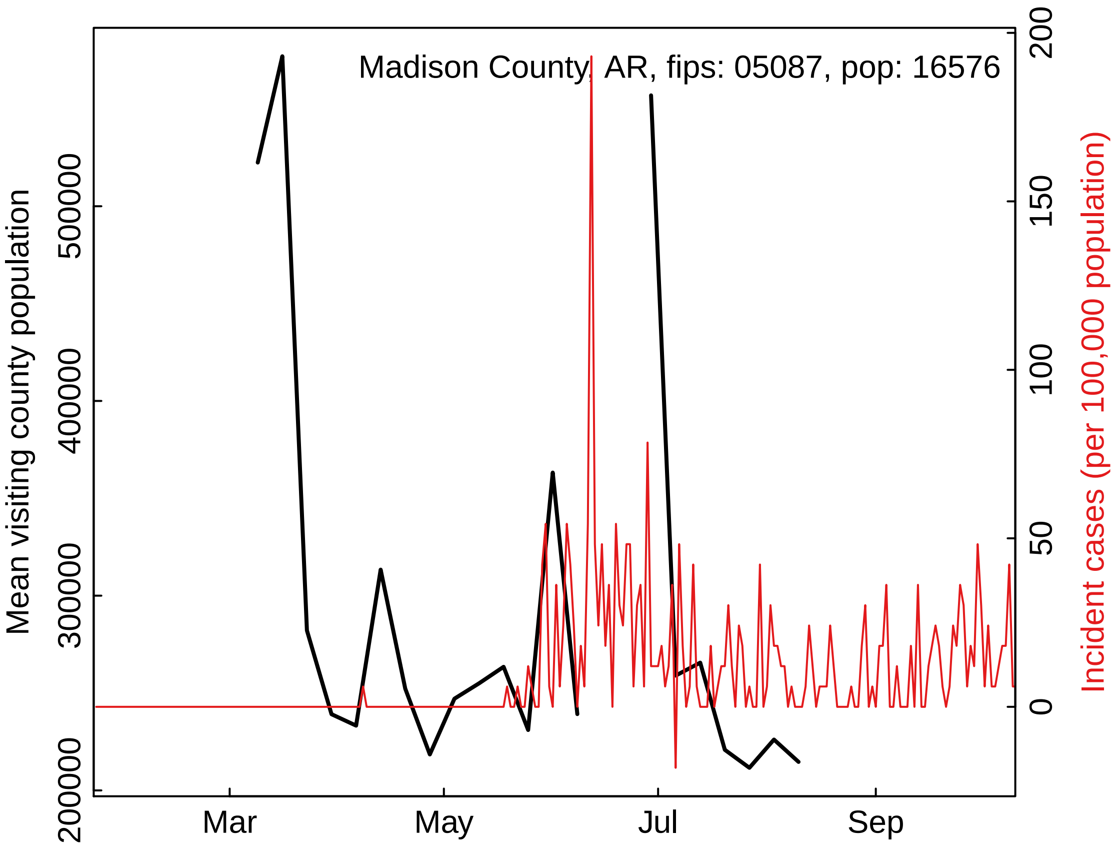

Madison County, AR, fips: 05087, pop: 16576
Mean visiting county population
200000
300000
400000
500000
Mar
May
Jul
Sep
0
50
100
150
200
Incident cases (per 100,000 population)

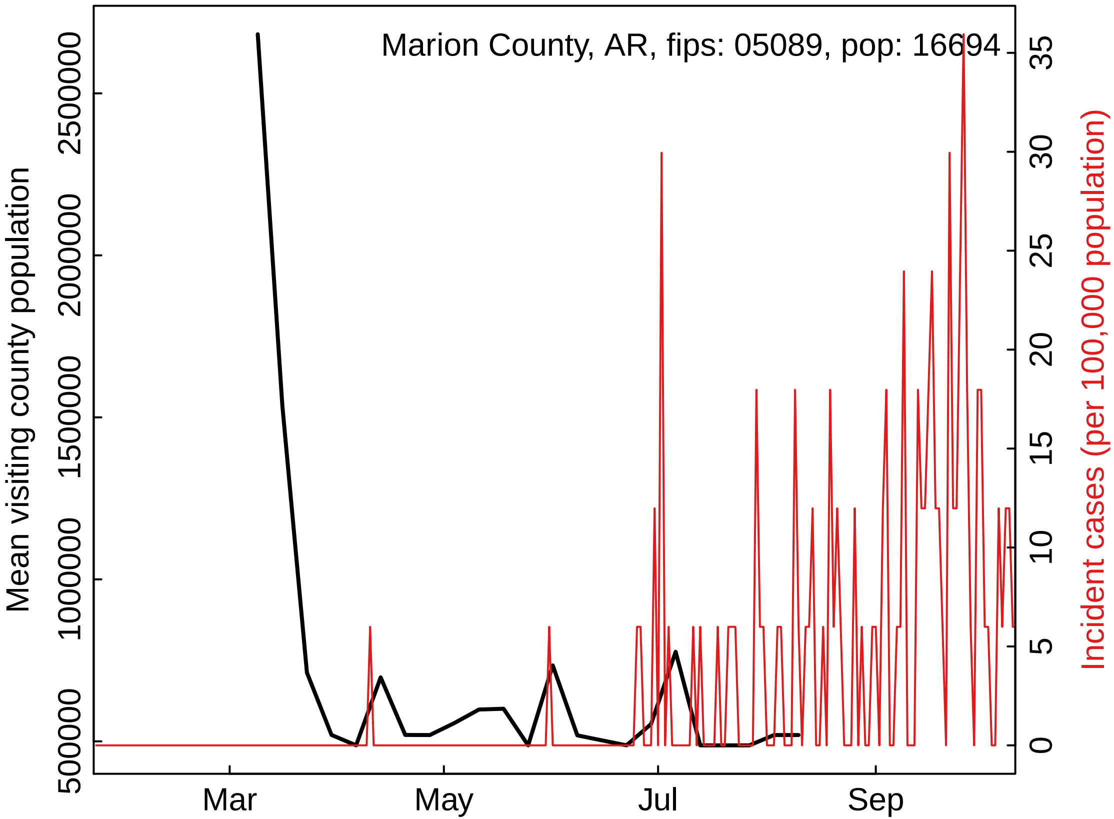

Marion County, AR, fips: 05089, pop: 16694
Mean visiting county population
500000
1000000
1500000
2000000
2500000
Incident cases (per 100,000 population)
0
5
10
15
20
25
30
35
Mar
May
Jul
Sep

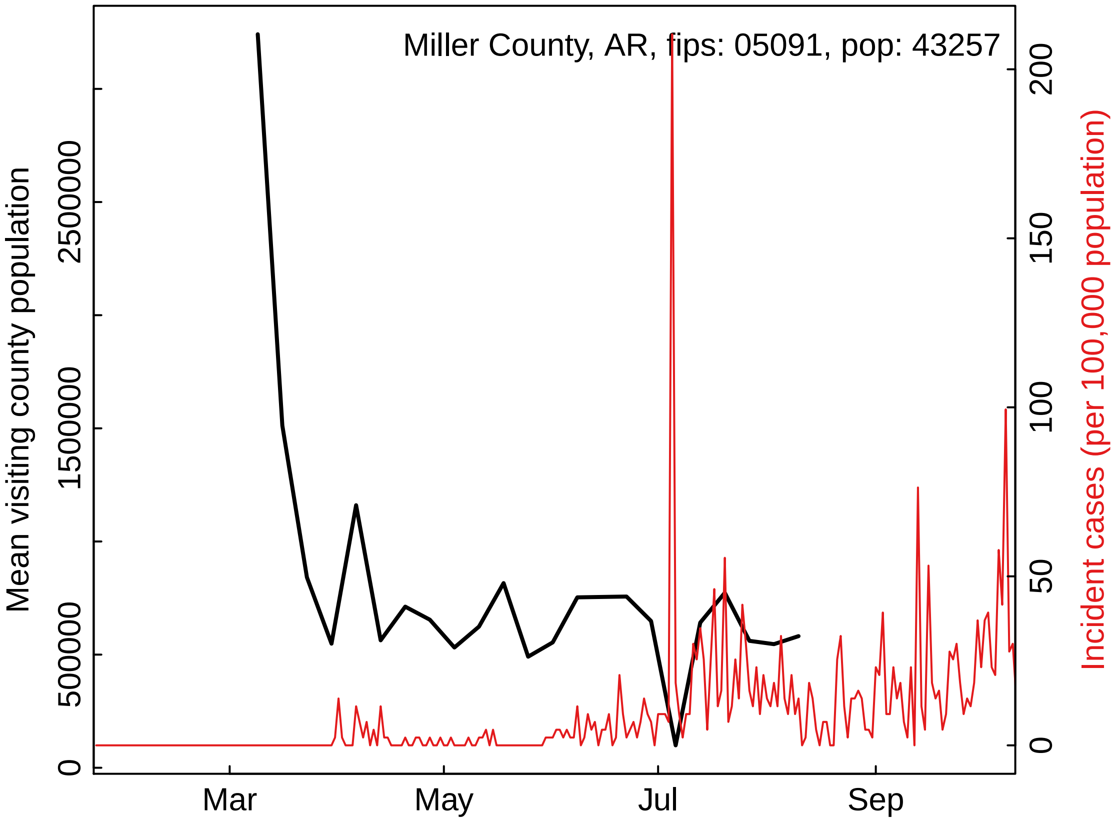
Miller County, AR, fips: 05091, pop: 43257
Mean visiting county population
0
500000
1500000
2500000
Incident cases (per 100,000 population)
0
50
100
150
200
Mar
May
Jul
Sep

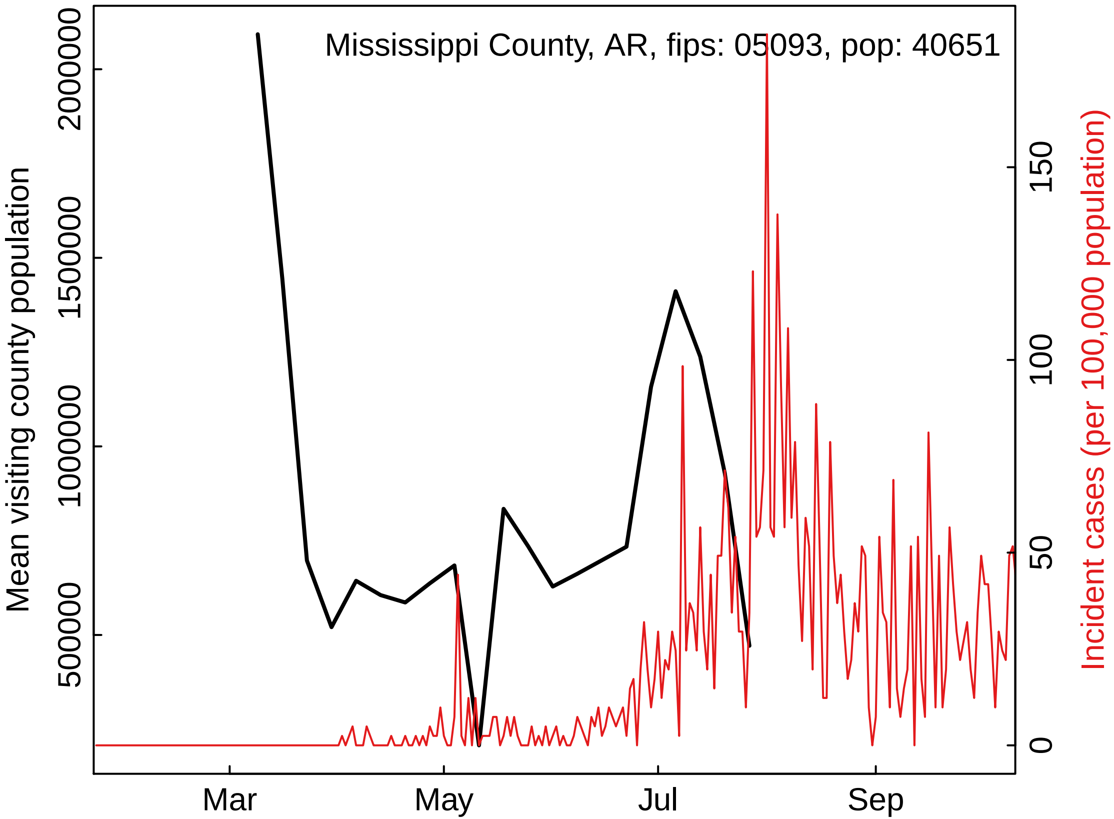
Mississippi County, AR, fips: 05093, pop: 40651
Mean visiting county population
500000
1000000
1500000
2000000
Incident cases (per 100,000 population)
0
50
100
150
Mar
May
Jul
Sep

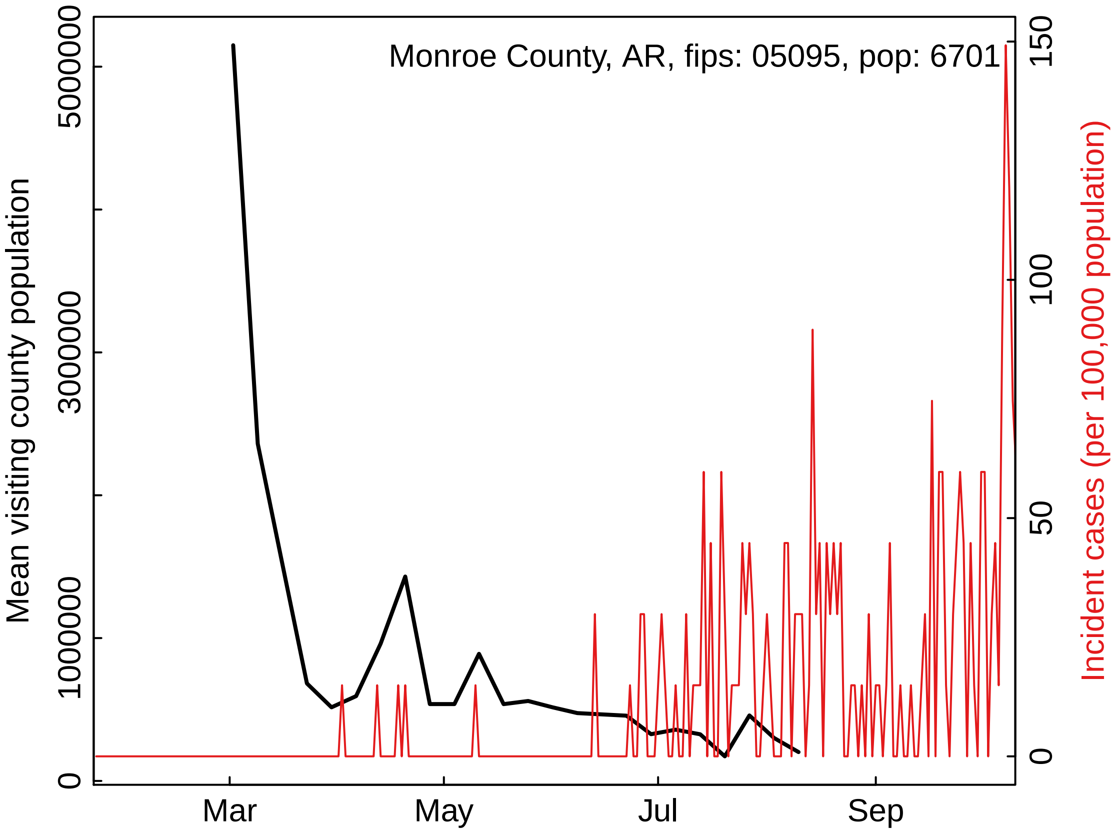

Monroe County, AR, fips: 05095, pop: 6701
Mean visiting county population
0
1000000
3000000
5000000
Incident cases (per 100,000 population)
0
50
100
150
Mar
May
Jul
Sep

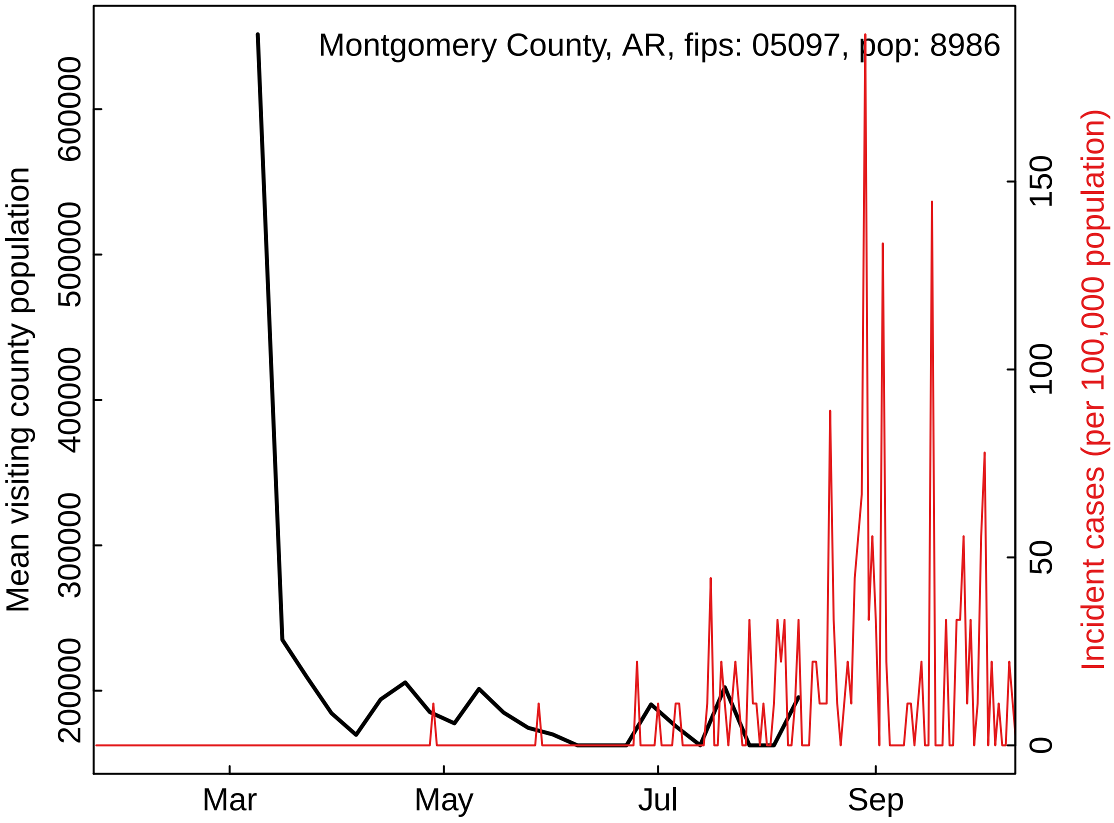

Montgomery County, AR, fips: 05097, pop: 8986
Mean visiting county population
200000
300000
400000
500000
600000
Incident cases (per 100,000 population)
0
50
100
150
Mar
May
Jul
Sep

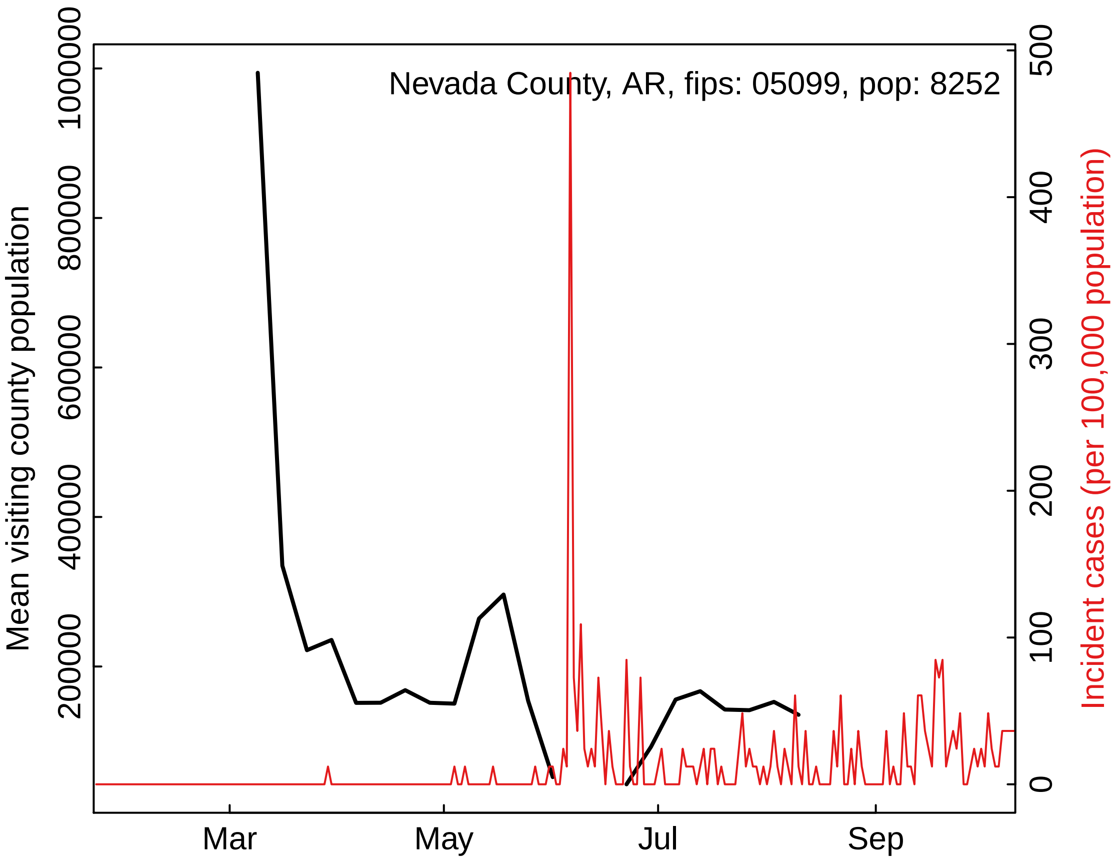

Nevada County, AR, fips: 05099, pop: 8252
Mean visiting county population
200000
400000
600000
800000
1000000
Incident cases (per 100,000 population)
0
100
200
300
400
500
Mar
May
Jul
Sep

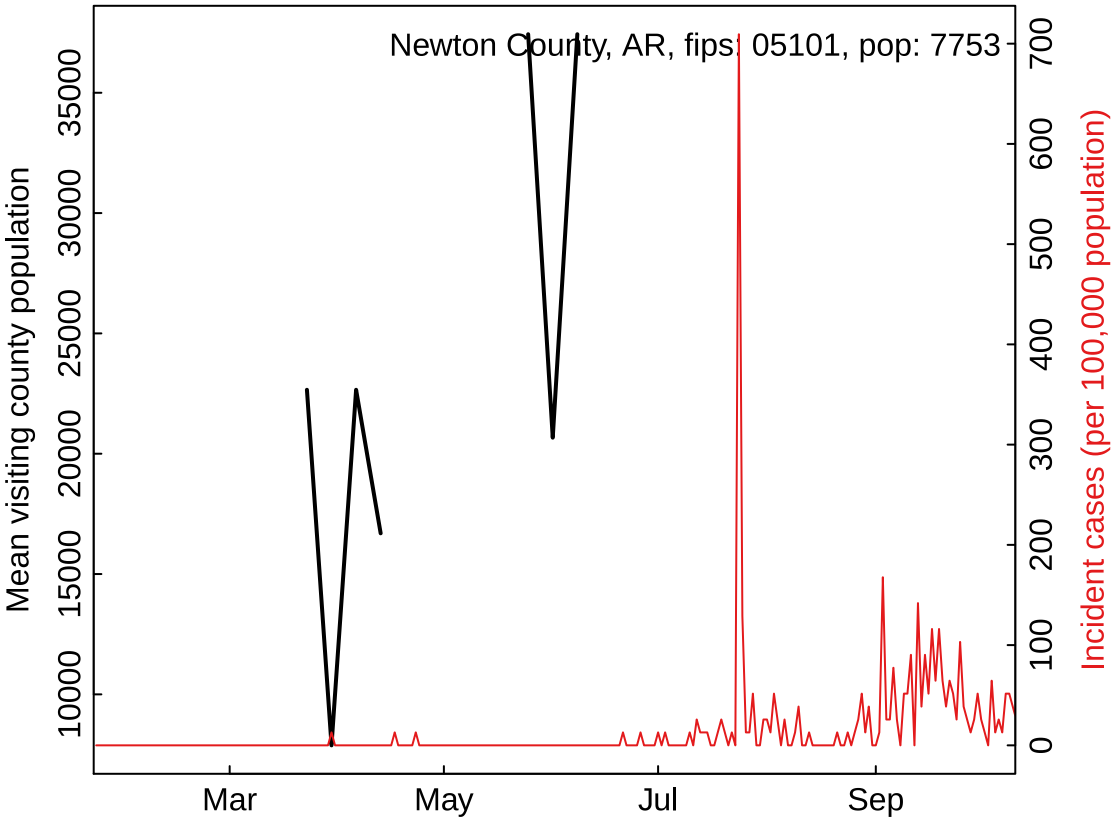
Newton County, AR, fips: 05101, pop: 7753
Mean visiting county population
10000
15000
20000
25000
30000
35000
Incident cases (per 100,000 population)
0
100
200
300
400
500
600
700
Mar
May
Jul
Sep

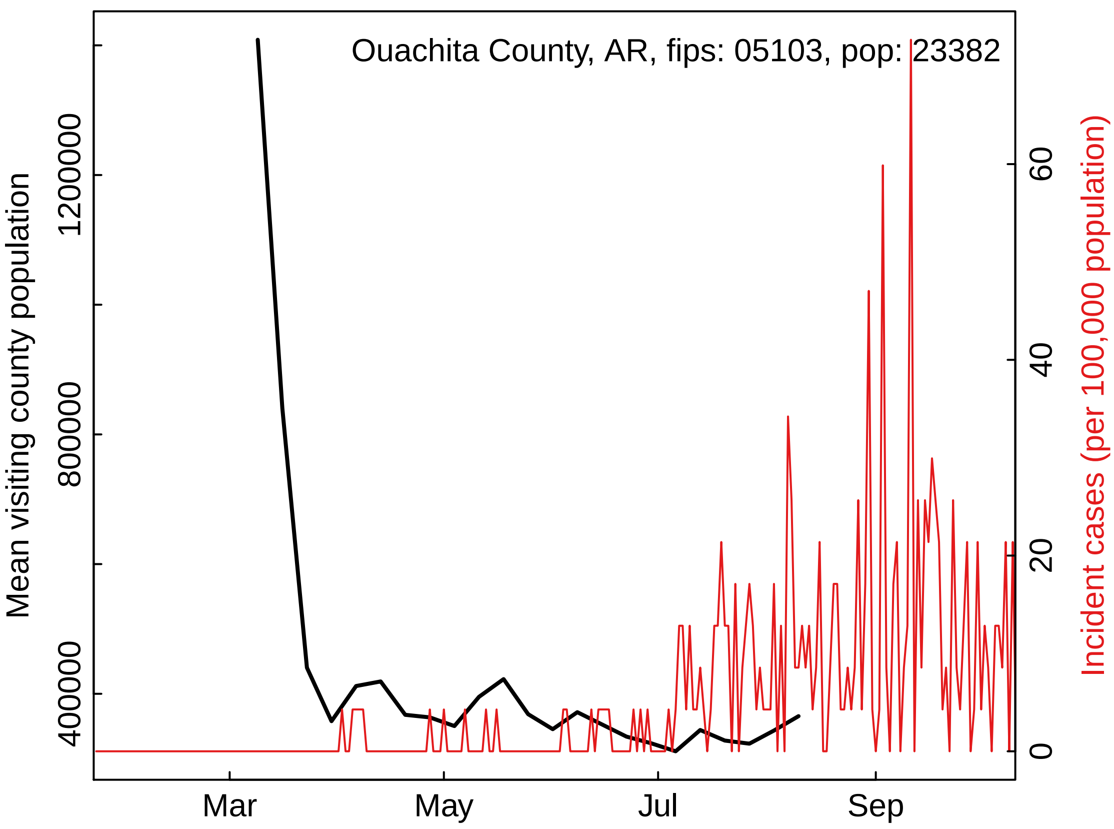
Ouachita County, AR, fips: 05103, pop: 23382
Mean visiting county population
400000
800000
1200000
Incident cases (per 100,000 population)
0
20
40
60
Mar
May
Jul
Sep

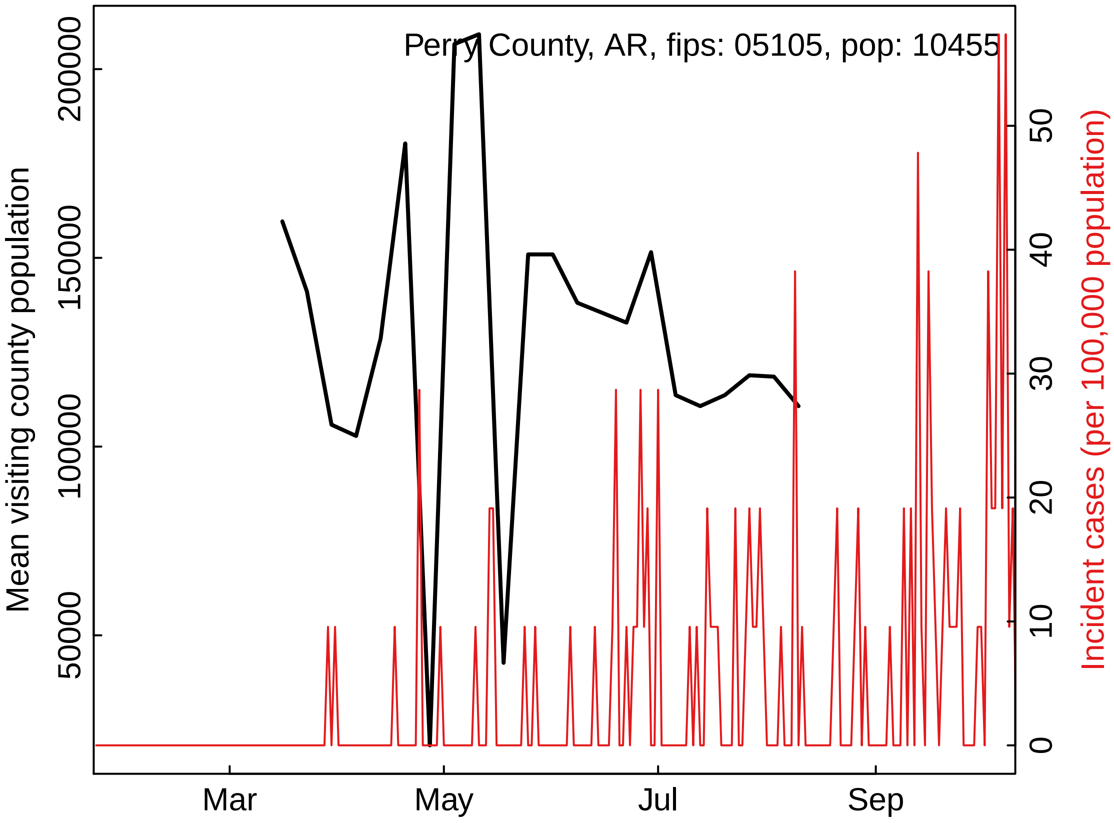

Perry County, AR, fips: 05105, pop: 10455
Mean visiting county population
50000
100000
150000
200000
Incident cases (per 100,000 population)
0
10
20
30
40
50
Mar
May
Jul
Sep

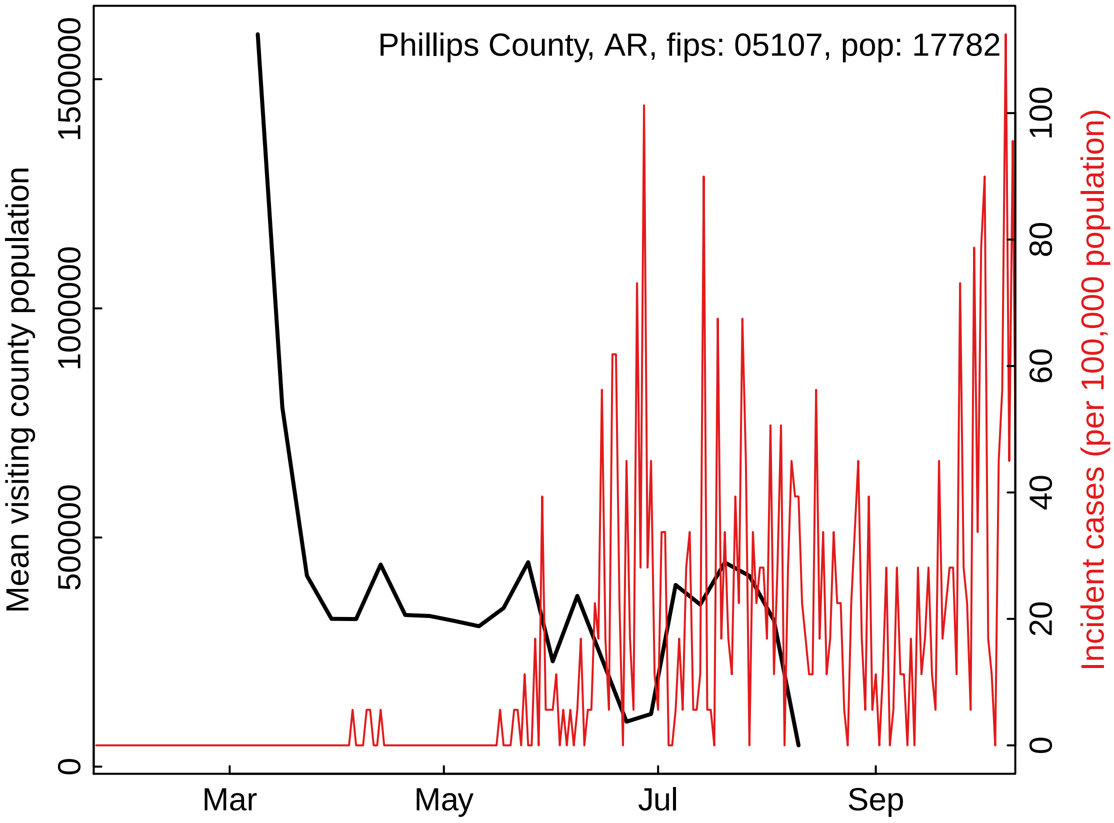
Phillips County, AR, fips: 05107, pop: 17782
Mean visiting county population
0
5000000
10000000
15000000
Incident cases (per 100,000 population)
0
20
40
60
80
100
Mar
May
Jul
Sep

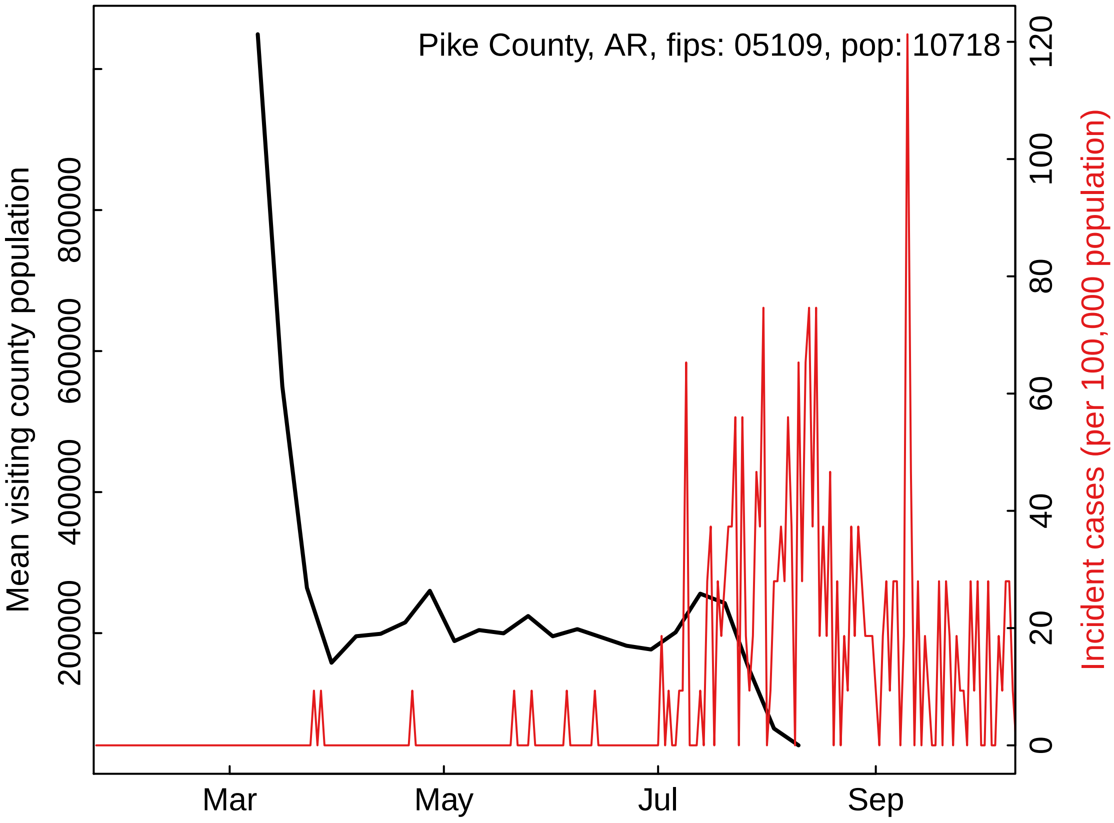

Pike County, AR, fips: 05109, pop: 10718
Mean visiting county population
200000
400000
600000
800000
Incident cases (per 100,000 population)
0
20
40
60
80
100
120
Mar
May
Jul
Sep

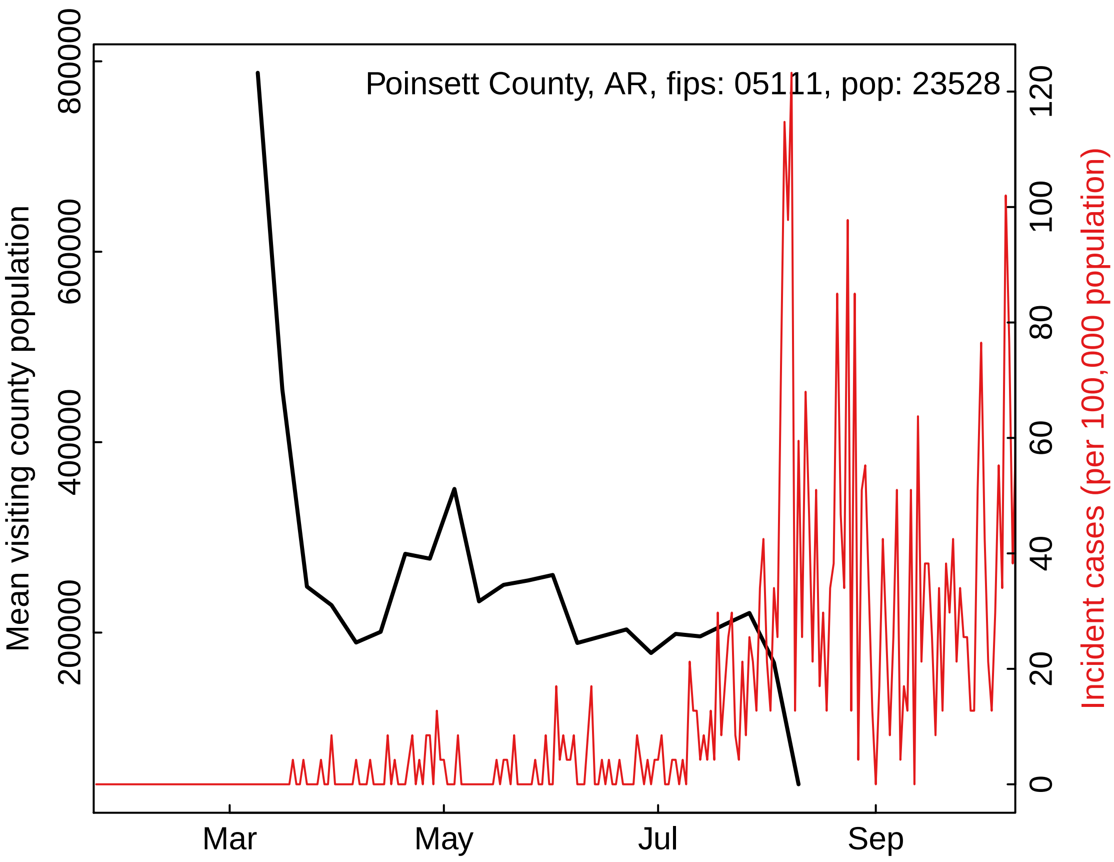

Poinsett County, AR, fips: 05111, pop: 23528
Mean visiting county population
200000
400000
600000
800000
Incident cases (per 100,000 population)
0
20
40
60
80
100
120
Mar
May
Jul
Sep

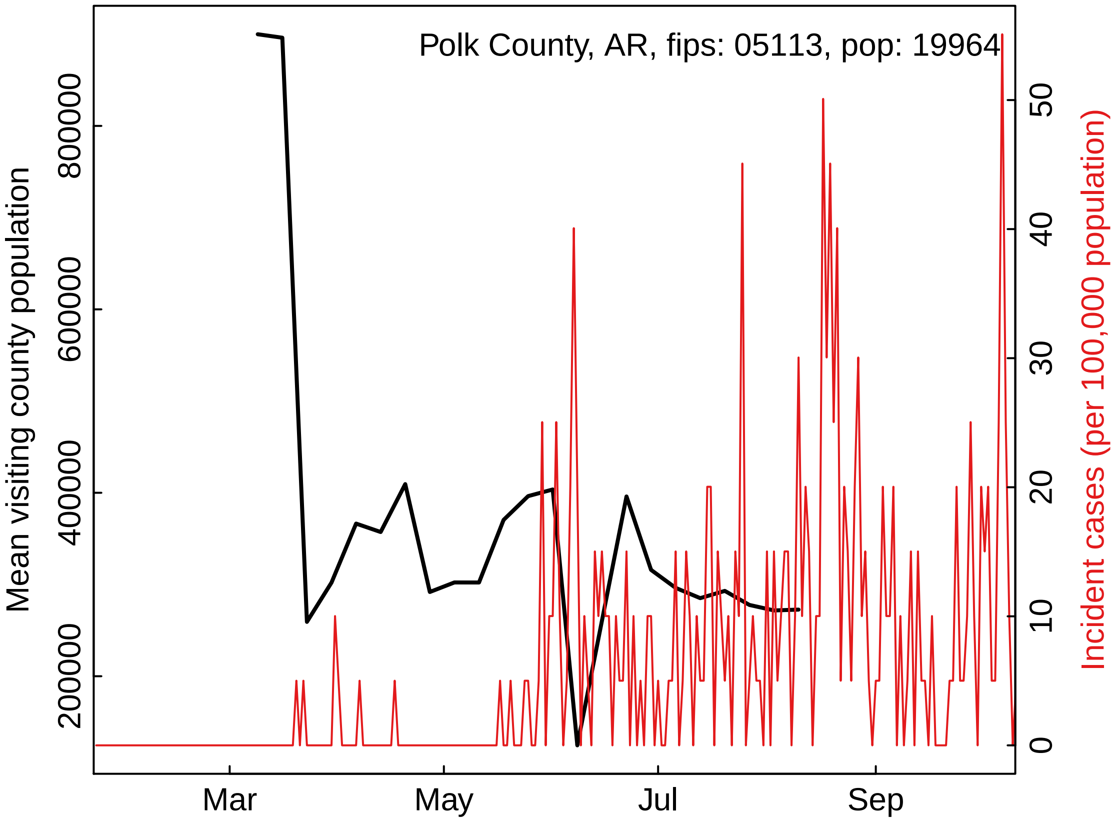

Polk County, AR, fips: 05113, pop: 19964
Mean visiting county population
200000
400000
600000
800000
Mar
May
Jul
Sep
0
10
20
30
40
50
Incident cases (per 100,000 population)

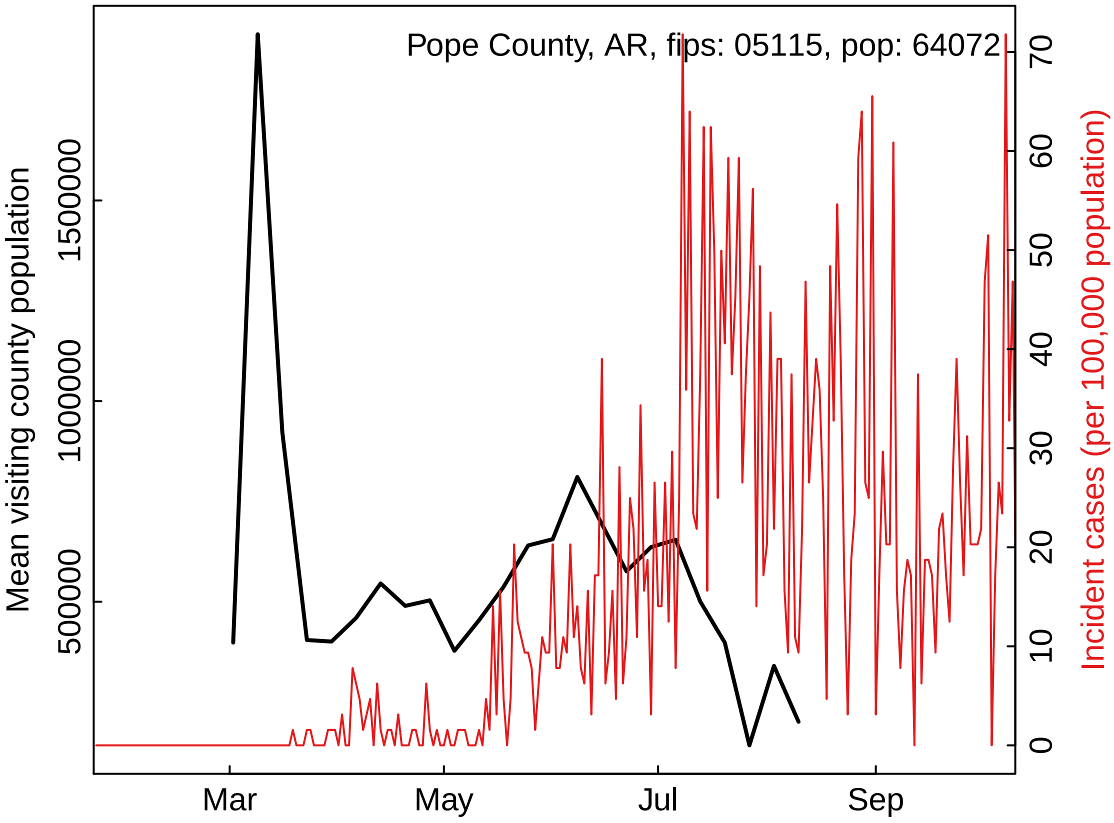

Pope County, AR, fips: 05115, pop: 64072
Mean visiting county population
500000
1000000
1500000
Incident cases (per 100,000 population)
0
10
20
30
40
50
60
70
Mar
May
Jul
Sep

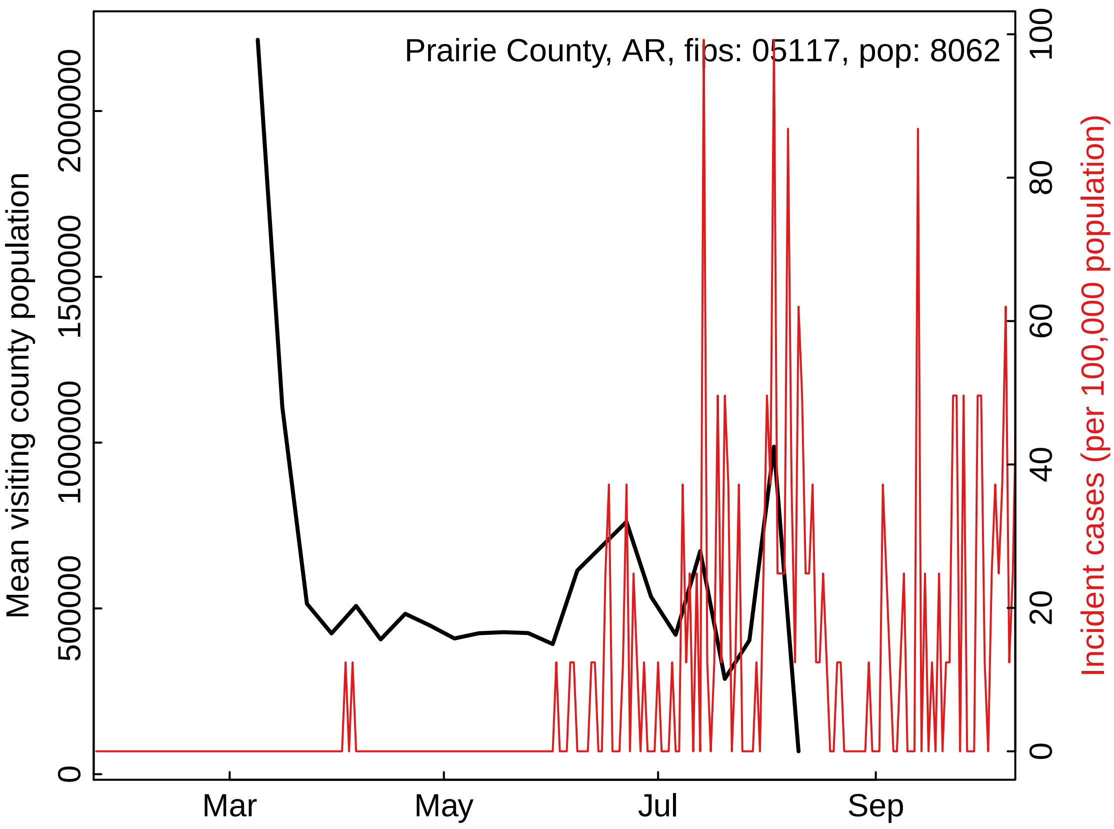

Prairie County, AR, fips: 05117, pop: 8062
Mean visiting county population
0
500000
1000000
1500000
2000000
Mar
May
Jul
Sep
0
20
40
60
80
100
Incident cases (per 100,000 population)

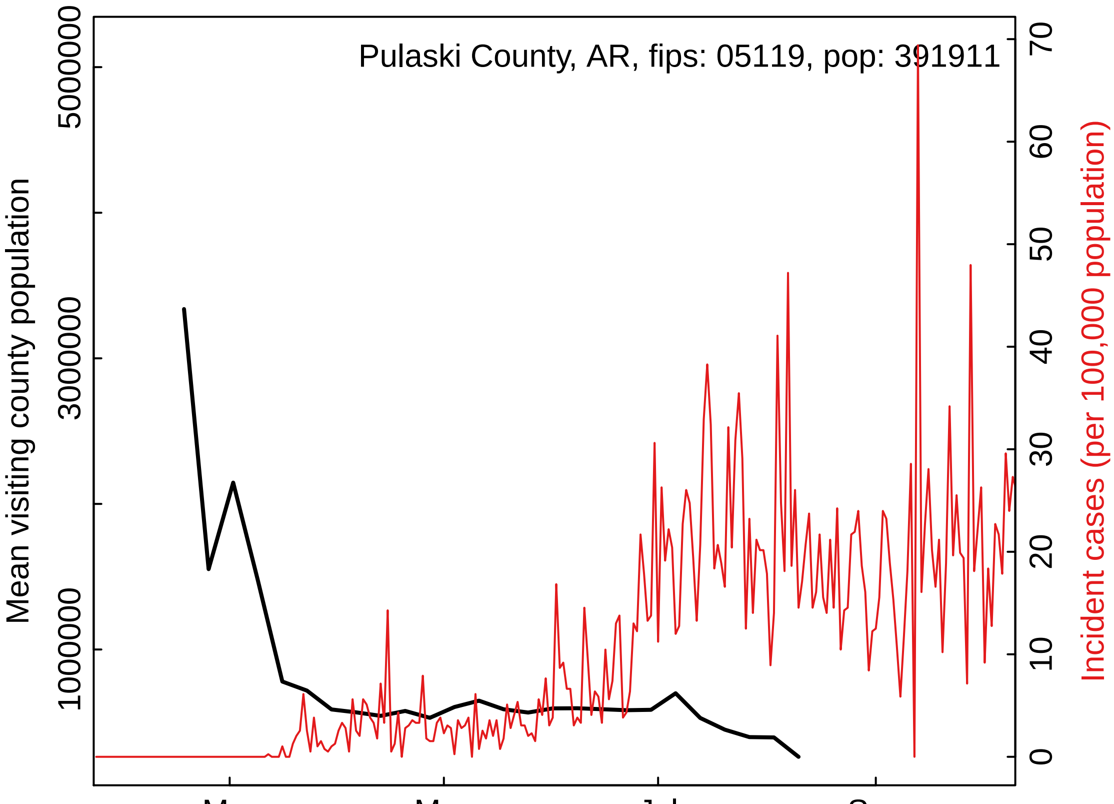

Pulaski County, AR, fips: 05119, pop: 391911
Mean visiting county population
Incident cases (per 100,000 population)
1000000
3000000
5000000
0
10
20
30
40
50
60
70
Mar
May
Jul
Sep

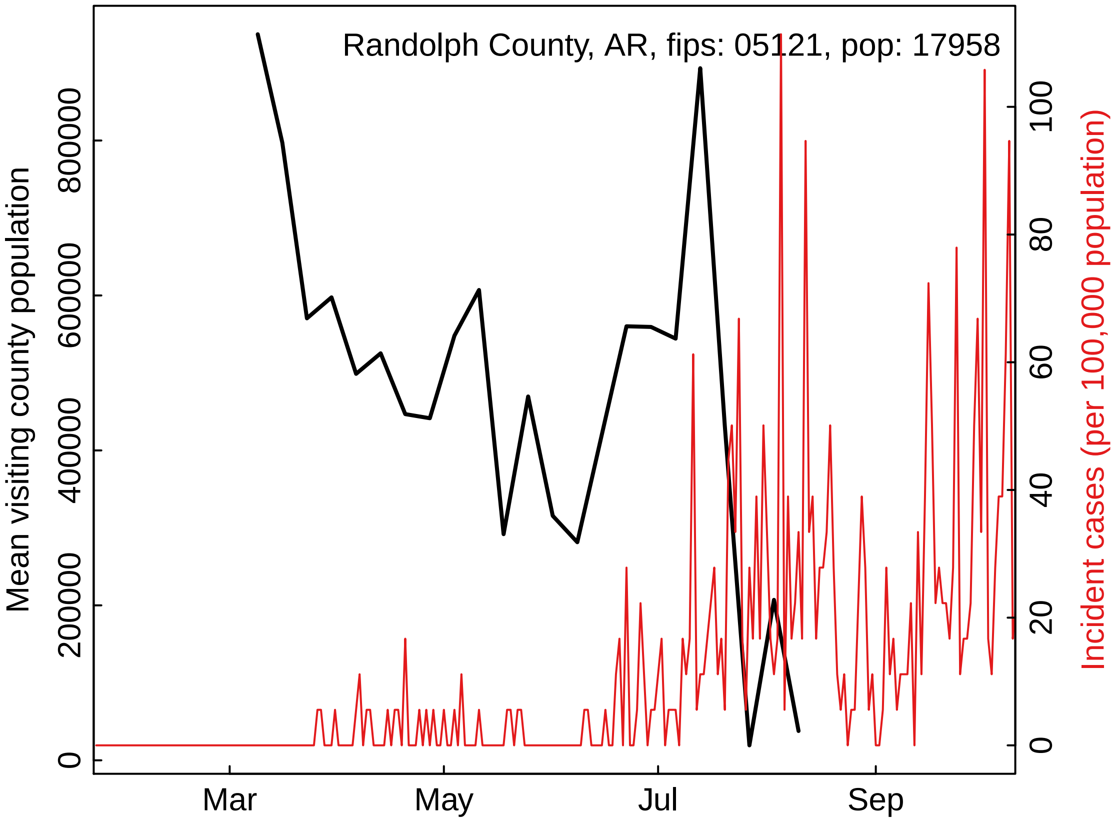

Randolph County, AR, fips: 05121, pop: 17958
Mean visiting county population
0
200000
400000
600000
800000
Mar
May
Jul
Sep
0
20
40
60
80
100
Incident cases (per 100,000 population)

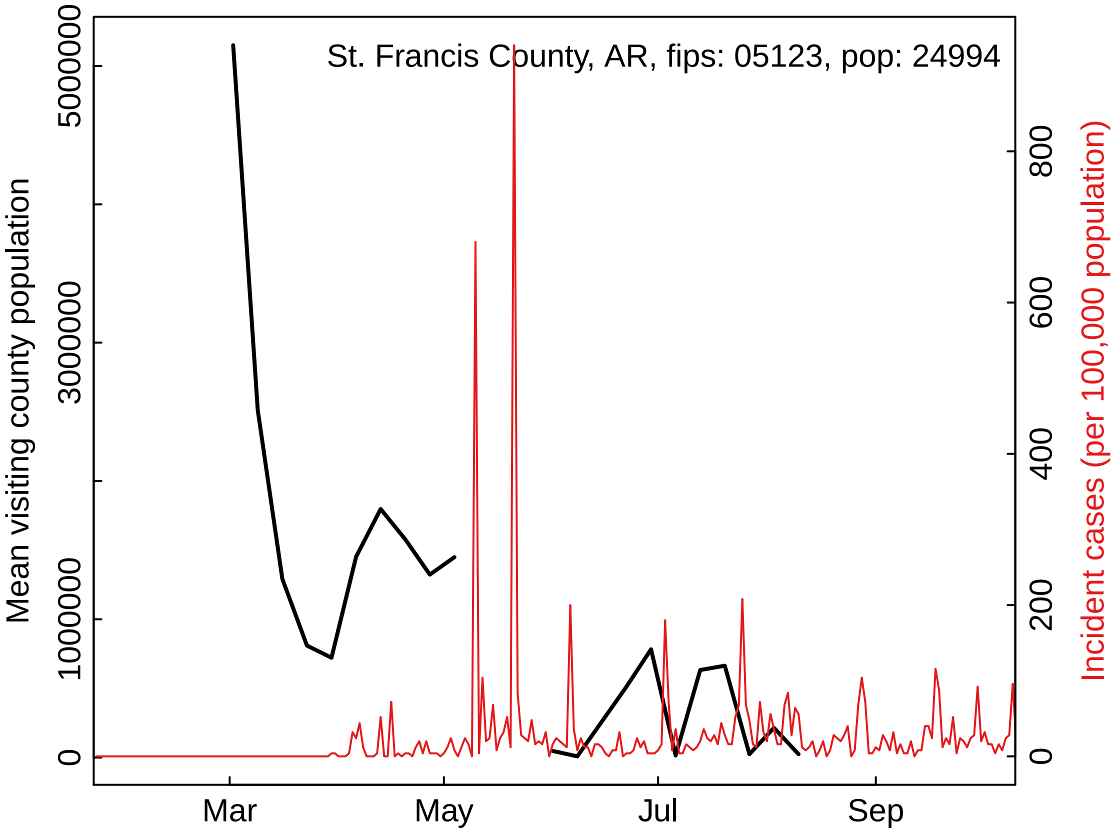

St. Francis County, AR, fips: 05123, pop: 24994
Mean visiting county population
0
1000000
3000000
5000000
Incident cases (per 100,000 population)
0
200
400
600
800
Mar
May
Jul
Sep

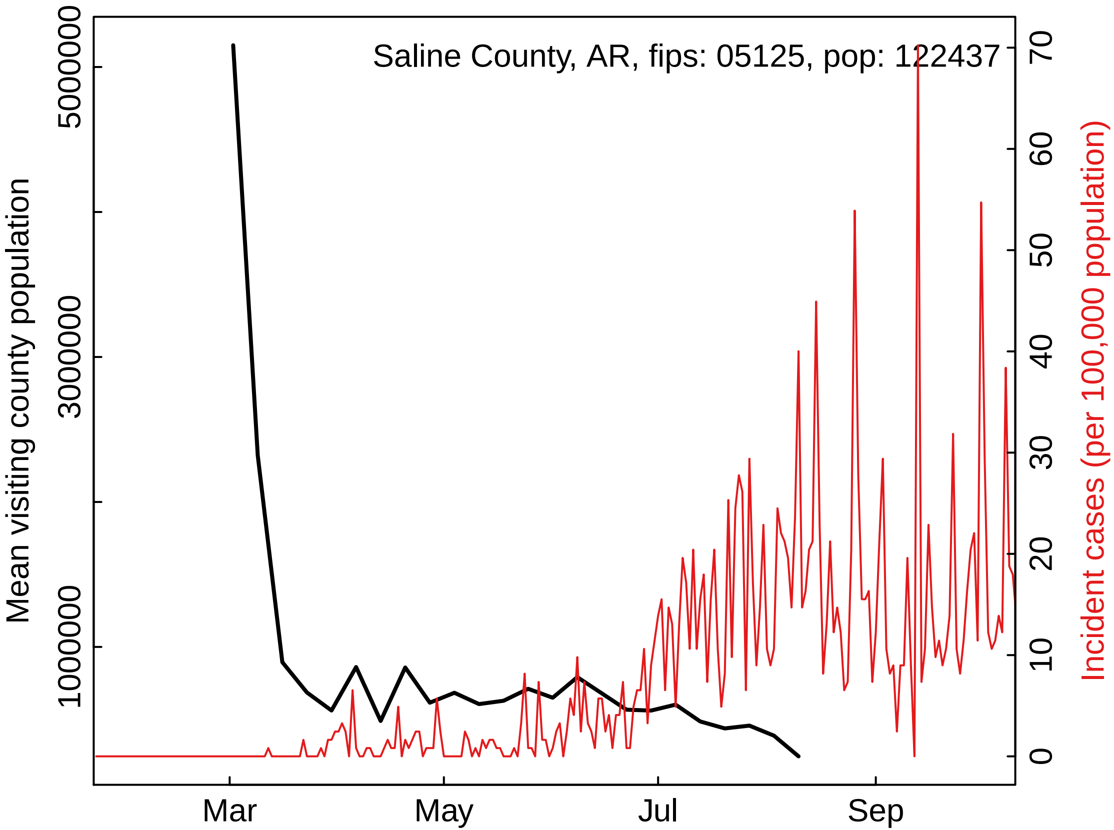

Saline County, AR, fips: 05125, pop: 122437
Mean visiting county population
Incident cases (per 100,000 population)
1000000
3000000
5000000
0
10
20
30
40
50
60
70
Mar
May
Jul
Sep

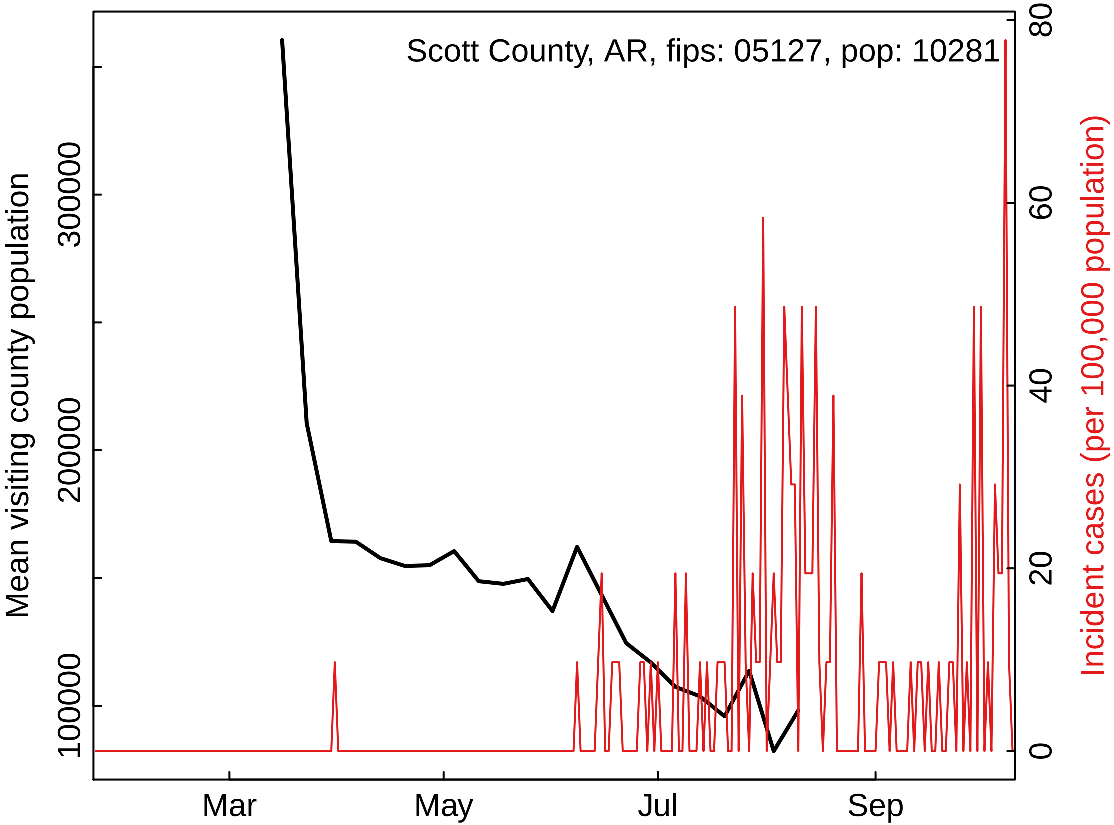
Scott County, AR, fips: 05127, pop: 10281
Mean visiting county population
100000
200000
300000
Incident cases (per 100,000 population)
0
20
40
60
80
Mar
May
Jul
Sep

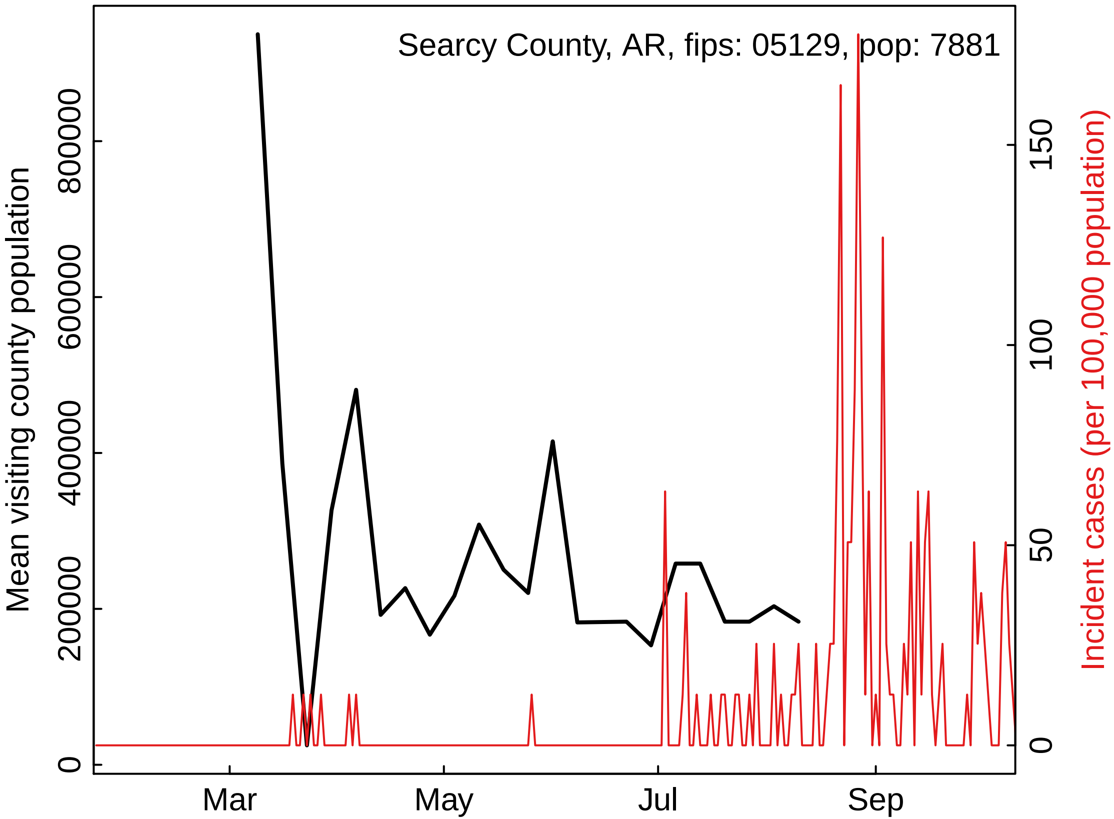

Searcy County, AR, fips: 05129, pop: 7881
Mean visiting county population
0
200000
400000
600000
800000
Incident cases (per 100,000 population)
0
50
100
150
Mar
May
Jul
Sep

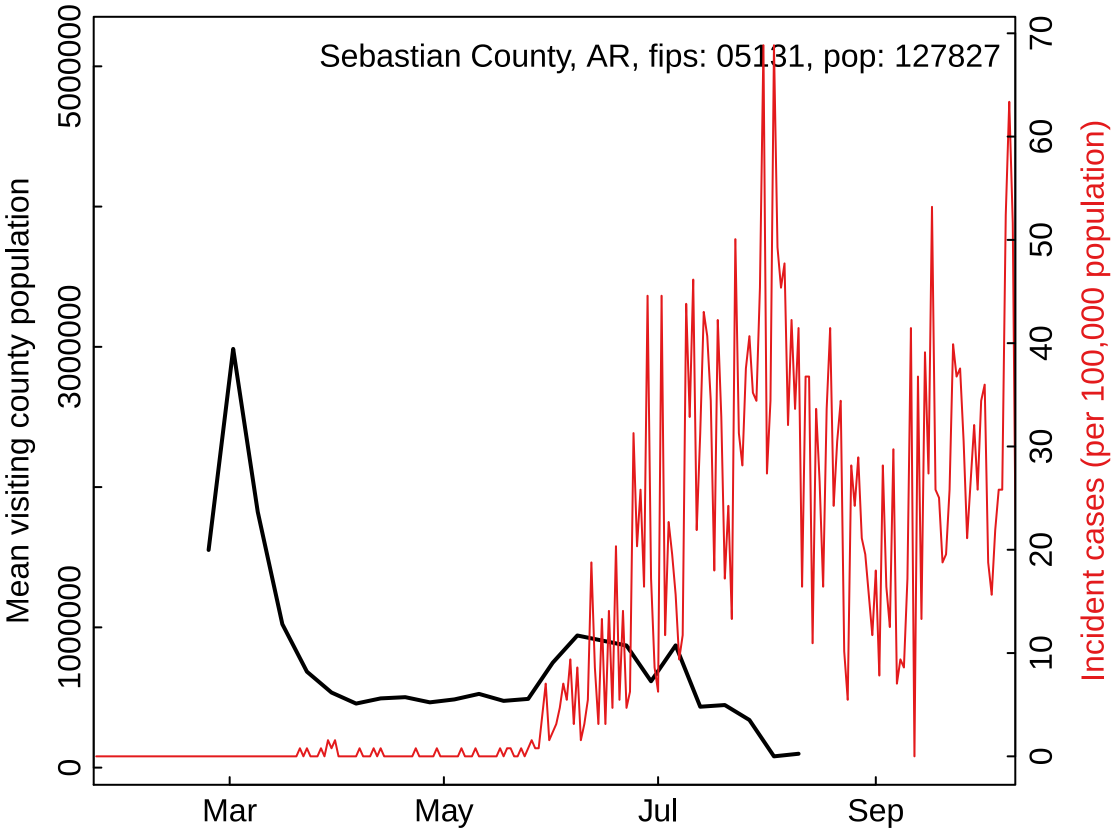

Sebastian County, AR, fips: 05131, pop: 127827
Mean visiting county population
Incident cases (per 100,000 population)
0
1000000
3000000
5000000
0
10
20
30
40
50
60
70
Mar
May
Jul
Sep

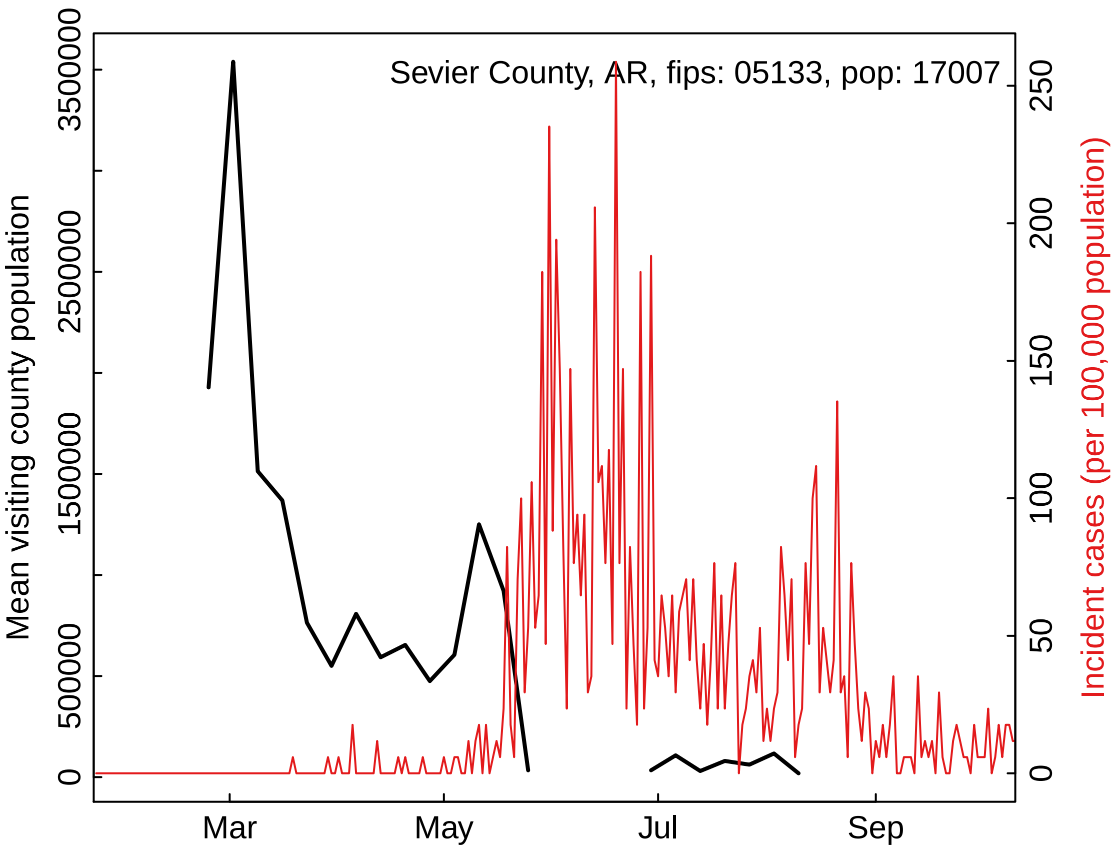

Sevier County, AR, fips: 05133, pop: 17007
Mean visiting county population
Incident cases (per 100,000 population)
0
500000
1500000
2500000
3500000
0
50
100
150
200
250
Mar
May
Jul
Sep

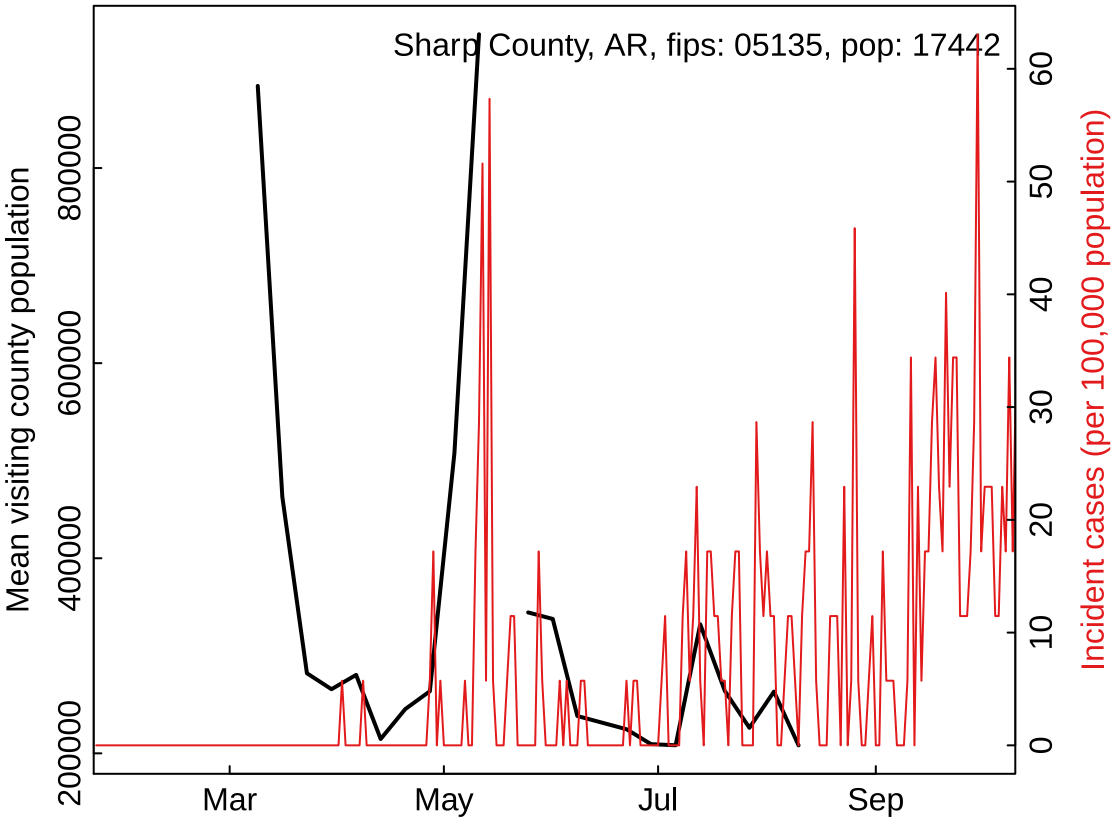
Sharp County, AR, fips: 05135, pop: 17442
Mean visiting county population
200000
400000
600000
800000
Incident cases (per 100,000 population)
0
10
20
30
40
50
60
Mar
May
Jul
Sep

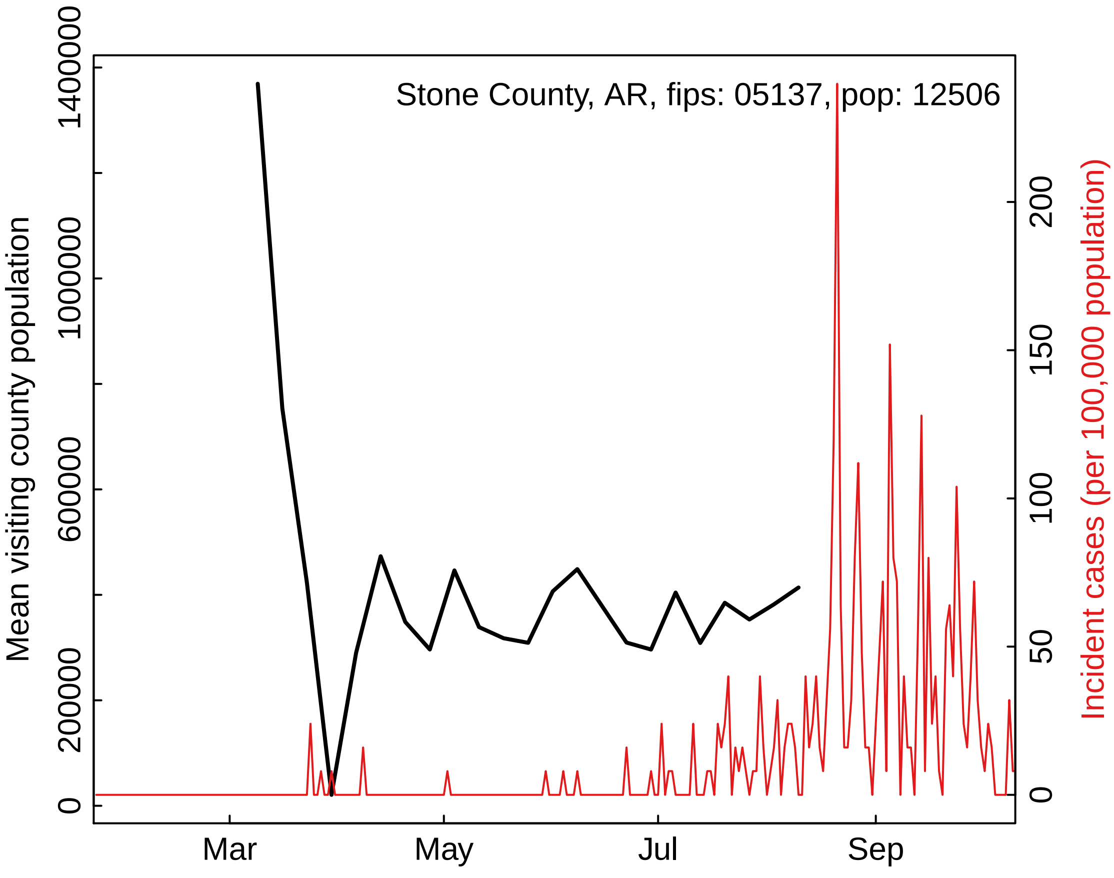

Stone County, AR, fips: 05137, pop: 12506
Mean visiting county population
0
200000
600000
1000000
1400000
Incident cases (per 100,000 population)
0
50
100
150
200
Mar
May
Jul
Sep

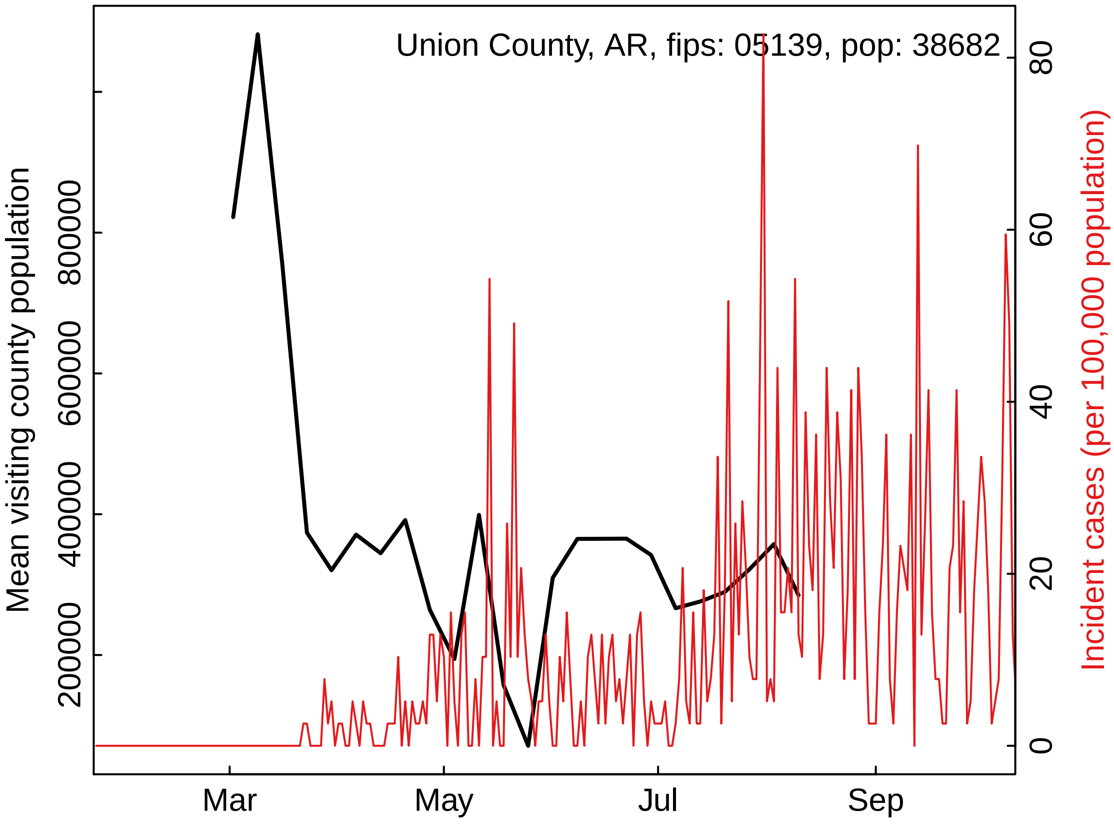

Union County, AR, fips: 05139, pop: 38682
Mean visiting county population
200000
400000
600000
800000
Incident cases (per 100,000 population)
0
20
40
60
80
Mar
May
Jul
Sep

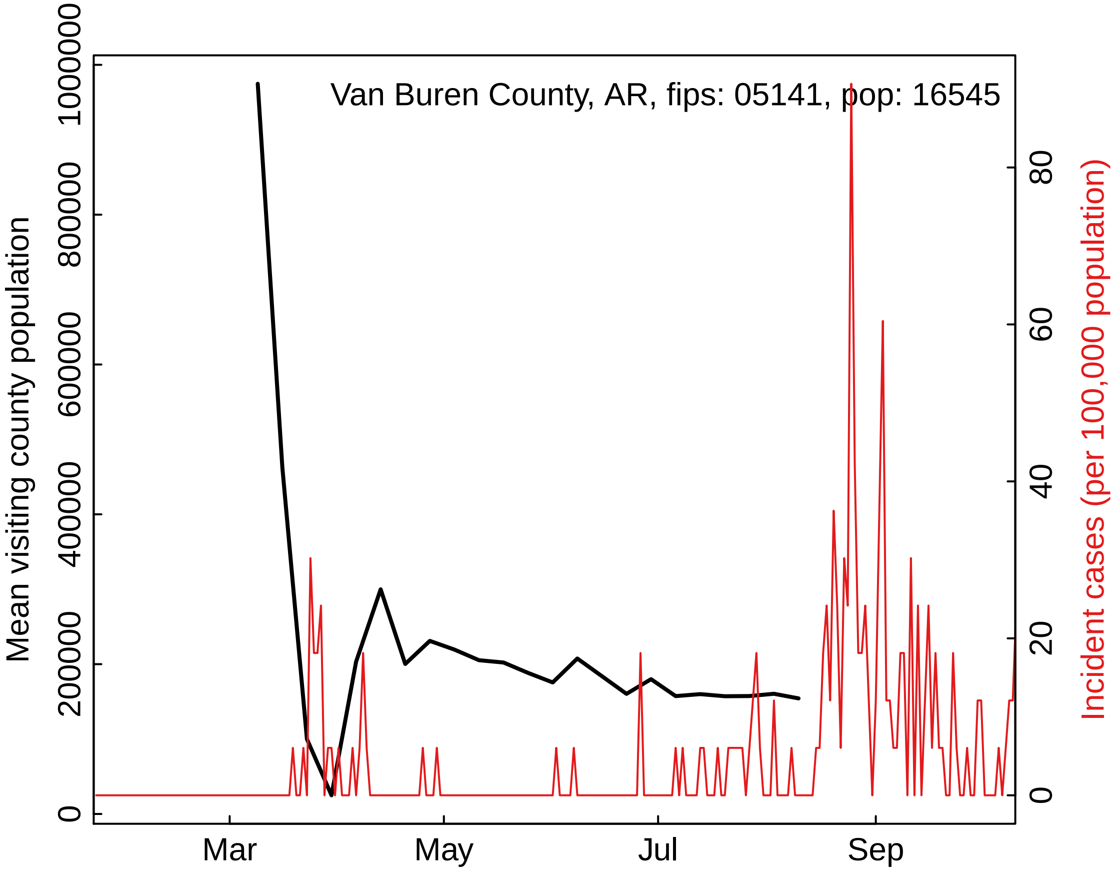
Van Buren County, AR, fips: 05141, pop: 16545
Mean visiting county population
0
200000
400000
600000
800000
1000000
Mar
May
Jul
Sep
0
20
40
60
80
Incident cases (per 100,000 population)

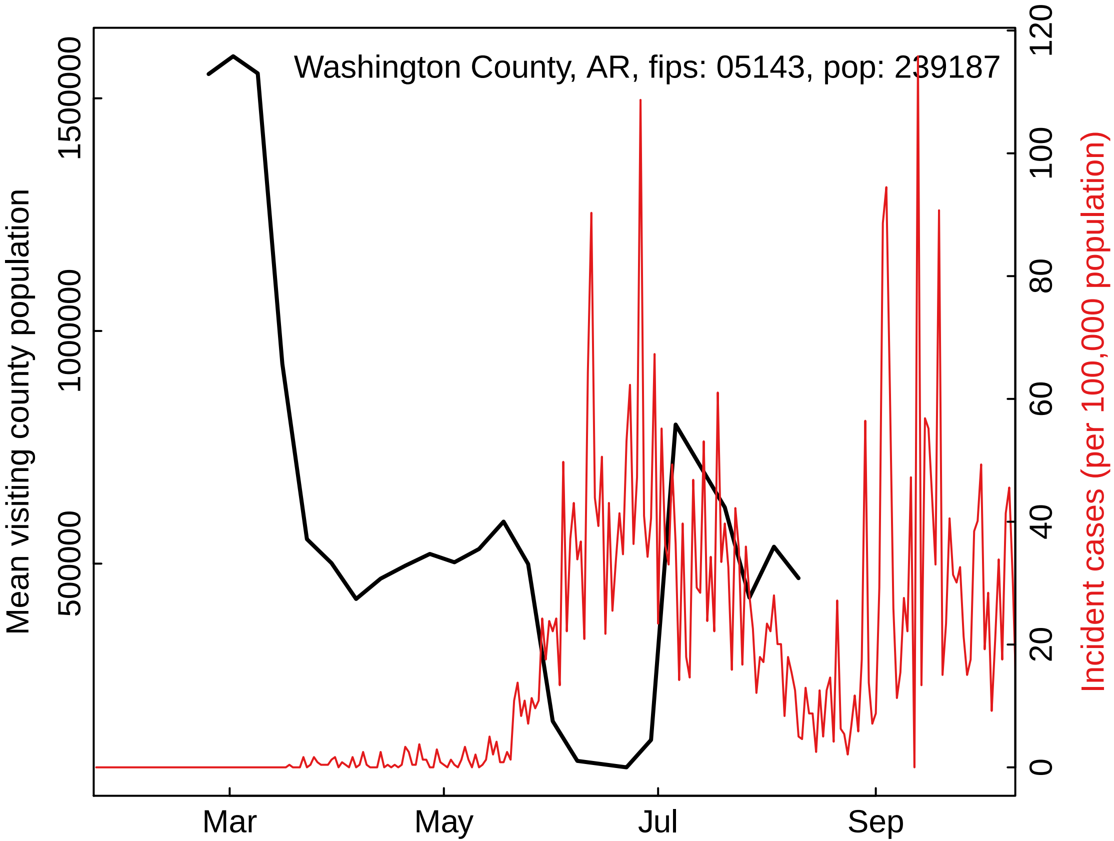

Washington County, AR, fips: 05143, pop: 239187
Mean visiting county population
500000
1000000
1500000
Incident cases (per 100,000 population)
0
20
40
60
80
100
120
Mar
May
Jul
Sep

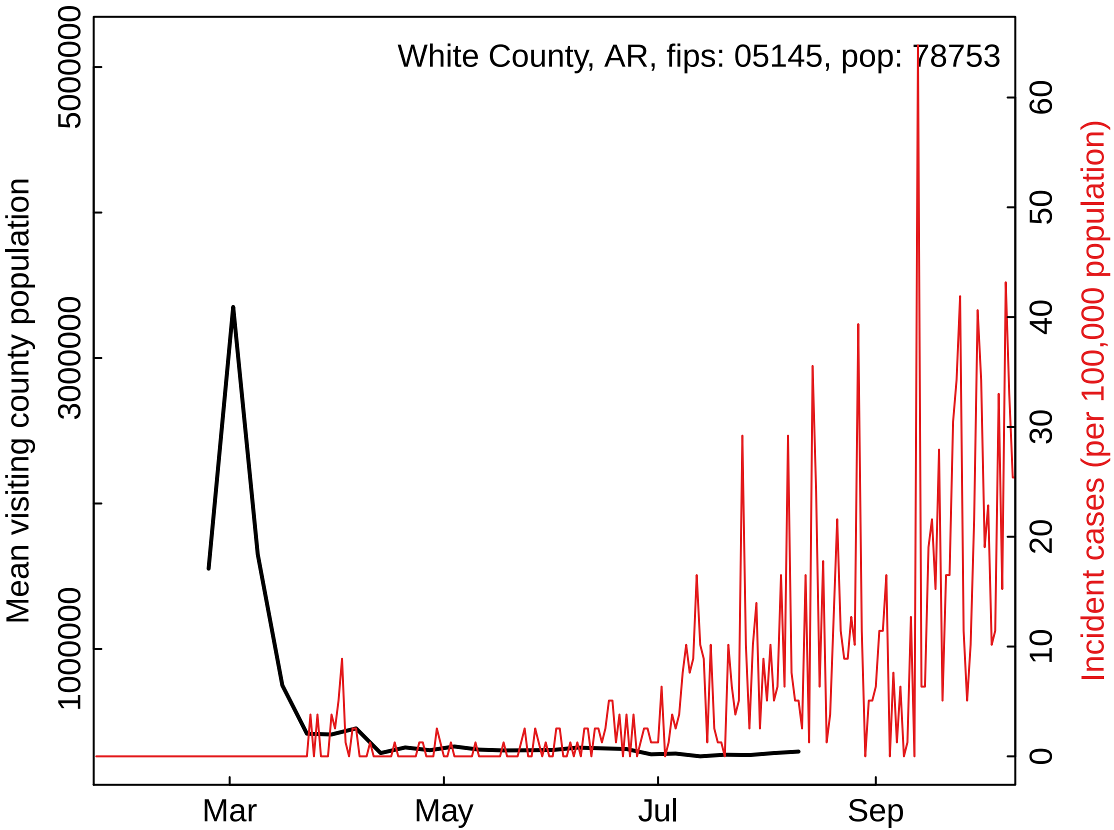

White County, AR, fips: 05145, pop: 78753
Mean visiting county population
1000000
3000000
5000000
Mar
May
Jul
Sep
0
10
20
30
40
50
60
Incident cases (per 100,000 population)

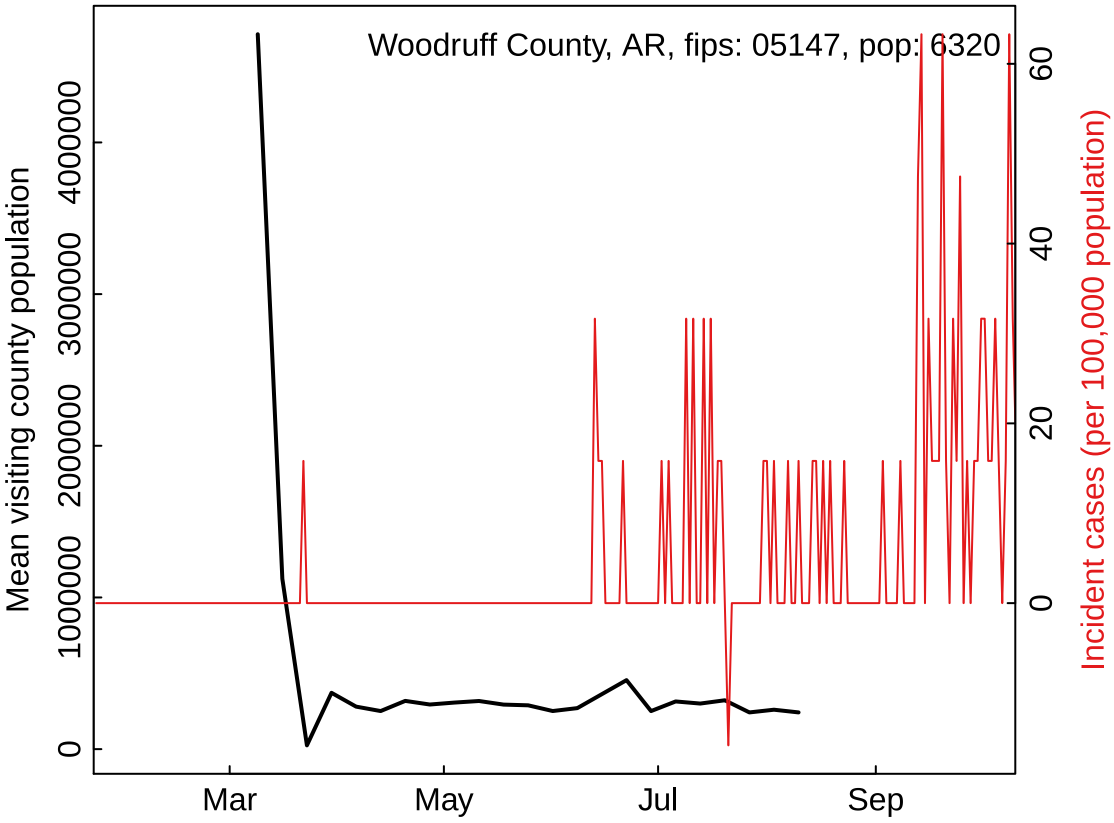
Woodruff County, AR, fips: 05147, pop: 6320
Mean visiting county population
0
1000000
2000000
3000000
4000000
Mar
May
Jul
Sep
0
20
40
60
Incident cases (per 100,000 population)

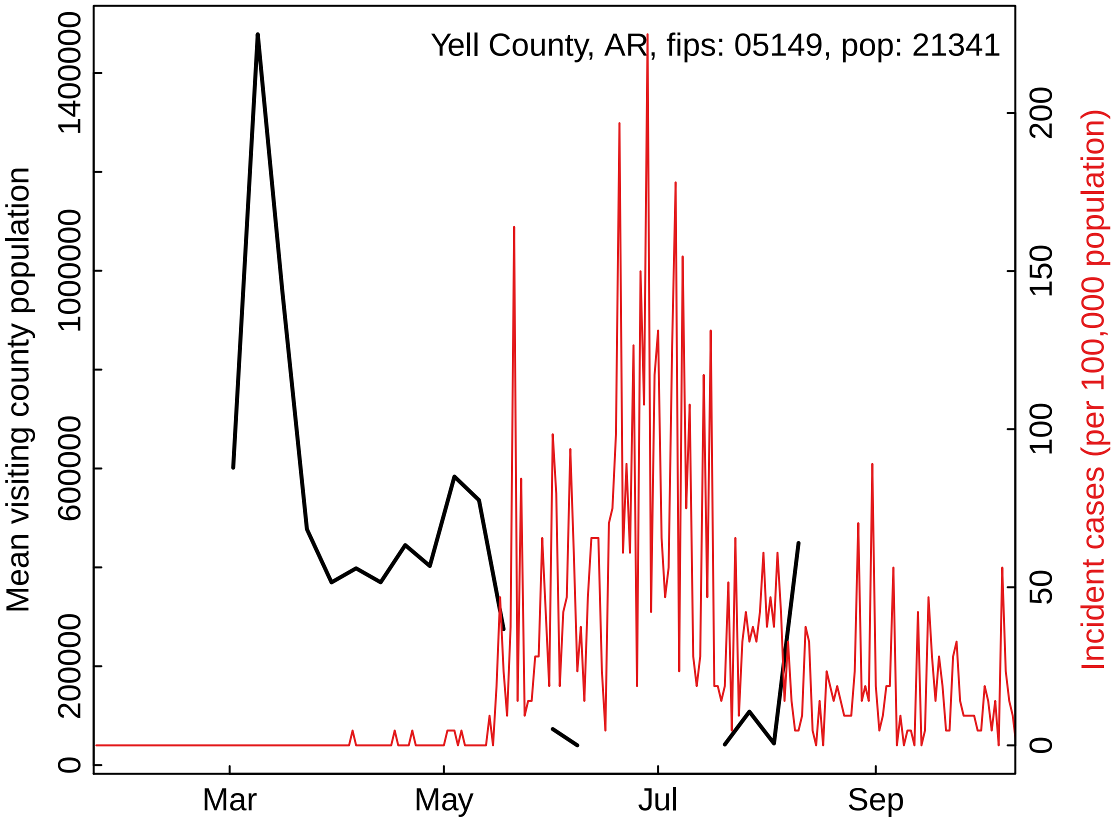
Yell County, AR, fips: 05149, pop: 21341
Mean visiting county population
0
200000
600000
1000000
1400000
Incident cases (per 100,000 population)
0
50
100
150
200
Mar
May
Jul
Sep

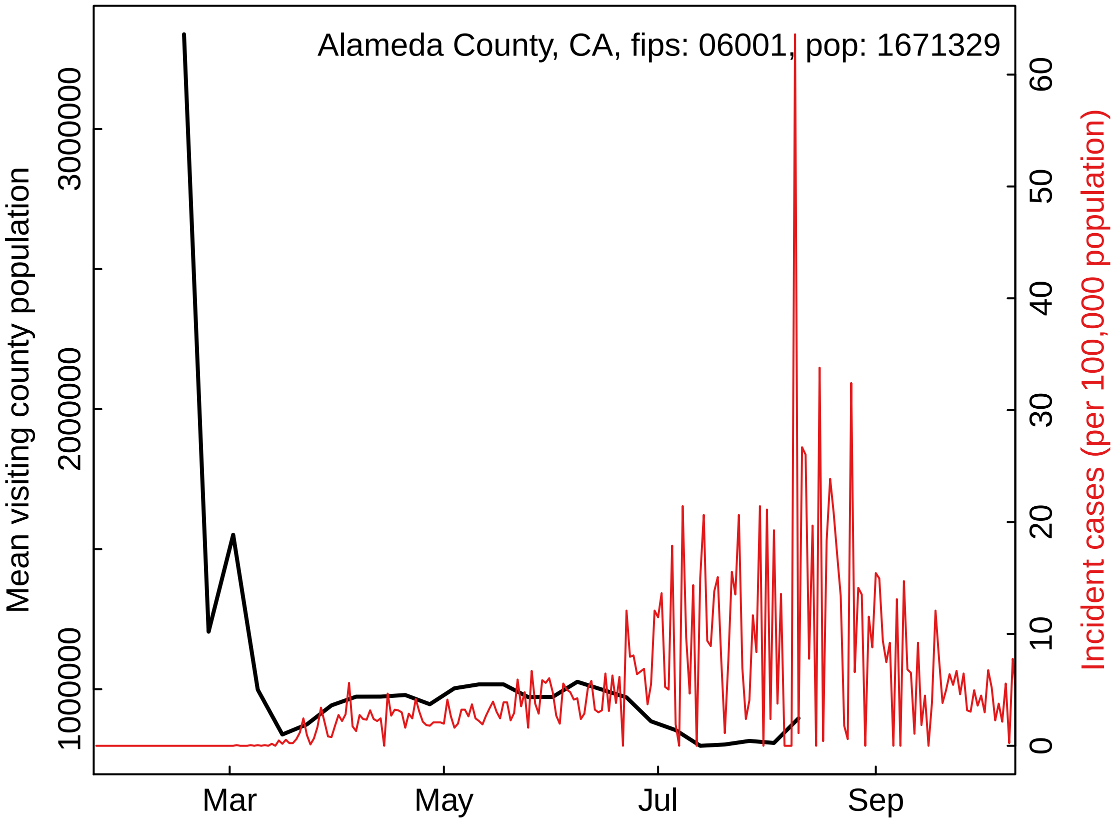

Alameda County, CA, fips: 06001, pop: 1671329
Mean visiting county population
1000000
2000000
3000000
Incident cases (per 100,000 population)
0
10
20
30
40
50
60
Mar
May
Jul
Sep

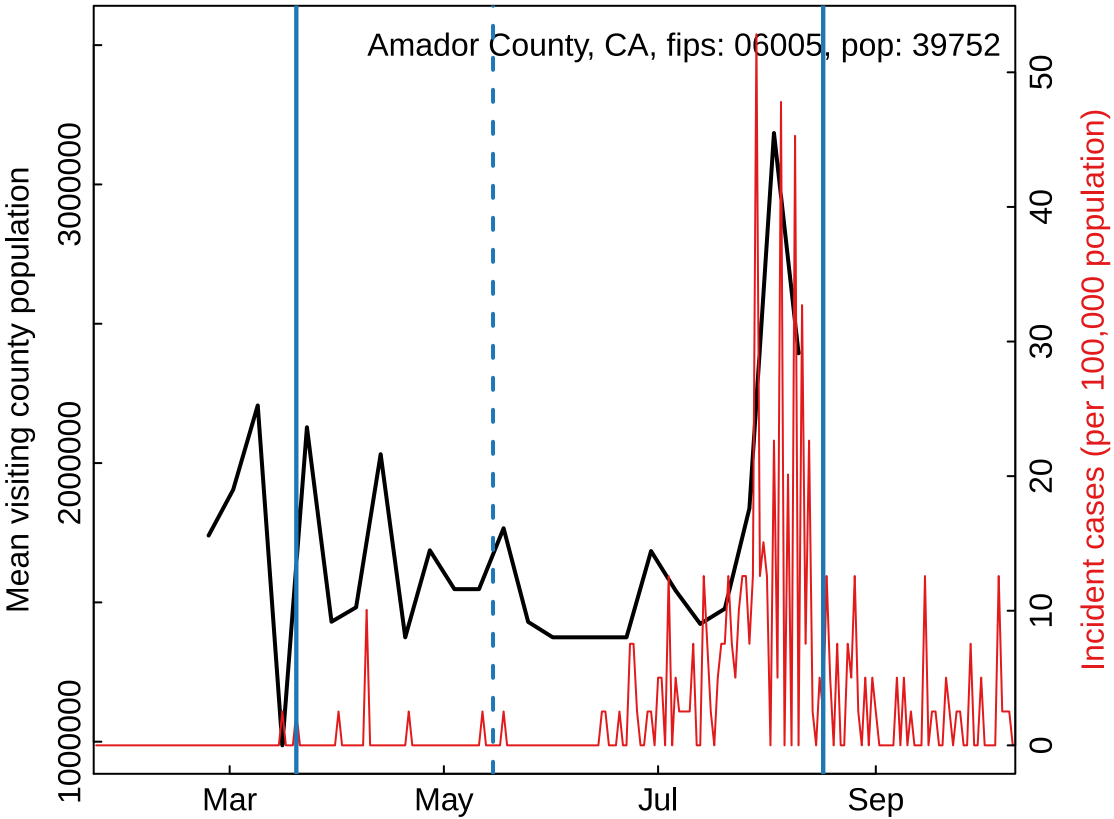

Amador County, CA, fips: 06005, pop: 39752
Mean visiting county population
10000000
20000000
30000000
Incident cases (per 100,000 population)
0
10
20
30
40
50
Mar
May
Jul
Sep

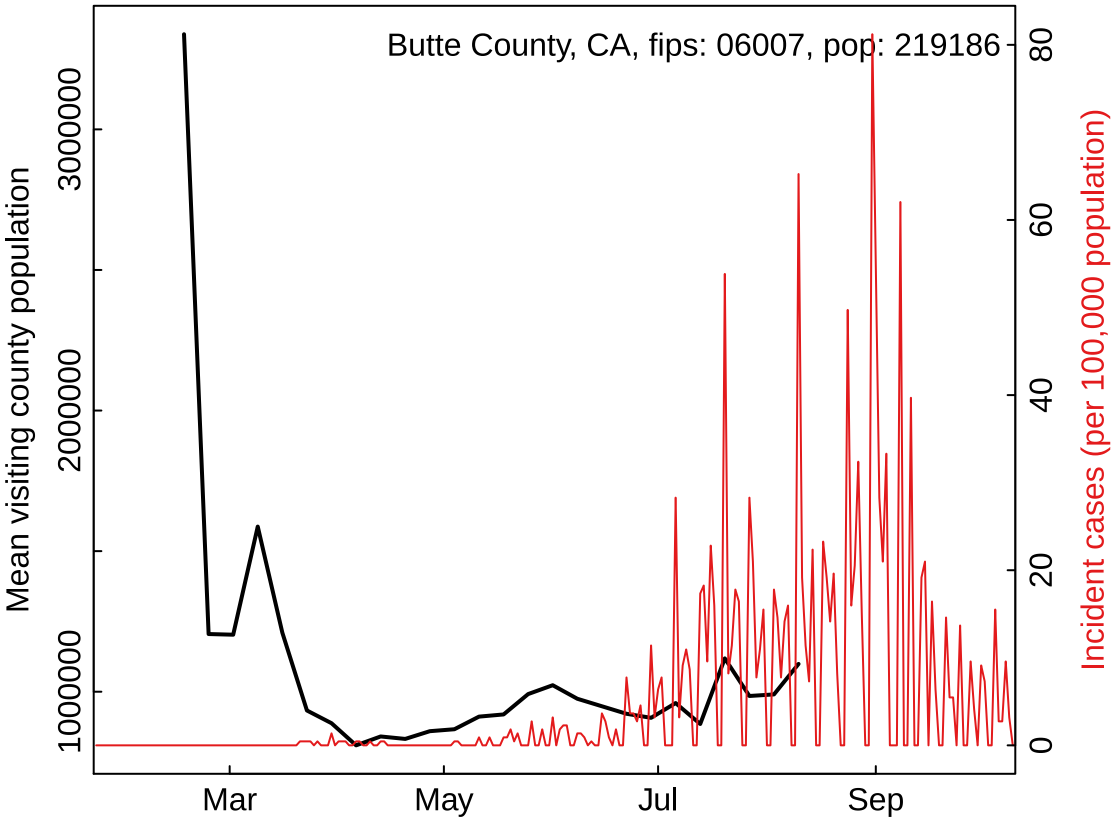

Butte County, CA, fips: 06007, pop: 219186
Mean visiting county population
10000000
20000000
30000000
Incident cases (per 100,000 population)
0
20
40
60
80
Mar
May
Jul
Sep

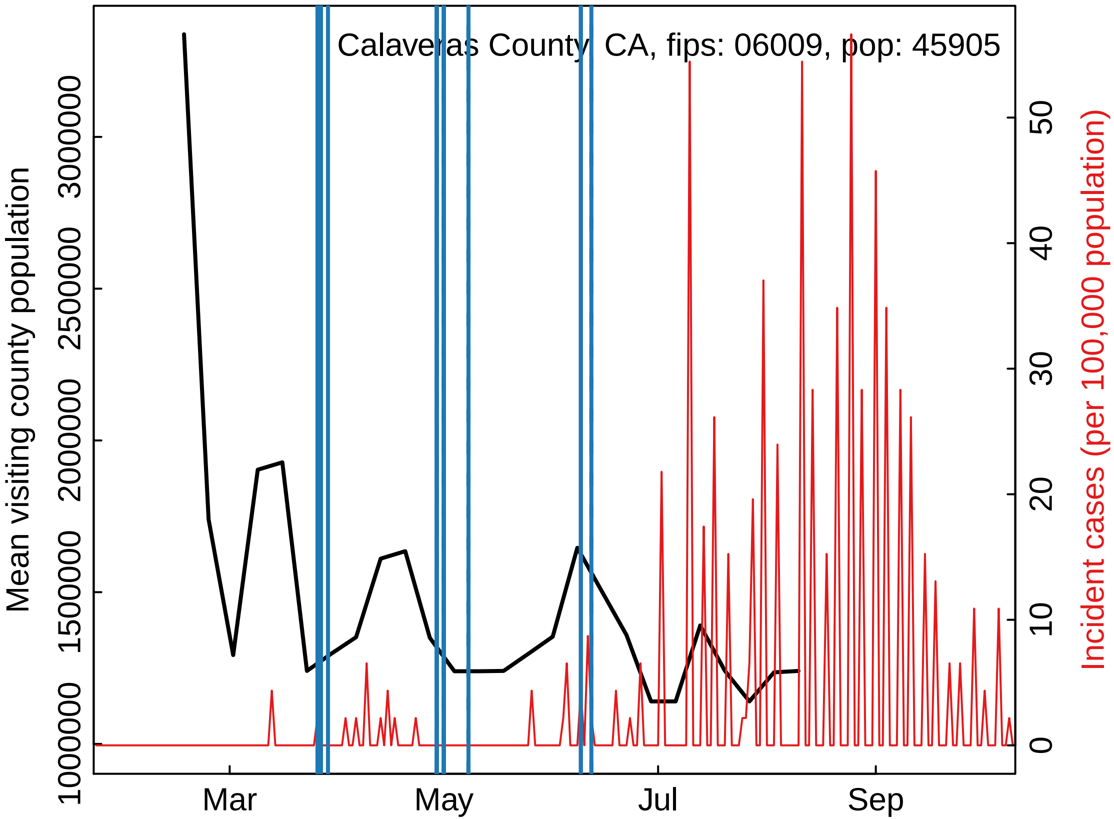
Calaveras County, CA, fips: 06009, pop: 45905
Mean visiting county population
1000000
1500000
2000000
2500000
3000000
Mar
May
Jul
Sep
Incident cases (per 100,000 population)
0
10
20
30
40
50

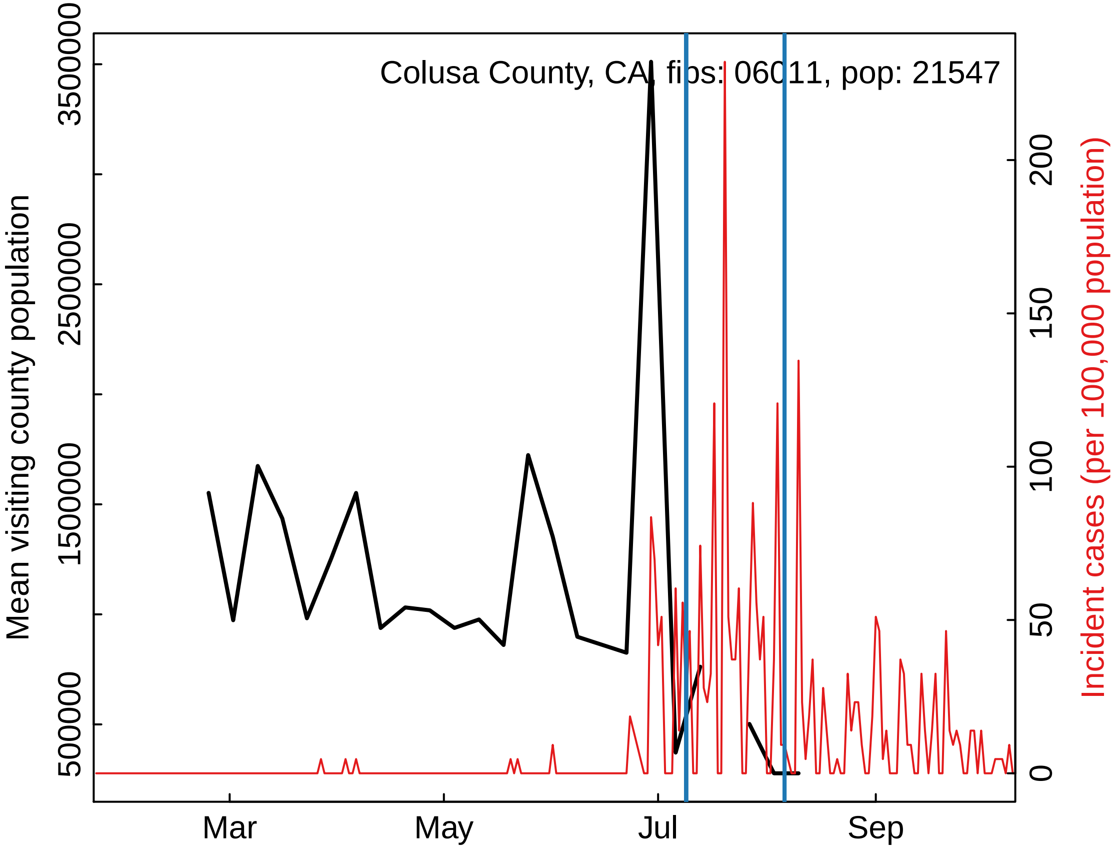
Colusa County, CA, fips: 06011, pop: 21547
Mean visiting county population
Incident cases (per 100,000 population)
500000
1500000
2500000
3500000
0
50
100
150
200
Mar
May
Jul
Sep

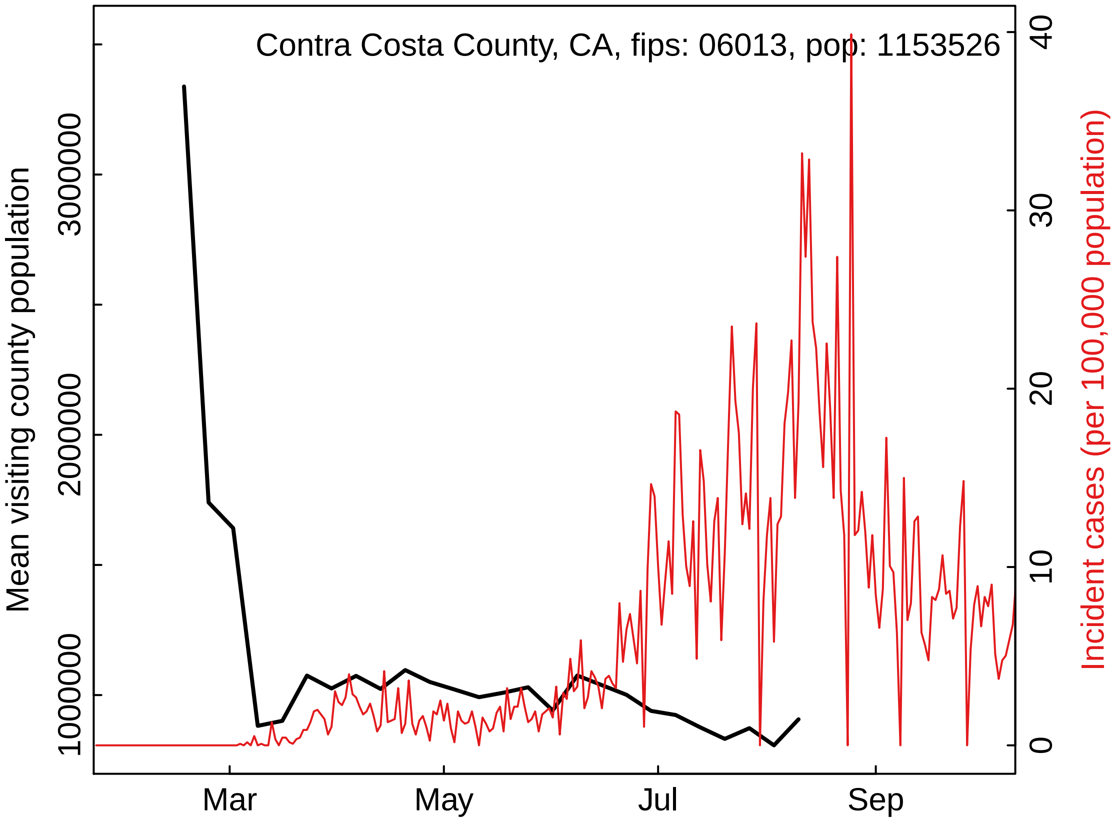

Contra Costa County, CA, fips: 06013, pop: 1153526
Mean visiting county population
Incident cases (per 100,000 population)
1000000
2000000
3000000
0
10
20
30
40
Mar
May
Jul
Sep

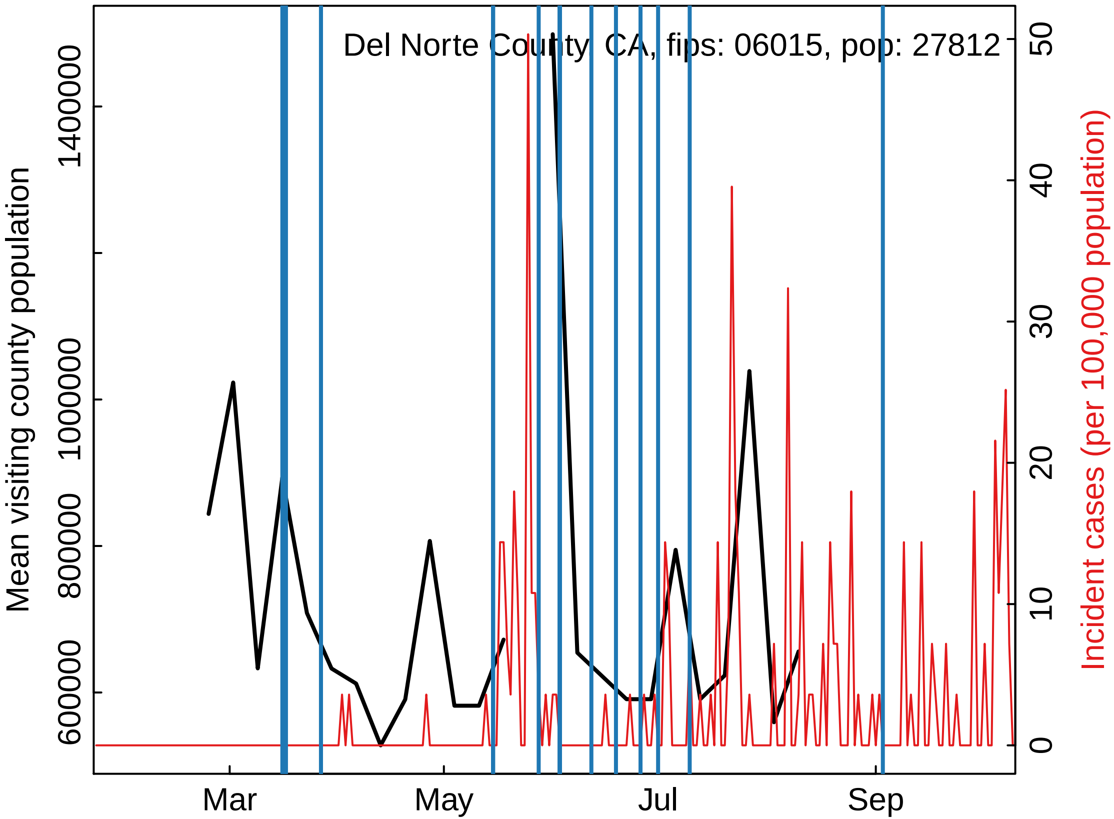
Del Norte County, CA, fips: 06015, pop: 27812
Mean visiting county population
600000
800000
1000000
1400000
Incident cases (per 100,000 population)
0
10
20
30
40
50
Mar
May
Jul
Sep

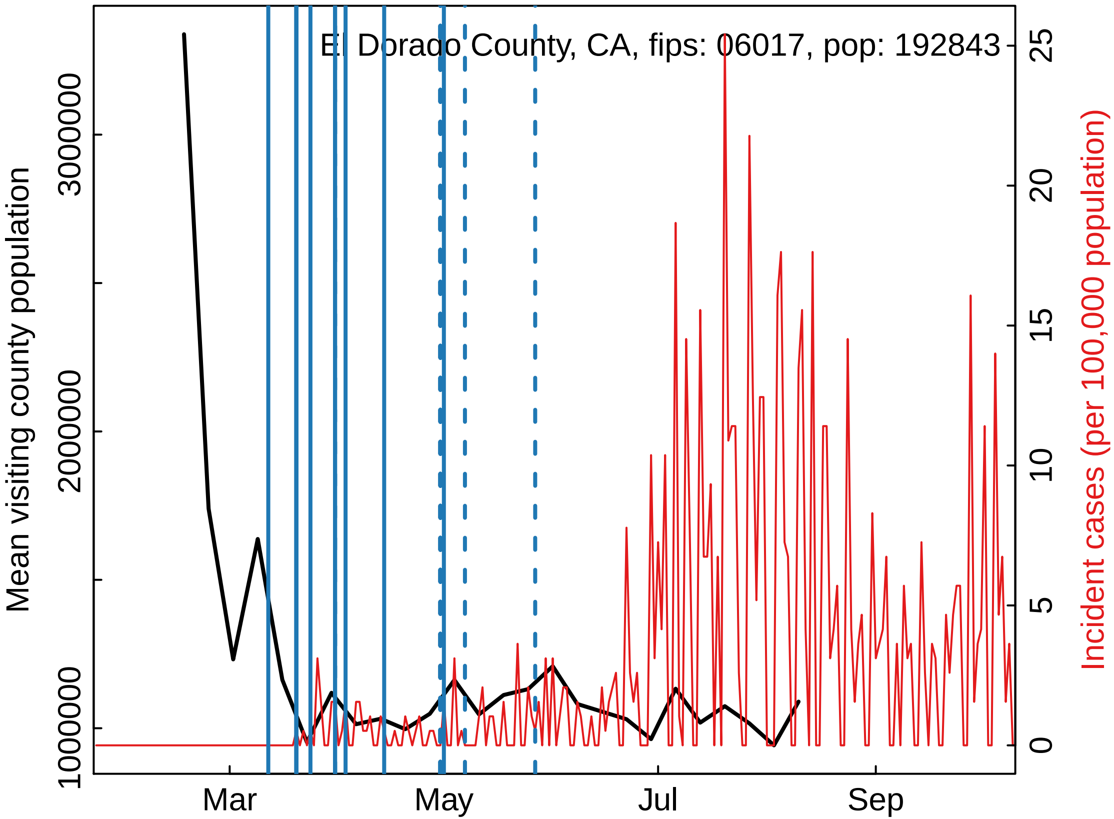

El Dorado County, CA, fips: 06017, pop: 192843
Mean visiting county population
1000000
2000000
3000000
Incident cases (per 100,000 population)
0
5
10
15
20
25
Mar
May
Jul
Sep

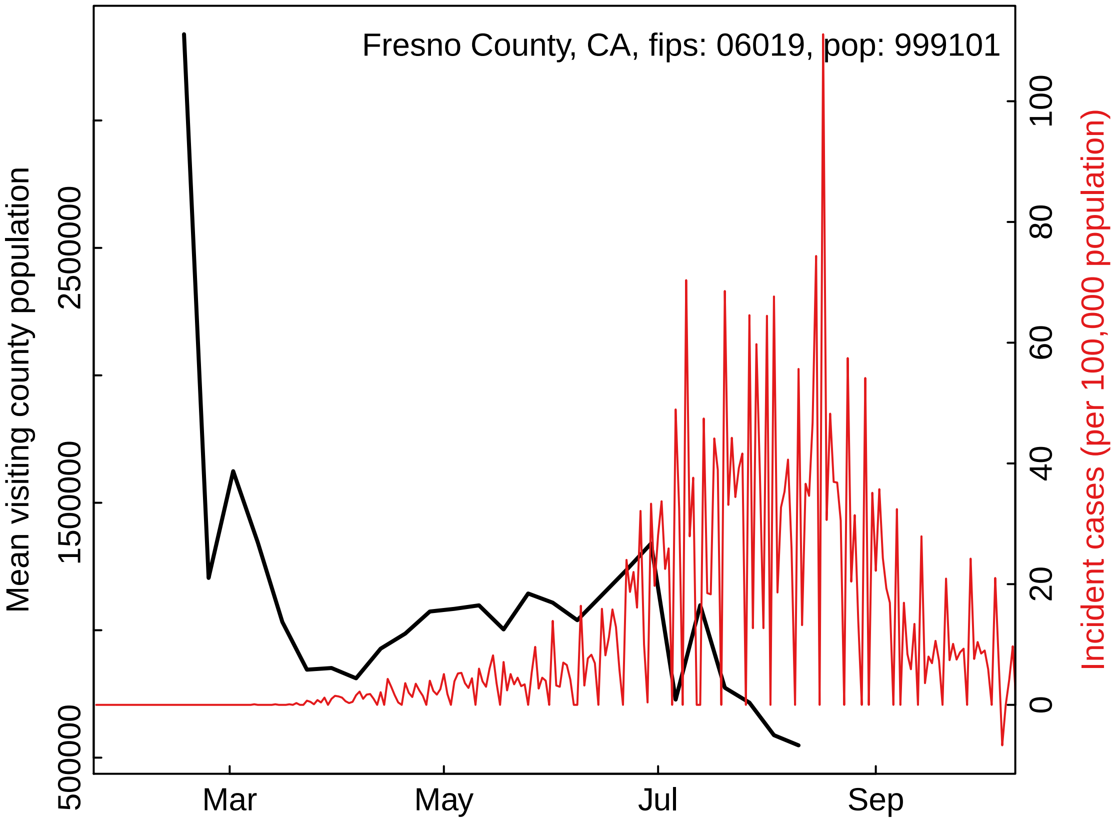

Fresno County, CA, fips: 06019, pop: 999101
Mean visiting county population
500000
1500000
2500000
Incident cases (per 100,000 population)
0
20
40
60
80
100
Mar
May
Jul
Sep

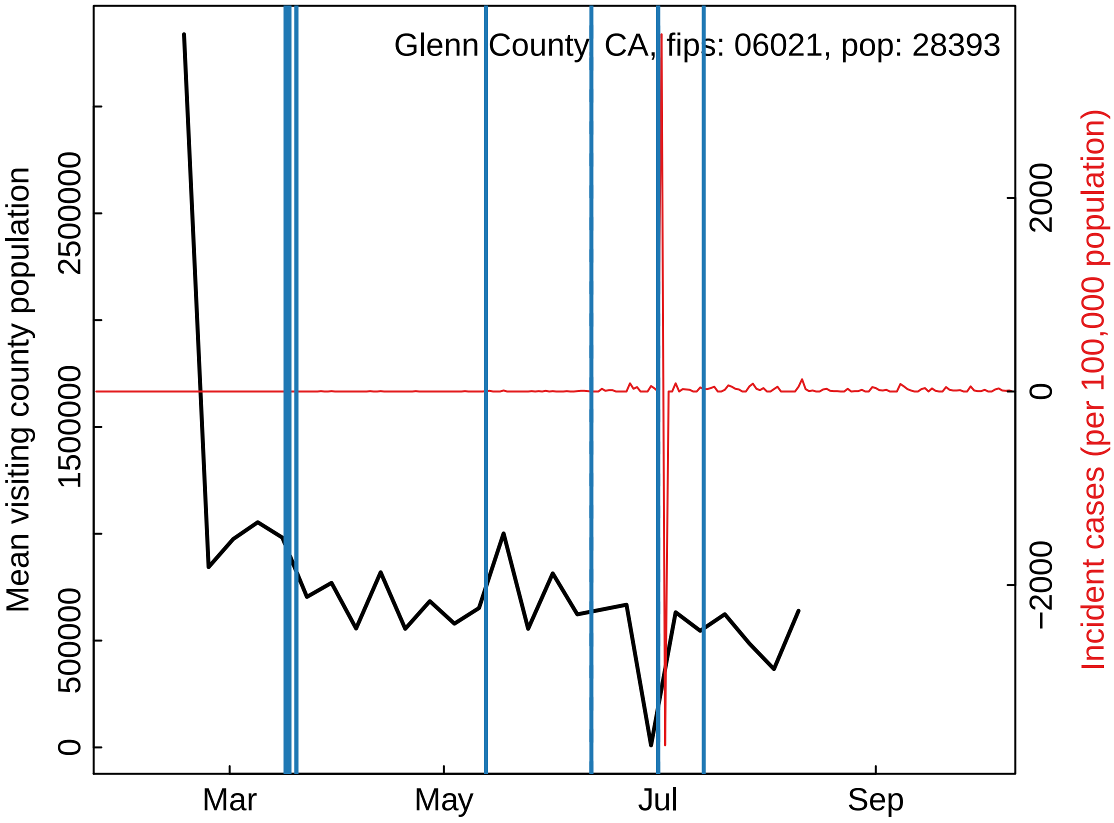

Glenn County, CA, fips: 06021, pop: 28393
Mean visiting county population
0
500000
1500000
2500000
Mar
May
Jul
Sep
Incident cases (per 100,000 population)
−2000
0
2000

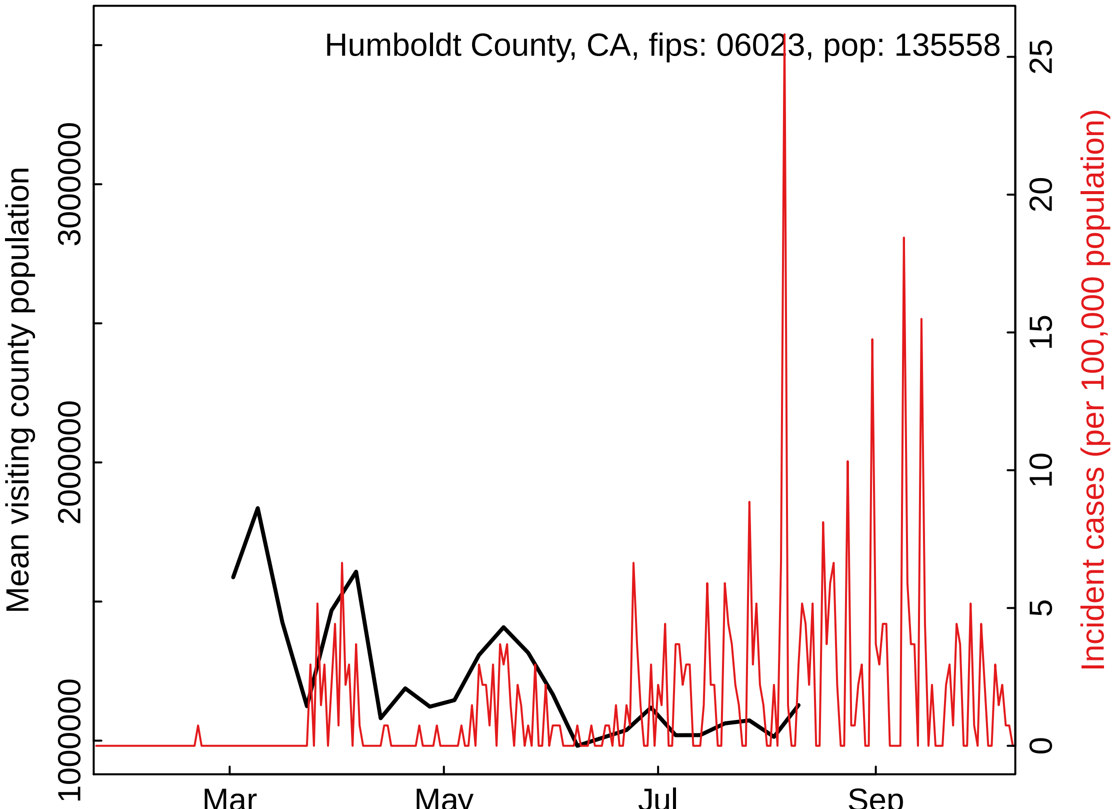
Humboldt County, CA, fips: 06023, pop: 135558
Mean visiting county population
10000000
20000000
30000000
Incident cases (per 100,000 population)
0
5
10
15
20
25
Mar
May
Jul
Sep

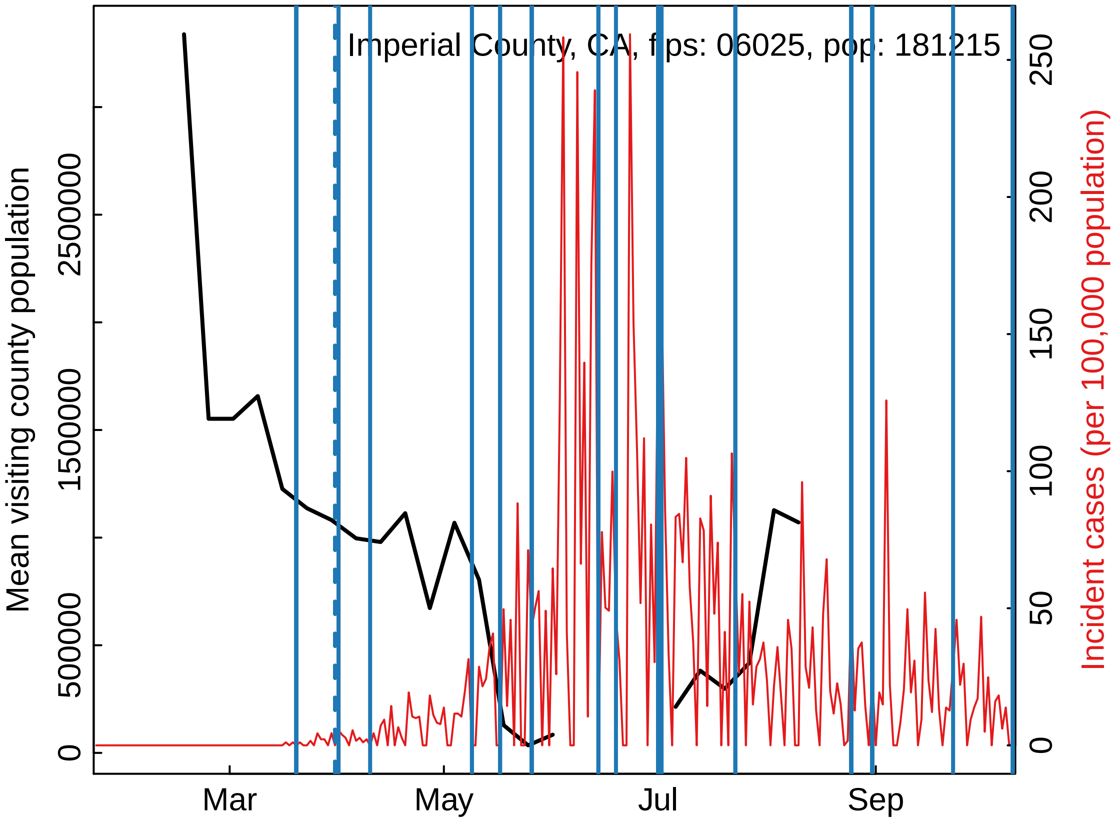

Imperial County, CA, fips: 06025, pop: 181215
Mean visiting county population
0
500000
1500000
2500000
Incident cases (per 100,000 population)
0
50
100
150
200
250
Mar
May
Jul
Sep

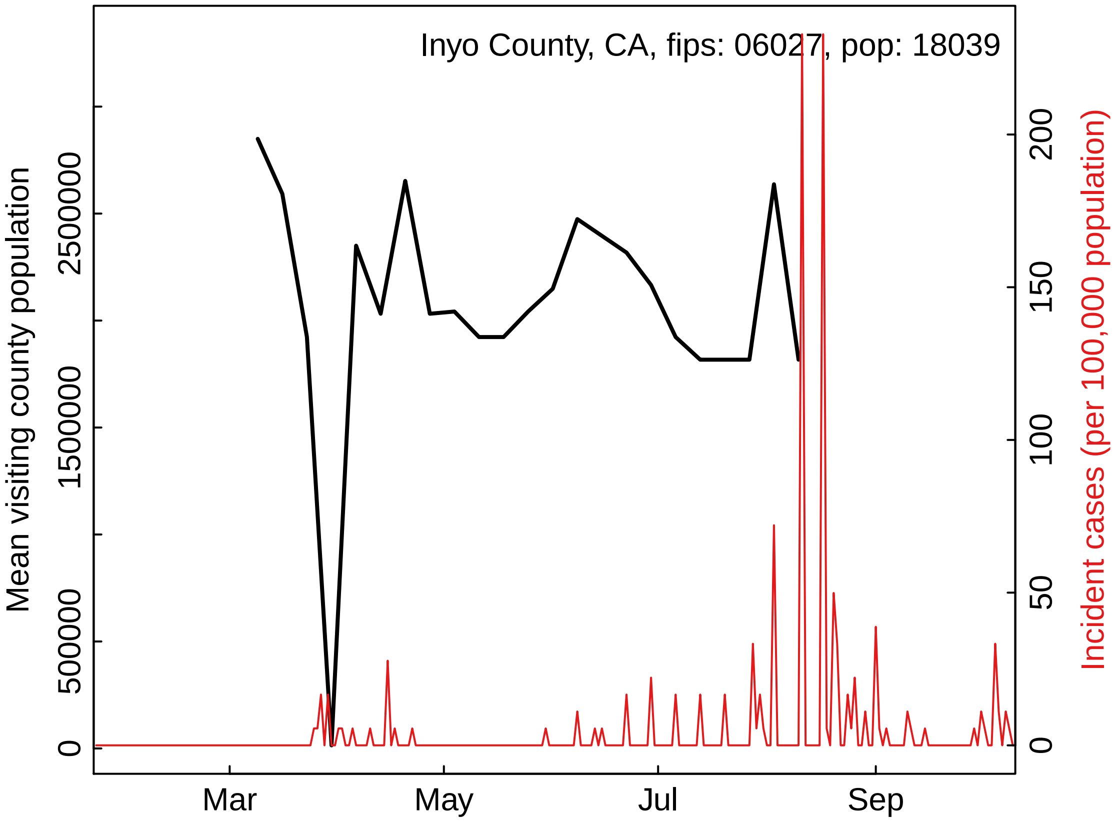

Inyo County, CA, fips: 06027, pop: 18039
Mean visiting county population
0
500000
1500000
2500000
Incident cases (per 100,000 population)
0
50
100
150
200
Mar
May
Jul
Sep

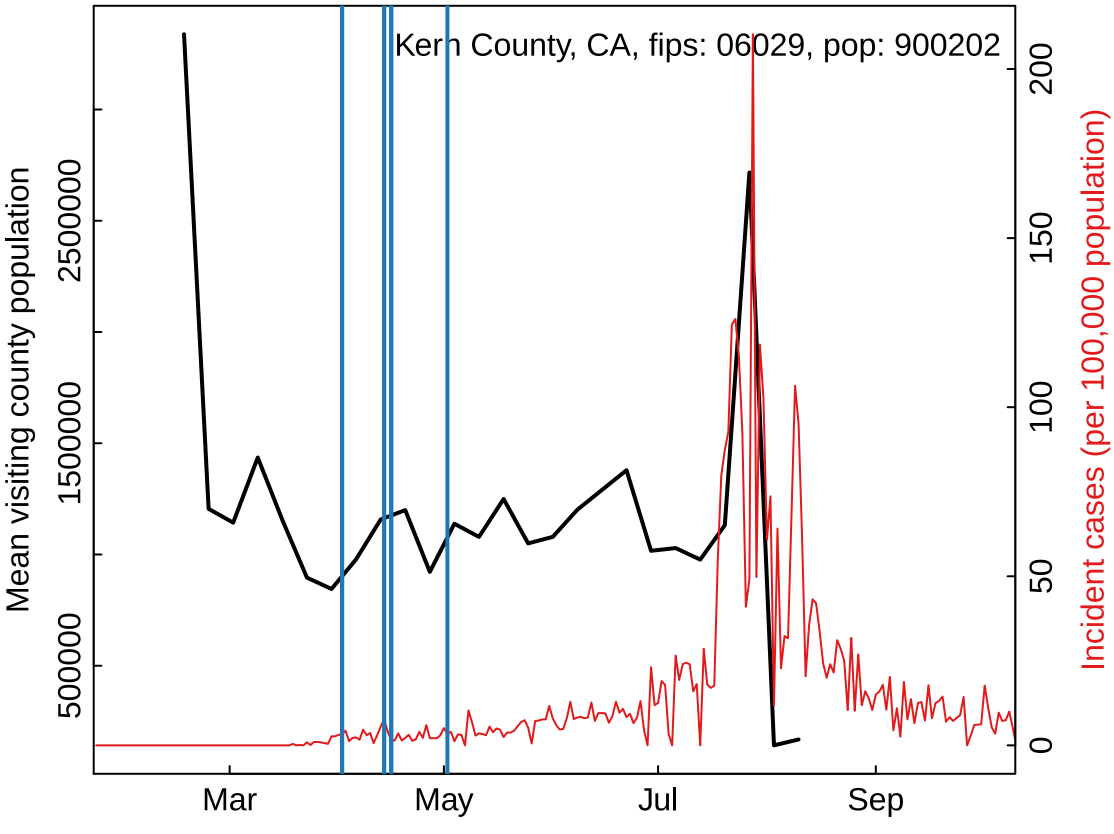

Kern County, CA, fips: 06029, pop: 900202
Mean visiting county population
500000
1500000
2500000
Incident cases (per 100,000 population)
0
50
100
150
200
Mar
May
Jul
Sep

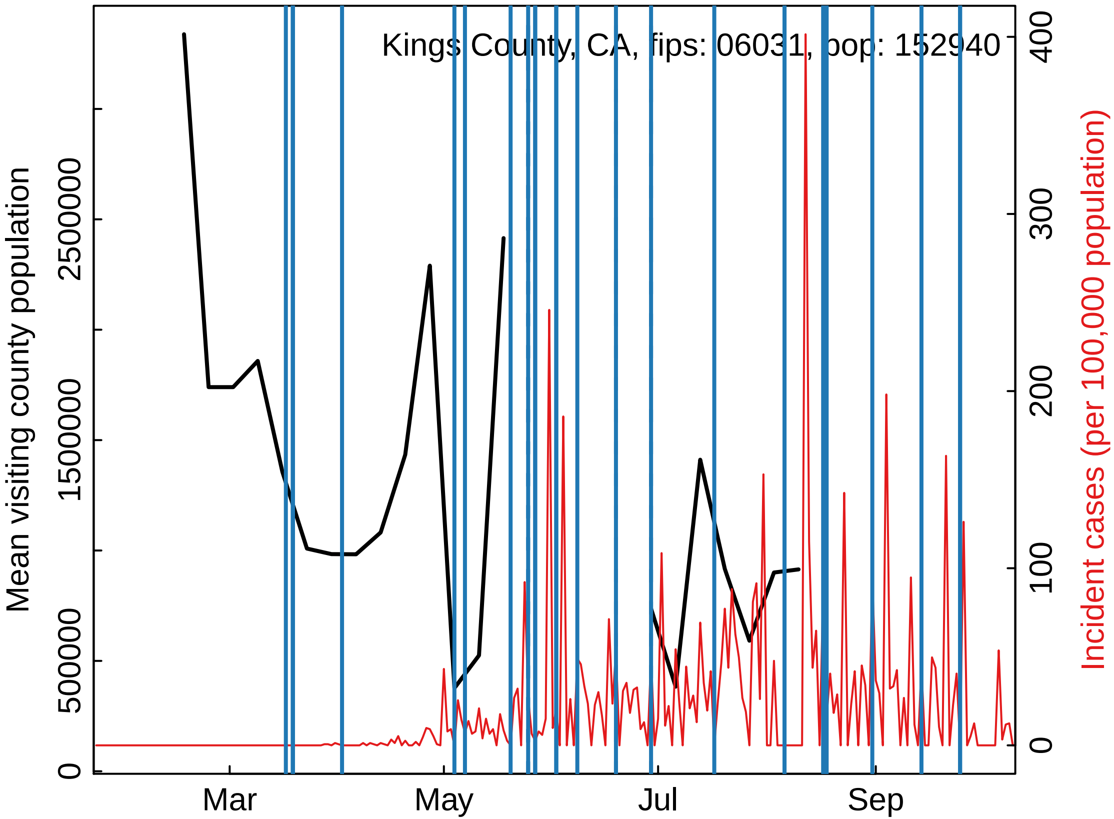

Kings County, CA, fips: 06031, pop: 152940
Mean visiting county population
0
500000
1500000
2500000
Mar
May
Jul
Sep
0
100
200
300
400
Incident cases (per 100,000 population)

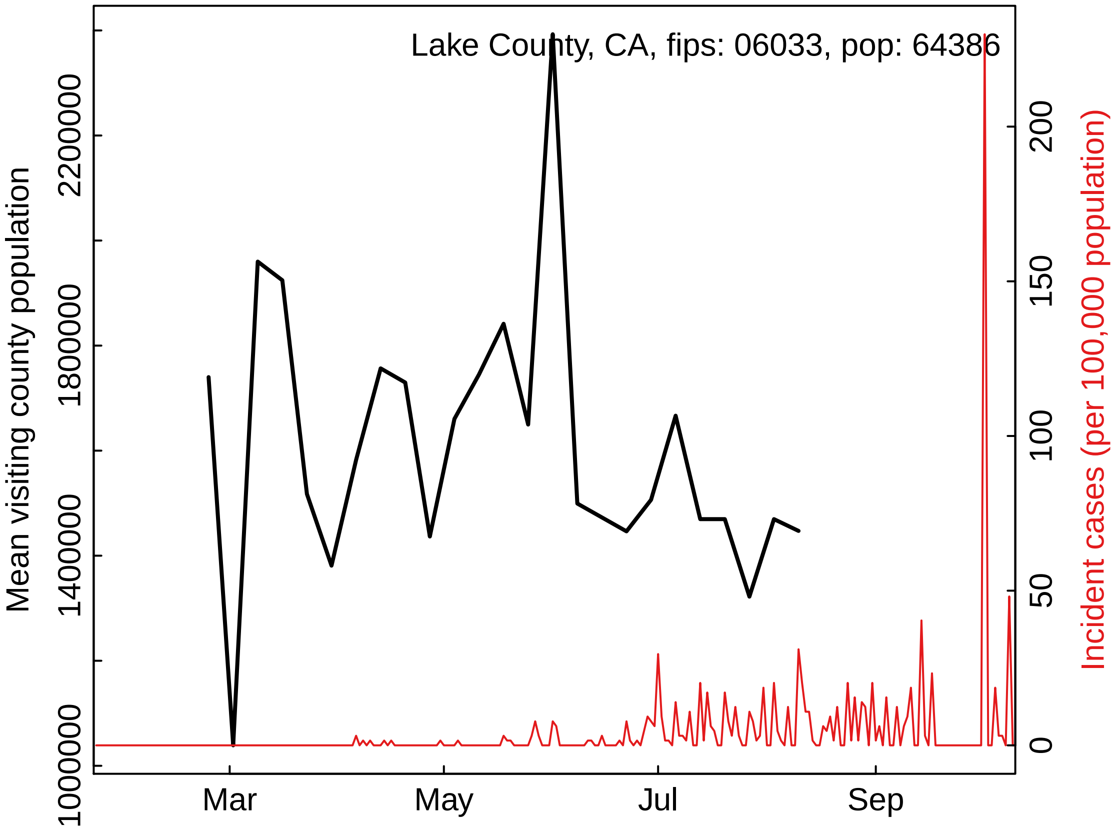
Lake County, CA, fips: 06033, pop: 64386
Mean visiting county population
10000000
14000000
18000000
22000000
Incident cases (per 100,000 population)
0
50
100
150
200
Mar
May
Jul
Sep

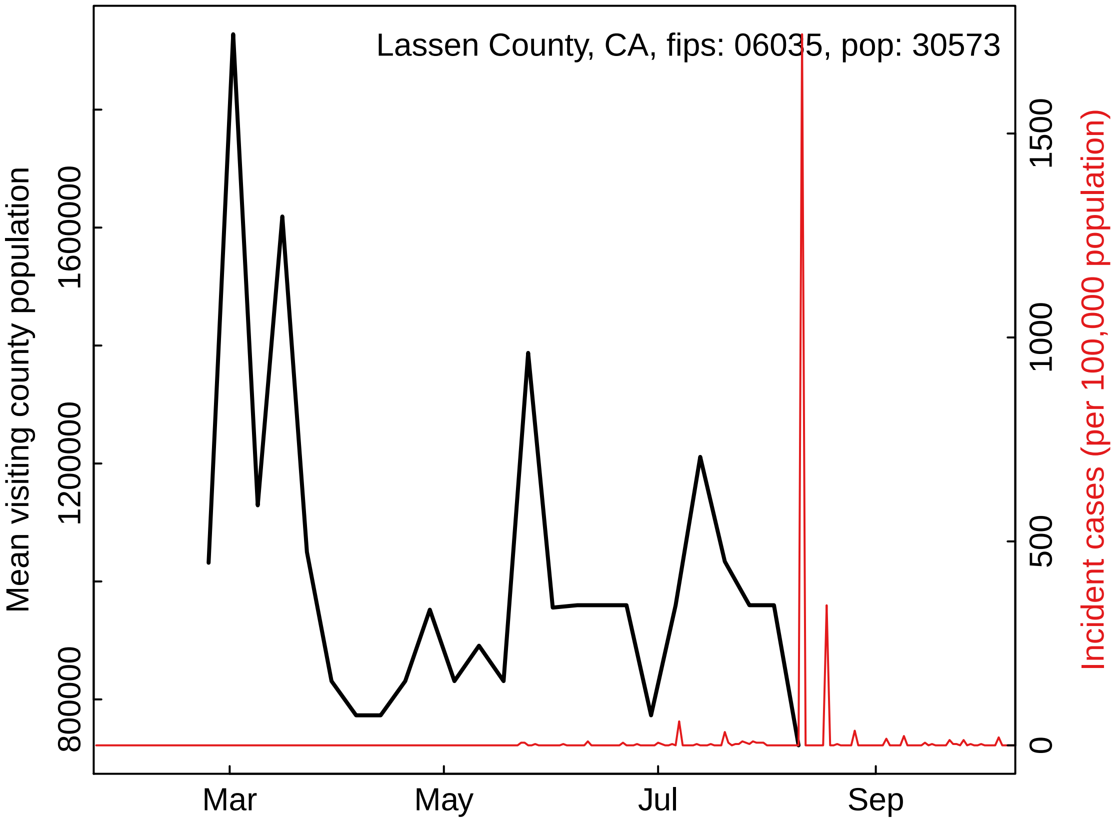

Lassen County, CA, fips: 06035, pop: 30573
Mean visiting county population
800000
1200000
1600000
Incident cases (per 100,000 population)
0
500
1000
1500
Mar
May
Jul
Sep

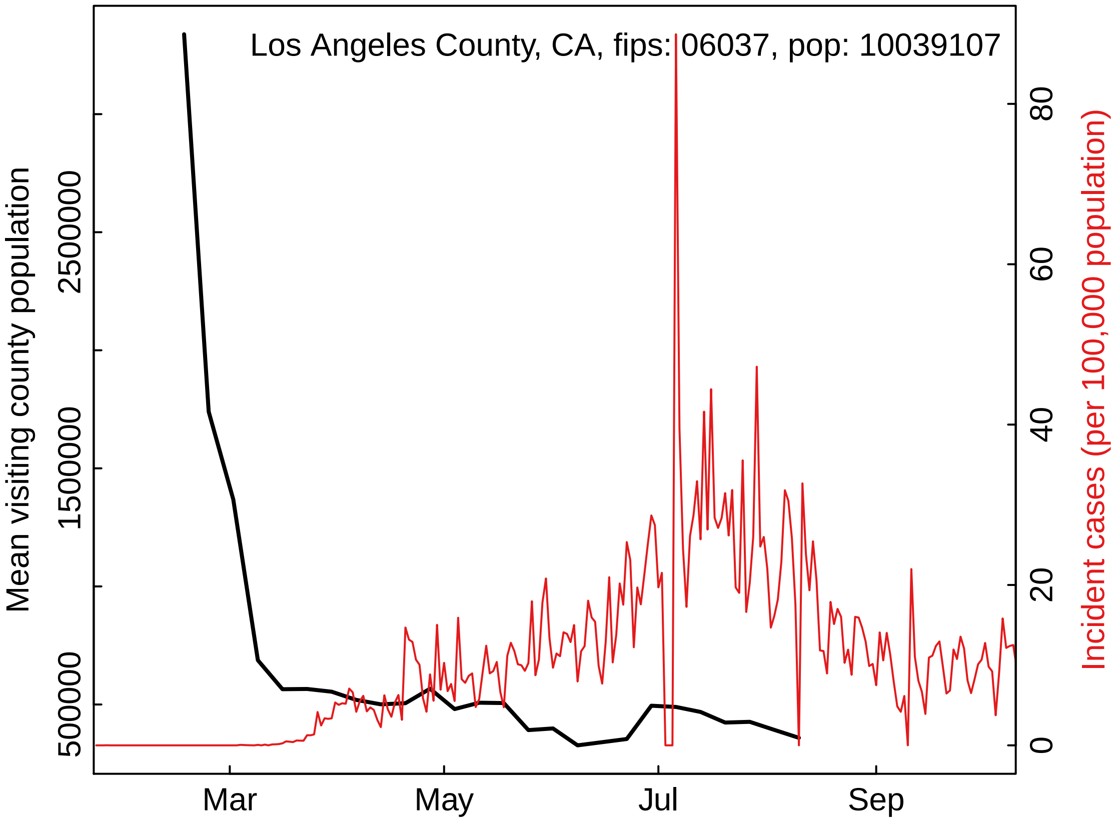
Los Angeles County, CA, fips: 06037, pop: 10039107
Mean visiting county population
500000
1500000
2500000
Mar
May
Jul
Sep
0
20
40
60
80
Incident cases (per 100,000 population)

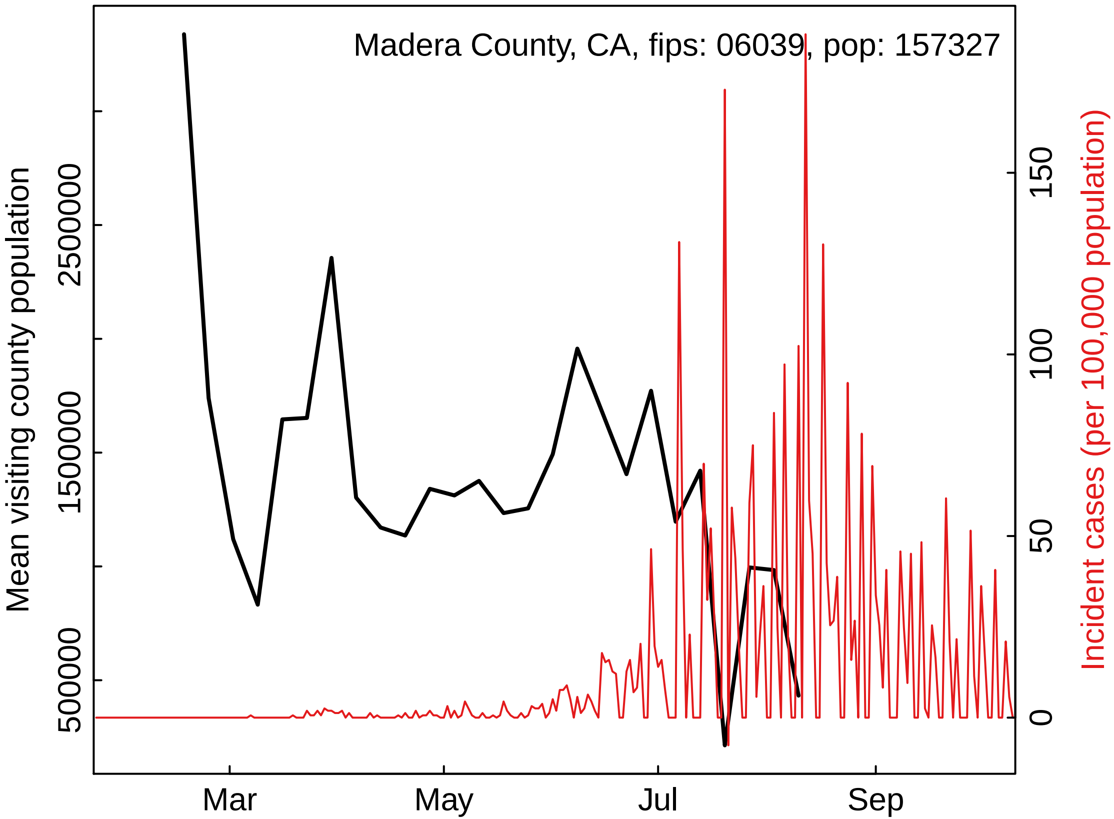

Madera County, CA, fips: 06039, pop: 157327
Mean visiting county population
500000
1500000
2500000
Incident cases (per 100,000 population)
0
50
100
150
Mar
May
Jul
Sep

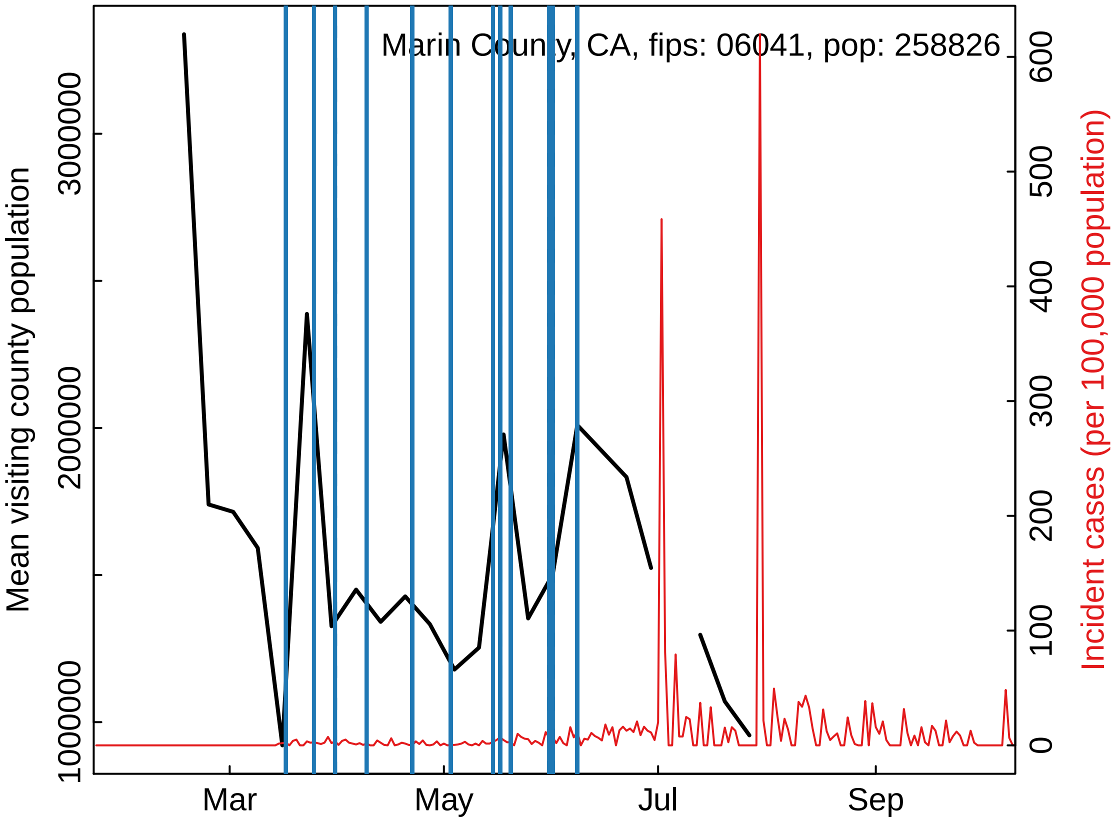

Marin County, CA, fips: 06041, pop: 258826
Mean visiting county population
1000000
2000000
3000000
Incident cases (per 100,000 population)
0
100
200
300
400
500
600
Mar
May
Jul
Sep

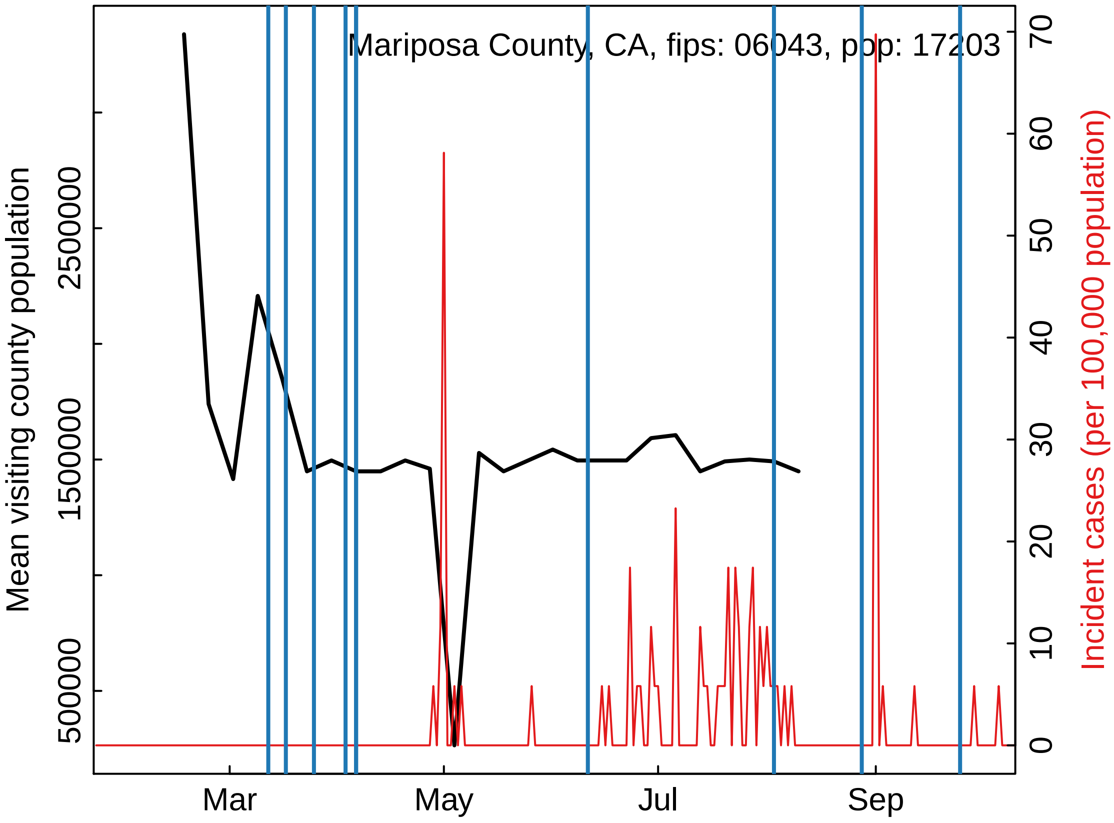

Mariposa County, CA, fips: 06043, pop: 17203
Mean visiting county population
500000
1500000
2500000
Mar
May
Jul
Sep
0
10
20
30
40
50
60
70
Incident cases (per 100,000 population)

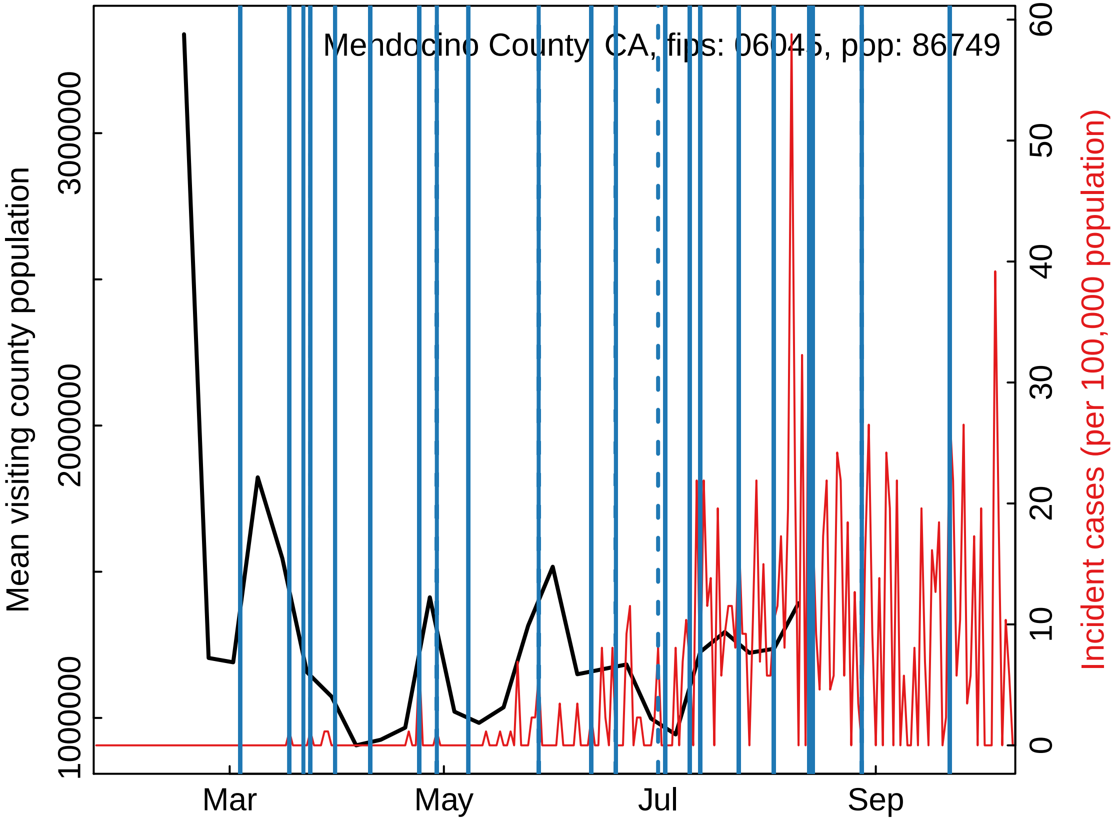

Mendocino County, CA, fips: 06045, pop: 86749
Mean visiting county population
Incident cases (per 100,000 population)
1000000
2000000
3000000
0
10
20
30
40
50
60
Mar
May
Jul
Sep

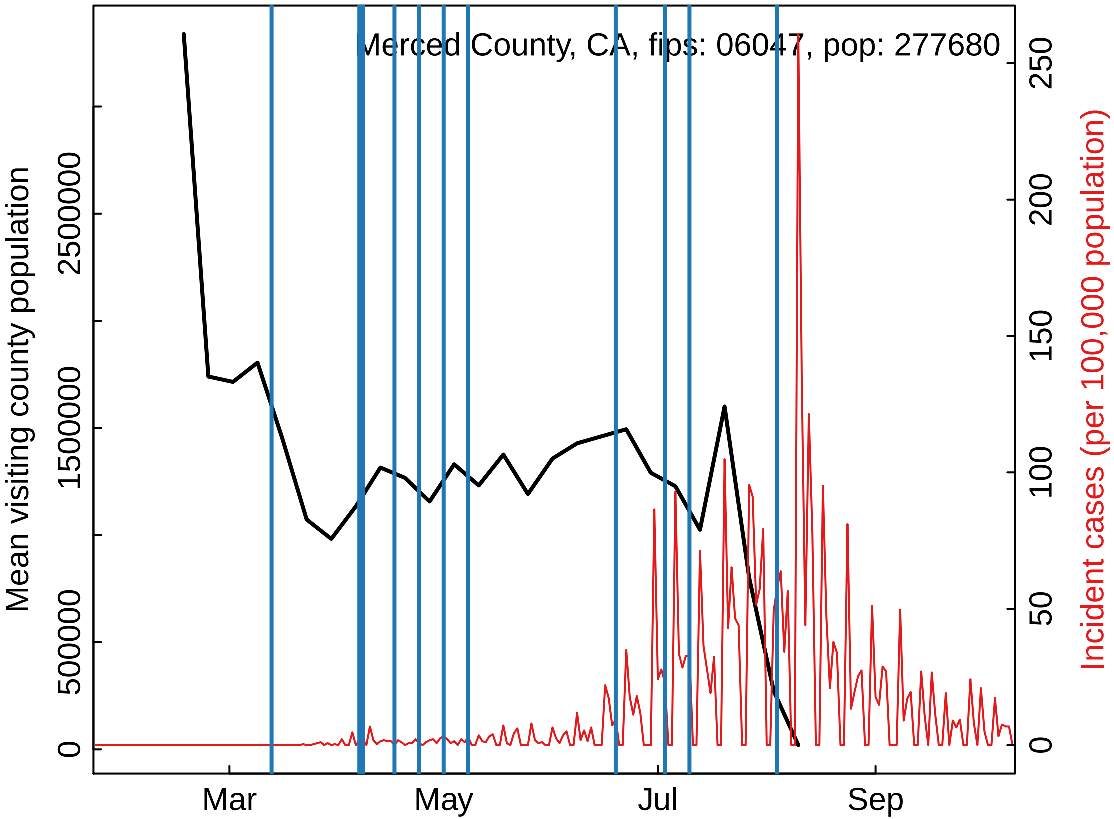

Merced County, CA, fips: 06047, pop: 277680
Mean visiting county population
0
500000
1500000
2500000
Incident cases (per 100,000 population)
0
50
100
150
200
250
Mar
May
Jul
Sep

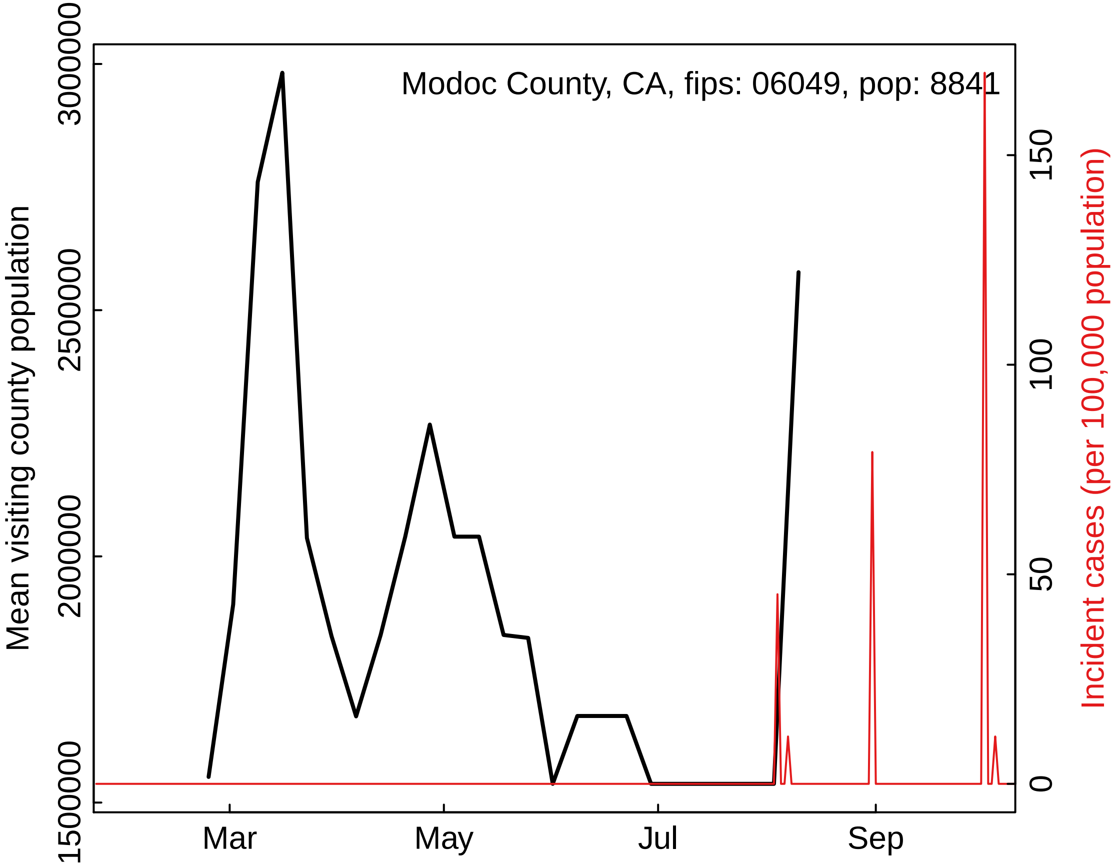
Modoc County, CA, fips: 06049, pop: 8841
Mean visiting county population
1500000
2000000
2500000
3000000
Incident cases (per 100,000 population)
0
50
100
150
Mar
May
Jul
Sep

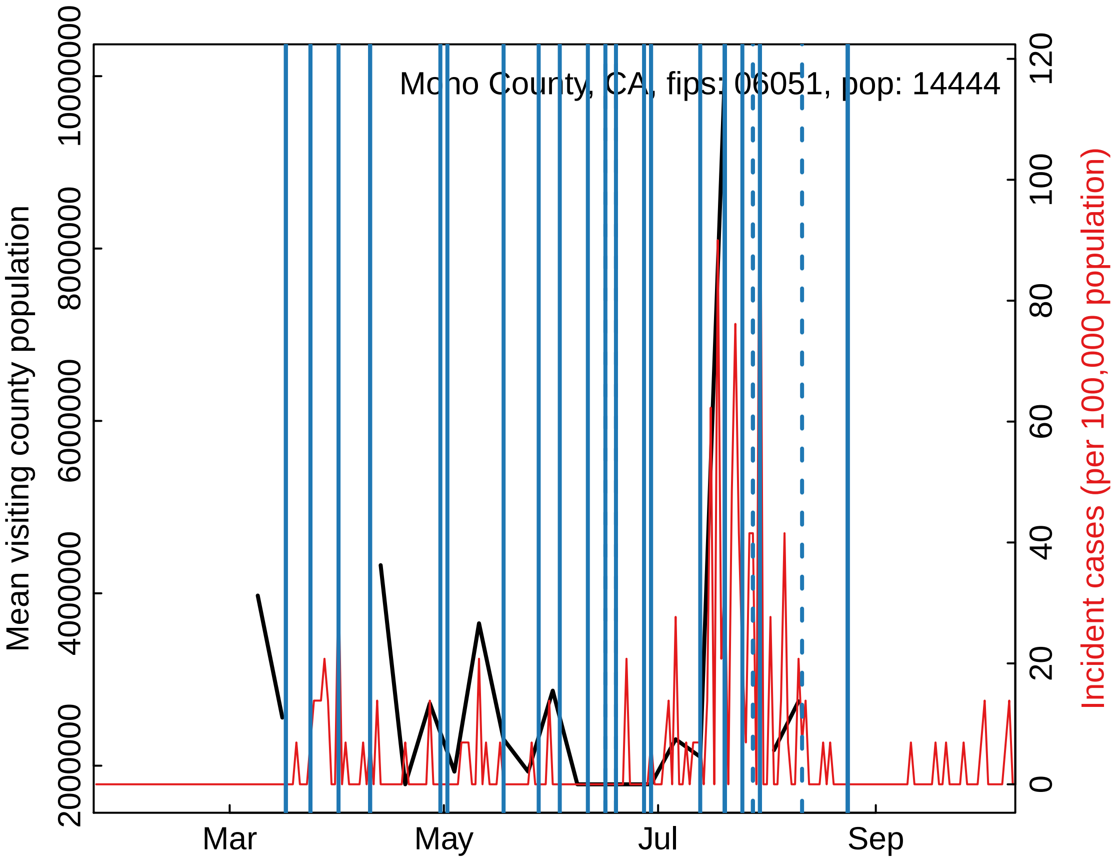
Mono County, CA, fips: 06051, pop: 14444
Mean visiting county population
Incident cases (per 100,000 population)
2000000
4000000
6000000
8000000
10000000
0
20
40
60
80
100
120
Mar
May
Jul
Sep

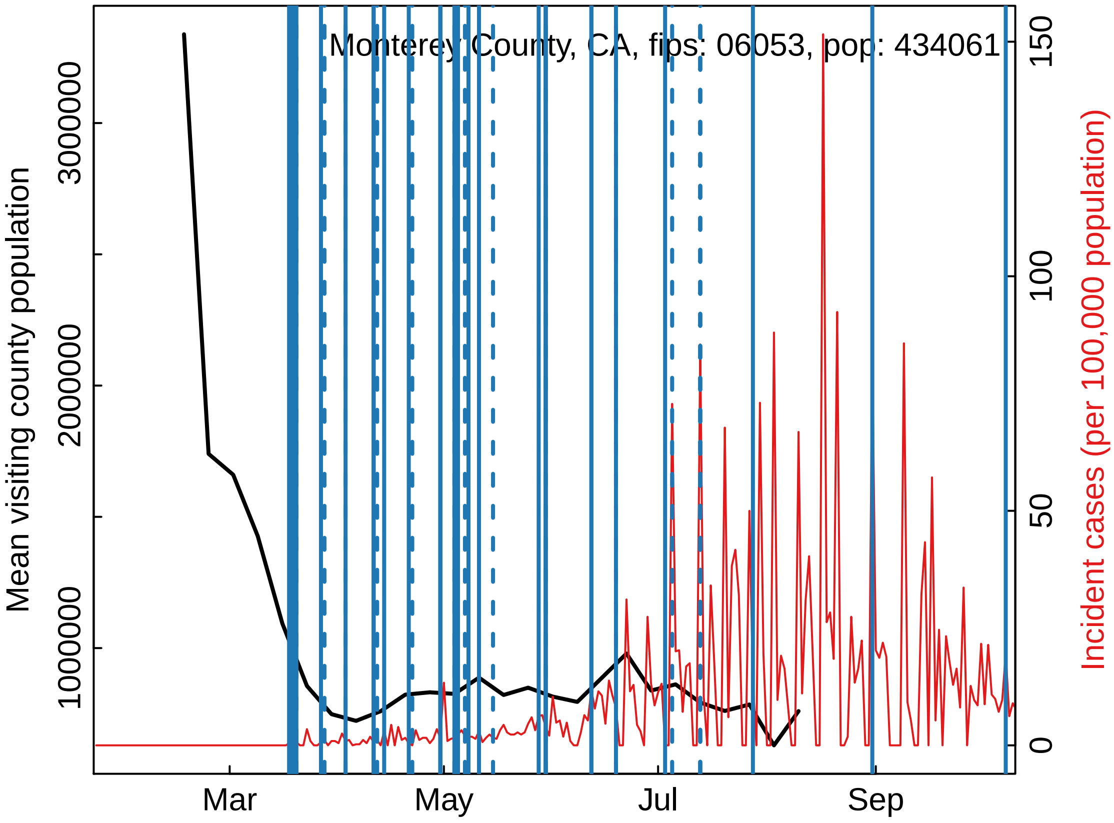

Monterey County, CA, fips: 06053, pop: 434061
Mean visiting county population
10000000
20000000
30000000
Incident cases (per 100,000 population)
0
50
100
150
Mar
May
Jul
Sep

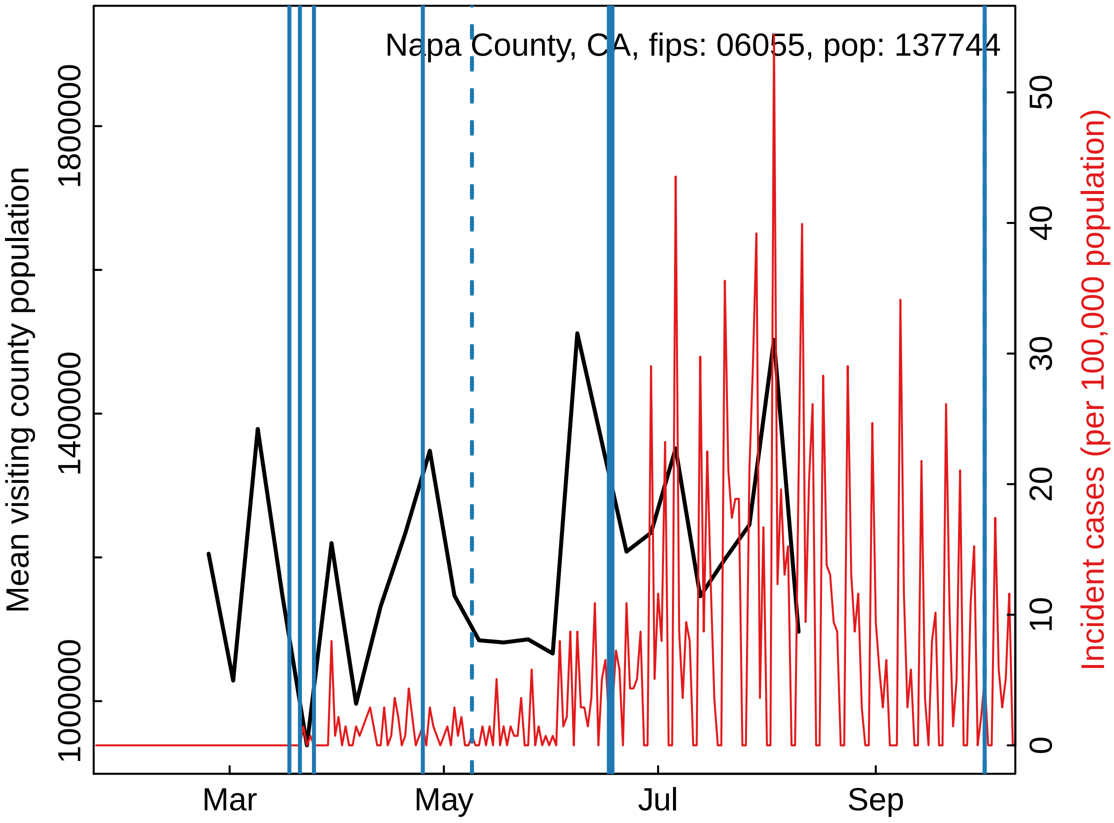

Napa County, CA, fips: 06055, pop: 137744
Mean visiting county population
1000000
1400000
1800000
Incident cases (per 100,000 population)
0
10
20
30
40
50
Mar
May
Jul
Sep

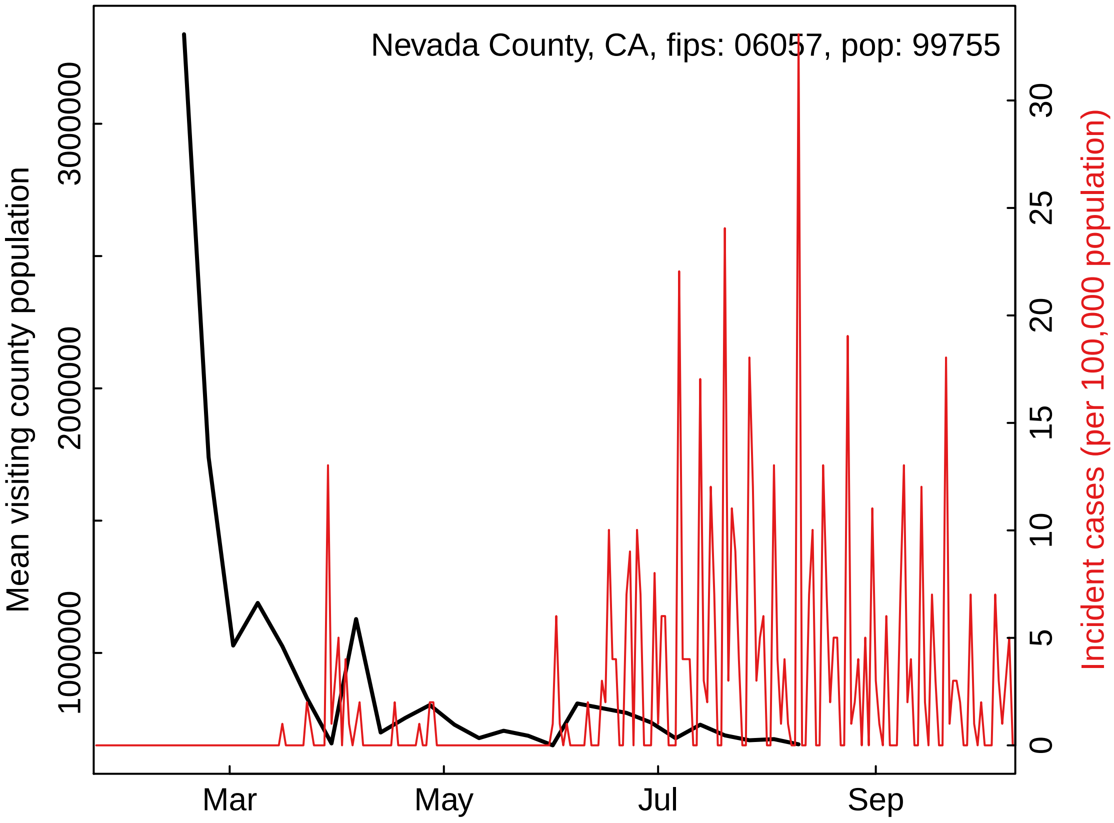

Nevada County, CA, fips: 06057, pop: 99755
Mean visiting county population
10000000
20000000
30000000
Incident cases (per 100,000 population)
0
5
10
15
20
25
30
Mar
May
Jul
Sep

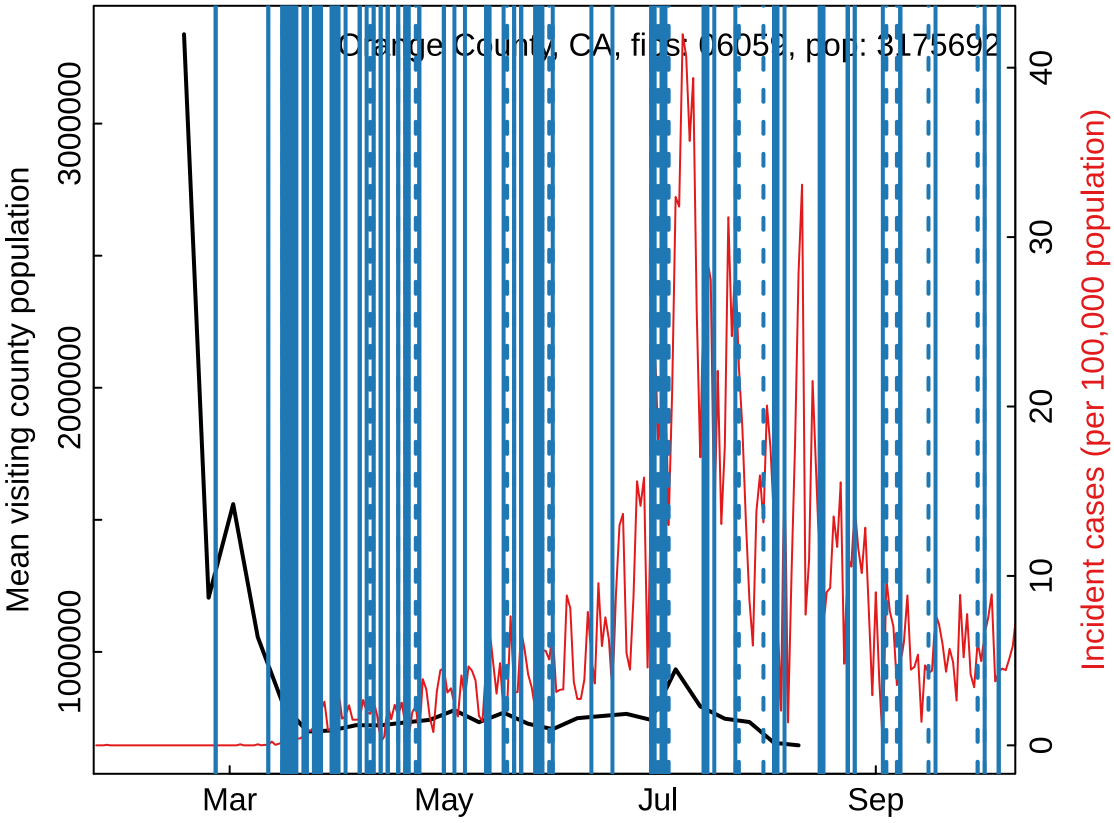

Orange County, CA, fips: 06059, pop: 3175692
Mean visiting county population
Incident cases (per 100,000 population)
10000000
20000000
30000000
0
10
20
30
40
Mar
May
Jul
Sep

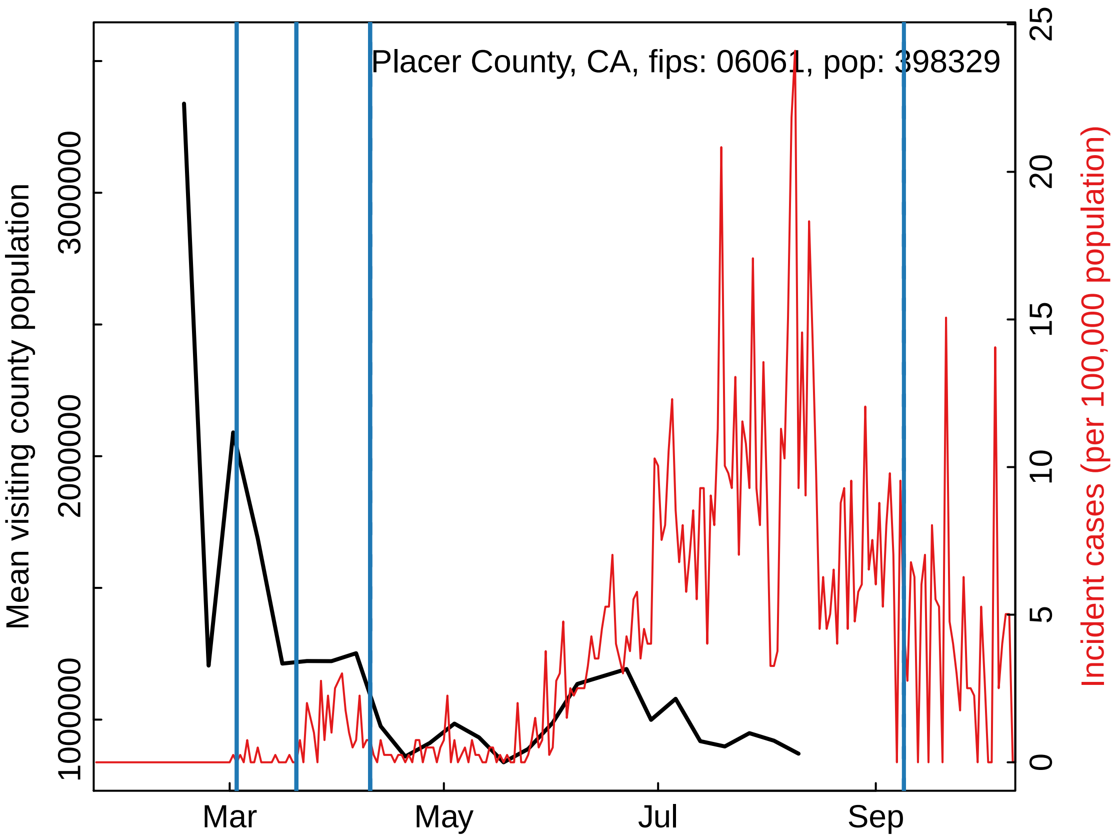

Placer County, CA, fips: 06061, pop: 398329
Mean visiting county population
1000000
2000000
3000000
Incident cases (per 100,000 population)
0
5
10
15
20
25
Mar
May
Jul
Sep

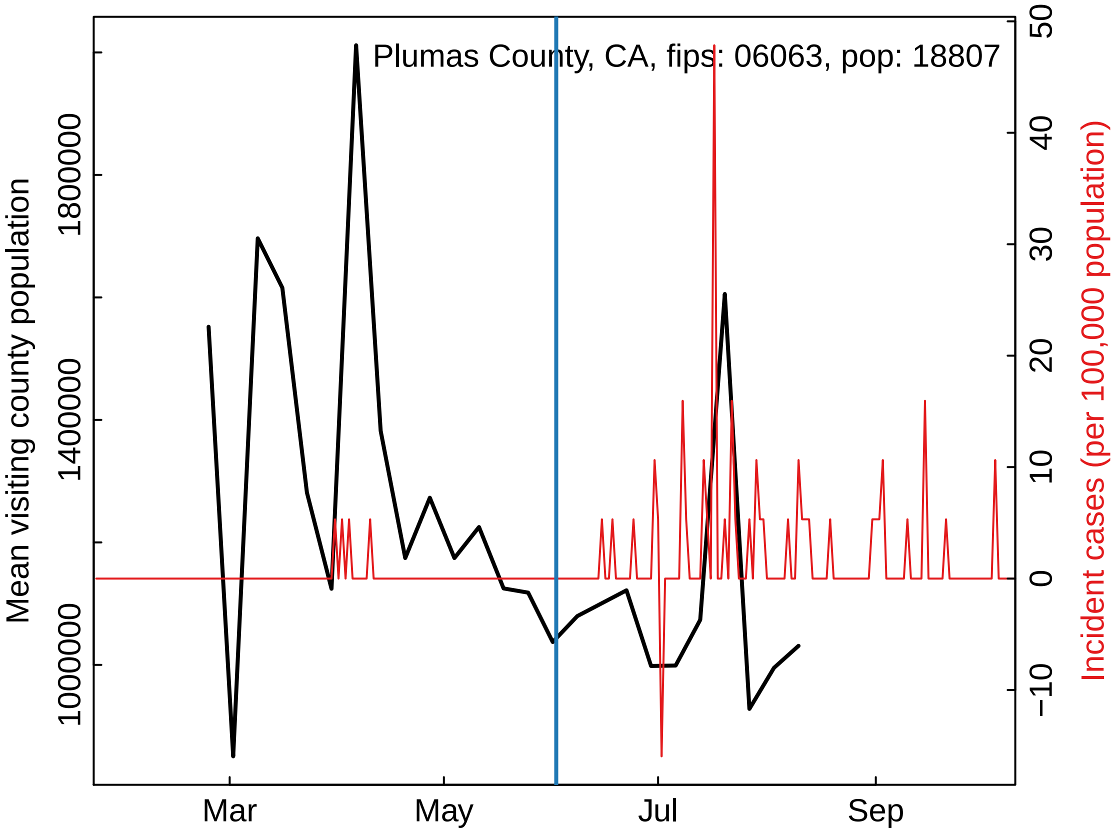

Plumas County, CA, fips: 06063, pop: 18807
Mean visiting county population
1000000
1400000
1800000
Mar
May
Jul
Sep
−10
0
10
20
30
40
50
Incident cases (per 100,000 population)

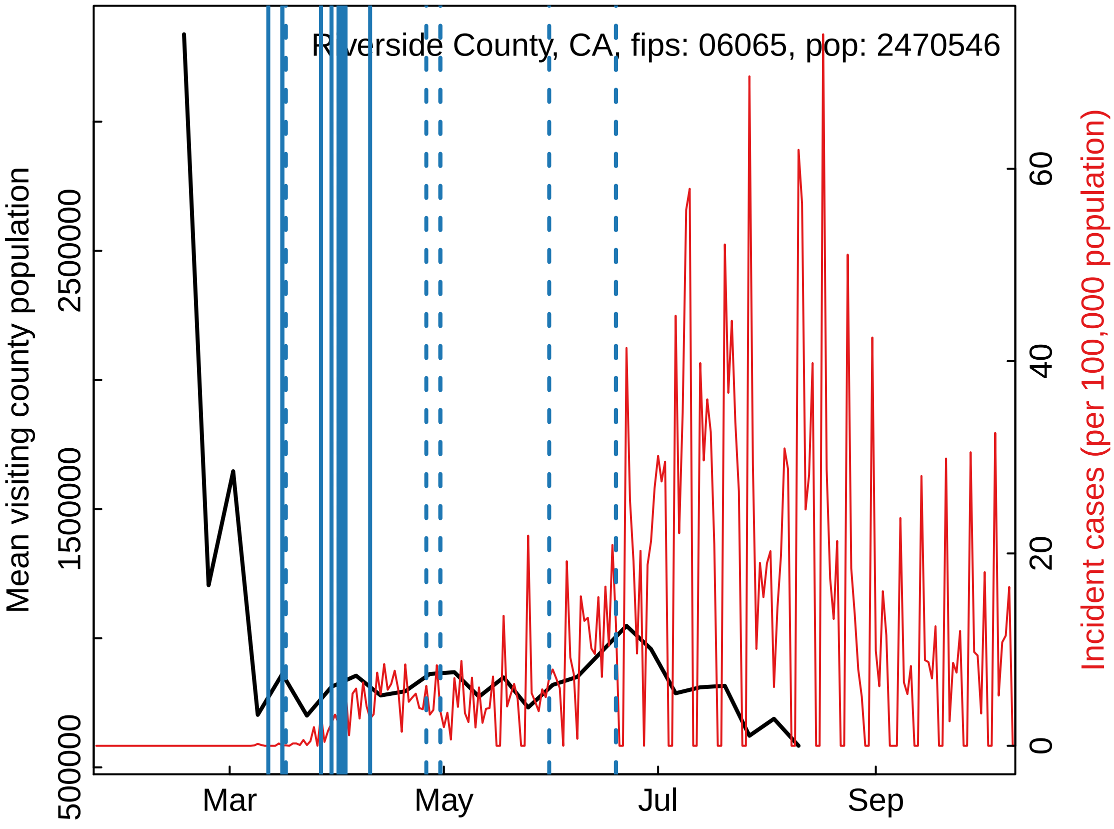
Riverside County, CA, fips: 06065, pop: 2470546
Mean visiting county population
Incident cases (per 100,000 population)
500000
1500000
2500000
0
20
40
60
Mar
May
Jul
Sep

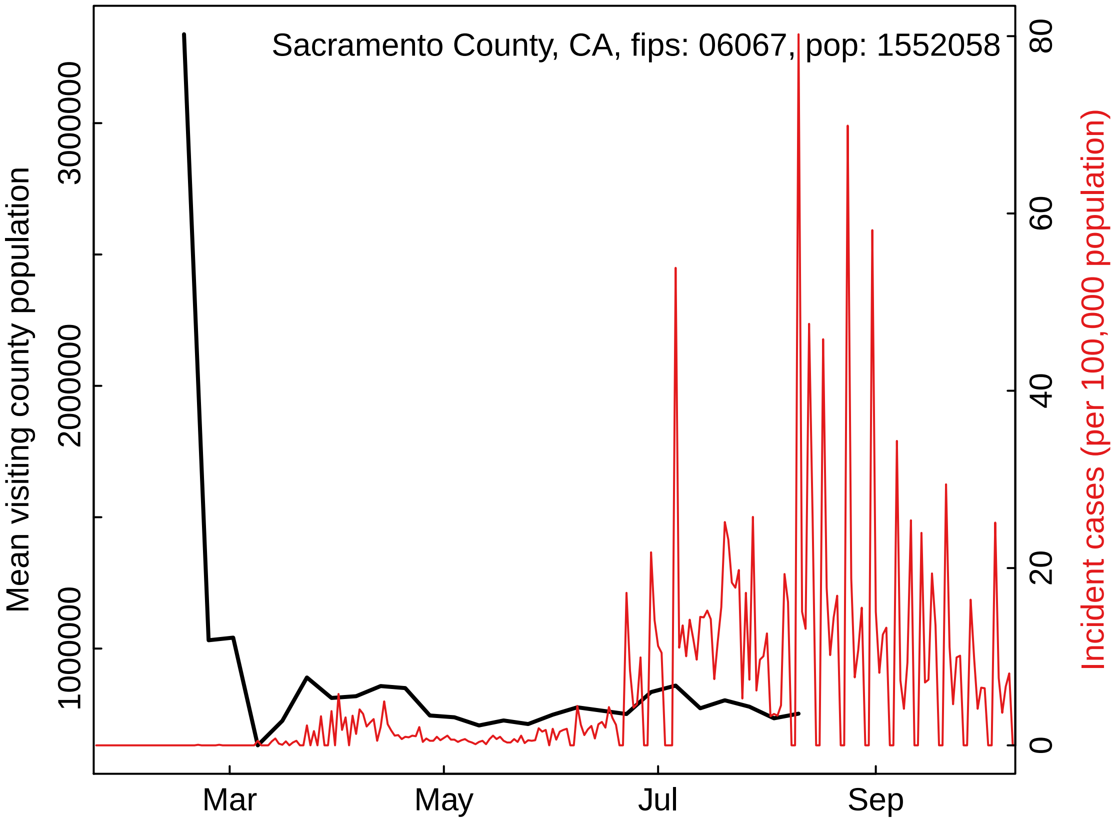

Sacramento County, CA, fips: 06067, pop: 1552058
Mean visiting county population
1000000
2000000
3000000
Incident cases (per 100,000 population)
0
20
40
60
80
Mar
May
Jul
Sep

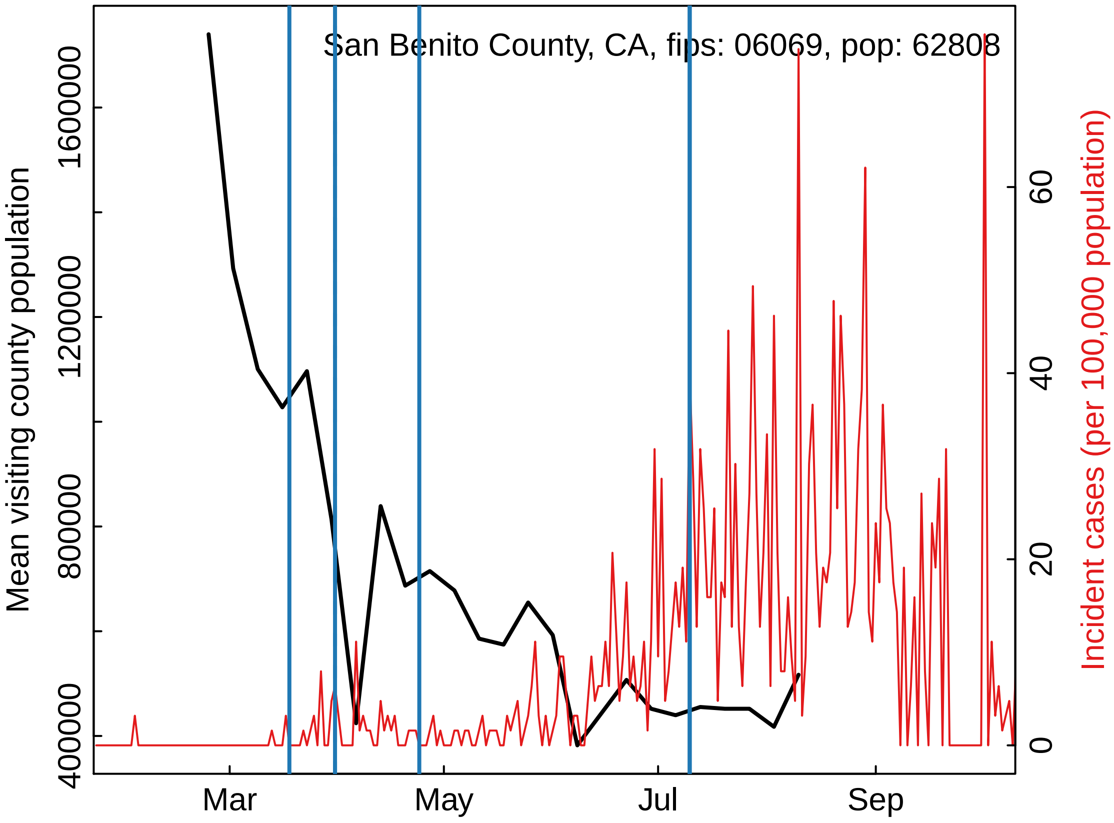

San Benito County, CA, fips: 06069, pop: 62808
Mean visiting county population
400000
800000
1200000
1600000
Incident cases (per 100,000 population)
0
20
40
60
Mar
May
Jul
Sep

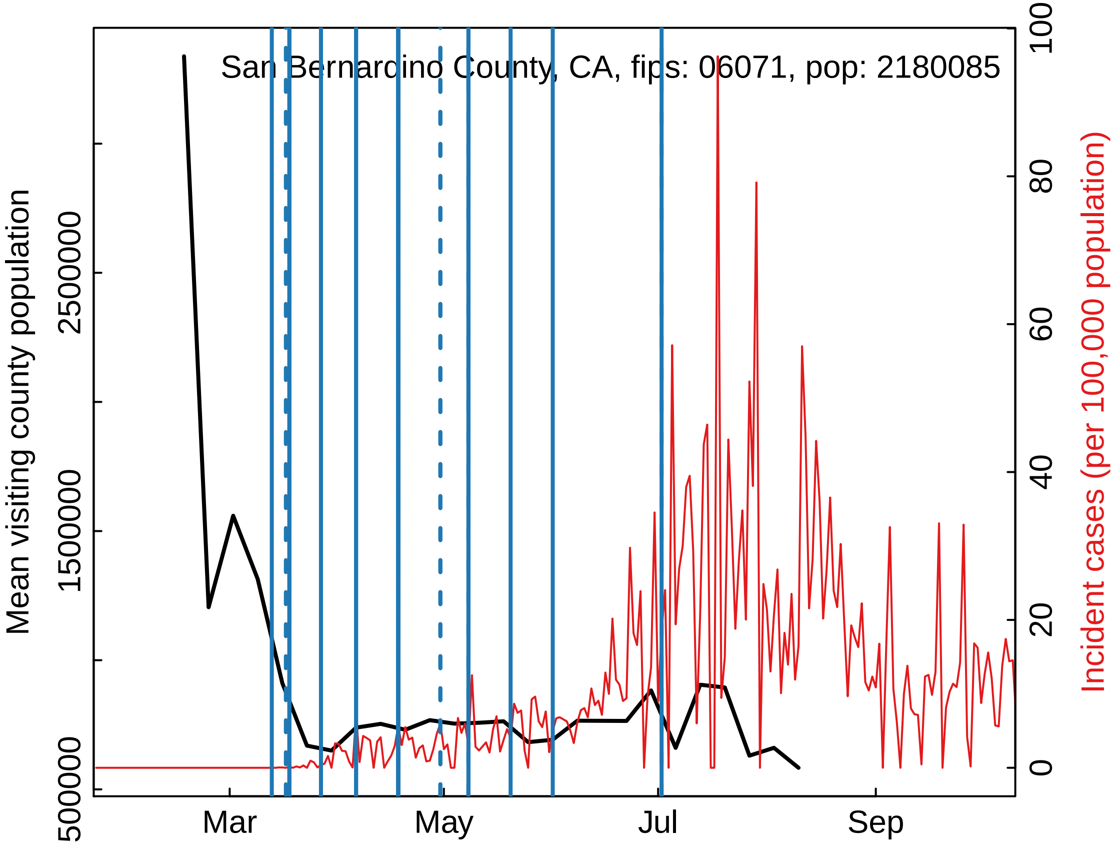
San Bernardino County, CA, fips: 06071, pop: 2180085
Mean visiting county population
500000
1500000
2500000
Incident cases (per 100,000 population)
0
20
40
60
80
100
Mar
May
Jul
Sep

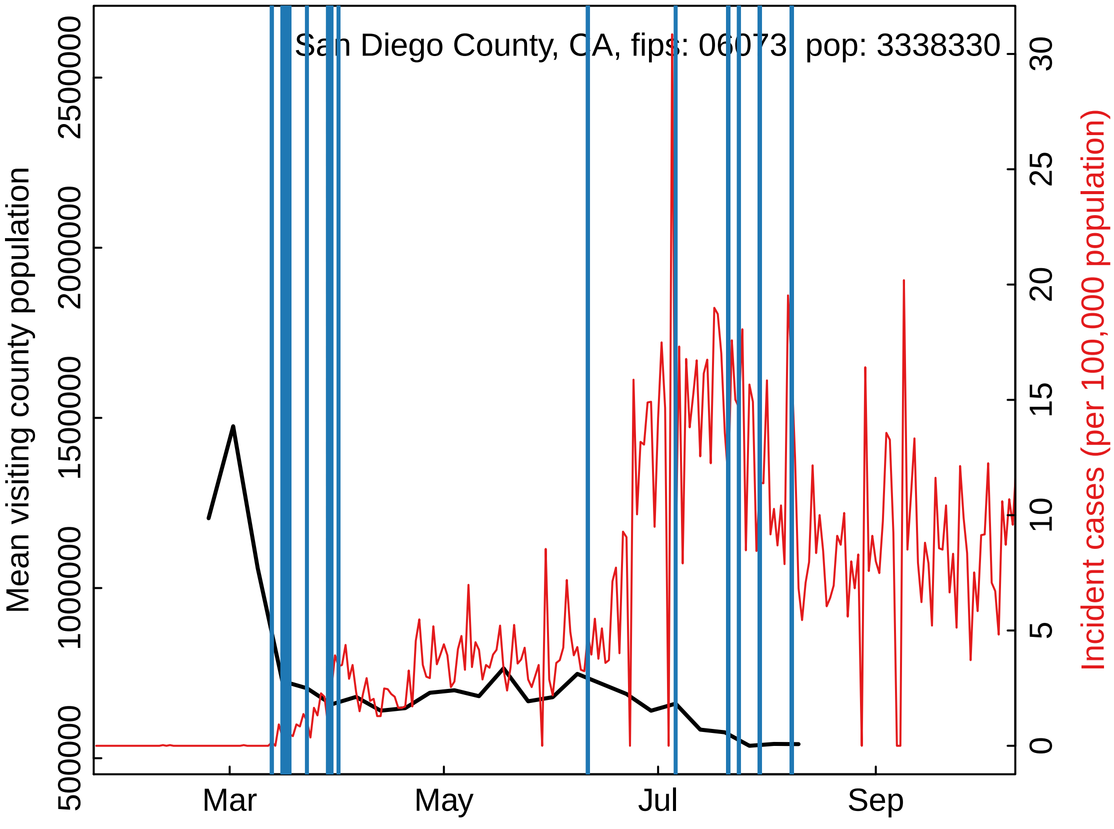
San Diego County, CA, fips: 06073, pop: 3338330
Mean visiting county population
500000
1000000
1500000
2000000
2500000
Incident cases (per 100,000 population)
0
5
10
15
20
25
30
Mar
May
Jul
Sep

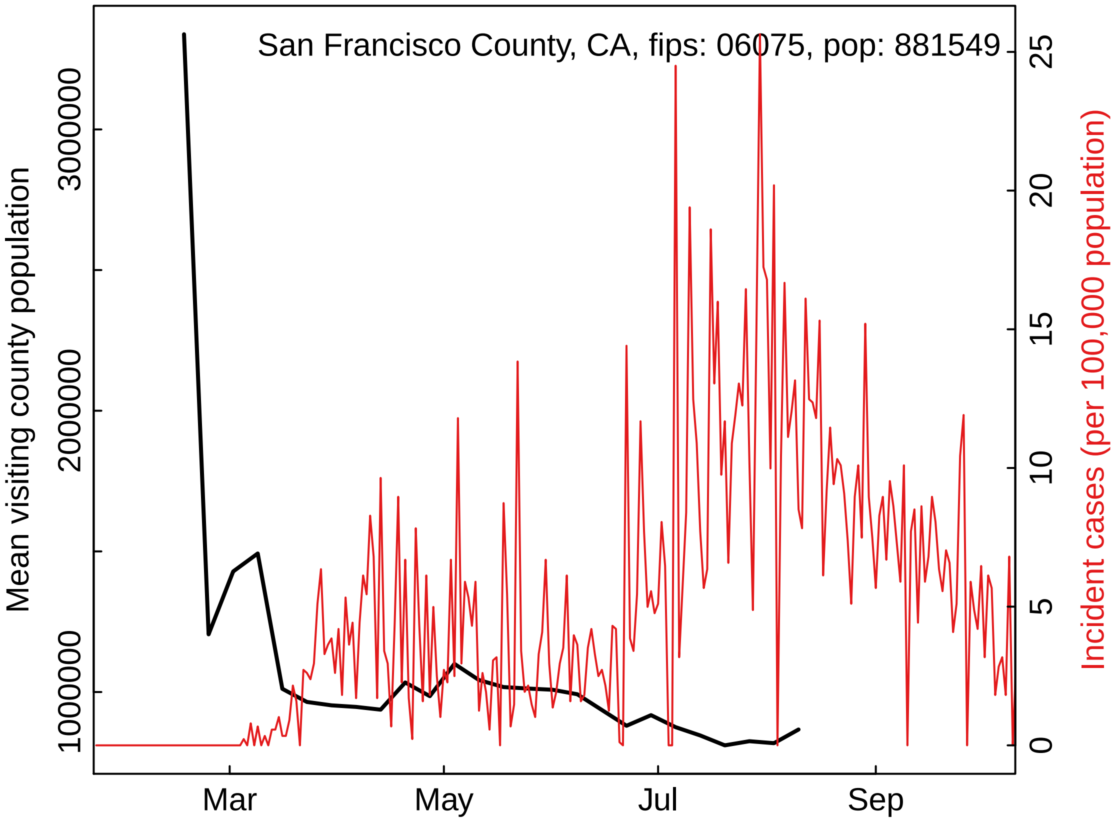
San Francisco County, CA, fips: 06075, pop: 881549
Mean visiting county population
10000000
20000000
30000000
Incident cases (per 100,000 population)
0
5
10
15
20
25
Mar
May
Jul
Sep

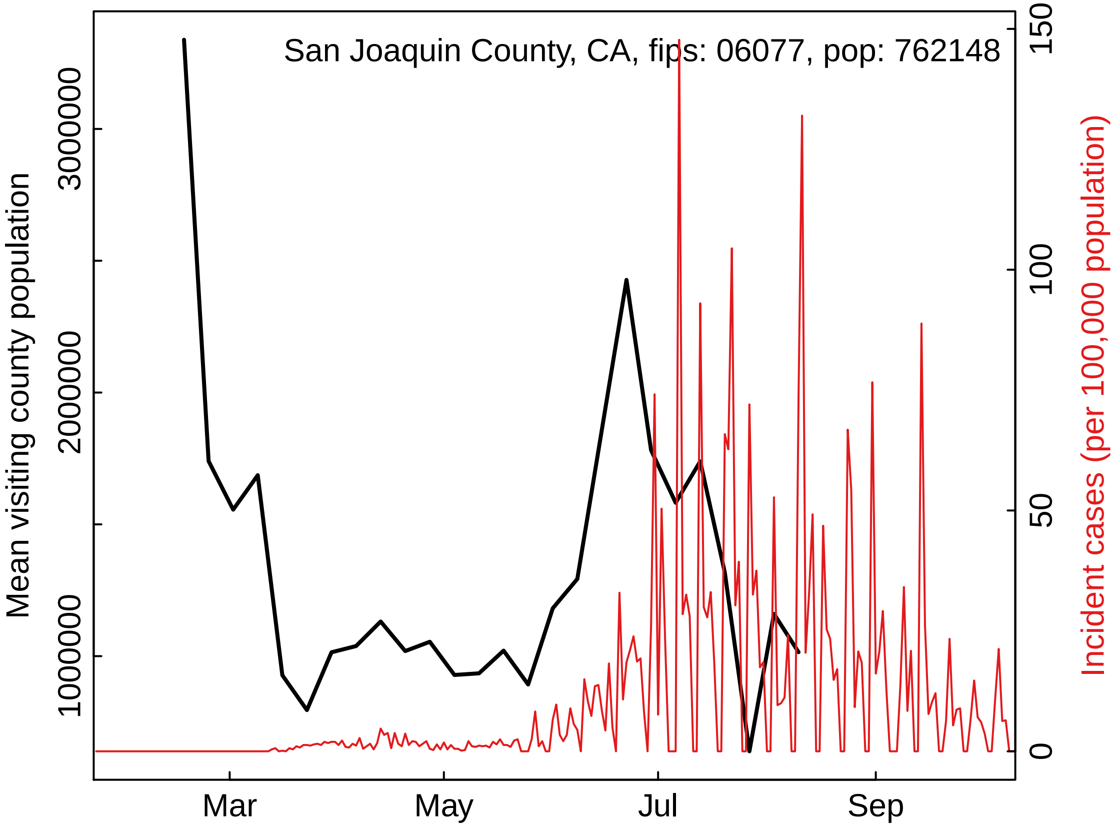
San Joaquin County, CA, fips: 06077, pop: 762148
Mean visiting county population
10000000
20000000
30000000
Incident cases (per 100,000 population)
0
50
100
150
Mar
May
Jul
Sep

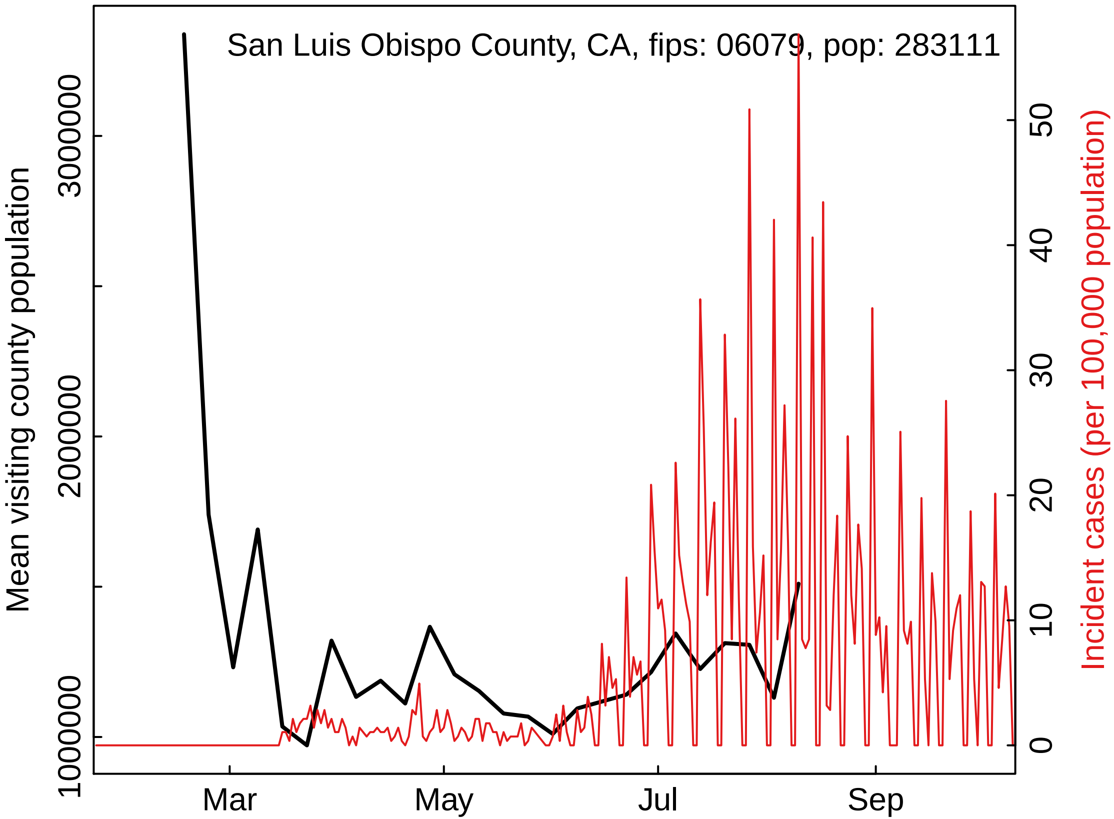
San Luis Obispo County, CA, fips: 06079, pop: 283111
Mean visiting county population
10000000
20000000
30000000
Incident cases (per 100,000 population)
0
10
20
30
40
50
Mar
May
Jul
Sep

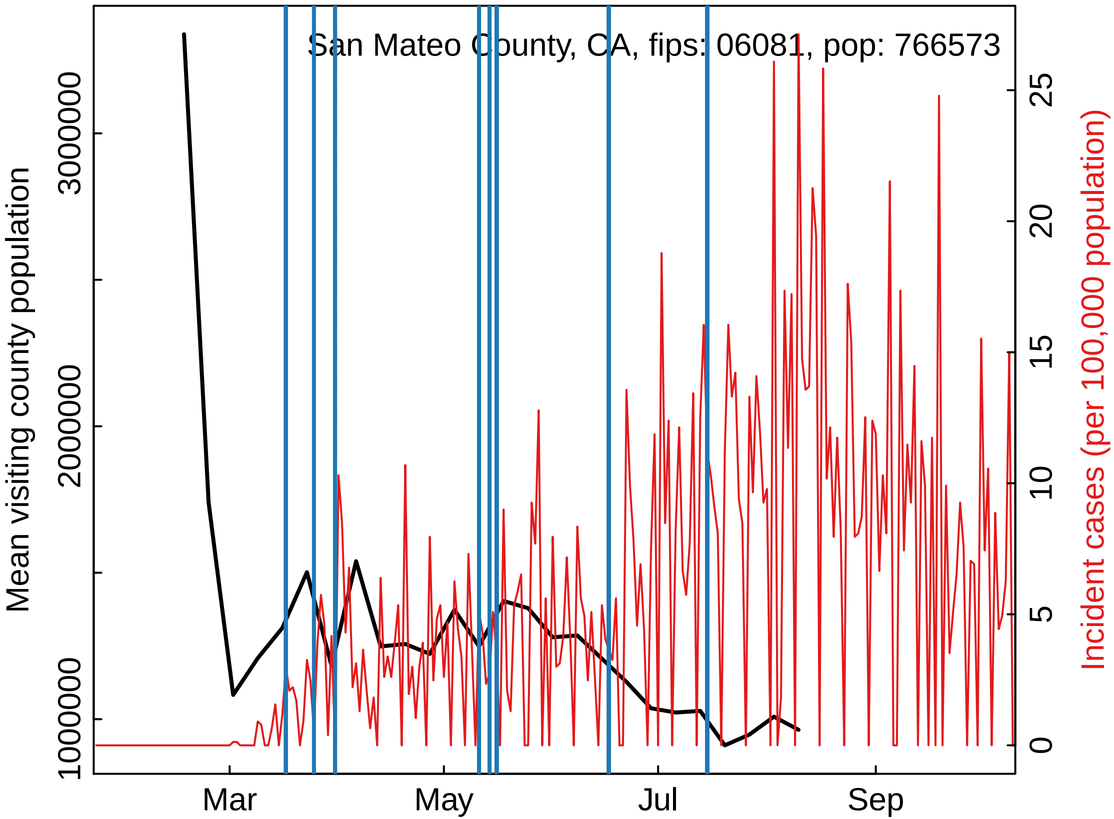

San Mateo County, CA, fips: 06081, pop: 766573
Mean visiting county population
1000000
2000000
3000000
Incident cases (per 100,000 population)
0
5
10
15
20
25
Mar
May
Jul
Sep

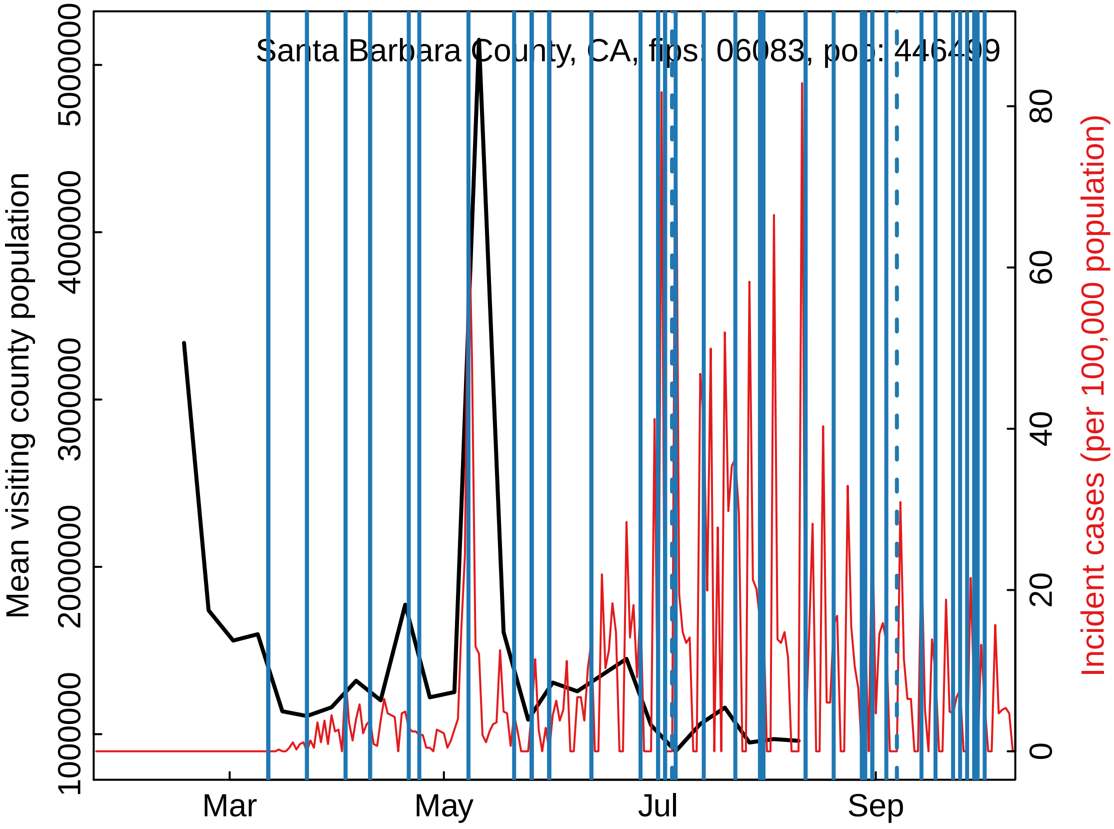

Santa Barbara County, CA, fips: 06083, pop: 446499
Mean visiting county population
Incident cases (per 100,000 population)
10000000
20000000
30000000
40000000
50000000
0
20
40
60
80
Mar
May
Jul
Sep

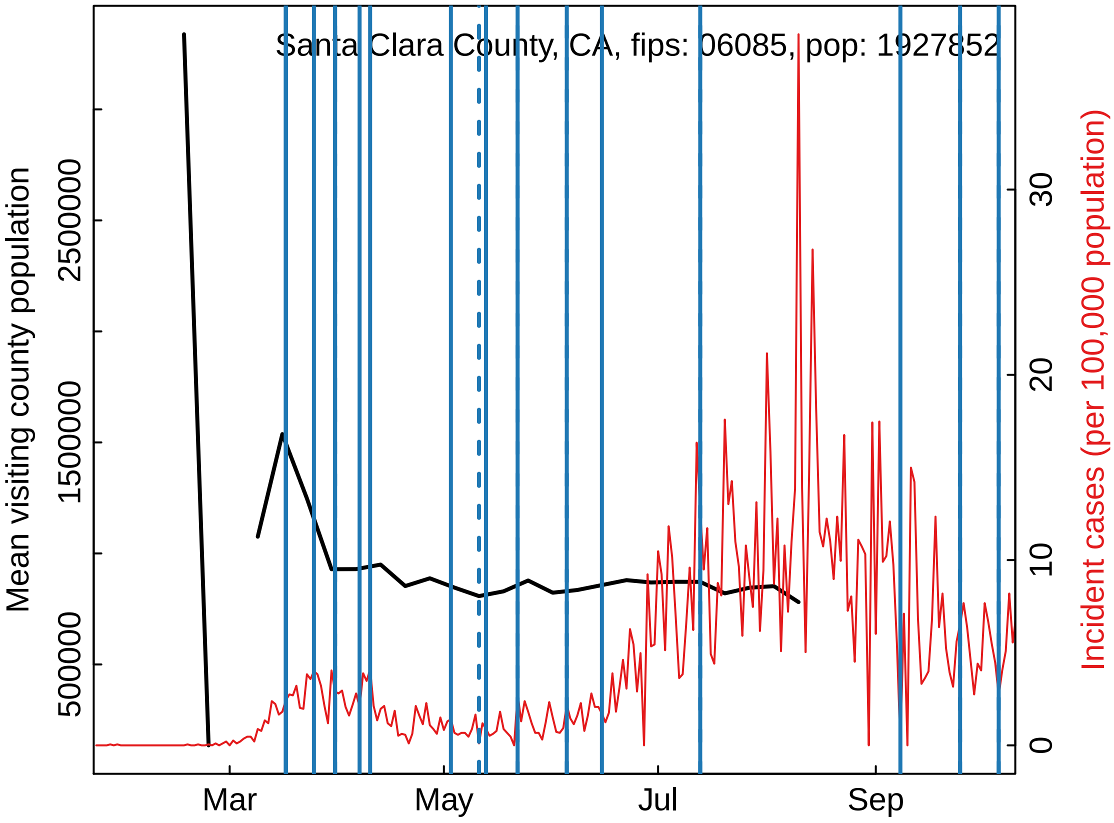
Santa Clara County, CA, fips: 06085, pop: 1927852
Mean visiting county population
500000
1500000
2500000
Incident cases (per 100,000 population)
0
10
20
30
Mar
May
Jul
Sep

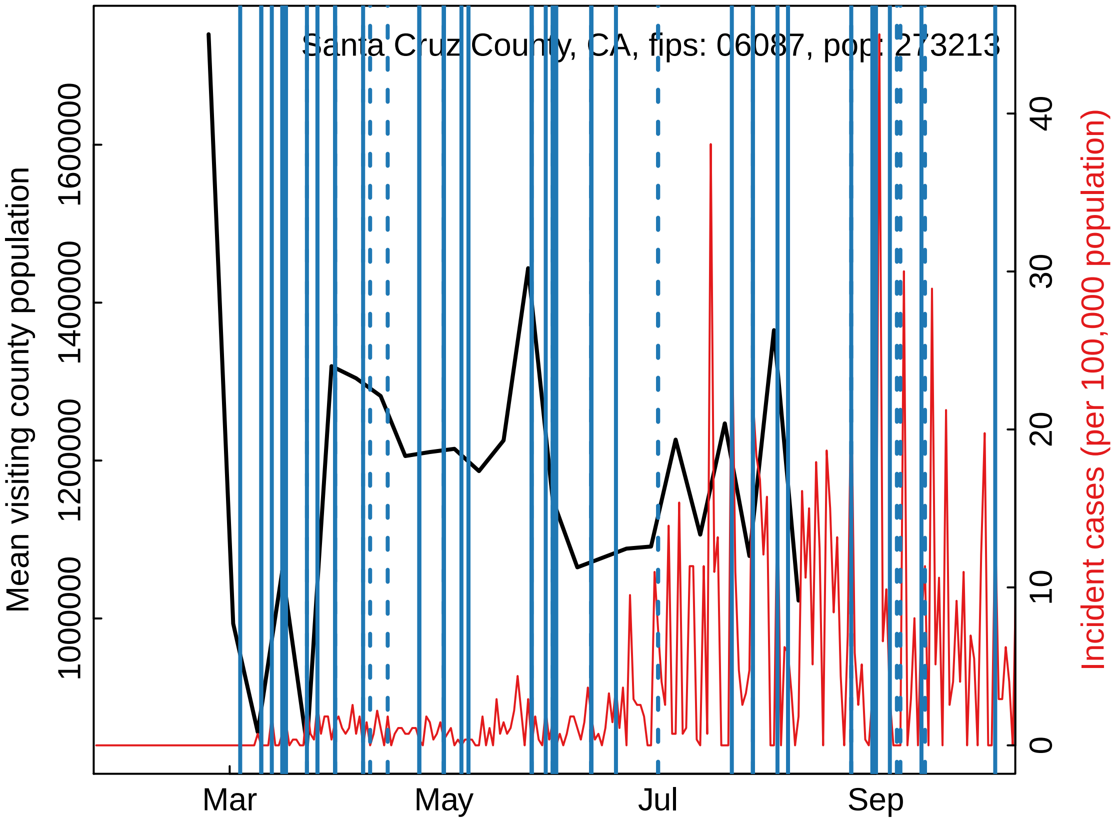

Santa Cruz County, CA, fips: 06087, pop: 273213
Mean visiting county population
Incident cases (per 100,000 population)
1000000
1200000
1400000
1600000
0
10
20
30
40
Mar
May
Jul
Sep

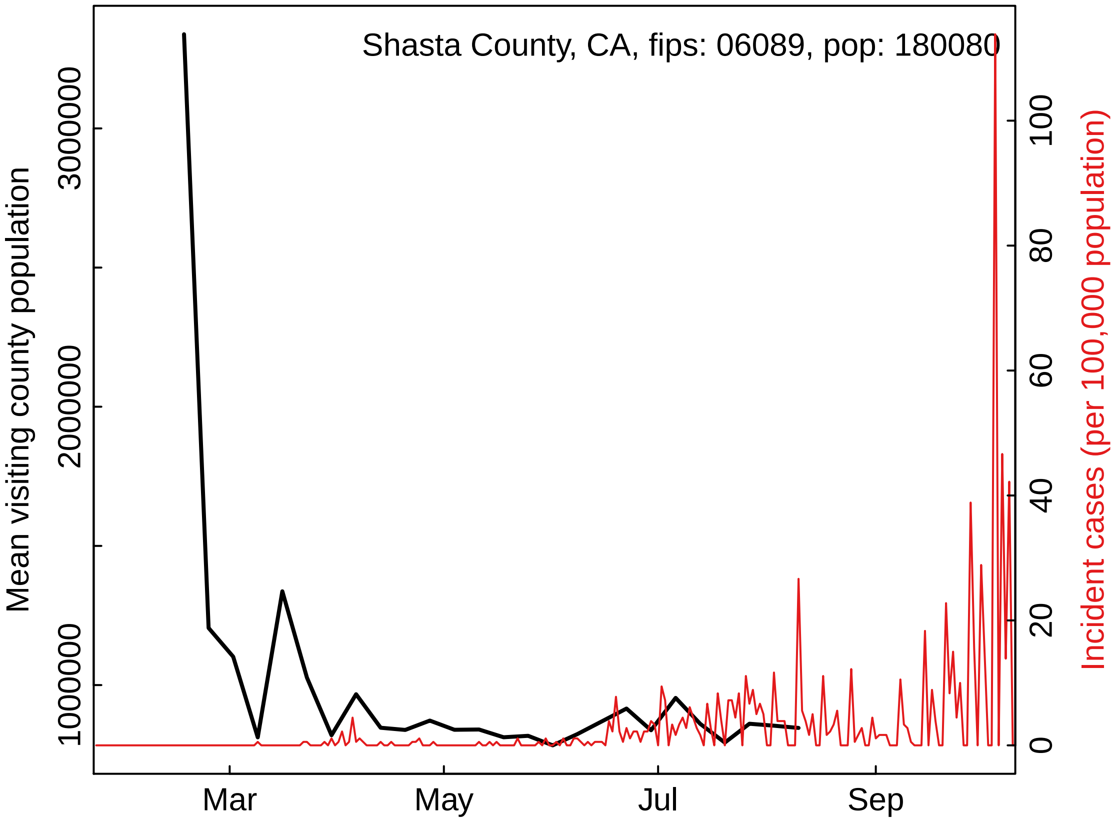

Shasta County, CA, fips: 06089, pop: 180080
Mean visiting county population
1000000
2000000
3000000
Incident cases (per 100,000 population)
0
20
40
60
80
100
Mar
May
Jul
Sep

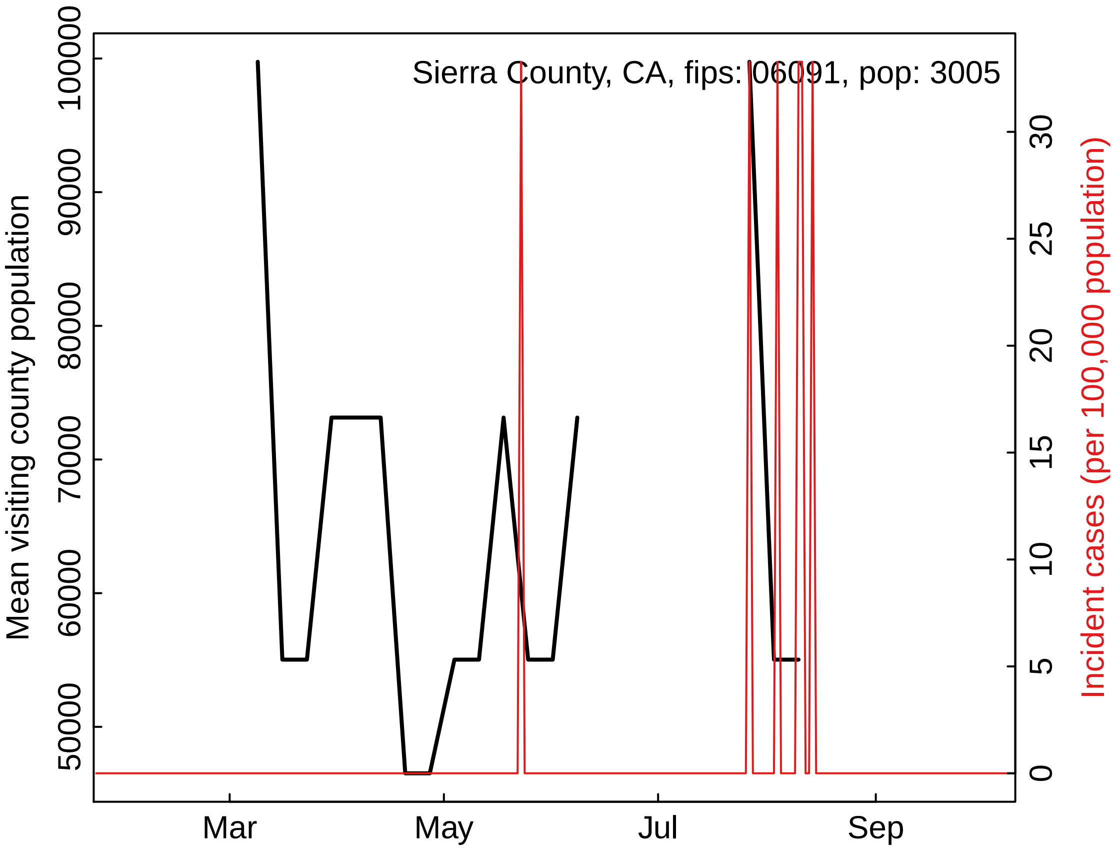

Sierra County, CA, fips: 06091, pop: 3005
Mean visiting county population
50000
60000
70000
80000
90000
100000
Incident cases (per 100,000 population)
0
5
10
15
20
25
30
Mar
May
Jul
Sep

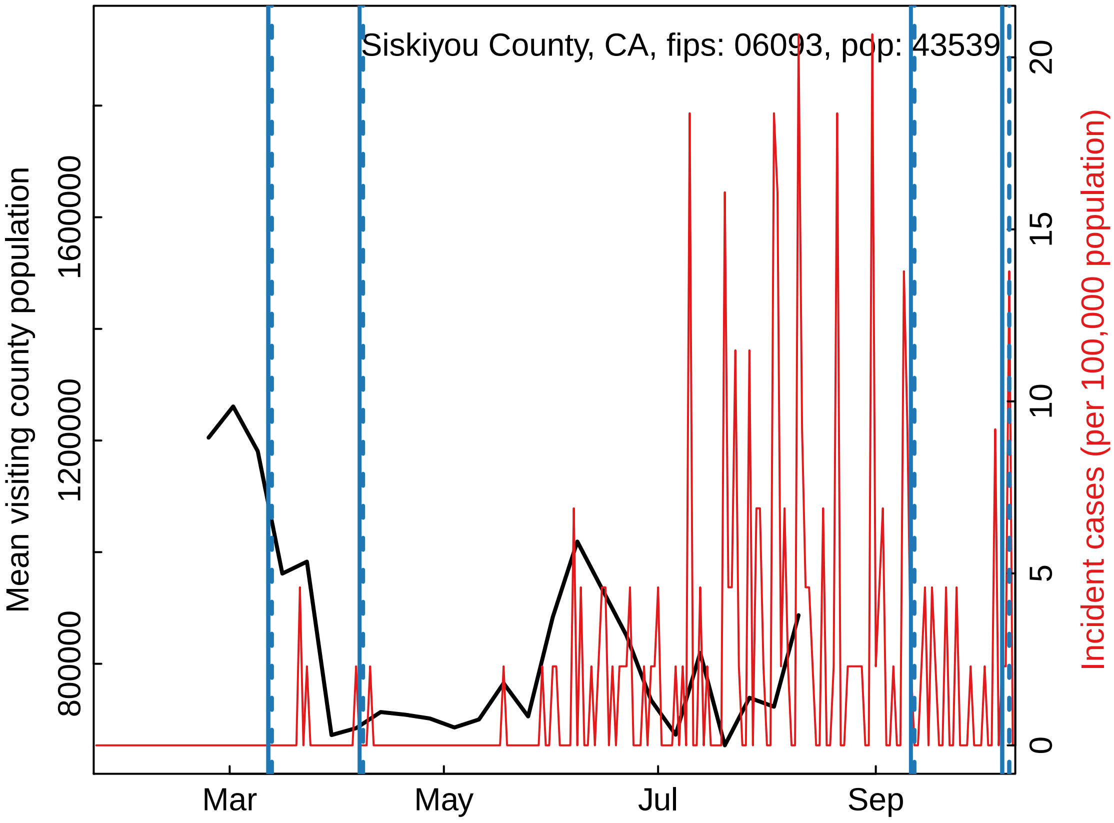

Siskiyou County, CA, fips: 06093, pop: 43539
Mean visiting county population
800000
1200000
1600000
Incident cases (per 100,000 population)
0
5
10
15
20
Mar
May
Jul
Sep

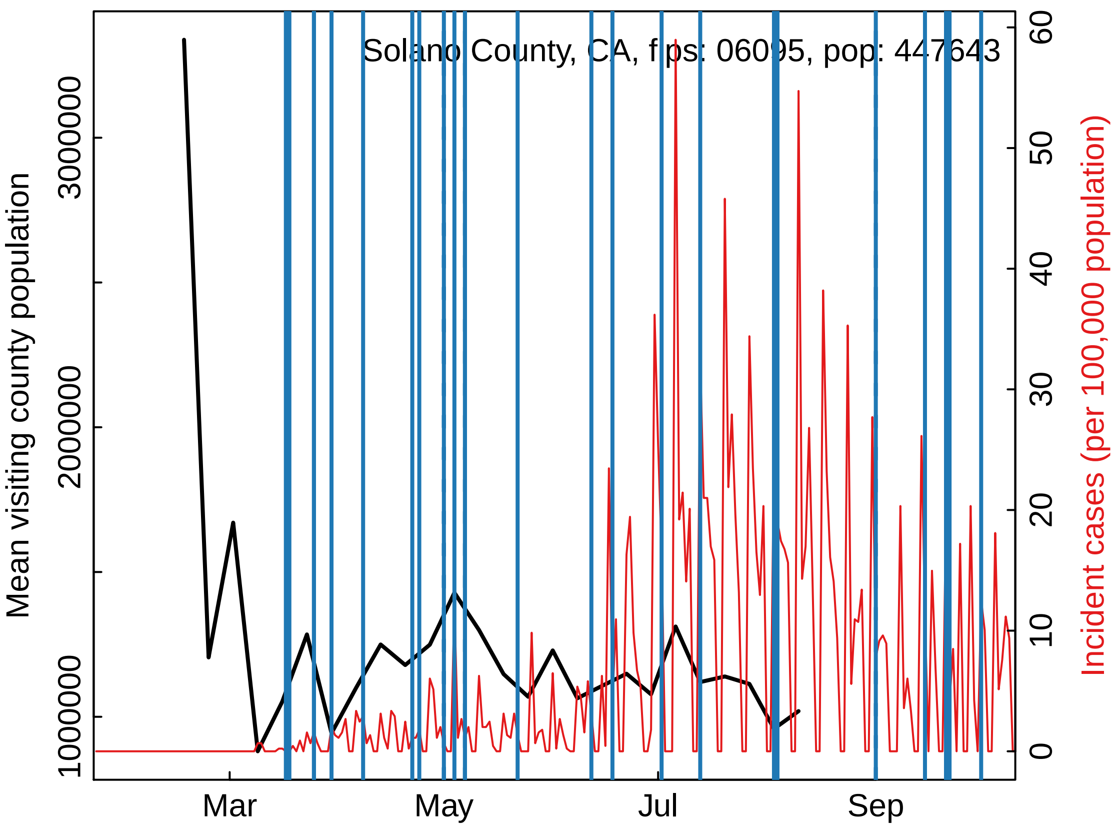

Solano County, CA, fips: 06095, pop: 447643
Mean visiting county population
10000000
20000000
30000000
Mar
May
Jul
Sep
0
10
20
30
40
50
60
Incident cases (per 100,000 population)

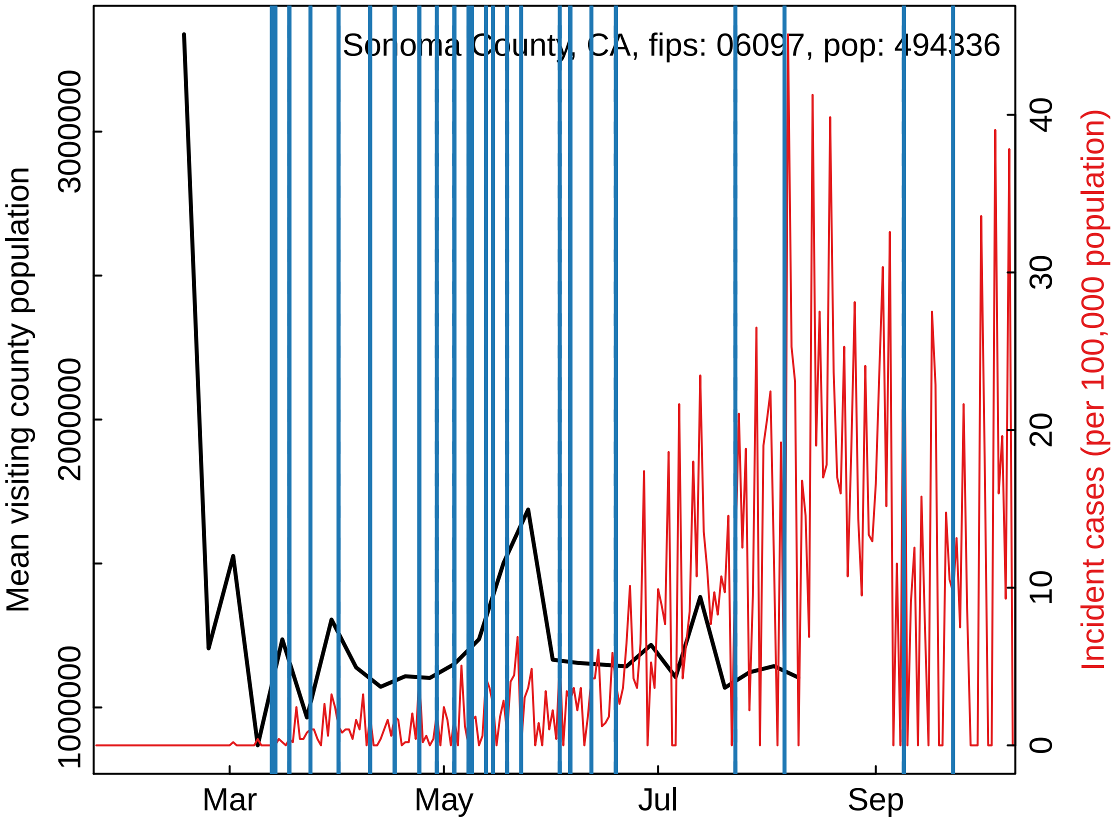
Sonoma County, CA, fips: 06097, pop: 494336
Mean visiting county population
1000000
2000000
3000000
Incident cases (per 100,000 population)
0
10
20
30
40
Mar
May
Jul
Sep

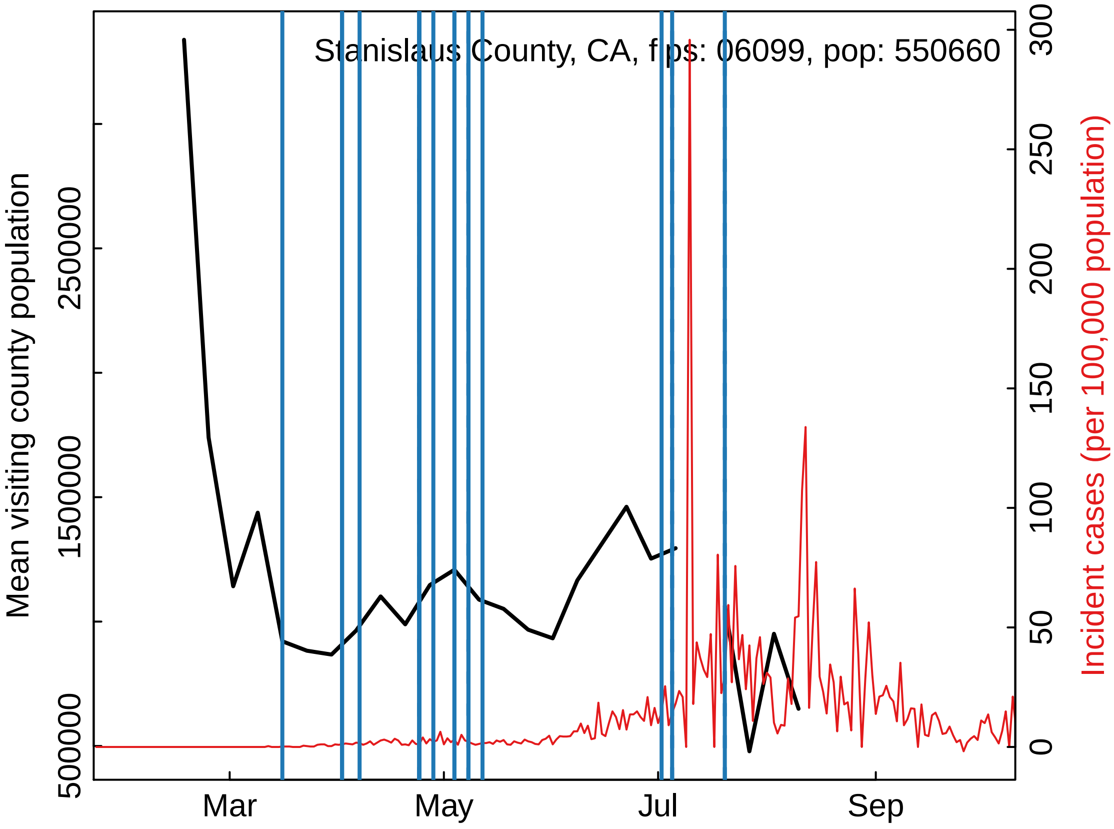

Stanislaus County, CA, fips: 06099, pop: 550660
Mean visiting county population
500000
1500000
2500000
Mar
May
Jul
Sep
Incident cases (per 100,000 population)
0
50
100
150
200
250
300

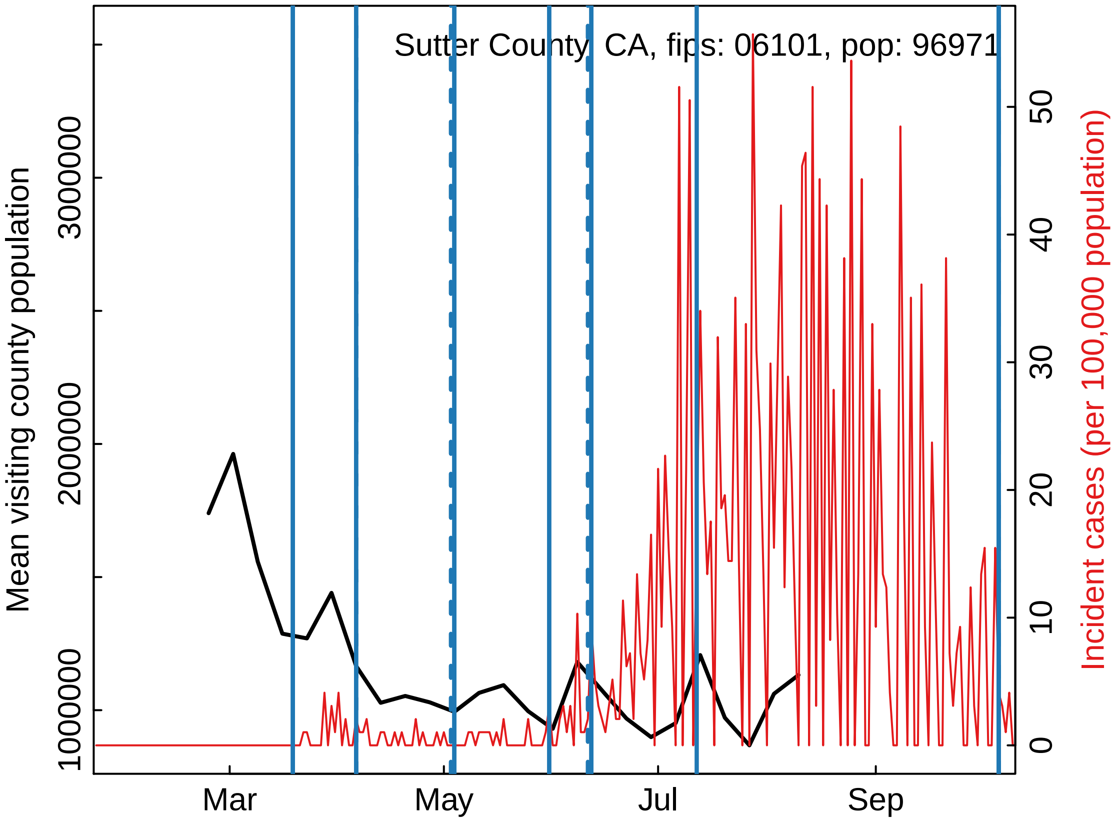

Sutter County CA, fips: 06101, pop: 96971
Mean visiting county population
Incident cases (per 100,000 population)
1000000
2000000
3000000
0
10
20
30
40
50
Mar
May
Jul
Sep

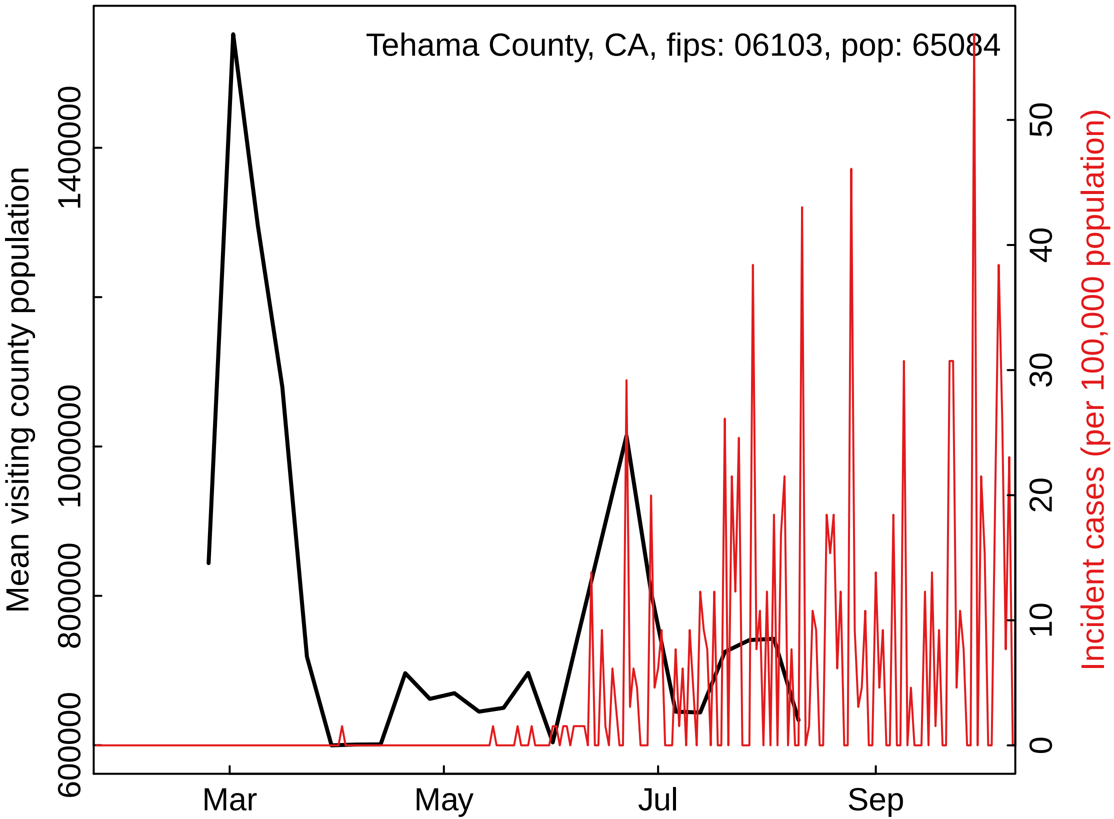

Tehama County, CA, fips: 06103, pop: 65084
Mean visiting county population
600000
800000
1000000
1400000
Mar
May
Jul
Sep
0
10
20
30
40
50
Incident cases (per 100,000 population)

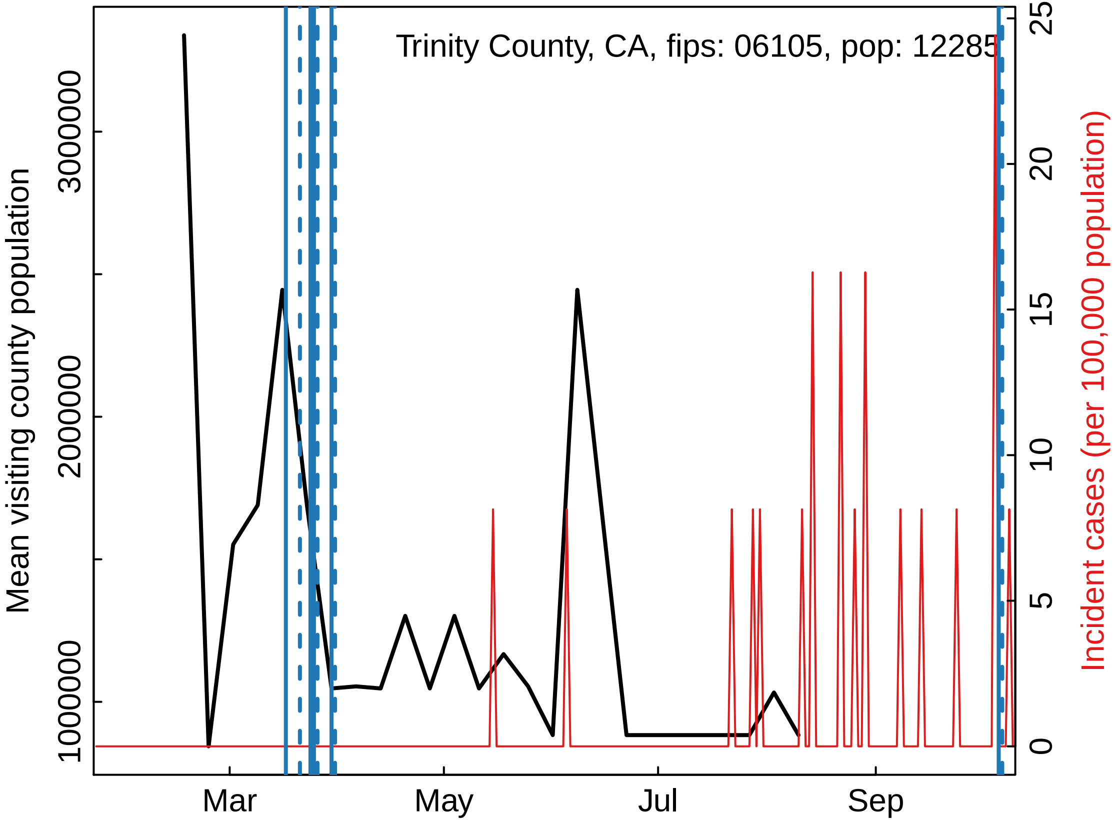
Trinity County, CA, fips: 06105, pop: 12285
Mean visiting county population
1000000
2000000
3000000
Mar
May
Jul
Sep
0
5
10
15
20
25
Incident cases (per 100,000 population)

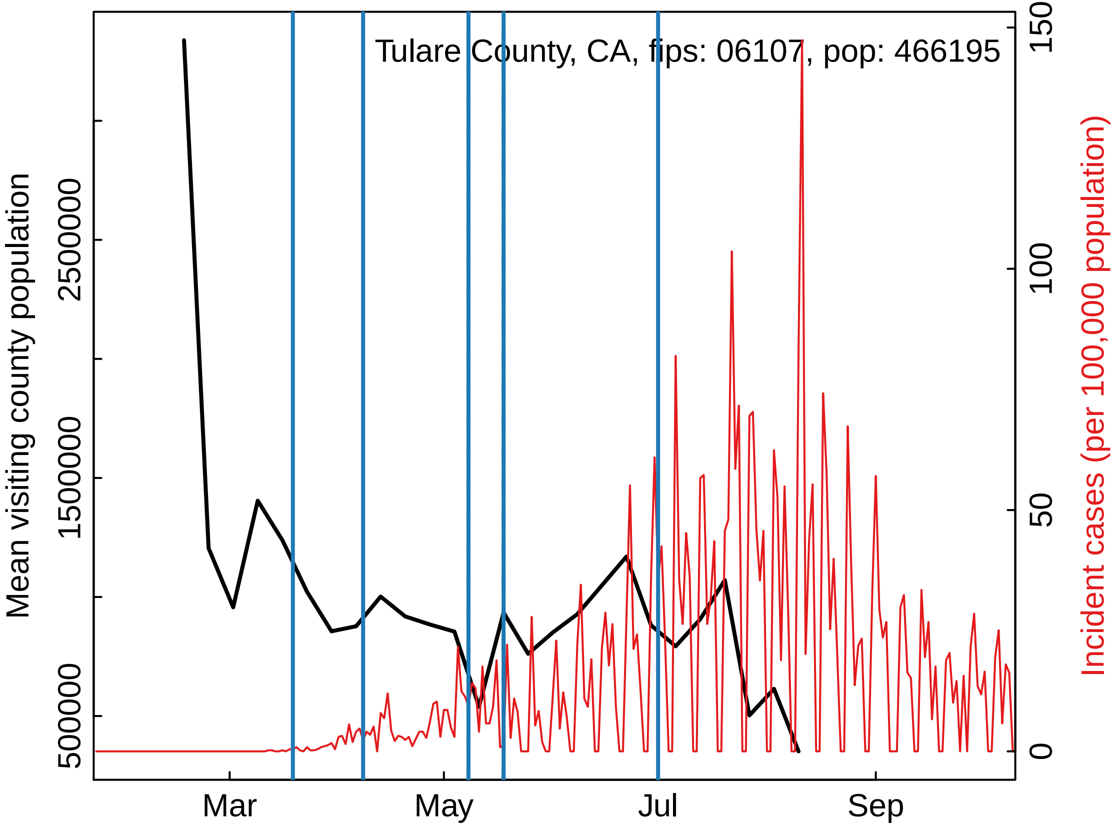
Tulare County, CA, fips: 06107, pop: 466195
Mean visiting county population
500000
1500000
2500000
Mar
May
Jul
Sep
0
50
100
150
Incident cases (per 100,000 population)

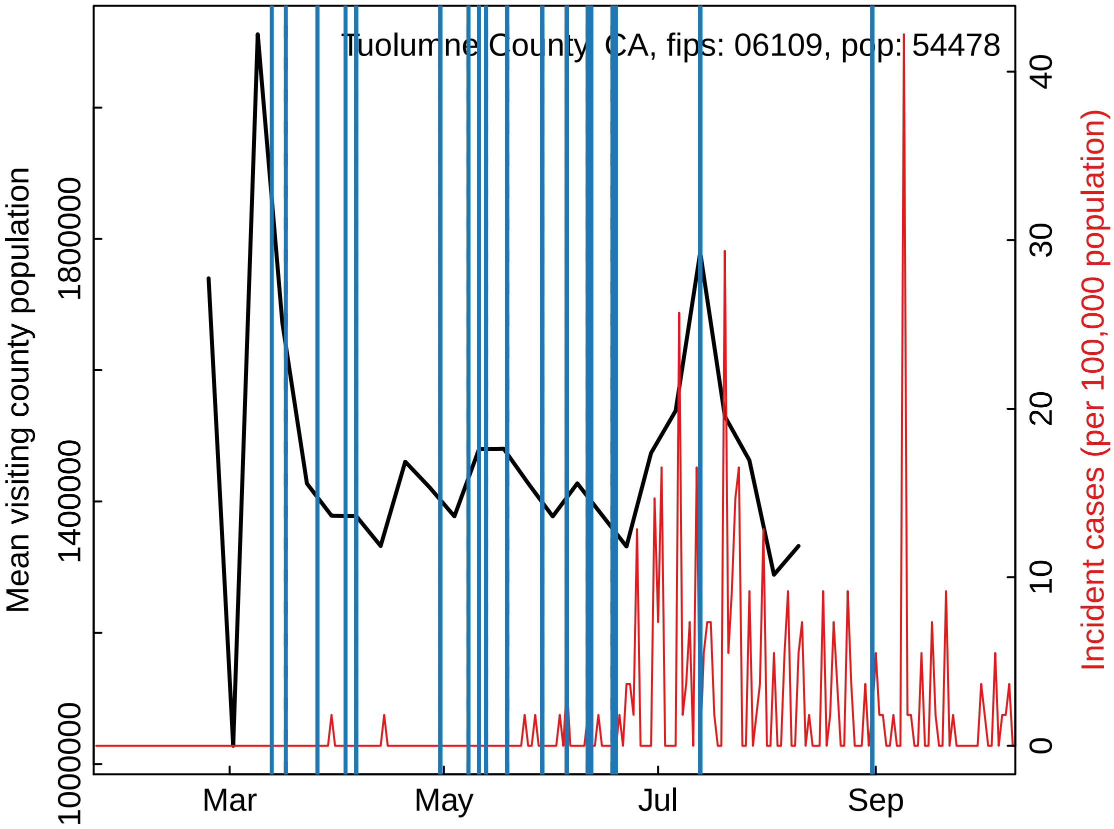

Tuolumne County CA, fips: 06109, pop: 54478
Mean visiting county population
1000000
1400000
1800000
Incident cases (per 100,000 population)
0
10
20
30
40
Mar
May
Jul
Sep

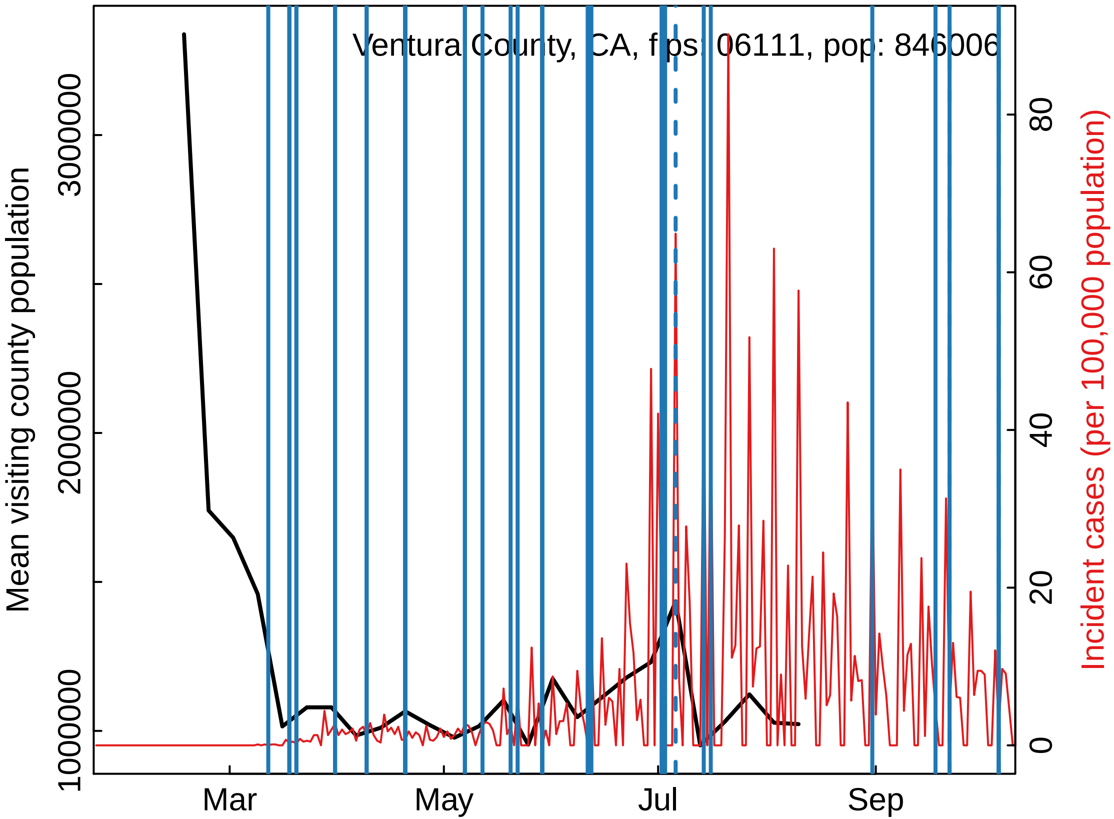
Ventura County, CA, fips: 06111, pop: 846006
Mean visiting county population
1000000
2000000
3000000
Mar
May
Jul
Sep
0
20
40
60
80
Incident cases (per 100,000 population)

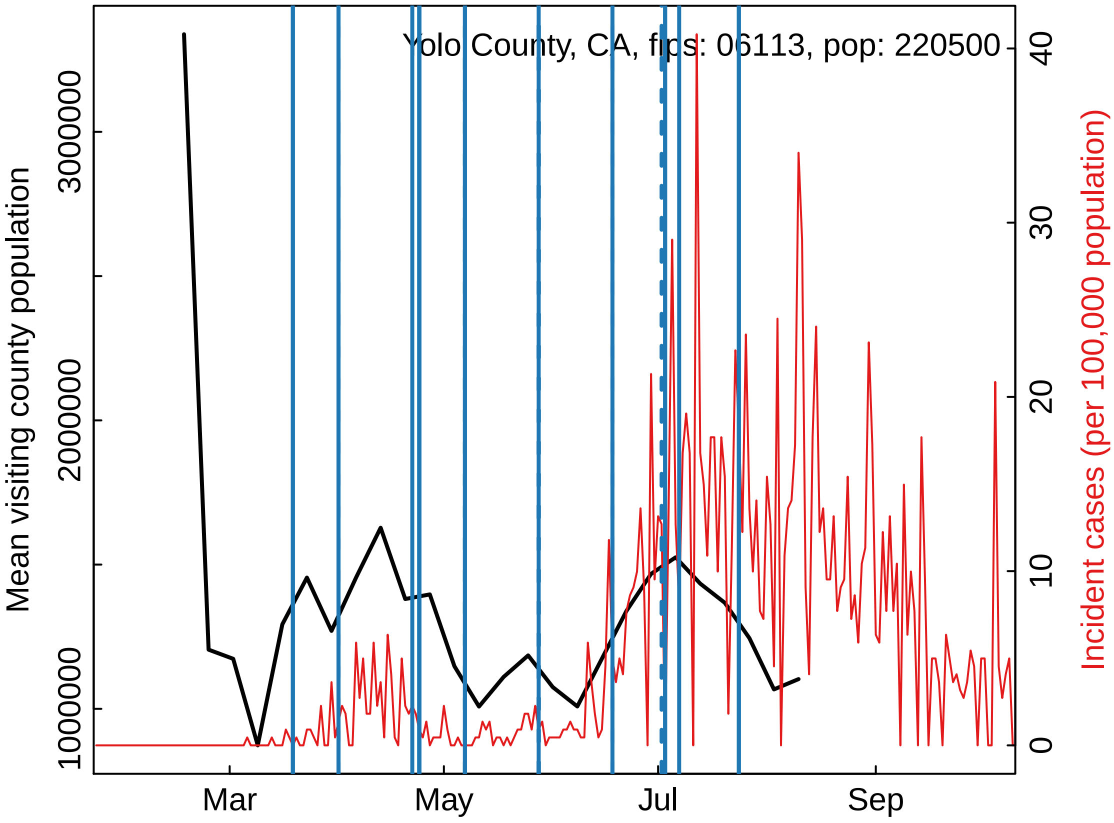

Yolo County, CA, fips: 06113, pop: 220500
Mean visiting county population
10000000
20000000
30000000
Incident cases (per 100,000 population)
0
10
20
30
40
Mar
May
Jul
Sep

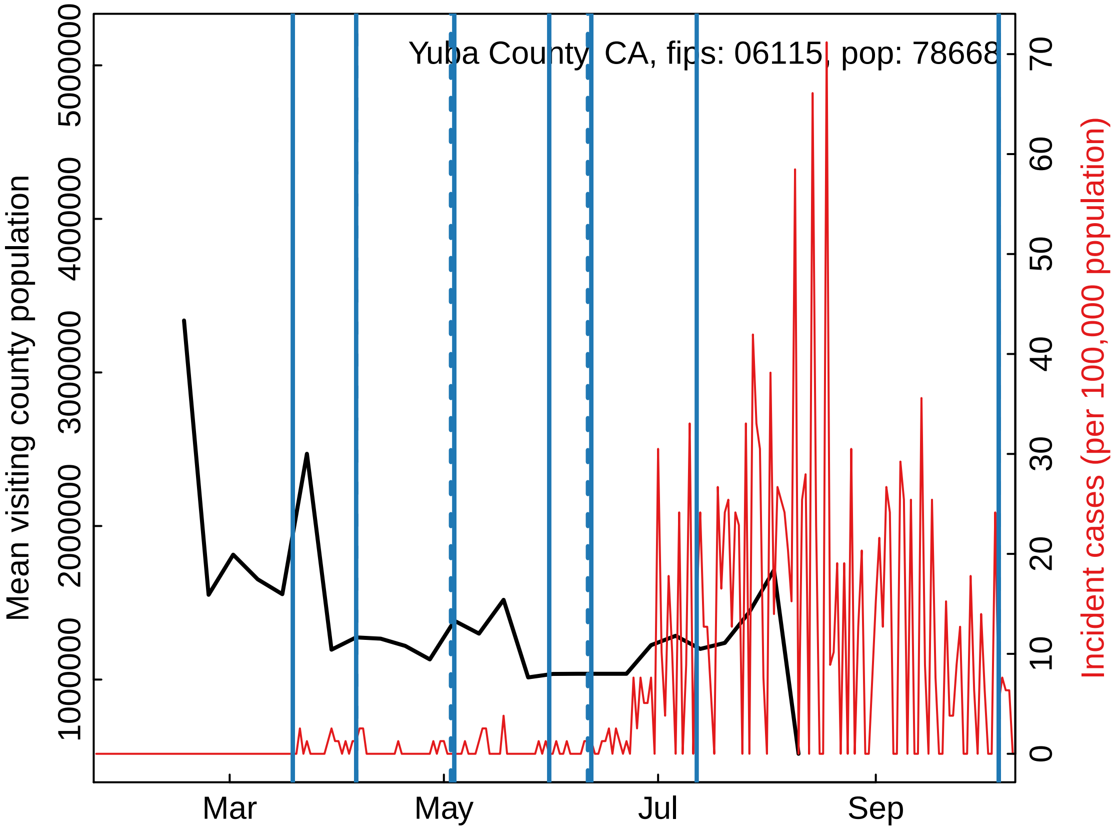

Yuba County, CA, fips: 06115, pop: 78668
Mean visiting county population
1000000
2000000
3000000
4000000
5000000
Incident cases (per 100,000 population)
0
10
20
30
40
50
60
70
Mar
May
Jul
Sep

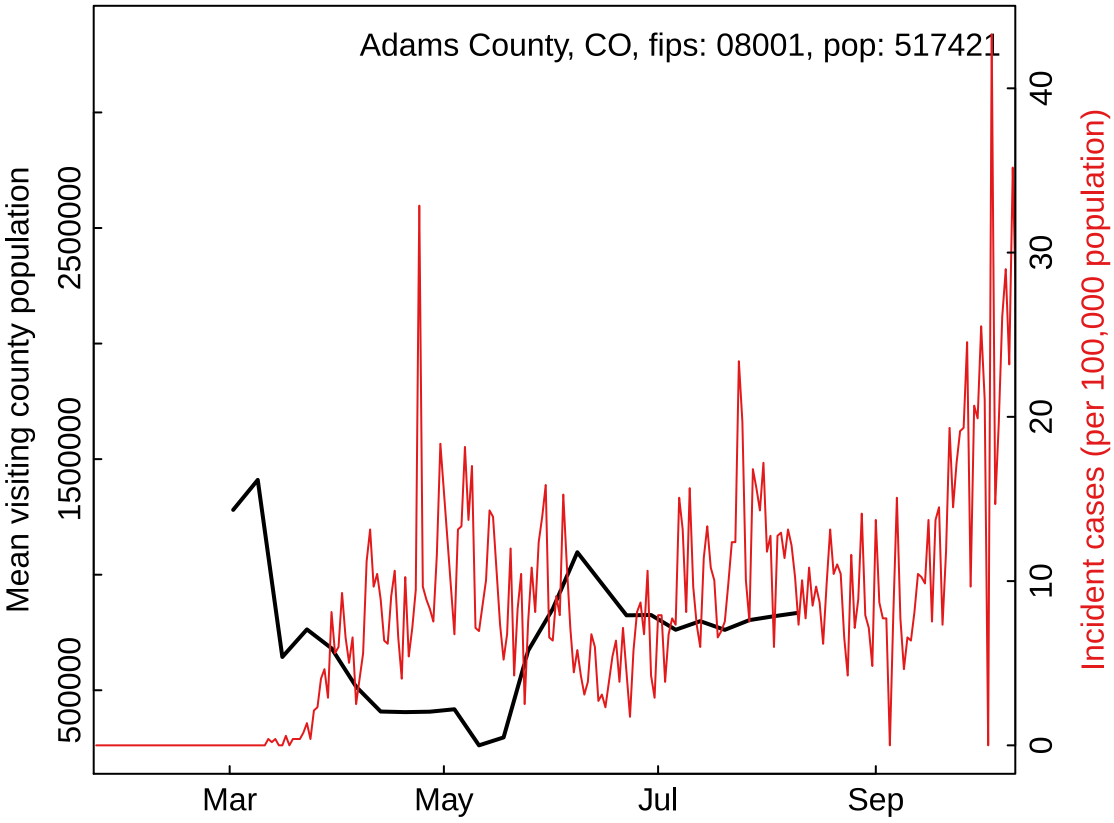
Adams County, CO, fips: 08001, pop: 517421
Mean visiting county population
500000
1500000
2500000
Incident cases (per 100,000 population)
0
10
20
30
40
Mar
May
Jul
Sep

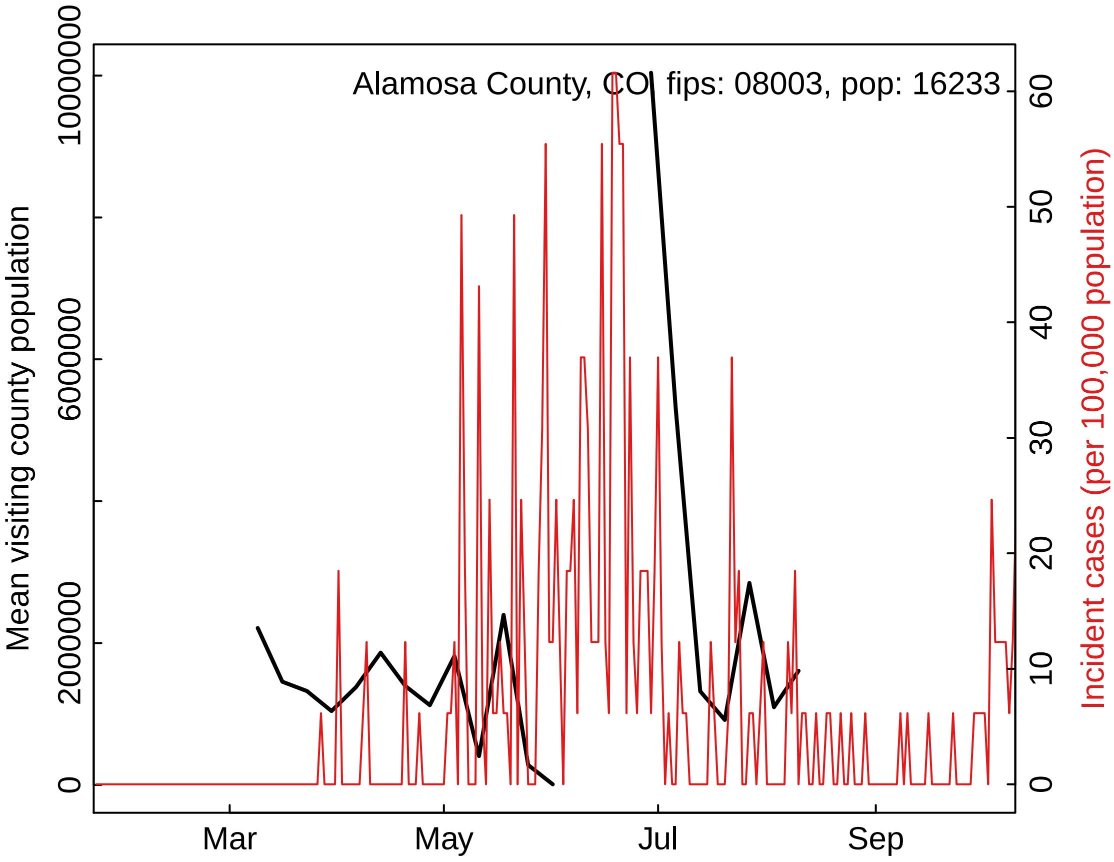

Alamosa County, CO fips: 08003, pop: 16233
Mean visiting county population
0
2000000
6000000
10000000
Incident cases (per 100,000 population)
0
10
20
30
40
50
60
Mar
May
Jul
Sep

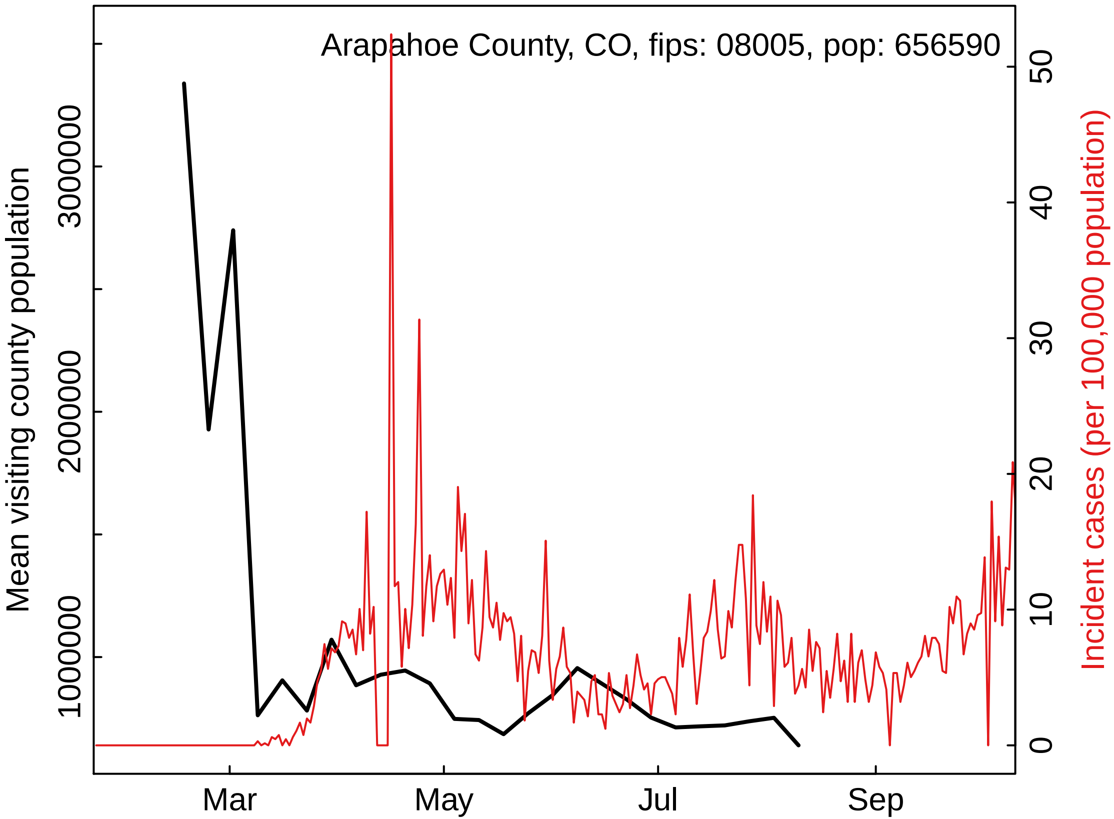

Arapahoe County, CO, fips: 08005, pop: 656590
Mean visiting county population
10000000
20000000
30000000
Incident cases (per 100,000 population)
0
10
20
30
40
50
Mar
May
Jul
Sep

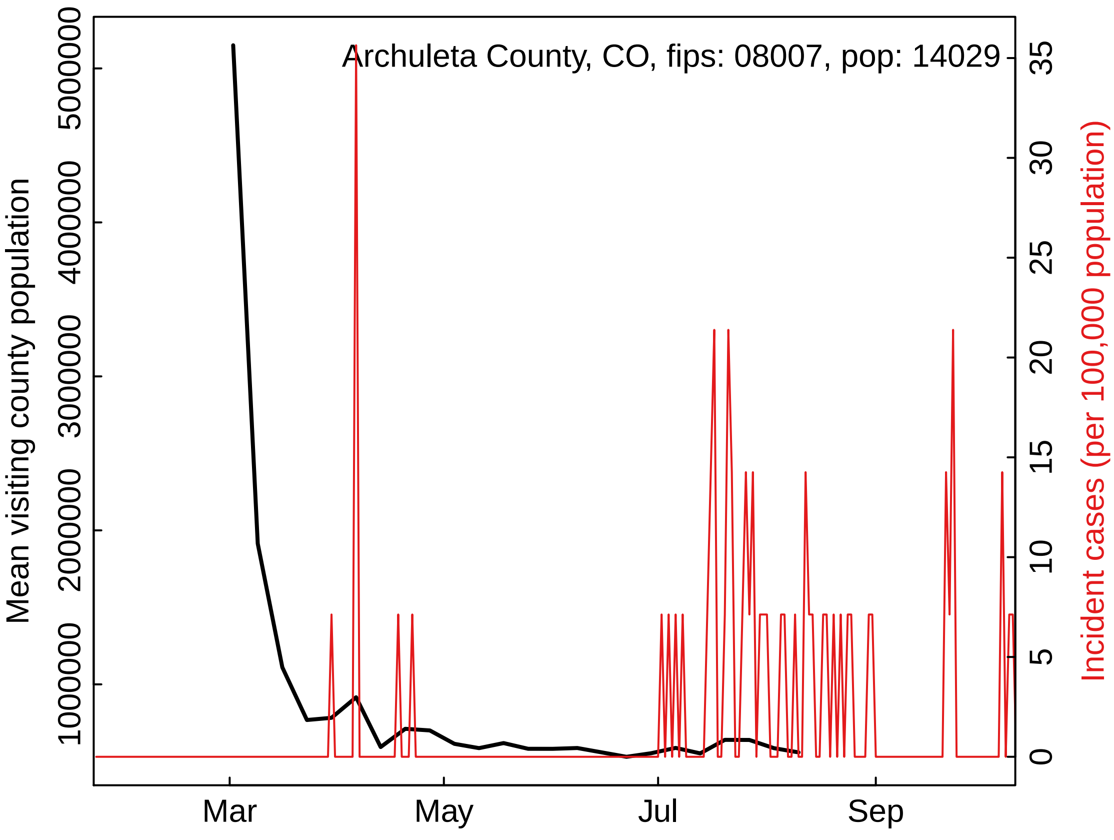

Archuleta County, CO, fips: 08007, pop: 14029
Mean visiting county population
1000000
2000000
3000000
4000000
5000000
Incident cases (per 100,000 population)
0
5
10
15
20
25
30
35
Mar
May
Jul
Sep

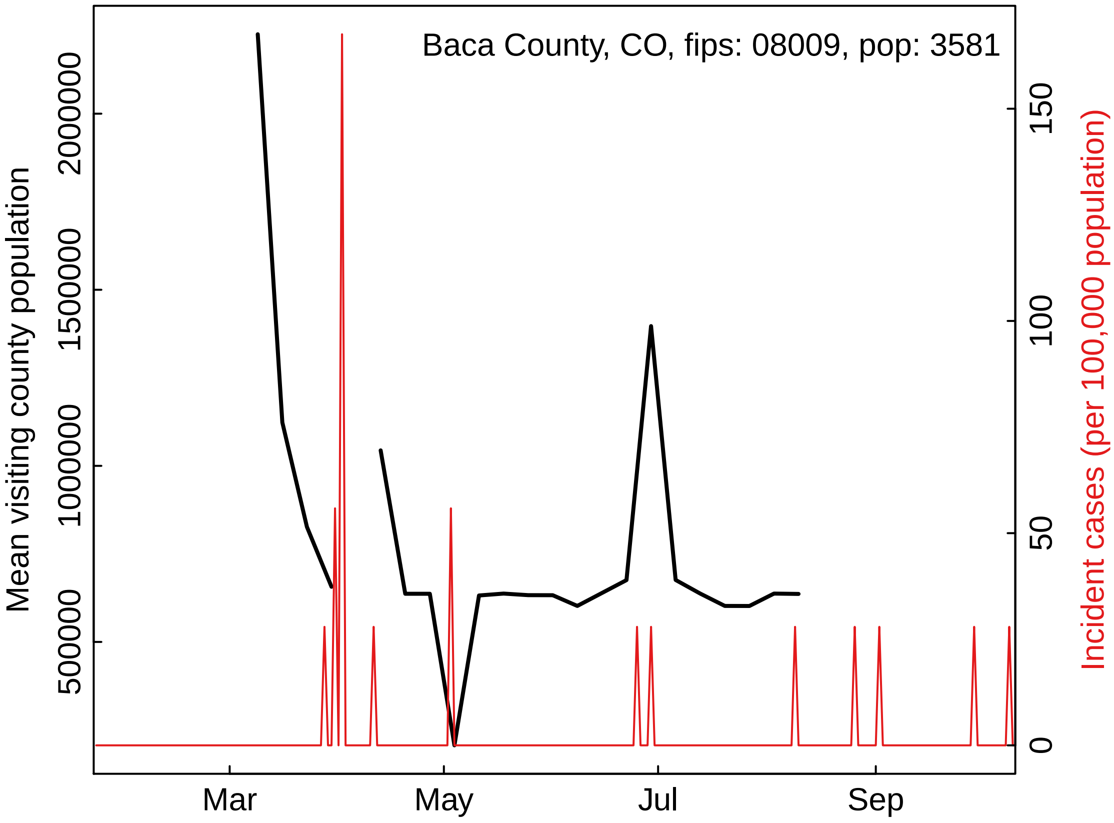

Baca County, CO, fips: 08009, pop: 3581
Mean visiting county population
500000
1000000
1500000
2000000
Incident cases (per 100,000 population)
0
50
100
150
Mar
May
Jul
Sep

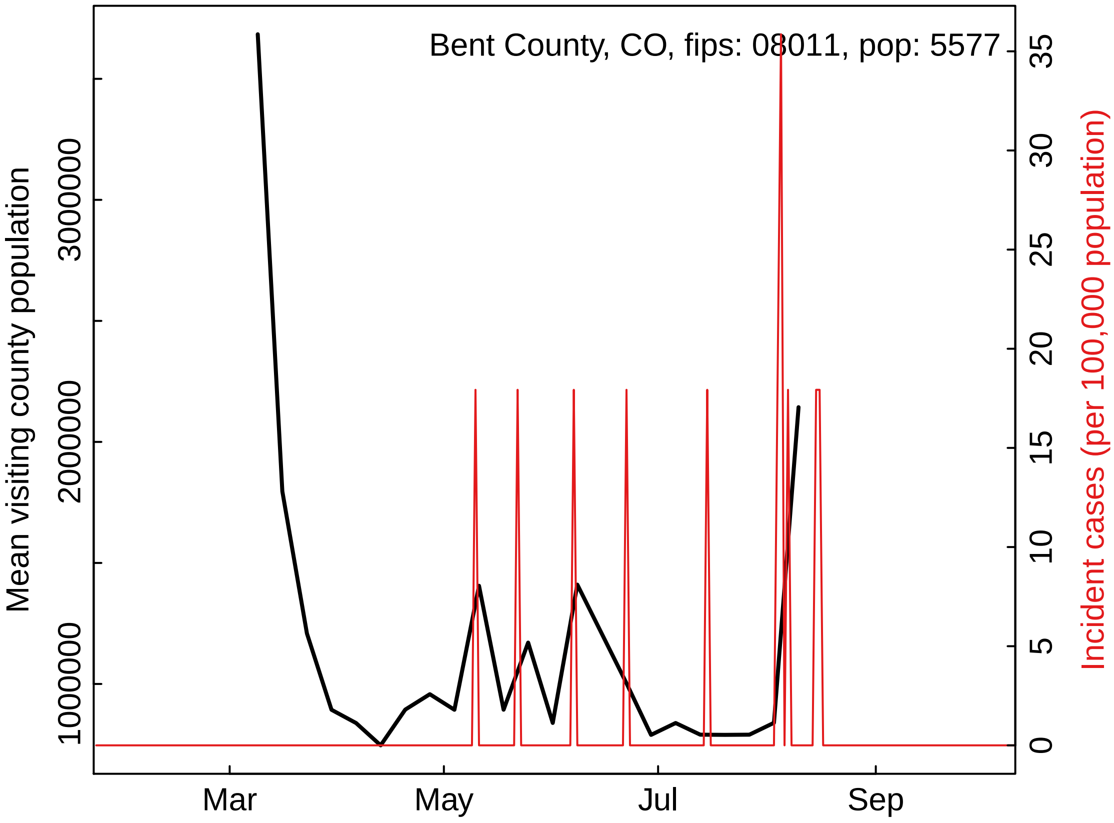

Bent County, CO, fips: 08011, pop: 5577
Mean visiting county population
10000000
20000000
30000000
Incident cases (per 100,000 population)
0
5
10
15
20
25
30
35
Mar
May
Jul
Sep

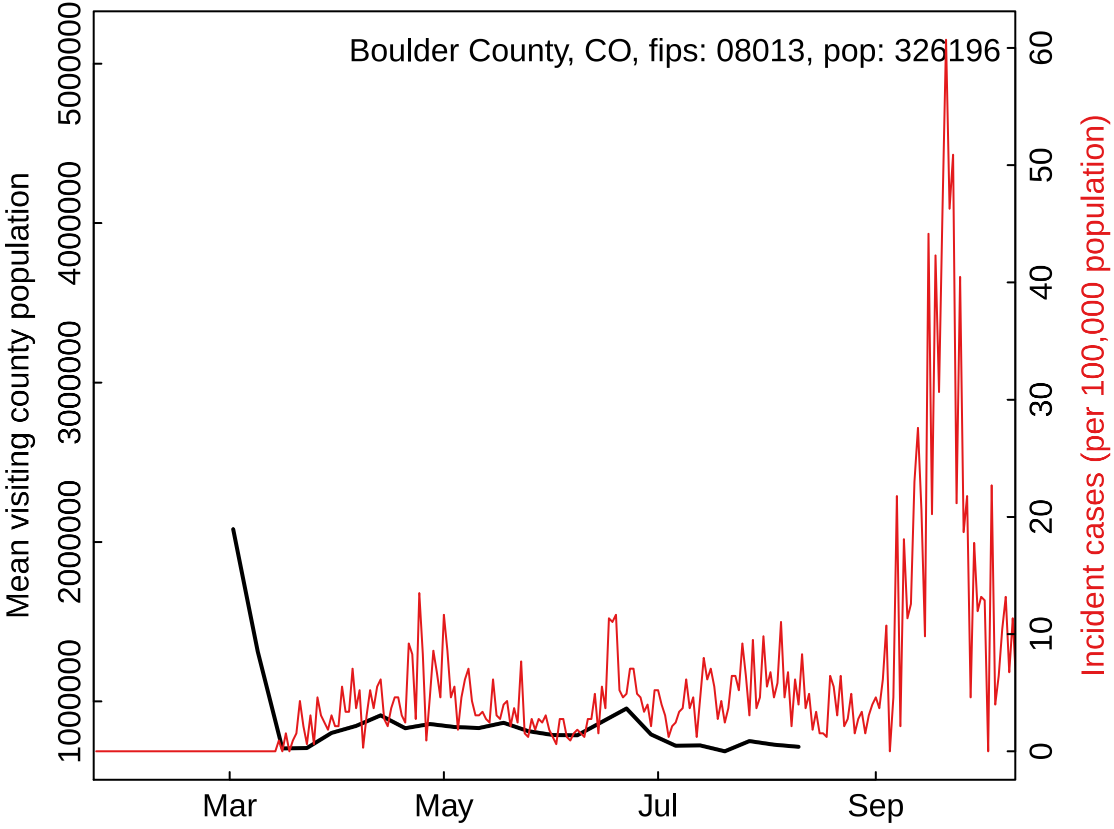
Boulder County, CO, fips: 08013, pop: 326196
Mean visiting county population
10000000
20000000
30000000
40000000
50000000
Incident cases (per 100,000 population)
0
10
20
30
40
50
60
Mar
May
Jul
Sep

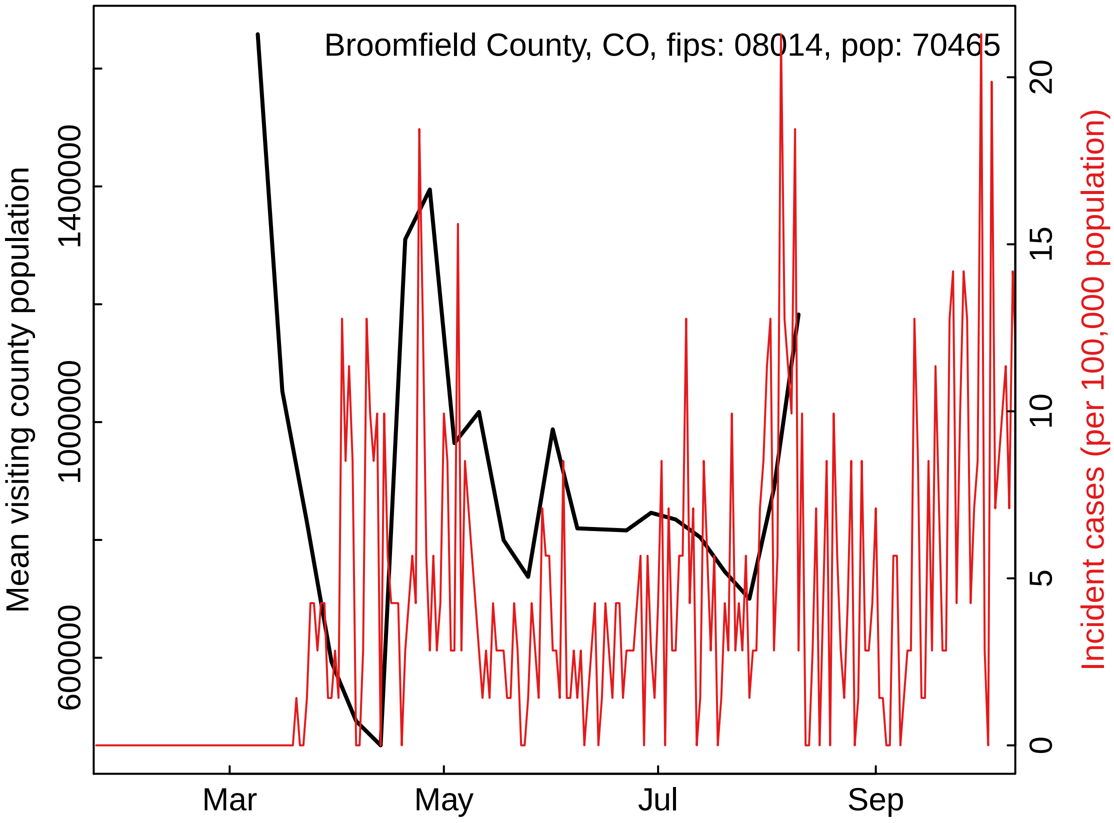

Broomfield County, CO, fips: 08014, pop: 70465
Mean visiting county population
600000
1000000
1400000
Incident cases (per 100,000 population)
0
5
10
15
20
Mar
May
Jul
Sep

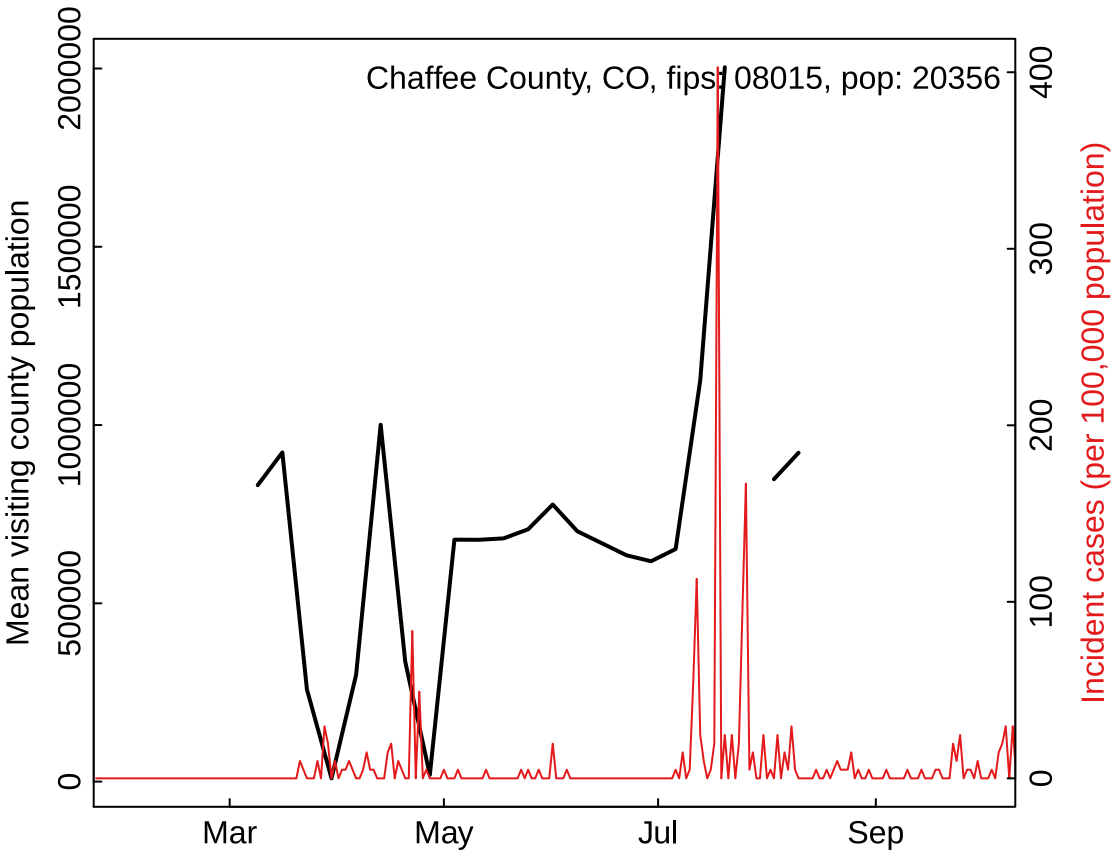
Chaffee County, CO, fips: 08015, pop: 20356
Mean visiting county population
Incident cases (per 100,000 population)
0
500000
1000000
1500000
2000000
0
100
200
300
400
Mar
May
Jul
Sep

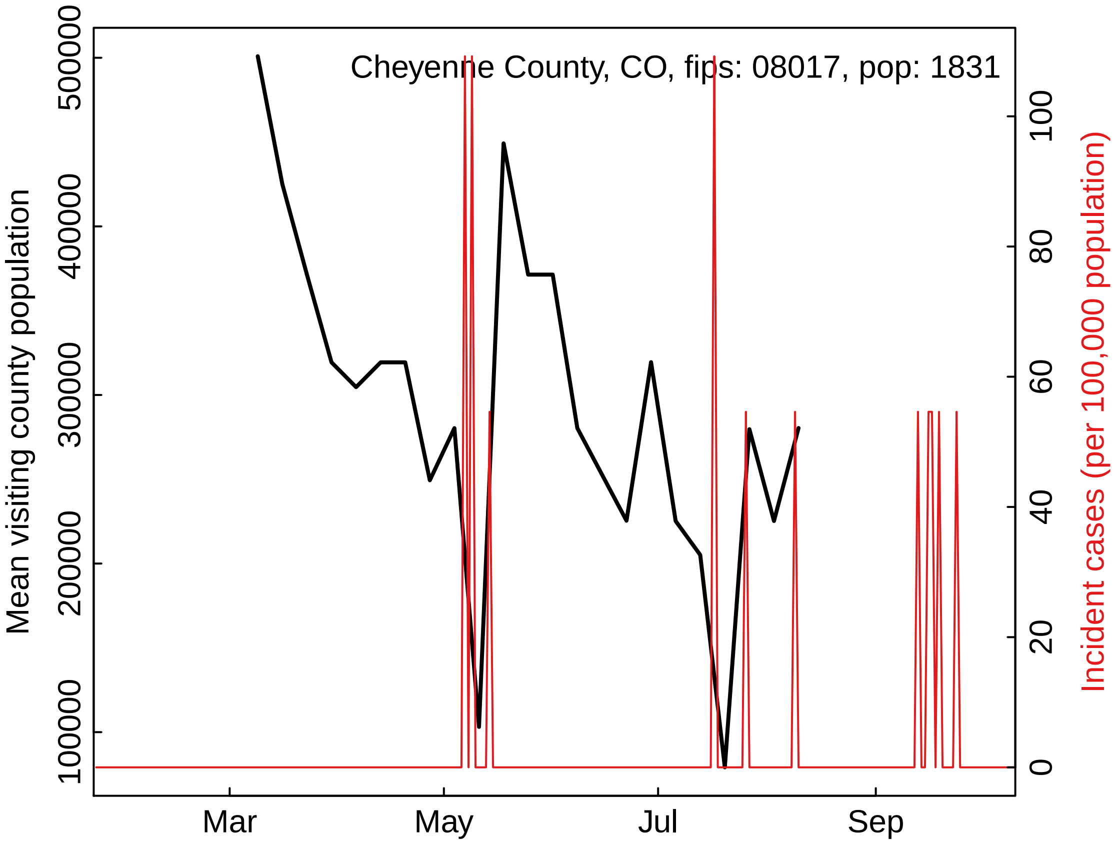
Cheyenne County, CO, fips: 08017, pop: 1831
Mean visiting county population
100000
200000
300000
400000
500000
Incident cases (per 100,000 population)
0
20
40
60
80
100
Mar
May
Jul
Sep

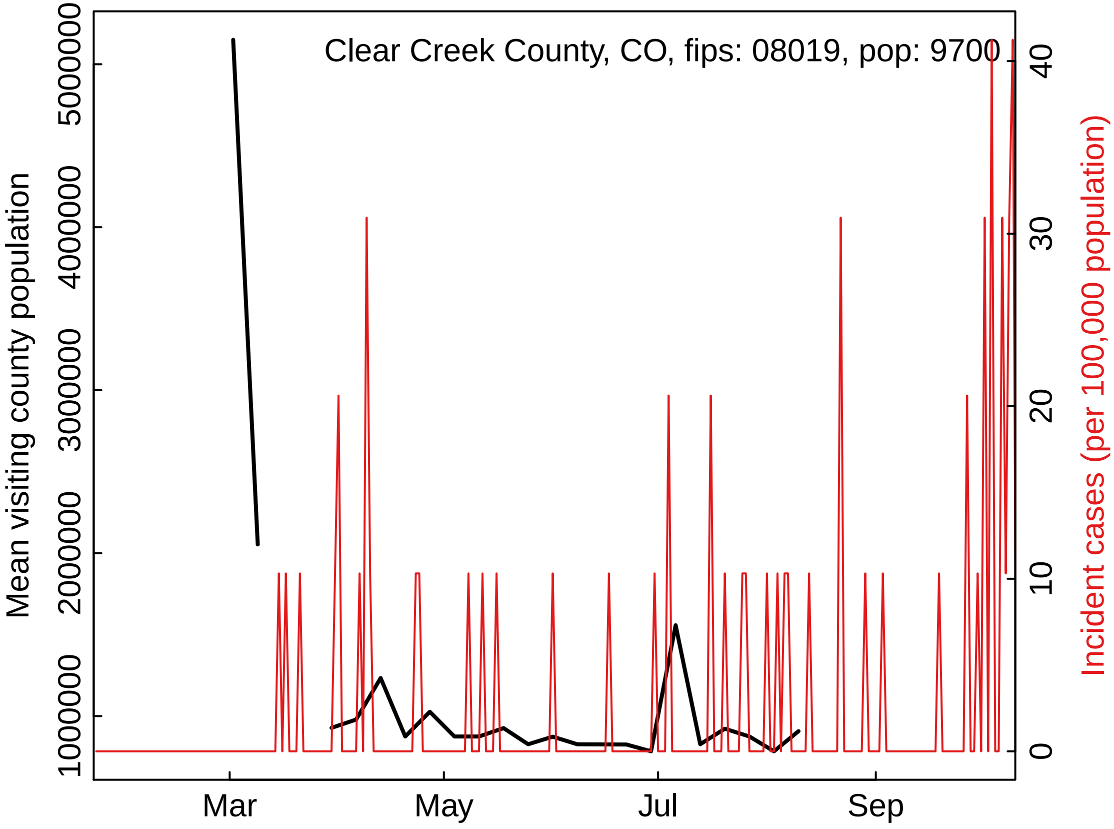

Clear Creek County, CO, fips: 08019, pop: 9700
Mean visiting county population
1000000
2000000
3000000
4000000
5000000
Incident cases (per 100,000 population)
0
10
20
30
40
Mar
May
Jul
Sep

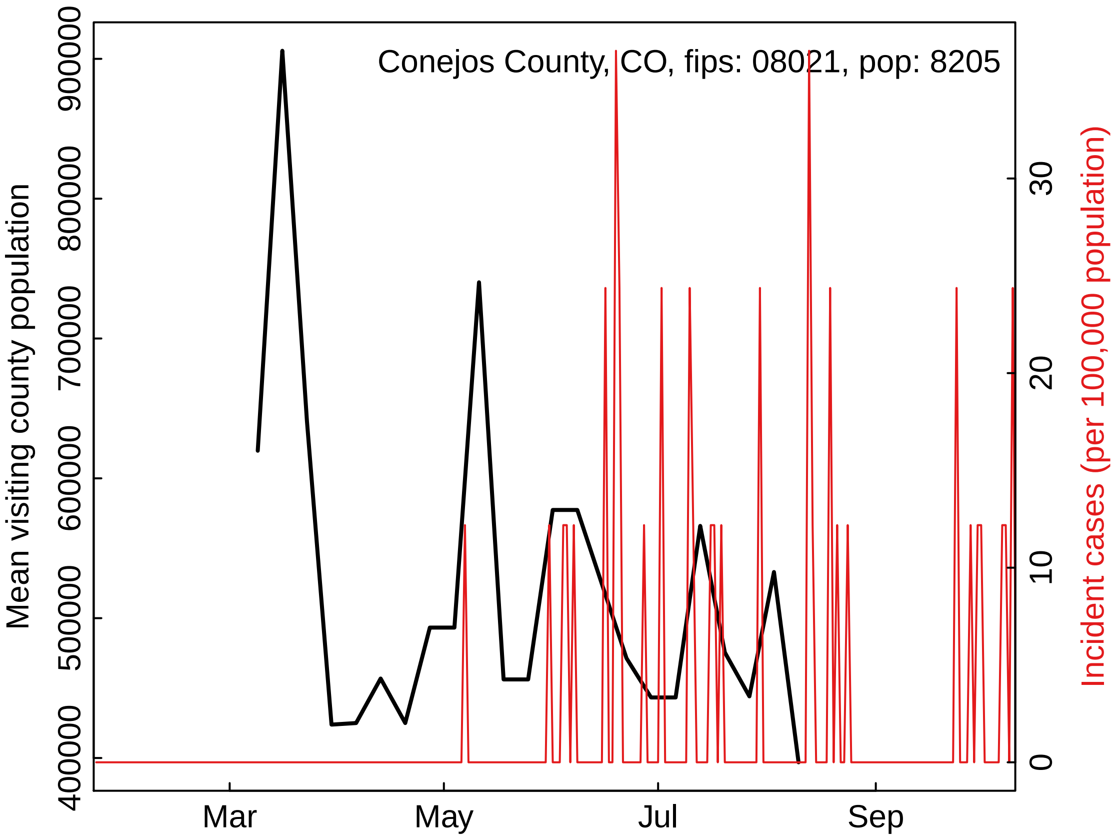

Conejos County, CO, fips: 08021, pop: 8205
Mean visiting county population
400000
500000
600000
700000
800000
900000
Mar
May
Jul
Sep
0
10
20
30
Incident cases (per 100,000 population)

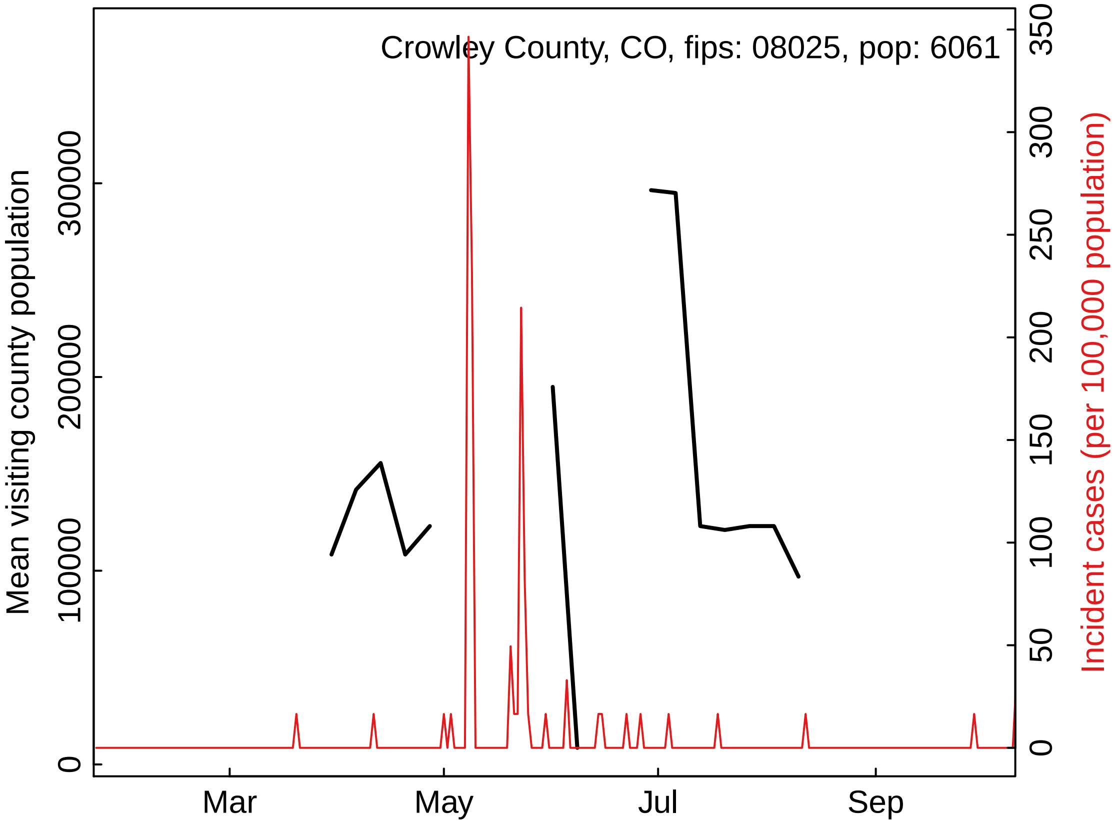

Crowley County, CO, fips: 08025, pop: 6061
Mean visiting county population
0
100000
200000
300000
Incident cases (per 100,000 population)
0
50
100
150
200
250
300
350
Mar
May
Jul
Sep

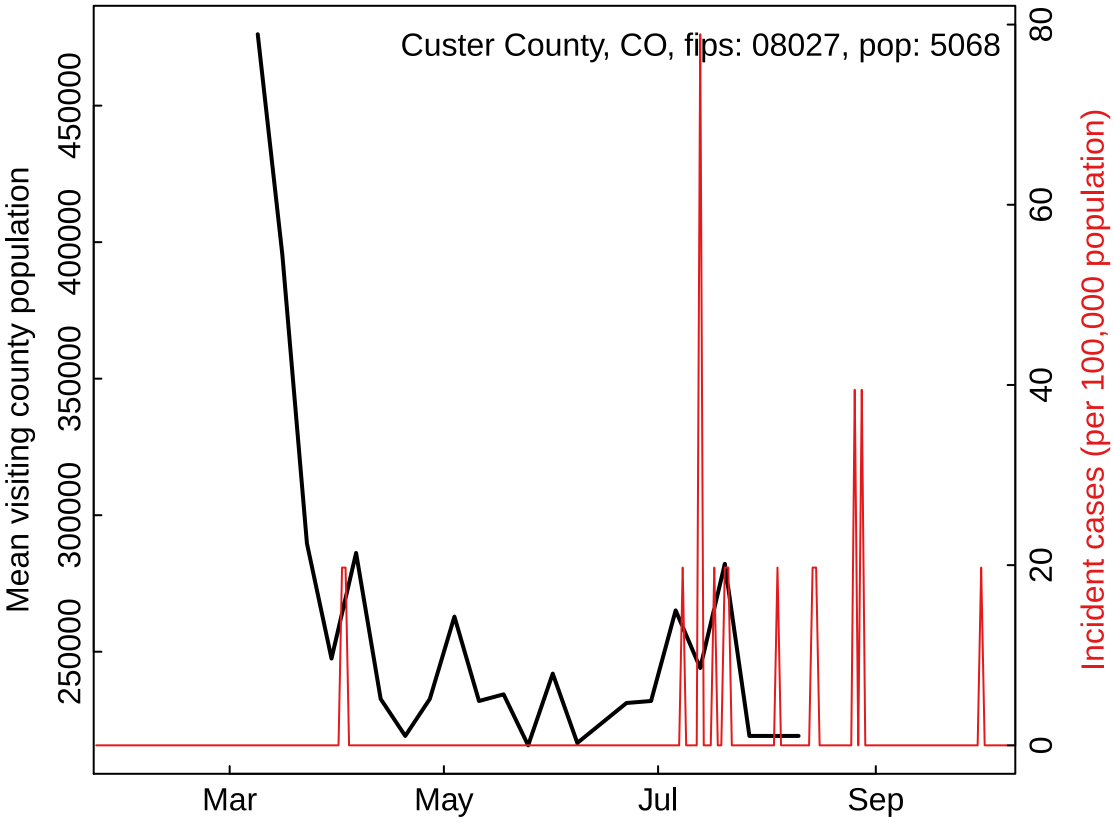

Custer County, CO, fips: 08027, pop: 5068
Mean visiting county population
250000
300000
350000
400000
450000
Incident cases (per 100,000 population)
0
20
40
60
80
Mar
May
Jul
Sep

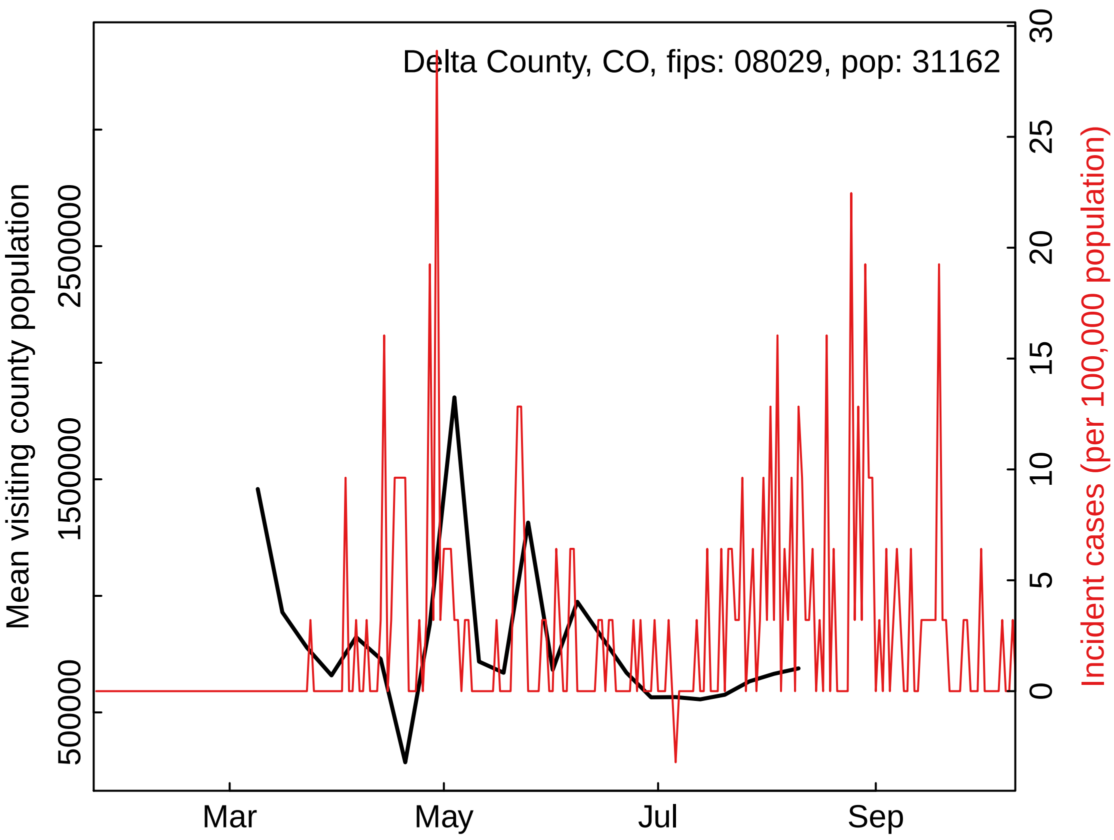

Delta County, CO, fips: 08029, pop: 31162
Mean visiting county population
500000
1500000
2500000
Incident cases (per 100,000 population)
0
5
10
15
20
25
30
Mar
May
Jul
Sep

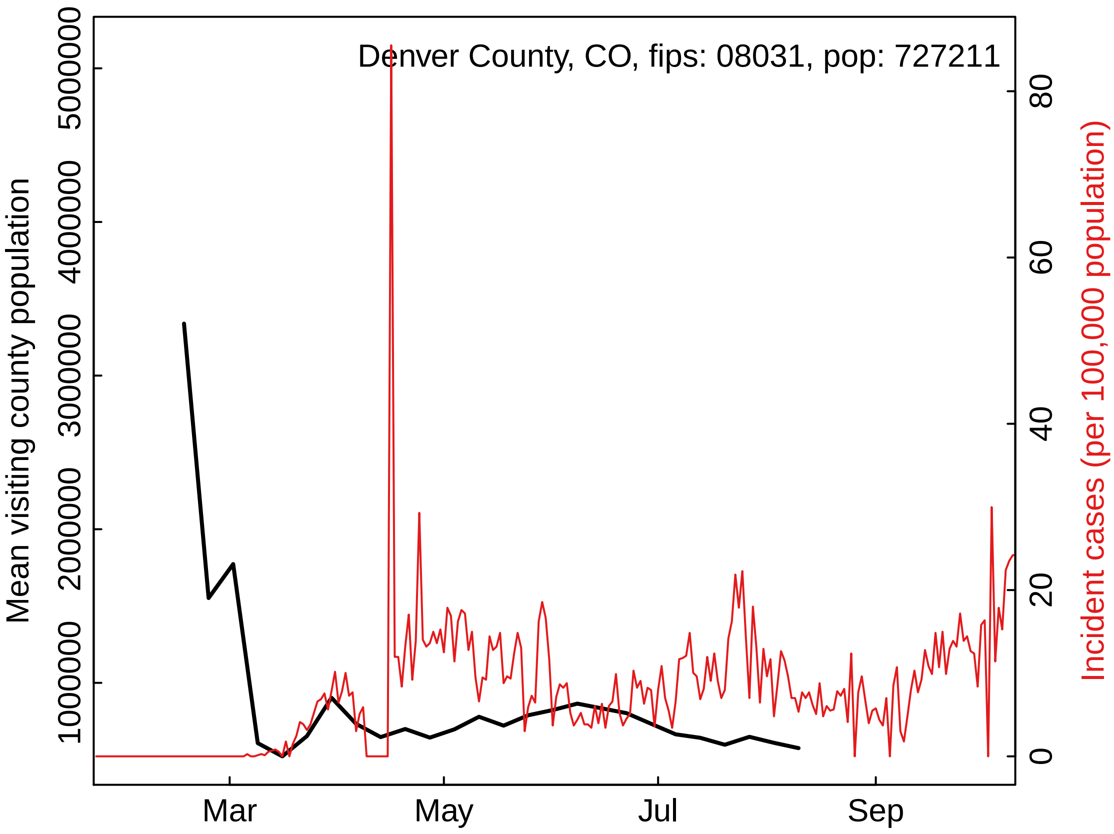

Denver County, CO, fips: 08031, pop: 727211
Mean visiting county population
1000000
2000000
3000000
4000000
5000000
Incident cases (per 100,000 population)
0
20
40
60
80
Mar
May
Jul
Sep

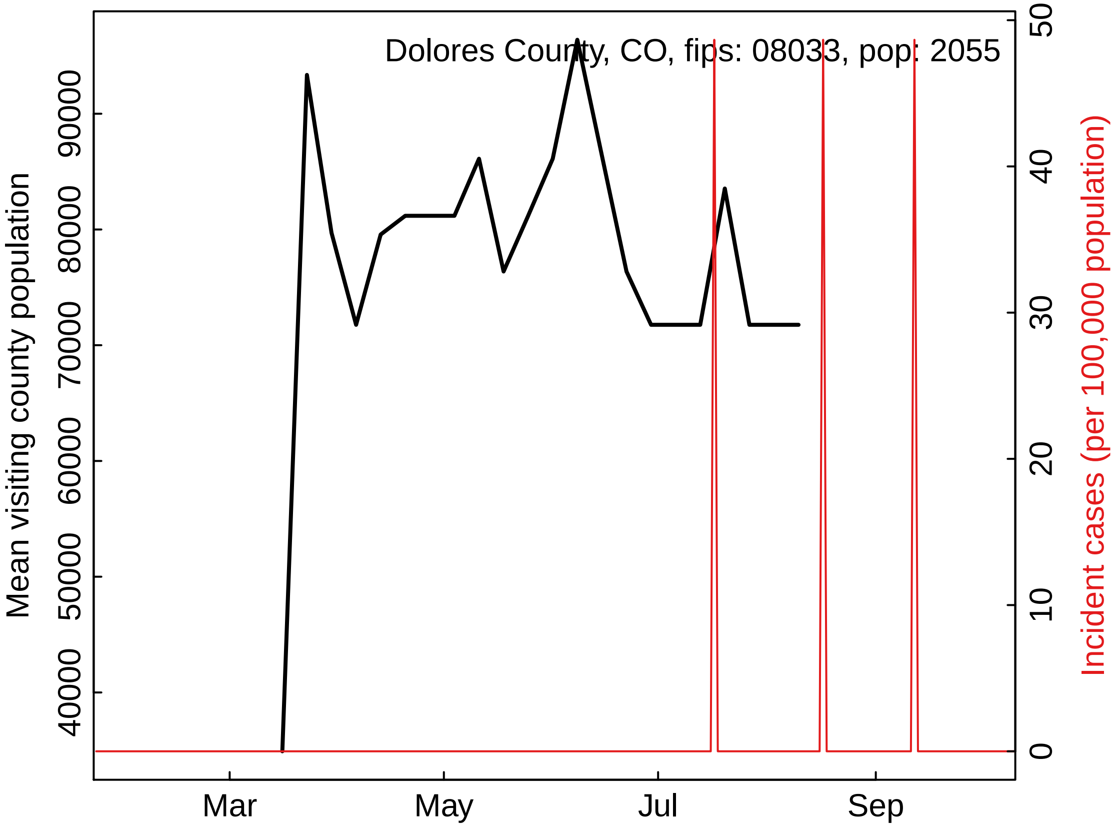

Dolores County, CO, fips: 08033, pop: 2055
Mean visiting county population
40000
50000
60000
70000
80000
90000
Incident cases (per 100,000 population)
0
10
20
30
40
50
Mar
May
Jul
Sep

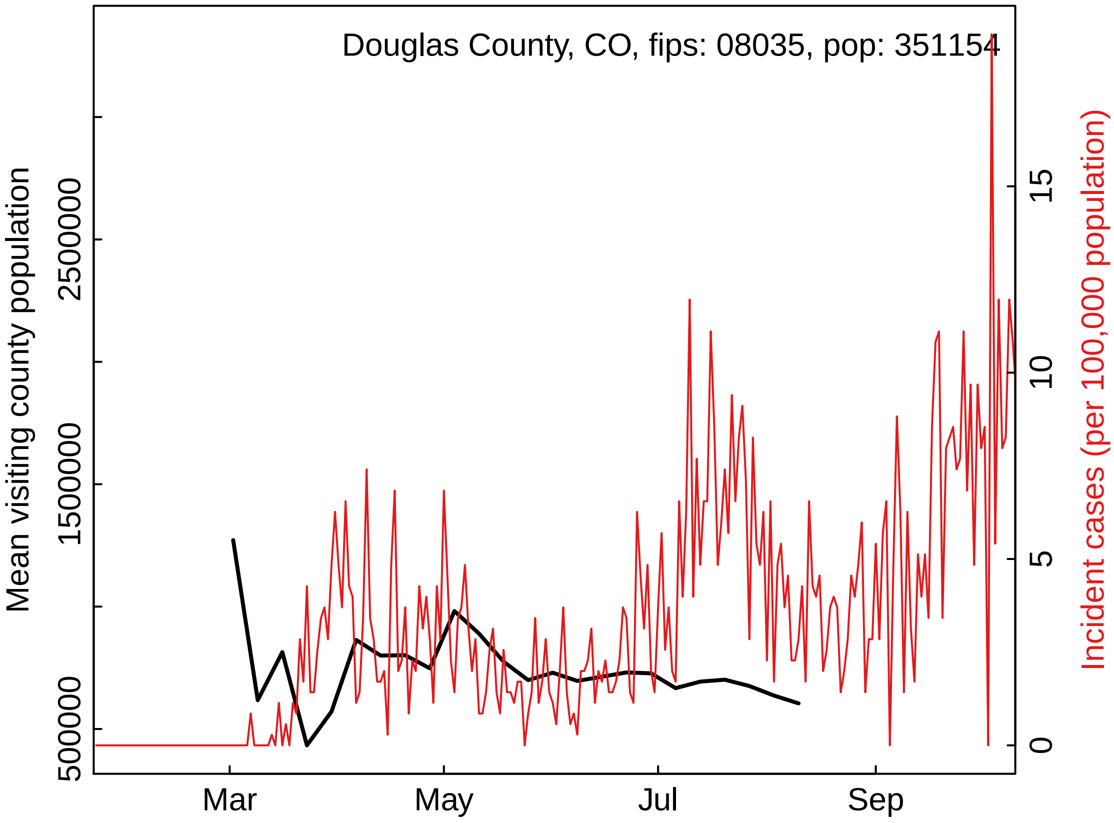
Douglas County, CO, fips: 08035, pop: 351154
Mean visiting county population
500000
1500000
2500000
Mar
May
Jul
Sep
0
5
10
15
Incident cases (per 100,000 population)

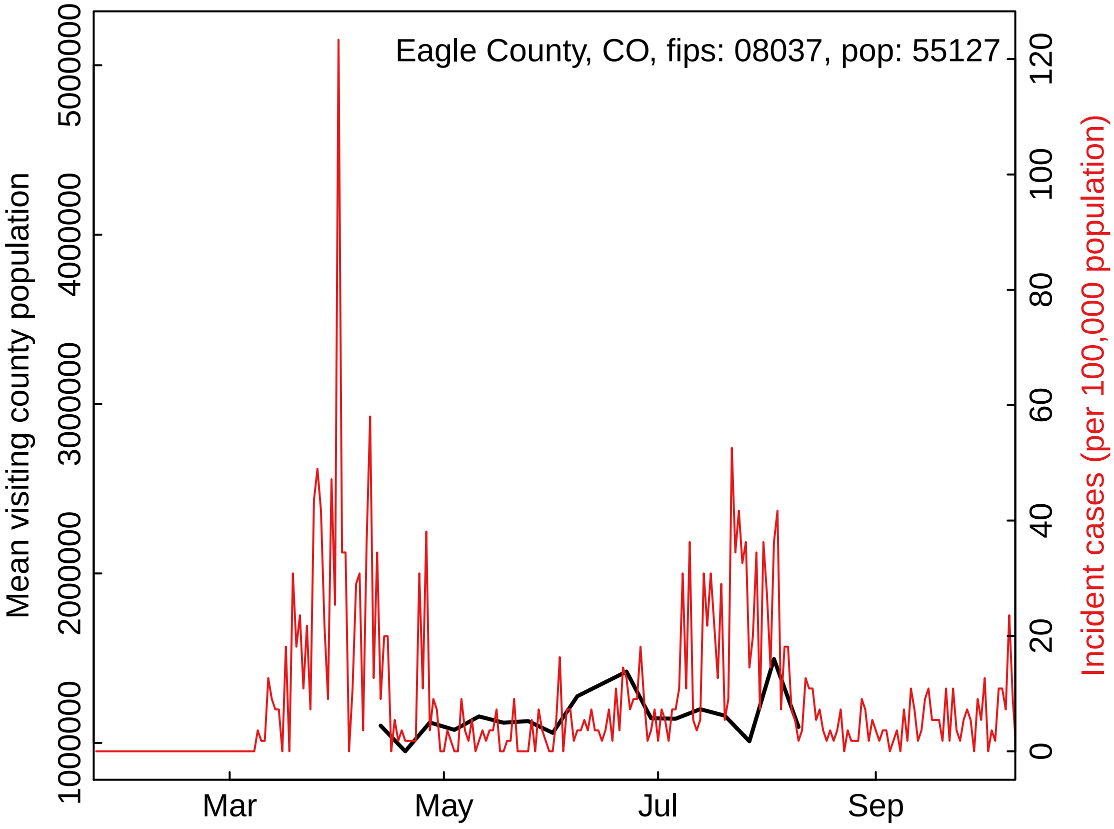

Eagle County, CO, fips: 08037, pop: 55127
Mean visiting county population
10000000
20000000
30000000
40000000
50000000
Incident cases (per 100,000 population)
0
20
40
60
80
100
120
Mar
May
Jul
Sep

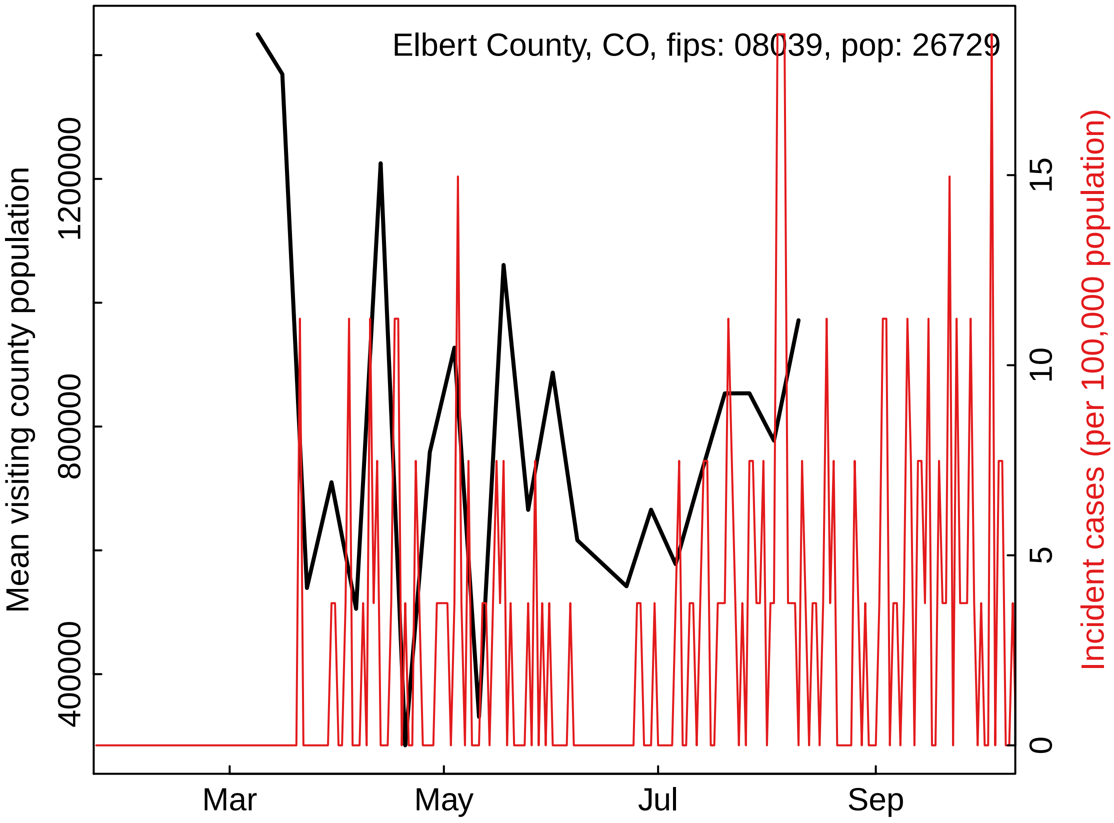

Elbert County, CO, fips: 08039, pop: 26729
Mean visiting county population
Incident cases (per 100,000 population)
400000
800000
1200000
0
5
10
15
Mar
May
Jul
Sep

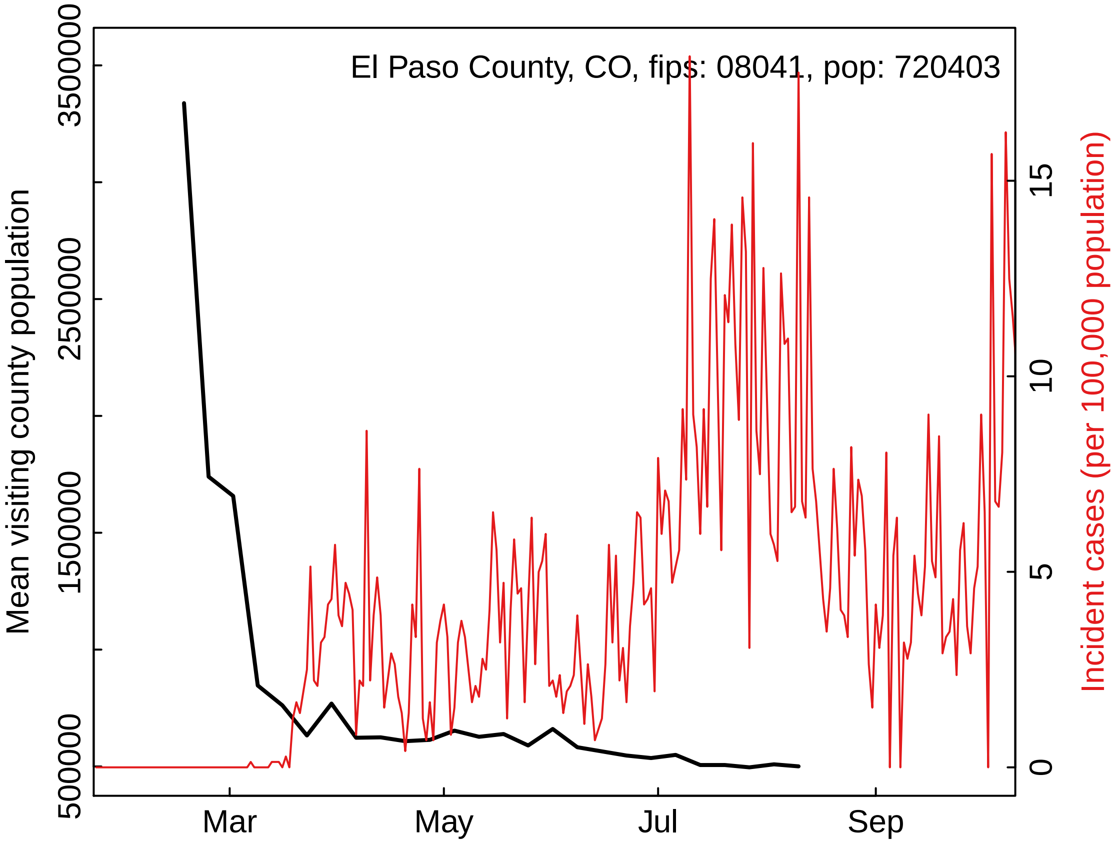

El Paso County, CO, fips: 08041, pop: 720403
Mean visiting county population
500000
1500000
2500000
3500000
Mar
May
Jul
Sep
0
5
10
15
Incident cases (per 100,000 population)

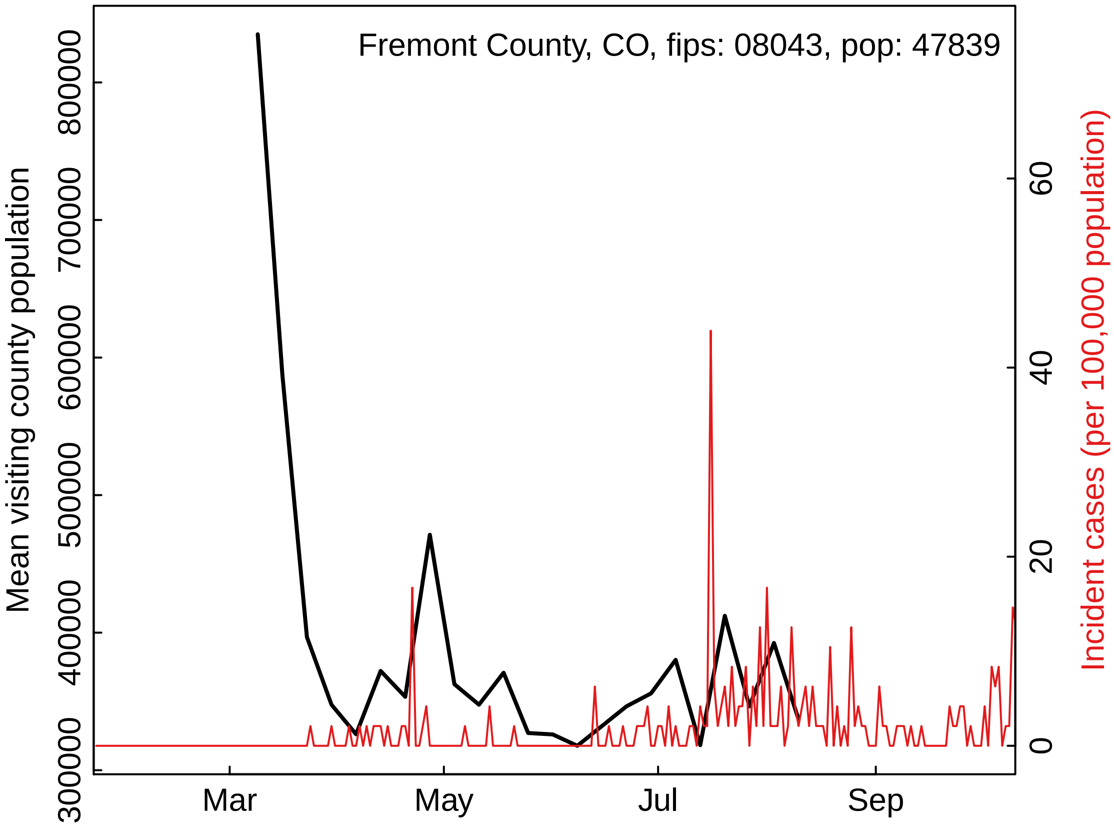

Fremont County, CO, fips: 08043, pop: 47839
Mean visiting county population
300000
400000
500000
600000
700000
800000
Incident cases (per 100,000 population)
0
20
40
60
Mar
May
Jul
Sep

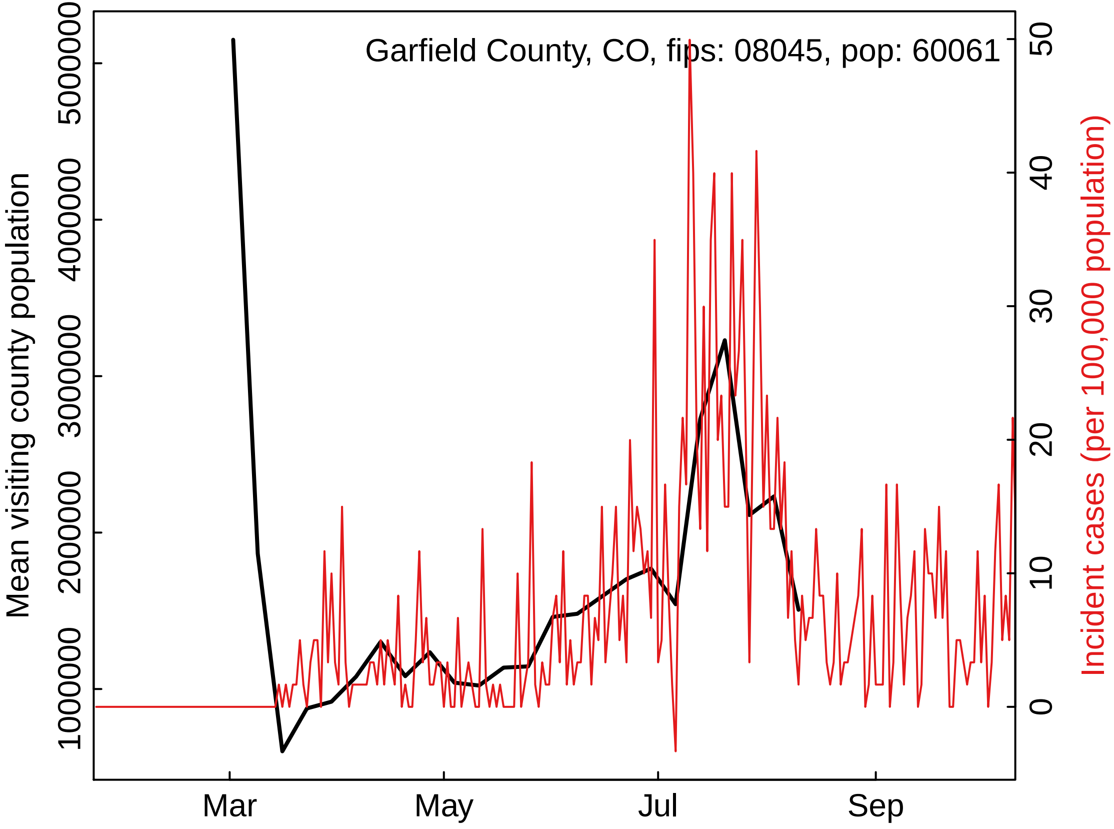

Garfield County, CO, fips: 08045, pop: 60061
Mean visiting county population
10000000
20000000
30000000
40000000
50000000
Incident cases (per 100,000 population)
0
10
20
30
40
50
Mar
May
Jul
Sep

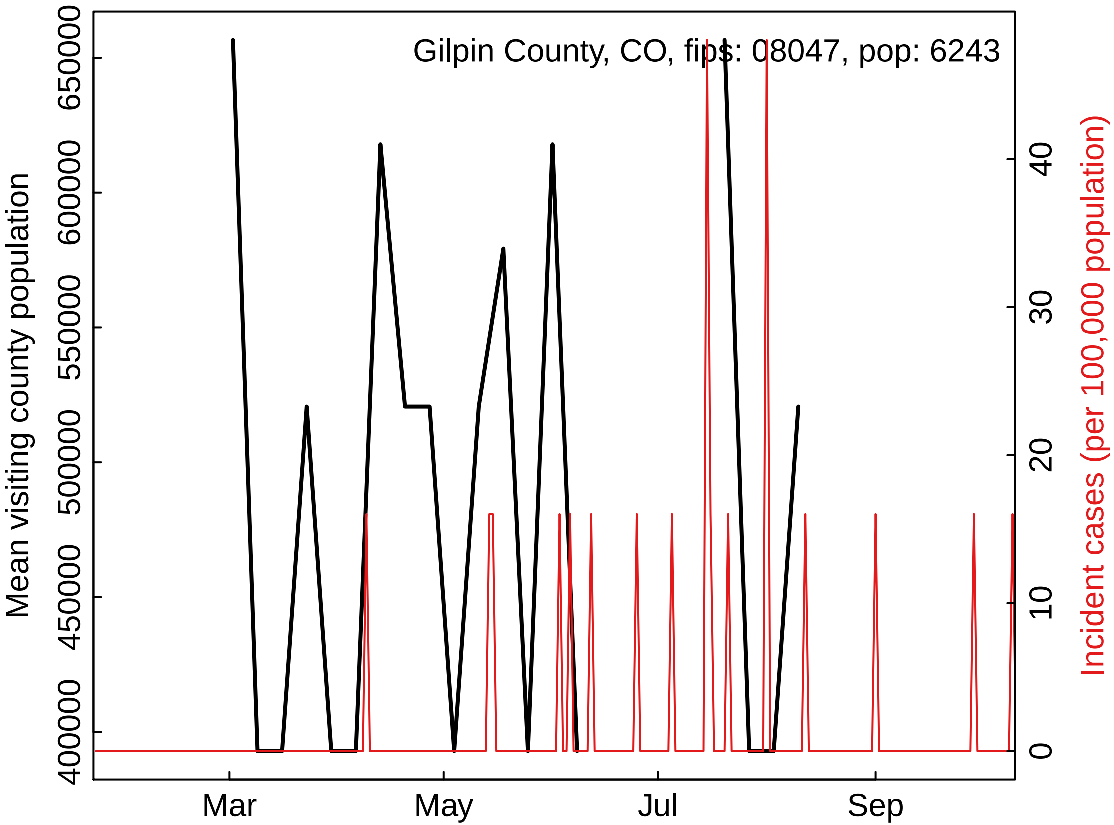

Gilpin County, CO, fips: 08047, pop: 6243
Mean visiting county population
400000
450000
500000
550000
600000
650000
Incident cases (per 100,000 population)
0
10
20
30
40
Mar
May
Jul
Sep

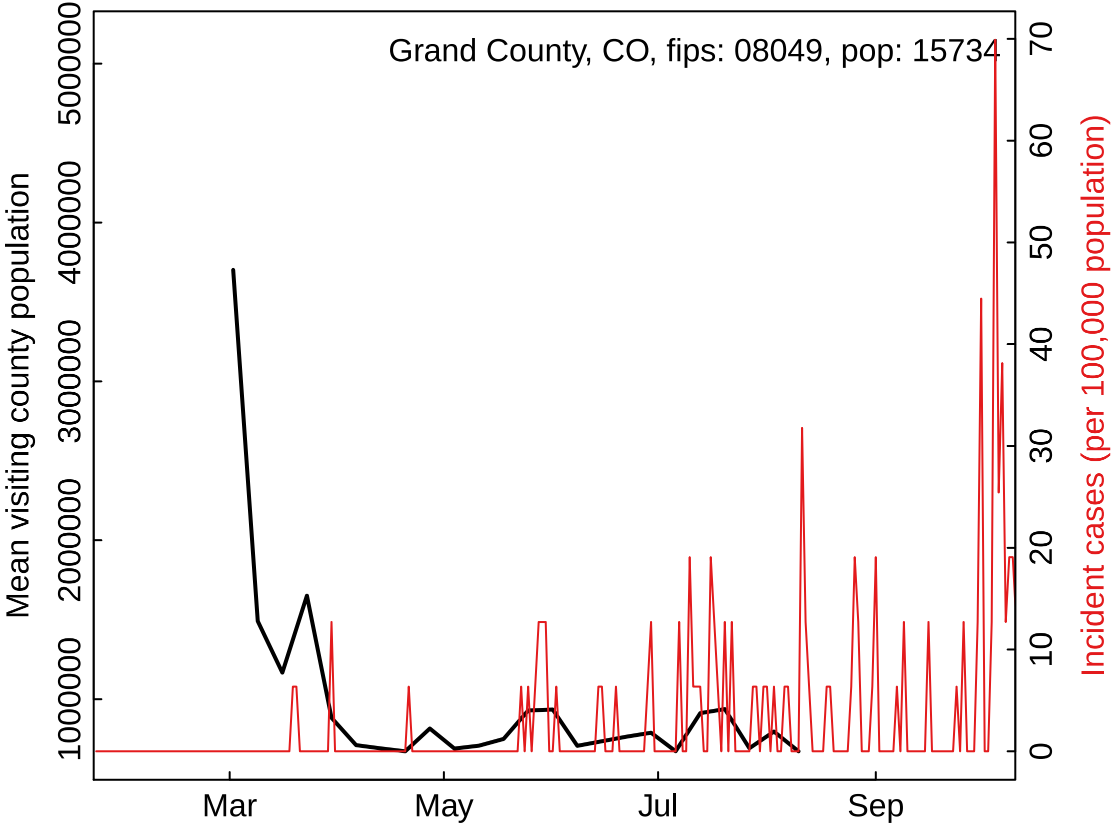

Grand County, CO, fips: 08049, pop: 15734
Mean visiting county population
Incident cases (per 100,000 population)
10000000
2000000
3000000
4000000
5000000
0
10
20
30
40
50
60
70
Mar
May
Jul
Sep

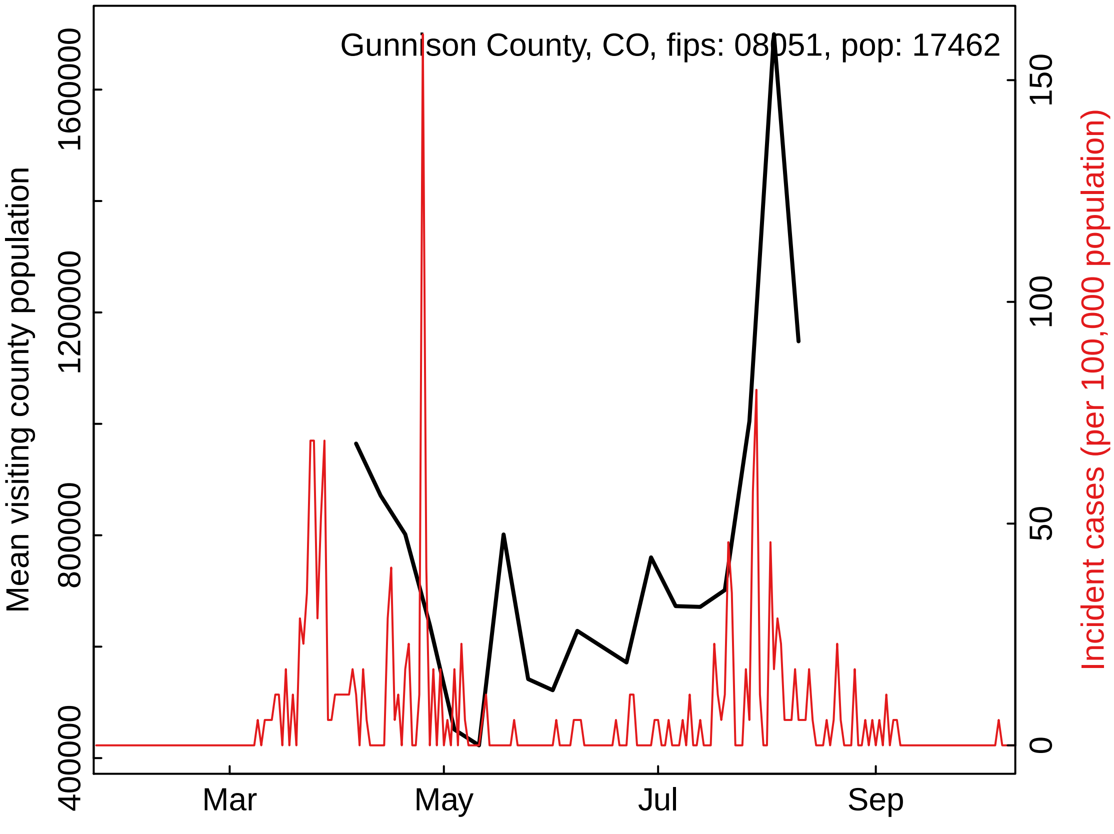

Gunnison County, CO, fips: 08051, pop: 17462
Mean visiting county population
400000
800000
1200000
1600000
Incident cases (per 100,000 population)
0
50
100
150
Mar
May
Jul
Sep

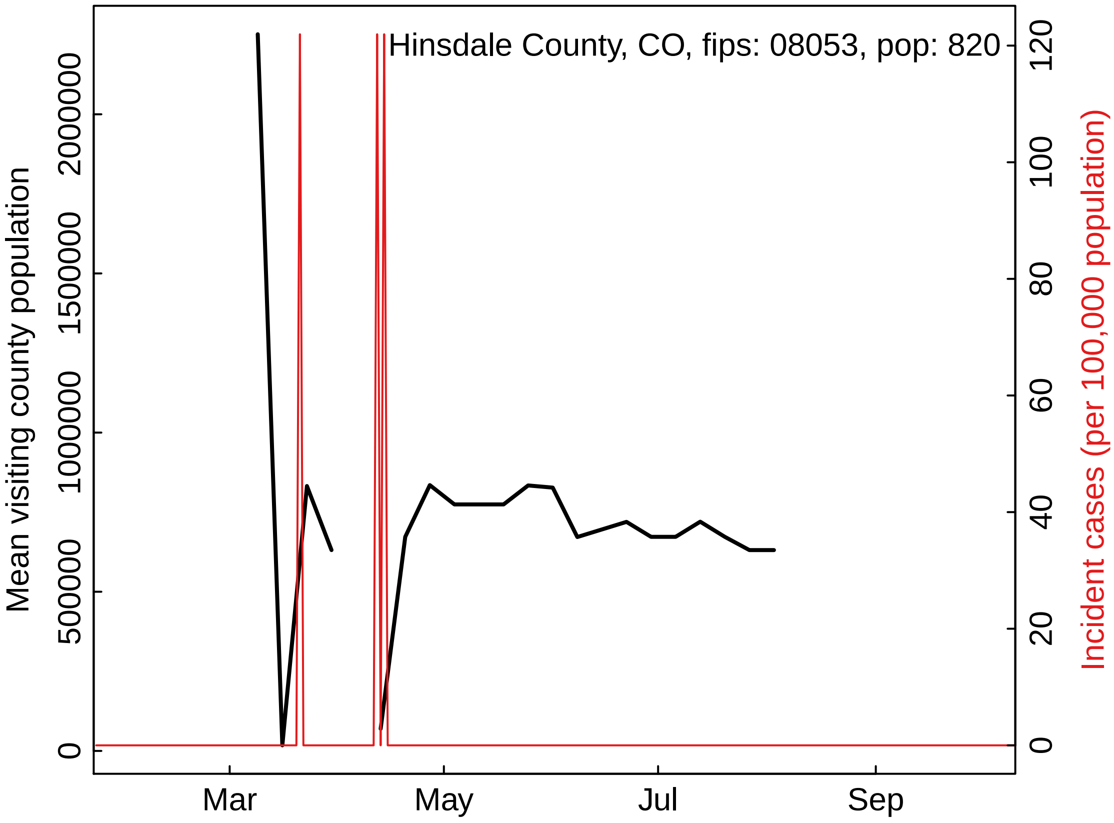
Hinsdale County, CO, fips: 08053, pop: 820
Mean visiting county population
0
500000
1000000
1500000
2000000
Incident cases (per 100,000 population)
0
20
40
60
80
100
120
Mar
May
Jul
Sep

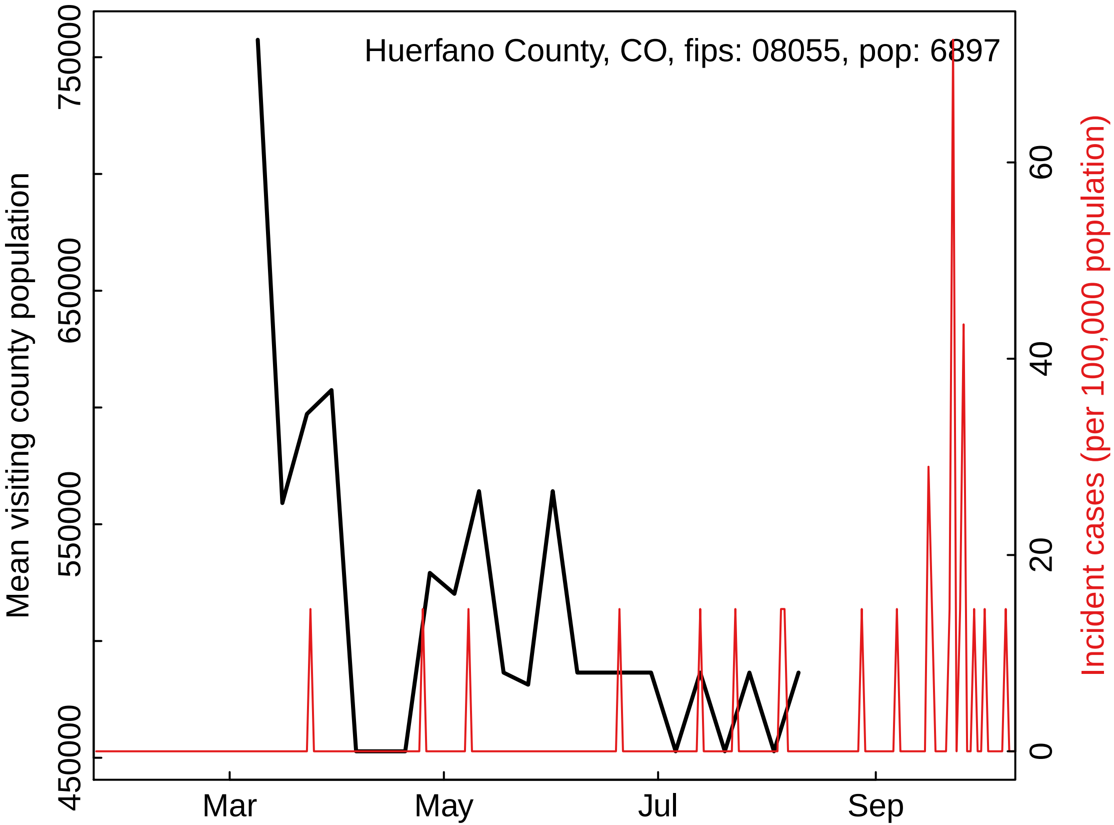

Huerfano County, CO, fips: 08055, pop: 6897
Mean visiting county population
Incident cases (per 100,000 population)
450000
550000
650000
750000
0
20
40
60
Mar
May
Jul
Sep

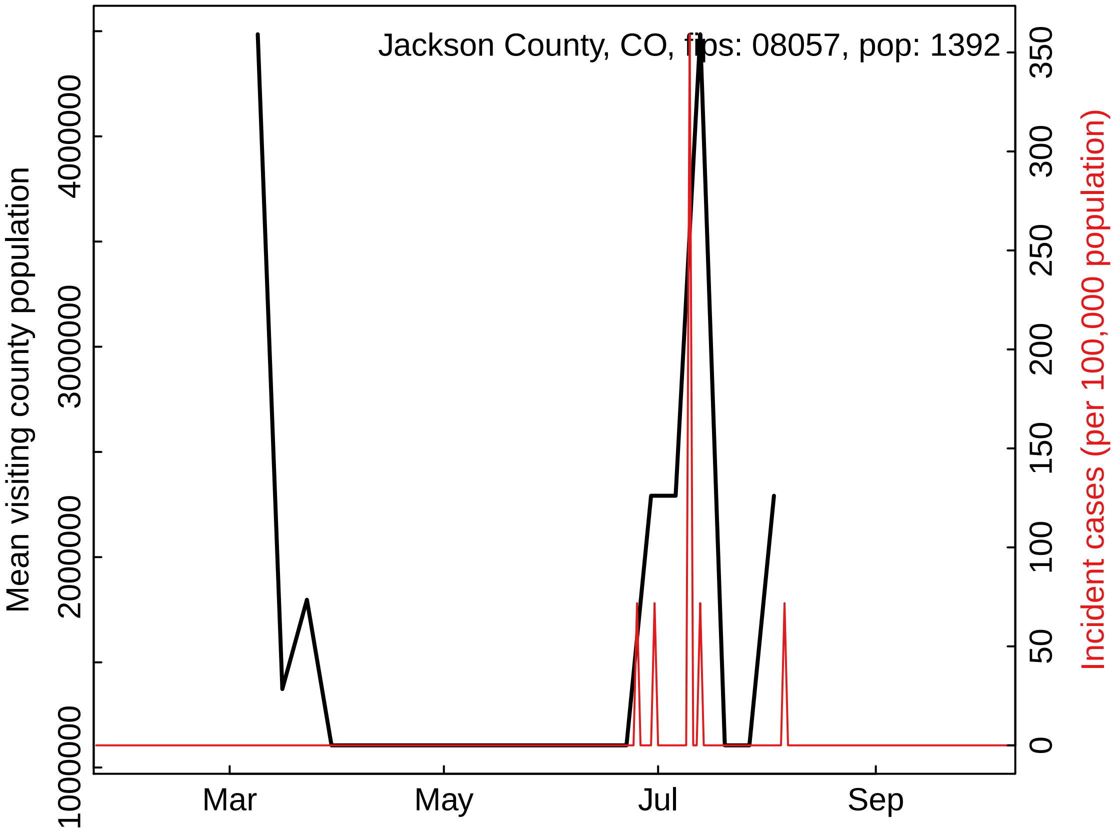

Jackson County, CO, fips: 08057, pop: 1392
Mean visiting county population
10000000
20000000
30000000
40000000
Mar
May
Jul
Sep
0
50
100
150
200
250
300
350
Incident cases (per 100,000 population)

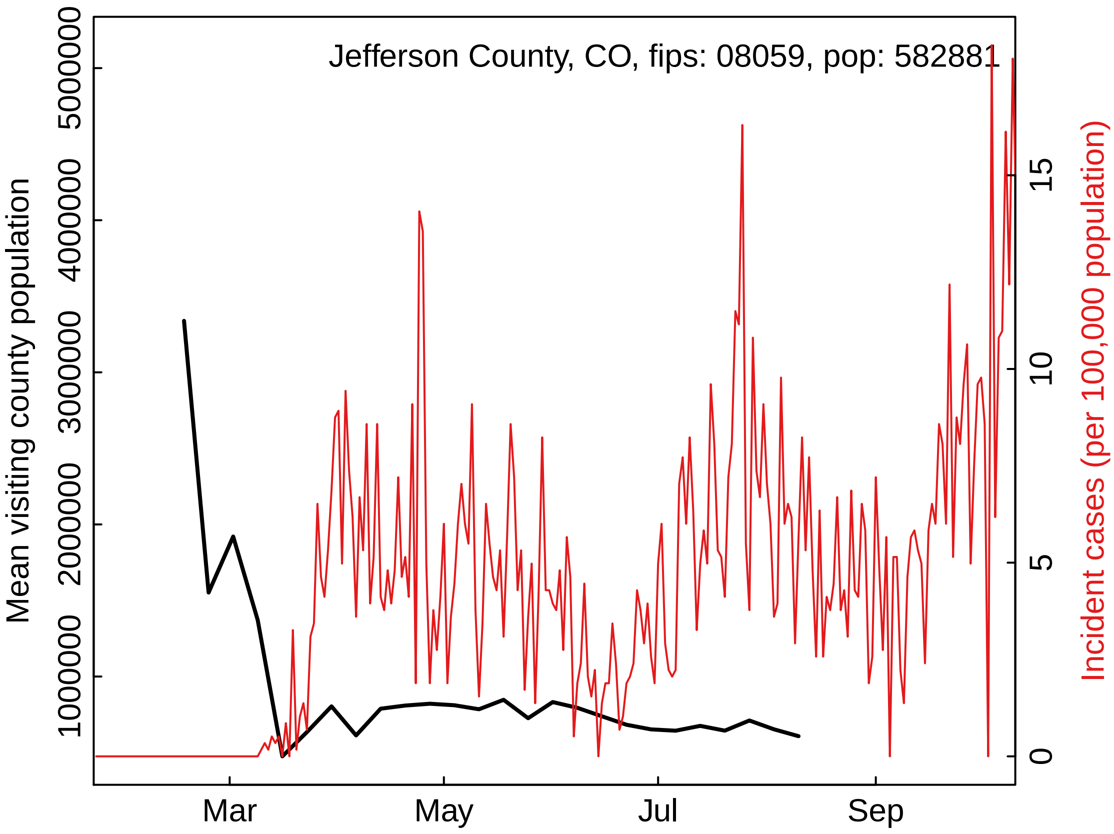

Jefferson County, CO, fips: 08059, pop: 582881
Mean visiting county population
10000000
20000000
30000000
40000000
50000000
Incident cases (per 100,000 population)
0
5
10
15
Mar
May
Jul
Sep

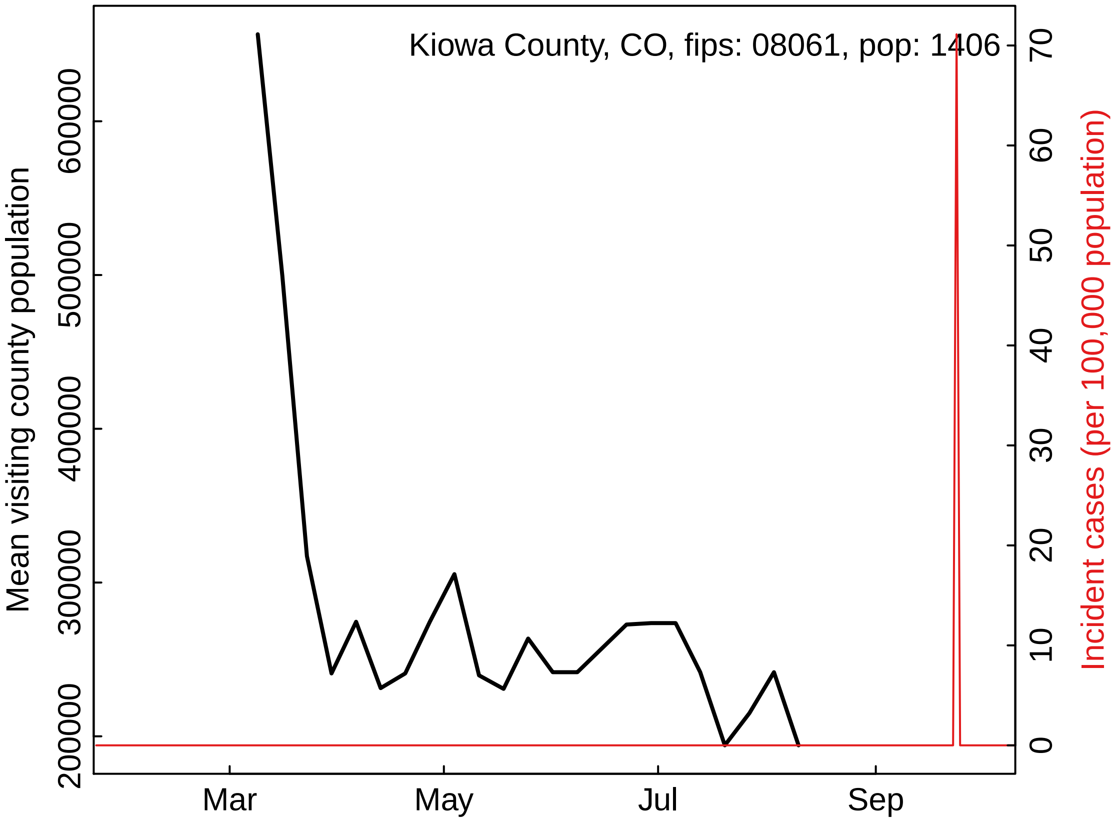
Kiowa County, CO, fips: 08061, pop: 1406
Mean visiting county population
200000
300000
400000
500000
600000
Incident cases (per 100,000 population)
0
10
20
30
40
50
60
70
Mar
May
Jul
Sep

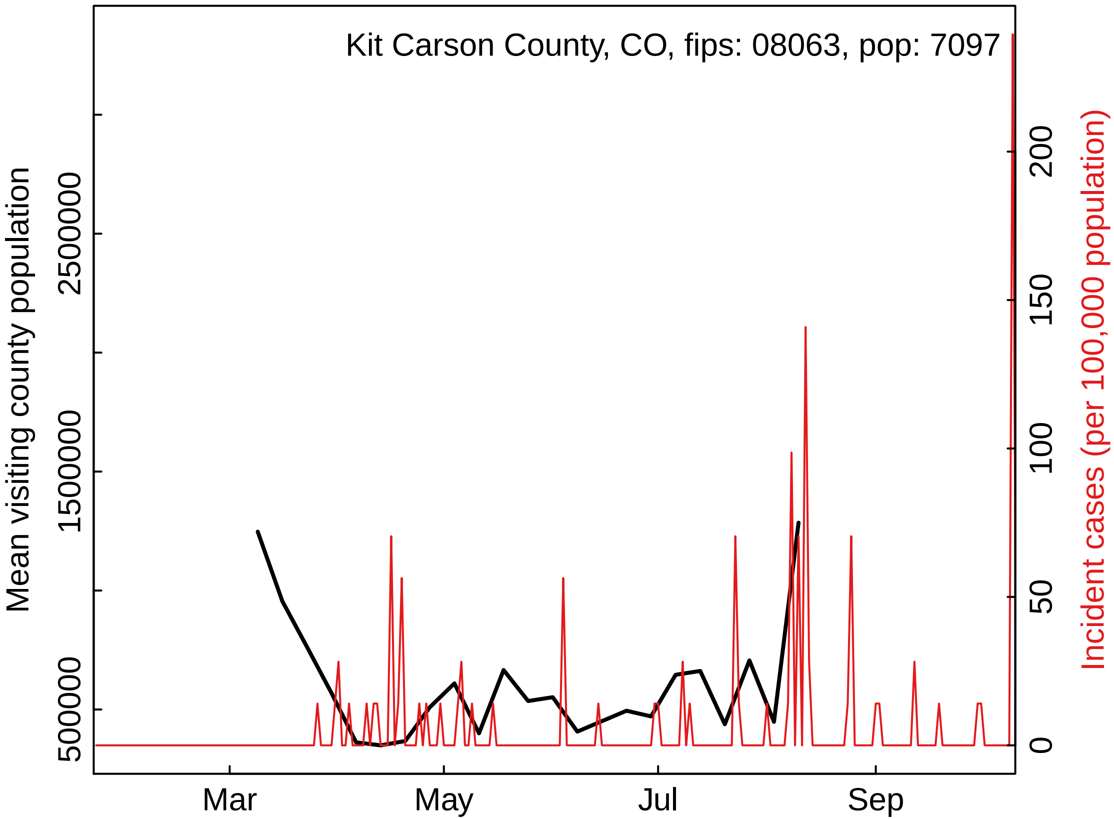

Kit Carson County, CO, fips: 08063, pop: 7097
Mean visiting county population
500000
1500000
2500000
Incident cases (per 100,000 population)
0
50
100
150
200
Mar
May
Jul
Sep

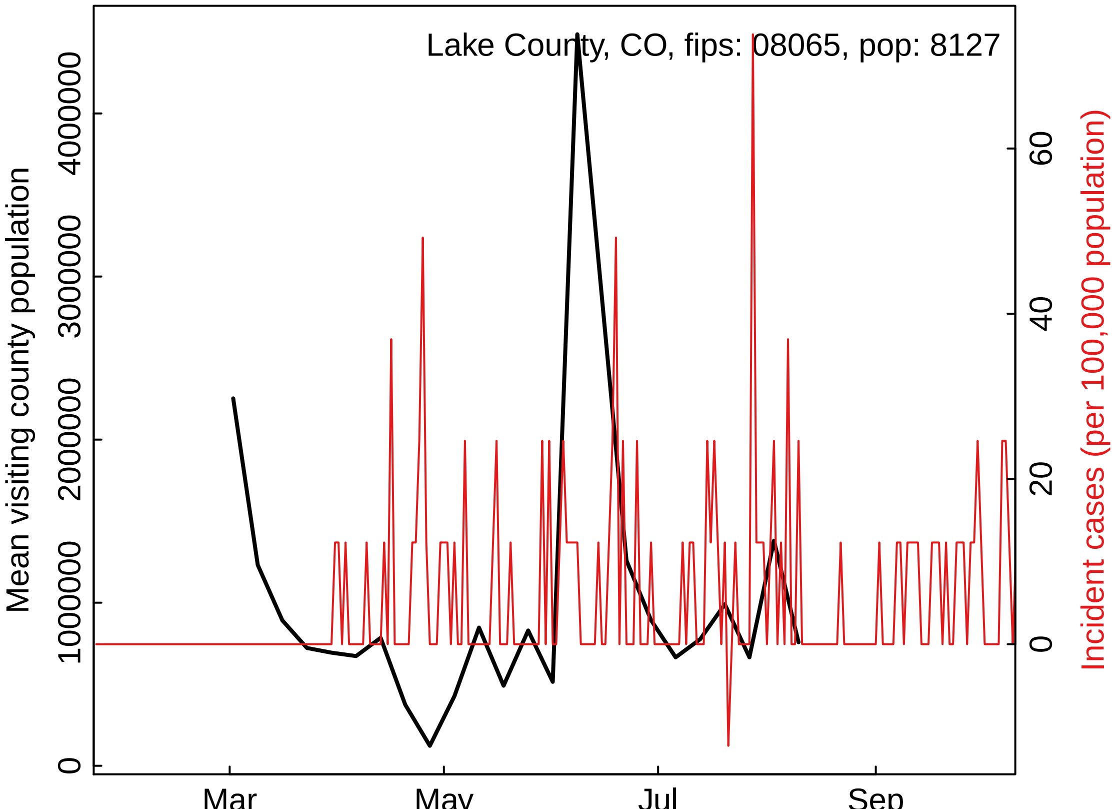

Lake County, CO, fips: 08065, pop: 8127
Mean visiting county population
0
1000000
2000000
3000000
4000000
Incident cases (per 100,000 population)
0
20
40
60
Mar
May
Jul
Sep

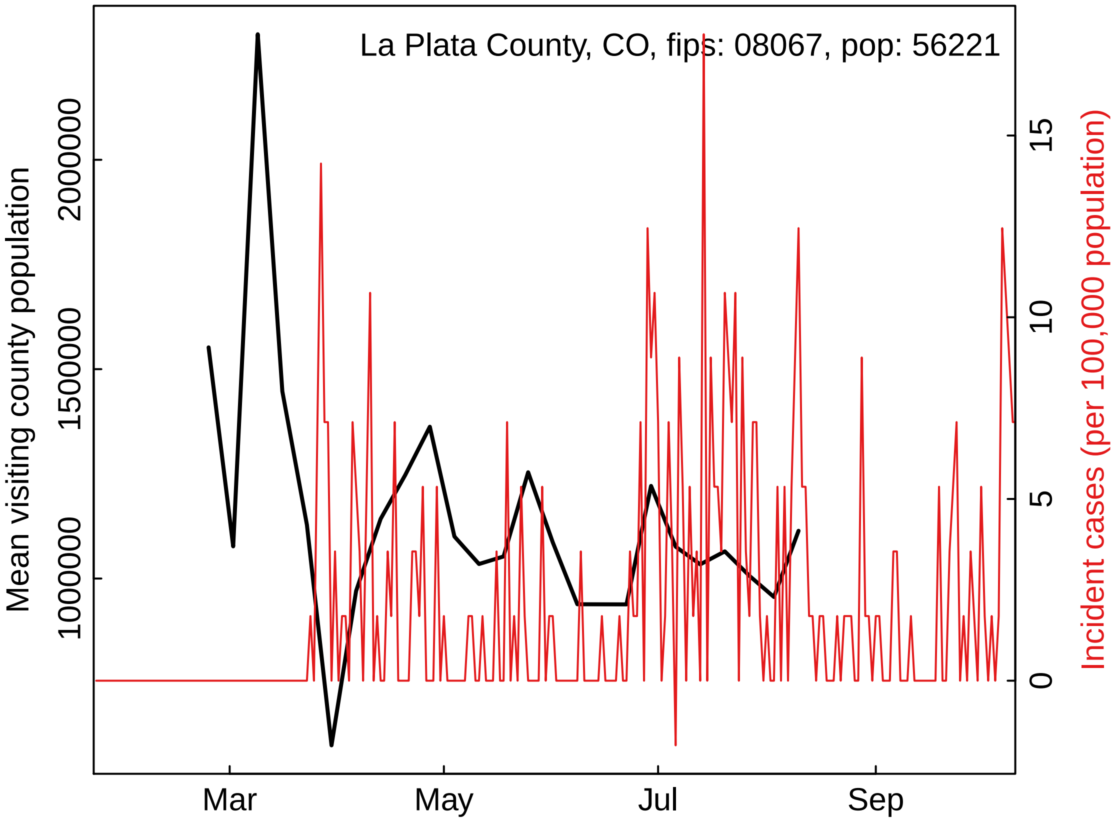

La Plata County, CO, fips: 08067, pop: 56221
Mean visiting county population
1000000
1500000
2000000
Incident cases (per 100,000 population)
0
5
10
15
Mar
May
Jul
Sep

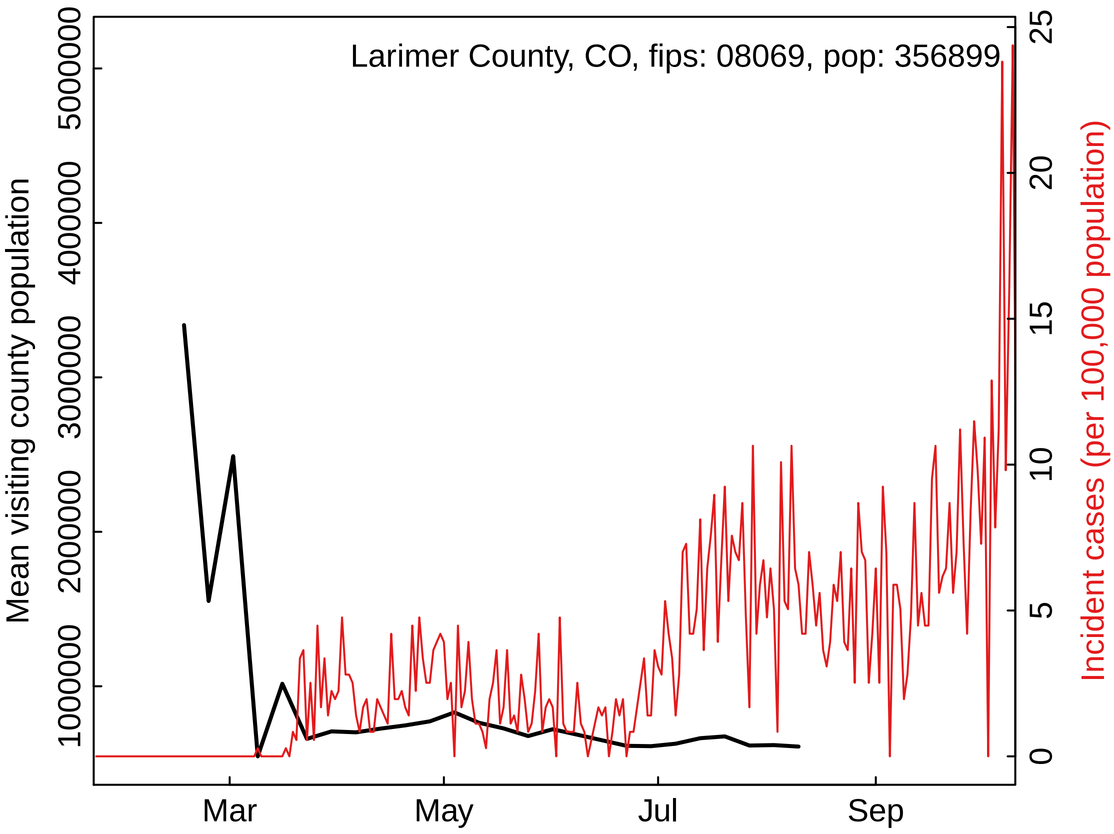

Larimer County, CO, fips: 08069, pop: 356899
Mean visiting county population
Incident cases (per 100,000 population)
1000000
2000000
3000000
4000000
5000000
0
5
10
15
20
25
Mar
May
Jul
Sep

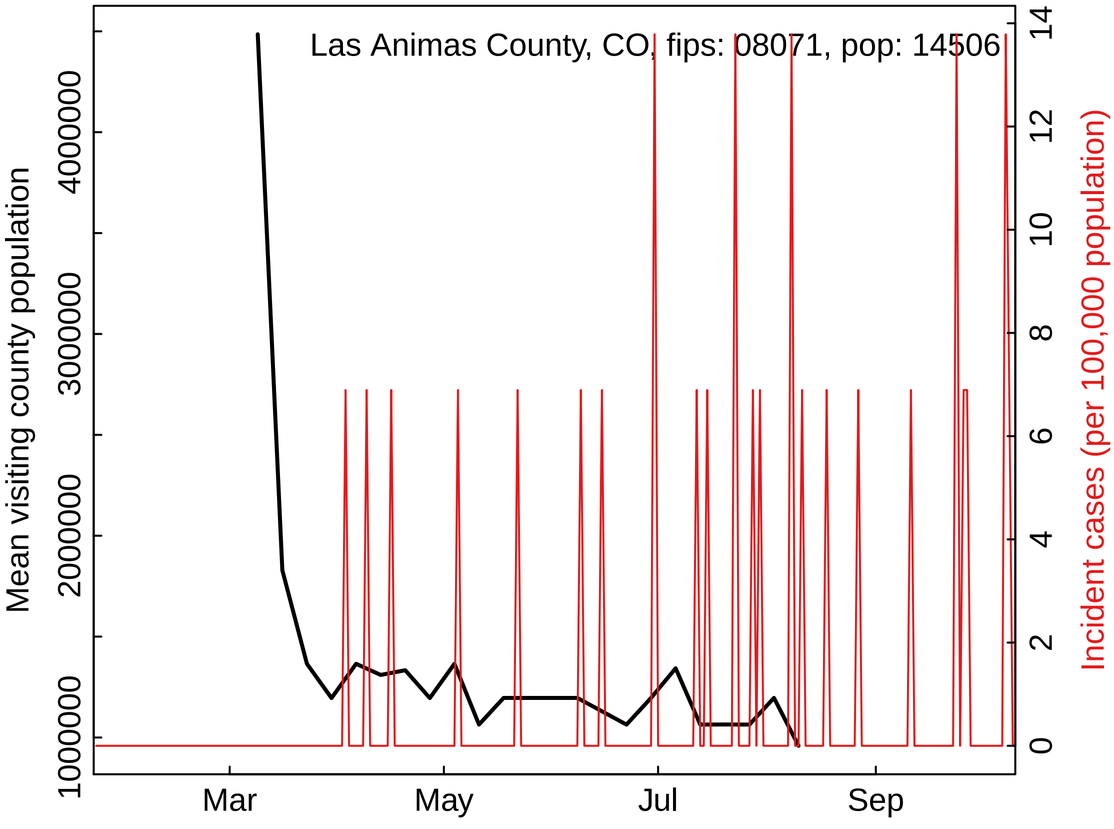

Las Animas County, CO, fips: 08071, pop: 14506
Mean visiting county population
10000000
20000000
30000000
40000000
Incident cases (per 100,000 population)
0
2
4
6
8
10
12
14
Mar
May
Jul
Sep

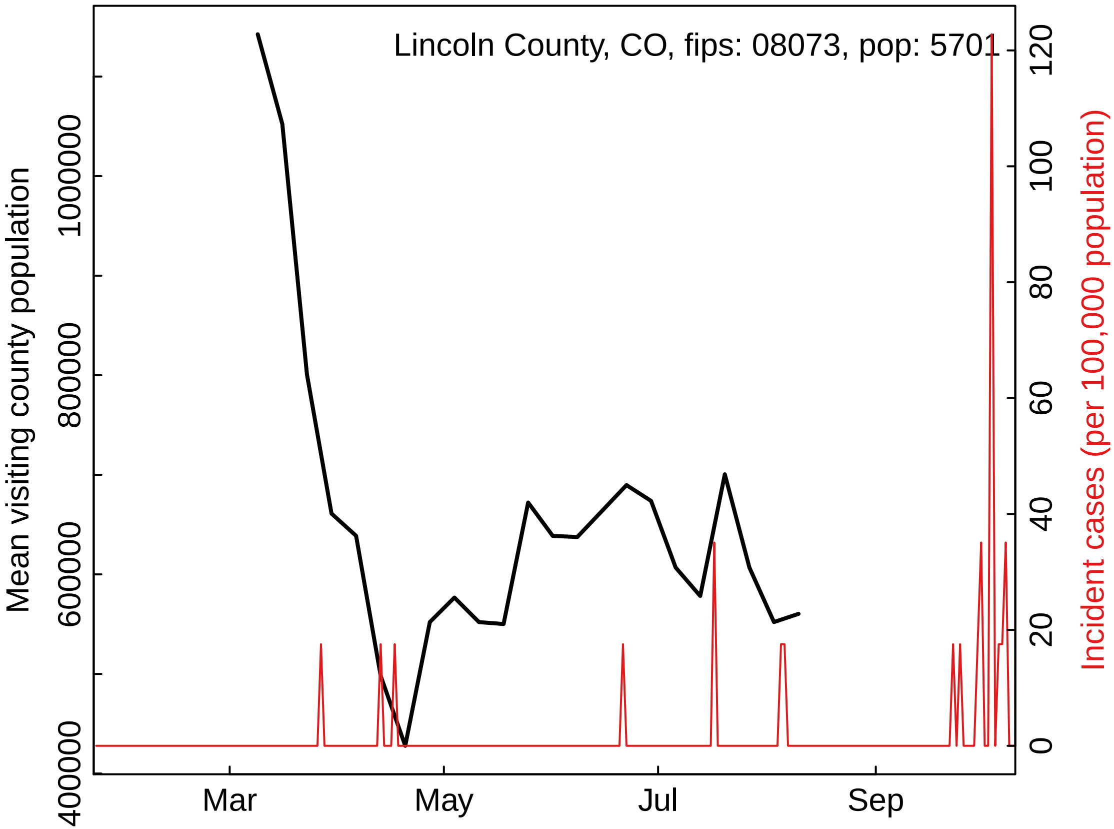

Lincoln County, CO, fips: 08073, pop: 5701
Mean visiting county population
400000
600000
800000
1000000
Incident cases (per 100,000 population)
0
20
40
60
80
100
120
Mar
May
Jul
Sep

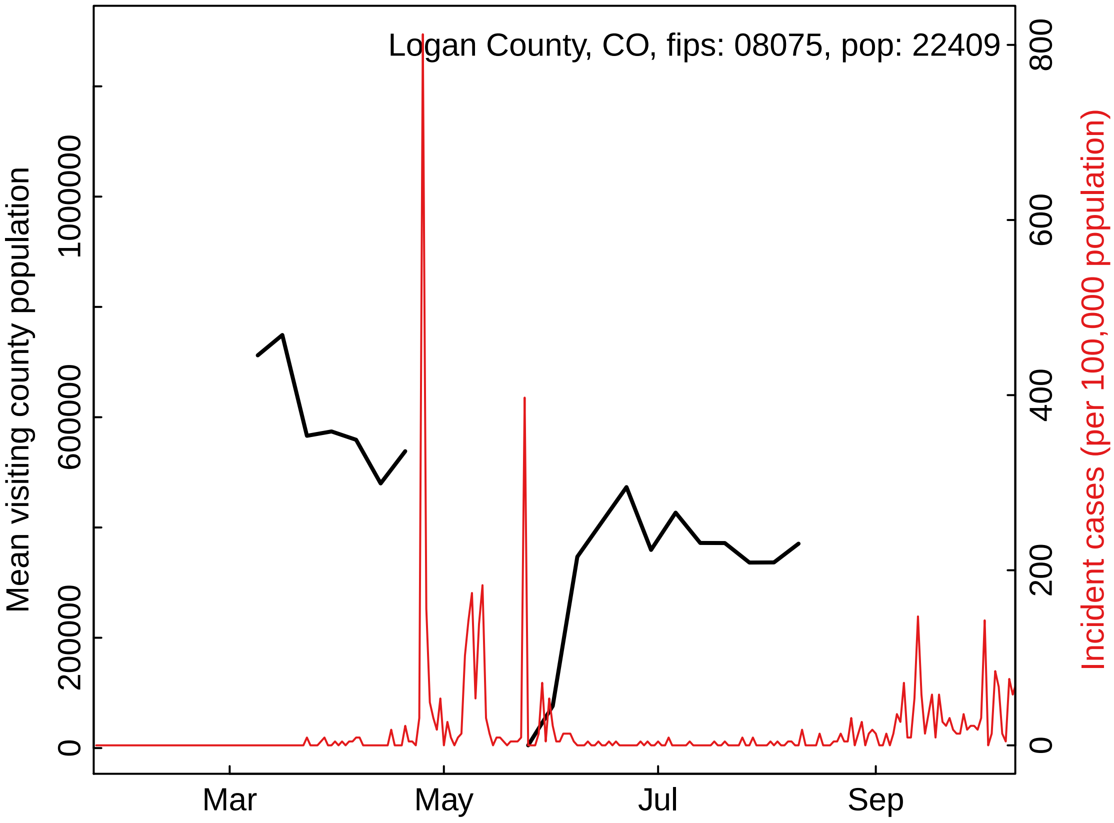

Logan County, CO, fips: 08075, pop: 22409
Mean visiting county population
0
200000
600000
1000000
Incident cases (per 100,000 population)
0
200
400
600
800
Mar
May
Jul
Sep

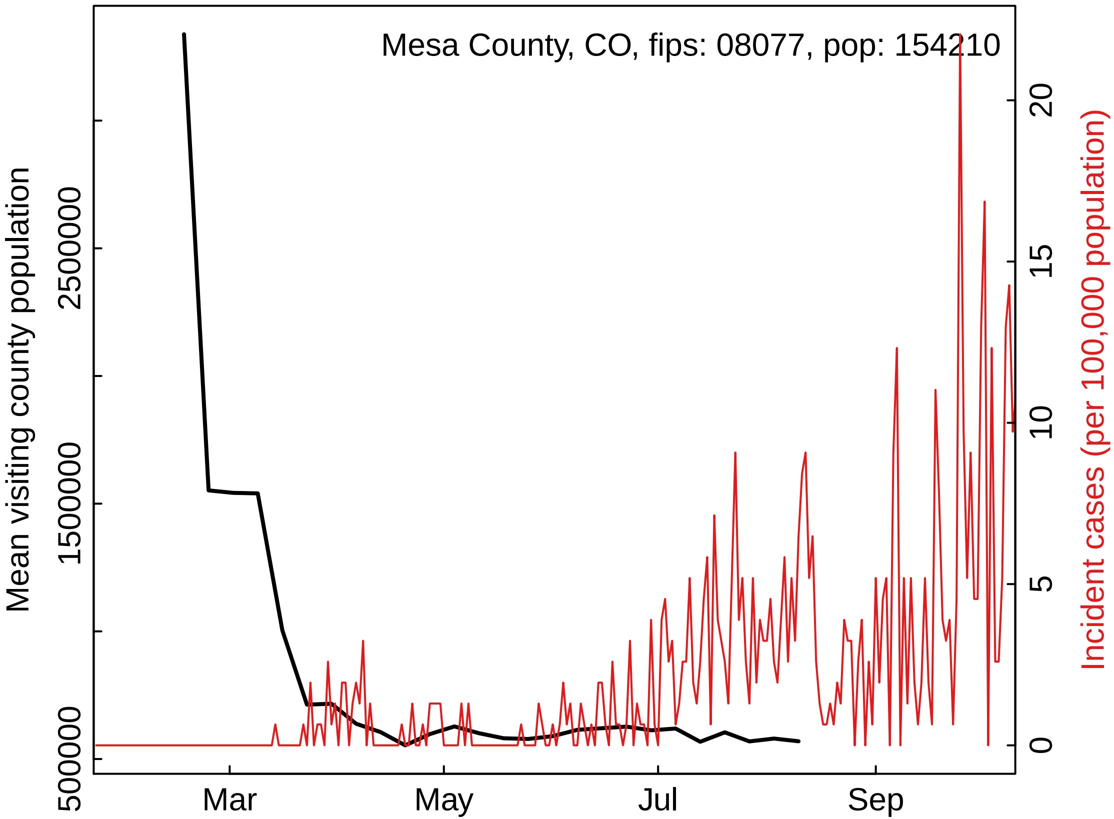

Mesa County, CO, fips: 08077, pop: 154210
Mean visiting county population
500000
1500000
2500000
Mar
May
Jul
Sep
0
5
10
15
20
Incident cases (per 100,000 population)

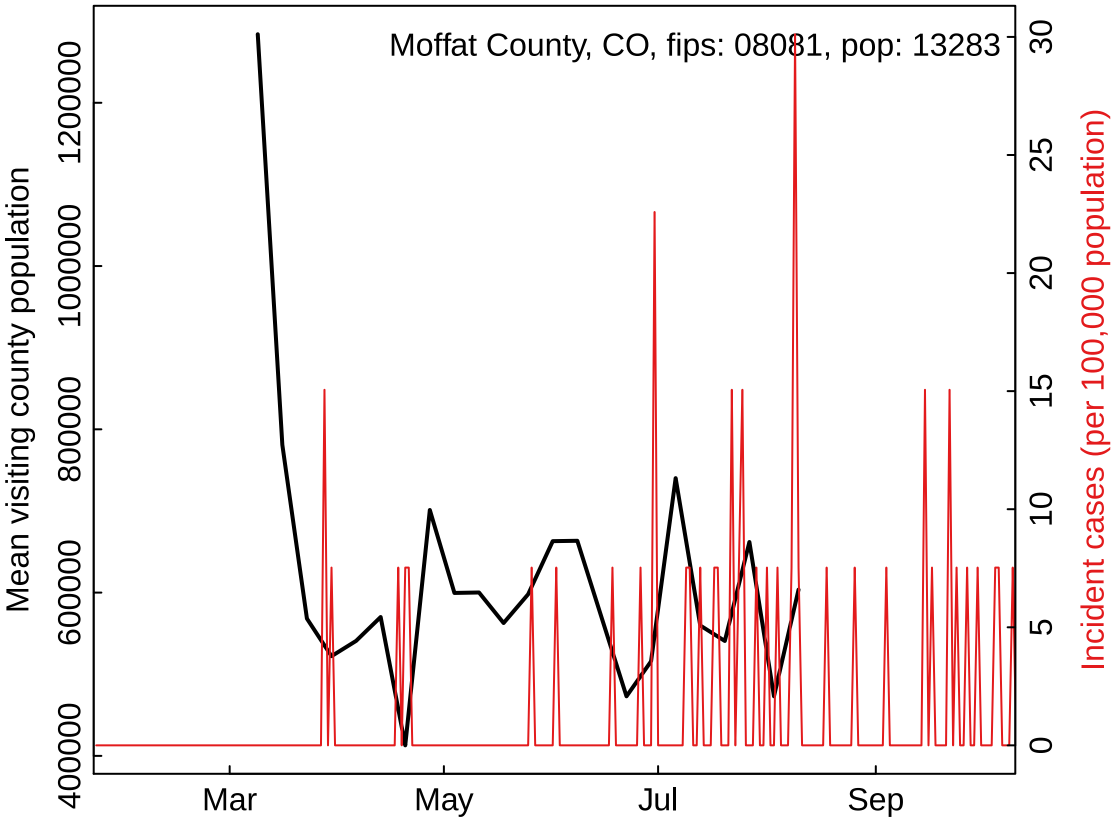

Moffat County, CO, fips: 08081, pop: 13283
Mean visiting county population
Incident cases (per 100,000 population)
400000
600000
800000
1000000
1200000
0
5
10
15
20
25
30
Mar
May
Jul
Sep

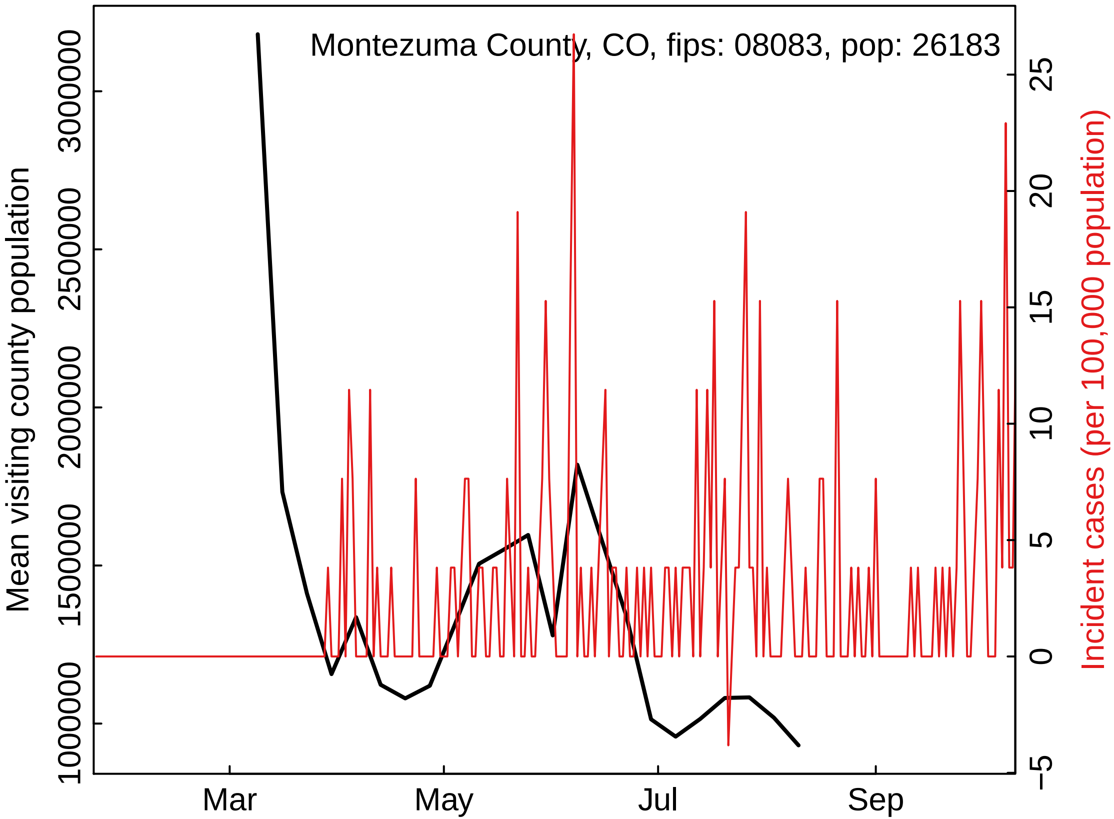

Montezuma County, CO, fips: 08083, pop: 26183
Mean visiting county population
10000000
15000000
20000000
25000000
30000000
Incident cases (per 100,000 population)
−5
0
5
10
15
20
25
Mar
May
Jul
Sep

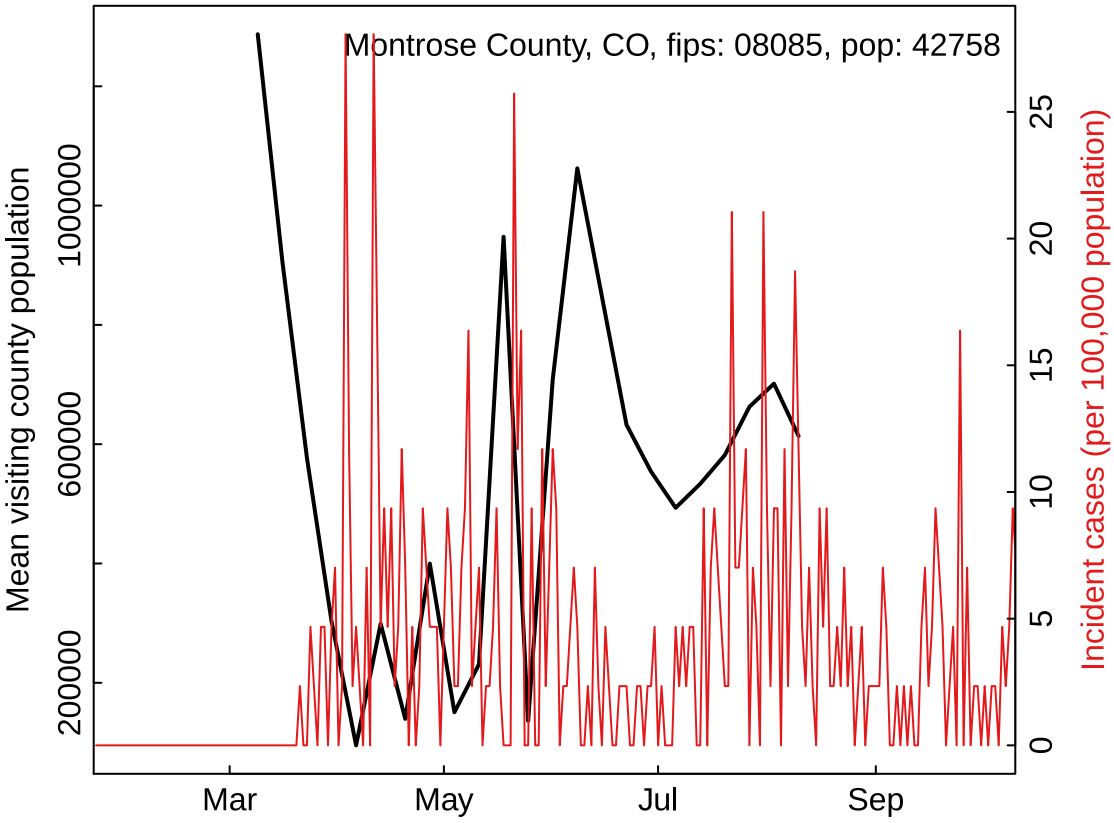

Montrose County, CO, fips: 08085, pop: 42758
Mean visiting county population
200000
600000
1000000
Incident cases (per 100,000 population)
0
5
10
15
20
25
Mar
May
Jul
Sep

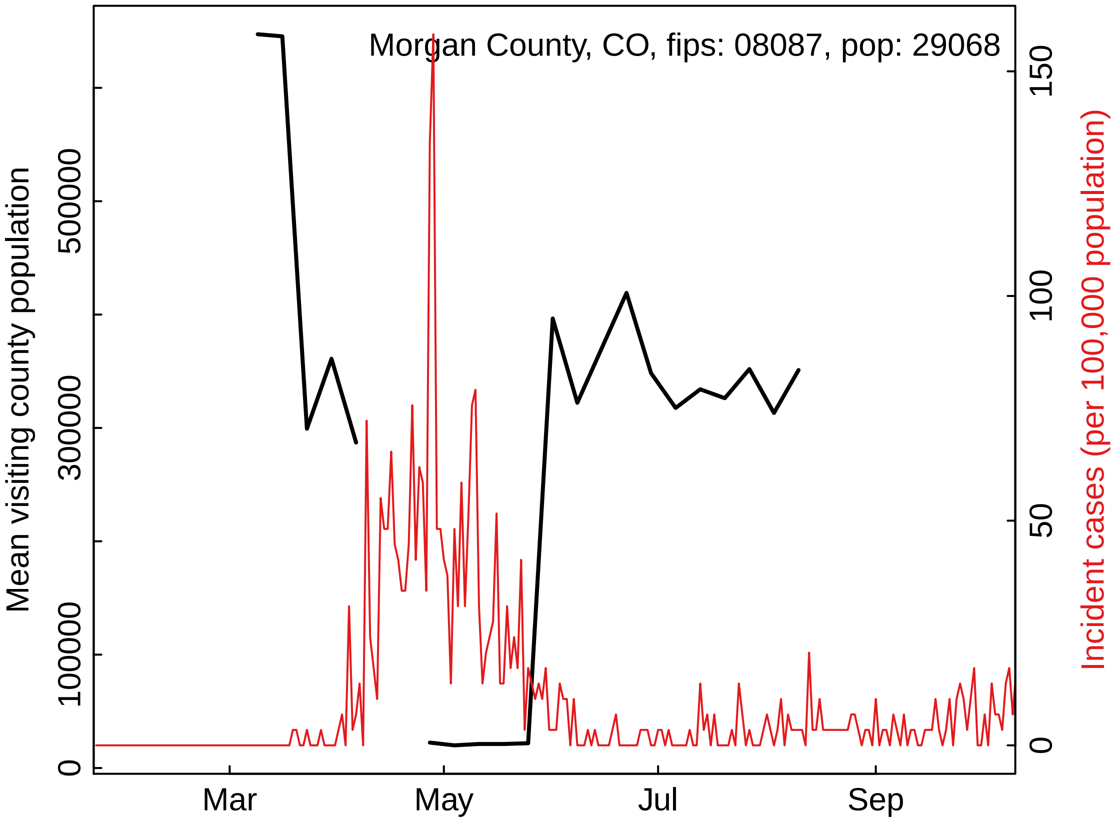

Morgan County, CO, fips: 08087, pop: 29068
Mean visiting county population
Incident cases (per 100,000 population)
0
100000
300000
500000
0
50
100
150
Mar
May
Jul
Sep

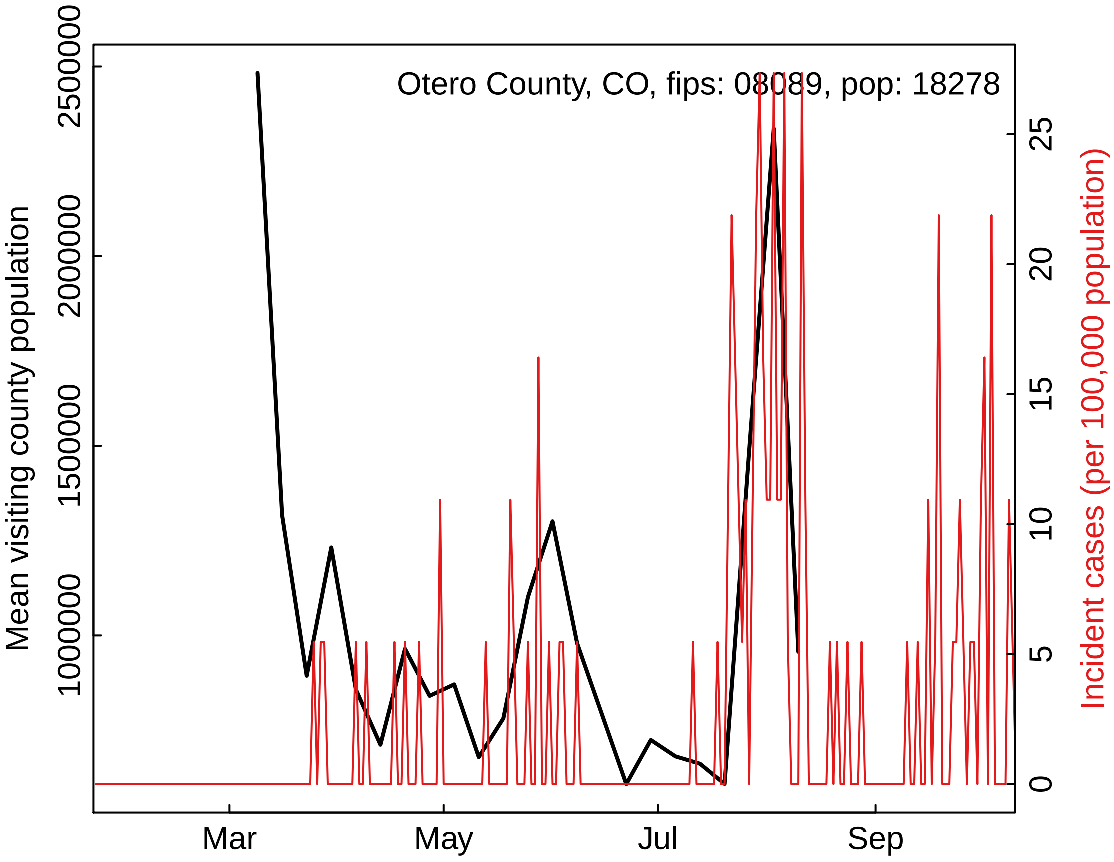

Otero County, CO, fips: 08089, pop: 18278
Mean visiting county population
1000000
1500000
2000000
2500000
Incident cases (per 100,000 population)
0
5
10
15
20
25
Mar
May
Jul
Sep

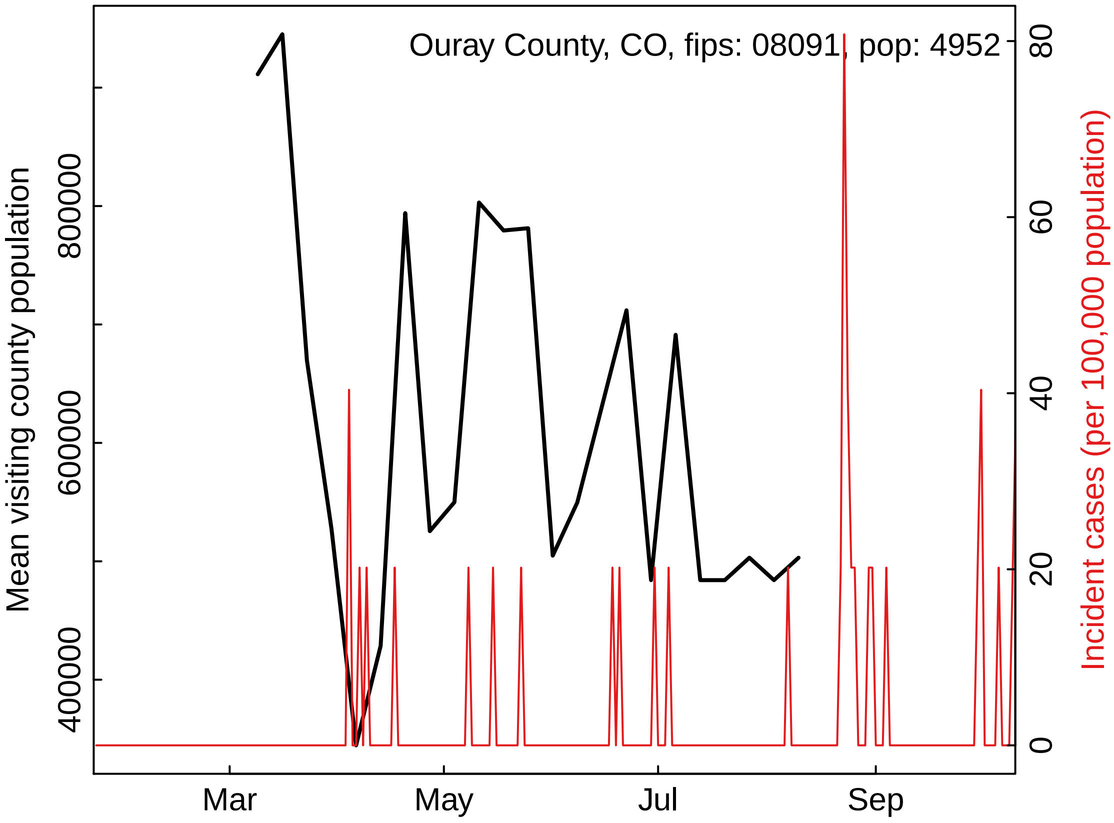

Ouray County, CO, fips: 08091, pop: 4952
Mean visiting county population
400000
600000
800000
Incident cases (per 100,000 population)
0
20
40
60
80
Mar
May
Jul
Sep

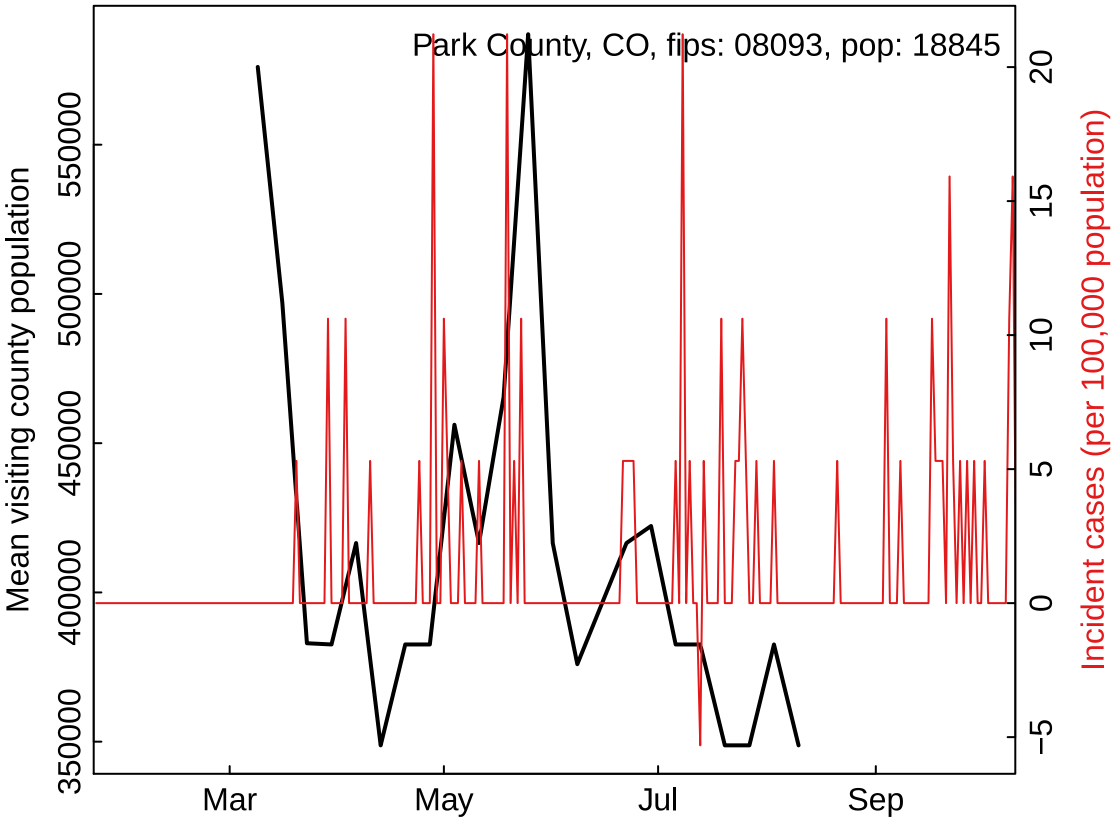
Park County, CO, fips: 08093, pop: 18845
Mean visiting county population
350000
400000
450000
500000
550000
Incident cases (per 100,000 population)
−5
0
5
10
15
20
Mar
May
Jul
Sep

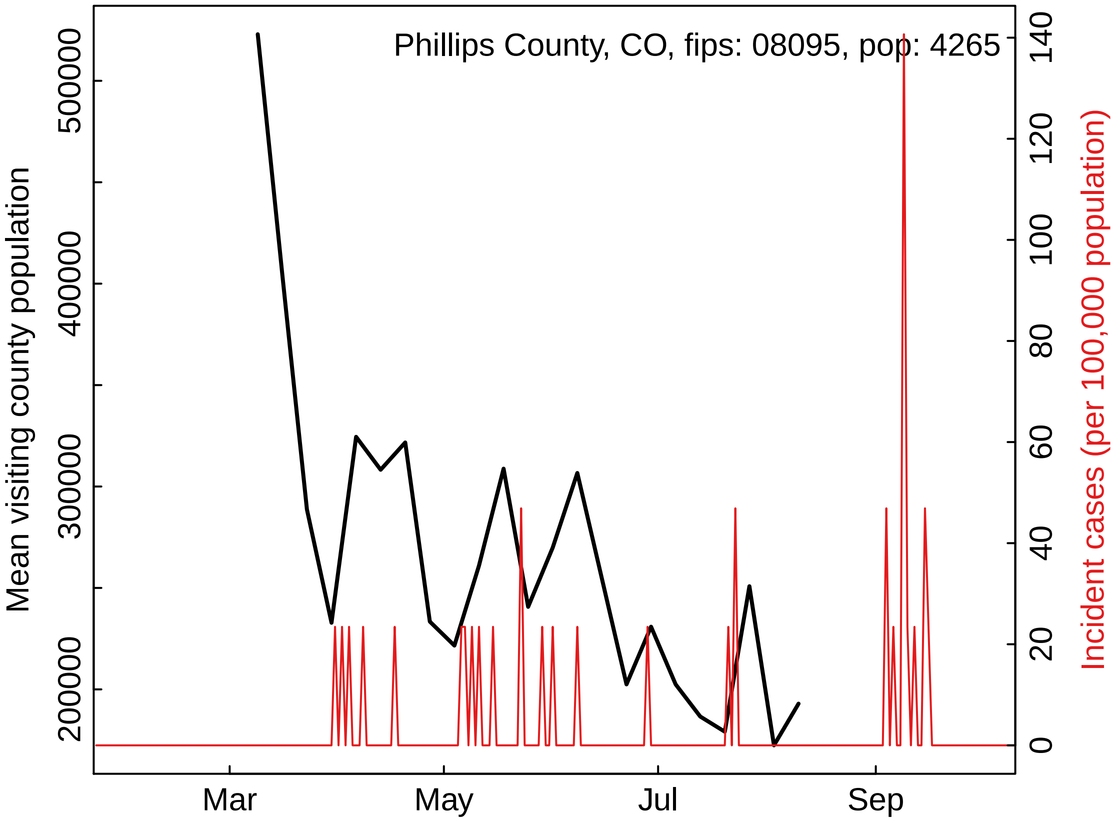

Phillips County, CO, fips: 08095, pop: 4265
Mean visiting county population
200000
300000
400000
500000
Incident cases (per 100,000 population)
0
20
40
60
80
100
120
140
Mar
May
Jul
Sep

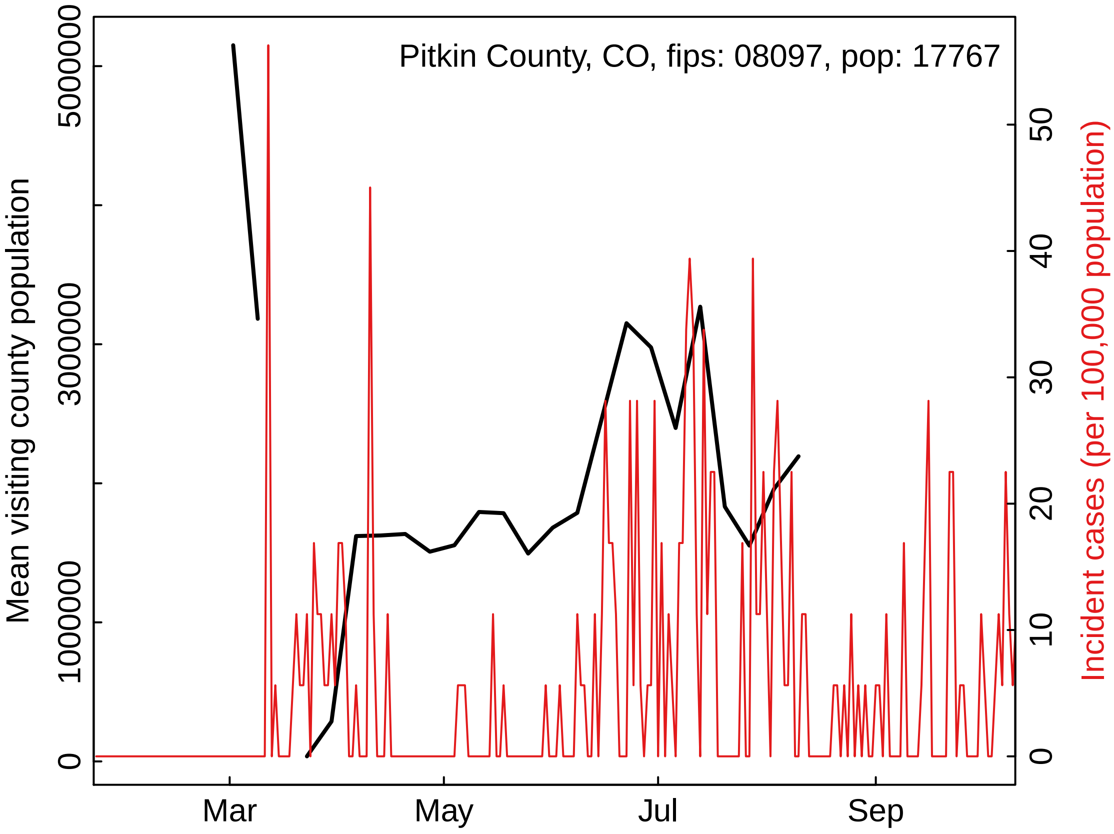

Pitkin County, CO, fips: 08097, pop: 17767
Mean visiting county population
0
1000000
3000000
5000000
Incident cases (per 100,000 population)
0
10
20
30
40
50
Mar
May
Jul
Sep

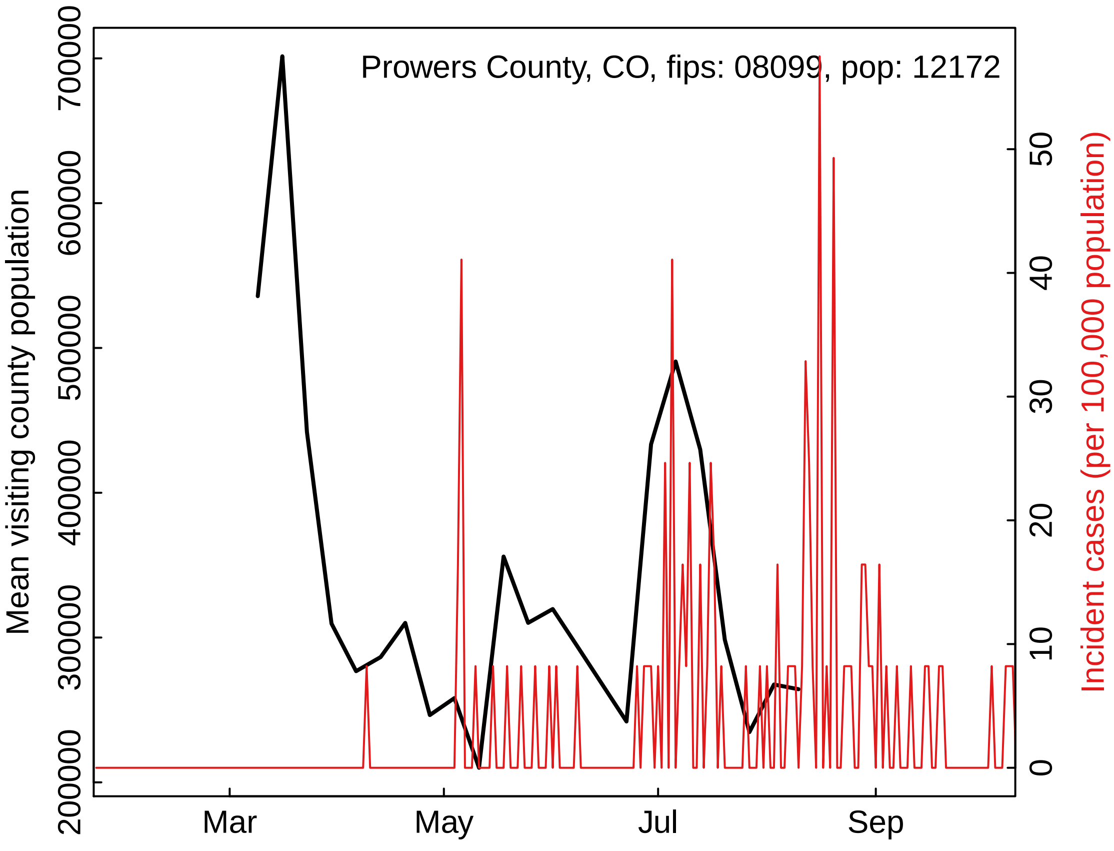

Prowers County, CO, fips: 08099, pop: 12172
Mean visiting county population
200000
300000
400000
500000
600000
700000
Incident cases (per 100,000 population)
0
10
20
30
40
50
Mar
May
Jul
Sep

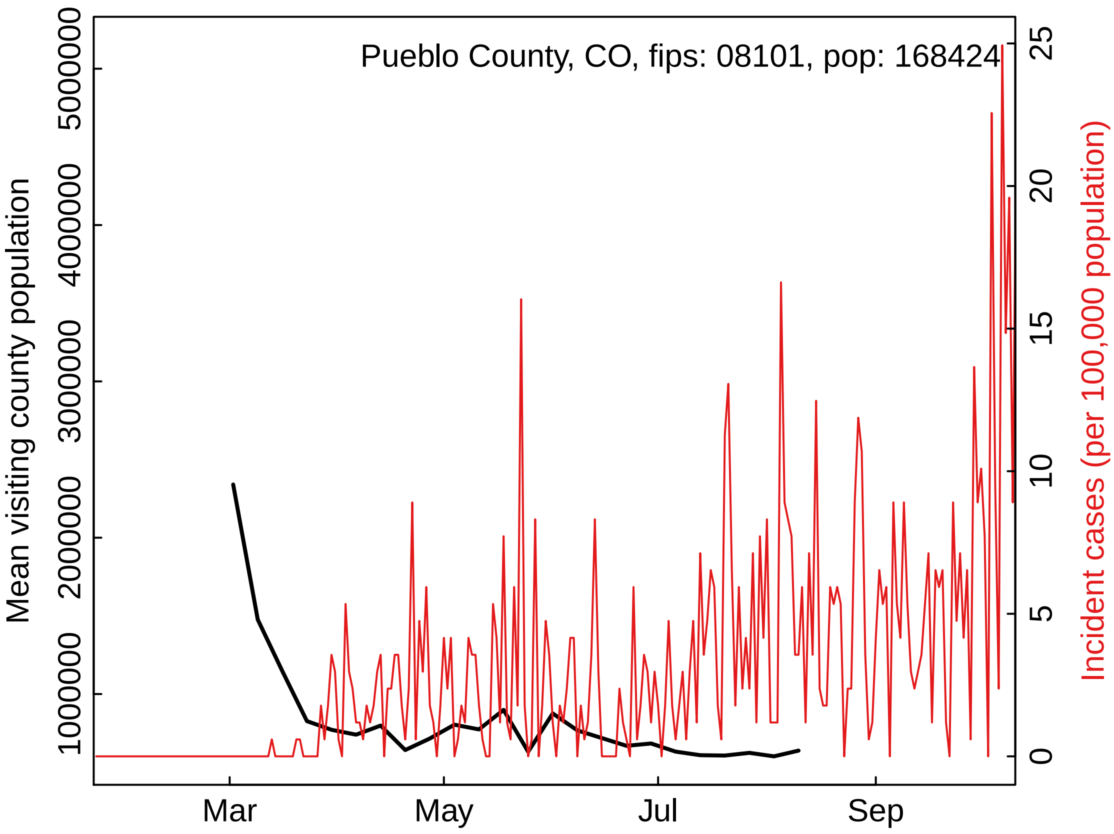

Pueblo County, CO, fips: 08101, pop: 168424
Mean visiting county population
10000000
20000000
30000000
40000000
50000000
Incident cases (per 100,000 population)
0
5
10
15
20
25
Mar
May
Jul
Sep

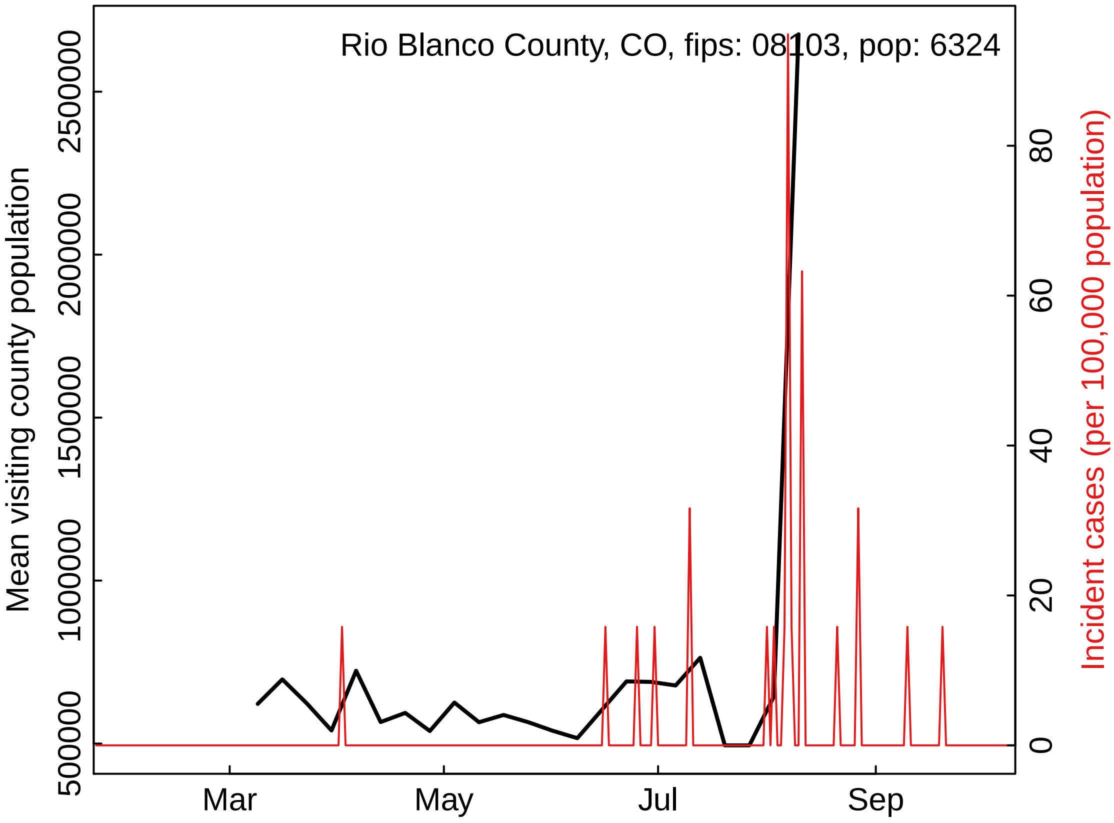
Rio Blanco County, CO, fips: 08103, pop: 6324
Mean visiting county population
500000
1000000
1500000
2000000
2500000
Incident cases (per 100,000 population)
0
20
40
60
80
Mar
May
Jul
Sep

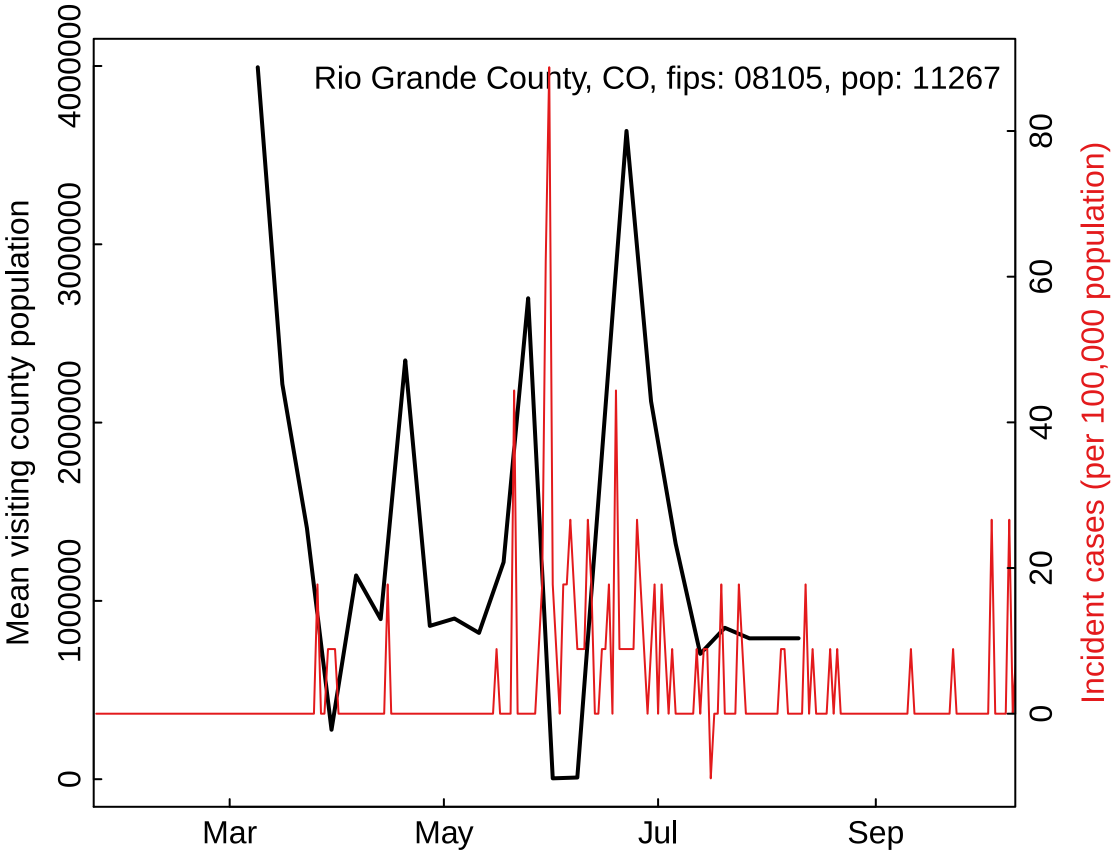

Rio Grande County, CO, fips: 08105, pop: 11267
Mean visiting county population
0
1000000
2000000
3000000
4000000
Incident cases (per 100,000 population)
0
20
40
60
80
Mar
May
Jul
Sep

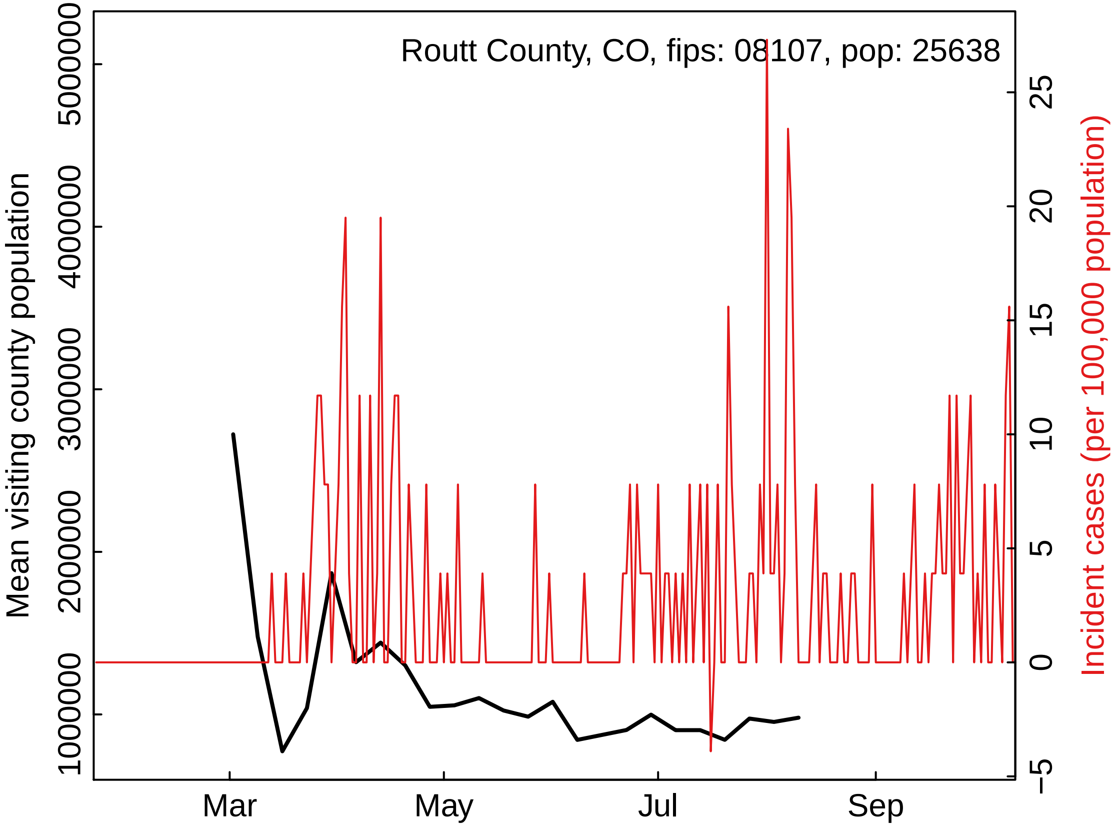

Routt County, CO, fips: 08107, pop: 25638
Mean visiting county population
10000000
20000000
30000000
40000000
50000000
Incident cases (per 100,000 population)
−5
0
5
10
15
20
25
Mar
May
Jul
Sep

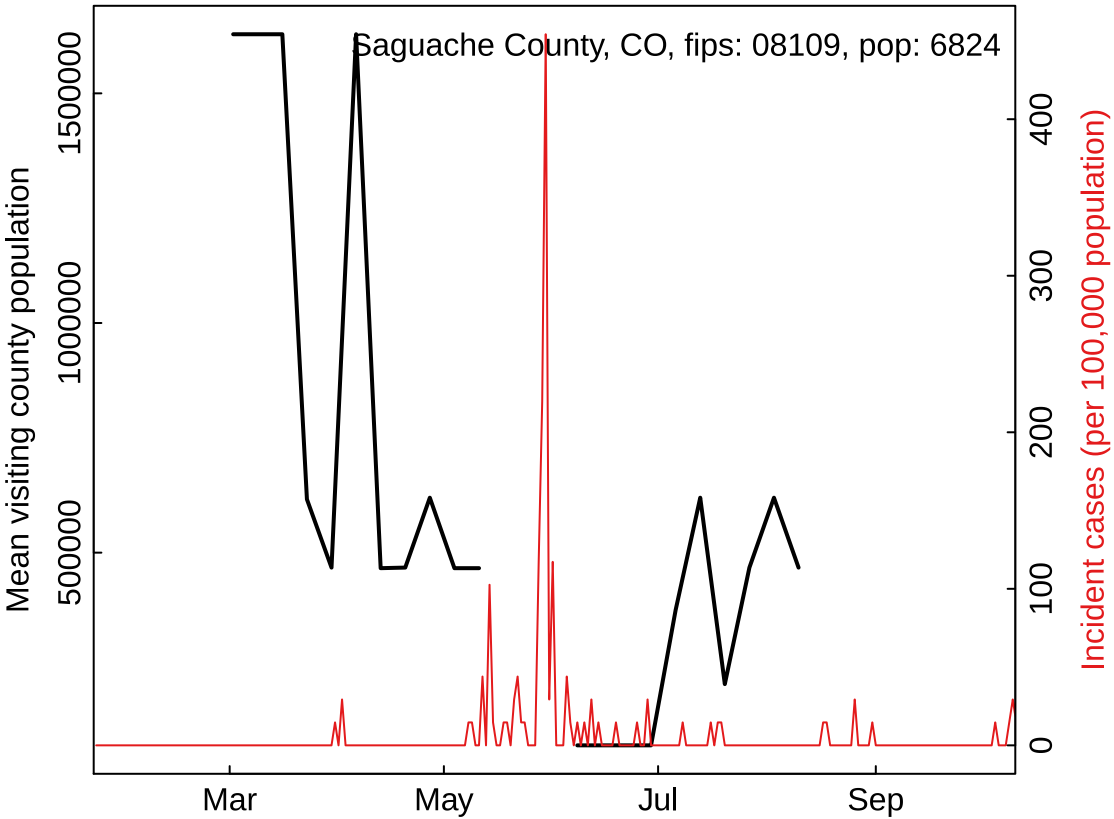

Saguache County, CO, fips: 08109, pop: 6824
Mean visiting county population
500000
1000000
1500000
Incident cases (per 100,000 population)
0
100
200
300
400
Mar
May
Jul
Sep

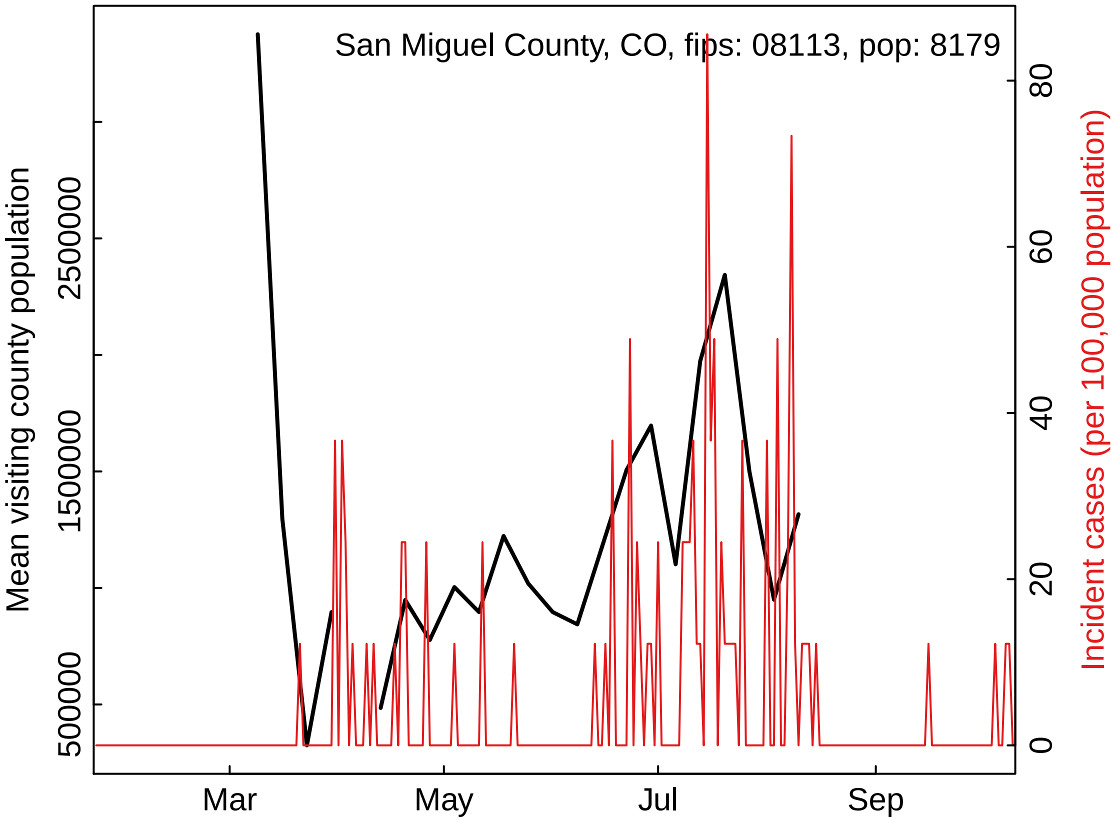
San Miguel County, CO, fips: 08113, pop: 8179
Mean visiting county population
500000
1500000
2500000
Incident cases (per 100,000 population)
0
20
40
60
80
Mar
May
Jul
Sep

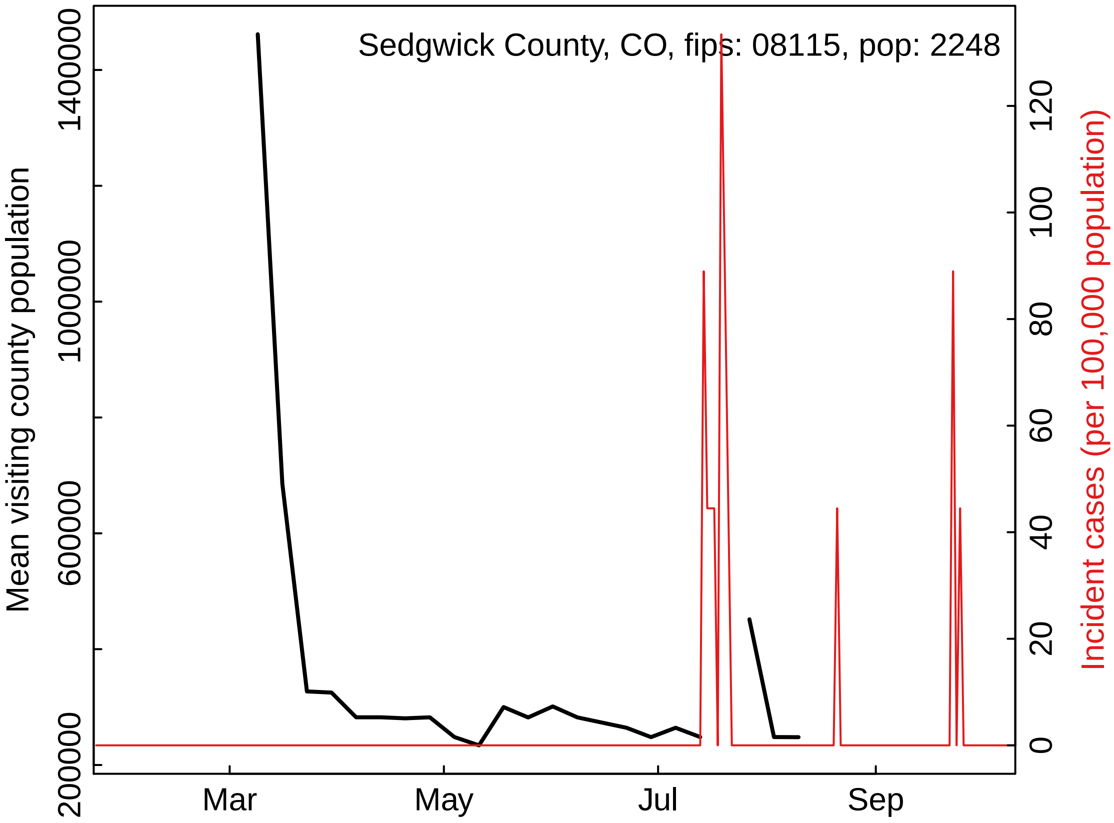

Sedgwick County, CO, fips: 08115, pop: 2248
Mean visiting county population
200000
600000
1000000
1400000
Incident cases (per 100,000 population)
0
20
40
60
80
100
120
Mar
May
Jul
Sep

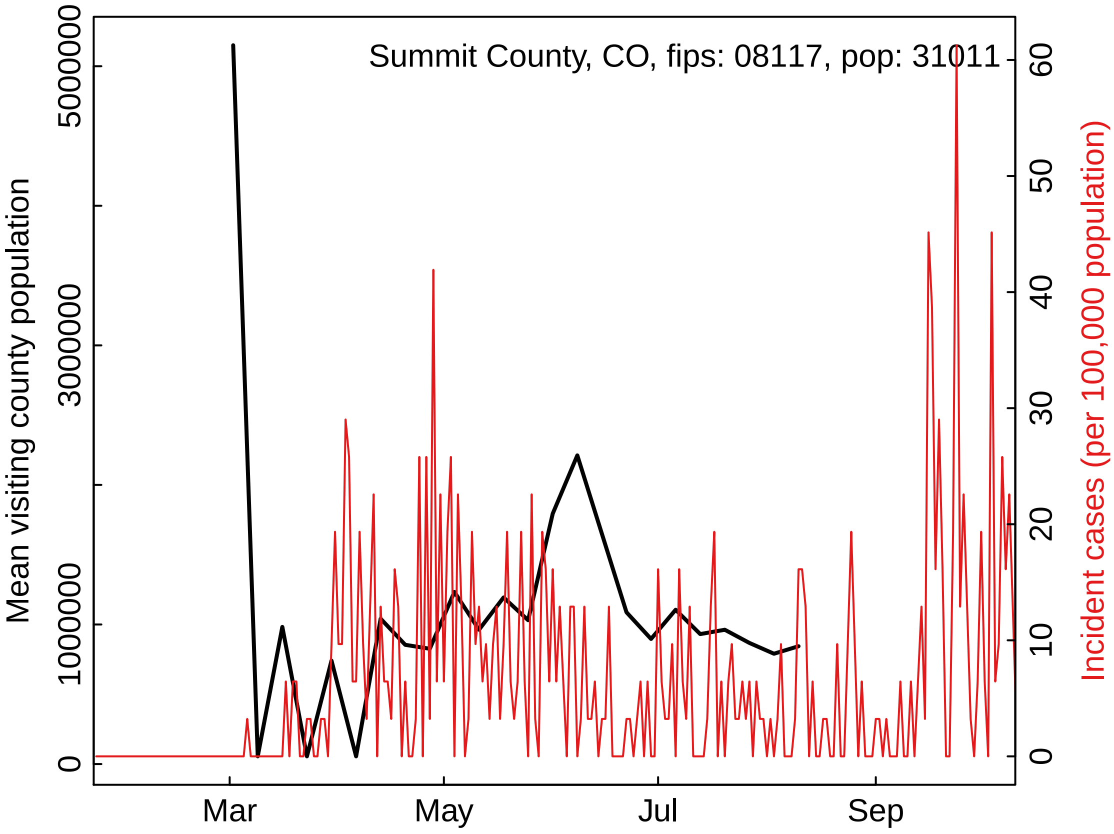

Summit County, CO, fips: 08117, pop: 31011
Mean visiting county population
0
1000000
3000000
5000000
Mar
May
Jul
Sep
Incident cases (per 100,000 population)
0
10
20
30
40
50
60

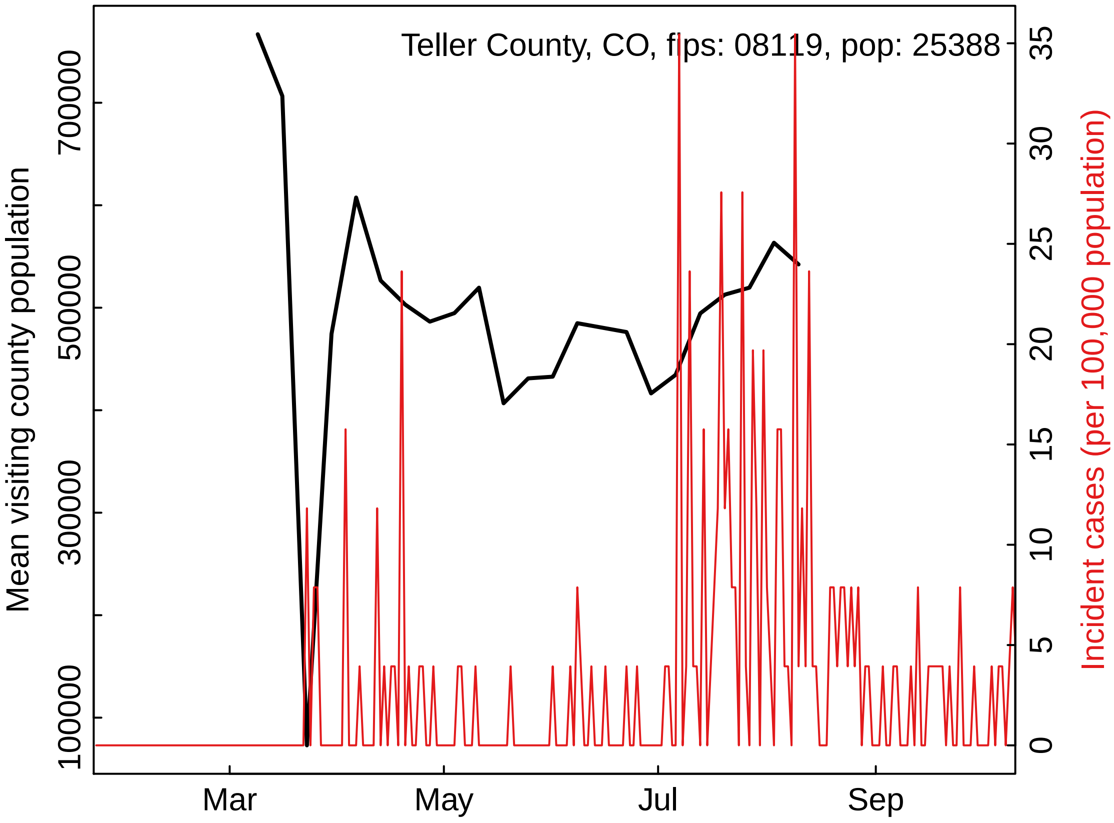

Teller County, CO, fips: 08119, pop: 25388
Mean visiting county population
100000
300000
500000
700000
Incident cases (per 100,000 population)
0
5
10
15
20
25
30
35
Mar
May
Jul
Sep

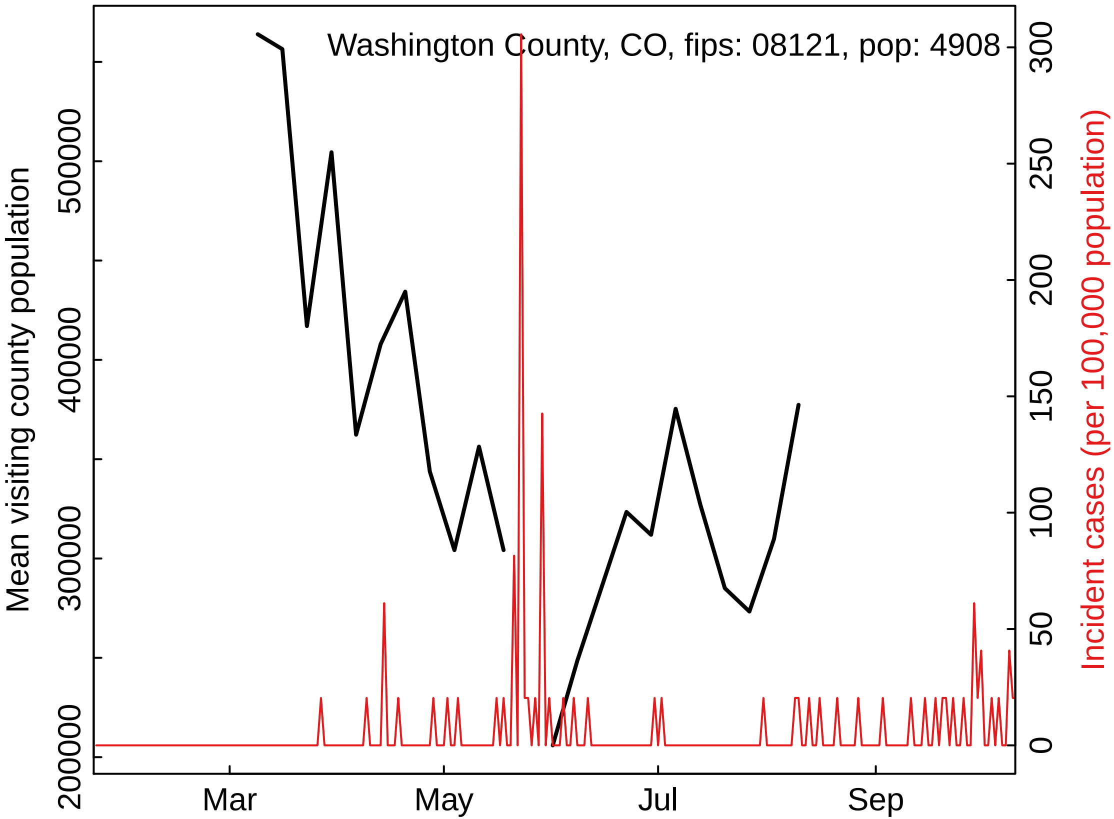

Washington County, CO, fips: 08121, pop: 4908
Mean visiting county population
200000
300000
400000
500000
Incident cases (per 100,000 population)
0
50
100
150
200
250
300
Mar
May
Jul
Sep

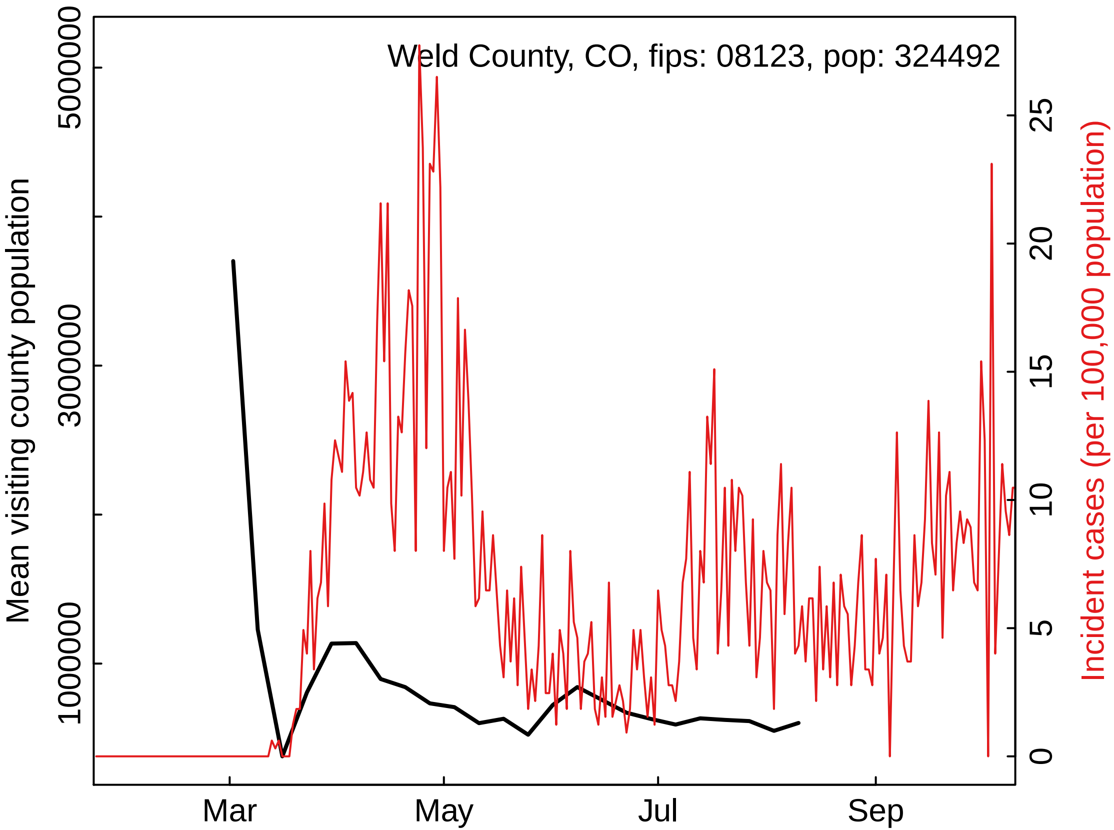

Weld County, CO, fips: 08123, pop: 324492
Mean visiting county population
1000000
3000000
5000000
Incident cases (per 100,000 population)
0
5
10
15
20
25
Mar
May
Jul
Sep

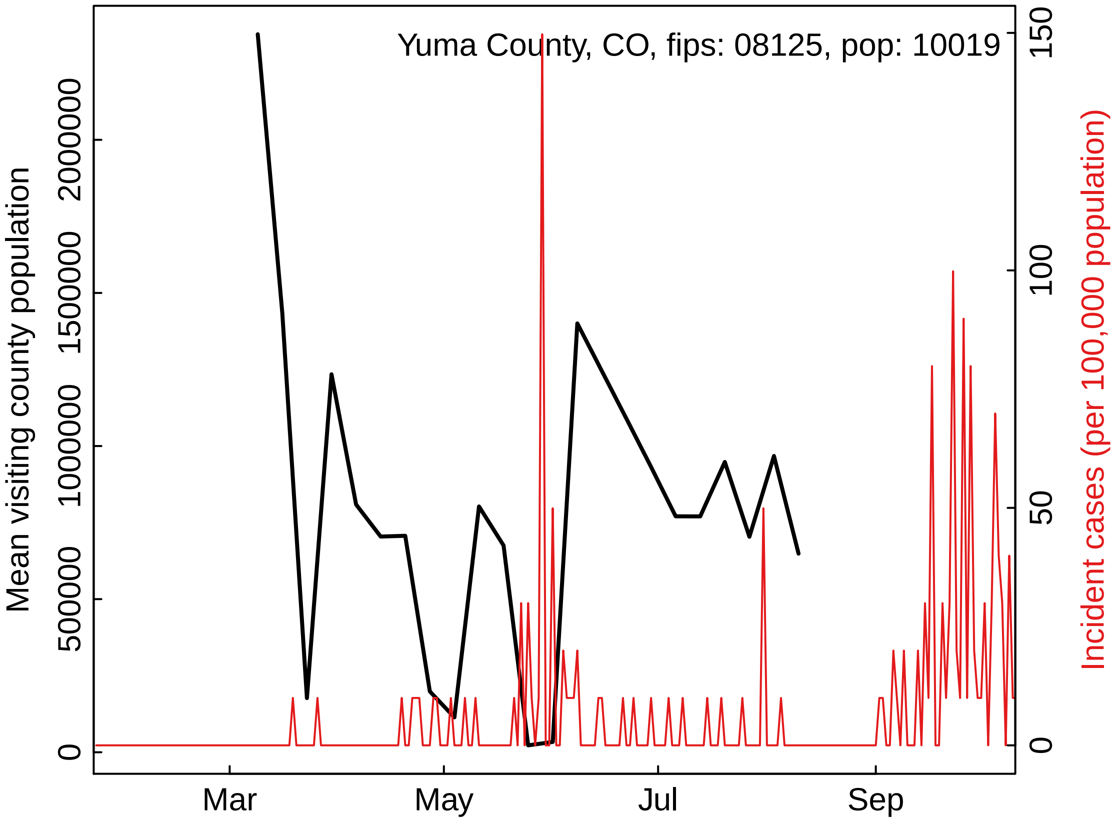

Yuma County, CO, fips: 08125, pop: 10019
Mean visiting county population
0
500000
1000000
1500000
2000000
Incident cases (per 100,000 population)
0
50
100
150
Mar
May
Jul
Sep

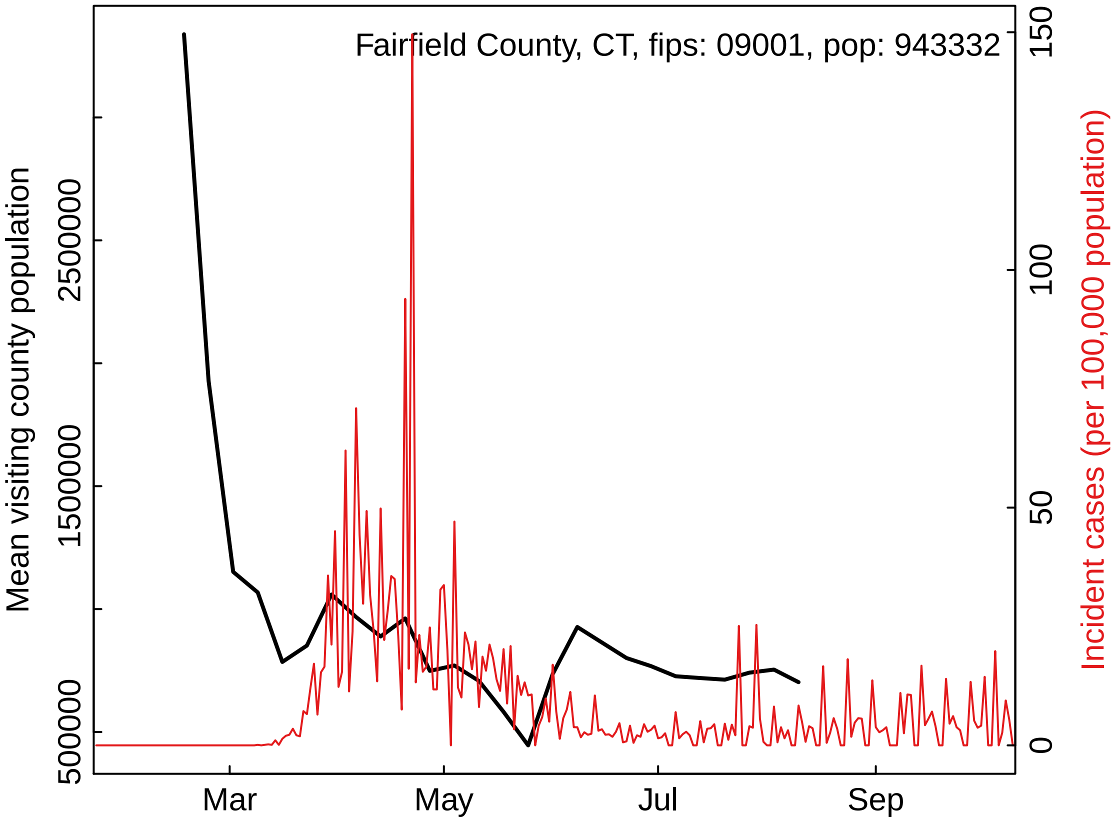
Fairfield County, CT, fips: 09001, pop: 943332
Mean visiting county population
500000
1500000
2500000
Incident cases (per 100,000 population)
0
50
100
150
Mar
May
Jul
Sep

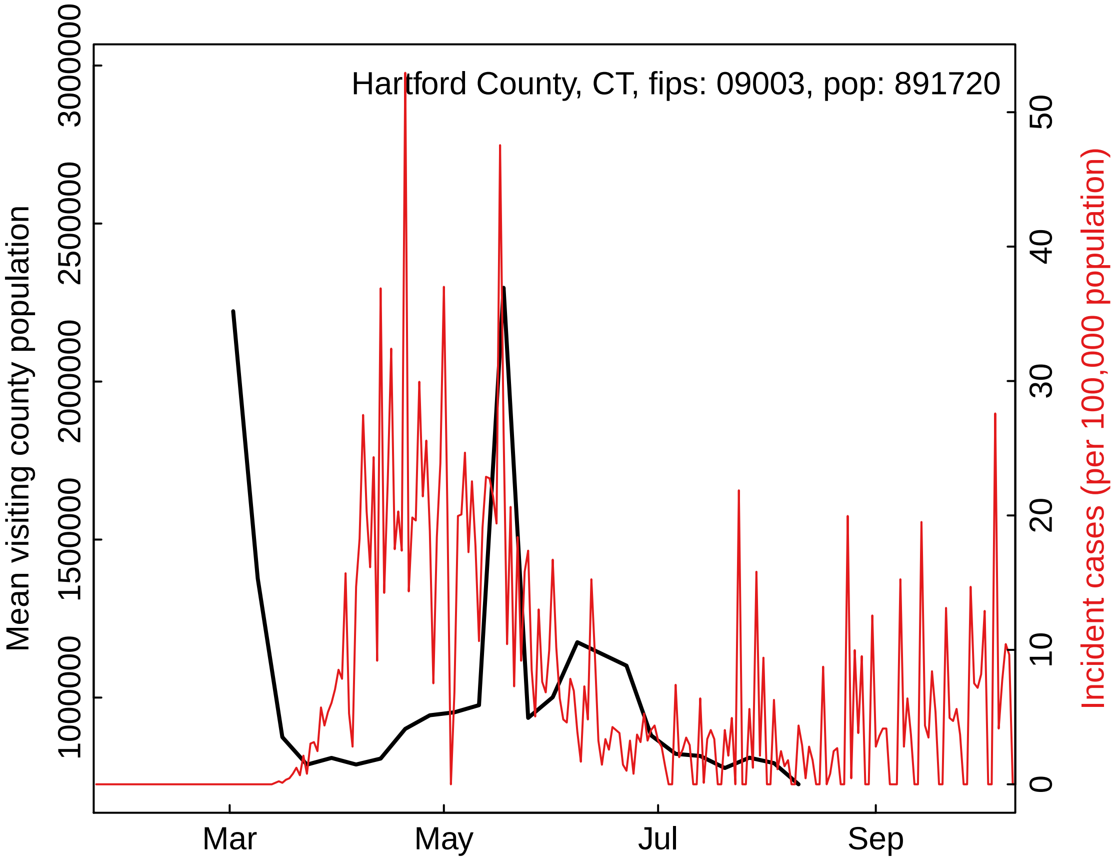
Hartford County, CT, fips: 09003, pop: 891720
Mean visiting county population
10000000
15000000
20000000
25000000
30000000
Incident cases (per 100,000 population)
0
10
20
30
40
50
Mar
May
Jul
Sep

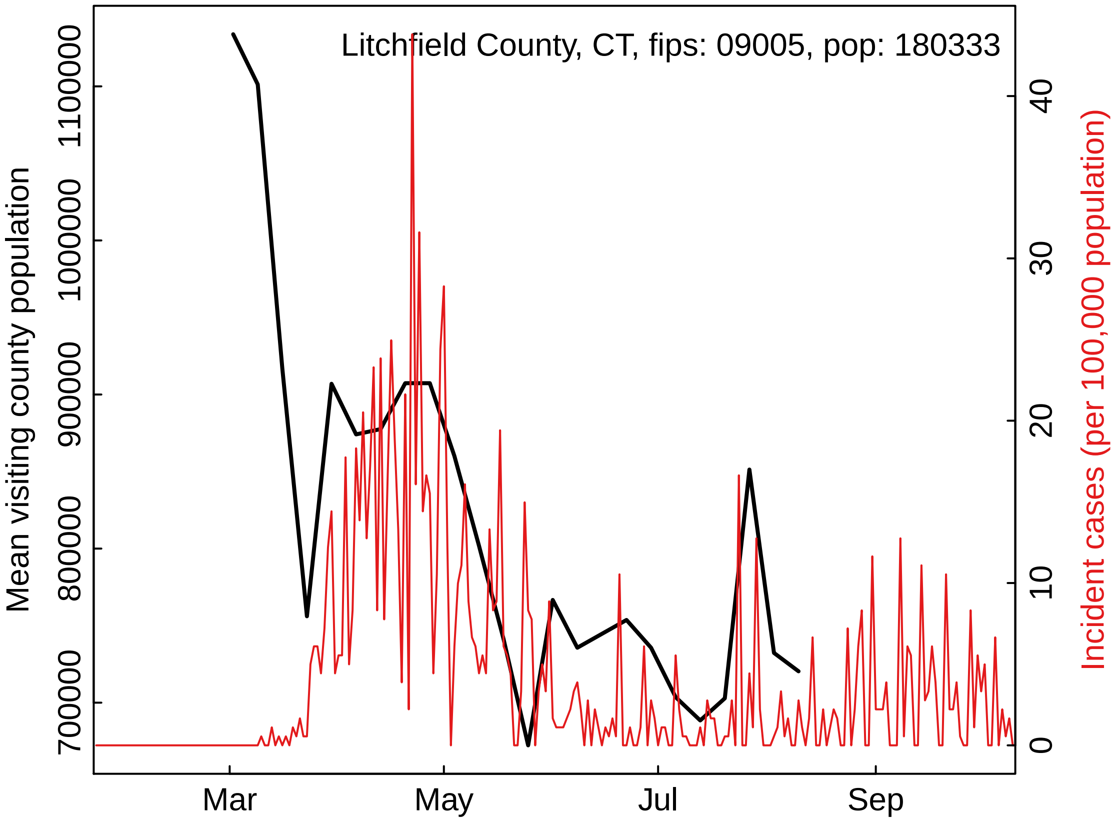

Litchfield County, CT, fips: 09005, pop: 180333
Mean visiting county population
Incident cases (per 100,000 population)
700000
800000
900000
1000000
1100000
0
10
20
30
40
Mar
May
Jul
Sep

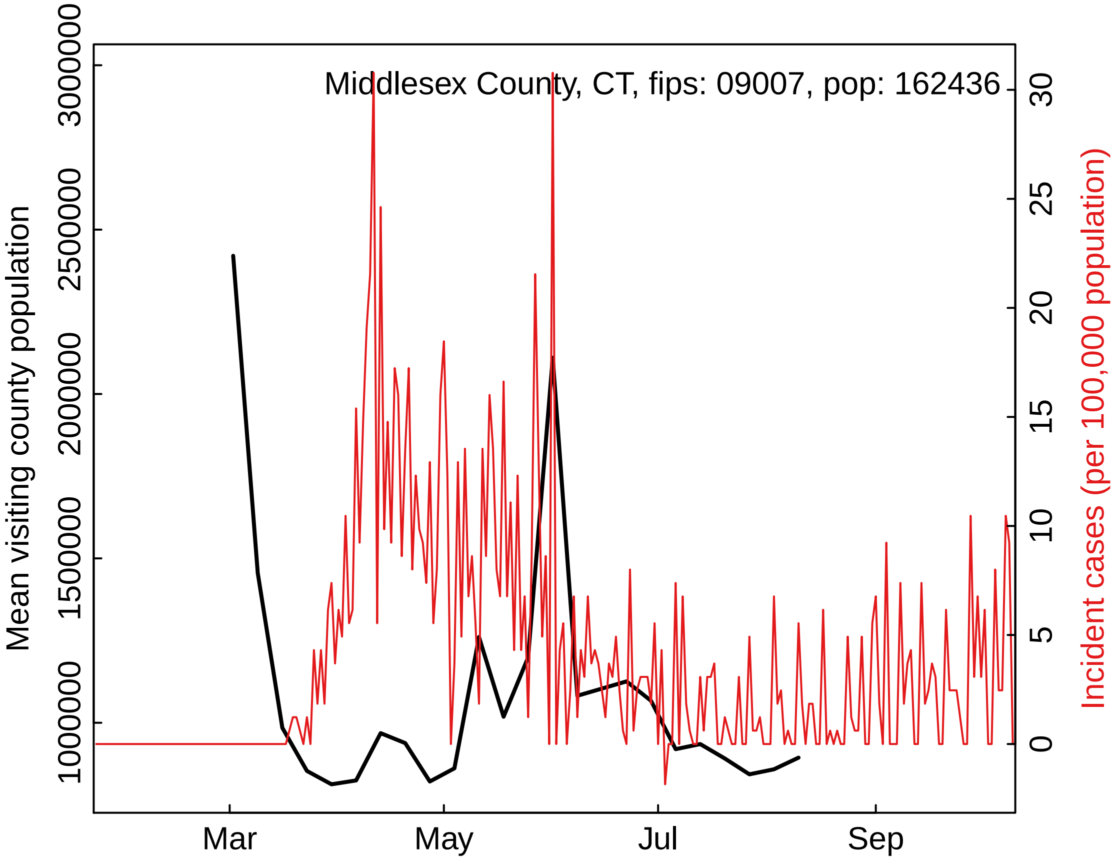

Middlesex County, CT, fips: 09007, pop: 162436
Mean visiting county population
Incident cases (per 100,000 population)
1000000
1500000
2000000
2500000
3000000
0
5
10
15
20
25
30
Mar
May
Jul
Sep

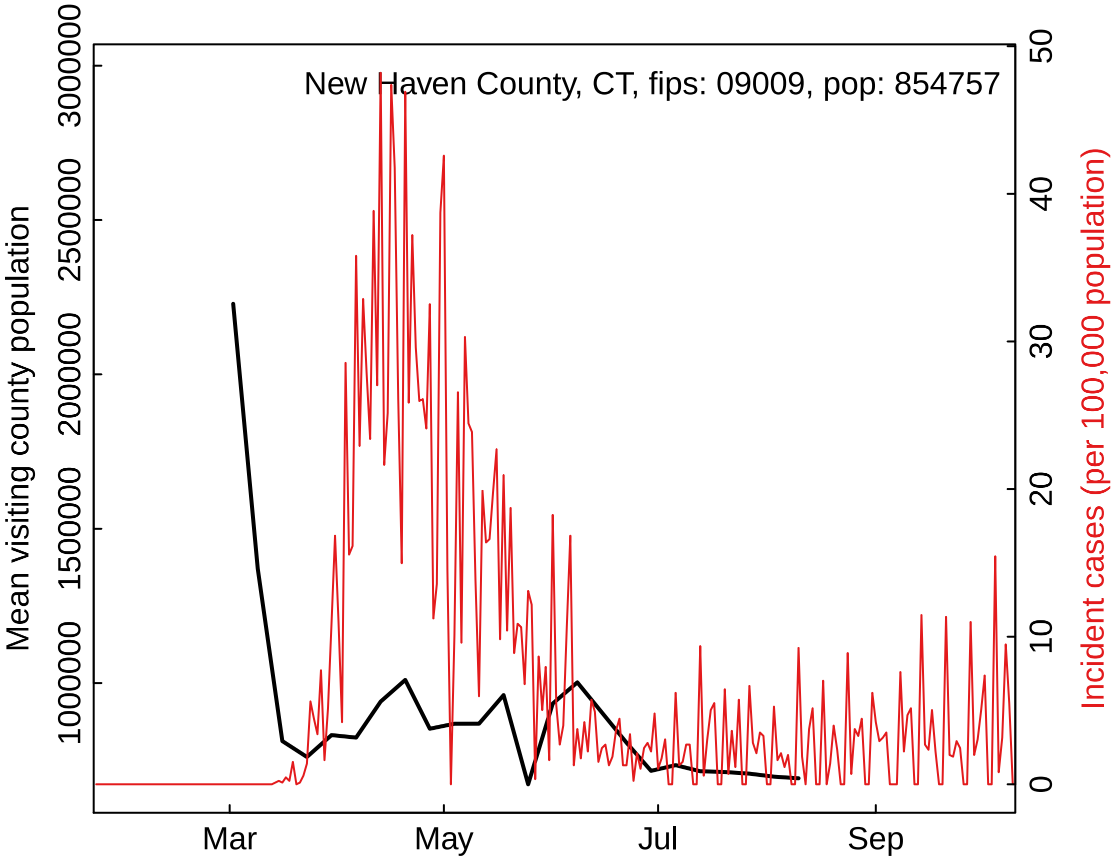

New Haven County, CT, fips: 09009, pop: 854757
Mean visiting county population
Incident cases (per 100,000 population)
1000000
1500000
2000000
2500000
3000000
0
10
20
30
40
50
Mar
May
Jul
Sep

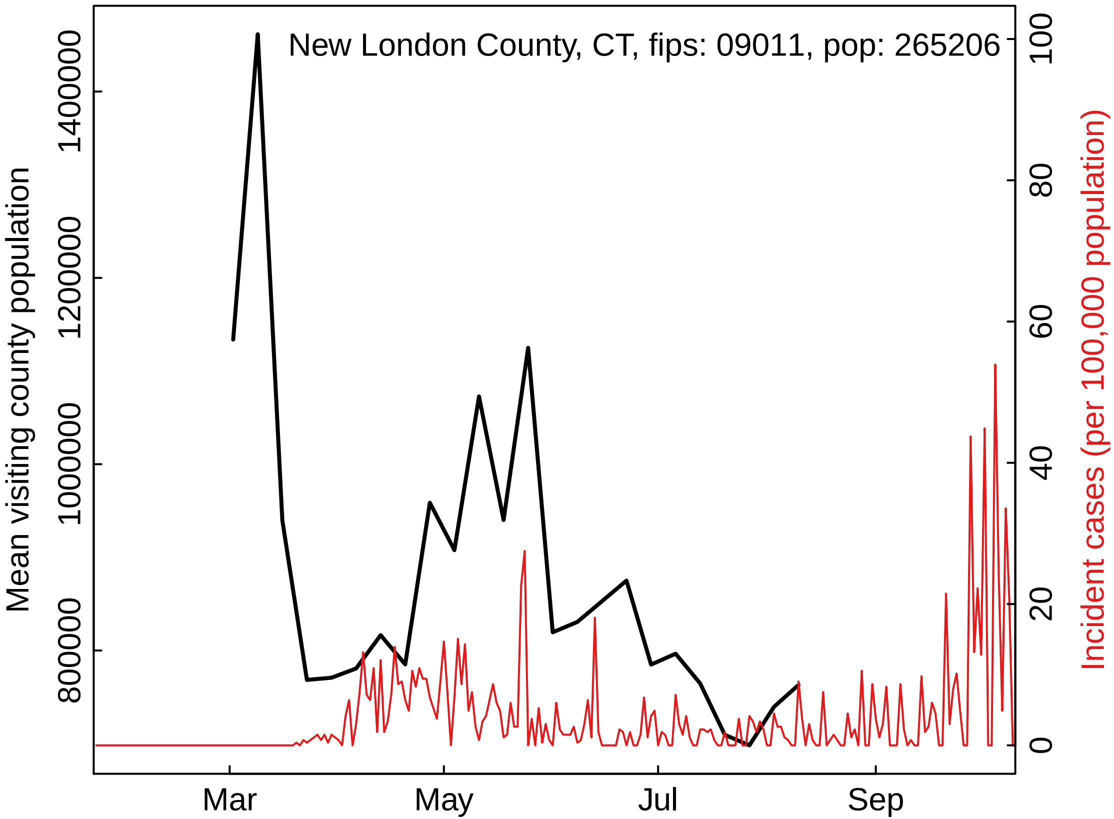

New London County, CT, fips: 09011, pop: 265206
Mean visiting county population
800000
1000000
1200000
1400000
Incident cases (per 100,000 population)
0
20
40
60
80
100
Mar
May
Jul
Sep

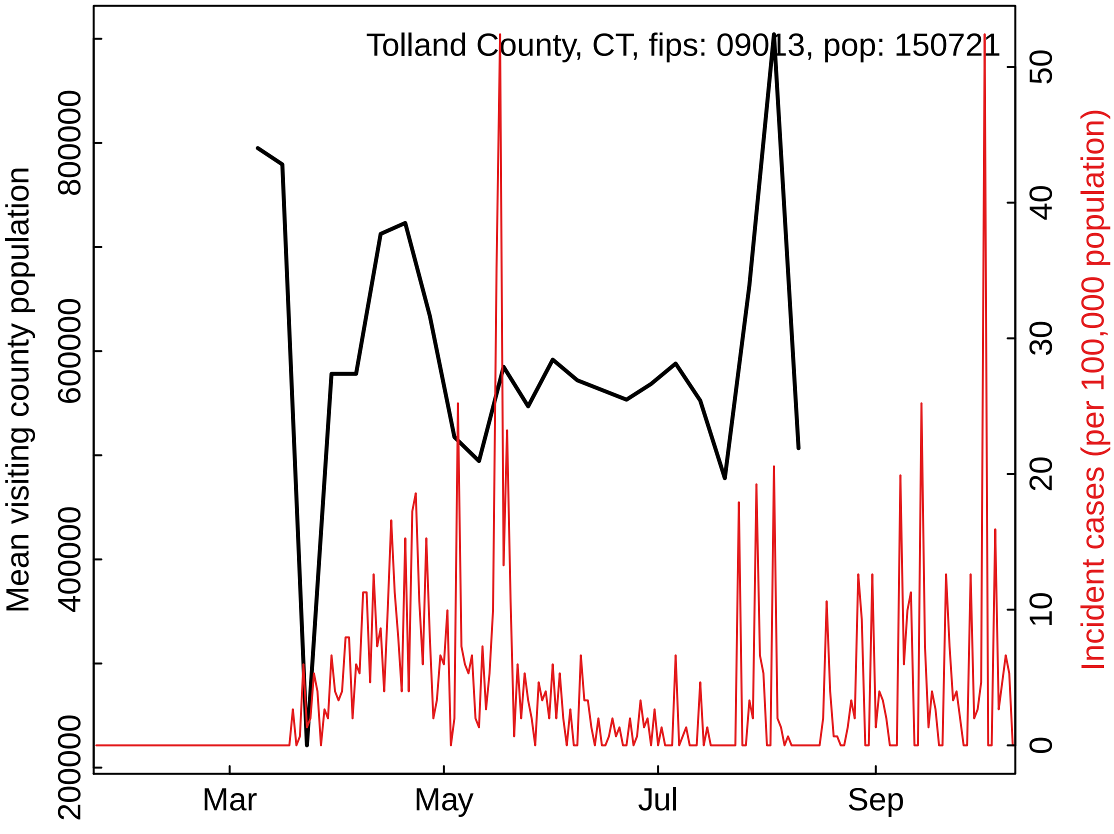
Tolland County, CT, fips: 09013, pop: 150721
Mean visiting county population
2000000
4000000
6000000
8000000
Incident cases (per 100,000 population)
0
10
20
30
40
50
Mar
May
Jul
Sep

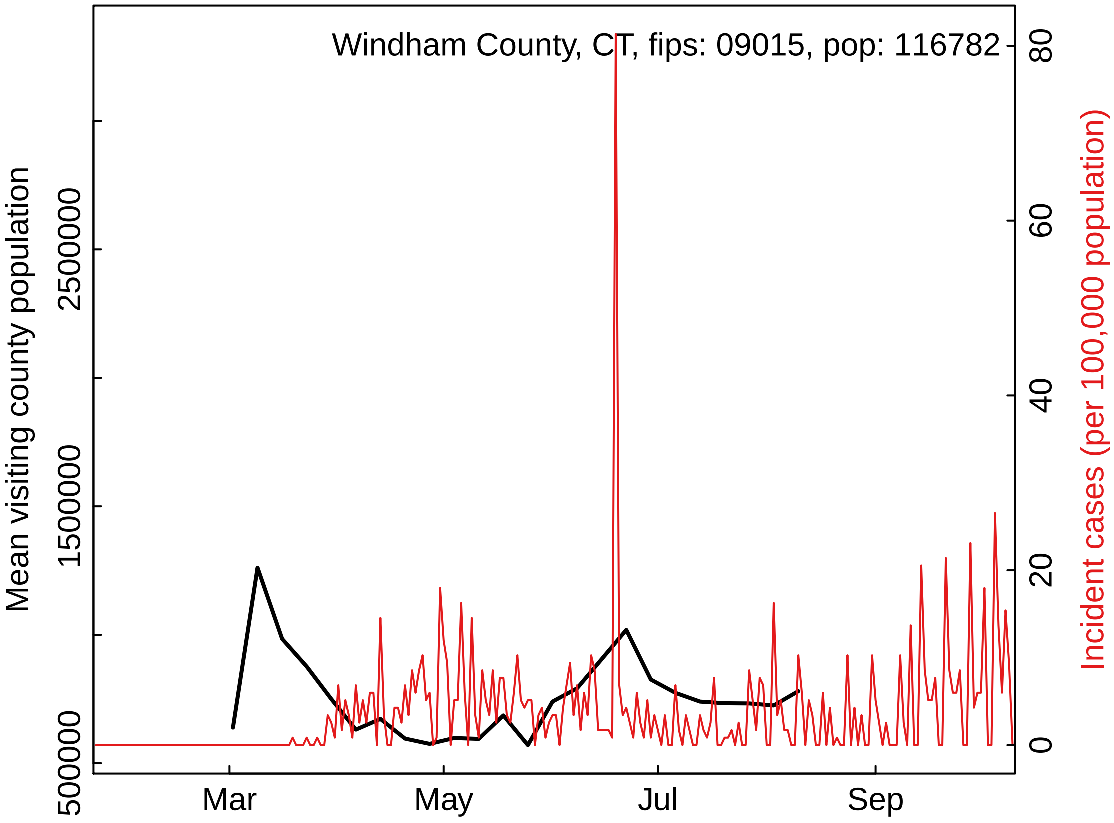

Windham County, CT, fips: 09015, pop: 116782
Mean visiting county population
500000
1500000
2500000
Incident cases (per 100,000 population)
0
20
40
60
80
Mar
May
Jul
Sep

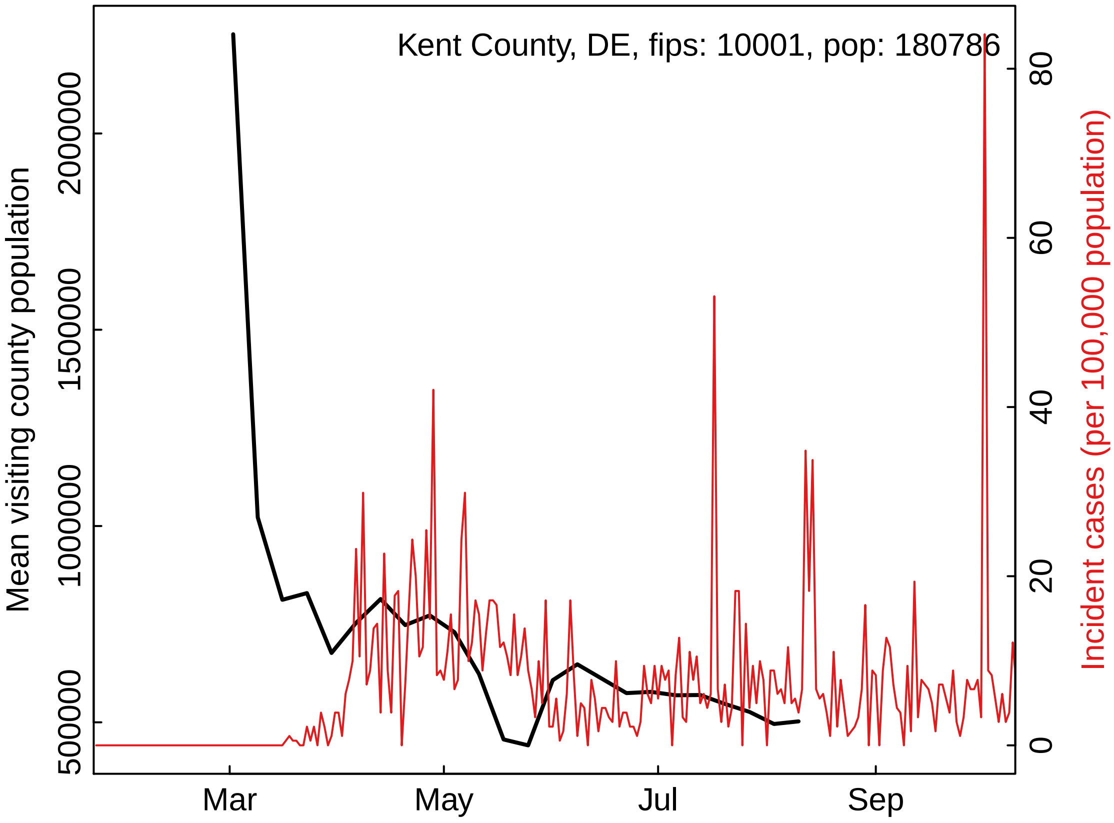
Kent County, DE, fips: 10001, pop: 180786
Mean visiting county population
500000
1000000
1500000
2000000
Incident cases (per 100,000 population)
0
20
40
60
80
Mar
May
Jul
Sep

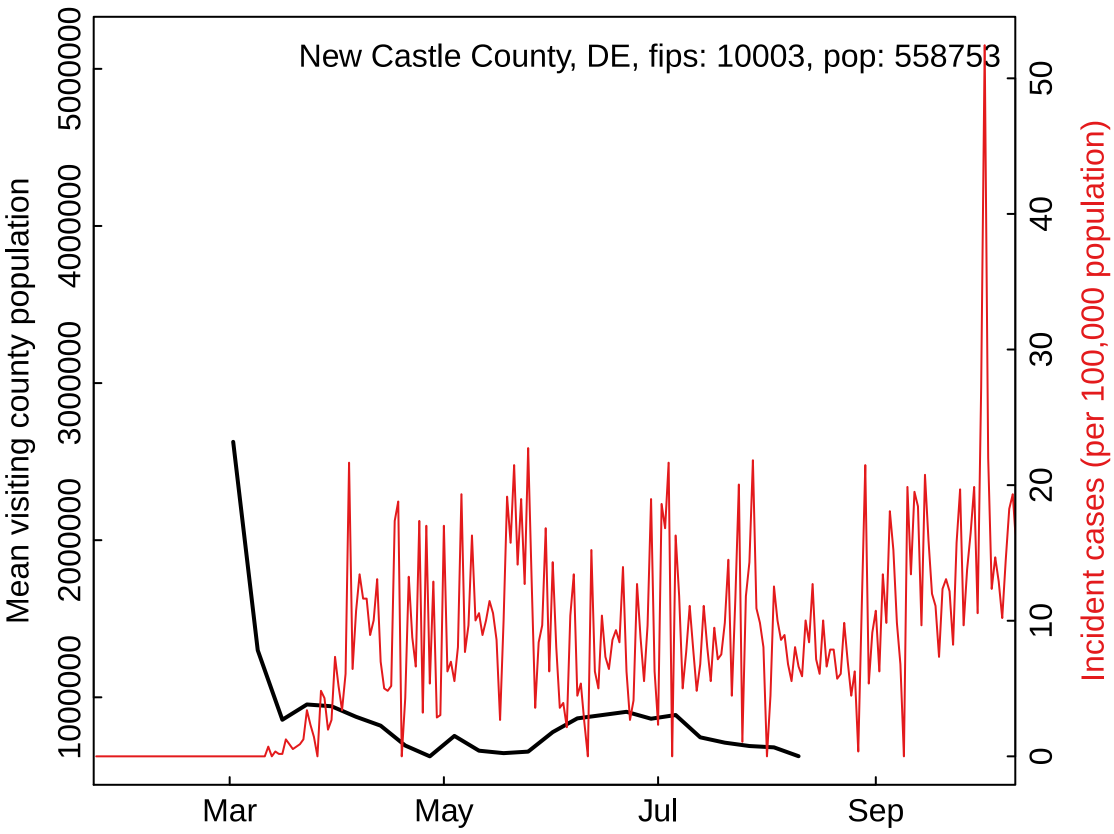

New Castle County, DE, fips: 10003, pop: 558753
Mean visiting county population
10000000
20000000
30000000
40000000
50000000
Incident cases (per 100,000 population)
0
10
20
30
40
50
Mar
May
Jul
Sep

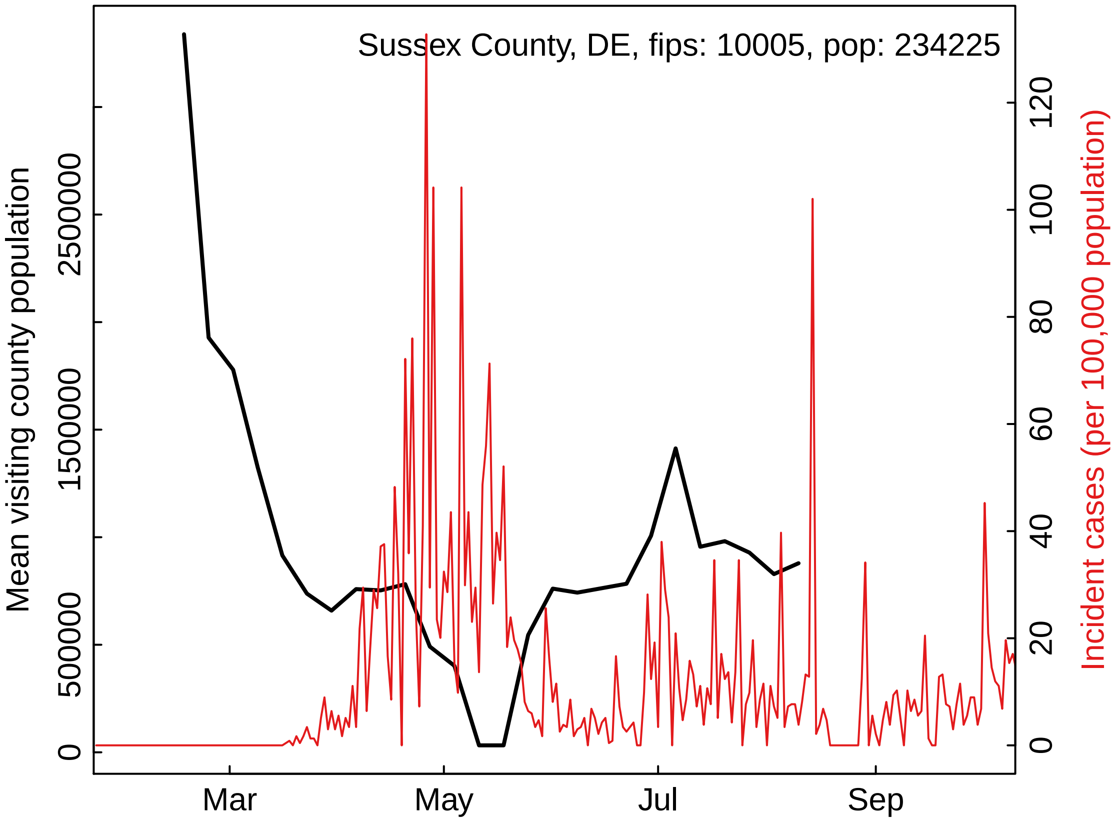

Sussex County, DE, fips: 10005, pop: 234225
Mean visiting county population
0
500000
1500000
2500000
Incident cases (per 100,000 population)
0
20
40
60
80
100
120
Mar
May
Jul
Sep

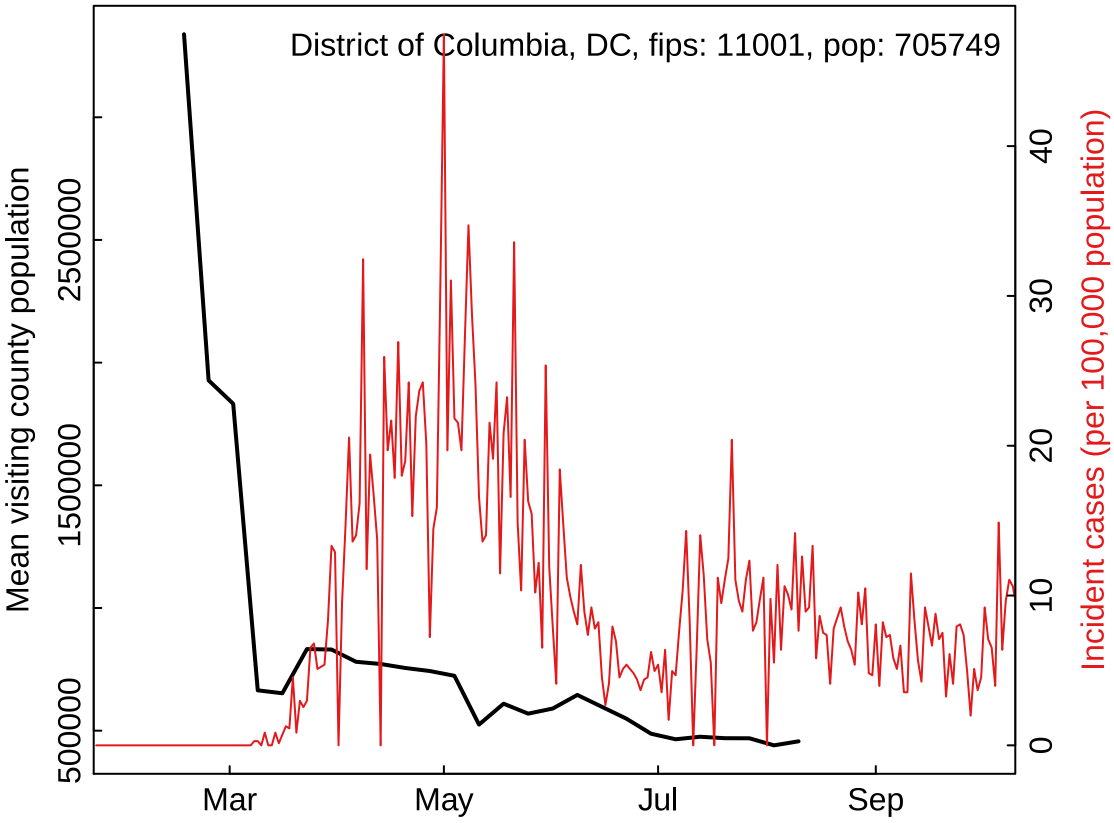
District of Columbia, DC, fips: 11001, pop: 705749
Mean visiting county population
500000
1500000
2500000
Incident cases (per 100,000 population)
0
10
20
30
40
Mar
May
Jul
Sep

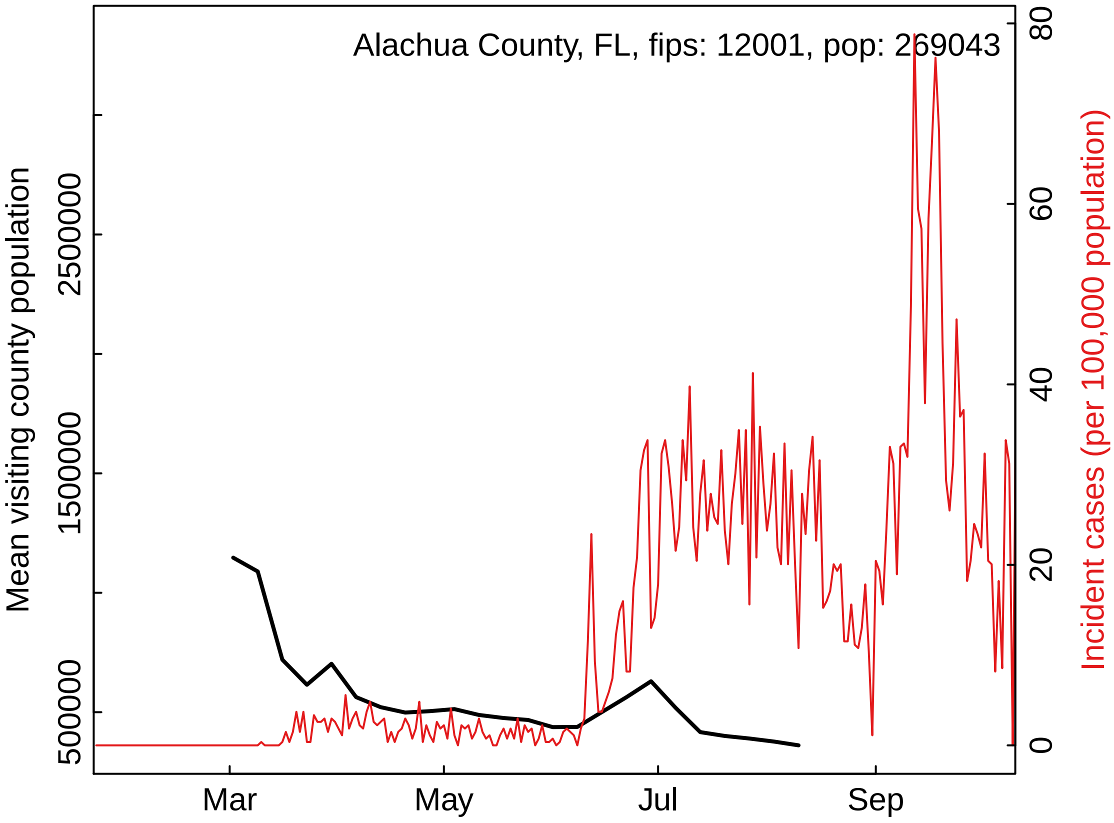
Alachua County, FL, fips: 12001, pop: 269043
Mean visiting county population
500000
1500000
2500000
Incident cases (per 100,000 population)
0
20
40
60
80
Mar
May
Jul
Sep

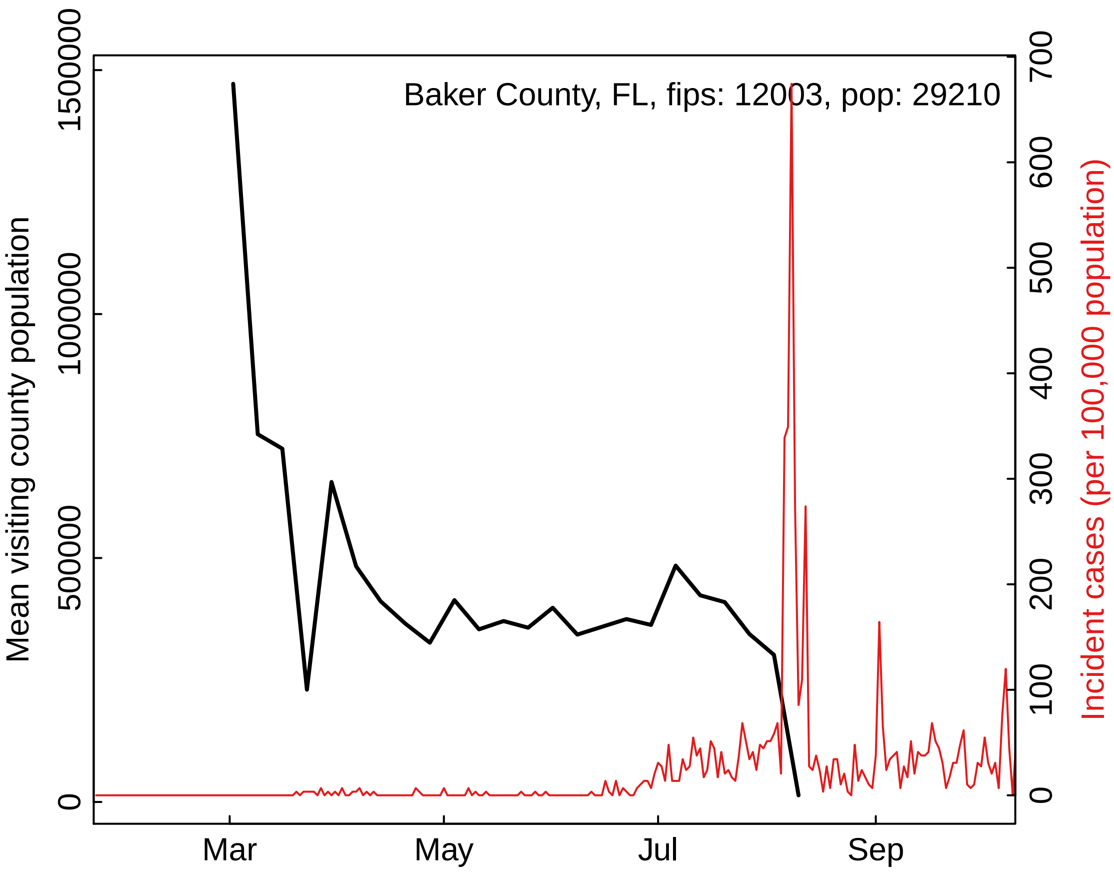

Baker County, FL, fips: 12003, pop: 29210
Mean visiting county population
0
500000
1000000
1500000
Incident cases (per 100,000 population)
0
100
200
300
400
500
600
700
Mar
May
Jul
Sep

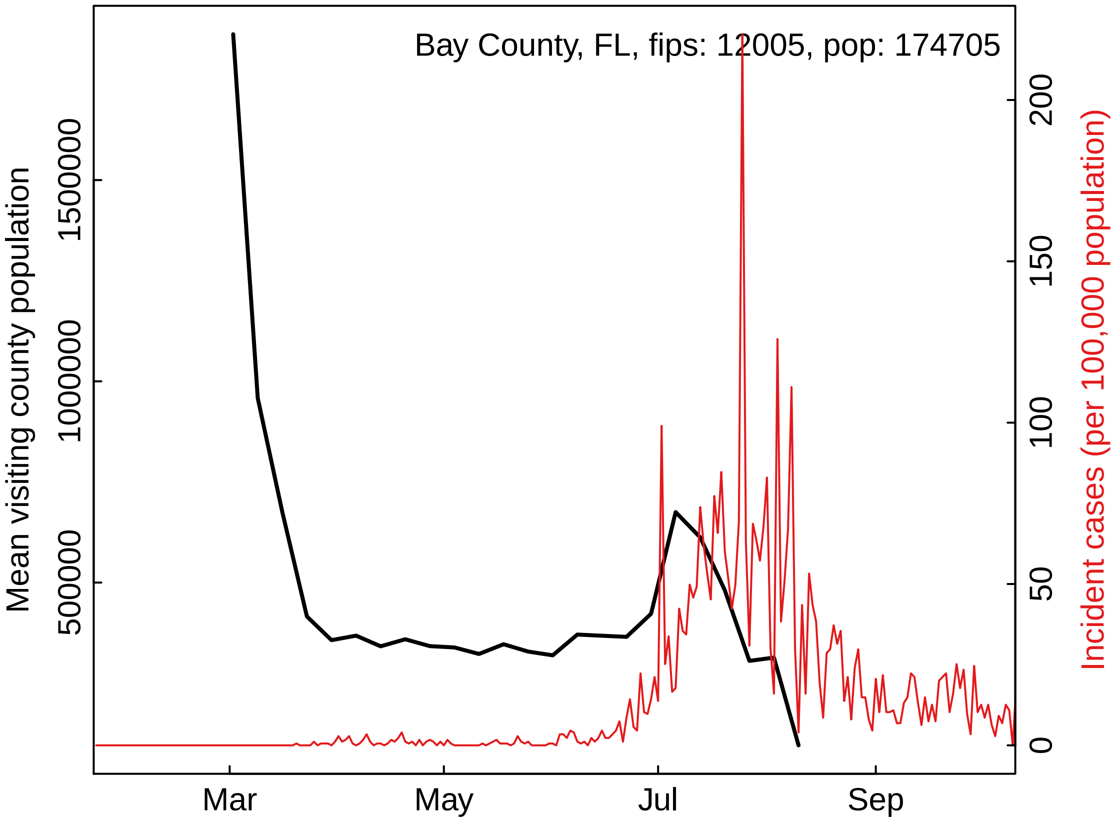
Bay County, FL, fips: 12005, pop: 174705
Mean visiting county population
500000
1000000
1500000
Incident cases (per 100,000 population)
0
50
100
150
200
Mar
May
Jul
Sep

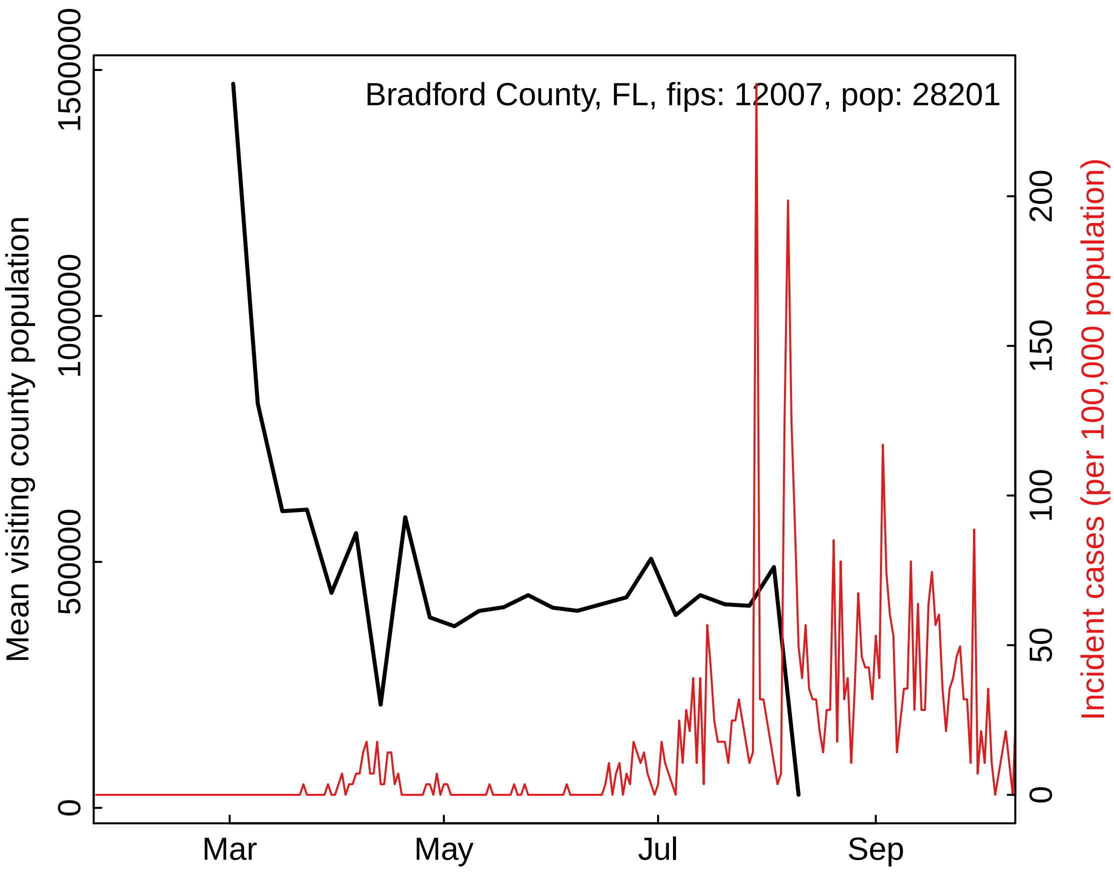

Bradford County, FL, fips: 12007, pop: 28201
Mean visiting county population
0
500000
1000000
1500000
Incident cases (per 100,000 population)
0
50
100
150
200
Mar
May
Jul
Sep

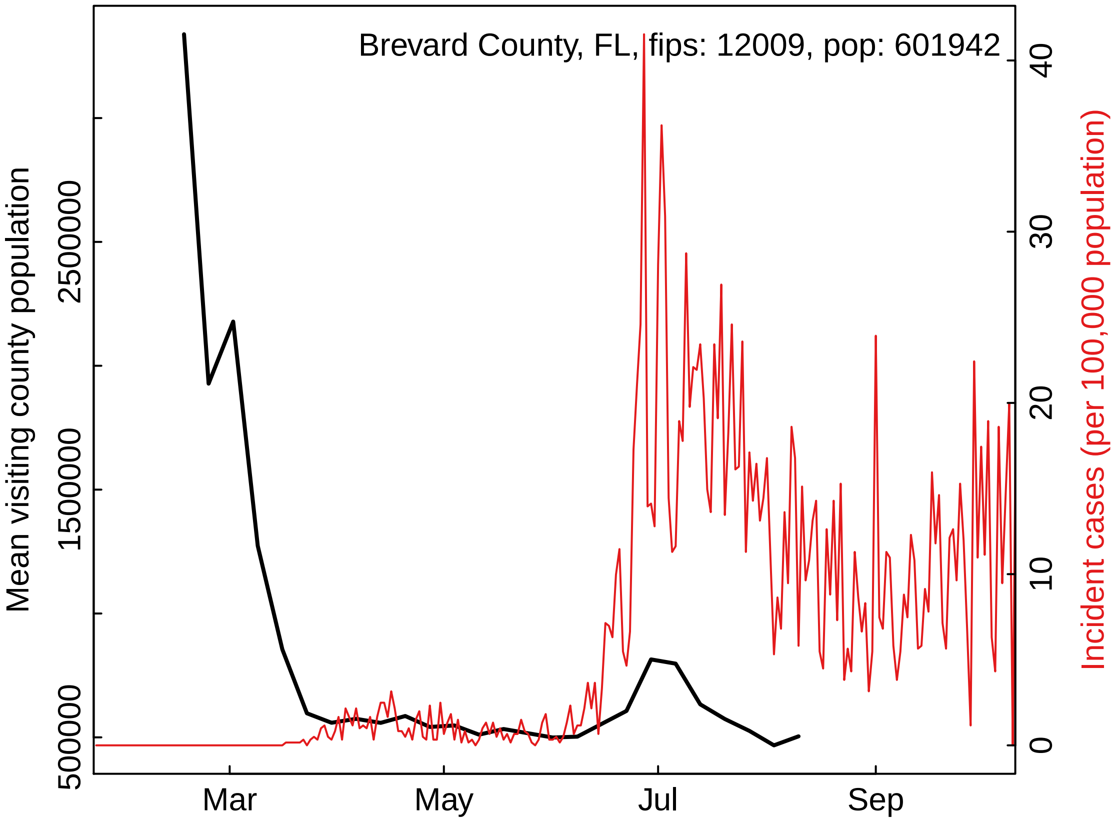

Brevard County, FL, fips: 12009, pop: 601942
Mean visiting county population
500000
1500000
2500000
Incident cases (per 100,000 population)
0
10
20
30
40
Mar
May
Jul
Sep

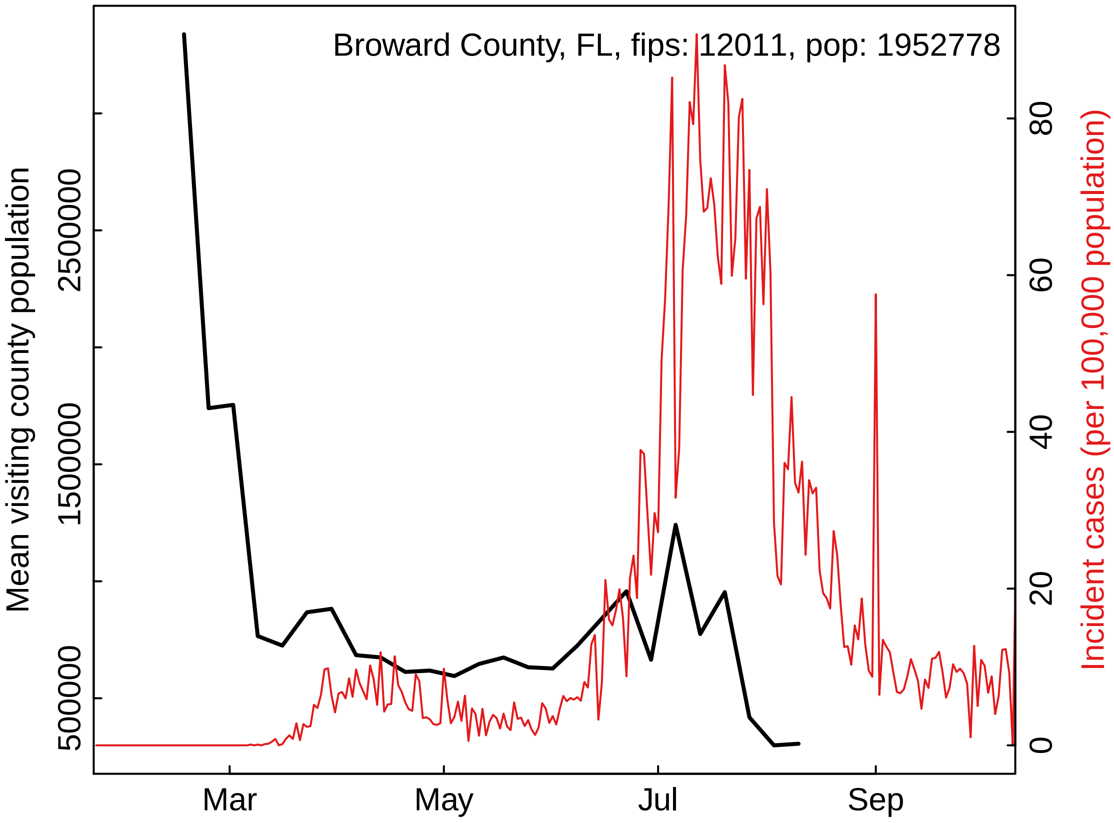

Broward County, FL, fips: 12011, pop: 1952778
Mean visiting county population
500000
1500000
2500000
Incident cases (per 100,000 population)
0
20
40
60
80
Mar
May
Jul
Sep

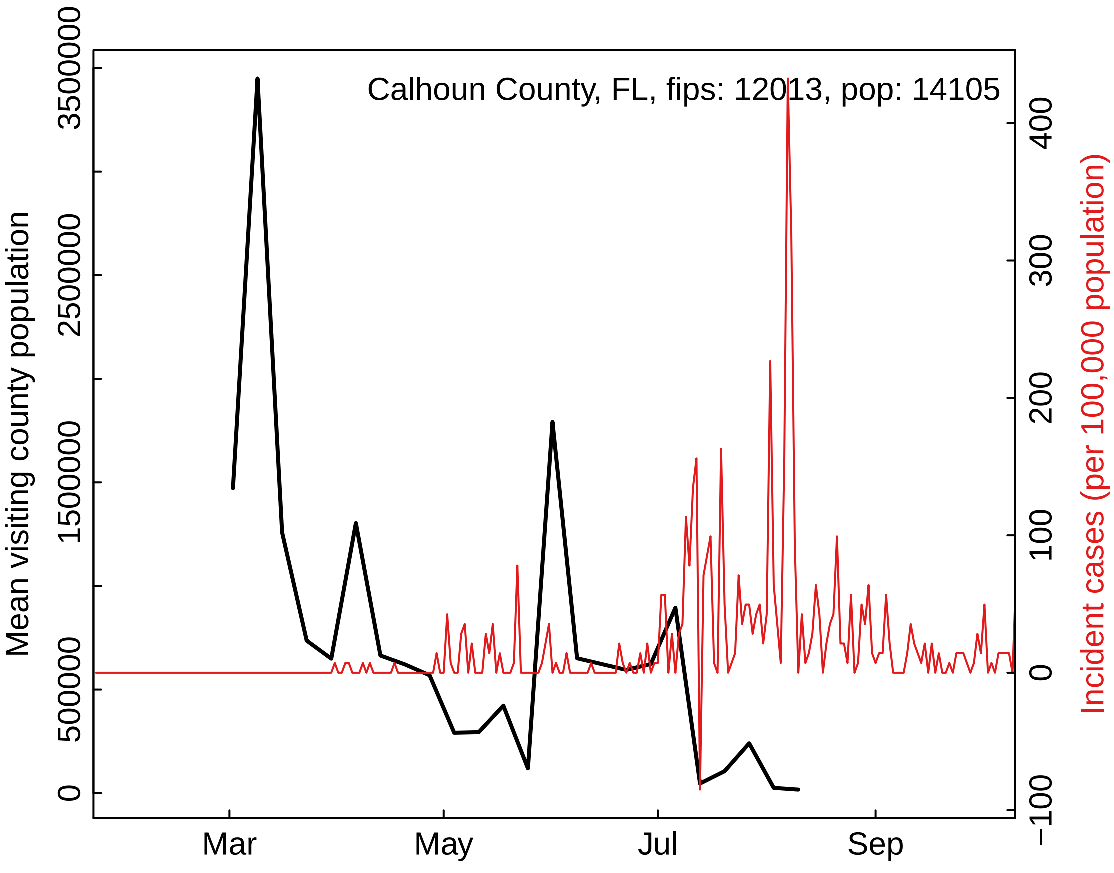

Calhoun County, FL, fips: 12013, pop: 14105
Mean visiting county population
0
500000
1500000
2500000
3500000
Incident cases (per 100,000 population)
−100
0
100
200
300
400
Mar
May
Jul
Sep

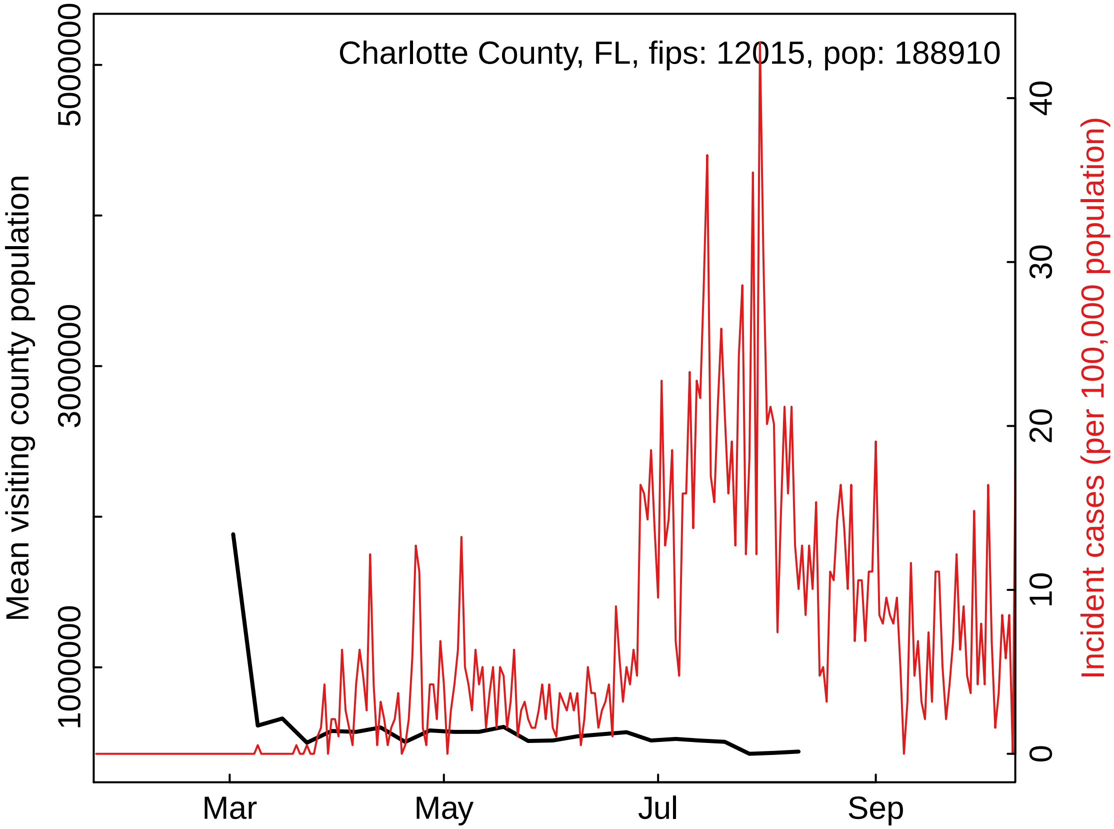

Charlotte County, FL, fips: 12015, pop: 188910
Mean visiting county population
1000000
3000000
5000000
Incident cases (per 100,000 population)
0
10
20
30
40
Mar
May
Jul
Sep

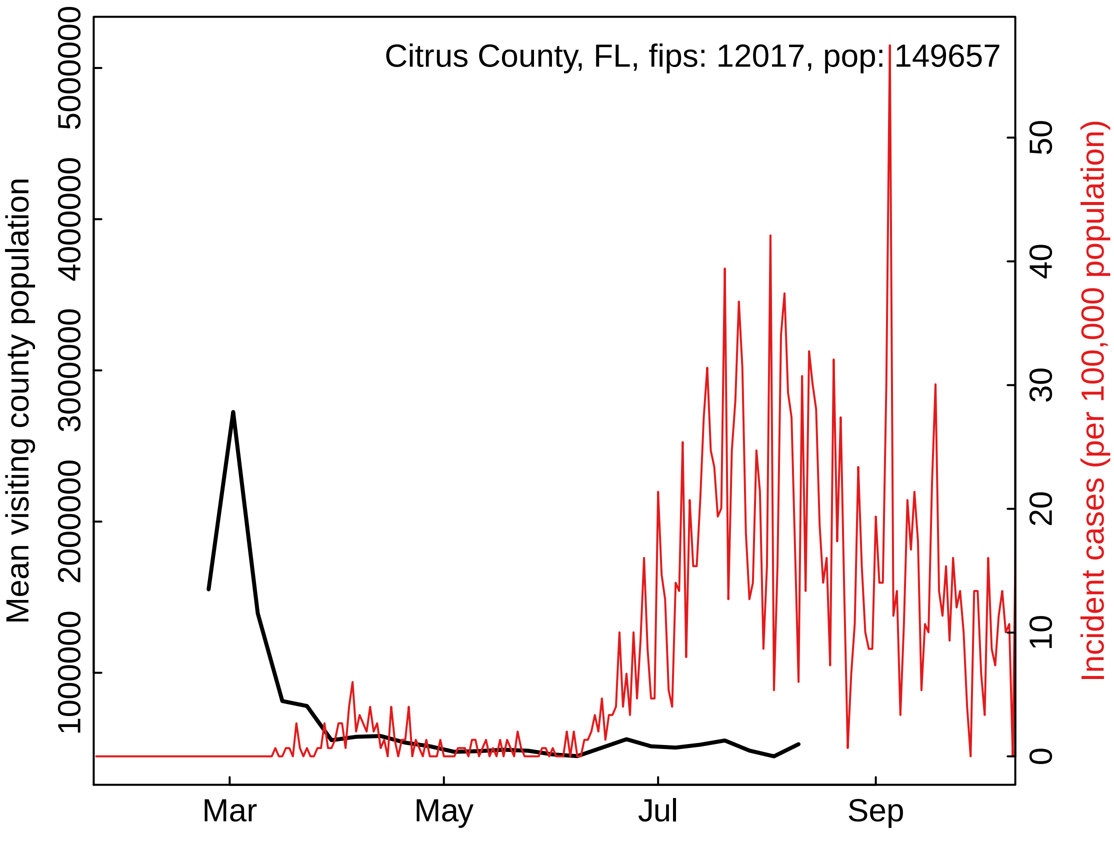

Citrus County, FL, fips: 12017, pop: 149657
Mean visiting county population
1000000
2000000
3000000
4000000
5000000
Incident cases (per 100,000 population)
0
10
20
30
40
50
Mar
May
Jul
Sep

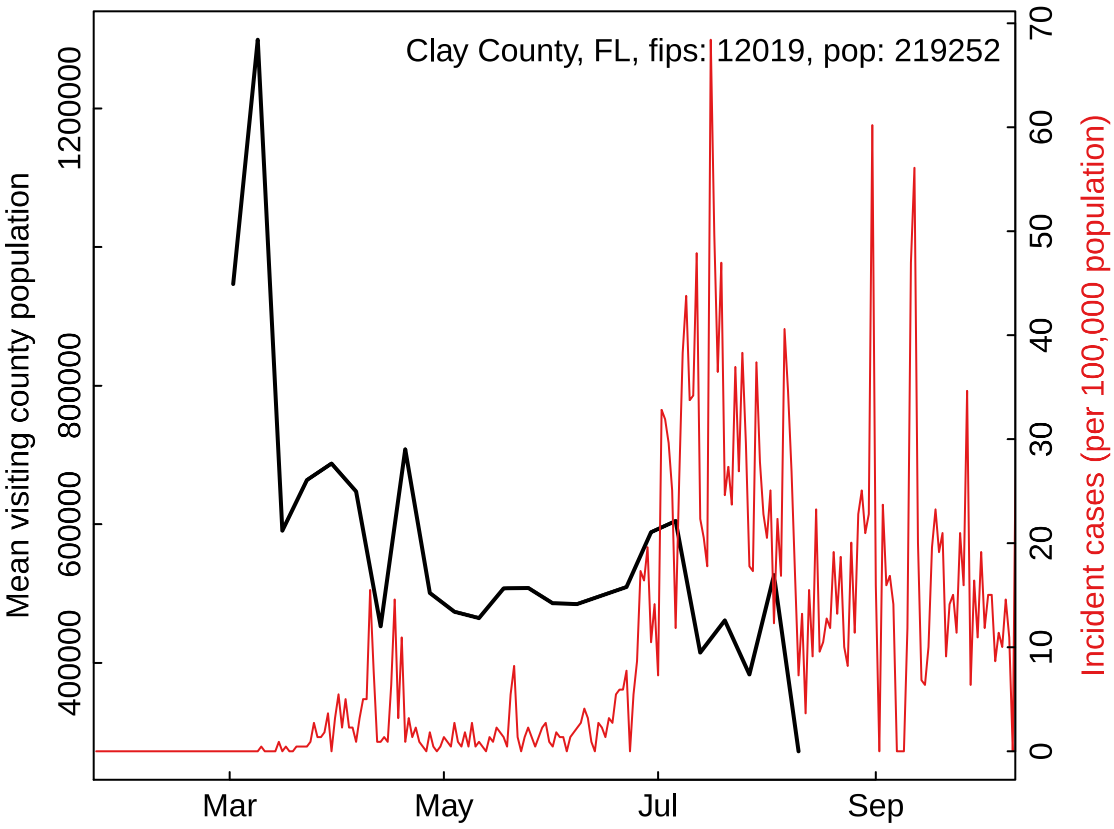
Clay County, FL, fips: 12019, pop: 219252
Mean visiting county population
400000
600000
800000
1200000
Incident cases (per 100,000 population)
0
10
20
30
40
50
60
70
Mar
May
Jul
Sep

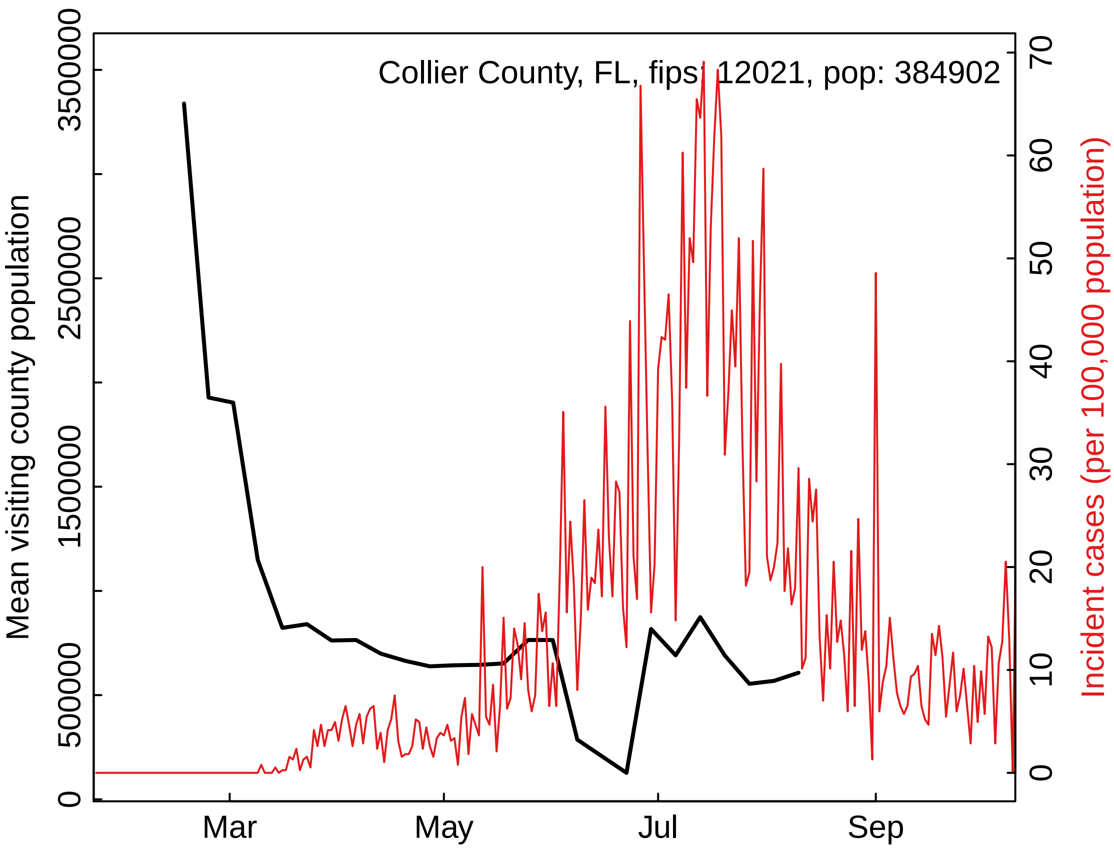

Collier County, FL, fips: 12021, pop: 384902
Mean visiting county population
0
500000
1500000
2500000
3500000
Incident cases (per 100,000 population)
0
10
20
30
40
50
60
70
Mar
May
Jul
Sep

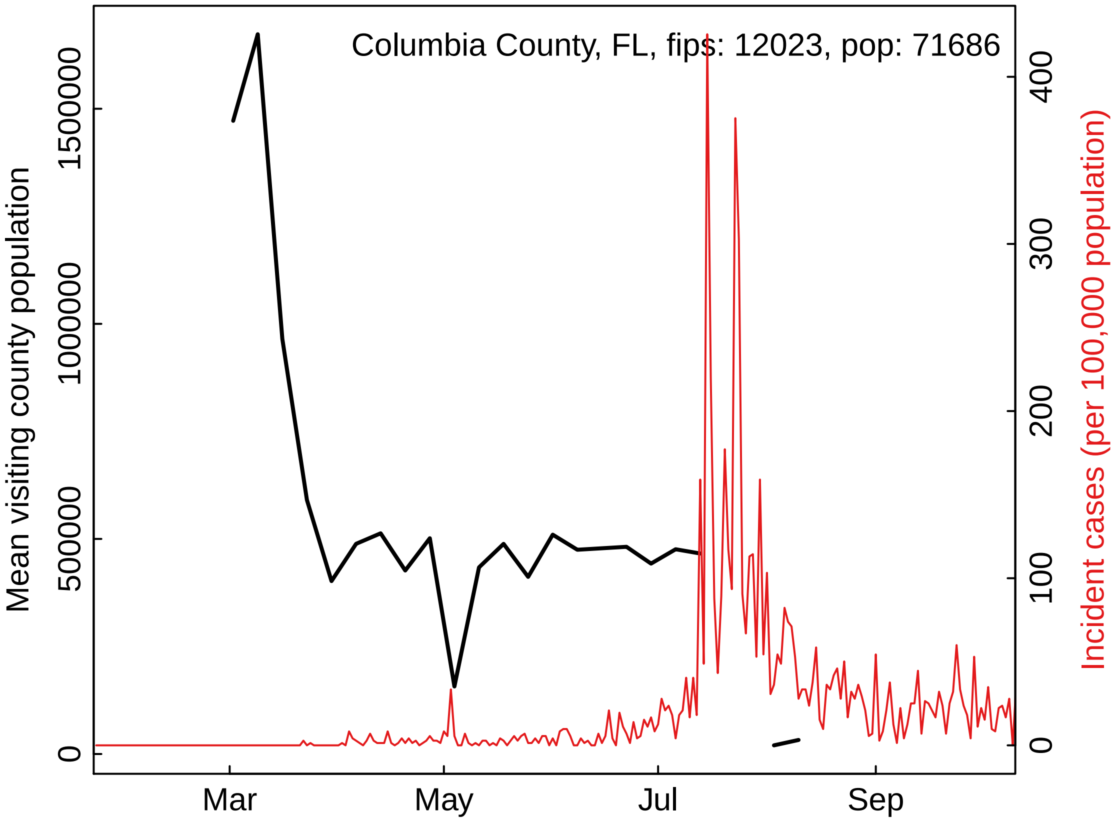
Columbia County, FL, fips: 12023, pop: 71686
Mean visiting county population
0
500000
1000000
1500000
Incident cases (per 100,000 population)
0
100
200
300
400
Mar
May
Jul
Sep

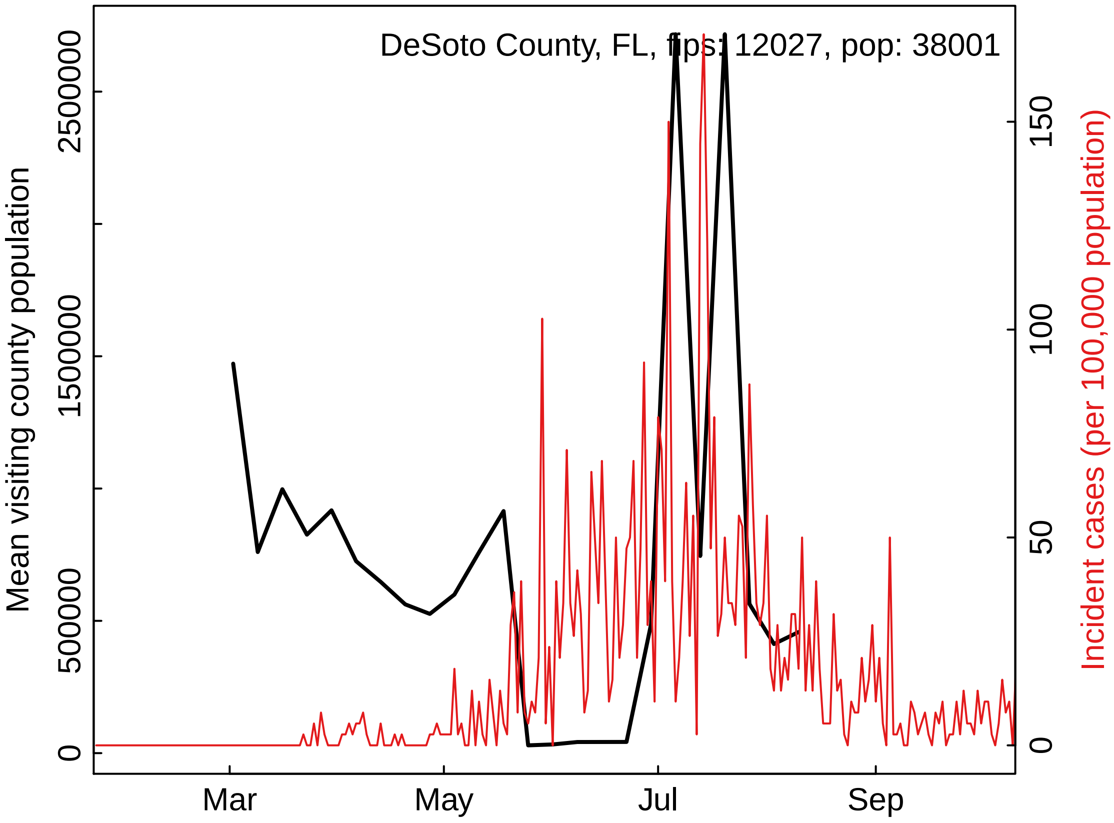

DeSoto County, FL, fips: 12027, pop: 38001
Mean visiting county population
0
500000
1500000
2500000
Incident cases (per 100,000 population)
0
50
100
150
Mar
May
Jul
Sep

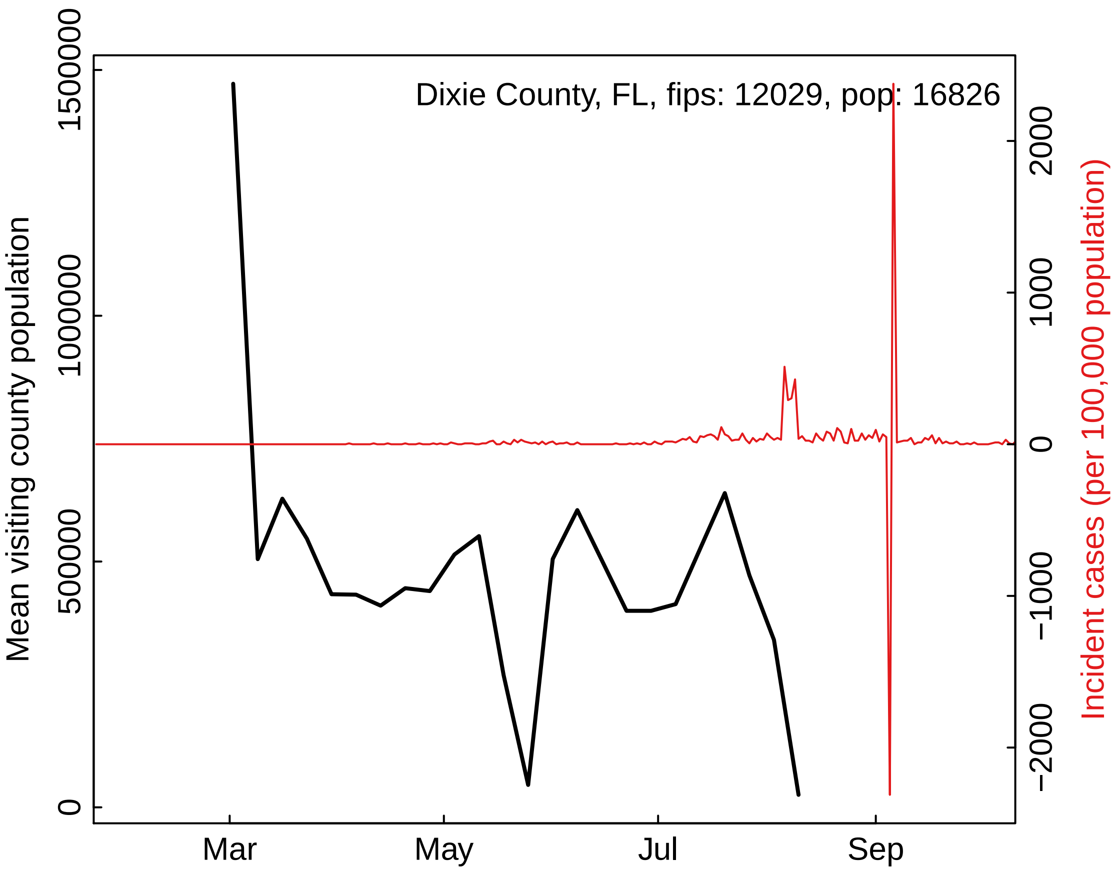
Dixie County, FL, fips: 12029, pop: 16826
Mean visiting county population
Incident cases (per 100,000 population)
0
500000
1000000
1500000
−2000
−1000
0
1000
2000
Mar
May
Jul
Sep

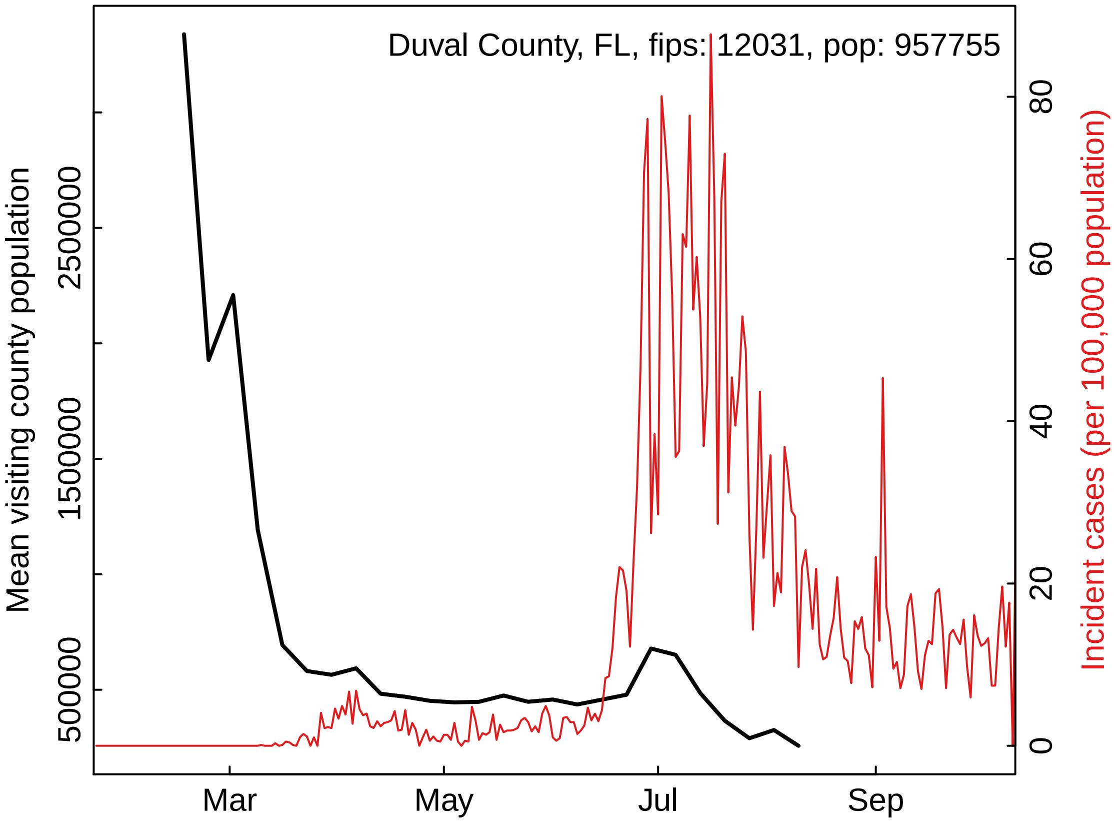

Duval County, FL, fips: 12031, pop: 957755
Mean visiting county population
500000
1500000
2500000
Incident cases (per 100,000 population)
0
20
40
60
80
Mar
May
Jul
Sep

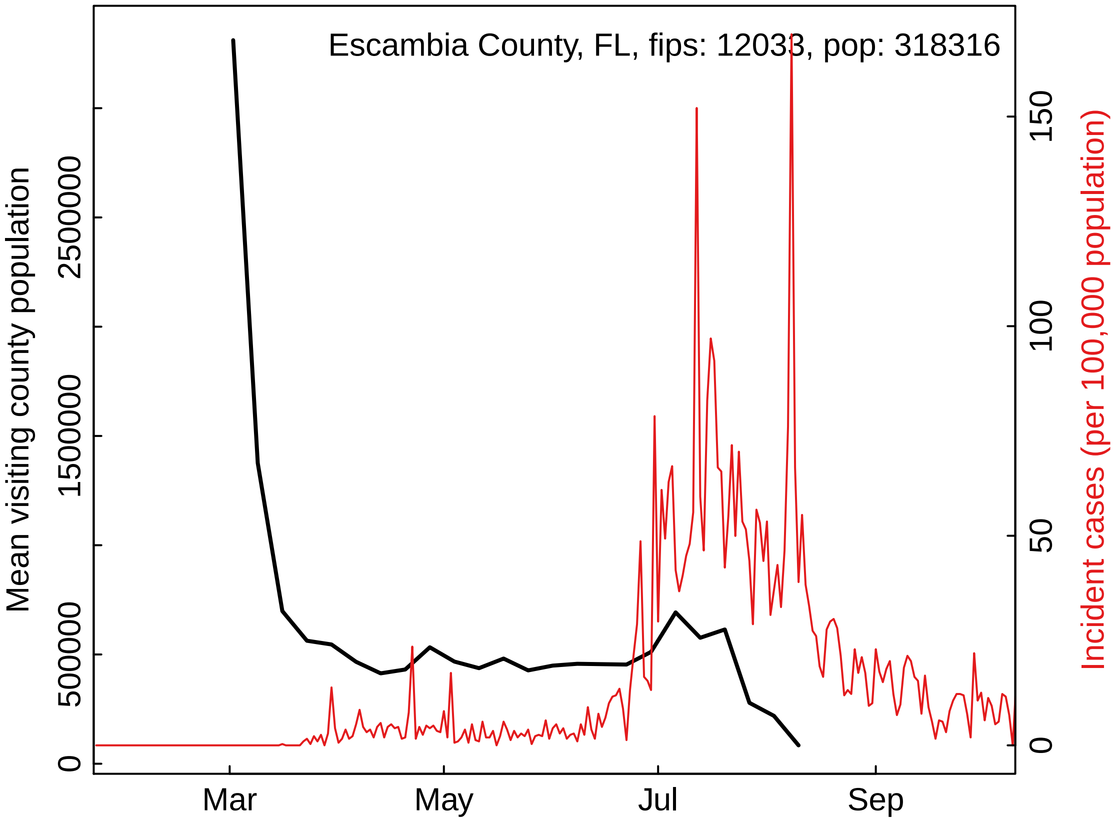

Escambia County, FL, fips: 12033, pop: 318316
Mean visiting county population
0
500000
1500000
2500000
Incident cases (per 100,000 population)
0
50
100
150
Mar
May
Jul
Sep

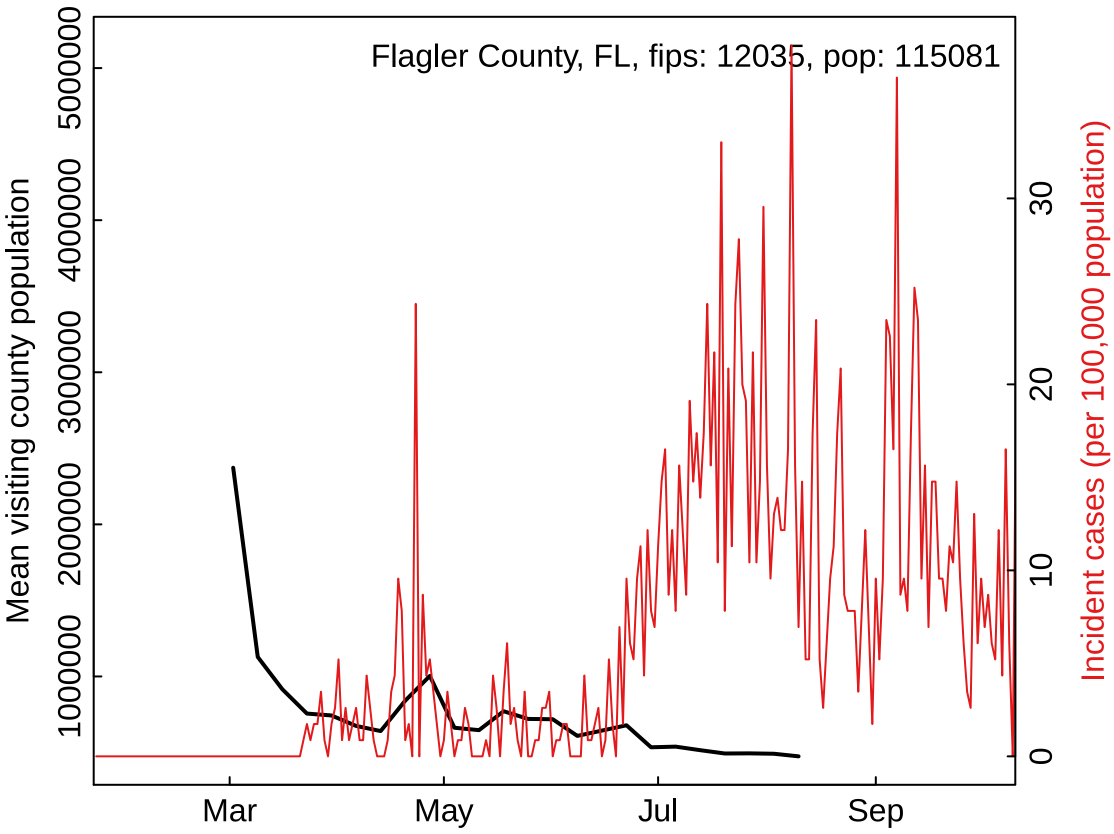

Flagler County, FL, fips: 12035, pop: 115081
Mean visiting county population
Incident cases (per 100,000 population)
1000000
2000000
3000000
4000000
5000000
0
10
20
30
Mar
May
Jul
Sep

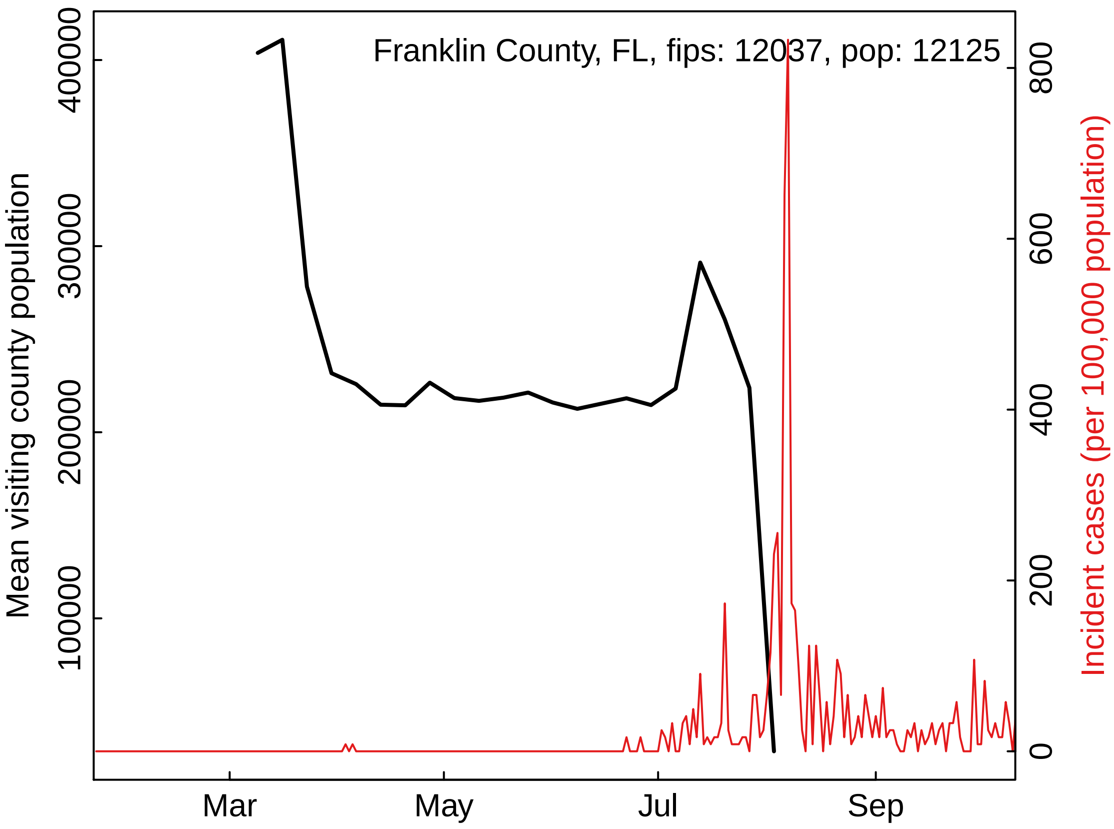

Franklin County, FL, fips: 12037, pop: 12125
Mean visiting county population
100000
200000
300000
400000
Incident cases (per 100,000 population)
0
200
400
600
800
Mar
May
Jul
Sep

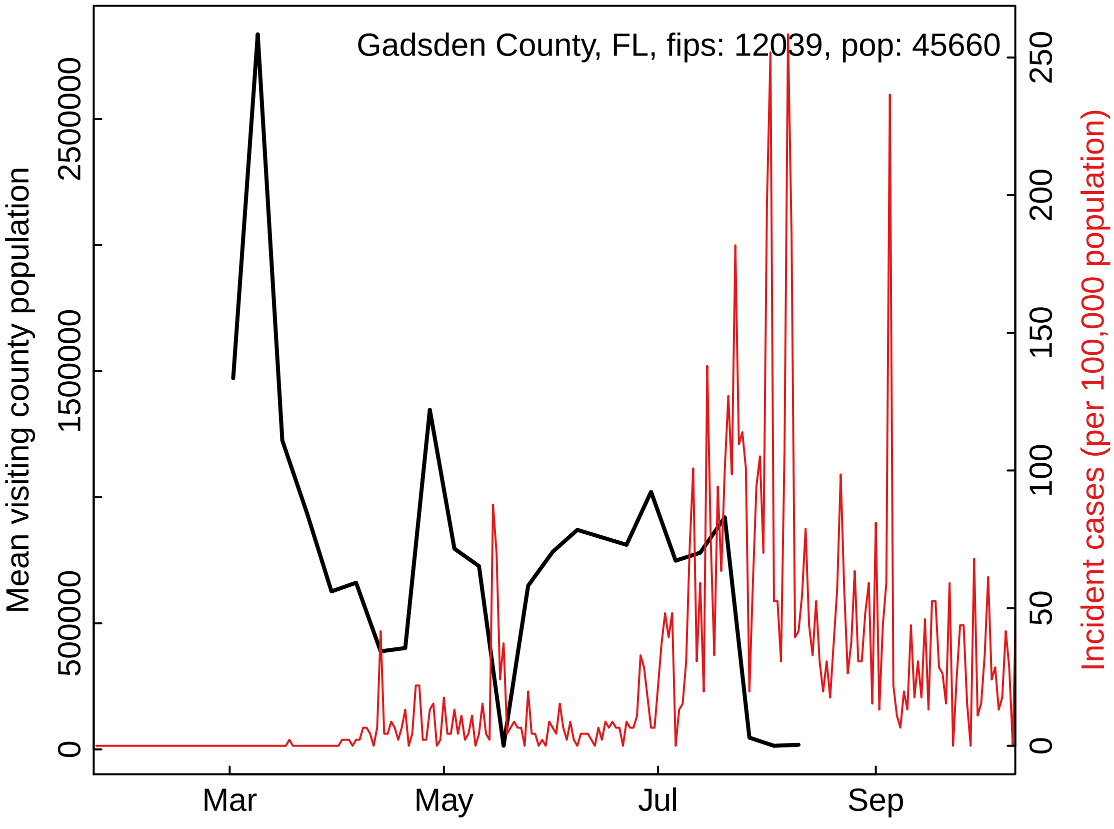
Gadsden County, FL, fips: 12039, pop: 45660
Mean visiting county population
0
500000
1500000
2500000
Incident cases (per 100,000 population)
0
50
100
150
200
250
Mar
May
Jul
Sep

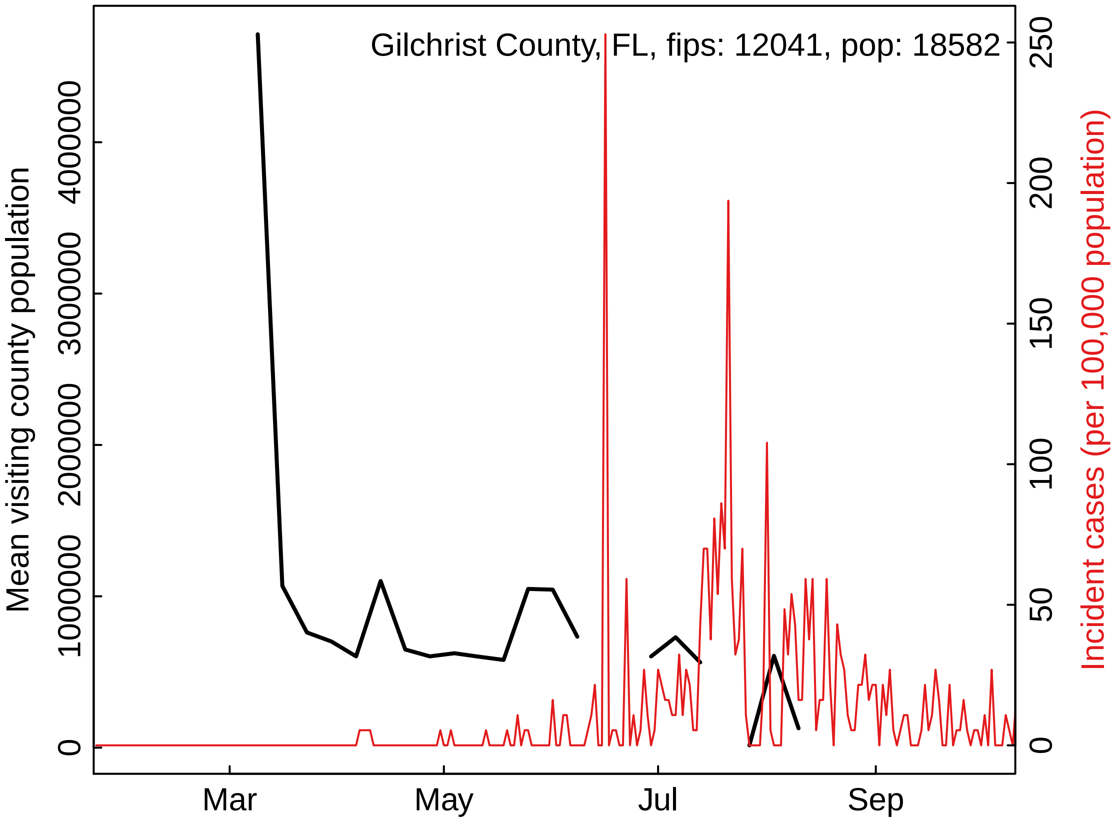
Gilchrist County, FL, fips: 12041, pop: 18582
Mean visiting county population
0
1000000
2000000
3000000
4000000
Incident cases (per 100,000 population)
0
50
100
150
200
250
Mar
May
Jul
Sep

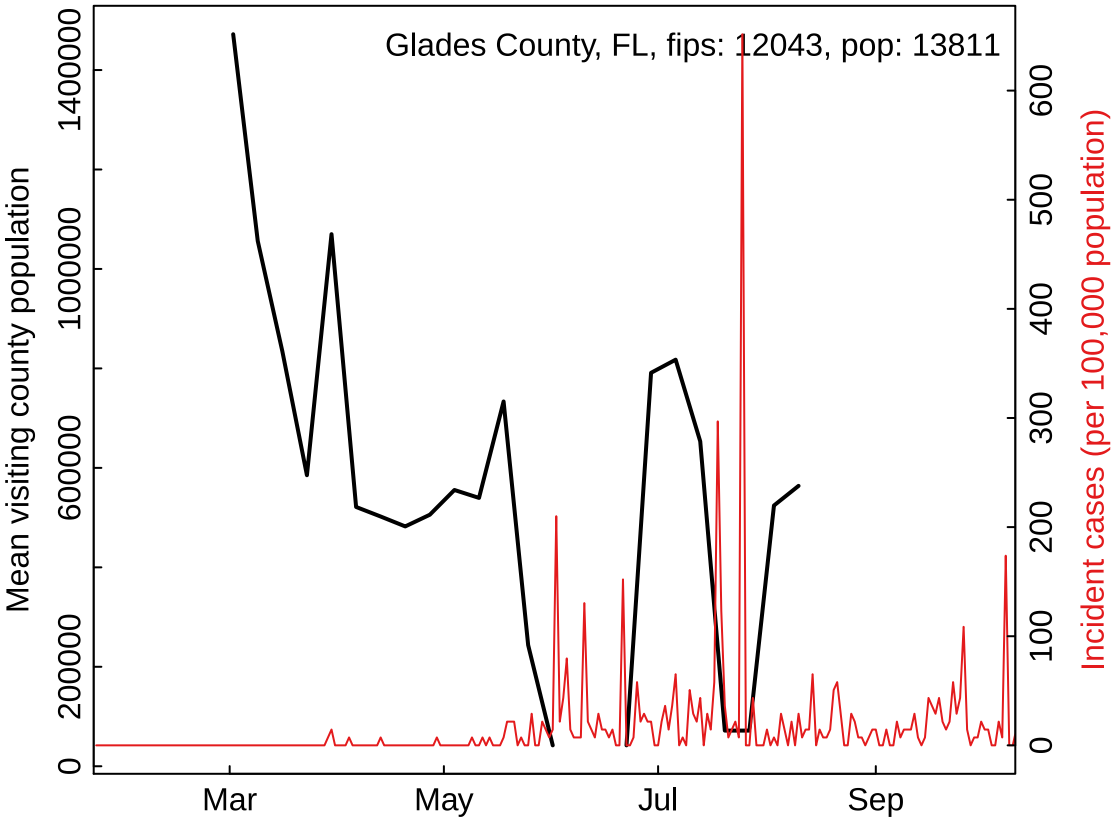

Glades County, FL, fips: 12043, pop: 13811
Mean visiting county population
0
200000
600000
1000000
1400000
Incident cases (per 100,000 population)
0
100
200
300
400
500
600
Mar
May
Jul
Sep

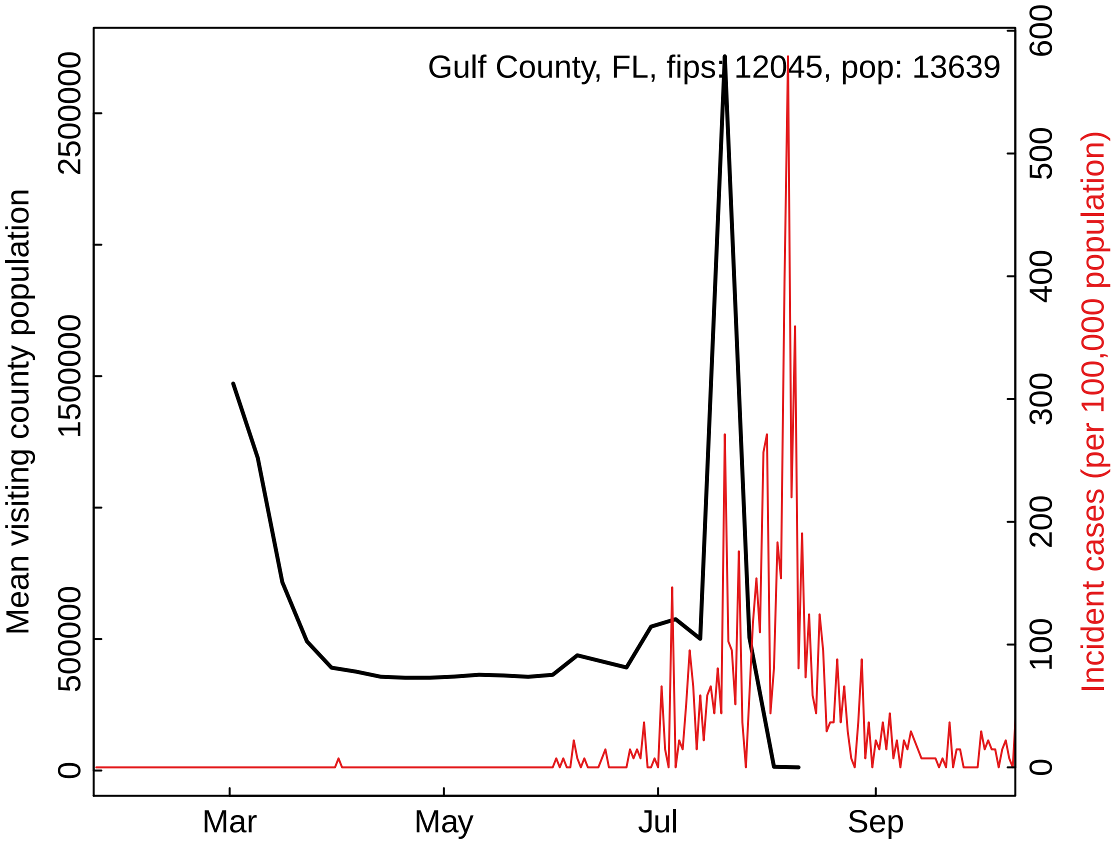
Gulf County, FL, fips: 12045, pop: 13639
Mean visiting county population
Incident cases (per 100,000 population)
0
500000
1500000
2500000
0
100
200
300
400
500
600
Mar
May
Jul
Sep

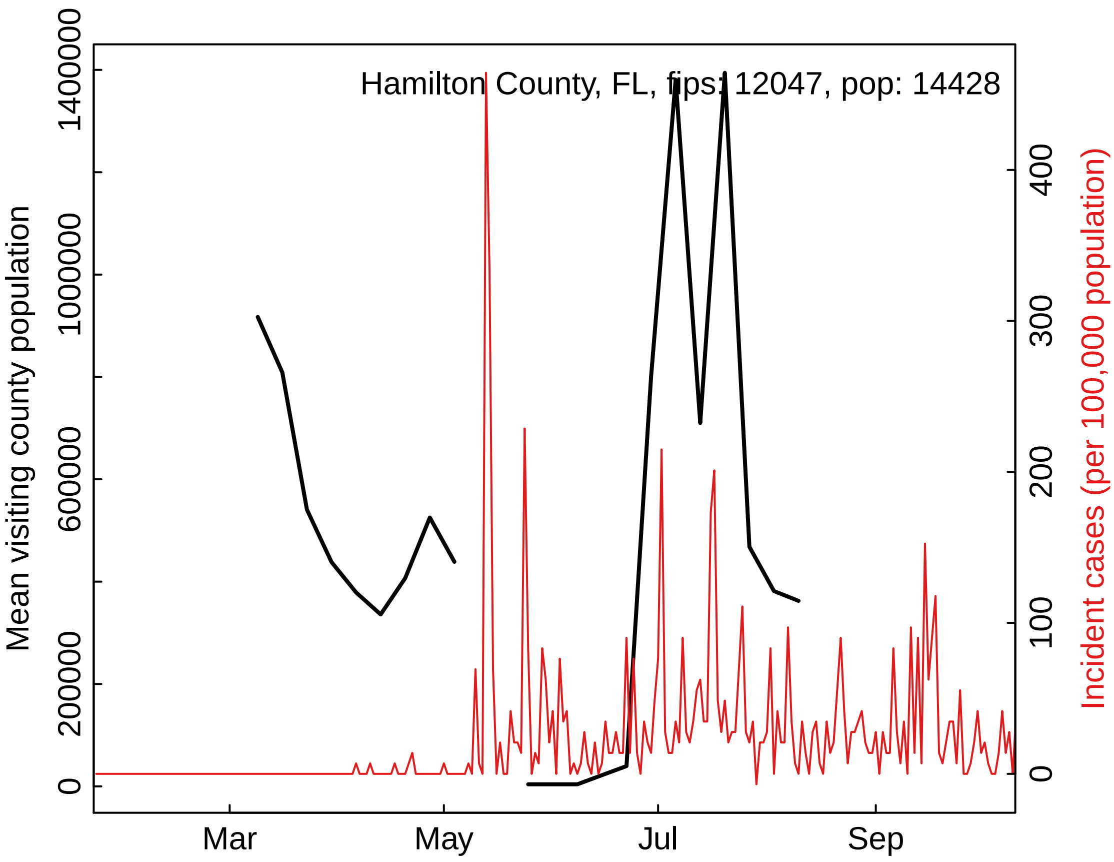

Hamilton County, FL, fips: 12047, pop: 14428
Mean visiting county population
0
2000000
6000000
10000000
14000000
Incident cases (per 100,000 population)
0
100
200
300
400
Mar
May
Jul
Sep

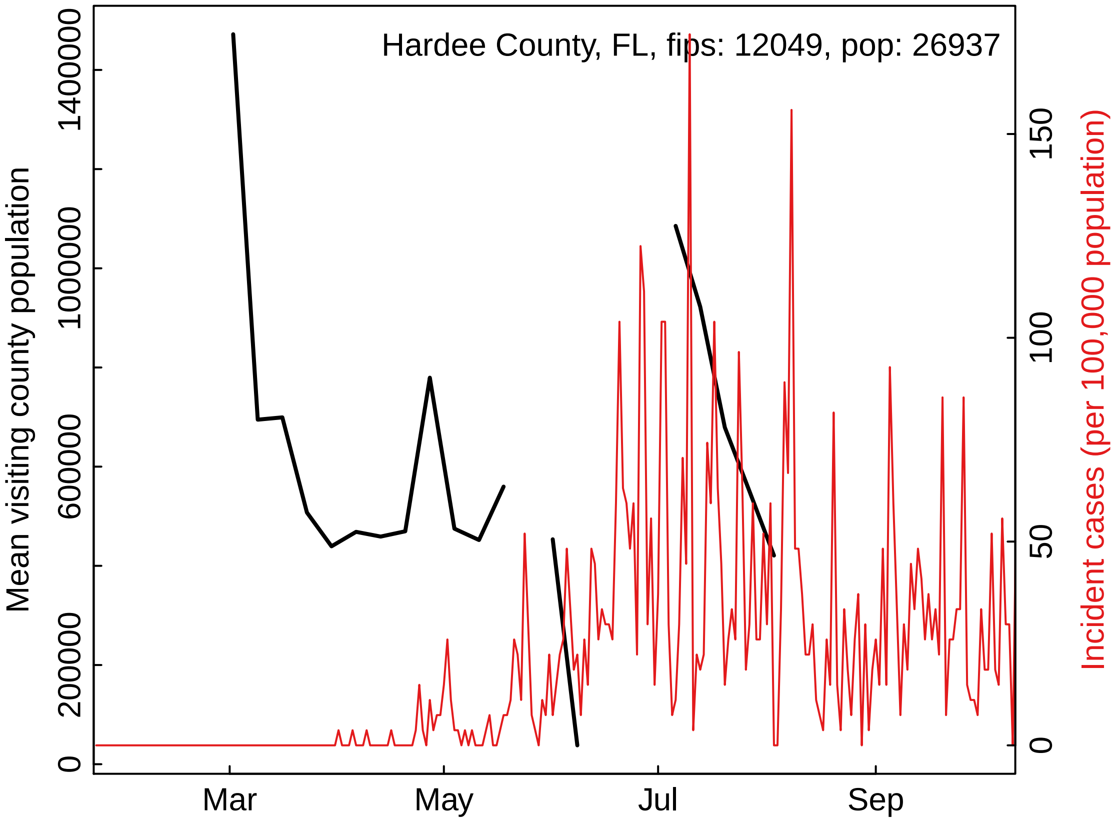

Hardee County, FL, fips: 12049, pop: 26937
Mean visiting county population
Incident cases (per 100,000 population)
0
200000
600000
1000000
1400000
0
50
100
150
Mar
May
Jul
Sep

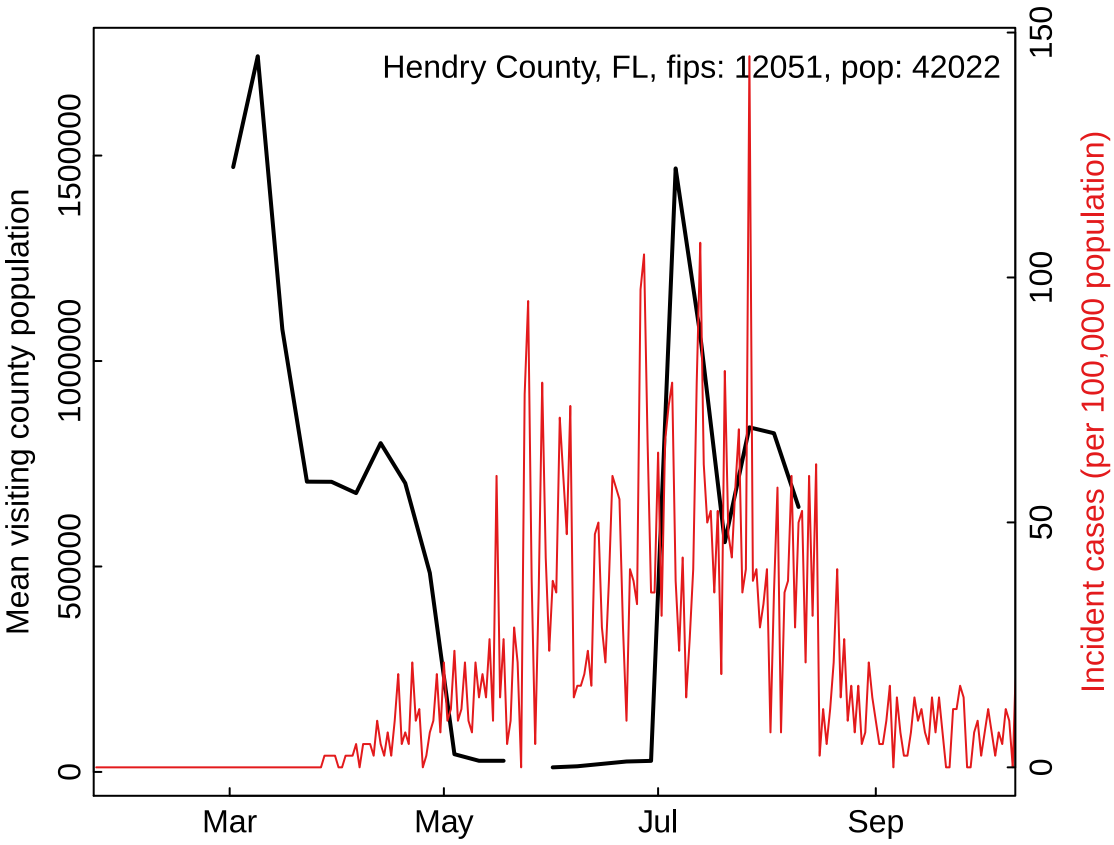

Hendry County, FL, fips: 12051, pop: 42022
Mean visiting county population
0
500000
1000000
1500000
Incident cases (per 100,000 population)
0
50
100
150
Mar
May
Jul
Sep

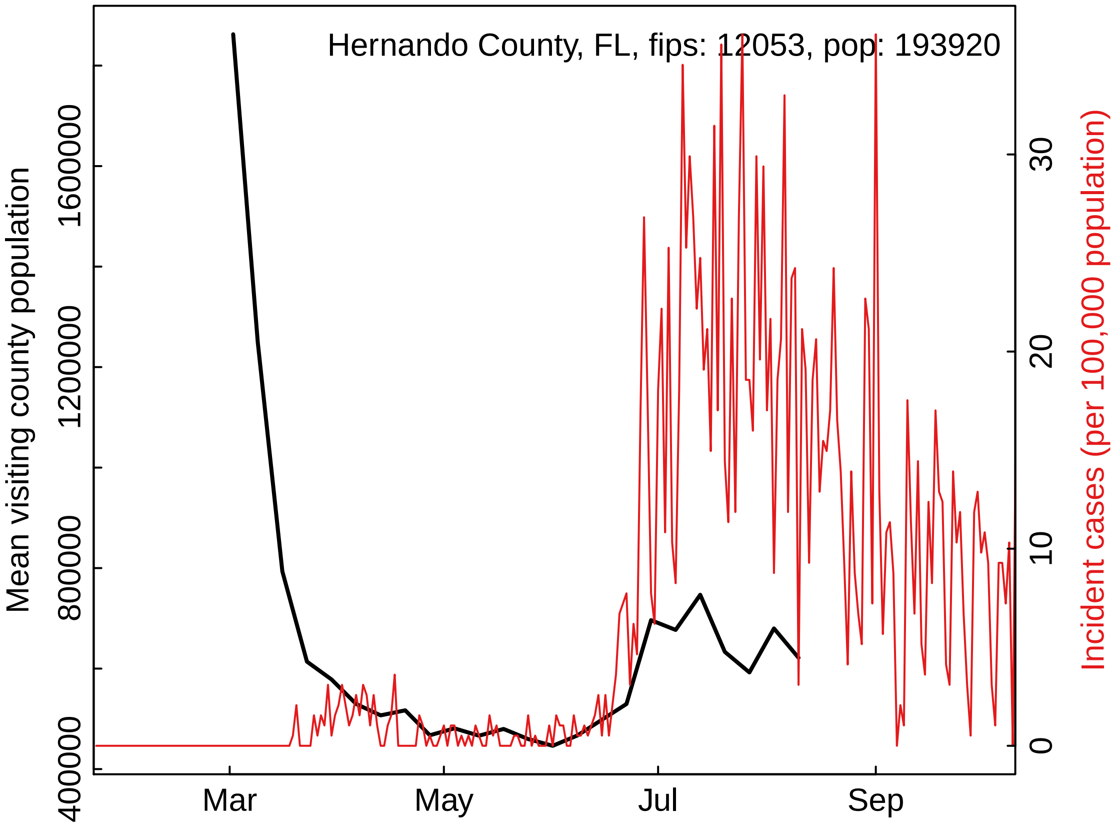
Hernando County, FL, fips: 12053, pop: 193920
Mean visiting county population
Incident cases (per 100,000 population)
400000
800000
1200000
1600000
0
10
20
30
Mar
May
Jul
Sep

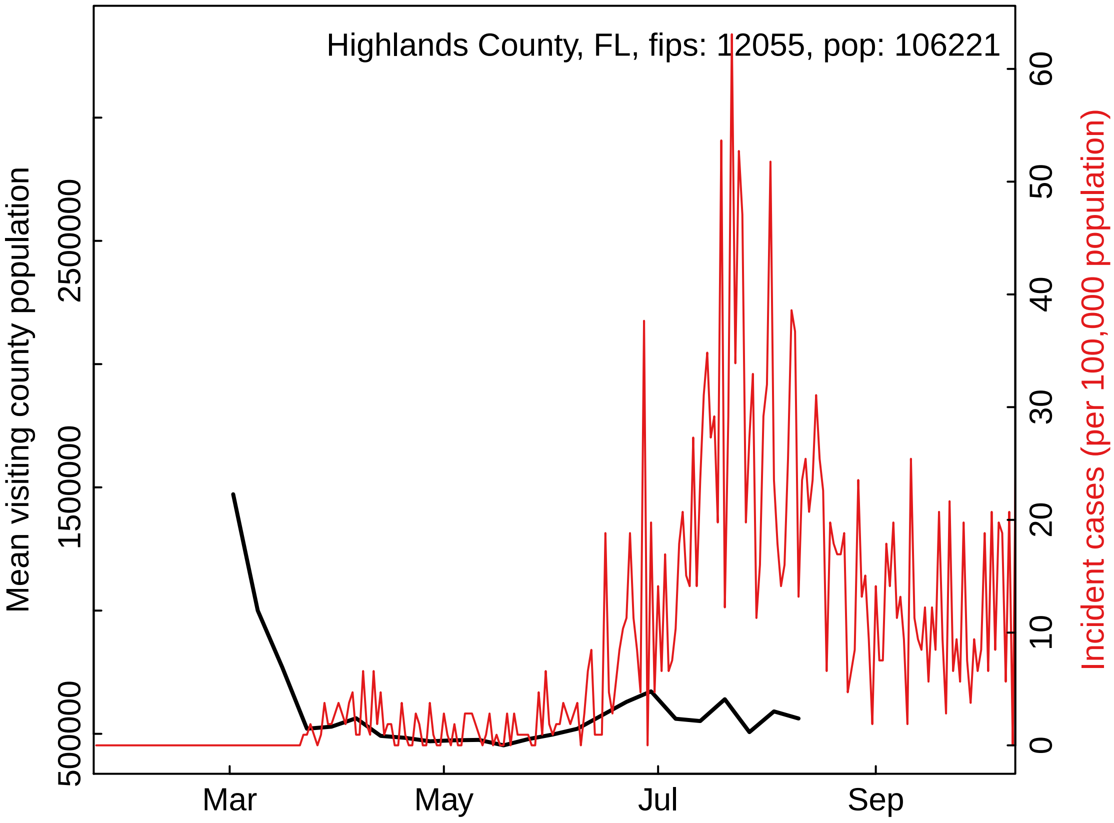

Highlands County, FL, fips: 12055, pop: 106221
Mean visiting county population
500000
1500000
2500000
Mar
May
Jul
Sep
0
10
20
30
40
50
60
Incident cases (per 100,000 population)

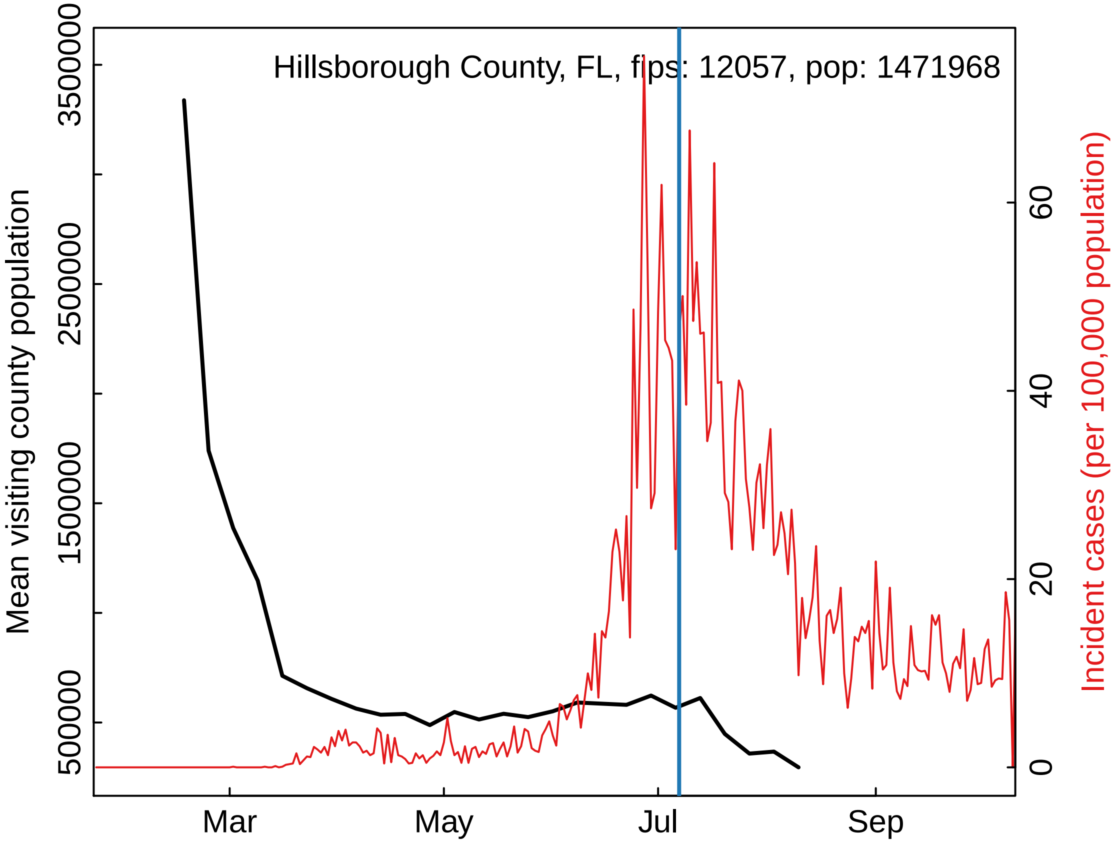

Hillsborough County, FL, fips: 12057, pop: 1471968
Mean visiting county population
500000
1500000
2500000
3500000
Incident cases (per 100,000 population)
0
20
40
60
Mar
May
Jul
Sep

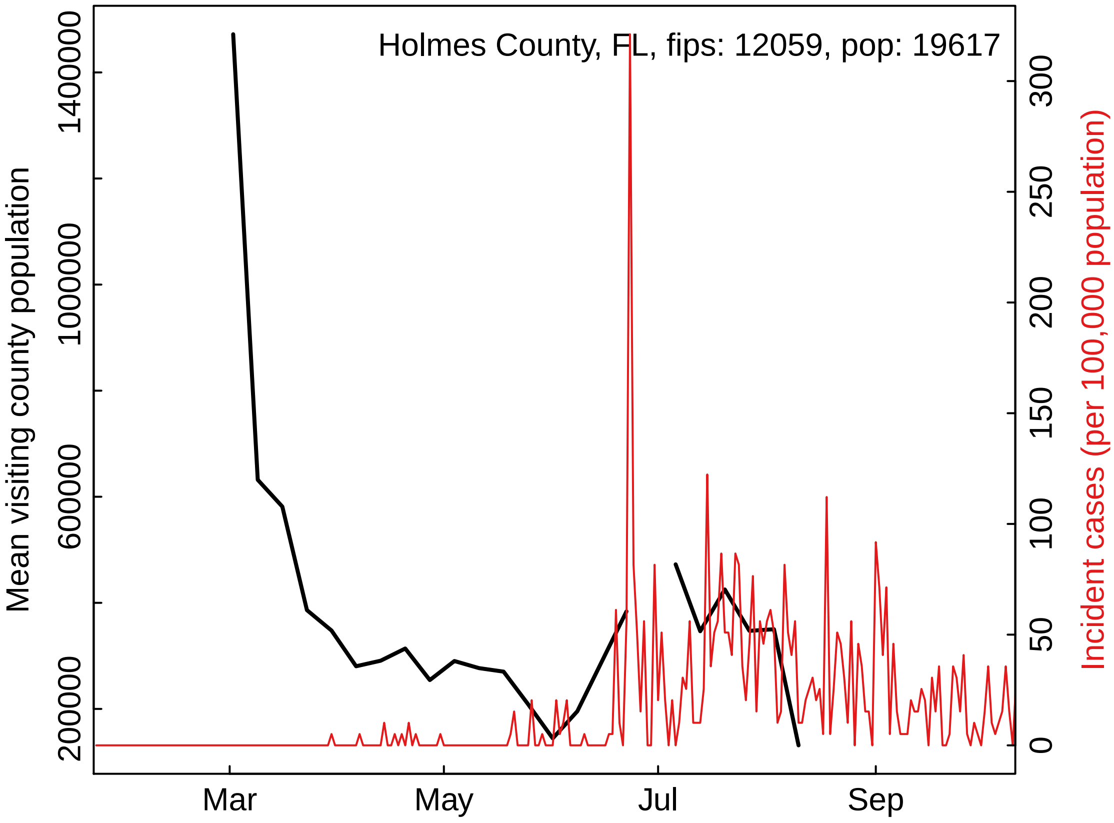

Holmes County, FL, fips: 12059, pop: 19617
Mean visiting county population
200000
600000
1000000
1400000
Incident cases (per 100,000 population)
0
50
100
150
200
250
300
Mar
May
Jul
Sep

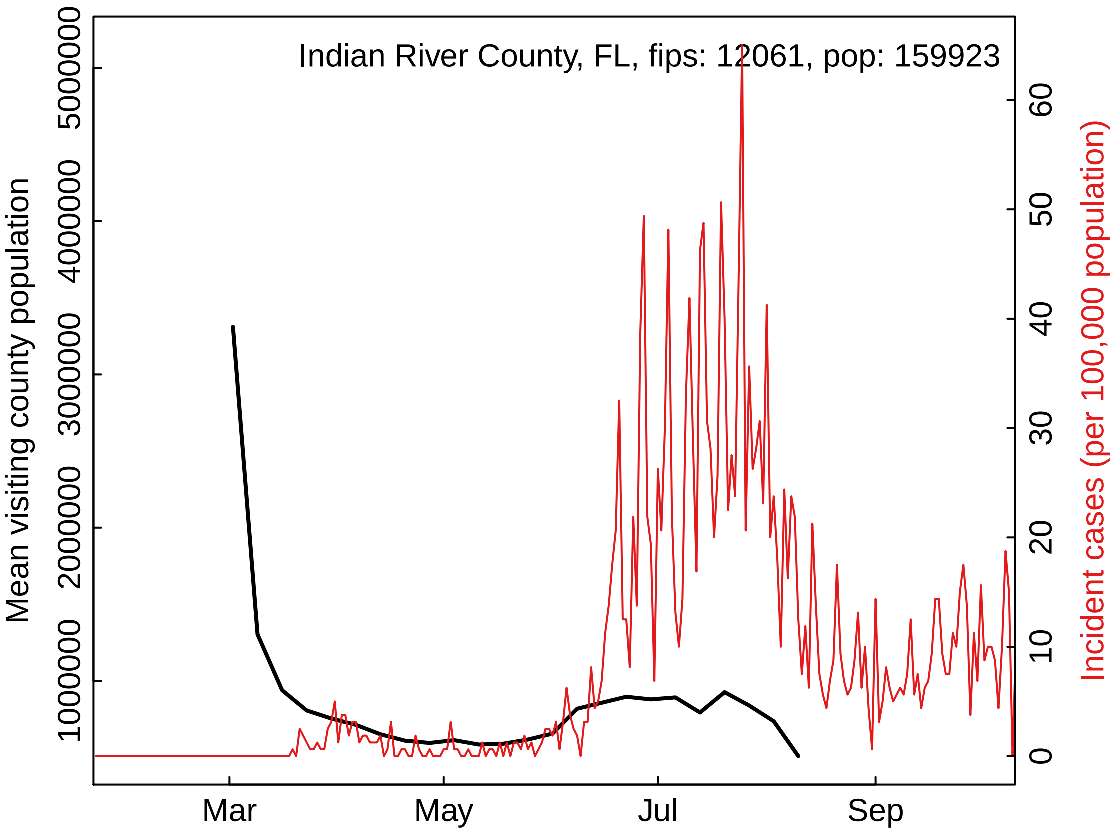

Indian River County, FL, fips: 12061, pop: 159923
Mean visiting county population
Incident cases (per 100,000 population)
1000000
2000000
3000000
4000000
5000000
0
10
20
30
40
50
60
Mar
May
Jul
Sep

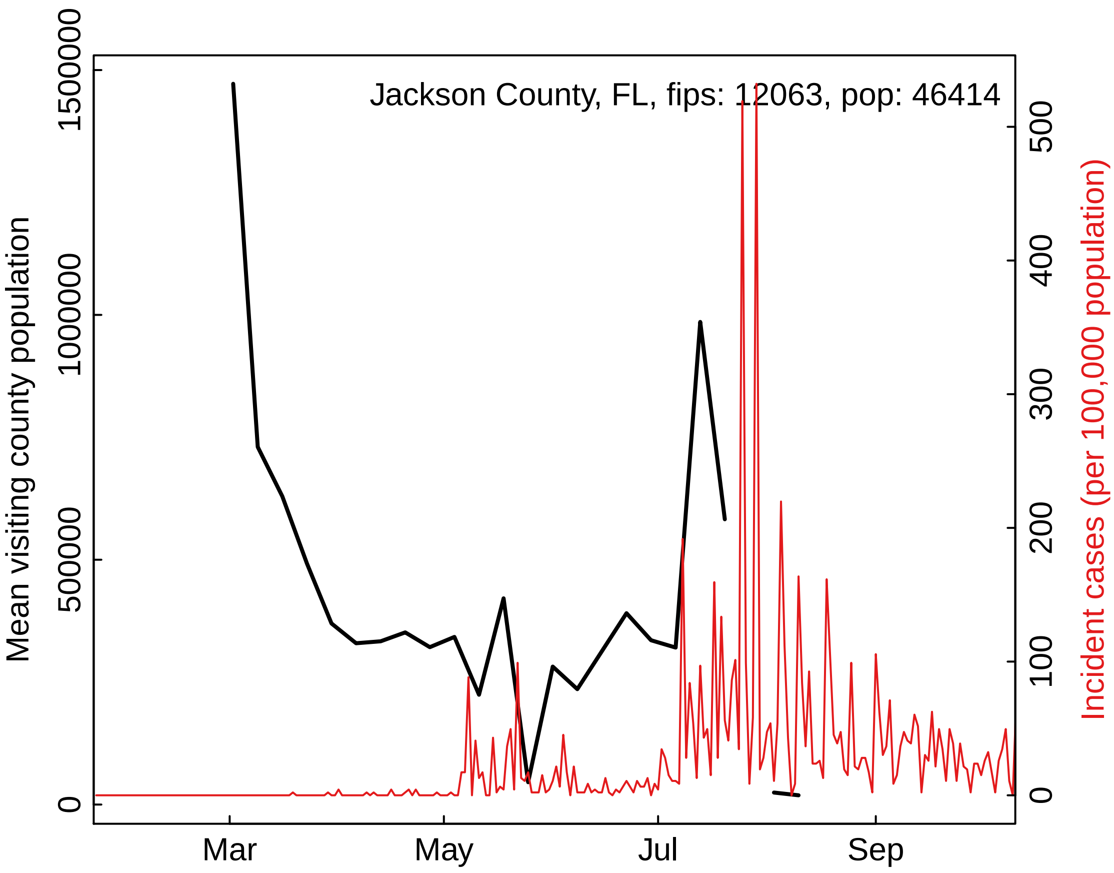
Jackson County, FL, fips: 12063, pop: 46414
Mean visiting county population
Incident cases (per 100,000 population)
0
500000
1000000
1500000
0
100
200
300
400
500
Mar
May
Jul
Sep

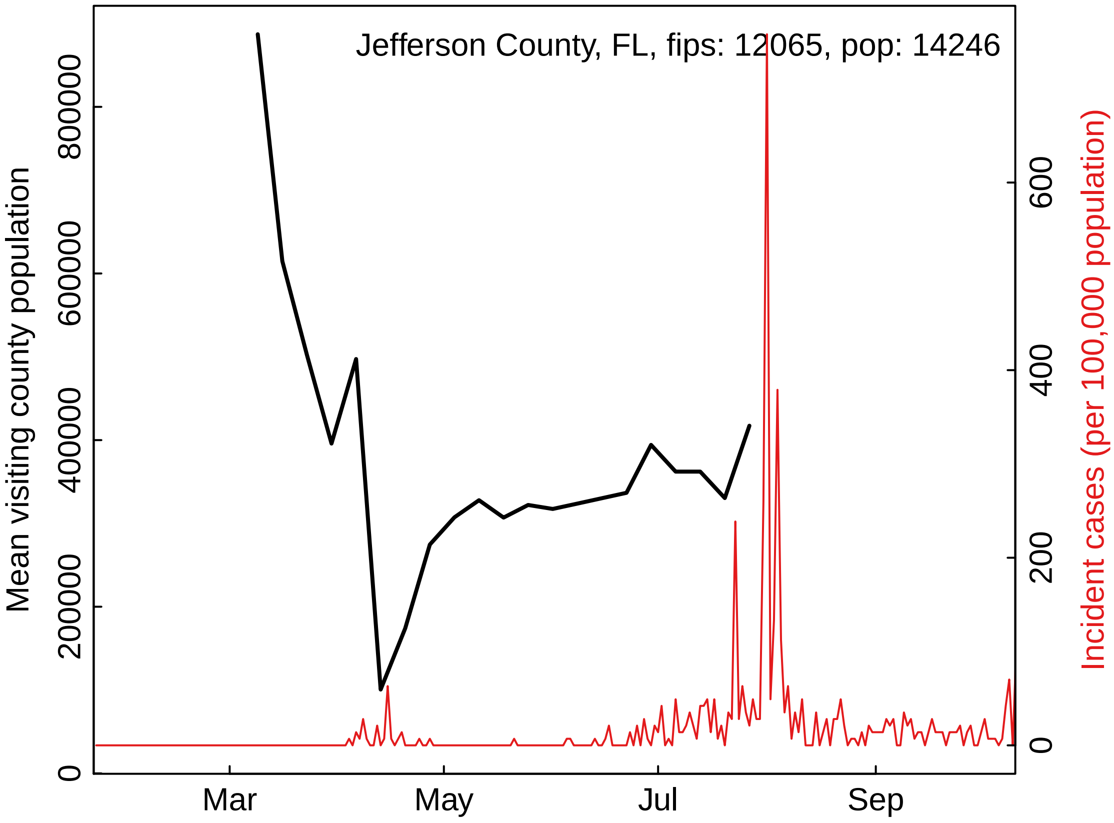

Jefferson County, FL, fips: 12065, pop: 14246
Mean visiting county population
0
200000
400000
600000
800000
Incident cases (per 100,000 population)
0
200
400
600
Mar
May
Jul
Sep

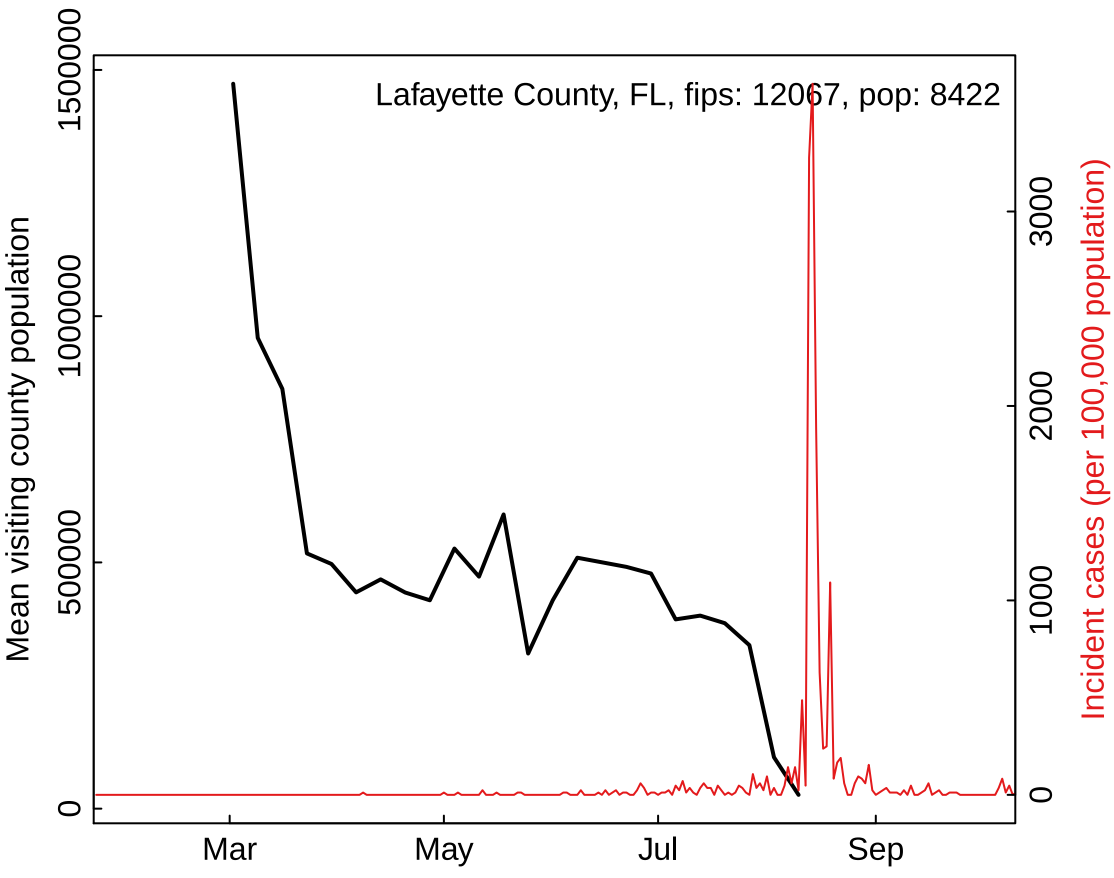

Lafayette County, FL, fips: 12067, pop: 8422
Mean visiting county population
0
500000
1000000
1500000
Incident cases (per 100,000 population)
0
1000
2000
3000
Mar
May
Jul
Sep

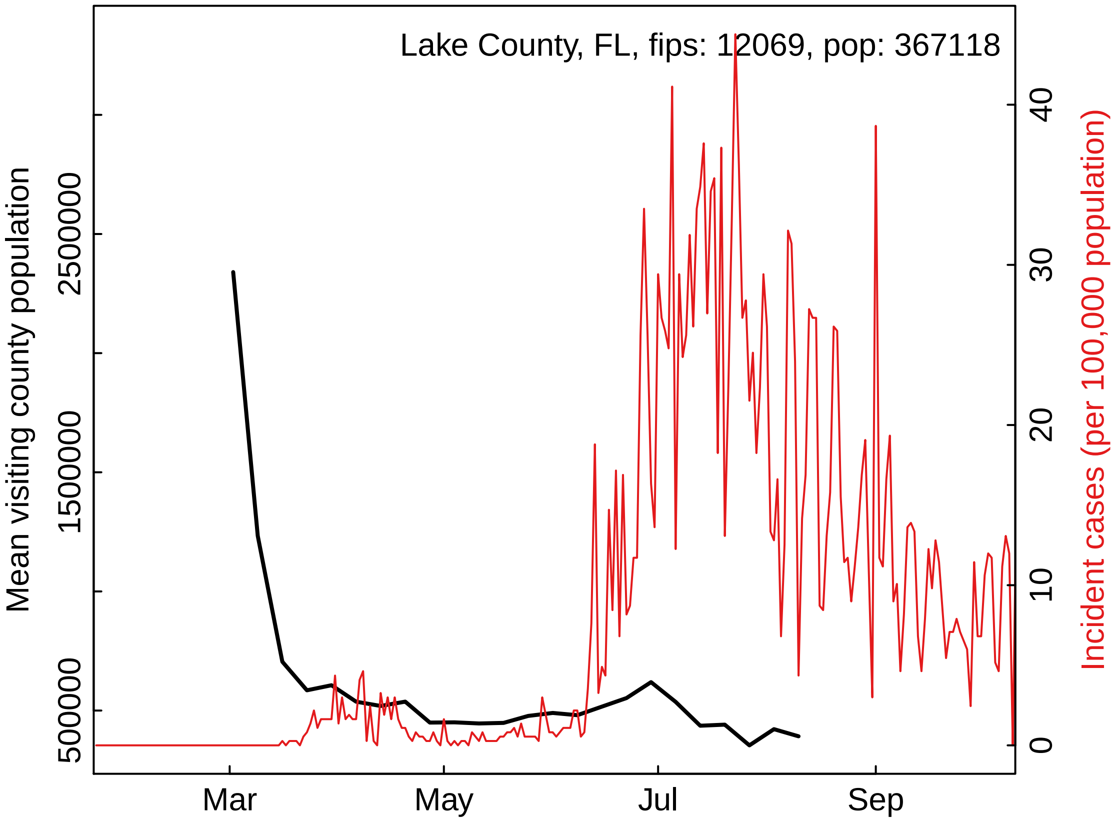

Lake County, FL, fips: 12069, pop: 367118
Mean visiting county population
500000
1500000
2500000
Incident cases (per 100,000 population)
0
10
20
30
40
Mar
May
Jul
Sep

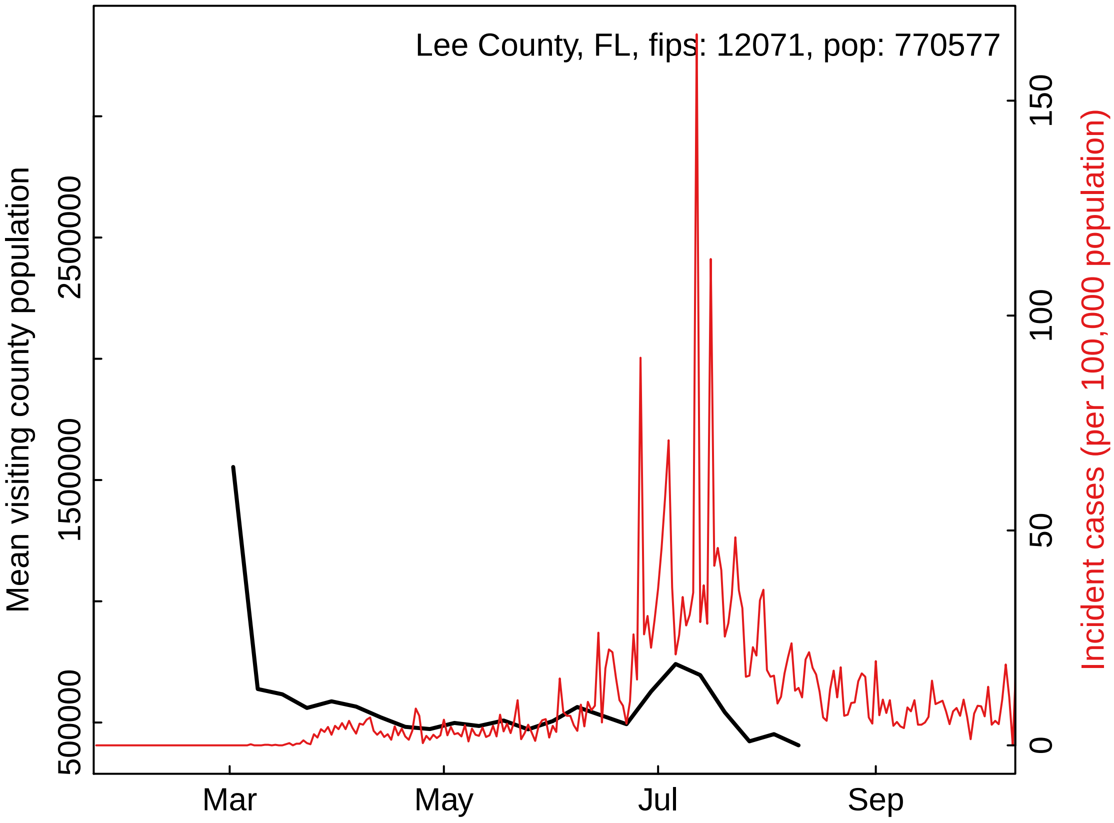

Lee County, FL, fips: 12071, pop: 770577
Mean visiting county population
500000
1500000
2500000
Mar
May
Jul
Sep
0
50
100
150
Incident cases (per 100,000 population)

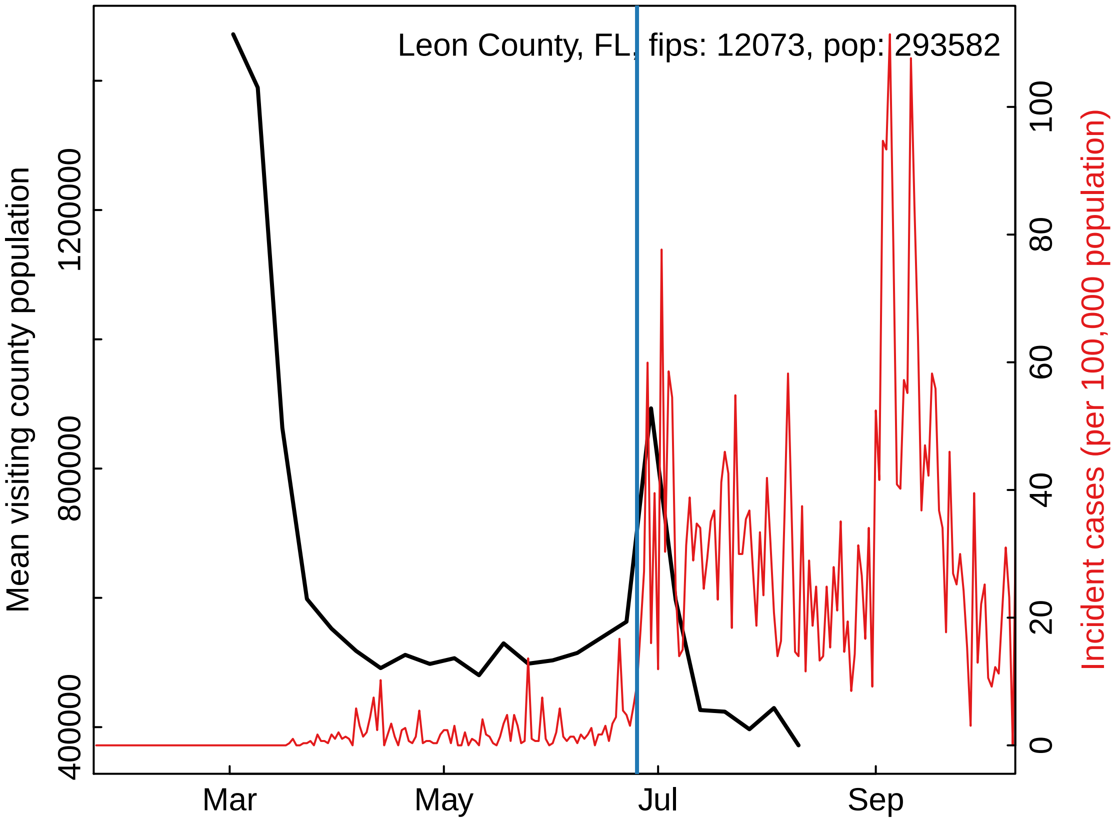

Leon County, FL, fips: 12073, pop: 293582
Mean visiting county population
400000
800000
1200000
Mar
May
Jul
Sep
0
20
40
60
80
100
Incident cases (per 100,000 population)

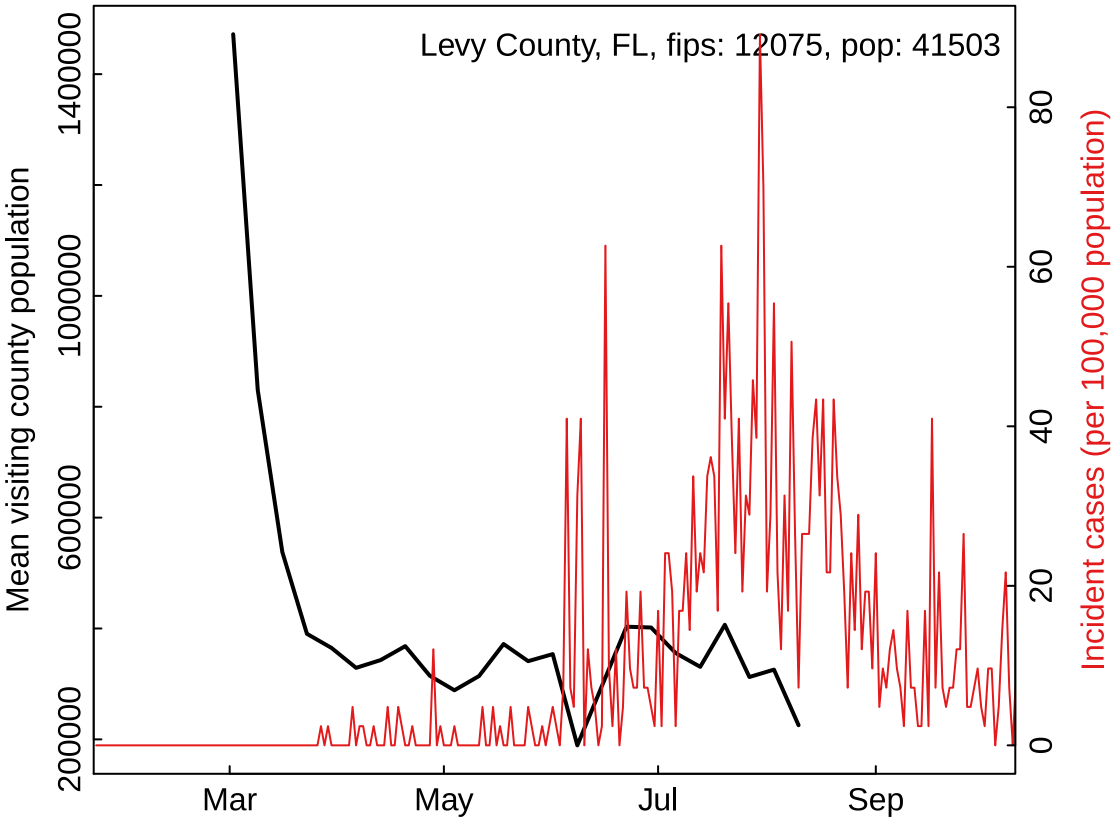

Levy County, FL, fips: 12075, pop: 41503
Mean visiting county population
200000
600000
1000000
1400000
Incident cases (per 100,000 population)
0
20
40
60
80
Mar
May
Jul
Sep

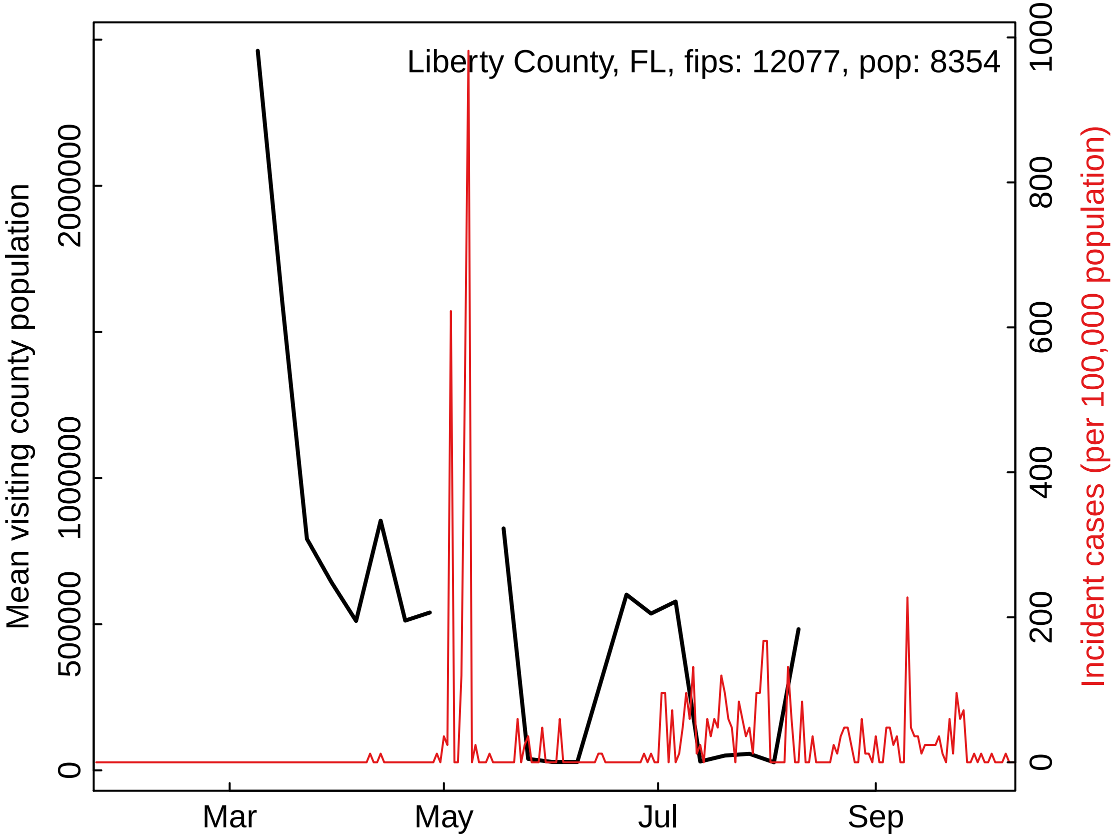

Liberty County, FL, fips: 12077, pop: 8354
Mean visiting county population
0
500000
1000000
2000000
Mar
May
Jul
Sep
0
200
400
600
800
1000
Incident cases (per 100,000 population)

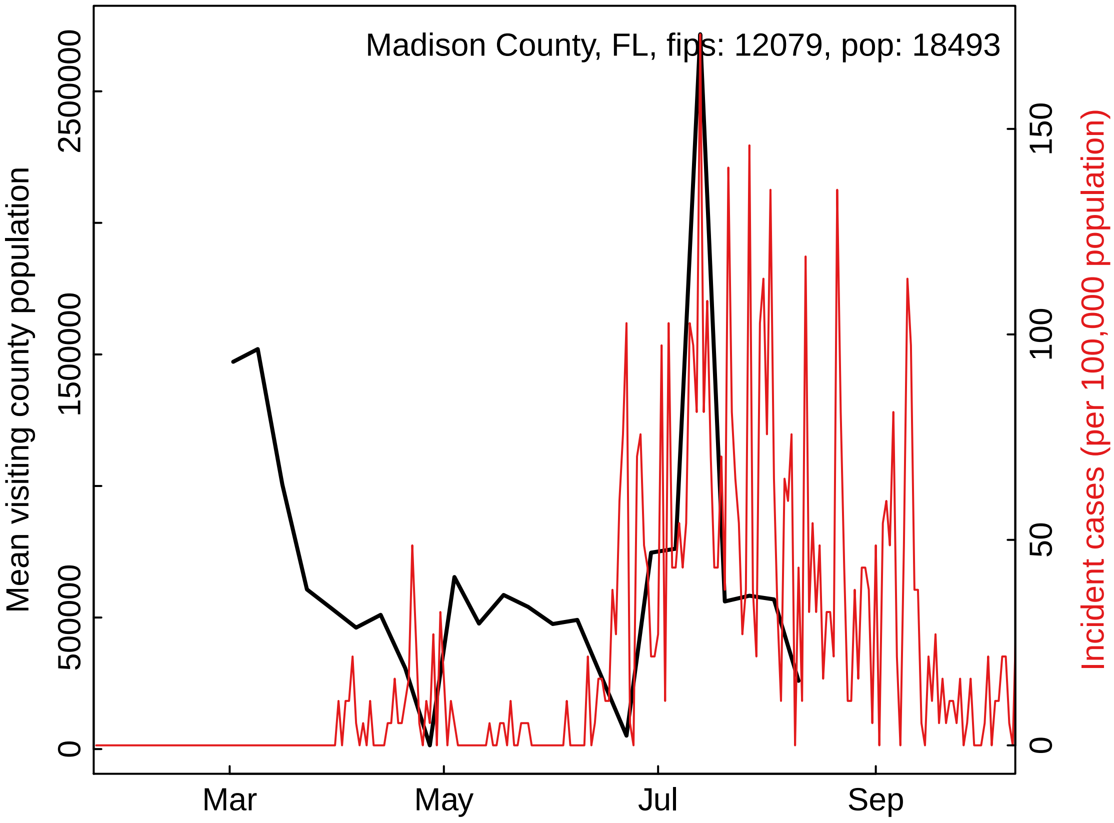

Madison County, FL, fips: 12079, pop: 18493
Mean visiting county population
0
500000
1500000
2500000
Incident cases (per 100,000 population)
0
50
100
150
Mar
May
Jul
Sep

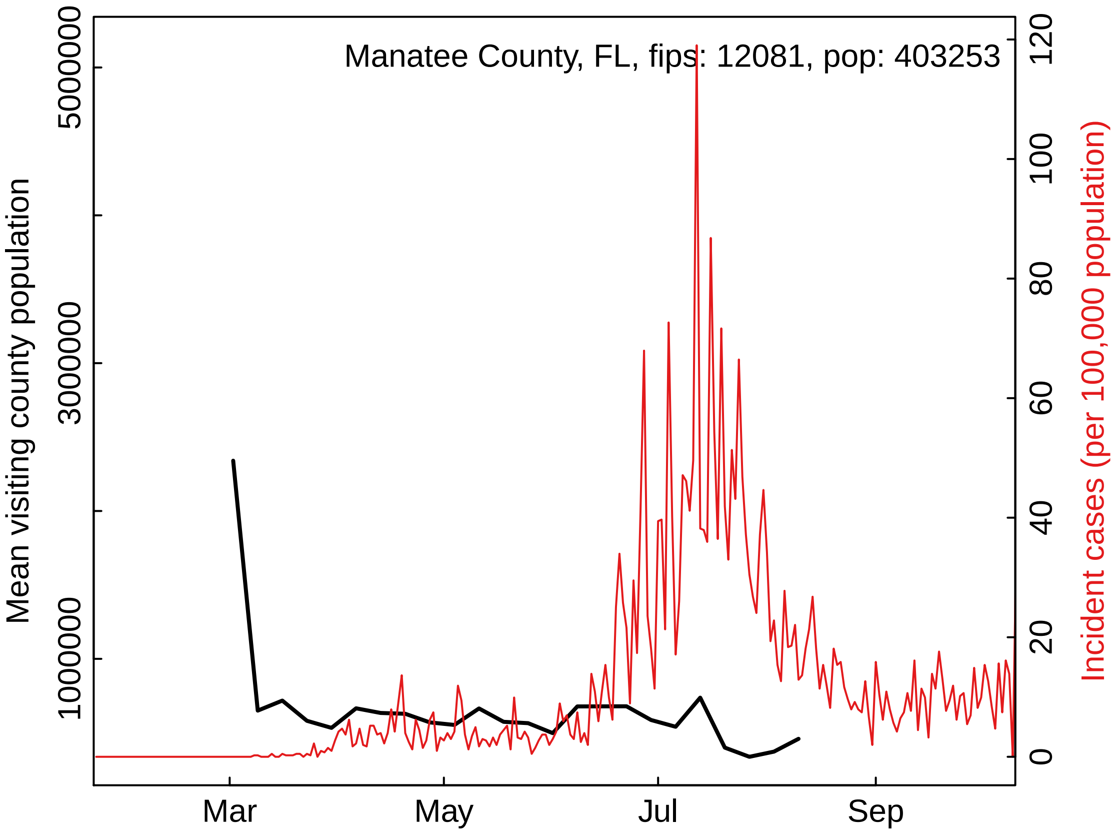

Manatee County, FL, fips: 12081, pop: 403253
Mean visiting county population
1000000
3000000
5000000
Incident cases (per 100,000 population)
0
20
40
60
80
100
120
Mar
May
Jul
Sep

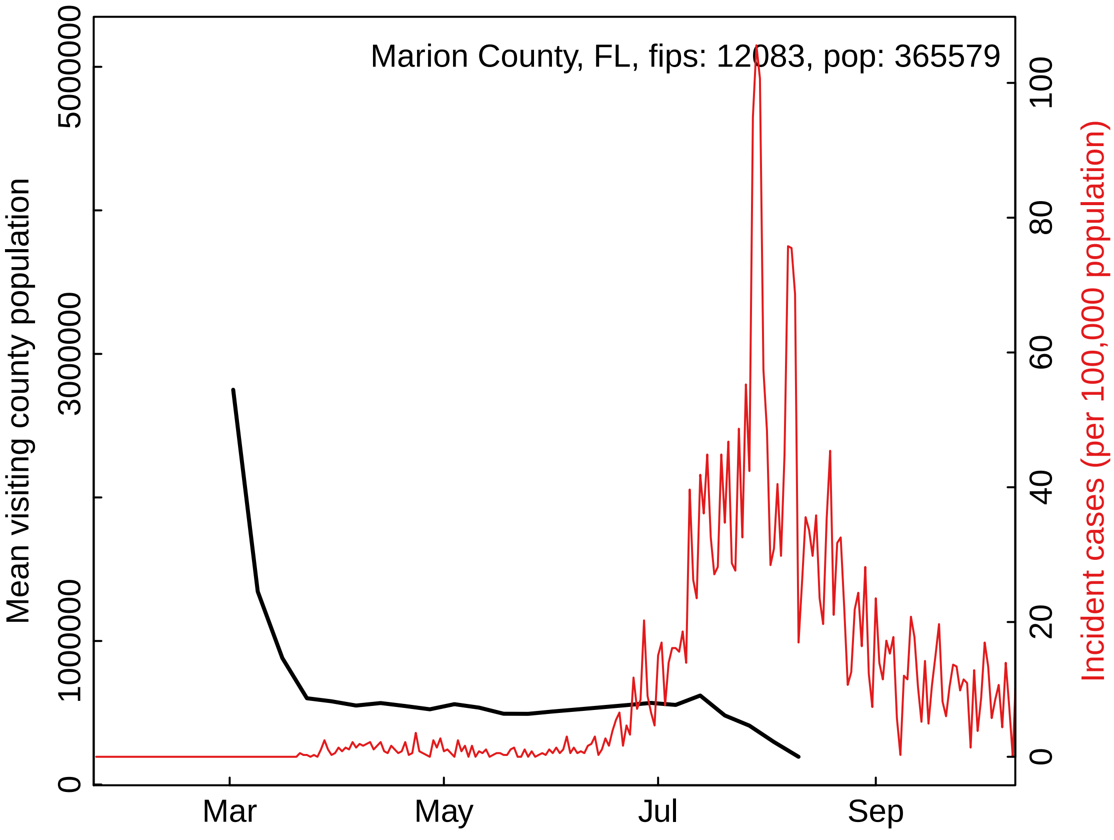

Marion County, FL, fips: 12083, pop: 365579
Mean visiting county population
0
1000000
3000000
5000000
Incident cases (per 100,000 population)
0
20
40
60
80
100
Mar
May
Jul
Sep

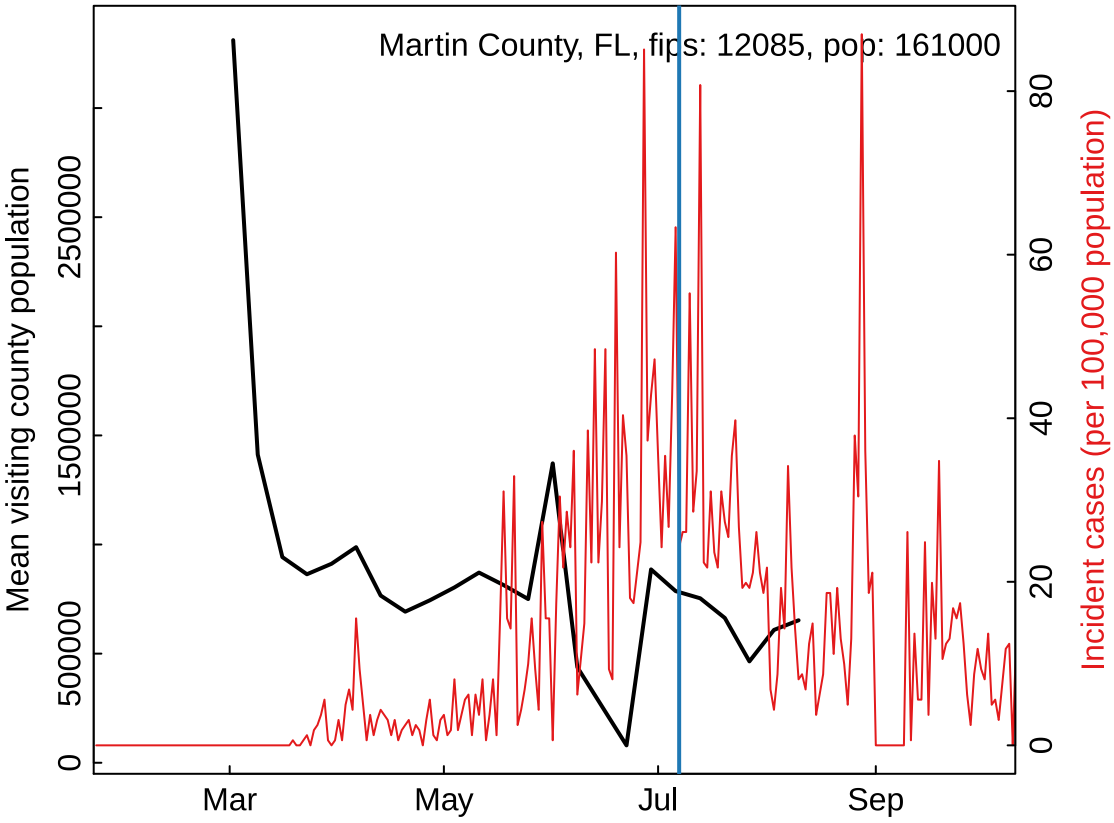

Martin County, FL, fips: 12085, pop: 161000
Mean visiting county population
0
500000
1500000
2500000
Incident cases (per 100,000 population)
0
20
40
60
80
Mar
May
Jul
Sep

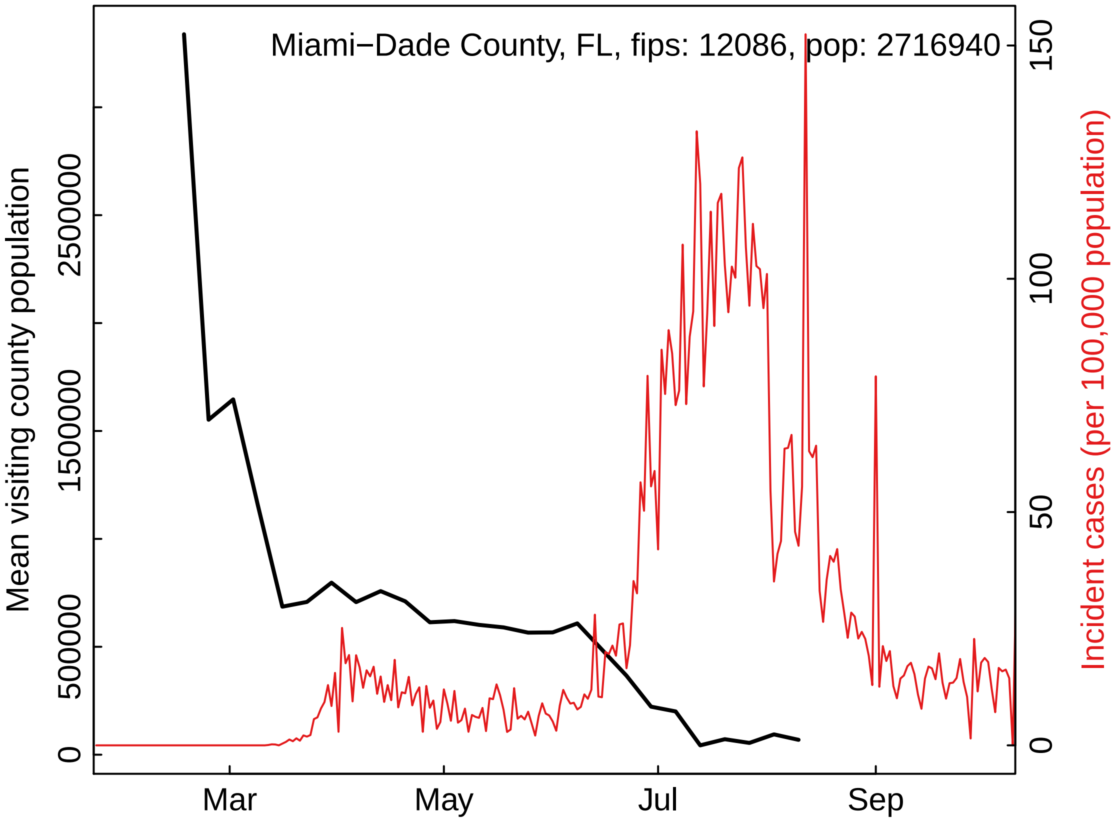

Miami−Dade County, FL, fips: 12086, pop: 2716940
Mean visiting county population
0
500000
1500000
2500000
Incident cases (per 100,000 population)
0
50
100
150
Mar
May
Jul
Sep

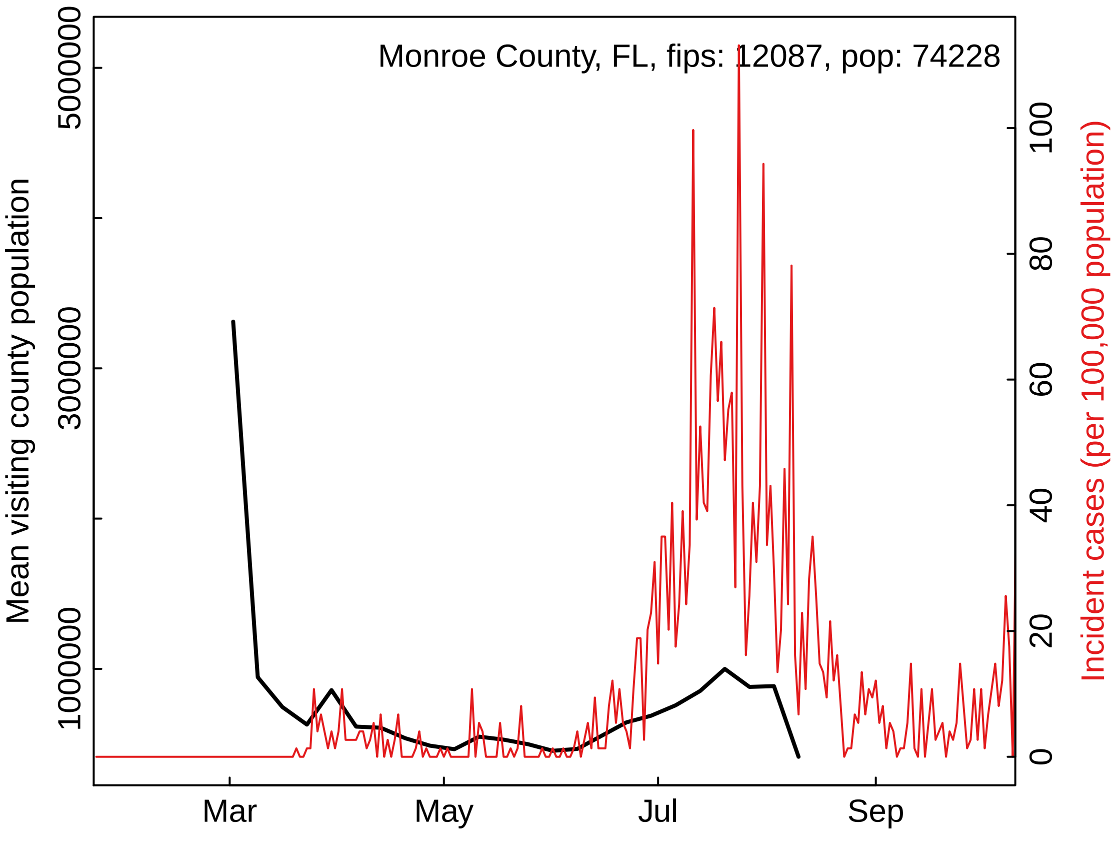

Monroe County, FL, fips: 12087, pop: 74228
Mean visiting county population
1000000
3000000
5000000
Mar
May
Jul
Sep
0
20
40
60
80
100
Incident cases (per 100,000 population)

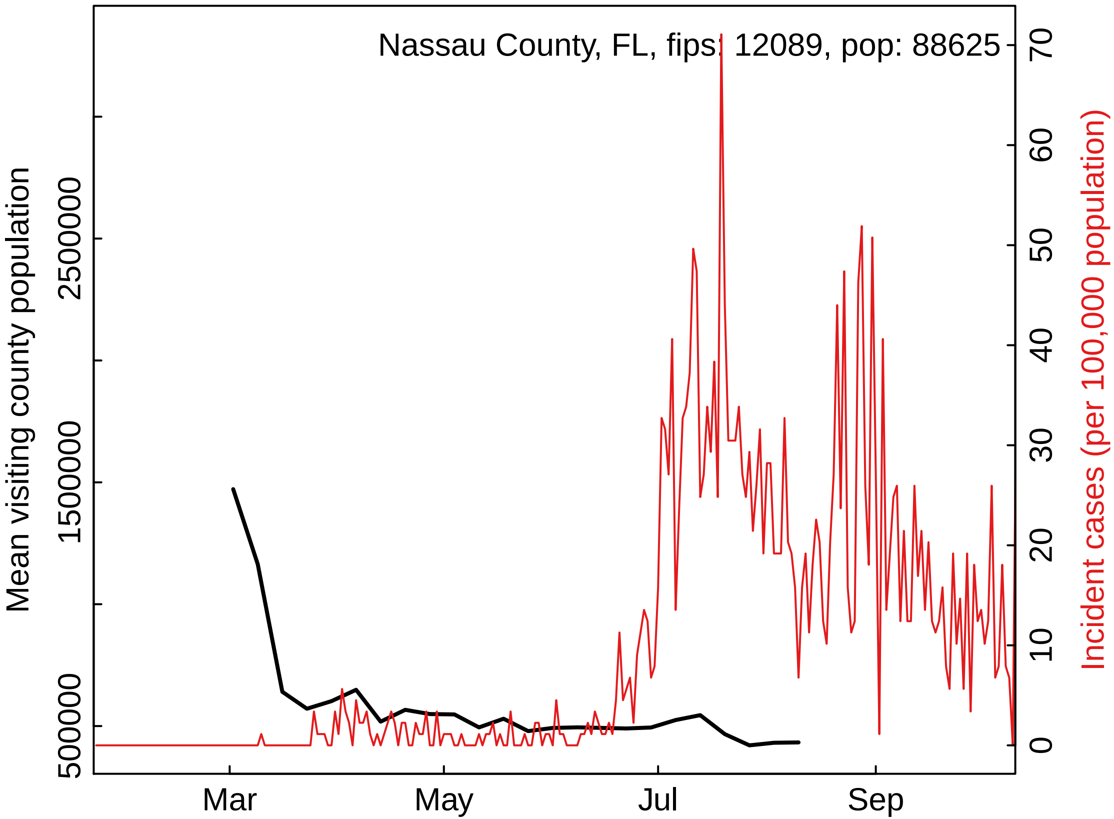

Nassau County, FL, fips: 12089, pop: 88625
Mean visiting county population
500000
1500000
2500000
Mar
May
Jul
Sep
0
10
20
30
40
50
60
70
Incident cases (per 100,000 population)

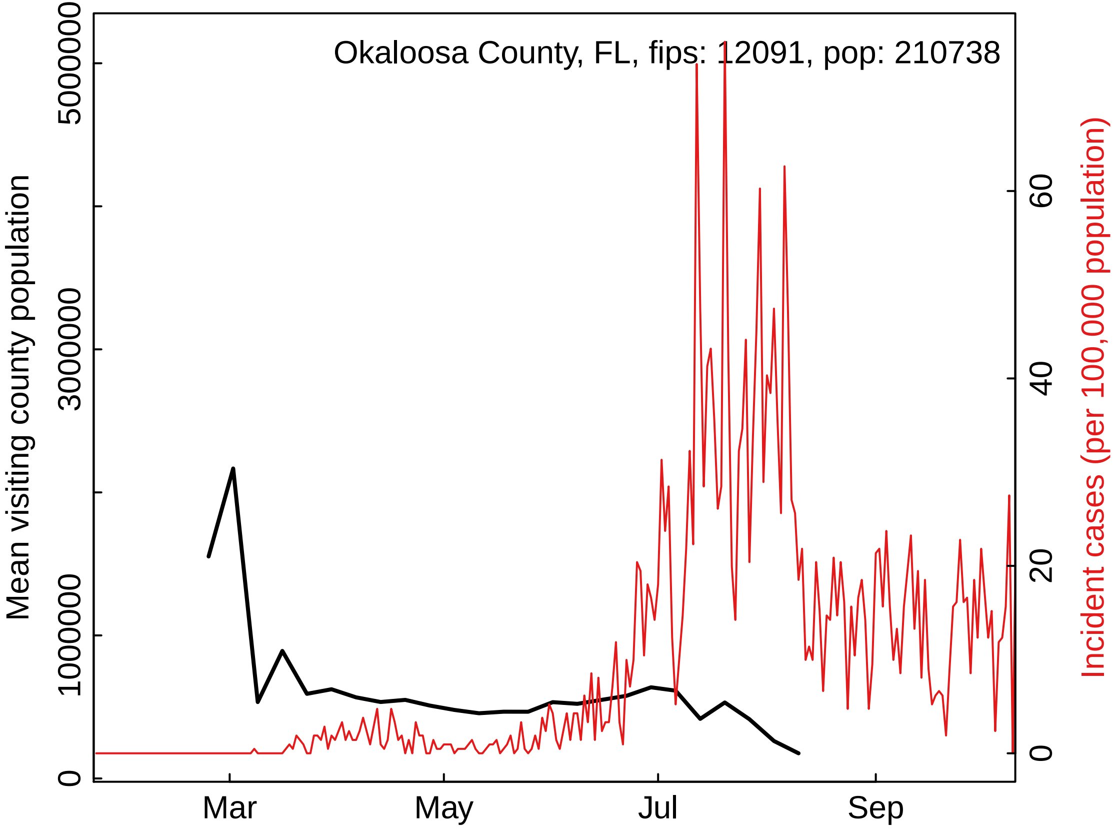

Okaloosa County, FL, fips: 12091, pop: 210738
Mean visiting county population
Incident cases (per 100,000 population)
0
1000000
3000000
5000000
0
20
40
60
Mar
May
Jul
Sep

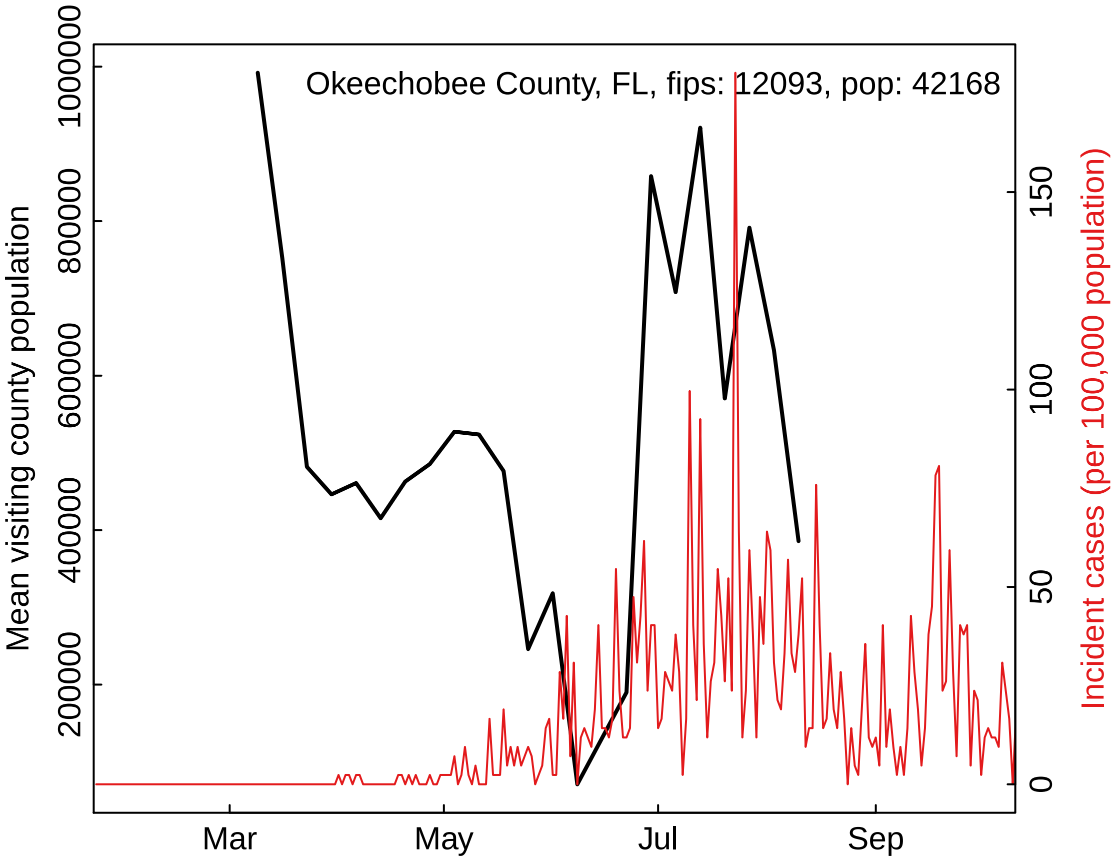

Okeechobee County, FL, fips: 12093, pop: 42168
Mean visiting county population
2000000
4000000
6000000
8000000
10000000
Incident cases (per 100,000 population)
0
50
100
150
Mar
May
Jul
Sep

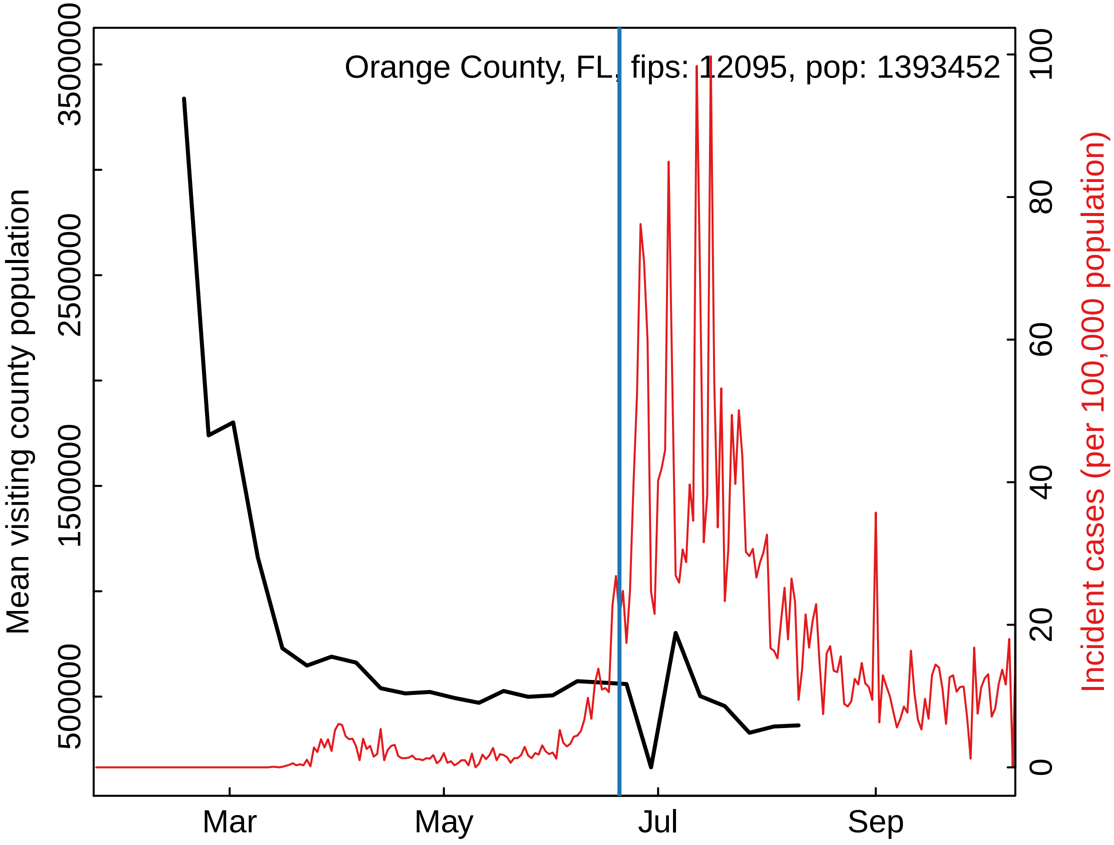

Orange County, FL, fips: 12095, pop: 1393452
Mean visiting county population
500000
1500000
2500000
3500000
Incident cases (per 100,000 population)
0
20
40
60
80
100
Mar
May
Jul
Sep

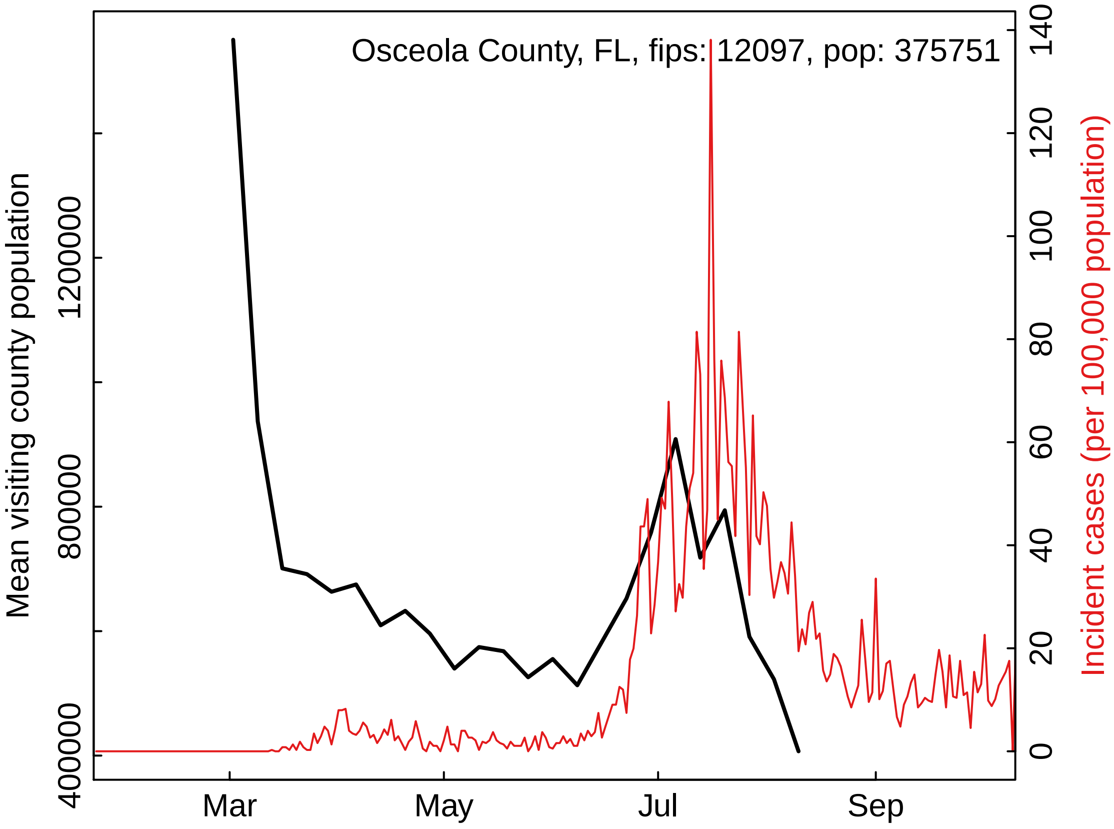

Osceola County, FL, fips: 12097, pop: 375751
Mean visiting county population
400000
800000
1200000
Incident cases (per 100,000 population)
0
20
40
60
80
100
120
140
Mar
May
Jul
Sep

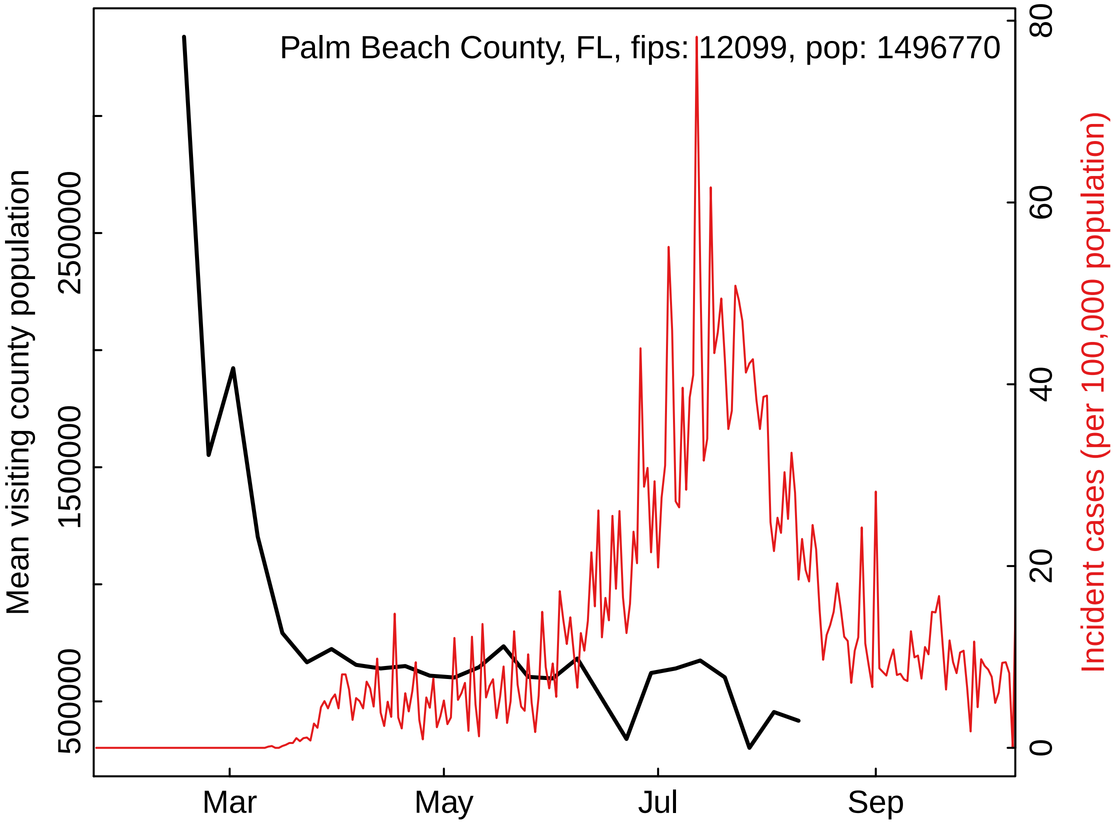

Palm Beach County, FL, fips: 12099, pop: 1496770
Mean visiting county population
500000
1500000
2500000
Incident cases (per 100,000 population)
0
20
40
60
80
Mar
May
Jul
Sep

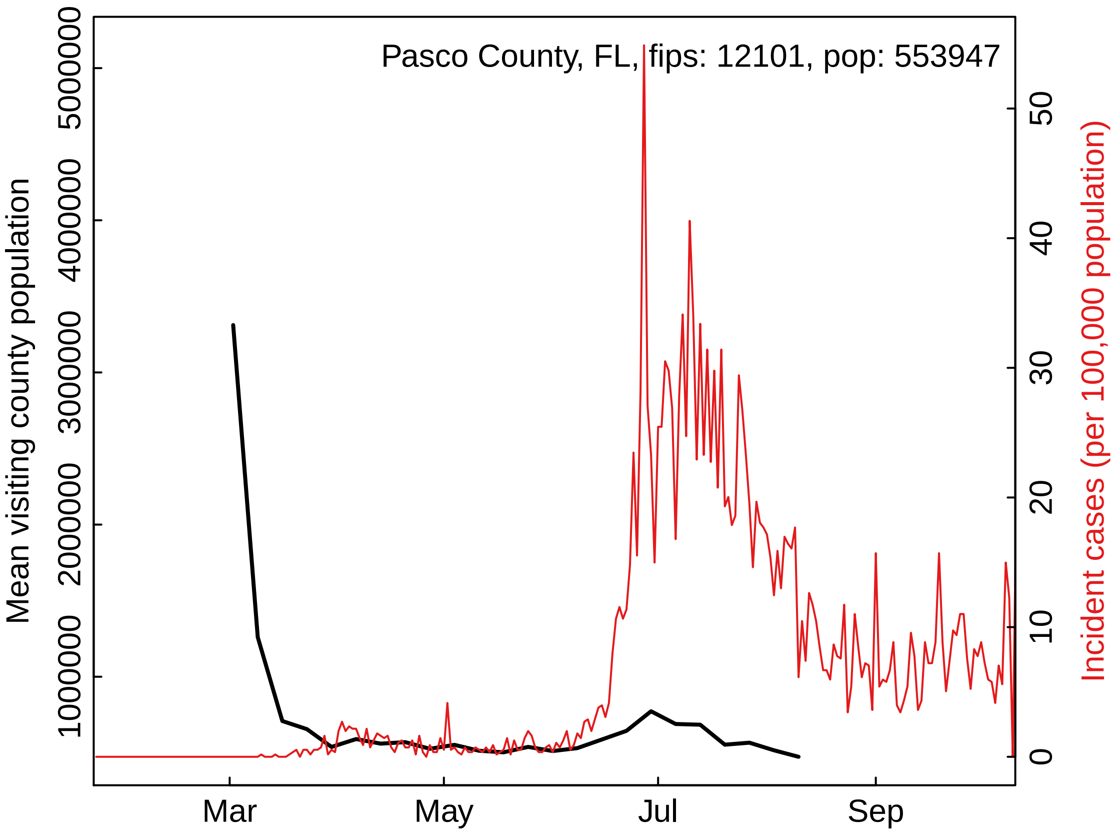

Pasco County, FL, fips: 12101, pop: 553947
Mean visiting county population
1000000
2000000
3000000
4000000
5000000
Incident cases (per 100,000 population)
0
10
20
30
40
50
Mar
May
Jul
Sep

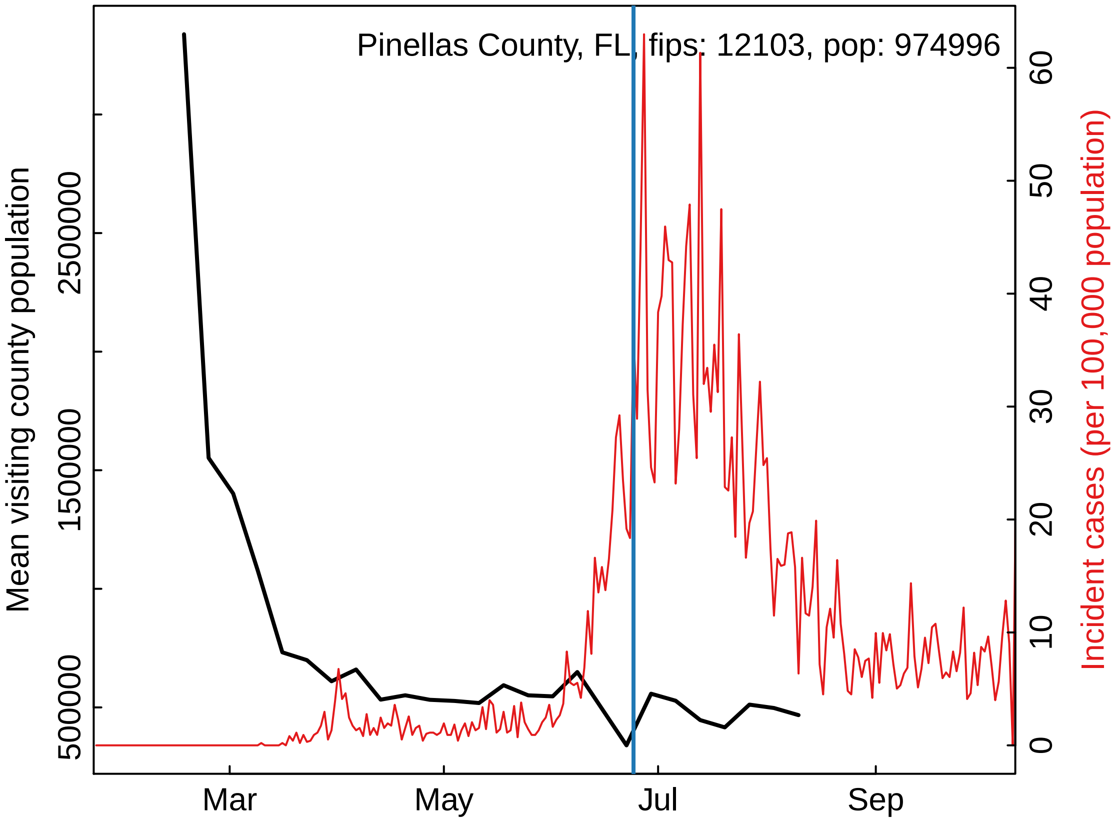
Pinellas County, FL, fips: 12103, pop: 974996
Mean visiting county population
500000
1500000
2500000
Incident cases (per 100,000 population)
0
10
20
30
40
50
60
Mar
May
Jul
Sep

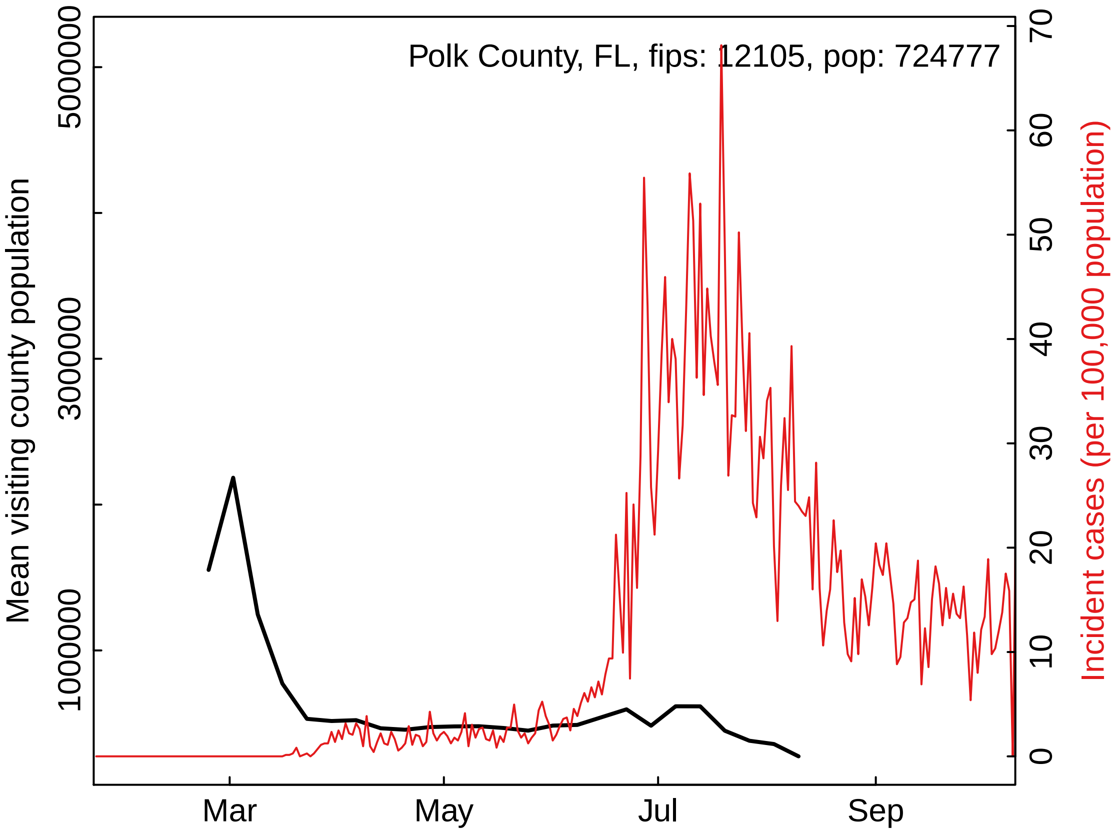
Polk County, FL, fips: 12105, pop: 724777
Mean visiting county population
1000000
3000000
5000000
Incident cases (per 100,000 population)
0
10
20
30
40
50
60
70
Mar
May
Jul
Sep

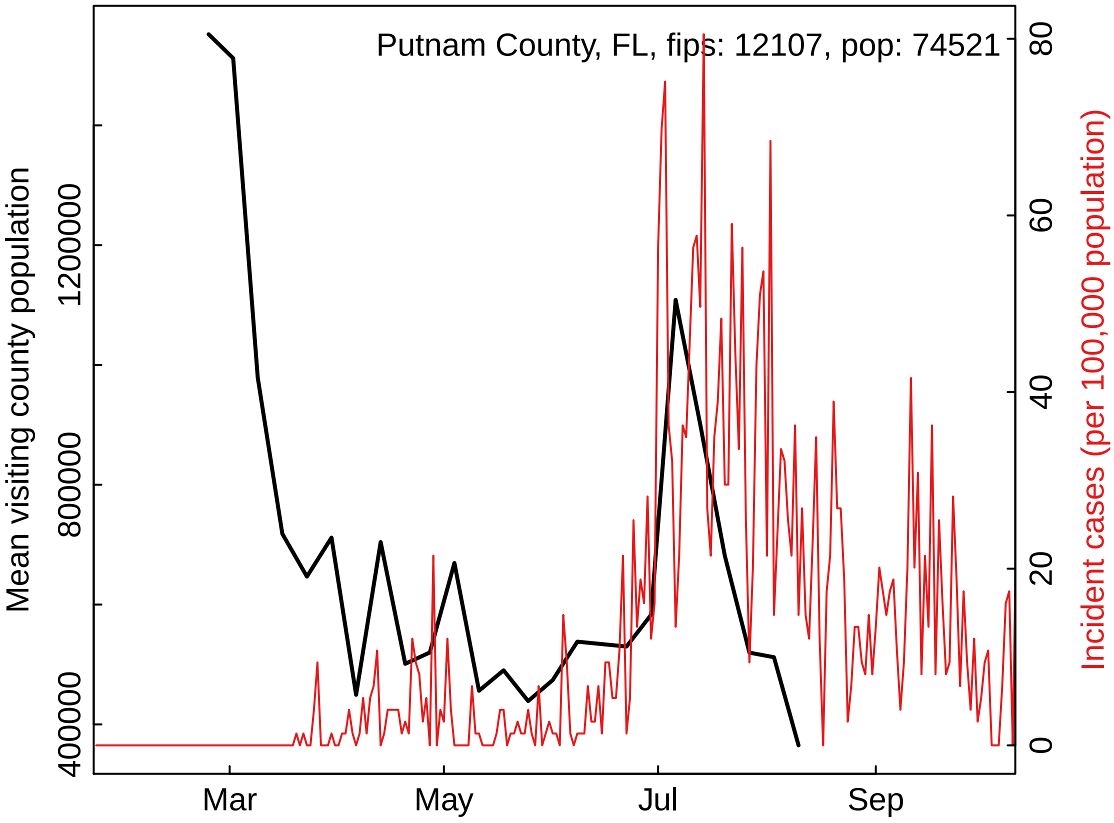
Putnam County, FL, fips: 12107, pop: 74521
Mean visiting county population
400000
800000
1200000
Incident cases (per 100,000 population)
0
20
40
60
80
Mar
May
Jul
Sep

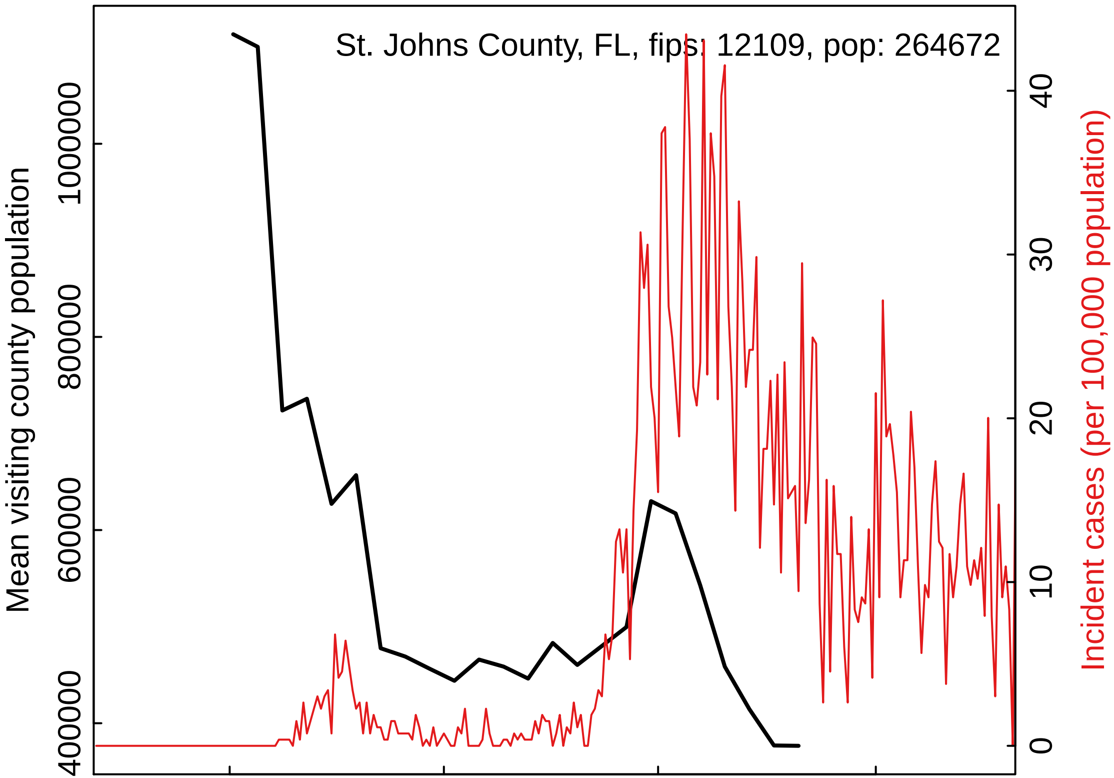
St. Johns County, FL, fips: 12109, pop: 264672
Mean visiting county population
4000000
6000000
8000000
10000000
Incident cases (per 100,000 population)
0
10
20
30
40
Mar
May
Jul
Sep

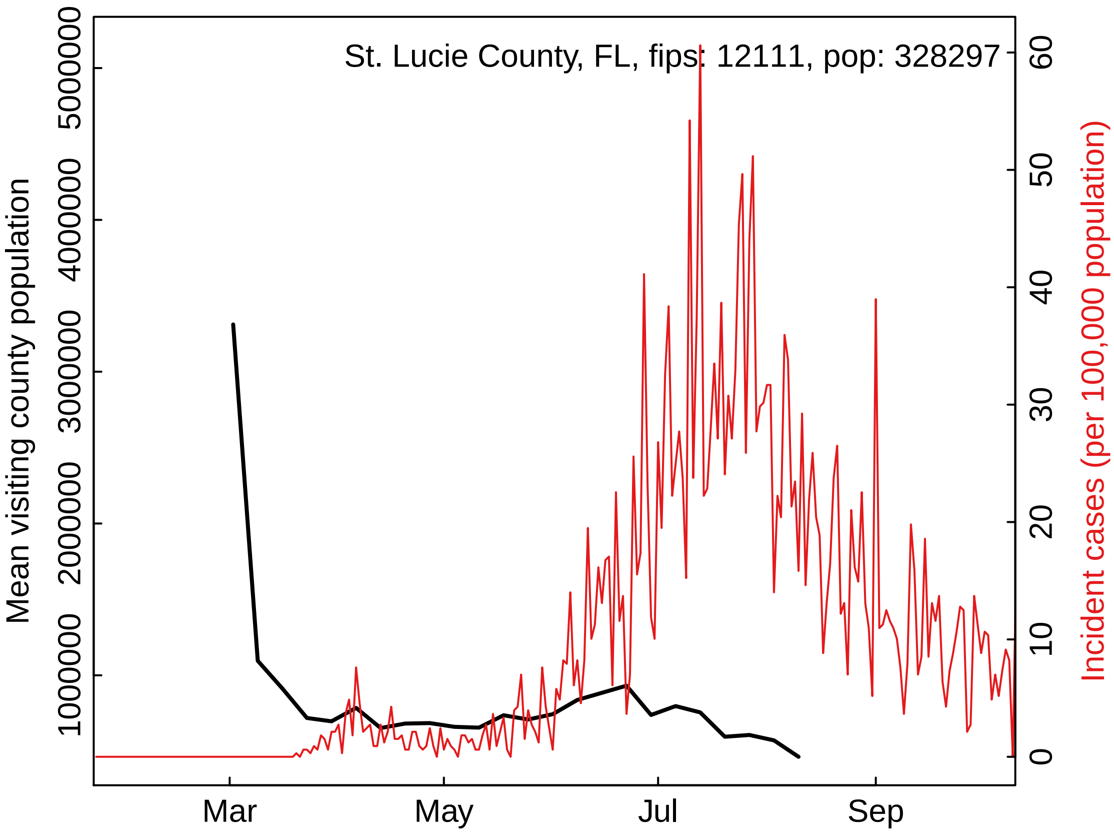
St. Lucie County, FL, fips: 12111, pop: 328297
Mean visiting county population
1000000
2000000
3000000
4000000
5000000
Incident cases (per 100,000 population)
0
10
20
30
40
50
60
Mar
May
Jul
Sep

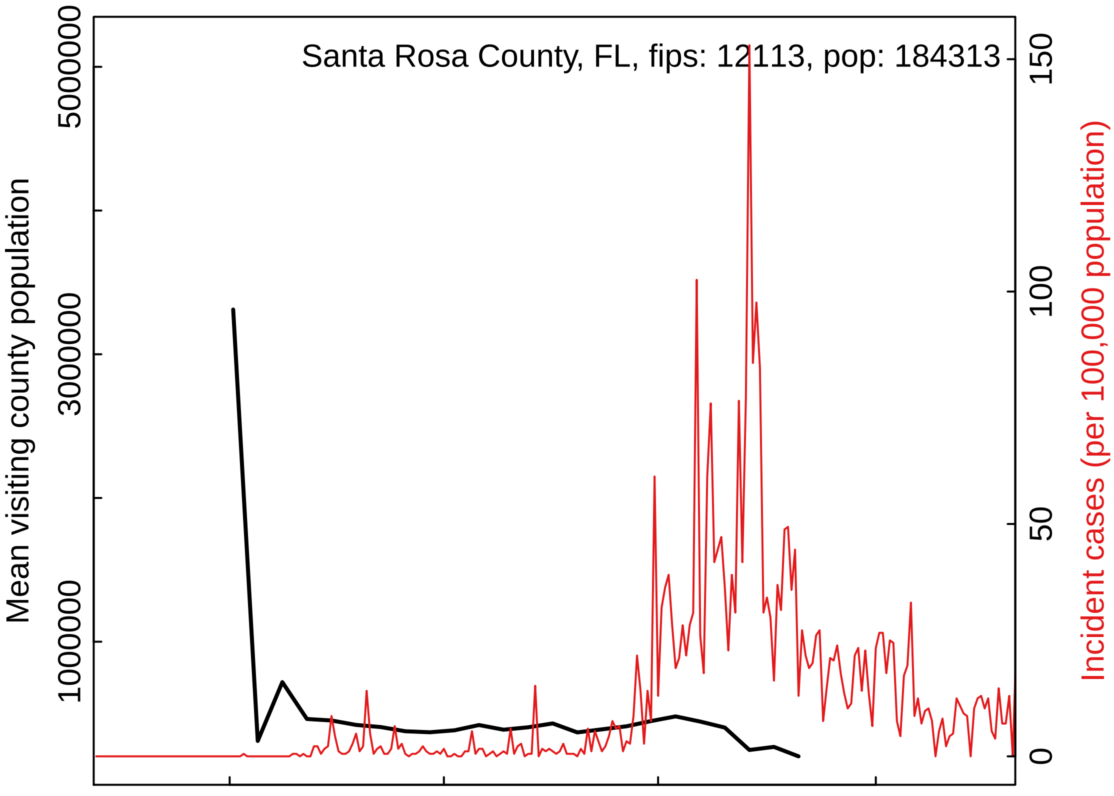

Santa Rosa County, FL, fips: 12113, pop: 184313
Mean visiting county population
1000000
3000000
5000000
Mar
May
Jul
Sep
Incident cases (per 100,000 population)
0
50
100
150

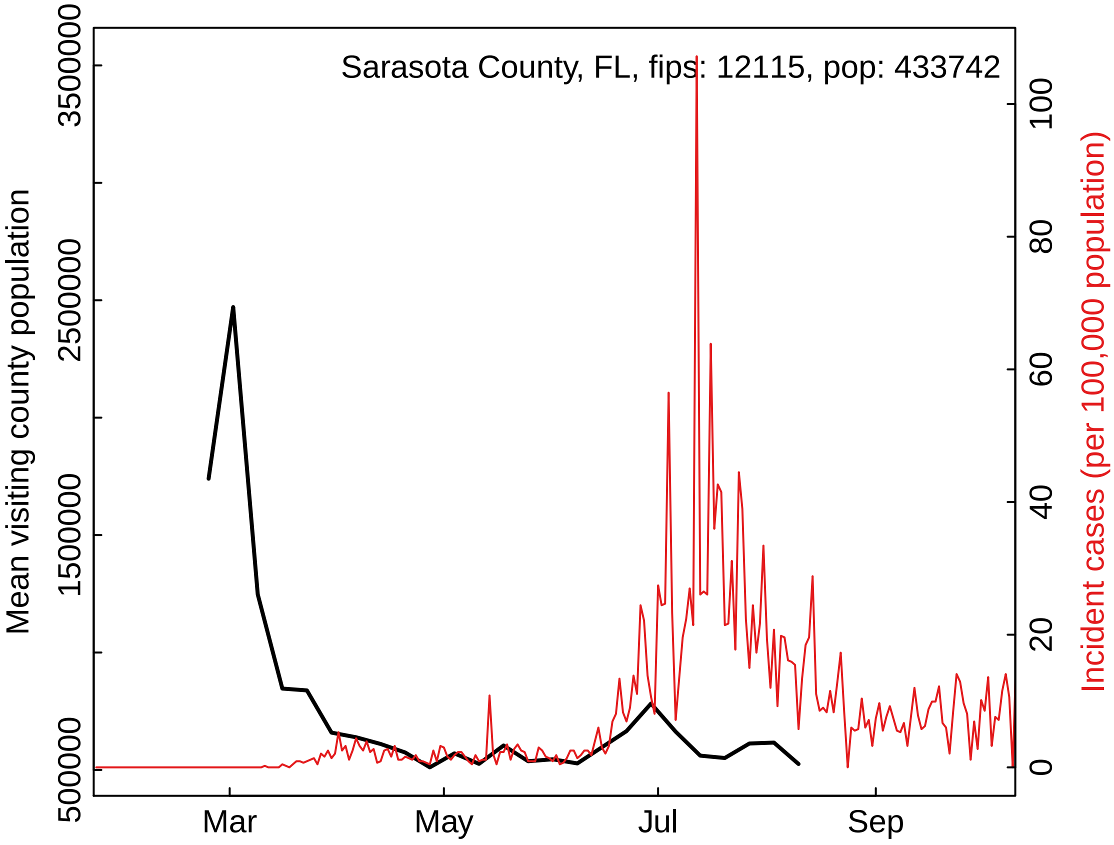

Sarasota County, FL, fips: 12115, pop: 433742
Mean visiting county population
Incident cases (per 100,000 population)
500000
1500000
2500000
3500000
0
20
40
60
80
100
Mar
May
Jul
Sep

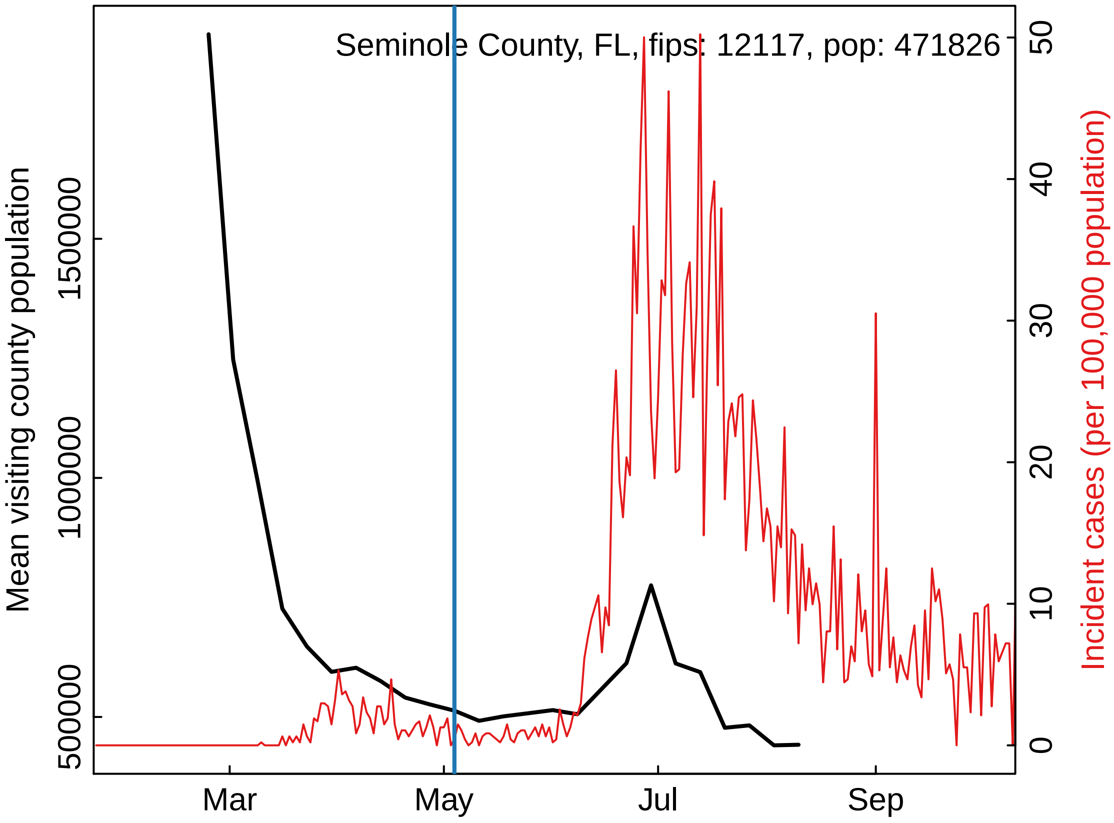

Seminole County, FL, fips: 12117, pop: 471826
Mean visiting county population
500000
1000000
1500000
Incident cases (per 100,000 population)
0
10
20
30
40
50
Mar
May
Jul
Sep

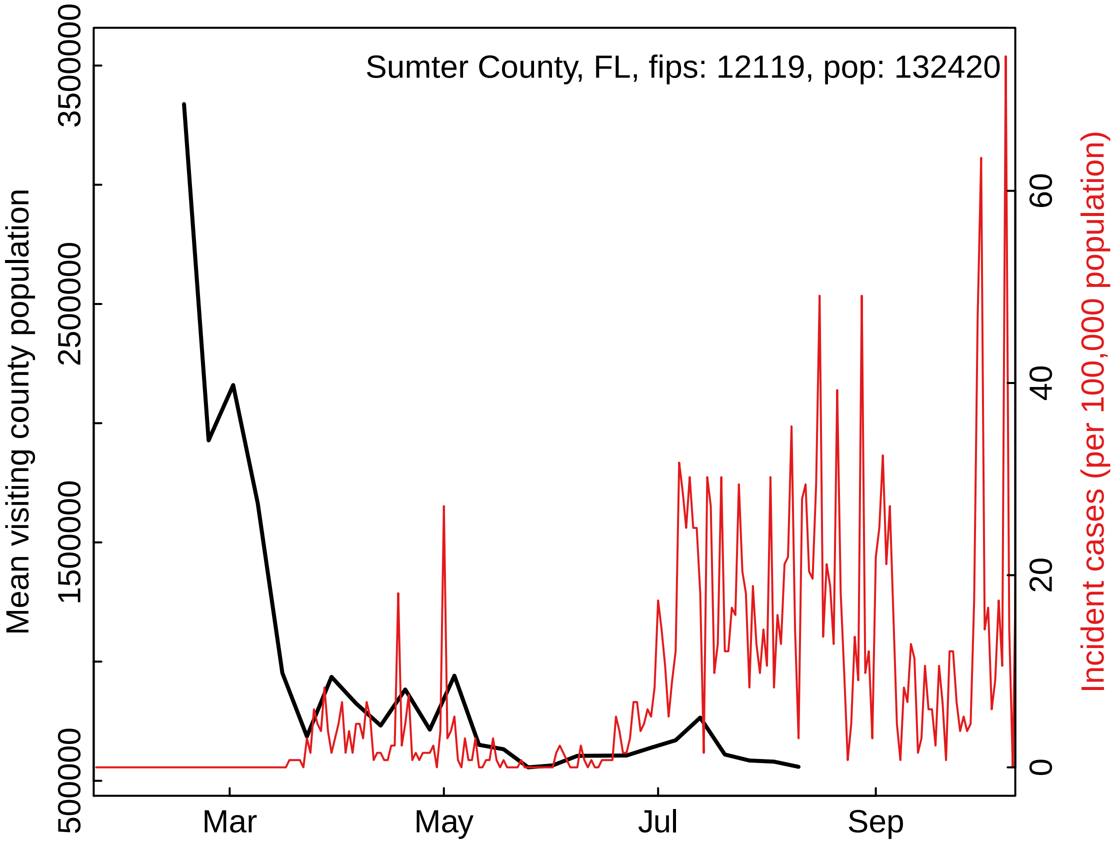

Sumter County, FL, fips: 12119, pop: 132420
Mean visiting county population
5000000
1500000
2500000
3500000
Mar
May
Jul
Sep
Incident cases (per 100,000 population)
0
20
40
60

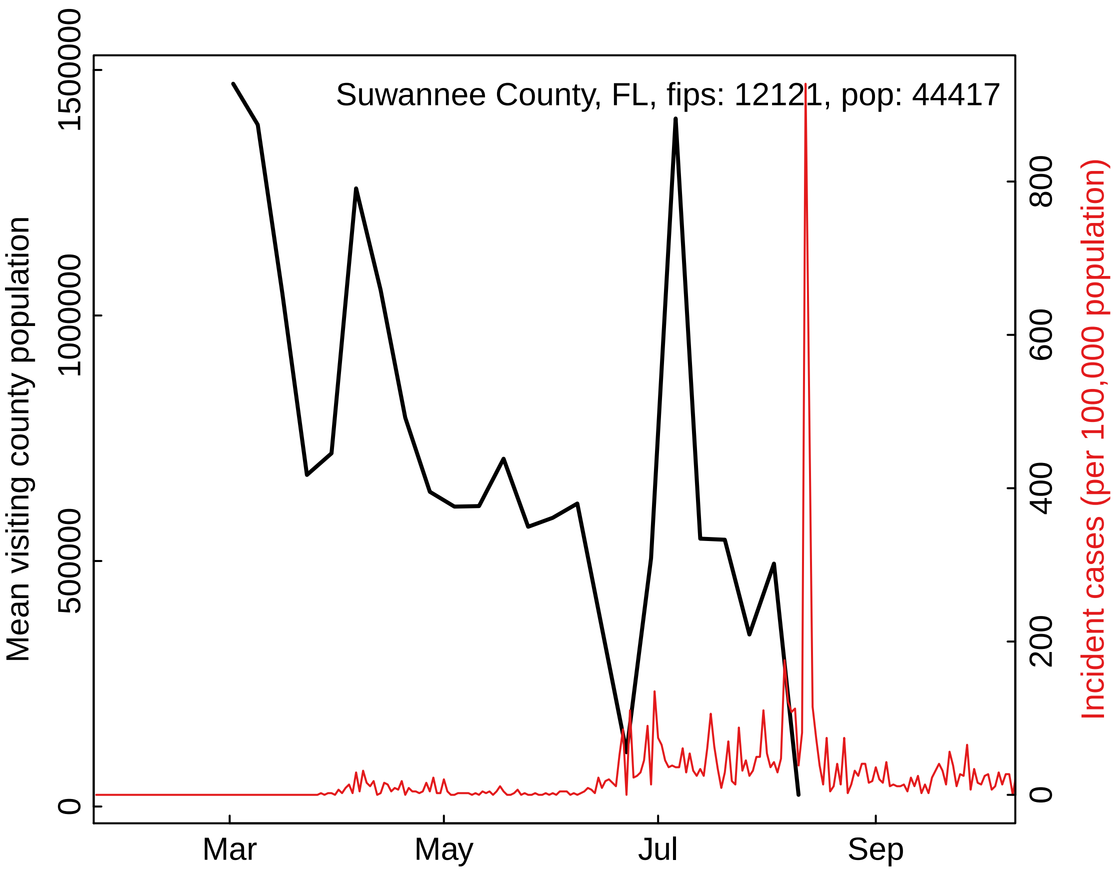

Suwannee County, FL, fips: 12121, pop: 44417
Mean visiting county population
0
500000
1000000
1500000
Incident cases (per 100,000 population)
0
200
400
600
800
Mar
May
Jul
Sep

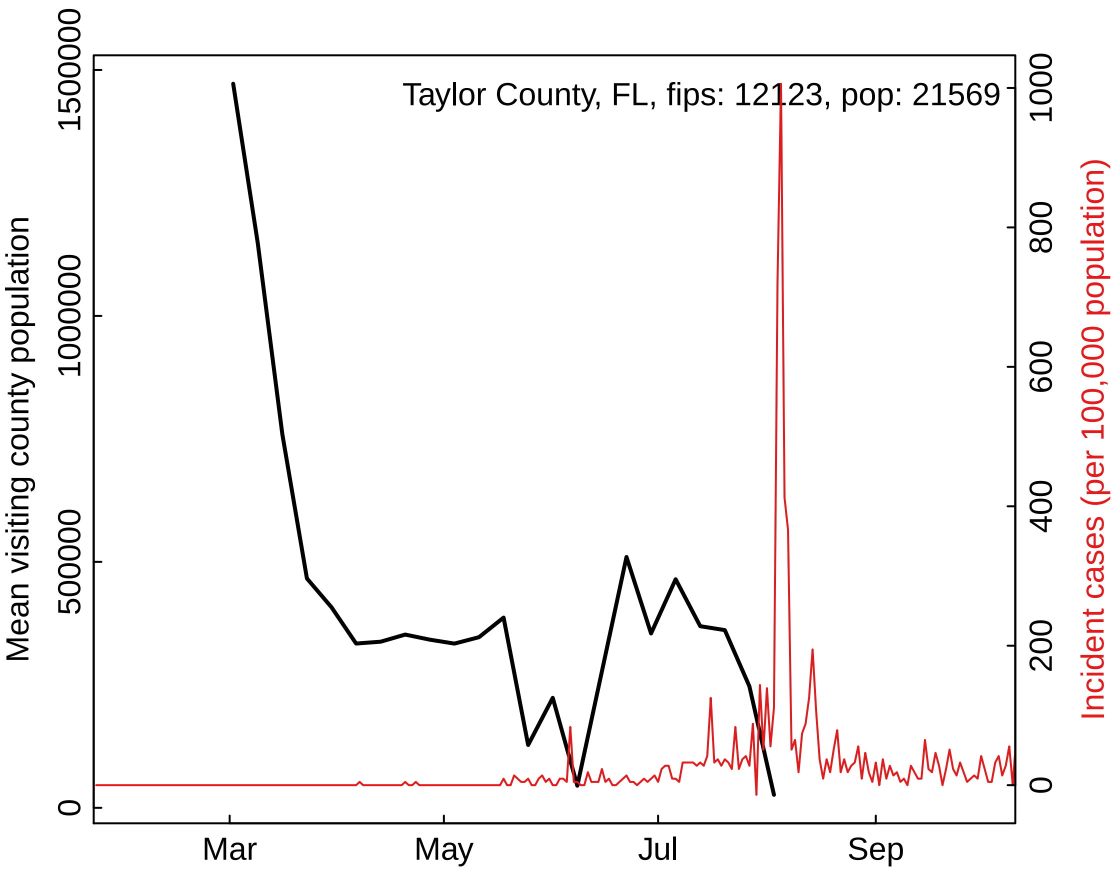
Taylor County, FL, fips: 12123, pop: 21569
Mean visiting county population
Incident cases (per 100,000 population)
0
500000
1000000
1500000
0
200
400
600
800
1000
Mar
May
Jul
Sep

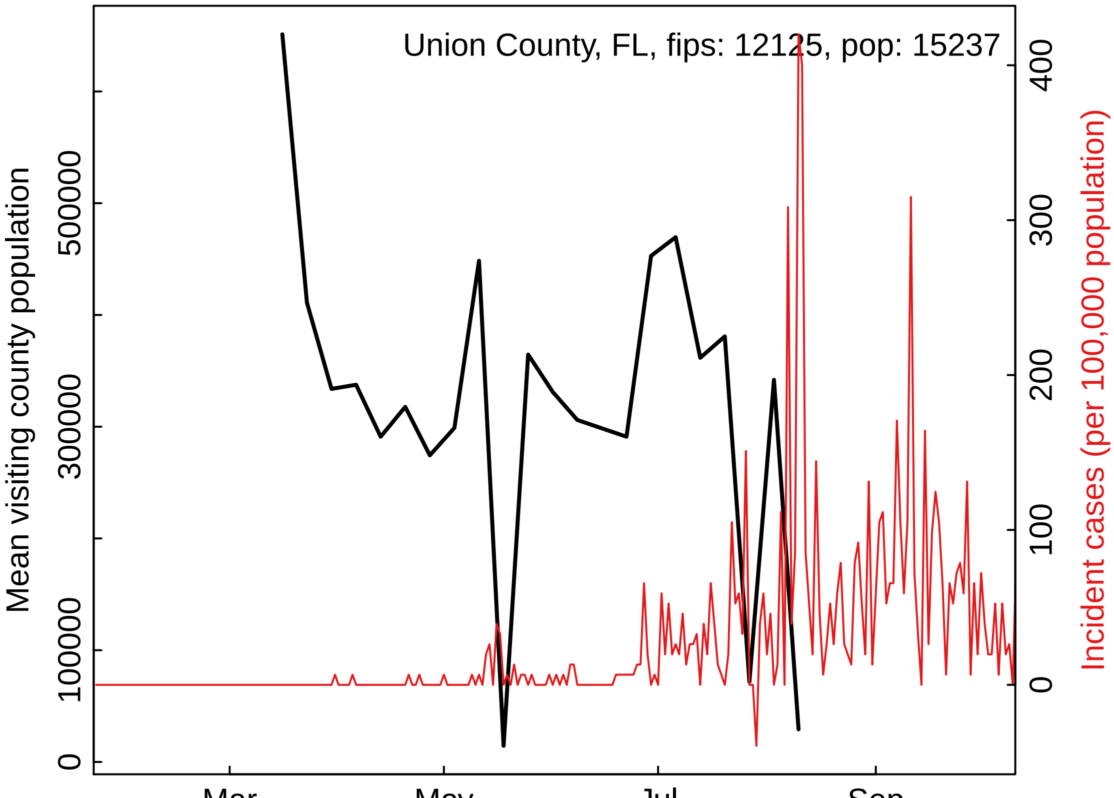
Union County, FL, fips: 12125, pop: 15237
Mean visiting county population
0
100000
300000
500000
Incident cases (per 100,000 population)
0
100
200
300
400
Mar
May
Jul
Sep

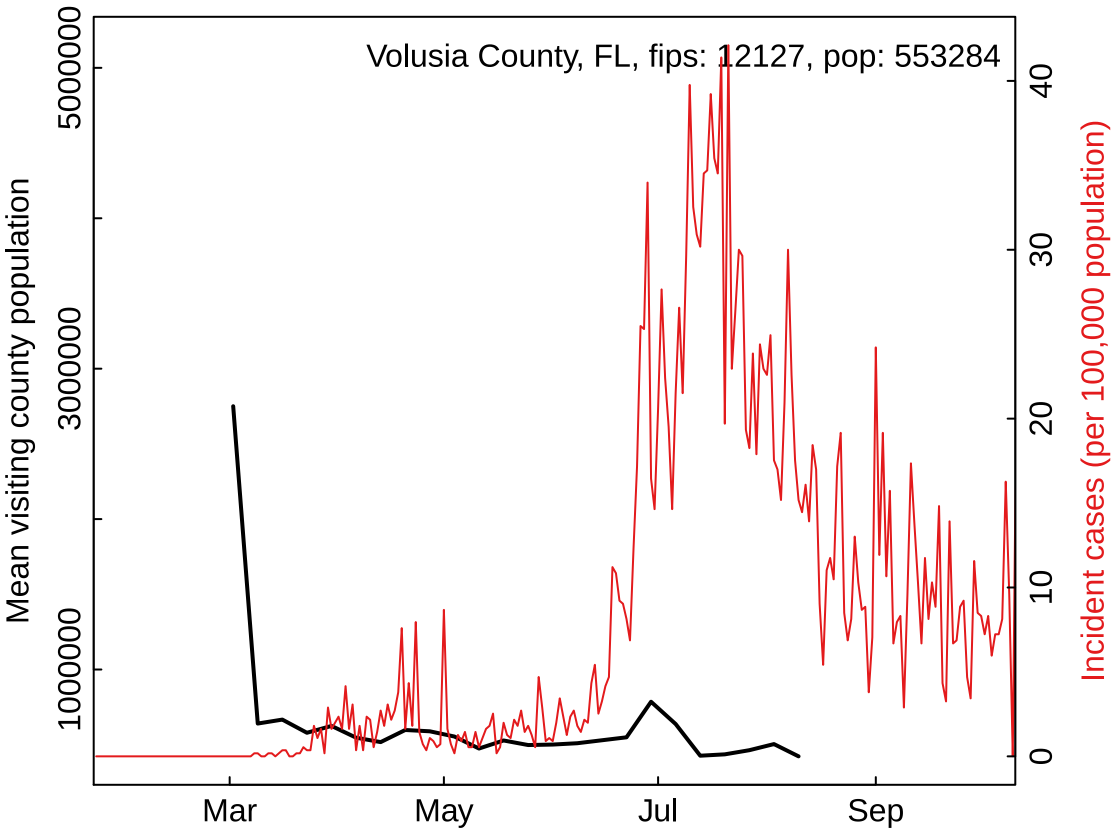

Volusia County, FL, fips: 12127, pop: 553284
Mean visiting county population
Incident cases (per 100,000 population)
1000000
3000000
5000000
0
10
20
30
40
Mar
May
Jul
Sep

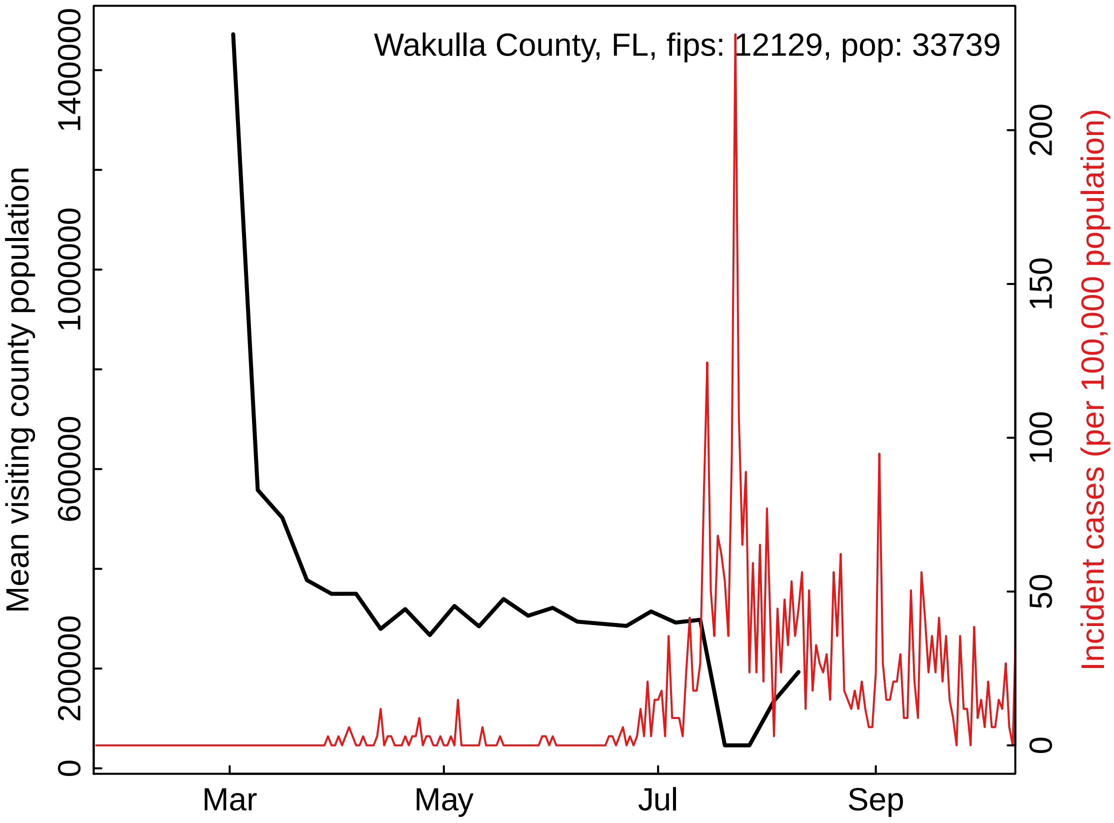
Wakulla County, FL, fips: 12129, pop: 33739
Mean visiting county population
0
200000
600000
1000000
1400000
Incident cases (per 100,000 population)
0
50
100
150
200
Mar
May
Jul
Sep

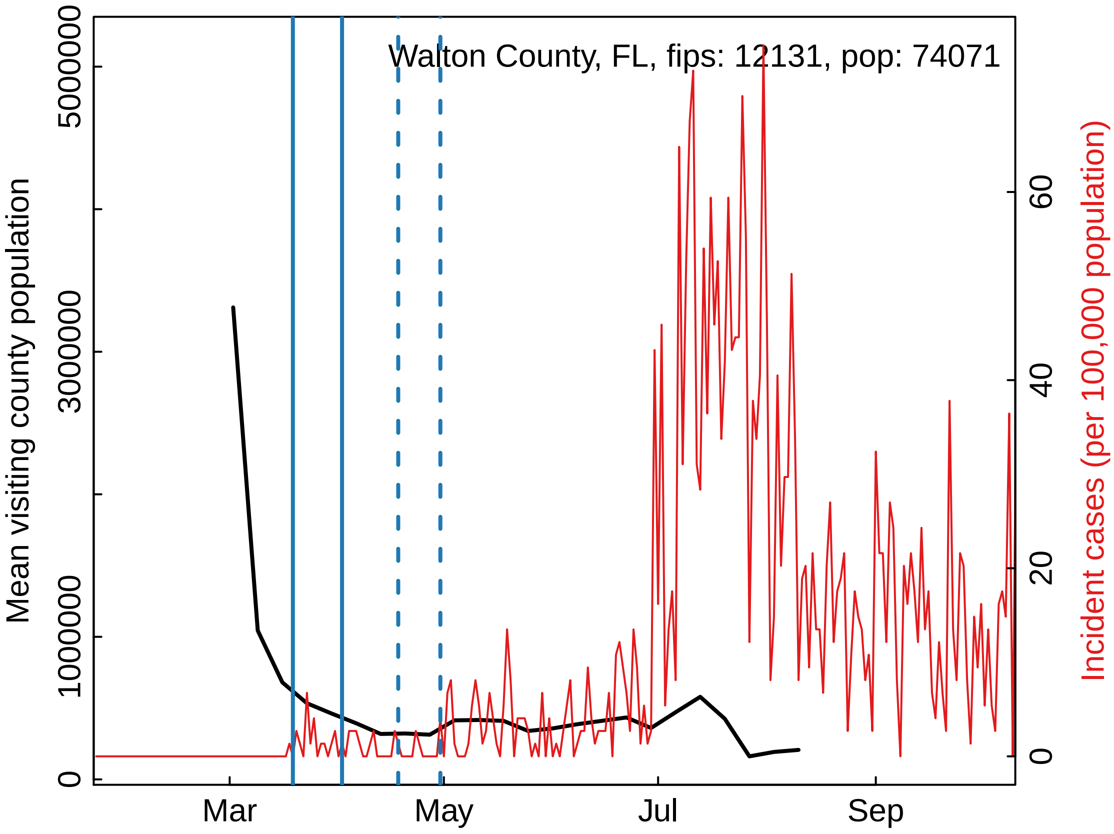

Walton County, FL, fips: 12131, pop: 74071
Mean visiting county population
0
1000000
3000000
5000000
Incident cases (per 100,000 population)
0
20
40
60
Mar
May
Jul
Sep

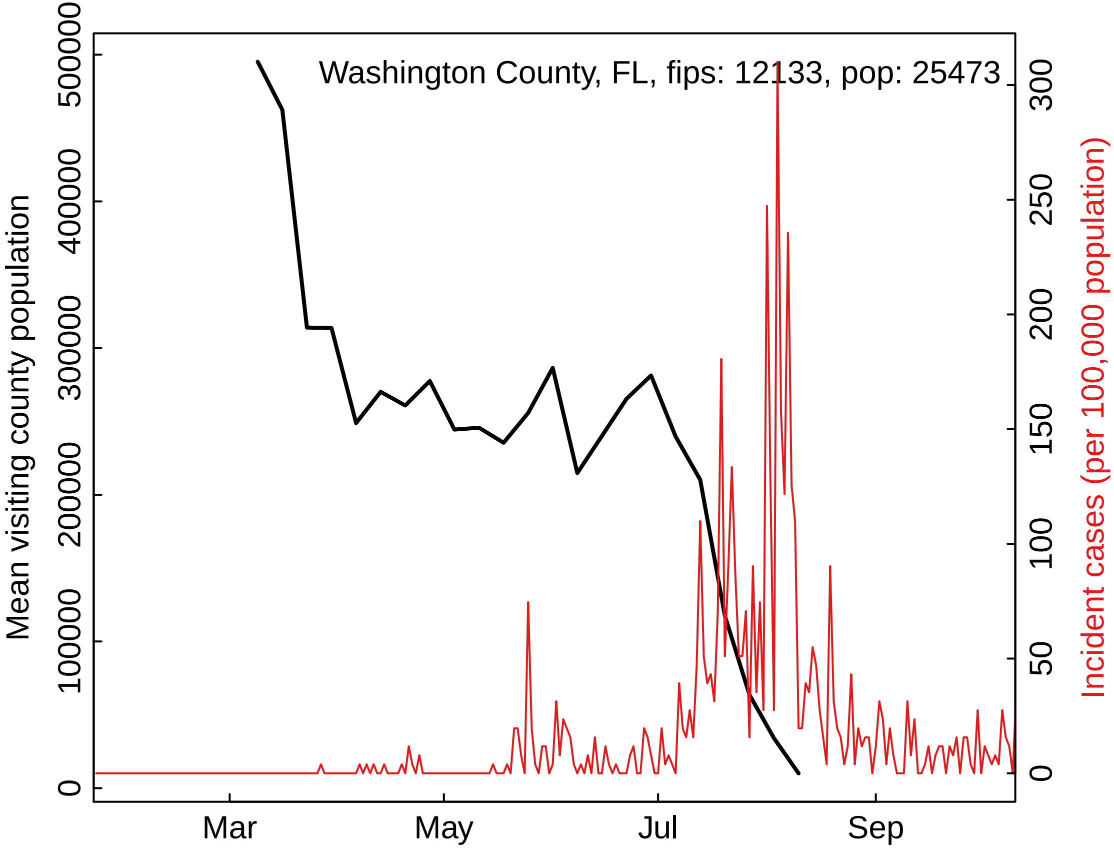

Washington County, FL, fips: 12133, pop: 25473
Mean visiting county population
Incident cases (per 100,000 population)
0
100000
200000
300000
400000
500000
0
50
100
150
200
250
300
Mar
May
Jul
Sep

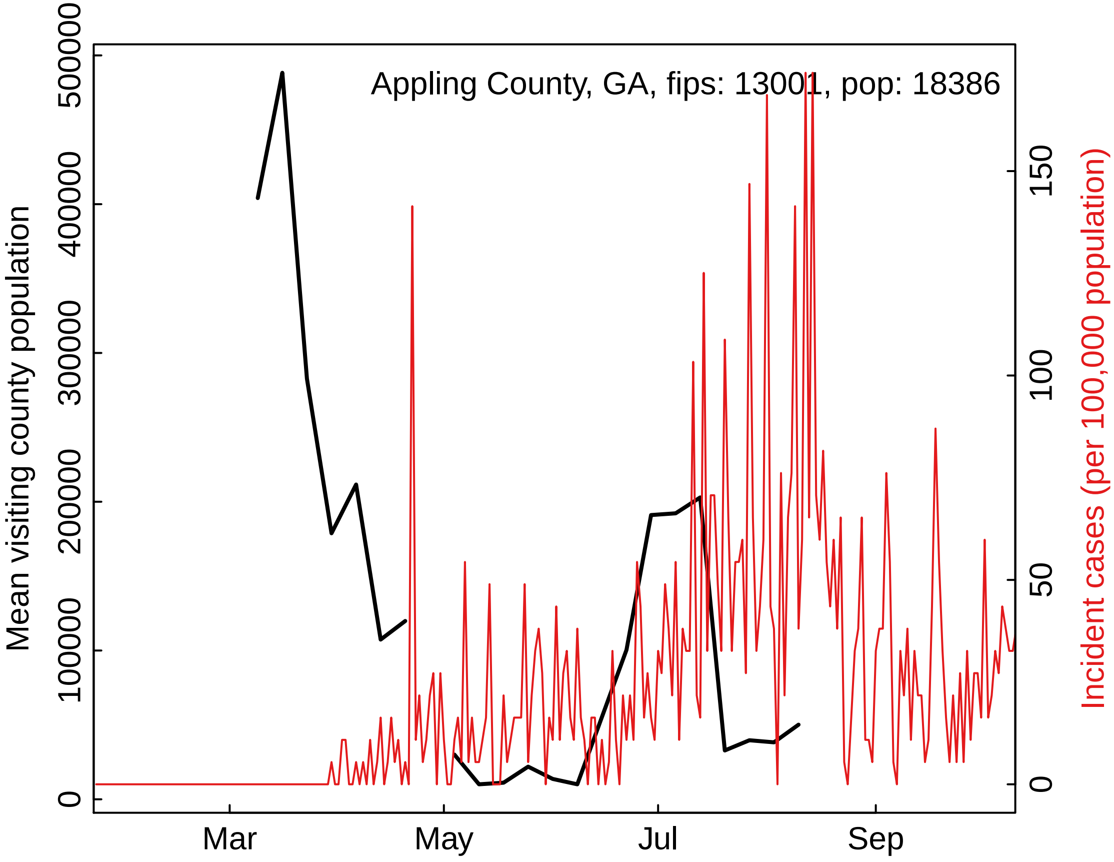

Appling County, GA, fips: 13001, pop: 18386
Mean visiting county population
Incident cases (per 100,000 population)
0
100000
200000
300000
400000
500000
0
50
100
150
Mar
May
Jul
Sep

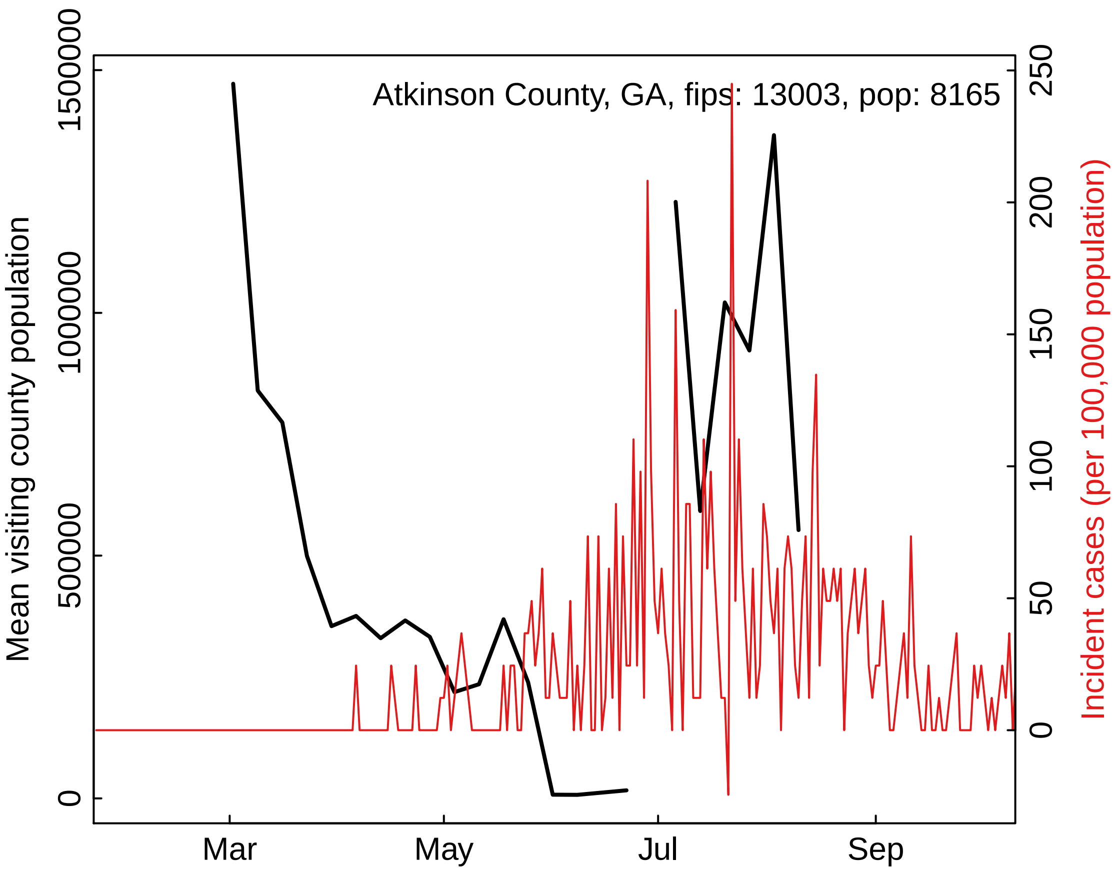

Atkinson County, GA, fips: 13003, pop: 8165
Mean visiting county population
0
500000
1000000
1500000
Incident cases (per 100,000 population)
0
50
100
150
200
250
Mar
May
Jul
Sep

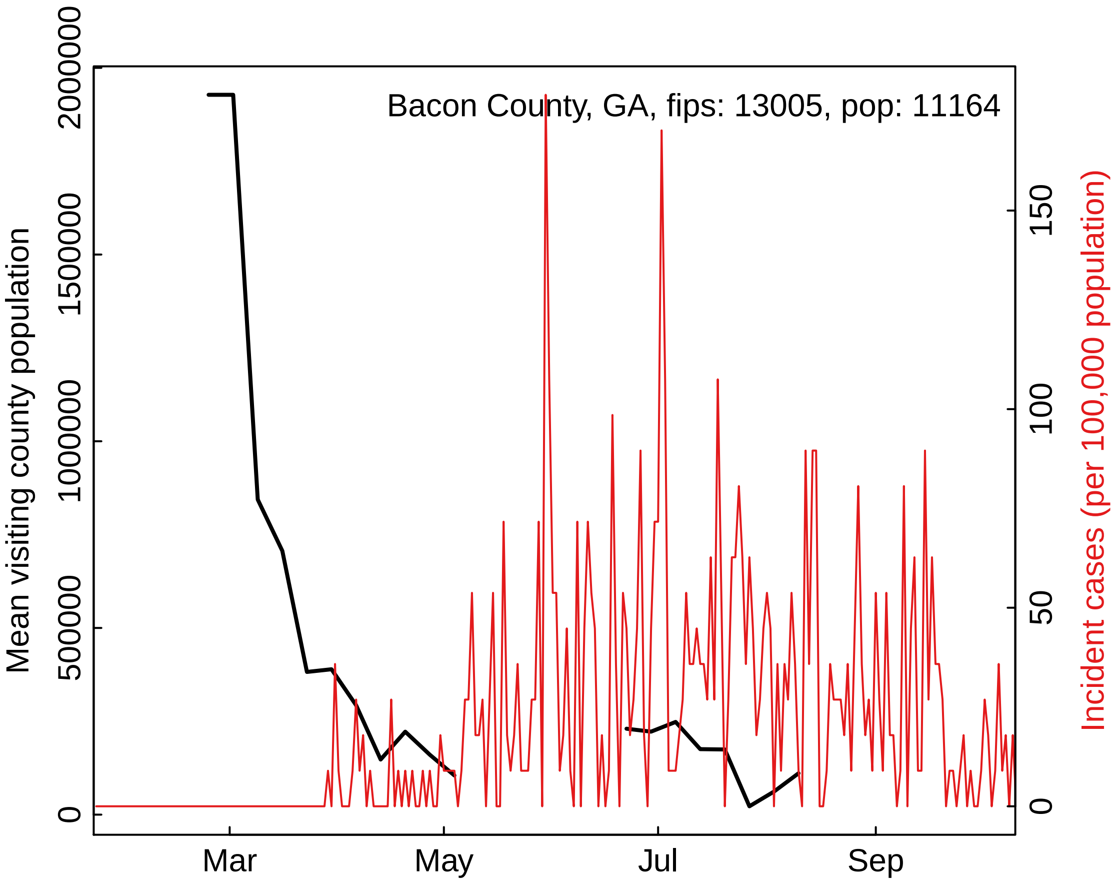

Bacon County, GA, fips: 13005, pop: 11164
Mean visiting county population
0
500000
1000000
1500000
2000000
Incident cases (per 100,000 population)
0
50
100
150
Mar
May
Jul
Sep

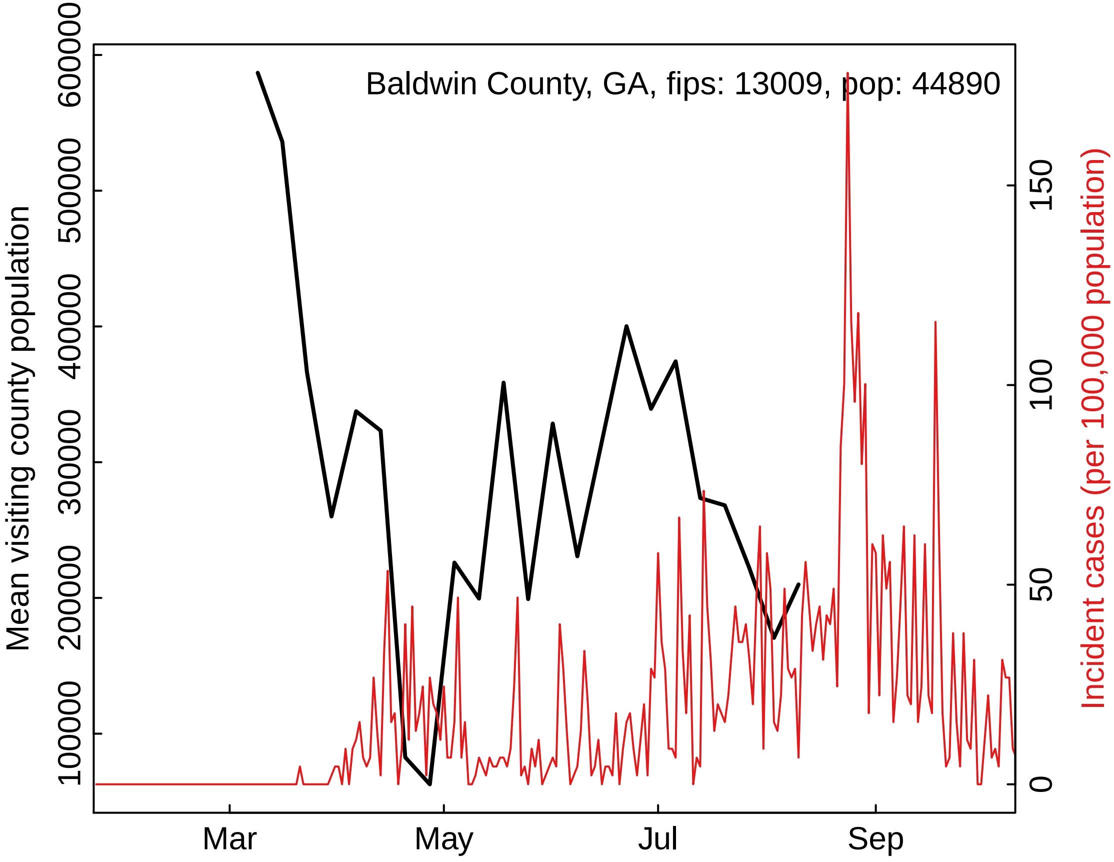

Baldwin County, GA, fips: 13009, pop: 44890
Mean visiting county population
100000
200000
300000
400000
500000
600000
Incident cases (per 100,000 population)
0
50
100
150
Mar
May
Jul
Sep

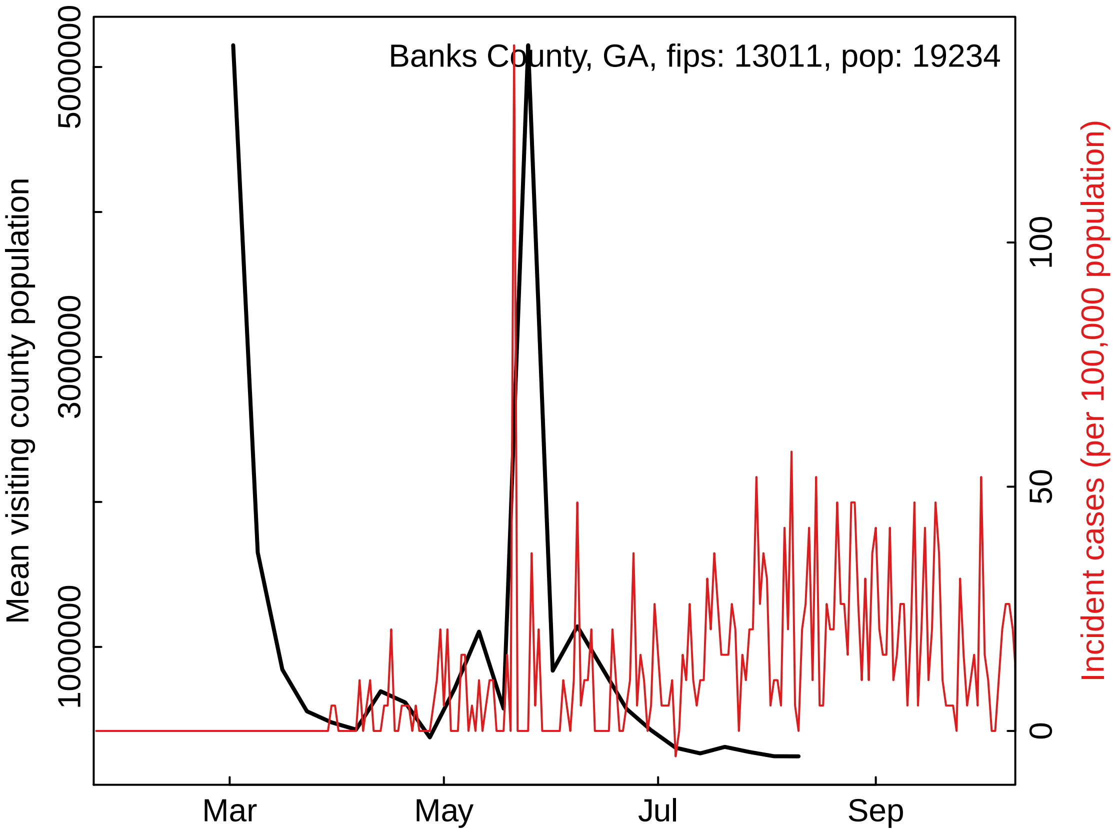

Banks County, GA, fips: 13011, pop: 19234
Mean visiting county population
Incident cases (per 100,000 population)
1000000
3000000
5000000
0
50
100
Mar
May
Jul
Sep

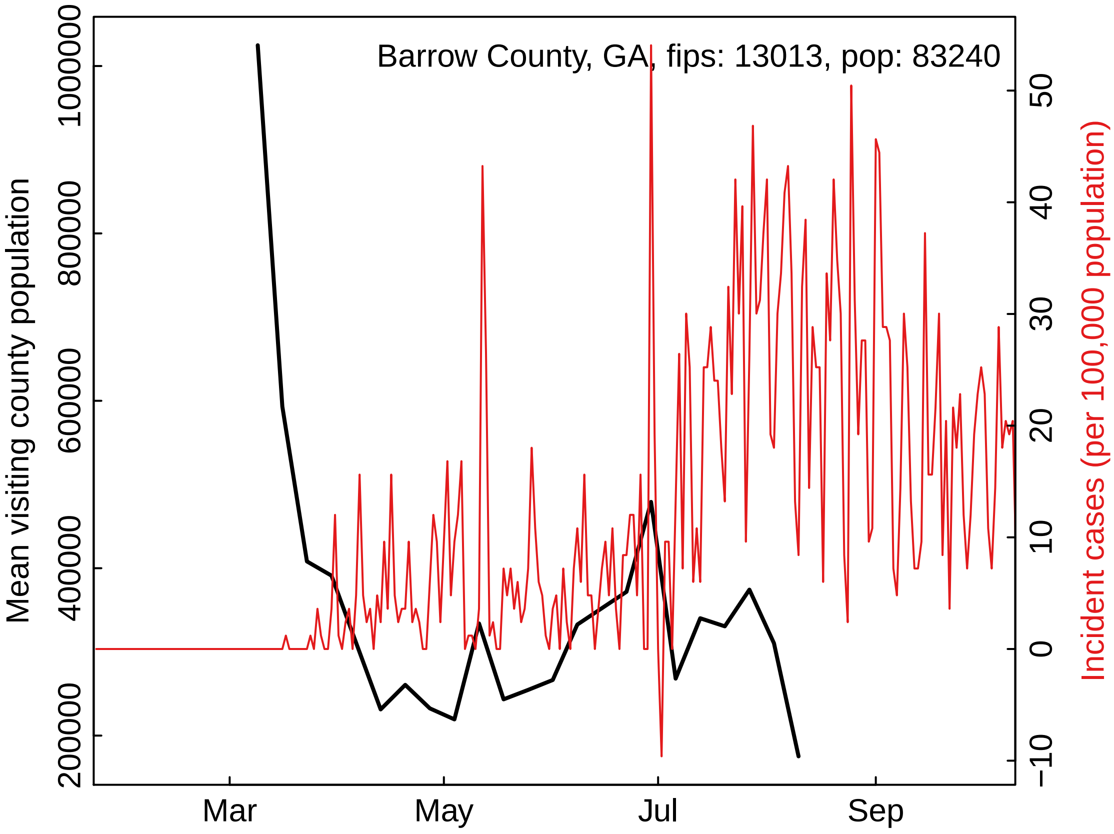
Barrow County, GA, fips: 13013, pop: 83240
Mean visiting county population
200000
400000
600000
800000
1000000
Incident cases (per 100,000 population)
−10
0
10
20
30
40
50
Mar
May
Jul
Sep

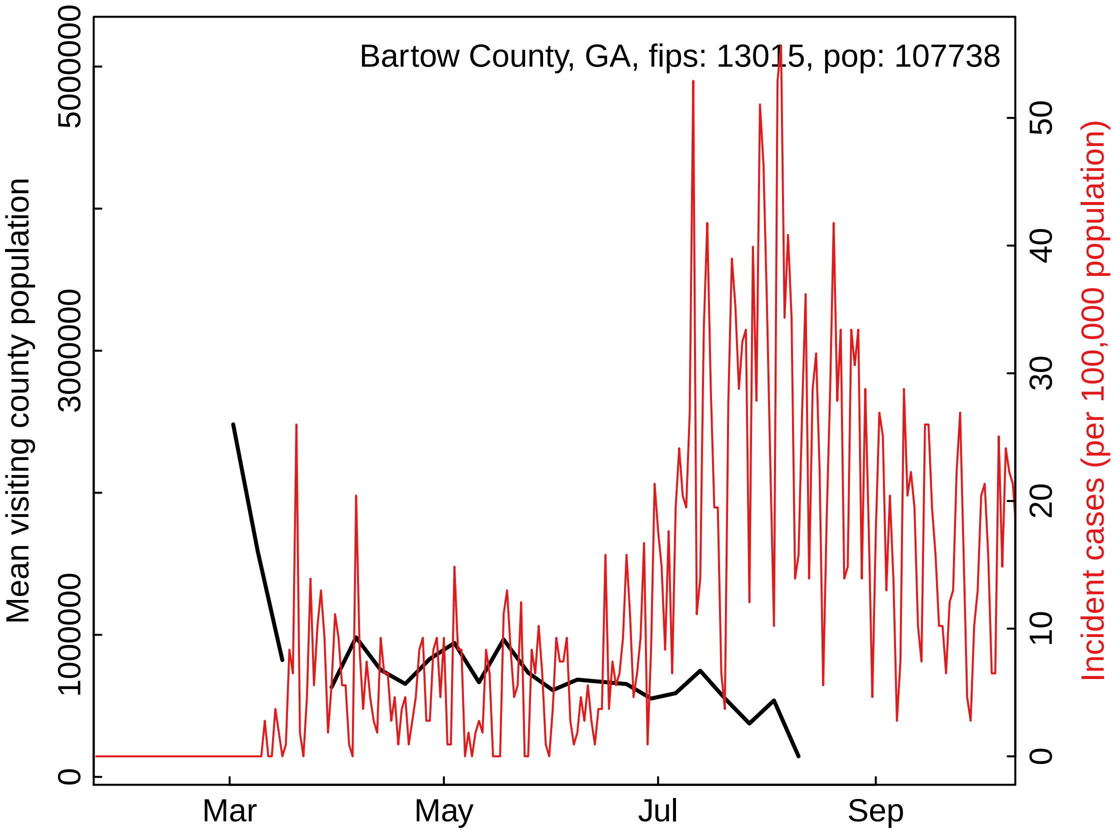

Bartow County, GA, fips: 13015, pop: 107738
Mean visiting county population
0
1000000
3000000
5000000
Incident cases (per 100,000 population)
0
10
20
30
40
50
Mar
May
Jul
Sep

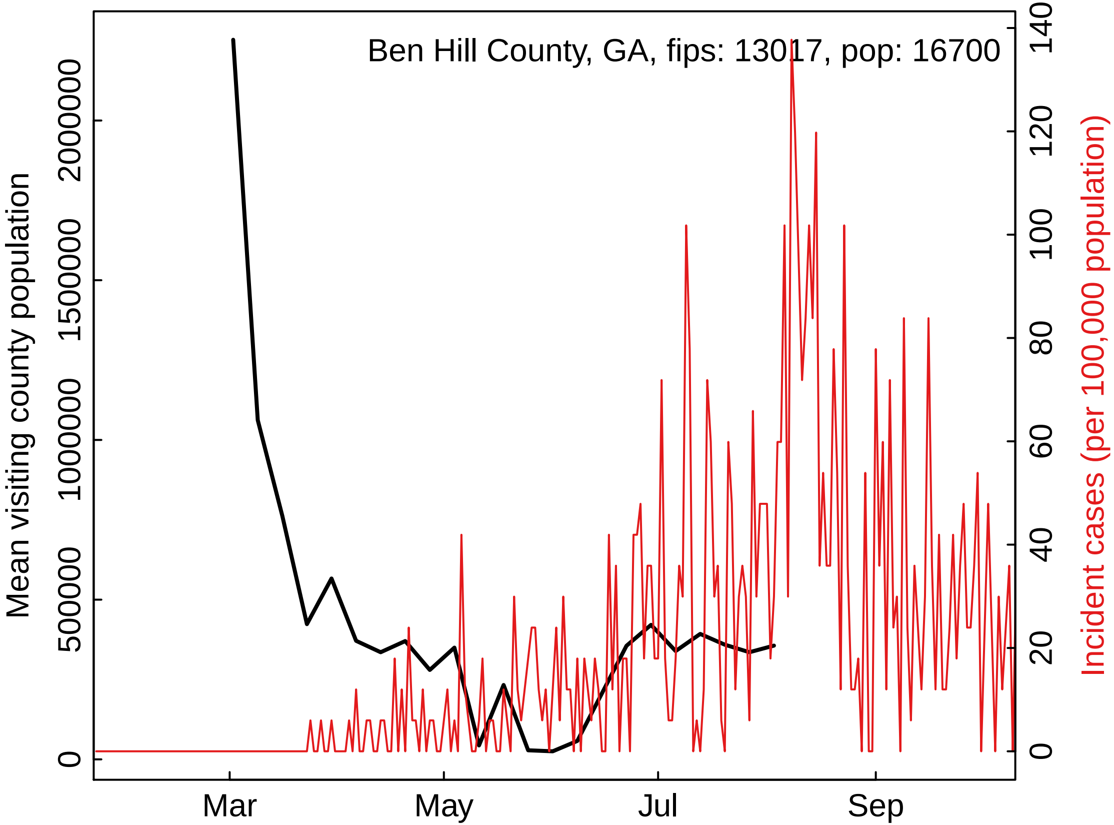

Ben Hill County, GA, fips: 13017, pop: 16700
Mean visiting county population
Incident cases (per 100,000 population)
0
500000
1000000
1500000
2000000
0
20
40
60
80
100
120
140
Mar
May
Jul
Sep

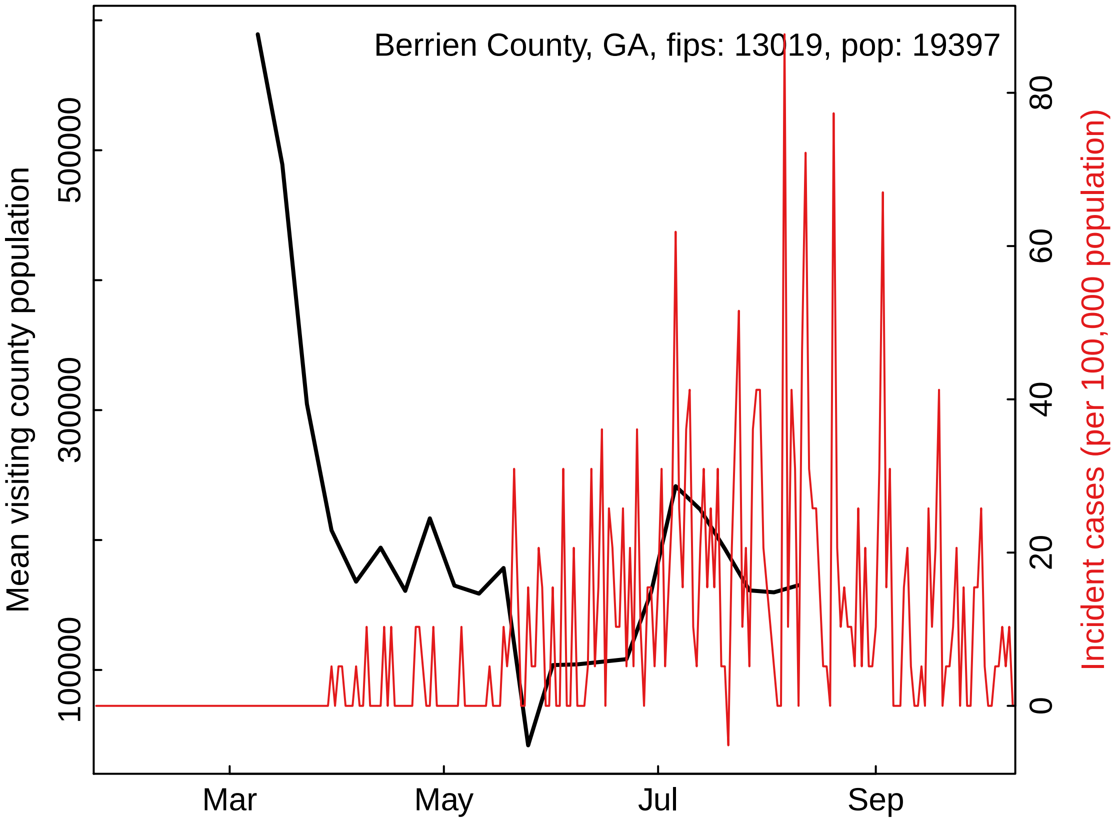

Berrien County, GA, fips: 13019, pop: 19397
Mean visiting county population
100000
300000
500000
Incident cases (per 100,000 population)
0
20
40
60
80
Mar
May
Jul
Sep

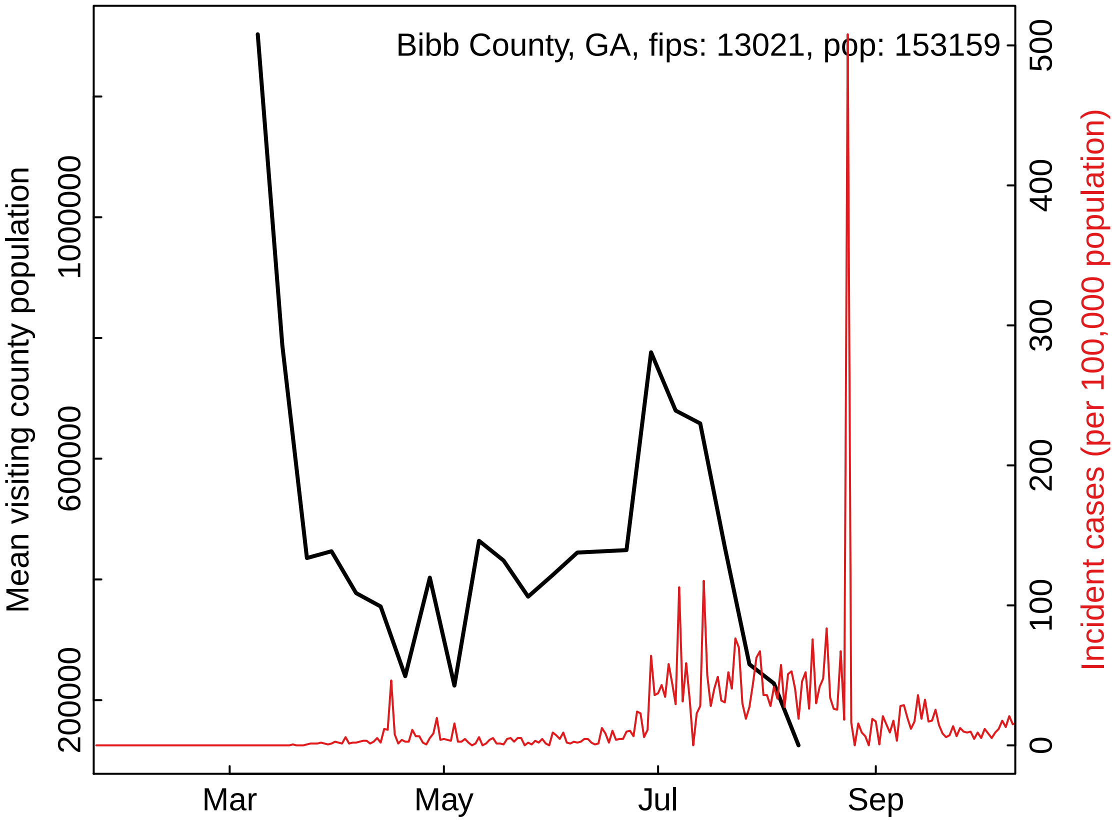

Bibb County, GA, fips: 13021, pop: 153159
Mean visiting county population
200000
600000
1000000
Incident cases (per 100,000 population)
0
100
200
300
400
500
Mar
May
Jul
Sep

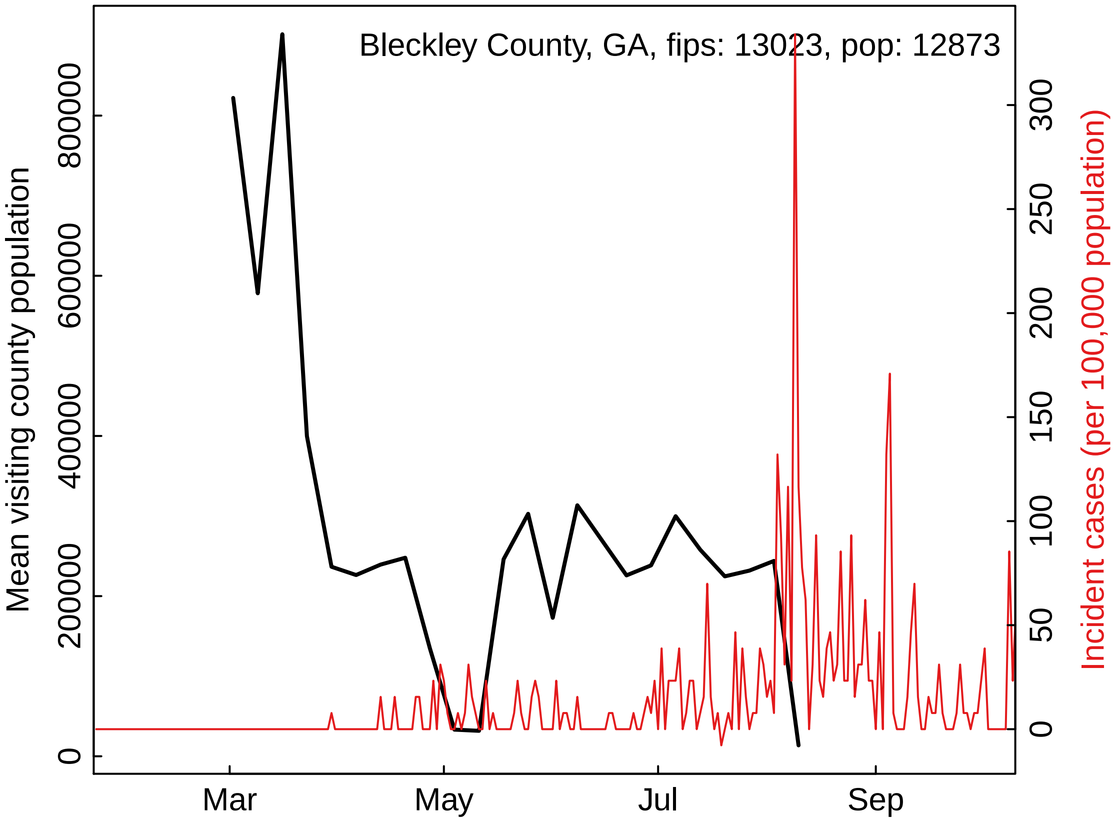

Bleckley County, GA, fips: 13023, pop: 12873
Mean visiting county population
0
200000
400000
600000
800000
Incident cases (per 100,000 population)
0
50
100
150
200
250
300
Mar
May
Jul
Sep

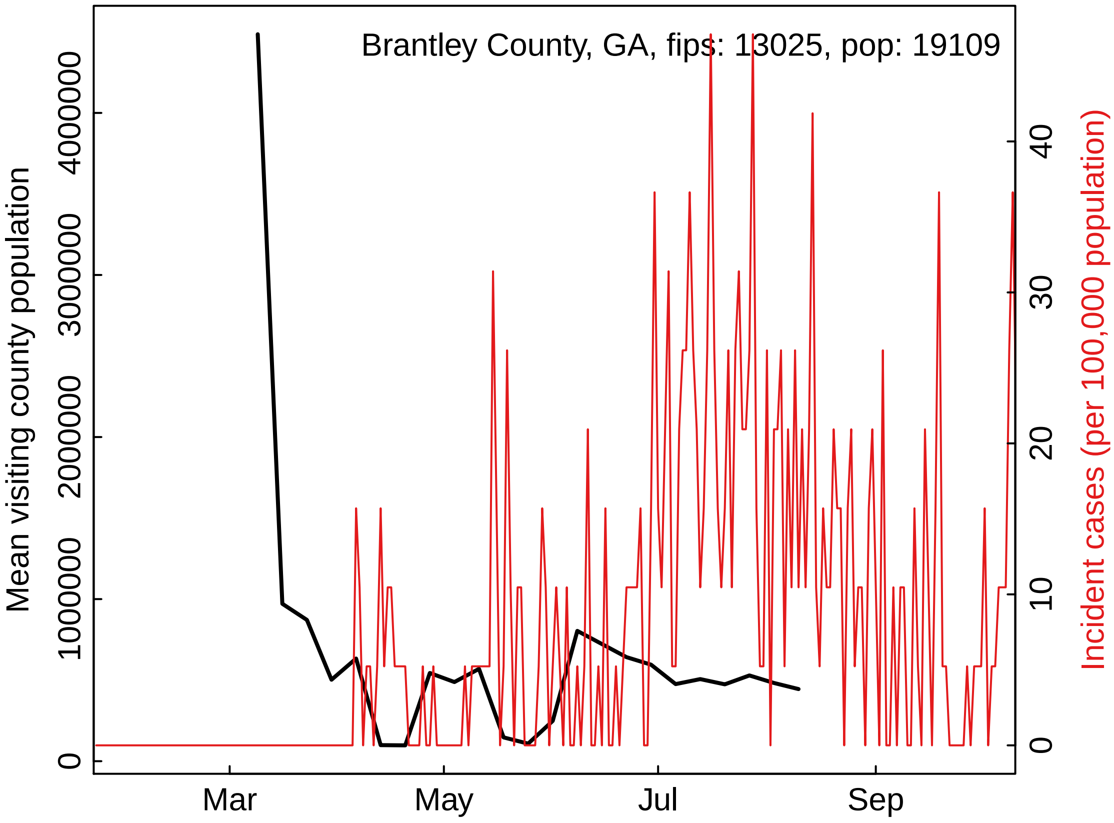
Brantley County, GA, fips: 13025, pop: 19109
Mean visiting county population
Incident cases (per 100,000 population)
0
1000000
2000000
3000000
4000000
0
10
20
30
40
Mar
May
Jul
Sep

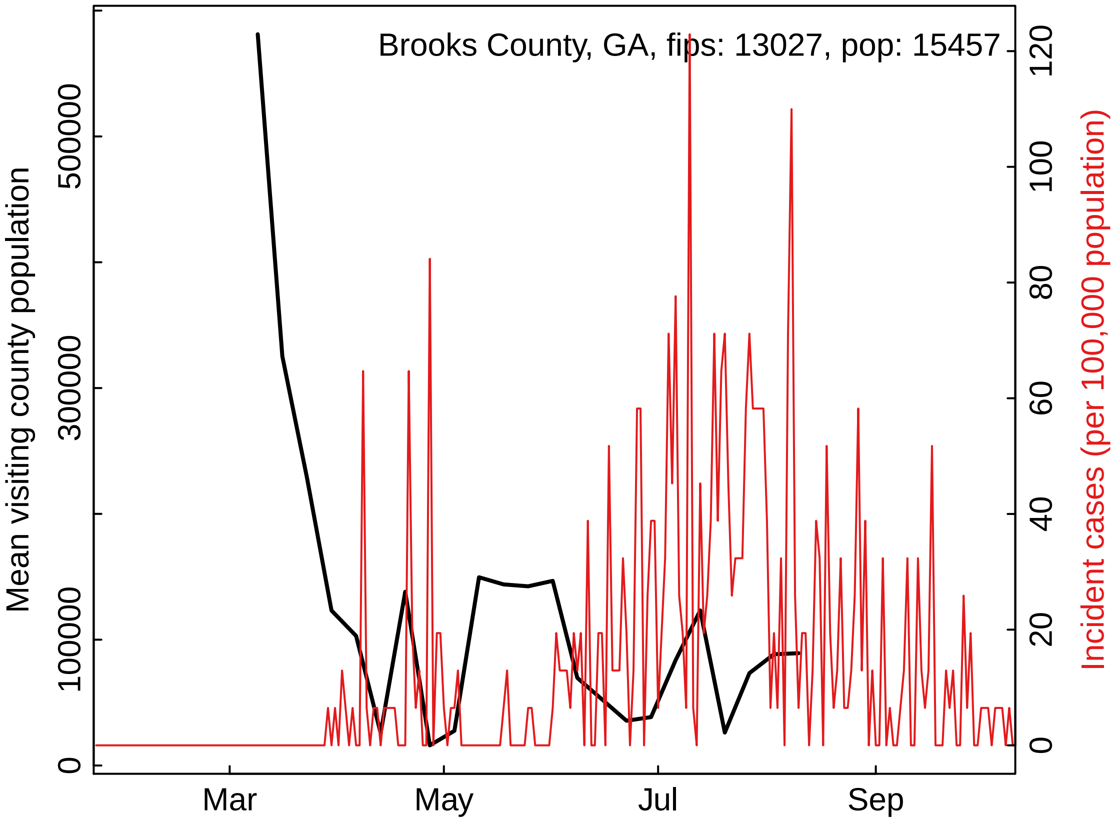

Brooks County, GA, fips: 13027, pop: 15457
Mean visiting county population
0
100000
300000
500000
Mar
May
Jul
Sep
Incident cases (per 100,000 population)
0
20
40
60
80
100
120

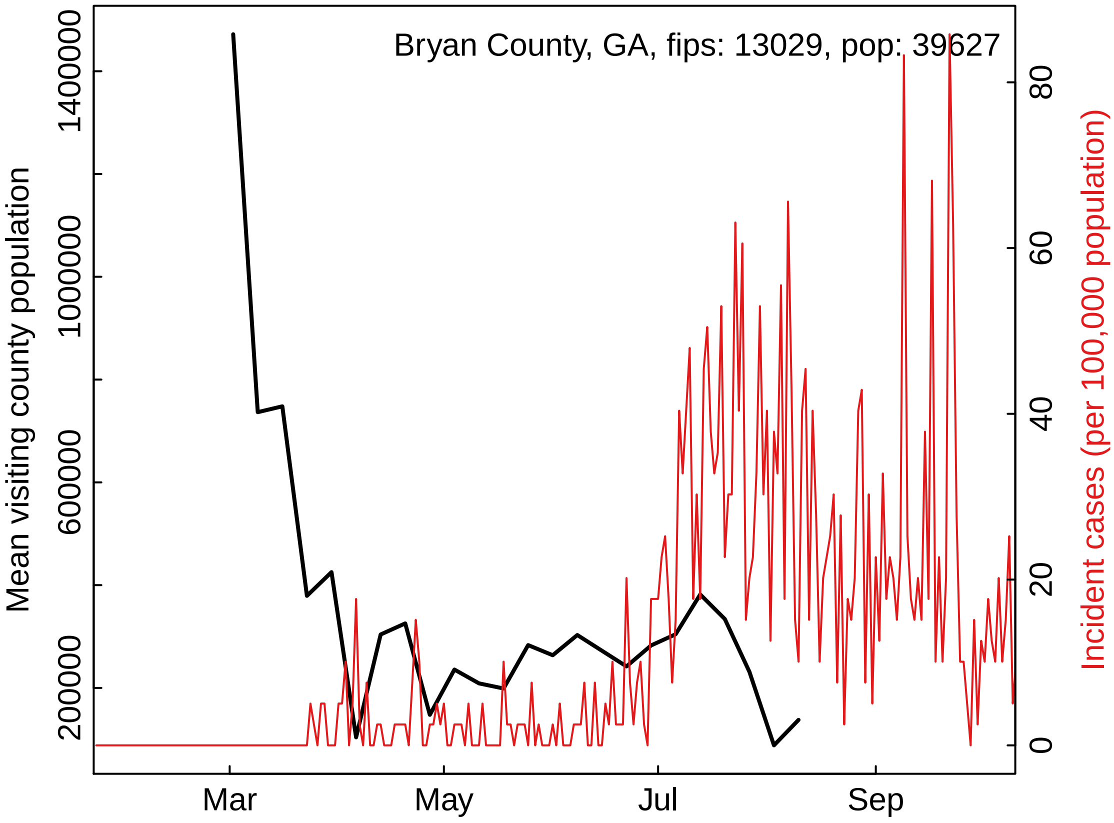

Bryan County, GA, fips: 13029, pop: 39627
Mean visiting county population
200000
600000
1000000
1400000
Incident cases (per 100,000 population)
0
20
40
60
80
Mar
May
Jul
Sep

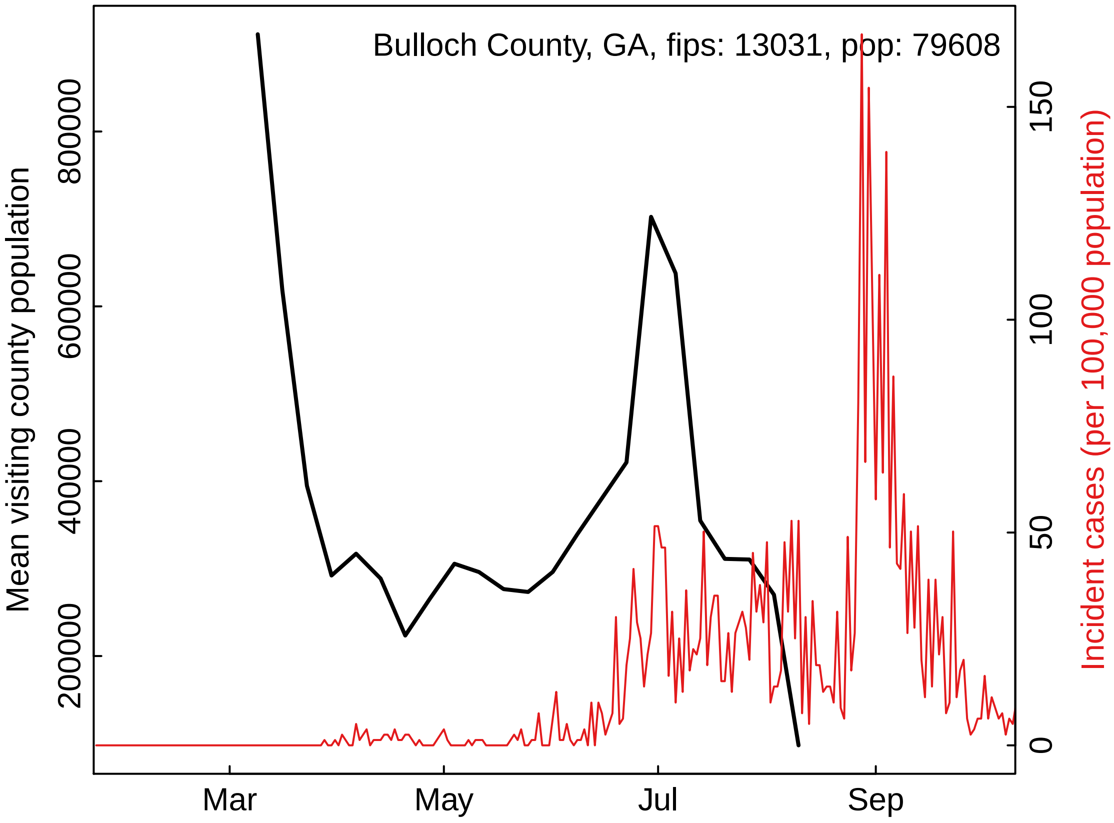

Bulloch County, GA, fips: 13031, pop: 79608
Mean visiting county population
200000
400000
600000
800000
Incident cases (per 100,000 population)
0
50
100
150
Mar
May
Jul
Sep

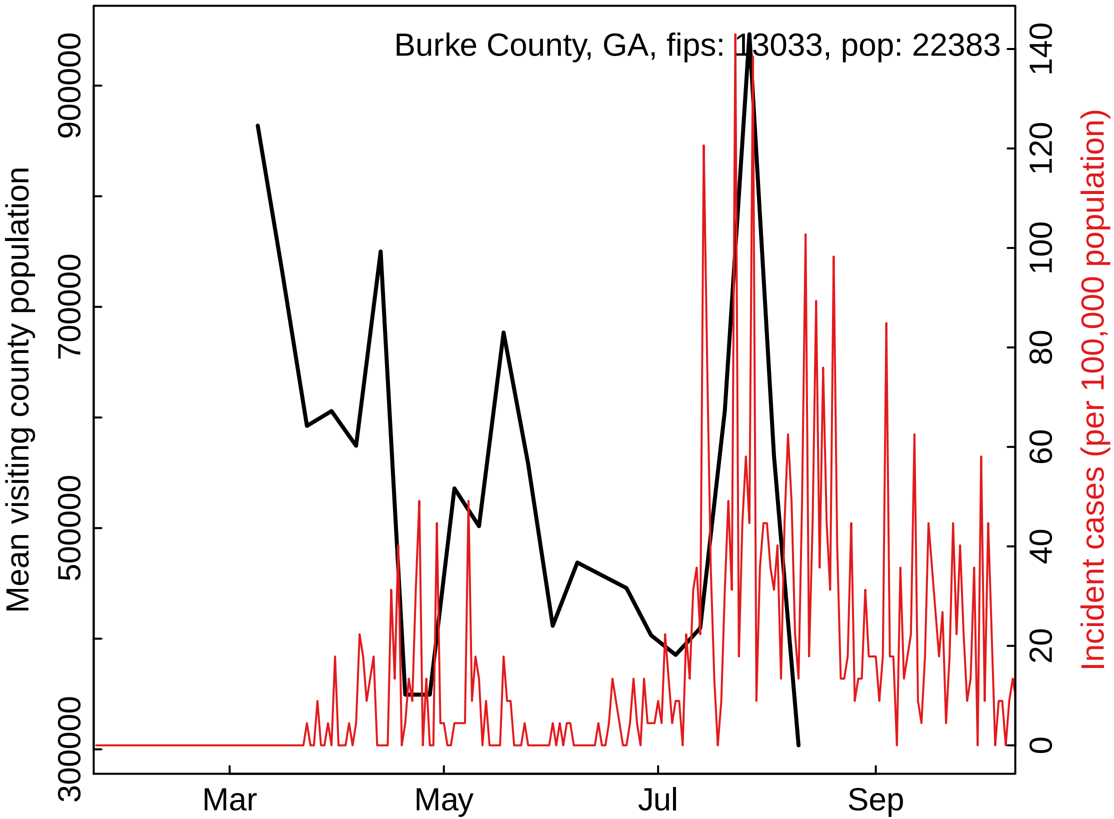

Burke County, GA, fips: 13033, pop: 22383
Mean visiting county population
Incident cases (per 100,000 population)
300000
500000
700000
900000
0
20
40
60
80
100
120
140
Mar
May
Jul
Sep

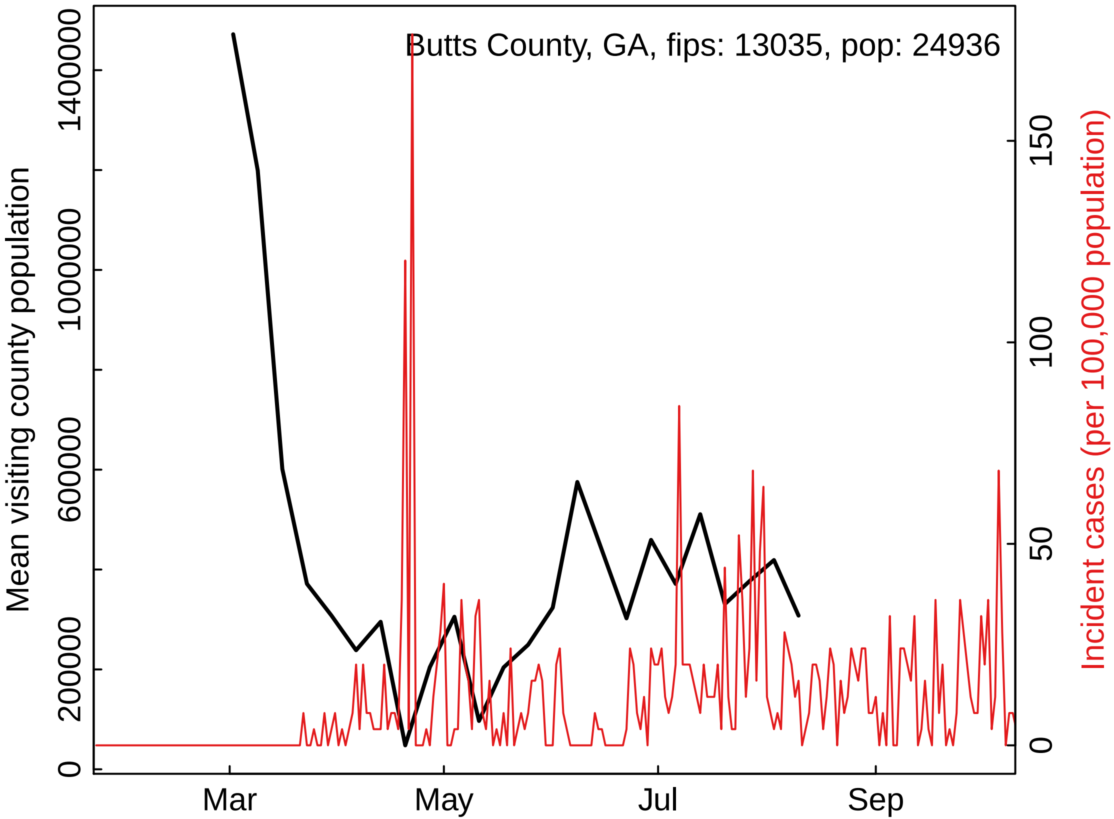

Butts County, GA, fips: 13035, pop: 24936
Mean visiting county population
0
200000
600000
1000000
1400000
Incident cases (per 100,000 population)
0
50
100
150
Mar
May
Jul
Sep

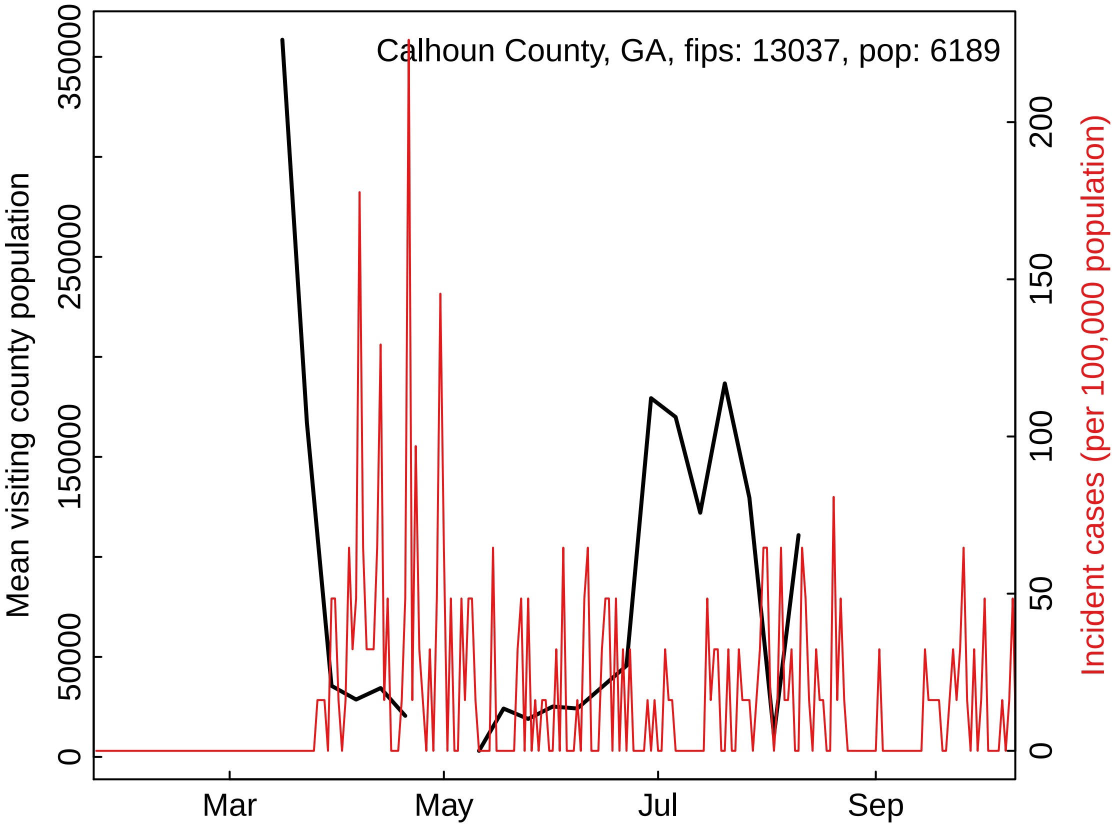

Calhoun County, GA, fips: 13037, pop: 6189
Mean visiting county population
0
50000
150000
250000
350000
Incident cases (per 100,000 population)
0
50
100
150
200
Mar
May
Jul
Sep

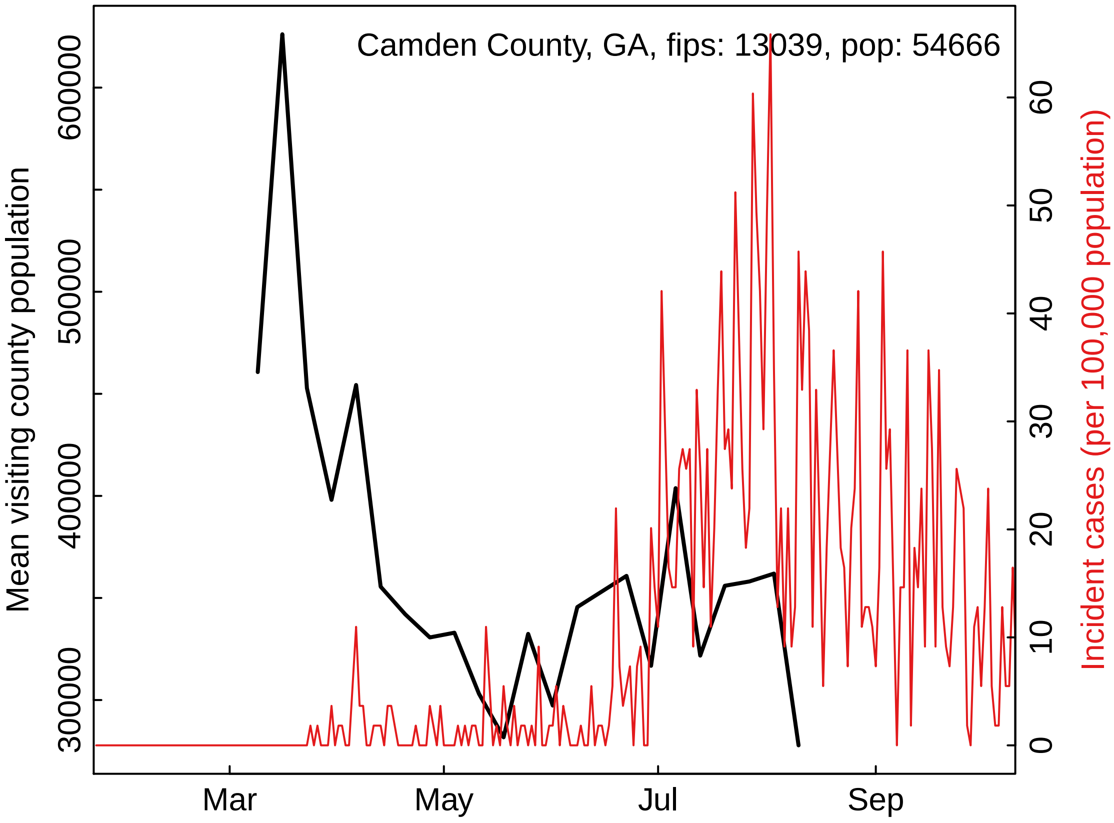
Camden County, GA, fips: 13039, pop: 54666
Mean visiting county population
Incident cases (per 100,000 population)
300000
400000
500000
600000
0
10
20
30
40
50
60
Mar
May
Jul
Sep

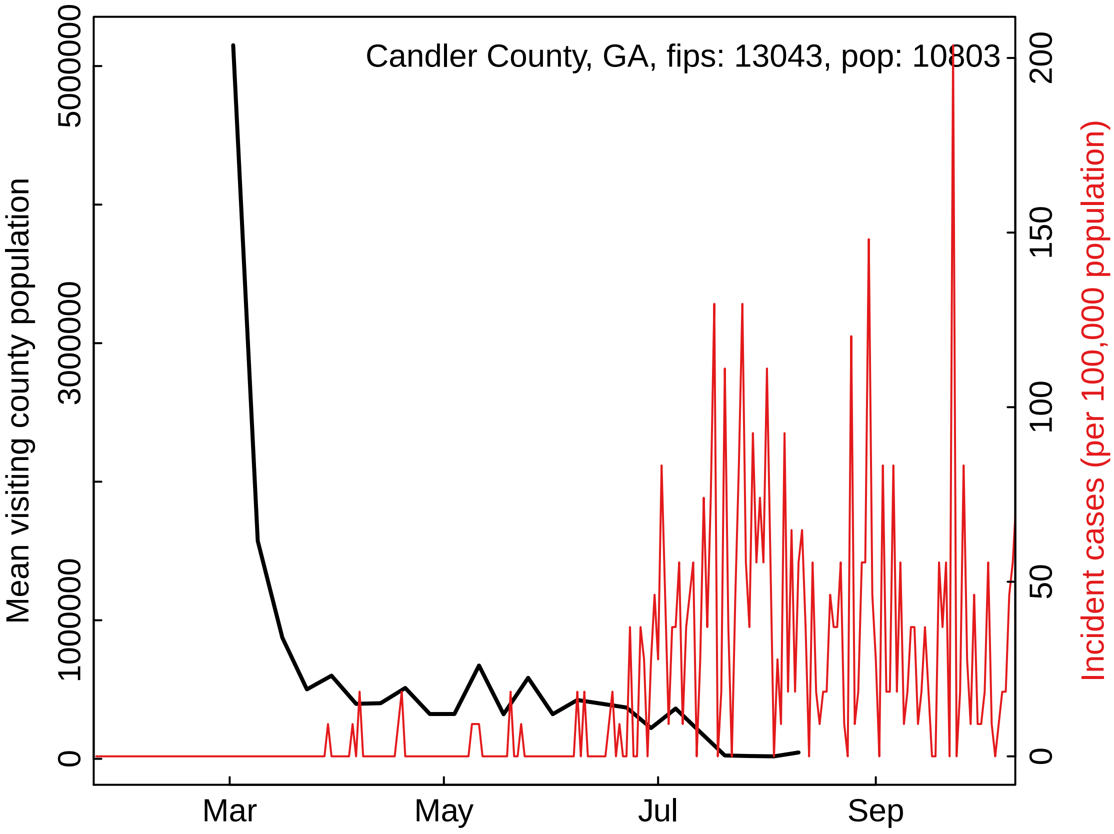
Candler County, GA, fips: 13043, pop: 10803
Mean visiting county population
Incident cases (per 100,000 population)
0
1000000
3000000
5000000
0
50
100
150
200
Mar
May
Jul
Sep

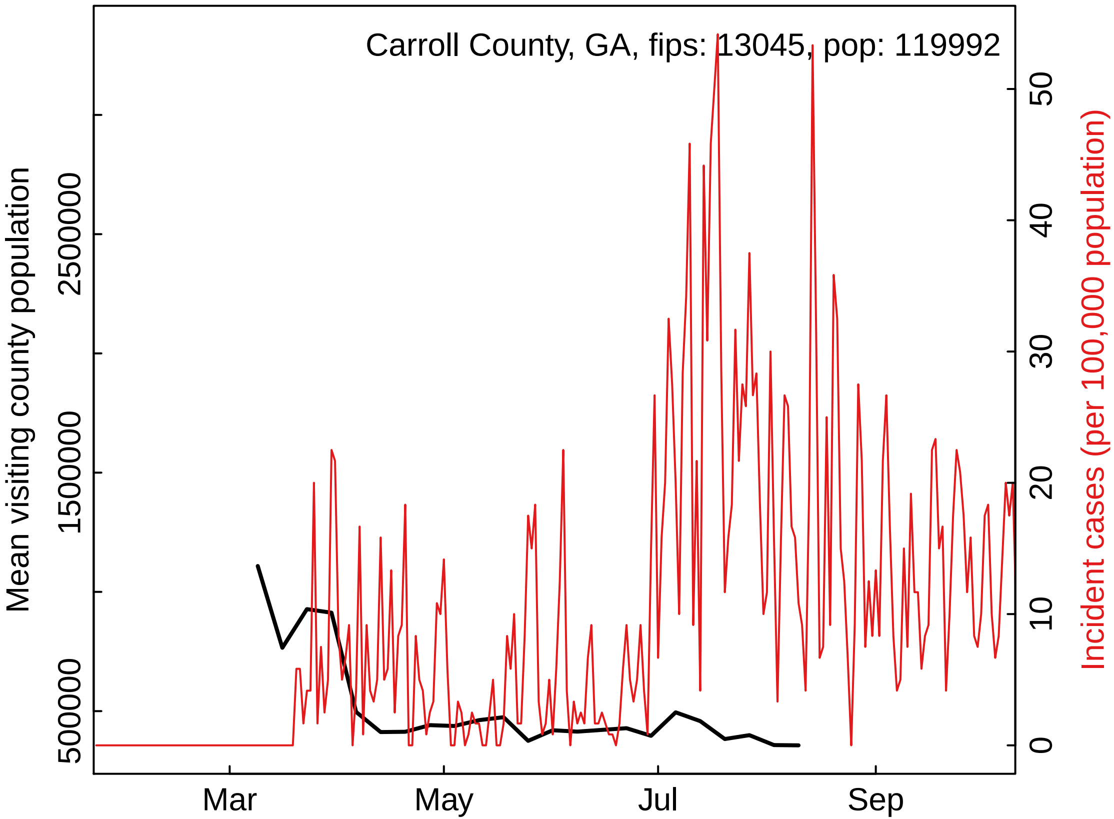

Carroll County, GA, fips: 13045, pop: 119992
Mean visiting county population
500000
1500000
2500000
Mar
May
Jul
Sep
0
10
20
30
40
50
Incident cases (per 100,000 population)

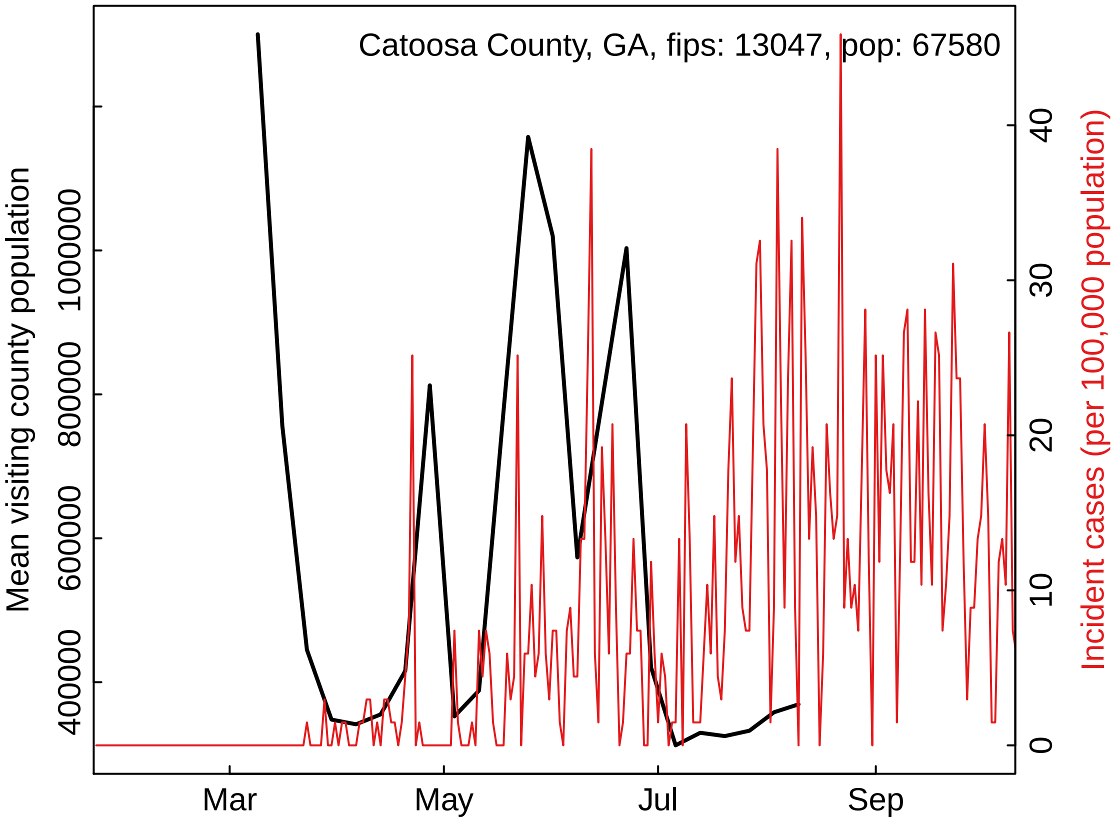
Catoosa County, GA, fips: 13047, pop: 67580
Mean visiting county population
400000
600000
800000
1000000
Incident cases (per 100,000 population)
0
10
20
30
40
Mar
May
Jul
Sep

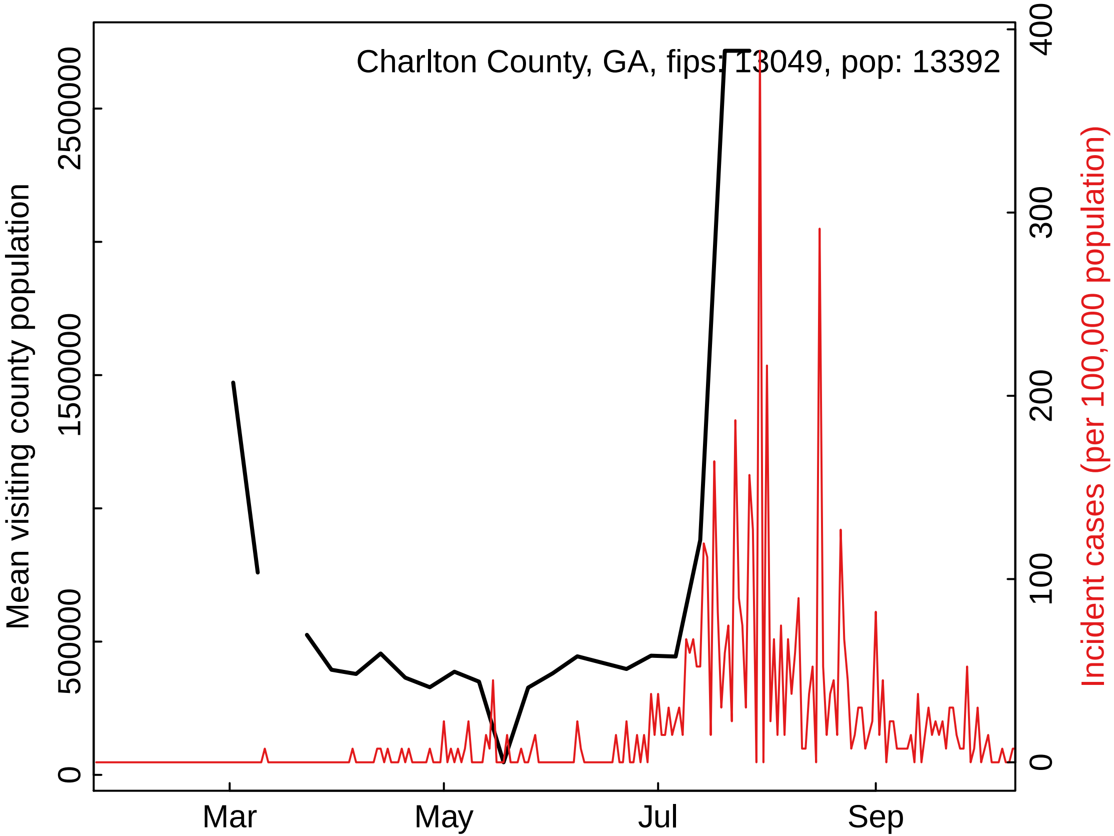

Charlton County, GA, fips: 13049, pop: 13392
Mean visiting county population
0
500000
1500000
2500000
Incident cases (per 100,000 population)
0
100
200
300
400
Mar
May
Jul
Sep

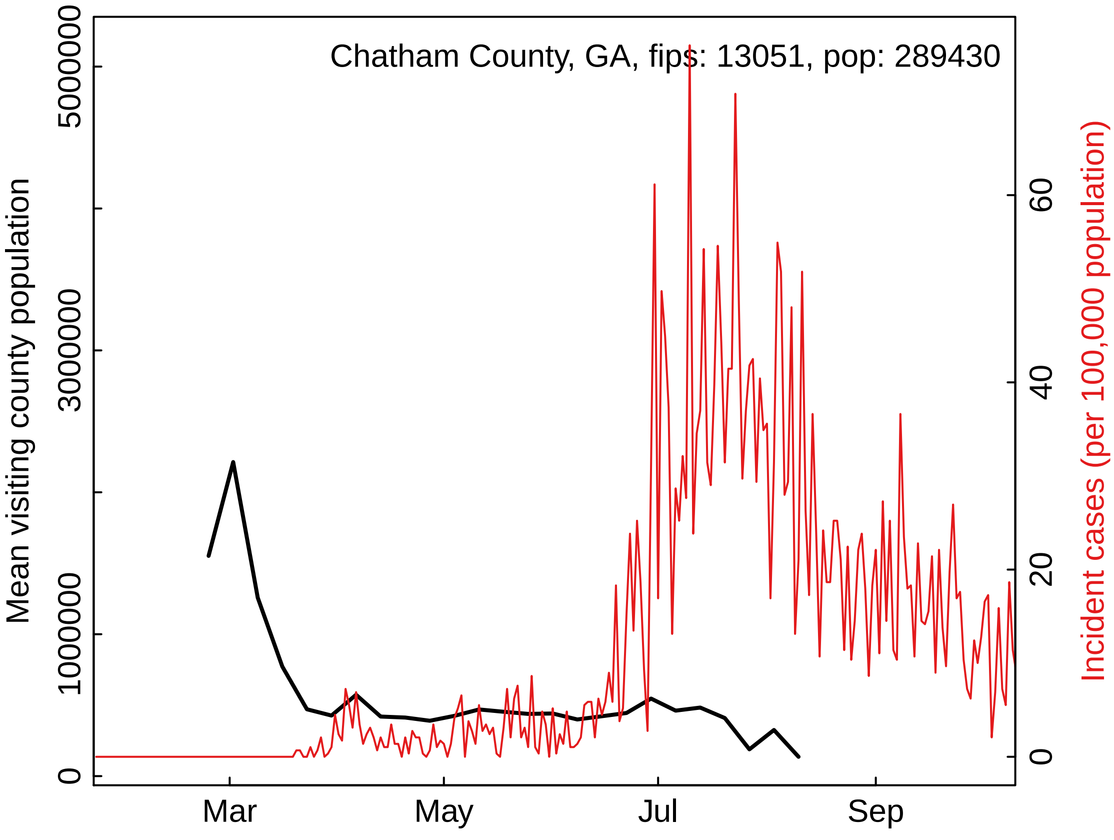

Chatham County, GA, fips: 13051, pop: 289430
Mean visiting county population
0
1000000
3000000
5000000
Incident cases (per 100,000 population)
0
20
40
60
Mar
May
Jul
Sep

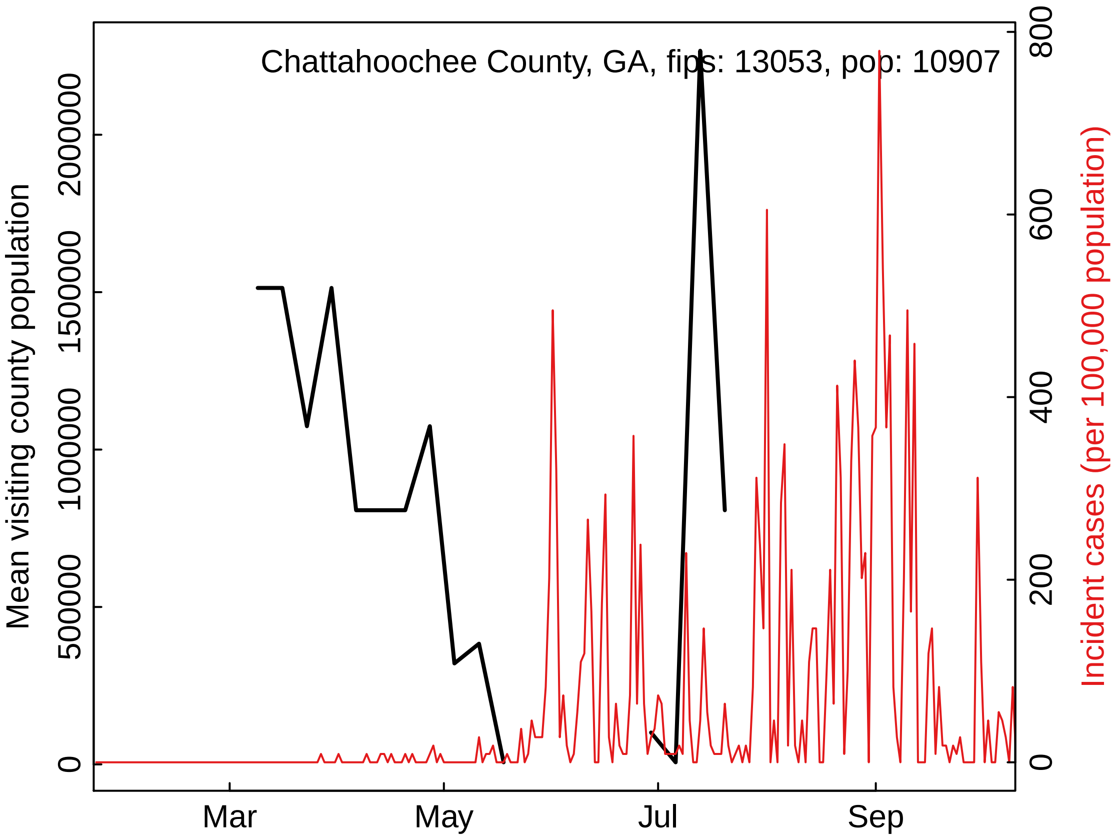

Chattahoochee County, GA, fips: 13053, pop: 10907
Mean visiting county population
0
500000
1000000
1500000
2000000
Incident cases (per 100,000 population)
0
200
400
600
800
Mar
May
Jul
Sep

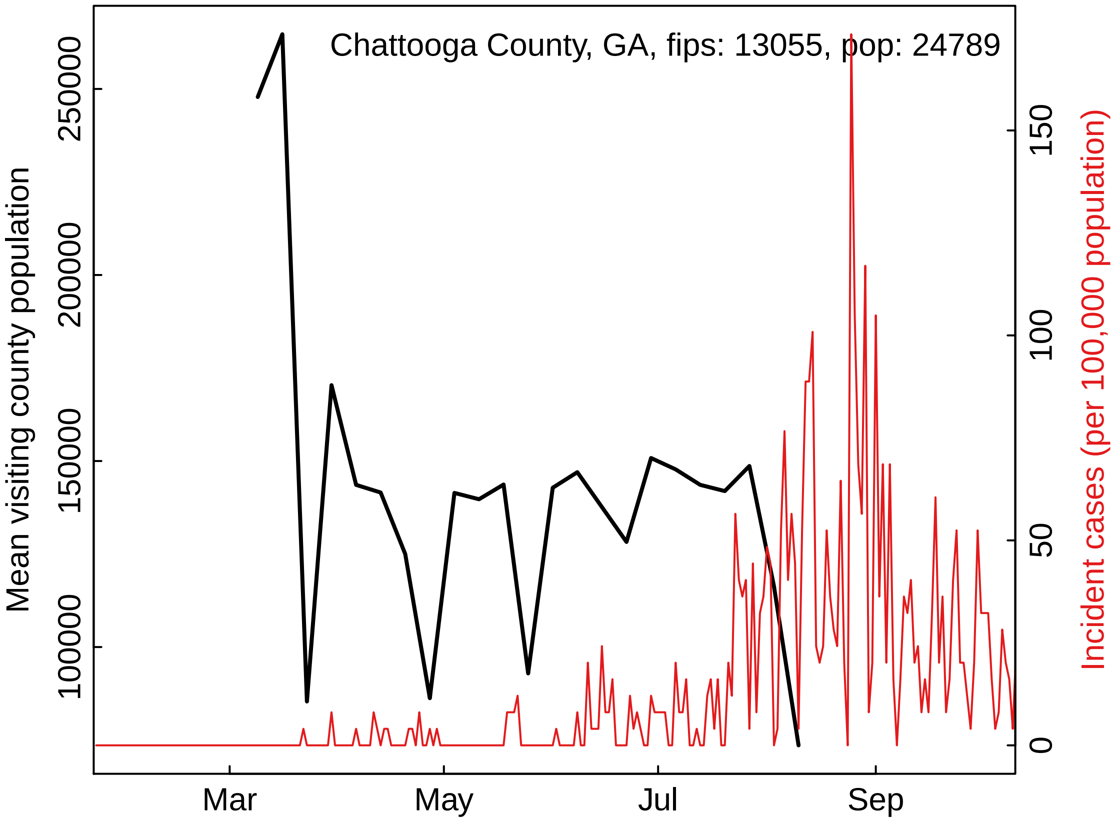

Chattooga County, GA, fips: 13055, pop: 24789
Mean visiting county population
100000
150000
200000
250000
Incident cases (per 100,000 population)
0
50
100
150
Mar
May
Jul
Sep

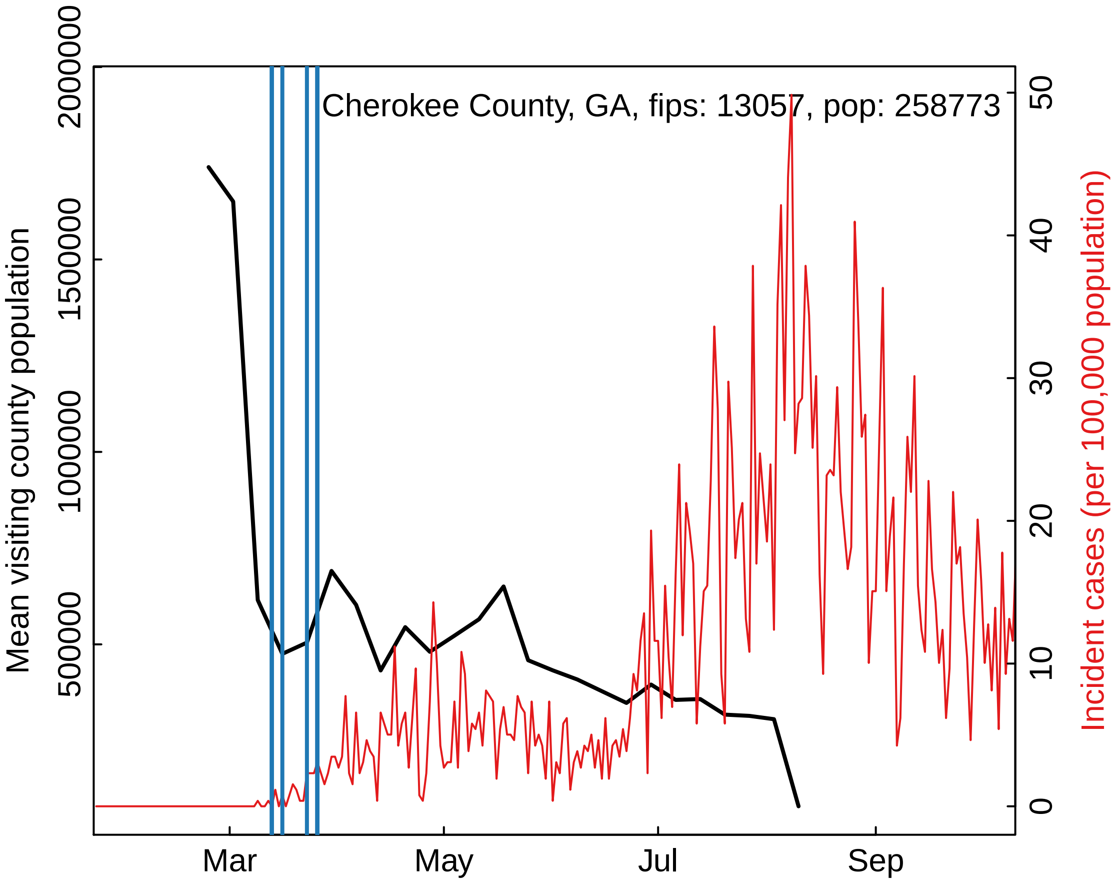

Cherokee County, GA, fips: 13057, pop: 258773
Mean visiting county population
Incident cases (per 100,000 population)
500000
1000000
1500000
2000000
0
10
20
30
40
50
Mar
May
Jul
Sep

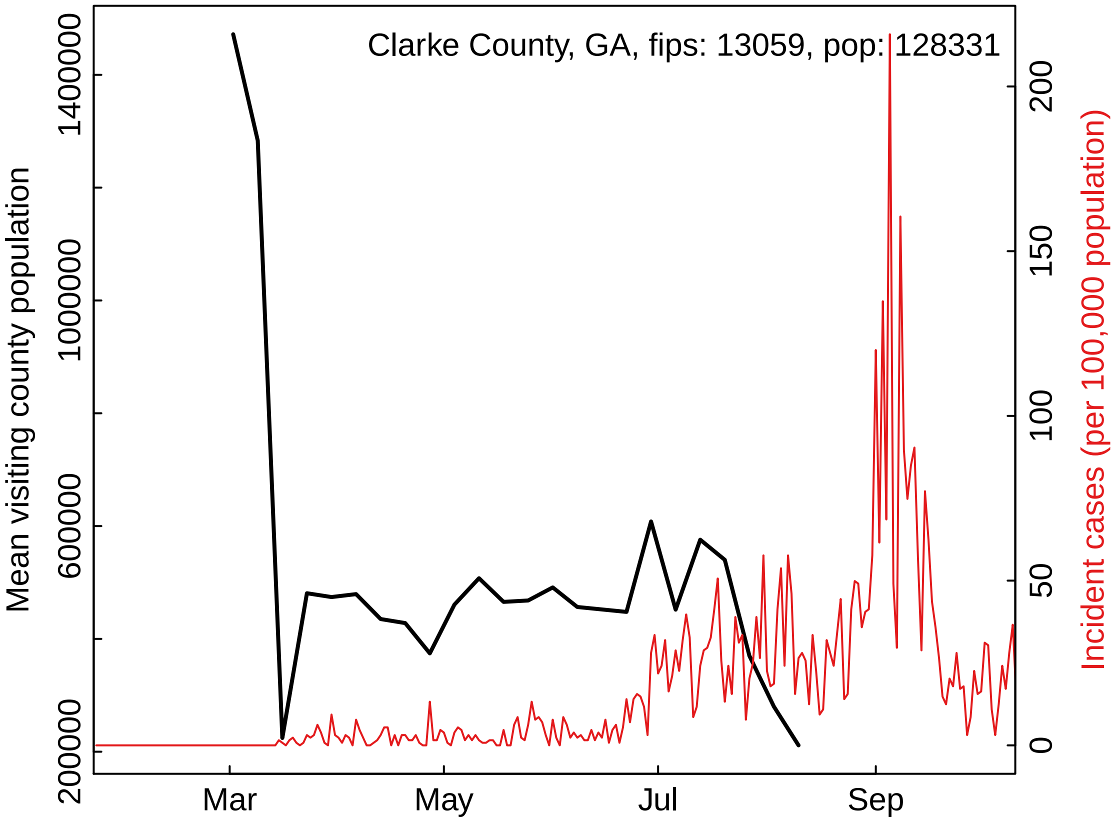

Clarke County, GA, fips: 13059, pop: 128331
Mean visiting county population
Incident cases (per 100,000 population)
2000000
600000
1000000
1400000
0
50
100
150
200
Mar
May
Jul
Sep

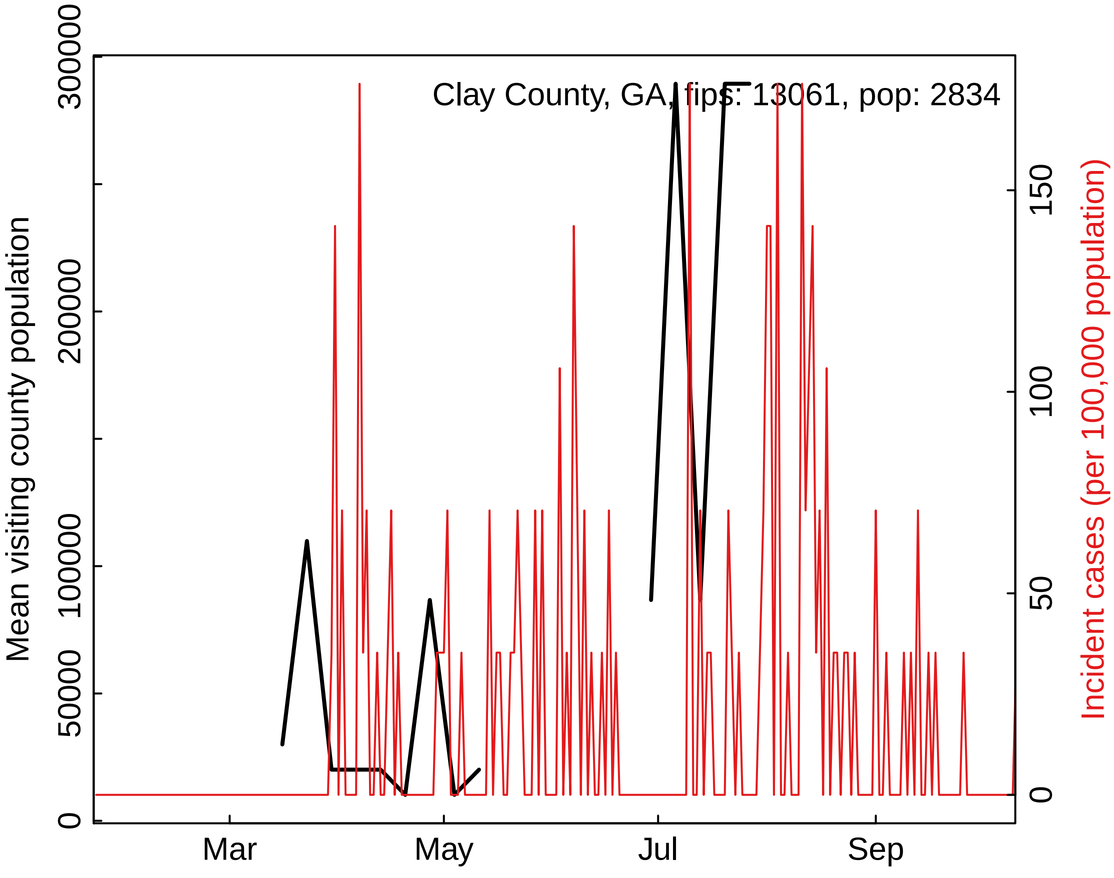
Clay County, GA, fips: 13061, pop: 2834
Mean visiting county population
0
50000
100000
200000
300000
Incident cases (per 100,000 population)
0
50
100
150
Mar
May
Jul
Sep

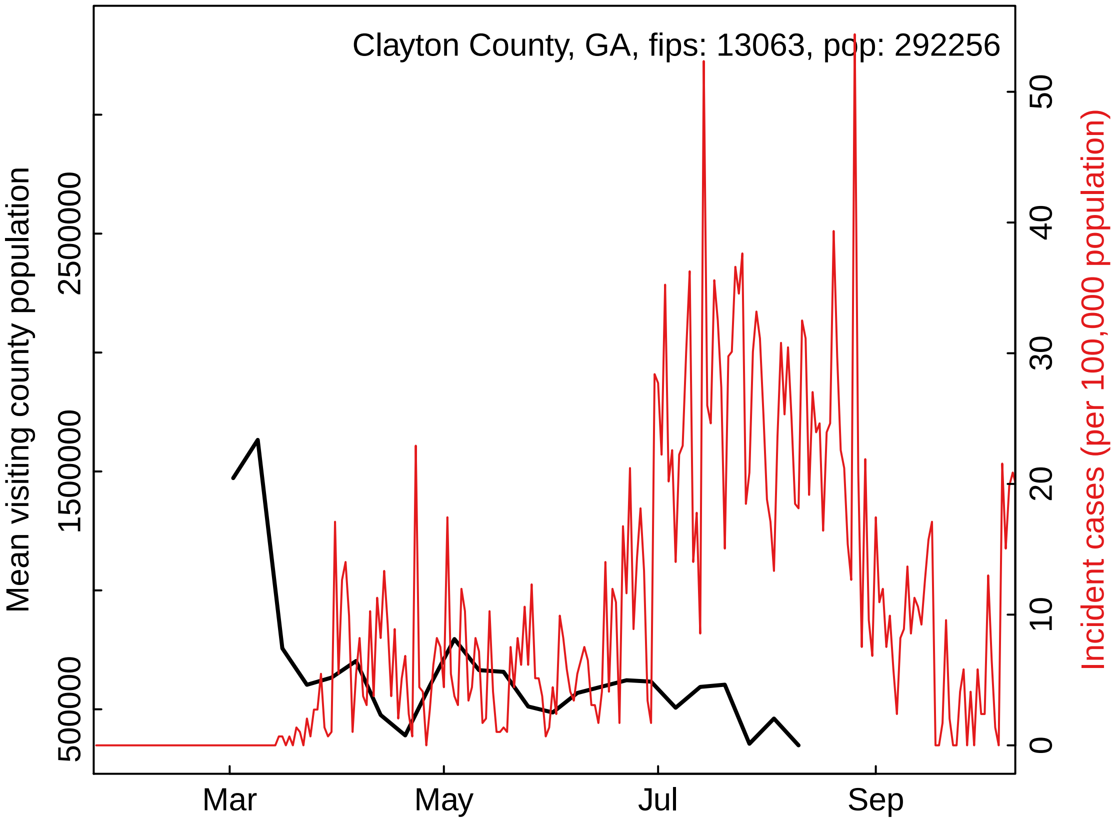

Clayton County, GA, fips: 13063, pop: 292256
Mean visiting county population
500000
1500000
2500000
Mar
May
Jul
Sep
0
10
20
30
40
50
Incident cases (per 100,000 population)

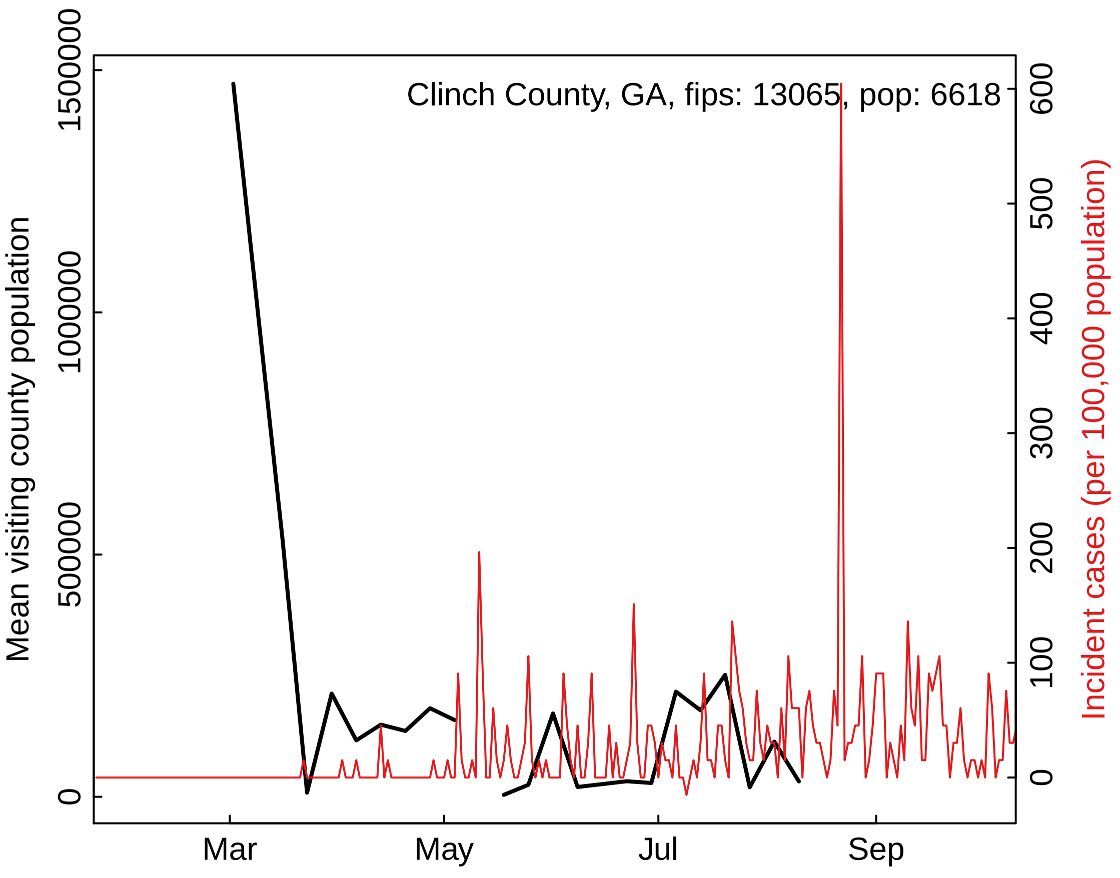

Clinch County, GA, fips: 13065, pop: 6618
Mean visiting county population
0
500000
1000000
1500000
Incident cases (per 100,000 population)
0
100
200
300
400
500
600
Mar
May
Jul
Sep

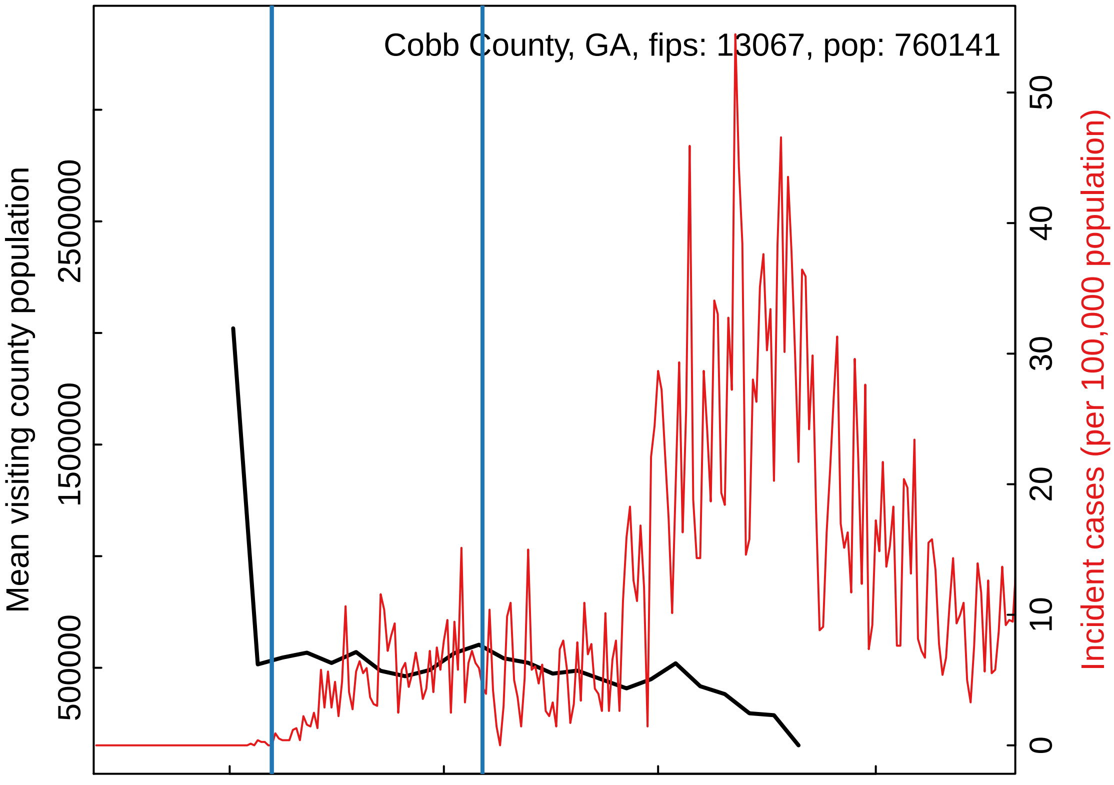
Cobb County, GA, fips: 13067, pop: 760141
Mean visiting county population
500000
1500000
2500000
Mar
May
Jul
Sep
0
10
20
30
40
50
Incident cases (per 100,000 population)

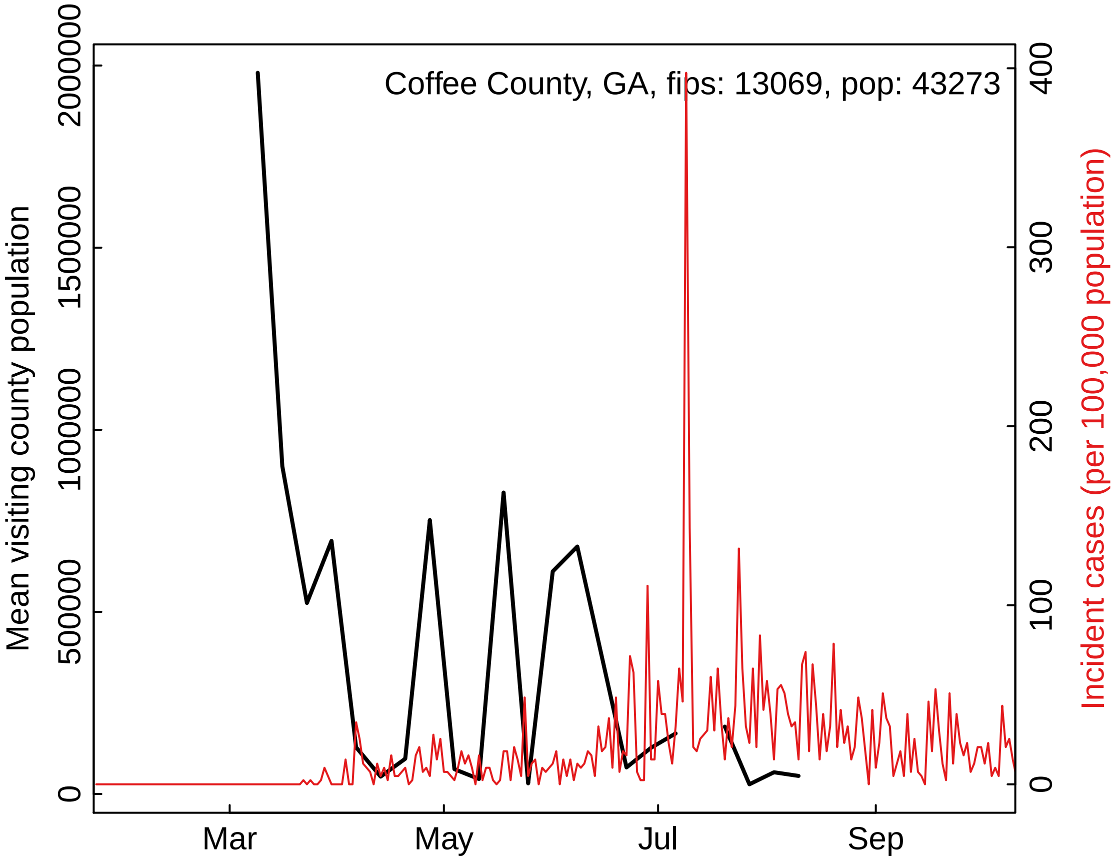

Coffee County, GA, fips: 13069, pop: 43273
Mean visiting county population
Incident cases (per 100,000 population)
0
500000
1000000
1500000
2000000
0
100
200
300
400
Mar
May
Jul
Sep

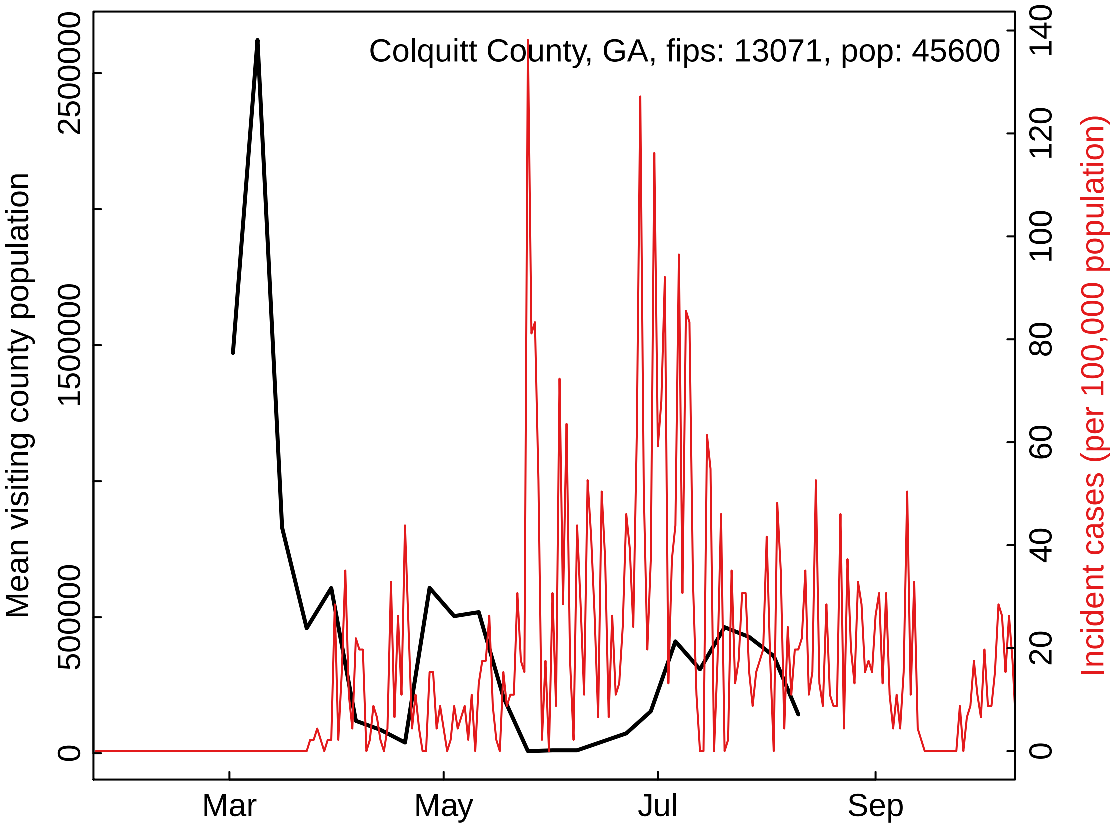

Colquitt County, GA, fips: 13071, pop: 45600
Mean visiting county population
Incident cases (per 100,000 population)
0
500000
1500000
2500000
0
20
40
60
80
100
120
140
Mar
May
Jul
Sep

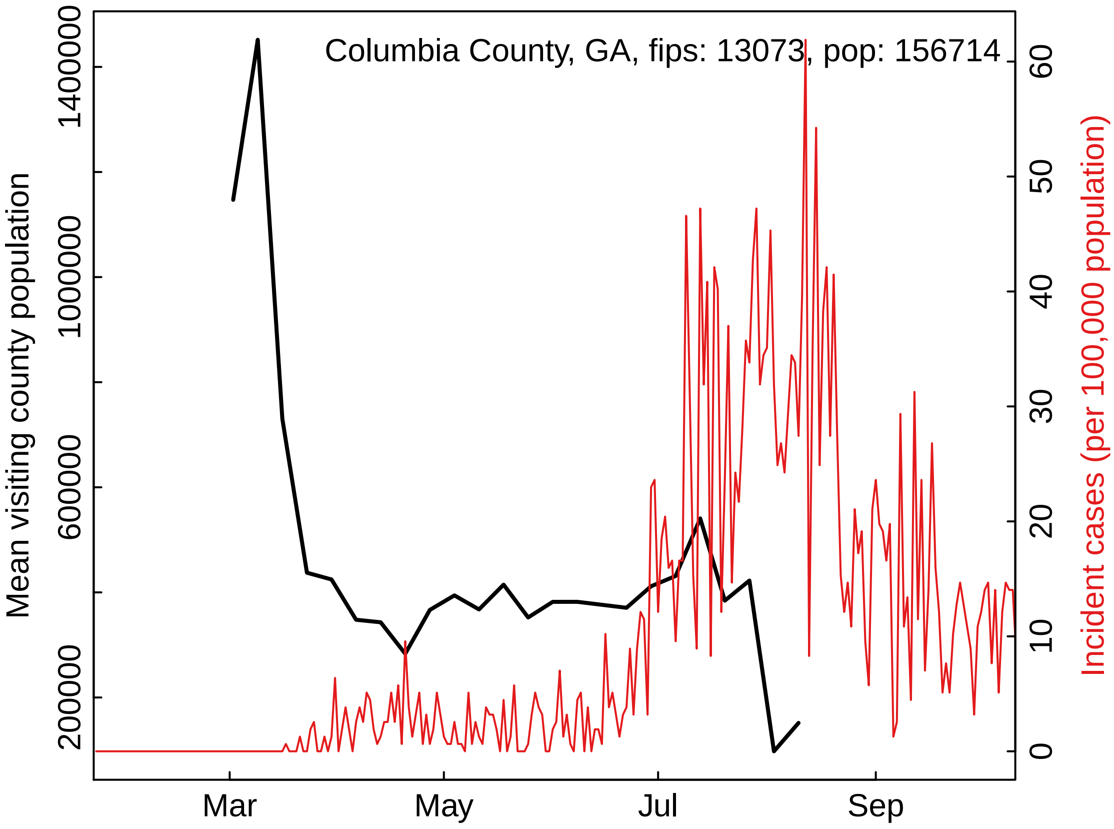
Columbia County, GA, fips: 13073, pop: 156714
Mean visiting county population
Incident cases (per 100,000 population)
200000
600000
1000000
1400000
0
10
20
30
40
50
60
Mar
May
Jul
Sep

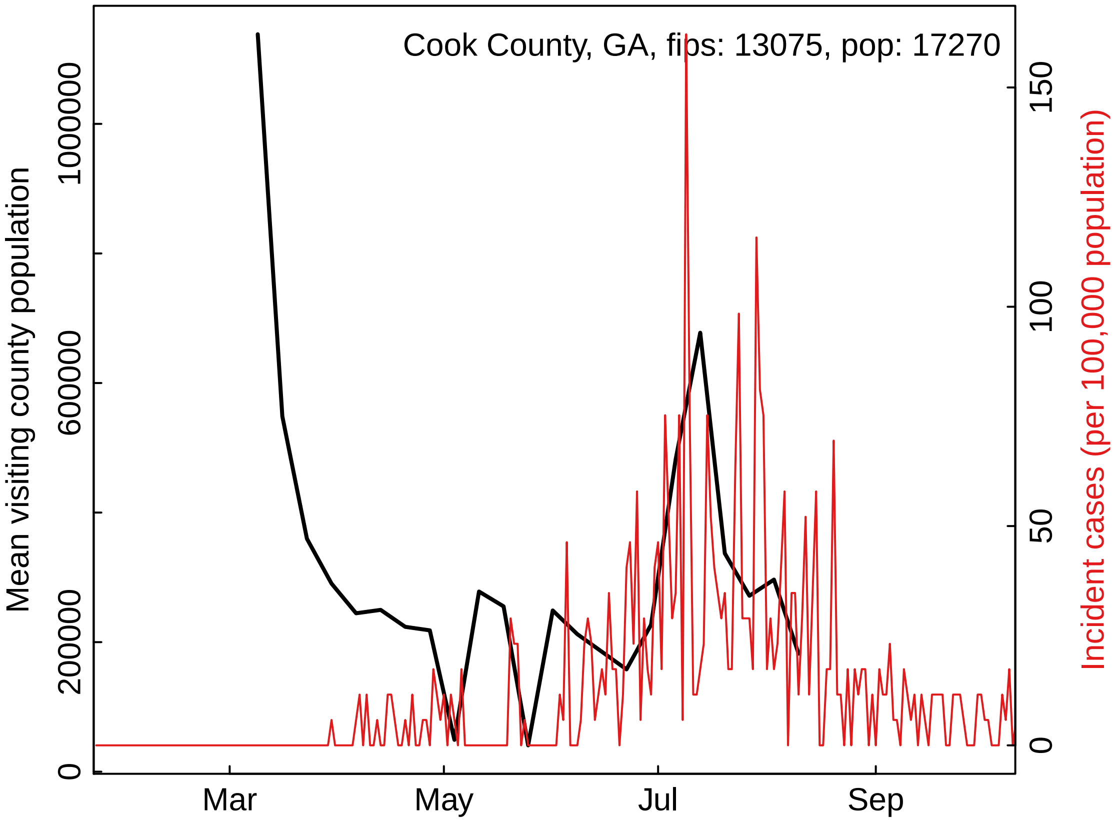

Cook County, GA, fips: 13075, pop: 17270
Mean visiting county population
0
200000
600000
1000000
Incident cases (per 100,000 population)
0
50
100
150
Mar
May
Jul
Sep

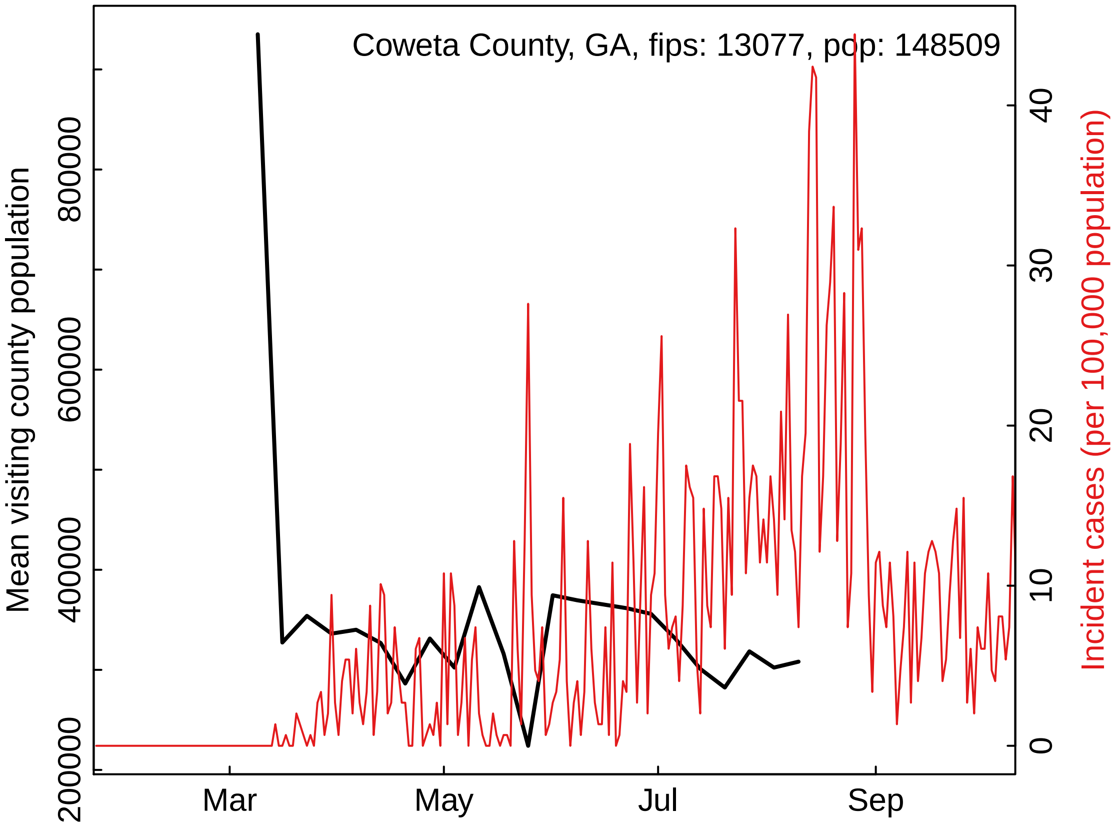
Coweta County, GA, fips: 13077, pop: 148509
Mean visiting county population
200000
400000
600000
800000
Incident cases (per 100,000 population)
0
10
20
30
40
Mar
May
Jul
Sep

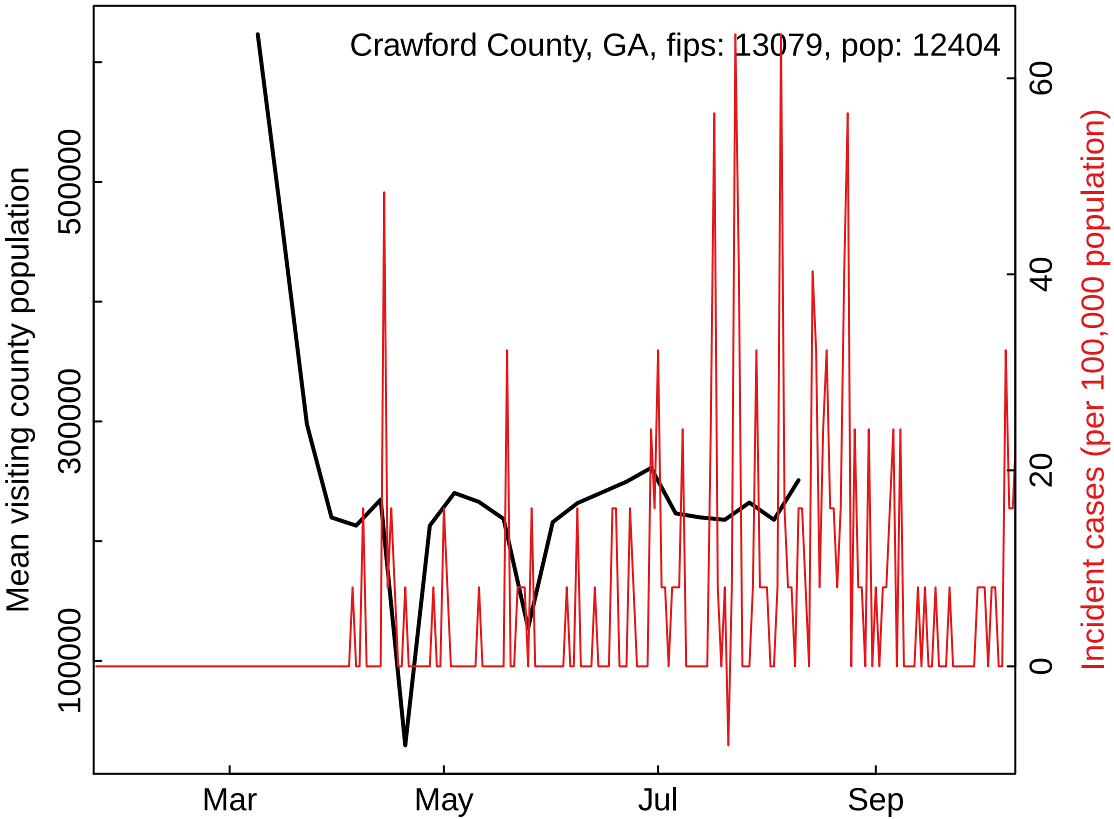

Crawford County, GA, fips: 13079, pop: 12404
Mean visiting county population
100000
300000
500000
Incident cases (per 100,000 population)
0
20
40
60
Mar
May
Jul
Sep

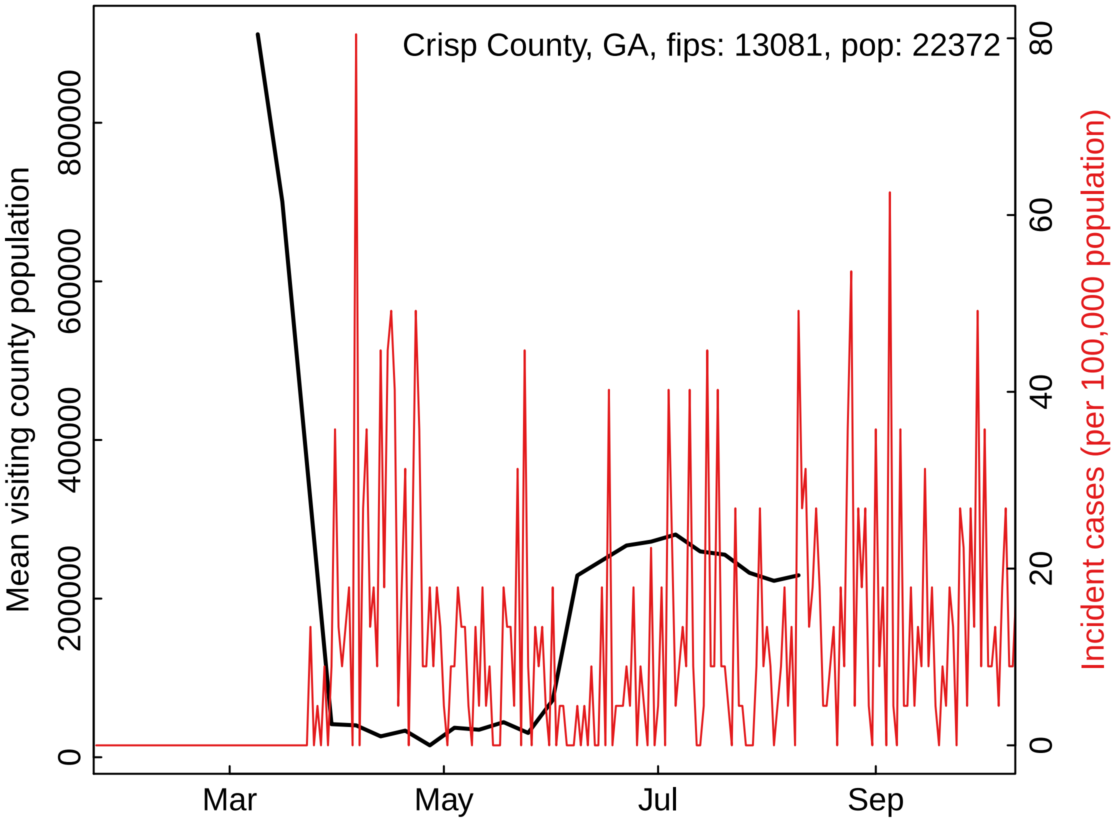

Crisp County, GA, fips: 13081, pop: 22372
Mean visiting county population
0
200000
400000
600000
800000
Incident cases (per 100,000 population)
0
20
40
60
80
Mar
May
Jul
Sep

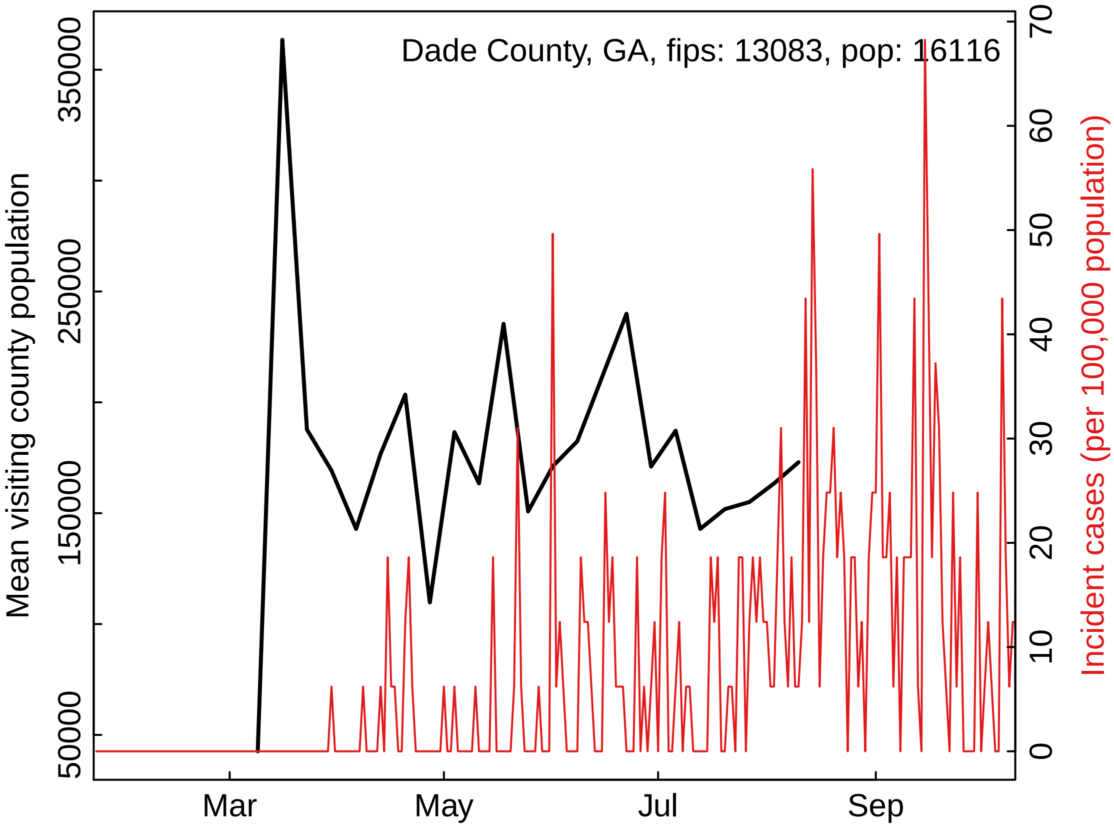
Dade County, GA, fips: 13083, pop: 16116
Mean visiting county population
50000
150000
250000
350000
Incident cases (per 100,000 population)
0
10
20
30
40
50
60
70
Mar
May
Jul
Sep

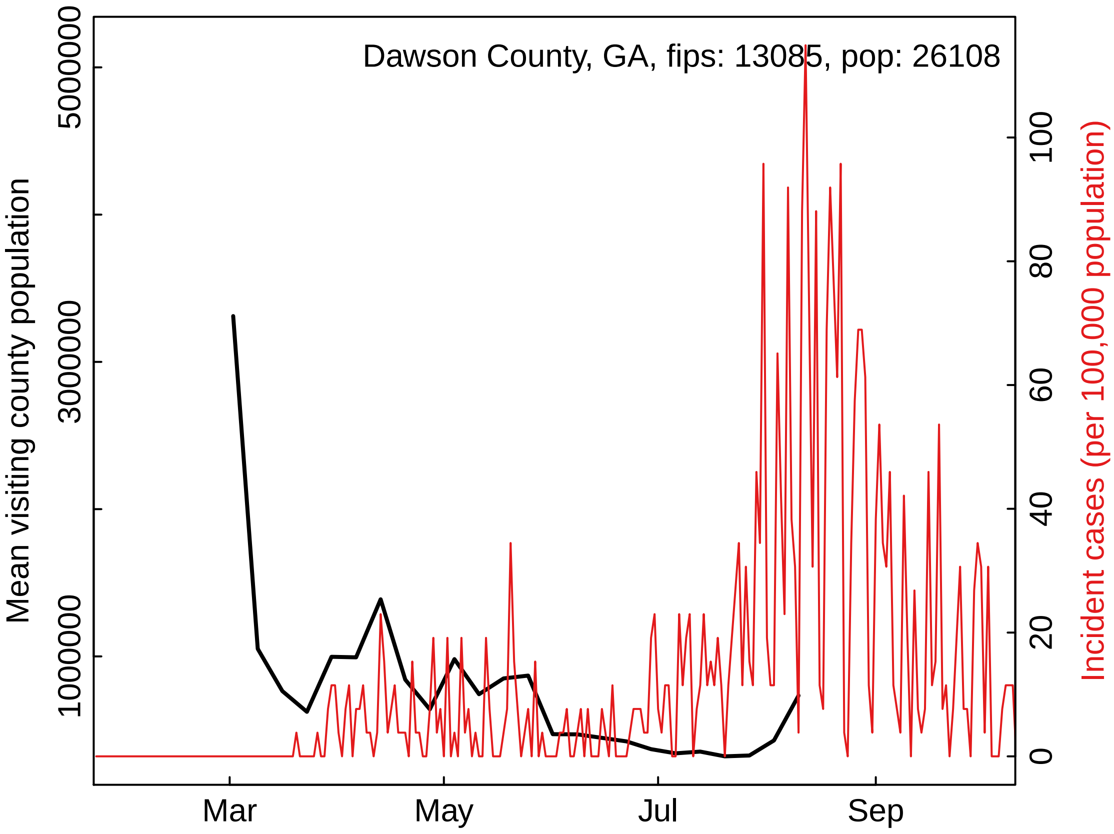

Dawson County, GA, fips: 13085, pop: 26108
Mean visiting county population
1000000
3000000
5000000
Mar
May
Jul
Sep
Incident cases (per 100,000 population)
0
20
40
60
80
100

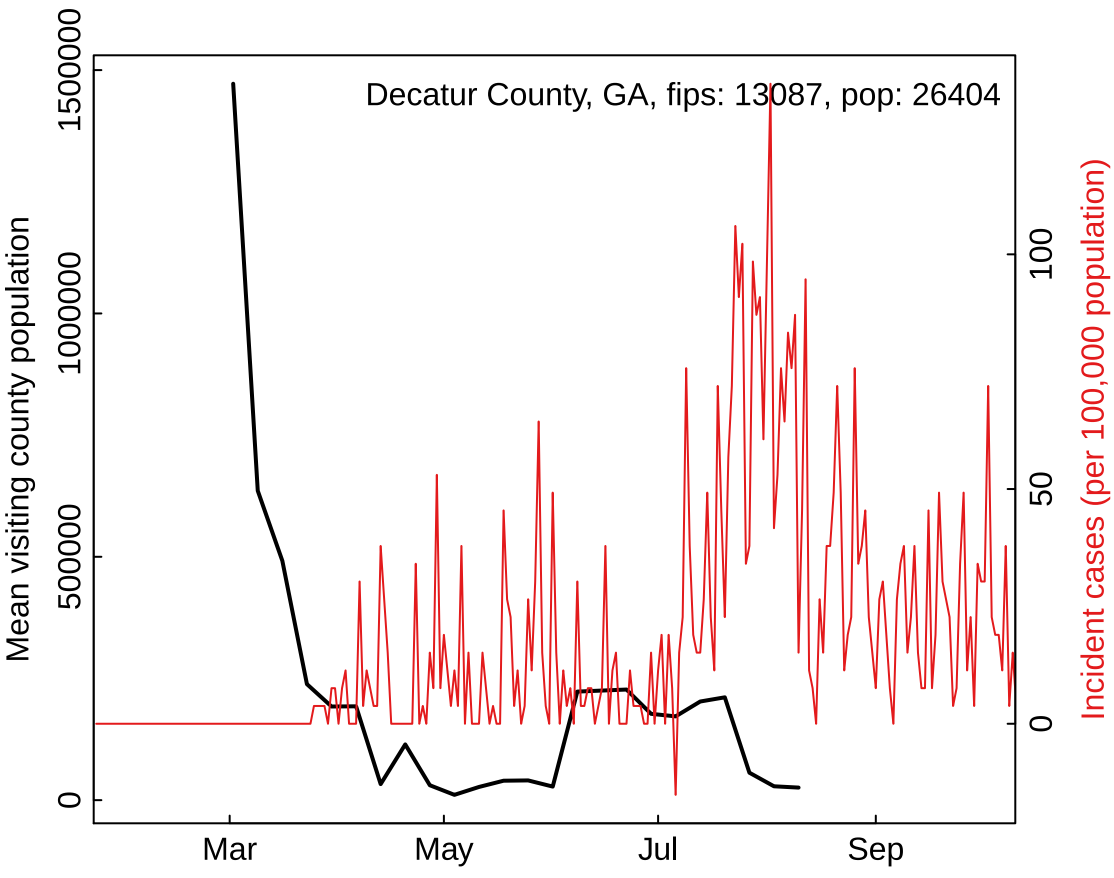

Decatur County, GA, fips: 13087, pop: 26404
Mean visiting county population
0
500000
1000000
1500000
Incident cases (per 100,000 population)
0
50
100
Mar
May
Jul
Sep

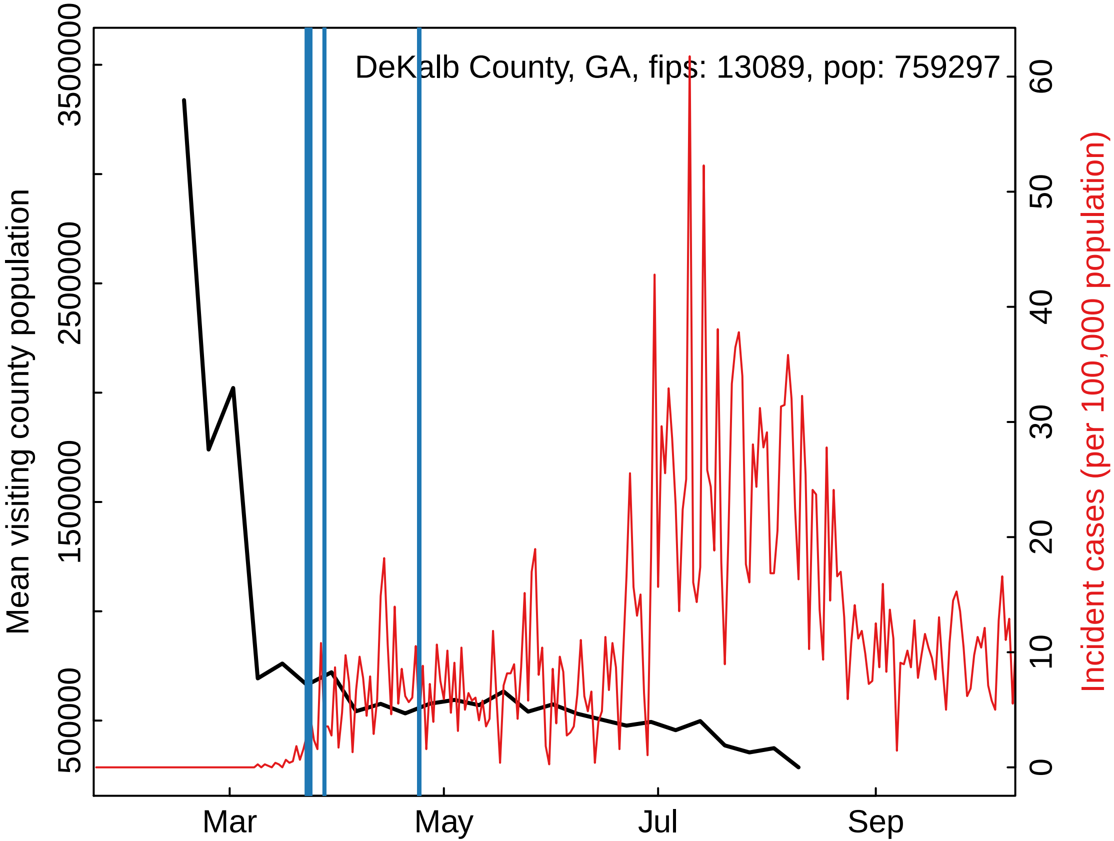
DeKalb County, GA, fips: 13089, pop: 759297
Mean visiting county population
500000
1500000
2500000
3500000
Incident cases (per 100,000 population)
0
10
20
30
40
50
60
Mar
May
Jul
Sep

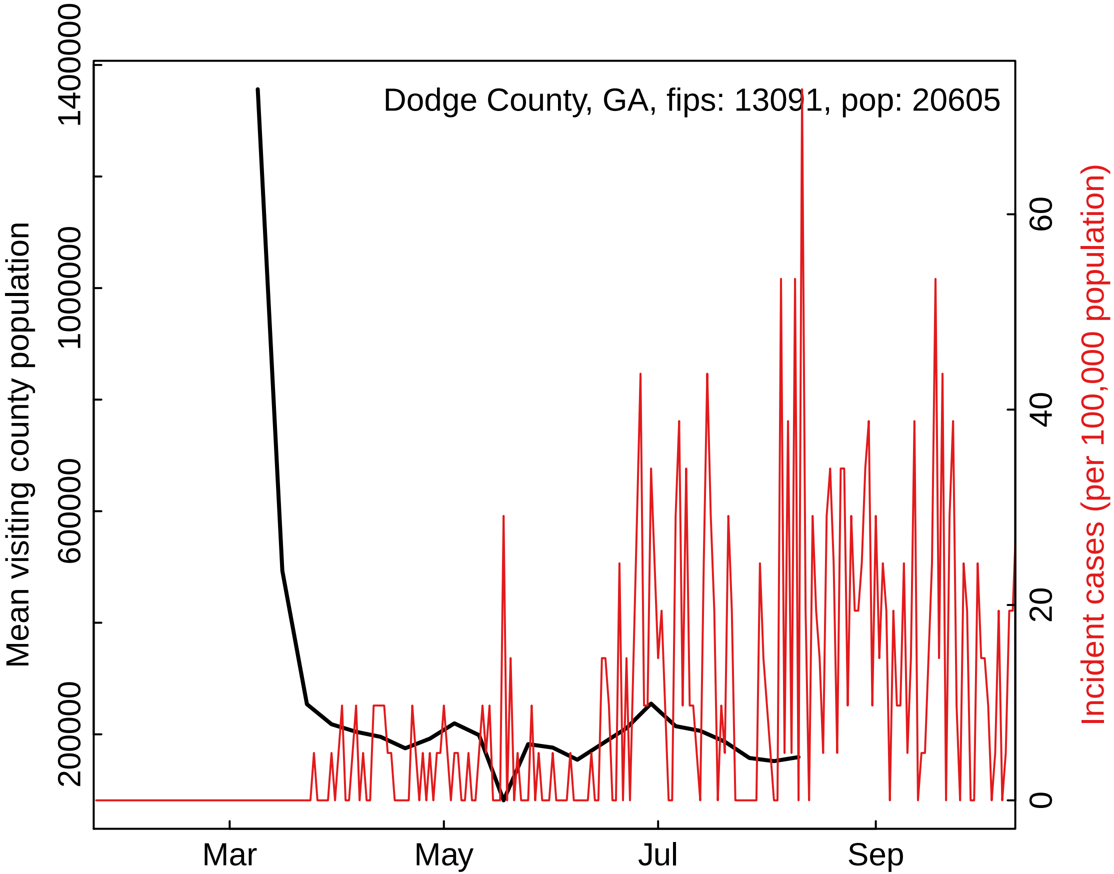

Dodge County, GA, fips: 13091, pop: 20605
Mean visiting county population
200000
600000
1000000
1400000
Incident cases (per 100,000 population)
0
20
40
60
Mar
May
Jul
Sep

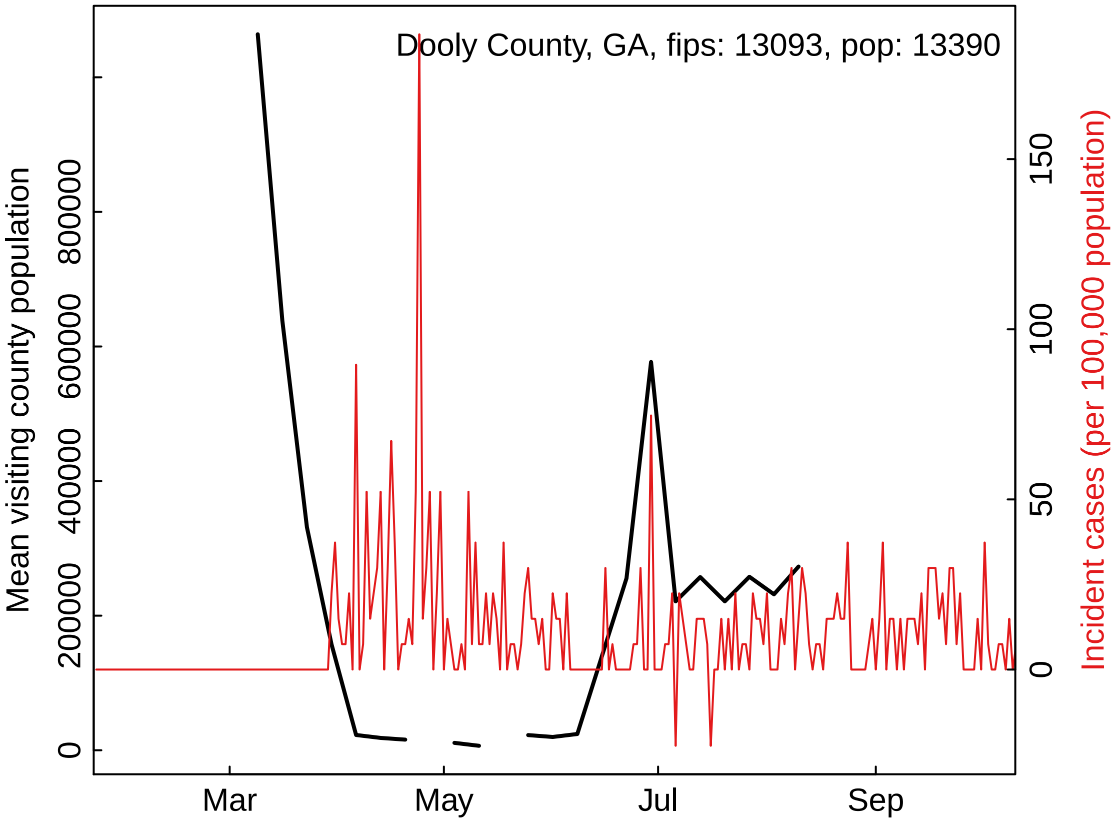

Dooly County, GA, fips: 13093, pop: 13390
Mean visiting county population
0
200000
400000
600000
800000
Incident cases (per 100,000 population)
0
50
100
150
Mar
May
Jul
Sep

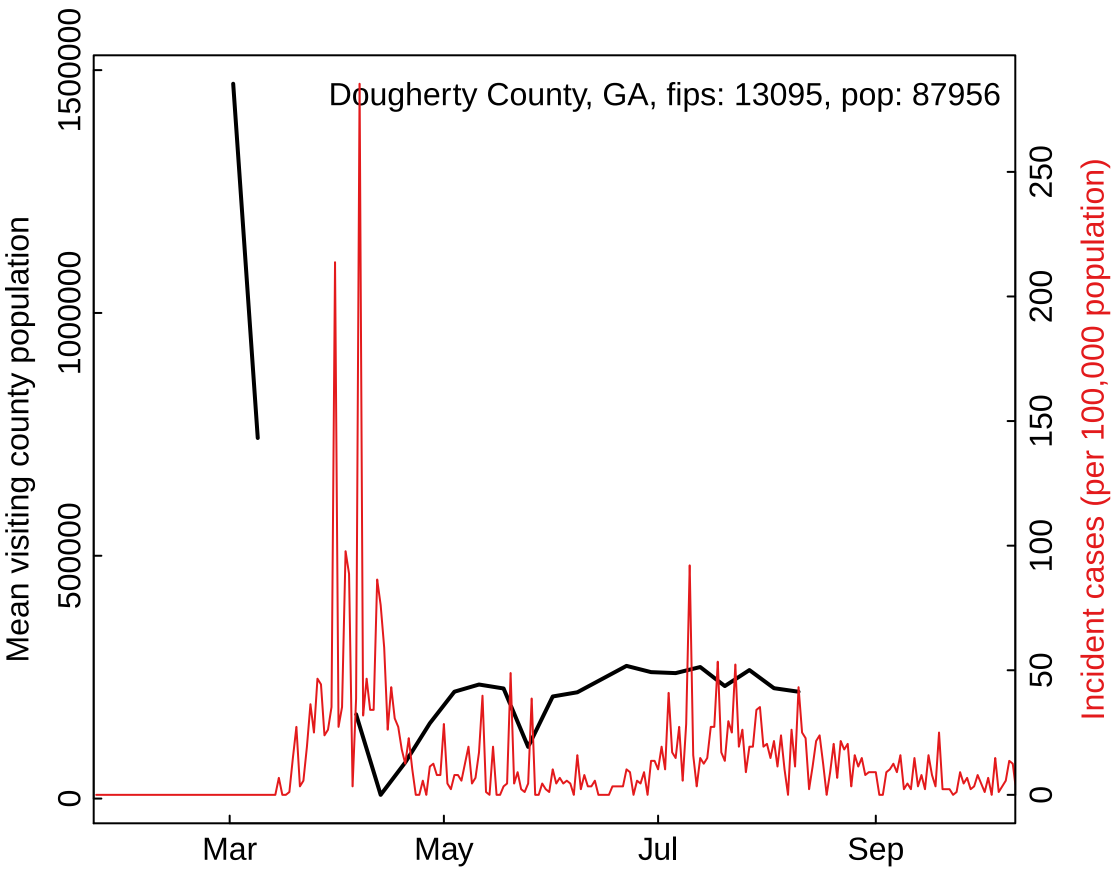

Dougherty County, GA, fips: 13095, pop: 87956
Mean visiting county population
0
500000
1000000
1500000
Incident cases (per 100,000 population)
0
50
100
150
200
250
Mar
May
Jul
Sep

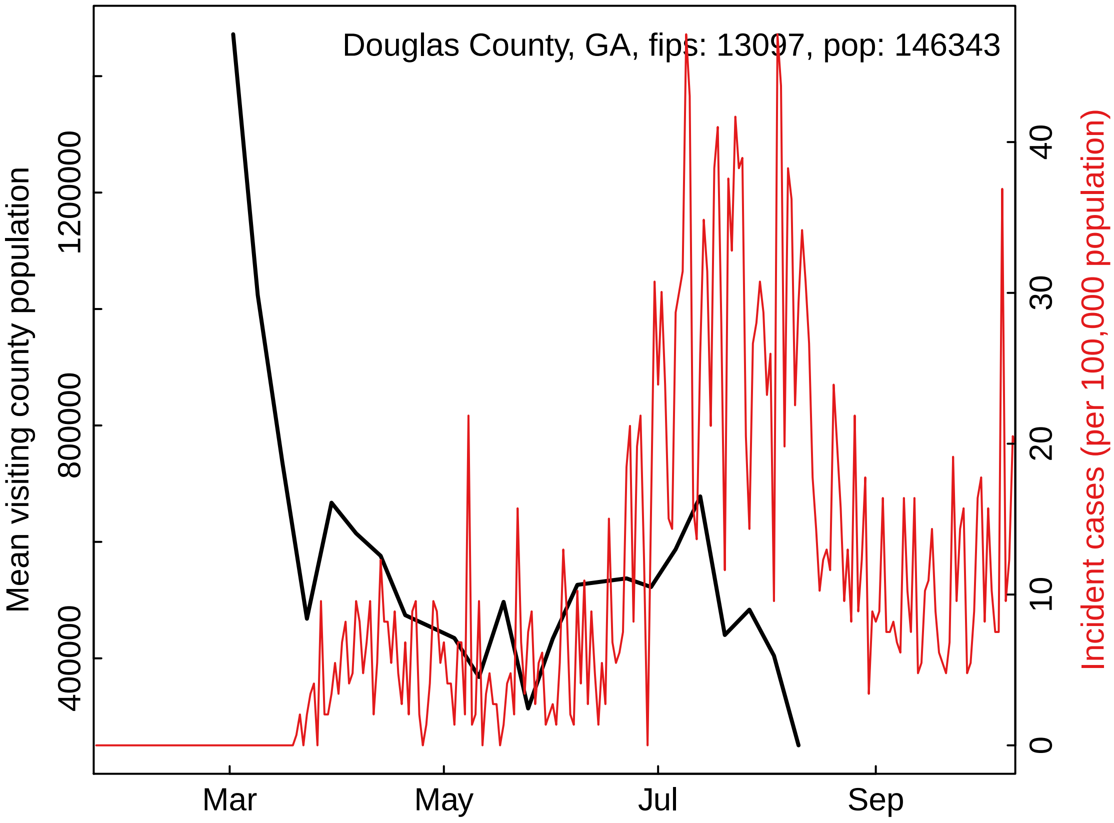
Douglas County, GA, fips: 13097, pop: 146343
Mean visiting county population
400000
800000
1200000
Incident cases (per 100,000 population)
0
10
20
30
40
Mar
May
Jul
Sep

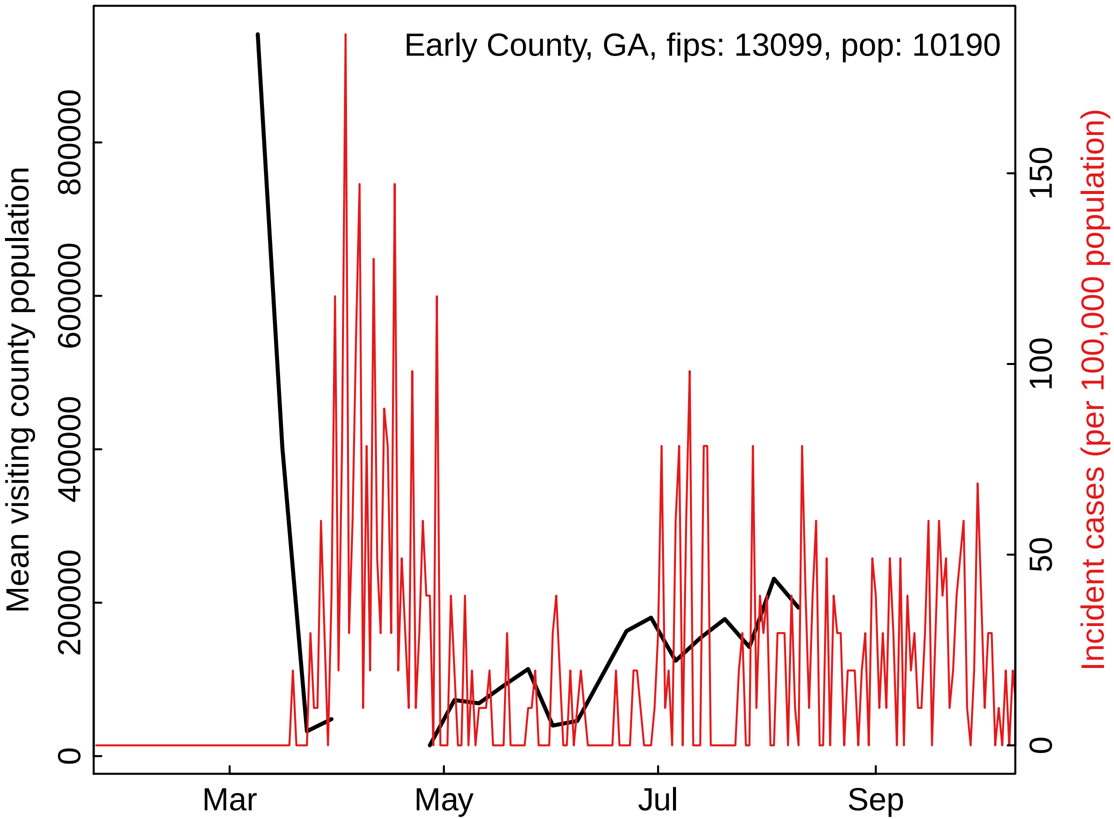

Early County, GA, fips: 13099, pop: 10190
Mean visiting county population
0
200000
400000
600000
800000
Incident cases (per 100,000 population)
0
50
100
150
Mar
May
Jul
Sep

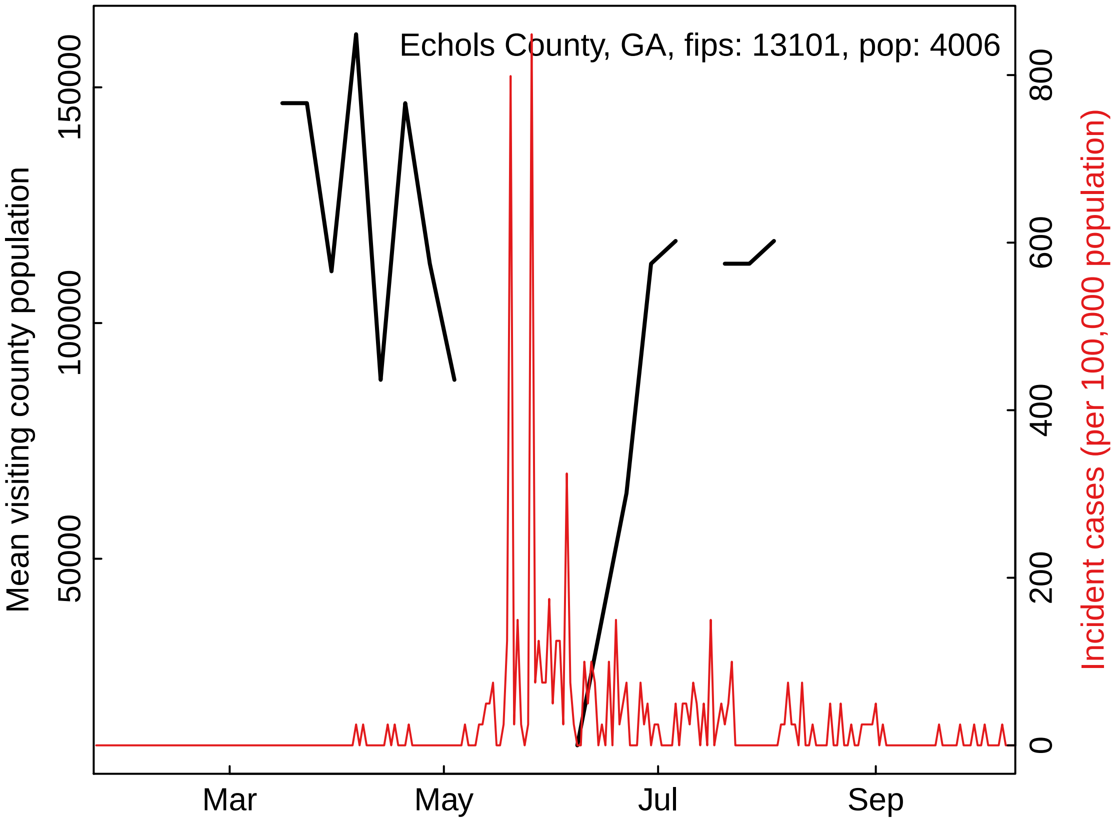

Echols County, GA, fips: 13101, pop: 4006
Mean visiting county population
150000
100000
50000
Incident cases (per 100,000 population)
800
600
400
200
0
Mar
May
Jul
Sep

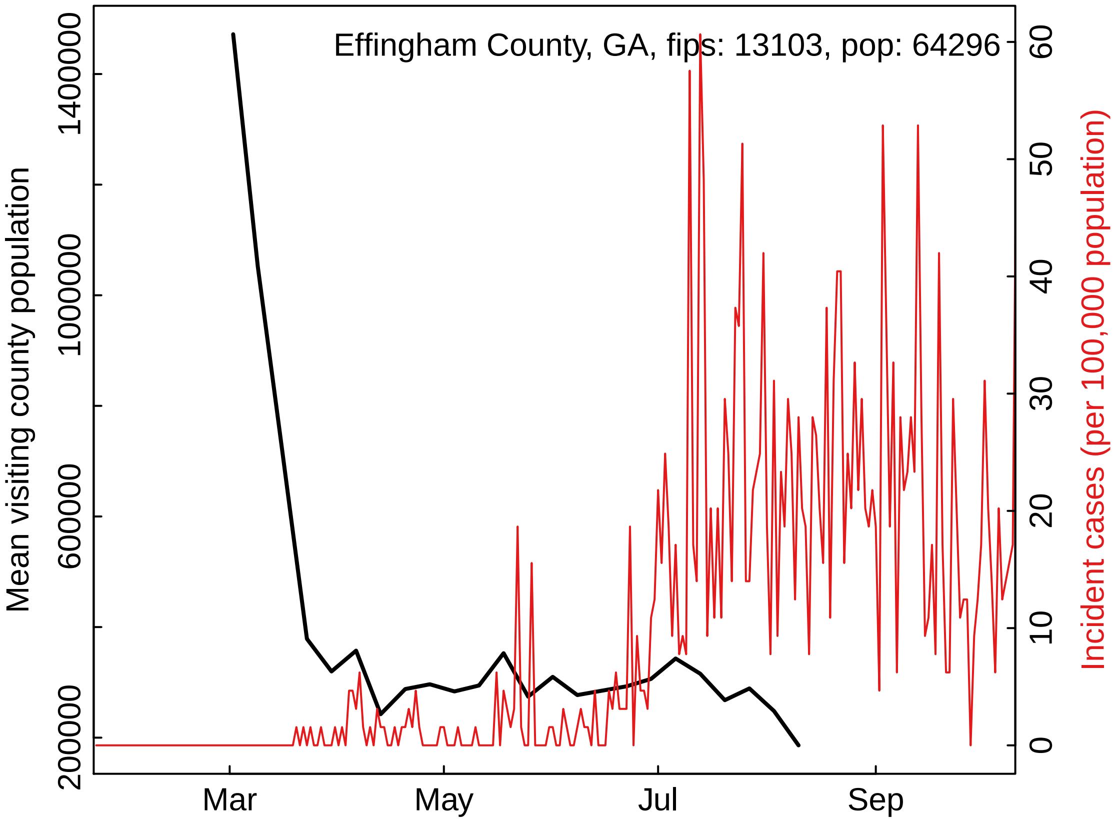

Effingham County, GA, fips: 13103, pop: 64296
Mean visiting county population
200000
600000
1000000
1400000
Incident cases (per 100,000 population)
0
10
20
30
40
50
60
Mar
May
Jul
Sep

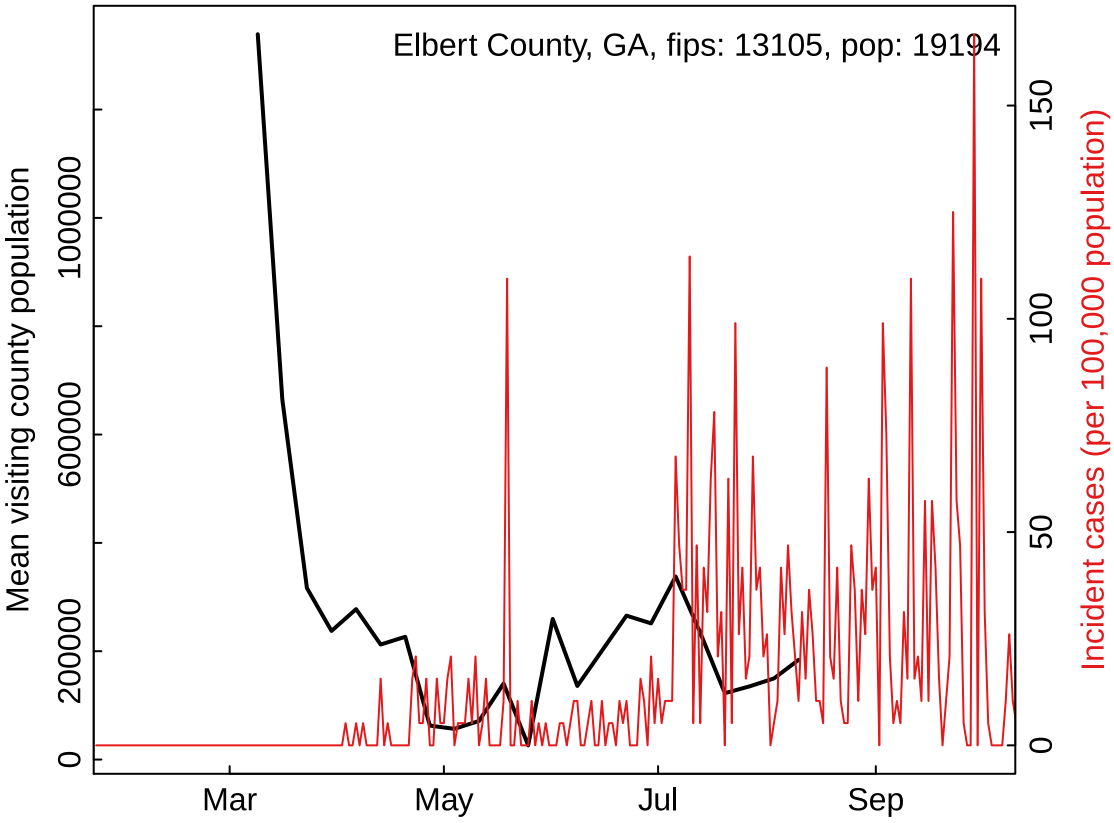
Elbert County, GA, fips: 13105, pop: 19194
Mean visiting county population
0
200000
600000
1000000
Incident cases (per 100,000 population)
0
50
100
150
Mar
May
Jul
Sep

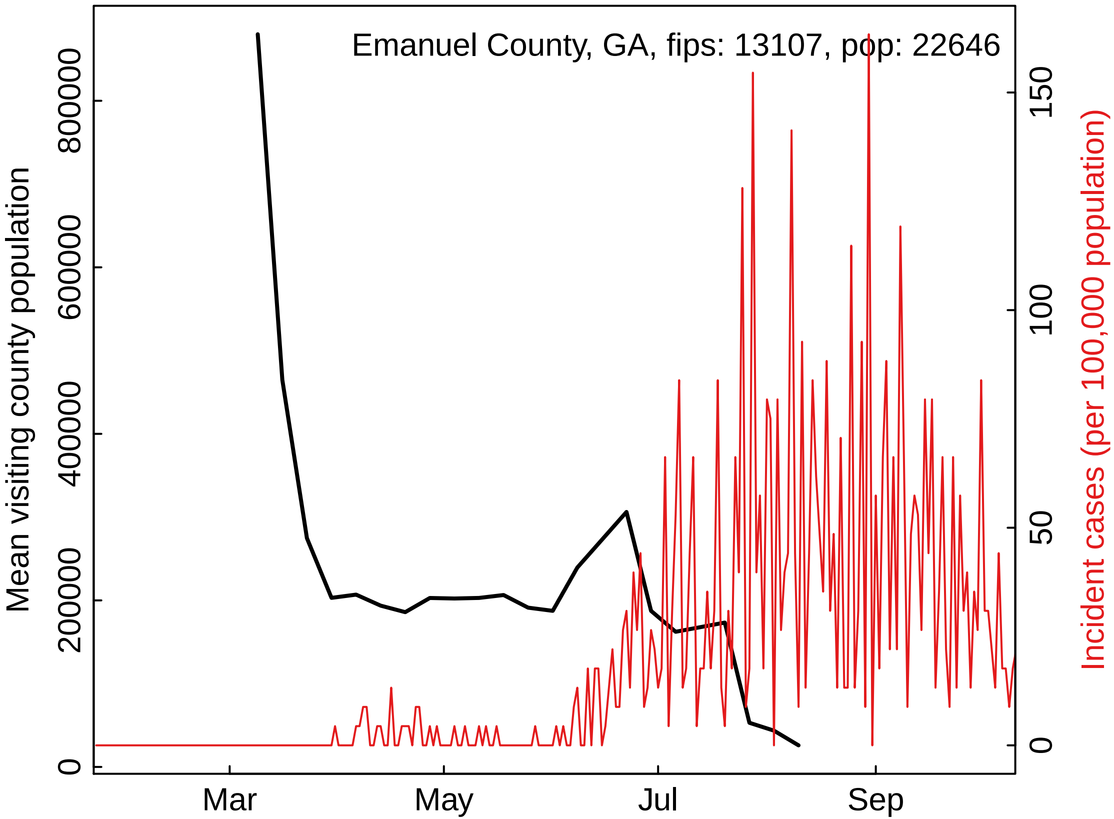

Emanuel County, GA, fips: 13107, pop: 22646
Mean visiting county population
0
200000
400000
600000
800000
Incident cases (per 100,000 population)
0
50
100
150
Mar
May
Jul
Sep

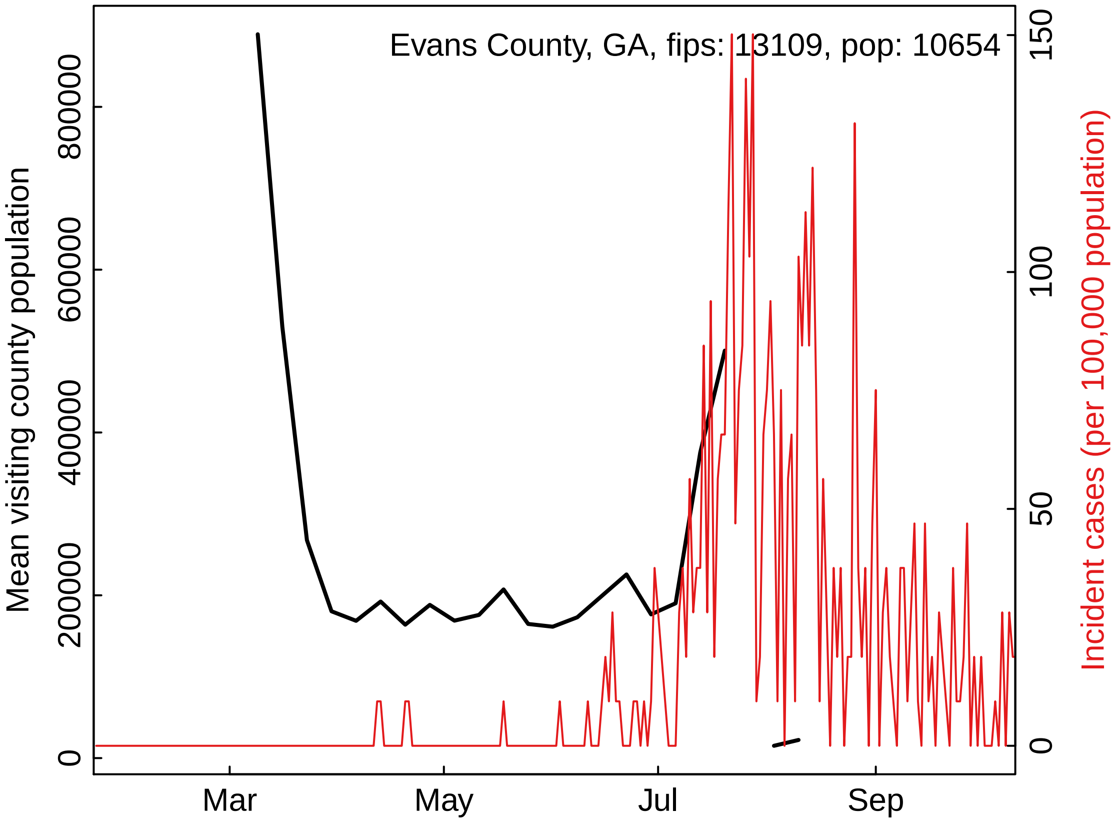

Evans County, GA, fips: 13109, pop: 10654
Mean visiting county population
Incident cases (per 100,000 population)
0
200000
400000
600000
800000
0
50
100
150
Mar
May
Jul
Sep

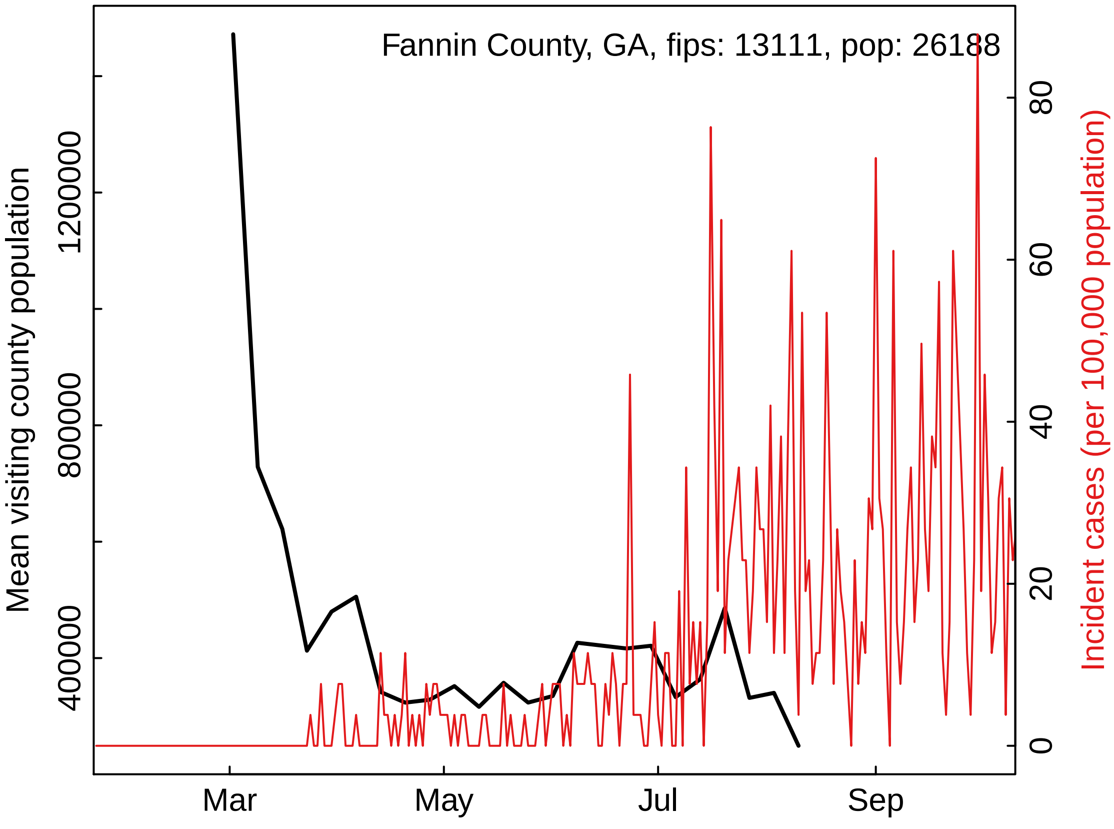

Fannin County, GA, fips: 13111, pop: 26188
Mean visiting county population
400000
800000
1200000
Mar
May
Jul
Sep
0
20
40
60
80
Incident cases (per 100,000 population)

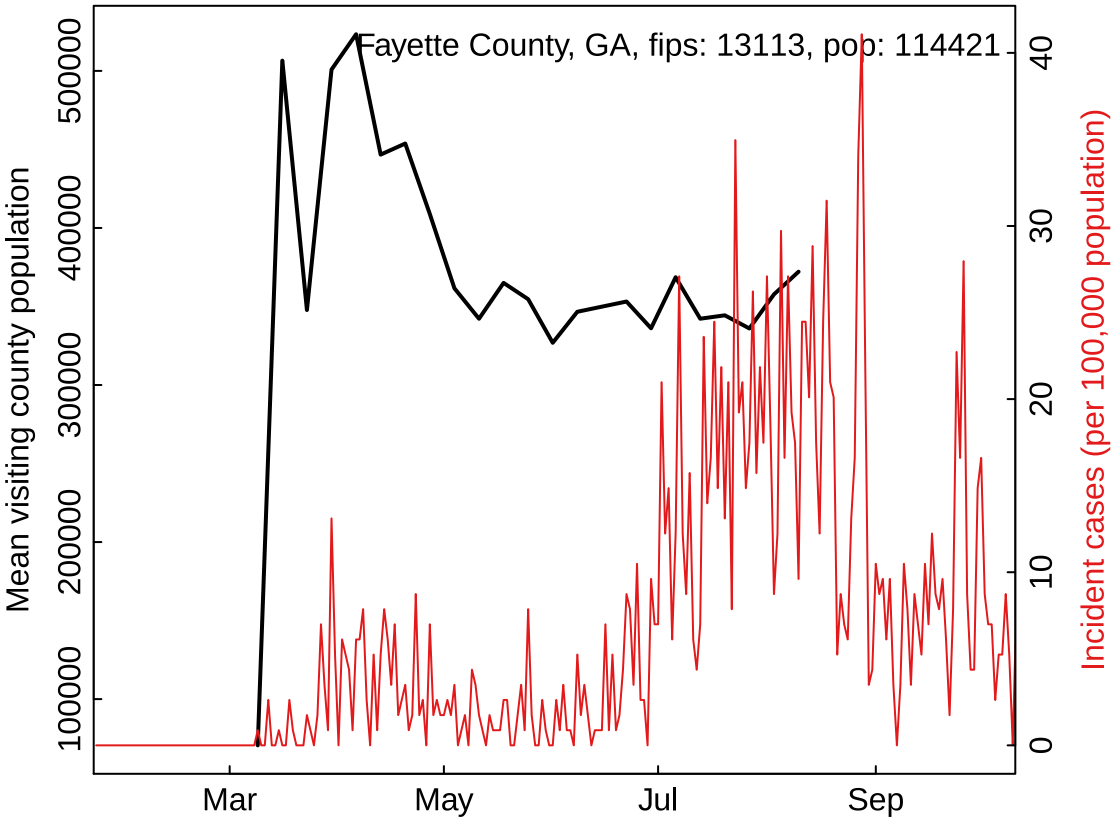

Fayette County, GA, fips: 13113, pop: 114421
Mean visiting county population
100000
200000
300000
400000
500000
Incident cases (per 100,000 population)
0
10
20
30
40
Mar
May
Jul
Sep

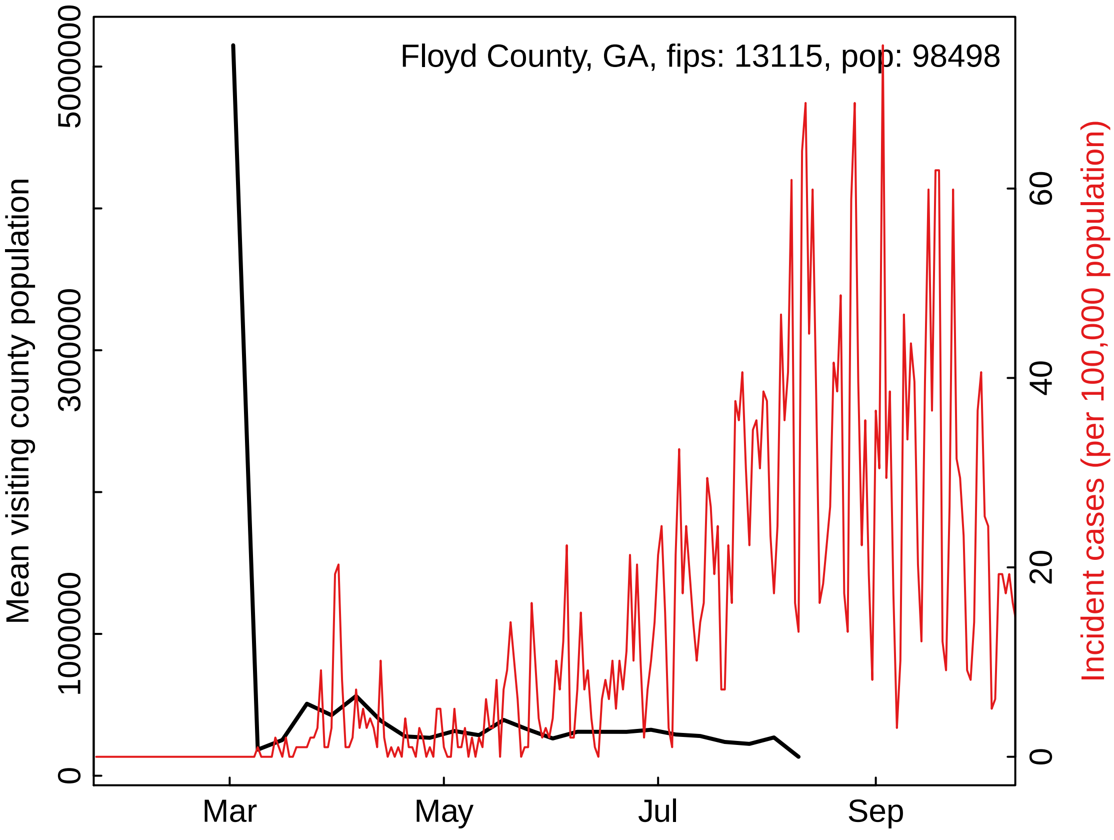

Floyd County, GA, fips: 13115, pop: 98498
Mean visiting county population
Incident cases (per 100,000 population)
0
1000000
3000000
5000000
0
20
40
60
Mar
May
Jul
Sep

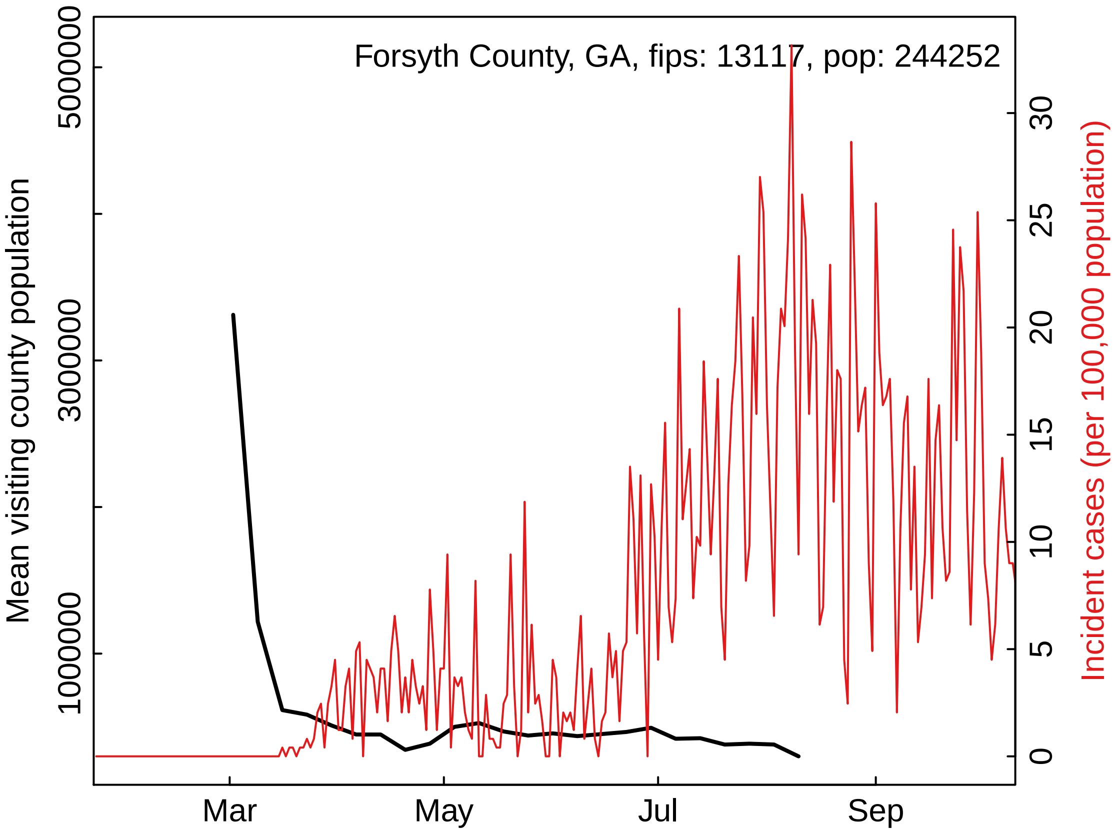
Forsyth County, GA, fips: 13117, pop: 244252
Mean visiting county population
Incident cases (per 100,000 population)
1000000
3000000
5000000
0
5
10
15
20
25
30
Mar
May
Jul
Sep

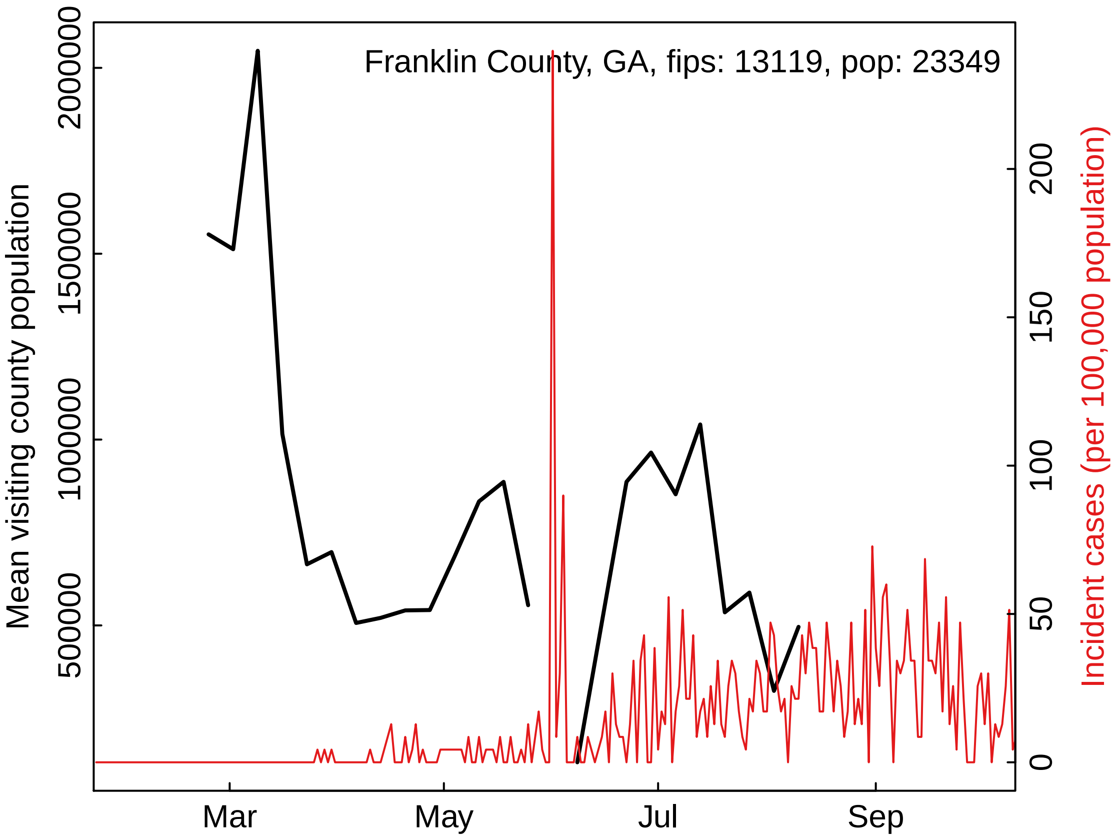
Franklin County, GA, fips: 13119, pop: 23349
Mean visiting county population
500000
1000000
1500000
2000000
Incident cases (per 100,000 population)
0
50
100
150
200
Mar
May
Jul
Sep

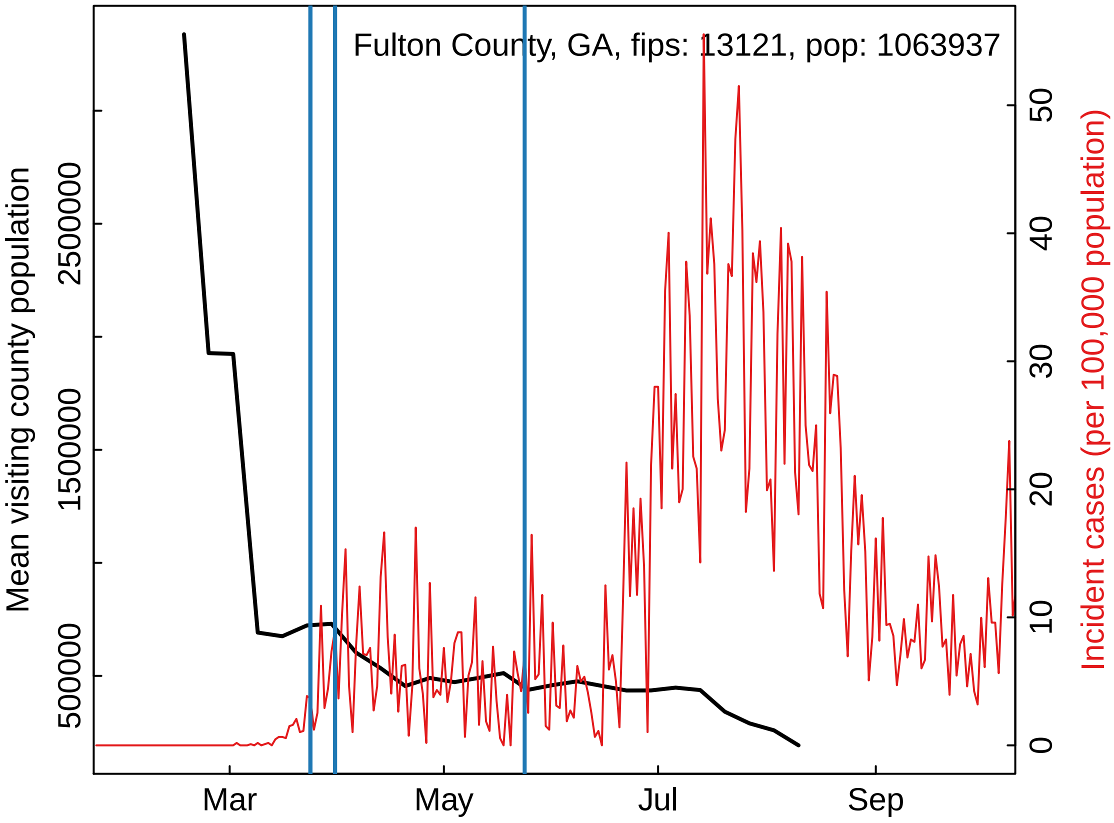

Fulton County, GA, fips: 13121, pop: 1063937
Mean visiting county population
500000
1500000
2500000
Incident cases (per 100,000 population)
0
10
20
30
40
50
Mar
May
Jul
Sep

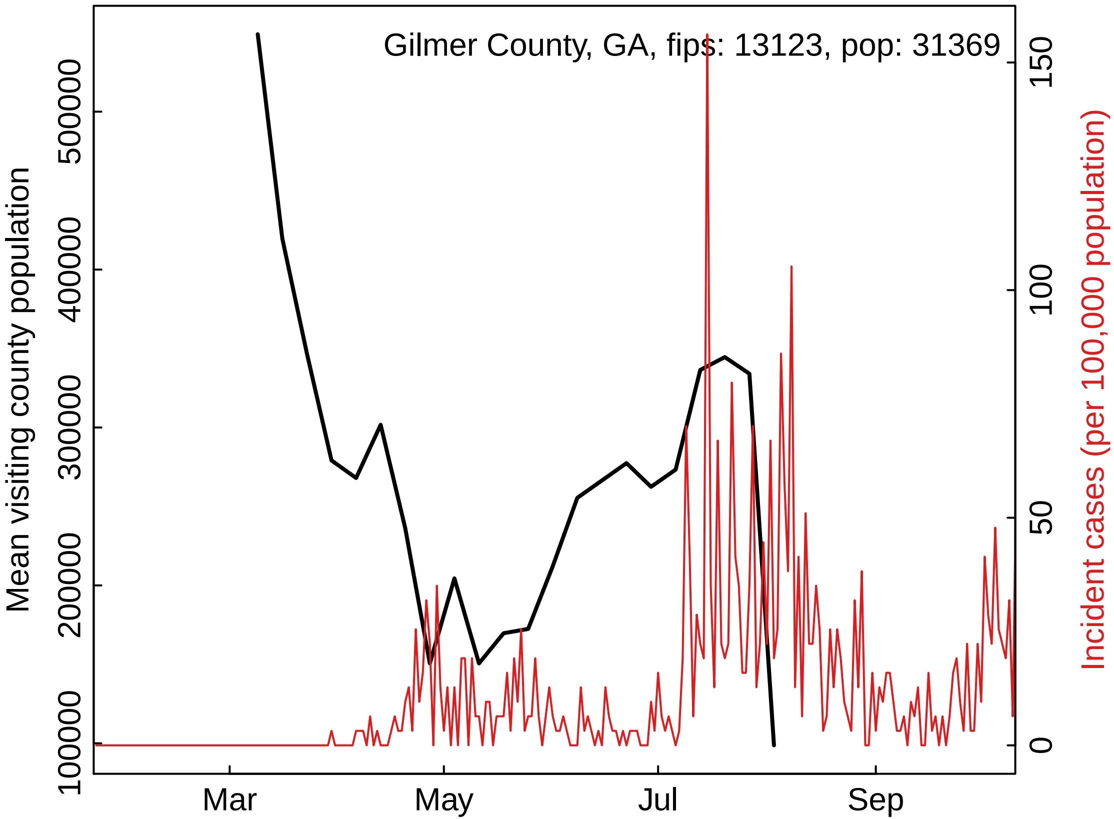
Gilmer County, GA, fips: 13123, pop: 31369
Mean visiting county population
Incident cases (per 100,000 population)
100000
200000
300000
400000
500000
0
50
100
150
Mar
May
Jul
Sep

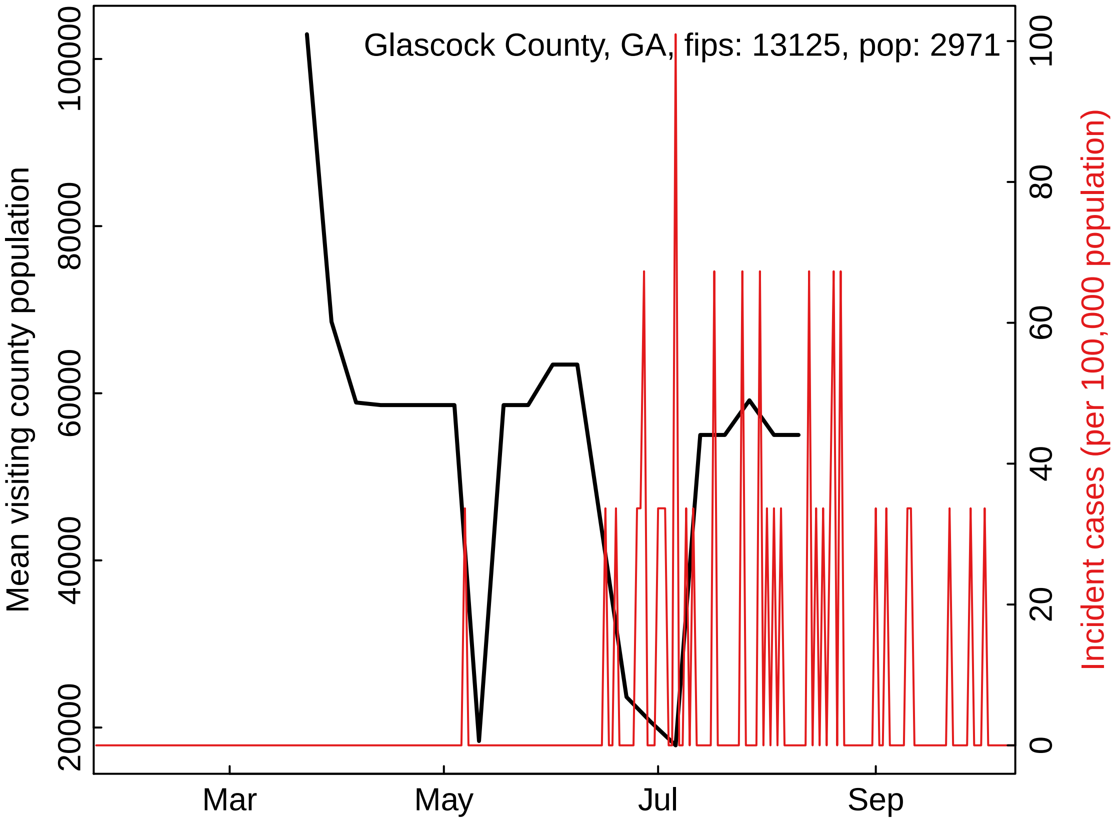

Glascock County, GA, fips: 13125, pop: 2971
Mean visiting county population
20000
40000
60000
80000
100000
Incident cases (per 100,000 population)
0
20
40
60
80
100
Mar
May
Jul
Sep

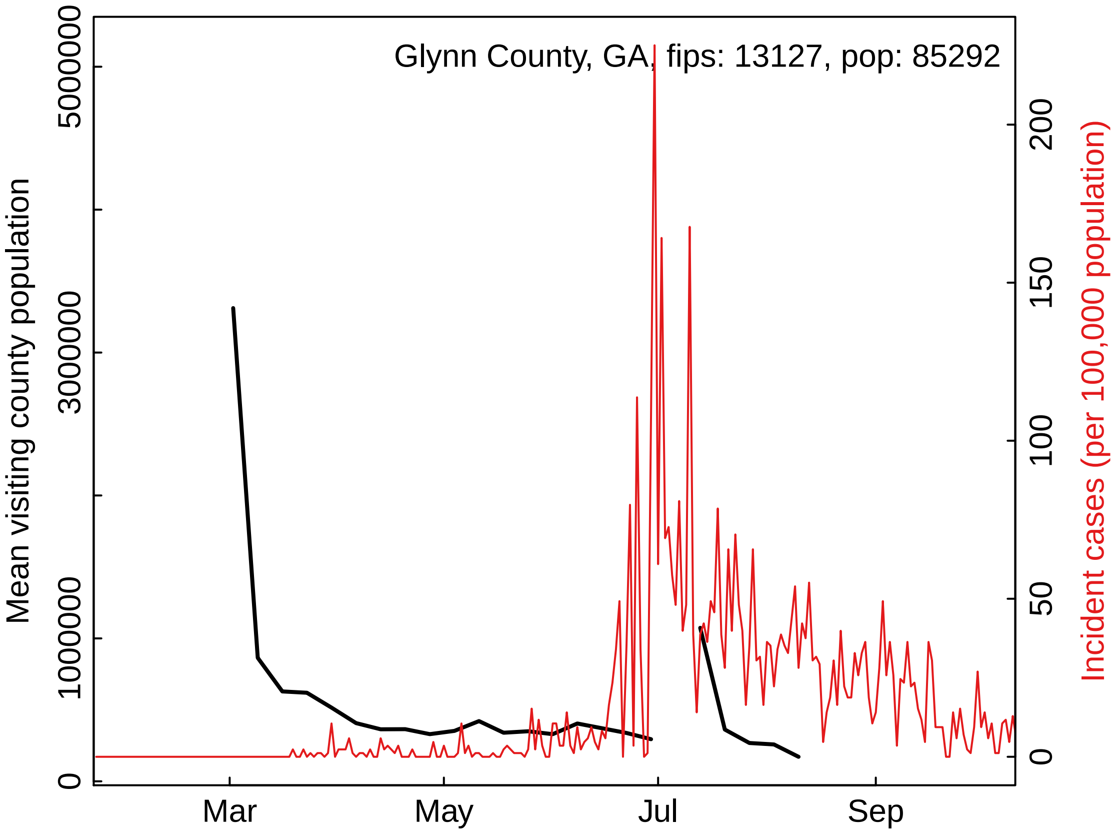

Glynn County, GA, fips: 13127, pop: 85292
Mean visiting county population
0
1000000
3000000
5000000
Incident cases (per 100,000 population)
0
50
100
150
200
Mar
May
Jul
Sep

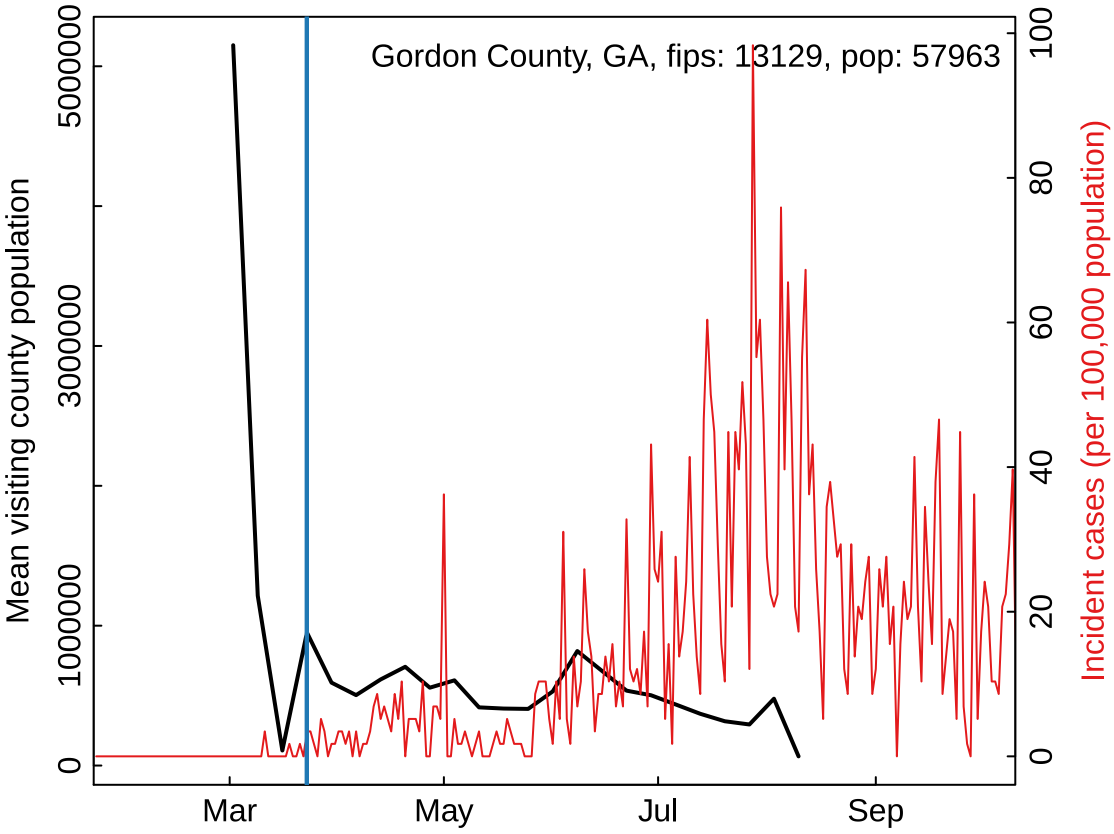

Gordon County, GA, fips: 13129, pop: 57963
Mean visiting county population
Incident cases (per 100,000 population)
0
1000000
3000000
5000000
0
20
40
60
80
100
Mar
May
Jul
Sep

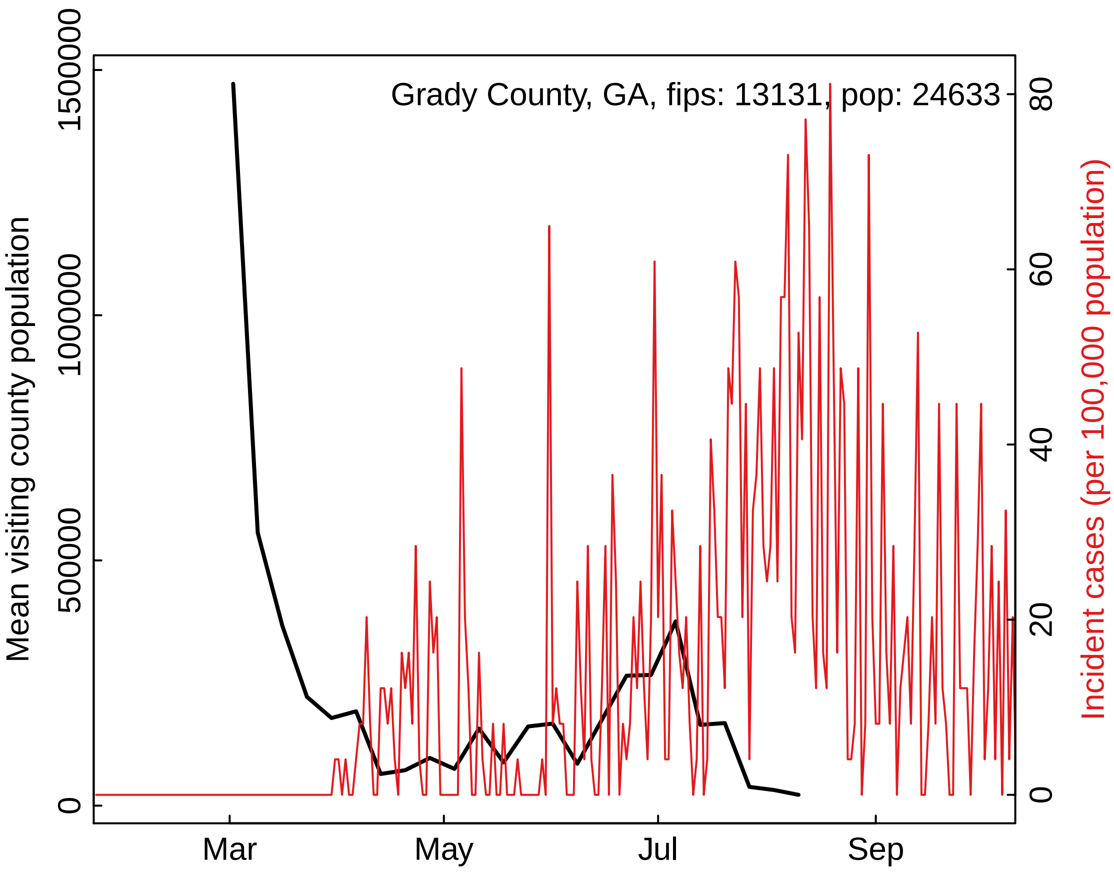

Grady County, GA, fips: 13131, pop: 24633
Mean visiting county population
0
500000
1000000
1500000
Incident cases (per 100,000 population)
0
20
40
60
80
Mar
May
Jul
Sep

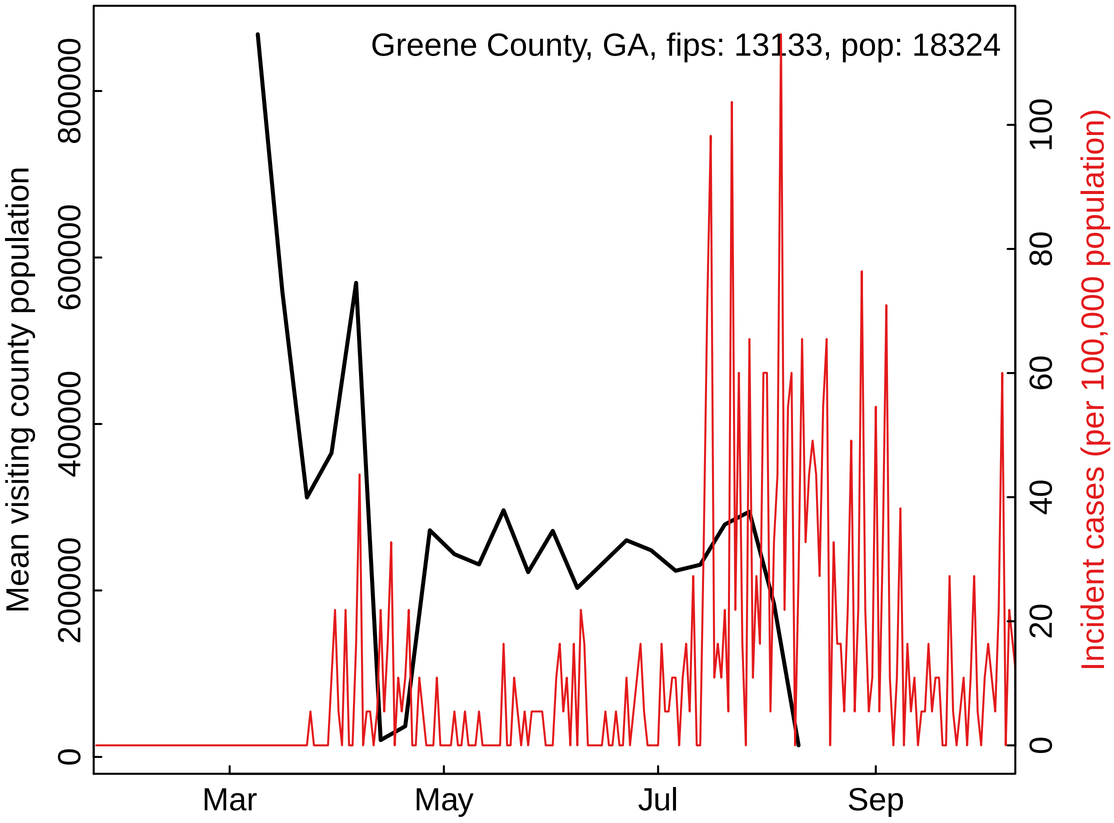

Greene County, GA, fips: 13133, pop: 18324
Mean visiting county population
0
200000
400000
600000
800000
Incident cases (per 100,000 population)
0
20
40
60
80
100
Mar
May
Jul
Sep

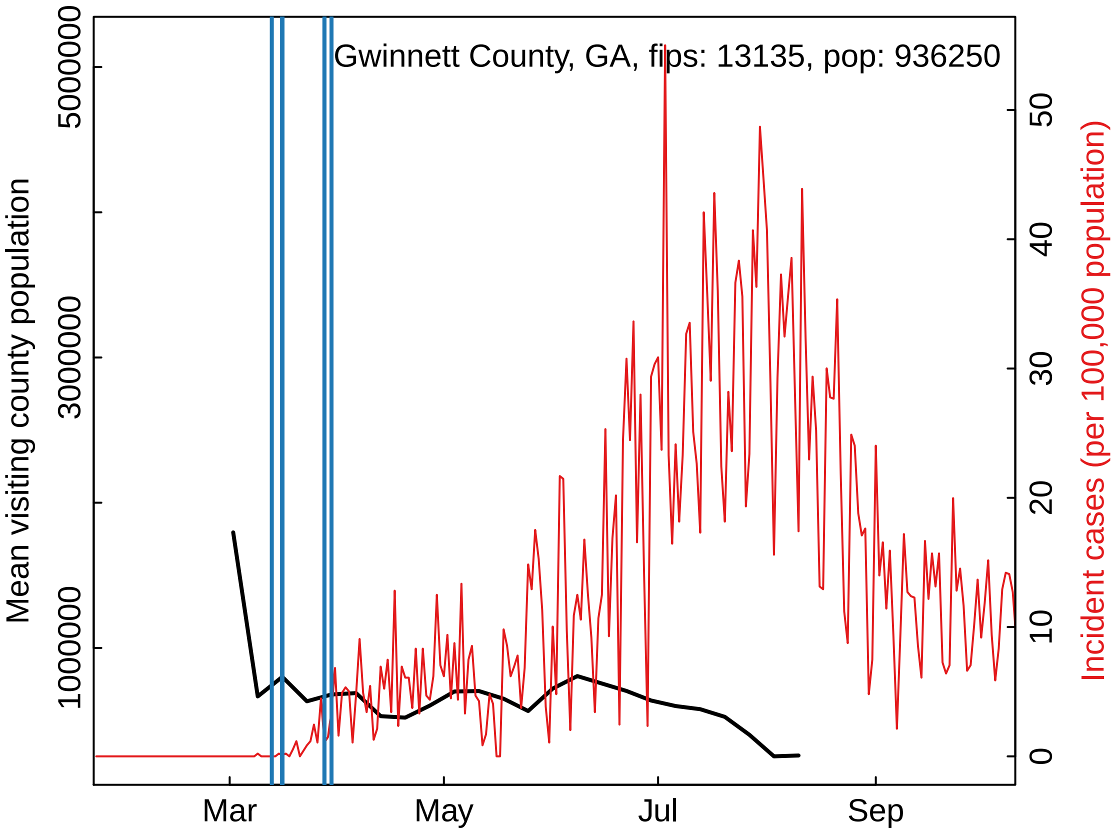

Gwinnett County, GA, fips: 13135, pop: 936250
Mean visiting county population
1000000
3000000
5000000
Incident cases (per 100,000 population)
0
10
20
30
40
50
Mar
May
Jul
Sep

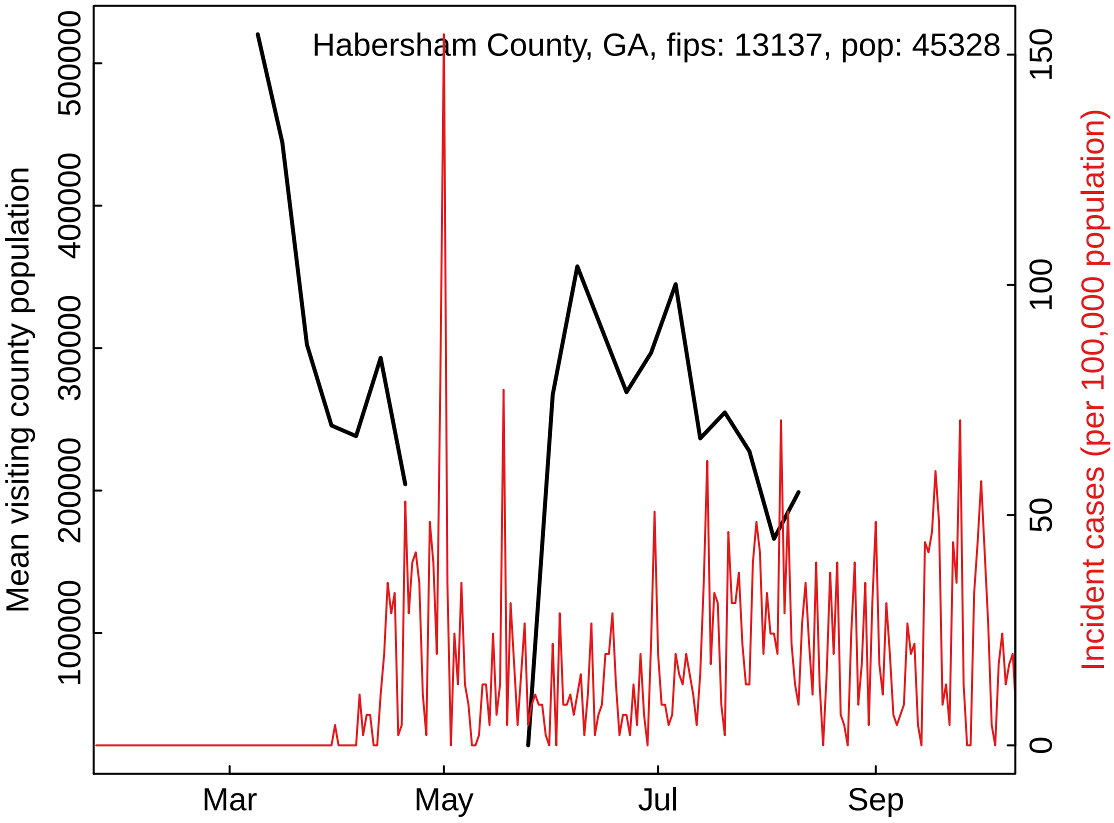

Habersham County, GA, fips: 13137, pop: 45328
Mean visiting county population
100000
200000
300000
400000
500000
Incident cases (per 100,000 population)
0
50
100
150
Mar
May
Jul
Sep

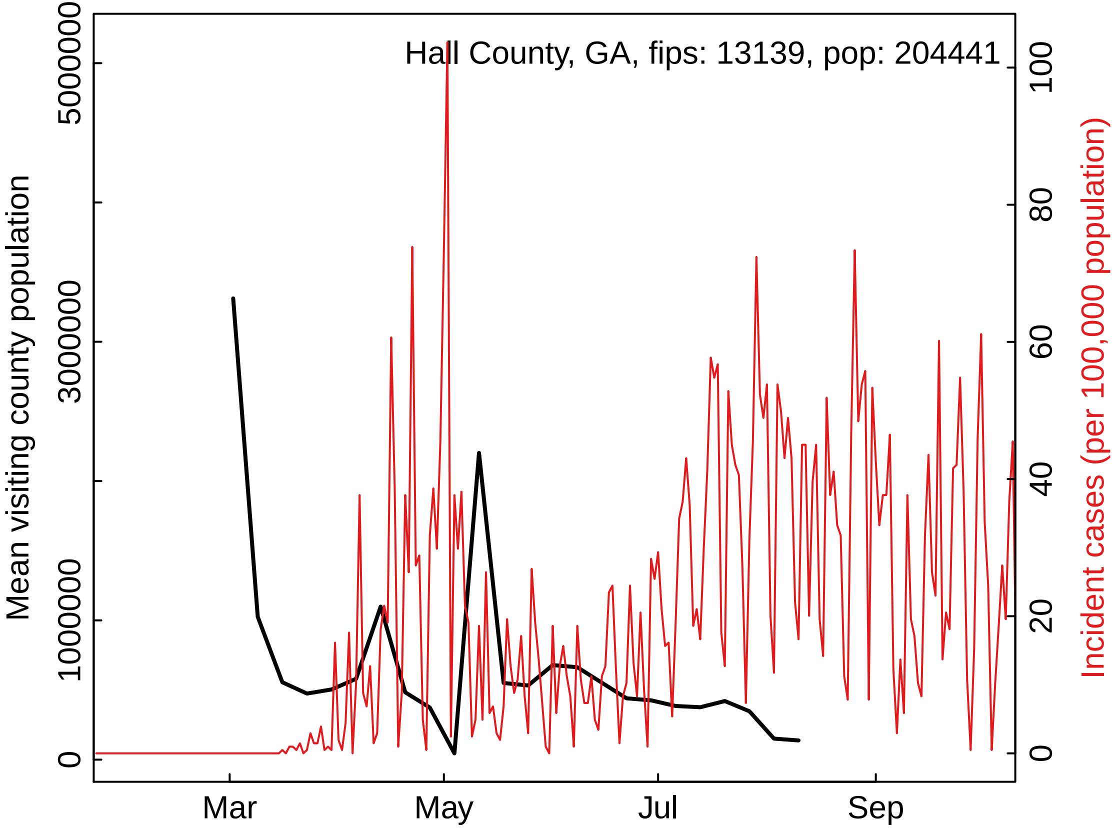

Hall County, GA, fips: 13139, pop: 204441
Mean visiting county population
0
1000000
3000000
5000000
Incident cases (per 100,000 population)
0
20
40
60
80
100
Mar
May
Jul
Sep

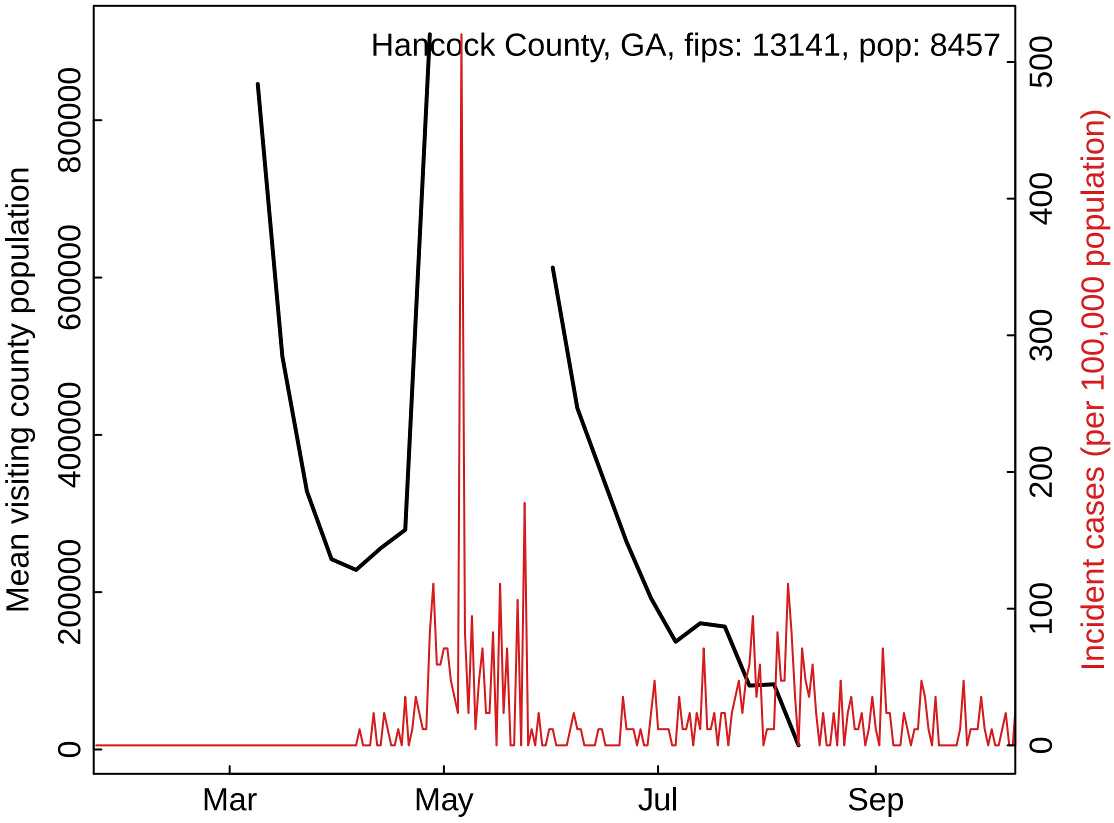

Hancock County, GA, fips: 13141, pop: 8457
Mean visiting county population
0
200000
400000
600000
800000
Incident cases (per 100,000 population)
0
100
200
300
400
500
Mar
May
Jul
Sep

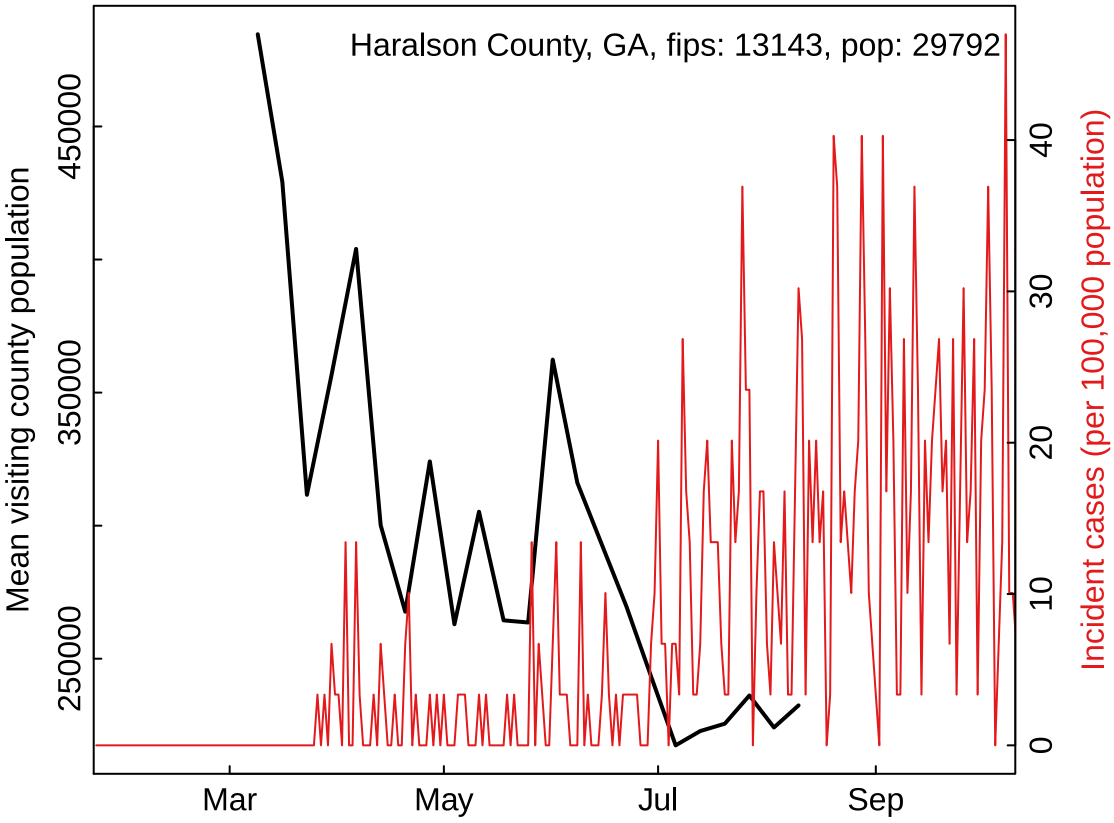

Haralson County, GA, fips: 13143, pop: 29792
Mean visiting county population
450000
350000
250000
Incident cases (per 100,000 population)
40
30
20
10
0
Mar
May
Jul
Sep

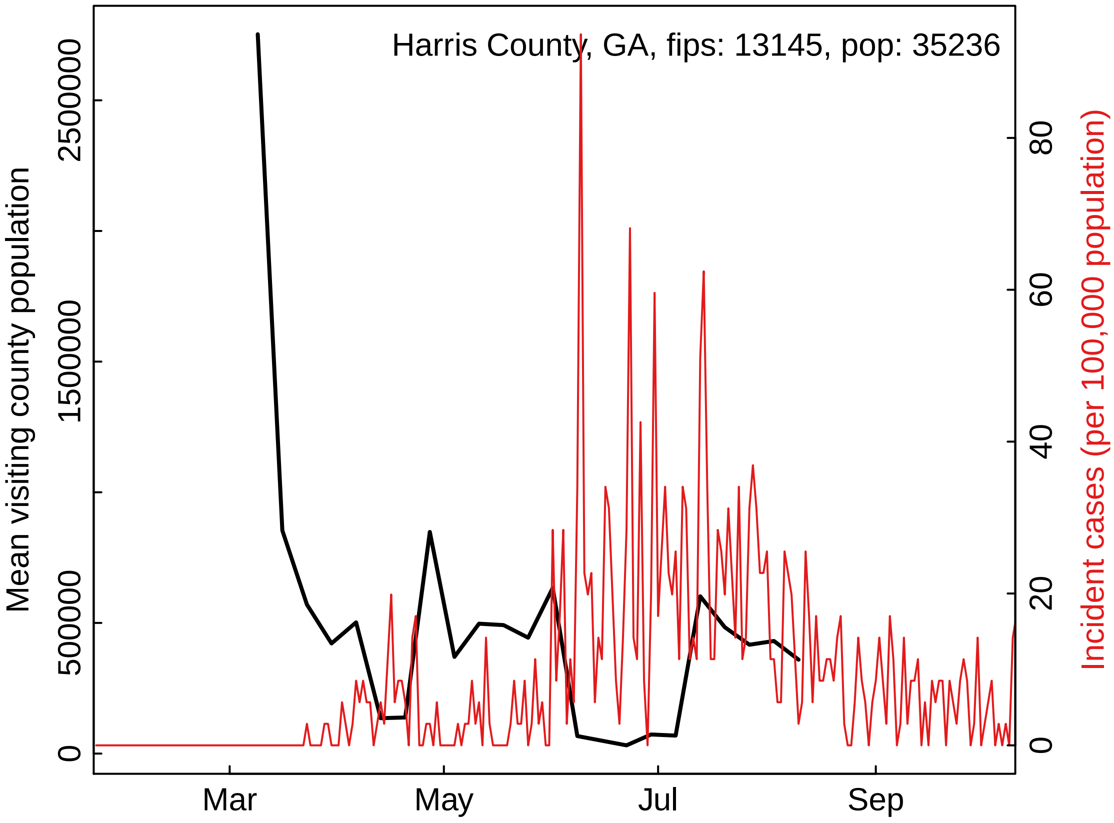

Harris County, GA, fips: 13145, pop: 35236
Mean visiting county population
0
500000
1500000
2500000
Incident cases (per 100,000 population)
0
20
40
60
80
Mar
May
Jul
Sep

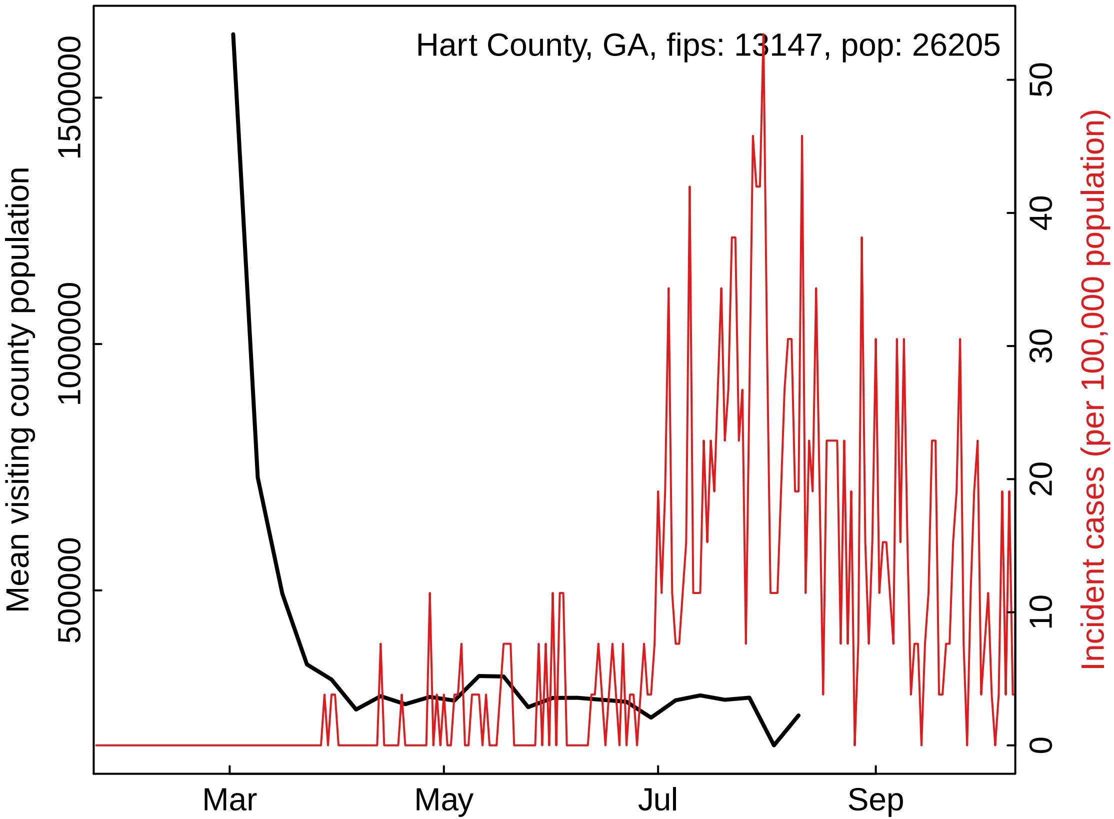

Hart County, GA, fips: 13147, pop: 26205
Mean visiting county population
500000
1000000
1500000
Incident cases (per 100,000 population)
0
10
20
30
40
50
Mar
May
Jul
Sep

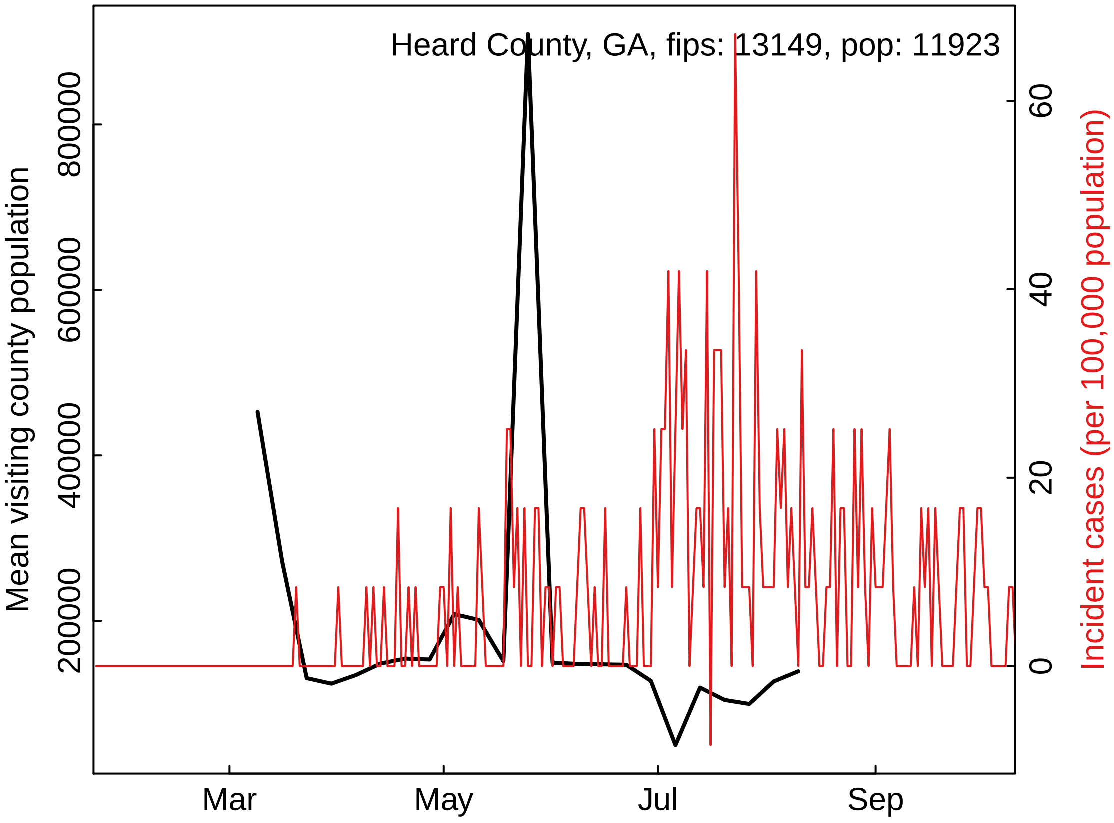

Heard County, GA, fips: 13149, pop: 11923
Mean visiting county population
200000
400000
600000
800000
Incident cases (per 100,000 population)
0
20
40
60
Mar
May
Jul
Sep

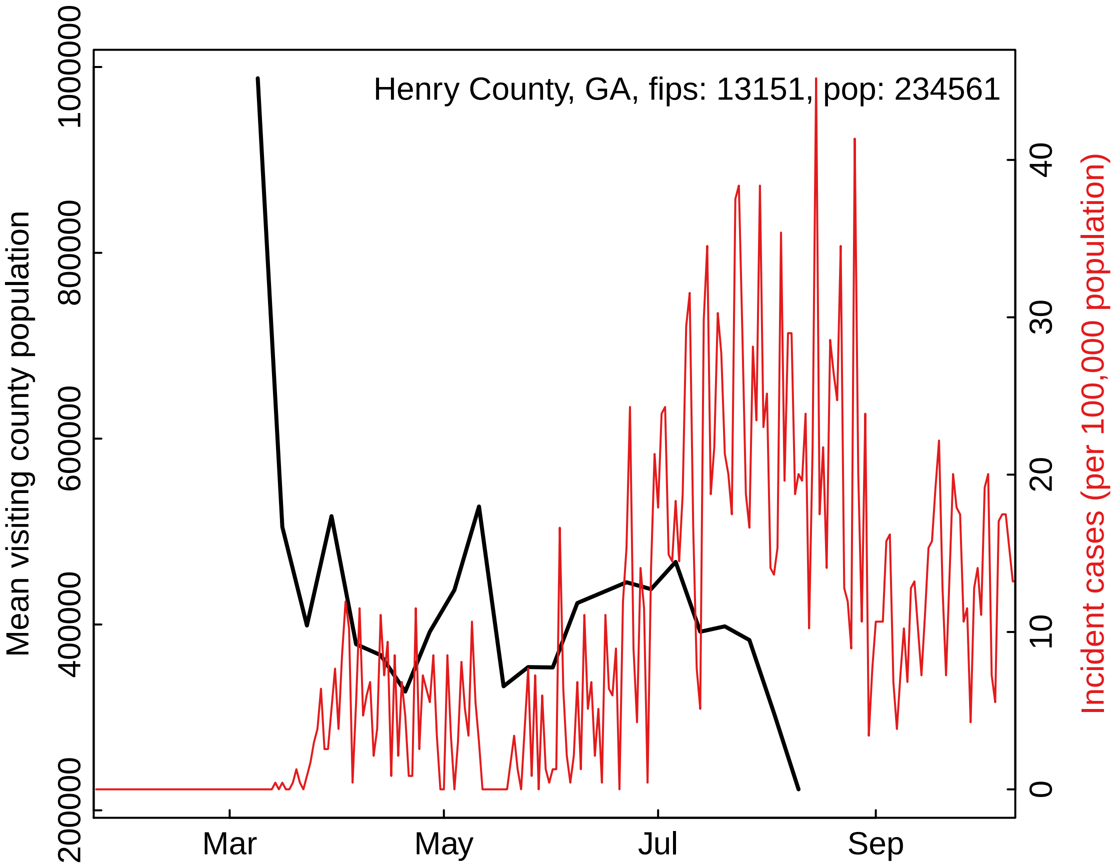

Henry County, GA, fips: 13151, pop: 234561
Mean visiting county population
Incident cases (per 100,000 population)
200000
400000
600000
800000
1000000
0
10
20
30
40
Mar
May
Jul
Sep

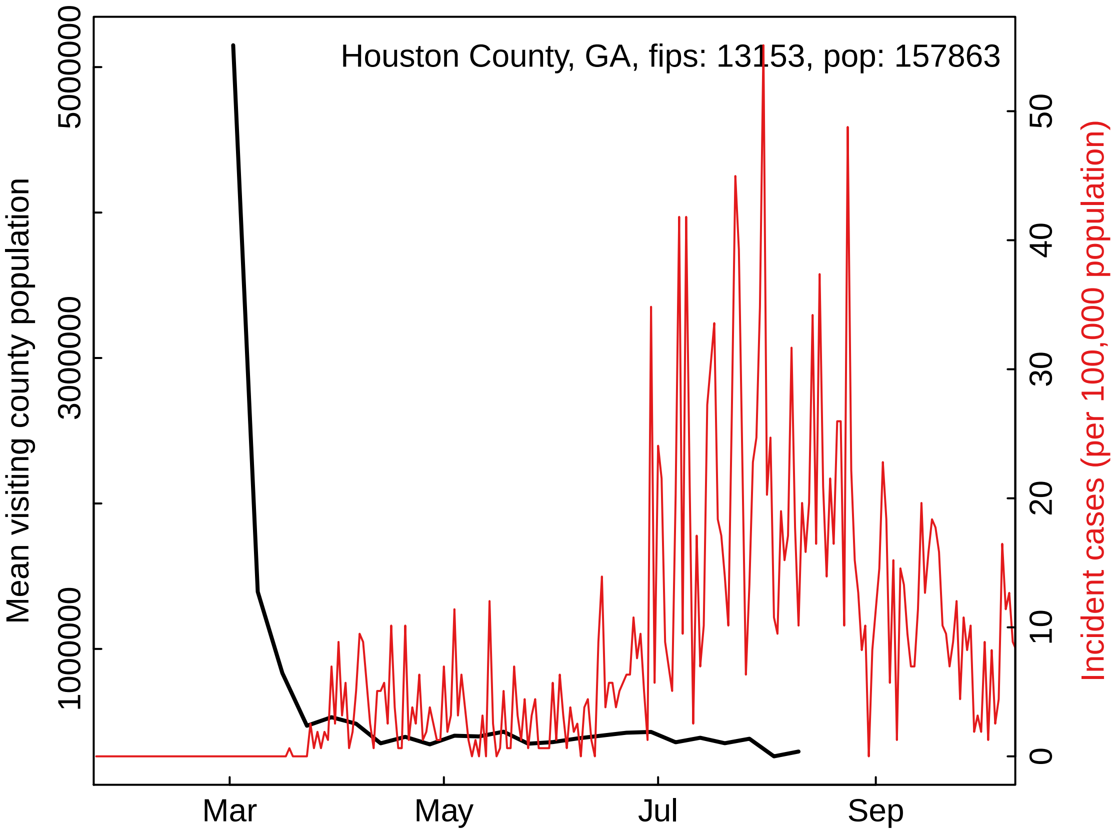

Houston County, GA, fips: 13153, pop: 157863
Mean visiting county population
1000000
3000000
5000000
Incident cases (per 100,000 population)
0
10
20
30
40
50
Mar
May
Jul
Sep

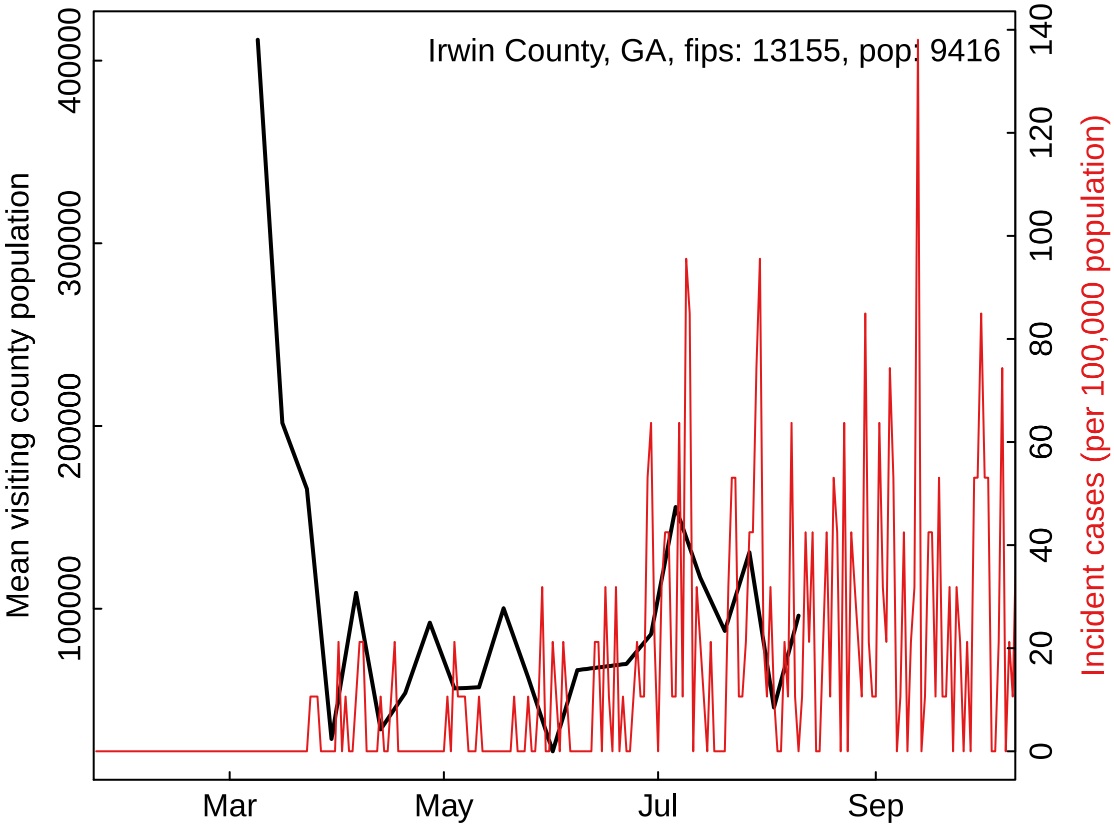

Irwin County, GA, fips: 13155, pop: 9416
Mean visiting county population
100000
200000
300000
400000
Incident cases (per 100,000 population)
0
20
40
60
80
100
120
140
Mar
May
Jul
Sep

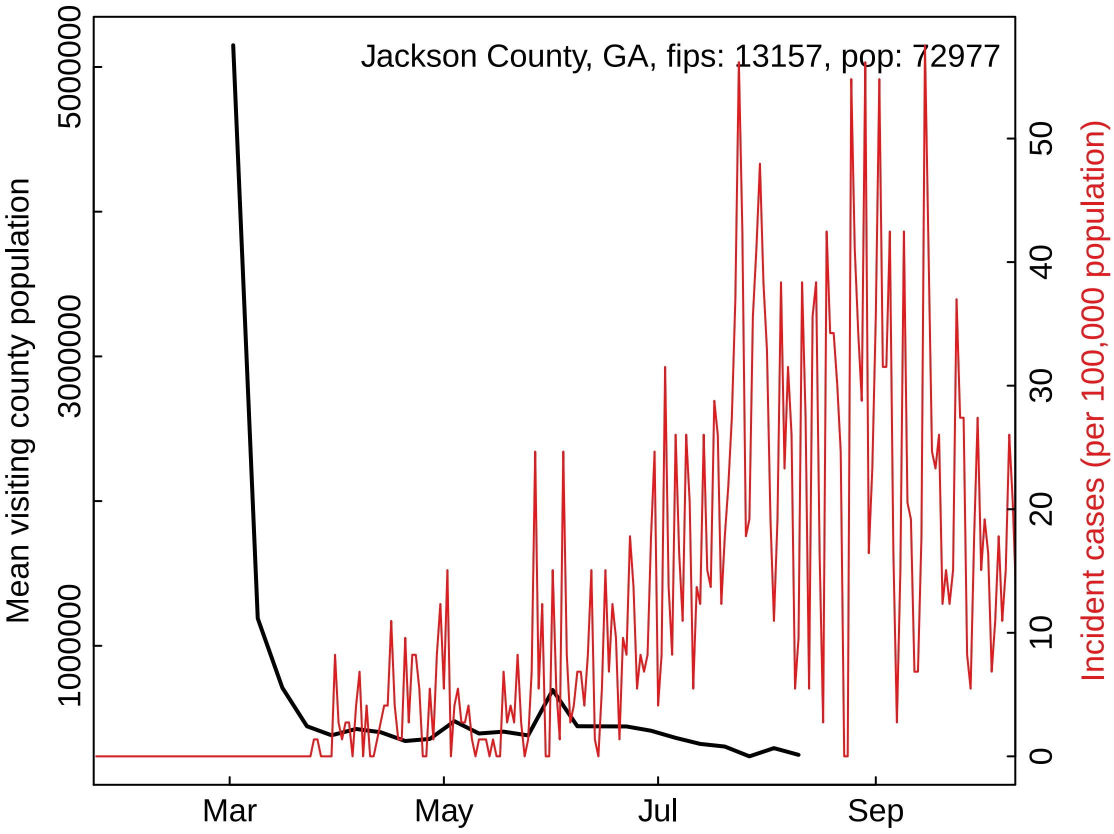

Jackson County, GA, fips: 13157, pop: 72977
Mean visiting county population
Incident cases (per 100,000 population)
1000000
3000000
5000000
0
10
20
30
40
50
Mar
May
Jul
Sep

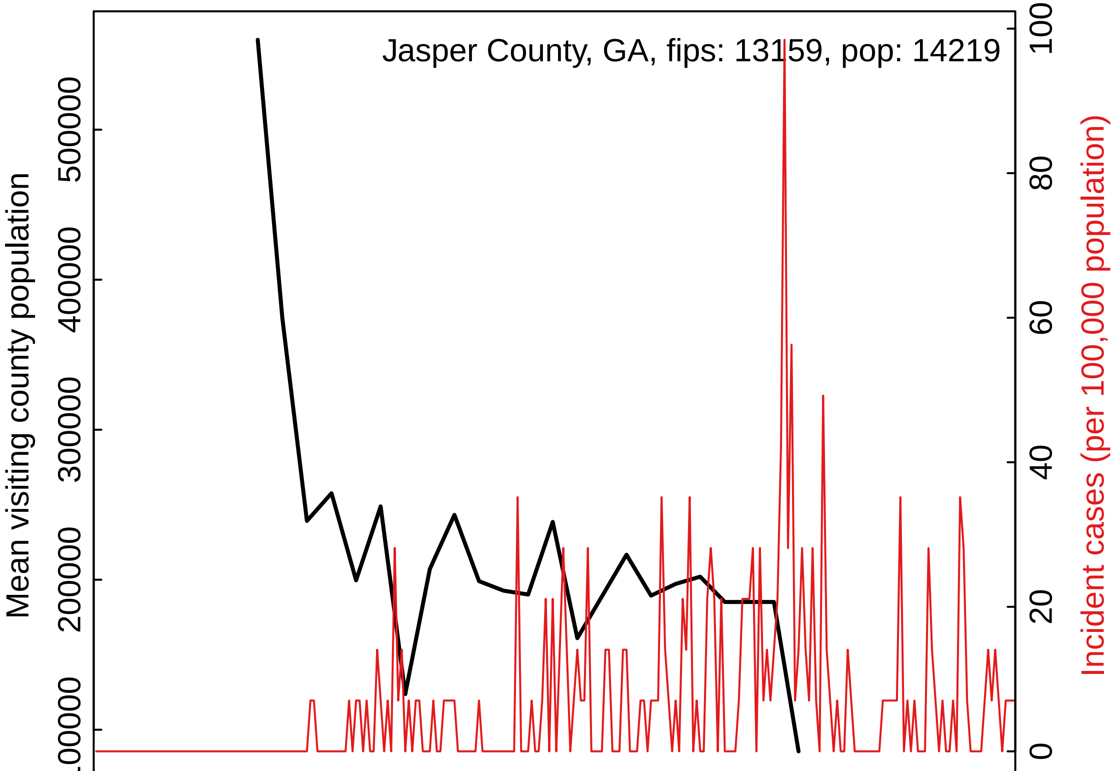

Jasper County, GA, fips: 13159, pop: 14219
Mean visiting county population
100000
200000
300000
400000
500000
Incident cases (per 100,000 population)
0
20
40
60
80
100
Mar
May
Jul
Sep

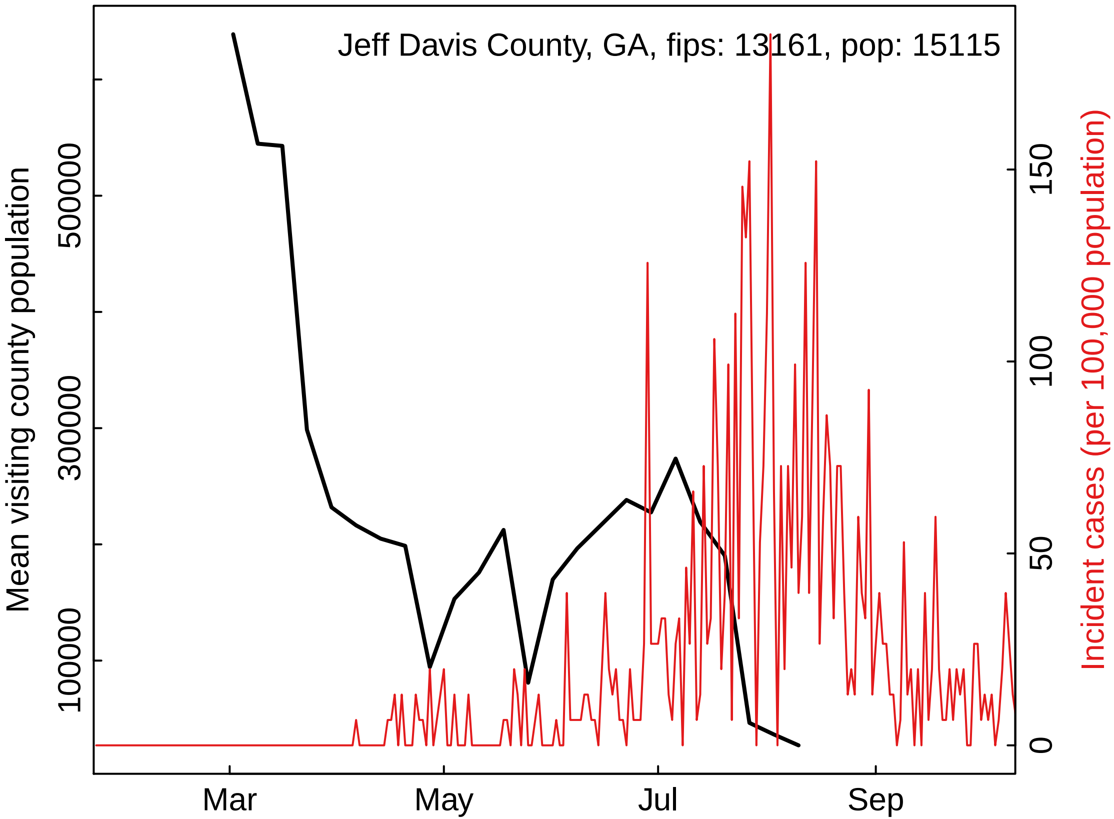

Jeff Davis County, GA, fips: 13161, pop: 15115
Mean visiting county population
100000
300000
500000
Incident cases (per 100,000 population)
0
50
100
150
Mar
May
Jul
Sep

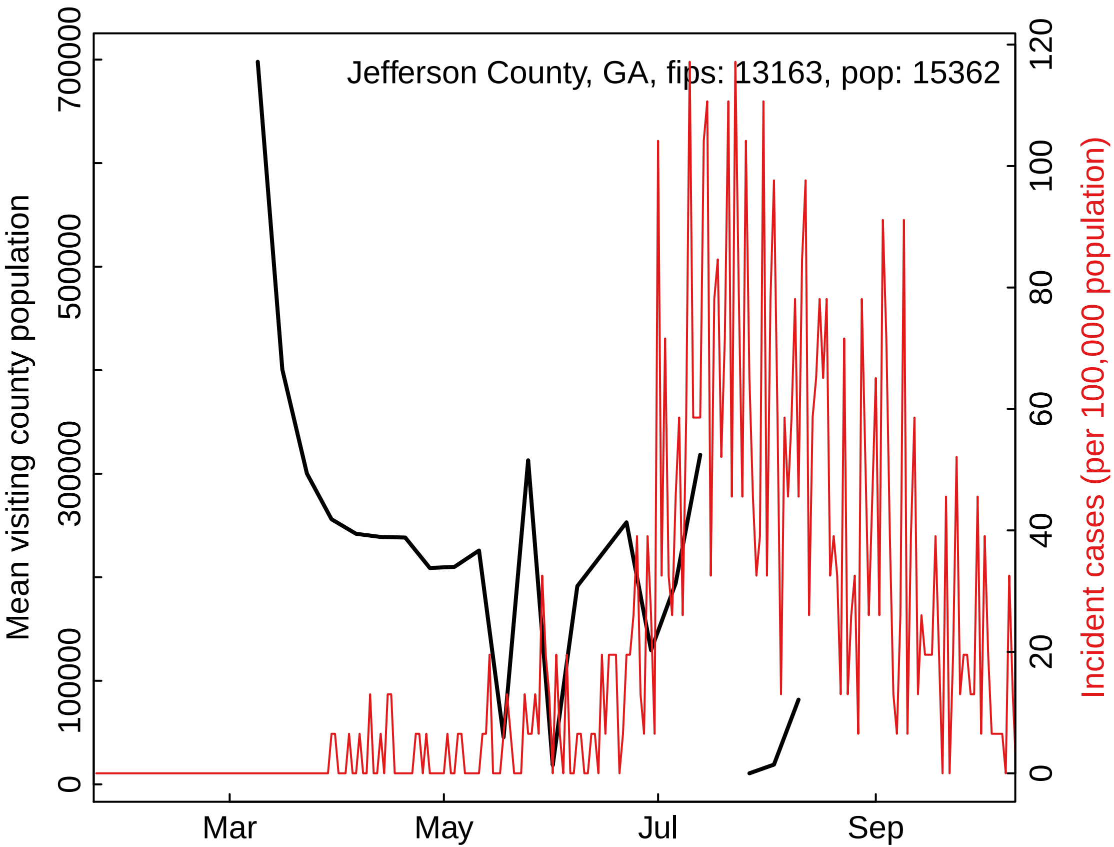
Jefferson County, GA, fips: 13163, pop: 15362
Mean visiting county population
Incident cases (per 100,000 population)
0
100000
300000
500000
700000
0
20
40
60
80
100
120
Mar
May
Jul
Sep

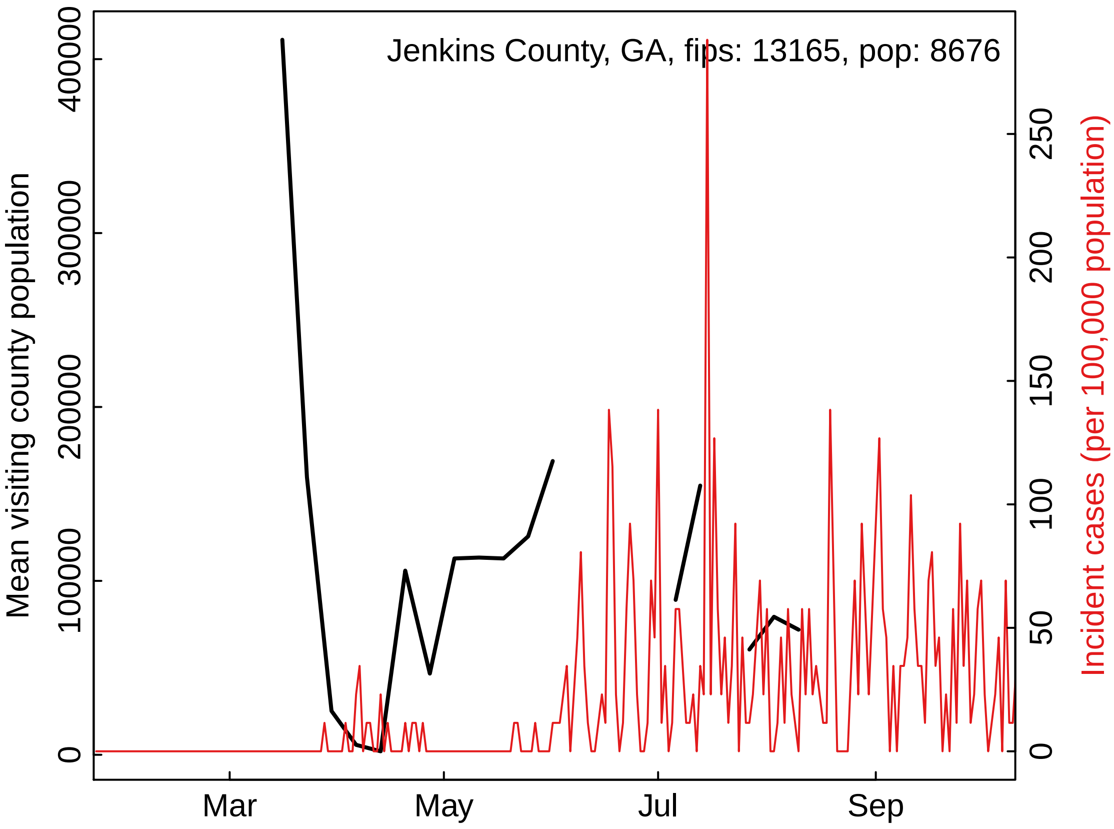

Jenkins County, GA, fips: 13165, pop: 8676
Mean visiting county population
0
100000
200000
300000
400000
Incident cases (per 100,000 population)
0
50
100
150
200
250
Mar
May
Jul
Sep

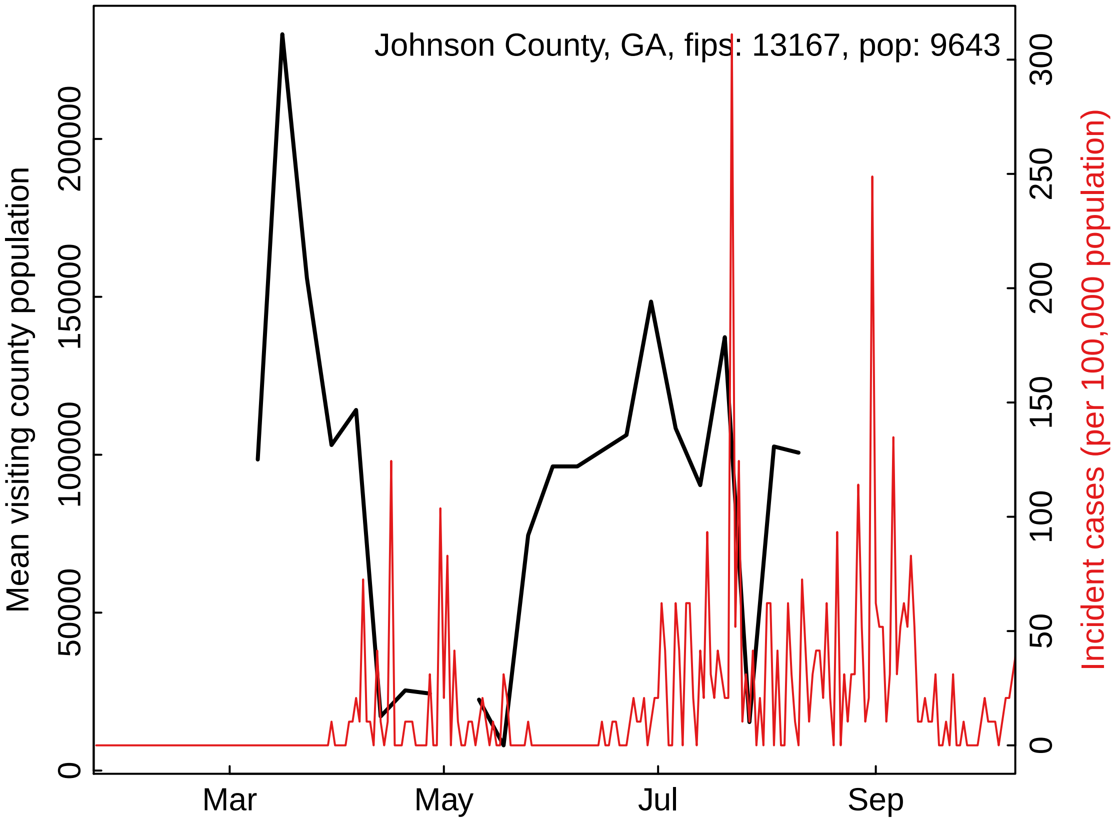

Johnson County, GA, fips: 13167, pop: 9643
Mean visiting county population
0
50000
100000
150000
200000
Incident cases (per 100,000 population)
0
50
100
150
200
250
300
Mar
May
Jul
Sep

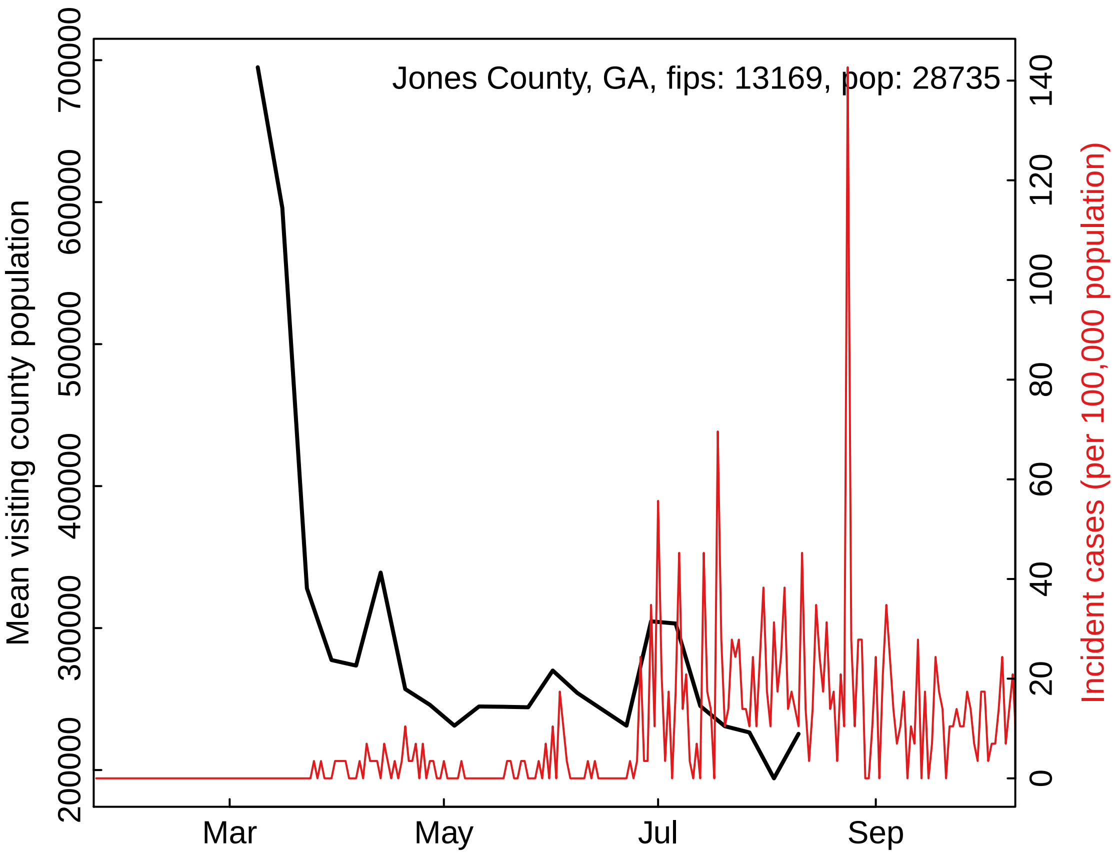

Jones County, GA, fips: 13169, pop: 28735
Mean visiting county population
Incident cases (per 100,000 population)
200000
300000
400000
500000
600000
700000
0
20
40
60
80
100
120
140
Mar
May
Jul
Sep

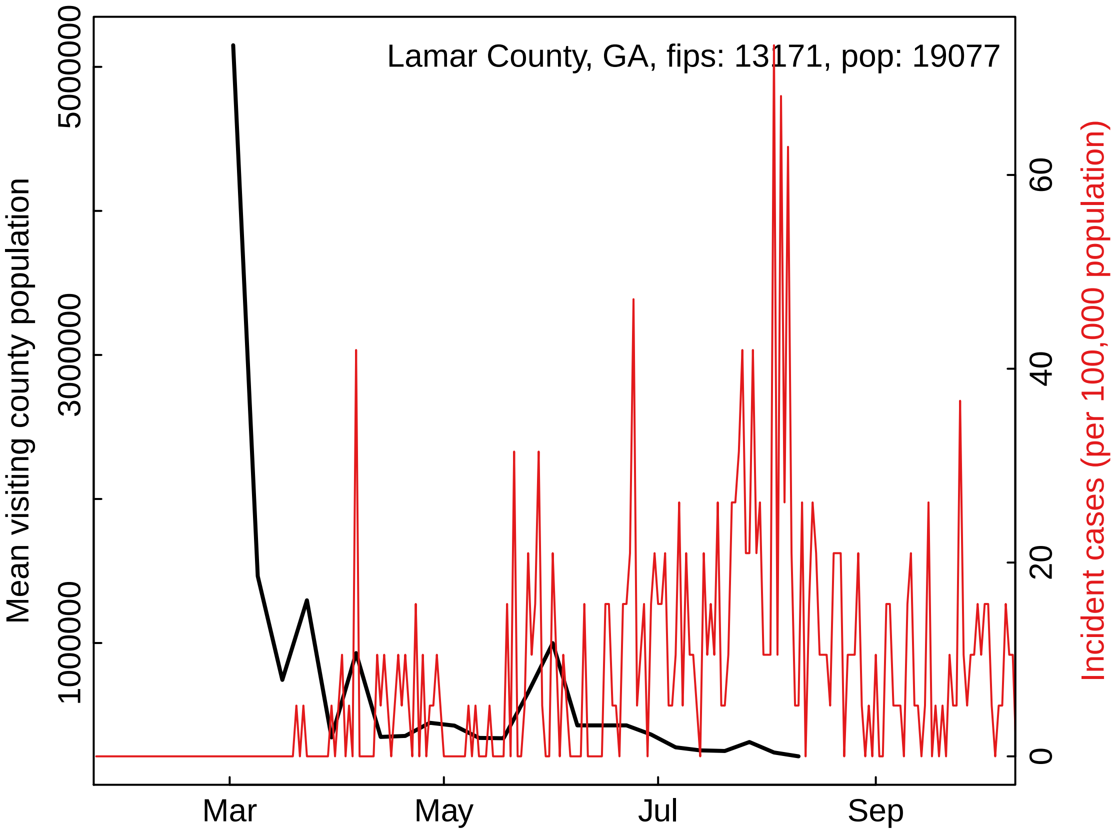

Lamar County, GA, fips: 13171, pop: 19077
Mean visiting county population
1000000
3000000
5000000
Incident cases (per 100,000 population)
0
20
40
60
Mar
May
Jul
Sep

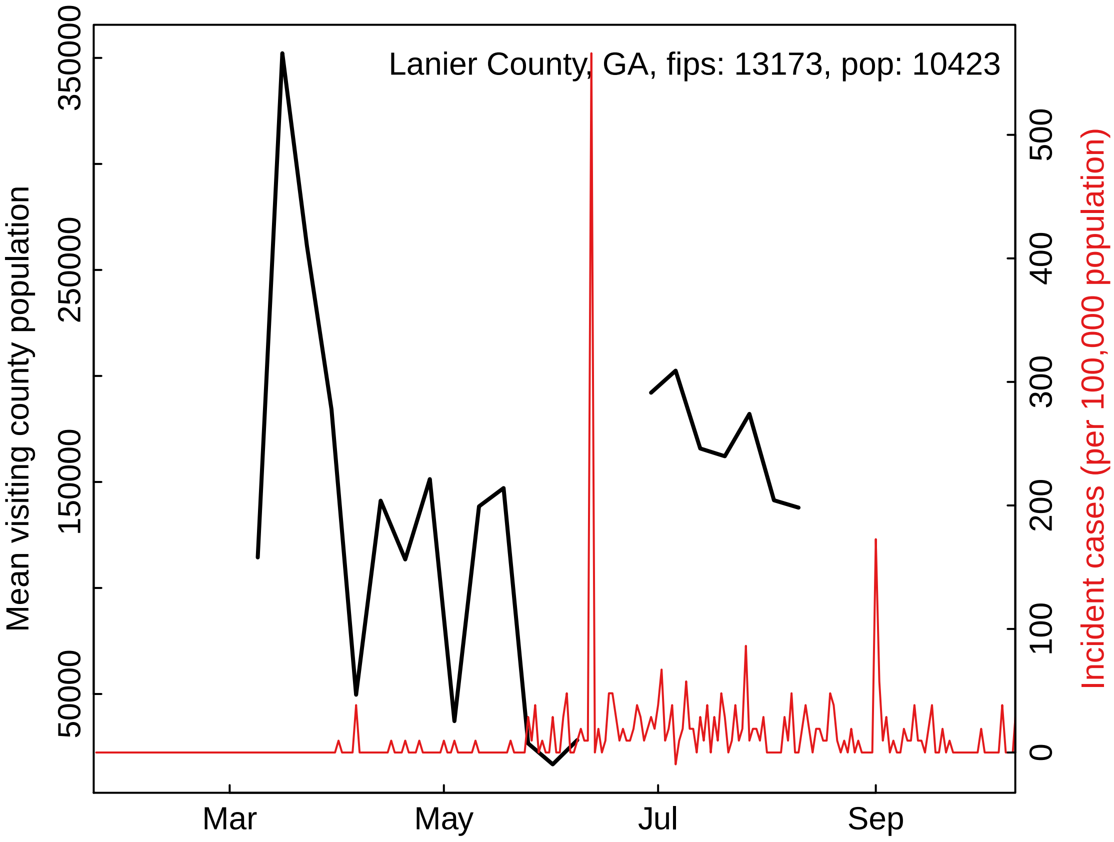

Lanier County, GA, fips: 13173, pop: 10423
Mean visiting county population
50000
150000
250000
350000
Incident cases (per 100,000 population)
0
100
200
300
400
500
Mar
May
Jul
Sep

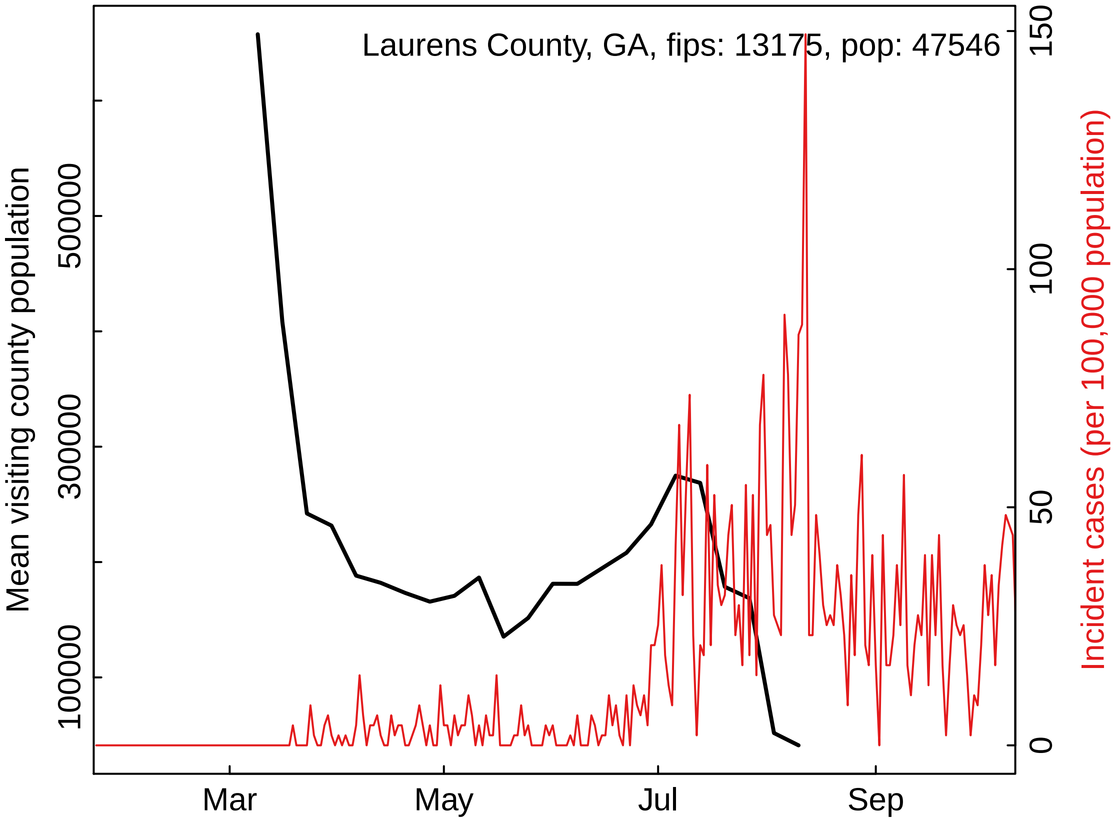

Laurens County, GA, fips: 13175, pop: 47546
Mean visiting county population
100000
300000
500000
Incident cases (per 100,000 population)
0
50
100
150
Mar
May
Jul
Sep

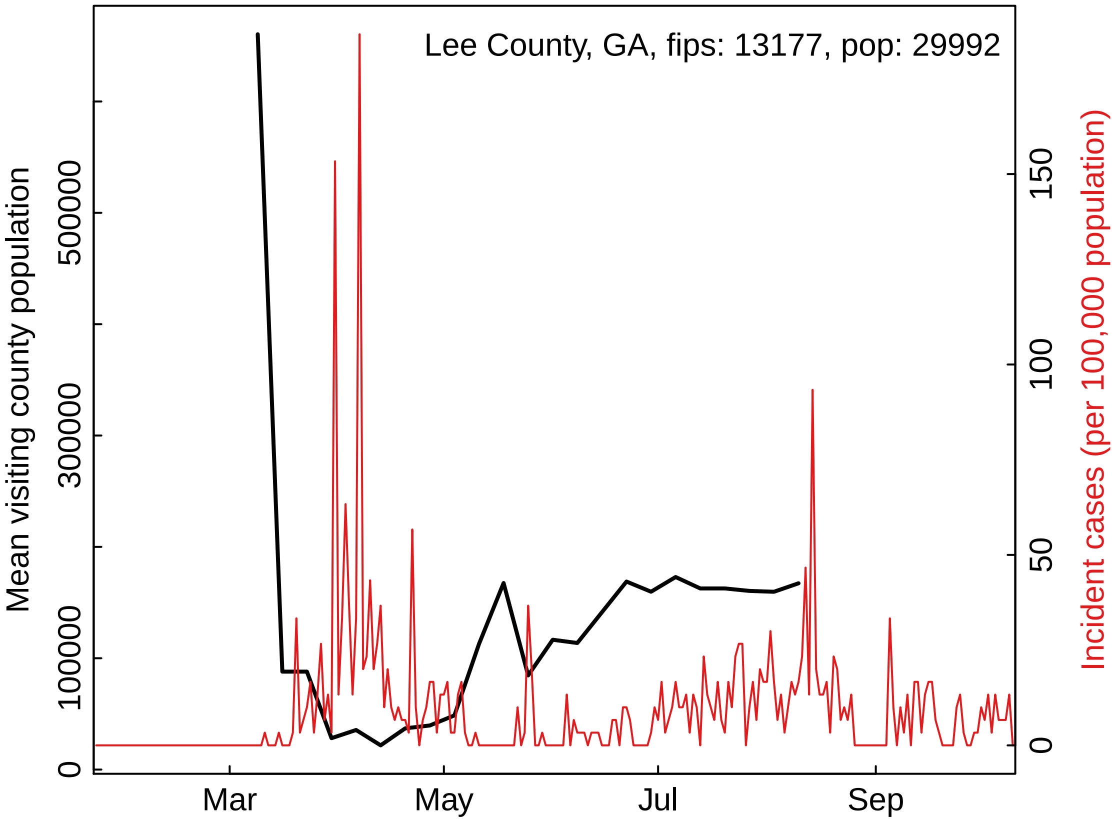

Lee County, GA, fips: 13177, pop: 29992
Mean visiting county population
0
100000
300000
500000
Mar
May
Jul
Sep
Incident cases (per 100,000 population)
0
50
100
150

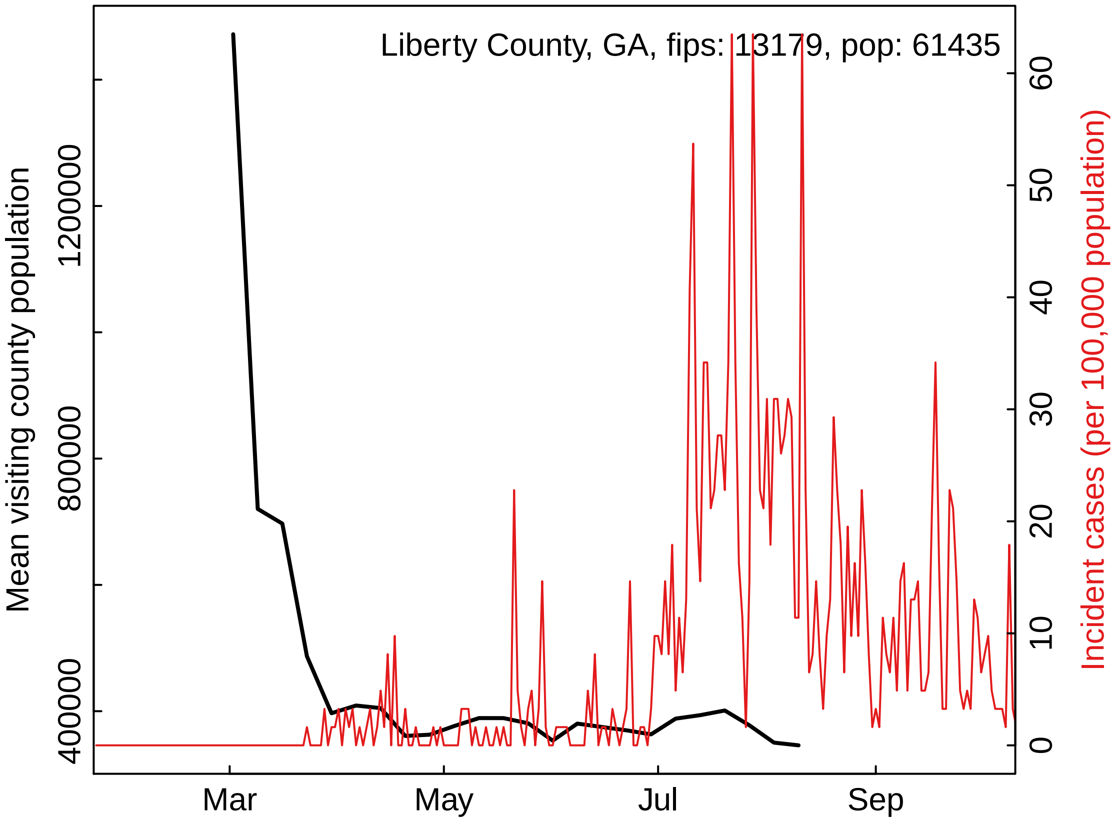
Liberty County, GA, fips: 13179, pop: 61435
Mean visiting county population
400000
800000
1200000
Incident cases (per 100,000 population)
0
10
20
30
40
50
60
Mar
May
Jul
Sep

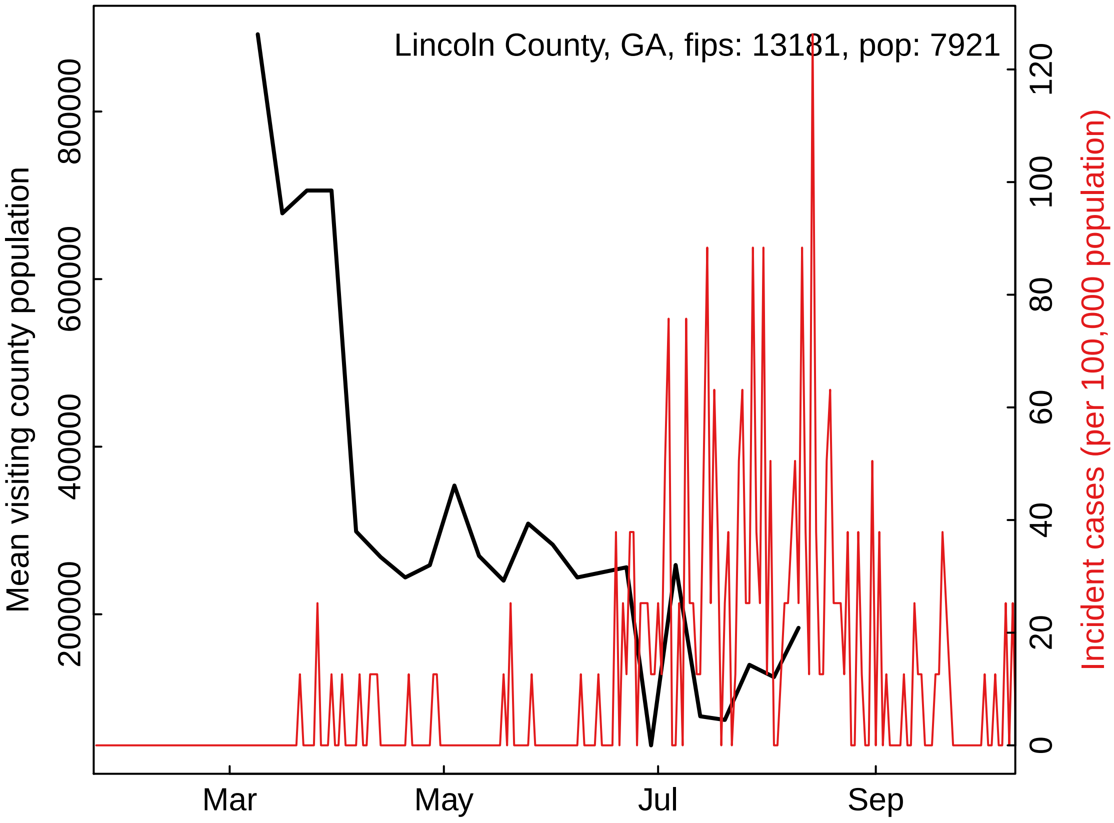

Lincoln County, GA, fips: 13181, pop: 7921
Mean visiting county population
200000
400000
600000
800000
Incident cases (per 100,000 population)
0
20
40
60
80
100
120
Mar
May
Jul
Sep

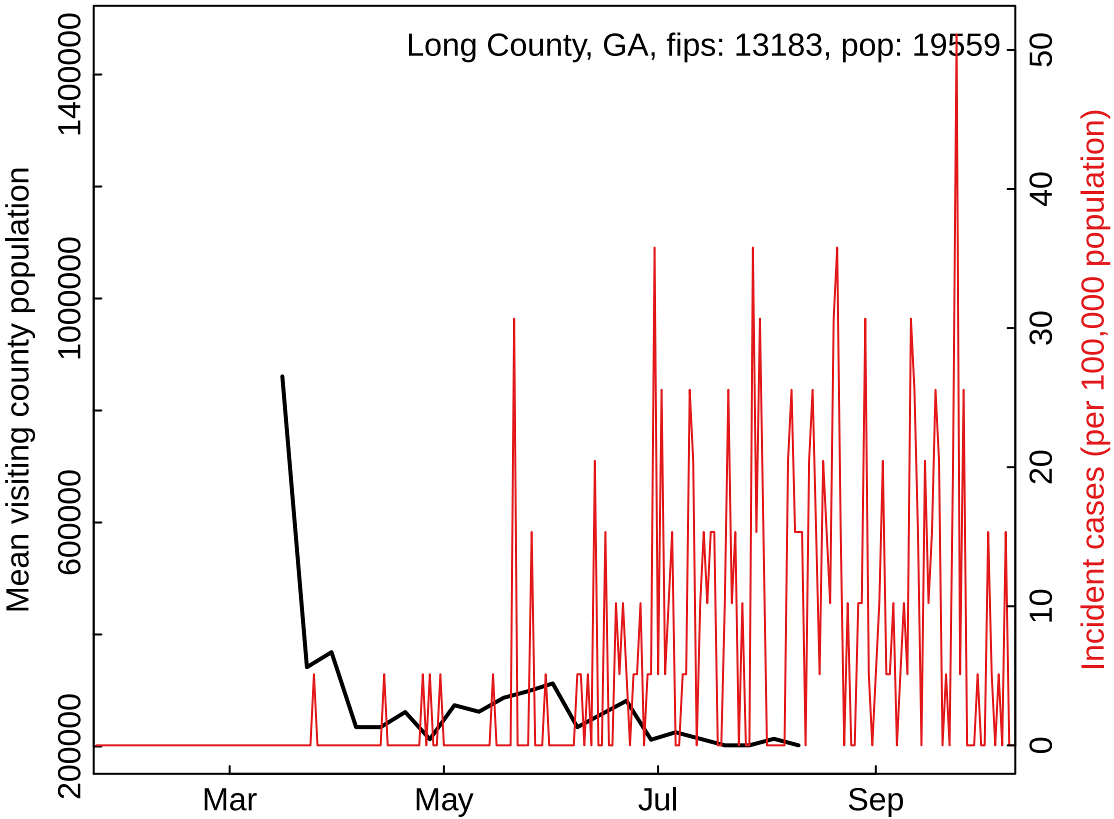
Long County, GA, fips: 13183, pop: 19559
Mean visiting county population
200000
600000
1000000
1400000
Incident cases (per 100,000 population)
0
10
20
30
40
50
Mar
May
Jul
Sep

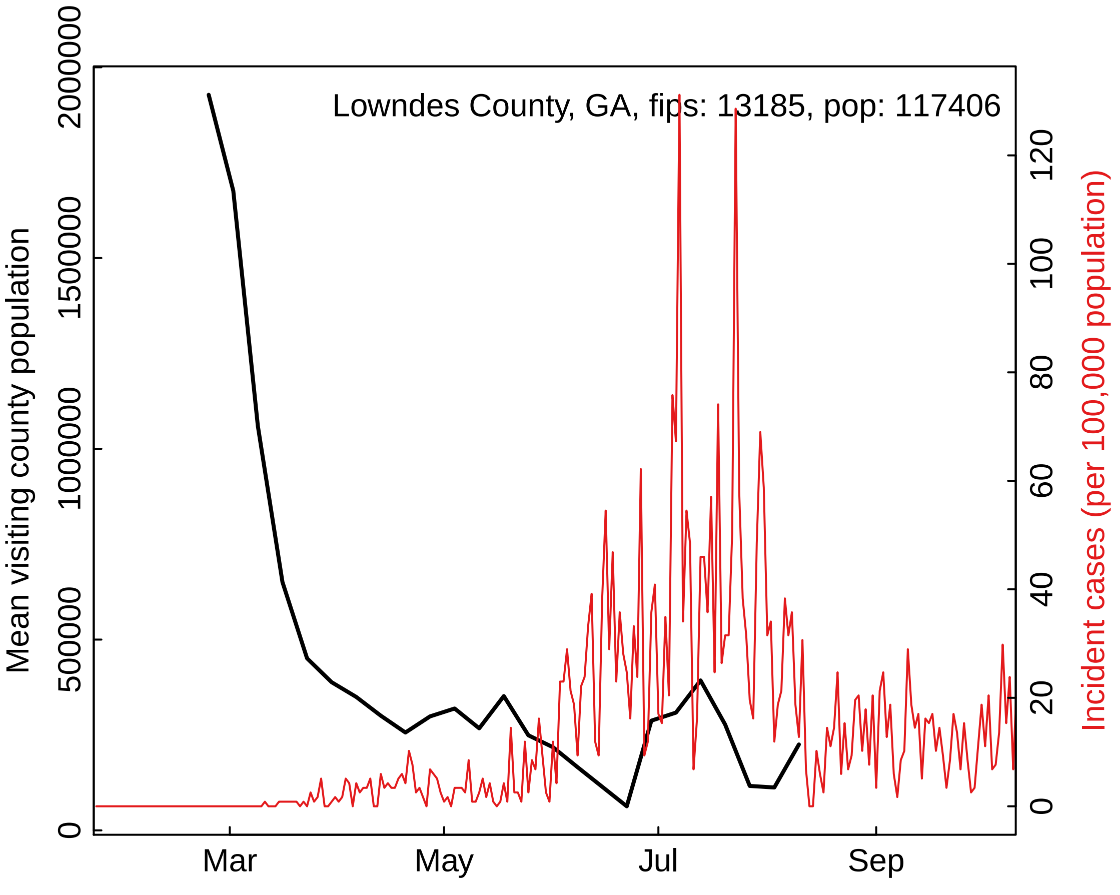

Lowndes County, GA, fips: 13185, pop: 117406
Mean visiting county population
0
500000
1000000
1500000
2000000
Incident cases (per 100,000 population)
0
20
40
60
80
100
120
Mar
May
Jul
Sep

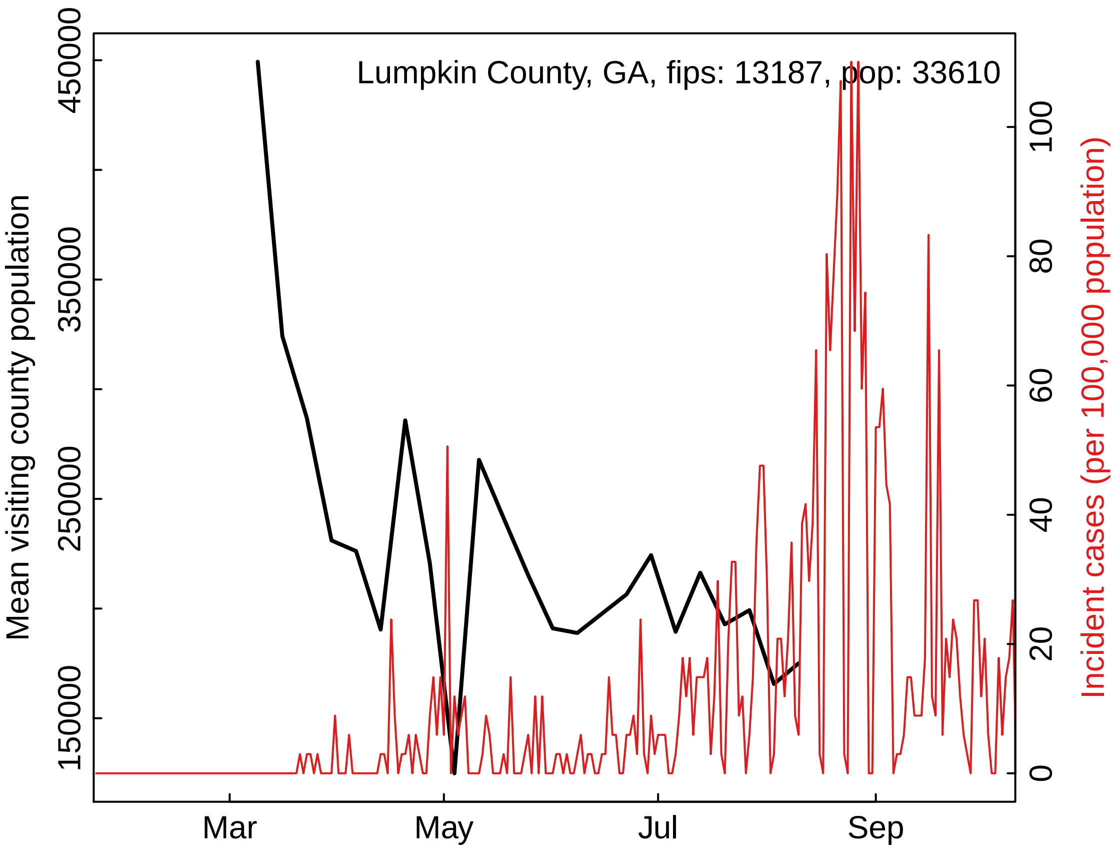

Lumpkin County, GA, fips: 13187, pop: 33610
Mean visiting county population
150000
250000
350000
450000
Incident cases (per 100,000 population)
0
20
40
60
80
100
Mar
May
Jul
Sep

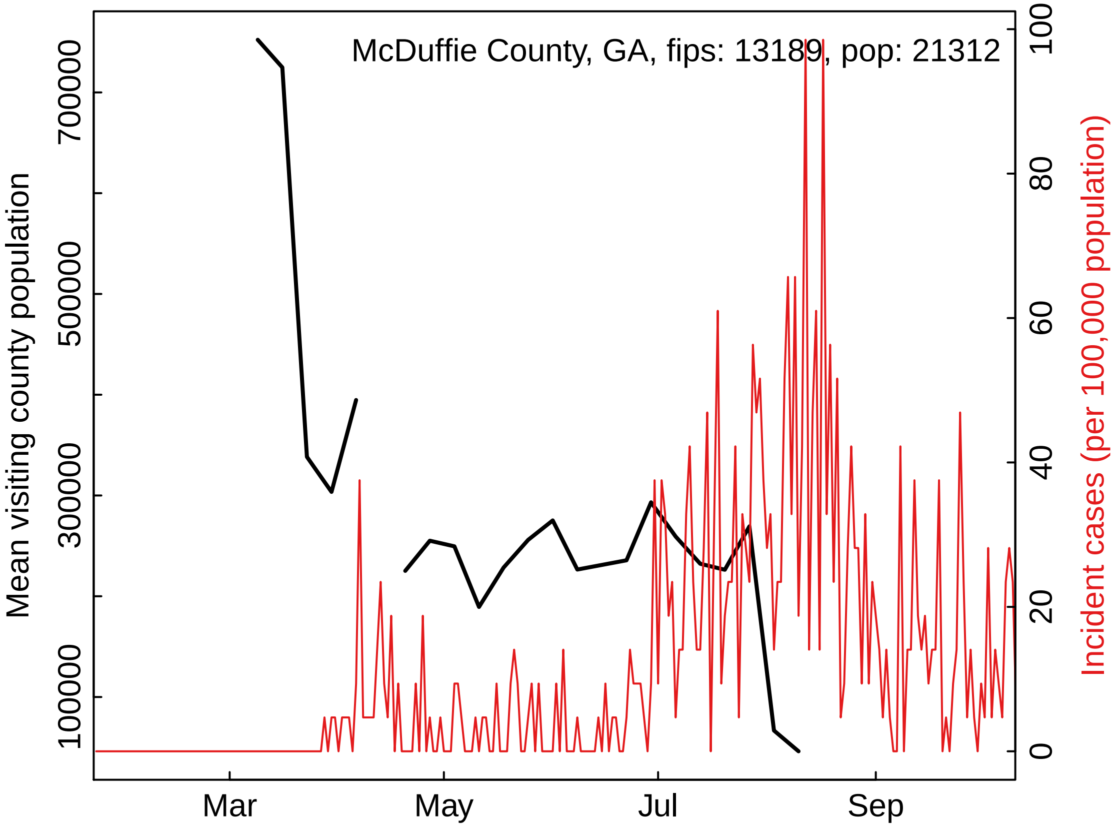
McDuffie County, GA, fips: 13189, pop: 21312
Mean visiting county population
100000
300000
500000
700000
Incident cases (per 100,000 population)
0
20
40
60
80
100
Mar
May
Jul
Sep

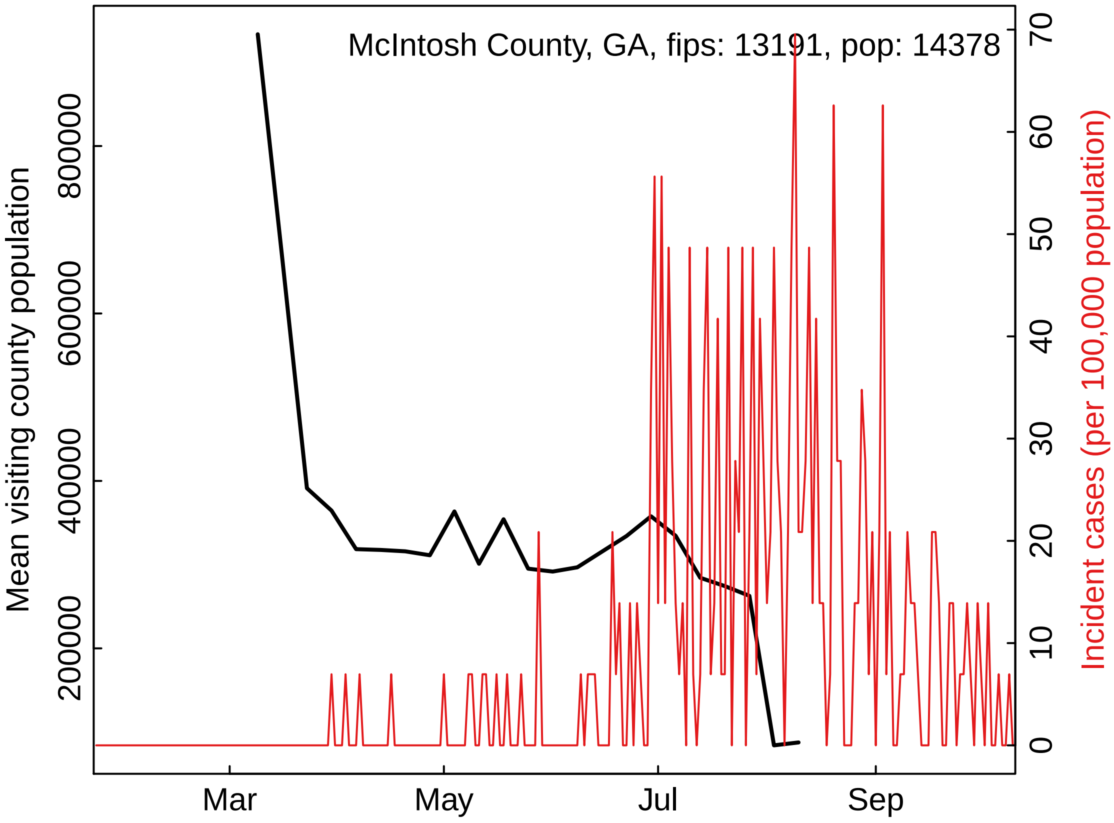

McIntosh County, GA, fips: 13191, pop: 14378
Mean visiting county population
Incident cases (per 100,000 population)
200000
400000
600000
800000
0
10
20
30
40
50
60
70
Mar
May
Jul
Sep

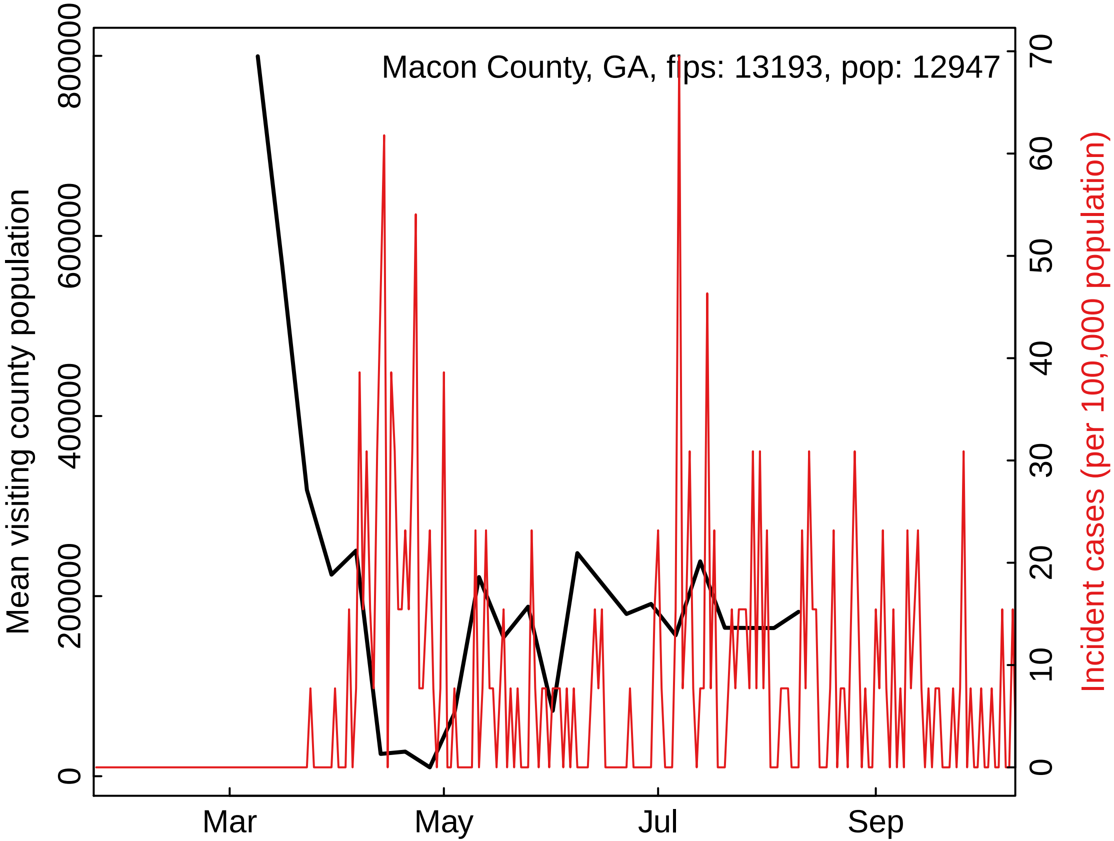

Macon County, GA, fips: 13193, pop: 12947
Mean visiting county population
0
200000
400000
600000
800000
Incident cases (per 100,000 population)
0
10
20
30
40
50
60
70
Mar
May
Jul
Sep

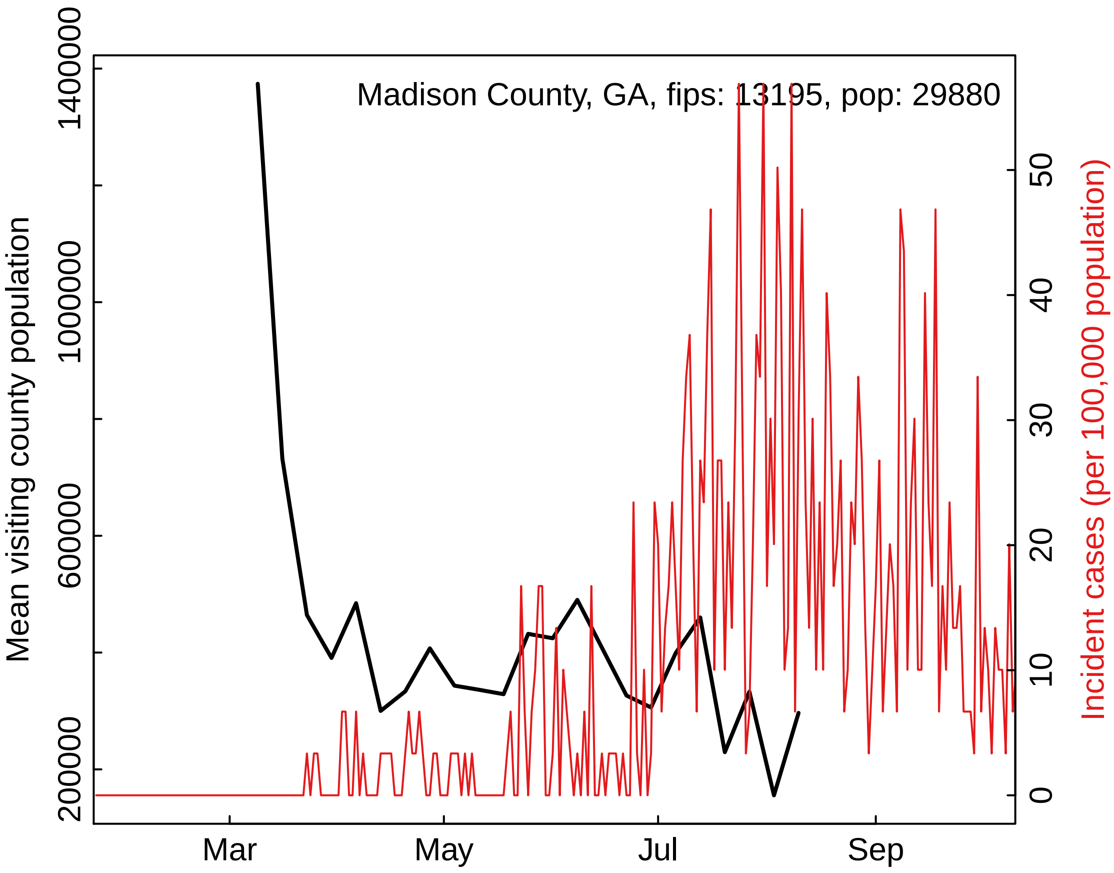

Madison County, GA, fips: 13195, pop: 29880
Mean visiting county population
Incident cases (per 100,000 population)
200000
600000
1000000
1400000
0
10
20
30
40
50
Mar
May
Jul
Sep

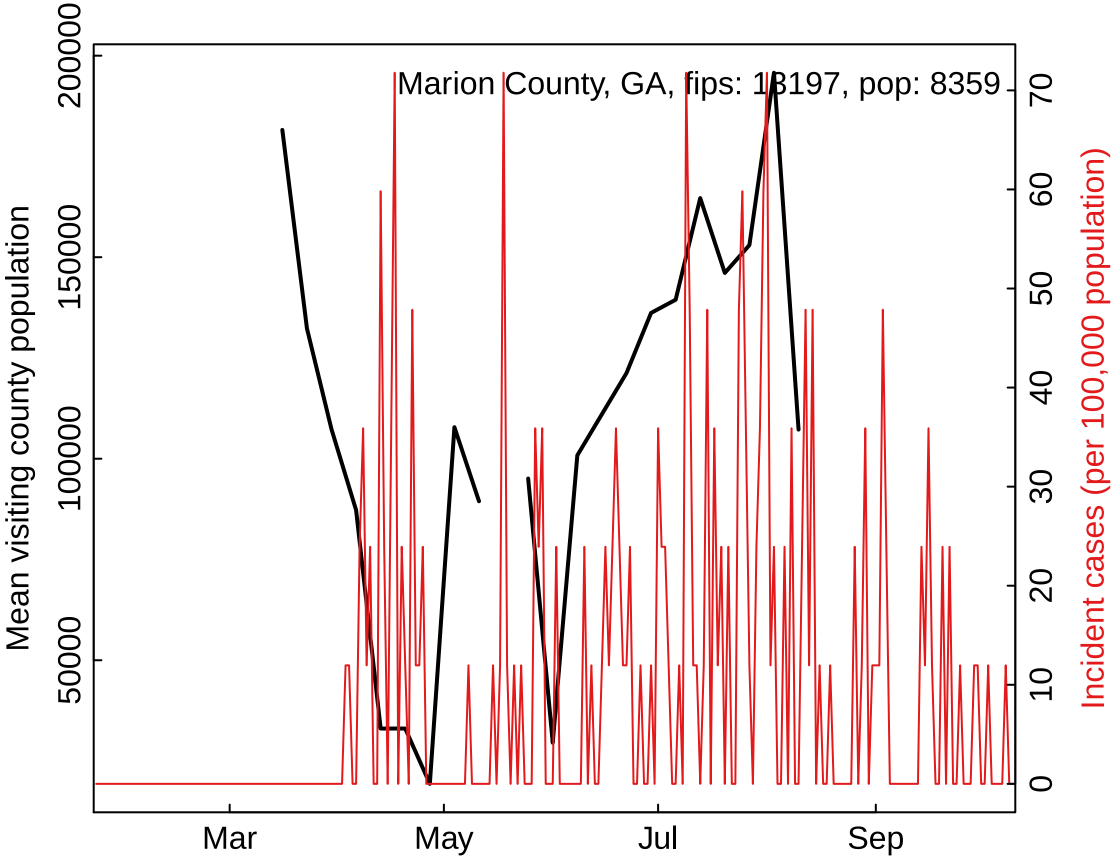
Marion County, GA, fips: 13197, pop: 8359
Mean visiting county population
Incident cases (per 100,000 population)
50000
100000
150000
200000
0
10
20
30
40
50
60
70
Mar
May
Jul
Sep

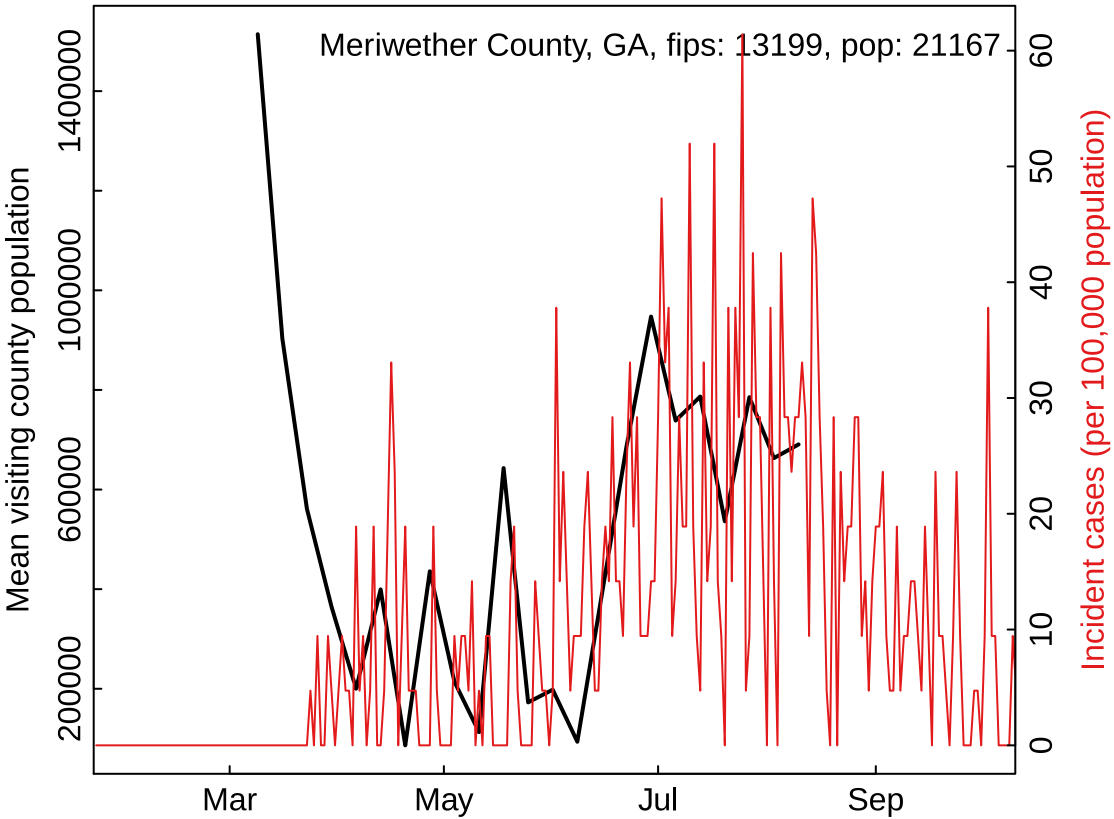

Meriwether County, GA, fips: 13199, pop: 21167
Mean visiting county population
200000
600000
1000000
1400000
Incident cases (per 100,000 population)
0
10
20
30
40
50
60
Mar
May
Jul
Sep

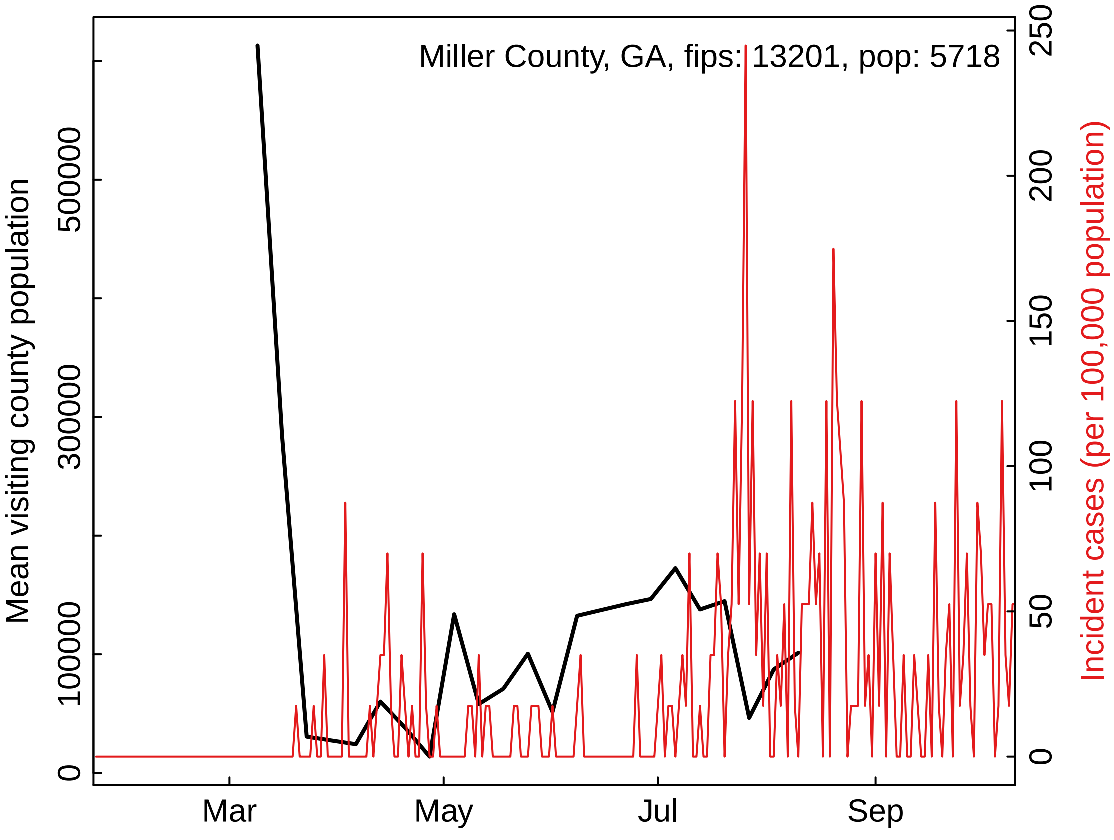

Miller County, GA, fips: 13201, pop: 5718
Mean visiting county population
0
100000
300000
500000
Mar
May
Jul
Sep
Incident cases (per 100,000 population)
0
50
100
150
200
250

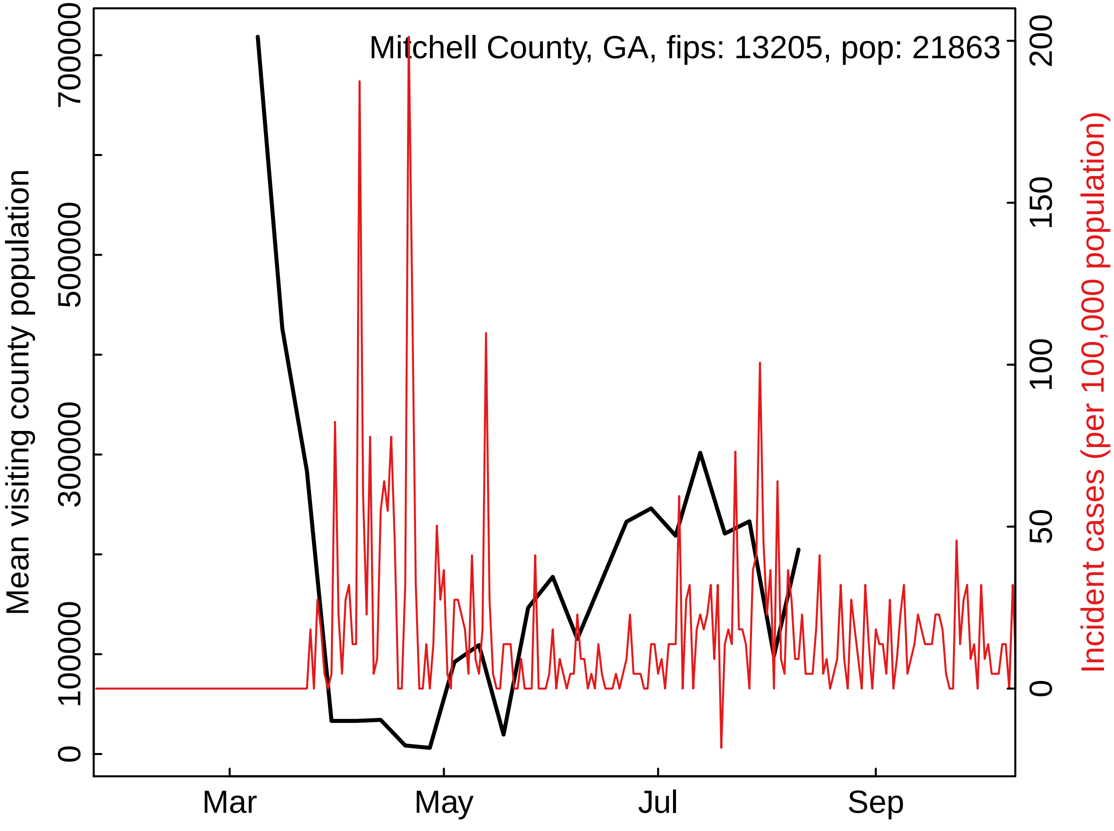

Mitchell County, GA, fips: 13205, pop: 21863
Mean visiting county population
Incident cases (per 100,000 population)
0
100000
300000
500000
700000
0
50
100
150
200
Mar
May
Jul
Sep

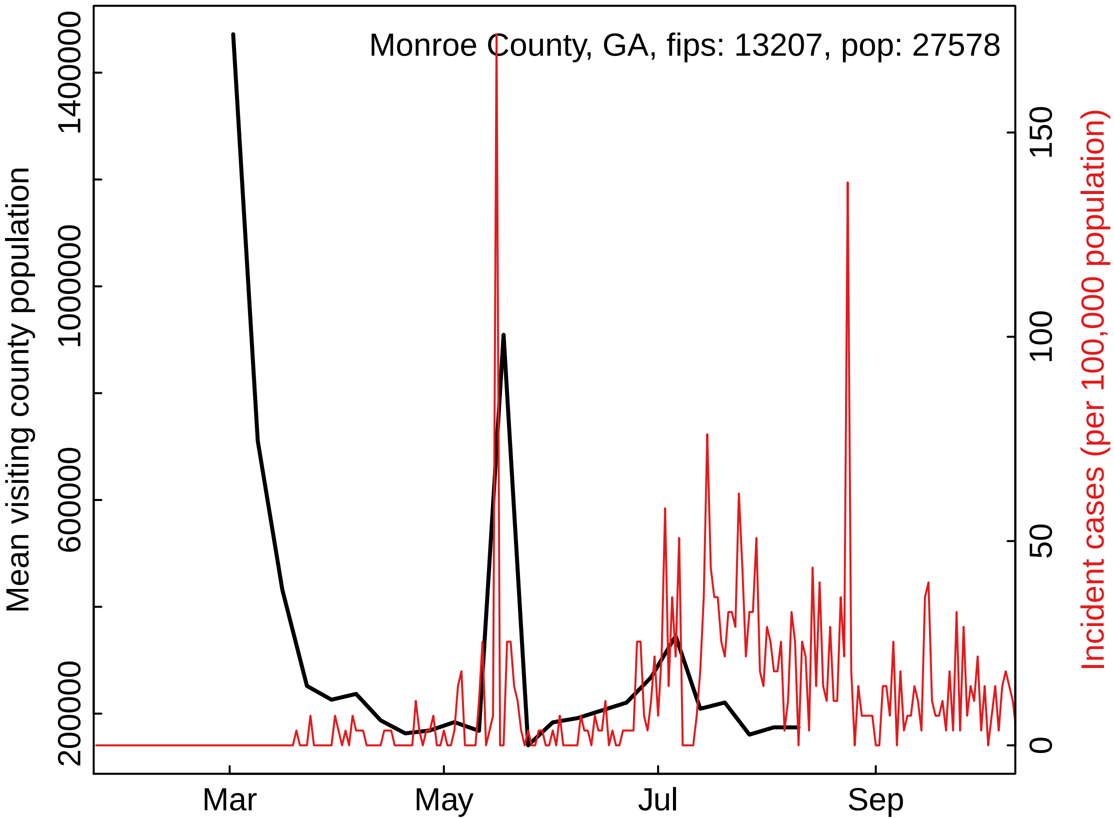
Monroe County, GA, fips: 13207, pop: 27578
Mean visiting county population
Incident cases (per 100,000 population)
200000
600000
1000000
1400000
0
50
100
150
Mar
May
Jul
Sep

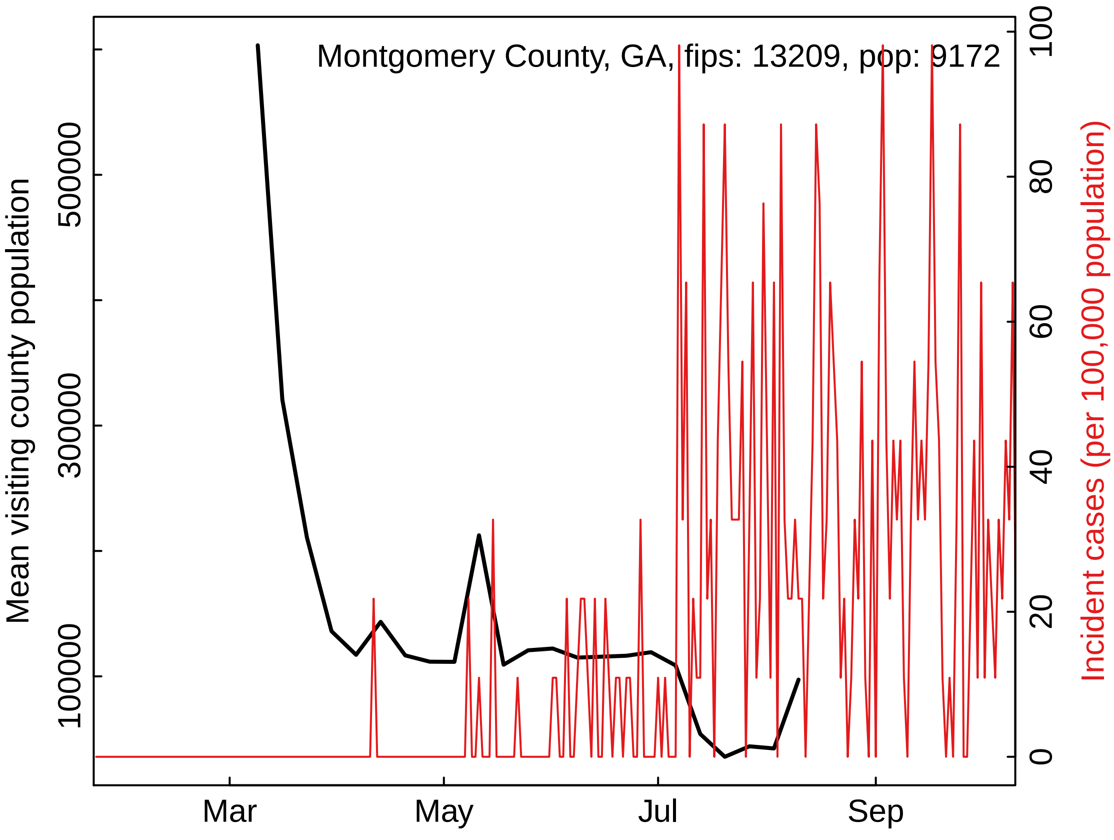

Montgomery County, GA, fips: 13209, pop: 9172
Mean visiting county population
100000
300000
500000
Mar
May
Jul
Sep
0
20
40
60
80
100
Incident cases (per 100,000 population)

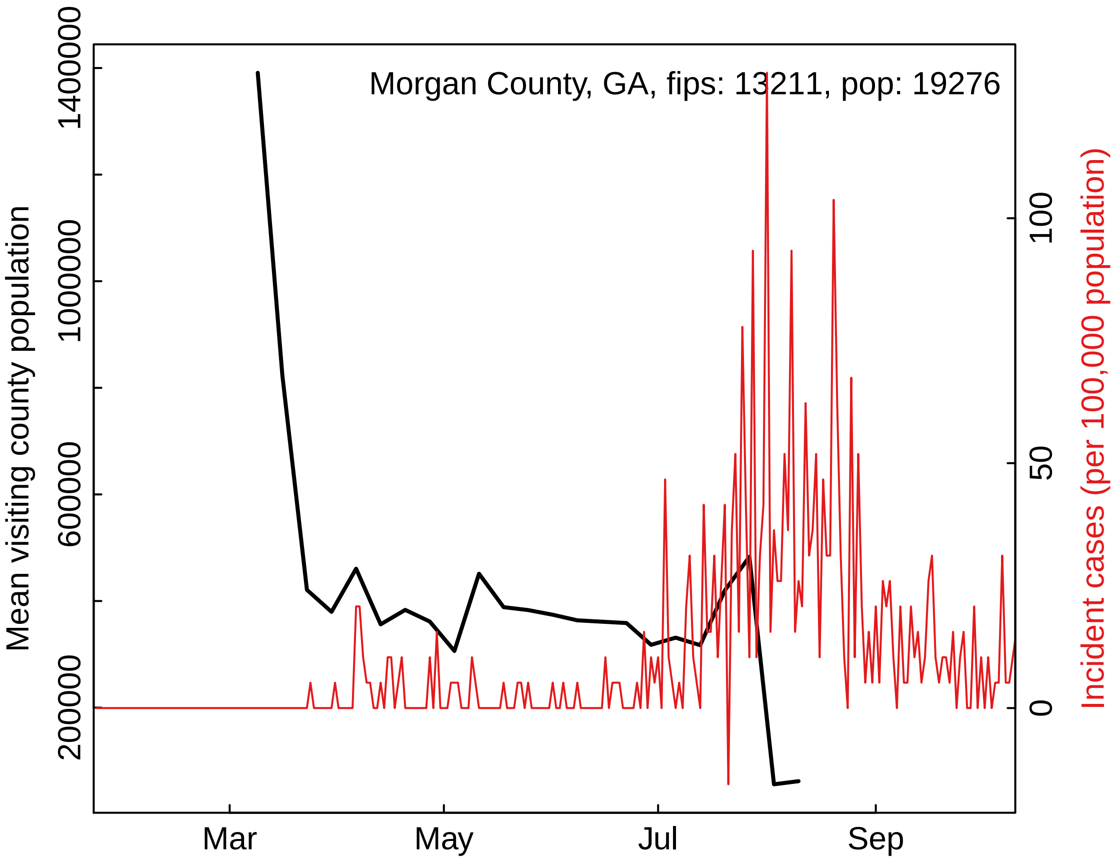

Morgan County, GA, fips: 13211, pop: 19276
Mean visiting county population
200000
600000
1000000
1400000
Incident cases (per 100,000 population)
0
50
100
Mar
May
Jul
Sep

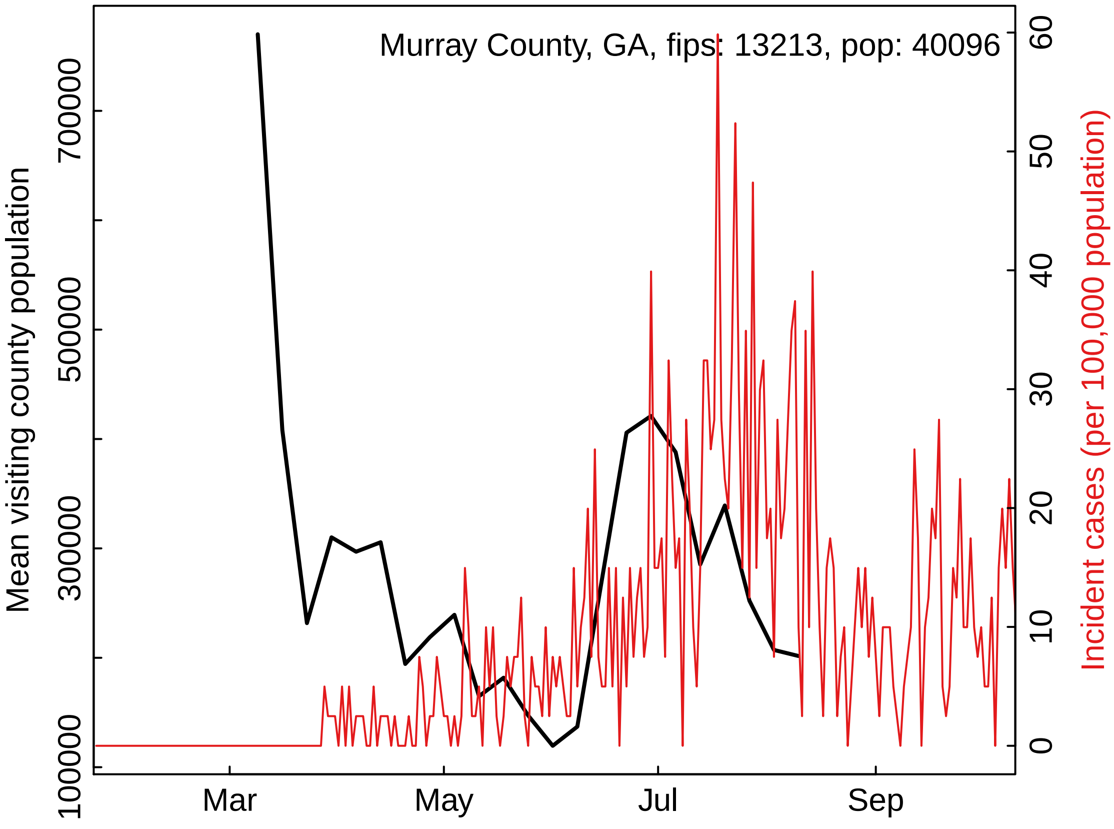

Murray County, GA, fips: 13213, pop: 40096
Mean visiting county population
100000
300000
500000
7000000
Mar
May
Jul
Sep
0
10
20
30
40
50
60
Incident cases (per 100,000 population)

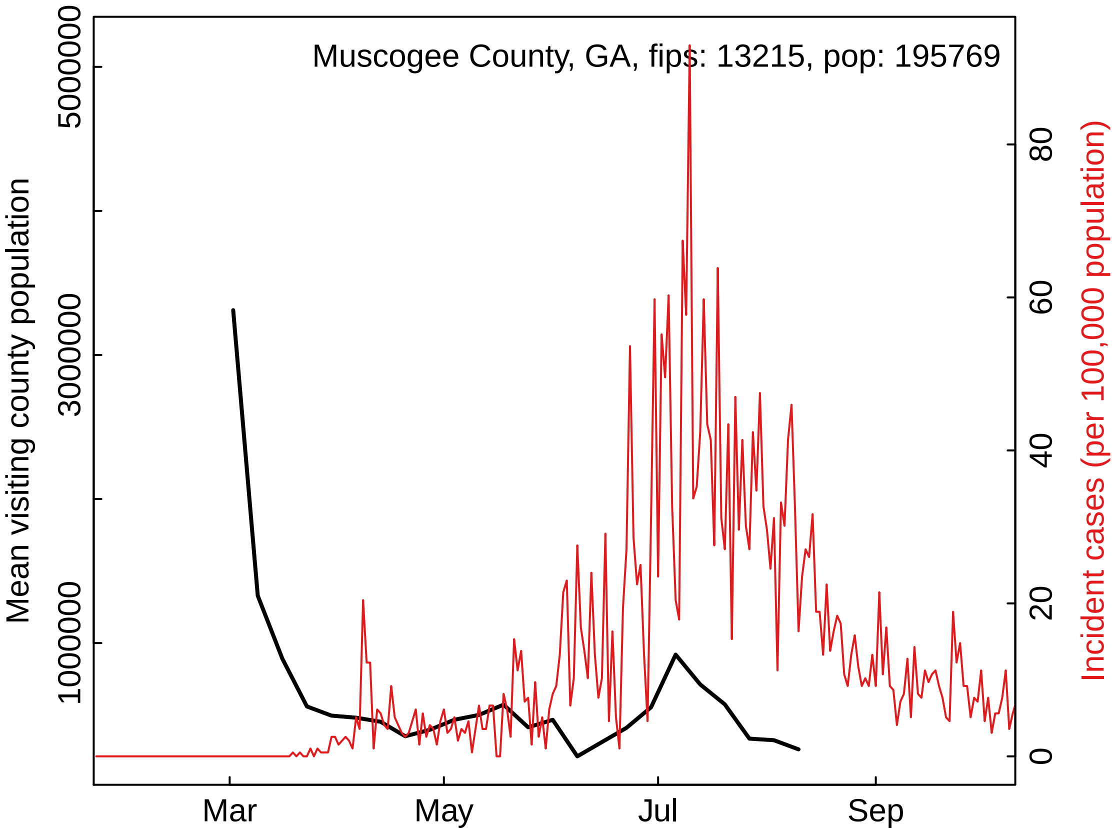

Muscogee County, GA, fips: 13215, pop: 195769
Mean visiting county population
1000000
3000000
5000000
Mar
May
Jul
Sep
0
20
40
60
80
Incident cases (per 100,000 population)

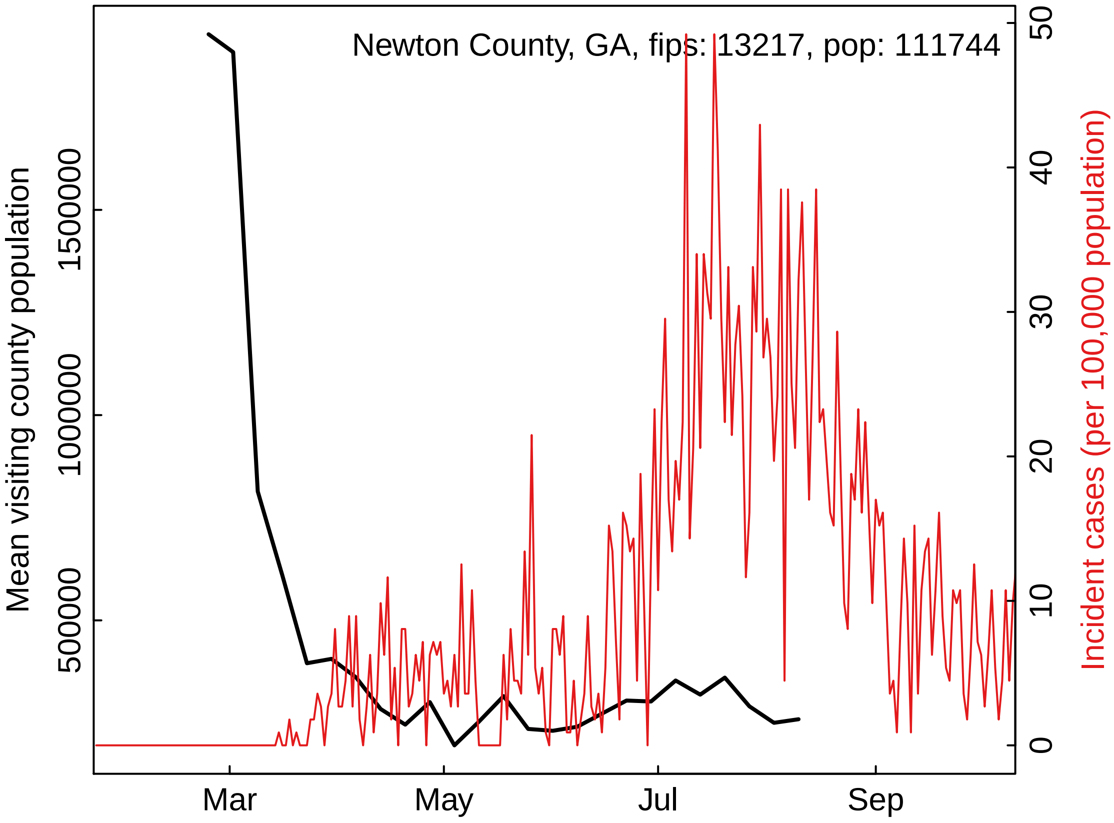
Newton County, GA, fips: 13217, pop: 111744
Mean visiting county population
500000
1000000
1500000
Incident cases (per 100,000 population)
0
10
20
30
40
50
Mar
May
Jul
Sep

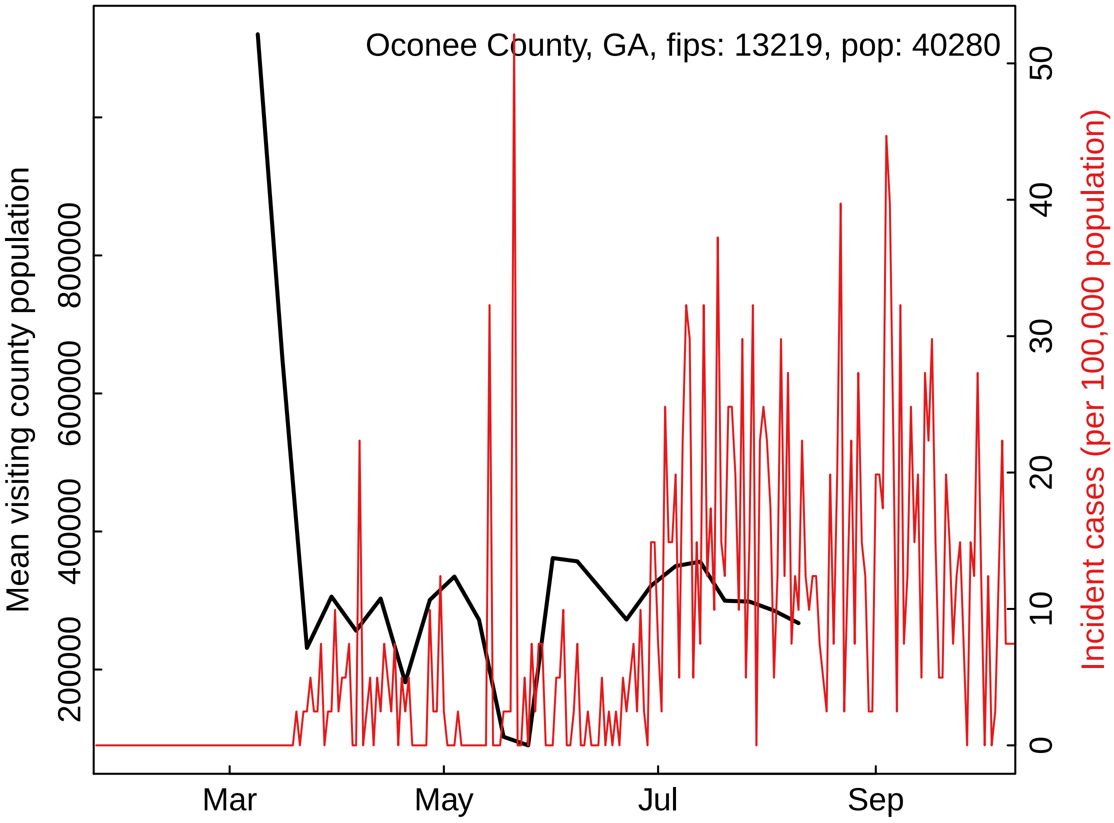

Oconee County, GA, fips: 13219, pop: 40280
Mean visiting county population
200000
400000
600000
800000
Incident cases (per 100,000 population)
0
10
20
30
40
50
Mar
May
Jul
Sep

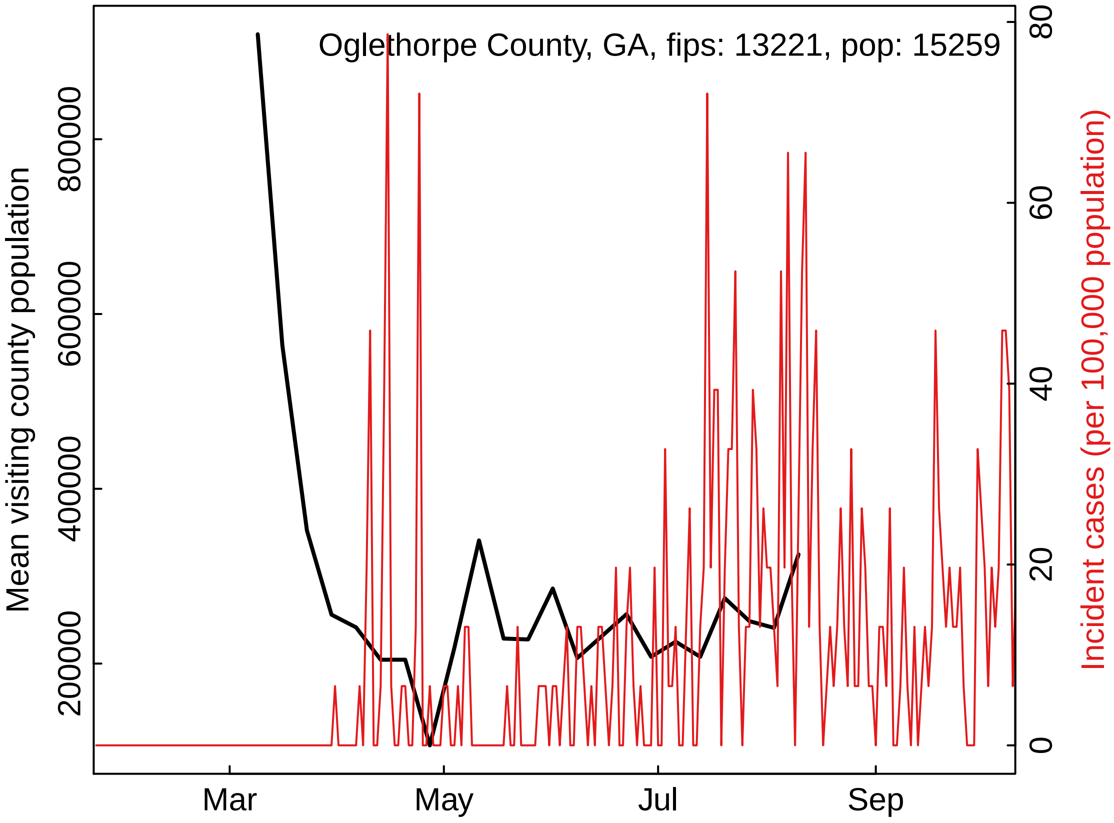

Oglethorpe County, GA, fips: 13221, pop: 15259
Mean visiting county population
200000
400000
600000
800000
Incident cases (per 100,000 population)
0
20
40
60
80
Mar
May
Jul
Sep

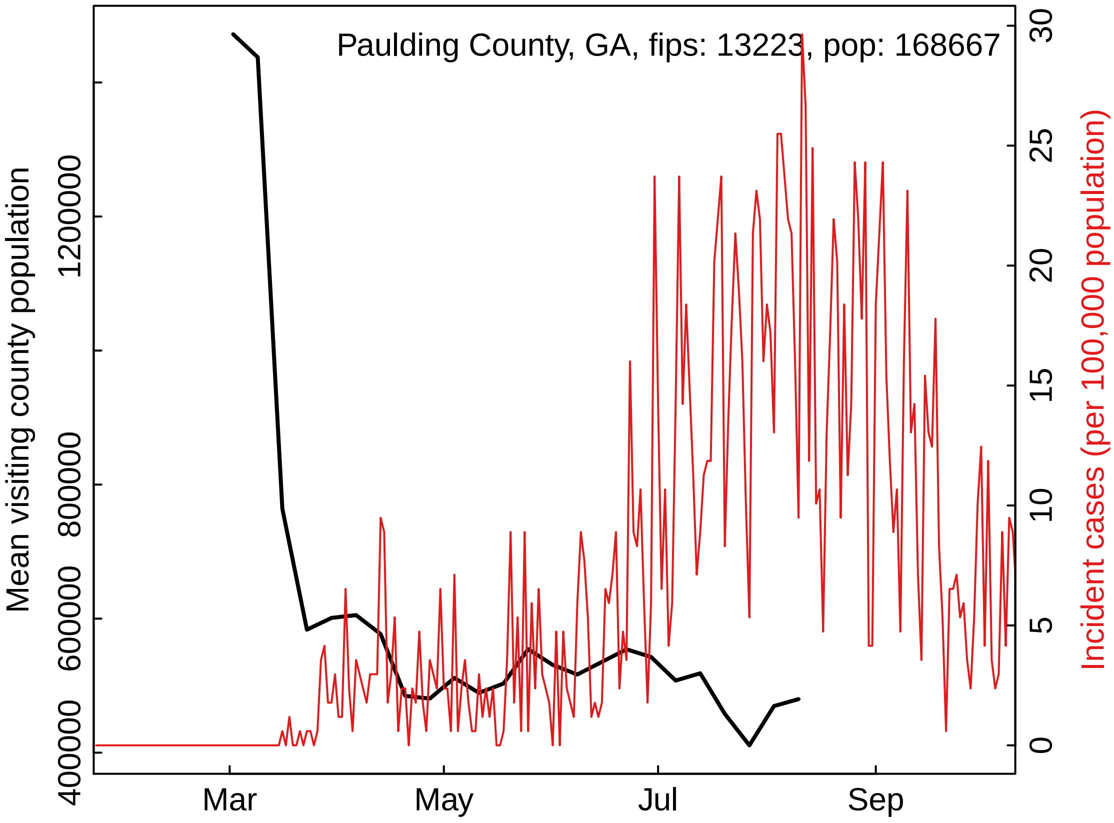

Paulding County, GA, fips: 13223, pop: 168667
Mean visiting county population
400000
600000
800000
1200000
Incident cases (per 100,000 population)
0
5
10
15
20
25
30
Mar
May
Jul
Sep

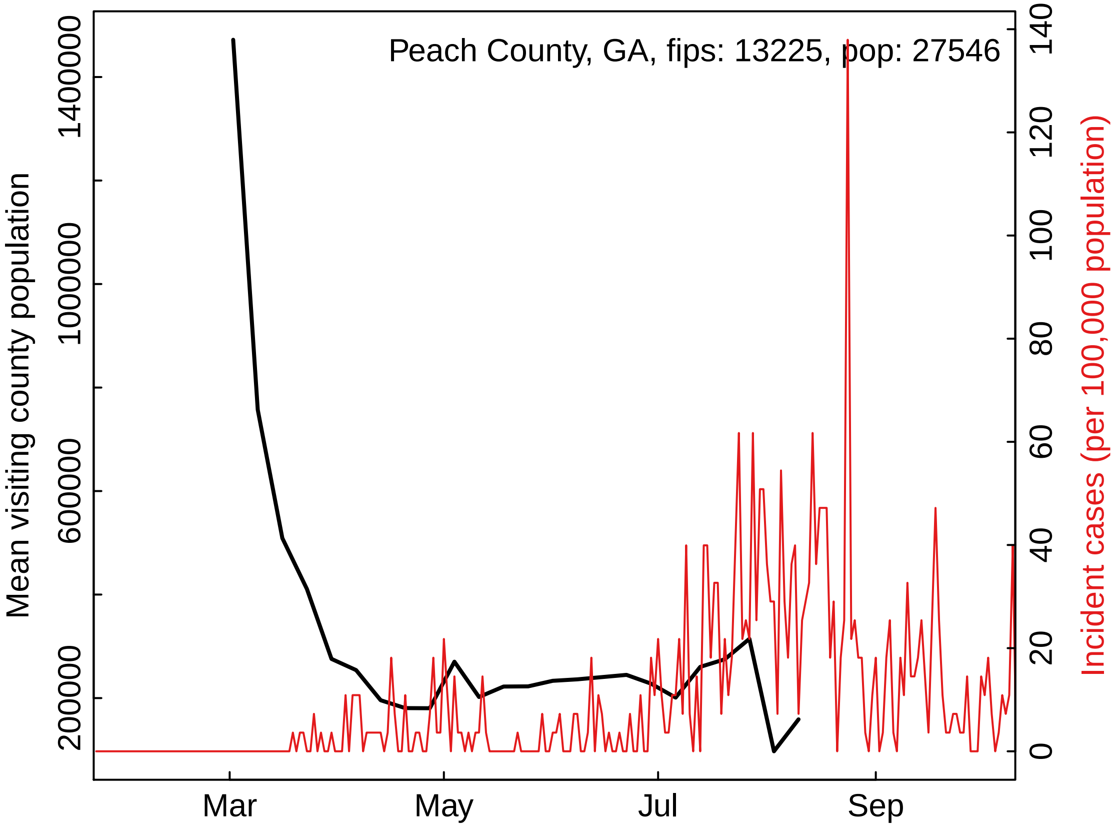
Peach County, GA, fips: 13225, pop: 27546
Mean visiting county population
200000
600000
1000000
1400000
0
20
40
60
80
100
120
140
Incident cases (per 100,000 population)
Mar
May
Jul
Sep

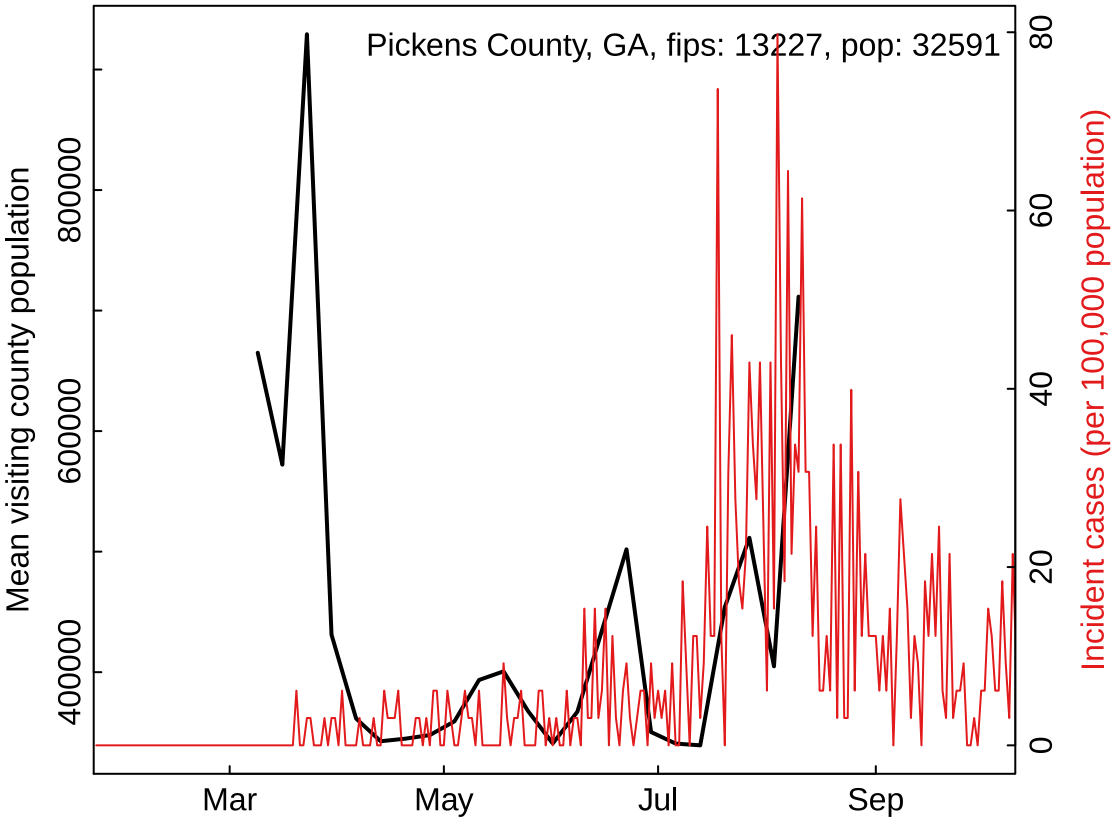

Pickens County, GA, fips: 13227, pop: 32591
Mean visiting county population
400000
600000
800000
Incident cases (per 100,000 population)
0
20
40
60
80
Mar
May
Jul
Sep

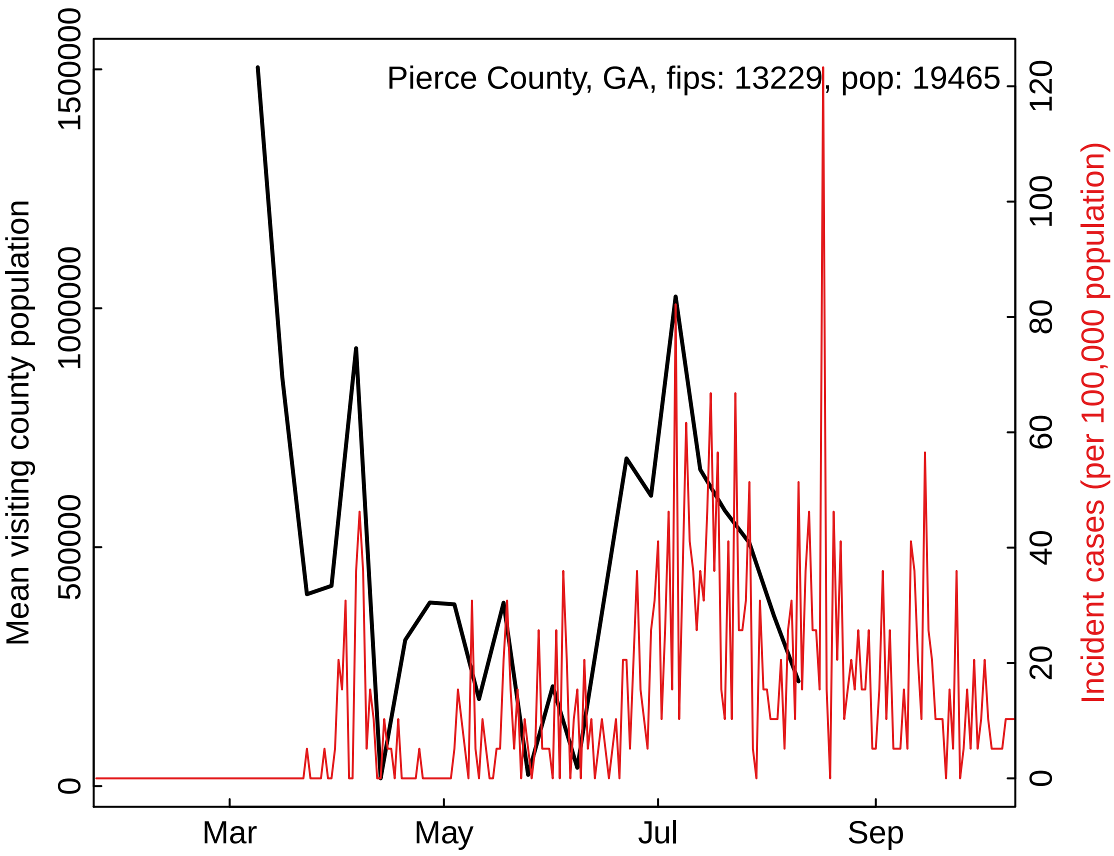

Pierce County, GA, fips: 13229, pop: 19465
Mean visiting county population
Incident cases (per 100,000 population)
0
500000
1000000
1500000
0
20
40
60
80
100
120
Mar
May
Jul
Sep

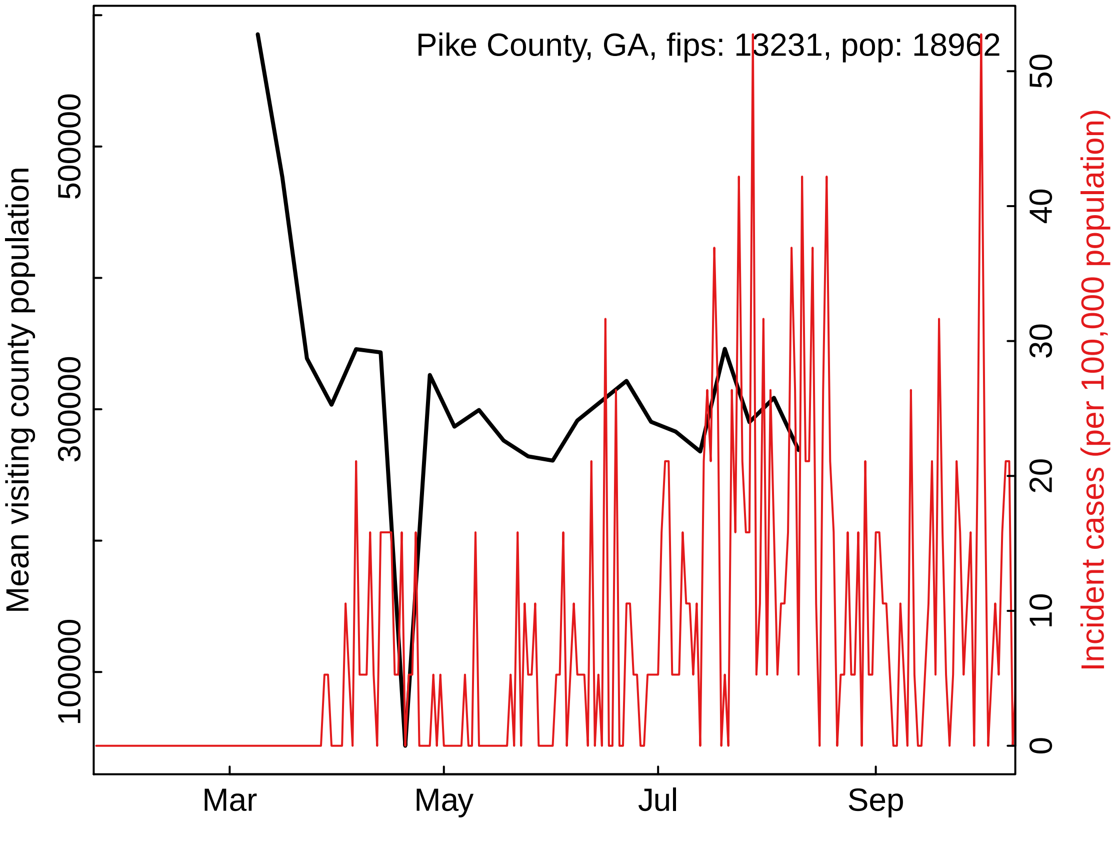

Pike County, GA, fips: 13231, pop: 18962
Mean visiting county population
100000
300000
500000
Incident cases (per 100,000 population)
0
10
20
30
40
50
Mar
May
Jul
Sep

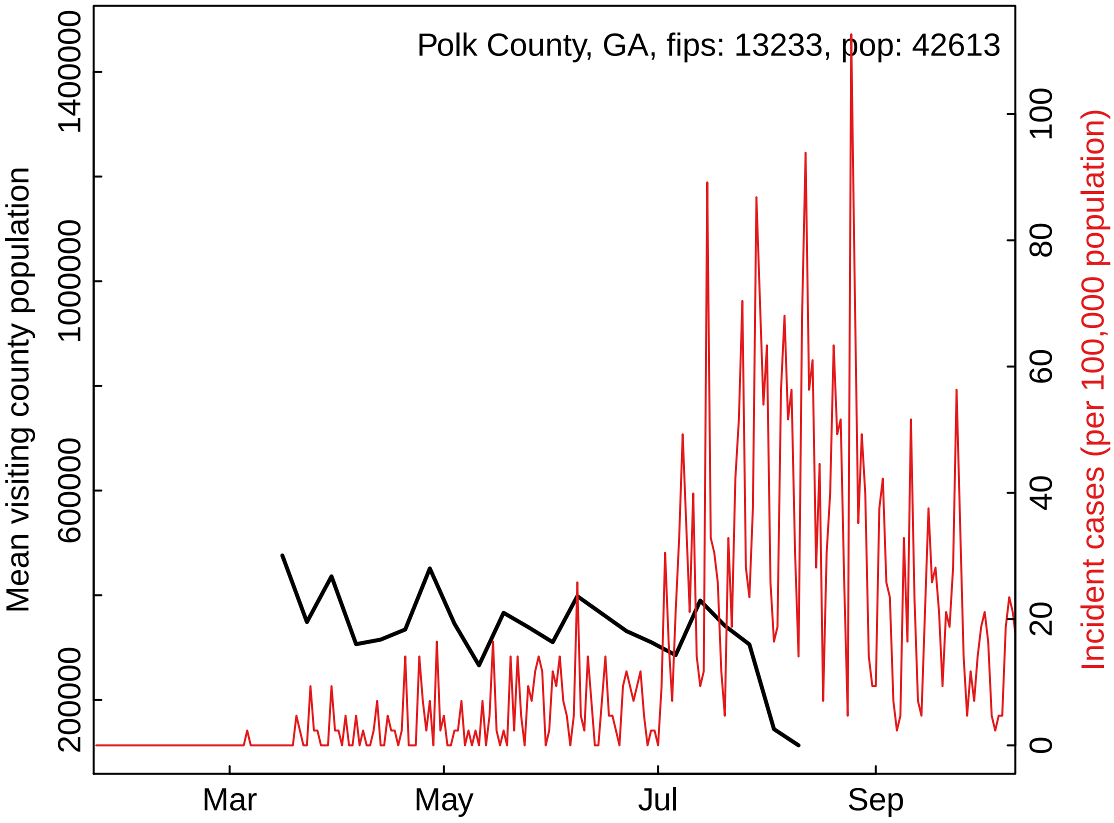
Polk County, GA, fips: 13233, pop: 42613
Mean visiting county population
200000
600000
1000000
1400000
Incident cases (per 100,000 population)
0
20
40
60
80
100
Mar
May
Jul
Sep

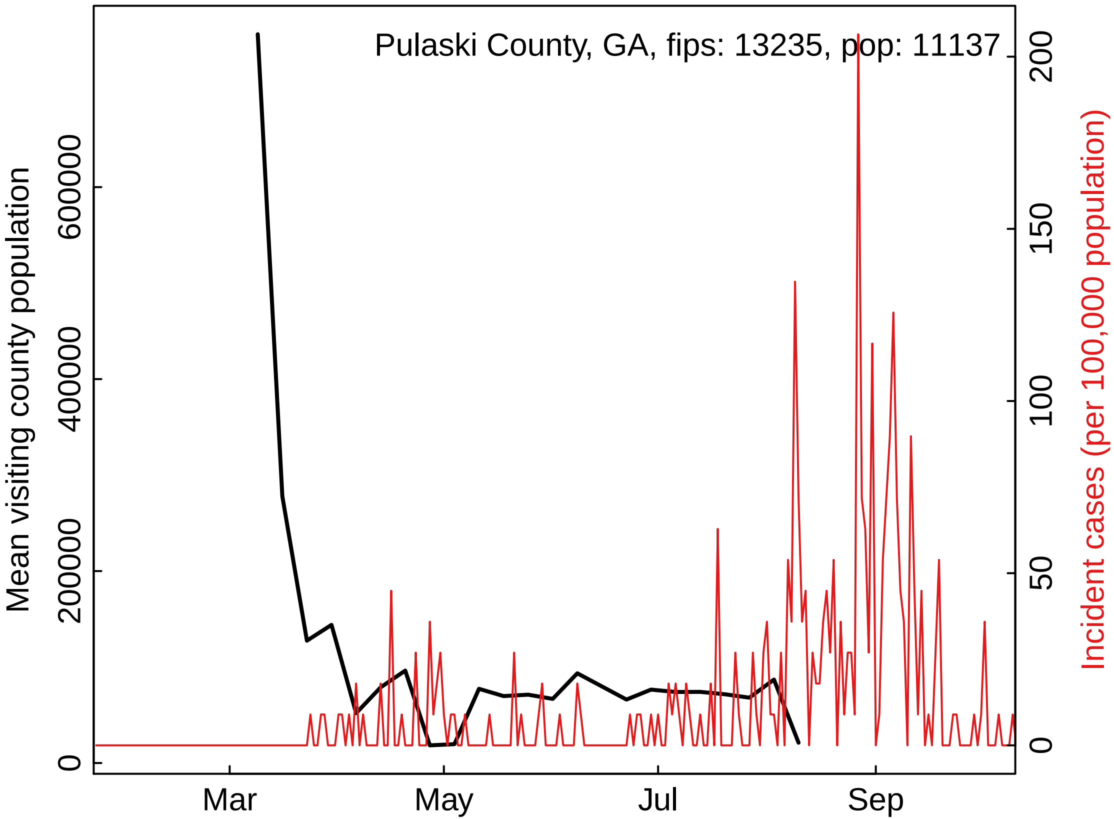

Pulaski County, GA, fips: 13235, pop: 11137
Mean visiting county population
0
200000
400000
600000
Incident cases (per 100,000 population)
0
50
100
150
200
Mar
May
Jul
Sep

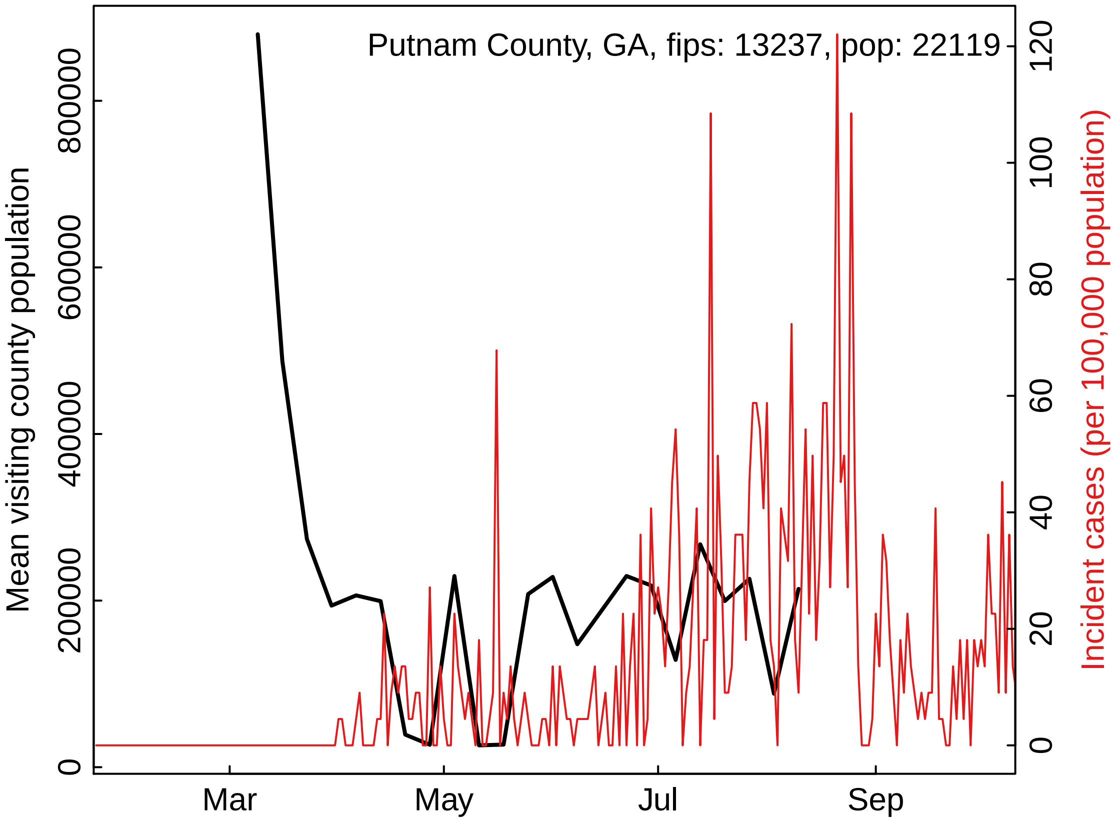
Putnam County, GA, fips: 13237, pop: 22119
Mean visiting county population
Incident cases (per 100,000 population)
0
200000
400000
600000
800000
0
20
40
60
80
100
120
Mar
May
Jul
Sep

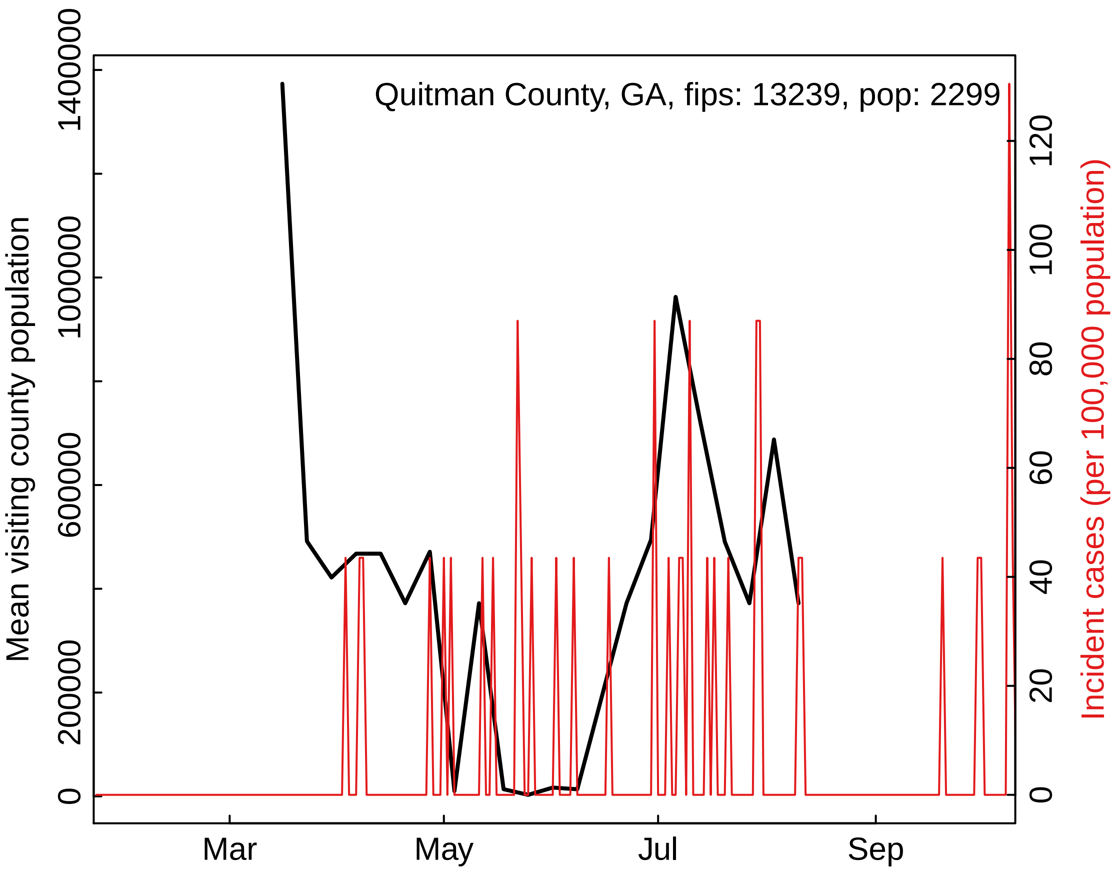

Quitman County, GA, fips: 13239, pop: 2299
Mean visiting county population
0
200000
600000
1000000
1400000
Incident cases (per 100,000 population)
0
20
40
60
80
100
120
Mar
May
Jul
Sep

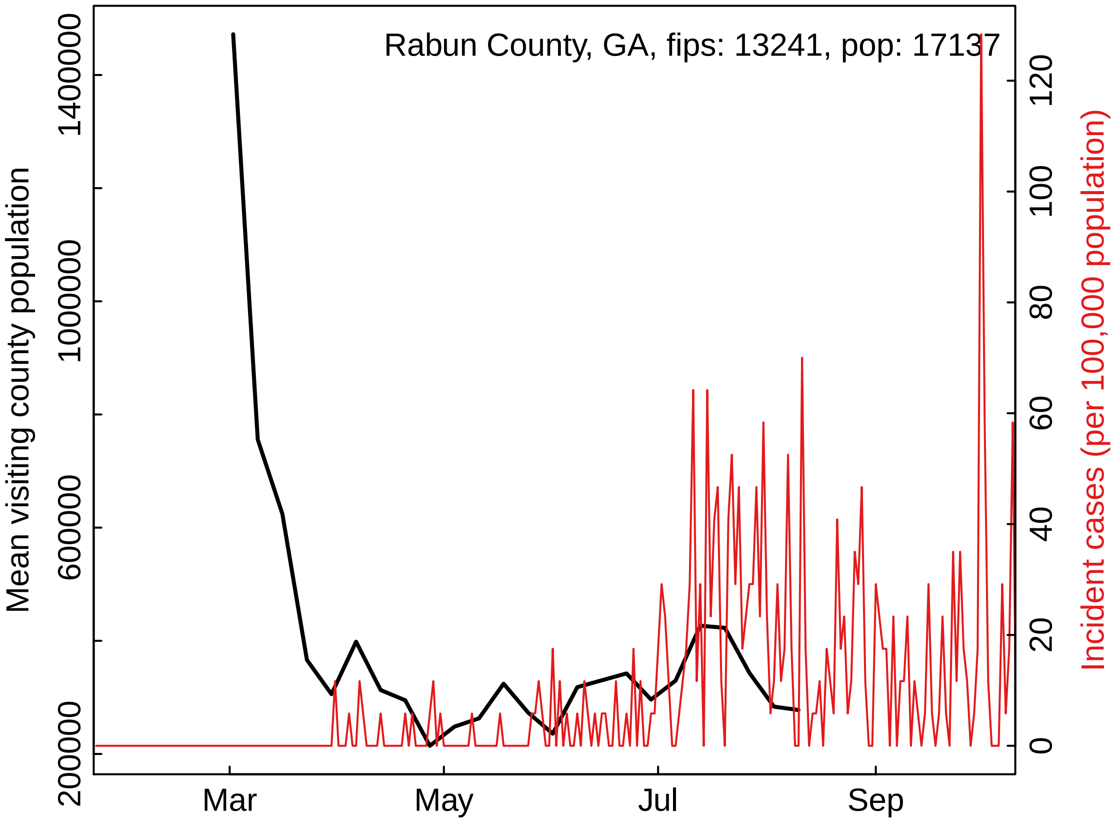

Rabun County, GA, fips: 13241, pop: 17137
Mean visiting county population
200000
600000
1000000
1400000
Mar
May
Jul
Sep
0
20
40
60
80
100
120
Incident cases (per 100,000 population)

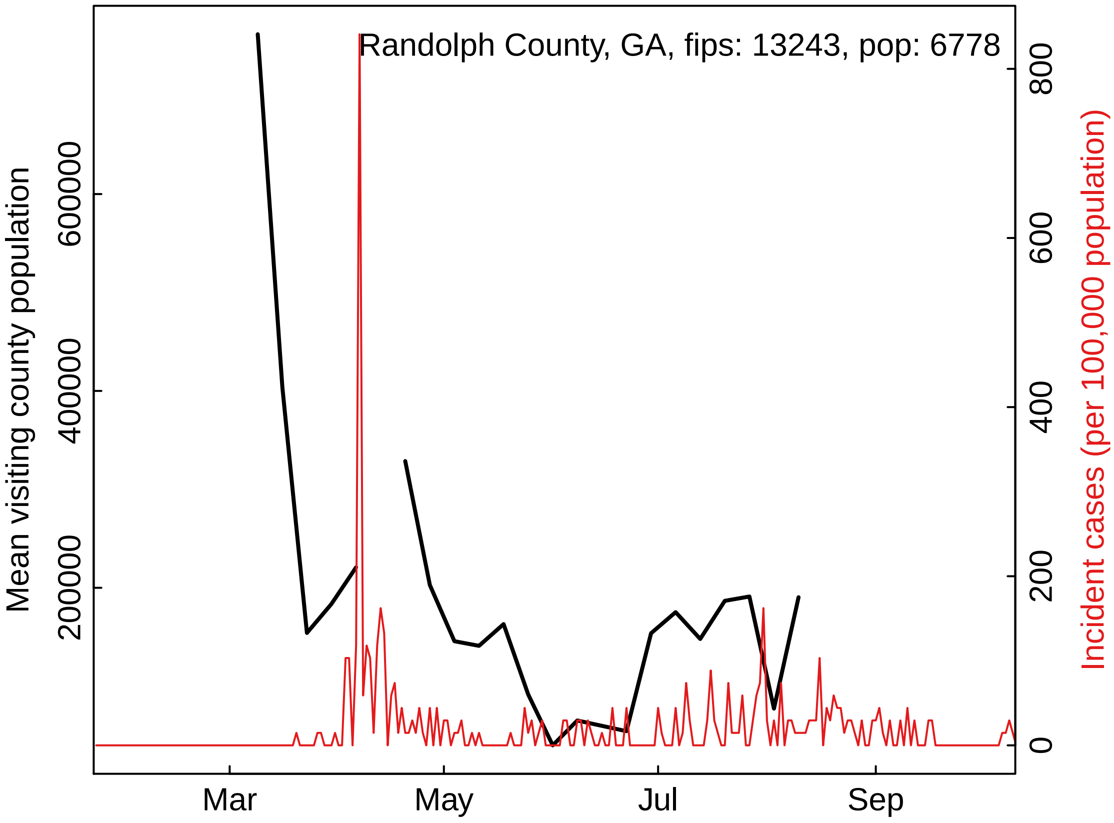

Randolph County, GA, fips: 13243, pop: 6778
Mean visiting county population
200000
400000
600000
Incident cases (per 100,000 population)
0
200
400
600
800
Mar
May
Jul
Sep

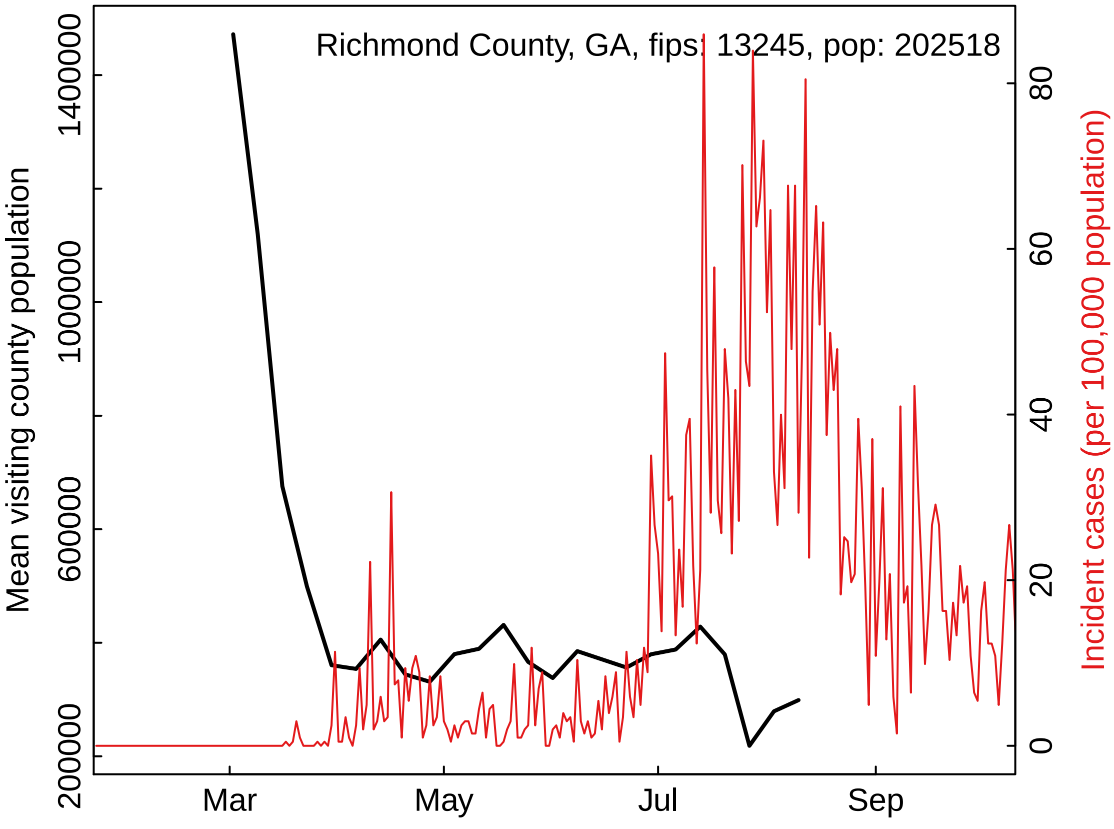

Richmond County, GA, fips: 13245, pop: 202518
Mean visiting county population
Incident cases (per 100,000 population)
200000
600000
1000000
1400000
0
20
40
60
80
Mar
May
Jul
Sep

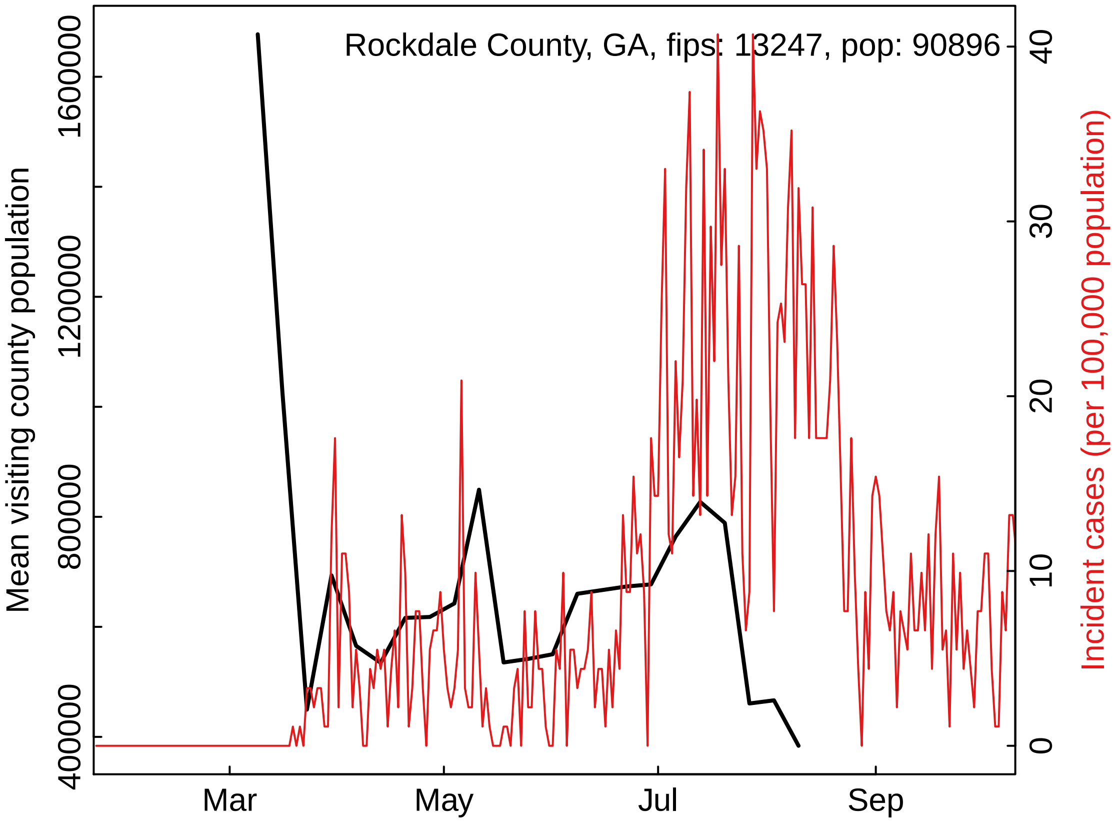

Rockdale County, GA, fips: 13247, pop: 90896
Mean visiting county population
400000
800000
1200000
1600000
Incident cases (per 100,000 population)
0
10
20
30
40
Mar
May
Jul
Sep

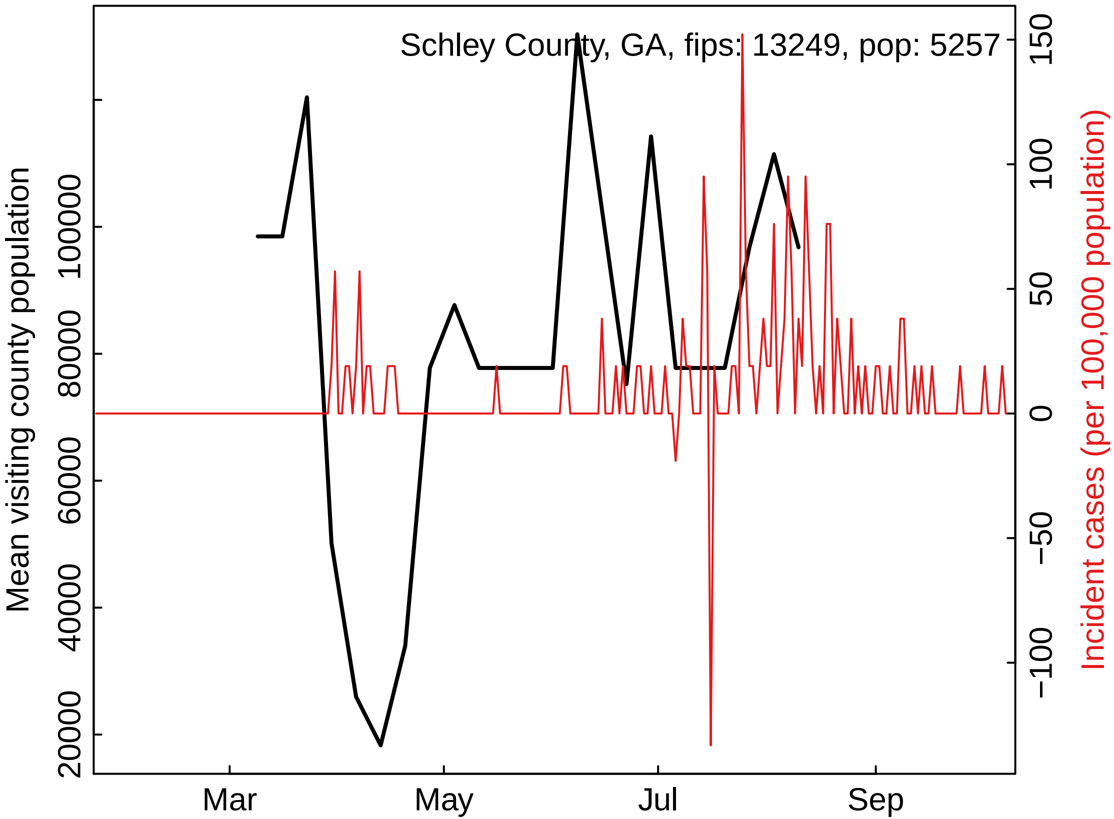

Schley County, GA, fips: 13249, pop: 5257
Mean visiting county population
20000
40000
60000
80000
100000
Mar
May
Jul
Sep
−100
−50
0
50
100
150
Incident cases (per 100,000 population)

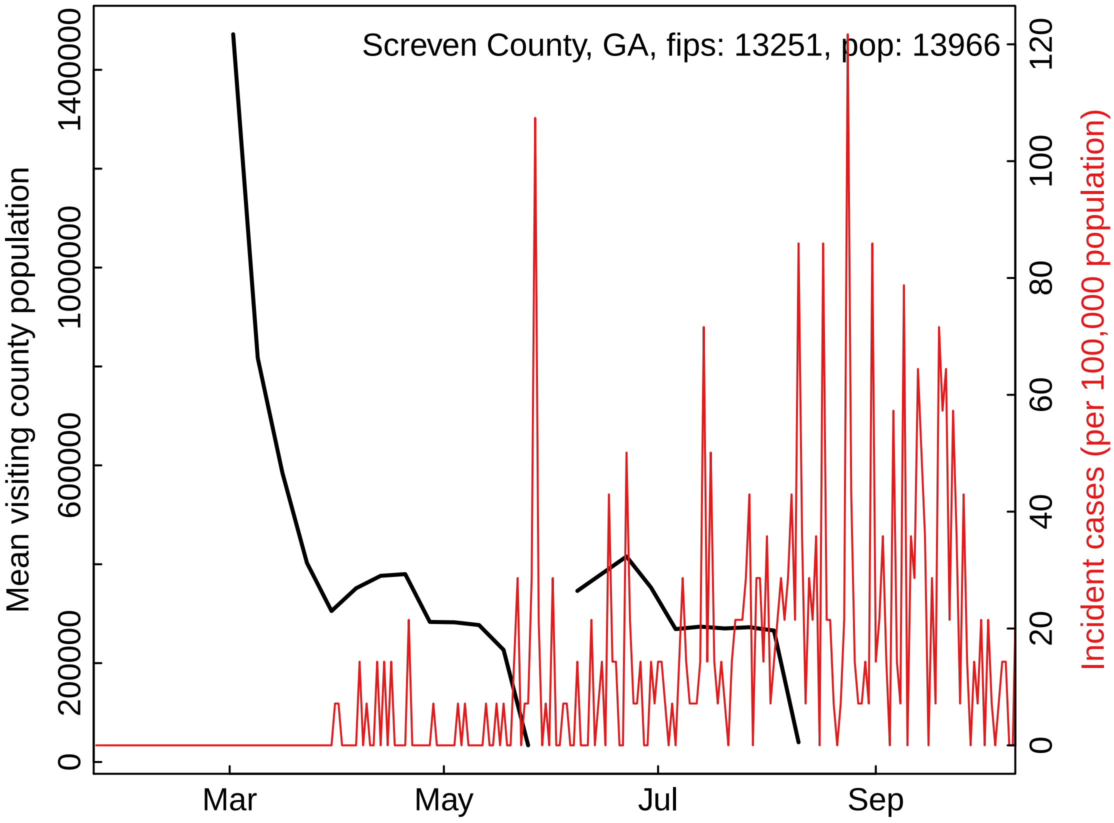

Screven County, GA, fips: 13251, pop: 13966
Mean visiting county population
0
200000
600000
1000000
1400000
Incident cases (per 100,000 population)
0
20
40
60
80
100
120
Mar
May
Jul
Sep

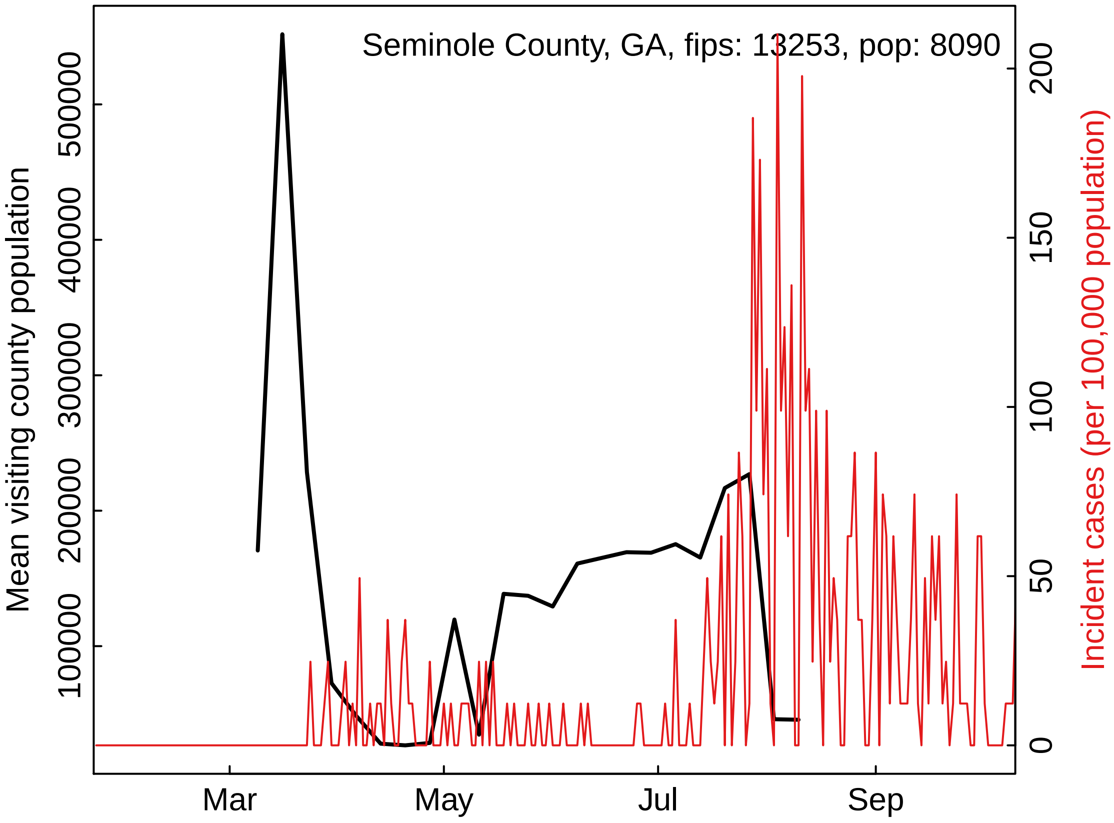
Seminole County, GA, fips: 13253, pop: 8090
Mean visiting county population
100000
200000
300000
400000
500000
Incident cases (per 100,000 population)
0
50
100
150
200
Mar
May
Jul
Sep

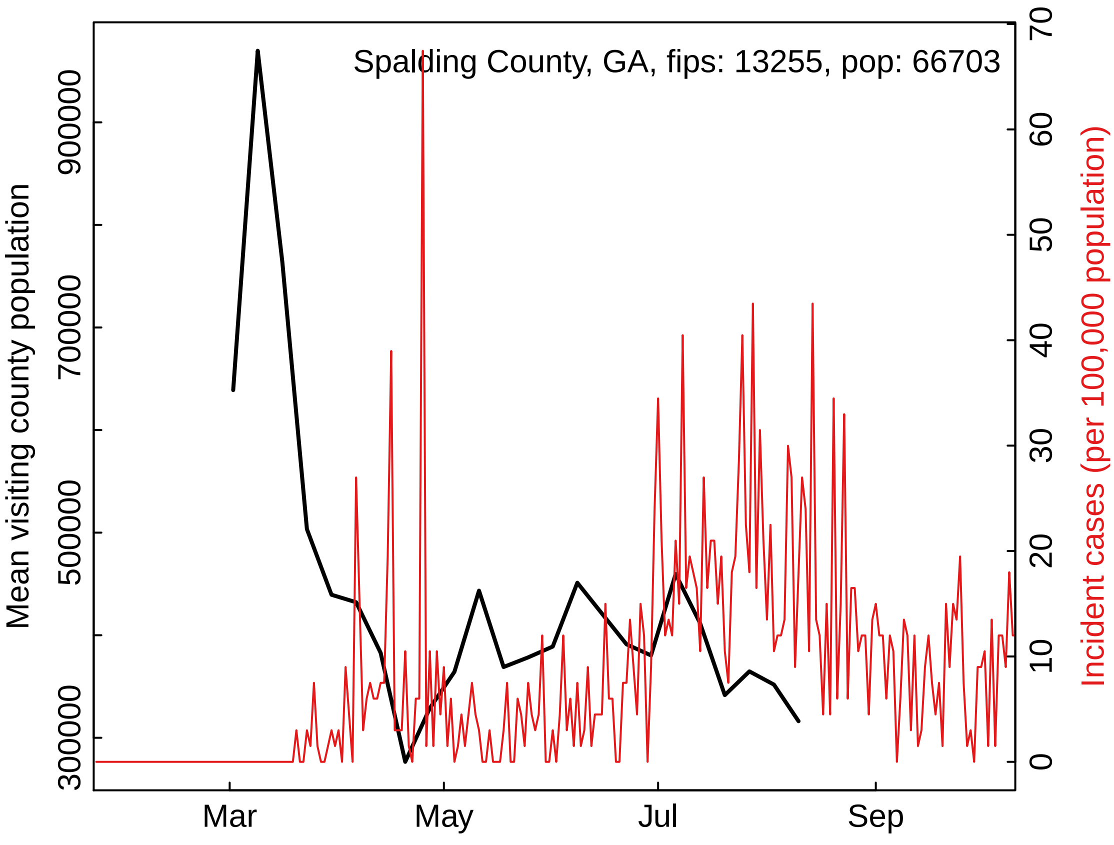
Spalding County, GA, fips: 13255, pop: 66703
Mean visiting county population
300000
500000
700000
900000
Incident cases (per 100,000 population)
0
10
20
30
40
50
60
70
Mar
May
Jul
Sep

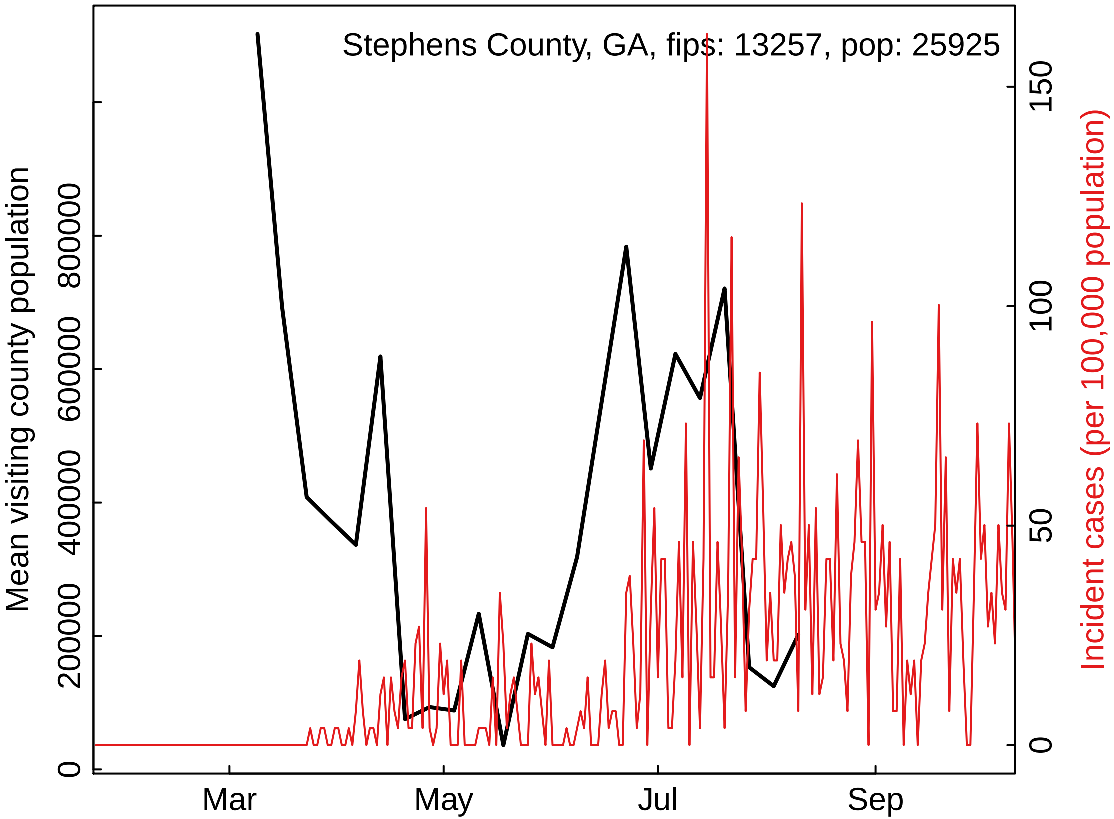

Stephens County, GA, fips: 13257, pop: 25925
Mean visiting county population
0
200000
400000
600000
800000
Incident cases (per 100,000 population)
0
50
100
150
Mar
May
Jul
Sep

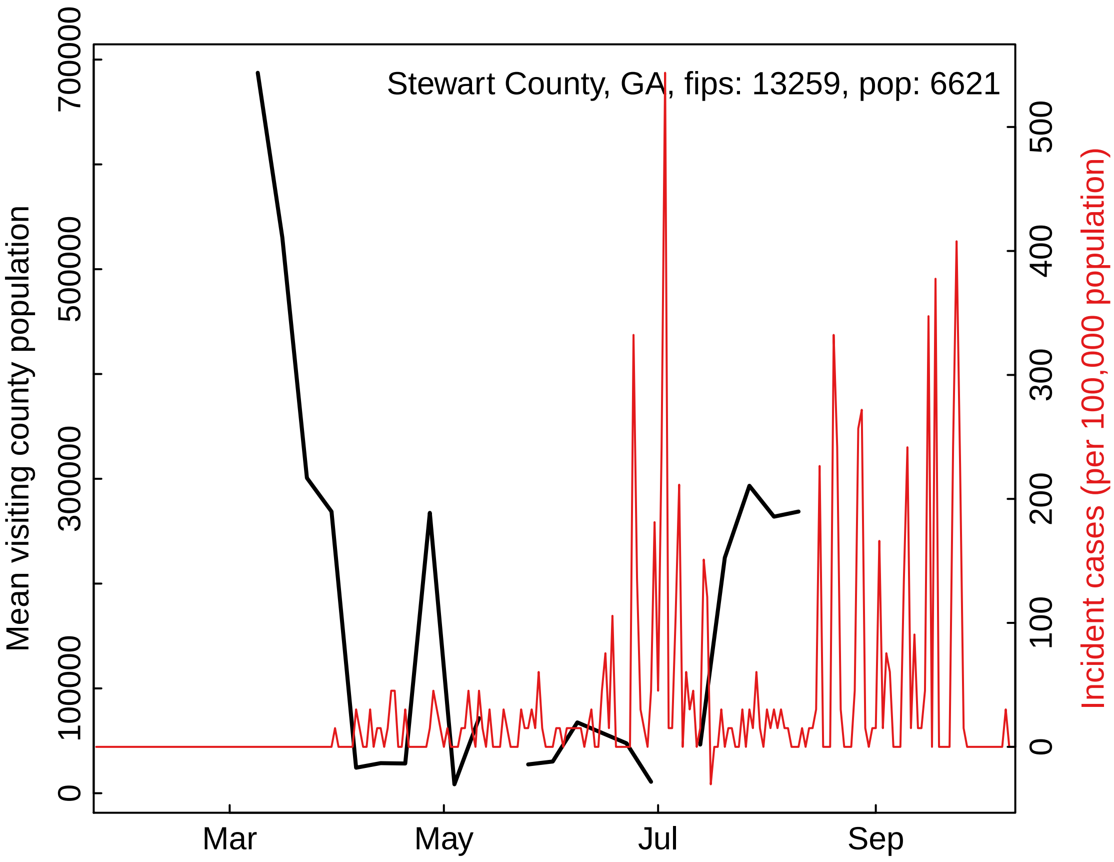

Stewart County, GA, fips: 13259, pop: 6621
Mean visiting county population
0
100000
300000
500000
700000
Incident cases (per 100,000 population)
0
100
200
300
400
500
Mar
May
Jul
Sep

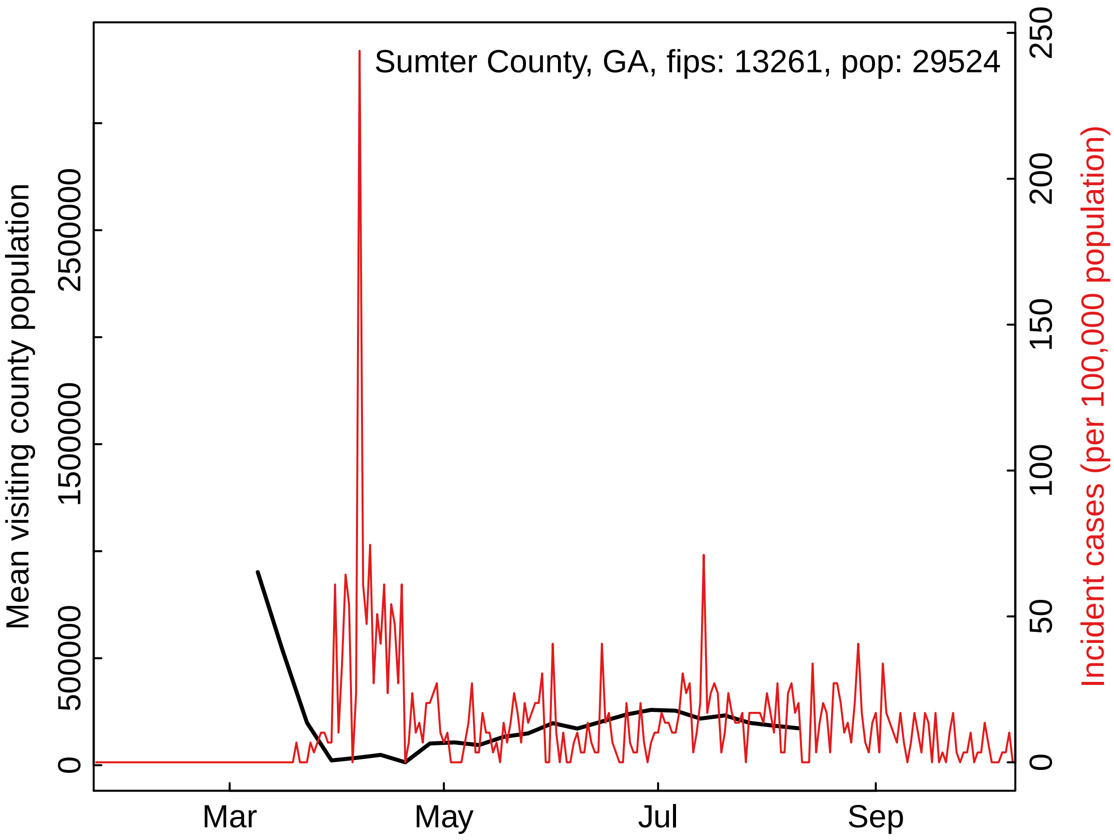

Sumter County, GA, fips: 13261, pop: 29524
Mean visiting county population
0
500000
1500000
2500000
Incident cases (per 100,000 population)
0
50
100
150
200
250
Mar
May
Jul
Sep

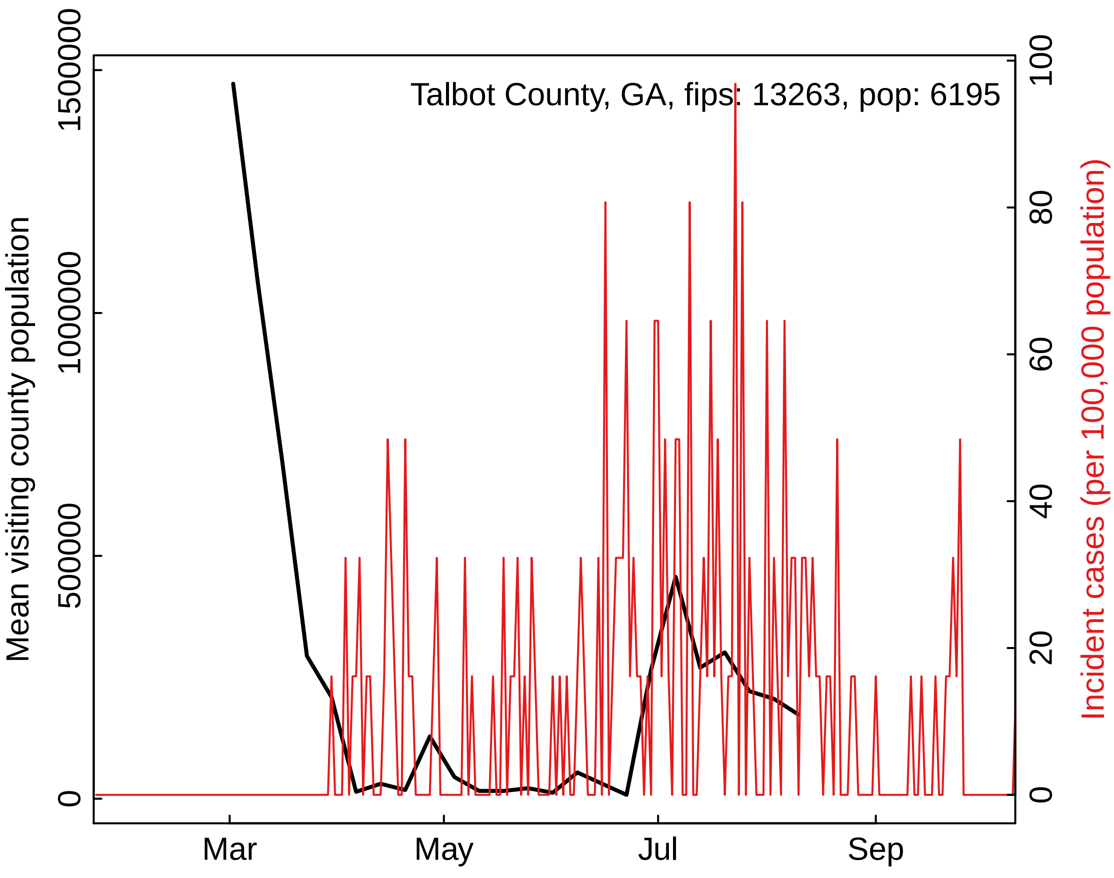

Talbot County, GA, fips: 13263, pop: 6195
Mean visiting county population
0
500000
1000000
1500000
Incident cases (per 100,000 population)
0
20
40
60
80
100
Mar
May
Jul
Sep

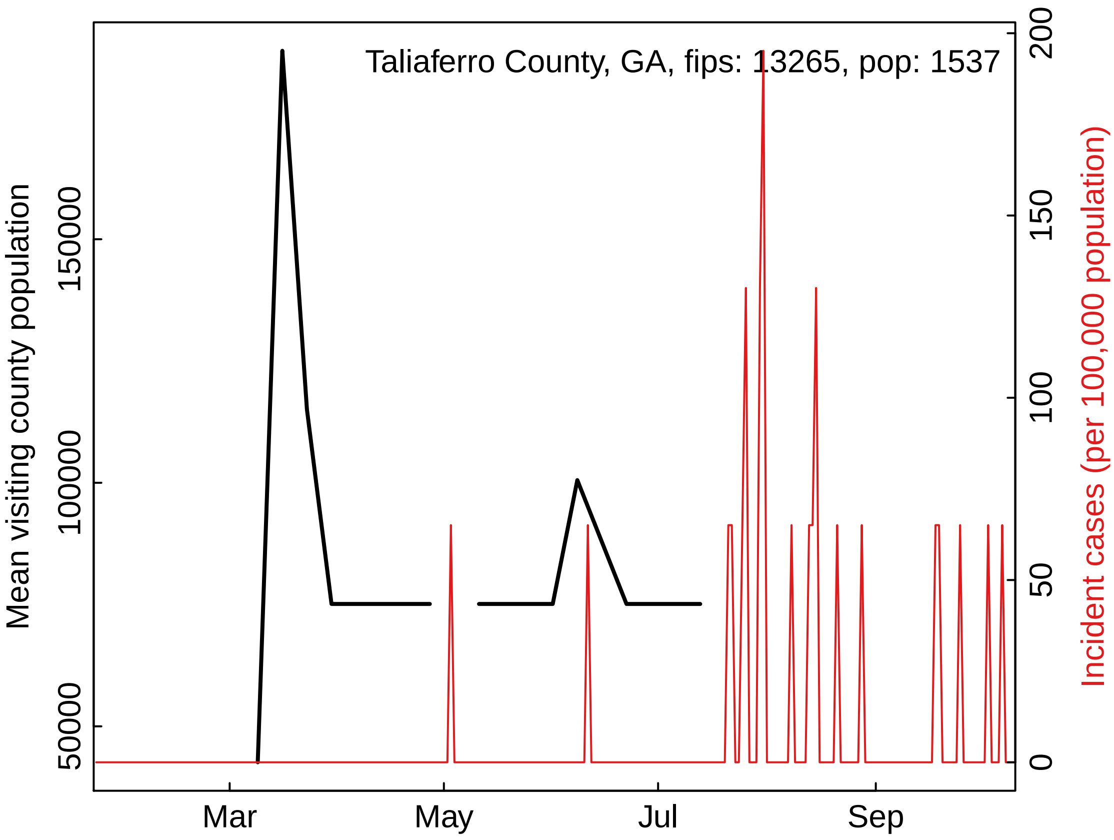

Taliaferro County, GA, fips: 13265, pop: 1537
Mean visiting county population
50000
100000
150000
Incident cases (per 100,000 population)
0
50
100
150
200
Mar
May
Jul
Sep

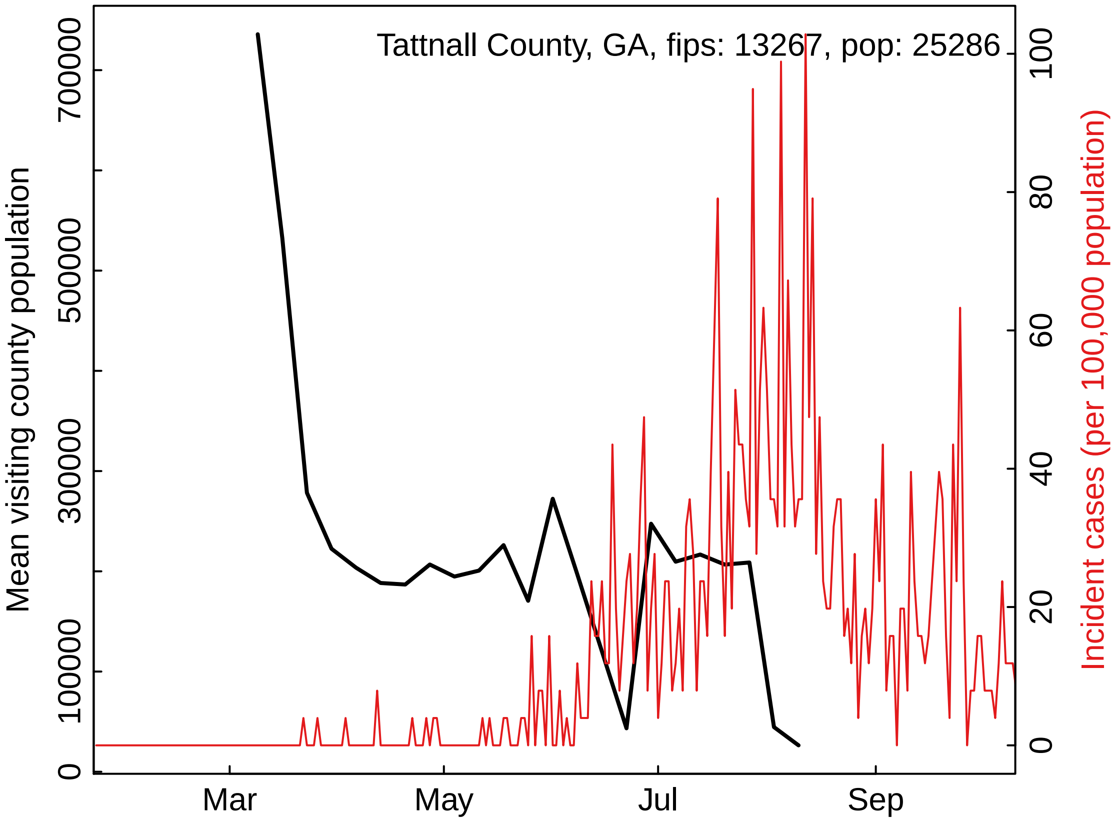
Tattnall County, GA, fips: 13267, pop: 25286
Mean visiting county population
0
100000
300000
500000
700000
Mar
May
Jul
Sep
Incident cases (per 100,000 population)
0
20
40
60
80
100

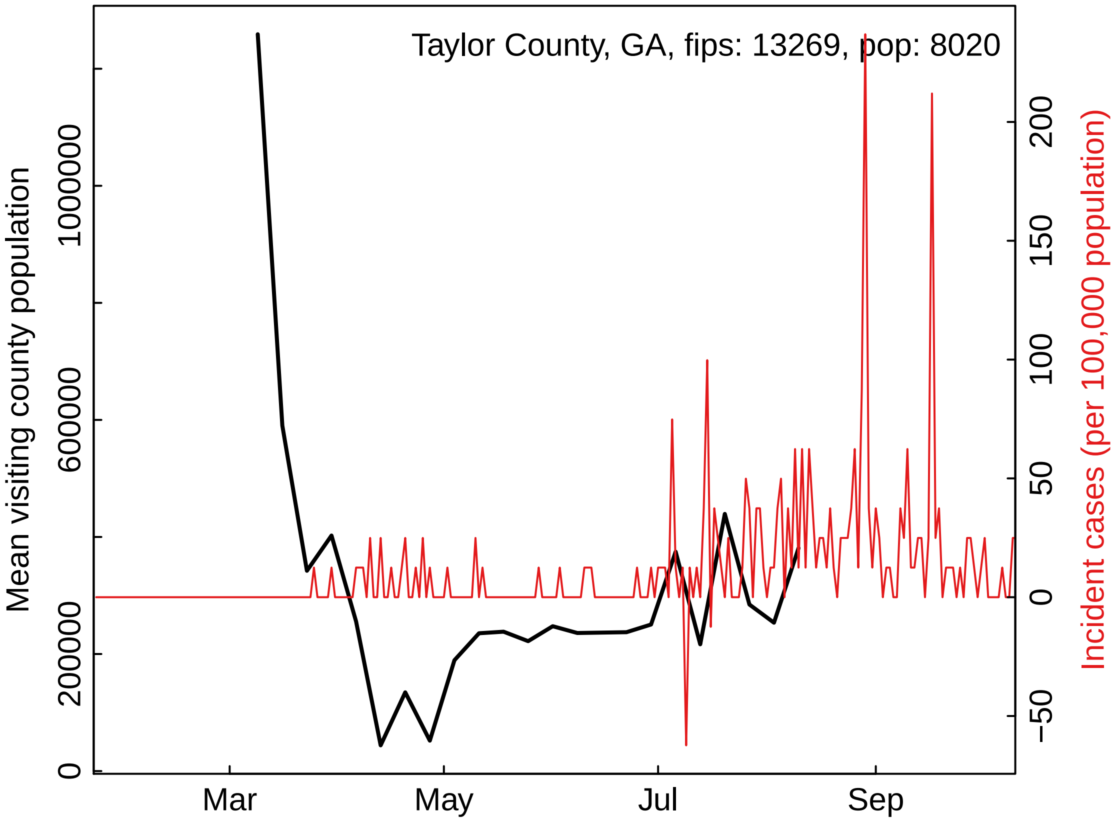
Taylor County, GA, fips: 13269, pop: 8020
Mean visiting county population
0
200000
600000
1000000
Incident cases (per 100,000 population)
−50
0
50
100
150
200
Mar
May
Jul
Sep

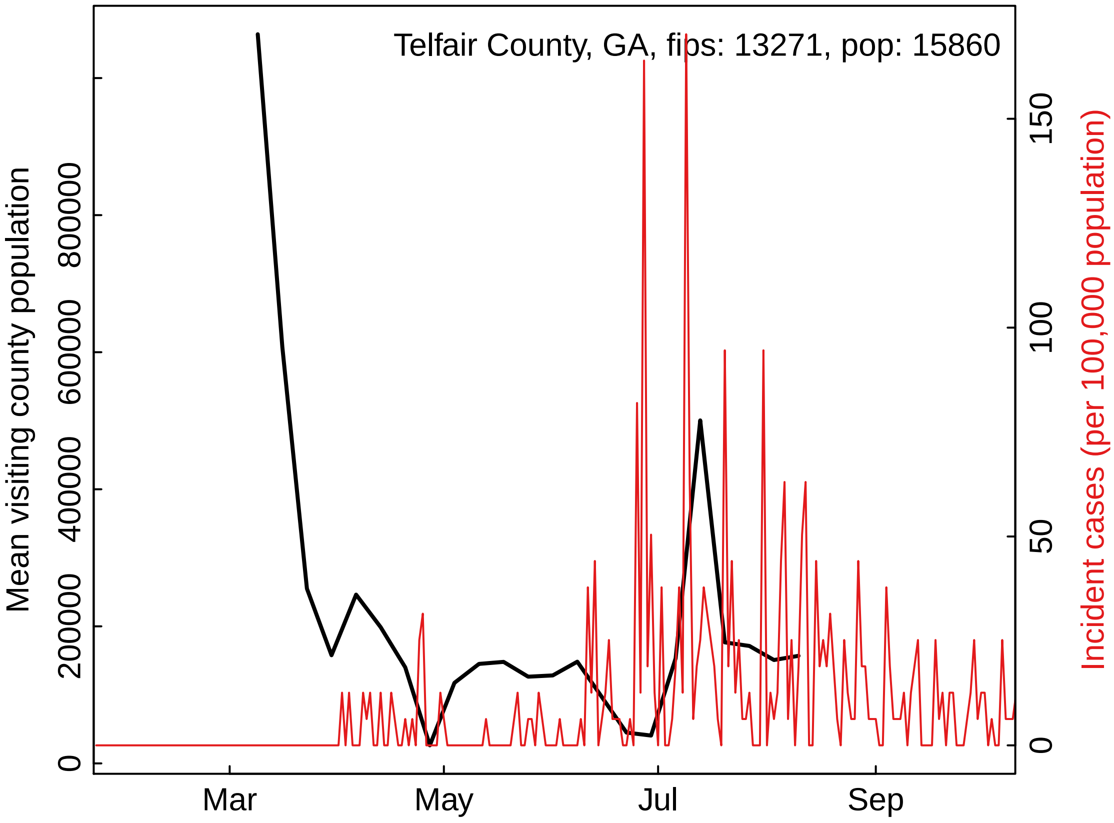

Telfair County, GA, fips: 13271, pop: 15860
Mean visiting county population
0
200000
400000
600000
800000
Mar
May
Jul
Sep
Incident cases (per 100,000 population)
0
50
100
150

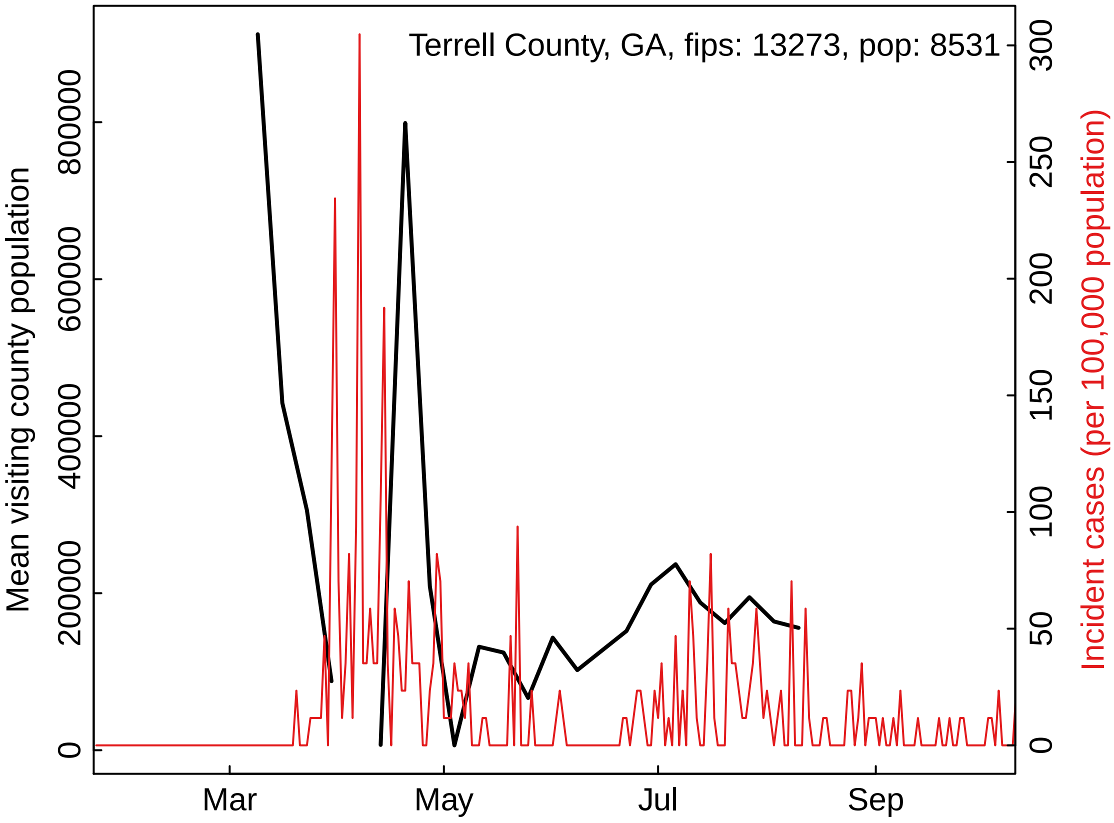

Terrell County, GA, fips: 13273, pop: 8531
Mean visiting county population
0
200000
400000
600000
800000
Incident cases (per 100,000 population)
0
50
100
150
200
250
300
Mar
May
Jul
Sep

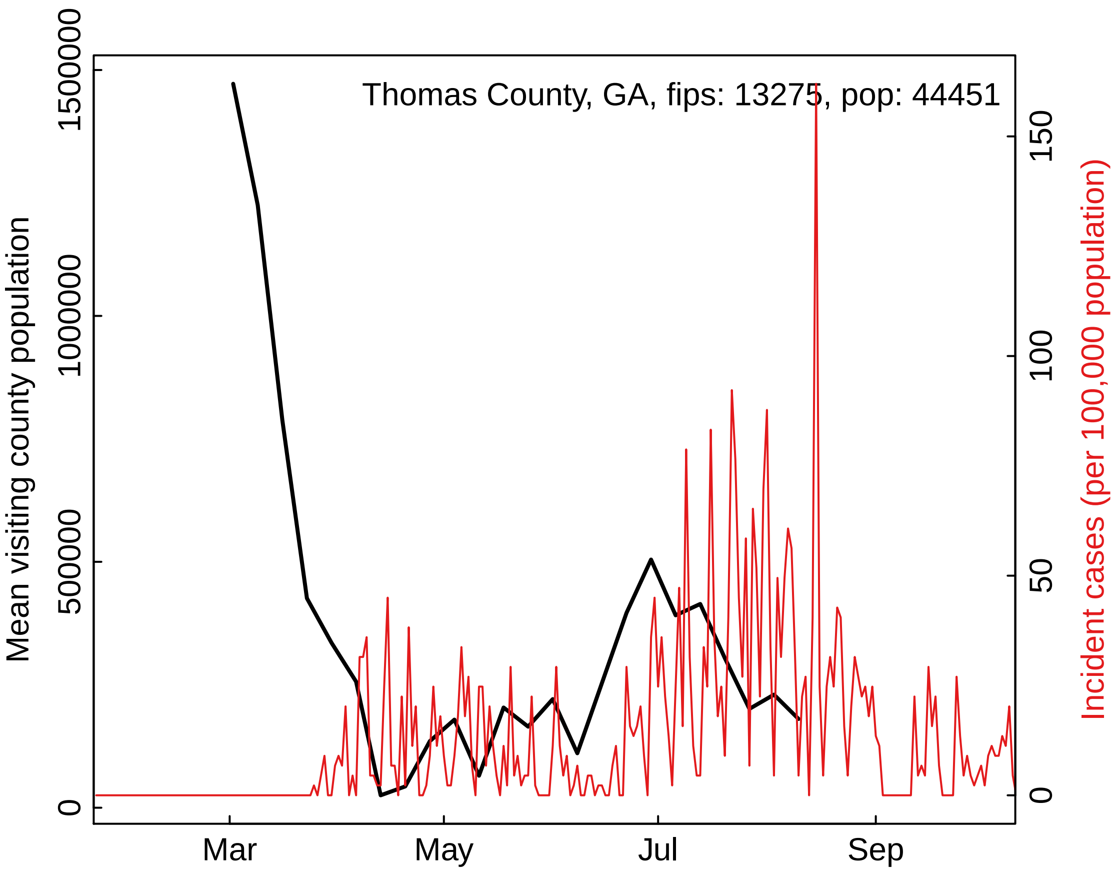

Thomas County, GA, fips: 13275, pop: 44451
Mean visiting county population
0
500000
1000000
1500000
Incident cases (per 100,000 population)
0
50
100
150
Mar
May
Jul
Sep

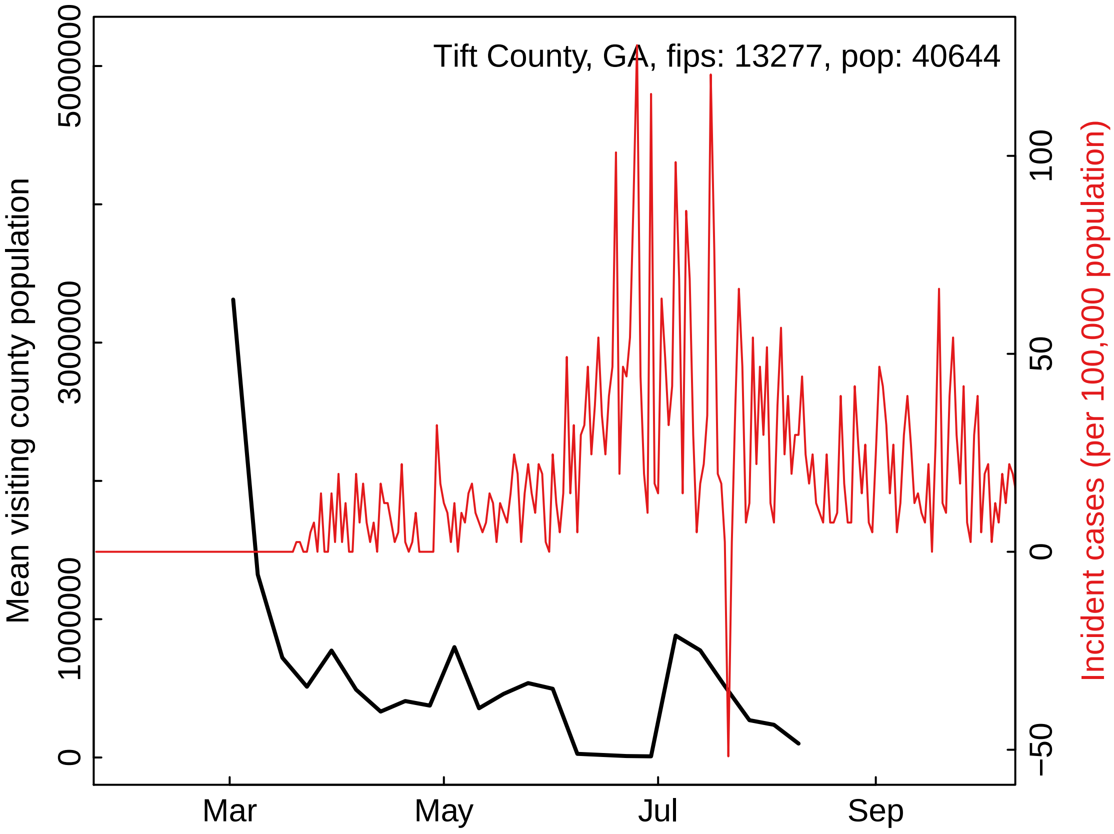
Tift County, GA, fips: 13277, pop: 40644
Mean visiting county population
Incident cases (per 100,000 population)
0
1000000
3000000
5000000
−50
0
50
100
Mar
May
Jul
Sep

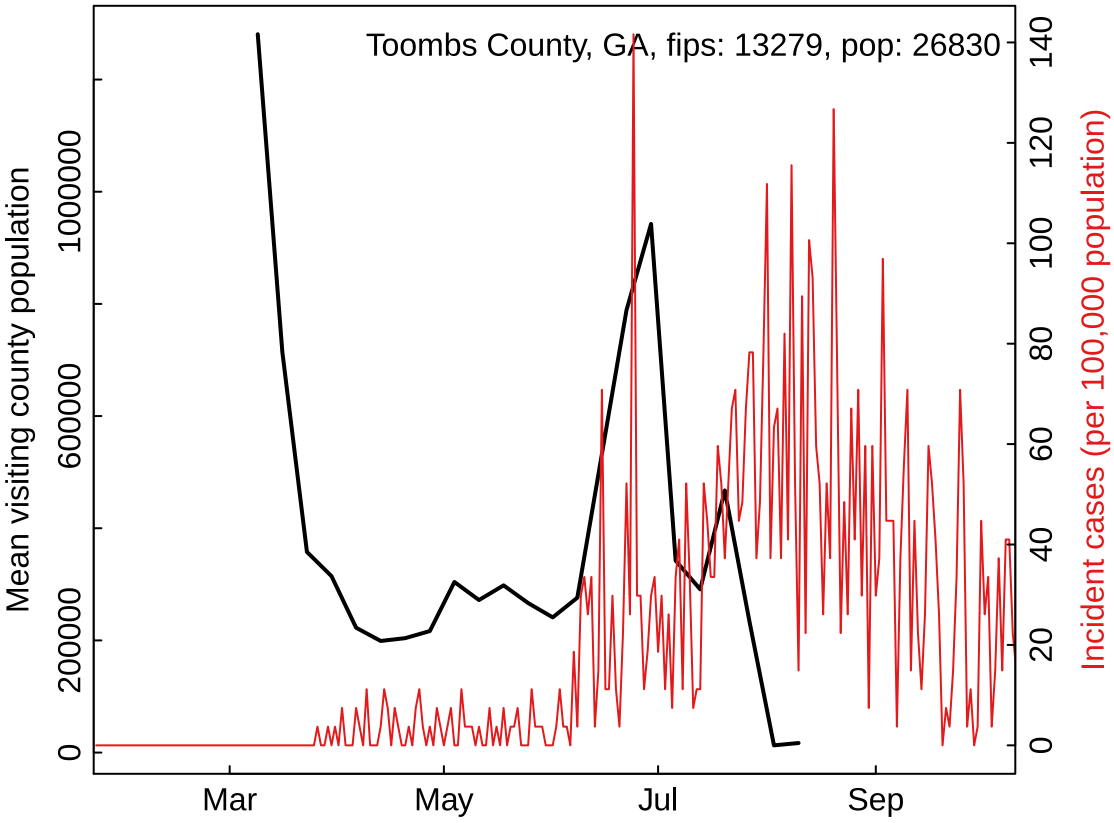

Toombs County, GA, fips: 13279, pop: 26830
Mean visiting county population
0
200000
600000
1000000
Incident cases (per 100,000 population)
0
20
40
60
80
100
120
140
Mar
May
Jul
Sep

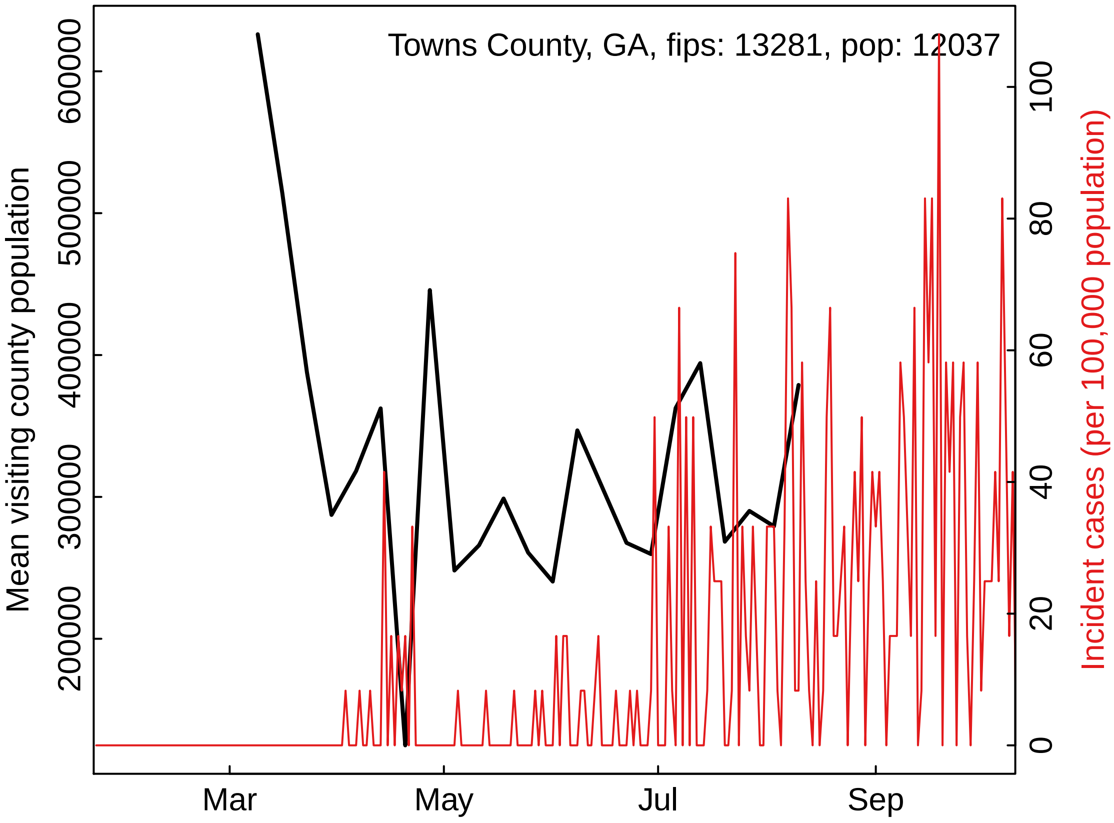

Towns County, GA, fips: 13281, pop: 12037
Mean visiting county population
200000
300000
400000
500000
600000
Incident cases (per 100,000 population)
0
20
40
60
80
100
Mar
May
Jul
Sep

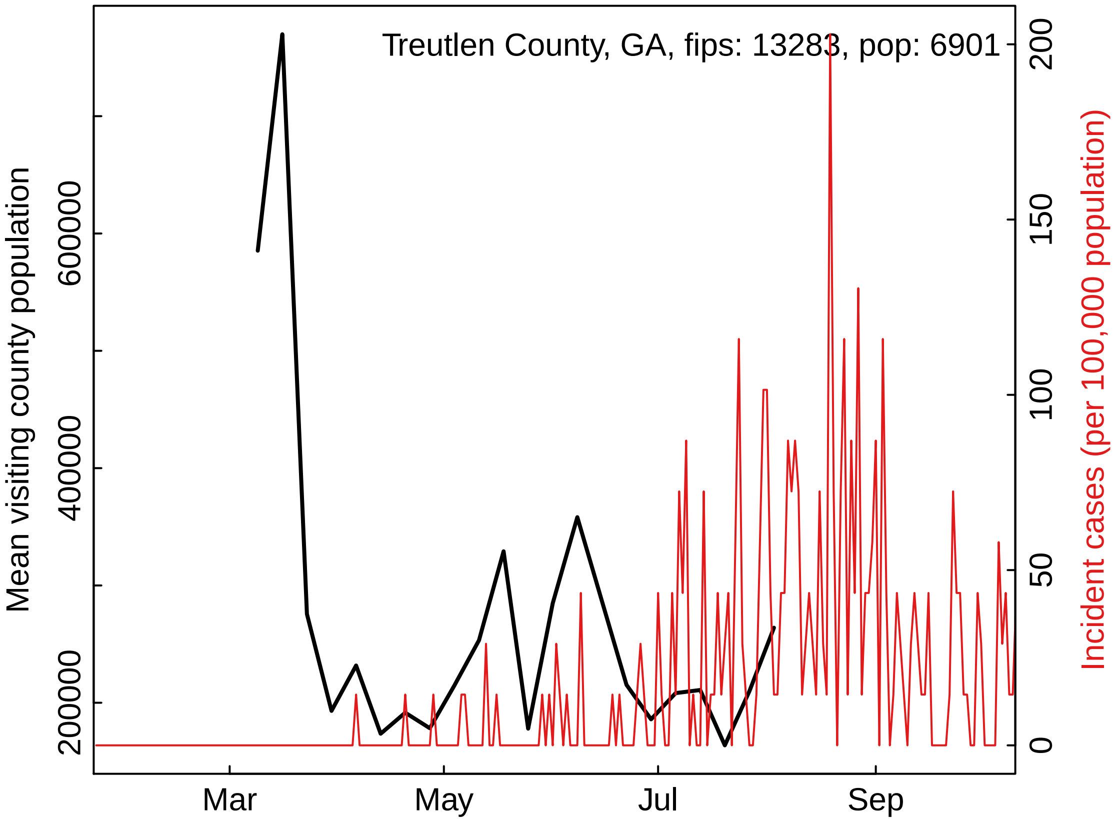

Treutlen County, GA, fips: 13283, pop: 6901
Mean visiting county population
200000
400000
600000
Incident cases (per 100,000 population)
0
50
100
150
200
Mar
May
Jul
Sep

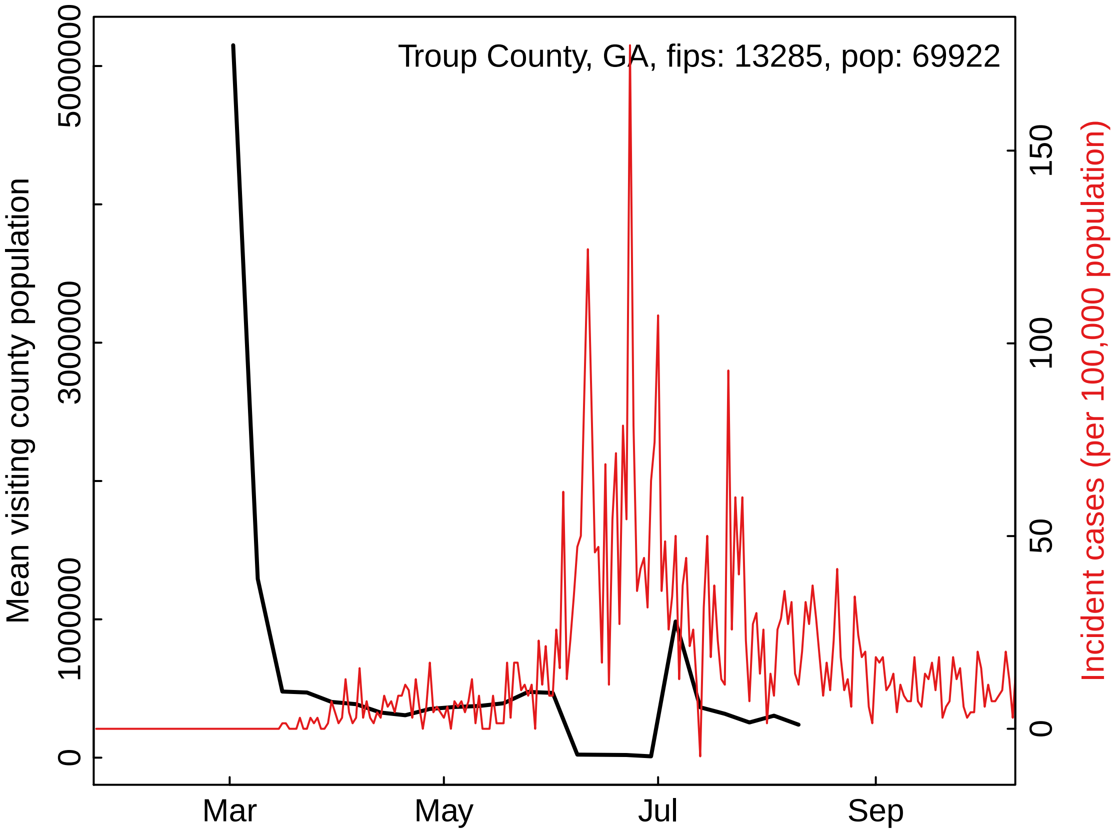

Troup County, GA, fips: 13285, pop: 69922
Mean visiting county population
0
1000000
3000000
5000000
Incident cases (per 100,000 population)
0
50
100
150
Mar
May
Jul
Sep

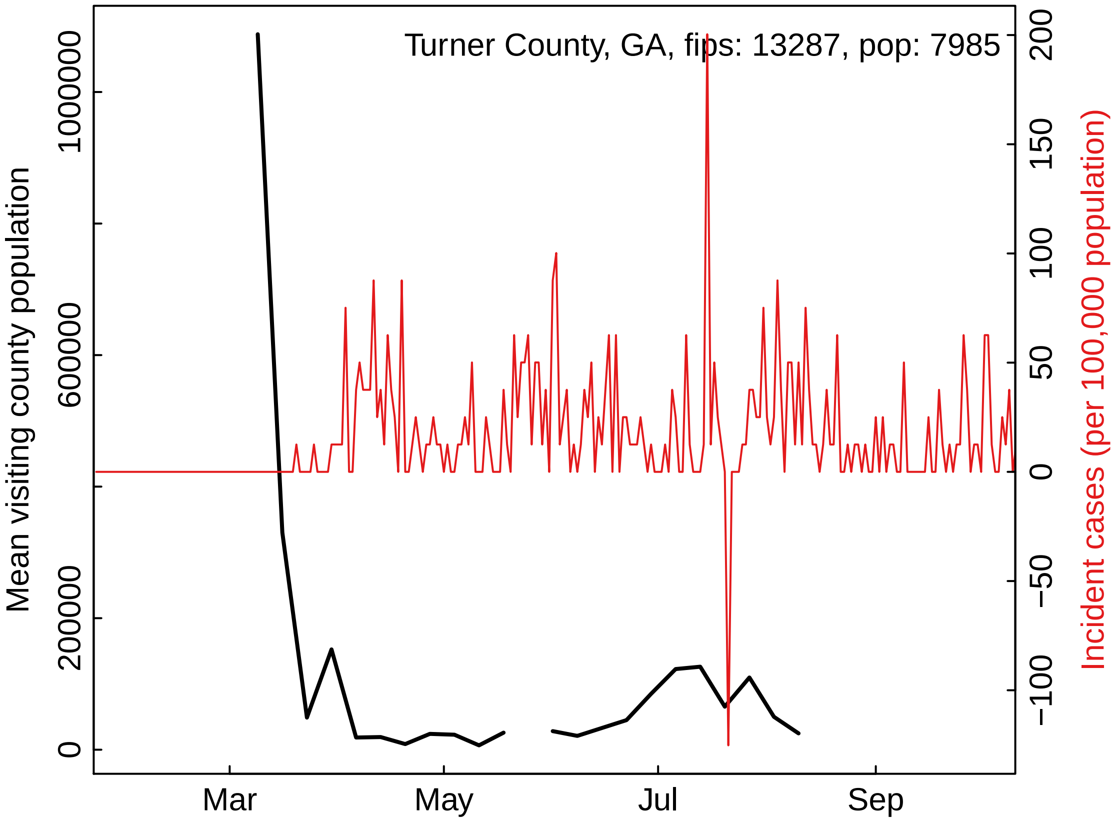

Turner County, GA, fips: 13287, pop: 7985
Mean visiting county population
0
200000
600000
1000000
Incident cases (per 100,000 population)
−100
−50
0
50
100
150
200
Mar
May
Jul
Sep

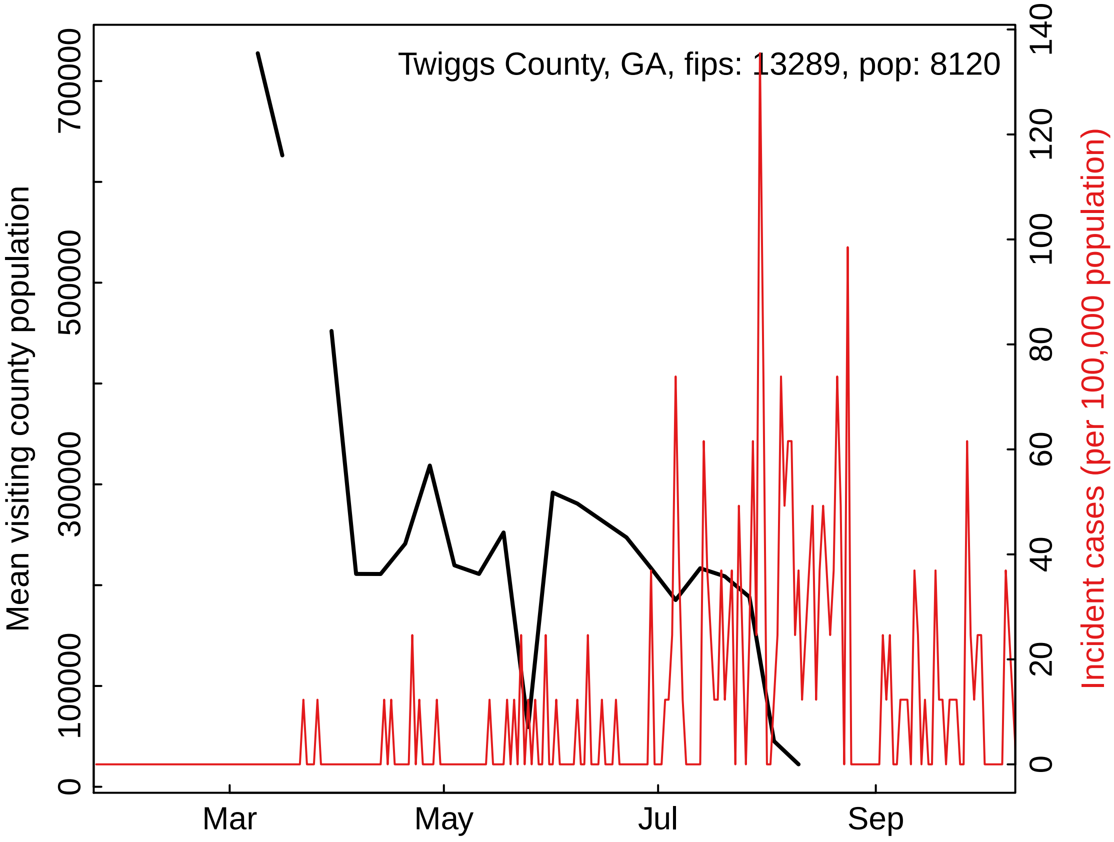

Twiggs County, GA, fips: 13289, pop: 8120
Mean visiting county population
0
100000
300000
500000
700000
Incident cases (per 100,000 population)
0
20
40
60
80
100
120
140
Mar
May
Jul
Sep

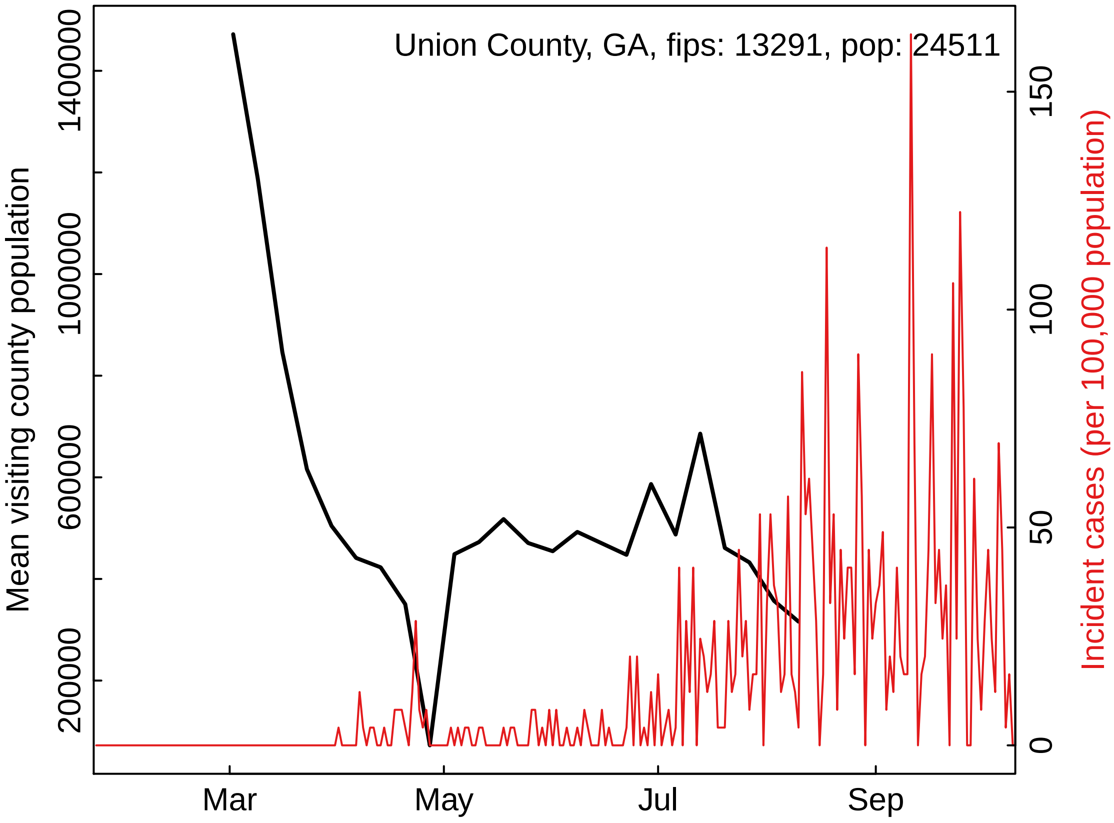

Union County, GA, fips: 13291, pop: 24511
Mean visiting county population
200000
600000
1000000
1400000
Incident cases (per 100,000 population)
0
50
100
150
Mar
May
Jul
Sep

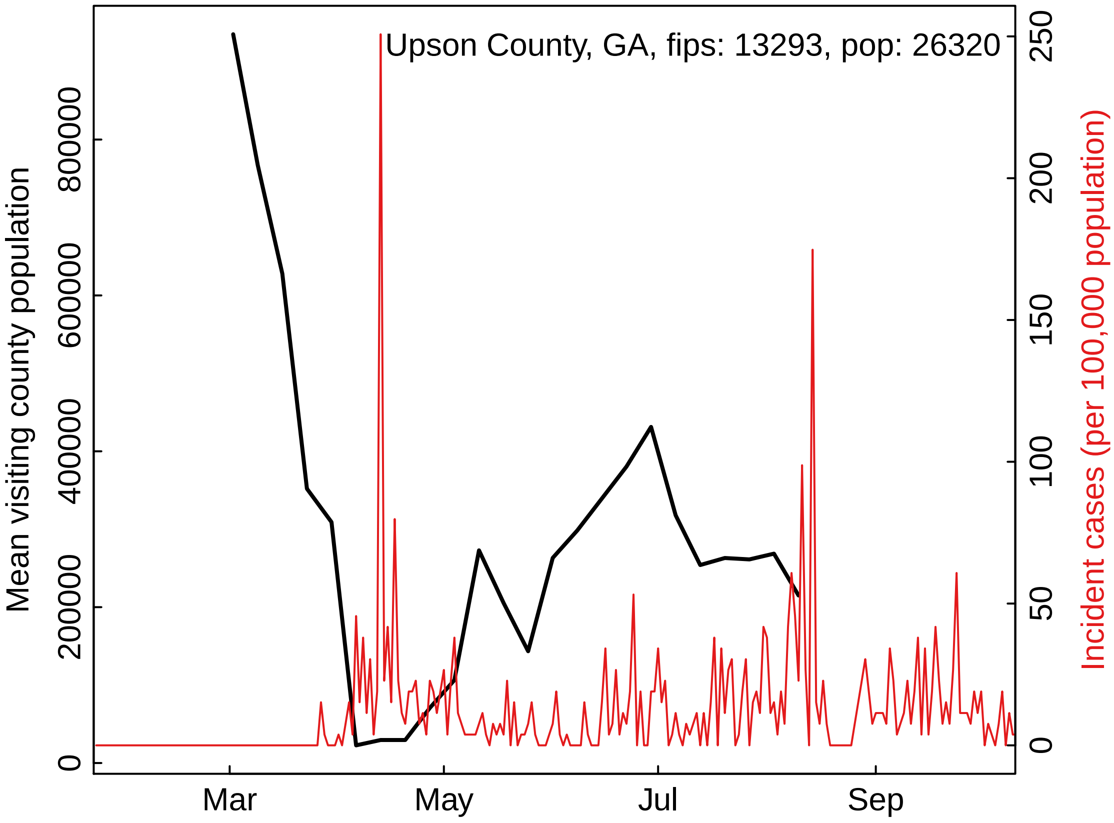

Upson County, GA, fips: 13293, pop: 26320
Mean visiting county population
0
200000
400000
600000
800000
Incident cases (per 100,000 population)
0
50
100
150
200
250
Mar
May
Jul
Sep

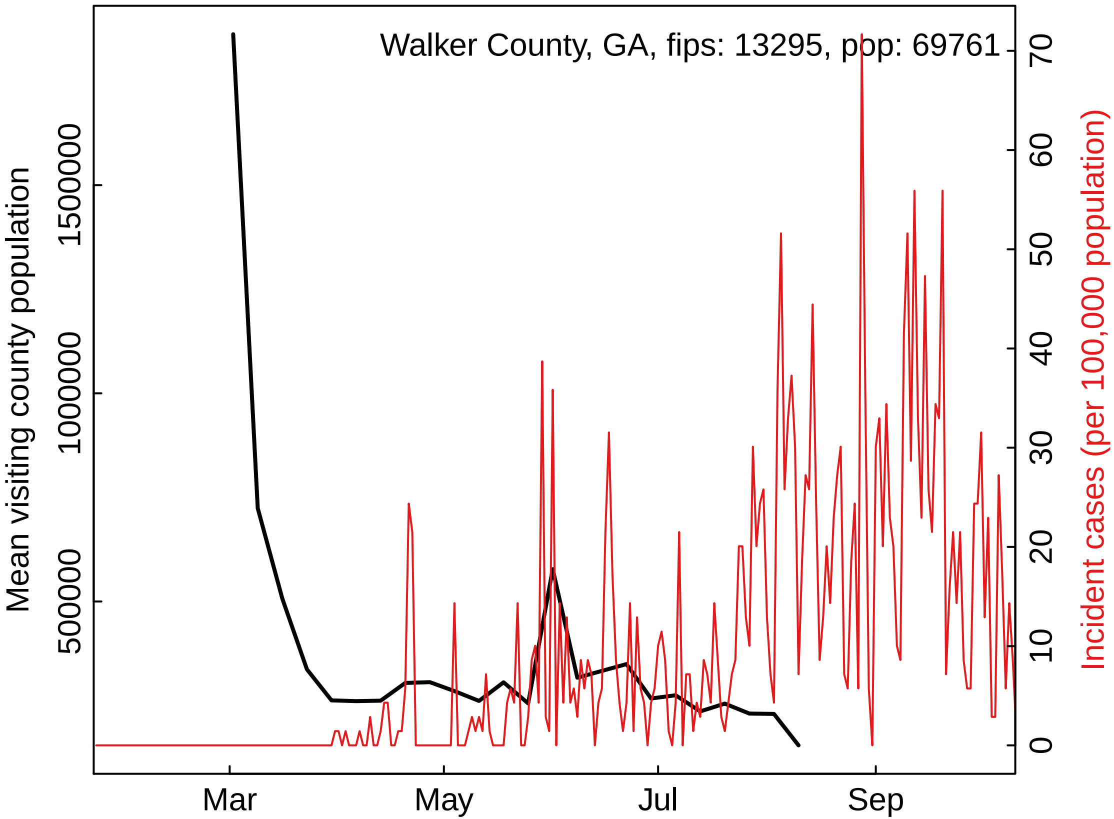

Walker County, GA, fips: 13295, pop: 69761
Mean visiting county population
500000
1000000
1500000
Incident cases (per 100,000 population)
0
10
20
30
40
50
60
70
Mar
May
Jul
Sep

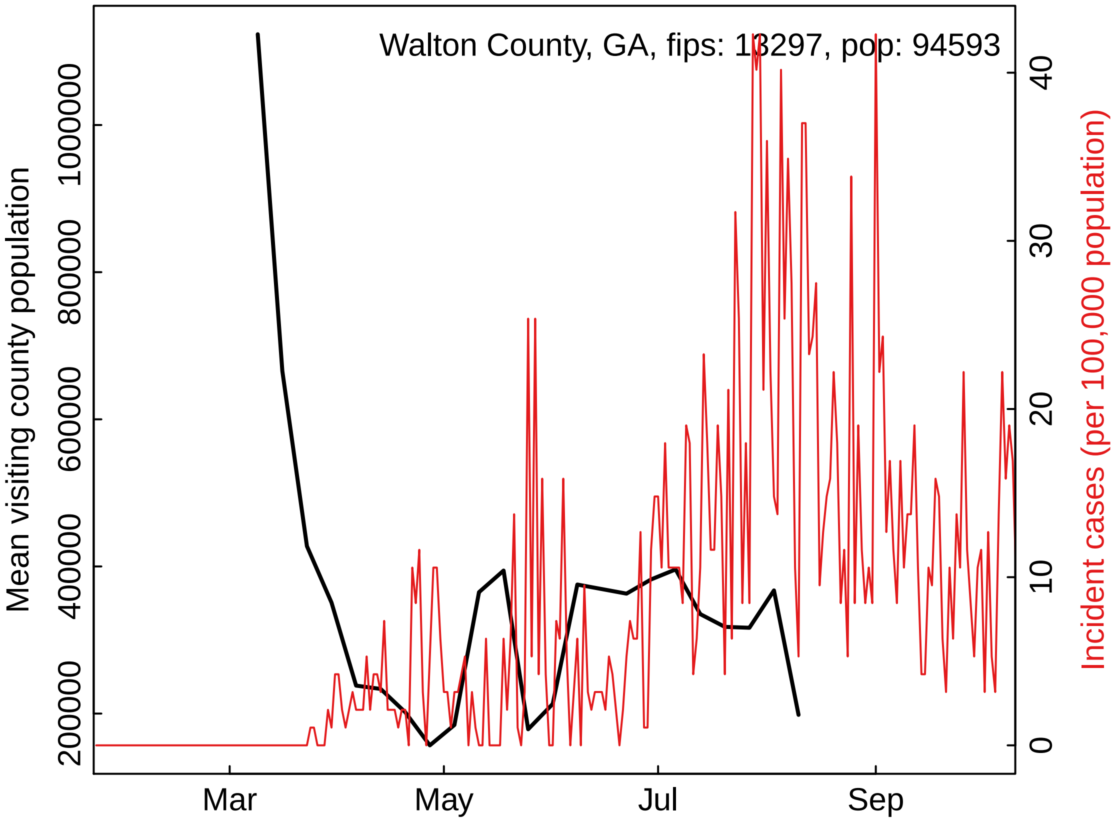

Walton County, GA, fips: 13297, pop: 94593
Mean visiting county population
200000
400000
600000
800000
1000000
Incident cases (per 100,000 population)
0
10
20
30
40
Mar
May
Jul
Sep

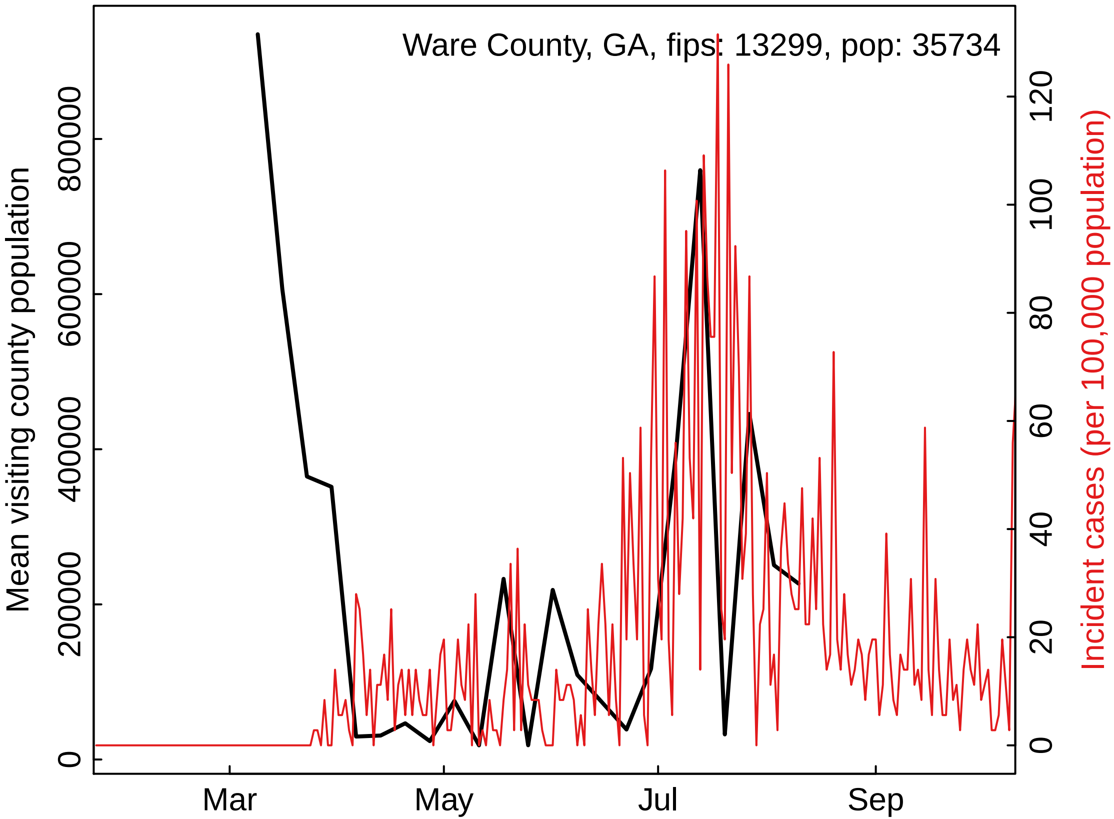

Ware County, GA, fips: 13299, pop: 35734
Mean visiting county population
0
200000
400000
600000
800000
Incident cases (per 100,000 population)
0
20
40
60
80
100
120
Mar
May
Jul
Sep

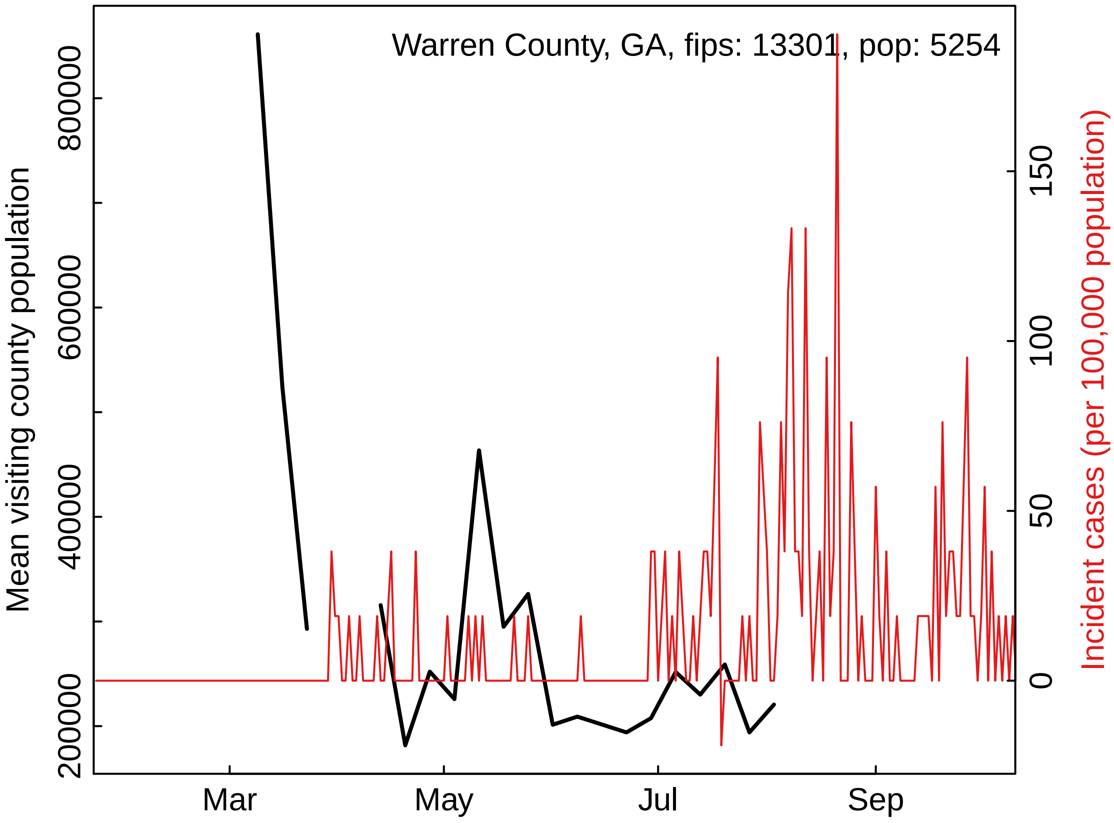

Warren County, GA, fips: 13301, pop: 5254
Mean visiting county population
200000
400000
600000
800000
Incident cases (per 100,000 population)
0
50
100
150
Mar
May
Jul
Sep

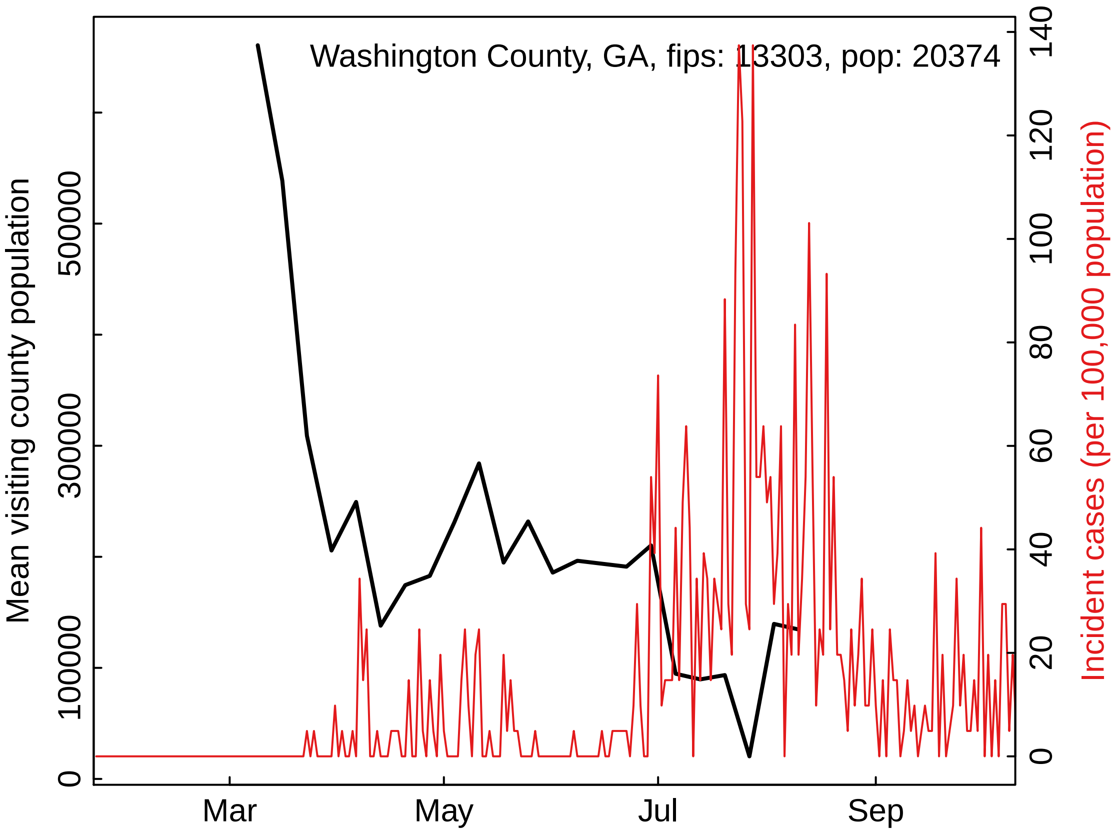

Washington County, GA, fips: 13303, pop: 20374
Mean visiting county population
Incident cases (per 100,000 population)
0
100000
300000
500000
0
20
40
60
80
100
120
140
Mar
May
Jul
Sep

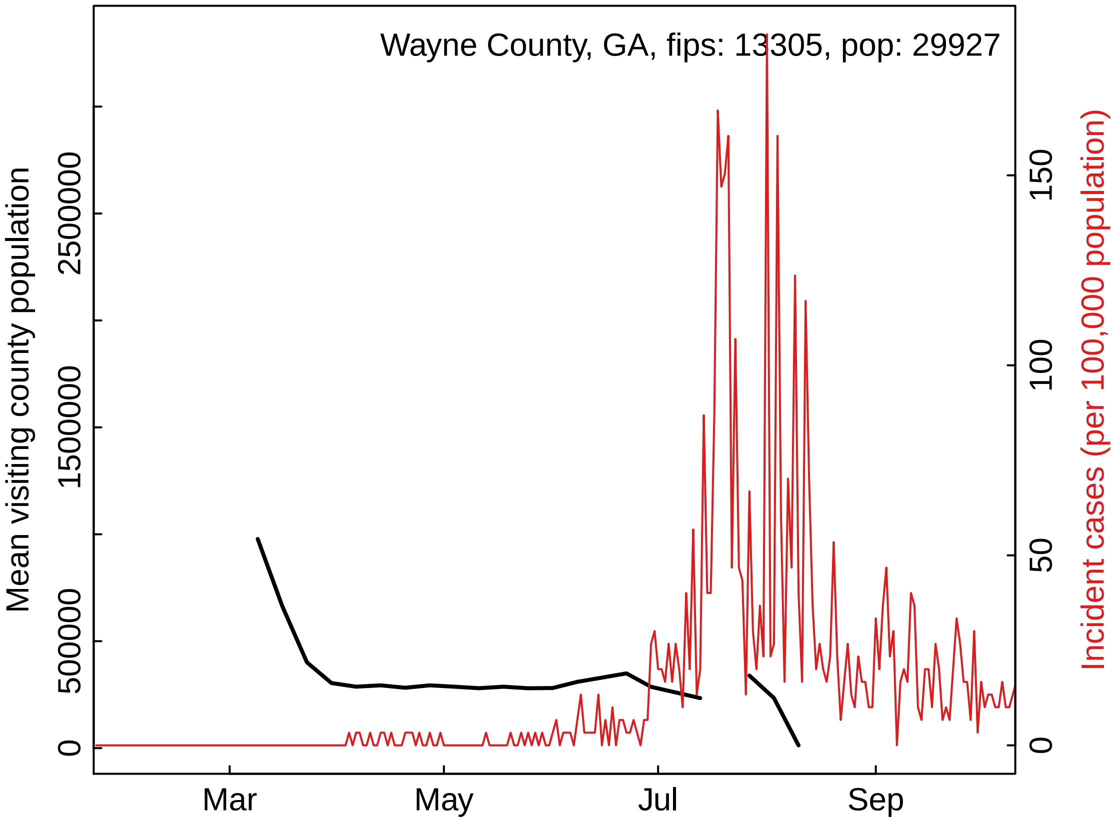
Wayne County, GA, fips: 13305, pop: 29927
Mean visiting county population
0
500000
1500000
2500000
Incident cases (per 100,000 population)
0
50
100
150
Mar
May
Jul
Sep

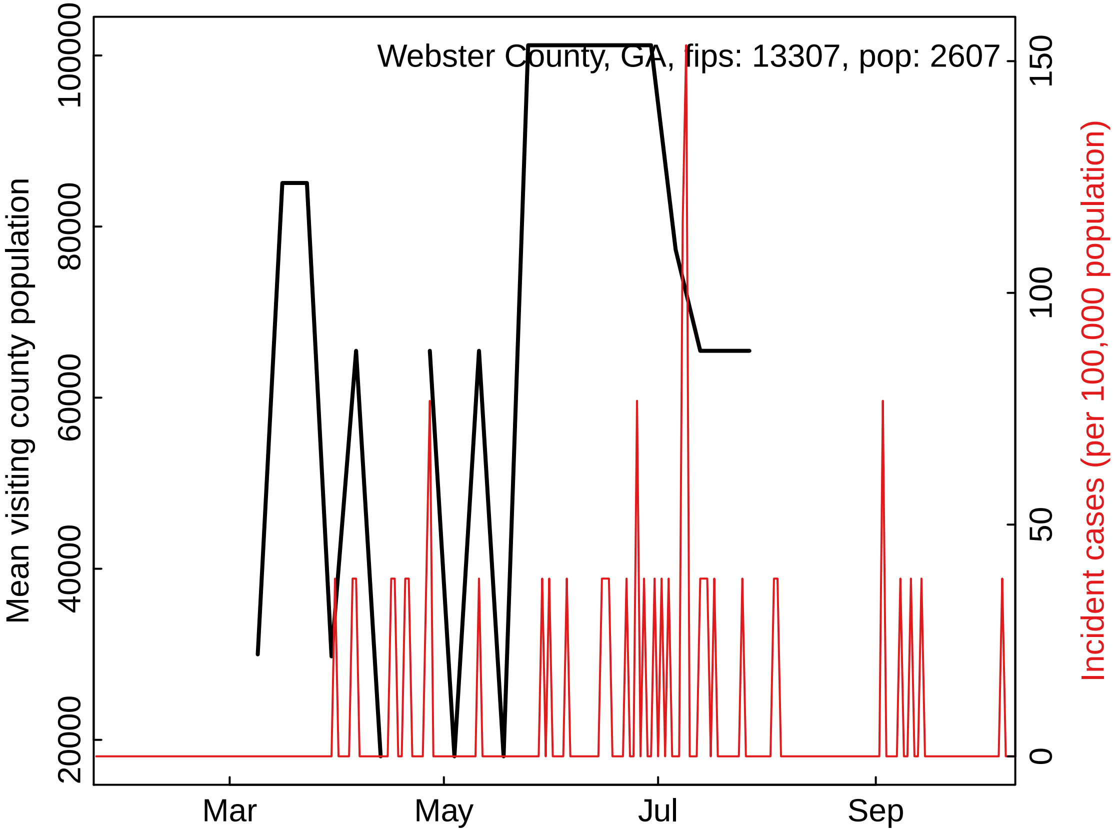
Webster County, GA, fips: 13307, pop: 2607
Mean visiting county population
Incident cases (per 100,000 population)
20000
40000
60000
80000
100000
0
50
100
150
Mar
May
Jul
Sep

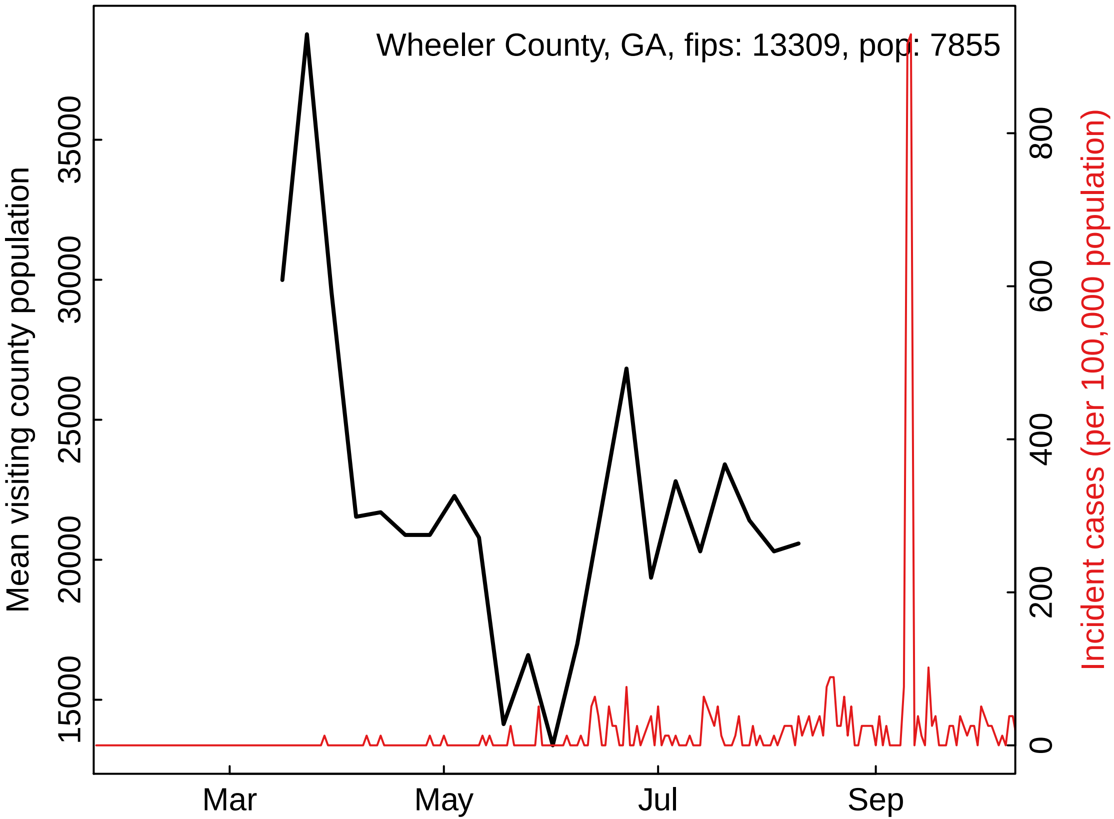
Wheeler County, GA, fips: 13309, pop: 7855
Mean visiting county population
15000
20000
25000
30000
35000
Incident cases (per 100,000 population)
0
200
400
600
800
Mar
May
Jul
Sep

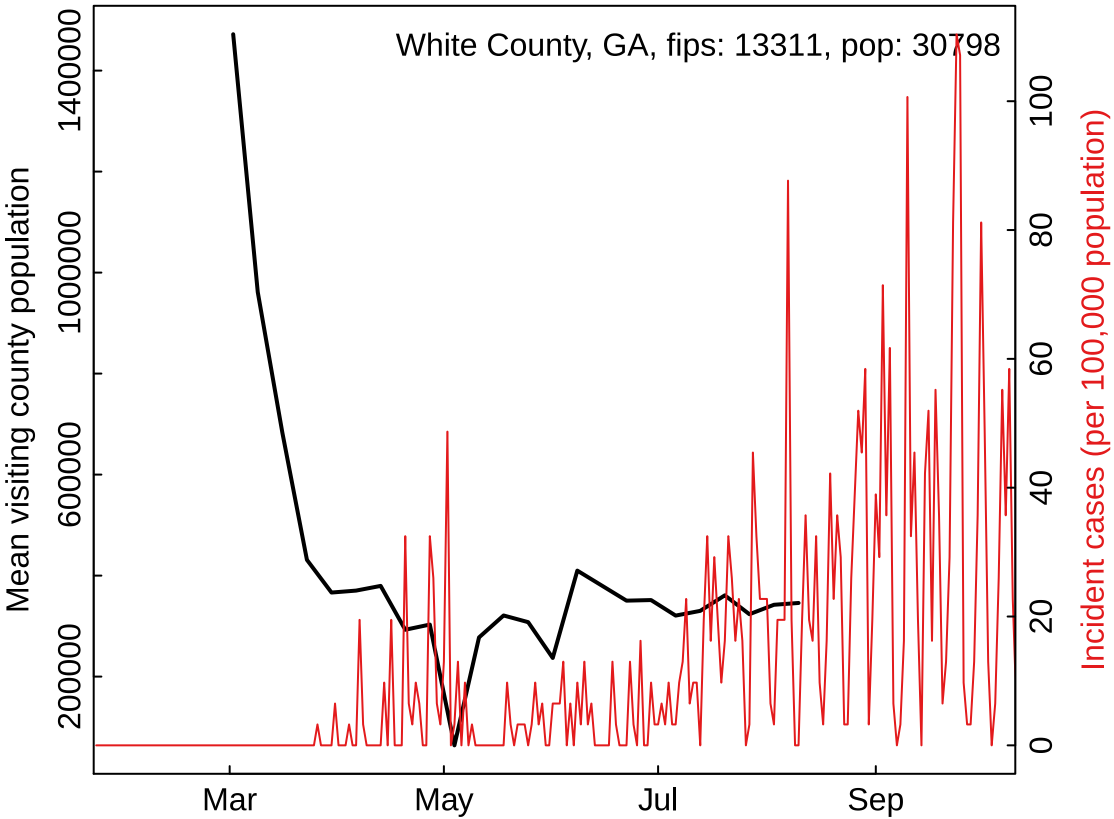
White County, GA, fips: 13311, pop: 30798
Mean visiting county population
200000
600000
1000000
1400000
Mar
May
Jul
Sep
0
20
40
60
80
100
Incident cases (per 100,000 population)

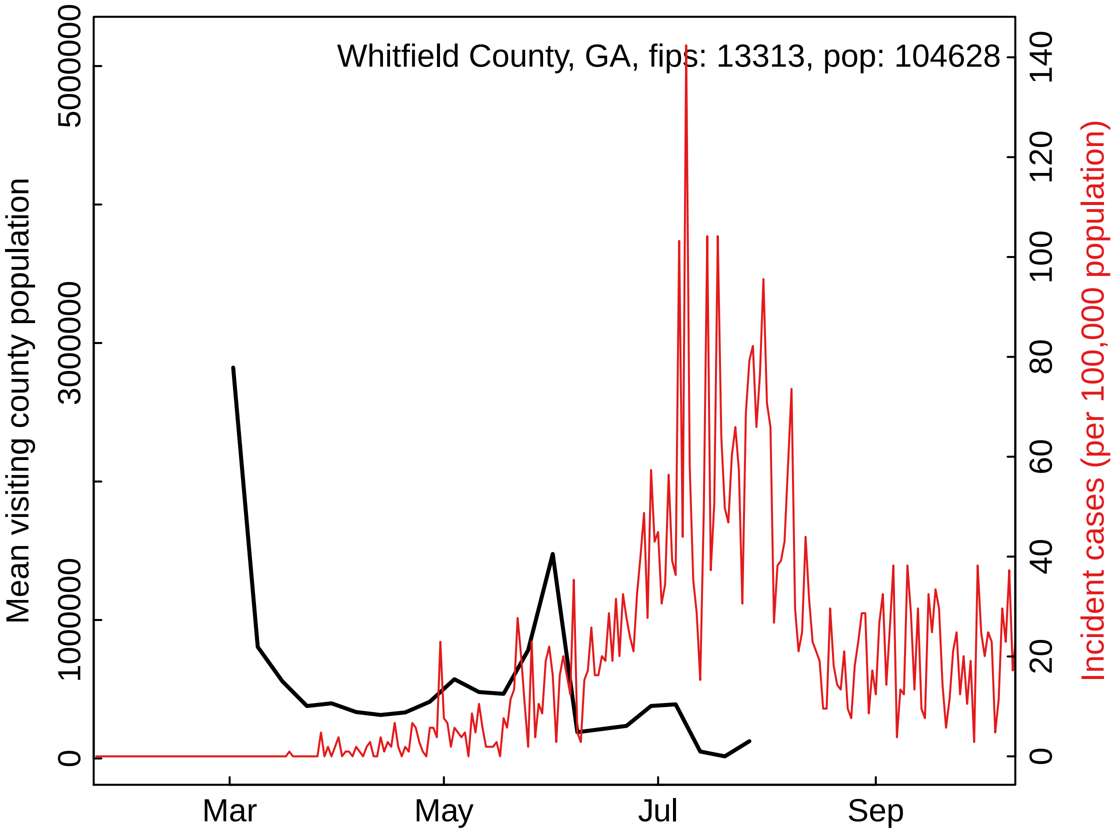

Whitfield County, GA, fips: 13313, pop: 104628
Mean visiting county population
0
1000000
3000000
5000000
Incident cases (per 100,000 population)
0
20
40
60
80
100
120
140
Mar
May
Jul
Sep

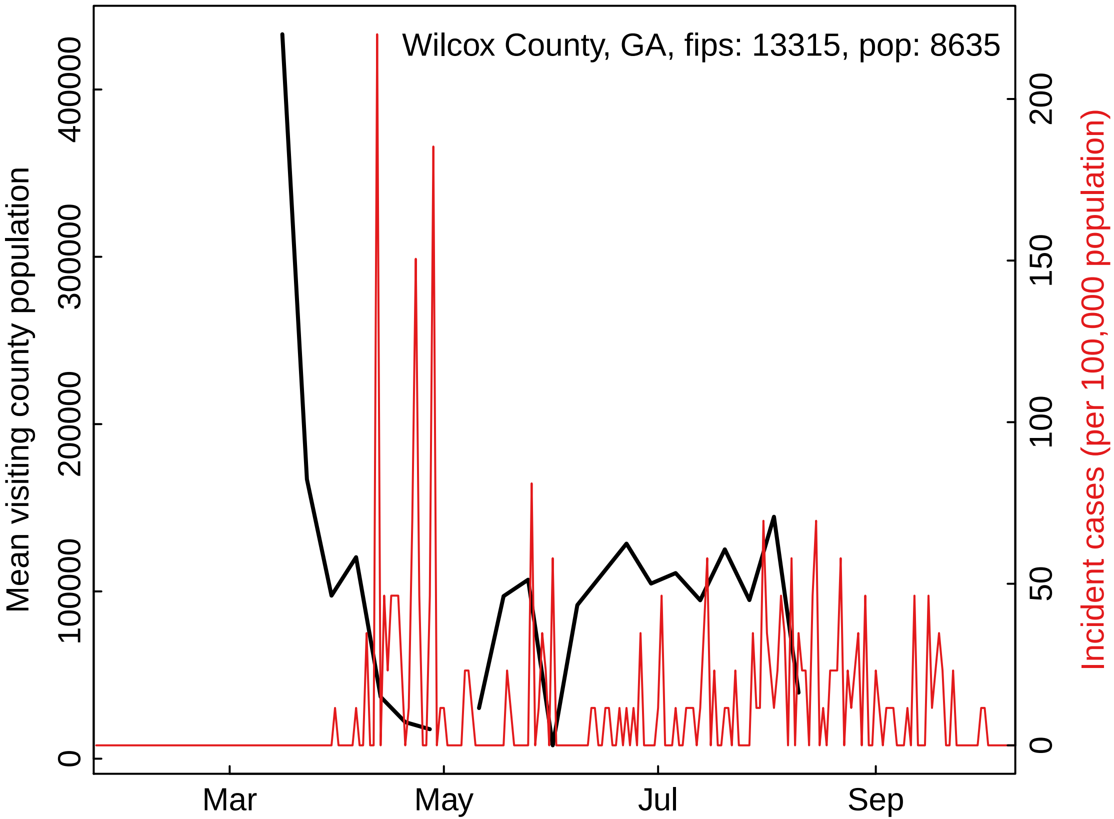
Wilcox County, GA, fips: 13315, pop: 8635
Mean visiting county population
Incident cases (per 100,000 population)
0
100000
200000
300000
400000
0
50
100
150
200
Mar
May
Jul
Sep

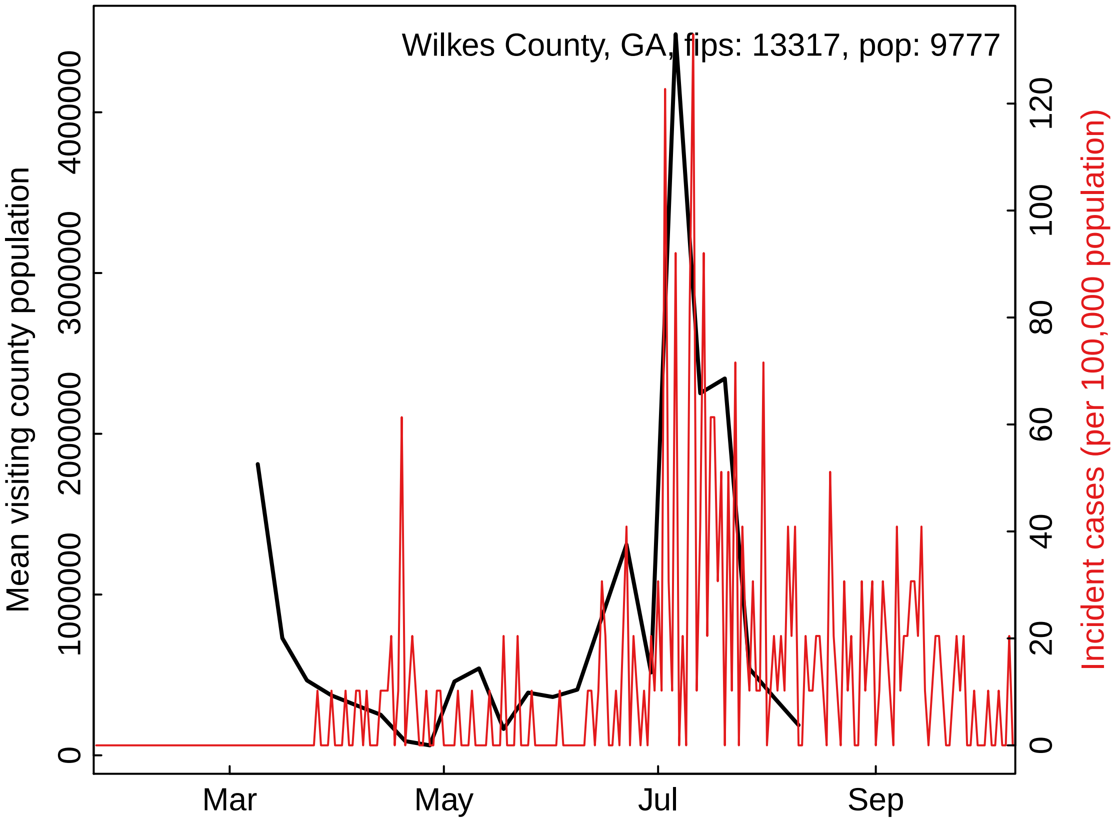

Wilkes County, GA, fips: 13317, pop: 9777
Mean visiting county population
0
1000000
2000000
3000000
4000000
Incident cases (per 100,000 population)
0
20
40
60
80
100
120
Mar
May
Jul
Sep

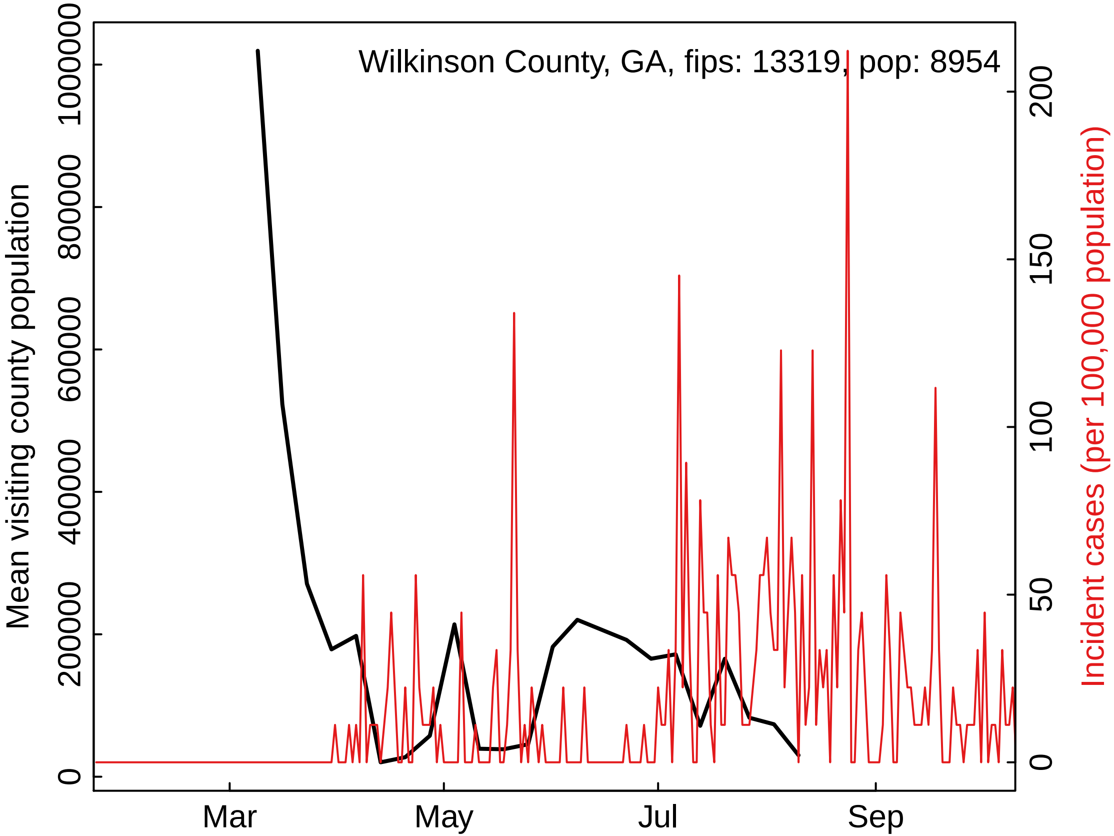

Wilkinson County, GA, fips: 13319, pop: 8954
Mean visiting county population
Incident cases (per 100,000 population)
0
200000
400000
600000
800000
1000000
0
50
100
150
200
Mar
May
Jul
Sep

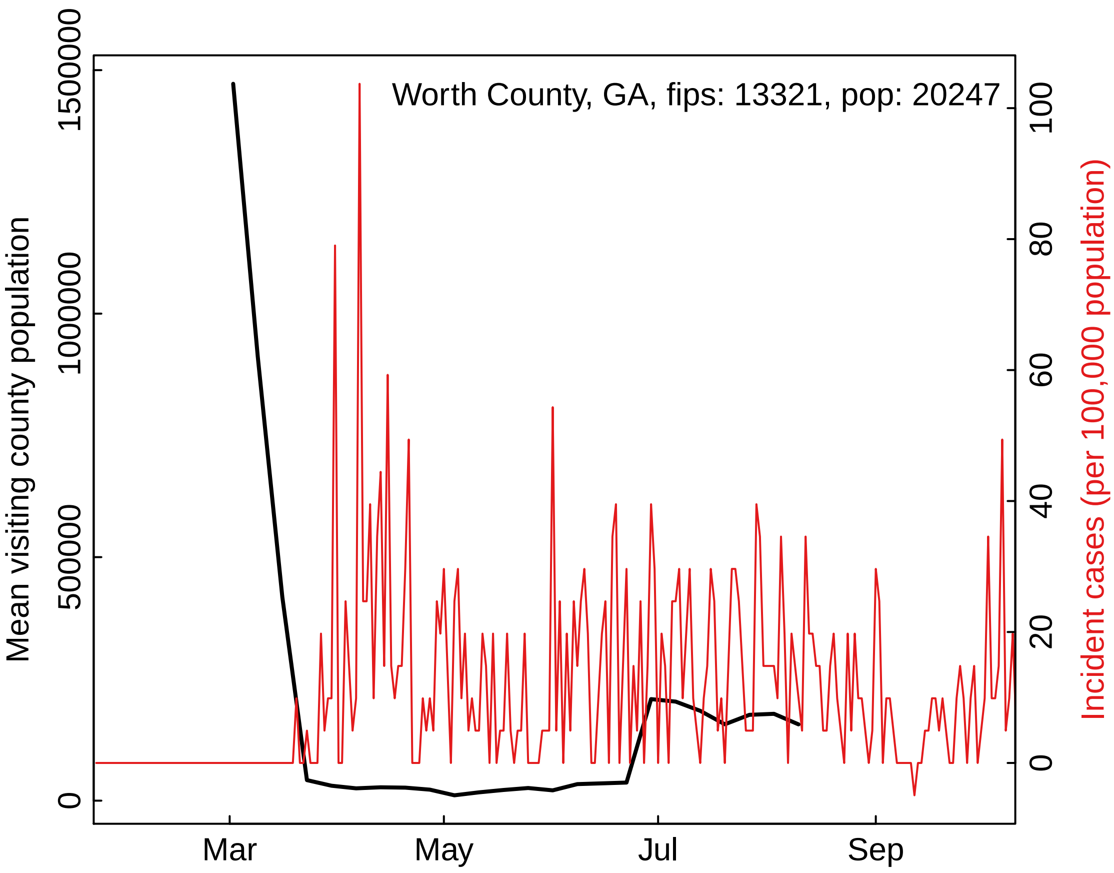

Worth County, GA, fips: 13321, pop: 20247
Mean visiting county population
0
500000
1000000
1500000
Incident cases (per 100,000 population)
0
20
40
60
80
100
Mar
May
Jul
Sep

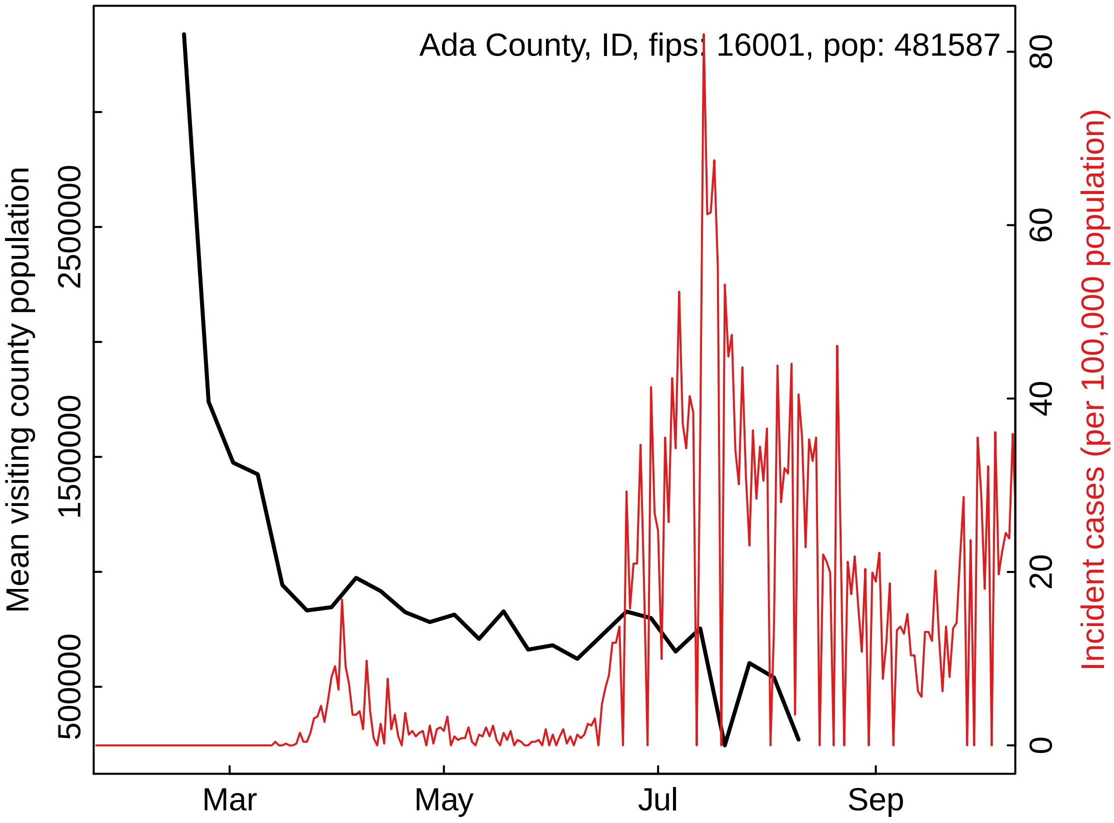

Ada County, ID, fips: 16001, pop: 481587
Mean visiting county population
500000
1500000
2500000
Mar
May
Jul
Sep
0
20
40
60
80
Incident cases (per 100,000 population)

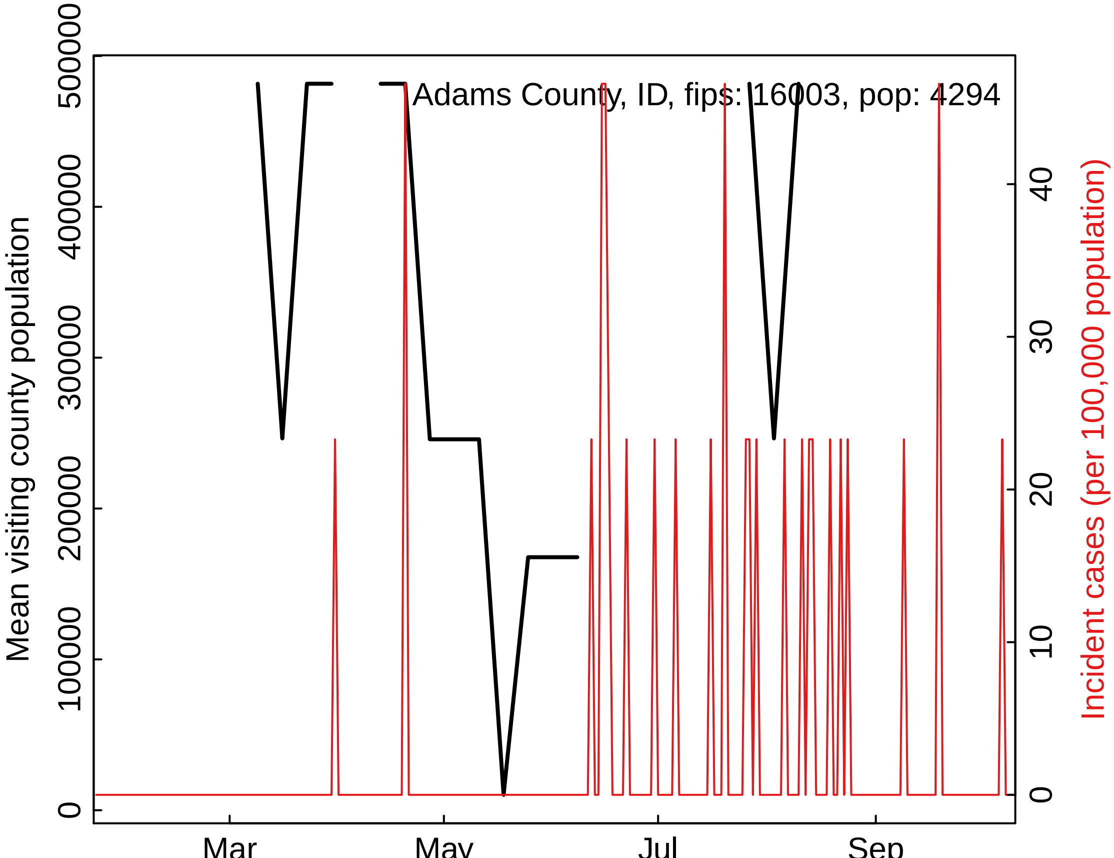

Adams County, ID, fips: 16003, pop: 4294
Mean visiting county population
Incident cases (per 100,000 population)
0
100000
200000
300000
400000
500000
0
10
20
30
40
Mar
May
Jul
Sep

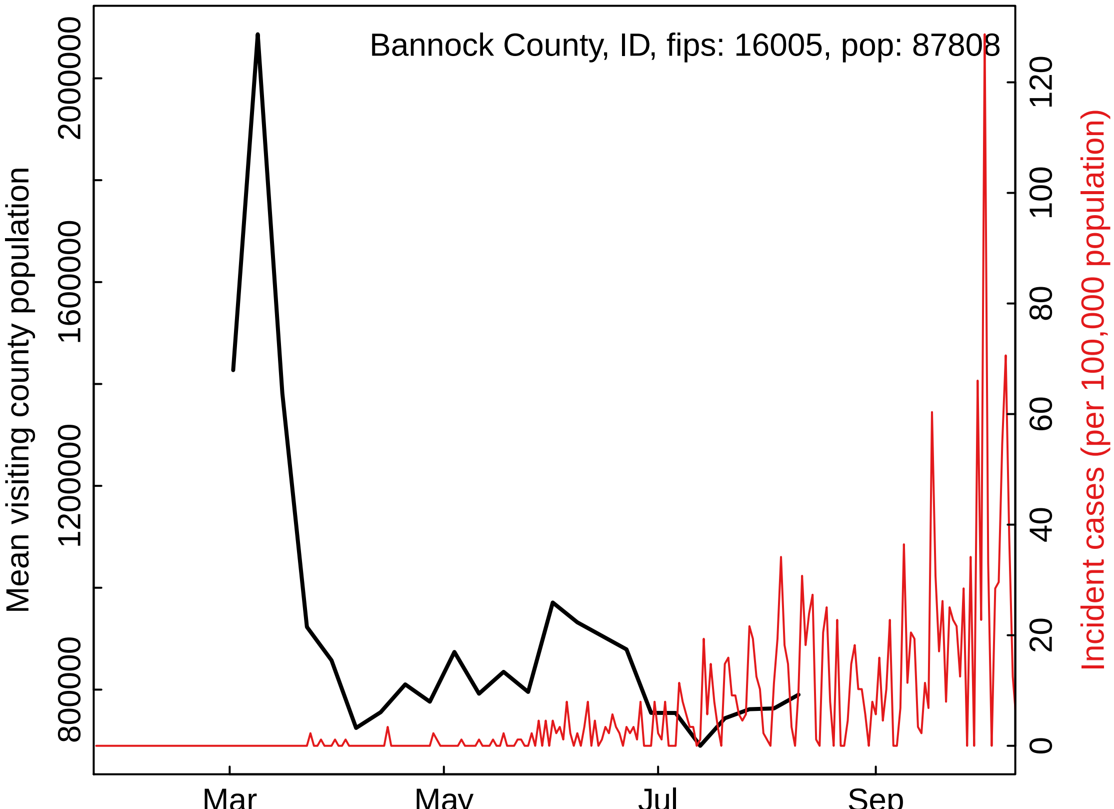

Bannock County, ID, fips: 16005, pop: 87808
Mean visiting county population
8000000
1200000
1600000
2000000
Incident cases (per 100,000 population)
0
20
40
60
80
100
120
Mar
May
Jul
Sep

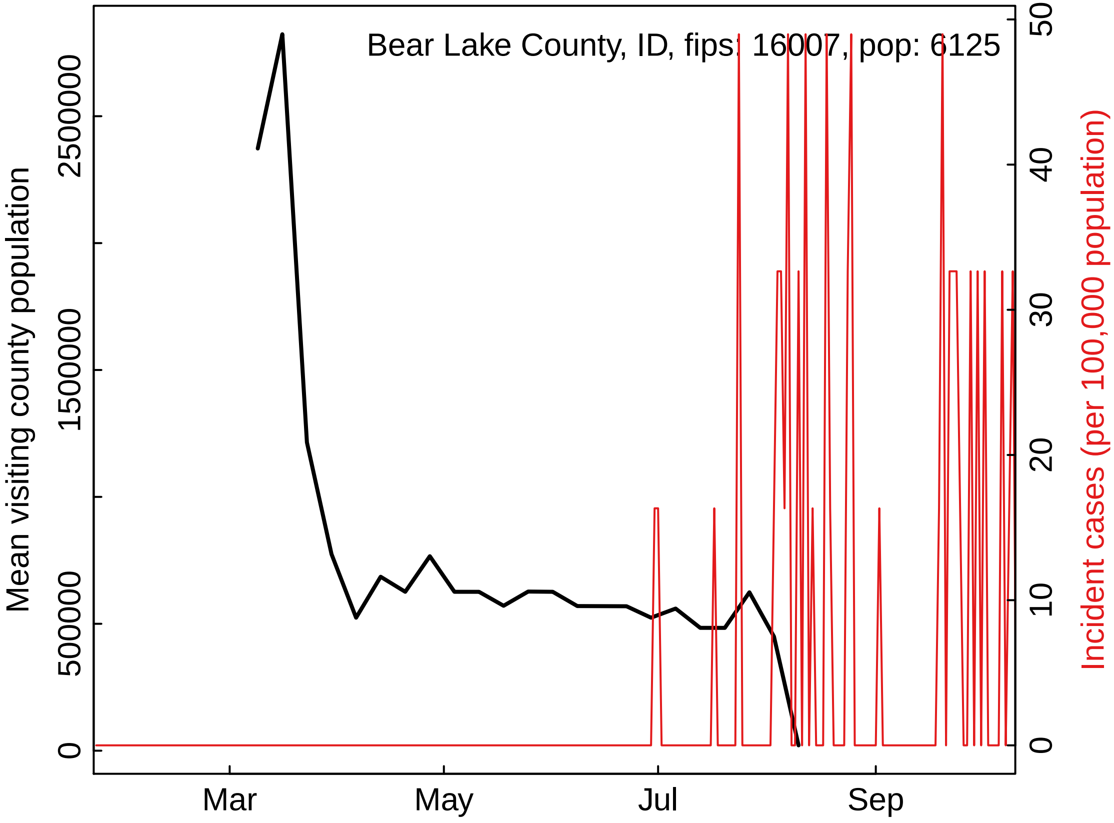

Bear Lake County, ID, fips: 16007, pop: 6125
Mean visiting county population
0
500000
1500000
2500000
Mar
May
Jul
Sep
0
10
20
30
40
50
Incident cases (per 100,000 population)

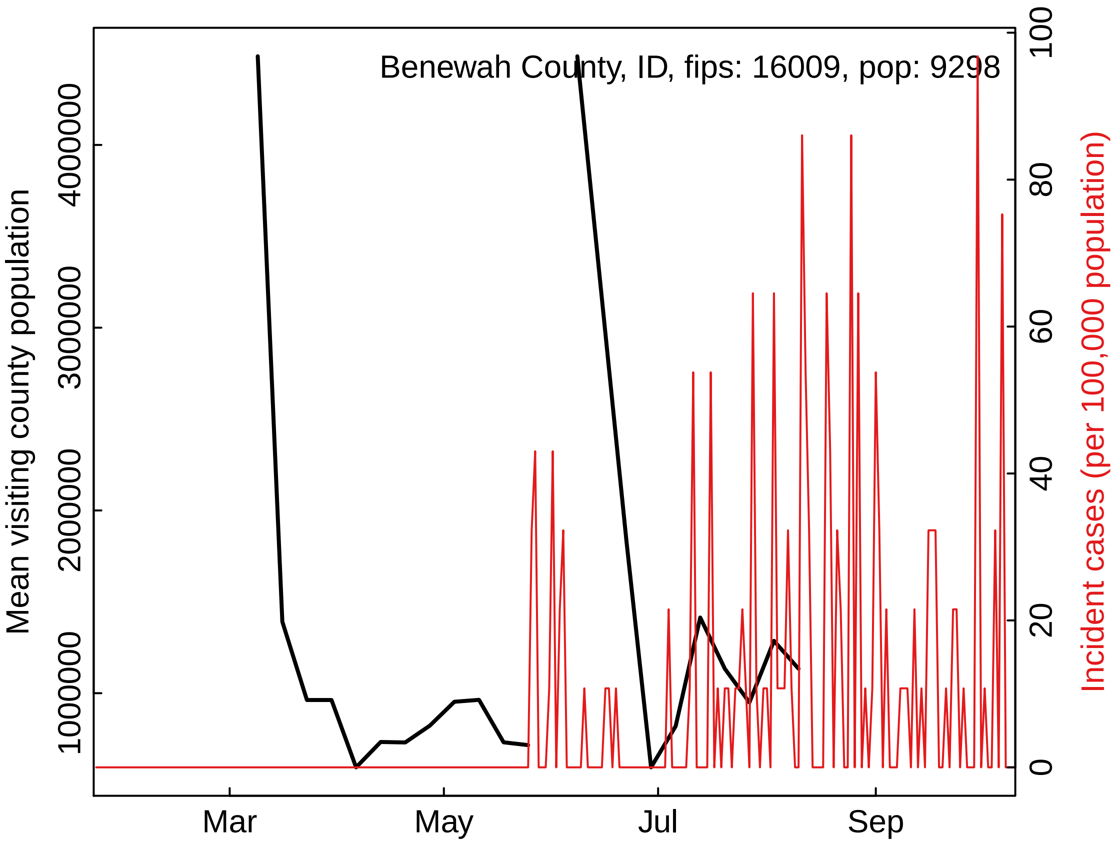

Benewah County, ID, fips: 16009, pop: 9298
Mean visiting county population
1000000
2000000
3000000
4000000
Incident cases (per 100,000 population)
0
20
40
60
80
100
Mar
May
Jul
Sep

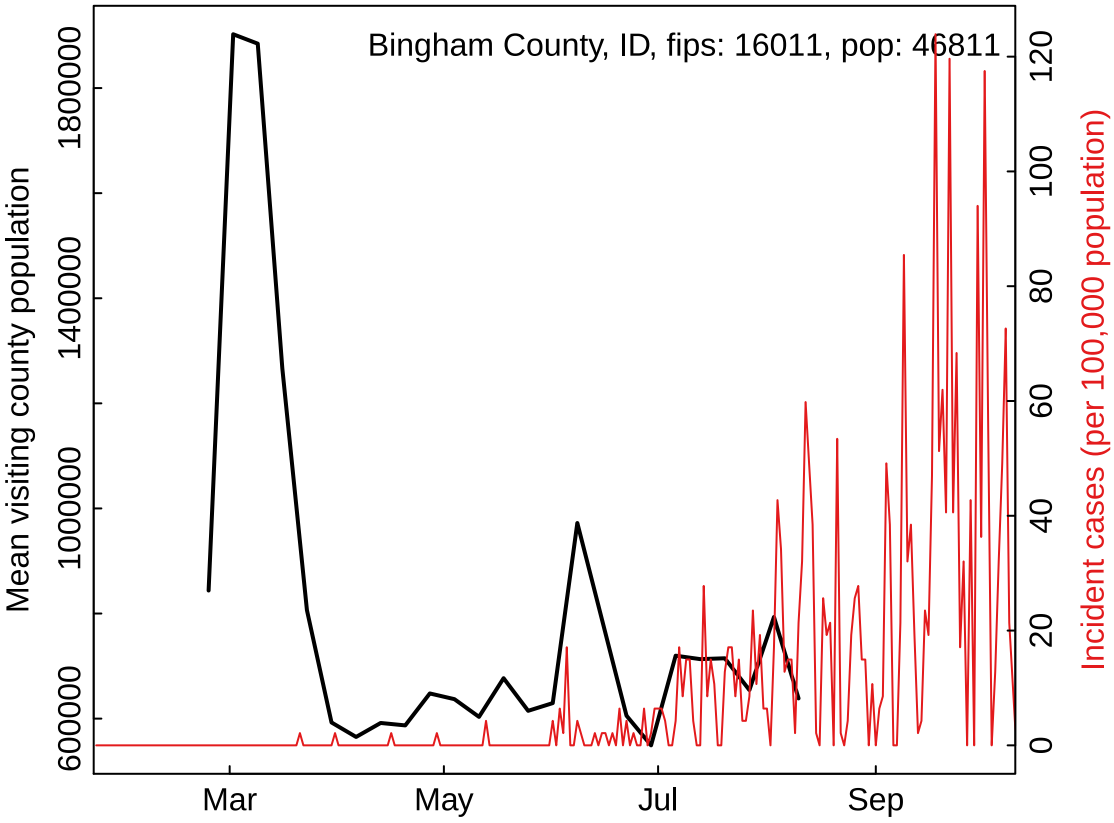

Bingham County, ID, fips: 16011, pop: 46811
Mean visiting county population
Incident cases (per 100,000 population)
600000
1000000
1400000
1800000
0
20
40
60
80
100
120
Mar
May
Jul
Sep

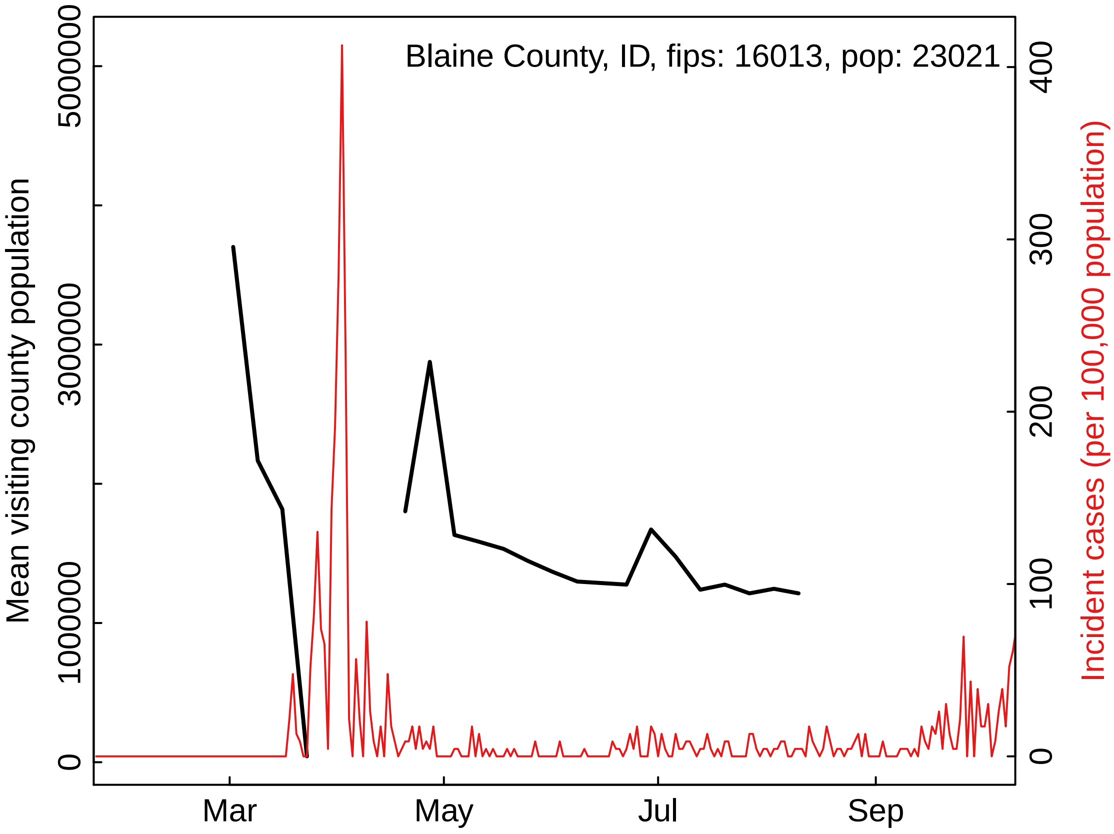

Blaine County, ID, fips: 16013, pop: 23021
Mean visiting county population
0
1000000
3000000
5000000
Incident cases (per 100,000 population)
0
100
200
300
400
Mar
May
Jul
Sep

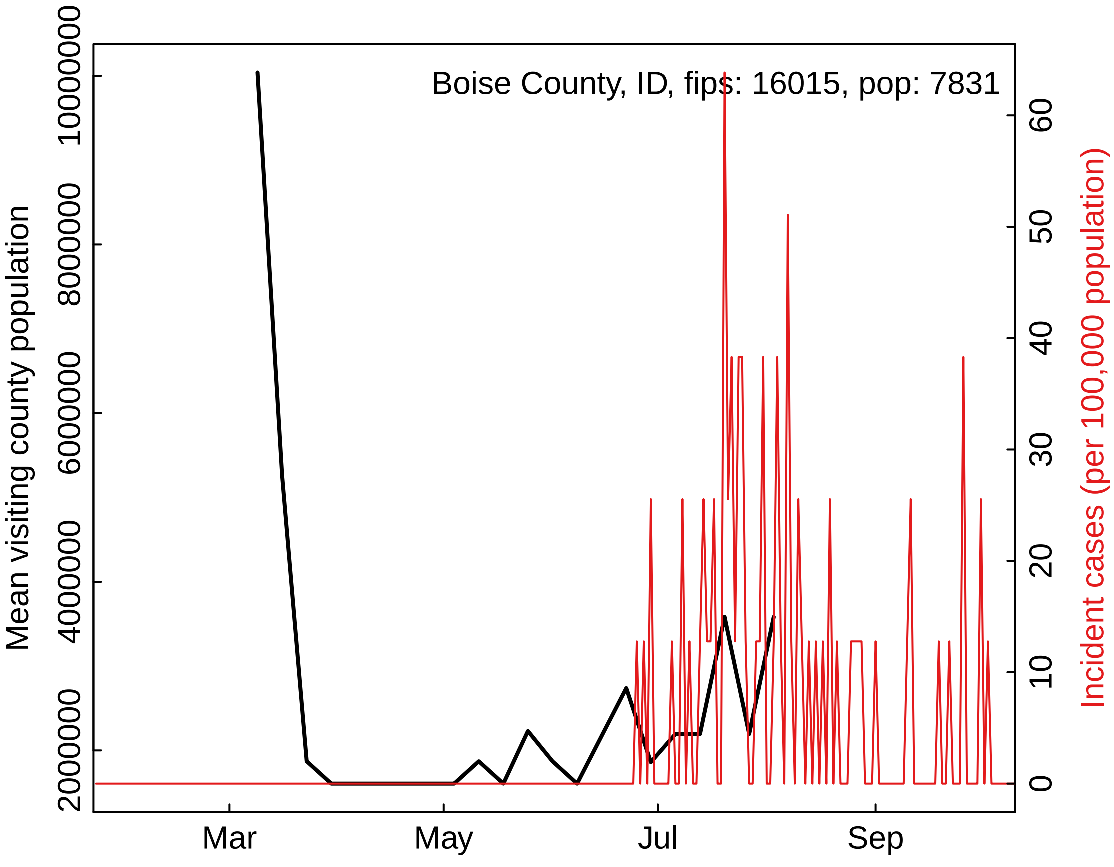

Boise County, ID, fips: 16015, pop: 7831
Mean visiting county population
20000000
40000000
60000000
80000000
100000000
Incident cases (per 100,000 population)
0
10
20
30
40
50
60
Mar
May
Jul
Sep

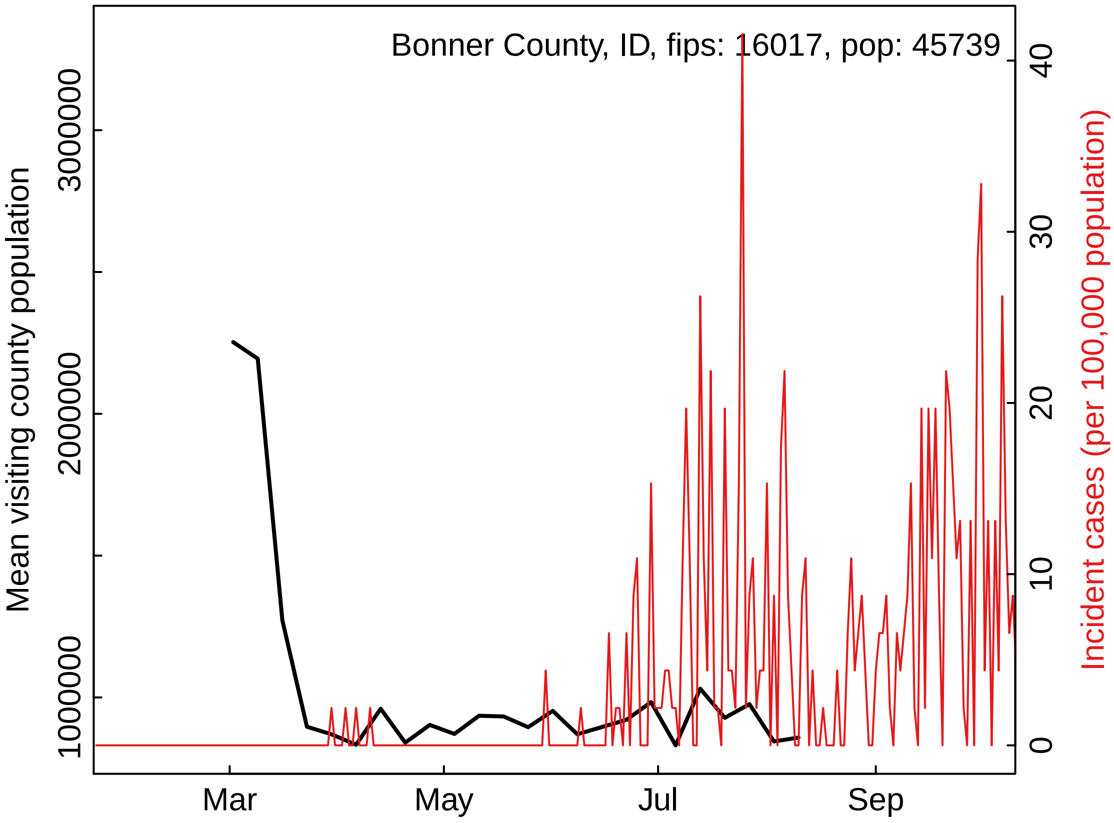

Bonner County, ID, fips: 16017, pop: 45739
Mean visiting county population
10000000
20000000
30000000
Incident cases (per 100,000 population)
0
10
20
30
40
Mar
May
Jul
Sep

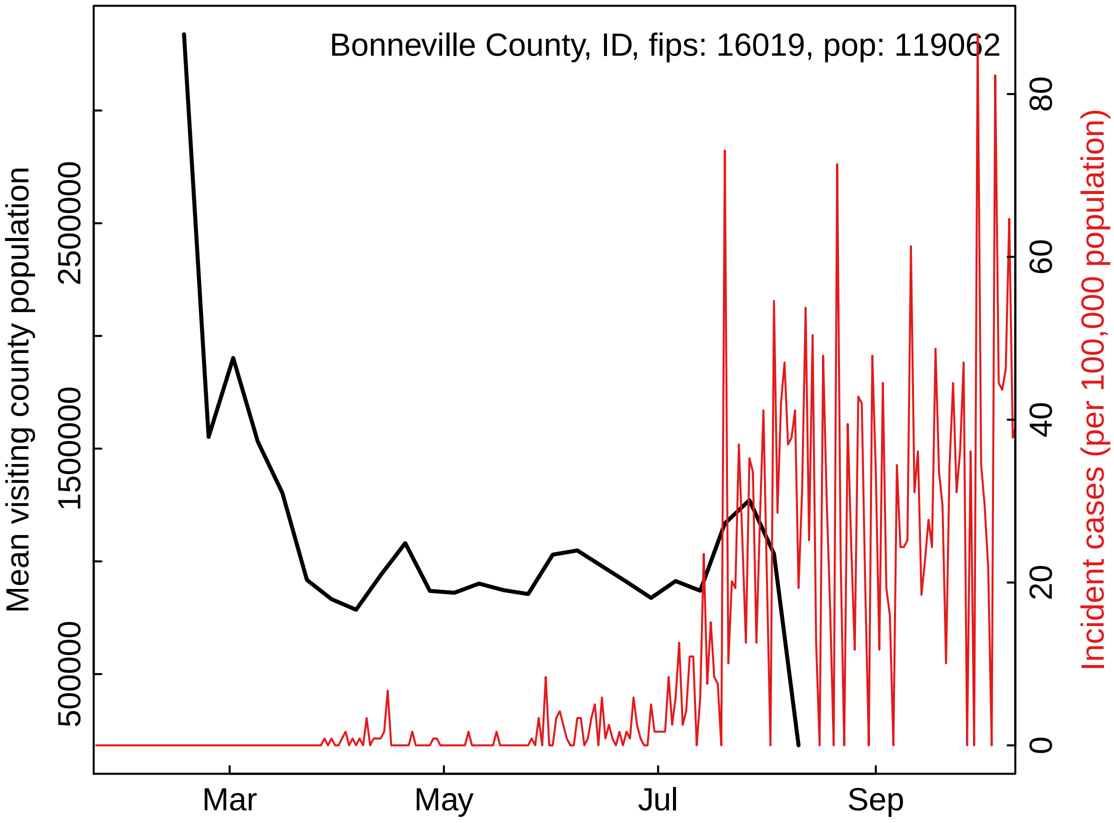

Bonneville County, ID, fips: 16019, pop: 119062
Mean visiting county population
500000
1500000
2500000
Mar
May
Jul
Sep
Incident cases (per 100,000 population)
0
20
40
60
80

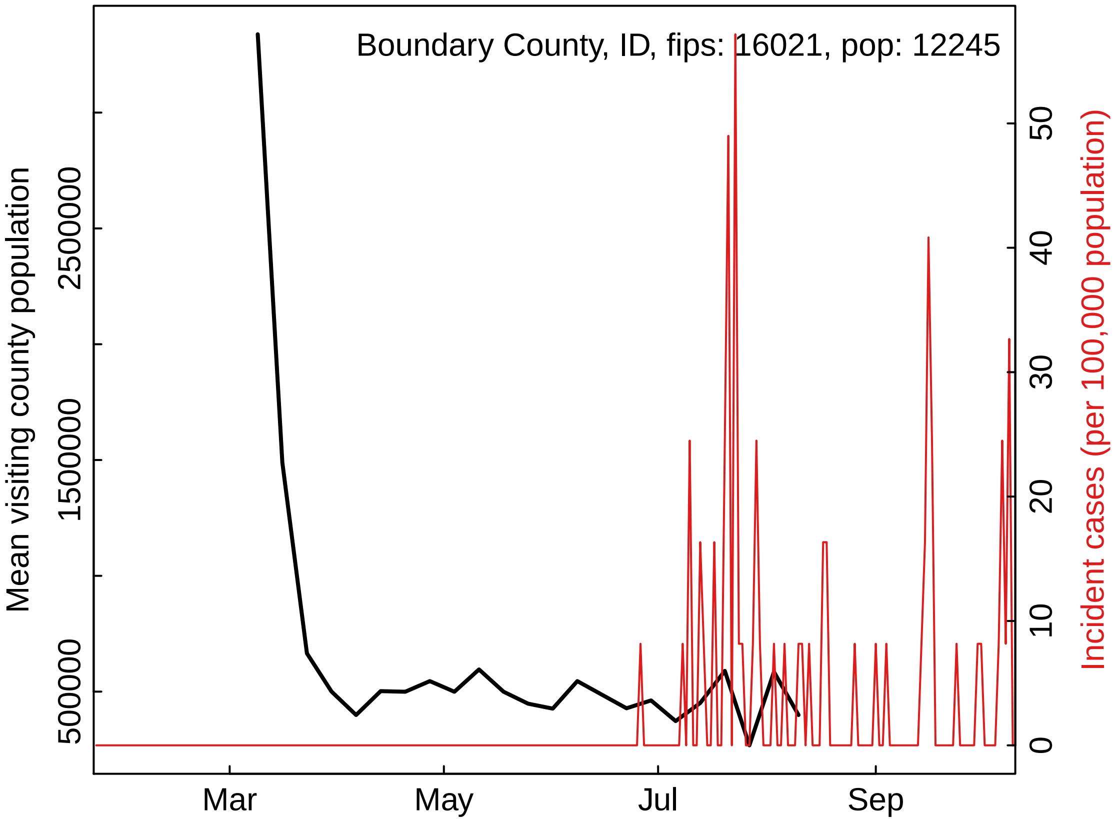
Boundary County, ID, fips: 16021, pop: 12245
Mean visiting county population
500000
1500000
2500000
Mar
May
Jul
Sep
0
10
20
30
40
50
Incident cases (per 100,000 population)

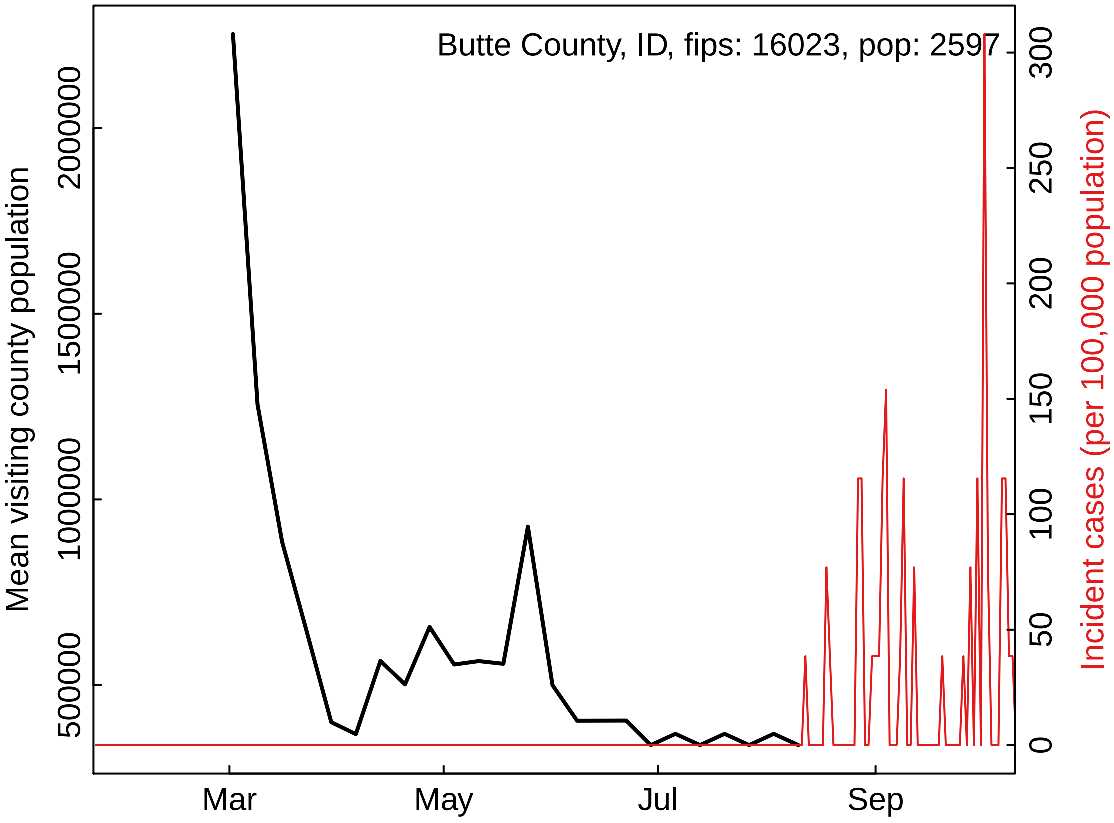

Butte County, ID, fips: 16023, pop: 2597
Mean visiting county population
500000
1000000
1500000
2000000
Incident cases (per 100,000 population)
0
50
100
150
200
250
300
Mar
May
Jul
Sep

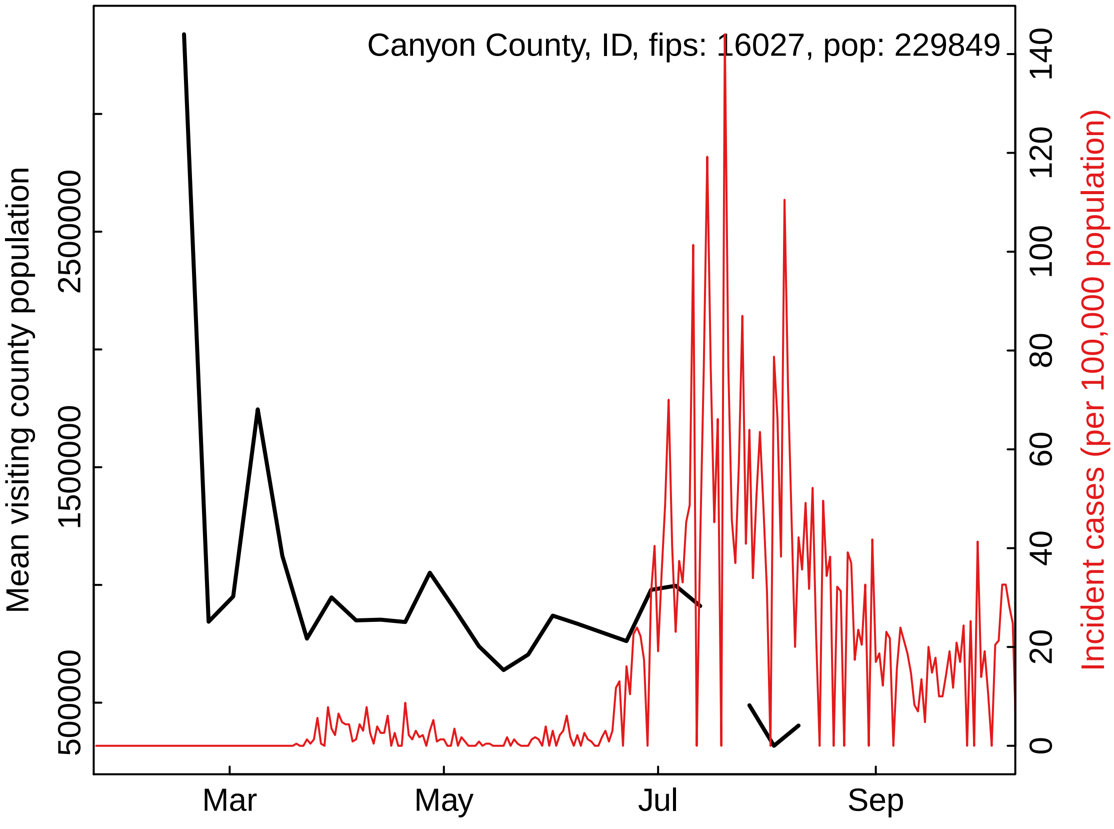
Canyon County, ID, fips: 16027, pop: 229849
Mean visiting county population
500000
1500000
2500000
Incident cases (per 100,000 population)
0
20
40
60
80
100
120
140
Mar
May
Jul
Sep

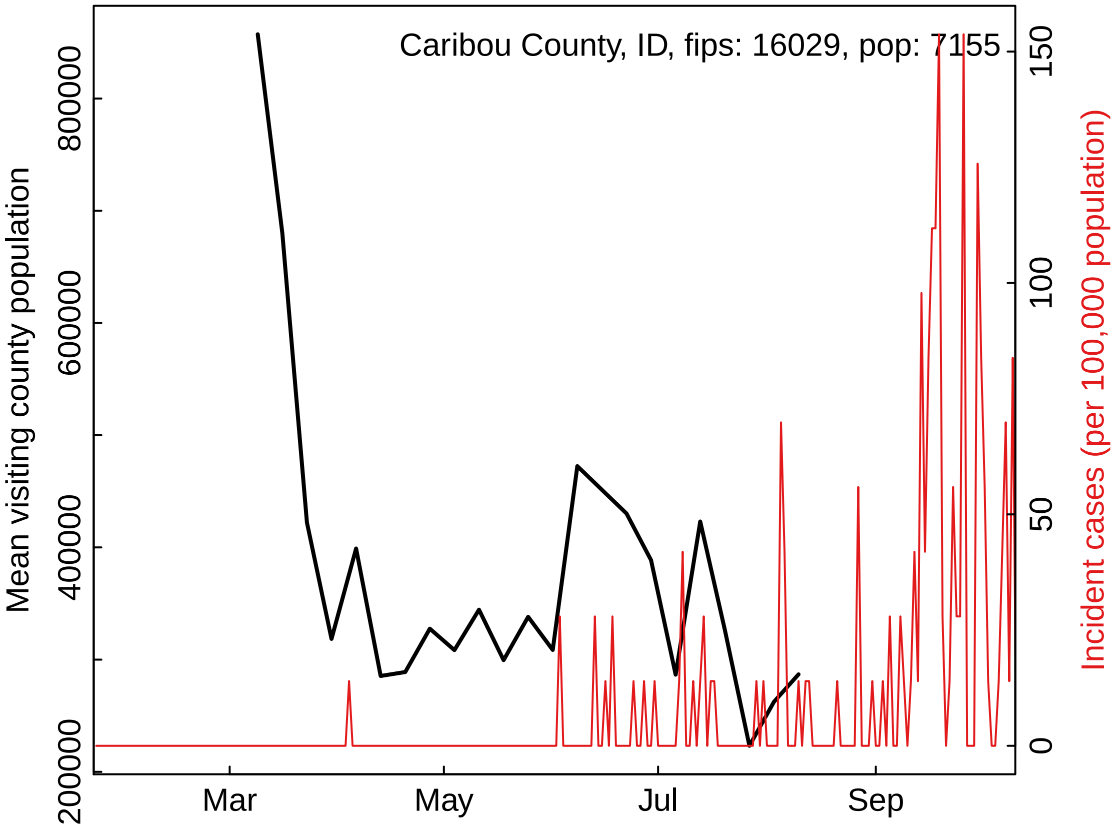

Caribou County, ID, fips: 16029, pop: 7155
Mean visiting county population
Incident cases (per 100,000 population)
200000
400000
600000
800000
0
50
100
150
Mar
May
Jul
Sep

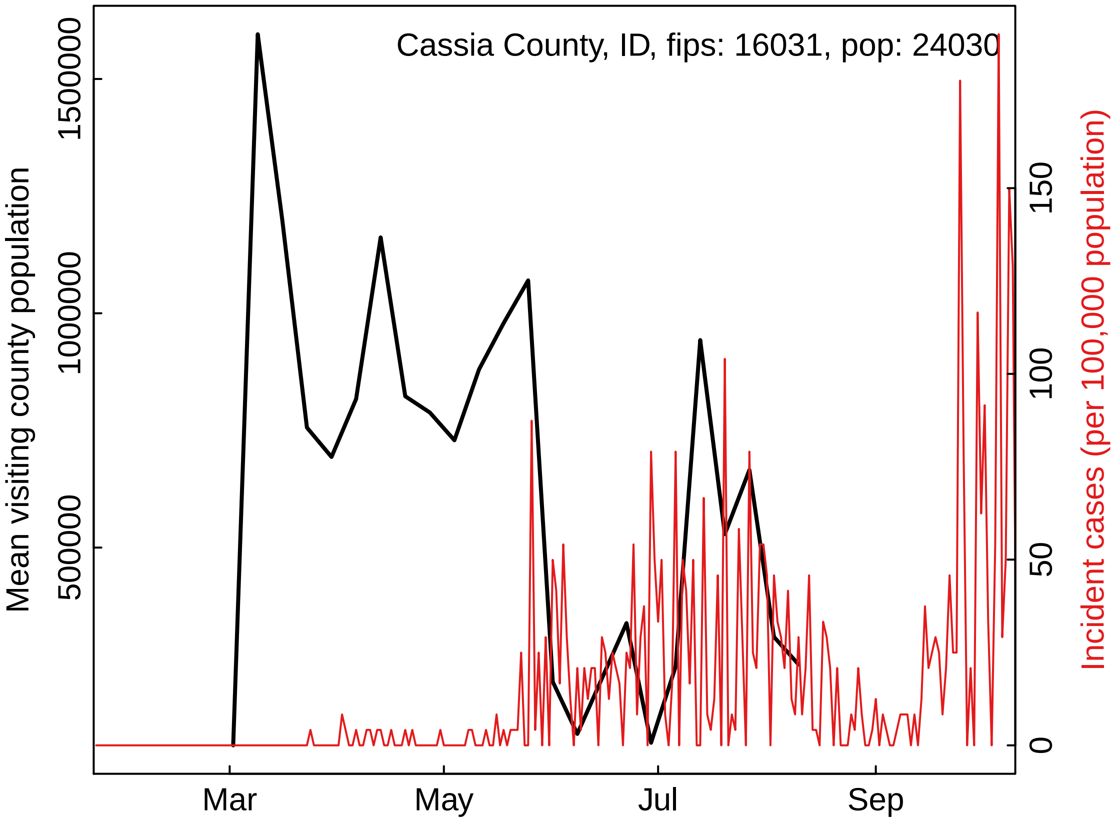
Cassia County, ID, fips: 16031, pop: 24030
Mean visiting county population
500000
1000000
1500000
Incident cases (per 100,000 population)
0
50
100
150
Mar
May
Jul
Sep

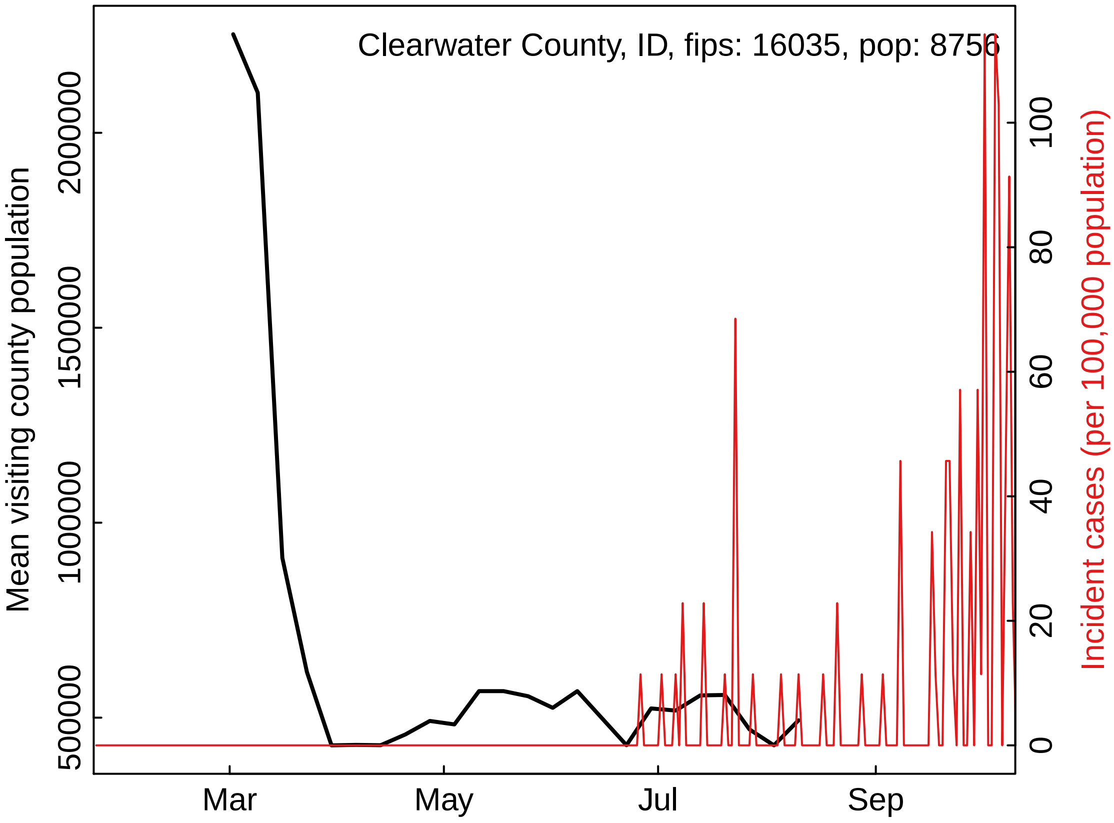

Clearwater County, ID, fips: 16035, pop: 8756
Mean visiting county population
500000
1000000
1500000
2000000
Mar
May
Jul
Sep
0
20
40
60
80
100
Incident cases (per 100,000 population)

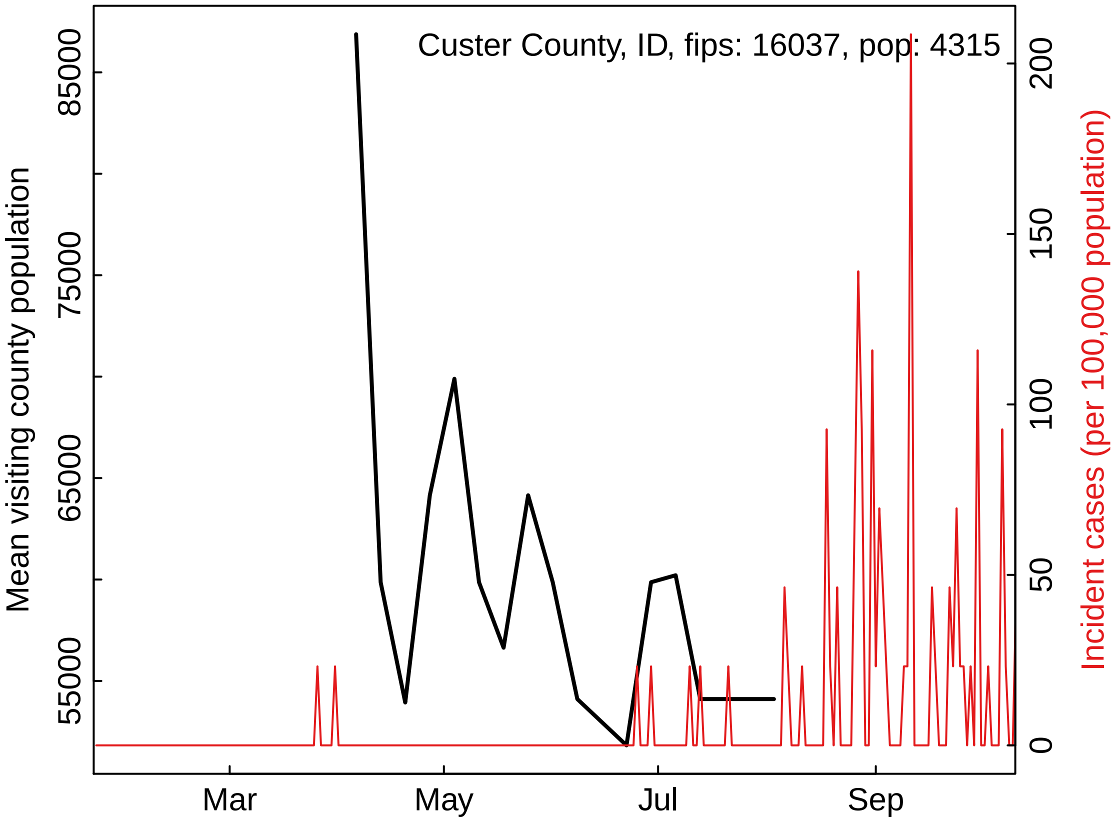

Custer County, ID, fips: 16037, pop: 4315
Mean visiting county population
55000
65000
75000
85000
Incident cases (per 100,000 population)
0
50
100
150
200
Mar
May
Jul
Sep

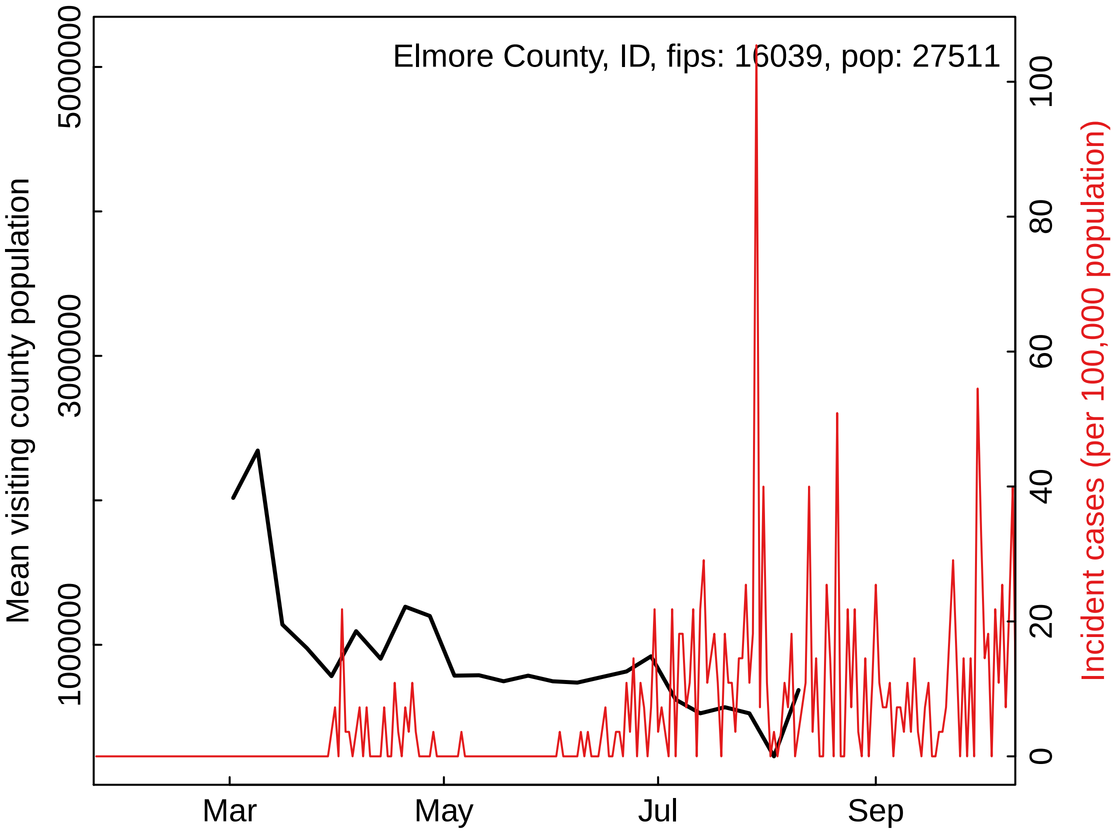
Elmore County, ID, fips: 16039, pop: 27511
Mean visiting county population
1000000
3000000
5000000
Incident cases (per 100,000 population)
0
20
40
60
80
100
Mar
May
Jul
Sep

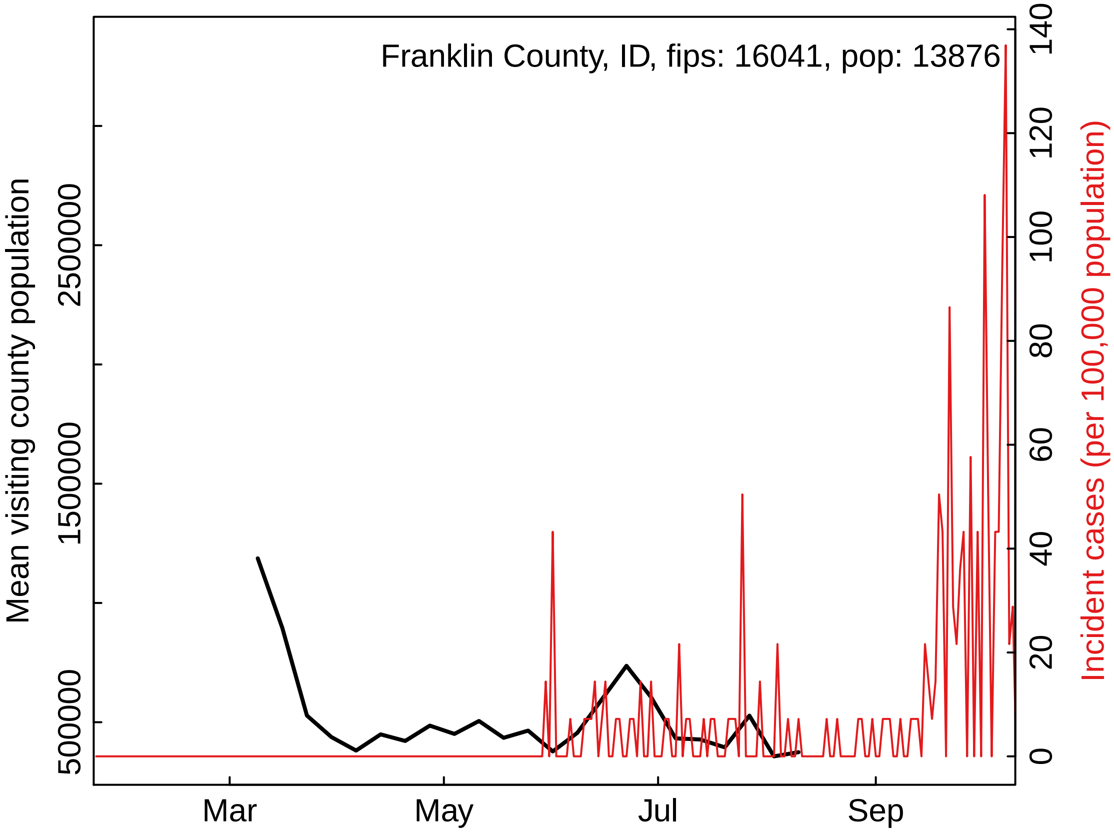

Franklin County, ID, fips: 16041, pop: 13876
Mean visiting county population
500000
1500000
2500000
Mar
May
Jul
Sep
0
20
40
60
80
100
120
140
Incident cases (per 100,000 population)

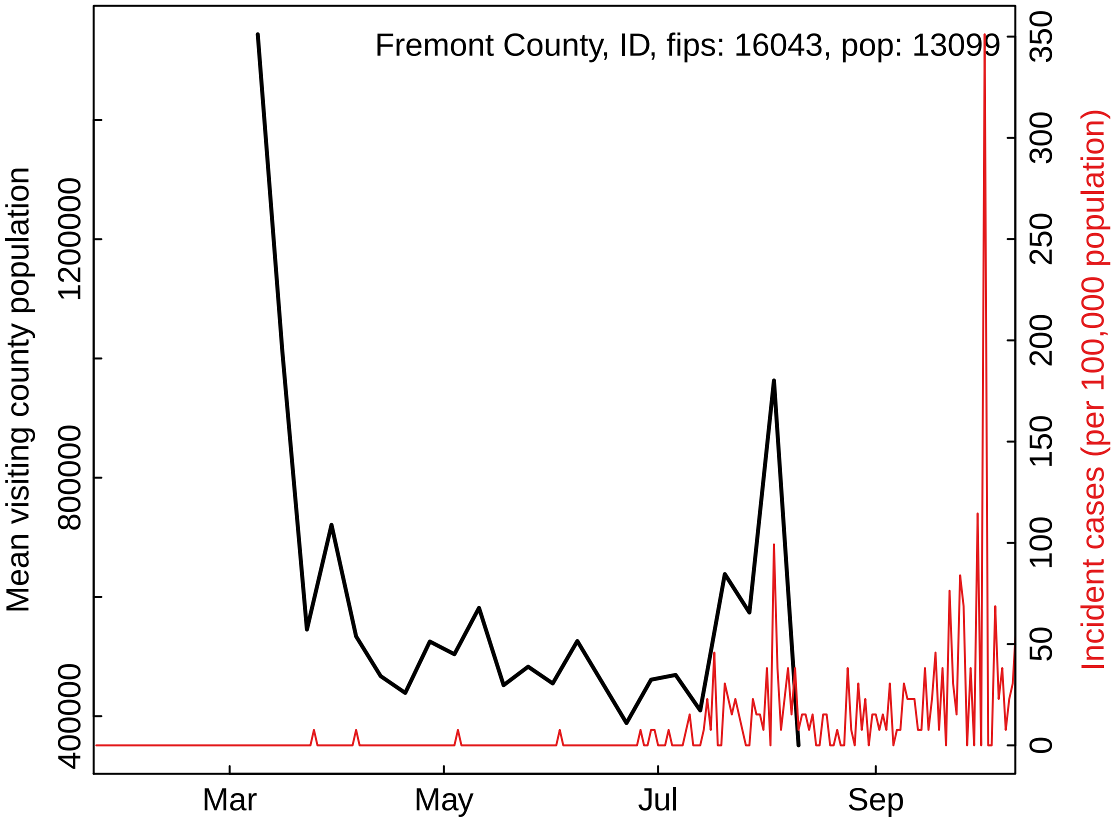

Fremont County, ID, fips: 16043, pop: 13099
Mean visiting county population
400000
800000
1200000
Incident cases (per 100,000 population)
0
50
100
150
200
250
300
350
Mar
May
Jul
Sep

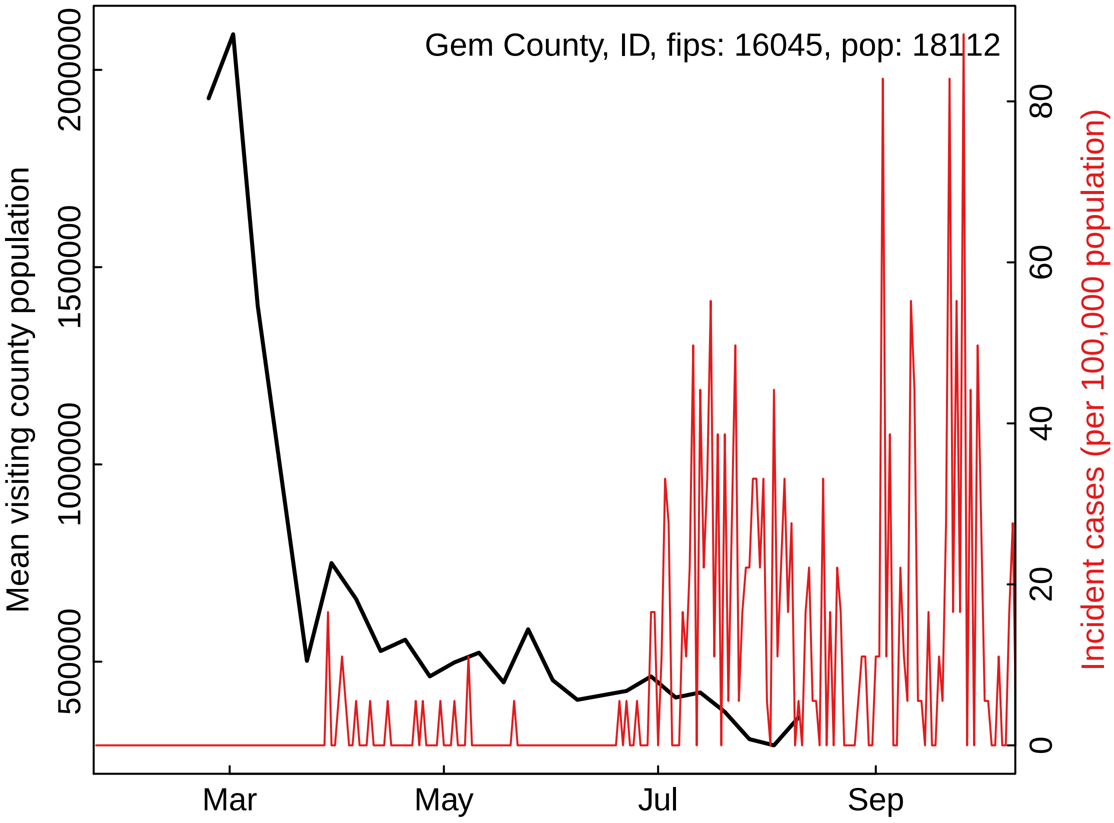

Gem County, ID, fips: 16045, pop: 18112
Mean visiting county population
500000
1000000
1500000
2000000
Incident cases (per 100,000 population)
0
20
40
60
80
Mar
May
Jul
Sep

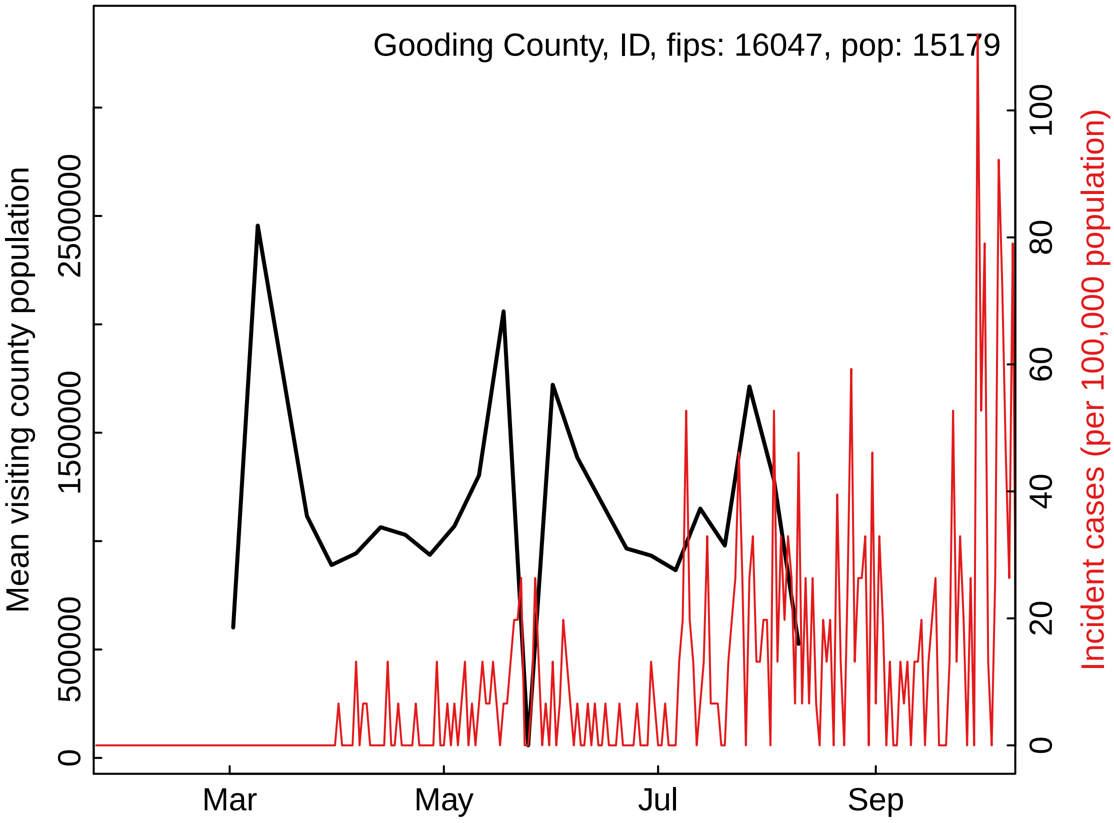
Gooding County, ID, fips: 16047, pop: 15179
Mean visiting county population
0
500000
1500000
2500000
Incident cases (per 100,000 population)
0
20
40
60
80
100
Mar
May
Jul
Sep

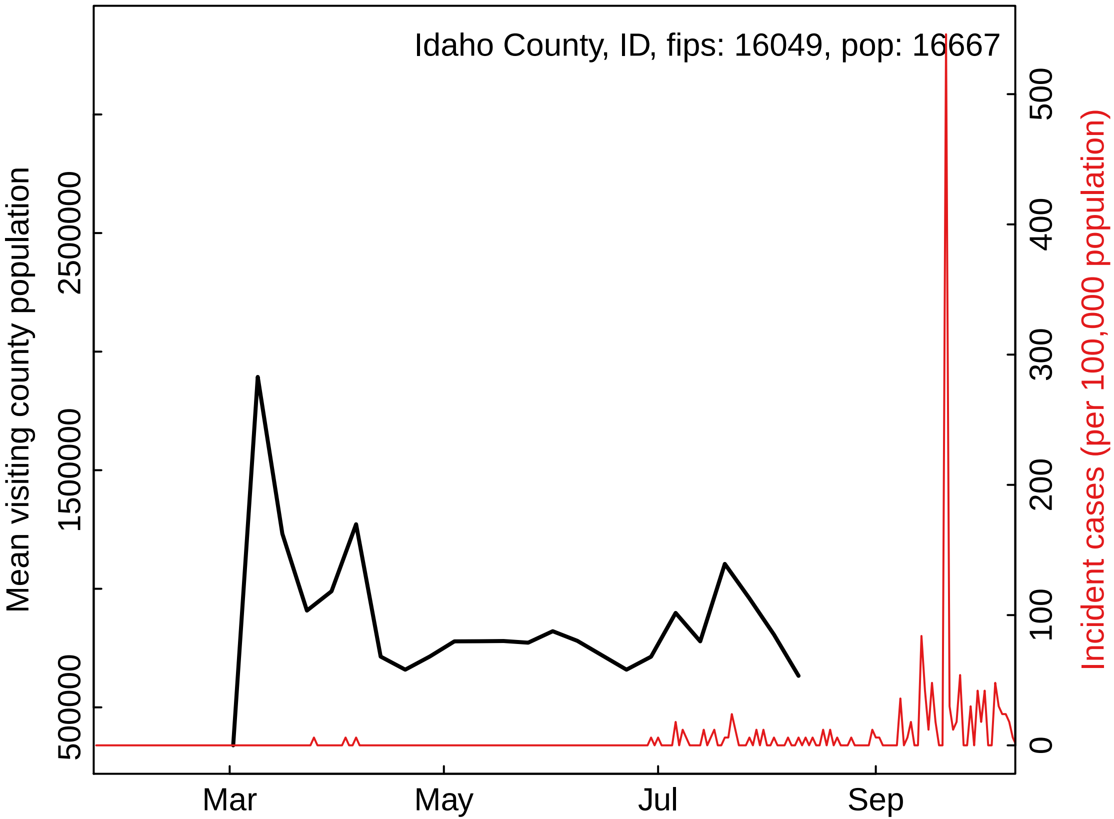
Idaho County, ID, fips: 16049, pop: 16667
Mean visiting county population
500000
1500000
2500000
Incident cases (per 100,000 population)
0
100
200
300
400
500
Mar
May
Jul
Sep

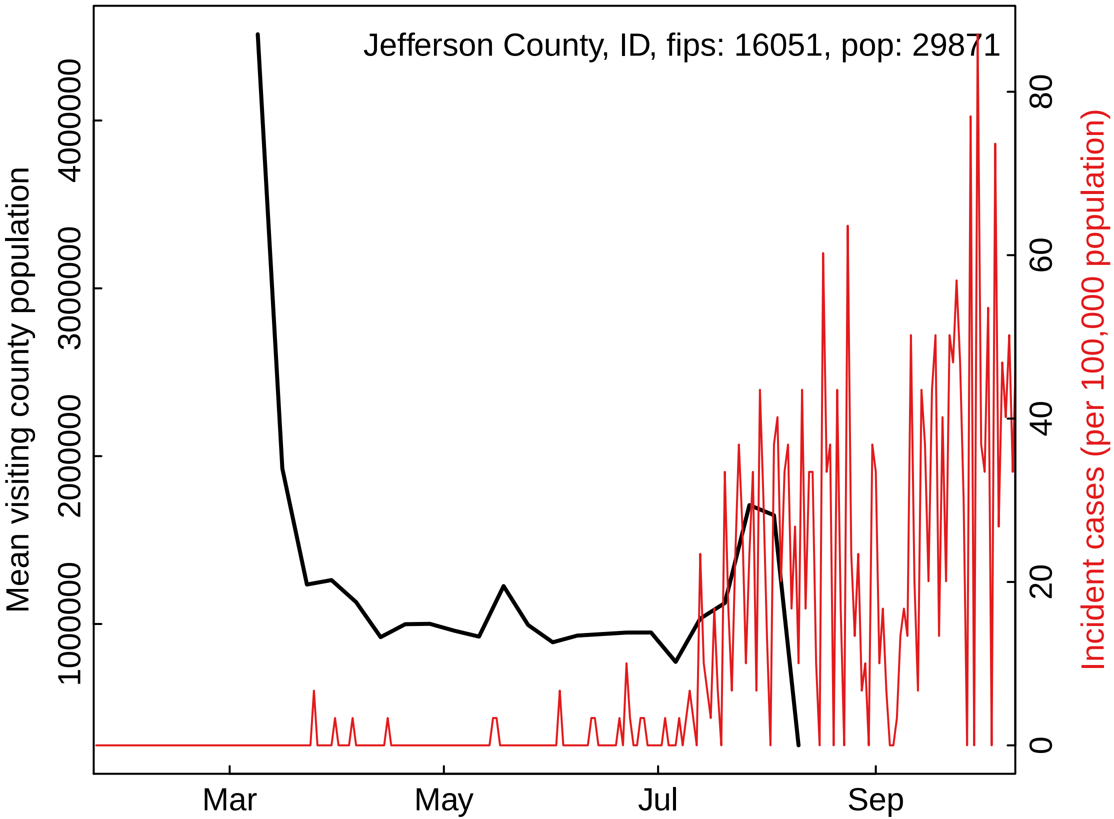

Jefferson County, ID, fips: 16051, pop: 29871
Mean visiting county population
1000000
2000000
3000000
4000000
Incident cases (per 100,000 population)
0
20
40
60
80
Mar
May
Jul
Sep

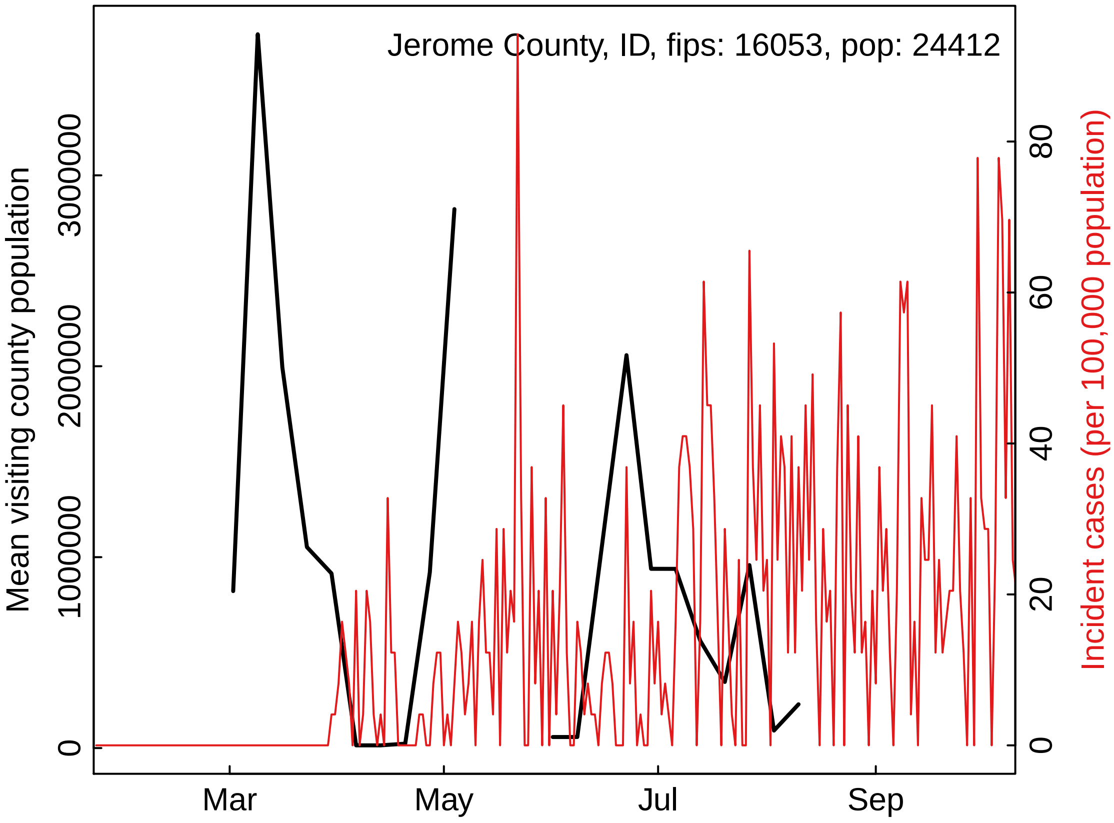

Jerome County, ID, fips: 16053, pop: 24412
Mean visiting county population
0
10000000
20000000
30000000
Incident cases (per 100,000 population)
0
20
40
60
80
Mar
May
Jul
Sep

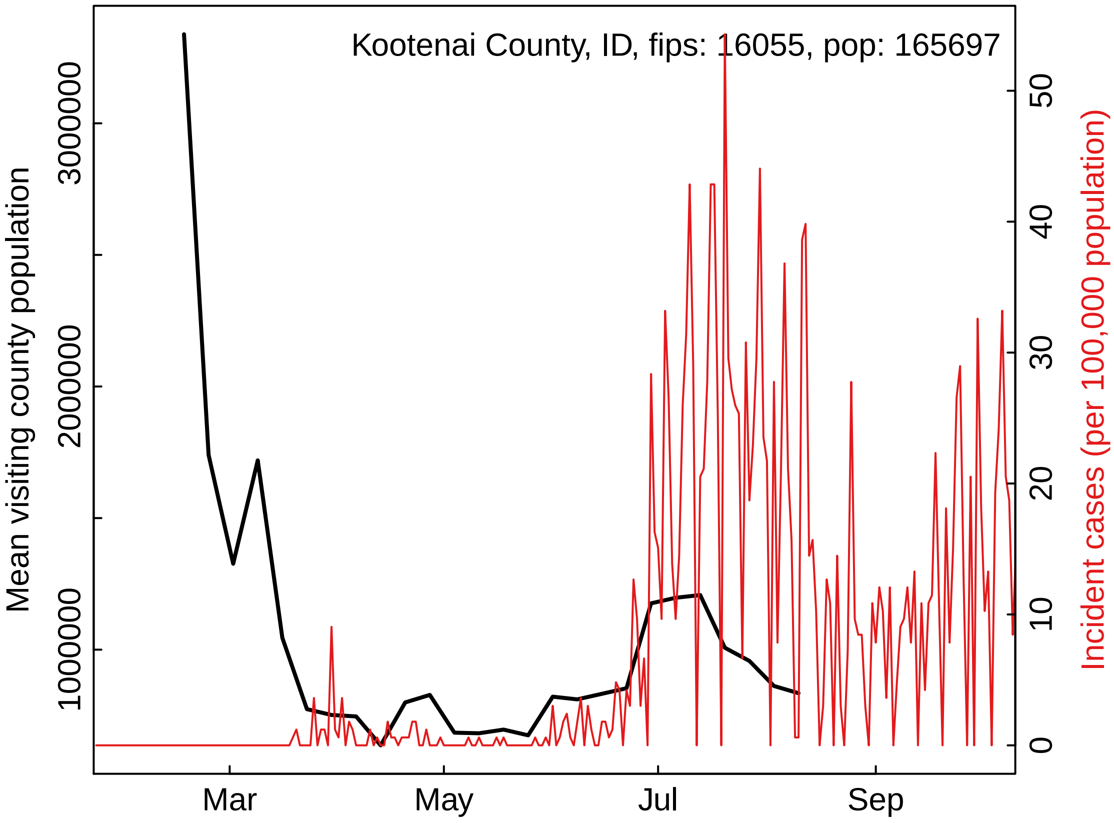

Kootenai County, ID, fips: 16055, pop: 165697
Mean visiting county population
1000000
2000000
3000000
Incident cases (per 100,000 population)
0
10
20
30
40
50
Mar
May
Jul
Sep

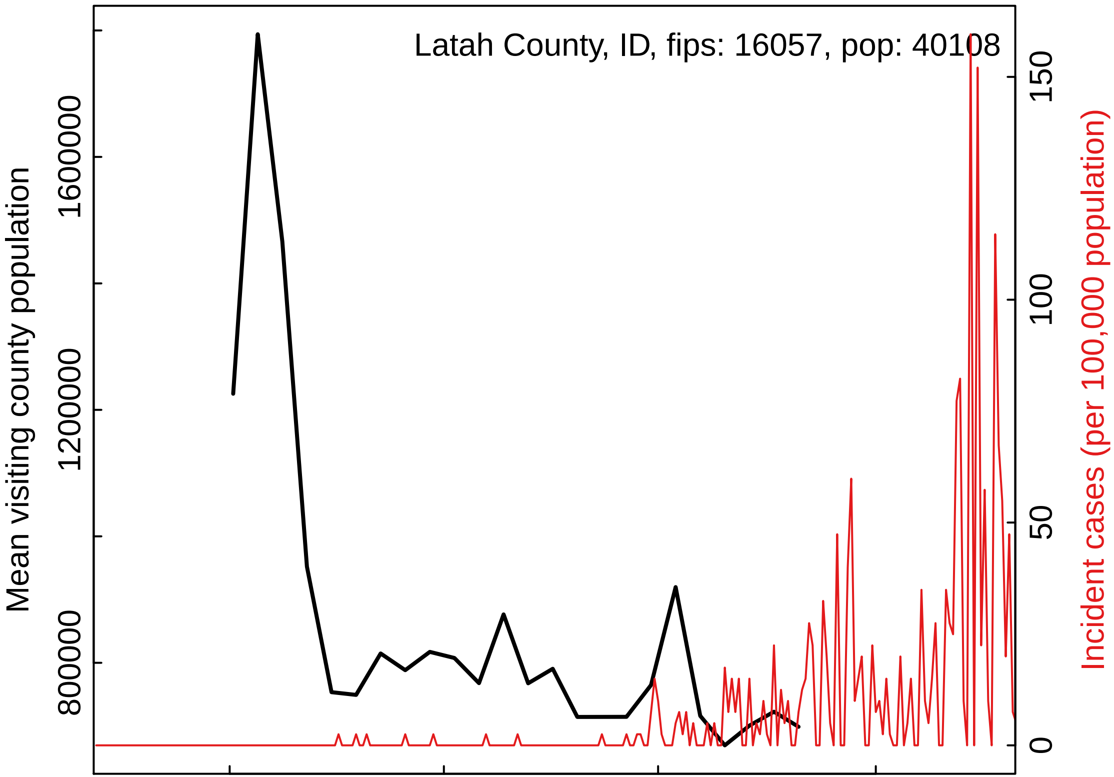

Latah County, ID, fips: 16057, pop: 40108
Mean visiting county population
800000
1200000
1600000
Incident cases (per 100,000 population)
0
50
100
150
Mar
May
Jul
Sep

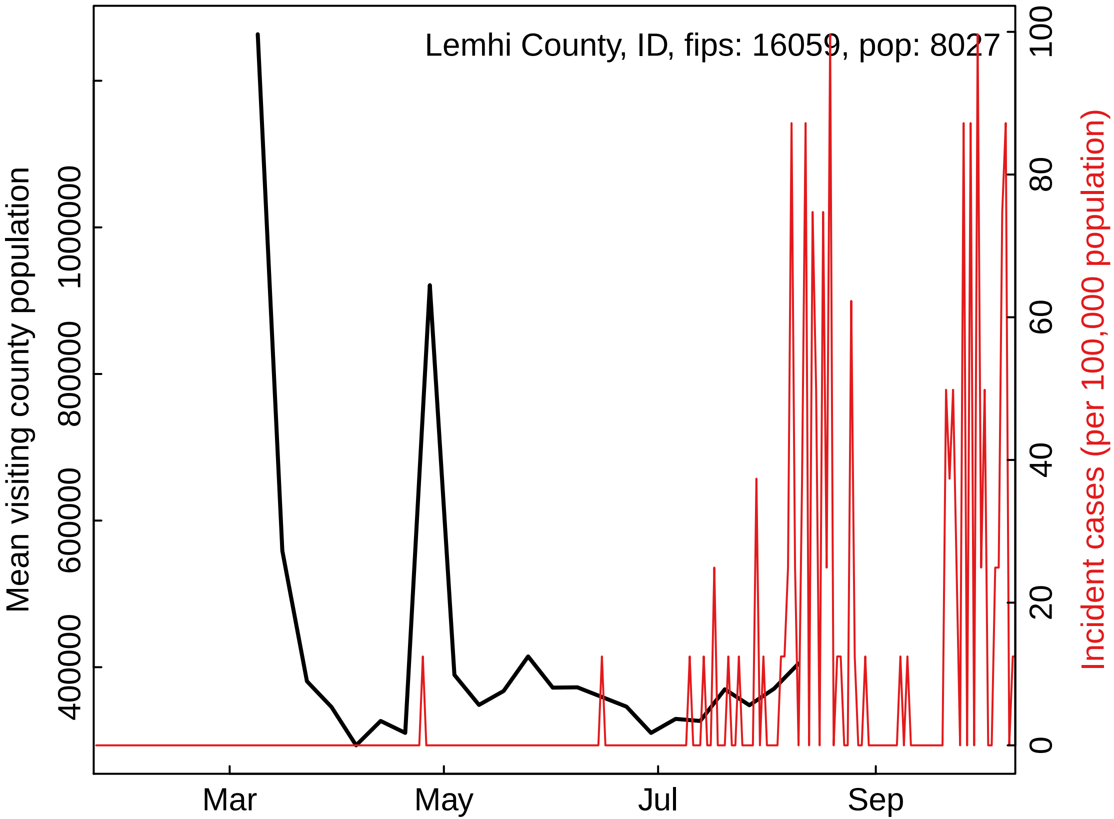
Lemhi County, ID, fips: 16059, pop: 8027
Mean visiting county population
400000
600000
800000
1000000
Incident cases (per 100,000 population)
0
20
40
60
80
100
Mar
May
Jul
Sep

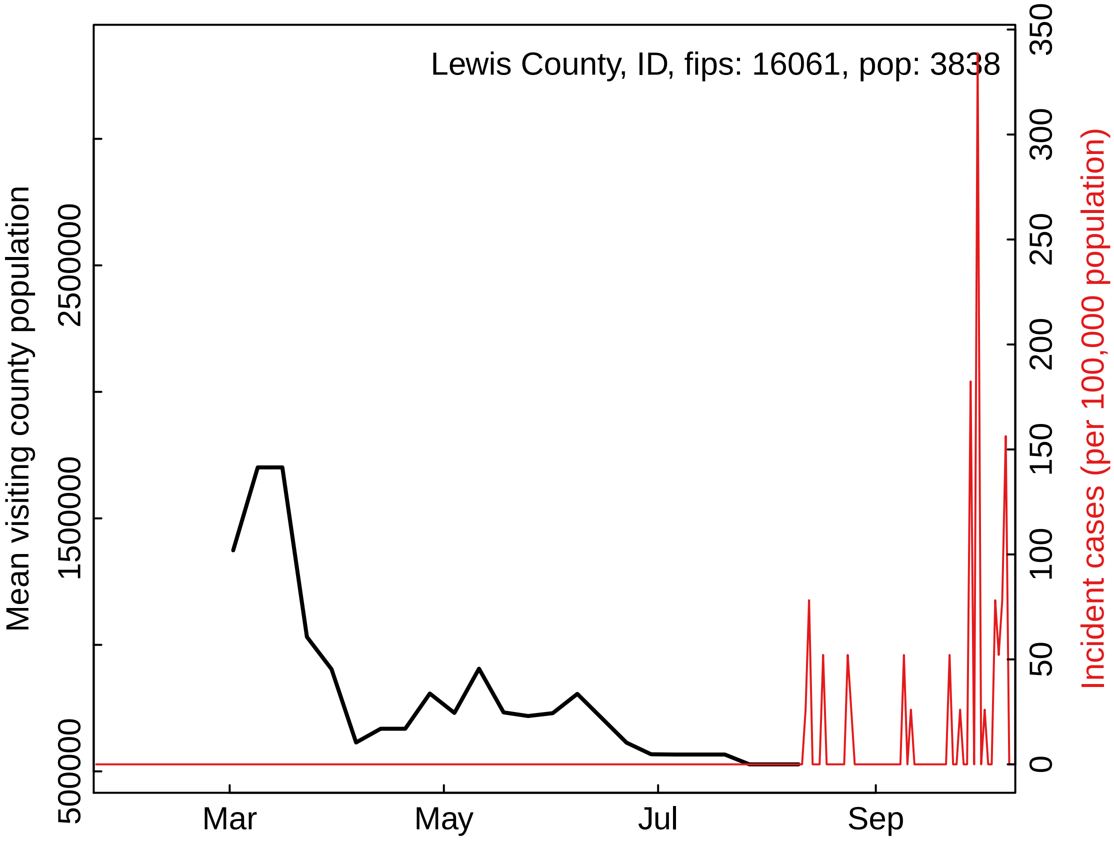

Lewis County, ID, fips: 16061, pop: 3838
Mean visiting county population
500000
1500000
2500000
Incident cases (per 100,000 population)
0
50
100
150
200
250
300
350
Mar
May
Jul
Sep

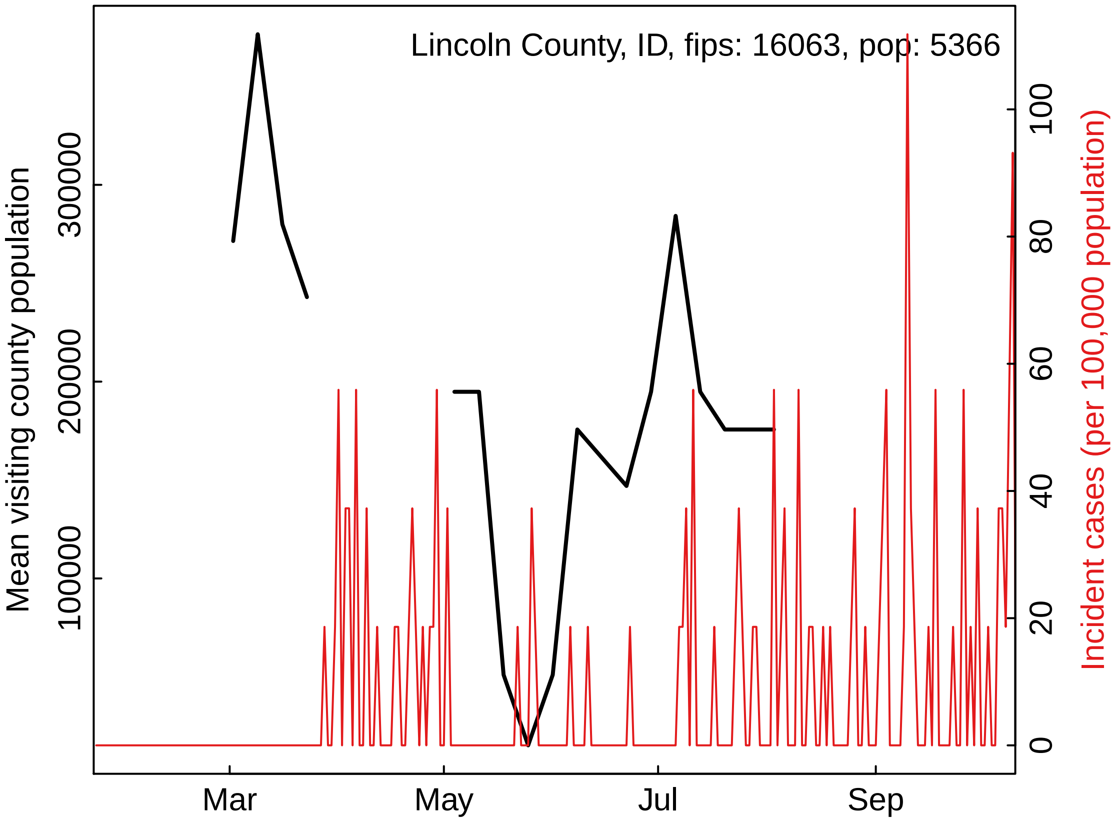

Lincoln County, ID, fips: 16063, pop: 5366
Mean visiting county population
100000
200000
300000
Mar
May
Jul
Sep
0
20
40
60
80
100
Incident cases (per 100,000 population)

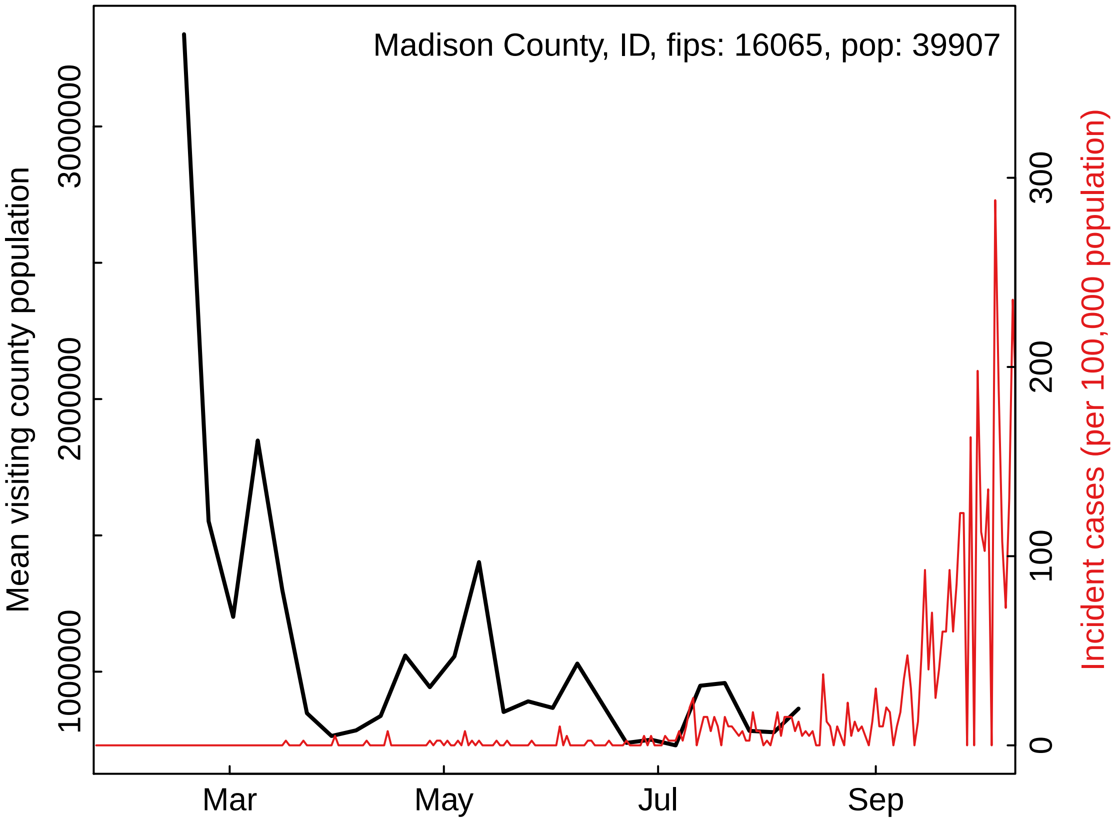
Madison County, ID, fips: 16065, pop: 39907
Mean visiting county population
1000000
2000000
3000000
Incident cases (per 100,000 population)
0
100
200
300
Mar
May
Jul
Sep

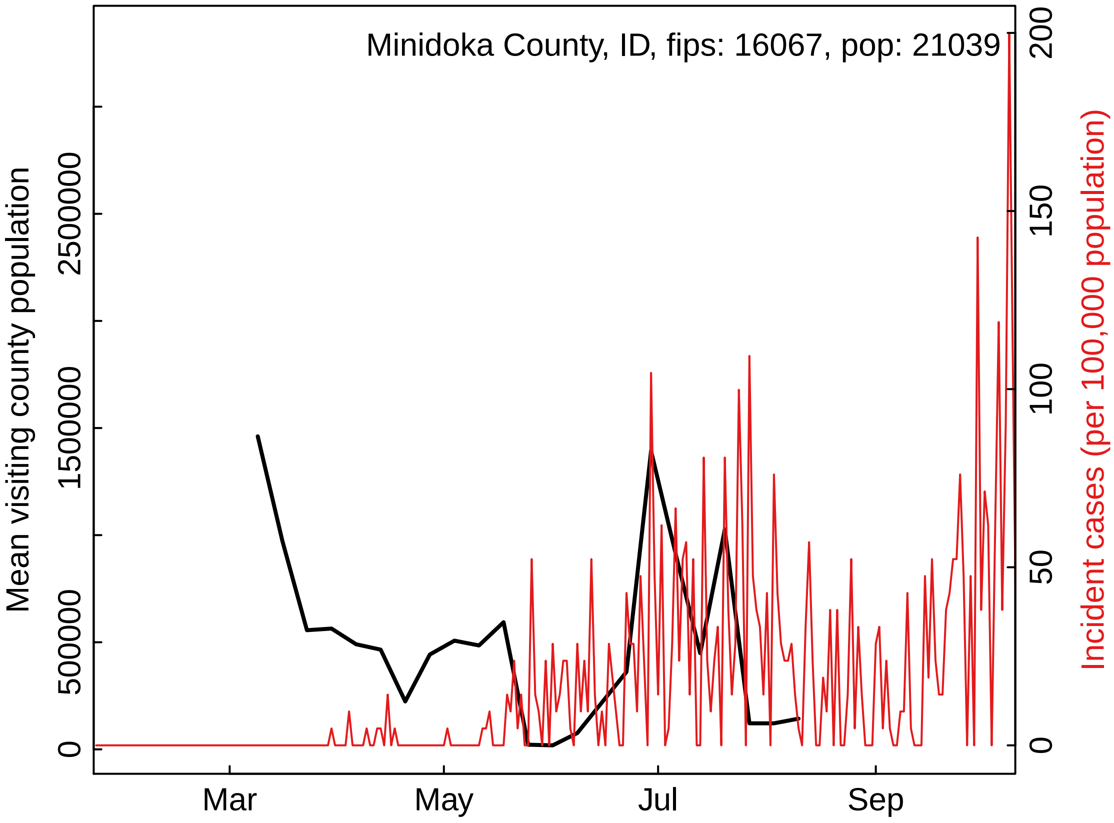

Minidoka County, ID, fips: 16067, pop: 21039
Mean visiting county population
0
500000
1500000
2500000
Incident cases (per 100,000 population)
0
50
100
150
200
Mar
May
Jul
Sep

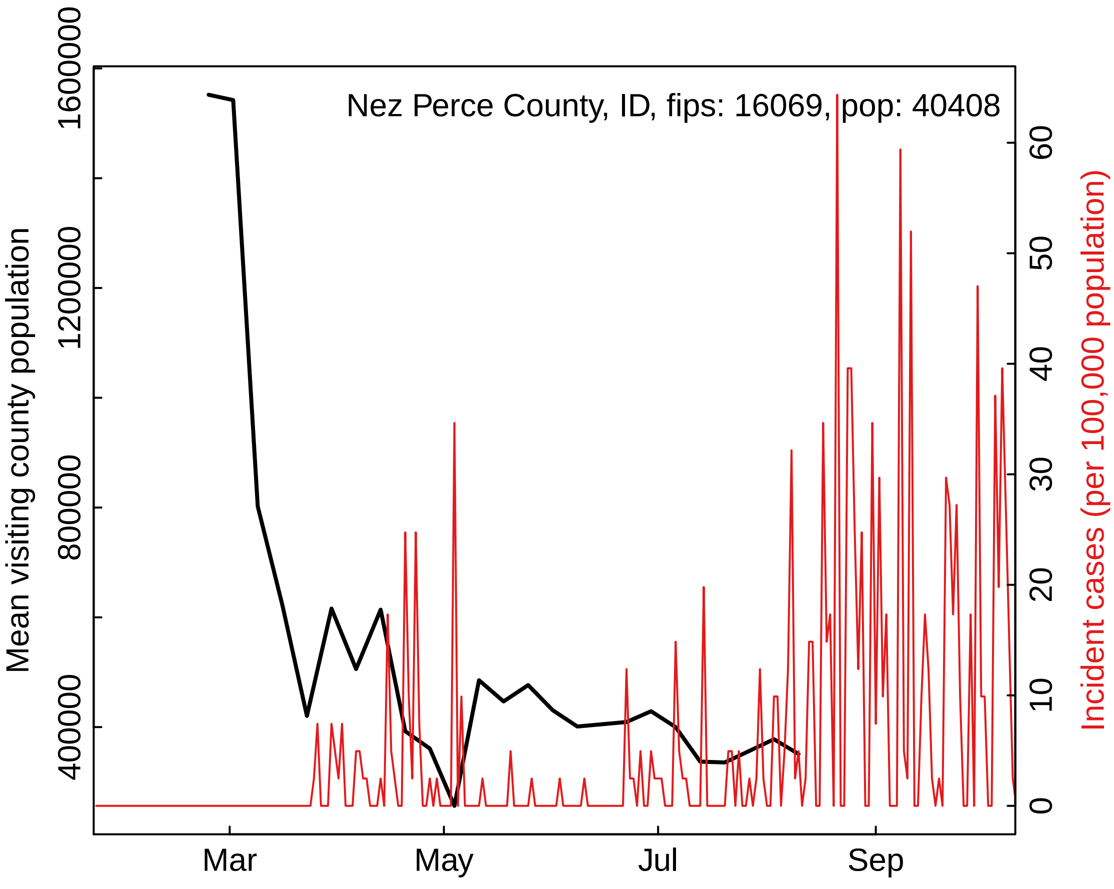

Nez Perce County, ID, fips: 16069, pop: 40408
Mean visiting county population
Incident cases (per 100,000 population)
400000
800000
1200000
1600000
0
10
20
30
40
50
60
Mar
May
Jul
Sep

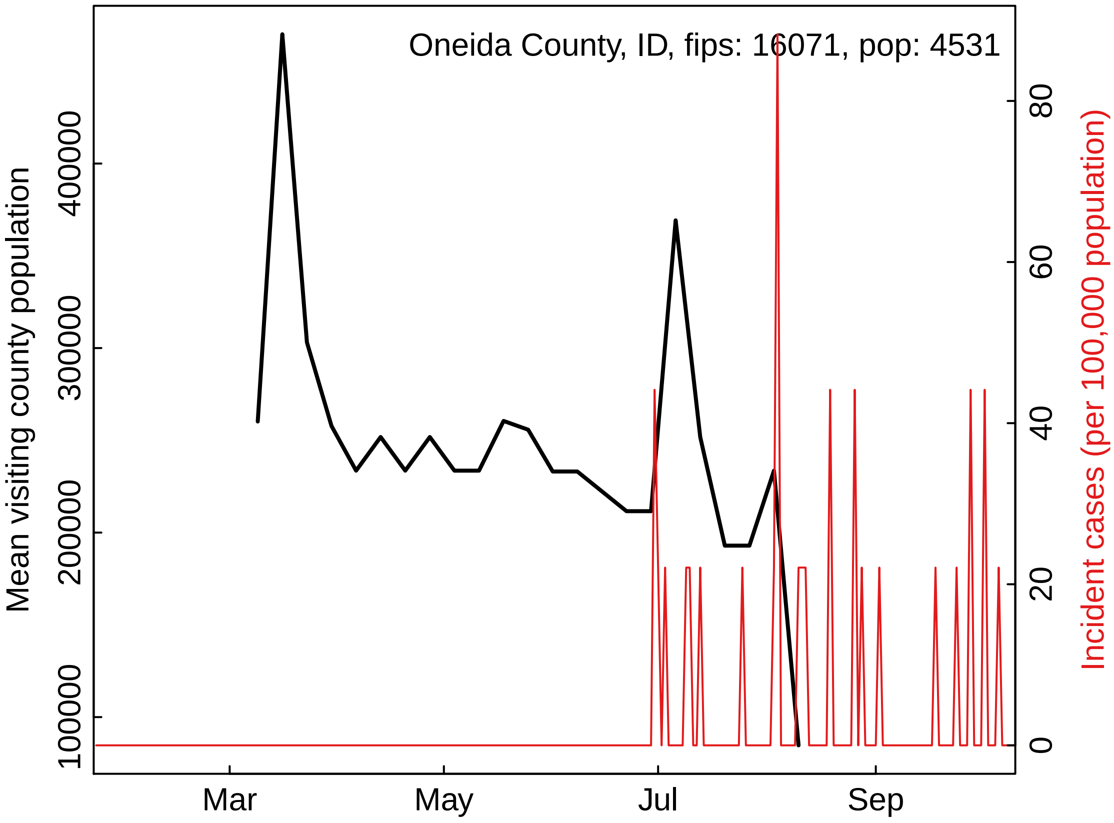

Oneida County, ID, fips: 16071, pop: 4531
Mean visiting county population
100000
200000
300000
400000
Incident cases (per 100,000 population)
0
20
40
60
80
Mar
May
Jul
Sep

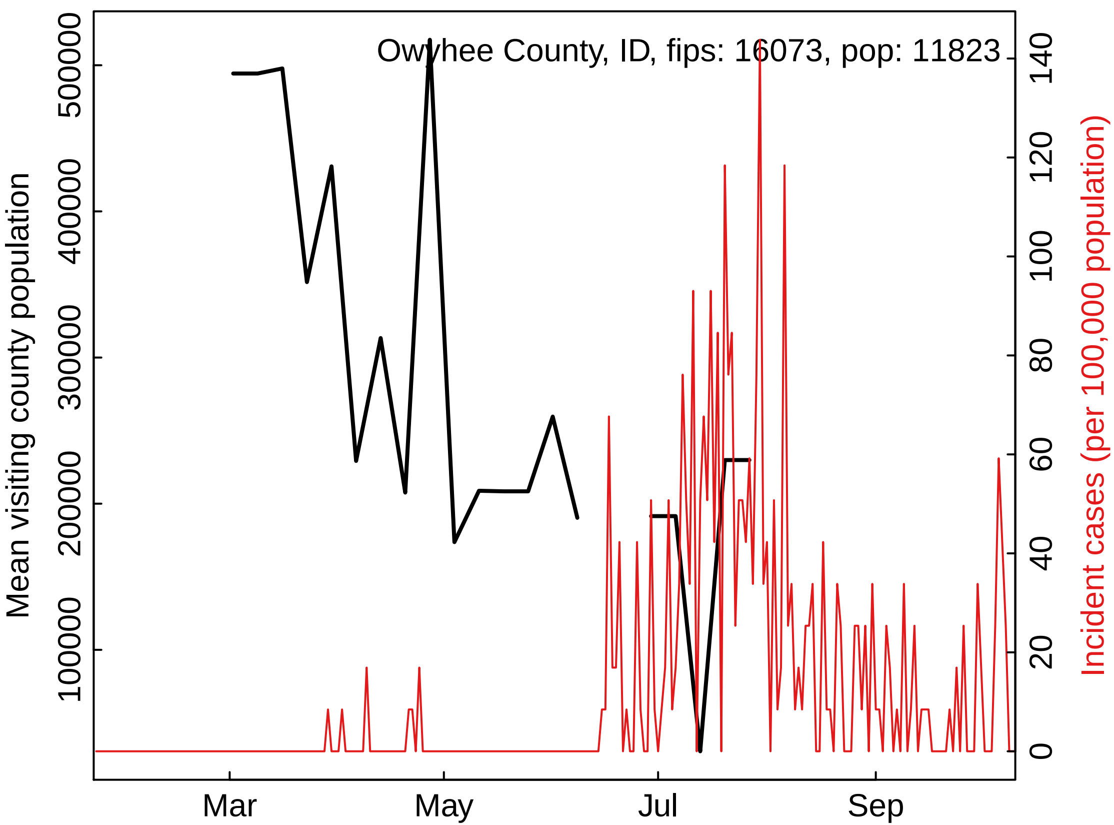
Owyhee County, ID, fips: 16073, pop: 11823
Mean visiting county population
Incident cases (per 100,000 population)
100000
200000
300000
400000
500000
0
20
40
60
80
100
120
140
Mar
May
Jul
Sep

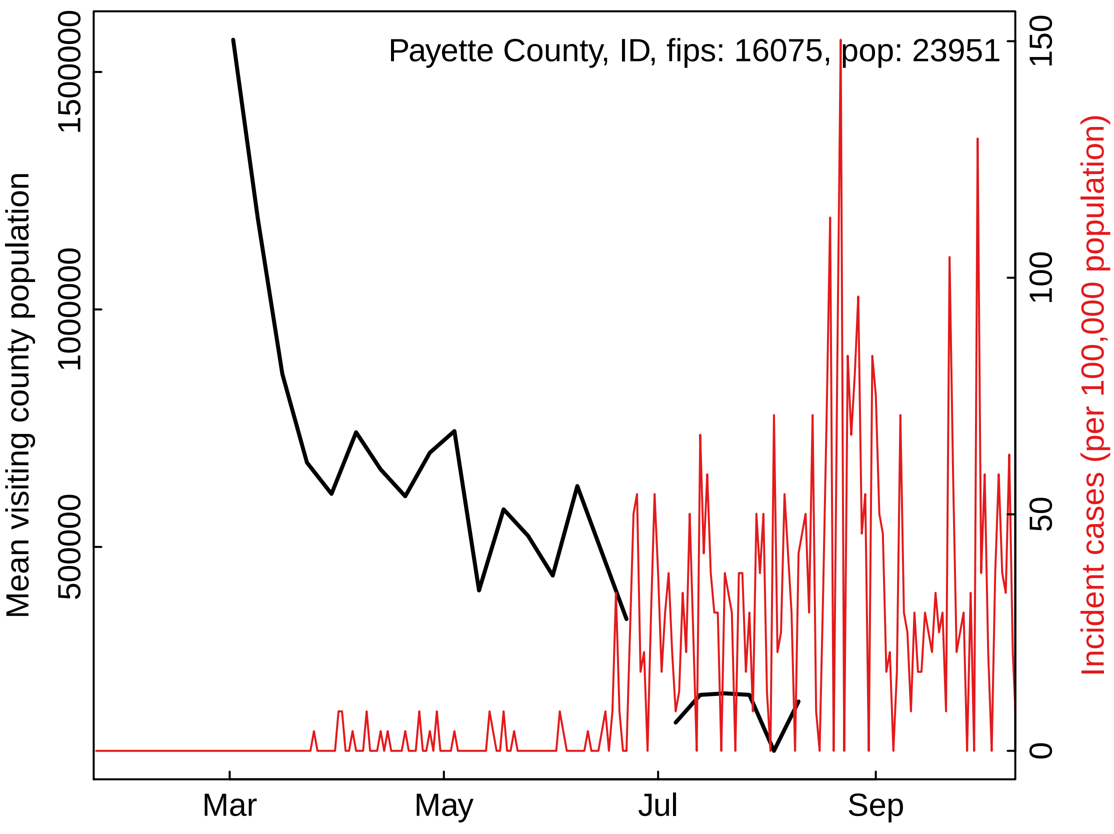
Payette County, ID, fips: 16075, pop: 23951
Mean visiting county population
500000
1000000
1500000
Incident cases (per 100,000 population)
0
50
100
150
Mar
May
Jul
Sep

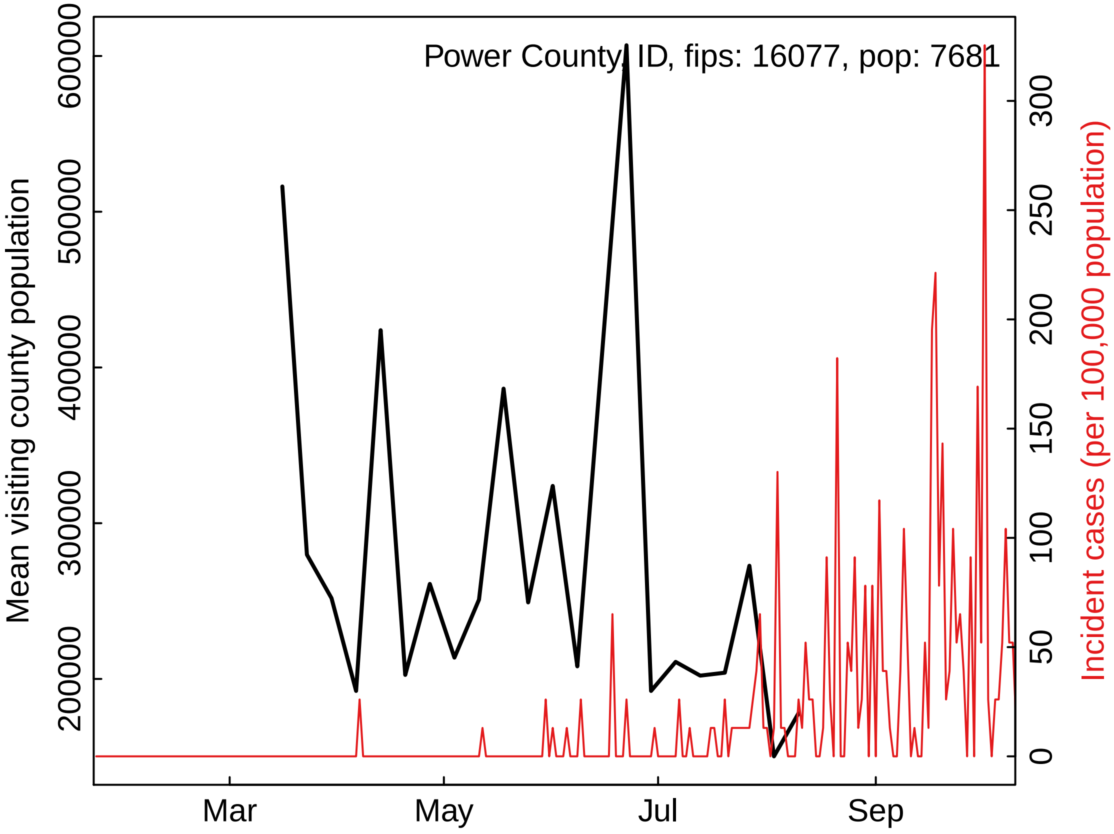

Power County, ID, fips: 16077, pop: 7681
Mean visiting county population
200000
300000
400000
500000
600000
Incident cases (per 100,000 population)
0
50
100
150
200
250
300
Mar
May
Jul
Sep

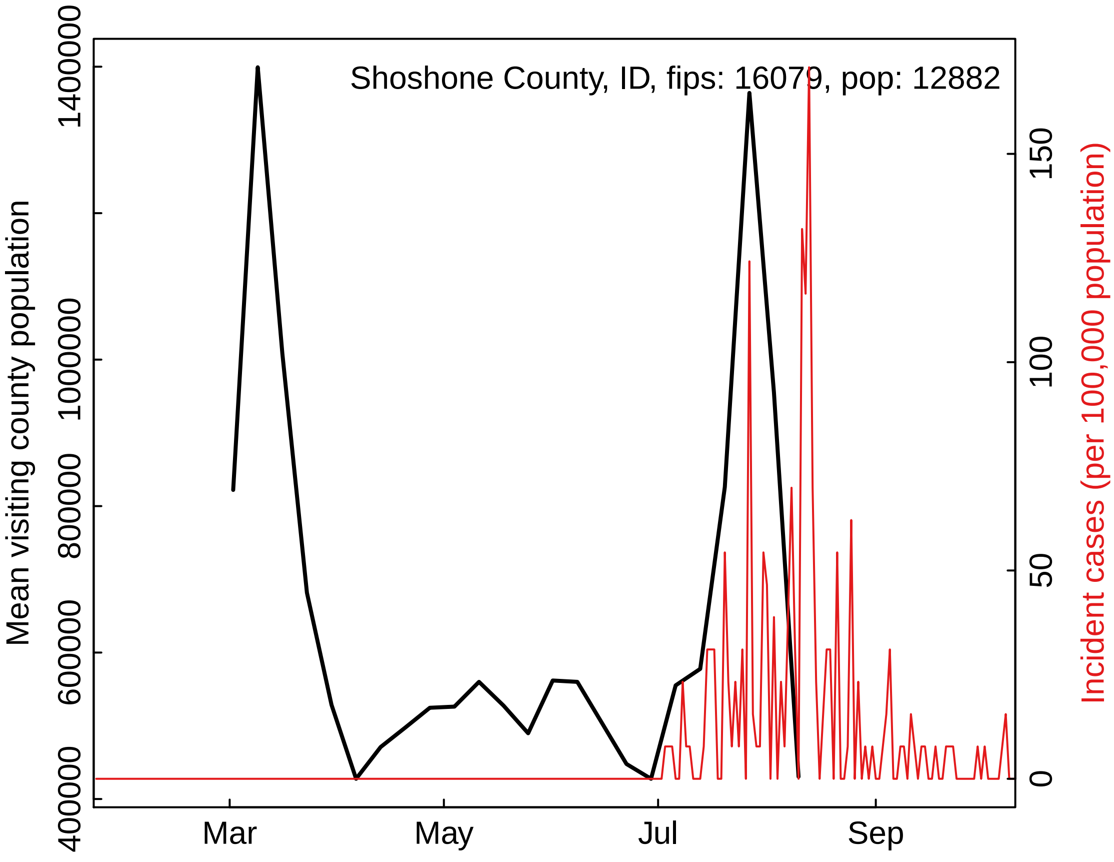

Shoshone County, ID, fips: 16079, pop: 12882
Mean visiting county population
Incident cases (per 100,000 population)
400000
600000
800000
1000000
1400000
0
50
100
150
Mar
May
Jul
Sep

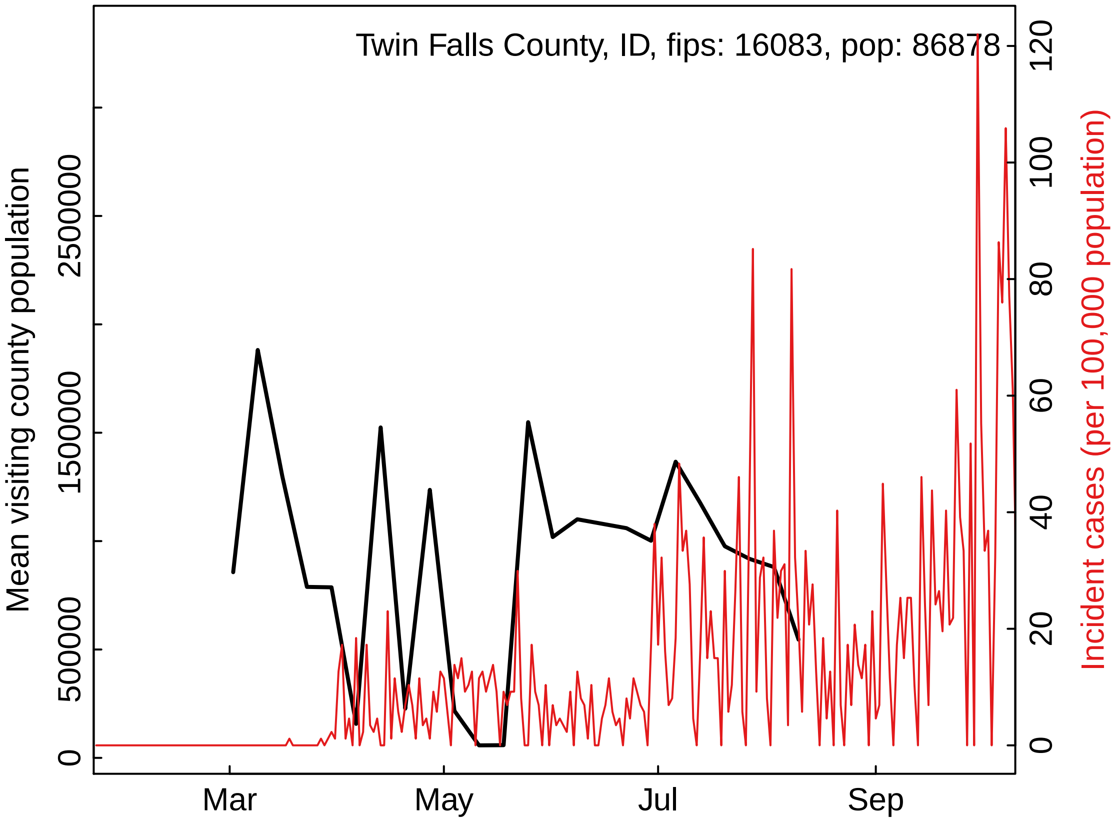

Twin Falls County, ID, fips: 16083, pop: 86878
Mean visiting county population
0
500000
1500000
2500000
Incident cases (per 100,000 population)
0
20
40
60
80
100
120
Mar
May
Jul
Sep

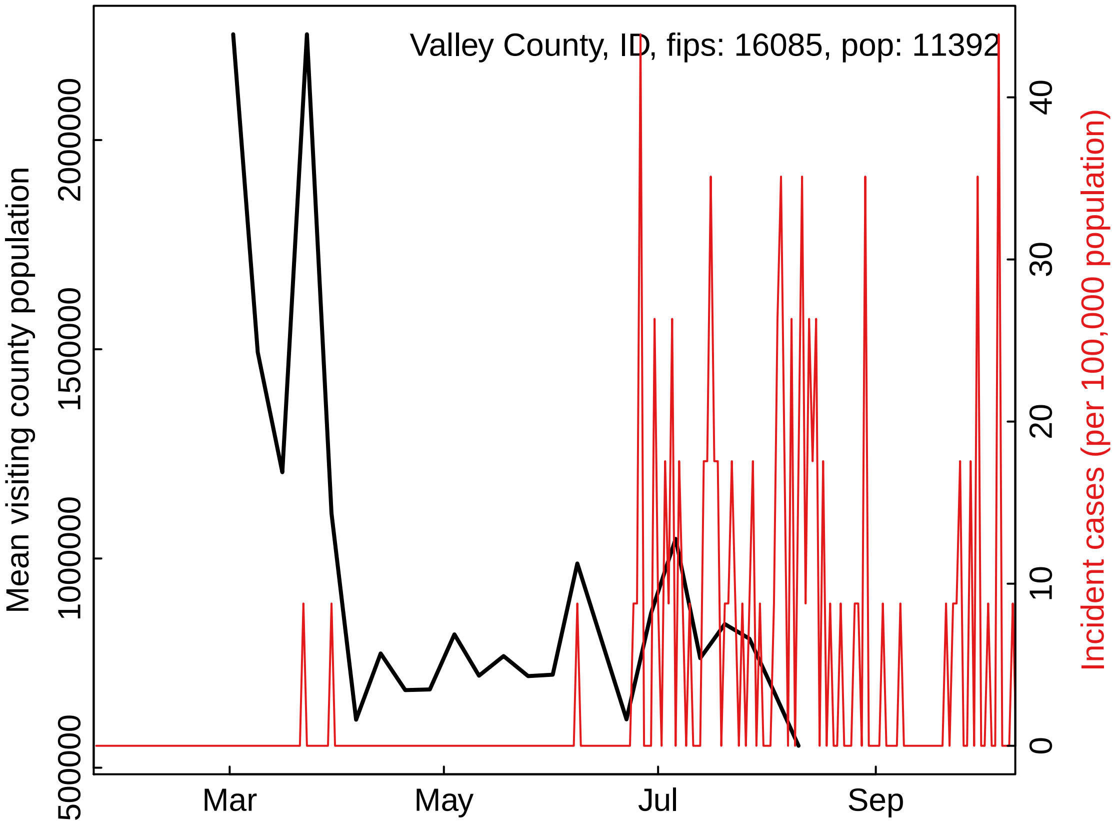

Valley County, ID, fips: 16085, pop: 11392
Mean visiting county population
5000000
10000000
15000000
20000000
Incident cases (per 100,000 population)
0
10
20
30
40
Mar
May
Jul
Sep

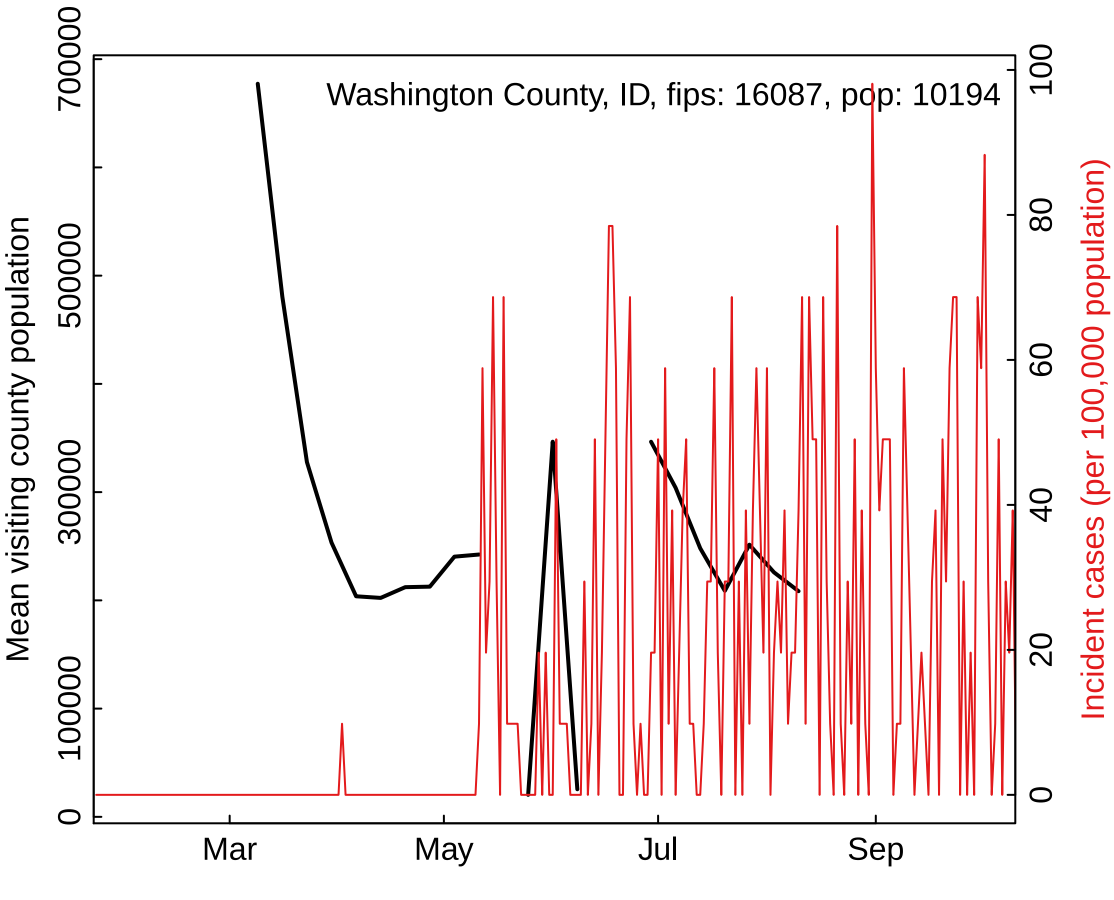

Washington County, ID, fips: 16087, pop: 10194
Mean visiting county population
Incident cases (per 100,000 population)
0
100000
300000
500000
700000
0
20
40
60
80
100
Mar
May
Jul
Sep

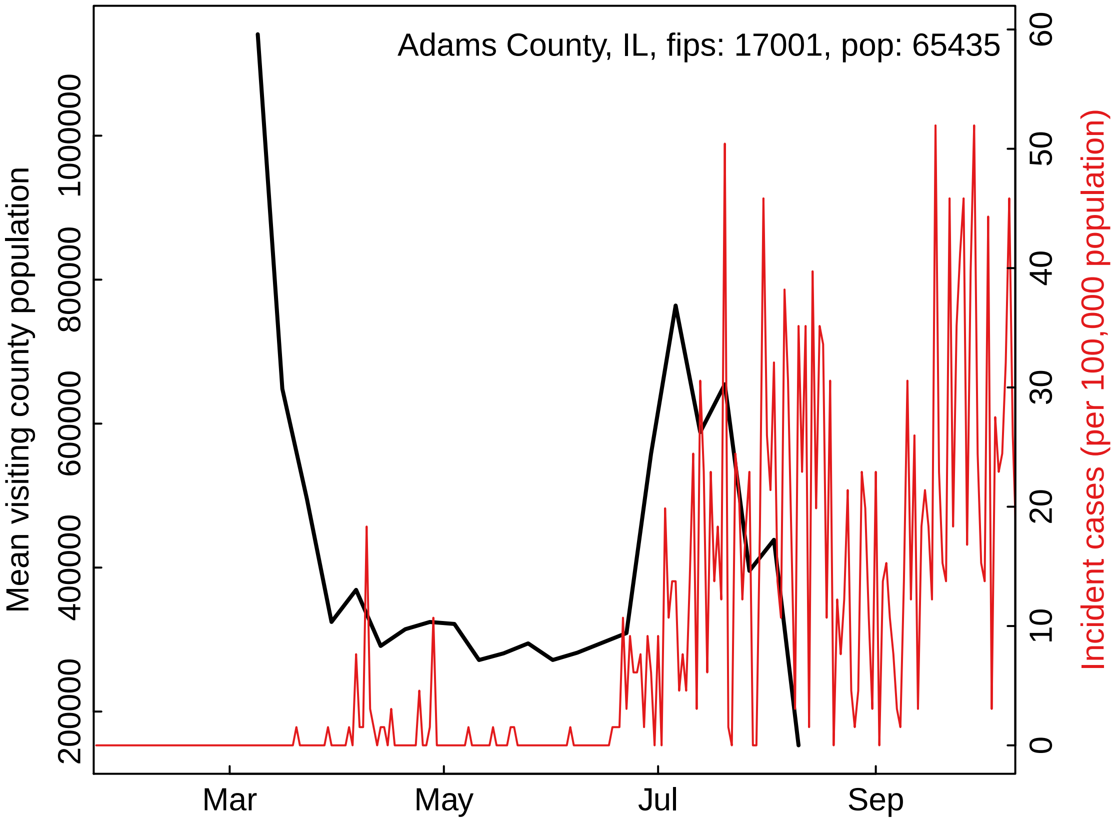
Adams County, IL, fips: 17001, pop: 65435
Mean visiting county population
200000
400000
600000
800000
1000000
Incident cases (per 100,000 population)
0
10
20
30
40
50
60
Mar
May
Jul
Sep

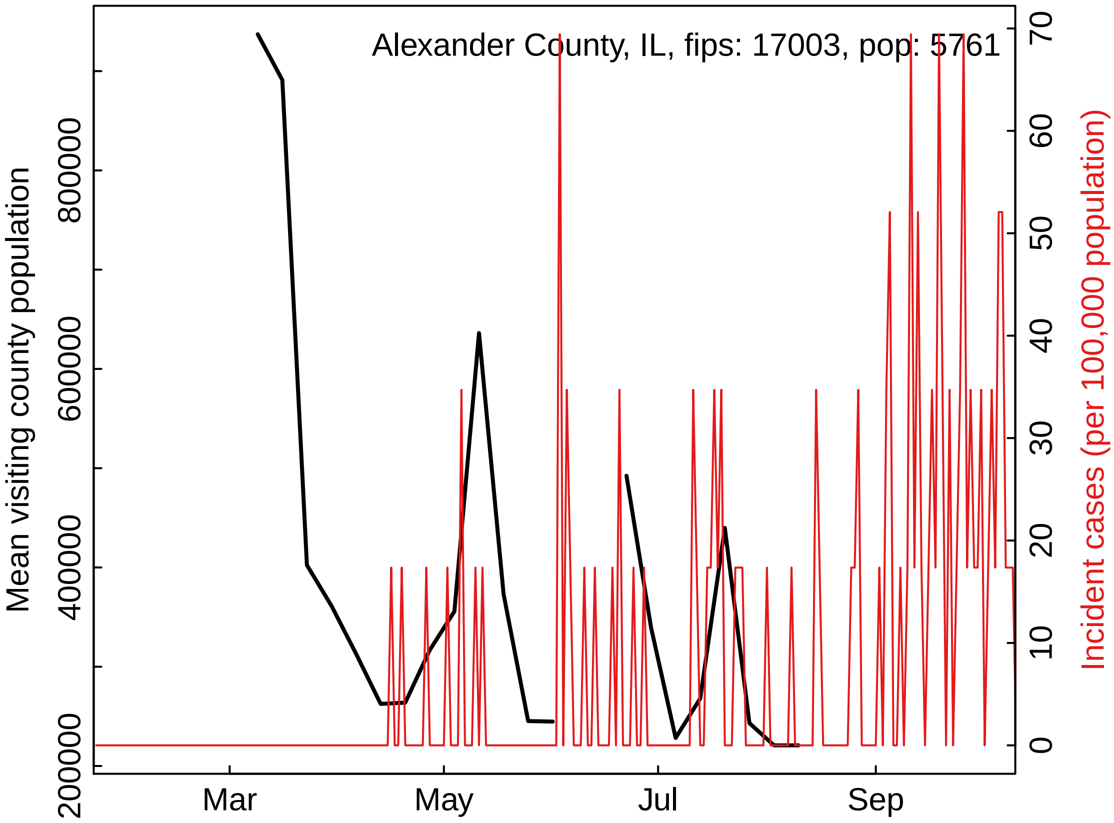

Alexander County, IL, fips: 17003, pop: 5761
Mean visiting county population
200000
400000
600000
800000
Mar
May
Jul
Sep
0
10
20
30
40
50
60
70
Incident cases (per 100,000 population)

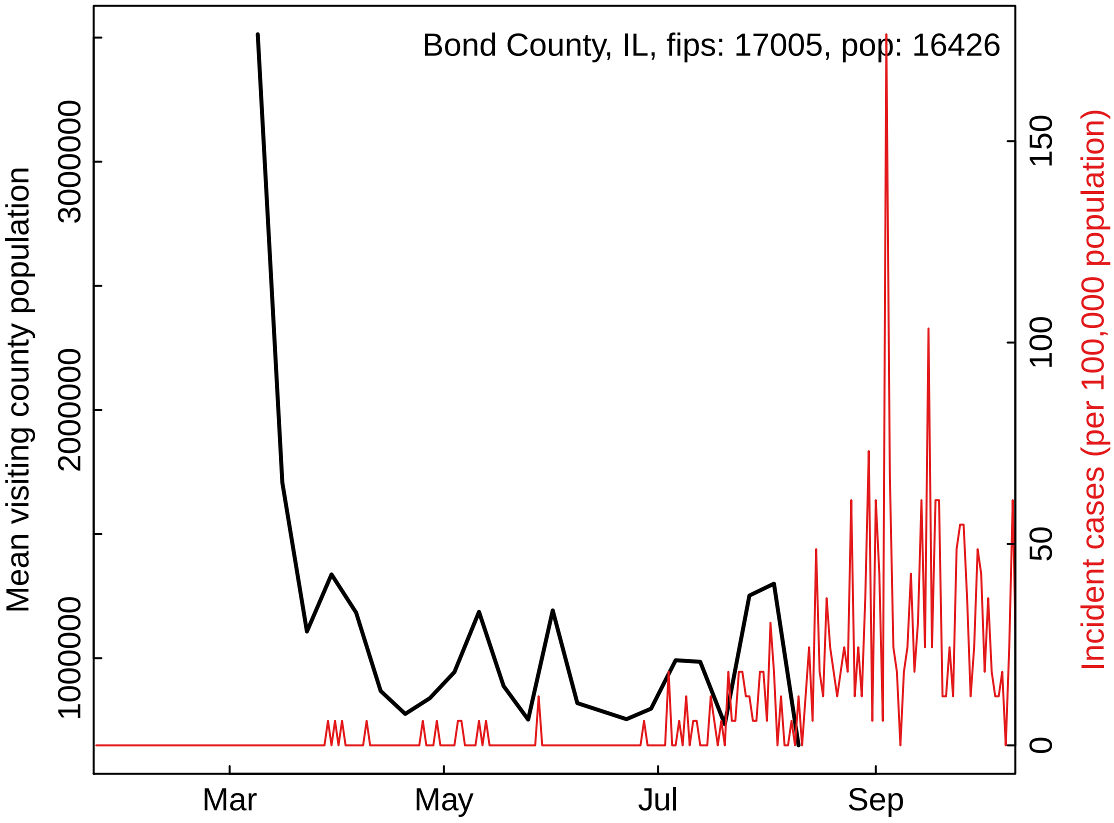
Bond County, IL, fips: 17005, pop: 16426
Mean visiting county population
10000000
20000000
30000000
Incident cases (per 100,000 population)
0
50
100
150
Mar
May
Jul
Sep

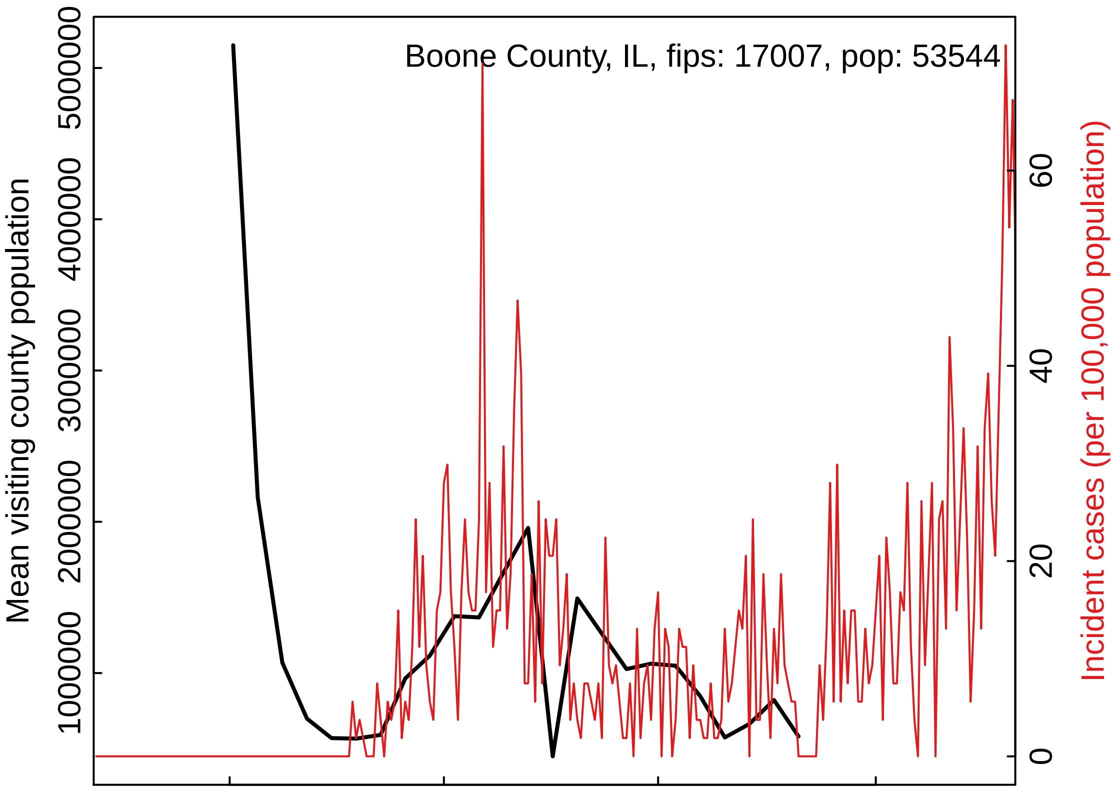

Boone County, IL, fips: 17007, pop: 53544
Mean visiting county population
1000000
2000000
3000000
4000000
5000000
Incident cases (per 100,000 population)
0
20
40
60
Mar
May
Jul
Sep

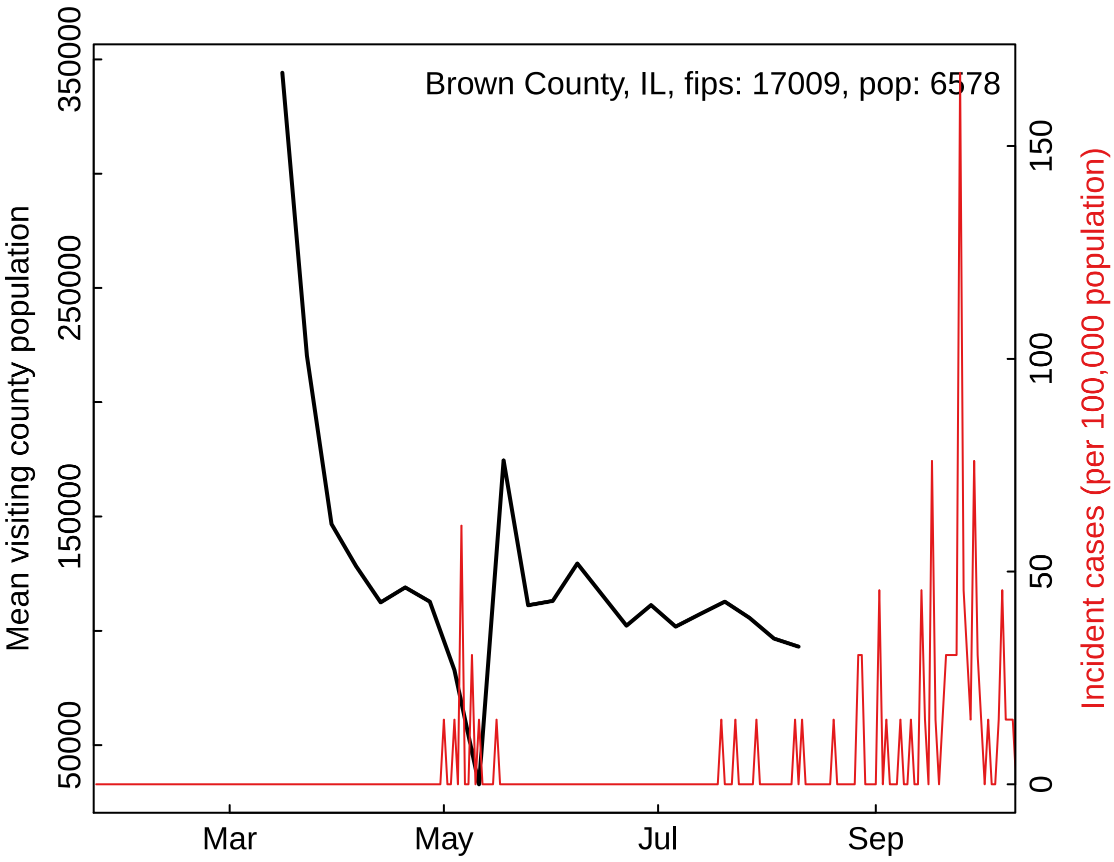

Brown County, IL, fips: 17009, pop: 6578
Mean visiting county population
350000
250000
150000
50000
Incident cases (per 100,000 population)
150
100
50
0
Mar
May
Jul
Sep

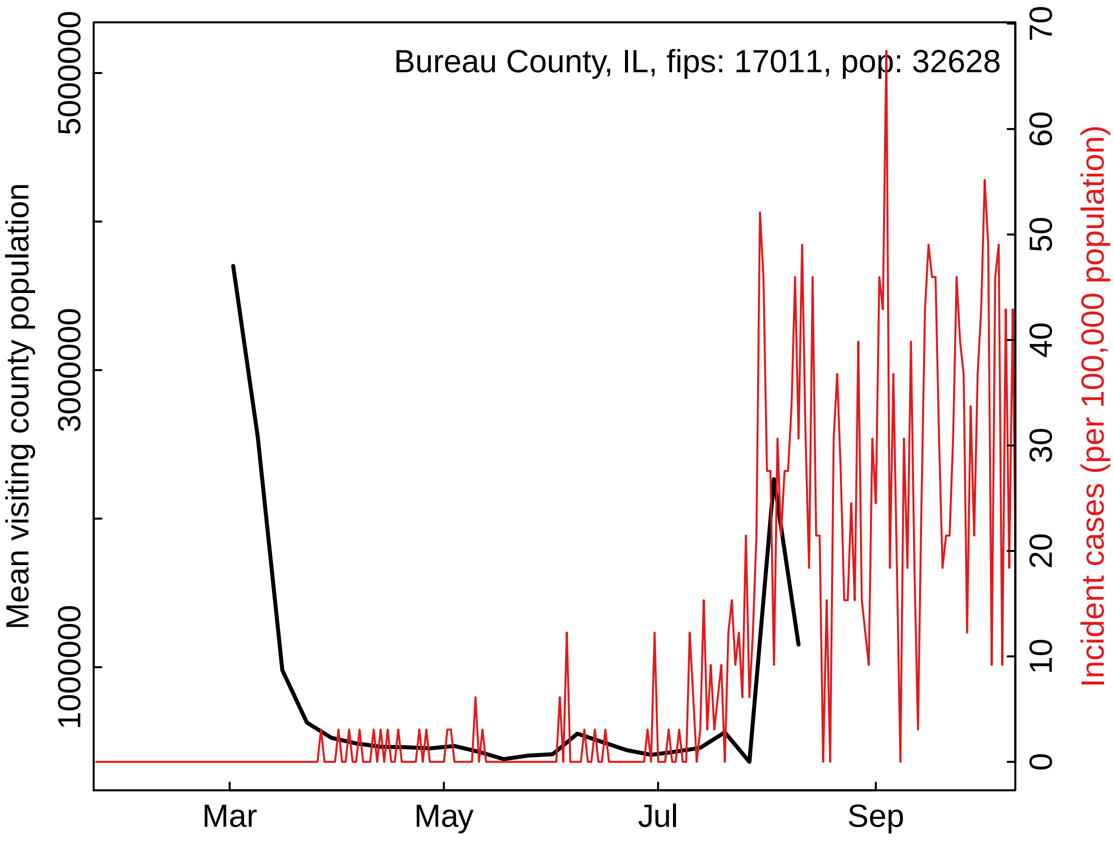

Bureau County, IL, fips: 17011, pop: 32628
Mean visiting county population
1000000
3000000
5000000
Mar
May
Jul
Sep
Incident cases (per 100,000 population)
0
10
20
30
40
50
60
70

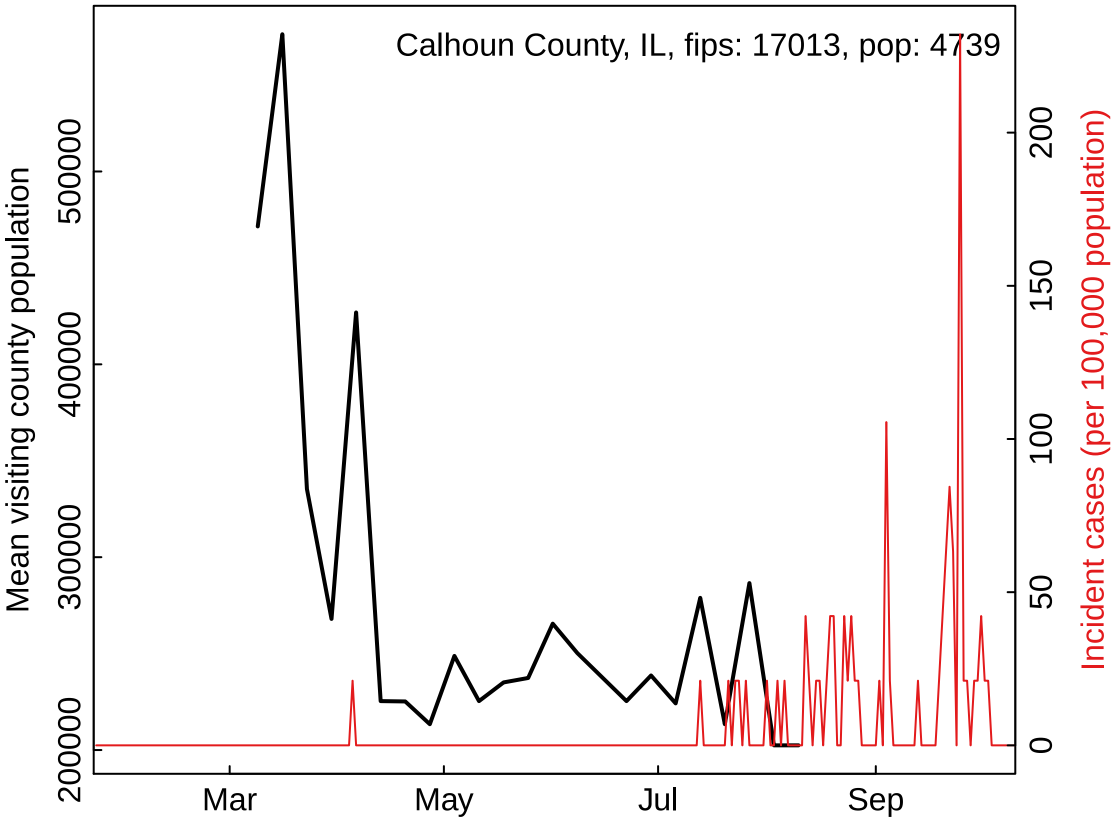

Calhoun County, IL, fips: 17013, pop: 4739
Mean visiting county population
200000
300000
400000
500000
Incident cases (per 100,000 population)
0
50
100
150
200
Mar
May
Jul
Sep

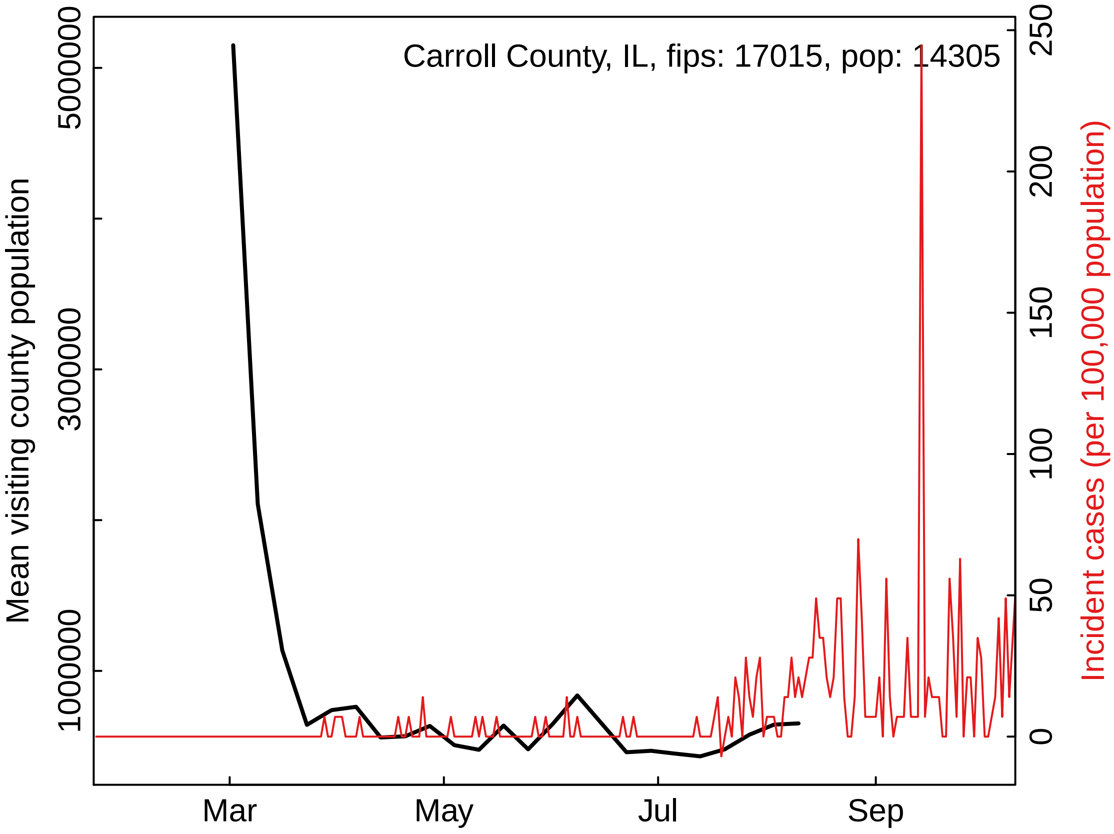

Carroll County, IL, fips: 17015, pop: 14305
Mean visiting county population
1000000
3000000
5000000
Incident cases (per 100,000 population)
0
50
100
150
200
250
Mar
May
Jul
Sep

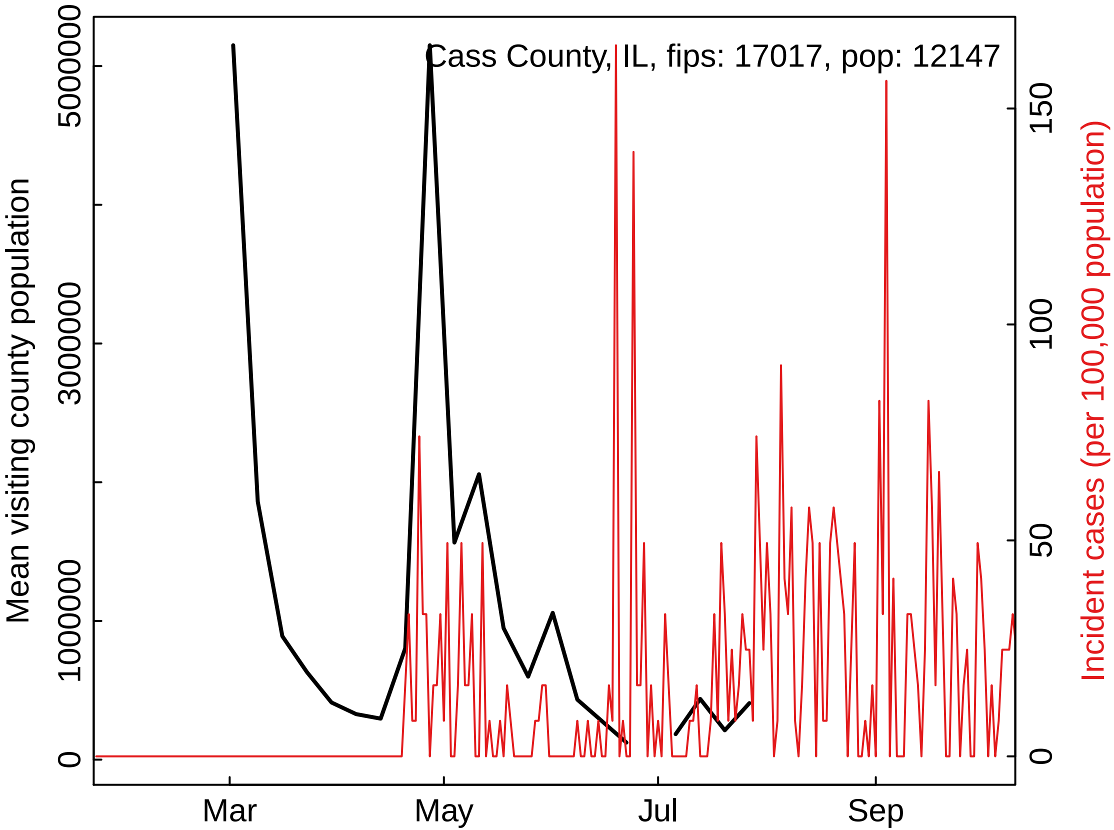

Cass County, IL, fips: 17017, pop: 12147
Mean visiting county population
0
1000000
3000000
5000000
Incident cases (per 100,000 population)
0
50
100
150
Mar
May
Jul
Sep

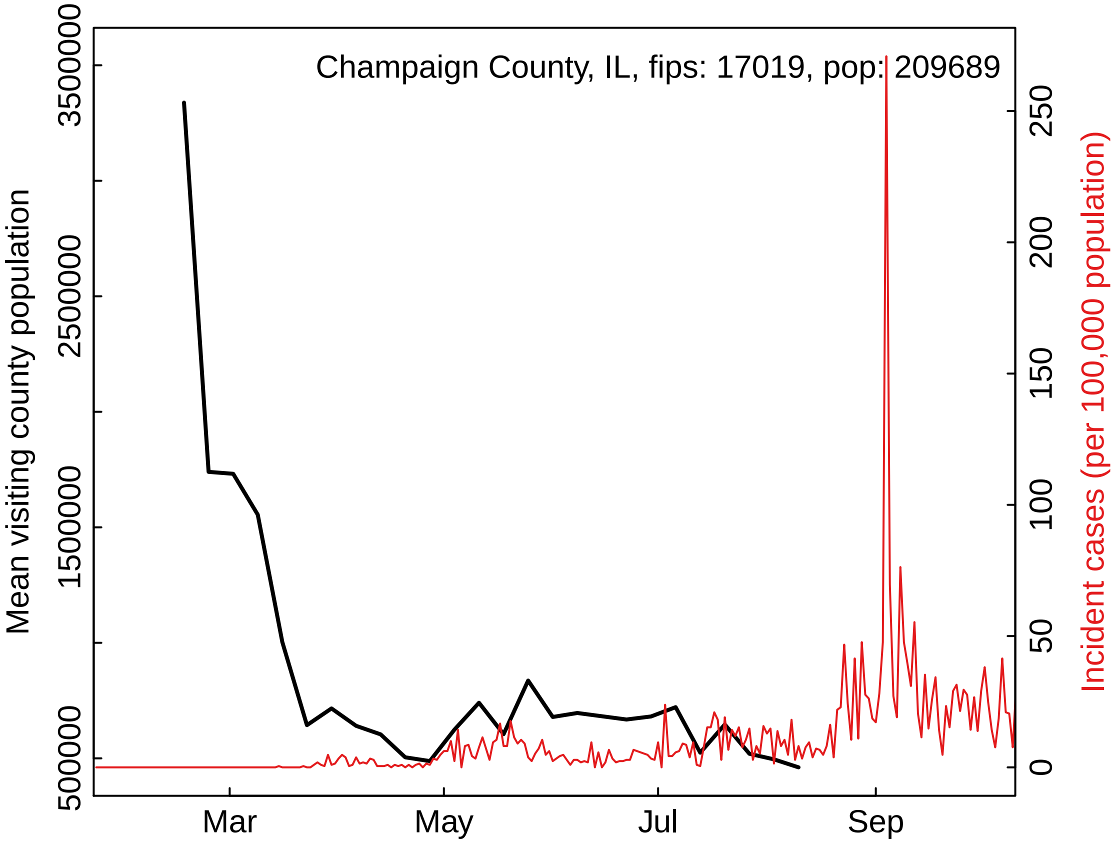

Champaign County, IL, fips: 17019, pop: 209689
Mean visiting county population
500000
1500000
2500000
3500000
Incident cases (per 100,000 population)
0
50
100
150
200
250
Mar
May
Jul
Sep

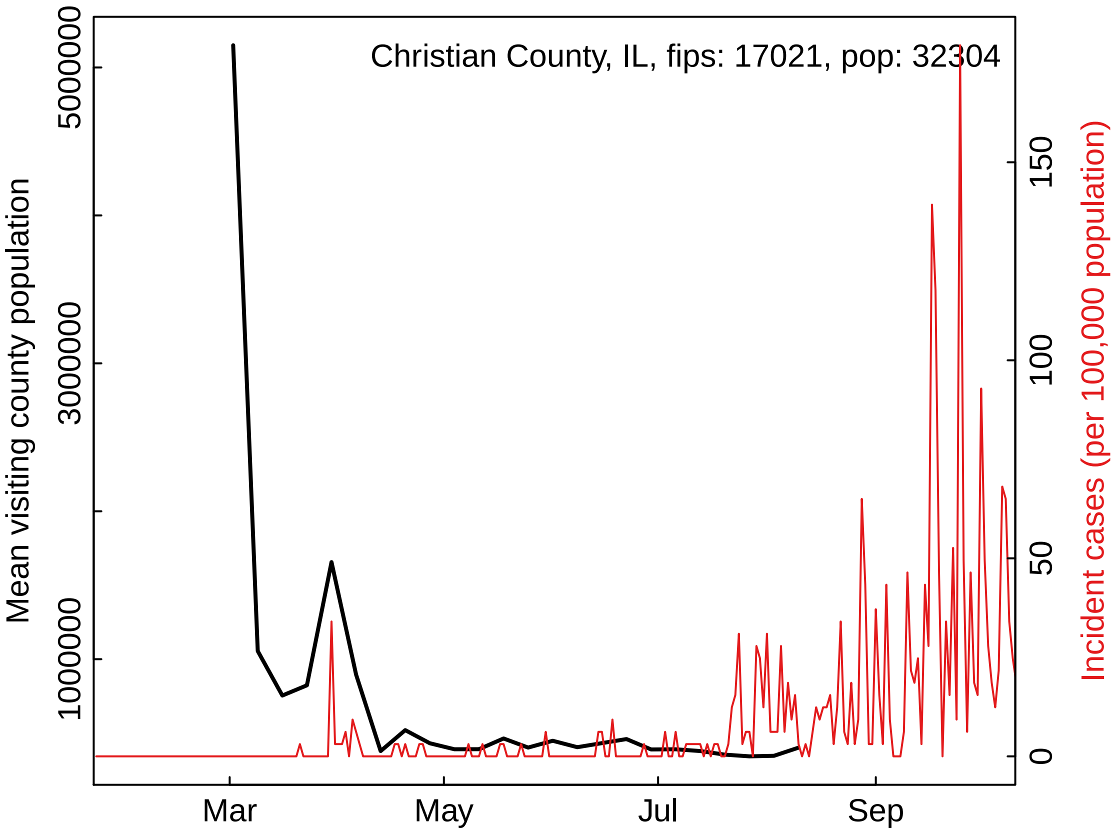

Christian County, IL, fips: 17021, pop: 32304
Mean visiting county population
1000000
3000000
5000000
Incident cases (per 100,000 population)
0
50
100
150
Mar
May
Jul
Sep

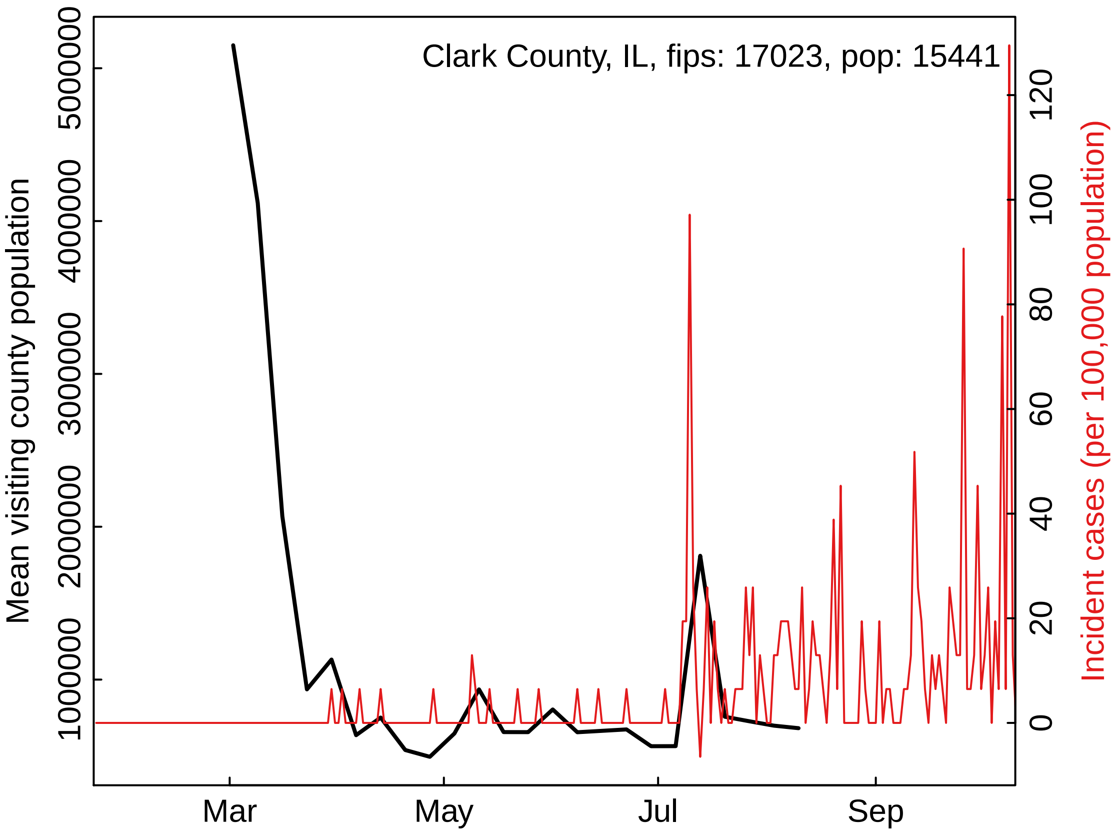

Clark County, IL, fips: 17023, pop: 15441
Mean visiting county population
Incident cases (per 100,000 population)
10000000
20000000
30000000
40000000
50000000
0
20
40
60
80
100
120
Mar
May
Jul
Sep

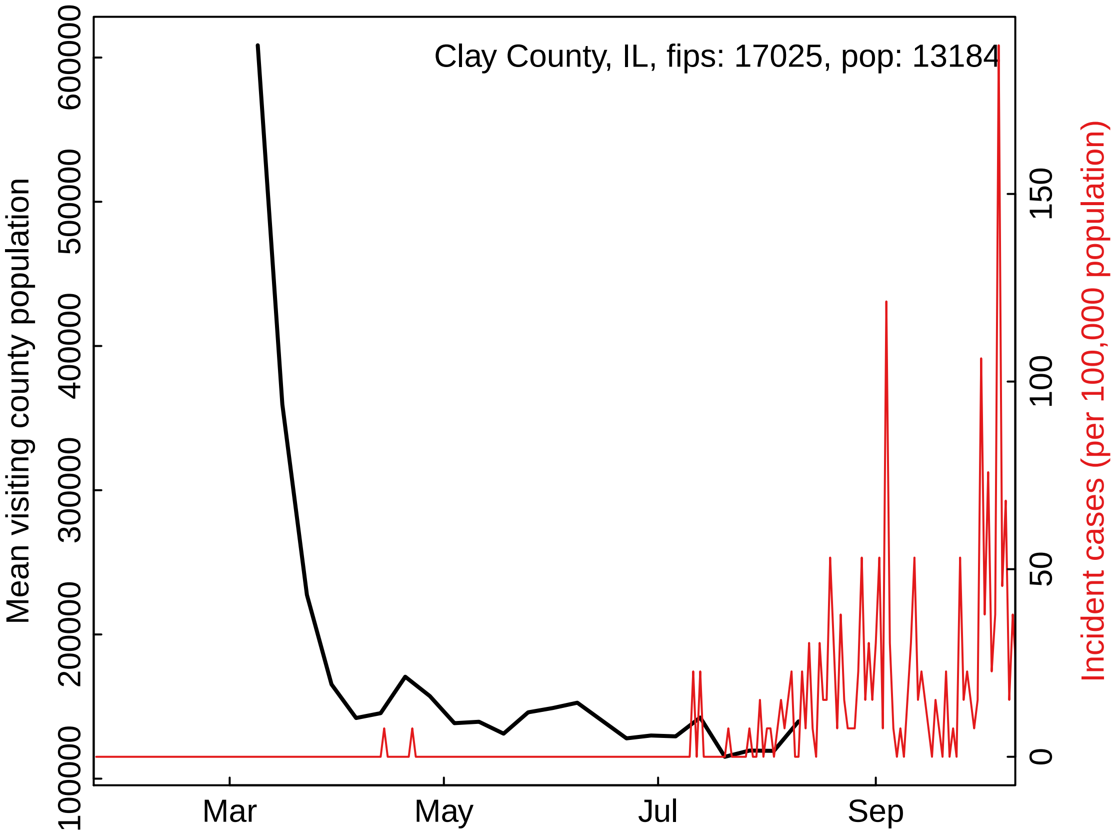
Clay County, IL, fips: 17025, pop: 13184
Mean visiting county population
100000
200000
300000
400000
500000
600000
Incident cases (per 100,000 population)
0
50
100
150
Mar
May
Jul
Sep

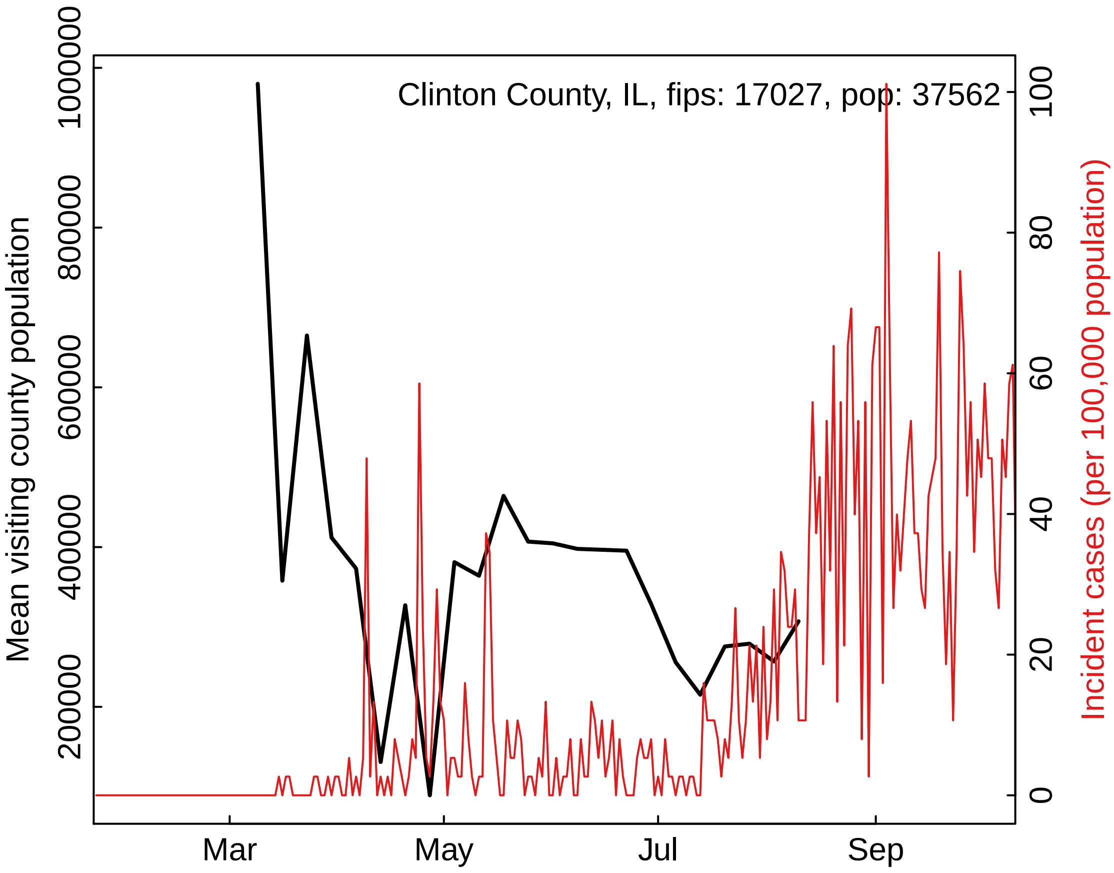
Clinton County, IL, fips: 17027, pop: 37562
Mean visiting county population
200000
400000
600000
800000
1000000
Incident cases (per 100,000 population)
0
20
40
60
80
100
Mar
May
Jul
Sep

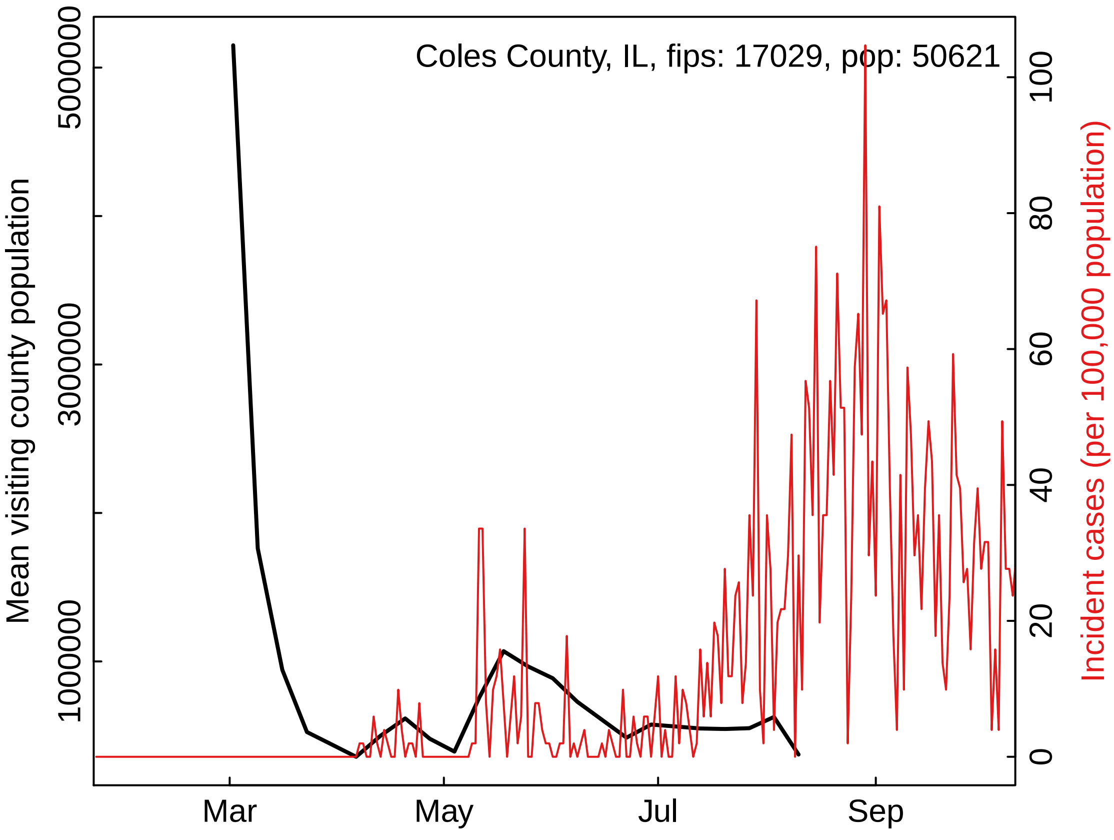

Coles County, IL, fips: 17029, pop: 50621
Mean visiting county population
1000000
3000000
5000000
Incident cases (per 100,000 population)
0
20
40
60
80
100
Mar
May
Jul
Sep

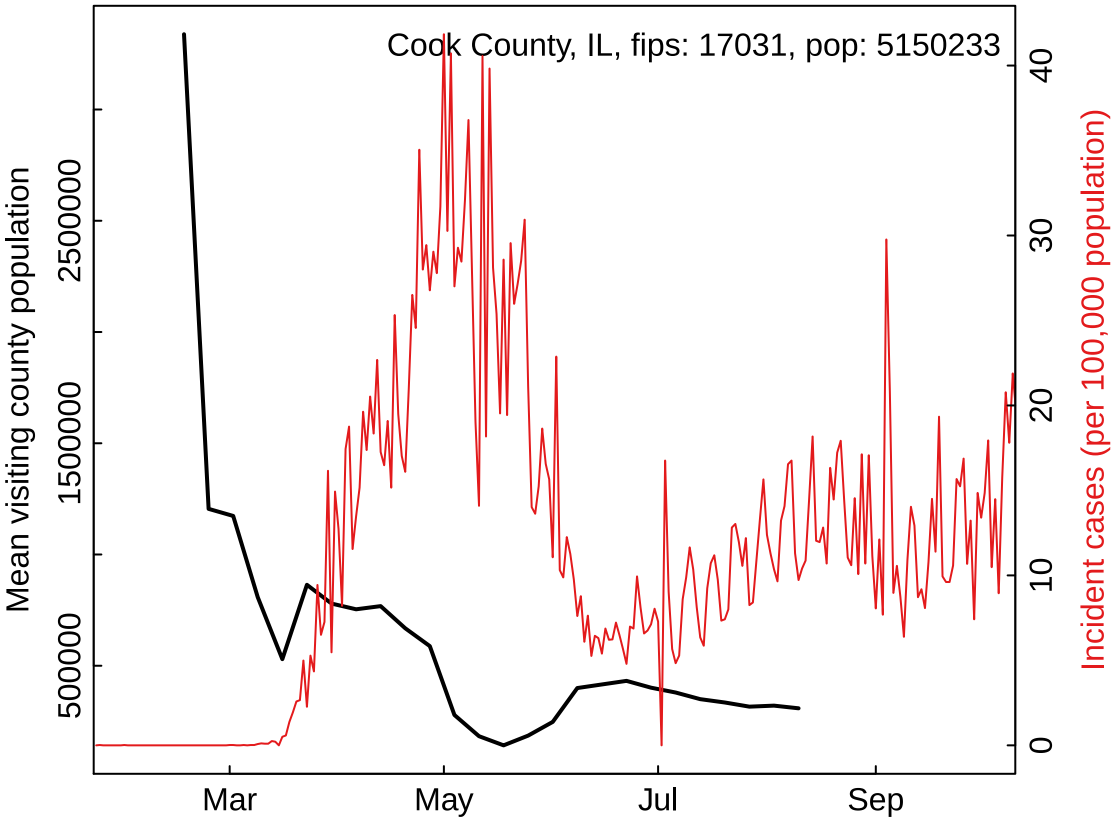

Cook County, IL, fips: 17031, pop: 5150233
Mean visiting county population
500000
1500000
2500000
Incident cases (per 100,000 population)
0
10
20
30
40
Mar
May
Jul
Sep

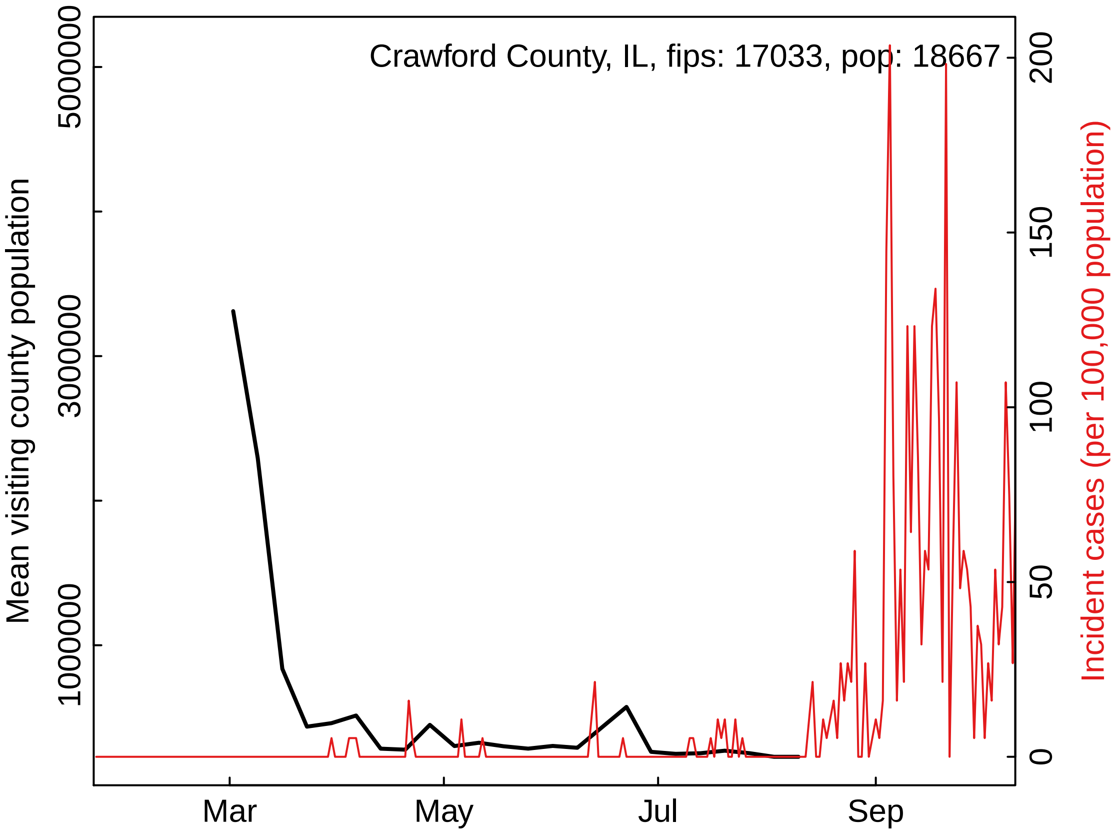
Crawford County, IL, fips: 17033, pop: 18667
Mean visiting county population
Incident cases (per 100,000 population)
1000000
3000000
5000000
0
50
100
150
200
Mar
May
Jul
Sep

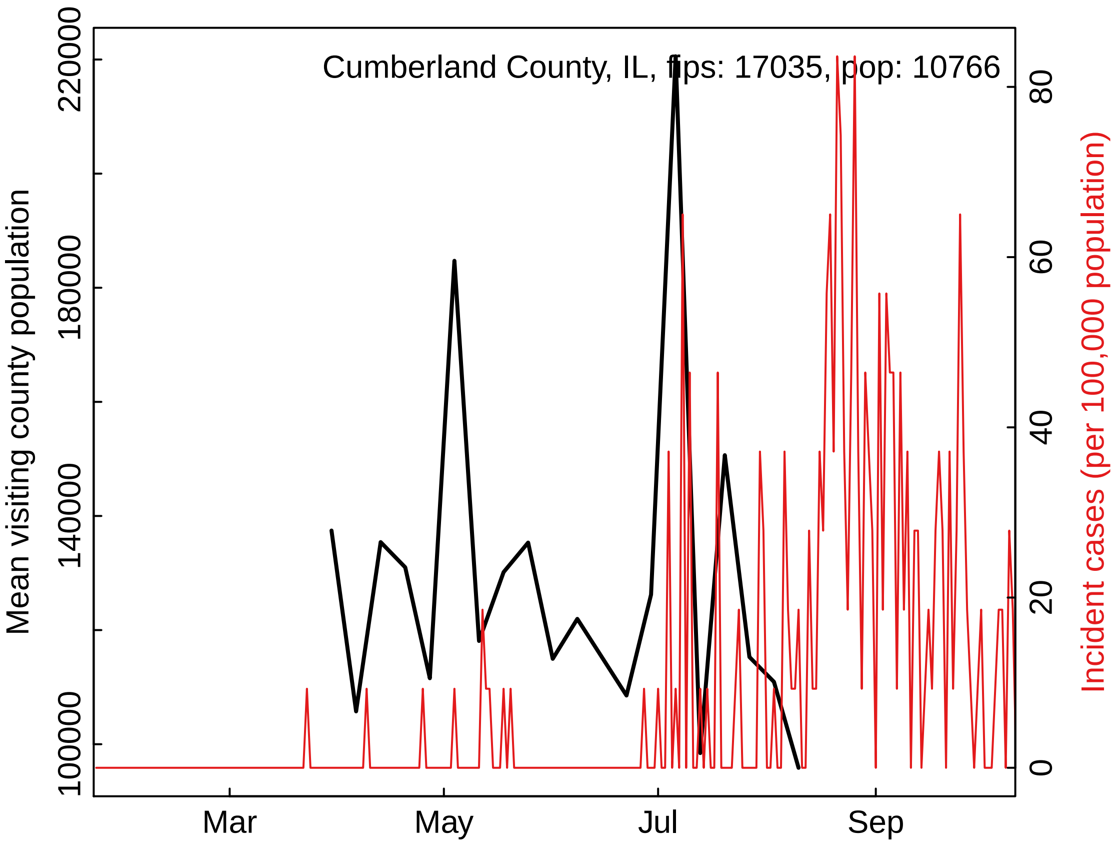

Cumberland County, IL, fips: 17035, pop: 10766
Mean visiting county population
100000
140000
180000
220000
Incident cases (per 100,000 population)
0
20
40
60
80
Mar
May
Jul
Sep

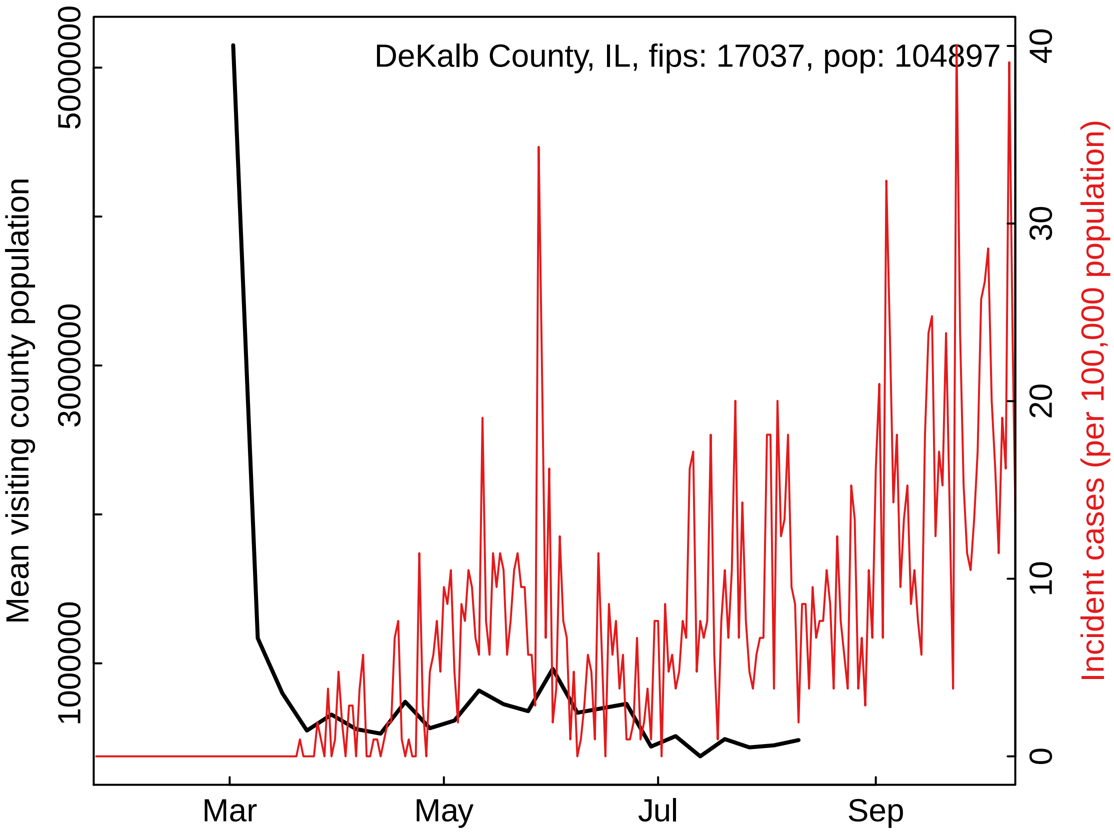

DeKalb County, IL, fips: 17037, pop: 104897
Mean visiting county population
1000000
3000000
5000000
Incident cases (per 100,000 population)
0
10
20
30
40
Mar
May
Jul
Sep

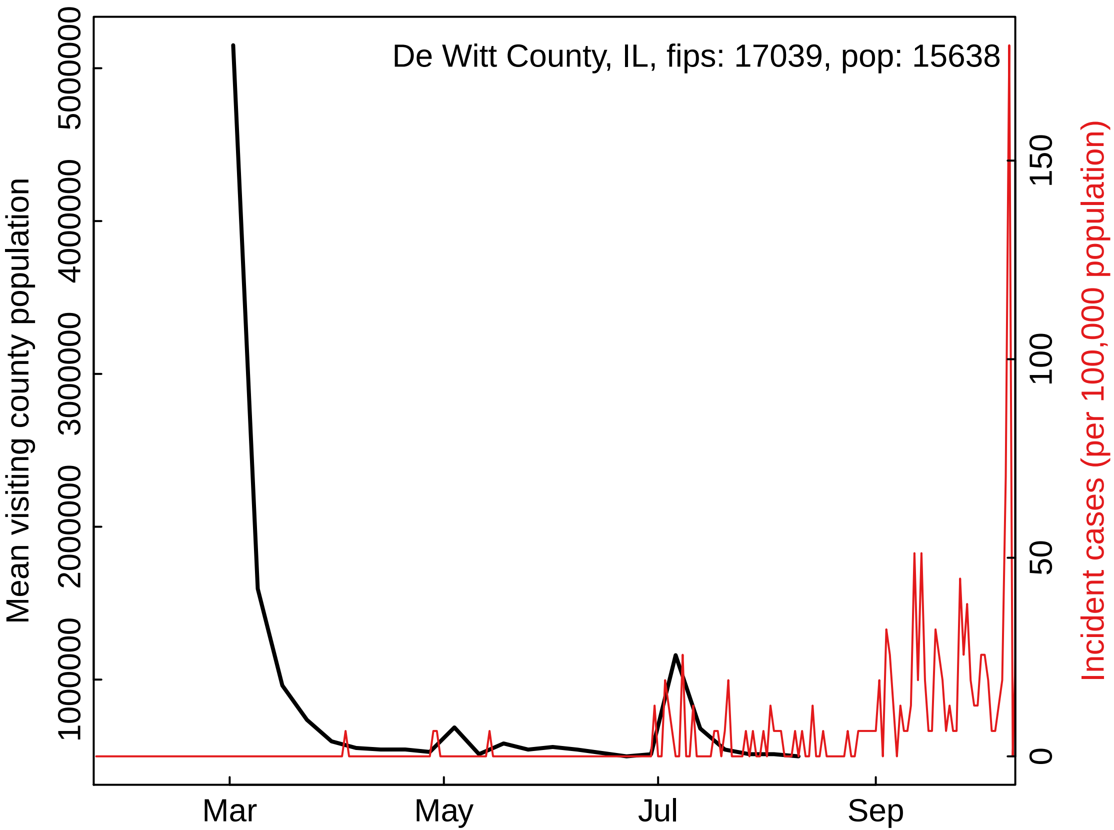

De Witt County, IL, fips: 17039, pop: 15638
Mean visiting county population
1000000
2000000
3000000
4000000
5000000
Incident cases (per 100,000 population)
0
50
100
150
Mar
May
Jul
Sep

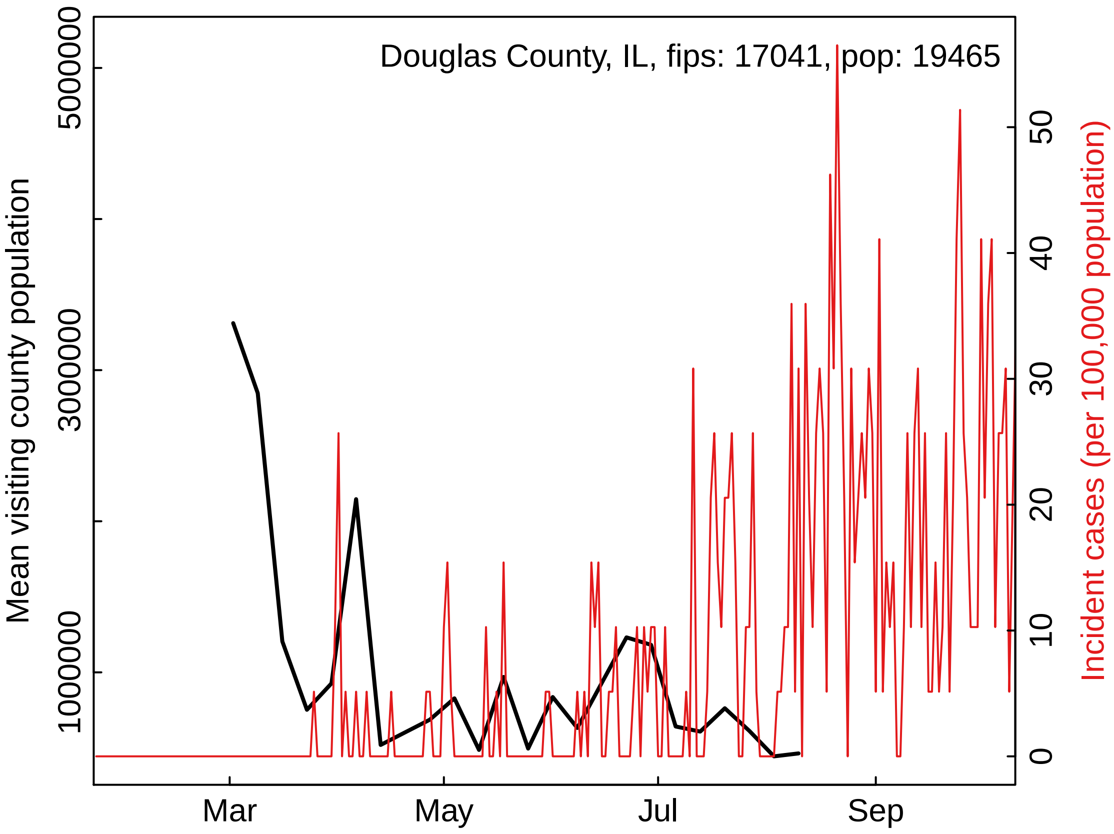
Douglas County, IL, fips: 17041, pop: 19465
Mean visiting county population
10000000
30000000
50000000
Incident cases (per 100,000 population)
0
10
20
30
40
50
Mar
May
Jul
Sep

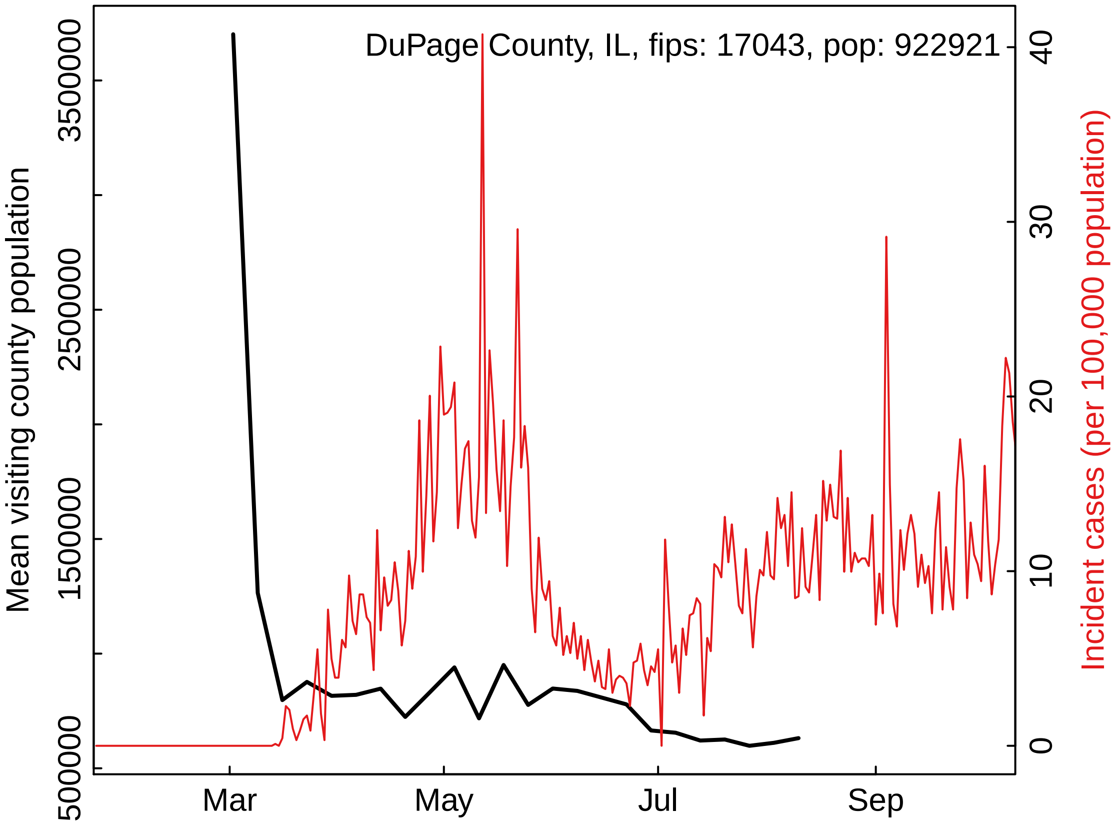

DuPage County, IL, fips: 17043, pop: 922921
Mean visiting county population
Incident cases (per 100,000 population)
5000000
15000000
25000000
35000000
0
10
20
30
40
Mar
May
Jul
Sep

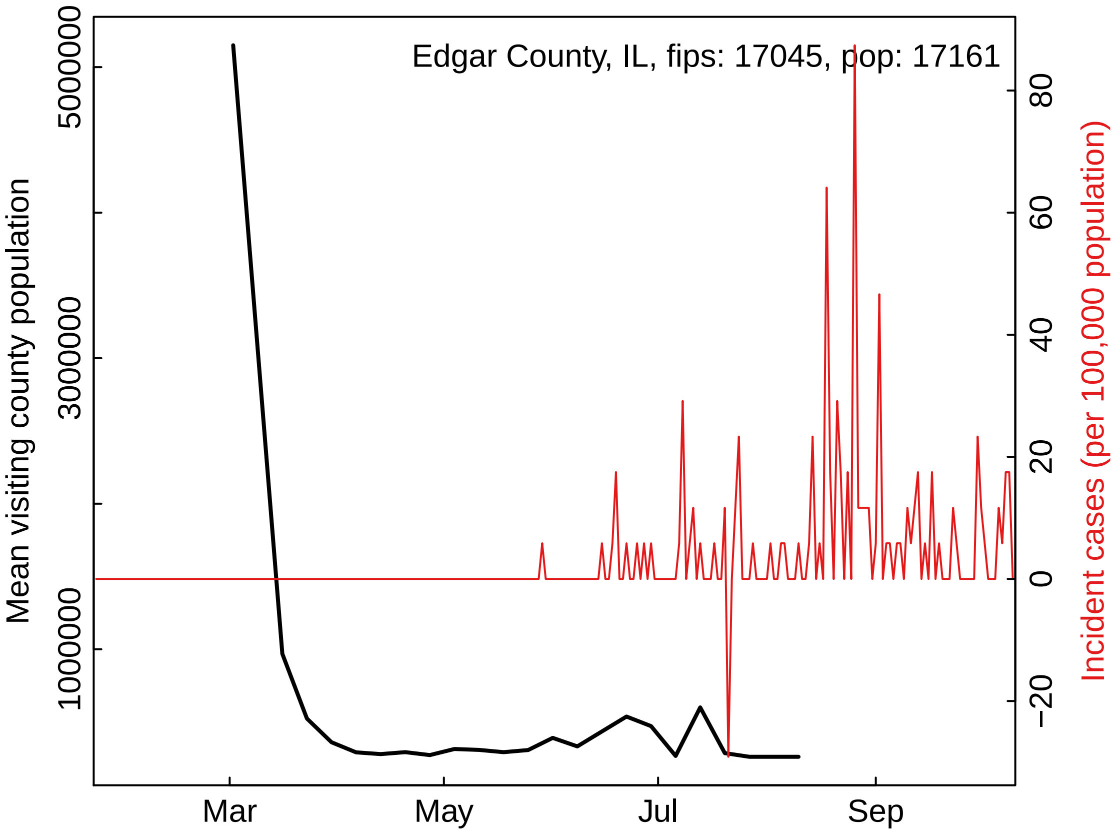

Edgar County, IL, fips: 17045, pop: 17161
Mean visiting county population
1000000
3000000
5000000
Incident cases (per 100,000 population)
−20
0
20
40
60
80
Mar
May
Jul
Sep

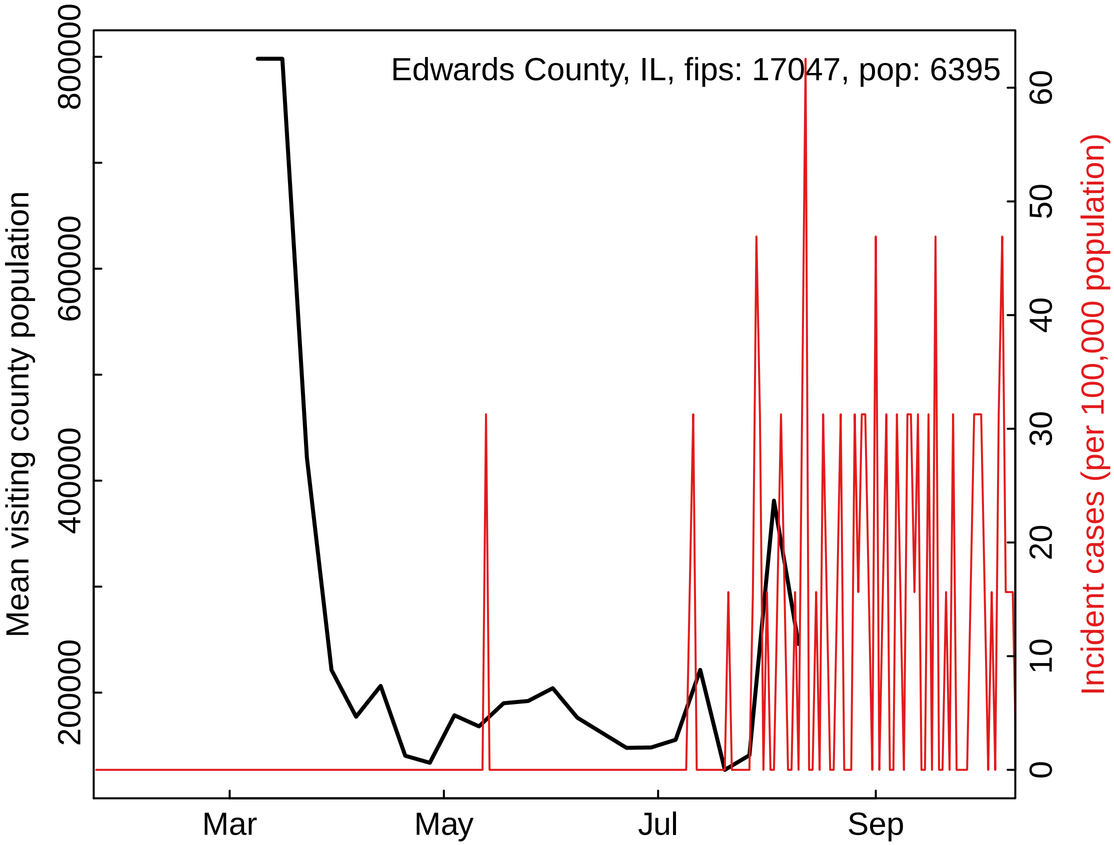

Edwards County, IL, fips: 17047, pop: 6395
Mean visiting county population
Incident cases (per 100,000 population)
200000
400000
600000
800000
0
10
20
30
40
50
60
Mar
May
Jul
Sep

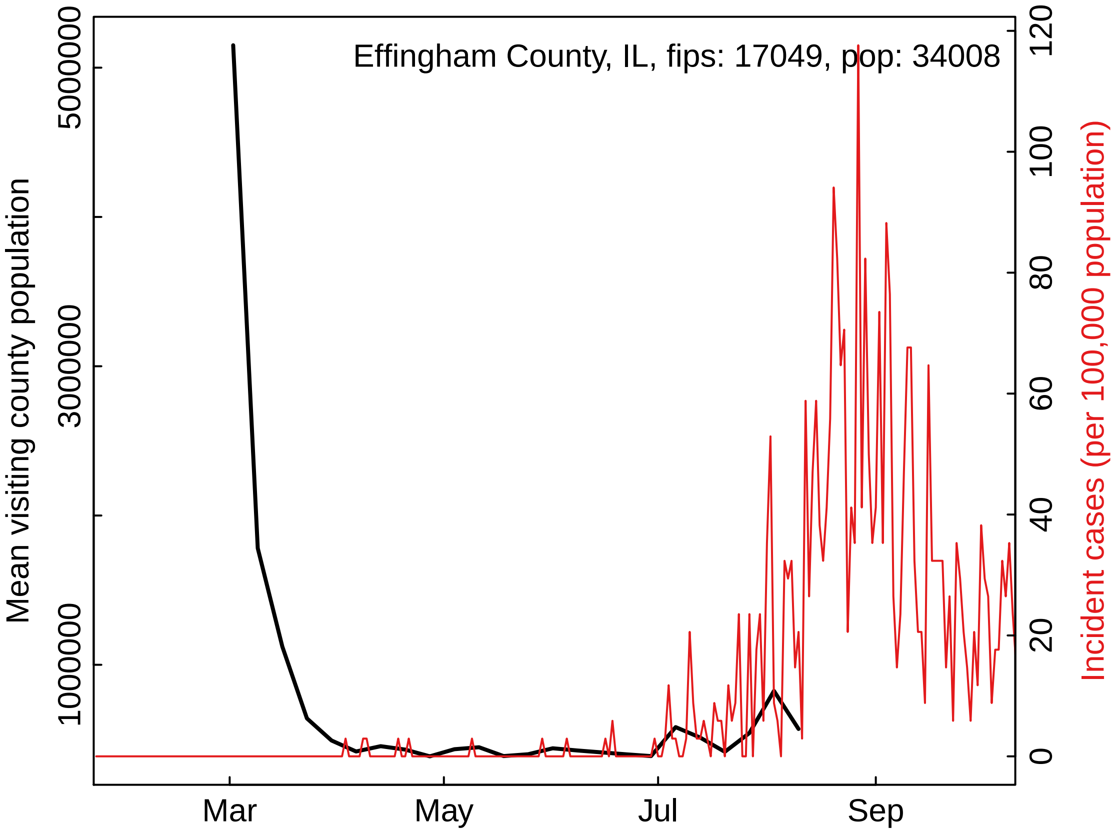

Effingham County, IL, fips: 17049, pop: 34008
Mean visiting county population
1000000
3000000
5000000
Incident cases (per 100,000 population)
0
20
40
60
80
100
120
Mar
May
Jul
Sep

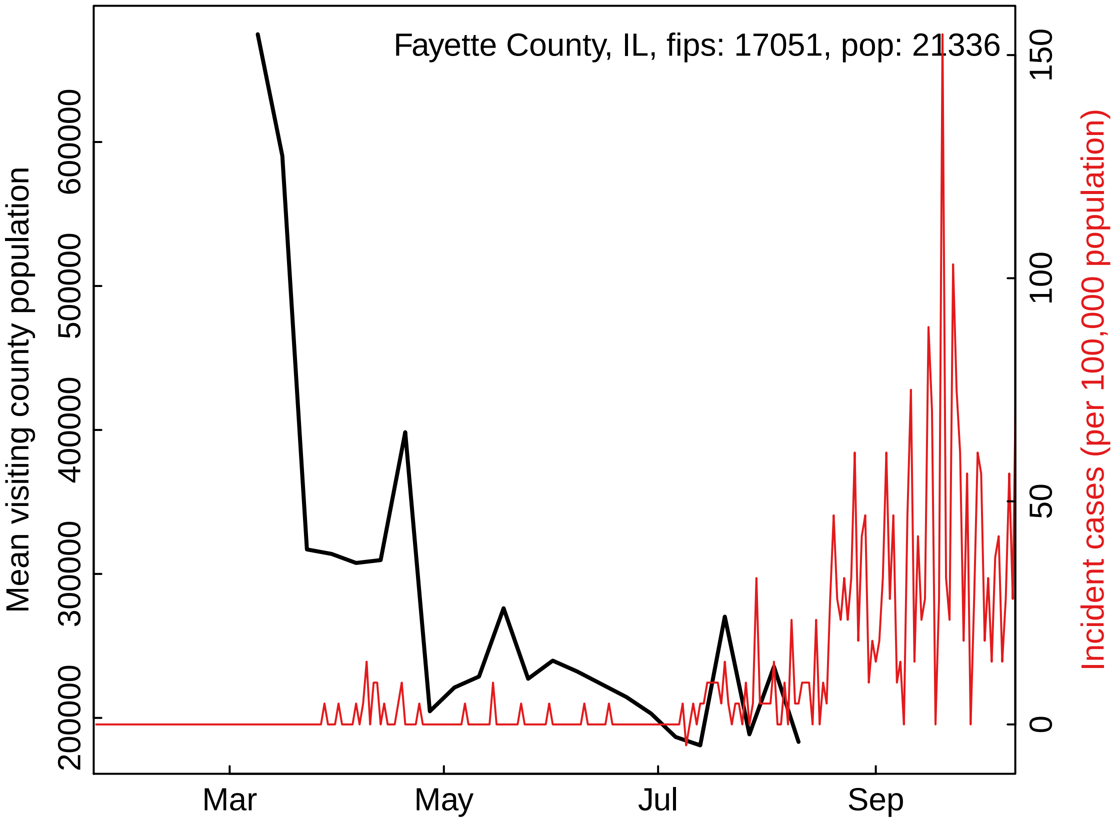

Fayette County, IL, fips: 17051, pop: 21336
Mean visiting county population
200000
300000
400000
500000
600000
Incident cases (per 100,000 population)
0
50
100
150
Mar
May
Jul
Sep

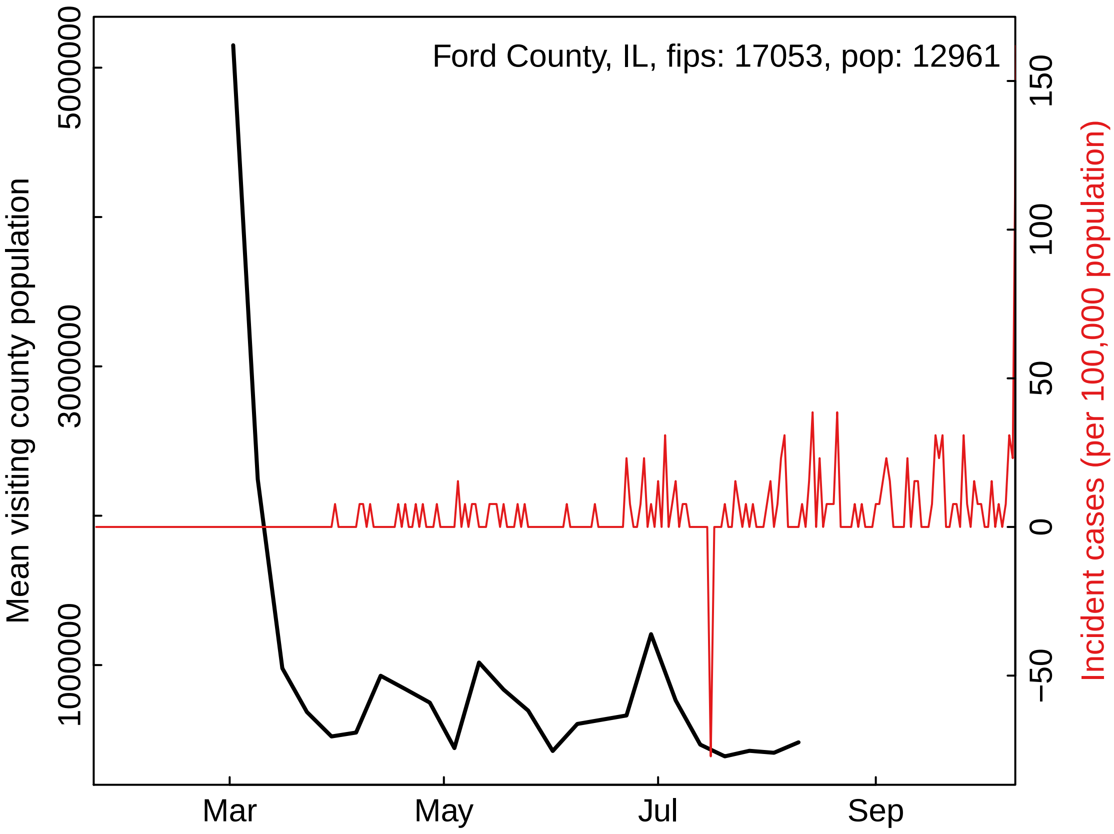

Ford County, IL, fips: 17053, pop: 12961
Mean visiting county population
1000000
3000000
5000000
Mar
May
Jul
Sep
Incident cases (per 100,000 population)
−50
0
50
100
150

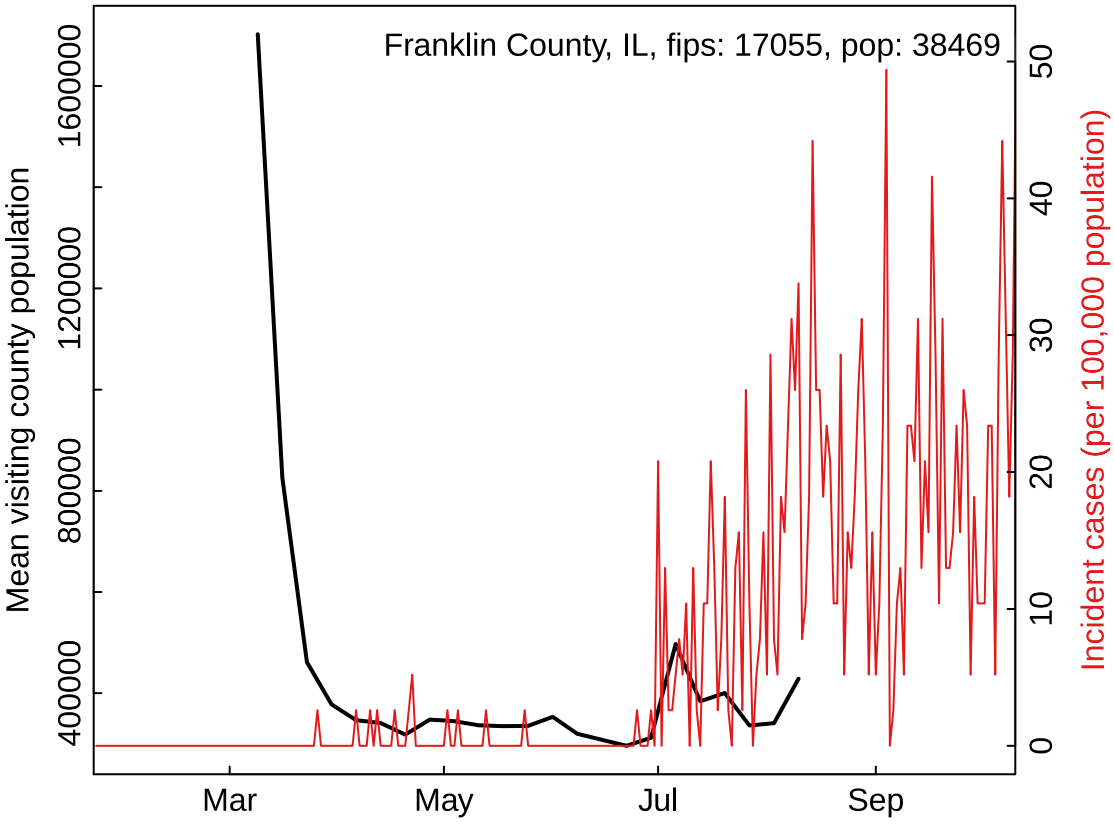

Franklin County, IL, fips: 17055, pop: 38469
Mean visiting county population
400000
800000
1200000
1600000
Incident cases (per 100,000 population)
0
10
20
30
40
50
Mar
May
Jul
Sep

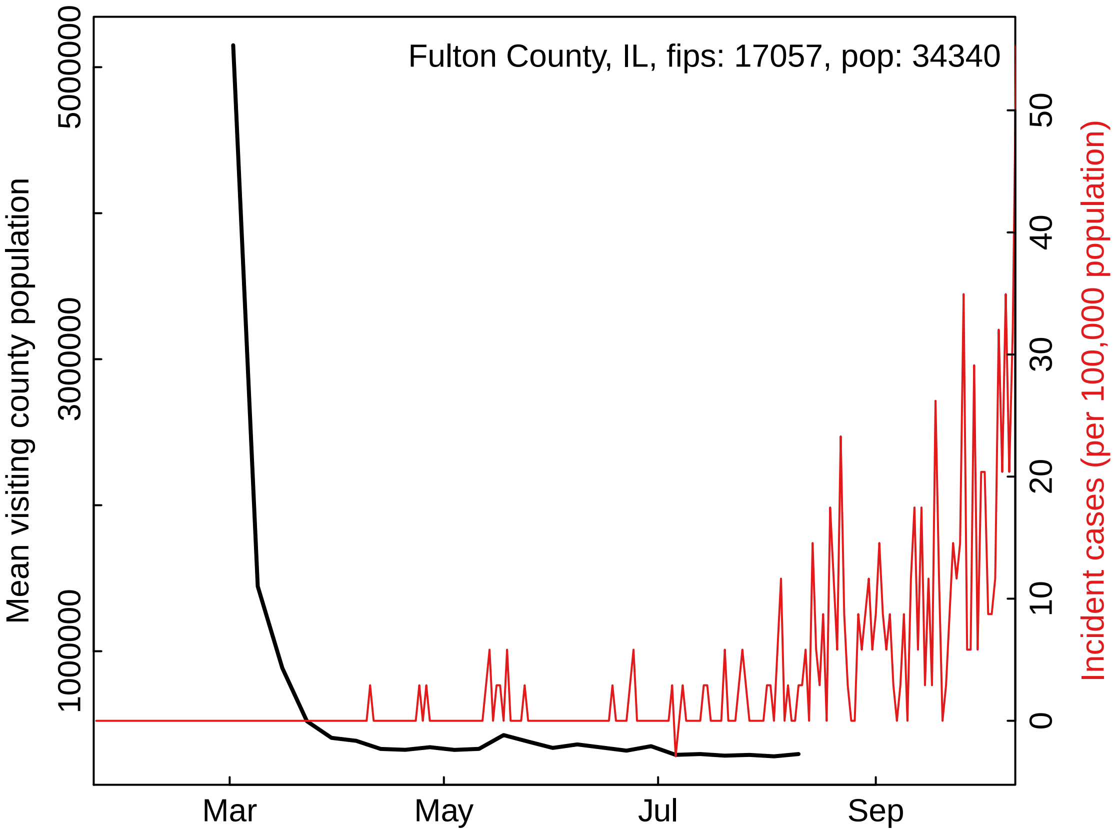

Fulton County, IL, fips: 17057, pop: 34340
Mean visiting county population
1000000
3000000
5000000
Incident cases (per 100,000 population)
0
10
20
30
40
50
Mar
May
Jul
Sep

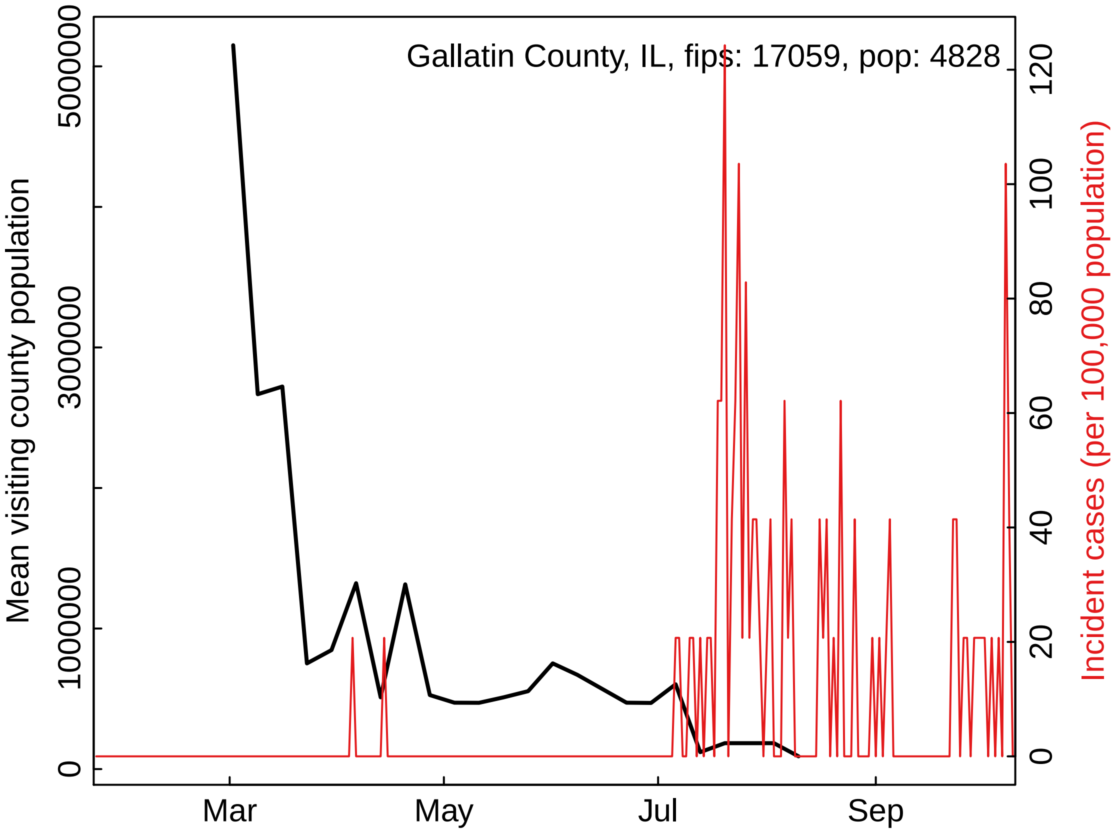

Gallatin County, IL, fips: 17059, pop: 4828
Mean visiting county population
Incident cases (per 100,000 population)
0
1000000
3000000
5000000
0
20
40
60
80
100
120
Mar
May
Jul
Sep

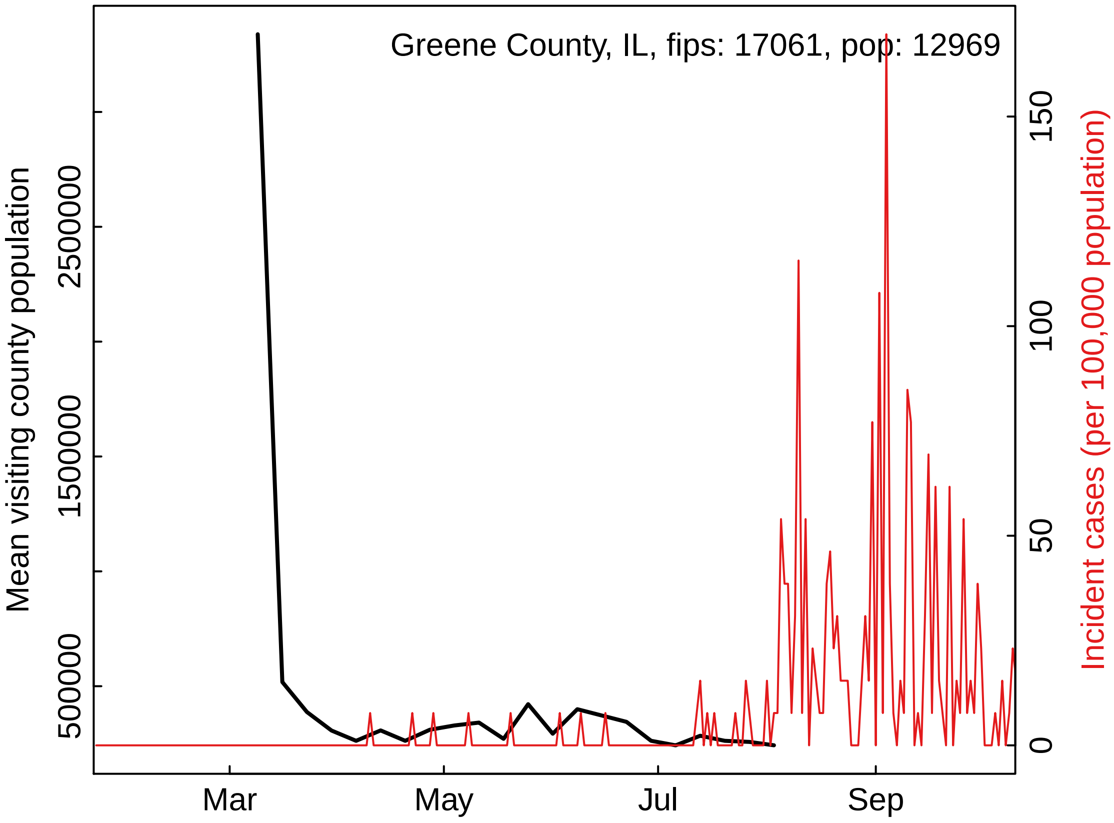

Greene County, IL, fips: 17061, pop: 12969
Mean visiting county population
500000
1500000
2500000
Incident cases (per 100,000 population)
0
50
100
150
Mar
May
Jul
Sep

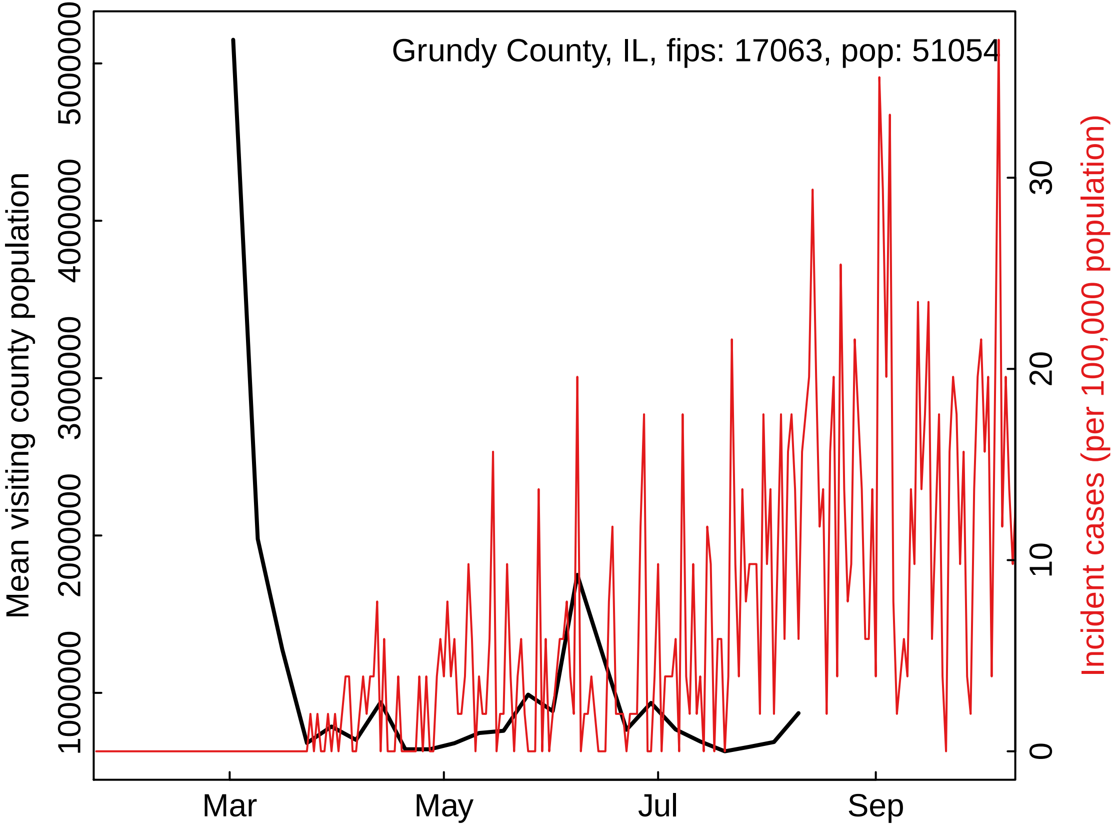

Grundy County, IL, fips: 17063, pop: 51054
Mean visiting county population
Incident cases (per 100,000 population)
10000000
20000000
30000000
40000000
50000000
0
10
20
30
Mar
May
Jul
Sep

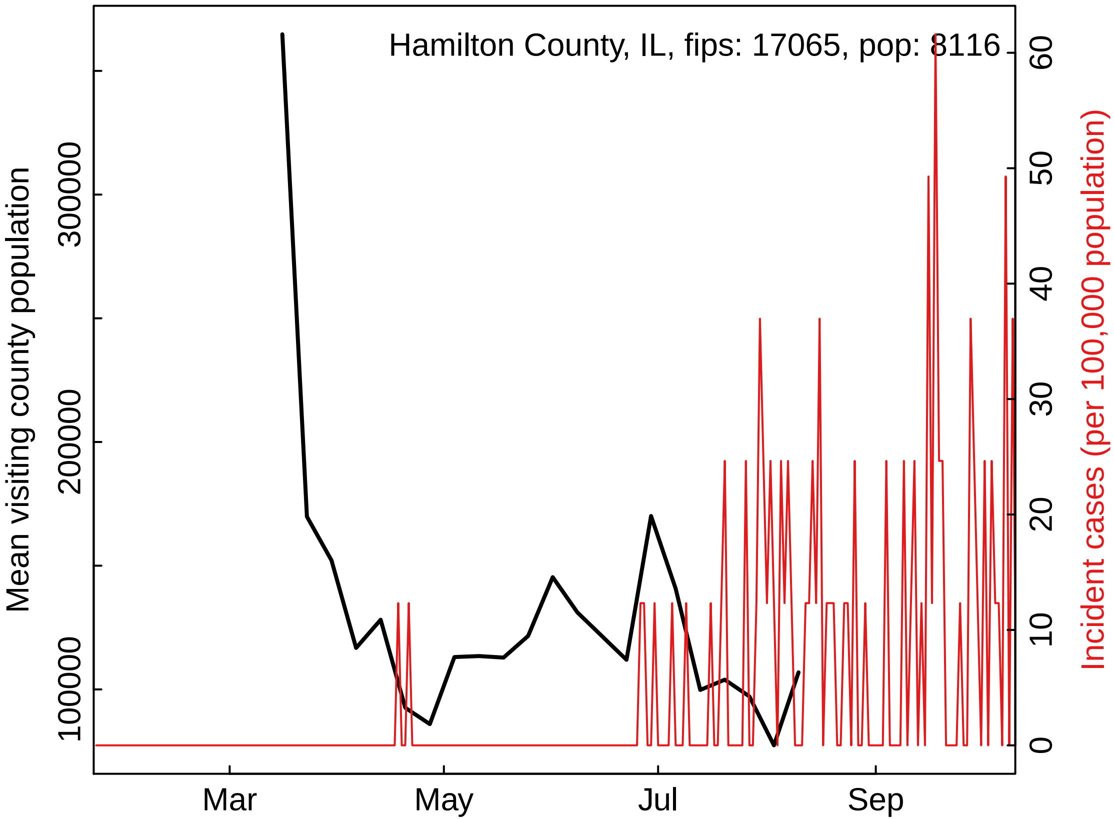

Hamilton County, IL, fips: 17065, pop: 8116
Mean visiting county population
100000
200000
300000
Incident cases (per 100,000 population)
0
10
20
30
40
50
60
Mar
May
Jul
Sep

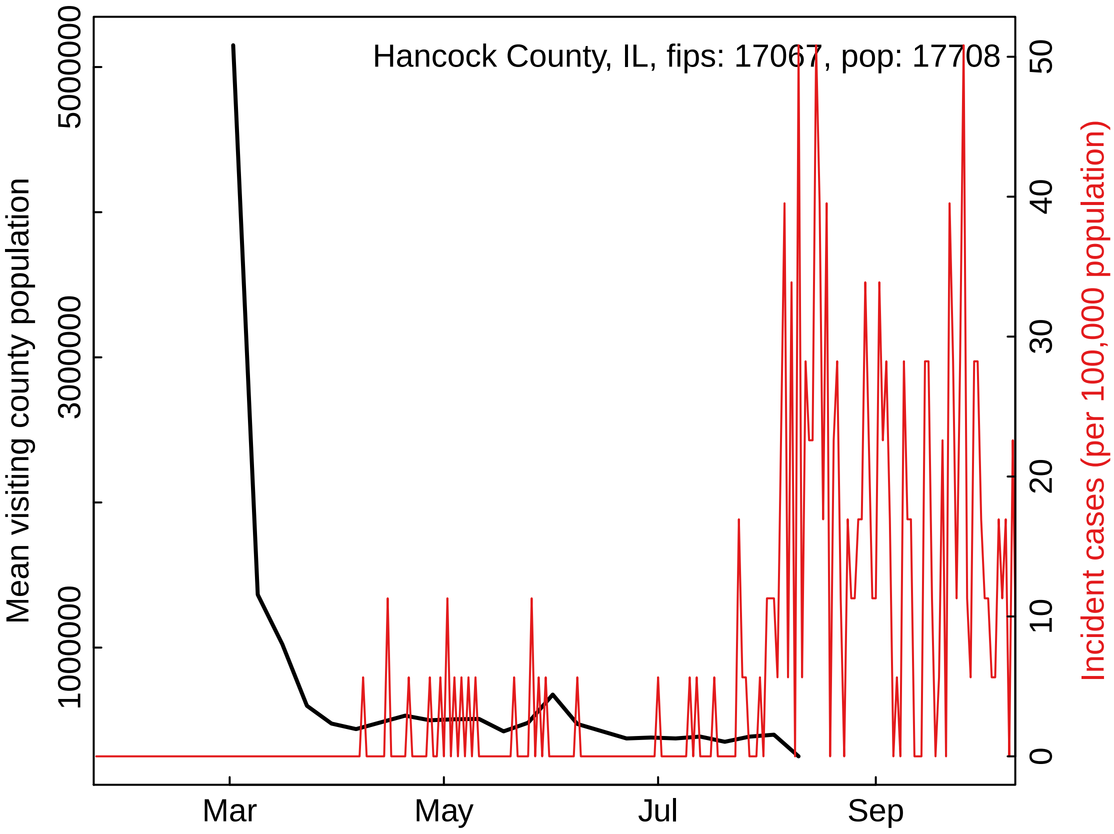

Hancock County, IL, fips: 17067, pop: 17708
Mean visiting county population
Incident cases (per 100,000 population)
1000000
3000000
5000000
0
10
20
30
40
50
Mar
May
Jul
Sep

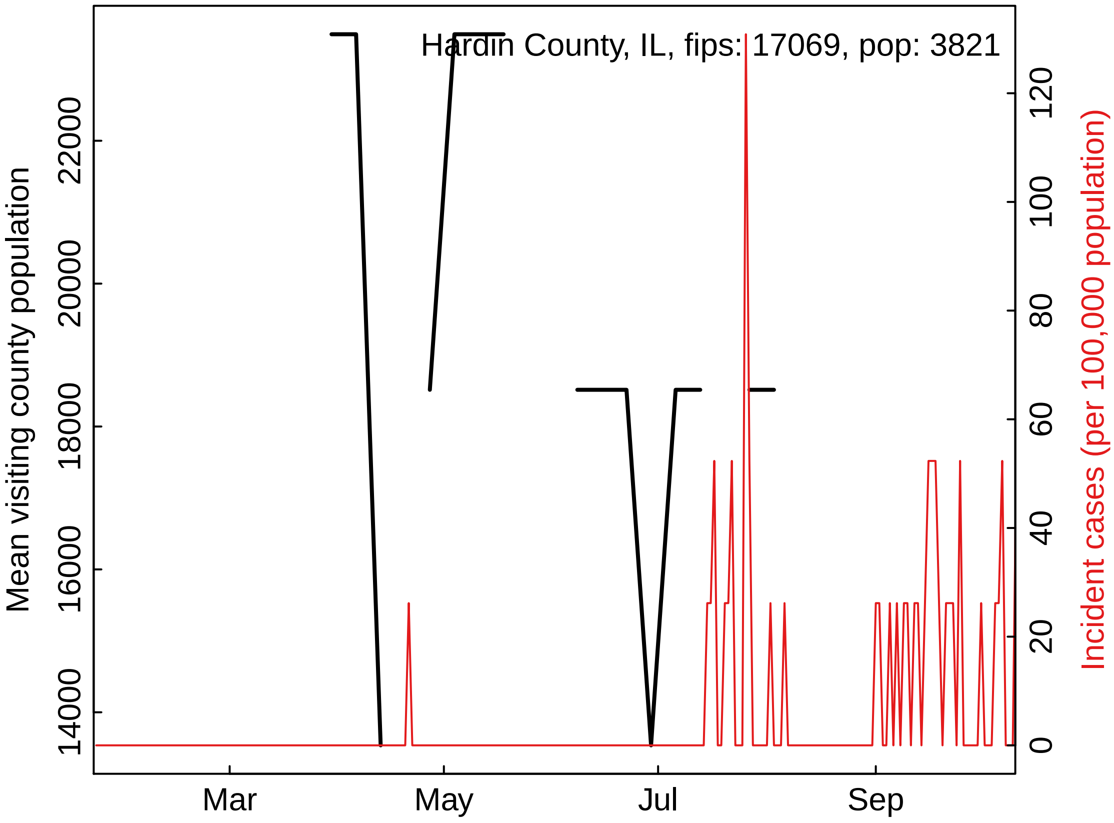

Hardin County, IL, fips: 17069, pop: 3821
Mean visiting county population
14000
16000
18000
20000
22000
Incident cases (per 100,000 population)
0
20
40
60
80
100
120
Mar
May
Jul
Sep

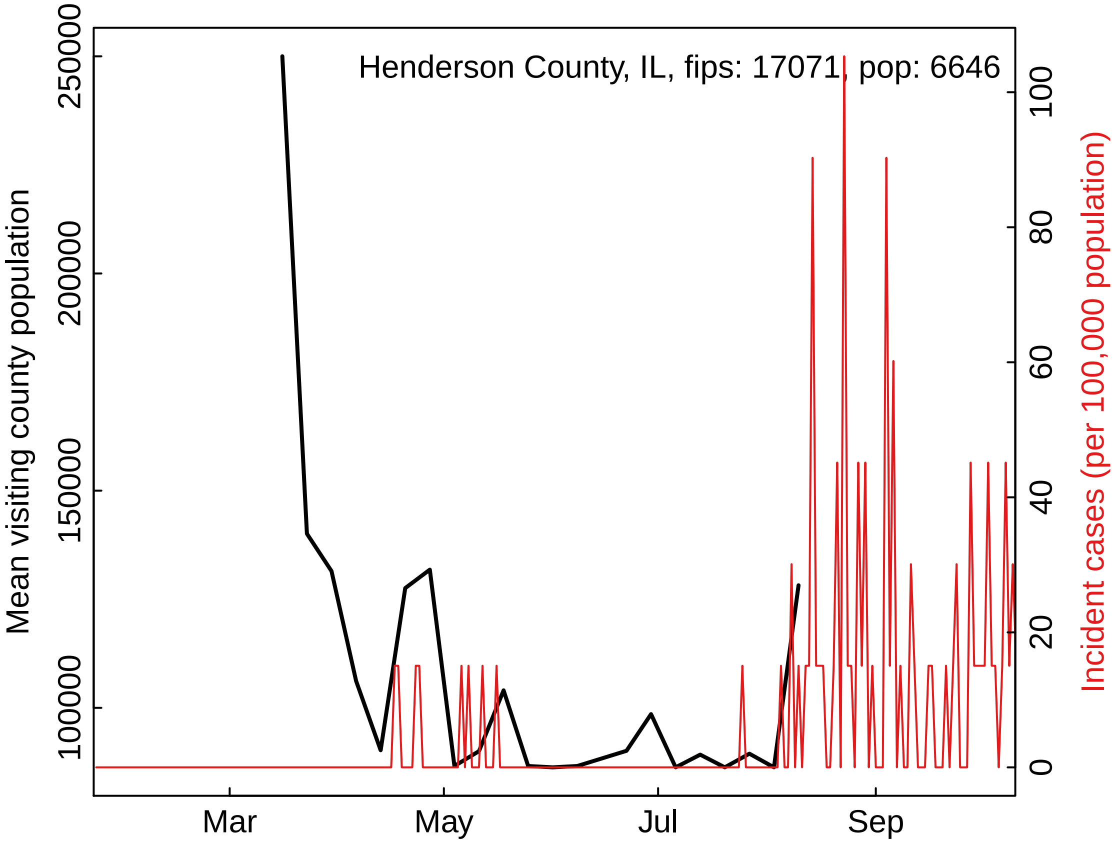

Henderson County, IL, fips: 17071, pop: 6646
Mean visiting county population
Incident cases (per 100,000 population)
100000
150000
200000
250000
0
20
40
60
80
100
Mar
May
Jul
Sep

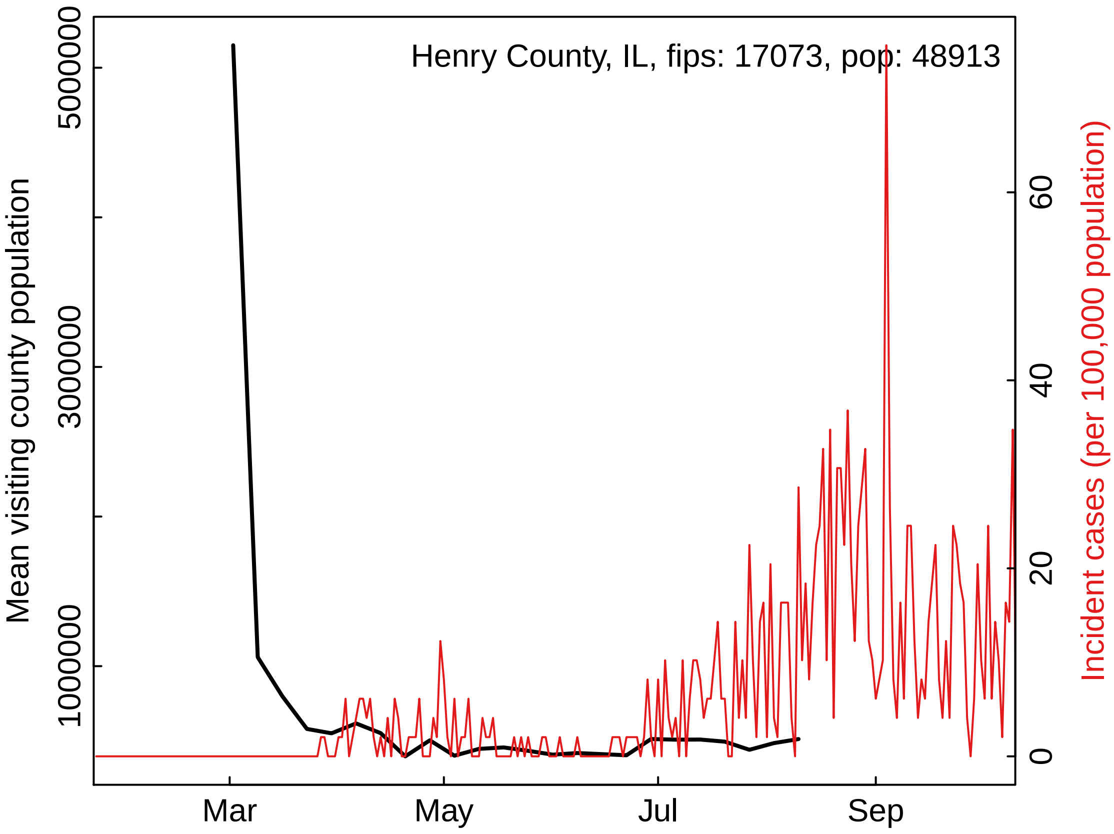

Henry County, IL, fips: 17073, pop: 48913
Mean visiting county population
1000000
3000000
5000000
Incident cases (per 100,000 population)
0
20
40
60
Mar
May
Jul
Sep

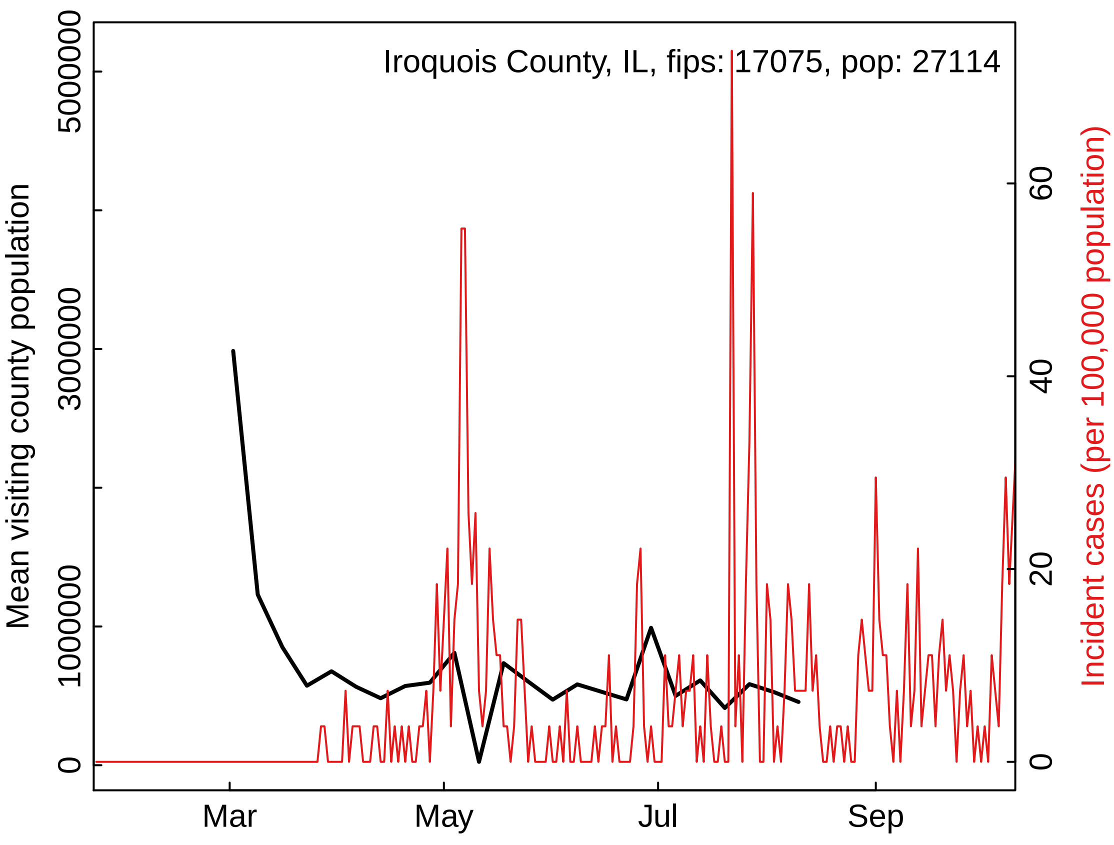

Iroquois County, IL, fips: 17075, pop: 27114
Mean visiting county population
0
1000000
3000000
5000000
Incident cases (per 100,000 population)
0
20
40
60
Mar
May
Jul
Sep

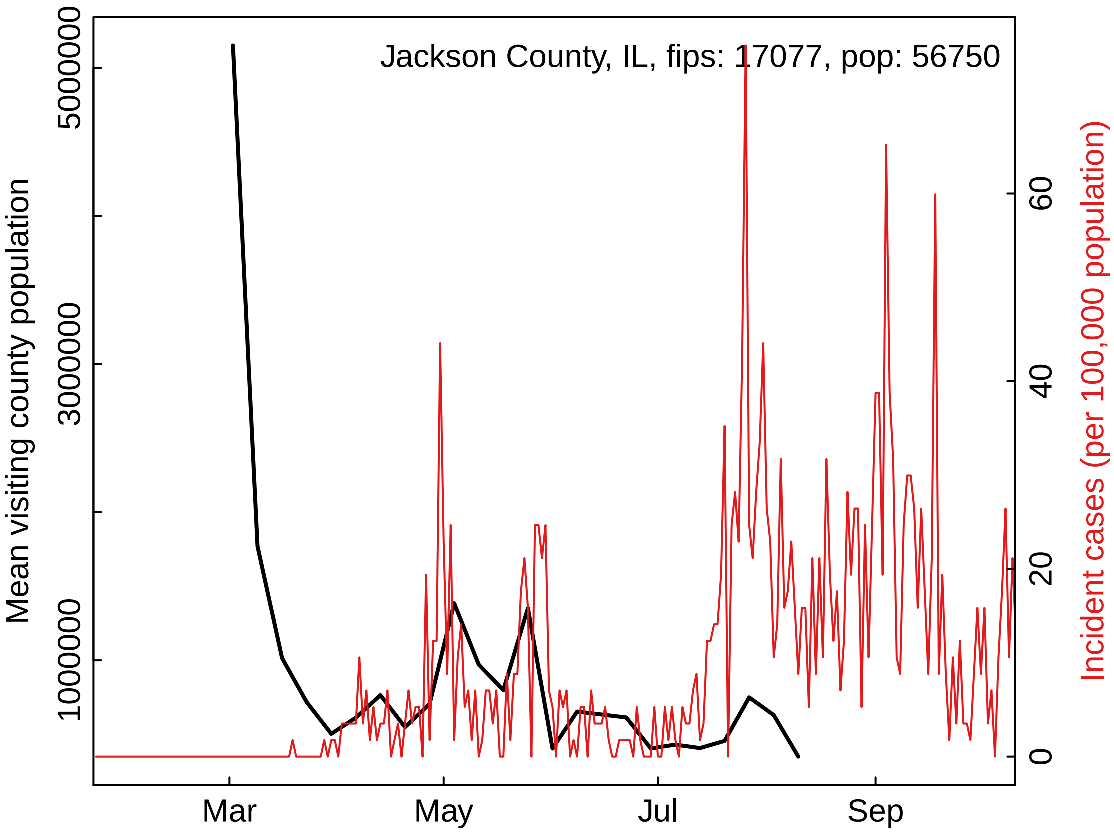

Jackson County, IL, fips: 17077, pop: 56750
Mean visiting county population
1000000
3000000
5000000
Incident cases (per 100,000 population)
0
20
40
60
Mar
May
Jul
Sep

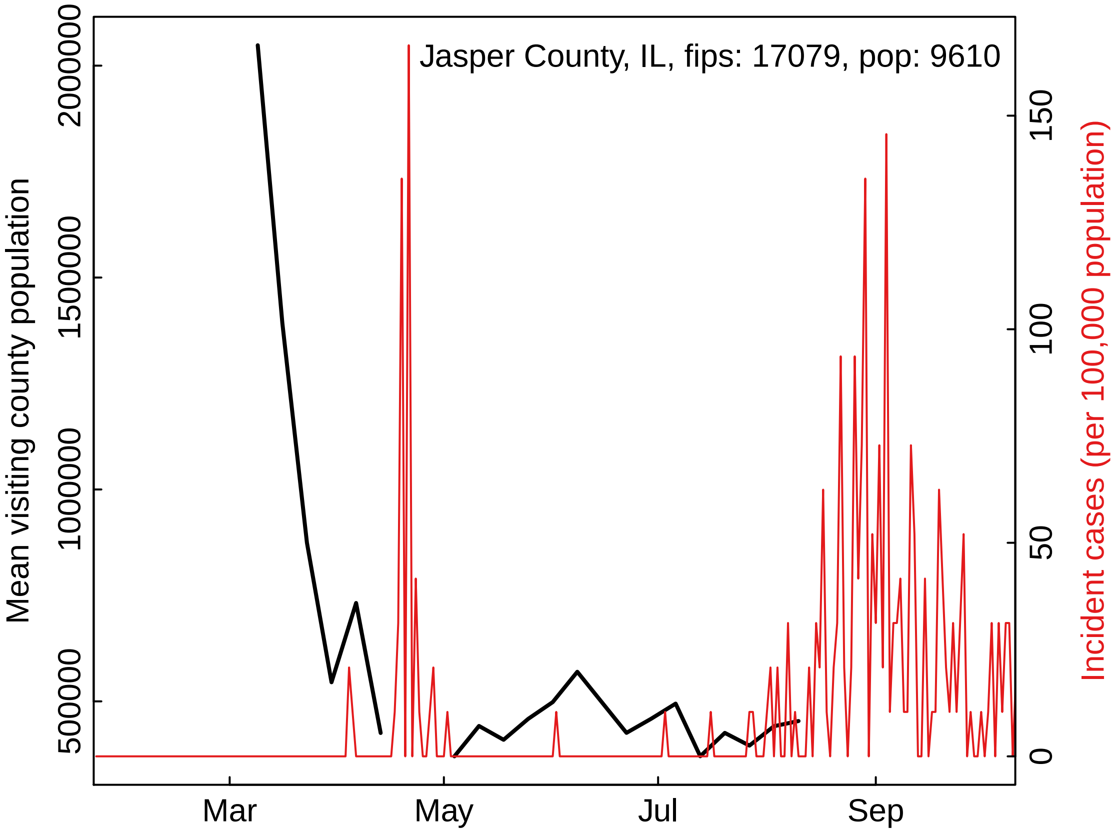
Jasper County, IL, fips: 17079, pop: 9610
Mean visiting county population
5000000
1000000
1500000
2000000
Incident cases (per 100,000 population)
0
50
100
150
Mar
May
Jul
Sep

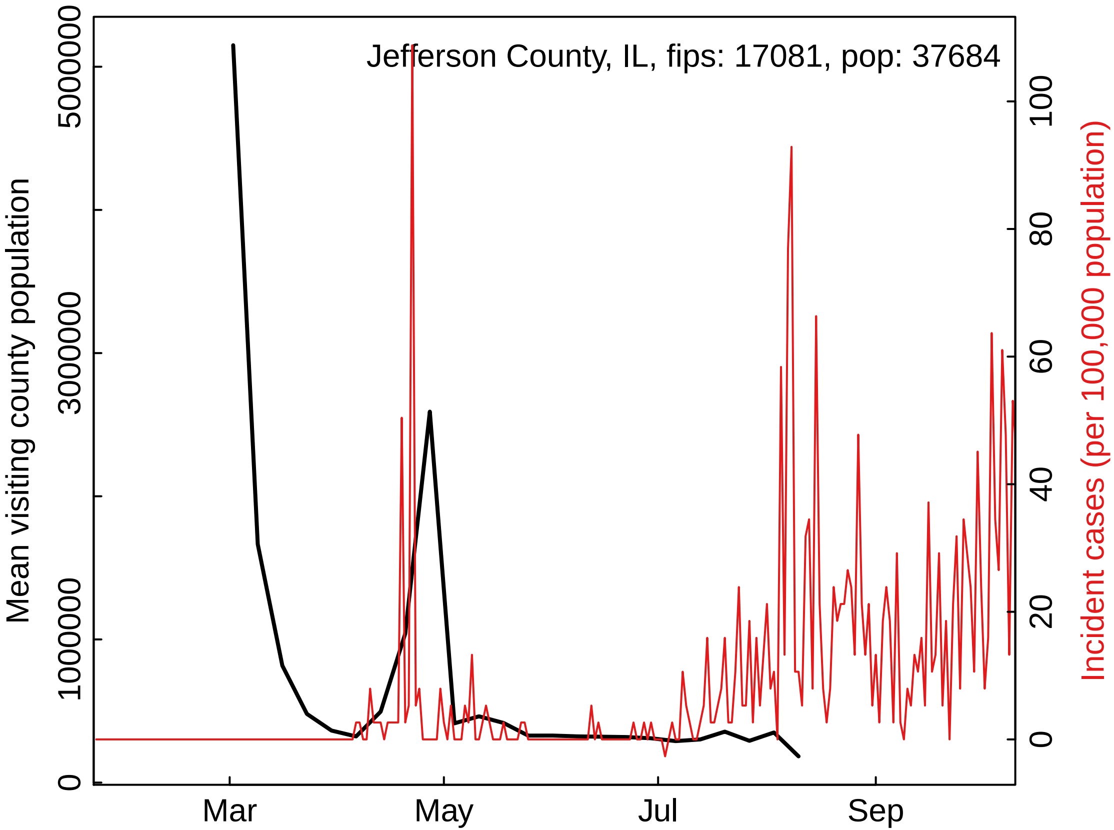
Jefferson County, IL, fips: 17081, pop: 37684
Mean visiting county population
0
1000000
3000000
5000000
Incident cases (per 100,000 population)
0
20
40
60
80
100
Mar
May
Jul
Sep

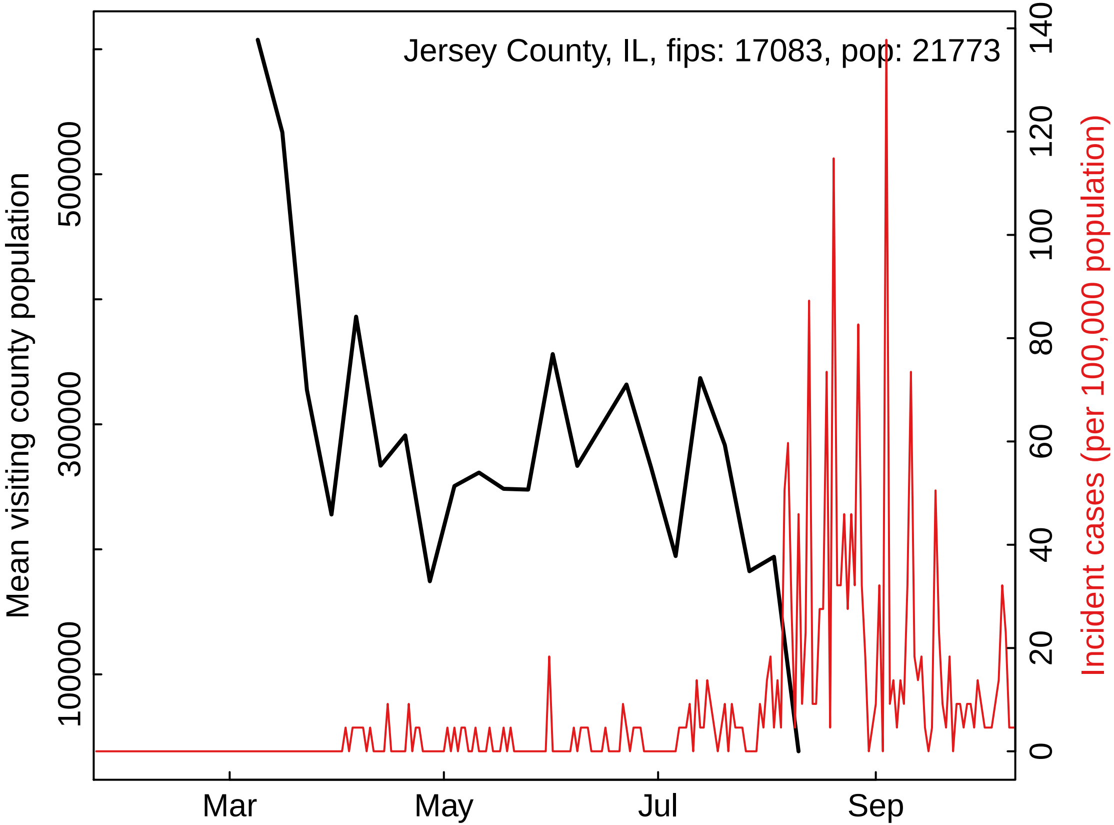
Jersey County, IL, fips: 17083, pop: 21773
Mean visiting county population
100000
300000
500000
Mar
May
Jul
Sep
0
20
40
60
80
100
120
140
Incident cases (per 100,000 population)

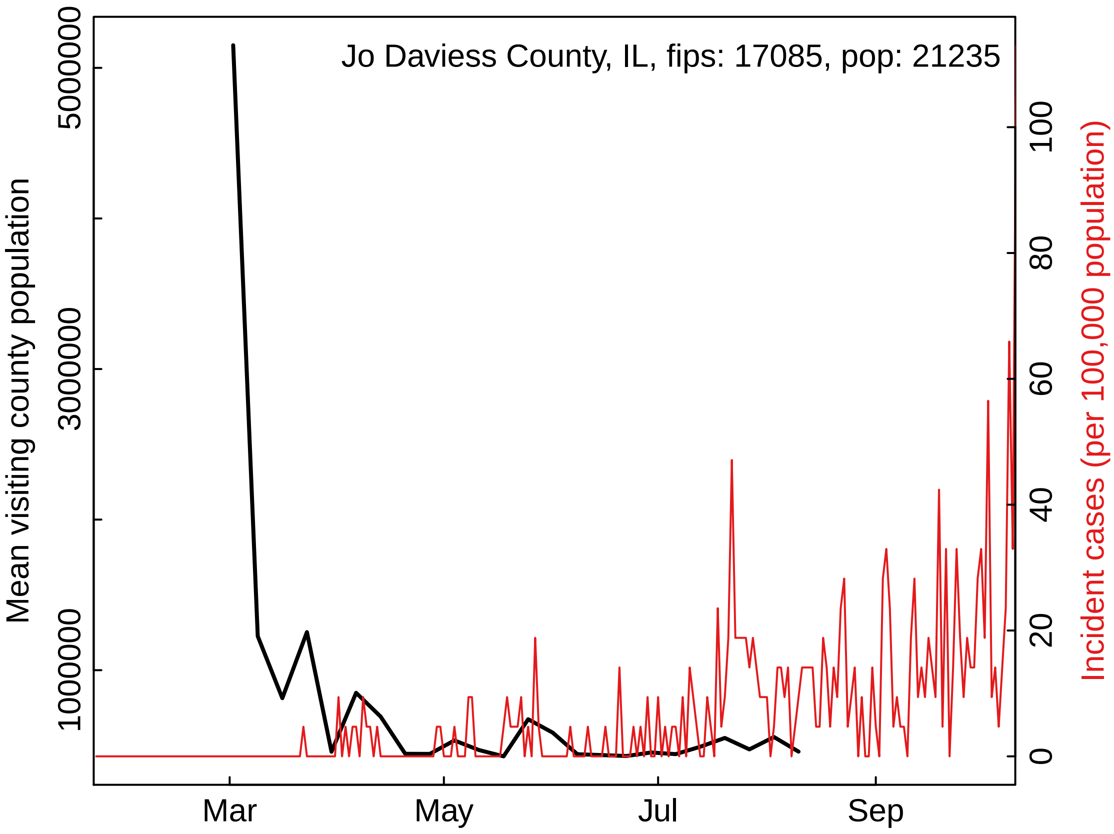

Jo Daviess County, IL, fips: 17085, pop: 21235
Mean visiting county population
Incident cases (per 100,000 population)
1000000
3000000
5000000
0
20
40
60
80
100
Mar
May
Jul
Sep

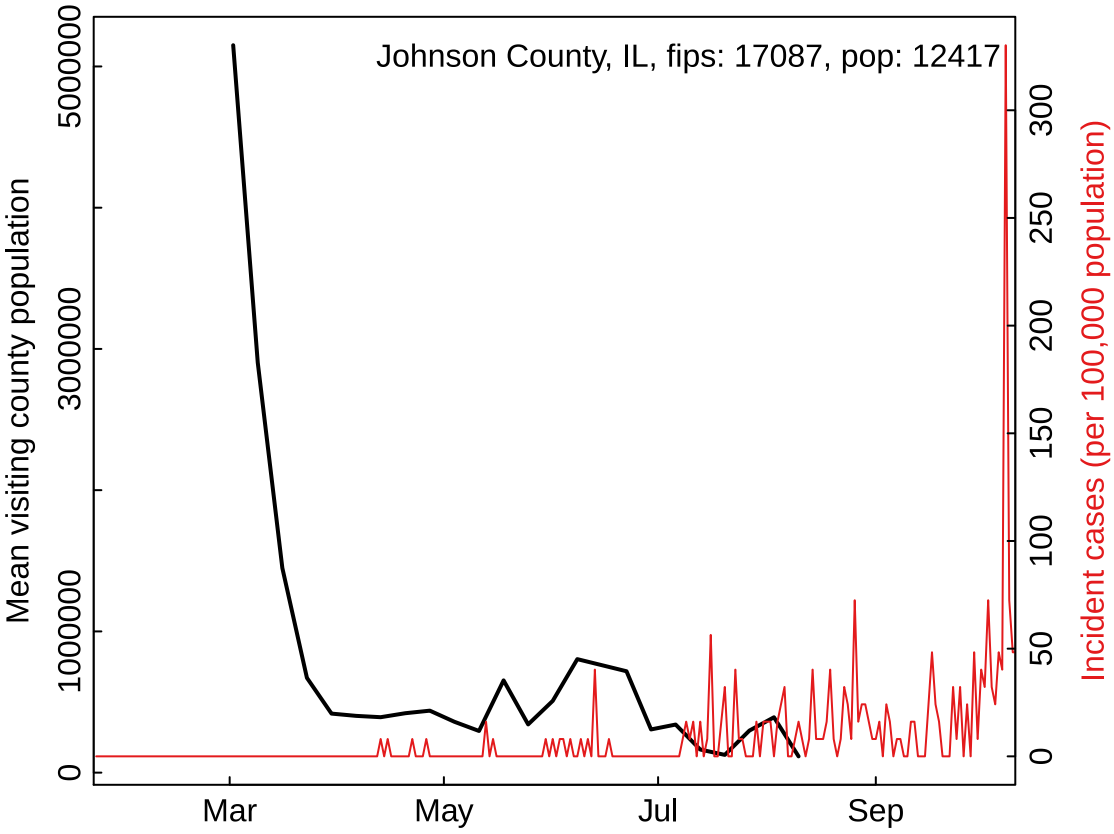

Johnson County, IL, fips: 17087, pop: 12417
Mean visiting county population
Incident cases (per 100,000 population)
0
1000000
3000000
5000000
0
50
100
150
200
250
300
Mar
May
Jul
Sep

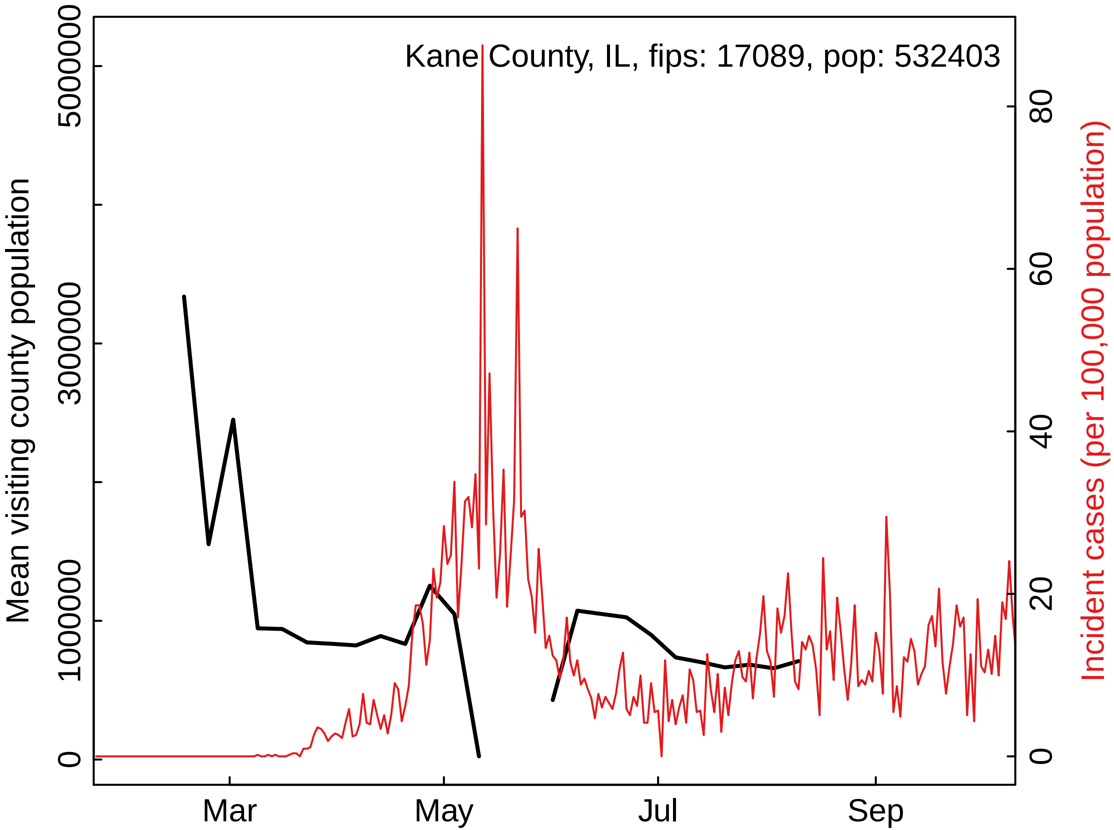

Kane County, IL, fips: 17089, pop: 532403
Mean visiting county population
Incident cases (per 100,000 population)
0
1000000
3000000
5000000
0
20
40
60
80
Mar
May
Jul
Sep

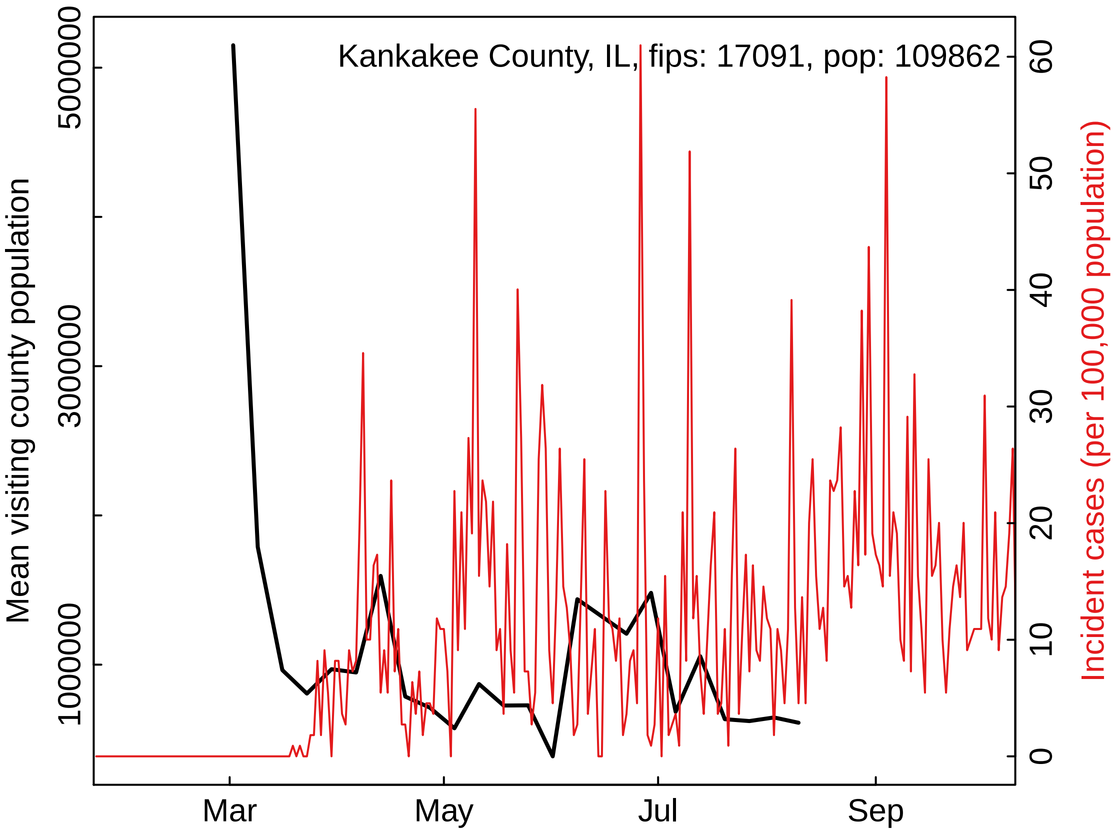

Kankakee County, IL, fips: 17091, pop: 109862
Mean visiting county population
Incident cases (per 100,000 population)
1000000
3000000
5000000
0
10
20
30
40
50
60
Mar
May
Jul
Sep

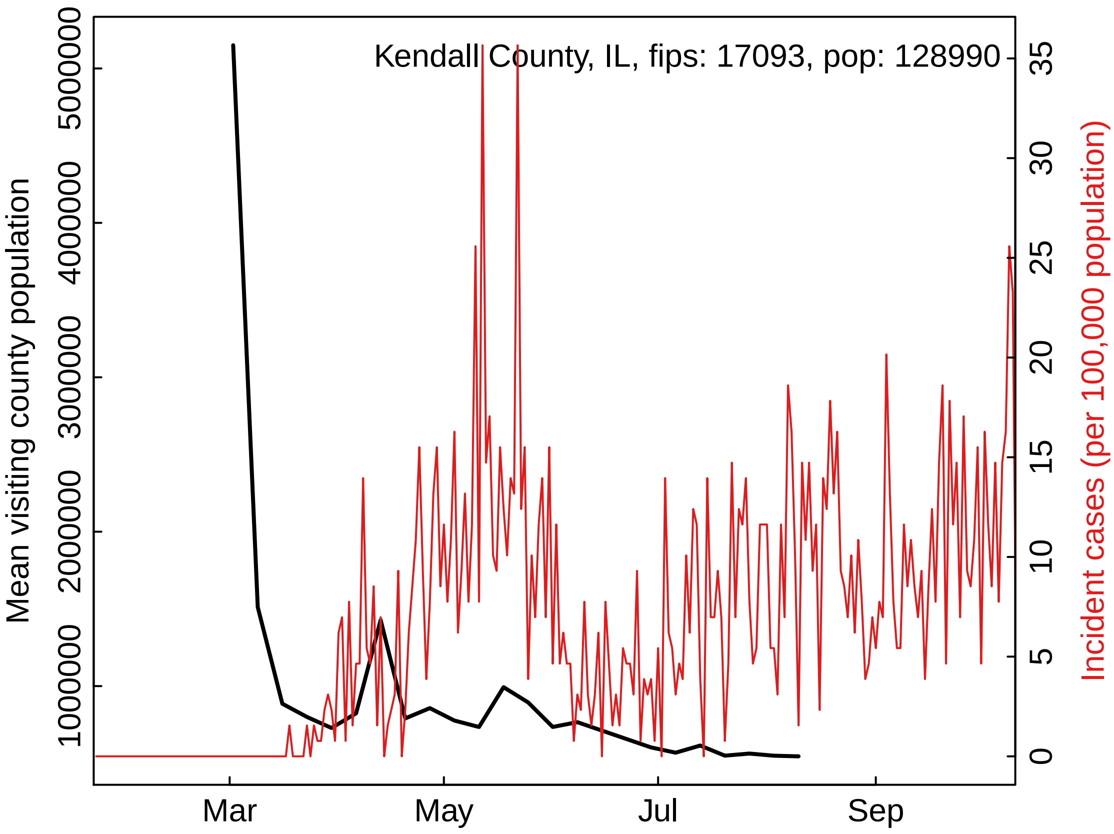

Kendall County, IL, fips: 17093, pop: 128990
Mean visiting county population
Incident cases (per 100,000 population)
1000000
2000000
3000000
4000000
5000000
0
5
10
15
20
25
30
35
Mar
May
Jul
Sep

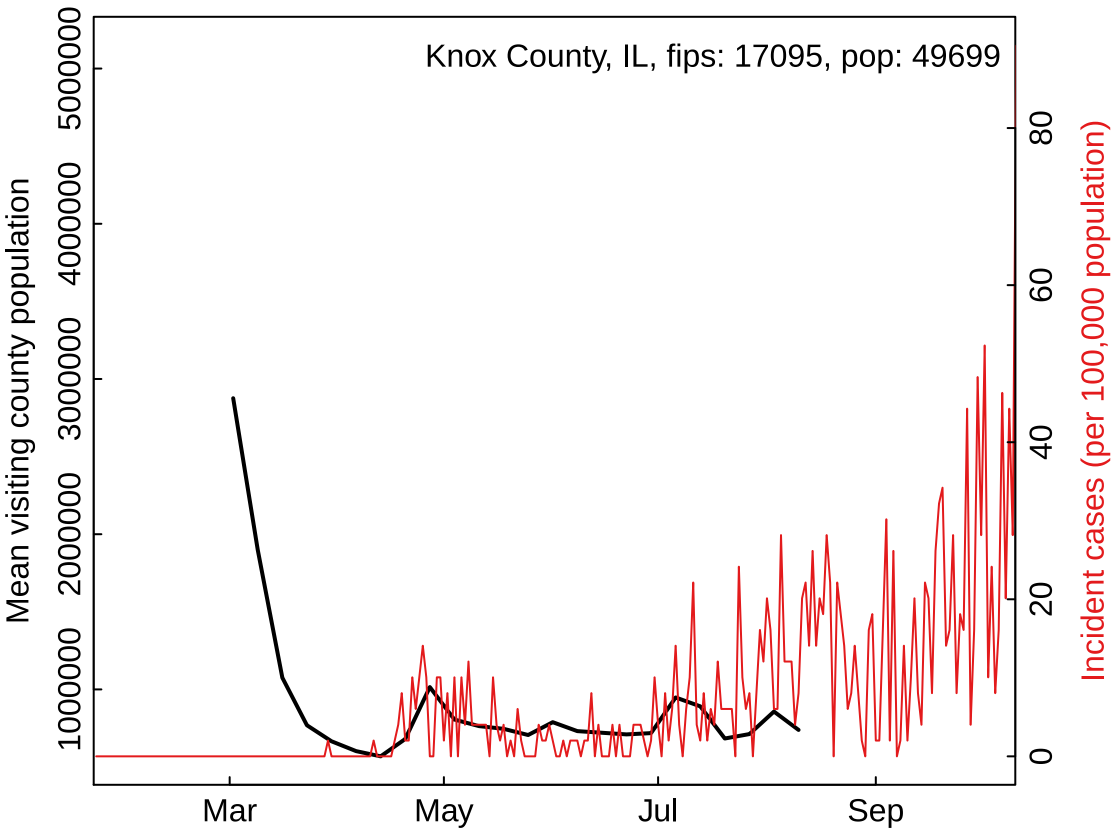

Knox County, IL, fips: 17095, pop: 49699
Mean visiting county population
10000000
20000000
30000000
40000000
50000000
Incident cases (per 100,000 population)
0
20
40
60
80
Mar
May
Jul
Sep

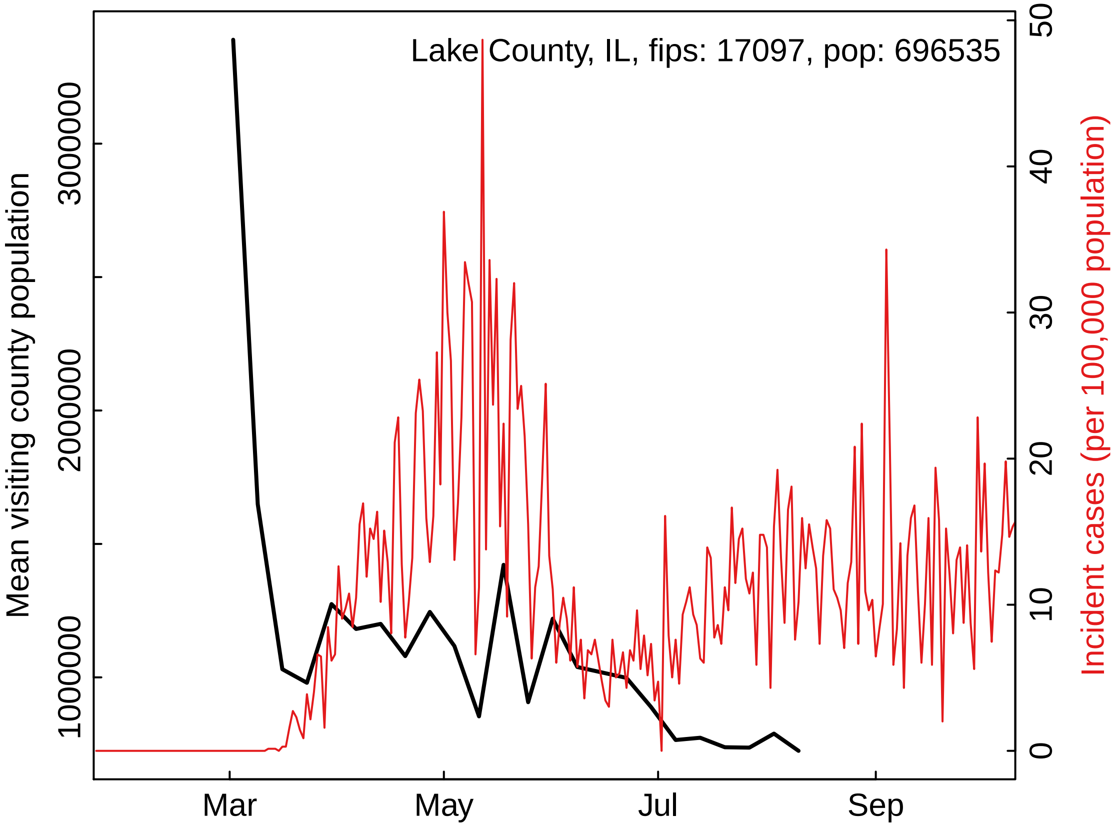

Lake County, IL, fips: 17097, pop: 696535
Mean visiting county population
Incident cases (per 100,000 population)
10000000
20000000
30000000
0
10
20
30
40
50
Mar
May
Jul
Sep

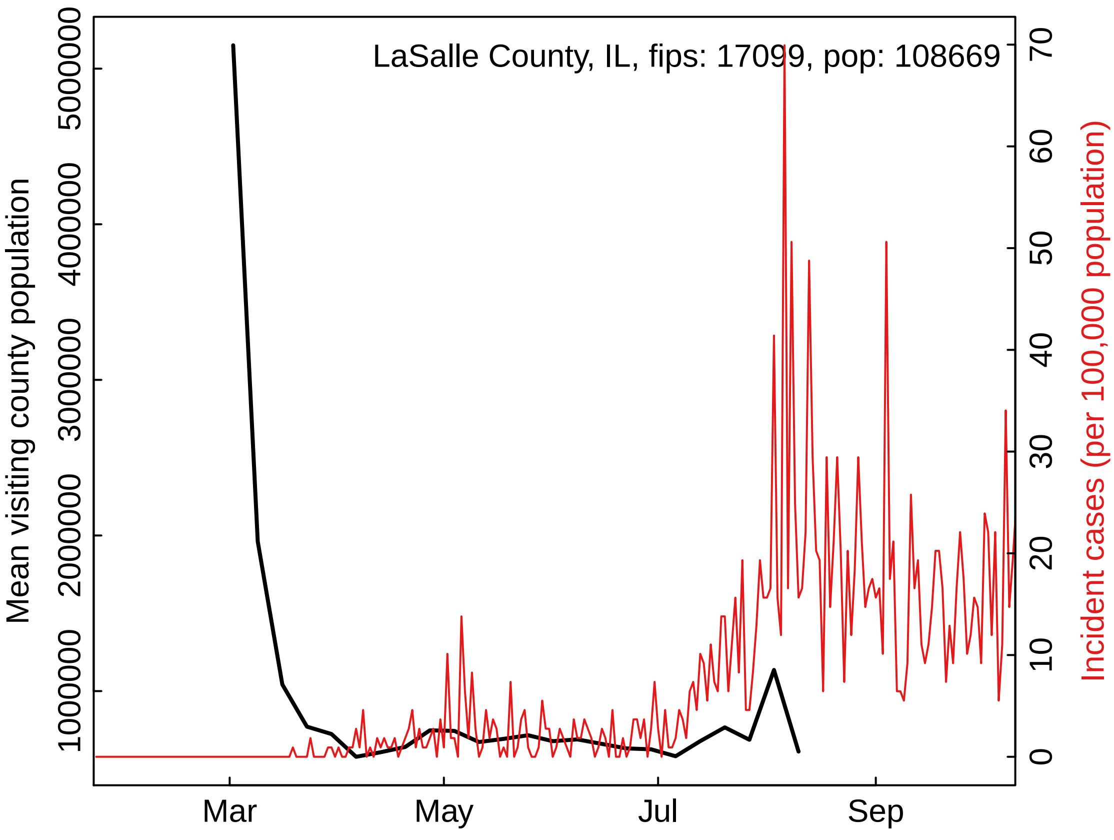
LaSalle County, IL, fips: 17099, pop: 108669
Mean visiting county population
Incident cases (per 100,000 population)
1000000
2000000
3000000
4000000
5000000
0
10
20
30
40
50
60
70
Mar
May
Jul
Sep

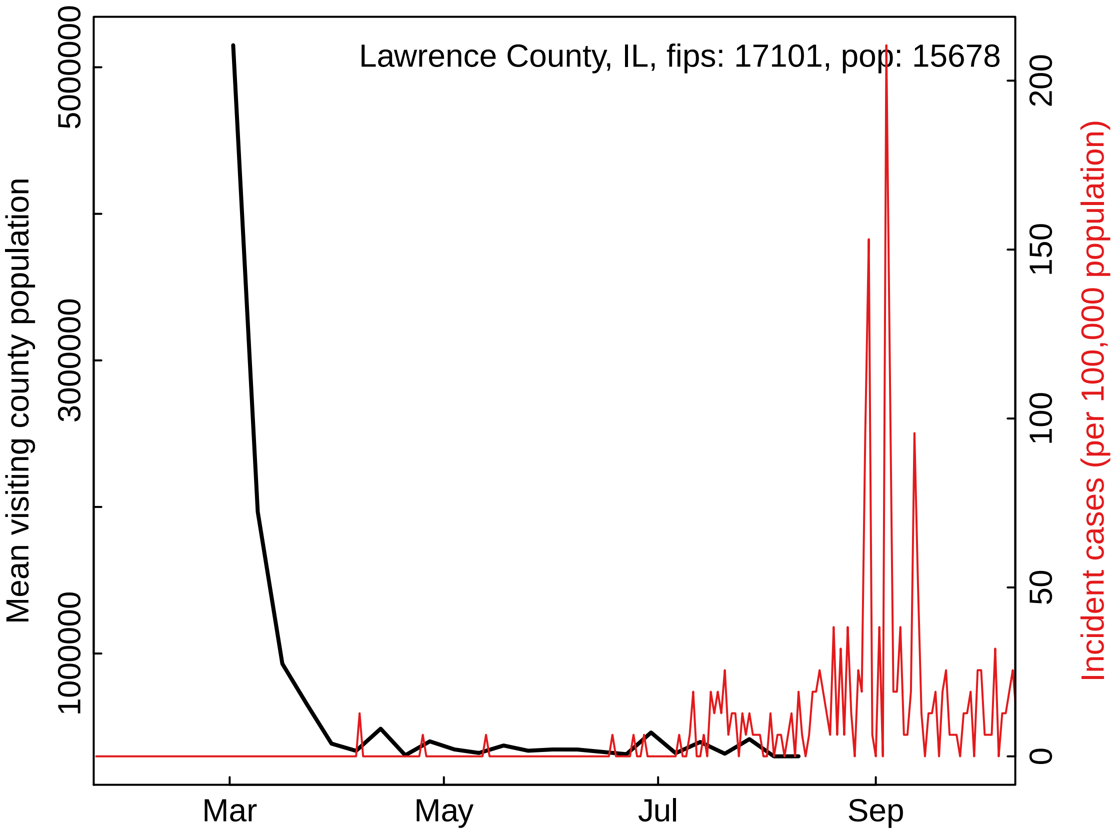
Lawrence County, IL, fips: 17101, pop: 15678
Mean visiting county population
1000000
3000000
5000000
Incident cases (per 100,000 population)
0
50
100
150
200
Mar
May
Jul
Sep

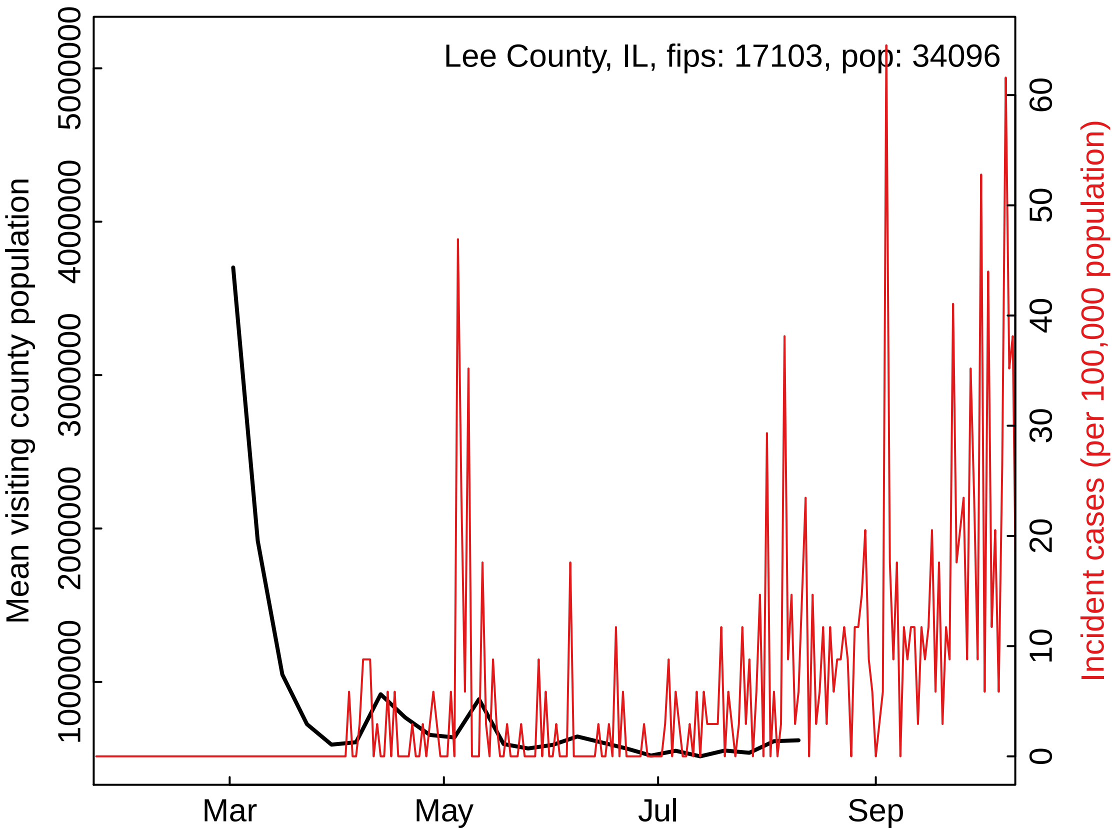

Lee County, IL, fips: 17103, pop: 34096
Mean visiting county population
Incident cases (per 100,000 population)
1000000
2000000
3000000
4000000
5000000
0
10
20
30
40
50
60
Mar
May
Jul
Sep

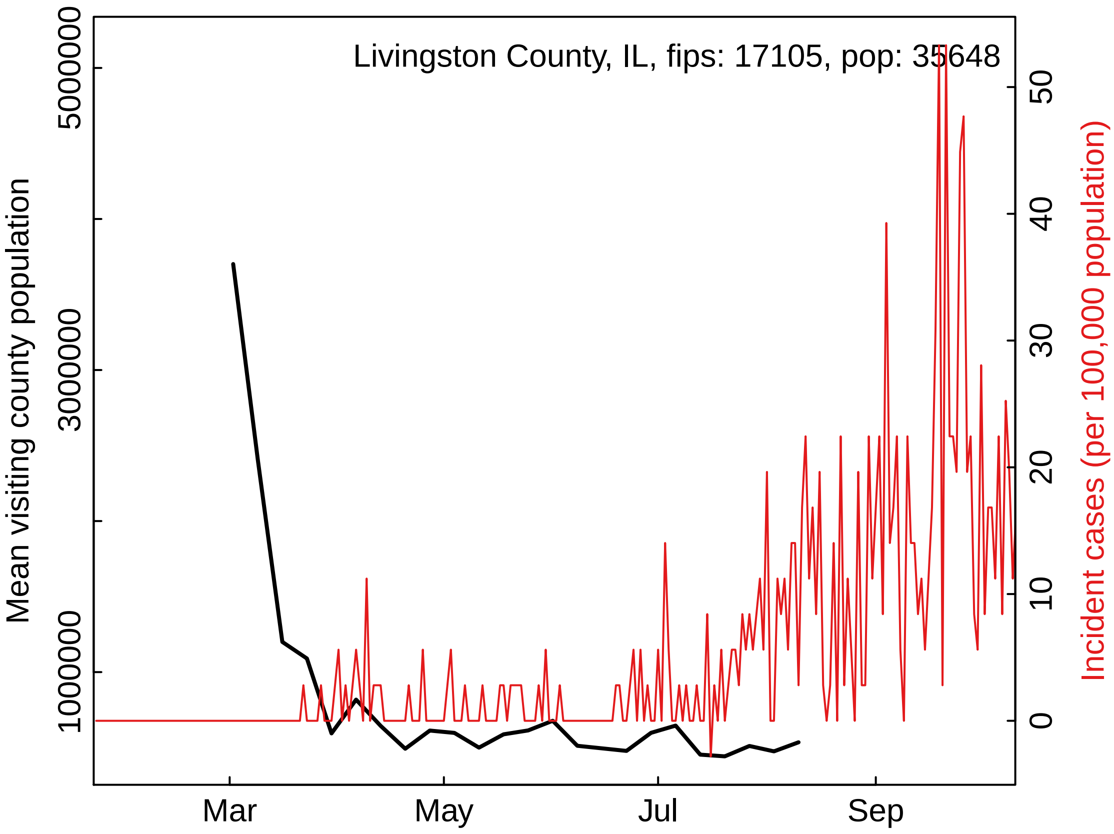

Livingston County, IL, fips: 17105, pop: 35648
Mean visiting county population
1000000
3000000
5000000
Incident cases (per 100,000 population)
0
10
20
30
40
50
Mar
May
Jul
Sep

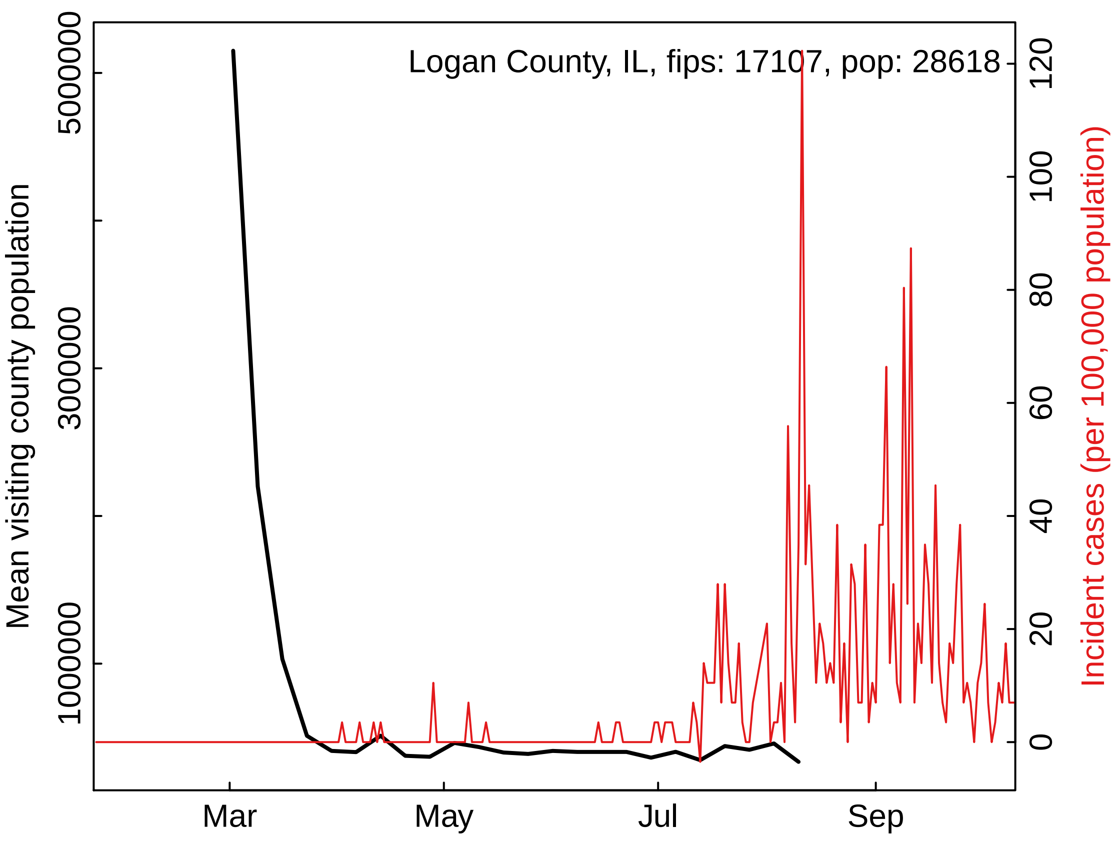

Logan County, IL, fips: 17107, pop: 28618
Mean visiting county population
1000000
3000000
5000000
Incident cases (per 100,000 population)
0
20
40
60
80
100
120
Mar
May
Jul
Sep

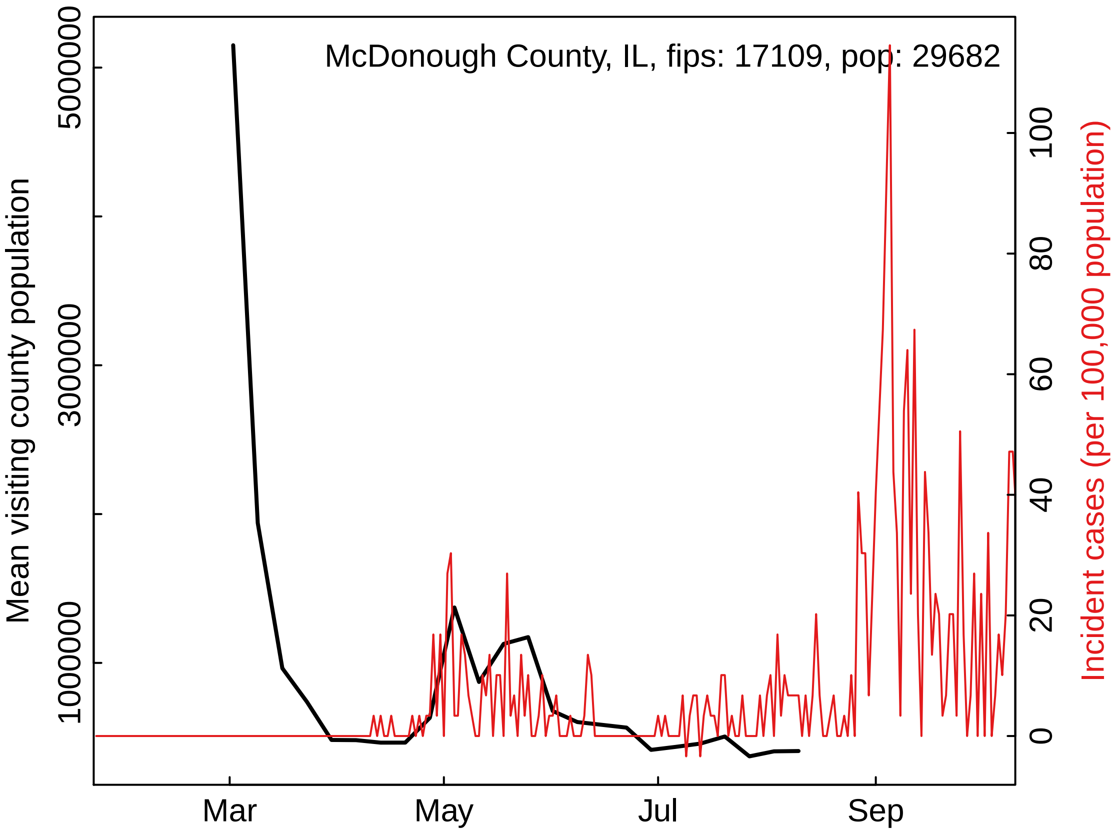
McDonough County, IL, fips: 17109, pop: 29682
Mean visiting county population
1000000
3000000
5000000
Incident cases (per 100,000 population)
0
20
40
60
80
100
Mar
May
Jul
Sep

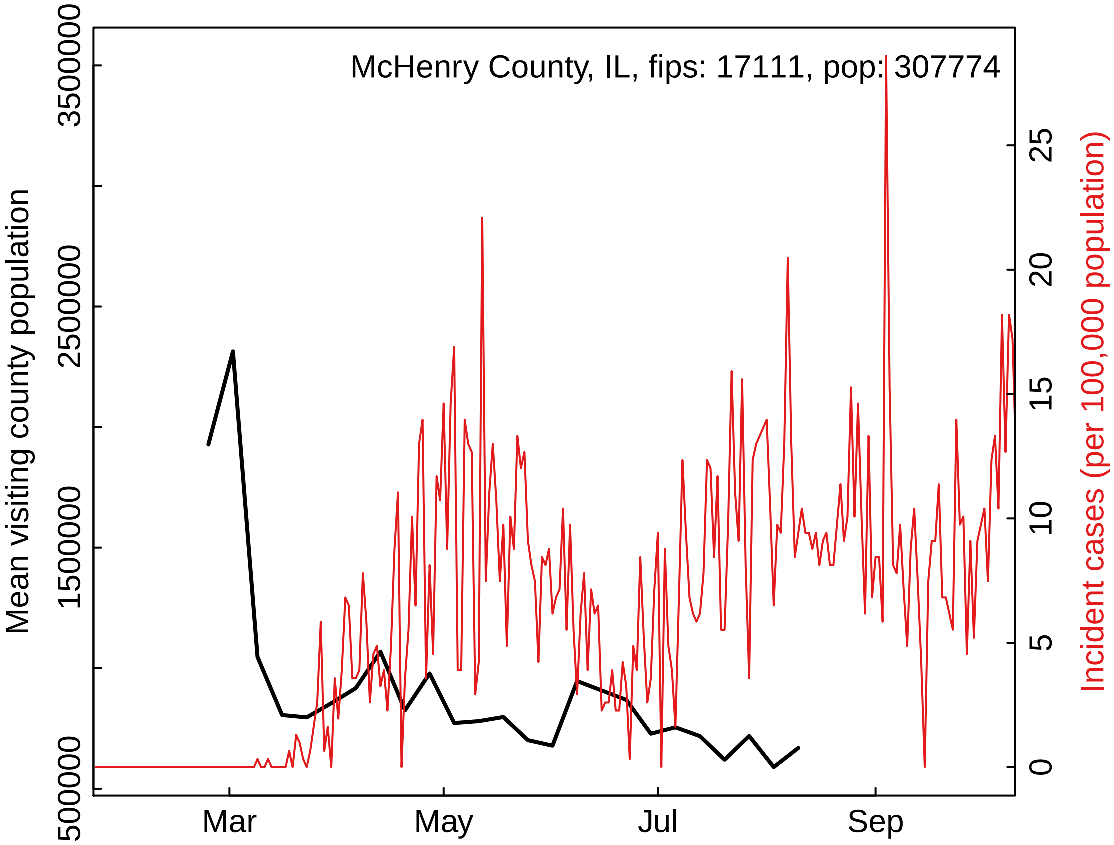

McHenry County, IL, fips: 17111, pop: 307774
Mean visiting county population
3500000
2500000
1500000
500000
Incident cases (per 100,000 population)
0
5
10
15
20
25
Mar
May
Jul
Sep

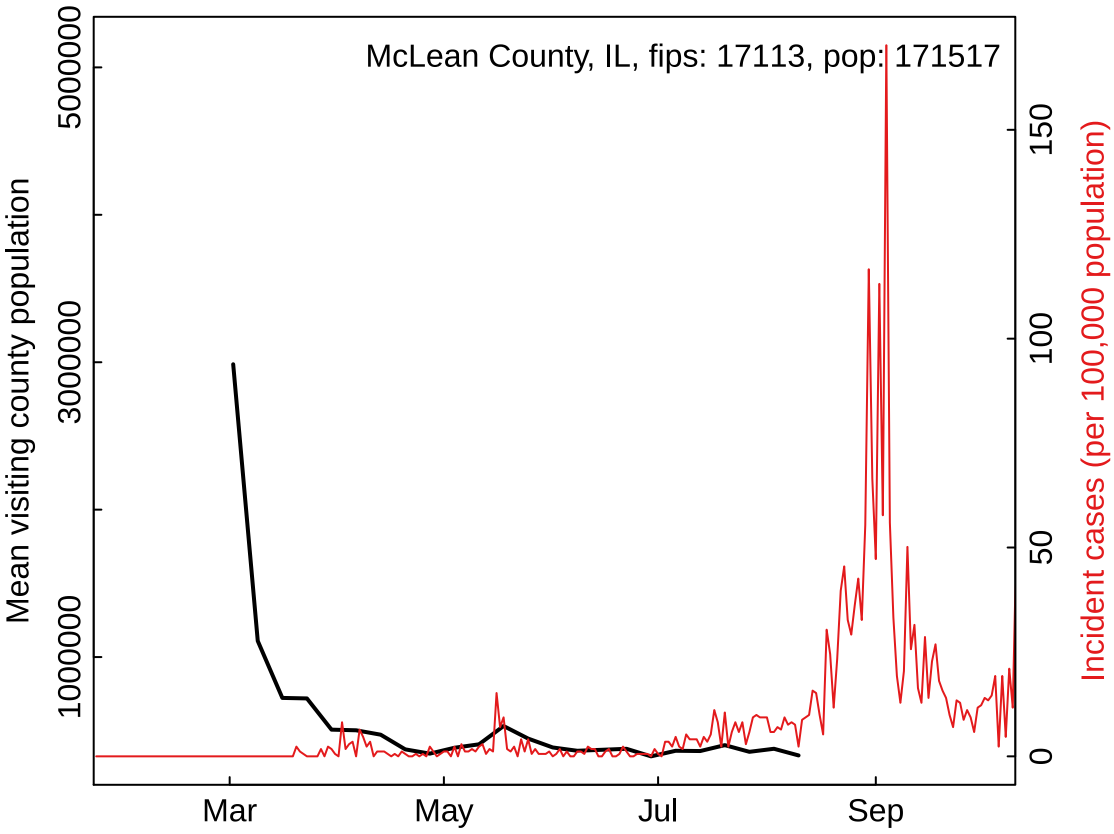

McLean County, IL, fips: 17113, pop: 171517
Mean visiting county population
1000000
3000000
5000000
Mar
May
Jul
Sep
0
50
100
150
Incident cases (per 100,000 population)

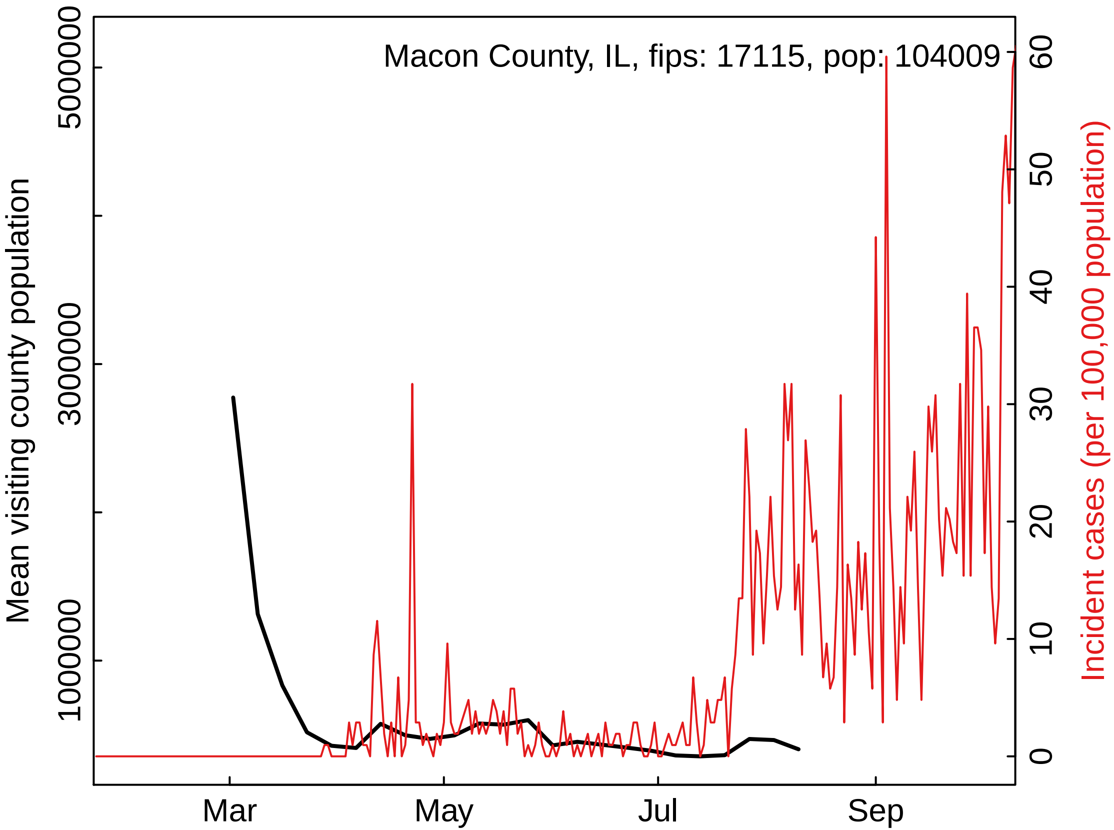

Macon County, IL, fips: 17115, pop: 104009
Mean visiting county population
1000000
3000000
5000000
Incident cases (per 100,000 population)
0
10
20
30
40
50
60
Mar
May
Jul
Sep

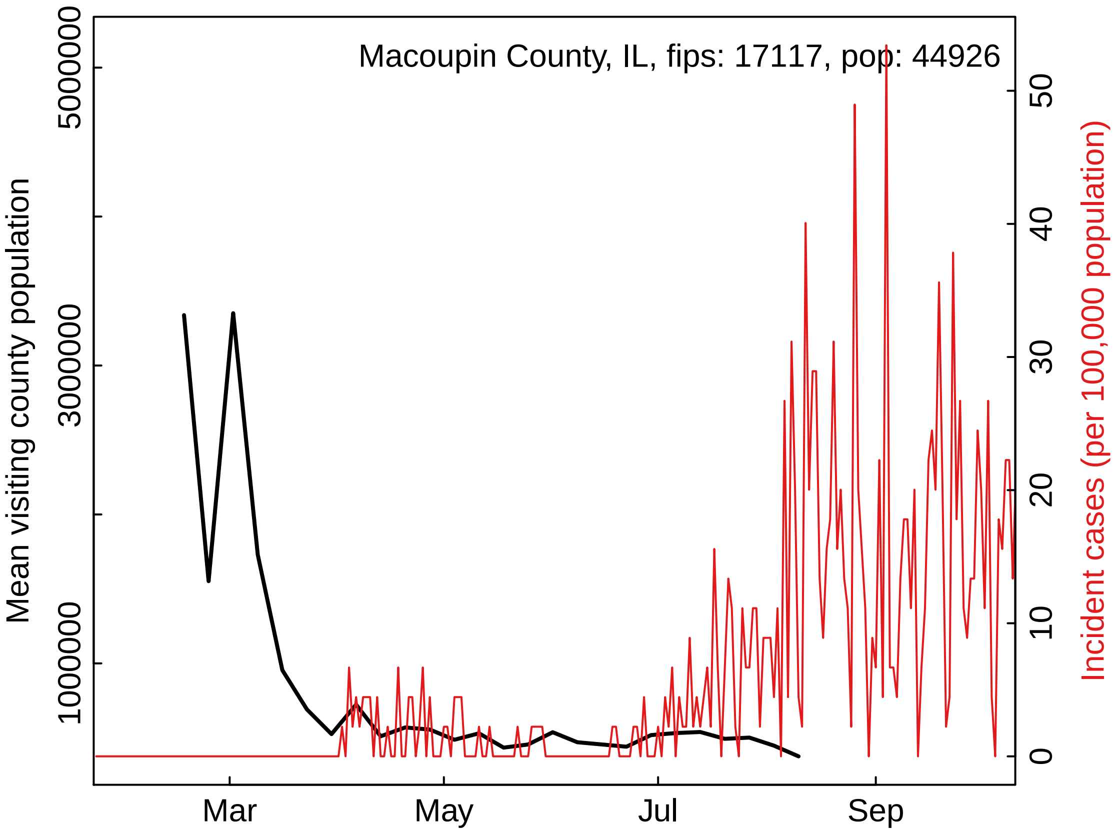
Macoupin County, IL, fips: 17117, pop: 44926
Mean visiting county population
1000000
3000000
5000000
Incident cases (per 100,000 population)
0
10
20
30
40
50
Mar
May
Jul
Sep

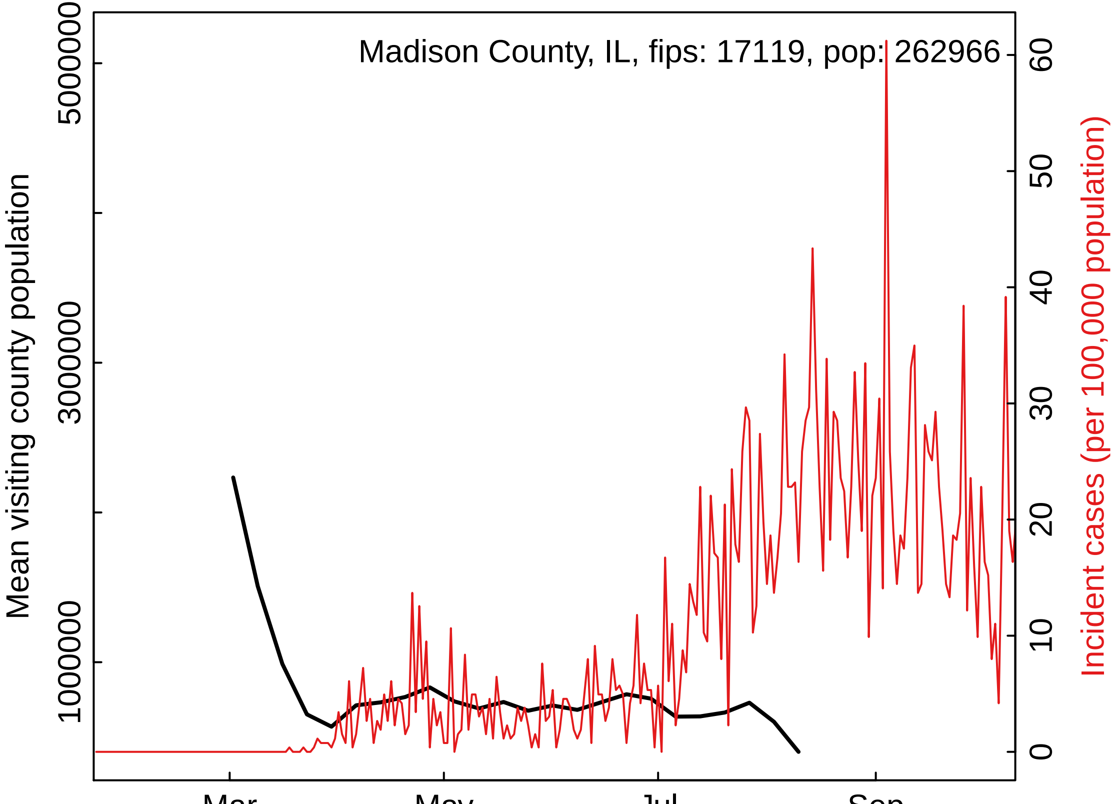

Madison County, IL, fips: 17119, pop: 262966
Mean visiting county population
1000000
3000000
5000000
Incident cases (per 100,000 population)
0
10
20
30
40
50
60
Mar
May
Jul
Sep

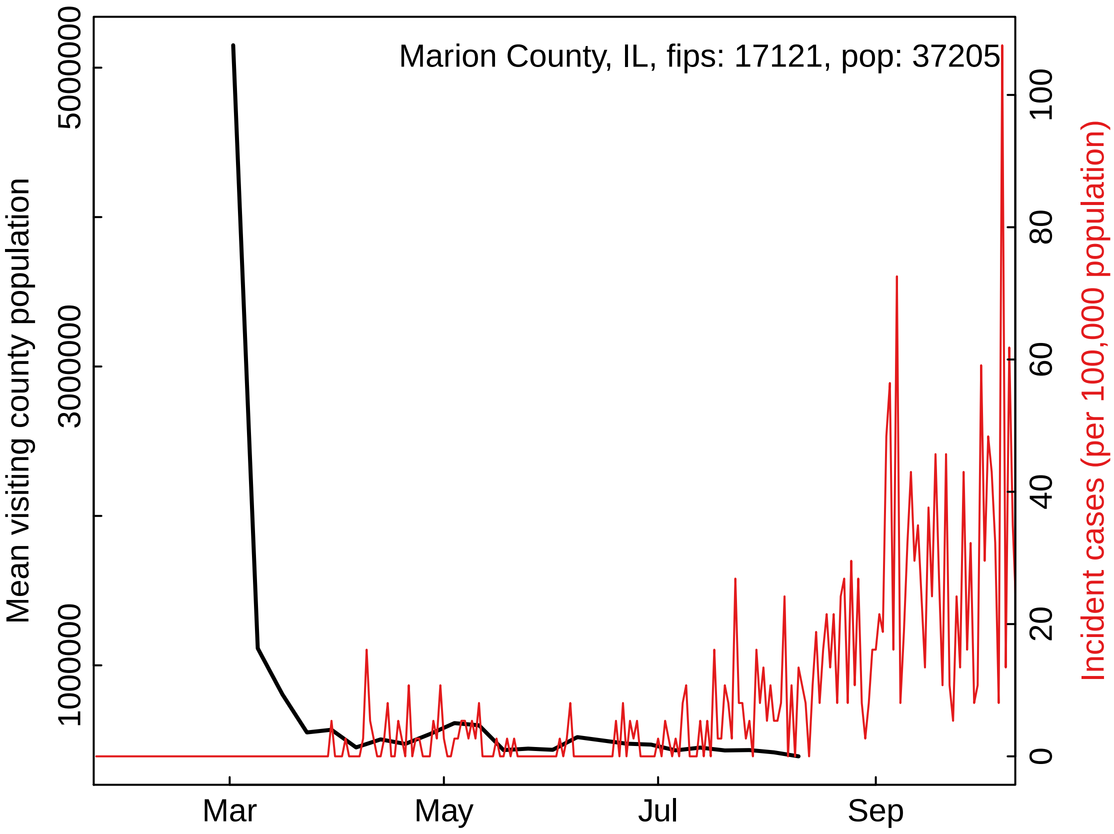

Marion County, IL, fips: 17121, pop: 37205
Mean visiting county population
1000000
3000000
5000000
Incident cases (per 100,000 population)
0
20
40
60
80
100
Mar
May
Jul
Sep

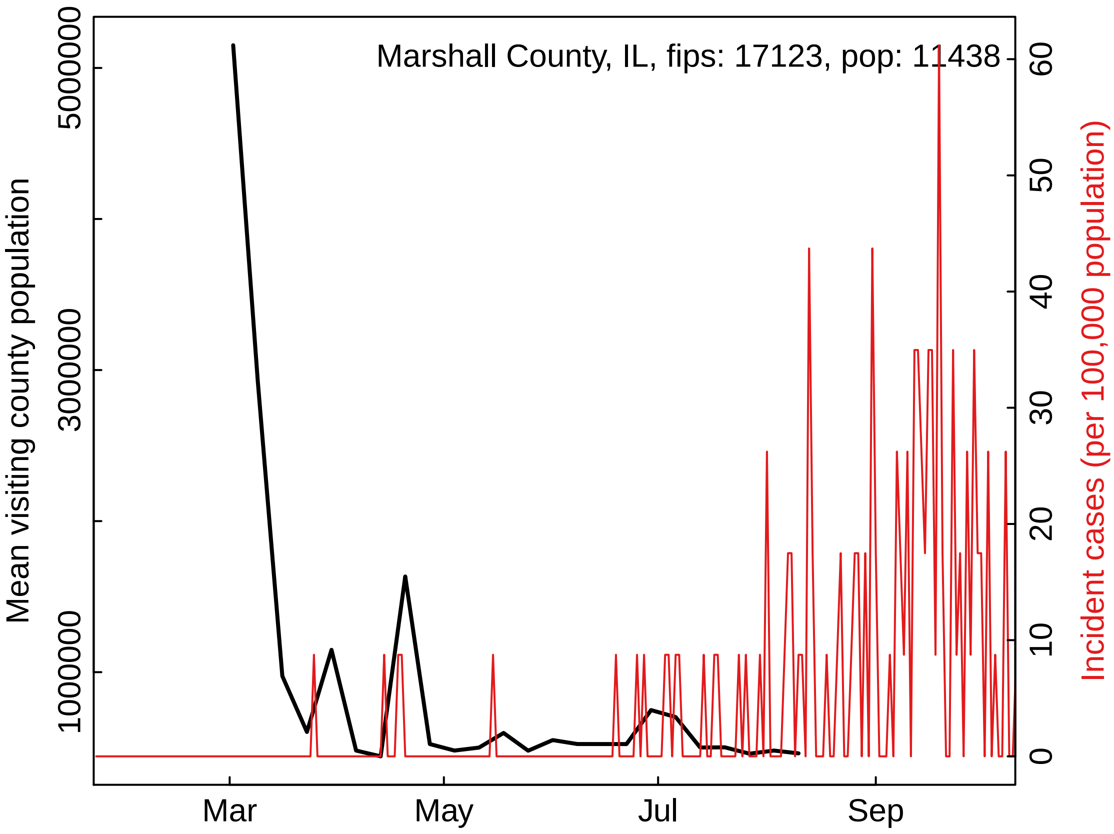
Marshall County, IL, fips: 17123, pop: 11438
Mean visiting county population
10000000
30000000
50000000
Incident cases (per 100,000 population)
0
10
20
30
40
50
60
Mar
May
Jul
Sep

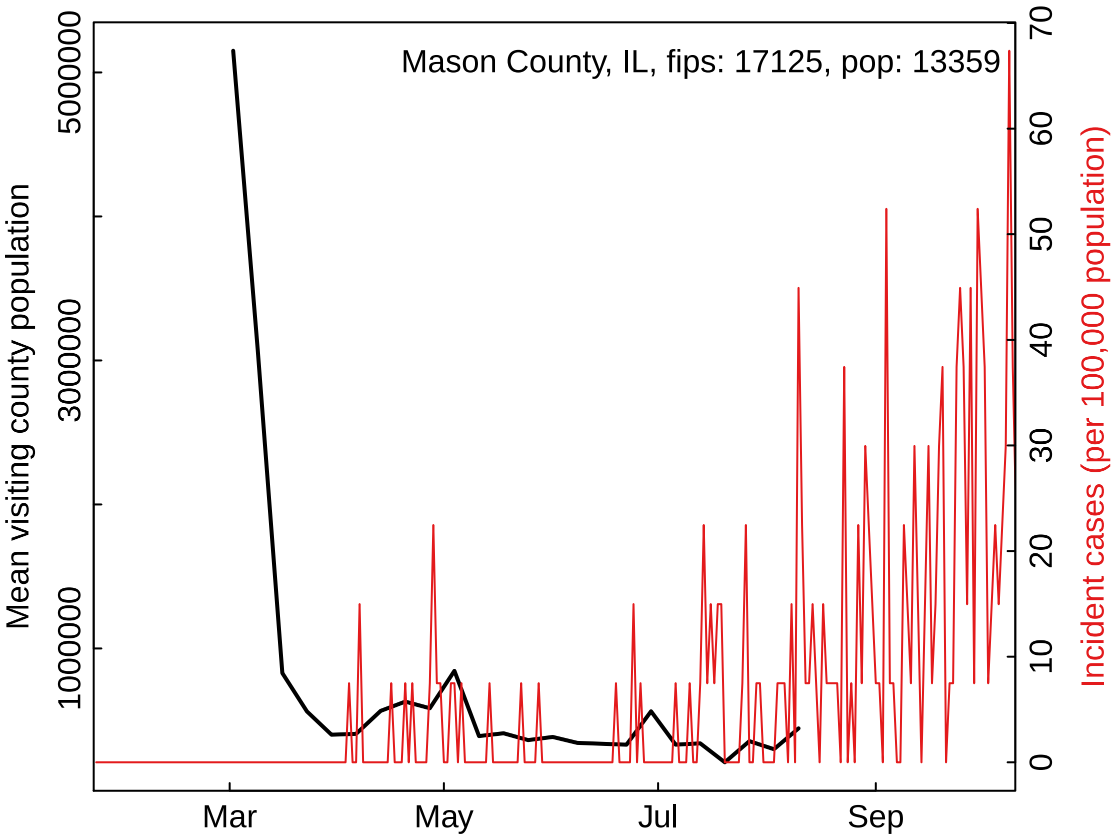

Mason County, IL, fips: 17125, pop: 13359
Mean visiting county population
1000000
3000000
5000000
Incident cases (per 100,000 population)
0
10
20
30
40
50
60
70
Mar
May
Jul
Sep

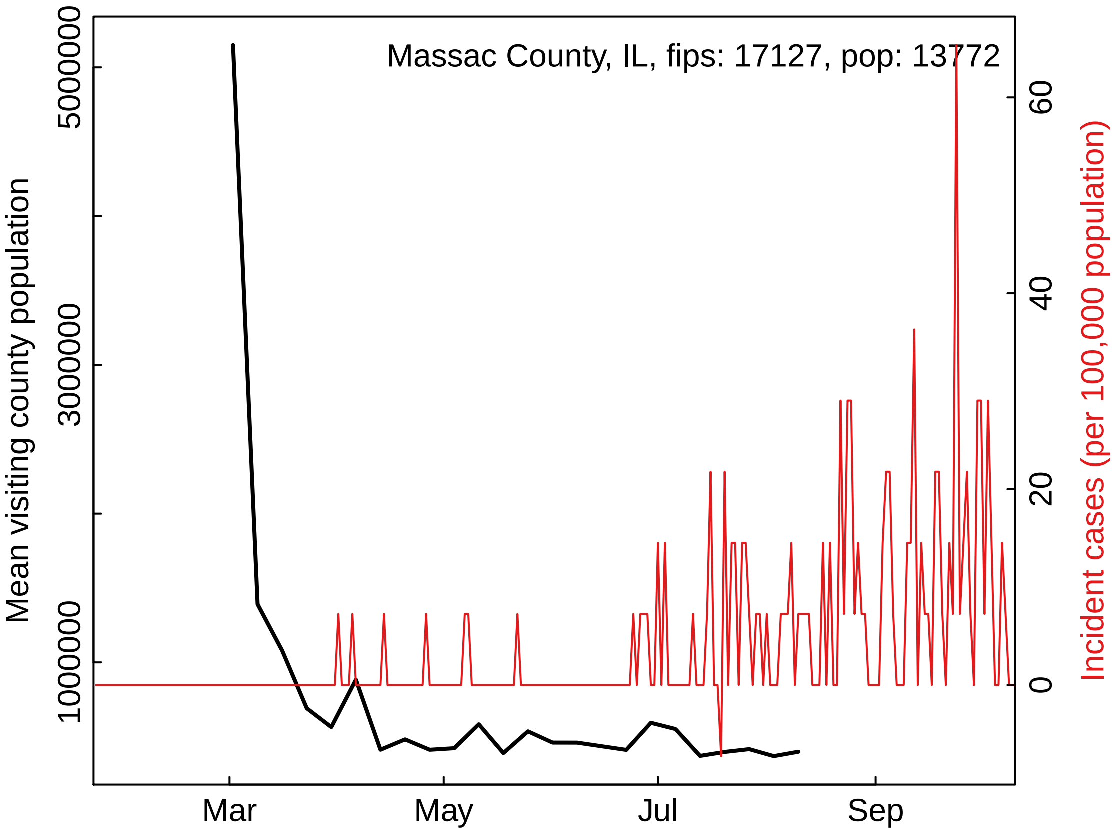

Massac County, IL, fips: 17127, pop: 13772
Mean visiting county population
1000000
3000000
5000000
Incident cases (per 100,000 population)
0
20
40
60
Mar
May
Jul
Sep

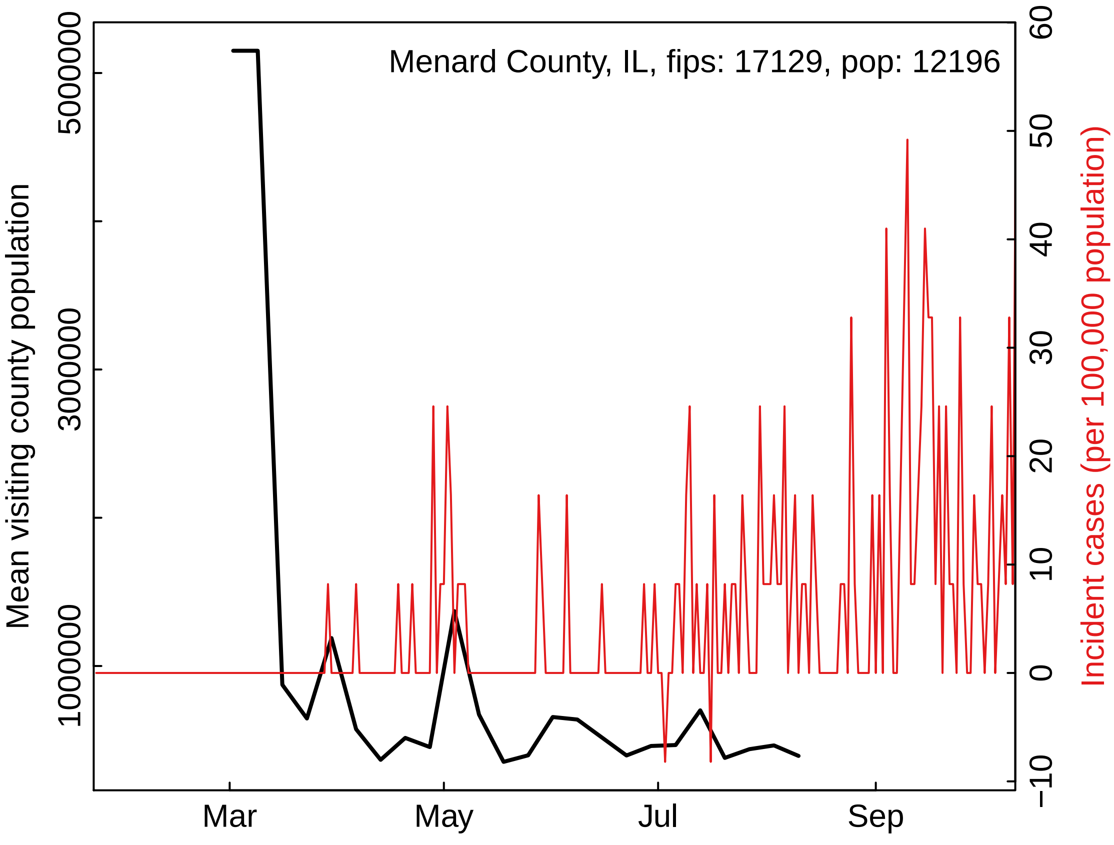
Menard County, IL, fips: 17129, pop: 12196
Mean visiting county population
1000000
3000000
5000000
Incident cases (per 100,000 population)
−10
0
10
20
30
40
50
60
Mar
May
Jul
Sep

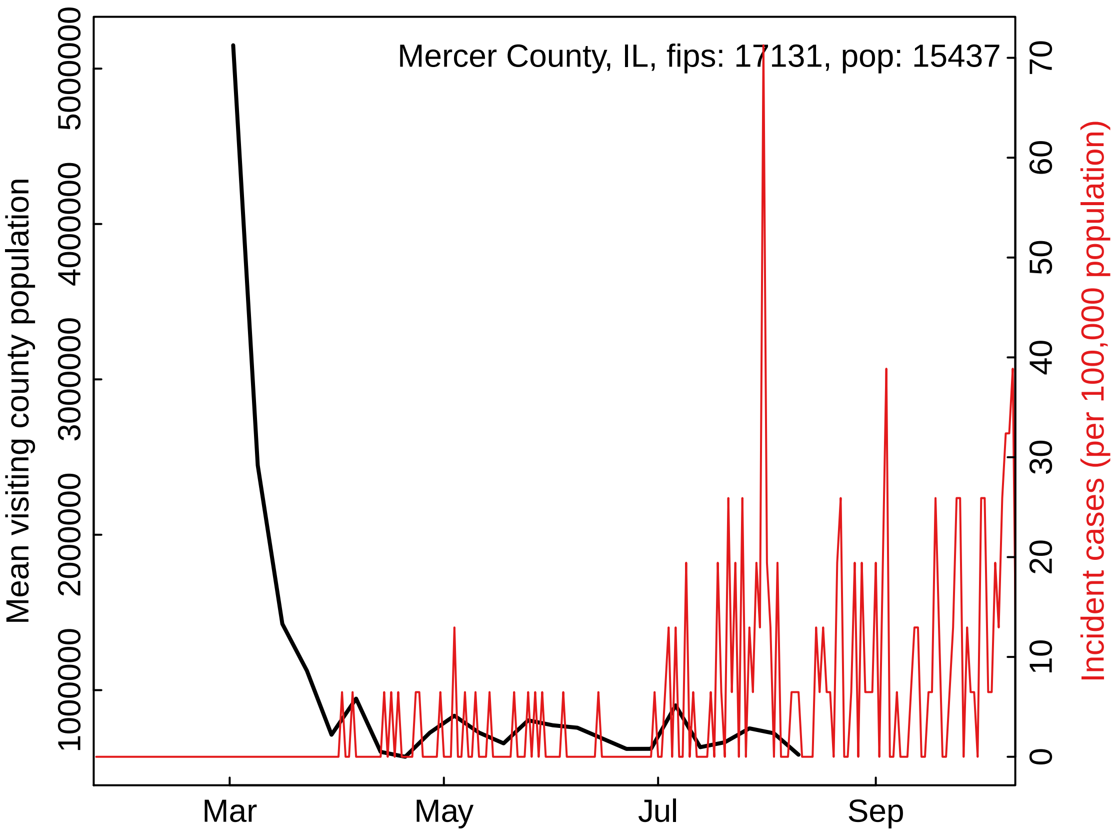

Mercer County, IL, fips: 17131, pop: 15437
Mean visiting county population
1000000
2000000
3000000
4000000
5000000
Incident cases (per 100,000 population)
0
10
20
30
40
50
60
70
Mar
May
Jul
Sep

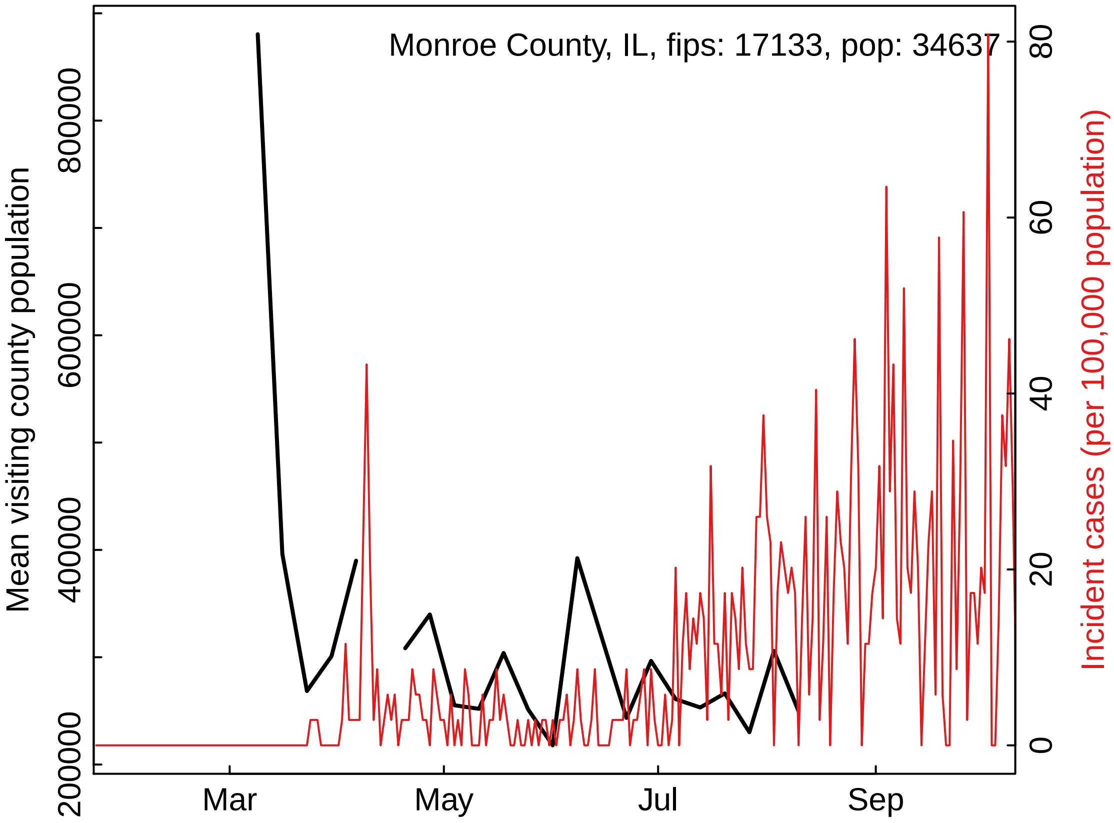

Monroe County, IL, fips: 17133, pop: 34637
Mean visiting county population
2000000
4000000
6000000
8000000
Mar
May
Jul
Sep
0
20
40
60
80
Incident cases (per 100,000 population)

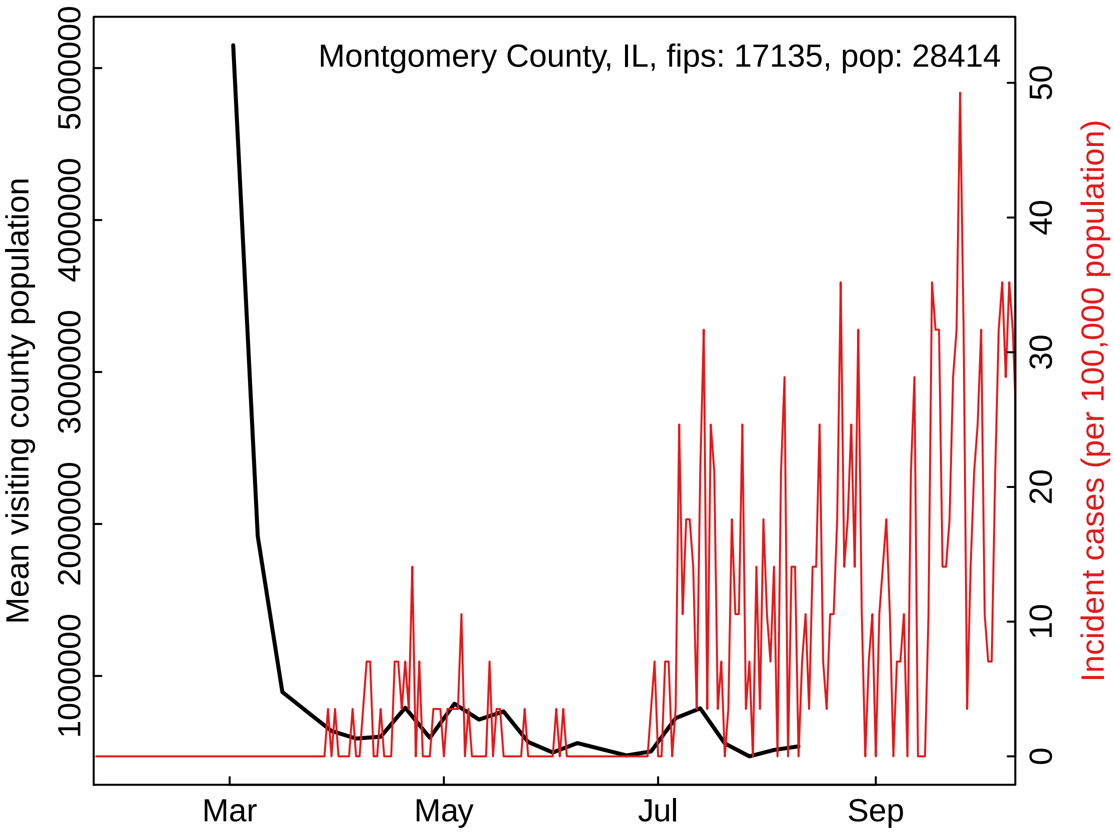

Montgomery County, IL, fips: 17135, pop: 28414
Mean visiting county population
5000000
4000000
3000000
2000000
1000000
Incident cases (per 100,000 population)
50
40
30
20
10
0
Mar
May
Jul
Sep

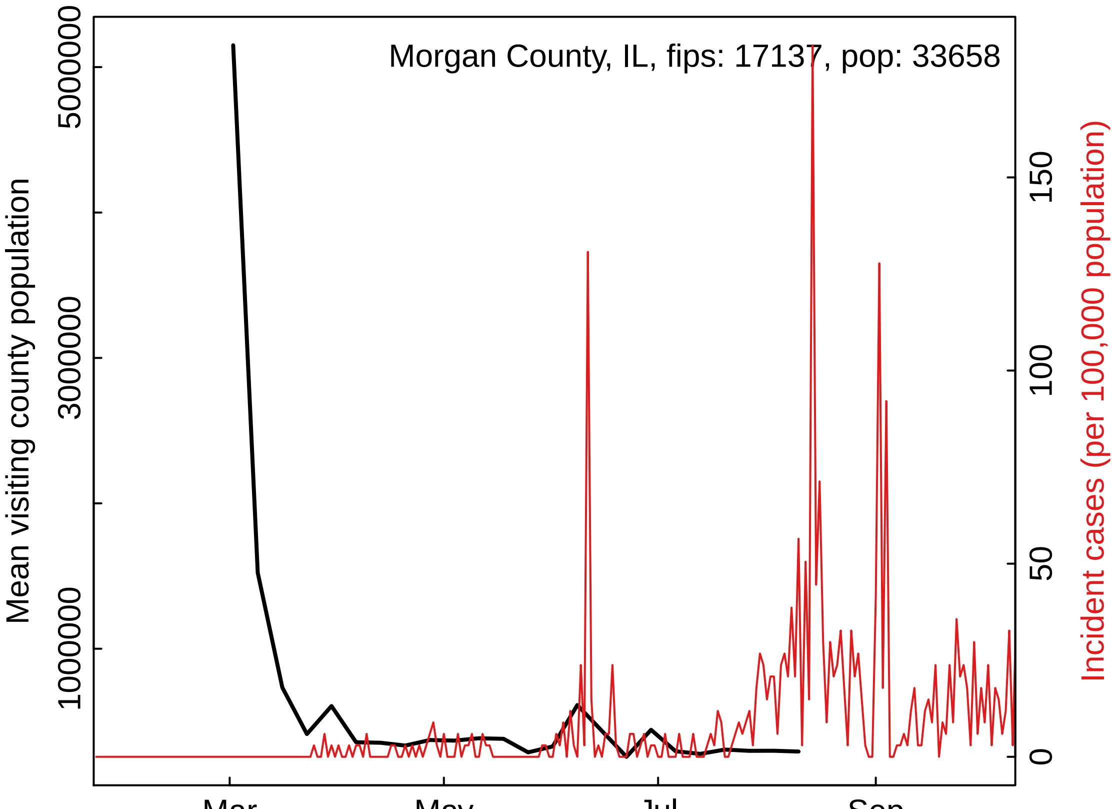

Morgan County, IL, fips: 17137, pop: 33658
Mean visiting county population
1000000
3000000
5000000
Incident cases (per 100,000 population)
0
50
100
150
Mar
May
Jul
Sep

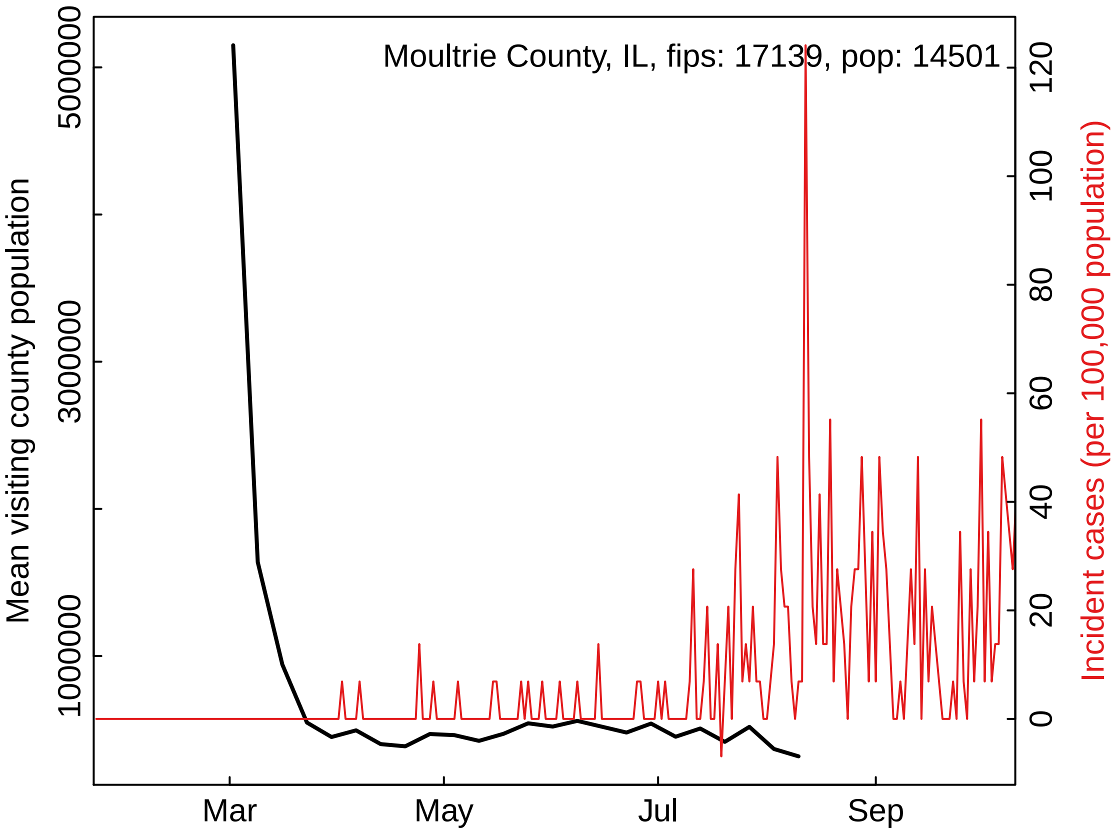

Moultrie County, IL, fips: 17139, pop: 14501
Mean visiting county population
1000000
3000000
5000000
Incident cases (per 100,000 population)
0
20
40
60
80
100
120
Mar
May
Jul
Sep

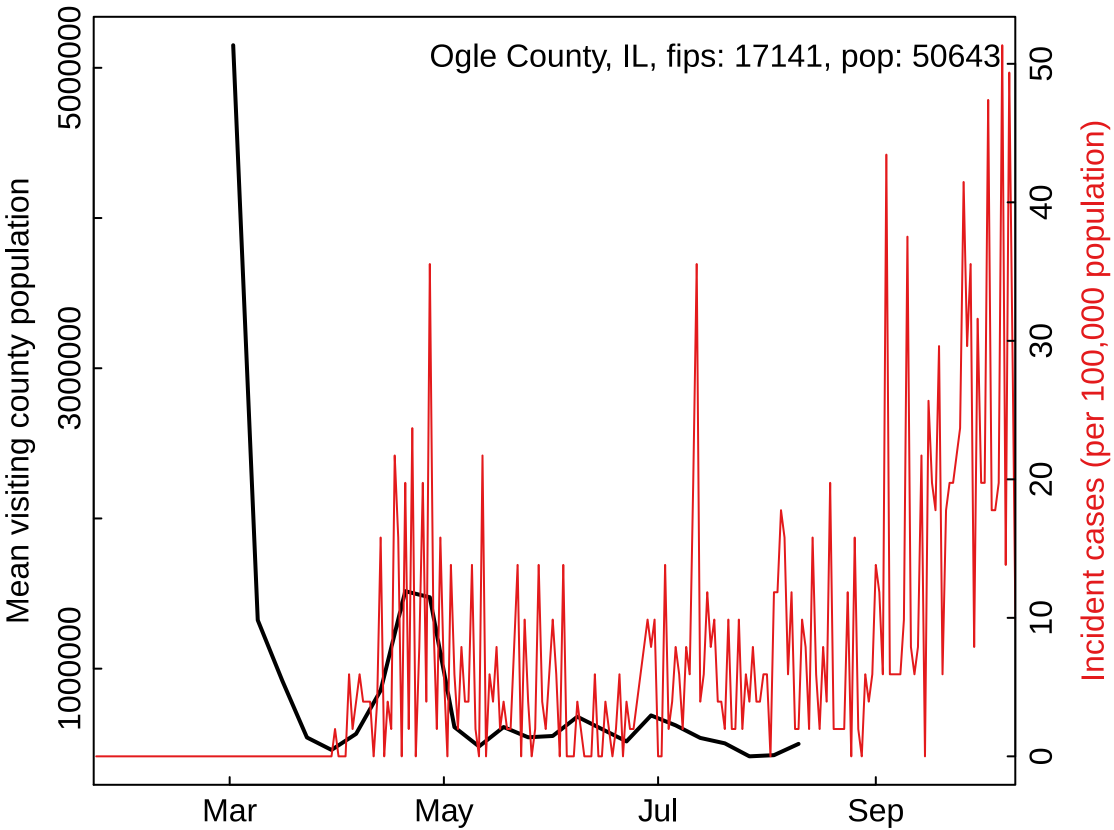

Ogle County, IL, fips: 17141, pop: 50643
Mean visiting county population
Incident cases (per 100,000 population)
1000000
3000000
5000000
0
10
20
30
40
50
Mar
May
Jul
Sep

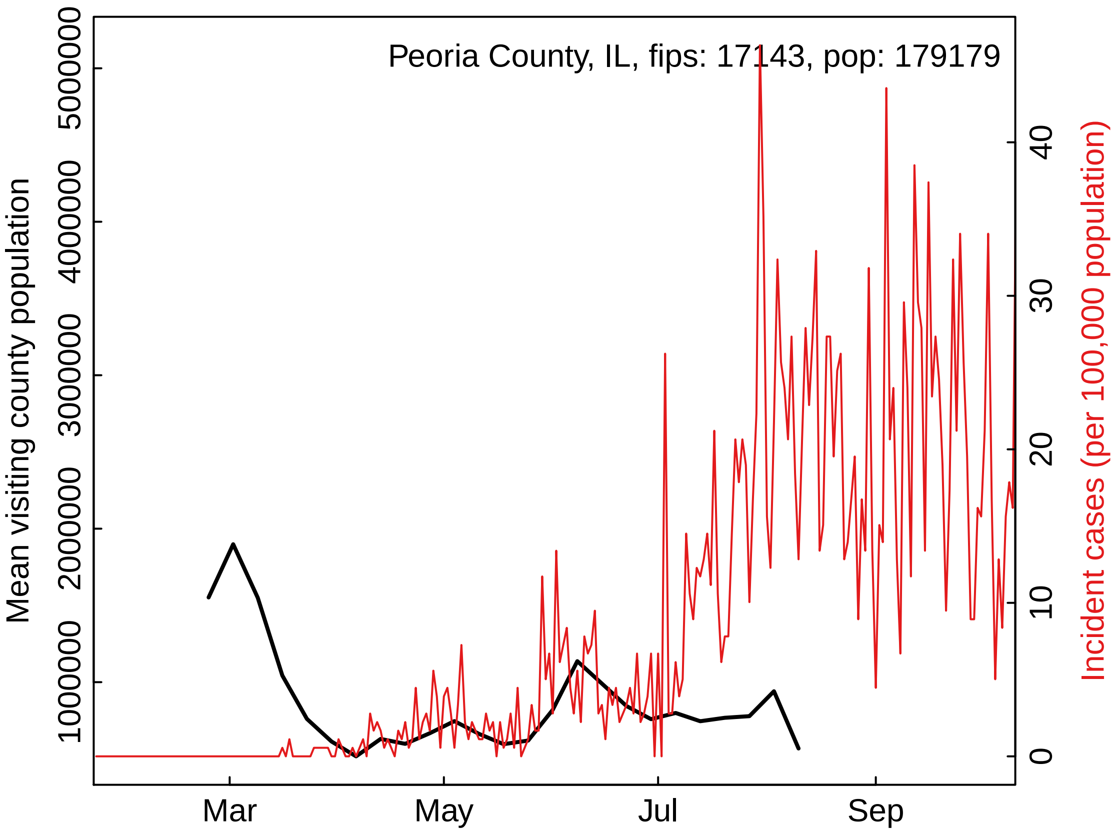

Peoria County, IL, fips: 17143, pop: 179179
Mean visiting county population
1000000
2000000
3000000
4000000
5000000
Mar
May
Jul
Sep
0
10
20
30
40
Incident cases (per 100,000 population)

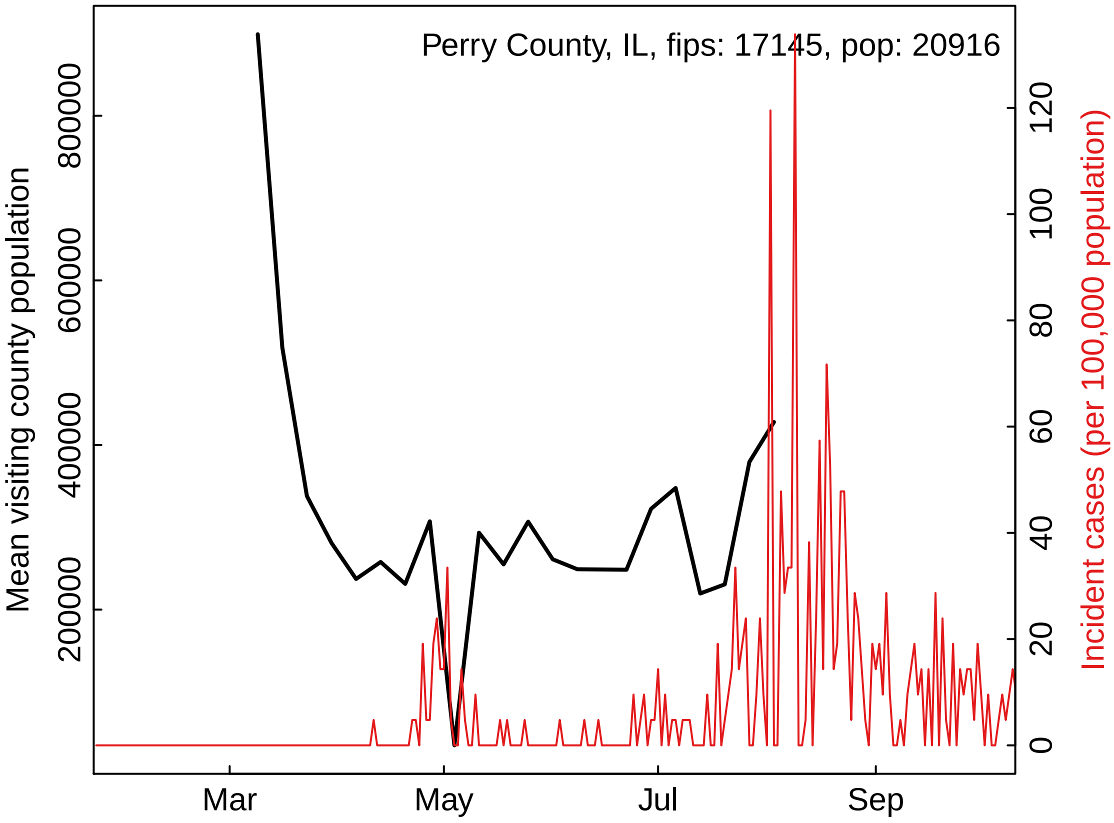

Perry County, IL, fips: 17145, pop: 20916
Mean visiting county population
200000
400000
600000
800000
Incident cases (per 100,000 population)
0
20
40
60
80
100
120
Mar
May
Jul
Sep

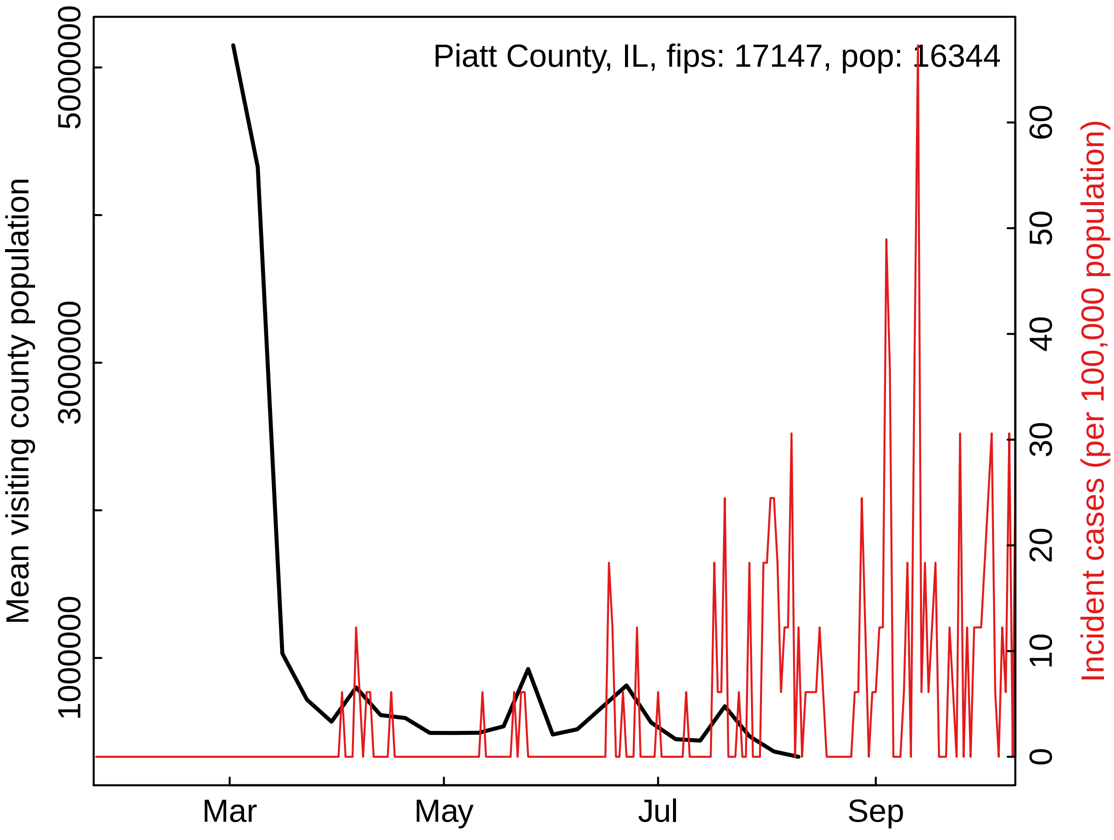
Piatt County, IL, fips: 17147, pop: 16344
Mean visiting county population
1000000
3000000
5000000
Mar
May
Jul
Sep
0
10
20
30
40
50
60
Incident cases (per 100,000 population)

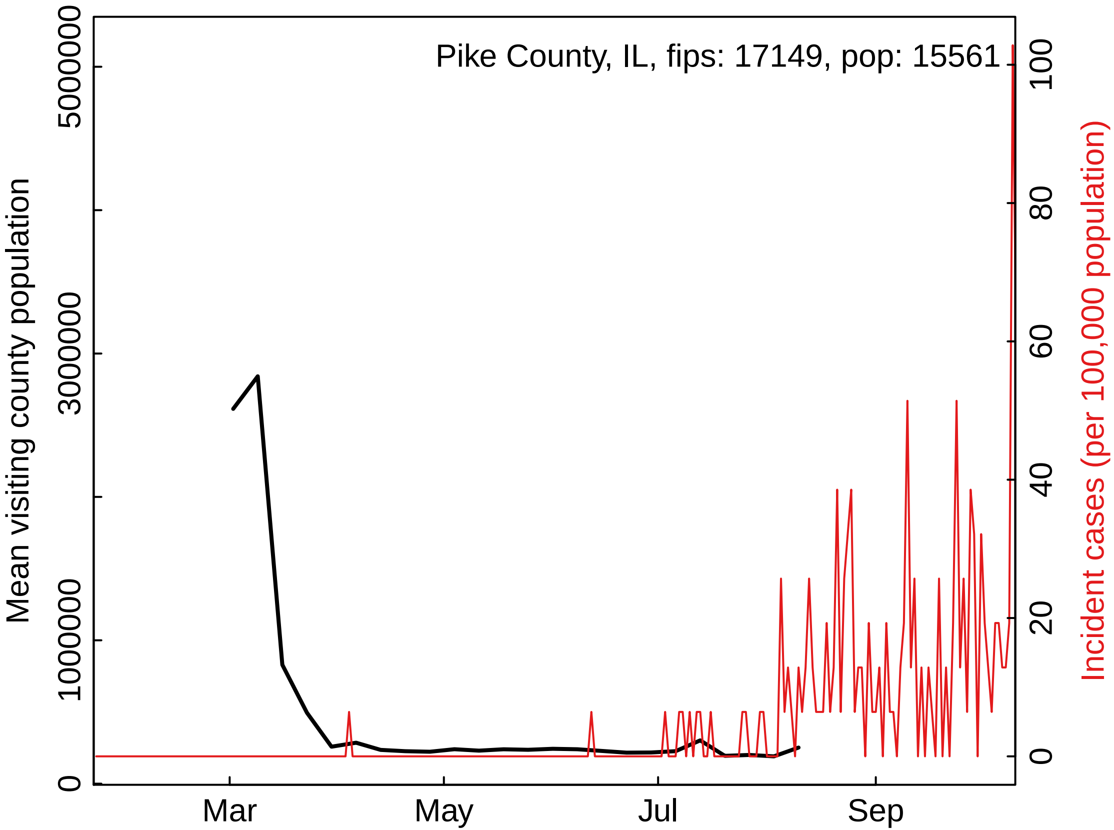

Pike County, IL, fips: 17149, pop: 15561
Mean visiting county population
Incident cases (per 100,000 population)
0
1000000
3000000
5000000
0
20
40
60
80
100
Mar
May
Jul
Sep

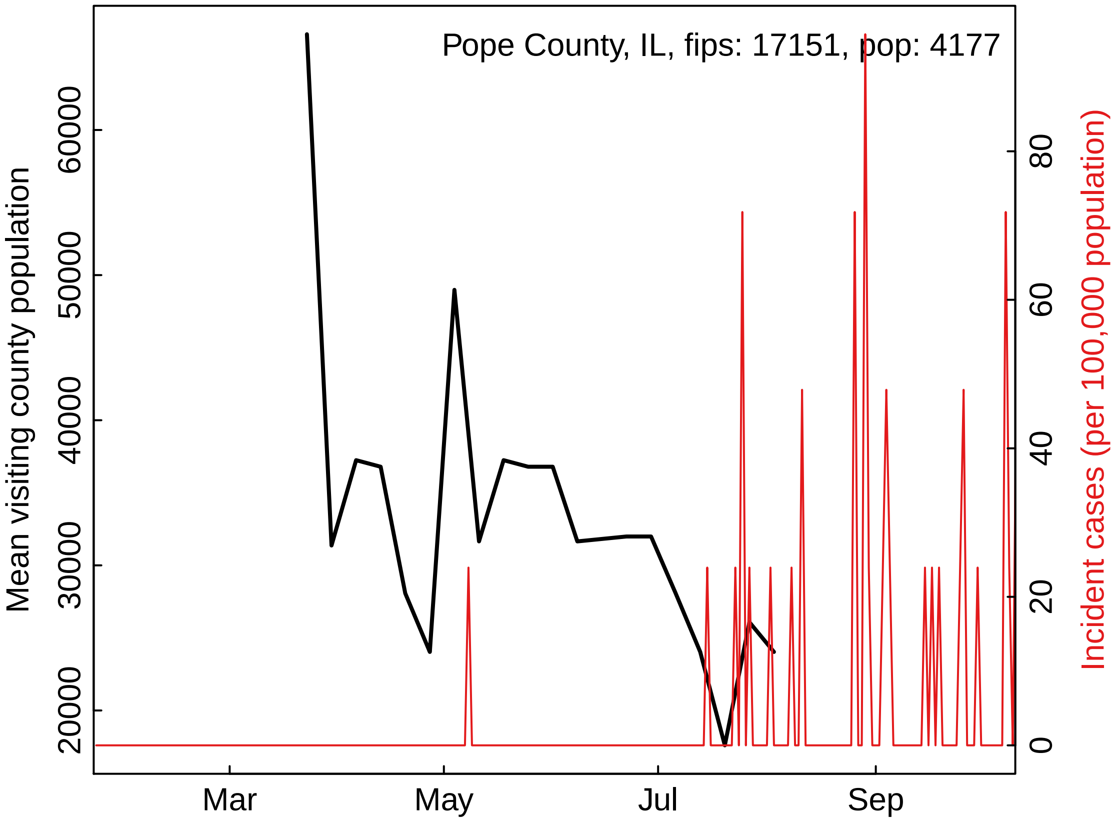
Pope County, IL, fips: 17151, pop: 4177
Mean visiting county population
20000
30000
40000
50000
60000
Incident cases (per 100,000 population)
0
20
40
60
80
Mar
May
Jul
Sep

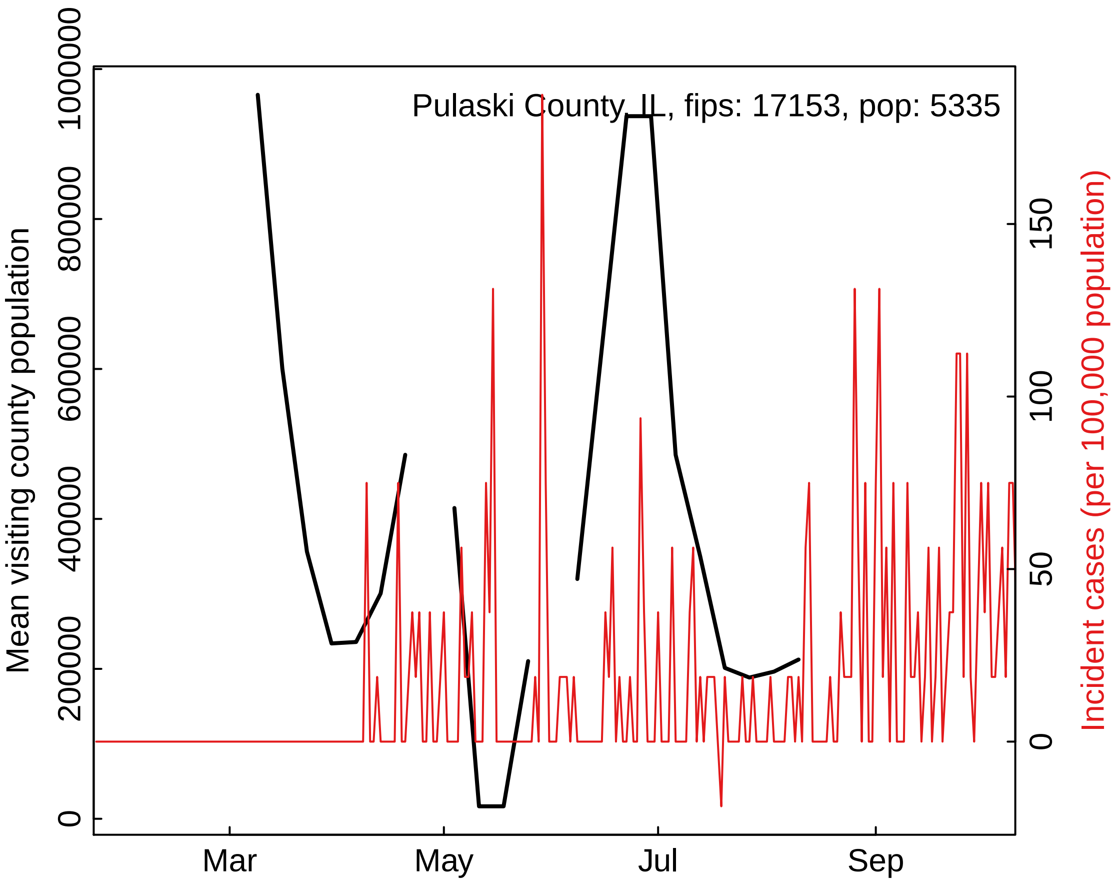

Pulaski County, IL, fips: 17153, pop: 5335
Mean visiting county population
Incident cases (per 100,000 population)
0
200000
400000
600000
800000
1000000
0
50
100
150
Mar
May
Jul
Sep

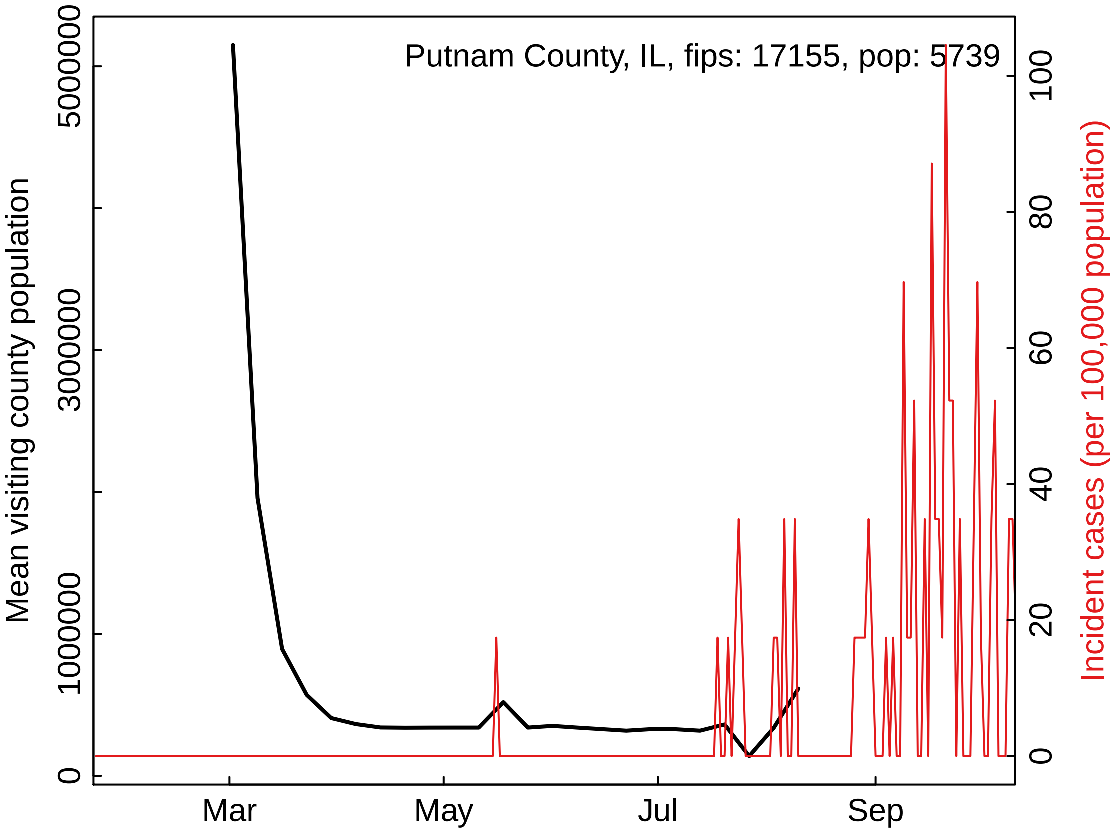
Putnam County, IL, fips: 17155, pop: 5739
Mean visiting county population
0
1000000
3000000
5000000
Incident cases (per 100,000 population)
0
20
40
60
80
100
Mar
May
Jul
Sep

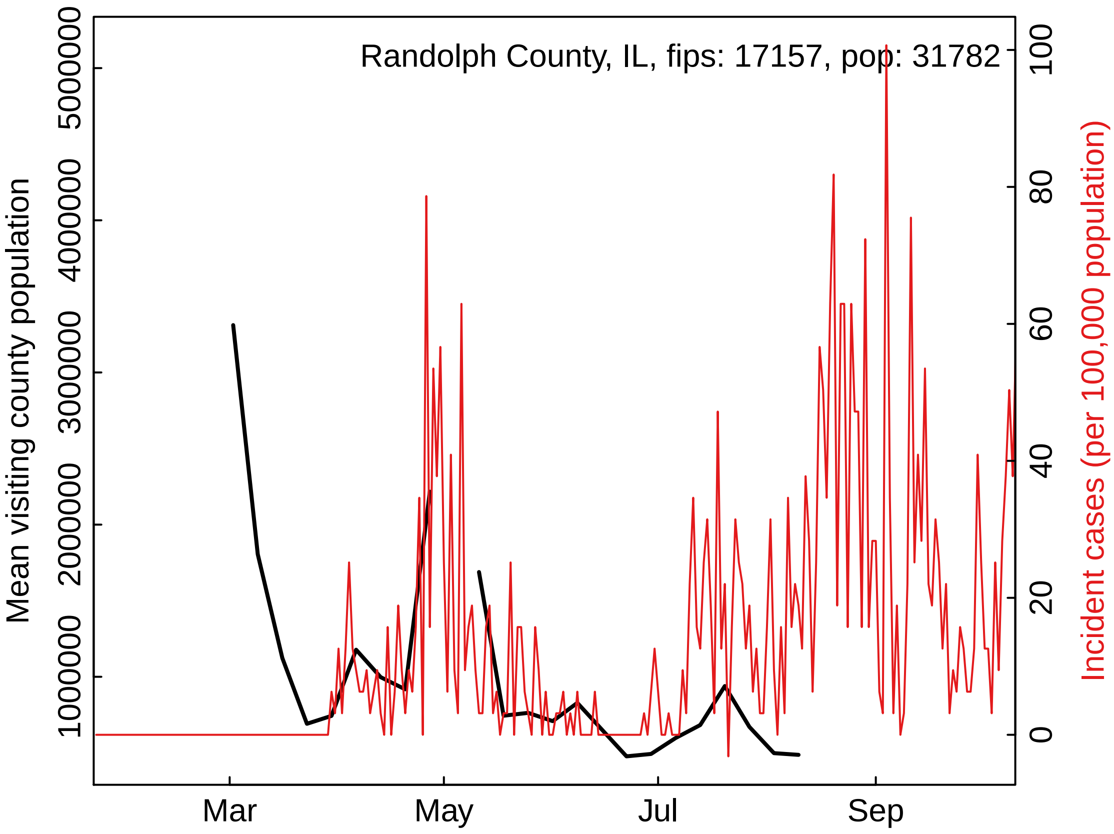

Randolph County, IL, fips: 17157, pop: 31782
Mean visiting county population
10000000
20000000
30000000
40000000
50000000
Incident cases (per 100,000 population)
0
20
40
60
80
100
Mar
May
Jul
Sep

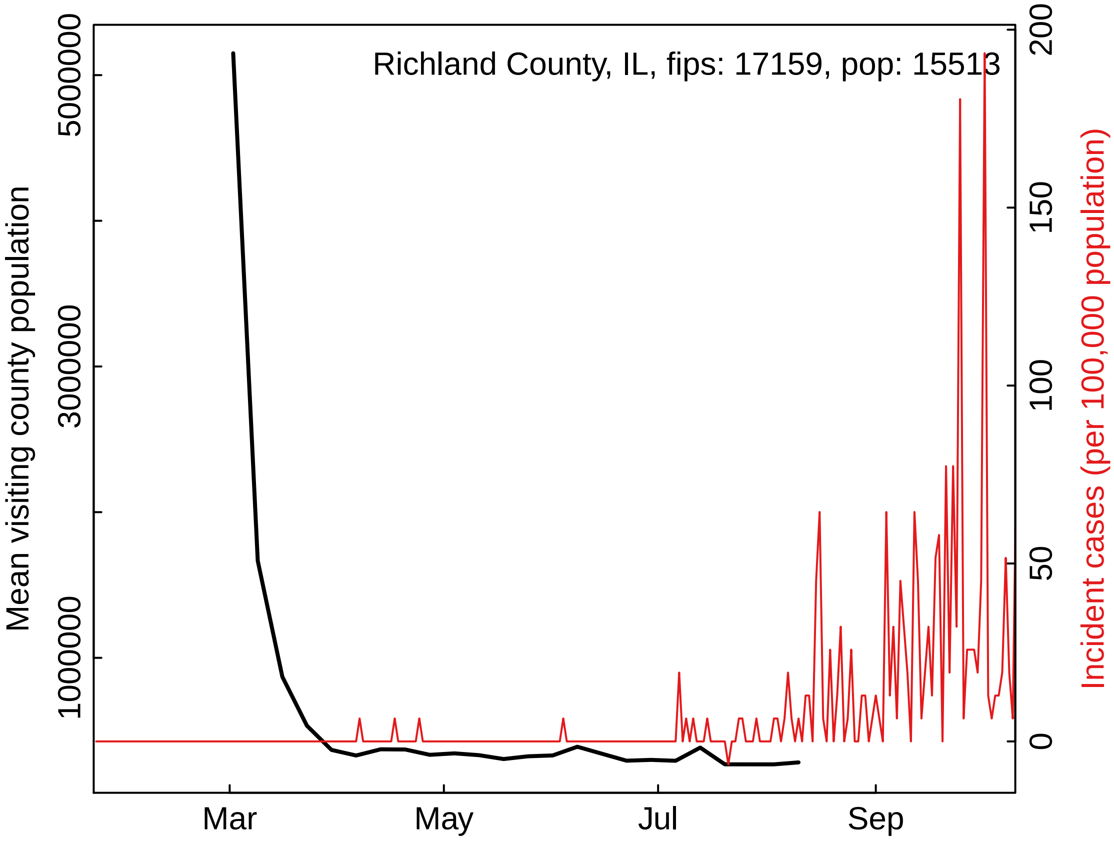

Richland County, IL, fips: 17159, pop: 15513
Mean visiting county population
1000000
3000000
5000000
Incident cases (per 100,000 population)
0
50
100
150
200
Mar
May
Jul
Sep

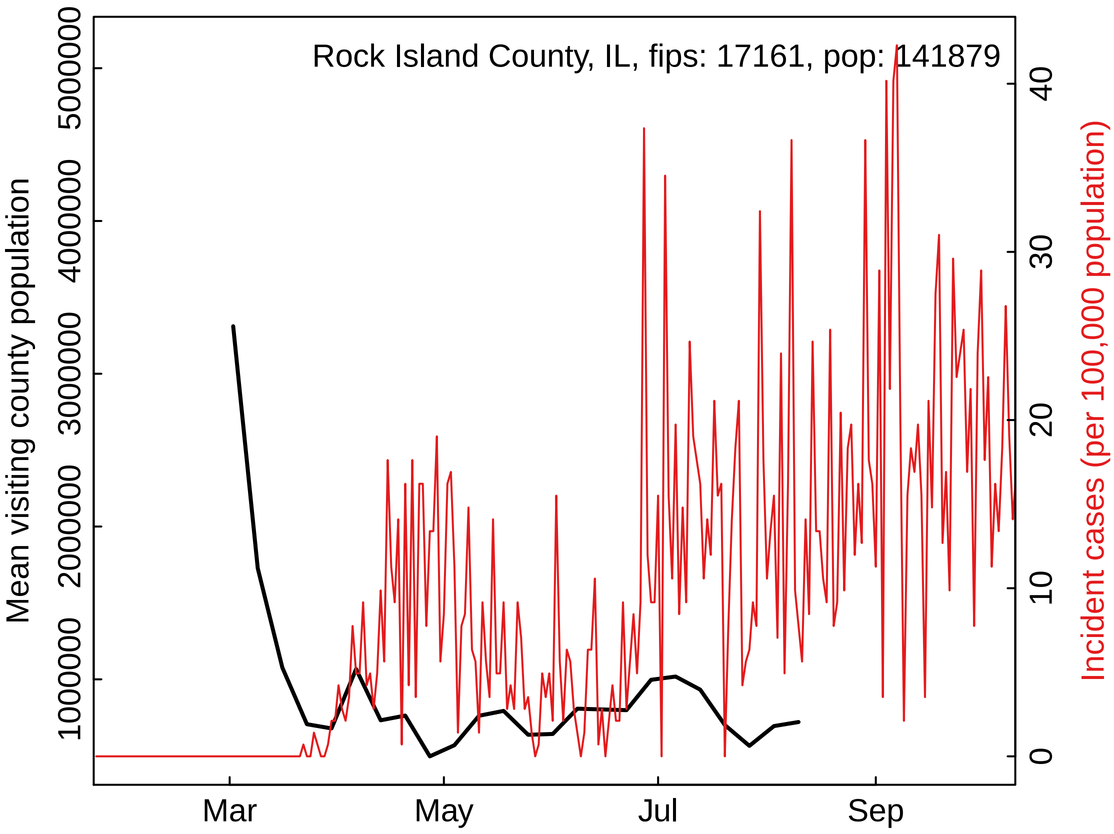

Rock Island County, IL, fips: 17161, pop: 141879
Mean visiting county population
10000000
20000000
30000000
40000000
50000000
Incident cases (per 100,000 population)
0
10
20
30
40
Mar
May
Jul
Sep

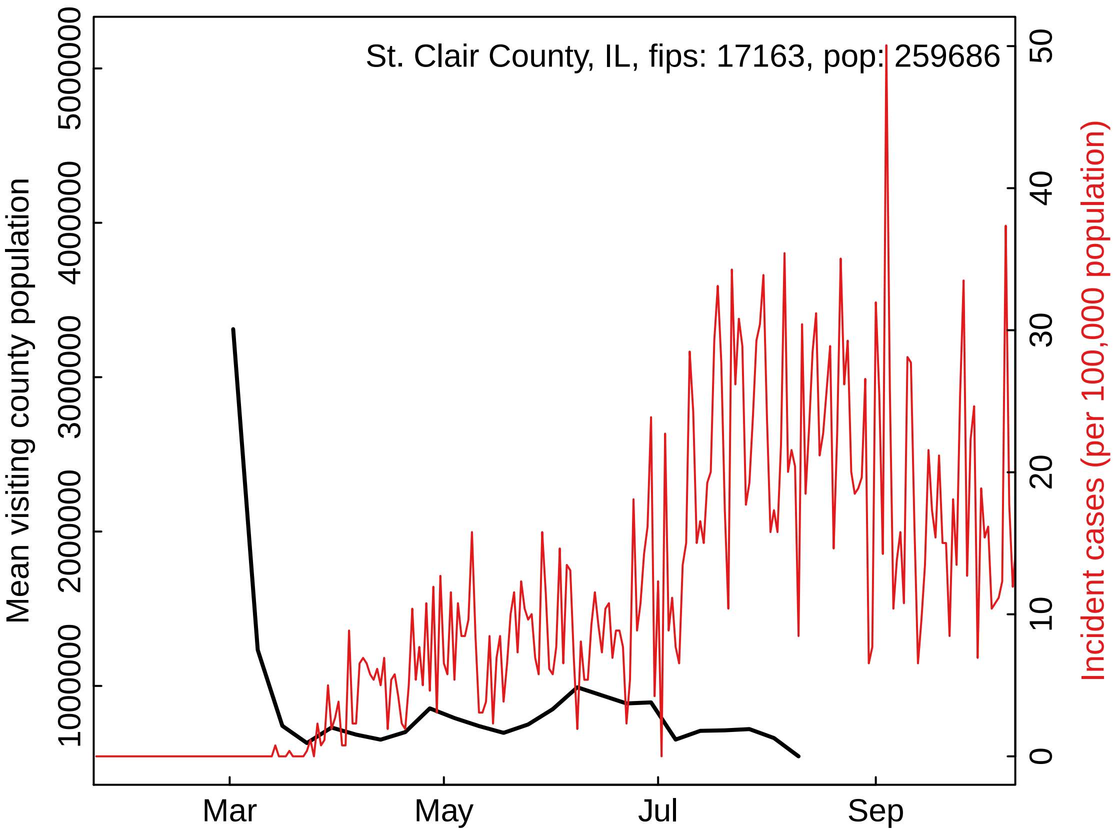

St. Clair County, IL, fips: 17163, pop: 259686
Mean visiting county population
10000000
20000000
30000000
40000000
50000000
Incident cases (per 100,000 population)
0
10
20
30
40
50
Mar
May
Jul
Sep

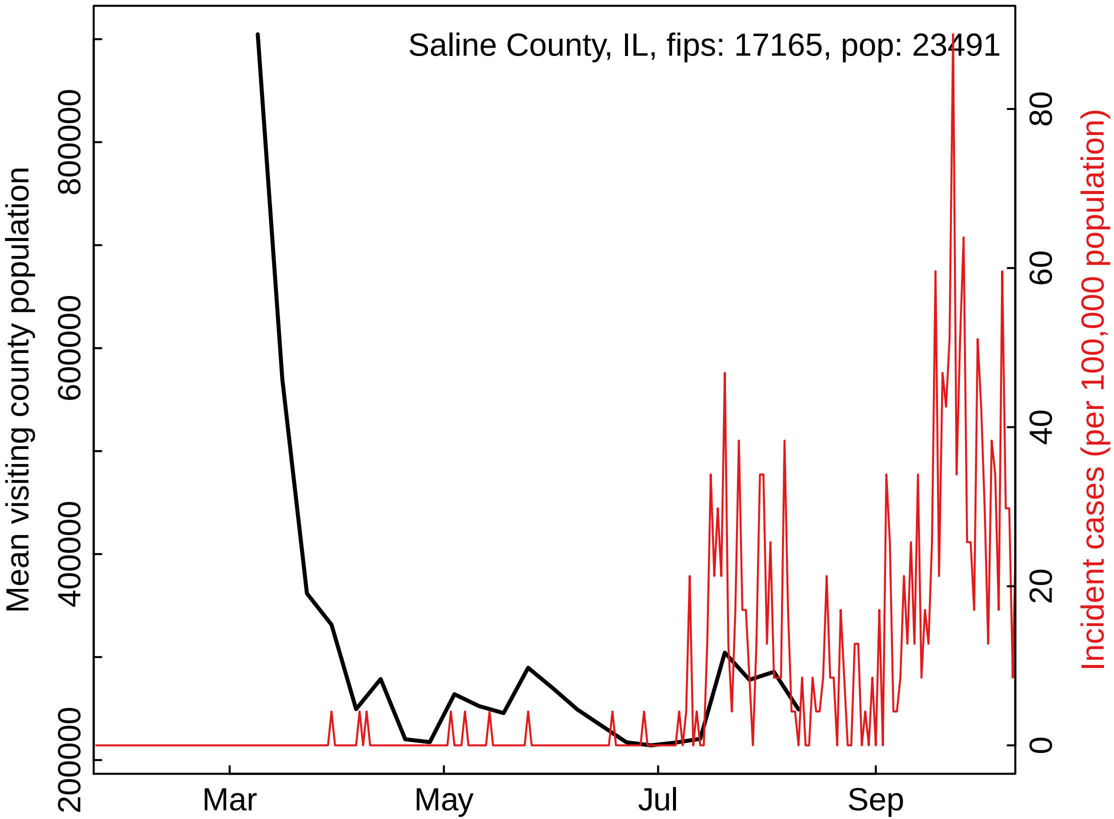

Saline County, IL, fips: 17165, pop: 23491
Mean visiting county population
200000
400000
600000
800000
Incident cases (per 100,000 population)
0
20
40
60
80
Mar
May
Jul
Sep

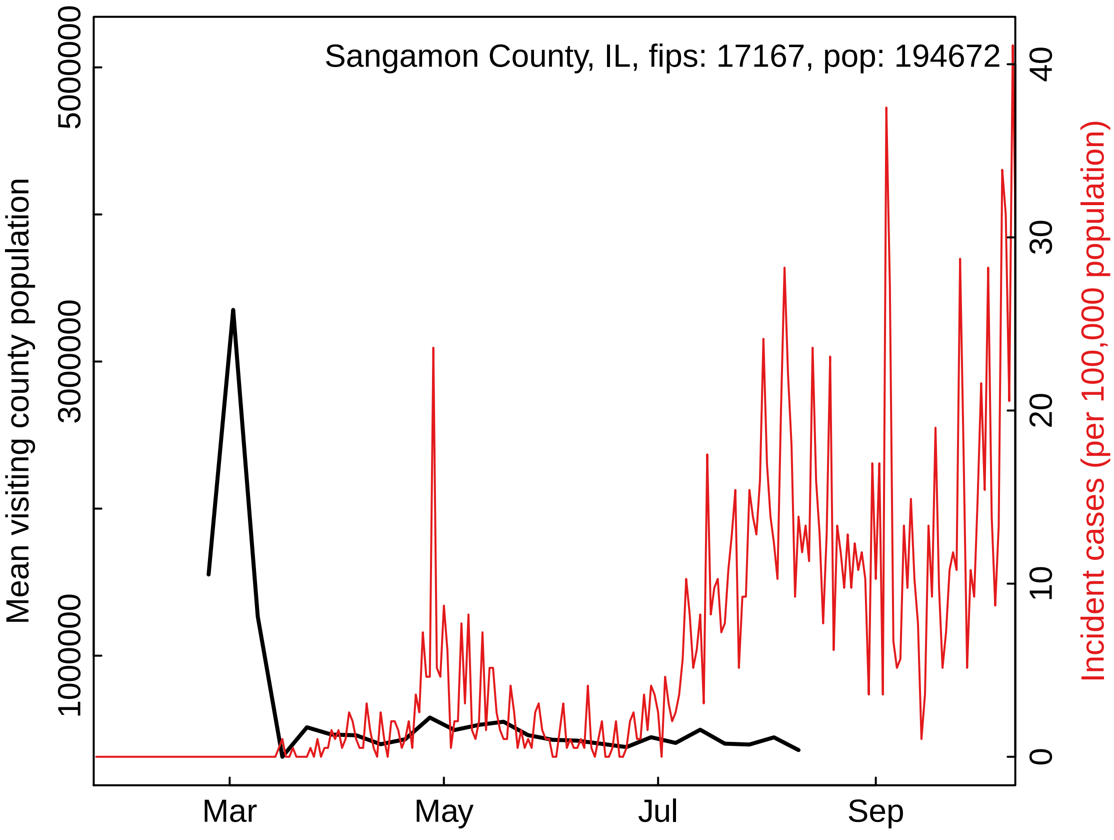
Sangamon County, IL, fips: 17167, pop: 194672
Mean visiting county population
Incident cases (per 100,000 population)
1000000
3000000
5000000
0
10
20
30
40
Mar
May
Jul
Sep

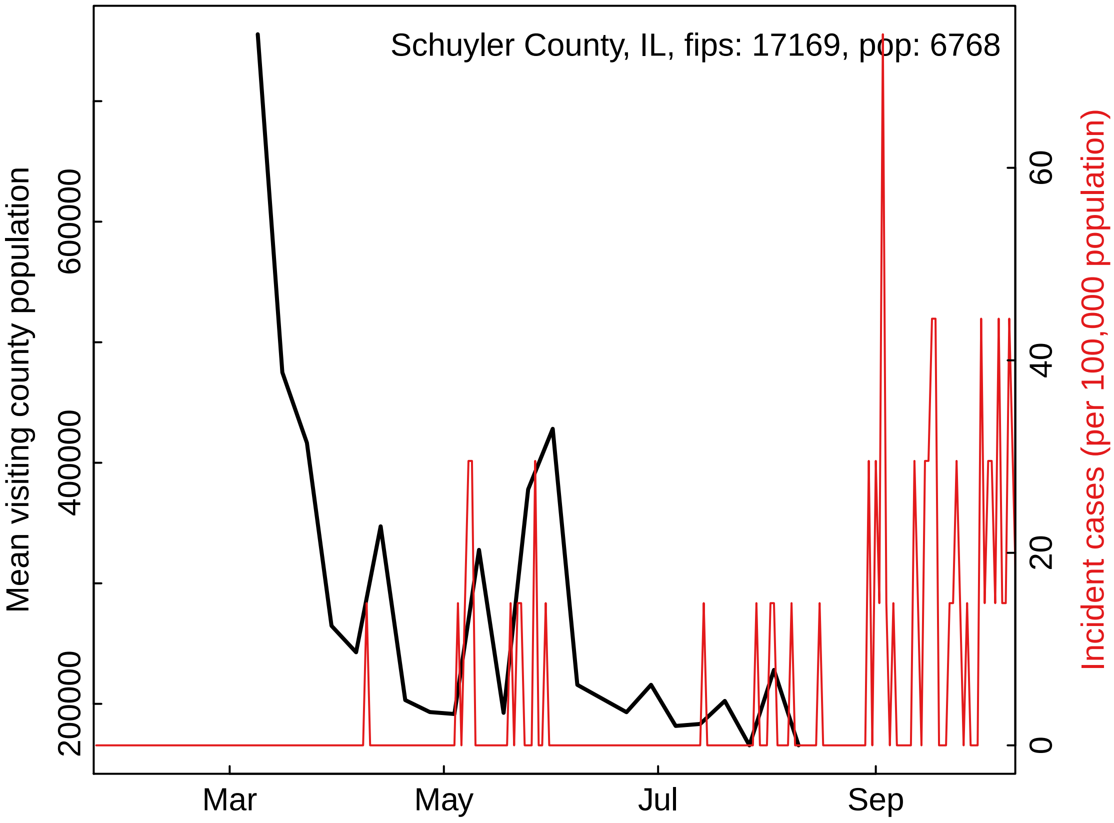

Schuyler County, IL, fips: 17169, pop: 6768
Mean visiting county population
200000
400000
600000
Incident cases (per 100,000 population)
0
20
40
60
Mar
May
Jul
Sep

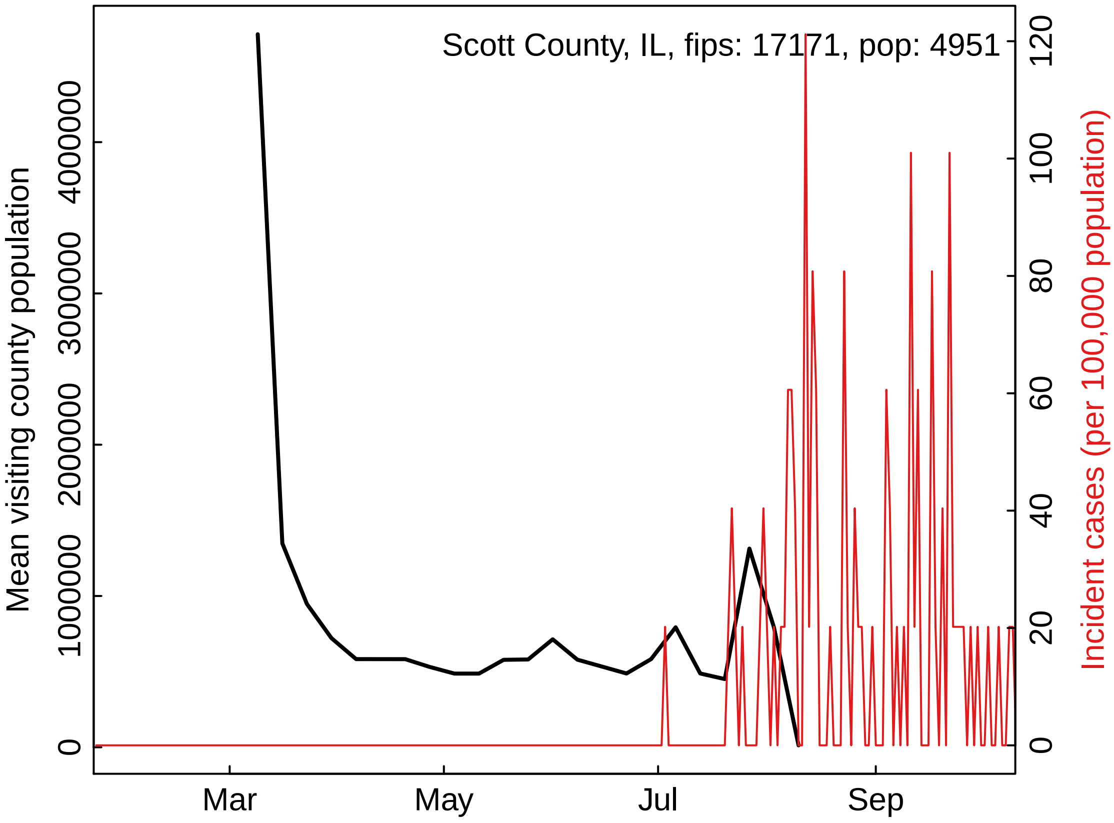
Scott County, IL, fips: 17171, pop: 4951
Mean visiting county population
0
1000000
2000000
3000000
4000000
Incident cases (per 100,000 population)
0
20
40
60
80
100
120
Mar
May
Jul
Sep

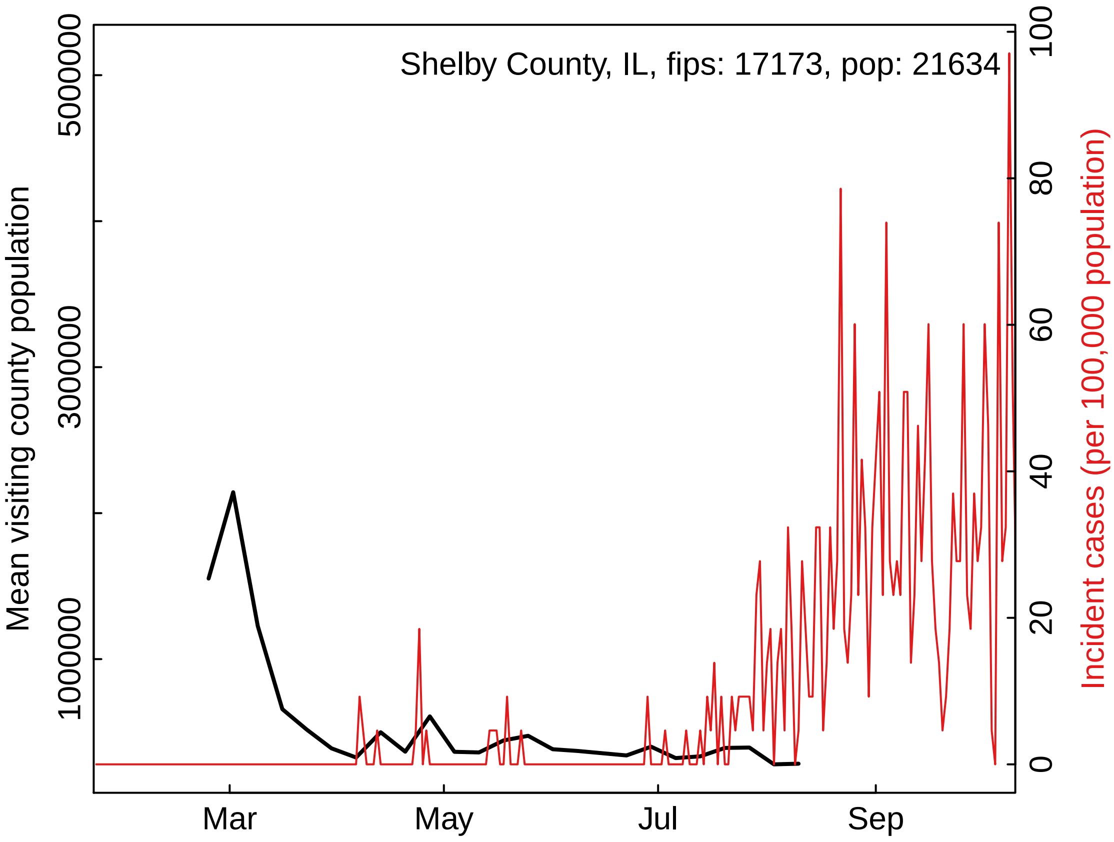

Shelby County, IL, fips: 17173, pop: 21634
Mean visiting county population
1000000
3000000
5000000
Incident cases (per 100,000 population)
0
20
40
60
80
100
Mar
May
Jul
Sep

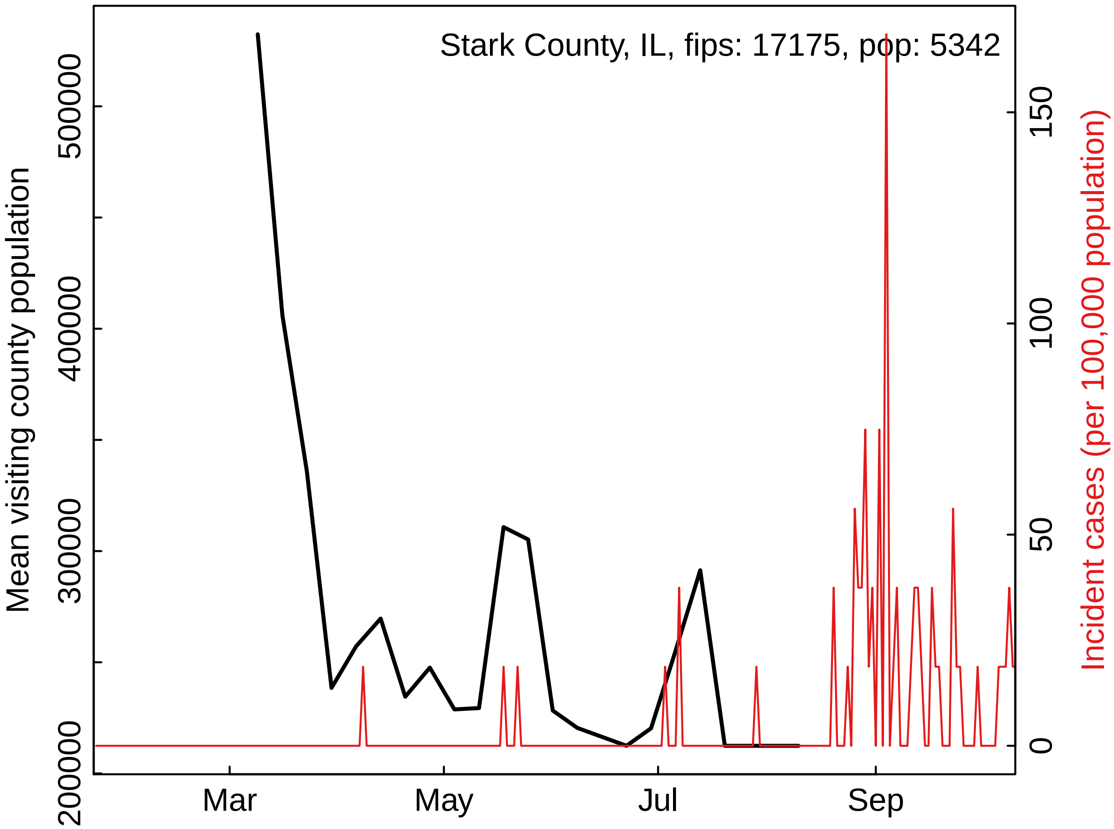
Stark County, IL, fips: 17175, pop: 5342
Mean visiting county population
200000
300000
400000
500000
Mar
May
Jul
Sep
0
50
100
150
Incident cases (per 100,000 population)

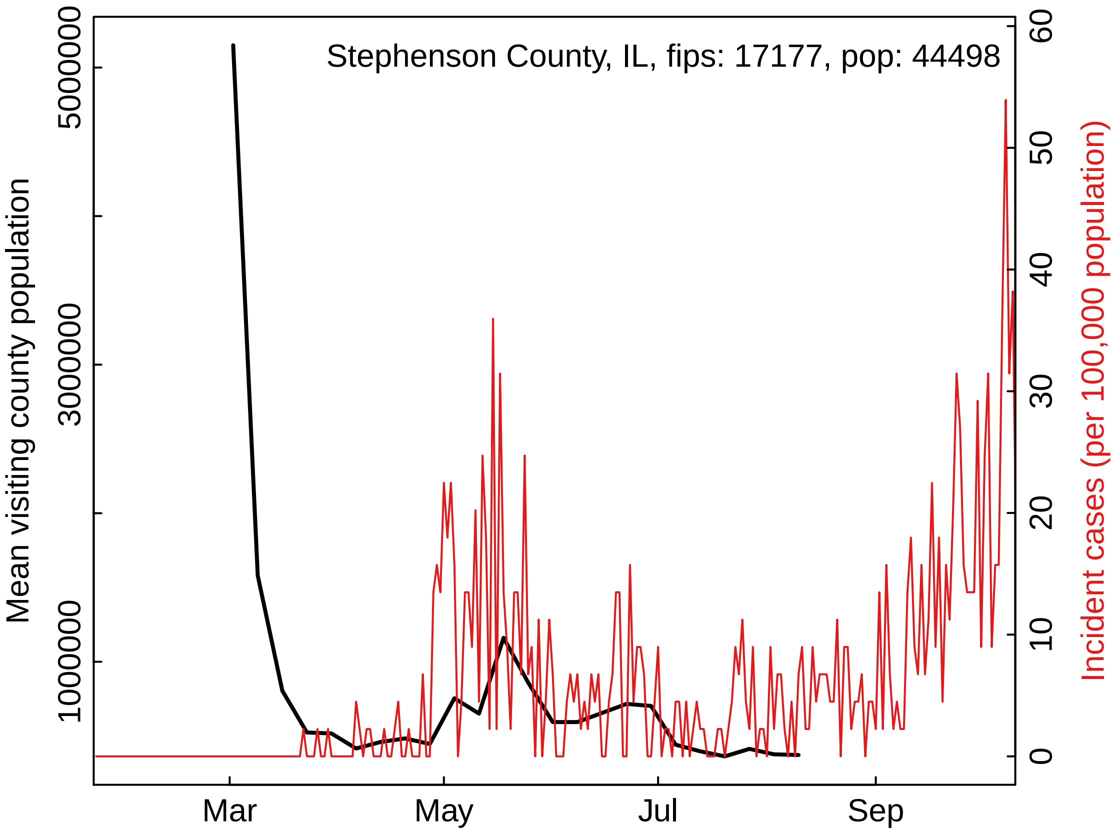

Stephenson County, IL, fips: 17177, pop: 44498
Mean visiting county population
10000000
30000000
50000000
Incident cases (per 100,000 population)
0
10
20
30
40
50
60
Mar
May
Jul
Sep

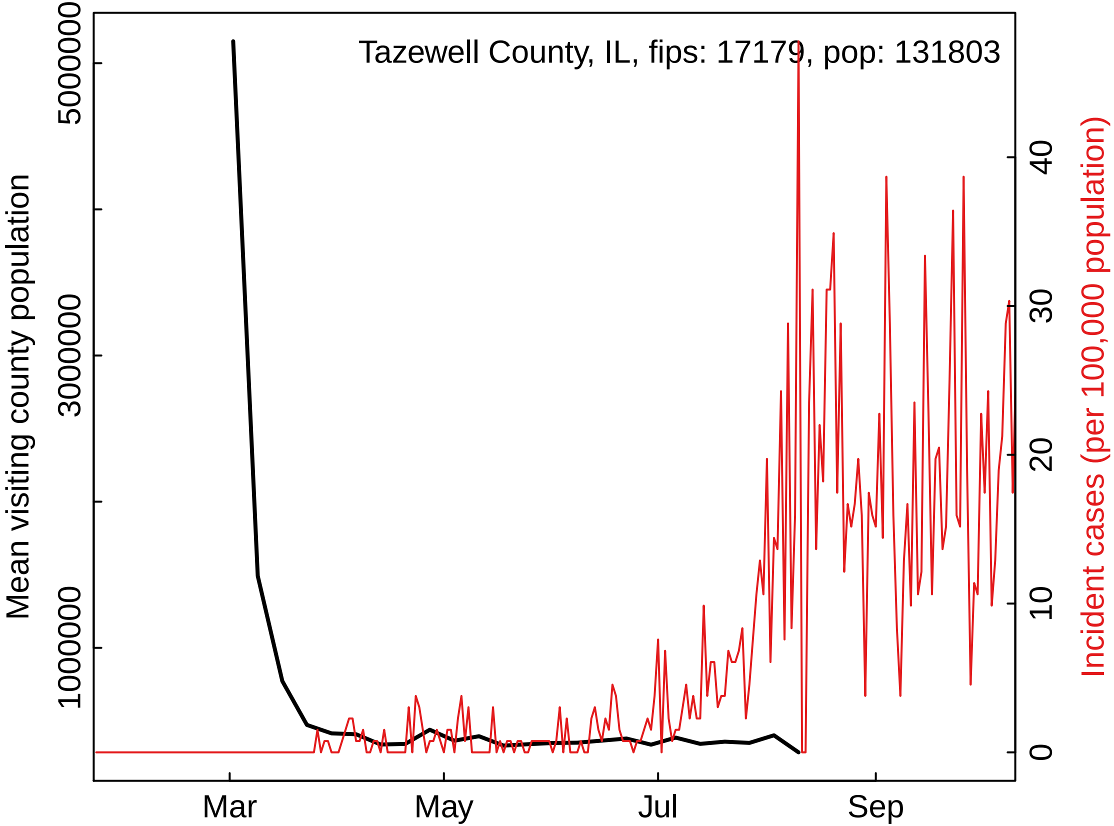

Tazewell County, IL, fips: 17179, pop: 131803
Mean visiting county population
1000000
3000000
5000000
Incident cases (per 100,000 population)
0
10
20
30
40
Mar
May
Jul
Sep

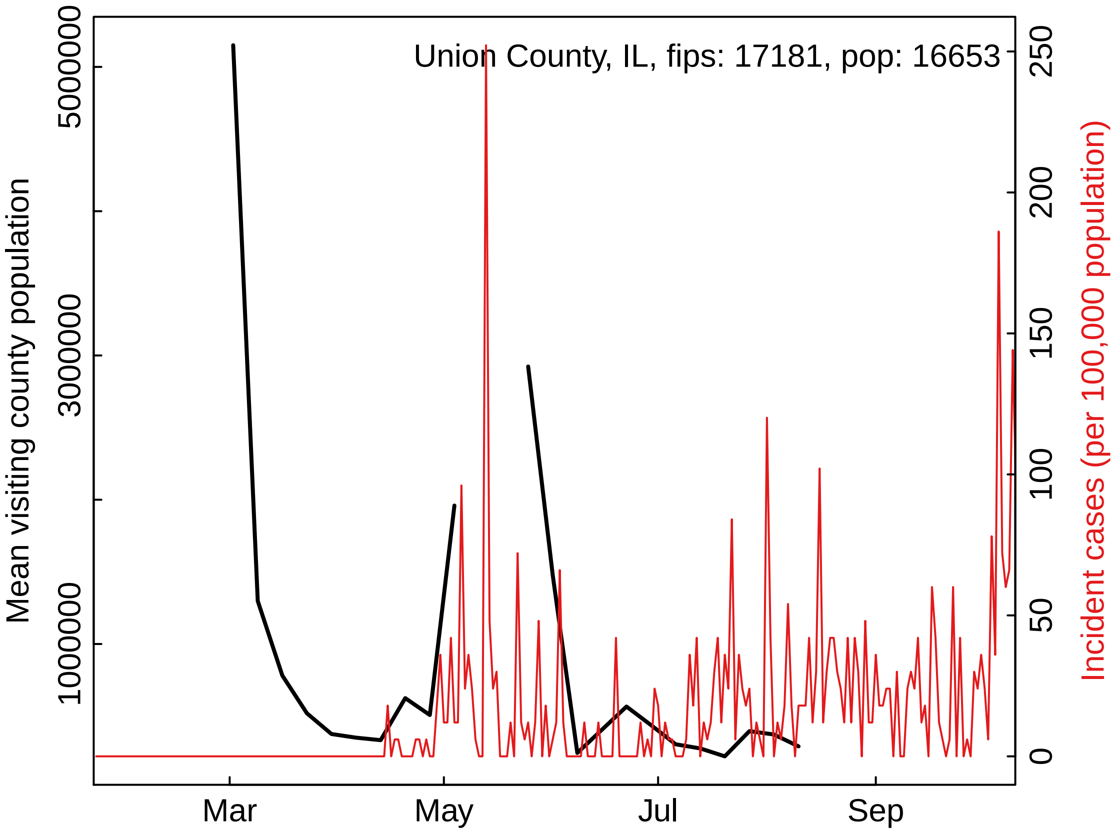
Union County, IL, fips: 17181, pop: 16653
Mean visiting county population
1000000
3000000
5000000
Incident cases (per 100,000 population)
0
50
100
150
200
250
Mar
May
Jul
Sep

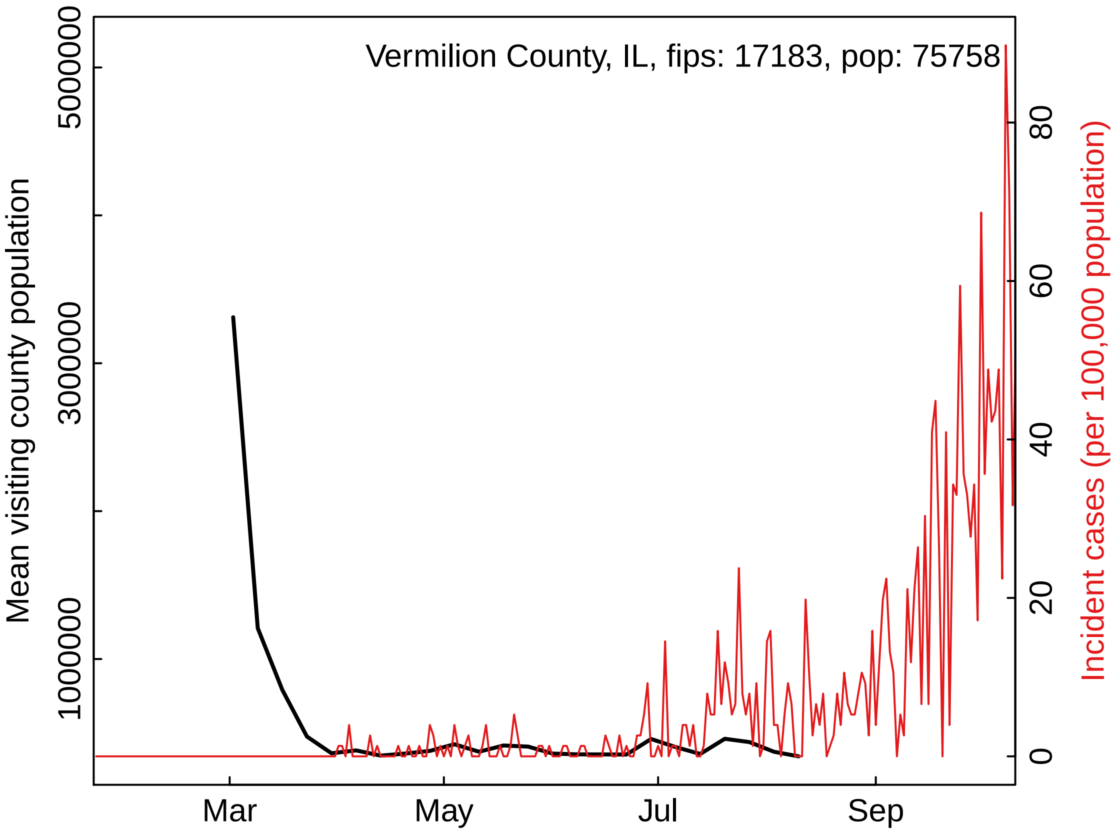
Vermilion County, IL, fips: 17183, pop: 75758
Mean visiting county population
1000000
3000000
5000000
Mar
May
Jul
Sep
0
20
40
60
80
Incident cases (per 100,000 population)

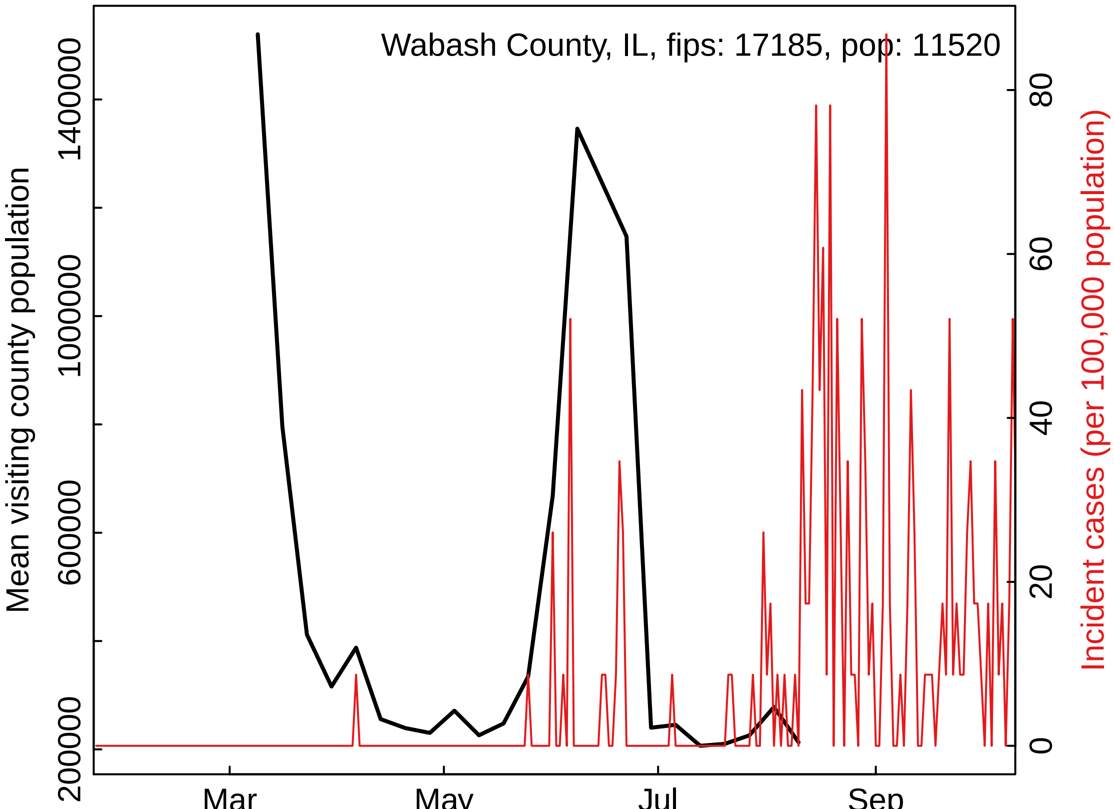

Wabash County, IL, fips: 17185, pop: 11520
Mean visiting county population
200000
600000
1000000
1400000
Incident cases (per 100,000 population)
0
20
40
60
80
Mar
May
Jul
Sep

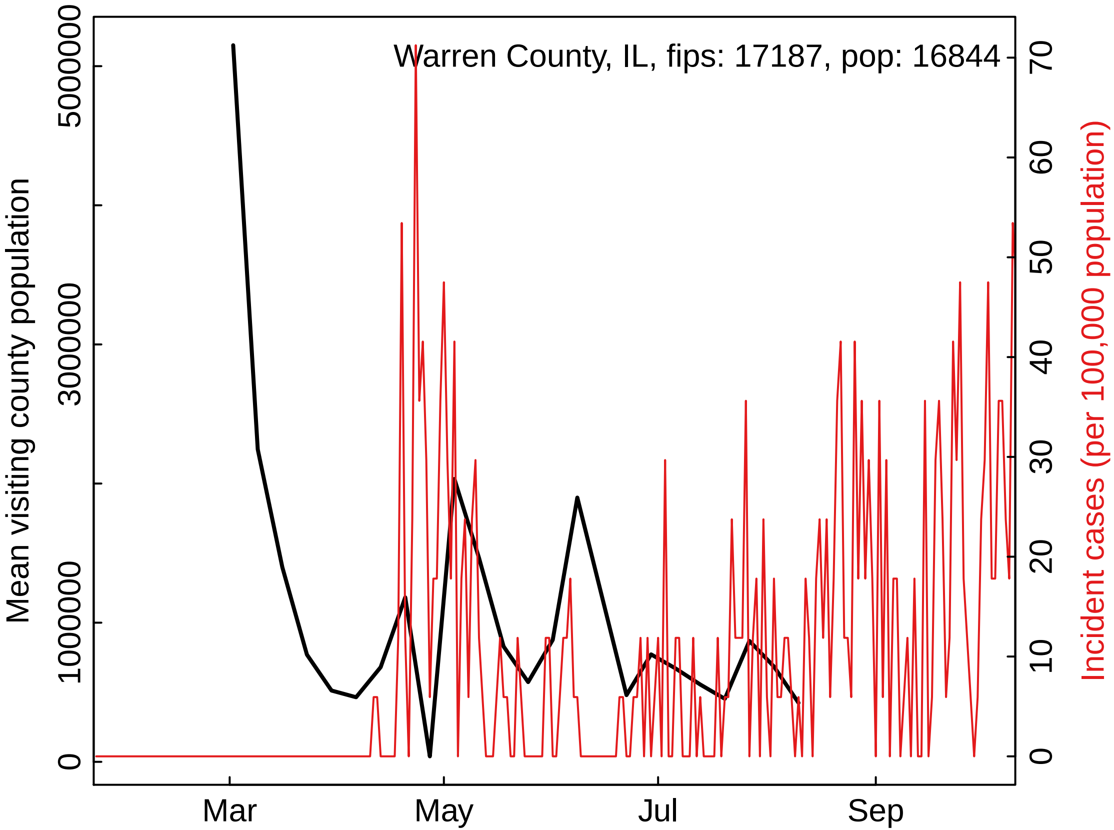

Warren County, IL, fips: 17187, pop: 16844
Mean visiting county population
Incident cases (per 100,000 population)
0
1000000
3000000
5000000
0
10
20
30
40
50
60
70
Mar
May
Jul
Sep

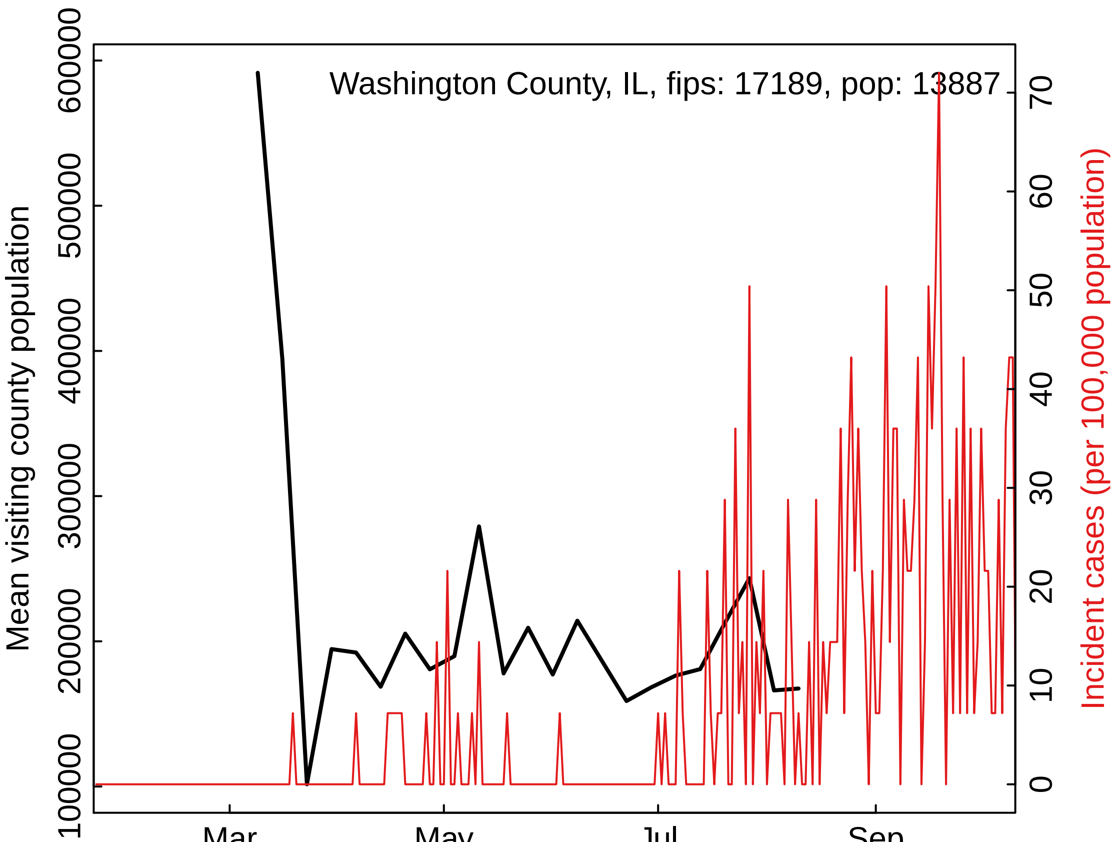
Washington County, IL, fips: 17189, pop: 13887
Mean visiting county population
Incident cases (per 100,000 population)
100000
200000
300000
400000
500000
600000
0
10
20
30
40
50
60
70
Mar
May
Jul
Sep

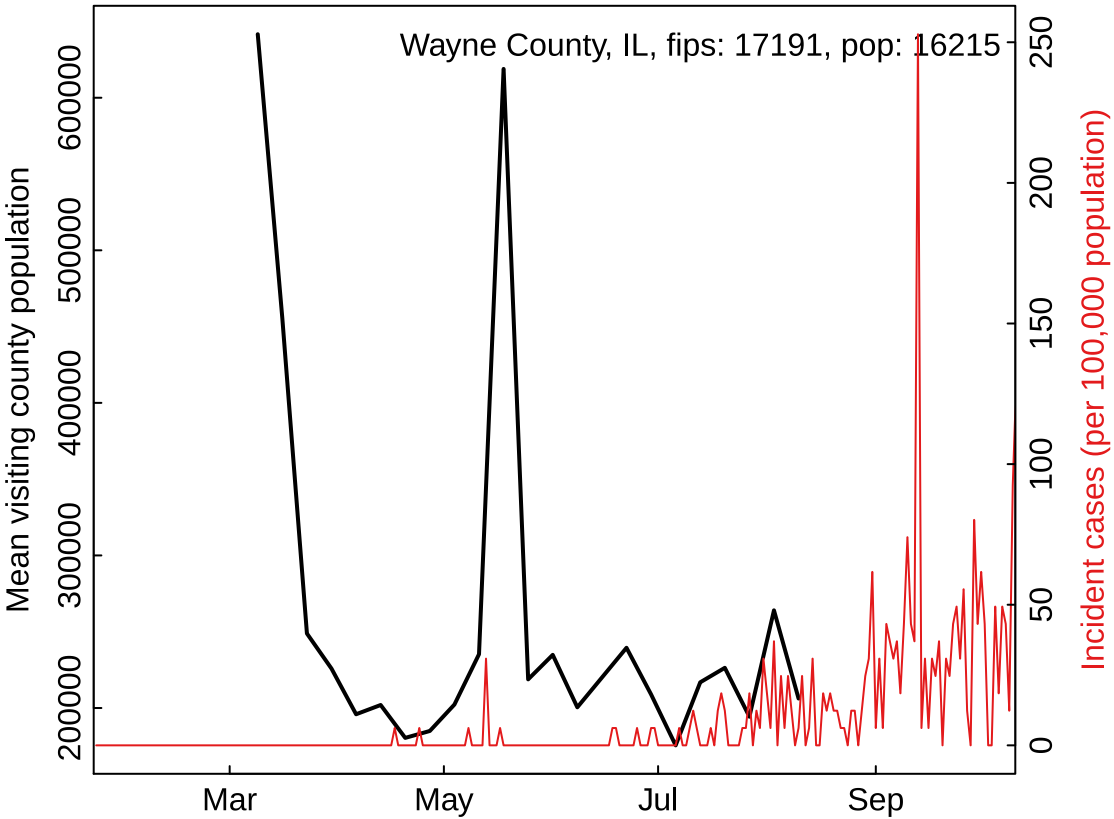

Wayne County, IL, fips: 17191, pop: 16215
Mean visiting county population
200000
300000
400000
500000
600000
Incident cases (per 100,000 population)
0
50
100
150
200
250
Mar
May
Jul
Sep

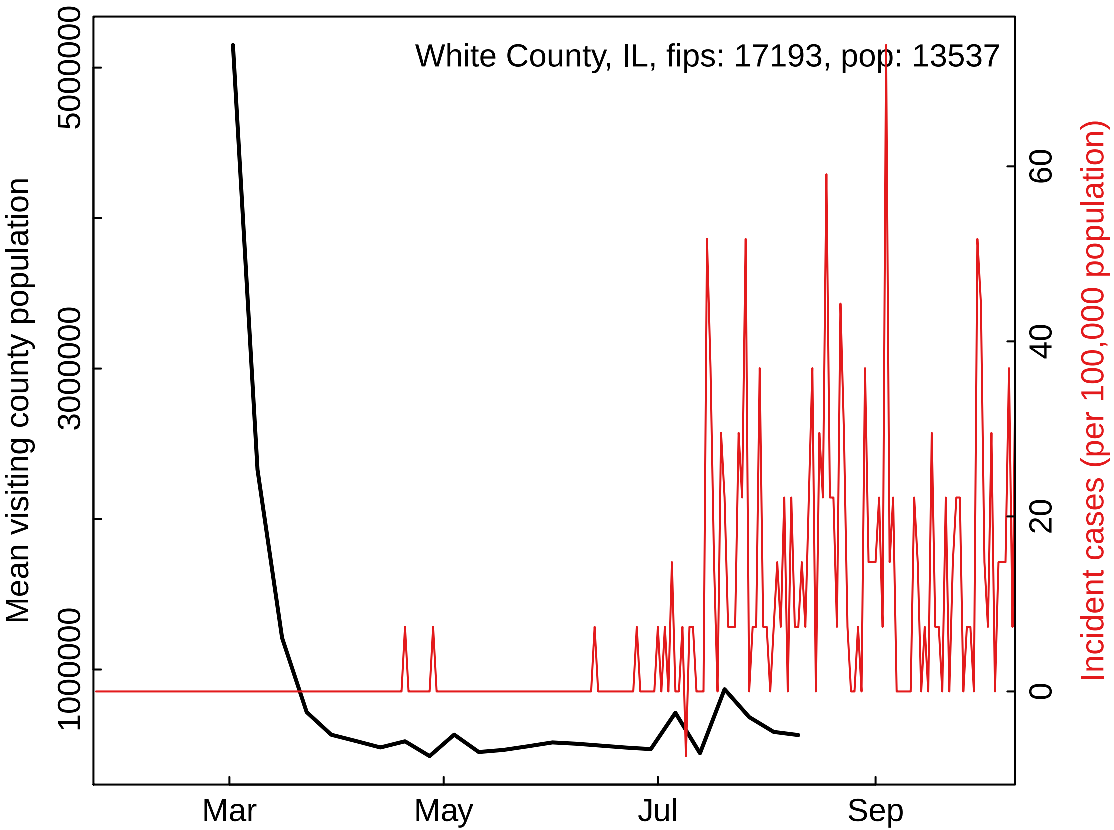

White County, IL, fips: 17193, pop: 13537
Mean visiting county population
1000000
3000000
5000000
Incident cases (per 100,000 population)
0
20
40
60
Mar
May
Jul
Sep

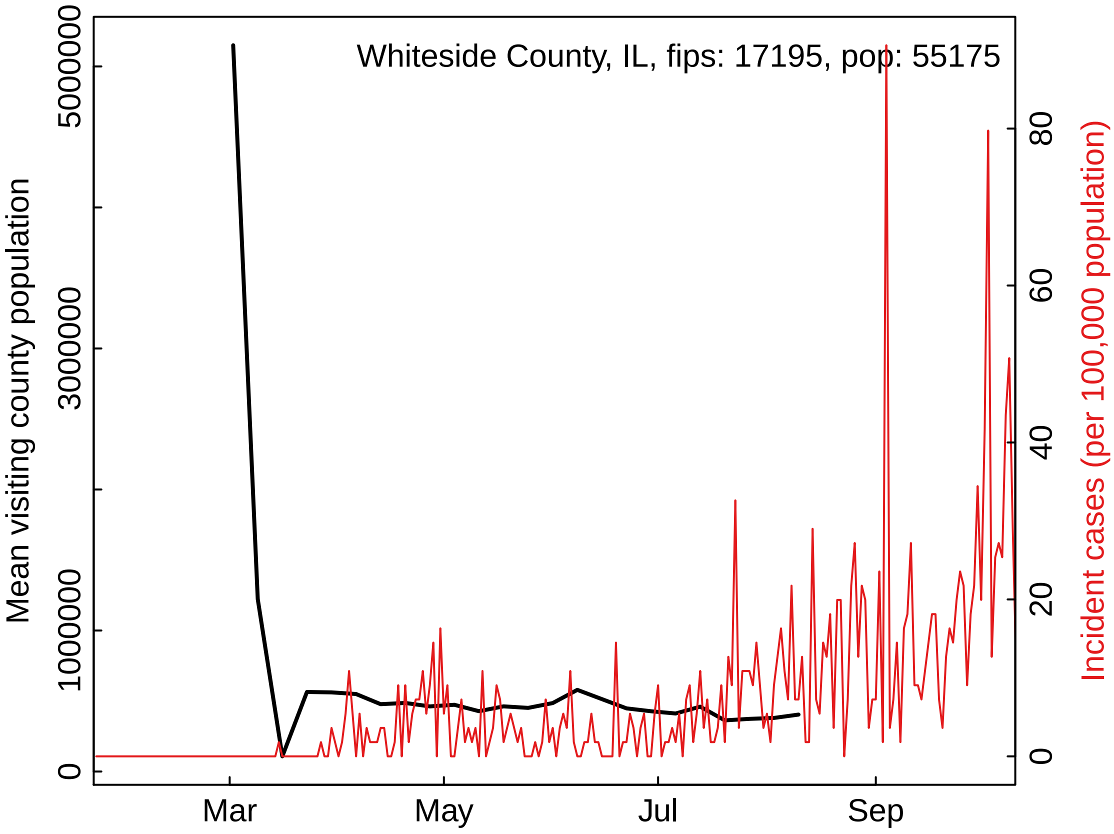

Whiteside County, IL, fips: 17195, pop: 55175
Mean visiting county population
0
1000000
3000000
5000000
Mar
May
Jul
Sep
0
20
40
60
80
Incident cases (per 100,000 population)

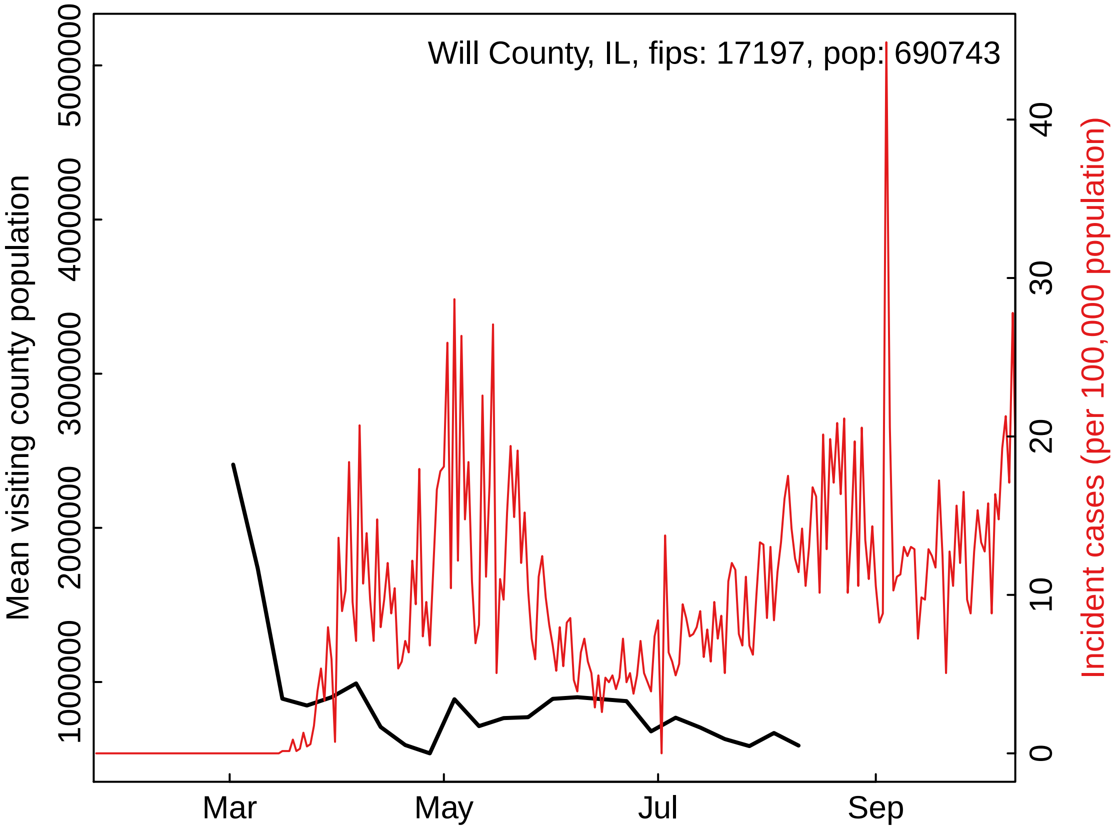
Will County, IL, fips: 17197, pop: 690743
Mean visiting county population
Incident cases (per 100,000 population)
10000000
20000000
30000000
40000000
50000000
0
10
20
30
40
Mar
May
Jul
Sep

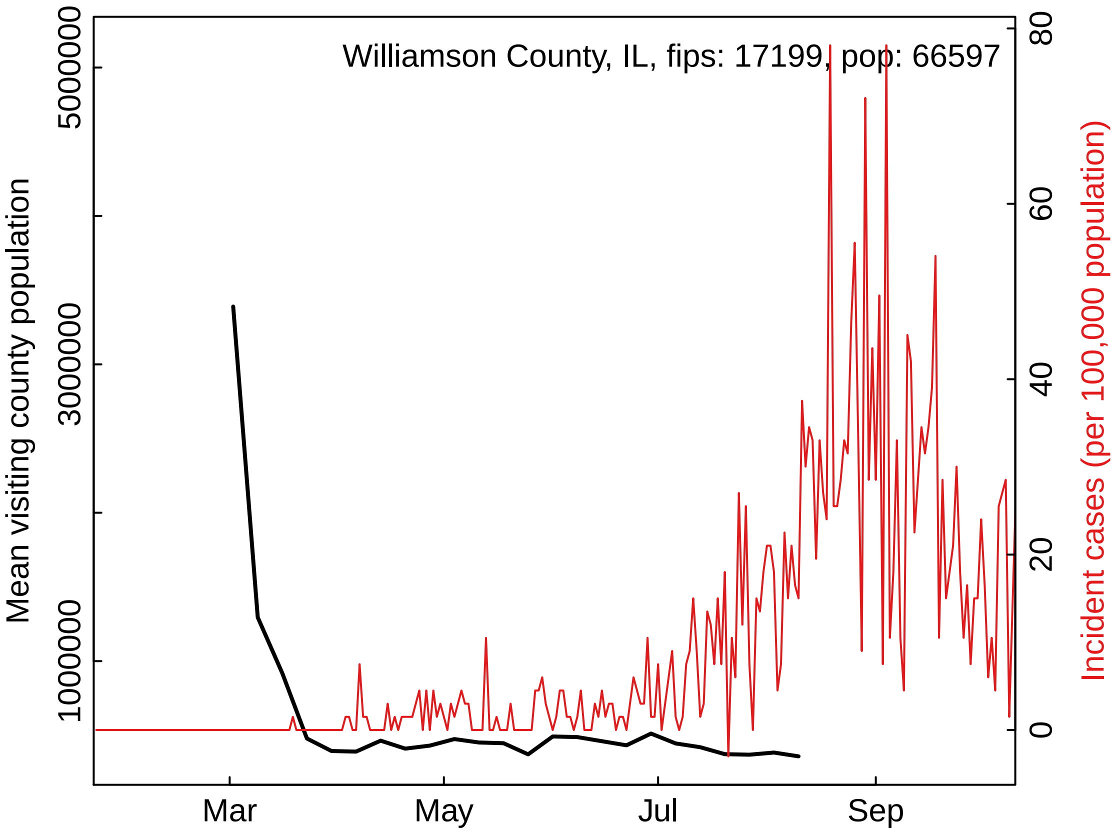

Williamson County, IL, fips: 17199, pop: 66597
Mean visiting county population
Incident cases (per 100,000 population)
1000000
3000000
5000000
0
20
40
60
80
Mar
May
Jul
Sep

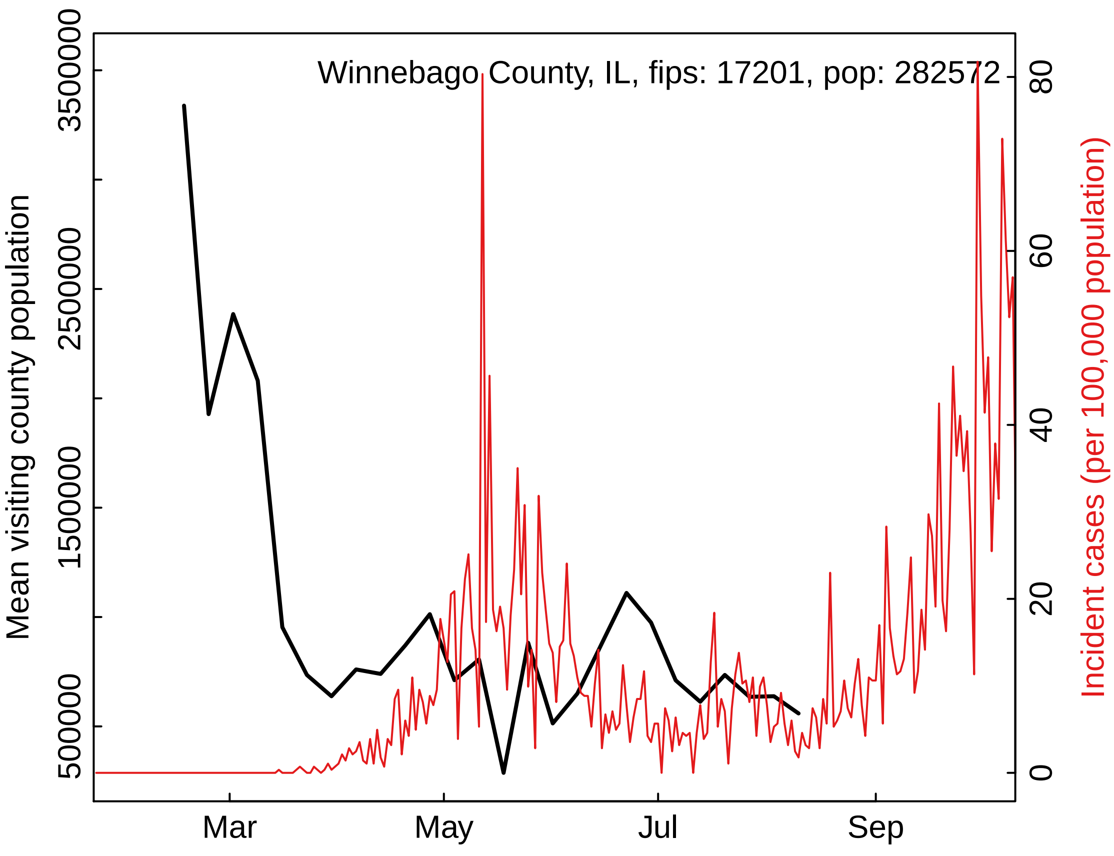

Winnebago County, IL, fips: 17201, pop: 282572
Mean visiting county population
Incident cases (per 100,000 population)
500000
1500000
2500000
3500000
0
20
40
60
80
Mar
May
Jul
Sep

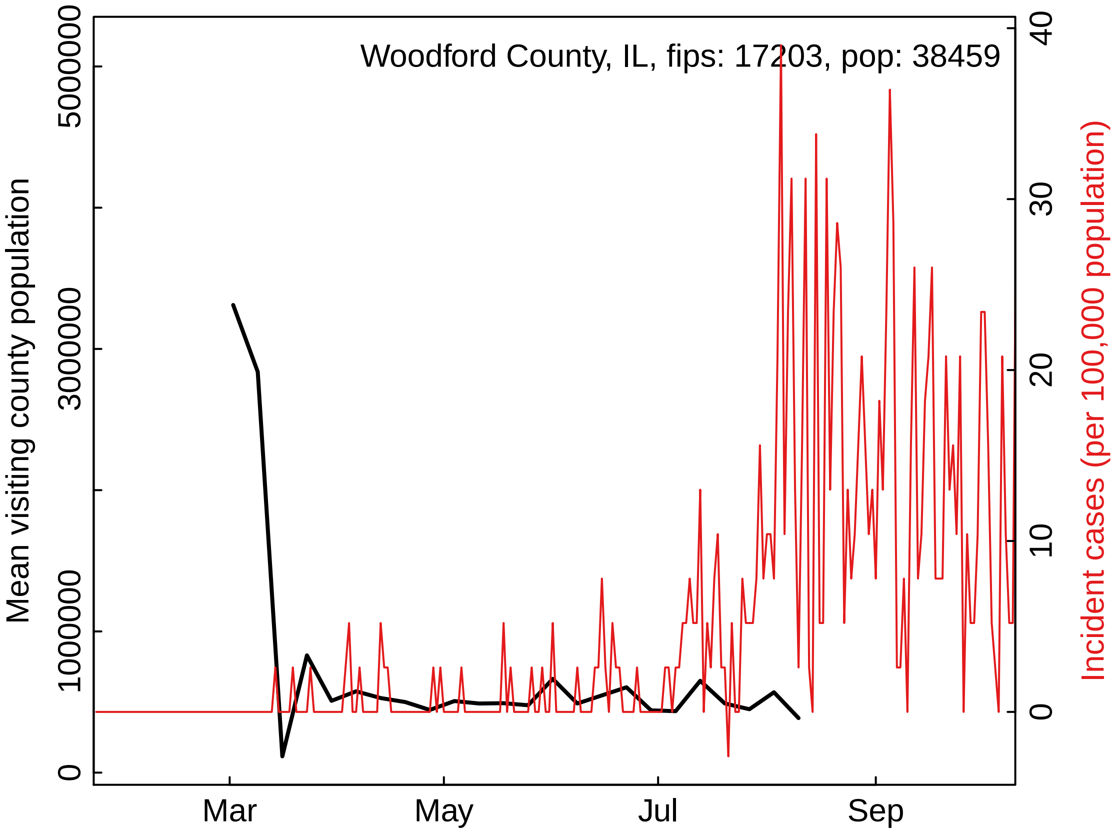
Woodford County, IL, fips: 17203, pop: 38459
Mean visiting county population
0
1000000
3000000
5000000
Incident cases (per 100,000 population)
0
10
20
30
40
Mar
May
Jul
Sep

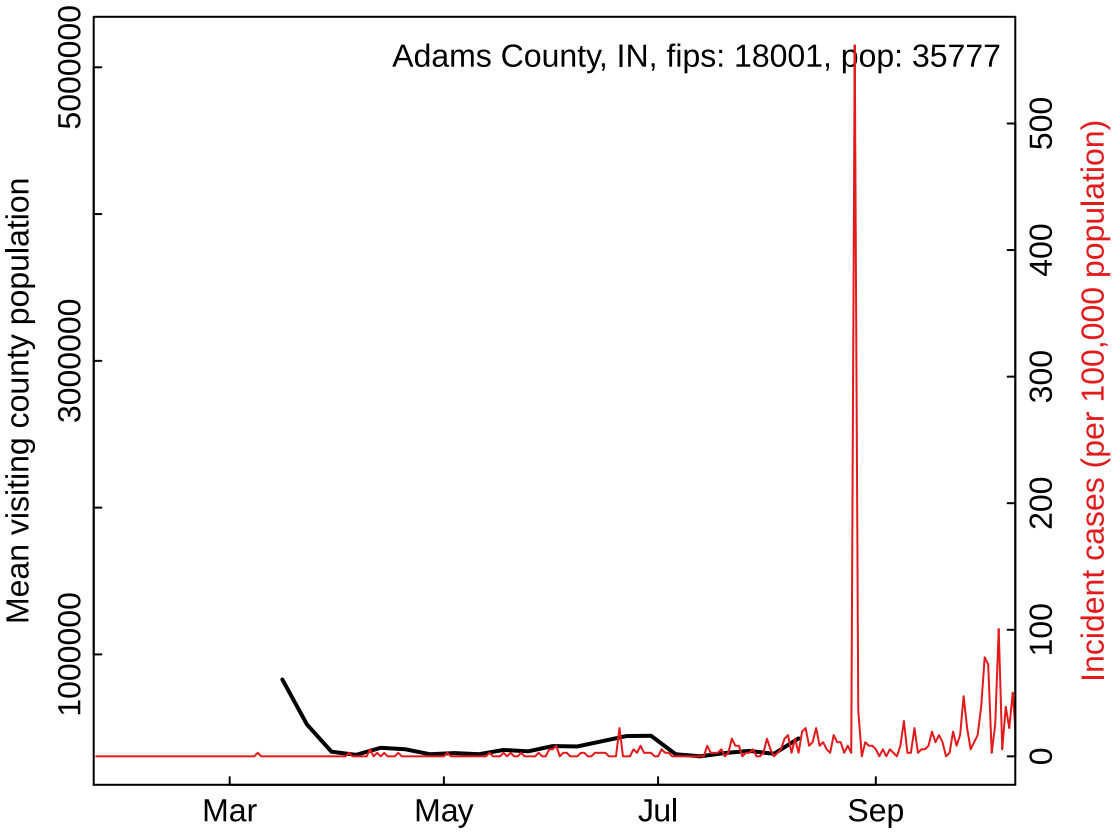

Adams County, IN, fips: 18001, pop: 35777
Mean visiting county population
1000000
3000000
5000000
Incident cases (per 100,000 population)
0
100
200
300
400
500
Mar
May
Jul
Sep

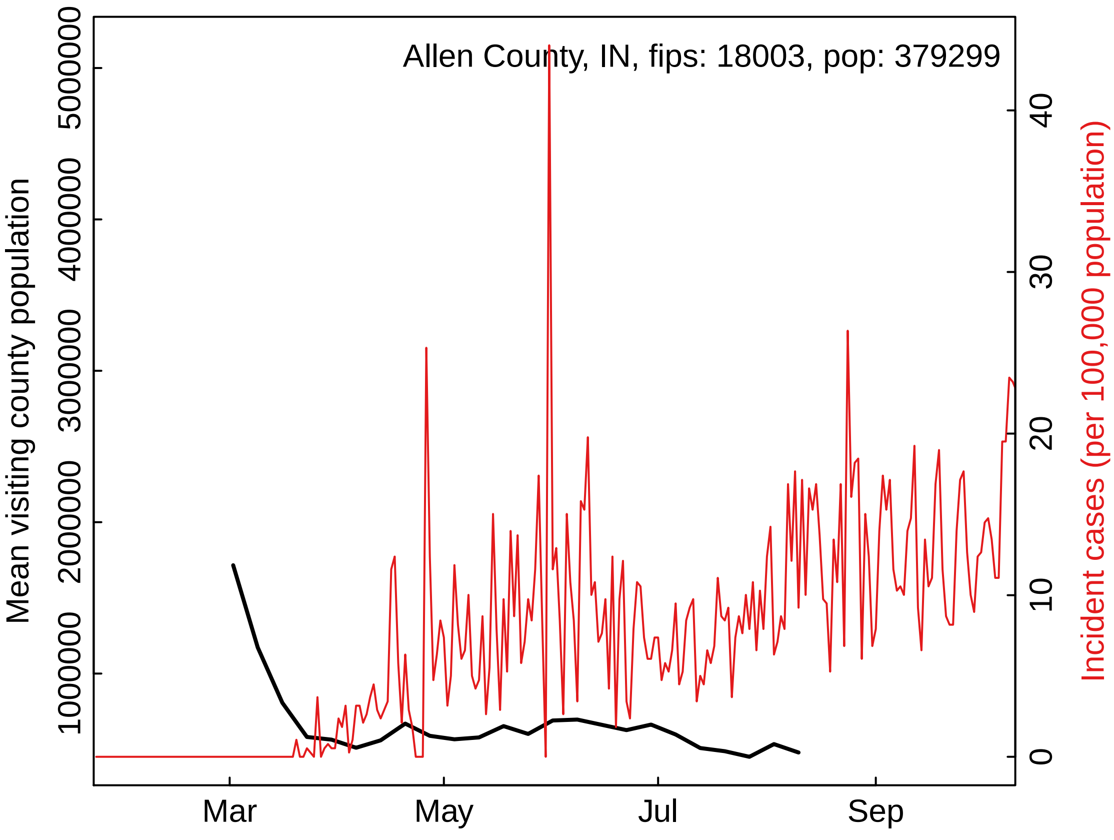
Allen County, IN, fips: 18003, pop: 379299
Mean visiting county population
1000000
2000000
3000000
4000000
5000000
Incident cases (per 100,000 population)
0
10
20
30
40
Mar
May
Jul
Sep

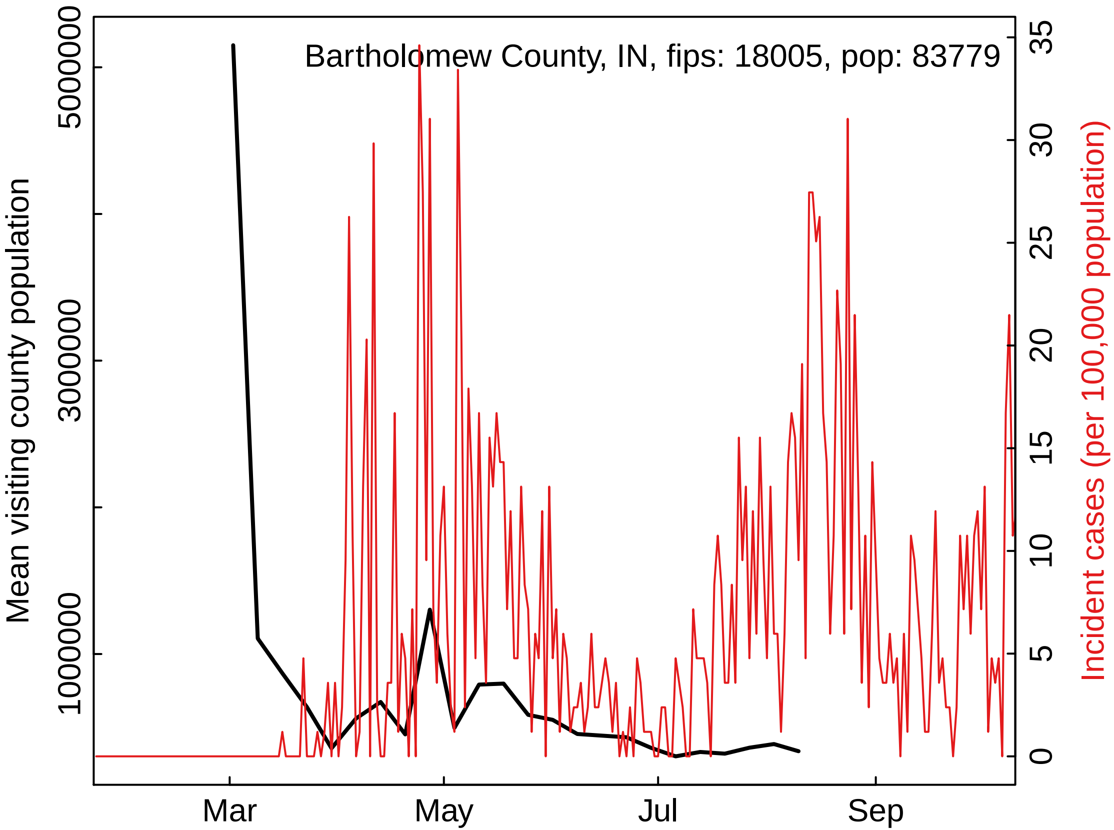
Bartholomew County, IN, fips: 18005, pop: 83779
Mean visiting county population
1000000
3000000
5000000
Incident cases (per 100,000 population)
0
5
10
15
20
25
30
35
Mar
May
Jul
Sep

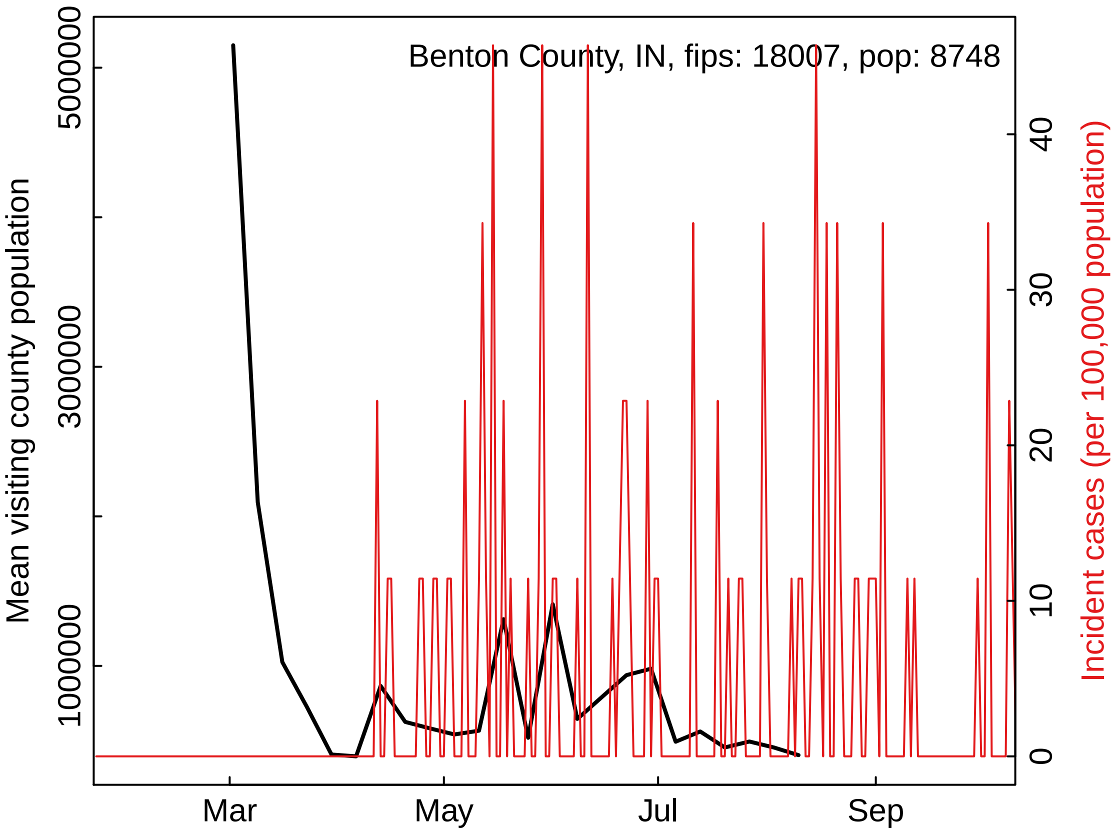
Benton County, IN, fips: 18007, pop: 8748
Mean visiting county population
1000000
3000000
5000000
Incident cases (per 100,000 population)
0
10
20
30
40
Mar
May
Jul
Sep

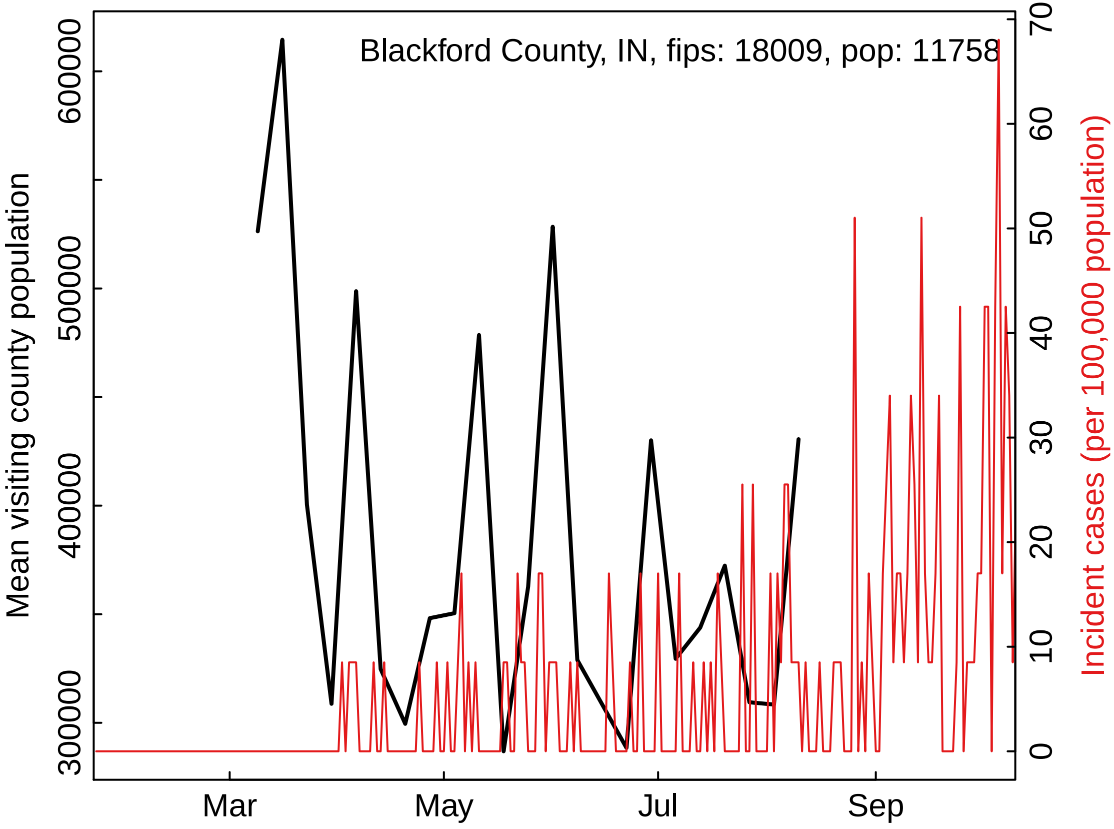

Blackford County, IN, fips: 18009, pop: 11758
Mean visiting county population
300000
400000
500000
600000
Incident cases (per 100,000 population)
0
10
20
30
40
50
60
70
Mar
May
Jul
Sep

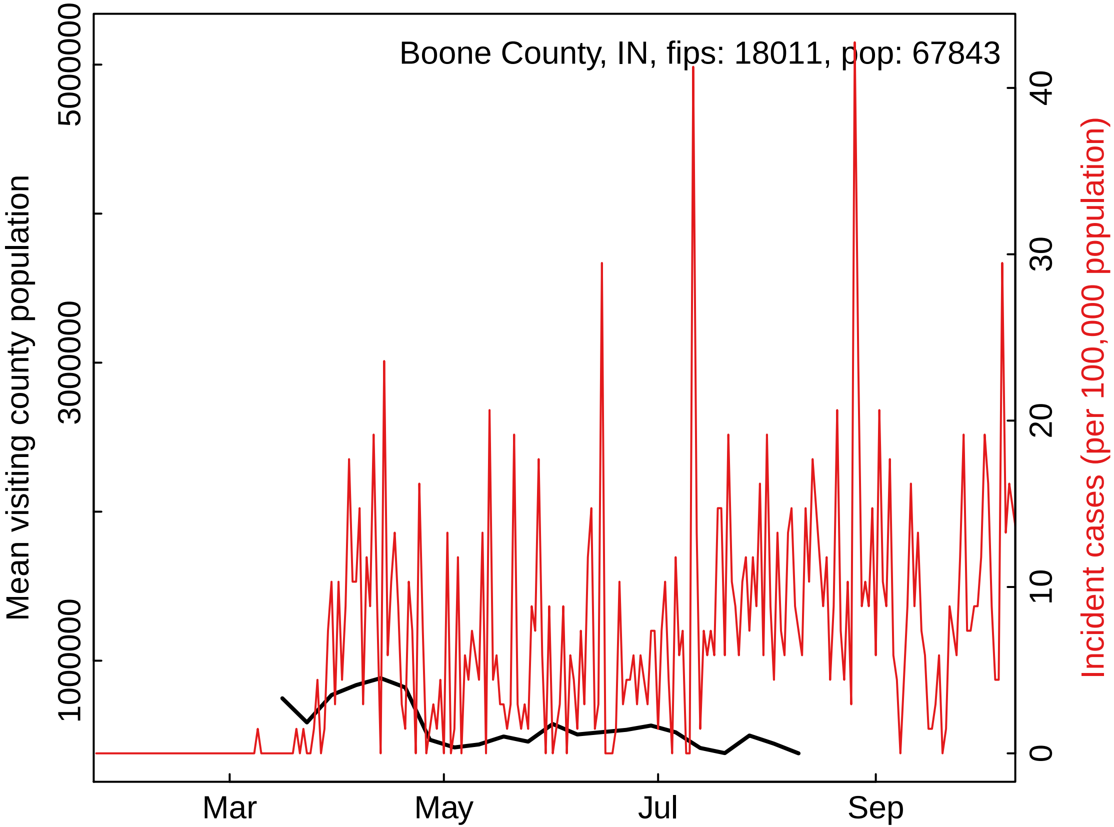

Boone County, IN, fips: 18011, pop: 67843
Mean visiting county population
10000000
30000000
50000000
Incident cases (per 100,000 population)
0
10
20
30
40
Mar
May
Jul
Sep

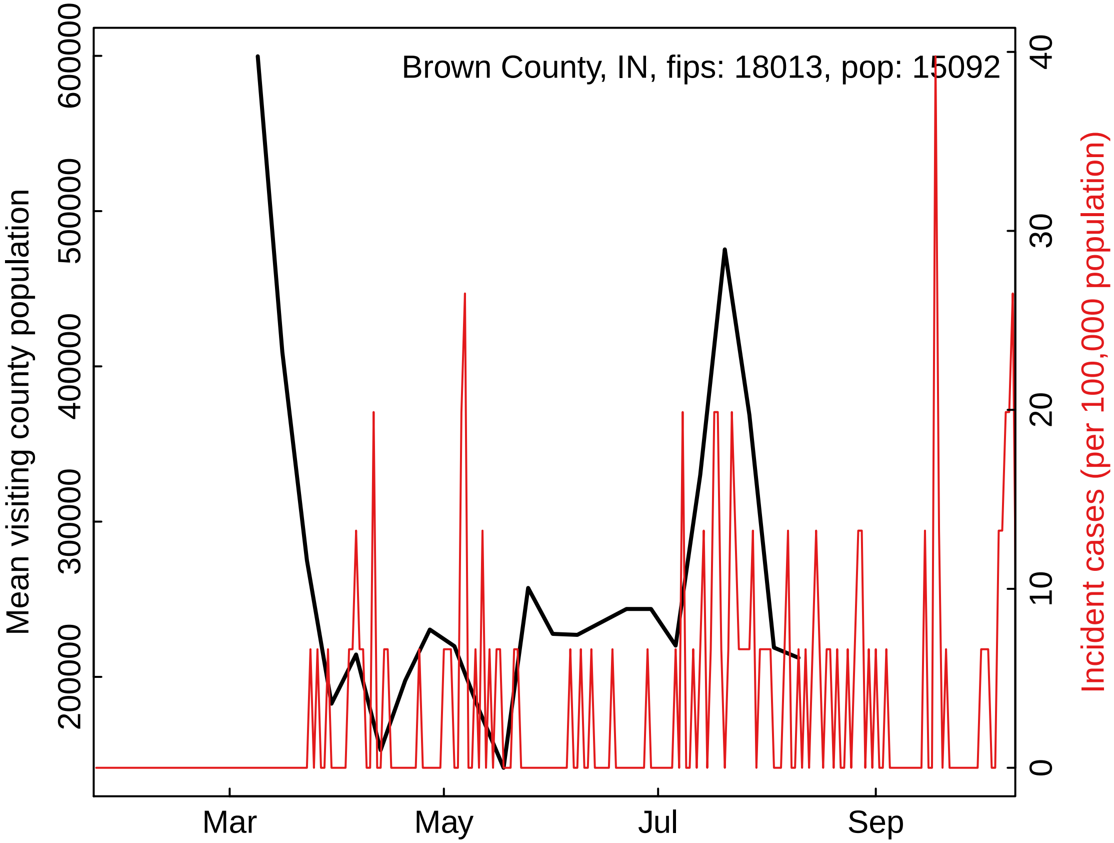

Brown County, IN, fips: 18013, pop: 15092
Mean visiting county population
200000
300000
400000
500000
600000
Incident cases (per 100,000 population)
0
10
20
30
40
Mar
May
Jul
Sep

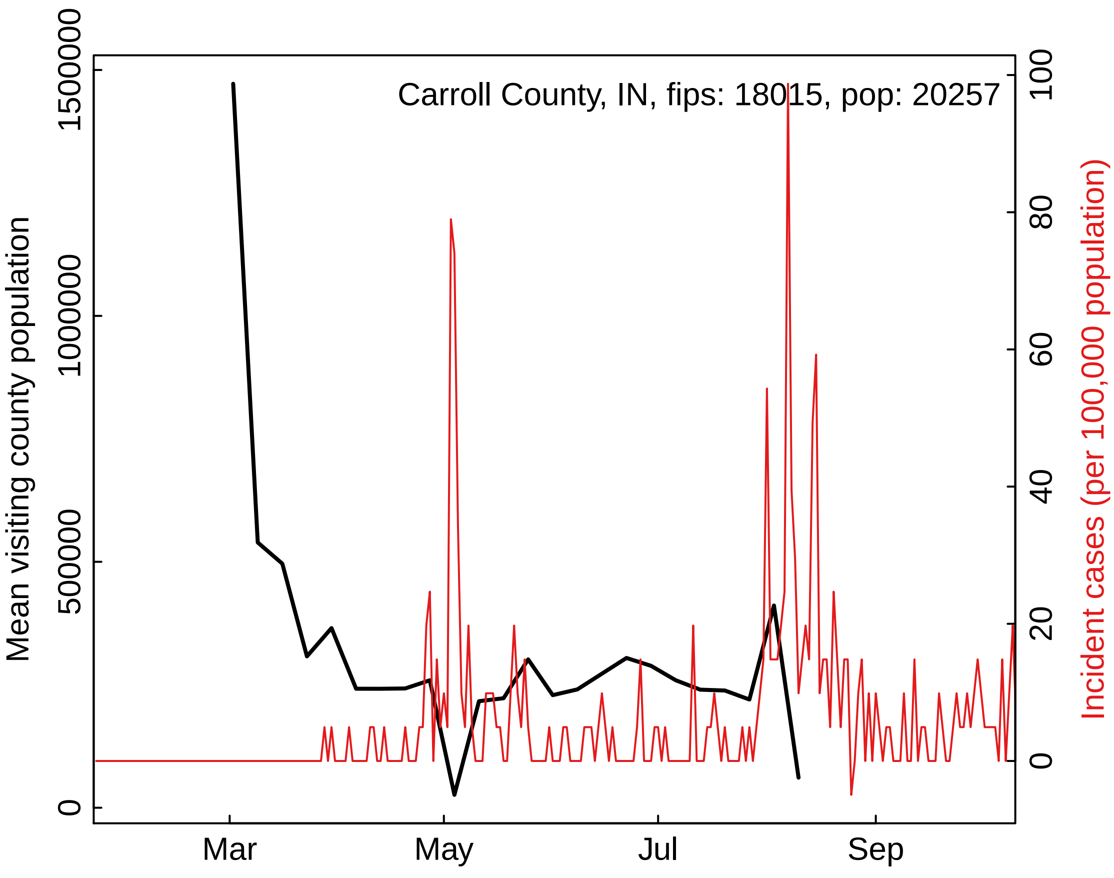

Carroll County, IN, fips: 18015, pop: 20257
Mean visiting county population
0
500000
1000000
1500000
Incident cases (per 100,000 population)
0
20
40
60
80
100
Mar
May
Jul
Sep

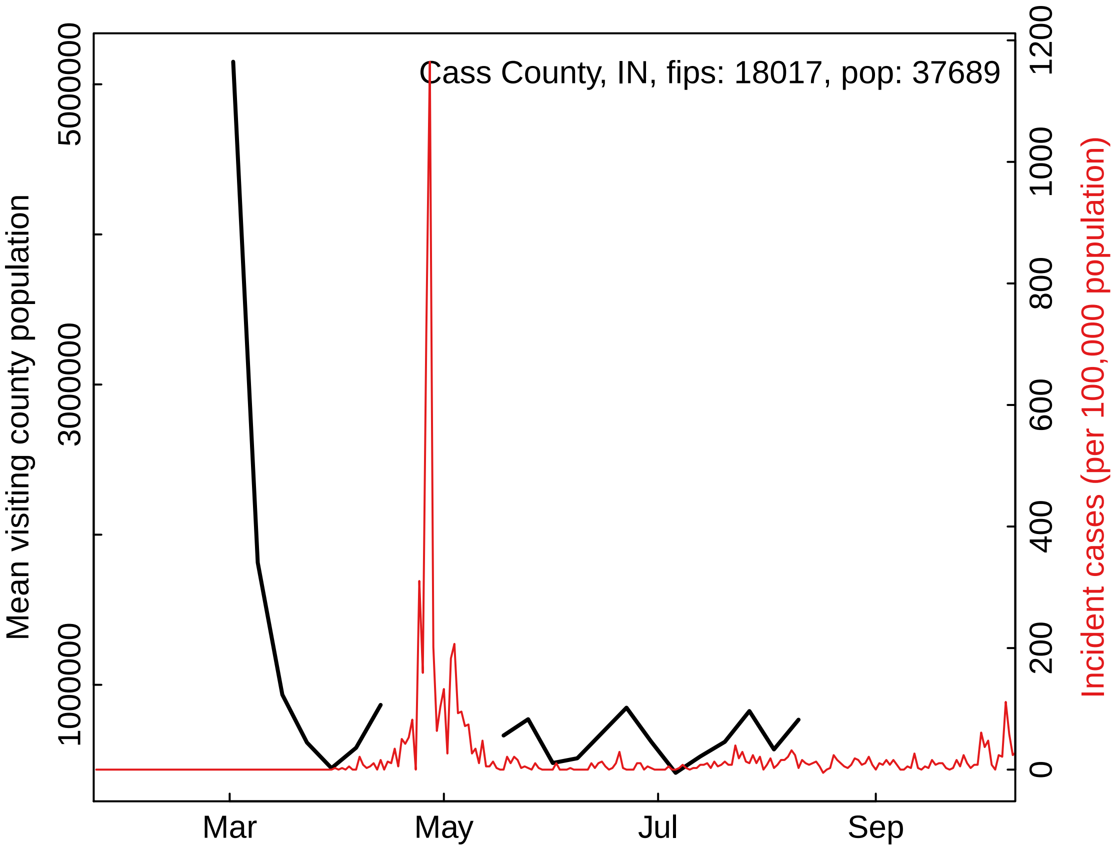

Cass County, IN, fips: 18017, pop: 37689
Mean visiting county population
Incident cases (per 100,000 population)
5000000
3000000
1000000
1200
1000
800
600
400
200
0
Mar
May
Jul
Sep

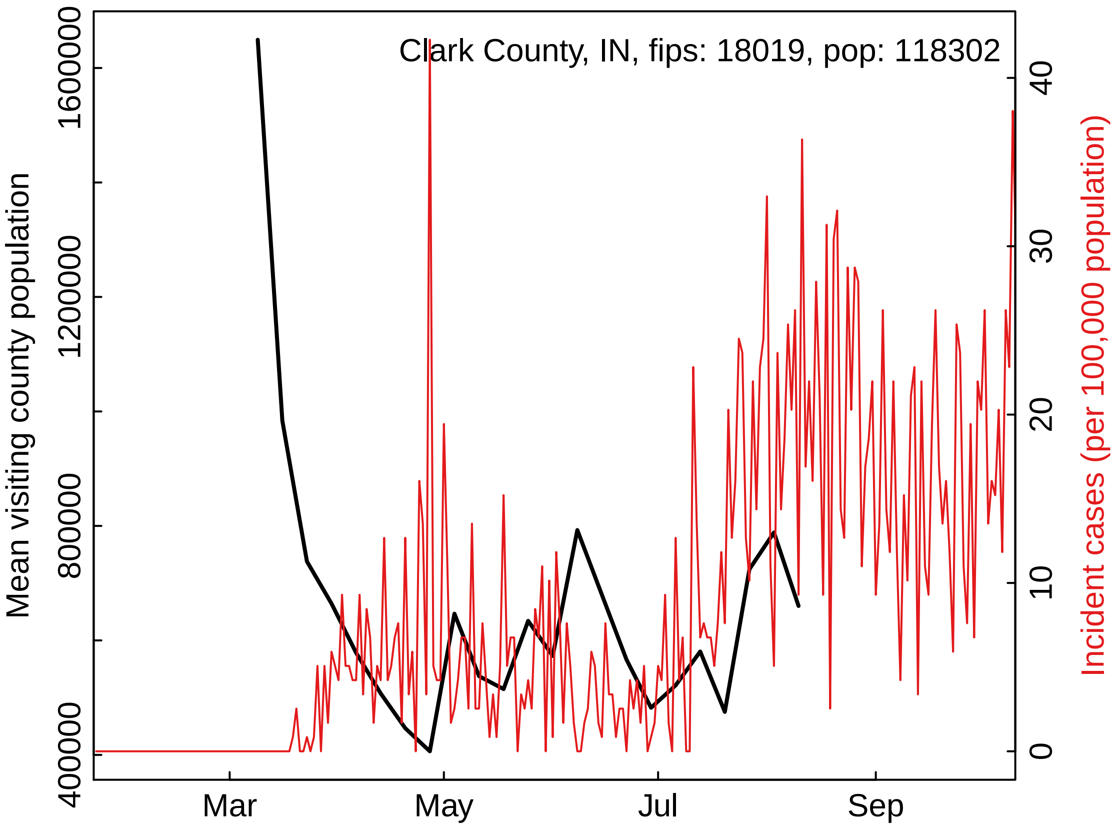

Clark County, IN, fips: 18019, pop: 118302
Mean visiting county population
4000000
8000000
12000000
16000000
Incident cases (per 100,000 population)
0
10
20
30
40
Mar
May
Jul
Sep

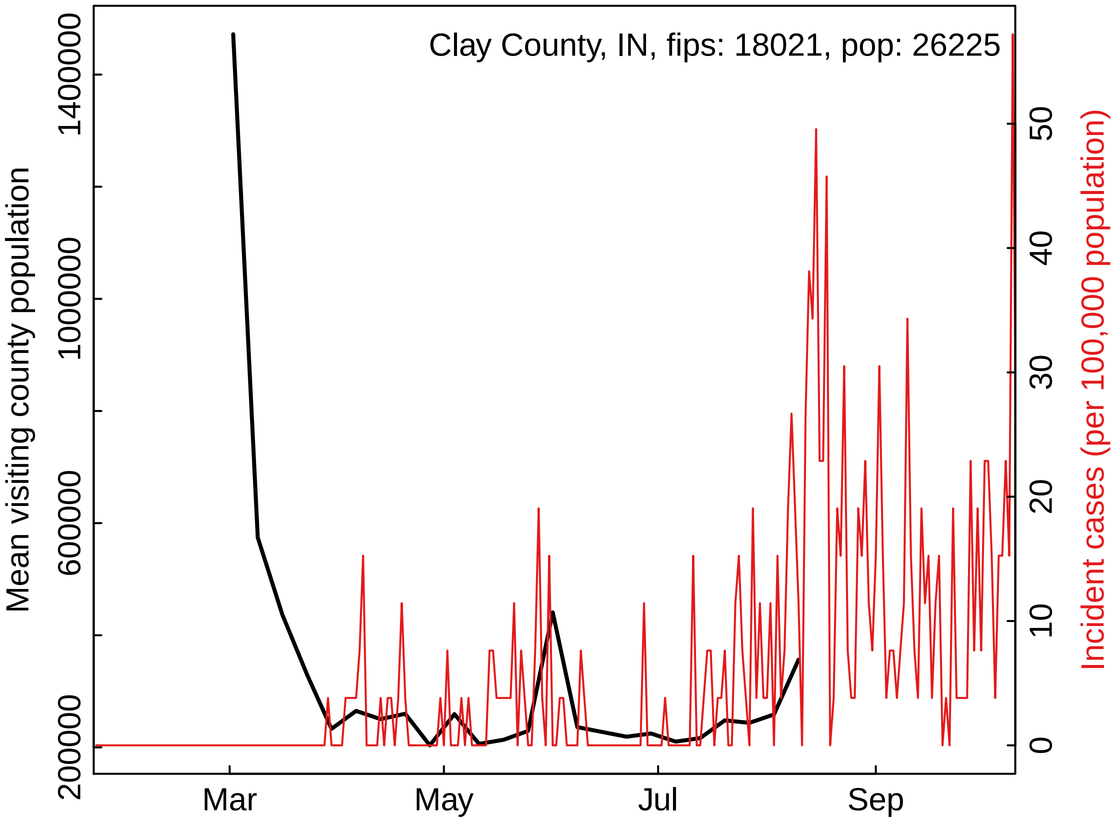
Clay County, IN, fips: 18021, pop: 26225
Mean visiting county population
200000
600000
1000000
1400000
Incident cases (per 100,000 population)
0
10
20
30
40
50
Mar
May
Jul
Sep

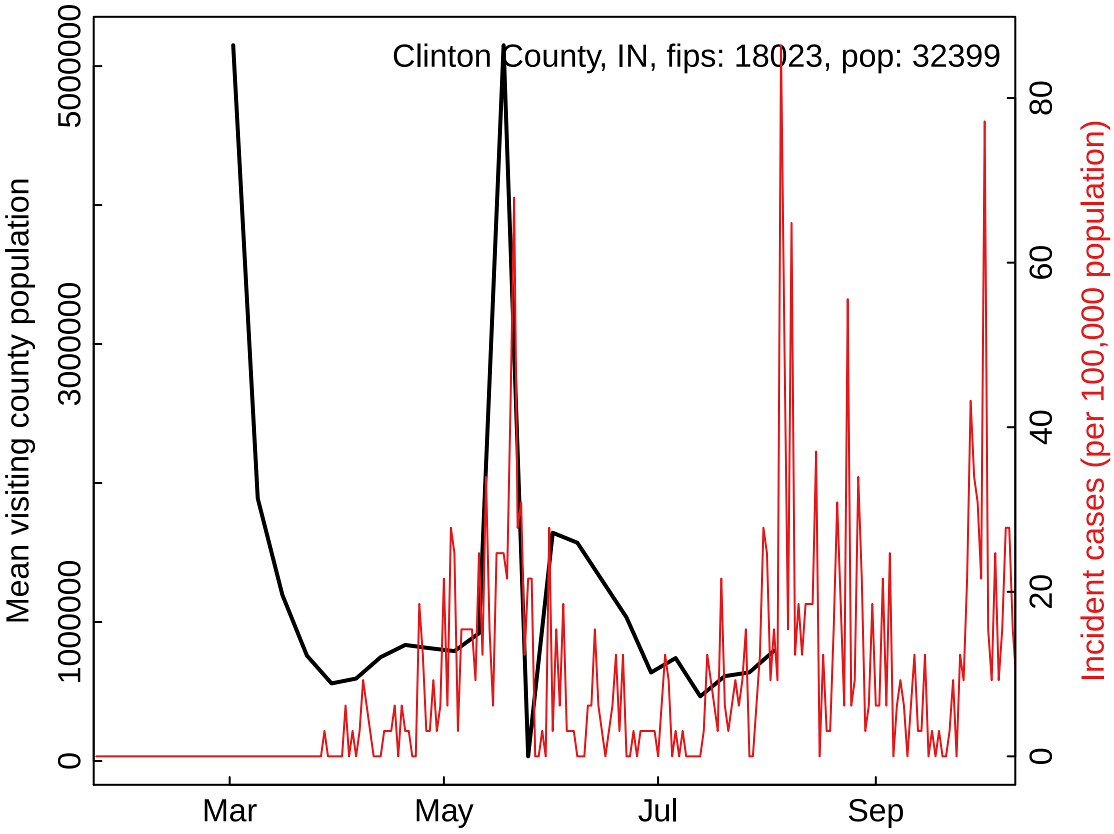

Clinton County, IN, fips: 18023, pop: 32399
Mean visiting county population
0
1000000
3000000
5000000
Incident cases (per 100,000 population)
0
20
40
60
80
Mar
May
Jul
Sep

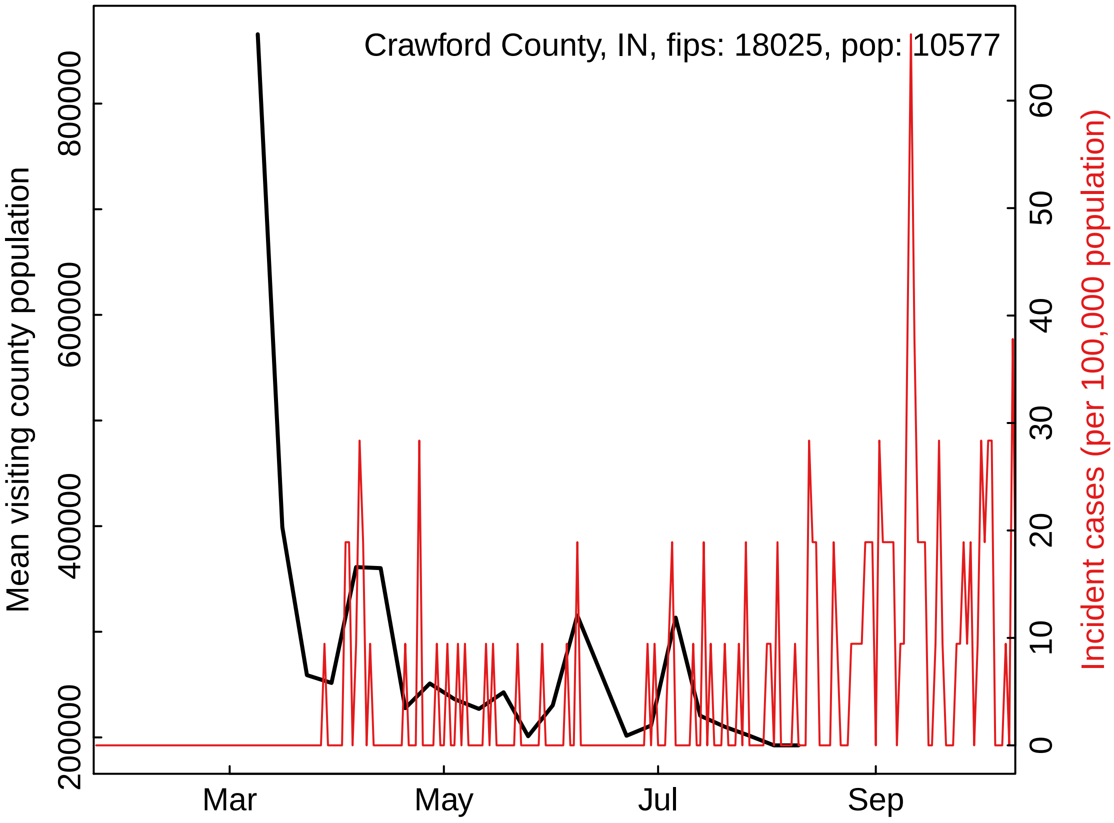

Crawford County, IN, fips: 18025, pop: 10577
Mean visiting county population
200000
400000
600000
800000
Mar
May
Jul
Sep
0
10
20
30
40
50
60
Incident cases (per 100,000 population)

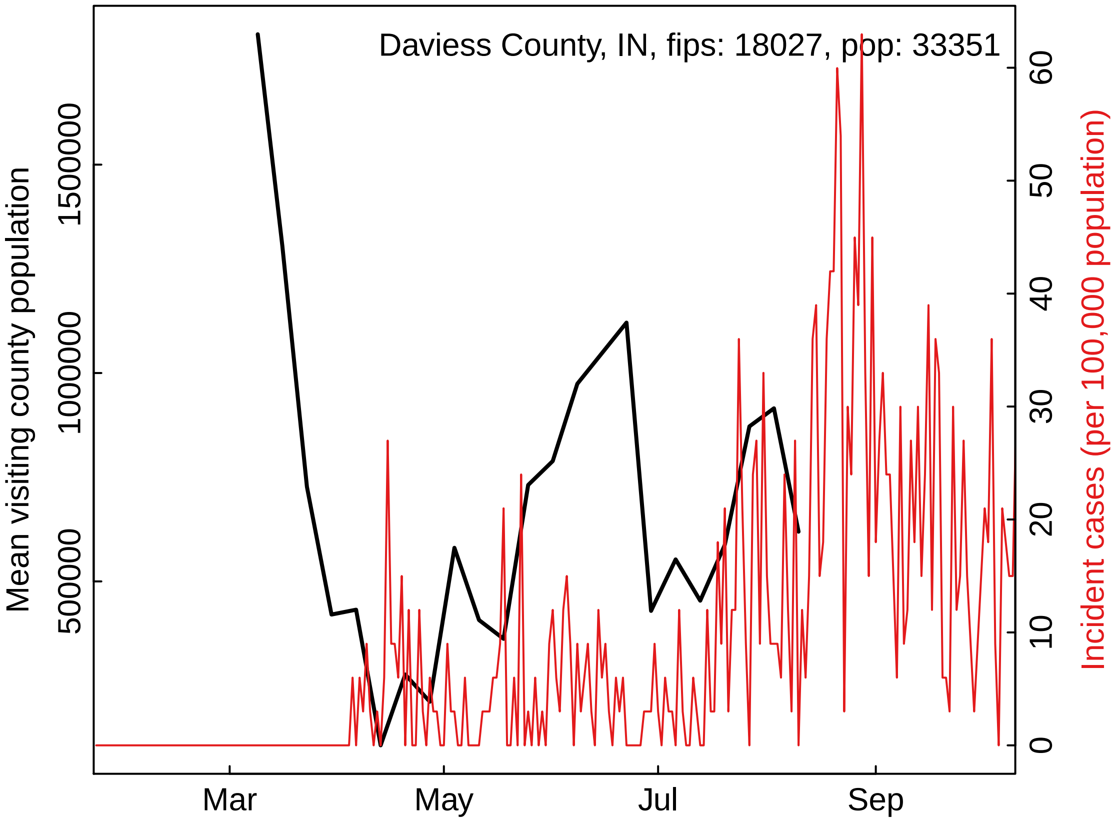
Daviess County, IN, fips: 18027, pop: 33351
Mean visiting county population
500000
1000000
1500000
Incident cases (per 100,000 population)
0
10
20
30
40
50
60
Mar
May
Jul
Sep

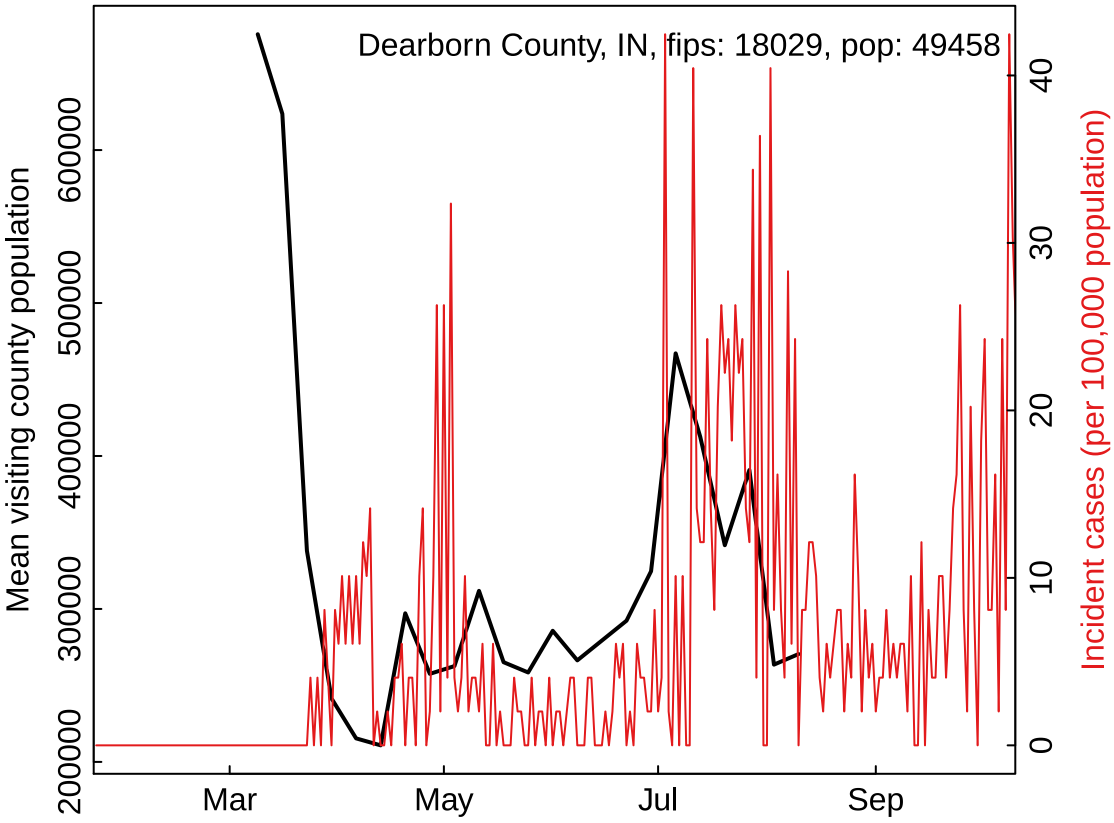

Dearborn County, IN, fips: 18029, pop: 49458
Mean visiting county population
200000
300000
400000
500000
600000
Incident cases (per 100,000 population)
0
10
20
30
40
Mar
May
Jul
Sep

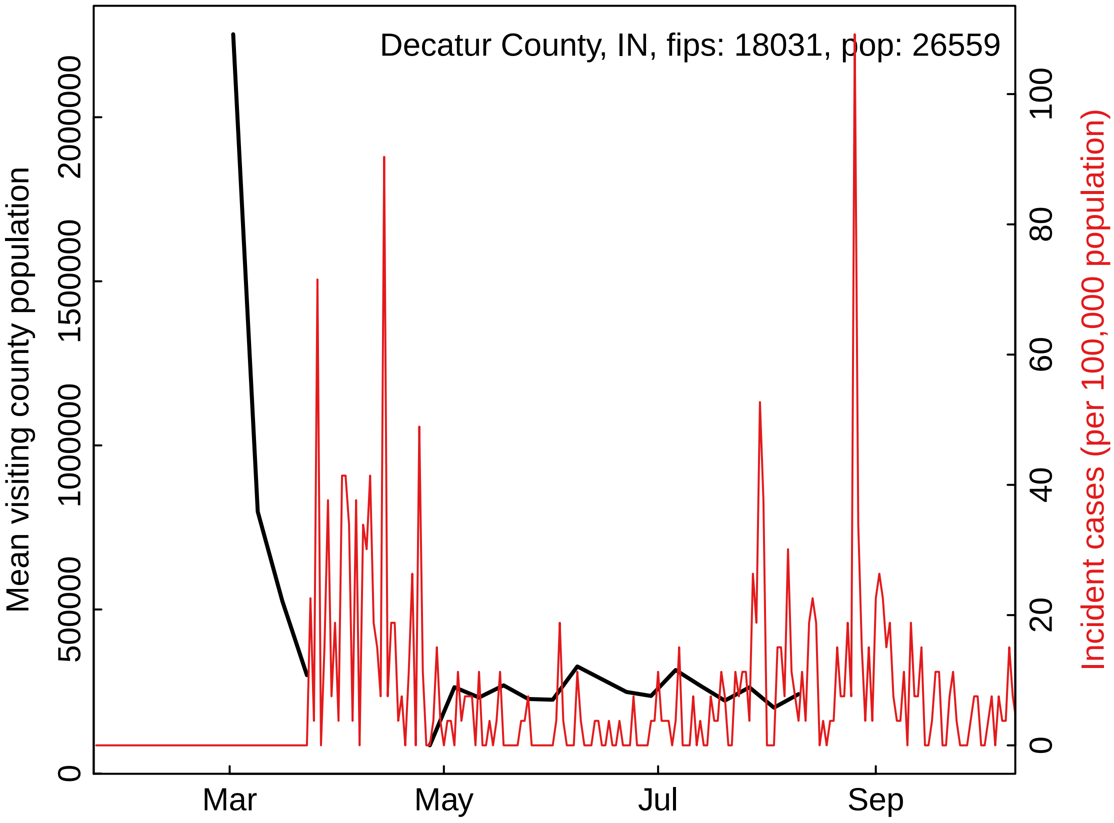

Decatur County, IN, fips: 18031, pop: 26559
Mean visiting county population
0
500000
1000000
1500000
2000000
Incident cases (per 100,000 population)
0
20
40
60
80
100
Mar
May
Jul
Sep

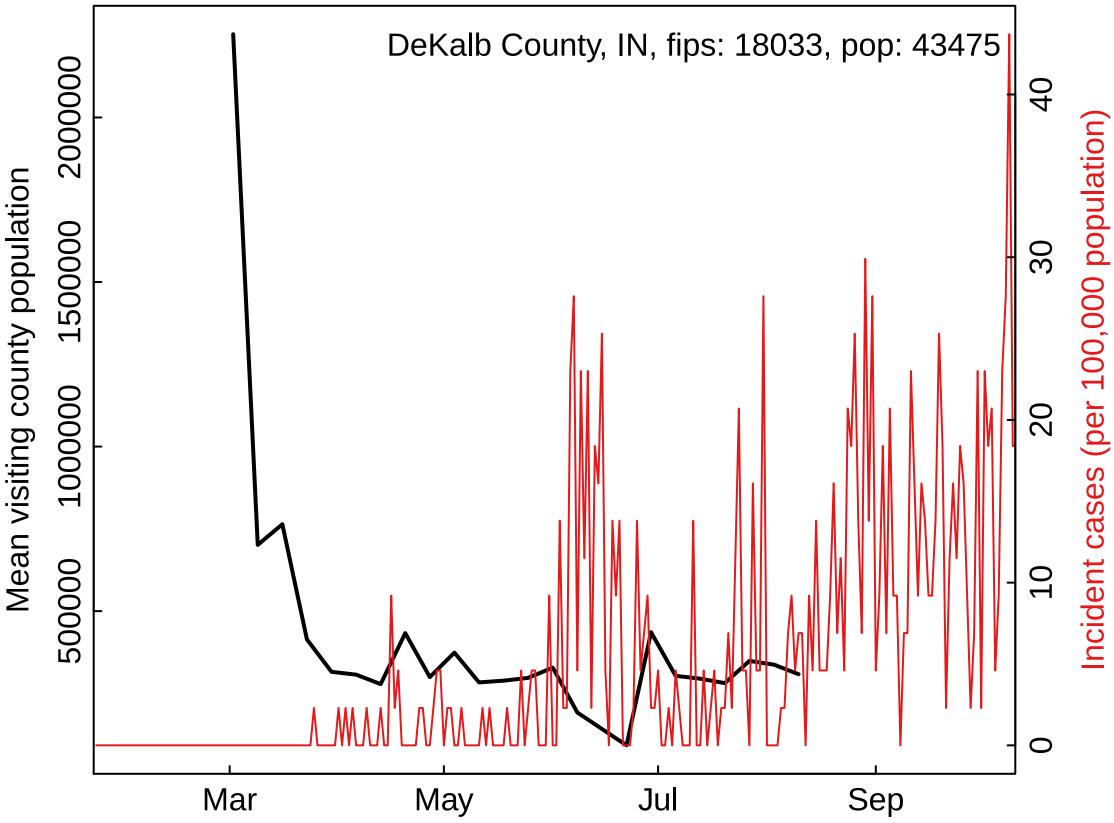
DeKalb County, IN, fips: 18033, pop: 43475
Mean visiting county population
500000
1000000
1500000
2000000
Incident cases (per 100,000 population)
0
10
20
30
40
Mar
May
Jul
Sep

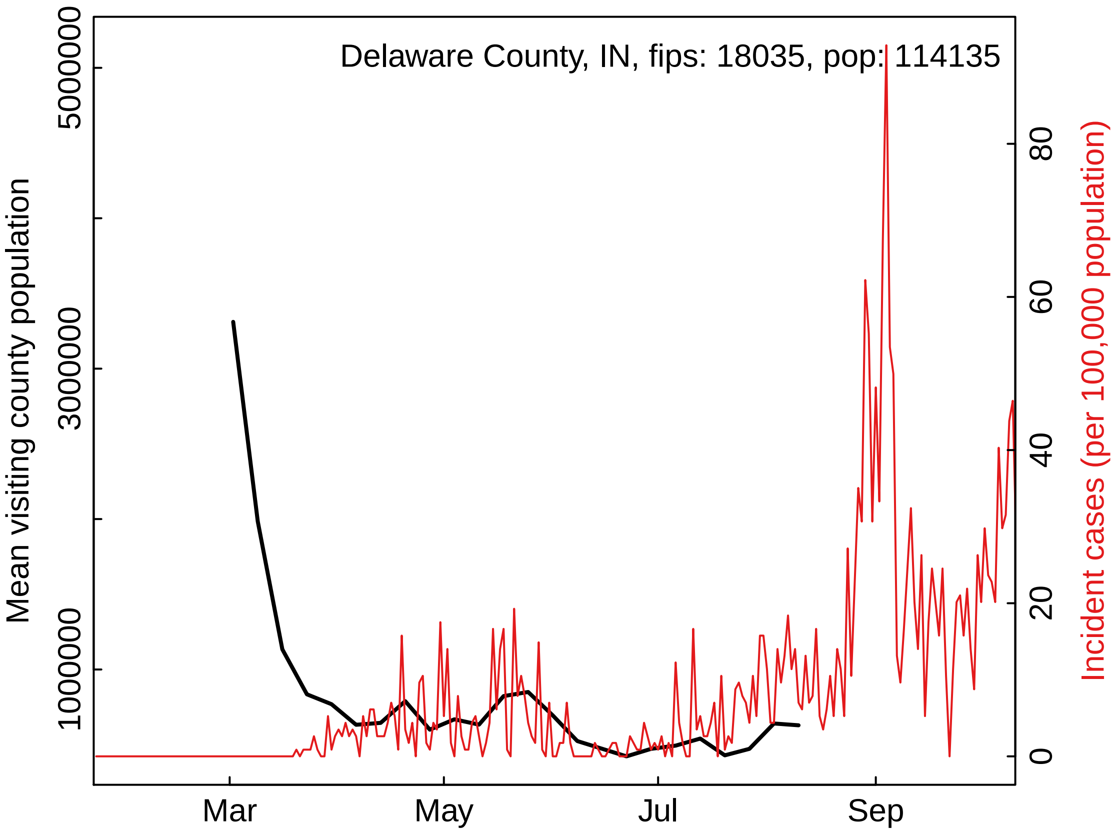

Delaware County, IN, fips: 18035, pop: 114135
Mean visiting county population
1000000
3000000
5000000
Incident cases (per 100,000 population)
0
20
40
60
80
Mar
May
Jul
Sep

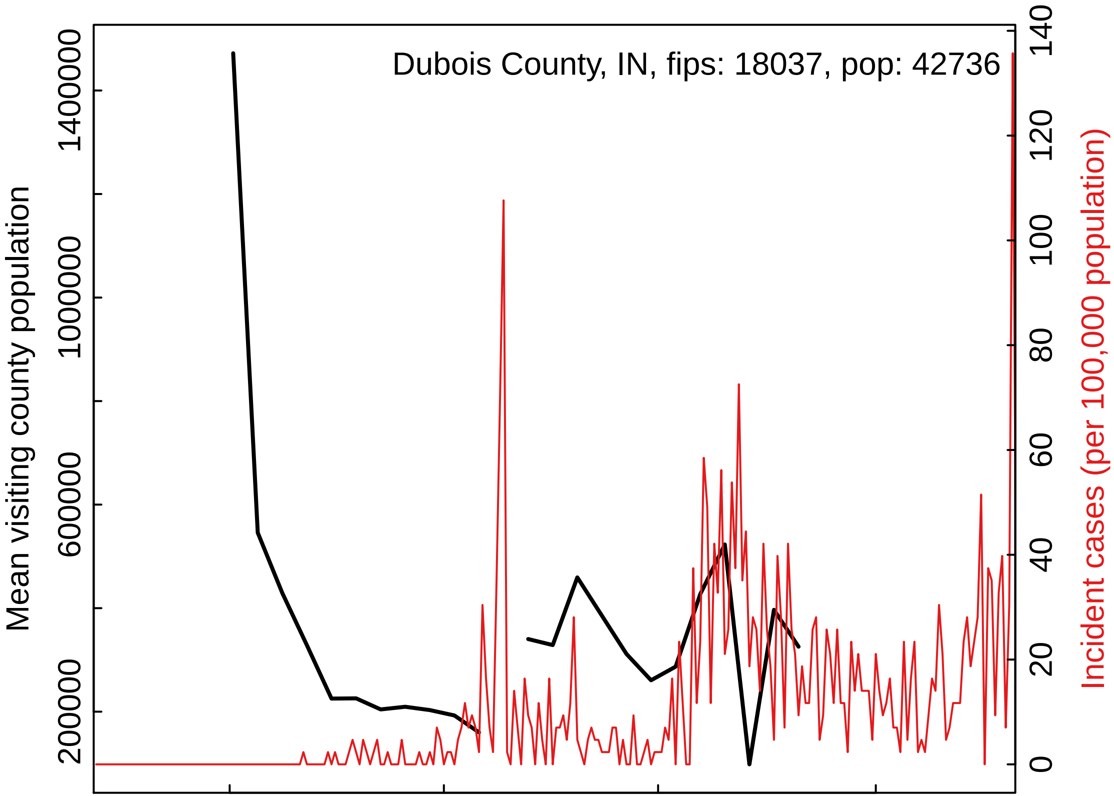

Dubois County, IN, fips: 18037, pop: 42736
Mean visiting county population
200000
600000
1000000
1400000
Incident cases (per 100,000 population)
0
20
40
60
80
100
120
140
Mar
May
Jul
Sep

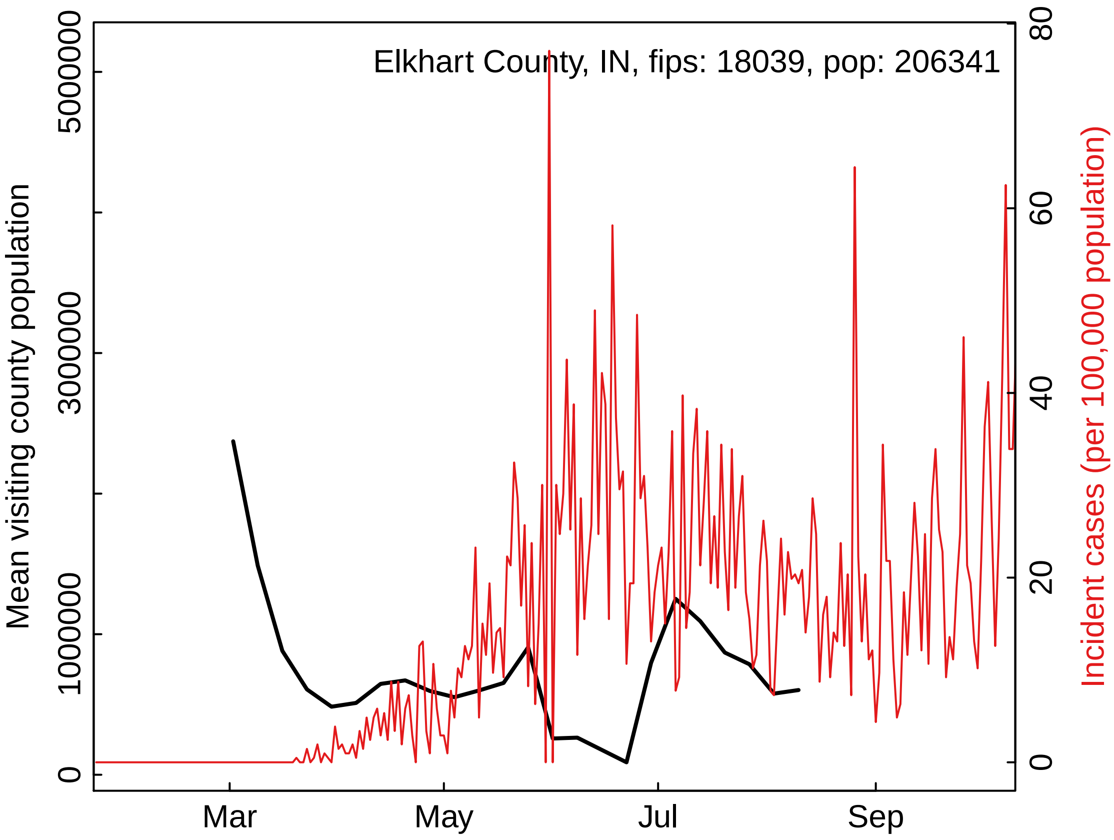

Elkhart County, IN, fips: 18039, pop: 206341
Mean visiting county population
0
1000000
3000000
5000000
Incident cases (per 100,000 population)
0
20
40
60
80
Mar
May
Jul
Sep

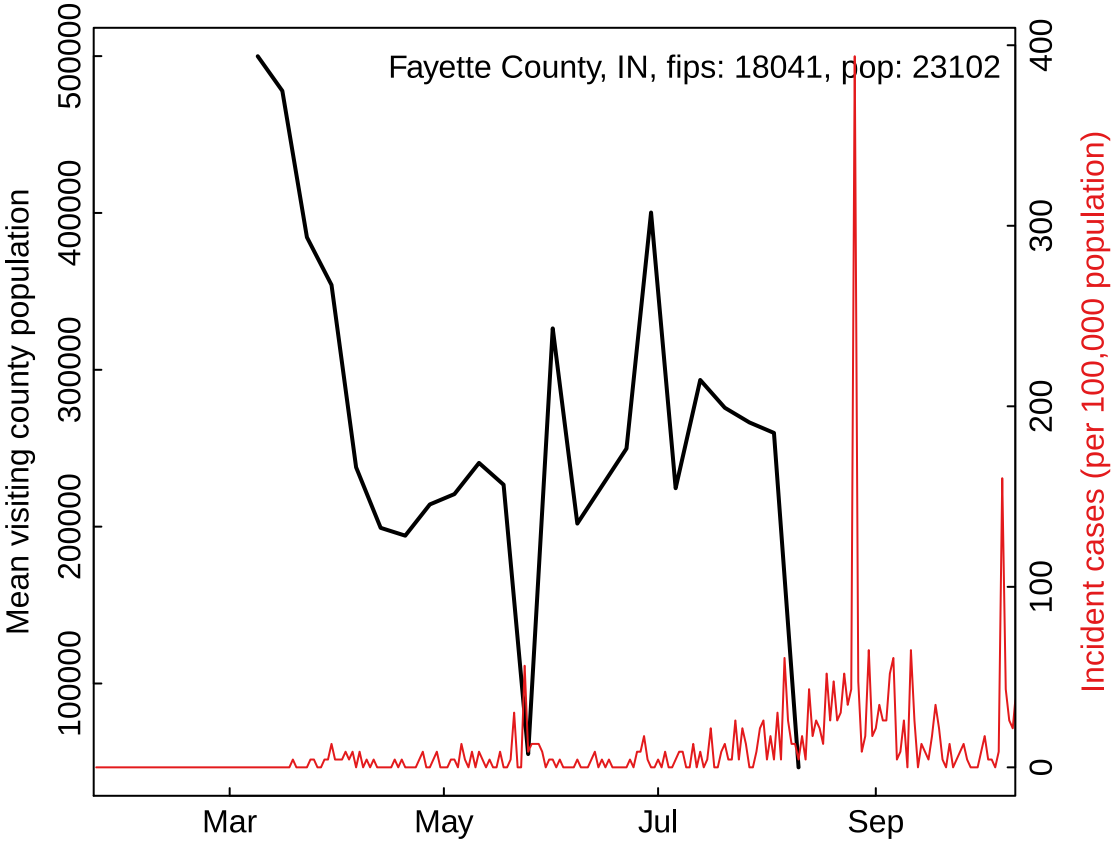

Fayette County, IN, fips: 18041, pop: 23102
Mean visiting county population
100000
200000
300000
400000
500000
Incident cases (per 100,000 population)
0
100
200
300
400
Mar
May
Jul
Sep

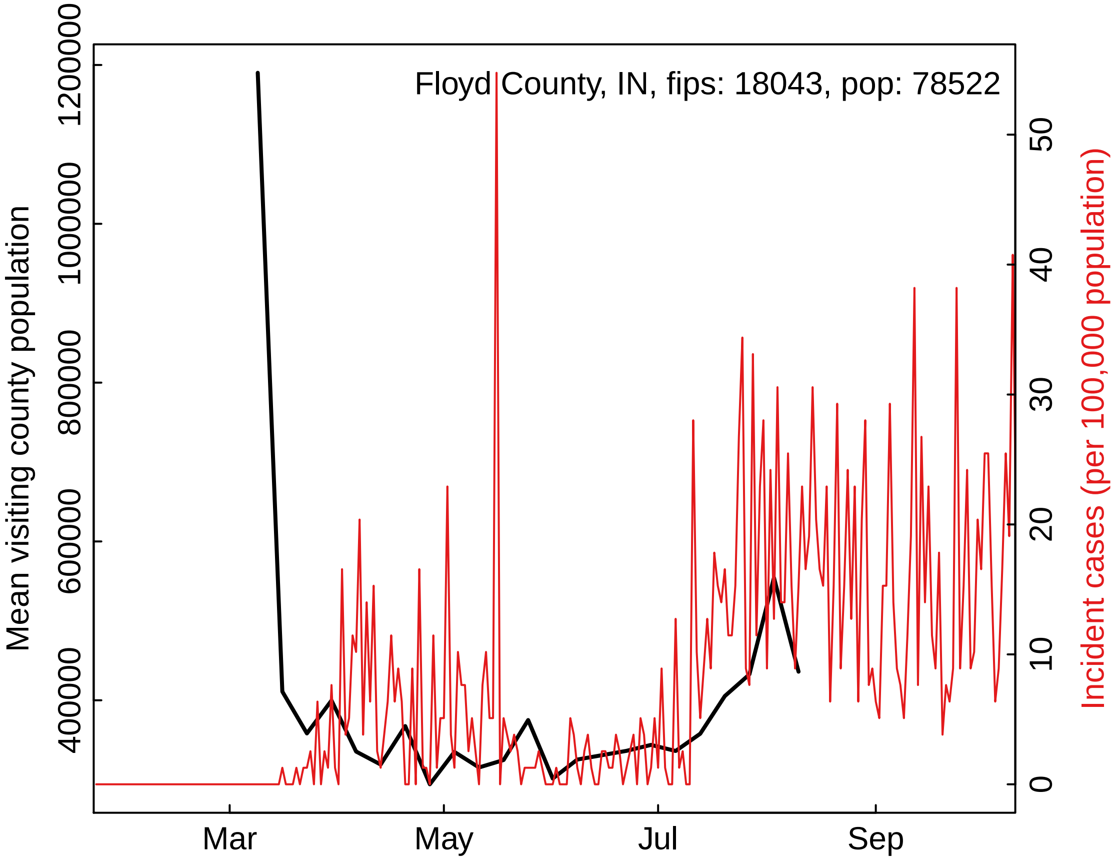

Floyd County, IN, fips: 18043, pop: 78522
Mean visiting county population
400000
600000
800000
1000000
1200000
Incident cases (per 100,000 population)
0
10
20
30
40
50
Mar
May
Jul
Sep

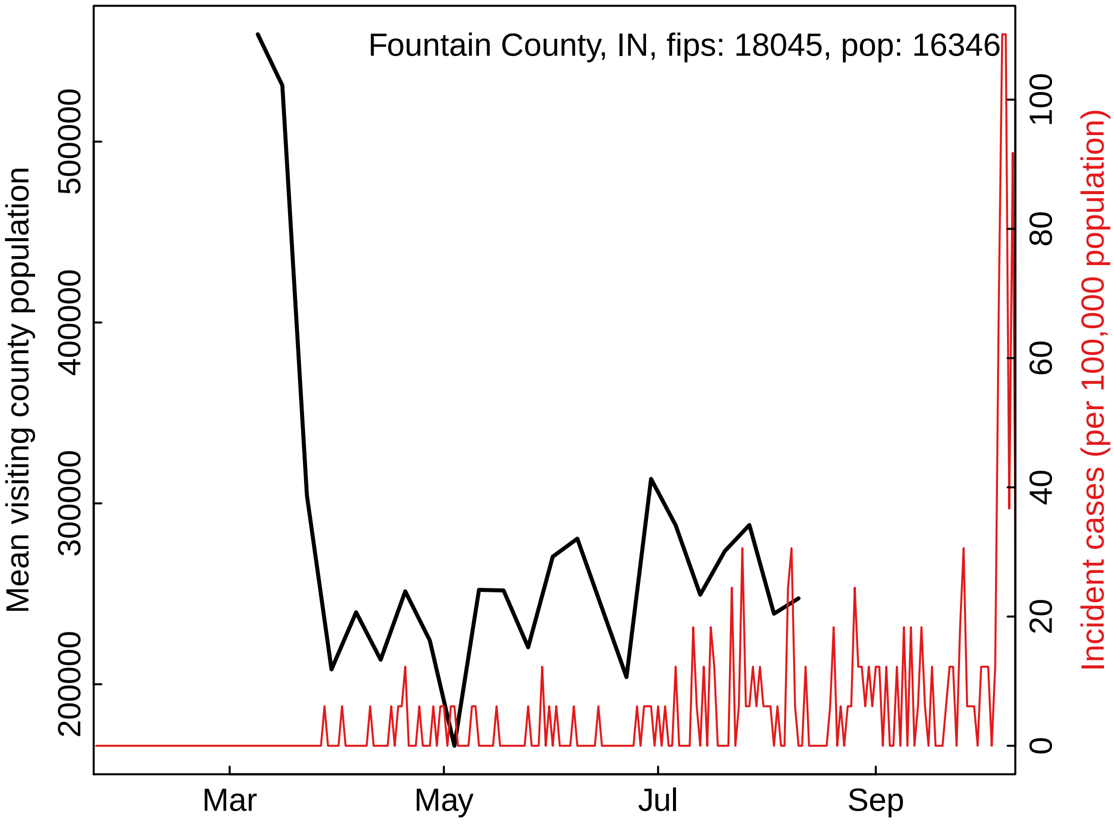

Fountain County, IN, fips: 18045, pop: 16346
Mean visiting county population
200000
300000
400000
500000
Incident cases (per 100,000 population)
0
20
40
60
80
100
Mar
May
Jul
Sep

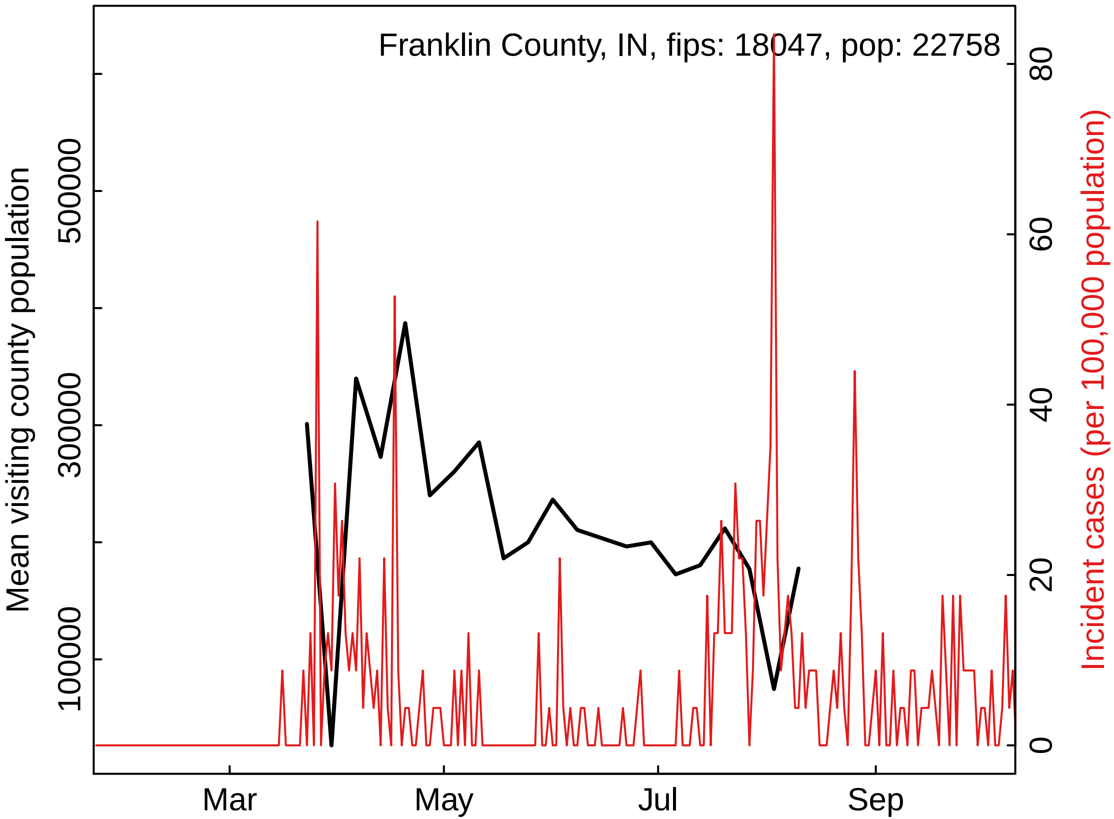

Franklin County, IN, fips: 18047, pop: 22758
Mean visiting county population
100000
300000
500000
Incident cases (per 100,000 population)
0
20
40
60
80
Mar
May
Jul
Sep

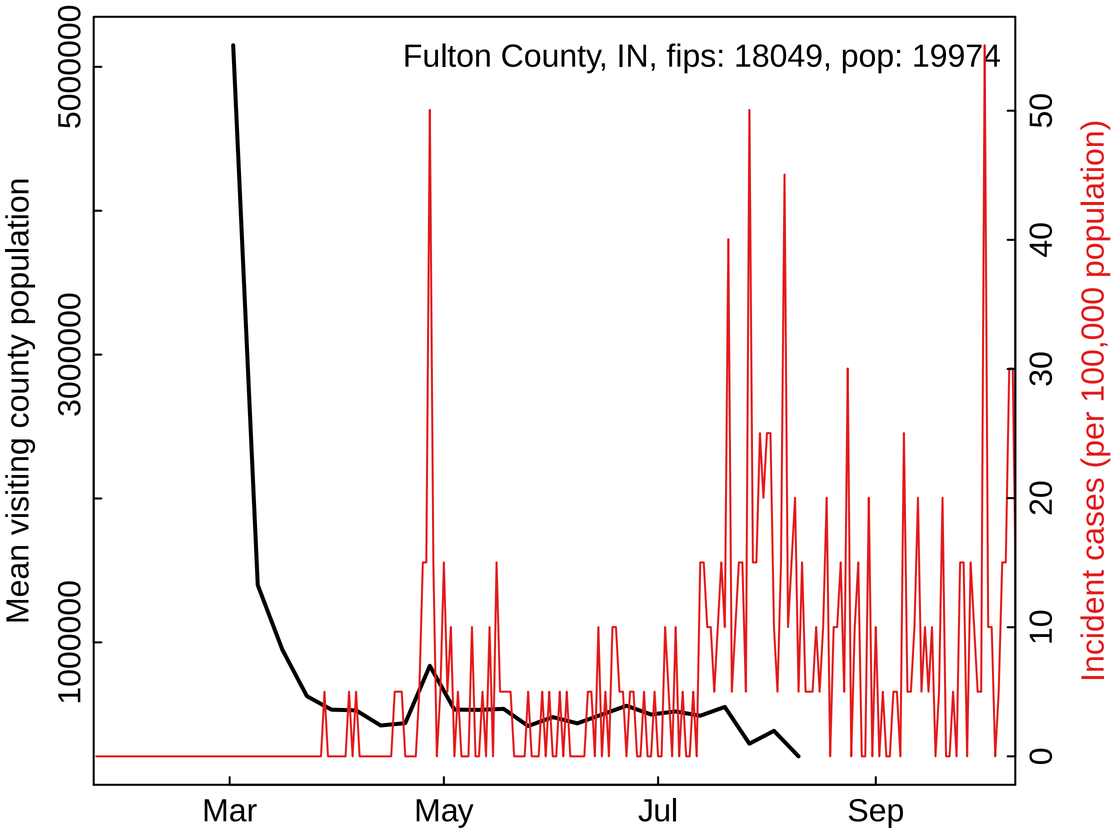

Fulton County, IN, fips: 18049, pop: 19974
Mean visiting county population
1000000
3000000
5000000
Incident cases (per 100,000 population)
0
10
20
30
40
50
Mar
May
Jul
Sep

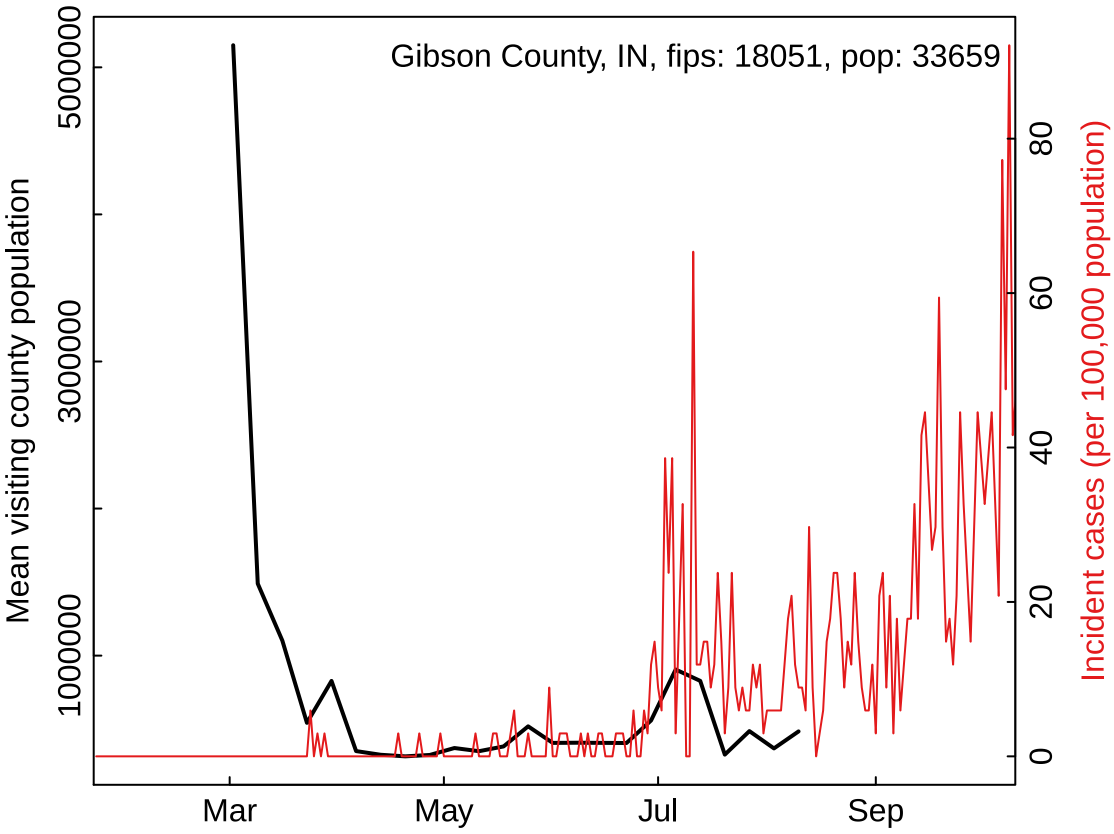
Gibson County, IN, fips: 18051, pop: 33659
Mean visiting county population
1000000
3000000
5000000
Incident cases (per 100,000 population)
0
20
40
60
80
Mar
May
Jul
Sep

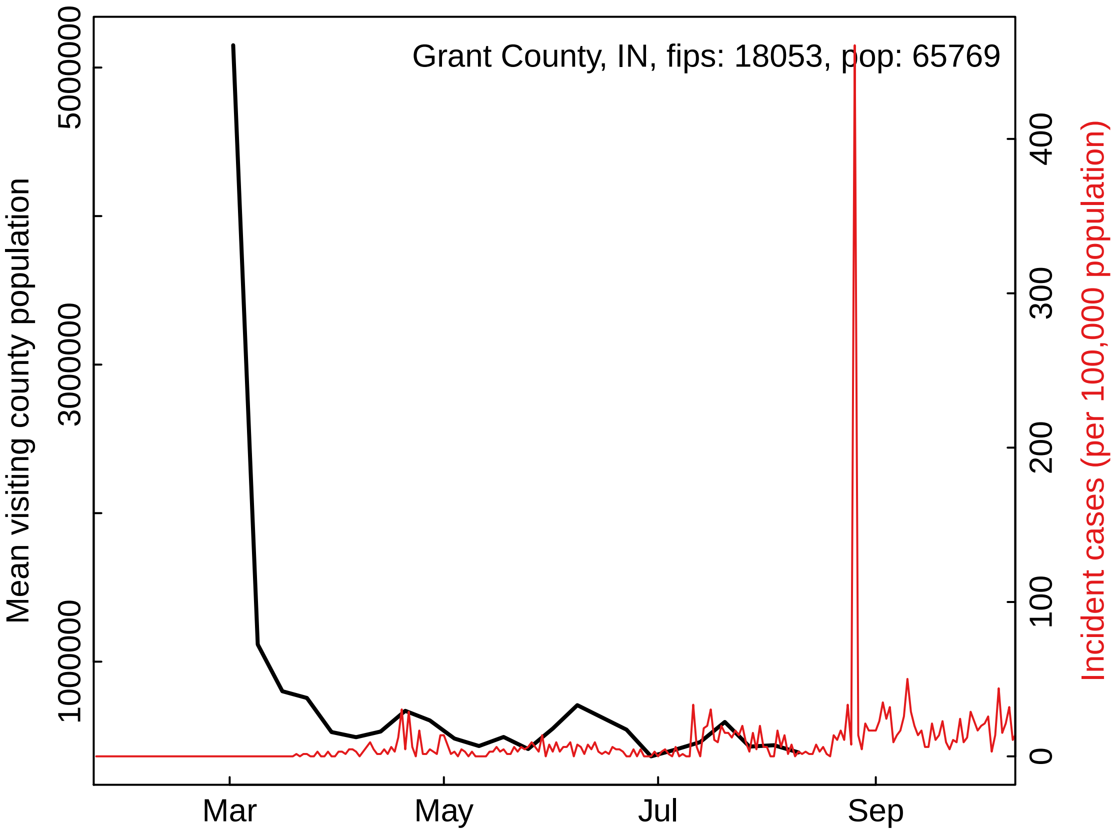
Grant County, IN, fips: 18053, pop: 65769
Mean visiting county population
1000000
3000000
5000000
Mar
May
Jul
Sep
0
100
200
300
400
Incident cases (per 100,000 population)

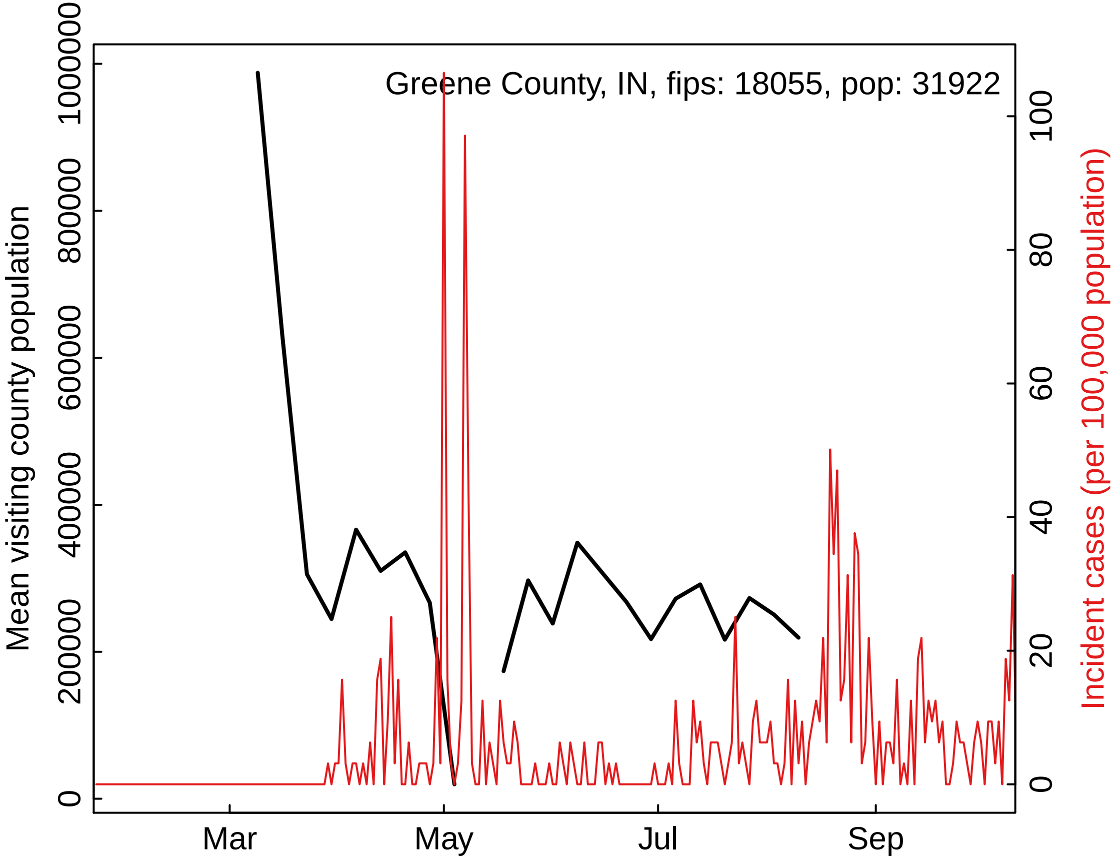
Greene County, IN, fips: 18055, pop: 31922
Mean visiting county population
0
200000
400000
600000
800000
1000000
Incident cases (per 100,000 population)
0
20
40
60
80
100
Mar
May
Jul
Sep

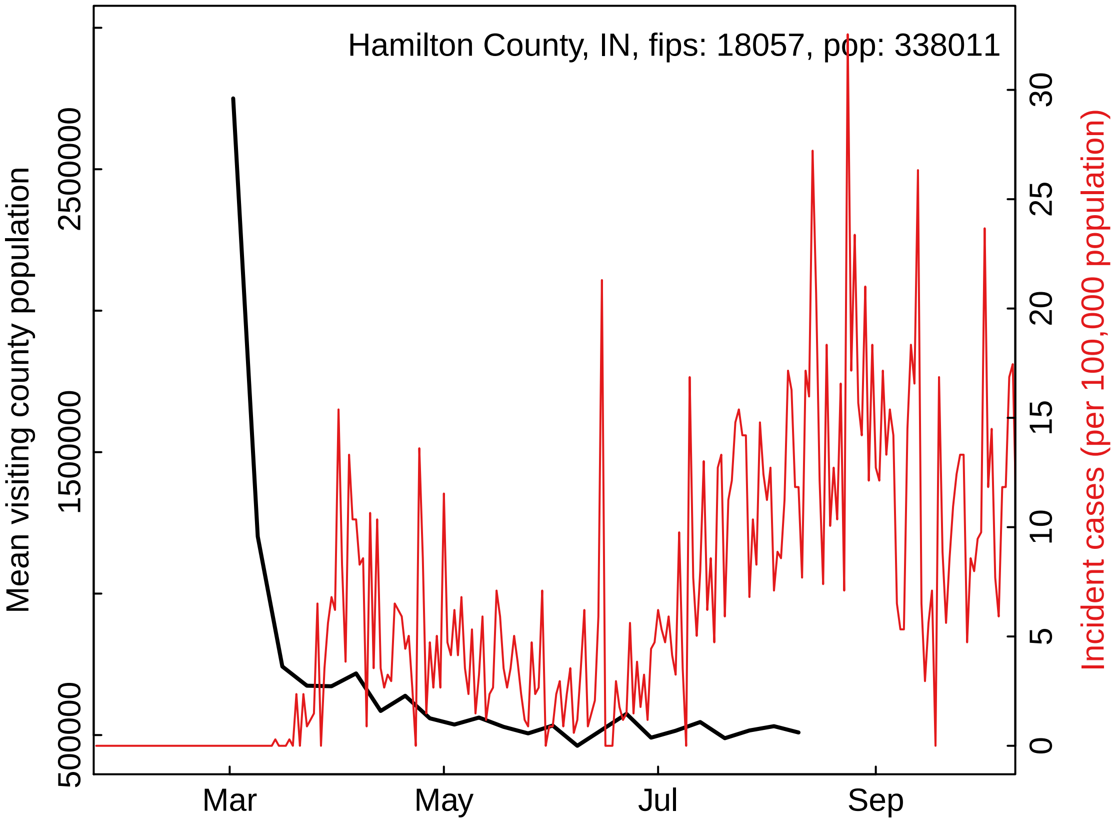

Hamilton County, IN, fips: 18057, pop: 338011
Mean visiting county population
500000
1500000
2500000
Mar
May
Jul
Sep
Incident cases (per 100,000 population)
0
5
10
15
20
25
30

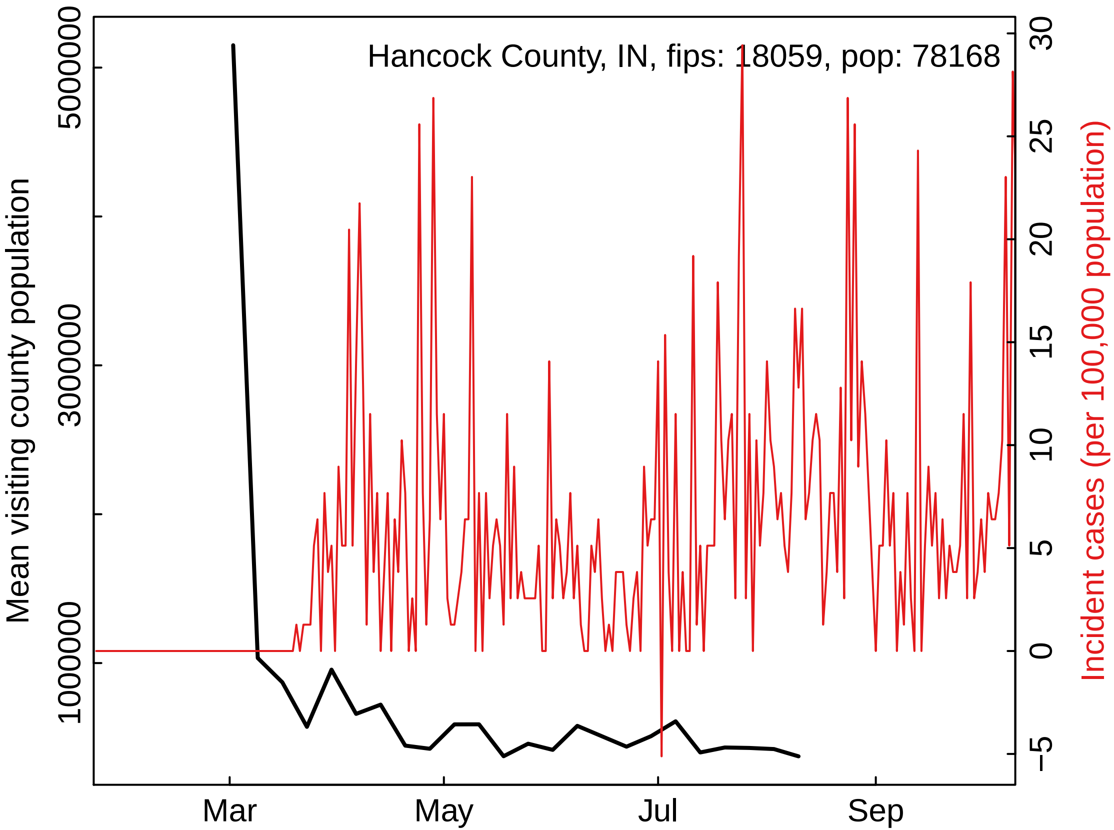

Hancock County, IN, fips: 18059, pop: 78168
Mean visiting county population
1000000
3000000
5000000
Incident cases (per 100,000 population)
−5
0
5
10
15
20
25
30
Mar
May
Jul
Sep

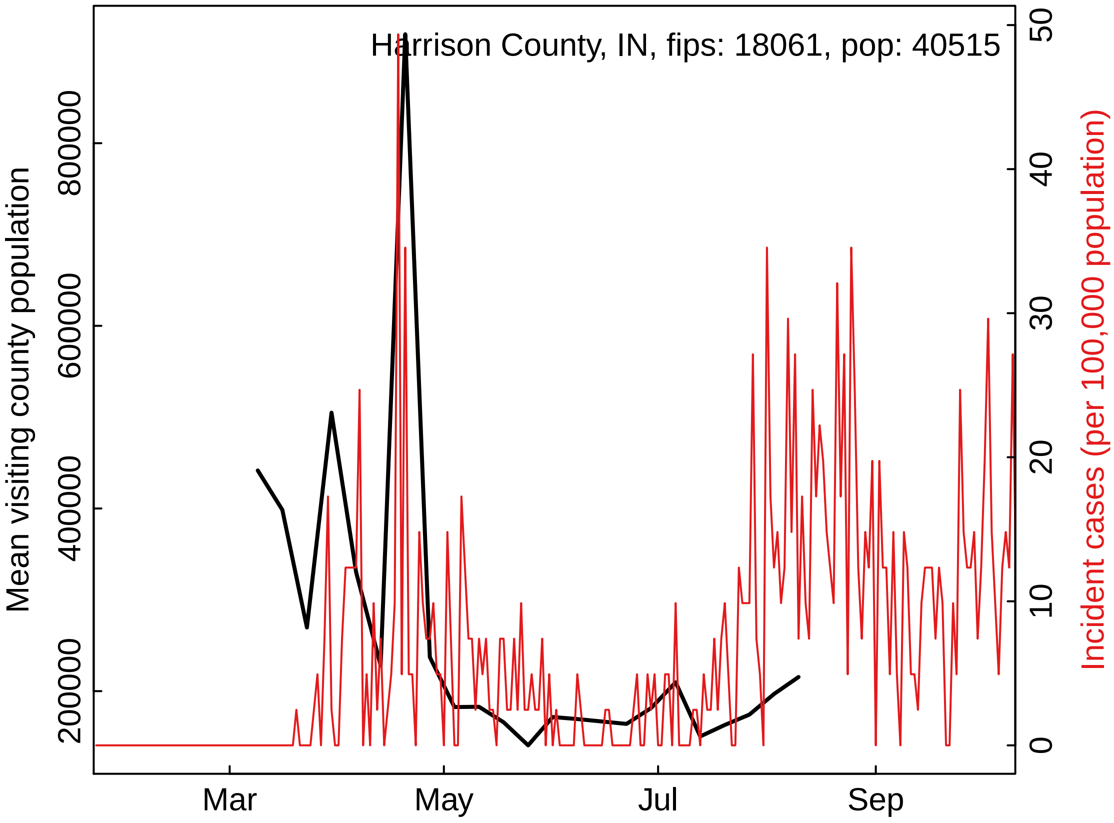
Harrison County, IN, fips: 18061, pop: 40515
Mean visiting county population
200000
400000
600000
800000
Incident cases (per 100,000 population)
0
10
20
30
40
50
Mar
May
Jul
Sep

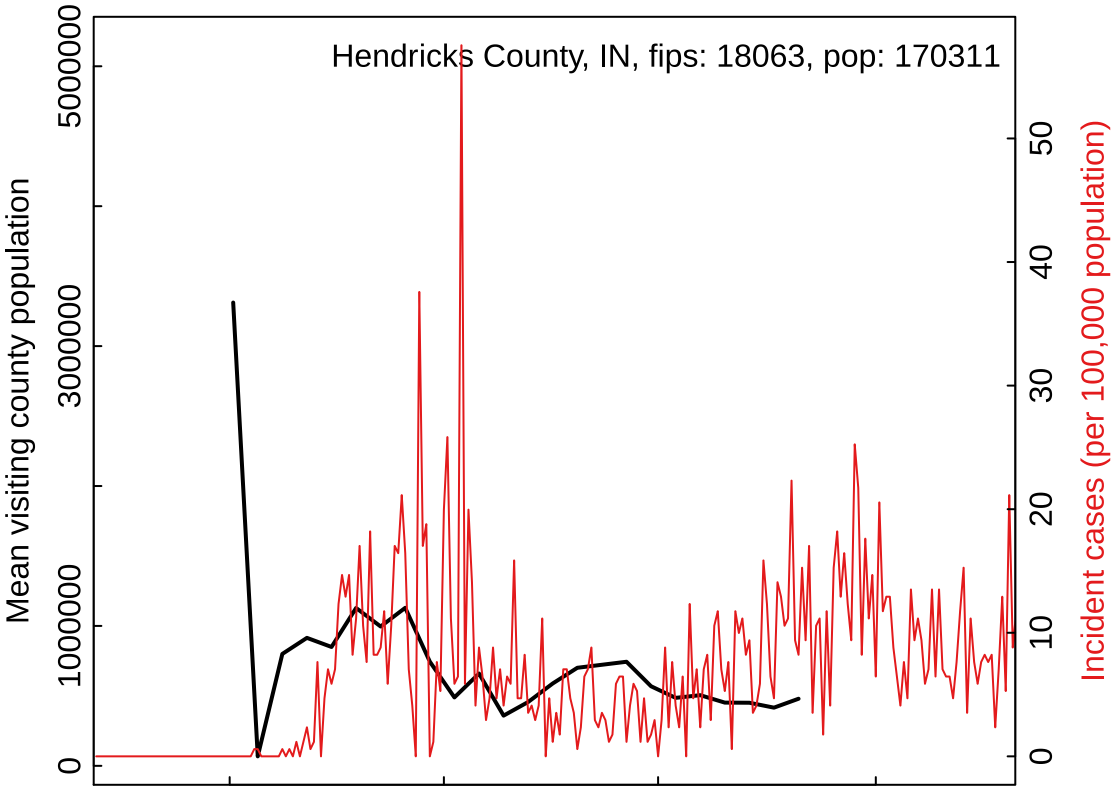
Hendricks County, IN, fips: 18063, pop: 170311
Mean visiting county population
0
1000000
3000000
5000000
Incident cases (per 100,000 population)
0
10
20
30
40
50
Mar
May
Jul
Sep

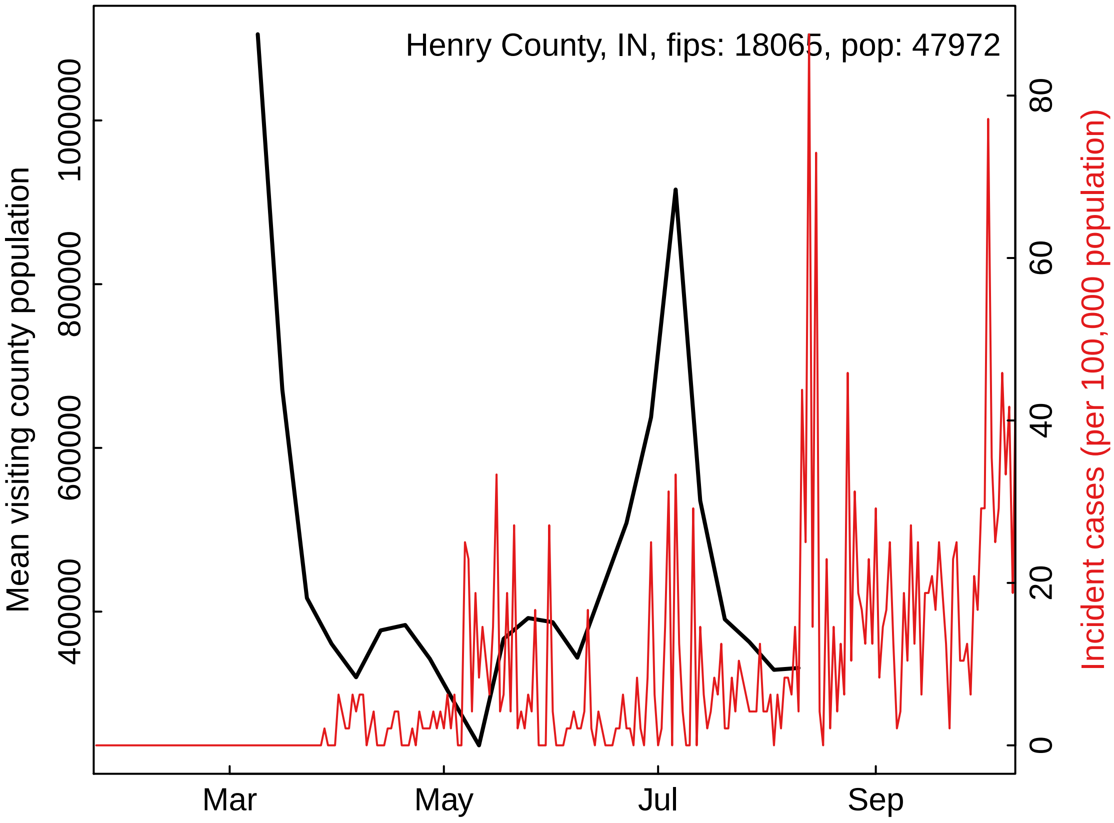

Henry County, IN, fips: 18065, pop: 47972
Mean visiting county population
400000
600000
800000
1000000
Incident cases (per 100,000 population)
0
20
40
60
80
Mar
May
Jul
Sep

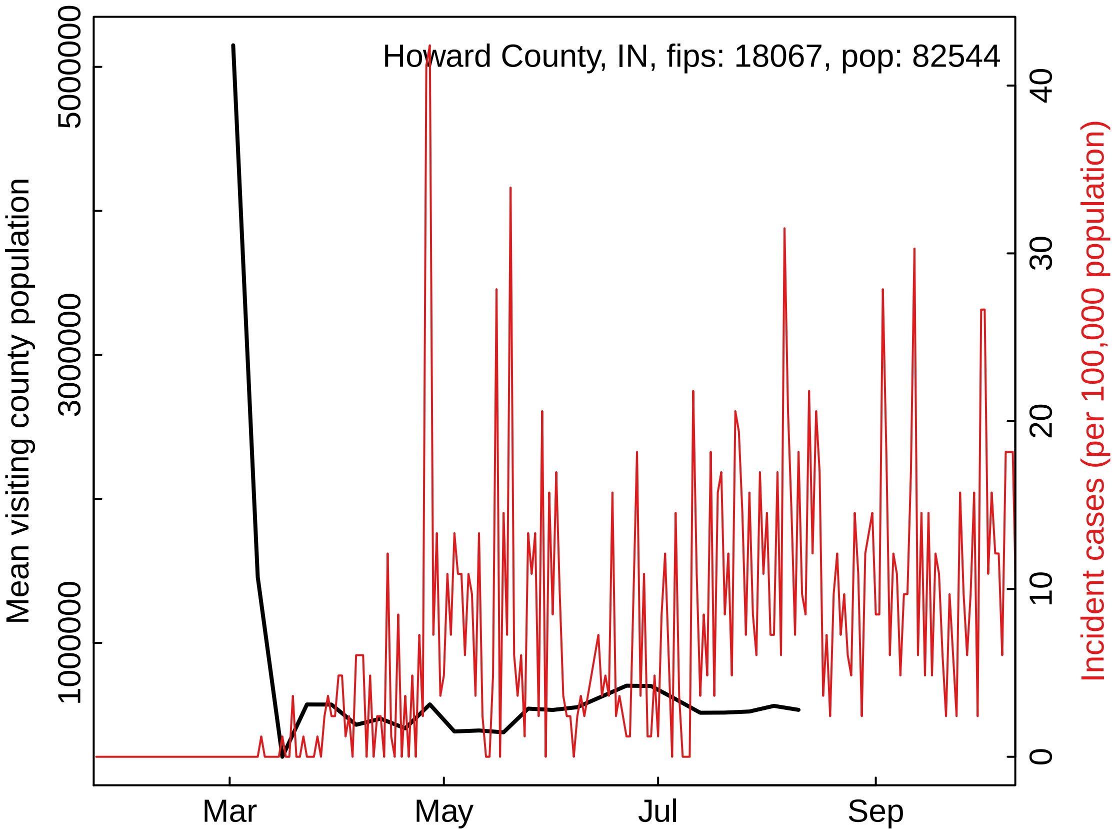

Howard County, IN, fips: 18067, pop: 82544
Mean visiting county population
1000000
3000000
5000000
Incident cases (per 100,000 population)
0
10
20
30
40
Mar
May
Jul
Sep

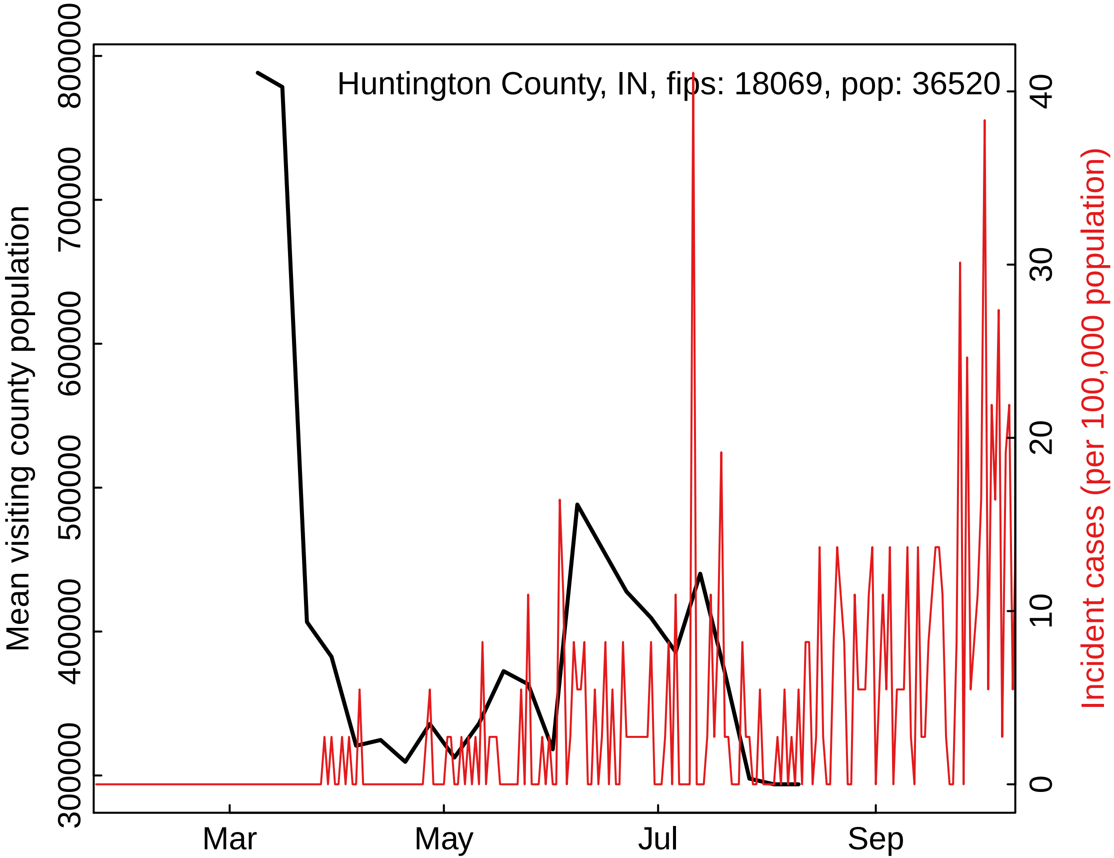

Huntington County, IN, fips: 18069, pop: 36520
Mean visiting county population
300000
400000
500000
600000
700000
800000
Incident cases (per 100,000 population)
0
10
20
30
40
Mar
May
Jul
Sep

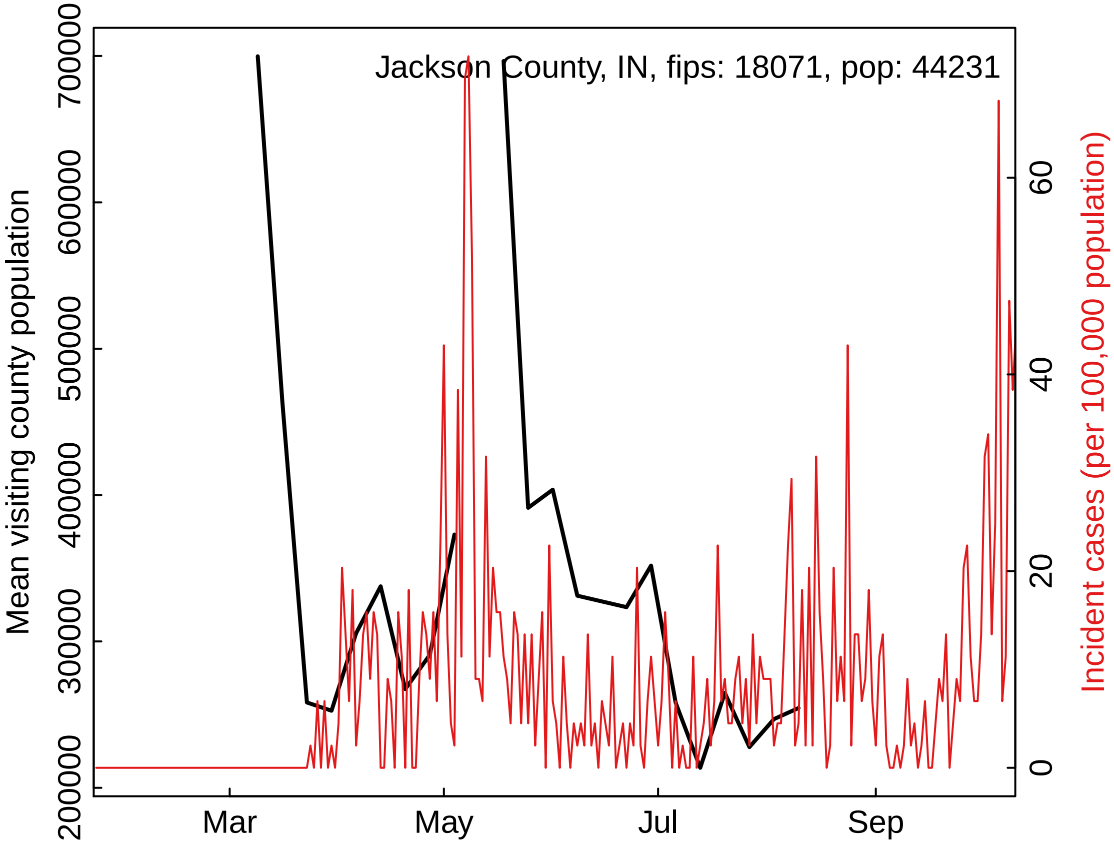

Jackson County, IN, fips: 18071, pop: 44231
Mean visiting county population
200000
300000
400000
500000
600000
700000
Mar
May
Jul
Sep
Incident cases (per 100,000 population)
0
20
40
60

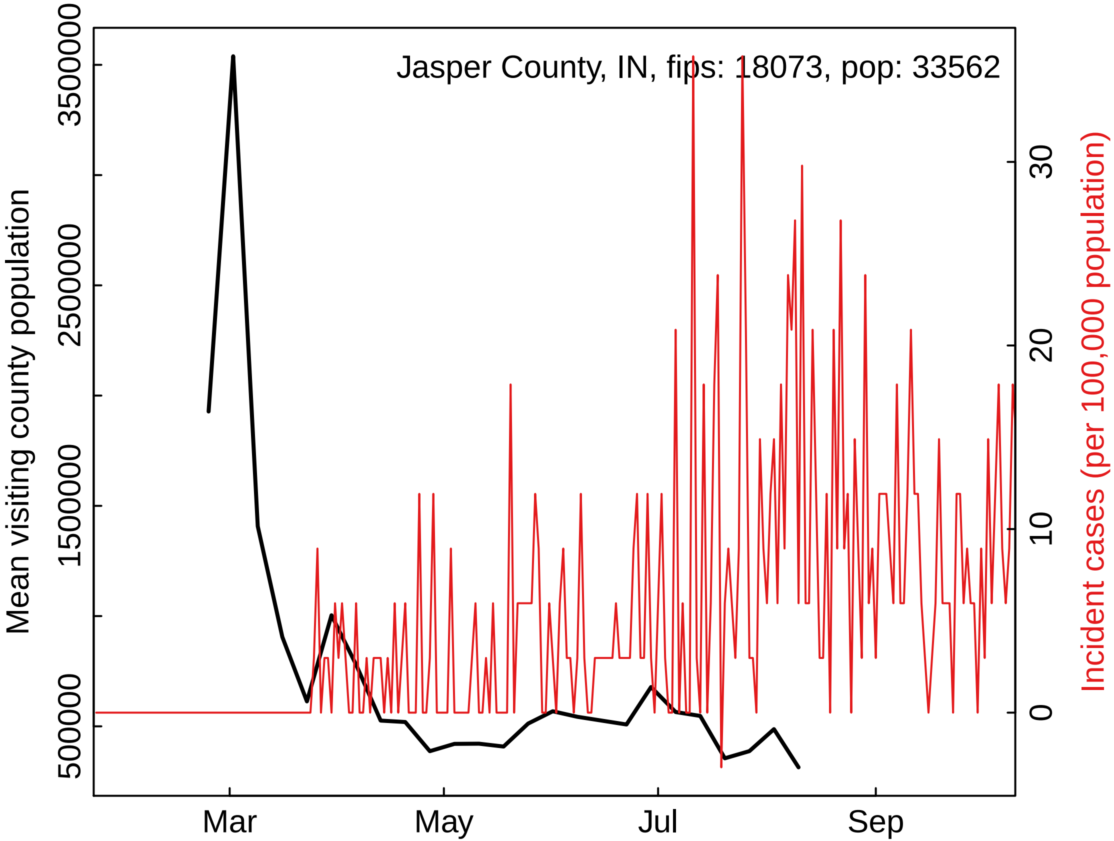

Jasper County, IN, fips: 18073, pop: 33562
Mean visiting county population
500000
1500000
2500000
3500000
Incident cases (per 100,000 population)
0
10
20
30
Mar
May
Jul
Sep

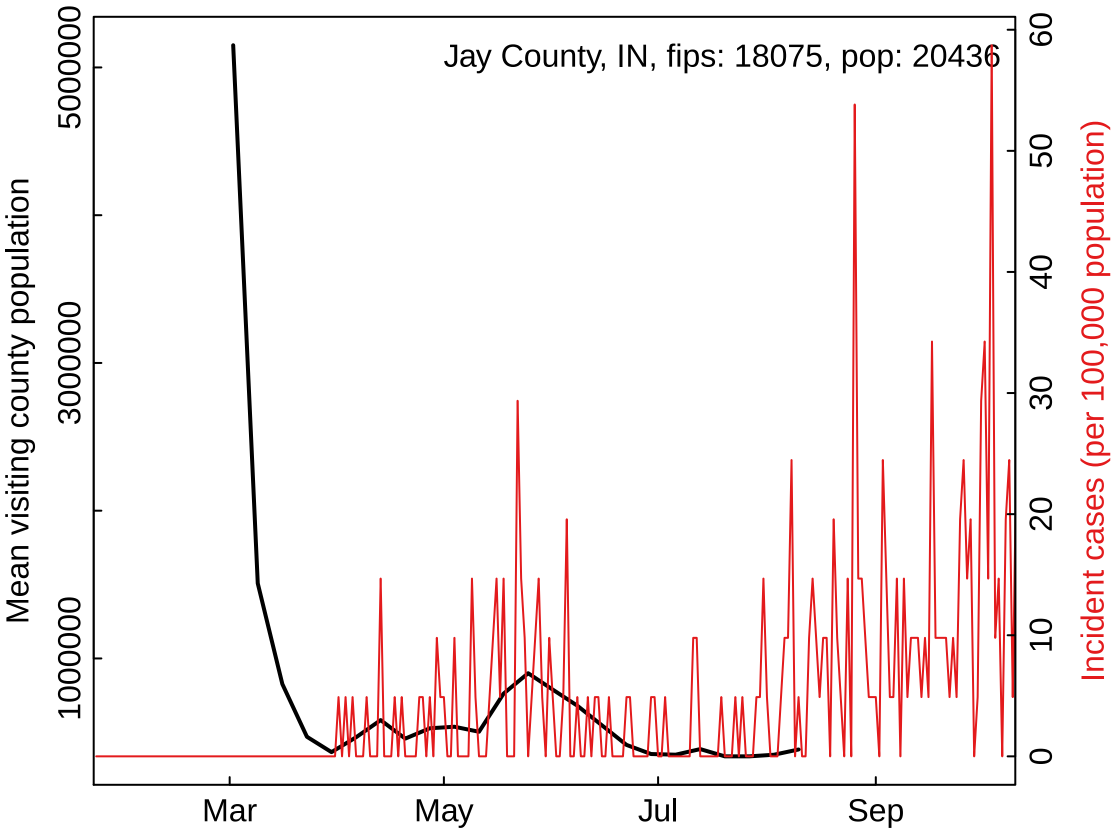

Jay County, IN, fips: 18075, pop: 20436
Mean visiting county population
Incident cases (per 100,000 population)
1000000
3000000
5000000
0
10
20
30
40
50
60
Mar
May
Jul
Sep

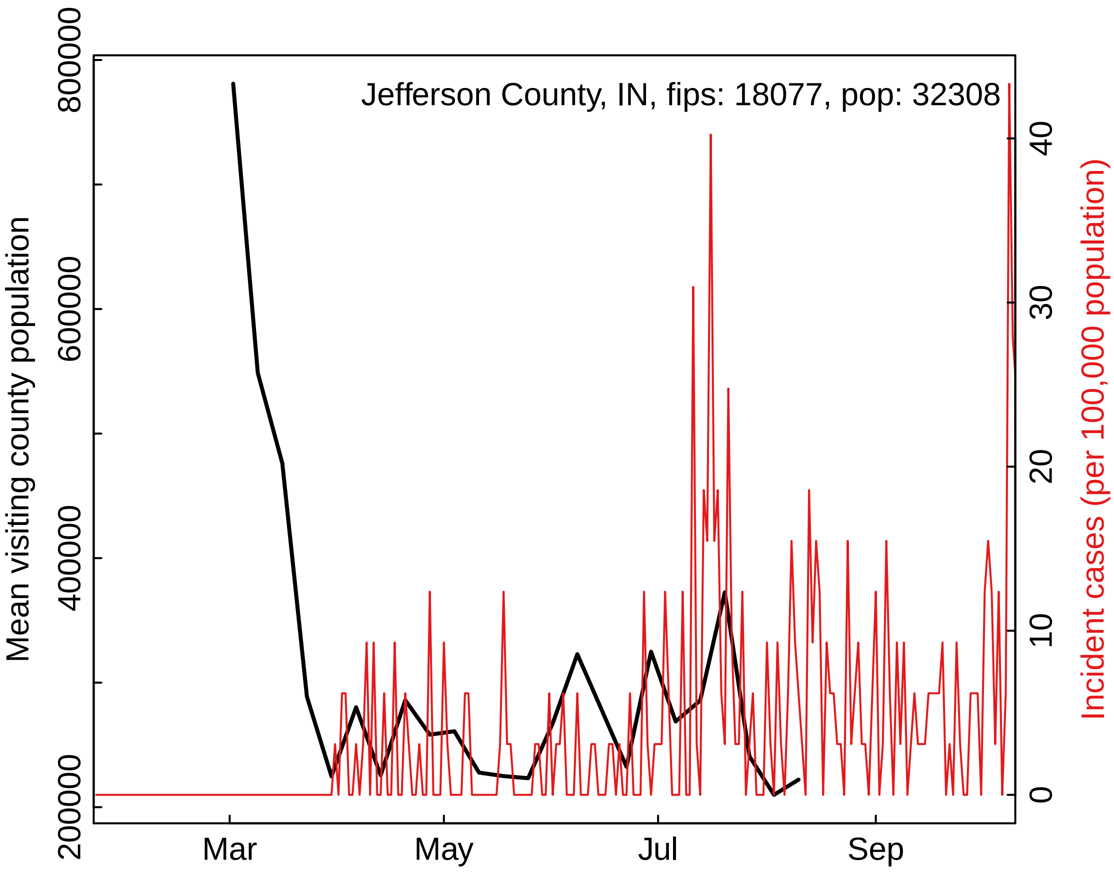

Jefferson County, IN, fips: 18077, pop: 32308
Mean visiting county population
Incident cases (per 100,000 population)
200000
400000
600000
800000
0
10
20
30
40
Mar
May
Jul
Sep

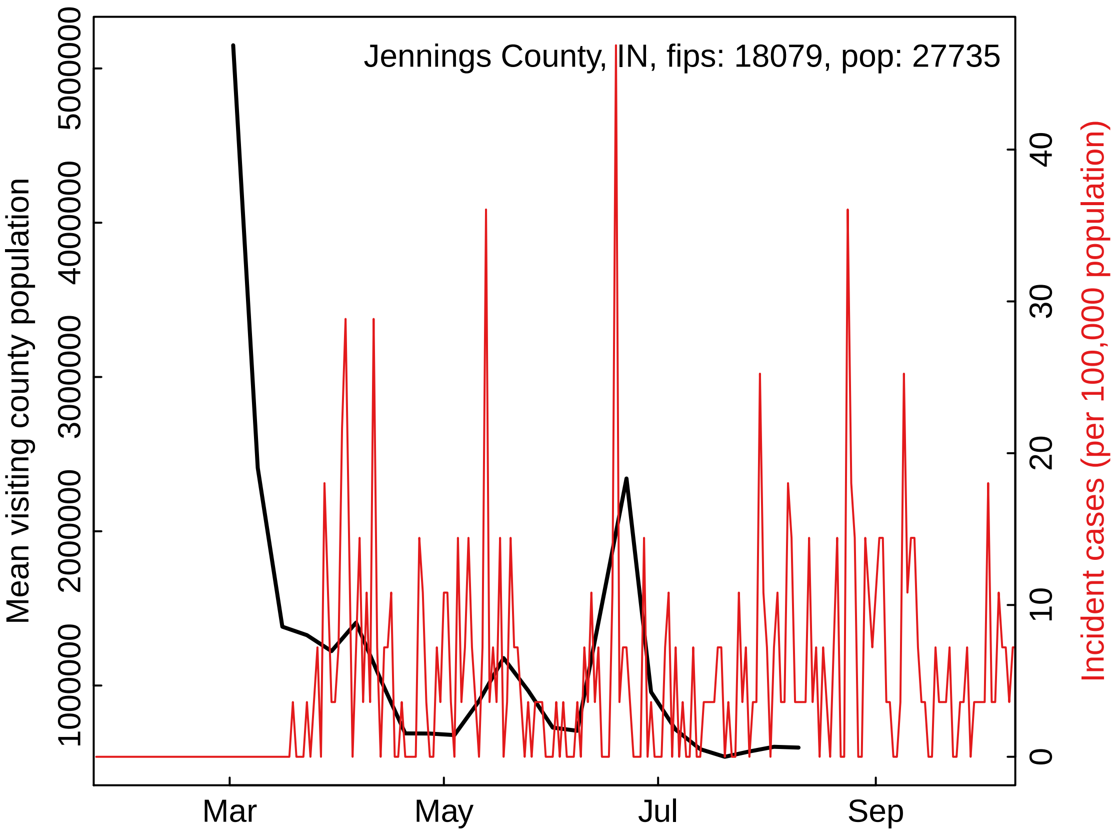

Jennings County, IN, fips: 18079, pop: 27735
Mean visiting county population
Incident cases (per 100,000 population)
10000000
20000000
30000000
40000000
50000000
0
10
20
30
40
Mar
May
Jul
Sep

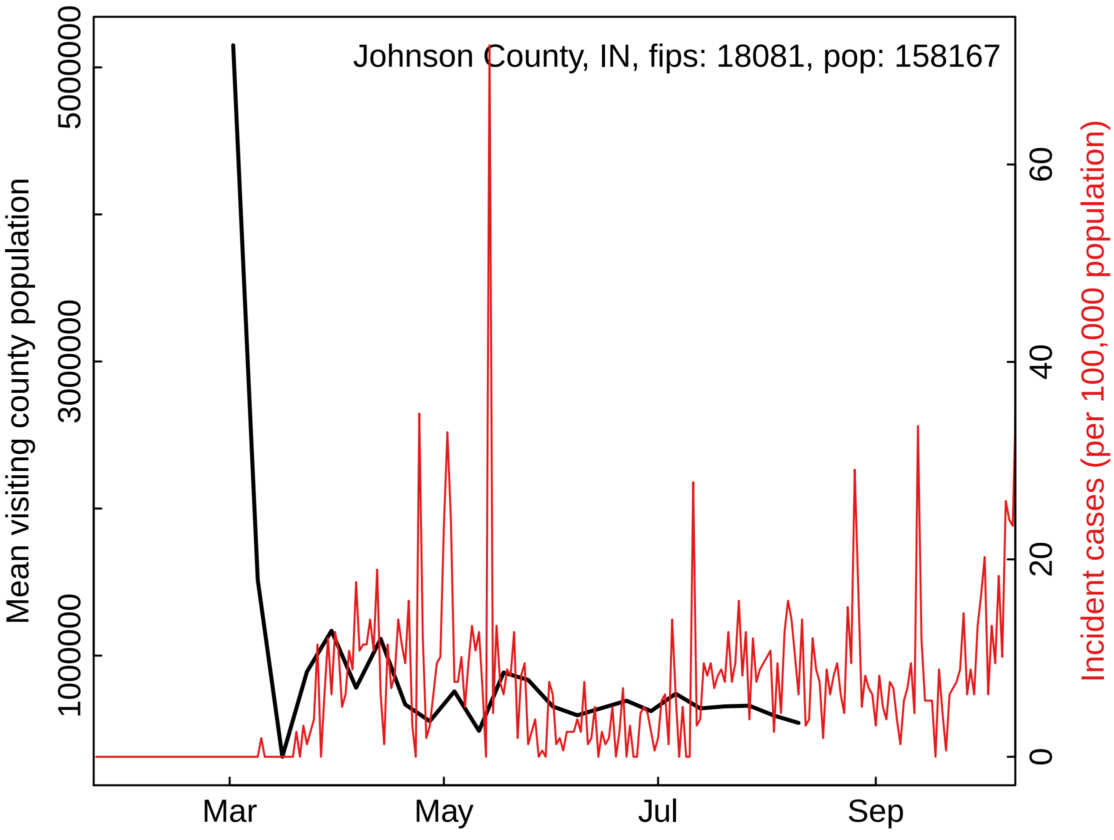

Johnson County, IN, fips: 18081, pop: 158167
Mean visiting county population
1000000
3000000
5000000
Incident cases (per 100,000 population)
0
20
40
60
Mar
May
Jul
Sep

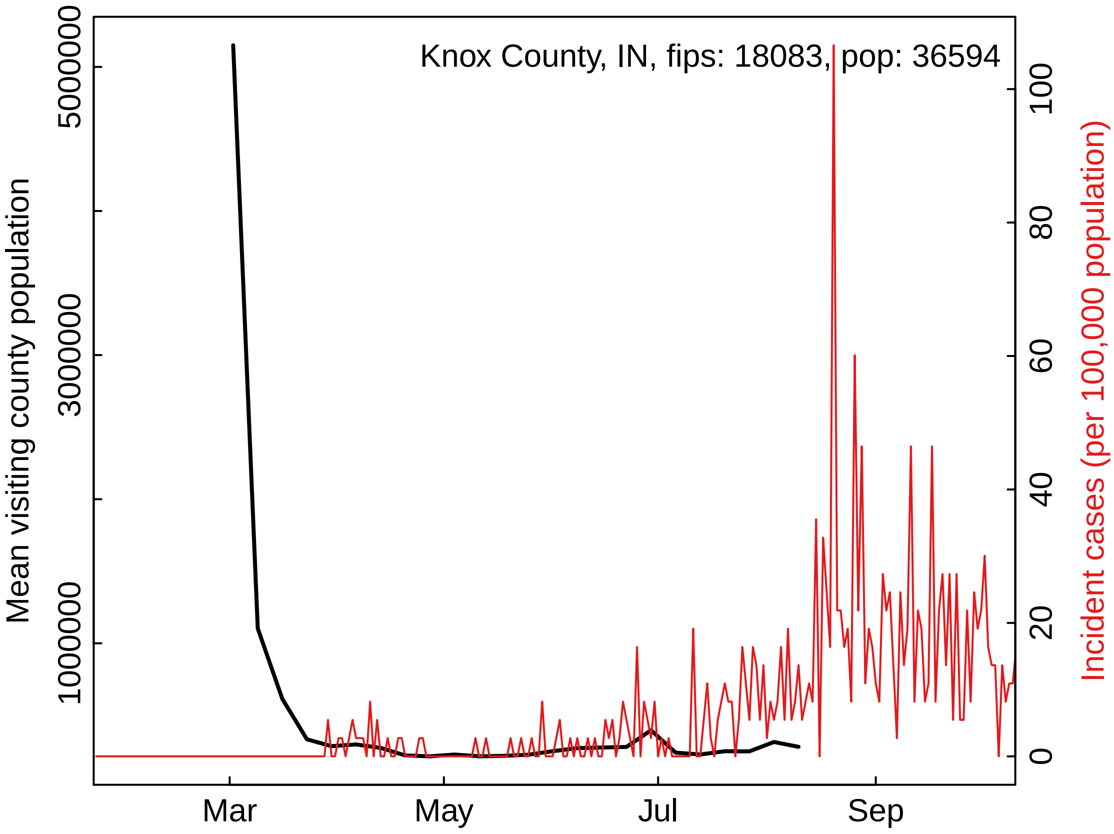
Knox County, IN, fips: 18083, pop: 36594
Mean visiting county population
1000000
3000000
5000000
Incident cases (per 100,000 population)
0
20
40
60
80
100
Mar
May
Jul
Sep

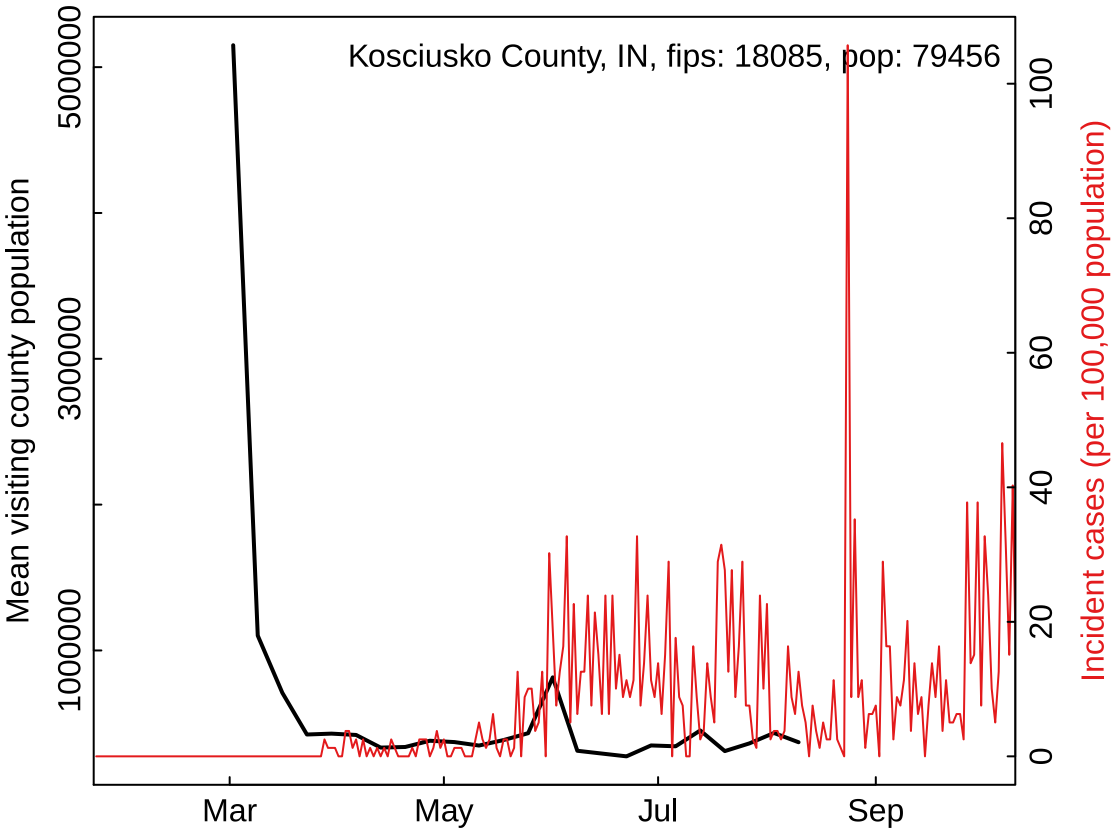

Kosciusko County, IN, fips: 18085, pop: 79456
Mean visiting county population
1000000
3000000
5000000
Incident cases (per 100,000 population)
0
20
40
60
80
100
Mar
May
Jul
Sep

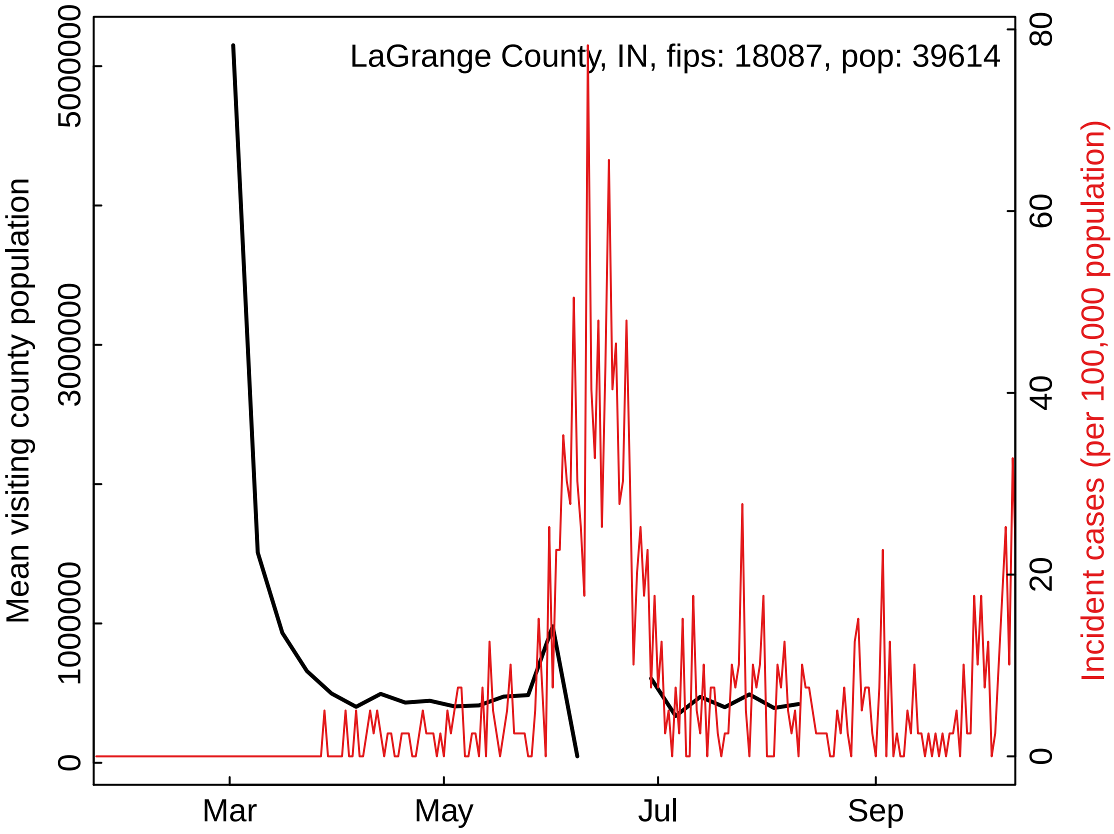

LaGrange County, IN, fips: 18087, pop: 39614
Mean visiting county population
0
1000000
3000000
5000000
Incident cases (per 100,000 population)
0
20
40
60
80
Mar
May
Jul
Sep

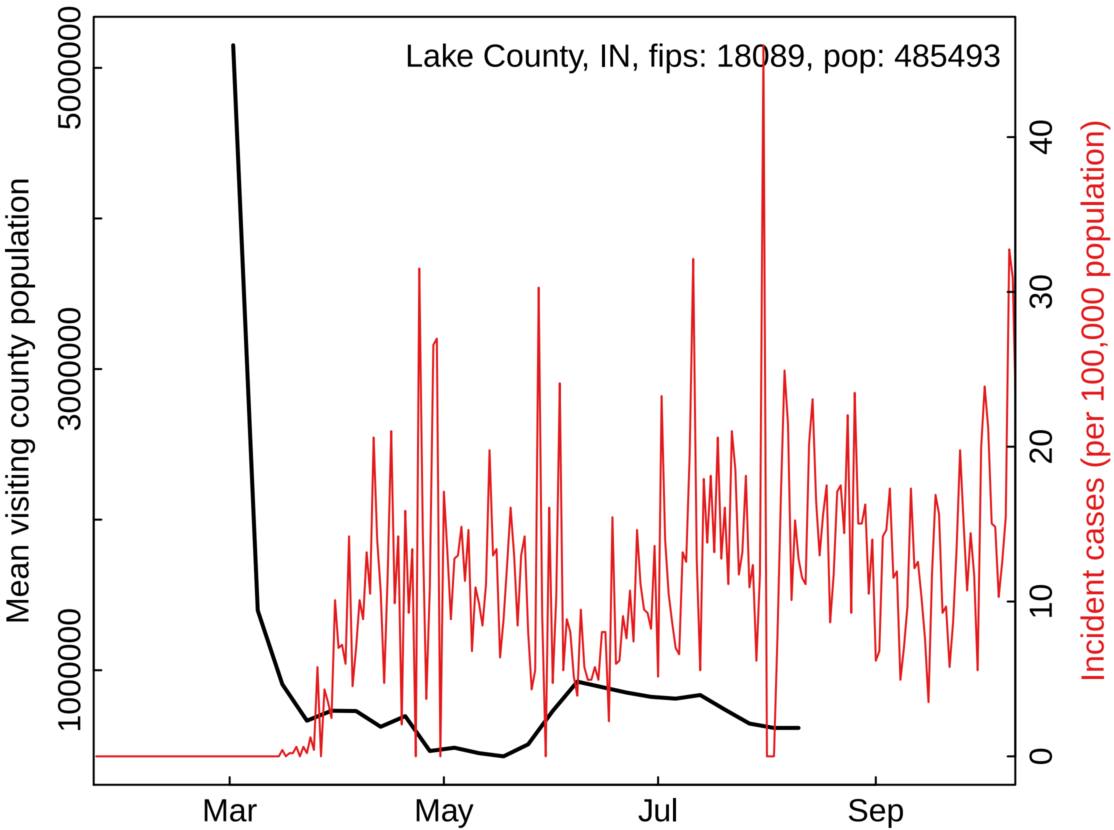

Lake County, IN, fips: 18089, pop: 485493
Mean visiting county population
1000000
3000000
5000000
Incident cases (per 100,000 population)
0
10
20
30
40
Mar
May
Jul
Sep

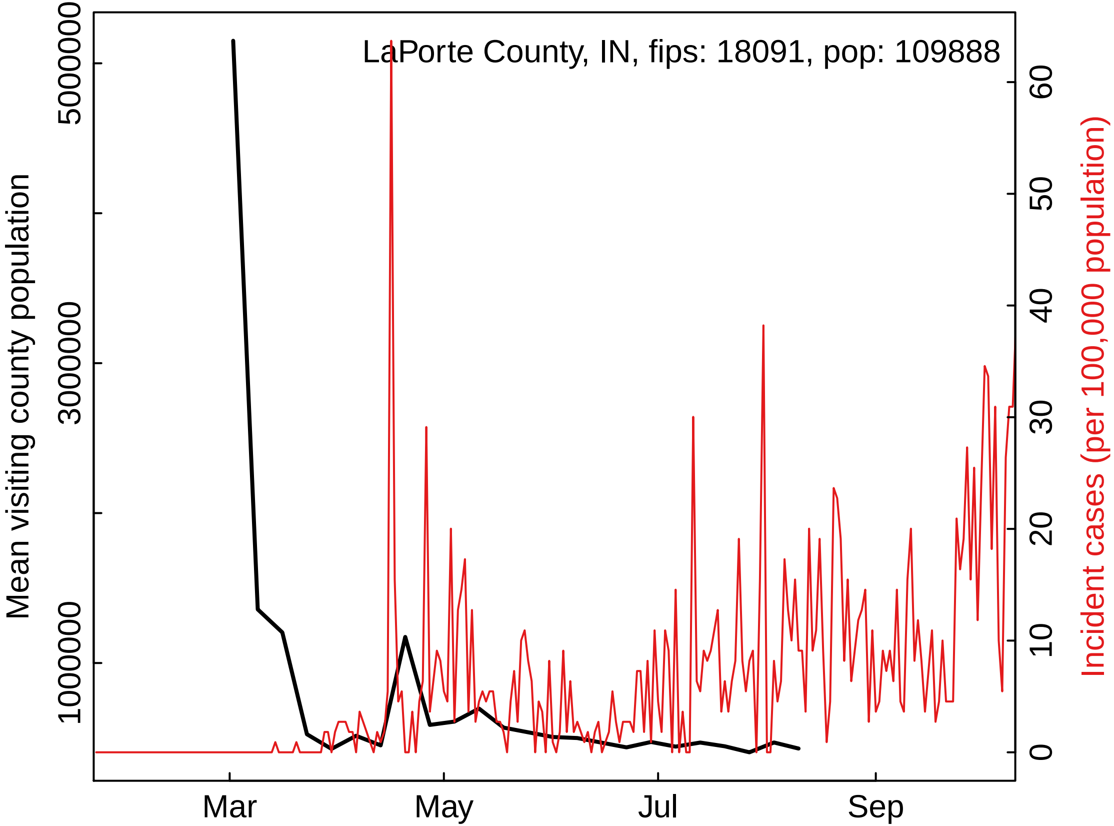

LaPorte County, IN, fips: 18091, pop: 109888
Mean visiting county population
1000000
3000000
5000000
Incident cases (per 100,000 population)
0
10
20
30
40
50
60
Mar
May
Jul
Sep

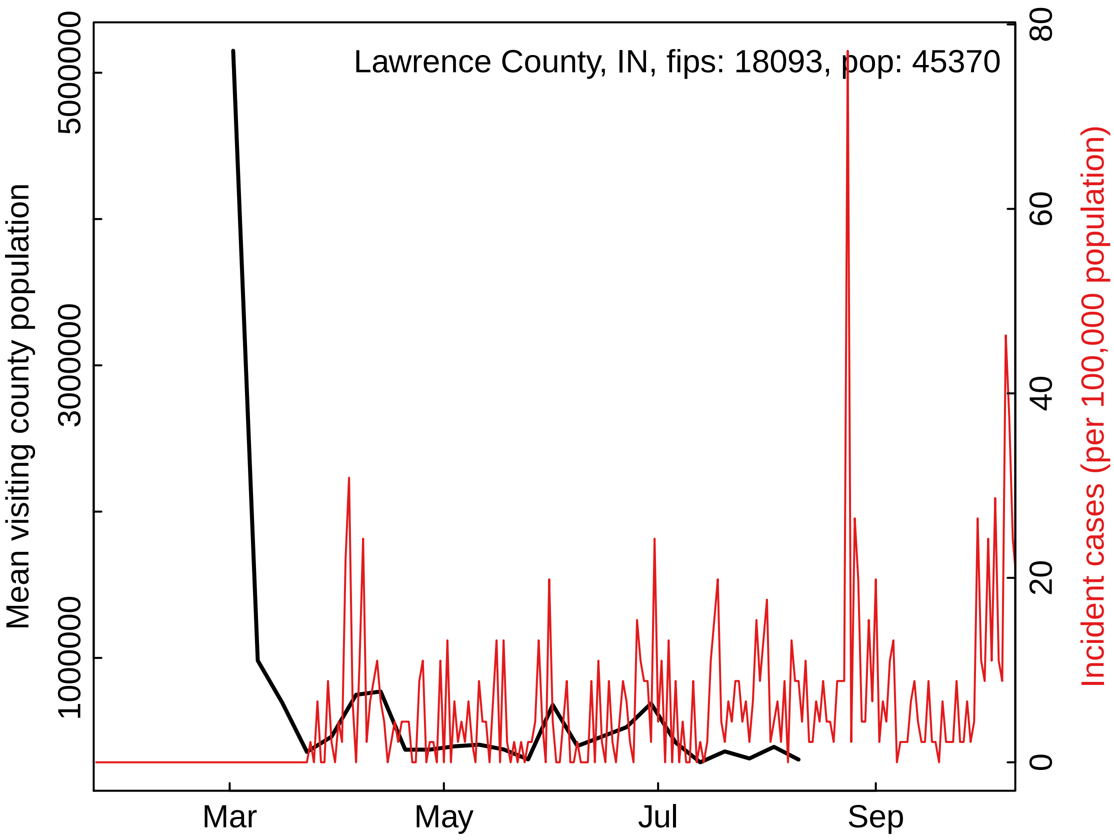

Lawrence County, IN, fips: 18093, pop: 45370
Mean visiting county population
1000000
3000000
5000000
Incident cases (per 100,000 population)
0
20
40
60
80
Mar
May
Jul
Sep

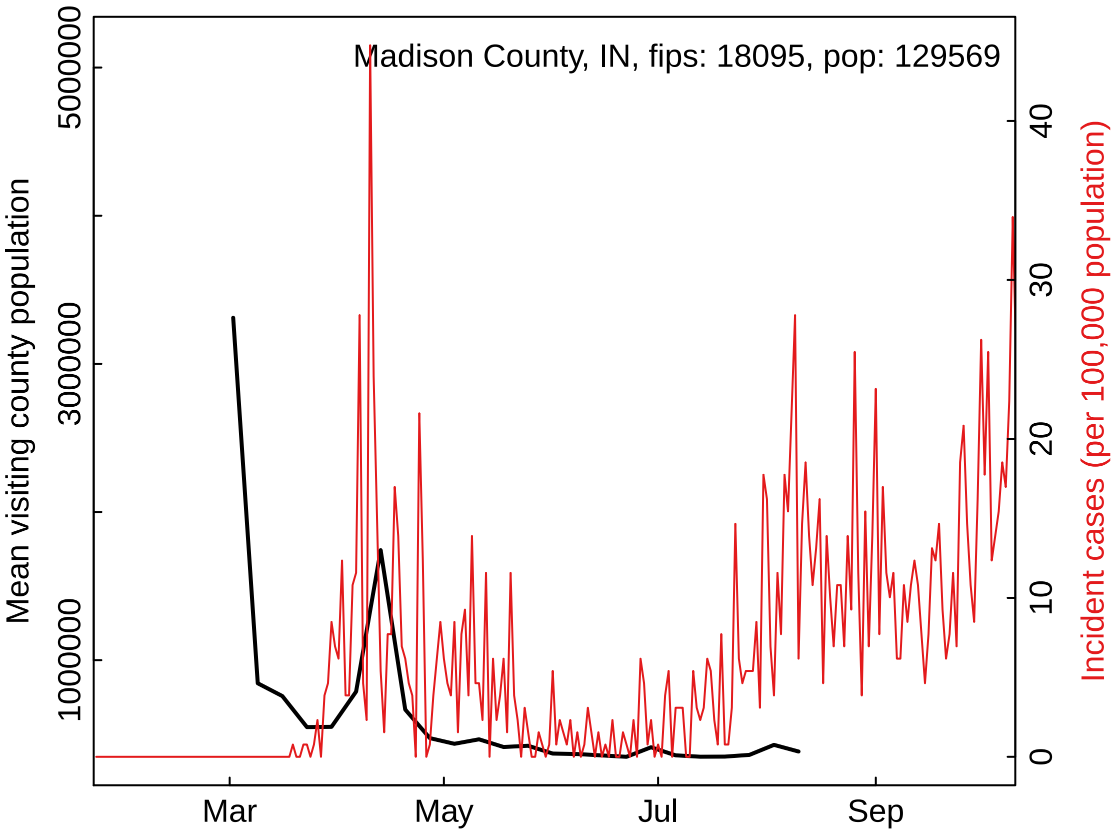
Madison County, IN, fips: 18095, pop: 129569
Mean visiting county population
1000000
3000000
5000000
Incident cases (per 100,000 population)
0
10
20
30
40
Mar
May
Jul
Sep

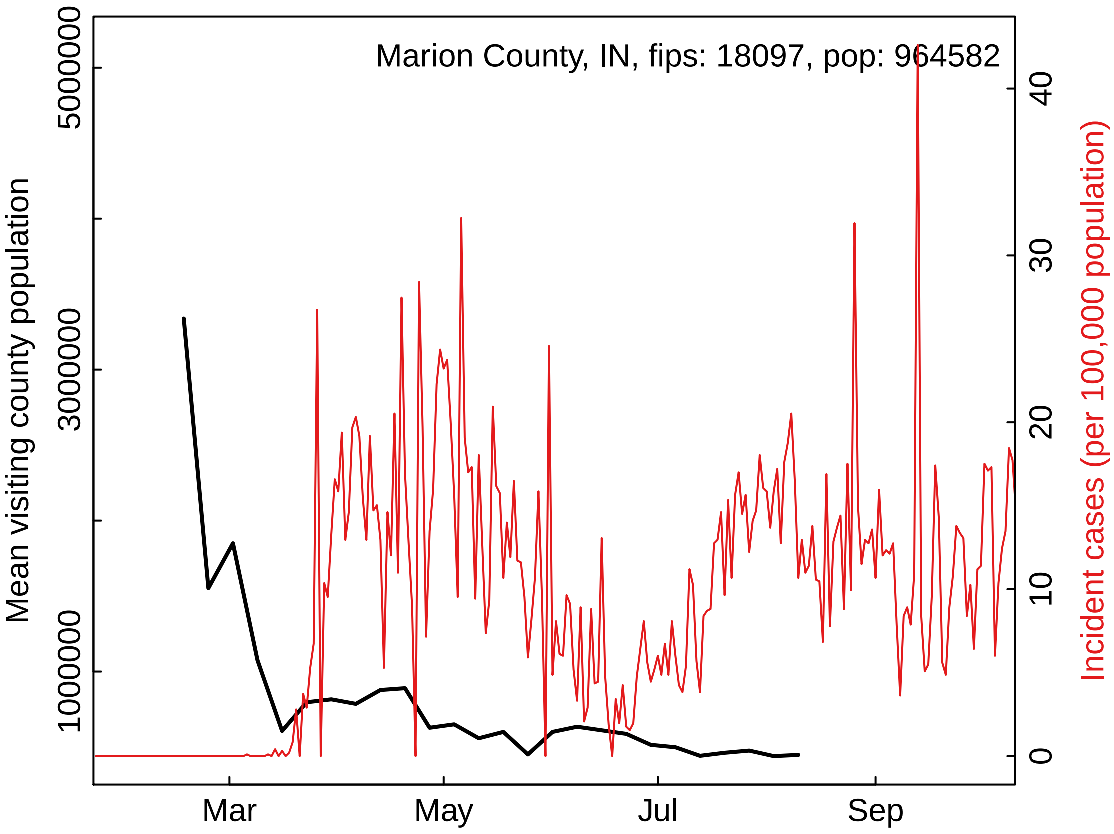
Marion County, IN, fips: 18097, pop: 964582
Mean visiting county population
1000000
3000000
5000000
Incident cases (per 100,000 population)
0
10
20
30
40
Mar
May
Jul
Sep

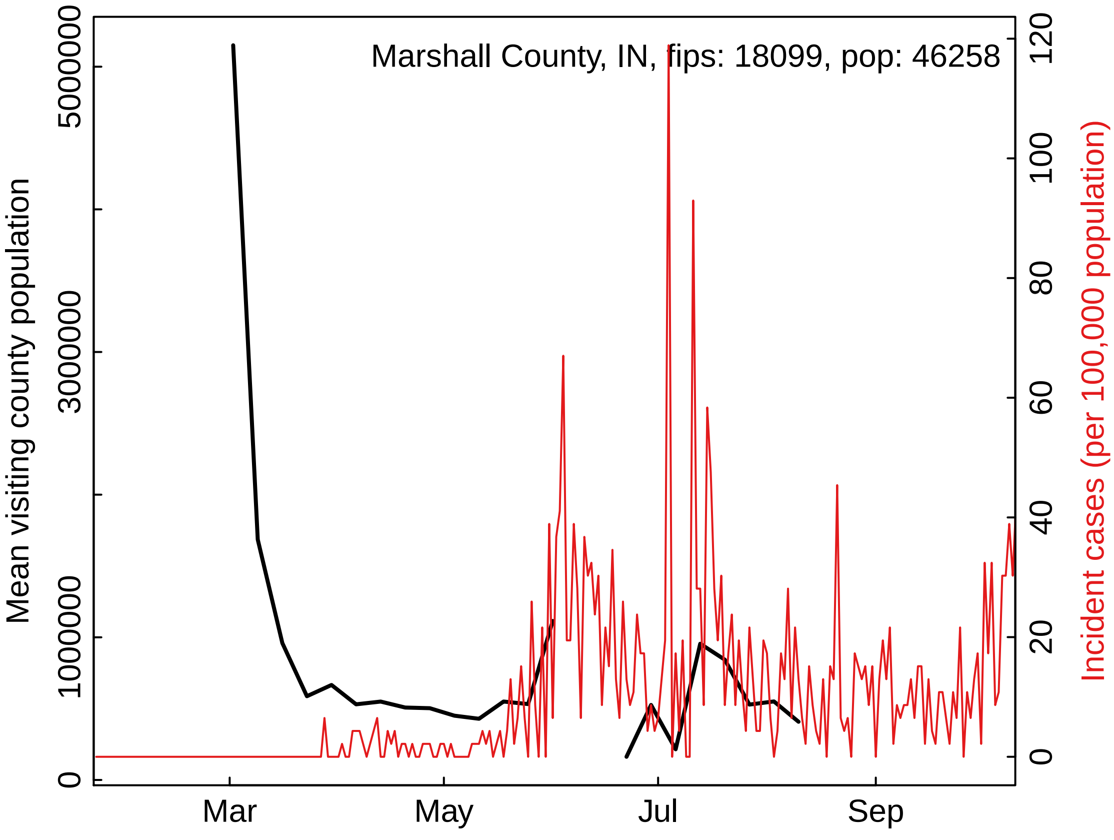

Marshall County, IN, fips: 18099, pop: 46258
Mean visiting county population
0
1000000
3000000
5000000
Incident cases (per 100,000 population)
0
20
40
60
80
100
120
Mar
May
Jul
Sep

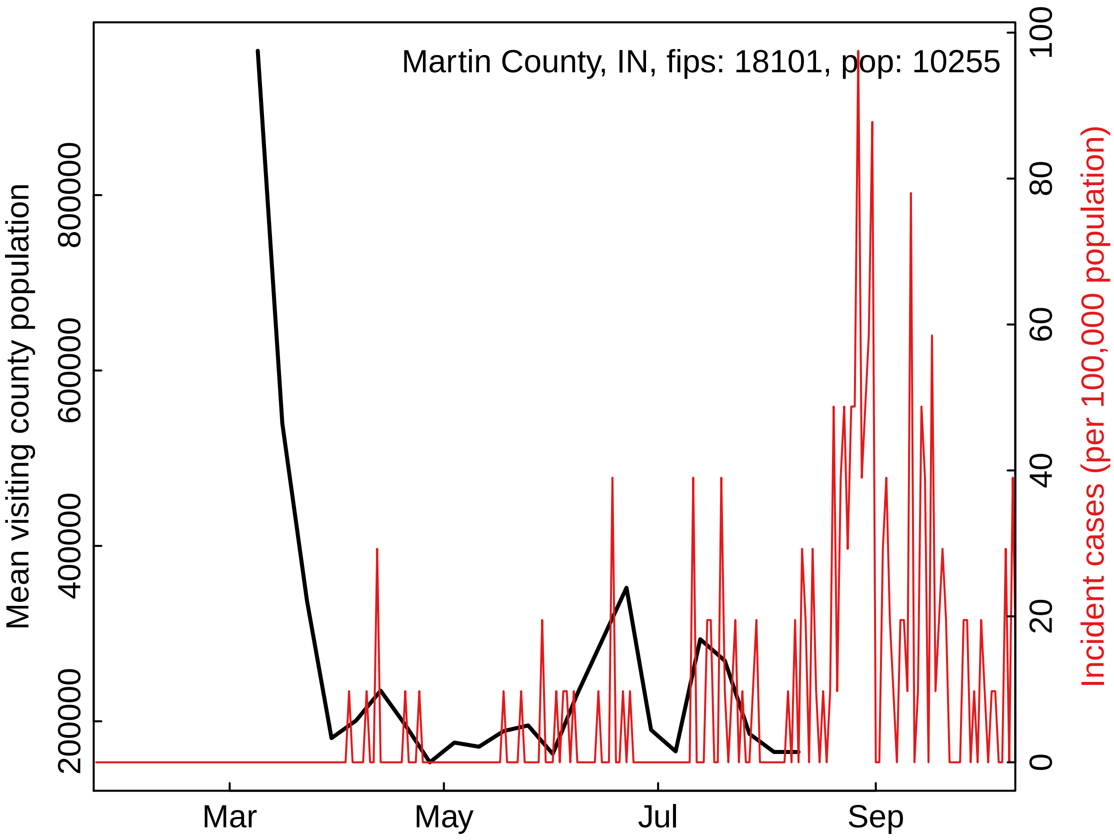

Martin County, IN, fips: 18101, pop: 10255
Mean visiting county population
200000
400000
600000
800000
Mar
May
Jul
Sep
0
20
40
60
80
100
Incident cases (per 100,000 population)

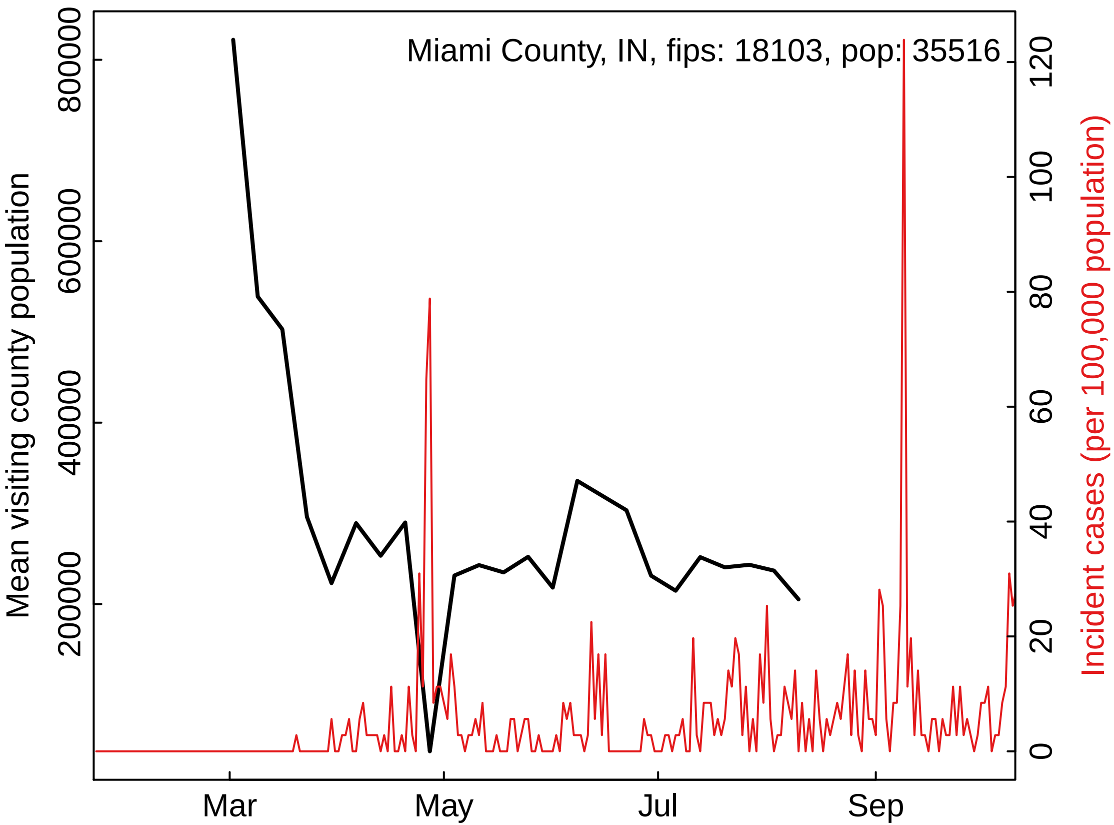
Miami County, IN, fips: 18103, pop: 35516
Mean visiting county population
200000
400000
600000
800000
Incident cases (per 100,000 population)
0
20
40
60
80
100
120
Mar
May
Jul
Sep

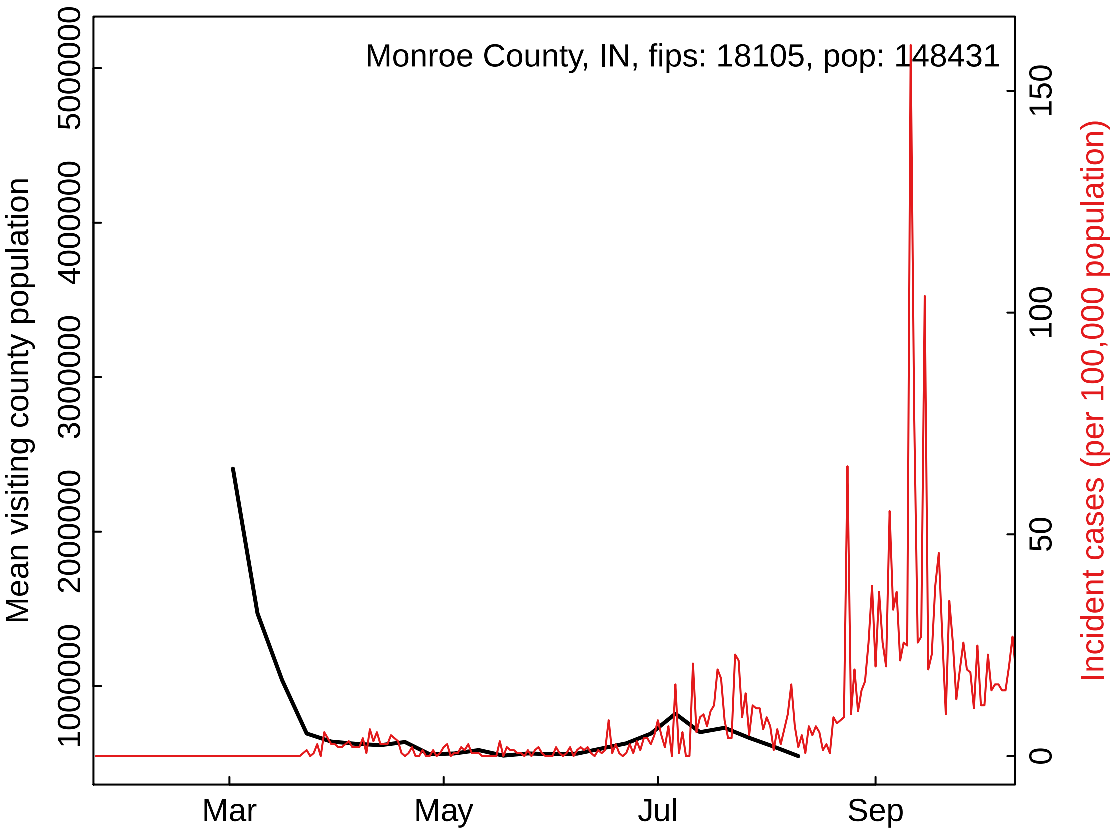

Monroe County, IN, fips: 18105, pop: 148431
Mean visiting county population
1000000
2000000
3000000
4000000
5000000
Incident cases (per 100,000 population)
0
50
100
150
Mar
May
Jul
Sep

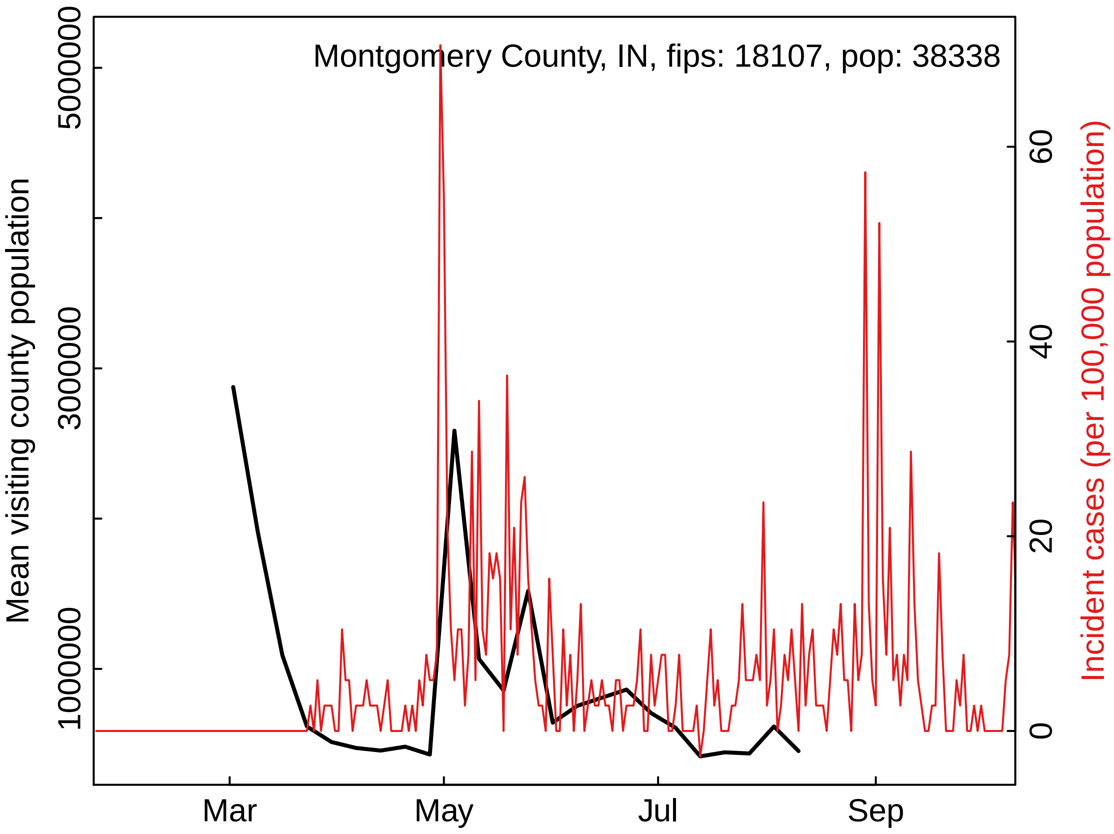
Montgomery County, IN, fips: 18107, pop: 38338
Mean visiting county population
1000000
3000000
5000000
Incident cases (per 100,000 population)
0
20
40
60
Mar
May
Jul
Sep

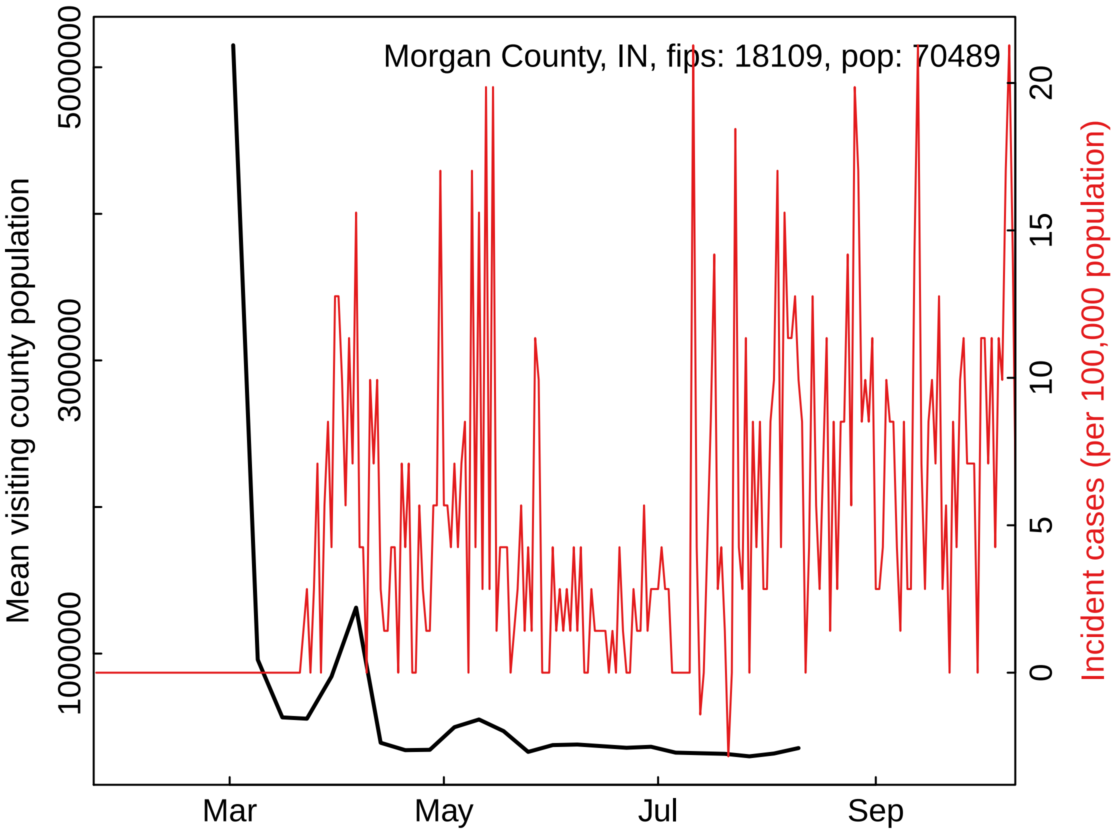

Morgan County, IN, fips: 18109, pop: 70489
Mean visiting county population
1000000
3000000
5000000
Incident cases (per 100,000 population)
0
5
10
15
20
Mar
May
Jul
Sep

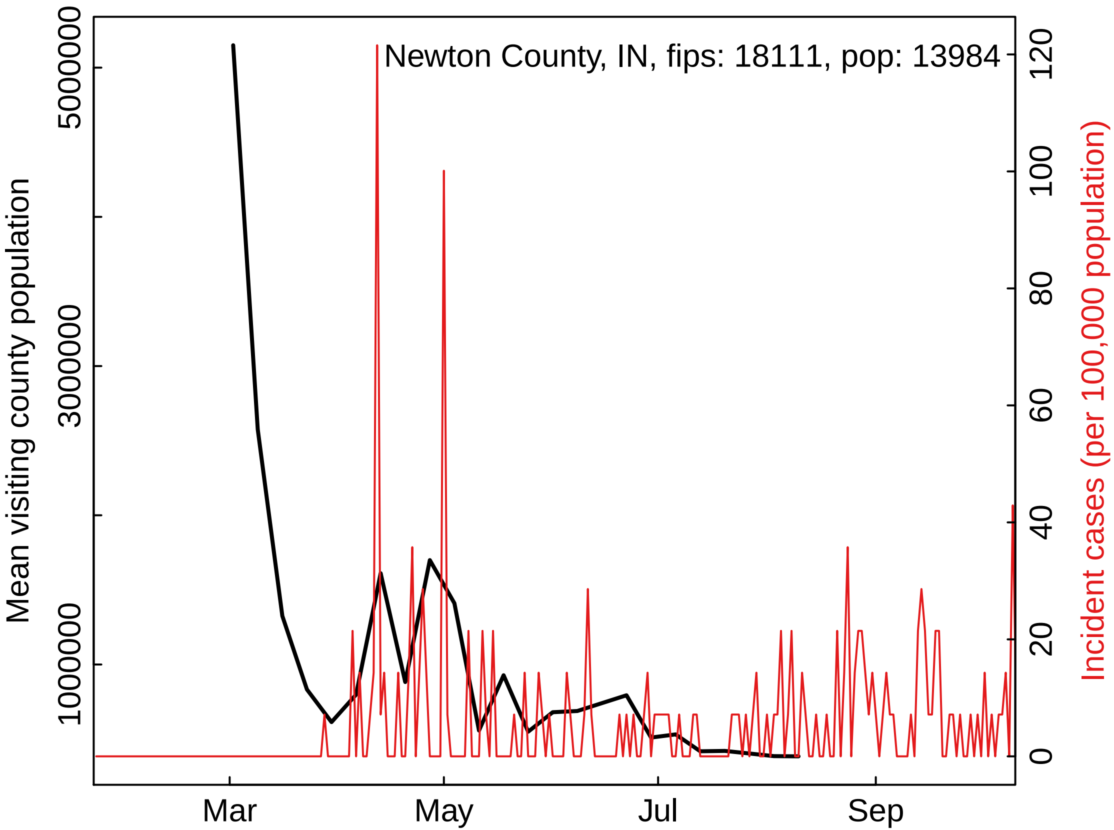

Newton County, IN, fips: 18111, pop: 13984
Mean visiting county population
1000000
3000000
5000000
Incident cases (per 100,000 population)
0
20
40
60
80
100
120
Mar
May
Jul
Sep

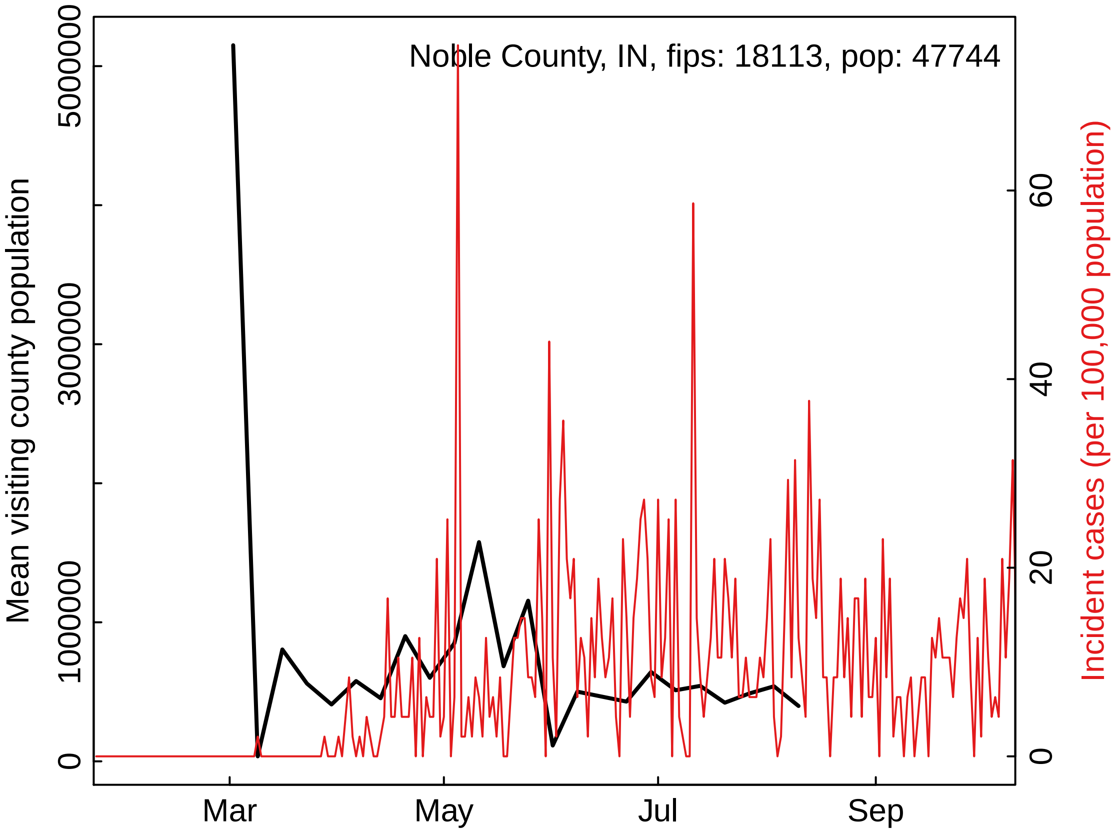

Noble County, IN, fips: 18113, pop: 47744
Mean visiting county population
Incident cases (per 100,000 population)
0
1000000
3000000
5000000
0
20
40
60
Mar
May
Jul
Sep

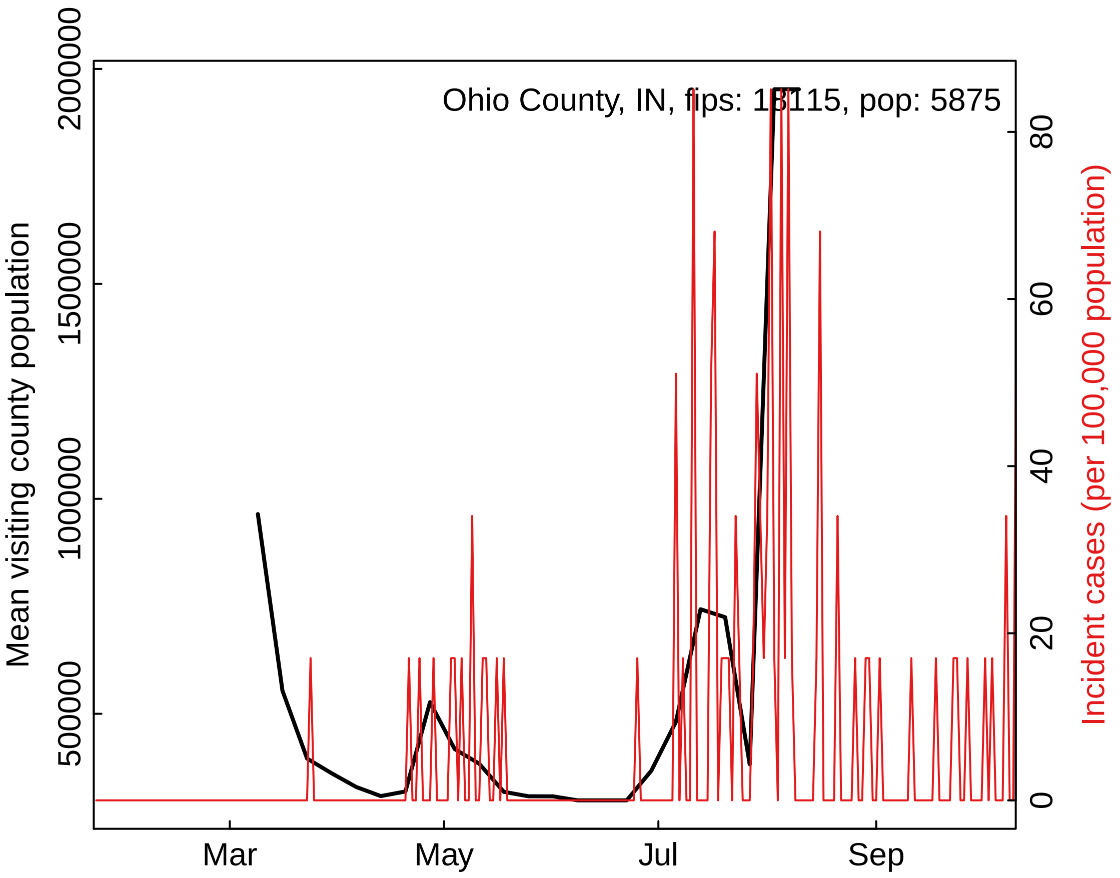
Ohio County, IN, fips: 18115, pop: 5875
Mean visiting county population
500000
1000000
1500000
2000000
Incident cases (per 100,000 population)
0
20
40
60
80
Mar
May
Jul
Sep

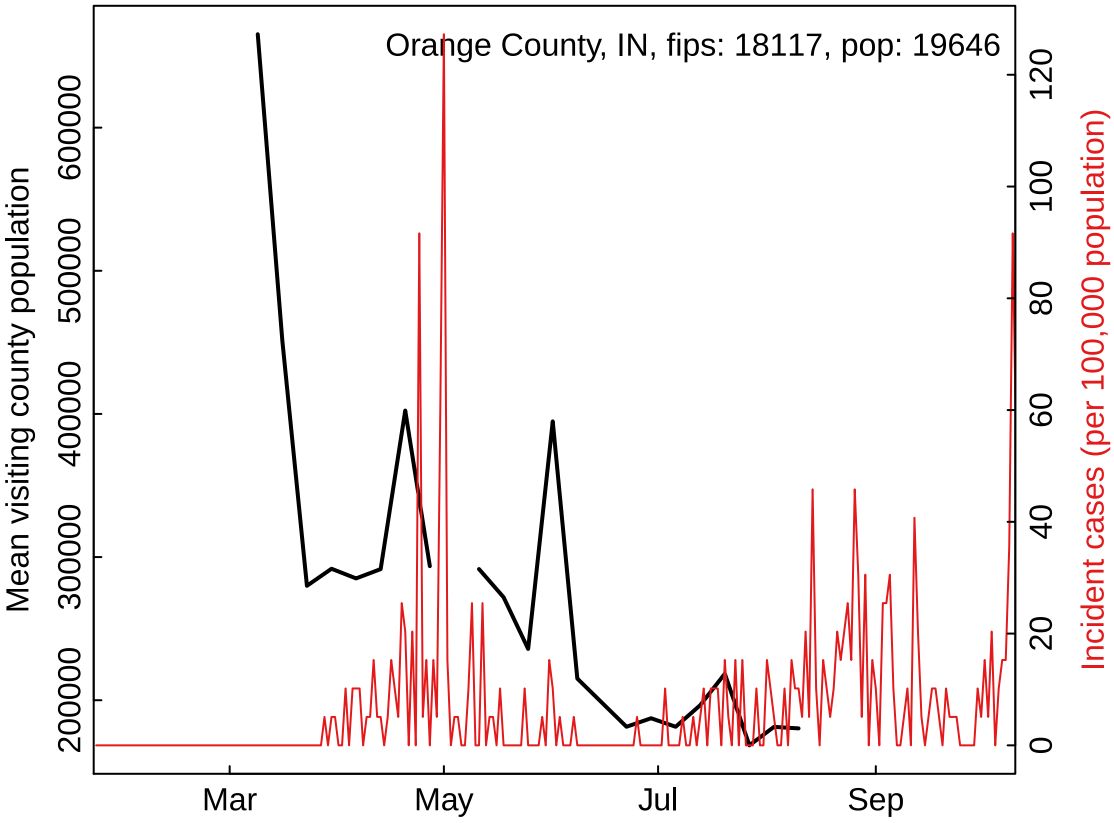

Orange County, IN, fips: 18117, pop: 19646
Mean visiting county population
200000
300000
400000
500000
600000
Incident cases (per 100,000 population)
0
20
40
60
80
100
120
Mar
May
Jul
Sep

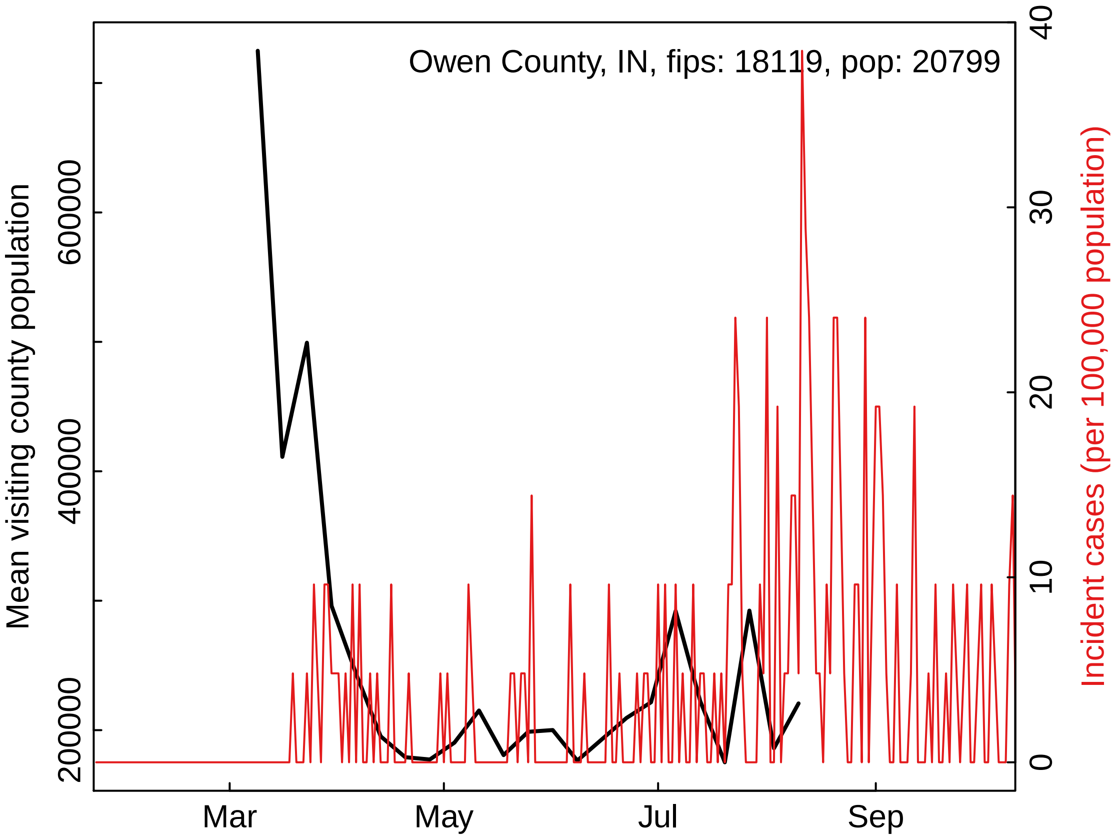

Owen County, IN, fips: 18119, pop: 20799
Mean visiting county population
200000
400000
600000
Incident cases (per 100,000 population)
0
10
20
30
40
Mar
May
Jul
Sep

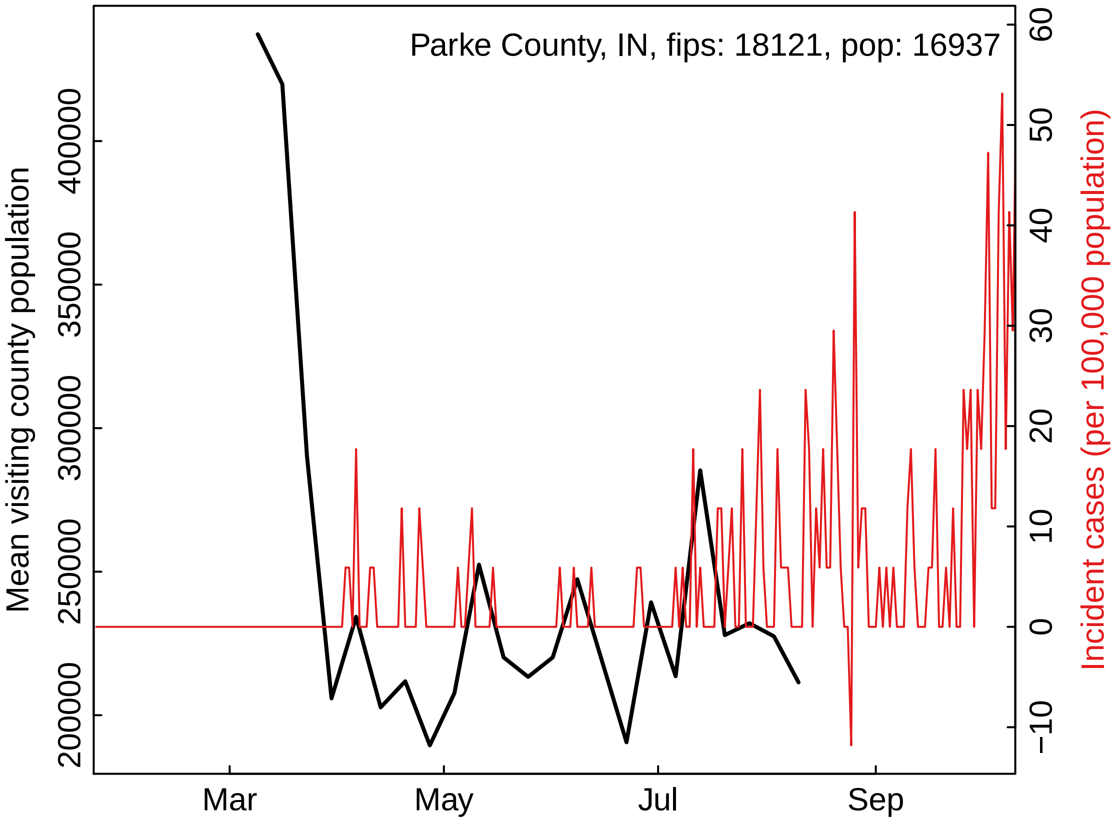

Parke County, IN, fips: 18121, pop: 16937
Mean visiting county population
Incident cases (per 100,000 population)
200000
250000
300000
350000
400000
−10
0
10
20
30
40
50
60
Mar
May
Jul
Sep

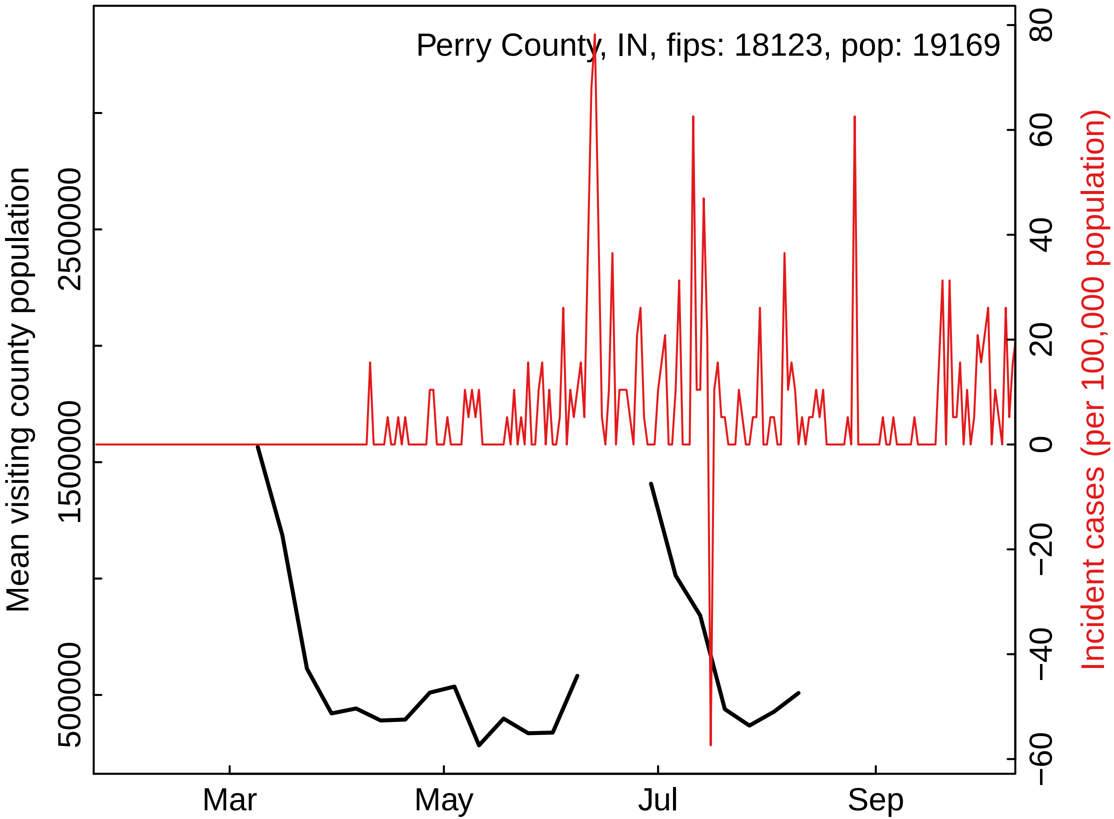

Perry County, IN, fips: 18123, pop: 19169
Mean visiting county population
500000
1500000
2500000
Mar
May
Jul
Sep
Incident cases (per 100,000 population)
−60
−40
−20
0
20
40
60
80

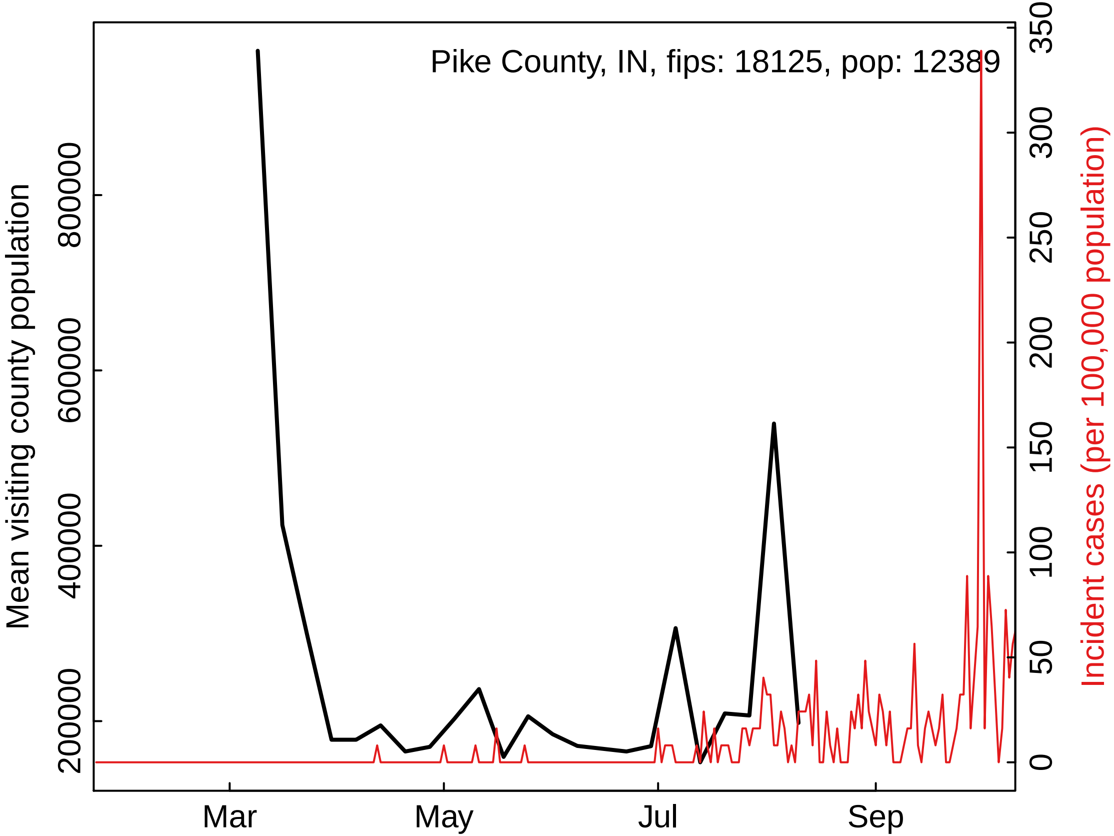
Pike County, IN, fips: 18125, pop: 12389
Mean visiting county population
200000
400000
600000
800000
Incident cases (per 100,000 population)
0
50
100
150
200
250
300
350
Mar
May
Jul
Sep

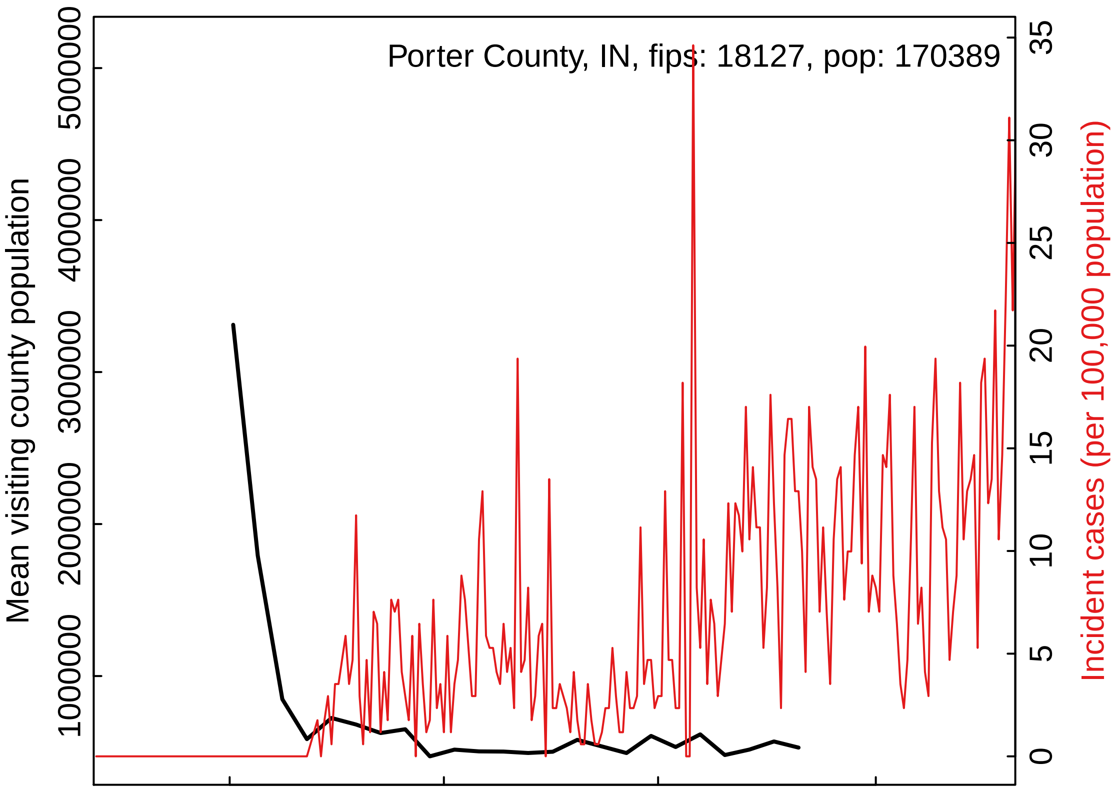

Porter County, IN, fips: 18127, pop: 170389
Mean visiting county population
1000000
2000000
3000000
4000000
5000000
Incident cases (per 100,000 population)
0
5
10
15
20
25
30
35
Mar
May
Jul
Sep

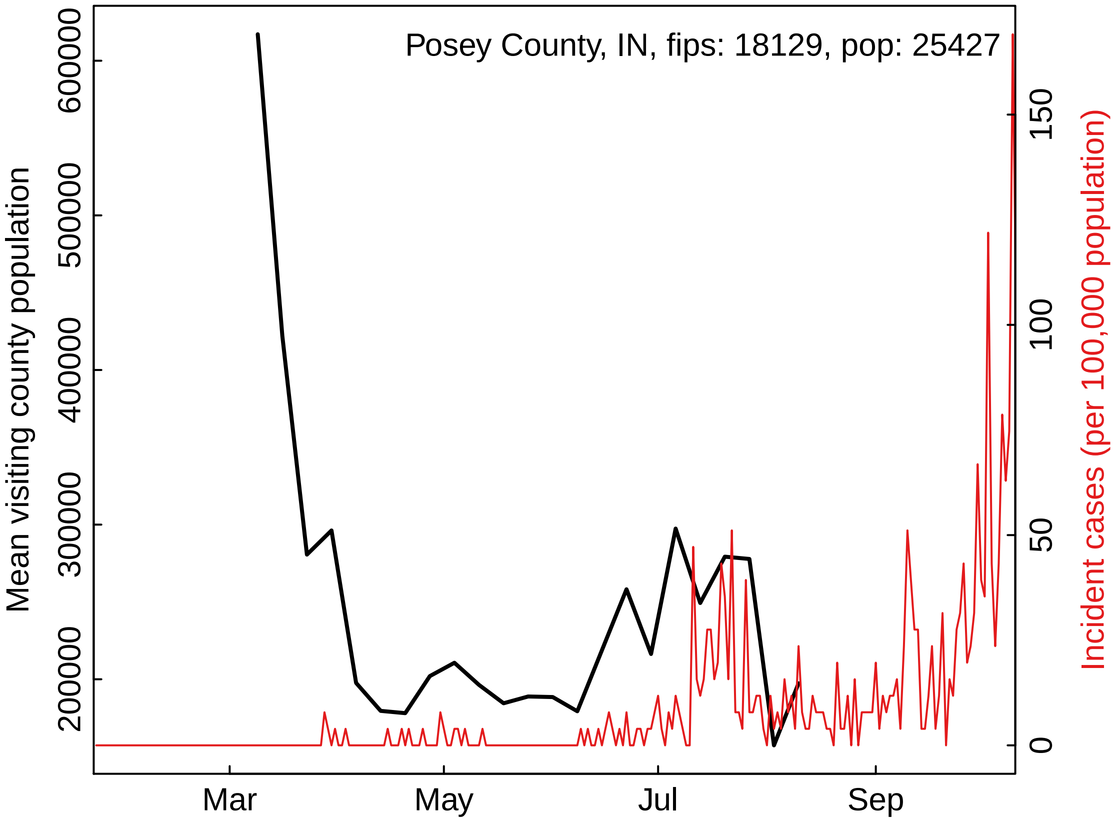

Posey County, IN, fips: 18129, pop: 25427
Mean visiting county population
200000
300000
400000
500000
600000
Incident cases (per 100,000 population)
0
50
100
150
Mar
May
Jul
Sep

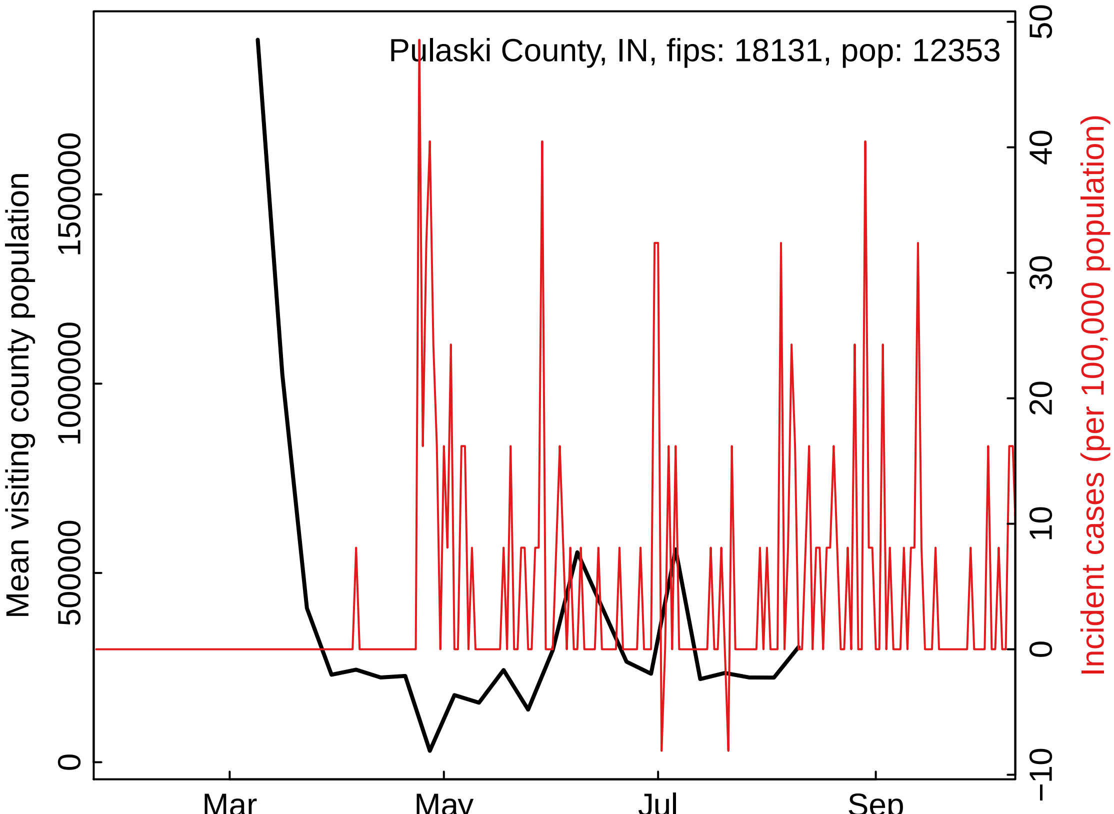

Pulaski County, IN, fips: 18131, pop: 12353
Mean visiting county population
0
500000
1000000
1500000
Mar
May
Jul
Sep
−10
0
10
20
30
40
50
Incident cases (per 100,000 population)

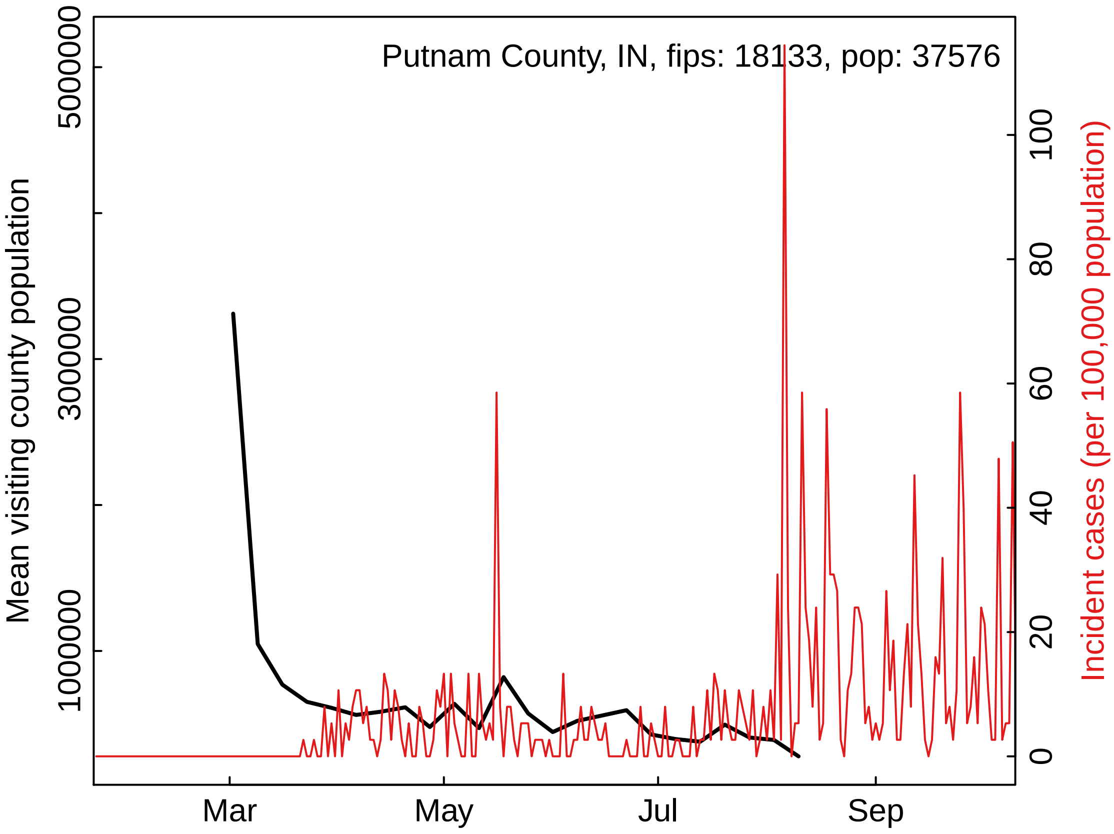

Putnam County, IN, fips: 18133, pop: 37576
Mean visiting county population
1000000
3000000
5000000
Incident cases (per 100,000 population)
0
20
40
60
80
100
Mar
May
Jul
Sep

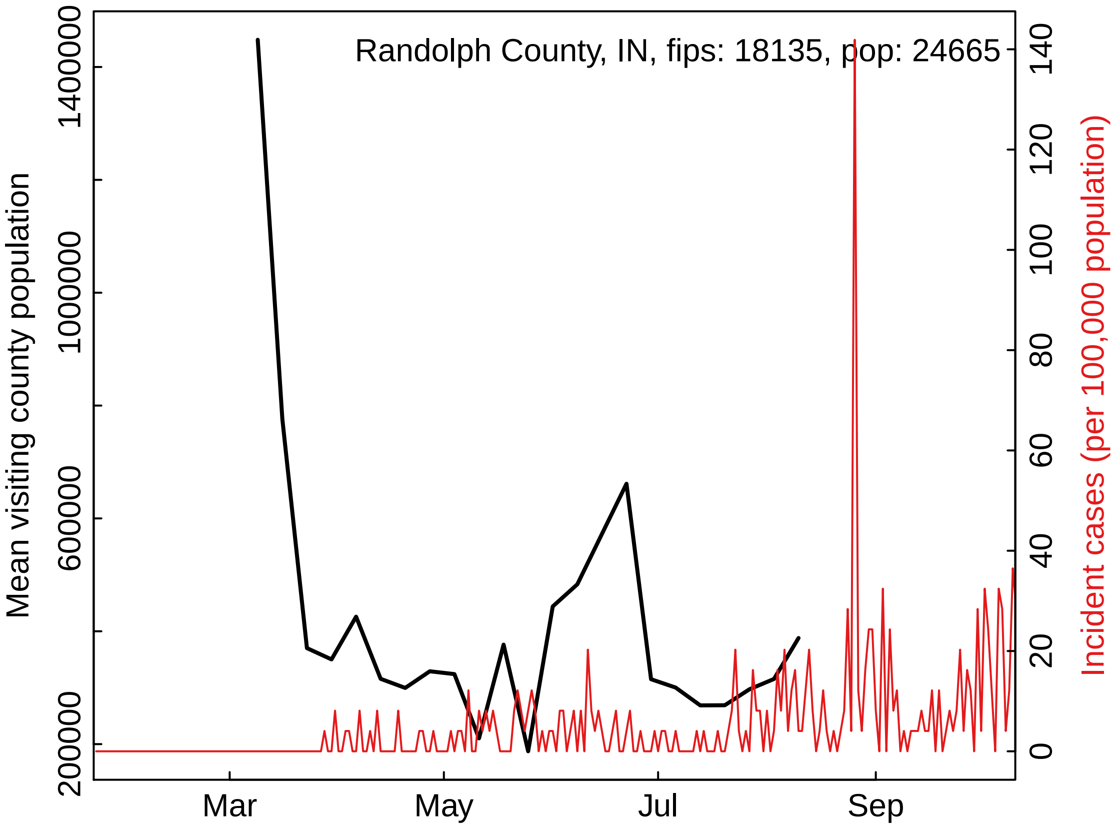
Randolph County, IN, fips: 18135, pop: 24665
Mean visiting county population
200000
600000
1000000
1400000
Incident cases (per 100,000 population)
0
20
40
60
80
100
120
140
Mar
May
Jul
Sep

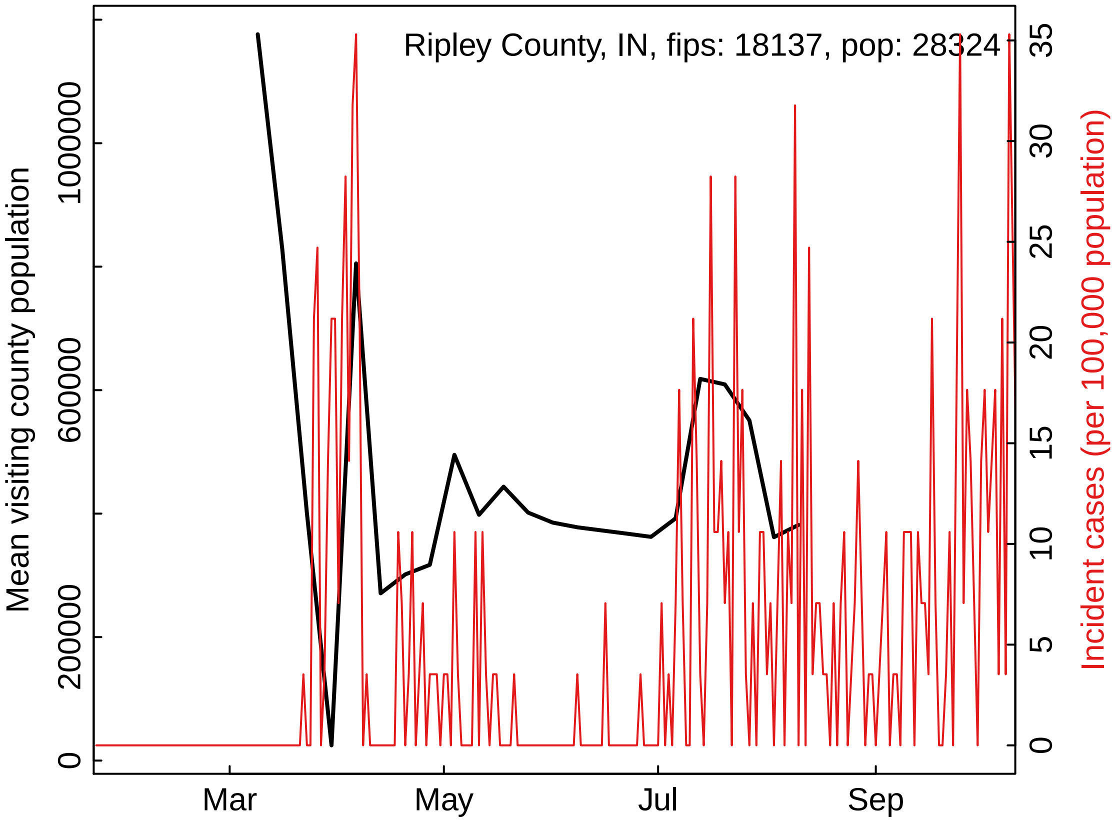

Ripley County, IN, fips: 18137, pop: 28324
Mean visiting county population
0
200000
600000
1000000
Incident cases (per 100,000 population)
0
5
10
15
20
25
30
35
Mar
May
Jul
Sep

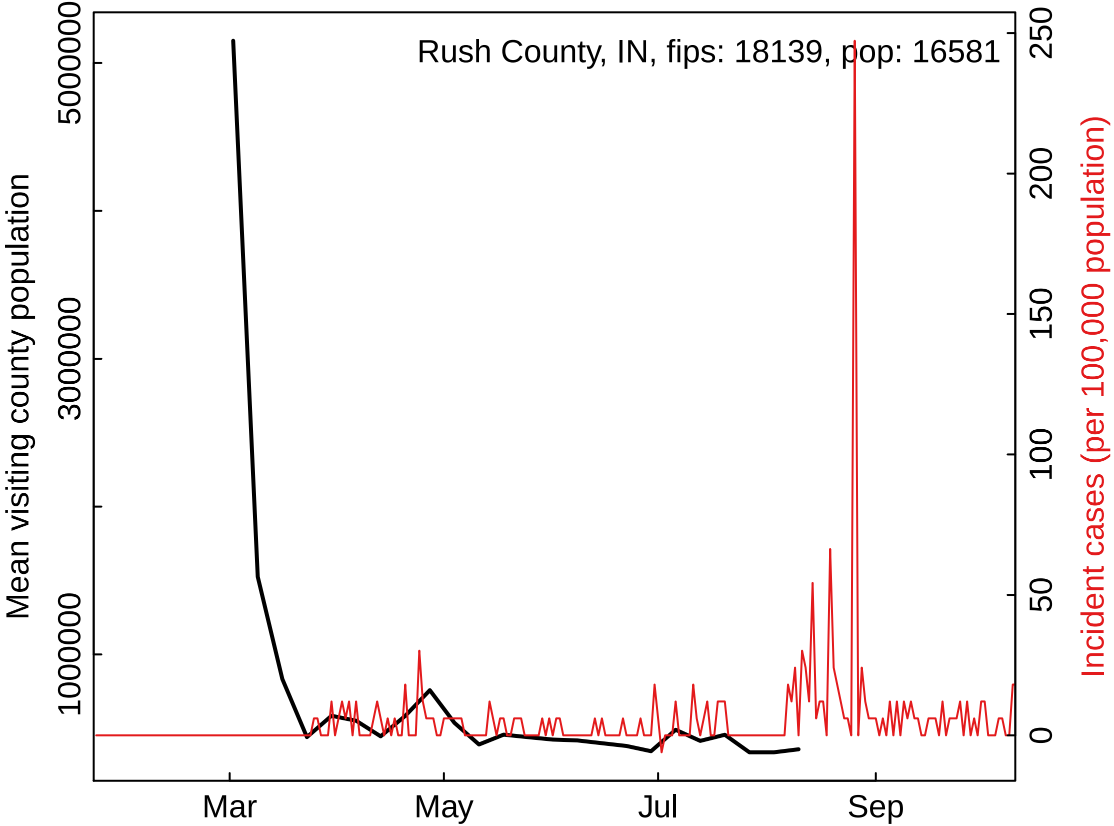
Rush County, IN, fips: 18139, pop: 16581
Mean visiting county population
1000000
3000000
5000000
Incident cases (per 100,000 population)
0
50
100
150
200
250
Mar
May
Jul
Sep

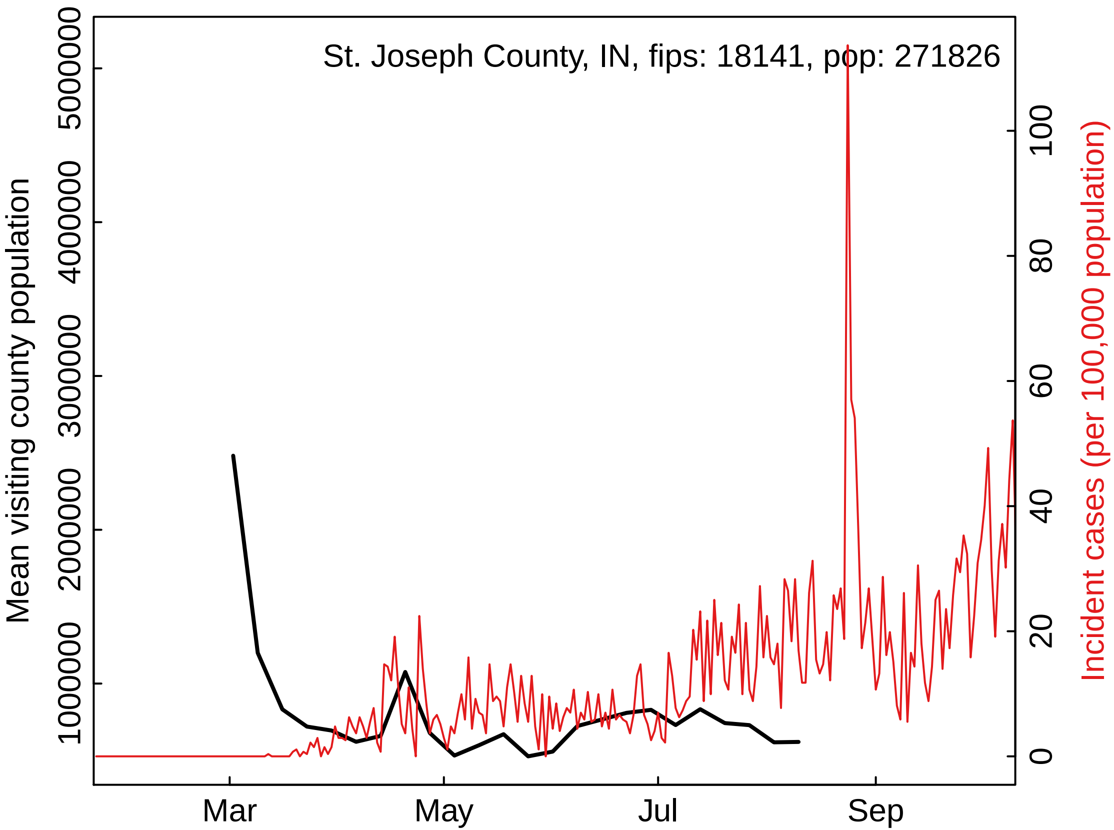
St. Joseph County, IN, fips: 18141, pop: 271826
Mean visiting county population
1000000
2000000
3000000
4000000
5000000
Incident cases (per 100,000 population)
0
20
40
60
80
100
Mar
May
Jul
Sep

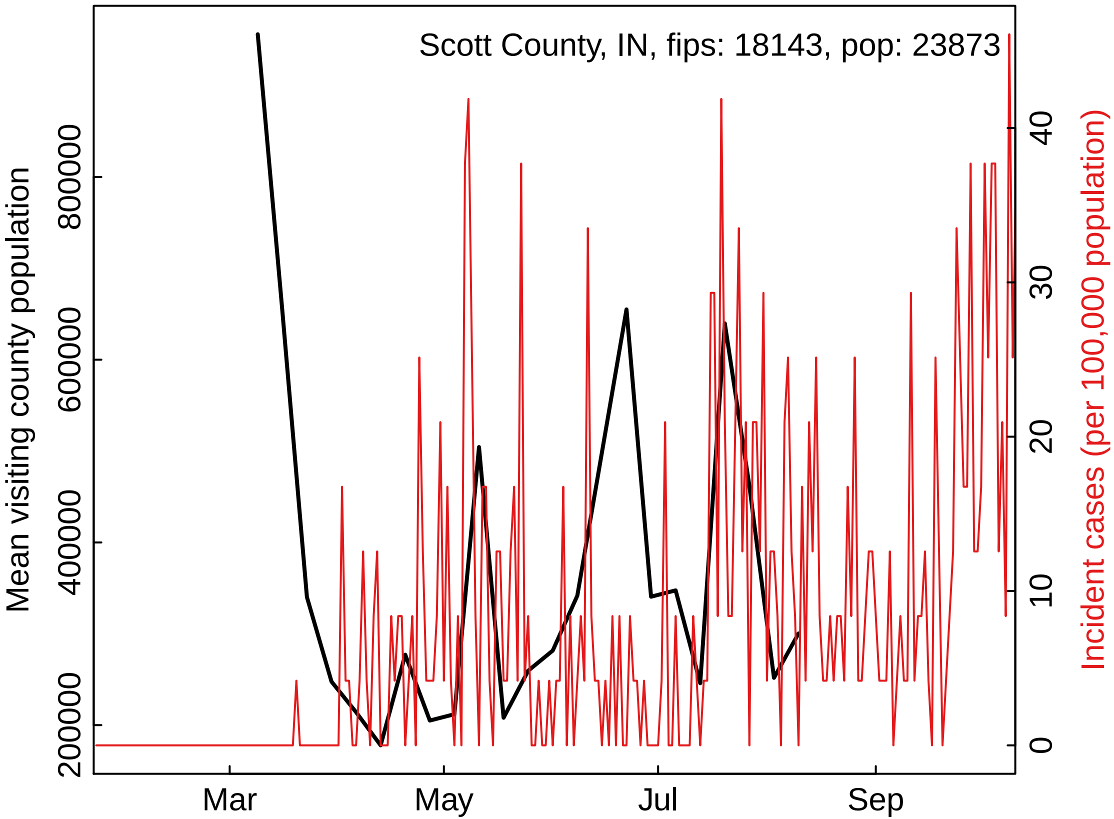

Scott County, IN, fips: 18143, pop: 23873
Mean visiting county population
200000
400000
600000
800000
Incident cases (per 100,000 population)
0
10
20
30
40
Mar
May
Jul
Sep

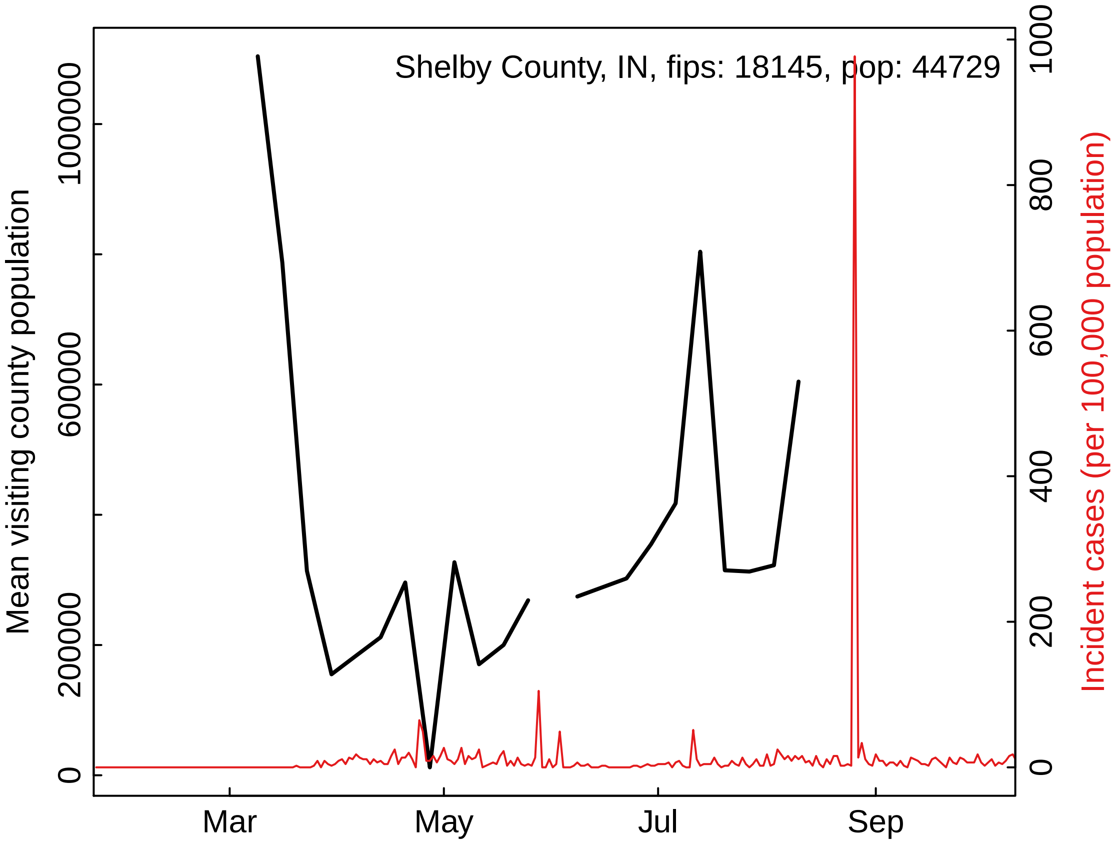

Shelby County, IN, fips: 18145, pop: 44729
Mean visiting county population
0
200000
600000
1000000
Mar
May
Jul
Sep
Incident cases (per 100,000 population)
0
200
400
600
800
1000

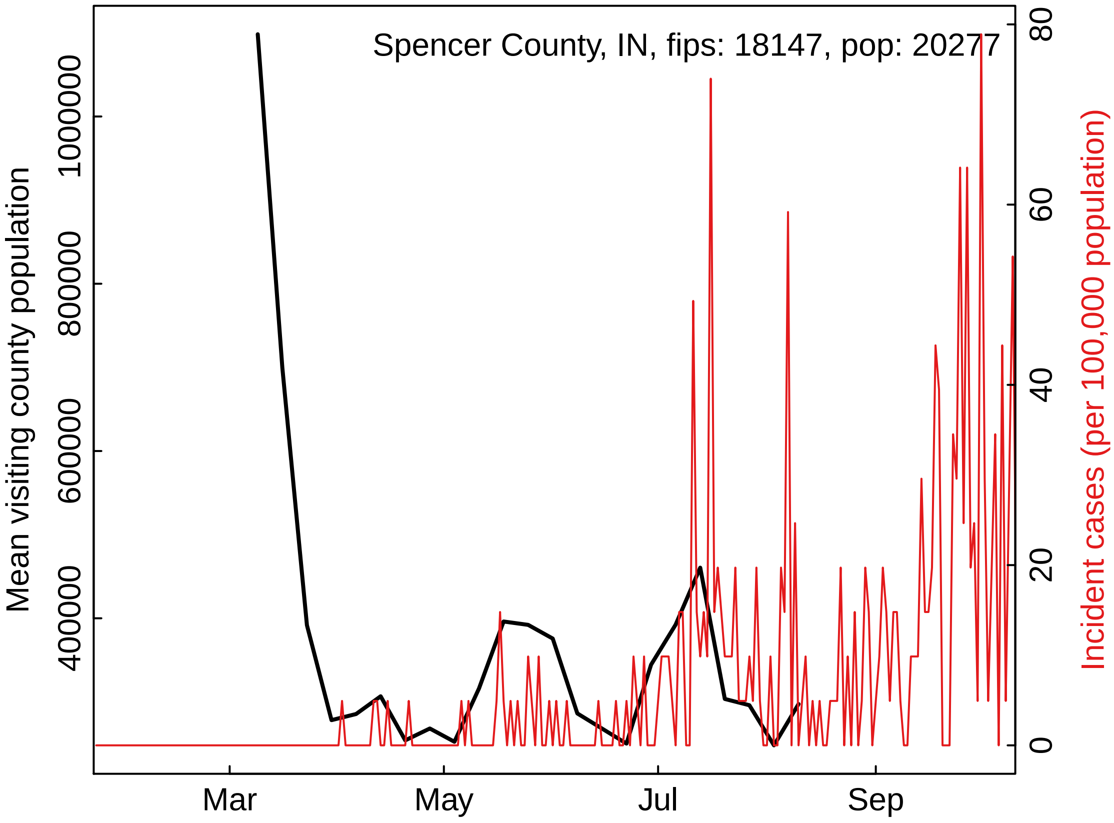

Spencer County, IN, fips: 18147, pop: 20277
Mean visiting county population
400000
600000
800000
1000000
Mar
May
Jul
Sep
0
20
40
60
80
Incident cases (per 100,000 population)

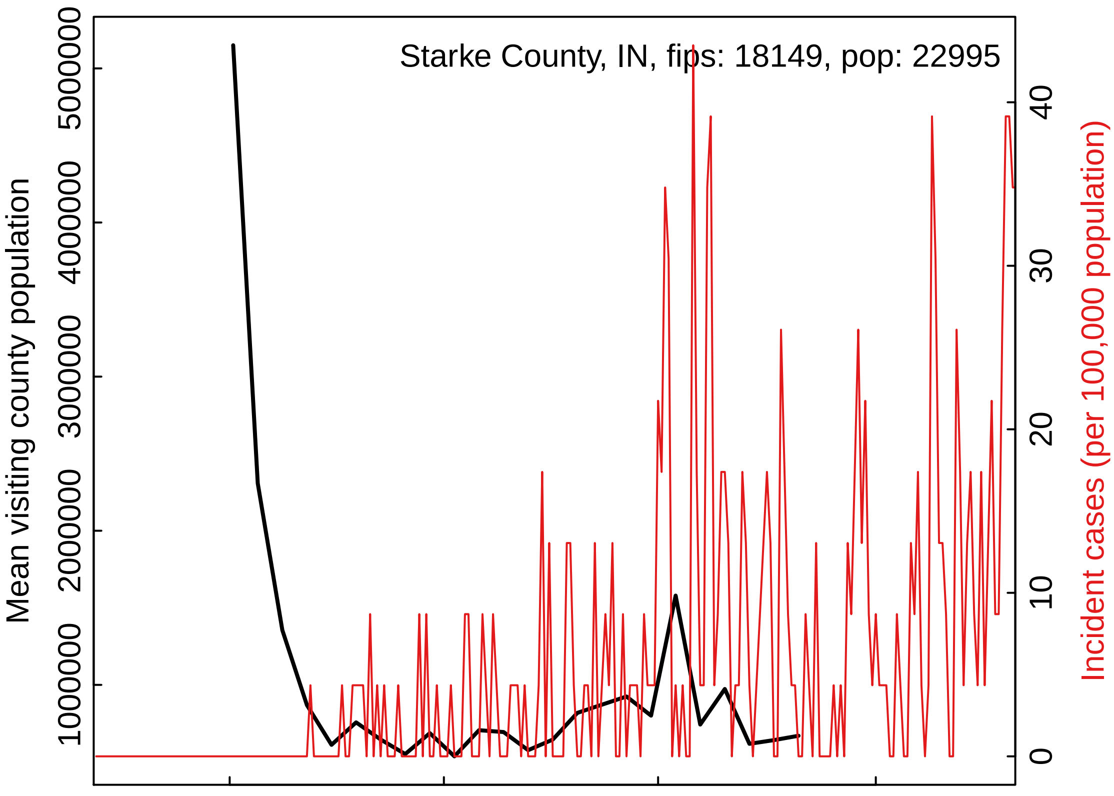
Starke County, IN, fips: 18149, pop: 22995
Mean visiting county population
1000000
2000000
3000000
4000000
5000000
Incident cases (per 100,000 population)
0
10
20
30
40
Mar
May
Jul
Sep

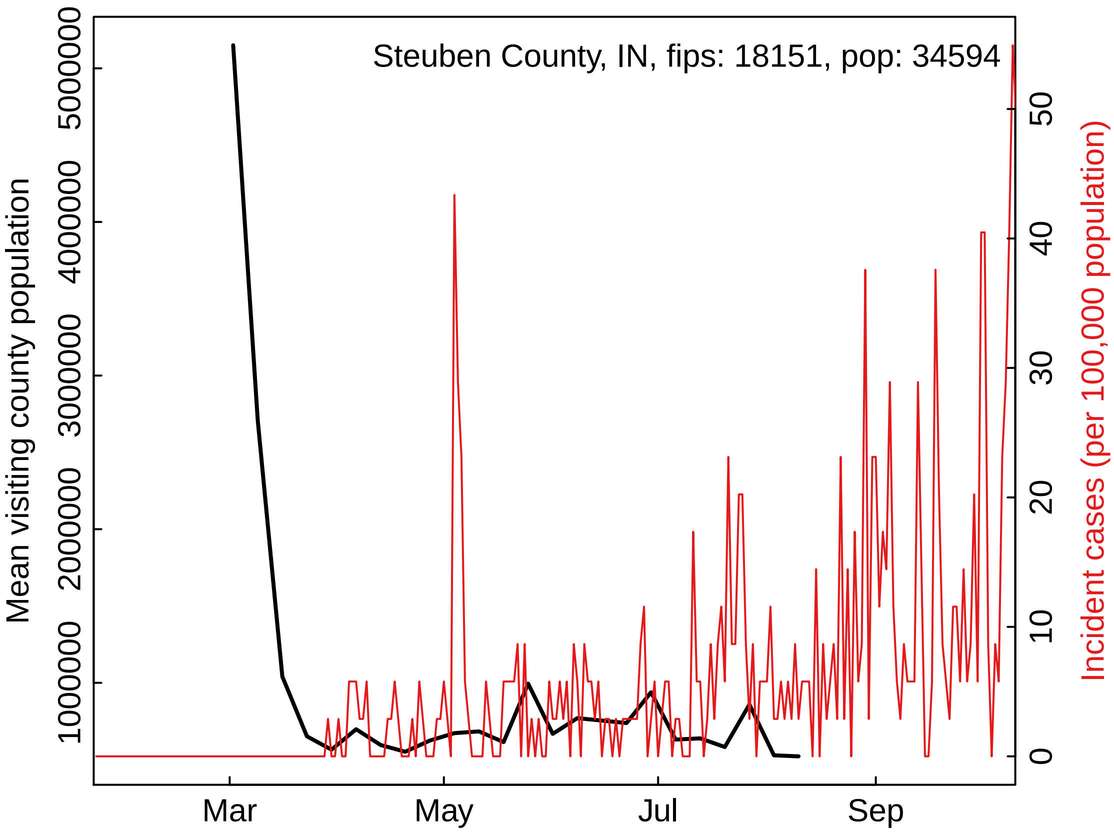

Steuben County, IN, fips: 18151, pop: 34594
Mean visiting county population
Incident cases (per 100,000 population)
1000000
2000000
3000000
4000000
5000000
0
10
20
30
40
50
Mar
May
Jul
Sep

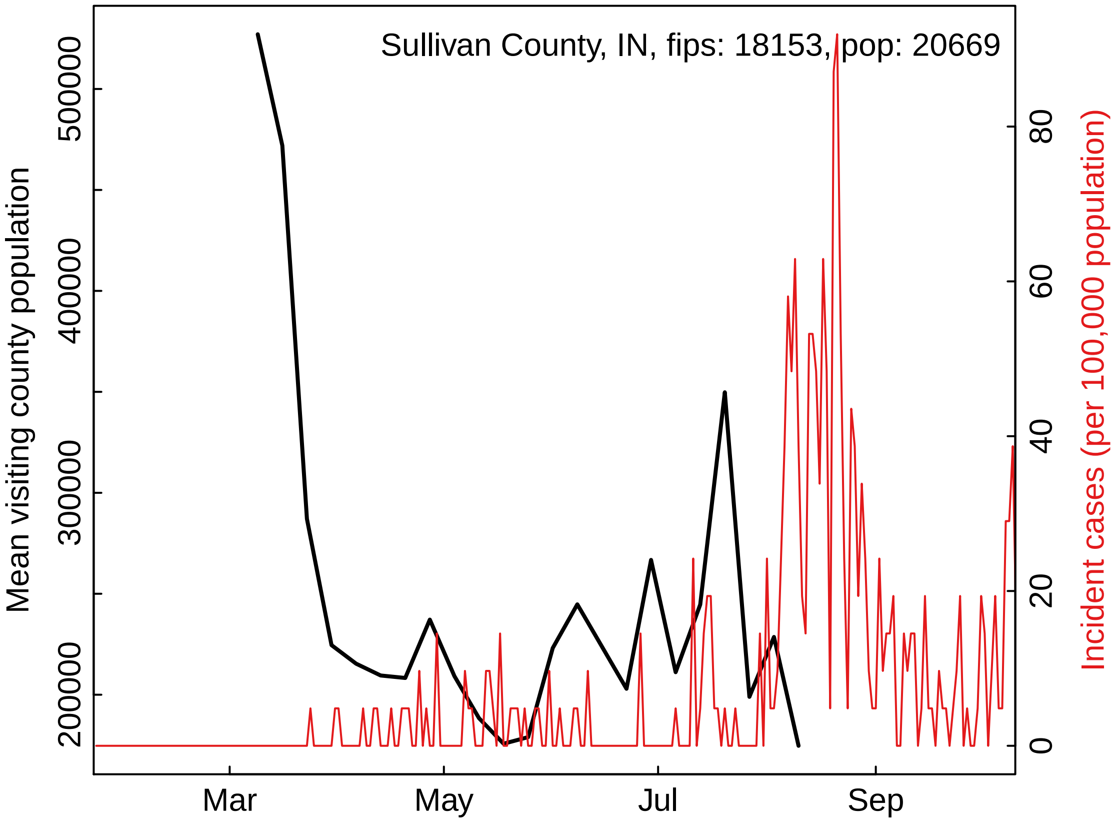

Sullivan County, IN, fips: 18153, pop: 20669
Mean visiting county population
200000
300000
400000
500000
Incident cases (per 100,000 population)
0
20
40
60
80
Mar
May
Jul
Sep

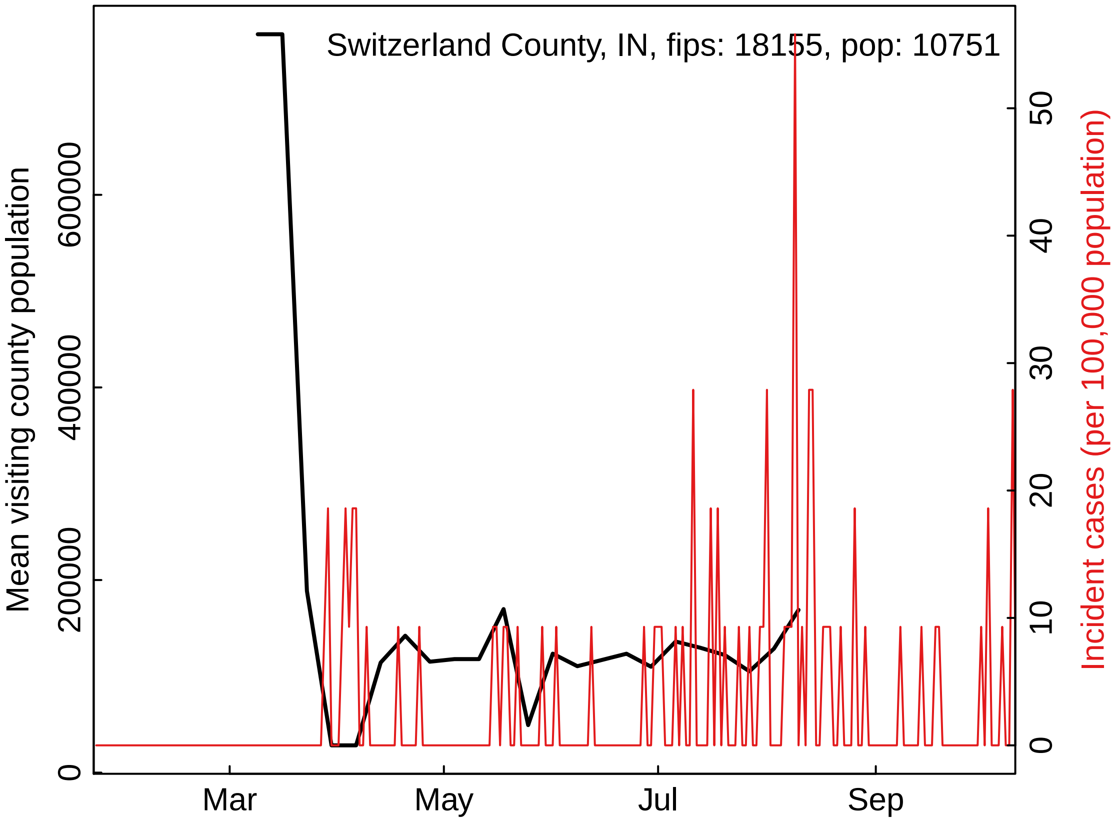
Switzerland County, IN, fips: 18155, pop: 10751
Mean visiting county population
0
200000
400000
600000
Incident cases (per 100,000 population)
0
10
20
30
40
50
Mar
May
Jul
Sep

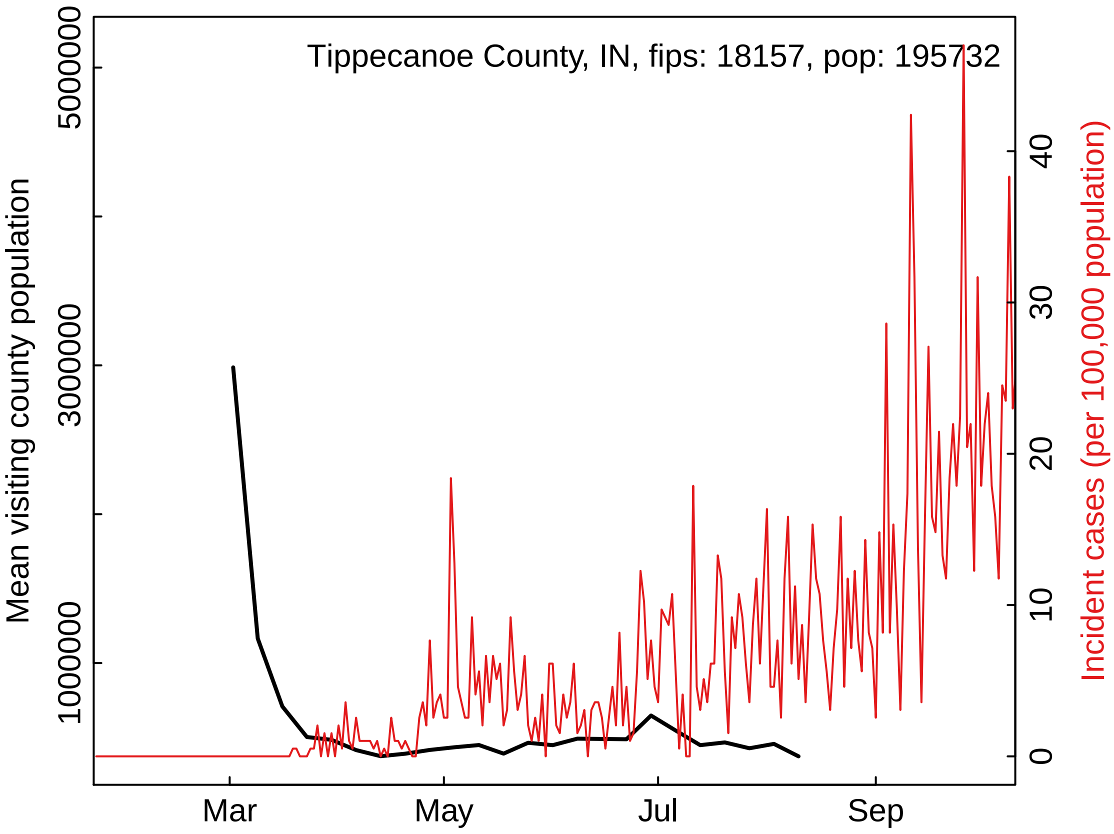

Tippecanoe County, IN, fips: 18157, pop: 195732
Mean visiting county population
5000000
3000000
1000000
Mar
May
Jul
Sep
0
10
20
30
40
Incident cases (per 100,000 population)

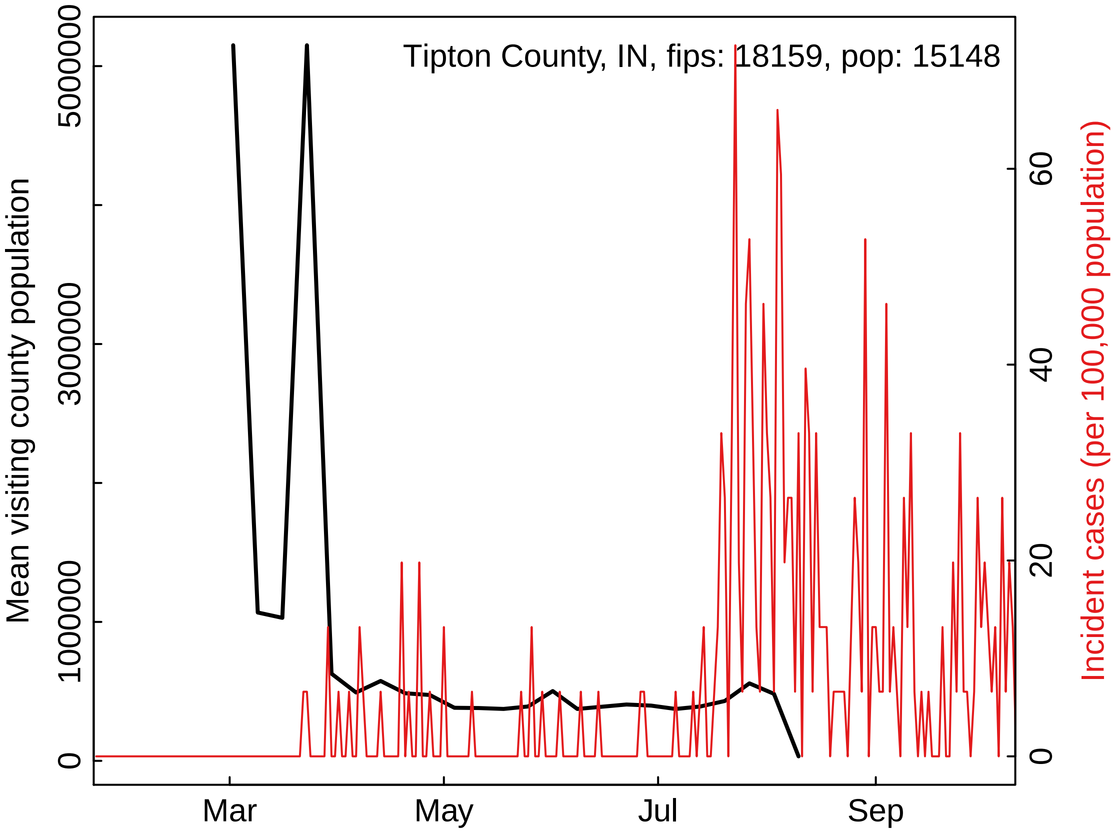
Tipton County, IN, fips: 18159, pop: 15148
Mean visiting county population
Incident cases (per 100,000 population)
0
1000000
3000000
5000000
0
20
40
60
Mar
May
Jul
Sep

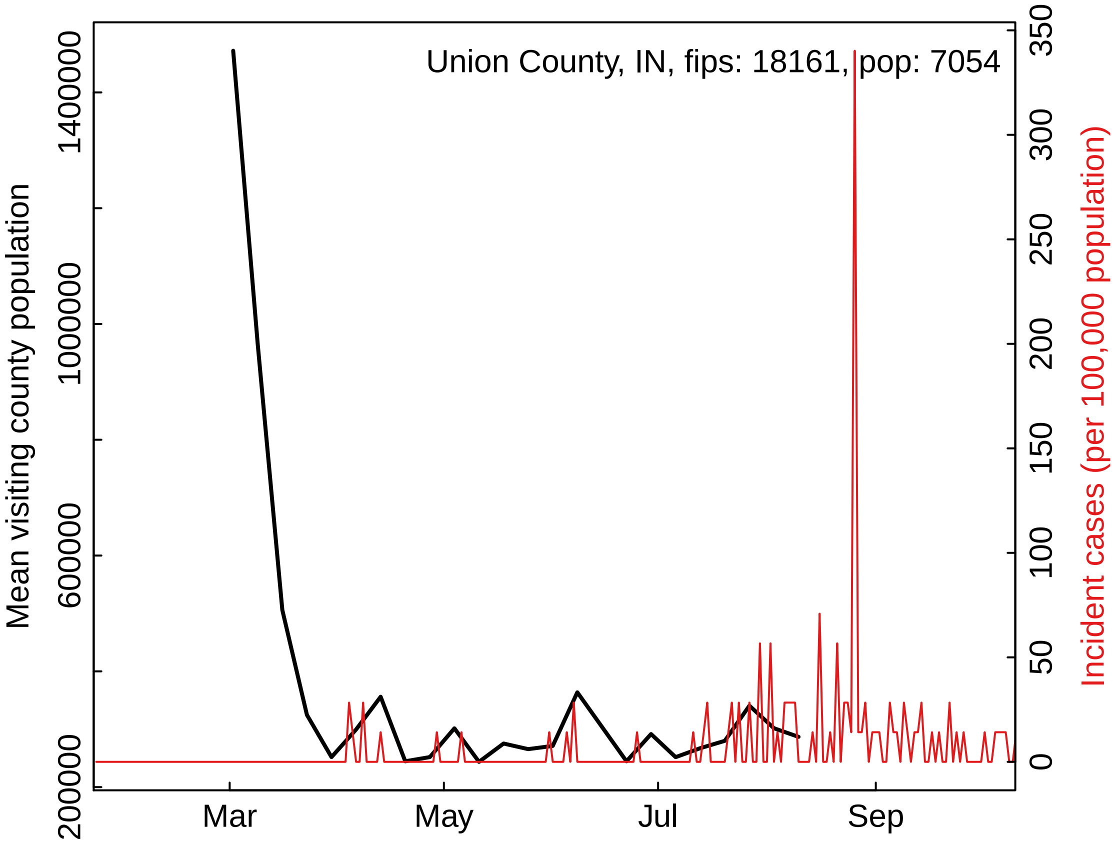

Union County, IN, fips: 18161, pop: 7054
Mean visiting county population
200000
600000
1000000
1400000
Incident cases (per 100,000 population)
0
50
100
150
200
250
300
350
Mar
May
Jul
Sep

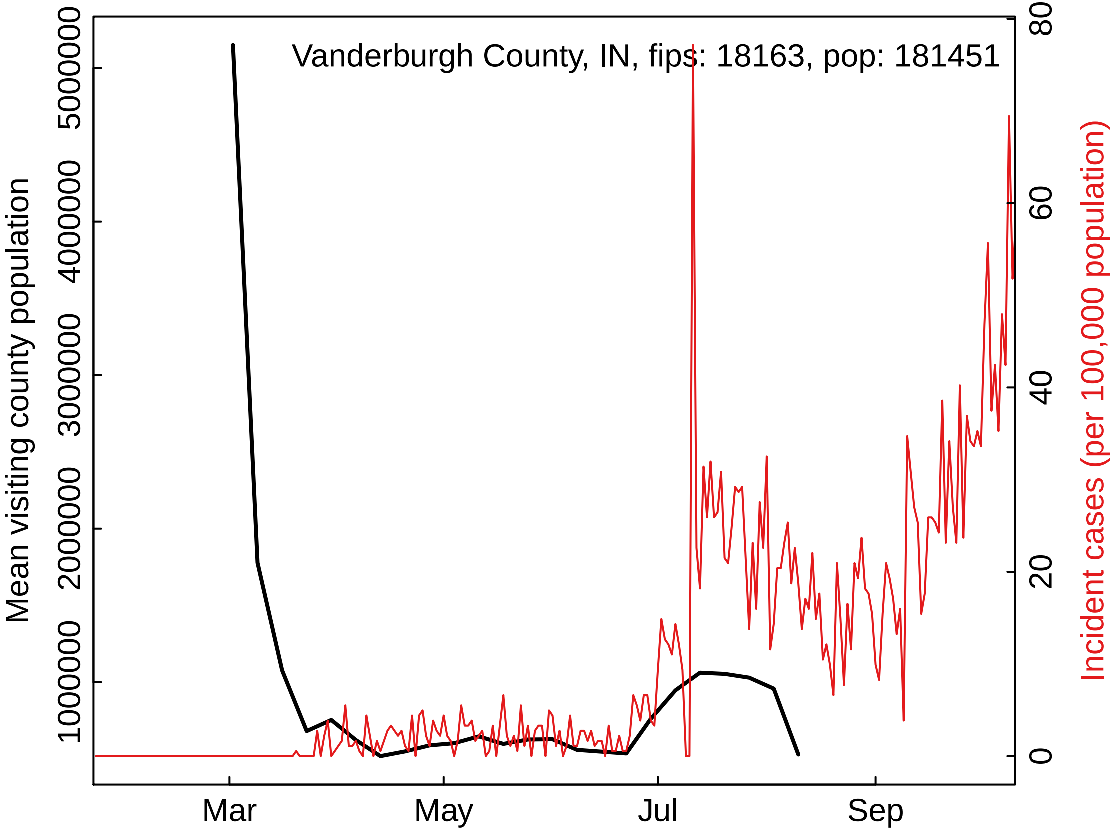

Vanderburgh County, IN, fips: 18163, pop: 181451
Mean visiting county population
1000000
2000000
3000000
4000000
5000000
Incident cases (per 100,000 population)
0
20
40
60
80
Mar
May
Jul
Sep

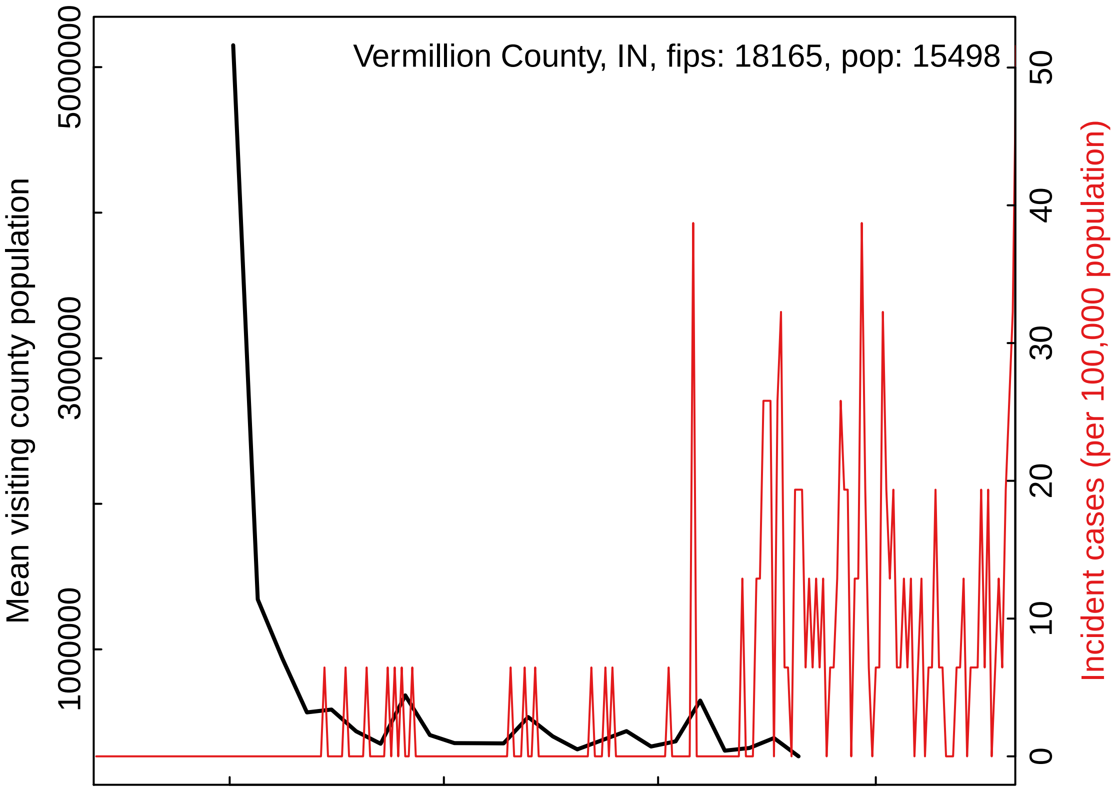

Vermillion County, IN, fips: 18165, pop: 15498
Mean visiting county population
Incident cases (per 100,000 population)
1000000
3000000
5000000
0
10
20
30
40
50
Mar
May
Jul
Sep

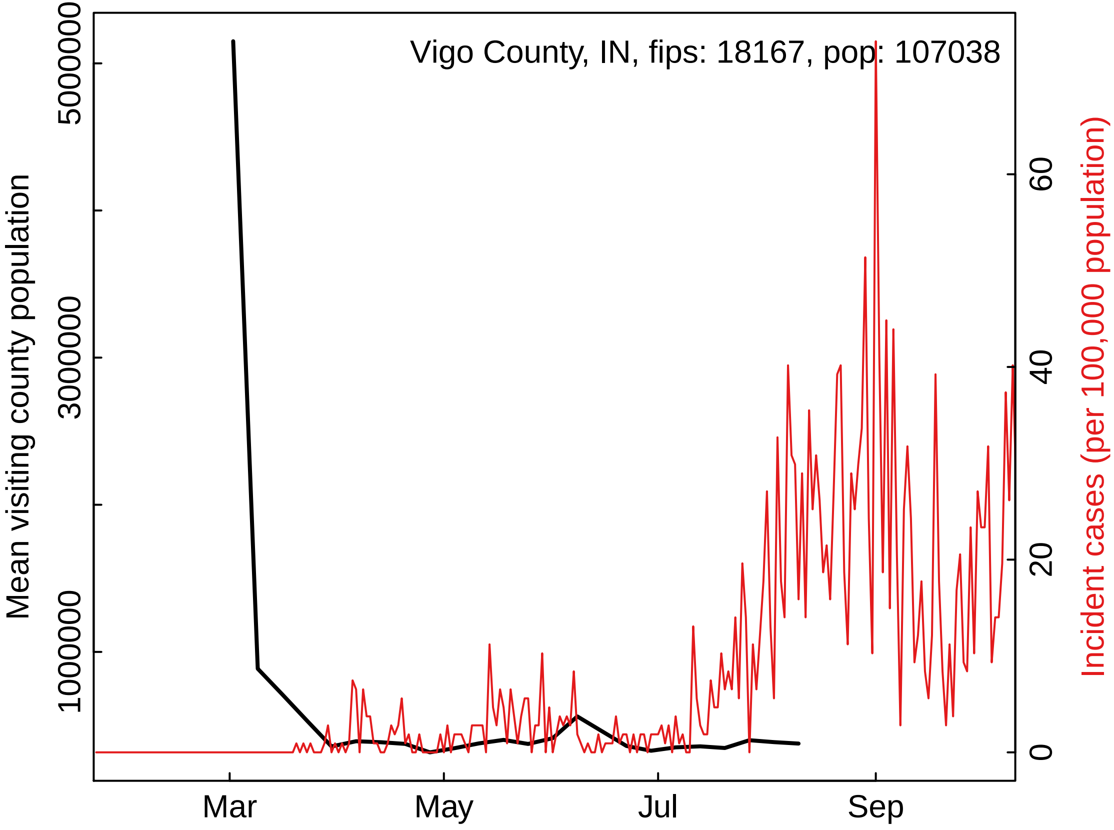
Vigo County, IN, fips: 18167, pop: 107038
Mean visiting county population
1000000
3000000
5000000
Mar
May
Jul
Sep
0
20
40
60
Incident cases (per 100,000 population)

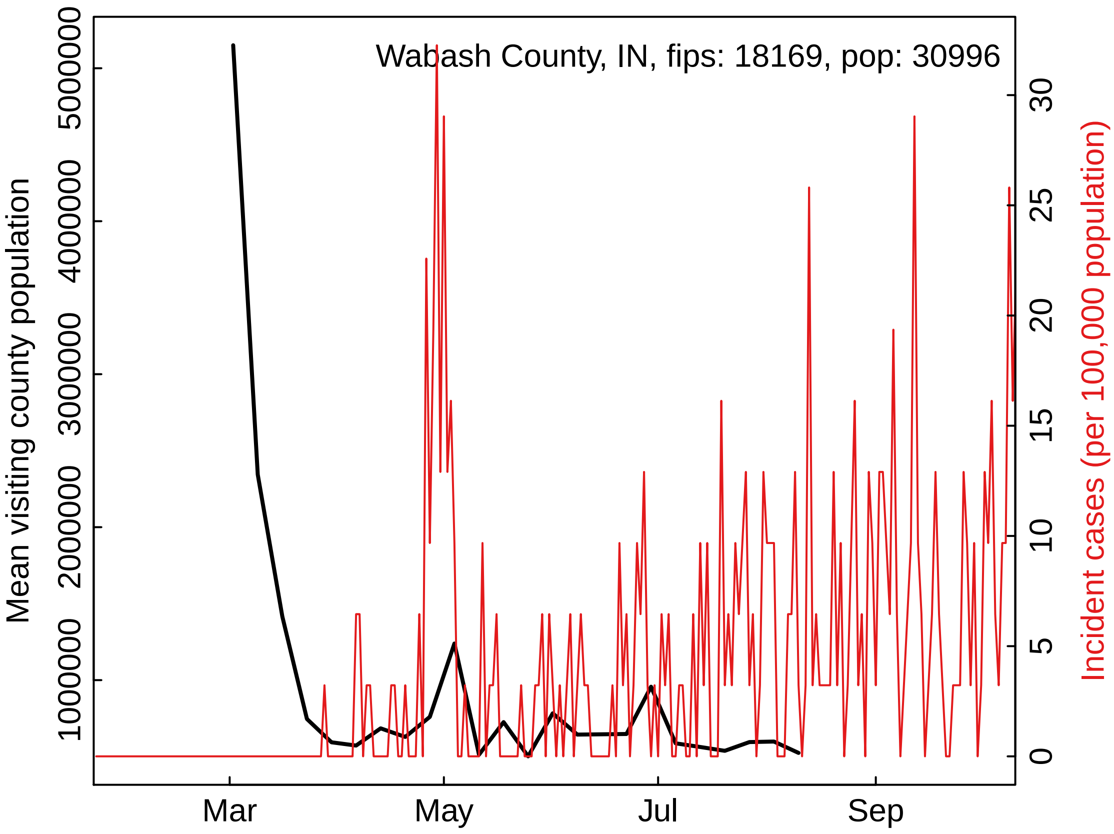

Wabash County, IN, fips: 18169, pop: 30996
Mean visiting county population
1000000
2000000
3000000
4000000
5000000
Incident cases (per 100,000 population)
0
5
10
15
20
25
30
Mar
May
Jul
Sep

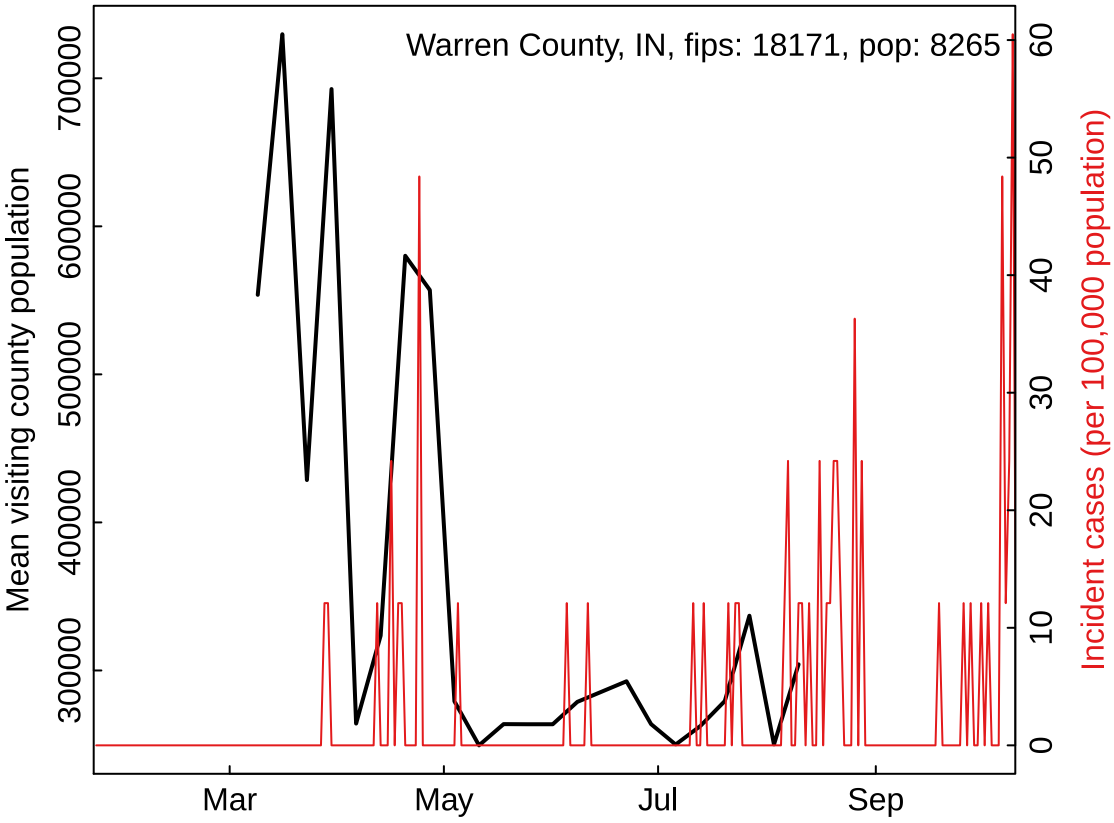

Warren County, IN, fips: 18171, pop: 8265
Mean visiting county population
300000
400000
500000
600000
700000
Incident cases (per 100,000 population)
0
10
20
30
40
50
60
Mar
May
Jul
Sep

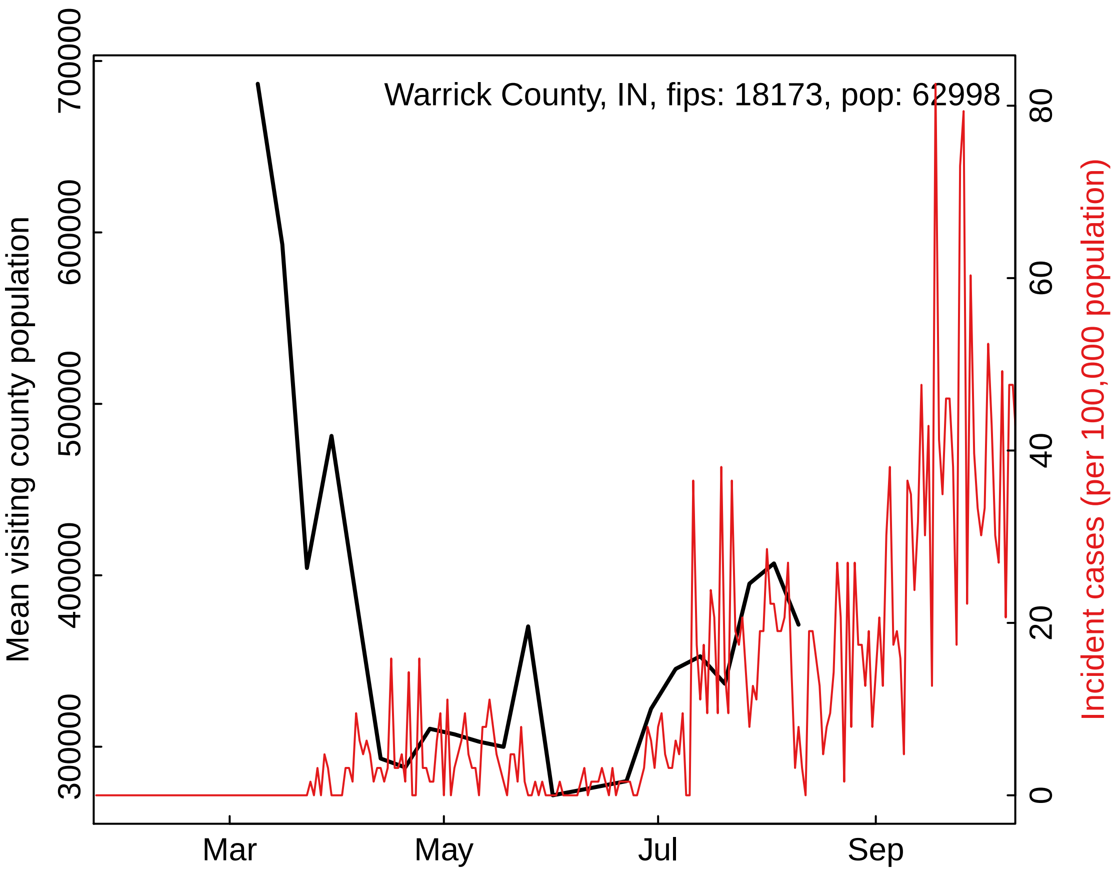

Warrick County, IN, fips: 18173, pop: 62998
Mean visiting county population
300000
400000
500000
600000
700000
Incident cases (per 100,000 population)
0
20
40
60
80
Mar
May
Jul
Sep

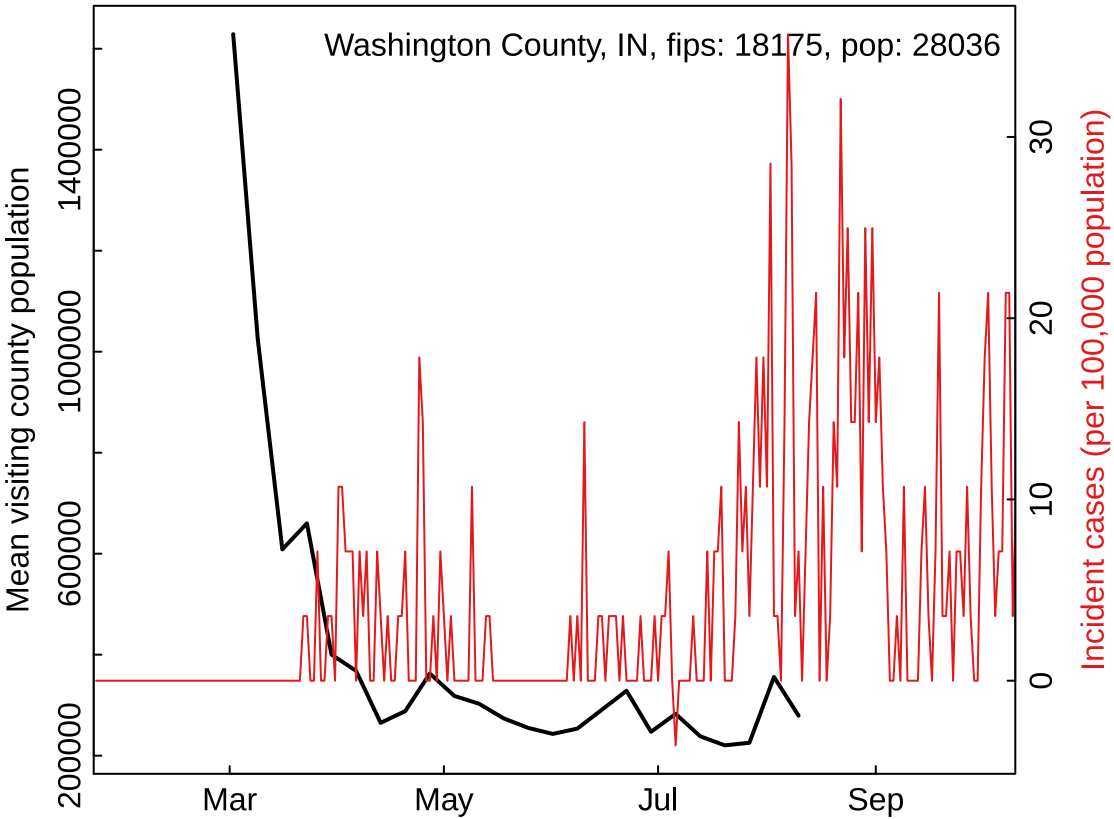
Washington County, IN, fips: 18175, pop: 28036
Mean visiting county population
Incident cases (per 100,000 population)
200000
600000
1000000
1400000
0
10
20
30
Mar
May
Jul
Sep

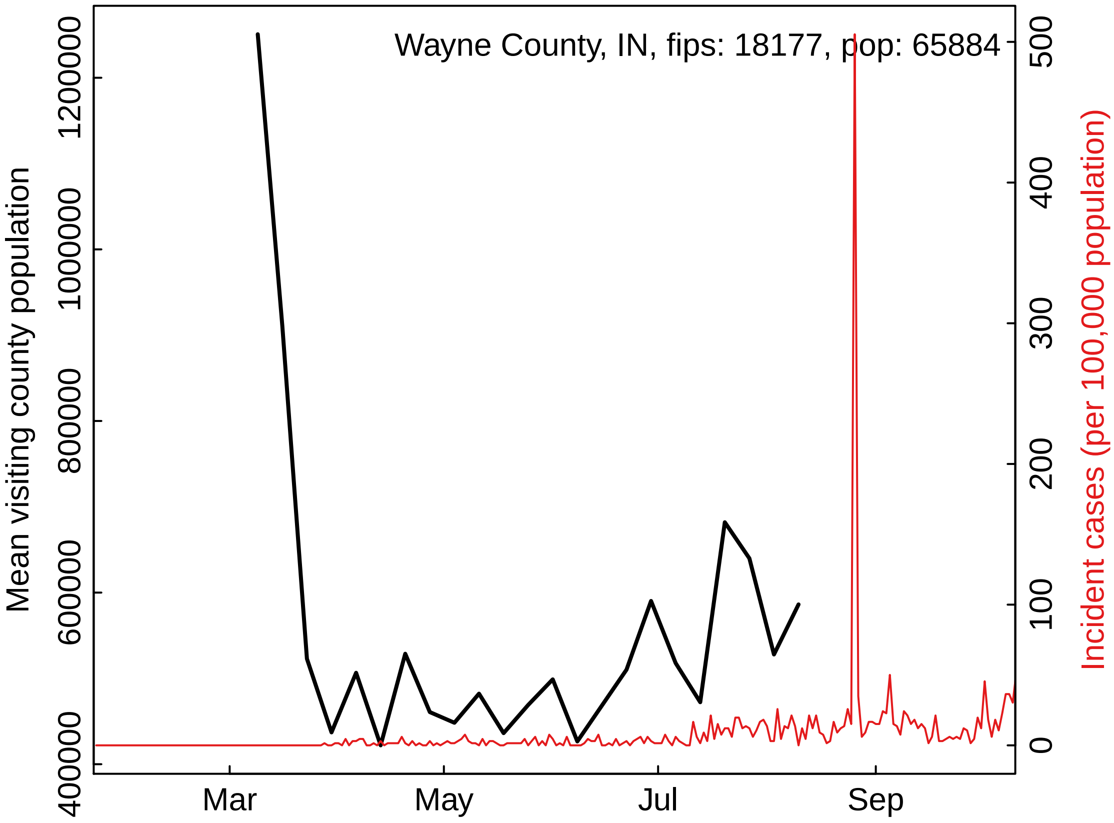

Wayne County, IN, fips: 18177, pop: 65884
Mean visiting county population
400000
600000
800000
1000000
1200000
Incident cases (per 100,000 population)
0
100
200
300
400
500
Mar
May
Jul
Sep

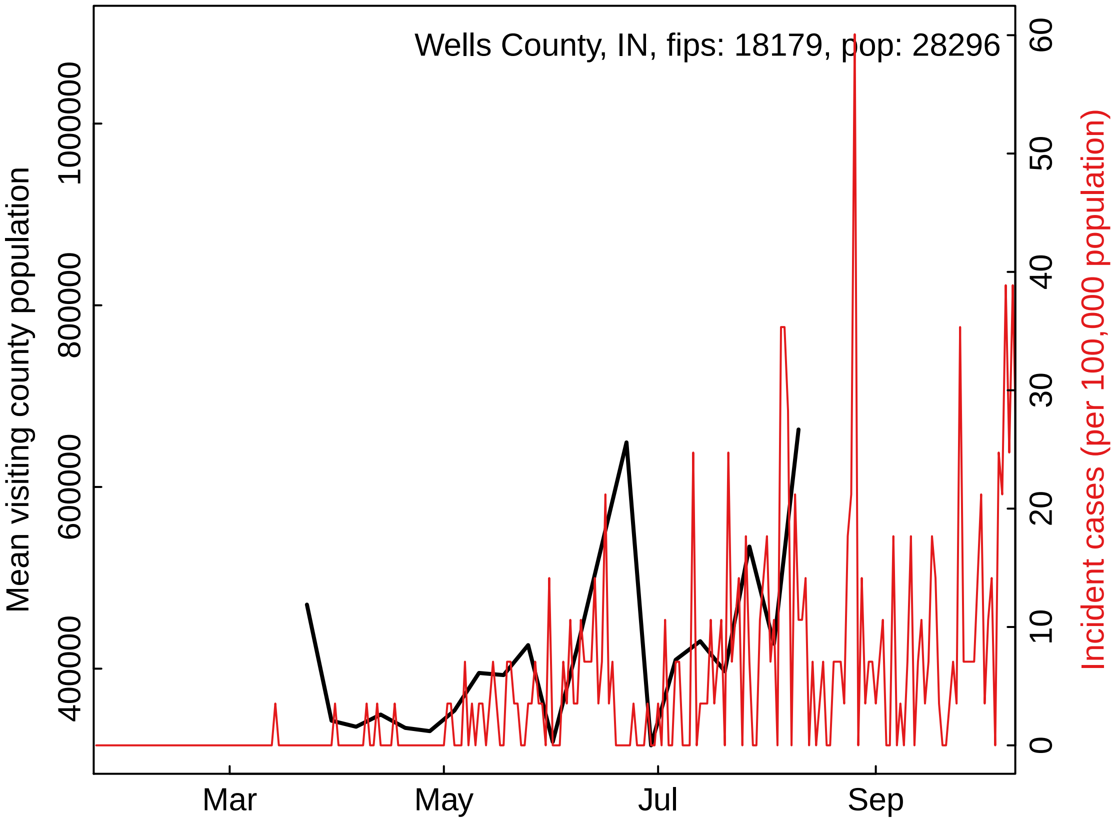

Wells County, IN, fips: 18179, pop: 28296
Mean visiting county population
400000
600000
800000
1000000
Mar
May
Jul
Sep
0
10
20
30
40
50
60
Incident cases (per 100,000 population)

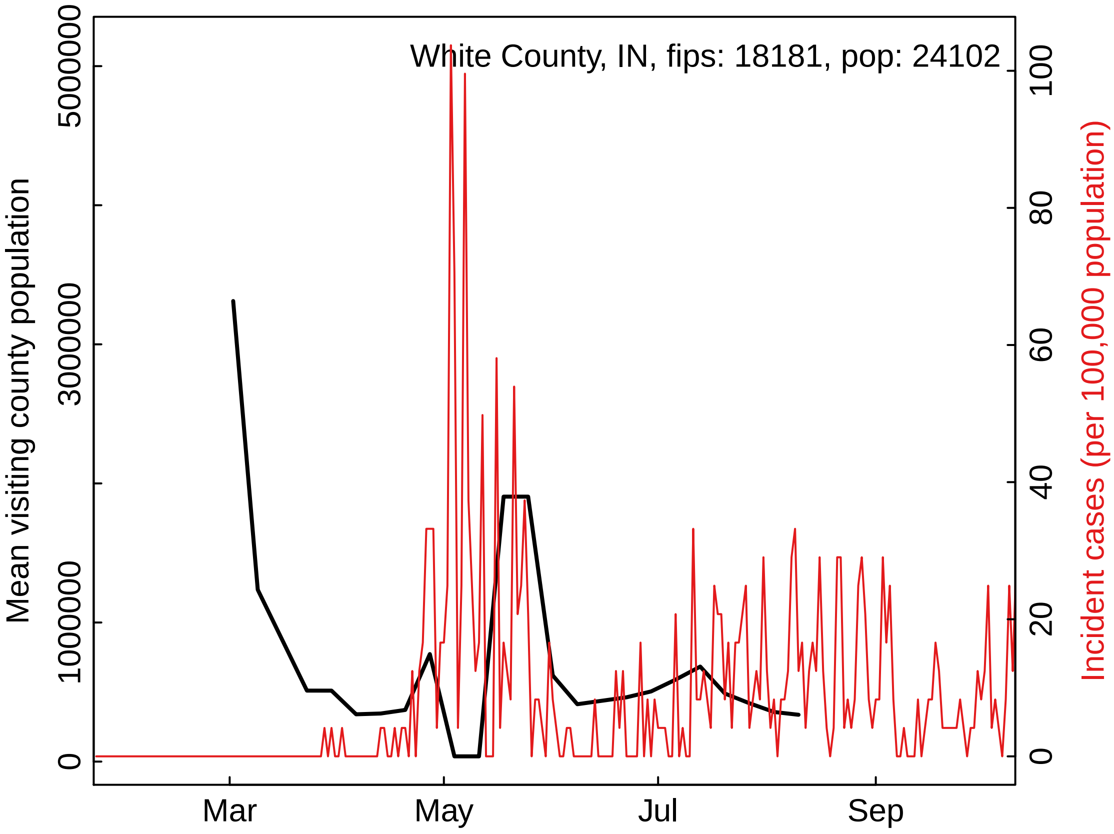

White County, IN, fips: 18181, pop: 24102
Mean visiting county population
0
1000000
3000000
5000000
Incident cases (per 100,000 population)
0
20
40
60
80
100
Mar
May
Jul
Sep

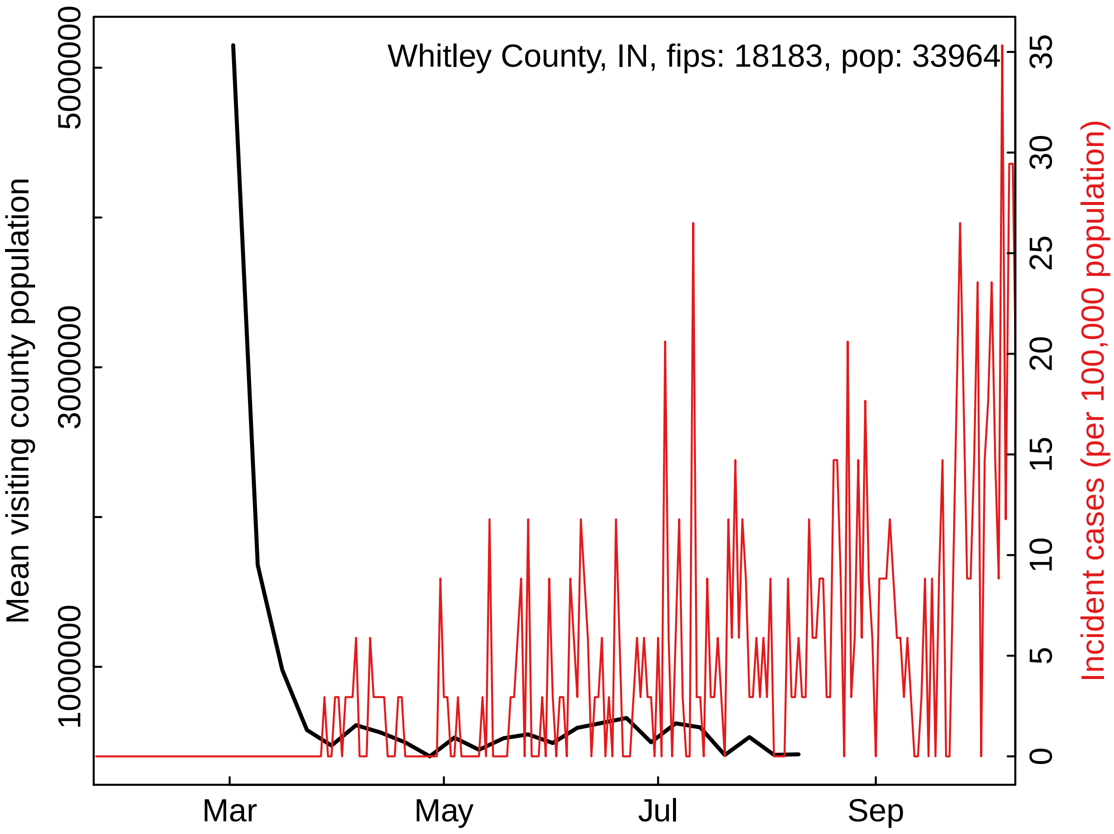

Whitley County, IN, fips: 18183, pop: 33964
Mean visiting county population
Incident cases (per 100,000 population)
1000000
3000000
5000000
0
5
10
15
20
25
30
35
Mar
May
Jul
Sep

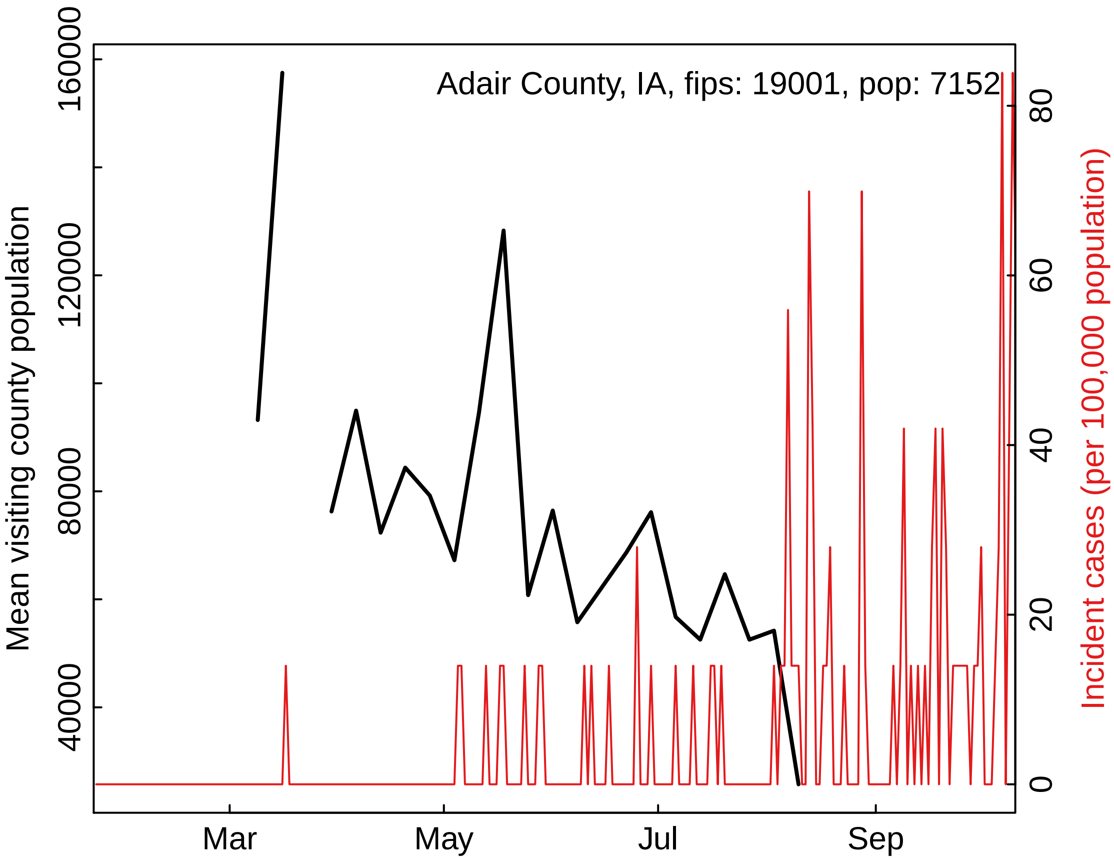
Adair County, IA, fips: 19001, pop: 7152
Mean visiting county population
40000
80000
120000
160000
Incident cases (per 100,000 population)
0
20
40
60
80
Mar
May
Jul
Sep

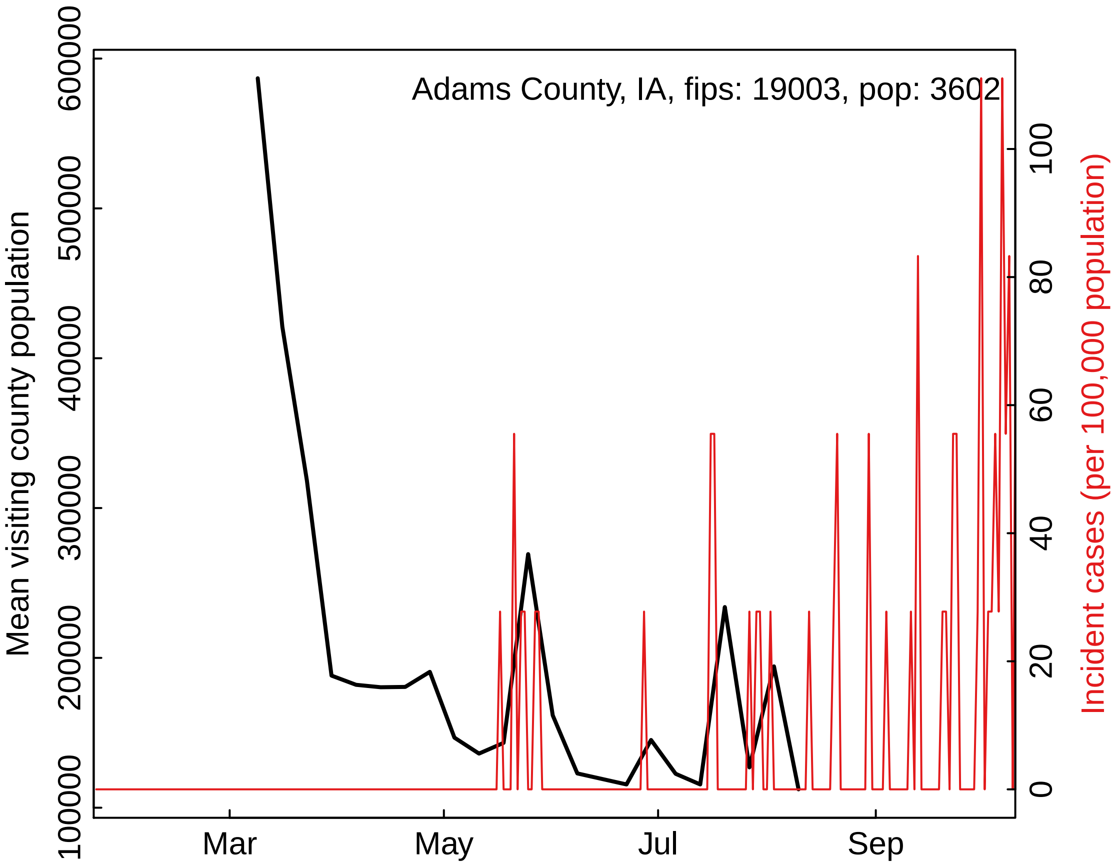

Adams County, IA, fips: 19003, pop: 3602
Mean visiting county population
Incident cases (per 100,000 population)
100000
200000
300000
400000
500000
600000
0
20
40
60
80
100
Mar
May
Jul
Sep

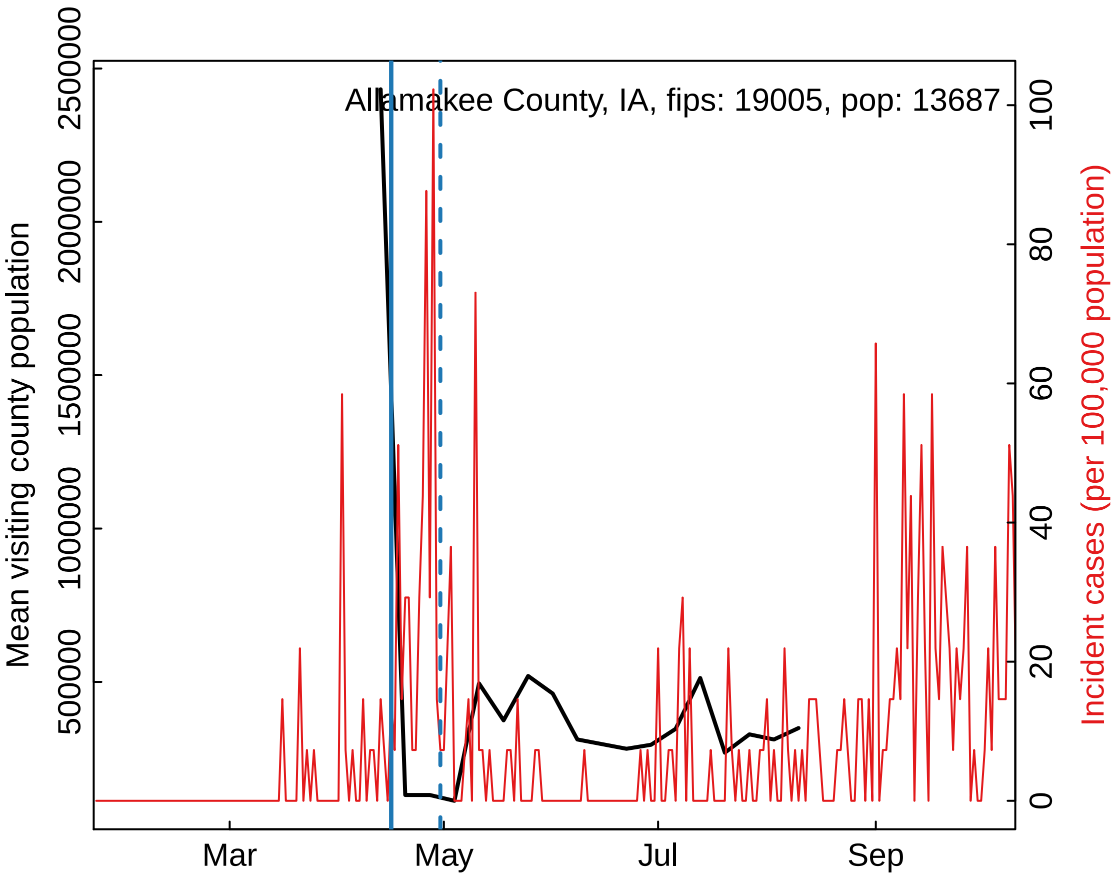

Allamakee County, IA, fips: 19005, pop: 13687
Mean visiting county population
500000
1000000
1500000
2000000
2500000
Incident cases (per 100,000 population)
0
20
40
60
80
100
Mar
May
Jul
Sep

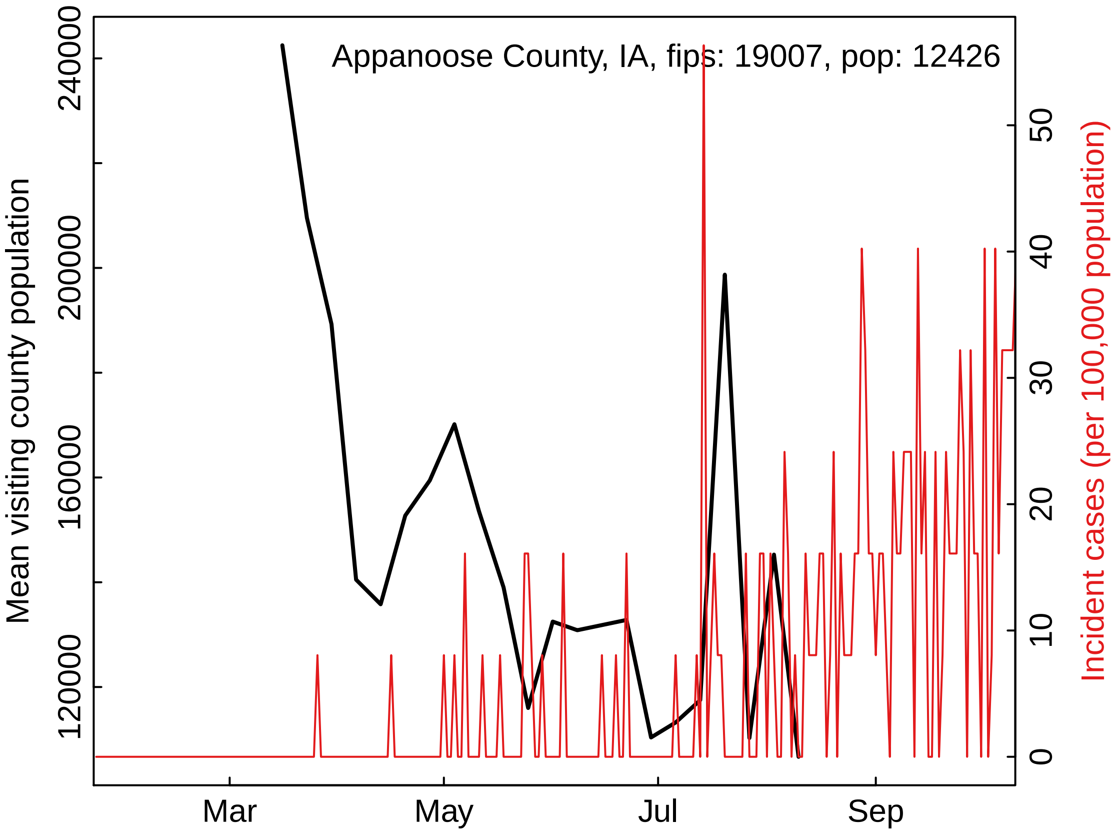

Appanoose County, IA, fips: 19007, pop: 12426
Mean visiting county population
Incident cases (per 100,000 population)
120000
160000
200000
240000
0
10
20
30
40
50
Mar
May
Jul
Sep

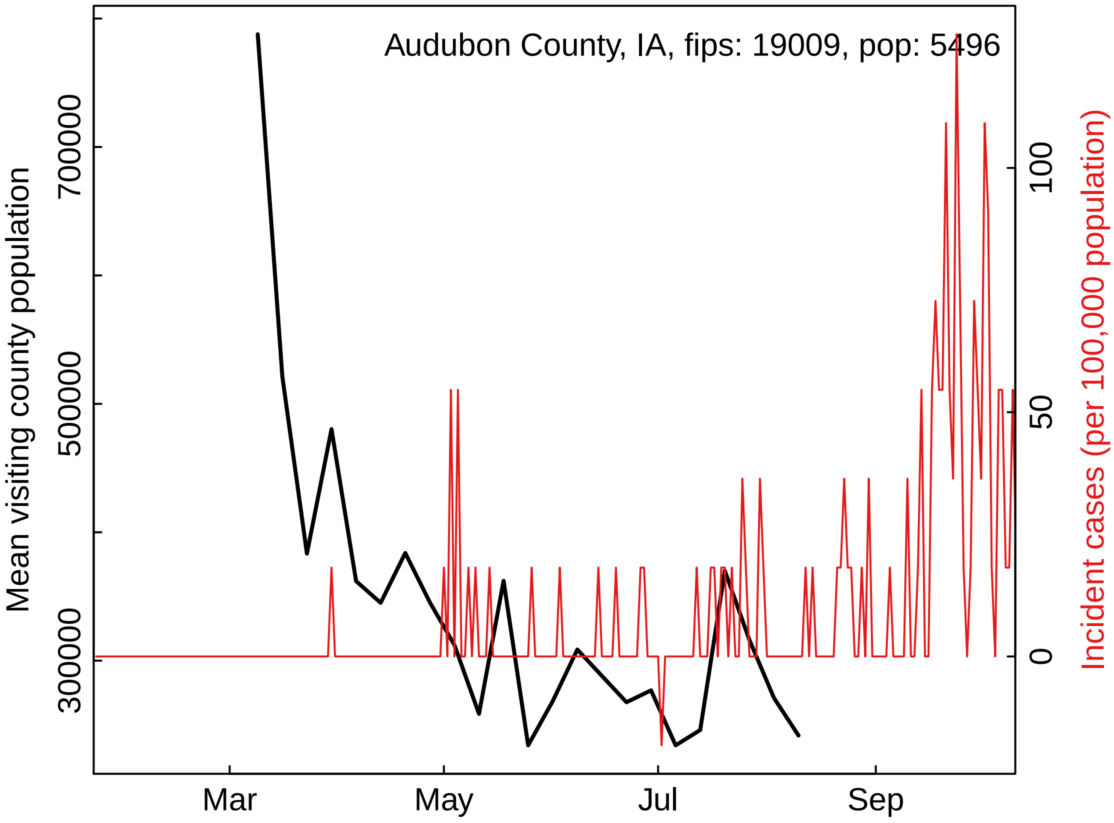

Audubon County, IA, fips: 19009, pop: 5496
Mean visiting county population
300000
500000
700000
Incident cases (per 100,000 population)
0
50
100
Mar
May
Jul
Sep

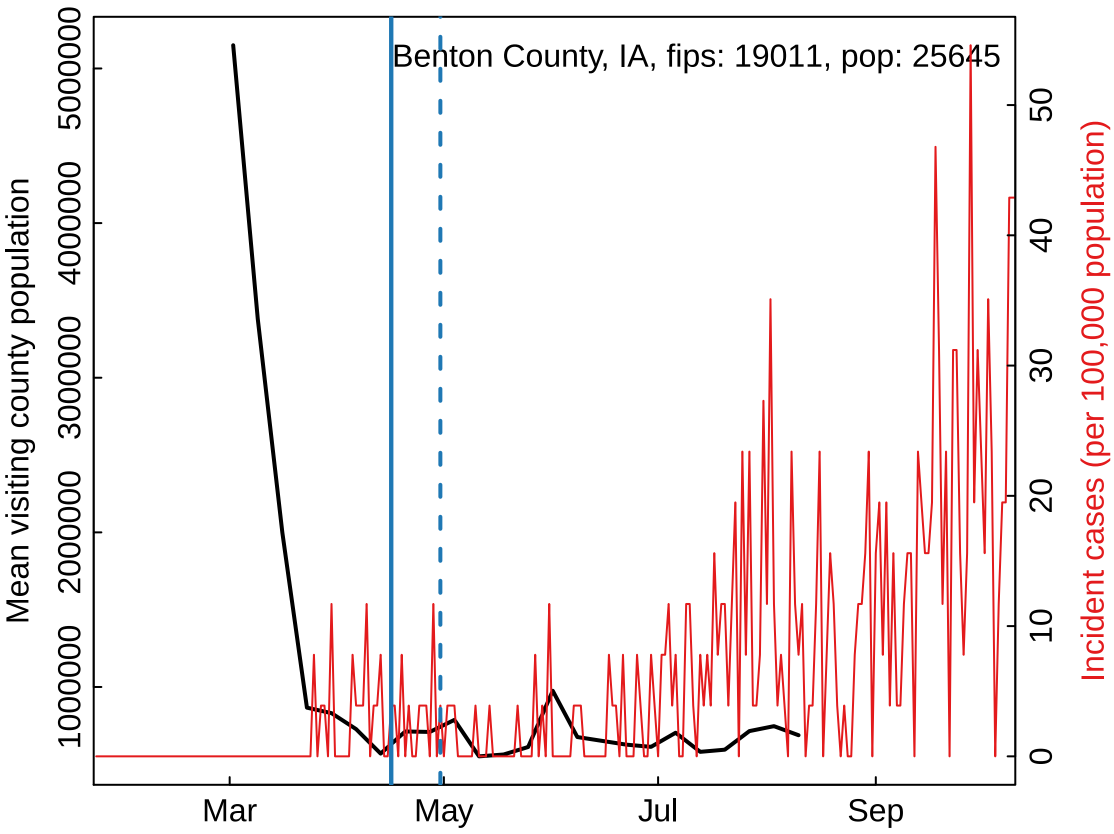

Benton County, IA, fips: 19011, pop: 25645
Mean visiting county population
10000000
20000000
30000000
40000000
50000000
Incident cases (per 100,000 population)
0
10
20
30
40
50
Mar
May
Jul
Sep

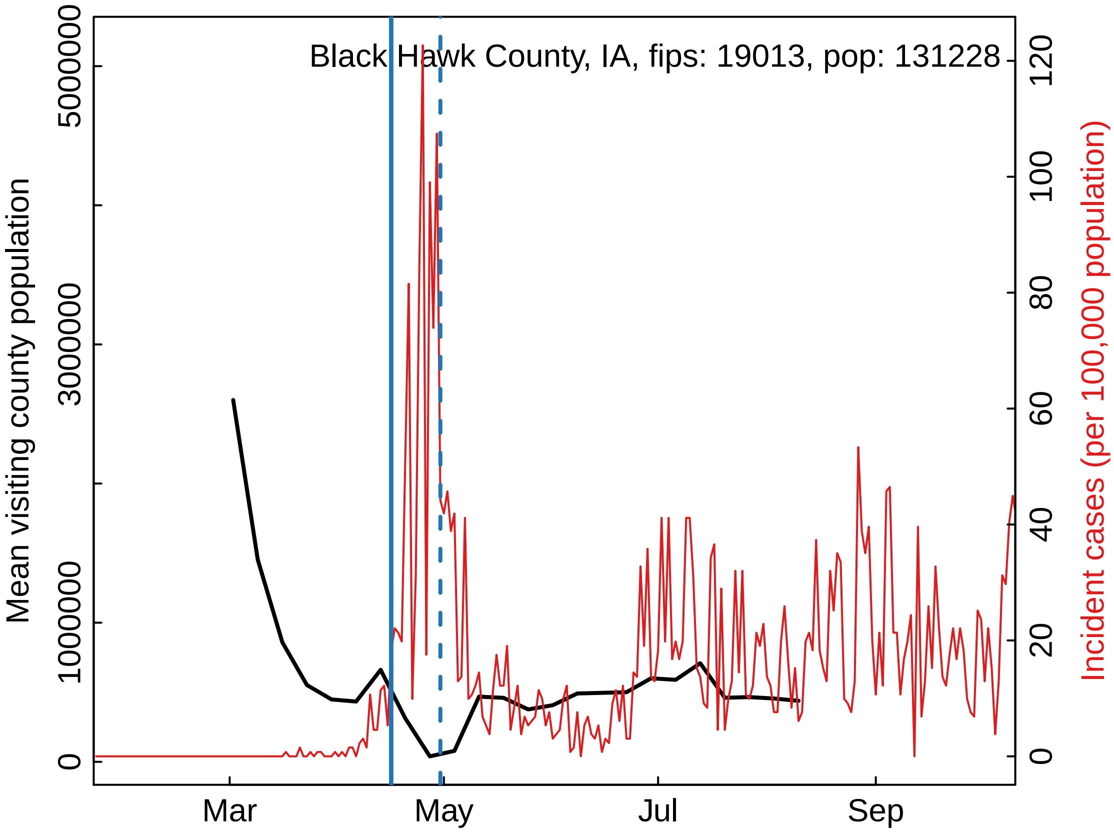

Black Hawk County, IA, fips: 19013, pop: 131228
Mean visiting county population
0
1000000
3000000
5000000
Incident cases (per 100,000 population)
0
20
40
60
80
100
120
Mar
May
Jul
Sep

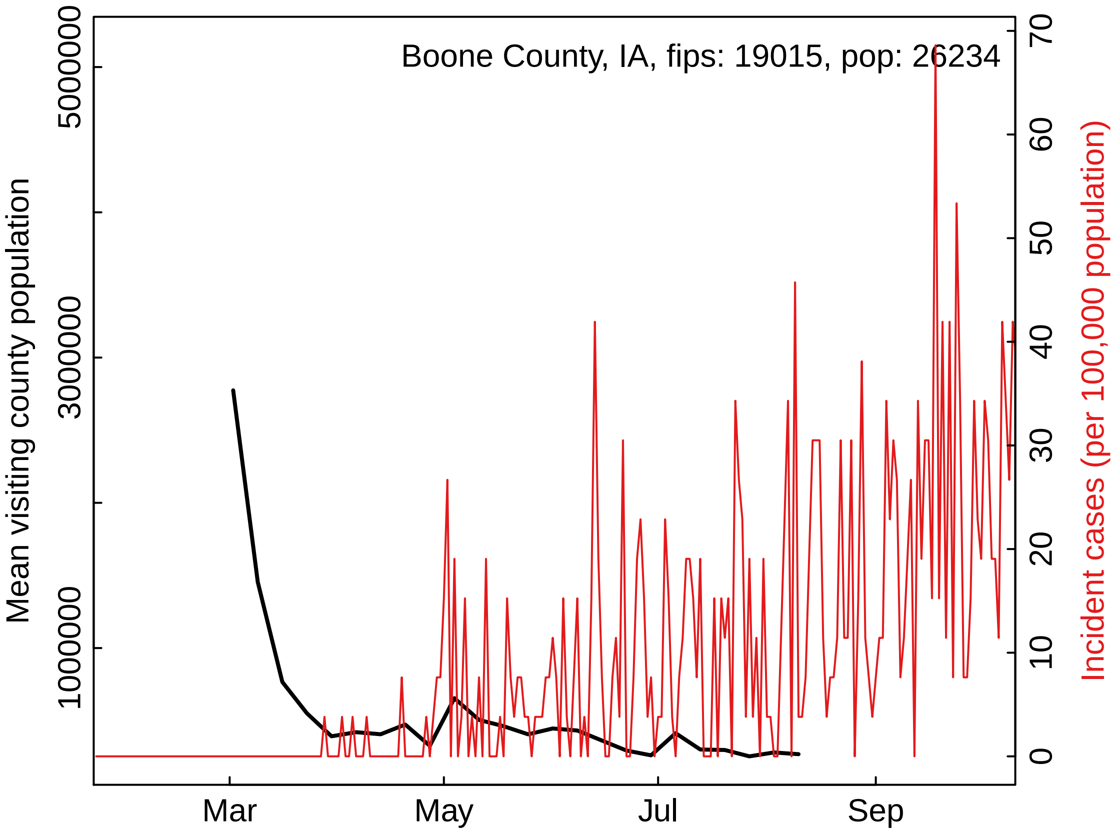

Boone County, IA, fips: 19015, pop: 26234
Mean visiting county population
1000000
3000000
5000000
Mar
May
Jul
Sep
0
10
20
30
40
50
60
70
Incident cases (per 100,000 population)

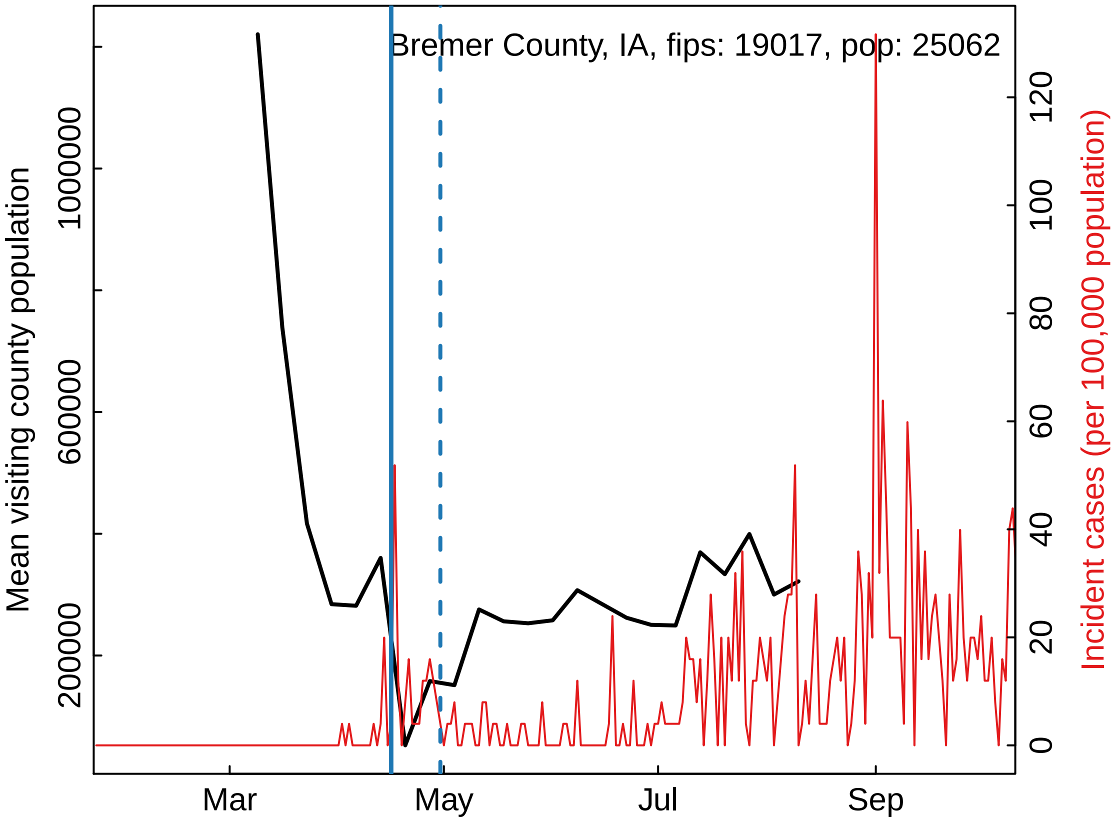

Bremer County, IA, fips: 19017, pop: 25062
Mean visiting county population
200000
600000
1000000
Incident cases (per 100,000 population)
0
20
40
60
80
100
120
Mar
May
Jul
Sep

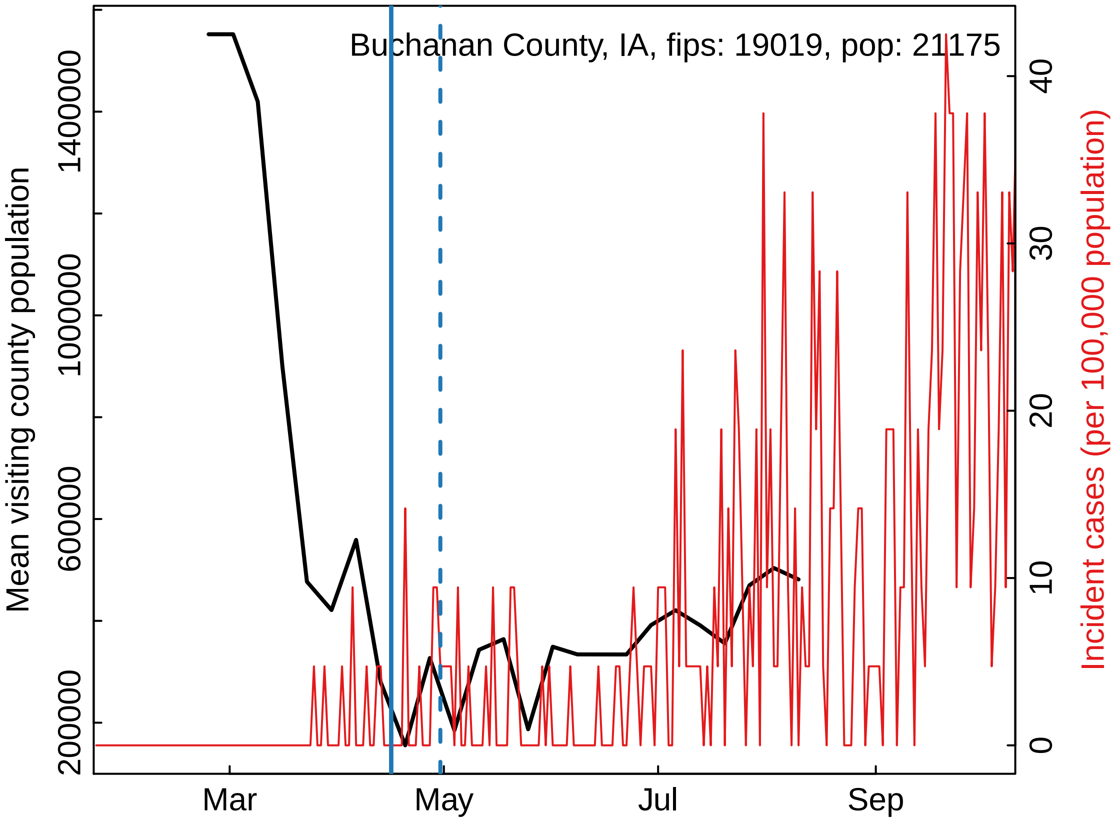
Buchanan County, IA, fips: 19019, pop: 21175
Mean visiting county population
200000
600000
1000000
1400000
Mar
May
Jul
Sep
0
10
20
30
40
Incident cases (per 100,000 population)

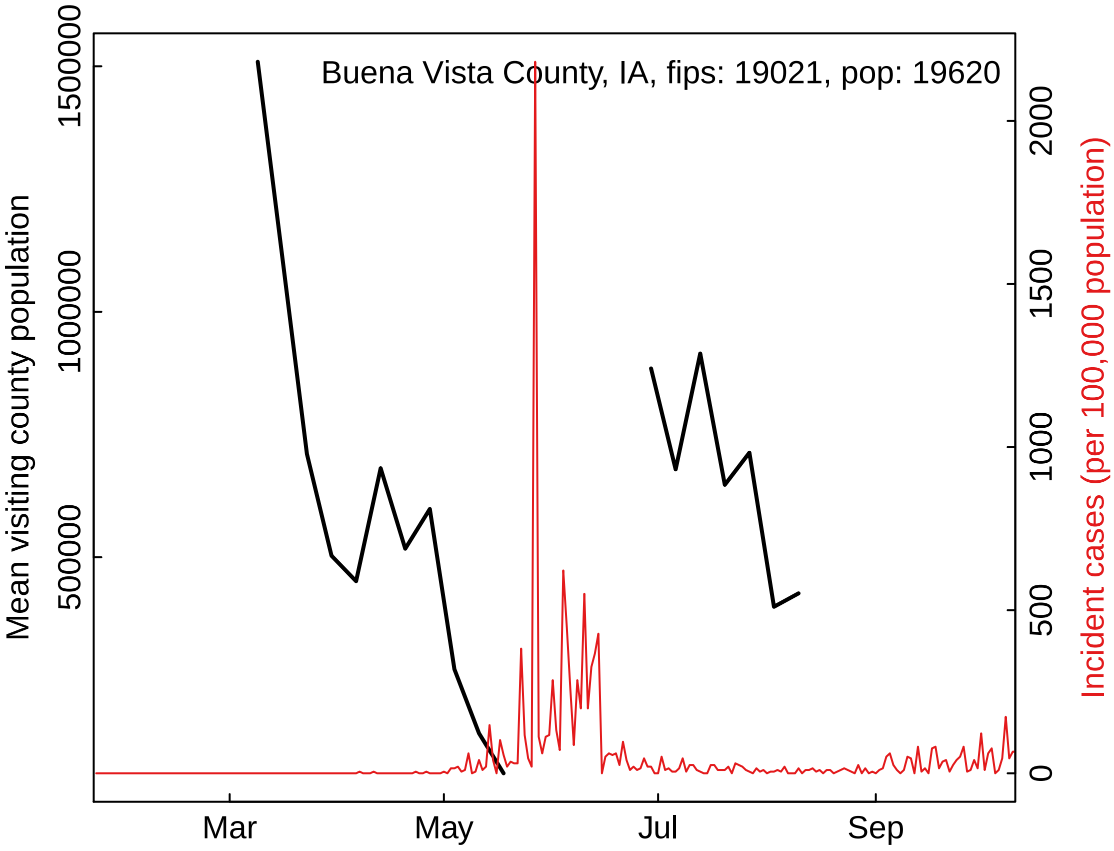

Buena Vista County, IA, fips: 19021, pop: 19620
Mean visiting county population
15000000
10000000
5000000
Incident cases (per 100,000 population)
2000
1500
1000
500
0
Mar
May
Jul
Sep

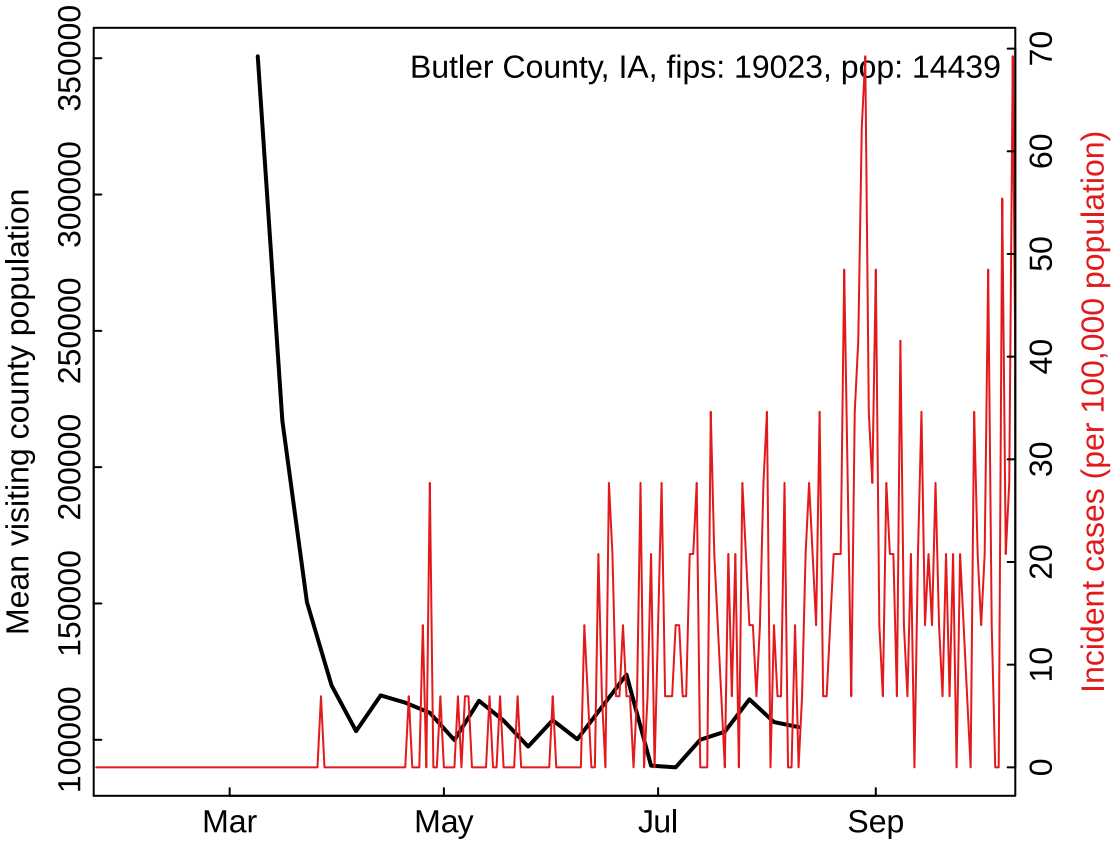

Butler County, IA, fips: 19023, pop: 14439
Mean visiting county population
100000
150000
200000
250000
300000
350000
Incident cases (per 100,000 population)
0
10
20
30
40
50
60
70
Mar
May
Jul
Sep

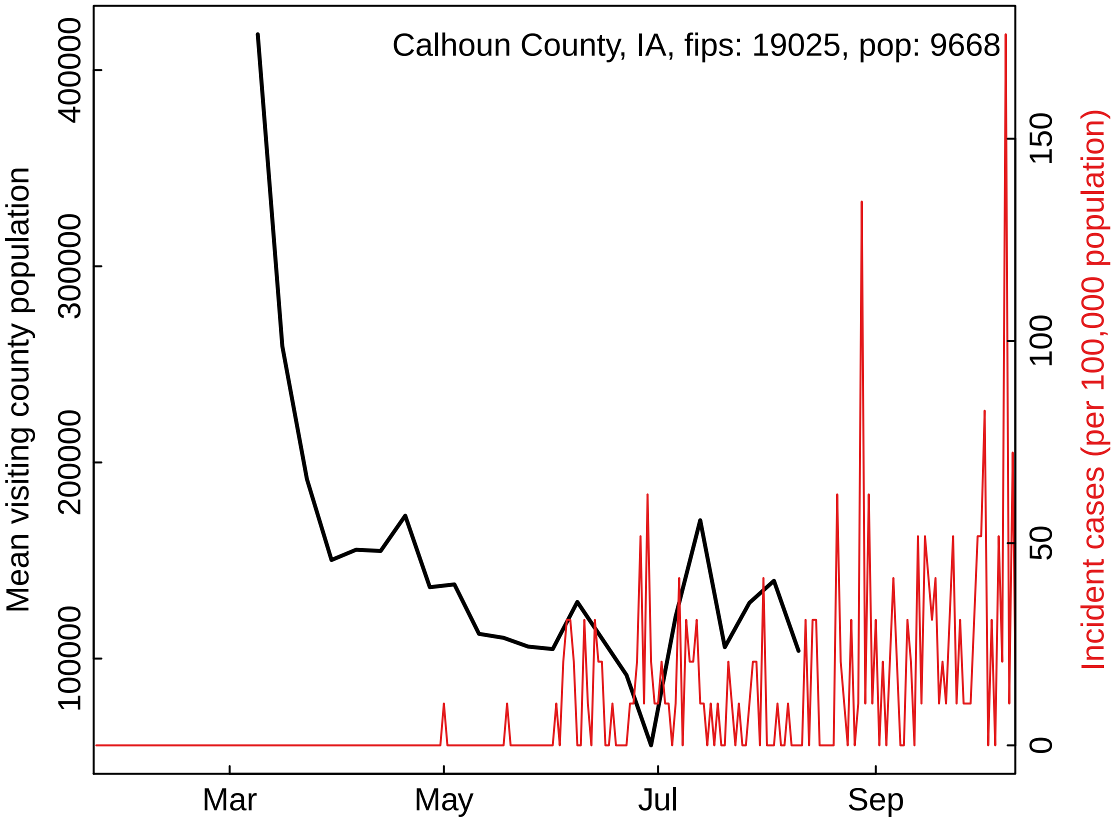

Calhoun County, IA, fips: 19025, pop: 9668
Mean visiting county population
Incident cases (per 100,000 population)
100000
200000
300000
400000
0
50
100
150
Mar
May
Jul
Sep

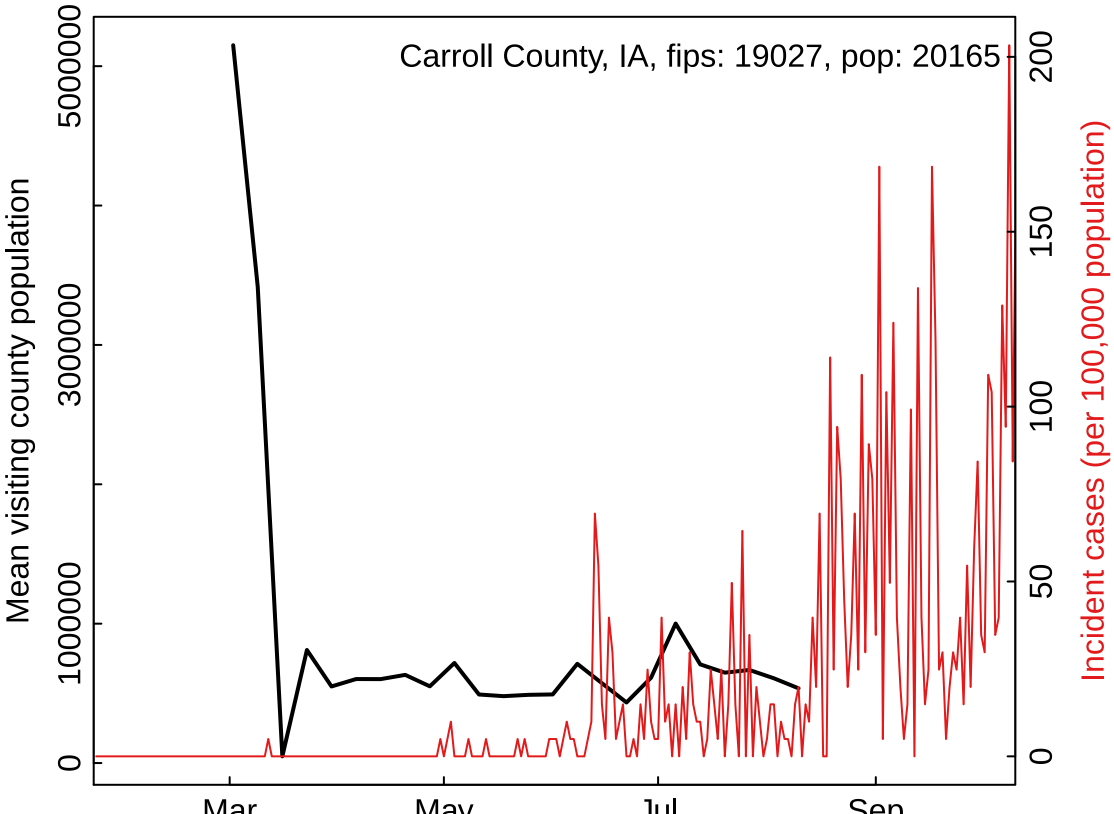

Carroll County, IA, fips: 19027, pop: 20165
Mean visiting county population
Incident cases (per 100,000 population)
0
1000000
3000000
5000000
0
50
100
150
200
Mar
May
Jul
Sep

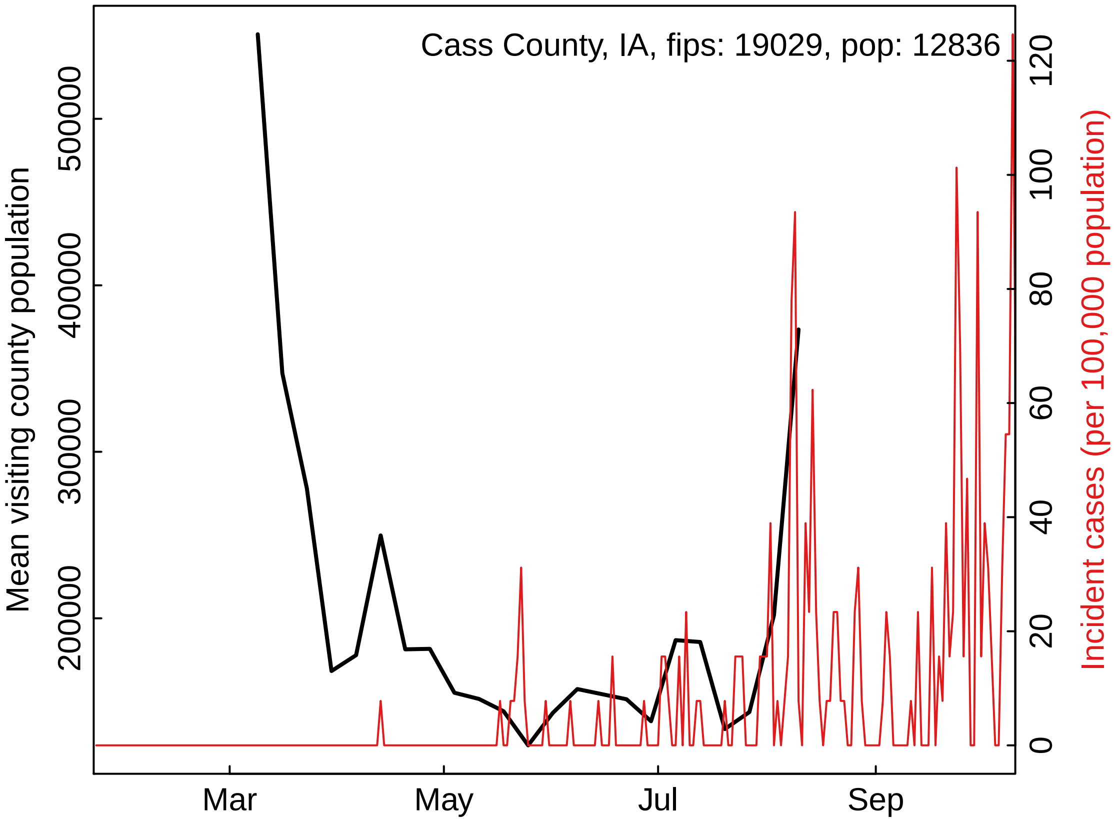
Cass County, IA, fips: 19029, pop: 12836
Mean visiting county population
200000
300000
400000
500000
Incident cases (per 100,000 population)
0
20
40
60
80
100
120
Mar
May
Jul
Sep

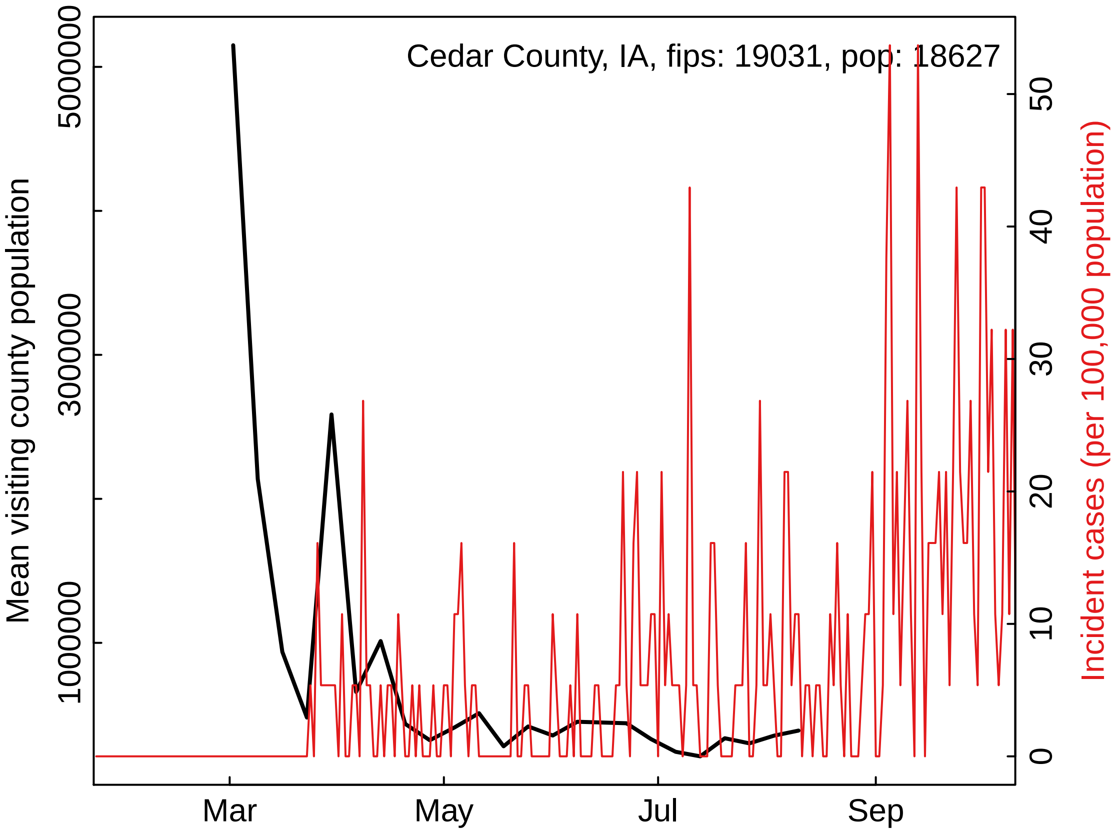

Cedar County, IA, fips: 19031, pop: 18627
Mean visiting county population
1000000
3000000
5000000
Incident cases (per 100,000 population)
0
10
20
30
40
50
Mar
May
Jul
Sep

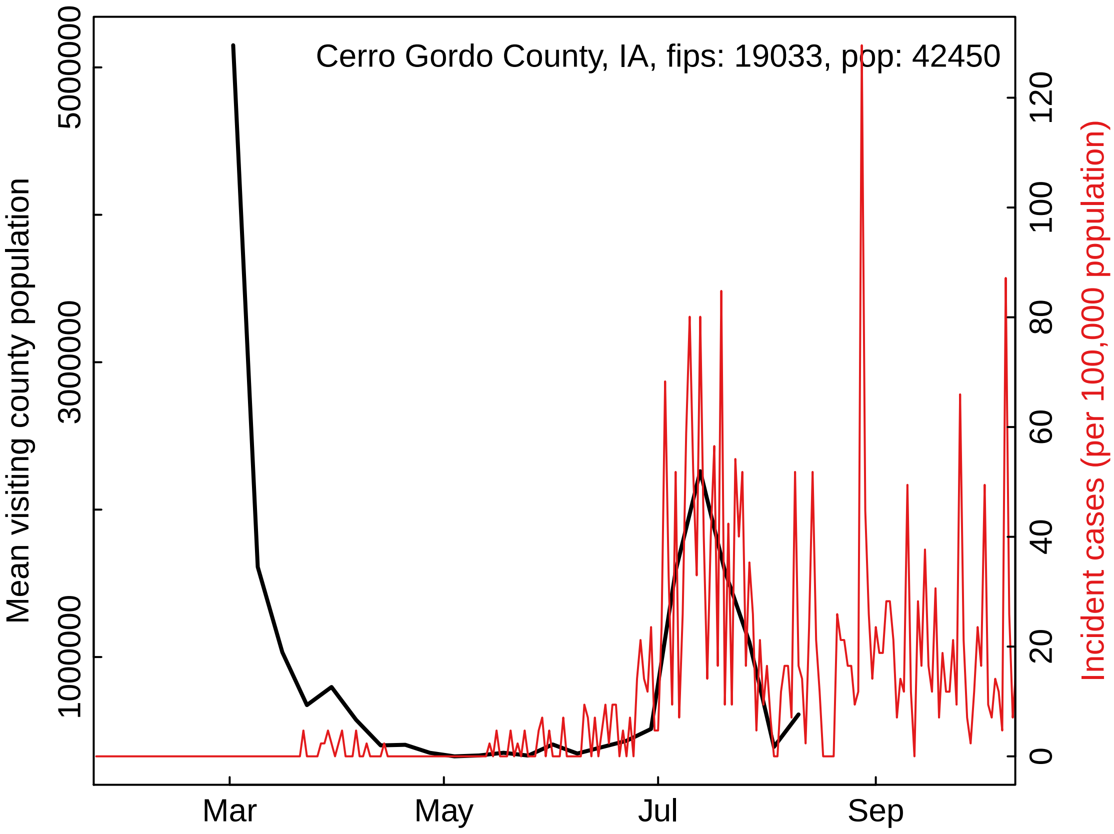
Cerro Gordo County, IA, fips: 19033, pop: 42450
Mean visiting county population
1000000
3000000
5000000
Incident cases (per 100,000 population)
0
20
40
60
80
100
120
Mar
May
Jul
Sep

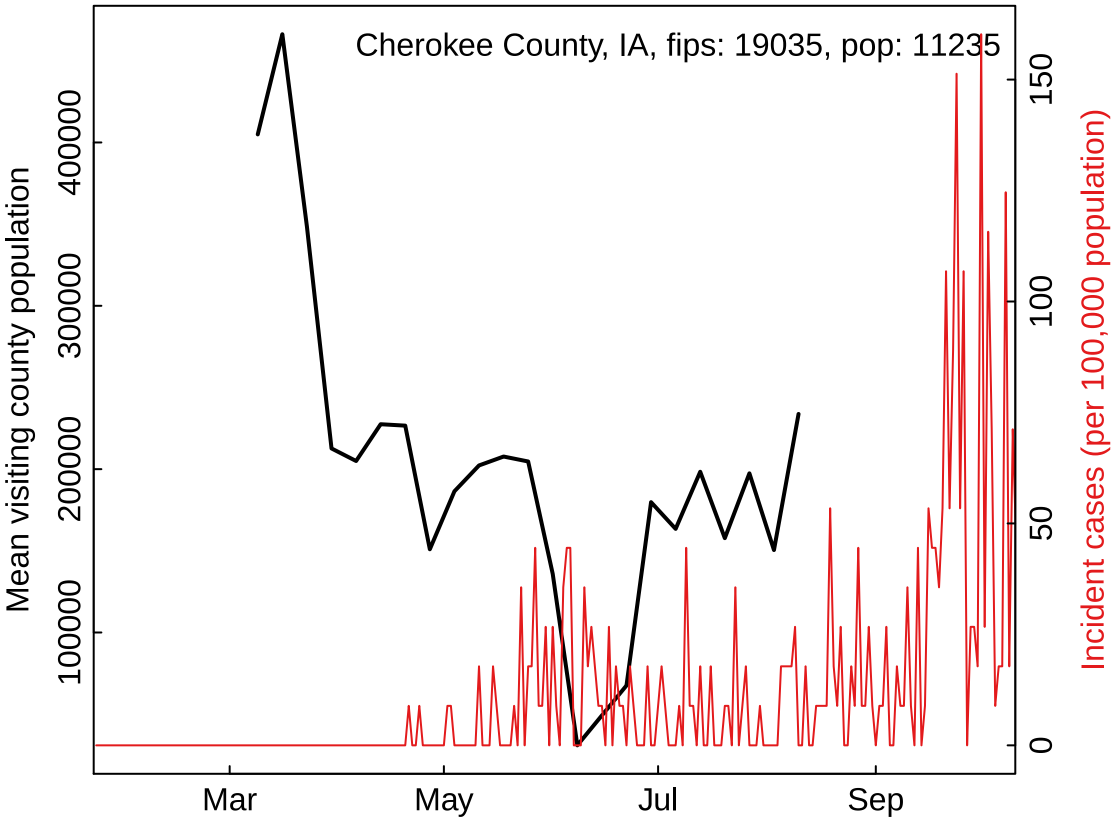
Cherokee County, IA, fips: 19035, pop: 11235
Mean visiting county population
100000
200000
300000
400000
Incident cases (per 100,000 population)
0
50
100
150
Mar
May
Jul
Sep

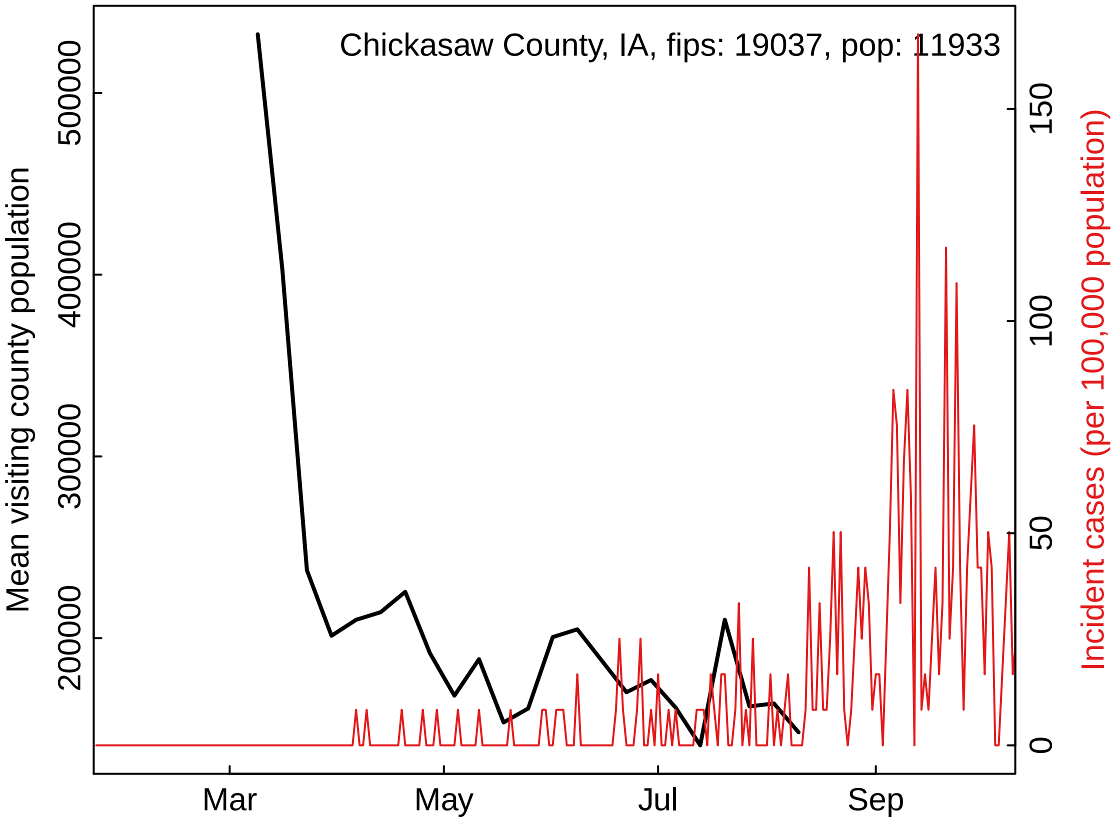

Chickasaw County, IA, fips: 19037, pop: 11933
Mean visiting county population
200000
300000
400000
500000
Incident cases (per 100,000 population)
0
50
100
150
Mar
May
Jul
Sep

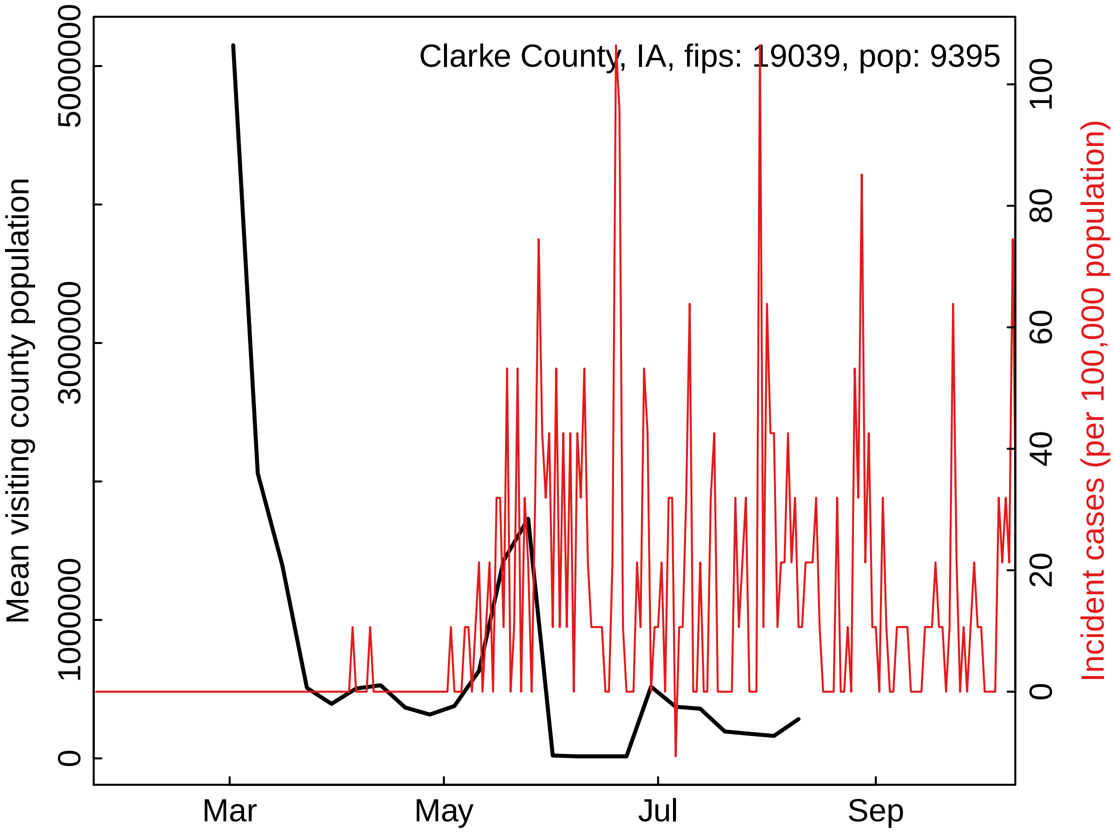

Clarke County, IA, fips: 19039, pop: 9395
Mean visiting county population
0
1000000
3000000
5000000
Incident cases (per 100,000 population)
0
20
40
60
80
100
Mar
May
Jul
Sep

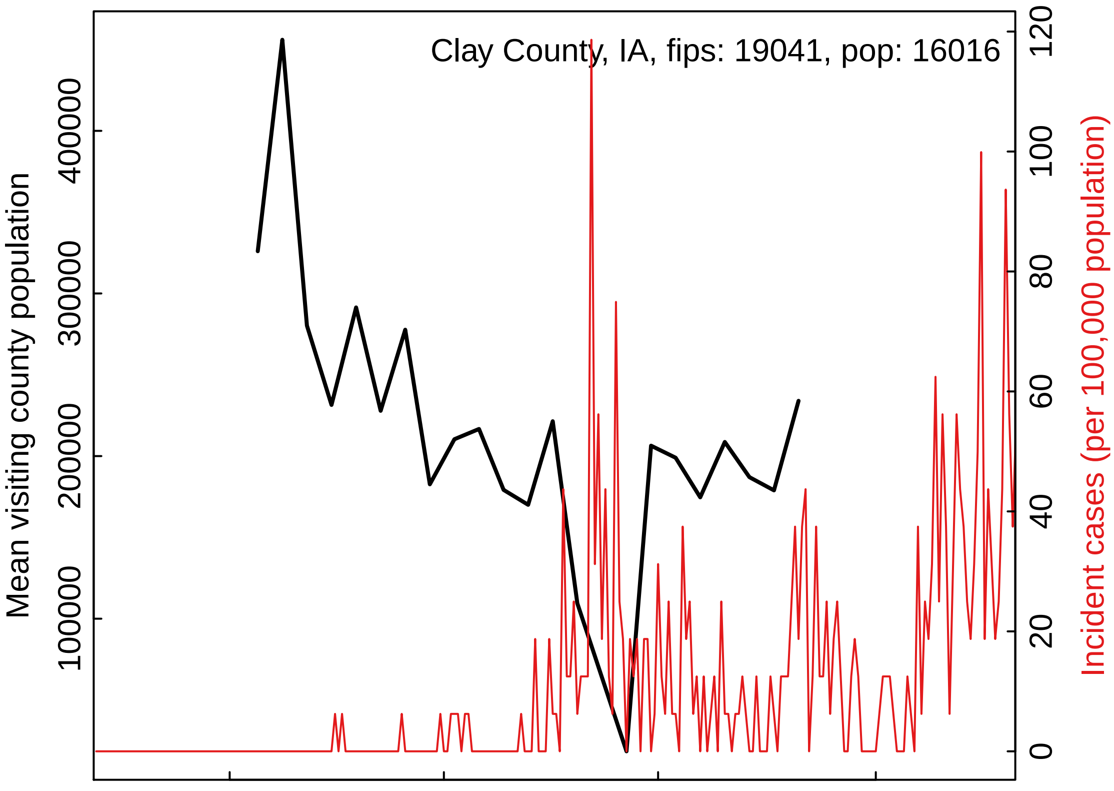
Clay County, IA, fips: 19041, pop: 16016
Mean visiting county population
100000
200000
300000
400000
Incident cases (per 100,000 population)
0
20
40
60
80
100
120
Mar
May
Jul
Sep

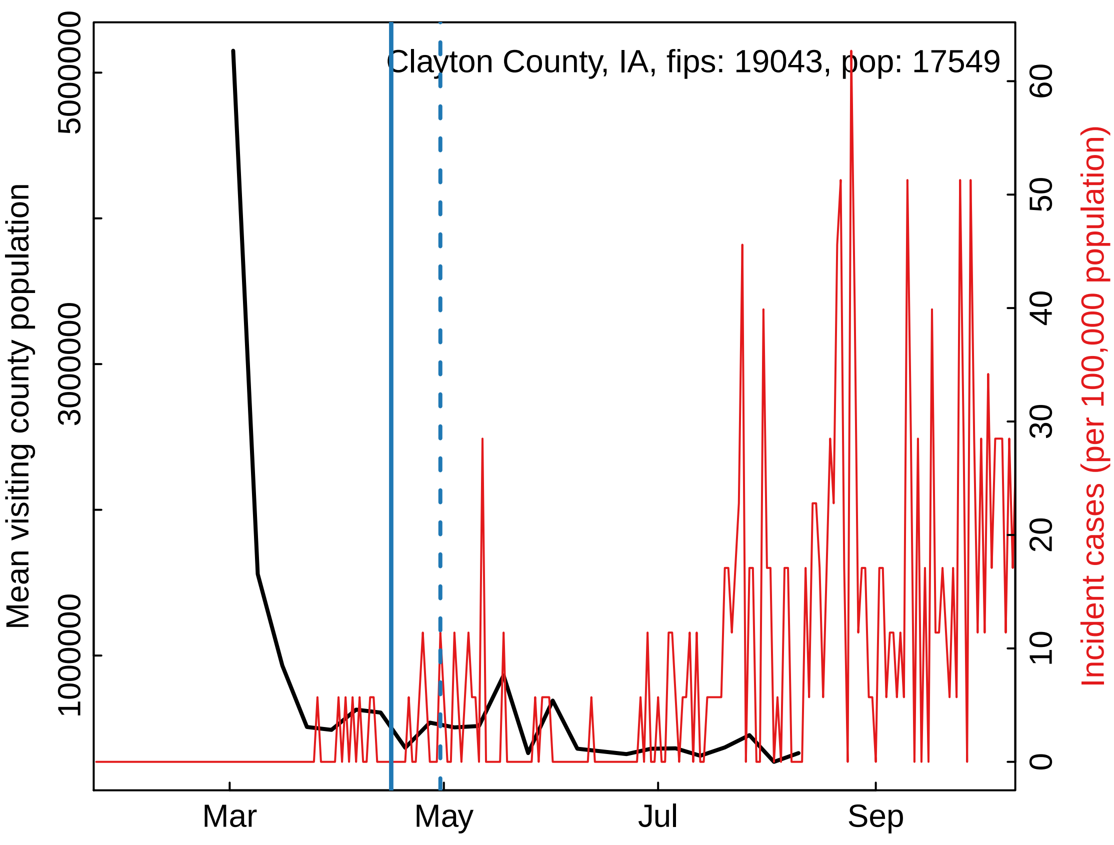
Clayton County, IA, fips: 19043, pop: 17549
Mean visiting county population
1000000
3000000
5000000
Incident cases (per 100,000 population)
0
10
20
30
40
50
60
Mar
May
Jul
Sep

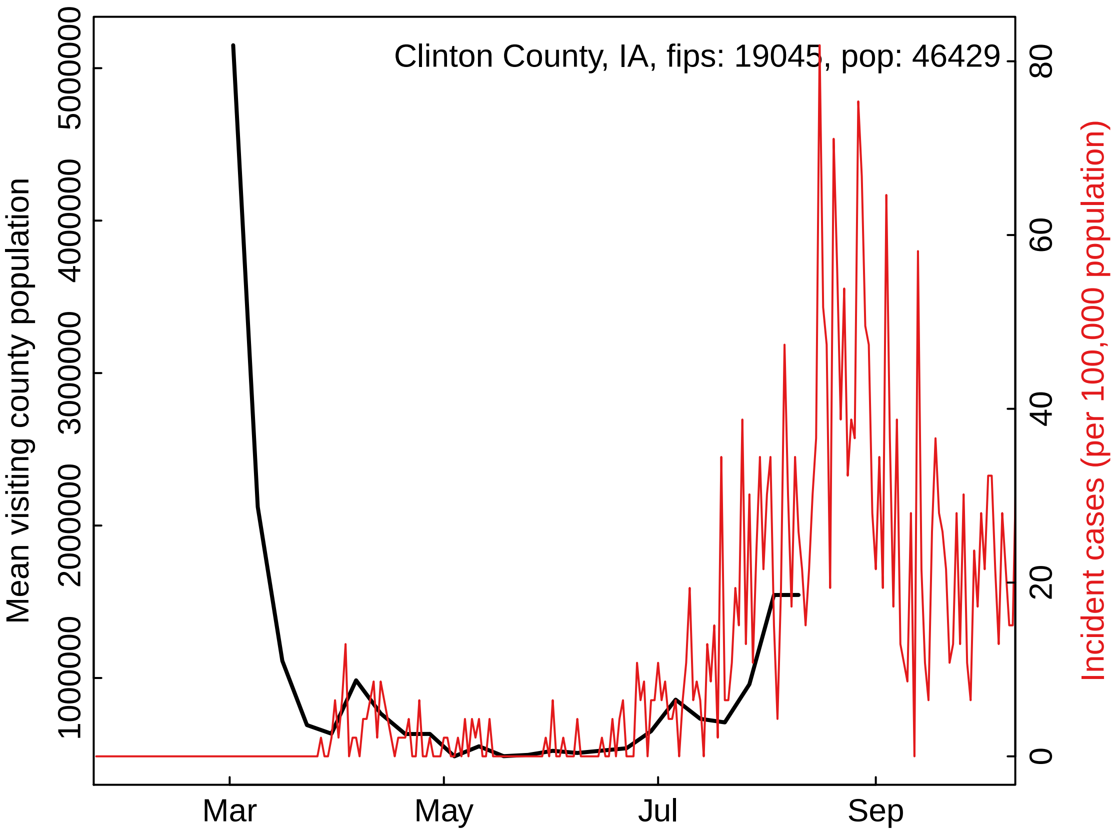

Clinton County, IA, fips: 19045, pop: 46429
Mean visiting county population
1000000
2000000
3000000
4000000
5000000
Mar
May
Jul
Sep
0
20
40
60
80
Incident cases (per 100,000 population)

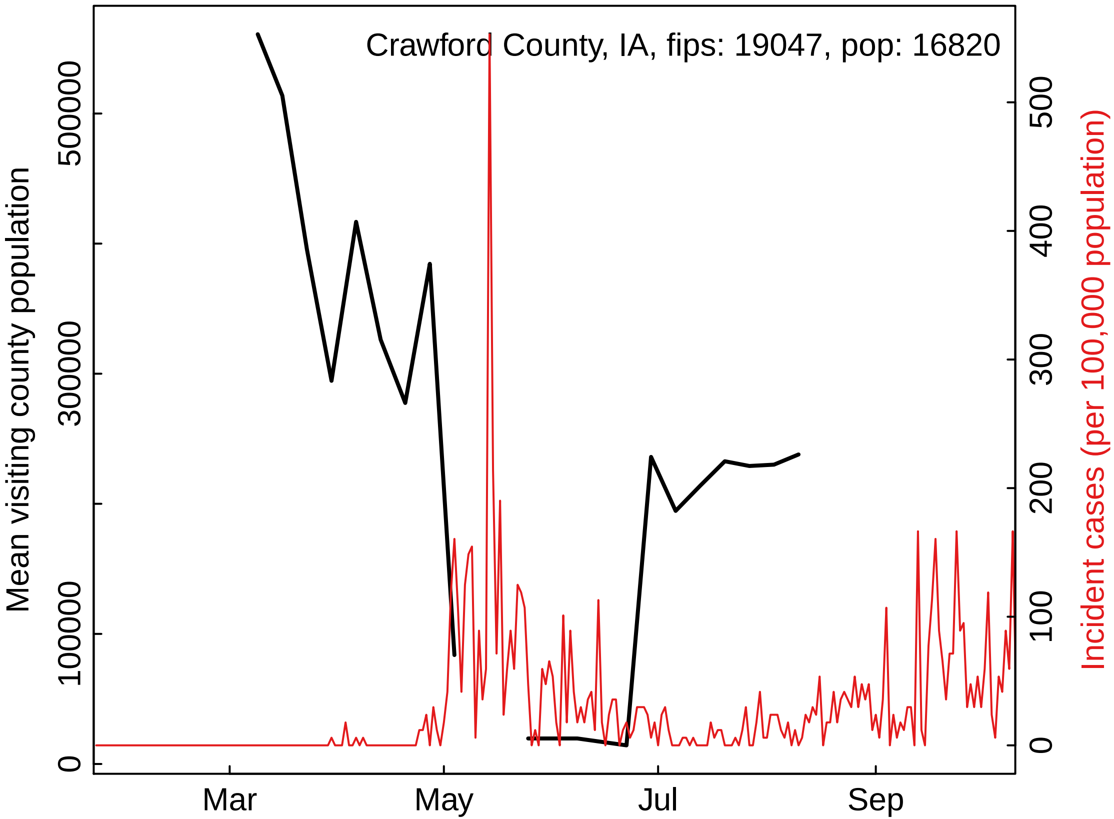

Crawford County, IA, fips: 19047, pop: 16820
Mean visiting county population
0
100000
300000
500000
Incident cases (per 100,000 population)
0
100
200
300
400
500
Mar
May
Jul
Sep

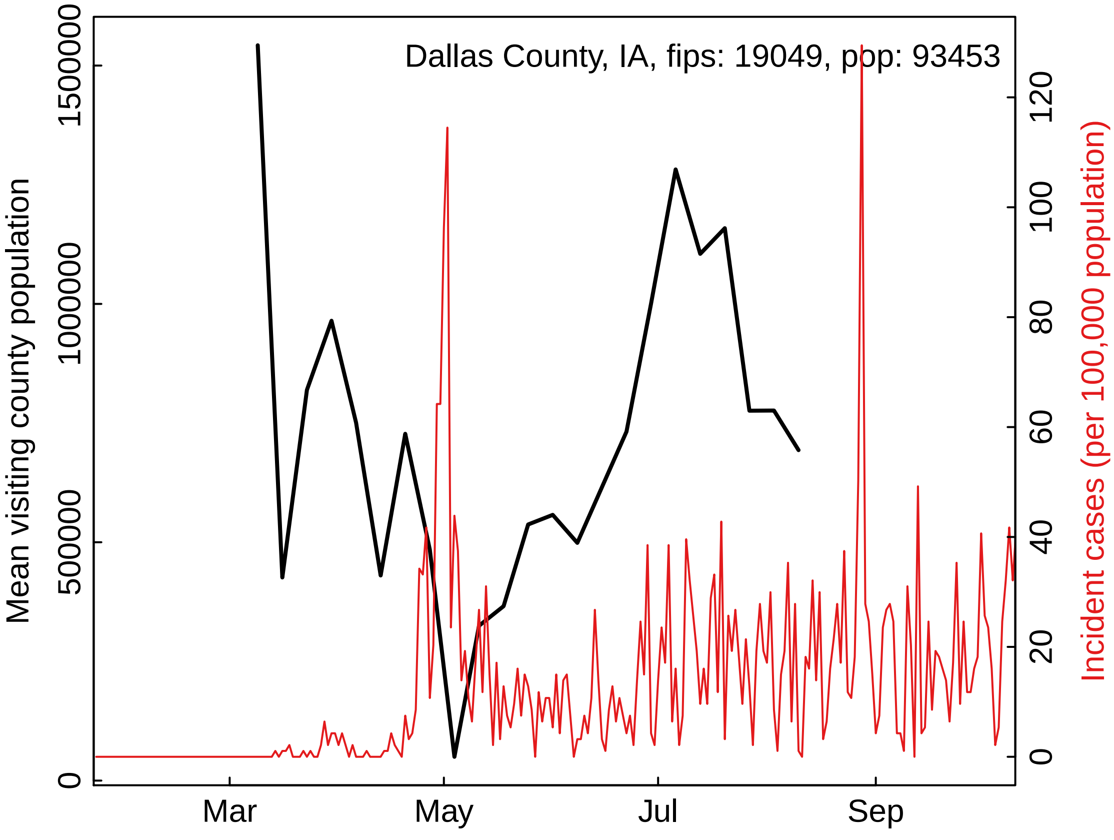

Dallas County, IA, fips: 19049, pop: 93453
Mean visiting county population
0
5000000
10000000
15000000
Incident cases (per 100,000 population)
0
20
40
60
80
100
120
Mar
May
Jul
Sep

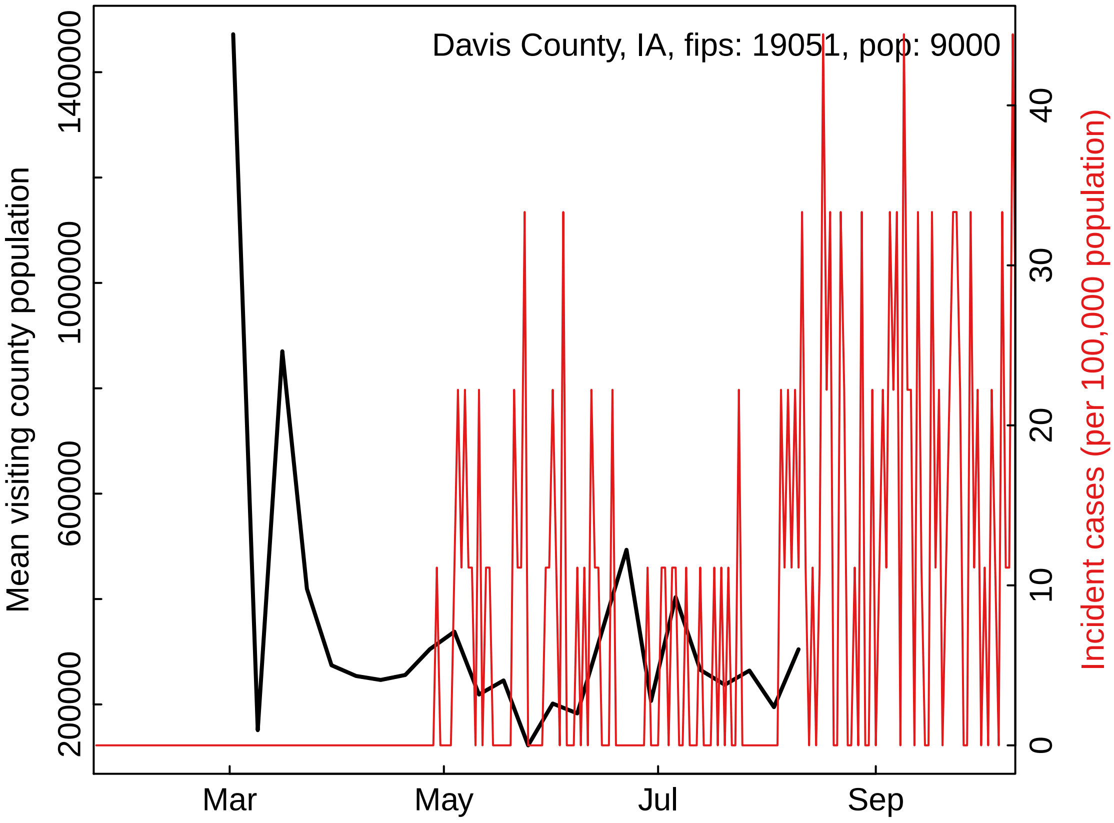

Davis County, IA, fips: 19051, pop: 9000
Mean visiting county population
200000
600000
1000000
1400000
Incident cases (per 100,000 population)
0
10
20
30
40
Mar
May
Jul
Sep

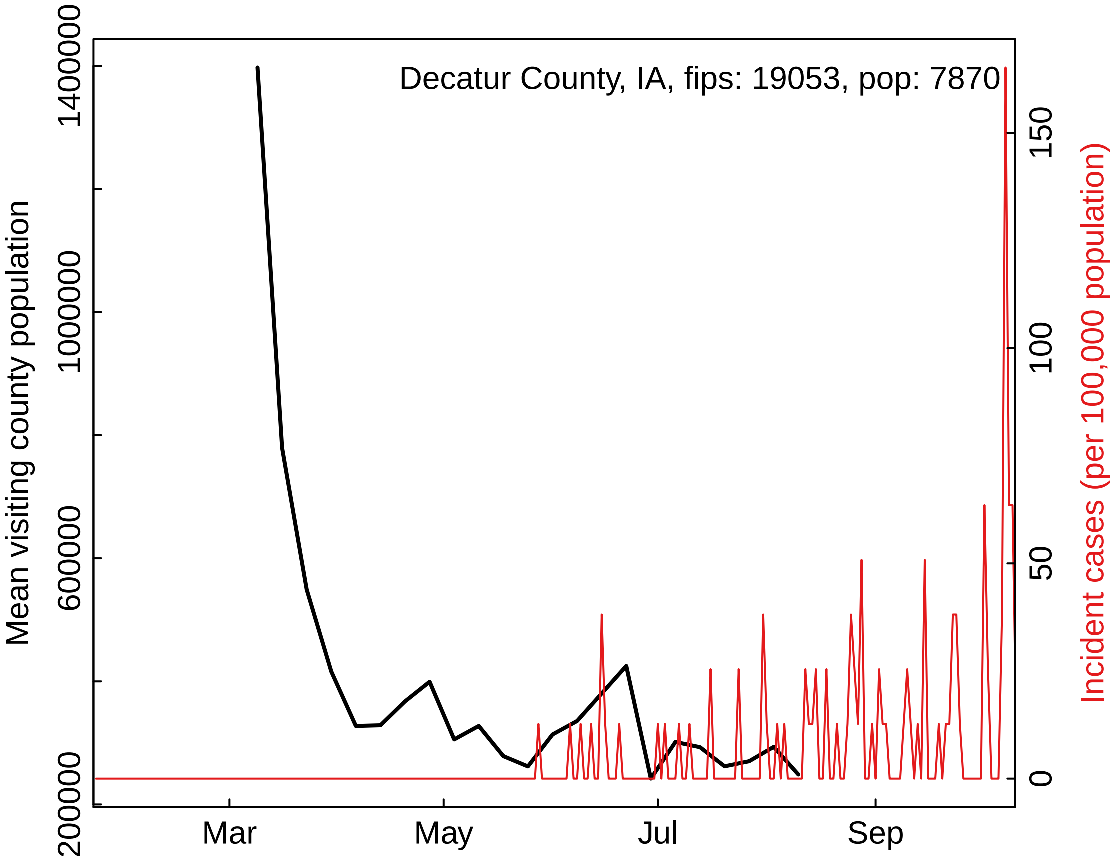
Decatur County, IA, fips: 19053, pop: 7870
Mean visiting county population
200000
600000
1000000
1400000
Incident cases (per 100,000 population)
0
50
100
150
Mar
May
Jul
Sep

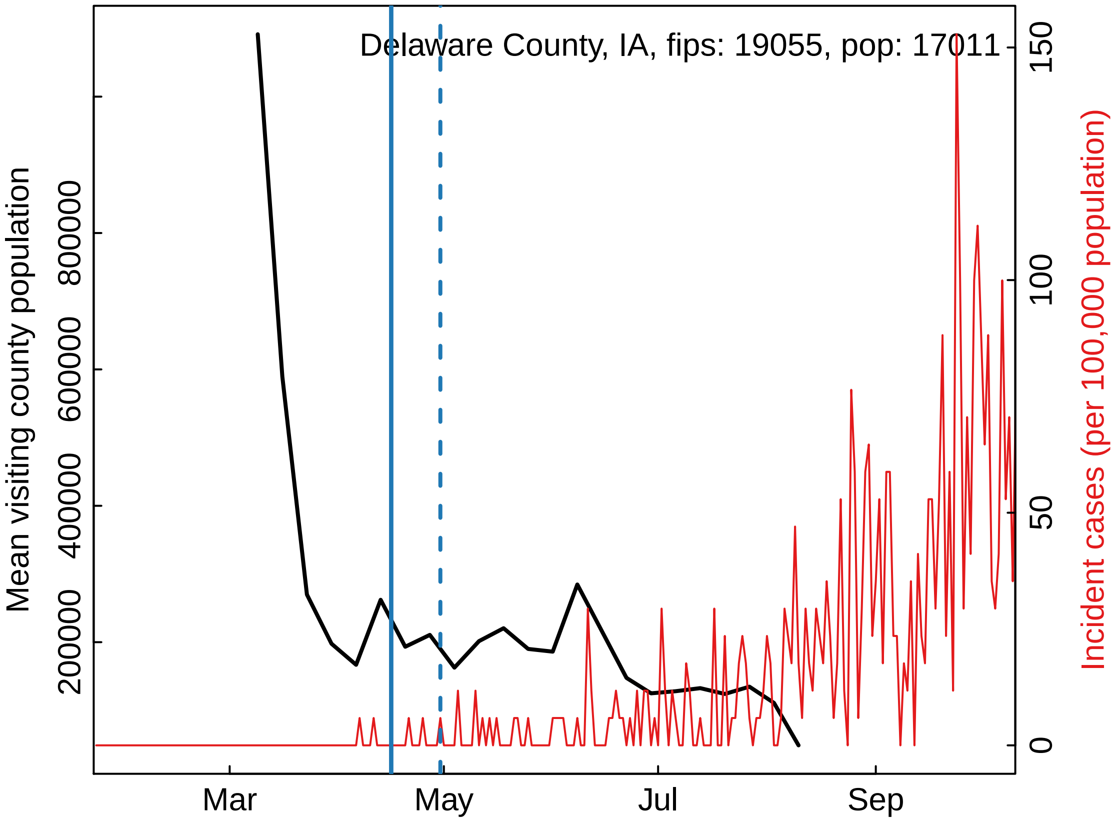

Delaware County, IA, fips: 19055, pop: 17011
Mean visiting county population
200000
400000
600000
800000
Incident cases (per 100,000 population)
0
50
100
150
Mar
May
Jul
Sep

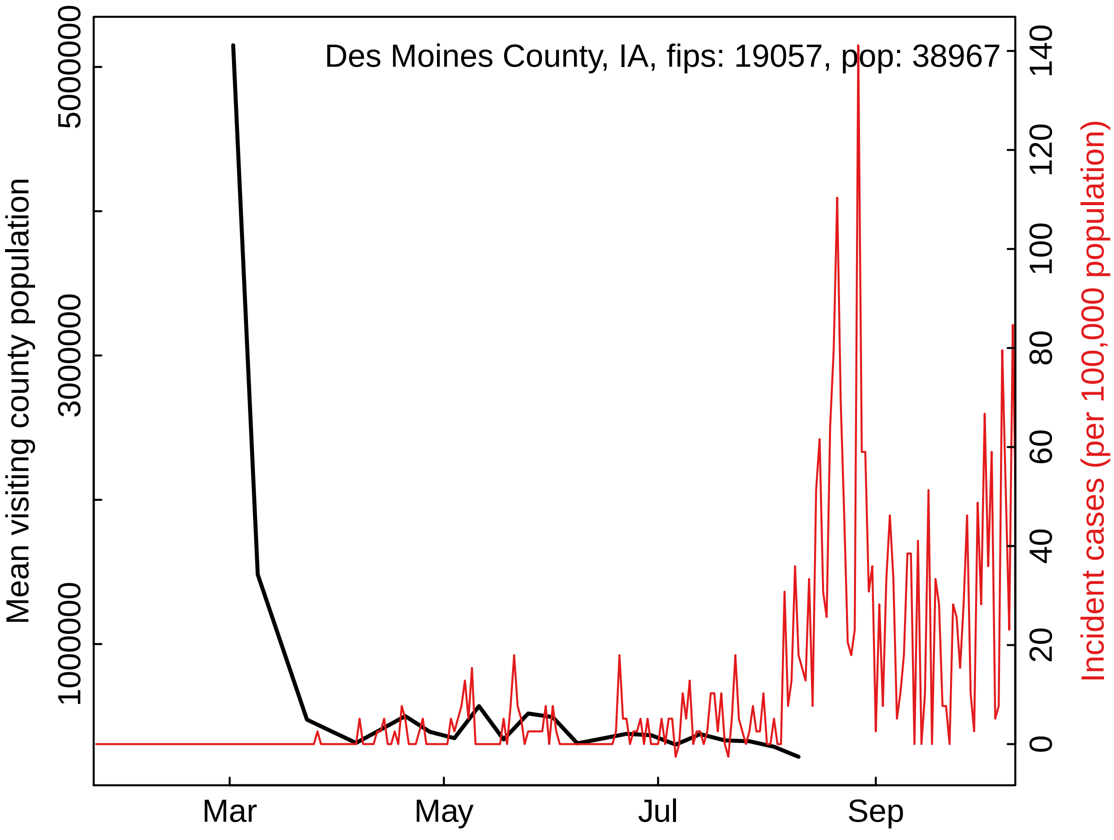
Des Moines County, IA, fips: 19057, pop: 38967
Mean visiting county population
1000000
3000000
5000000
Mar
May
Jul
Sep
0
20
40
60
80
100
120
140
Incident cases (per 100,000 population)

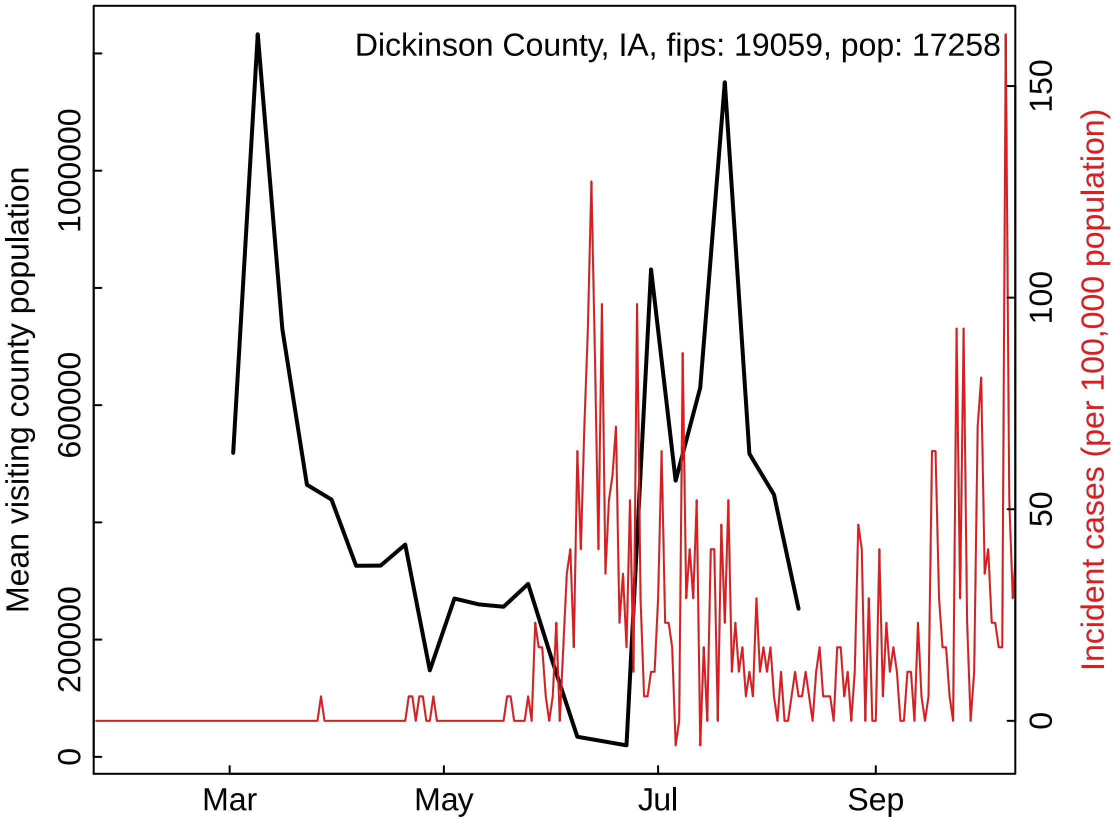
Dickinson County, IA, fips: 19059, pop: 17258
Mean visiting county population
0
200000
600000
1000000
Incident cases (per 100,000 population)
0
50
100
150
Mar
May
Jul
Sep

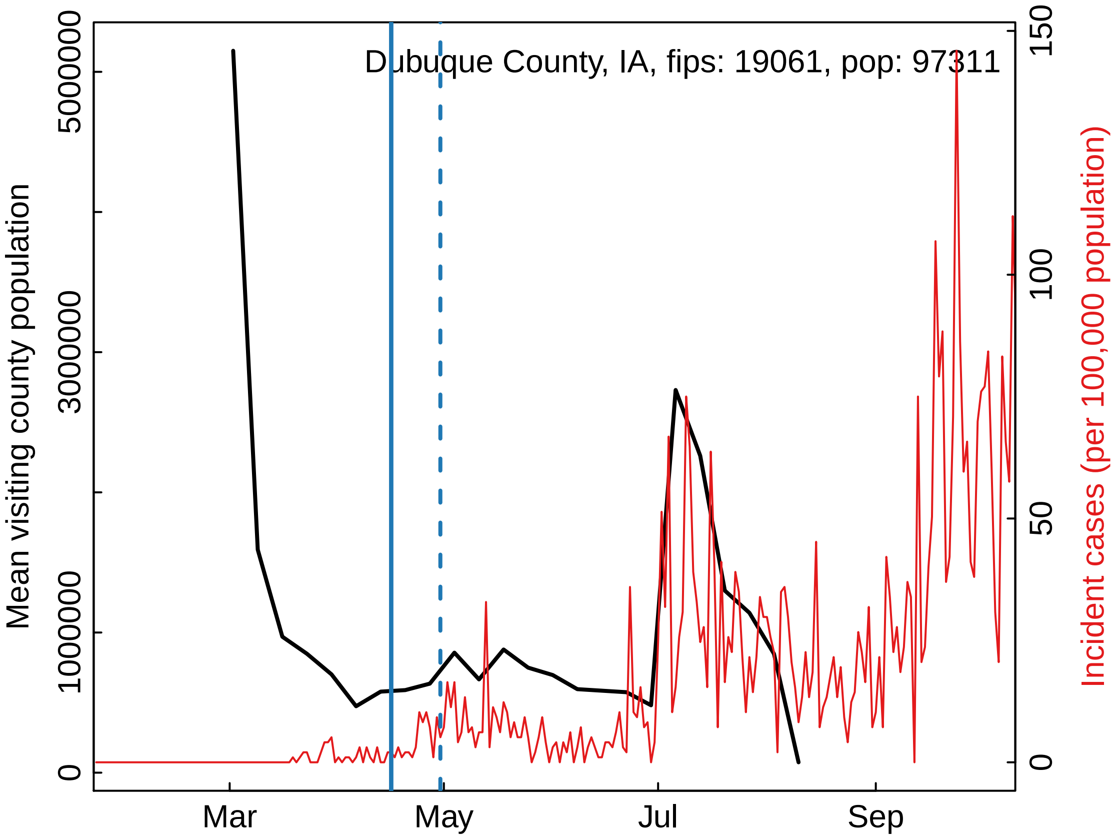

Dubuque County, IA, fips: 19061, pop: 97311
Mean visiting county population
Incident cases (per 100,000 population)
0
1000000
3000000
5000000
0
50
100
150
Mar
May
Jul
Sep

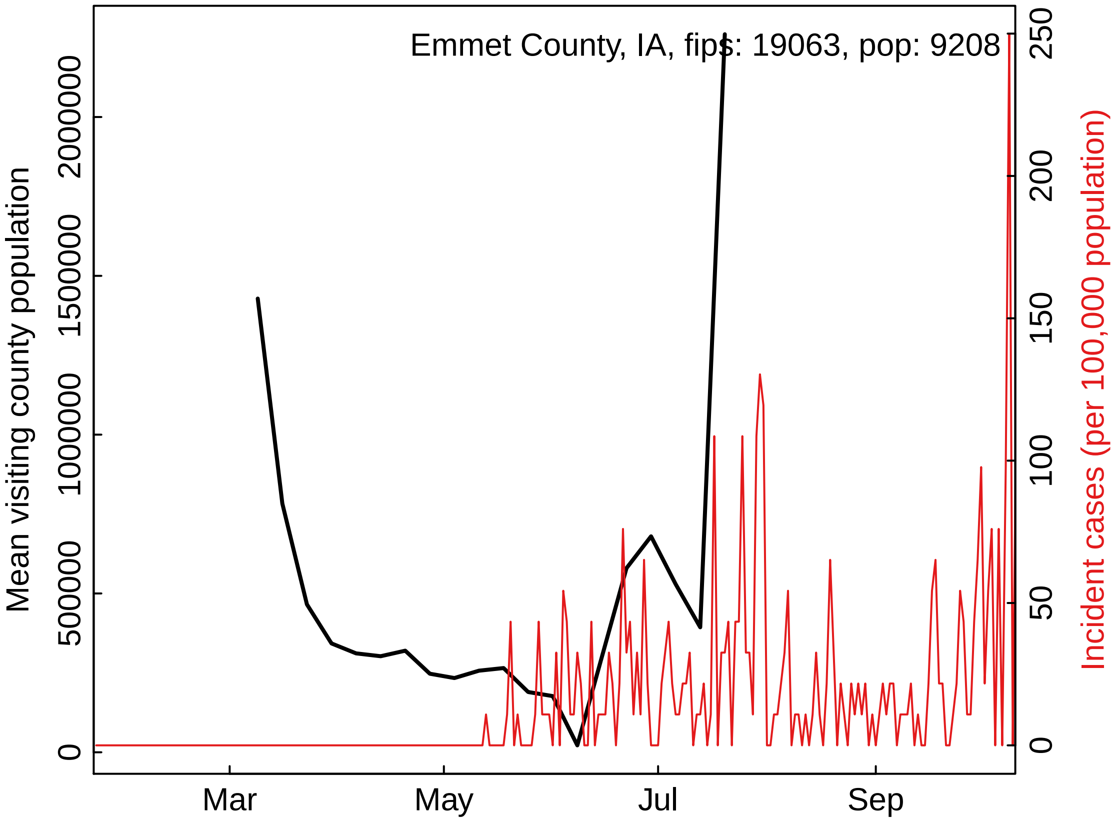
Emmet County, IA, fips: 19063, pop: 9208
Mean visiting county population
0
500000
1000000
1500000
2000000
Incident cases (per 100,000 population)
0
50
100
150
200
250
Mar
May
Jul
Sep

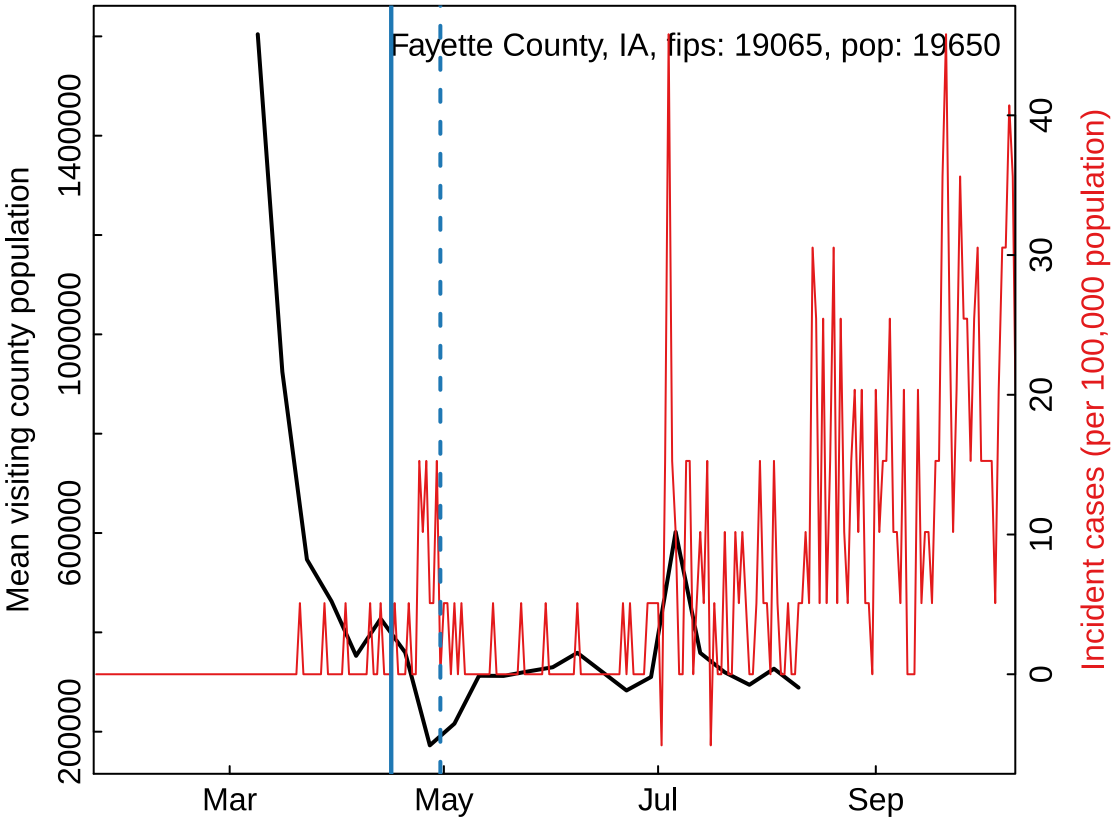

Fayette County, IA, fips: 19065, pop: 19650
Mean visiting county population
200000
600000
1000000
1400000
Incident cases (per 100,000 population)
0
10
20
30
40
Mar
May
Jul
Sep

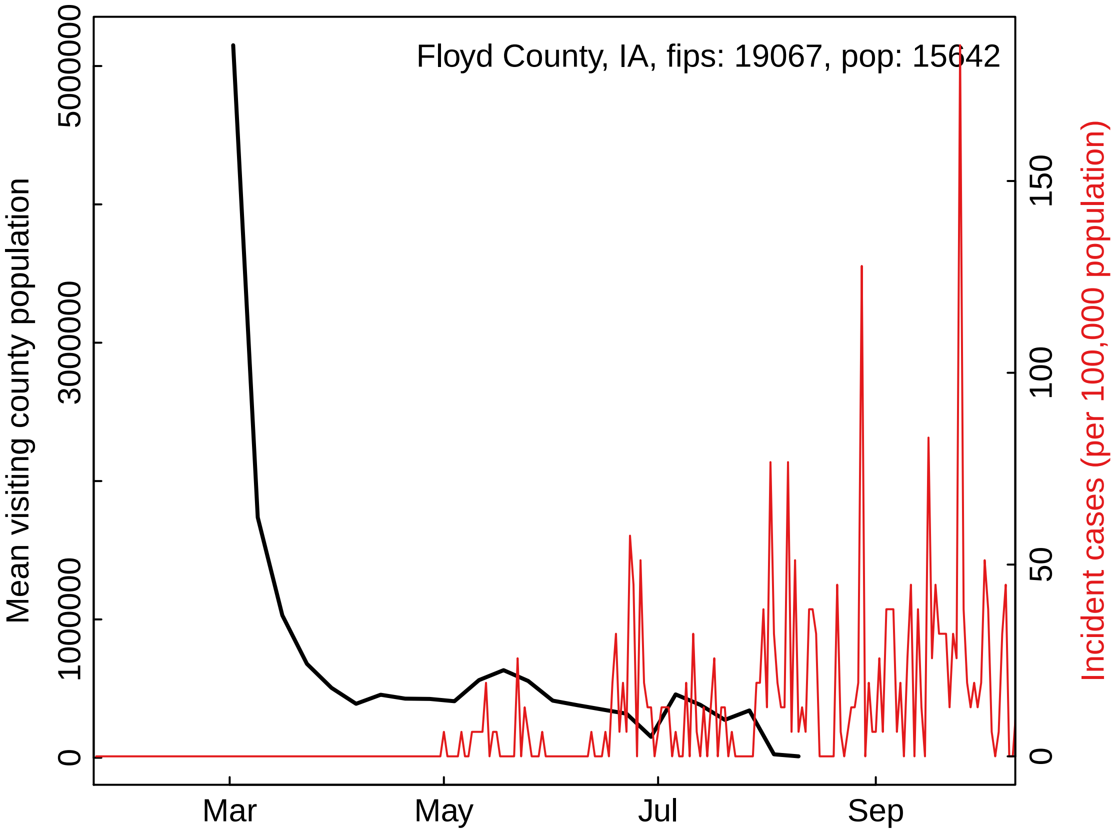

Floyd County, IA, fips: 19067, pop: 15642
Mean visiting county population
Incident cases (per 100,000 population)
0
1000000
3000000
5000000
0
50
100
150
Mar
May
Jul
Sep

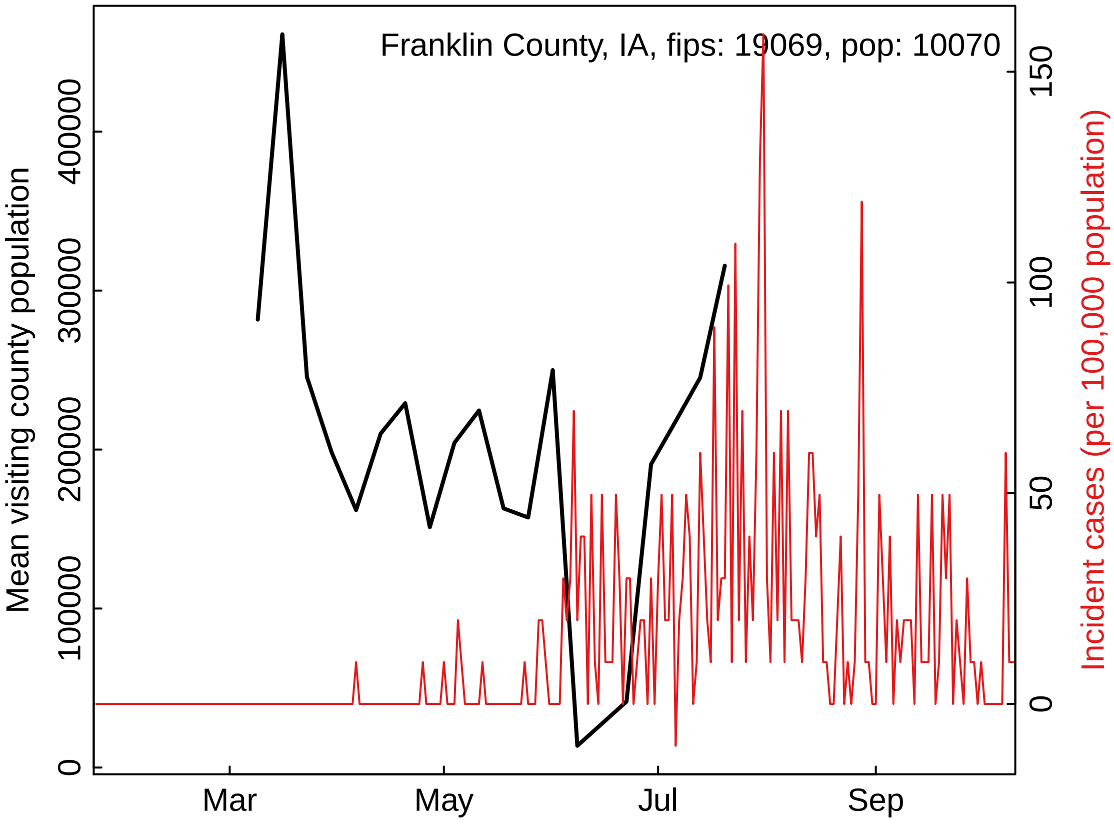

Franklin County, IA, fips: 19069, pop: 10070
Mean visiting county population
Incident cases (per 100,000 population)
0
100000
200000
300000
400000
0
50
100
150
Mar
May
Jul
Sep

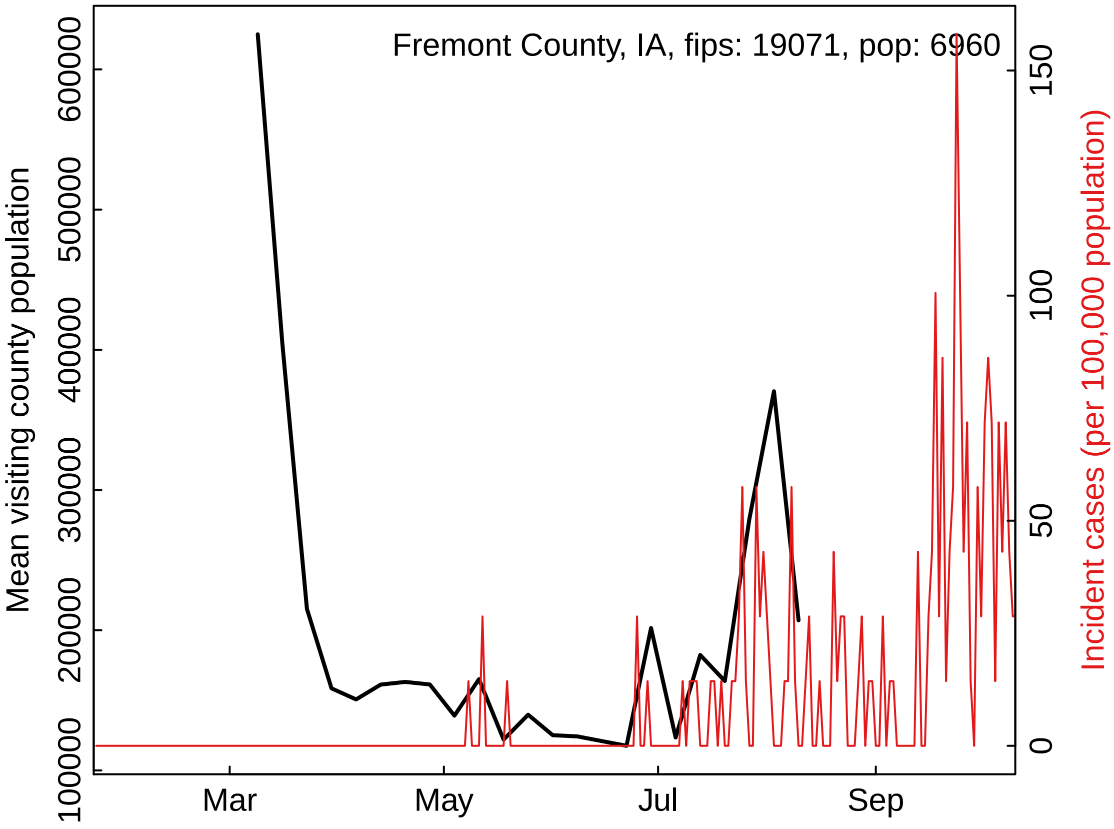

Fremont County, IA, fips: 19071, pop: 6960
Mean visiting county population
100000
200000
300000
400000
500000
600000
Incident cases (per 100,000 population)
0
50
100
150
Mar
May
Jul
Sep

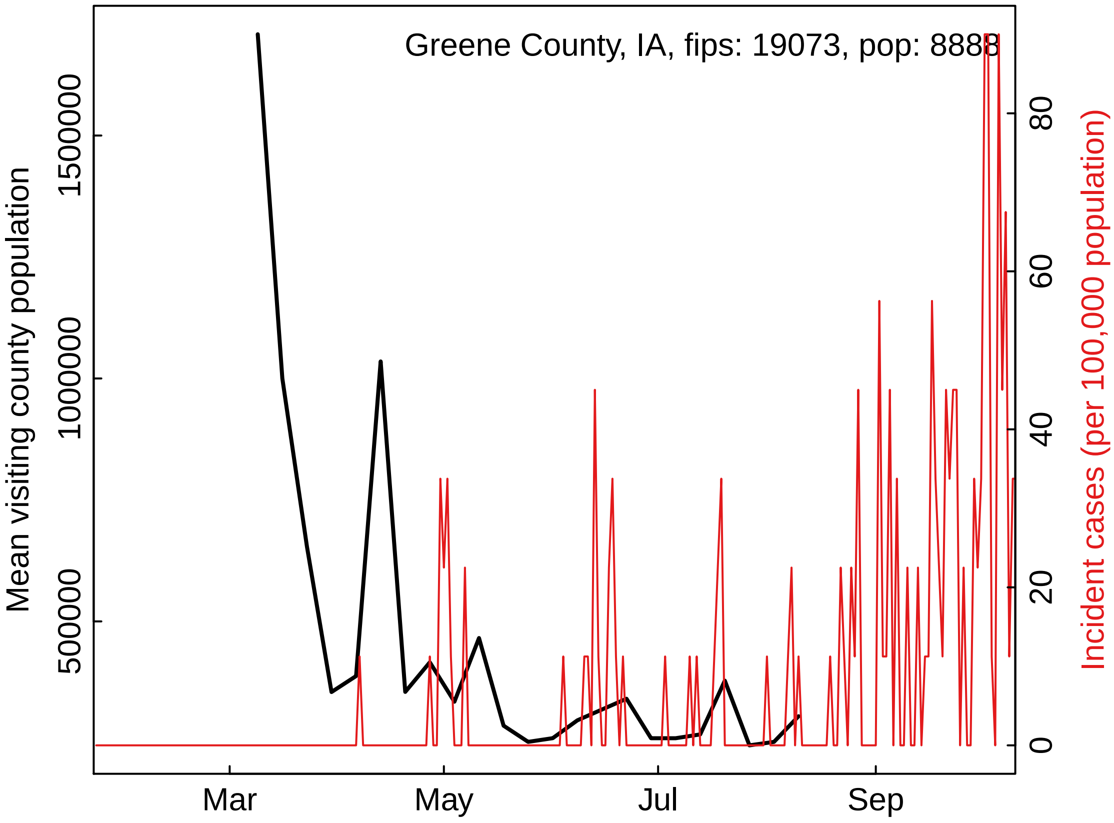
Greene County, IA, fips: 19073, pop: 8888
Mean visiting county population
500000
1000000
1500000
Mar
May
Jul
Sep
0
20
40
60
80
Incident cases (per 100,000 population)

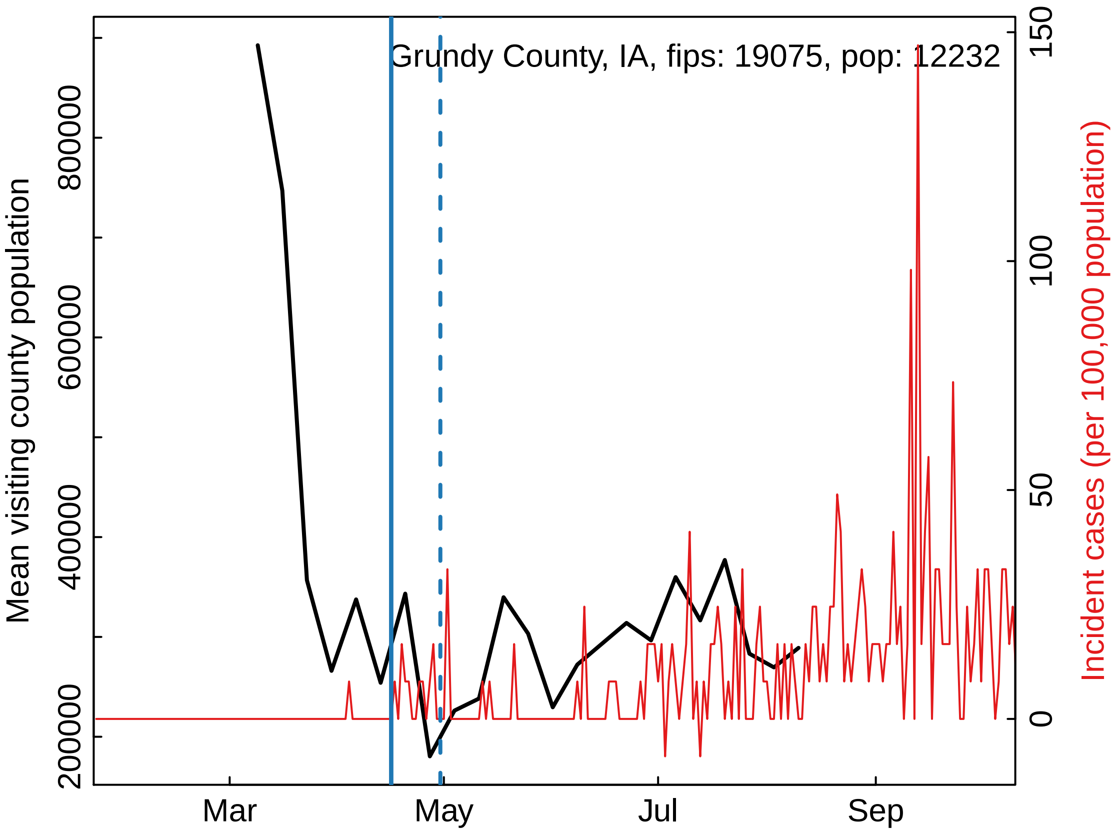

Grundy County, IA, fips: 19075, pop: 12232
Mean visiting county population
200000
400000
600000
800000
Incident cases (per 100,000 population)
0
50
100
150
Mar
May
Jul
Sep

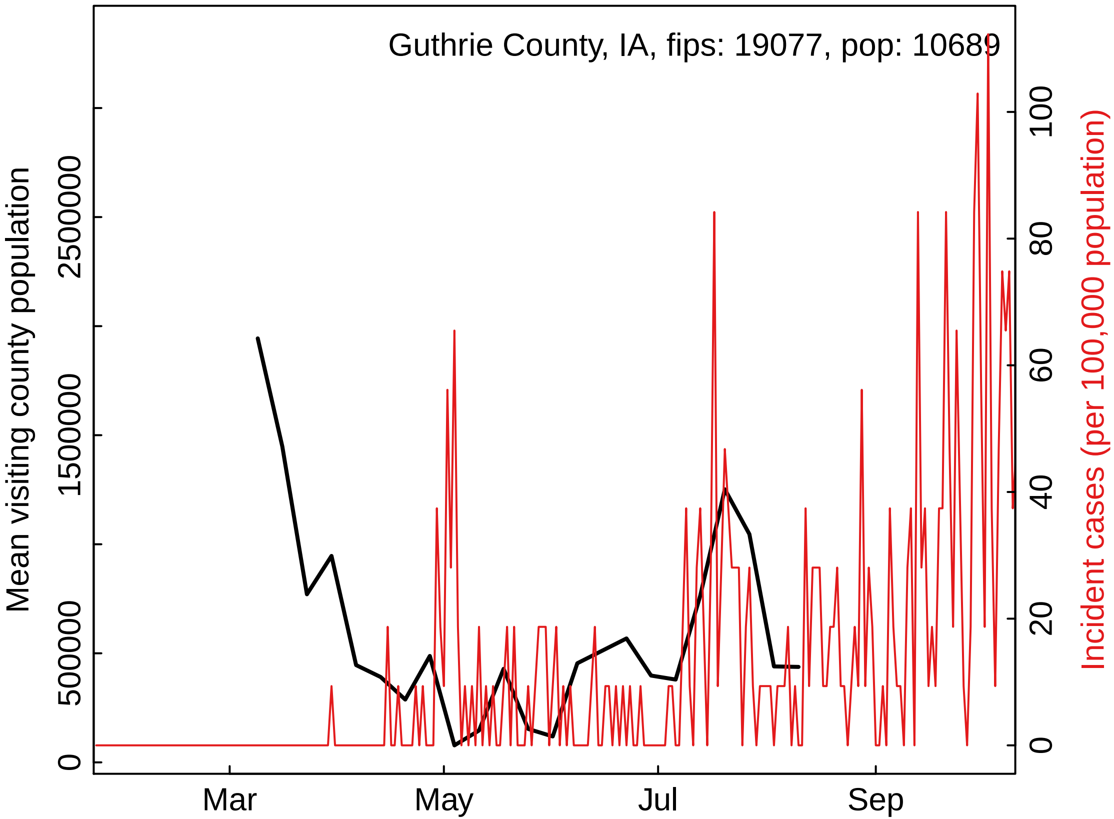

Guthrie County, IA, fips: 19077, pop: 10689
Mean visiting county population
0
500000
1500000
2500000
Mar
May
Jul
Sep
0
20
40
60
80
100
Incident cases (per 100,000 population)

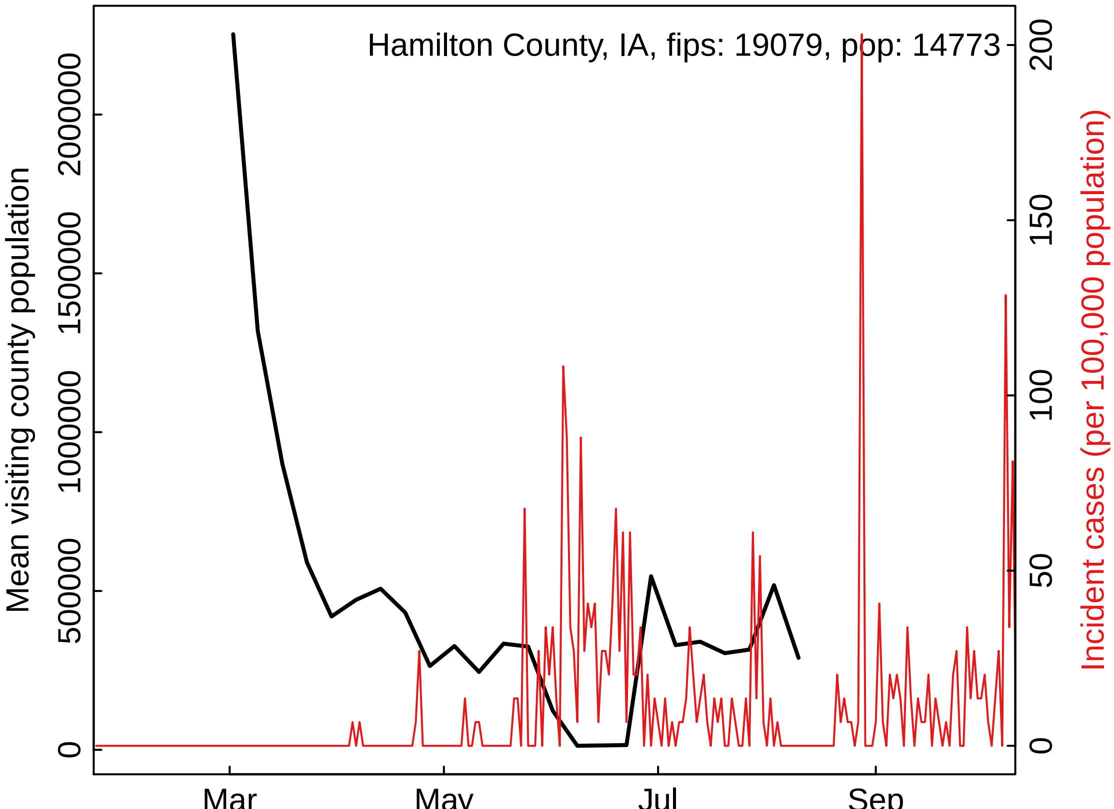

Hamilton County, IA, fips: 19079, pop: 14773
Mean visiting county population
0
500000
1000000
1500000
2000000
Incident cases (per 100,000 population)
0
50
100
150
200
Mar
May
Jul
Sep

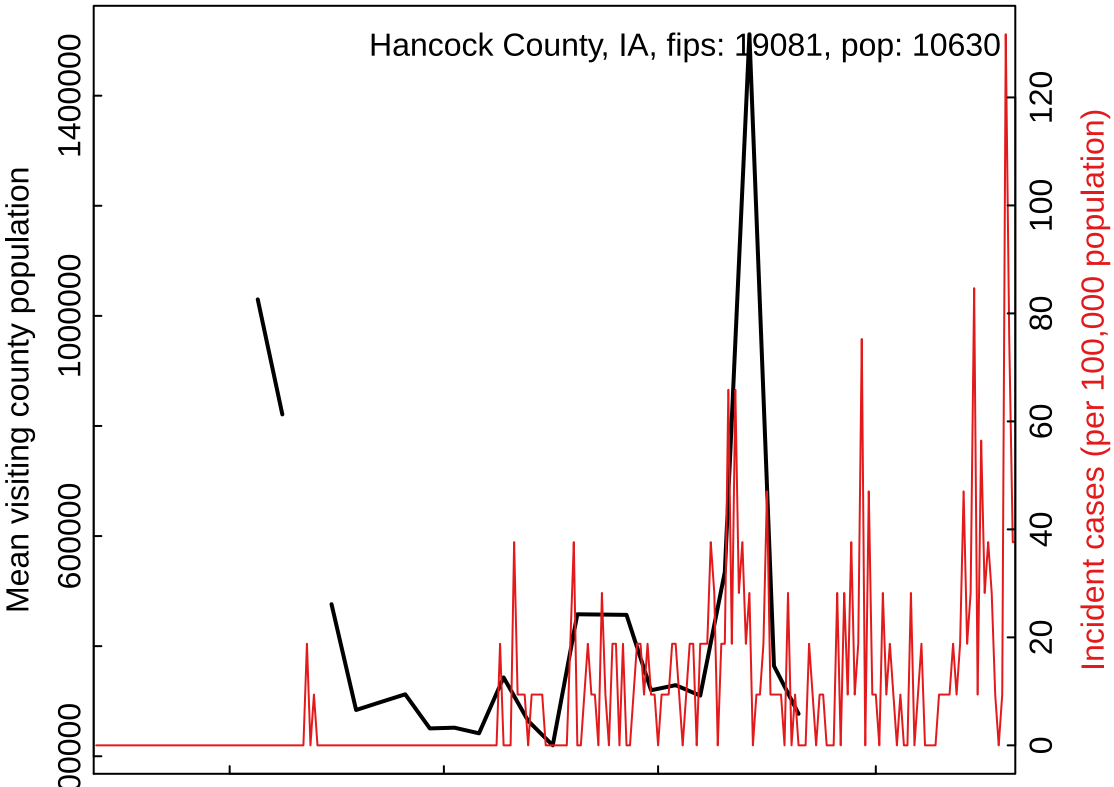

Hancock County, IA, fips: 19081, pop: 10630
Mean visiting county population
200000
600000
1000000
1400000
Incident cases (per 100,000 population)
0
20
40
60
80
100
120
Mar
May
Jul
Sep

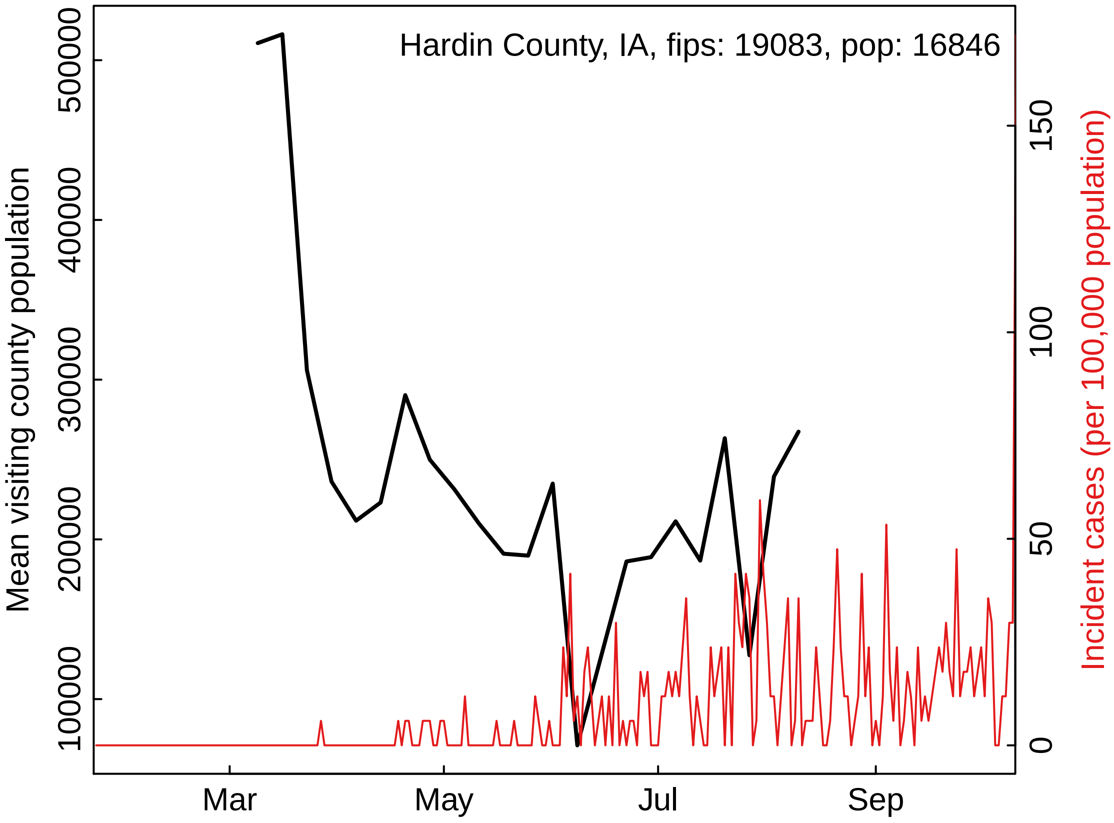

Hardin County, IA, fips: 19083, pop: 16846
Mean visiting county population
Incident cases (per 100,000 population)
100000
200000
300000
400000
500000
0
50
100
150
Mar
May
Jul
Sep

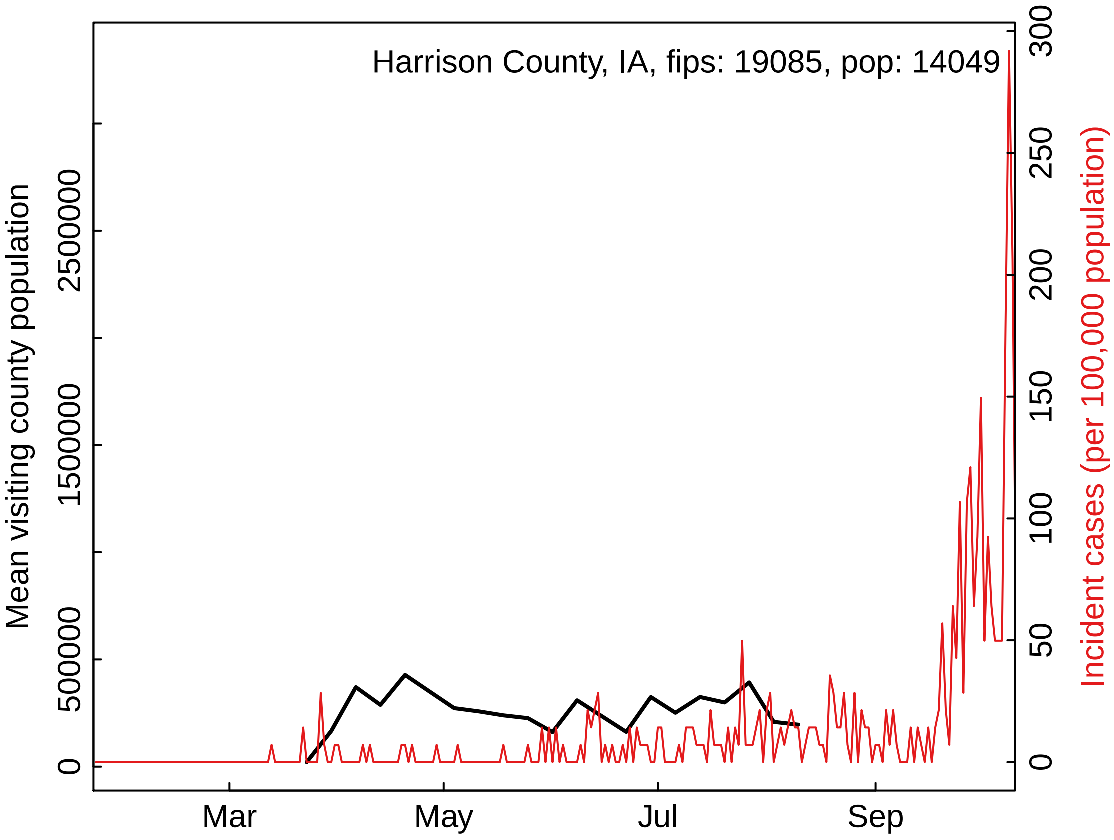
Harrison County, IA, fips: 19085, pop: 14049
Mean visiting county population
0
500000
1500000
2500000
Incident cases (per 100,000 population)
0
50
100
150
200
250
300
Mar
May
Jul
Sep

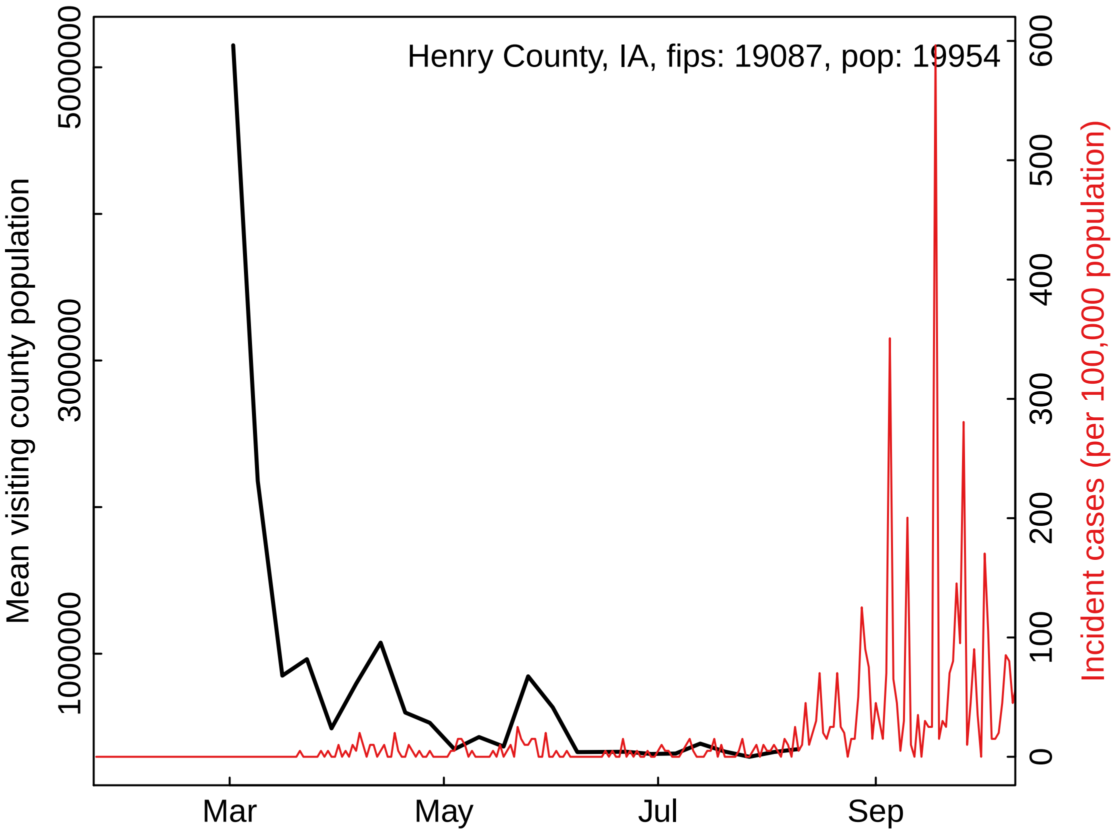
Henry County, IA, fips: 19087, pop: 19954
Mean visiting county population
Incident cases (per 100,000 population)
1000000
3000000
5000000
0
100
200
300
400
500
600
Mar
May
Jul
Sep

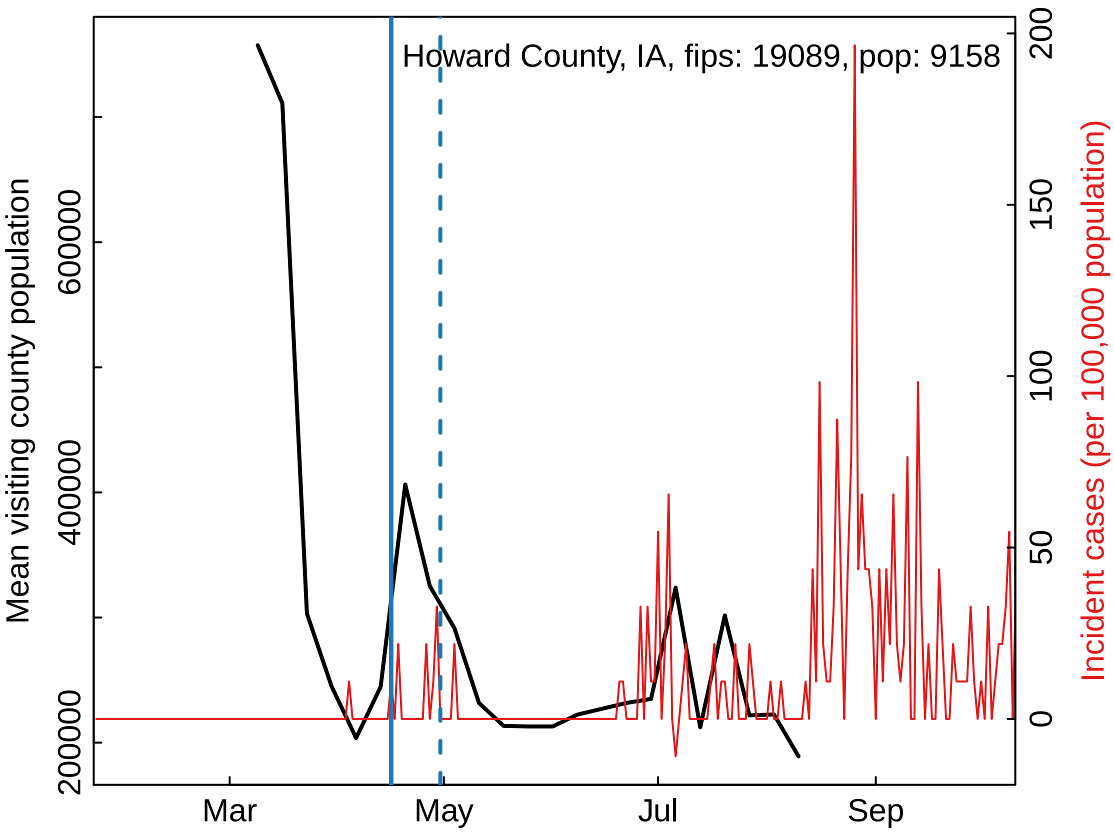

Howard County, IA, fips: 19089, pop: 9158
Mean visiting county population
200000
400000
600000
Incident cases (per 100,000 population)
0
50
100
150
200
Mar
May
Jul
Sep

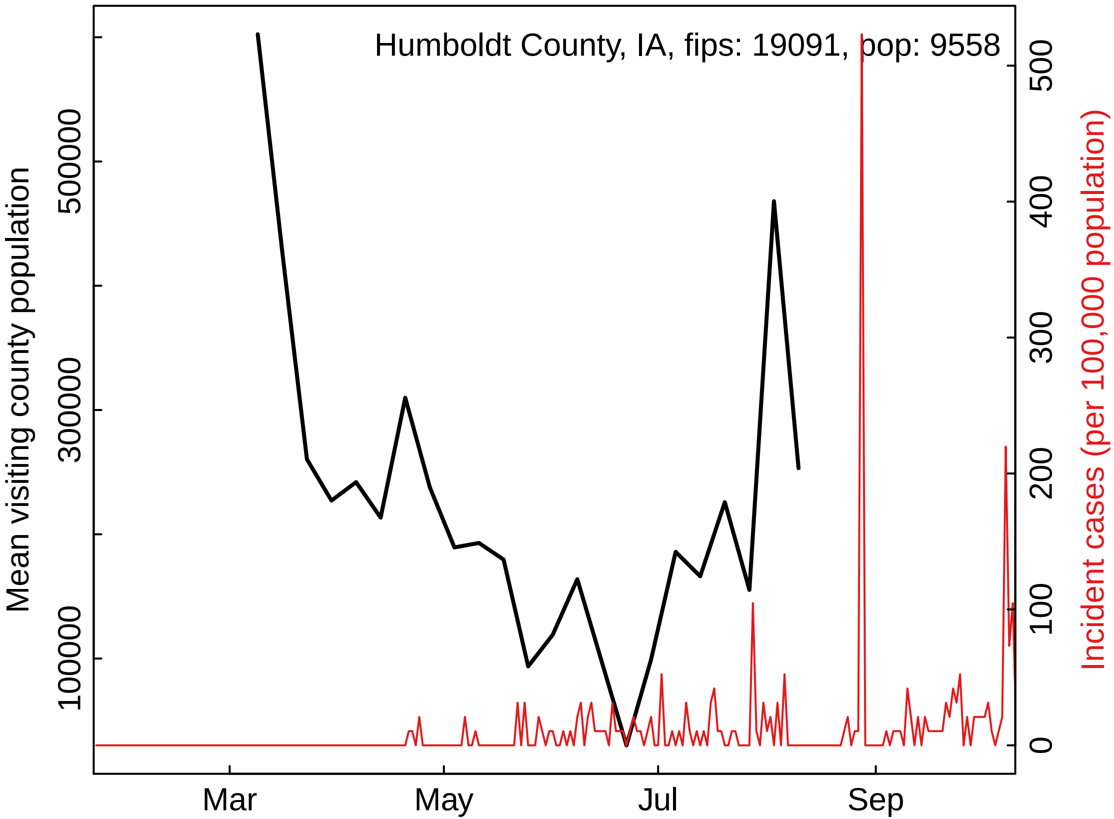

Humboldt County, IA, fips: 19091, pop: 9558
Mean visiting county population
100000
300000
500000
Incident cases (per 100,000 population)
0
100
200
300
400
500
Mar
May
Jul
Sep

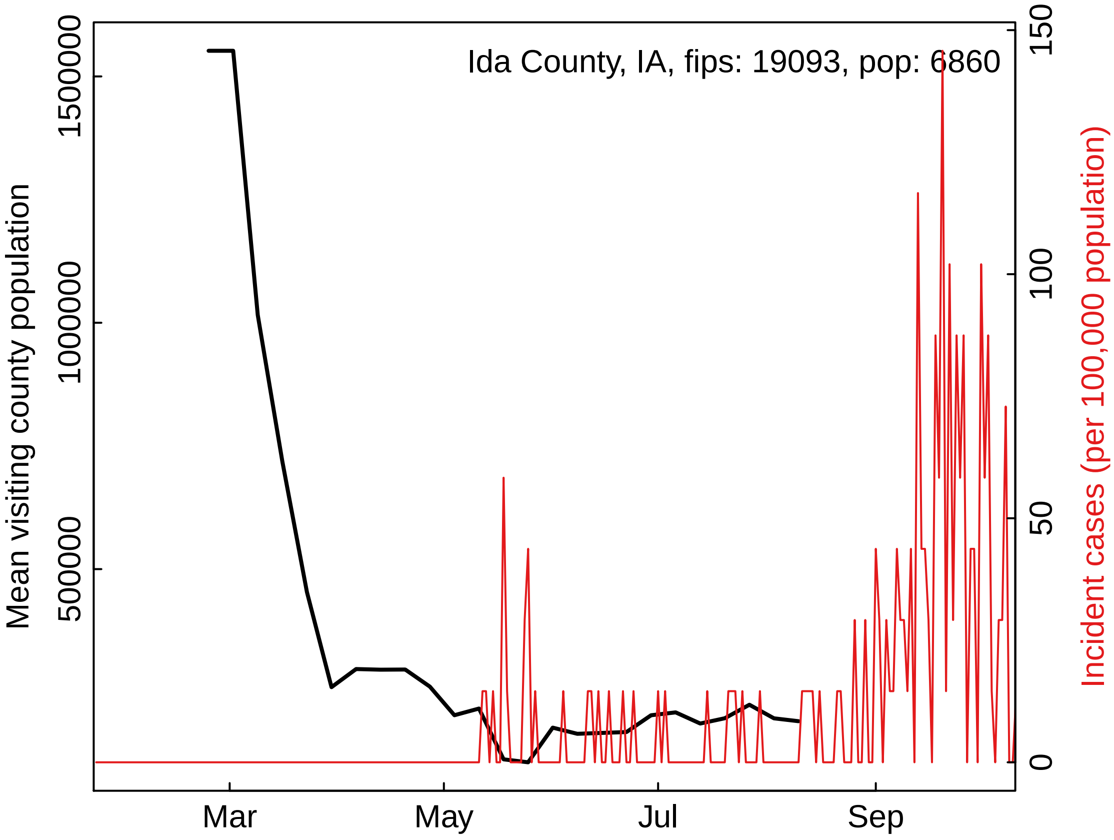

Ida County, IA, fips: 19093, pop: 6860
Mean visiting county population
500000
1000000
1500000
Incident cases (per 100,000 population)
0
50
100
150
Mar
May
Jul
Sep

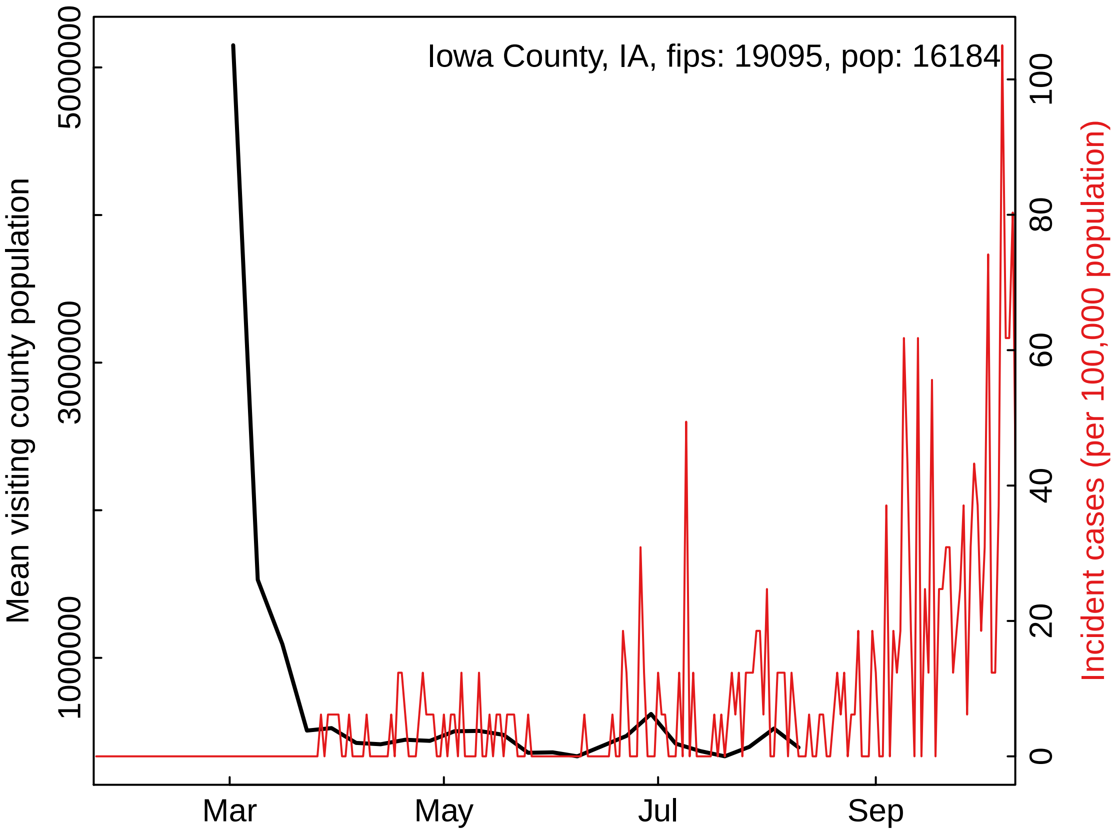
Iowa County, IA, fips: 19095, pop: 16184
Mean visiting county population
1000000
3000000
5000000
Mar
May
Jul
Sep
0
20
40
60
80
100
Incident cases (per 100,000 population)

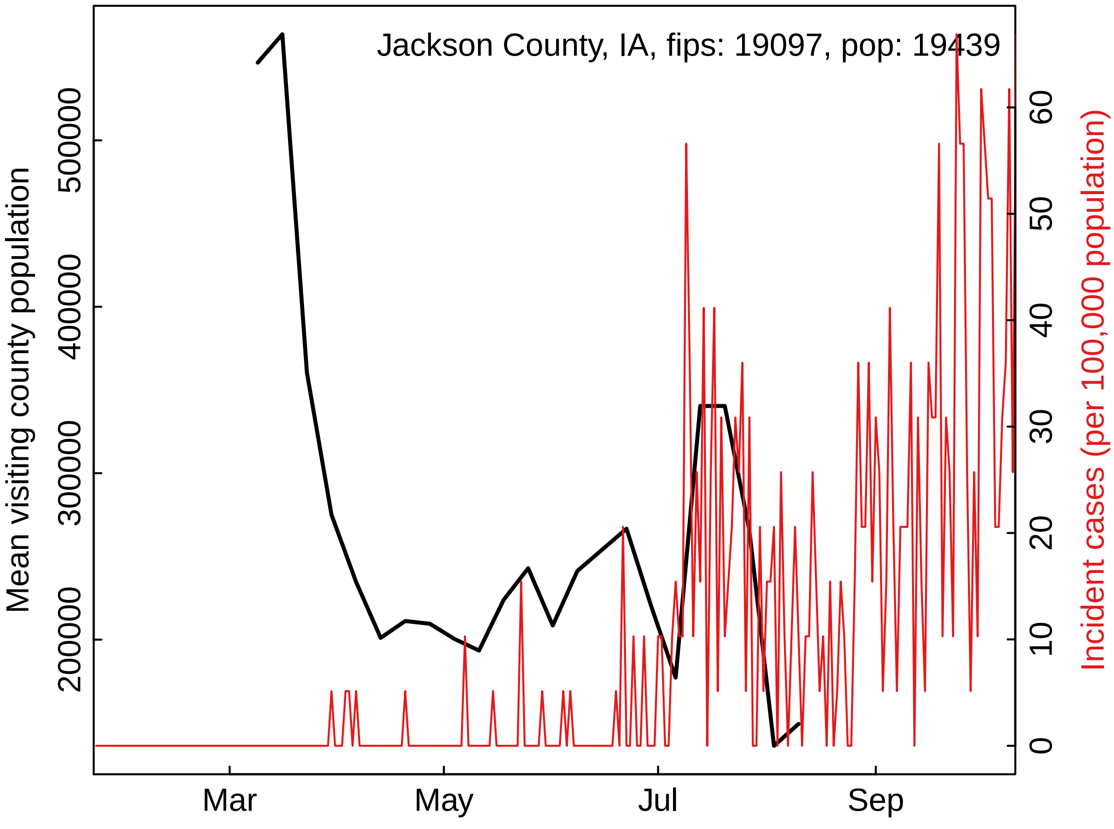

Jackson County, IA, fips: 19097, pop: 19439
Mean visiting county population
200000
300000
400000
500000
Incident cases (per 100,000 population)
0
10
20
30
40
50
60
Mar
May
Jul
Sep

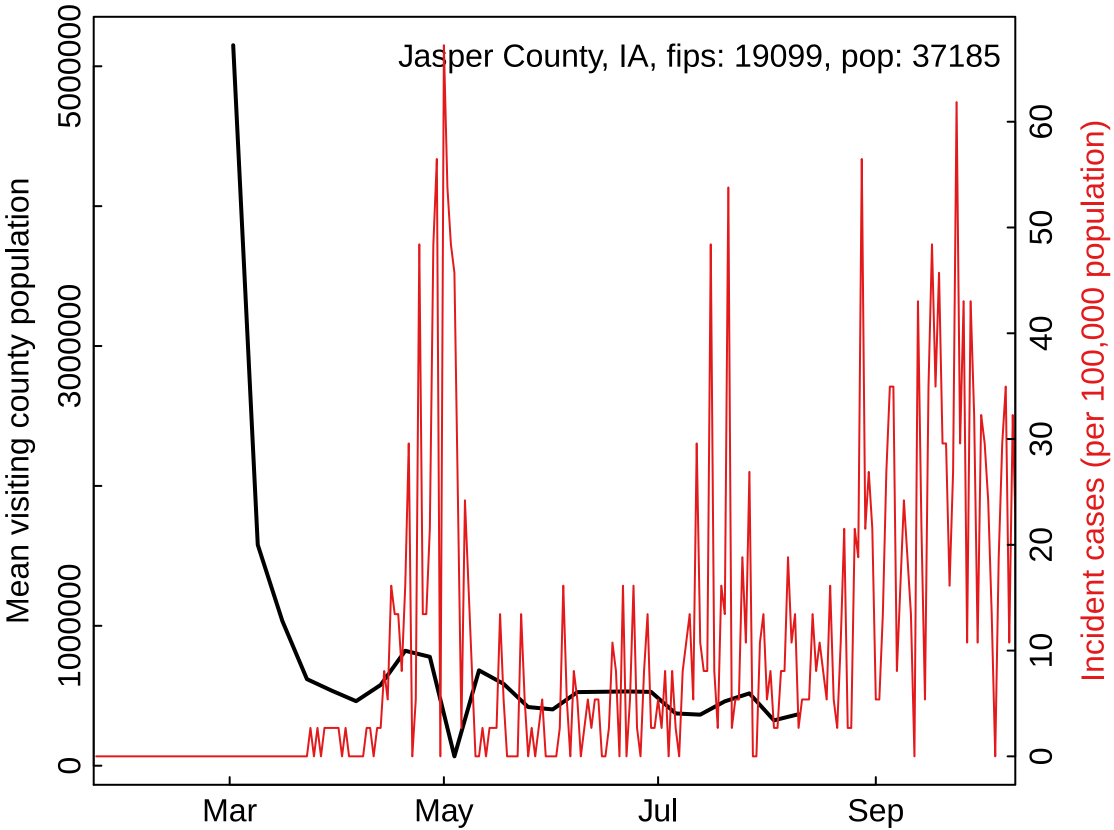

Jasper County, IA, fips: 19099, pop: 37185
Mean visiting county population
0
1000000
3000000
5000000
Incident cases (per 100,000 population)
0
10
20
30
40
50
60
Mar
May
Jul
Sep

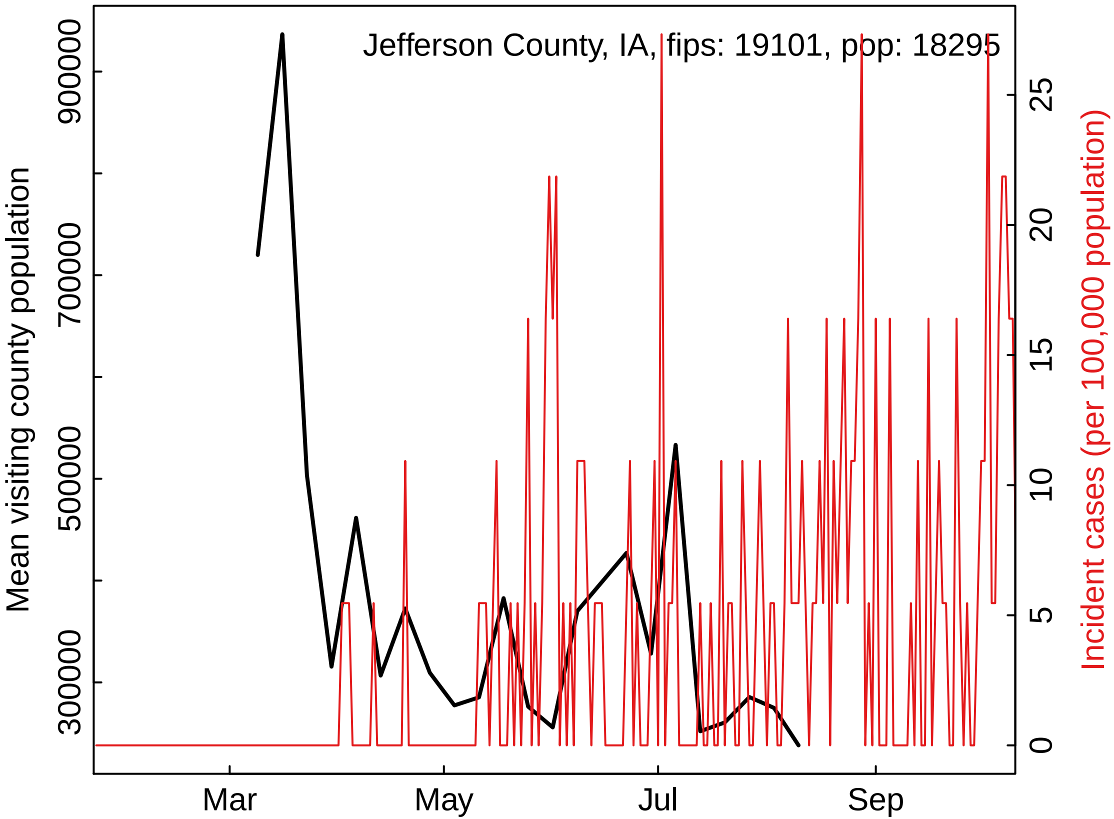
Jefferson County, IA, fips: 19101, pop: 18295
Mean visiting county population
300000
500000
700000
900000
Incident cases (per 100,000 population)
0
5
10
15
20
25
Mar
May
Jul
Sep

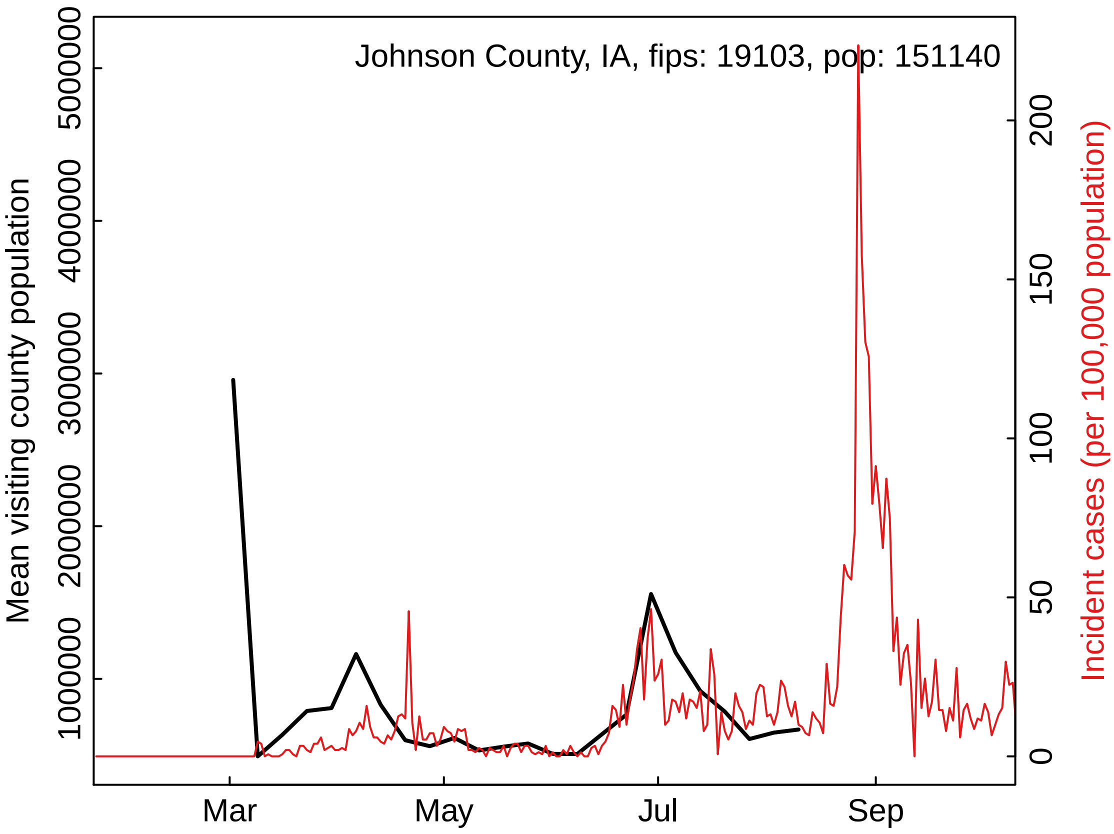

Johnson County, IA, fips: 19103, pop: 151140
Mean visiting county population
1000000
2000000
3000000
4000000
5000000
Incident cases (per 100,000 population)
0
50
100
150
200
Mar
May
Jul
Sep

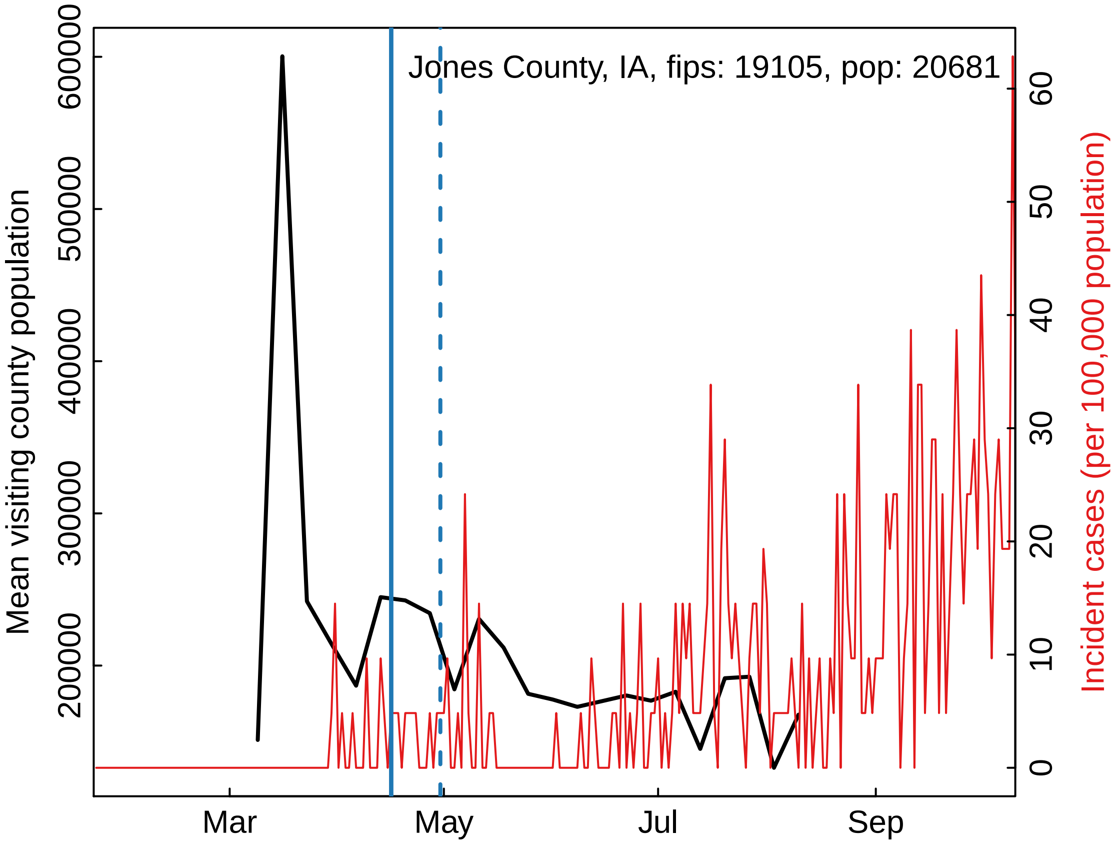

Jones County, IA, fips: 19105, pop: 20681
Mean visiting county population
200000
300000
400000
500000
600000
Incident cases (per 100,000 population)
0
10
20
30
40
50
60
Mar
May
Jul
Sep

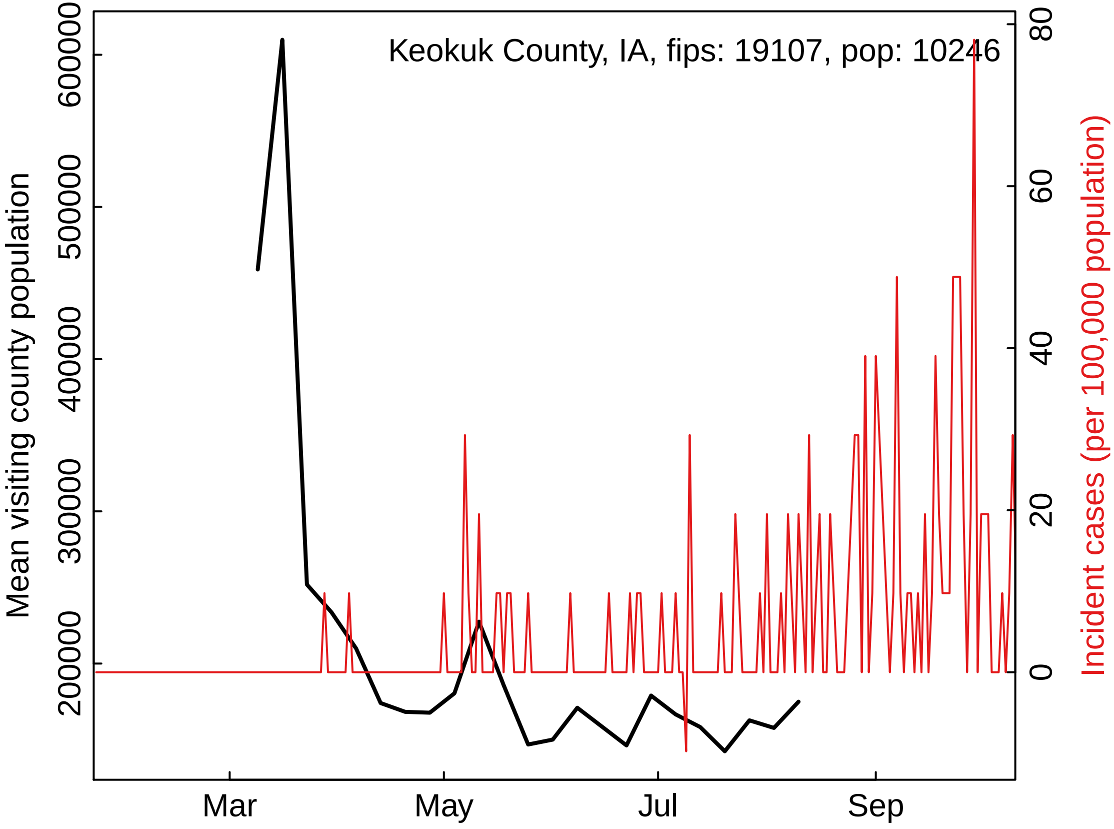

Keokuk County, IA, fips: 19107, pop: 10246
Mean visiting county population
200000
300000
400000
500000
600000
Incident cases (per 100,000 population)
0
20
40
60
80
Mar
May
Jul
Sep

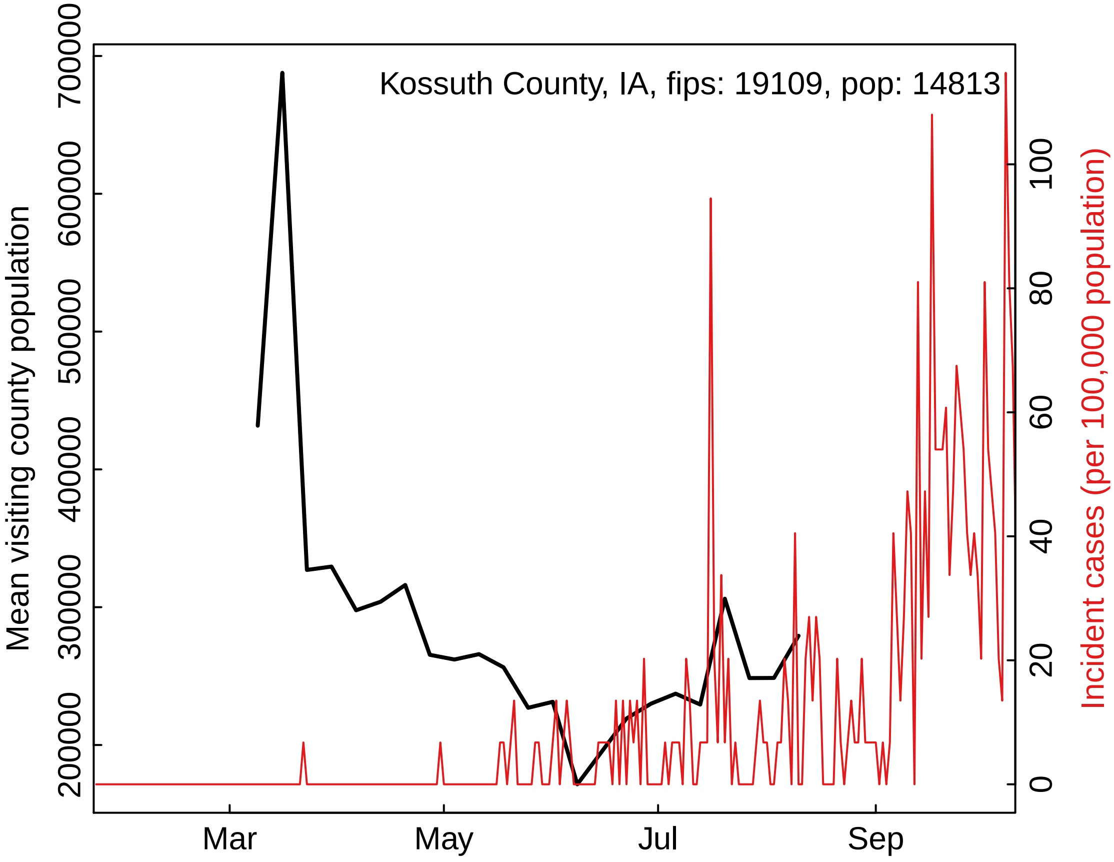

Kossuth County, IA, fips: 19109, pop: 14813
Mean visiting county population
200000
300000
400000
500000
600000
700000
Incident cases (per 100,000 population)
0
20
40
60
80
100
Mar
May
Jul
Sep

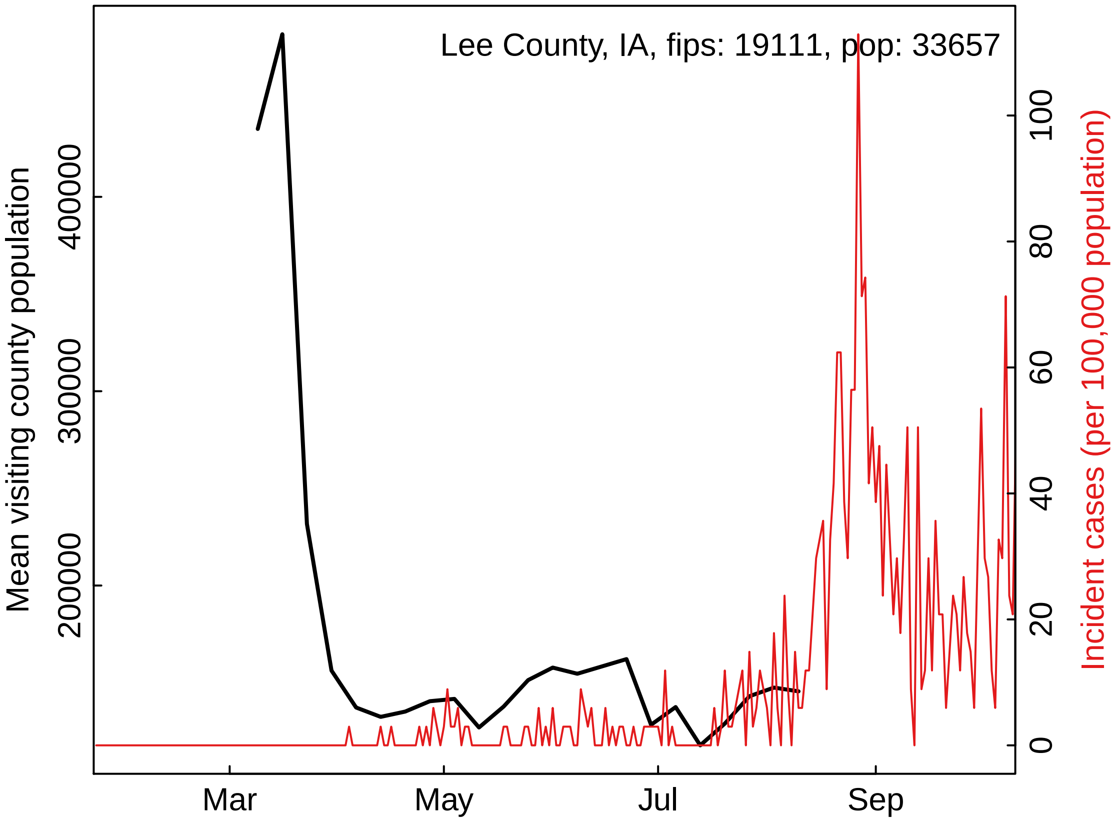

Lee County, IA, fips: 19111, pop: 33657
Mean visiting county population
200000
300000
400000
Incident cases (per 100,000 population)
0
20
40
60
80
100
Mar
May
Jul
Sep

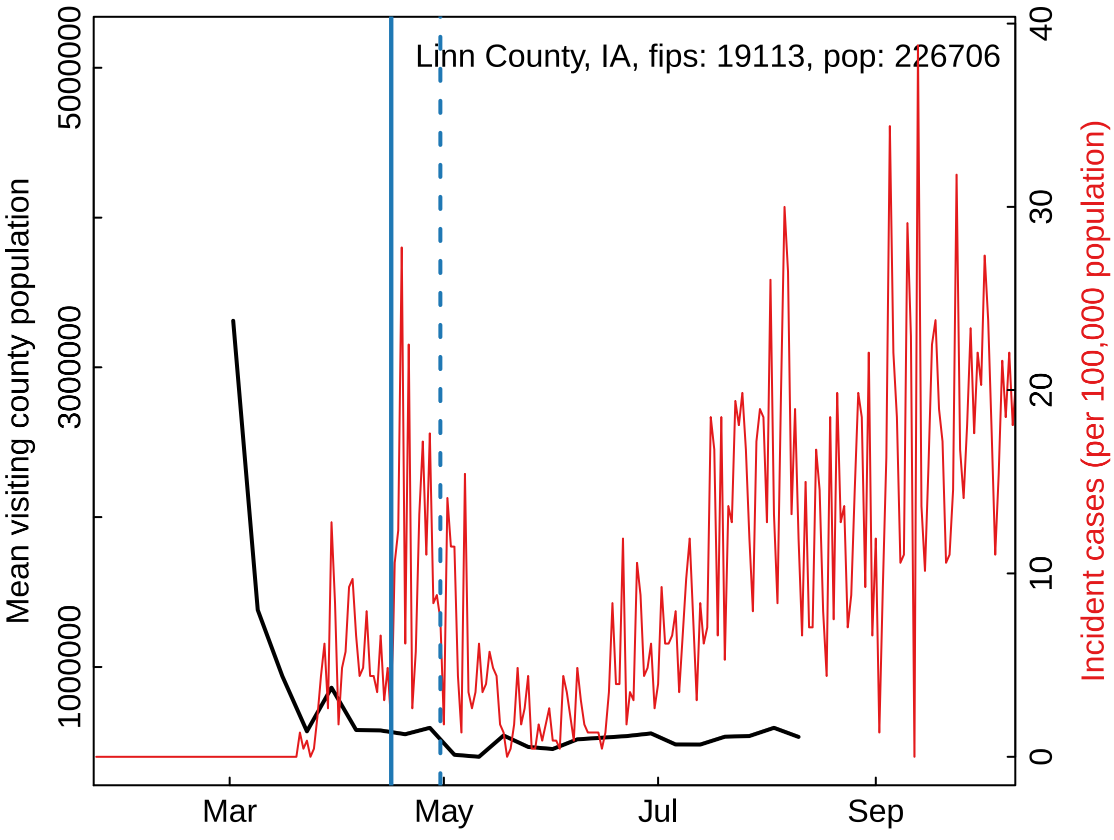

Linn County, IA, fips: 19113, pop: 226706
Mean visiting county population
1000000
3000000
5000000
Incident cases (per 100,000 population)
0
10
20
30
40
Mar
May
Jul
Sep

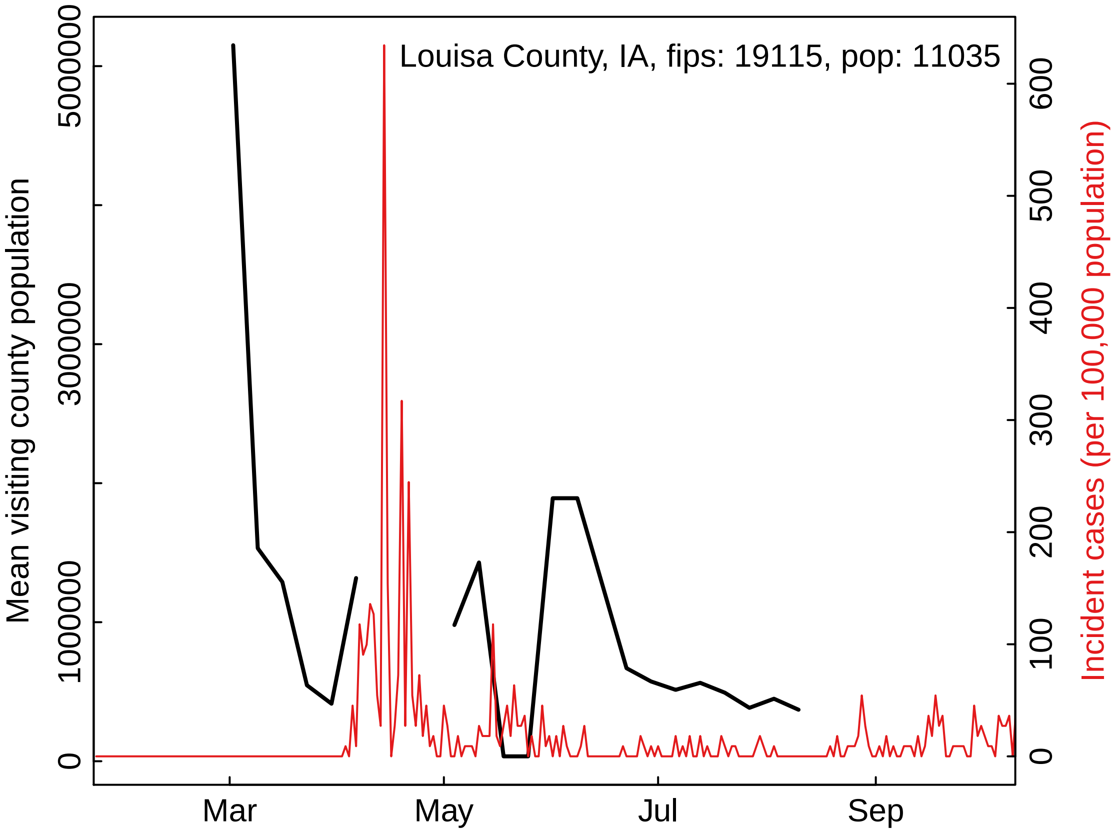

Louisa County, IA, fips: 19115, pop: 11035
Mean visiting county population
Incident cases (per 100,000 population)
0
1000000
3000000
5000000
0
100
200
300
400
500
600
Mar
May
Jul
Sep

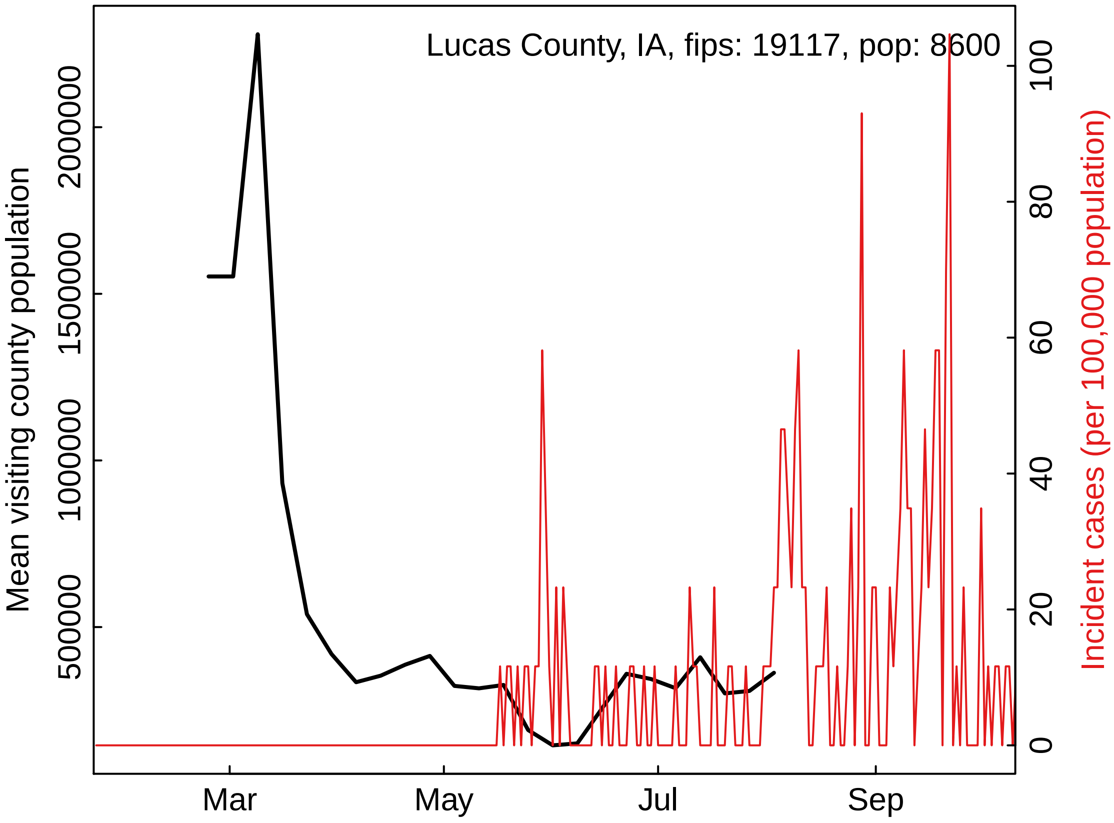

Lucas County, IA, fips: 19117, pop: 8600
Mean visiting county population
500000
1000000
1500000
2000000
Incident cases (per 100,000 population)
0
20
40
60
80
100
Mar
May
Jul
Sep

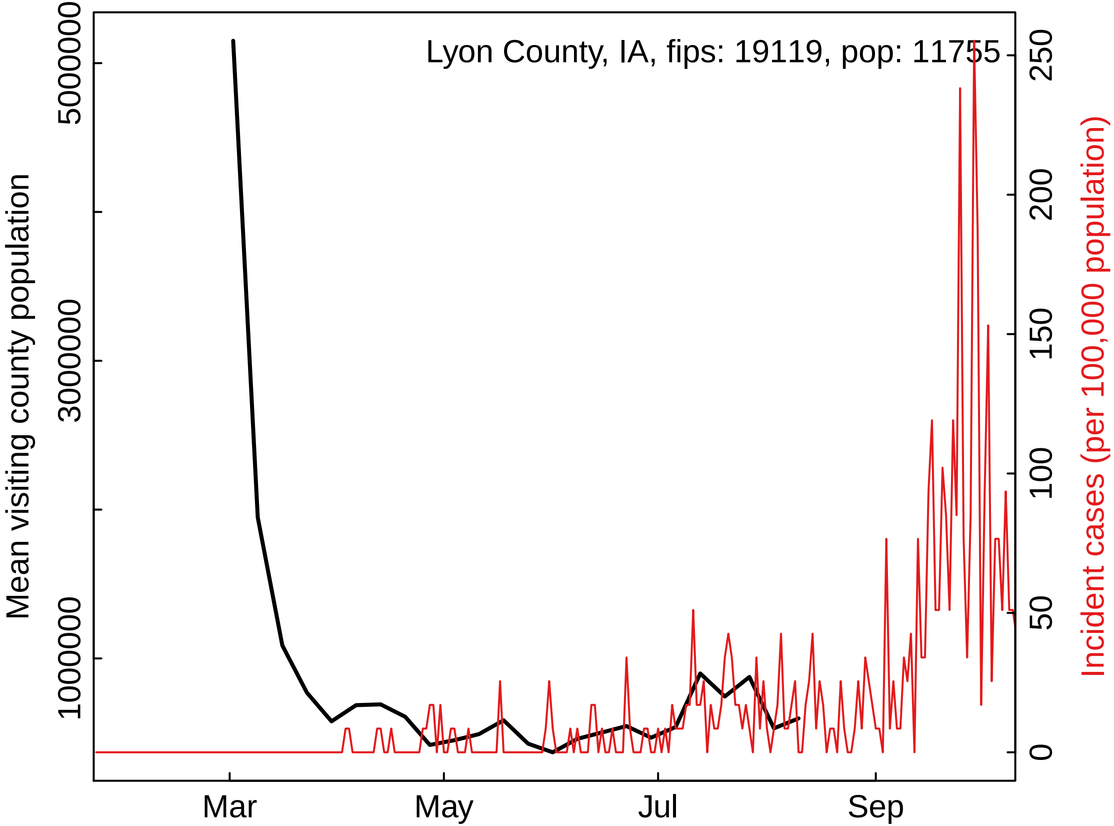

Lyon County, IA, fips: 19119, pop: 11755
Mean visiting county population
10000000
30000000
50000000
Incident cases (per 100,000 population)
0
50
100
150
200
250
Mar
May
Jul
Sep

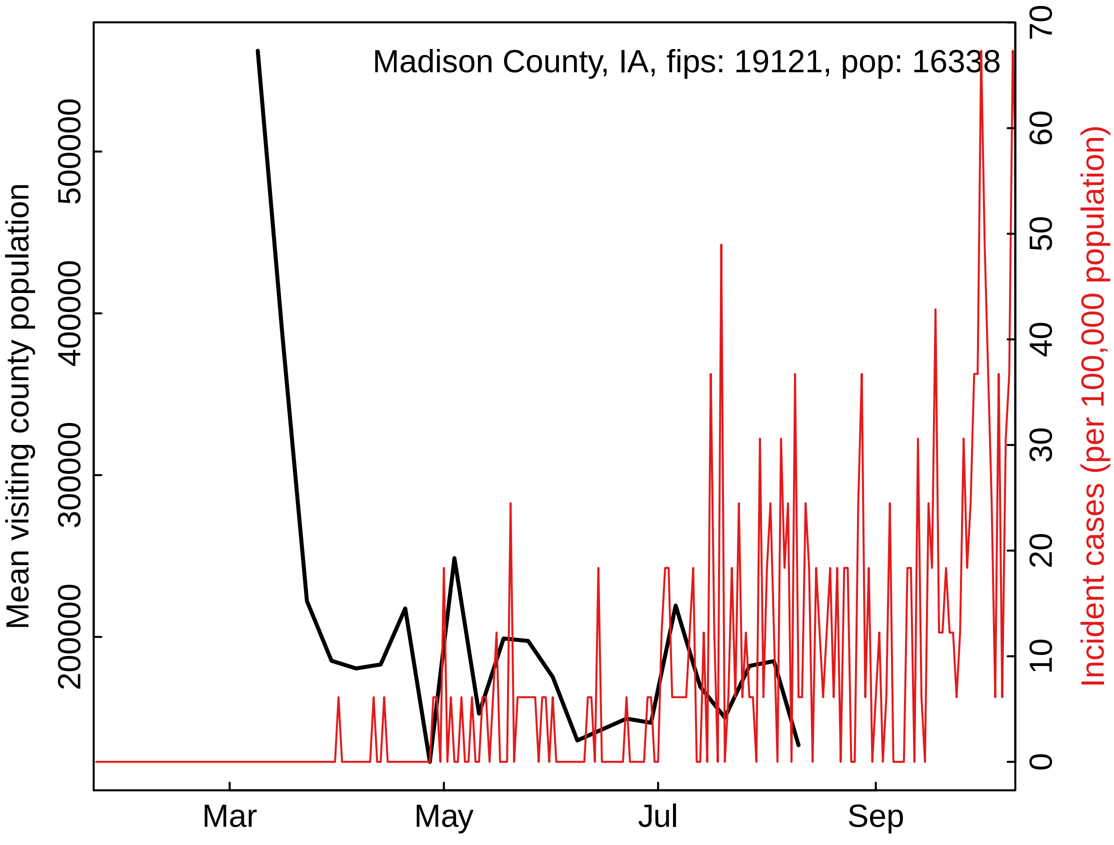

Madison County, IA, fips: 19121, pop: 16338
Mean visiting county population
200000
300000
400000
500000
Incident cases (per 100,000 population)
0
10
20
30
40
50
60
70
Mar
May
Jul
Sep

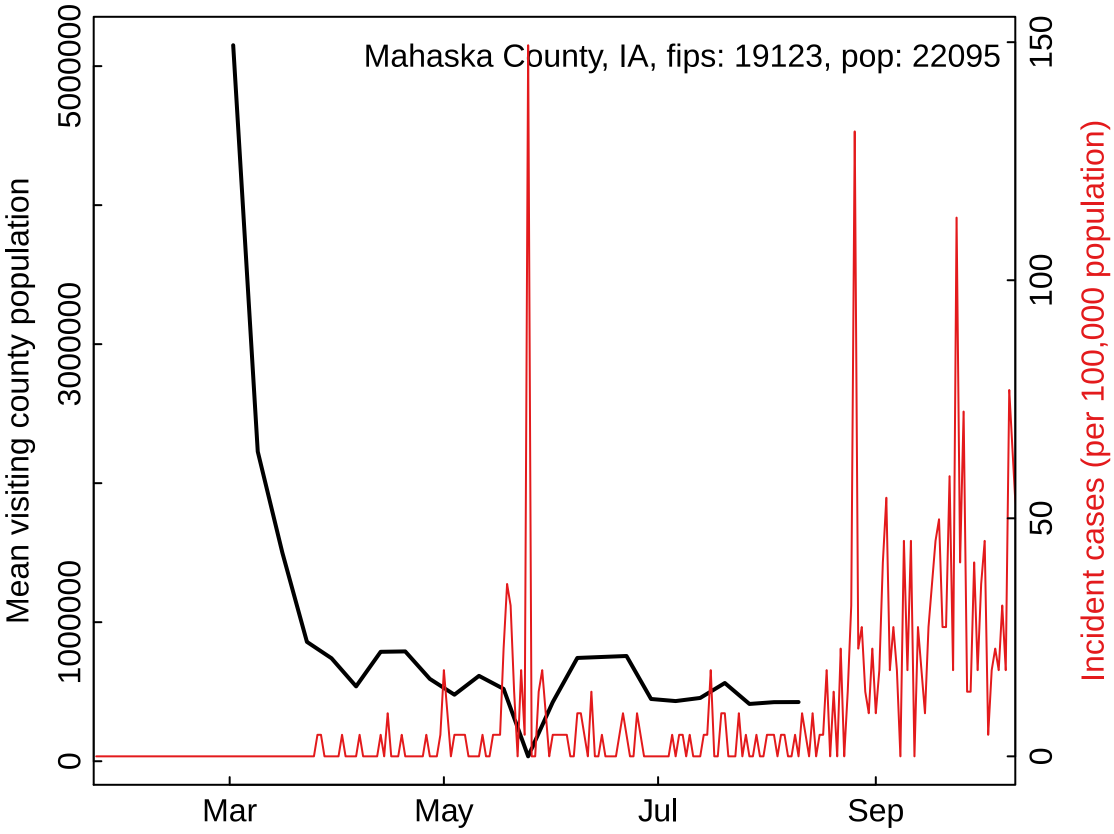

Mahaska County, IA, fips: 19123, pop: 22095
Mean visiting county population
Incident cases (per 100,000 population)
0
1000000
3000000
5000000
0
50
100
150
Mar
May
Jul
Sep

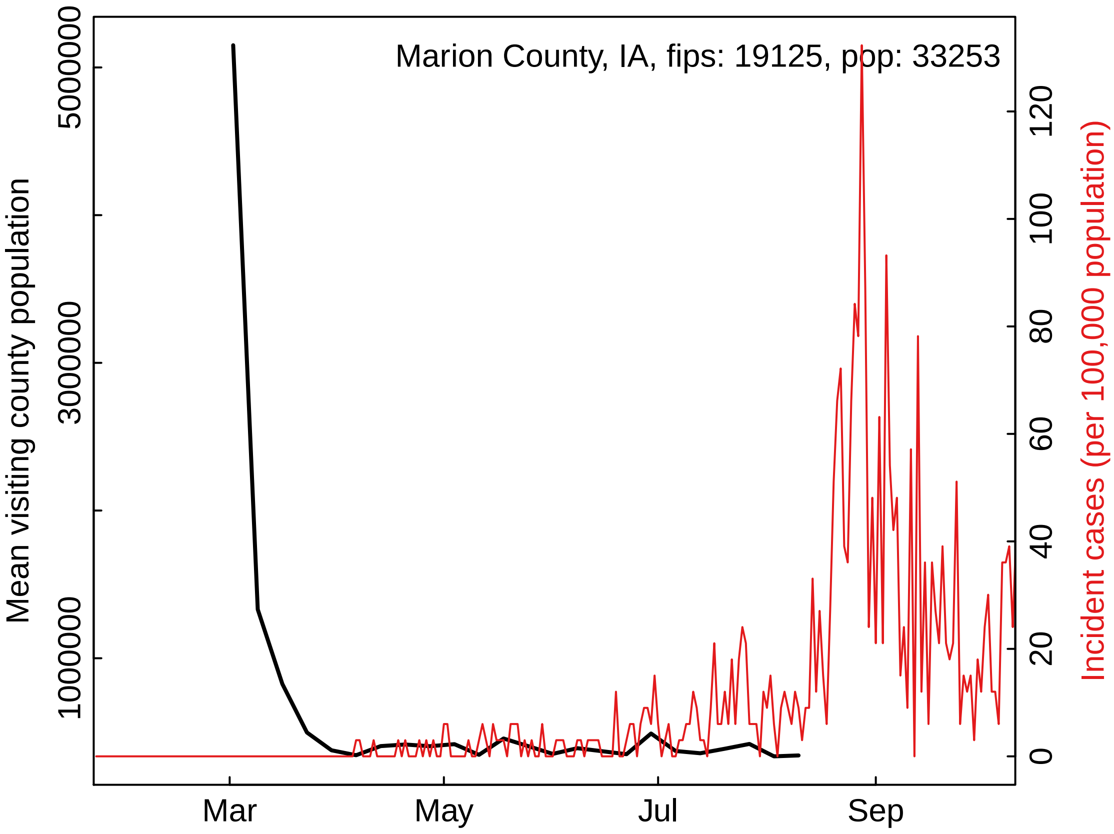

Marion County, IA, fips: 19125, pop: 33253
Mean visiting county population
1000000
3000000
5000000
Incident cases (per 100,000 population)
0
20
40
60
80
100
120
Mar
May
Jul
Sep

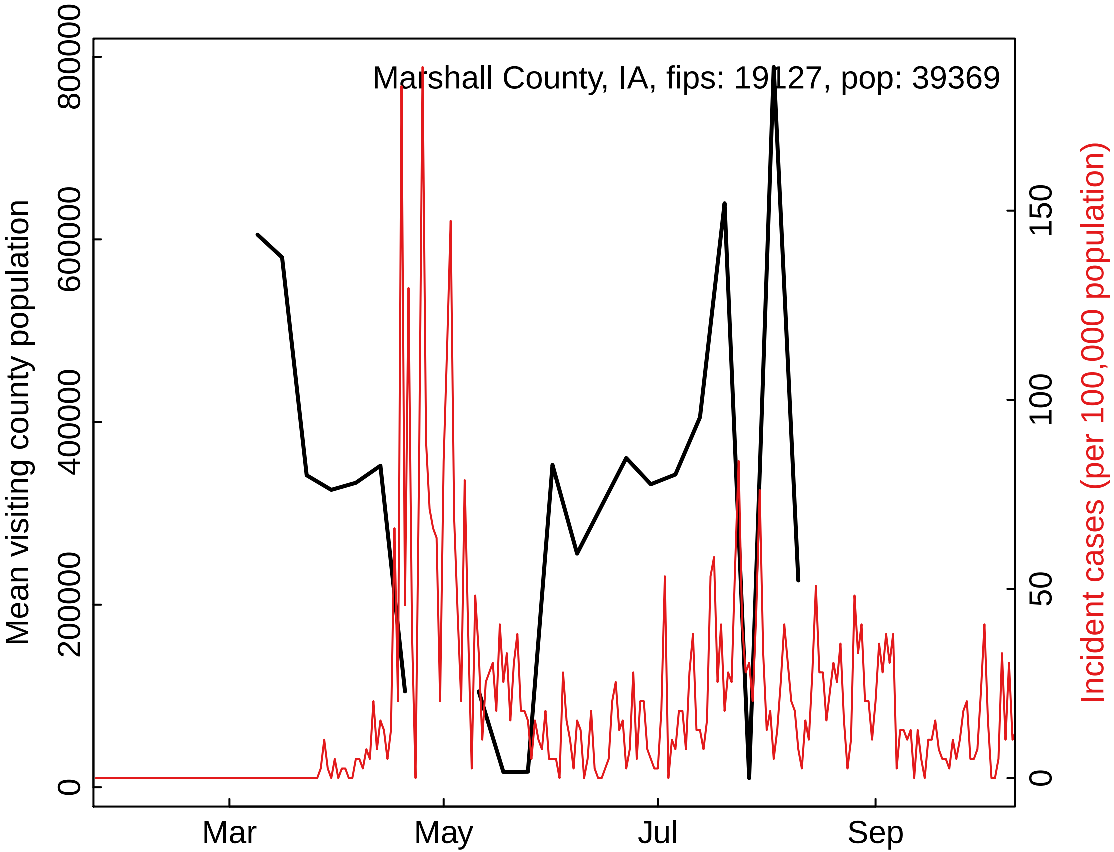

Marshall County, IA, fips: 19127, pop: 39369
Mean visiting county population
Incident cases (per 100,000 population)
0
200000
400000
600000
800000
0
50
100
150
Mar
May
Jul
Sep

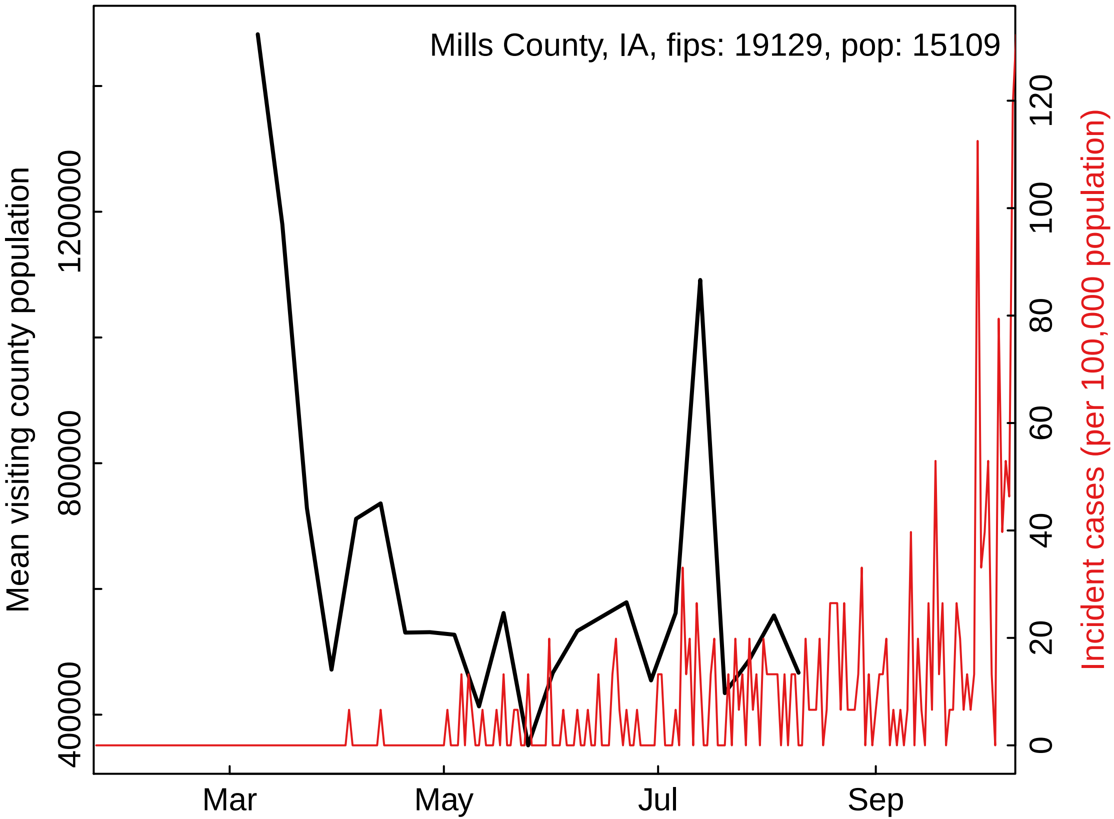
Mills County, IA, fips: 19129, pop: 15109
Mean visiting county population
400000
800000
1200000
Incident cases (per 100,000 population)
0
20
40
60
80
100
120
Mar
May
Jul
Sep

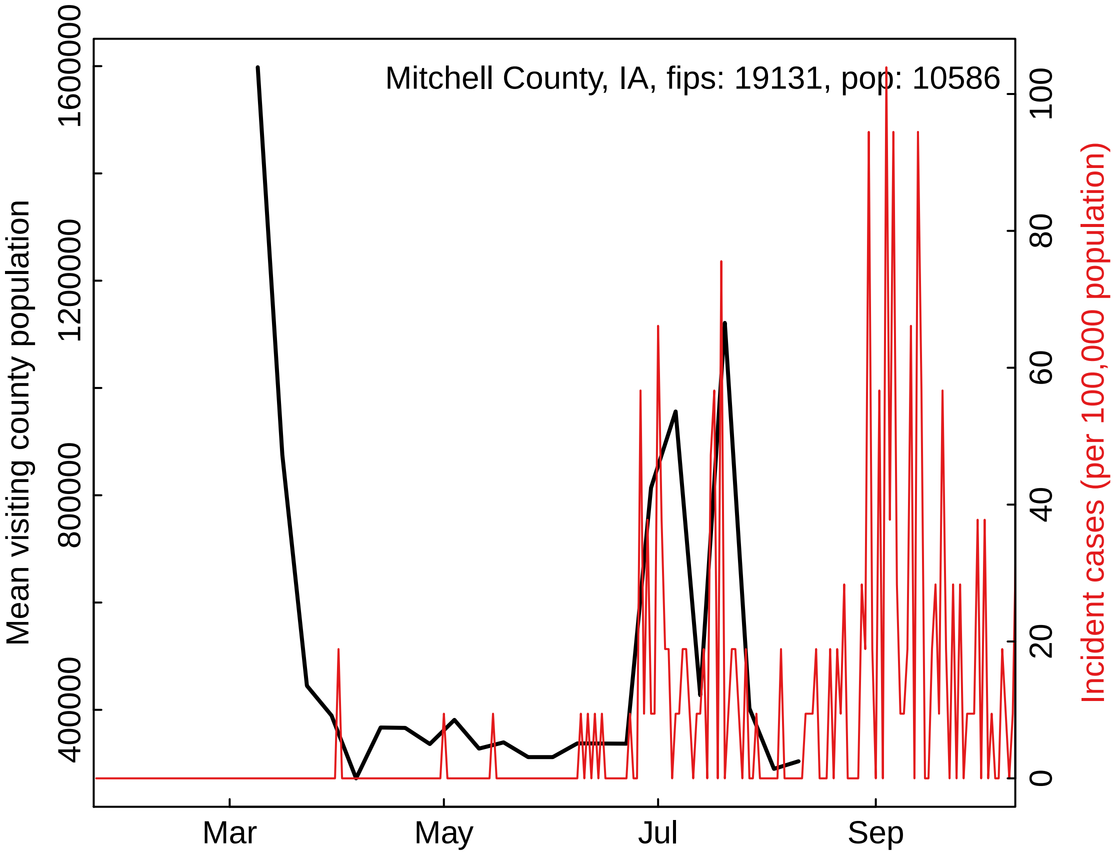

Mitchell County, IA, fips: 19131, pop: 10586
Mean visiting county population
400000
800000
1200000
1600000
Mar
May
Jul
Sep
0
20
40
60
80
100
Incident cases (per 100,000 population)

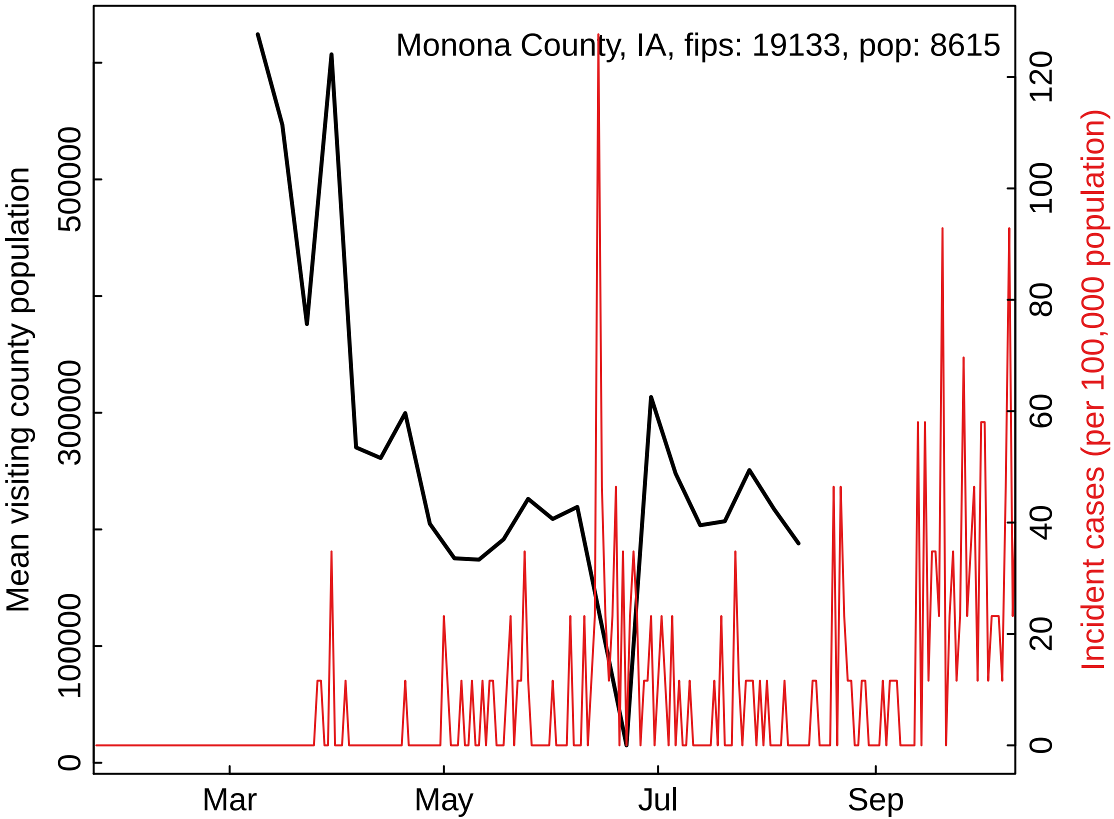
Monona County, IA, fips: 19133, pop: 8615
Mean visiting county population
0
100000
300000
500000
Incident cases (per 100,000 population)
0
20
40
60
80
100
120
Mar
May
Jul
Sep

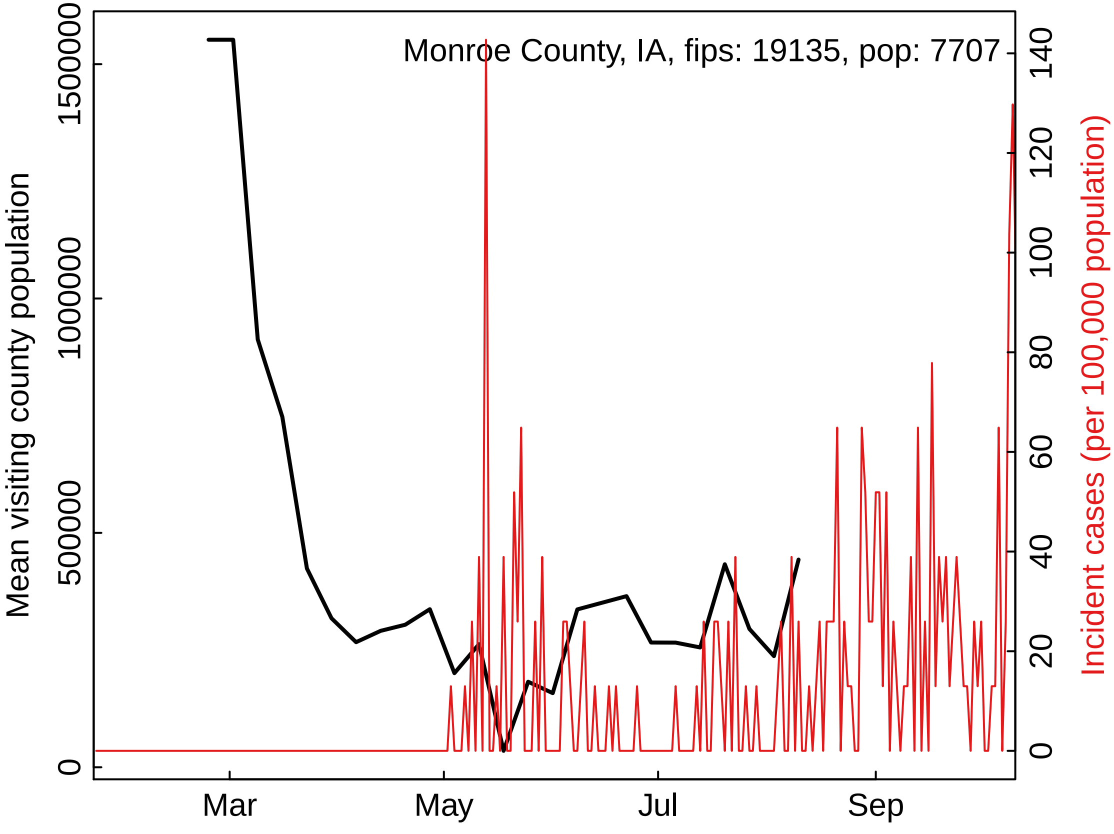

Monroe County, IA, fips: 19135, pop: 7707
Mean visiting county population
0
500000
1000000
1500000
Incident cases (per 100,000 population)
0
20
40
60
80
100
120
140
Mar
May
Jul
Sep

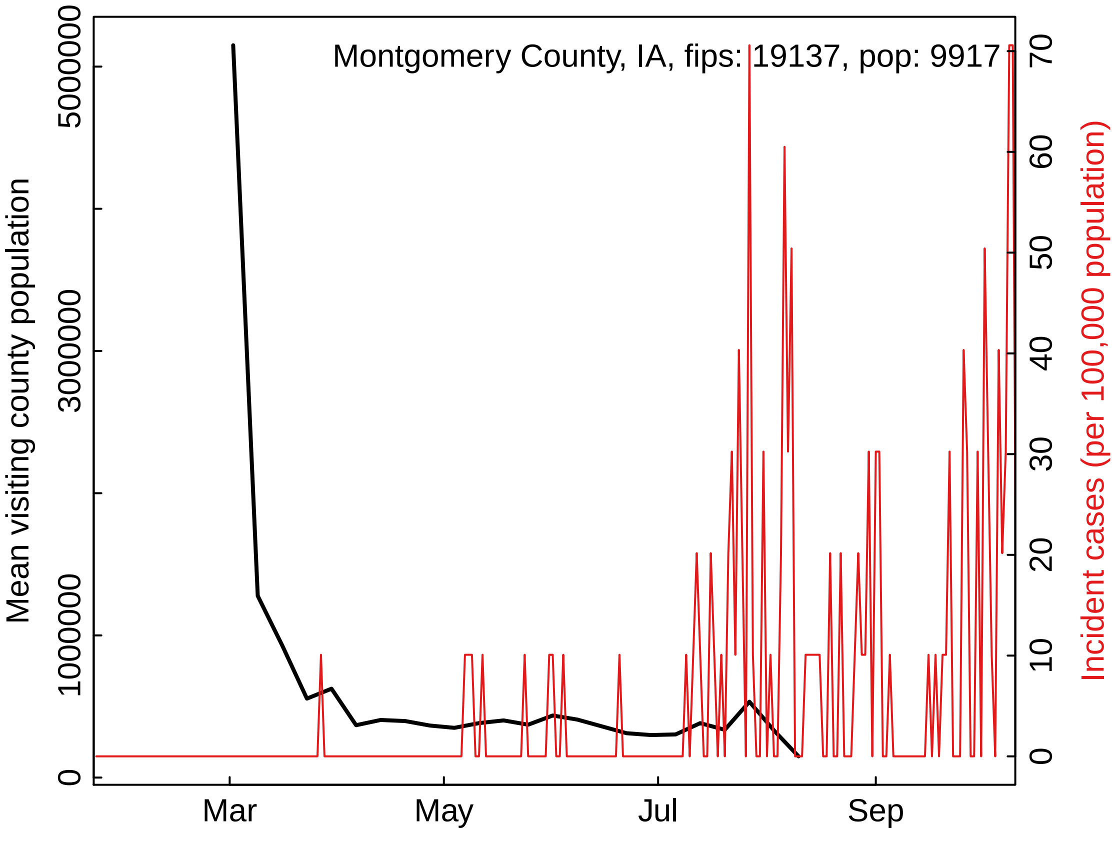

Montgomery County, IA, fips: 19137, pop: 9917
Mean visiting county population
Incident cases (per 100,000 population)
0
1000000
3000000
5000000
0
10
20
30
40
50
60
70
Mar
May
Jul
Sep

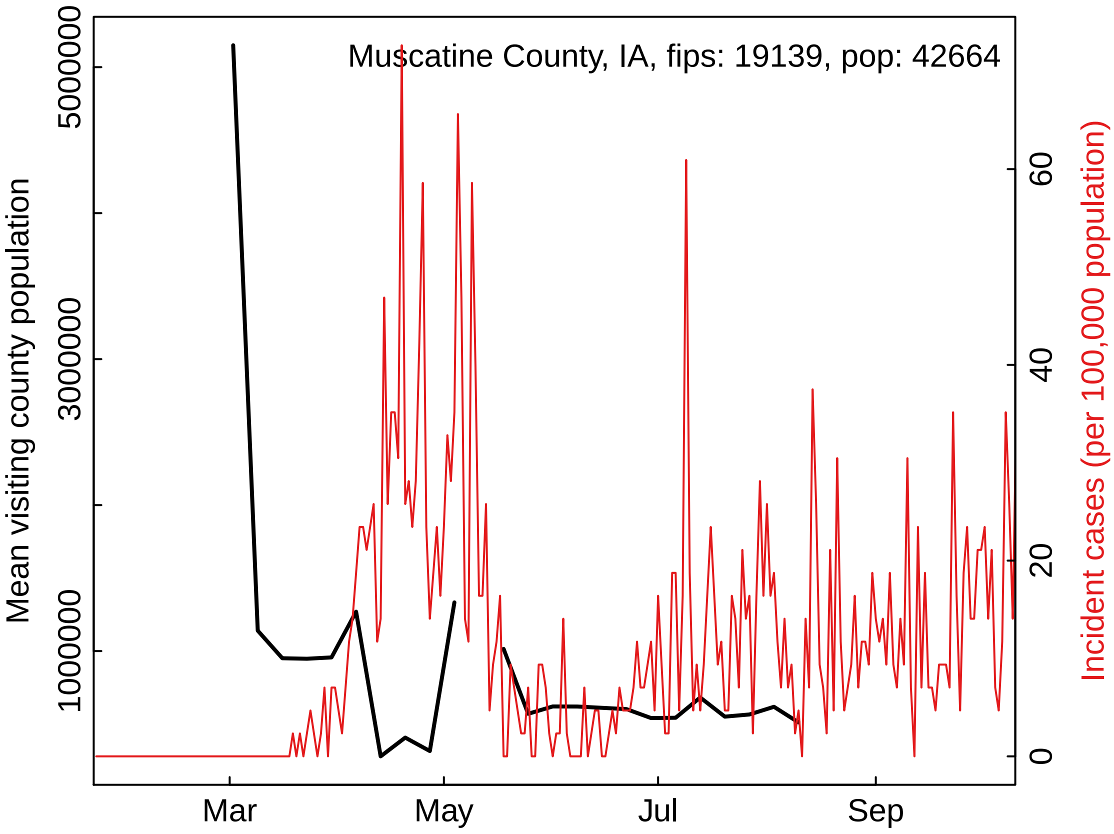
Muscatine County, IA, fips: 19139, pop: 42664
Mean visiting county population
1000000
3000000
5000000
Incident cases (per 100,000 population)
0
20
40
60
Mar
May
Jul
Sep

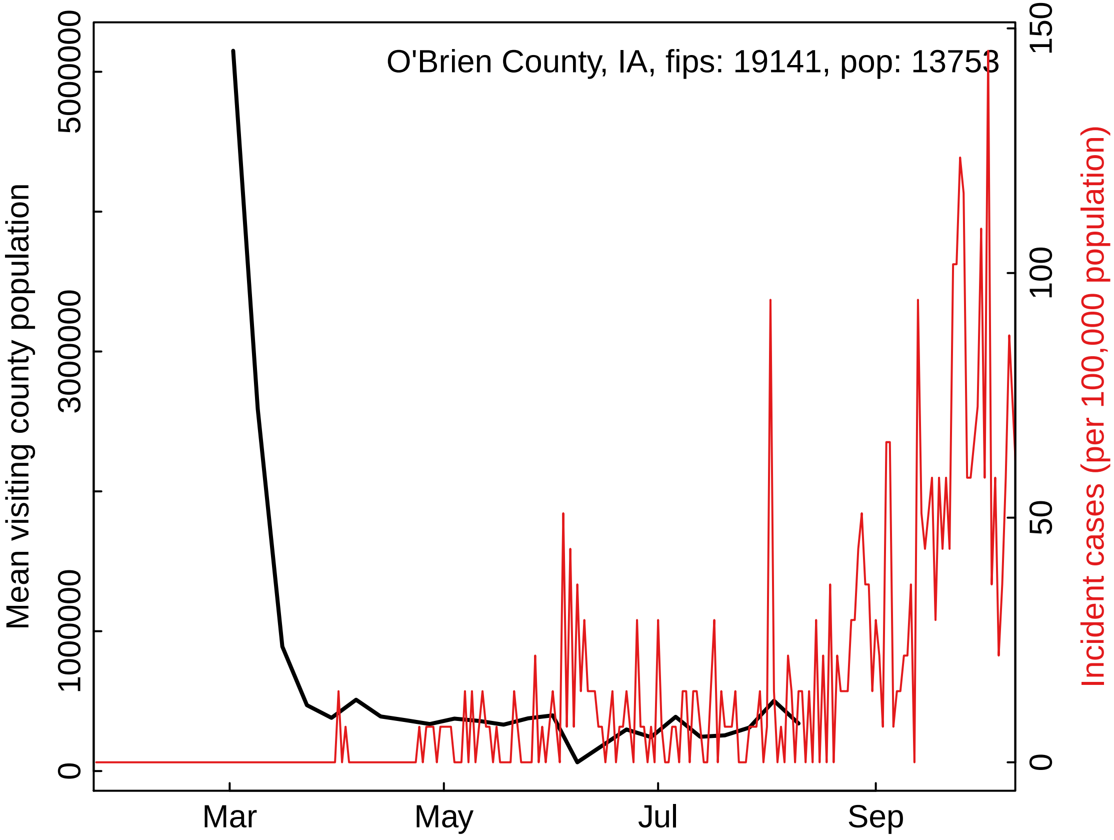

O'Brien County, IA, fips: 19141, pop: 13753
Mean visiting county population
0
1000000
3000000
5000000
Incident cases (per 100,000 population)
0
50
100
150
Mar
May
Jul
Sep

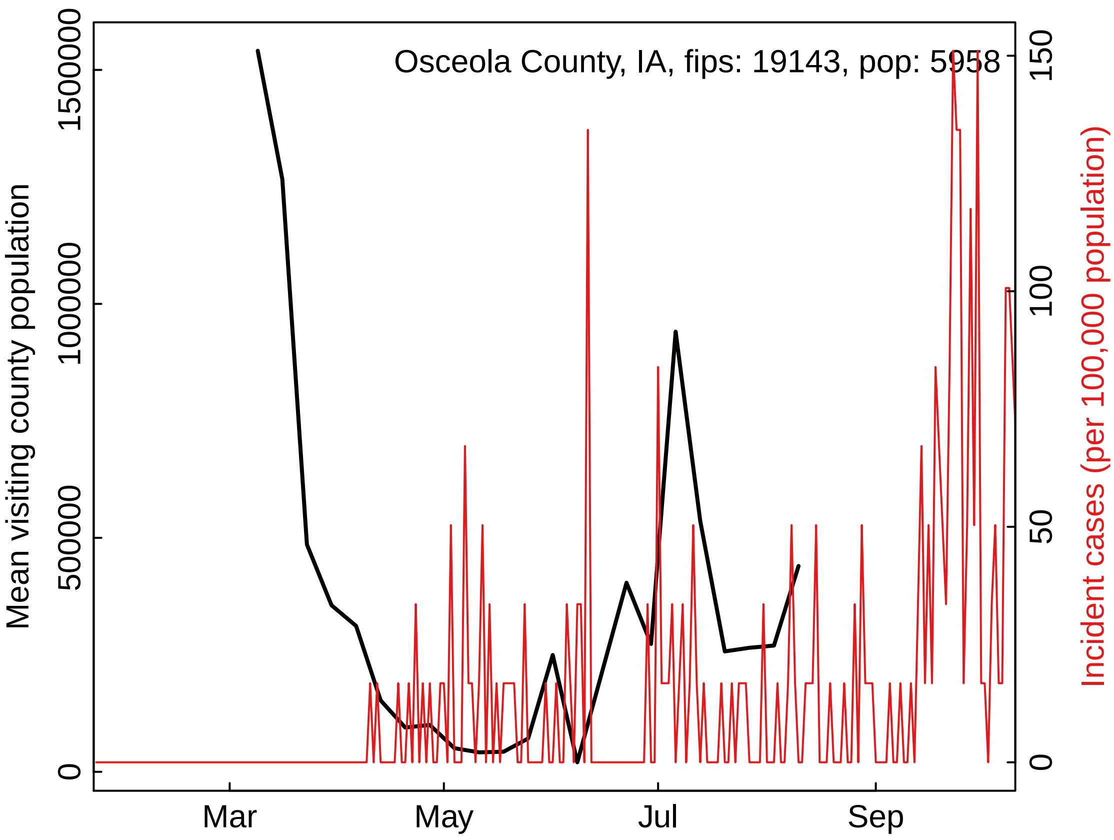
Osceola County, IA, fips: 19143, pop: 5958
Mean visiting county population
Incident cases (per 100,000 population)
0
500000
1000000
1500000
0
50
100
150
Mar
May
Jul
Sep

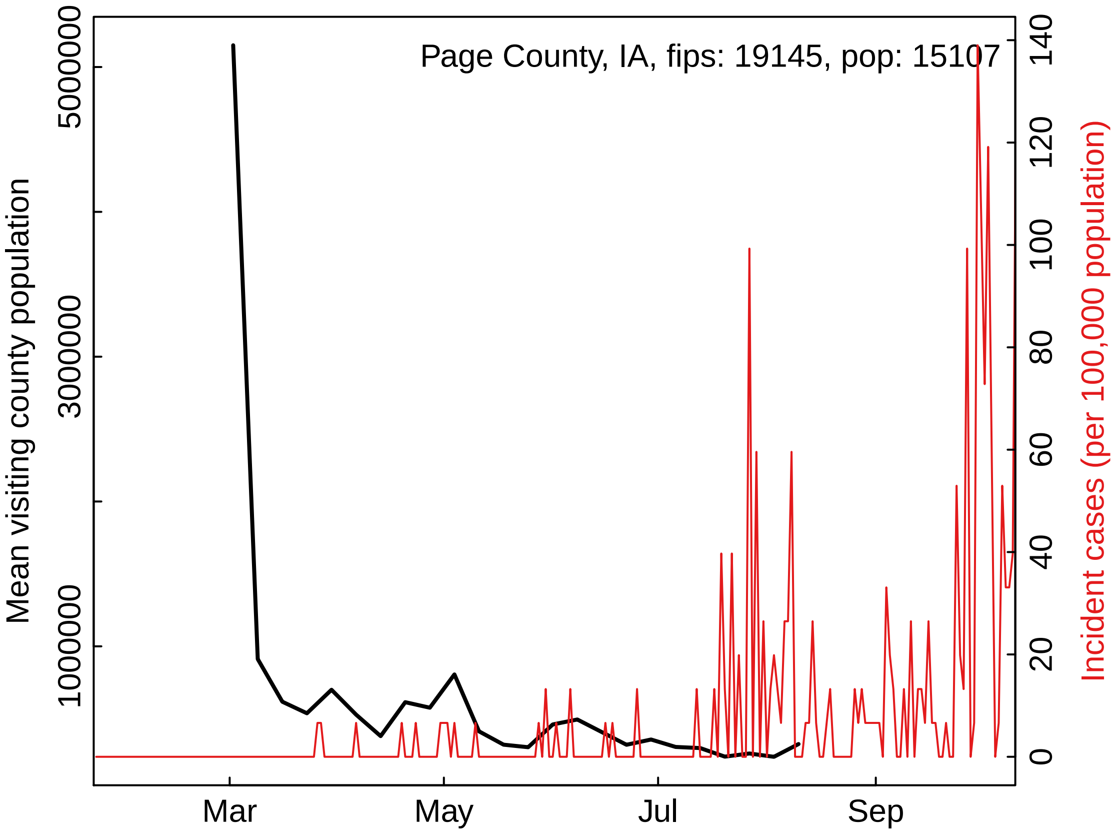

Page County, IA, fips: 19145, pop: 15107
Mean visiting county population
1000000
3000000
5000000
Mar
May
Jul
Sep
0
20
40
60
80
100
120
140
Incident cases (per 100,000 population)

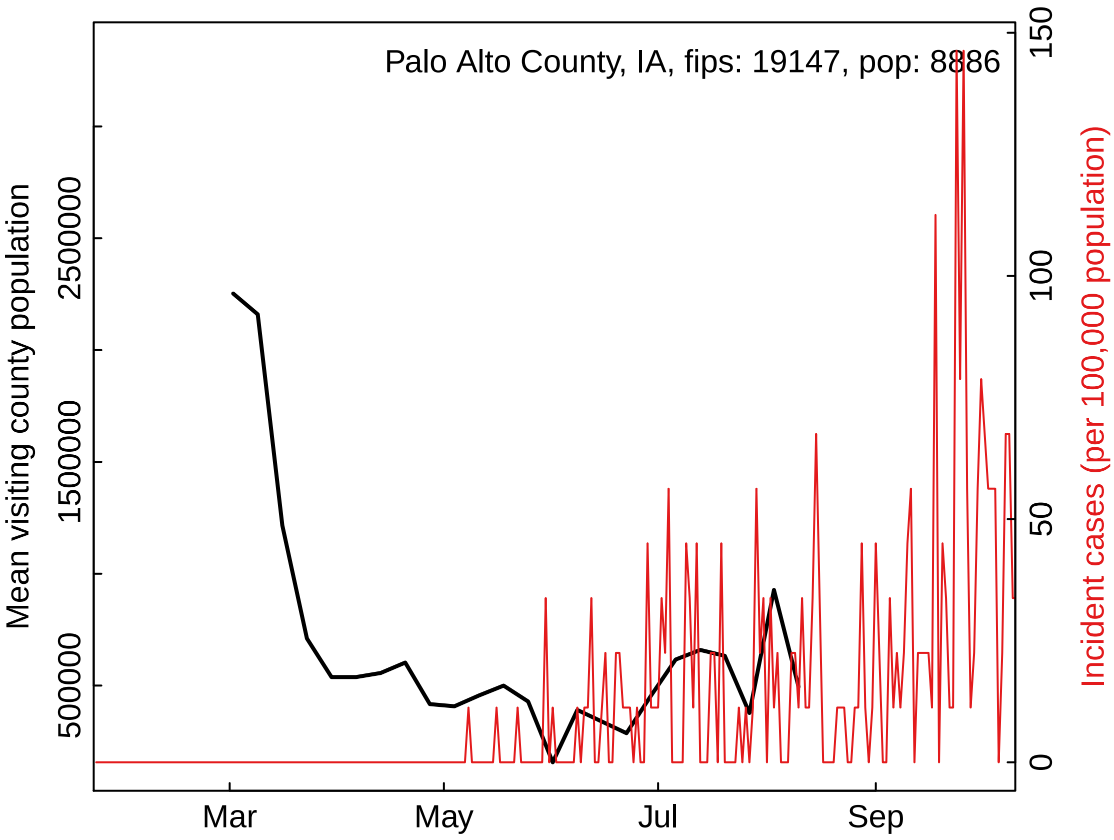

Palo Alto County, IA, fips: 19147, pop: 8886
Mean visiting county population
500000
1500000
2500000
Mar
May
Jul
Sep
0
50
100
150
Incident cases (per 100,000 population)

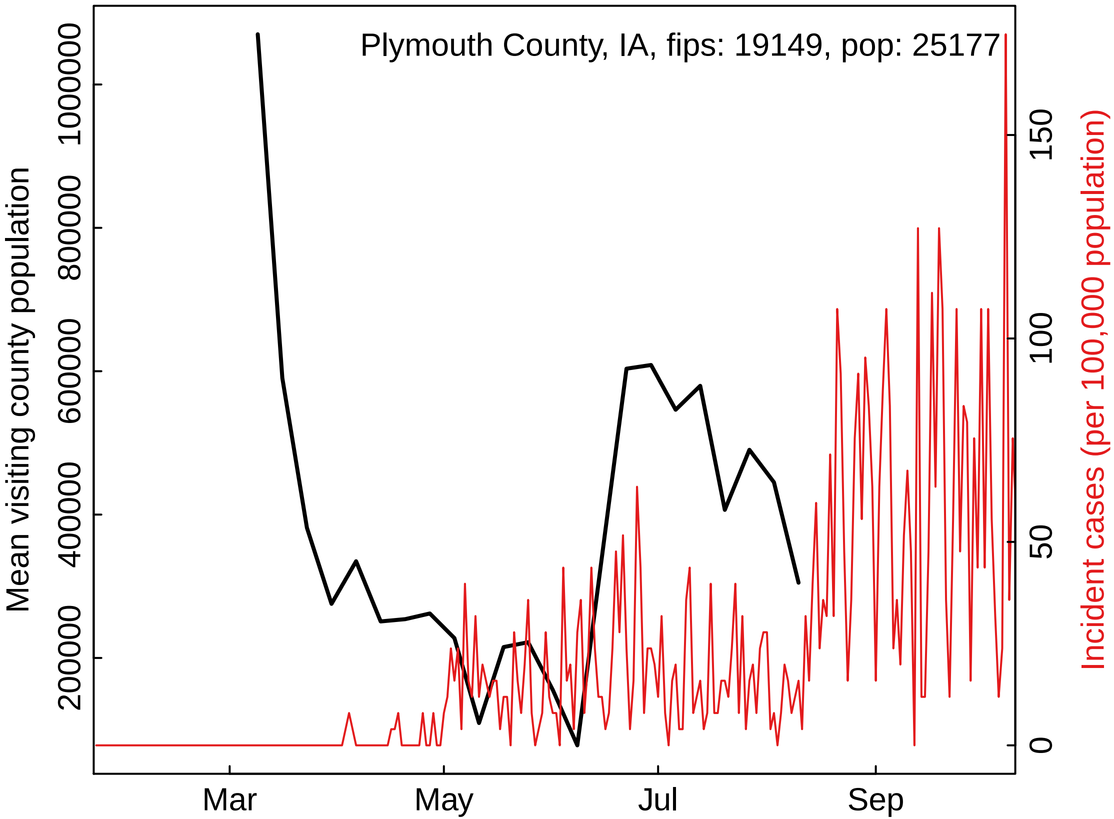

Plymouth County, IA, fips: 19149, pop: 25177
Mean visiting county population
Incident cases (per 100,000 population)
200000
400000
600000
800000
1000000
0
50
100
150
Mar
May
Jul
Sep

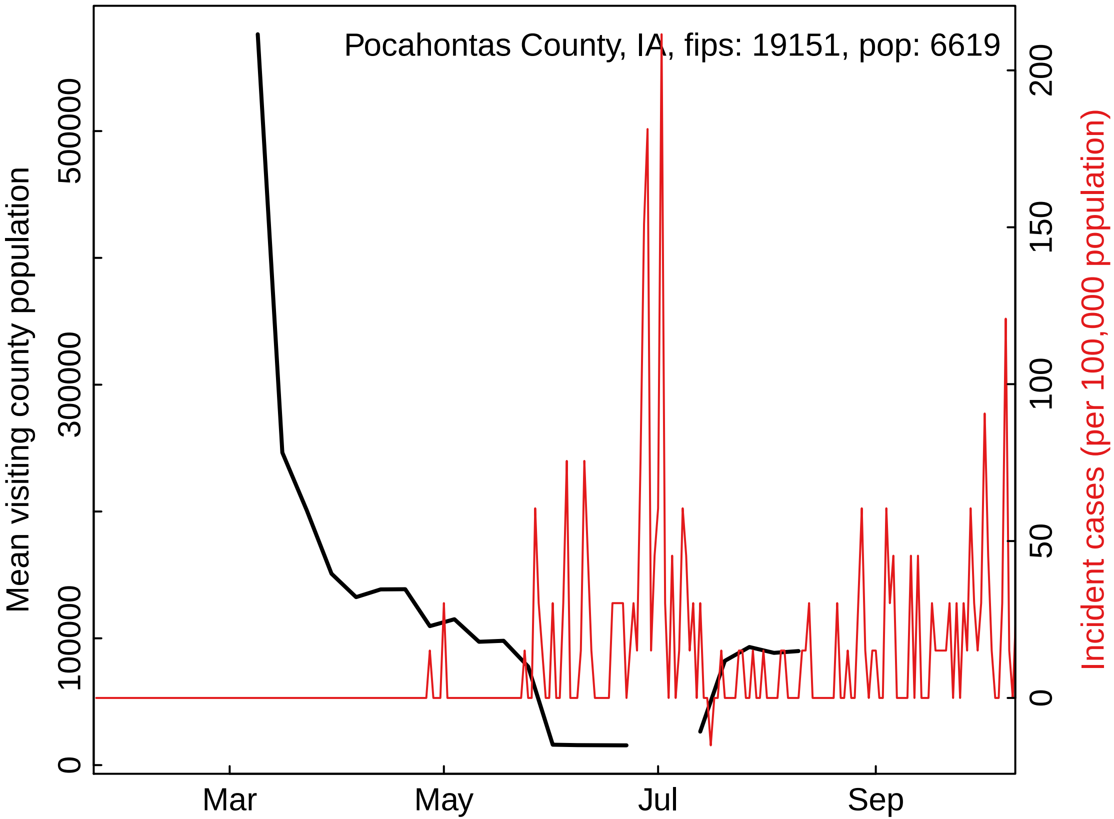

Pocahontas County, IA, fips: 19151, pop: 6619
Mean visiting county population
0
100000
300000
500000
Incident cases (per 100,000 population)
0
50
100
150
200
Mar
May
Jul
Sep

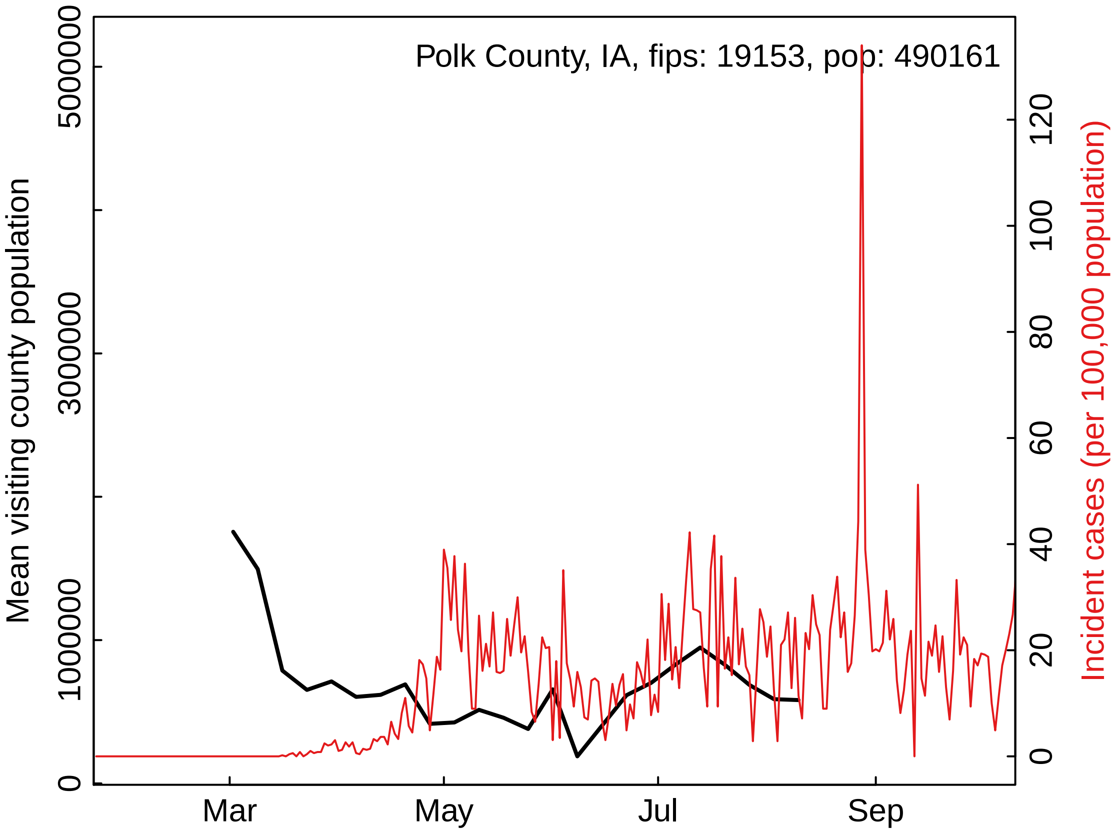

Polk County, IA, fips: 19153, pop: 490161
Mean visiting county population
Incident cases (per 100,000 population)
0
1000000
3000000
5000000
0
20
40
60
80
100
120
Mar
May
Jul
Sep

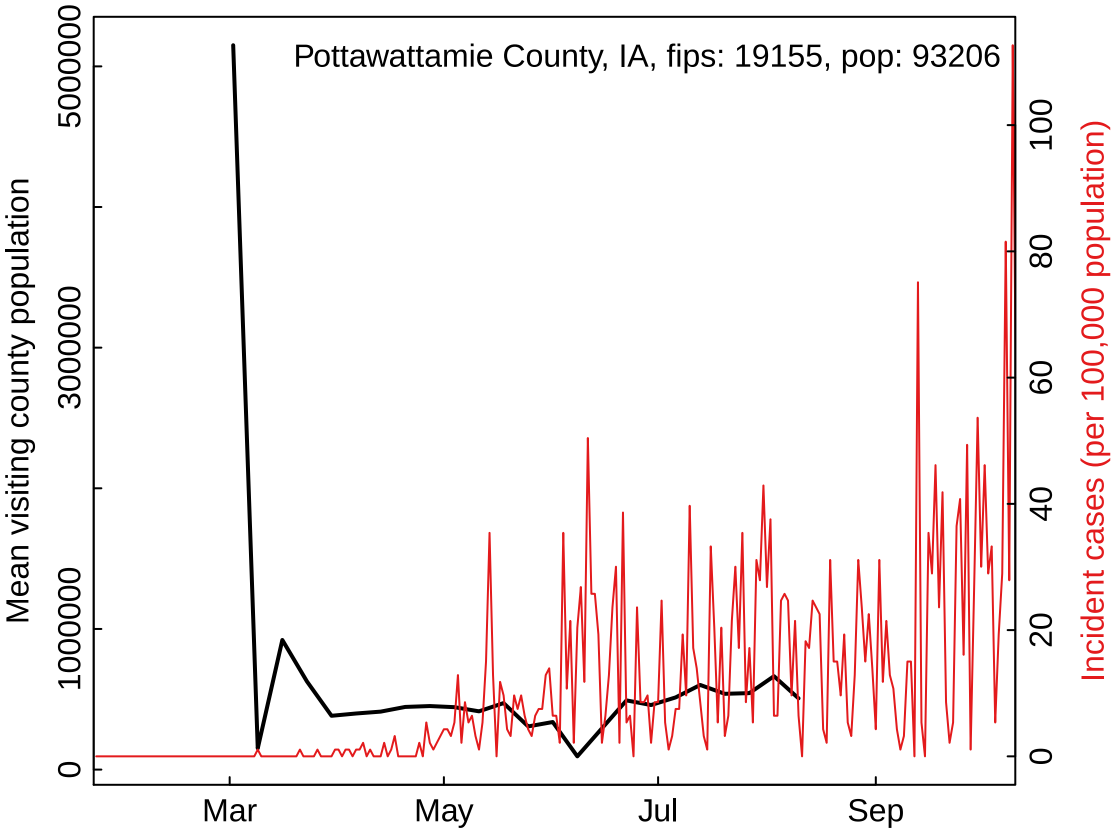
Pottawattamie County, IA, fips: 19155, pop: 93206
Mean visiting county population
0
1000000
3000000
5000000
Mar
May
Jul
Sep
0
20
40
60
80
100
Incident cases (per 100,000 population)

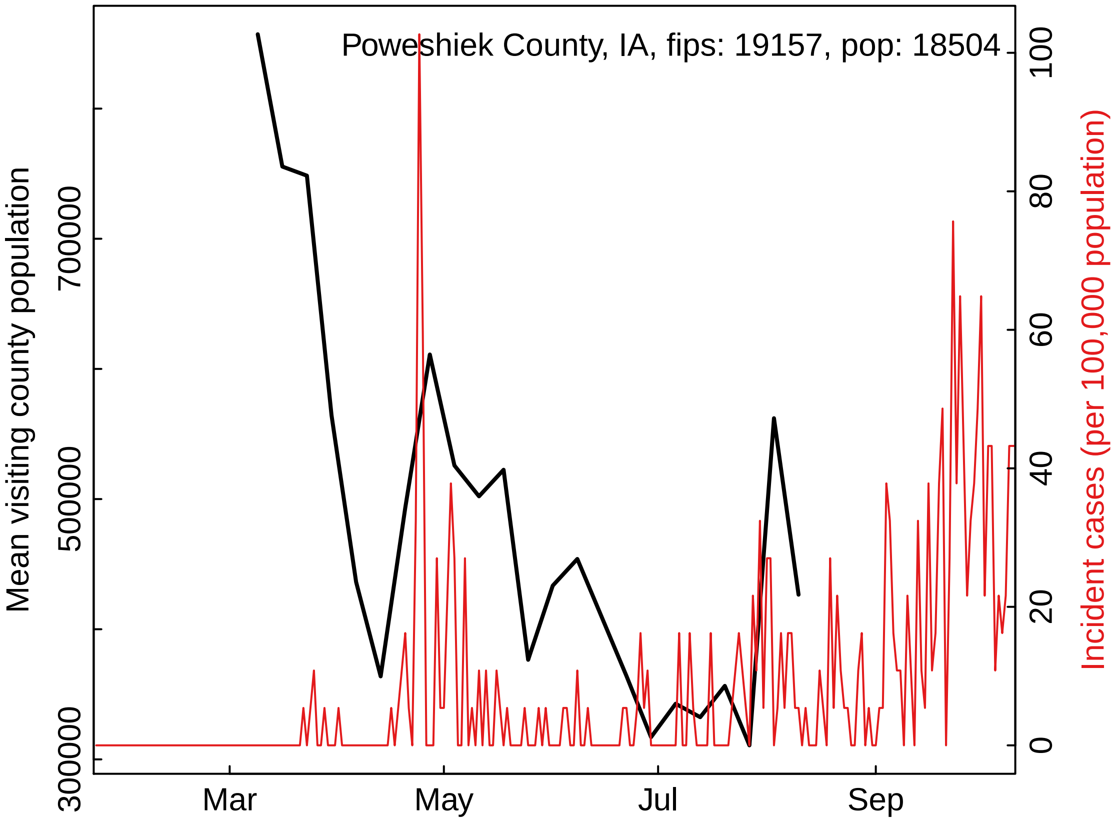

Poweshiek County, IA, fips: 19157, pop: 18504
Mean visiting county population
300000
500000
700000
Mar
May
Jul
Sep
0
20
40
60
80
100
Incident cases (per 100,000 population)

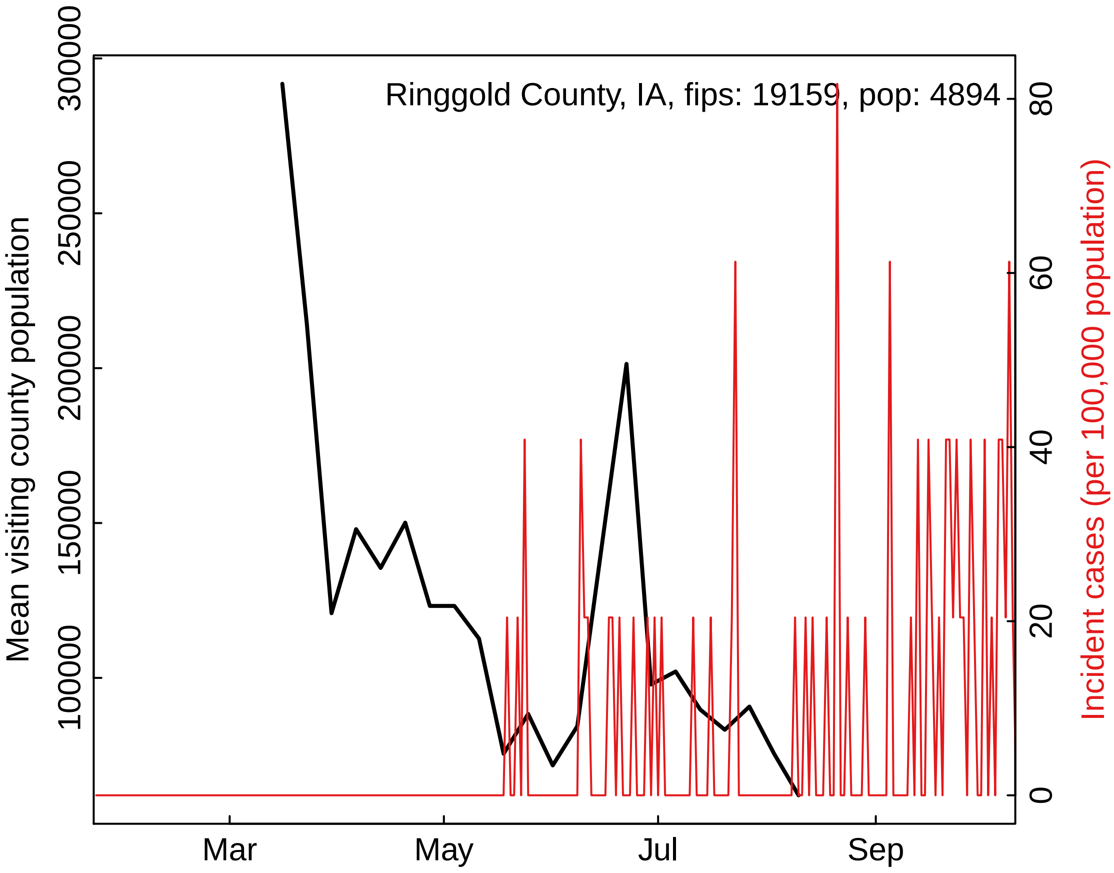

Ringgold County, IA, fips: 19159, pop: 4894
Mean visiting county population
100000
150000
200000
250000
300000
Incident cases (per 100,000 population)
0
20
40
60
80
Mar
May
Jul
Sep

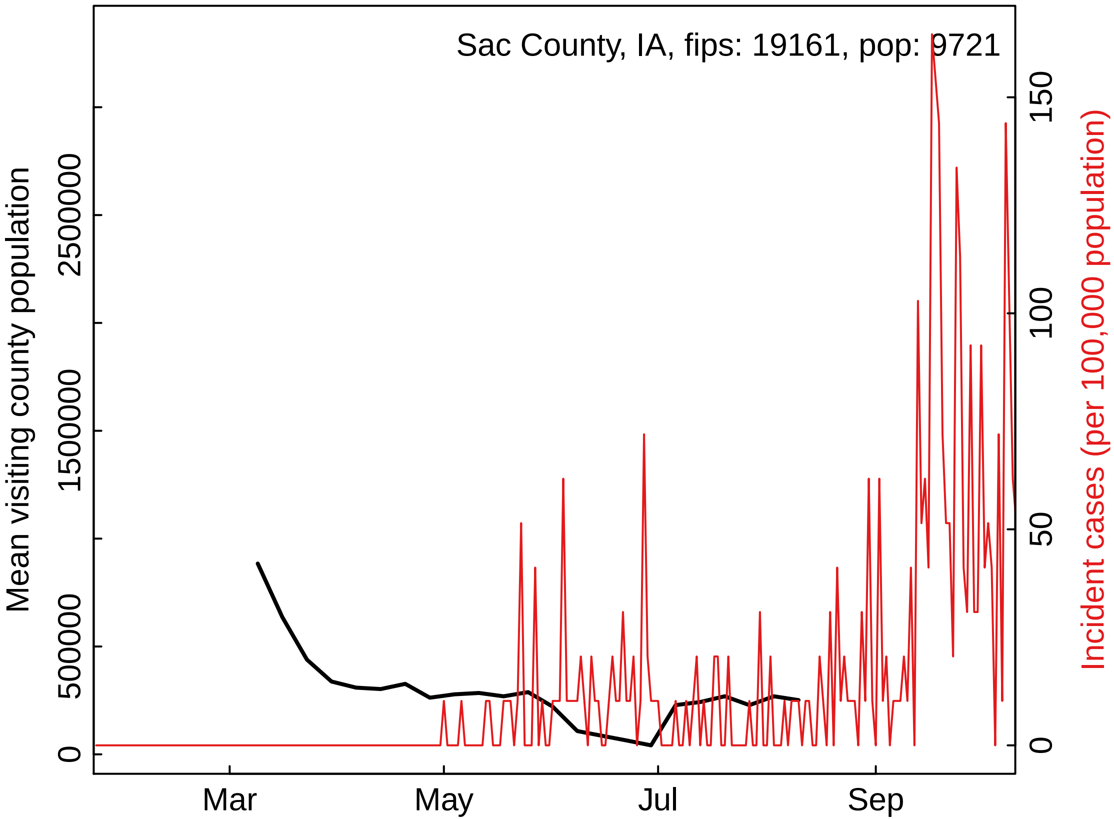

Sac County, IA, fips: 19161, pop: 9721
Mean visiting county population
0
500000
1500000
2500000
Incident cases (per 100,000 population)
0
50
100
150
Mar
May
Jul
Sep

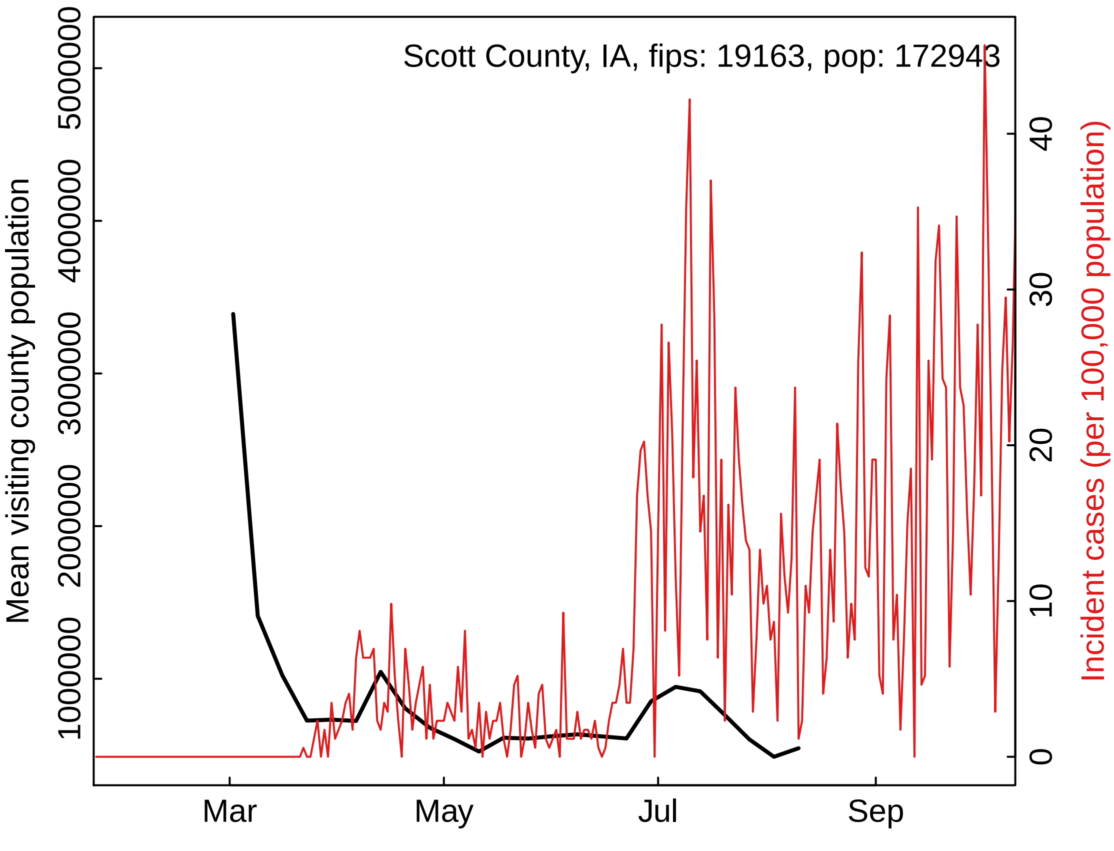

Scott County, IA, fips: 19163, pop: 172943
Mean visiting county population
1000000
2000000
3000000
4000000
5000000
Incident cases (per 100,000 population)
0
10
20
30
40
Mar
May
Jul
Sep

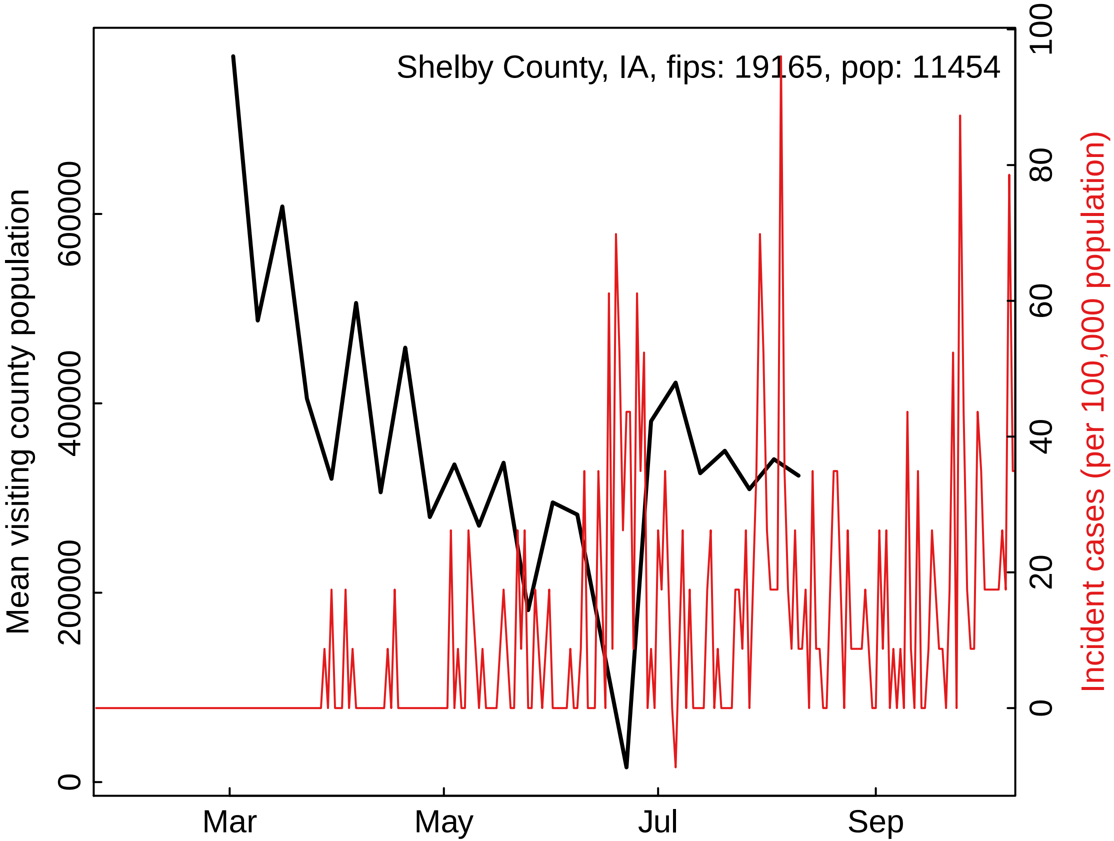

Shelby County, IA, fips: 19165, pop: 11454
Mean visiting county population
0
200000
400000
600000
Incident cases (per 100,000 population)
0
20
40
60
80
100
Mar
May
Jul
Sep

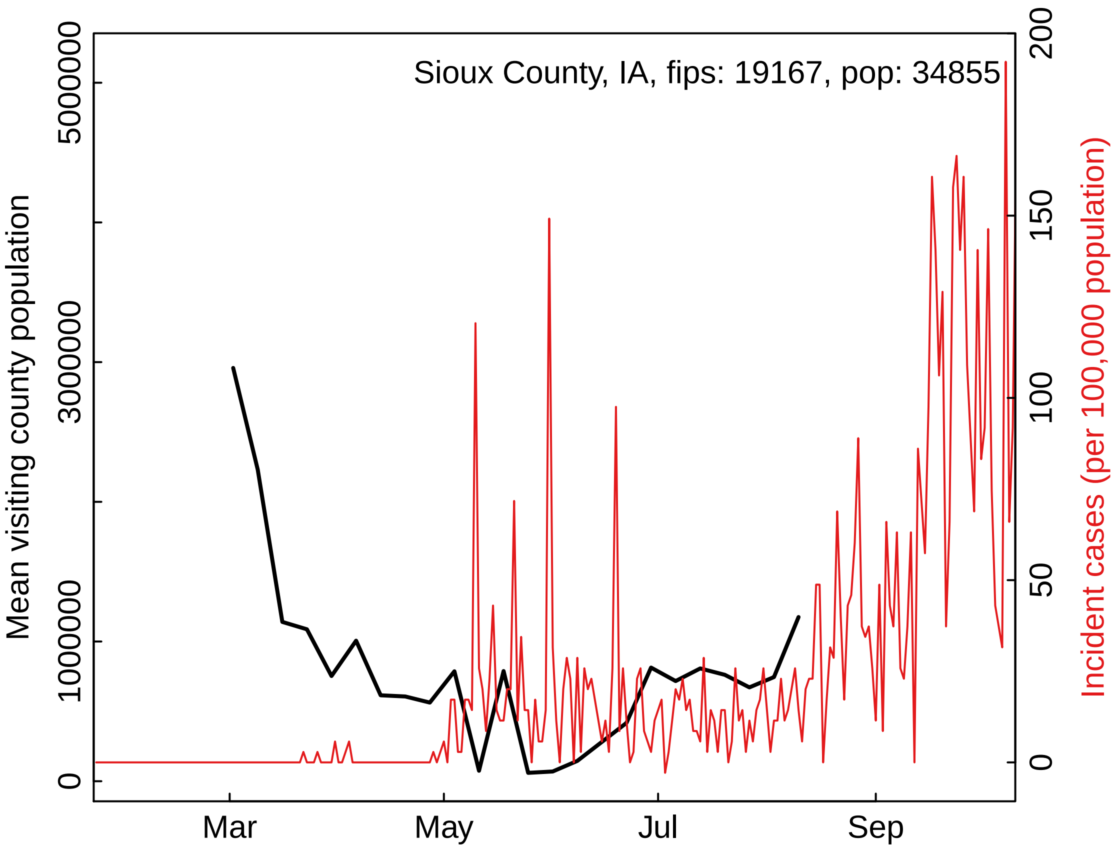

Sioux County, IA, fips: 19167, pop: 34855
Mean visiting county population
Incident cases (per 100,000 population)
0
1000000
3000000
5000000
0
50
100
150
200
Mar
May
Jul
Sep

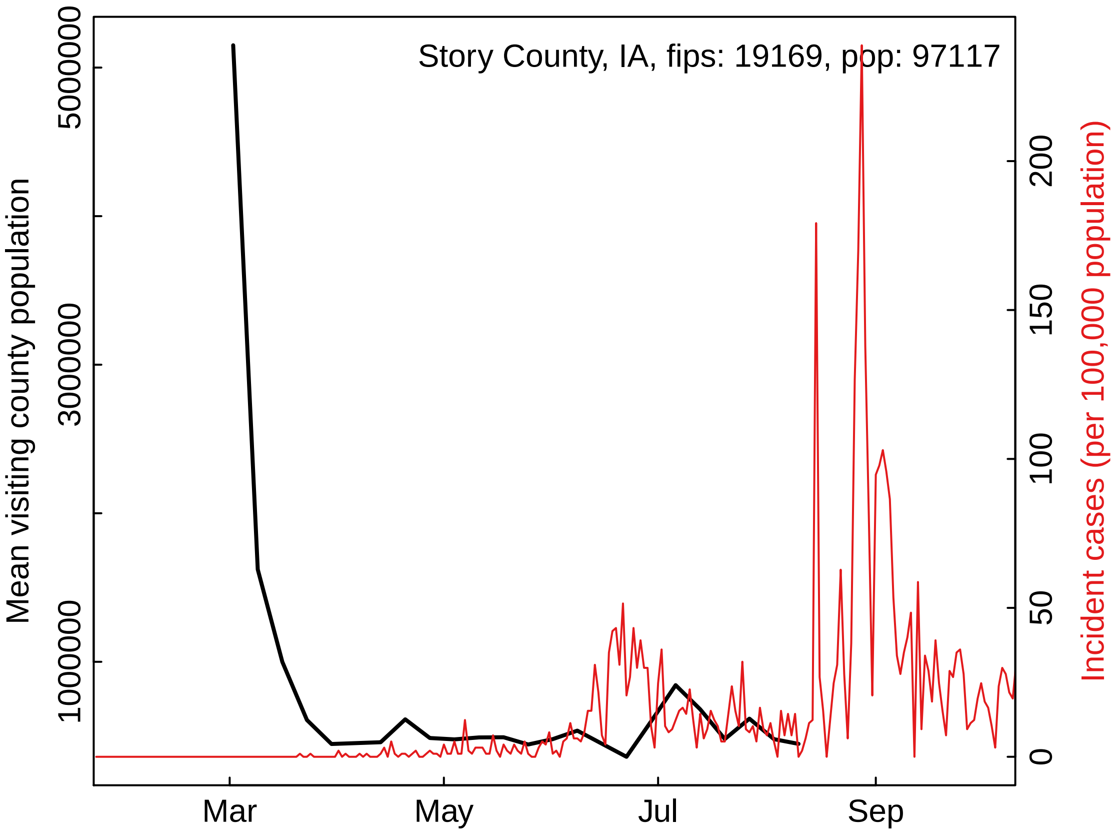

Story County, IA, fips: 19169, pop: 97117
Mean visiting county population
Incident cases (per 100,000 population)
1000000
3000000
5000000
0
50
100
150
200
Mar
May
Jul
Sep

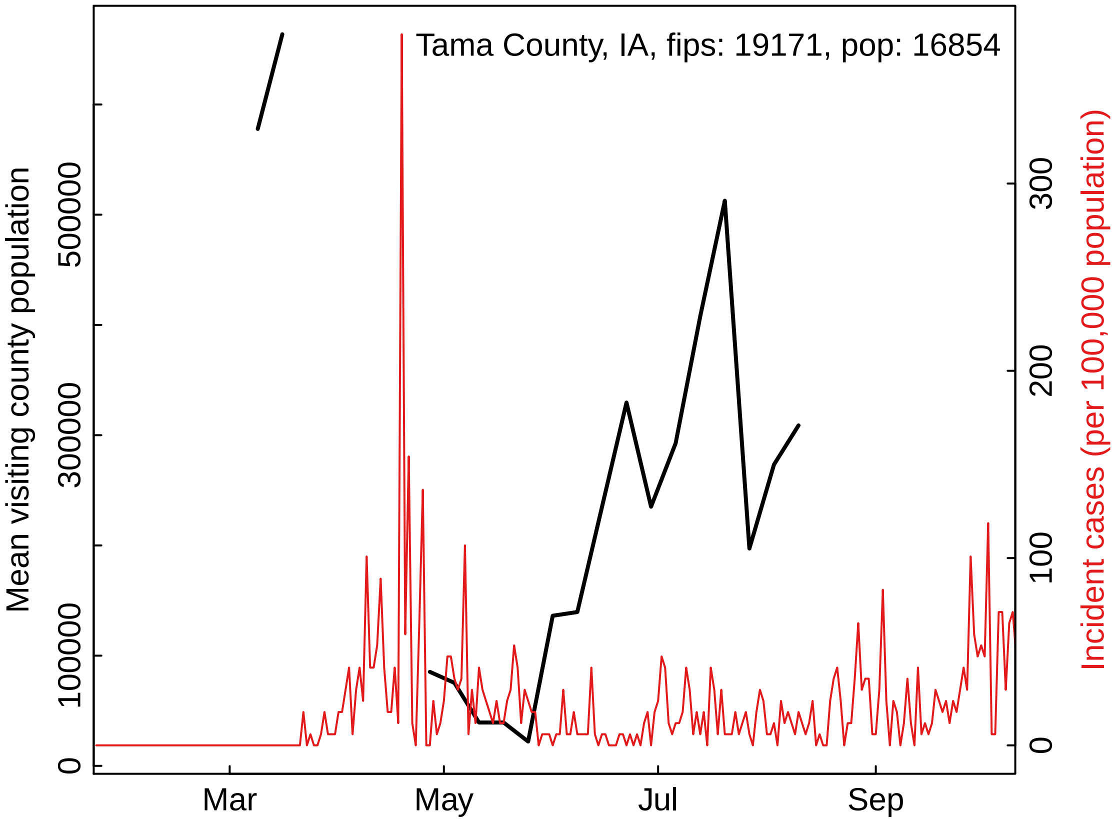
Tama County, IA, fips: 19171, pop: 16854
Mean visiting county population
0
100000
300000
500000
Incident cases (per 100,000 population)
0
100
200
300
Mar
May
Jul
Sep

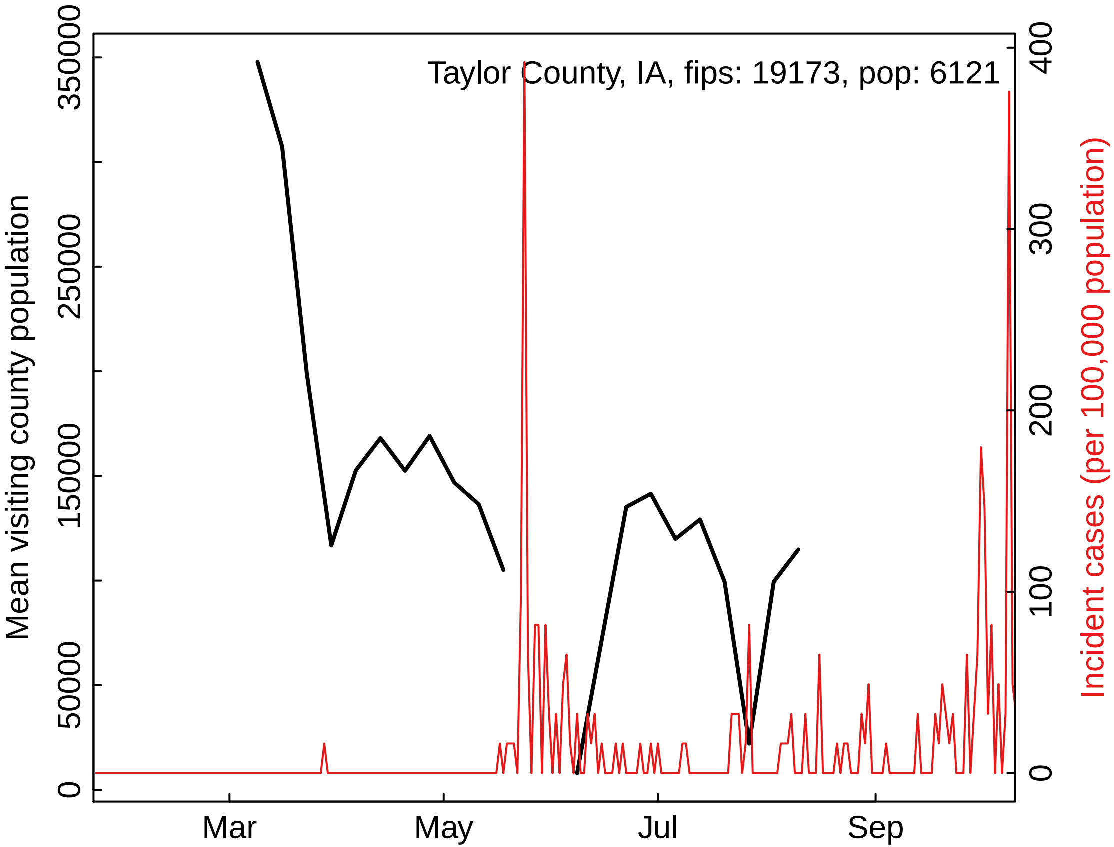

Taylor County, IA, fips: 19173, pop: 6121
Mean visiting county population
0
50000
150000
250000
350000
Incident cases (per 100,000 population)
0
100
200
300
400
Mar
May
Jul
Sep

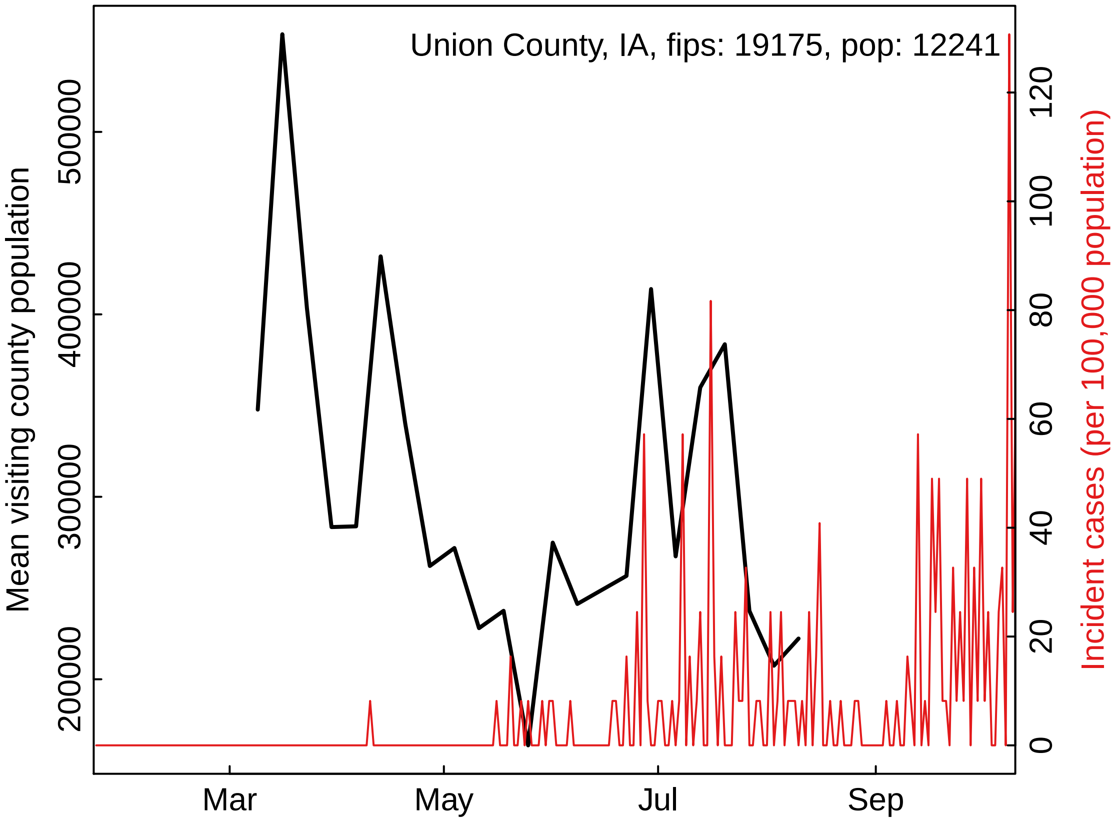

Union County, IA, fips: 19175, pop: 12241
Mean visiting county population
200000
300000
400000
5000000
Incident cases (per 100,000 population)
0
20
40
60
80
100
120
Mar
May
Jul
Sep

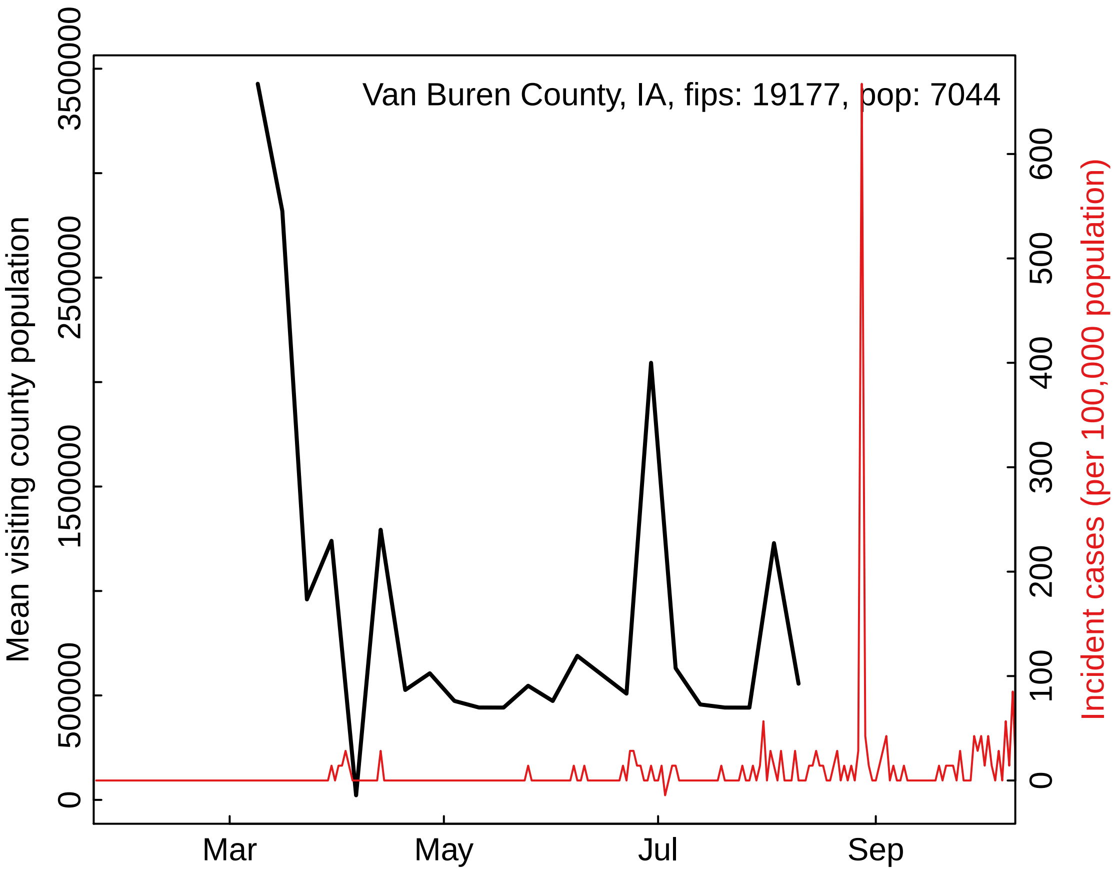

Van Buren County, IA, fips: 19177, pop: 7044
Mean visiting county population
0
500000
1500000
2500000
3500000
Incident cases (per 100,000 population)
0
100
200
300
400
500
600
Mar
May
Jul
Sep

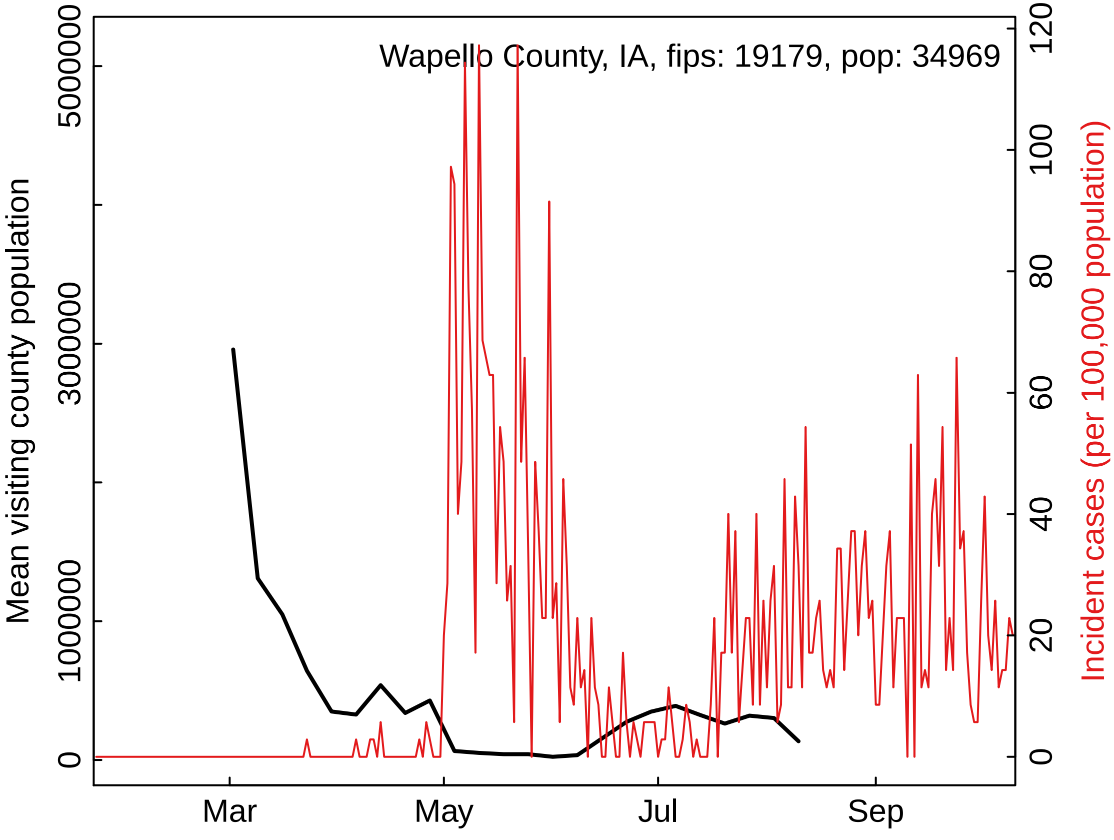

Wapello County, IA, fips: 19179, pop: 34969
Mean visiting county population
0
1000000
3000000
5000000
Incident cases (per 100,000 population)
0
20
40
60
80
100
120
Mar
May
Jul
Sep

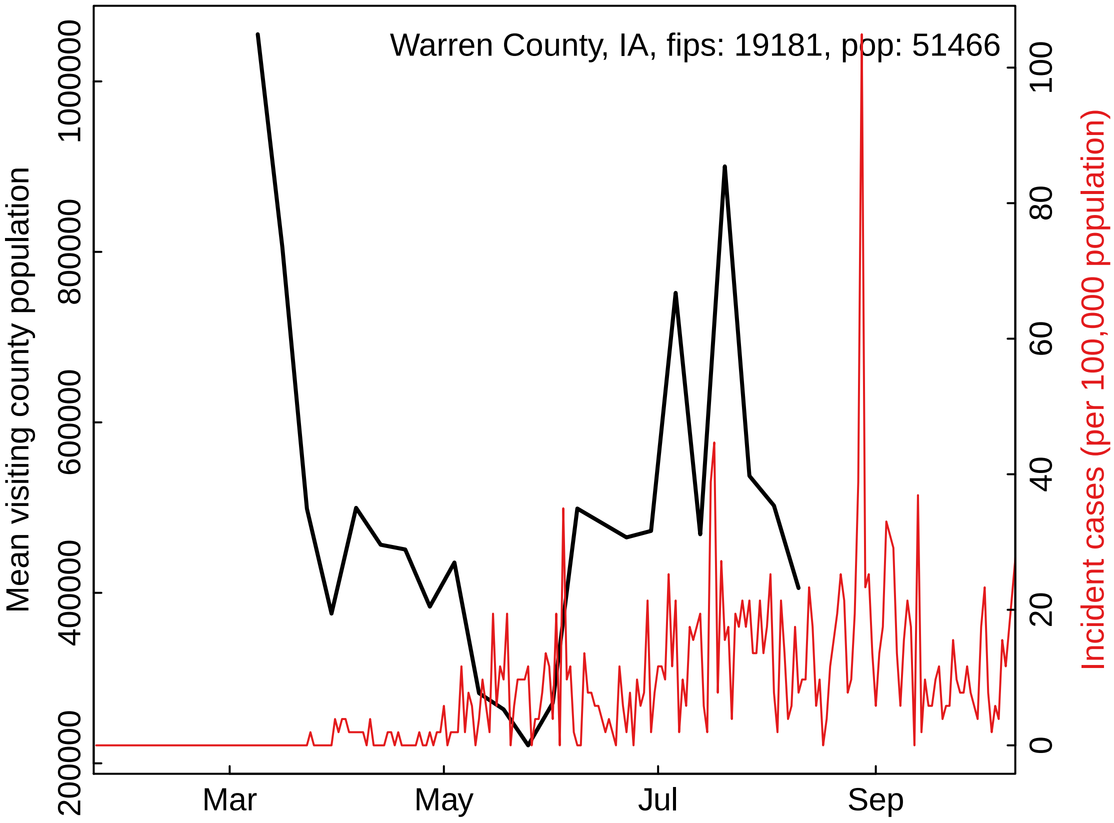

Warren County, IA, fips: 19181, pop: 51466
Mean visiting county population
200000
400000
600000
800000
1000000
Incident cases (per 100,000 population)
0
20
40
60
80
100
Mar
May
Jul
Sep

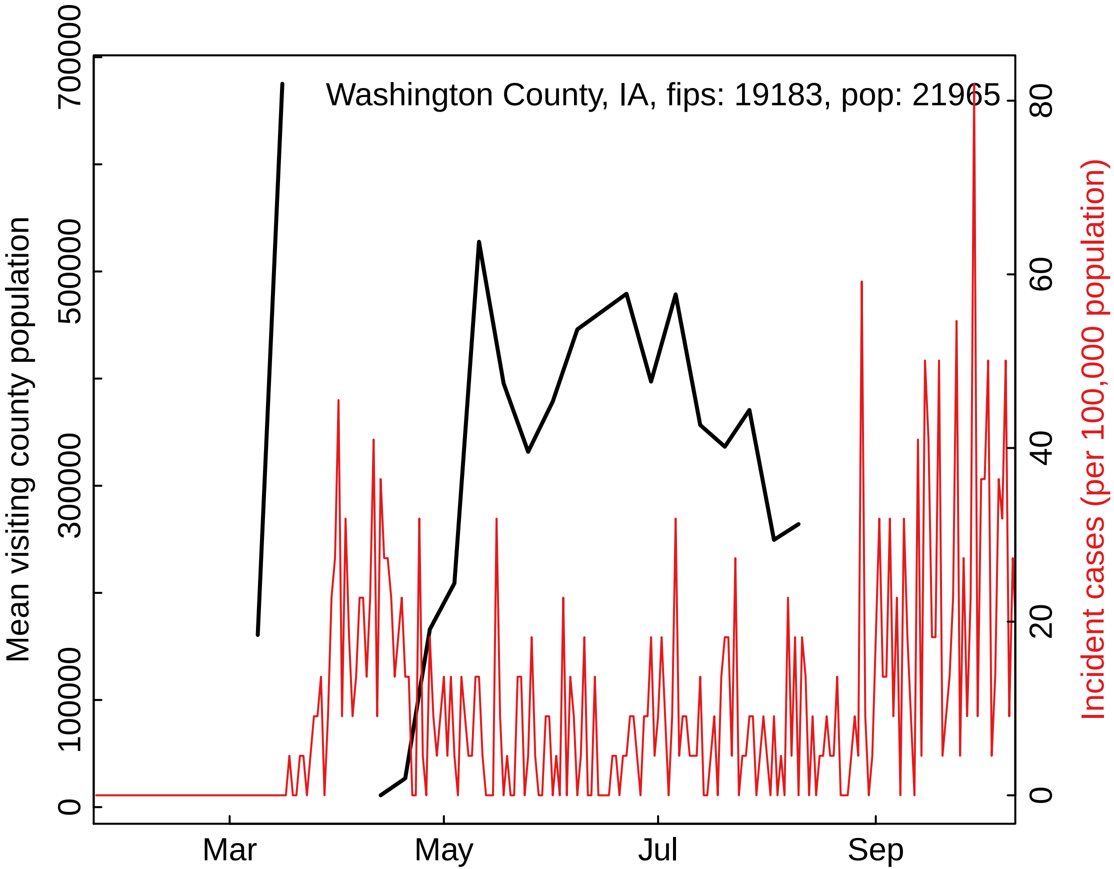
Washington County, IA, fips: 19183, pop: 21965
Mean visiting county population
Incident cases (per 100,000 population)
0
100000
300000
500000
700000
0
20
40
60
80
Mar
May
Jul
Sep

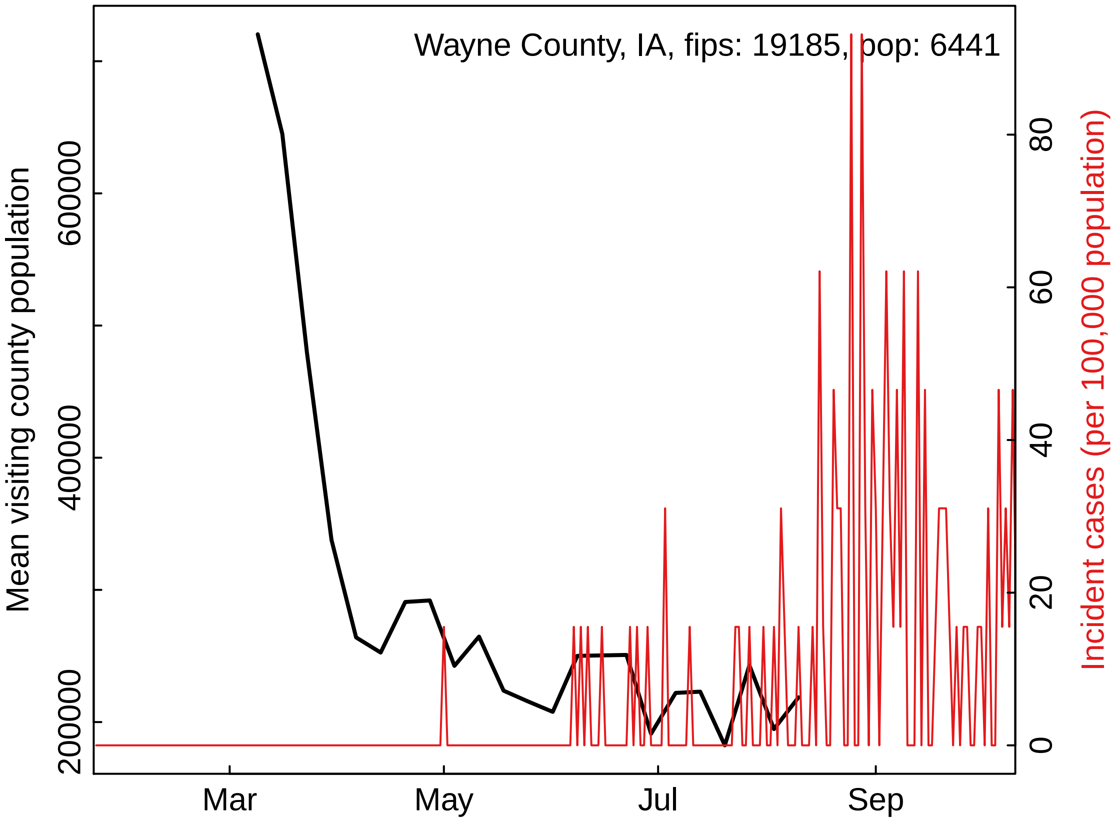

Wayne County, IA, fips: 19185, pop: 6441
Mean visiting county population
200000
400000
600000
Mar
May
Jul
Sep
0
20
40
60
80
Incident cases (per 100,000 population)

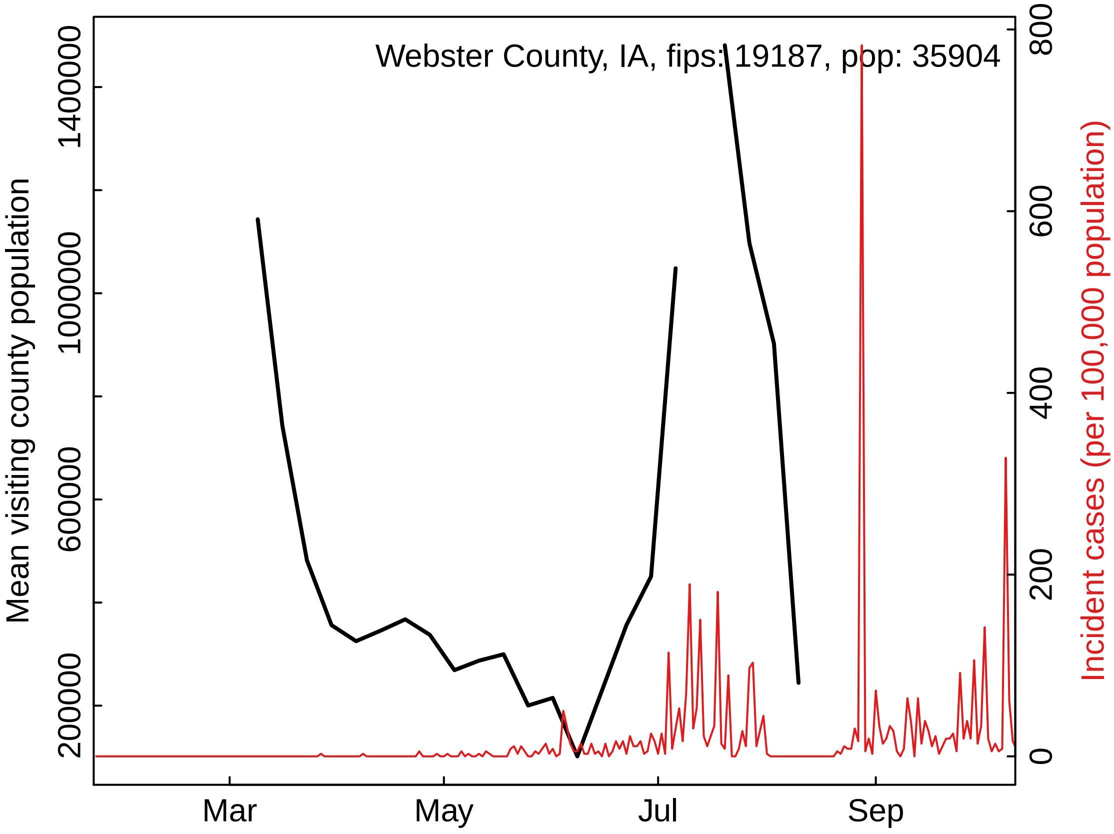

Webster County, IA, fips: 19187, pop: 35904
Mean visiting county population
200000
600000
1000000
1400000
Incident cases (per 100,000 population)
0
200
400
600
800
Mar
May
Jul
Sep

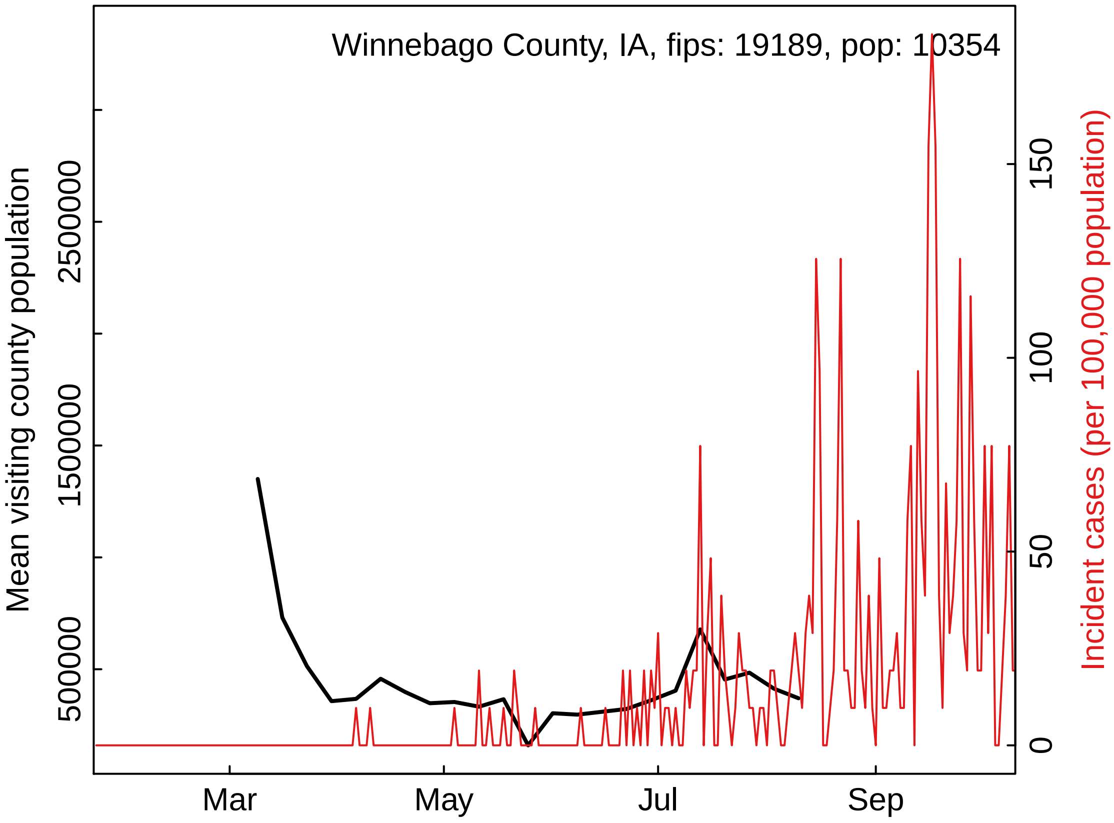

Winnebago County, IA, fips: 19189, pop: 10354
Mean visiting county population
500000
1500000
2500000
Incident cases (per 100,000 population)
0
50
100
150
Mar
May
Jul
Sep

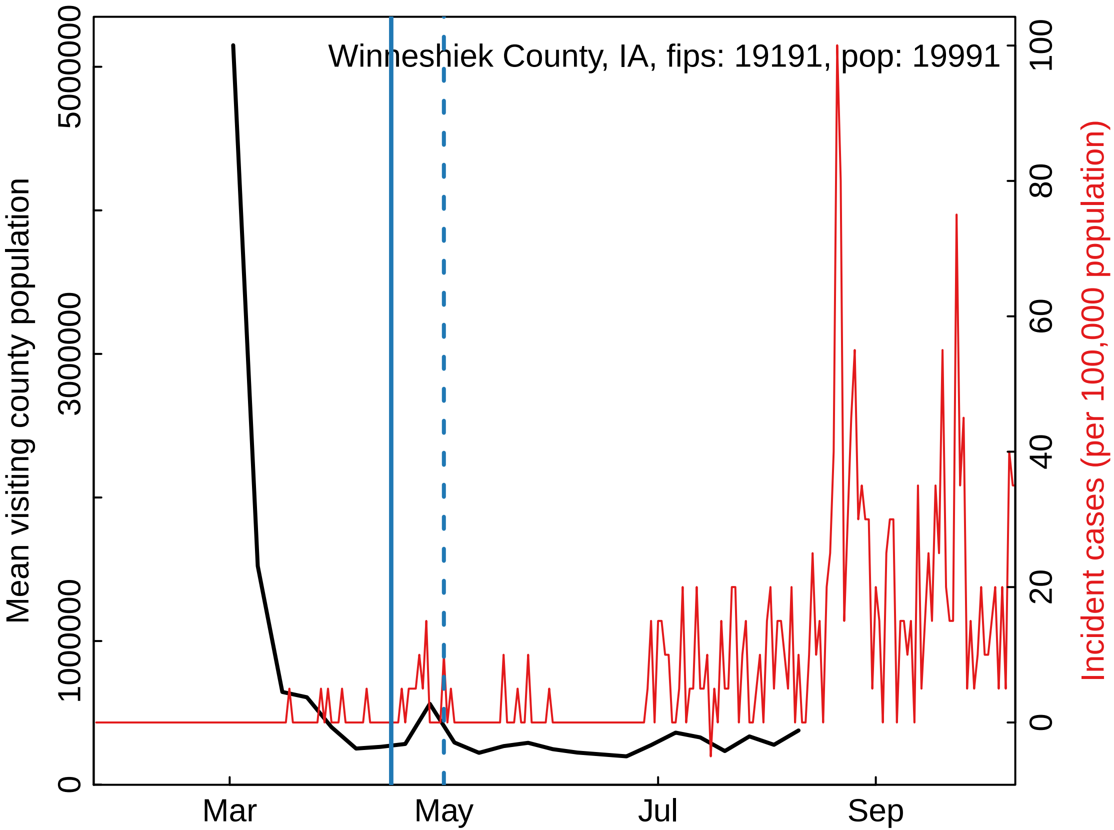

Winneshiek County, IA, fips: 19191, pop: 19991
Mean visiting county population
Incident cases (per 100,000 population)
0
1000000
3000000
5000000
0
20
40
60
80
100
Mar
May
Jul
Sep

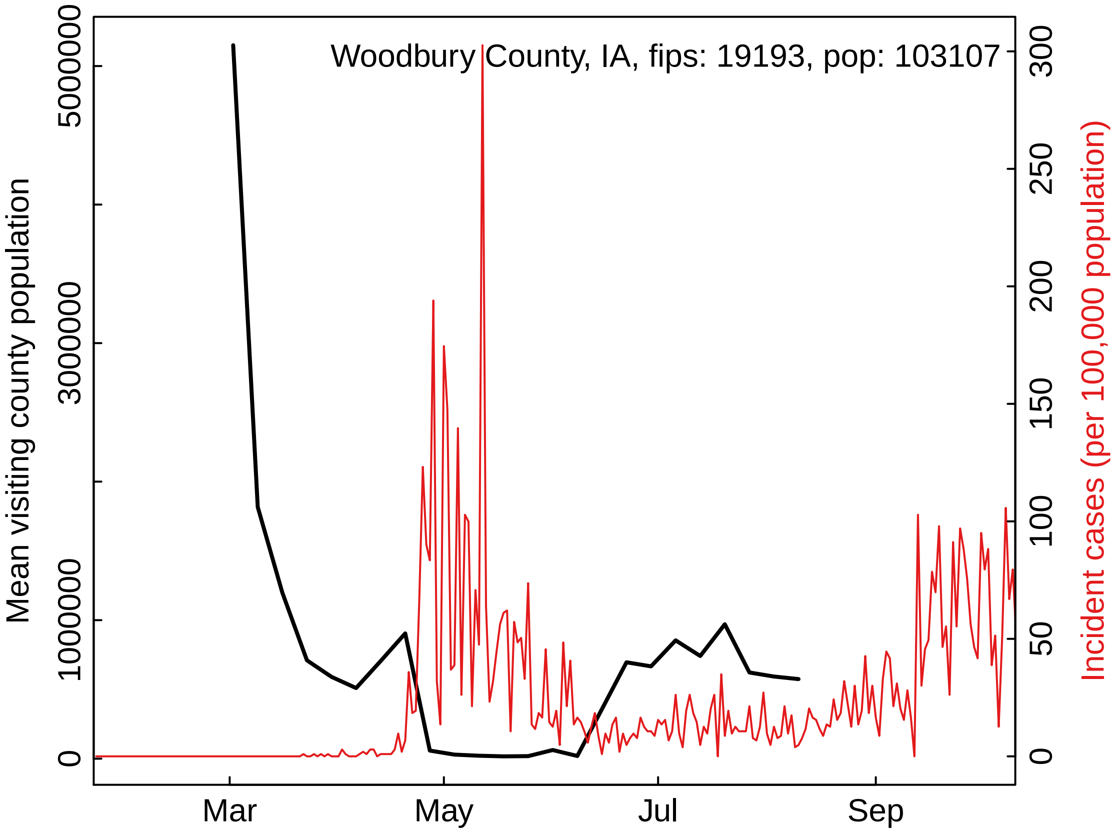
Woodbury County, IA, fips: 19193, pop: 103107
Mean visiting county population
Incident cases (per 100,000 population)
0
1000000
3000000
5000000
0
50
100
150
200
250
300
Mar
May
Jul
Sep

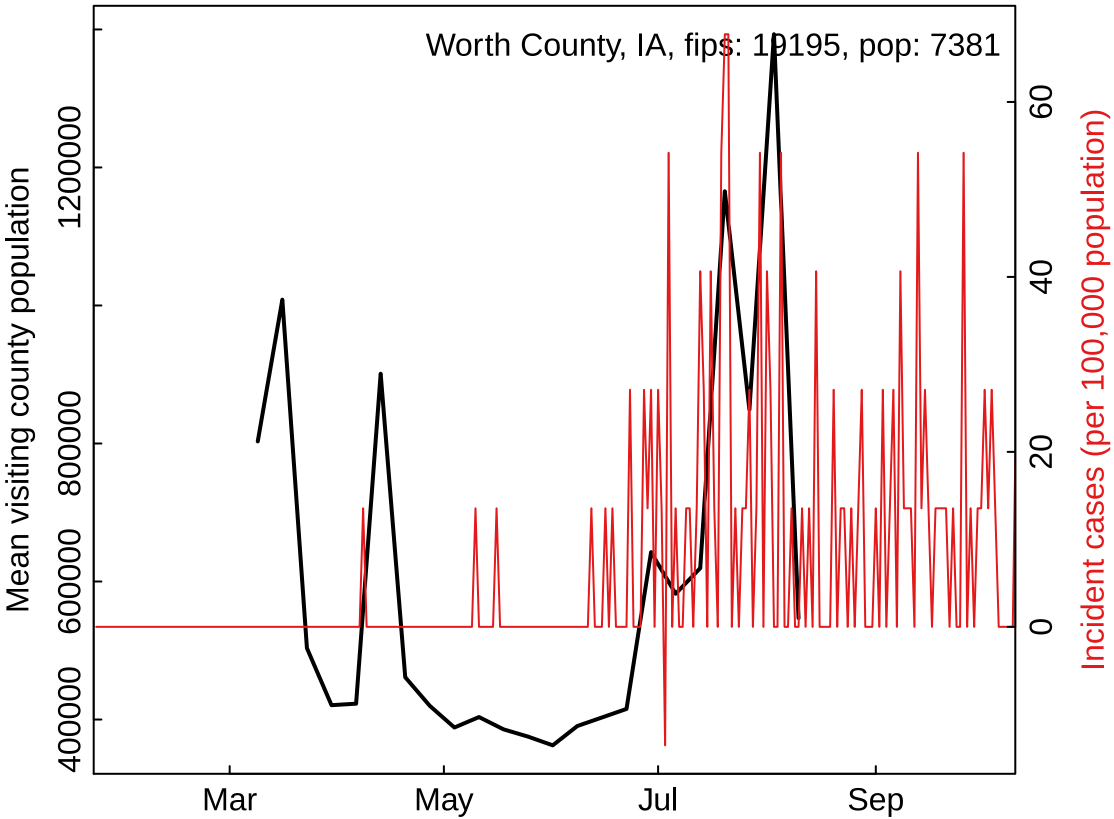

Worth County, IA, fips: 19195, pop: 7381
Mean visiting county population
400000
600000
800000
1200000
Incident cases (per 100,000 population)
0
20
40
60
Mar
May
Jul
Sep

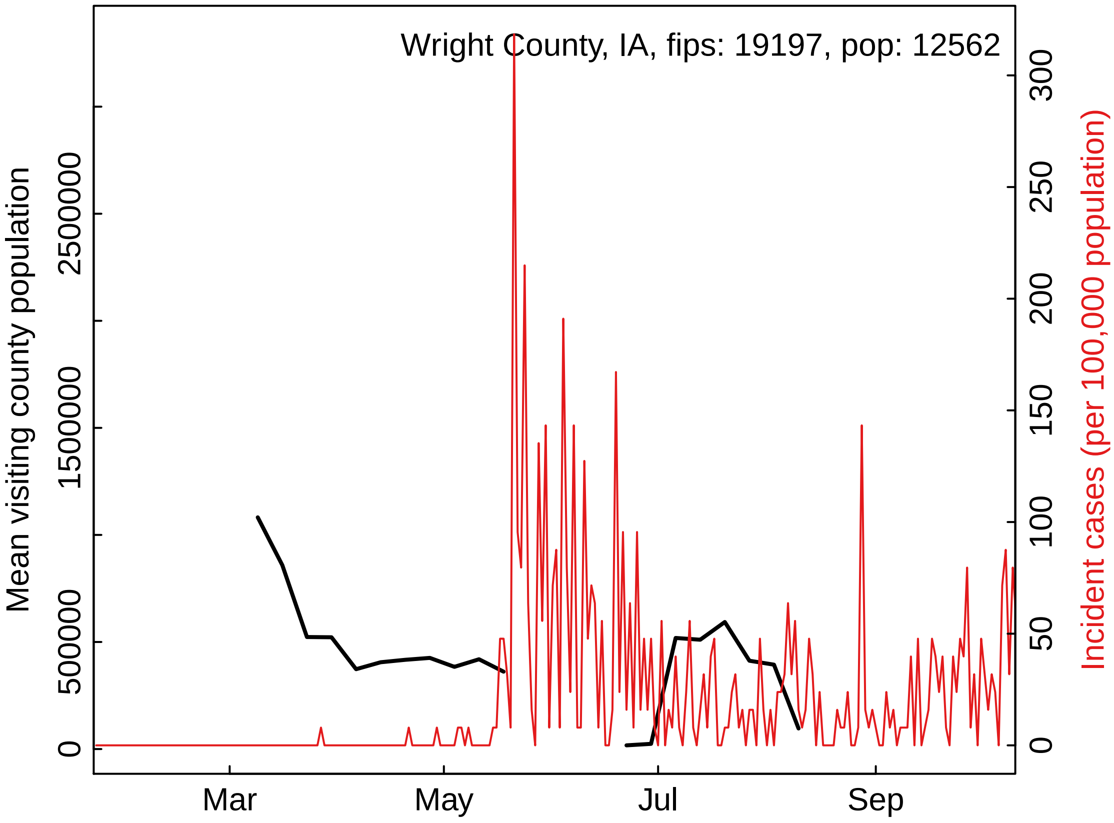

Wright County, IA, fips: 19197, pop: 12562
Mean visiting county population
0
500000
1500000
2500000
Incident cases (per 100,000 population)
0
50
100
150
200
250
300
Mar
May
Jul
Sep

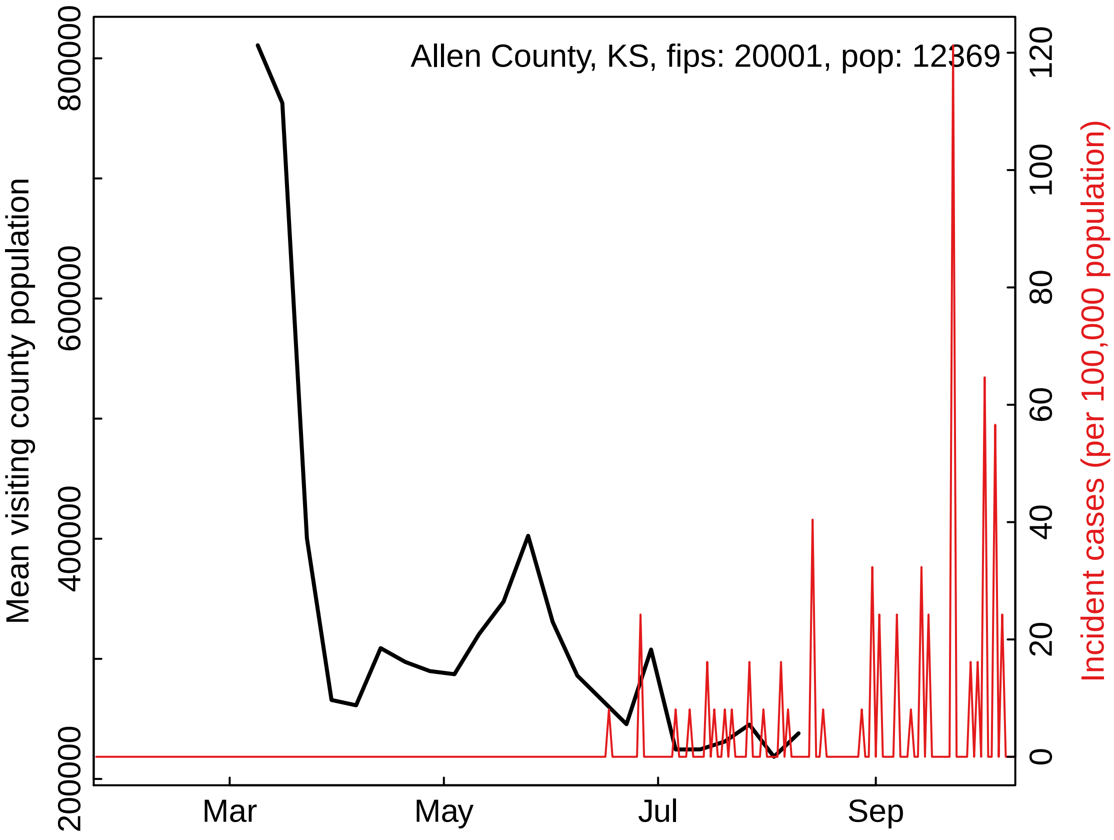
Allen County, KS, fips: 20001, pop: 12369
Mean visiting county population
200000
400000
600000
800000
Incident cases (per 100,000 population)
0
20
40
60
80
100
120
Mar
May
Jul
Sep

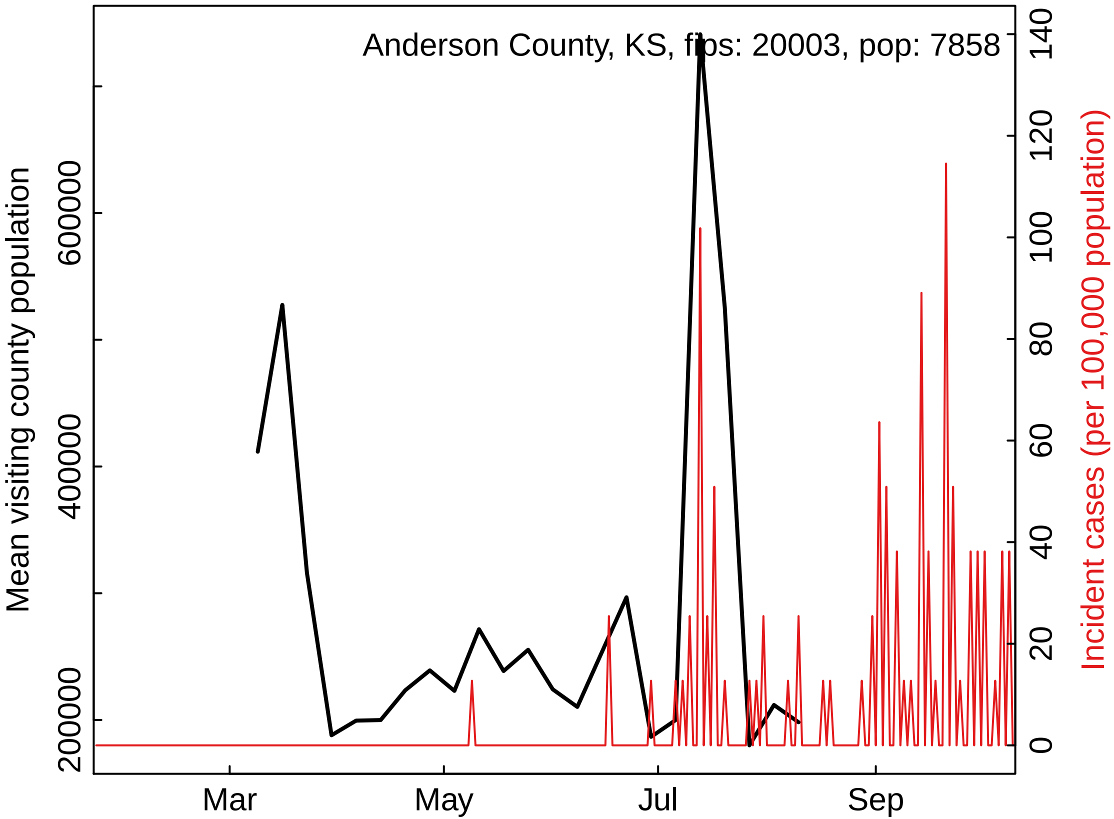

Anderson County, KS, fips: 20003, pop: 7858
Mean visiting county population
200000
400000
600000
Incident cases (per 100,000 population)
0
20
40
60
80
100
120
140
Mar
May
Jul
Sep

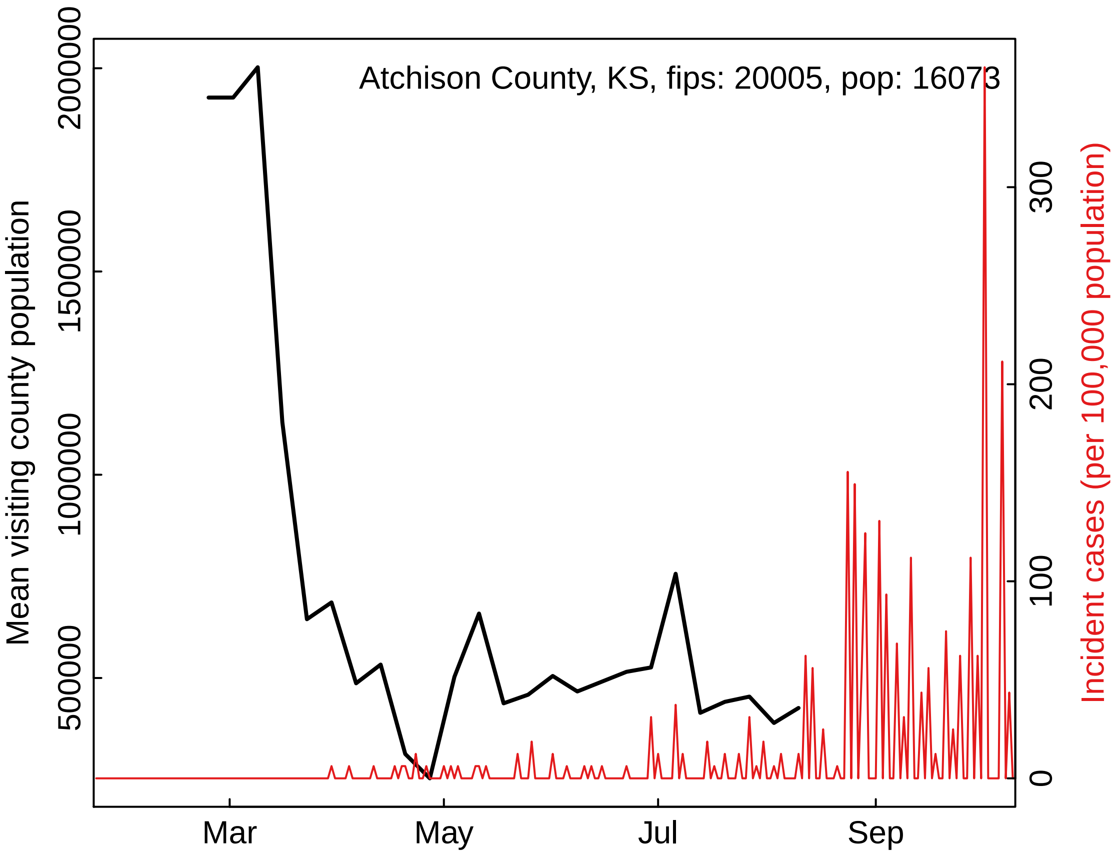

Atchison County, KS, fips: 20005, pop: 16073
Mean visiting county population
Incident cases (per 100,000 population)
2000000
1500000
1000000
500000
300
200
100
0
Mar
May
Jul
Sep

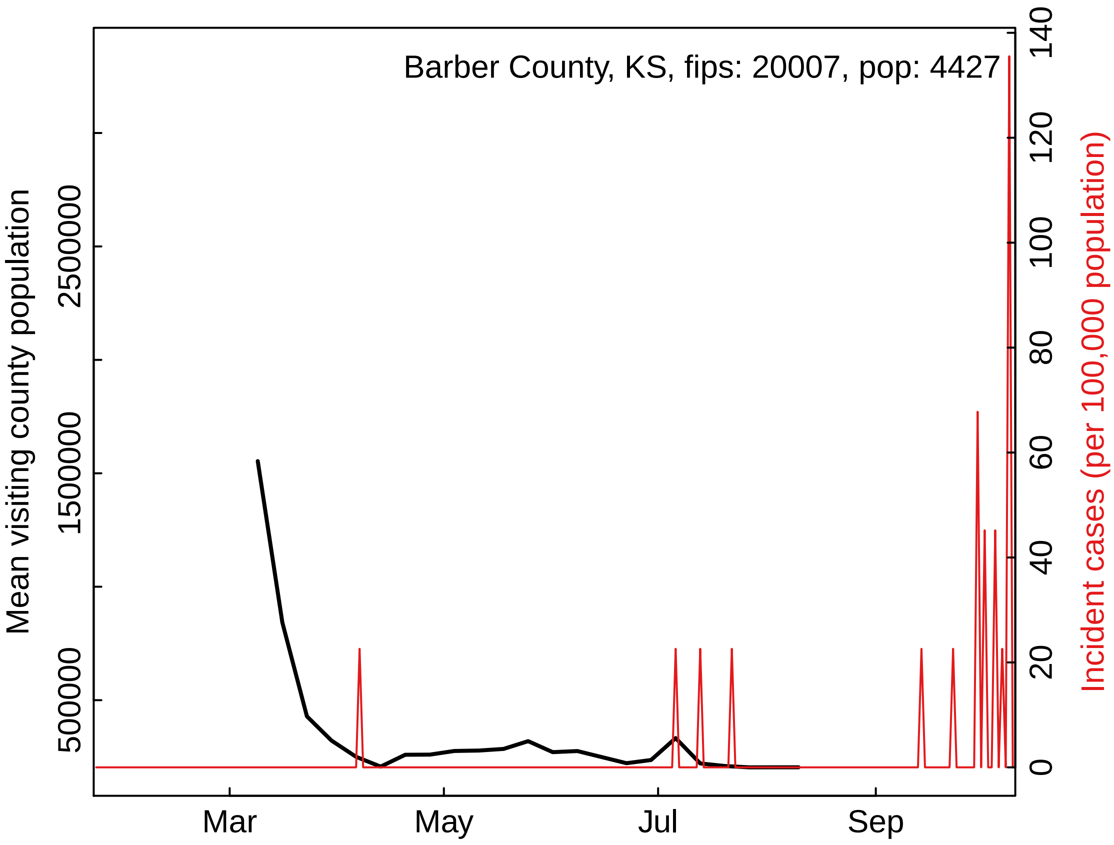

Barber County, KS, fips: 20007, pop: 4427
Mean visiting county population
500000
1500000
2500000
Incident cases (per 100,000 population)
0
20
40
60
80
100
120
140
Mar
May
Jul
Sep

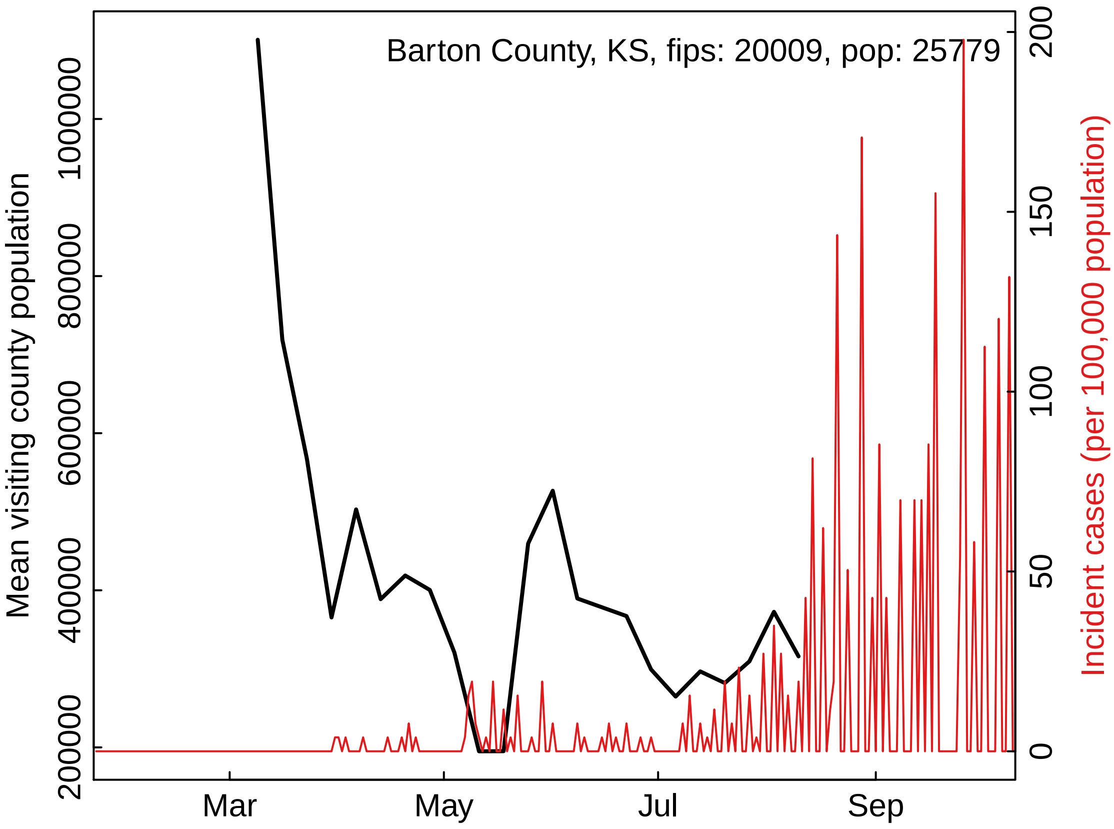

Barton County, KS, fips: 20009, pop: 25779
Mean visiting county population
200000
400000
600000
800000
1000000
Incident cases (per 100,000 population)
0
50
100
150
200
Mar
May
Jul
Sep

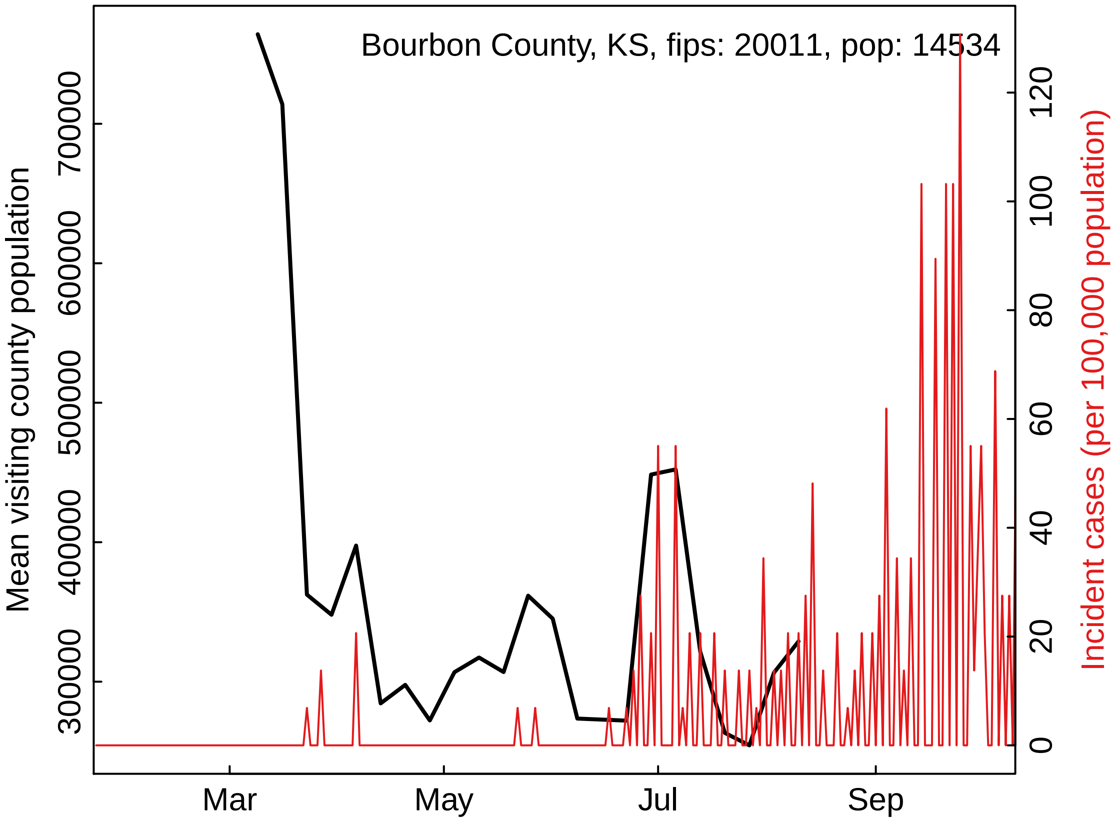
Bourbon County, KS, fips: 20011, pop: 14534
Mean visiting county population
300000
400000
500000
600000
700000
Incident cases (per 100,000 population)
0
20
40
60
80
100
120
Mar
May
Jul
Sep

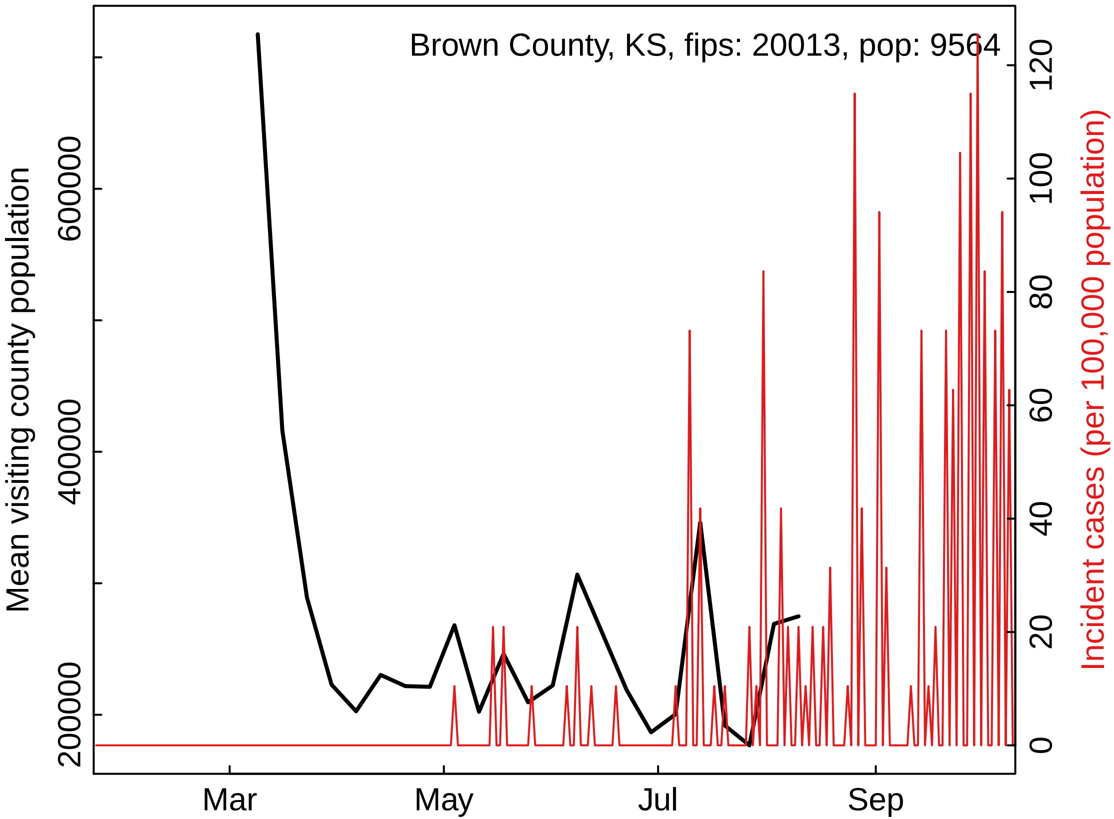
Brown County, KS, fips: 20013, pop: 9564
Mean visiting county population
200000
400000
600000
Incident cases (per 100,000 population)
0
20
40
60
80
100
120
Mar
May
Jul
Sep

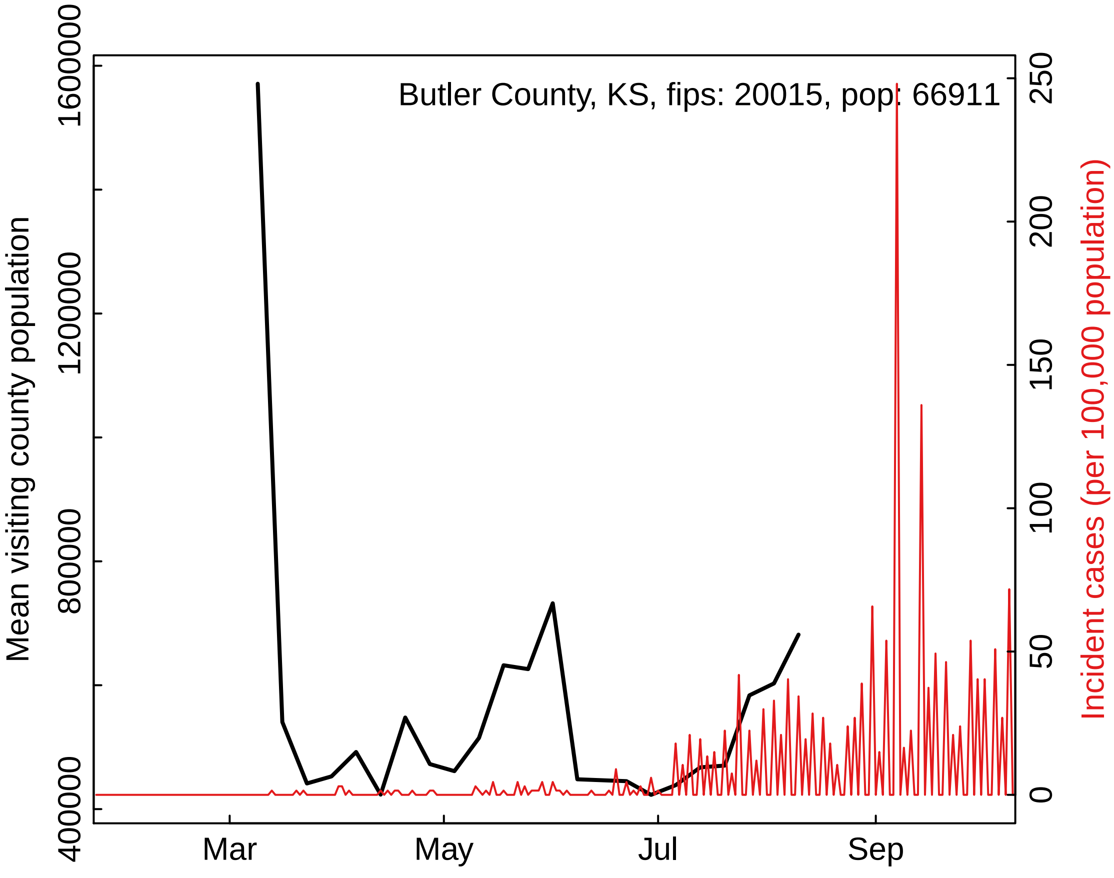

Butler County, KS, fips: 20015, pop: 66911
Mean visiting county population
Incident cases (per 100,000 population)
400000
800000
1200000
1600000
0
50
100
150
200
250
Mar
May
Jul
Sep

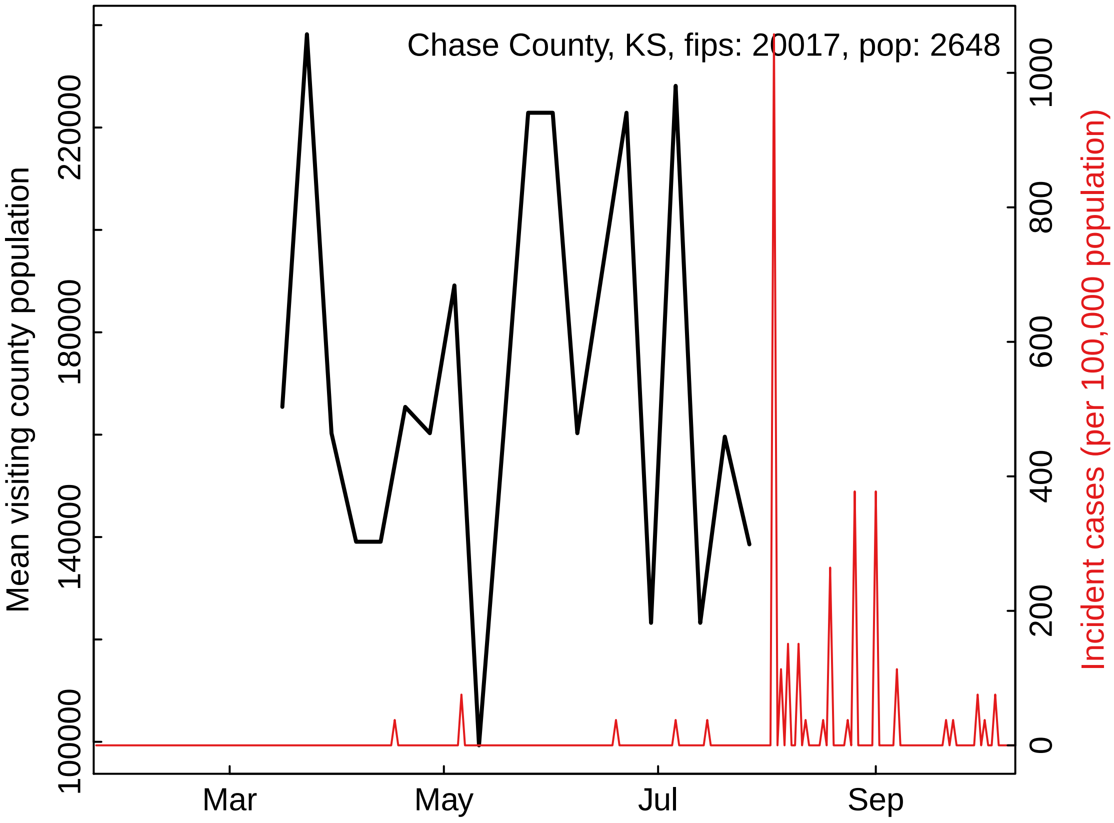

Chase County, KS, fips: 20017, pop: 2648
Mean visiting county population
100000
1400000
1800000
2200000
Incident cases (per 100,000 population)
0
200
400
600
800
1000
Mar
May
Jul
Sep

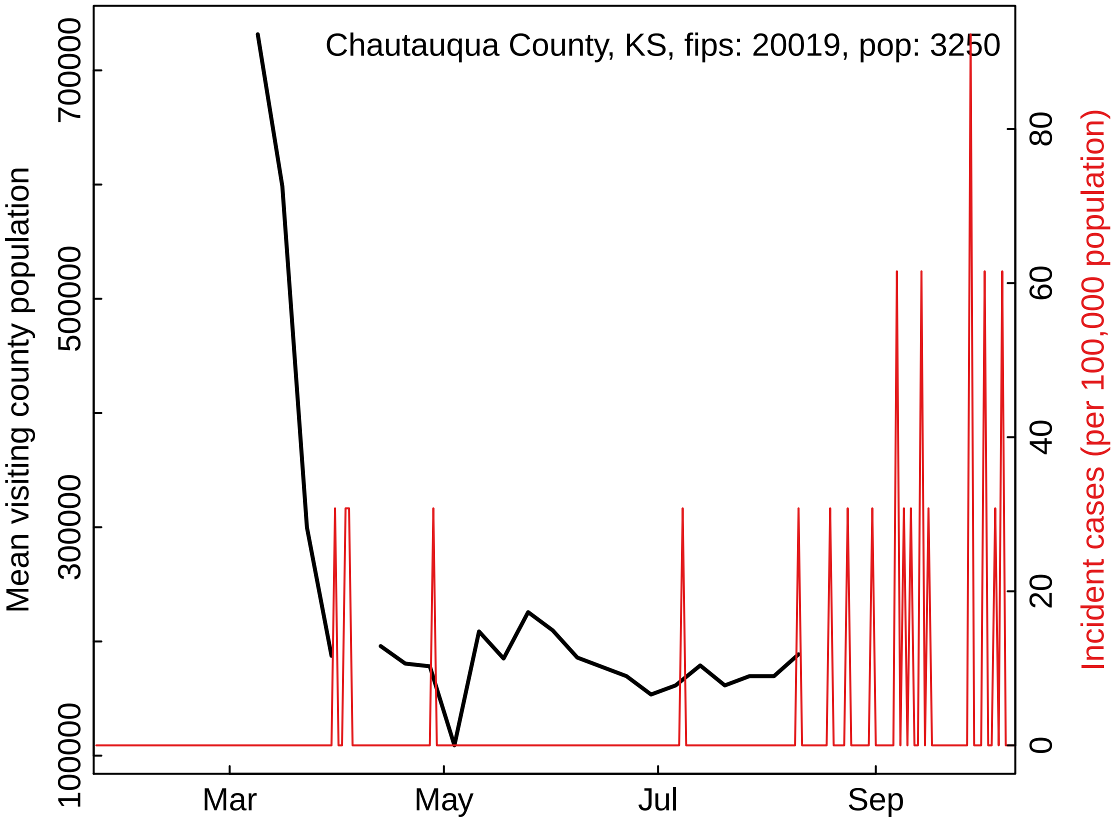

Chautauqua County, KS, fips: 20019, pop: 3250
Mean visiting county population
100000
300000
500000
700000
Mar
May
Jul
Sep
0
20
40
60
80
Incident cases (per 100,000 population)

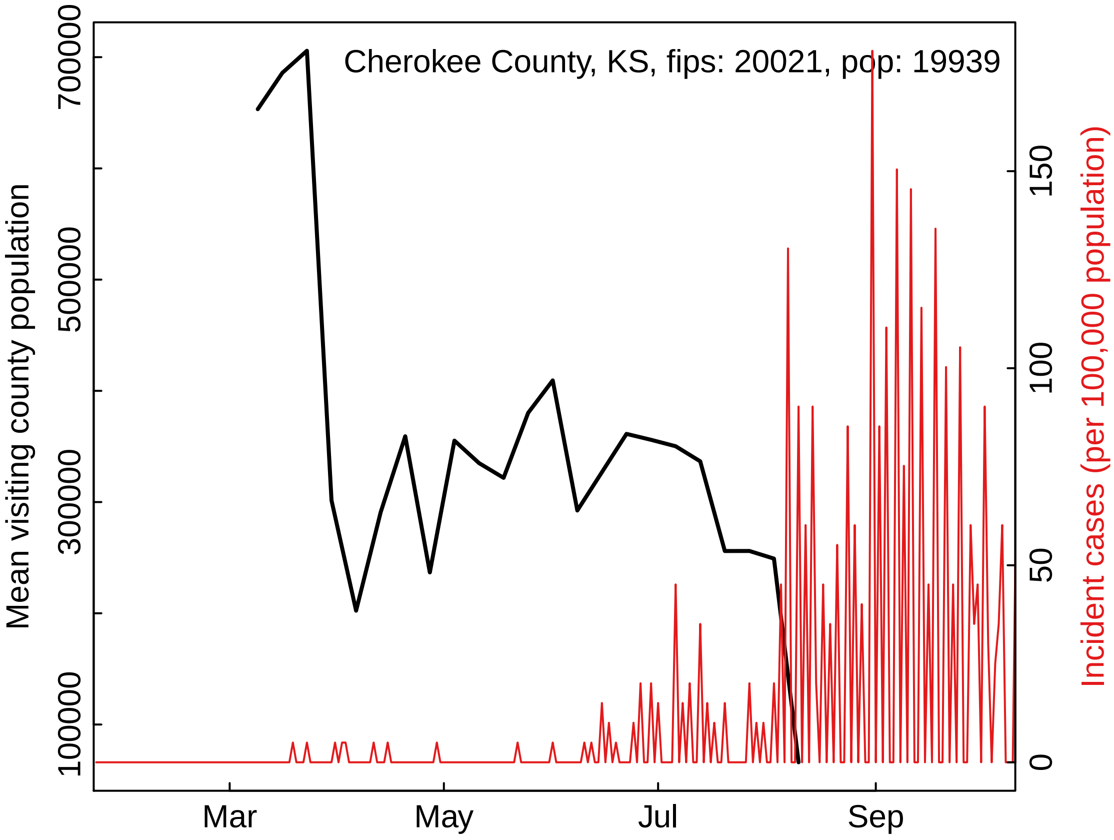

Cherokee County, KS, fips: 20021, pop: 19939
Mean visiting county population
100000
300000
500000
700000
Incident cases (per 100,000 population)
0
50
100
150
Mar
May
Jul
Sep

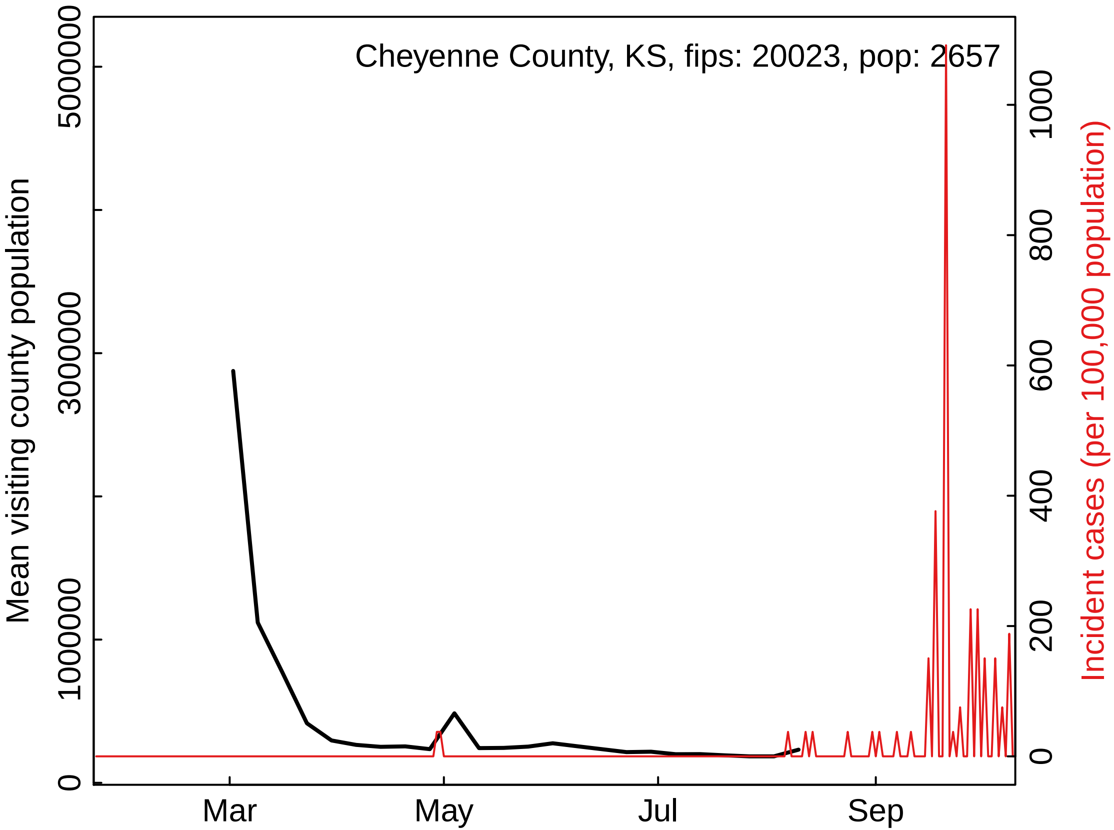
Cheyenne County, KS, fips: 20023, pop: 2657
Mean visiting county population
0
1000000
3000000
5000000
Incident cases (per 100,000 population)
0
200
400
600
800
1000
Mar
May
Jul
Sep

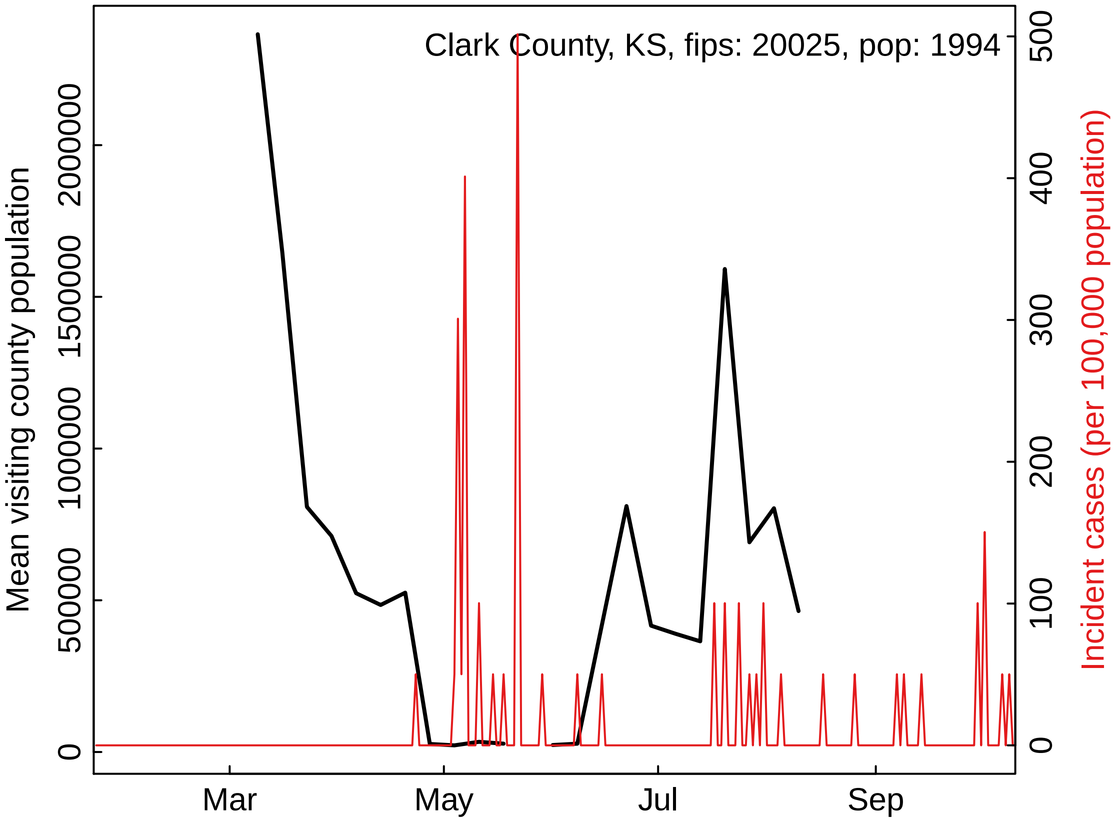

Clark County, KS, fips: 20025, pop: 1994
Mean visiting county population
Incident cases (per 100,000 population)
0
500000
1000000
1500000
2000000
0
100
200
300
400
500
Mar
May
Jul
Sep

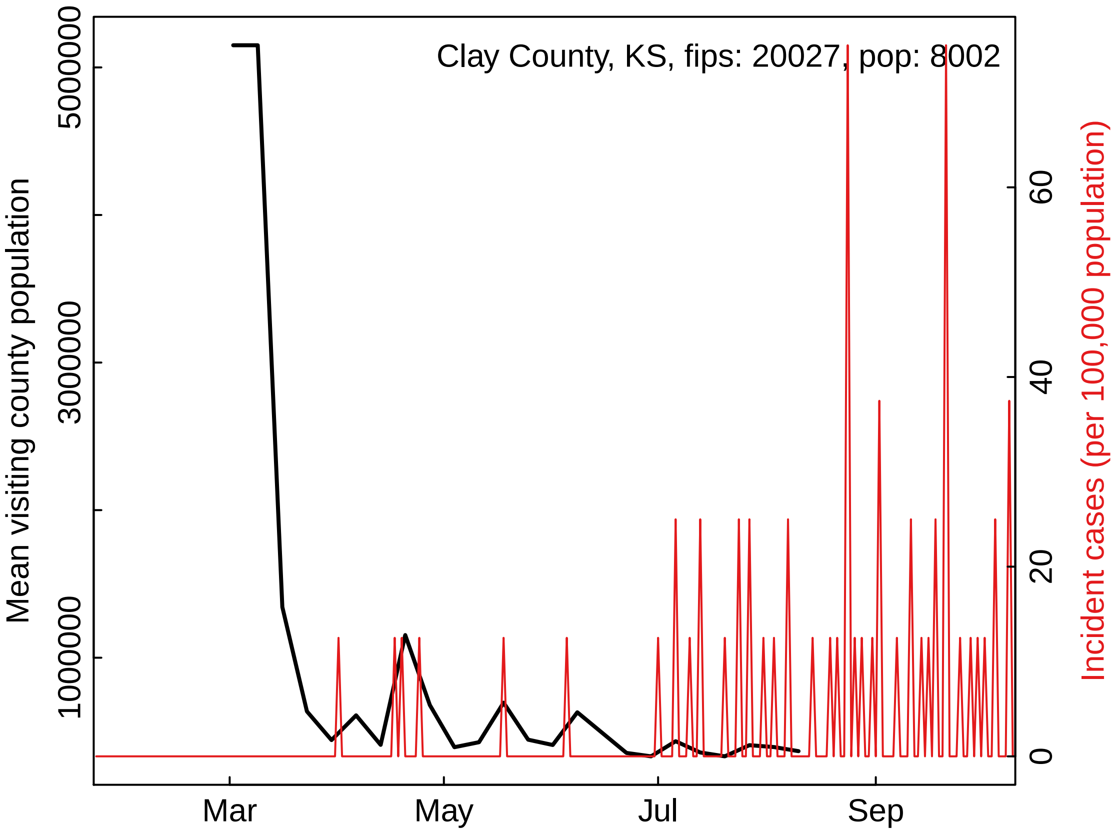
Clay County, KS, fips: 20027, pop: 8002
Mean visiting county population
1000000
3000000
5000000
Incident cases (per 100,000 population)
0
20
40
60
Mar
May
Jul
Sep

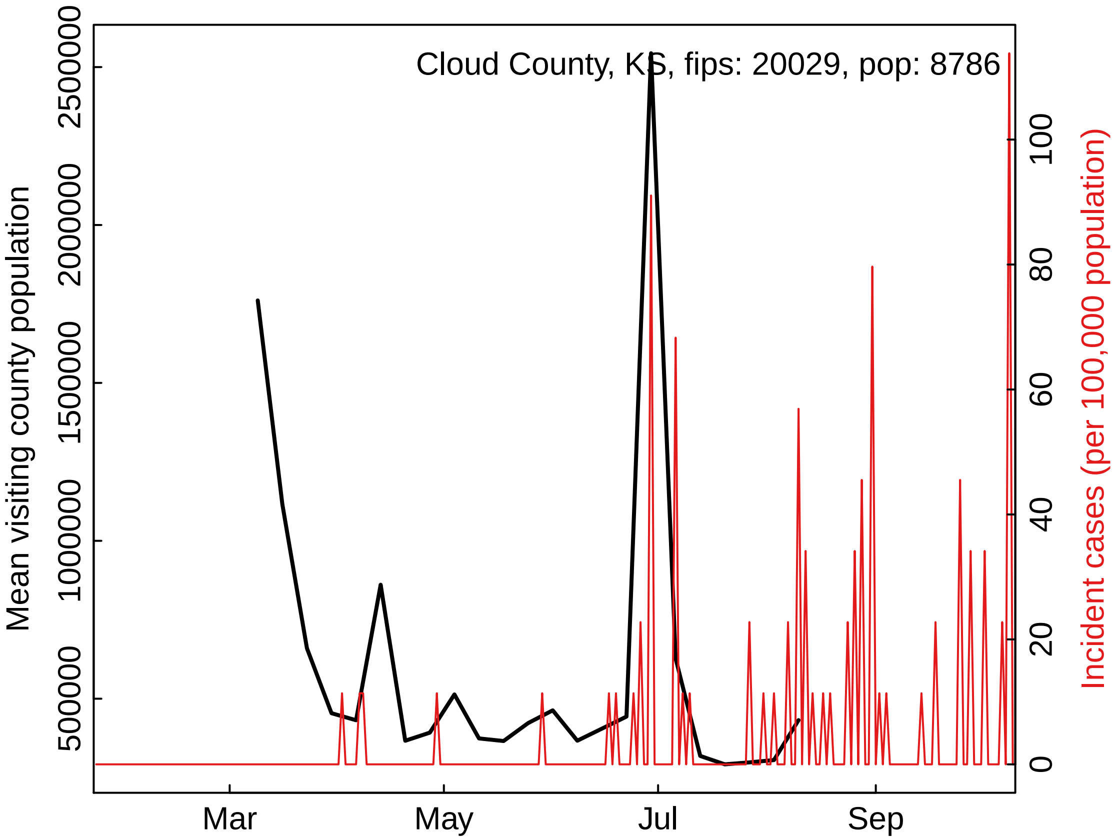

Cloud County, KS, fips: 20029, pop: 8786
Mean visiting county population
Incident cases (per 100,000 population)
500000
1000000
1500000
2000000
2500000
0
20
40
60
80
100
Mar
May
Jul
Sep

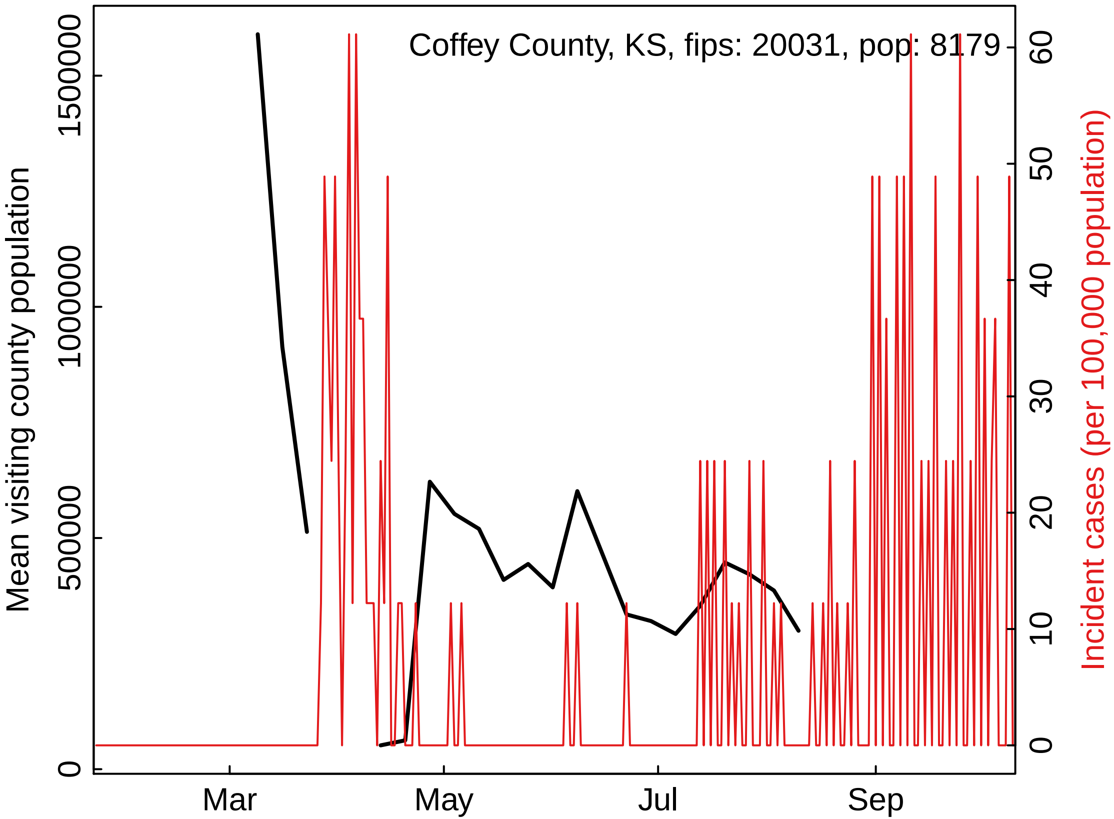

Coffey County, KS, fips: 20031, pop: 8179
Mean visiting county population
0
500000
1000000
1500000
Incident cases (per 100,000 population)
0
10
20
30
40
50
60
Mar
May
Jul
Sep

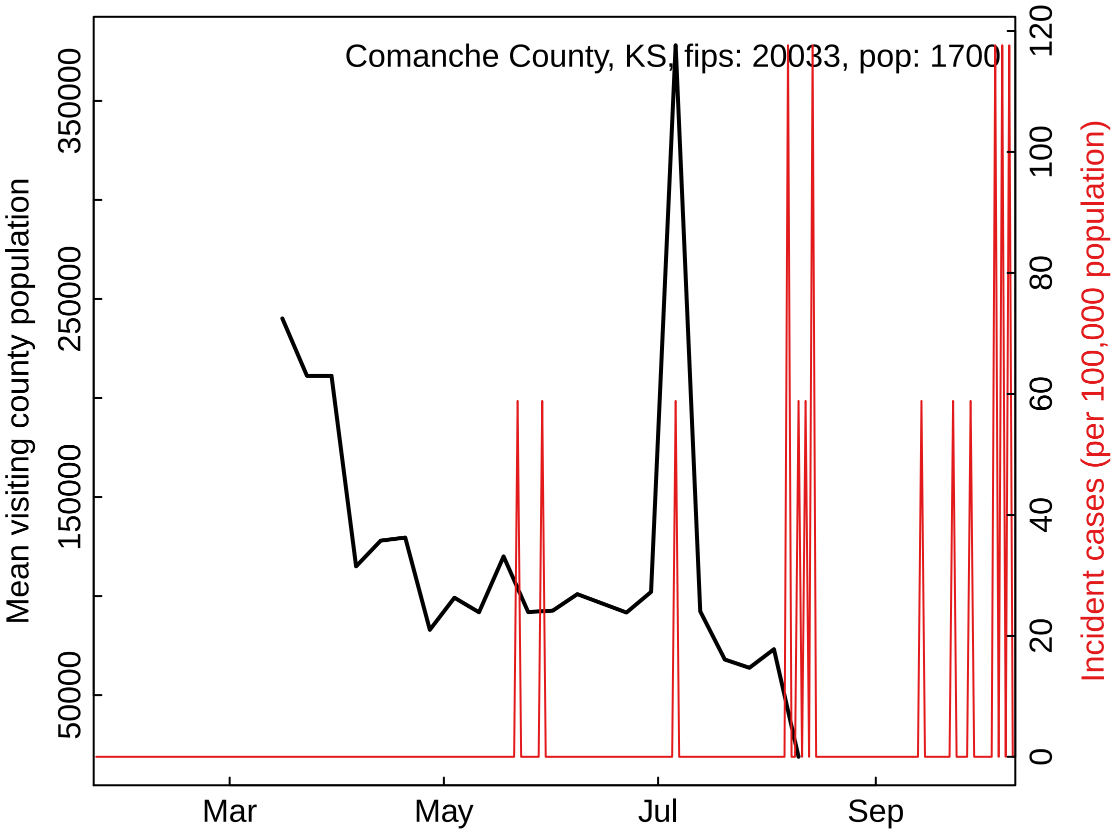
Comanche County, KS, fips: 20033, pop: 1700
Mean visiting county population
50000
150000
250000
350000
Incident cases (per 100,000 population)
0
20
40
60
80
100
120
Mar
May
Jul
Sep

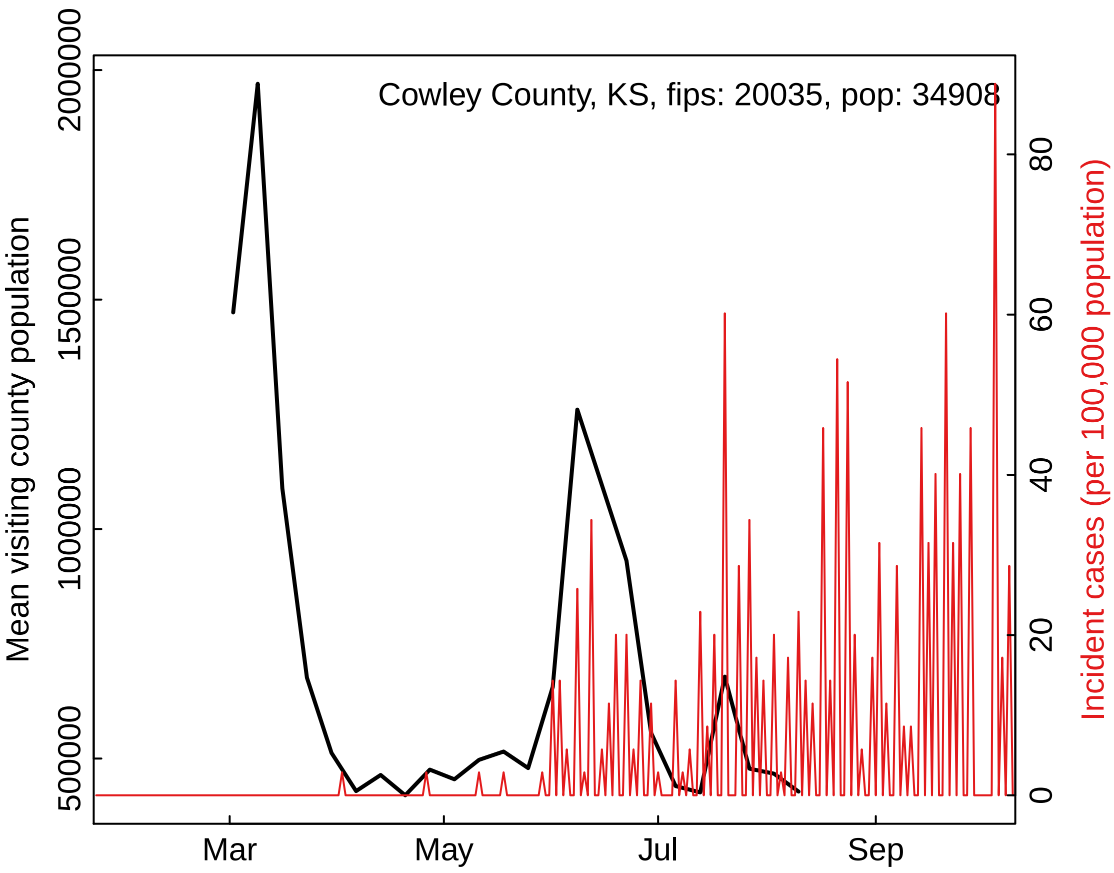
Cowley County, KS, fips: 20035, pop: 34908
Mean visiting county population
500000
1000000
1500000
2000000
Incident cases (per 100,000 population)
0
20
40
60
80
Mar
May
Jul
Sep

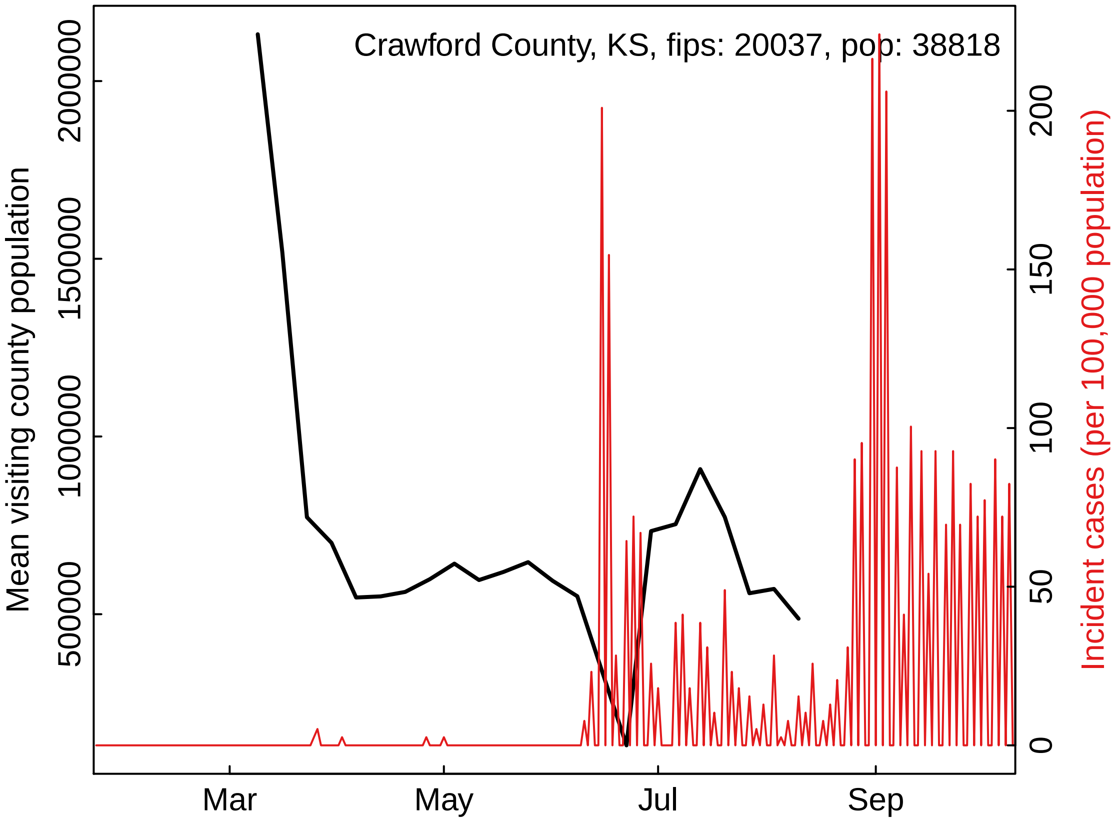

Crawford County, KS, fips: 20037, pop: 38818
Mean visiting county population
500000
1000000
1500000
2000000
Incident cases (per 100,000 population)
0
50
100
150
200
Mar
May
Jul
Sep

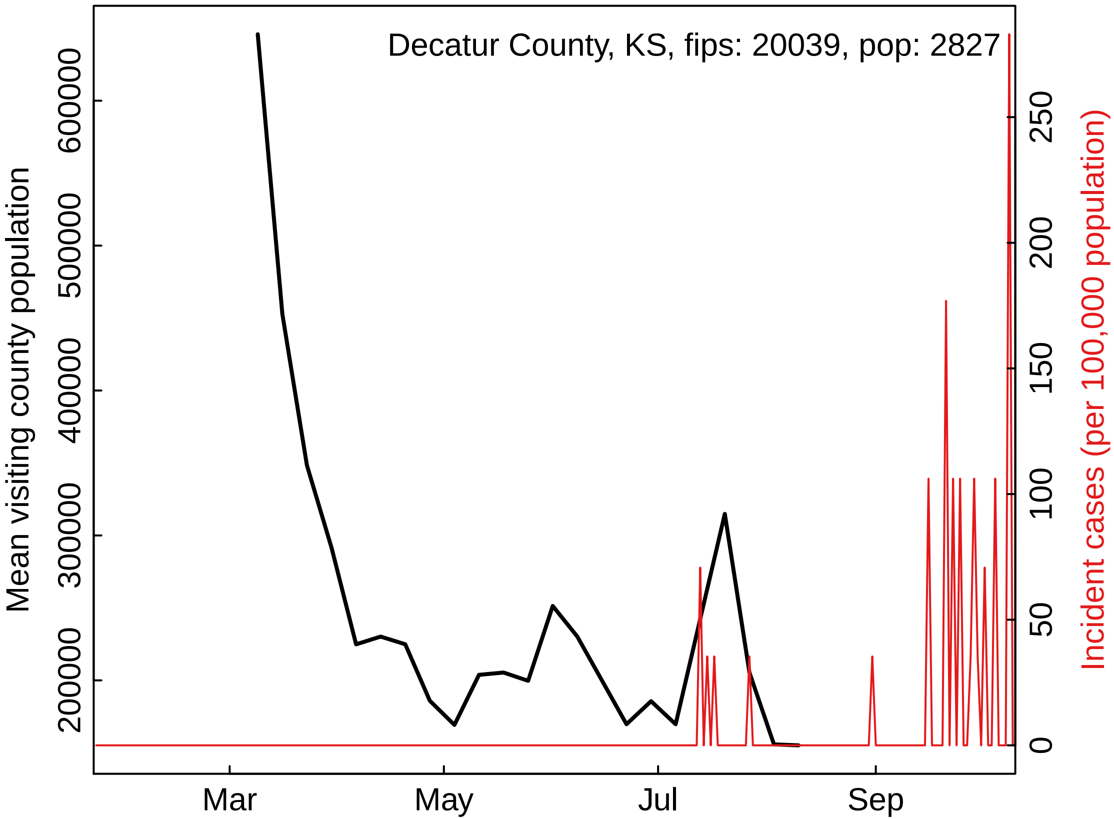

Decatur County, KS, fips: 20039, pop: 2827
Mean visiting county population
200000
300000
400000
500000
600000
Incident cases (per 100,000 population)
0
50
100
150
200
250
Mar
May
Jul
Sep

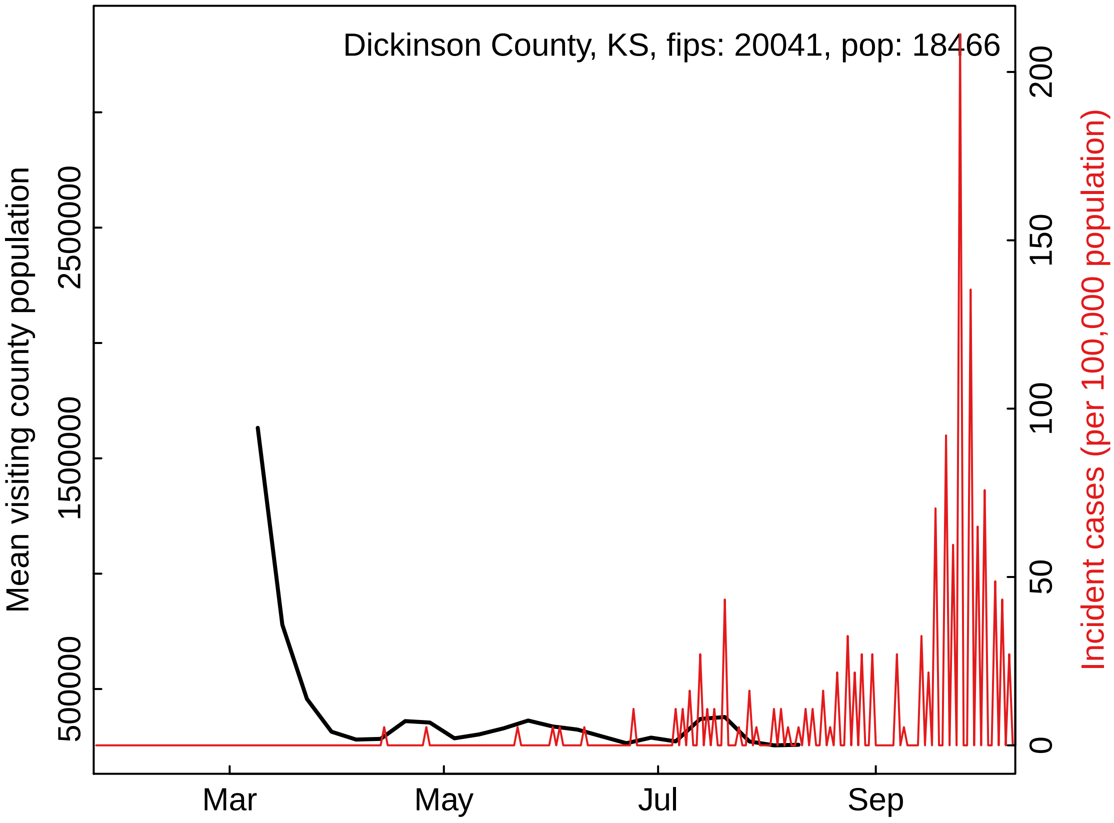

Dickinson County, KS, fips: 20041, pop: 18466
Mean visiting county population
500000
1500000
2500000
Incident cases (per 100,000 population)
0
50
100
150
200
Mar
May
Jul
Sep

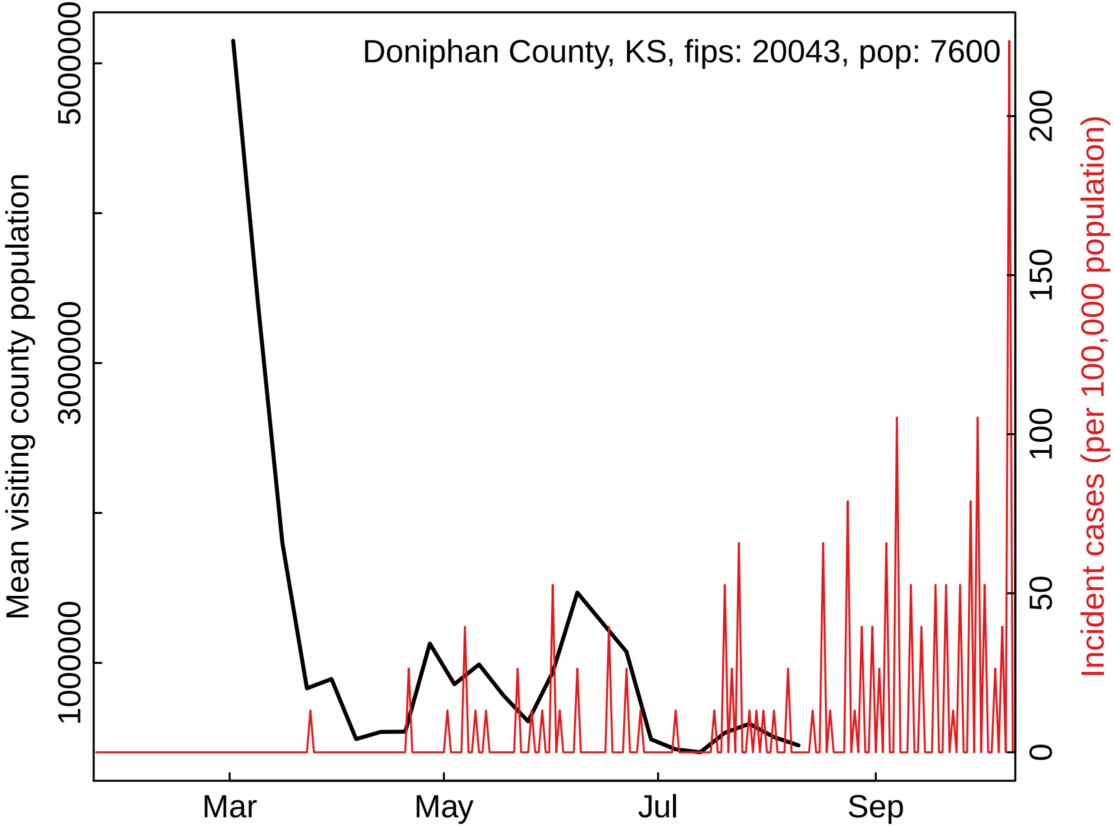

Doniphan County, KS, fips: 20043, pop: 7600
Mean visiting county population
1000000
3000000
5000000
Mar
May
Jul
Sep
0
50
100
150
200
Incident cases (per 100,000 population)

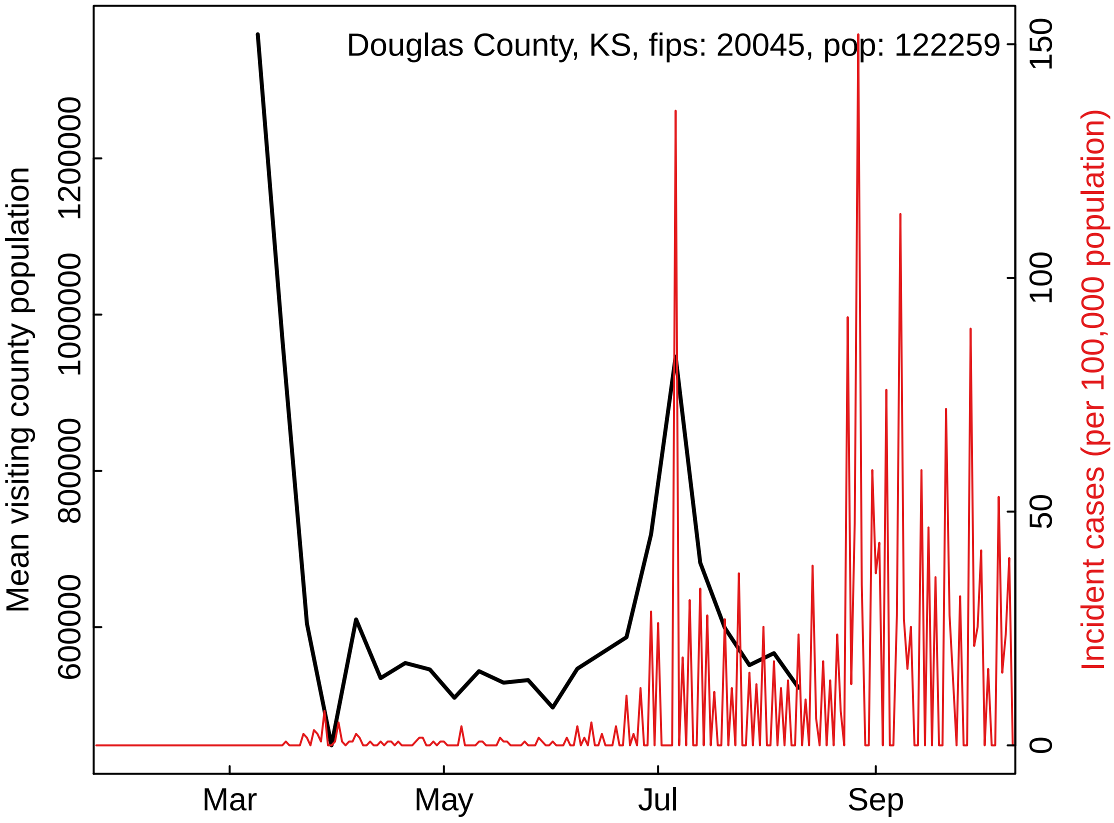

Douglas County, KS, fips: 20045, pop: 122259
Mean visiting county population
600000
800000
1000000
1200000
Incident cases (per 100,000 population)
0
50
100
150
Mar
May
Jul
Sep

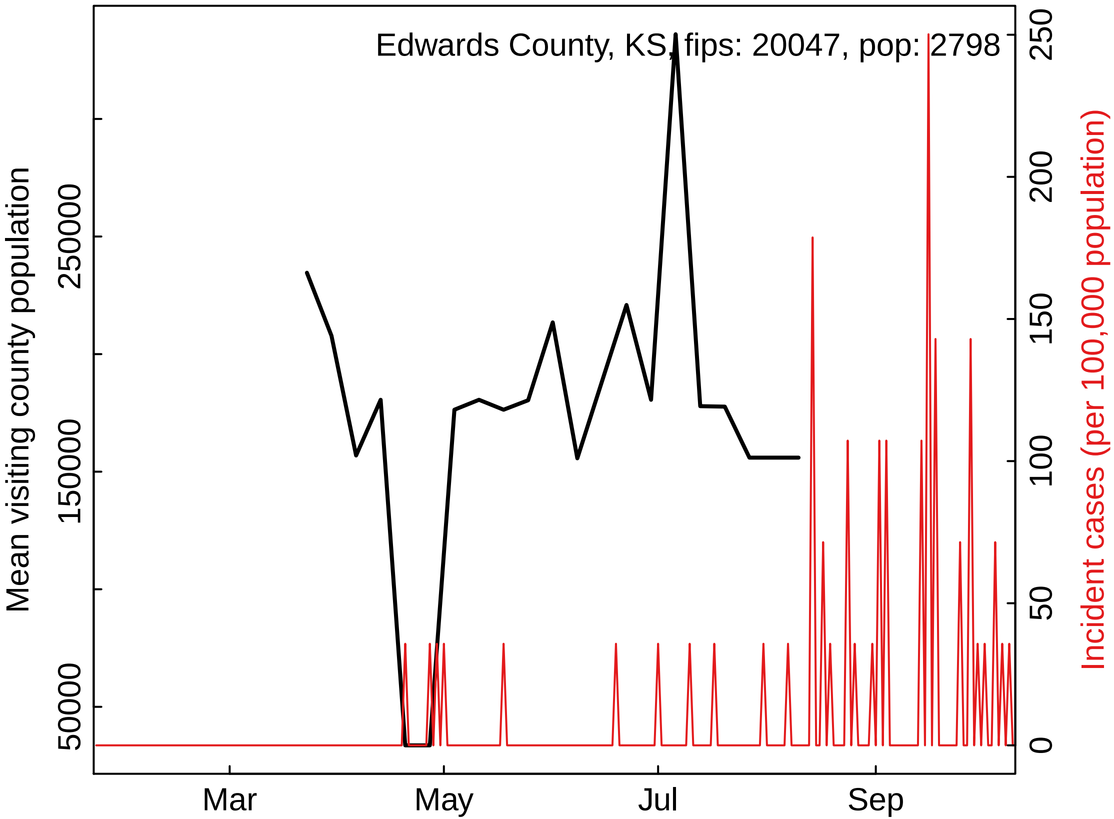

Edwards County, KS, fips: 20047, pop: 2798
Mean visiting county population
50000
150000
250000
Incident cases (per 100,000 population)
0
50
100
150
200
250
Mar
May
Jul
Sep

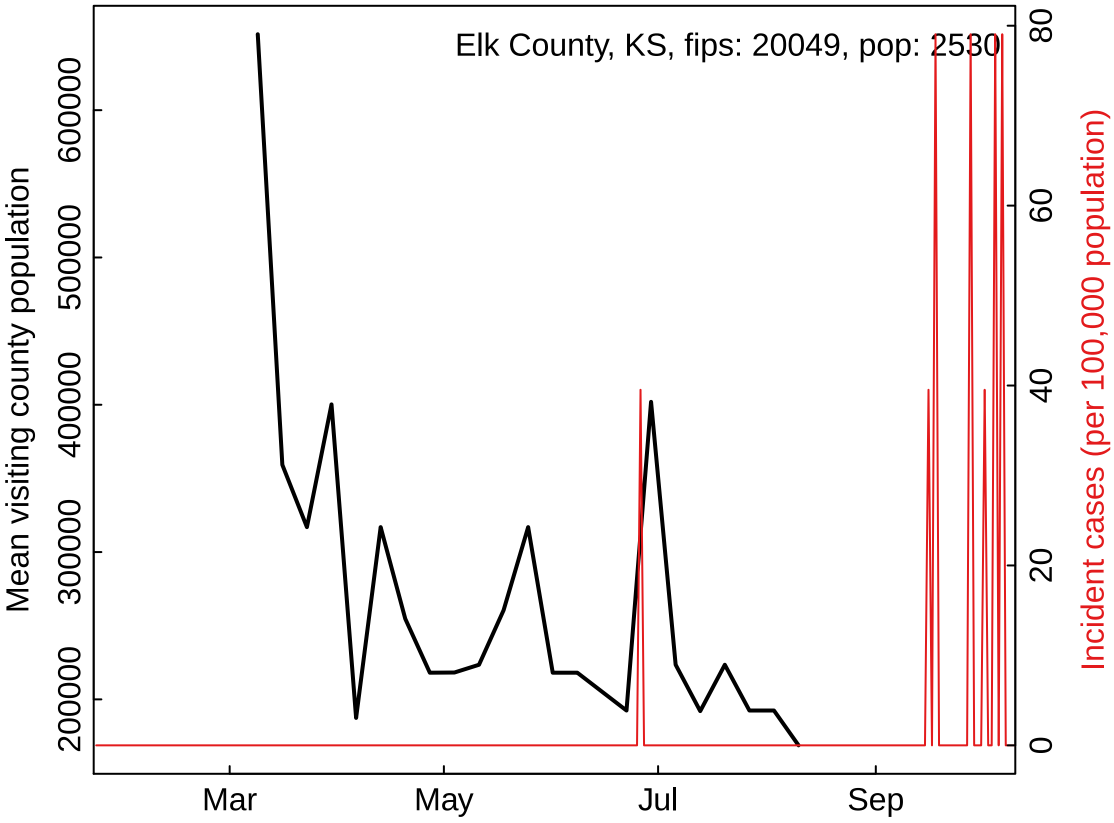
Elk County, KS, fips: 20049, pop: 2530
Mean visiting county population
200000
300000
400000
500000
600000
Incident cases (per 100,000 population)
0
20
40
60
80
Mar
May
Jul
Sep

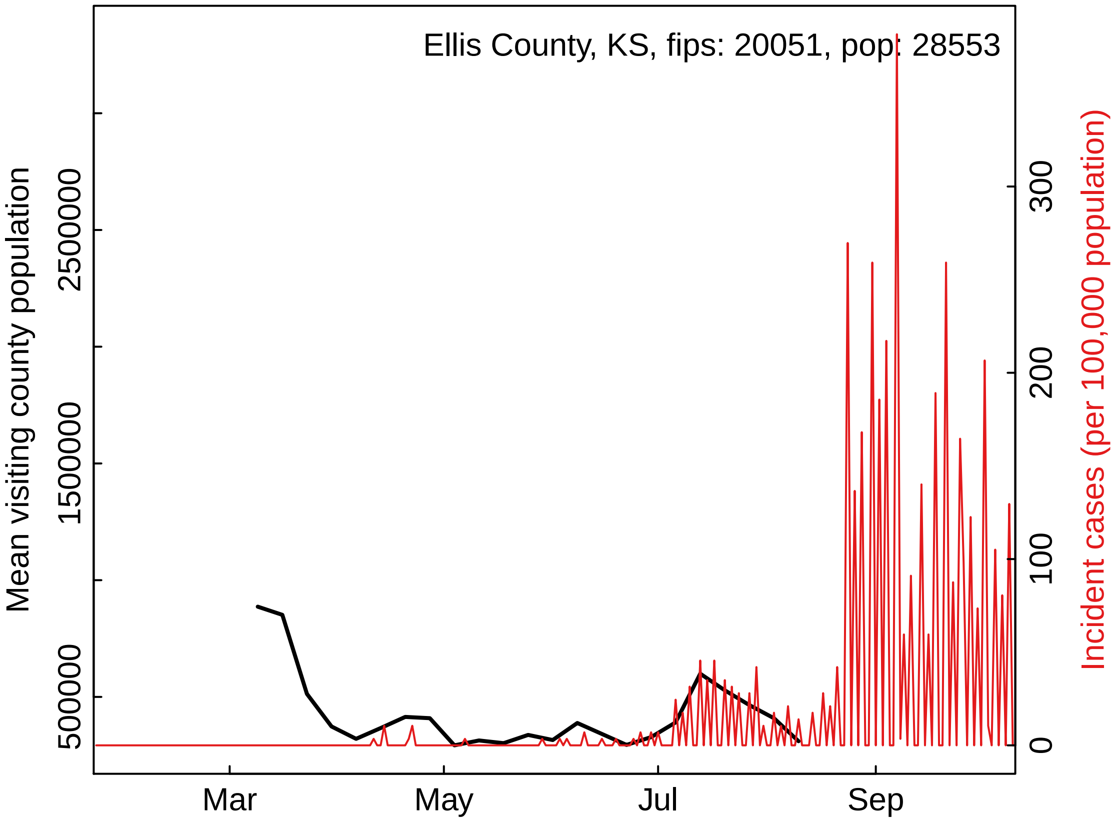

Ellis County, KS, fips: 20051, pop: 28553
Mean visiting county population
500000
1500000
2500000
Mar
May
Jul
Sep
Incident cases (per 100,000 population)
0
100
200
300

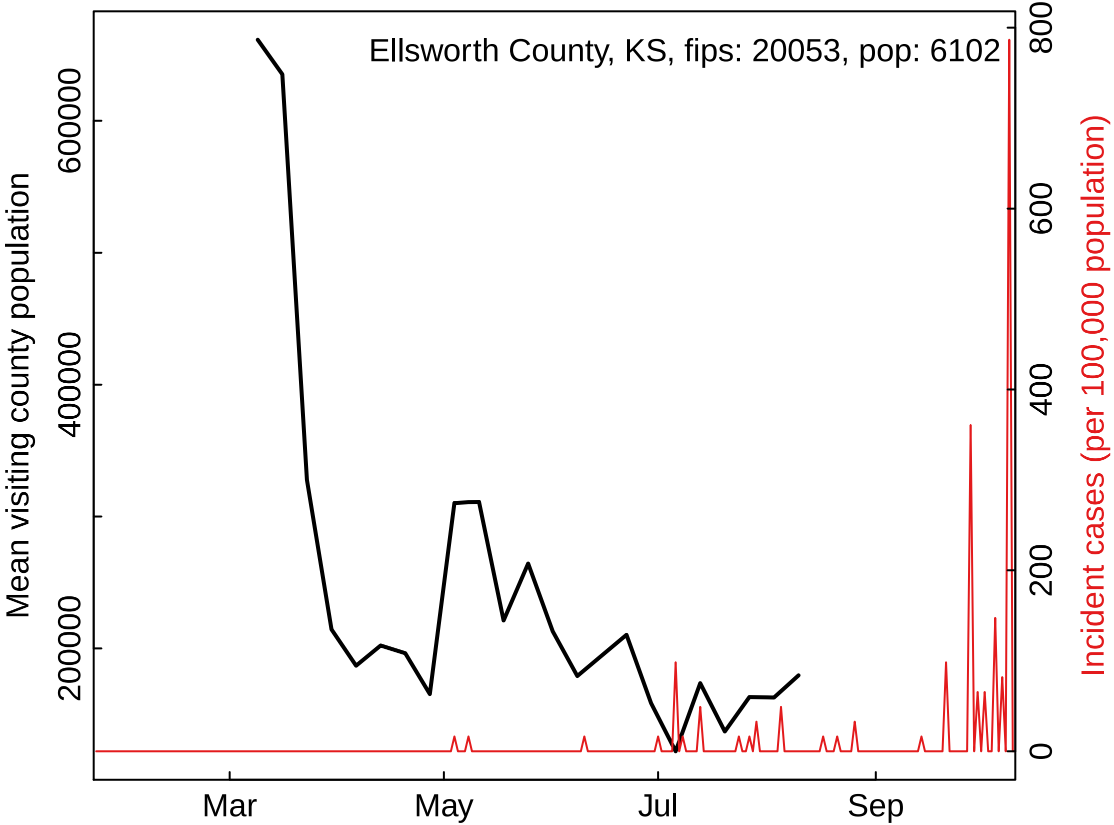

Ellsworth County, KS, fips: 20053, pop: 6102
Mean visiting county population
200000
400000
600000
Incident cases (per 100,000 population)
0
200
400
600
800
Mar
May
Jul
Sep

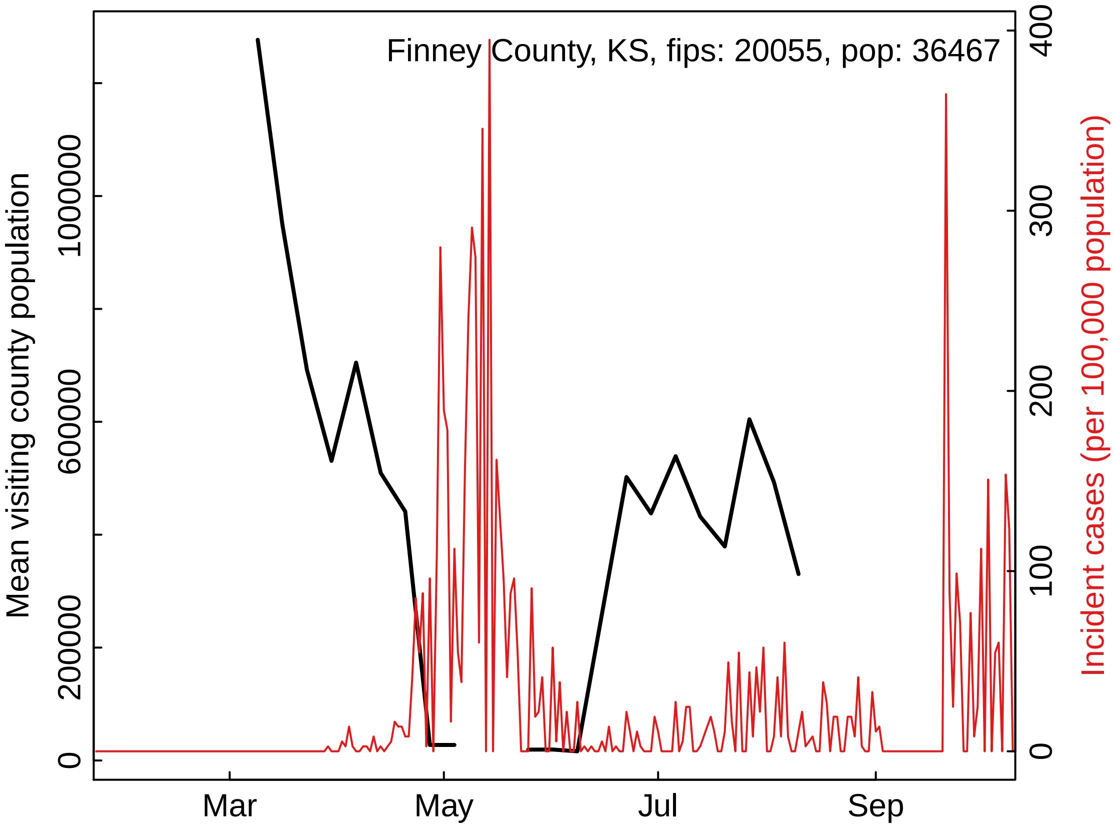

Finney County, KS, fips: 20055, pop: 36467
Mean visiting county population
0
200000
600000
1000000
Incident cases (per 100,000 population)
0
100
200
300
400
Mar
May
Jul
Sep

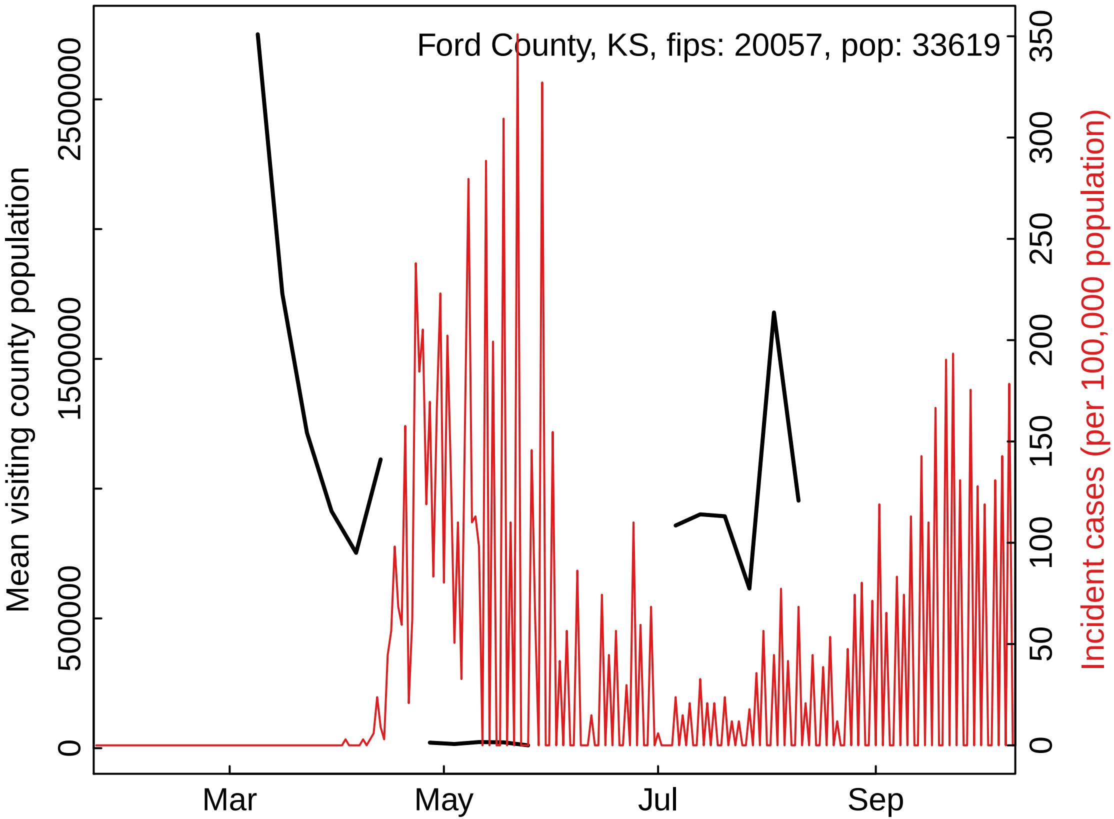

Ford County, KS, fips: 20057, pop: 33619
Mean visiting county population
0
500000
1500000
2500000
Incident cases (per 100,000 population)
0
50
100
150
200
250
300
350
Mar
May
Jul
Sep

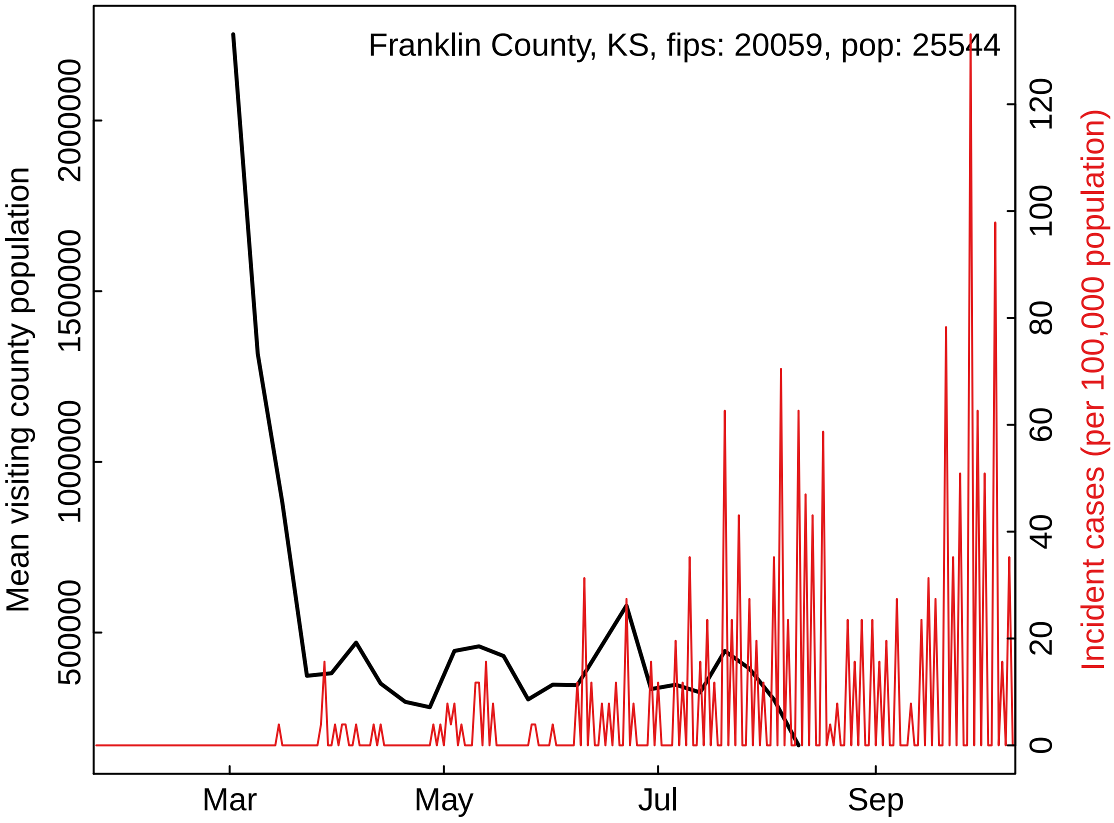

Franklin County, KS, fips: 20059, pop: 25544
Mean visiting county population
500000
1000000
1500000
2000000
Incident cases (per 100,000 population)
0
20
40
60
80
100
120
Mar
May
Jul
Sep

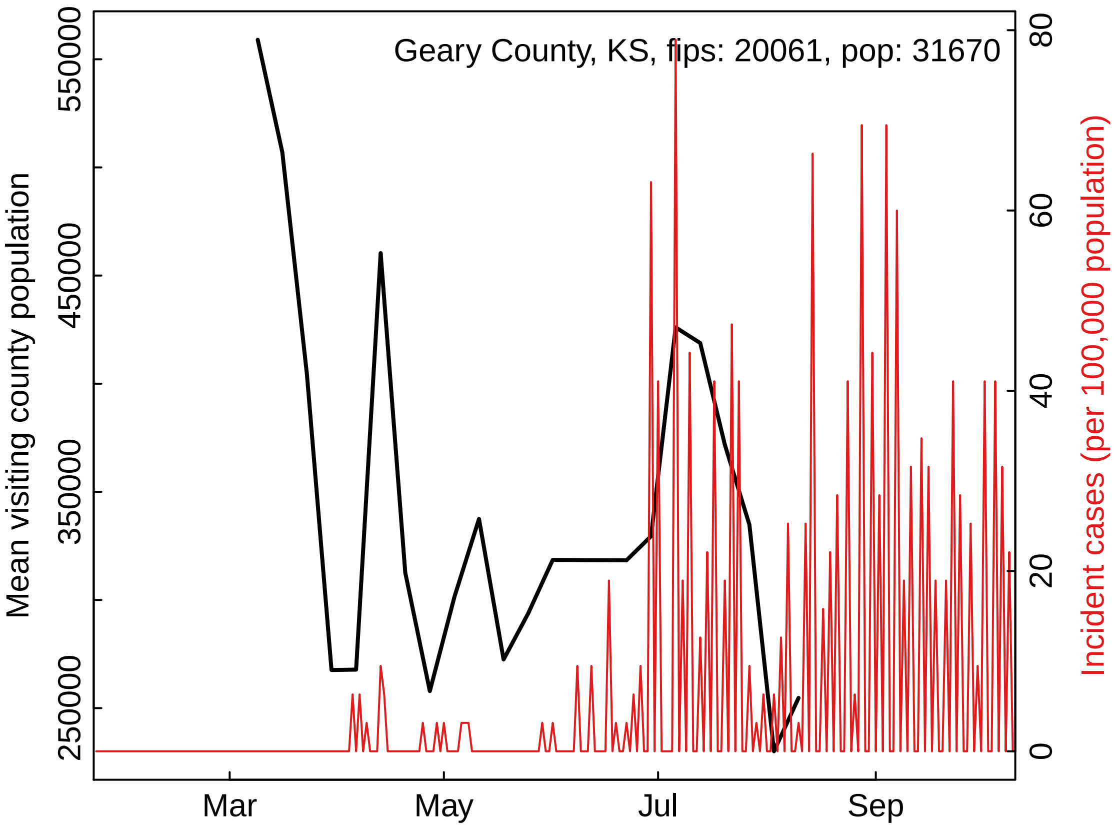

Geary County, KS, fips: 20061, pop: 31670
Mean visiting county population
250000
350000
450000
550000
Incident cases (per 100,000 population)
0
20
40
60
80
Mar
May
Jul
Sep

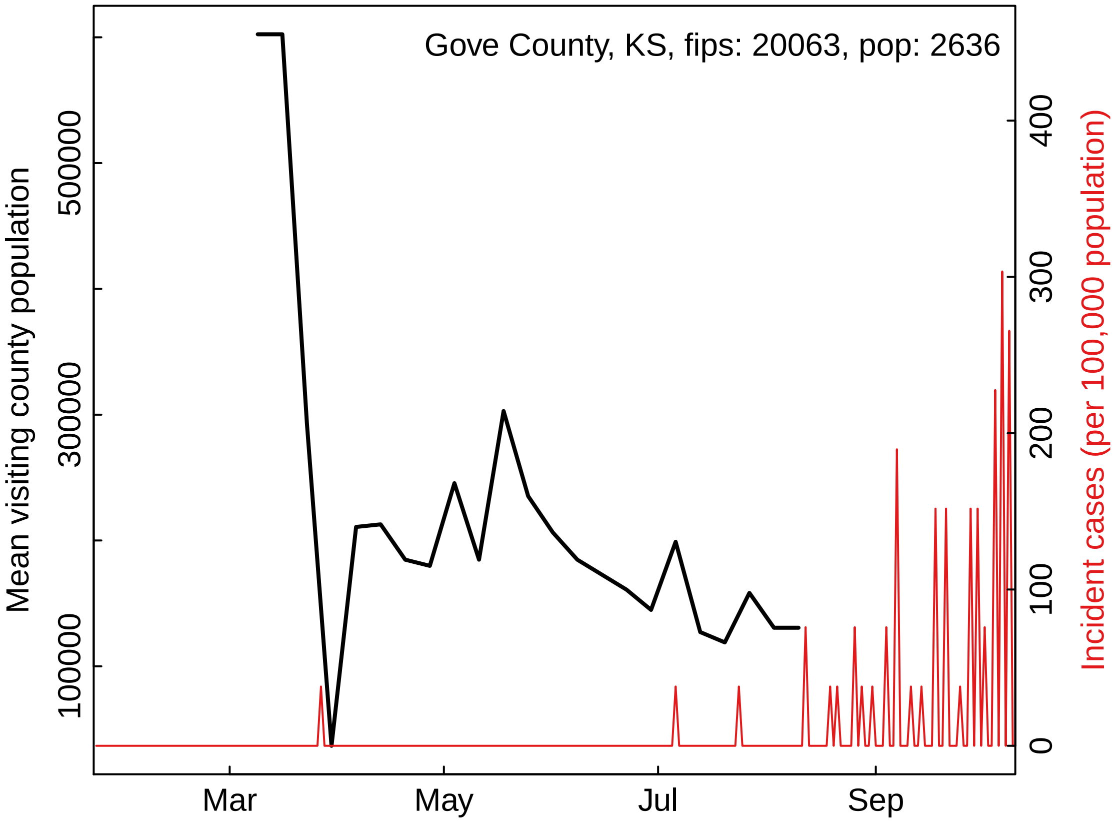

Gove County, KS, fips: 20063, pop: 2636
Mean visiting county population
Incident cases (per 100,000 population)
100000
300000
500000
0
100
200
300
400
Mar
May
Jul
Sep

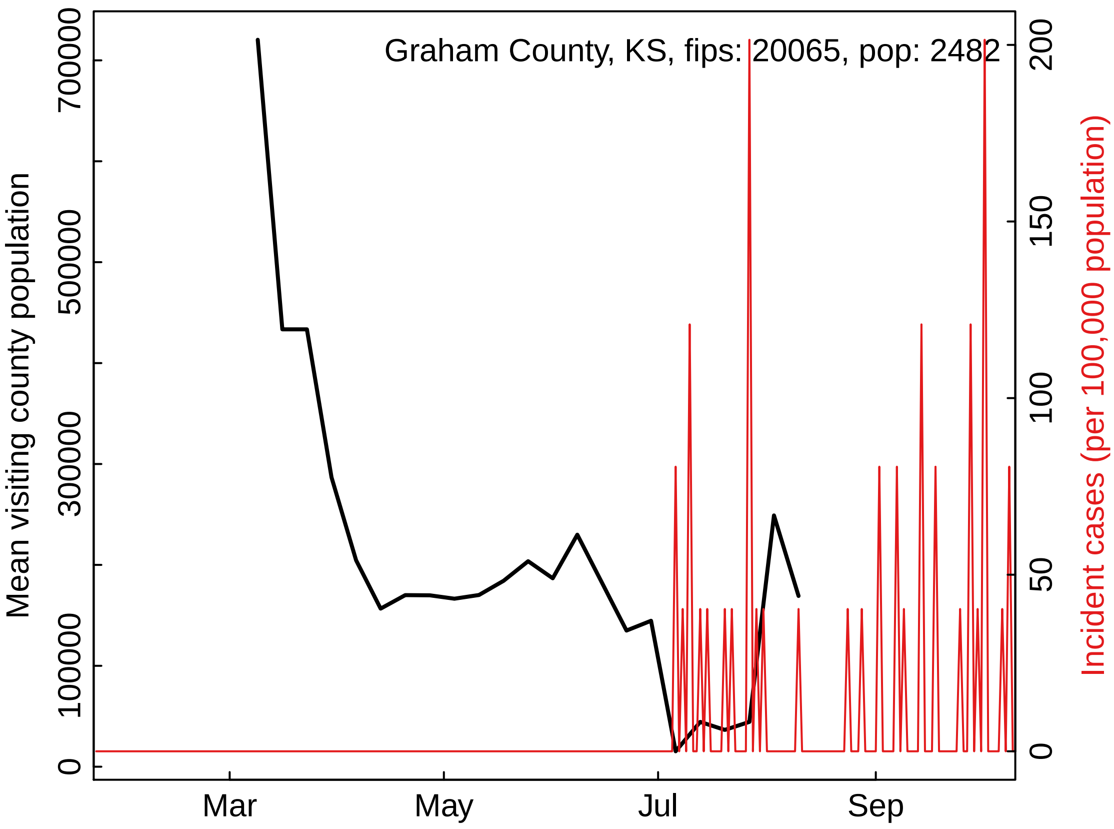
Graham County, KS, fips: 20065, pop: 2482
Mean visiting county population
0
100000
300000
500000
700000
Incident cases (per 100,000 population)
0
50
100
150
200
Mar
May
Jul
Sep

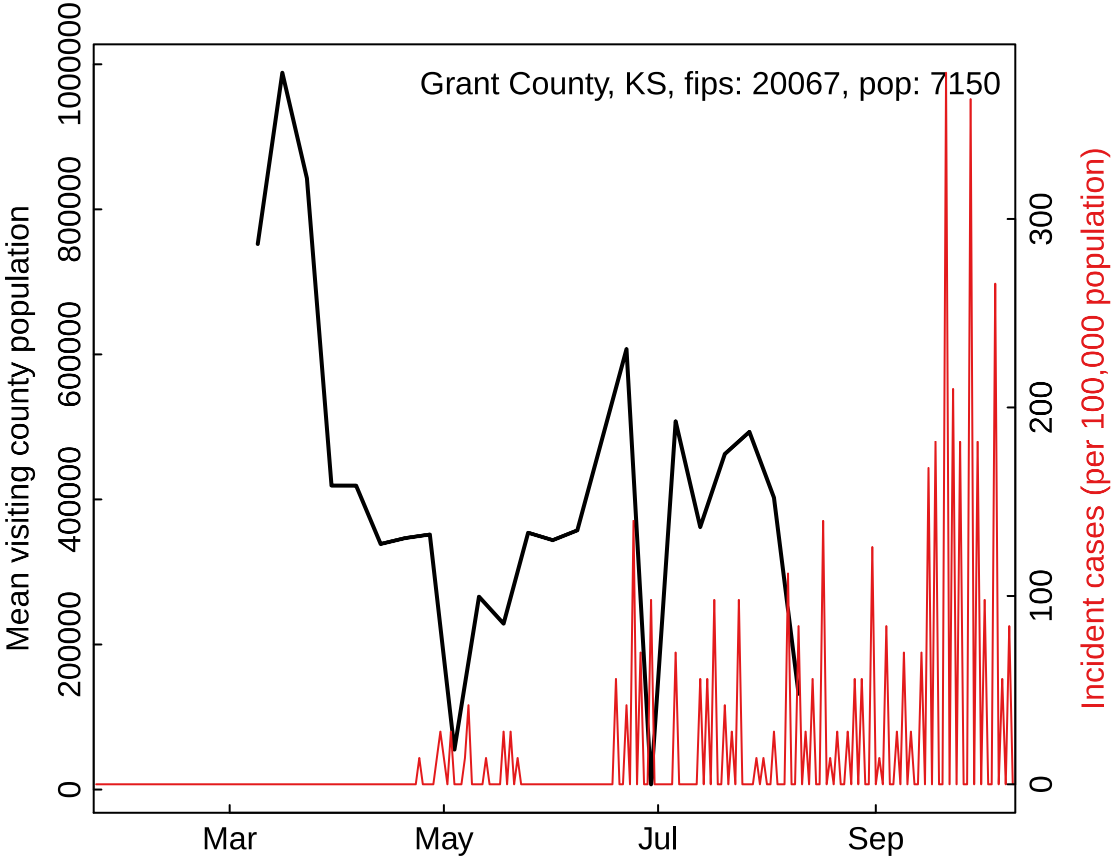

Grant County, KS, fips: 20067, pop: 7150
Mean visiting county population
0
200000
400000
600000
800000
1000000
Incident cases (per 100,000 population)
0
100
200
300
Mar
May
Jul
Sep

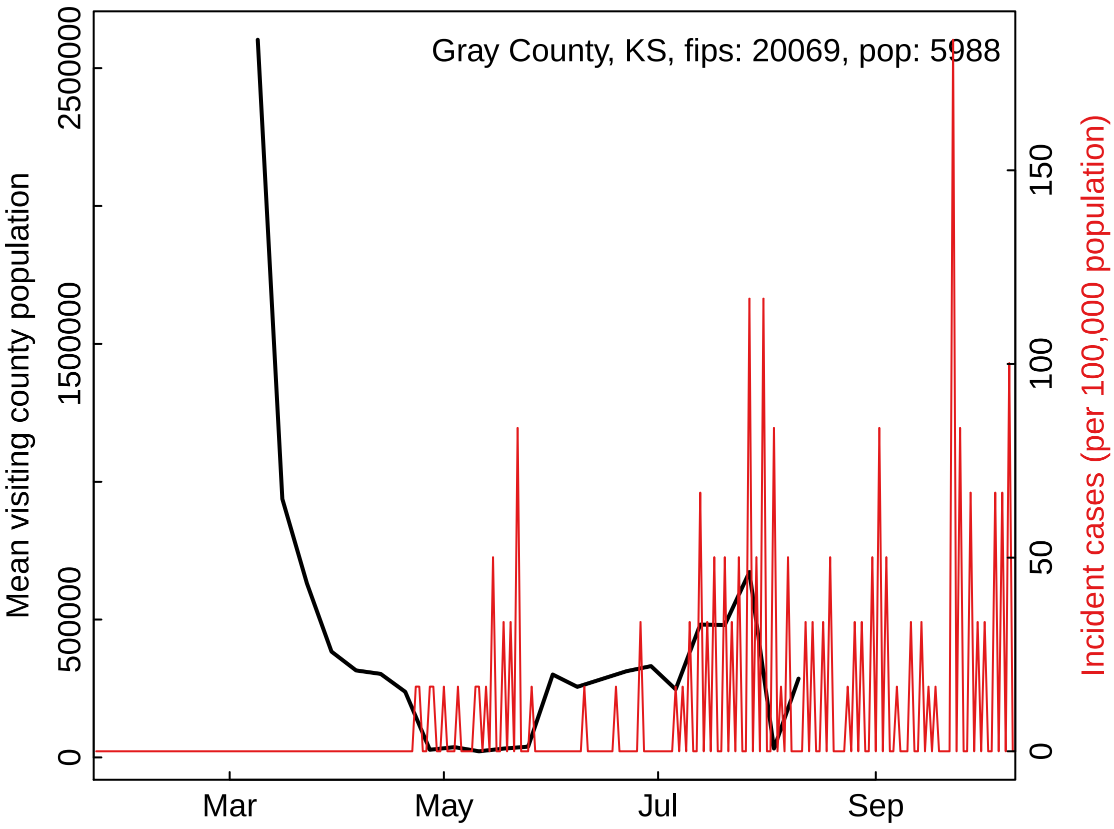

Gray County, KS, fips: 20069, pop: 5988
Mean visiting county population
0
500000
1500000
2500000
Incident cases (per 100,000 population)
0
50
100
150
Mar
May
Jul
Sep

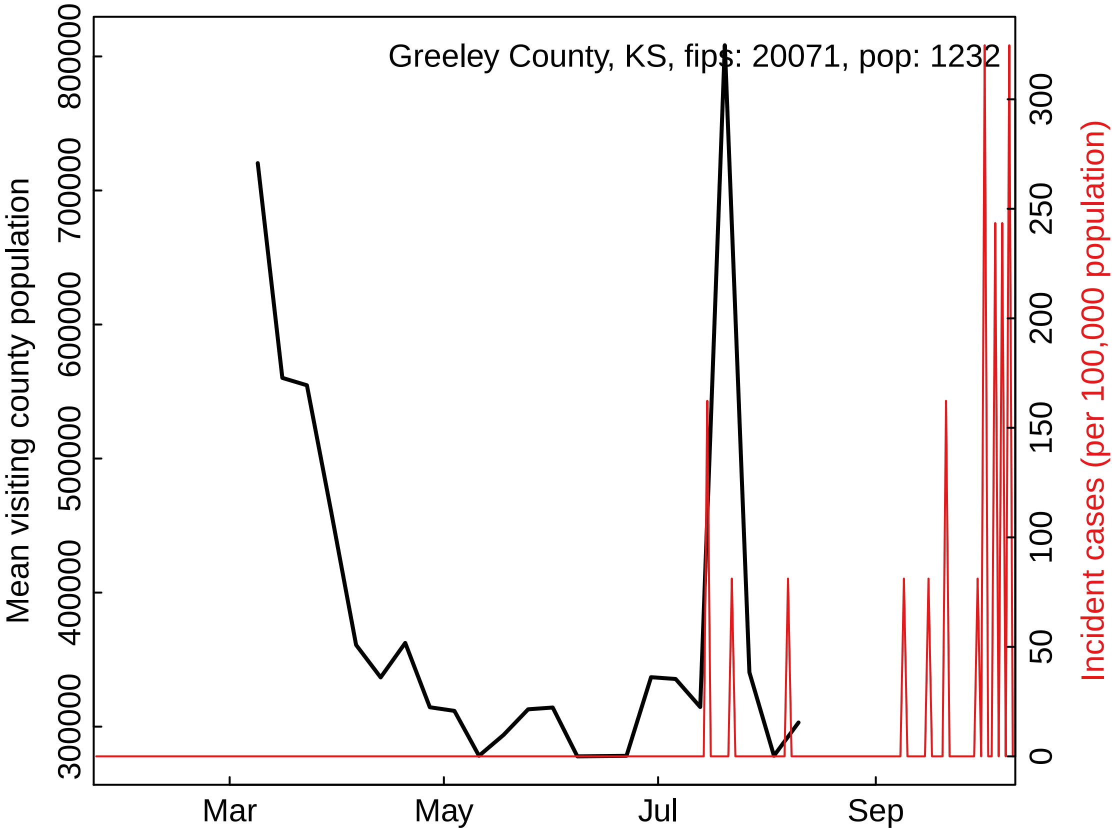
Greeley County, KS, fips: 20071, pop: 1232
Mean visiting county population
300000
400000
500000
600000
700000
800000
Incident cases (per 100,000 population)
0
50
100
150
200
250
300
Mar
May
Jul
Sep

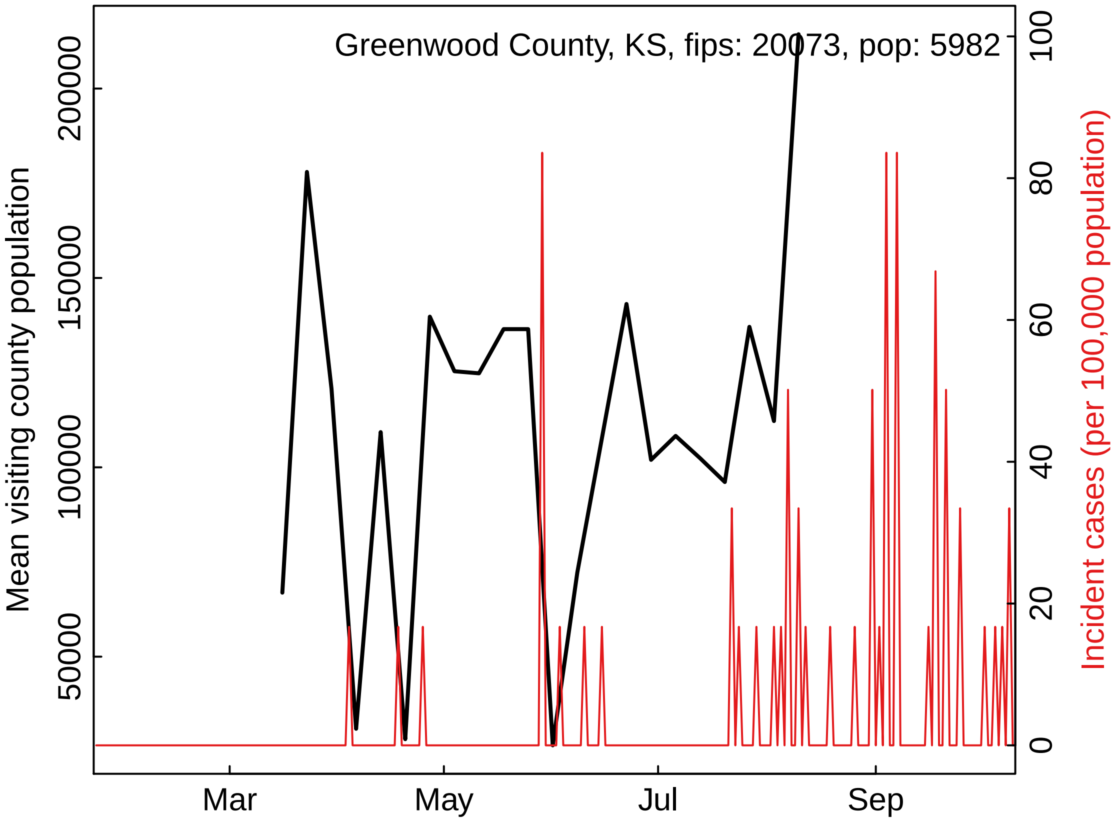

Greenwood County, KS, fips: 20073, pop: 5982
Mean visiting county population
50000
100000
150000
200000
Incident cases (per 100,000 population)
0
20
40
60
80
100
Mar
May
Jul
Sep

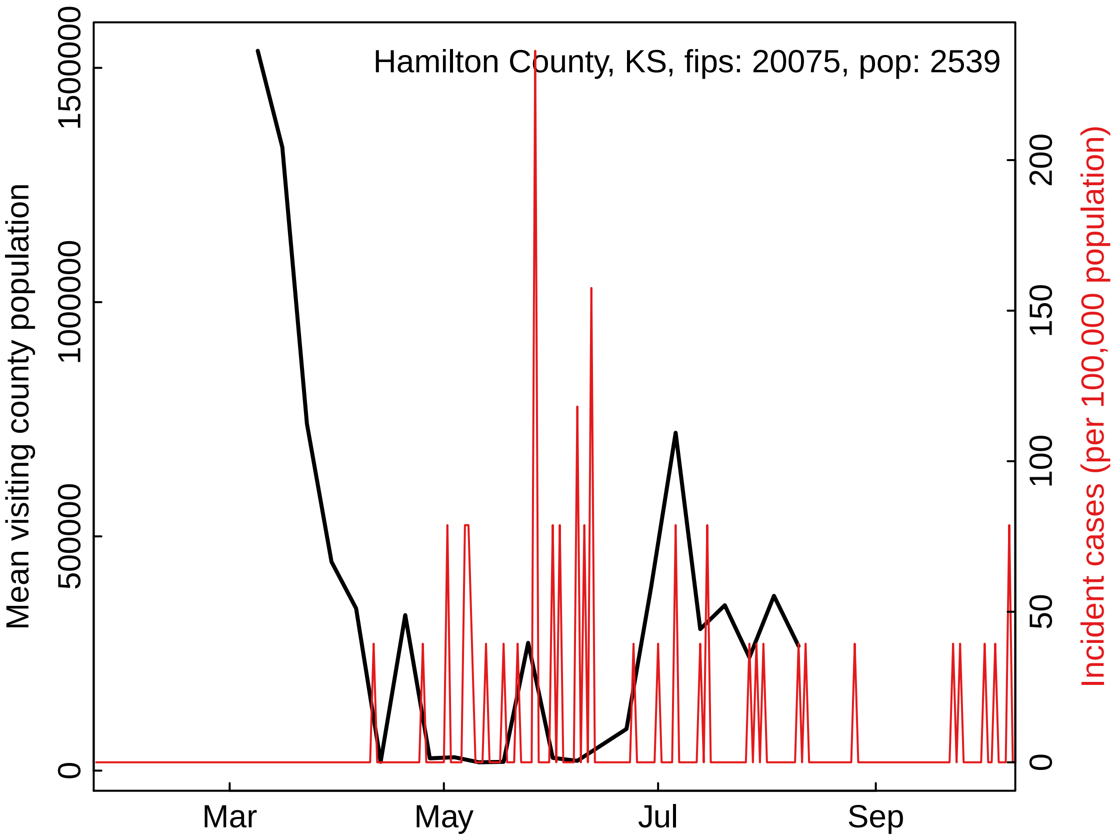

Hamilton County, KS, fips: 20075, pop: 2539
Mean visiting county population
0
500000
1000000
1500000
Incident cases (per 100,000 population)
0
50
100
150
200
Mar
May
Jul
Sep

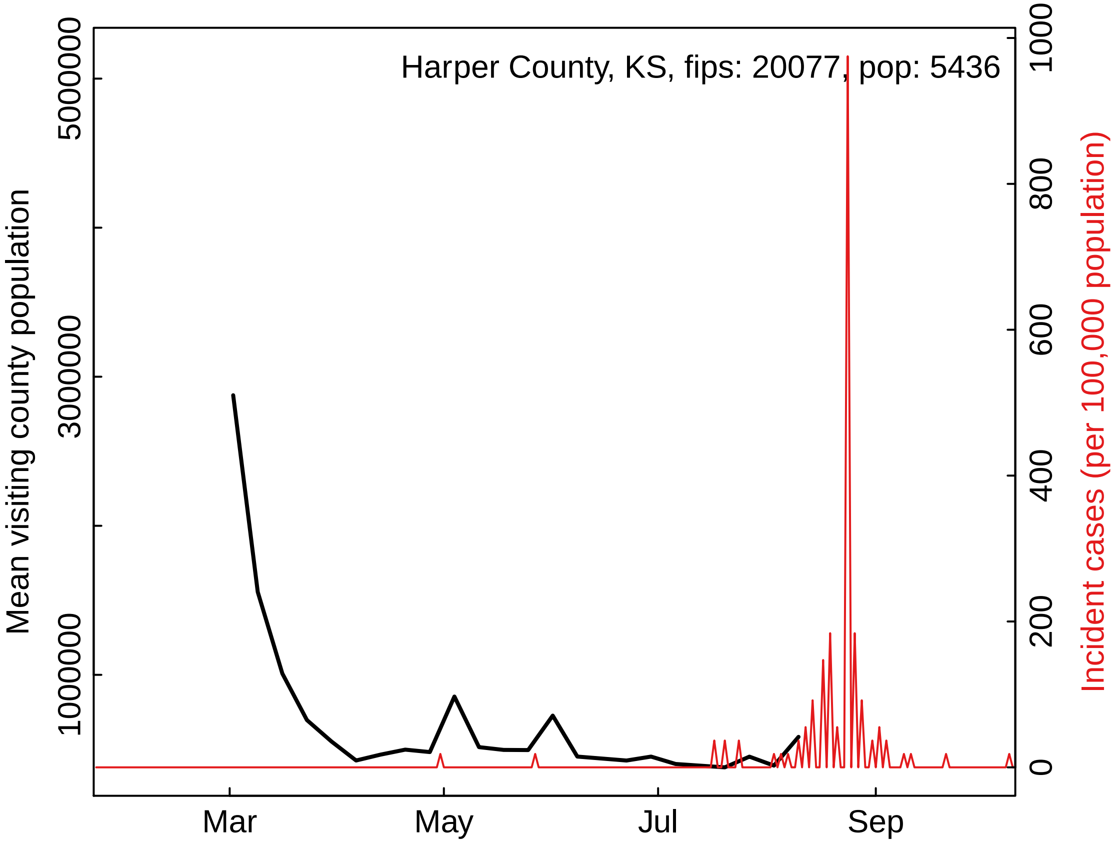

Harper County, KS, fips: 20077, pop: 5436
Mean visiting county population
1000000
3000000
5000000
Incident cases (per 100,000 population)
0
200
400
600
800
1000
Mar
May
Jul
Sep

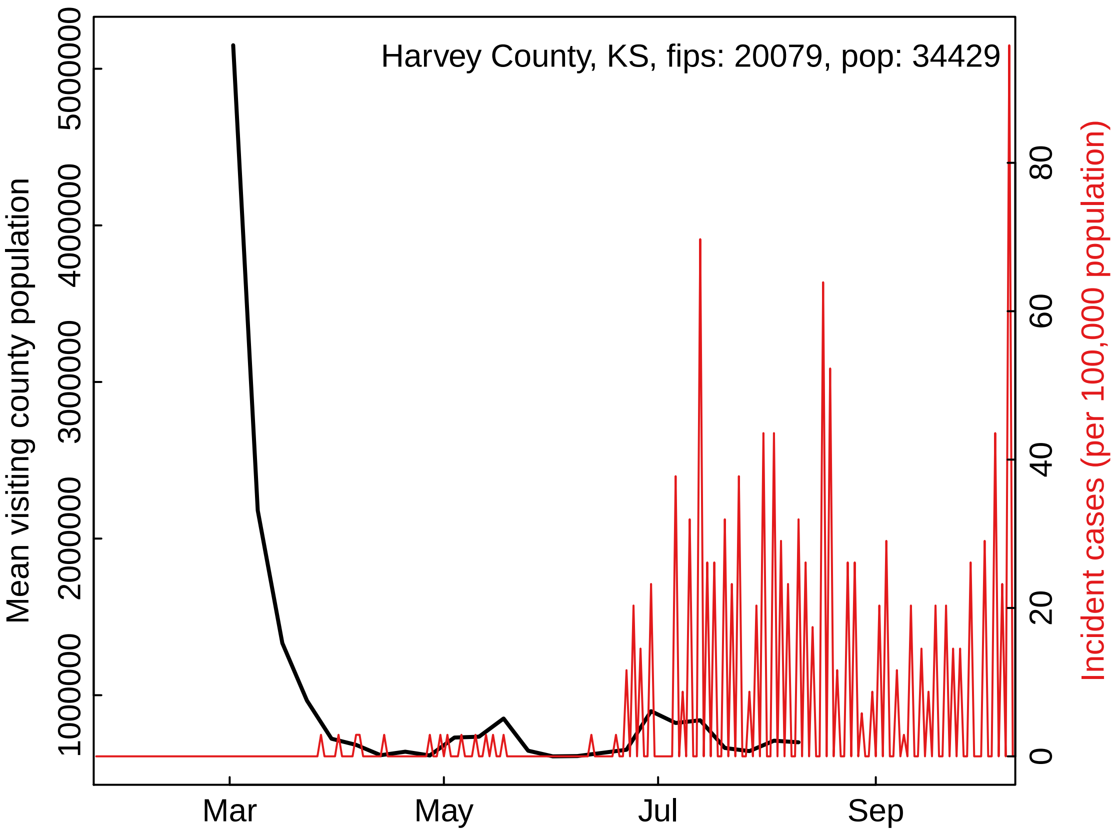

Harvey County, KS, fips: 20079, pop: 34429
Mean visiting county population
Incident cases (per 100,000 population)
1000000
2000000
3000000
4000000
5000000
0
20
40
60
80
Mar
May
Jul
Sep

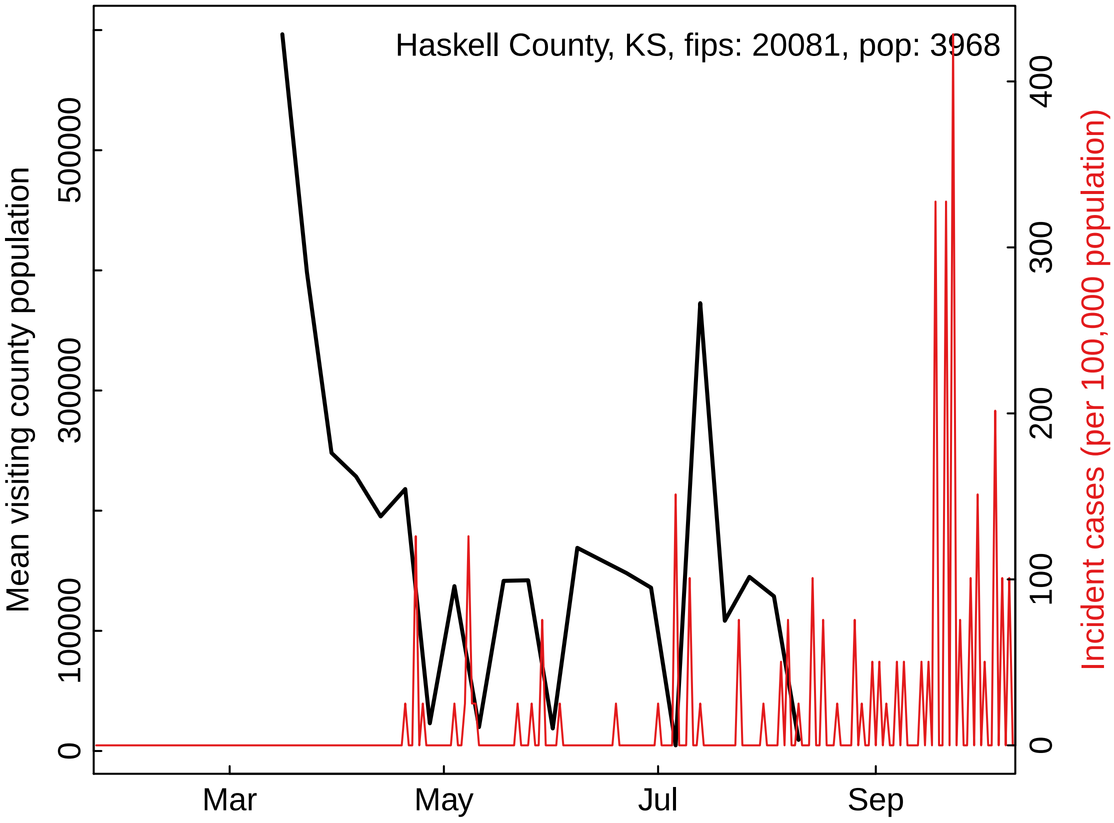

Haskell County, KS, fips: 20081, pop: 3968
Mean visiting county population
0
100000
300000
500000
Incident cases (per 100,000 population)
0
100
200
300
400
Mar
May
Jul
Sep

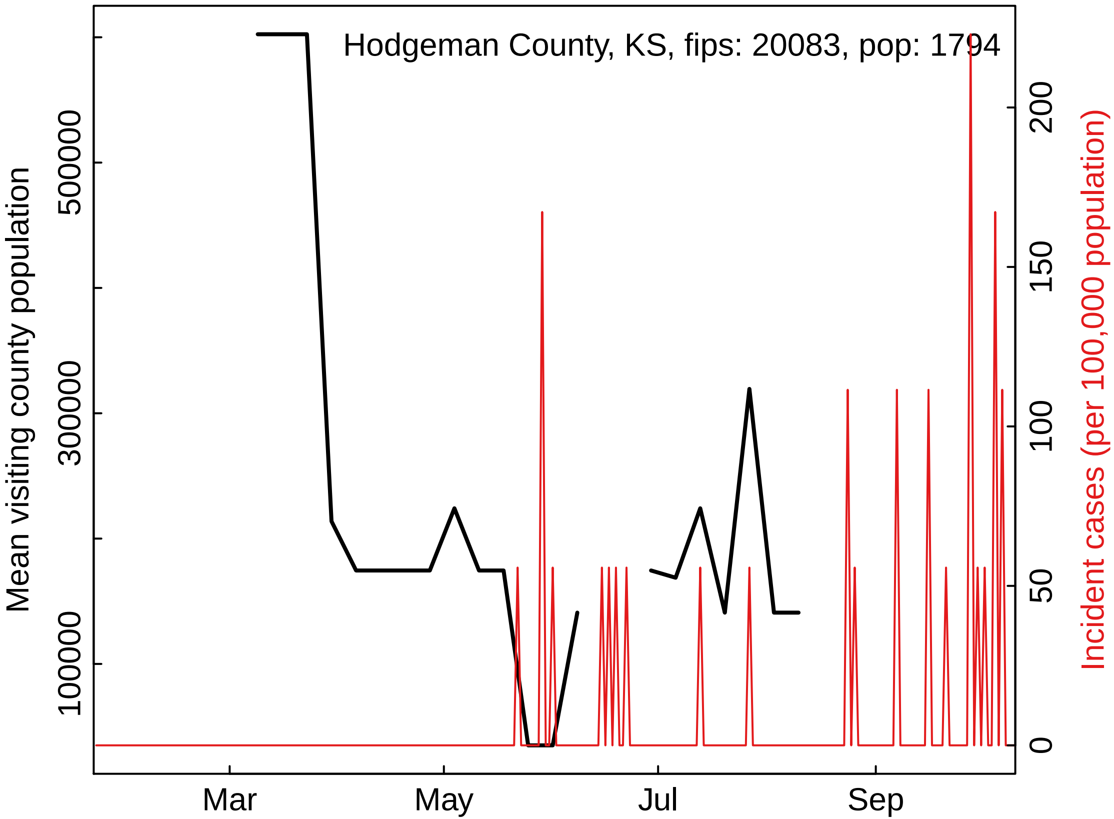

Hodgeman County, KS, fips: 20083, pop: 1794
Mean visiting county population
100000
300000
500000
Incident cases (per 100,000 population)
0
50
100
150
200
Mar
May
Jul
Sep

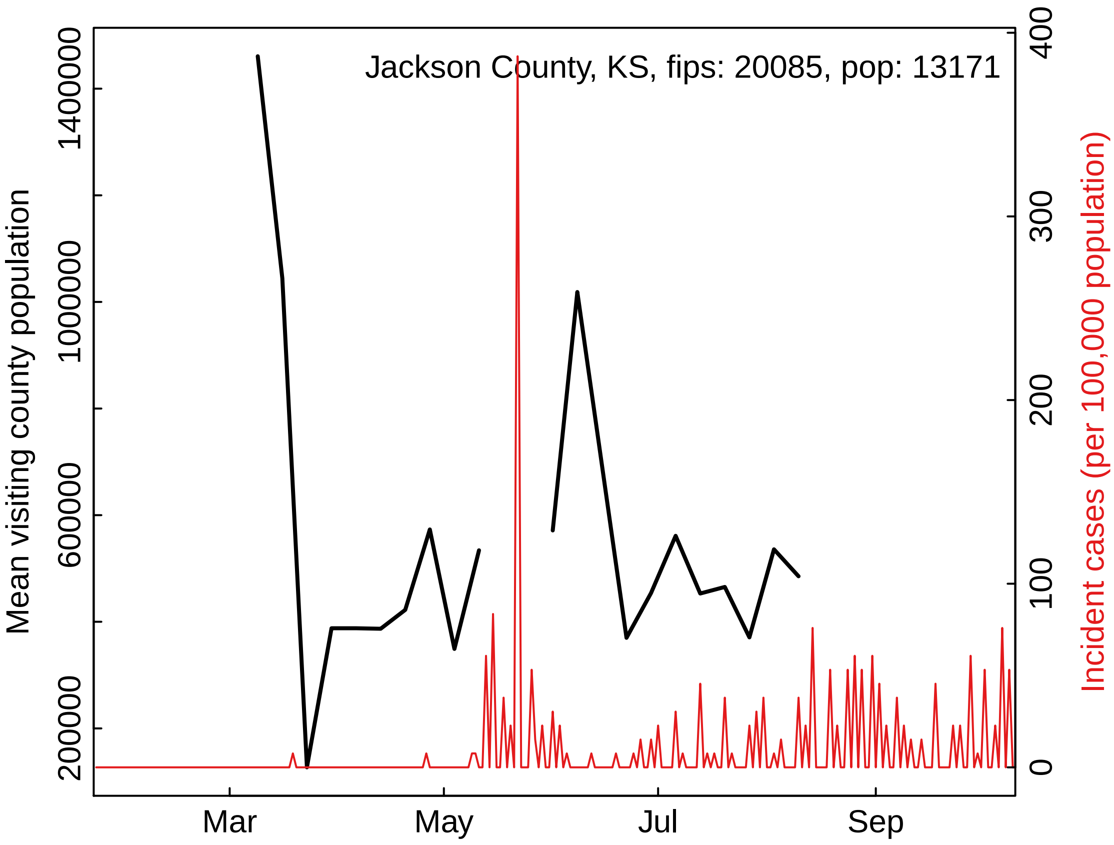

Jackson County, KS, fips: 20085, pop: 13171
Mean visiting county population
200000
600000
1000000
1400000
Incident cases (per 100,000 population)
0
100
200
300
400
Mar
May
Jul
Sep

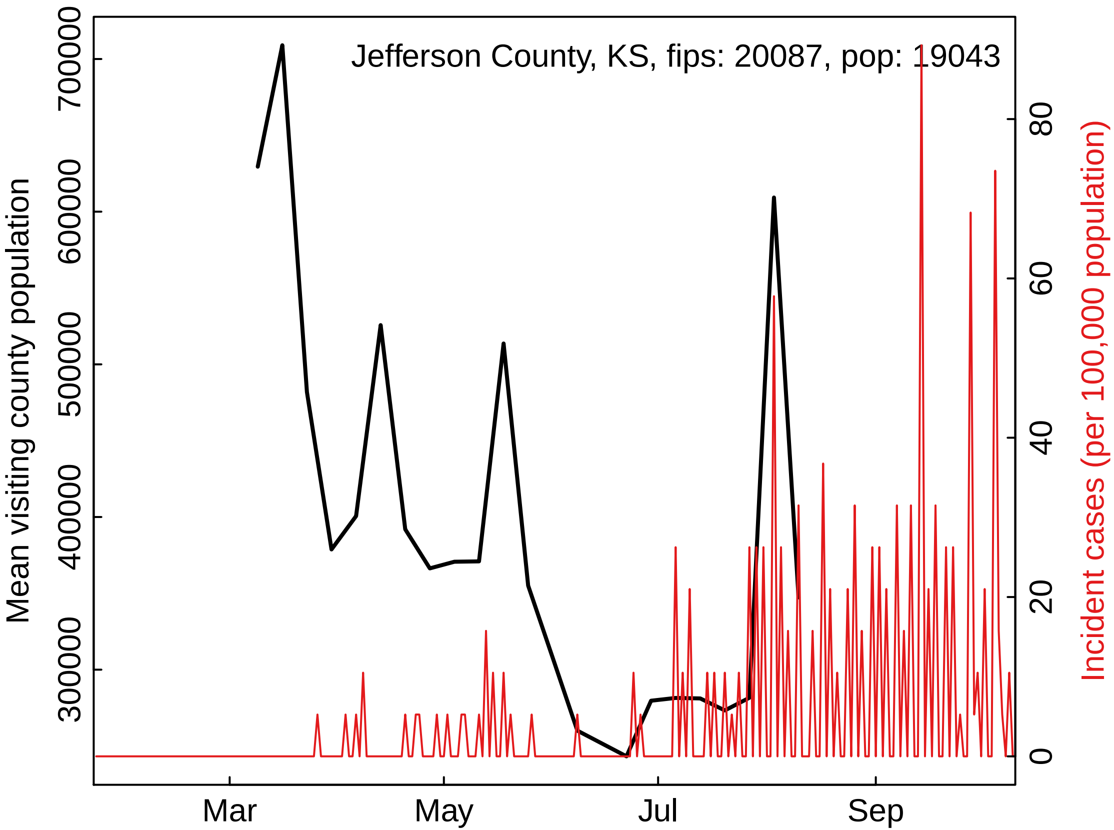
Jefferson County, KS, fips: 20087, pop: 19043
Mean visiting county population
300000
400000
500000
600000
700000
Mar
May
Jul
Sep
0
20
40
60
80
Incident cases (per 100,000 population)

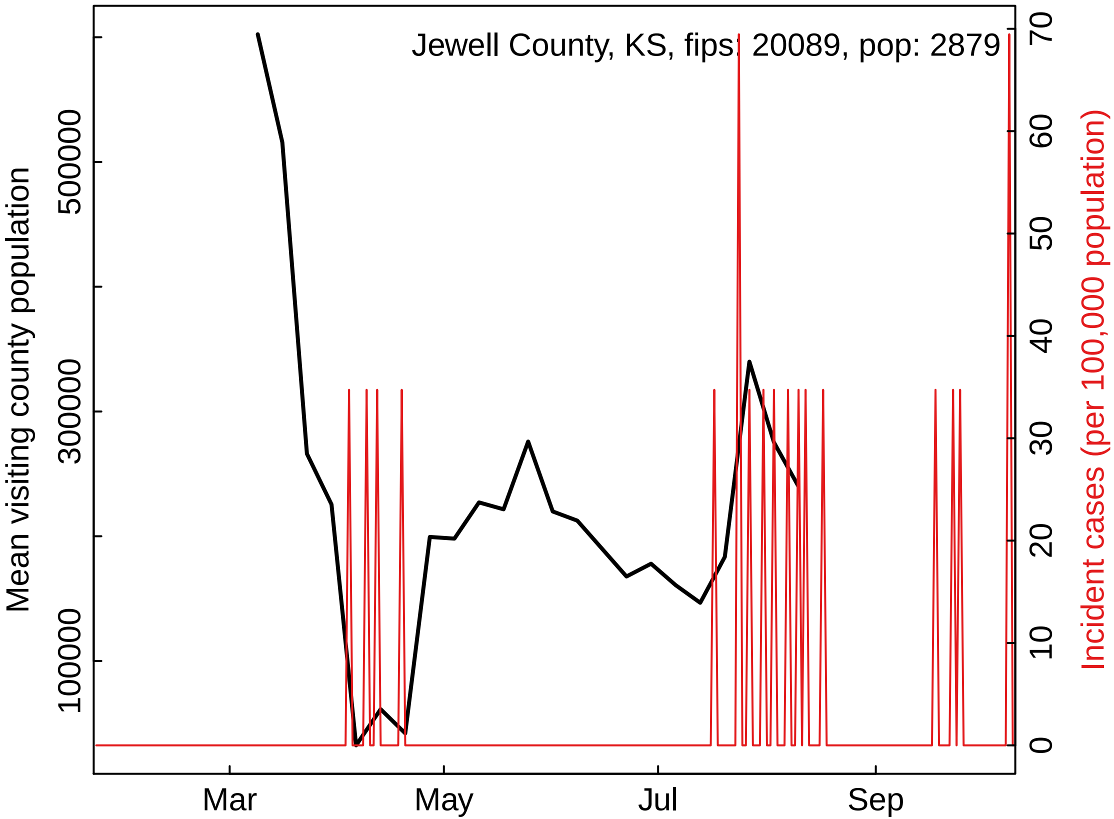

Jewell County, KS, fips: 20089, pop: 2879
Mean visiting county population
100000
300000
500000
Incident cases (per 100,000 population)
0
10
20
30
40
50
60
70
Mar
May
Jul
Sep

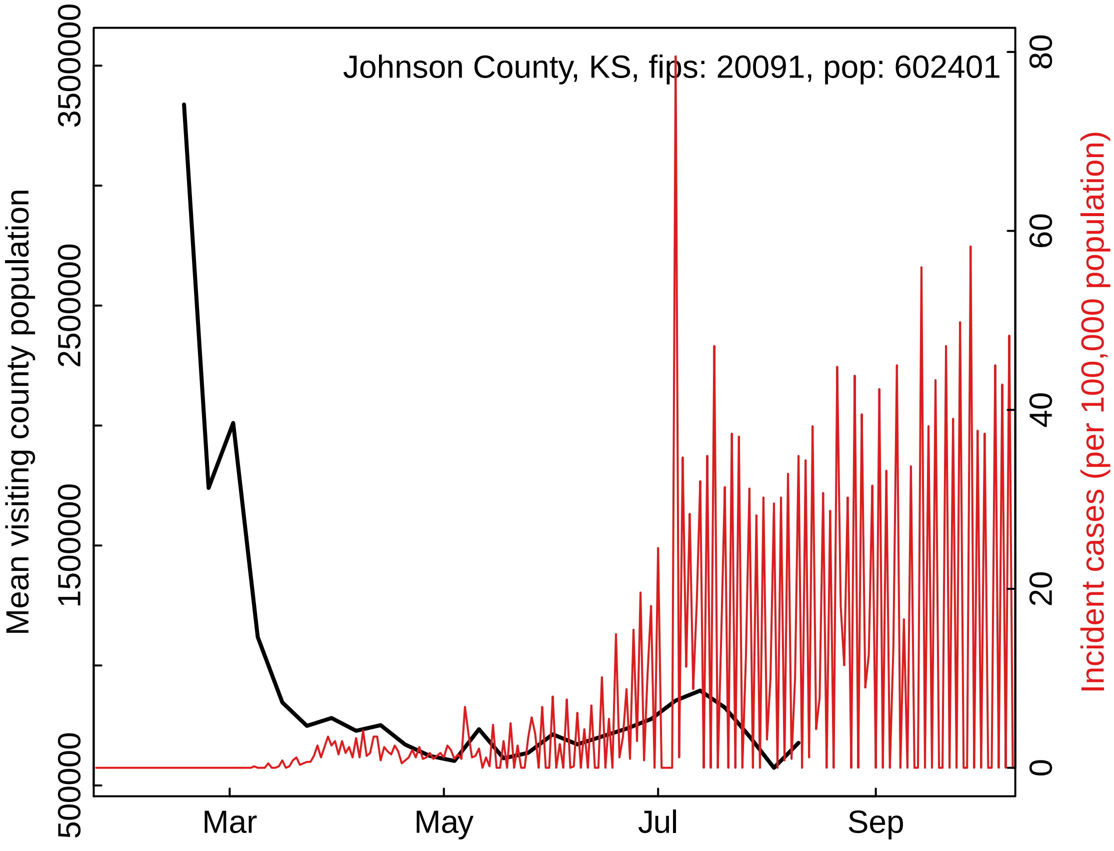

Johnson County, KS, fips: 20091, pop: 602401
Mean visiting county population
500000
1500000
2500000
3500000
Incident cases (per 100,000 population)
0
20
40
60
80
Mar
May
Jul
Sep

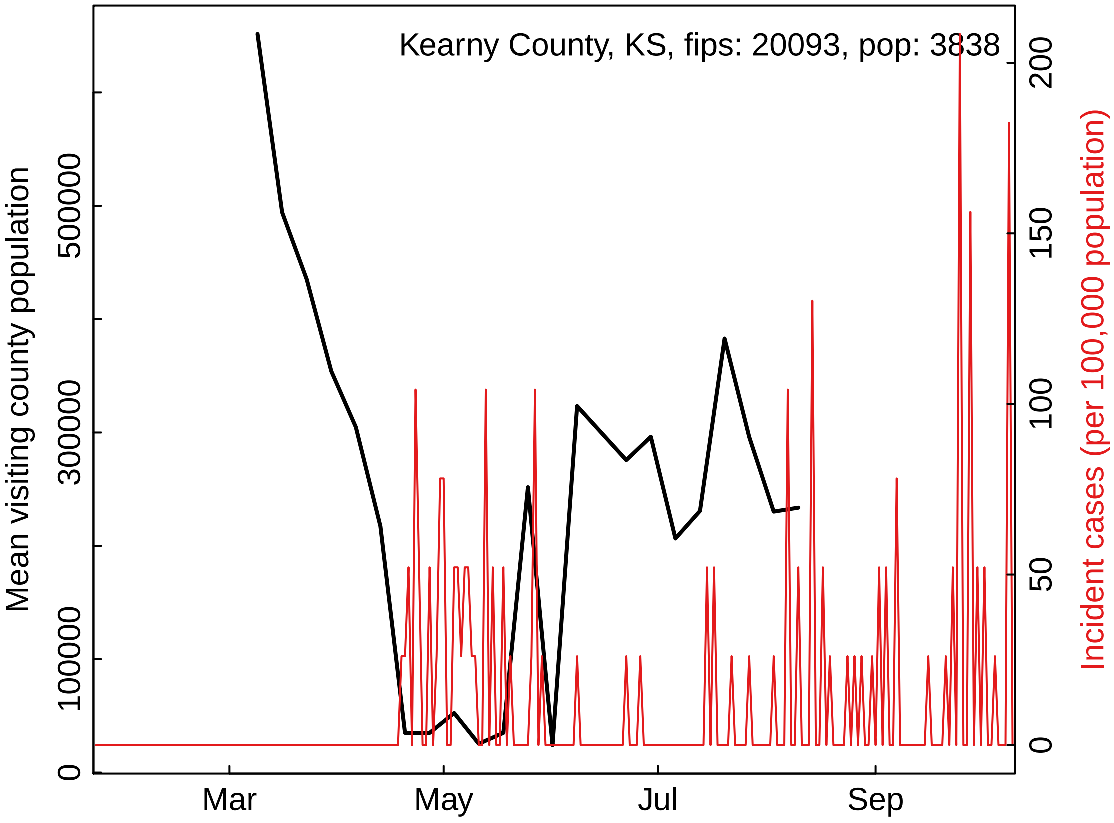

Kearny County, KS, fips: 20093, pop: 3838
Mean visiting county population
0
100000
300000
500000
Incident cases (per 100,000 population)
0
50
100
150
200
Mar
May
Jul
Sep

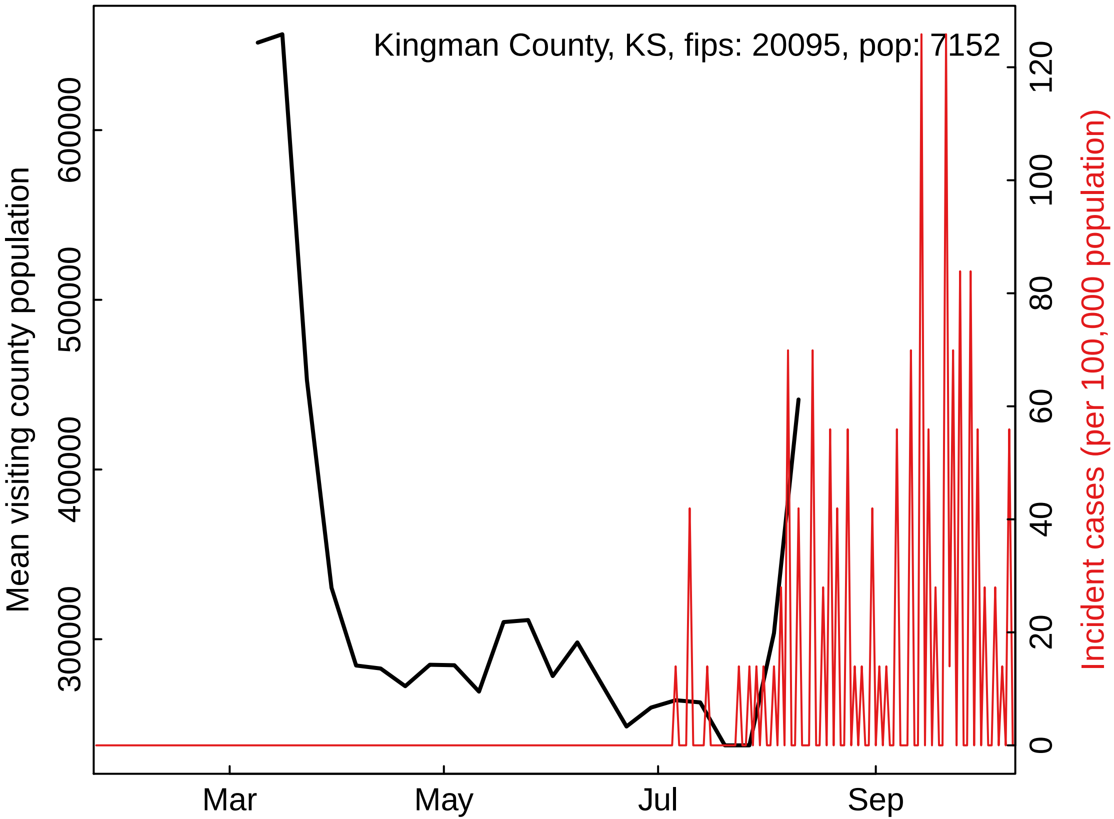

Kingman County, KS, fips: 20095, pop: 7152
Mean visiting county population
300000
400000
500000
600000
Incident cases (per 100,000 population)
0
20
40
60
80
100
120
Mar
May
Jul
Sep

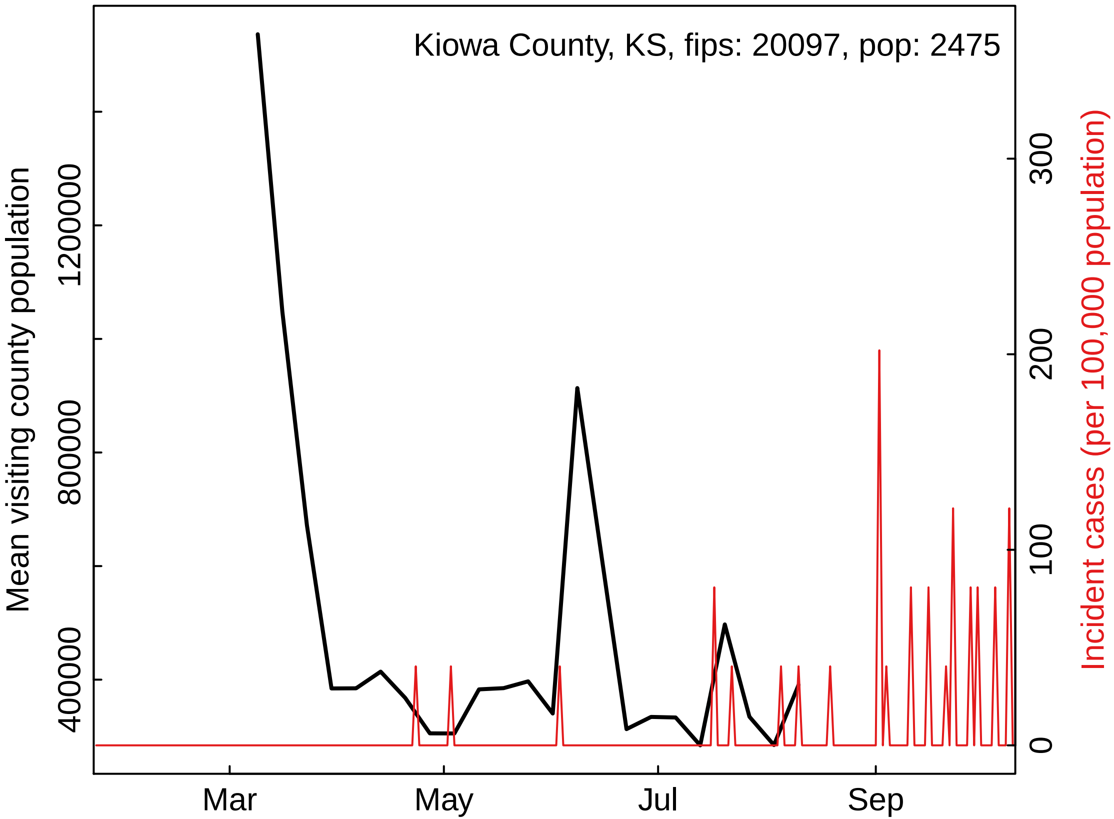
Kiowa County, KS, fips: 20097, pop: 2475
Mean visiting county population
400000
800000
1200000
Incident cases (per 100,000 population)
0
100
200
300
Mar
May
Jul
Sep

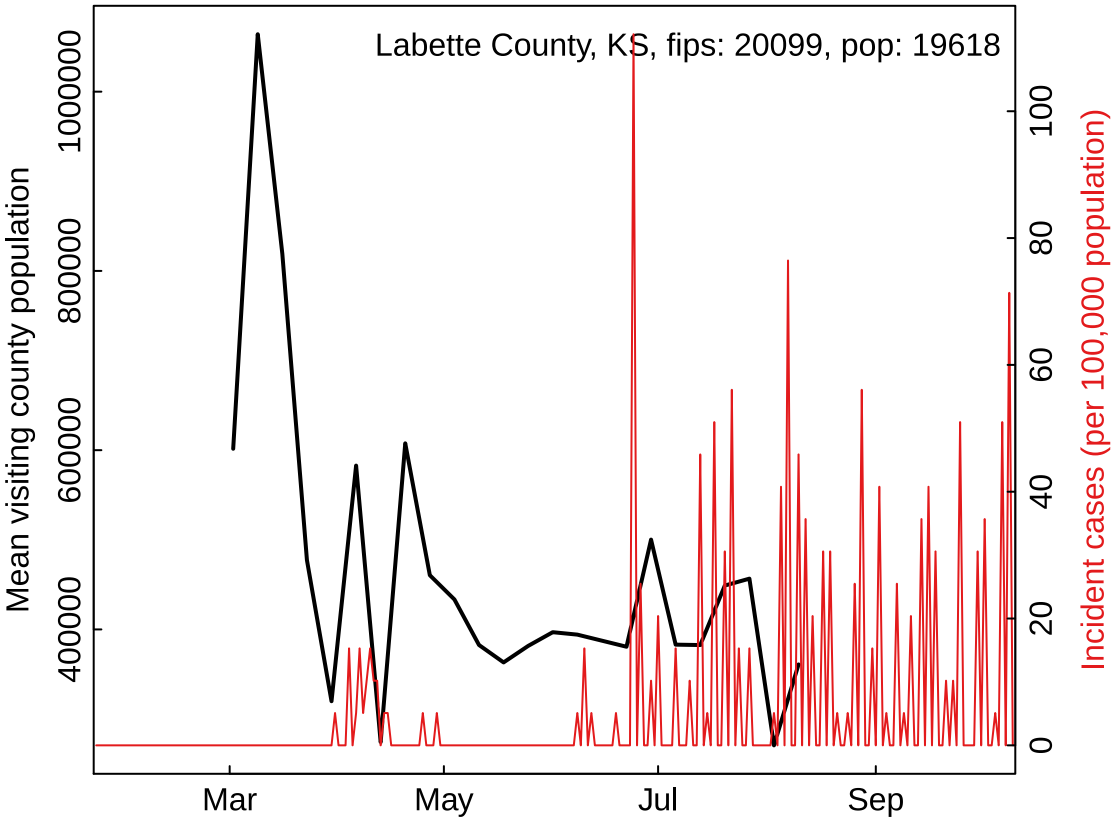

Labette County, KS, fips: 20099, pop: 19618
Mean visiting county population
400000
600000
800000
1000000
Incident cases (per 100,000 population)
0
20
40
60
80
100
Mar
May
Jul
Sep

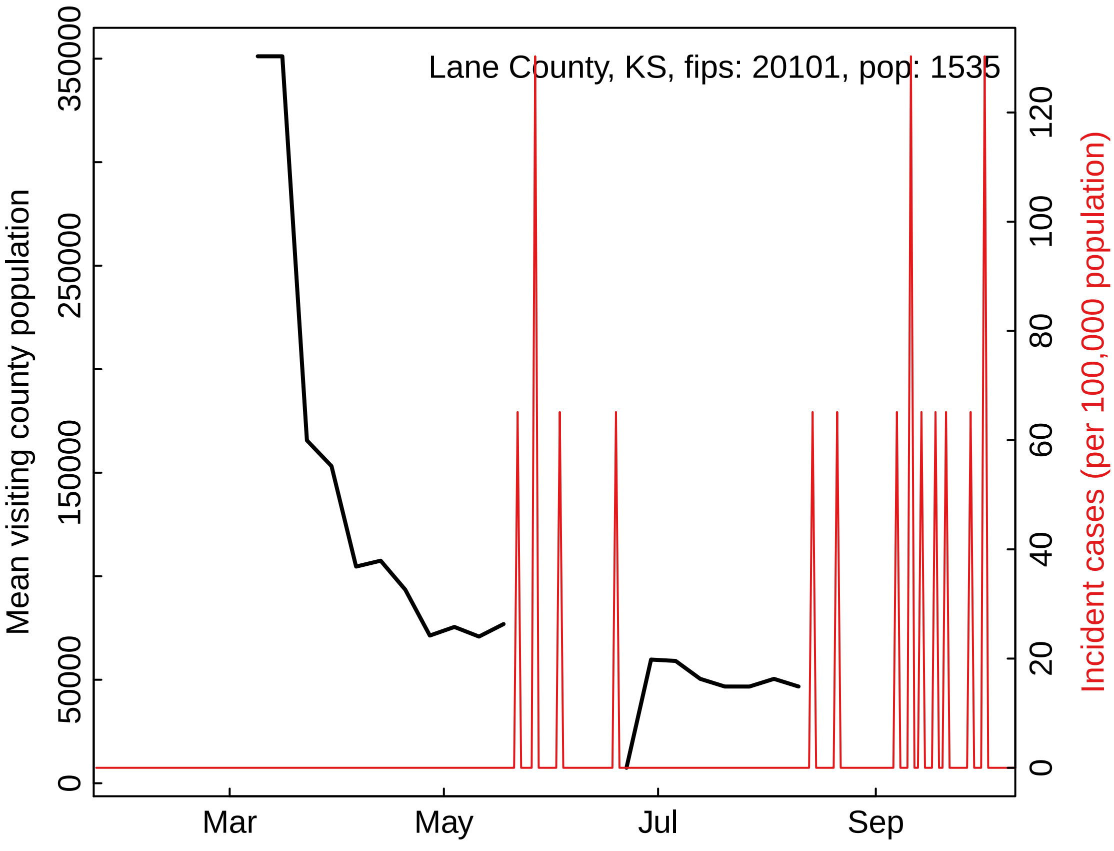

Lane County, KS, fips: 20101, pop: 1535
Mean visiting county population
Incident cases (per 100,000 population)
0
50000
150000
250000
350000
0
20
40
60
80
100
120
Mar
May
Jul
Sep

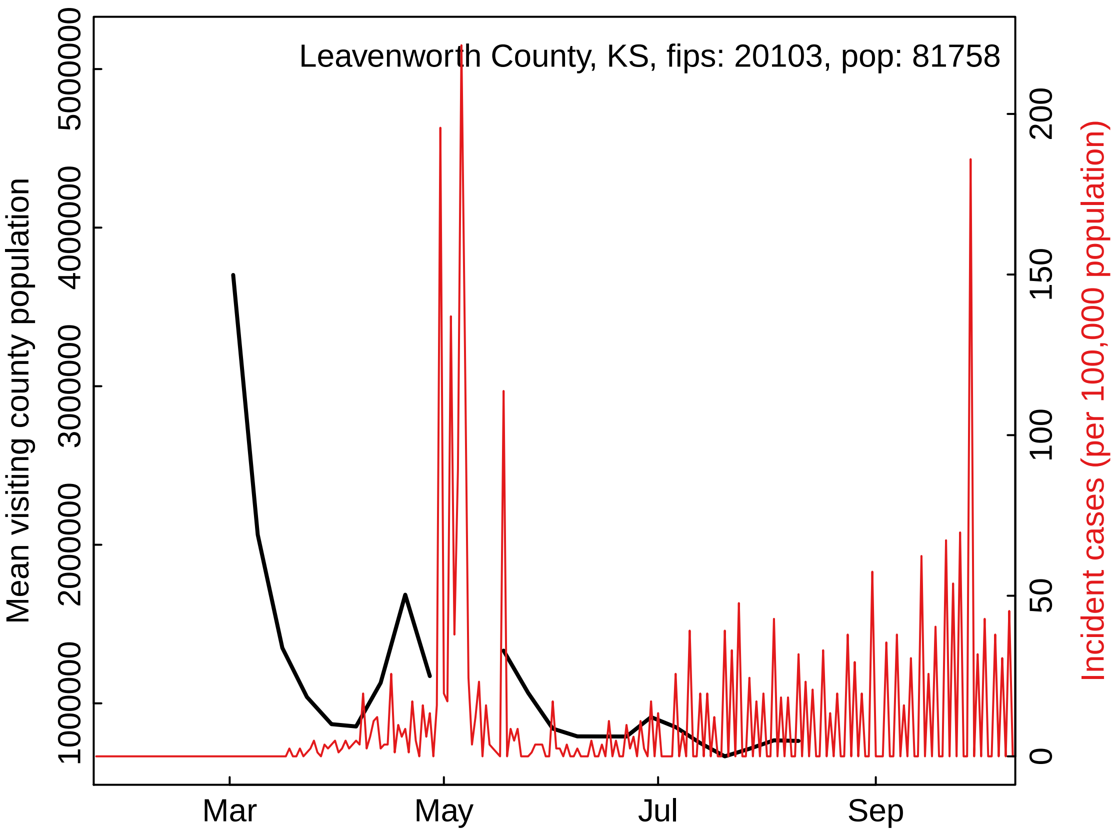

Leavenworth County, KS, fips: 20103, pop: 81758
Mean visiting county population
10000000
20000000
30000000
40000000
50000000
Incident cases (per 100,000 population)
0
50
100
150
200
Mar
May
Jul
Sep

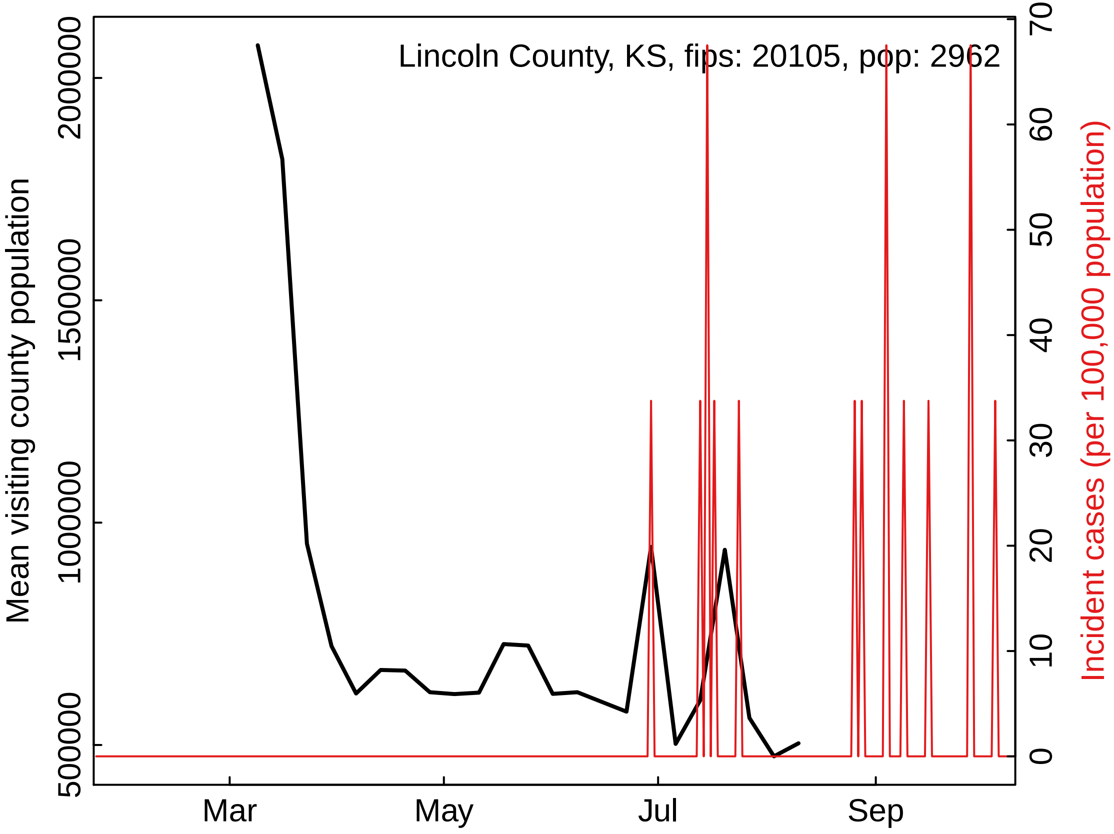
Lincoln County, KS, fips: 20105, pop: 2962
Mean visiting county population
500000
1000000
1500000
2000000
Incident cases (per 100,000 population)
0
10
20
30
40
50
60
70
Mar
May
Jul
Sep

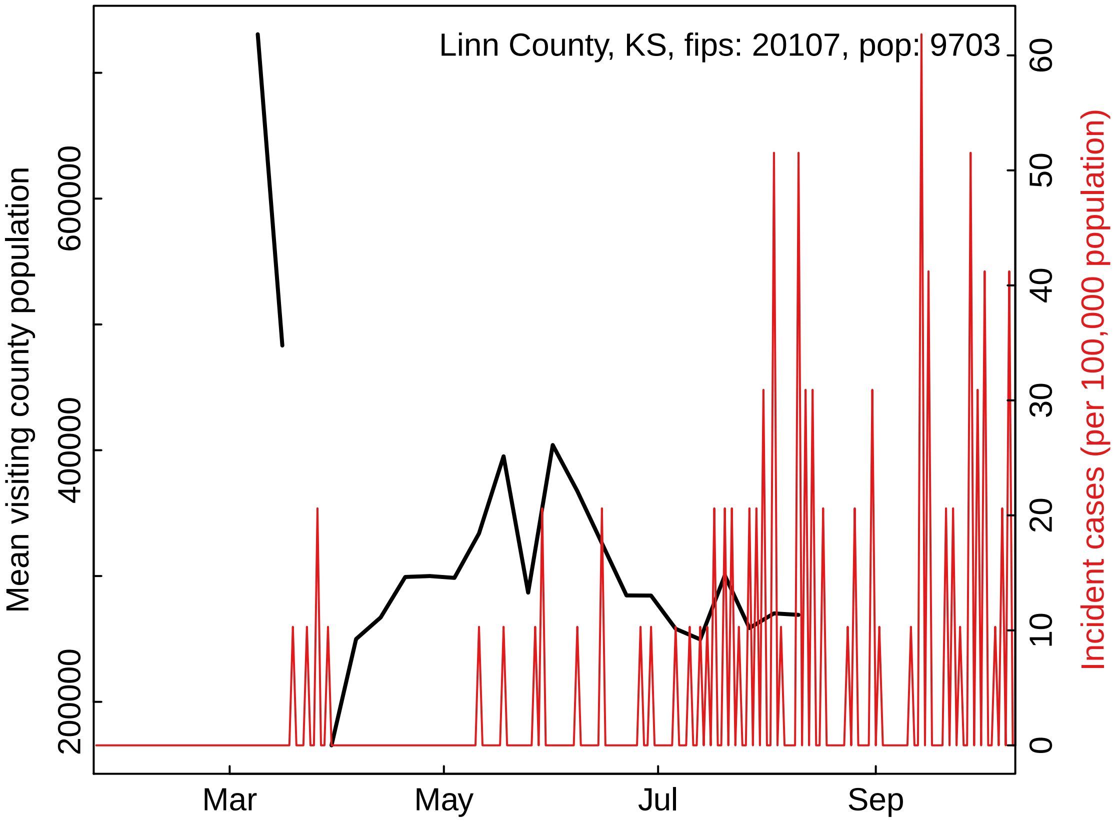

Linn County, KS, fips: 20107, pop: 9703
Mean visiting county population
200000
400000
600000
Incident cases (per 100,000 population)
0
10
20
30
40
50
60
Mar
May
Jul
Sep

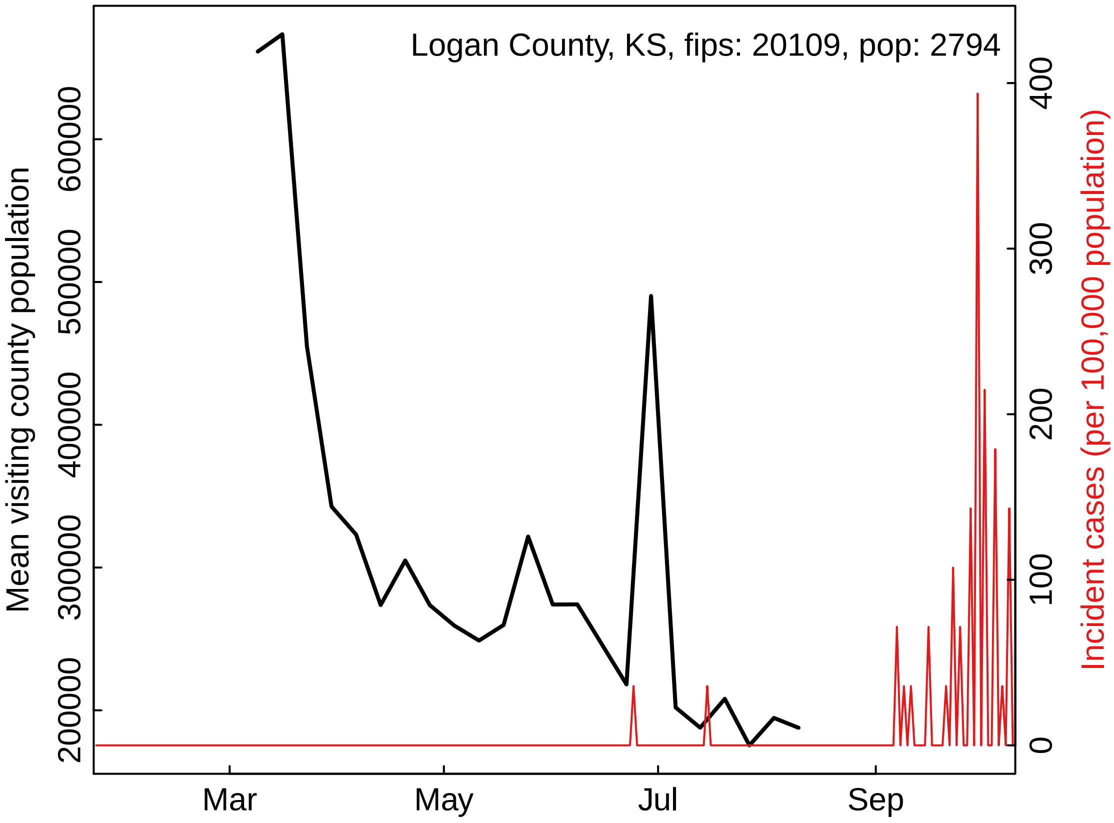

Logan County, KS, fips: 20109, pop: 2794
Mean visiting county population
200000
300000
400000
500000
600000
Incident cases (per 100,000 population)
0
100
200
300
400
Mar
May
Jul
Sep

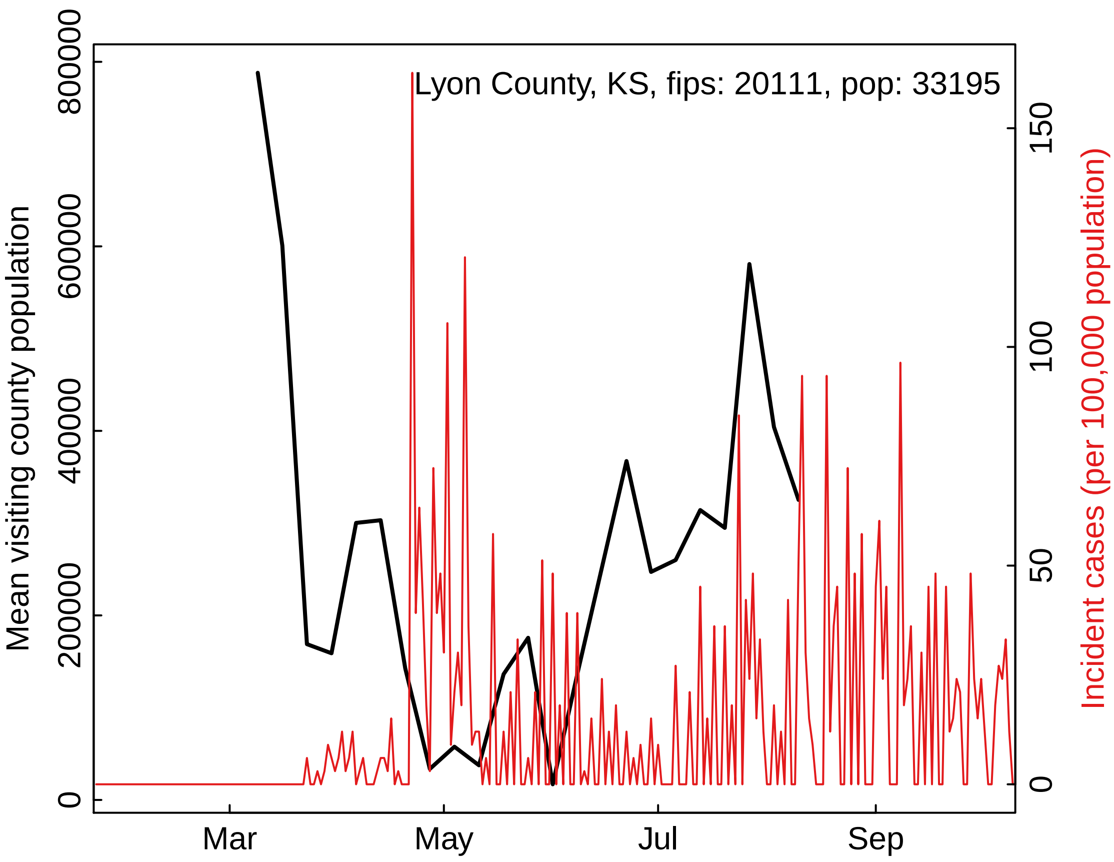

Lyon County, KS, fips: 20111, pop: 33195
Mean visiting county population
0
200000
400000
600000
800000
Incident cases (per 100,000 population)
0
50
100
150
Mar
May
Jul
Sep

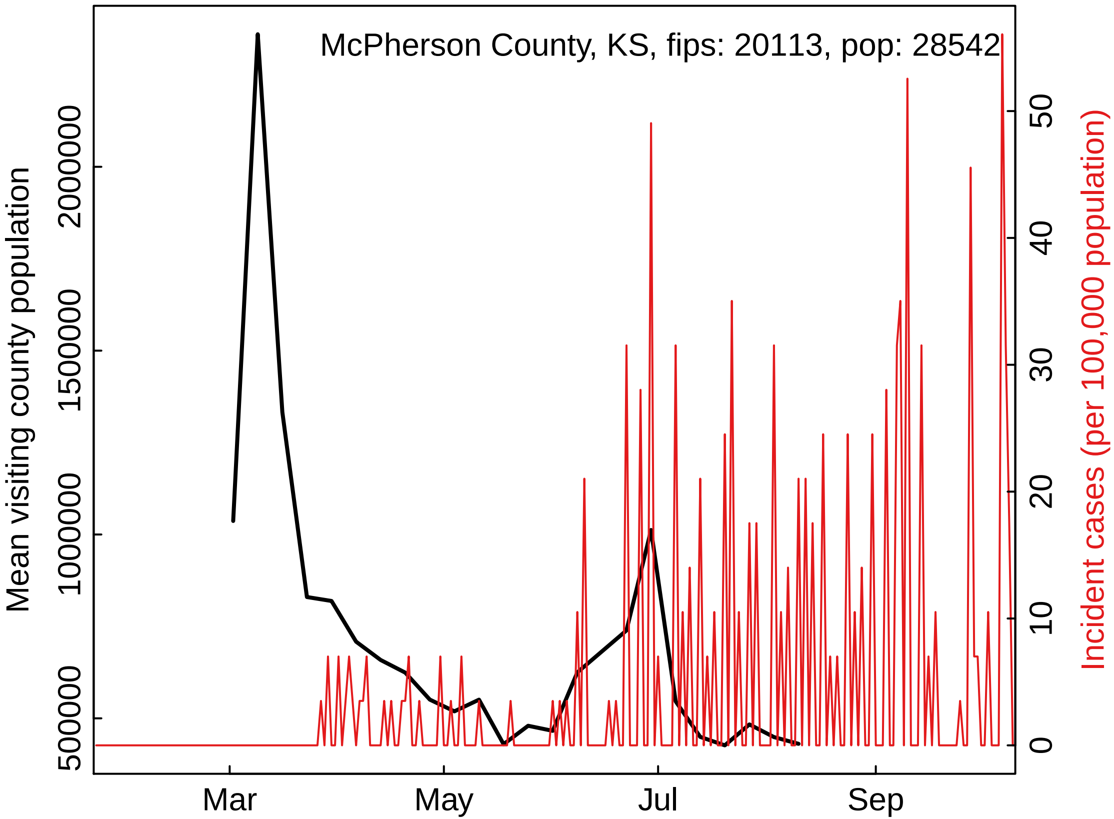

McPherson County, KS, fips: 20113, pop: 28542
Mean visiting county population
500000
1000000
1500000
2000000
Incident cases (per 100,000 population)
0
10
20
30
40
50
Mar
May
Jul
Sep

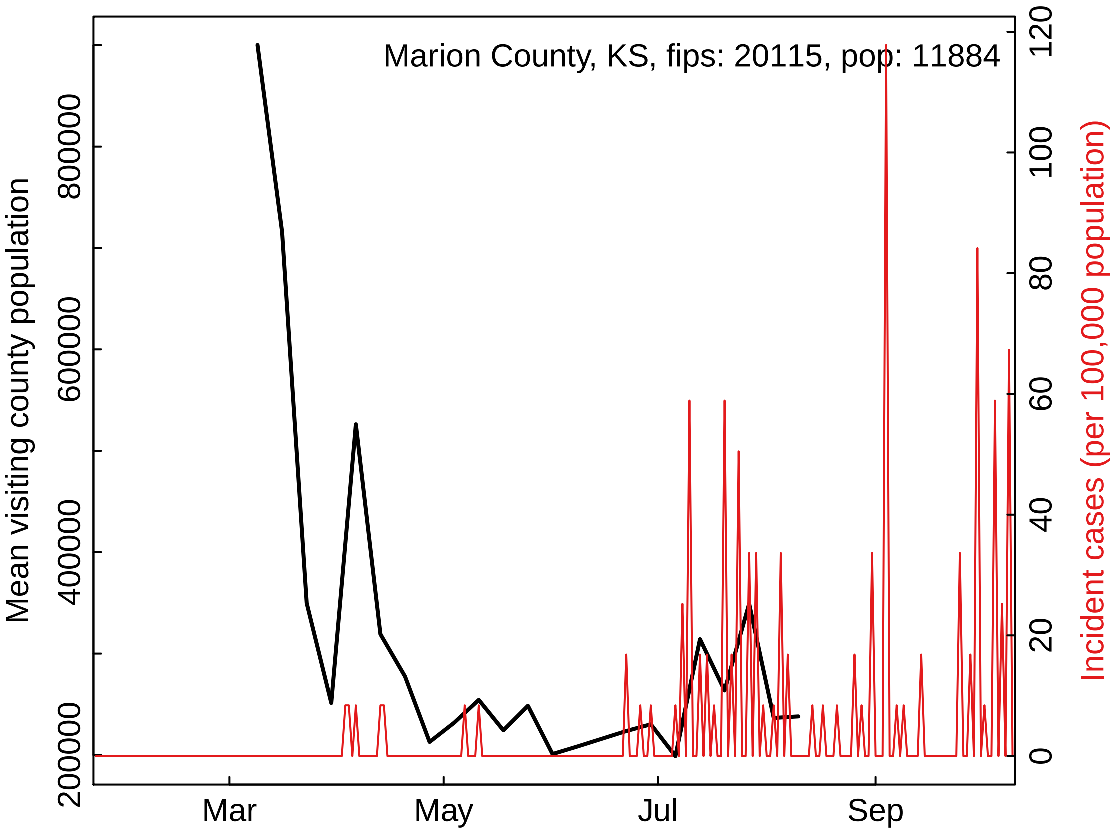

Marion County, KS, fips: 20115, pop: 11884
Mean visiting county population
200000
400000
600000
800000
Incident cases (per 100,000 population)
0
20
40
60
80
100
120
Mar
May
Jul
Sep

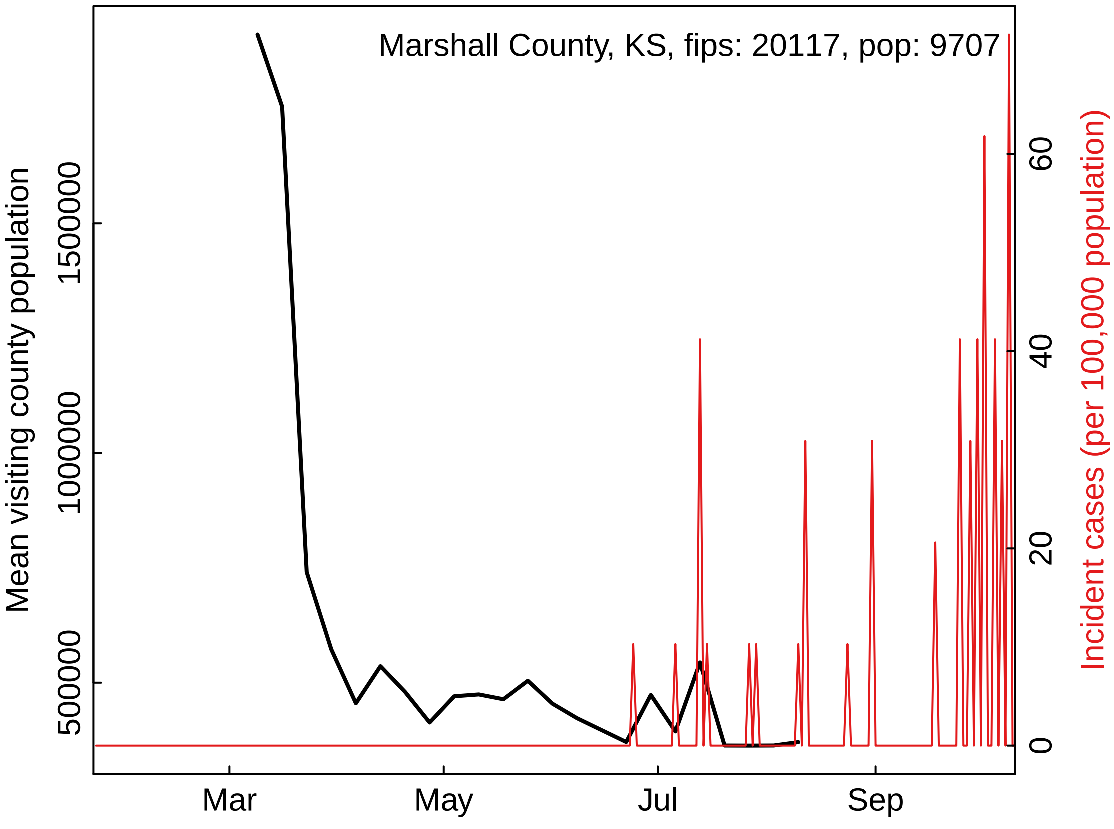

Marshall County, KS, fips: 20117, pop: 9707
Mean visiting county population
Incident cases (per 100,000 population)
500000
1000000
1500000
0
20
40
60
Mar
May
Jul
Sep

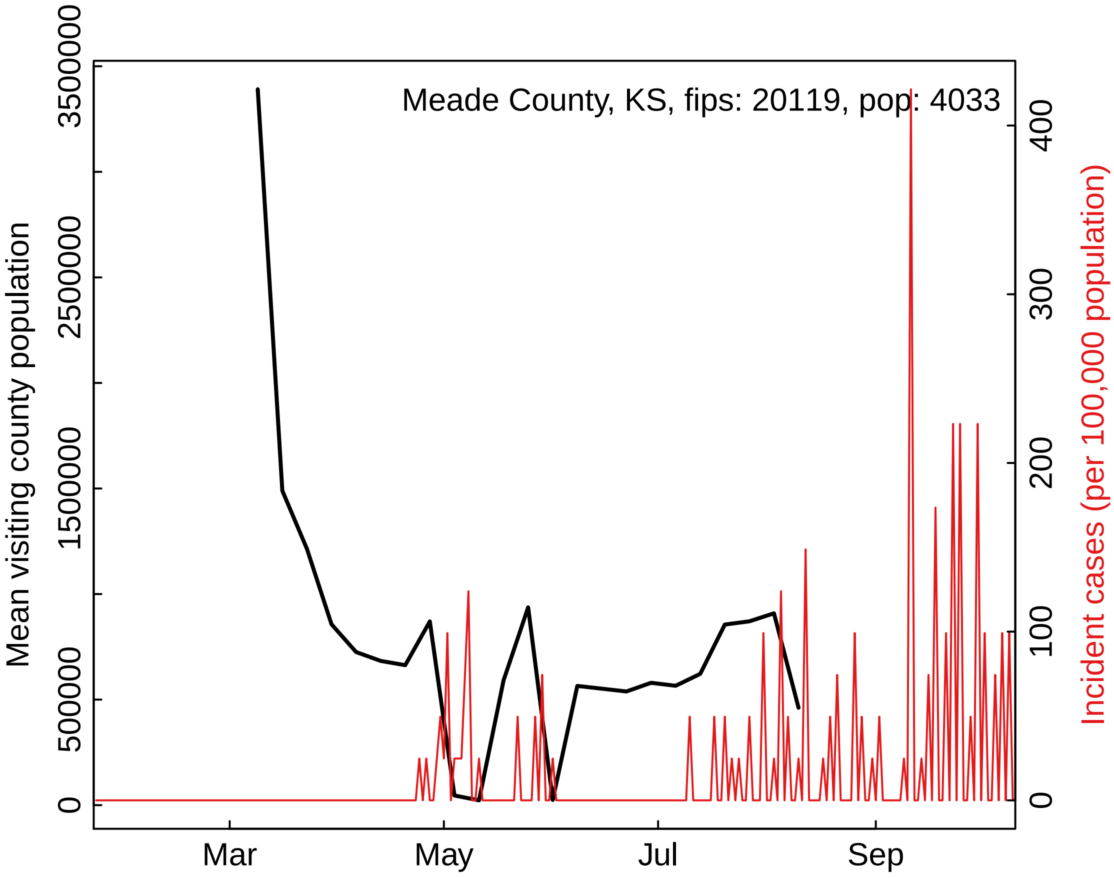

Meade County, KS, fips: 20119, pop: 4033
Mean visiting county population
Incident cases (per 100,000 population)
0
500000
1500000
2500000
3500000
0
100
200
300
400
Mar
May
Jul
Sep

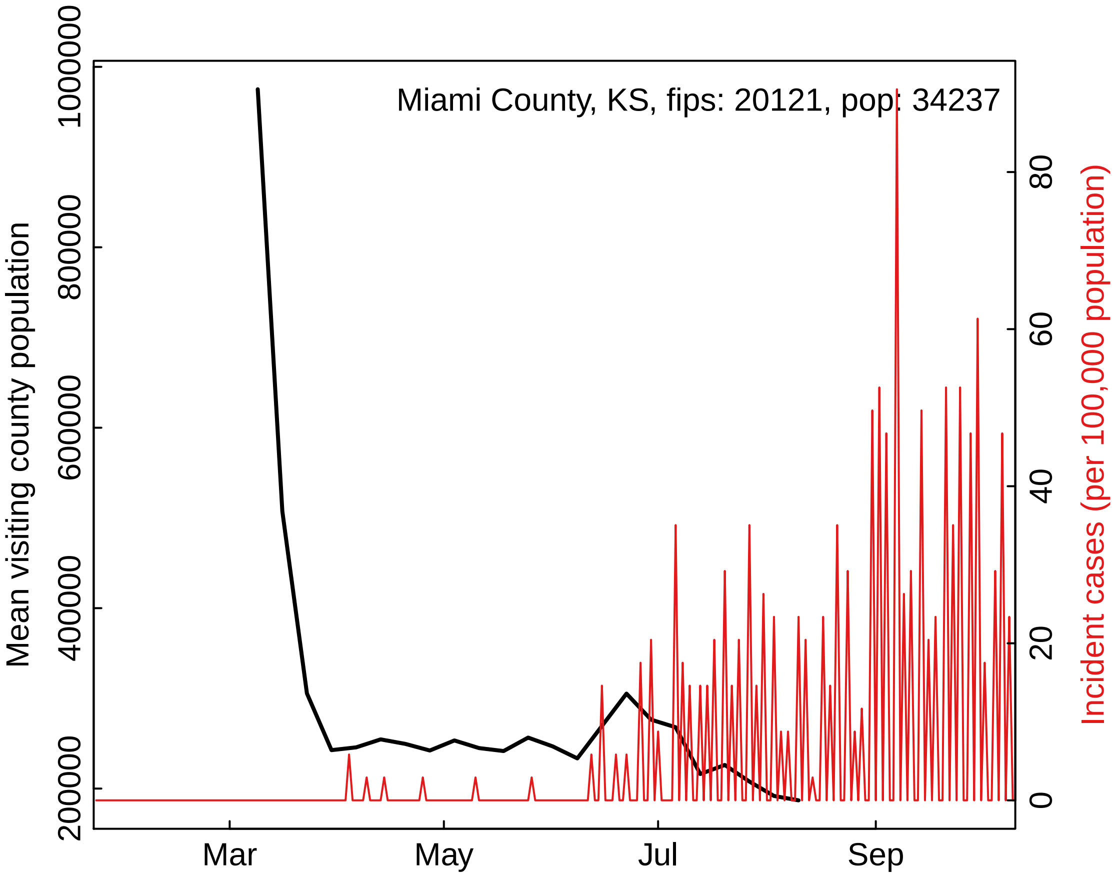

Miami County, KS, fips: 20121, pop: 34237
Mean visiting county population
200000
400000
600000
800000
1000000
Mar
May
Jul
Sep
0
20
40
60
80
Incident cases (per 100,000 population)

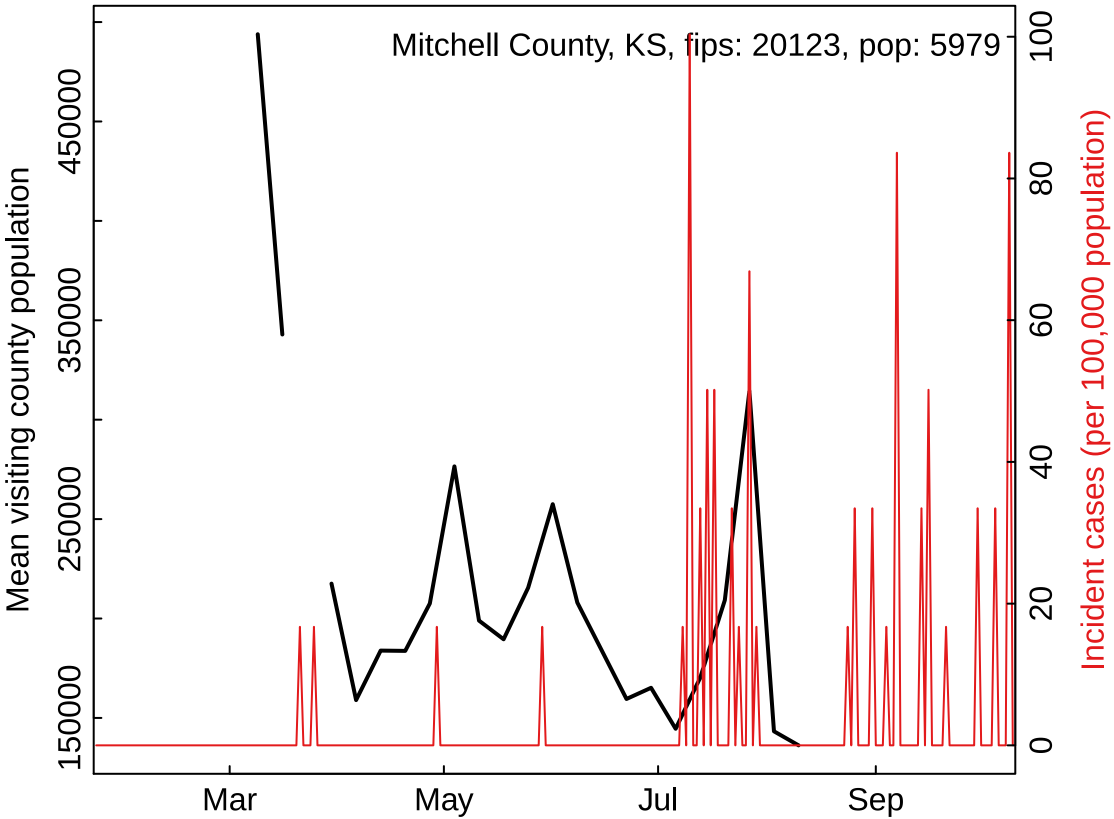
Mitchell County, KS, fips: 20123, pop: 5979
Mean visiting county population
150000
250000
350000
450000
Incident cases (per 100,000 population)
0
20
40
60
80
100
Mar
May
Jul
Sep

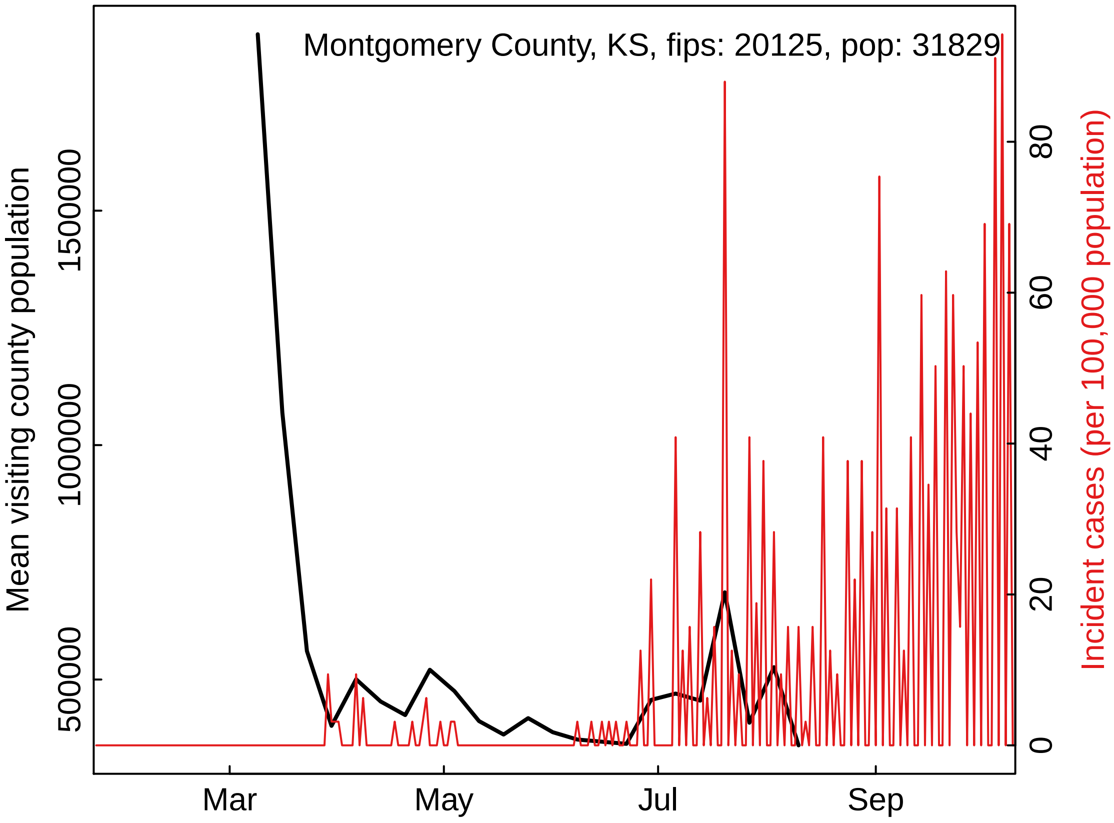

Montgomery County, KS, fips: 20125, pop: 31829
Mean visiting county population
500000
1000000
1500000
Mar
May
Jul
Sep
0
20
40
60
80
Incident cases (per 100,000 population)

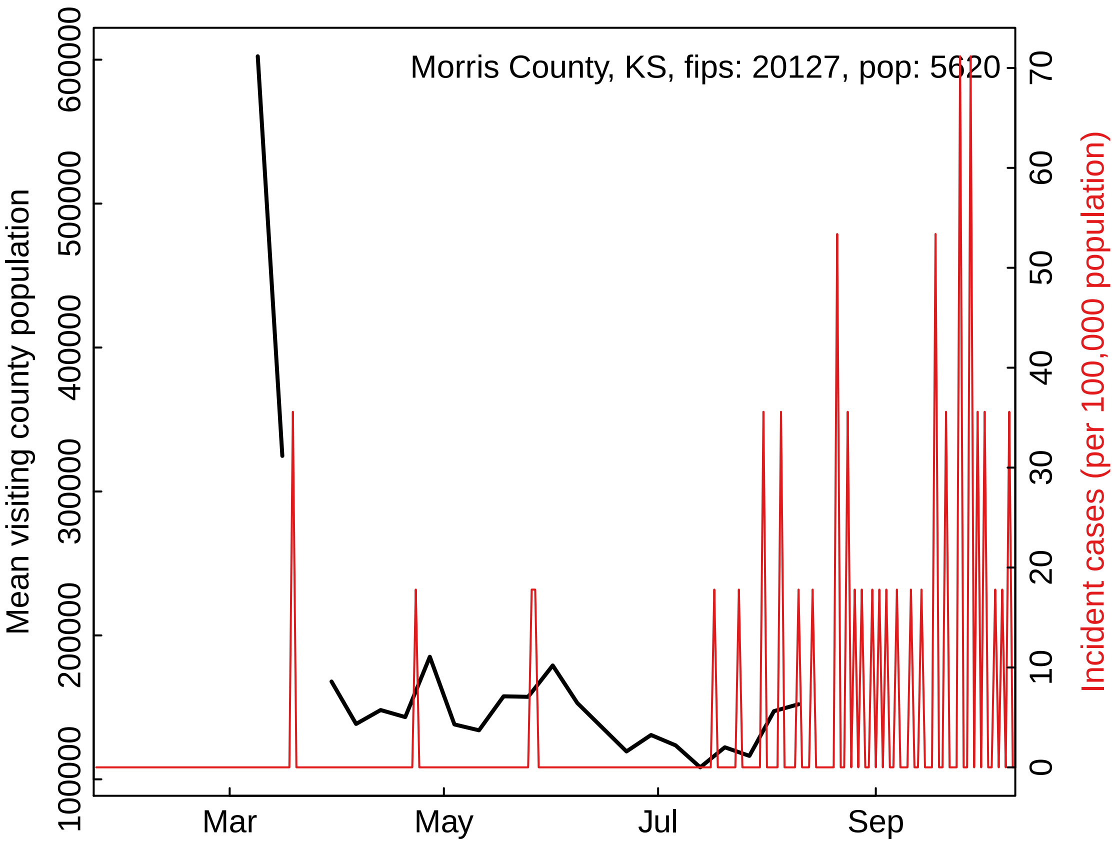

Morris County, KS, fips: 20127, pop: 5620
Mean visiting county population
Incident cases (per 100,000 population)
100000
200000
300000
400000
500000
600000
0
10
20
30
40
50
60
70
Mar
May
Jul
Sep

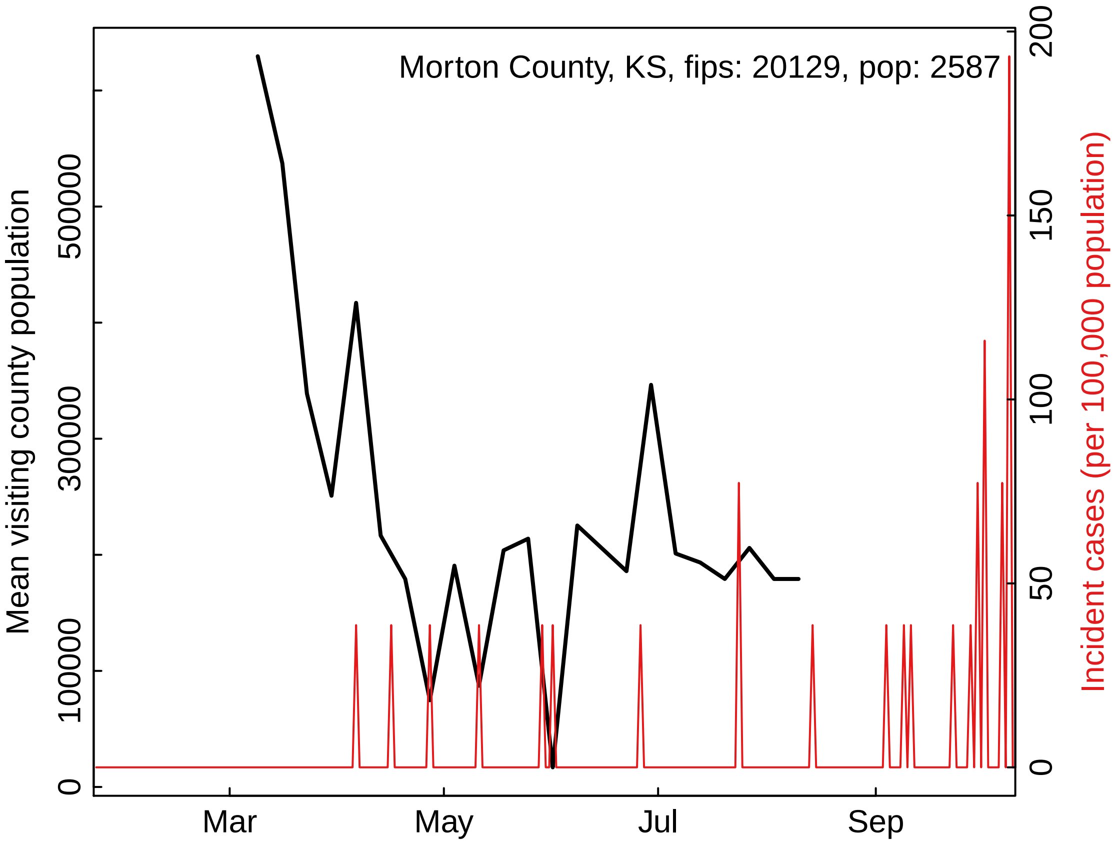

Morton County, KS, fips: 20129, pop: 2587
Mean visiting county population
0
100000
300000
500000
Incident cases (per 100,000 population)
0
50
100
150
200
Mar
May
Jul
Sep

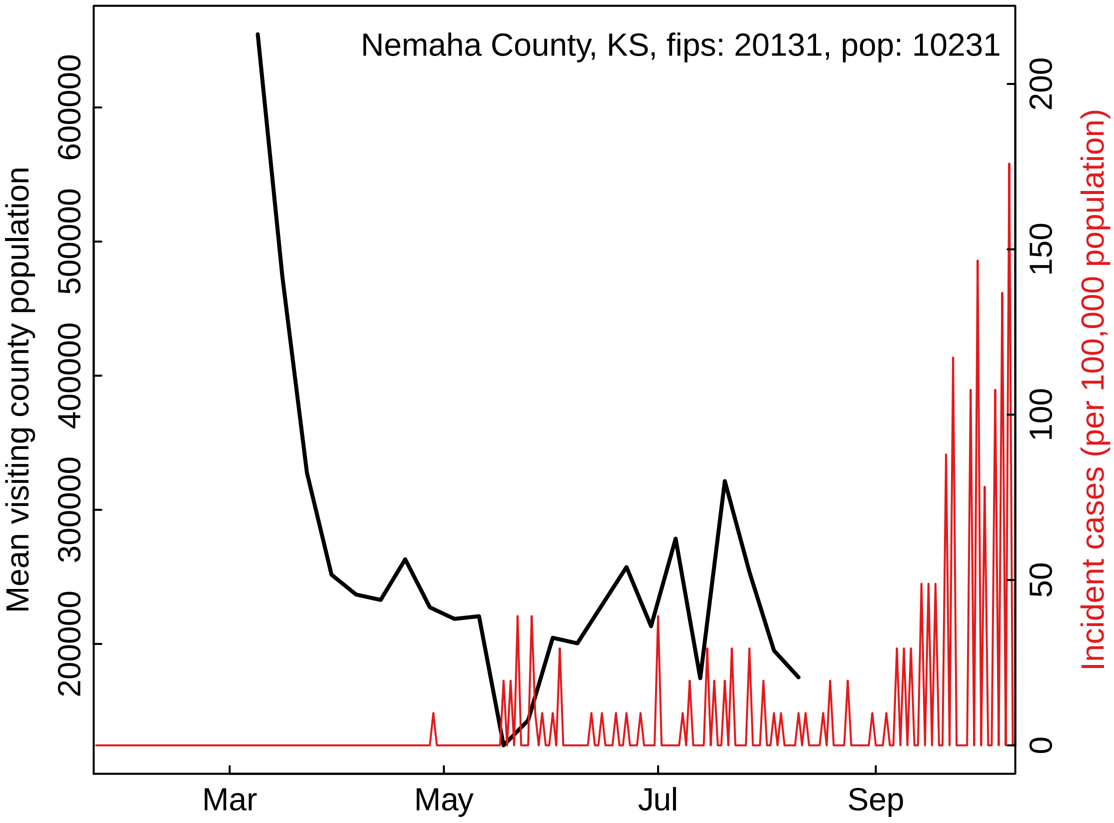

Nemaha County, KS, fips: 20131, pop: 10231
Mean visiting county population
200000
300000
400000
500000
600000
Incident cases (per 100,000 population)
0
50
100
150
200
Mar
May
Jul
Sep

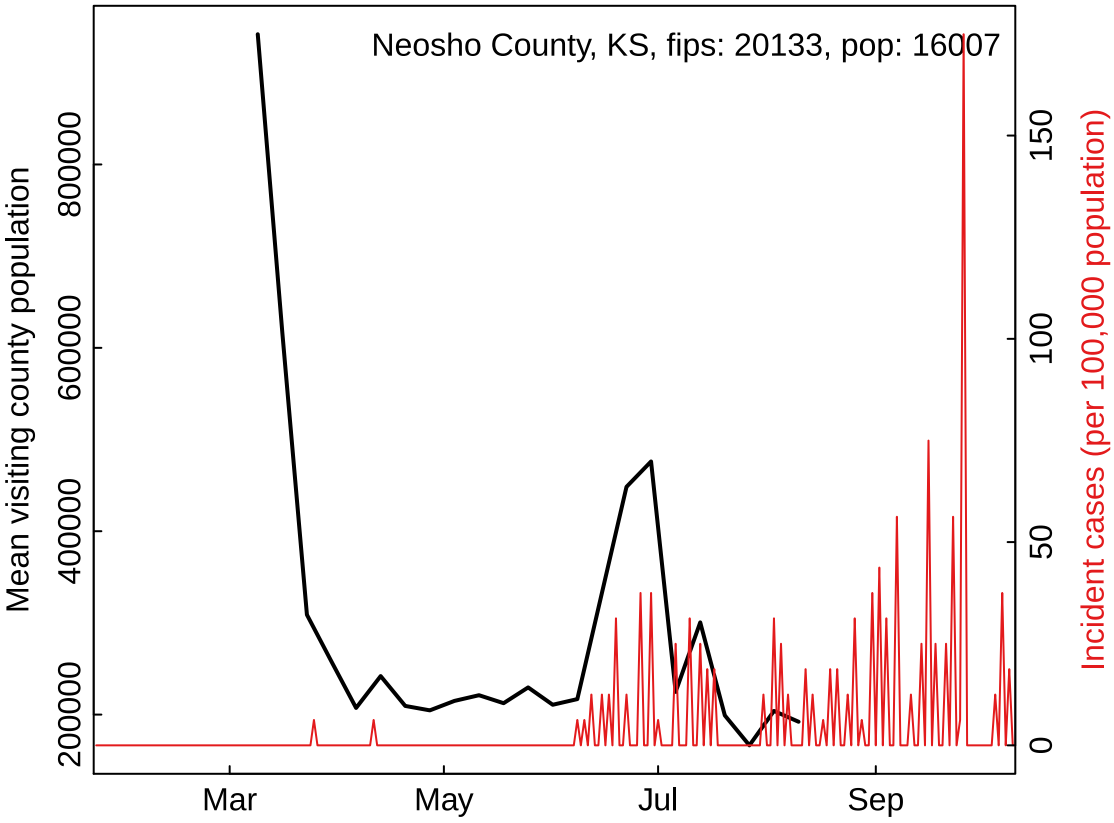

Neosho County, KS, fips: 20133, pop: 16007
Mean visiting county population
200000
400000
600000
800000
Incident cases (per 100,000 population)
0
50
100
150
Mar
May
Jul
Sep

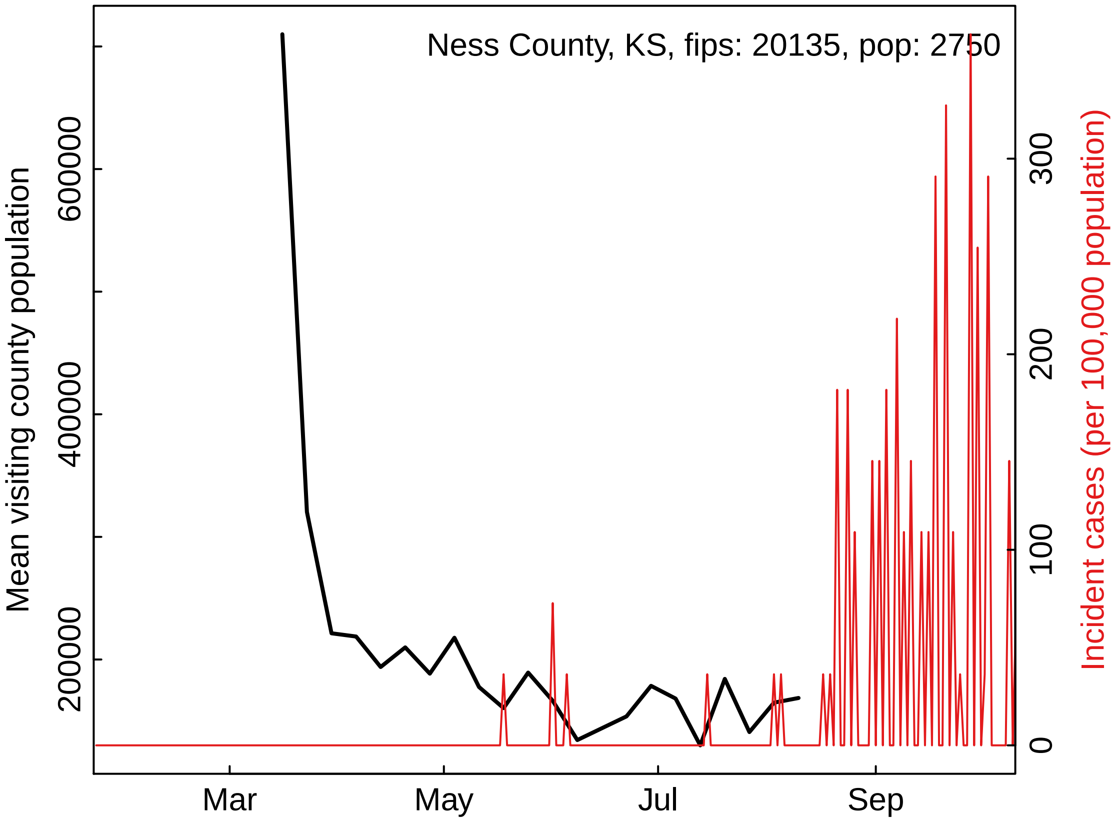

Ness County, KS, fips: 20135, pop: 2750
Mean visiting county population
200000
400000
600000
Incident cases (per 100,000 population)
0
100
200
300
Mar
May
Jul
Sep

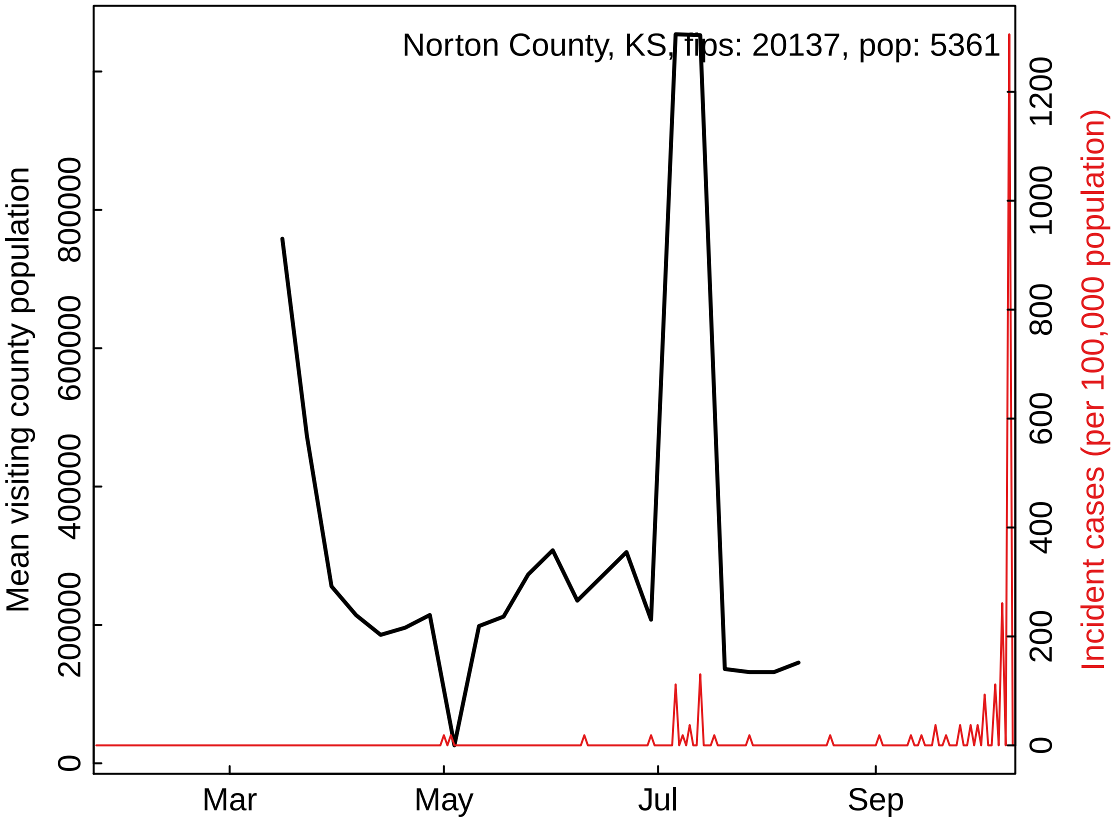

Norton County, KS, fips: 20137, pop: 5361
Mean visiting county population
0
200000
400000
600000
800000
Incident cases (per 100,000 population)
0
200
400
600
800
1000
1200
Mar
May
Jul
Sep

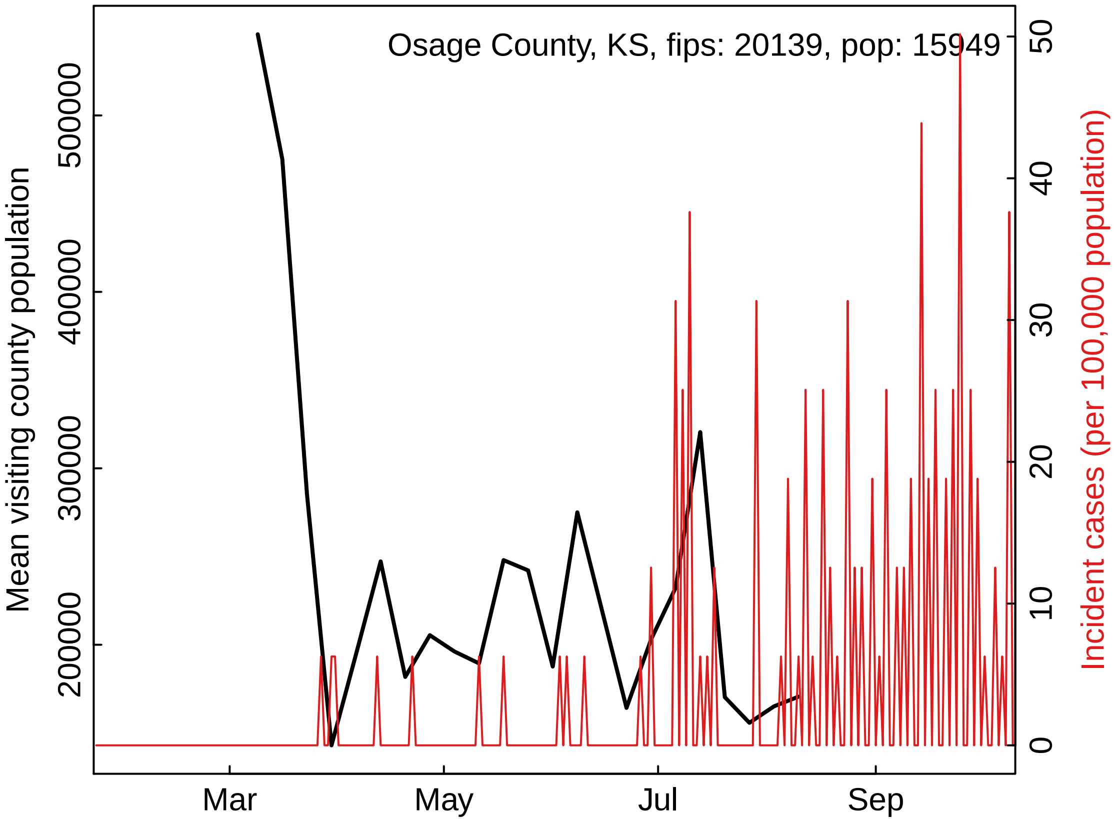

Osage County, KS, fips: 20139, pop: 15949
Mean visiting county population
200000
300000
400000
500000
Incident cases (per 100,000 population)
0
10
20
30
40
50
Mar
May
Jul
Sep

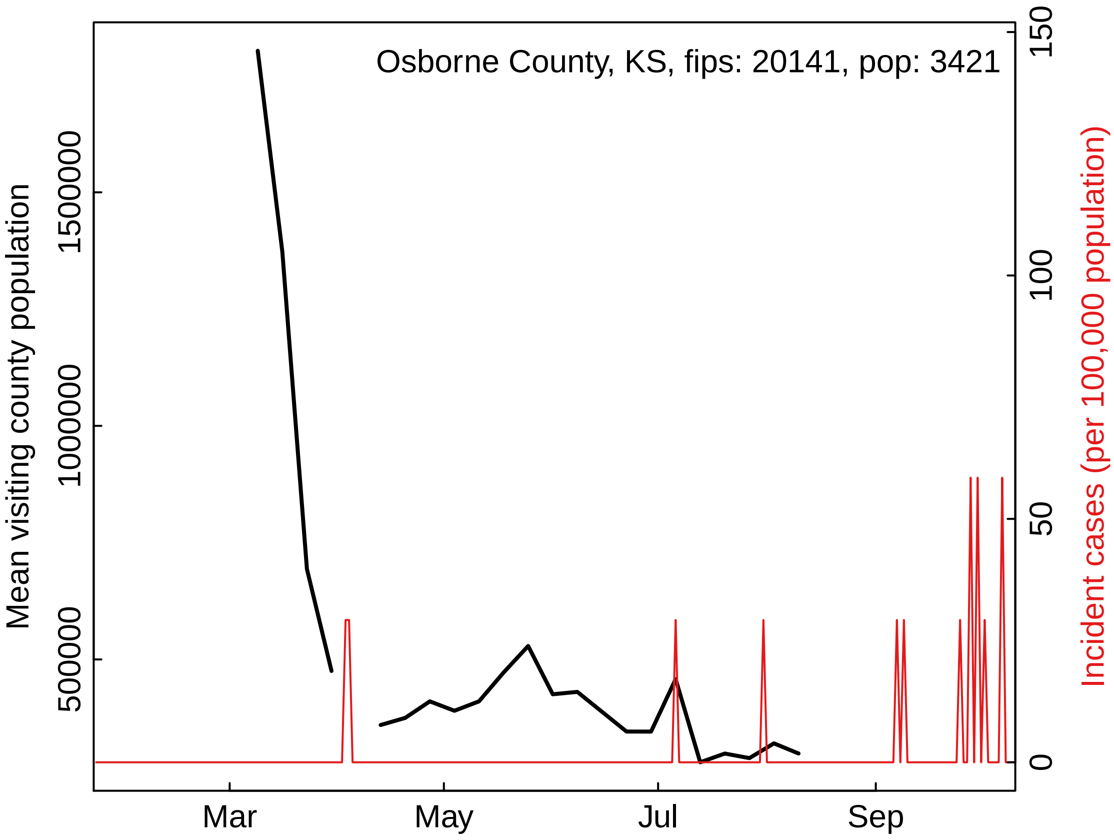

Osborne County, KS, fips: 20141, pop: 3421
Mean visiting county population
500000
1000000
1500000
Incident cases (per 100,000 population)
0
50
100
150
Mar
May
Jul
Sep

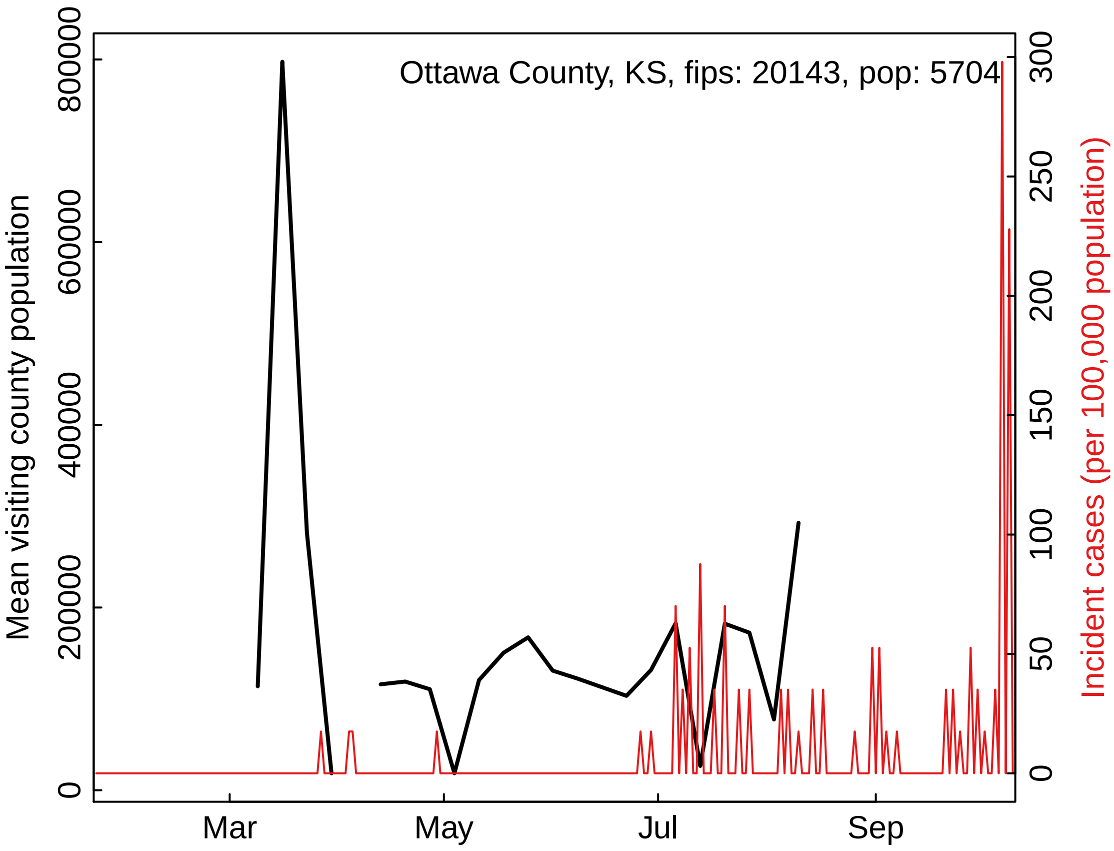
Ottawa County, KS, fips: 20143, pop: 5704
Mean visiting county population
0
200000
400000
600000
800000
Incident cases (per 100,000 population)
0
50
100
150
200
250
300
Mar
May
Jul
Sep

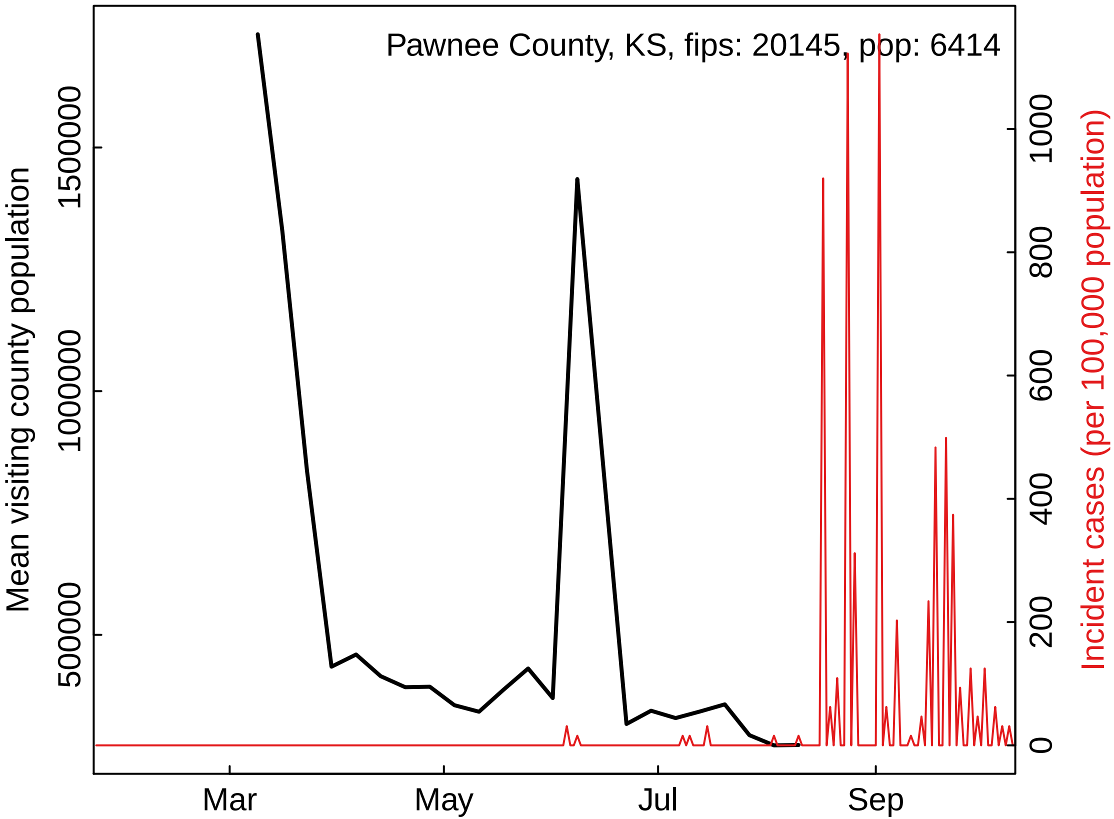

Pawnee County, KS, fips: 20145, pop: 6414
Mean visiting county population
500000
1000000
1500000
Incident cases (per 100,000 population)
0
200
400
600
800
1000
Mar
May
Jul
Sep

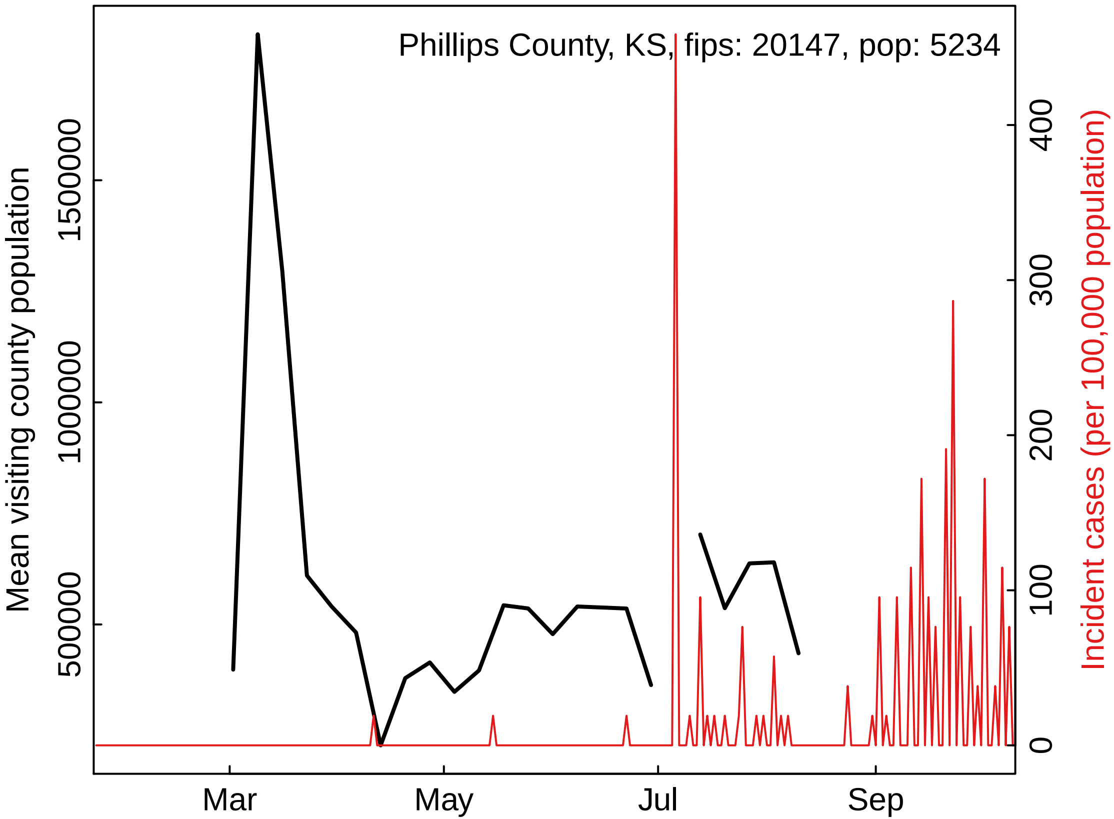

Phillips County, KS, fips: 20147, pop: 5234
Mean visiting county population
500000
1000000
1500000
Incident cases (per 100,000 population)
0
100
200
300
400
Mar
May
Jul
Sep

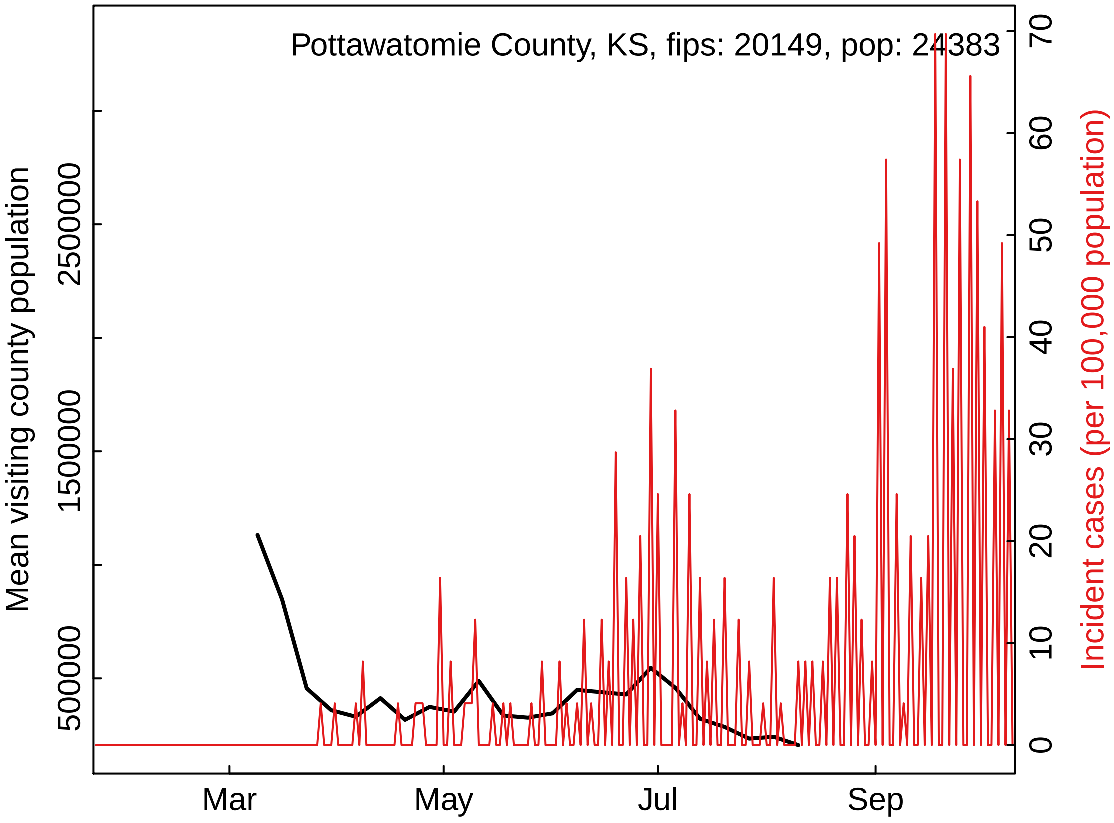

Pottawatomie County, KS, fips: 20149, pop: 24383
Mean visiting county population
500000
1500000
2500000
Incident cases (per 100,000 population)
0
10
20
30
40
50
60
70
Mar
May
Jul
Sep

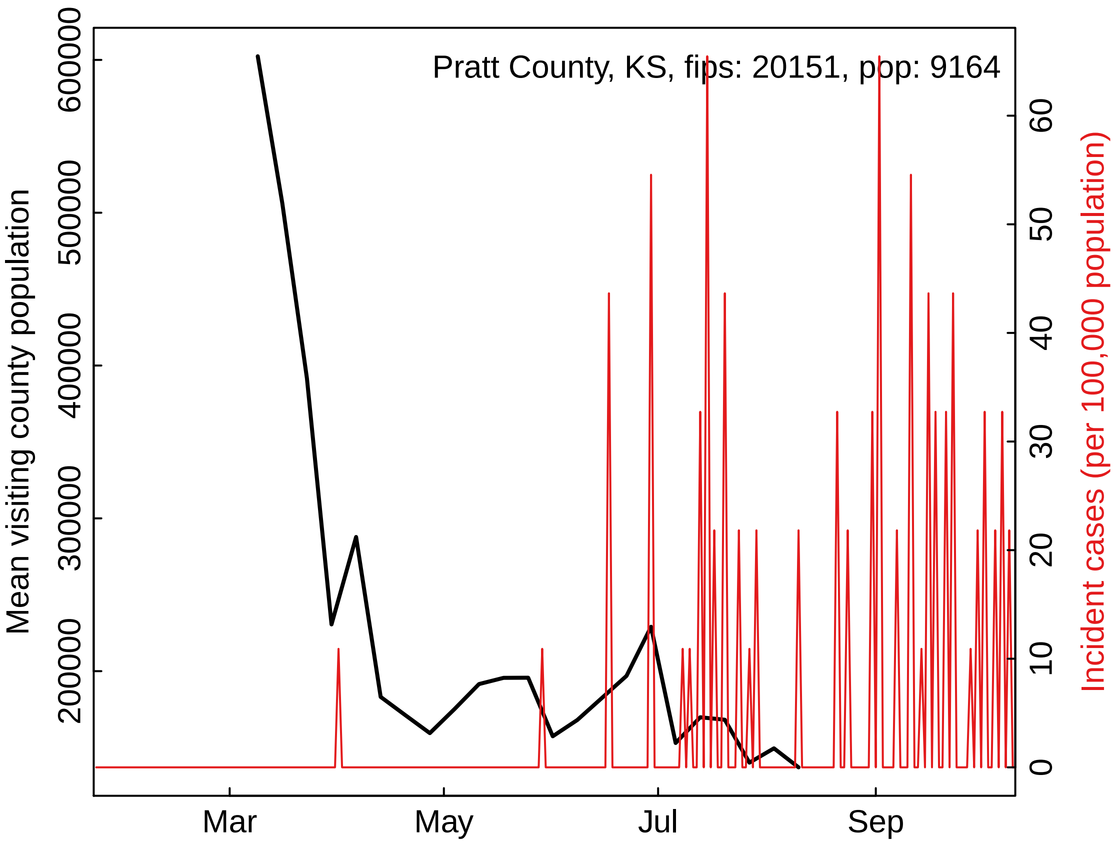

Pratt County, KS, fips: 20151, pop: 9164
Mean visiting county population
200000
300000
400000
500000
600000
Incident cases (per 100,000 population)
0
10
20
30
40
50
60
Mar
May
Jul
Sep

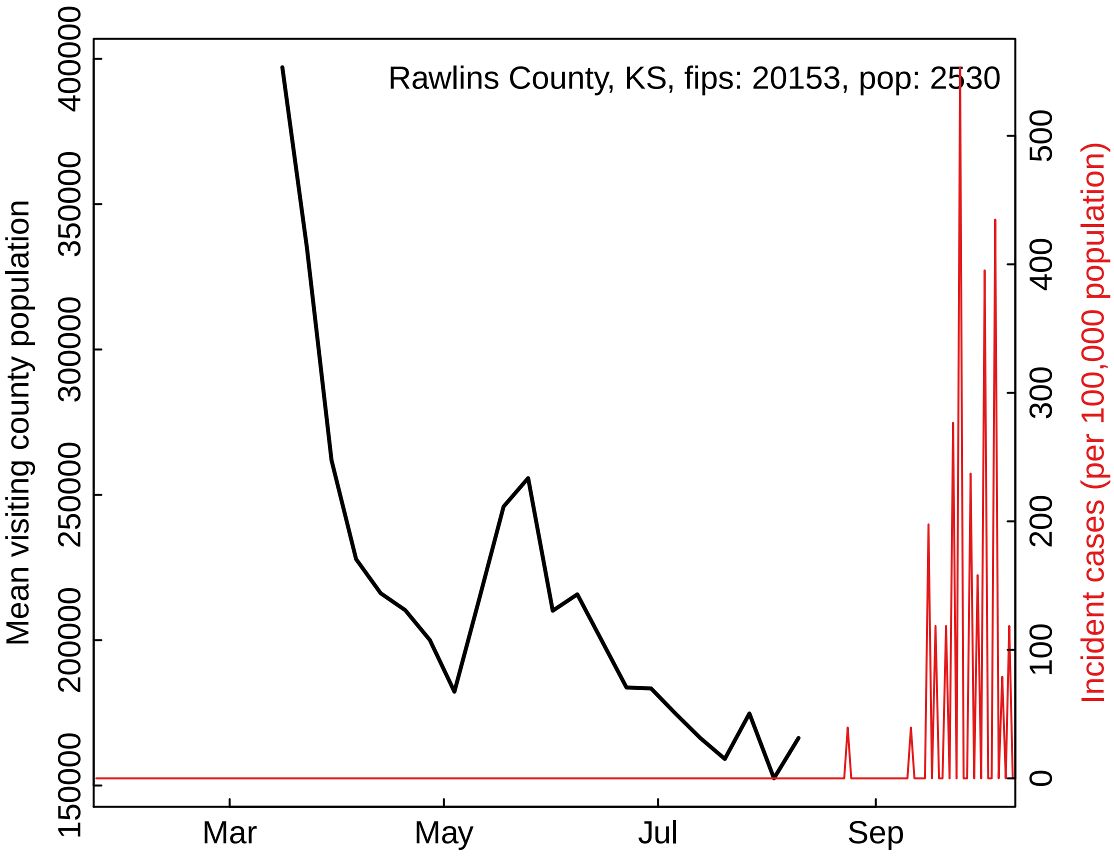
Rawlins County, KS, fips: 20153, pop: 2530
Mean visiting county population
150000
200000
250000
300000
350000
400000
Mar
May
Jul
Sep
0
100
200
300
400
500
Incident cases (per 100,000 population)

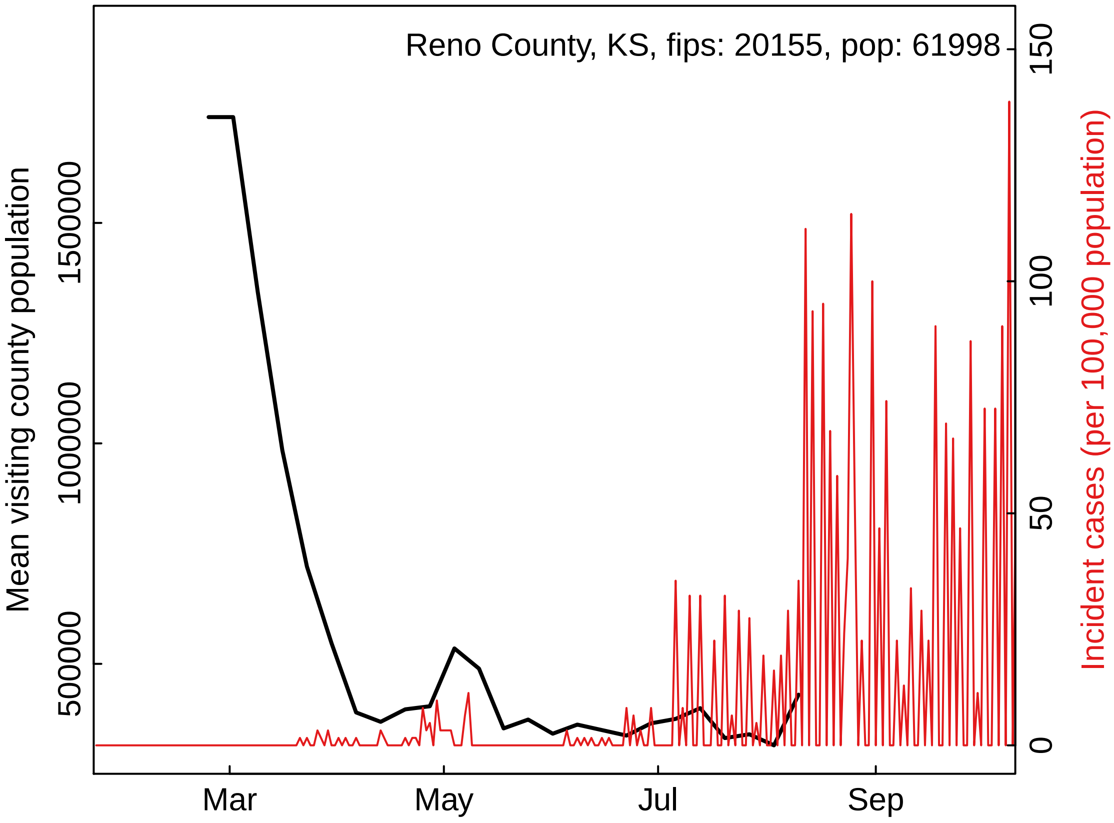

Reno County, KS, fips: 20155, pop: 61998
Mean visiting county population
500000
1000000
1500000
Incident cases (per 100,000 population)
0
50
100
150
Mar
May
Jul
Sep

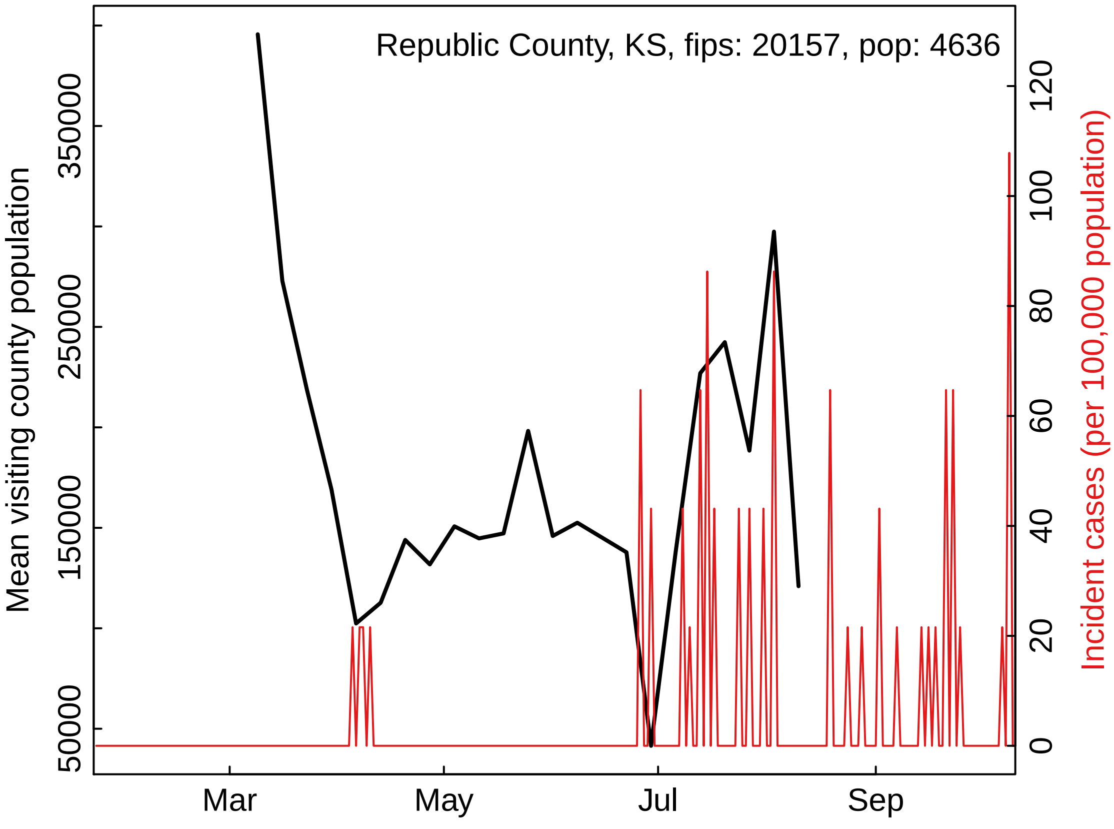

Republic County, KS, fips: 20157, pop: 4636
Mean visiting county population
50000
150000
250000
350000
Mar
May
Jul
Sep
0
20
40
60
80
100
120
Incident cases (per 100,000 population)

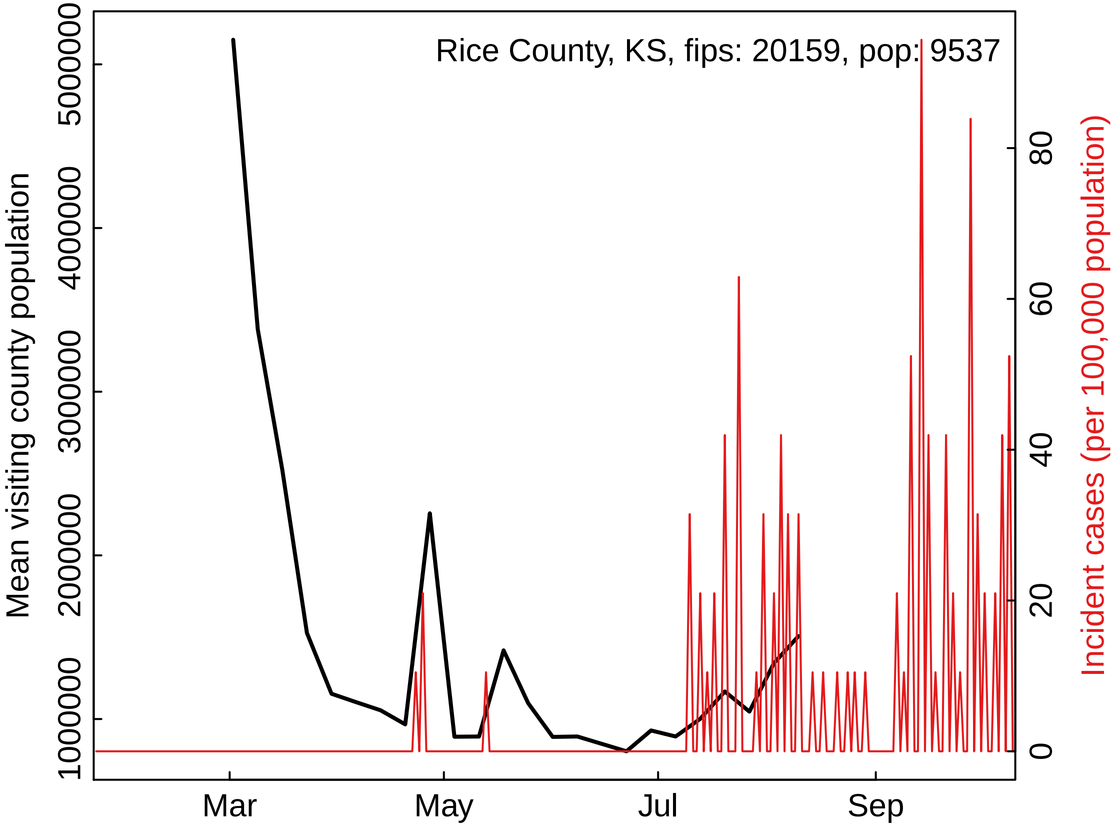
Rice County, KS, fips: 20159, pop: 9537
Mean visiting county population
1000000
2000000
3000000
4000000
5000000
Incident cases (per 100,000 population)
0
20
40
60
80
Mar
May
Jul
Sep

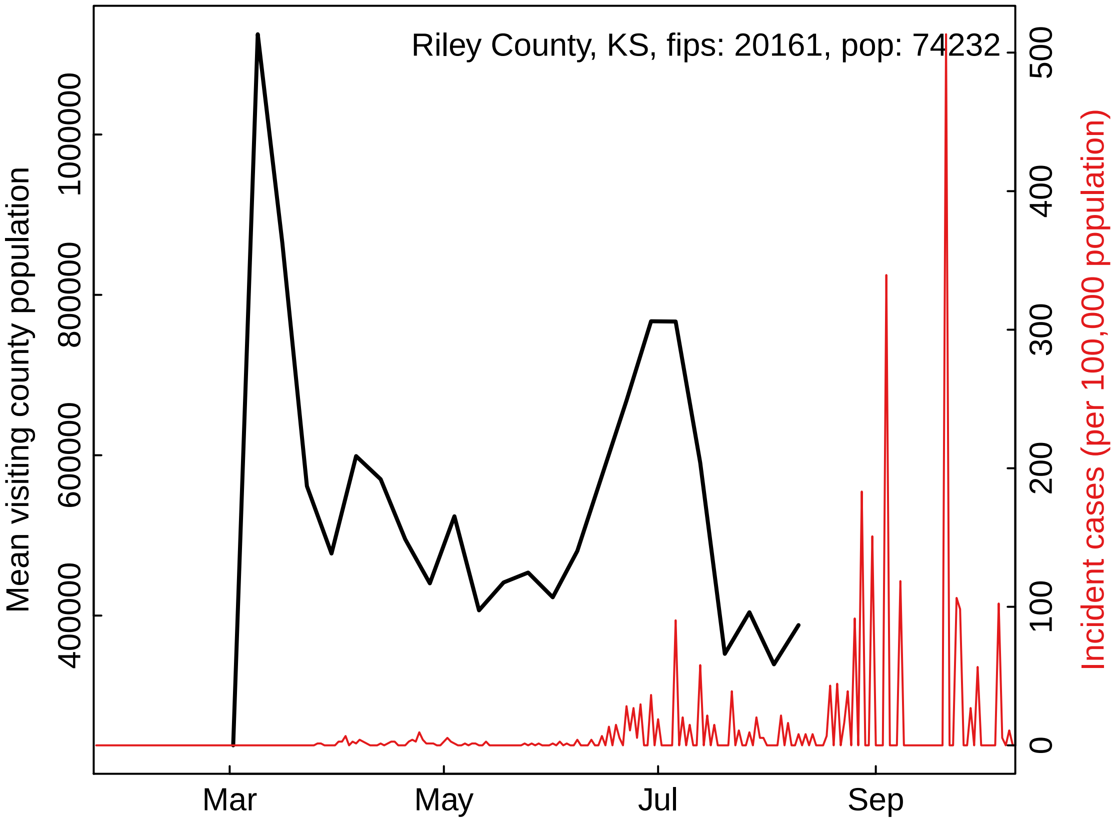
Riley County, KS, fips: 20161, pop: 74232
Mean visiting county population
400000
600000
800000
1000000
Mar
May
Jul
Sep
0
100
200
300
400
500
Incident cases (per 100,000 population)

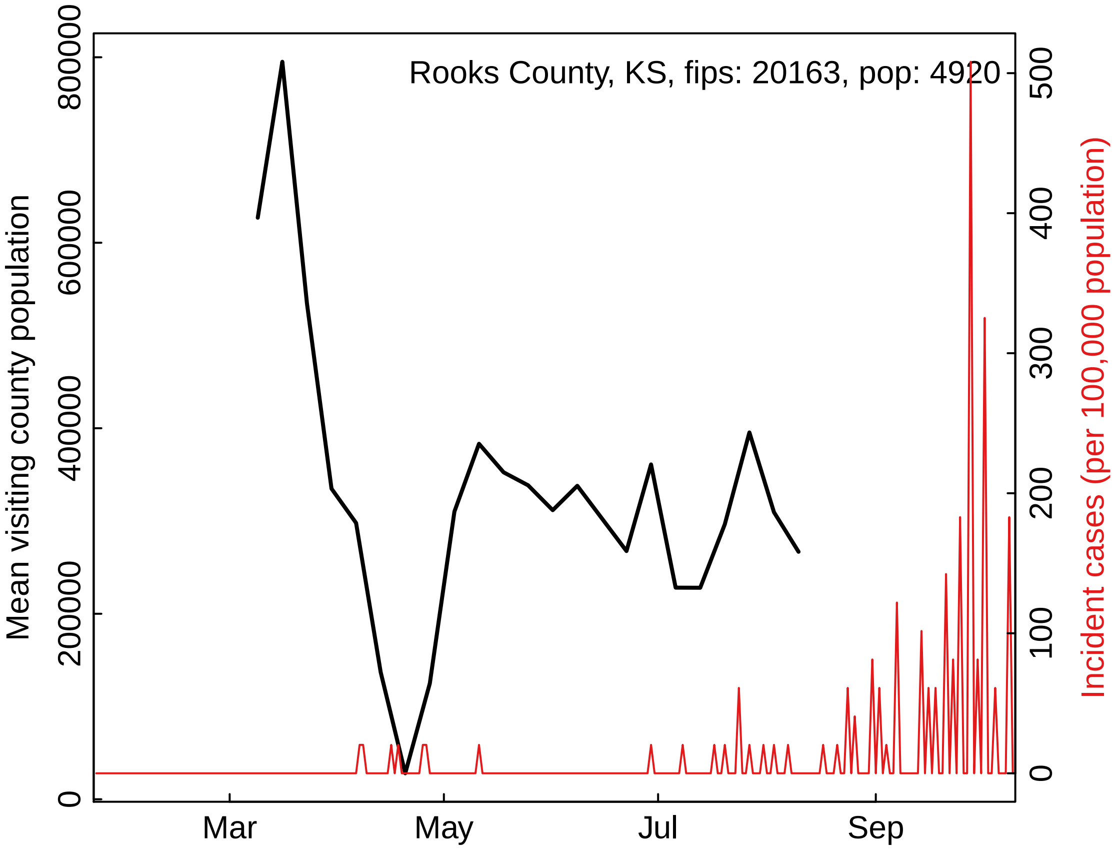

Rooks County, KS, fips: 20163, pop: 4920
Mean visiting county population
0
200000
400000
600000
800000
Incident cases (per 100,000 population)
0
100
200
300
400
500
Mar
May
Jul
Sep

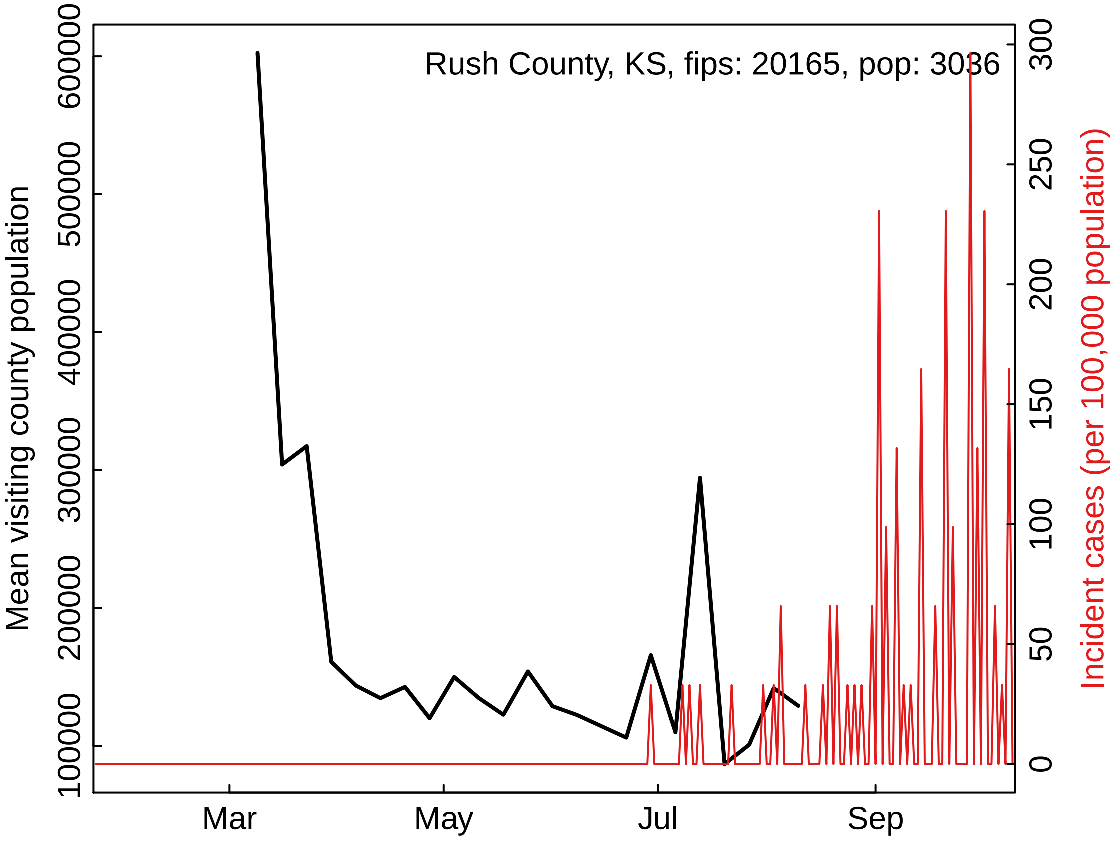

Rush County, KS, fips: 20165, pop: 3036
Mean visiting county population
100000
200000
300000
400000
500000
600000
Incident cases (per 100,000 population)
0
50
100
150
200
250
300
Mar
May
Jul
Sep

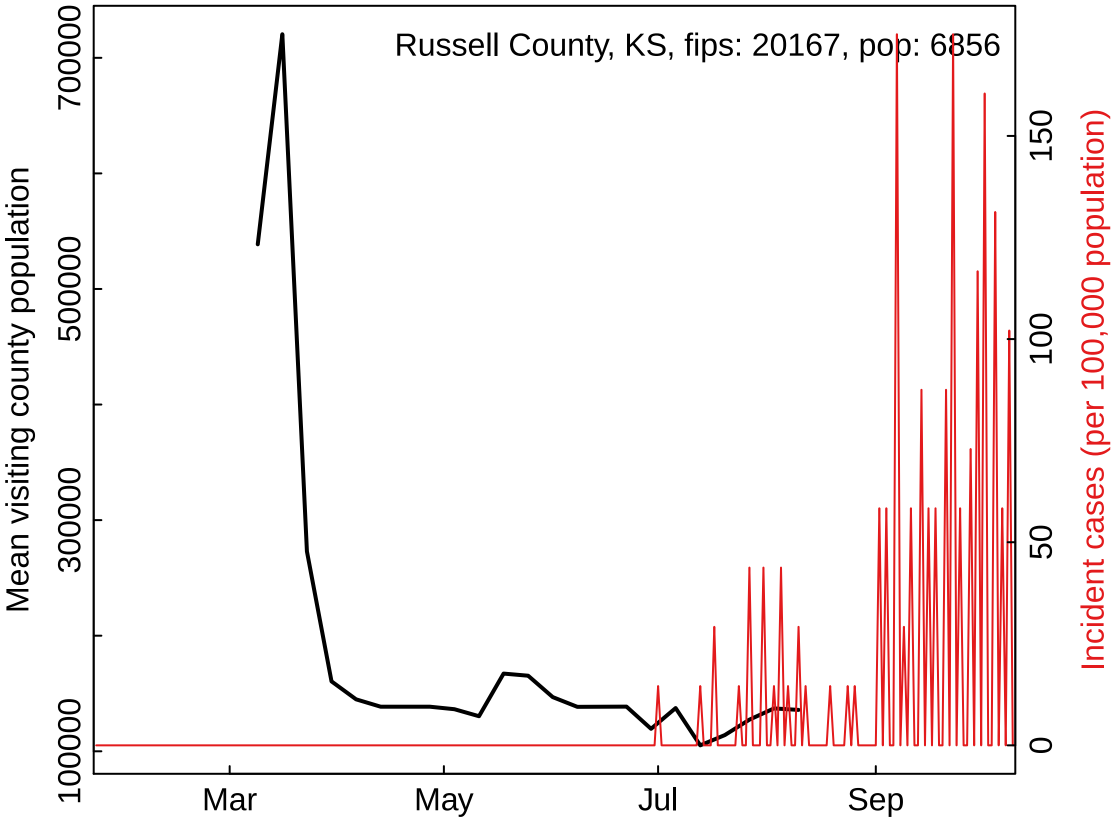

Russell County, KS, fips: 20167, pop: 6856
Mean visiting county population
100000
300000
500000
700000
Incident cases (per 100,000 population)
0
50
100
150
Mar
May
Jul
Sep

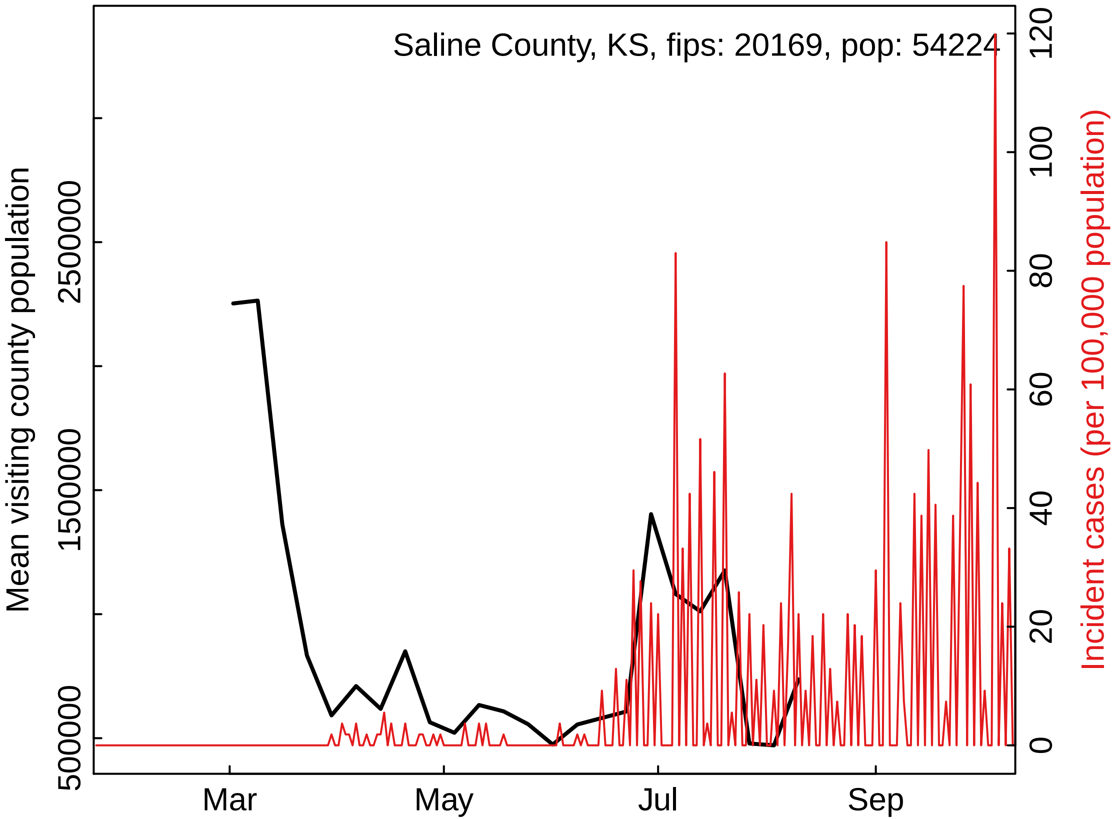

Saline County, KS, fips: 20169, pop: 54224
Mean visiting county population
500000
1500000
2500000
Mar
May
Jul
Sep
0
20
40
60
80
100
120
Incident cases (per 100,000 population)

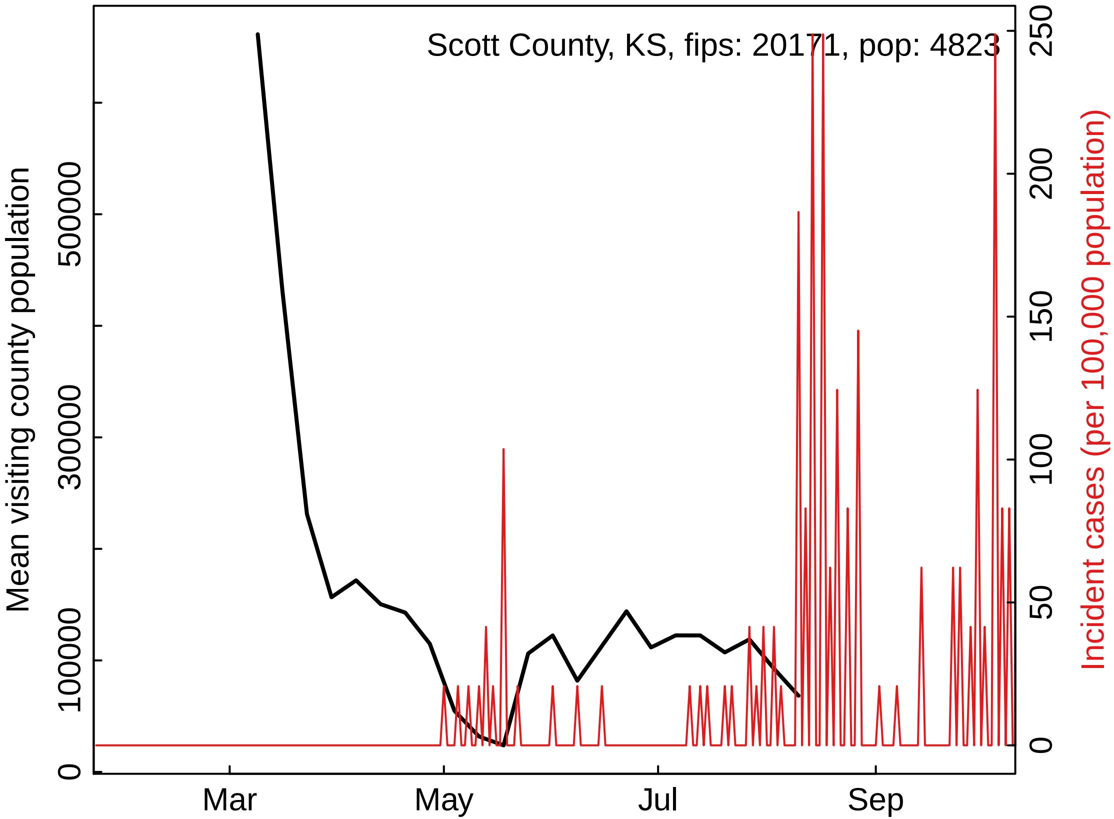

Scott County, KS, fips: 20171, pop: 4823
Mean visiting county population
0
100000
300000
500000
Mar
May
Jul
Sep
0
50
100
150
200
250
Incident cases (per 100,000 population)

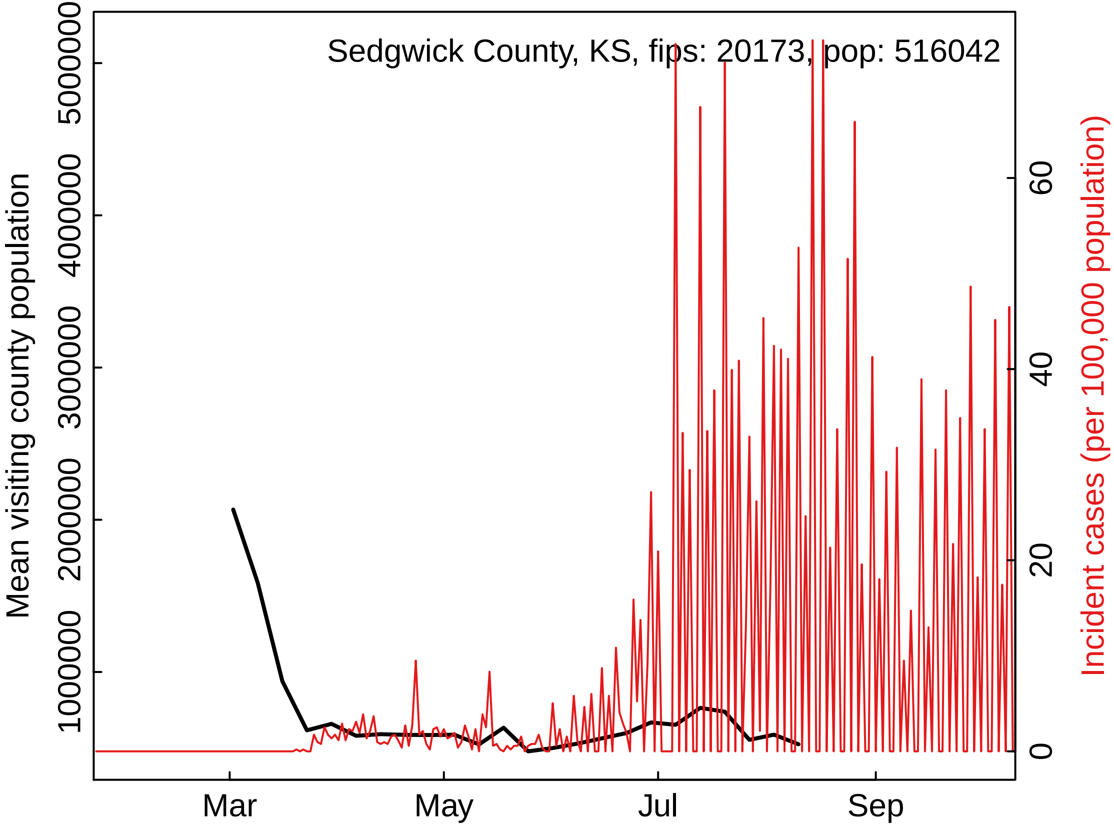
Sedgwick County, KS, fips: 20173, pop: 516042
Mean visiting county population
1000000
2000000
3000000
4000000
5000000
Incident cases (per 100,000 population)
0
20
40
60
Mar
May
Jul
Sep

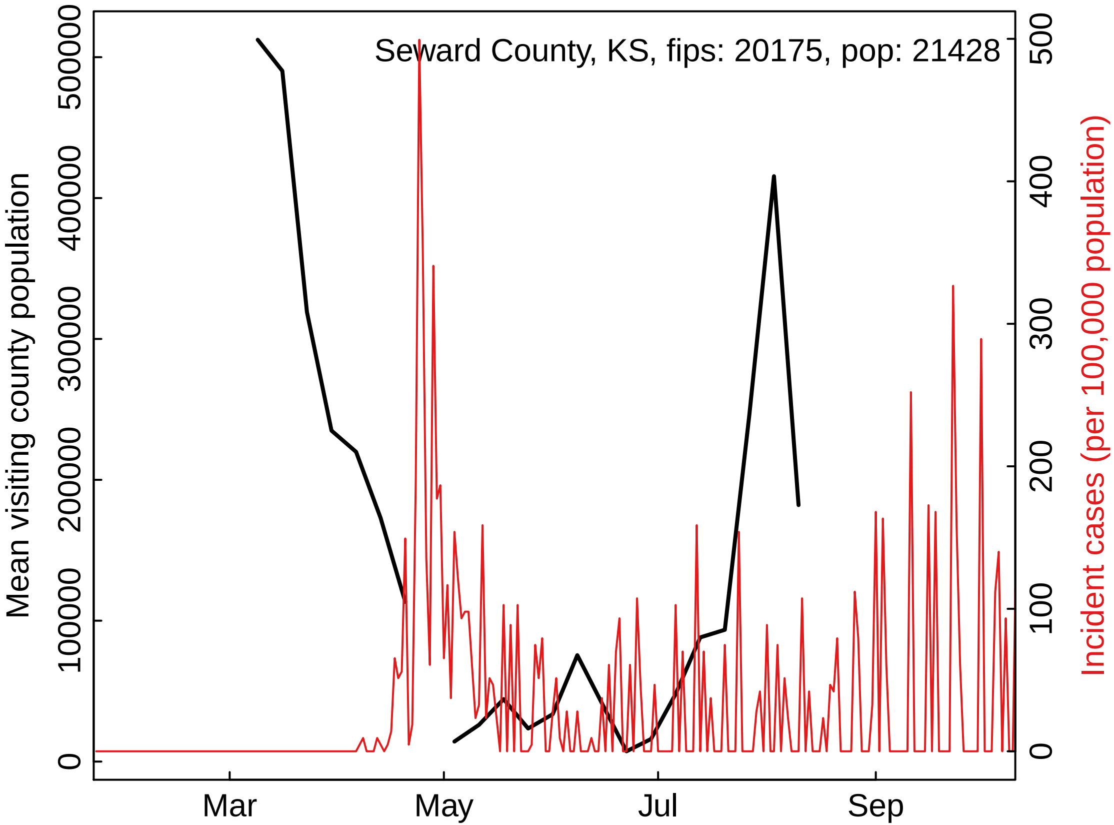

Seward County, KS, fips: 20175, pop: 21428
Mean visiting county population
Incident cases (per 100,000 population)
0
100000
200000
300000
400000
500000
0
100
200
300
400
500
Mar
May
Jul
Sep

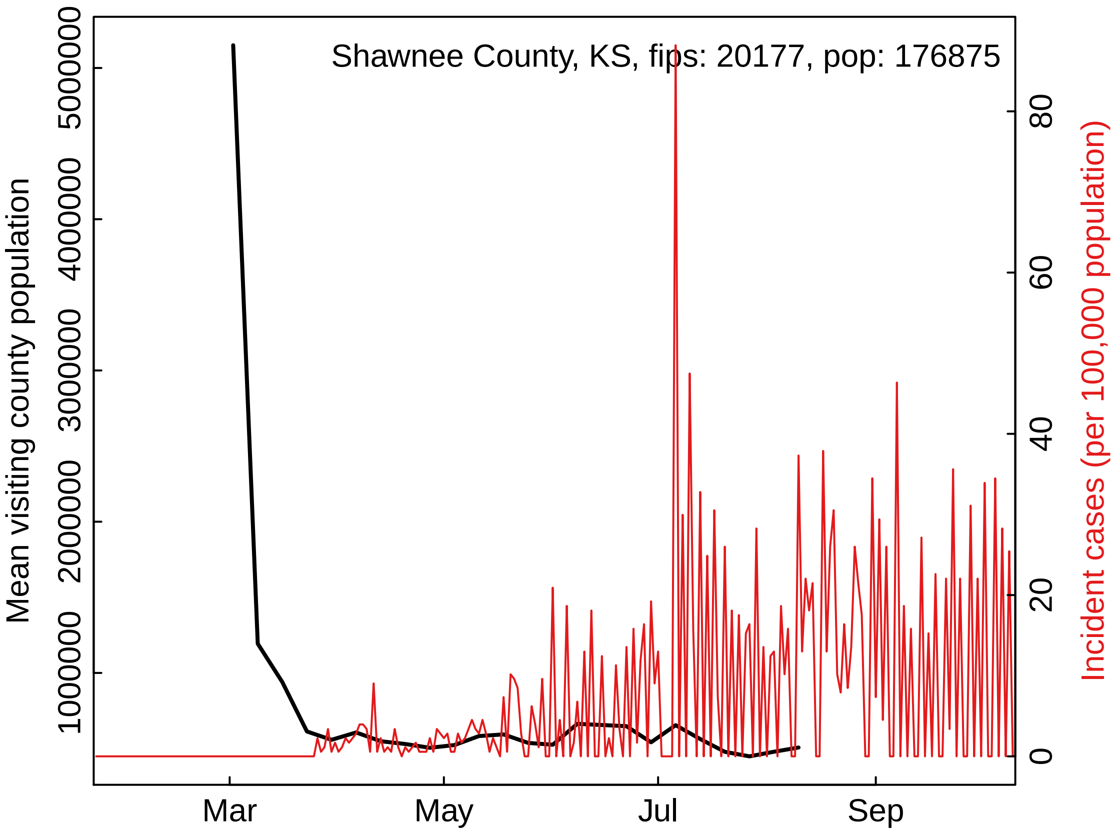

Shawnee County, KS, fips: 20177, pop: 176875
Mean visiting county population
1000000
2000000
3000000
4000000
5000000
Incident cases (per 100,000 population)
0
20
40
60
80
Mar
May
Jul
Sep

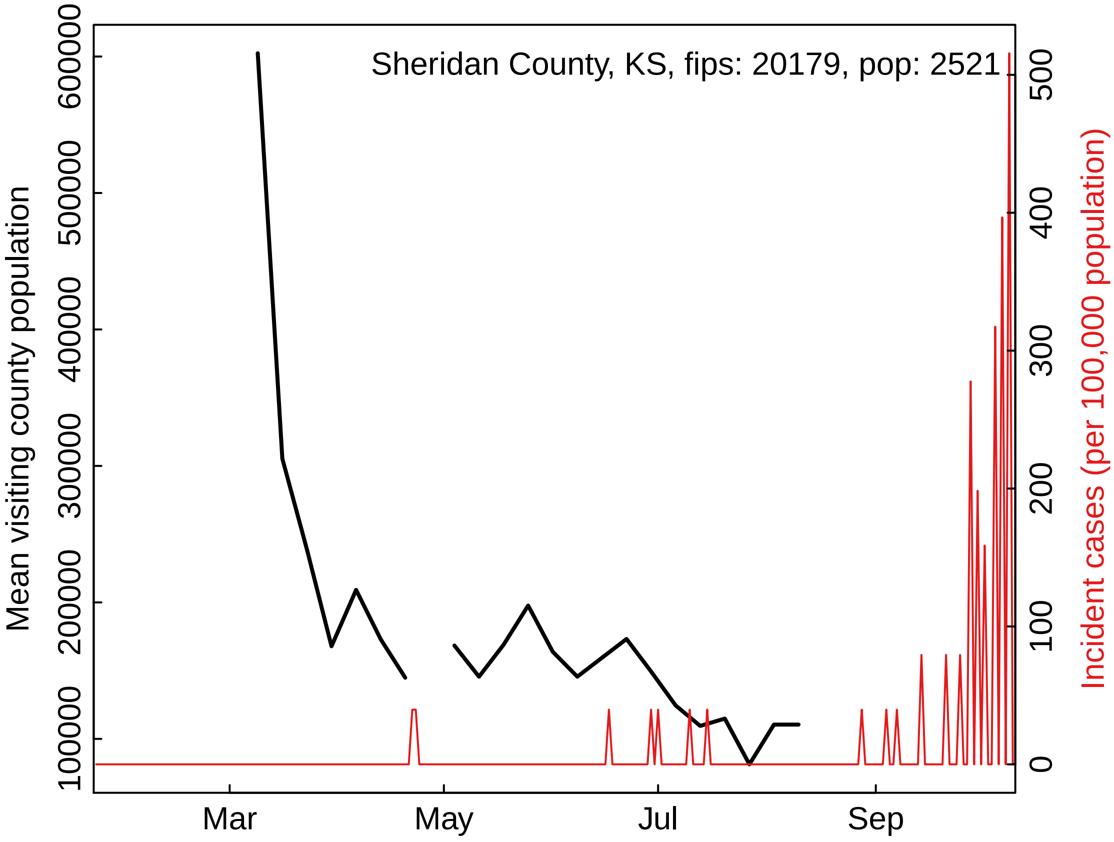

Sheridan County, KS, fips: 20179, pop: 2521
Mean visiting county population
Incident cases (per 100,000 population)
100000
200000
300000
400000
500000
600000
0
100
200
300
400
500
Mar
May
Jul
Sep

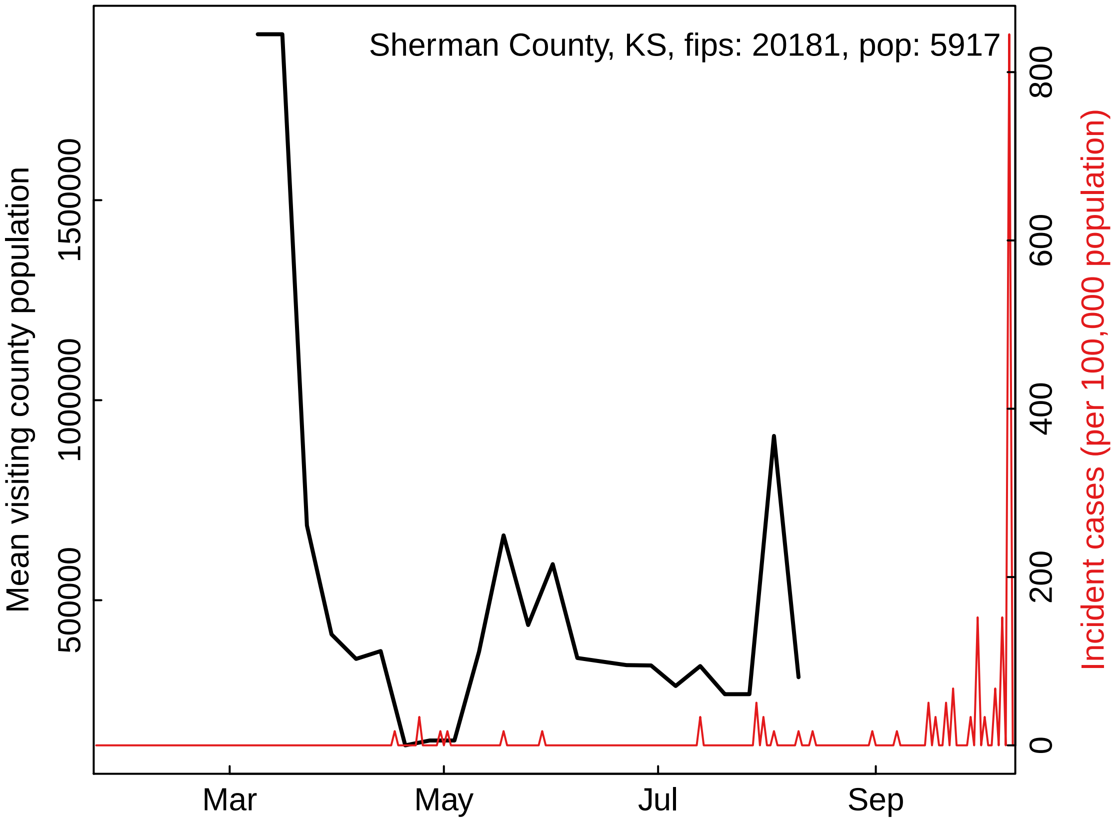

Sherman County, KS, fips: 20181, pop: 5917
Mean visiting county population
500000
1000000
1500000
Incident cases (per 100,000 population)
0
200
400
600
800
Mar
May
Jul
Sep

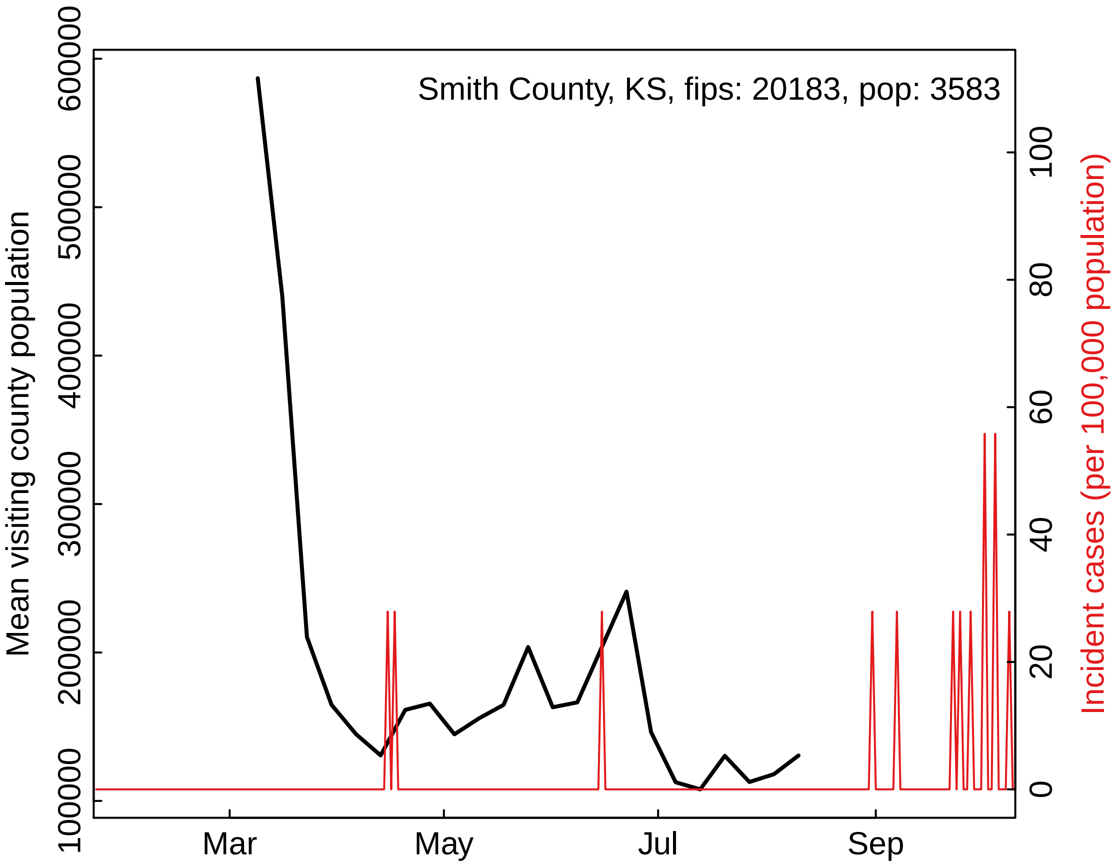

Smith County, KS, fips: 20183, pop: 3583
Mean visiting county population
100000
200000
300000
400000
500000
600000
0
20
40
60
80
100
Incident cases (per 100,000 population)
Mar
May
Jul
Sep

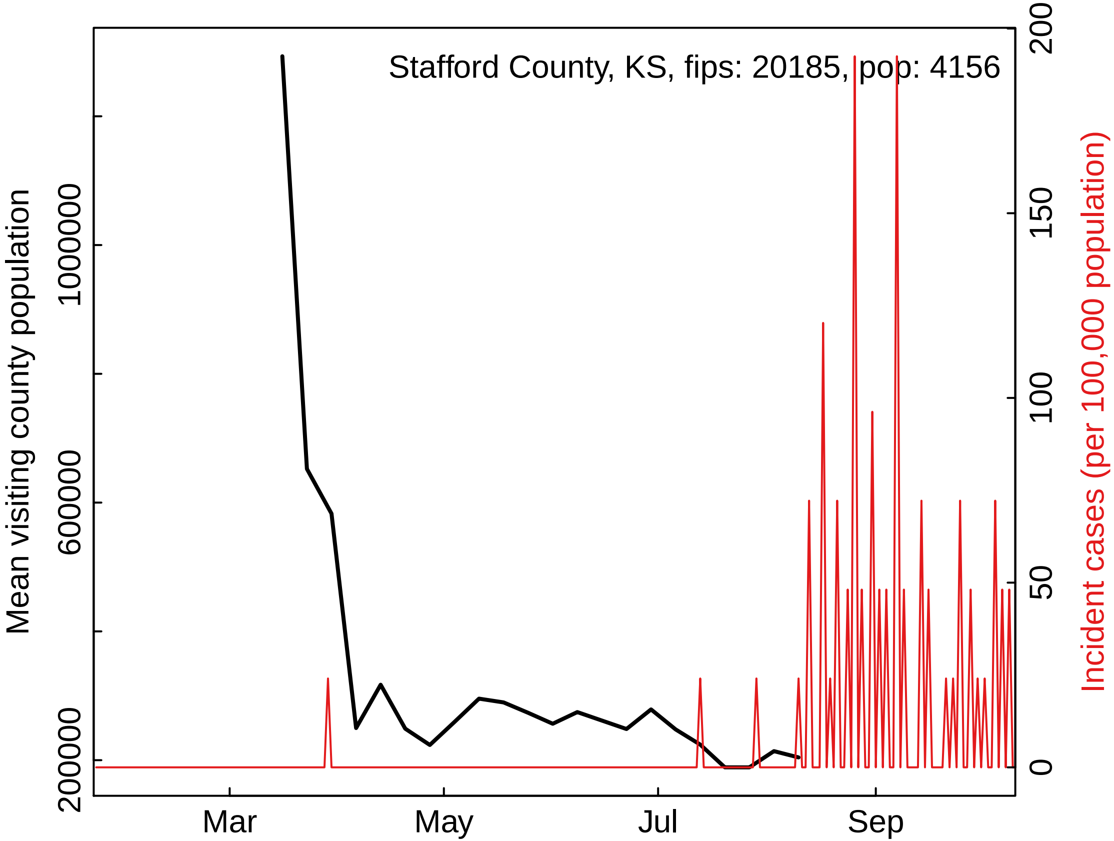
Stafford County, KS, fips: 20185, pop: 4156
Mean visiting county population
200000
600000
1000000
Incident cases (per 100,000 population)
0
50
100
150
200
Mar
May
Jul
Sep

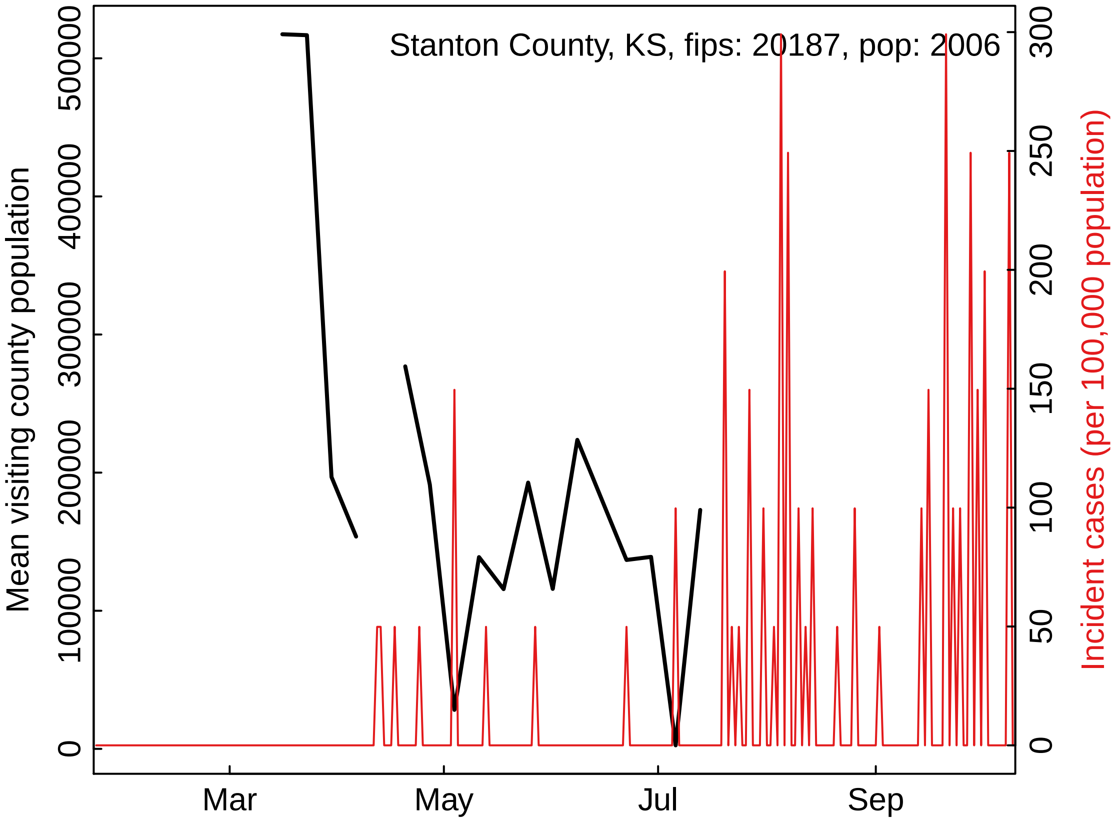

Stanton County, KS, fips: 20187, pop: 2006
Mean visiting county population
0
100000
200000
300000
400000
500000
Incident cases (per 100,000 population)
0
50
100
150
200
250
300
Mar
May
Jul
Sep

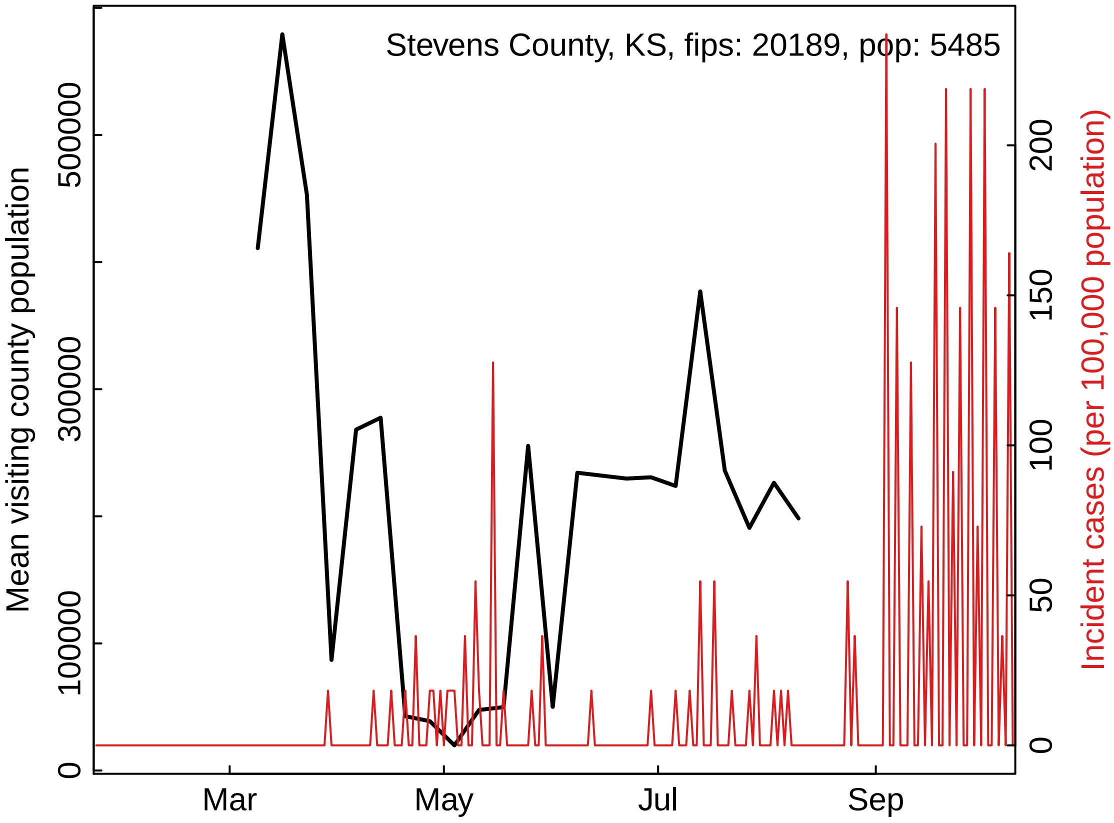

Stevens County, KS, fips: 20189, pop: 5485
Mean visiting county population
Incident cases (per 100,000 population)
0
100000
300000
500000
0
50
100
150
200
Mar
May
Jul
Sep

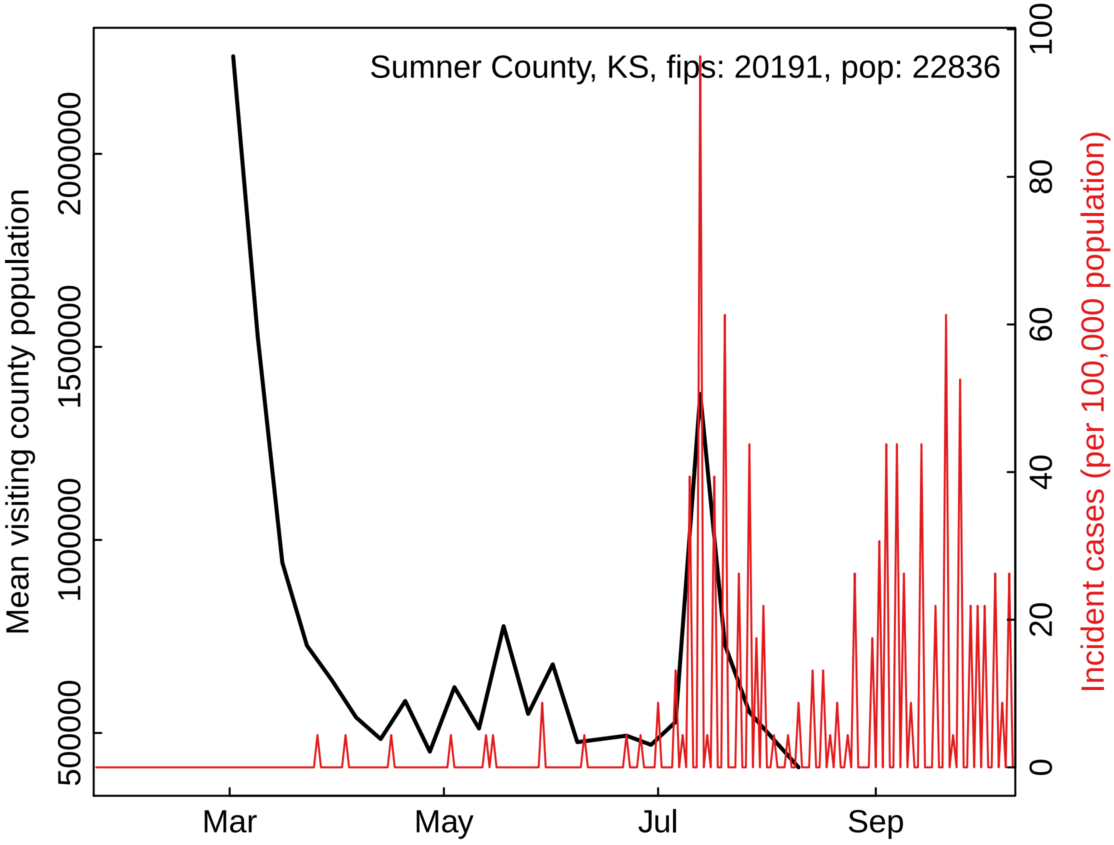

Sumner County, KS, fips: 20191, pop: 22836
Mean visiting county population
500000
1000000
1500000
2000000
Incident cases (per 100,000 population)
0
20
40
60
80
100
Mar
May
Jul
Sep

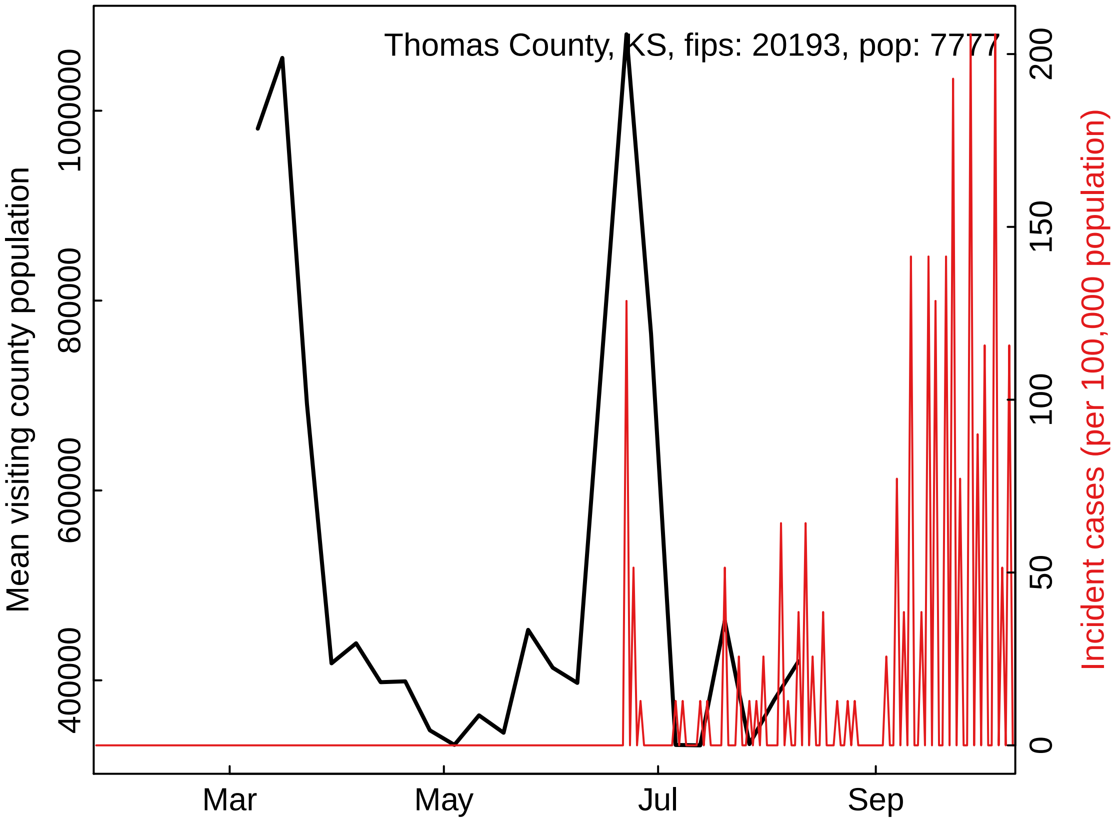

Thomas County, KS, fips: 20193, pop: 7777
Mean visiting county population
400000
600000
800000
1000000
Incident cases (per 100,000 population)
0
50
100
150
200
Mar
May
Jul
Sep

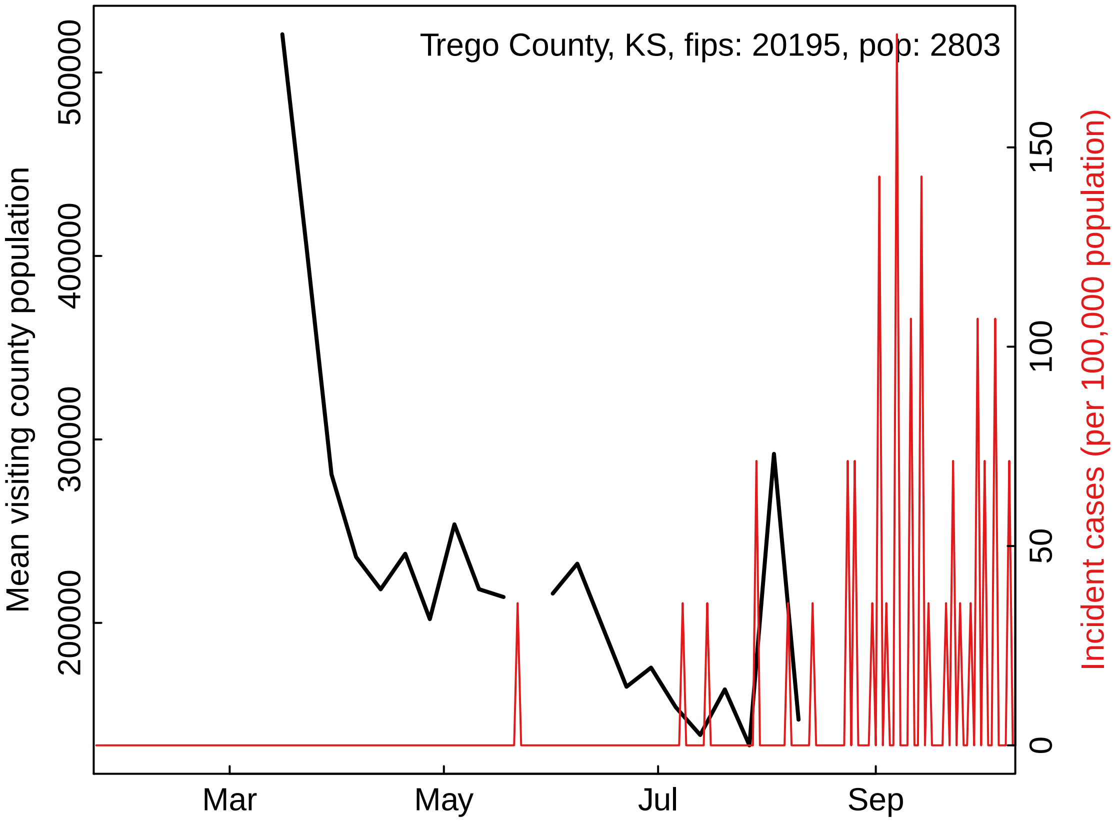

Trego County, KS, fips: 20195, pop: 2803
Mean visiting county population
200000
300000
400000
500000
Incident cases (per 100,000 population)
0
50
100
150
Mar
May
Jul
Sep

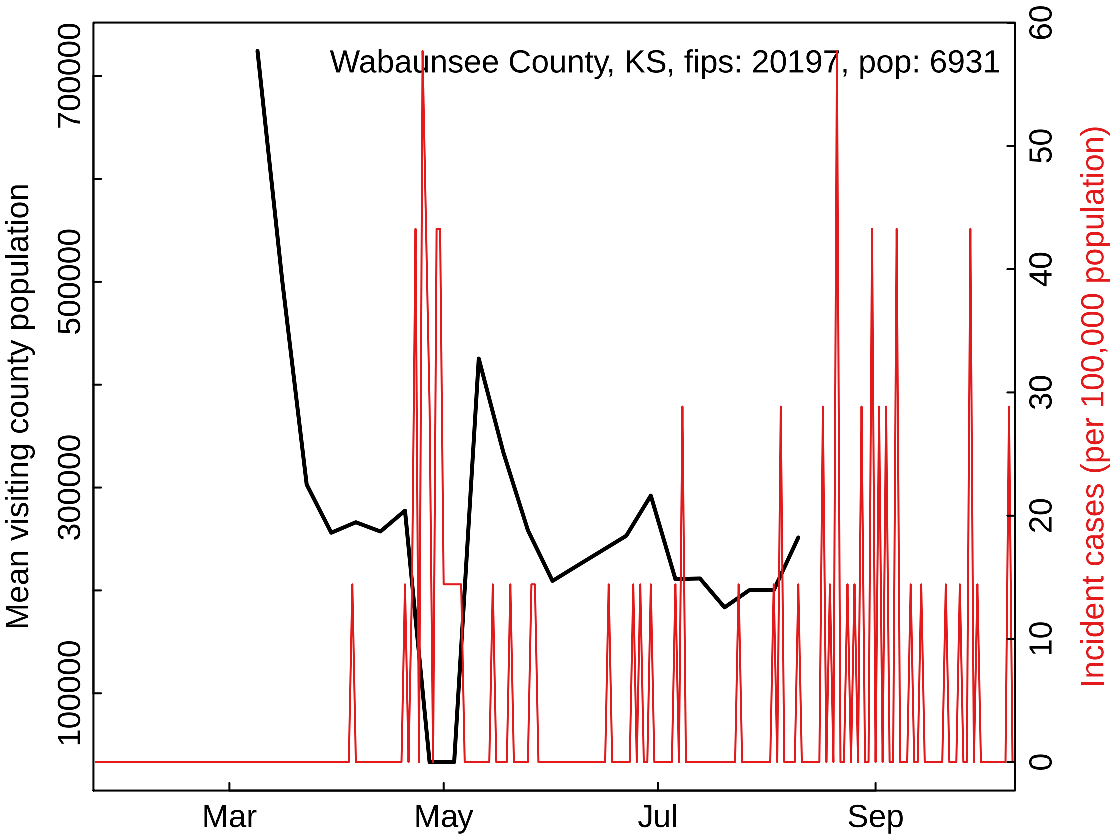

Wabaunsee County, KS, fips: 20197, pop: 6931
Mean visiting county population
100000
300000
500000
700000
Incident cases (per 100,000 population)
0
10
20
30
40
50
60
Mar
May
Jul
Sep

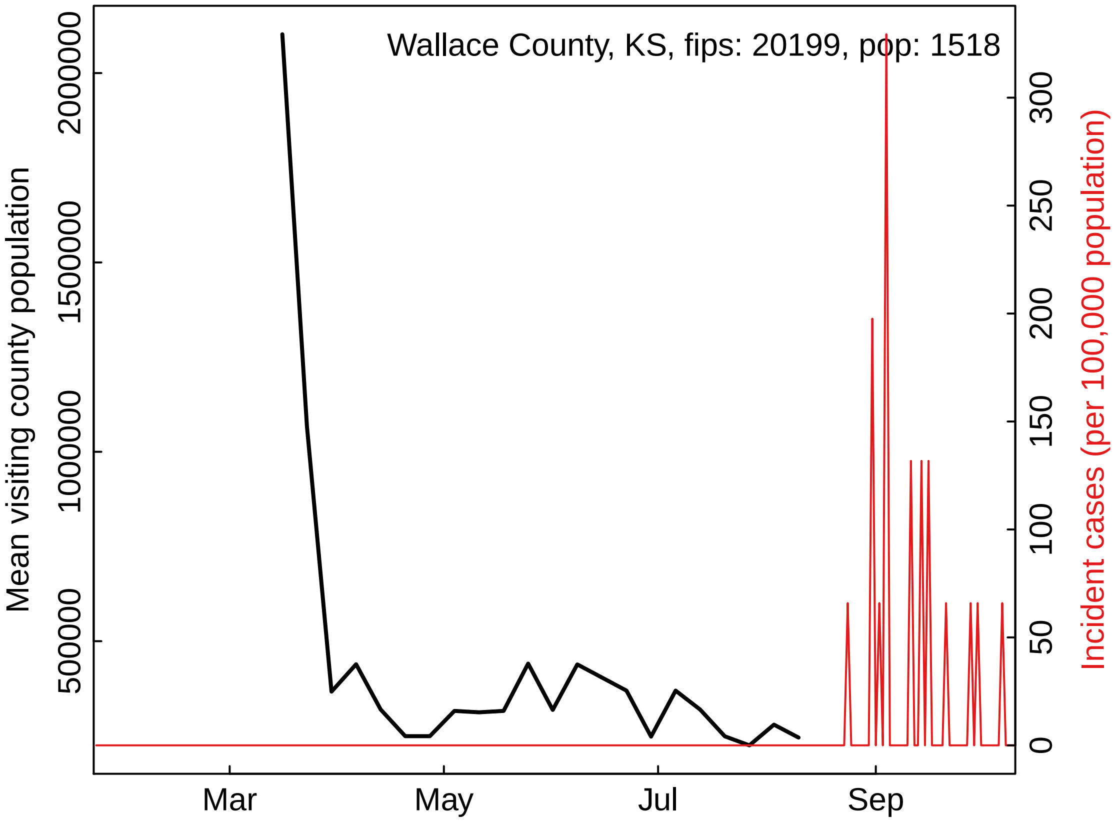

Wallace County, KS, fips: 20199, pop: 1518
Mean visiting county population
500000
1000000
1500000
2000000
Incident cases (per 100,000 population)
0
50
100
150
200
250
300
Mar
May
Jul
Sep

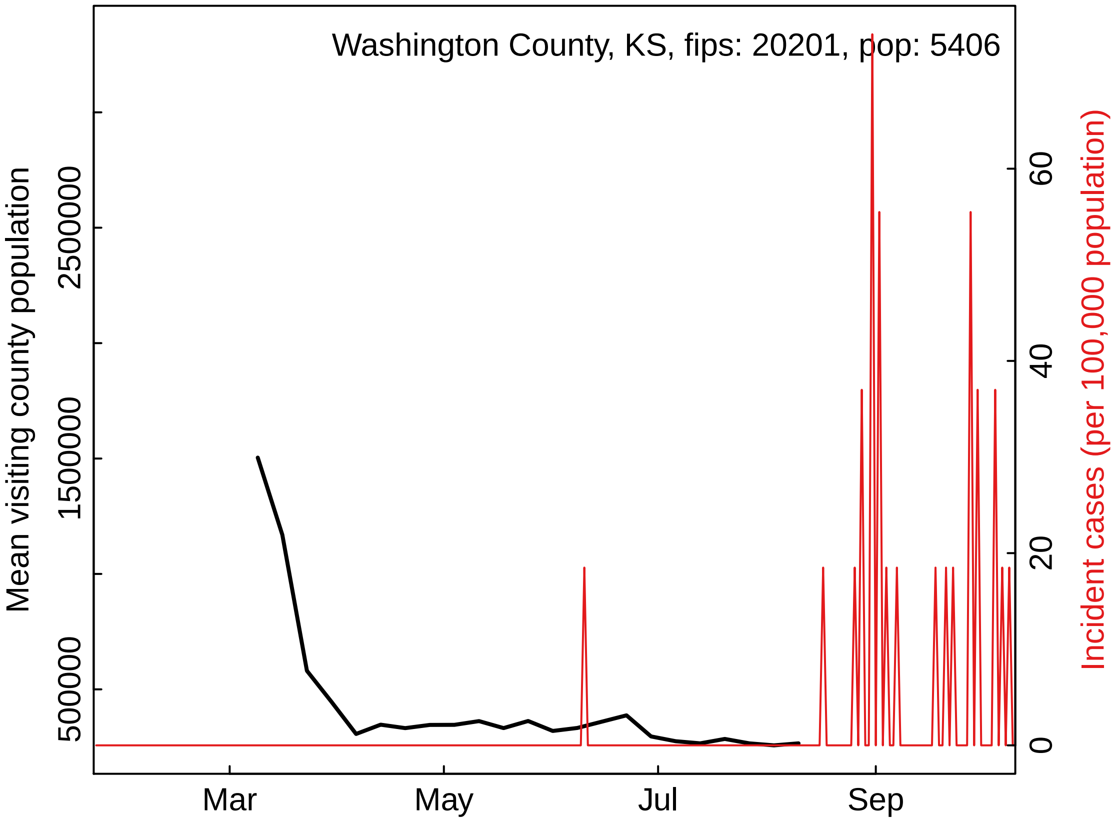

Washington County, KS, fips: 20201, pop: 5406
Mean visiting county population
500000
1500000
2500000
Mar
May
Jul
Sep
0
20
40
60
Incident cases (per 100,000 population)

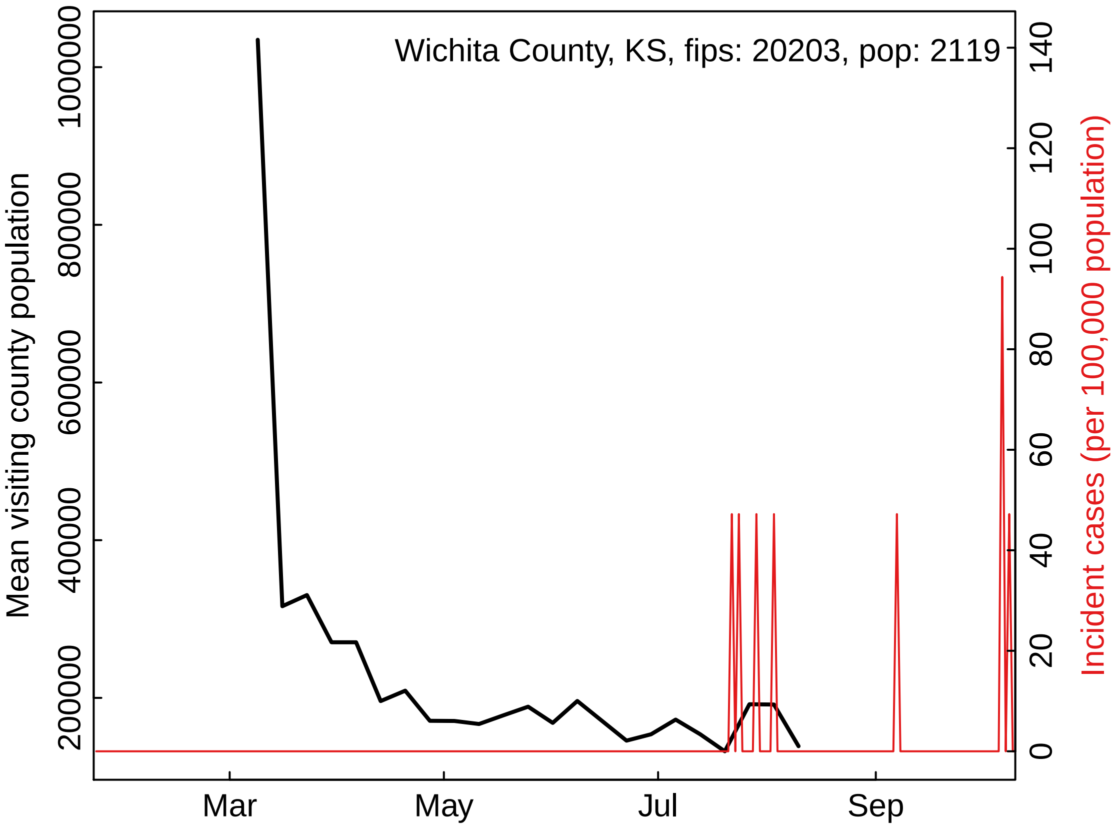

Wichita County, KS, fips: 20203, pop: 2119
Mean visiting county population
200000
400000
600000
800000
1000000
Incident cases (per 100,000 population)
0
20
40
60
80
100
120
140
Mar
May
Jul
Sep

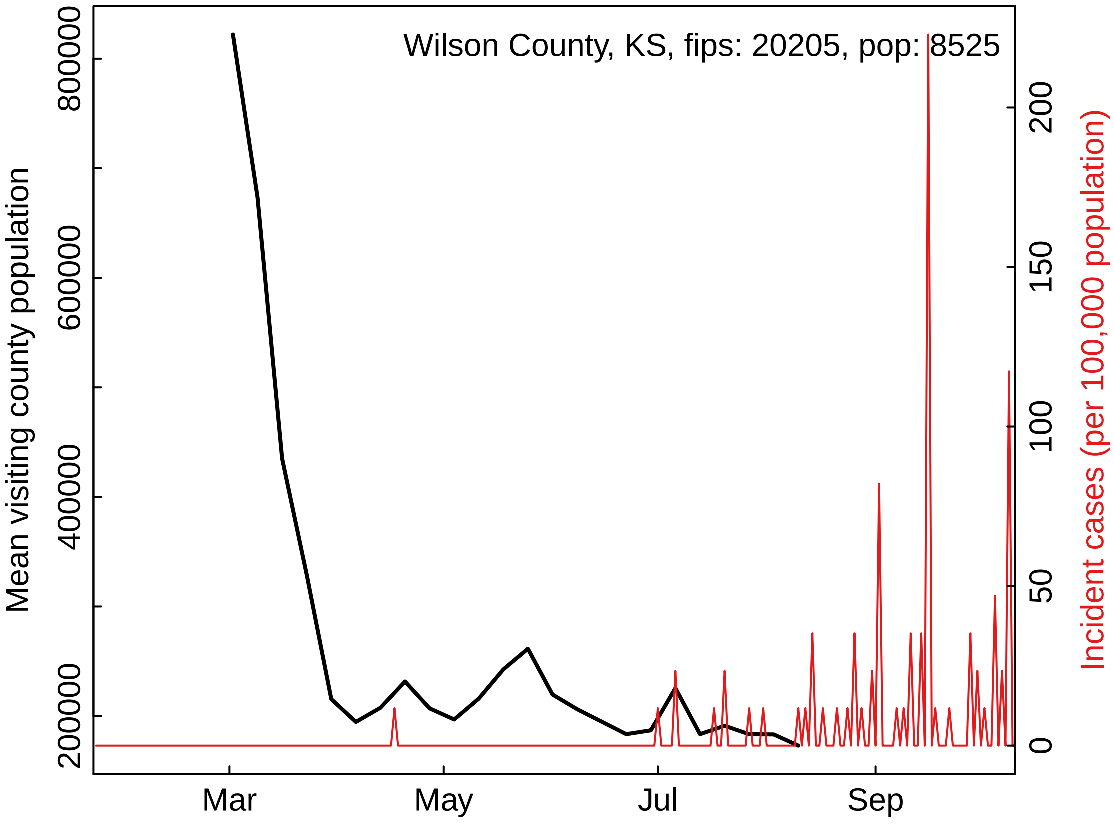
Wilson County, KS, fips: 20205, pop: 8525
Mean visiting county population
Incident cases (per 100,000 population)
200000
400000
600000
800000
0
50
100
150
200
Mar
May
Jul
Sep

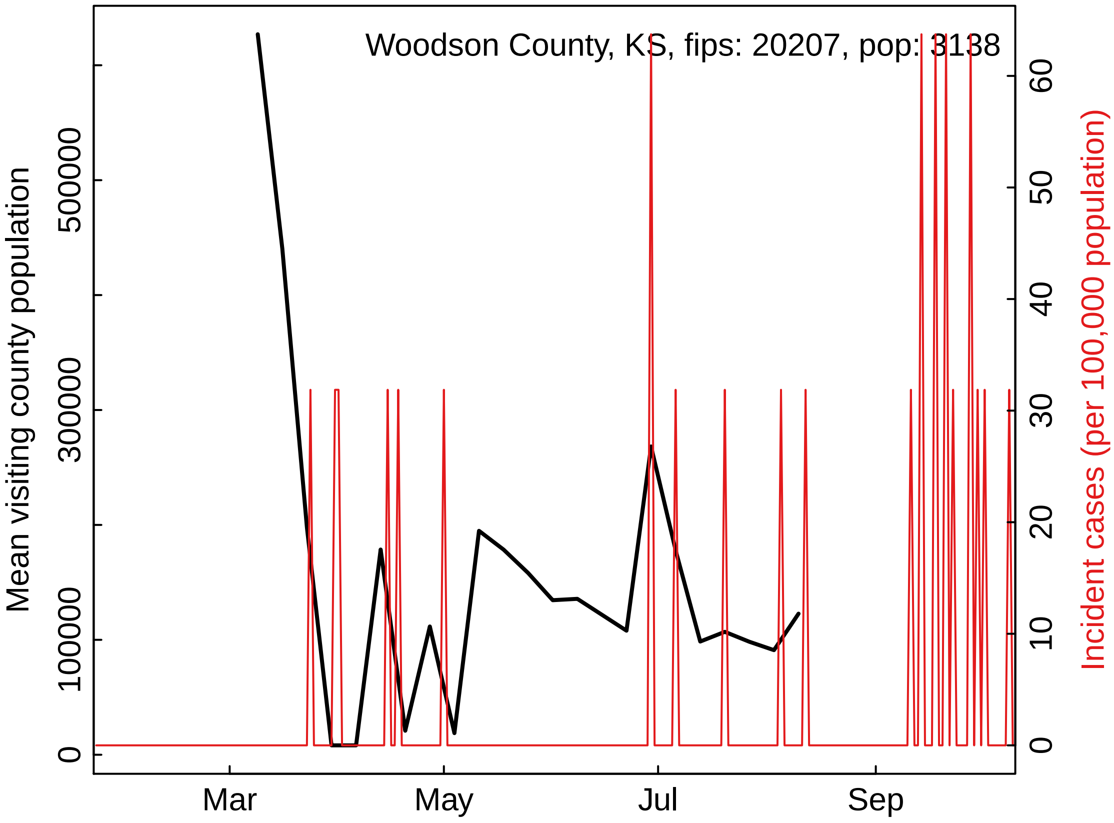

Woodson County, KS, fips: 20207, pop: 3138
Mean visiting county population
0
100000
300000
500000
Incident cases (per 100,000 population)
0
10
20
30
40
50
60
Mar
May
Jul
Sep

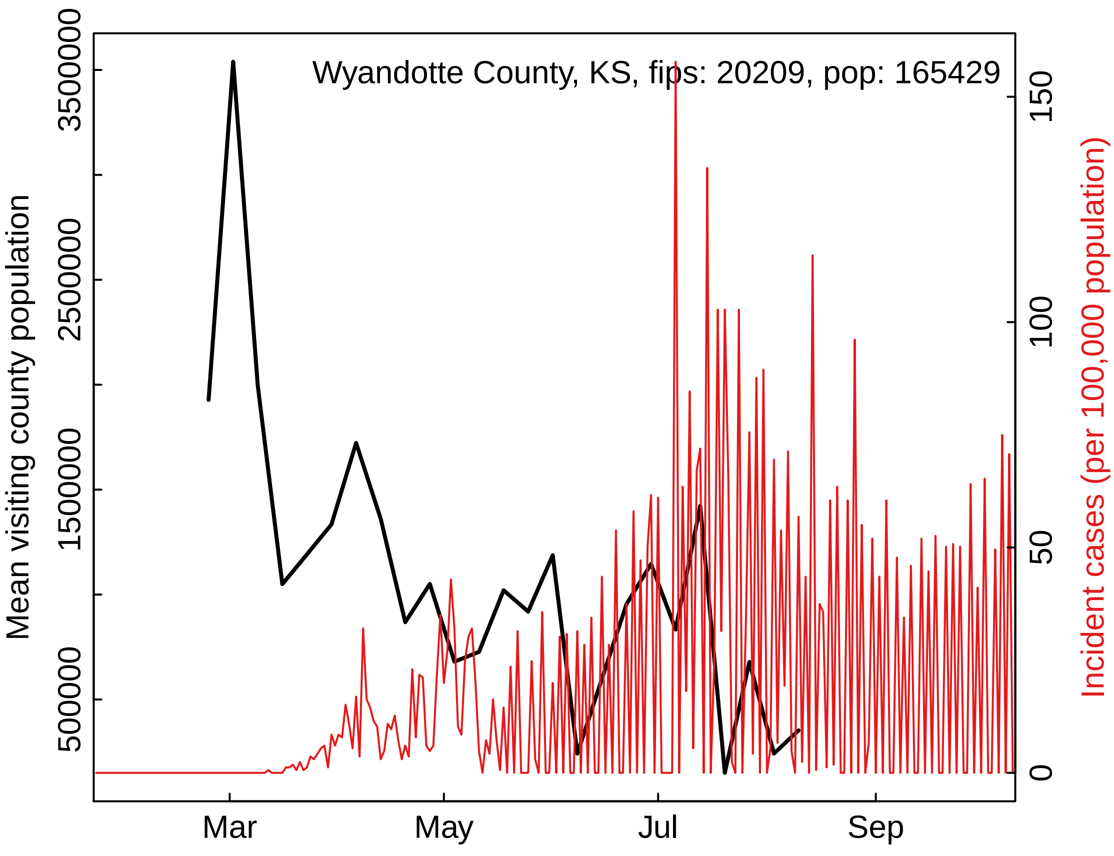

Wyandotte County, KS, fips: 20209, pop: 165429
Mean visiting county population
500000
1500000
2500000
3500000
Incident cases (per 100,000 population)
0
50
100
150
Mar
May
Jul
Sep

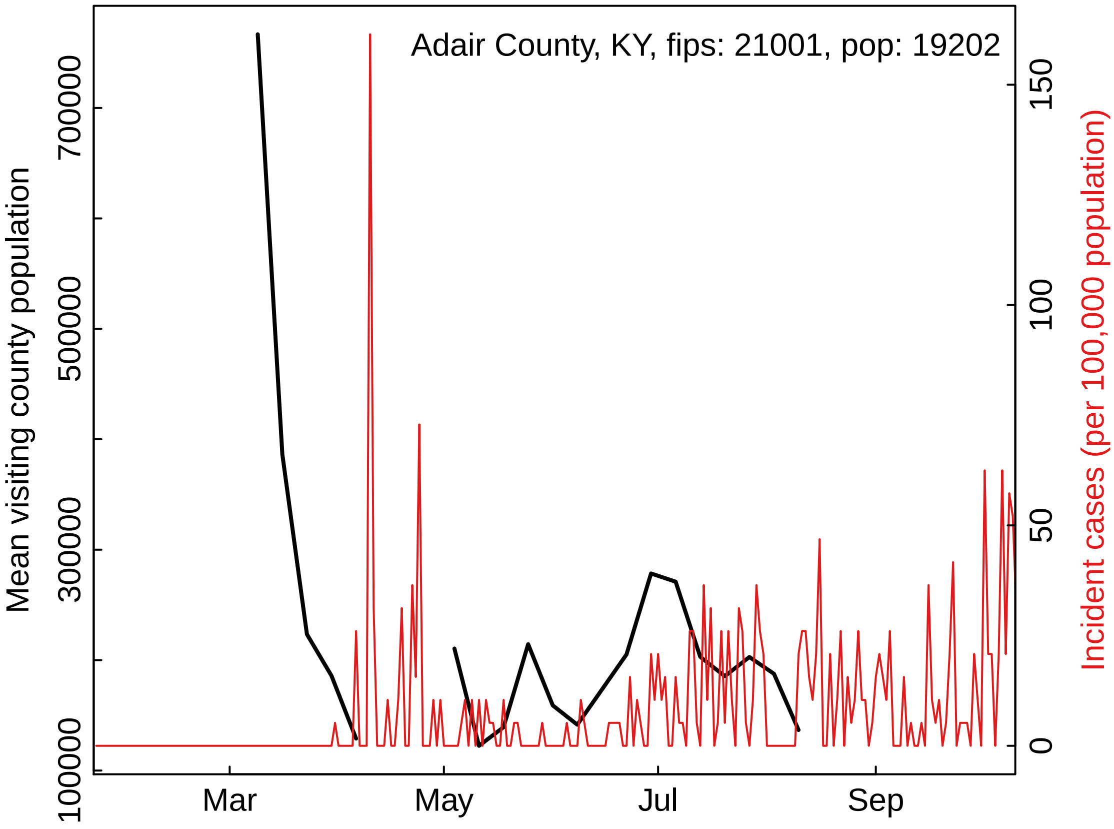

Adair County, KY, fips: 21001, pop: 19202
Mean visiting county population
100000
300000
500000
700000
Incident cases (per 100,000 population)
0
50
100
150
Mar
May
Jul
Sep

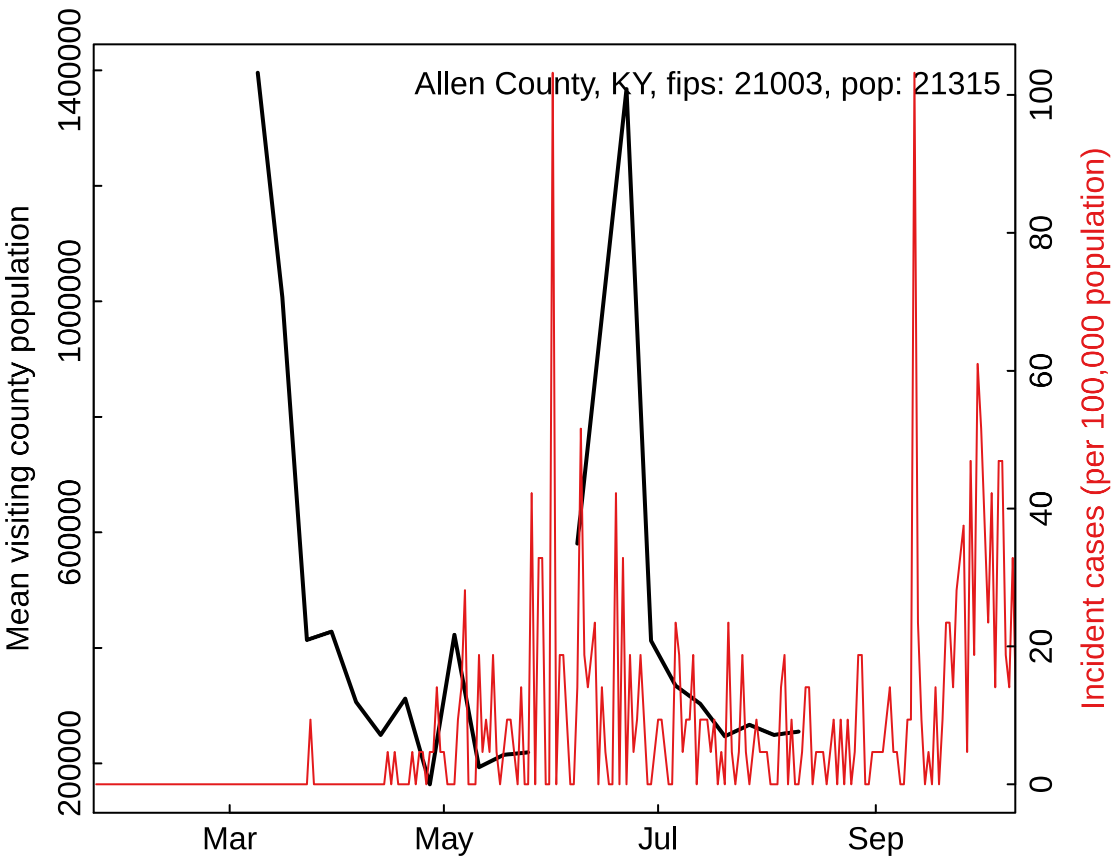
Allen County, KY, fips: 21003, pop: 21315
Mean visiting county population
Incident cases (per 100,000 population)
200000
600000
1000000
1400000
0
20
40
60
80
100
Mar
May
Jul
Sep

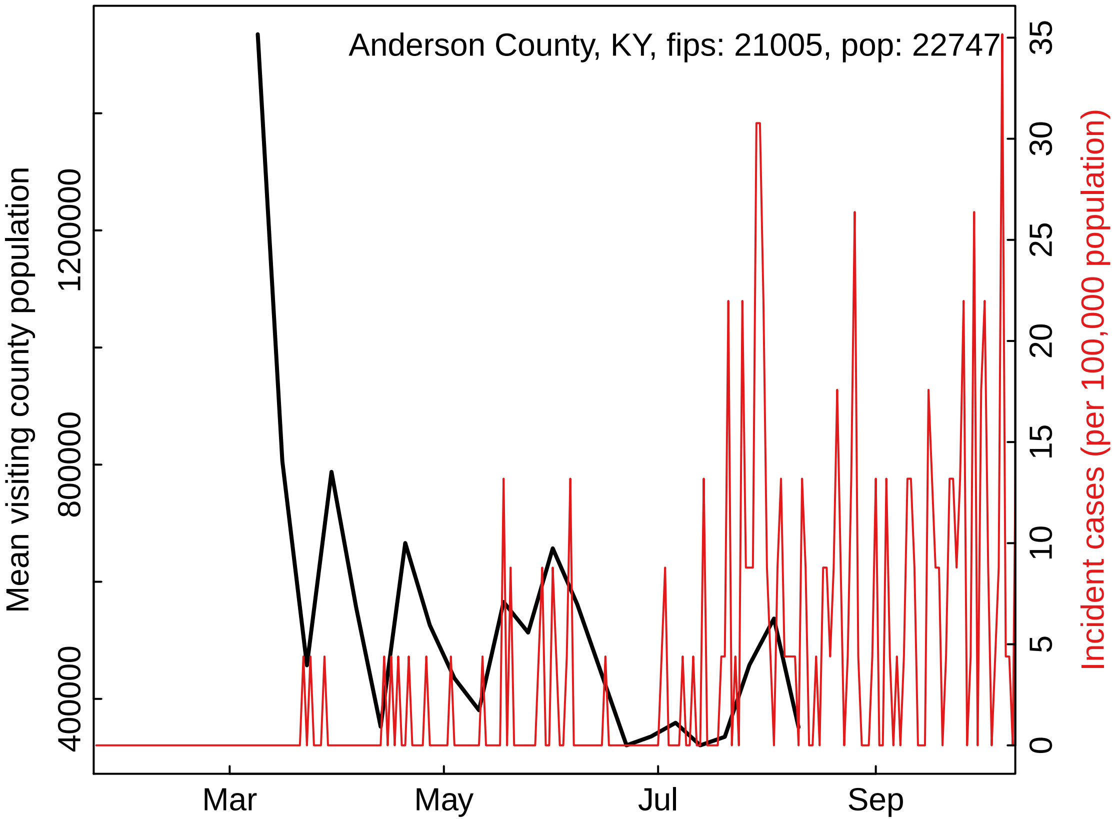

Anderson County, KY, fips: 21005, pop: 22747
Mean visiting county population
400000
800000
1200000
Incident cases (per 100,000 population)
0
5
10
15
20
25
30
35
Mar
May
Jul
Sep

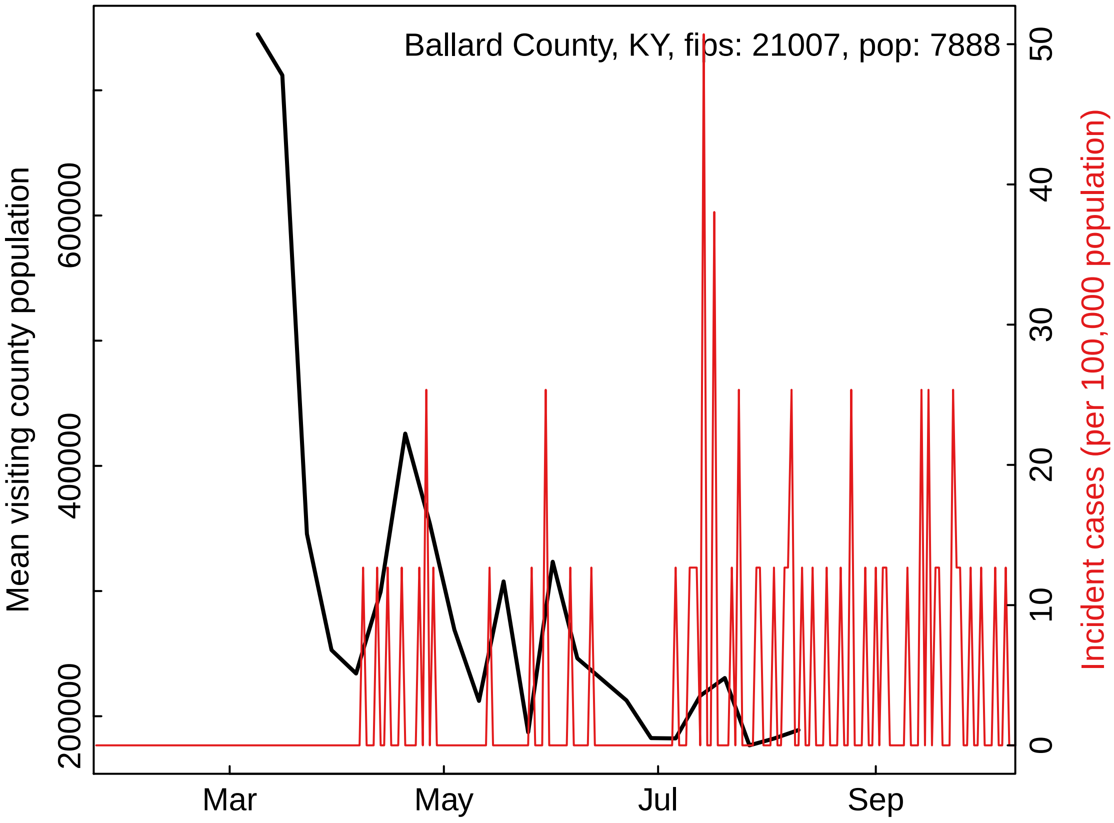

Ballard County, KY, fips: 21007, pop: 7888
Mean visiting county population
200000
400000
600000
Incident cases (per 100,000 population)
0
10
20
30
40
50
Mar
May
Jul
Sep

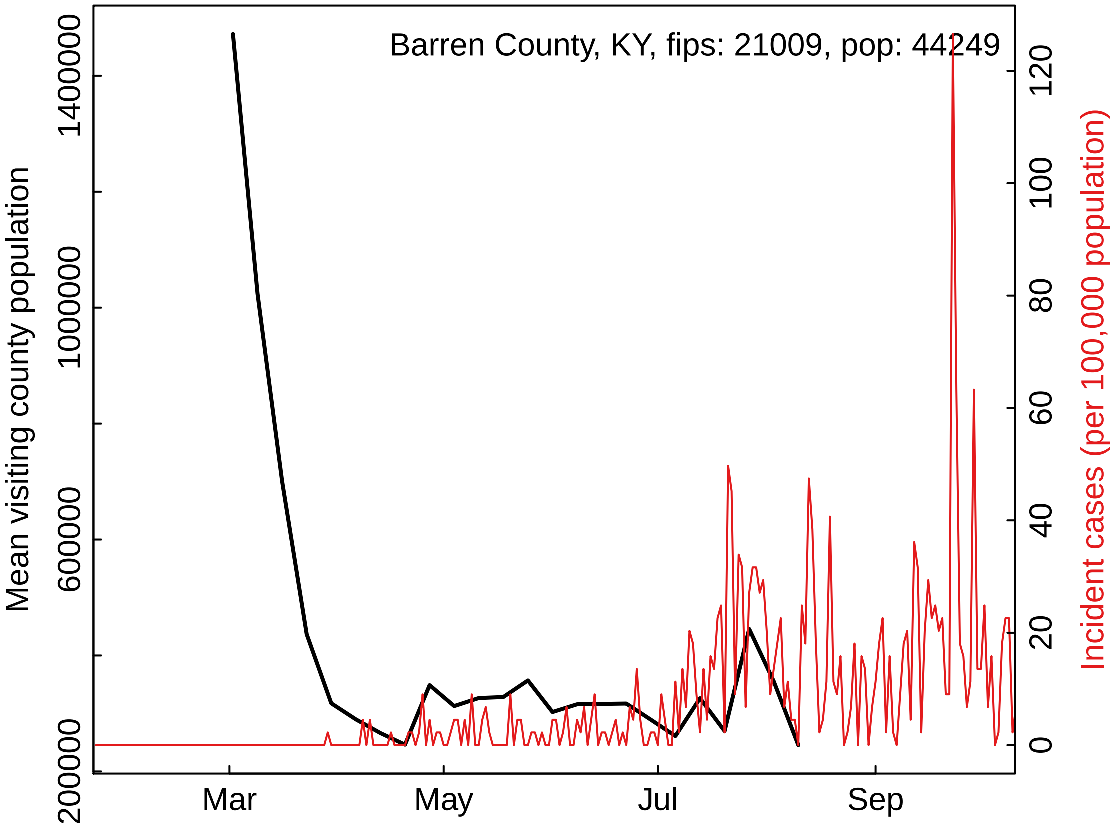

Barren County, KY, fips: 21009, pop: 44249
Mean visiting county population
200000
600000
1000000
1400000
Incident cases (per 100,000 population)
0
20
40
60
80
100
120
Mar
May
Jul
Sep

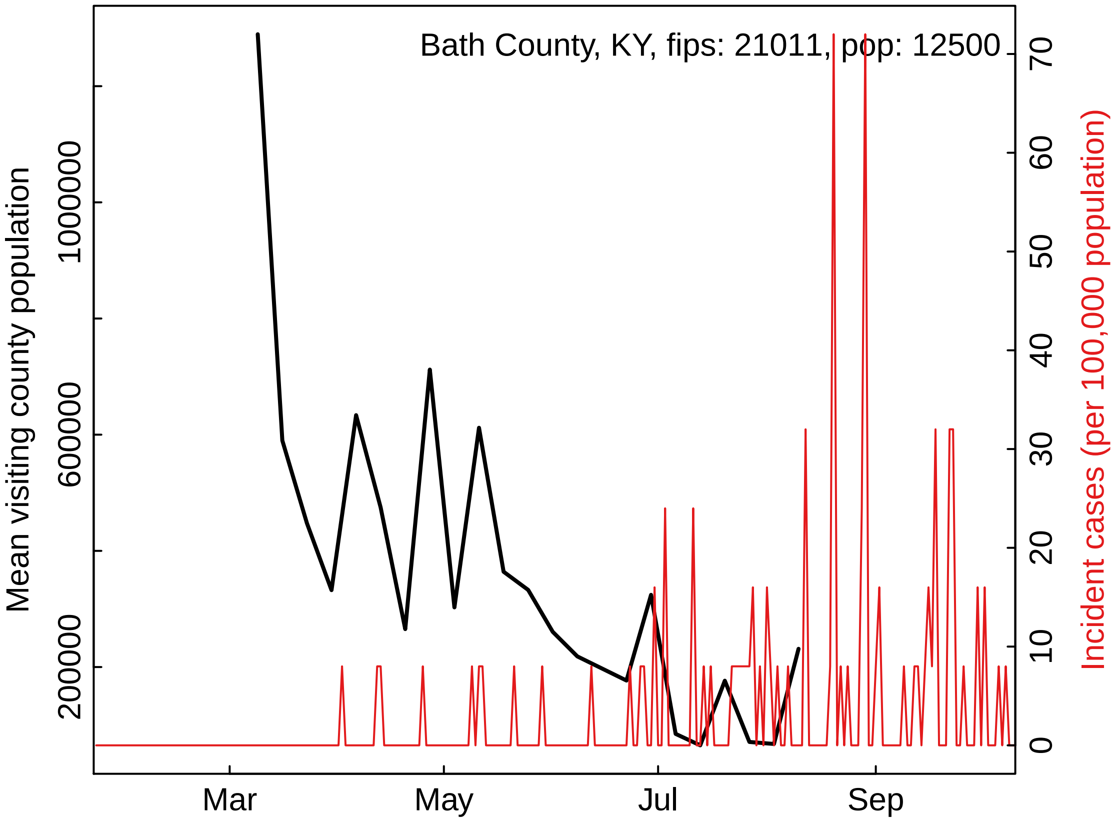

Bath County, KY, fips: 21011, pop: 12500
Mean visiting county population
200000
600000
1000000
Mar
May
Jul
Sep
0
10
20
30
40
50
60
70
Incident cases (per 100,000 population)

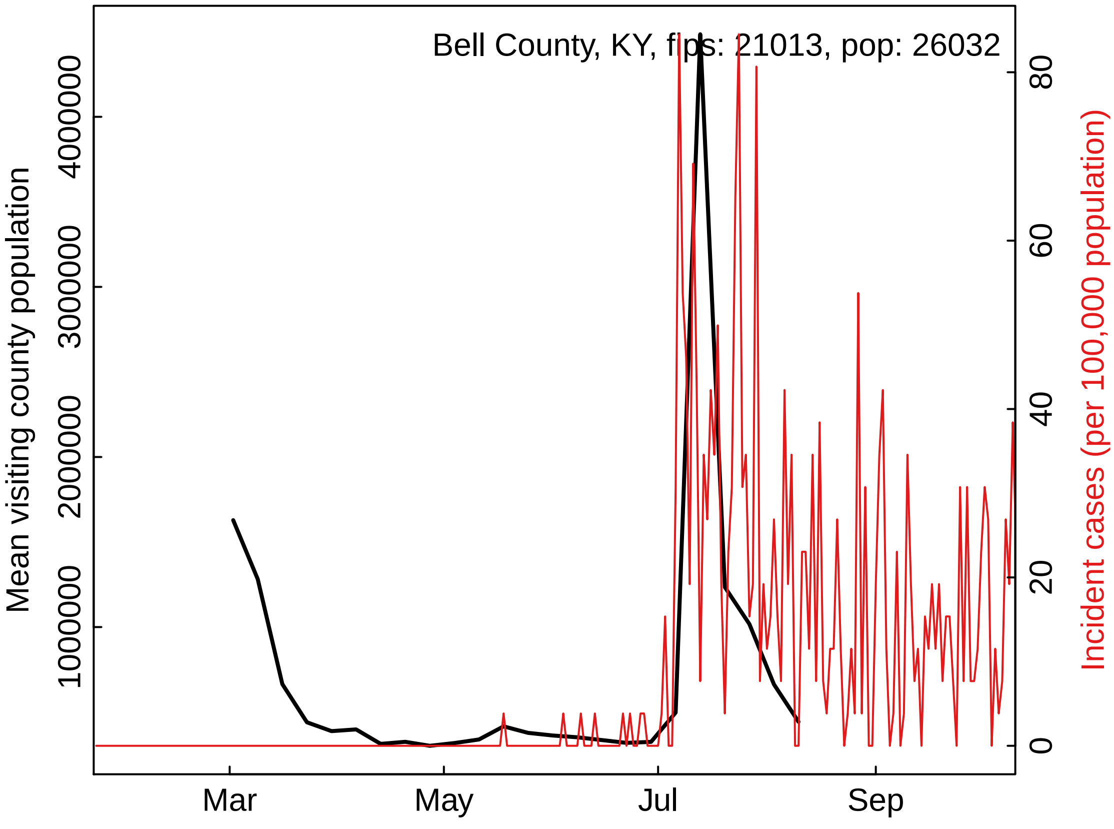

Bell County, KY, fips: 21013, pop: 26032
Mean visiting county population
1000000
2000000
3000000
4000000
Incident cases (per 100,000 population)
0
20
40
60
80
Mar
May
Jul
Sep

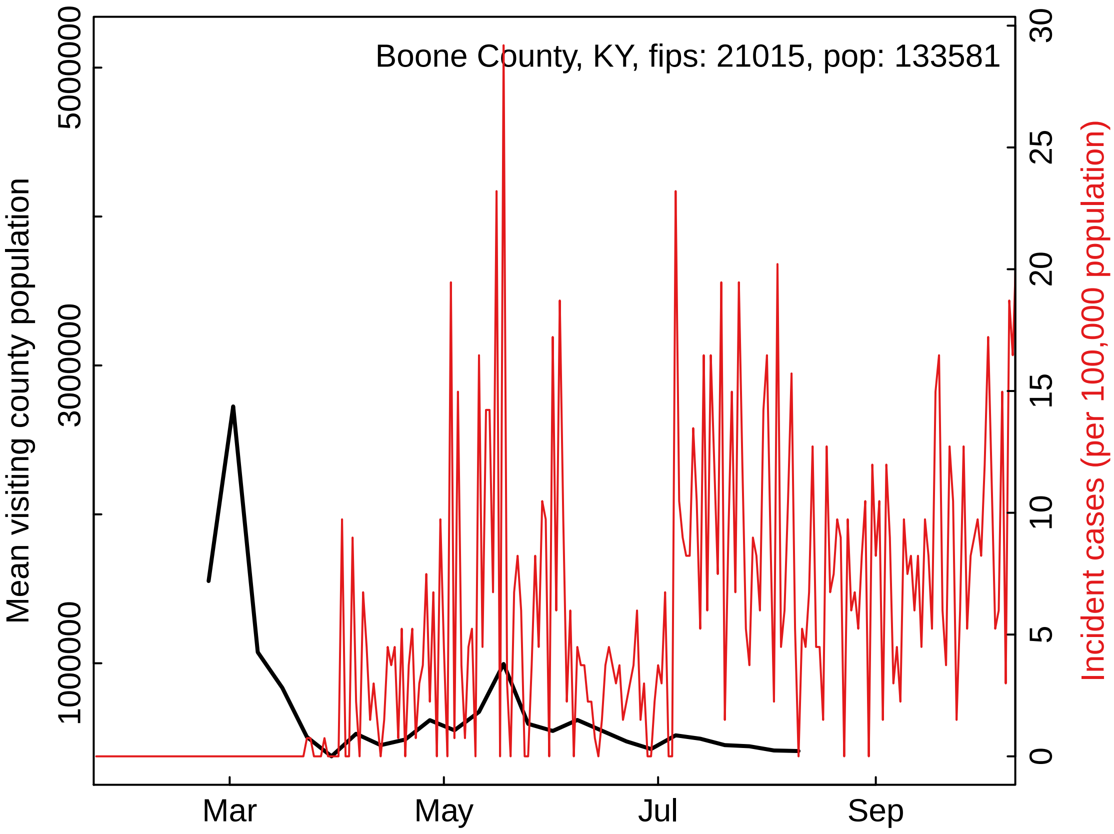

Boone County, KY, fips: 21015, pop: 133581
Mean visiting county population
Incident cases (per 100,000 population)
1000000
3000000
5000000
0
5
10
15
20
25
30
Mar
May
Jul
Sep

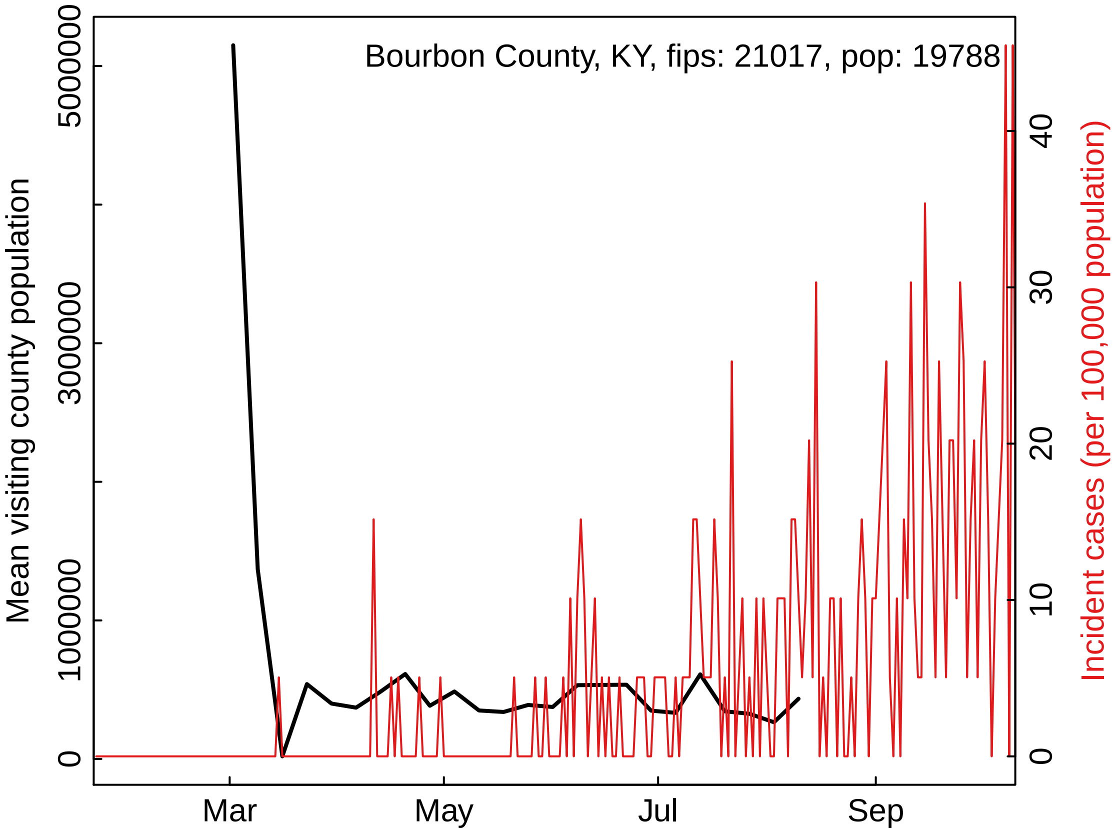

Bourbon County, KY, fips: 21017, pop: 19788
Mean visiting county population
0
1000000
3000000
5000000
Incident cases (per 100,000 population)
0
10
20
30
40
Mar
May
Jul
Sep

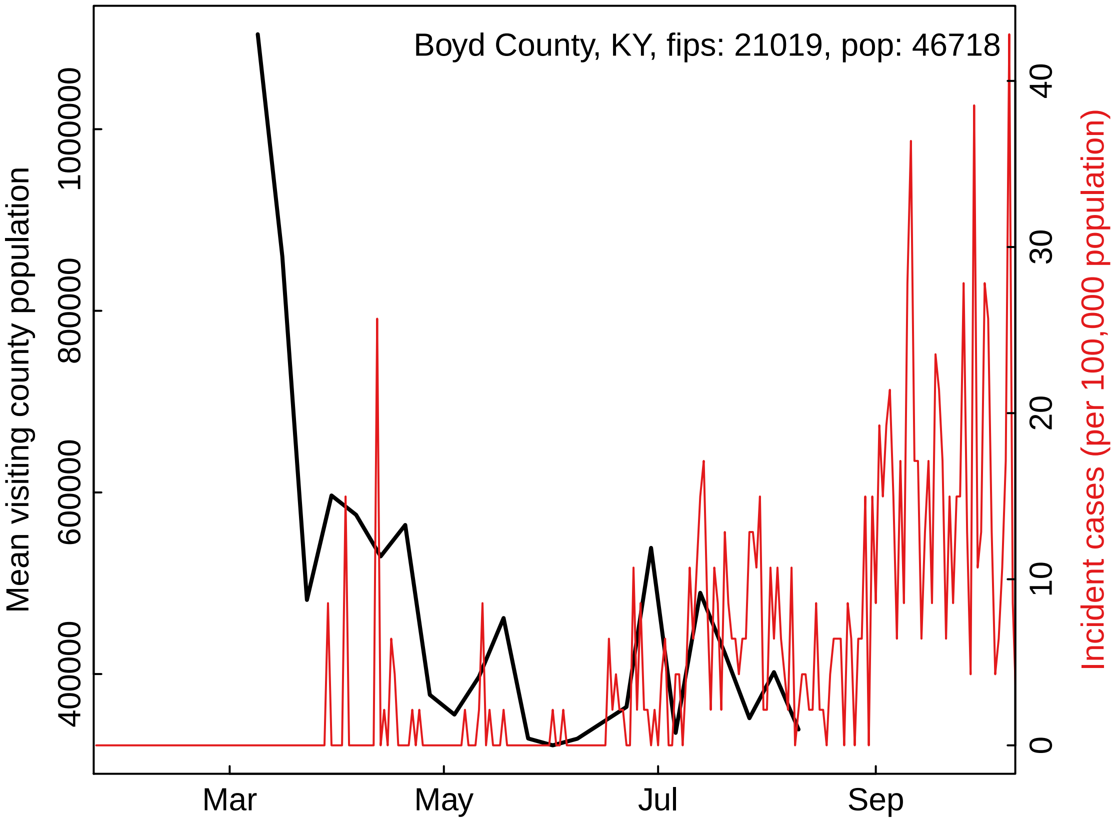
Boyd County, KY, fips: 21019, pop: 46718
Mean visiting county population
400000
600000
800000
1000000
Incident cases (per 100,000 population)
0
10
20
30
40
Mar
May
Jul
Sep

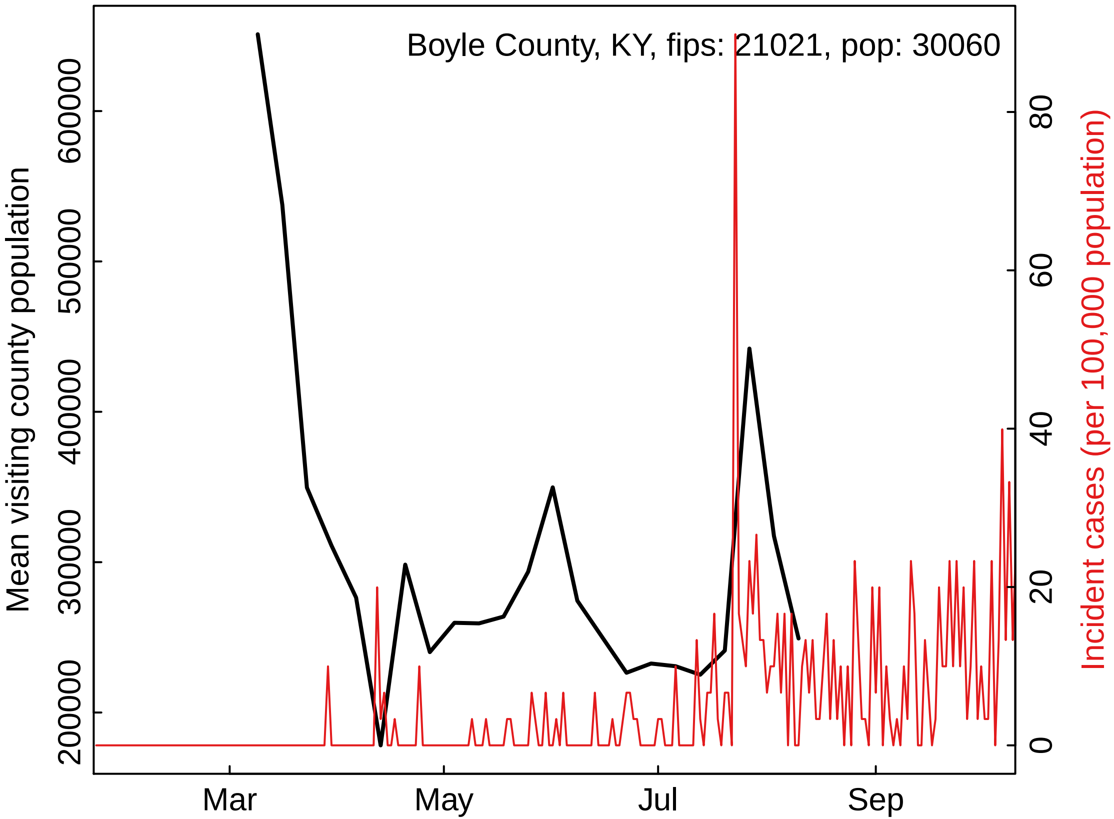

Boyle County, KY, fips: 21021, pop: 30060
Mean visiting county population
200000
300000
400000
500000
600000
Mar
May
Jul
Sep
0
20
40
60
80
Incident cases (per 100,000 population)

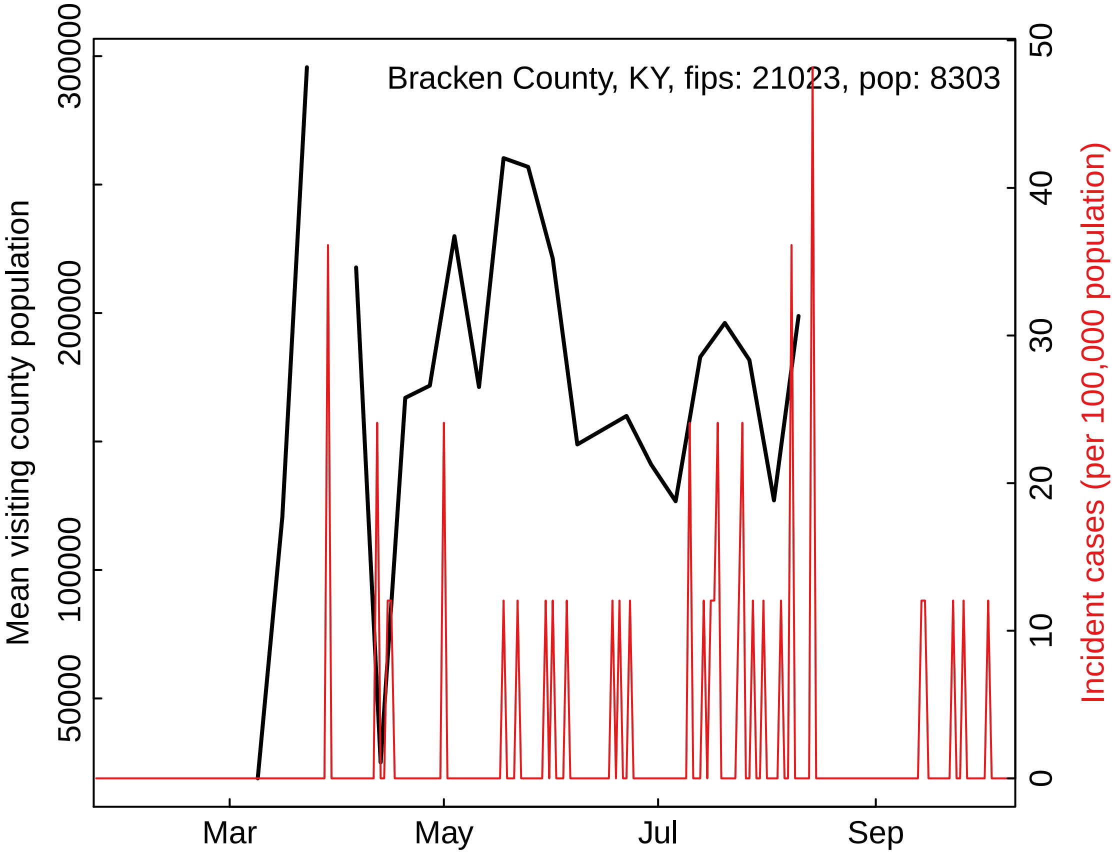

Bracken County, KY, fips: 21023, pop: 8303
Mean visiting county population
300000
200000
100000
50000
Incident cases (per 100,000 population)
50
40
30
20
10
0
Mar
May
Jul
Sep

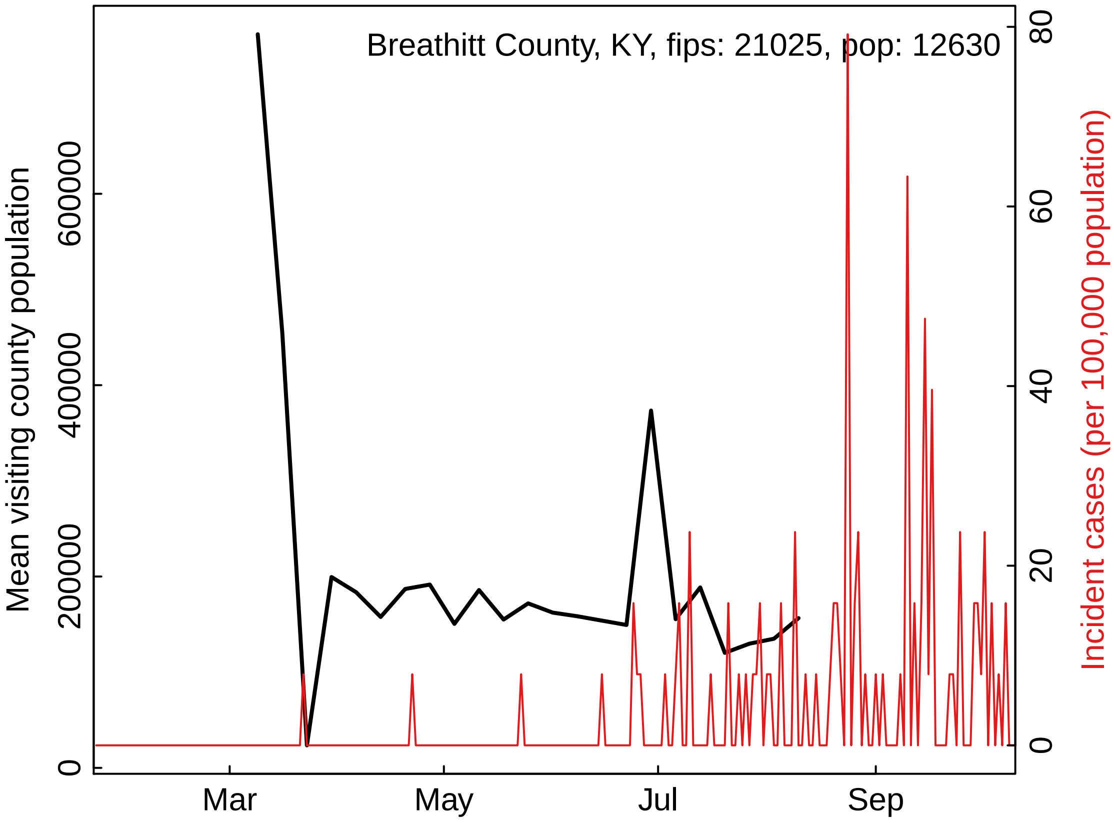

Breathitt County, KY, fips: 21025, pop: 12630
Mean visiting county population
0
200000
400000
600000
Incident cases (per 100,000 population)
0
20
40
60
80
Mar
May
Jul
Sep

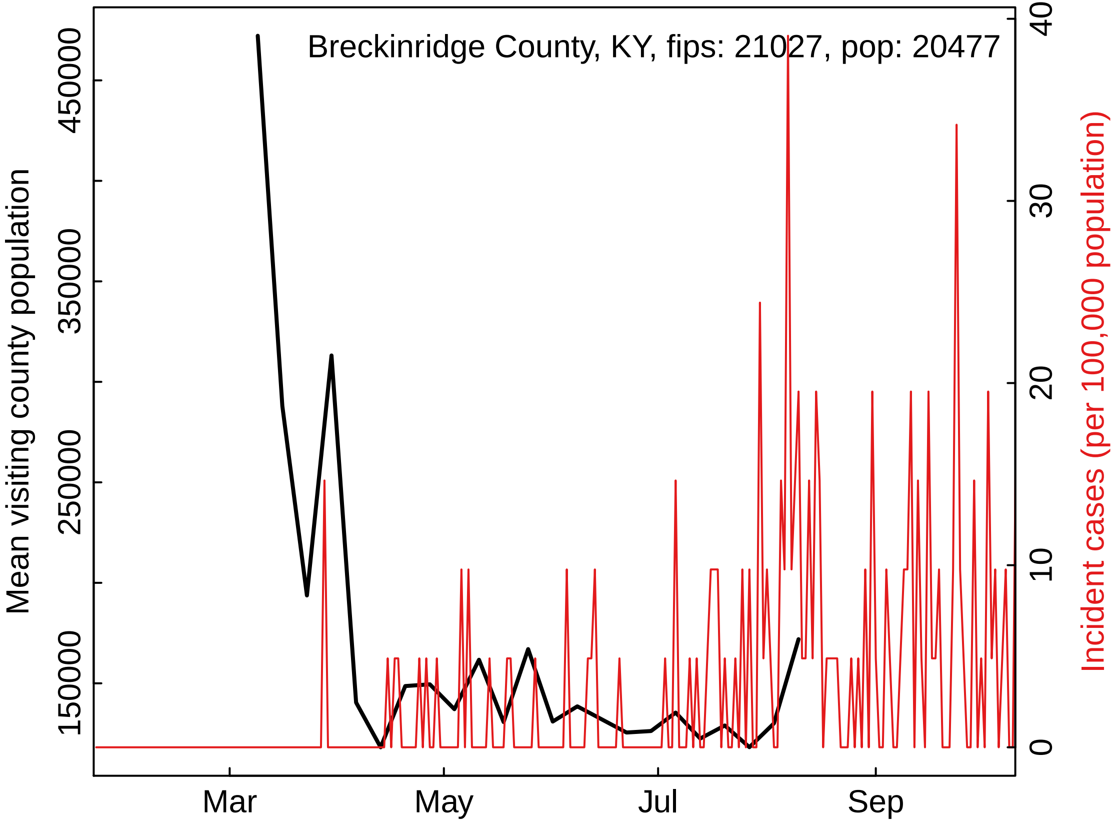
Breckinridge County, KY, fips: 21027, pop: 20477
Mean visiting county population
150000
250000
350000
450000
Incident cases (per 100,000 population)
0
10
20
30
40
Mar
May
Jul
Sep

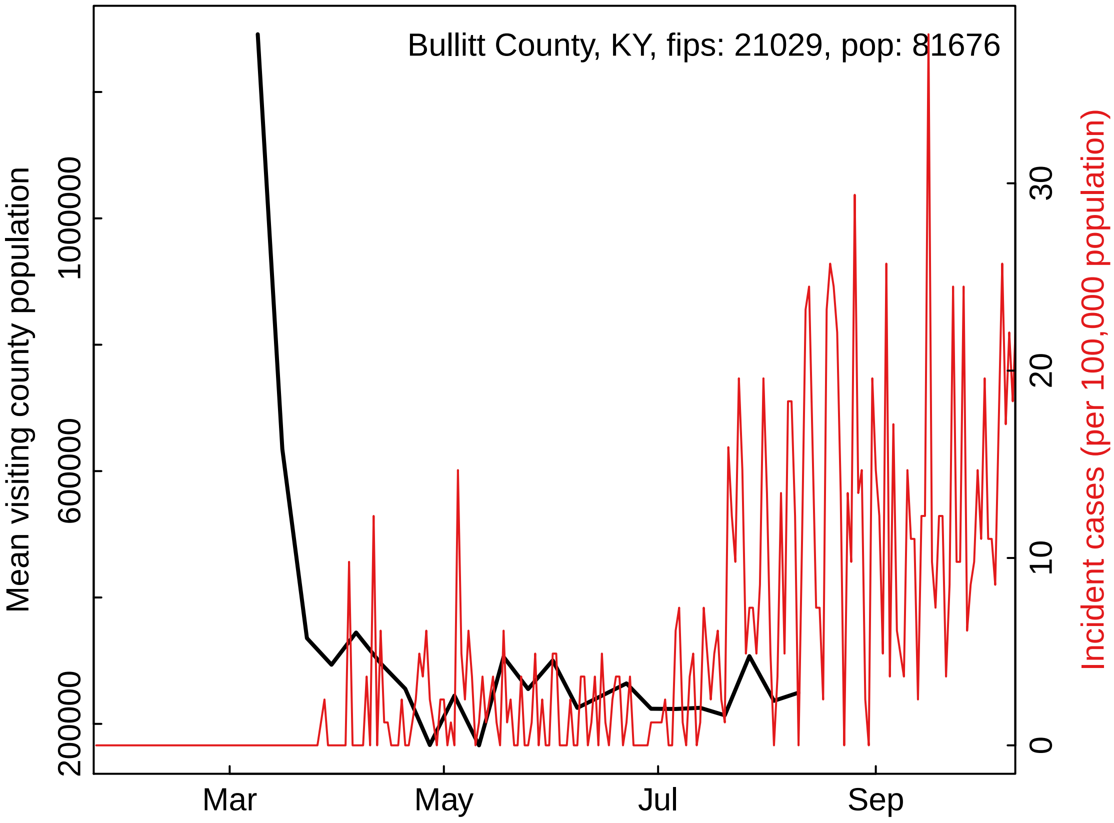

Bullitt County, KY, fips: 21029, pop: 81676
Mean visiting county population
200000
600000
1000000
Incident cases (per 100,000 population)
0
10
20
30
Mar
May
Jul
Sep

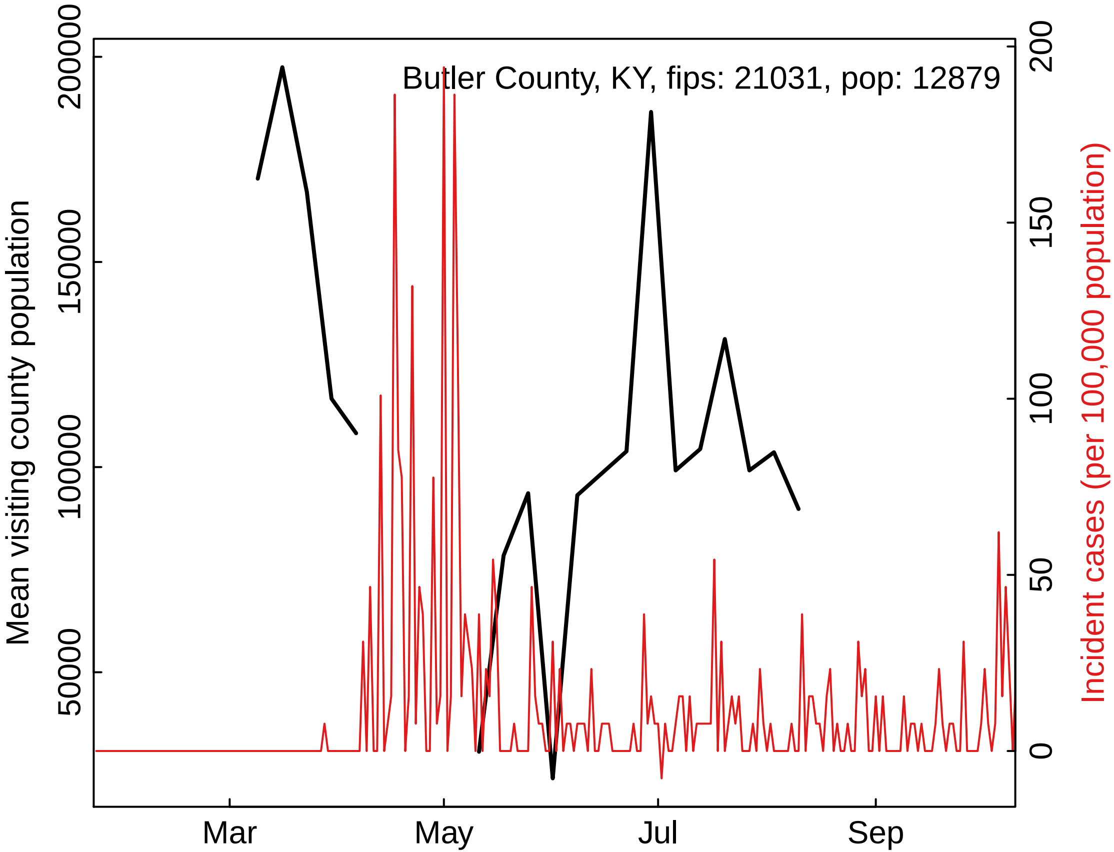

Butler County, KY, fips: 21031, pop: 12879
Mean visiting county population
50000
100000
150000
200000
Incident cases (per 100,000 population)
0
50
100
150
200
Mar
May
Jul
Sep

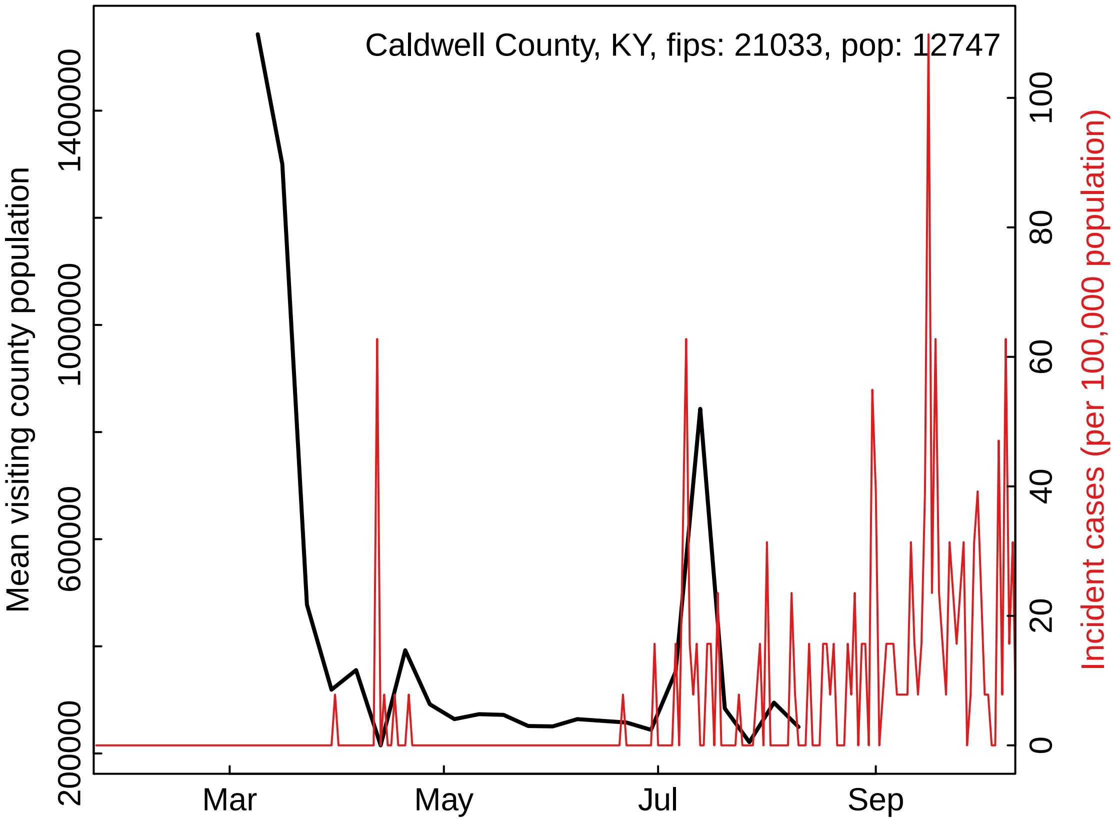
Caldwell County, KY, fips: 21033, pop: 12747
Mean visiting county population
200000
600000
1000000
1400000
Incident cases (per 100,000 population)
0
20
40
60
80
100
Mar
May
Jul
Sep

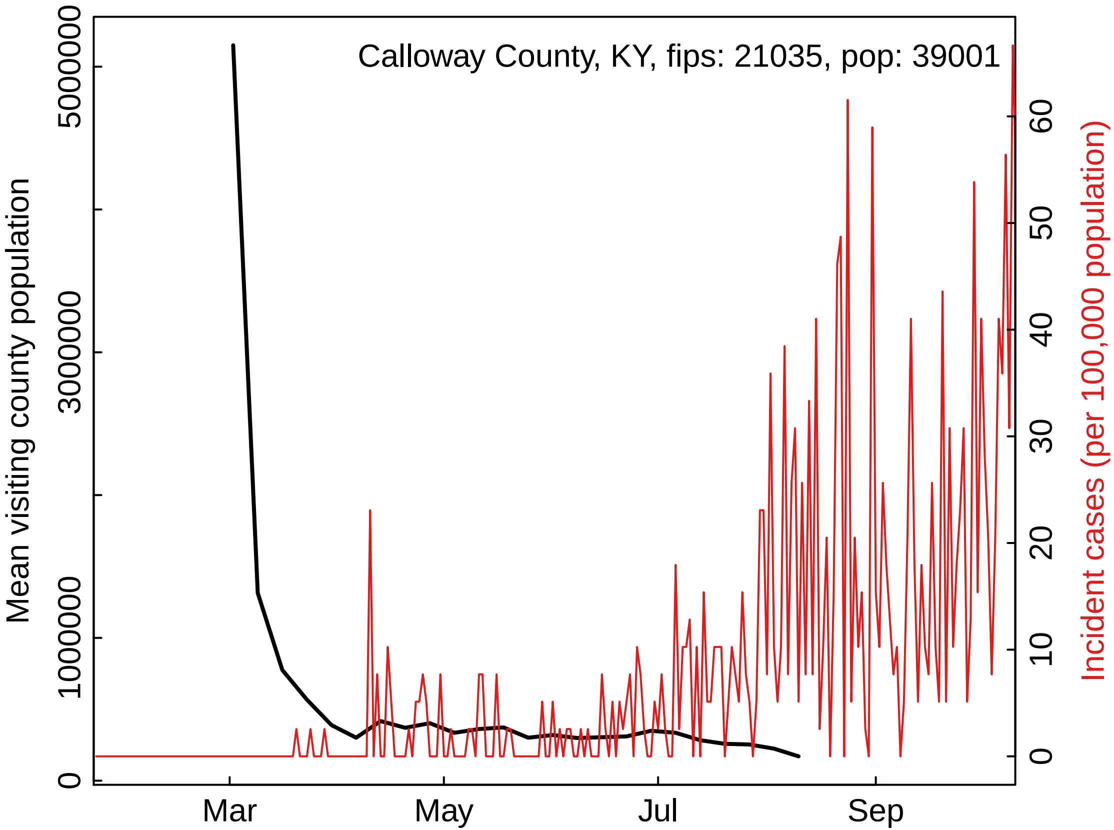
Calloway County, KY, fips: 21035, pop: 39001
Mean visiting county population
0
1000000
3000000
5000000
Incident cases (per 100,000 population)
0
10
20
30
40
50
60
Mar
May
Jul
Sep

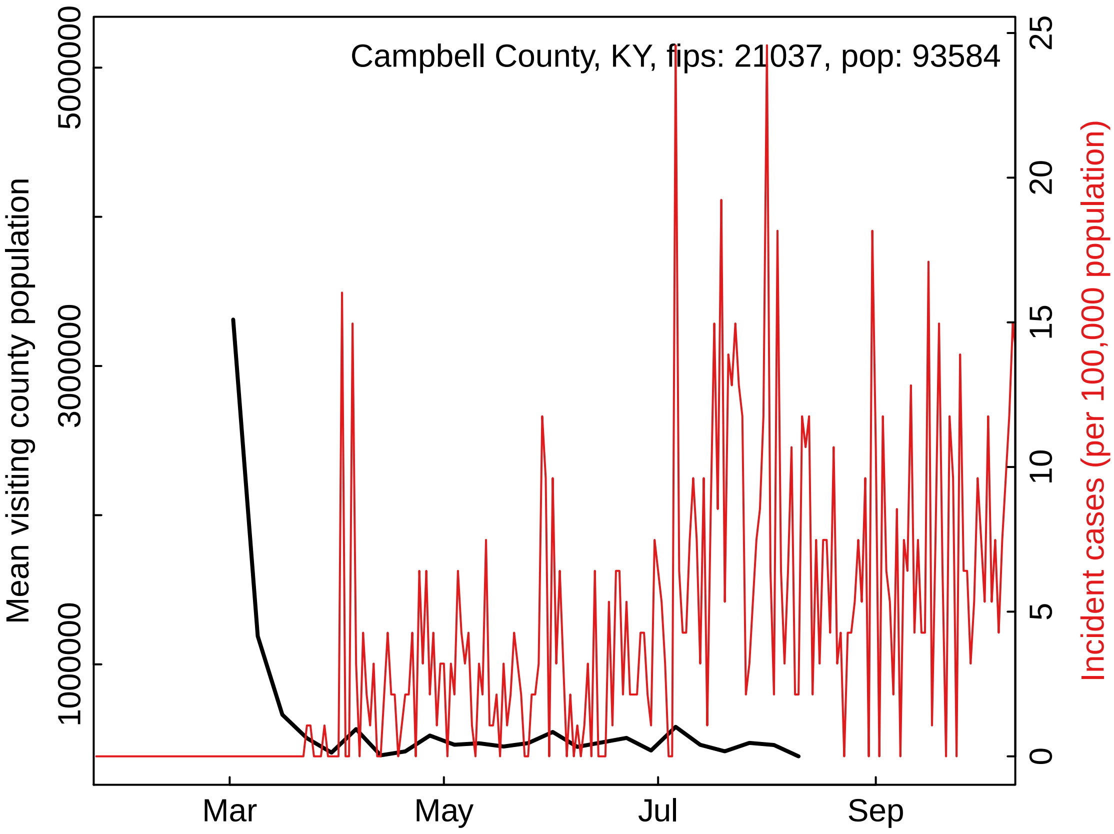
Campbell County, KY, fips: 21037, pop: 93584
Mean visiting county population
10000000
30000000
50000000
Incident cases (per 100,000 population)
0
5
10
15
20
25
Mar
May
Jul
Sep

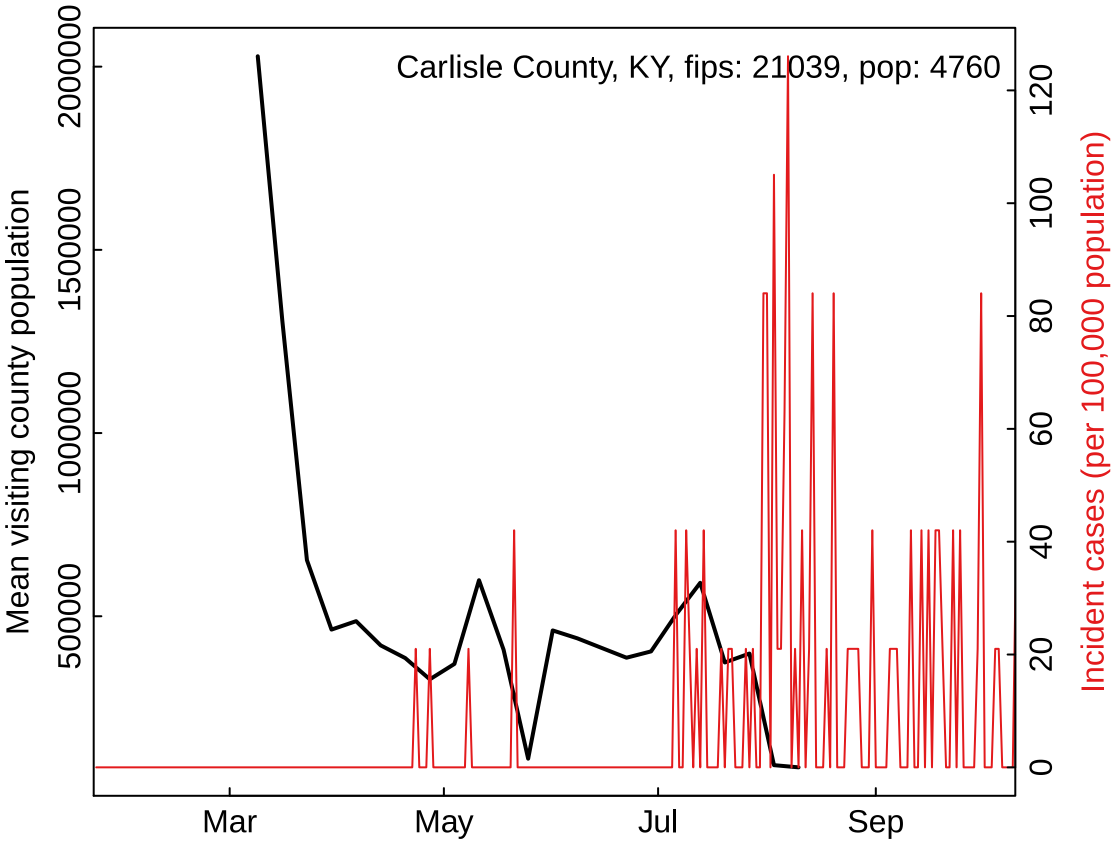

Carlisle County, KY, fips: 21039, pop: 4760
Mean visiting county population
500000
1000000
1500000
2000000
Incident cases (per 100,000 population)
0
20
40
60
80
100
120
Mar
May
Jul
Sep

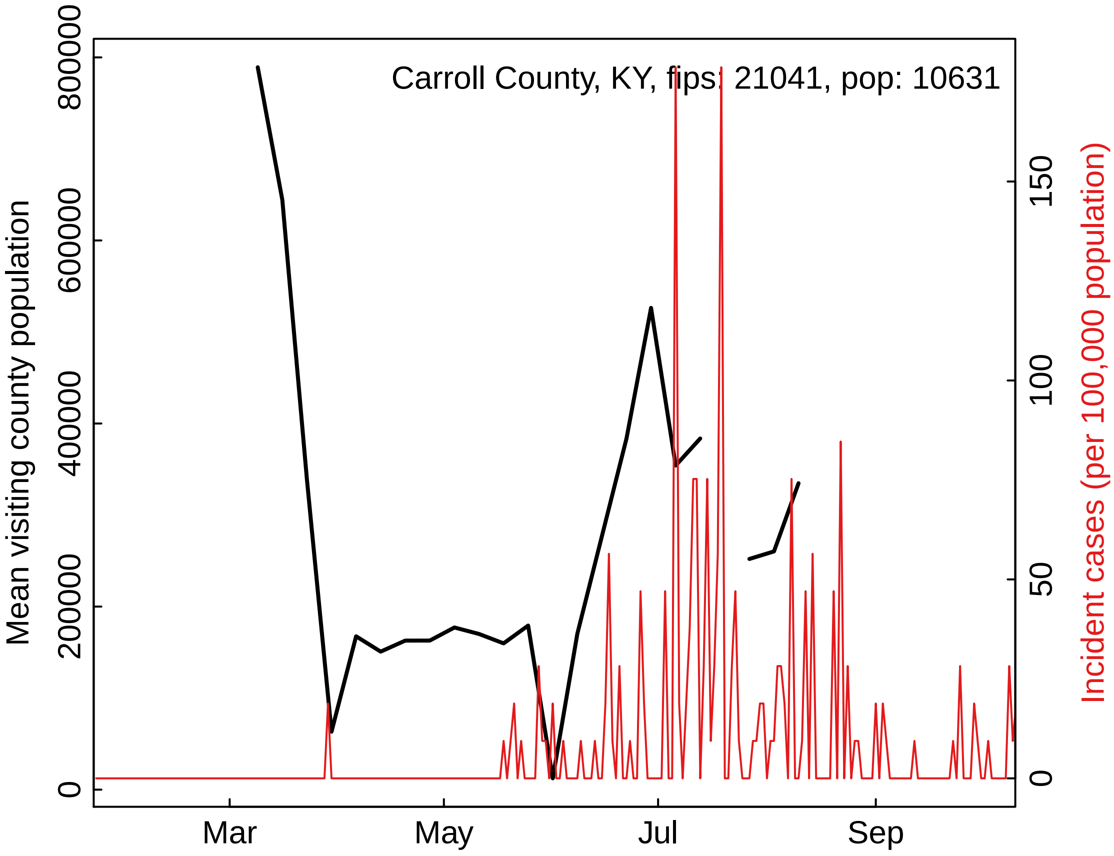

Carroll County, KY, fips: 21041, pop: 10631
Mean visiting county population
0
200000
400000
600000
800000
Incident cases (per 100,000 population)
0
50
100
150
Mar
May
Jul
Sep

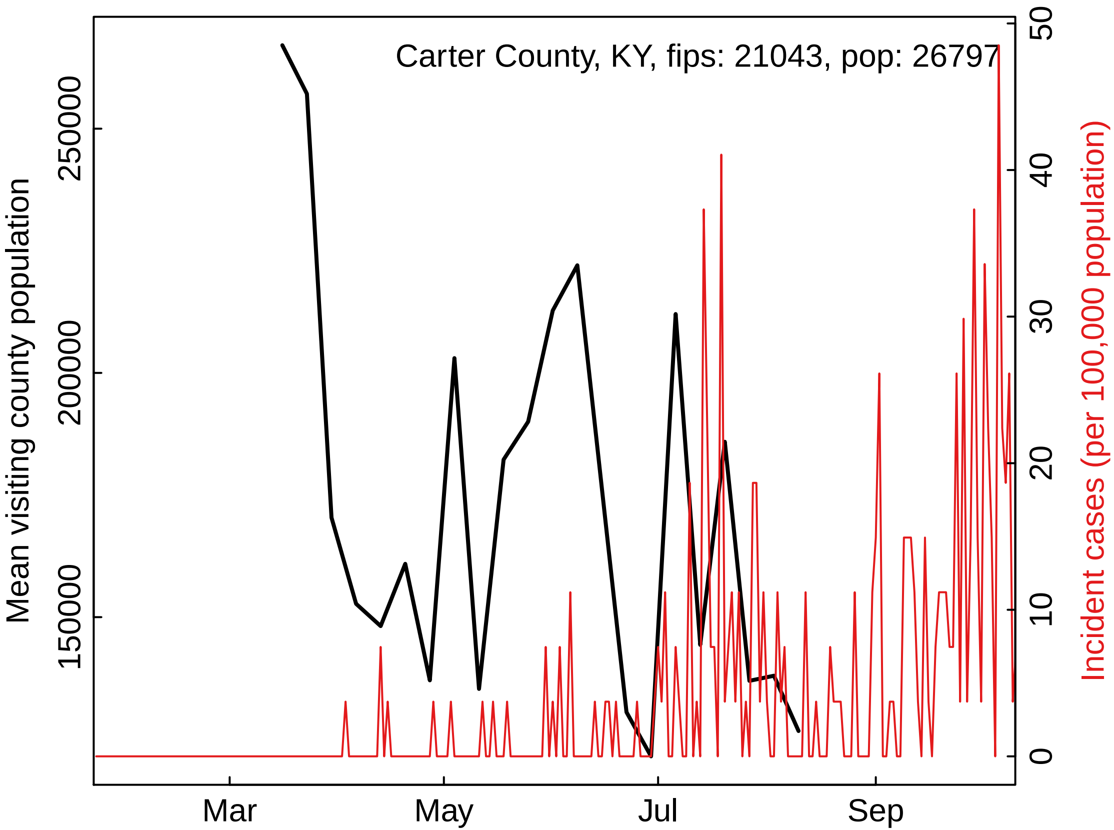

Carter County, KY, fips: 21043, pop: 26797
Mean visiting county population
150000
200000
250000
Incident cases (per 100,000 population)
0
10
20
30
40
50
Mar
May
Jul
Sep

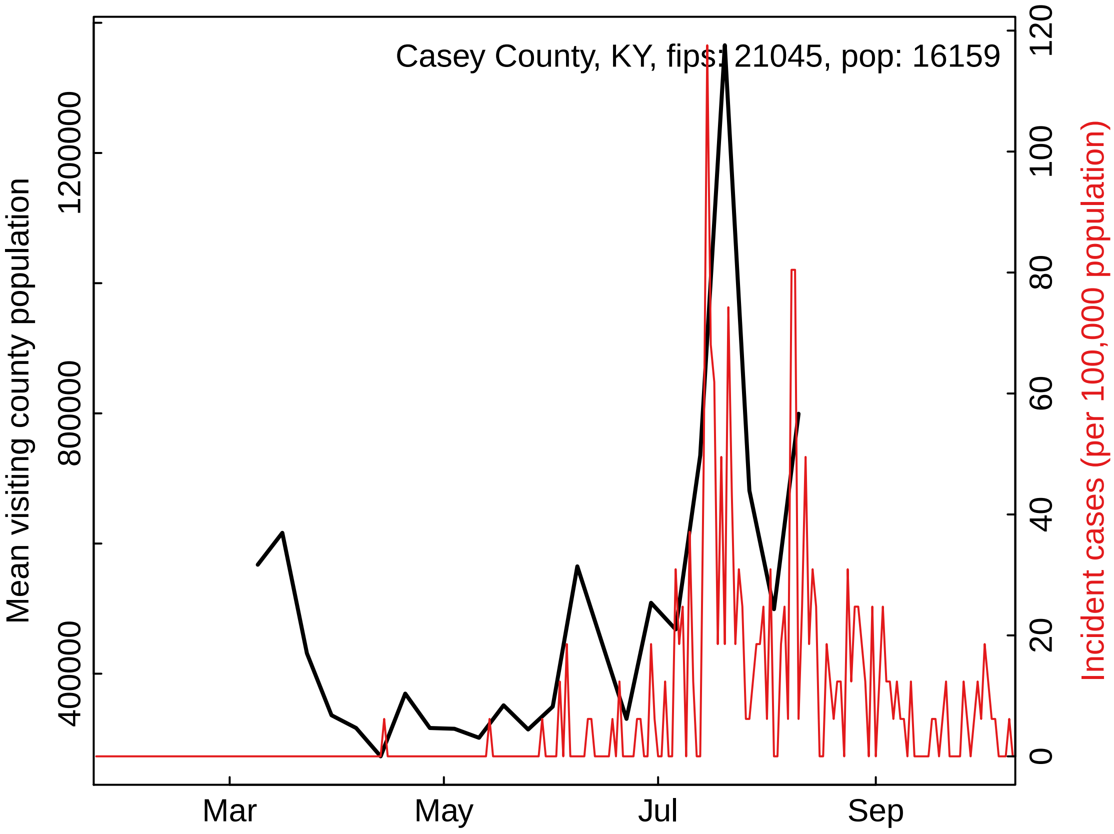

Casey County, KY, fips: 21045, pop: 16159
Mean visiting county population
400000
800000
1200000
Incident cases (per 100,000 population)
0
20
40
60
80
100
120
Mar
May
Jul
Sep

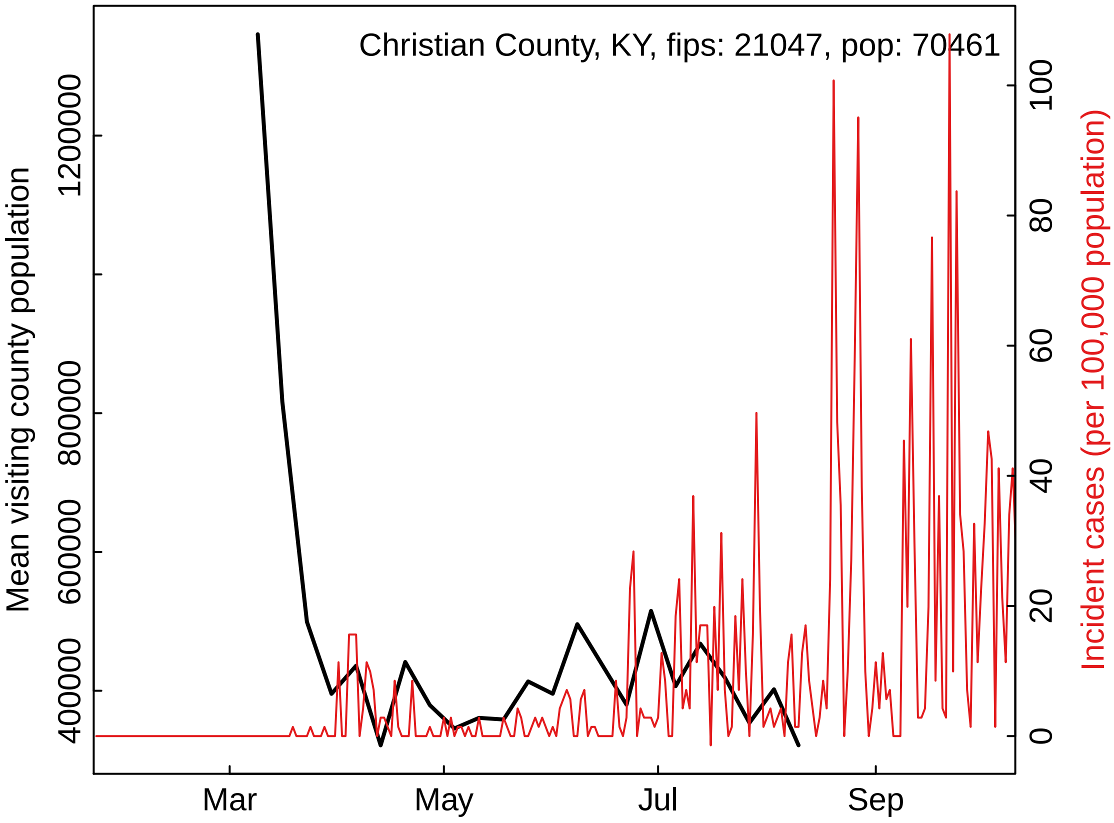

Christian County, KY, fips: 21047, pop: 70461
Mean visiting county population
400000
600000
800000
1200000
Incident cases (per 100,000 population)
0
20
40
60
80
100
Mar
May
Jul
Sep

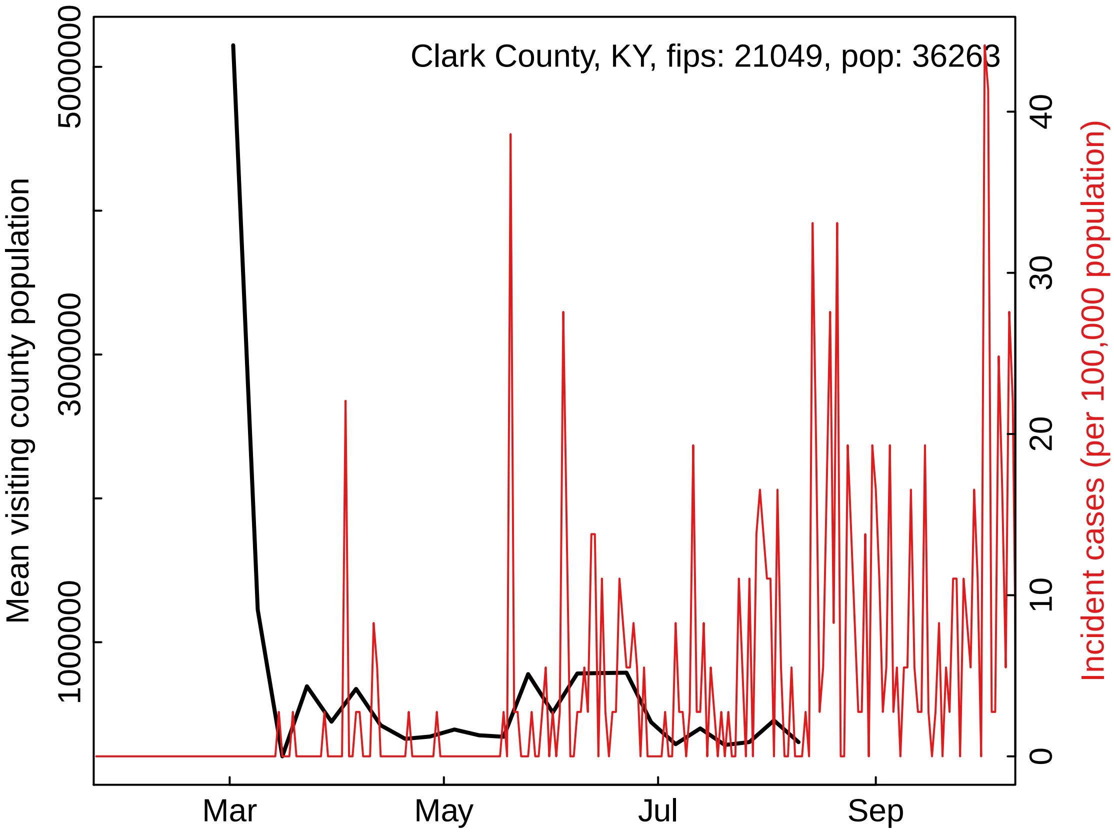

Clark County, KY, fips: 21049, pop: 36263
Mean visiting county population
10000000
30000000
50000000
Incident cases (per 100,000 population)
0
10
20
30
40
Mar
May
Jul
Sep

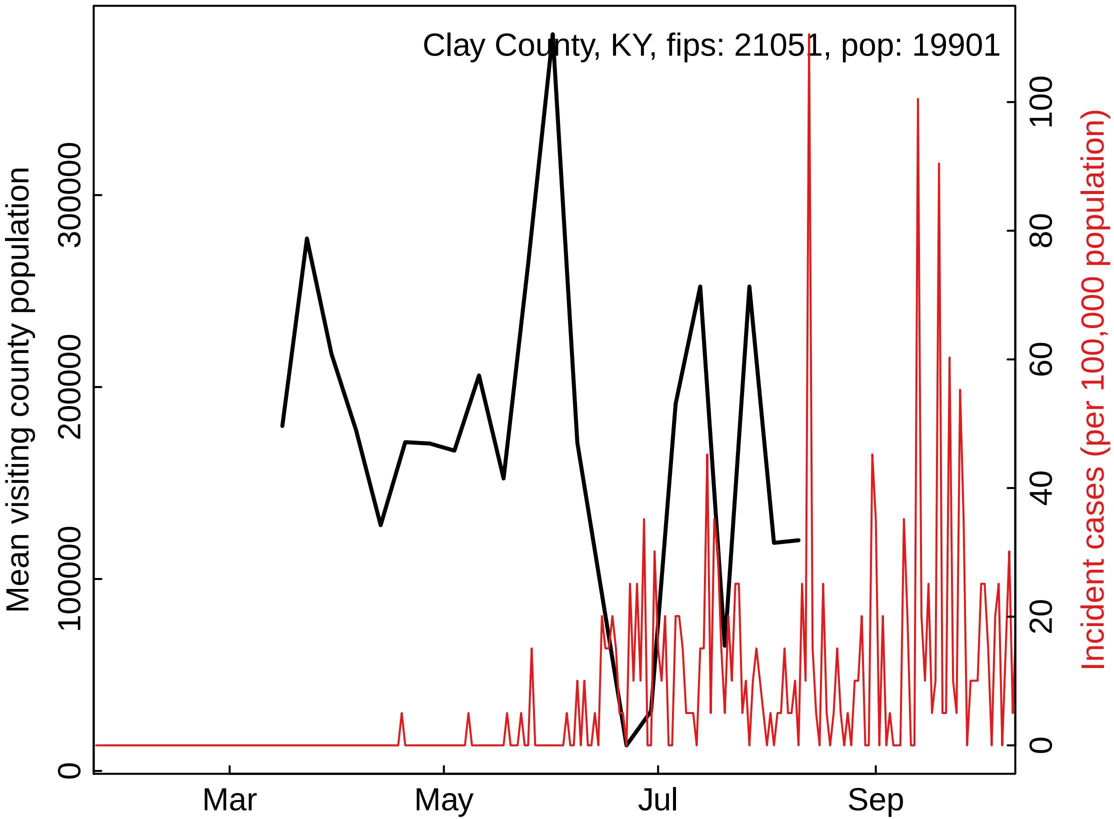

Clay County, KY, fips: 21051, pop: 19901
Mean visiting county population
0
100000
200000
300000
Mar
May
Jul
Sep
0
20
40
60
80
100
Incident cases (per 100,000 population)

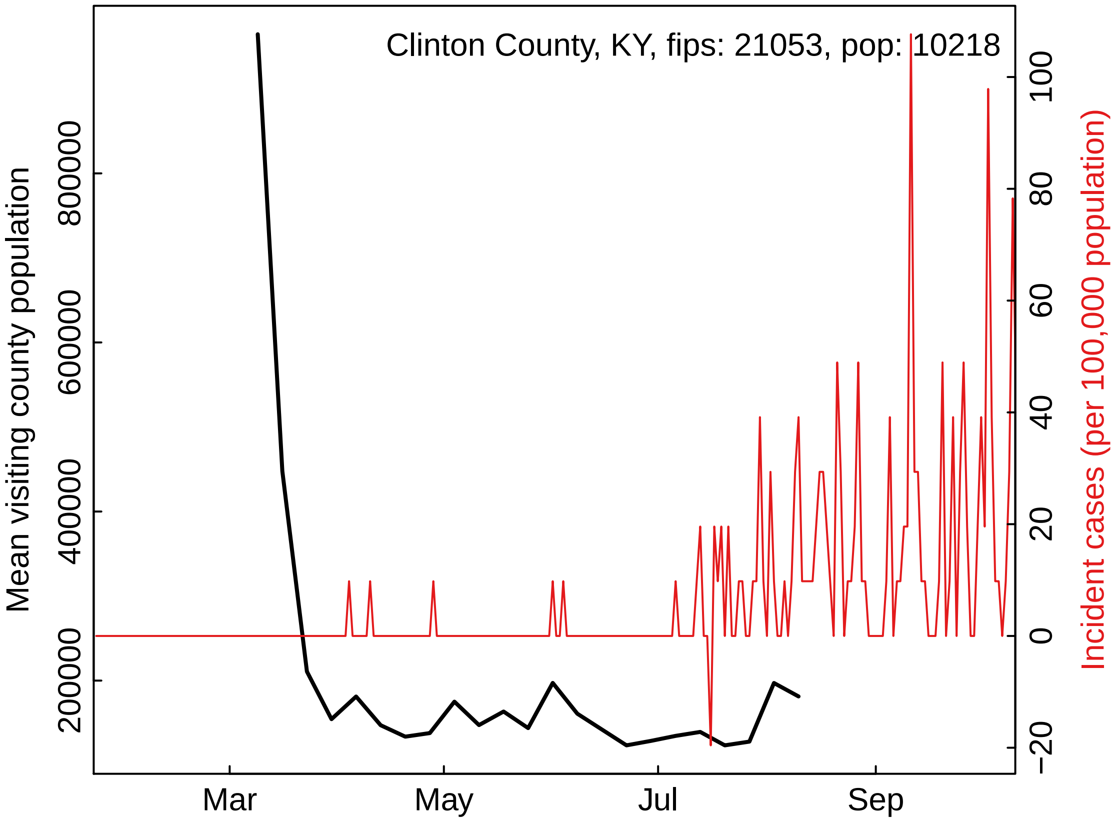

Clinton County, KY, fips: 21053, pop: 10218
Mean visiting county population
200000
400000
600000
800000
Incident cases (per 100,000 population)
−20
0
20
40
60
80
100
Mar
May
Jul
Sep

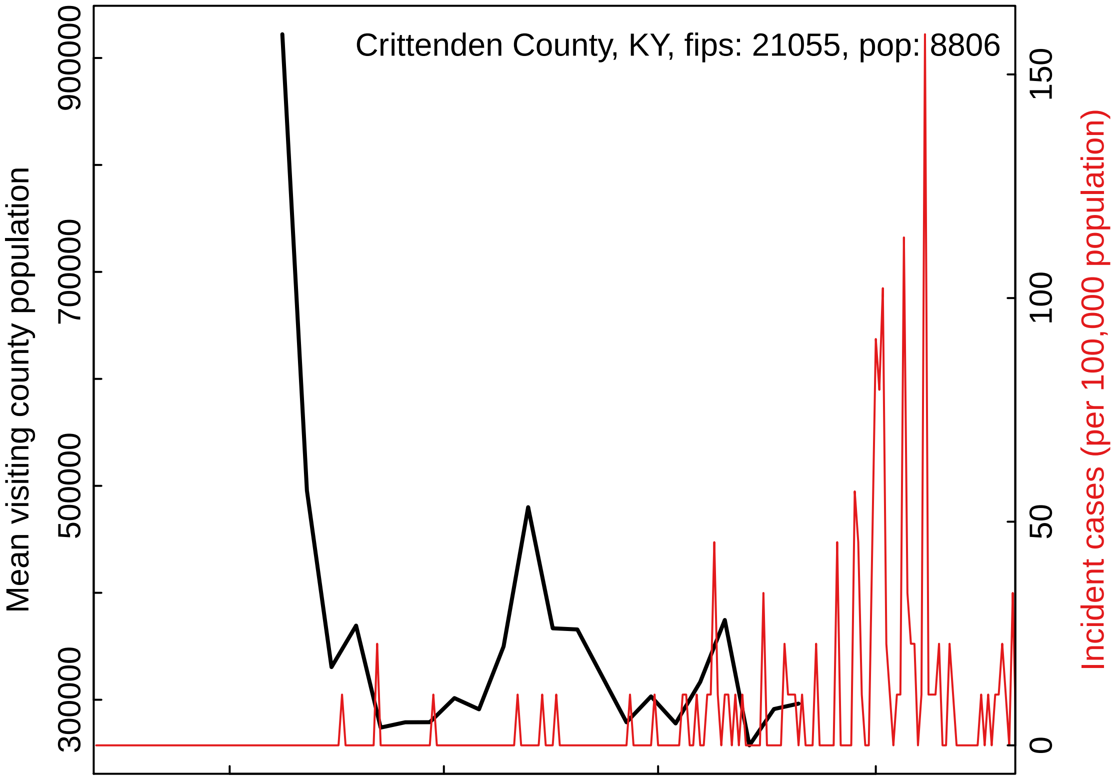

Crittenden County, KY, fips: 21055, pop: 8806
Mean visiting county population
300000
500000
700000
900000
Incident cases (per 100,000 population)
0
50
100
150
Mar
May
Jul
Sep

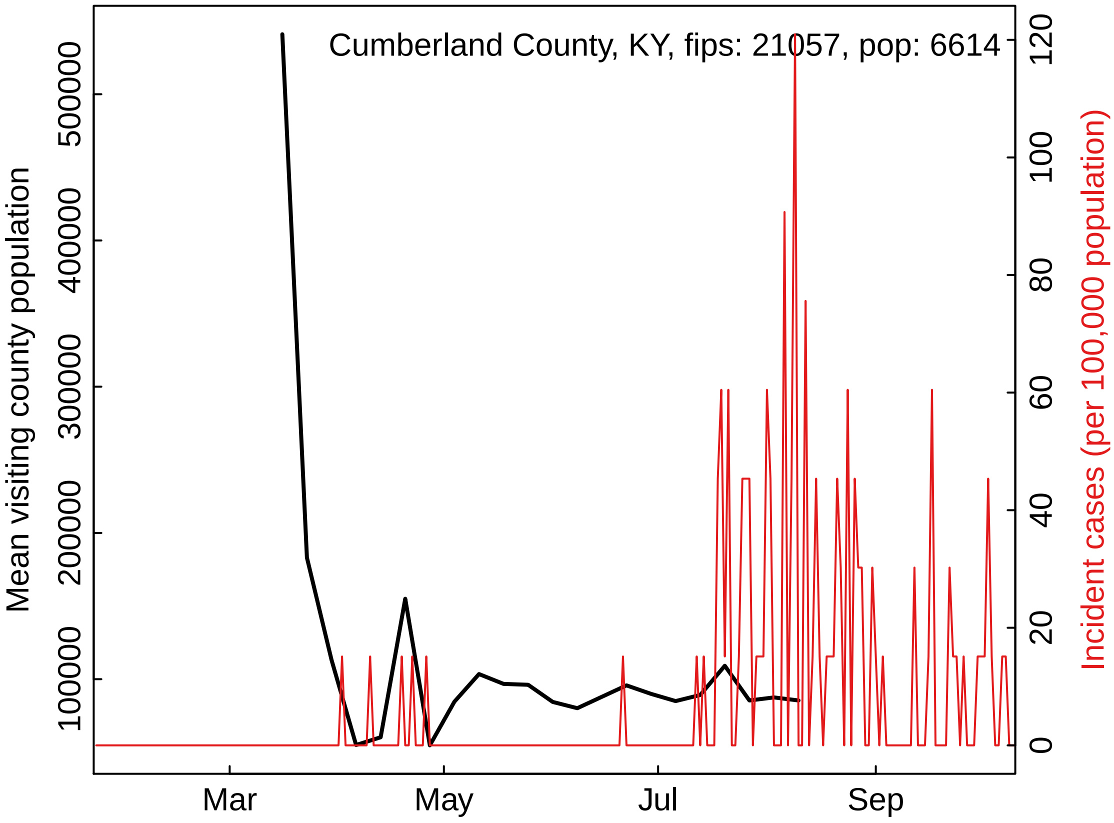

Cumberland County, KY, fips: 21057, pop: 6614
Mean visiting county population
100000
200000
300000
400000
500000
Incident cases (per 100,000 population)
0
20
40
60
80
100
120
Mar
May
Jul
Sep

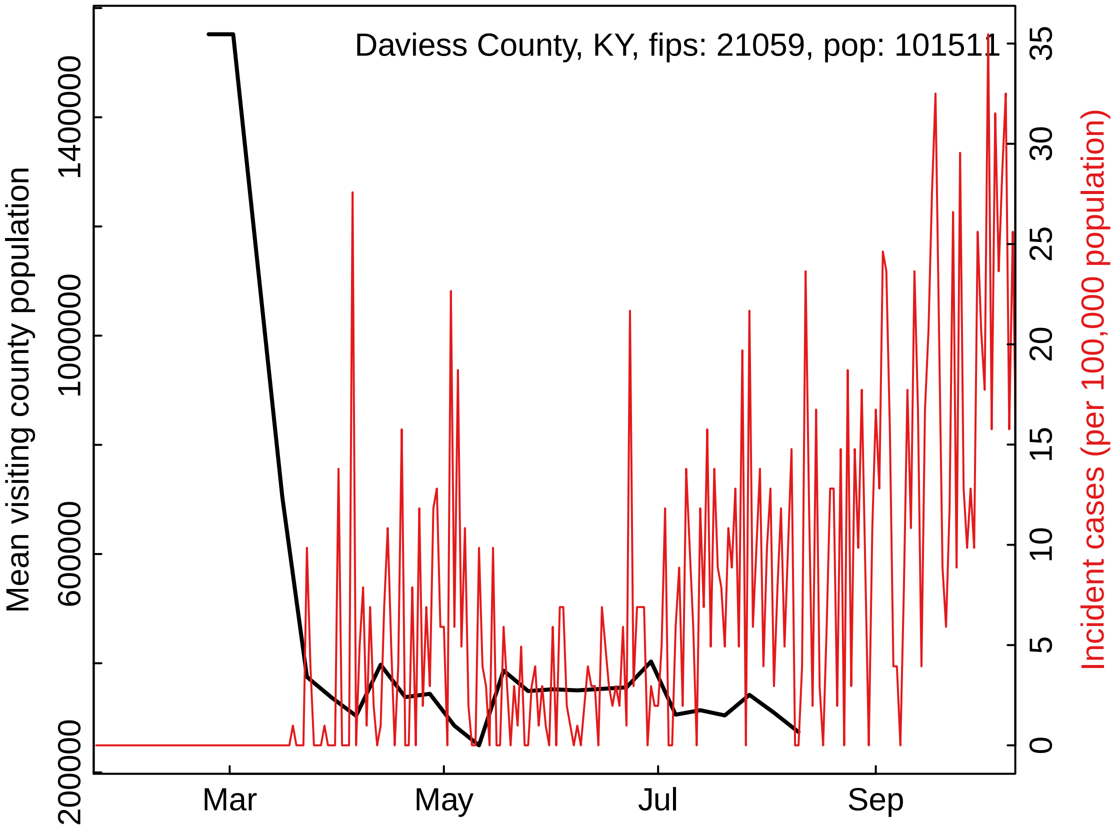

Daviess County, KY, fips: 21059, pop: 101511
Mean visiting county population
Incident cases (per 100,000 population)
200000
600000
1000000
1400000
0
5
10
15
20
25
30
35
Mar
May
Jul
Sep

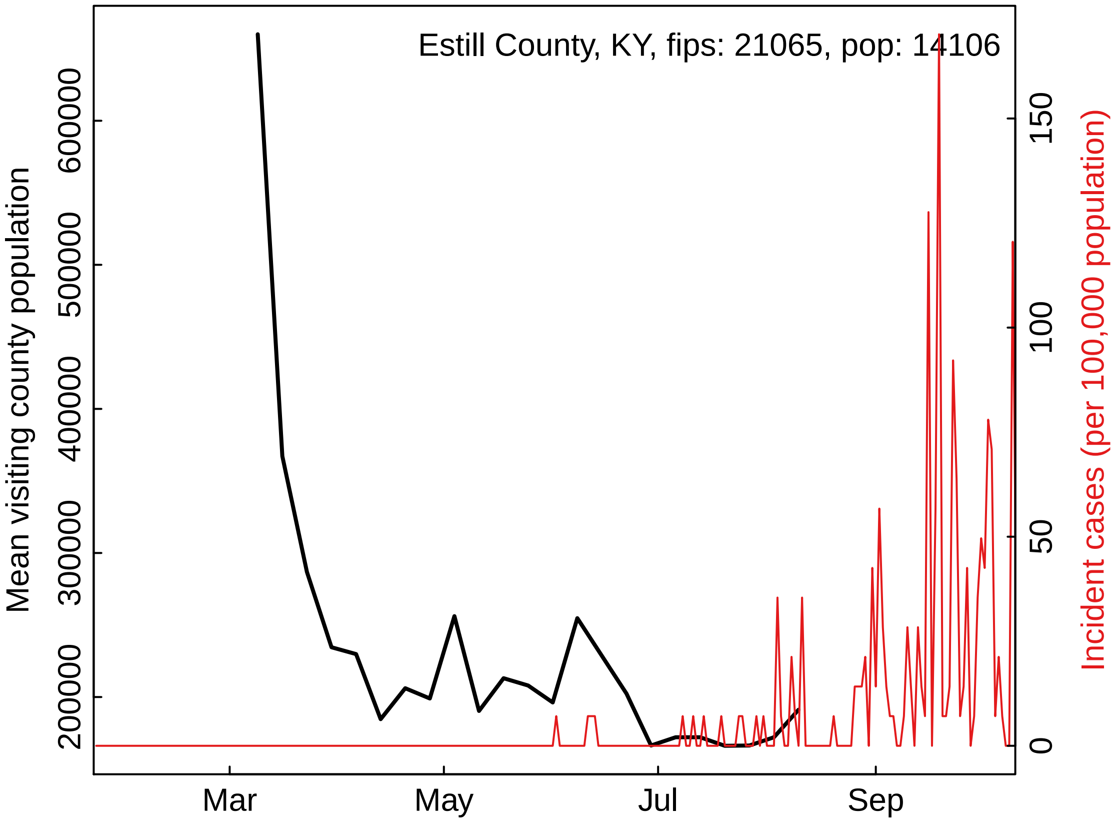
Estill County, KY, fips: 21065, pop: 14106
Mean visiting county population
200000
300000
400000
500000
600000
Incident cases (per 100,000 population)
0
50
100
150
Mar
May
Jul
Sep

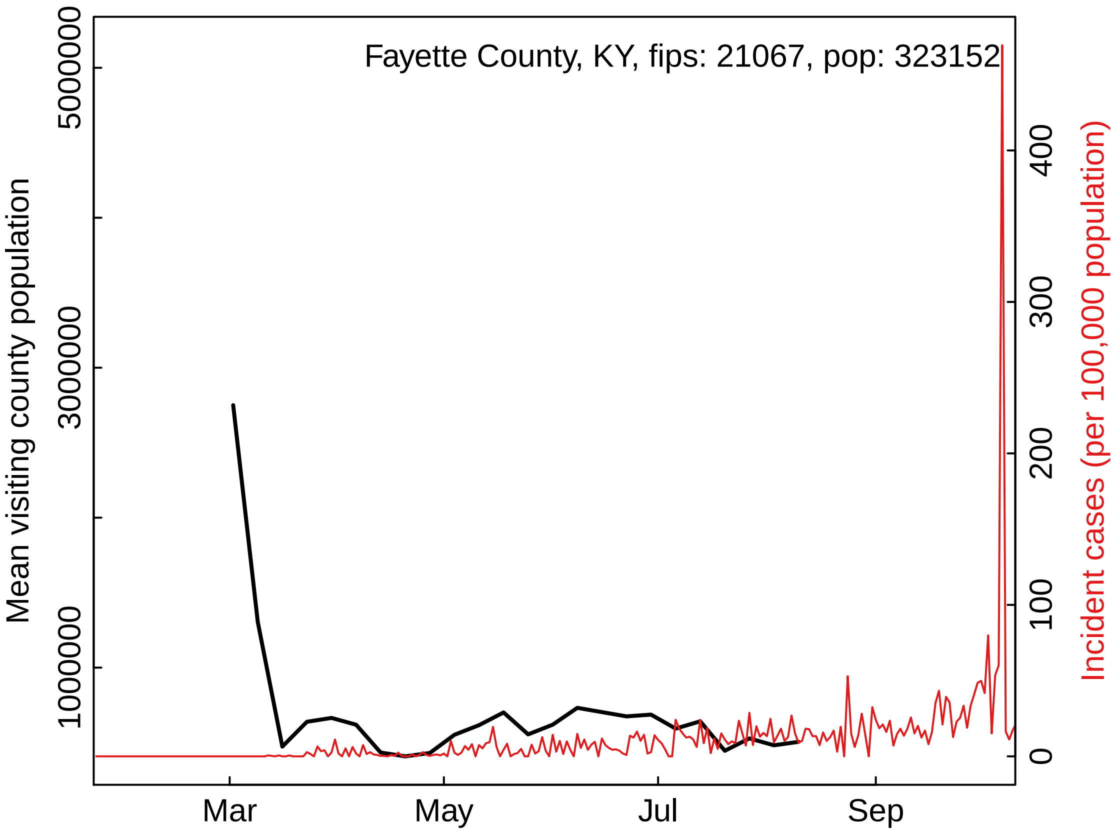

Fayette County, KY, fips: 21067, pop: 323152
Mean visiting county population
1000000
3000000
5000000
Incident cases (per 100,000 population)
0
100
200
300
400
Mar
May
Jul
Sep

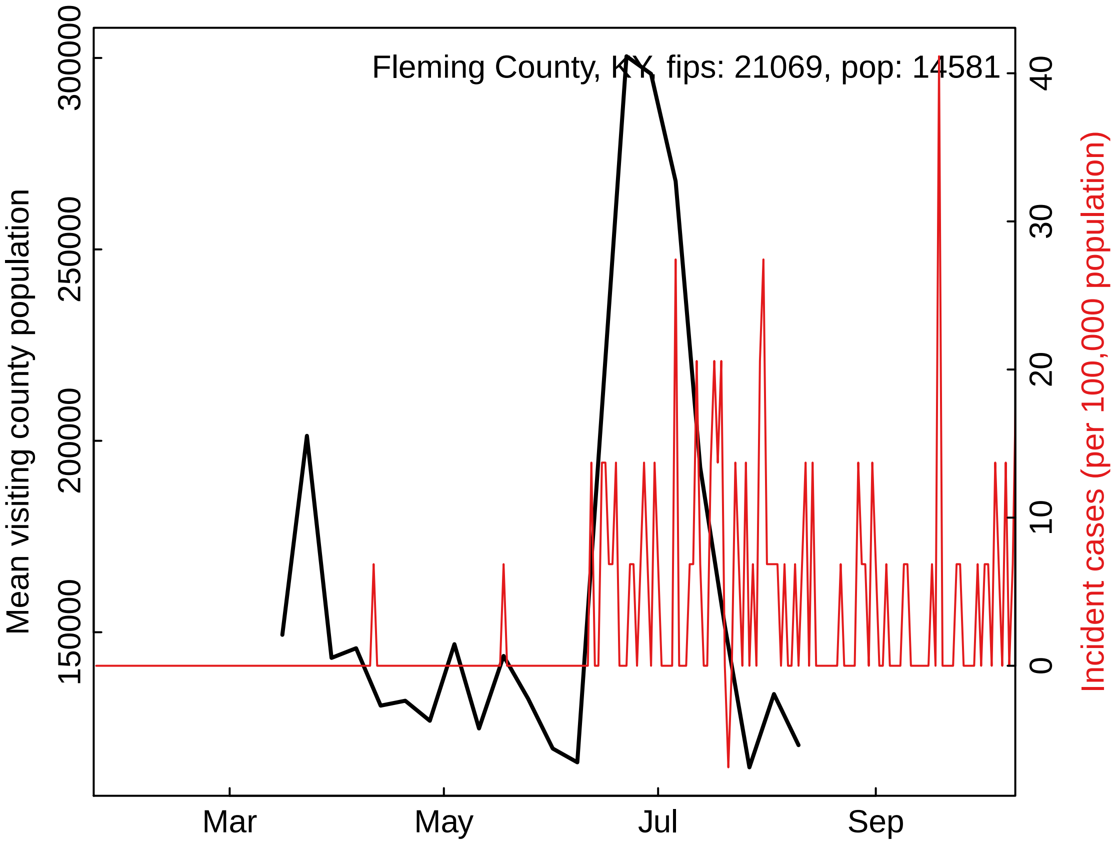

Fleming County, KY, fips: 21069, pop: 14581
Mean visiting county population
Incident cases (per 100,000 population)
300000
250000
200000
150000
40
30
20
10
0
Mar
May
Jul
Sep

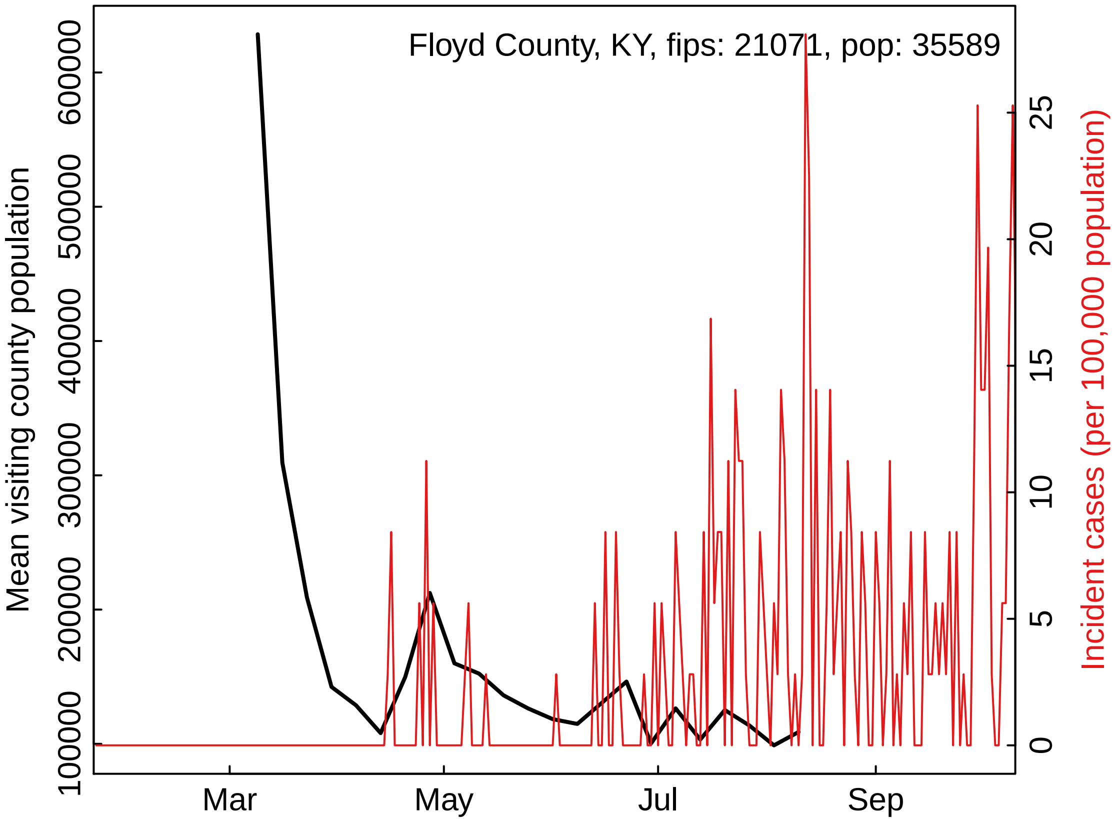

Floyd County, KY, fips: 21071, pop: 35589
Mean visiting county population
100000
200000
300000
400000
500000
600000
Mar
May
Jul
Sep
0
5
10
15
20
25
Incident cases (per 100,000 population)

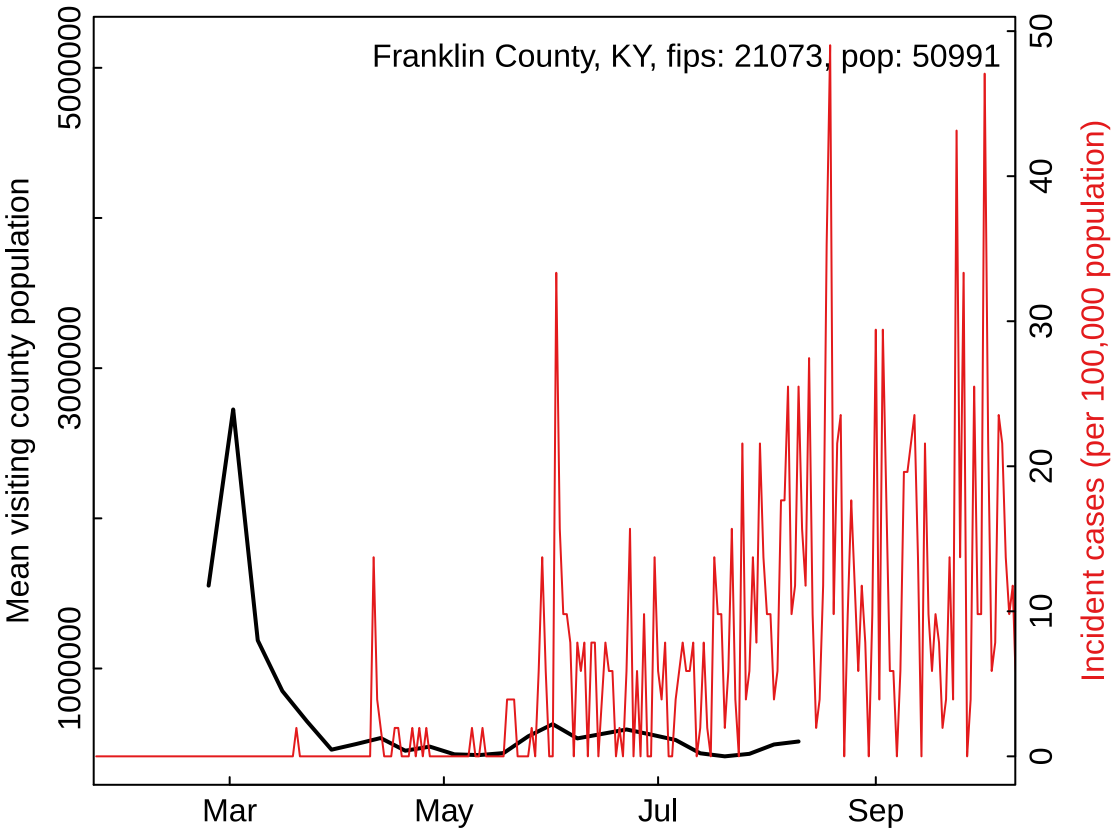

Franklin County, KY, fips: 21073, pop: 50991
Mean visiting county population
1000000
3000000
5000000
Incident cases (per 100,000 population)
0
10
20
30
40
50
Mar
May
Jul
Sep

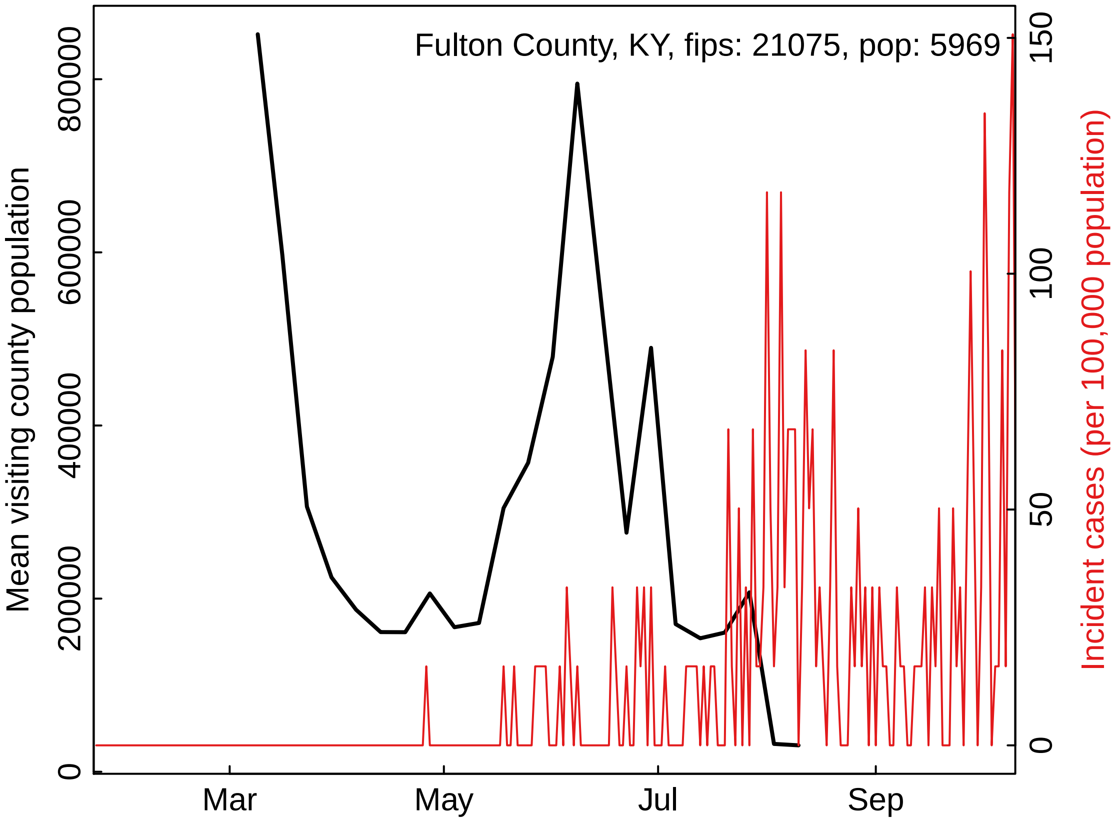

Fulton County, KY, fips: 21075, pop: 5969
Mean visiting county population
0
200000
400000
600000
800000
Incident cases (per 100,000 population)
0
50
100
150
Mar
May
Jul
Sep

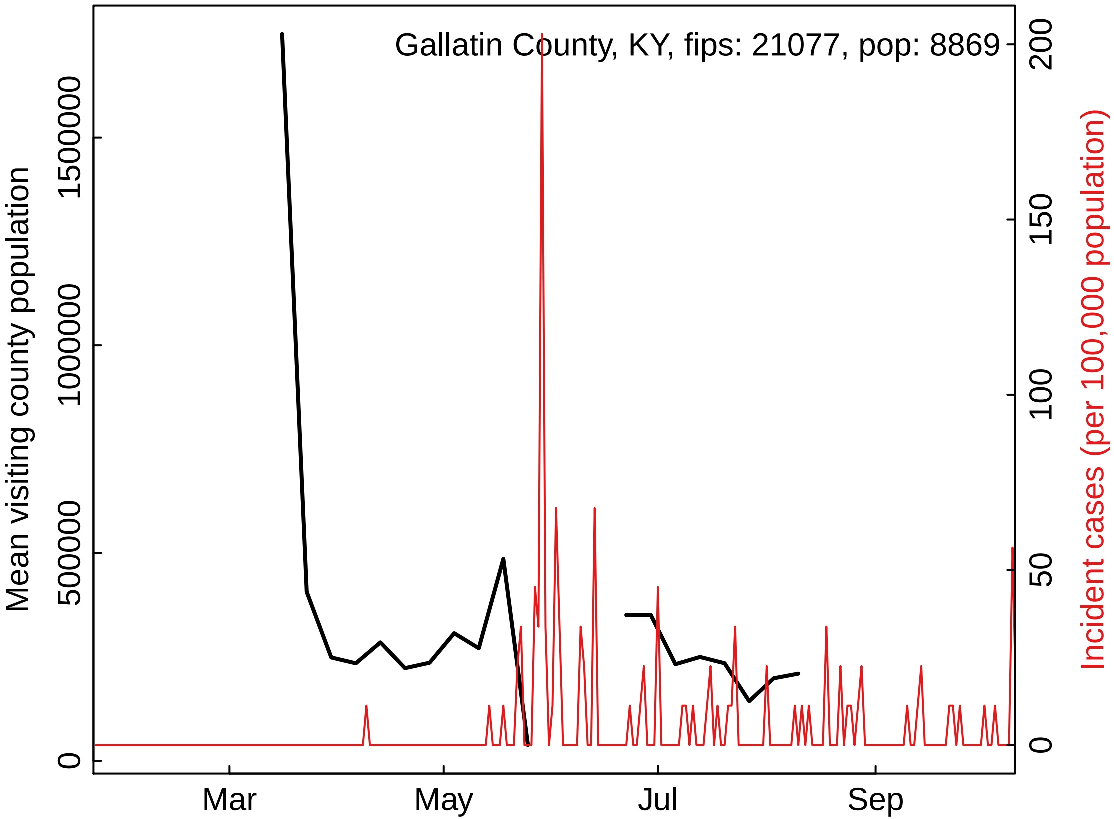

Gallatin County, KY, fips: 21077, pop: 8869
Mean visiting county population
0
500000
1000000
1500000
Incident cases (per 100,000 population)
0
50
100
150
200
Mar
May
Jul
Sep

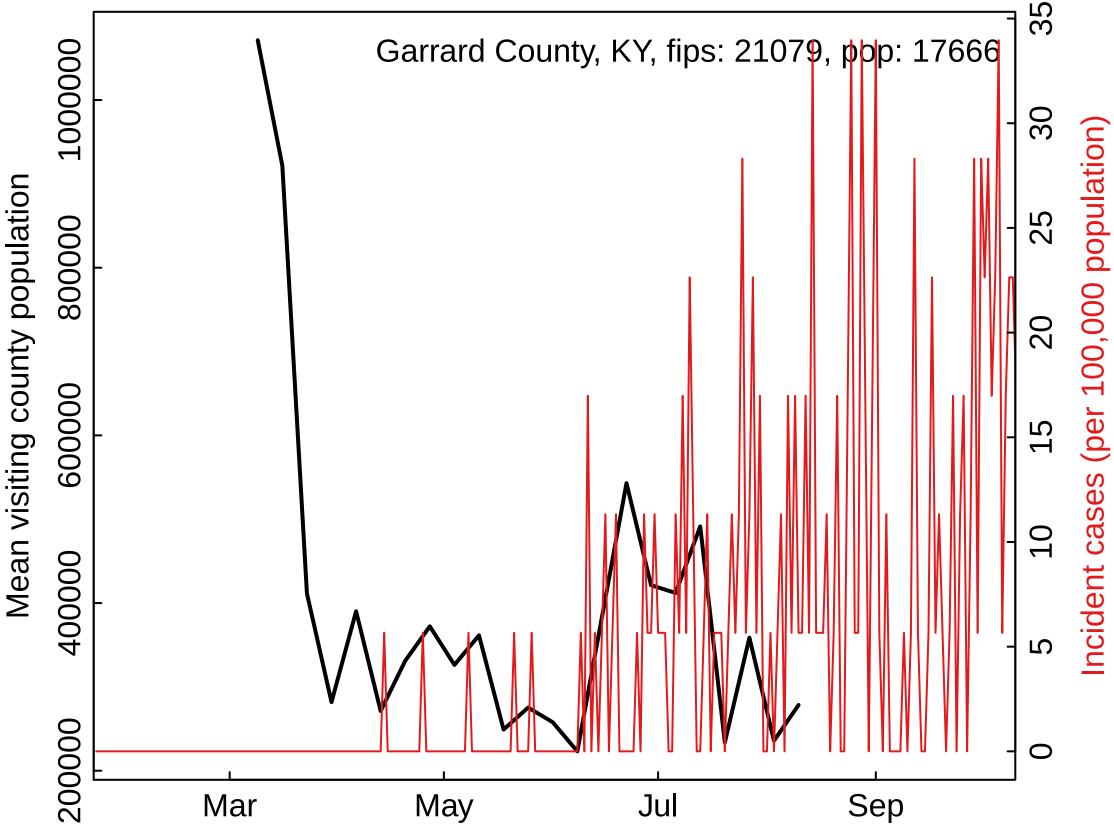
Garrard County, KY, fips: 21079, pop: 17666
Mean visiting county population
200000
400000
600000
800000
1000000
Mar
May
Jul
Sep
Incident cases (per 100,000 population)
0
5
10
15
20
25
30
35

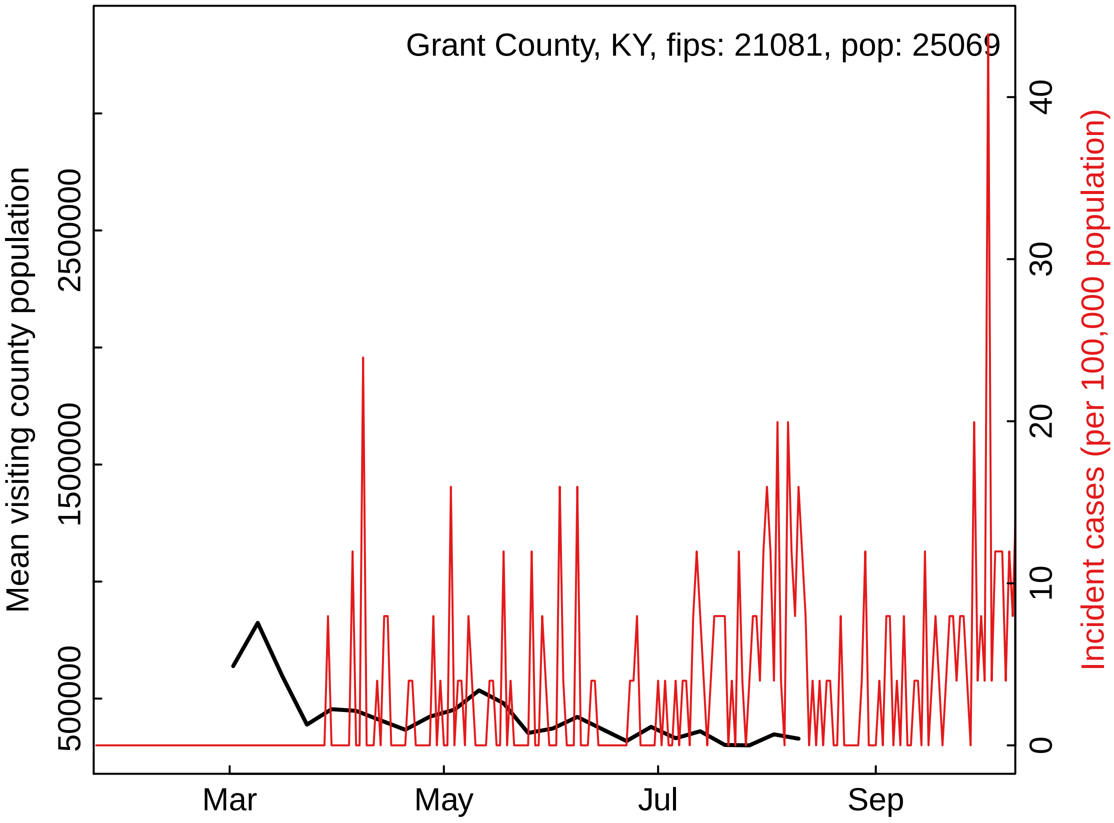

Grant County, KY, fips: 21081, pop: 25069
Mean visiting county population
500000
1500000
2500000
Mar
May
Jul
Sep
0
10
20
30
40
Incident cases (per 100,000 population)

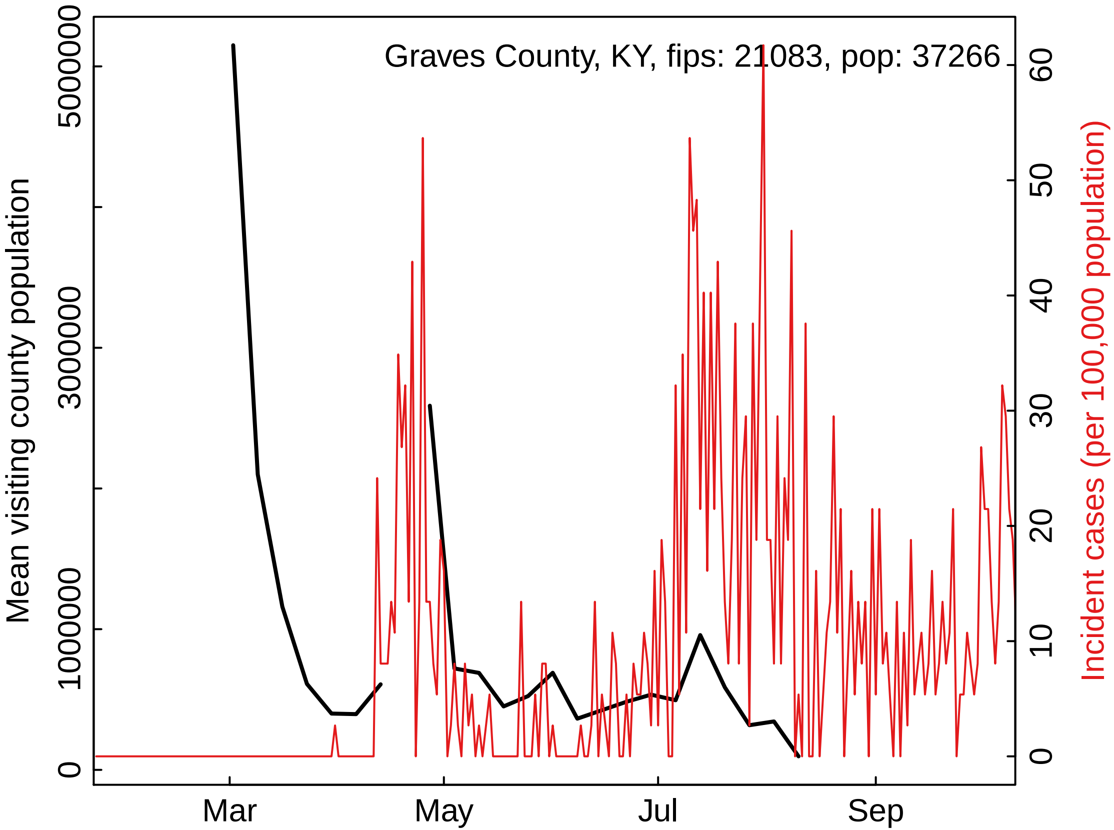

Graves County, KY, fips: 21083, pop: 37266
Mean visiting county population
0
1000000
3000000
5000000
Incident cases (per 100,000 population)
0
10
20
30
40
50
60
Mar
May
Jul
Sep

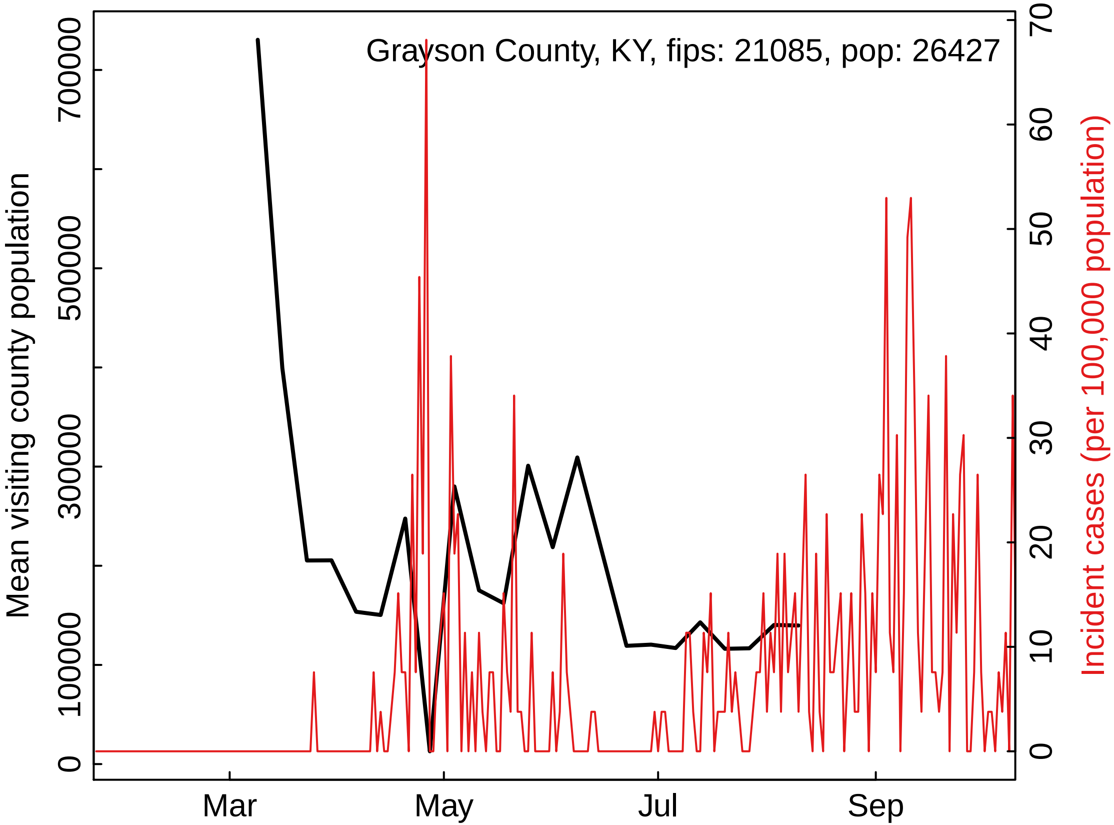

Grayson County, KY, fips: 21085, pop: 26427
Mean visiting county population
0
100000
300000
500000
700000
Incident cases (per 100,000 population)
0
10
20
30
40
50
60
70
Mar
May
Jul
Sep

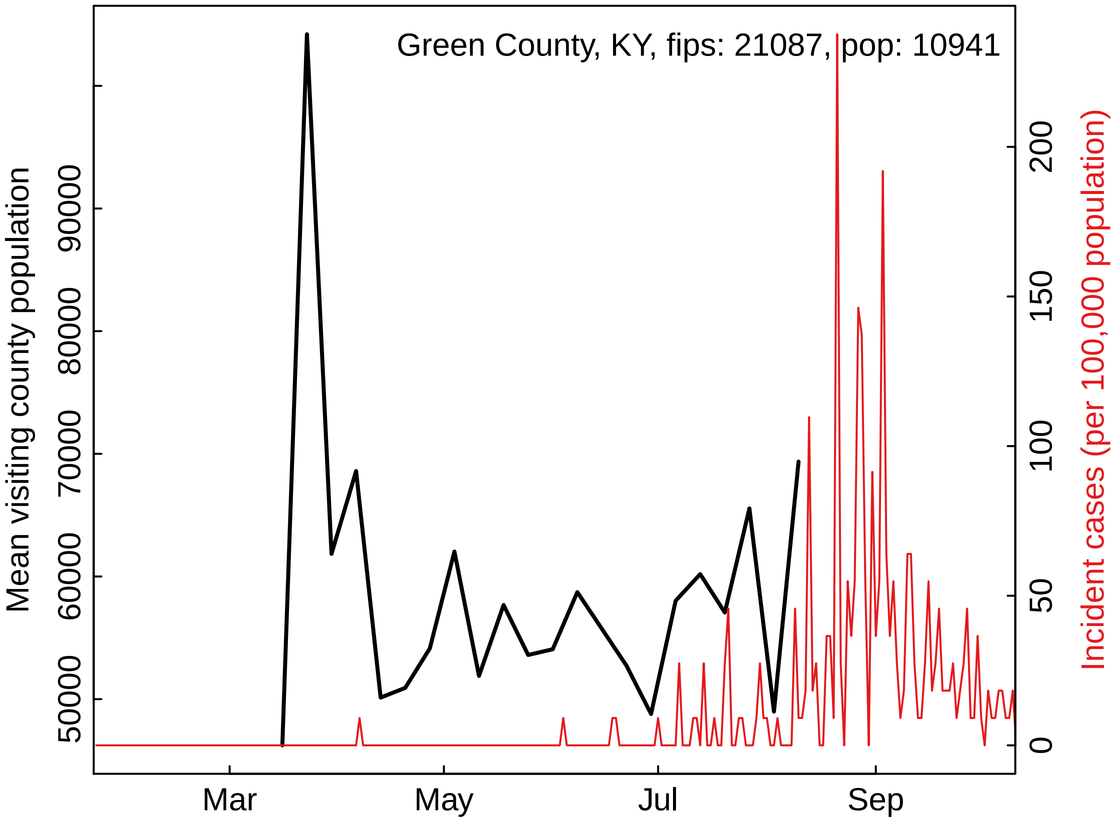

Green County, KY, fips: 21087, pop: 10941
Mean visiting county population
50000
60000
70000
80000
90000
Incident cases (per 100,000 population)
0
50
100
150
200
Mar
May
Jul
Sep

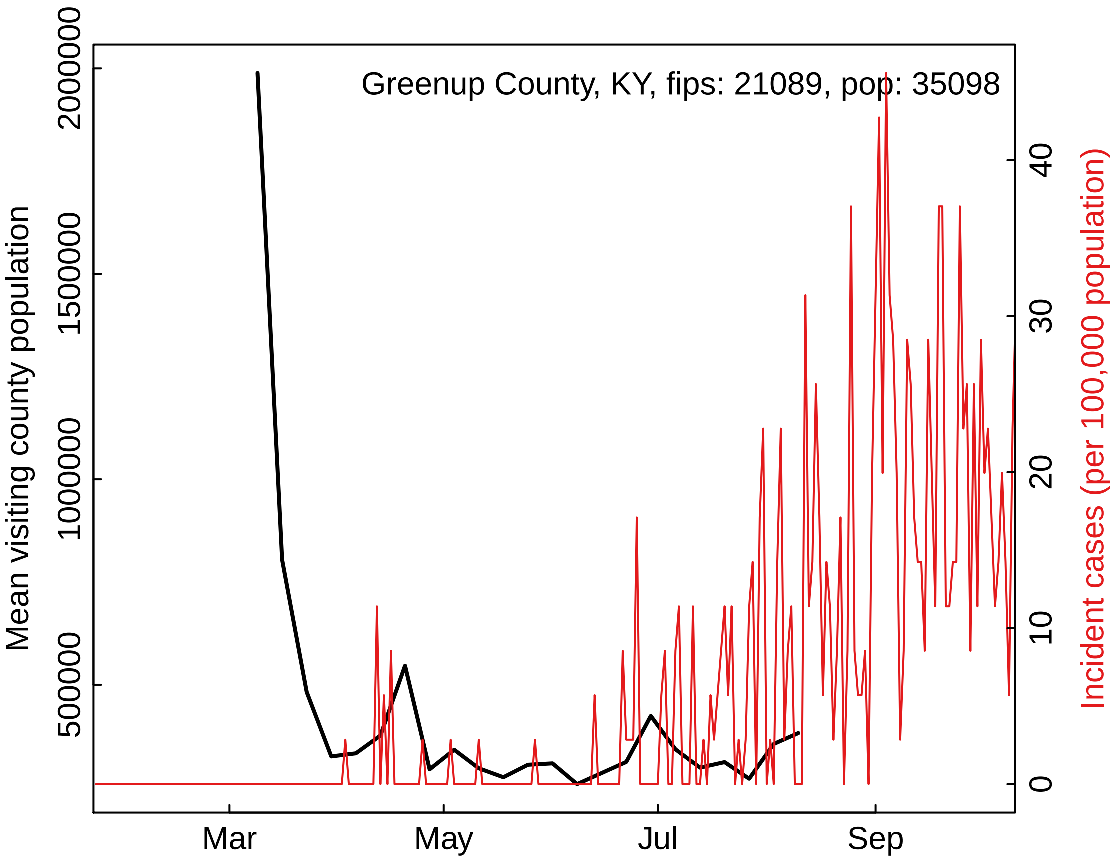
Greenup County, KY, fips: 21089, pop: 35098
Mean visiting county population
Incident cases (per 100,000 population)
500000
1000000
1500000
2000000
0
10
20
30
40
Mar
May
Jul
Sep

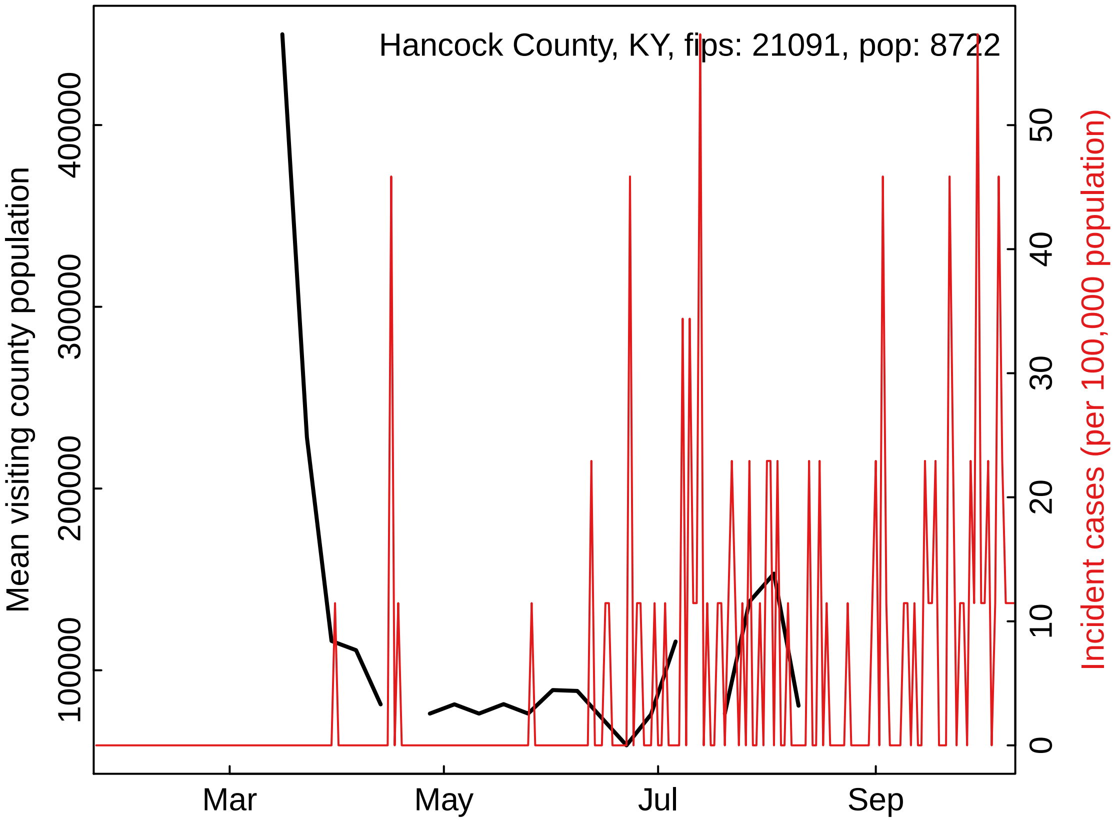
Hancock County, KY, fips: 21091, pop: 8722
Mean visiting county population
100000
200000
300000
400000
Incident cases (per 100,000 population)
0
10
20
30
40
50
Mar
May
Jul
Sep

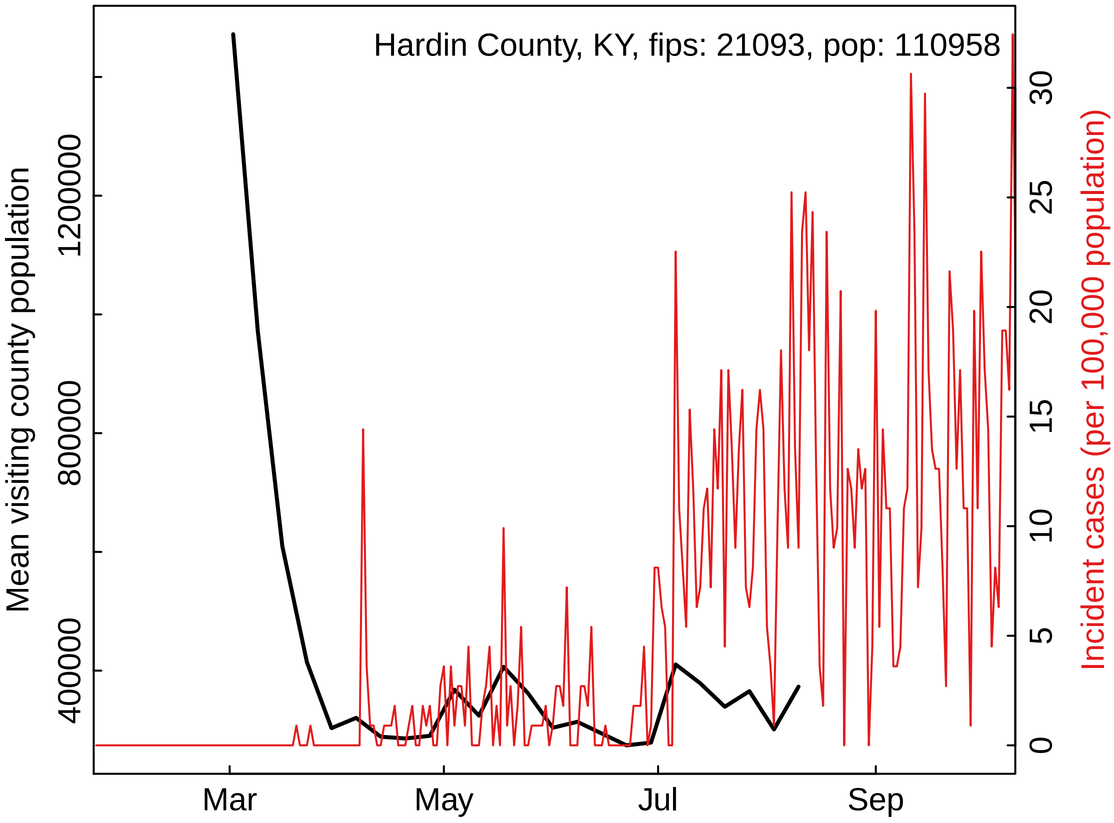

Hardin County, KY, fips: 21093, pop: 110958
Mean visiting county population
400000
800000
1200000
Incident cases (per 100,000 population)
0
5
10
15
20
25
30
Mar
May
Jul
Sep

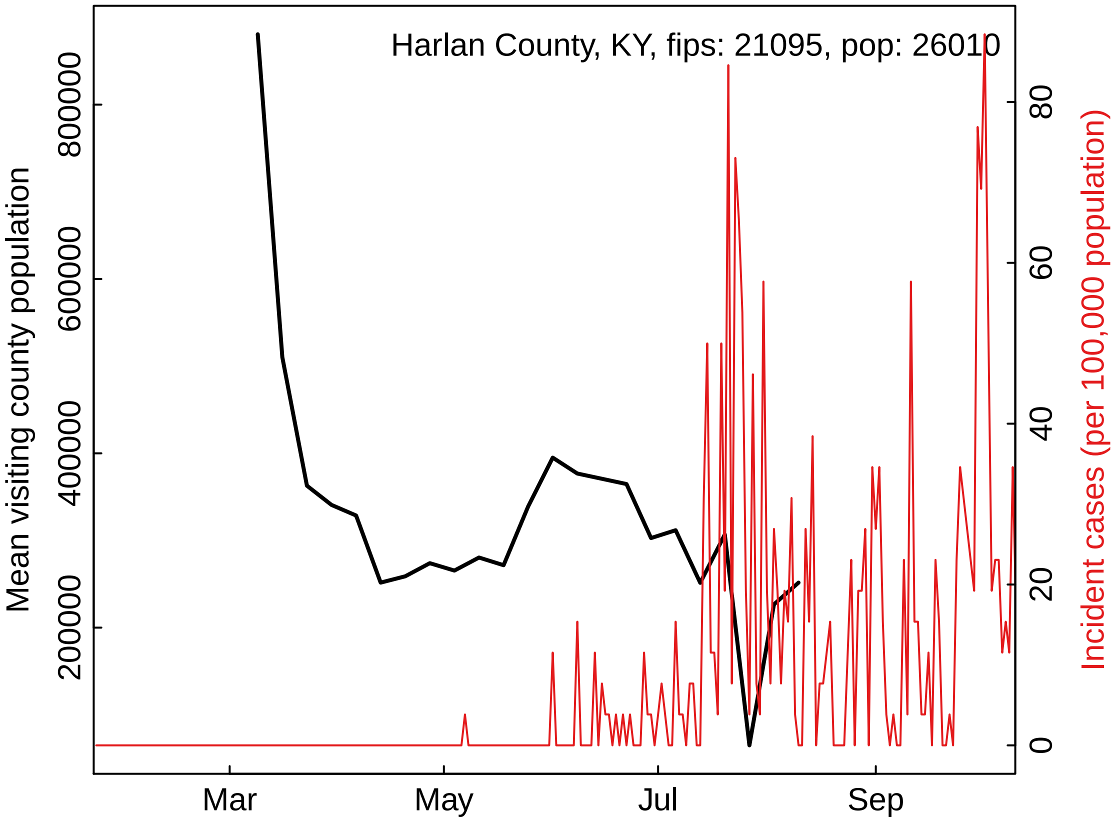

Harlan County, KY, fips: 21095, pop: 26010
Mean visiting county population
200000
400000
600000
800000
Incident cases (per 100,000 population)
0
20
40
60
80
Mar
May
Jul
Sep

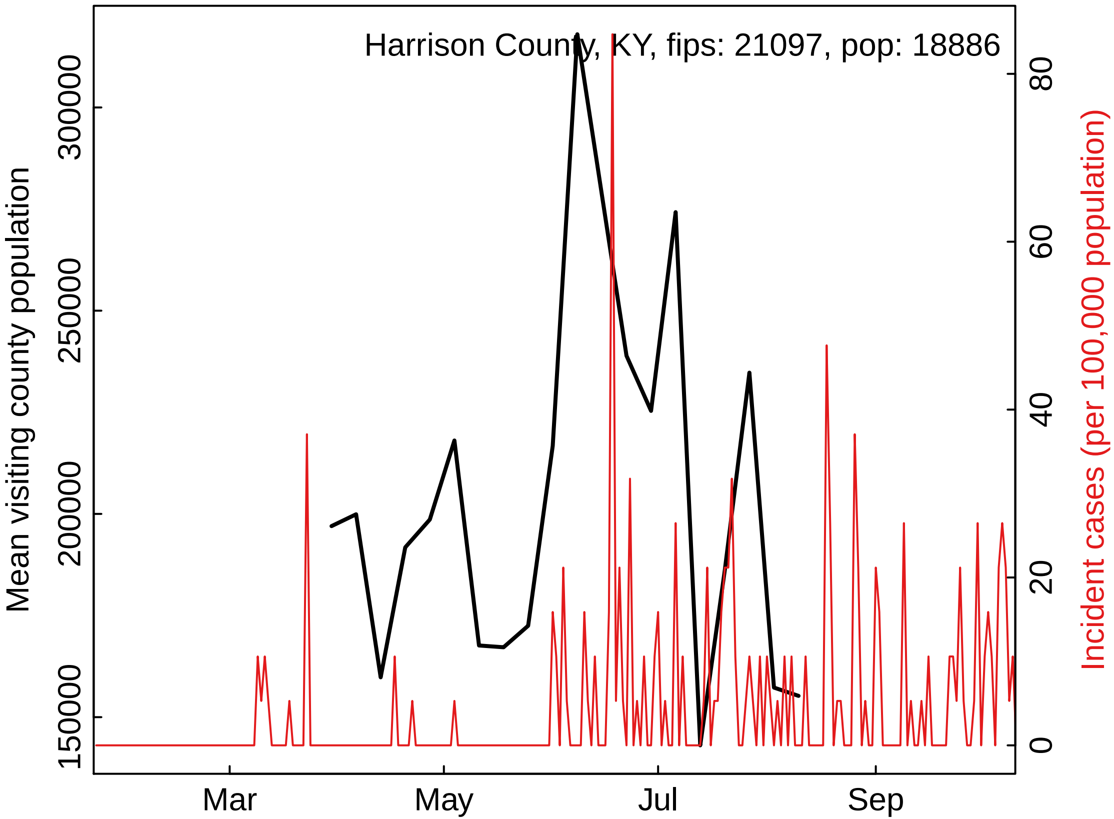

Harrison County, KY, fips: 21097, pop: 18886
Mean visiting county population
150000
200000
250000
300000
Incident cases (per 100,000 population)
0
20
40
60
80
Mar
May
Jul
Sep

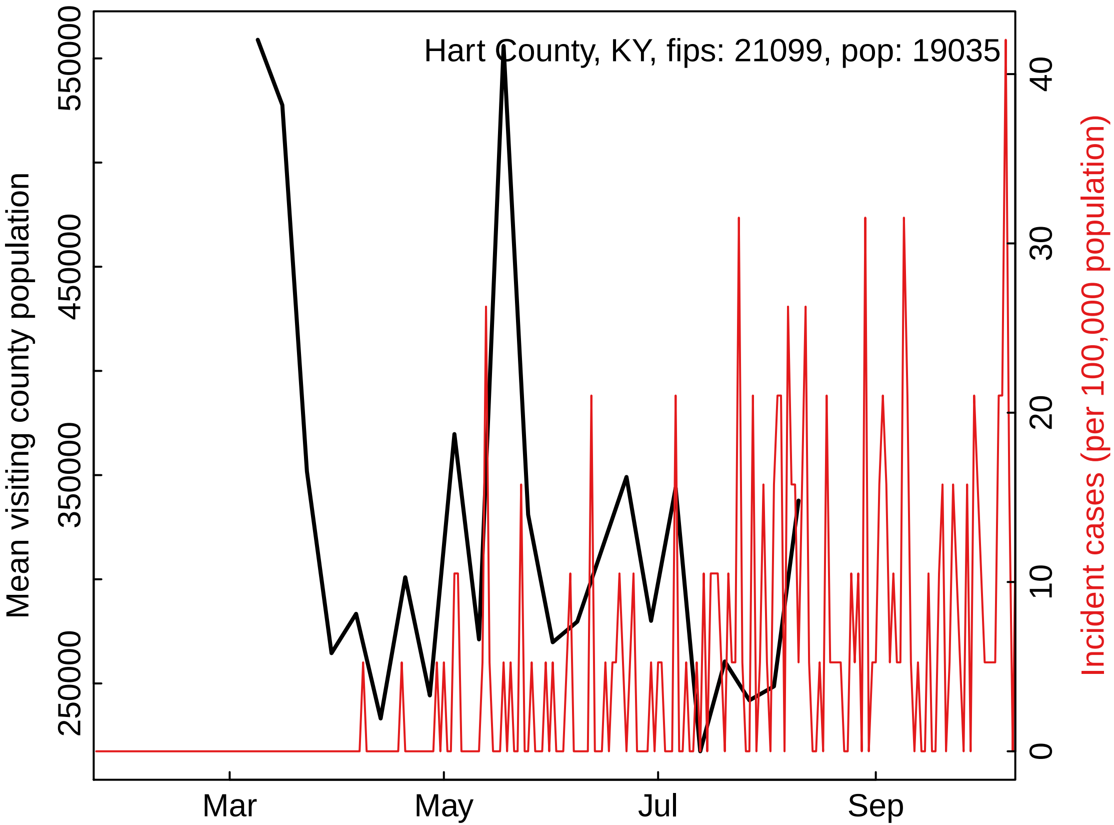

Hart County, KY, fips: 21099, pop: 19035
Mean visiting county population
250000
350000
450000
550000
Incident cases (per 100,000 population)
0
10
20
30
40
Mar
May
Jul
Sep

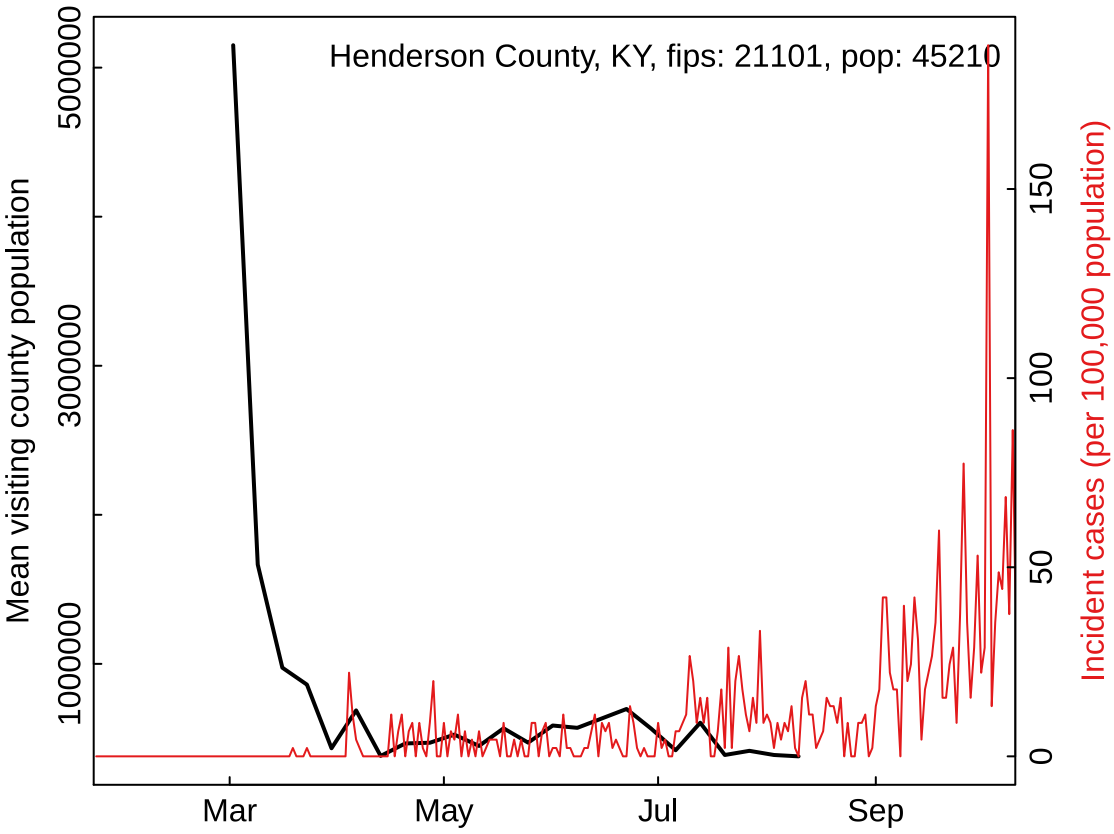
Henderson County, KY, fips: 21101, pop: 45210
Mean visiting county population
1000000
3000000
5000000
Incident cases (per 100,000 population)
0
50
100
150
Mar
May
Jul
Sep

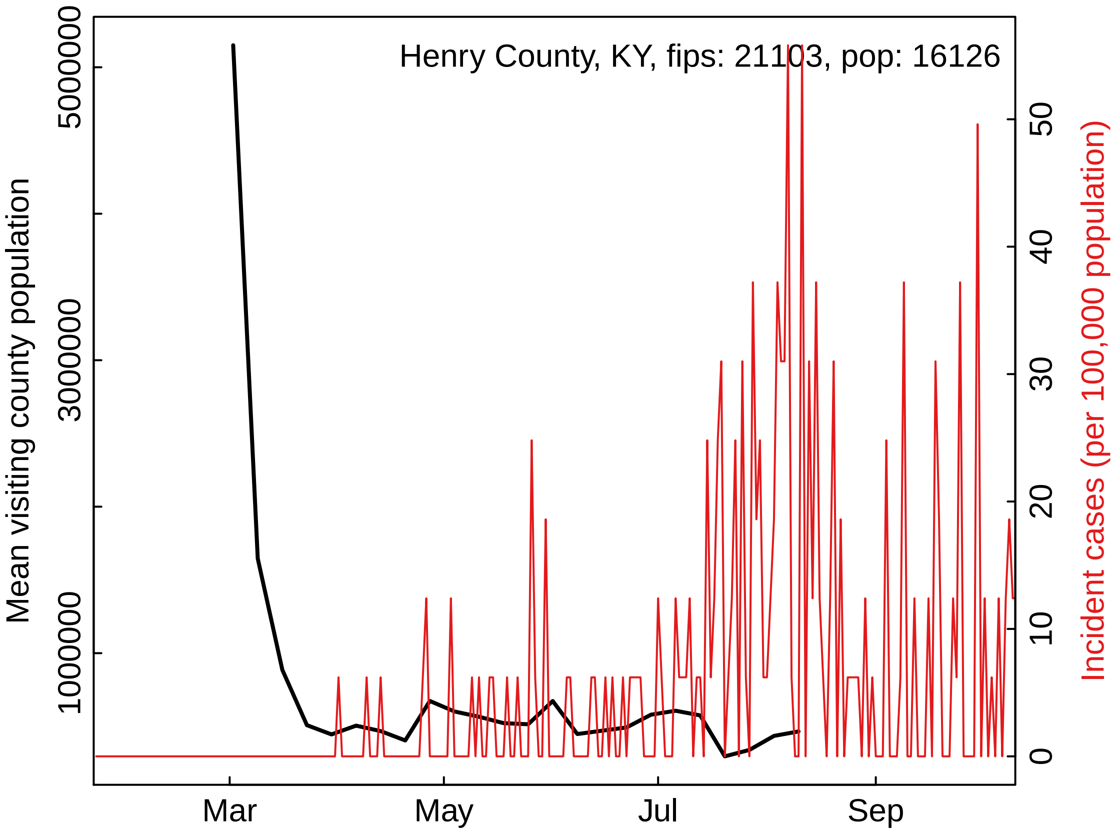

Henry County, KY, fips: 21103, pop: 16126
Mean visiting county population
5000000
3000000
1000000
Mar
May
Jul
Sep
0
10
20
30
40
50
Incident cases (per 100,000 population)

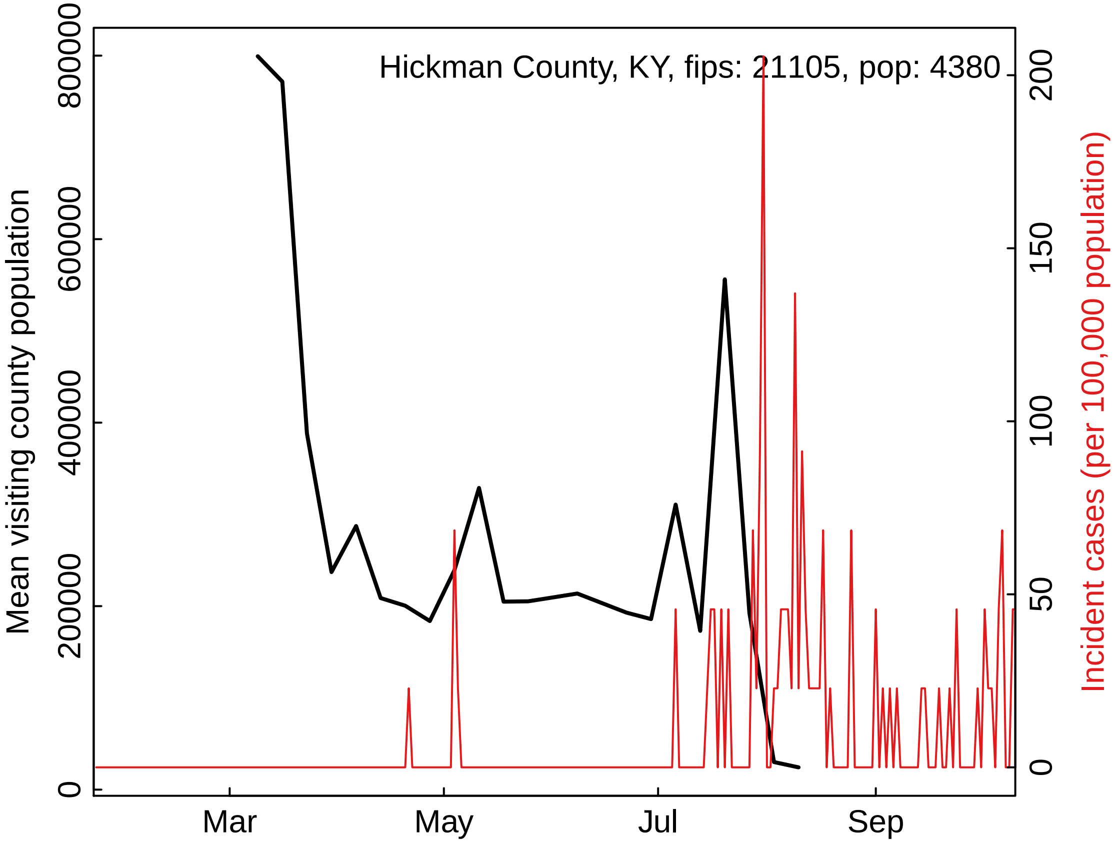

Hickman County, KY, fips: 21105, pop: 4380
Mean visiting county population
Incident cases (per 100,000 population)
0
200000
400000
600000
800000
0
50
100
150
200
Mar
May
Jul
Sep

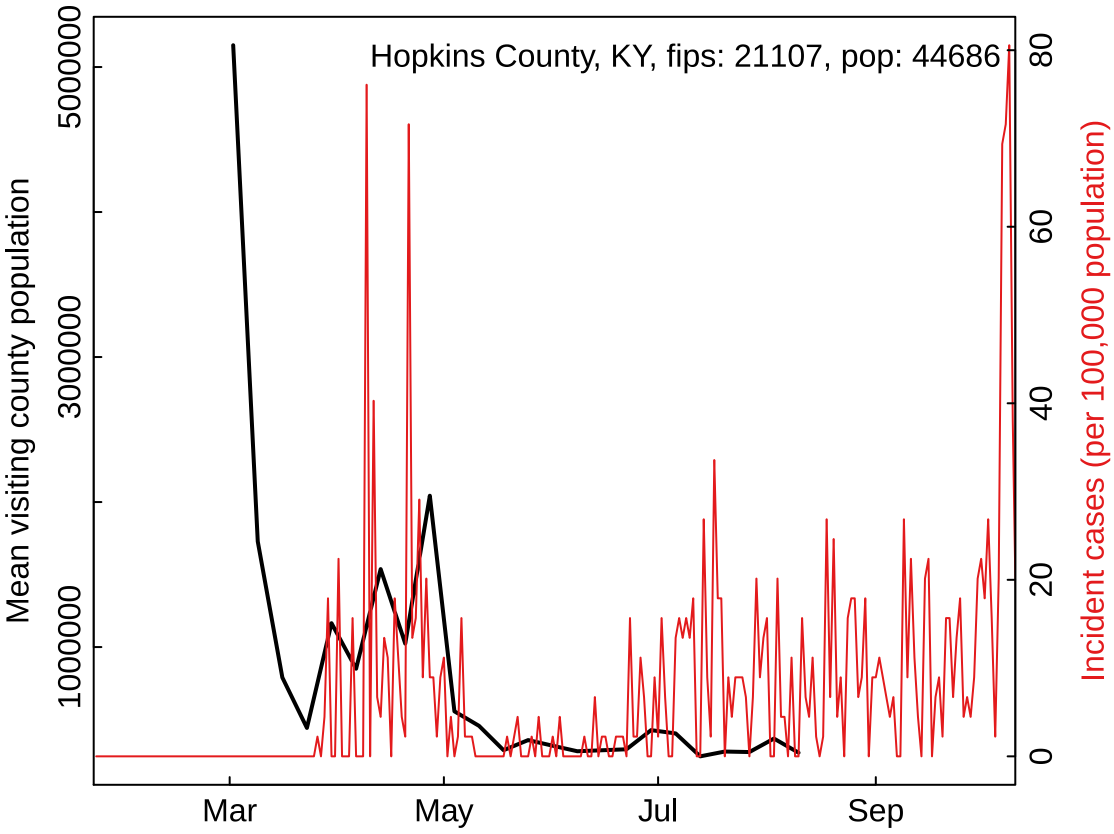

Hopkins County, KY, fips: 21107, pop: 44686
Mean visiting county population
1000000
3000000
5000000
Incident cases (per 100,000 population)
0
20
40
60
80
Mar
May
Jul
Sep

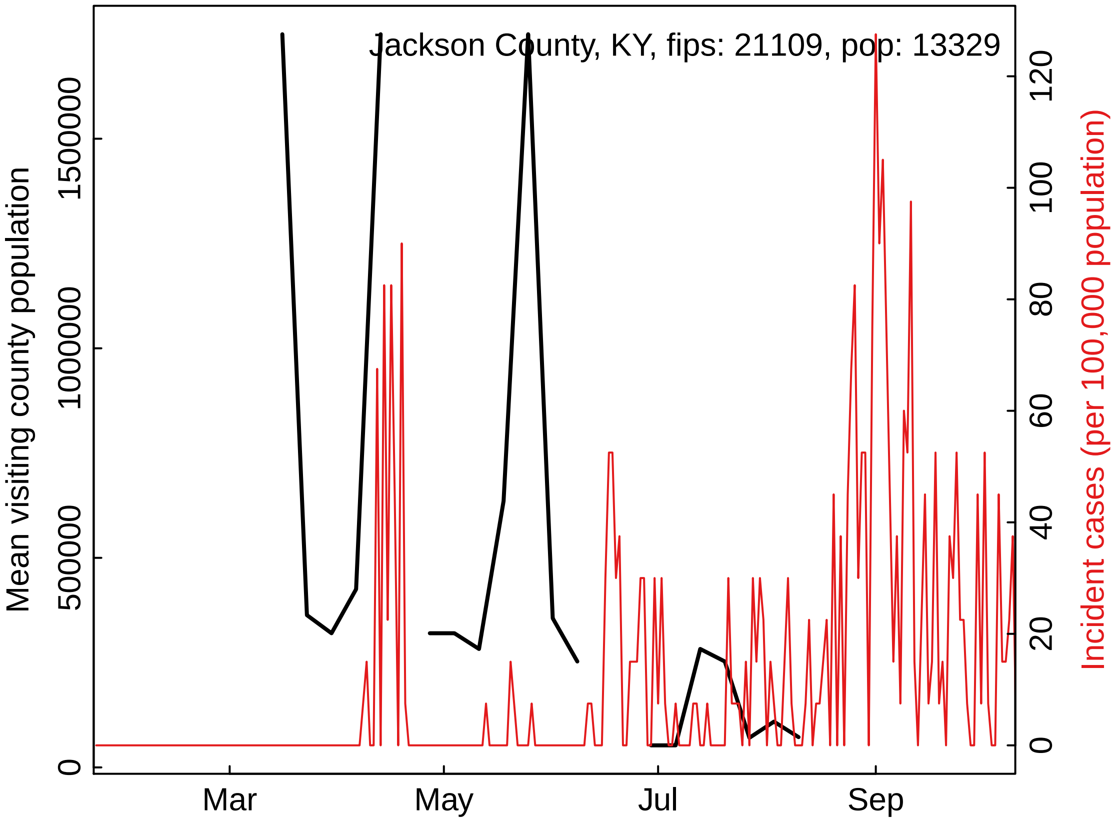

Jackson County, KY, fips: 21109, pop: 13329
Mean visiting county population
0
500000
1000000
1500000
Incident cases (per 100,000 population)
0
20
40
60
80
100
120
Mar
May
Jul
Sep

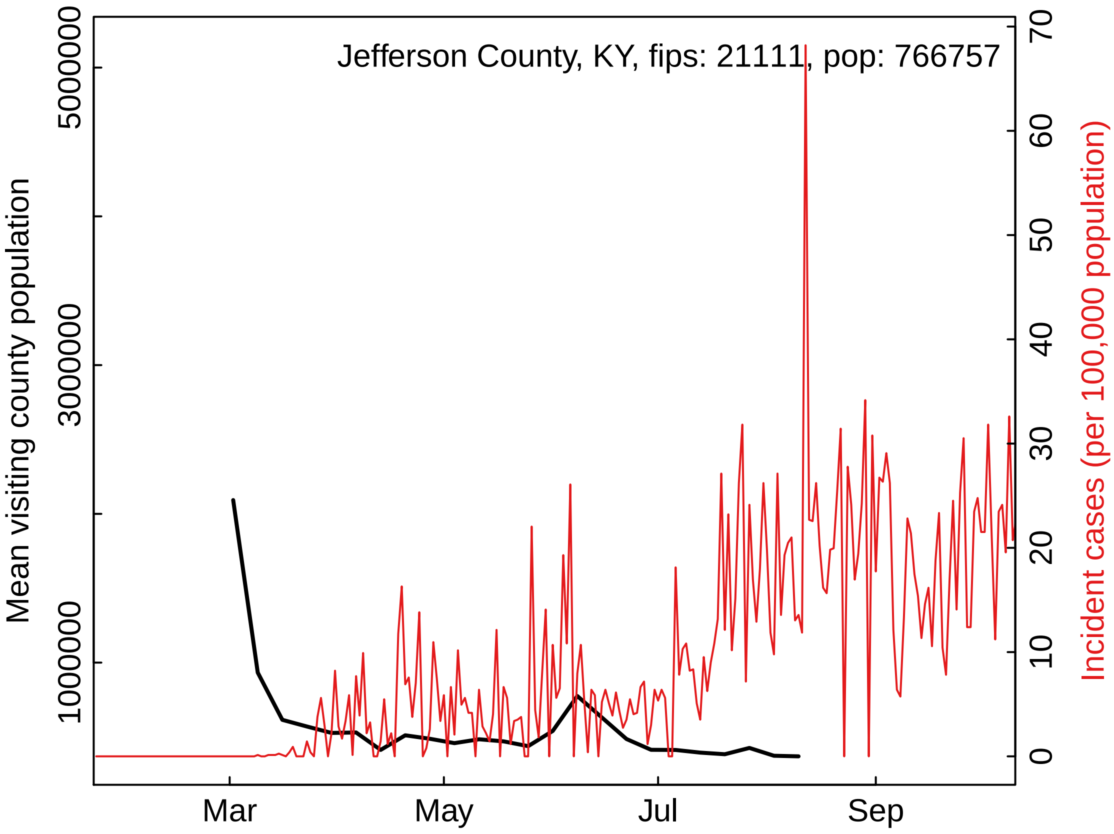

Jefferson County, KY, fips: 21111, pop: 766757
Mean visiting county population
1000000
3000000
5000000
Incident cases (per 100,000 population)
0
10
20
30
40
50
60
70
Mar
May
Jul
Sep

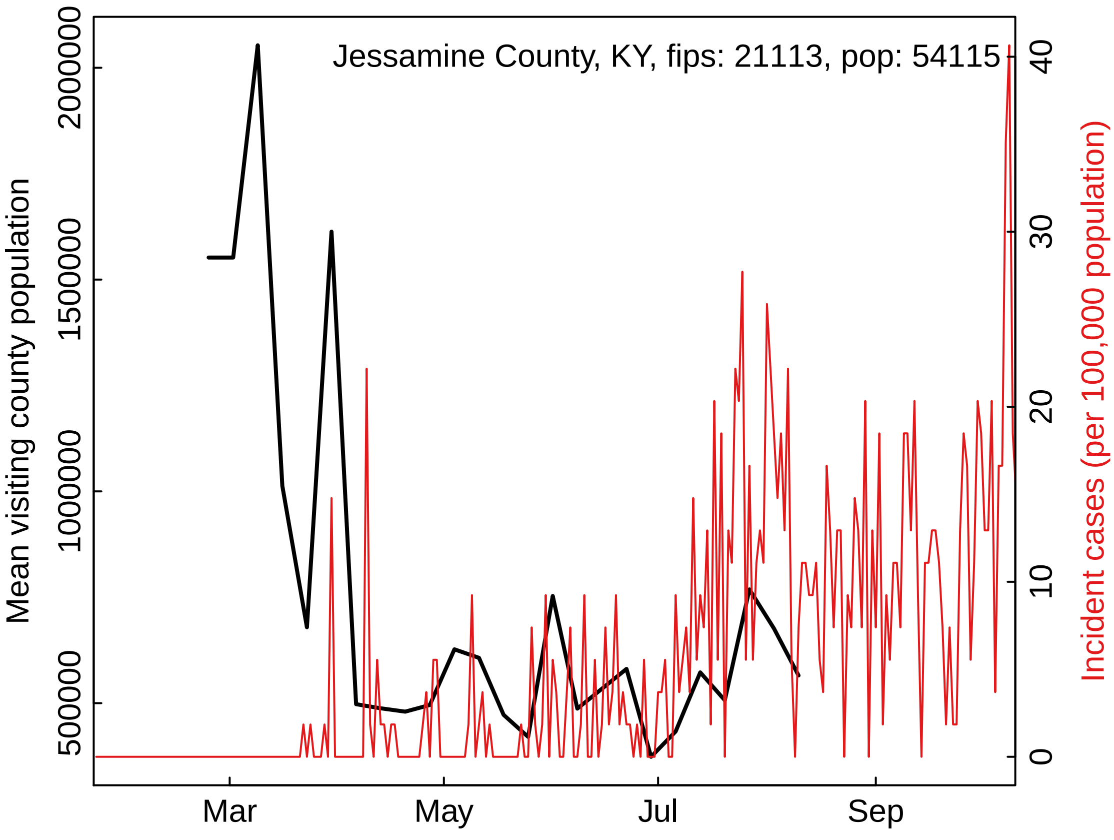

Jessamine County, KY, fips: 21113, pop: 54115
Mean visiting county population
Incident cases (per 100,000 population)
500000
1000000
1500000
2000000
0
10
20
30
40
Mar
May
Jul
Sep

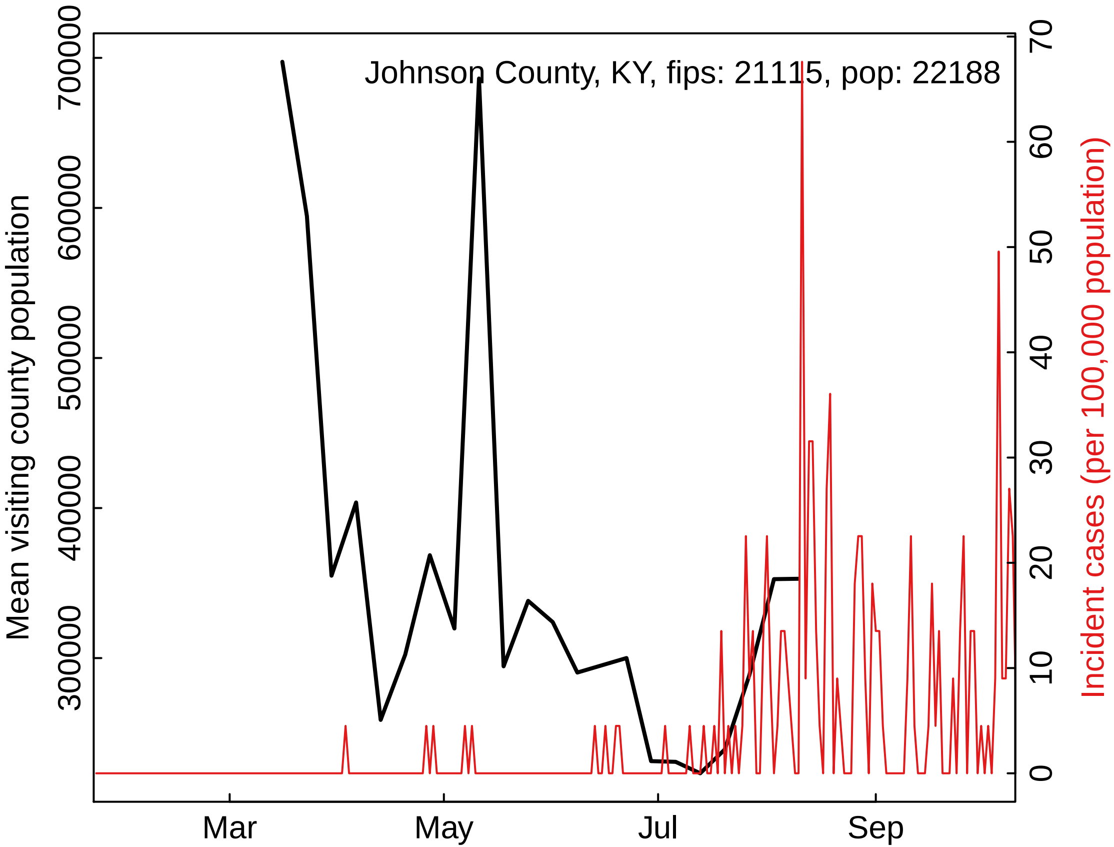

Johnson County, KY, fips: 21115, pop: 22188
Mean visiting county population
300000
400000
500000
600000
700000
Incident cases (per 100,000 population)
0
10
20
30
40
50
60
70
Mar
May
Jul
Sep

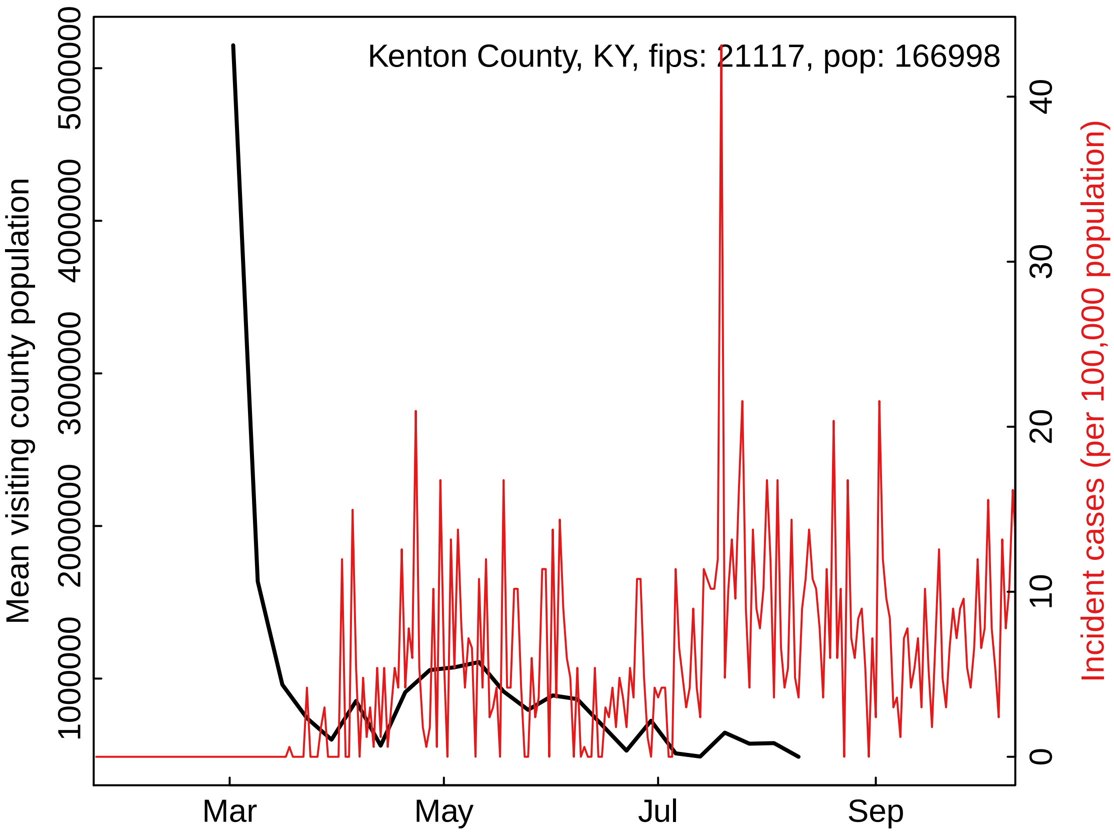

Kenton County, KY, fips: 21117, pop: 166998
Mean visiting county population
10000000
20000000
30000000
40000000
50000000
Incident cases (per 100,000 population)
0
10
20
30
40
Mar
May
Jul
Sep

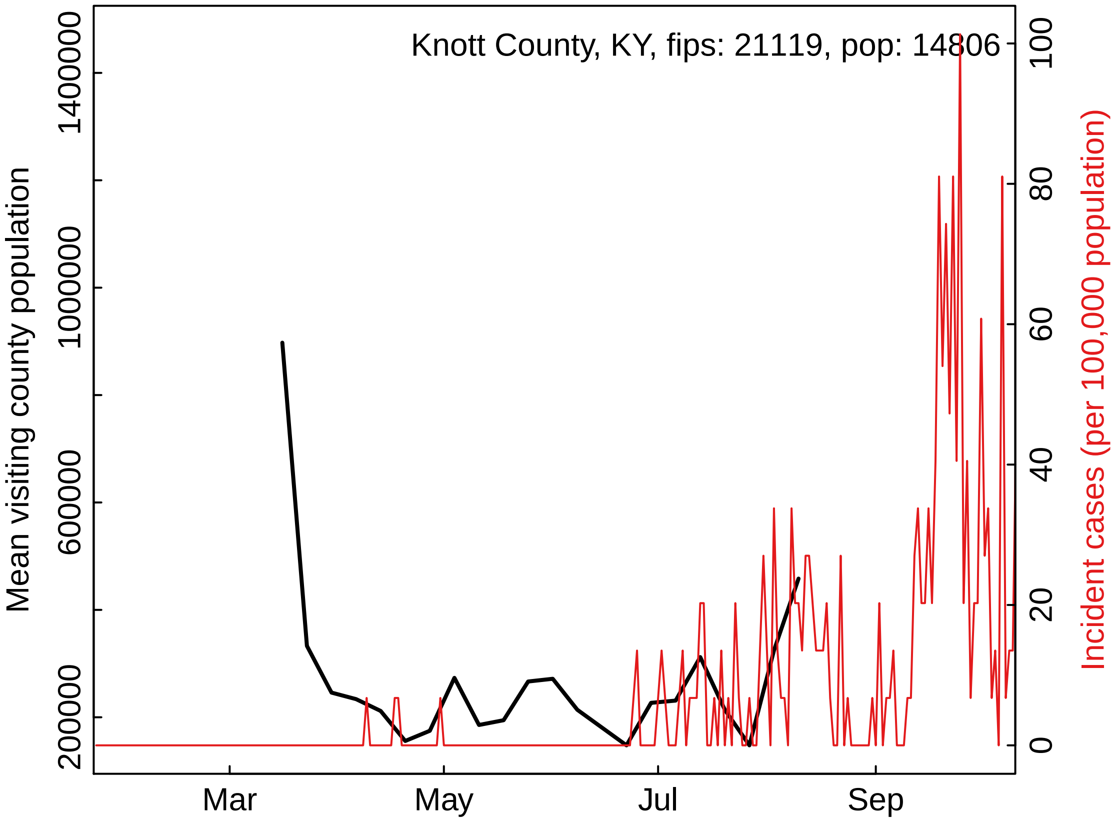

Knott County, KY, fips: 21119, pop: 14806
Mean visiting county population
200000
600000
1000000
1400000
Incident cases (per 100,000 population)
0
20
40
60
80
100
Mar
May
Jul
Sep

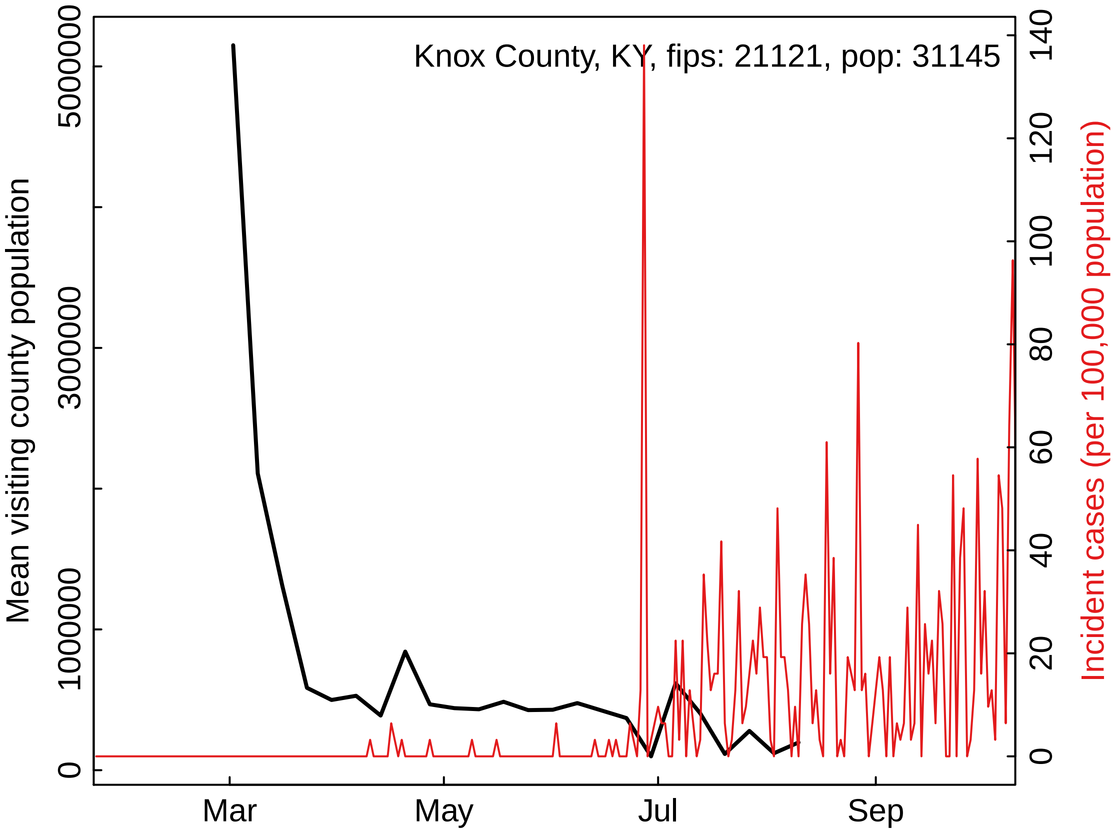

Knox County, KY, fips: 21121, pop: 31145
Mean visiting county population
Incident cases (per 100,000 population)
0
1000000
3000000
5000000
0
20
40
60
80
100
120
140
Mar
May
Jul
Sep

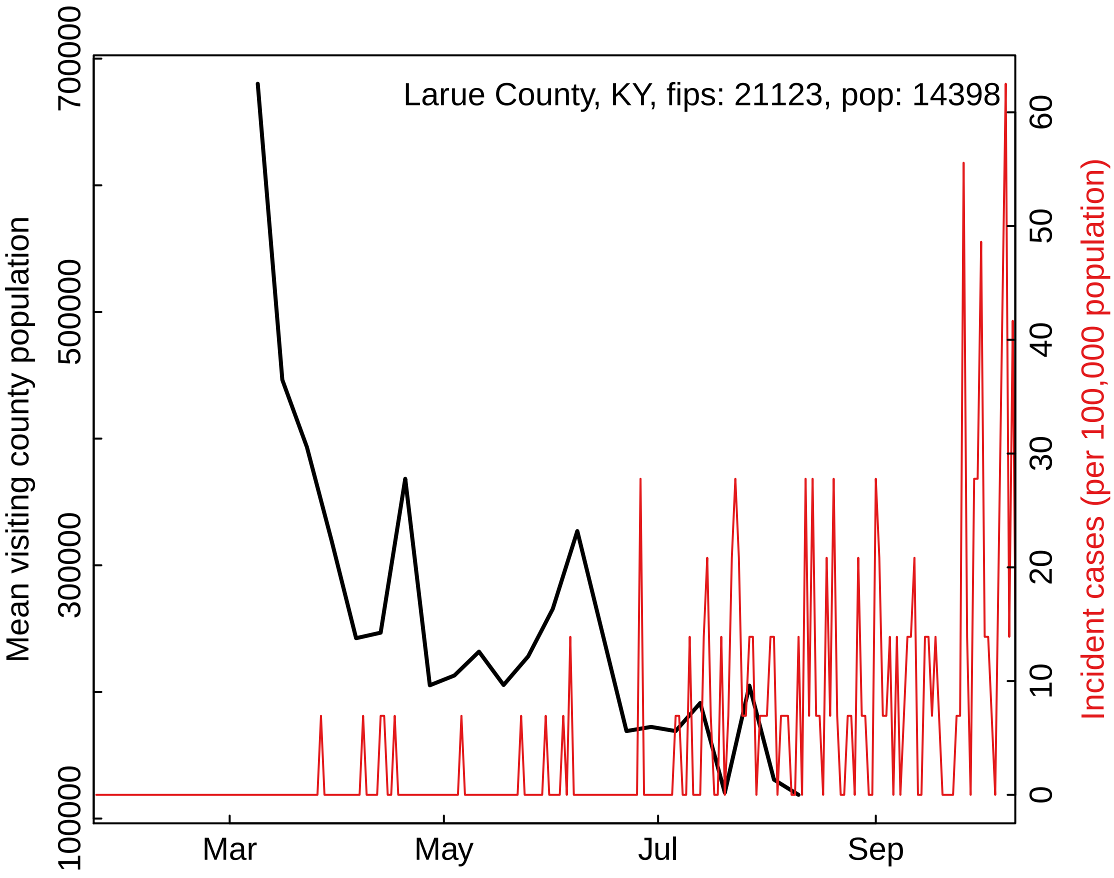

Larue County, KY, fips: 21123, pop: 14398
Mean visiting county population
100000
300000
500000
700000
Incident cases (per 100,000 population)
0
10
20
30
40
50
60
Mar
May
Jul
Sep

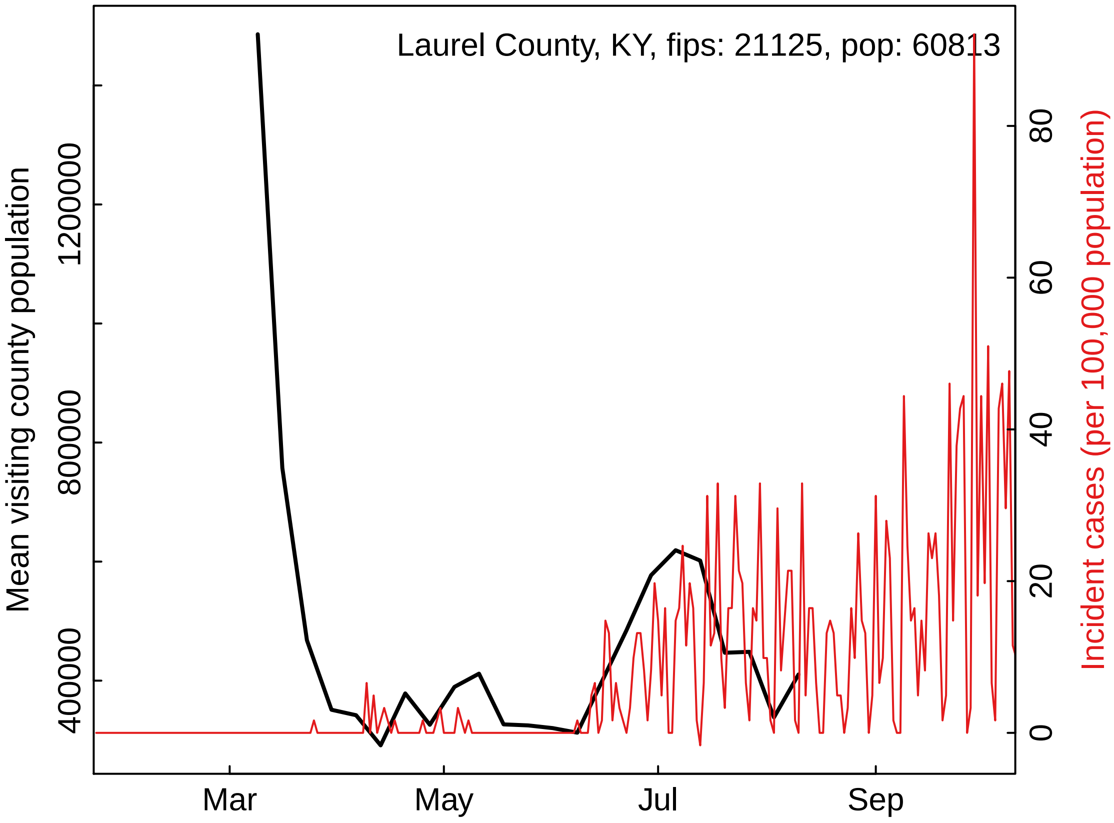

Laurel County, KY, fips: 21125, pop: 60813
Mean visiting county population
400000
800000
1200000
Incident cases (per 100,000 population)
0
20
40
60
80
Mar
May
Jul
Sep

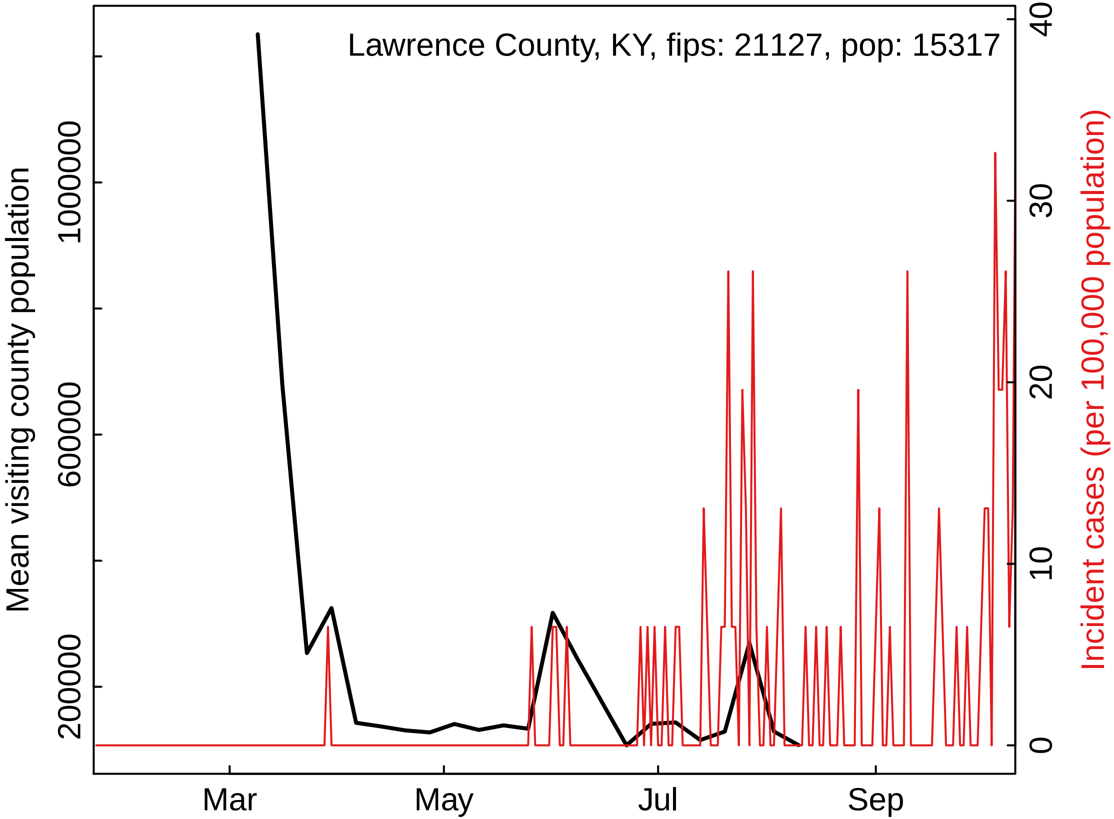
Lawrence County, KY, fips: 21127, pop: 15317
Mean visiting county population
200000
600000
1000000
Mar
May
Jul
Sep
0
10
20
30
40
Incident cases (per 100,000 population)

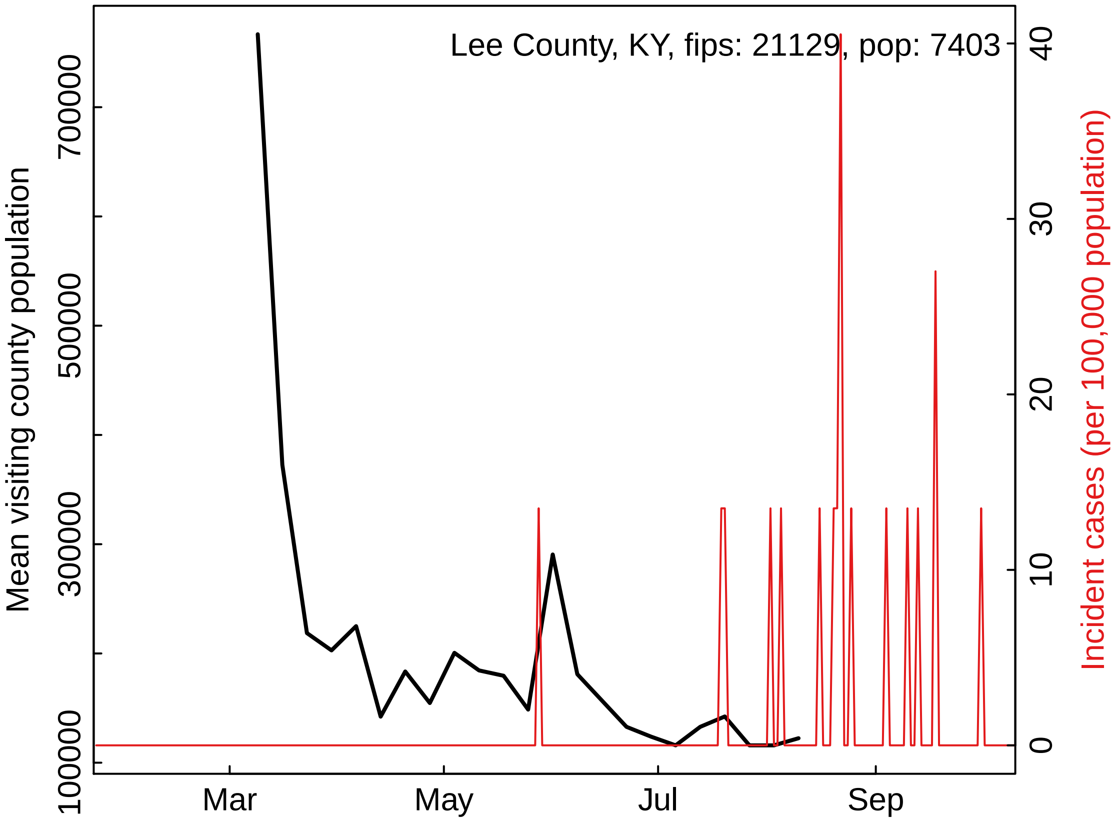

Lee County, KY, fips: 21129, pop: 7403
Mean visiting county population
100000
300000
500000
700000
Incident cases (per 100,000 population)
0
10
20
30
40
Mar
May
Jul
Sep

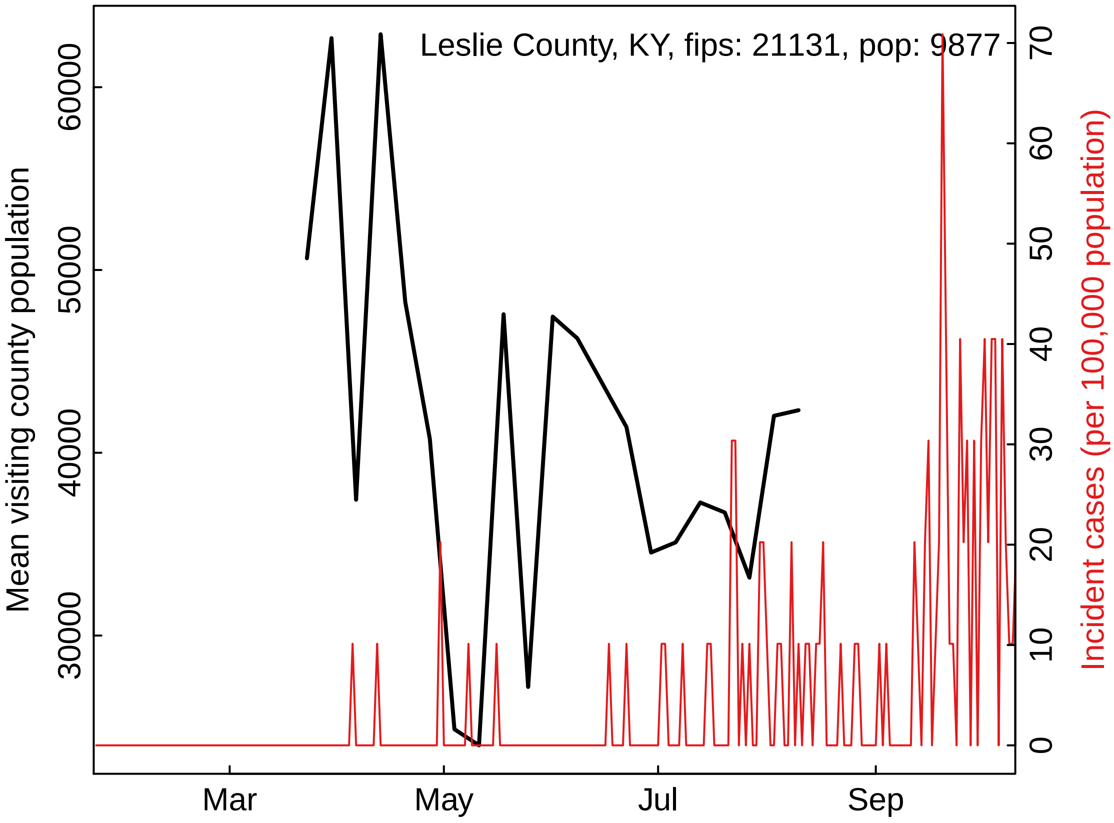

Leslie County, KY, fips: 21131, pop: 9877
Mean visiting county population
30000
40000
50000
60000
Incident cases (per 100,000 population)
0
10
20
30
40
50
60
70
Mar
May
Jul
Sep

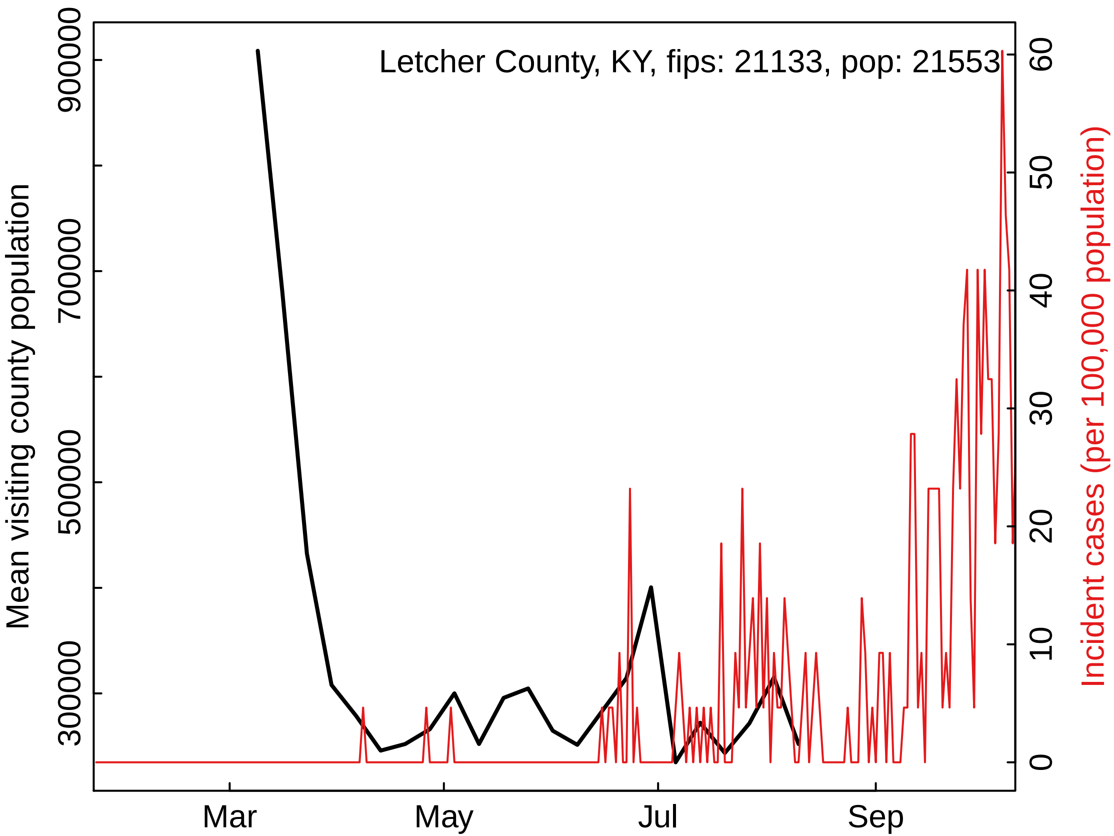

Letcher County, KY, fips: 21133, pop: 21553
Mean visiting county population
300000
500000
700000
900000
Incident cases (per 100,000 population)
0
10
20
30
40
50
60
Mar
May
Jul
Sep

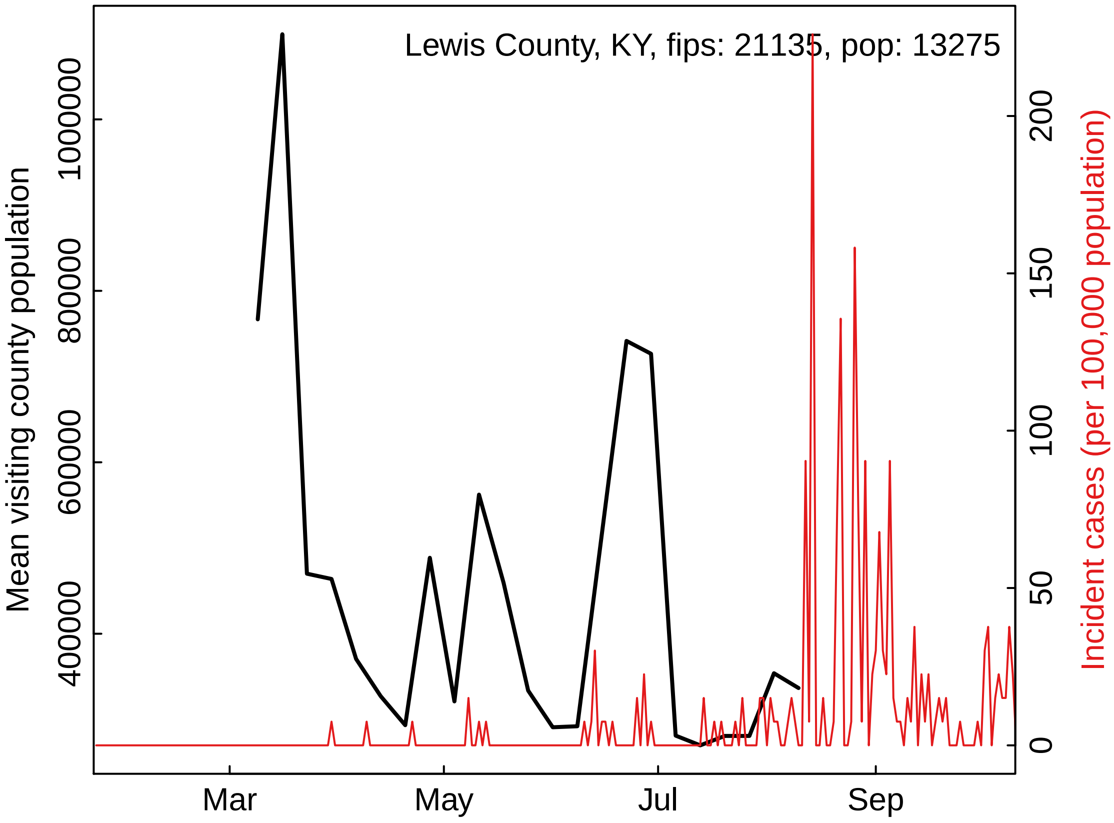

Lewis County, KY, fips: 21135, pop: 13275
Mean visiting county population
400000
600000
800000
1000000
Incident cases (per 100,000 population)
0
50
100
150
200
Mar
May
Jul
Sep

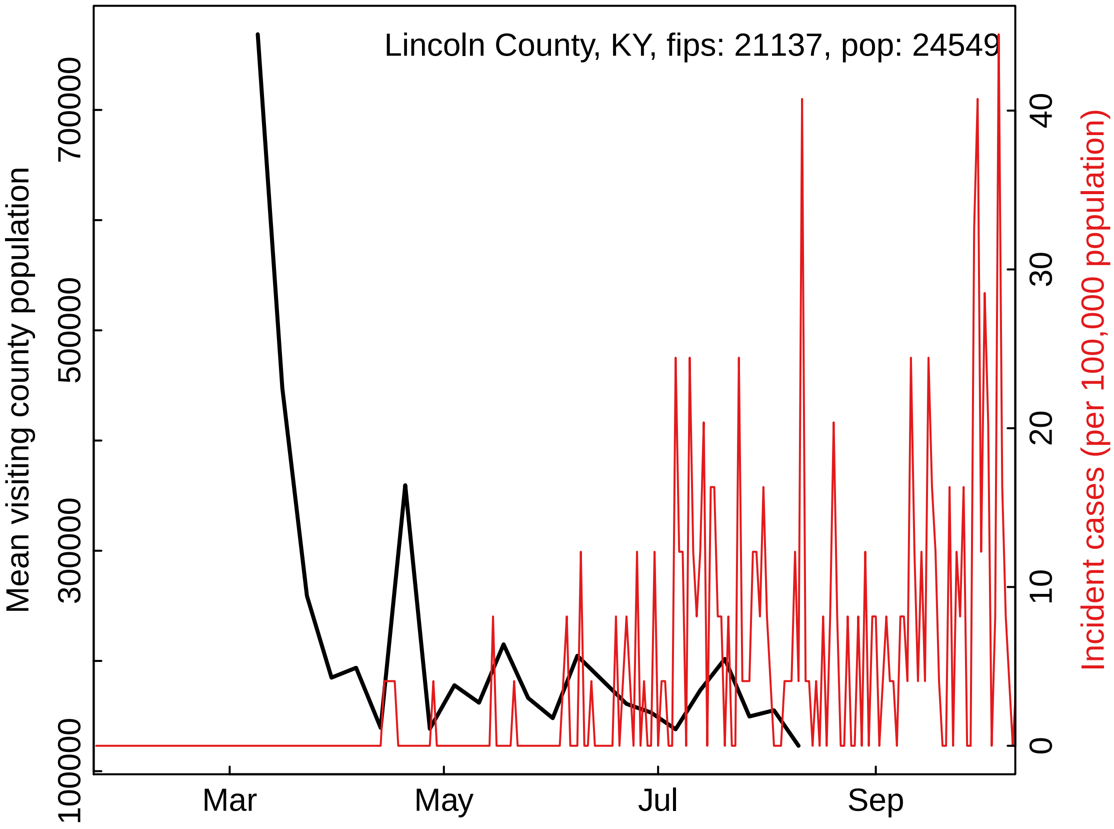

Lincoln County, KY, fips: 21137, pop: 24549
Mean visiting county population
100000
300000
500000
700000
Incident cases (per 100,000 population)
0
10
20
30
40
Mar
May
Jul
Sep

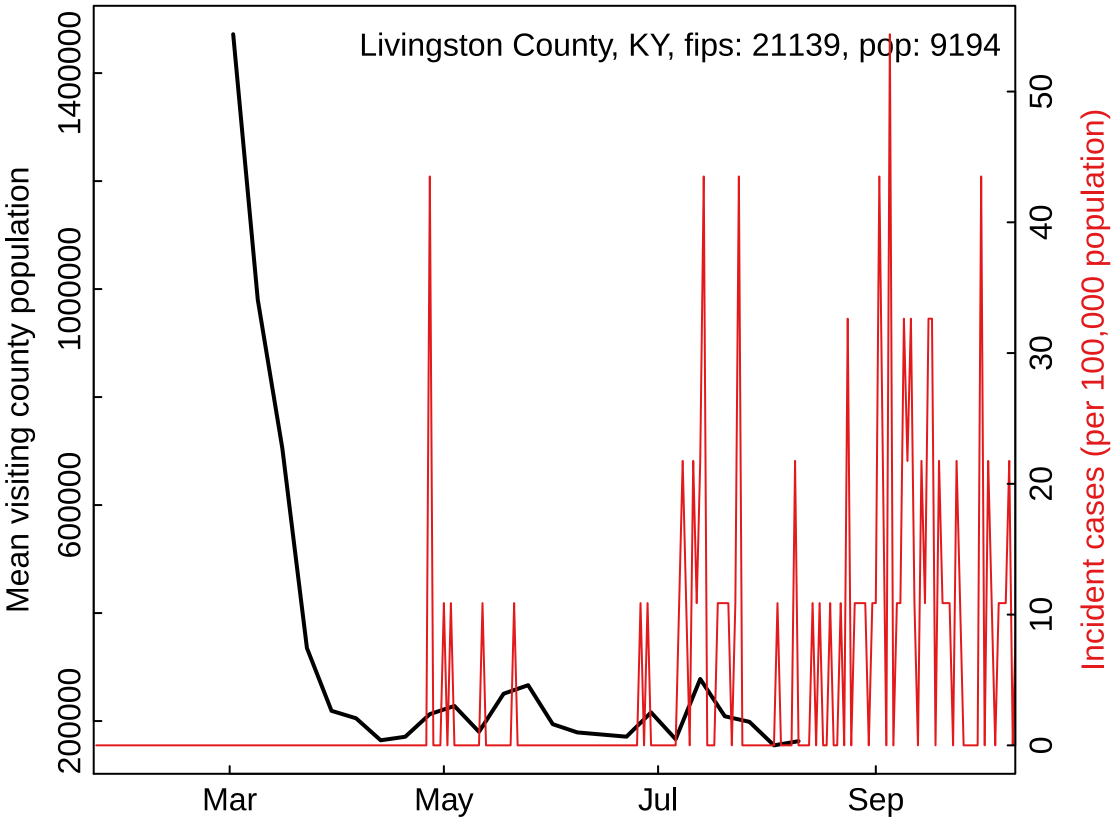

Livingston County, KY, fips: 21139, pop: 9194
Mean visiting county population
200000
600000
1000000
1400000
Incident cases (per 100,000 population)
0
10
20
30
40
50
Mar
May
Jul
Sep

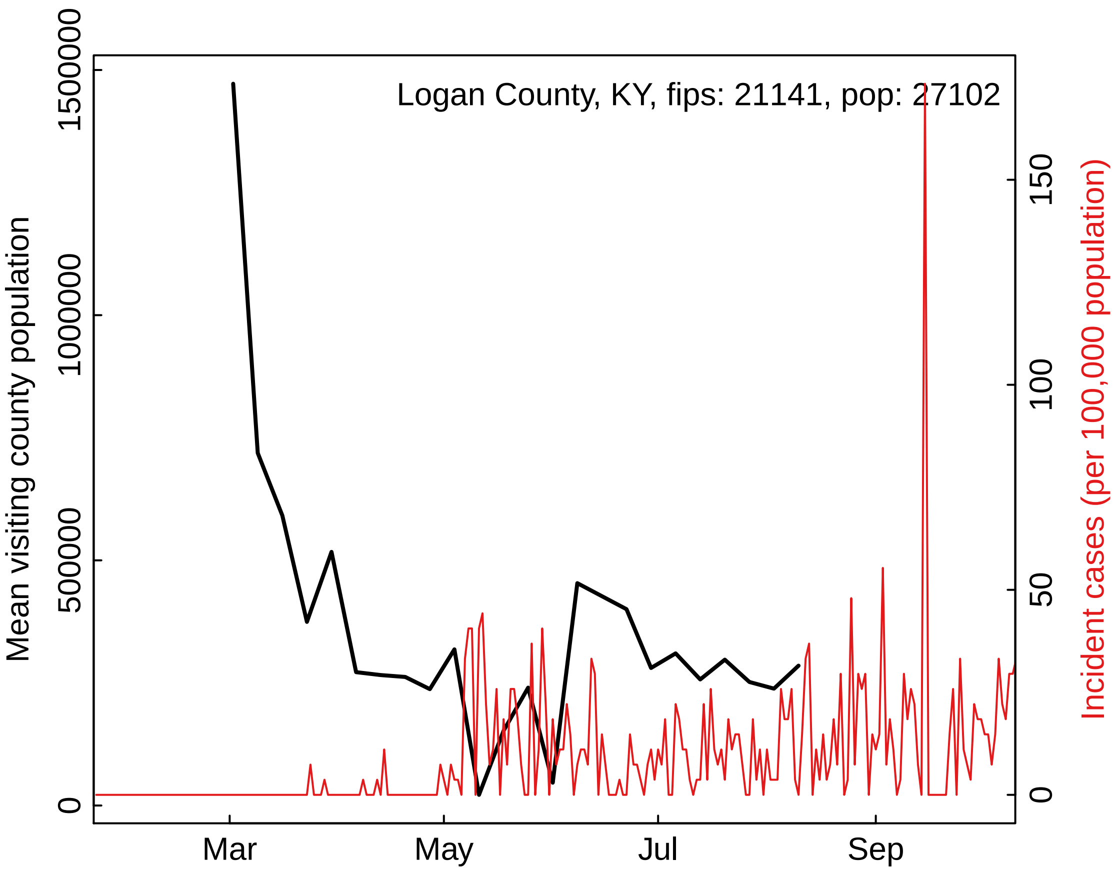

Logan County, KY, fips: 21141, pop: 27102
Mean visiting county population
0
500000
1000000
1500000
Mar
May
Jul
Sep
Incident cases (per 100,000 population)
0
50
100
150

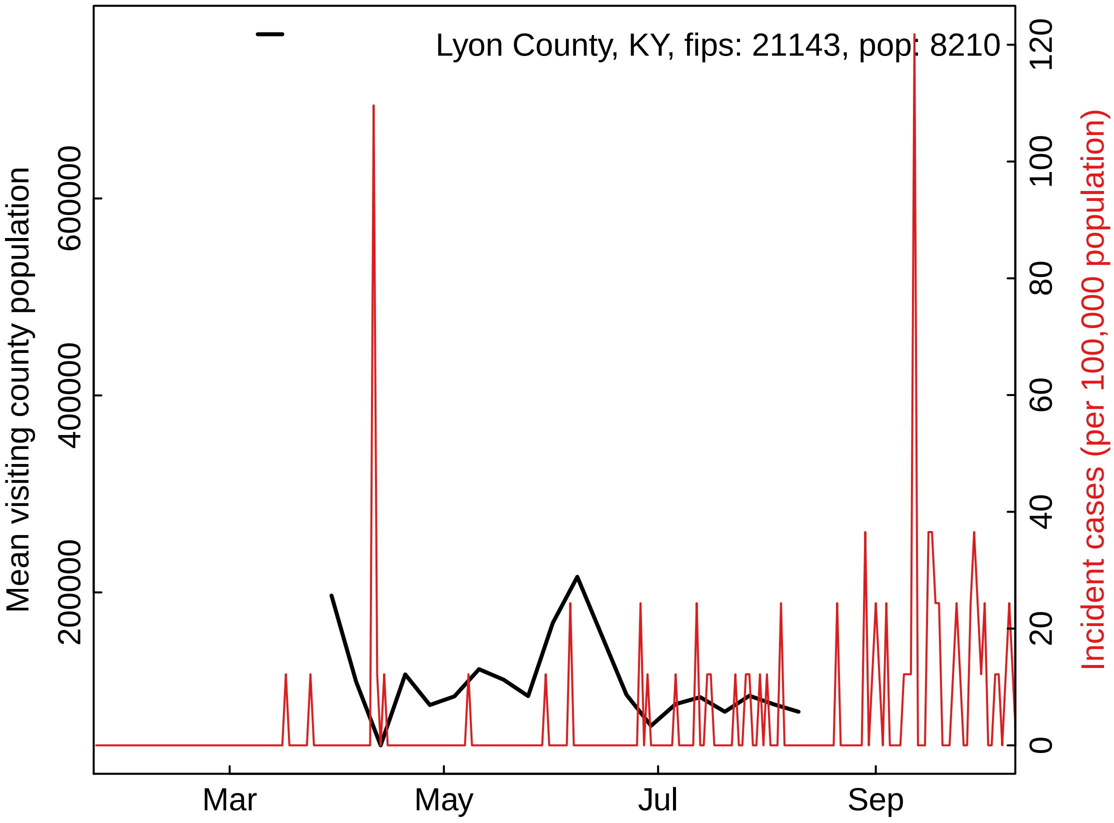

Lyon County, KY, fips: 21143, pop: 8210
Mean visiting county population
200000
400000
600000
Incident cases (per 100,000 population)
0
20
40
60
80
100
120
Mar
May
Jul
Sep

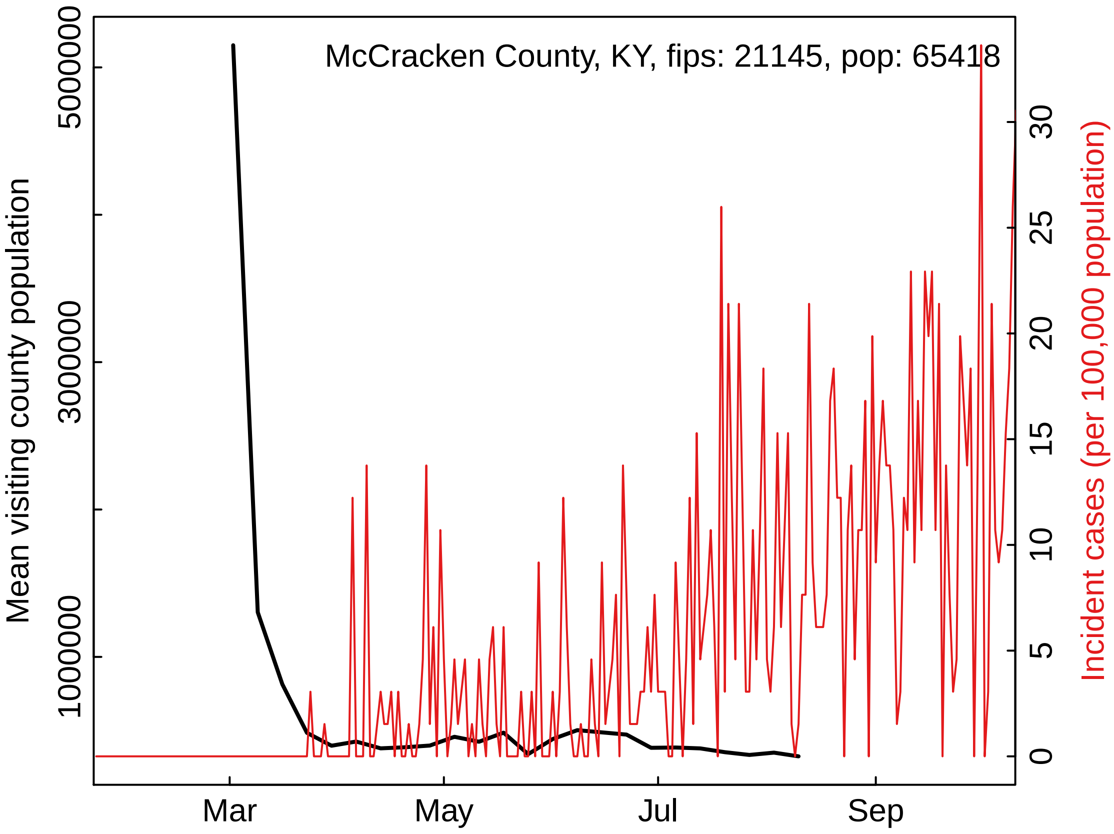

McCracken County, KY, fips: 21145, pop: 65418
Mean visiting county population
1000000
3000000
5000000
Incident cases (per 100,000 population)
0
5
10
15
20
25
30
Mar
May
Jul
Sep

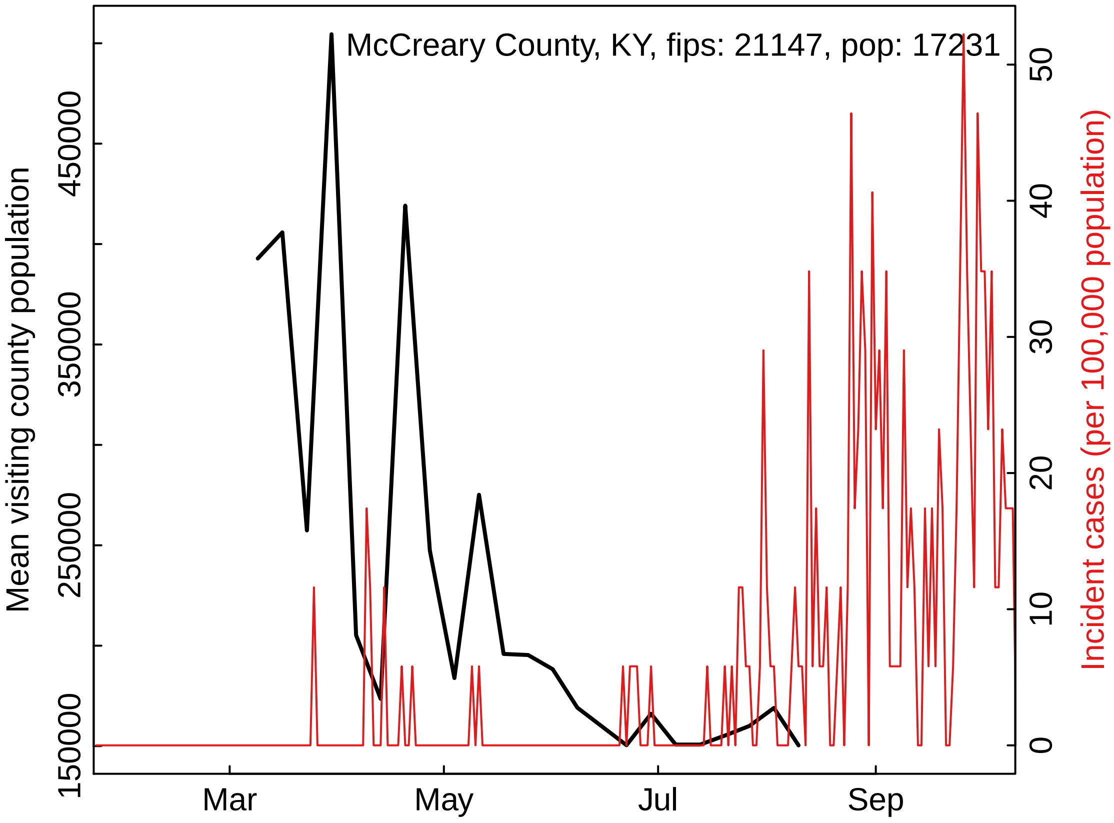

McCreary County, KY, fips: 21147, pop: 17231
Mean visiting county population
150000
250000
350000
450000
Incident cases (per 100,000 population)
0
10
20
30
40
50
Mar
May
Jul
Sep

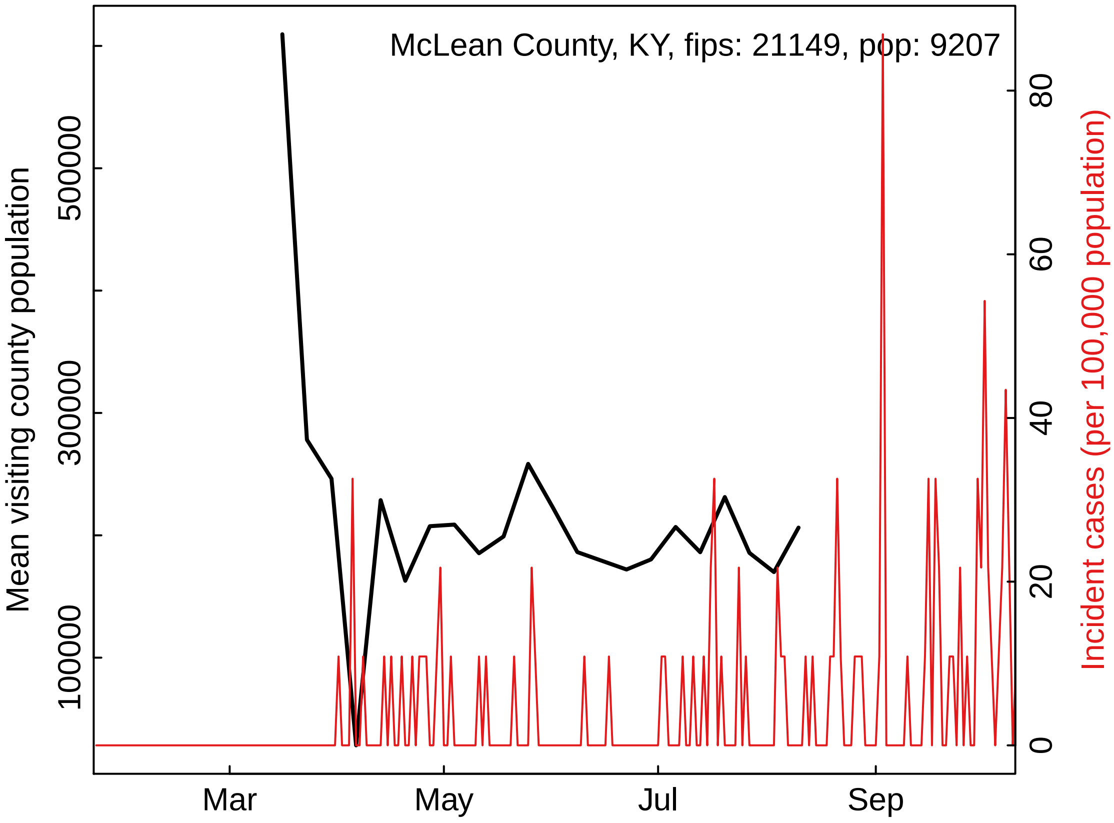
McLean County, KY, fips: 21149, pop: 9207
Mean visiting county population
100000
300000
500000
Incident cases (per 100,000 population)
0
20
40
60
80
Mar
May
Jul
Sep

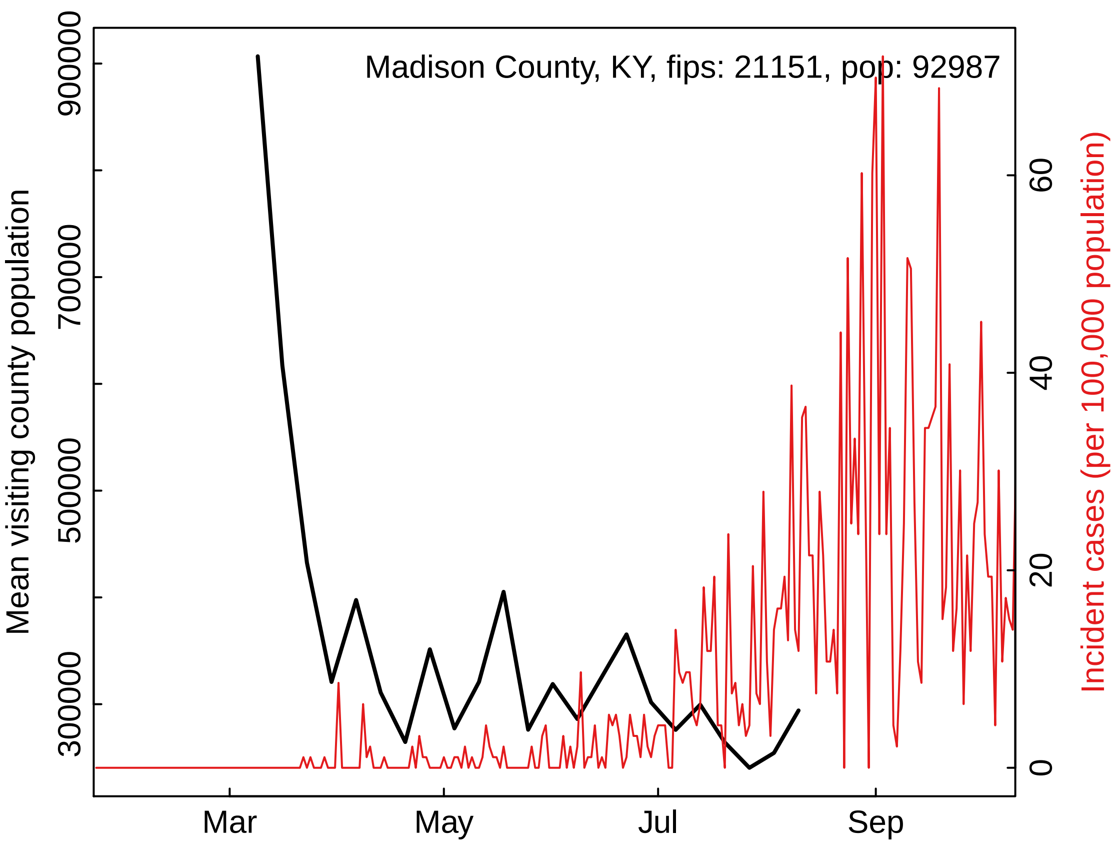

Madison County, KY, fips: 21151, pop: 92987
Mean visiting county population
300000
500000
700000
900000
Incident cases (per 100,000 population)
0
20
40
60
Mar
May
Jul
Sep

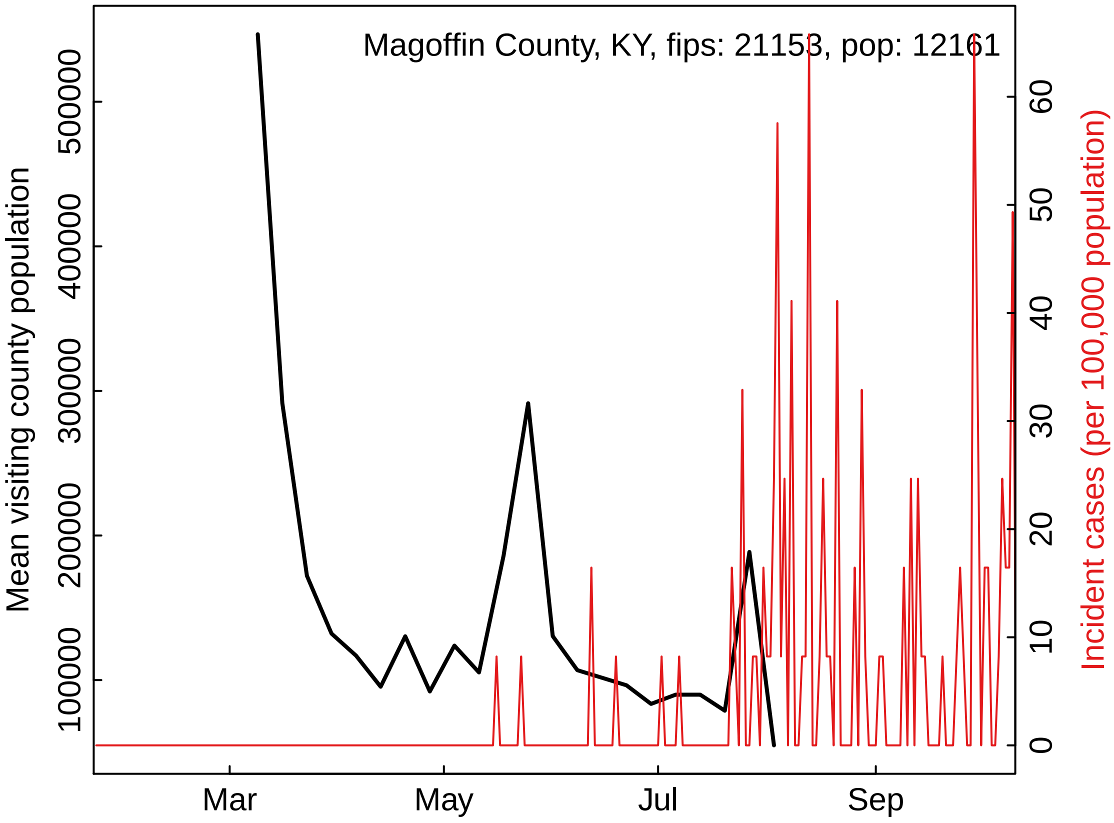

Magoffin County, KY, fips: 21153, pop: 12161
Mean visiting county population
100000
200000
300000
400000
500000
Incident cases (per 100,000 population)
0
10
20
30
40
50
60
Mar
May
Jul
Sep

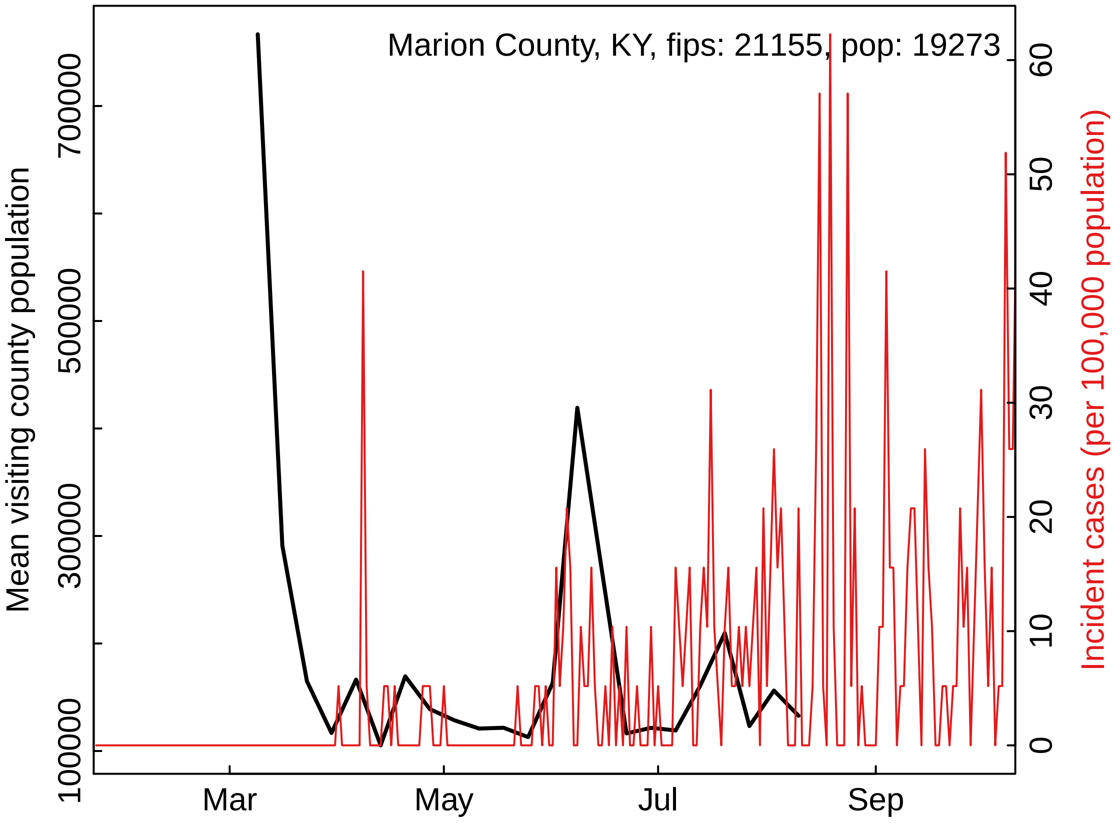

Marion County, KY, fips: 21155, pop: 19273
Mean visiting county population
Incident cases (per 100,000 population)
100000
300000
500000
700000
0
10
20
30
40
50
60
Mar
May
Jul
Sep

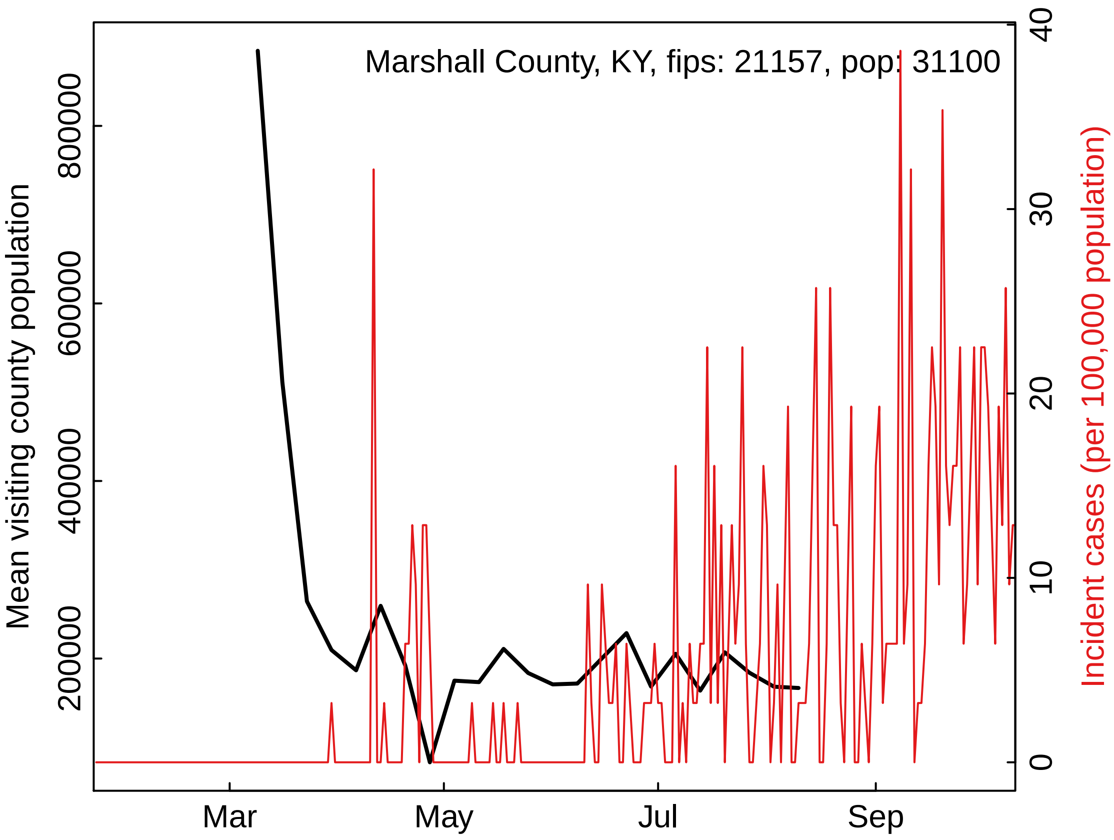

Marshall County, KY, fips: 21157, pop: 31100
Mean visiting county population
200000
400000
600000
800000
Mar
May
Jul
Sep
0
10
20
30
40
Incident cases (per 100,000 population)

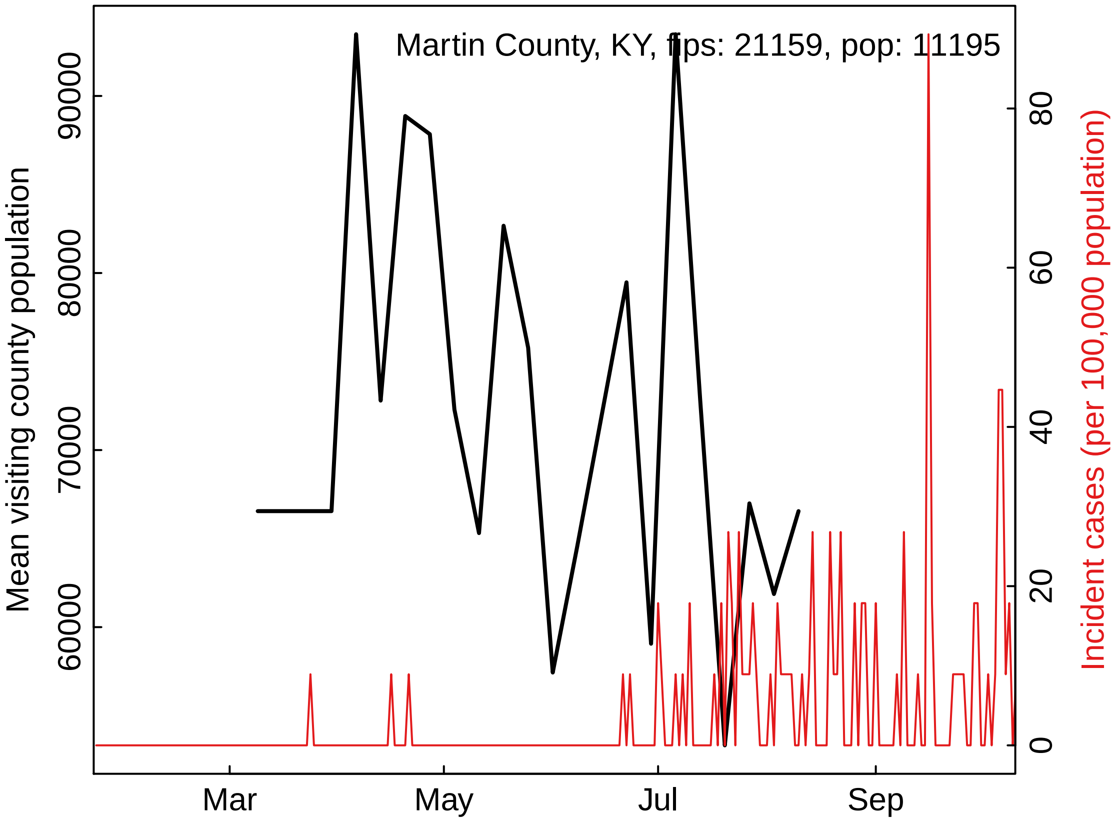

Martin County, KY, fips: 21159, pop: 11195
Mean visiting county population
60000
70000
80000
90000
Incident cases (per 100,000 population)
0
20
40
60
80
Mar
May
Jul
Sep

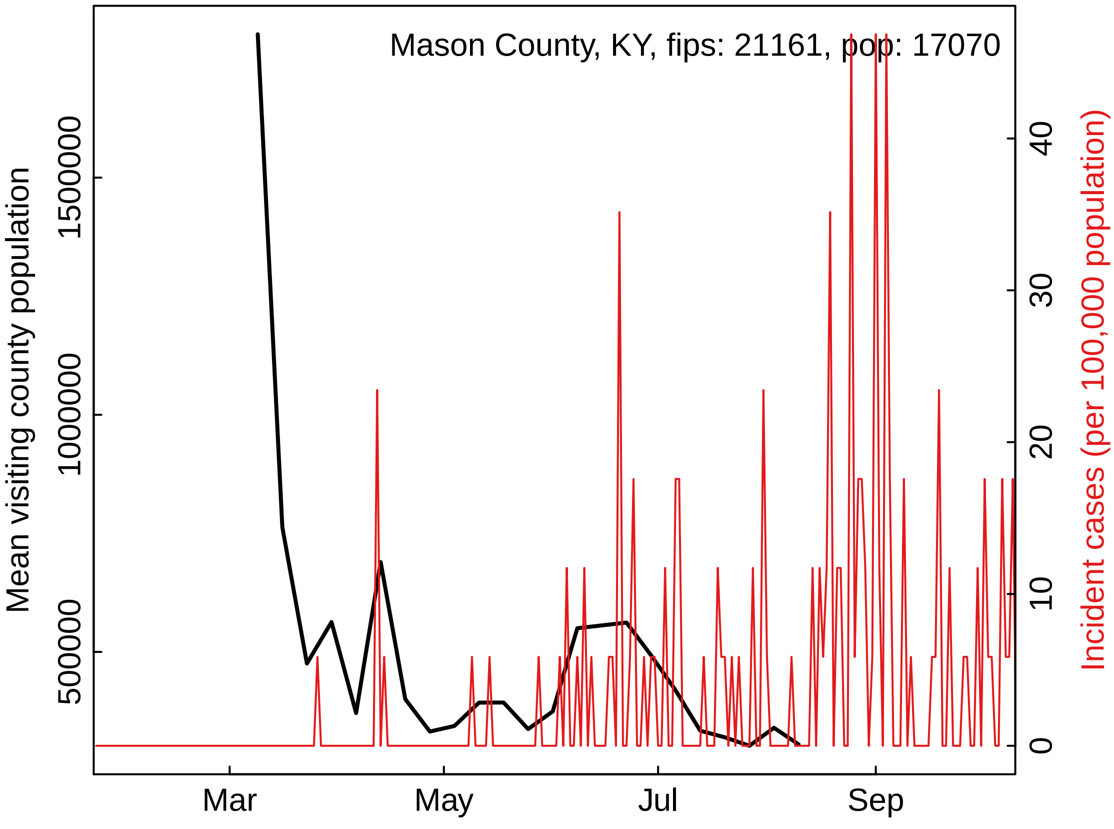
Mason County, KY, fips: 21161, pop: 17070
Mean visiting county population
500000
1000000
1500000
Incident cases (per 100,000 population)
0
10
20
30
40
Mar
May
Jul
Sep

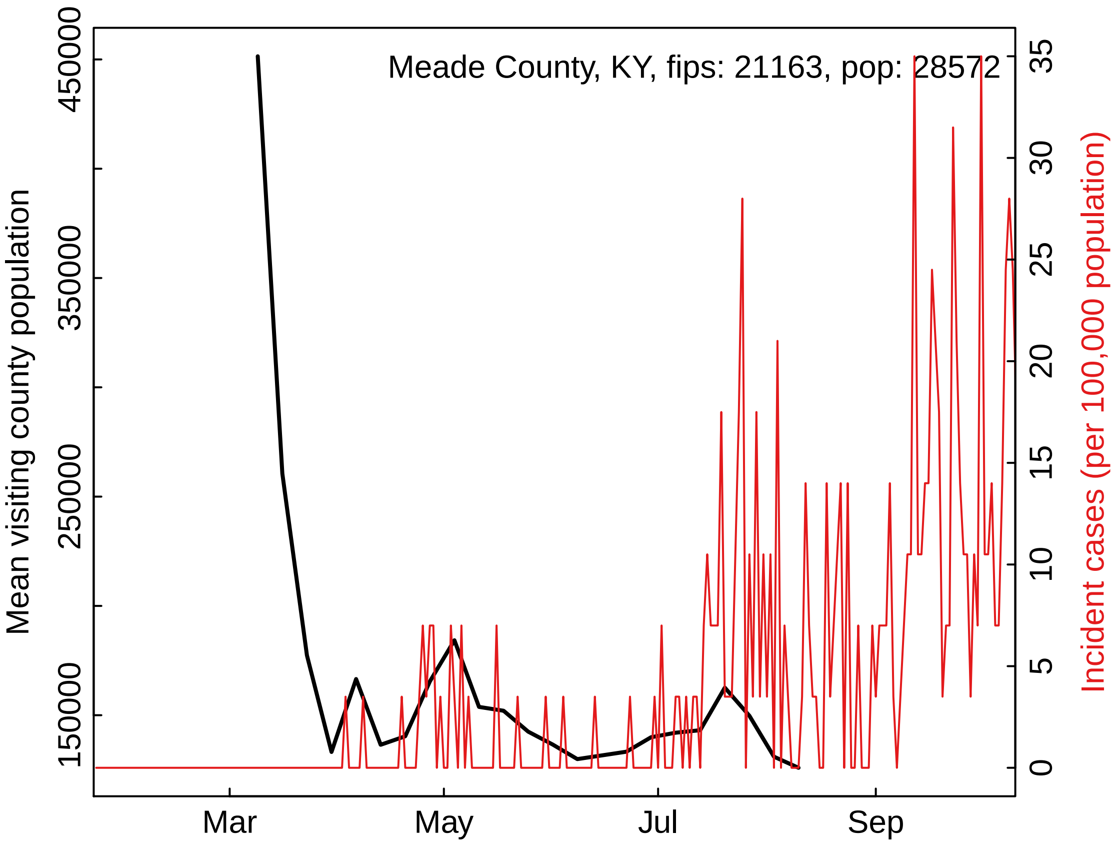
Meade County, KY, fips: 21163, pop: 28572
Mean visiting county population
150000
250000
350000
450000
Incident cases (per 100,000 population)
0
5
10
15
20
25
30
35
Mar
May
Jul
Sep

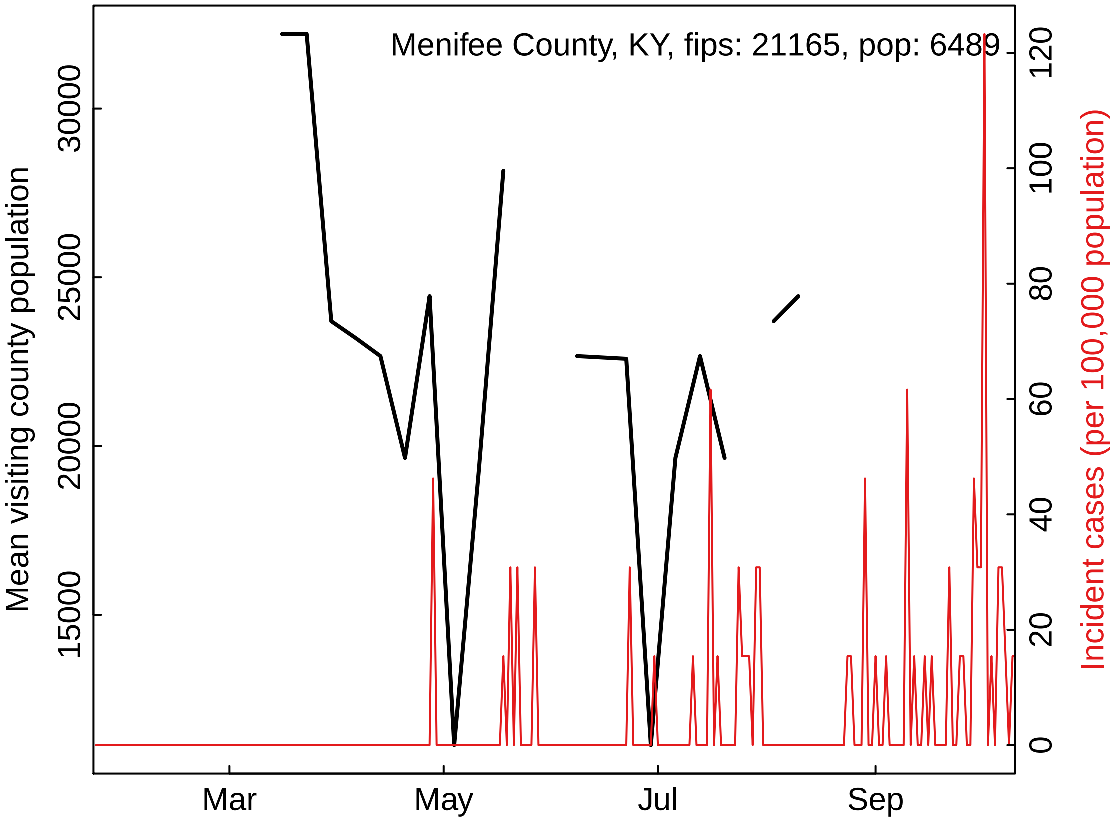

Menifee County, KY, fips: 21165, pop: 6489
Mean visiting county population
15000
20000
25000
30000
Incident cases (per 100,000 population)
0
20
40
60
80
100
120
Mar
May
Jul
Sep

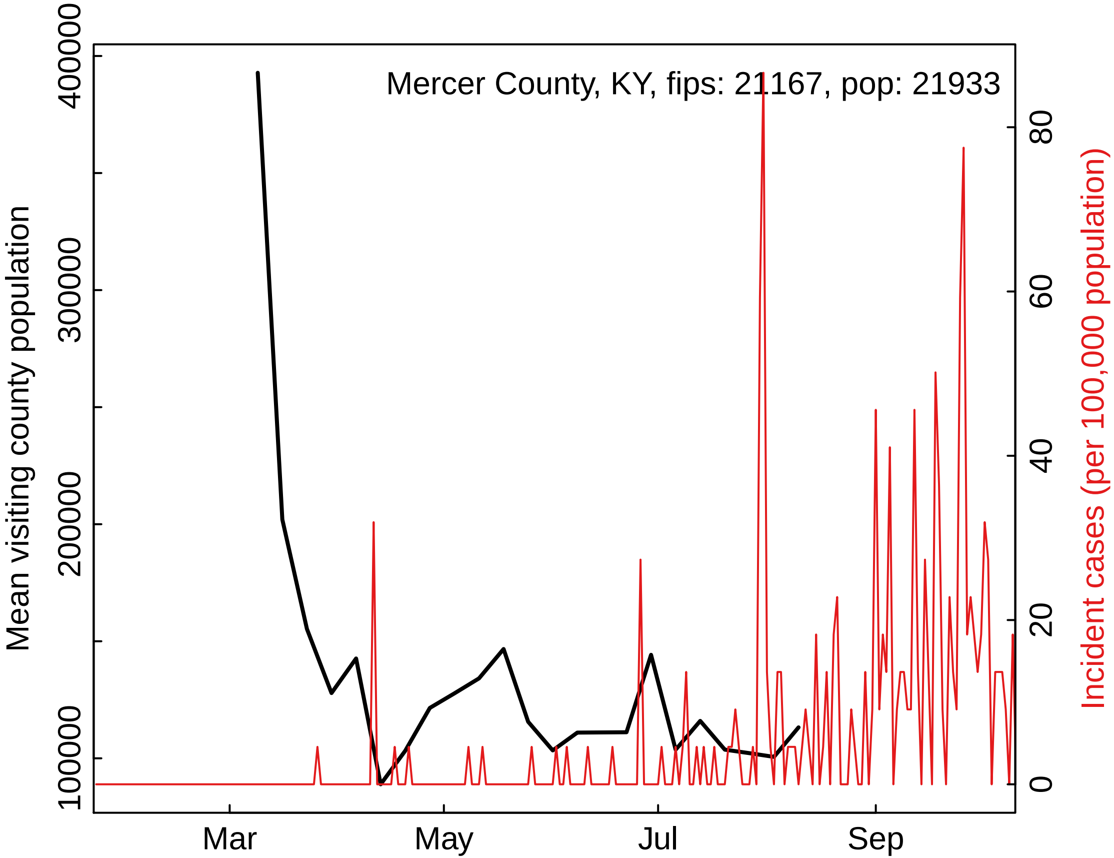

Mercer County, KY, fips: 21167, pop: 21933
Mean visiting county population
100000
200000
300000
400000
Incident cases (per 100,000 population)
0
20
40
60
80
Mar
May
Jul
Sep

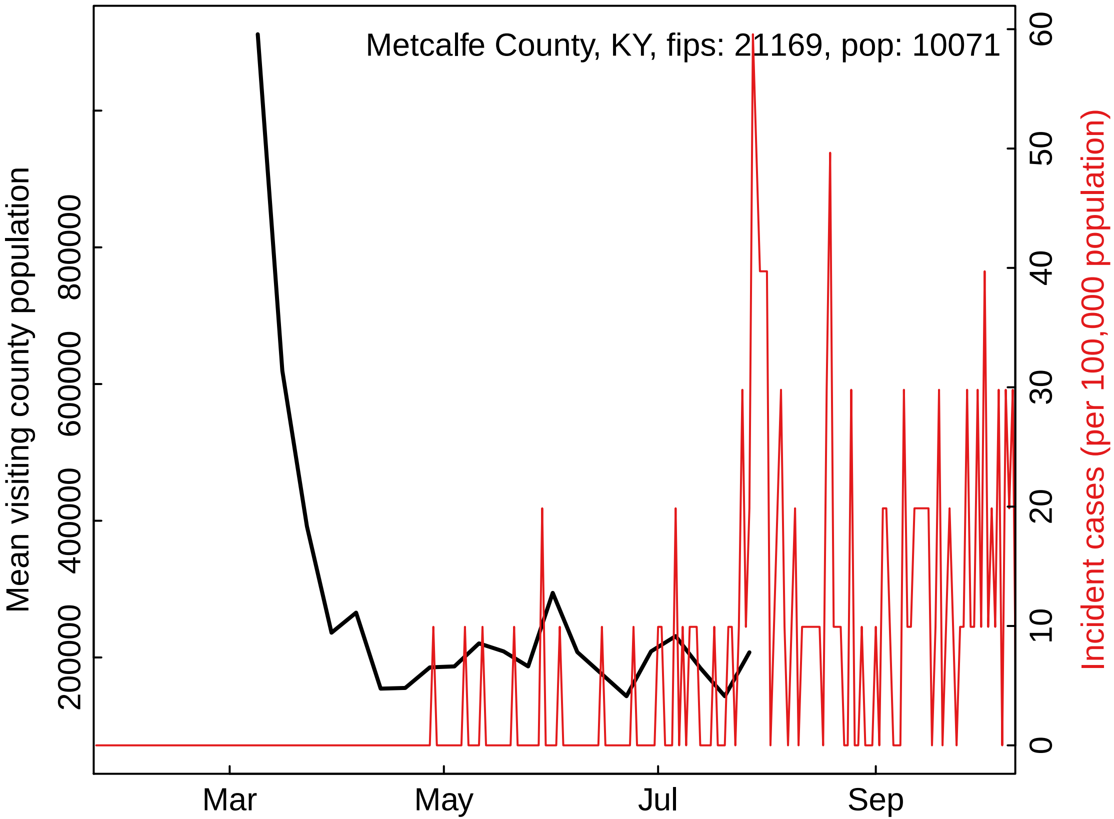
Metcalfe County, KY, fips: 21169, pop: 10071
Mean visiting county population
200000
400000
600000
800000
Incident cases (per 100,000 population)
0
10
20
30
40
50
60
Mar
May
Jul
Sep

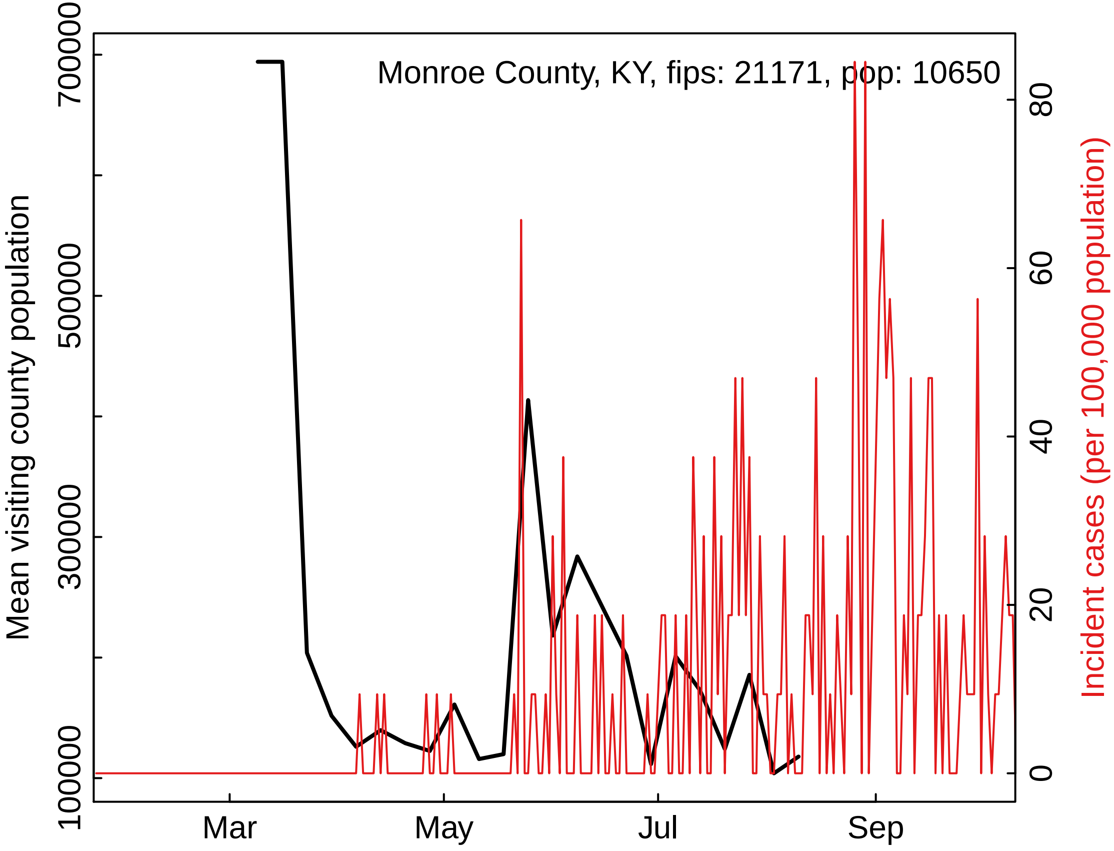

Monroe County, KY, fips: 21171, pop: 10650
Mean visiting county population
100000
300000
500000
700000
Incident cases (per 100,000 population)
0
20
40
60
80
Mar
May
Jul
Sep

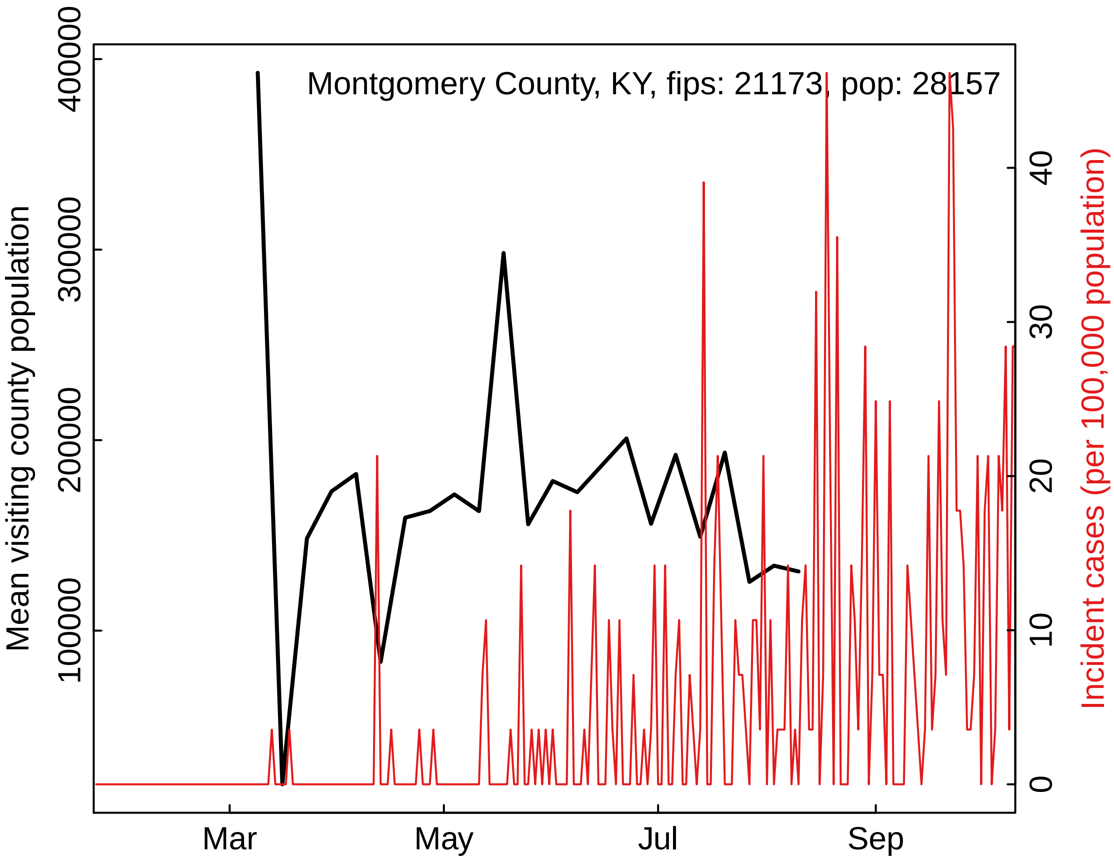

Montgomery County, KY, fips: 21173, pop: 28157
Mean visiting county population
100000
200000
300000
400000
Incident cases (per 100,000 population)
0
10
20
30
40
Mar
May
Jul
Sep

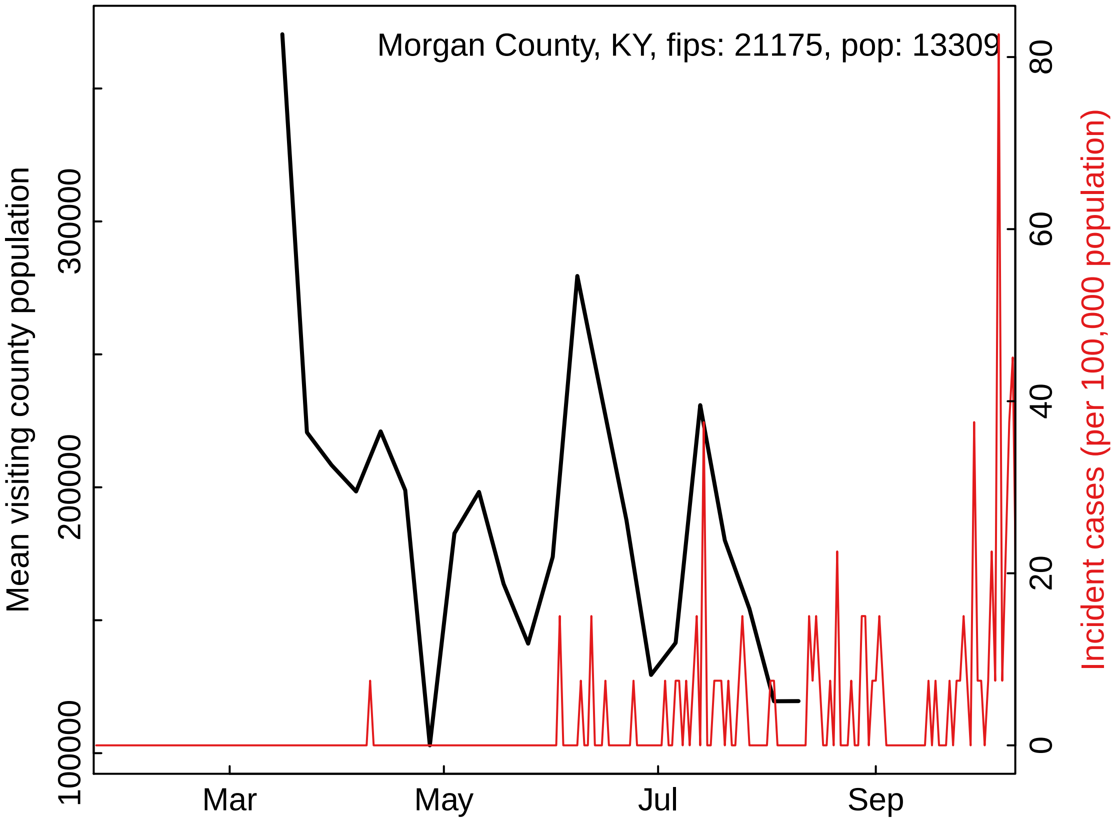

Morgan County, KY, fips: 21175, pop: 13309
Mean visiting county population
100000
200000
300000
Mar
May
Jul
Sep
0
20
40
60
80
Incident cases (per 100,000 population)

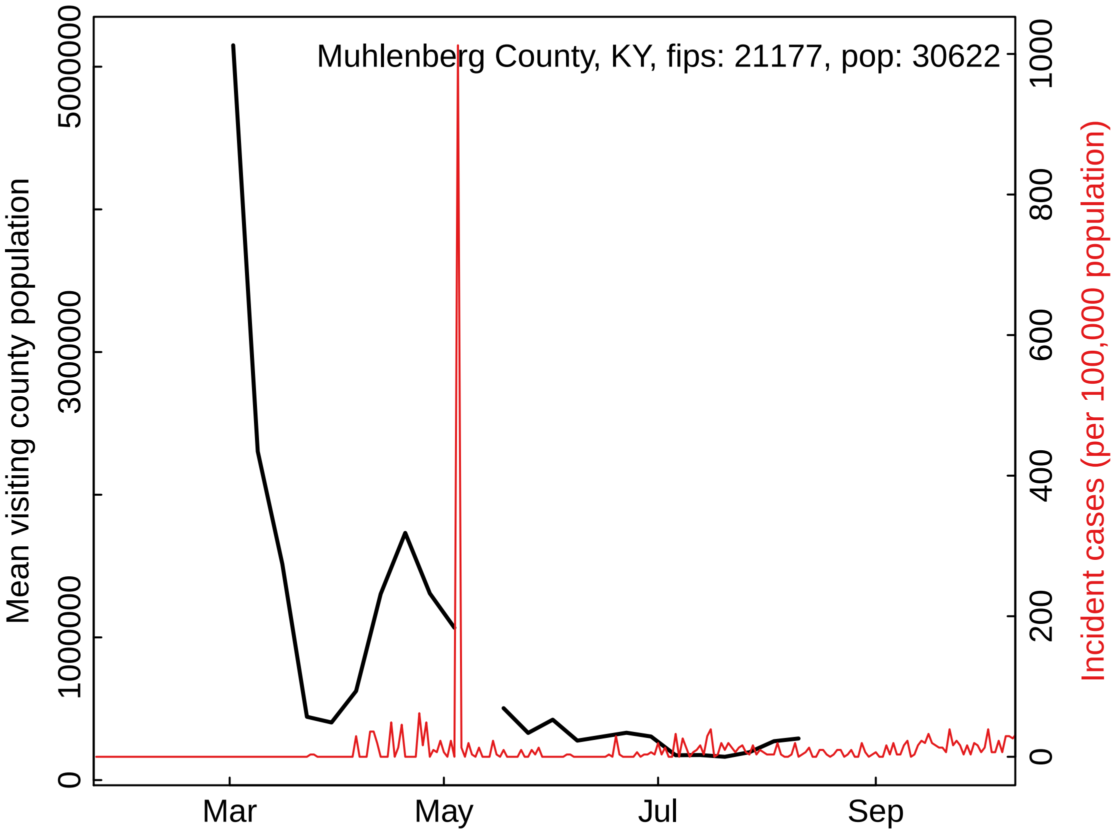
Muhlenberg County, KY, fips: 21177, pop: 30622
Mean visiting county population
0
1000000
3000000
5000000
Incident cases (per 100,000 population)
0
200
400
600
800
1000
Mar
May
Jul
Sep

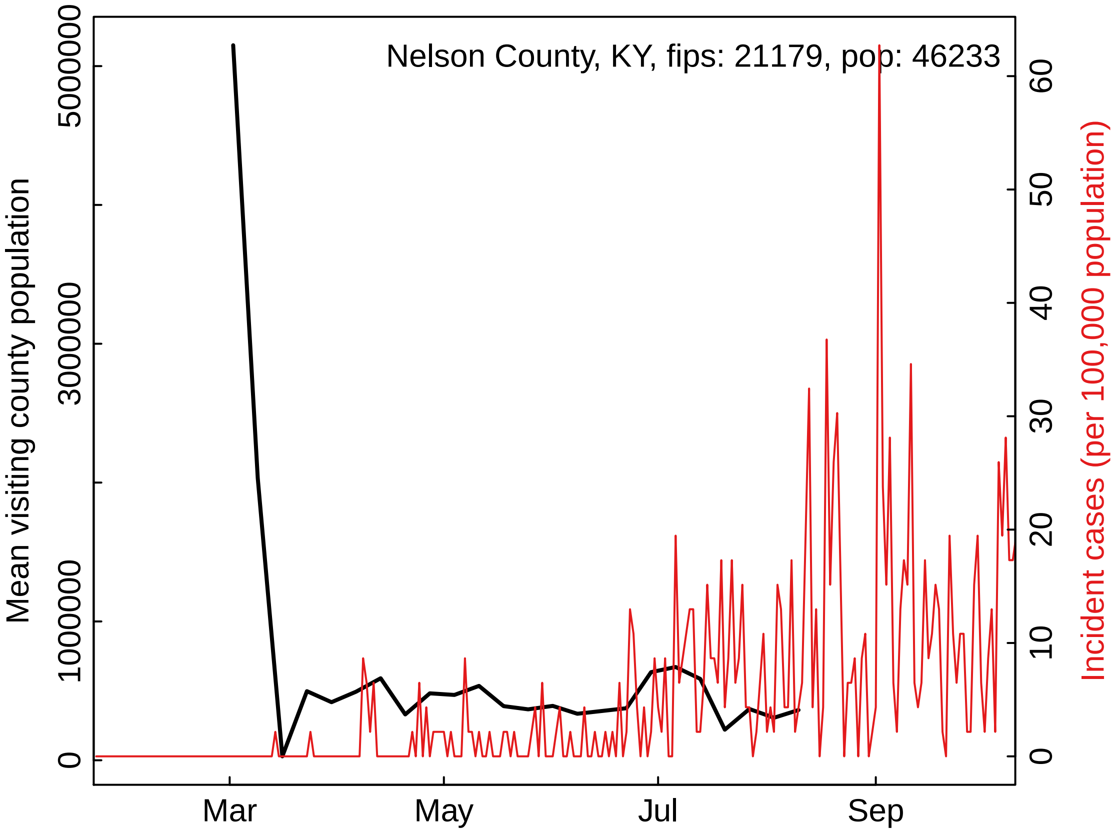

Nelson County, KY, fips: 21179, pop: 46233
Mean visiting county population
0
1000000
3000000
5000000
Incident cases (per 100,000 population)
0
10
20
30
40
50
60
Mar
May
Jul
Sep

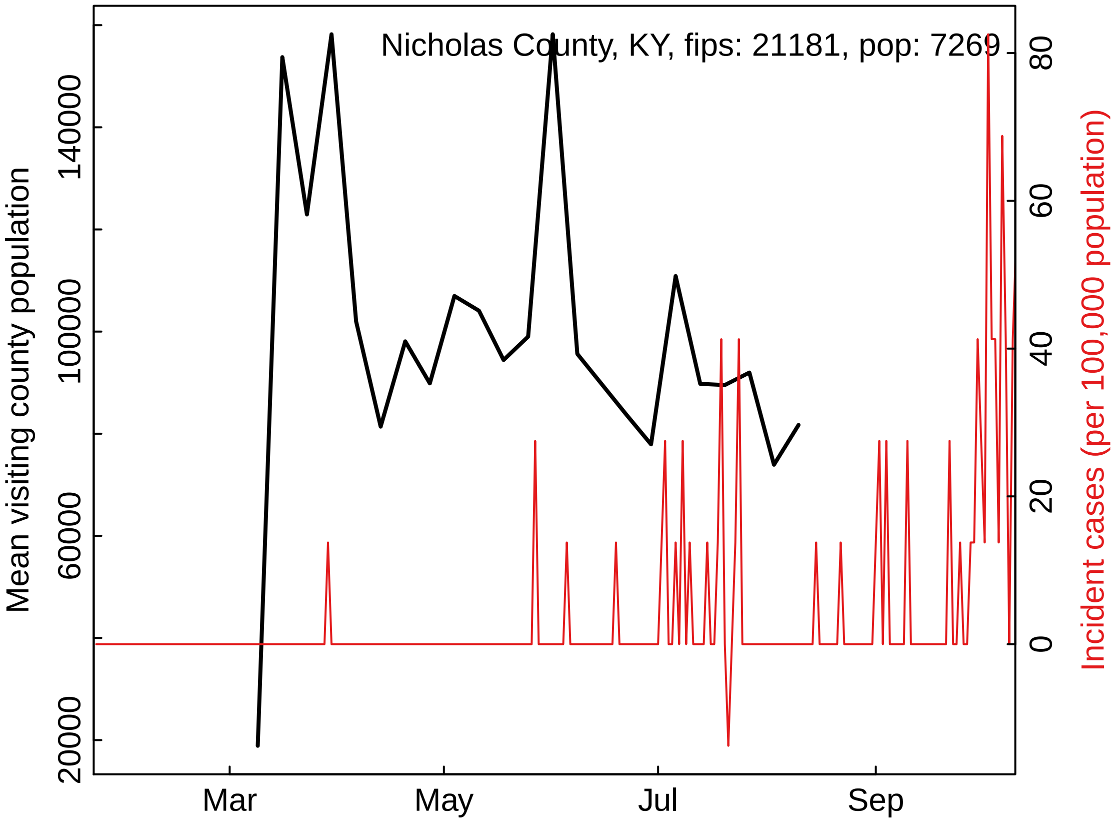

Nicholas County, KY, fips: 21181, pop: 7269
Mean visiting county population
20000
60000
100000
140000
Mar
May
Jul
Sep
0
20
40
60
80
Incident cases (per 100,000 population)

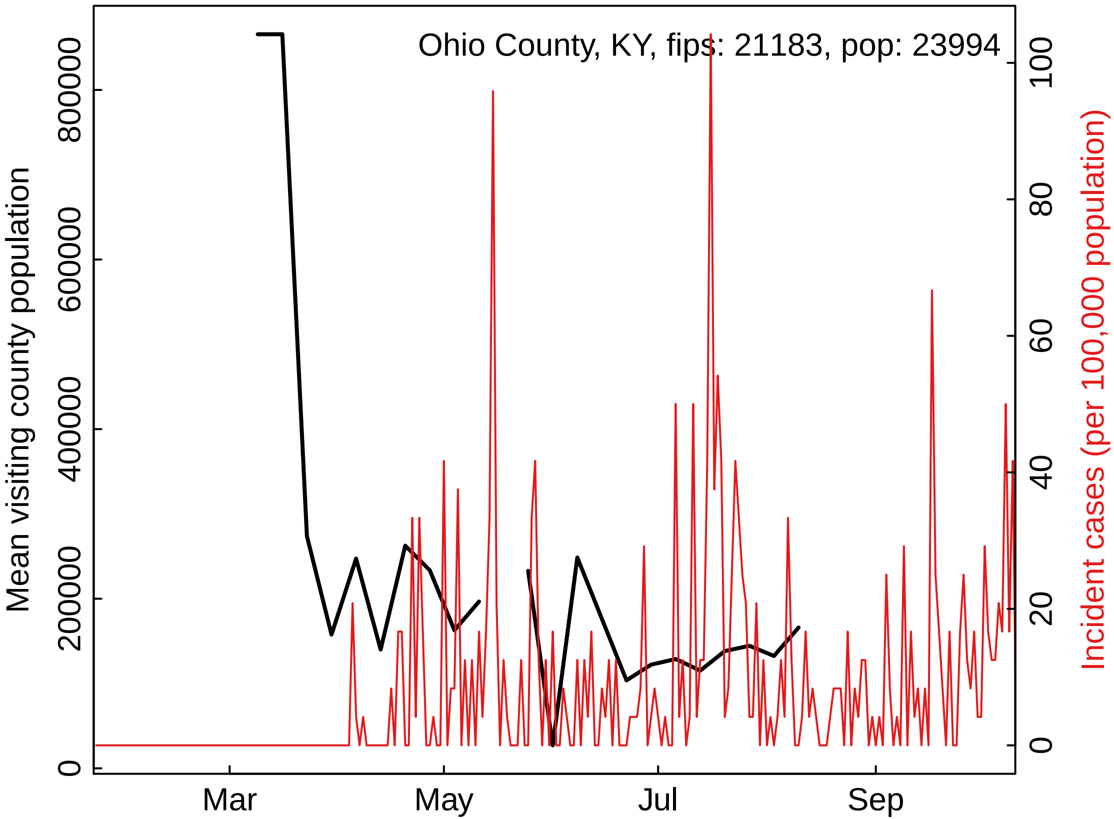
Ohio County, KY, fips: 21183, pop: 23994
Mean visiting county population
0
200000
400000
600000
800000
Incident cases (per 100,000 population)
0
20
40
60
80
100
Mar
May
Jul
Sep

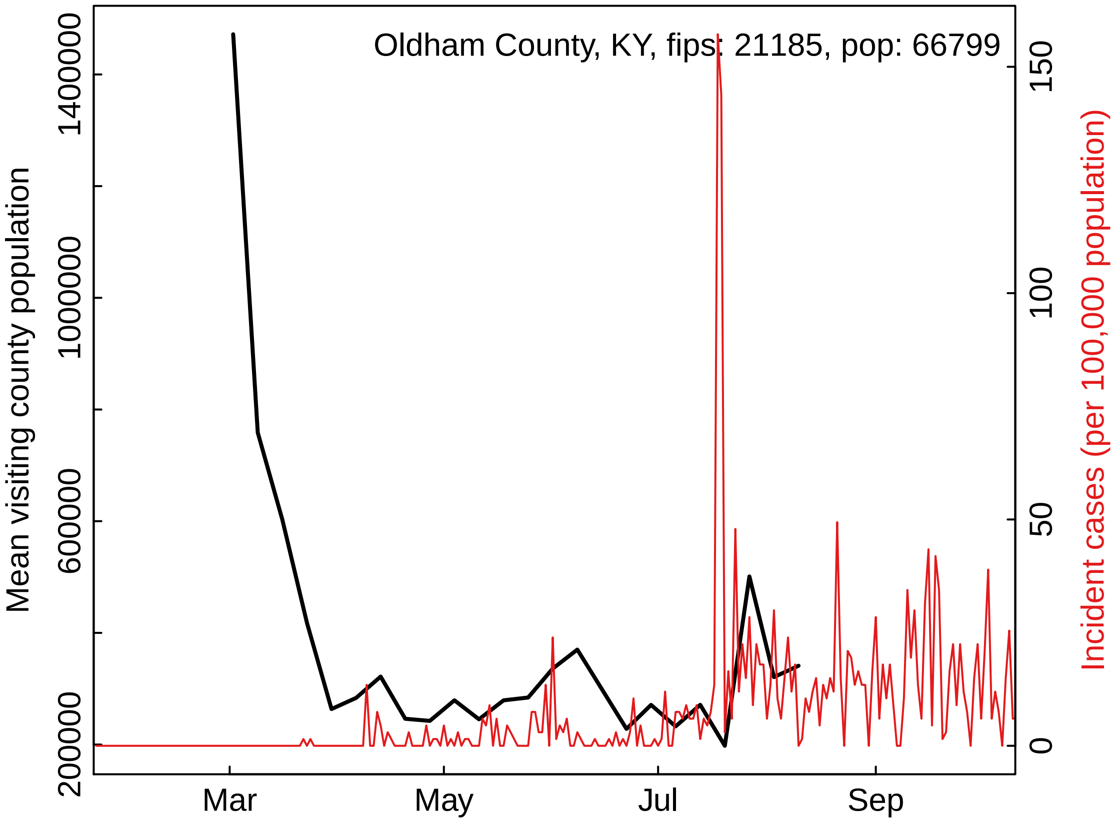

Oldham County, KY, fips: 21185, pop: 66799
Mean visiting county population
200000
600000
1000000
1400000
Incident cases (per 100,000 population)
0
50
100
150
Mar
May
Jul
Sep

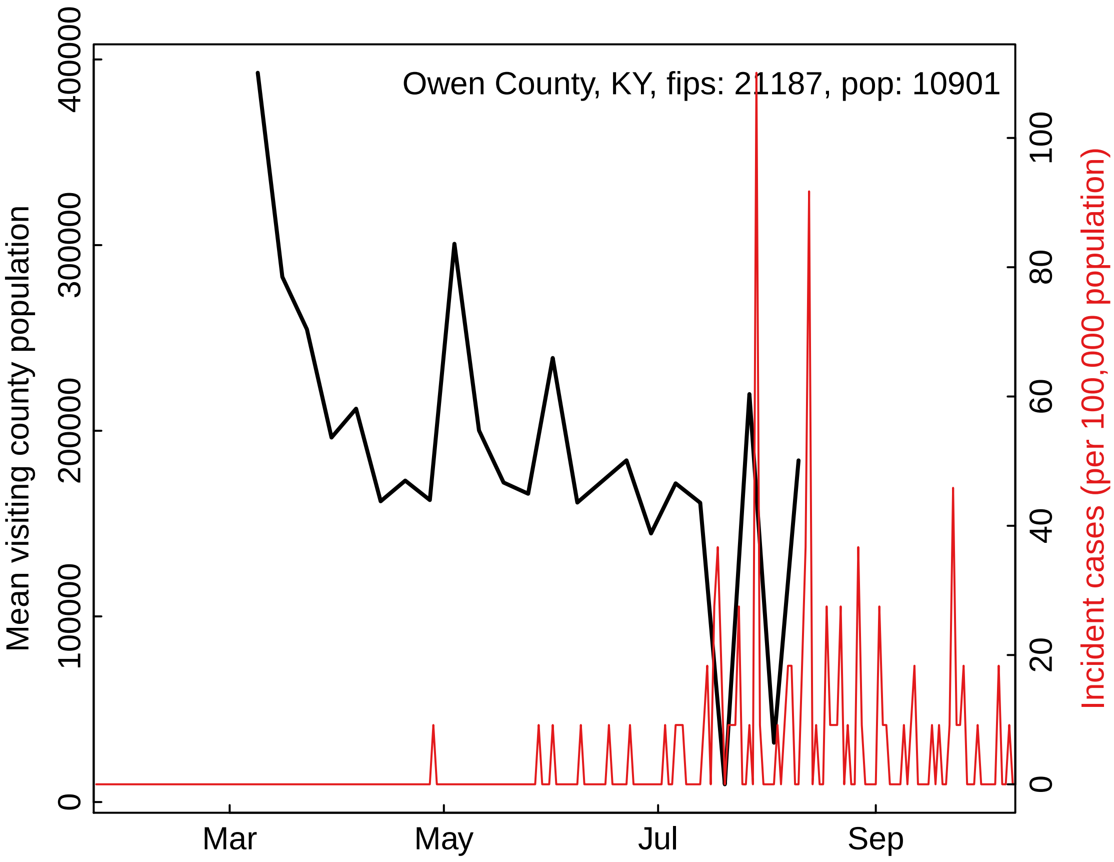

Owen County, KY, fips: 21187, pop: 10901
Mean visiting county population
0
100000
200000
300000
400000
Incident cases (per 100,000 population)
0
20
40
60
80
100
Mar
May
Jul
Sep

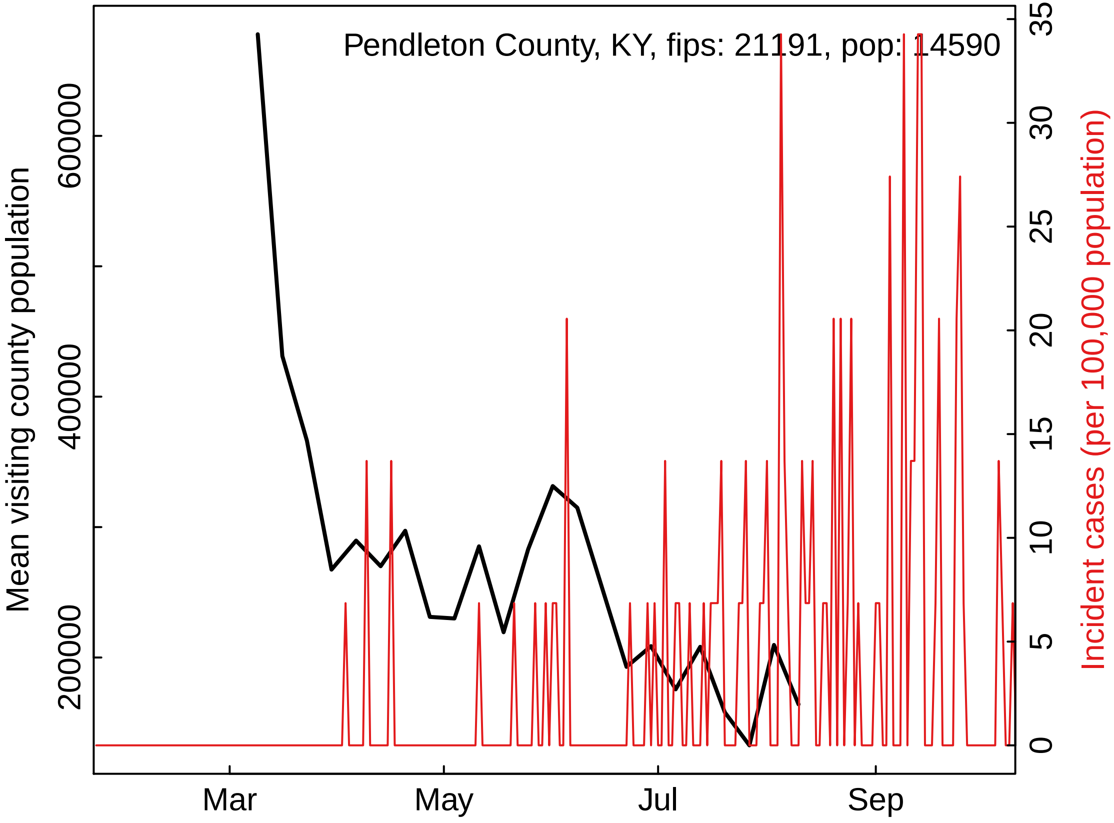

Pendleton County, KY, fips: 21191, pop: 14590
Mean visiting county population
200000
400000
600000
Incident cases (per 100,000 population)
0
5
10
15
20
25
30
35
Mar
May
Jul
Sep

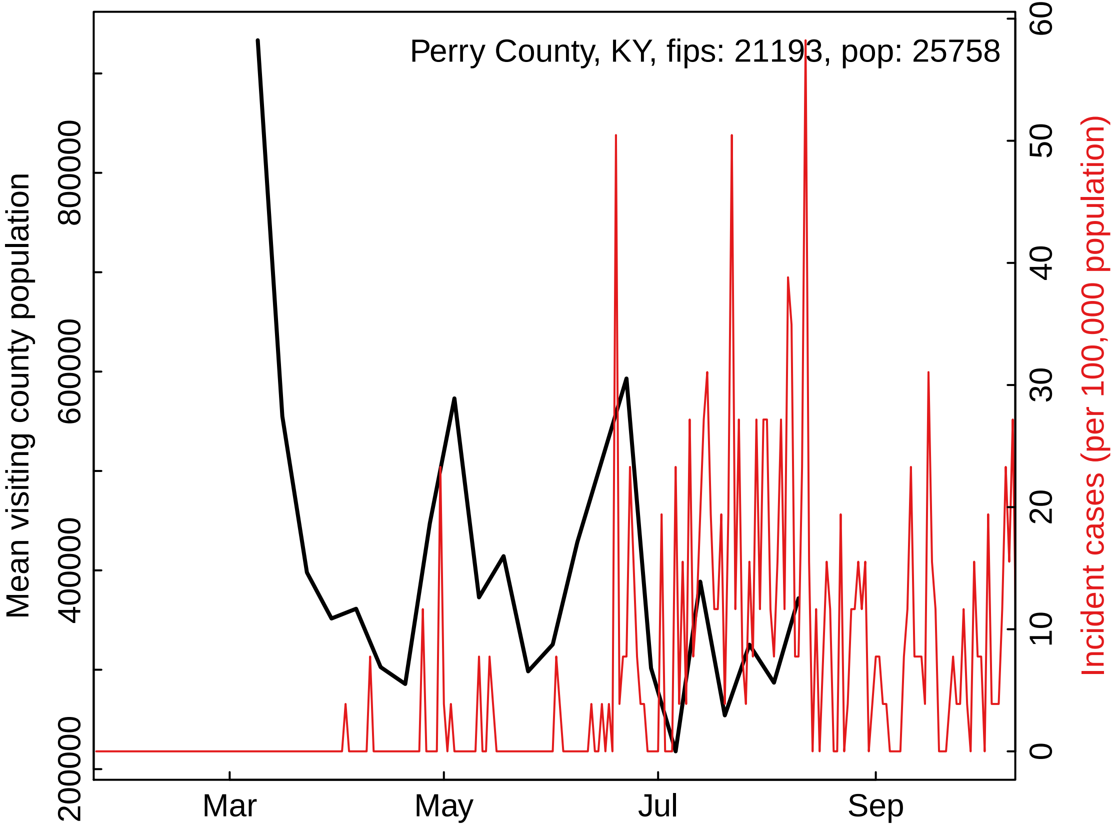

Perry County, KY, fips: 21193, pop: 25758
Mean visiting county population
200000
400000
600000
800000
Mar
May
Jul
Sep
Incident cases (per 100,000 population)
0
10
20
30
40
50
60

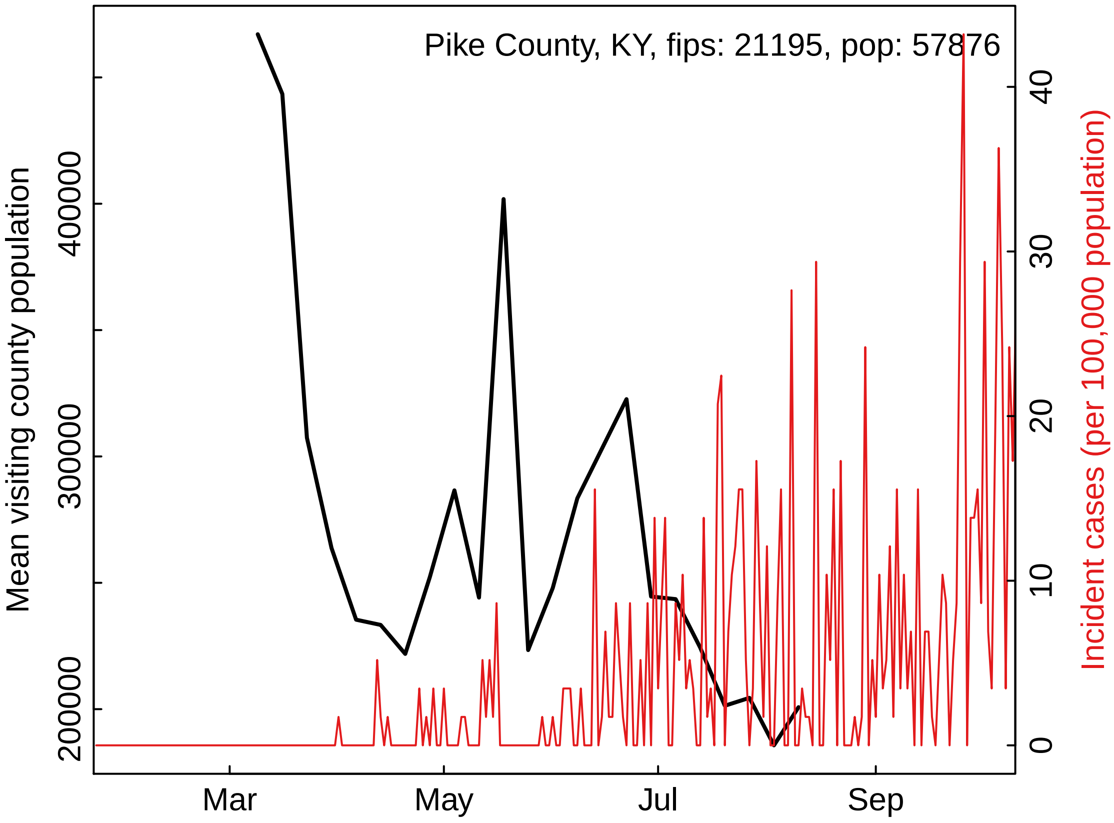

Pike County, KY, fips: 21195, pop: 57876
Mean visiting county population
200000
300000
400000
Incident cases (per 100,000 population)
0
10
20
30
40
Mar
May
Jul
Sep

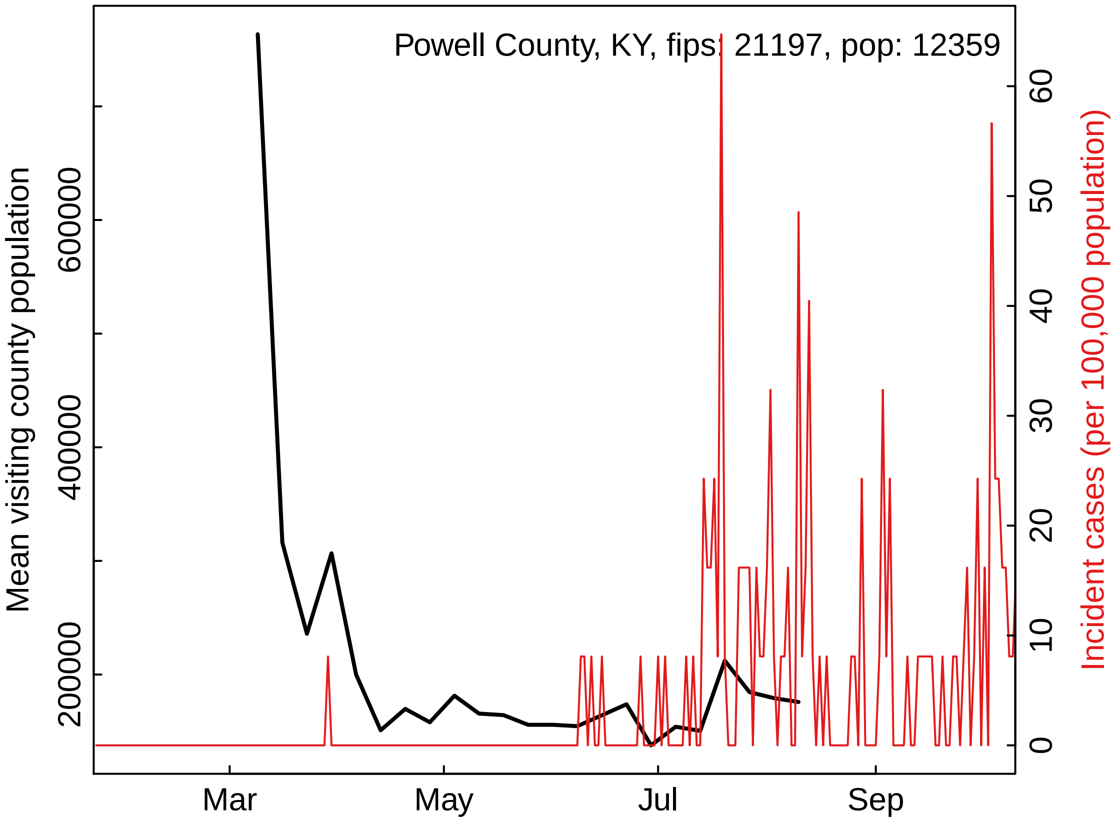
Powell County, KY, fips: 21197, pop: 12359
Mean visiting county population
200000
400000
600000
Incident cases (per 100,000 population)
0
10
20
30
40
50
60
Mar
May
Jul
Sep

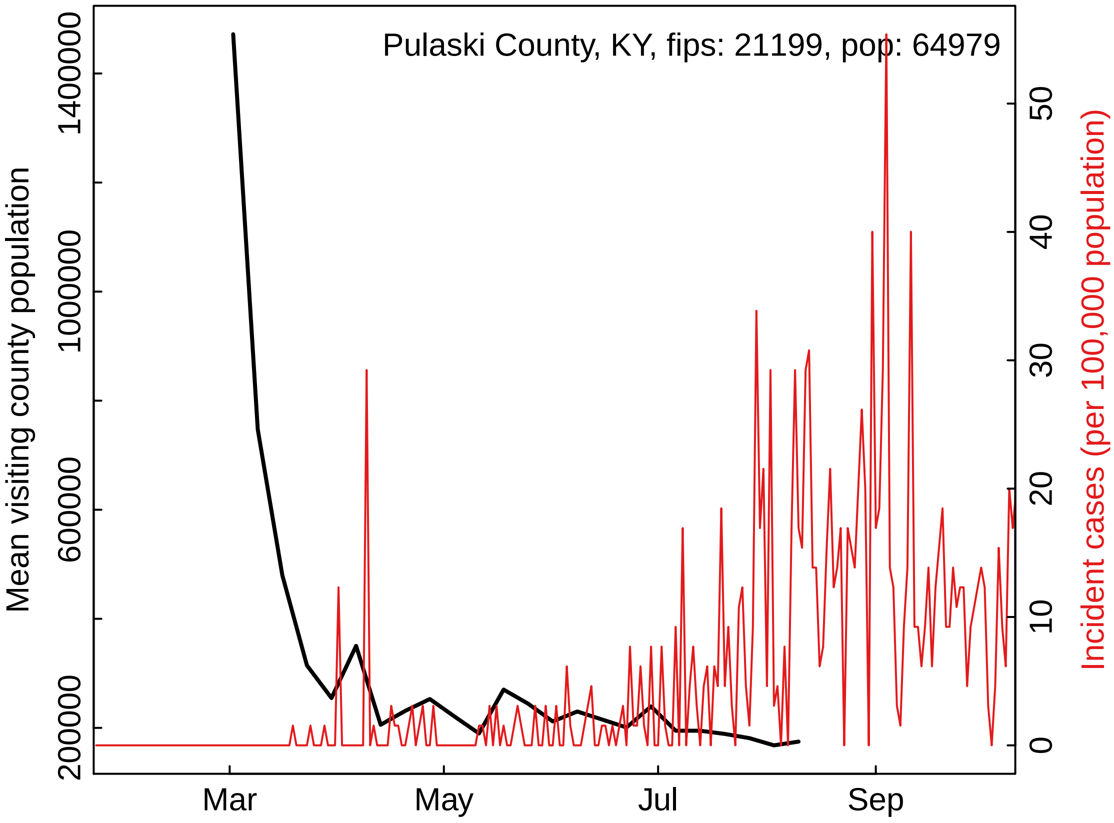

Pulaski County, KY, fips: 21199, pop: 64979
Mean visiting county population
200000
600000
1000000
1400000
Incident cases (per 100,000 population)
0
10
20
30
40
50
Mar
May
Jul
Sep

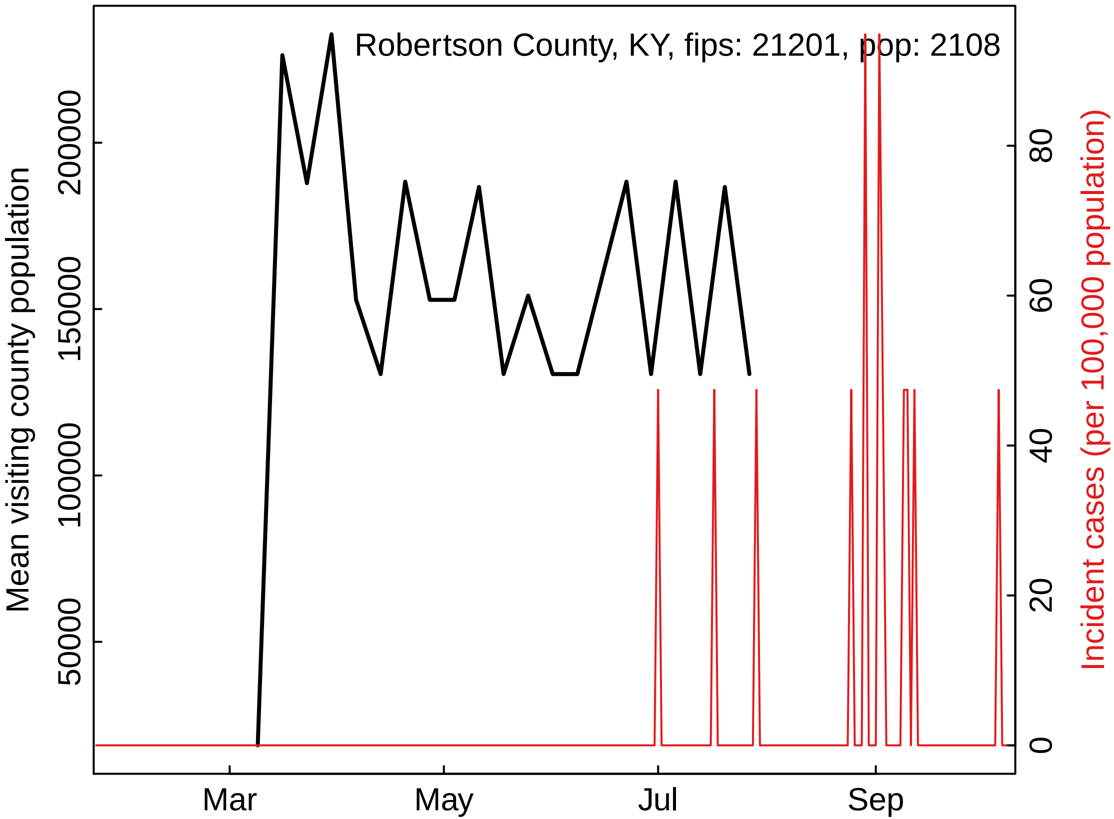
Robertson County, KY, fips: 21201, pop: 2108
Mean visiting county population
50000
100000
150000
200000
Incident cases (per 100,000 population)
0
20
40
60
80
Mar
May
Jul
Sep

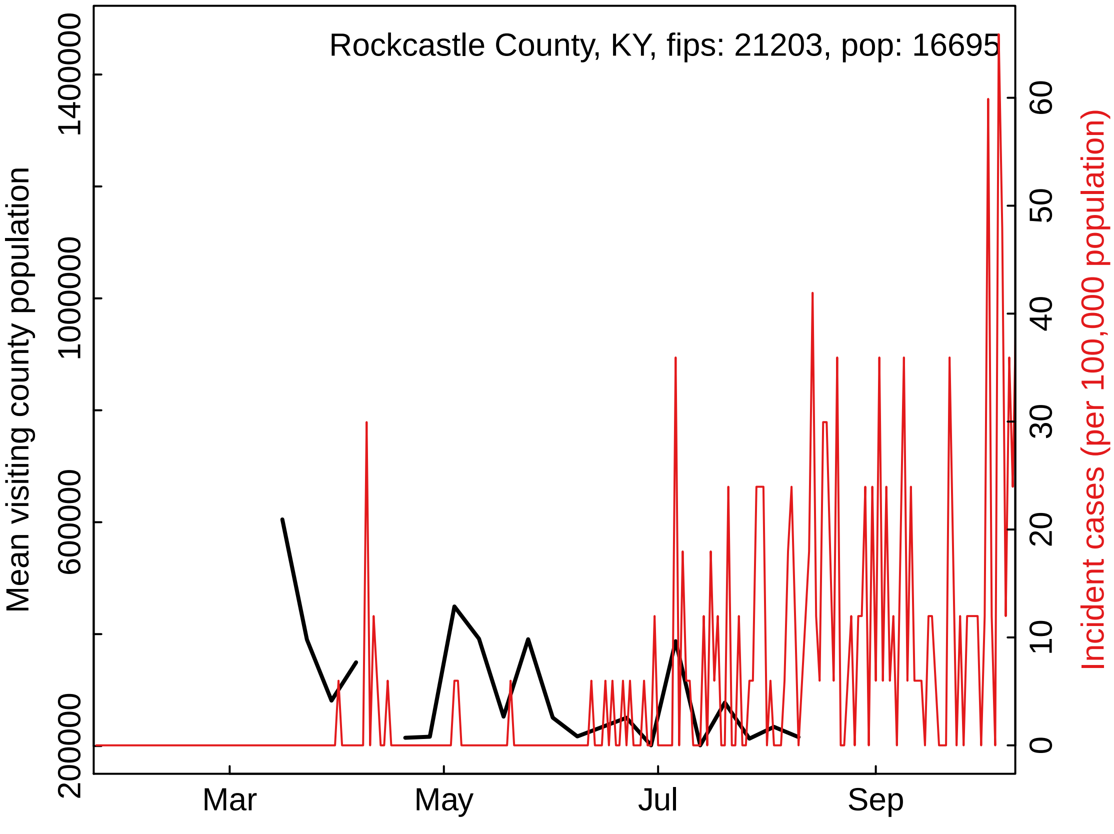
Rockcastle County, KY, fips: 21203, pop: 16695
Mean visiting county population
200000
600000
1000000
1400000
Mar
May
Jul
Sep
0
10
20
30
40
50
60
Incident cases (per 100,000 population)

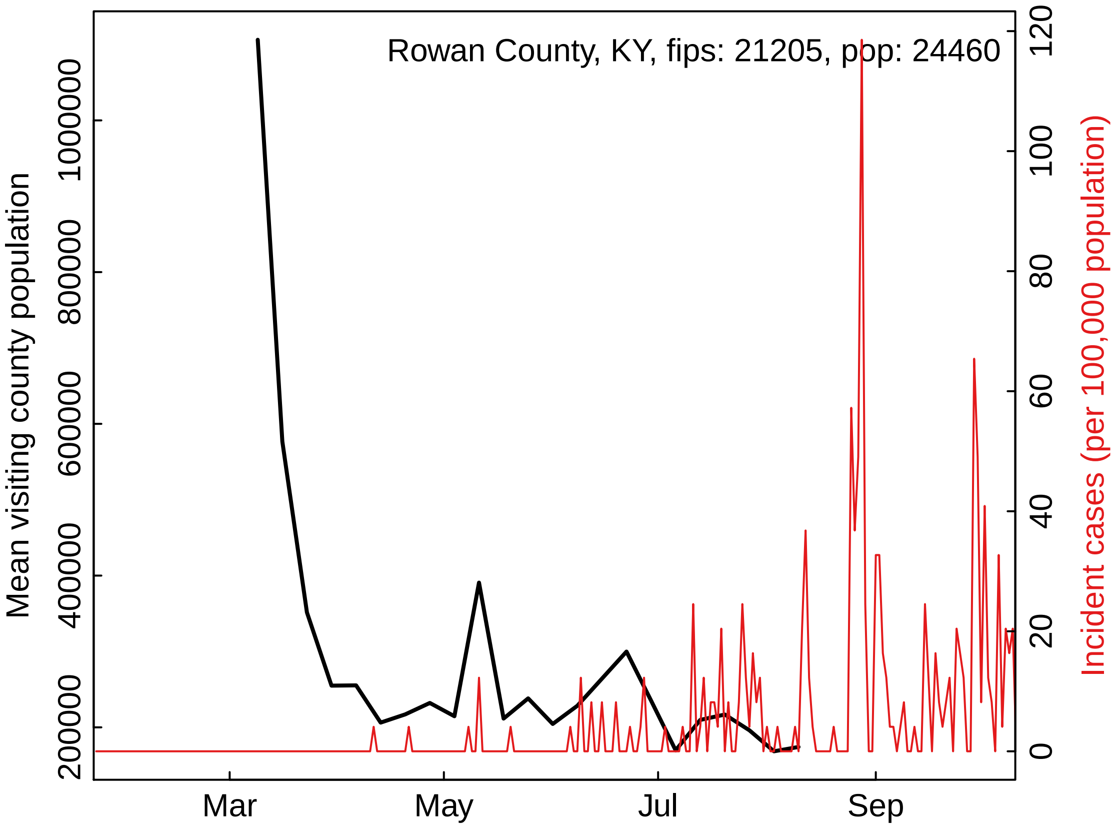

Rowan County, KY, fips: 21205, pop: 24460
Mean visiting county population
200000
400000
600000
800000
1000000
Incident cases (per 100,000 population)
0
20
40
60
80
100
120
Mar
May
Jul
Sep

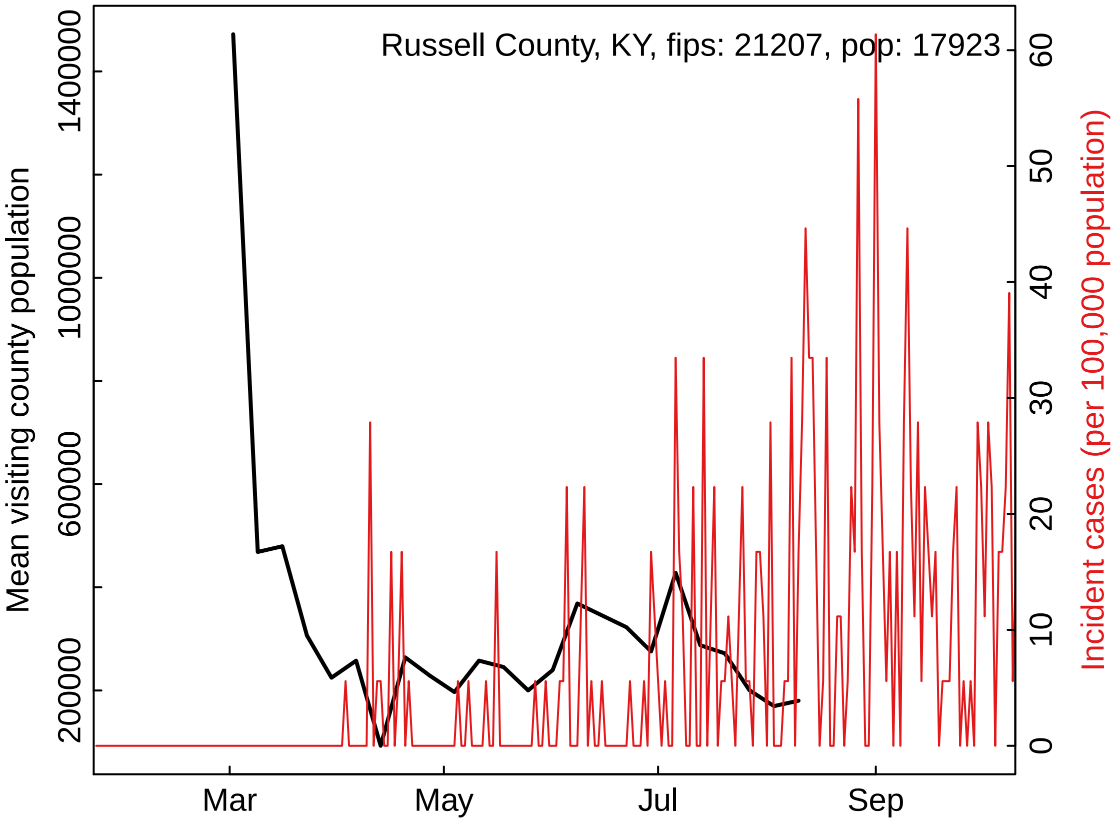

Russell County, KY, fips: 21207, pop: 17923
Mean visiting county population
Incident cases (per 100,000 population)
200000
600000
1000000
1400000
0
10
20
30
40
50
60
Mar
May
Jul
Sep

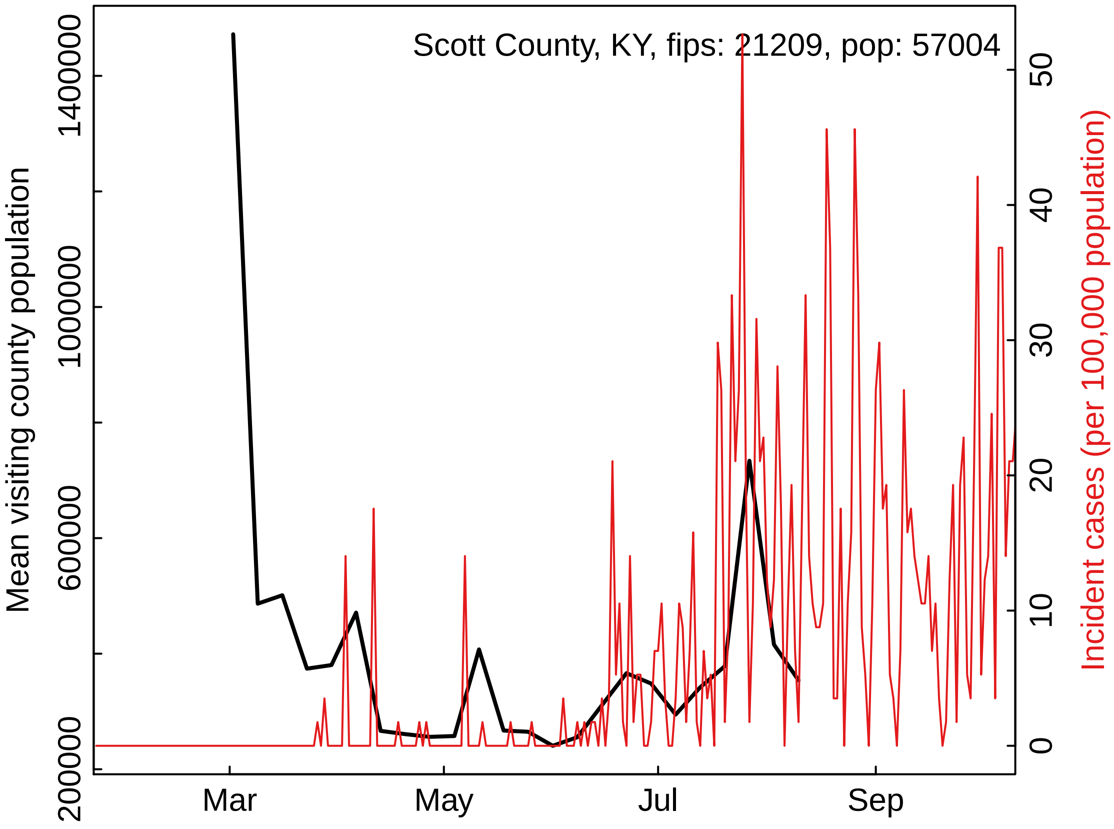

Scott County, KY, fips: 21209, pop: 57004
Mean visiting county population
200000
600000
1000000
1400000
Mar
May
Jul
Sep
0
10
20
30
40
50
Incident cases (per 100,000 population)

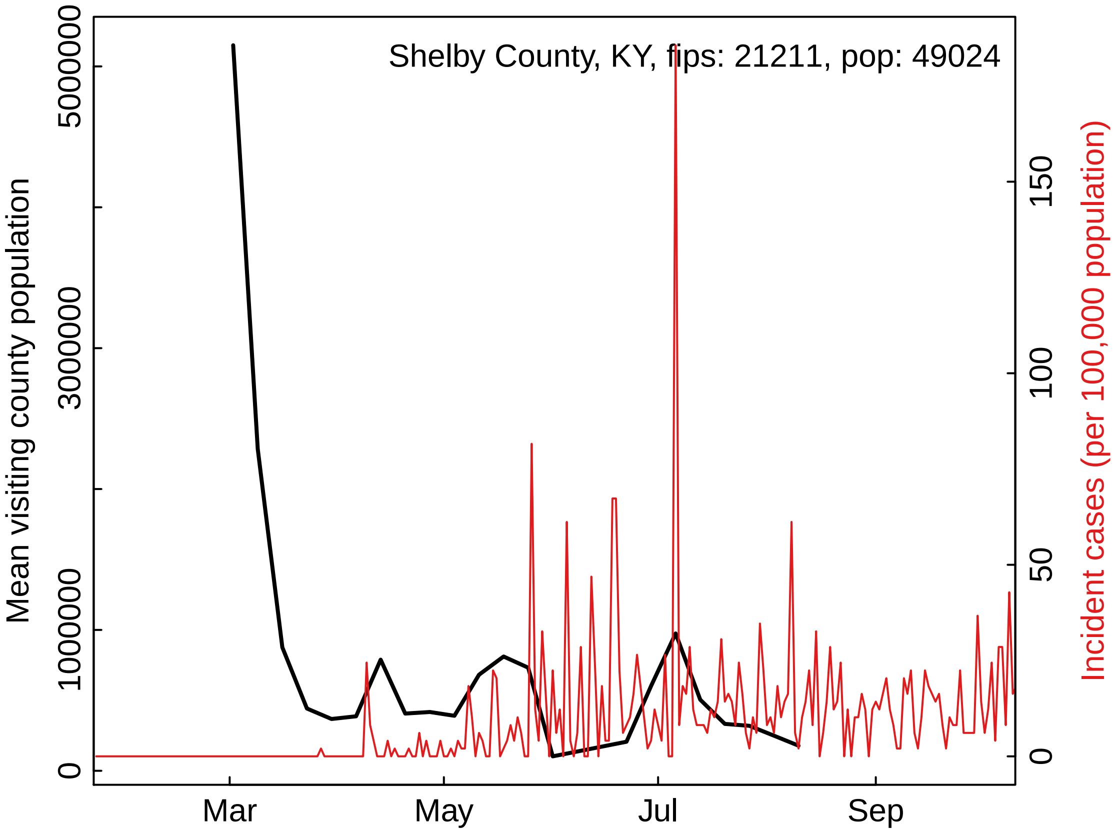

Shelby County, KY, fips: 21211, pop: 49024
Mean visiting county population
0
1000000
3000000
5000000
Incident cases (per 100,000 population)
0
50
100
150
Mar
May
Jul
Sep

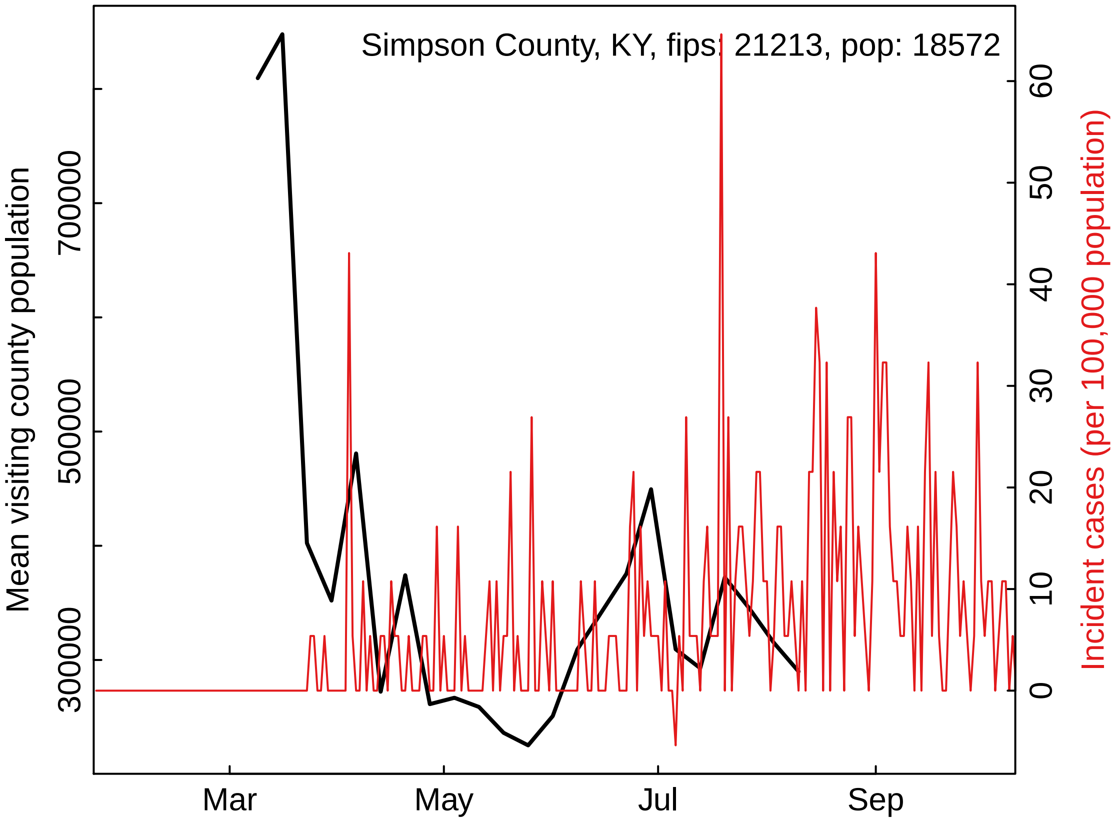

Simpson County, KY, fips: 21213, pop: 18572
Mean visiting county population
300000
500000
700000
Incident cases (per 100,000 population)
0
10
20
30
40
50
60
Mar
May
Jul
Sep

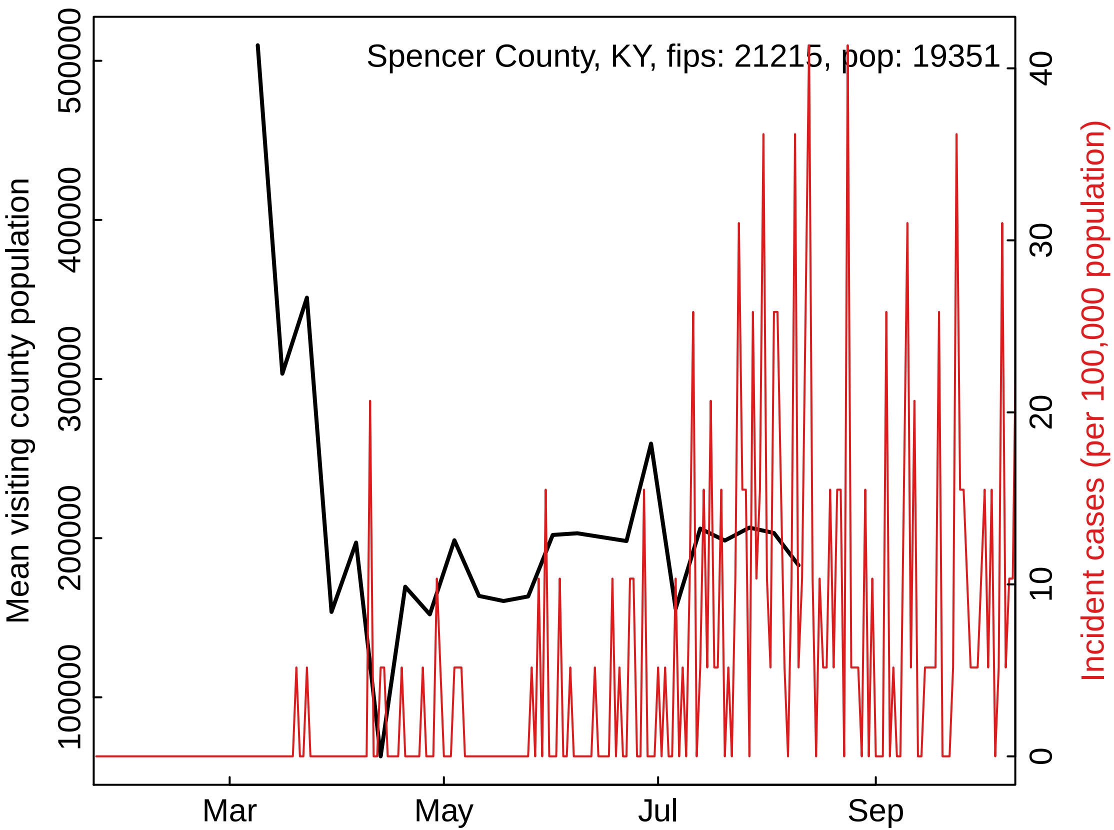

Spencer County, KY, fips: 21215, pop: 19351
Mean visiting county population
100000
200000
300000
400000
500000
Incident cases (per 100,000 population)
0
10
20
30
40
Mar
May
Jul
Sep

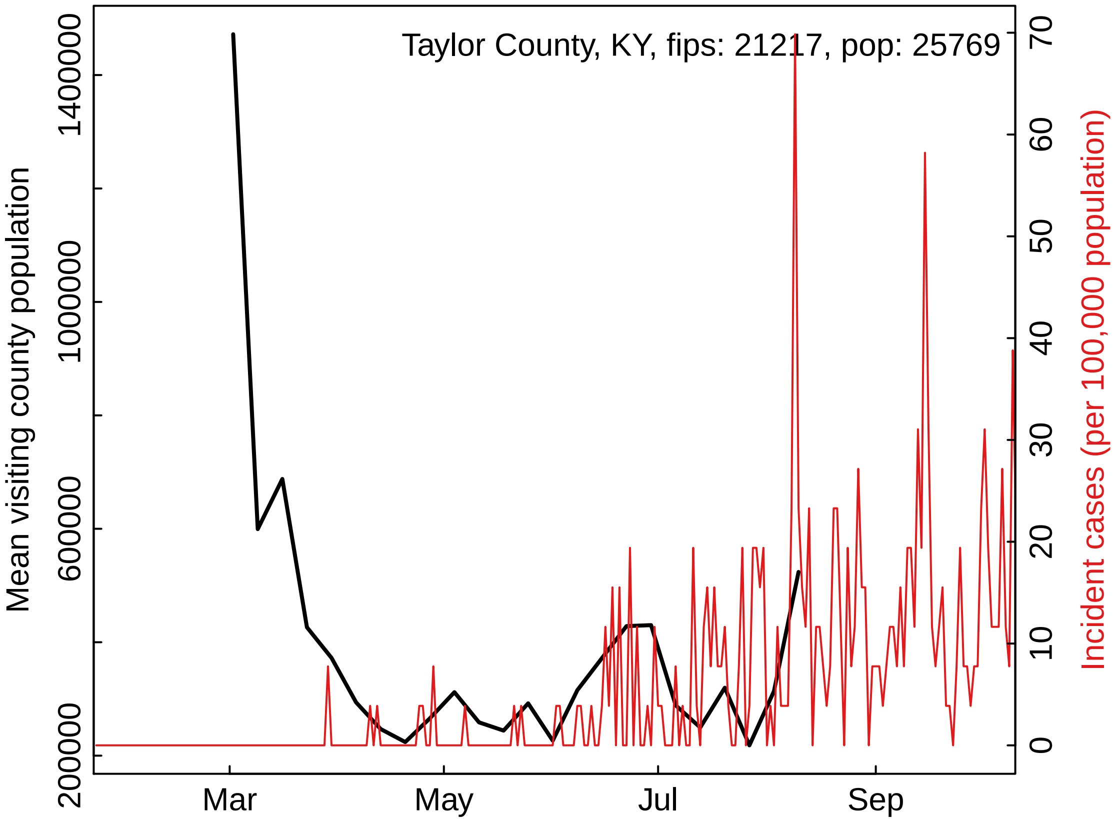

Taylor County, KY, fips: 21217, pop: 25769
Mean visiting county population
200000
600000
1000000
1400000
Incident cases (per 100,000 population)
0
10
20
30
40
50
60
70
Mar
May
Jul
Sep

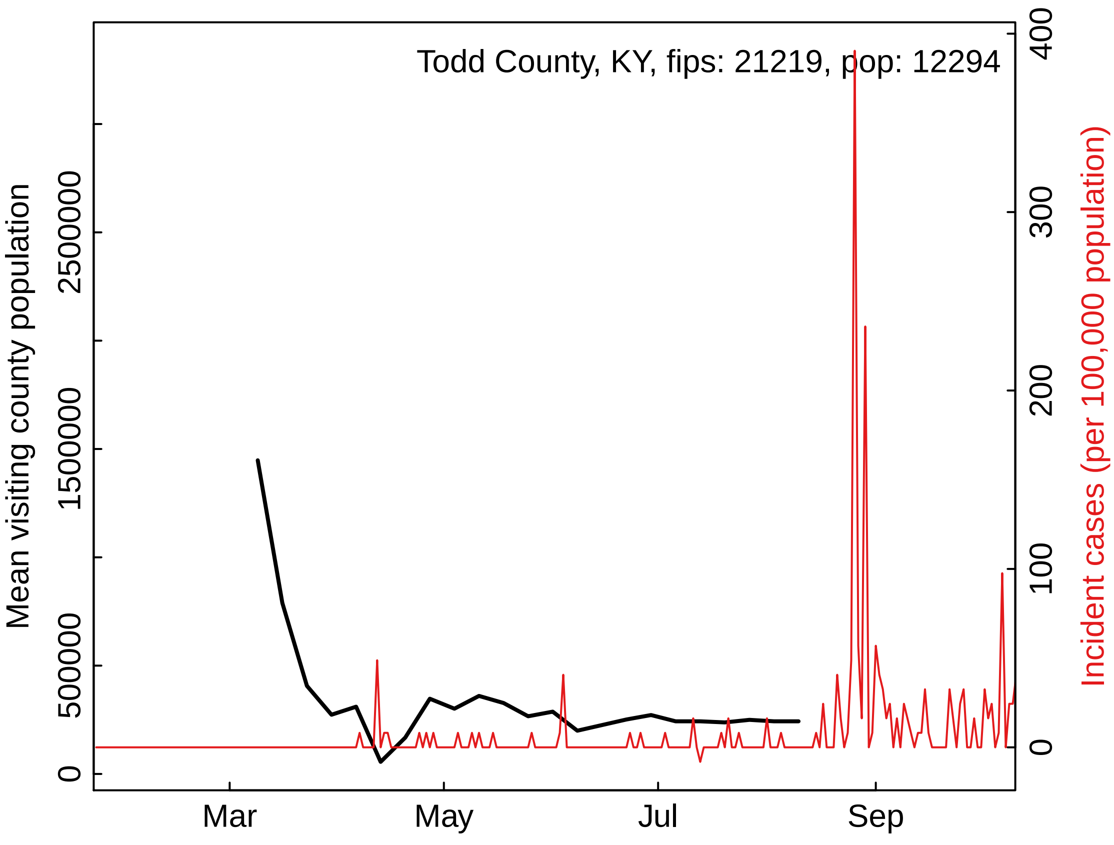

Todd County, KY, fips: 21219, pop: 12294
Mean visiting county population
0
500000
1500000
2500000
Incident cases (per 100,000 population)
0
100
200
300
400
Mar
May
Jul
Sep

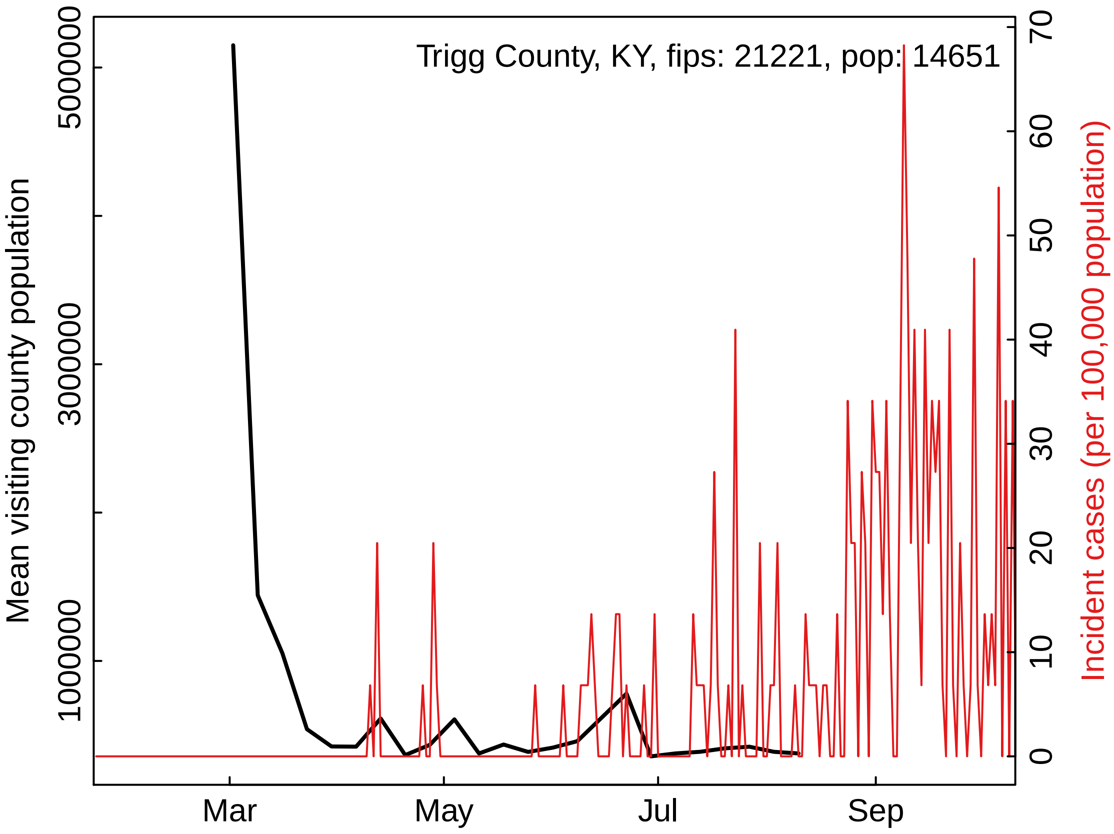

Trigg County, KY, fips: 21221, pop: 14651
Mean visiting county population
1000000
3000000
5000000
Incident cases (per 100,000 population)
0
10
20
30
40
50
60
70
Mar
May
Jul
Sep

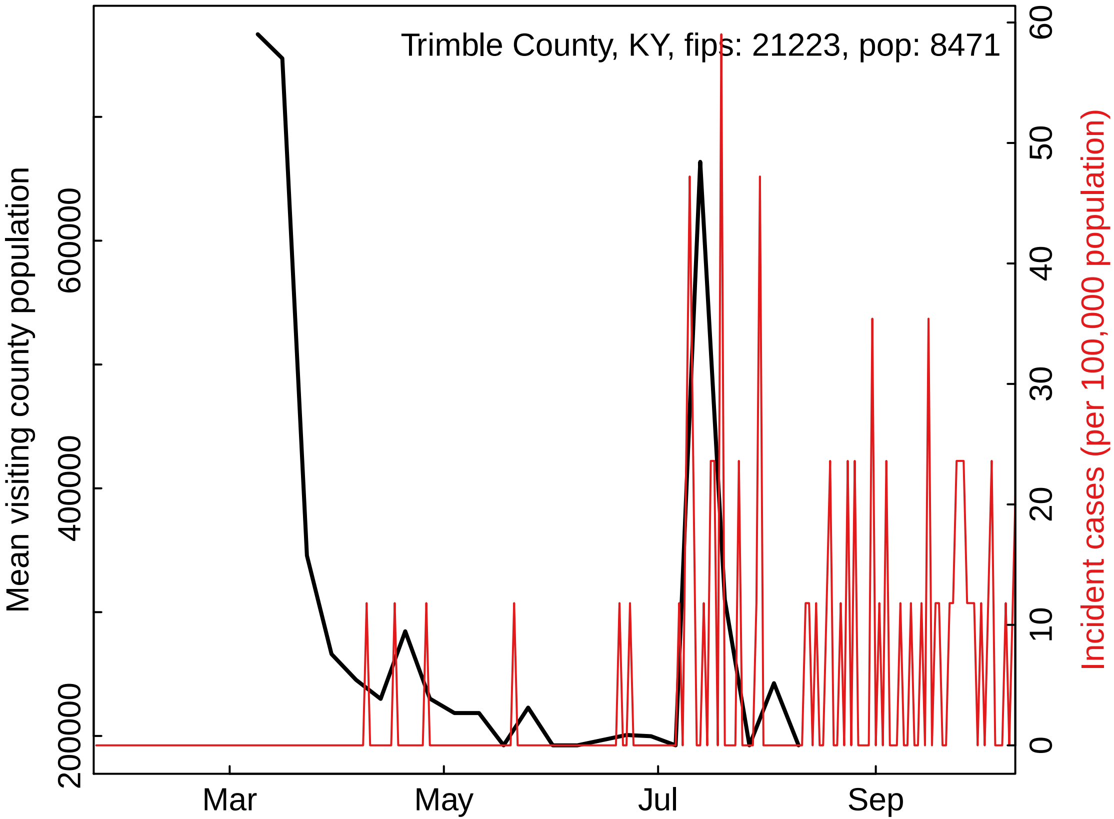

Trimble County, KY, fips: 21223, pop: 8471
Mean visiting county population
200000
400000
600000
Incident cases (per 100,000 population)
0
10
20
30
40
50
60
Mar
May
Jul
Sep

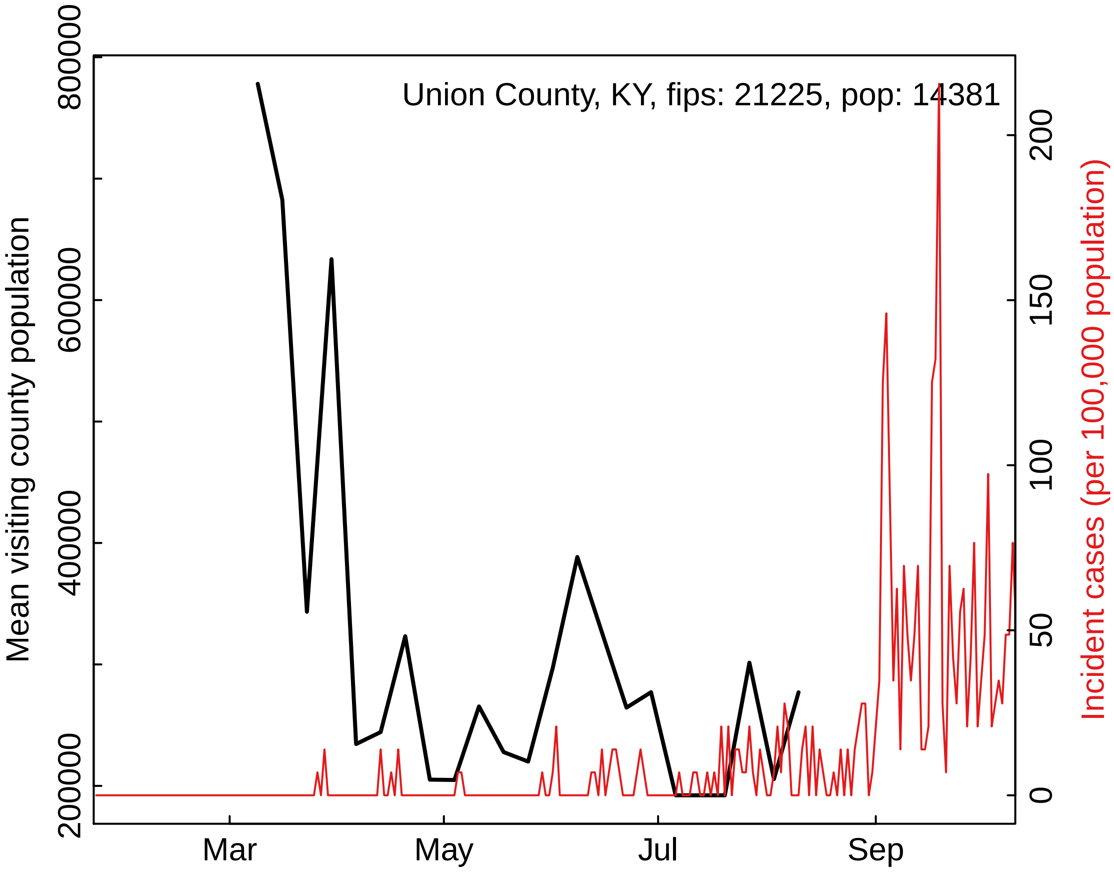

Union County, KY, fips: 21225, pop: 14381
Mean visiting county population
200000
400000
600000
800000
Mar
May
Jul
Sep
0
50
100
150
200
Incident cases (per 100,000 population)

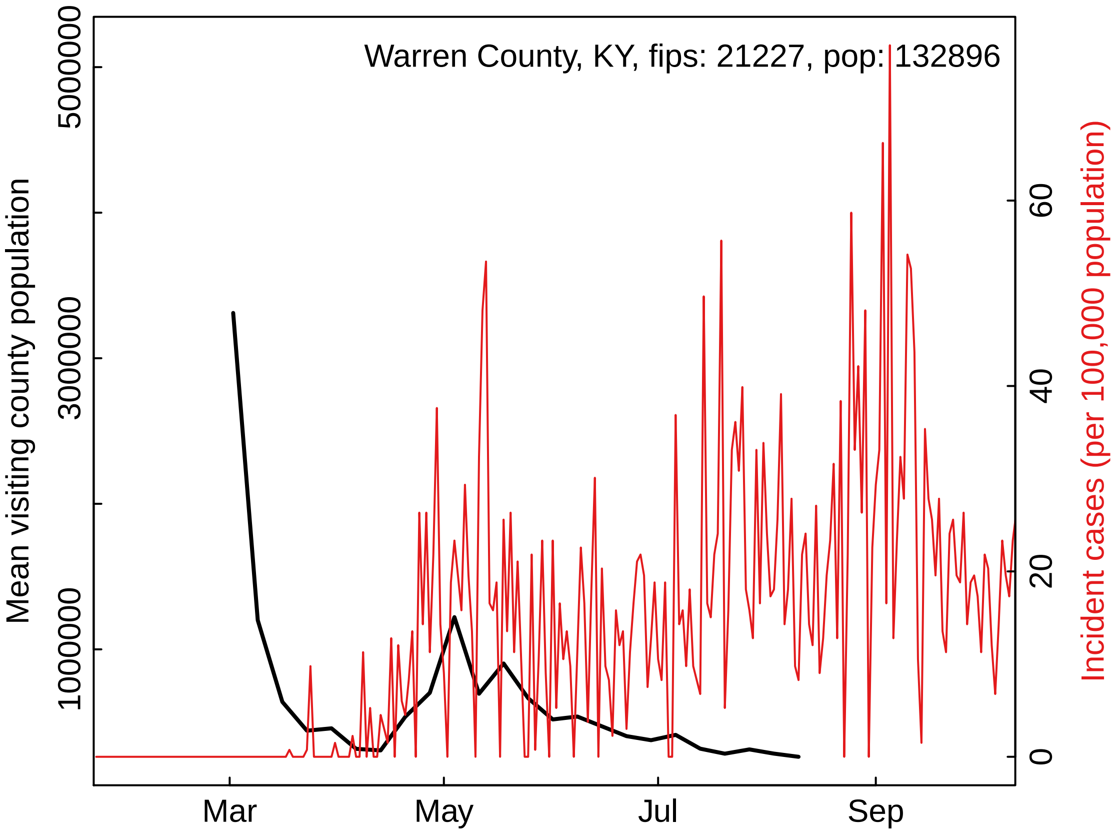

Warren County, KY, fips: 21227, pop: 132896
Mean visiting county population
1000000
3000000
5000000
Incident cases (per 100,000 population)
0
20
40
60
Mar
May
Jul
Sep

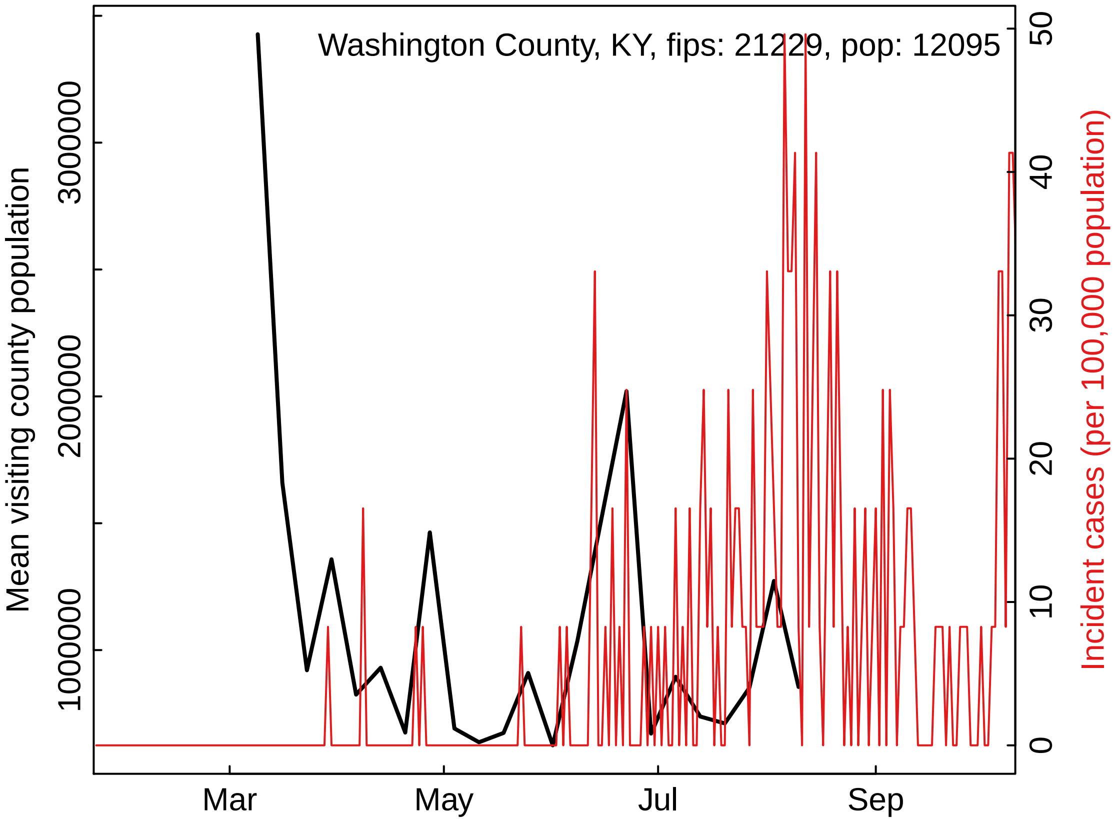

Washington County, KY, fips: 21229, pop: 12095
Mean visiting county population
10000000
20000000
30000000
Incident cases (per 100,000 population)
0
10
20
30
40
50
Mar
May
Jul
Sep

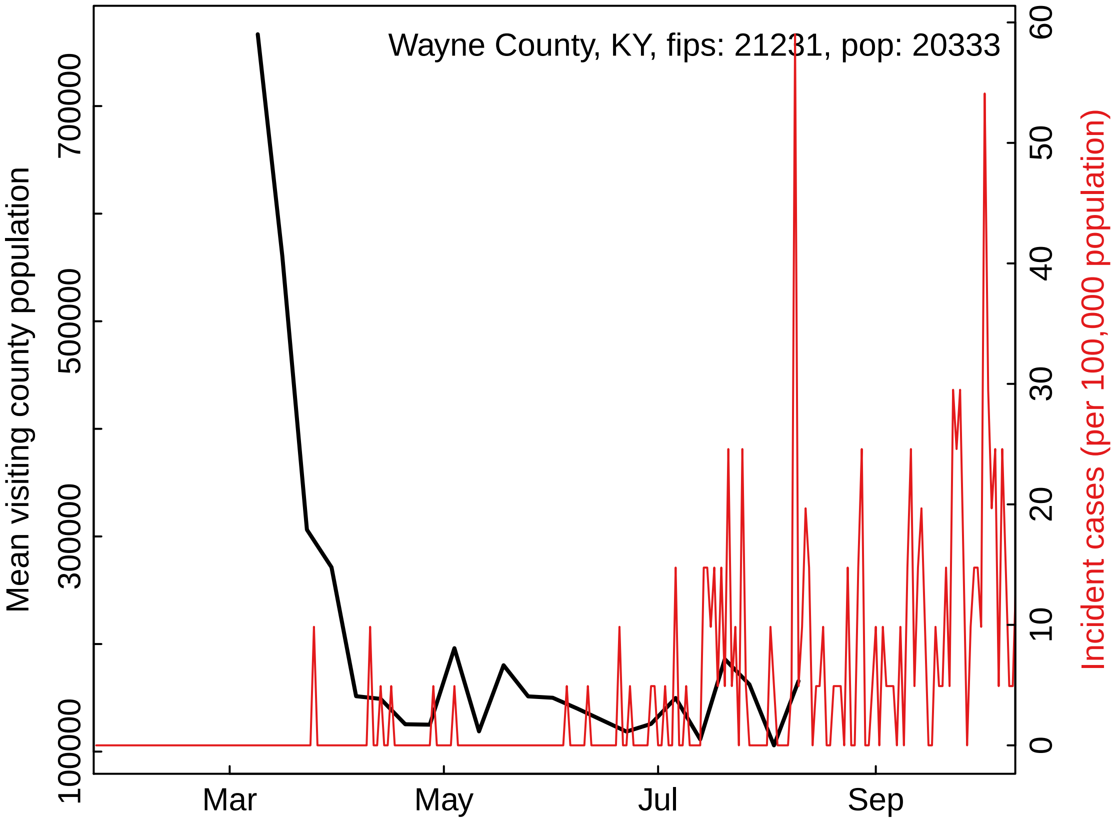

Wayne County, KY, fips: 21231, pop: 20333
Mean visiting county population
100000
300000
500000
700000
Incident cases (per 100,000 population)
0
10
20
30
40
50
60
Mar
May
Jul
Sep

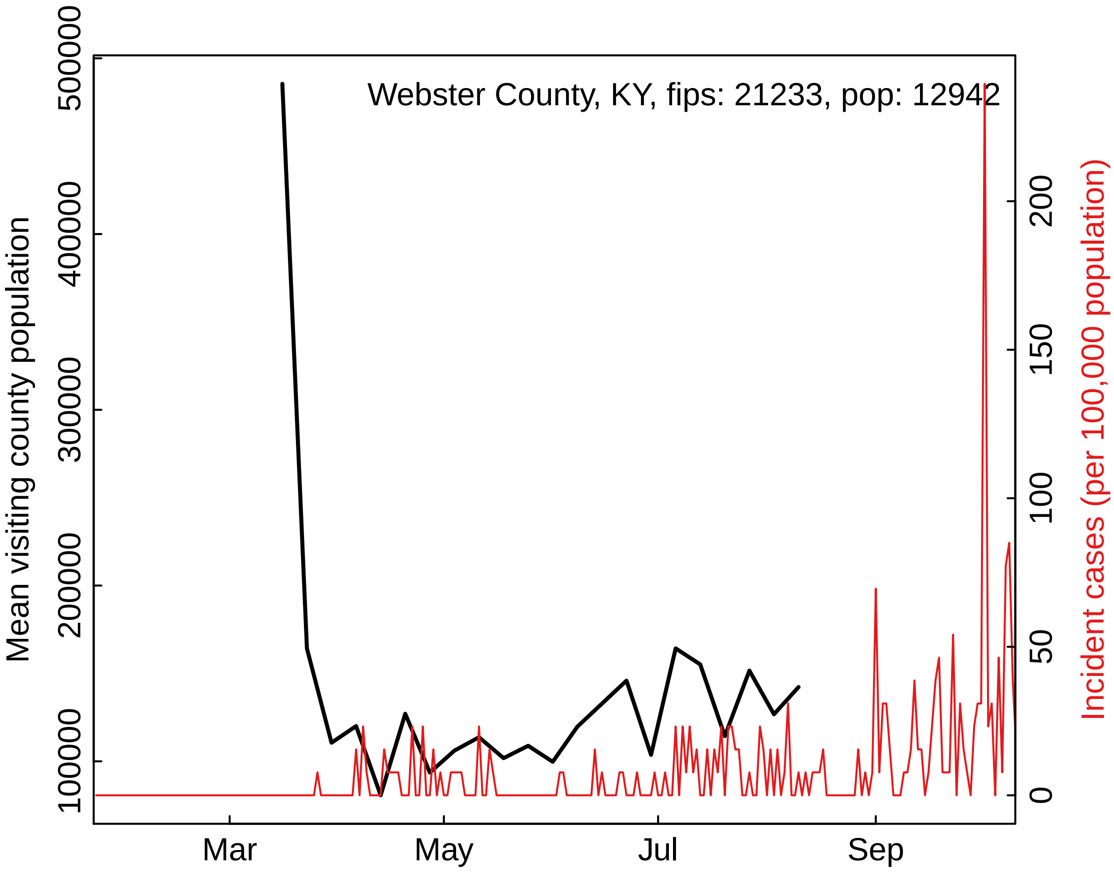
Webster County, KY, fips: 21233, pop: 12942
Mean visiting county population
Incident cases (per 100,000 population)
100000
200000
300000
400000
500000
0
50
100
150
200
Mar
May
Jul
Sep

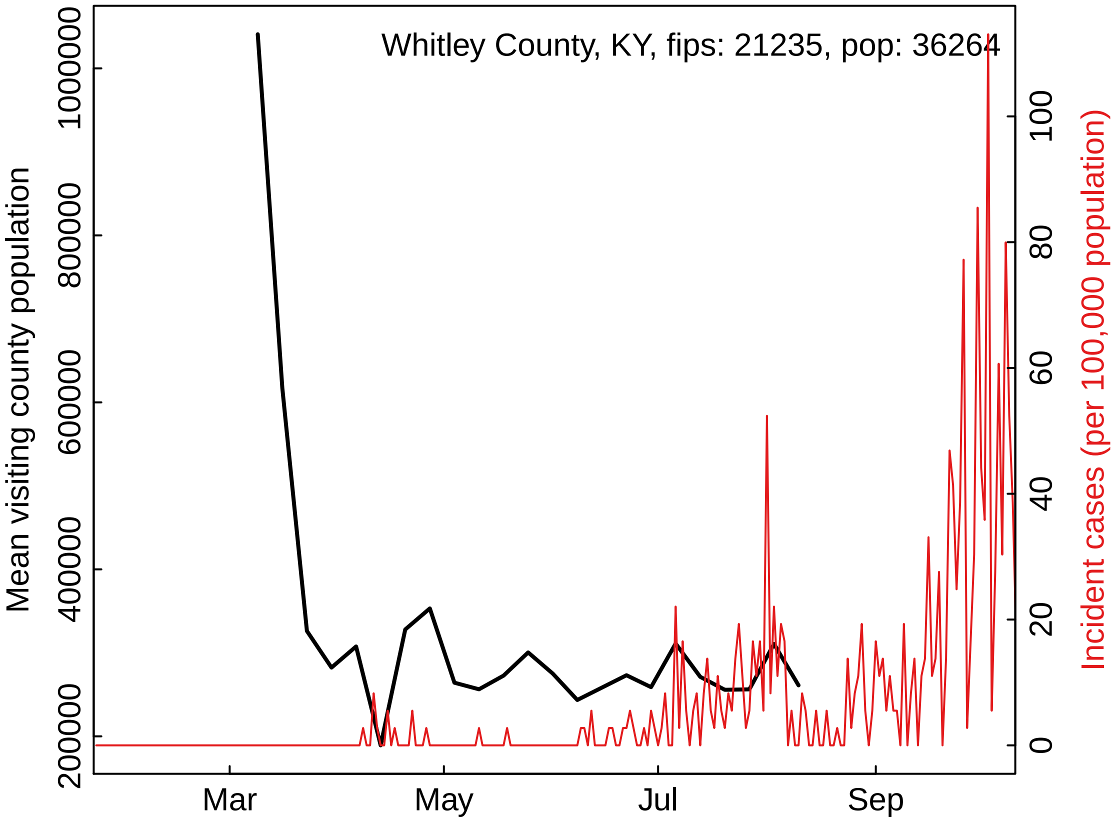

Whitley County, KY, fips: 21235, pop: 36264
Mean visiting county population
200000
400000
600000
800000
1000000
Incident cases (per 100,000 population)
0
20
40
60
80
100
Mar
May
Jul
Sep

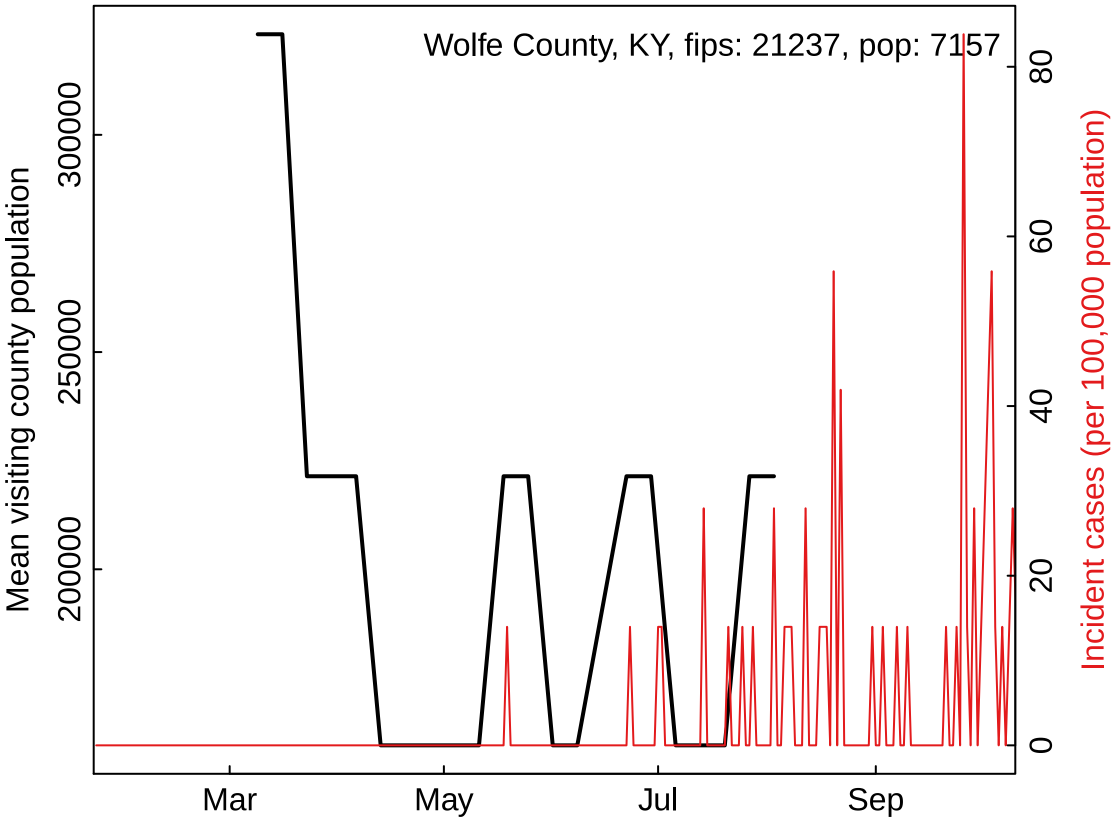
Wolfe County, KY, fips: 21237, pop: 7157
Mean visiting county population
200000
250000
300000
Incident cases (per 100,000 population)
0
20
40
60
80
Mar
May
Jul
Sep

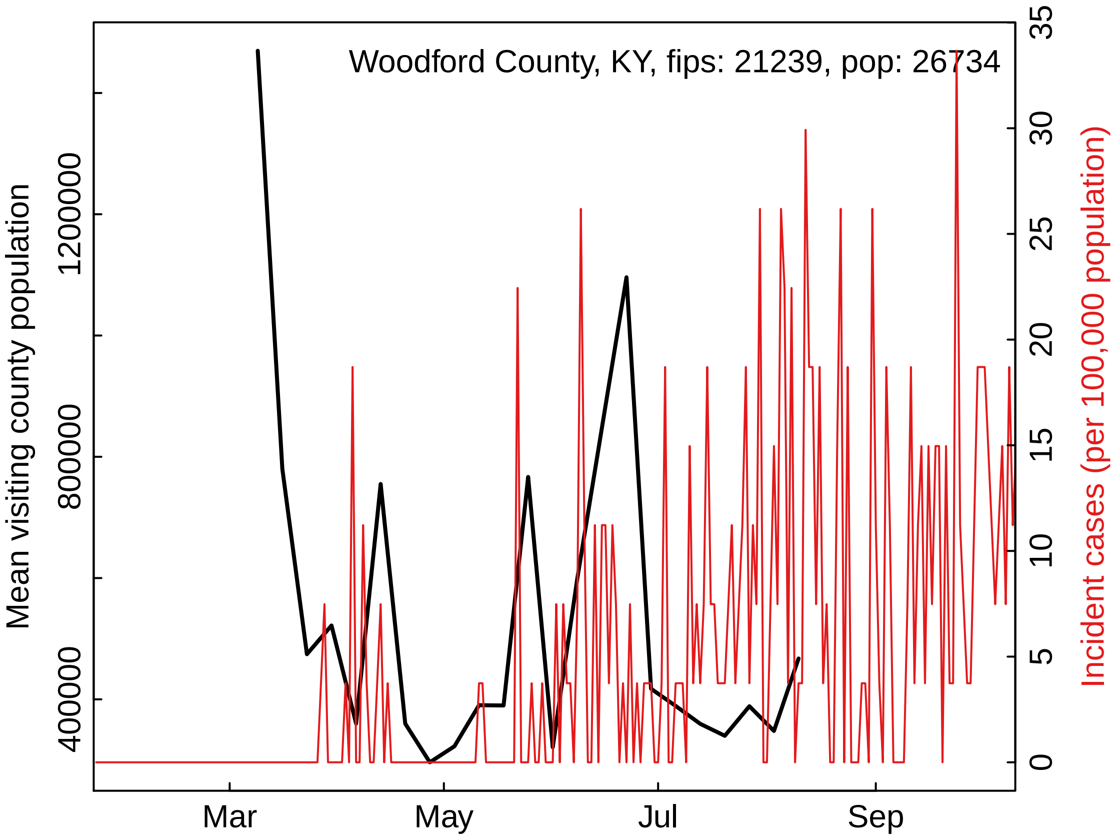

Woodford County, KY, fips: 21239, pop: 26734
Mean visiting county population
400000
800000
1200000
Incident cases (per 100,000 population)
0
5
10
15
20
25
30
35
Mar
May
Jul
Sep

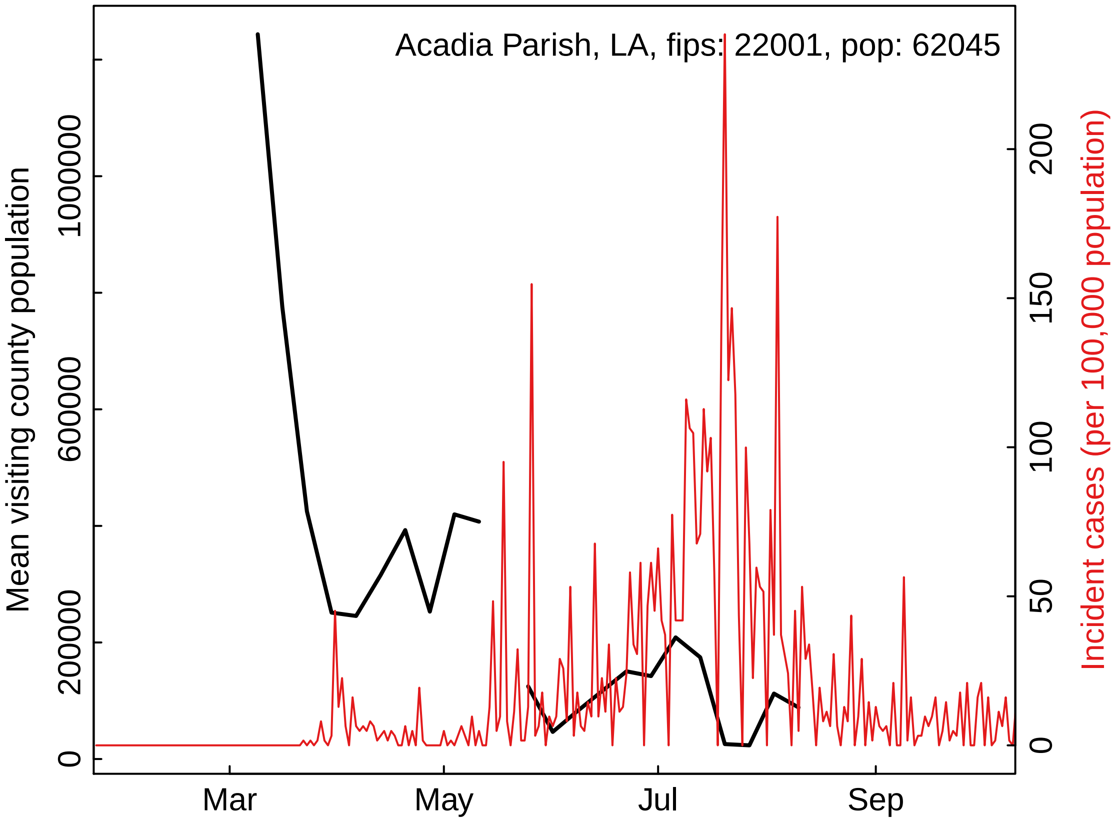

Acadia Parish, LA, fips: 22001, pop: 62045
Mean visiting county population
0
200000
600000
1000000
Incident cases (per 100,000 population)
0
50
100
150
200
Mar
May
Jul
Sep

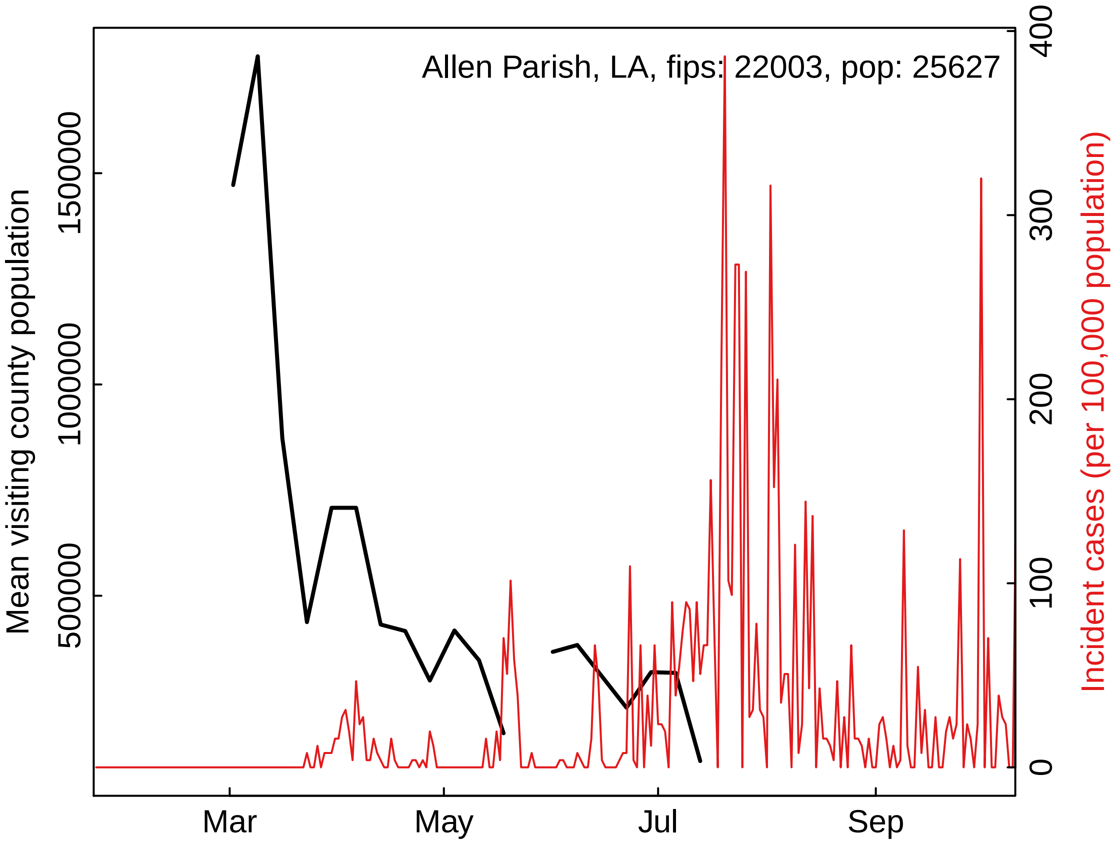

Allen Parish, LA, fips: 22003, pop: 25627
Mean visiting county population
500000
1000000
1500000
Incident cases (per 100,000 population)
0
100
200
300
400
Mar
May
Jul
Sep

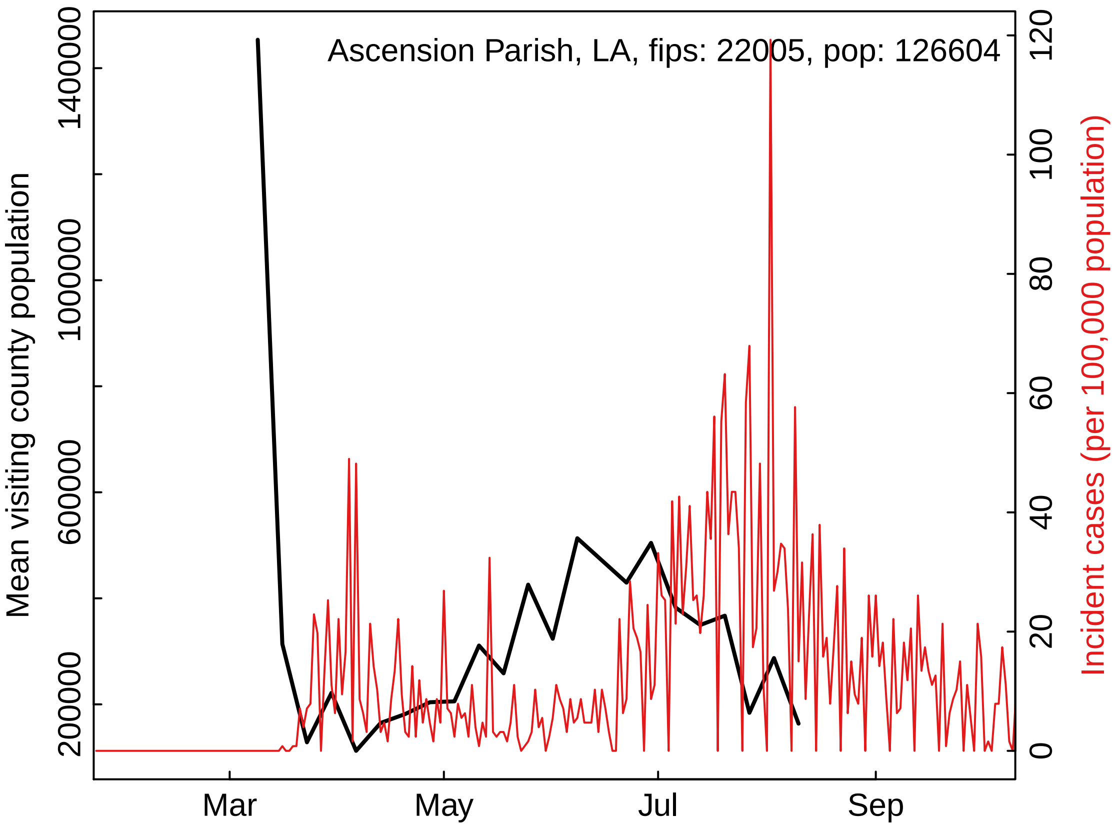
Ascension Parish, LA, fips: 22005, pop: 126604
Mean visiting county population
200000
600000
1000000
1400000
Incident cases (per 100,000 population)
0
20
40
60
80
100
120
Mar
May
Jul
Sep

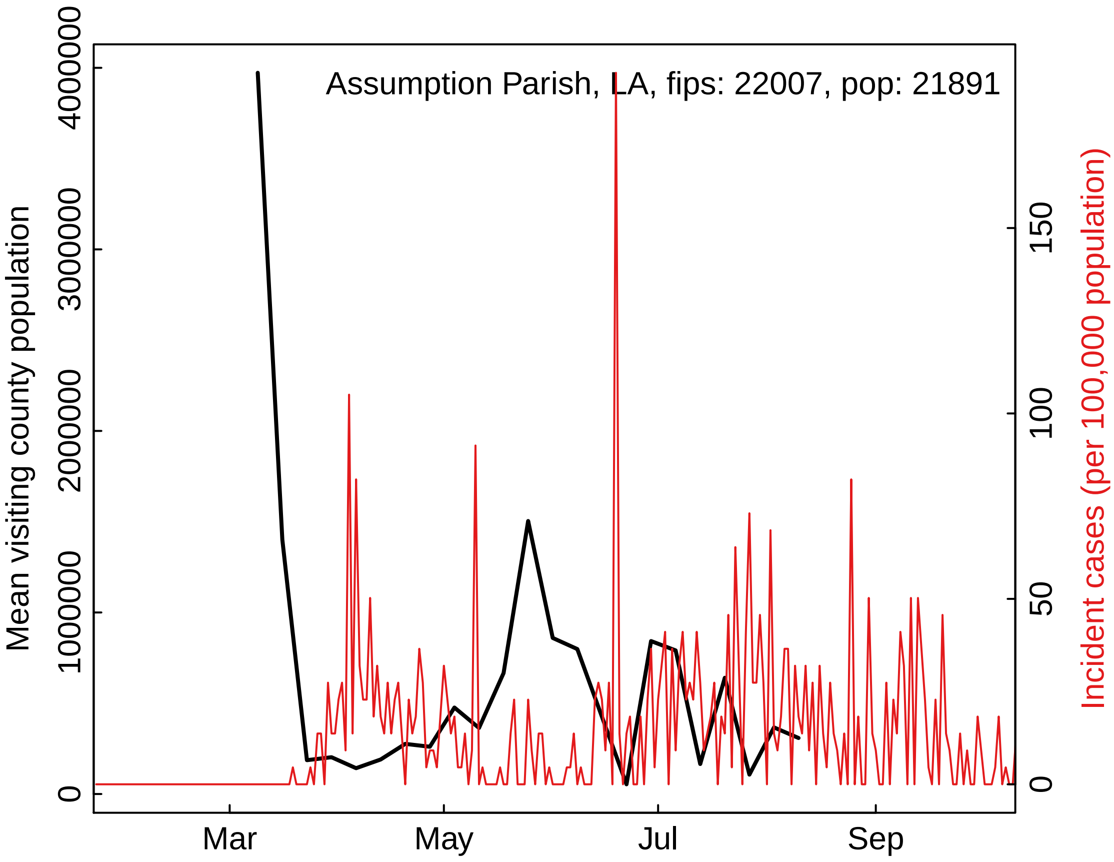

Assumption Parish, LA, fips: 22007, pop: 21891
Mean visiting county population
0
1000000
2000000
3000000
4000000
Incident cases (per 100,000 population)
0
50
100
150
Mar
May
Jul
Sep

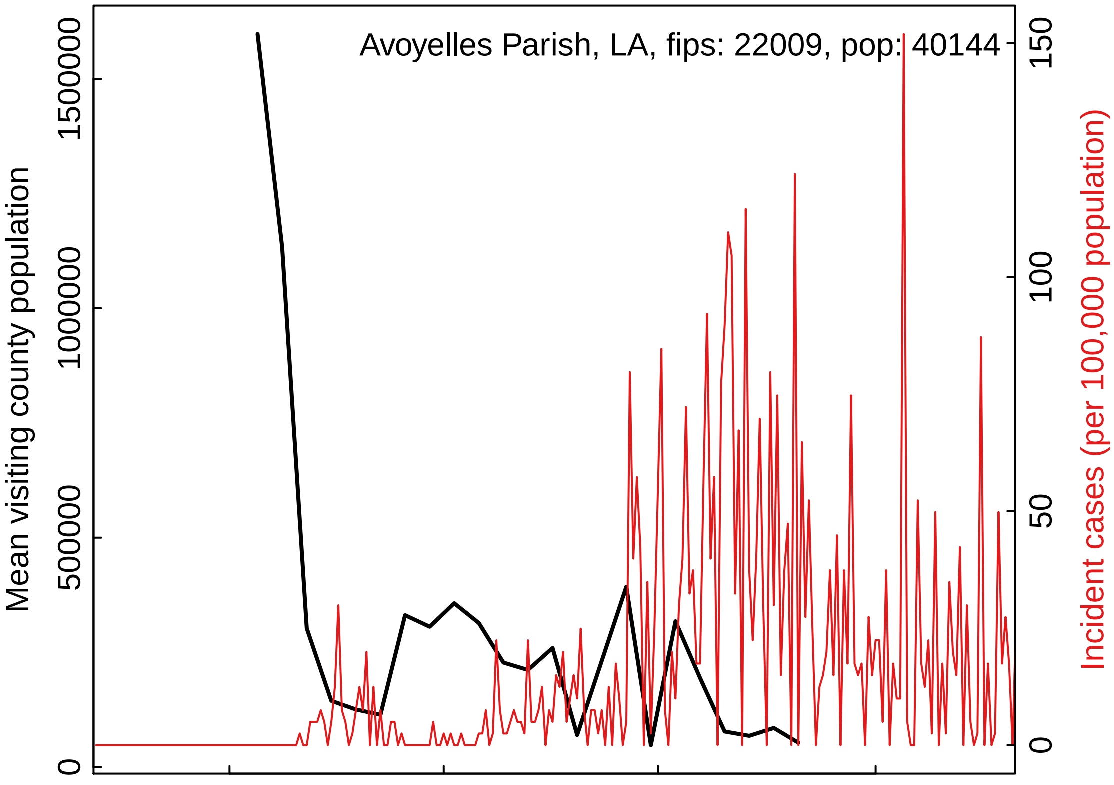
Avoyelles Parish, LA, fips: 22009, pop: 40144
Mean visiting county population
0
500000
1000000
1500000
Incident cases (per 100,000 population)
0
50
100
150
Mar
May
Jul
Sep

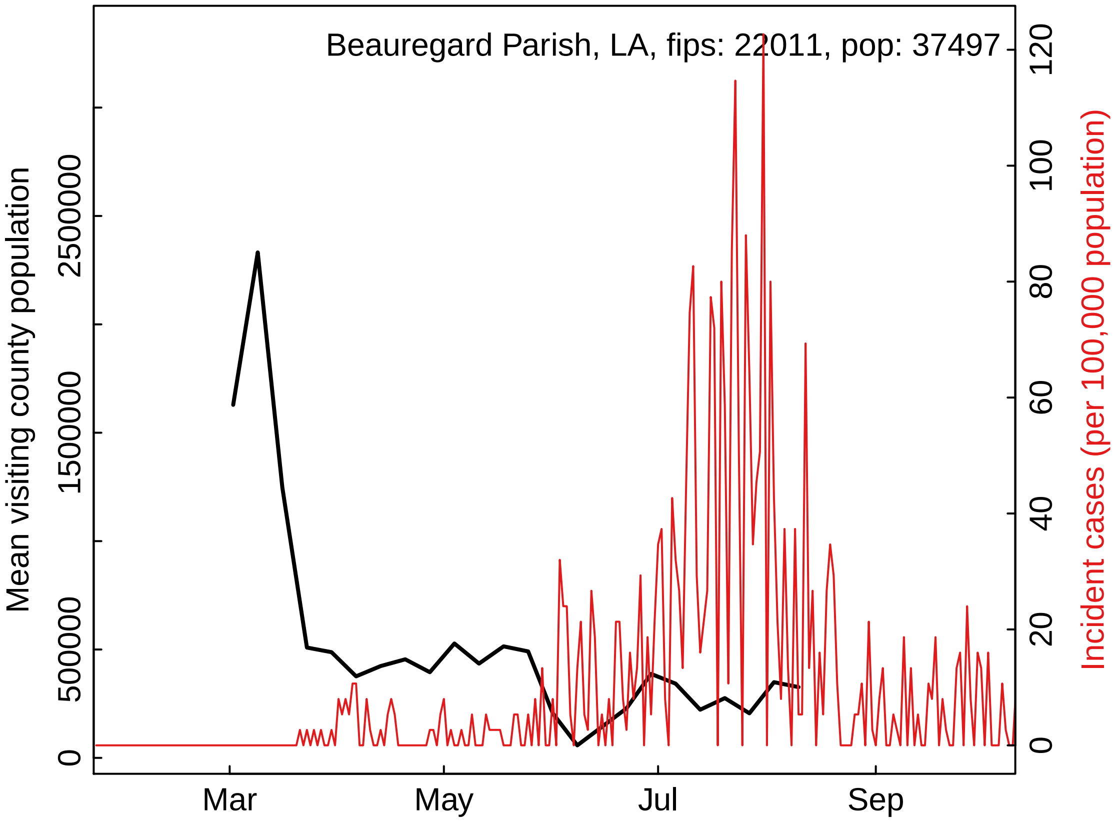
Beauregard Parish, LA, fips: 22011, pop: 37497
Mean visiting county population
0
500000
1500000
2500000
Incident cases (per 100,000 population)
0
20
40
60
80
100
120
Mar
May
Jul
Sep

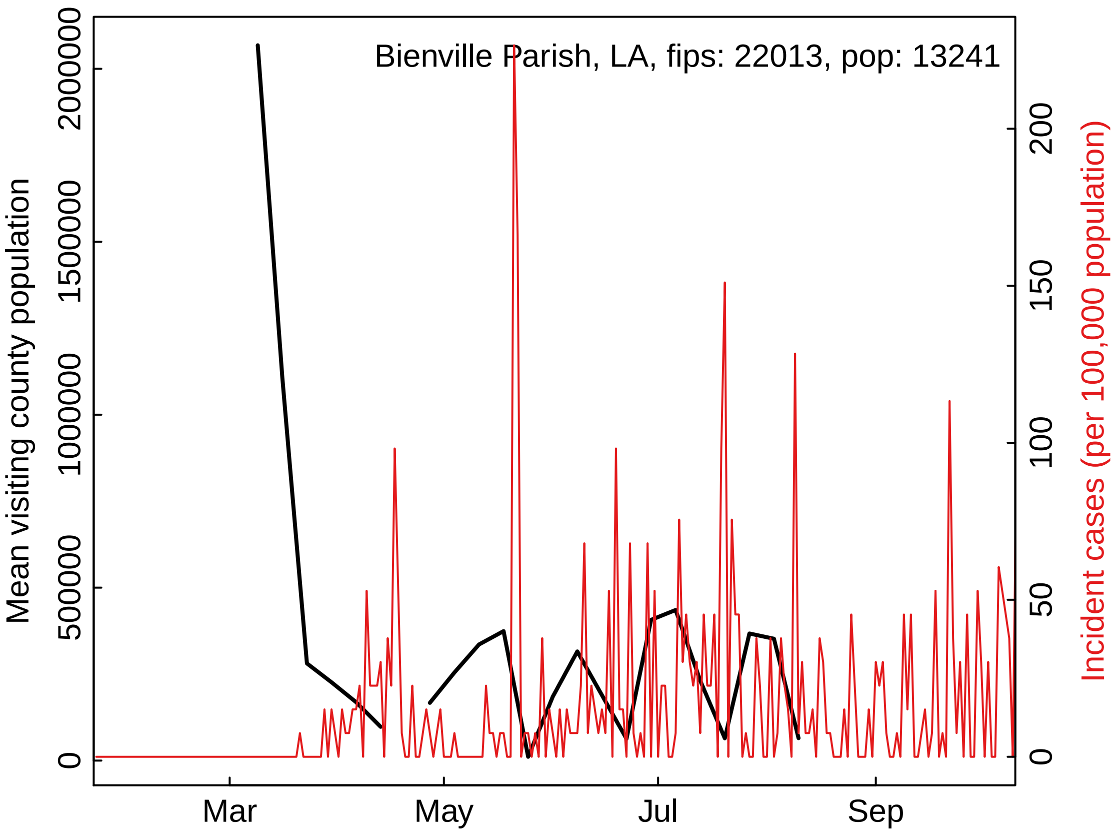
Bienville Parish, LA, fips: 22013, pop: 13241
Mean visiting county population
0
500000
1000000
1500000
2000000
Incident cases (per 100,000 population)
0
50
100
150
200
Mar
May
Jul
Sep

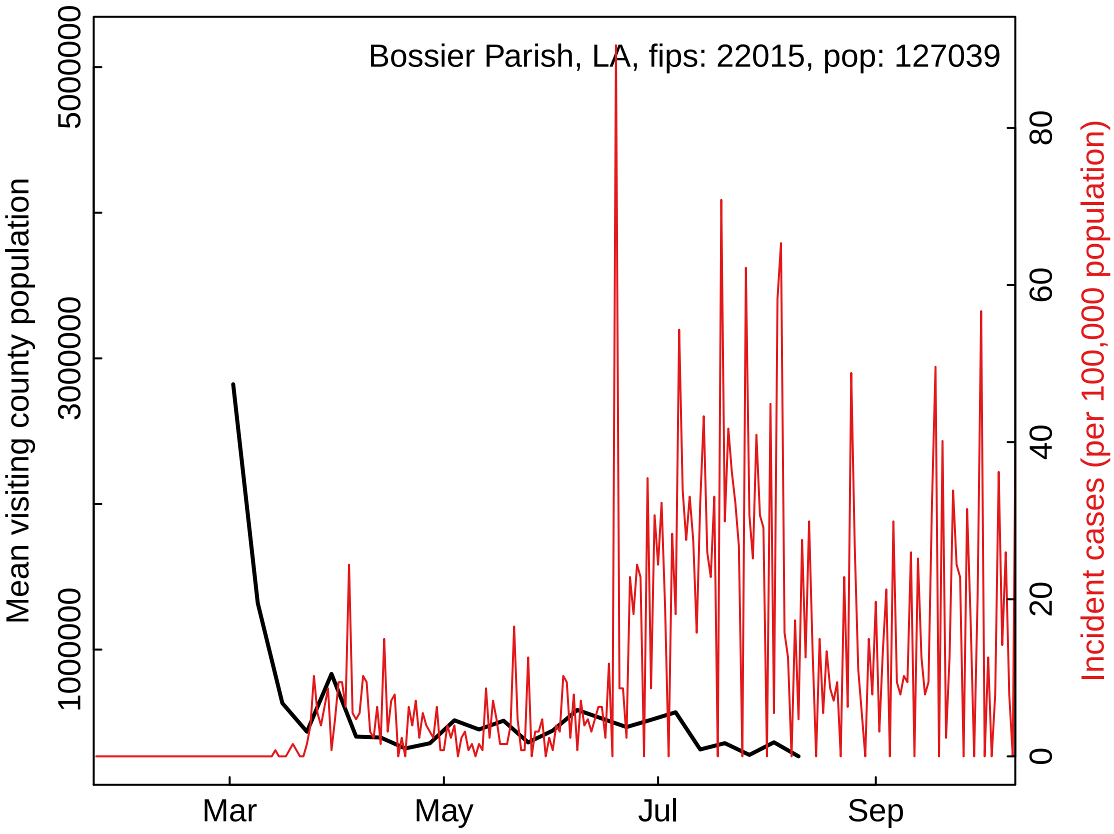

Bossier Parish, LA, fips: 22015, pop: 127039
Mean visiting county population
1000000
3000000
5000000
Incident cases (per 100,000 population)
0
20
40
60
80
Mar
May
Jul
Sep

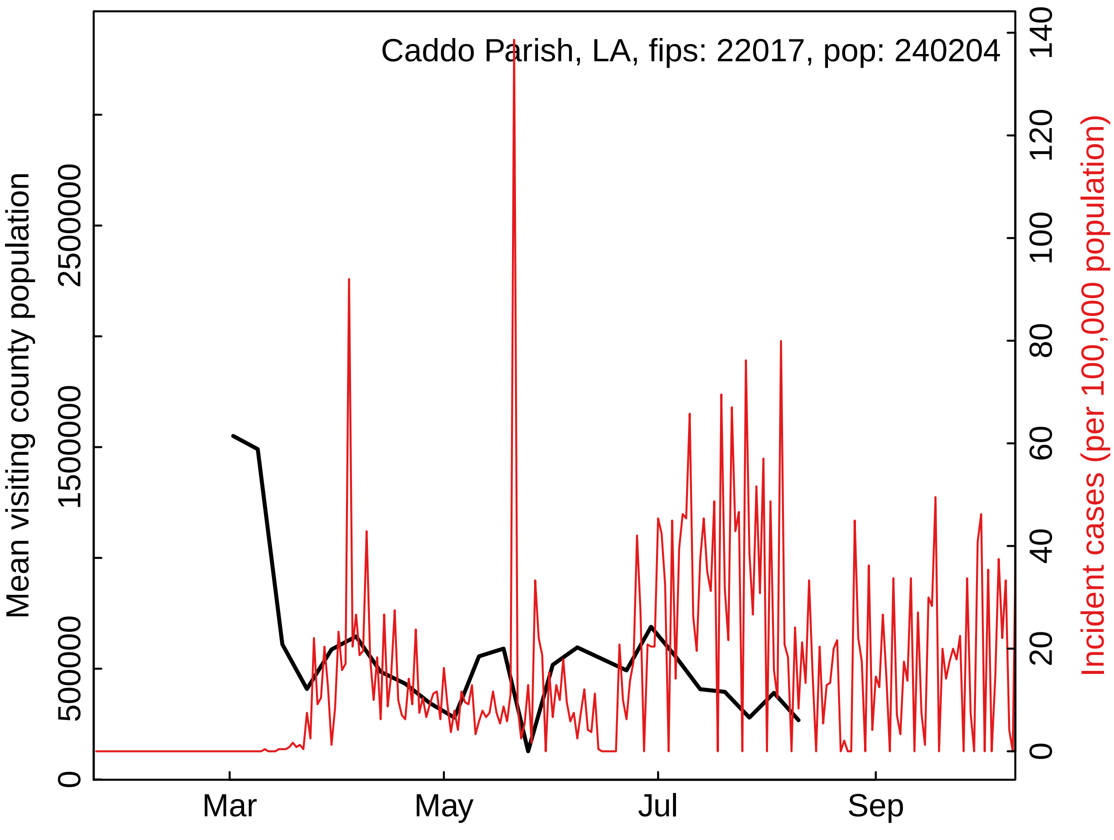

Caddo Parish, LA, fips: 22017, pop: 240204
Mean visiting county population
0
500000
1500000
2500000
Mar
May
Jul
Sep
0
20
40
60
80
100
120
140
Incident cases (per 100,000 population)

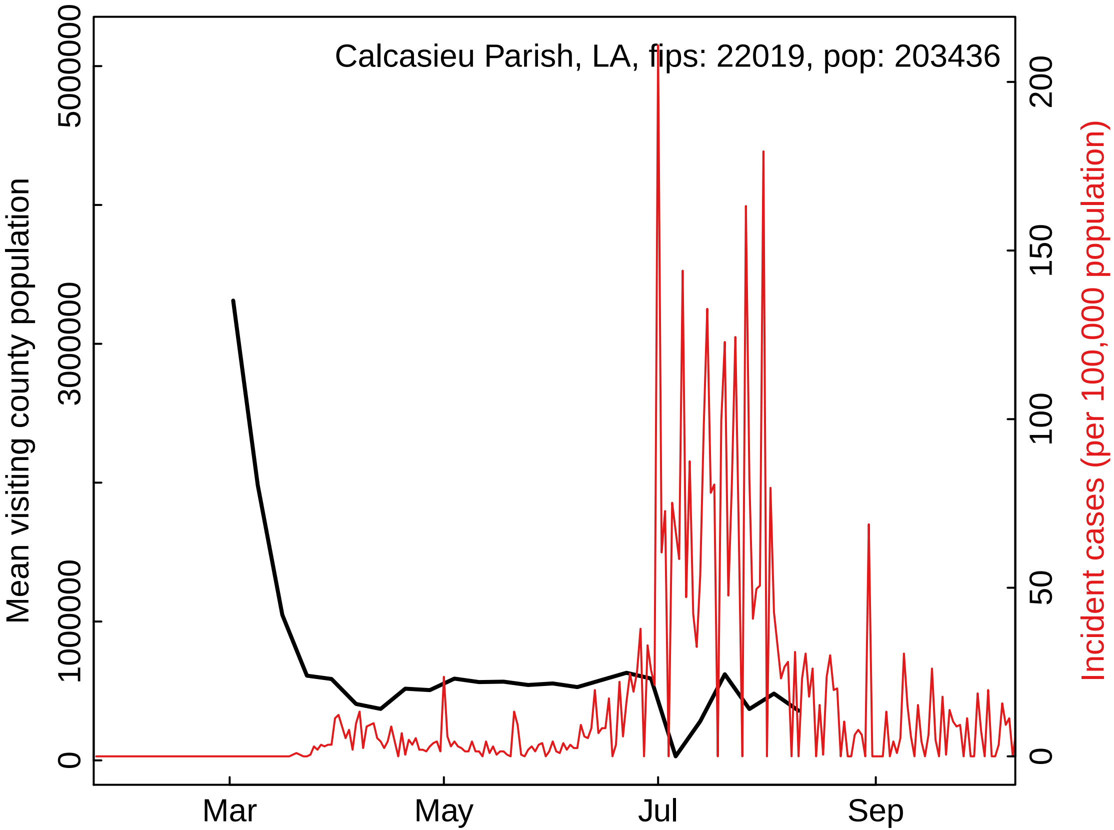

Calcasieu Parish, LA, fips: 22019, pop: 203436
Mean visiting county population
0
1000000
3000000
5000000
Incident cases (per 100,000 population)
0
50
100
150
200
Mar
May
Jul
Sep

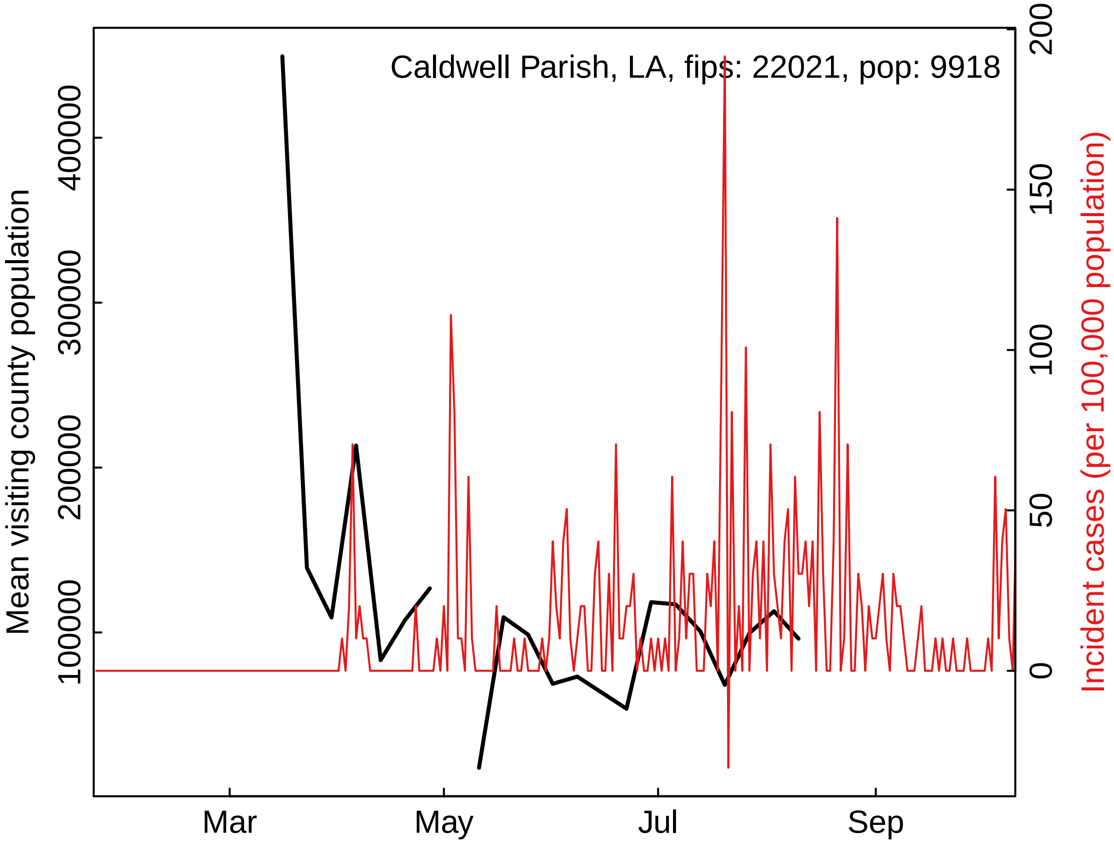

Caldwell Parish, LA, fips: 22021, pop: 9918
Mean visiting county population
100000
200000
300000
400000
Incident cases (per 100,000 population)
0
50
100
150
200
Mar
May
Jul
Sep

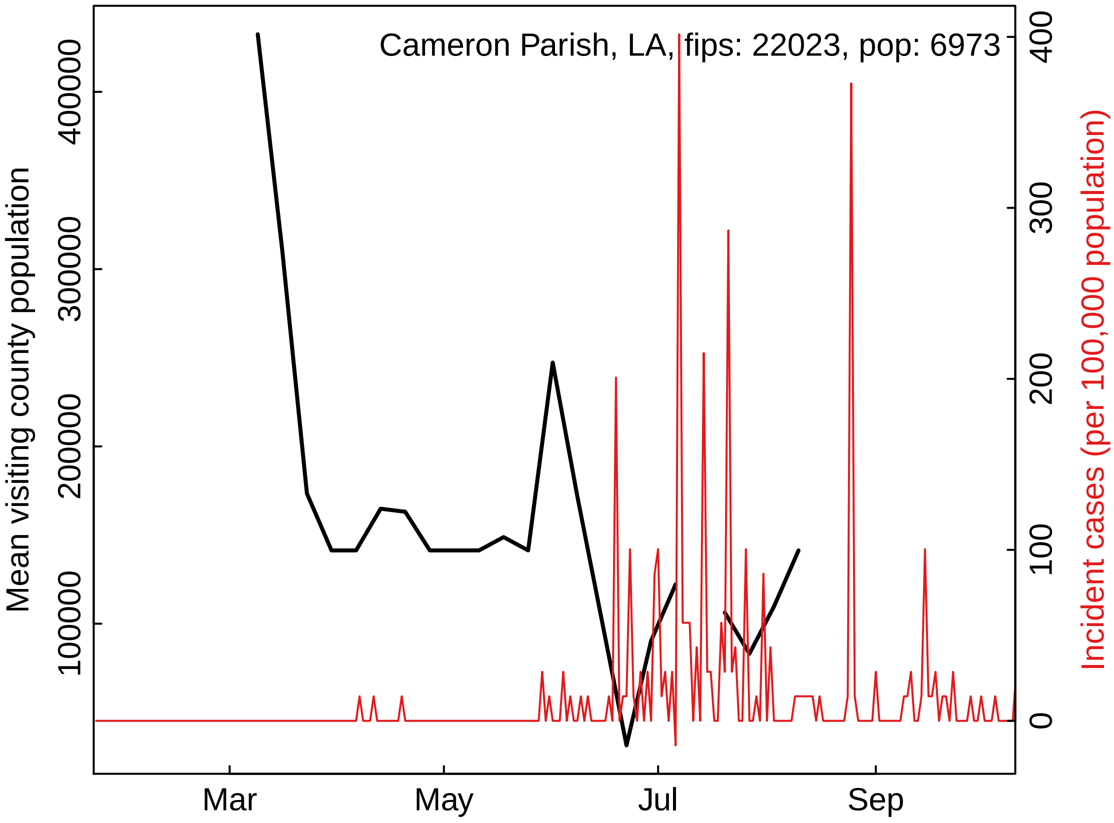

Cameron Parish, LA, fips: 22023, pop: 6973
Mean visiting county population
100000
200000
300000
400000
Incident cases (per 100,000 population)
0
100
200
300
400
Mar
May
Jul
Sep

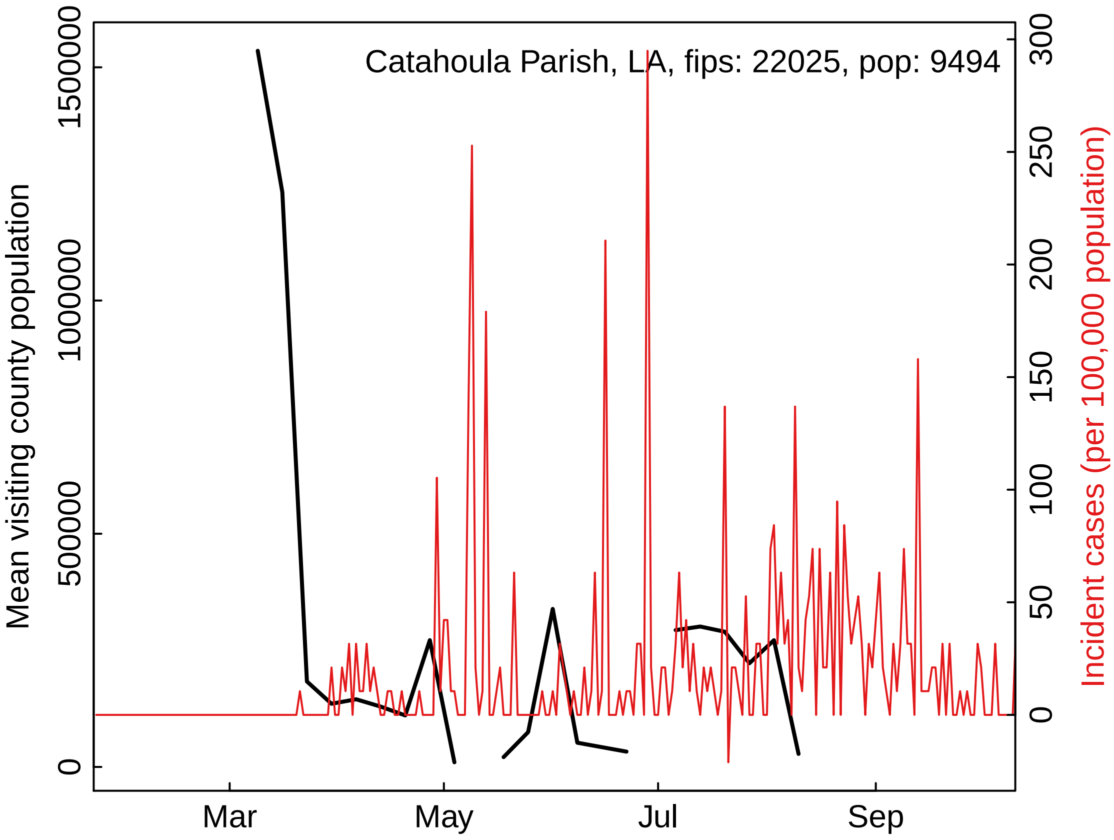
Catahoula Parish, LA, fips: 22025, pop: 9494
Mean visiting county population
0
500000
1000000
1500000
Incident cases (per 100,000 population)
0
50
100
150
200
250
300
Mar
May
Jul
Sep

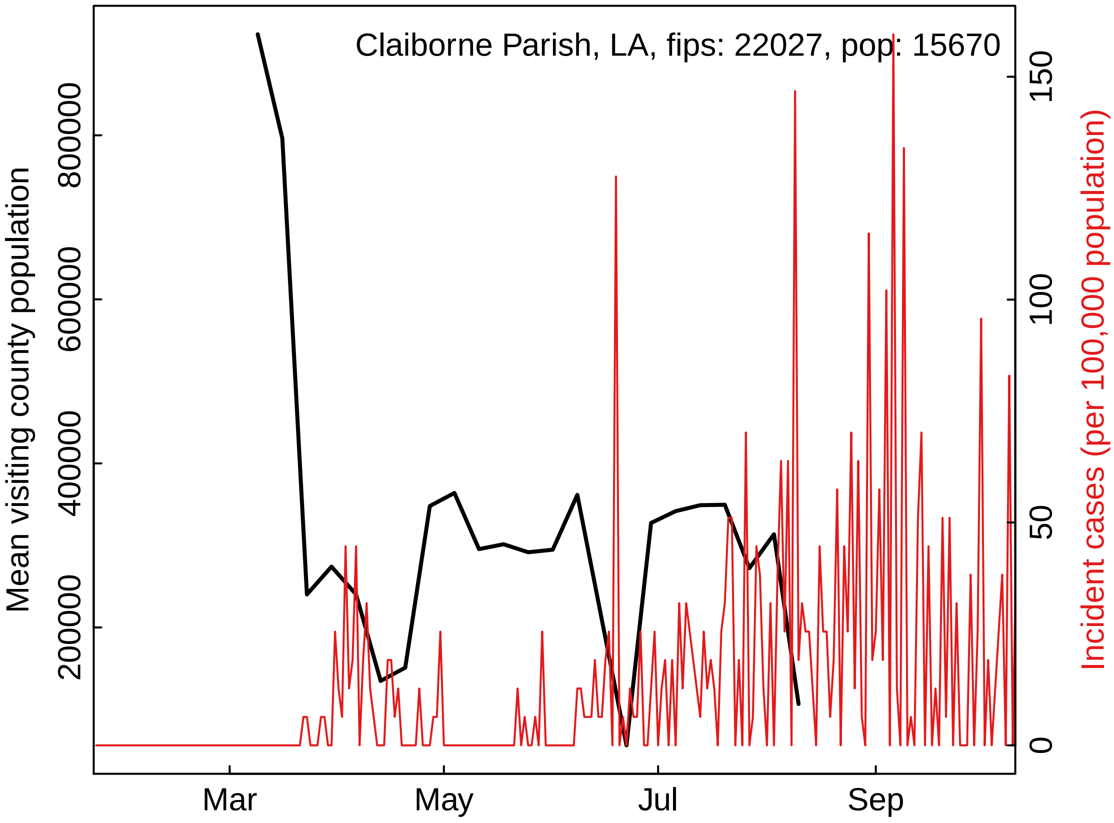

Claiborne Parish, LA, fips: 22027, pop: 15670
Mean visiting county population
200000
400000
600000
800000
Incident cases (per 100,000 population)
0
50
100
150
Mar
May
Jul
Sep

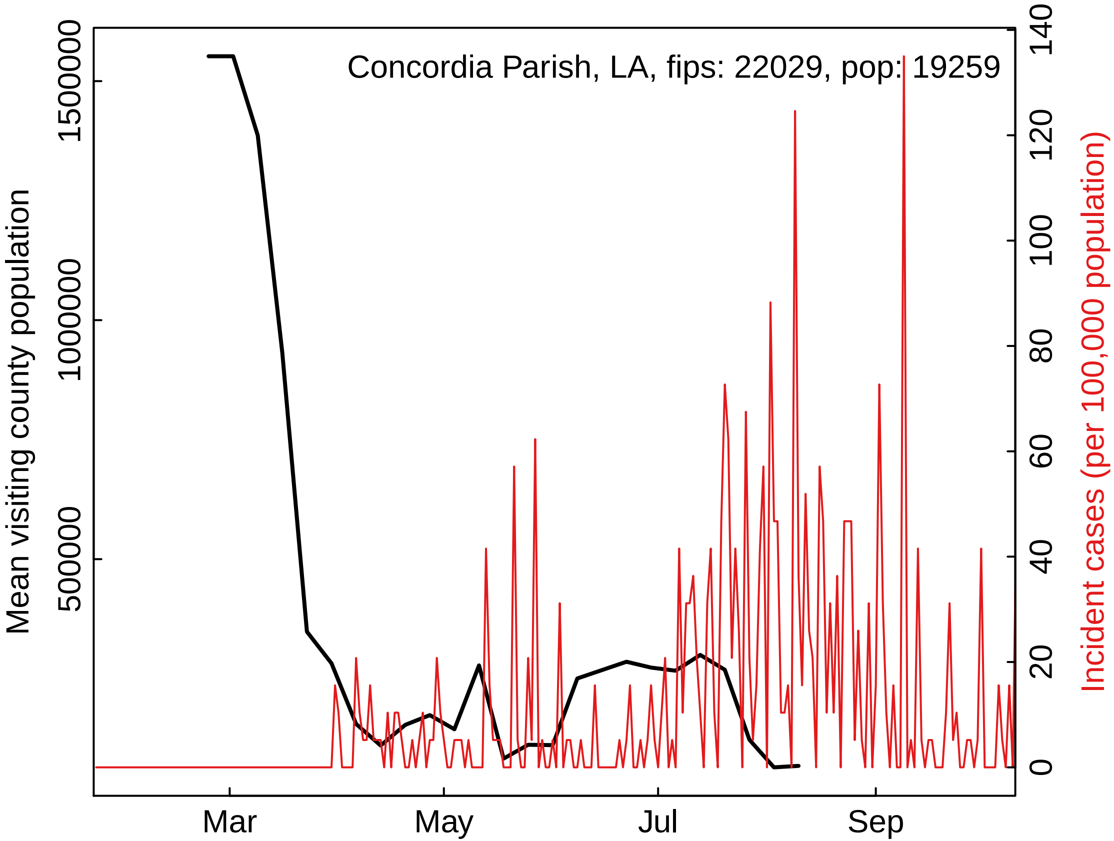

Concordia Parish, LA, fips: 22029, pop: 19259
Mean visiting county population
500000
1000000
1500000
Incident cases (per 100,000 population)
0
20
40
60
80
100
120
140
Mar
May
Jul
Sep

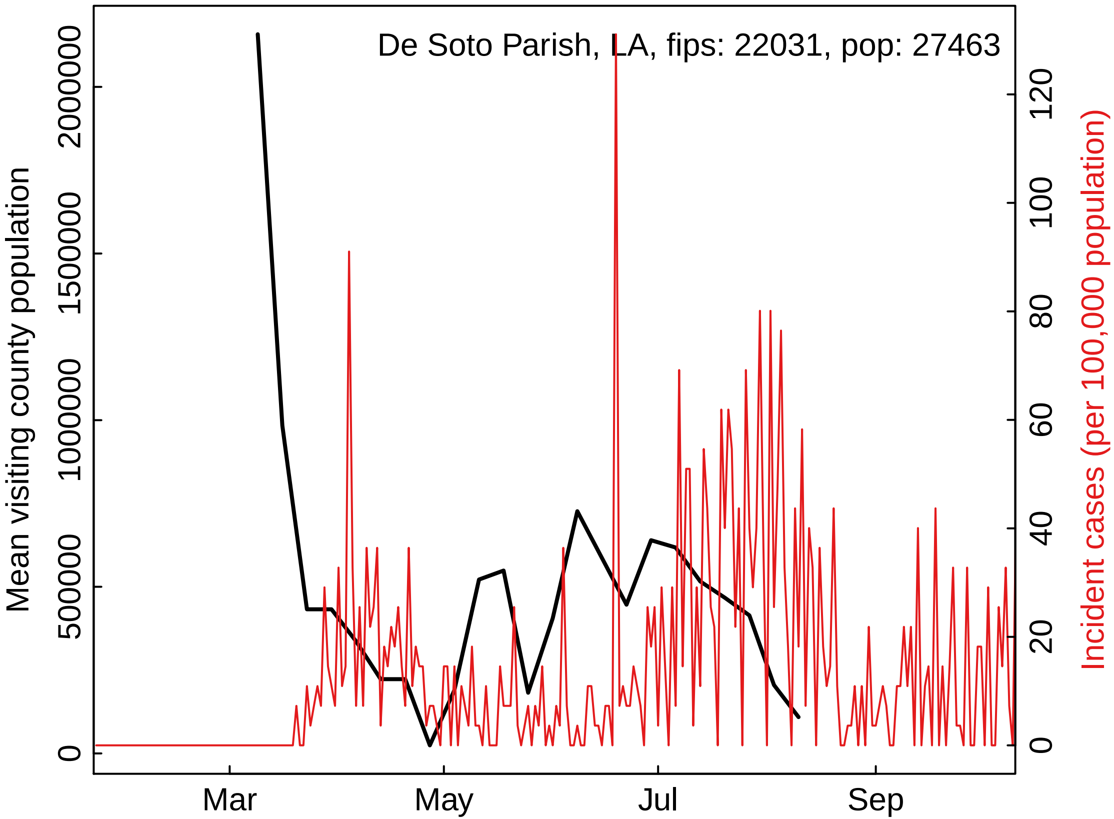

De Soto Parish, LA, fips: 22031, pop: 27463
Mean visiting county population
0
500000
1000000
1500000
2000000
Incident cases (per 100,000 population)
0
20
40
60
80
100
120
Mar
May
Jul
Sep

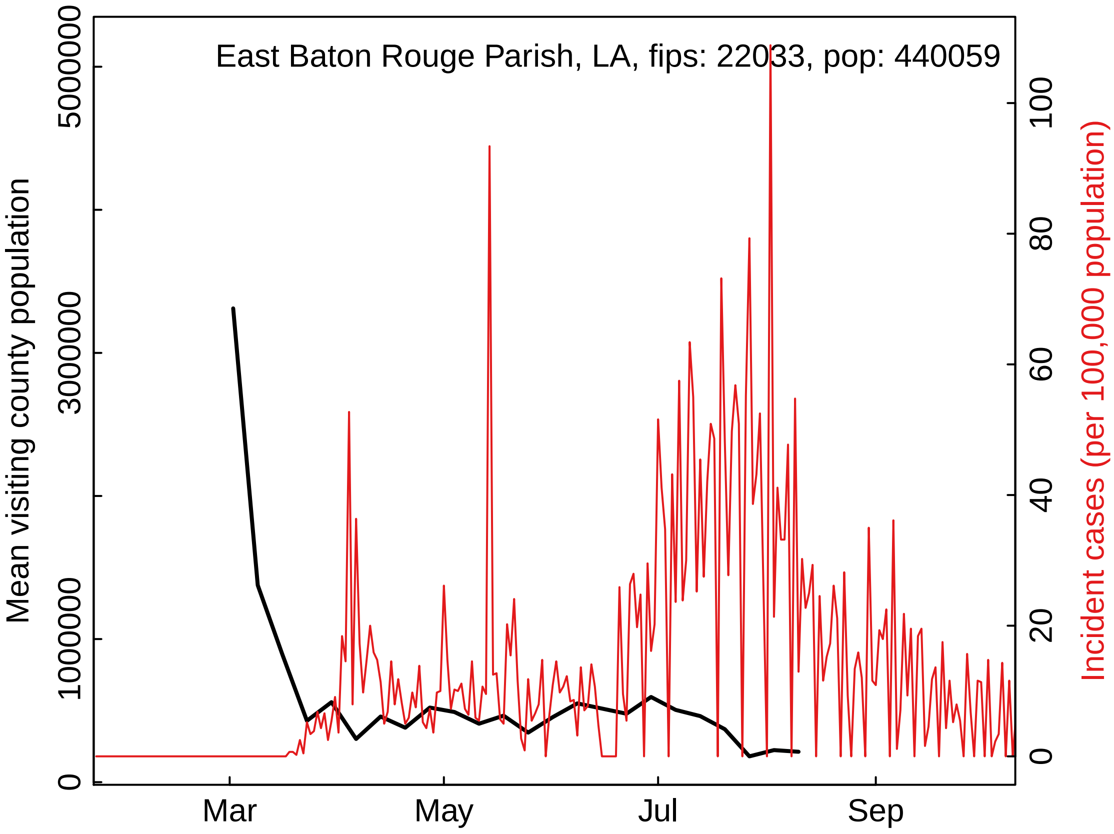

East Baton Rouge Parish, LA, fips: 22033, pop: 440059
Mean visiting county population
0
1000000
3000000
5000000
Mar
May
Jul
Sep
0
20
40
60
80
100
Incident cases (per 100,000 population)

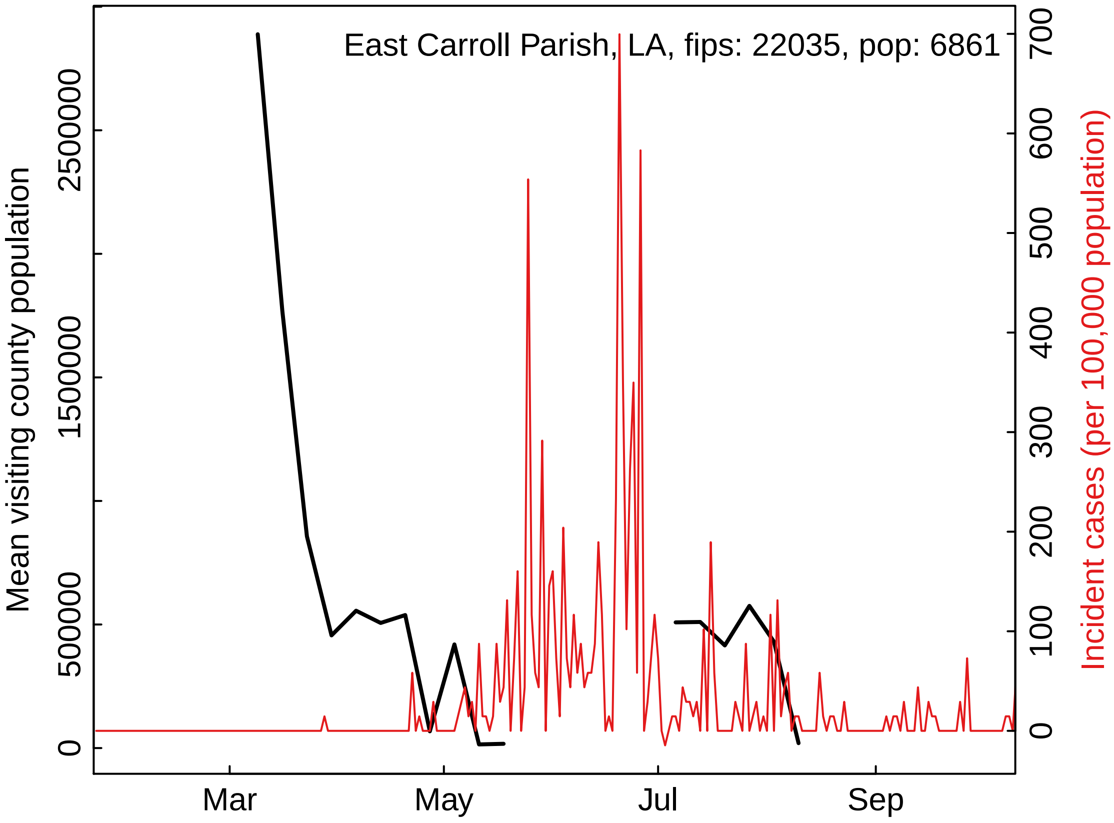

East Carroll Parish, LA, fips: 22035, pop: 6861
Mean visiting county population
0
500000
1500000
2500000
Incident cases (per 100,000 population)
0
100
200
300
400
500
600
700
Mar
May
Jul
Sep

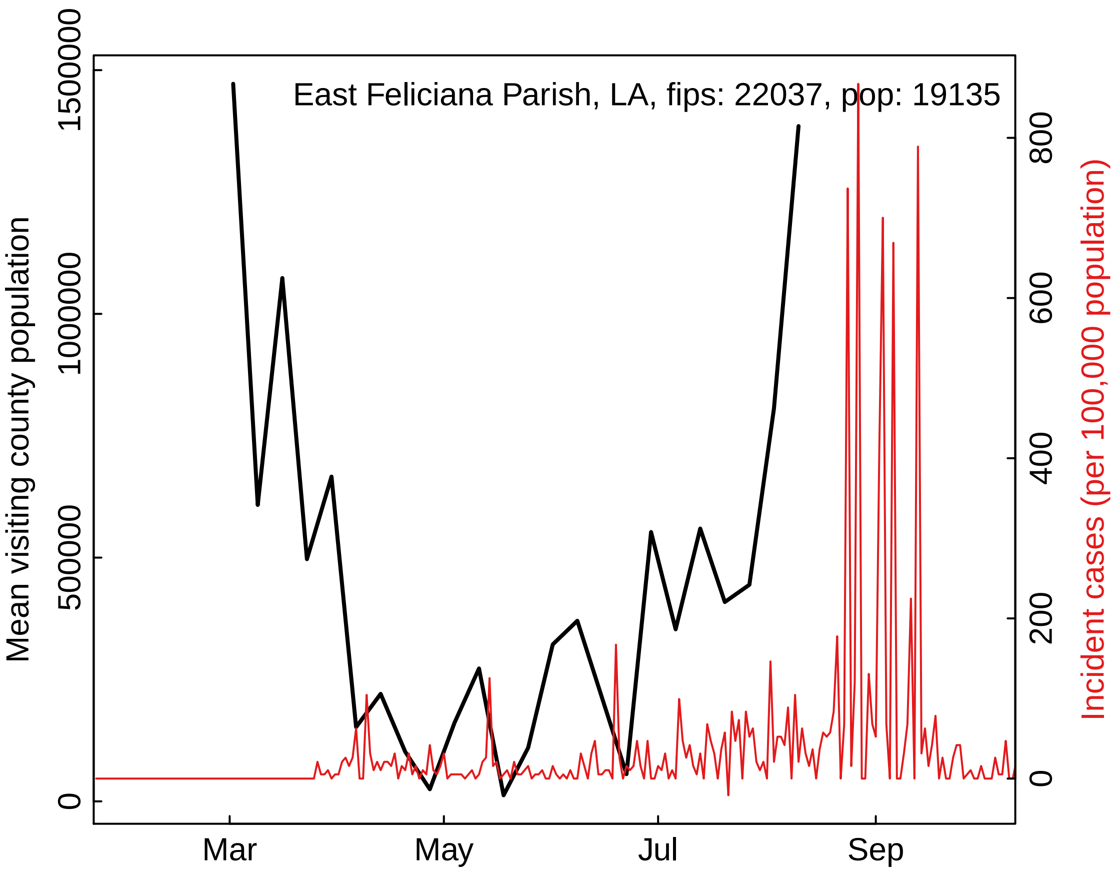

East Feliciana Parish, LA, fips: 22037, pop: 19135
Mean visiting county population
0
500000
1000000
1500000
Incident cases (per 100,000 population)
0
200
400
600
800
Mar
May
Jul
Sep

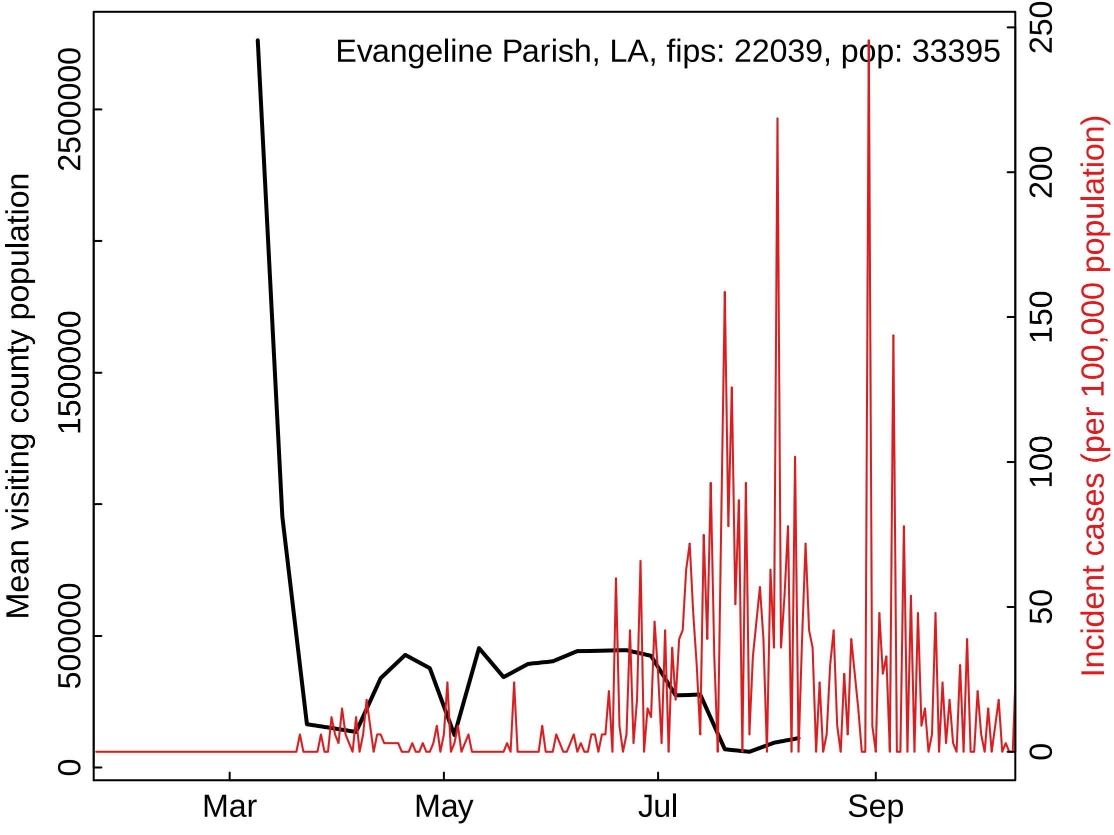

Evangeline Parish, LA, fips: 22039, pop: 33395
Mean visiting county population
Incident cases (per 100,000 population)
0
500000
1500000
2500000
0
50
100
150
200
250
Mar
May
Jul
Sep

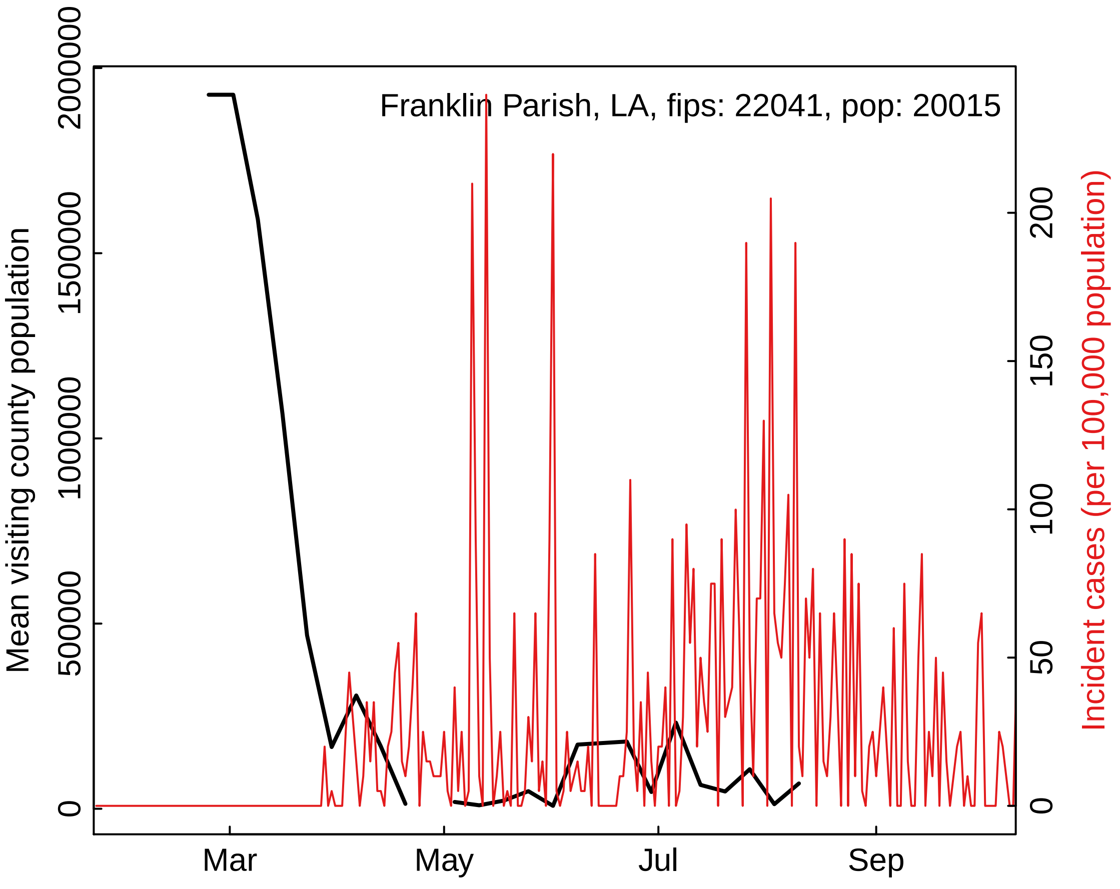
Franklin Parish, LA, fips: 22041, pop: 20015
Mean visiting county population
Incident cases (per 100,000 population)
0
500000
1000000
1500000
2000000
0
50
100
150
200
Mar
May
Jul
Sep

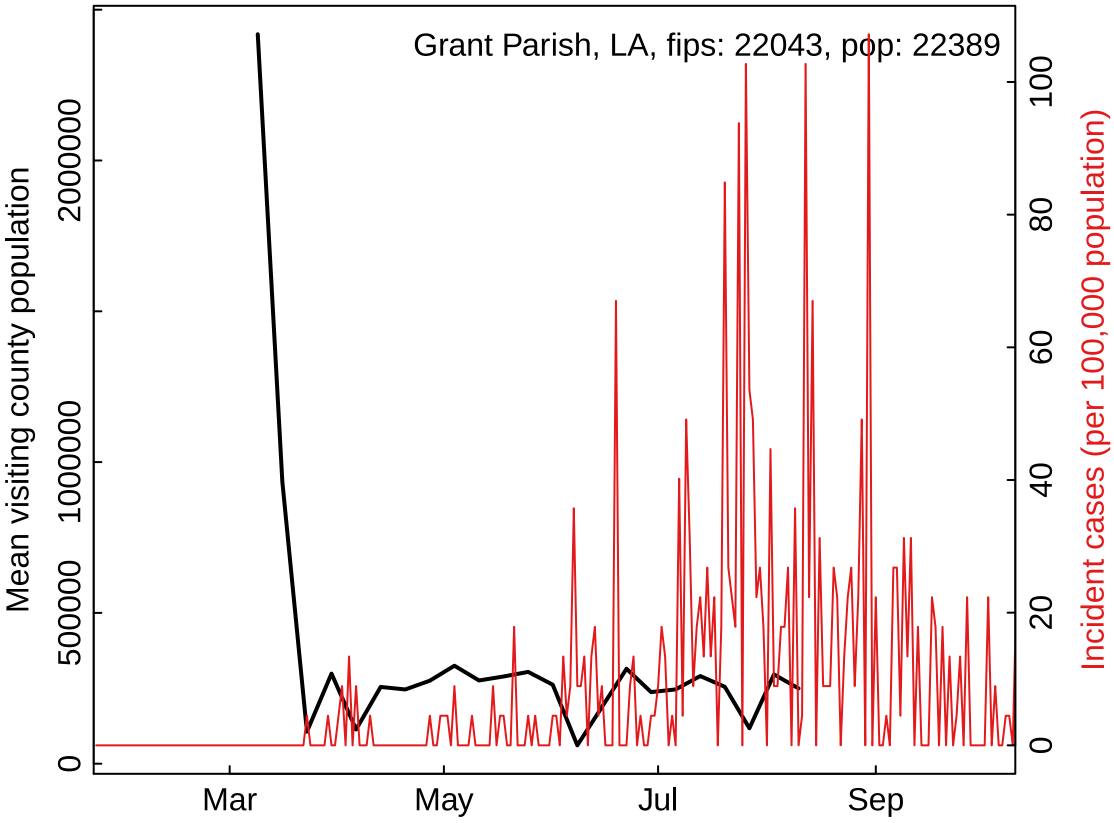

Grant Parish, LA, fips: 22043, pop: 22389
Mean visiting county population
Incident cases (per 100,000 population)
0
500000
1000000
2000000
0
20
40
60
80
100
Mar
May
Jul
Sep

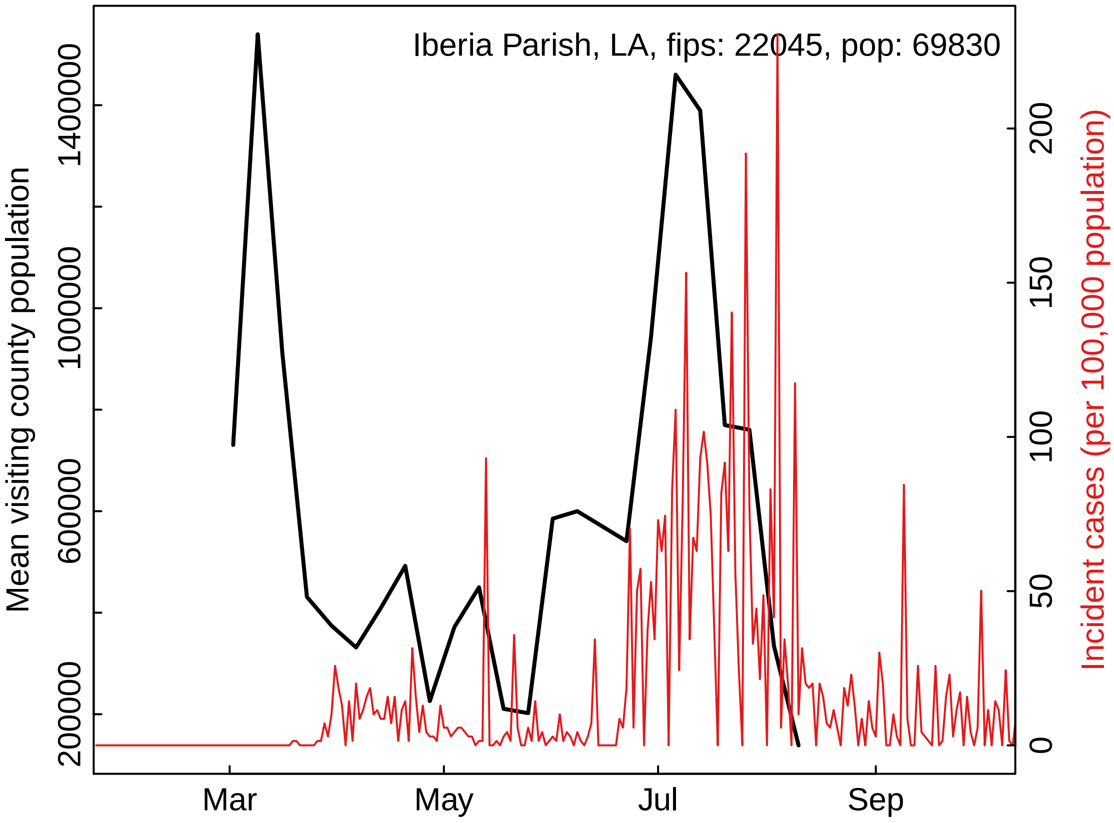

Iberia Parish, LA, fips: 22045, pop: 69830
Mean visiting county population
200000
600000
1000000
1400000
Incident cases (per 100,000 population)
0
50
100
150
200
Mar
May
Jul
Sep

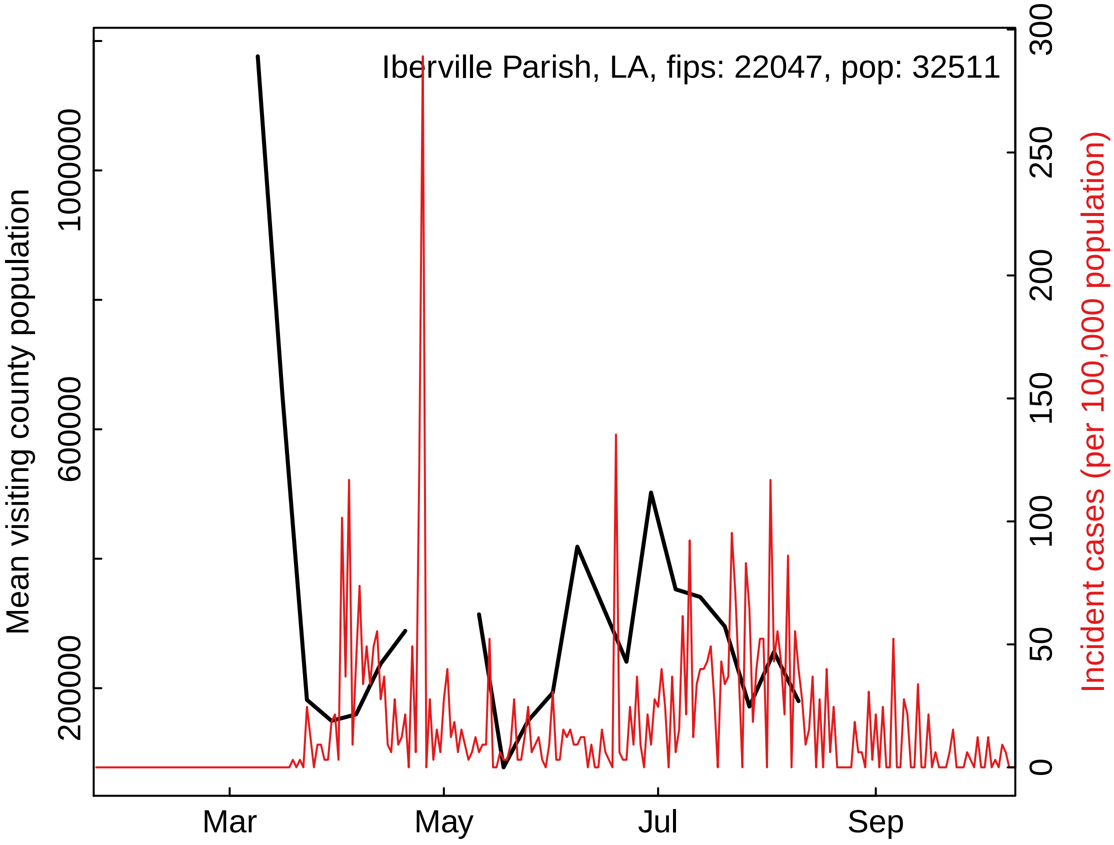

Iberville Parish, LA, fips: 22047, pop: 32511
Mean visiting county population
200000
600000
1000000
Incident cases (per 100,000 population)
0
50
100
150
200
250
300
Mar
May
Jul
Sep

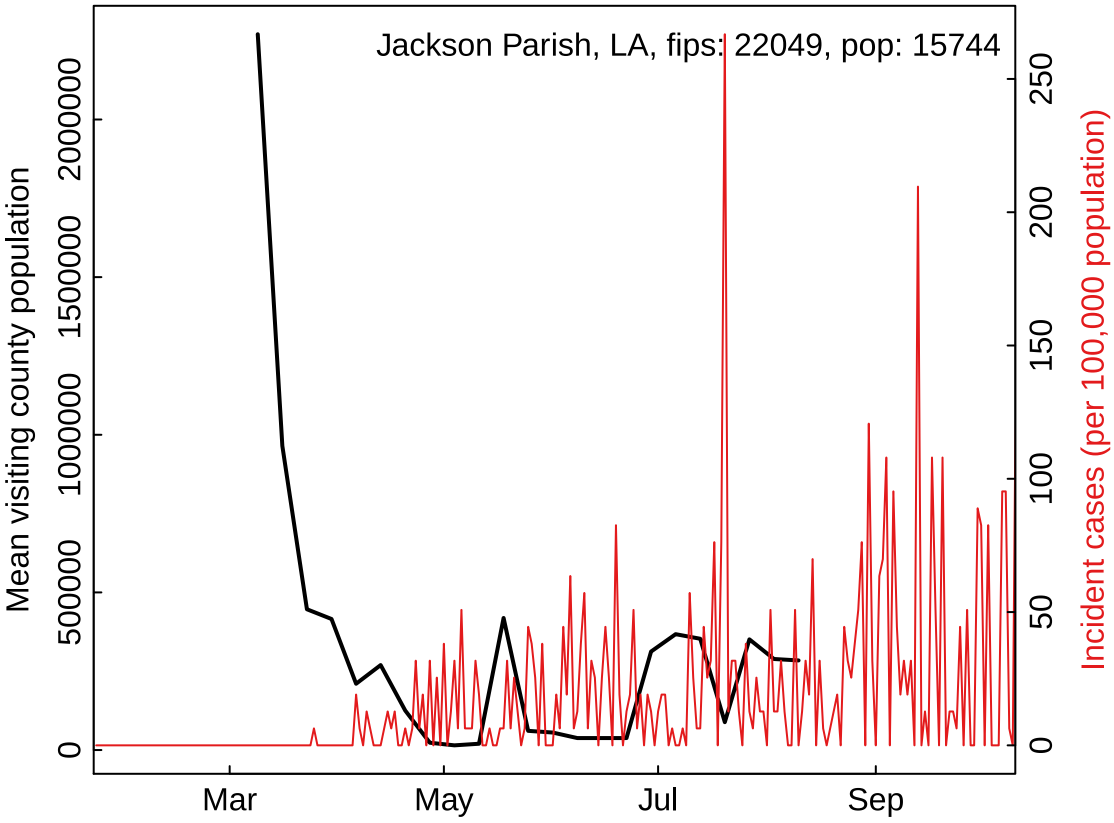

Jackson Parish, LA, fips: 22049, pop: 15744
Mean visiting county population
0
500000
1000000
1500000
2000000
Incident cases (per 100,000 population)
0
50
100
150
200
250
Mar
May
Jul
Sep

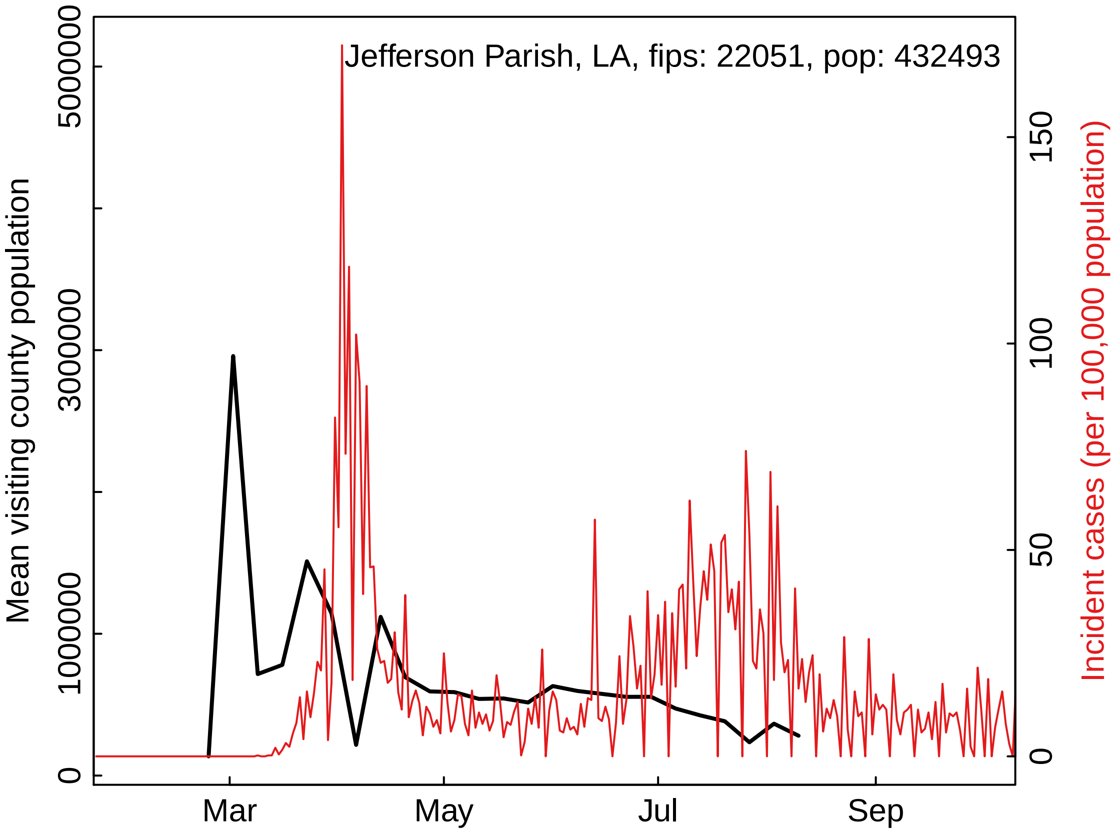

Jefferson Parish, LA, fips: 22051, pop: 432493
Mean visiting county population
Incident cases (per 100,000 population)
0
1000000
3000000
5000000
0
50
100
150
Mar
May
Jul
Sep

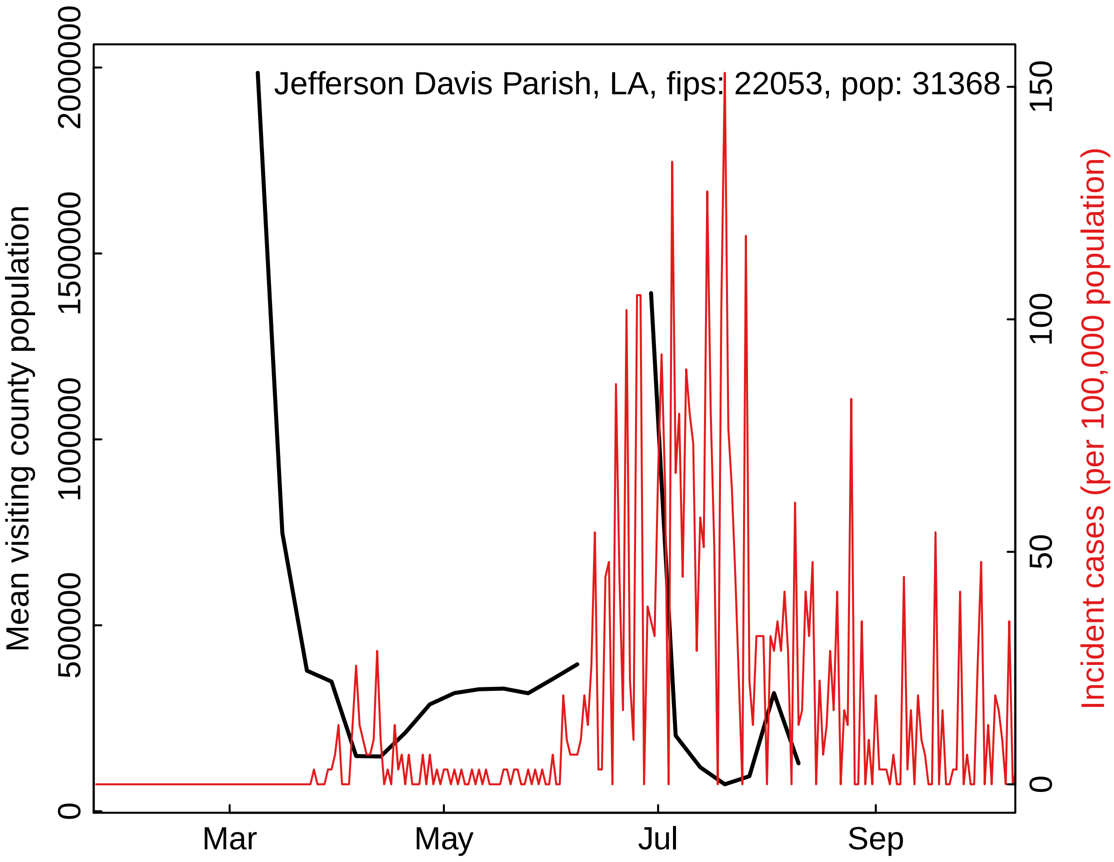
Jefferson Davis Parish, LA, fips: 22053, pop: 31368
Mean visiting county population
0
500000
1000000
1500000
2000000
Mar
May
Jul
Sep
Incident cases (per 100,000 population)
0
50
100
150

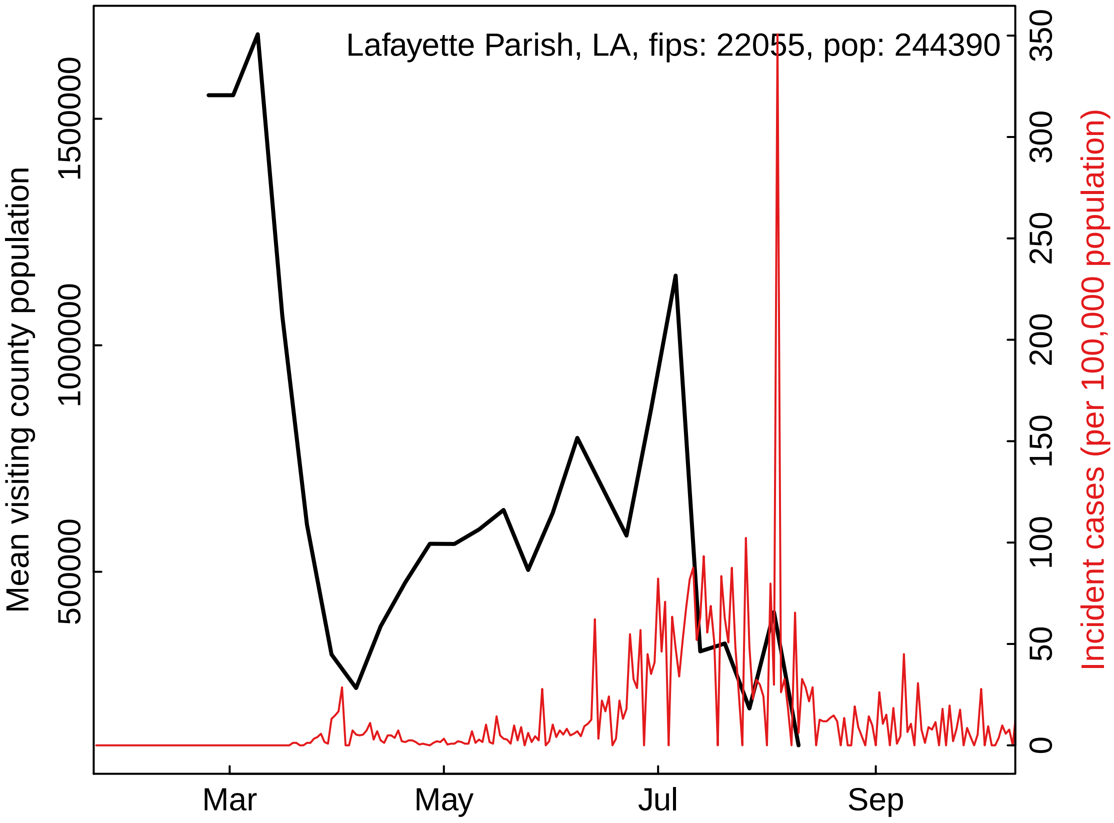

Lafayette Parish, LA, fips: 22055, pop: 244390
Mean visiting county population
500000
1000000
1500000
Incident cases (per 100,000 population)
0
50
100
150
200
250
300
350
Mar
May
Jul
Sep

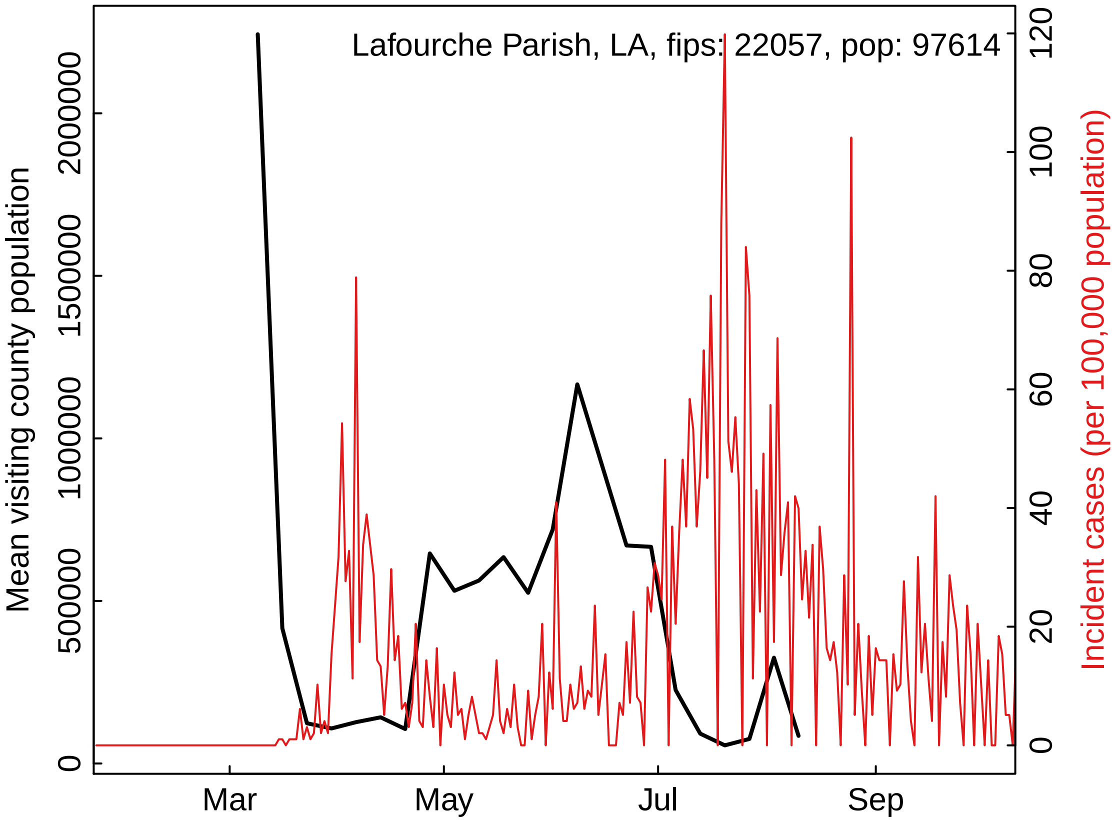
Lafourche Parish, LA, fips: 22057, pop: 97614
Mean visiting county population
0
500000
1000000
1500000
2000000
Incident cases (per 100,000 population)
0
20
40
60
80
100
120
Mar
May
Jul
Sep

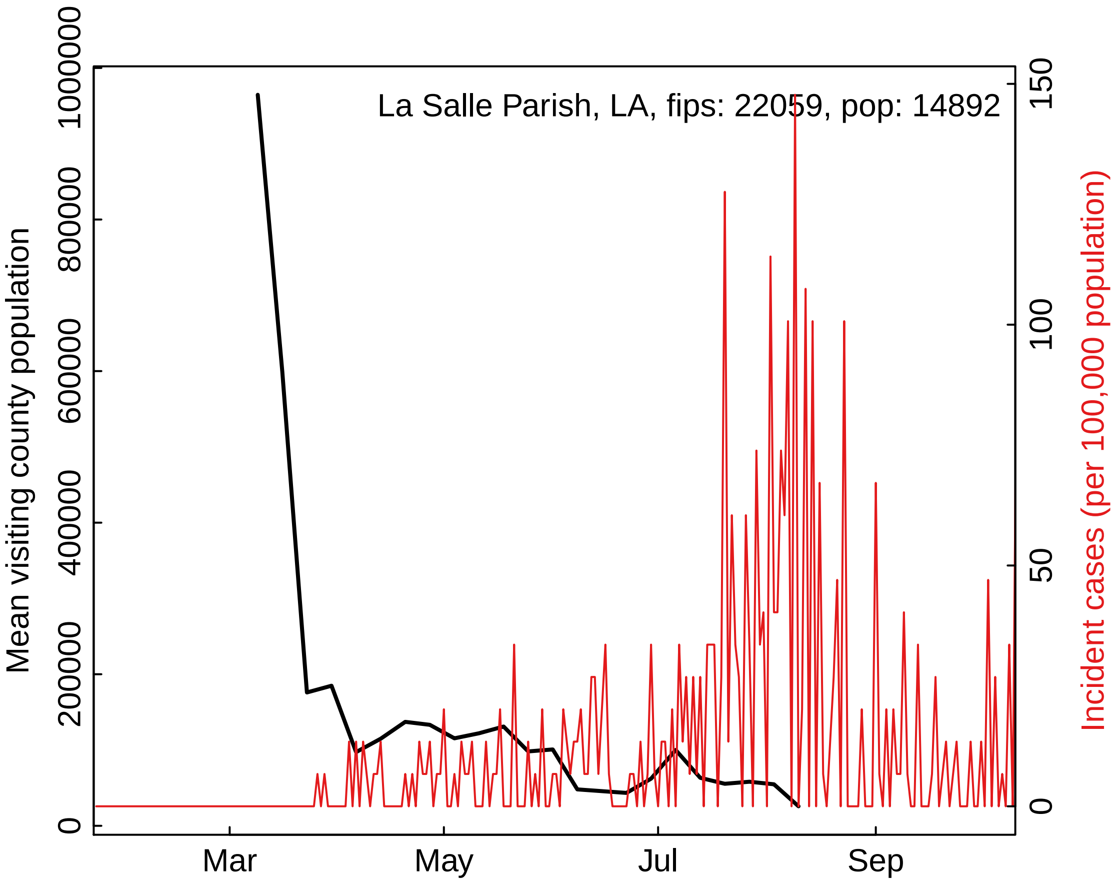
La Salle Parish, LA, fips: 22059, pop: 14892
Mean visiting county population
Incident cases (per 100,000 population)
0
200000
400000
600000
800000
1000000
0
50
100
150
Mar
May
Jul
Sep

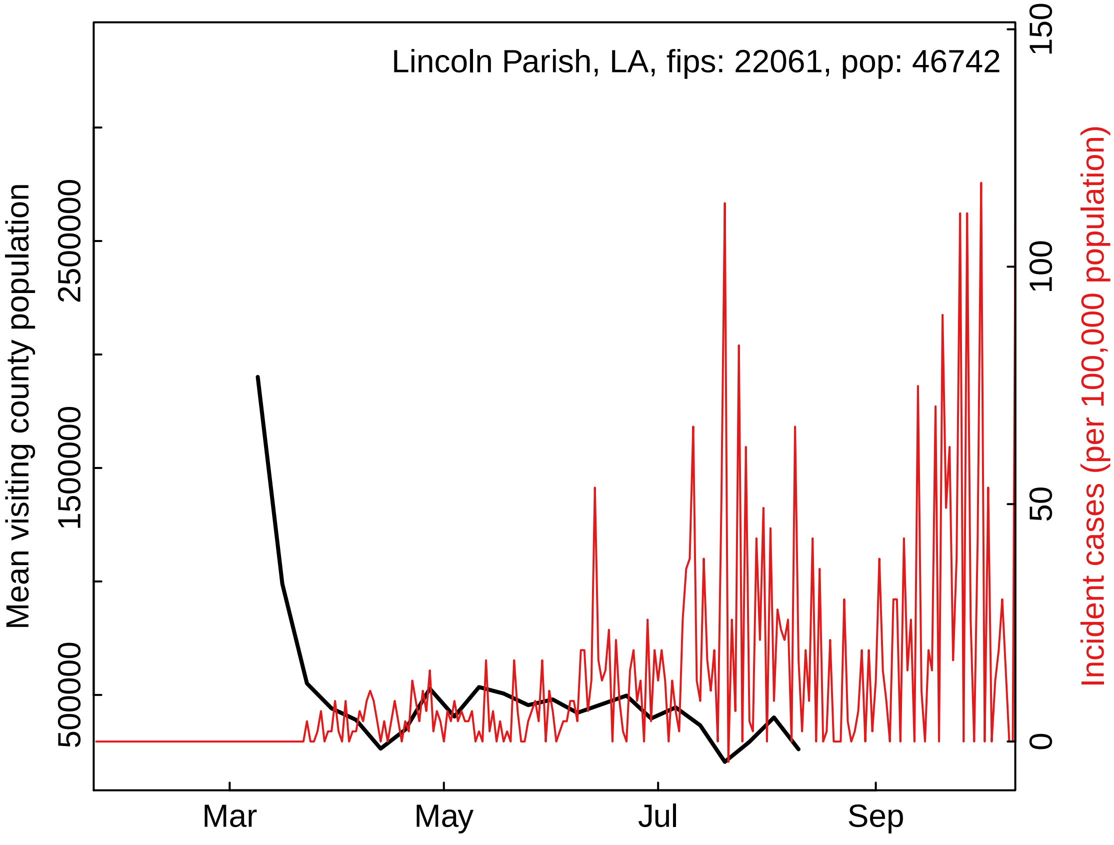

Lincoln Parish, LA, fips: 22061, pop: 46742
Mean visiting county population
500000
1500000
2500000
Mar
May
Jul
Sep
0
50
100
150
Incident cases (per 100,000 population)

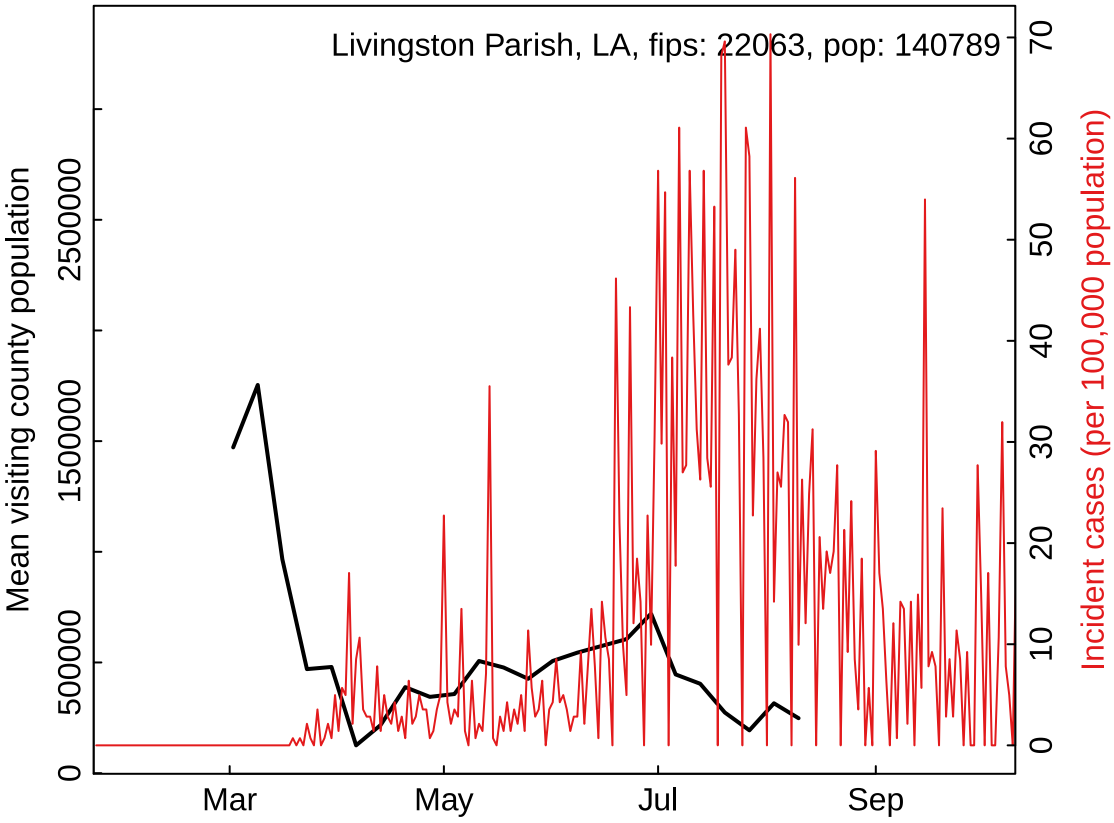

Livingston Parish, LA, fips: 22063, pop: 140789
Mean visiting county population
Incident cases (per 100,000 population)
0
500000
1500000
2500000
0
10
20
30
40
50
60
70
Mar
May
Jul
Sep

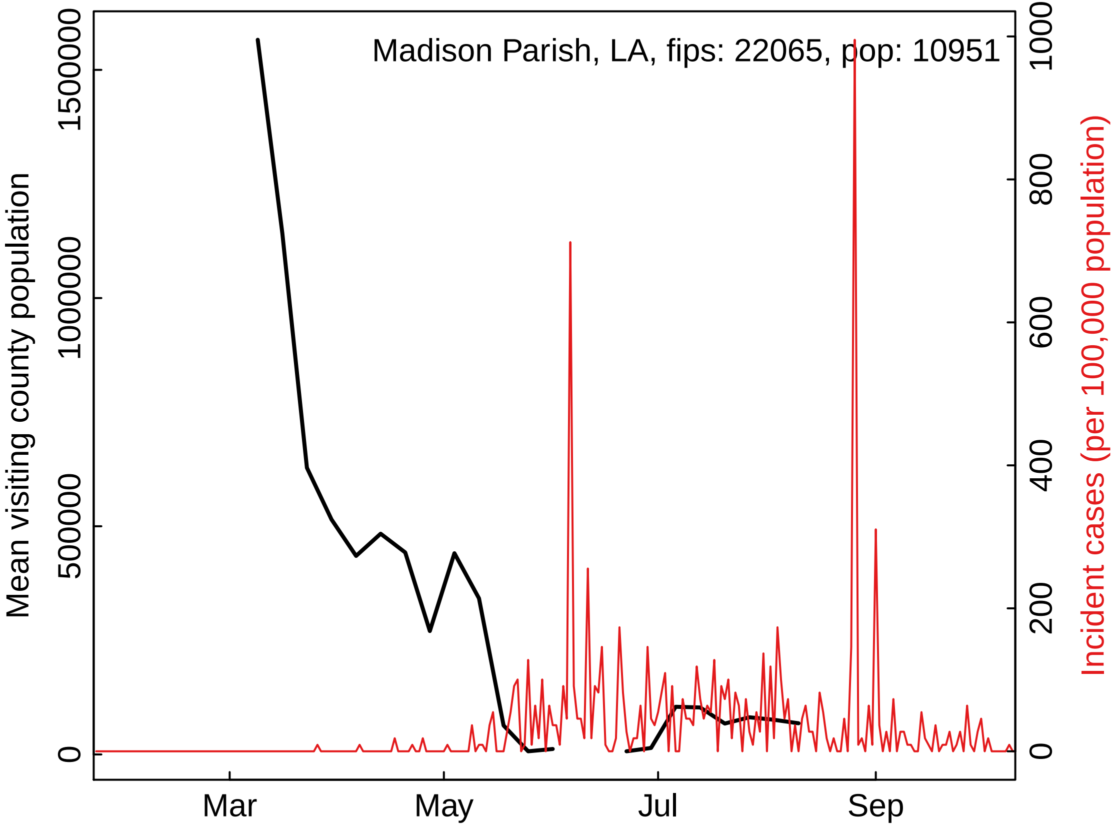
Madison Parish, LA, fips: 22065, pop: 10951
Mean visiting county population
0
500000
1000000
1500000
Incident cases (per 100,000 population)
0
200
400
600
800
1000
Mar
May
Jul
Sep

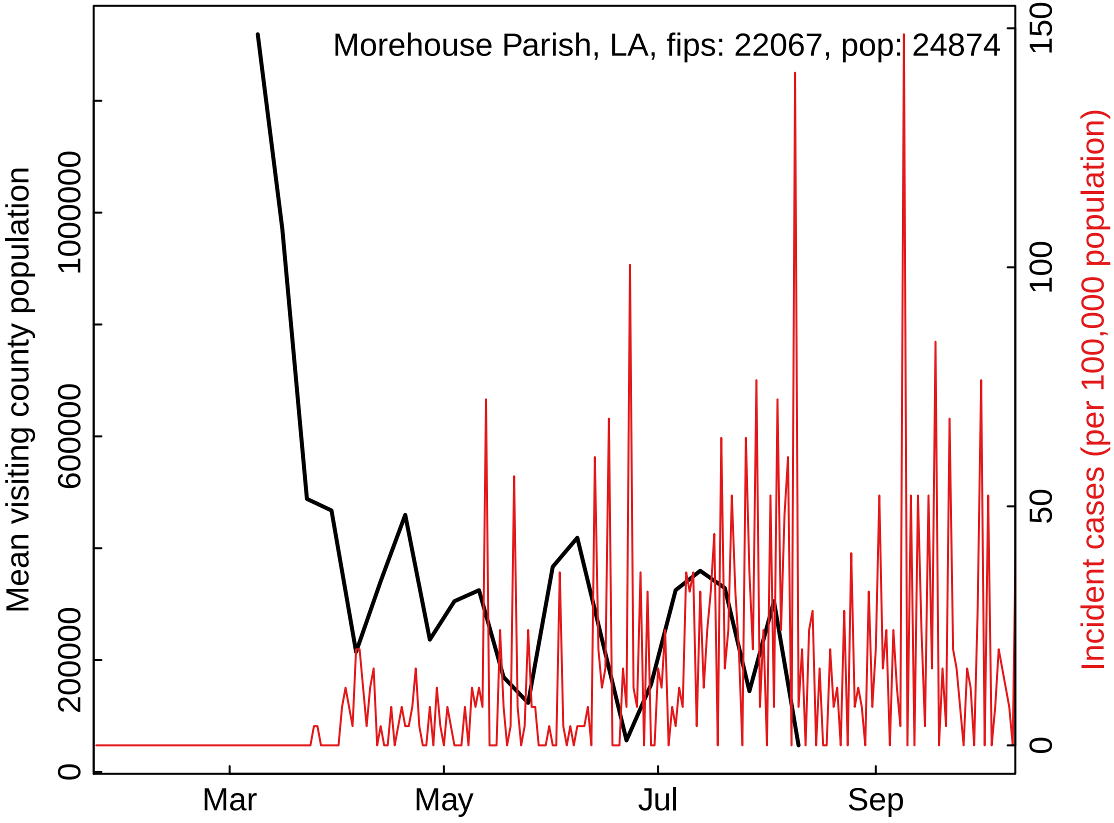

Morehouse Parish, LA, fips: 22067, pop: 24874
Mean visiting county population
Incident cases (per 100,000 population)
0
200000
600000
1000000
0
50
100
150
Mar
May
Jul
Sep

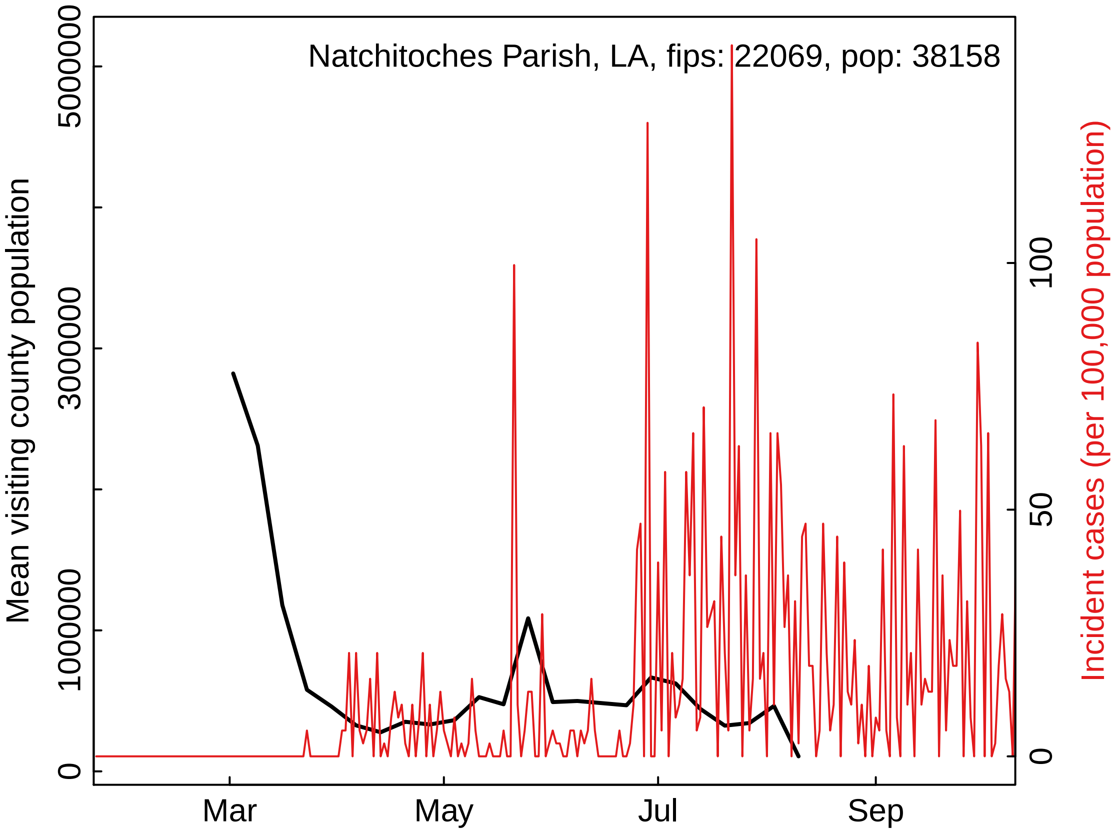

Natchitoches Parish, LA, fips: 22069, pop: 38158
Mean visiting county population
Incident cases (per 100,000 population)
0
1000000
3000000
5000000
0
50
100
Mar
May
Jul
Sep

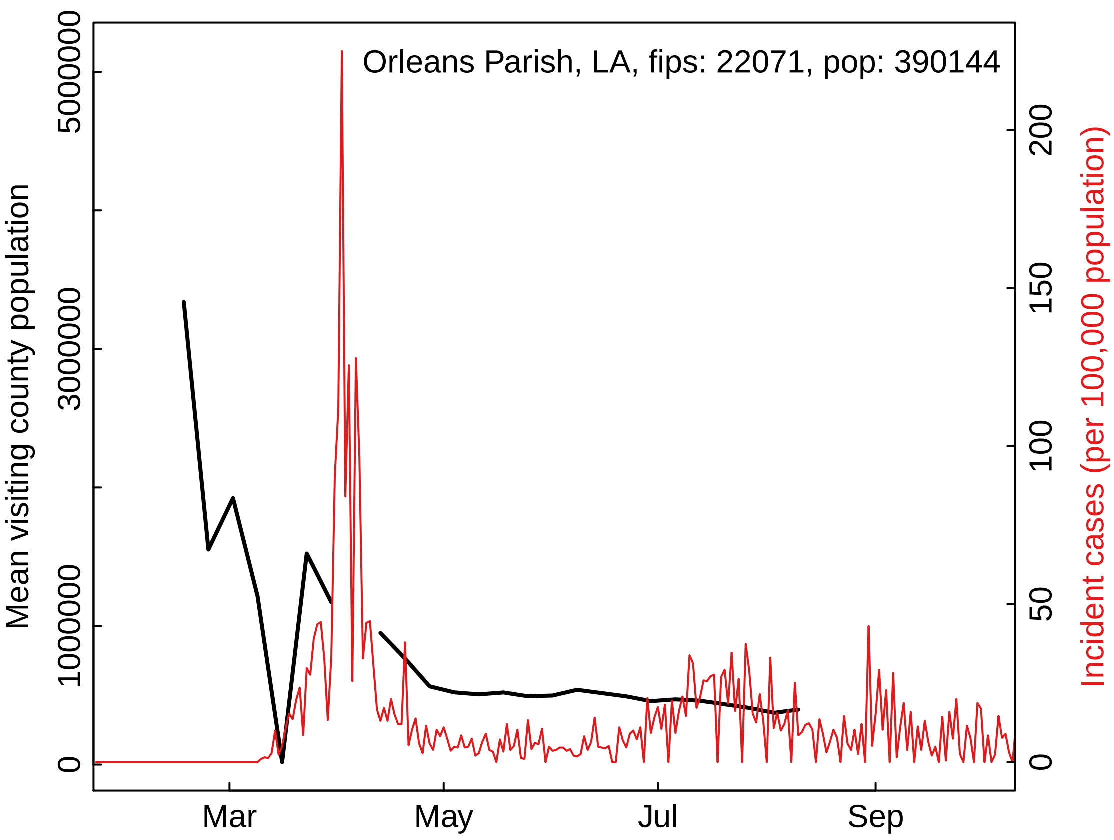

Orleans Parish, LA, fips: 22071, pop: 390144
Mean visiting county population
Incident cases (per 100,000 population)
0
1000000
3000000
5000000
0
50
100
150
200
Mar
May
Jul
Sep

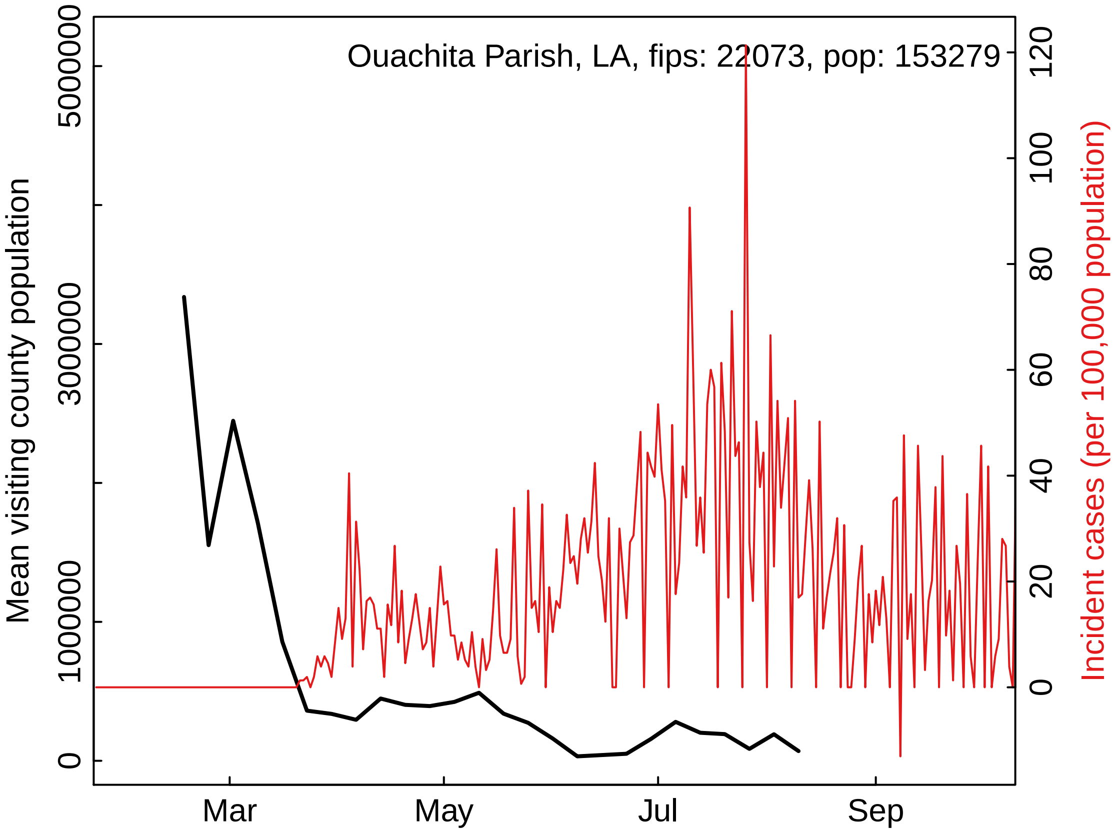

Ouachita Parish, LA, fips: 22073, pop: 153279
Mean visiting county population
0
1000000
3000000
5000000
Incident cases (per 100,000 population)
0
20
40
60
80
100
120
Mar
May
Jul
Sep

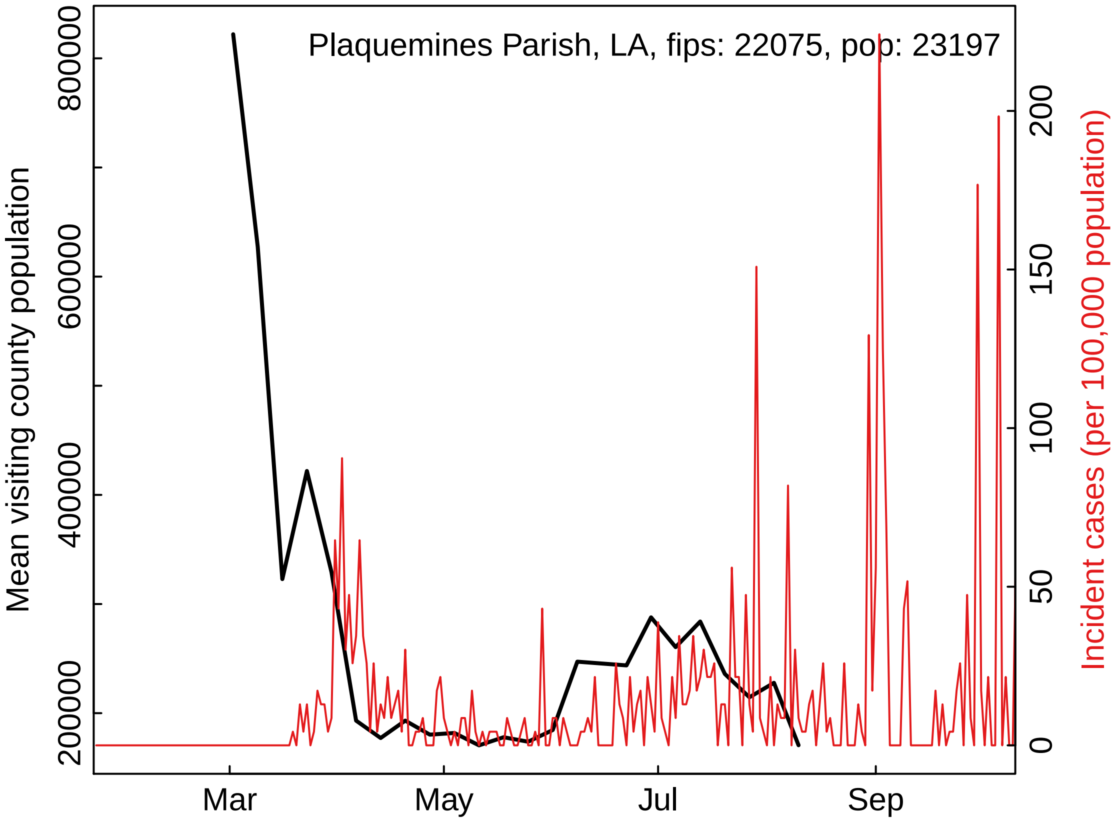

Plaquemines Parish, LA, fips: 22075, pop: 23197
Mean visiting county population
200000
400000
600000
800000
Incident cases (per 100,000 population)
0
50
100
150
200
Mar
May
Jul
Sep

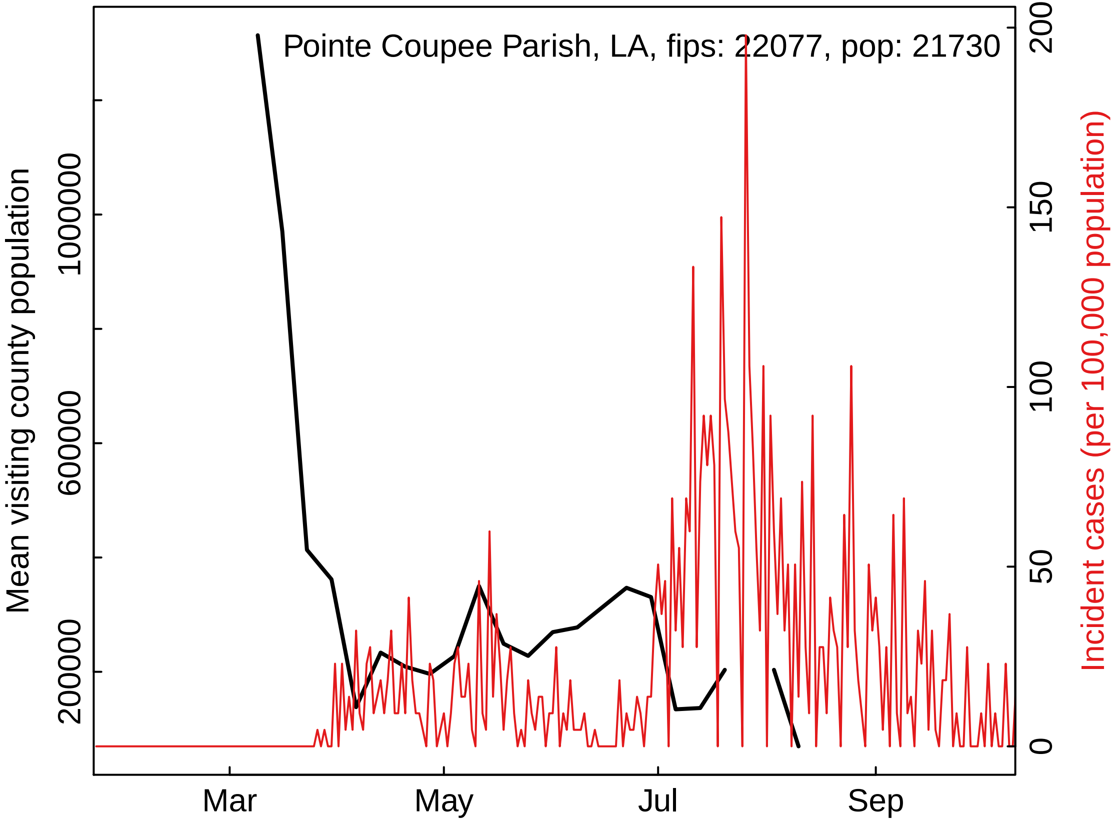

Pointe Coupee Parish, LA, fips: 22077, pop: 21730
Mean visiting county population
200000
600000
1000000
Incident cases (per 100,000 population)
0
50
100
150
200
Mar
May
Jul
Sep

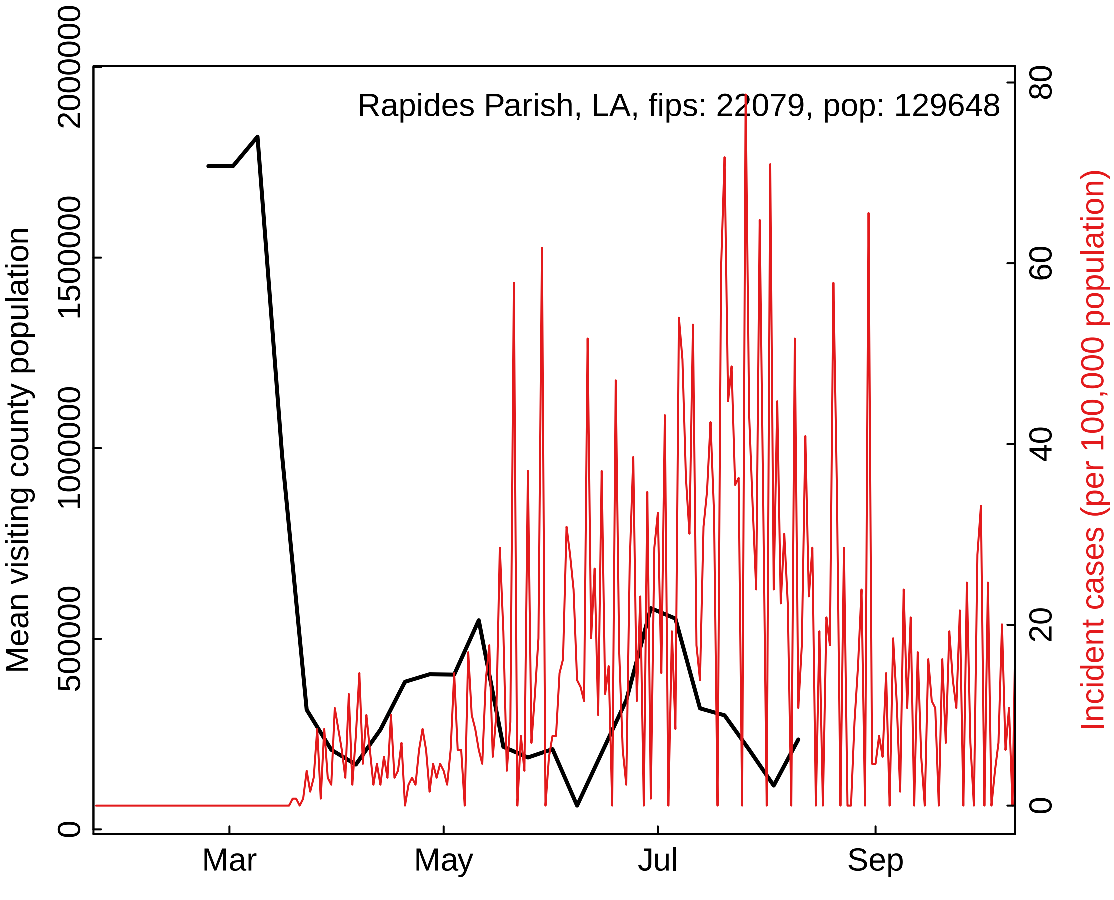

Rapides Parish, LA, fips: 22079, pop: 129648
Mean visiting county population
0
500000
1000000
1500000
2000000
Incident cases (per 100,000 population)
0
20
40
60
80
Mar
May
Jul
Sep

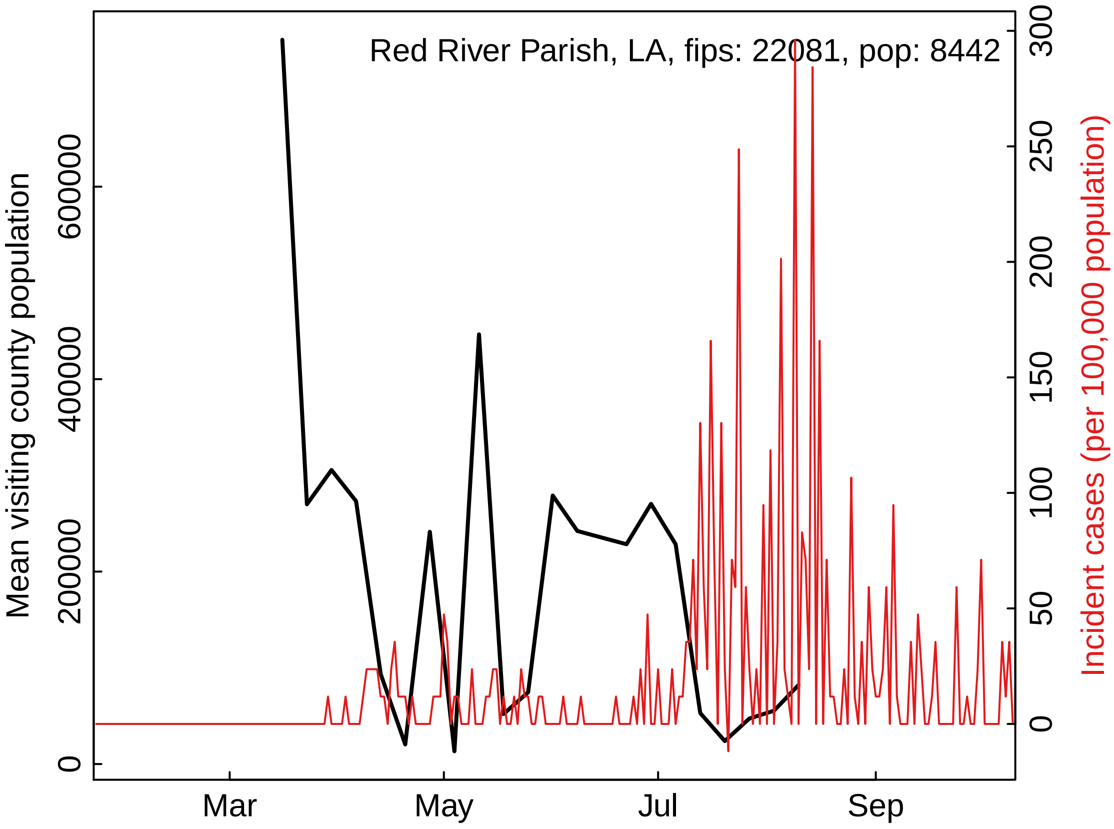

Red River Parish, LA, fips: 22081, pop: 8442
Mean visiting county population
0
200000
400000
600000
Incident cases (per 100,000 population)
0
50
100
150
200
250
300
Mar
May
Jul
Sep

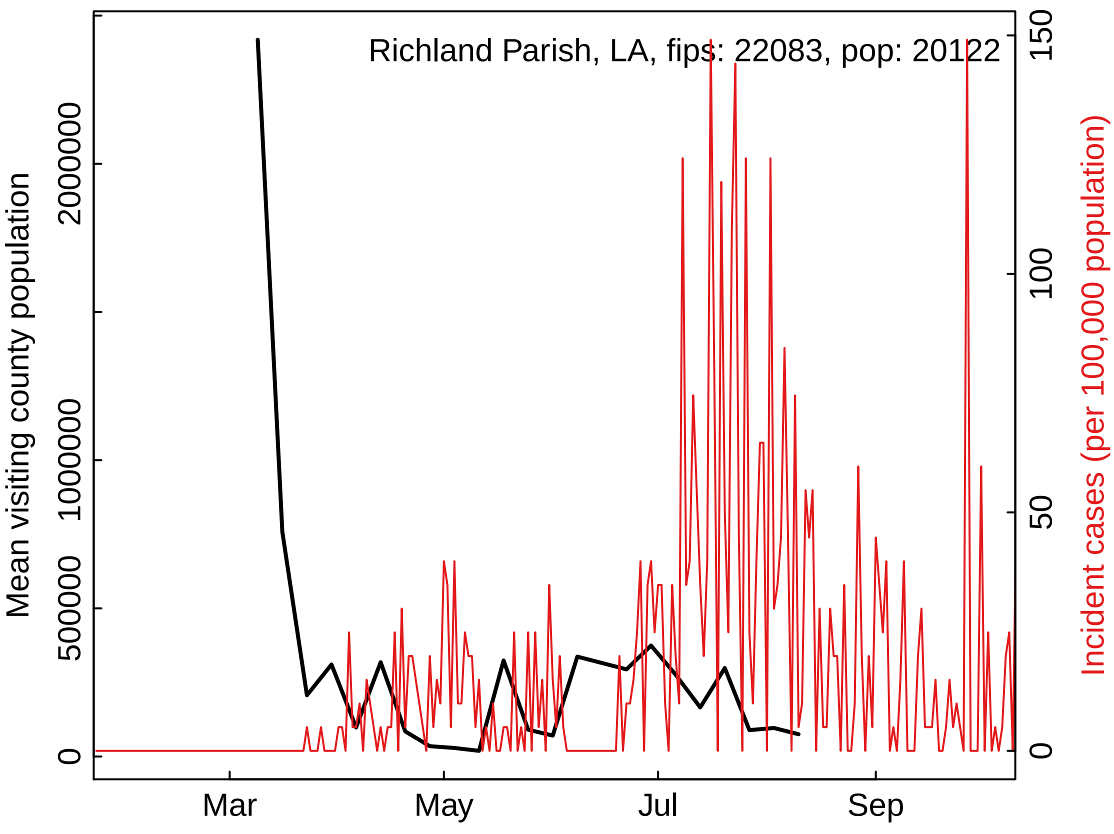

Richland Parish, LA, fips: 22083, pop: 20122
Mean visiting county population
0
500000
1000000
2000000
Incident cases (per 100,000 population)
0
50
100
150
Mar
May
Jul
Sep

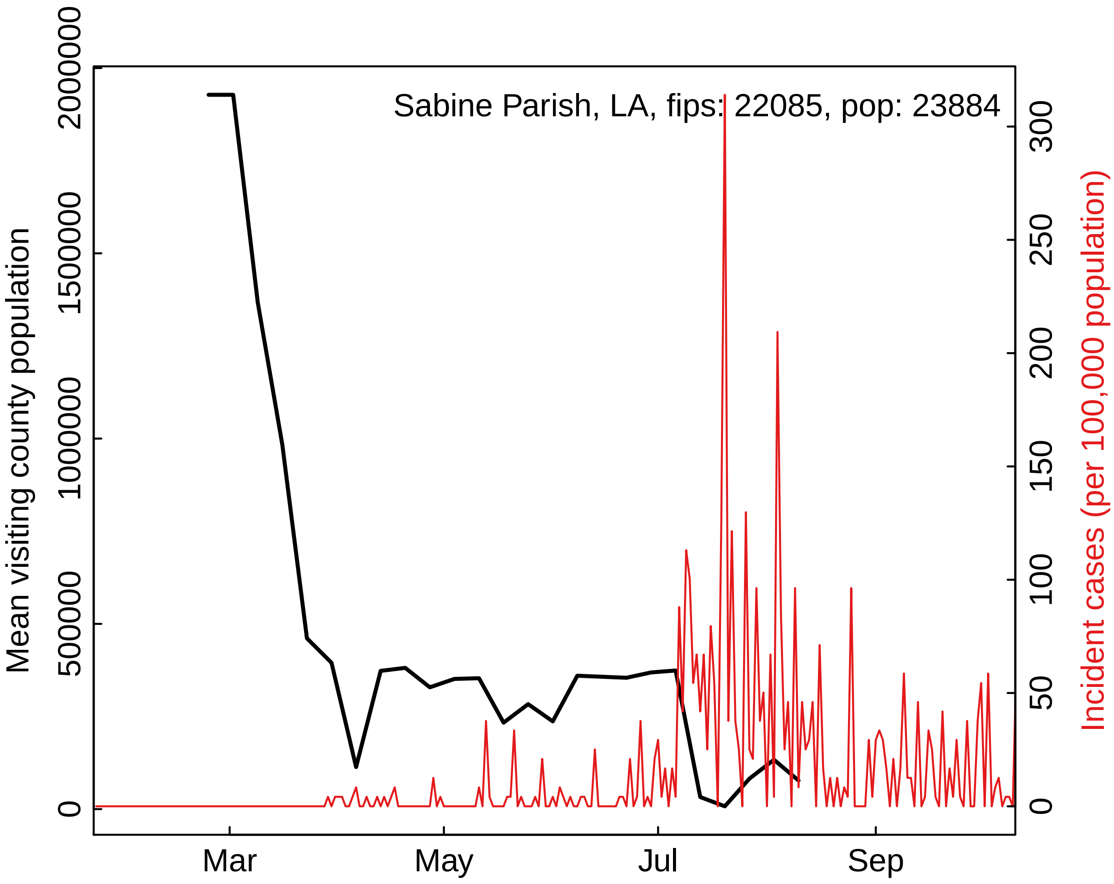
Sabine Parish, LA, fips: 22085, pop: 23884
Mean visiting county population
Incident cases (per 100,000 population)
0
500000
1000000
1500000
2000000
0
50
100
150
200
250
300
Mar
May
Jul
Sep

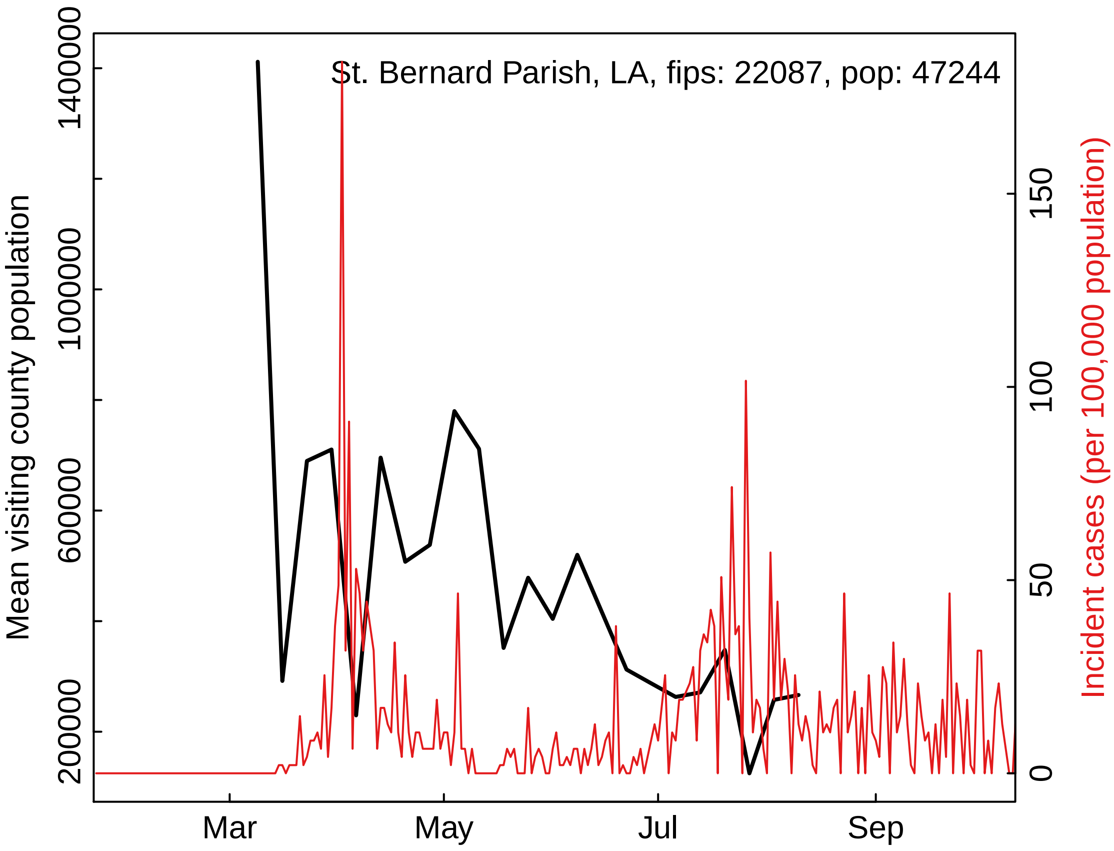
St. Bernard Parish, LA, fips: 22087, pop: 47244
Mean visiting county population
200000
600000
1000000
1400000
Incident cases (per 100,000 population)
0
50
100
150
Mar
May
Jul
Sep

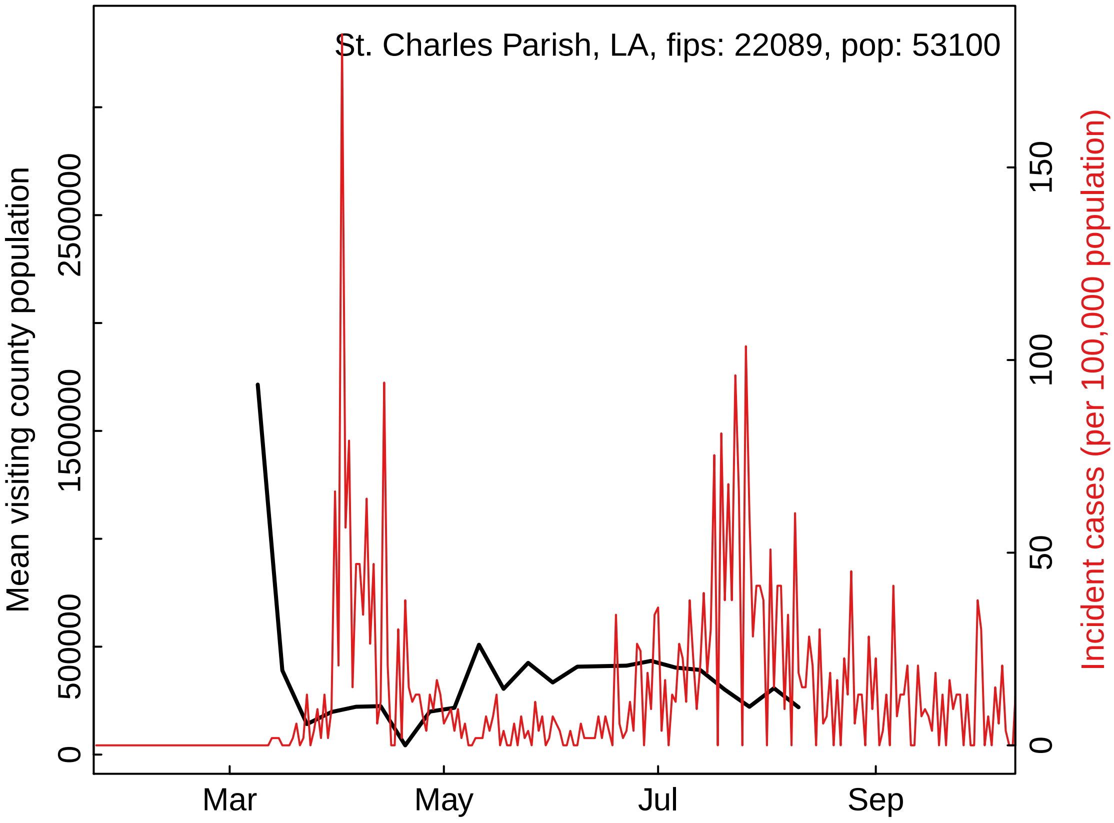

St. Charles Parish, LA, fips: 22089, pop: 53100
Mean visiting county population
0
500000
1500000
2500000
Incident cases (per 100,000 population)
0
50
100
150
Mar
May
Jul
Sep

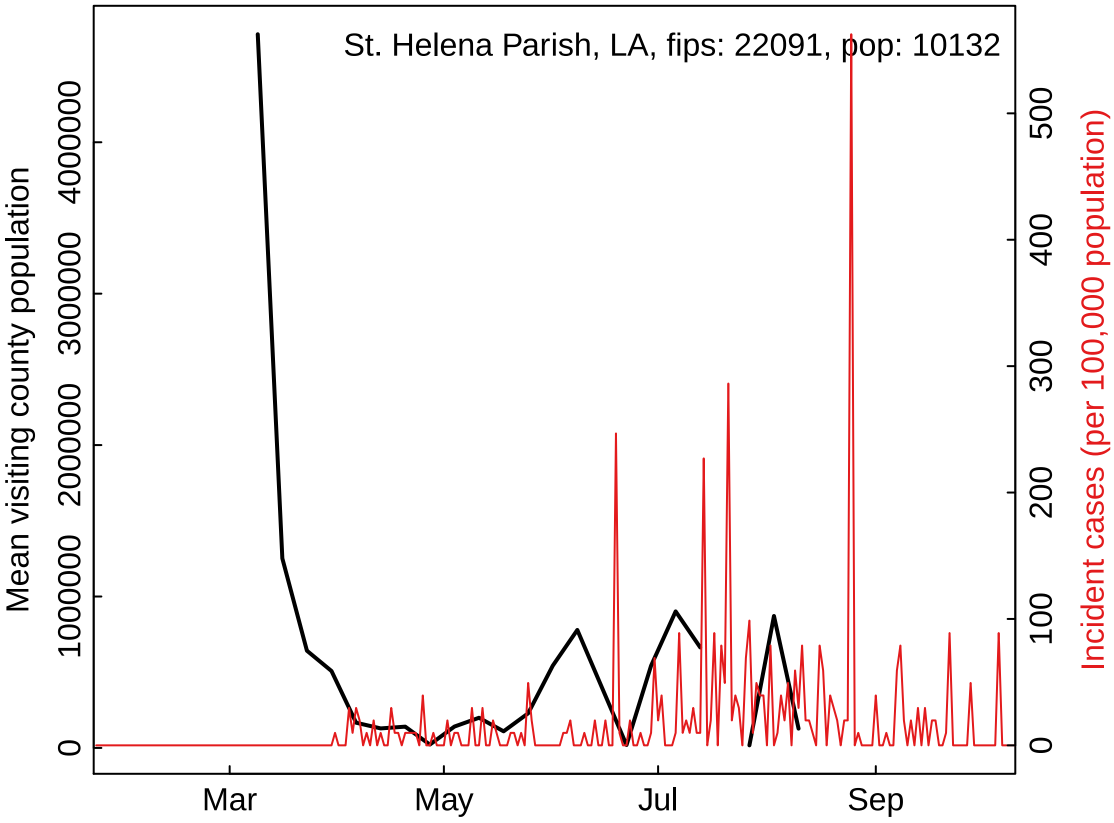
St. Helena Parish, LA, fips: 22091, pop: 10132
Mean visiting county population
0
1000000
2000000
3000000
4000000
Incident cases (per 100,000 population)
0
100
200
300
400
500
Mar
May
Jul
Sep

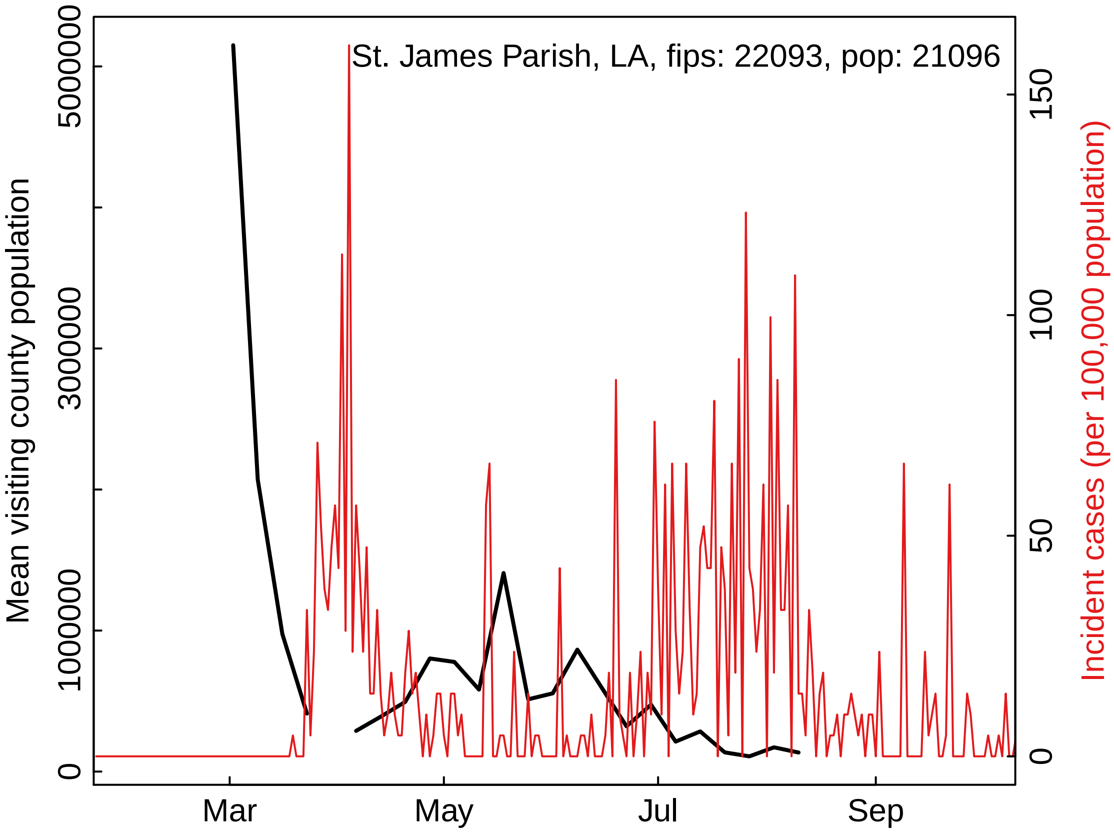

St. James Parish, LA, fips: 22093, pop: 21096
Mean visiting county population
Incident cases (per 100,000 population)
0
1000000
3000000
5000000
0
50
100
150
Mar
May
Jul
Sep

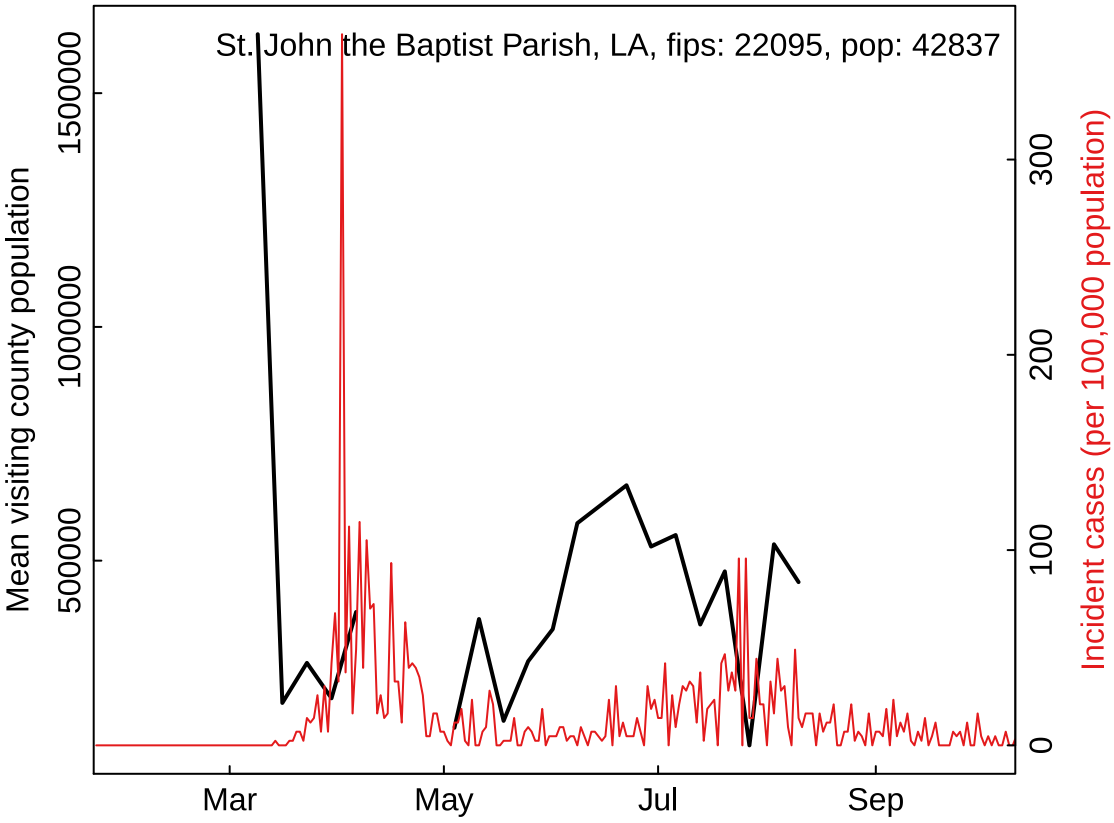
St. John the Baptist Parish, LA, fips: 22095, pop: 42837
Mean visiting county population
500000
1000000
1500000
Incident cases (per 100,000 population)
0
100
200
300
Mar
May
Jul
Sep

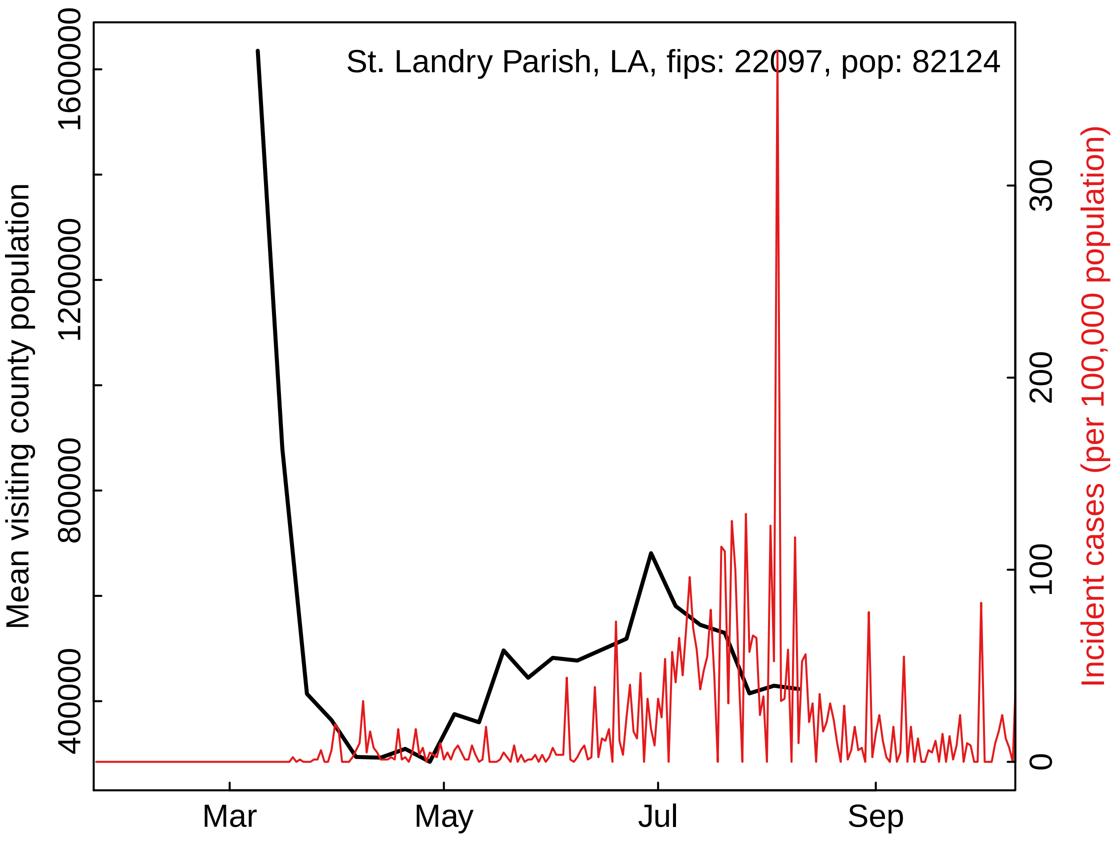

St. Landry Parish, LA, fips: 22097, pop: 82124
Mean visiting county population
400000
800000
1200000
1600000
Incident cases (per 100,000 population)
0
100
200
300
Mar
May
Jul
Sep

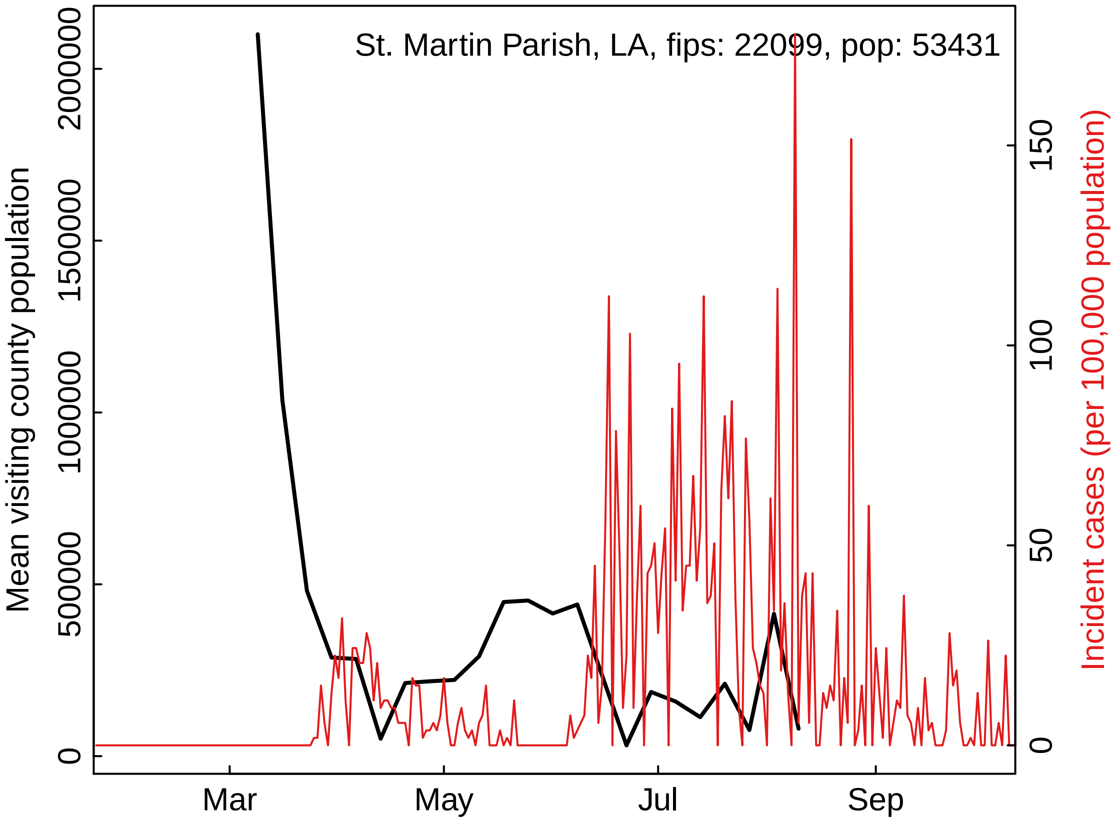
St. Martin Parish, LA, fips: 22099, pop: 53431
Mean visiting county population
0
500000
1000000
1500000
2000000
Incident cases (per 100,000 population)
0
50
100
150
Mar
May
Jul
Sep

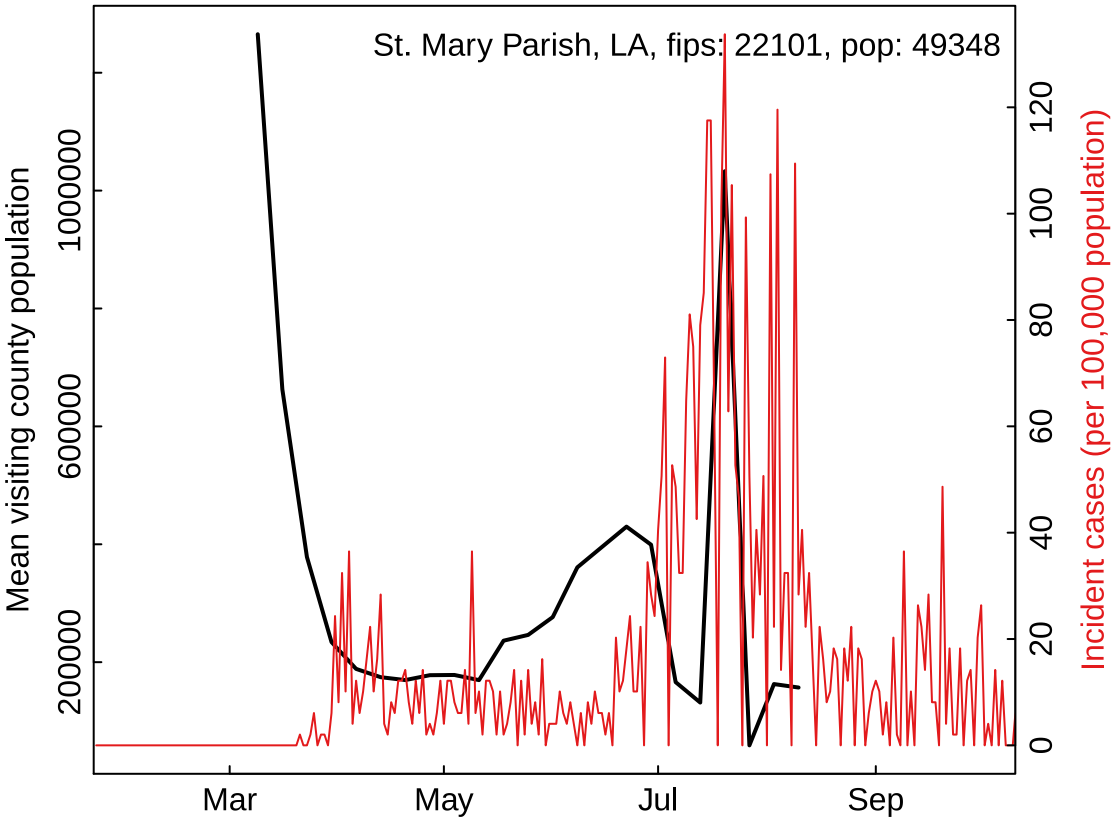

St. Mary Parish, LA, fips: 22101, pop: 49348
Mean visiting county population
200000
600000
1000000
Incident cases (per 100,000 population)
0
20
40
60
80
100
120
Mar
May
Jul
Sep

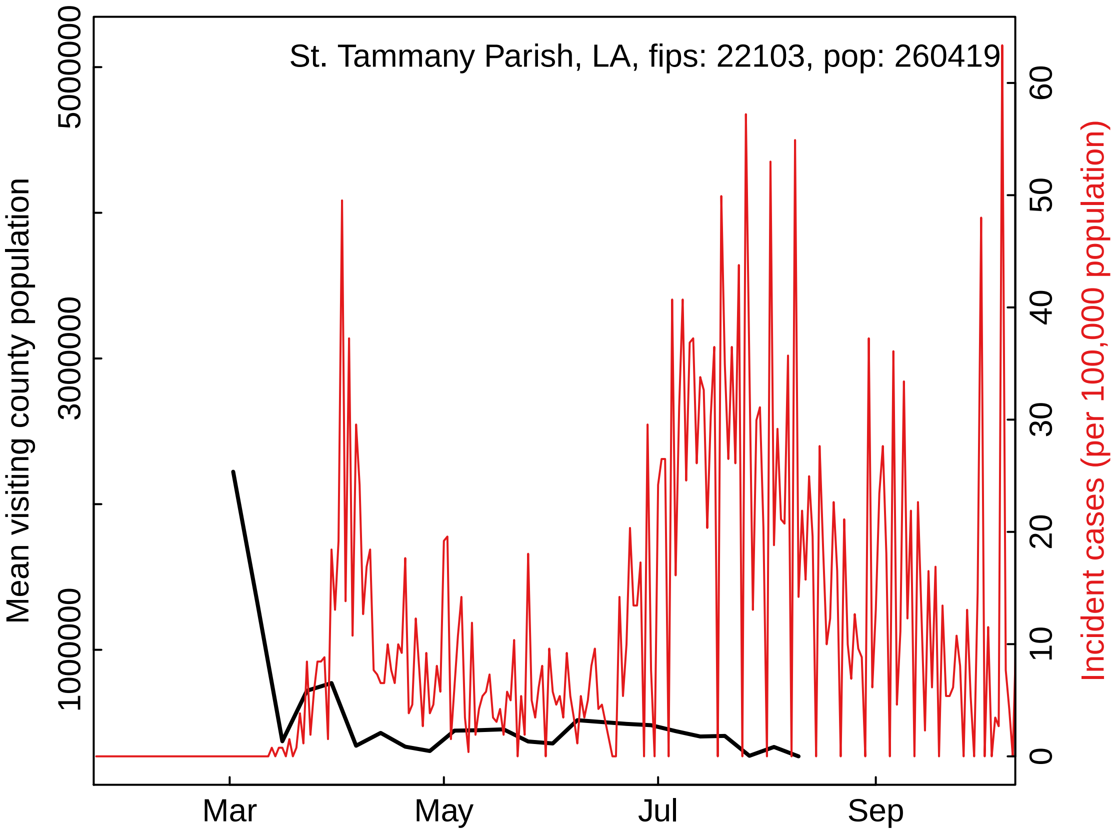

St. Tammany Parish, LA, fips: 22103, pop: 260419
Mean visiting county population
1000000
3000000
5000000
Incident cases (per 100,000 population)
0
10
20
30
40
50
60
Mar
May
Jul
Sep

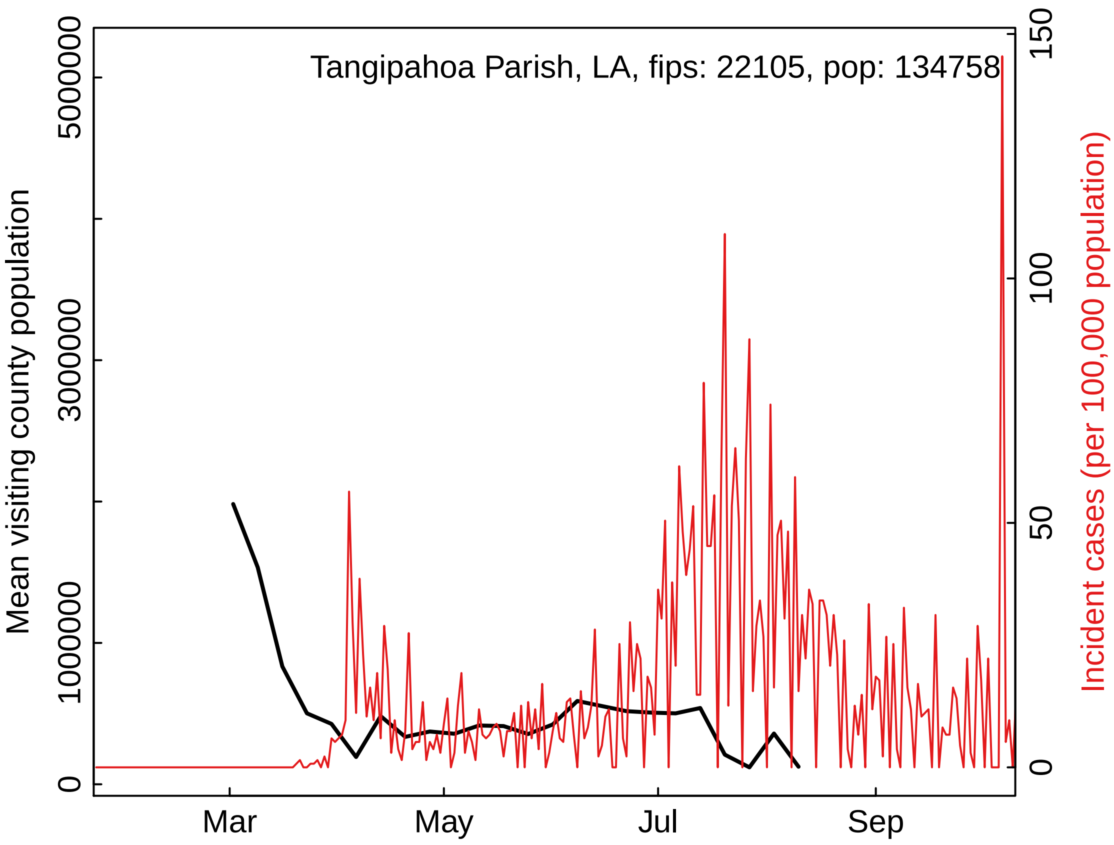

Tangipahoa Parish, LA, fips: 22105, pop: 134758
Mean visiting county population
0
1000000
3000000
5000000
Incident cases (per 100,000 population)
0
50
100
150
Mar
May
Jul
Sep

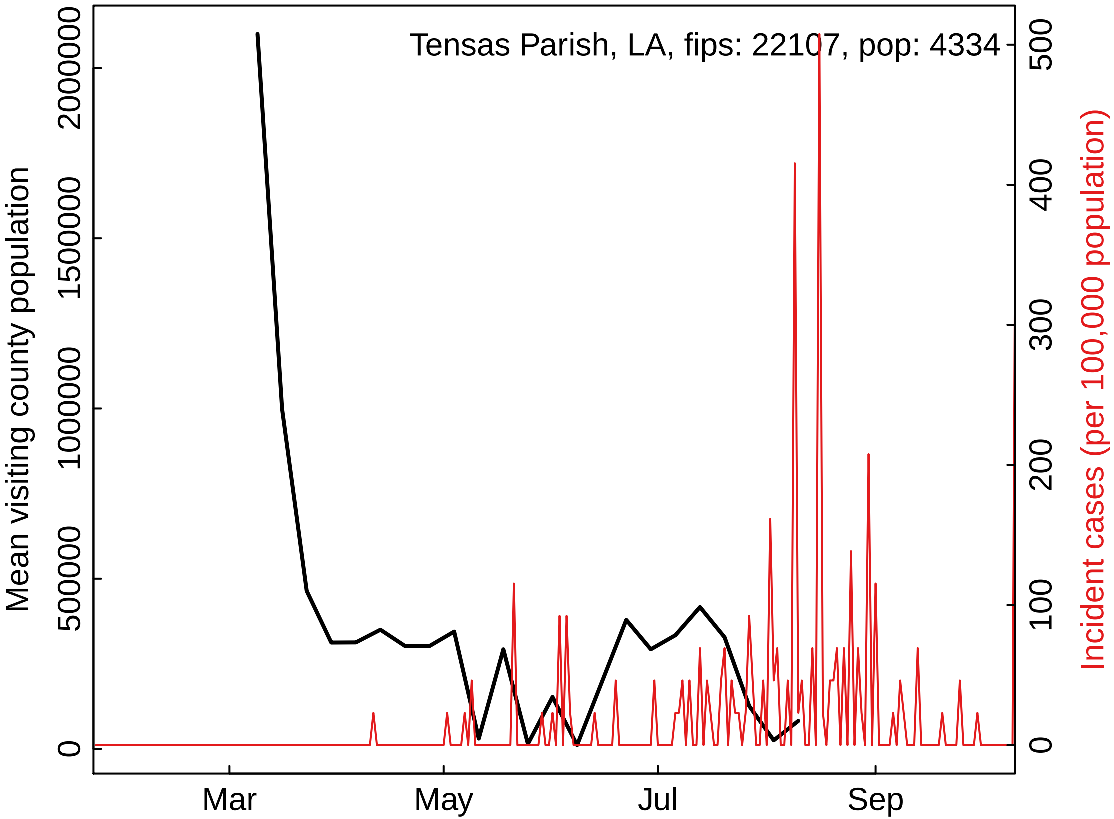
Tensas Parish, LA, fips: 22107, pop: 4334
Mean visiting county population
0
500000
1000000
1500000
2000000
Incident cases (per 100,000 population)
0
100
200
300
400
500
Mar
May
Jul
Sep

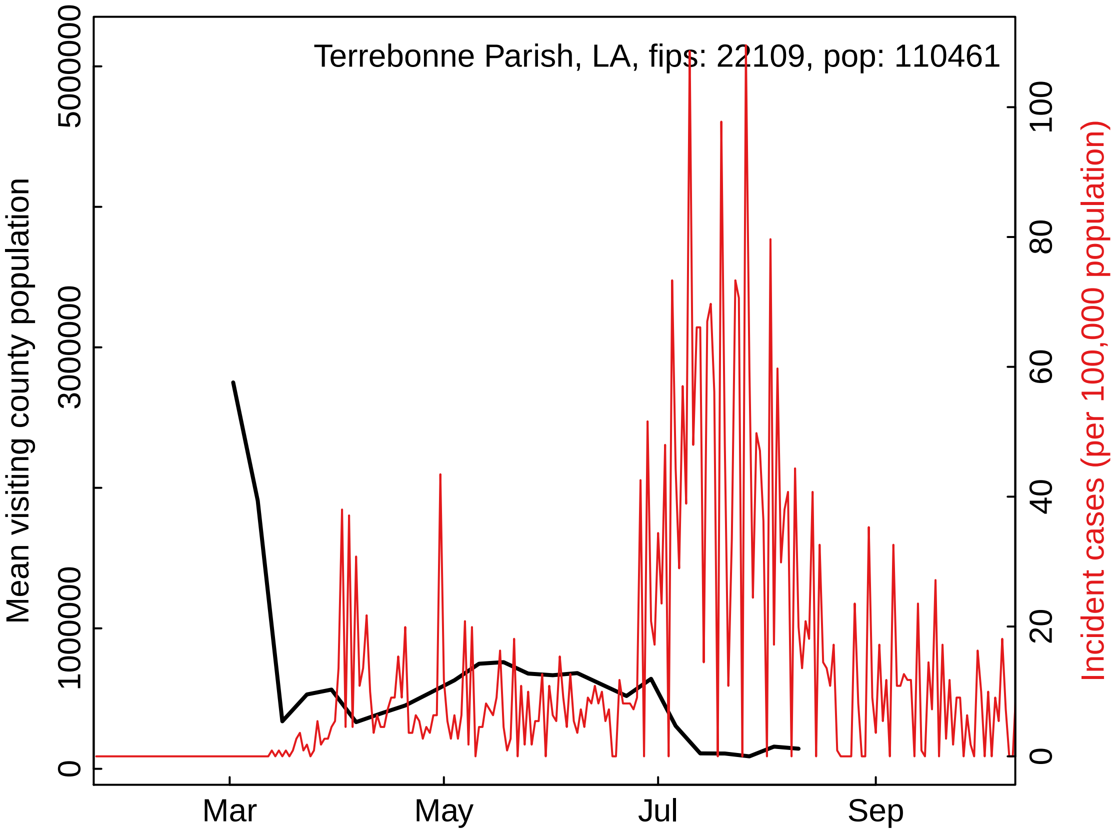
Terrebonne Parish, LA, fips: 22109, pop: 110461
Mean visiting county population
0
1000000
3000000
5000000
Incident cases (per 100,000 population)
0
20
40
60
80
100
Mar
May
Jul
Sep

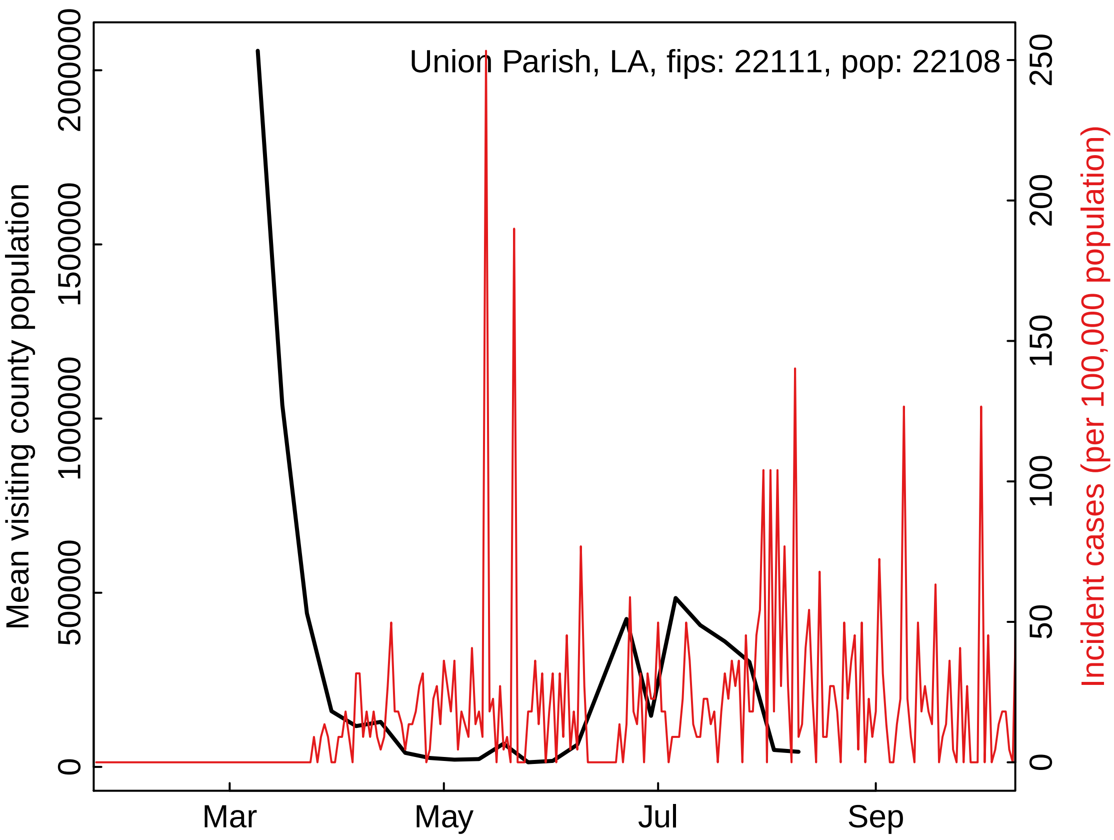

Union Parish, LA, fips: 22111, pop: 22108
Mean visiting county population
Incident cases (per 100,000 population)
0
500000
1000000
1500000
2000000
0
50
100
150
200
250
Mar
May
Jul
Sep

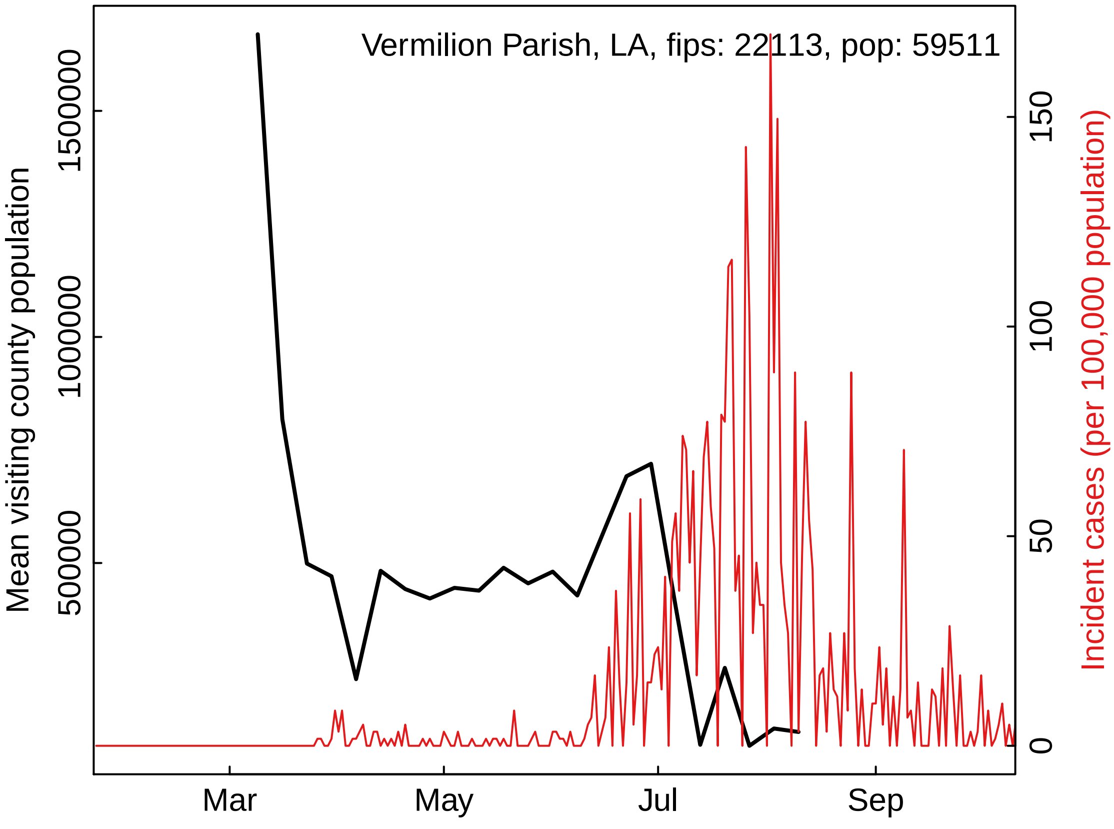

Vermilion Parish, LA, fips: 22113, pop: 59511
Mean visiting county population
500000
1000000
1500000
Incident cases (per 100,000 population)
0
50
100
150
Mar
May
Jul
Sep

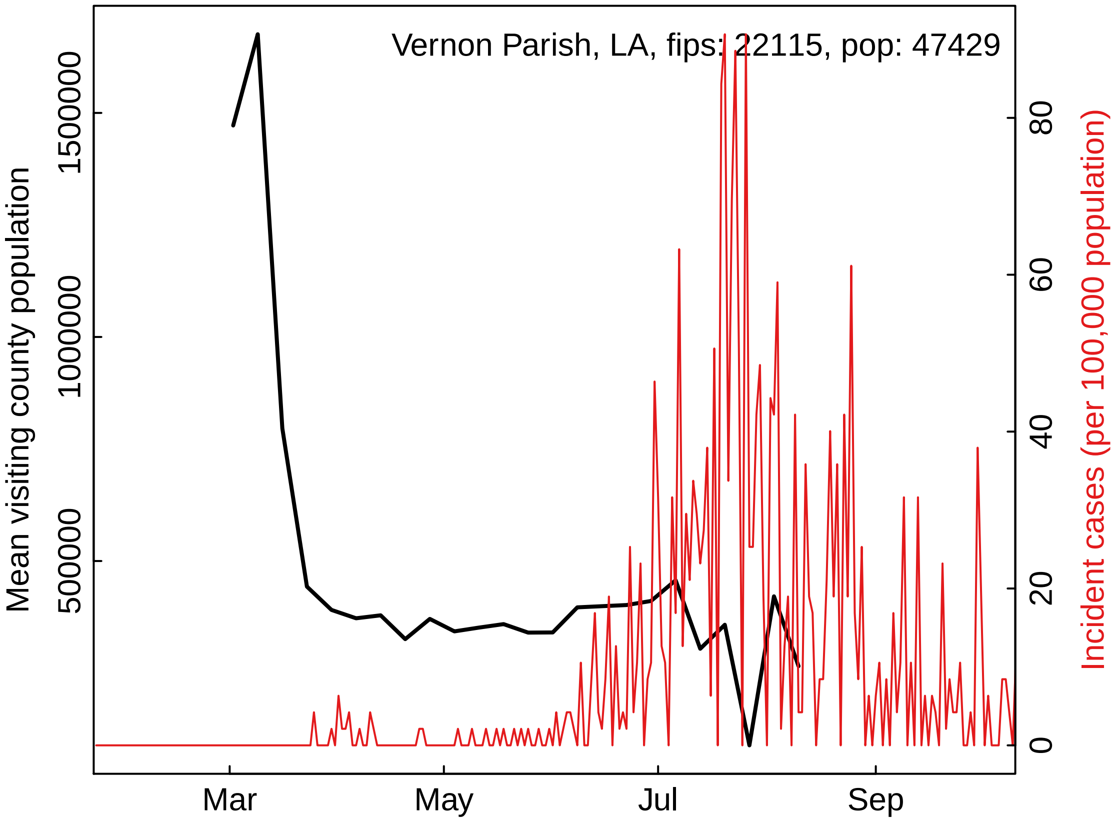

Vernon Parish, LA, fips: 22115, pop: 47429
Mean visiting county population
500000
1000000
1500000
Incident cases (per 100,000 population)
0
20
40
60
80
Mar
May
Jul
Sep

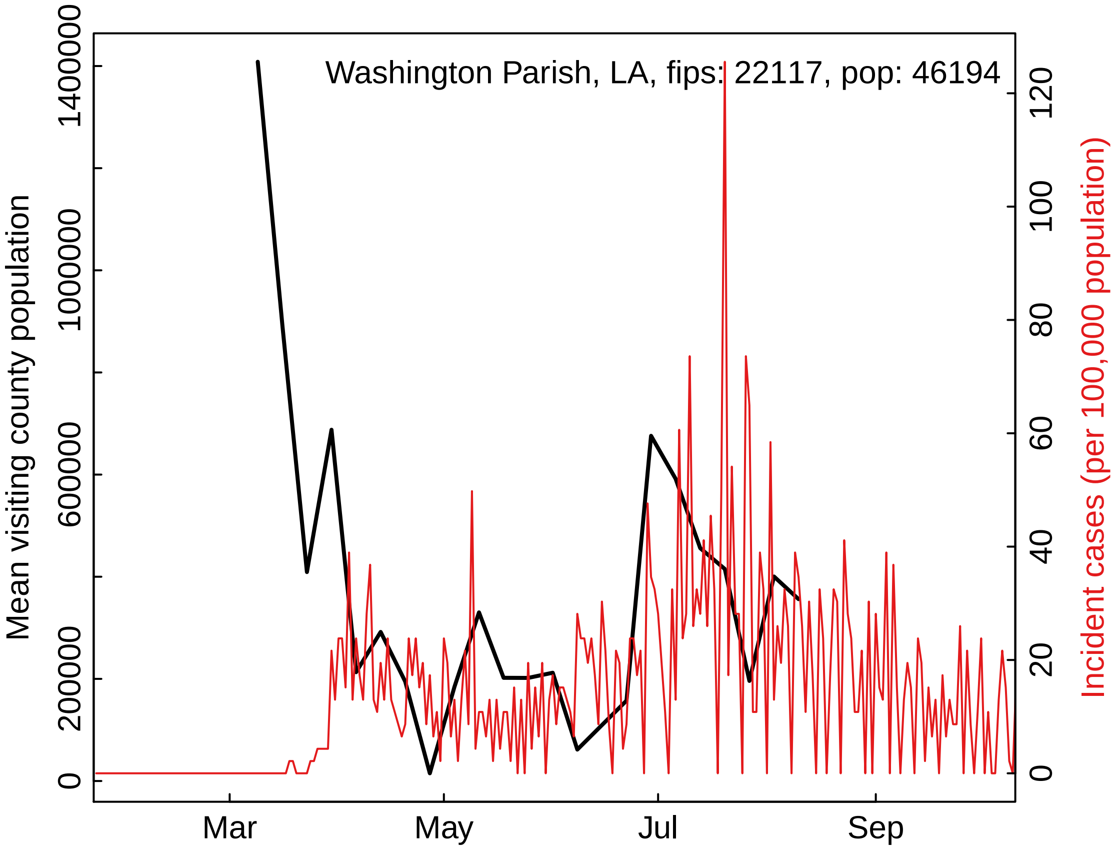

Washington Parish, LA, fips: 22117, pop: 46194
Mean visiting county population
0
200000
600000
1000000
1400000
Incident cases (per 100,000 population)
0
20
40
60
80
100
120
Mar
May
Jul
Sep

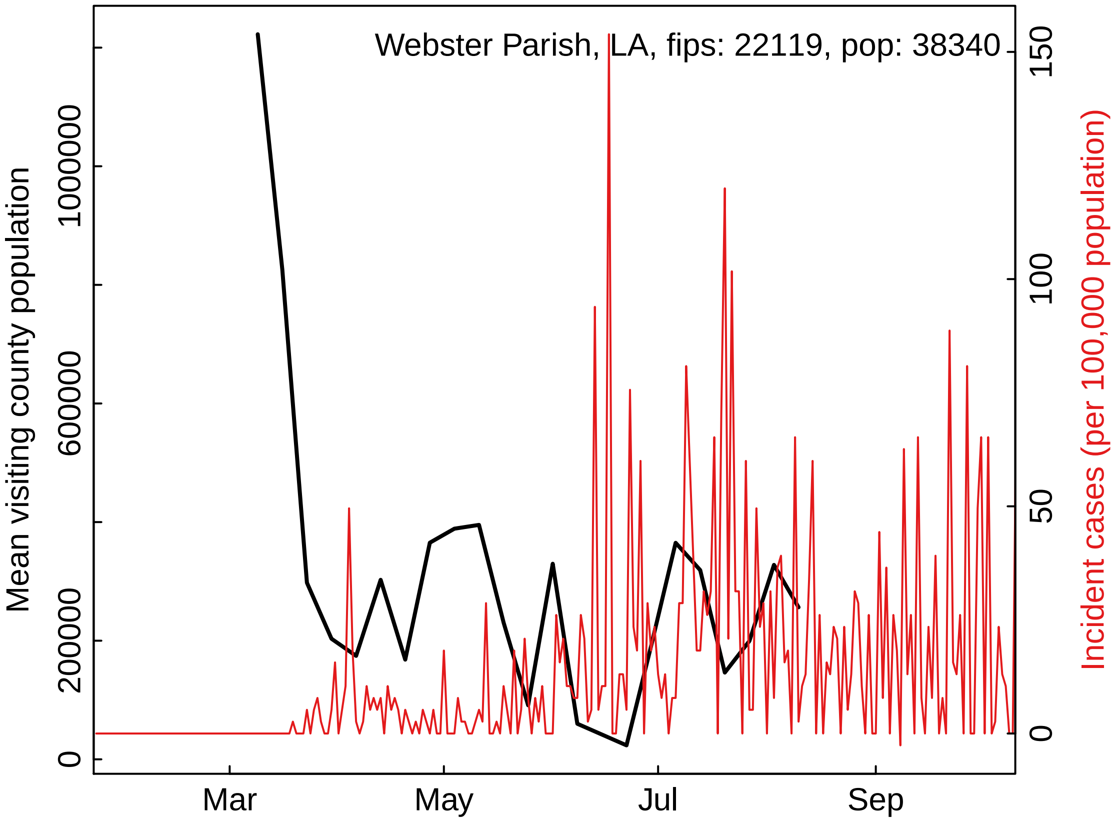

Webster Parish, LA, fips: 22119, pop: 38340
Mean visiting county population
0
200000
600000
1000000
Incident cases (per 100,000 population)
0
50
100
150
Mar
May
Jul
Sep

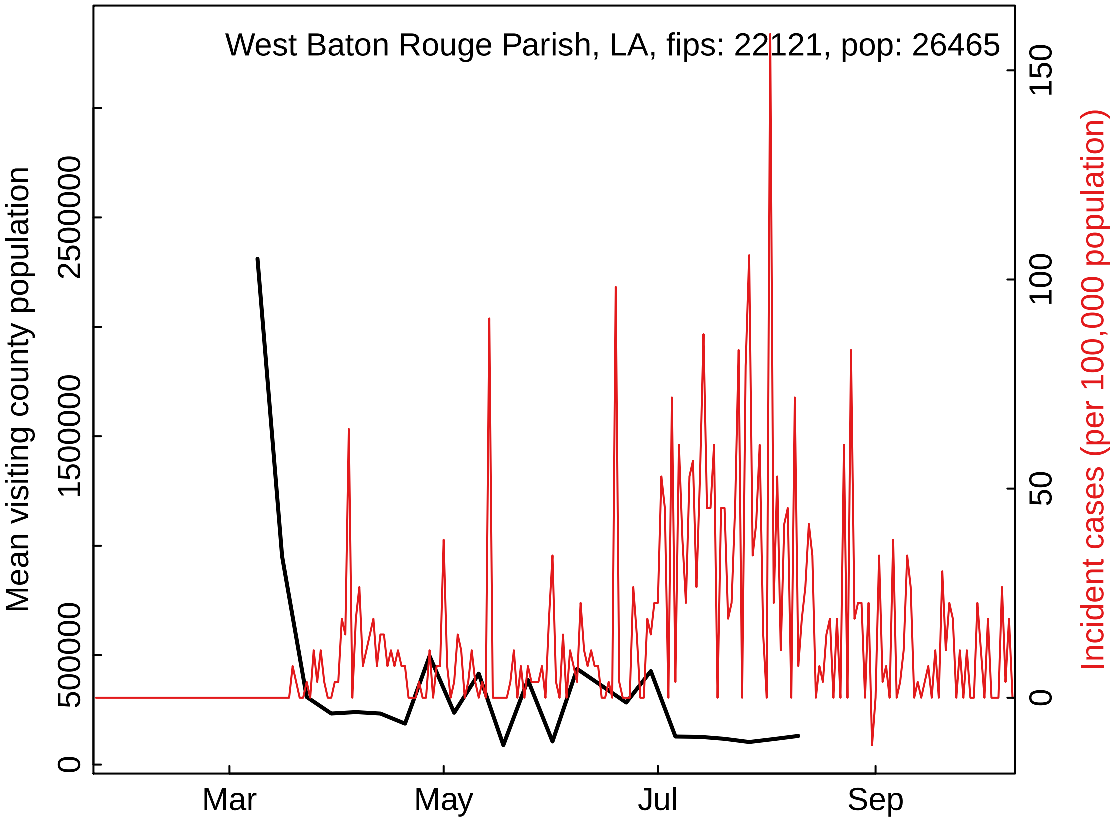

West Baton Rouge Parish, LA, fips: 22121, pop: 26465
Mean visiting county population
0
500000
1500000
2500000
Incident cases (per 100,000 population)
0
50
100
150
Mar
May
Jul
Sep

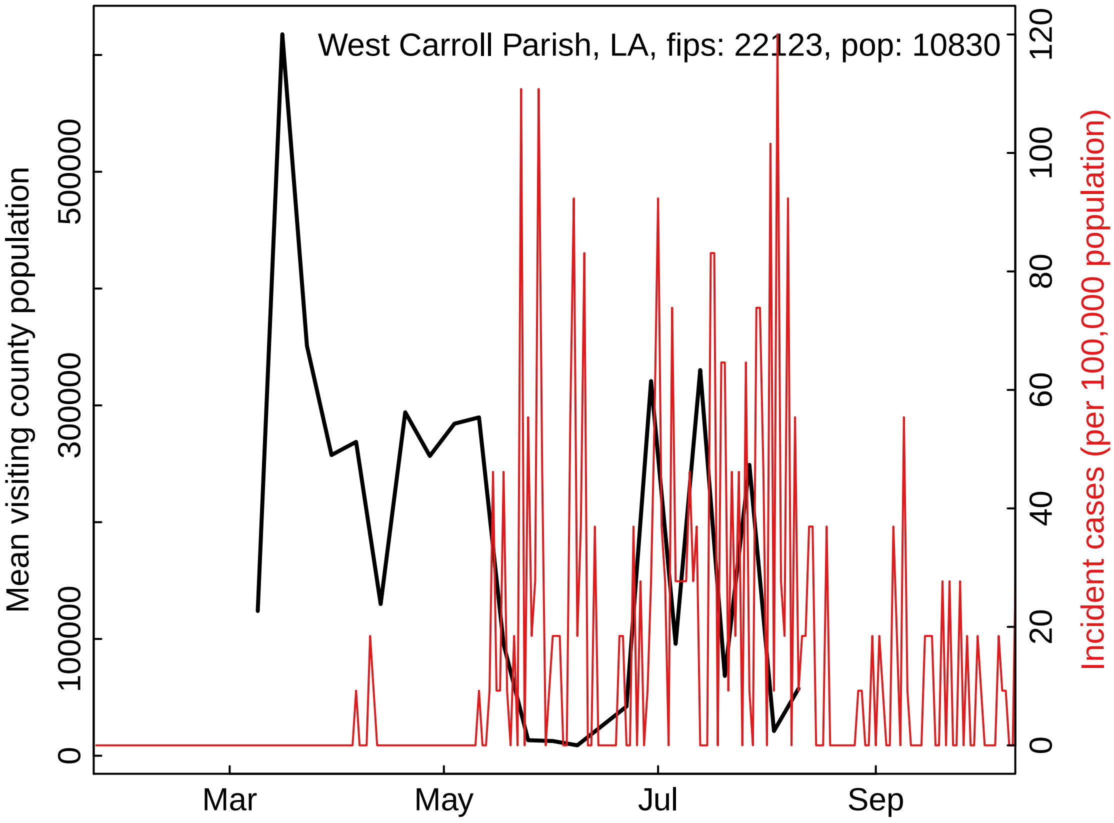

West Carroll Parish, LA, fips: 22123, pop: 10830
Mean visiting county population
0
100000
300000
500000
Incident cases (per 100,000 population)
0
20
40
60
80
100
120
Mar
May
Jul
Sep

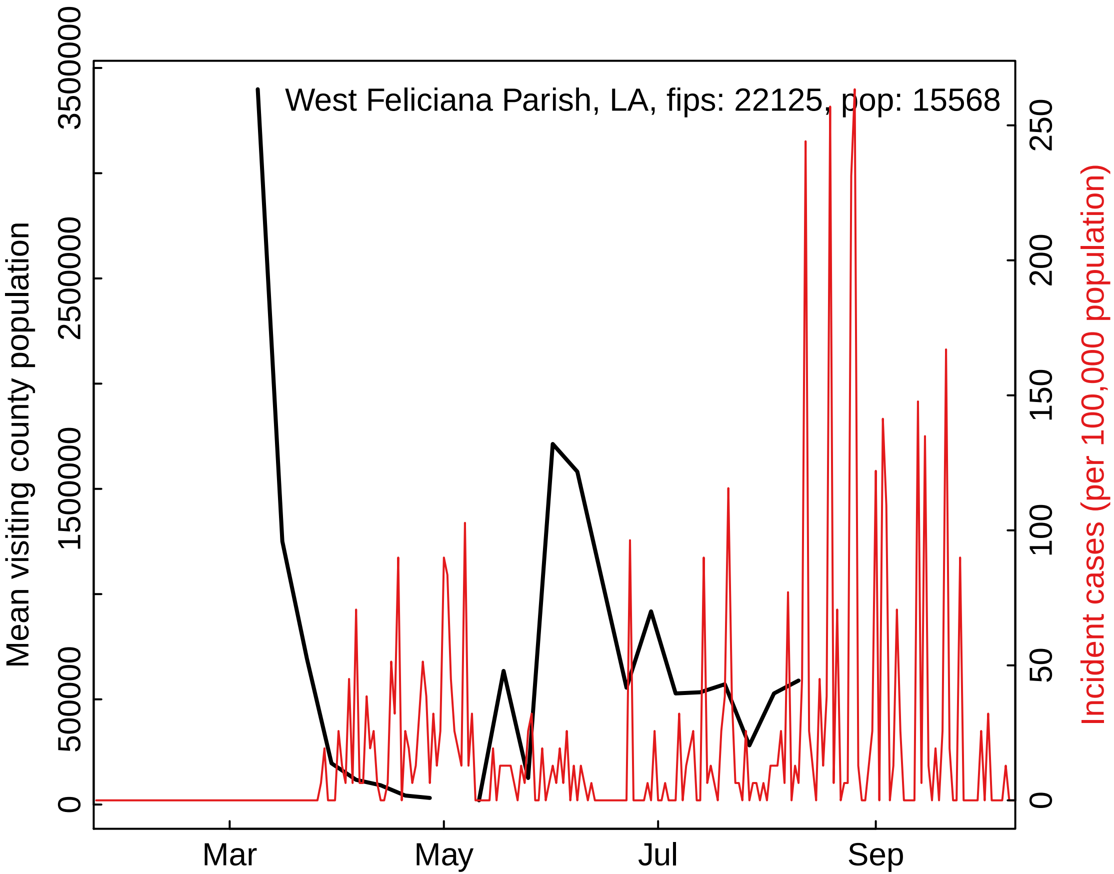

West Feliciana Parish, LA, fips: 22125, pop: 15568
Mean visiting county population
0
5000000
1500000
2500000
3500000
Incident cases (per 100,000 population)
0
50
100
150
200
250
Mar
May
Jul
Sep

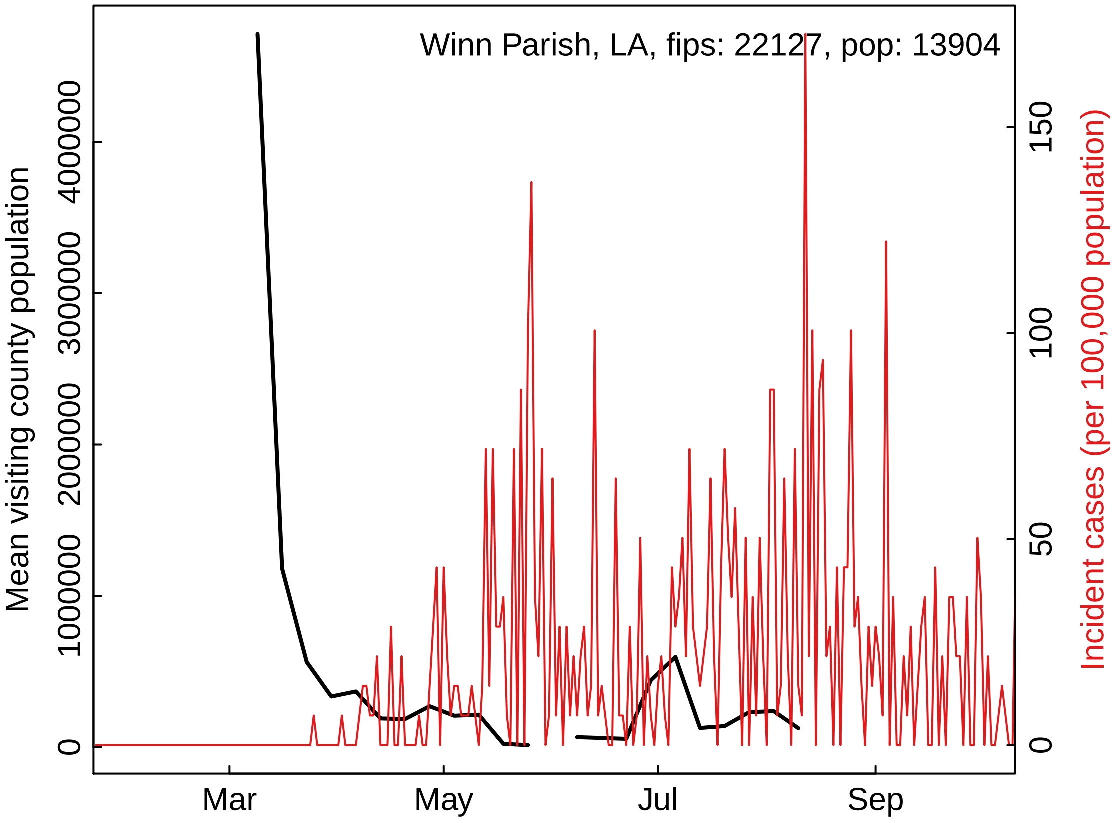

Winn Parish, LA, fips: 22127, pop: 13904
Mean visiting county population
0
1000000
2000000
3000000
4000000
Incident cases (per 100,000 population)
0
50
100
150
Mar
May
Jul
Sep

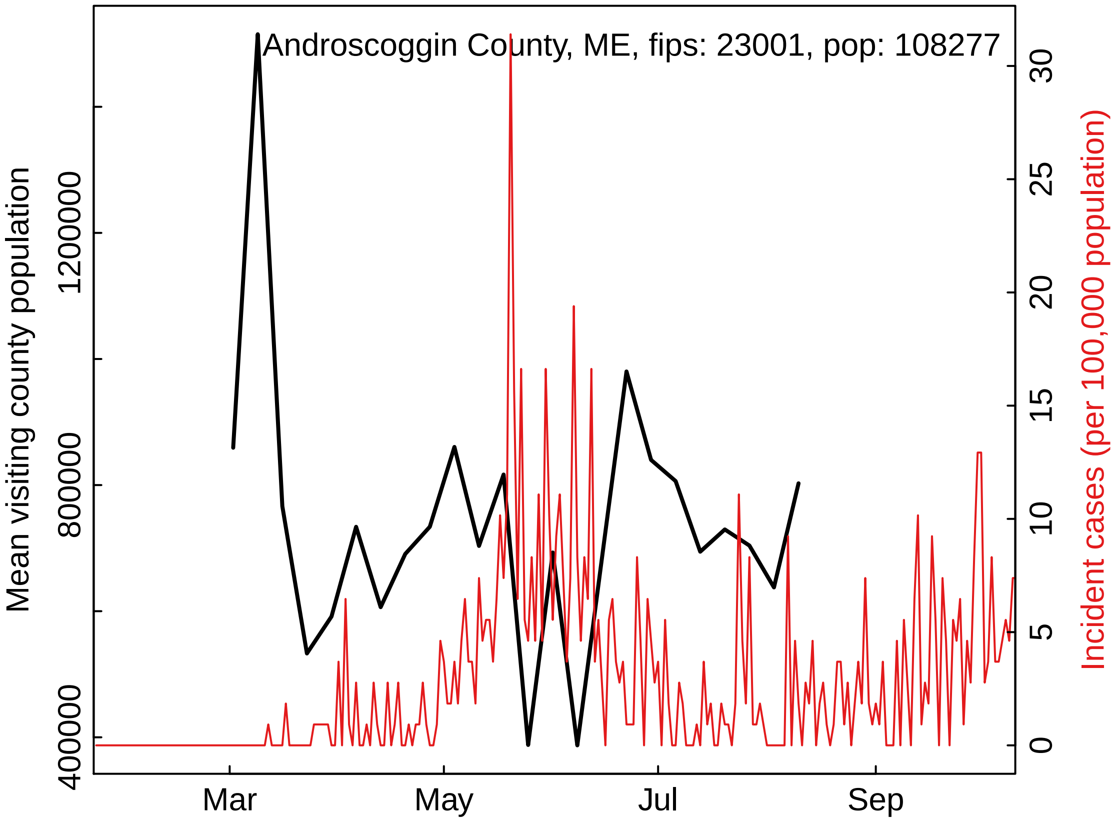

Androscoggin County, ME, fips: 23001, pop: 108277
Mean visiting county population
400000
800000
1200000
Incident cases (per 100,000 population)
0
5
10
15
20
25
30
Mar
May
Jul
Sep

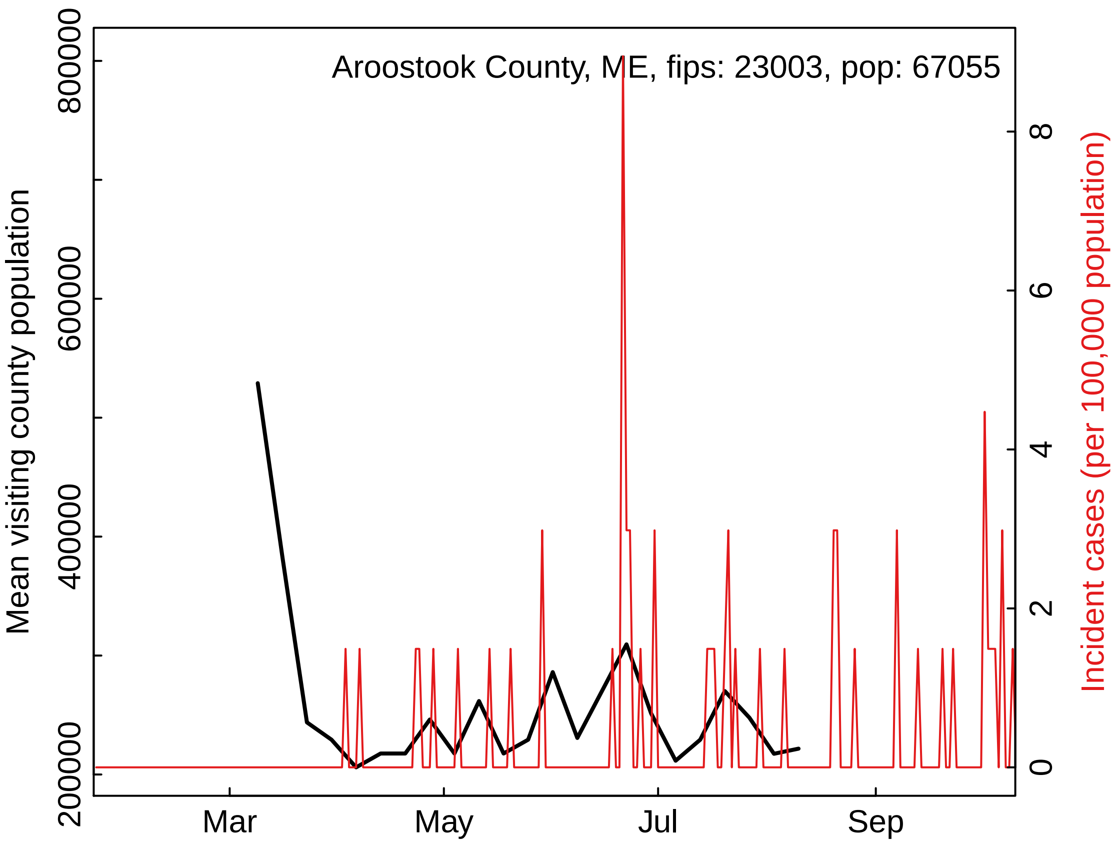

Aroostook County, ME, fips: 23003, pop: 67055
Mean visiting county population
200000
400000
600000
800000
Incident cases (per 100,000 population)
0
2
4
6
8
Mar
May
Jul
Sep

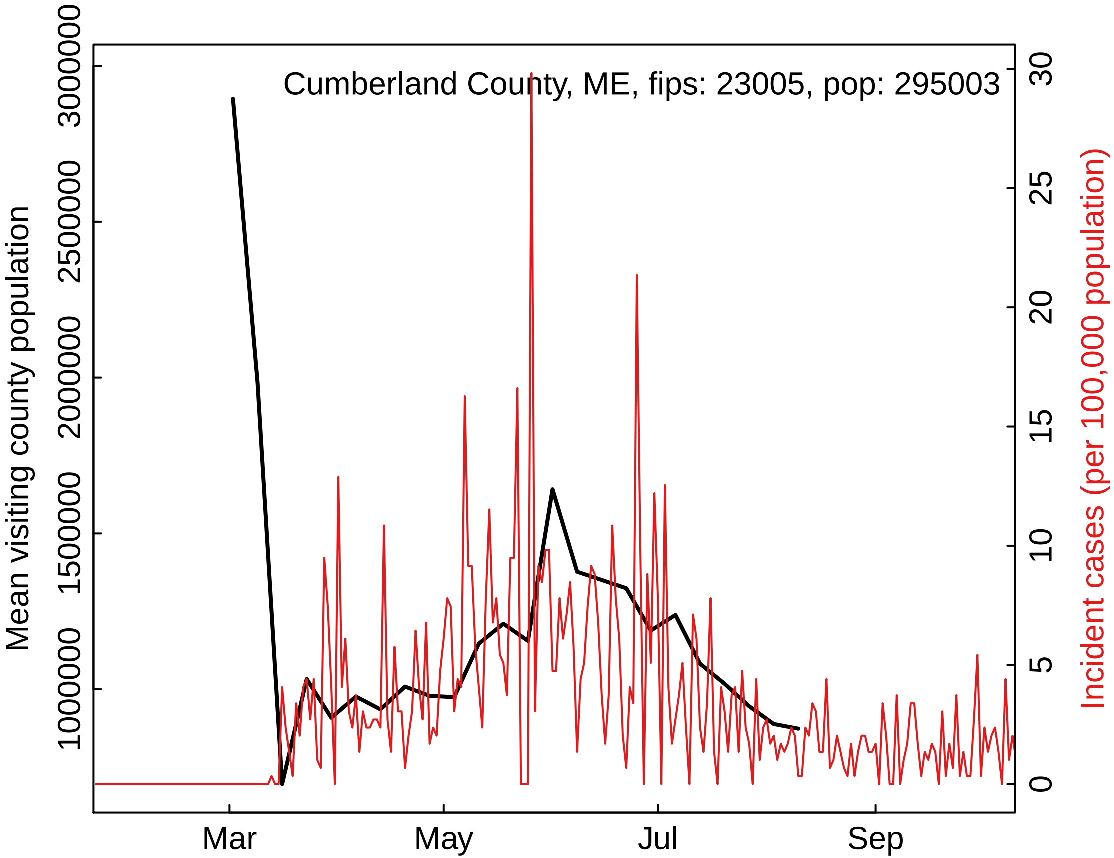

Cumberland County, ME, fips: 23005, pop: 295003
Mean visiting county population
30000000
25000000
20000000
15000000
10000000
Incident cases (per 100,000 population)
30
25
20
15
10
5
0
Mar
May
Jul
Sep

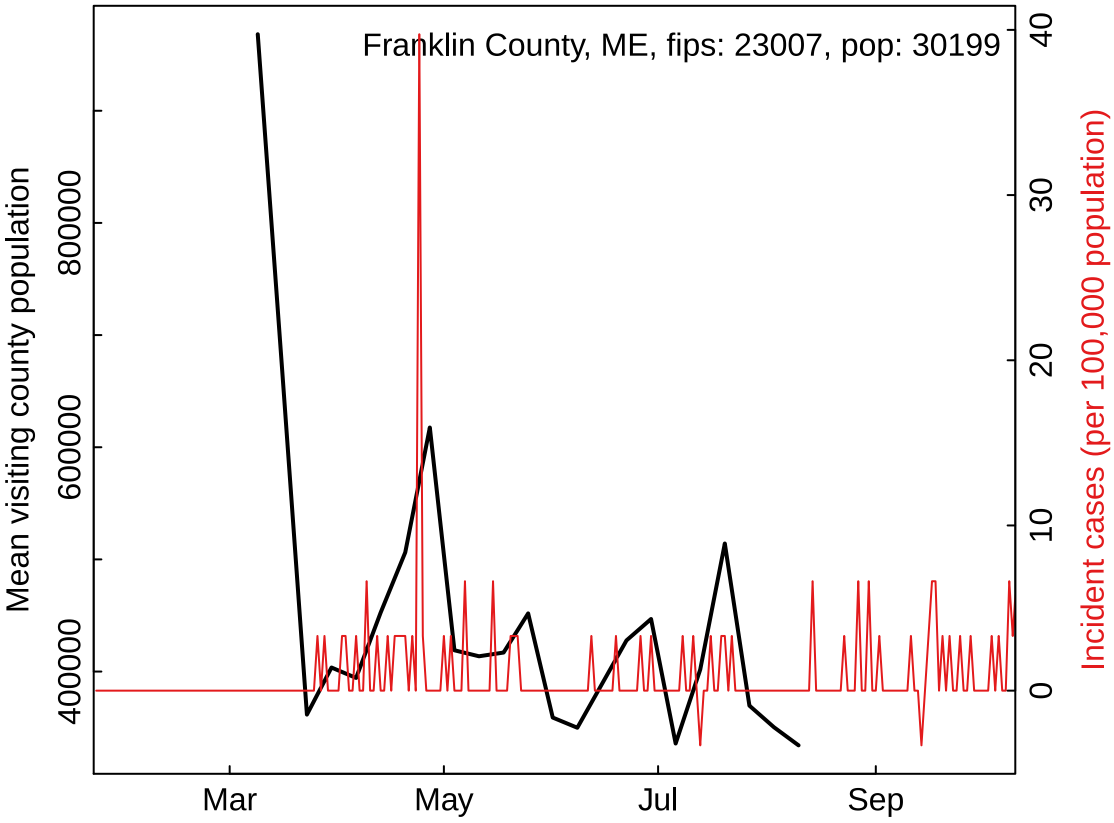
Franklin County, ME, fips: 23007, pop: 30199
Mean visiting county population
400000
600000
800000
Incident cases (per 100,000 population)
0
10
20
30
40
Mar
May
Jul
Sep

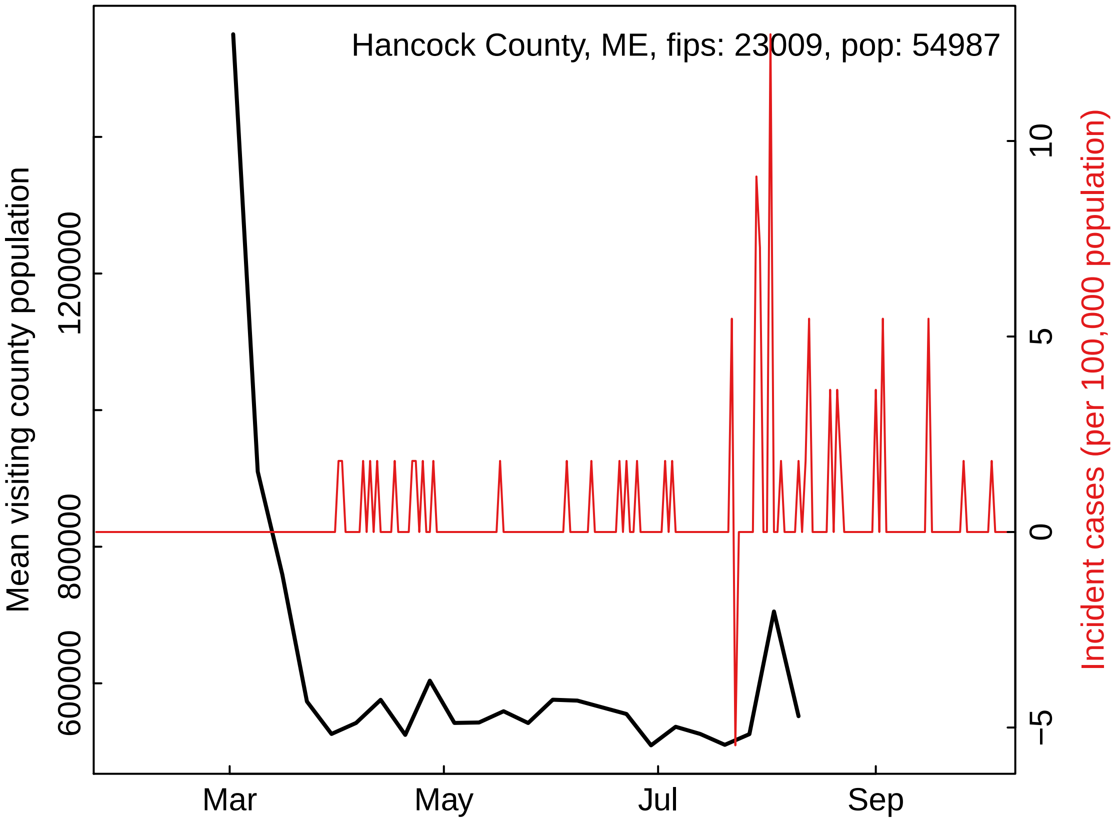
Hancock County, ME, fips: 23009, pop: 54987
Mean visiting county population
600000
800000
1200000
Incident cases (per 100,000 population)
−5
0
5
10
Mar
May
Jul
Sep

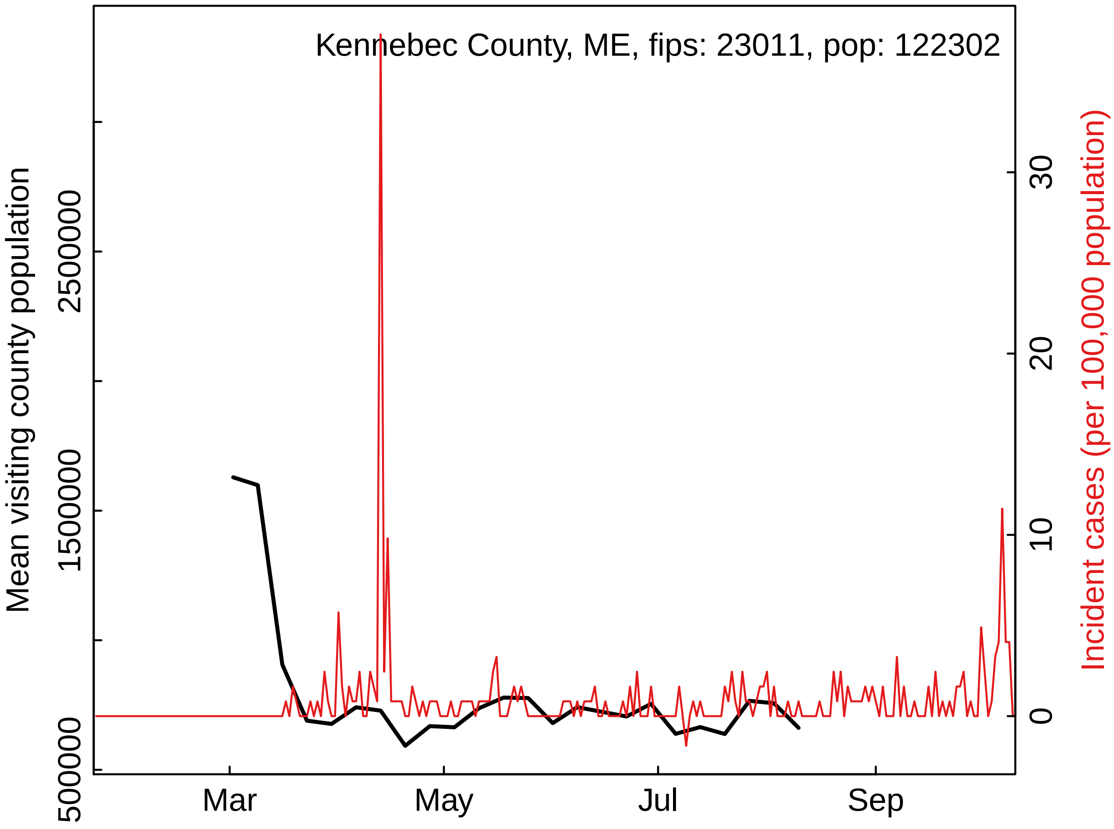

Kennebec County, ME, fips: 23011, pop: 122302
Mean visiting county population
500000
1500000
2500000
Incident cases (per 100,000 population)
0
10
20
30
Mar
May
Jul
Sep

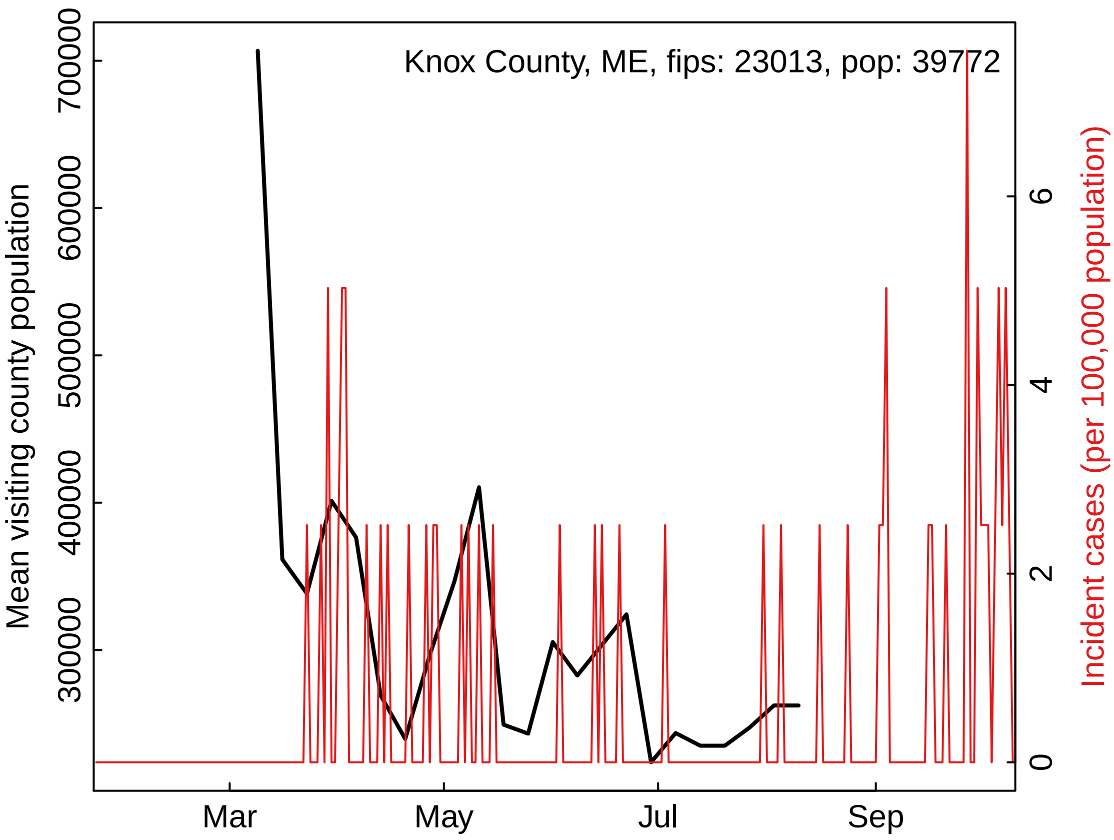
Knox County, ME, fips: 23013, pop: 39772
Mean visiting county population
Incident cases (per 100,000 population)
300000
400000
500000
600000
700000
0
2
4
6
Mar
May
Jul
Sep

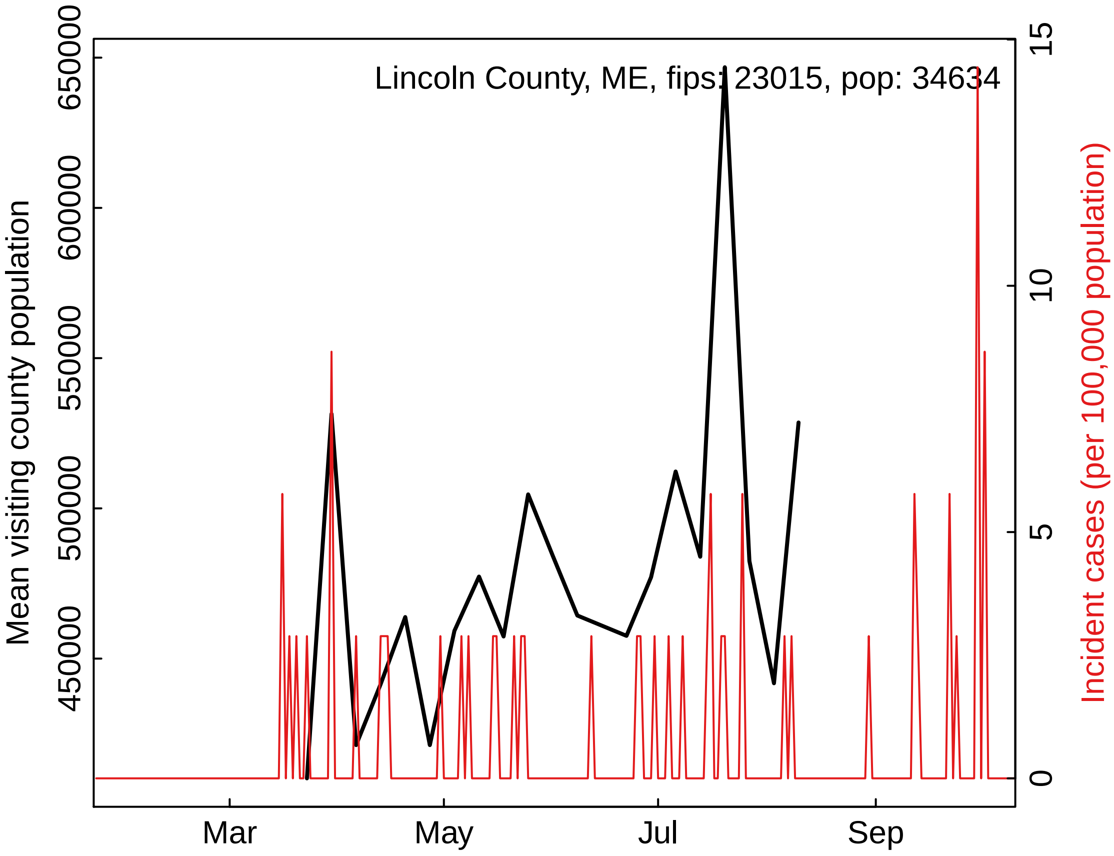

Lincoln County, ME, fips: 23015, pop: 34634
Mean visiting county population
450000
500000
550000
600000
650000
Incident cases (per 100,000 population)
0
5
10
15
Mar
May
Jul
Sep

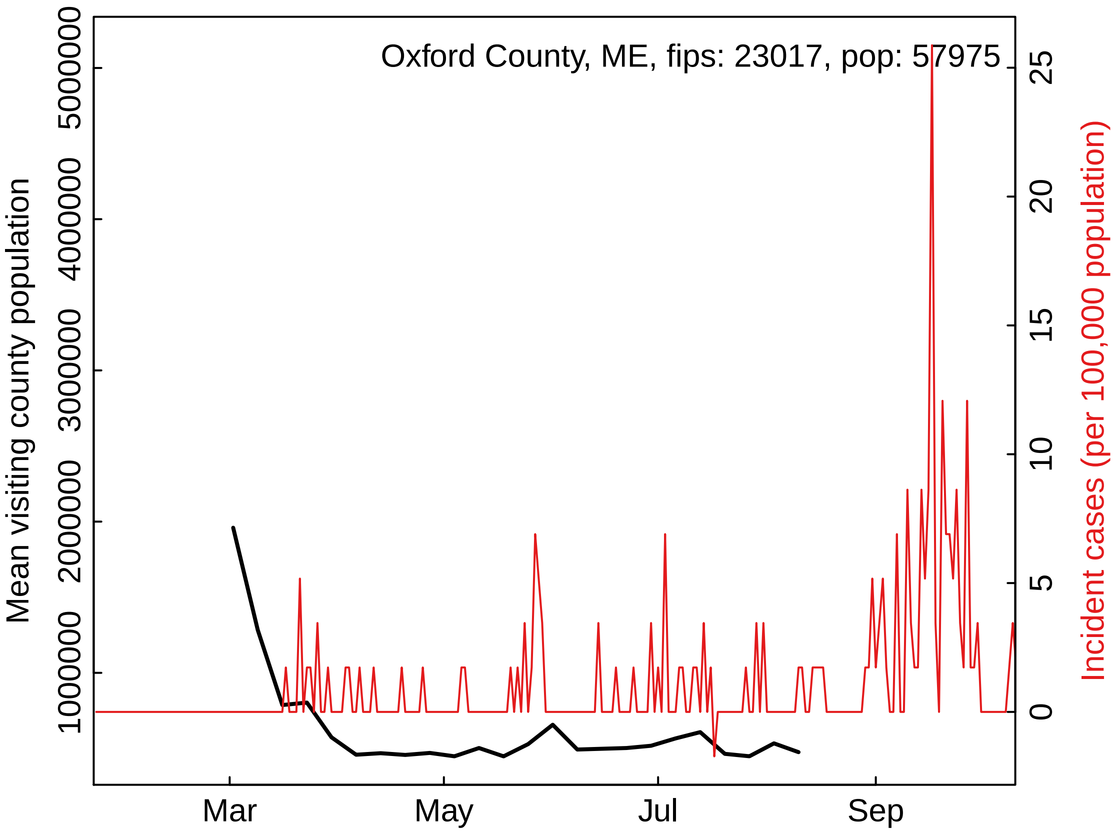

Oxford County, ME, fips: 23017, pop: 57975
Mean visiting county population
1000000
2000000
3000000
4000000
5000000
Incident cases (per 100,000 population)
0
5
10
15
20
25
Mar
May
Jul
Sep

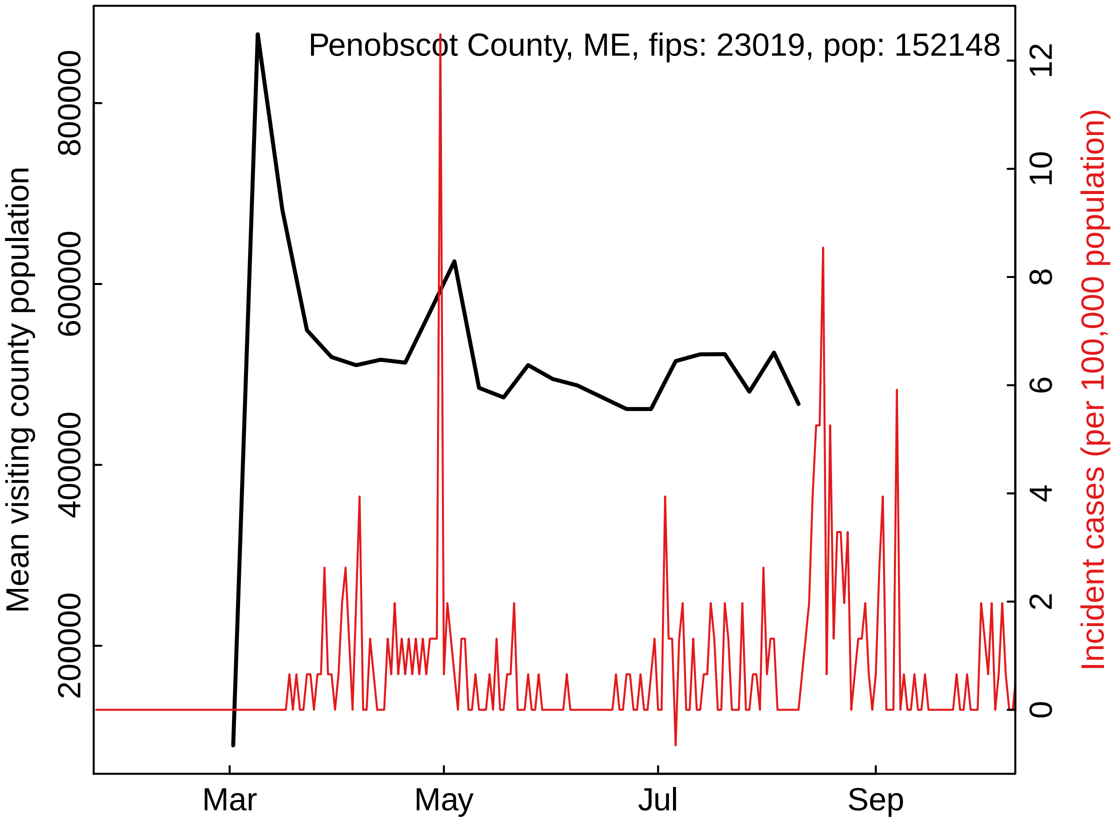
Penobscot County, ME, fips: 23019, pop: 152148
Mean visiting county population
200000
400000
600000
800000
Incident cases (per 100,000 population)
0
2
4
6
8
10
12
Mar
May
Jul
Sep

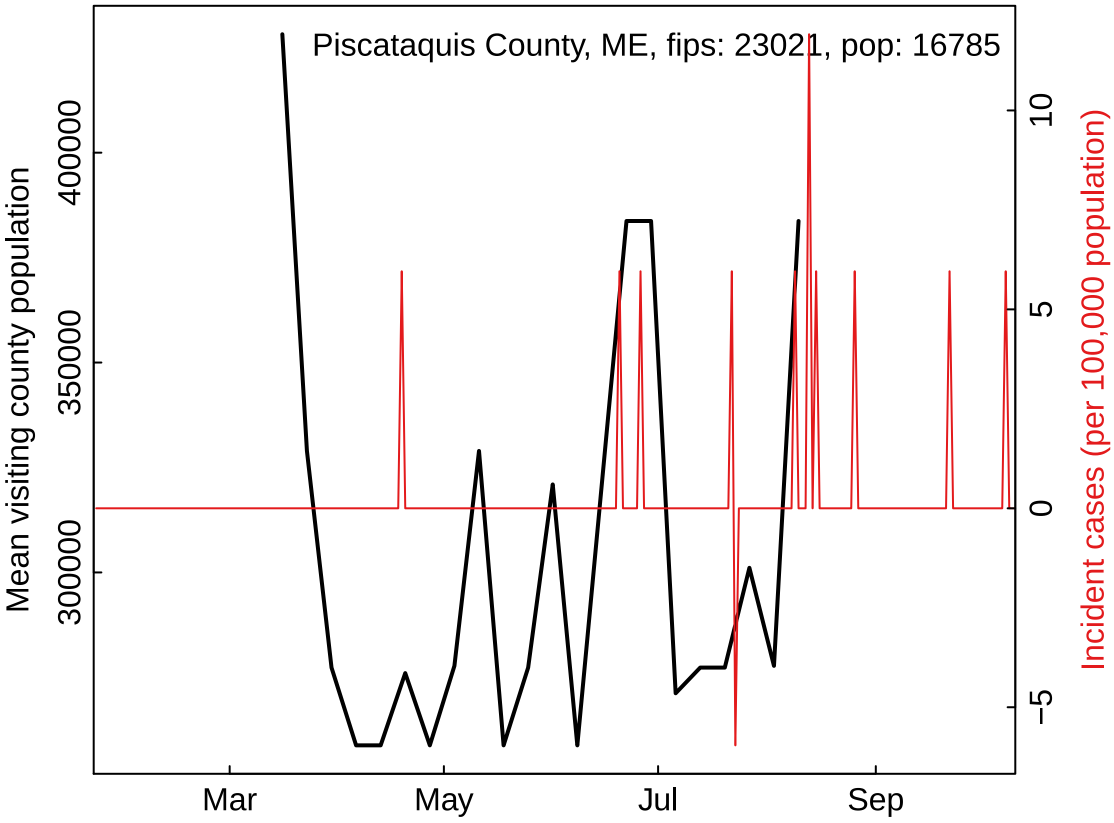

Piscataquis County, ME, fips: 23021, pop: 16785
Mean visiting county population
300000
350000
400000
Incident cases (per 100,000 population)
−5
0
5
10
Mar
May
Jul
Sep

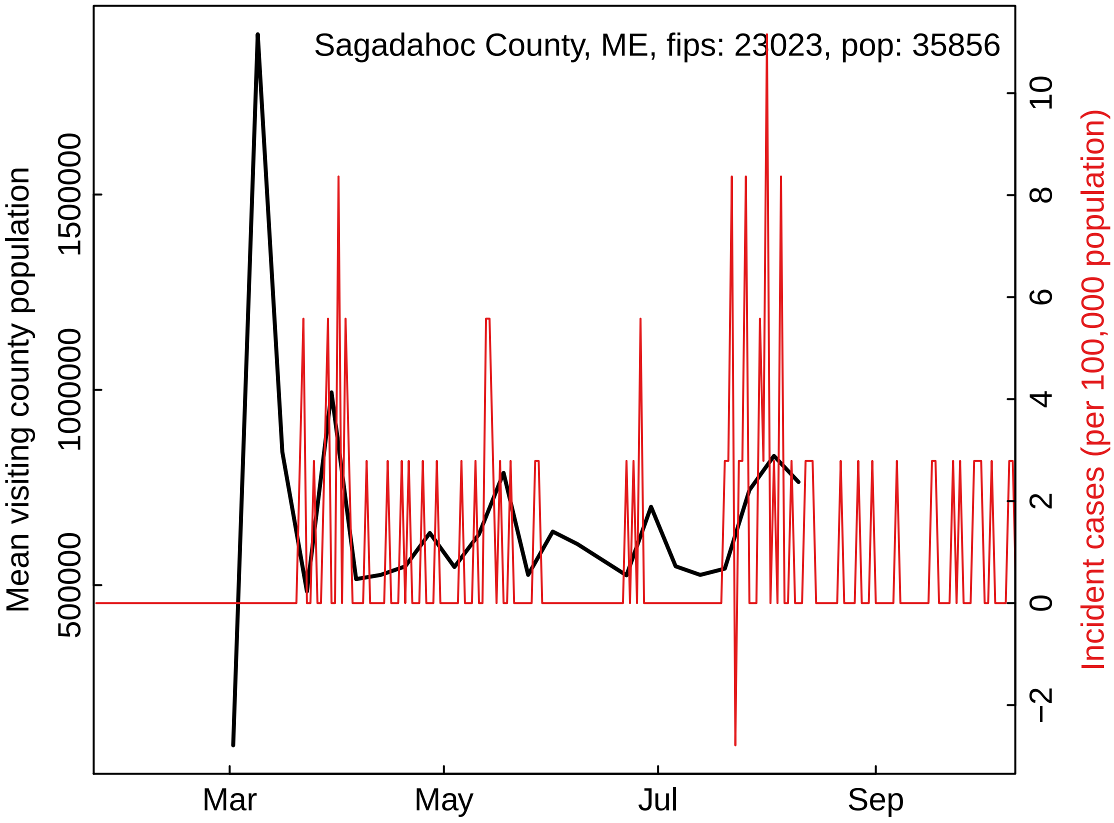

Sagadahoc County, ME, fips: 23023, pop: 35856
Mean visiting county population
500000
1000000
1500000
Incident cases (per 100,000 population)
−2
0
2
4
6
8
10
Mar
May
Jul
Sep

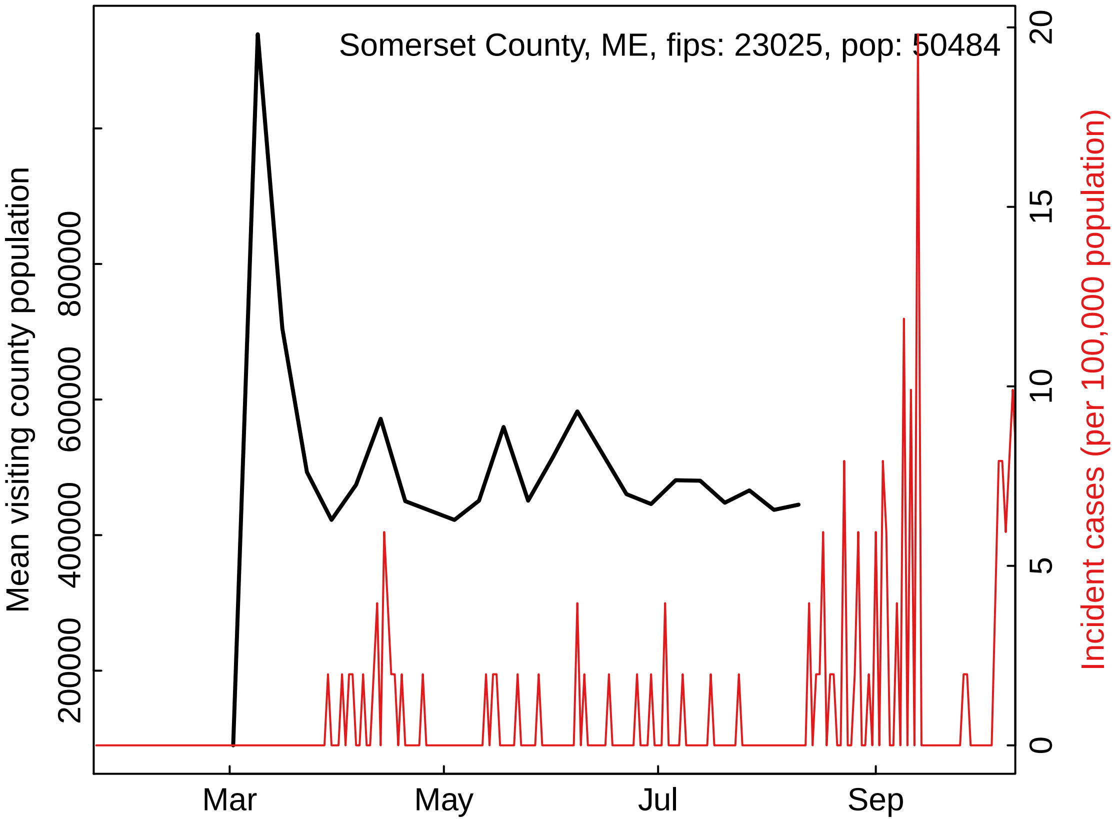

Somerset County, ME, fips: 23025, pop: 50484
Mean visiting county population
200000
400000
600000
800000
Incident cases (per 100,000 population)
0
5
10
15
20
Mar
May
Jul
Sep

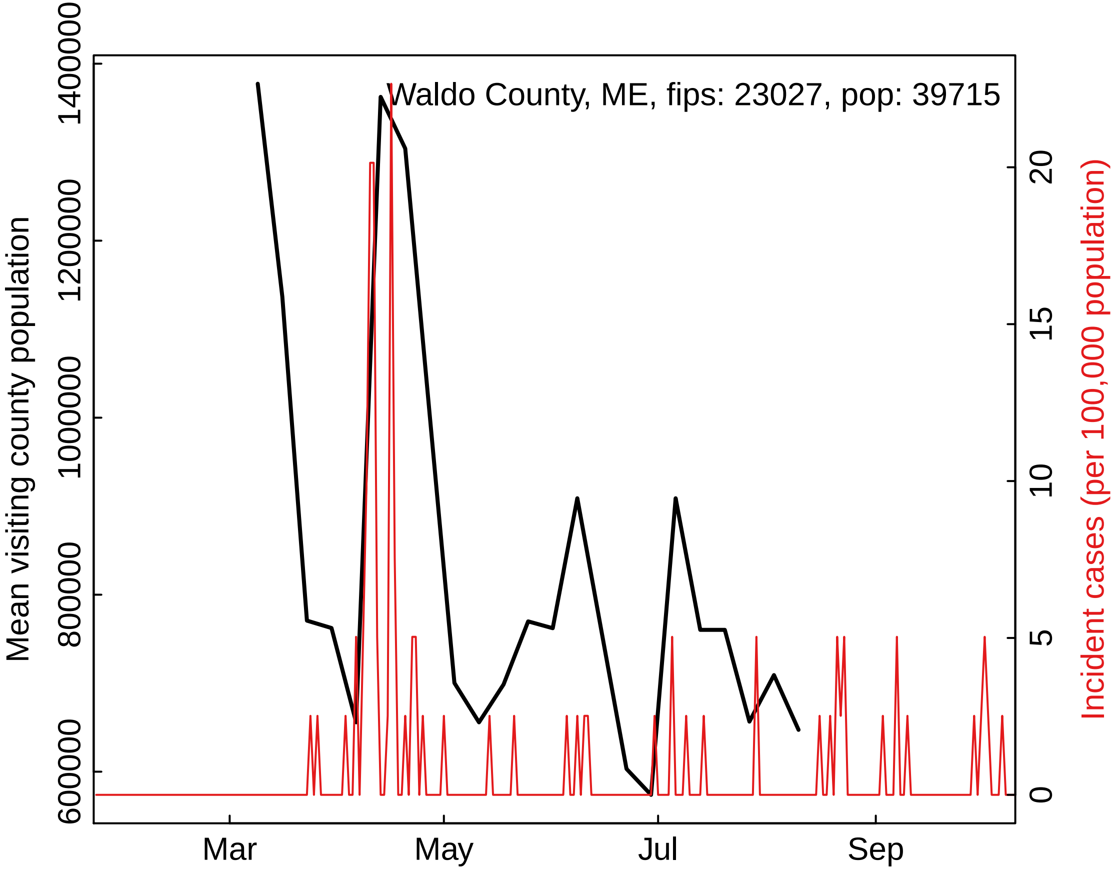
Waldo County, ME, fips: 23027, pop: 39715
Mean visiting county population
600000
800000
1000000
1200000
1400000
Incident cases (per 100,000 population)
0
5
10
15
20
Mar
May
Jul
Sep

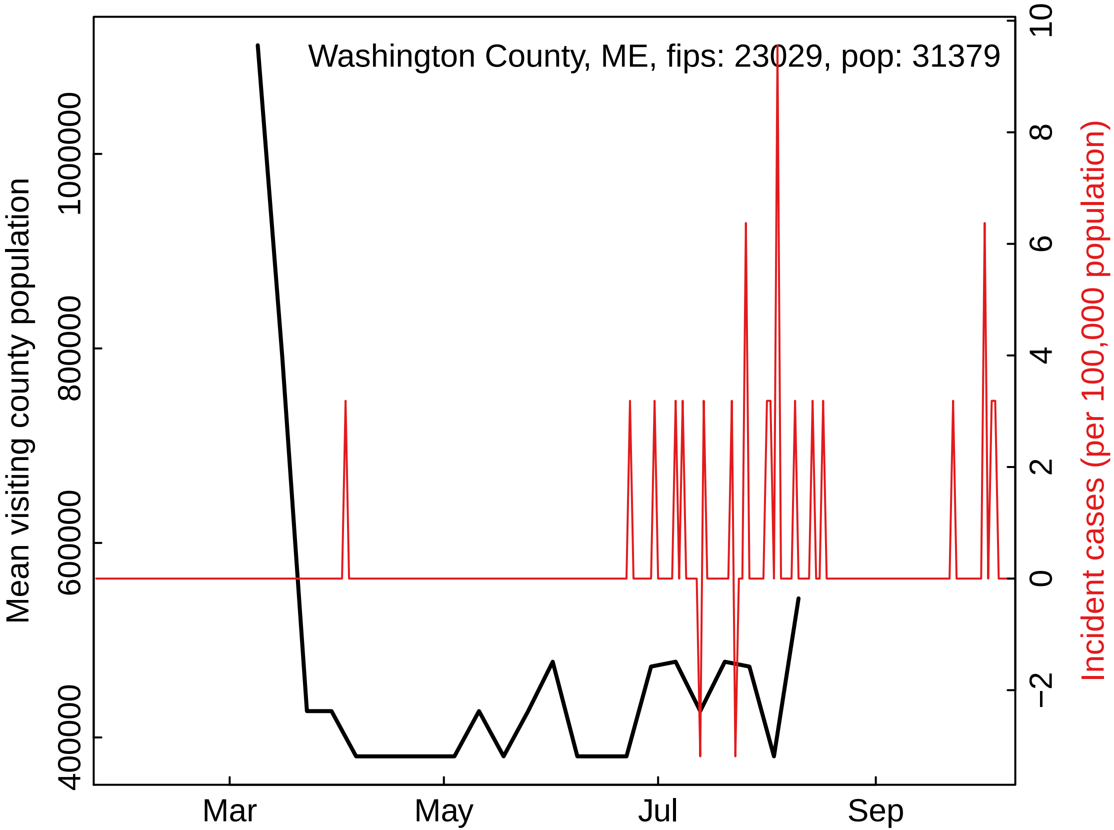

Washington County, ME, fips: 23029, pop: 31379
Mean visiting county population
400000
600000
8000000
10000000
Incident cases (per 100,000 population)
−2
0
2
4
6
8
10
Mar
May
Jul
Sep

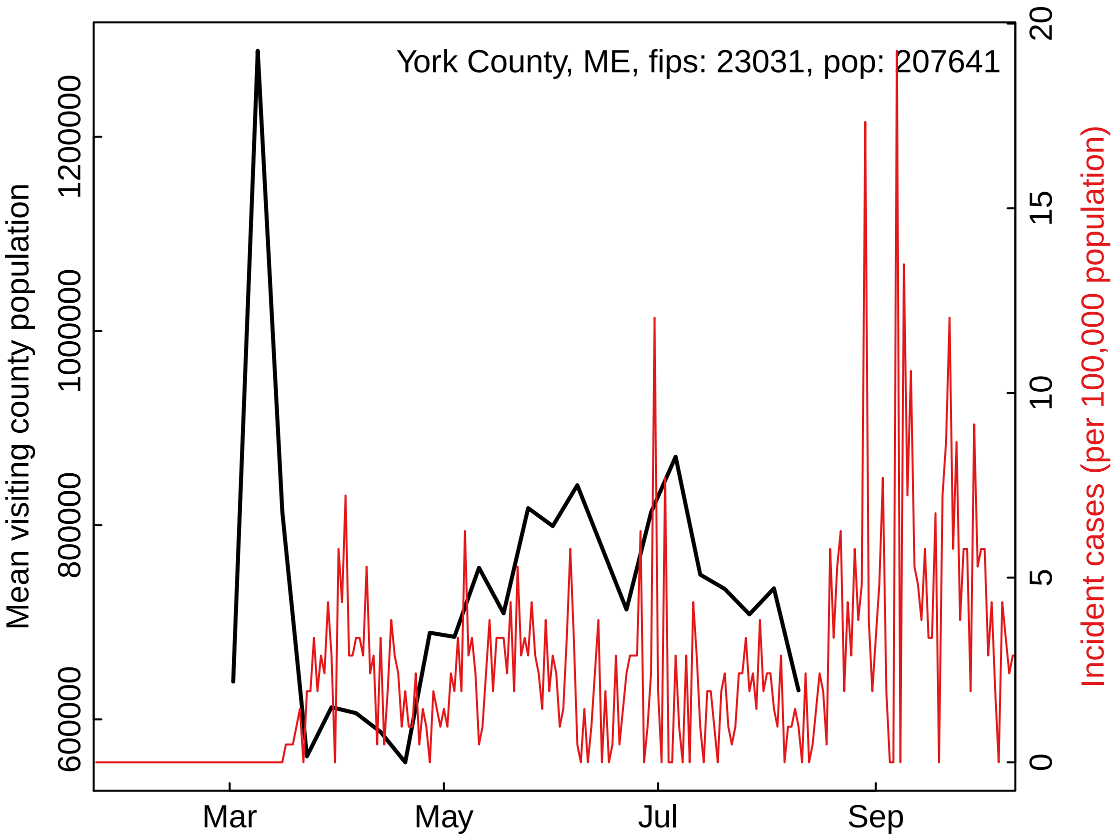

York County, ME, fips: 23031, pop: 207641
Mean visiting county population
600000
800000
1000000
1200000
Incident cases (per 100,000 population)
0
5
10
15
20
Mar
May
Jul
Sep

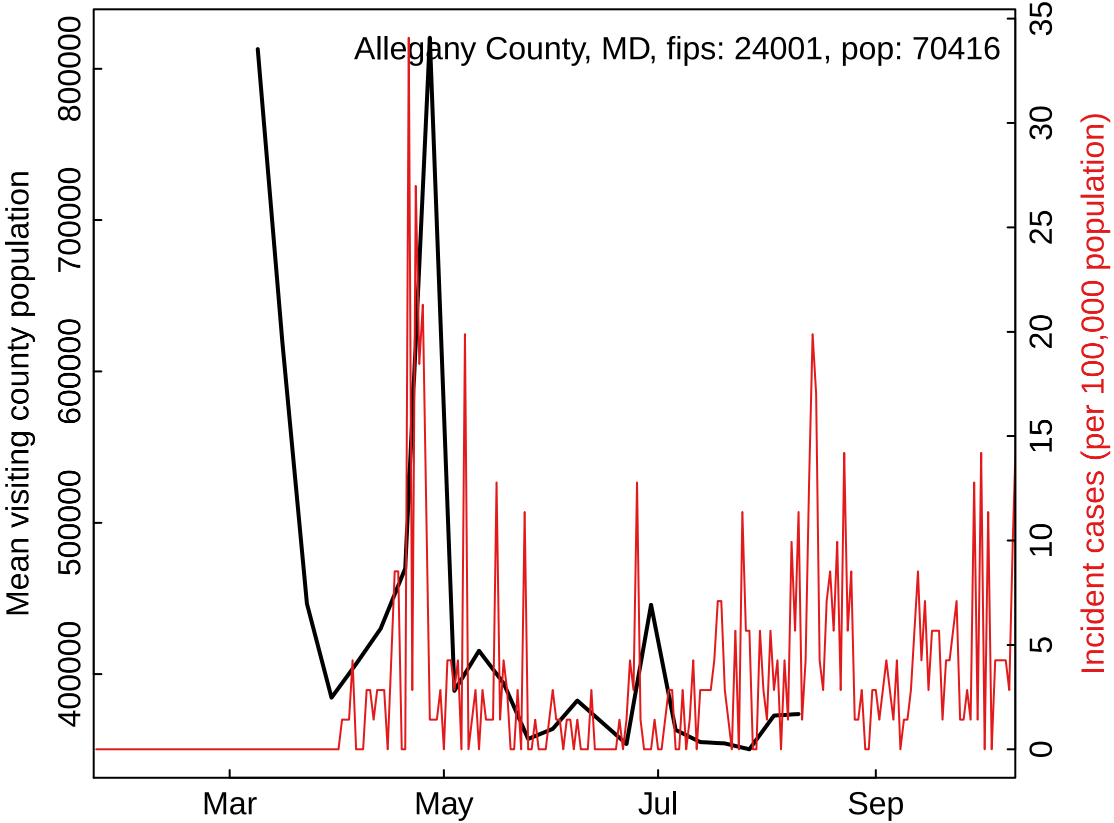
Allegany County, MD, fips: 24001, pop: 70416
Mean visiting county population
400000
500000
600000
700000
800000
Incident cases (per 100,000 population)
0
5
10
15
20
25
30
35
Mar
May
Jul
Sep

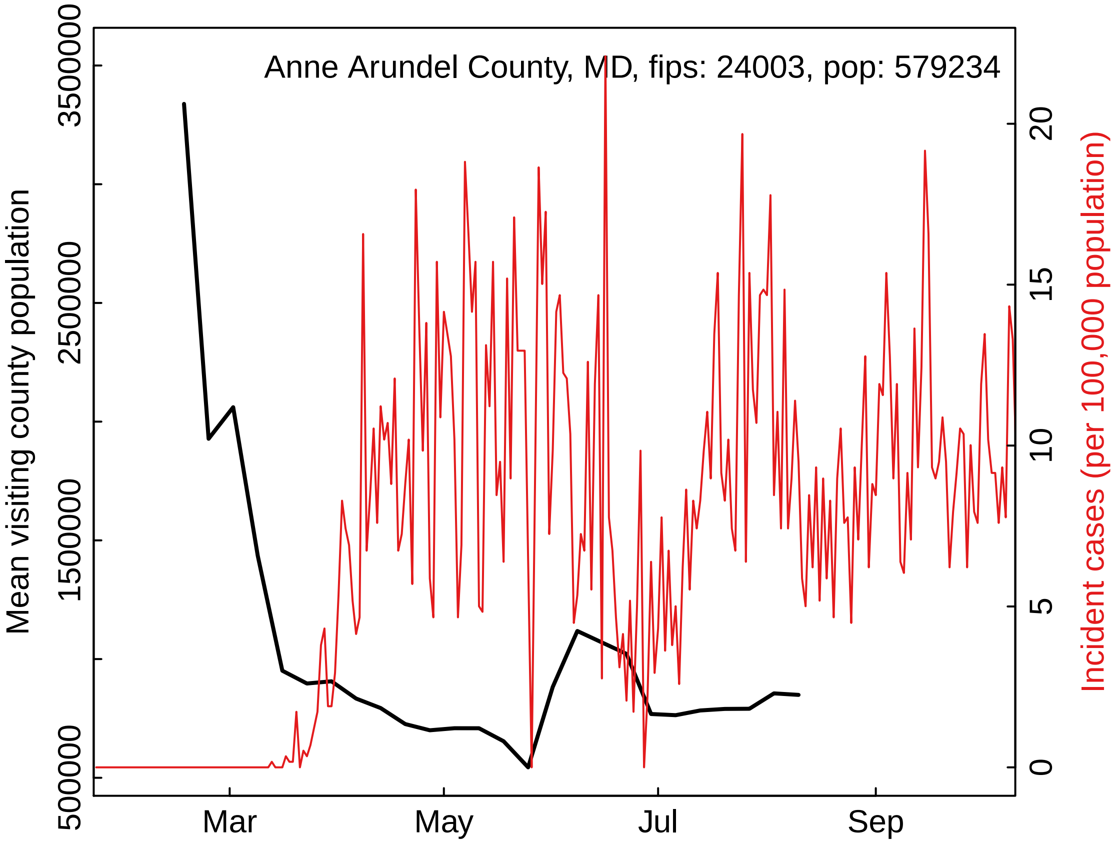

Anne Arundel County, MD, fips: 24003, pop: 579234
Mean visiting county population
Incident cases (per 100,000 population)
500000
1500000
2500000
3500000
0
5
10
15
20
Mar
May
Jul
Sep

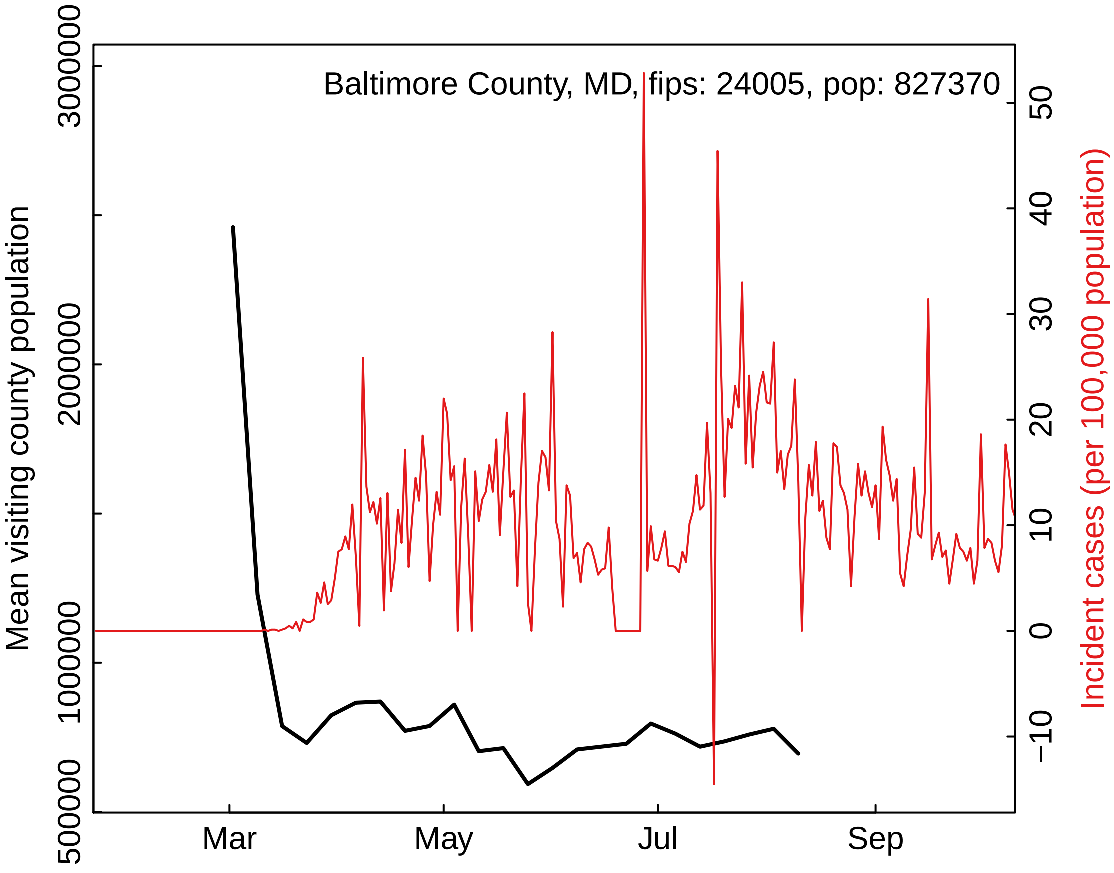

Baltimore County, MD, fips: 24005, pop: 827370
Mean visiting county population
Incident cases (per 100,000 population)
500000
1000000
2000000
3000000
−10
0
10
20
30
40
50
Mar
May
Jul
Sep

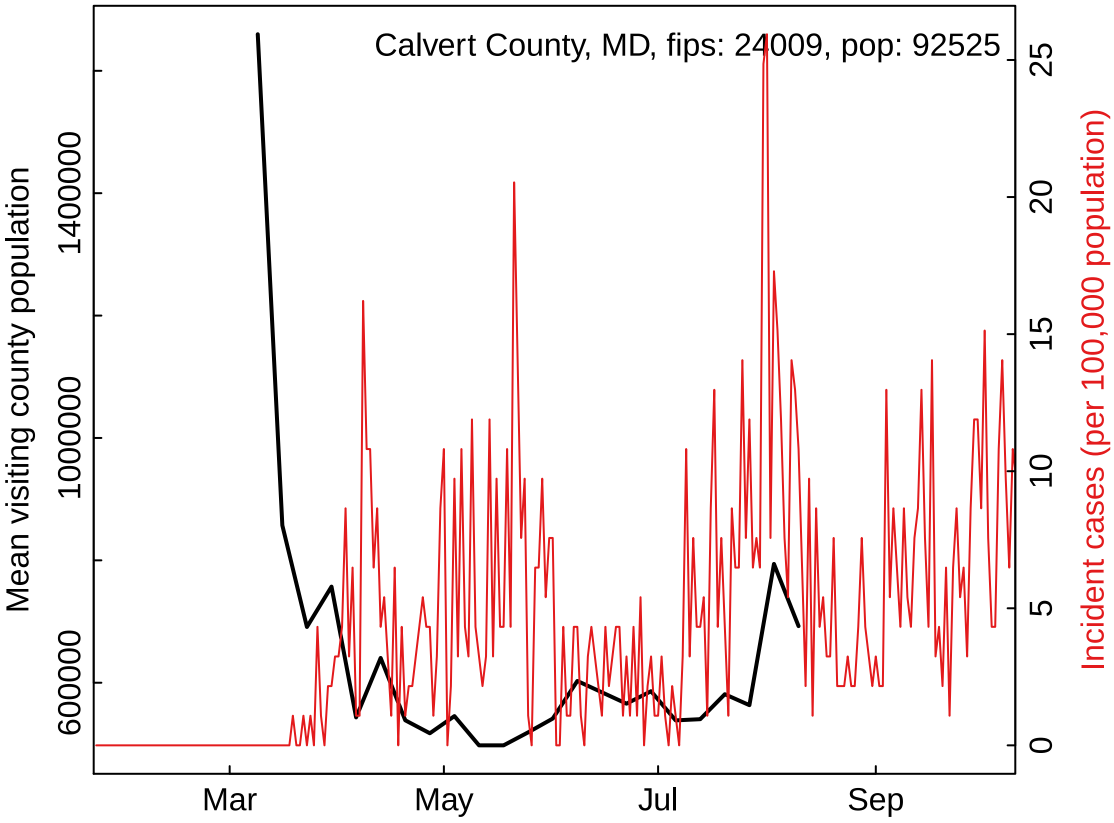
Calvert County, MD, fips: 24009, pop: 92525
Mean visiting county population
600000
1000000
1400000
Incident cases (per 100,000 population)
0
5
10
15
20
25
Mar
May
Jul
Sep

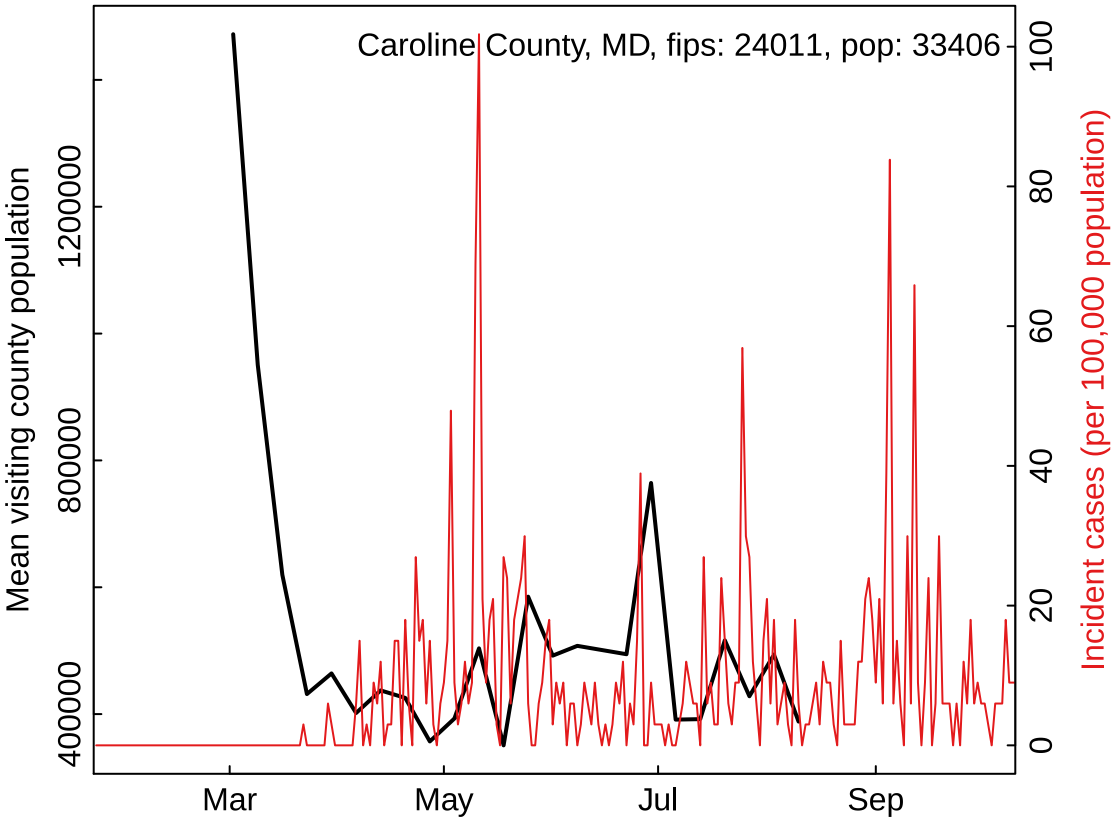
Caroline County, MD, fips: 24011, pop: 33406
Mean visiting county population
400000
800000
1200000
Incident cases (per 100,000 population)
0
20
40
60
80
100
Mar
May
Jul
Sep

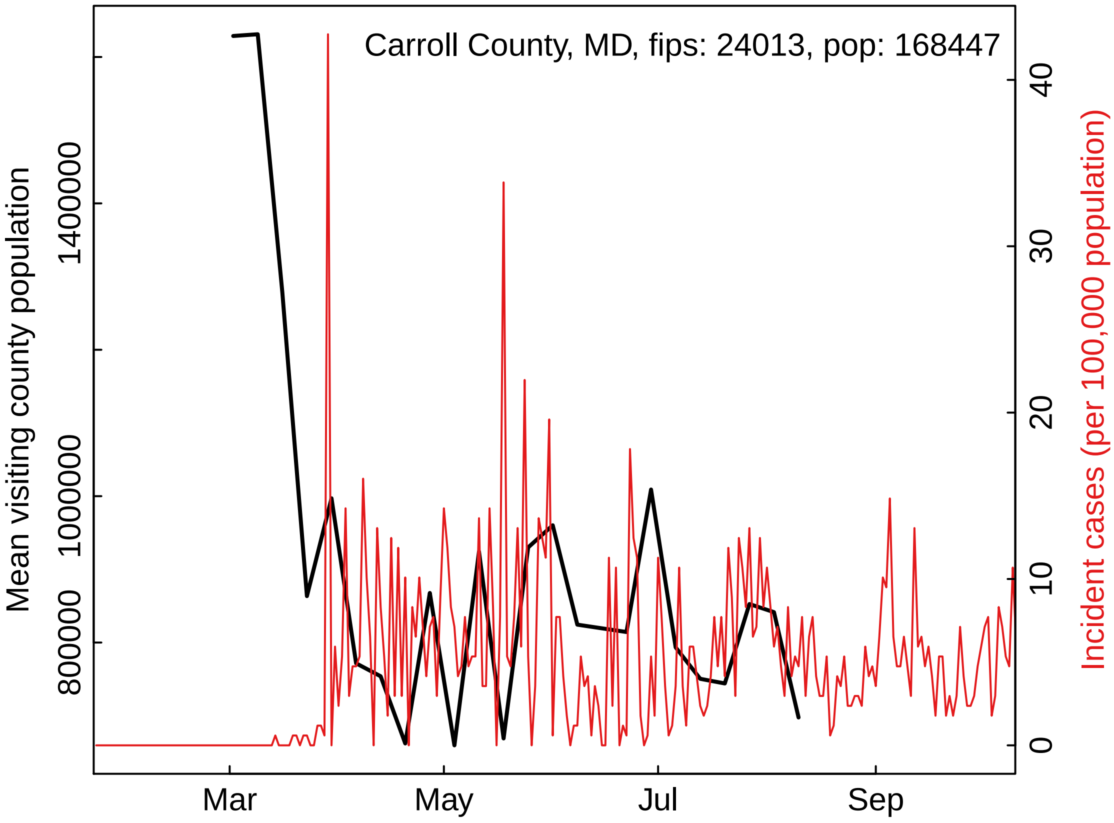
Carroll County, MD, fips: 24013, pop: 168447
Mean visiting county population
800000
1000000
1400000
Incident cases (per 100,000 population)
0
10
20
30
40
Mar
May
Jul
Sep

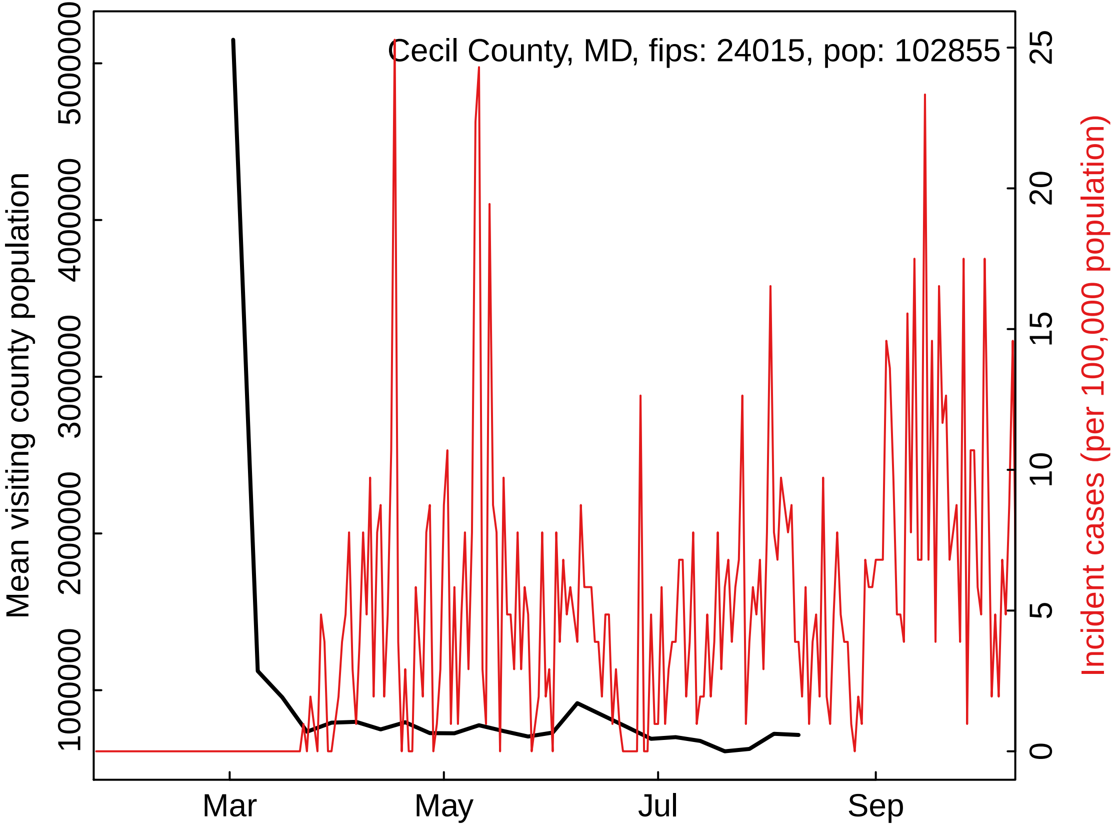

Cecil County, MD, fips: 24015, pop: 102855
Mean visiting county population
10000000
20000000
30000000
40000000
50000000
Incident cases (per 100,000 population)
0
5
10
15
20
25
Mar
May
Jul
Sep

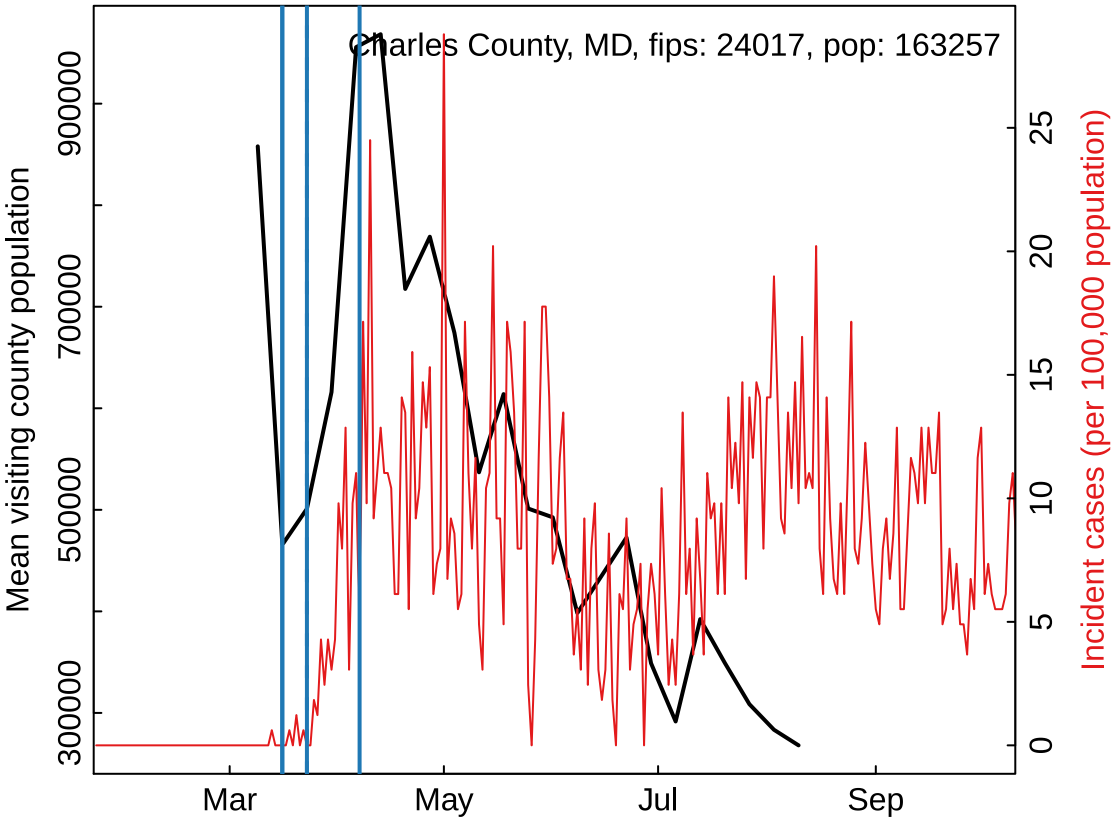

Charles County, MD, fips: 24017, pop: 163257
Mean visiting county population
300000
500000
700000
900000
Incident cases (per 100,000 population)
0
5
10
15
20
25
Mar
May
Jul
Sep

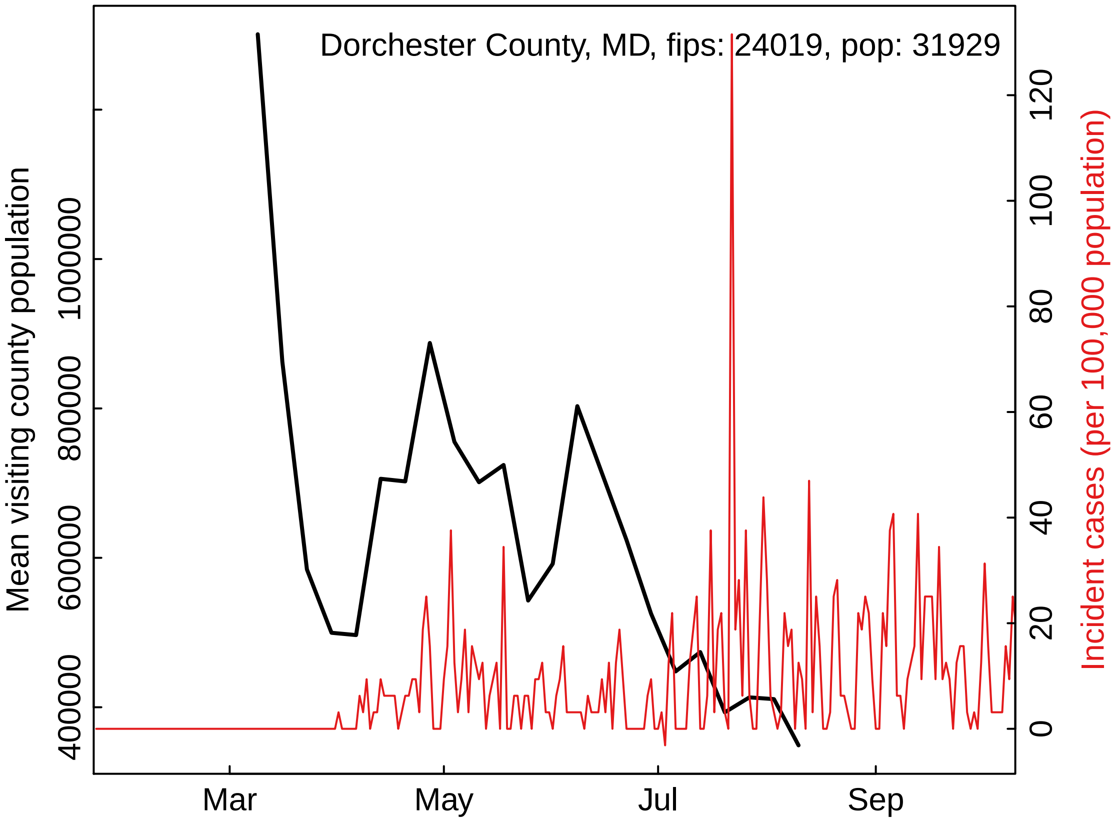

Dorchester County, MD, fips: 24019, pop: 31929
Mean visiting county population
400000
600000
800000
1000000
Incident cases (per 100,000 population)
0
20
40
60
80
100
120
Mar
May
Jul
Sep

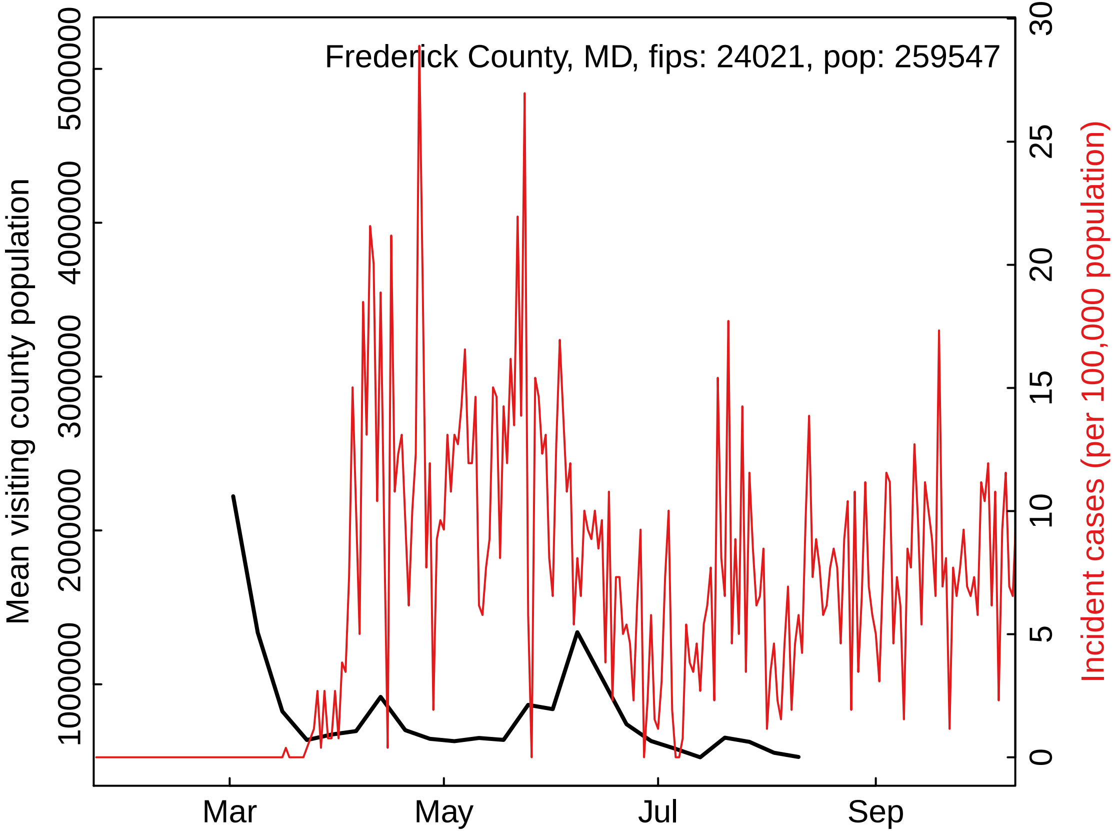

Frederick County, MD, fips: 24021, pop: 259547
Mean visiting county population
Incident cases (per 100,000 population)
1000000
2000000
3000000
4000000
5000000
0
5
10
15
20
25
30
Mar
May
Jul
Sep

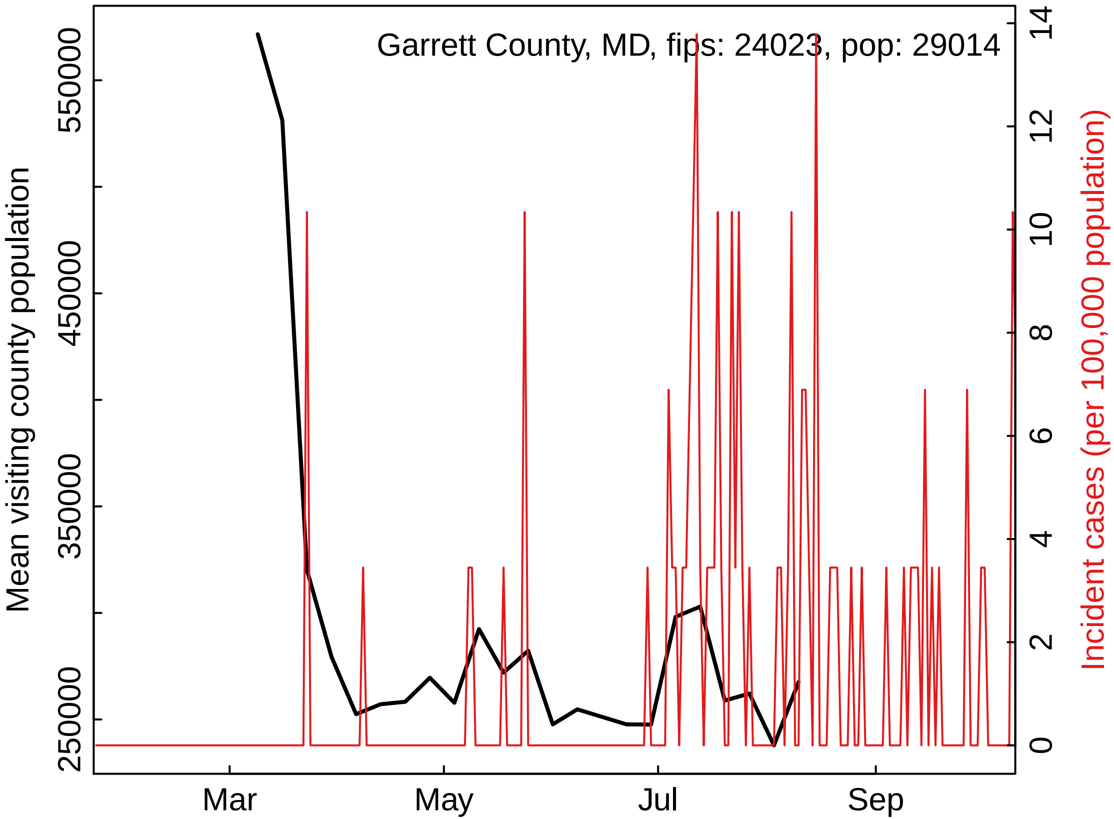
Garrett County, MD, fips: 24023, pop: 29014
Mean visiting county population
250000
350000
450000
550000
Mar
May
Jul
Sep
0
2
4
6
8
10
12
14
Incident cases (per 100,000 population)

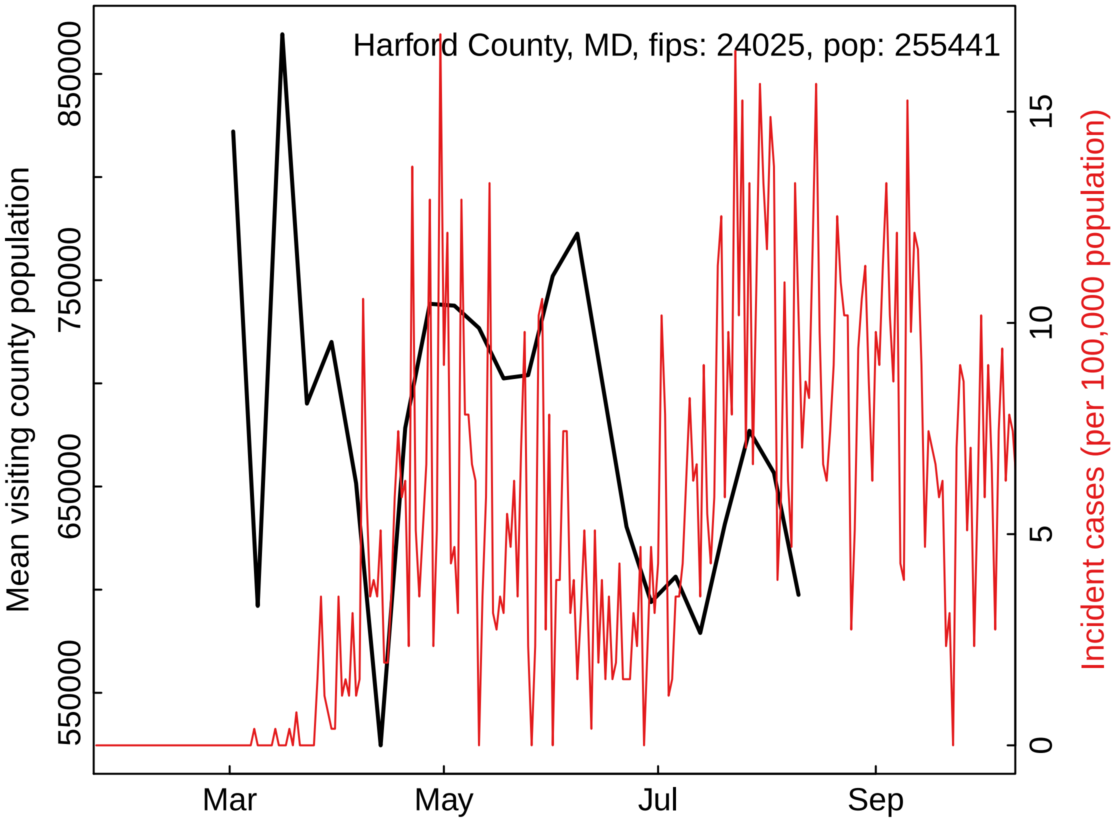

Harford County, MD, fips: 24025, pop: 255441
Mean visiting county population
550000
650000
750000
850000
Incident cases (per 100,000 population)
0
5
10
15
Mar
May
Jul
Sep

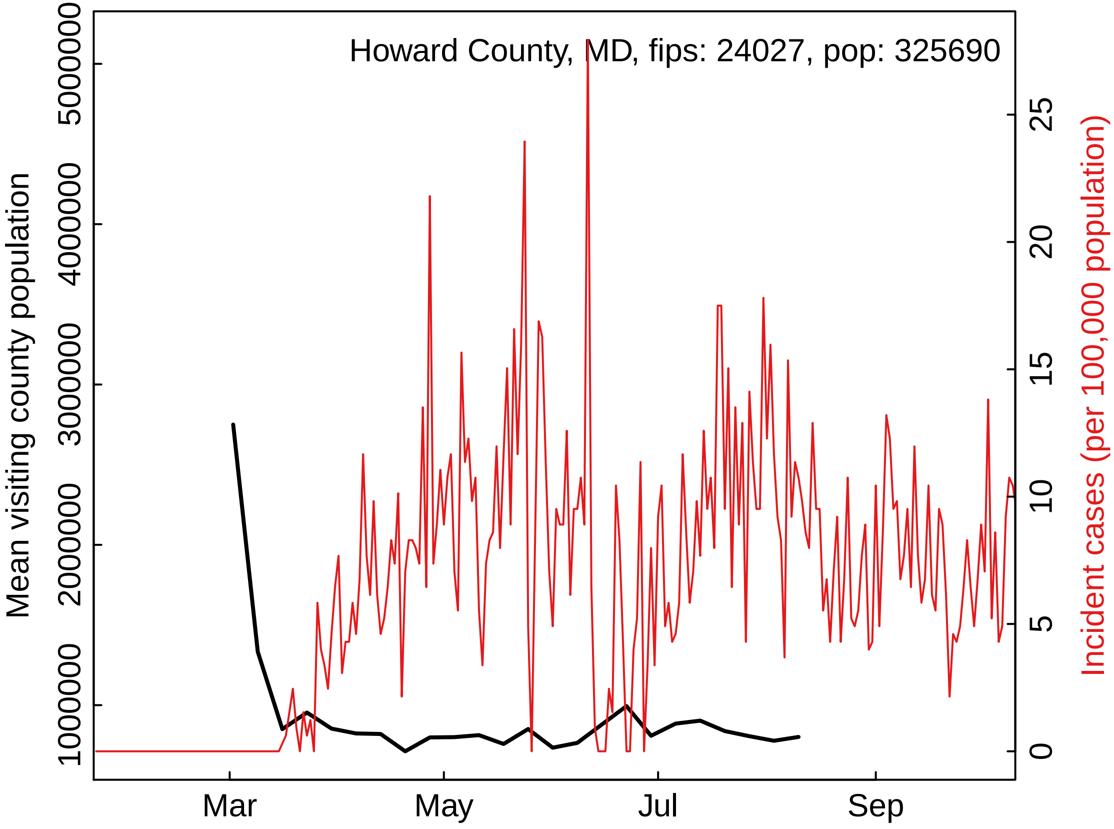

Howard County, MD, fips: 24027, pop: 325690
Mean visiting county population
10000000
20000000
30000000
40000000
50000000
Incident cases (per 100,000 population)
0
5
10
15
20
25
Mar
May
Jul
Sep

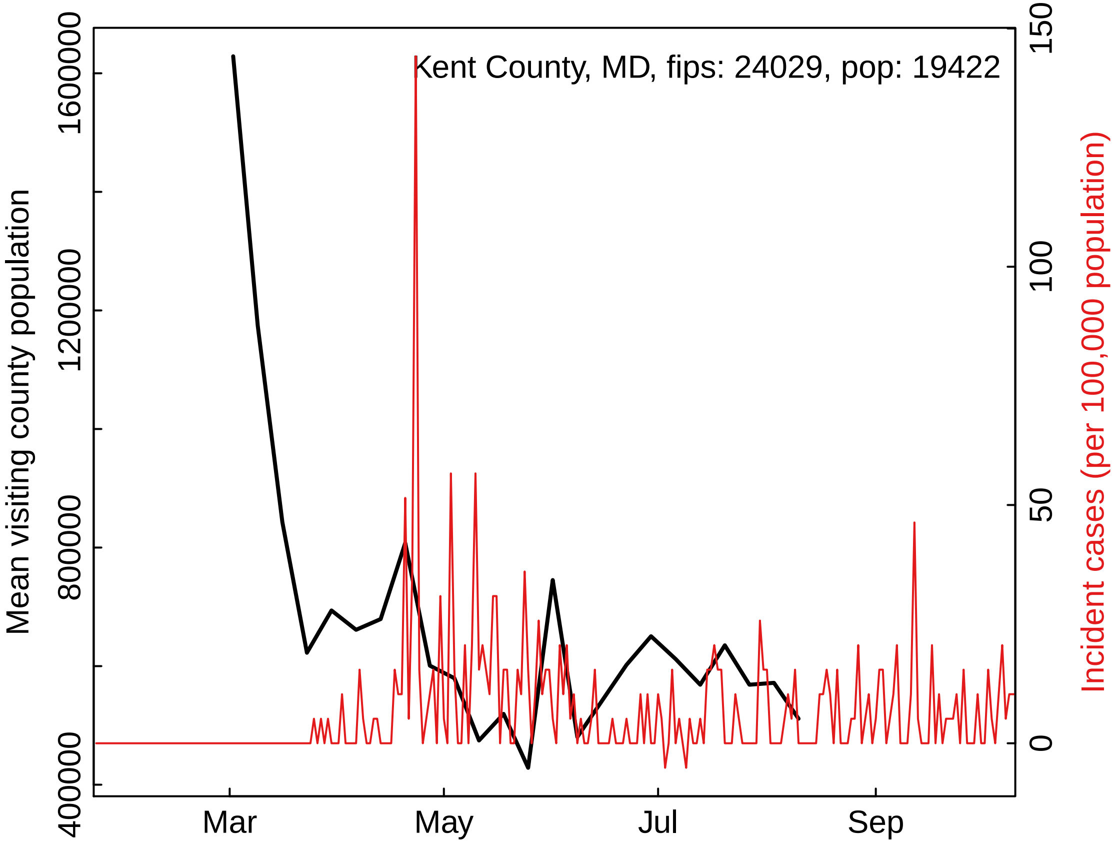

Kent County, MD, fips: 24029, pop: 19422
Mean visiting county population
400000
800000
1200000
1600000
Incident cases (per 100,000 population)
0
50
100
150
Mar
May
Jul
Sep

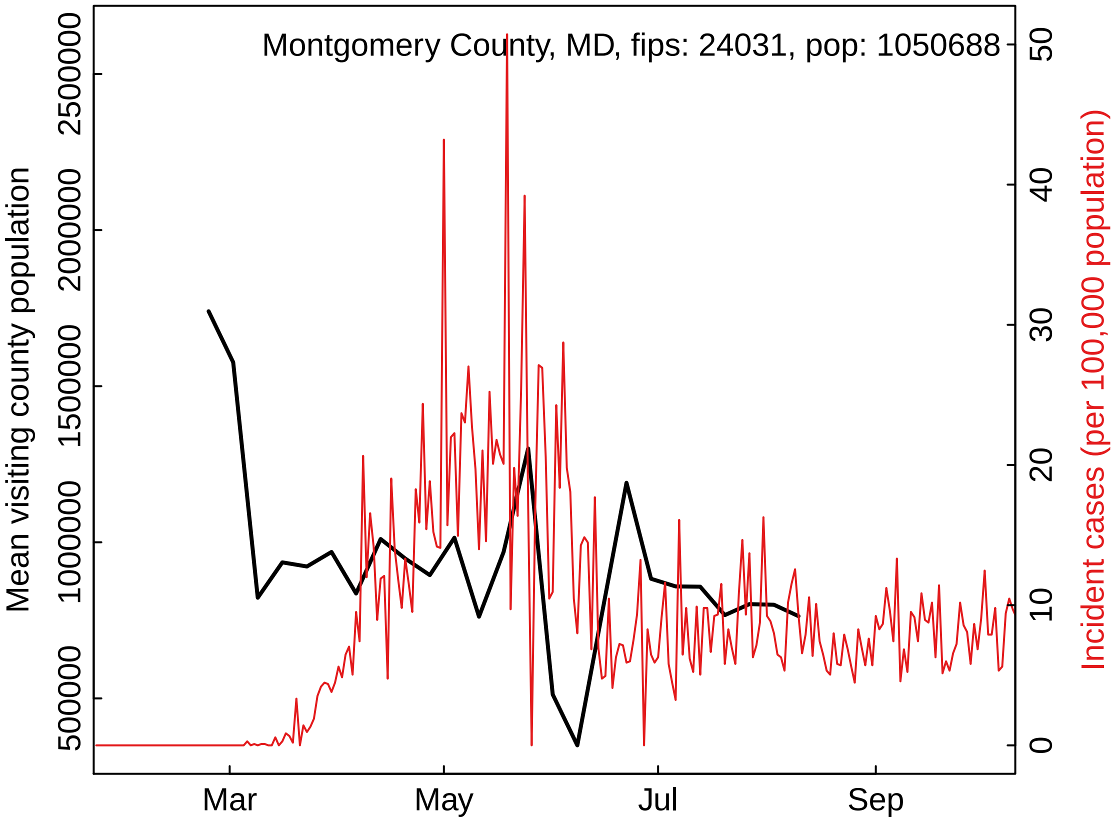

Montgomery County, MD, fips: 24031, pop: 1050688
Mean visiting county population
500000
1000000
1500000
2000000
2500000
Incident cases (per 100,000 population)
0
10
20
30
40
50
Mar
May
Jul
Sep

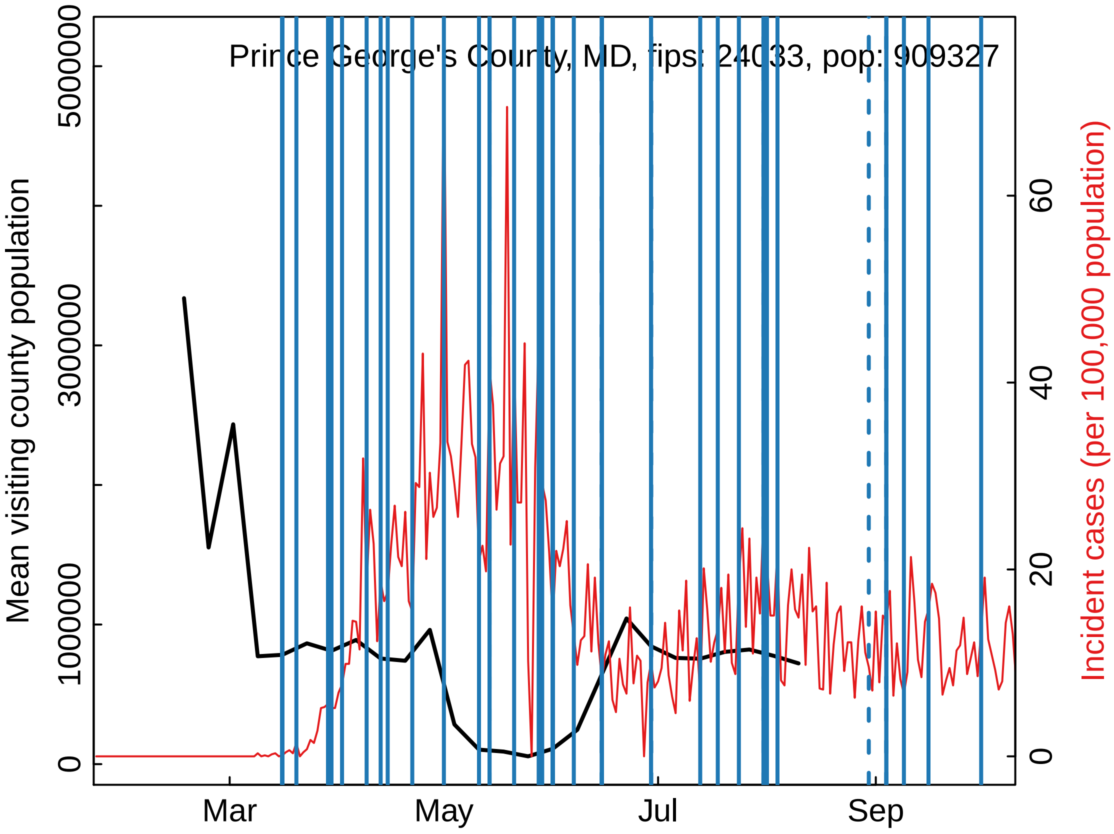

Prince George's County, MD, fips: 24033, pop: 909327
Mean visiting county population
0
1000000
3000000
5000000
Incident cases (per 100,000 population)
0
20
40
60
Mar
May
Jul
Sep

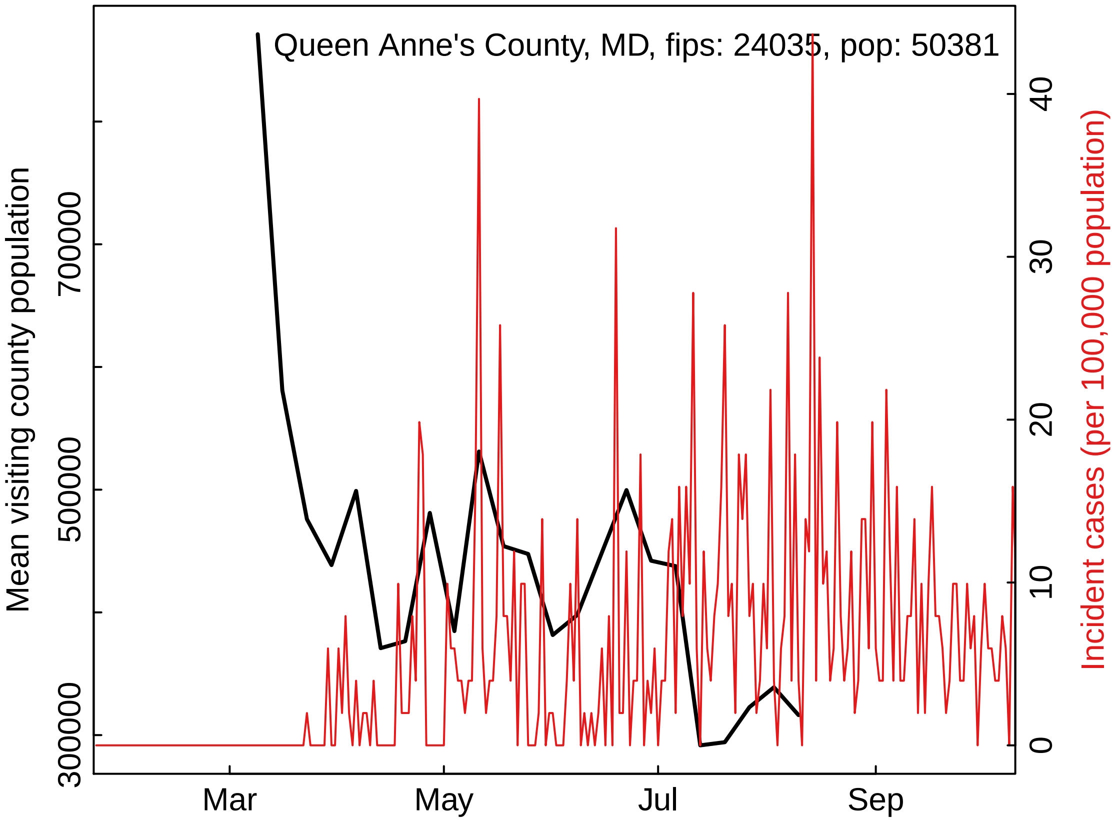

Queen Anne's County, MD, fips: 24035, pop: 50381
Mean visiting county population
300000
500000
700000
Incident cases (per 100,000 population)
0
10
20
30
40
Mar
May
Jul
Sep

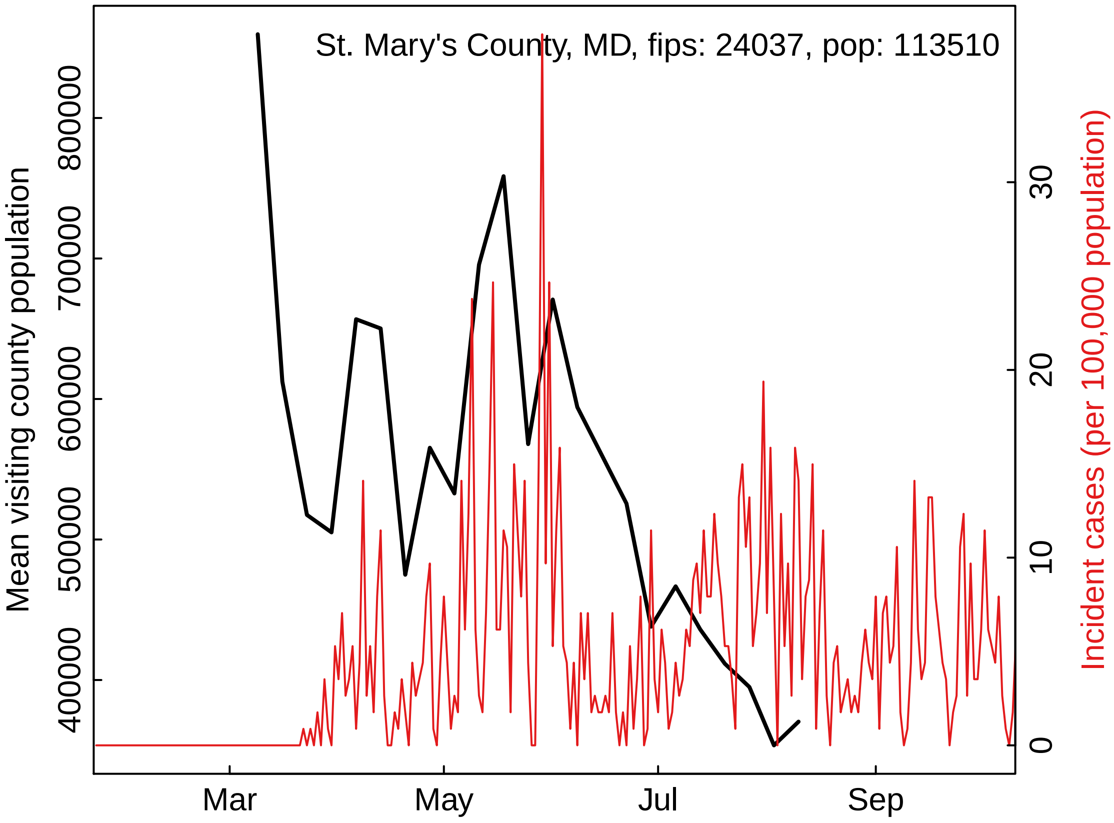

St. Mary's County, MD, fips: 24037, pop: 113510
Mean visiting county population
Incident cases (per 100,000 population)
400000
500000
600000
700000
800000
0
10
20
30
Mar
May
Jul
Sep

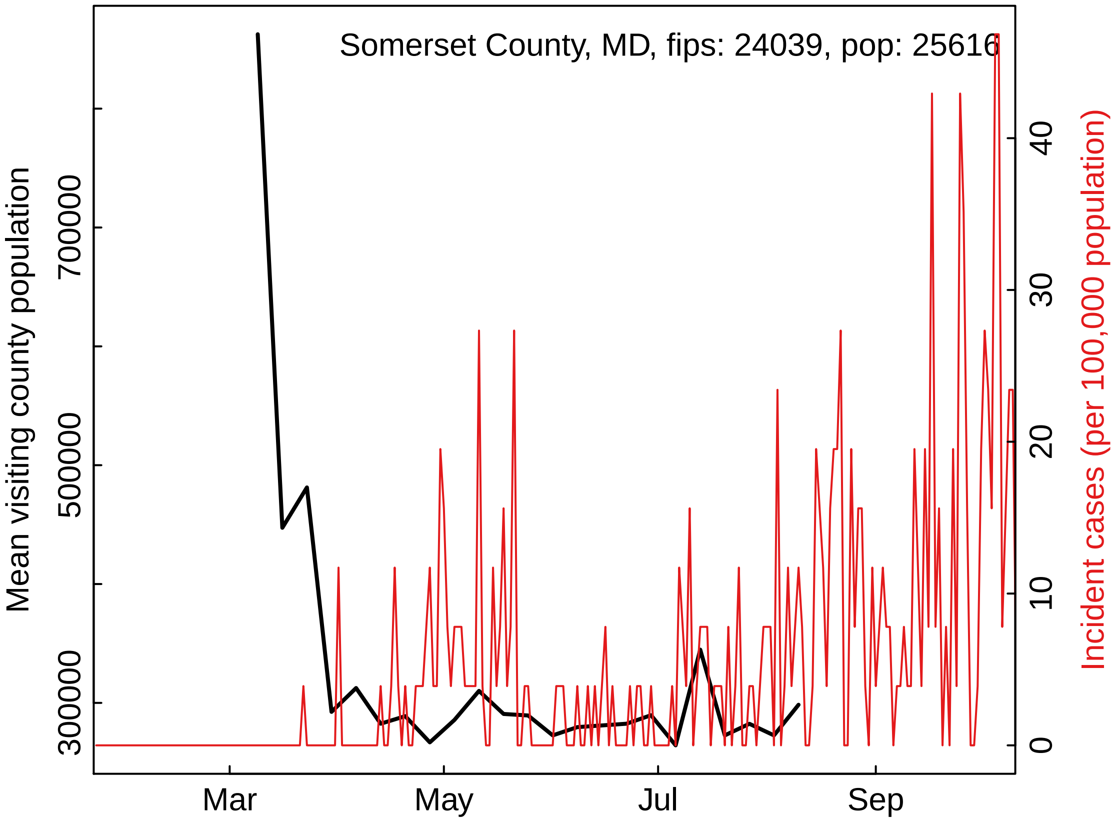
Somerset County, MD, fips: 24039, pop: 25616
Mean visiting county population
Incident cases (per 100,000 population)
300000
500000
7000000
0
10
20
30
40
Mar
May
Jul
Sep

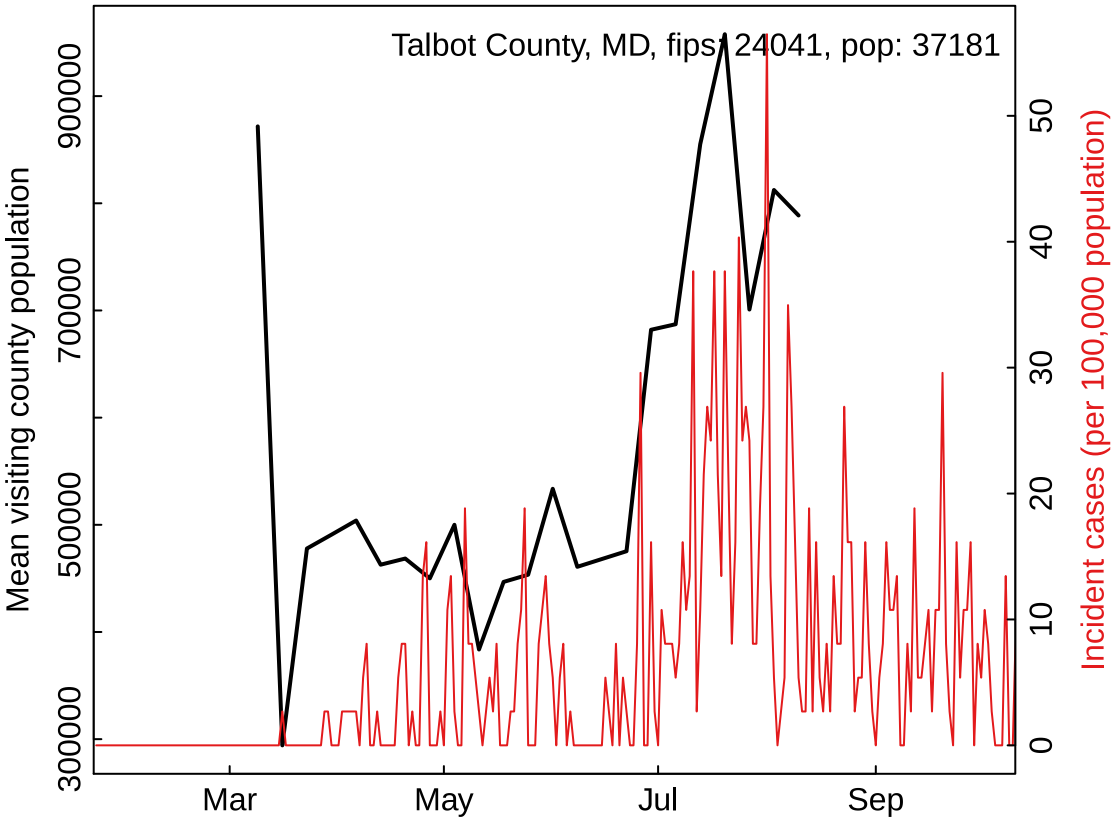
Talbot County, MD, fips: 24041, pop: 37181
Mean visiting county population
300000
500000
700000
900000
Incident cases (per 100,000 population)
0
10
20
30
40
50
Mar
May
Jul
Sep

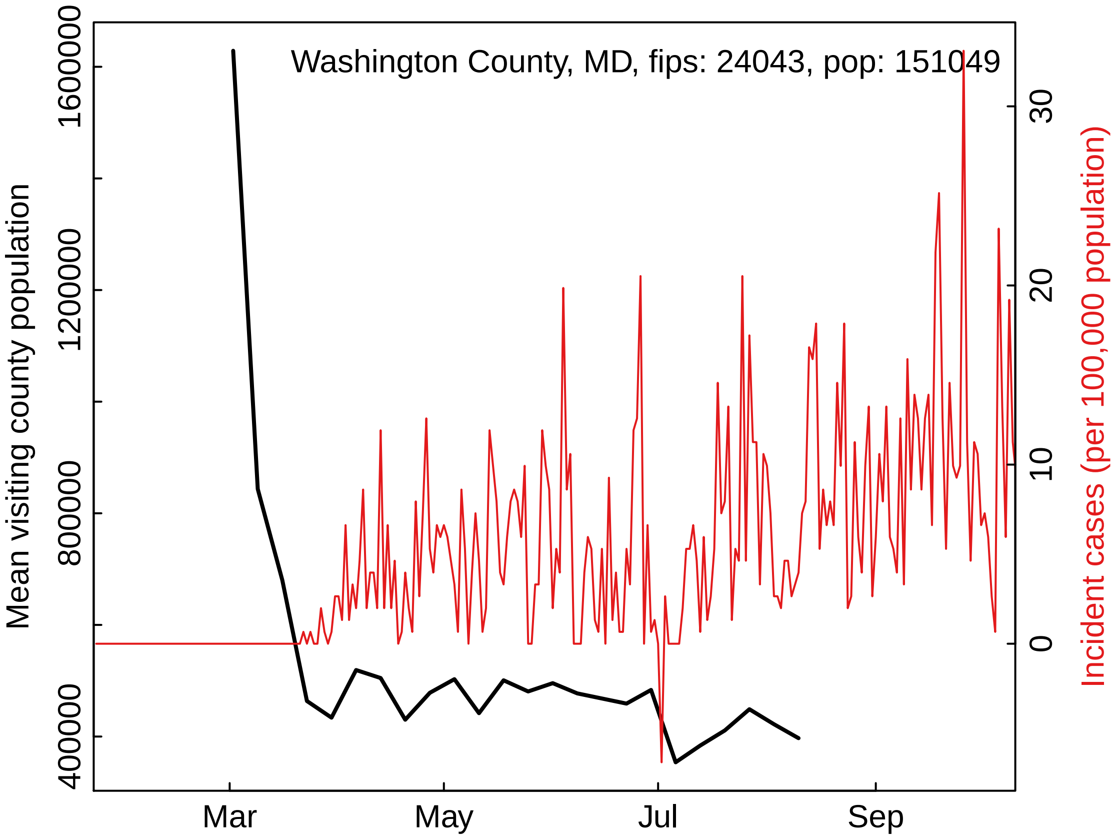

Washington County, MD, fips: 24043, pop: 151049
Mean visiting county population
400000
800000
1200000
1600000
Mar
May
Jul
Sep
0
10
20
30
Incident cases (per 100,000 population)

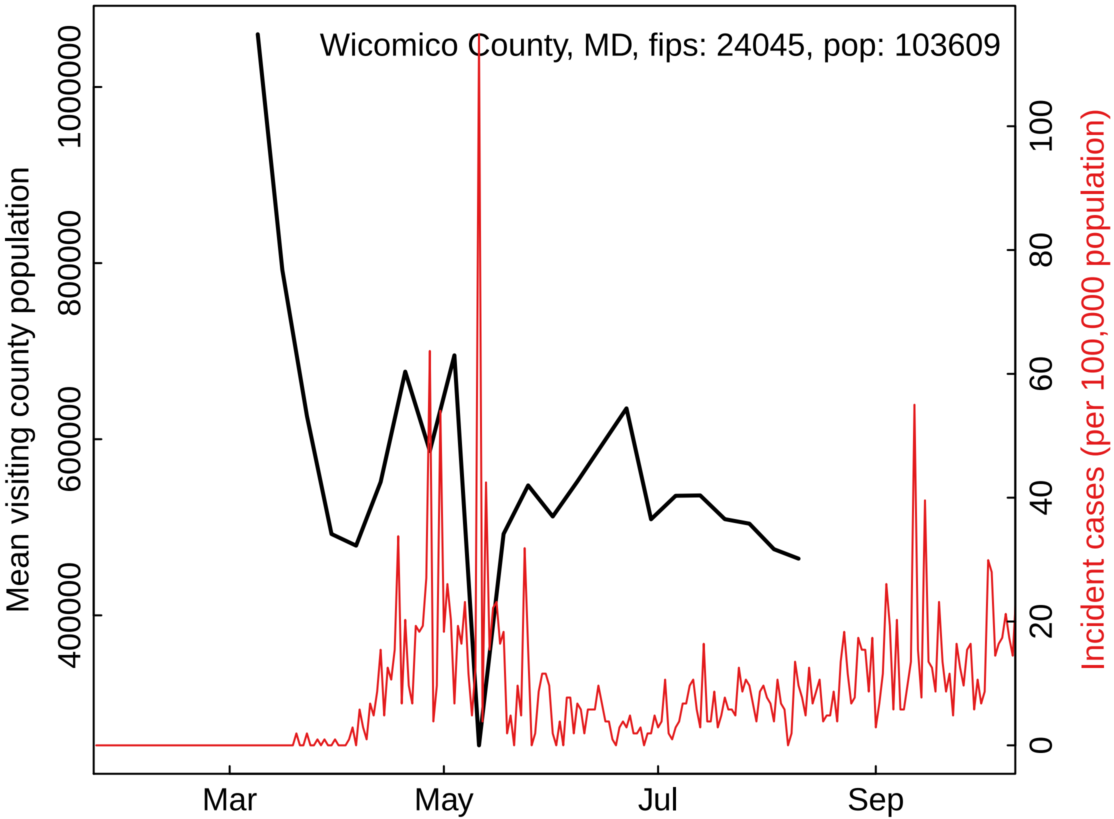
Wicomico County, MD, fips: 24045, pop: 103609
Mean visiting county population
400000
600000
800000
1000000
Incident cases (per 100,000 population)
0
20
40
60
80
100
Mar
May
Jul
Sep

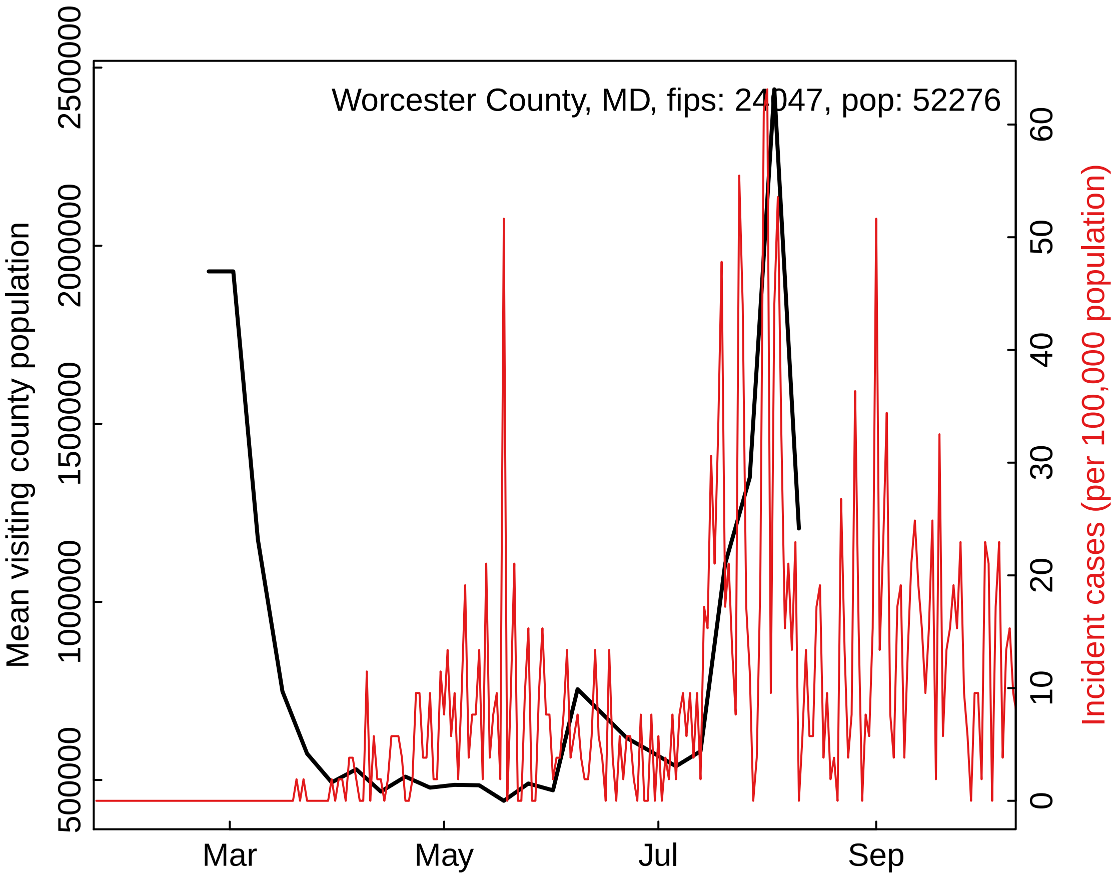

Worcester County, MD, fips: 24047, pop: 52276
Mean visiting county population
Incident cases (per 100,000 population)
500000
1000000
1500000
2000000
2500000
0
10
20
30
40
50
60
Mar
May
Jul
Sep

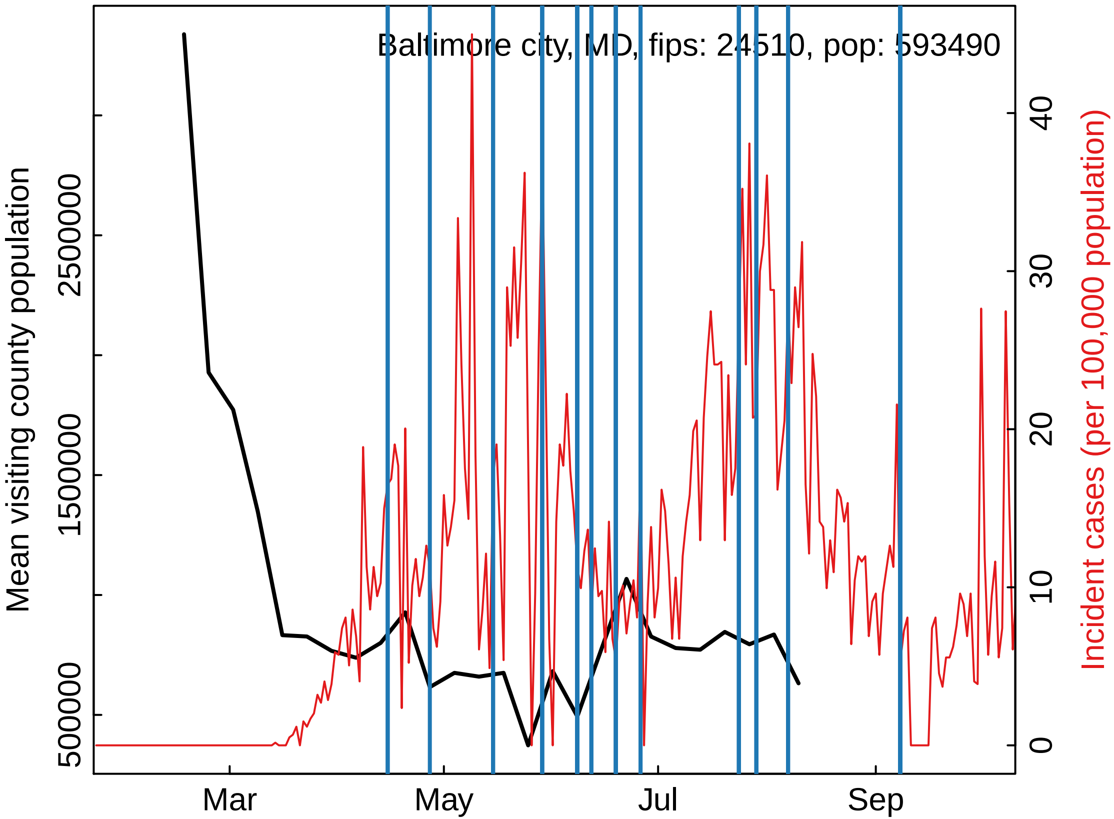

Baltimore city, MD, fips: 24510, pop: 593490
Mean visiting county population
500000
1500000
2500000
Incident cases (per 100,000 population)
0
10
20
30
40
Mar
May
Jul
Sep

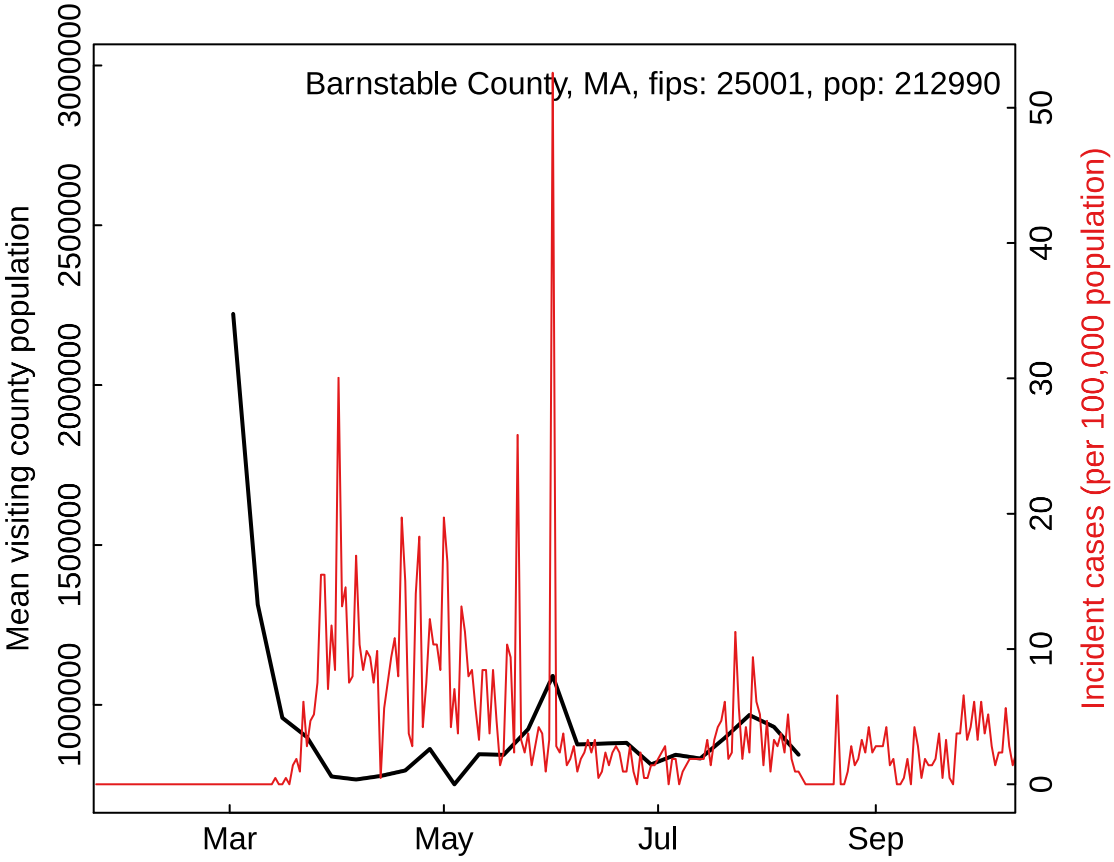

Barnstable County, MA, fips: 25001, pop: 212990
Mean visiting county population
10000000
15000000
20000000
25000000
30000000
Incident cases (per 100,000 population)
0
10
20
30
40
50
Mar
May
Jul
Sep

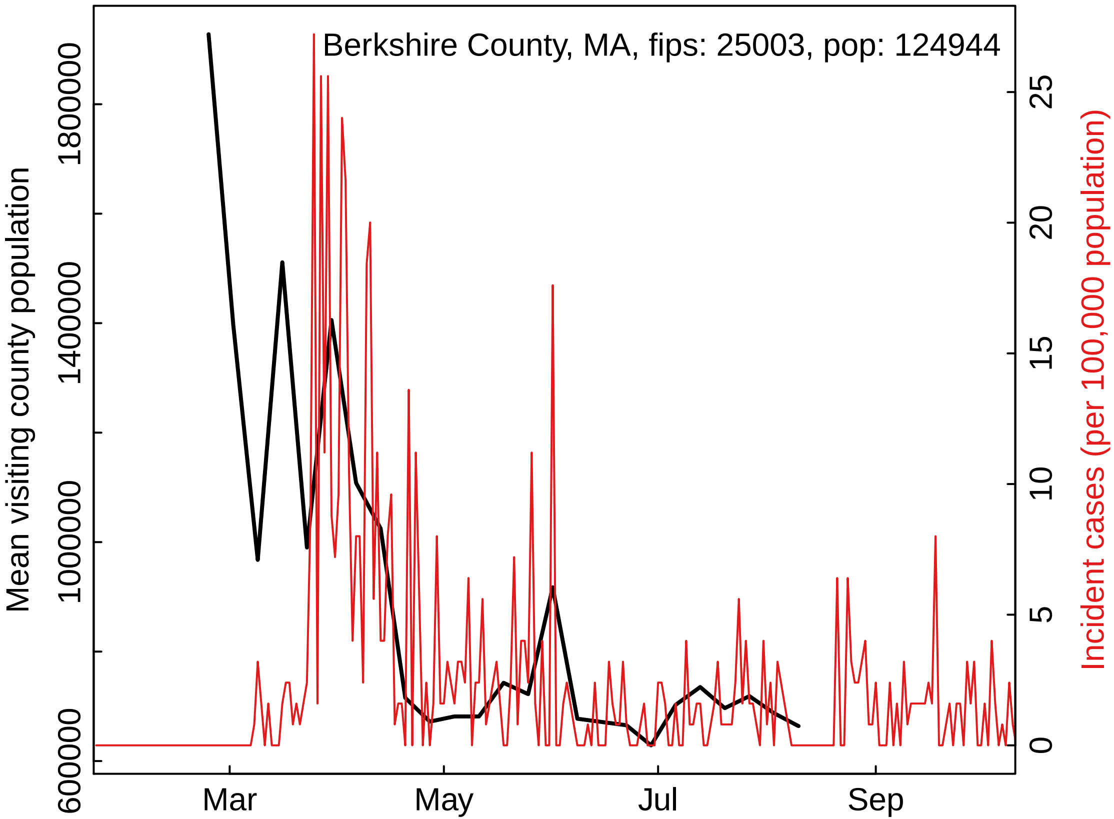

Berkshire County, MA, fips: 25003, pop: 124944
Mean visiting county population
6000000
10000000
1400000
18000000
Mar
May
Jul
Sep
0
5
10
15
20
25
Incident cases (per 100,000 population)

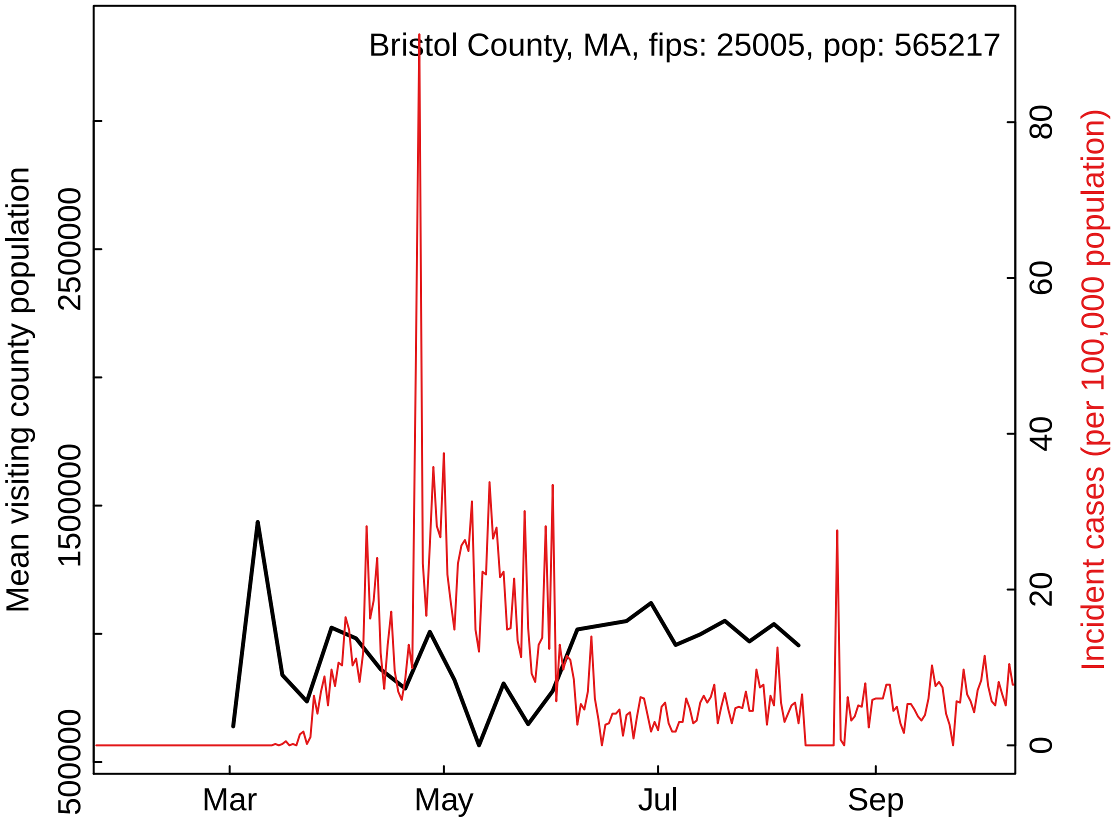

Bristol County, MA, fips: 25005, pop: 565217
Mean visiting county population
500000
1500000
2500000
Mar
May
Jul
Sep
0
20
40
60
80
Incident cases (per 100,000 population)

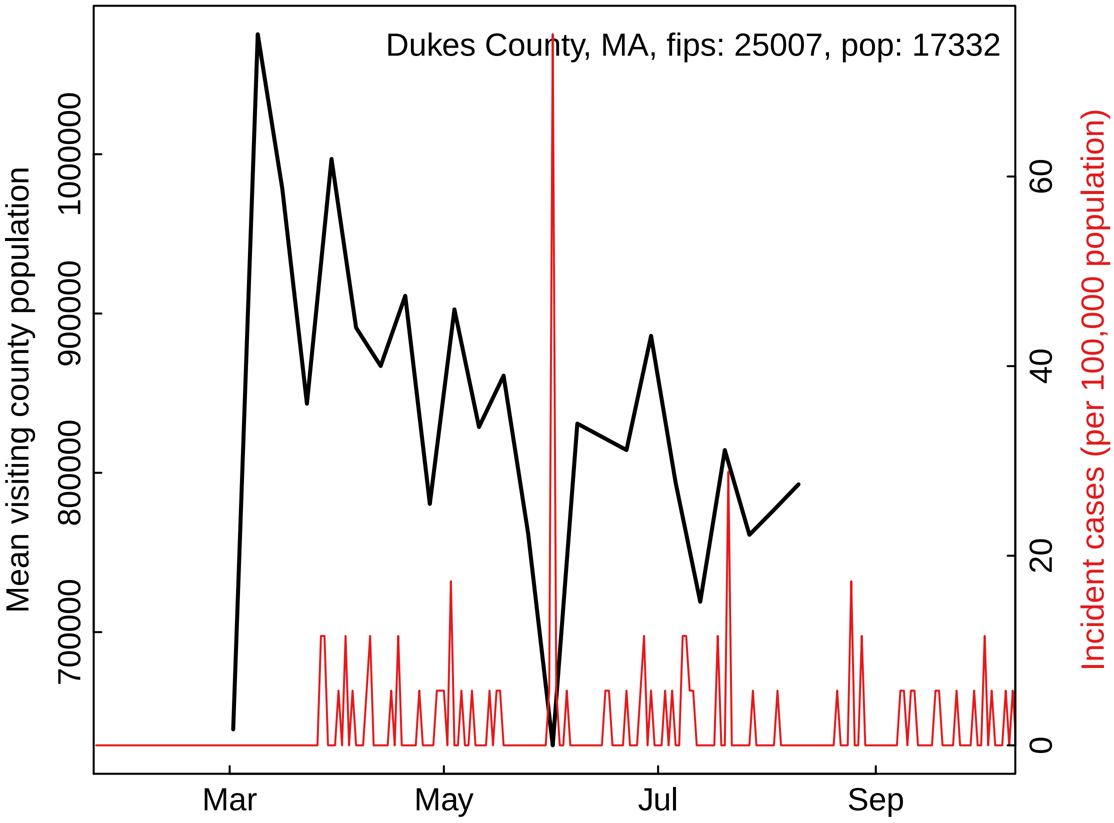

Dukes County, MA, fips: 25007, pop: 17332
Mean visiting county population
700000
800000
900000
1000000
Incident cases (per 100,000 population)
0
20
40
60
Mar
May
Jul
Sep

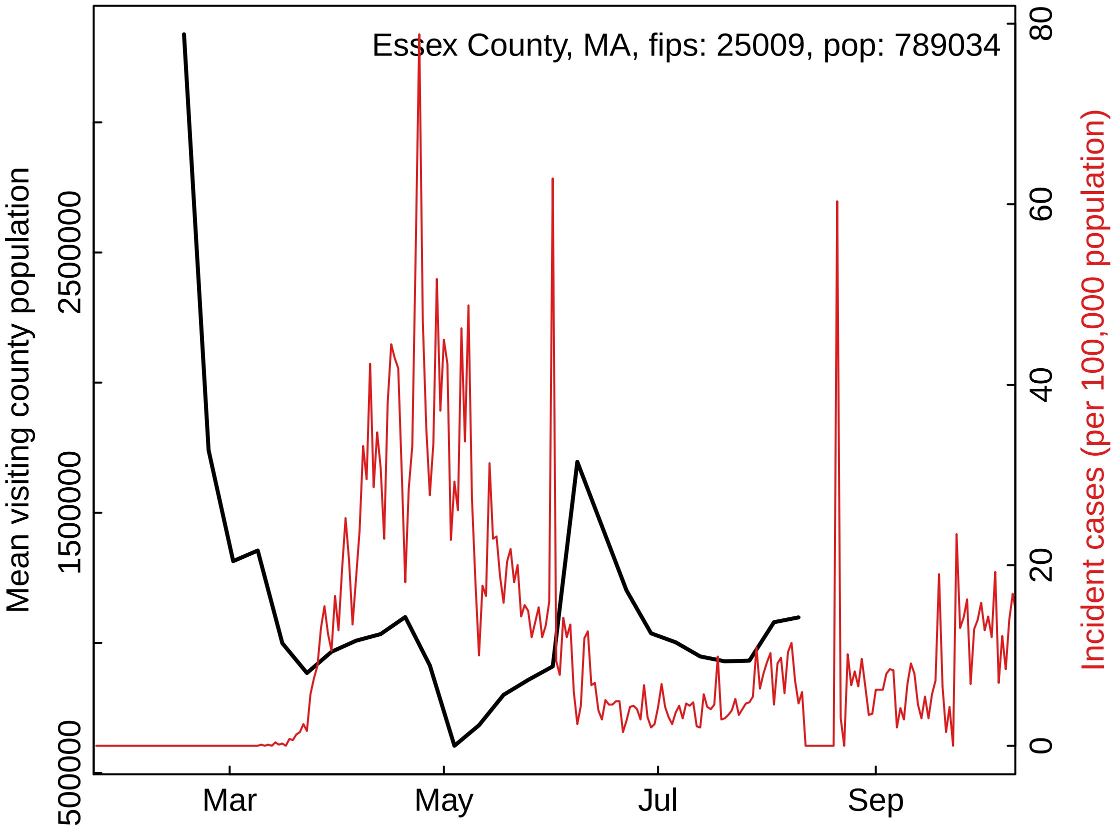

Essex County, MA, fips: 25009, pop: 789034
Mean visiting county population
500000
1500000
2500000
Mar
May
Jul
Sep
0
20
40
60
80
Incident cases (per 100,000 population)

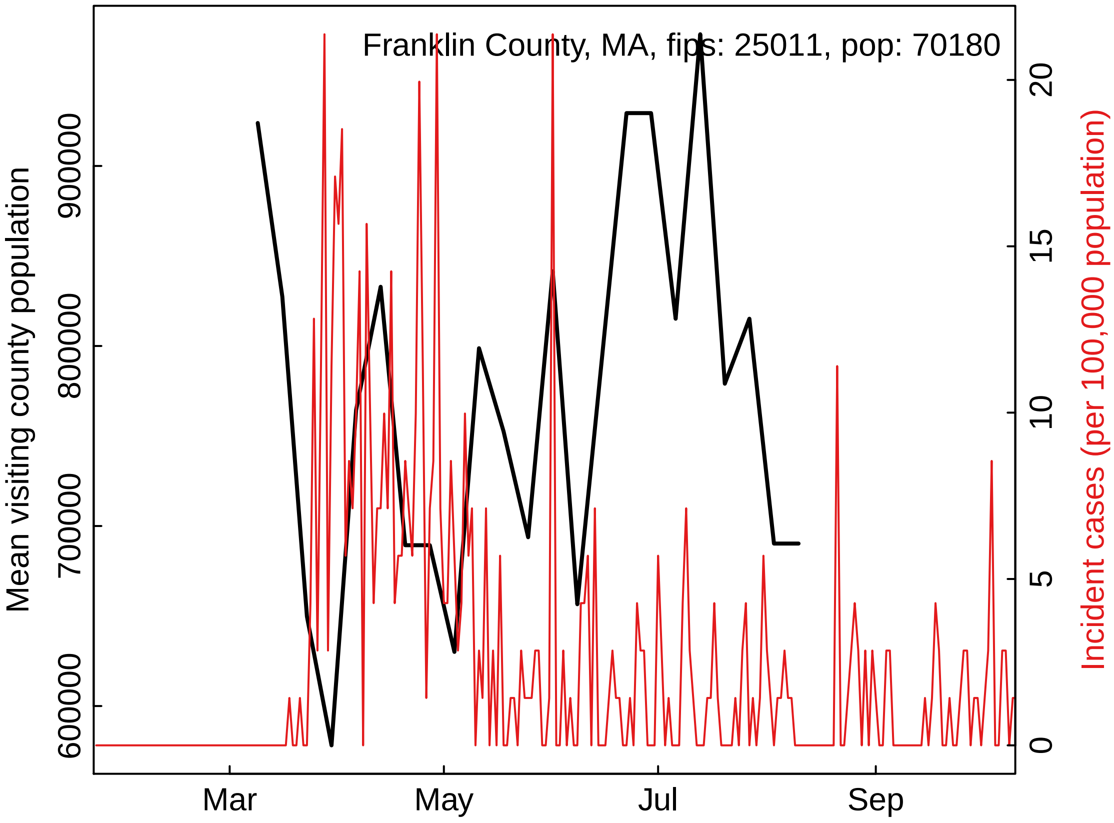

Franklin County, MA, fips: 25011, pop: 70180
Mean visiting county population
600000
700000
800000
900000
Incident cases (per 100,000 population)
0
5
10
15
20
Mar
May
Jul
Sep

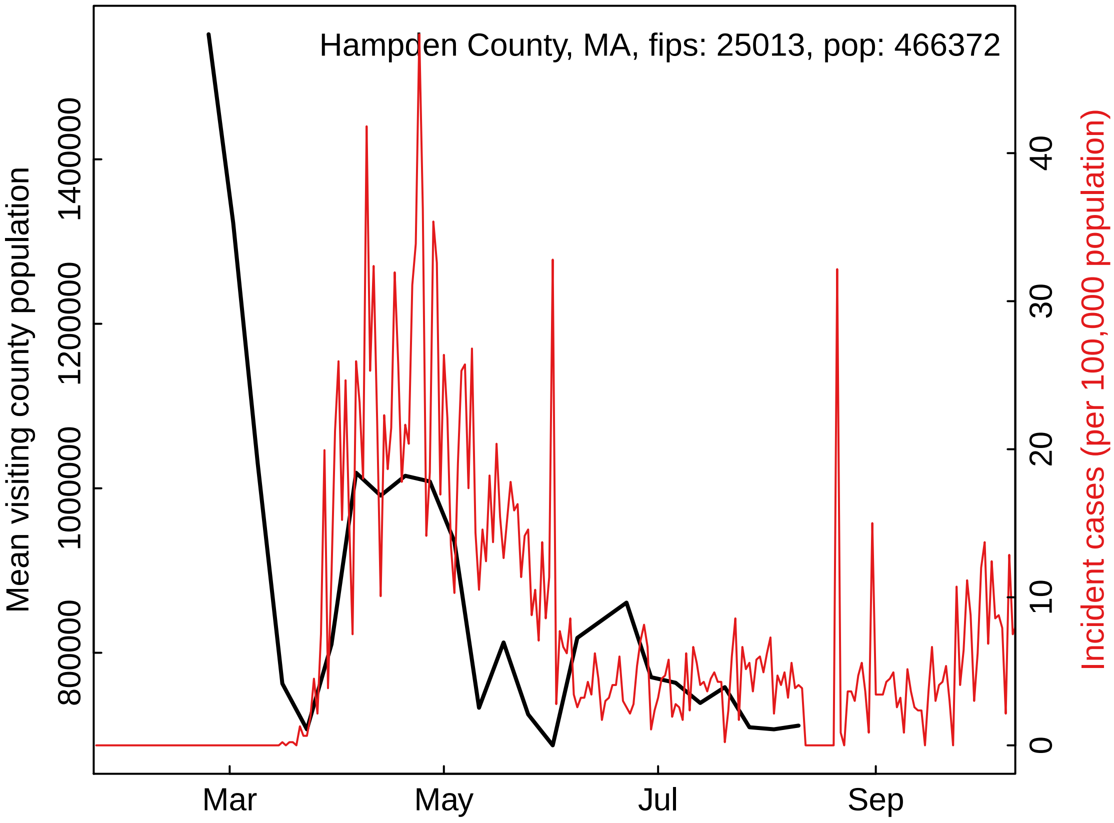
Hampden County, MA, fips: 25013, pop: 466372
Mean visiting county population
800000
1000000
1200000
1400000
Incident cases (per 100,000 population)
0
10
20
30
40
Mar
May
Jul
Sep

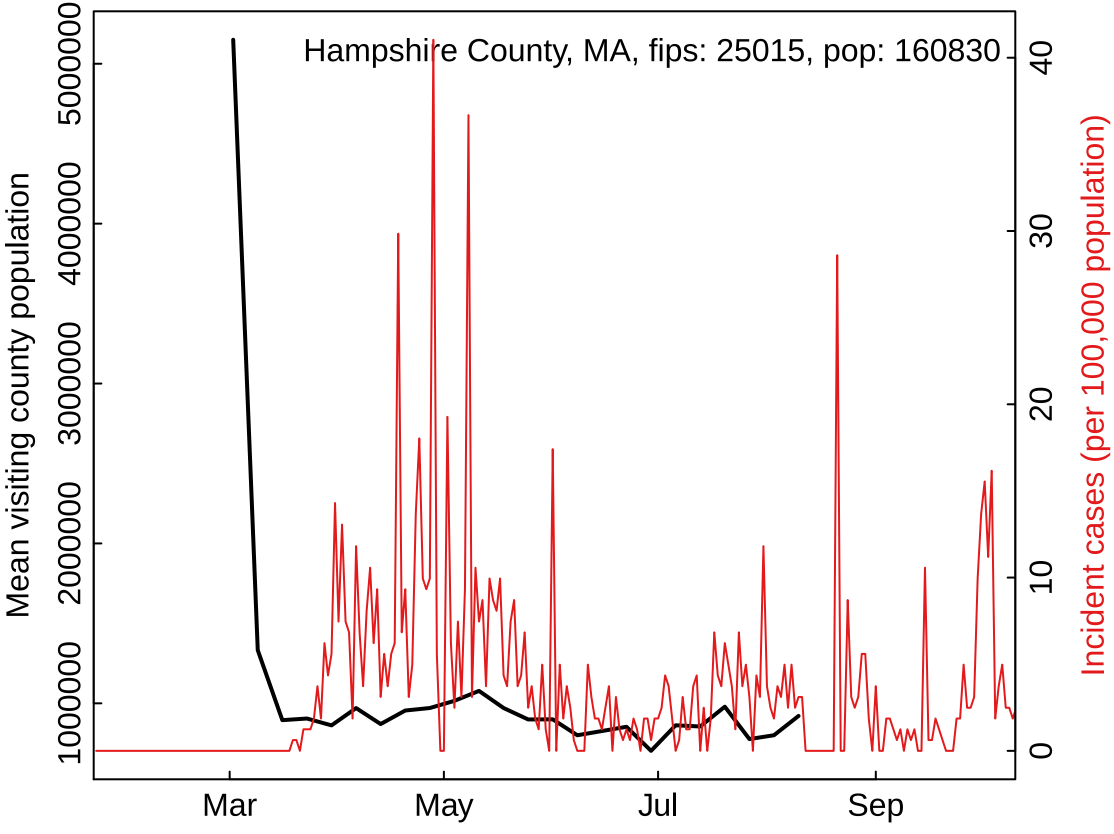
Hampshire County, MA, fips: 25015, pop: 160830
Mean visiting county population
10000000
20000000
30000000
40000000
50000000
Incident cases (per 100,000 population)
0
10
20
30
40
Mar
May
Jul
Sep

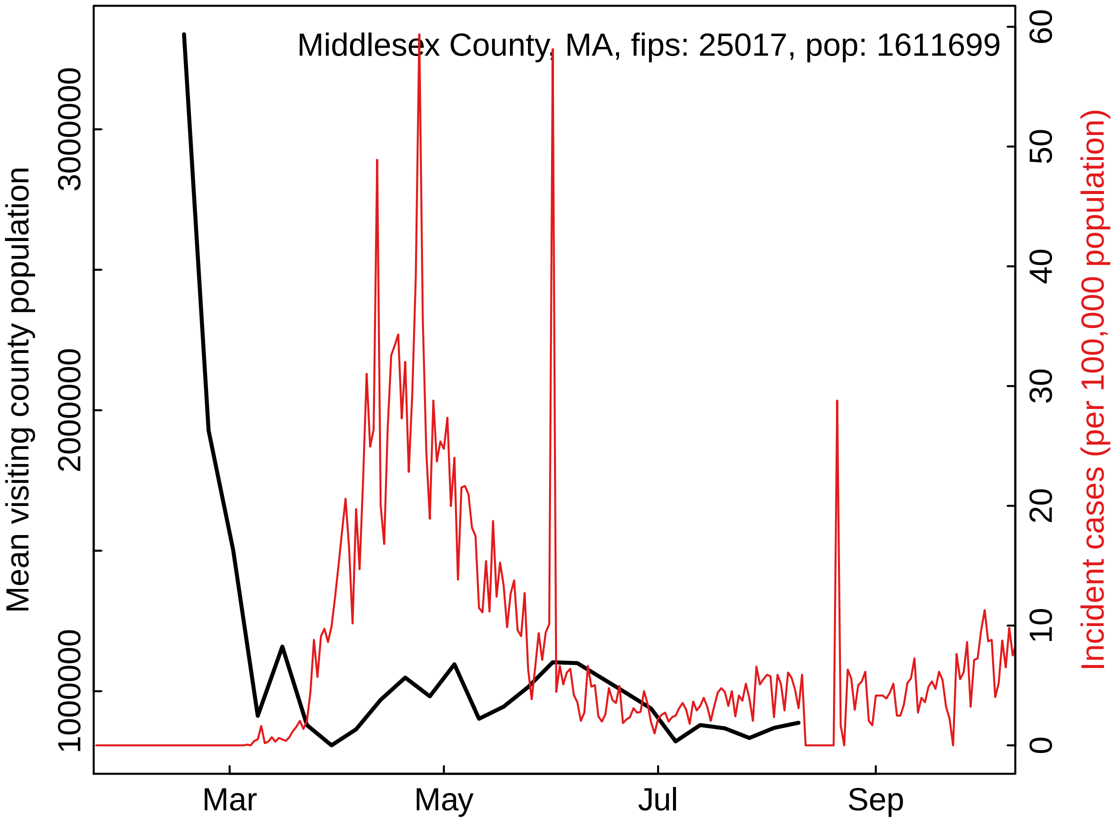

Middlesex County, MA, fips: 25017, pop: 1611699
Mean visiting county population
10000000
20000000
30000000
Incident cases (per 100,000 population)
0
10
20
30
40
50
60
Mar
May
Jul
Sep

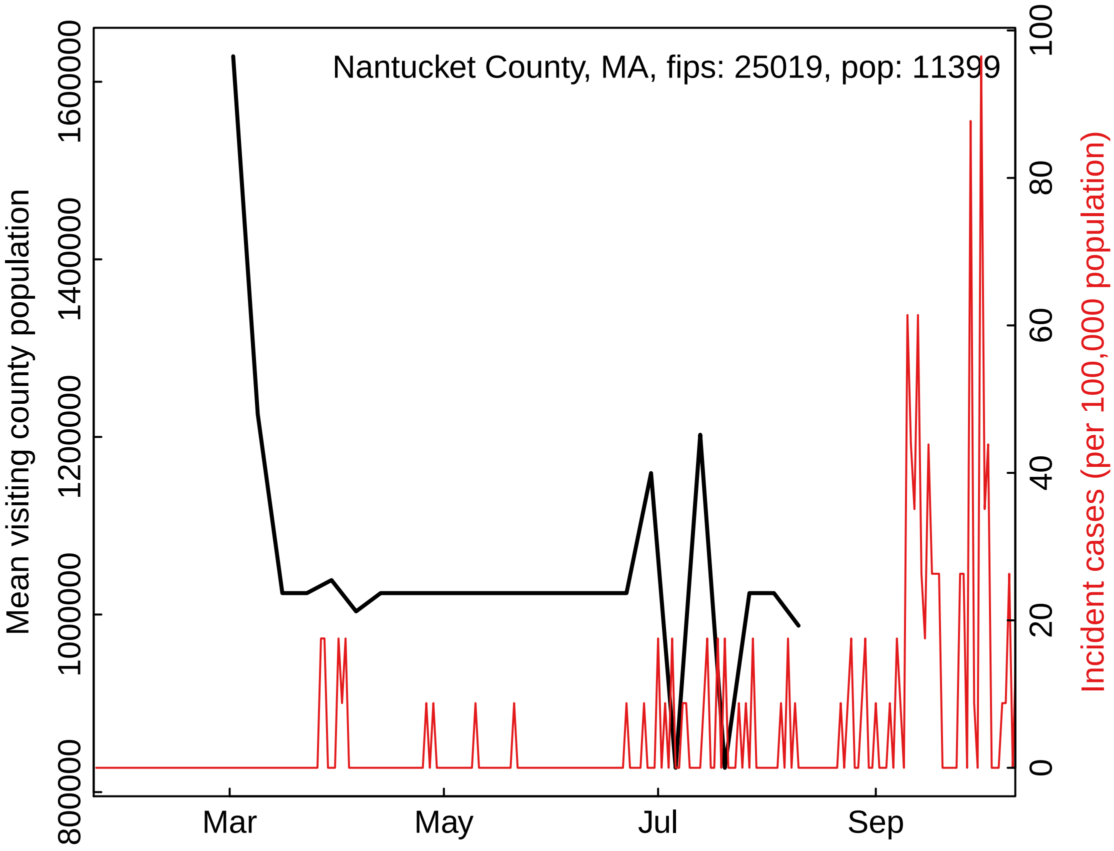

Nantucket County, MA, fips: 25019, pop: 11399
Mean visiting county population
Incident cases (per 100,000 population)
800000
1000000
1200000
1400000
1600000
0
20
40
60
80
100
Mar
May
Jul
Sep

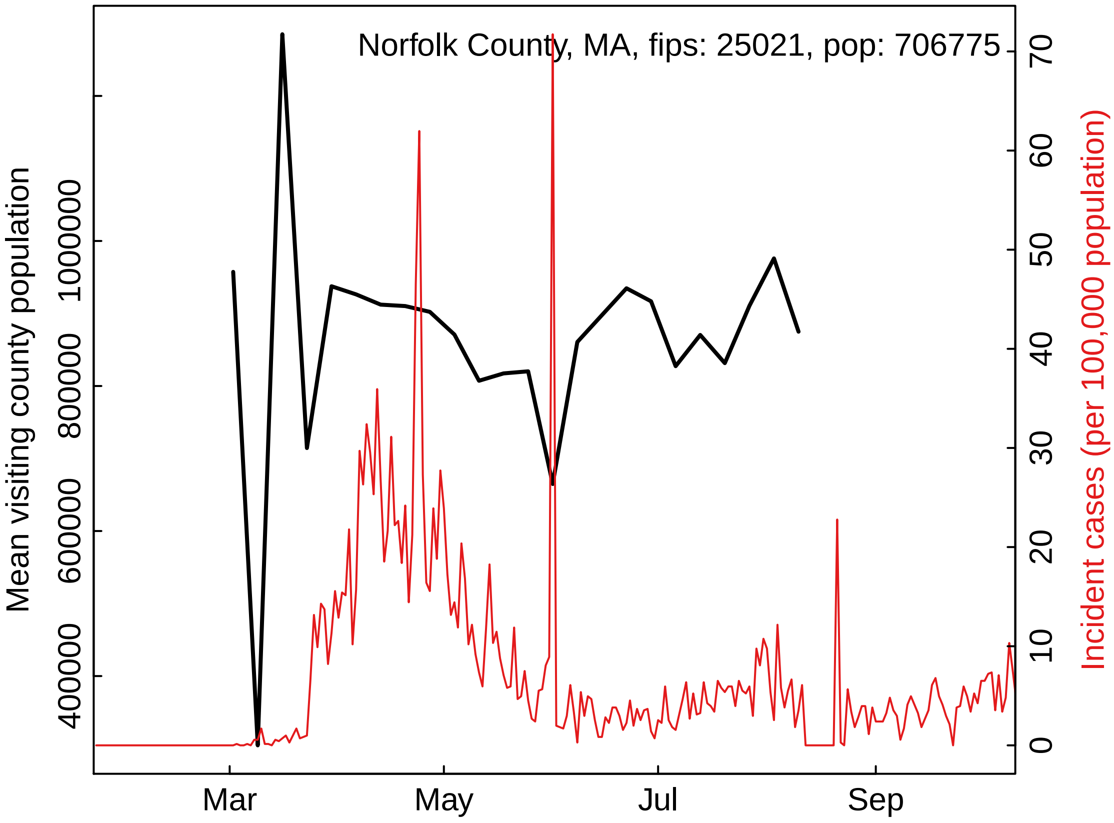

Norfolk County, MA, fips: 25021, pop: 706775
Mean visiting county population
400000
600000
800000
1000000
Incident cases (per 100,000 population)
0
10
20
30
40
50
60
70
Mar
May
Jul
Sep

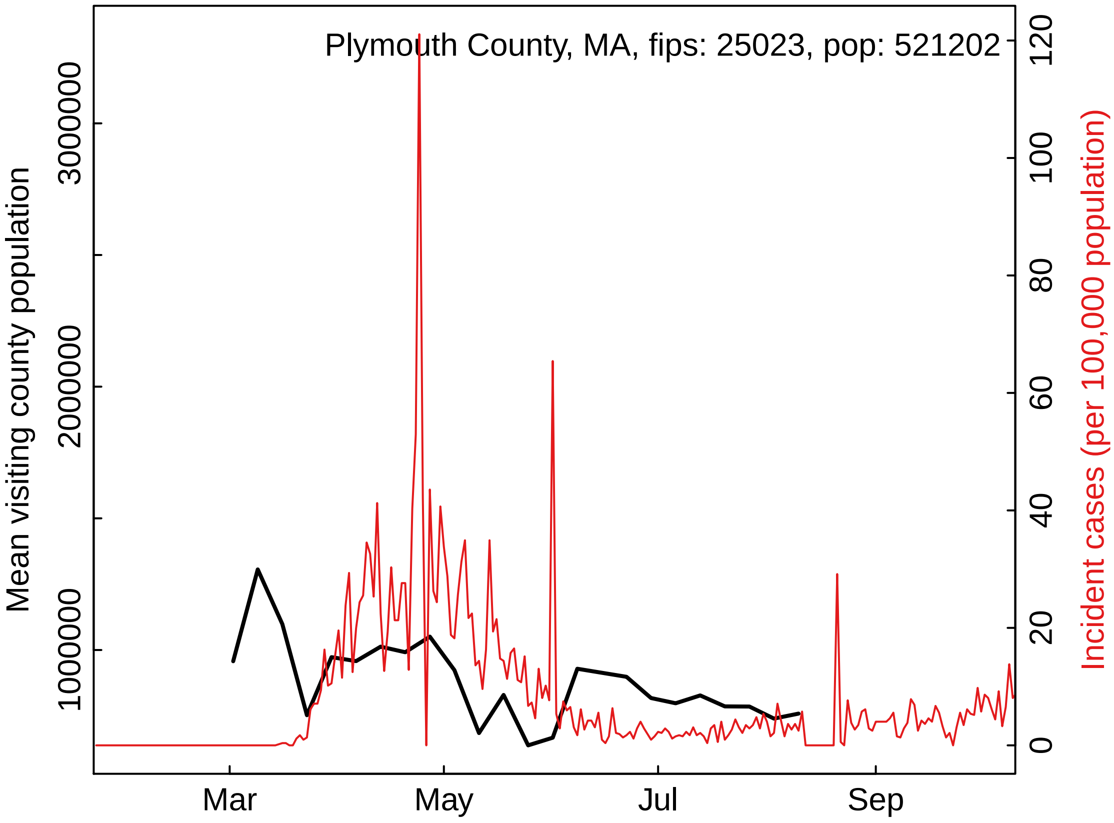

Plymouth County, MA, fips: 25023, pop: 521202
Mean visiting county population
10000000
20000000
30000000
Incident cases (per 100,000 population)
0
20
40
60
80
100
120
Mar
May
Jul
Sep

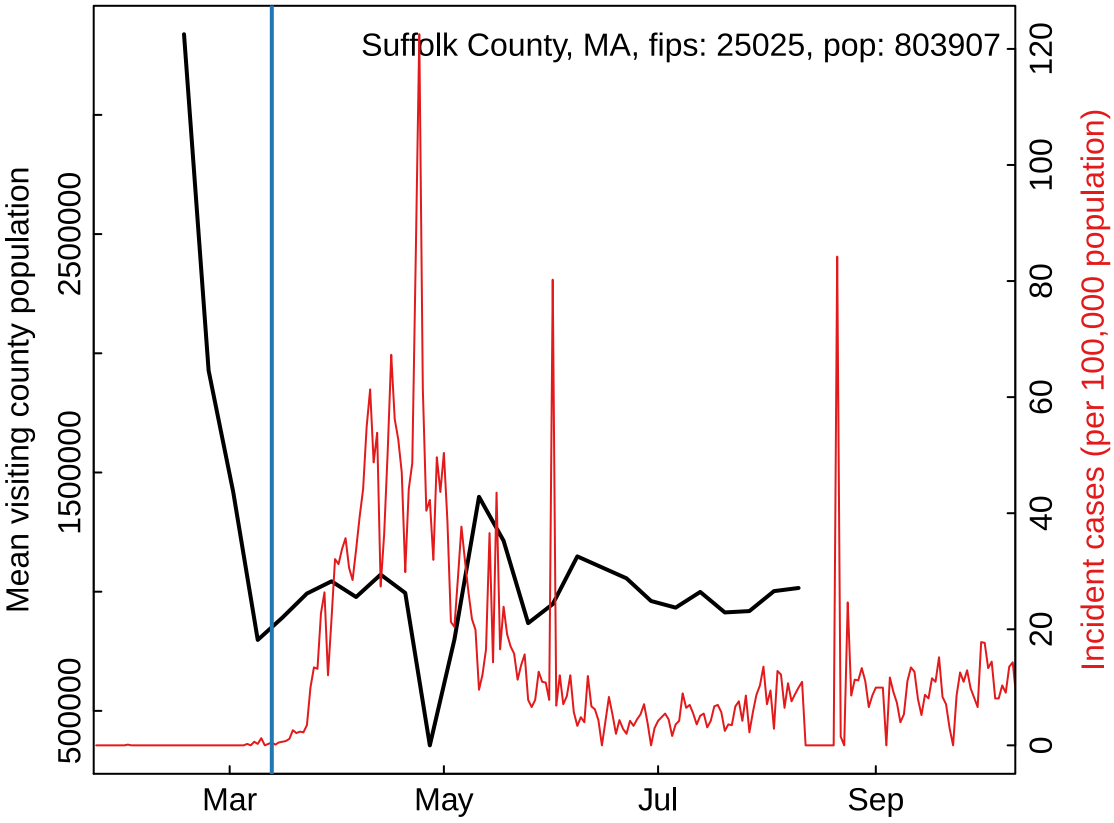

Suffolk County, MA, fips: 25025, pop: 803907
Mean visiting county population
500000
1500000
2500000
Incident cases (per 100,000 population)
0
20
40
60
80
100
120
Mar
May
Jul
Sep

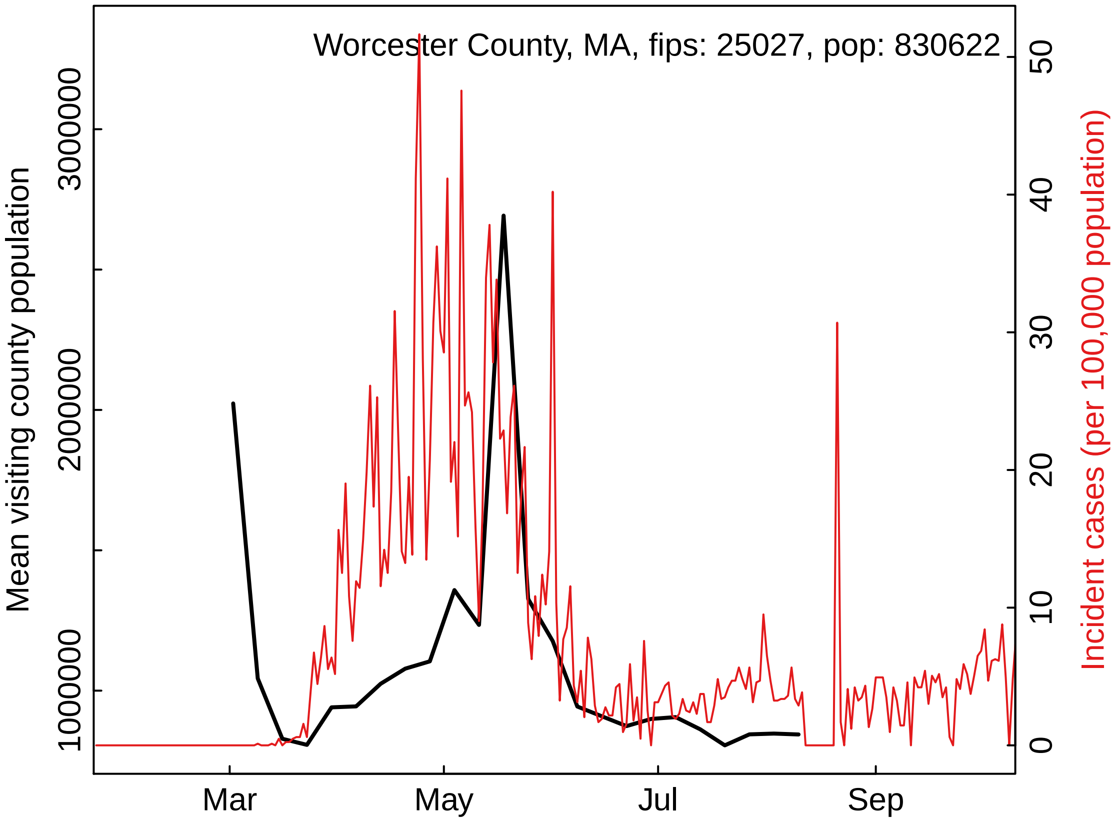

Worcester County, MA, fips: 25027, pop: 830622
Mean visiting county population
1000000
2000000
3000000
Incident cases (per 100,000 population)
0
10
20
30
40
50
Mar
May
Jul
Sep

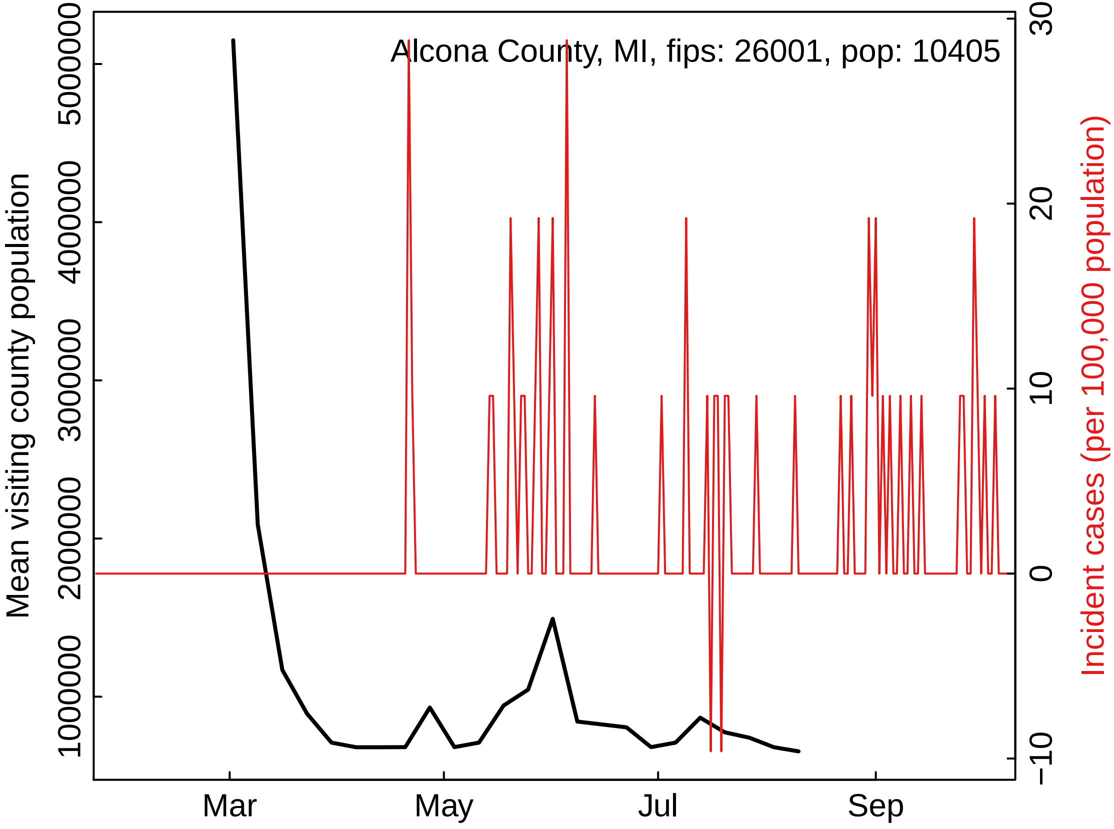

Alcona County, MI, fips: 26001, pop: 10405
Mean visiting county population
Incident cases (per 100,000 population)
10000000
20000000
30000000
40000000
50000000
−10
0
10
20
30
Mar
May
Jul
Sep

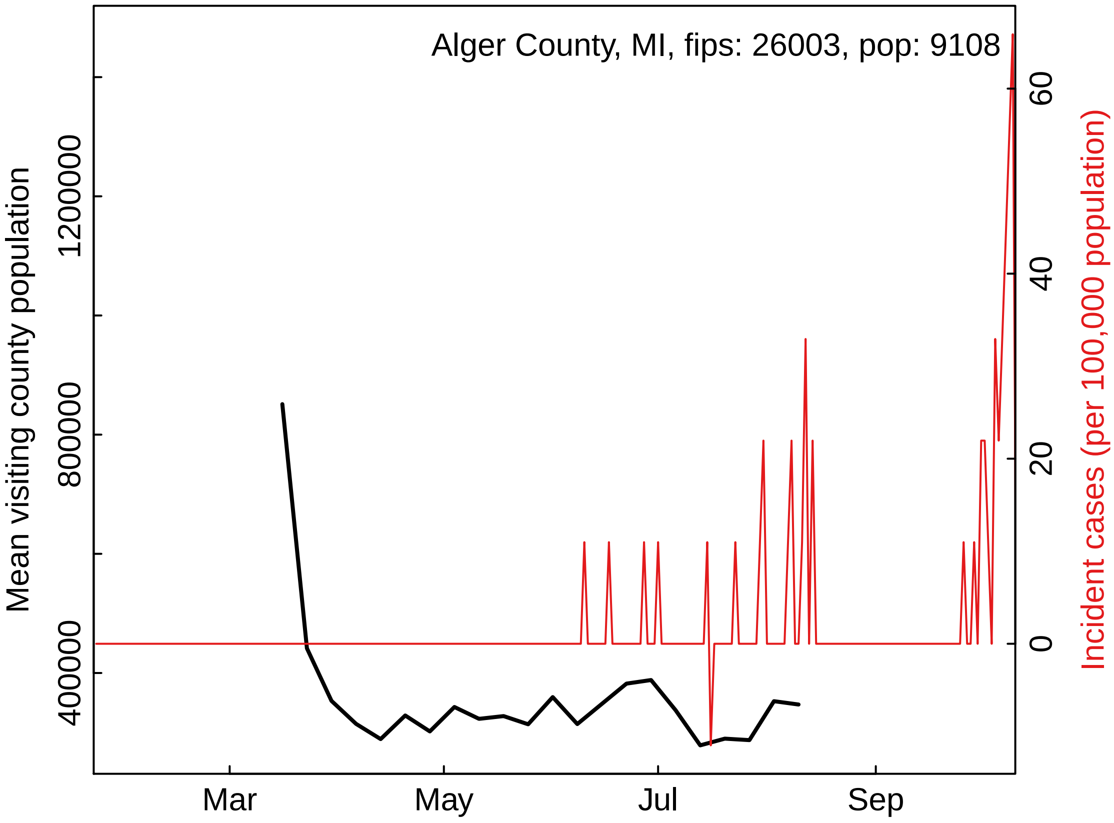

Alger County, MI, fips: 26003, pop: 9108
Mean visiting county population
400000
800000
1200000
Incident cases (per 100,000 population)
0
20
40
60
Mar
May
Jul
Sep

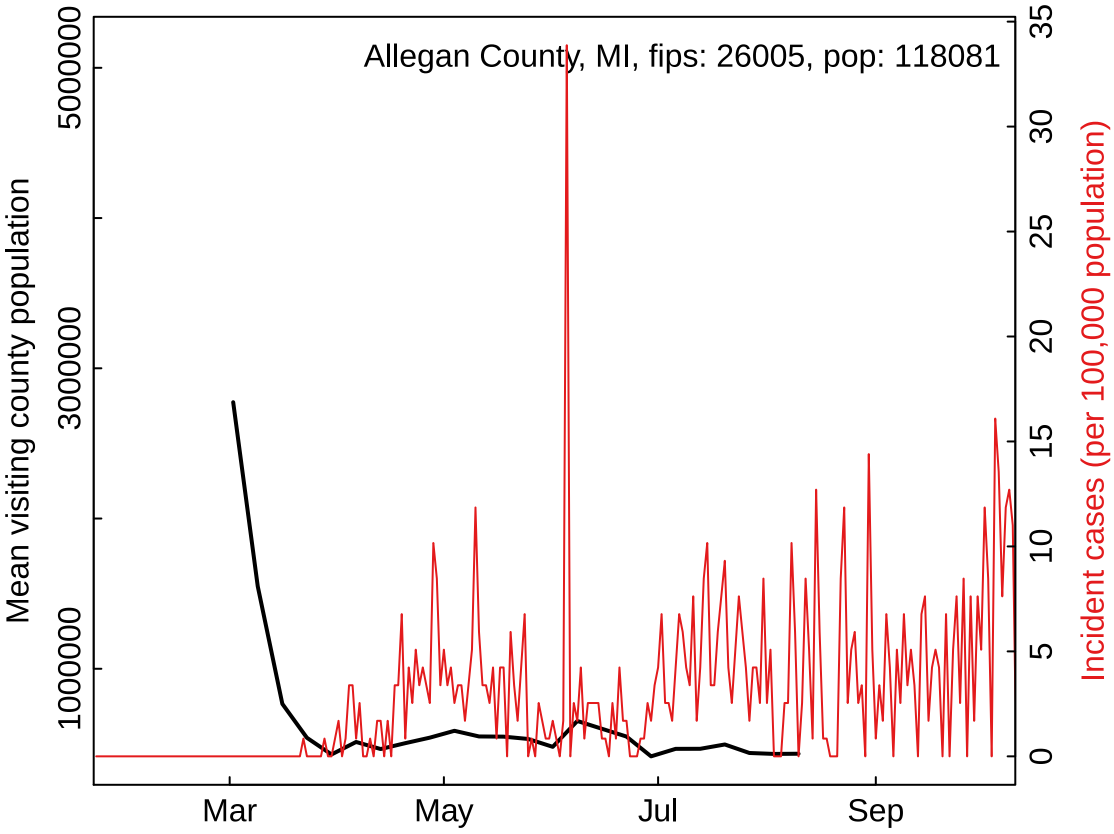

Allegan County, MI, fips: 26005, pop: 118081
Mean visiting county population
1000000
3000000
5000000
Incident cases (per 100,000 population)
0
5
10
15
20
25
30
35
Mar
May
Jul
Sep

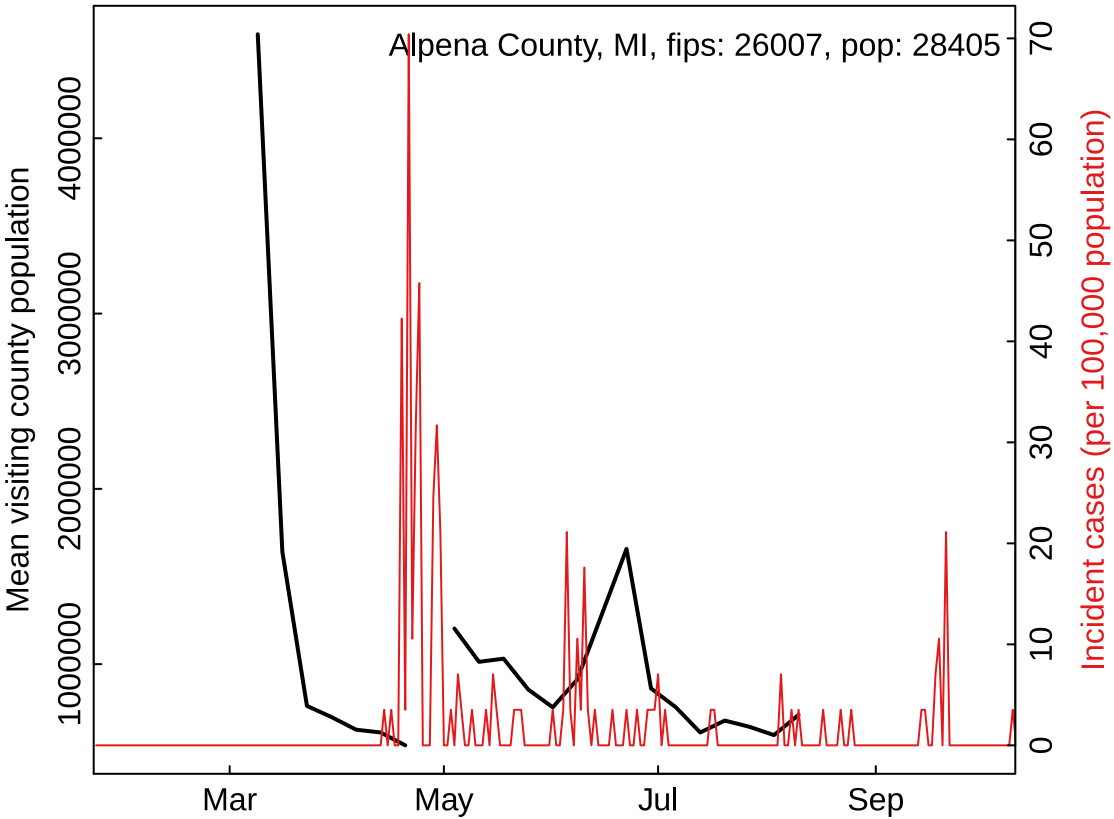

Alpena County, MI, fips: 26007, pop: 28405
Mean visiting county population
1000000
2000000
3000000
4000000
Incident cases (per 100,000 population)
0
10
20
30
40
50
60
70
Mar
May
Jul
Sep

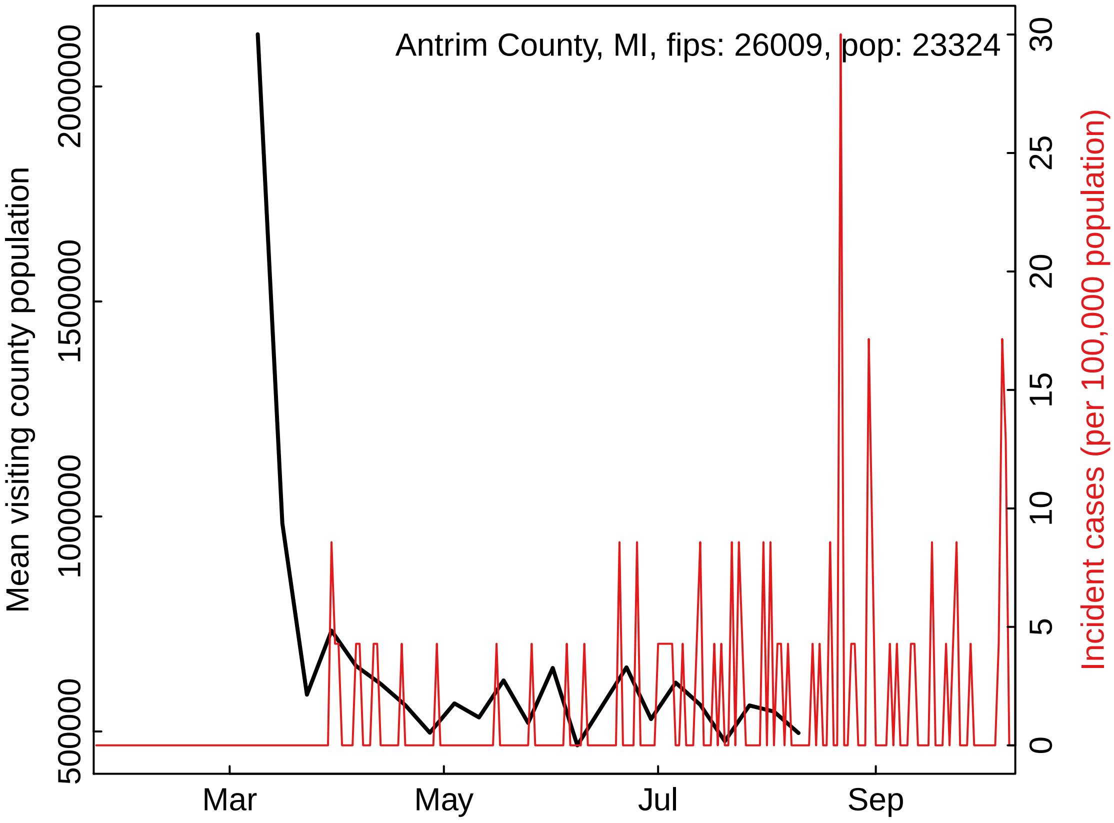

Antrim County, MI, fips: 26009, pop: 23324
Mean visiting county population
500000
1000000
1500000
2000000
Incident cases (per 100,000 population)
0
5
10
15
20
25
30
Mar
May
Jul
Sep

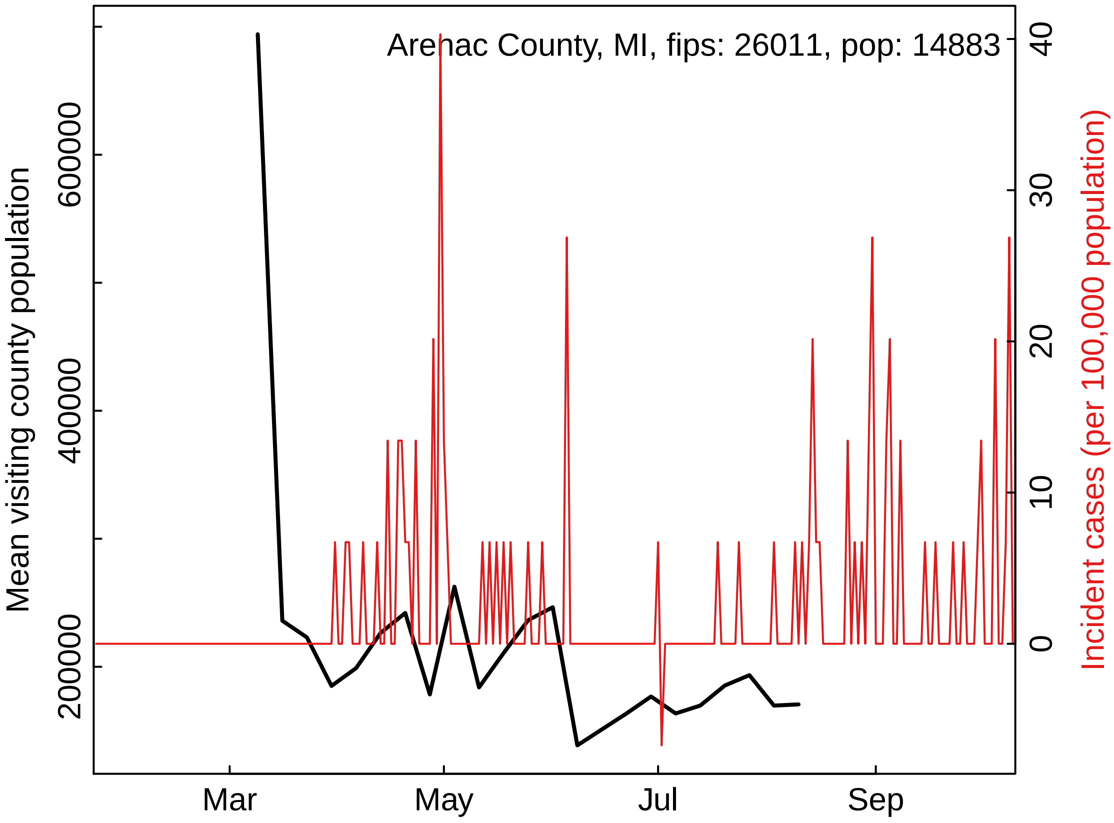

Arenac County, MI, fips: 26011, pop: 14883
Mean visiting county population
200000
400000
600000
Incident cases (per 100,000 population)
0
10
20
30
40
Mar
May
Jul
Sep

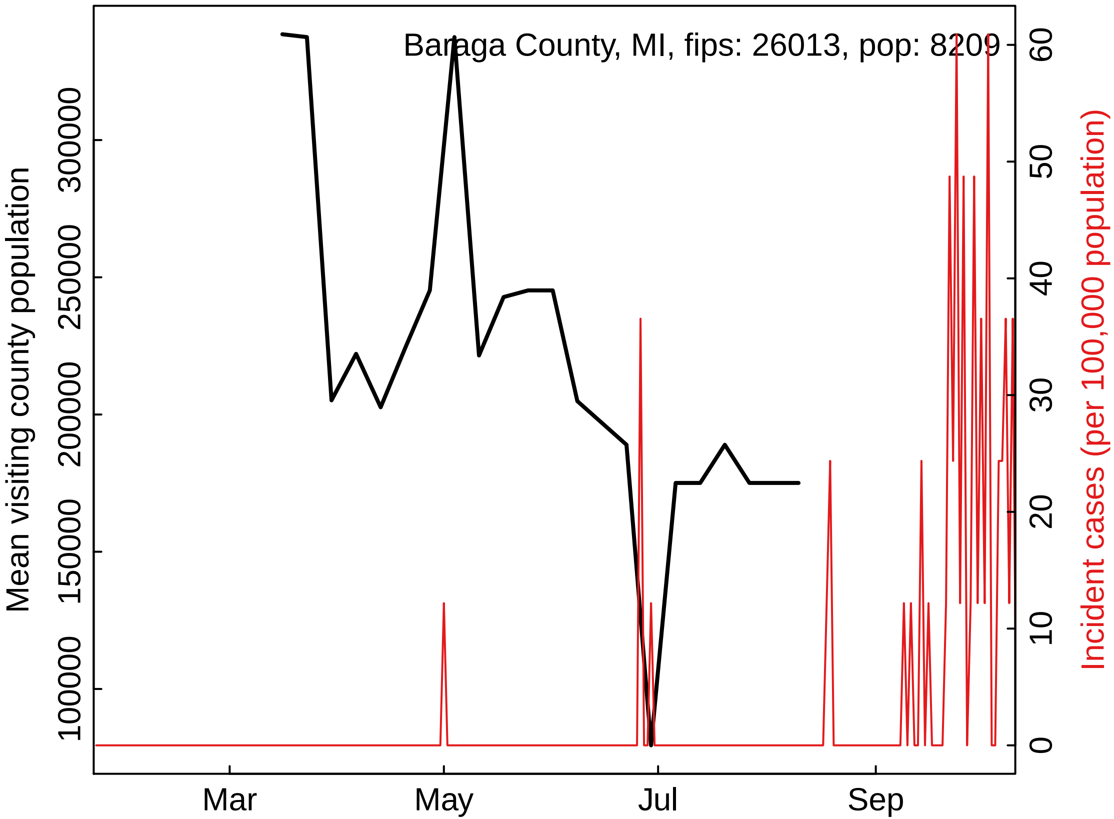

Baraga County, MI, fips: 26013, pop: 8209
Mean visiting county population
100000
150000
200000
250000
300000
Incident cases (per 100,000 population)
0
10
20
30
40
50
60
Mar
May
Jul
Sep

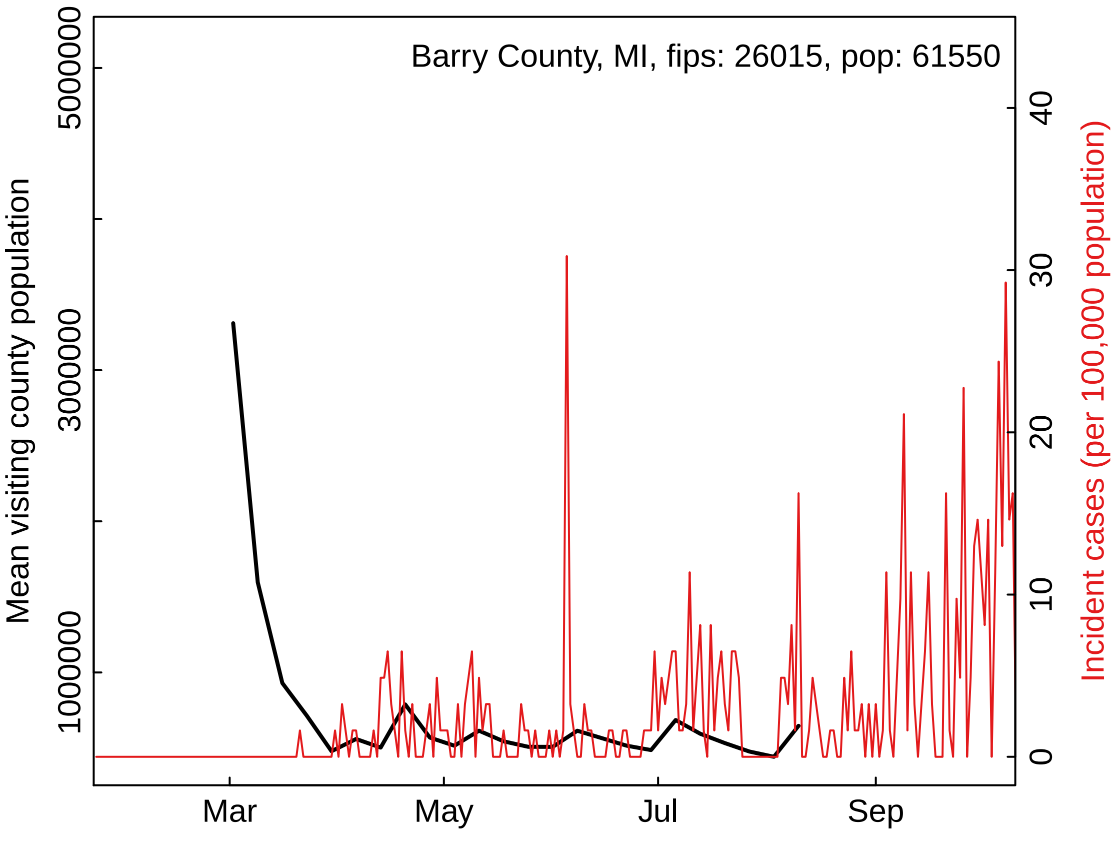

Barry County, MI, fips: 26015, pop: 61550
Mean visiting county population
Incident cases (per 100,000 population)
1000000
3000000
5000000
0
10
20
30
40
Mar
May
Jul
Sep

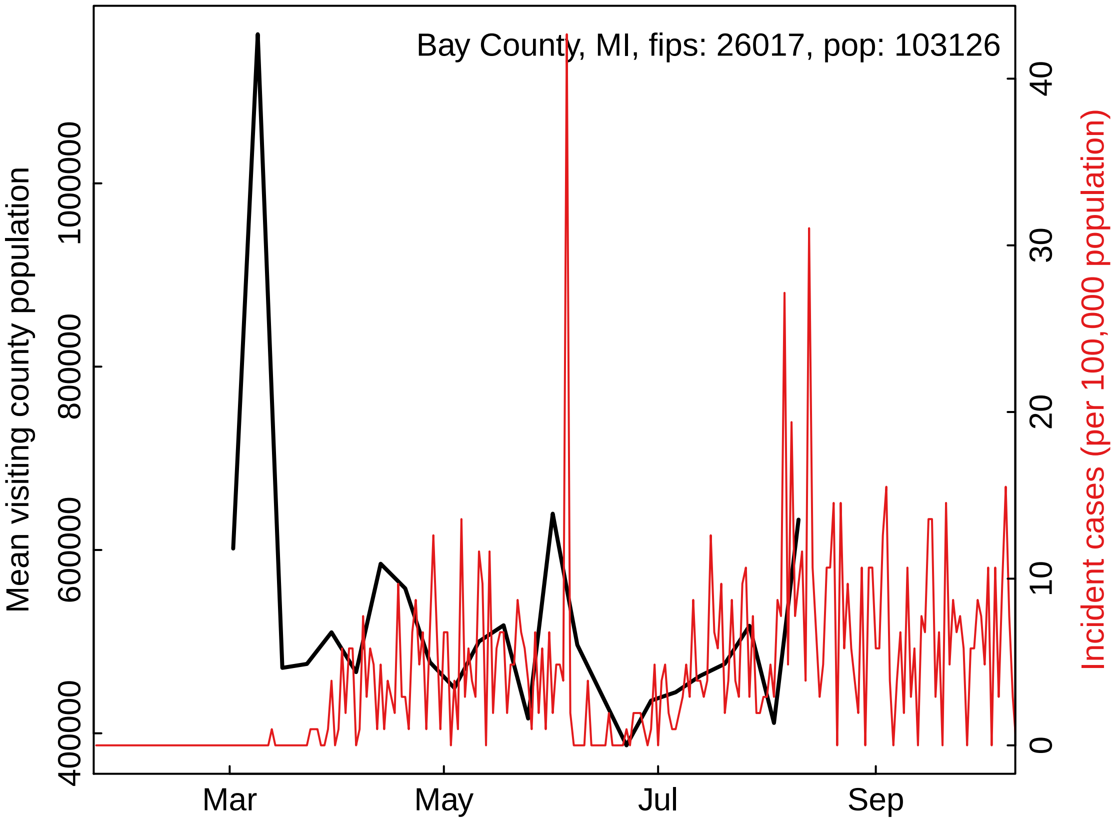

Bay County, MI, fips: 26017, pop: 103126
Mean visiting county population
400000
600000
800000
1000000
Incident cases (per 100,000 population)
0
10
20
30
40
Mar
May
Jul
Sep

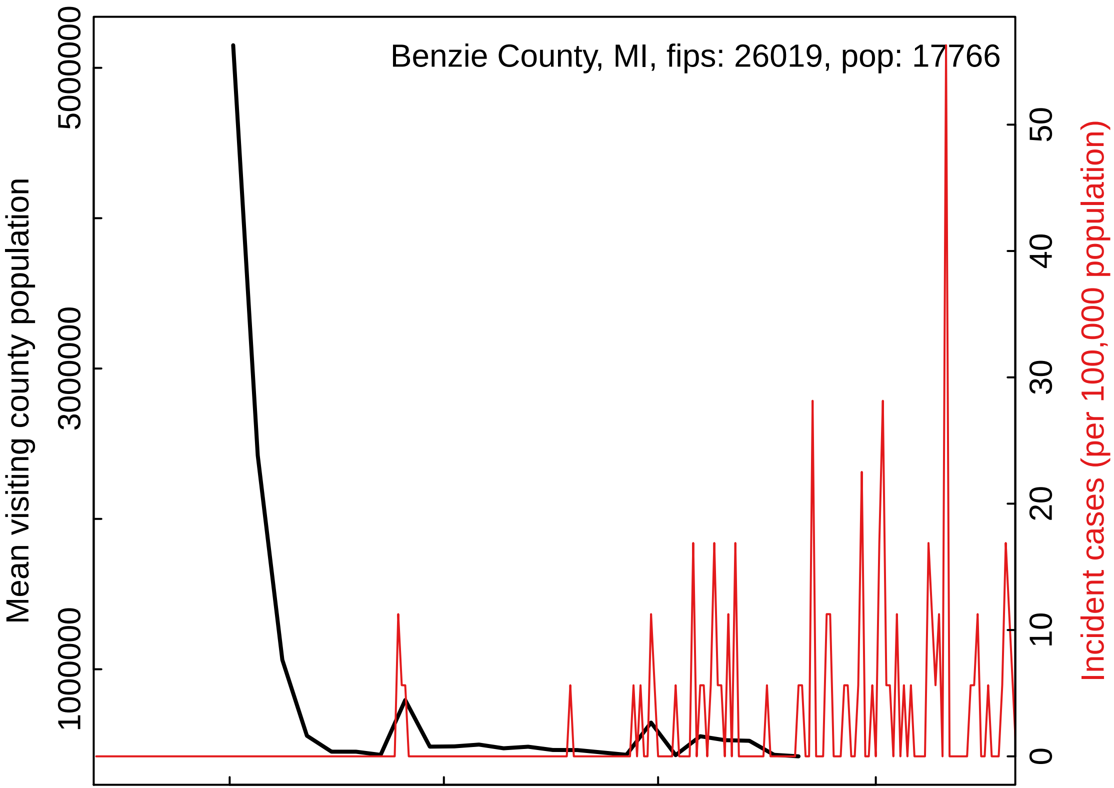

Benzie County, MI, fips: 26019, pop: 17766
Mean visiting county population
1000000
3000000
5000000
Mar
May
Jul
Sep
0
10
20
30
40
50
Incident cases (per 100,000 population)

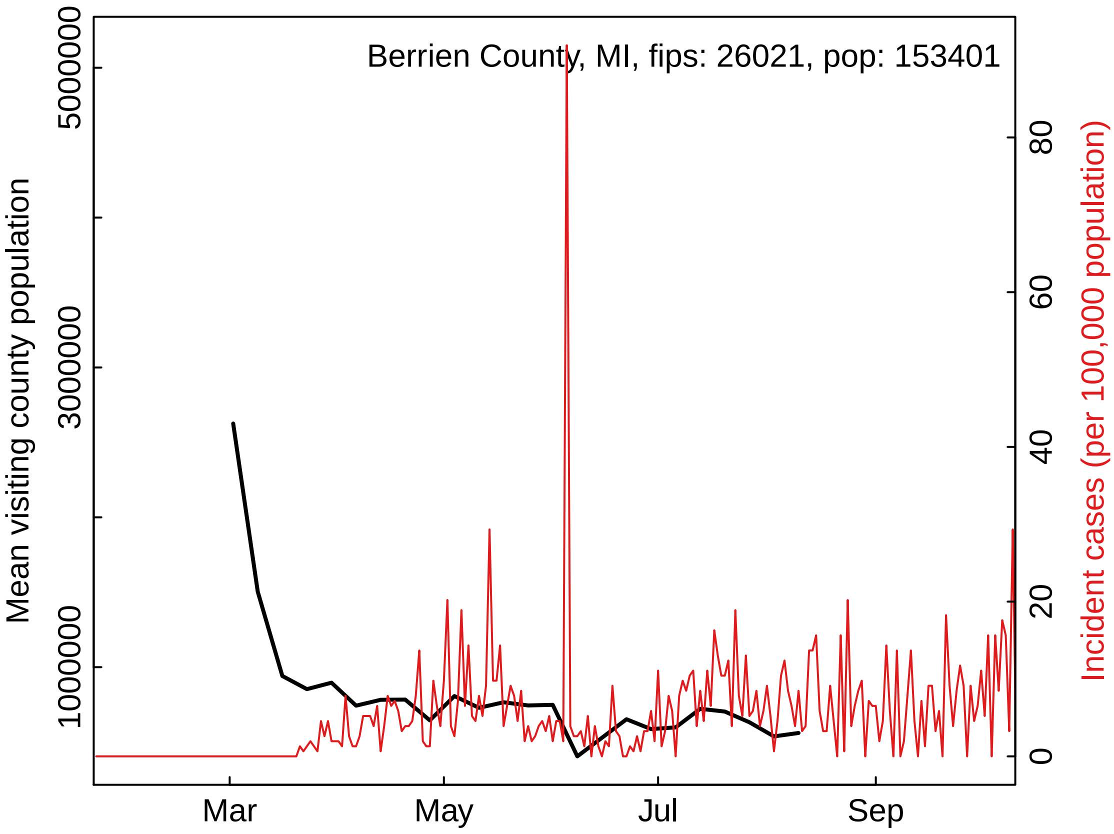
Berrien County, MI, fips: 26021, pop: 153401
Mean visiting county population
5000000
3000000
1000000
Mar
May
Jul
Sep
0
20
40
60
80
Incident cases (per 100,000 population)

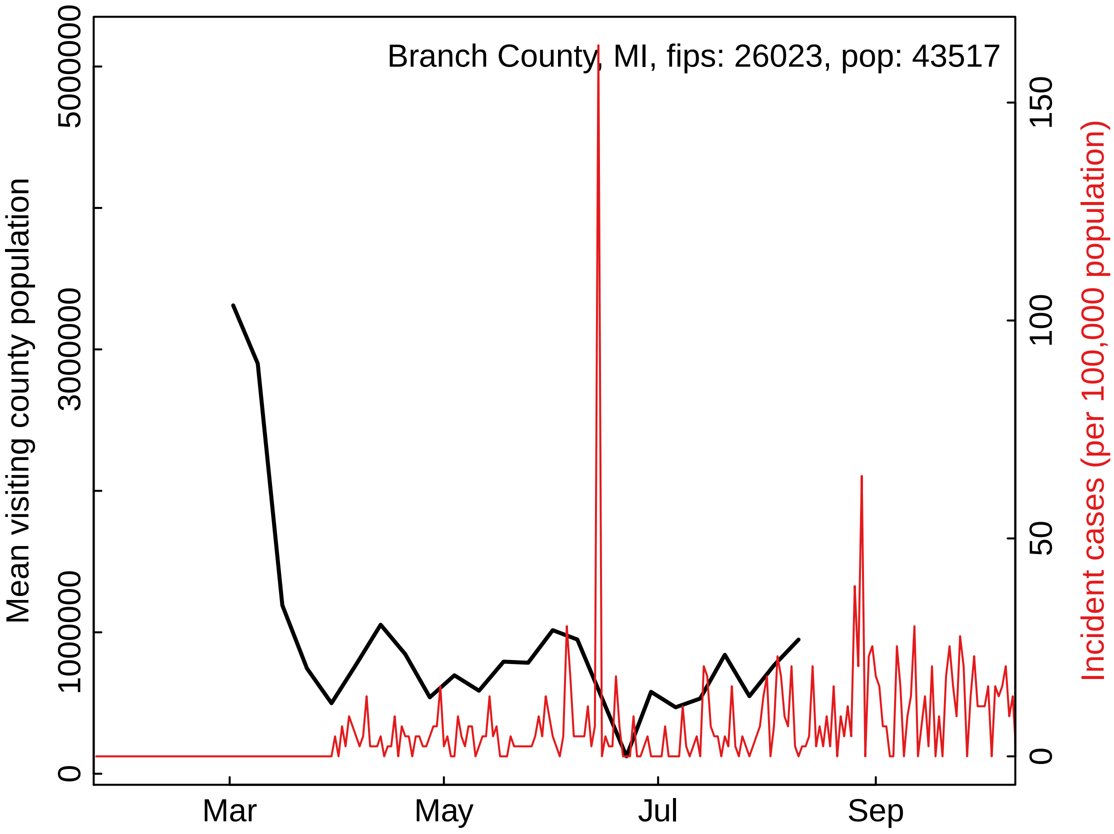

Branch County, MI, fips: 26023, pop: 43517
Mean visiting county population
0
1000000
3000000
5000000
Incident cases (per 100,000 population)
0
50
100
150
Mar
May
Jul
Sep

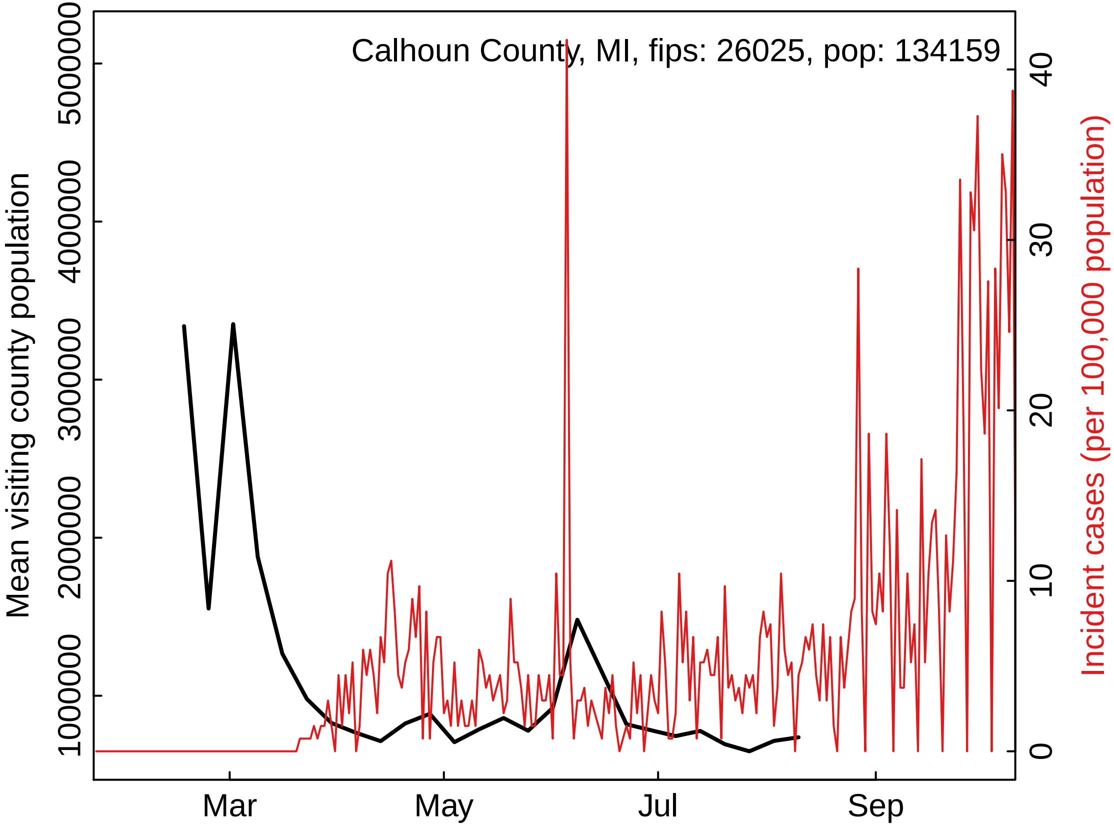

Calhoun County, MI, fips: 26025, pop: 134159
Mean visiting county population
1000000
2000000
3000000
4000000
5000000
Incident cases (per 100,000 population)
0
10
20
30
40
Mar
May
Jul
Sep

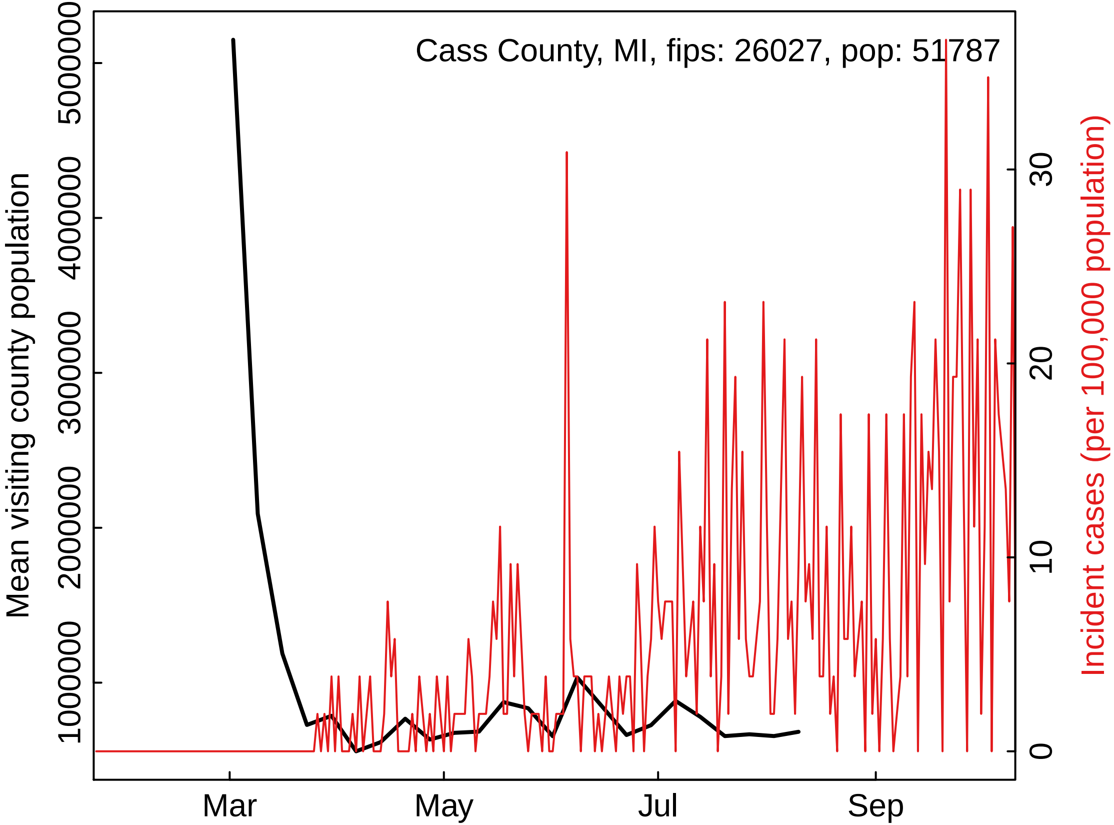

Cass County, MI, fips: 26027, pop: 51787
Mean visiting county population
10000000
20000000
30000000
40000000
50000000
Incident cases (per 100,000 population)
0
10
20
30
Mar
May
Jul
Sep

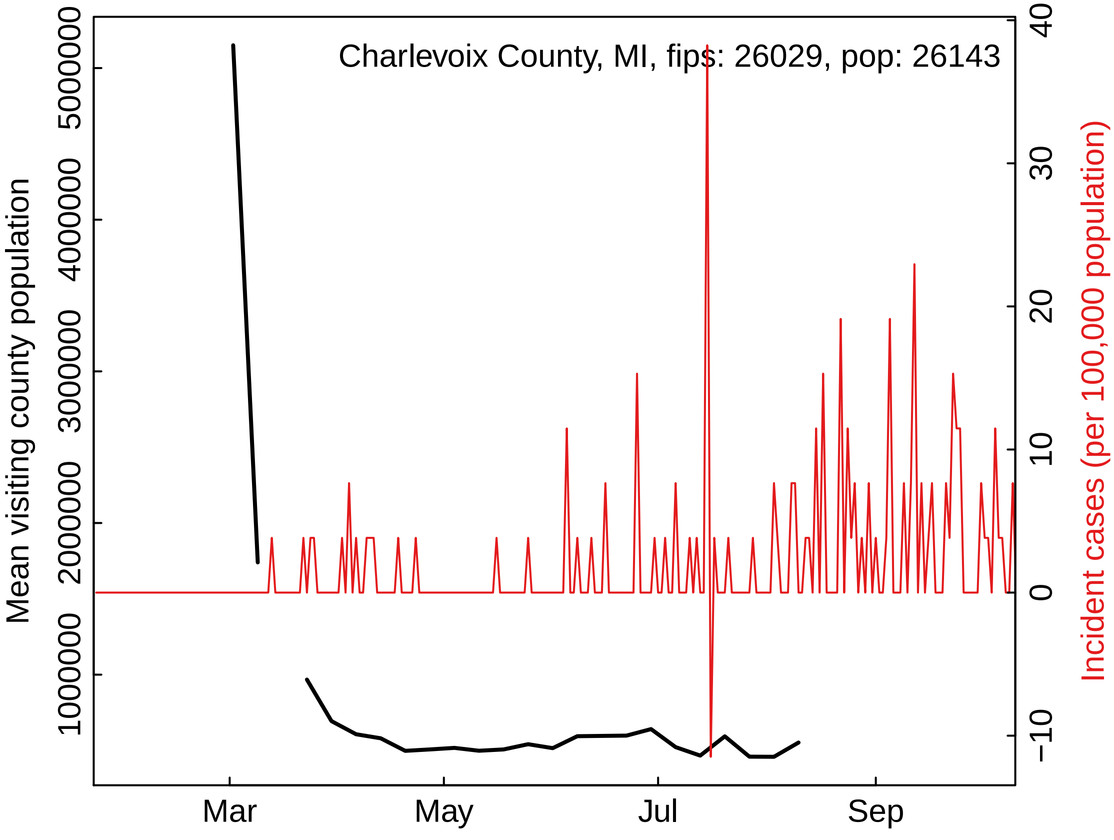

Charlevoix County, MI, fips: 26029, pop: 26143
Mean visiting county population
Incident cases (per 100,000 population)
1000000
2000000
3000000
4000000
5000000
−10
0
10
20
30
40
Mar
May
Jul
Sep

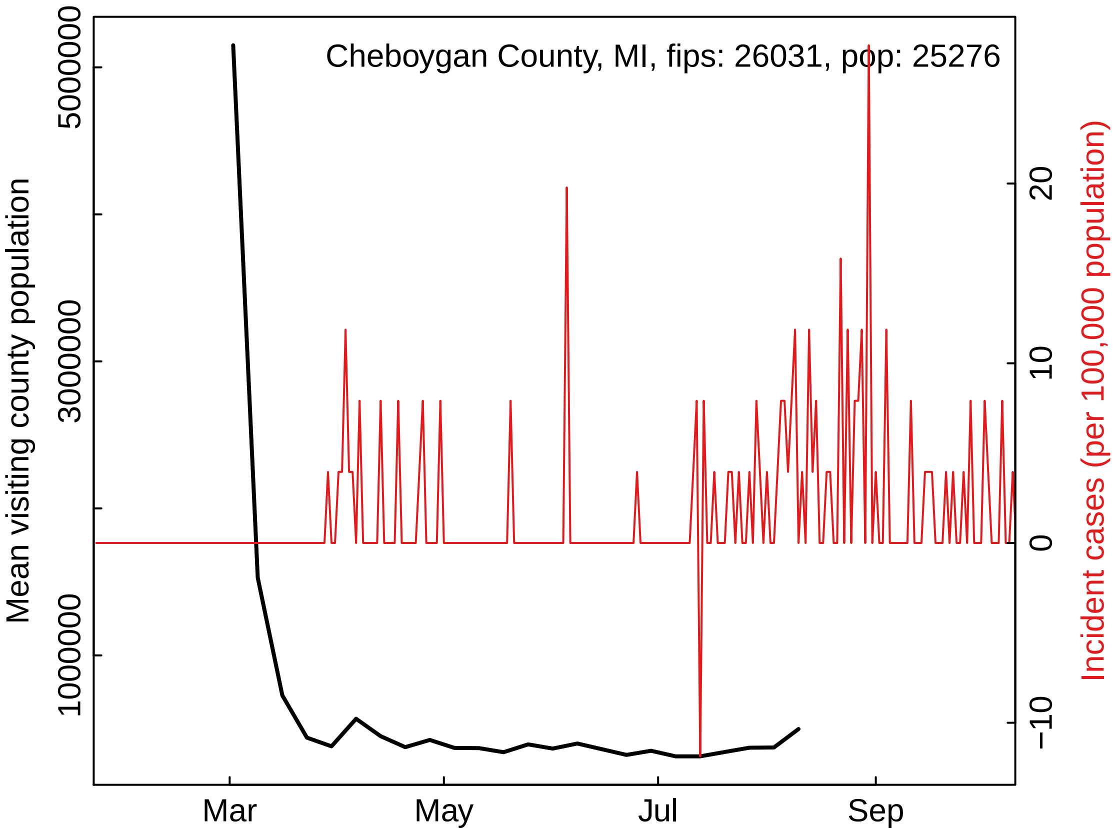
Cheboygan County, MI, fips: 26031, pop: 25276
Mean visiting county population
1000000
3000000
5000000
Incident cases (per 100,000 population)
−10
0
10
20
Mar
May
Jul
Sep

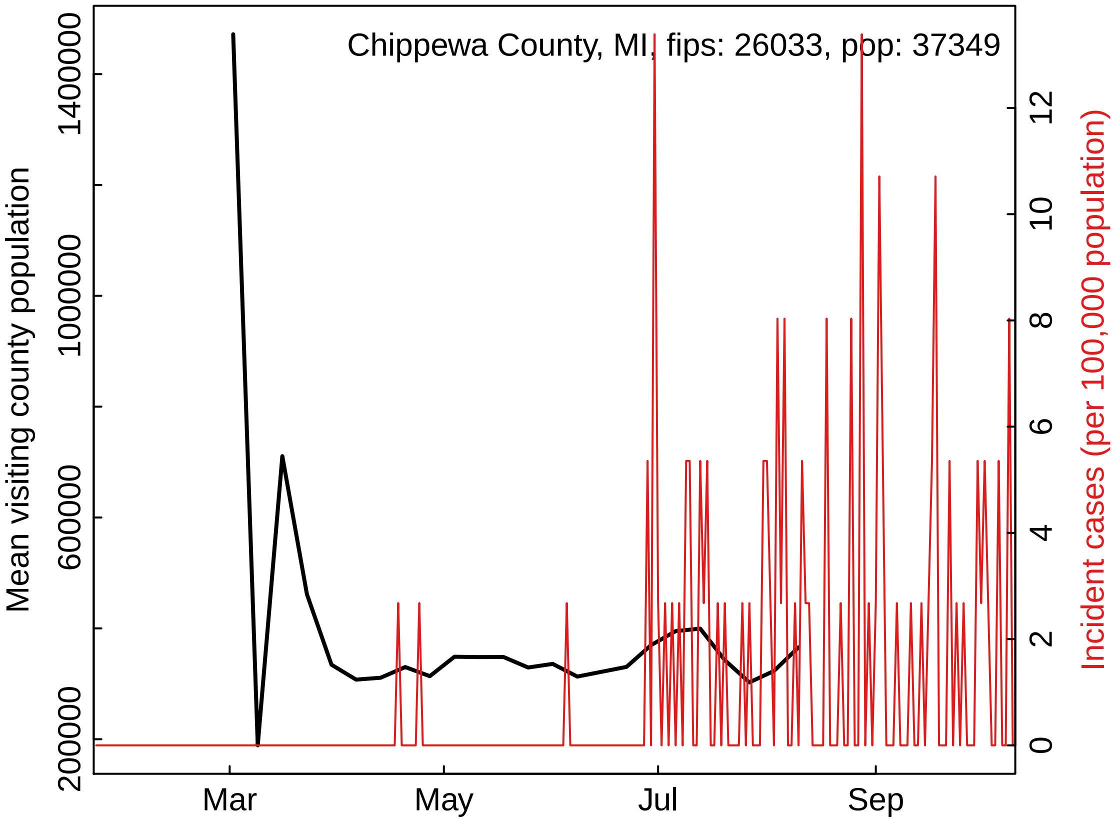

Chippewa County, MI, fips: 26033, pop: 37349
Mean visiting county population
200000
600000
1000000
1400000
Incident cases (per 100,000 population)
0
2
4
6
8
10
12
Mar
May
Jul
Sep

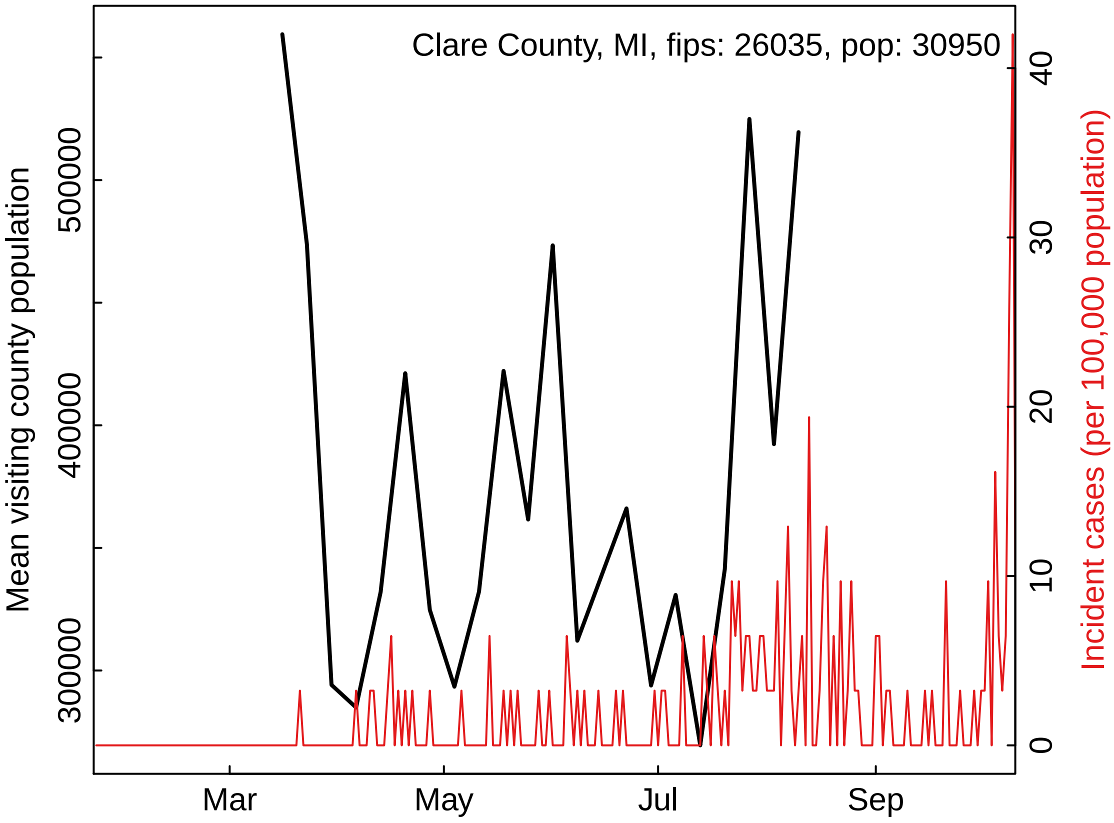

Clare County, MI, fips: 26035, pop: 30950
Mean visiting county population
300000
400000
500000
Incident cases (per 100,000 population)
0
10
20
30
40
Mar
May
Jul
Sep

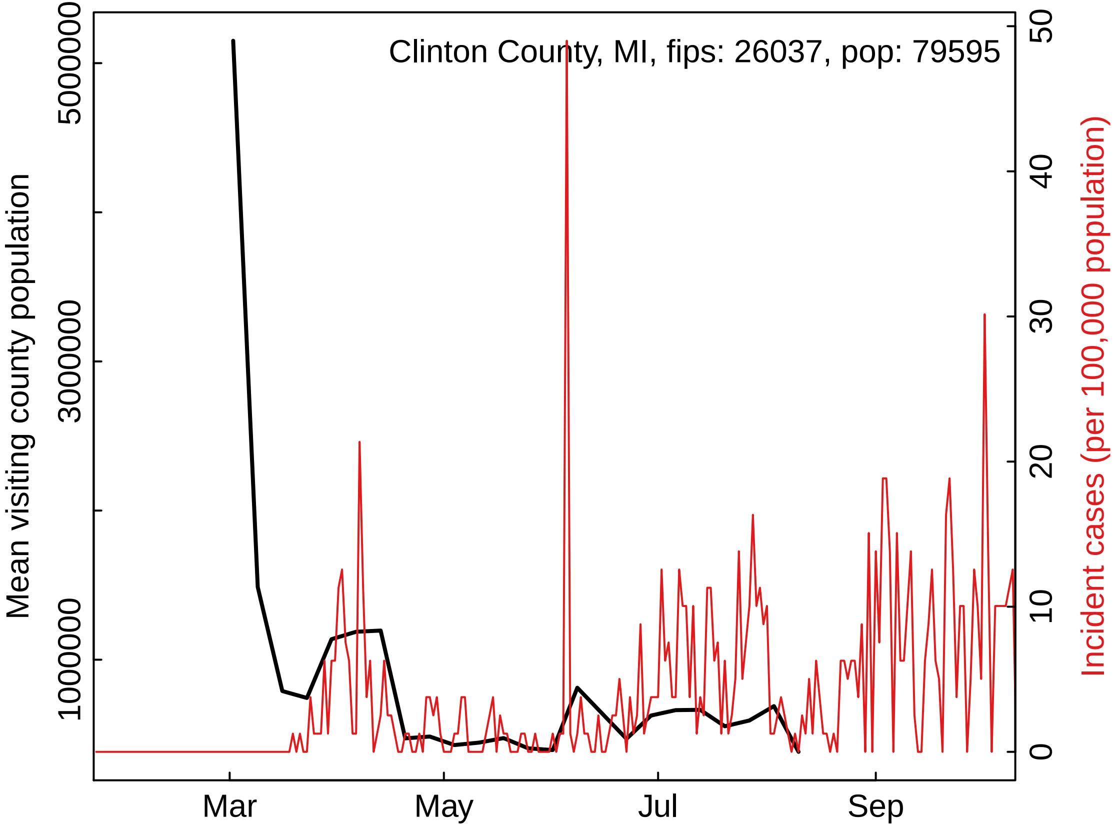

Clinton County, MI, fips: 26037, pop: 79595
Mean visiting county population
10000000
30000000
50000000
Incident cases (per 100,000 population)
0
10
20
30
40
50
Mar
May
Jul
Sep

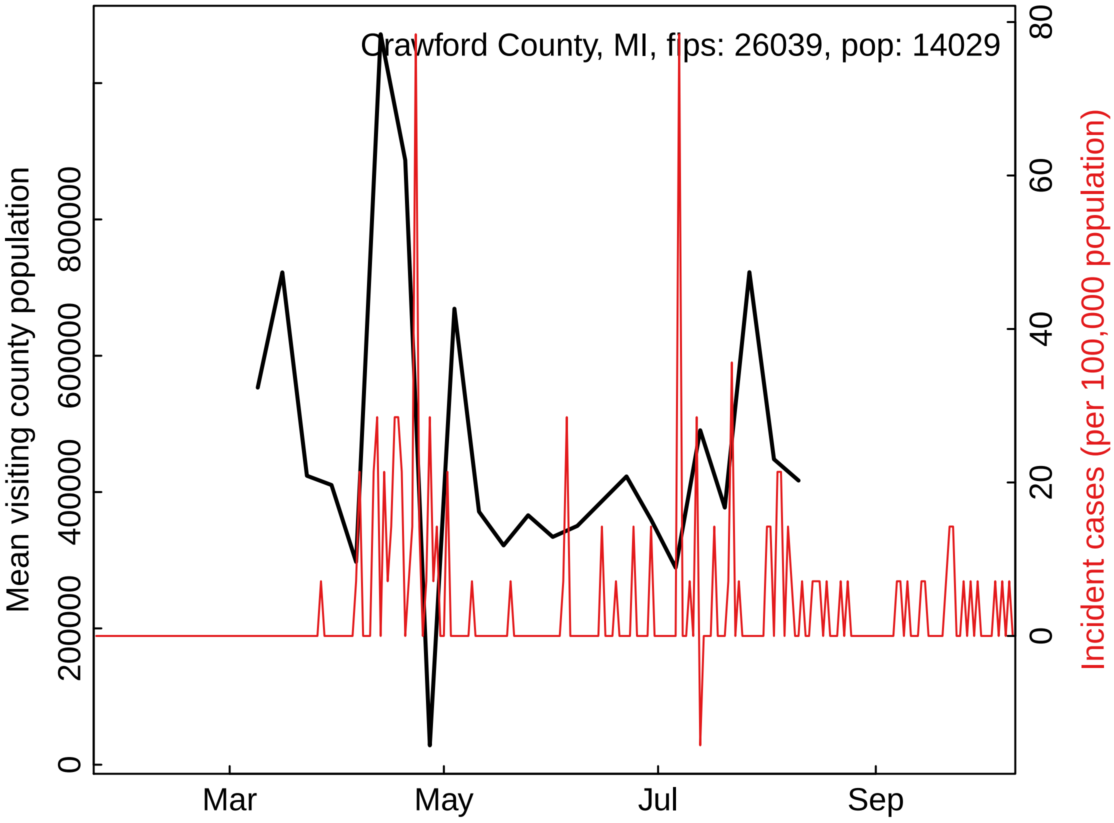

Crawford County, MI, flips: 26039, pop: 14029
Mean visiting county population
0
200000
400000
600000
800000
Mar
May
Jul
Sep
0
20
40
60
80
Incident cases (per 100,000 population)

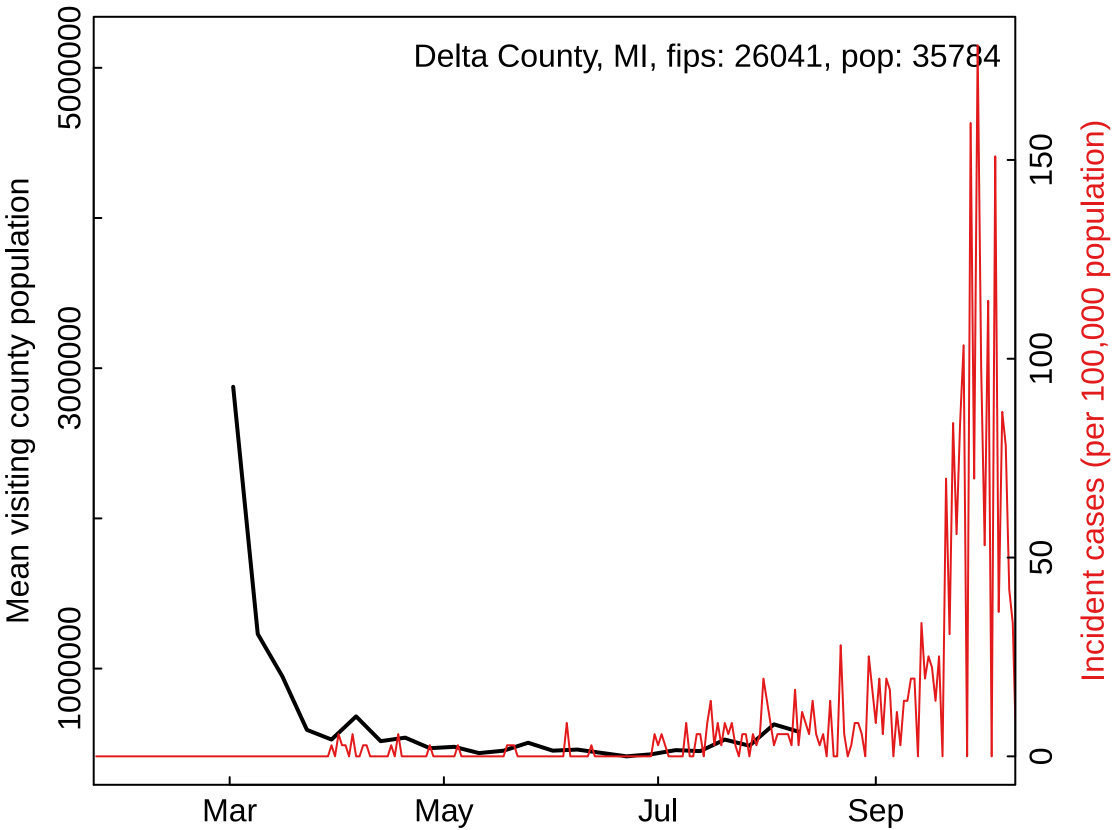

Delta County, MI, fips: 26041, pop: 35784
Mean visiting county population
1000000
3000000
5000000
Incident cases (per 100,000 population)
0
50
100
150
Mar
May
Jul
Sep

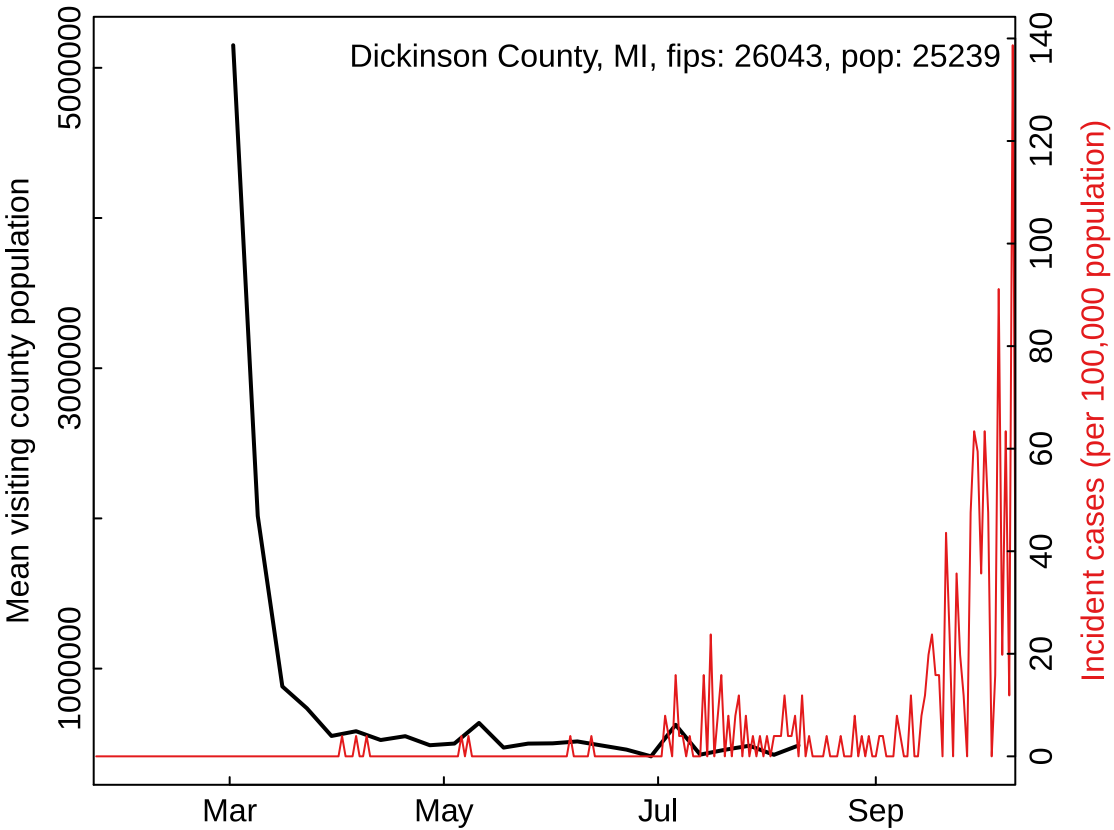
Dickinson County, MI, fips: 26043, pop: 25239
Mean visiting county population
1000000
3000000
5000000
Incident cases (per 100,000 population)
0
20
40
60
80
100
120
140
Mar
May
Jul
Sep

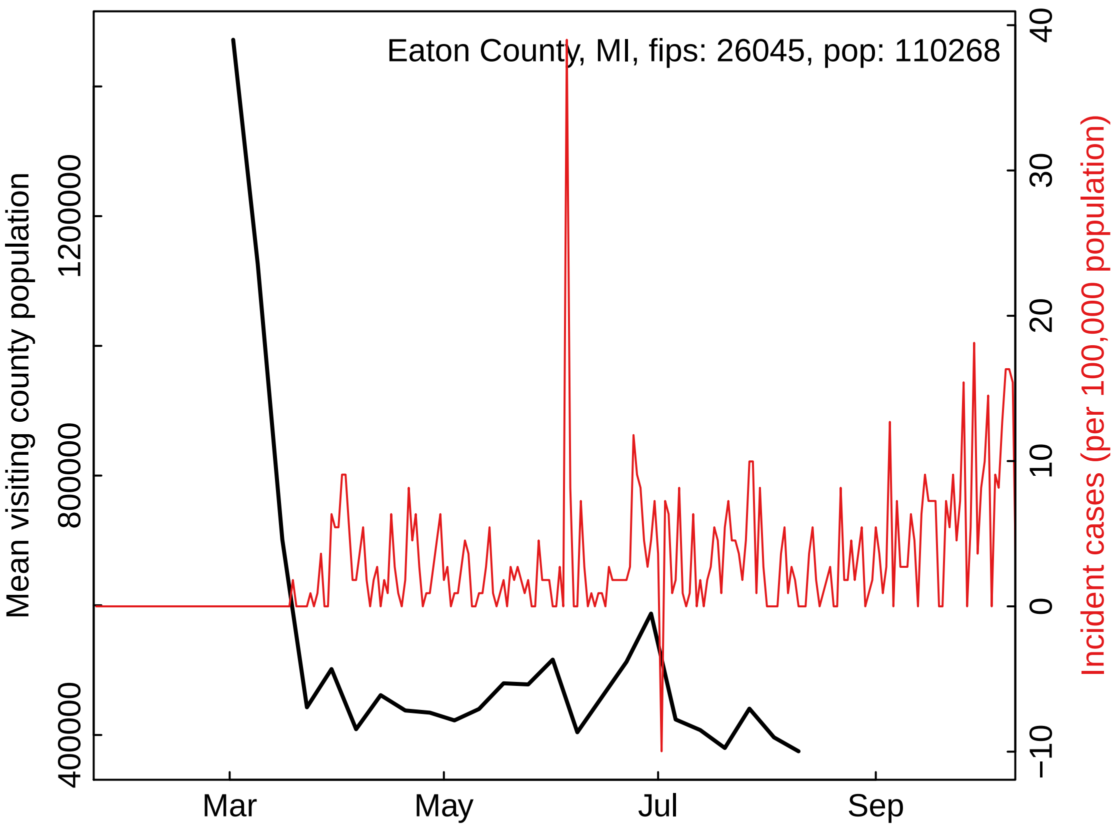

Eaton County, MI, fips: 26045, pop: 110268
Mean visiting county population
400000
800000
1200000
Mar
May
Jul
Sep
Incident cases (per 100,000 population)
−10
0
10
20
30
40

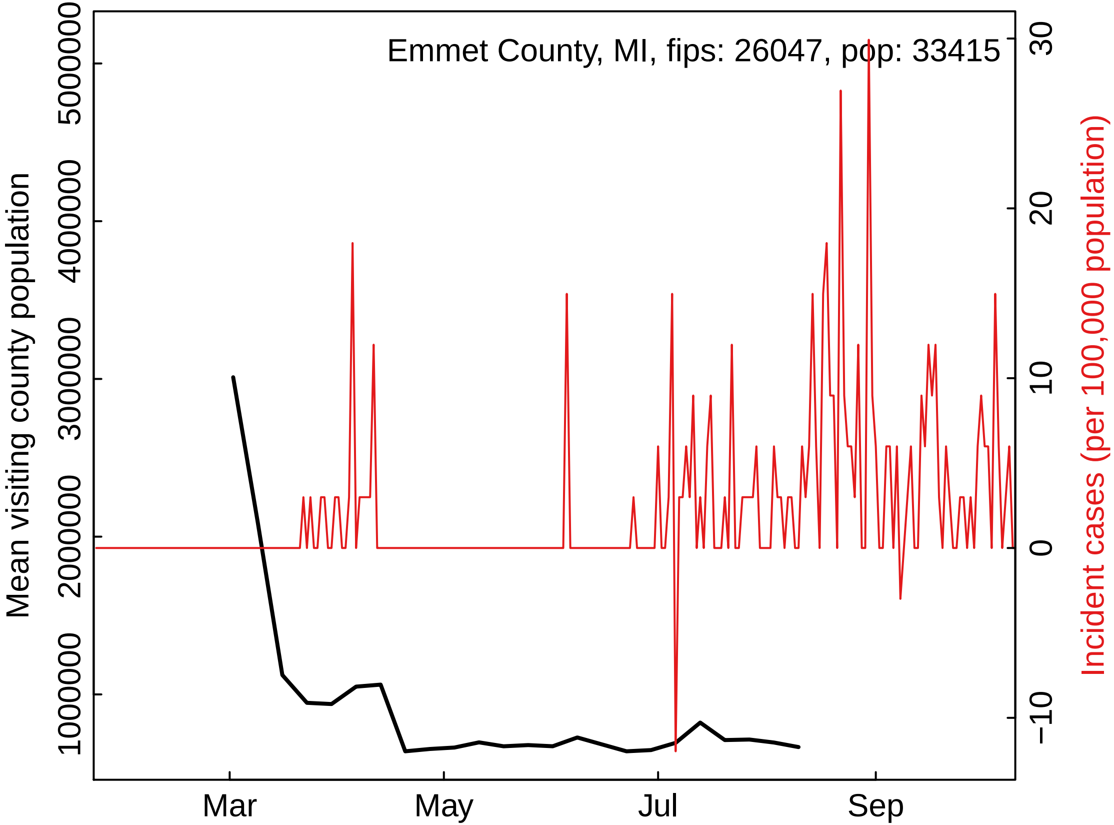

Emmet County, MI, fips: 26047, pop: 33415
Mean visiting county population
1000000
2000000
3000000
4000000
5000000
Incident cases (per 100,000 population)
−10
0
10
20
30
Mar
May
Jul
Sep

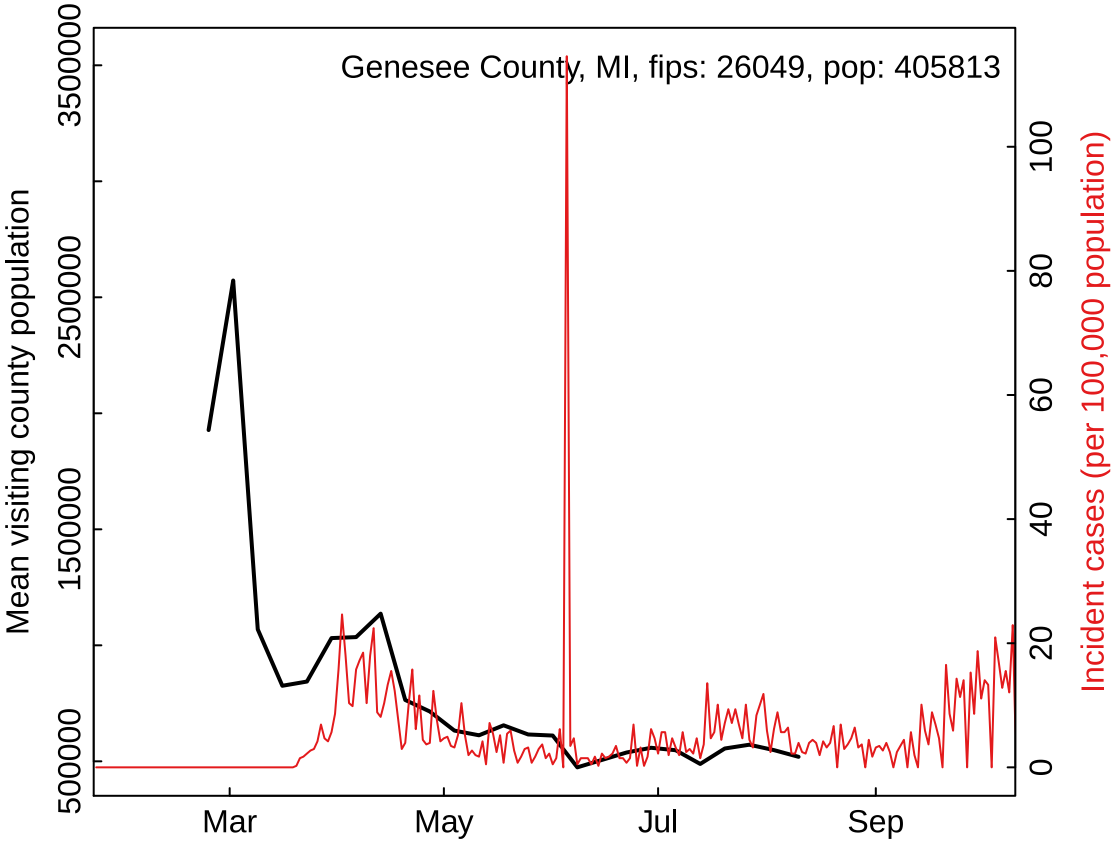

Genesee County, MI, fips: 26049, pop: 405813
Mean visiting county population
500000
1500000
2500000
3500000
Incident cases (per 100,000 population)
0
20
40
60
80
100
Mar
May
Jul
Sep

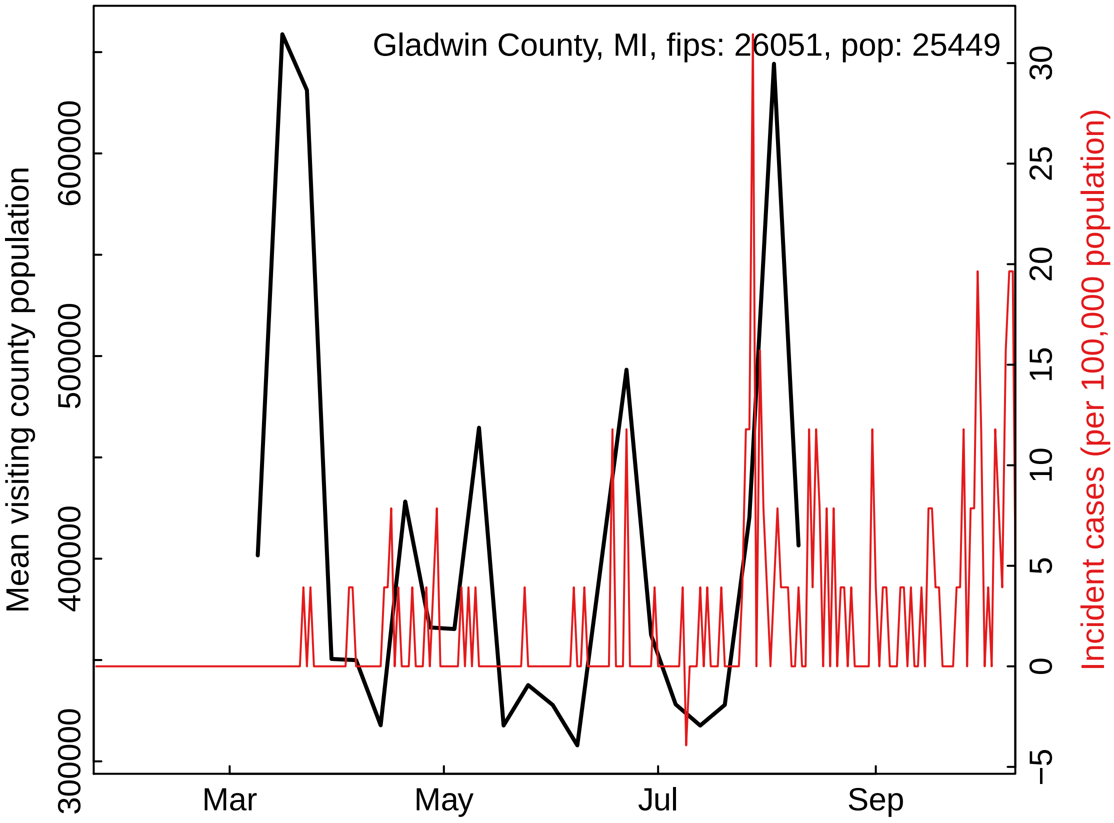
Gladwin County, MI, fips: 26051, pop: 25449
Mean visiting county population
300000
400000
500000
600000
Incident cases (per 100,000 population)
−5
0
5
10
15
20
25
30
Mar
May
Jul
Sep

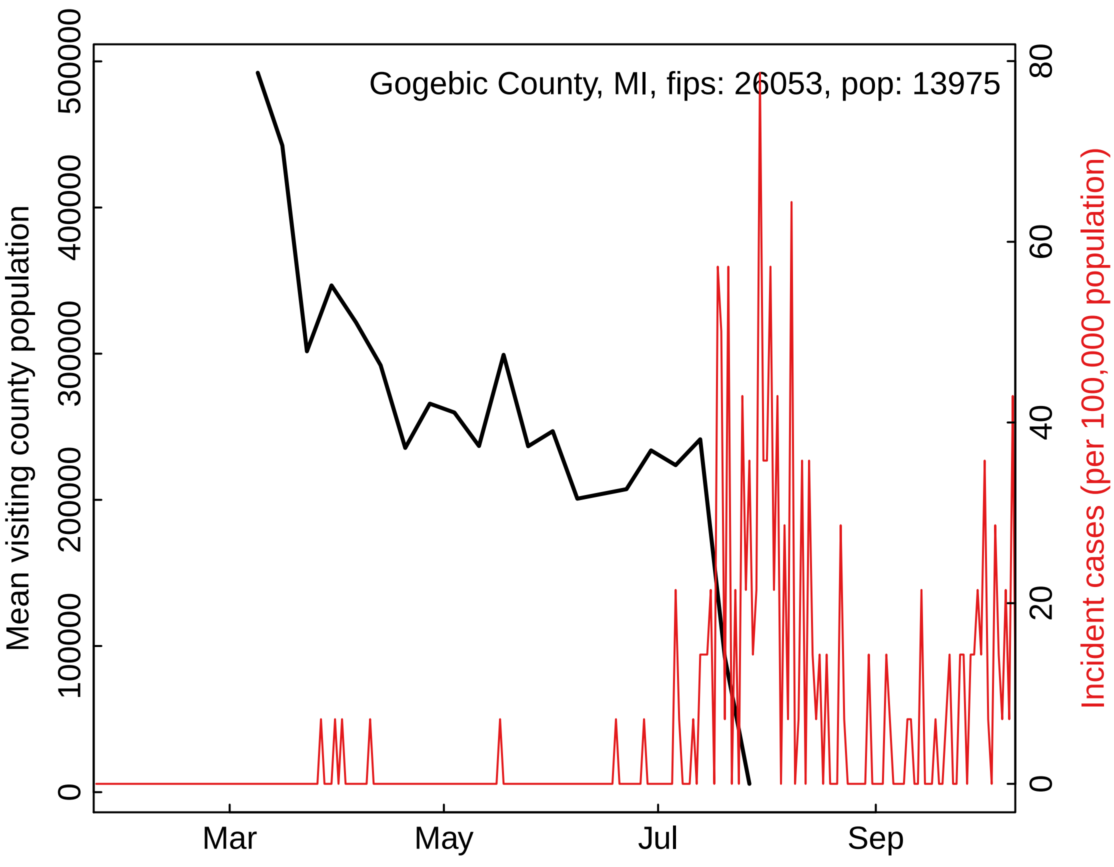
Gogebic County, MI, fips: 26053, pop: 13975
Mean visiting county population
0
100000
200000
300000
400000
500000
Incident cases (per 100,000 population)
0
20
40
60
80
Mar
May
Jul
Sep

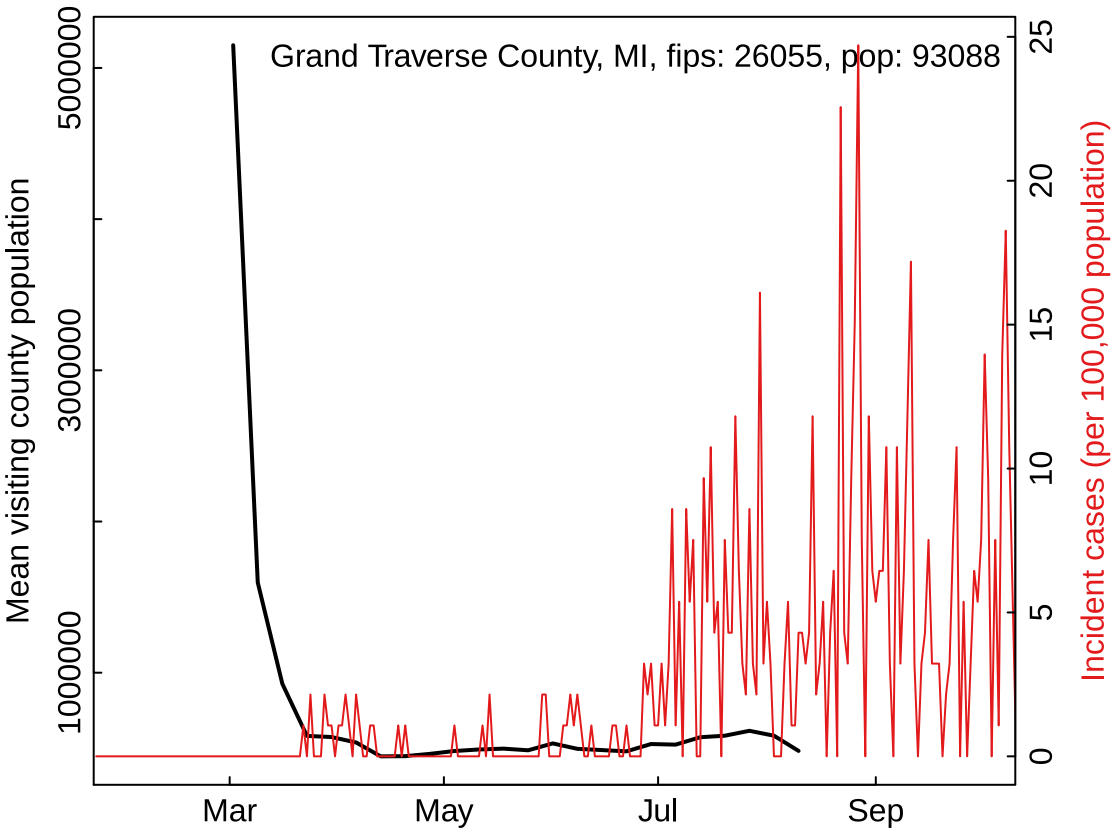

Grand Traverse County, MI, fips: 26055, pop: 93088
Mean visiting county population
Incident cases (per 100,000 population)
1000000
3000000
5000000
0
5
10
15
20
25
Mar
May
Jul
Sep

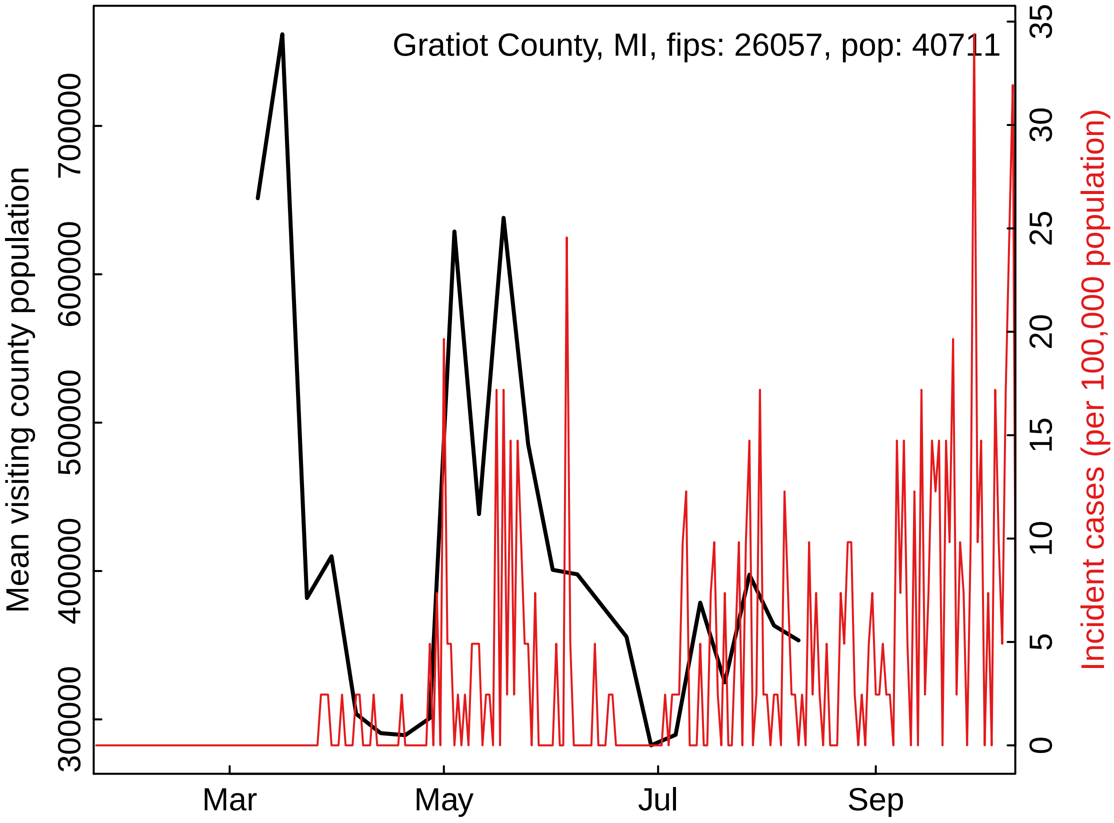
Gratiot County, MI, fips: 26057, pop: 40711
Mean visiting county population
300000
400000
500000
600000
700000
Incident cases (per 100,000 population)
0
5
10
15
20
25
30
35
Mar
May
Jul
Sep

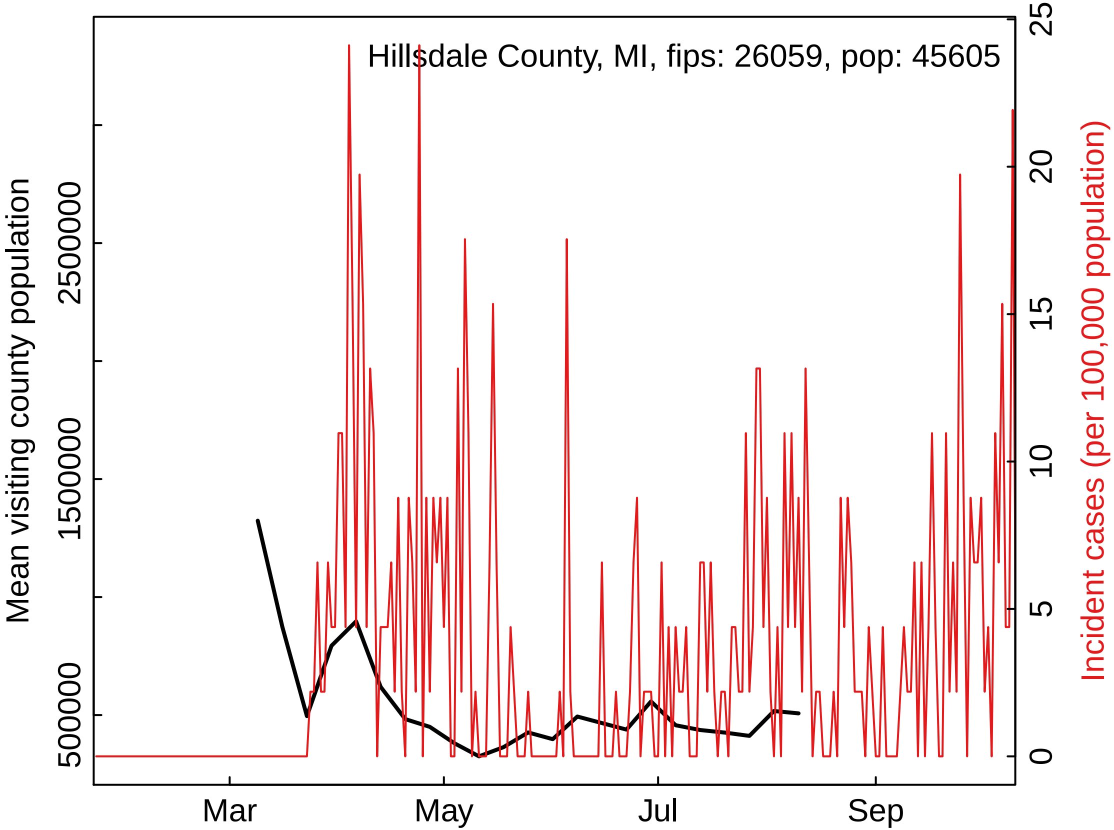

Hillsdale County, MI, fips: 26059, pop: 45605
Mean visiting county population
500000
1500000
2500000
Mar
May
Jul
Sep
0
5
10
15
20
25
Incident cases (per 100,000 population)

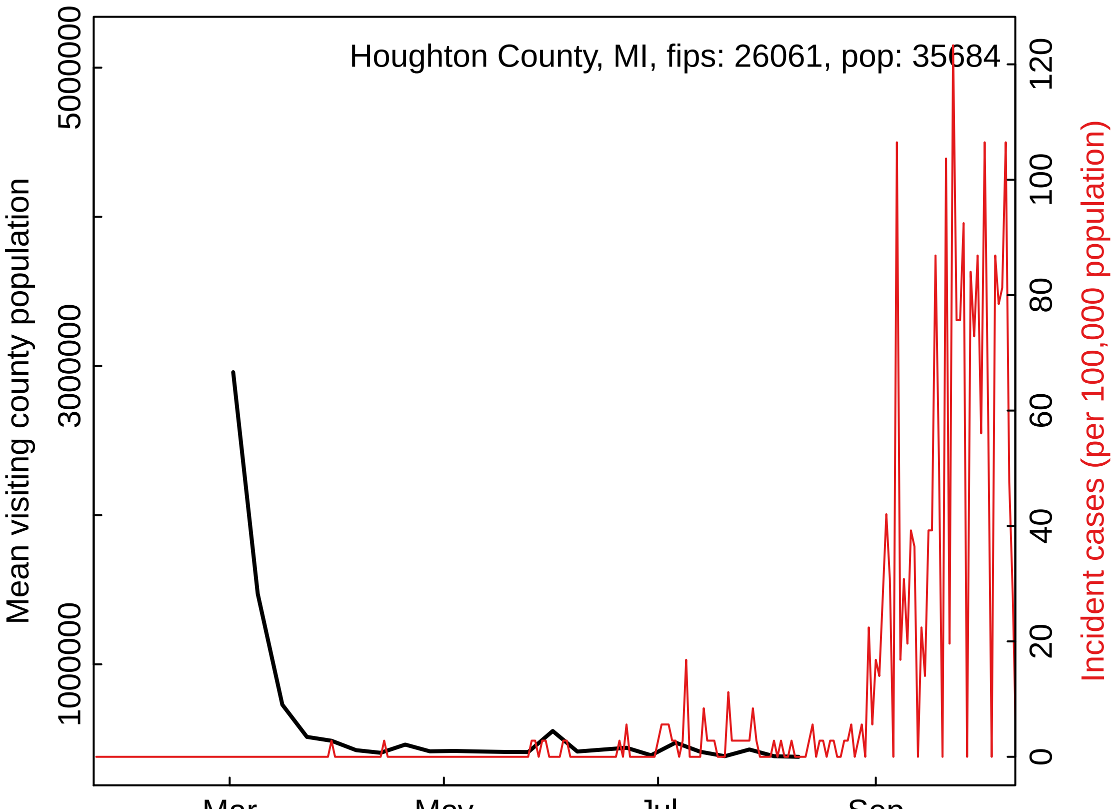

Houghton County, MI, fips: 26061, pop: 35684
Mean visiting county population
1000000
3000000
5000000
Mar
May
Jul
Sep
0
20
40
60
80
100
120
Incident cases (per 100,000 population)

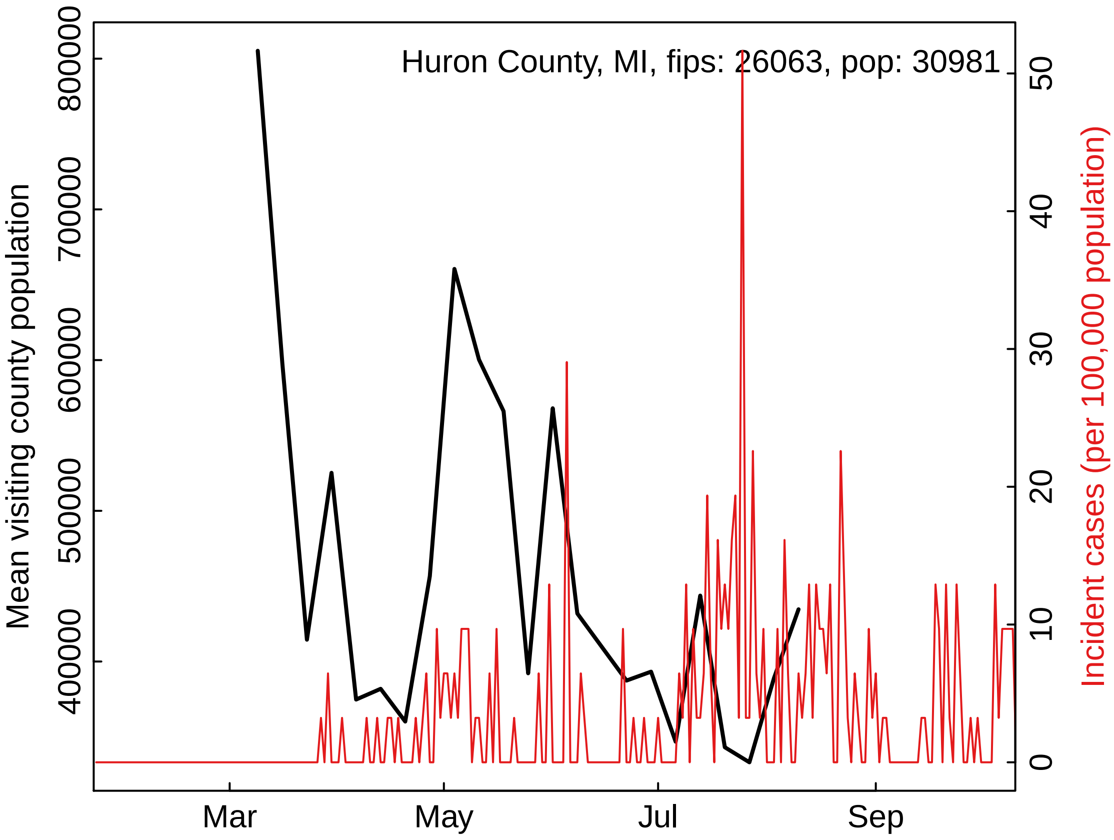

Huron County, MI, fips: 26063, pop: 30981
Mean visiting county population
400000
500000
600000
700000
800000
Incident cases (per 100,000 population)
0
10
20
30
40
50
Mar
May
Jul
Sep

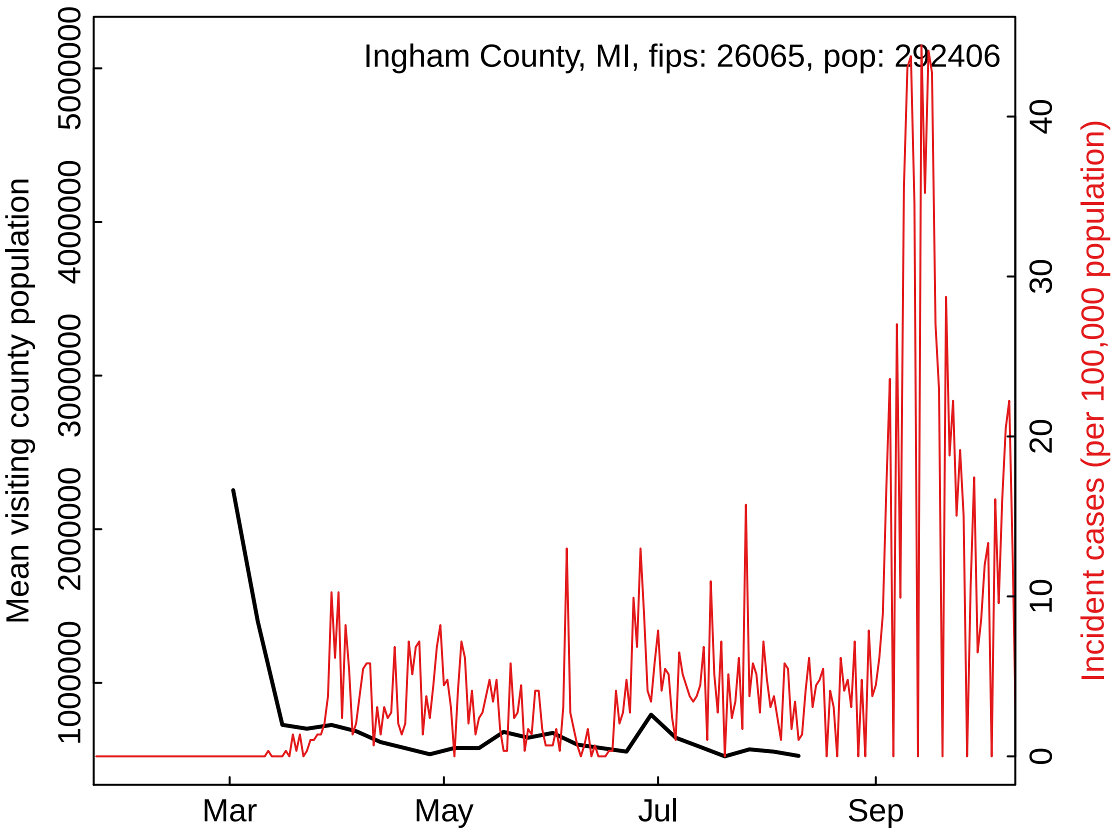
Ingham County, MI, fips: 26065, pop: 292406
Mean visiting county population
1000000
2000000
3000000
4000000
5000000
Incident cases (per 100,000 population)
0
10
20
30
40
Mar
May
Jul
Sep

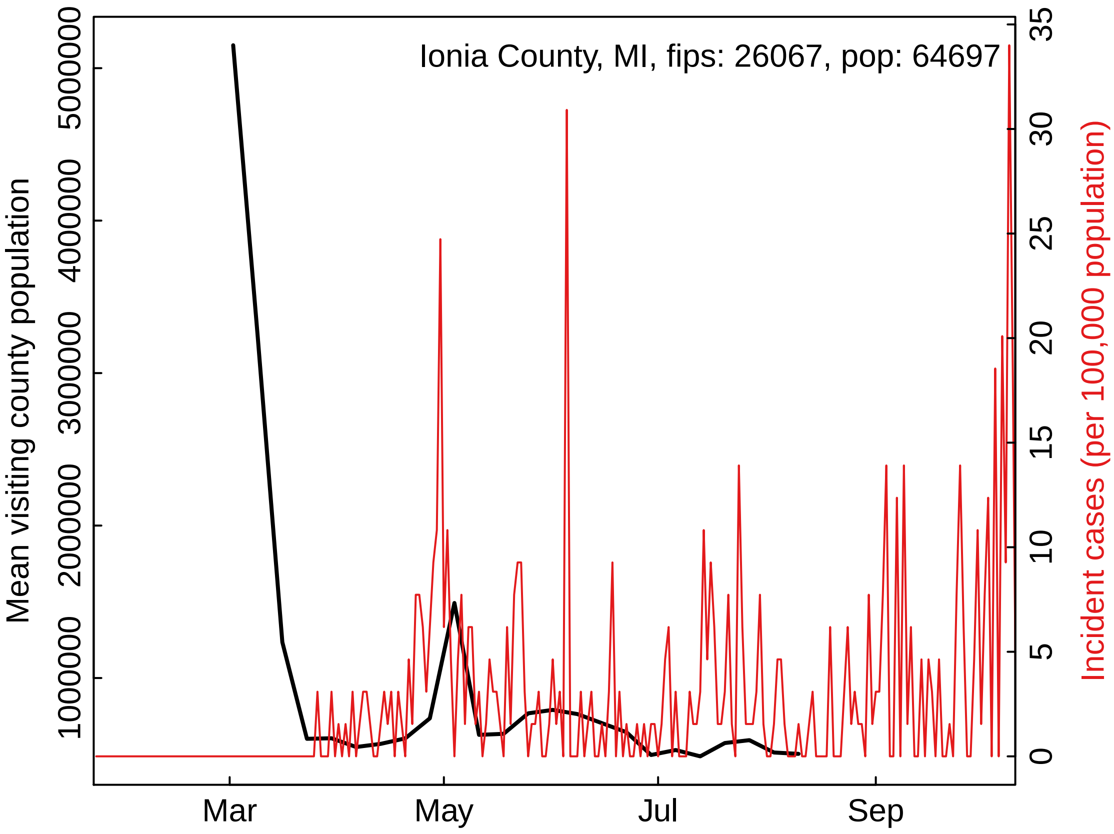

Ionia County, MI, fips: 26067, pop: 64697
Mean visiting county population
Incident cases (per 100,000 population)
1000000
2000000
3000000
4000000
5000000
0
5
10
15
20
25
30
35
Mar
May
Jul
Sep

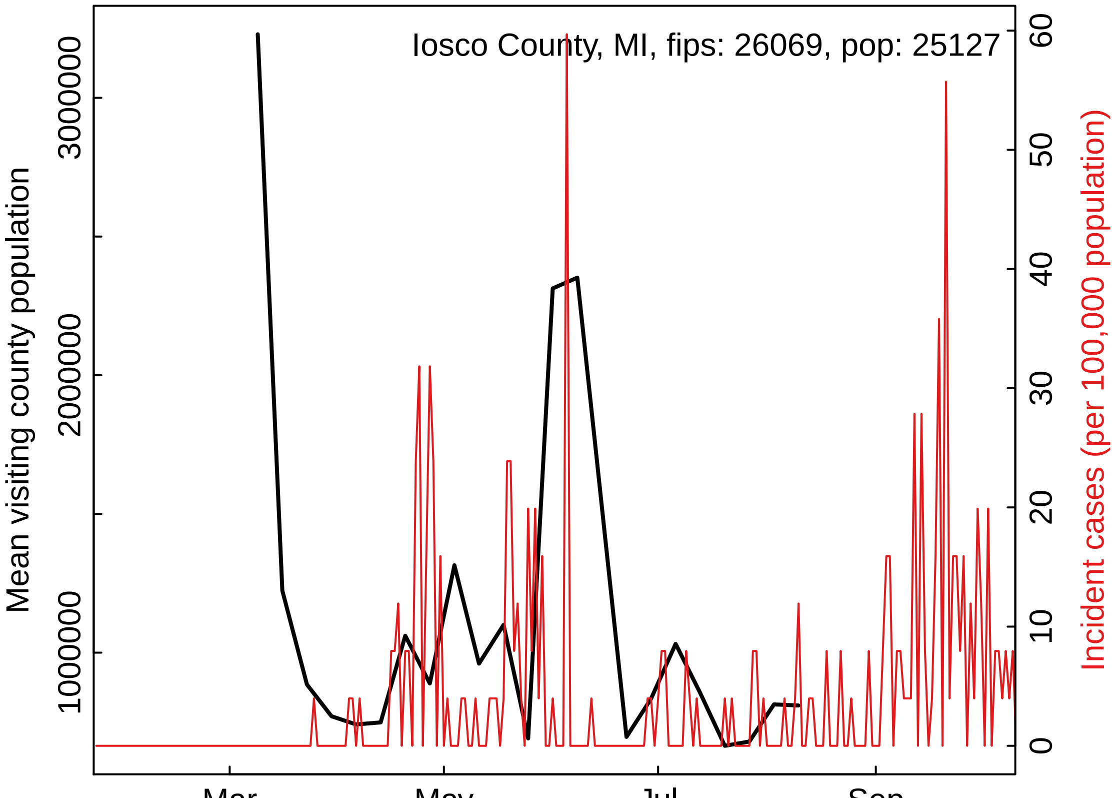

Iosco County, MI, fips: 26069, pop: 25127
Mean visiting county population
1000000
2000000
3000000
Incident cases (per 100,000 population)
0
10
20
30
40
50
60
Mar
May
Jul
Sep

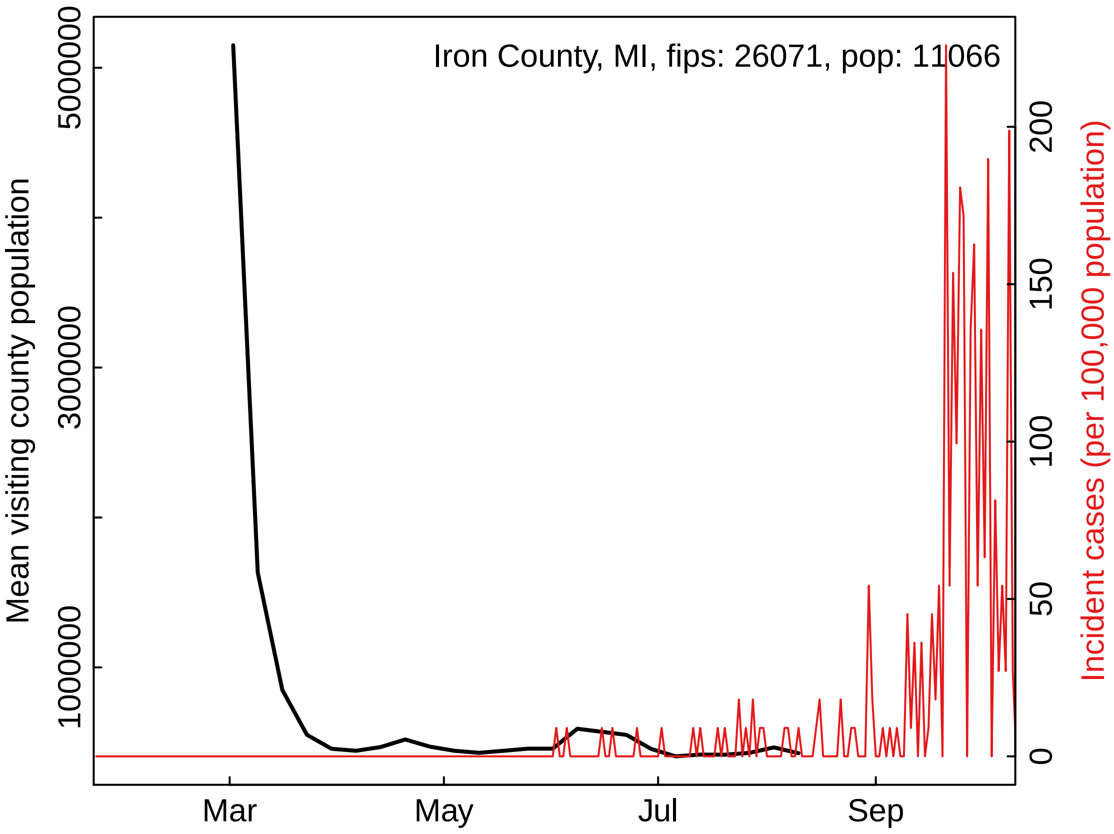

Iron County, MI, fips: 26071, pop: 11066
Mean visiting county population
1000000
3000000
5000000
Incident cases (per 100,000 population)
0
50
100
150
200
Mar
May
Jul
Sep

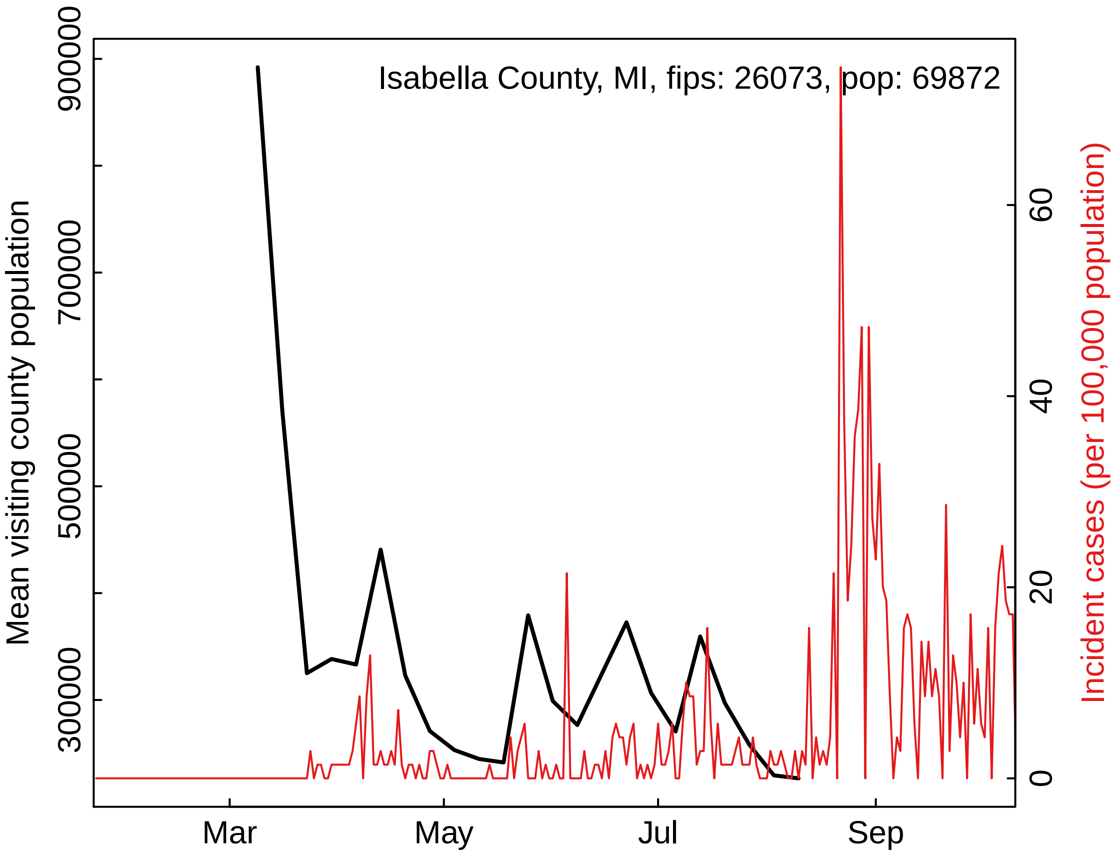
Isabella County, MI, fips: 26073, pop: 69872
Mean visiting county population
300000
500000
700000
900000
Incident cases (per 100,000 population)
0
20
40
60
Mar
May
Jul
Sep

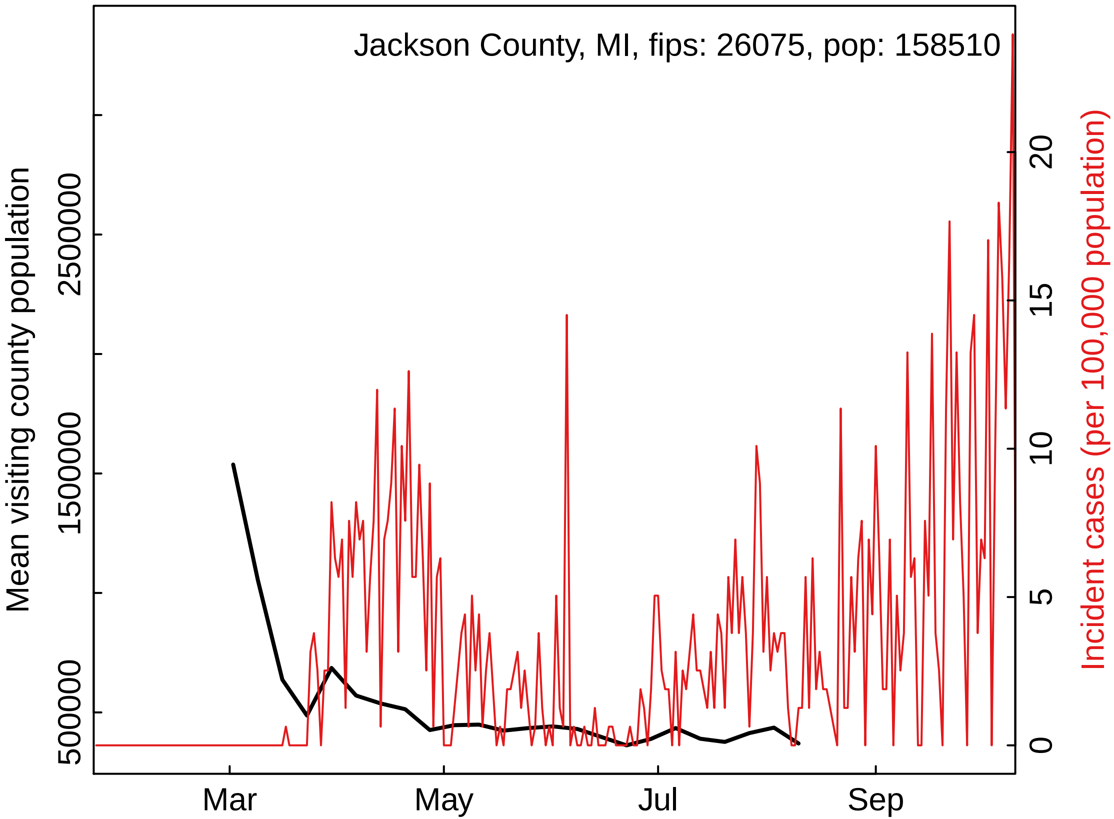

Jackson County, MI, fips: 26075, pop: 158510
Mean visiting county population
500000
1500000
2500000
Incident cases (per 100,000 population)
0
5
10
15
20
Mar
May
Jul
Sep

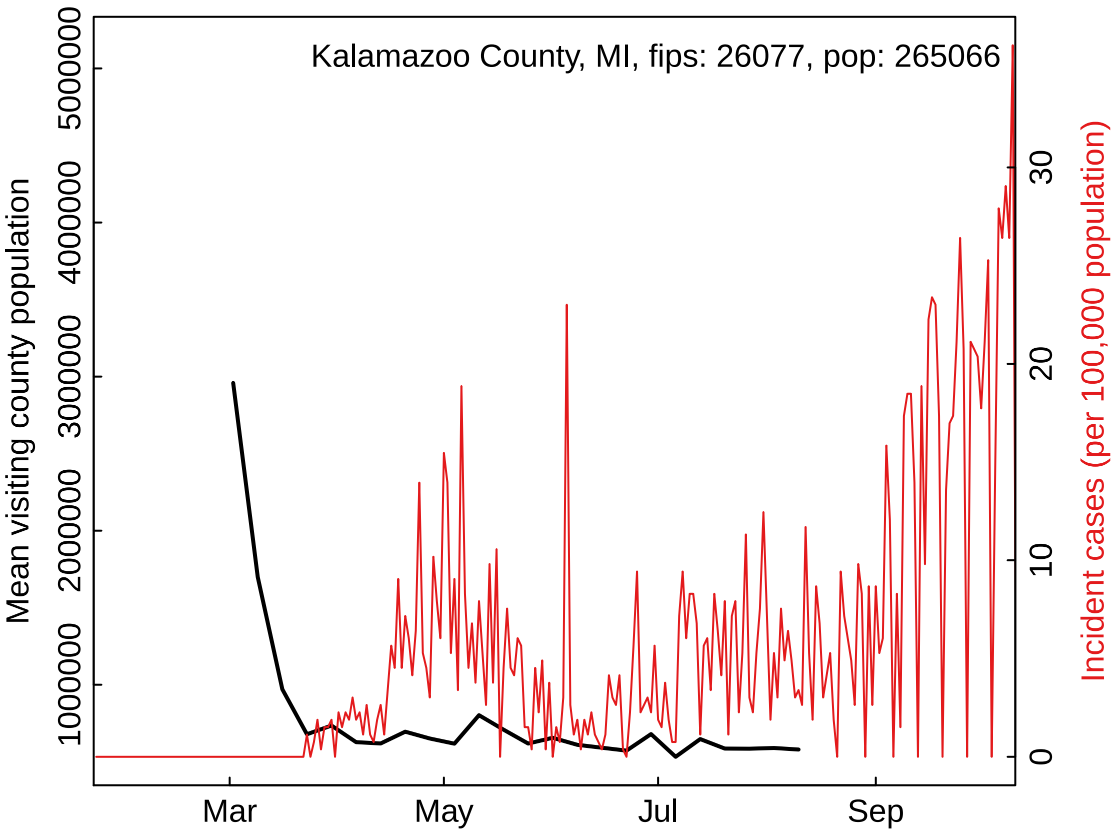

Kalamazoo County, MI, fips: 26077, pop: 265066
Mean visiting county population
1000000
2000000
3000000
4000000
5000000
Incident cases (per 100,000 population)
0
10
20
30
Mar
May
Jul
Sep

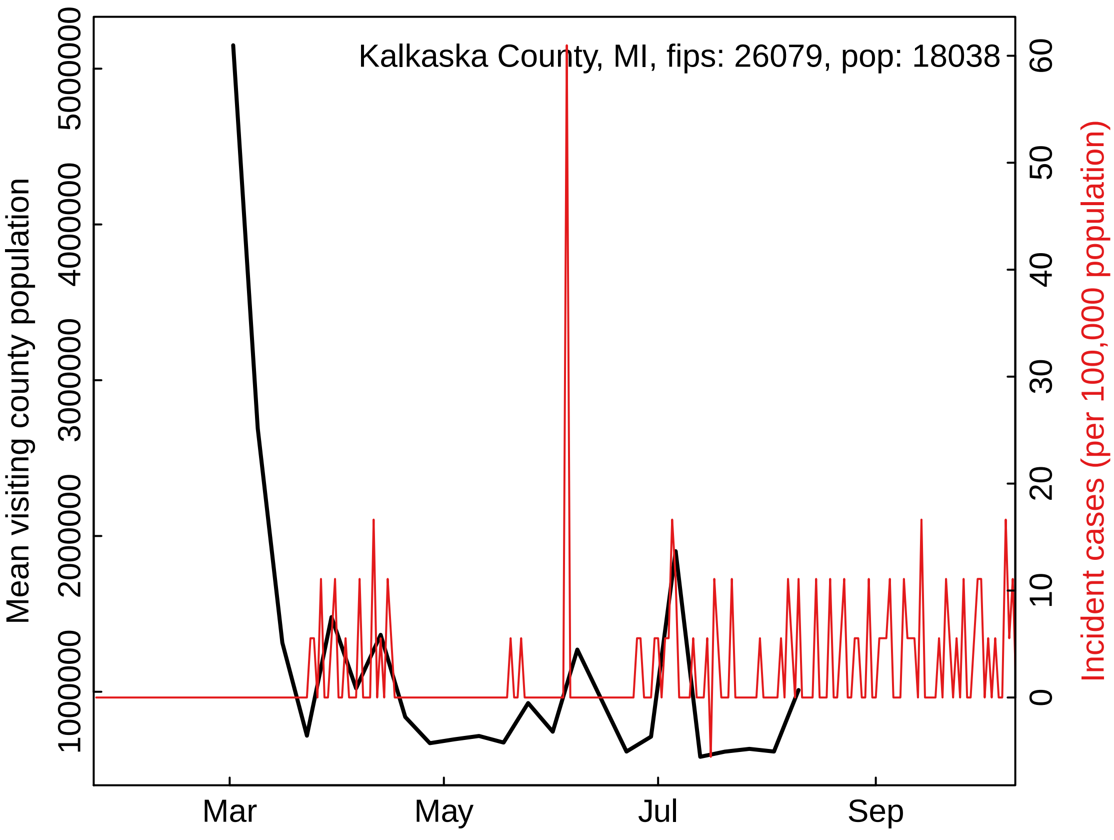

Kalkaska County, MI, fips: 26079, pop: 18038
Mean visiting county population
1000000
2000000
3000000
4000000
5000000
Incident cases (per 100,000 population)
0
10
20
30
40
50
60
Mar
May
Jul
Sep

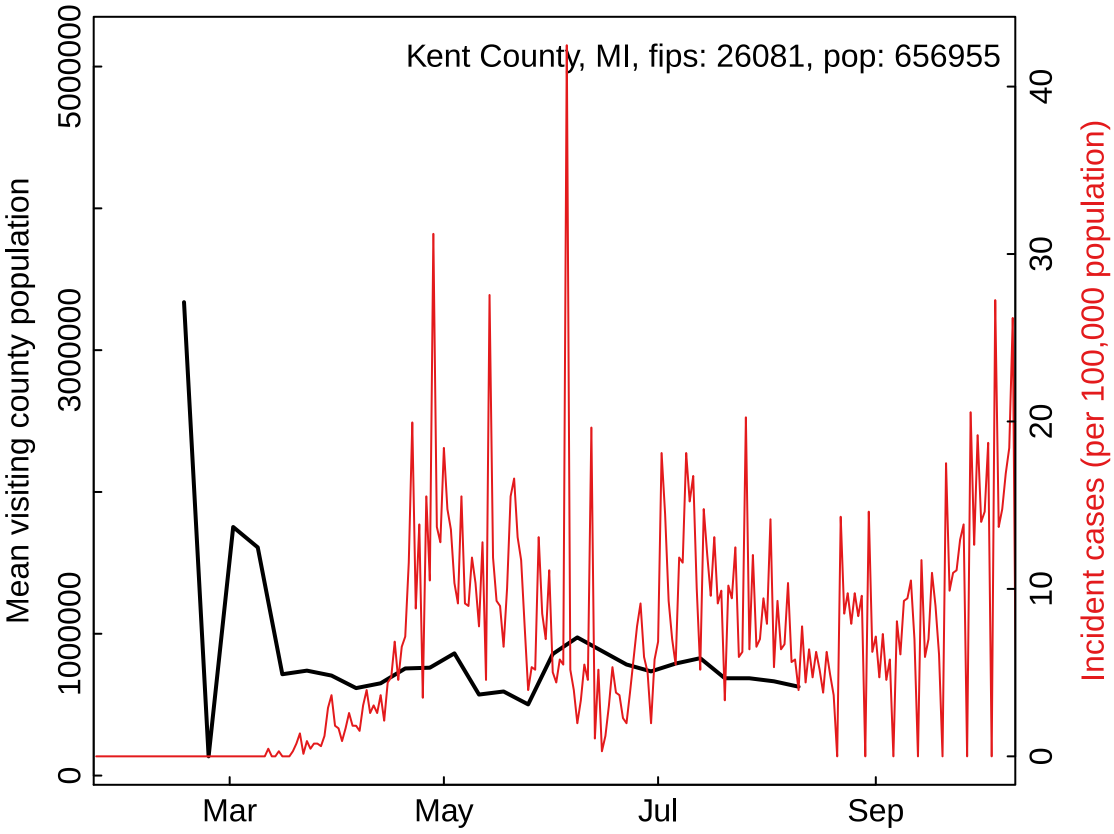

Kent County, MI, fips: 26081, pop: 656955
Mean visiting county population
0
1000000
3000000
5000000
Incident cases (per 100,000 population)
0
10
20
30
40
Mar
May
Jul
Sep

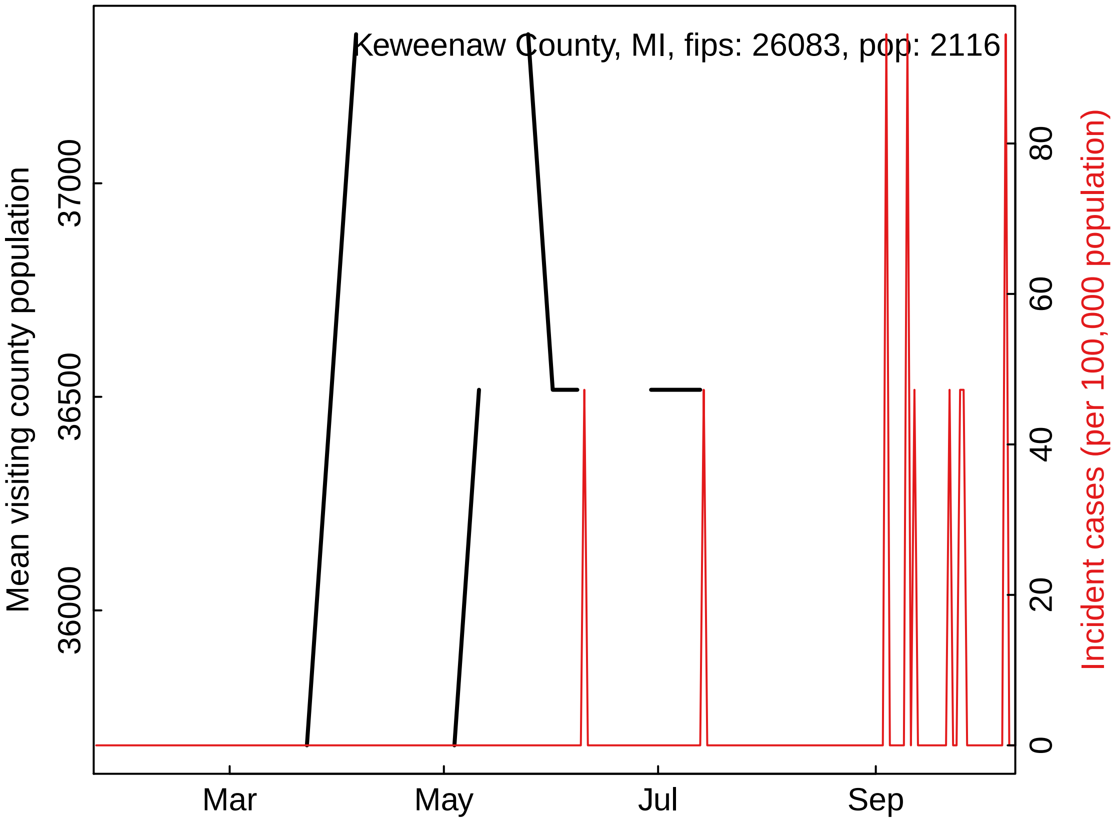

Keweenaw County, MI, fips: 26083, pop: 2116
Mean visiting county population
36000
36500
37000
Incident cases (per 100,000 population)
0
20
40
60
80
Mar
May
Jul
Sep

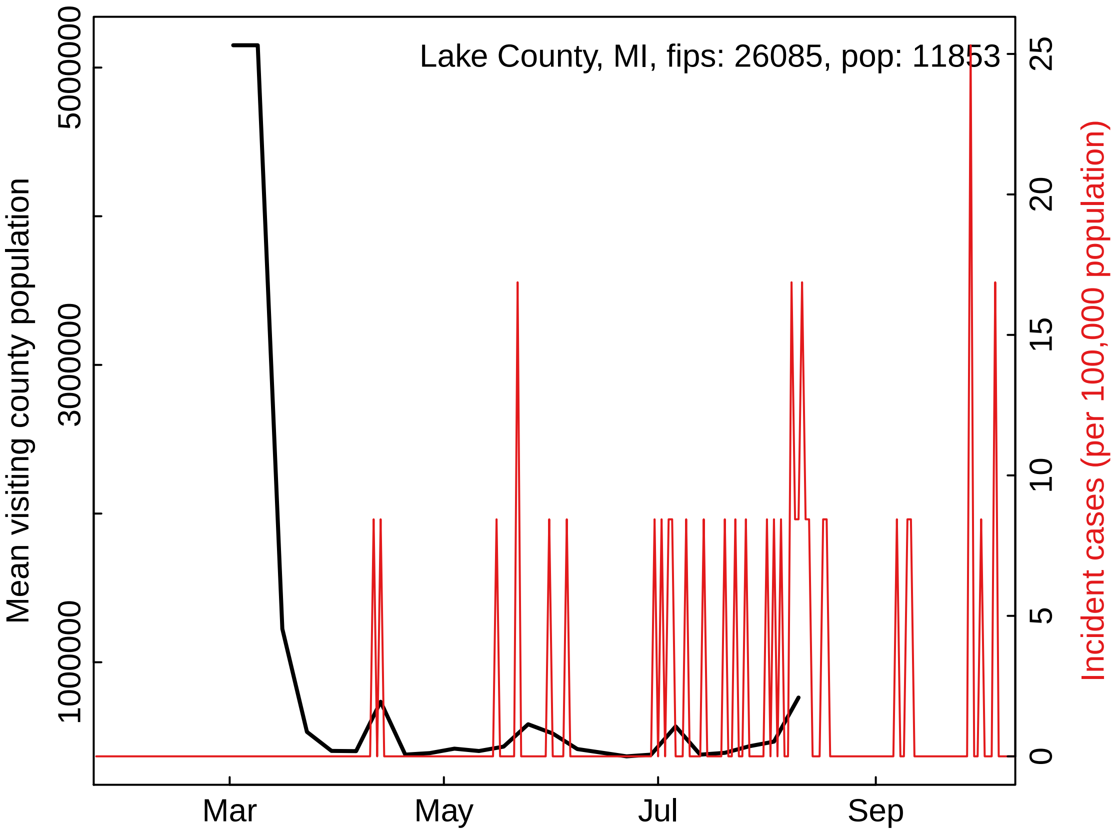

Lake County, MI, fips: 26085, pop: 11853
Mean visiting county population
10000000
30000000
50000000
Incident cases (per 100,000 population)
0
5
10
15
20
25
Mar
May
Jul
Sep

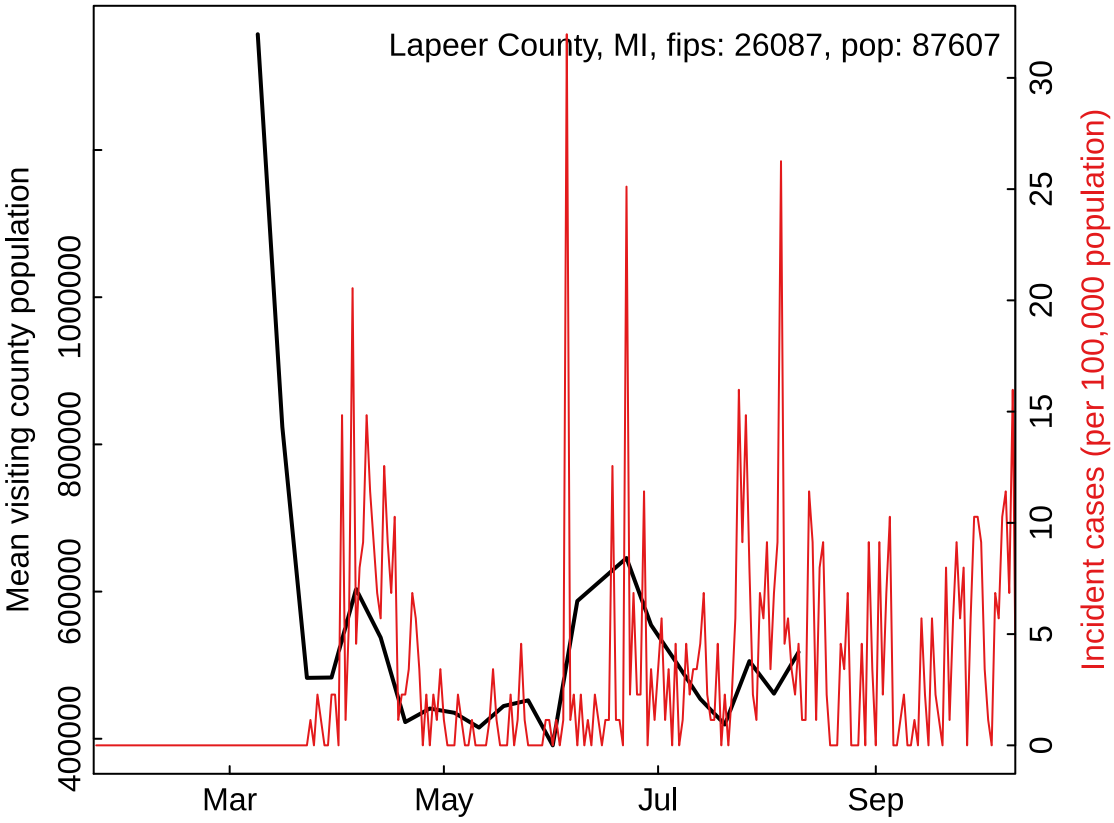

Lapeer County, MI, fips: 26087, pop: 87607
Mean visiting county population
400000
600000
800000
1000000
Incident cases (per 100,000 population)
0
5
10
15
20
25
30
Mar
May
Jul
Sep

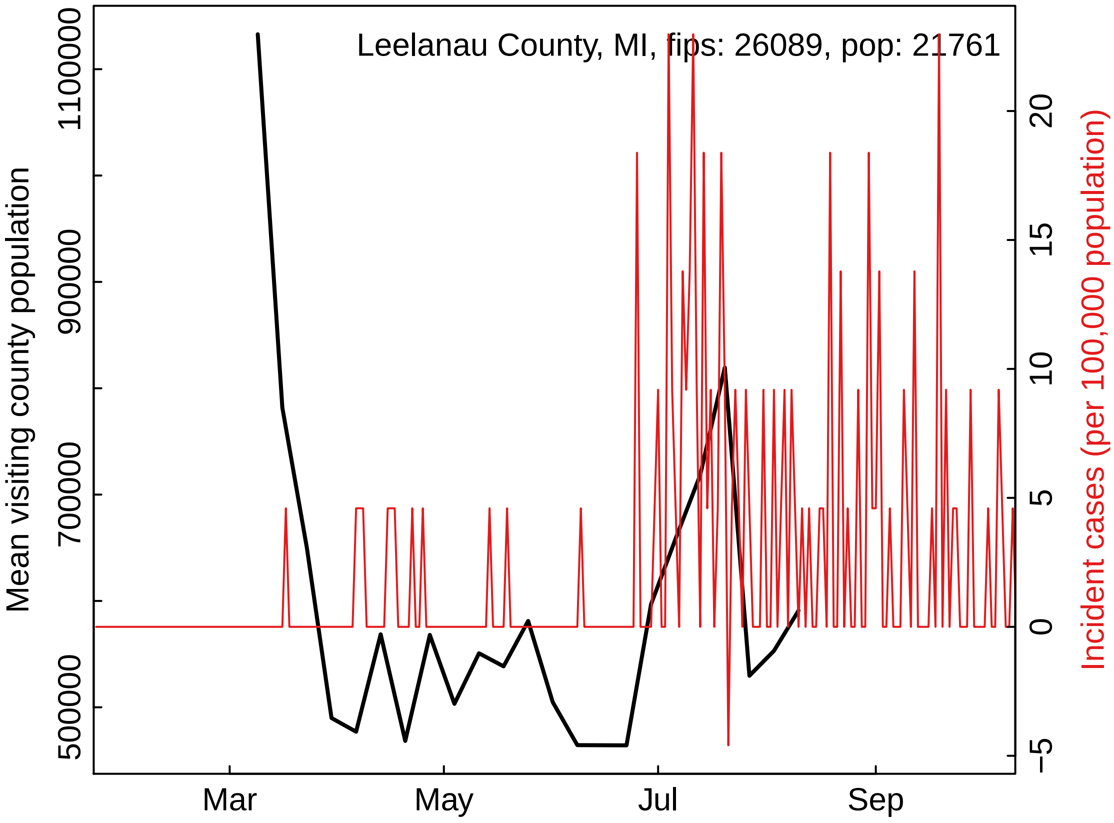

Leelanau County, MI, fips: 26089, pop: 21761
Mean visiting county population
500000
700000
900000
1100000
Incident cases (per 100,000 population)
−5
0
5
10
15
20
Mar
May
Jul
Sep

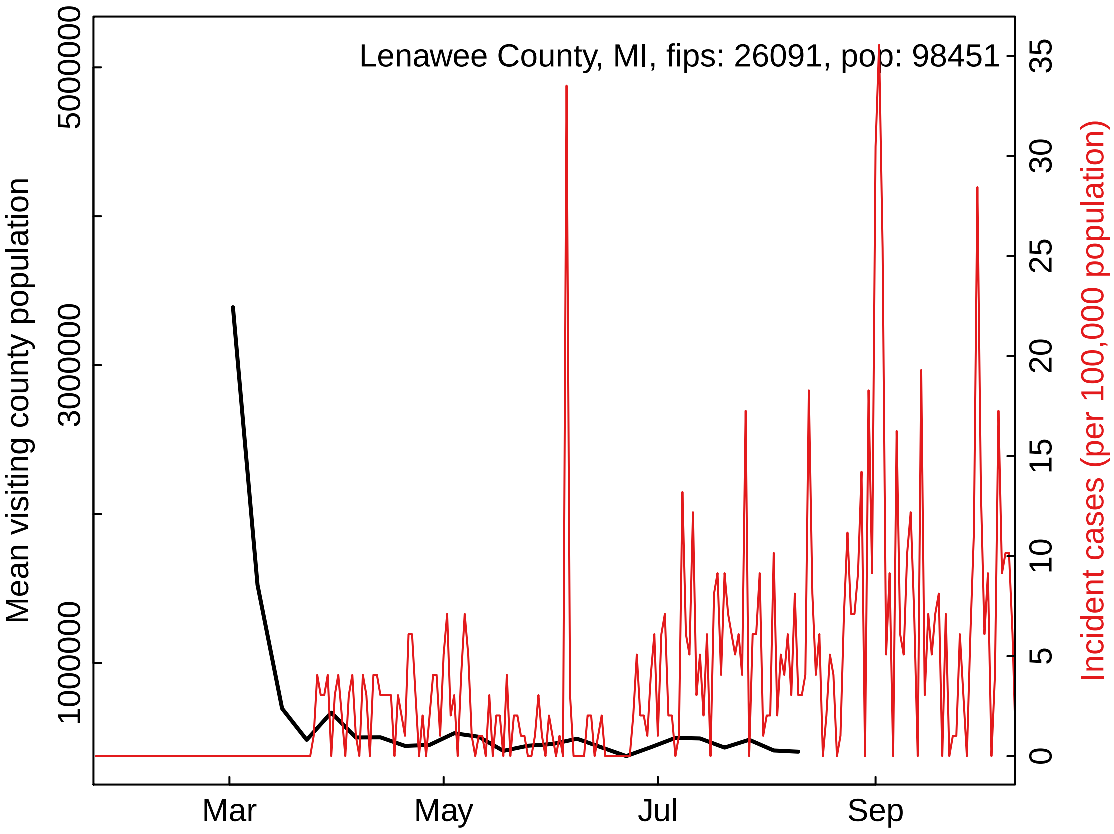

Lenawee County, MI, fips: 26091, pop: 98451
Mean visiting county population
1000000
3000000
5000000
Mar
May
Jul
Sep
Incident cases (per 100,000 population)
0
5
10
15
20
25
30
35

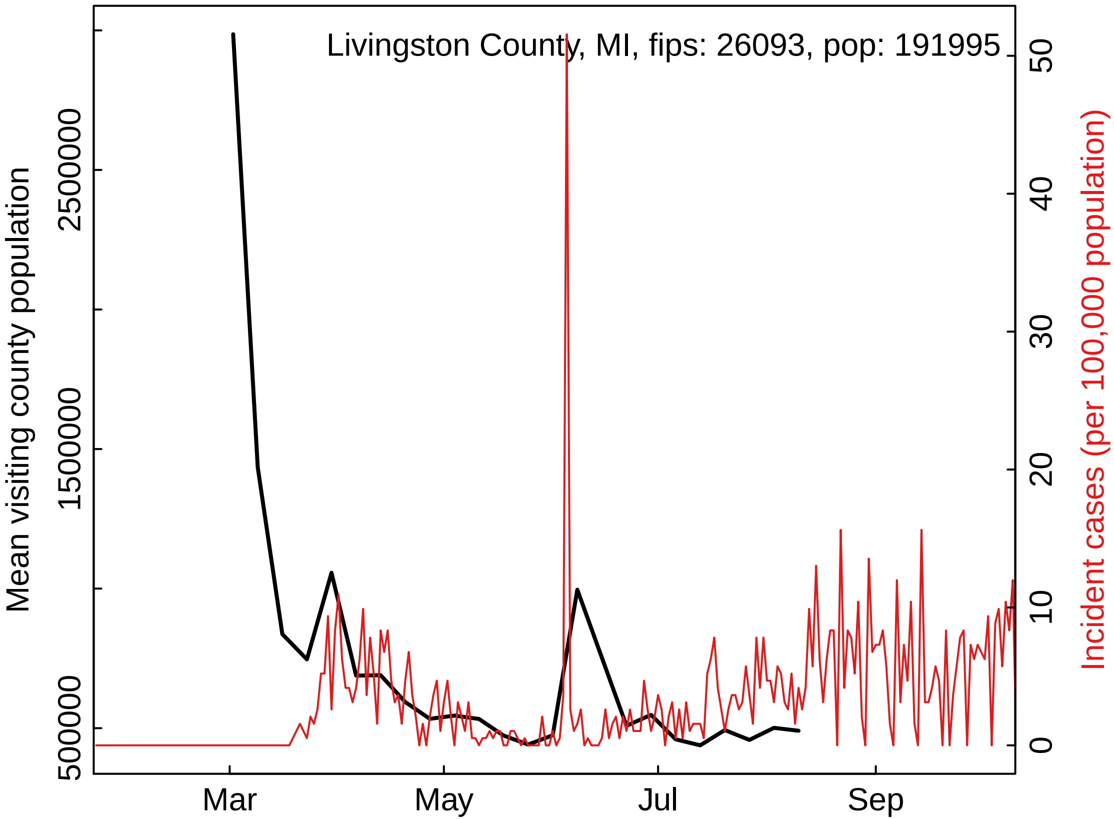

Livingston County, MI, fips: 26093, pop: 191995
Mean visiting county population
500000
1500000
2500000
Incident cases (per 100,000 population)
0
10
20
30
40
50
Mar
May
Jul
Sep

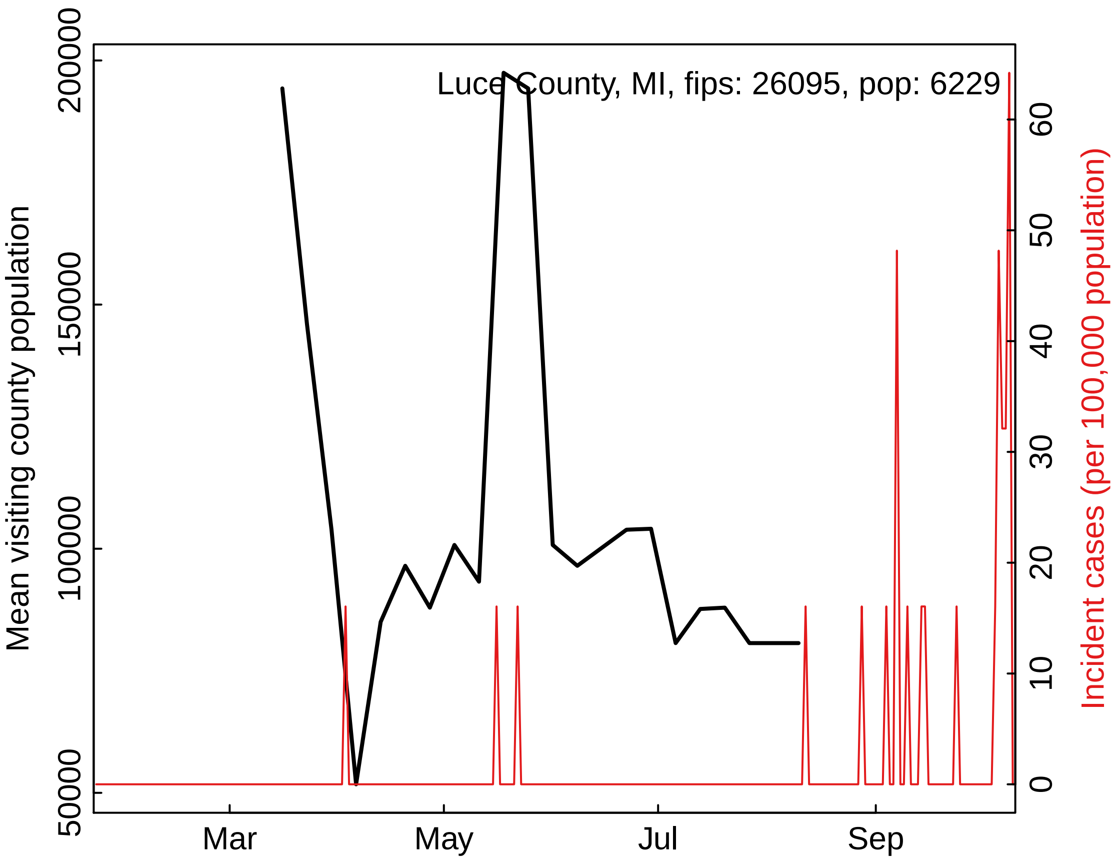
Luce County, MI, fips: 26095, pop: 6229
Mean visiting county population
50000
100000
150000
200000
Incident cases (per 100,000 population)
0
10
20
30
40
50
60
Mar
May
Jul
Sep

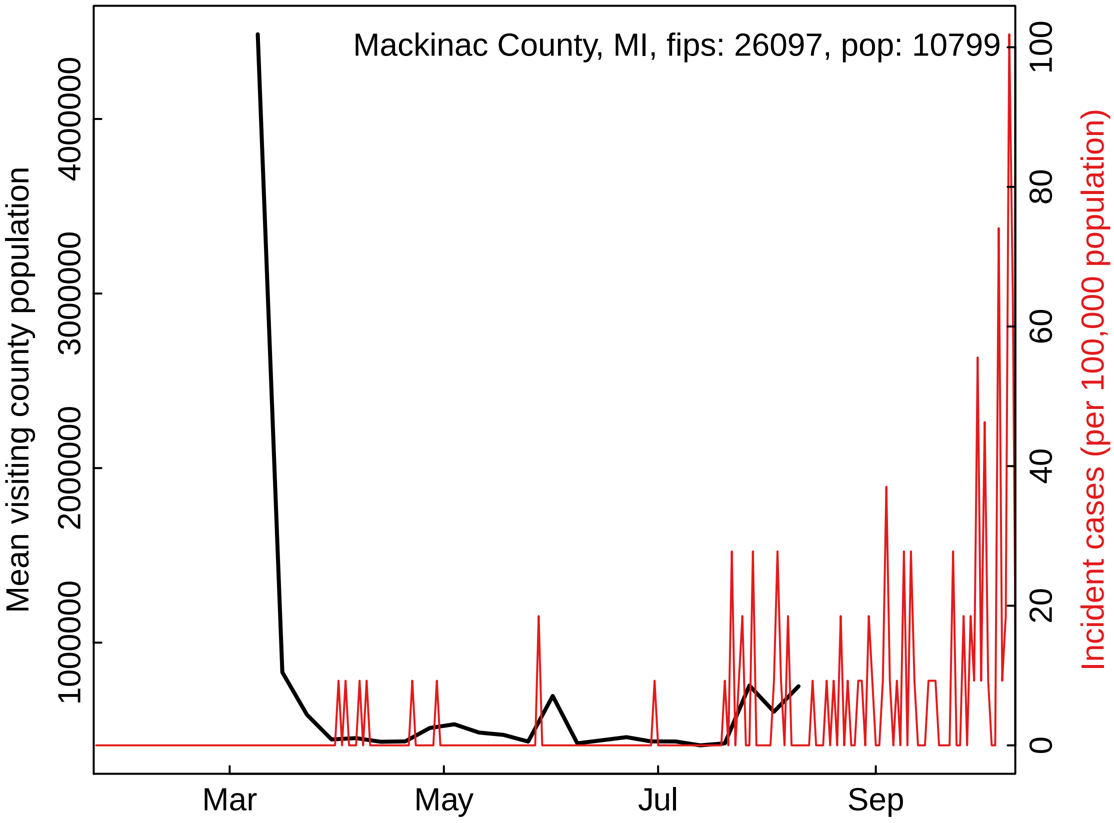

Mackinac County, MI, fips: 26097, pop: 10799
Mean visiting county population
1000000
2000000
3000000
4000000
Incident cases (per 100,000 population)
0
20
40
60
80
100
Mar
May
Jul
Sep

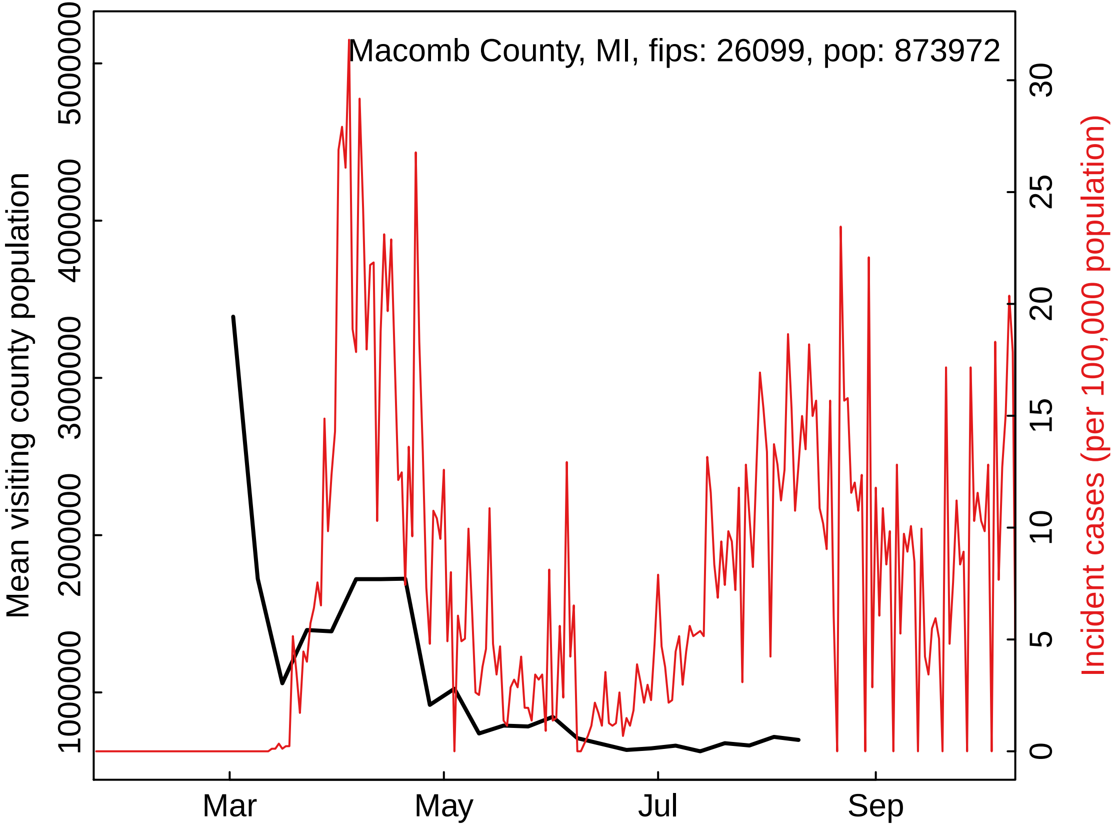

Macomb County, MI, fips: 26099, pop: 873972
Mean visiting county population
1000000
2000000
3000000
4000000
5000000
Incident cases (per 100,000 population)
0
5
10
15
20
25
30
Mar
May
Jul
Sep

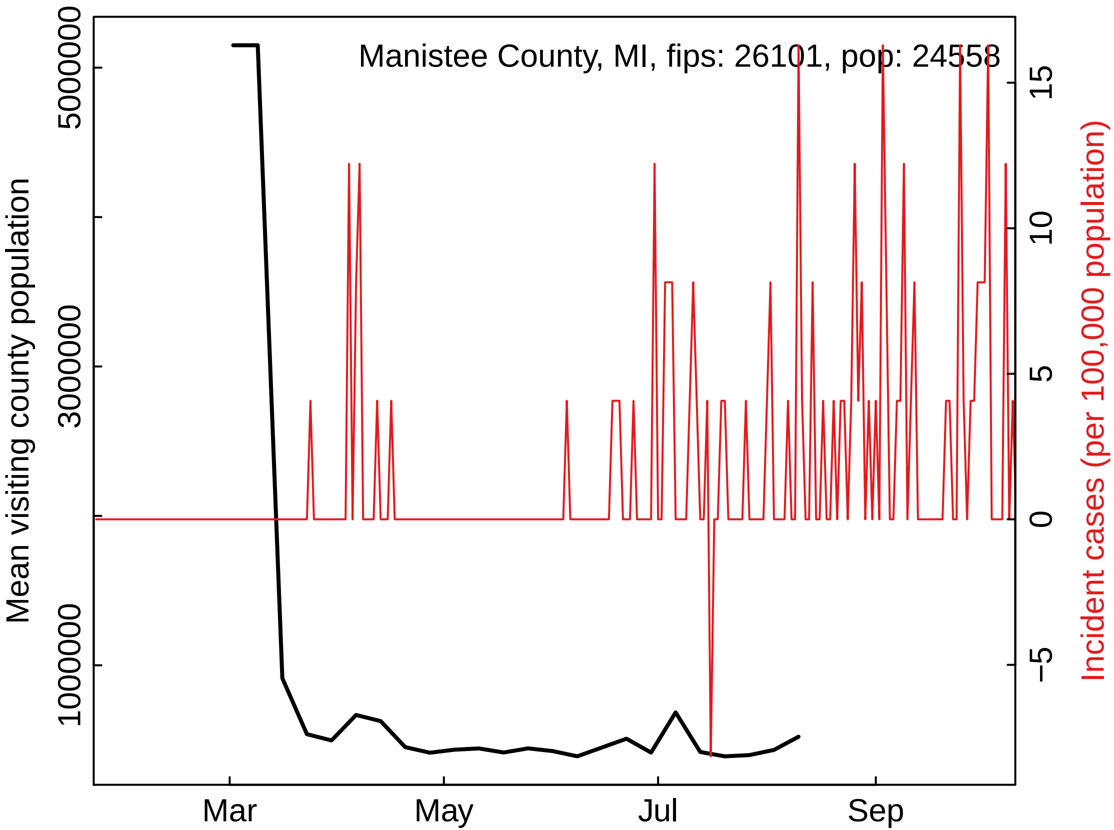

Manistee County, MI, fips: 26101, pop: 24558
Mean visiting county population
1000000
3000000
5000000
Incident cases (per 100,000 population)
−5
0
5
10
15
Mar
May
Jul
Sep

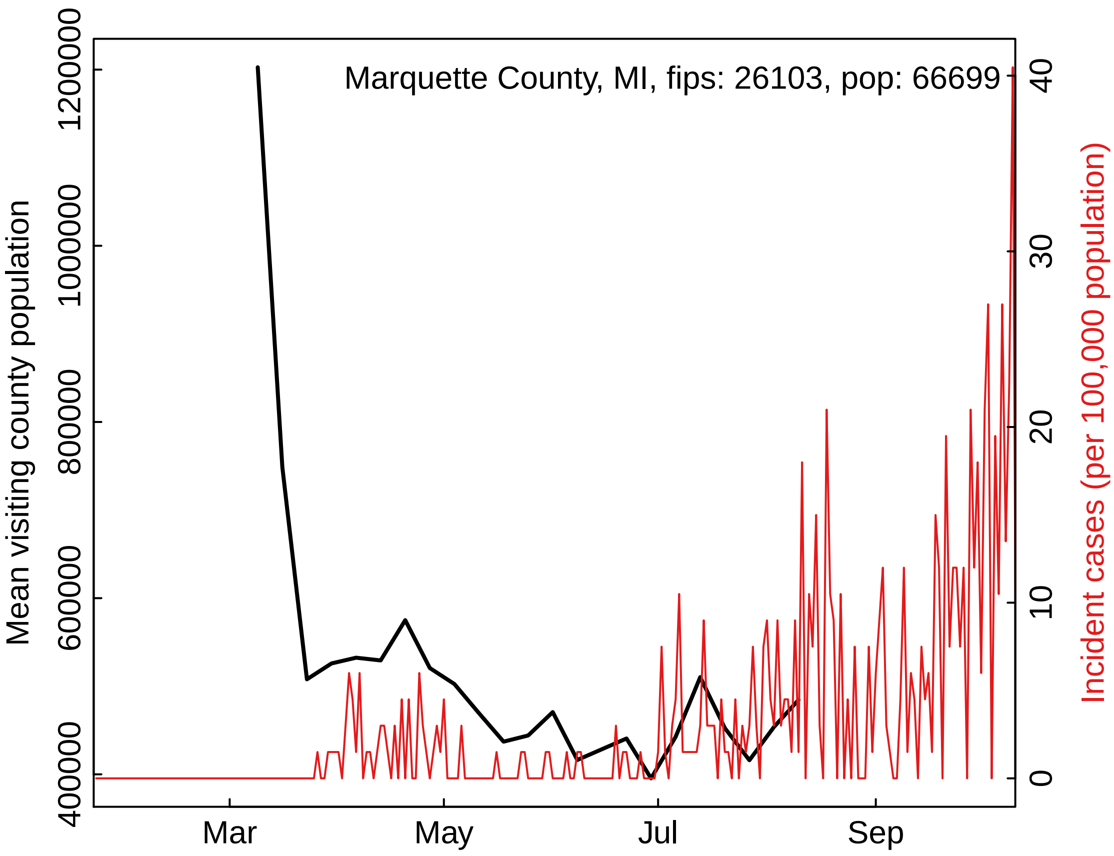

Marquette County, MI, fips: 26103, pop: 66699
Mean visiting county population
Incident cases (per 100,000 population)
400000
600000
800000
1000000
1200000
0
10
20
30
40
Mar
May
Jul
Sep

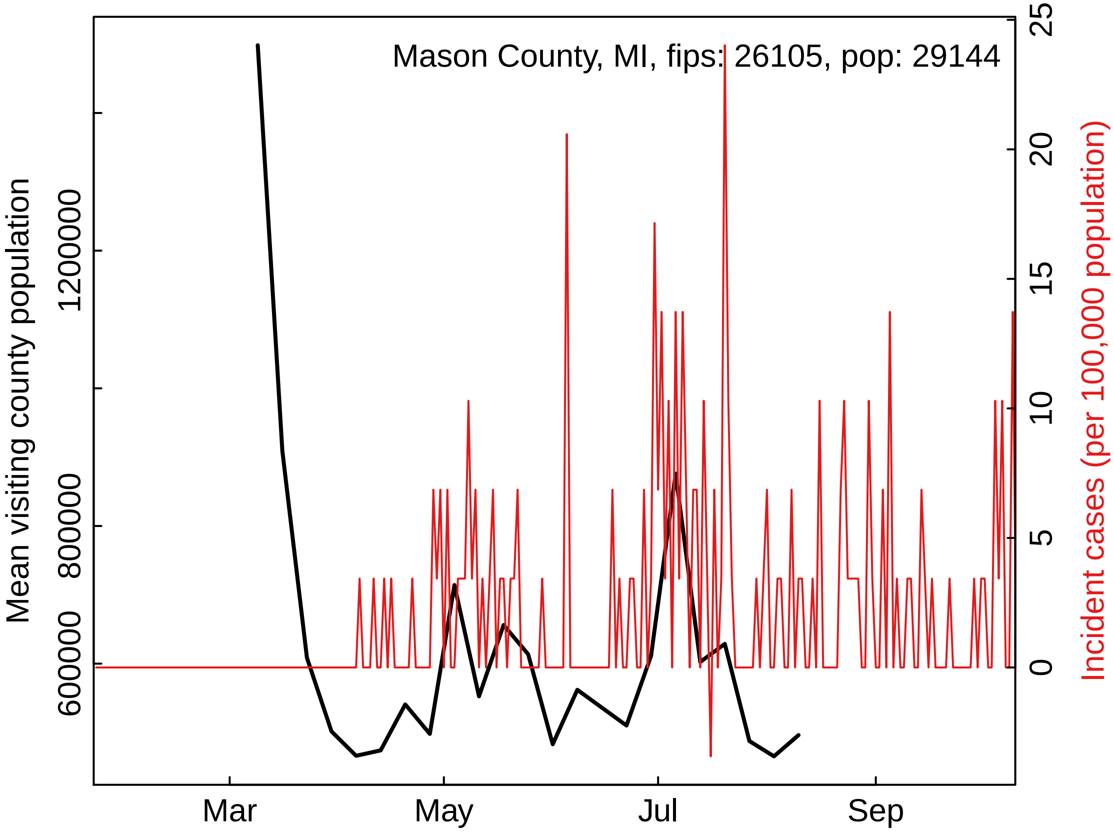

Mason County, MI, fips: 26105, pop: 29144
Mean visiting county population
600000
800000
1200000
Incident cases (per 100,000 population)
0
5
10
15
20
25
Mar
May
Jul
Sep

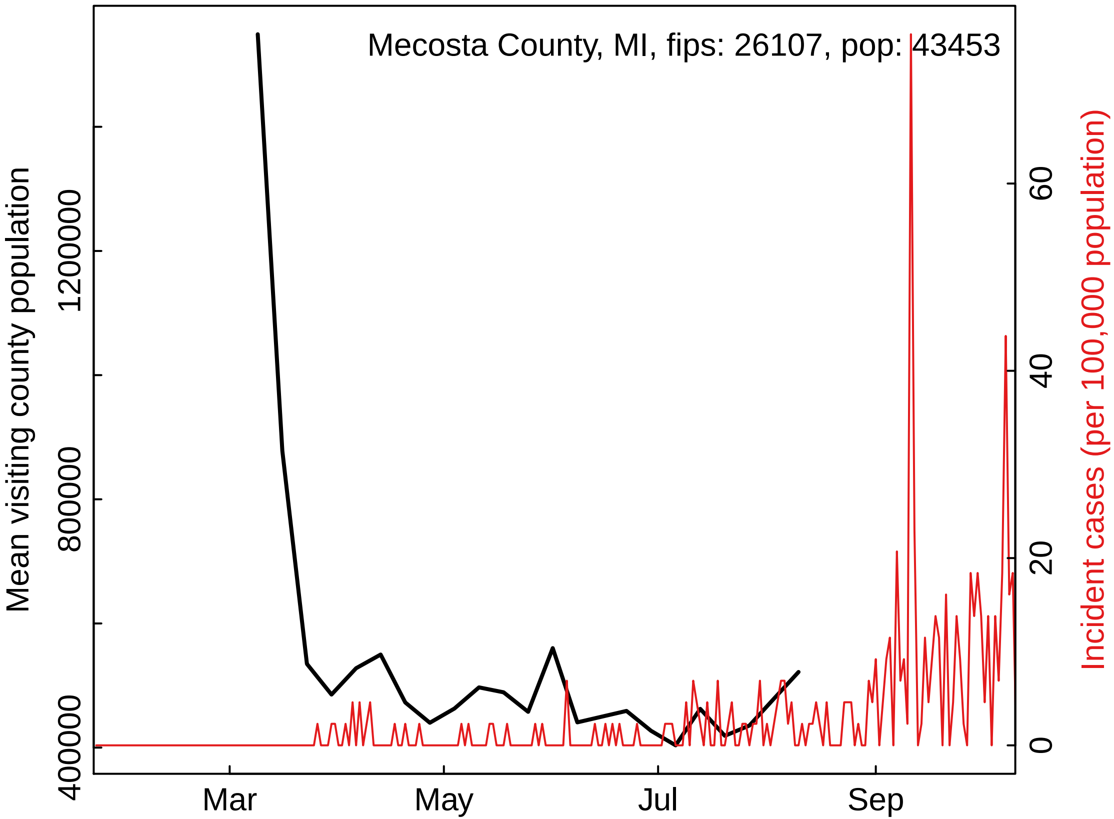

Mecosta County, MI, fips: 26107, pop: 43453
Mean visiting county population
400000
800000
1200000
Mar
May
Jul
Sep
0
20
40
60
Incident cases (per 100,000 population)

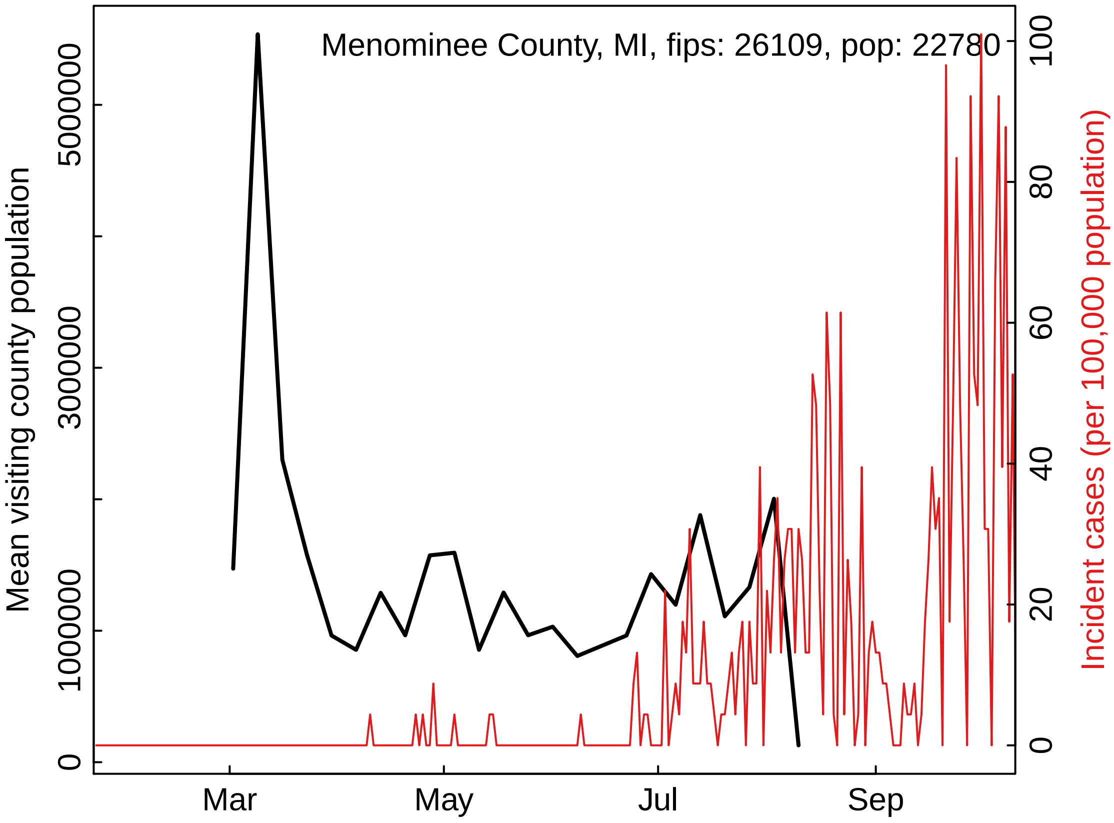

Menominee County, MI, fips: 26109, pop: 22780
Mean visiting county population
0
1000000
3000000
5000000
Incident cases (per 100,000 population)
0
20
40
60
80
100
Mar
May
Jul
Sep

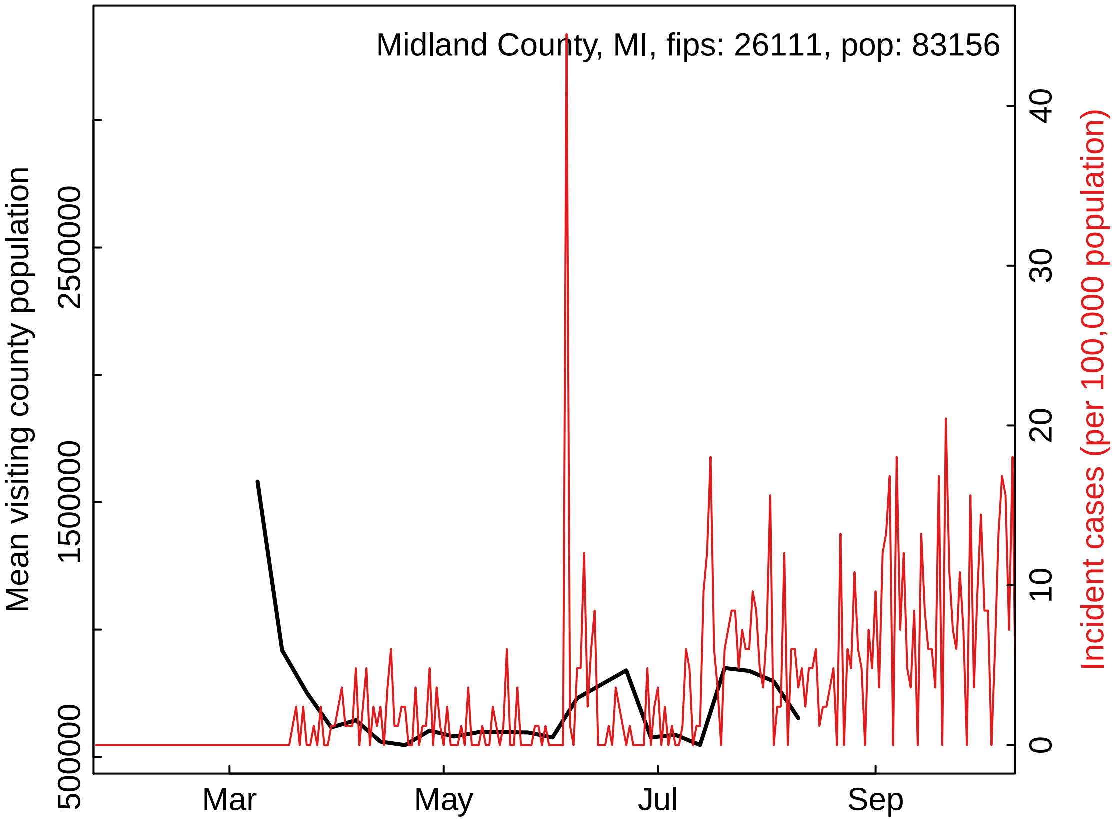
Midland County, MI, fips: 26111, pop: 83156
Mean visiting county population
500000
1500000
2500000
Mar
May
Jul
Sep
0
10
20
30
40
Incident cases (per 100,000 population)

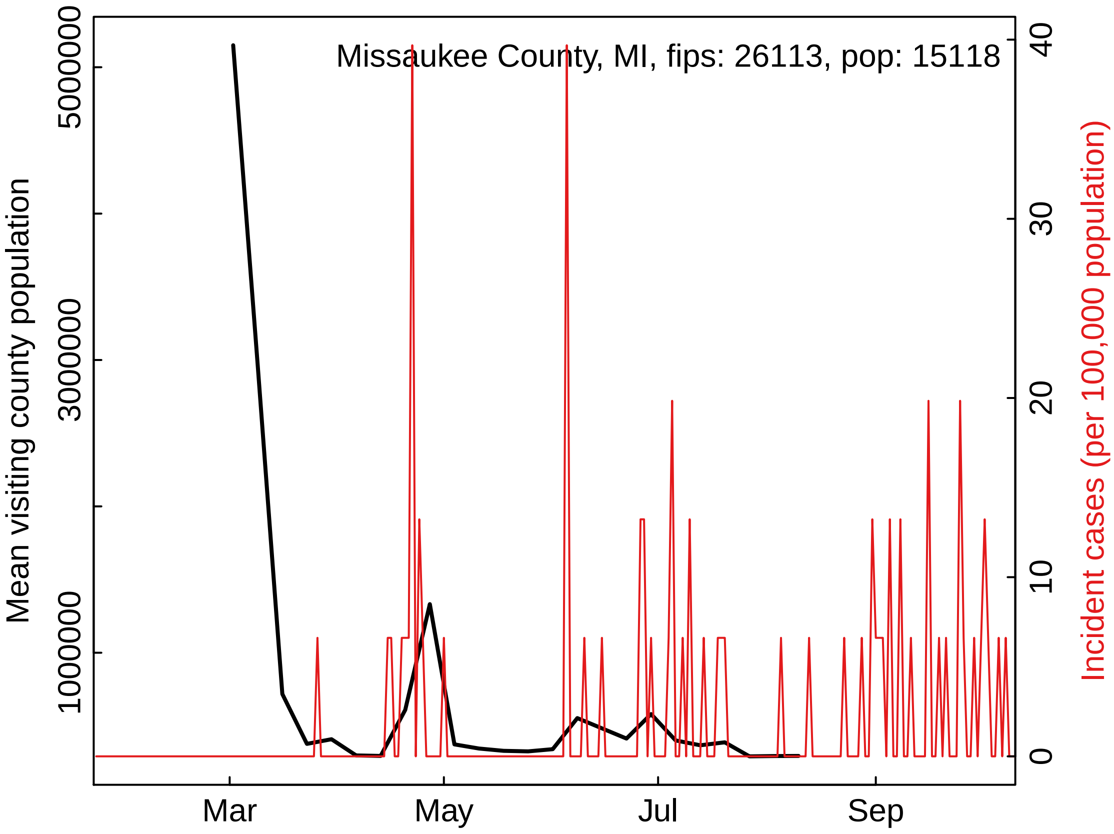

Missaukee County, MI, fips: 26113, pop: 15118
Mean visiting county population
1000000
3000000
5000000
Incident cases (per 100,000 population)
0
10
20
30
40
Mar
May
Jul
Sep

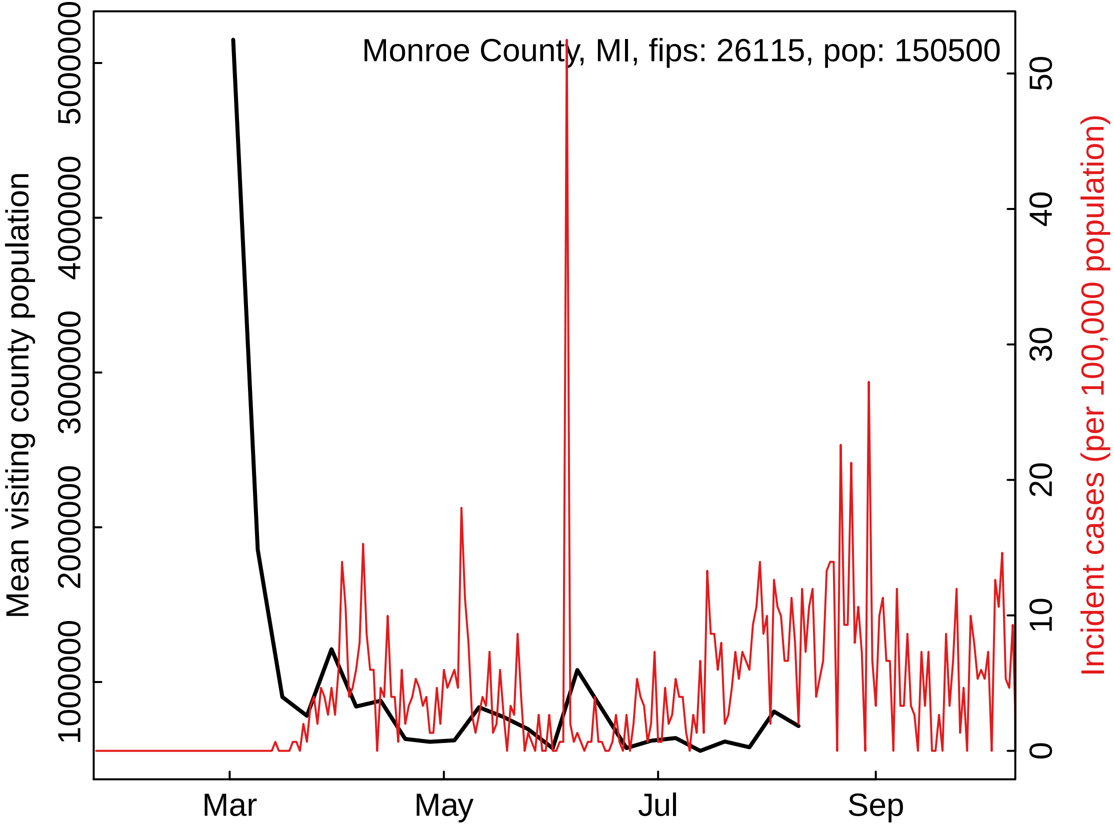
Monroe County, MI, fips: 26115, pop: 150500
Mean visiting county population
10000000
20000000
30000000
40000000
50000000
Incident cases (per 100,000 population)
0
10
20
30
40
50
Mar
May
Jul
Sep

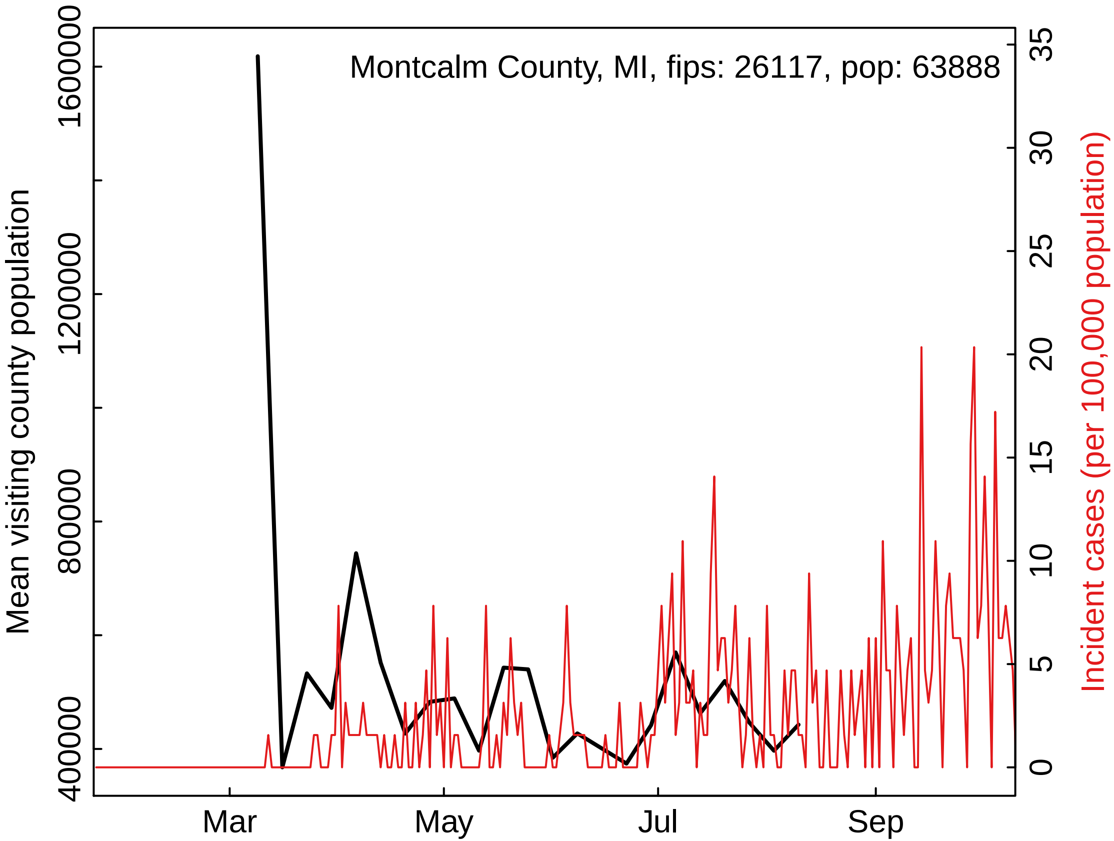

Montcalm County, MI, fips: 26117, pop: 63888
Mean visiting county population
400000
800000
1200000
1600000
Incident cases (per 100,000 population)
0
5
10
15
20
25
30
35
Mar
May
Jul
Sep

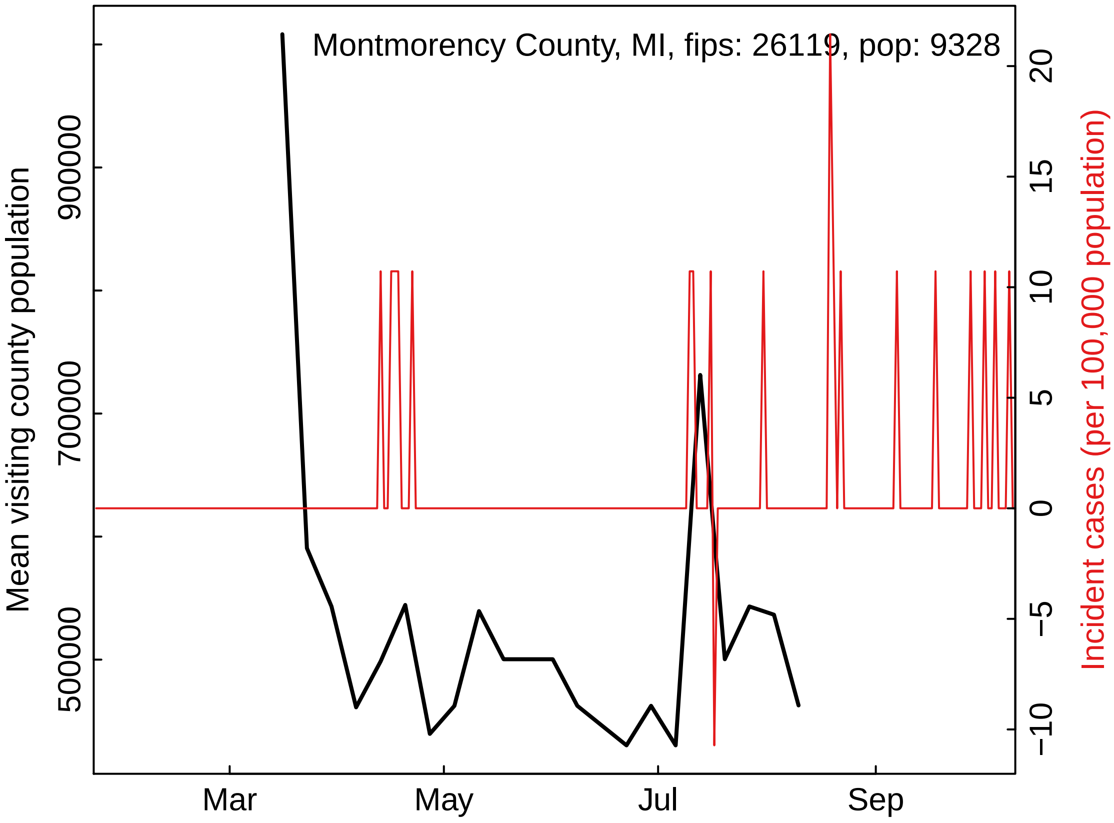
Montmorency County, MI, fips: 26119, pop: 9328
Mean visiting county population
500000
700000
900000
Incident cases (per 100,000 population)
−10
−5
0
5
10
15
20
Mar
May
Jul
Sep

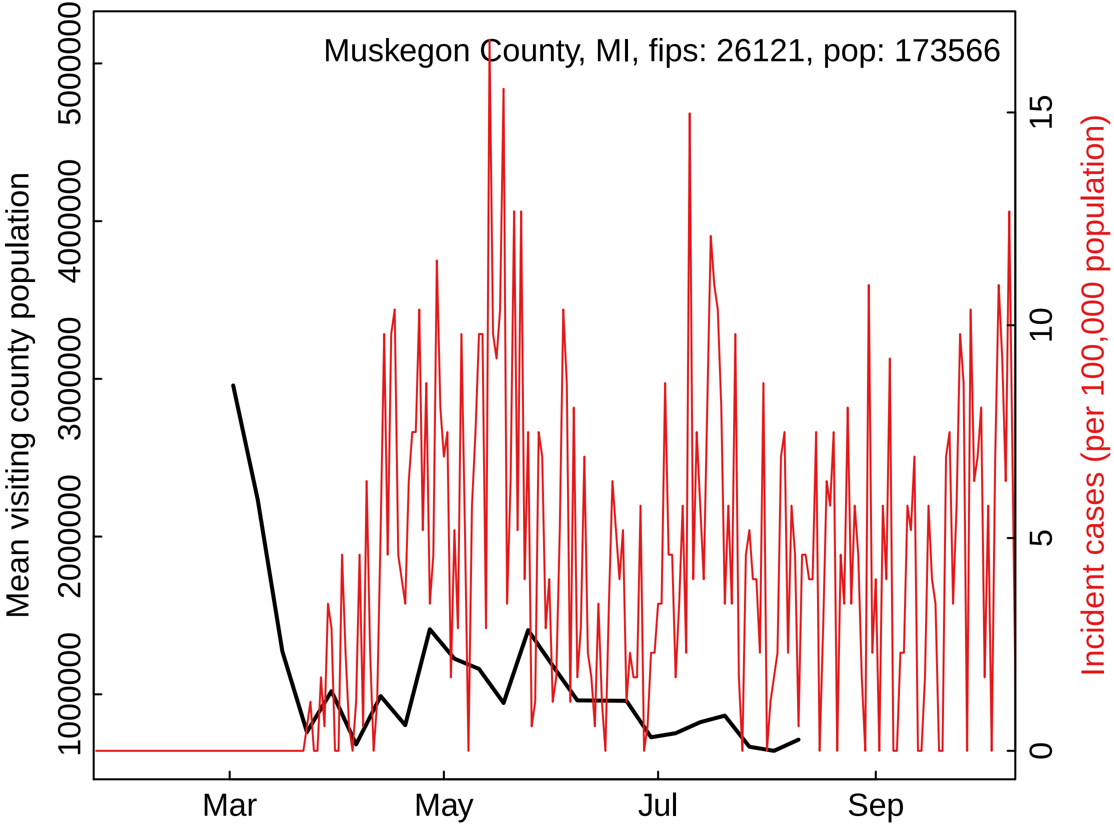

Muskegon County, MI, fips: 26121, pop: 173566
Mean visiting county population
10000000
20000000
30000000
40000000
50000000
Incident cases (per 100,000 population)
0
5
10
15
Mar
May
Jul
Sep

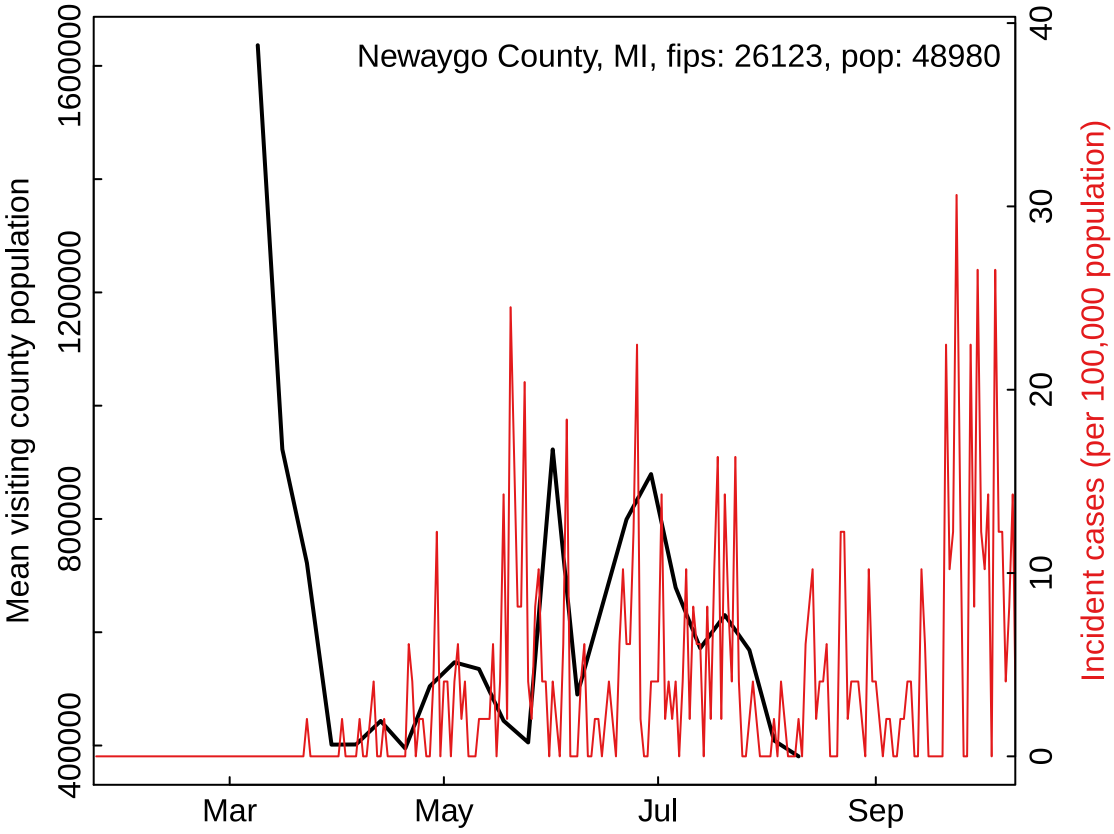

Newaygo County, MI, fips: 26123, pop: 48980
Mean visiting county population
400000
800000
1200000
1600000
Incident cases (per 100,000 population)
0
10
20
30
40
Mar
May
Jul
Sep

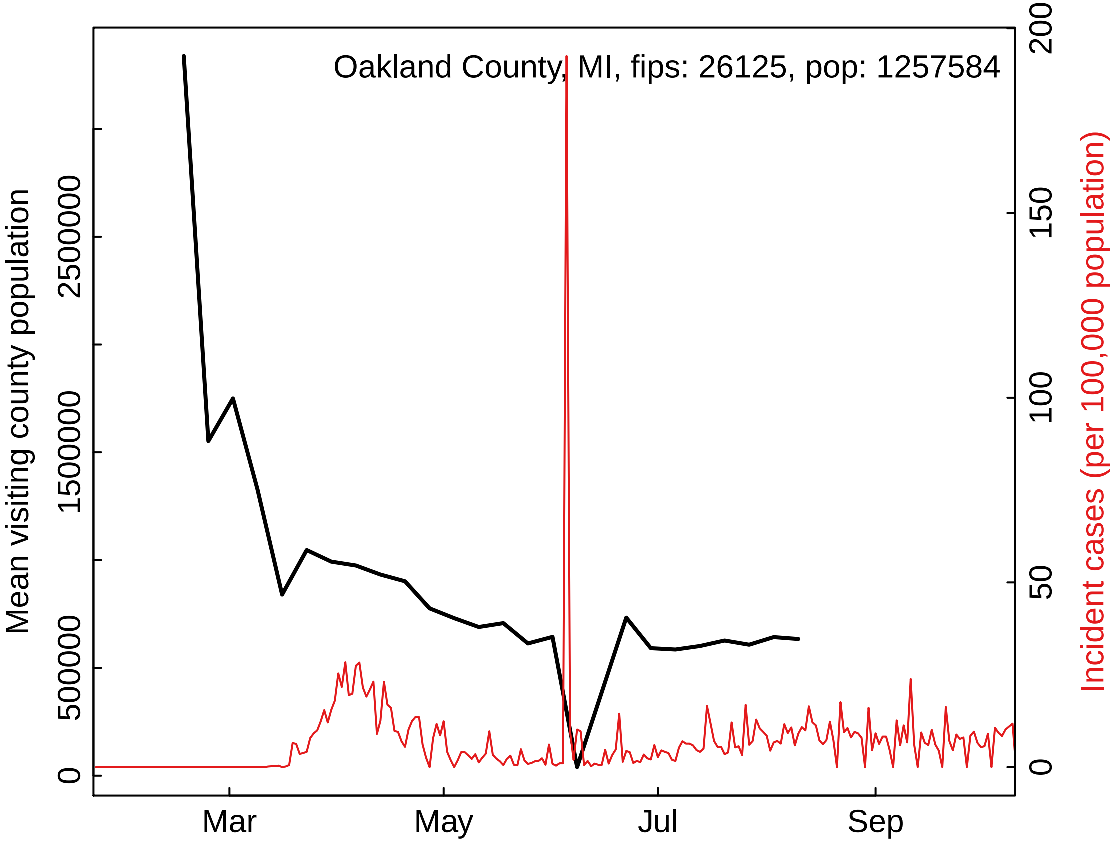

Oakland County, MI, fips: 26125, pop: 1257584
Mean visiting county population
0
500000
1500000
2500000
Incident cases (per 100,000 population)
0
50
100
150
200
Mar
May
Jul
Sep

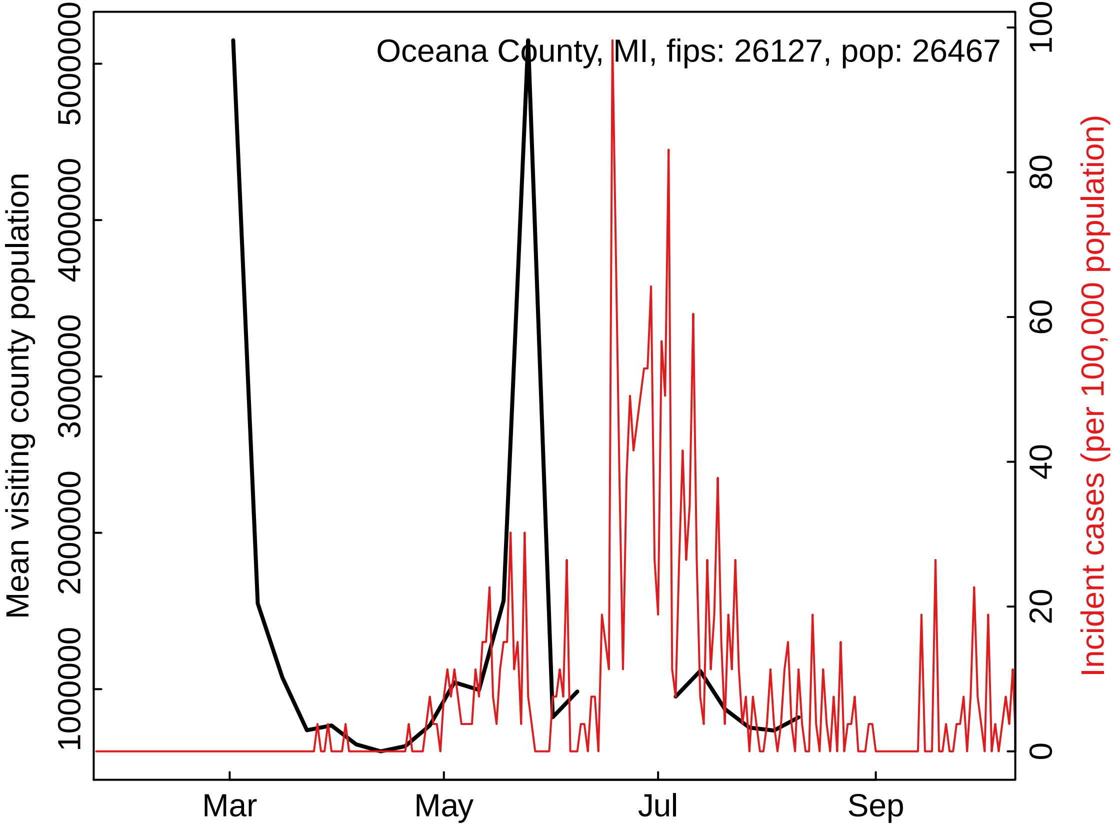

Oceana County, MI, fips: 26127, pop: 26467
Mean visiting county population
Incident cases (per 100,000 population)
1000000
2000000
3000000
4000000
5000000
0
20
40
60
80
100
Mar
May
Jul
Sep

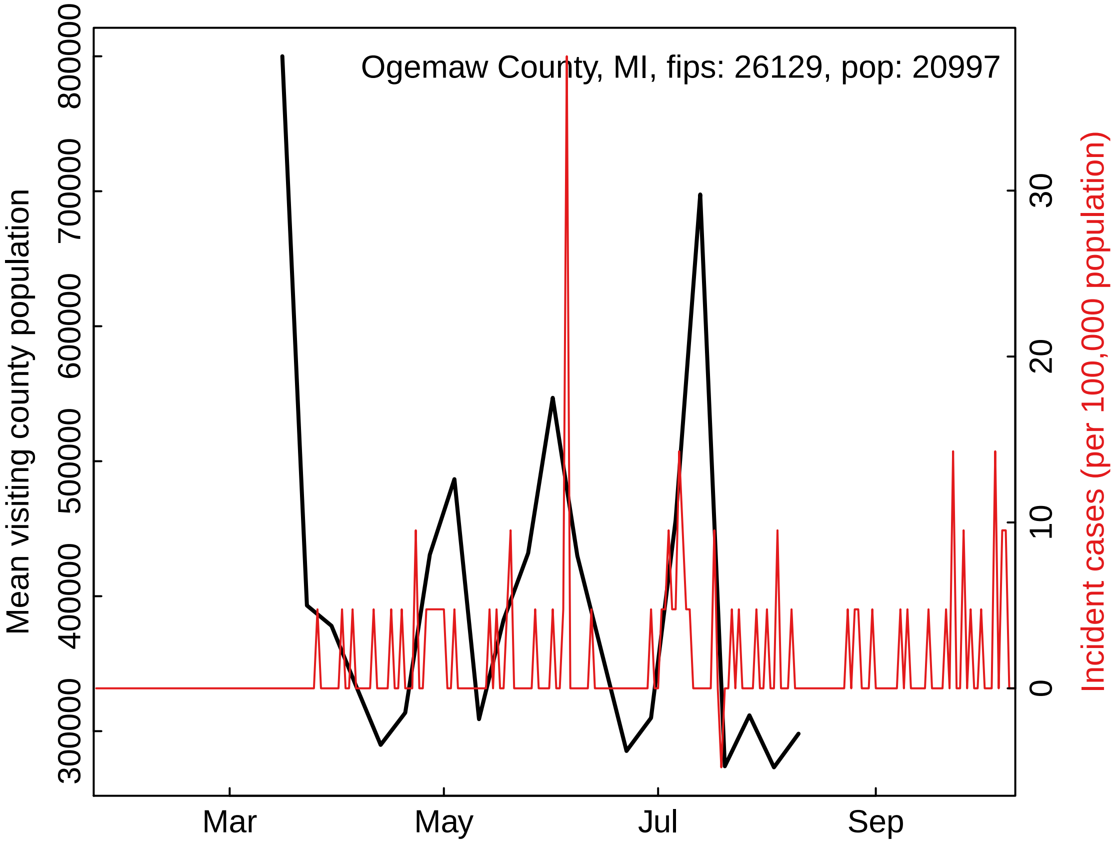

Ogemaw County, MI, fips: 26129, pop: 20997
Mean visiting county population
300000
400000
500000
600000
700000
800000
Incident cases (per 100,000 population)
0
10
20
30
Mar
May
Jul
Sep

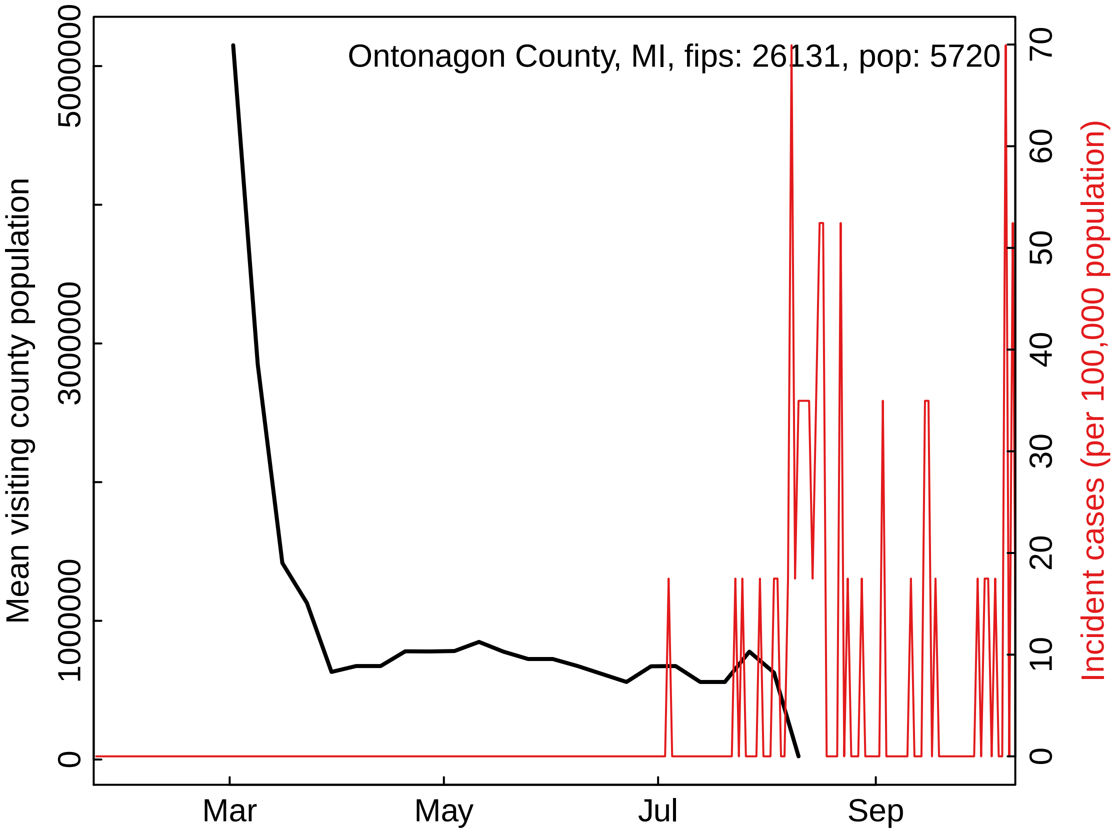

Ontonagon County, MI, fips: 26131, pop: 5720
Mean visiting county population
0
1000000
3000000
5000000
Incident cases (per 100,000 population)
0
10
20
30
40
50
60
70
Mar
May
Jul
Sep

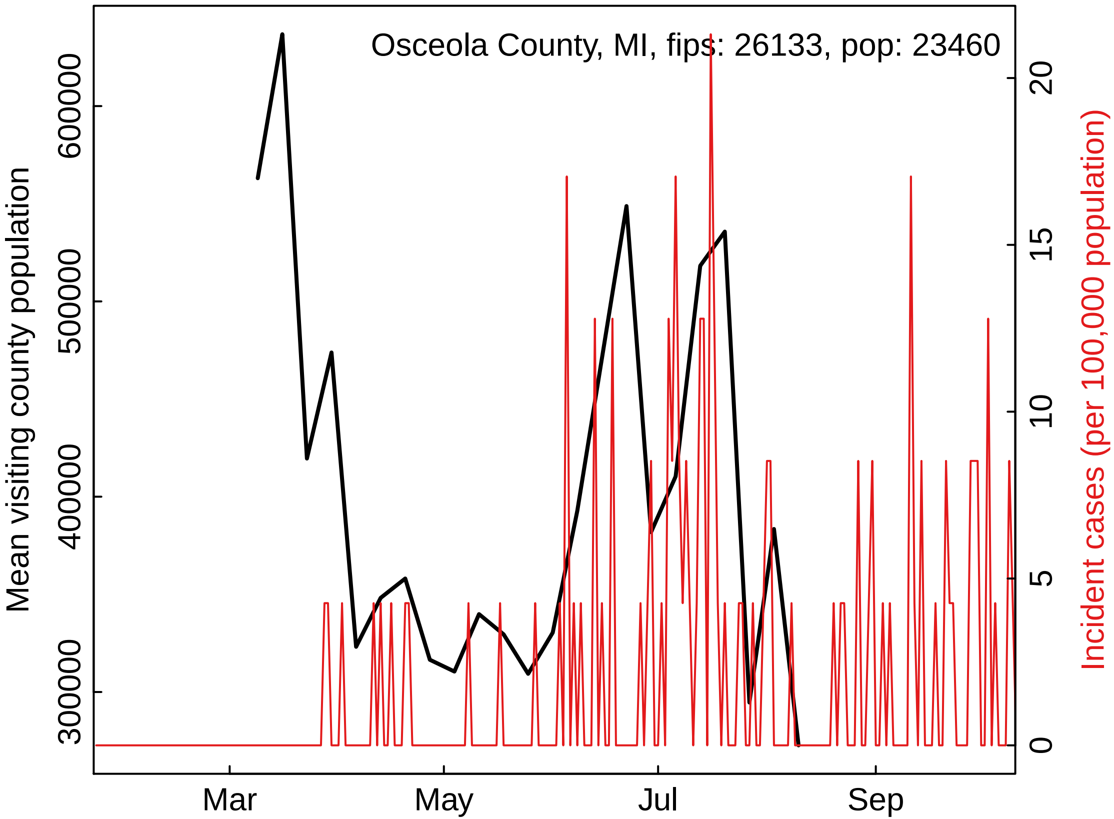

Osceola County, MI, fips: 26133, pop: 23460
Mean visiting county population
300000
400000
500000
600000
Incident cases (per 100,000 population)
0
5
10
15
20
Mar
May
Jul
Sep

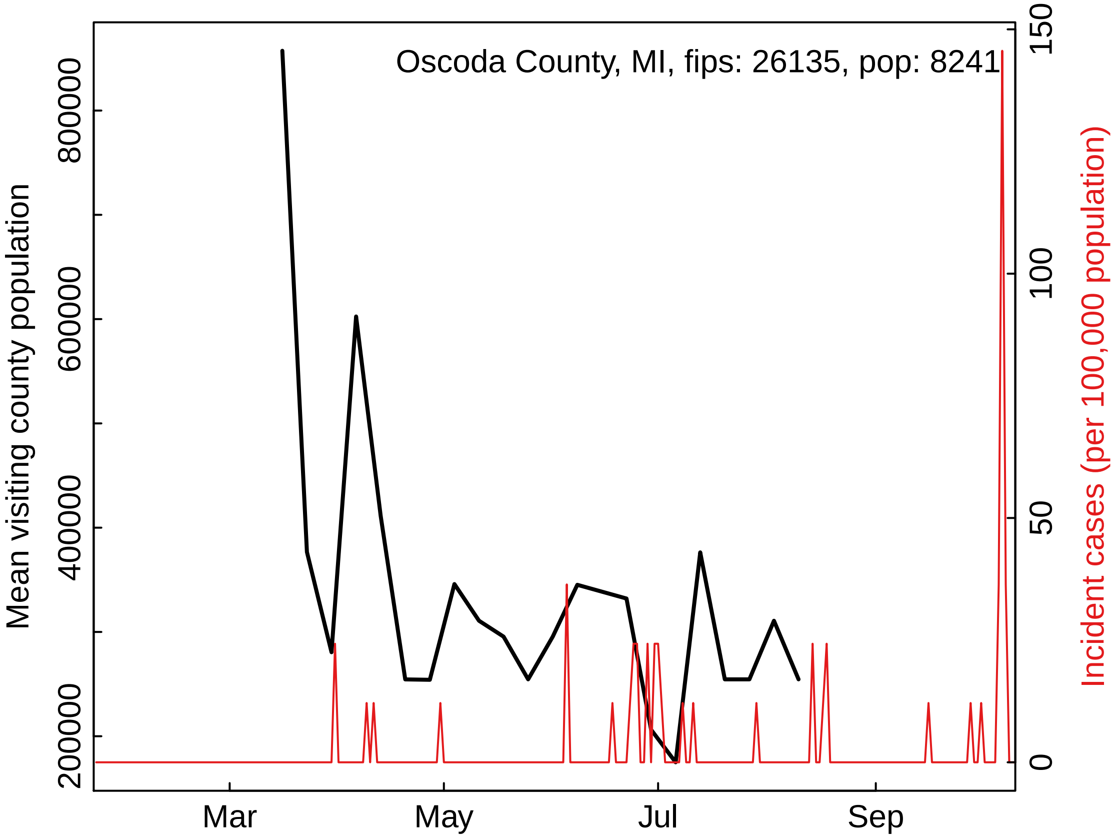

Oscoda County, MI, fips: 26135, pop: 8241
Mean visiting county population
200000
400000
600000
800000
Incident cases (per 100,000 population)
0
50
100
150
Mar
May
Jul
Sep

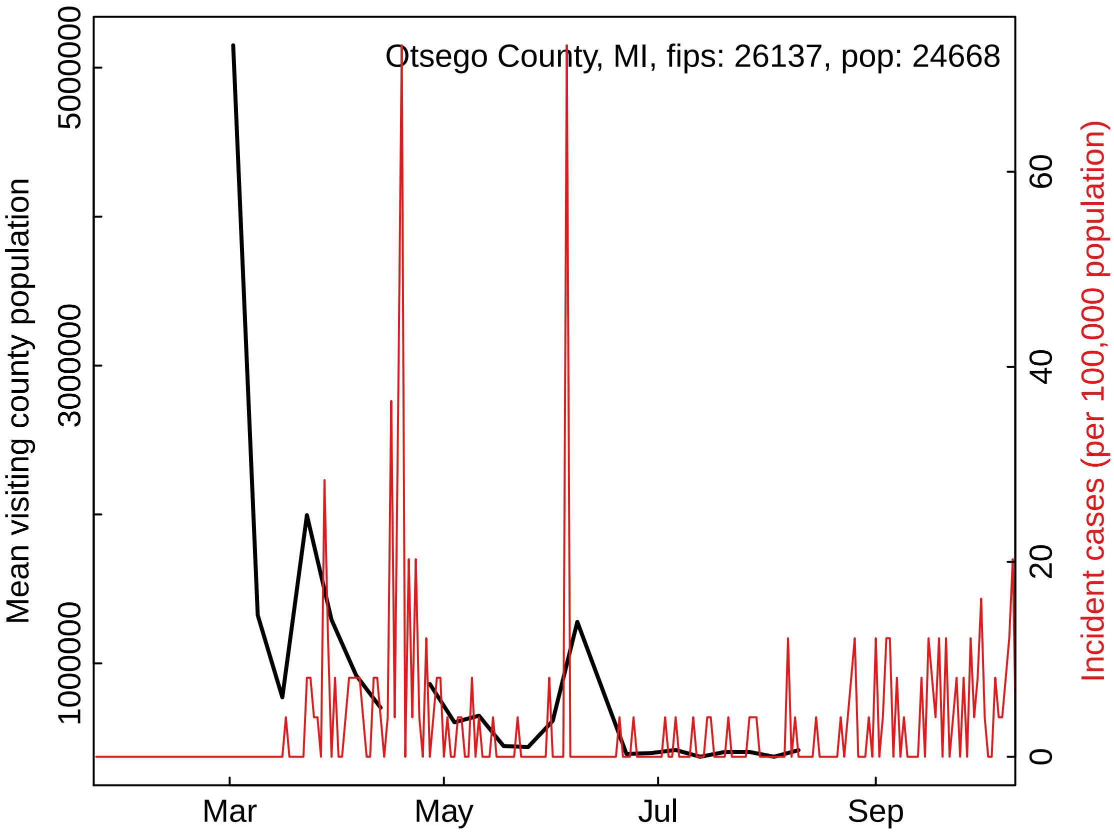

Otsego County, MI, fips: 26137, pop: 24668
Mean visiting county population
10000000
30000000
50000000
Incident cases (per 100,000 population)
0
20
40
60
Mar
May
Jul
Sep

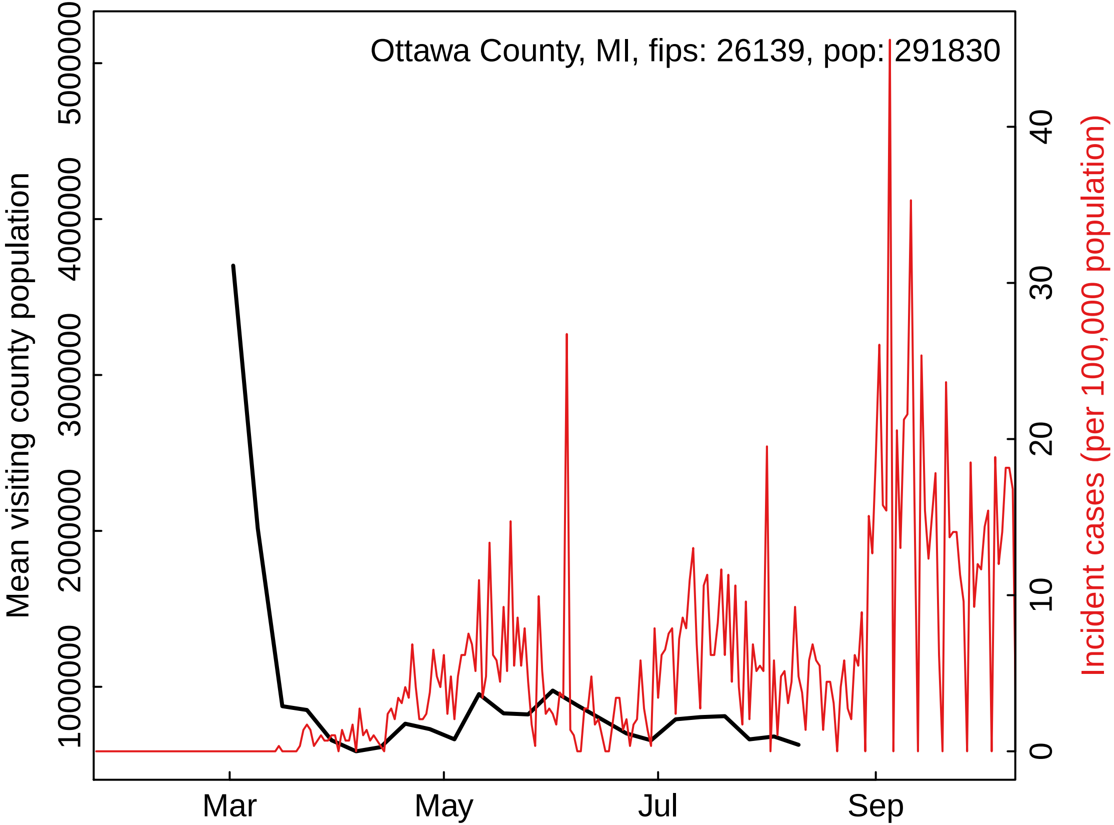
Ottawa County, MI, fips: 26139, pop: 291830
Mean visiting county population
10000000
20000000
30000000
40000000
50000000
Incident cases (per 100,000 population)
0
10
20
30
40
Mar
May
Jul
Sep

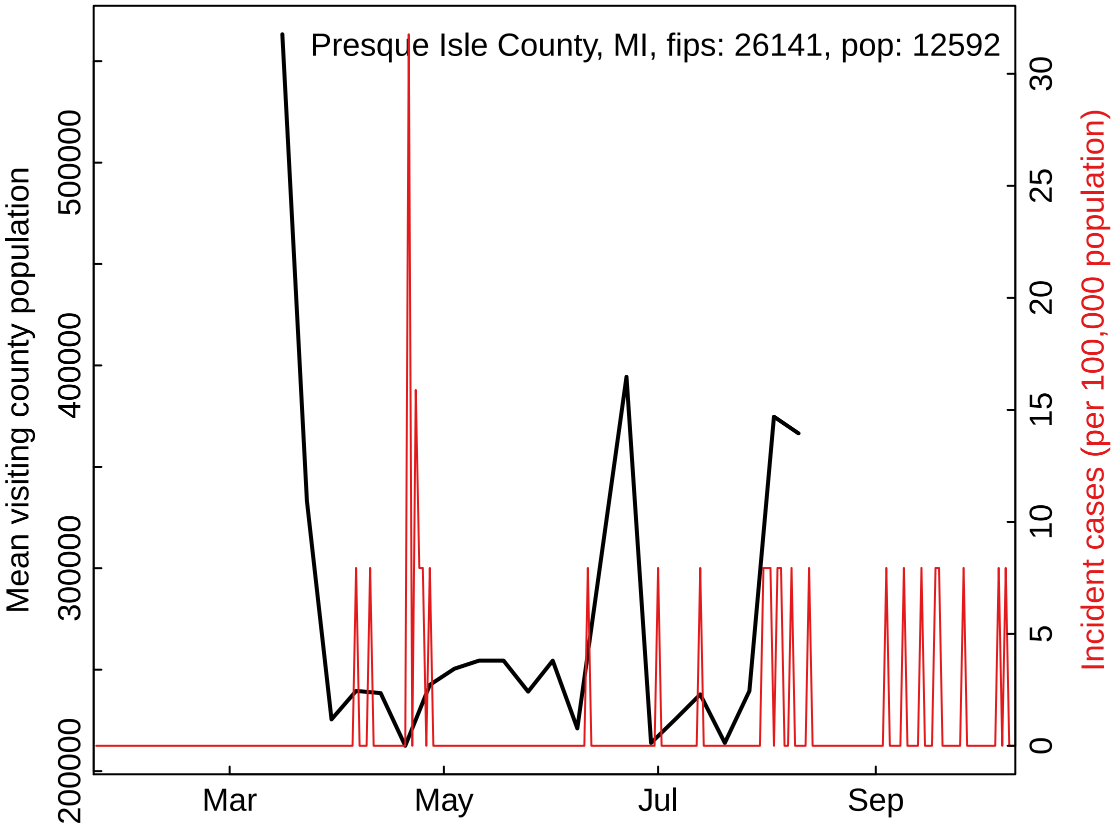

Presque Isle County, MI, fips: 26141, pop: 12592
Mean visiting county population
200000
300000
400000
500000
Incident cases (per 100,000 population)
0
5
10
15
20
25
30
Mar
May
Jul
Sep

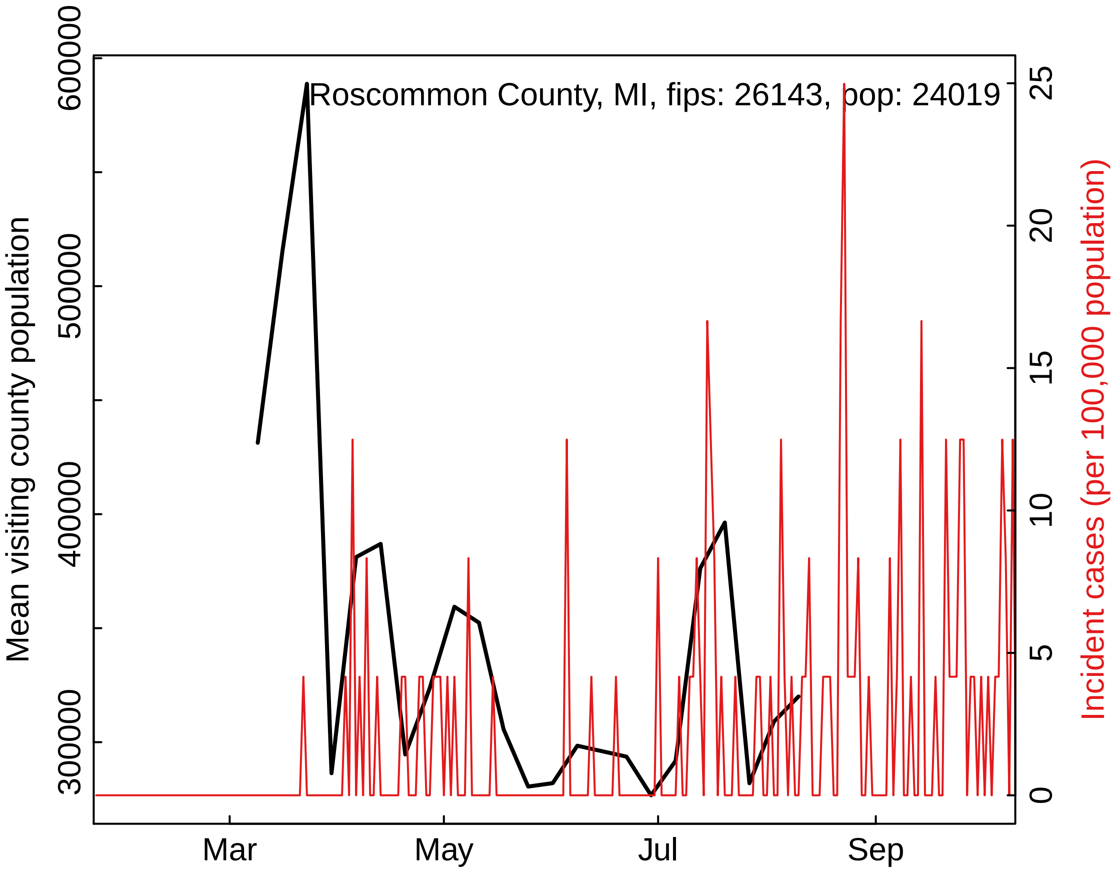

Roscommon County, MI, fips: 26143, pop: 24019
Mean visiting county population
300000
400000
500000
600000
Incident cases (per 100,000 population)
0
5
10
15
20
25
Mar
May
Jul
Sep

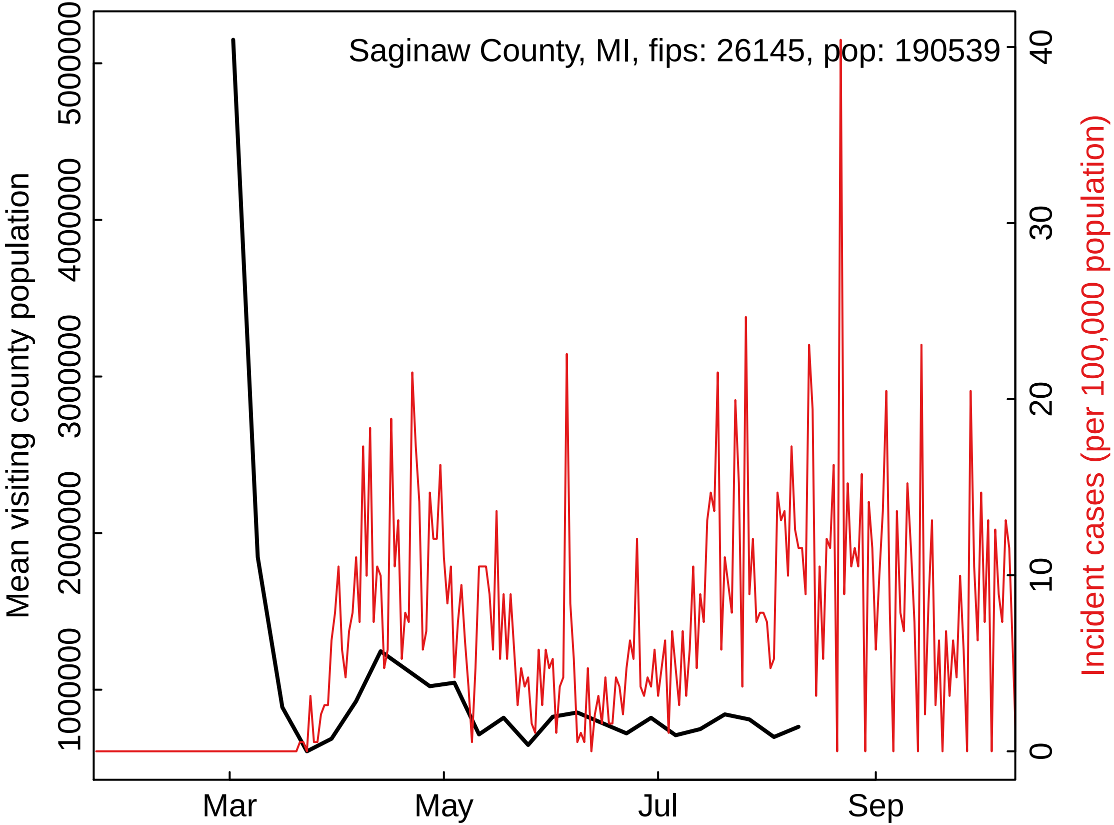

Saginaw County, MI, fips: 26145, pop: 190539
Mean visiting county population
10000000
20000000
30000000
40000000
50000000
Incident cases (per 100,000 population)
0
10
20
30
40
Mar
May
Jul
Sep

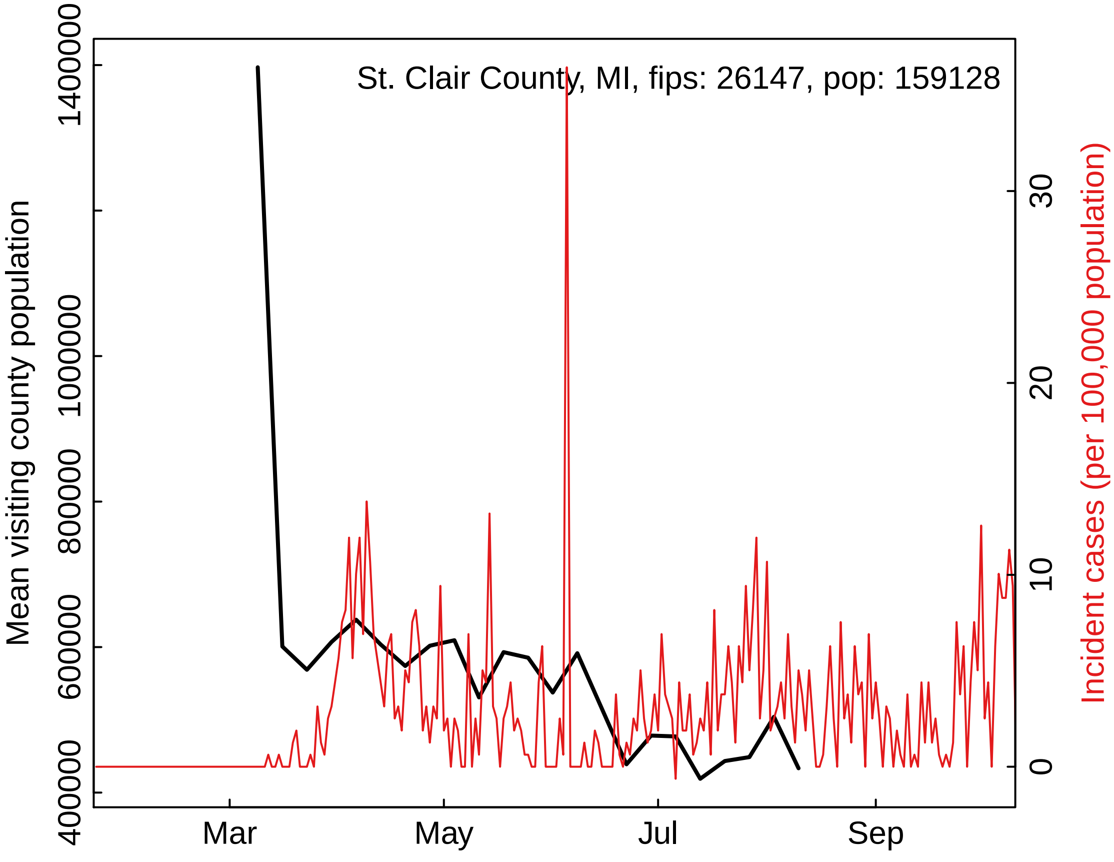

St. Clair County, MI, fips: 26147, pop: 159128
Mean visiting county population
400000
600000
800000
1000000
1400000
Incident cases (per 100,000 population)
0
10
20
30
Mar
May
Jul
Sep

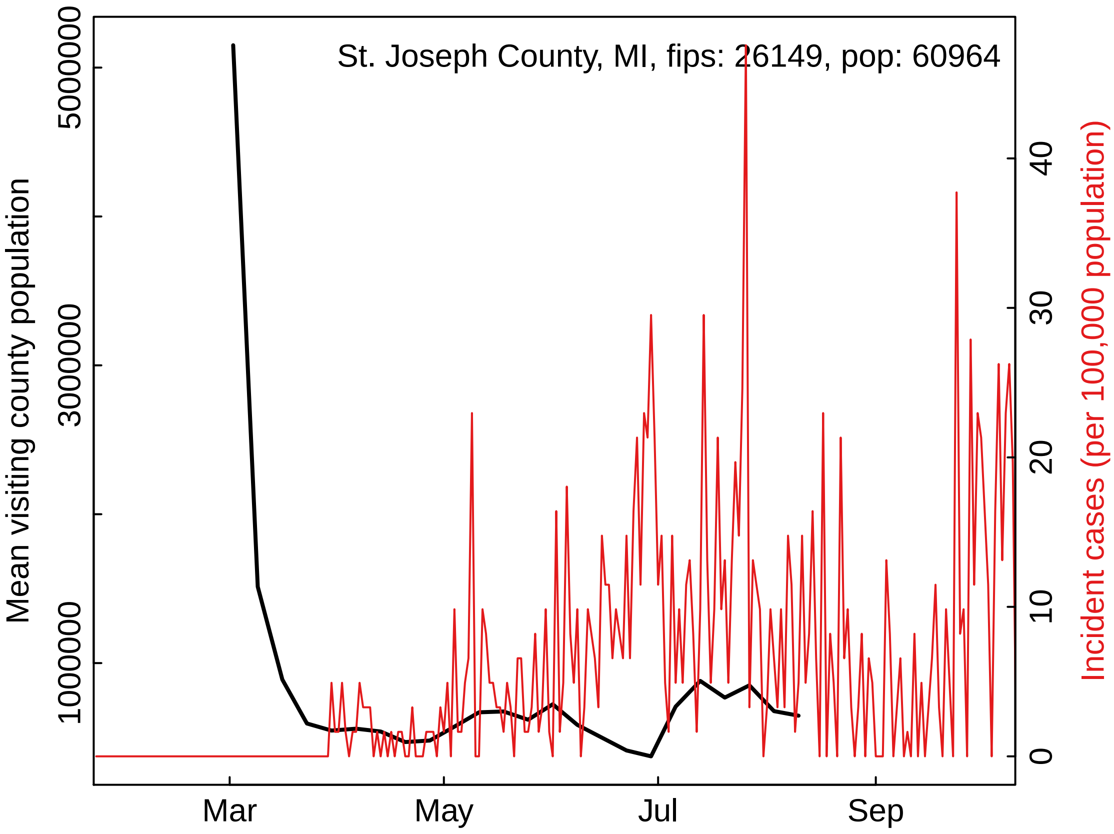

St. Joseph County, MI, fips: 26149, pop: 60964
Mean visiting county population
1000000
3000000
5000000
Incident cases (per 100,000 population)
0
10
20
30
40
Mar
May
Jul
Sep

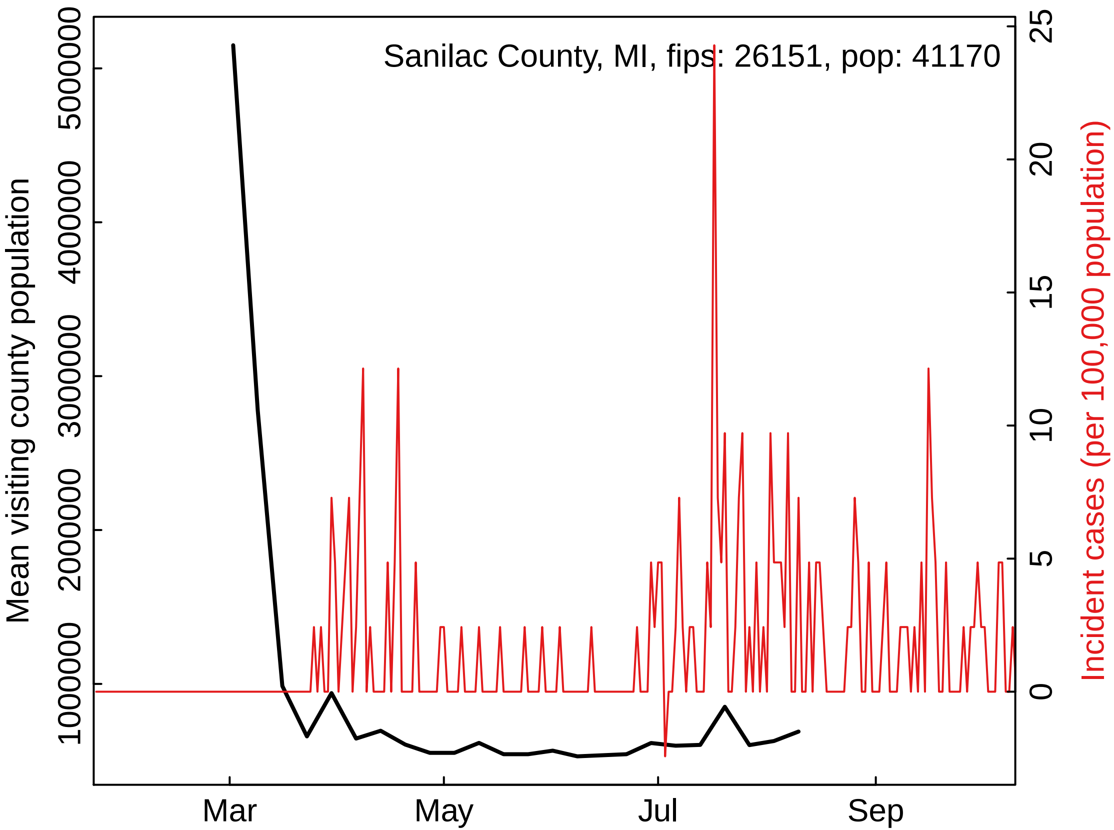

Sanilac County, MI, fips: 26151, pop: 41170
Mean visiting county population
1000000
2000000
3000000
4000000
5000000
Incident cases (per 100,000 population)
0
5
10
15
20
25
Mar
May
Jul
Sep

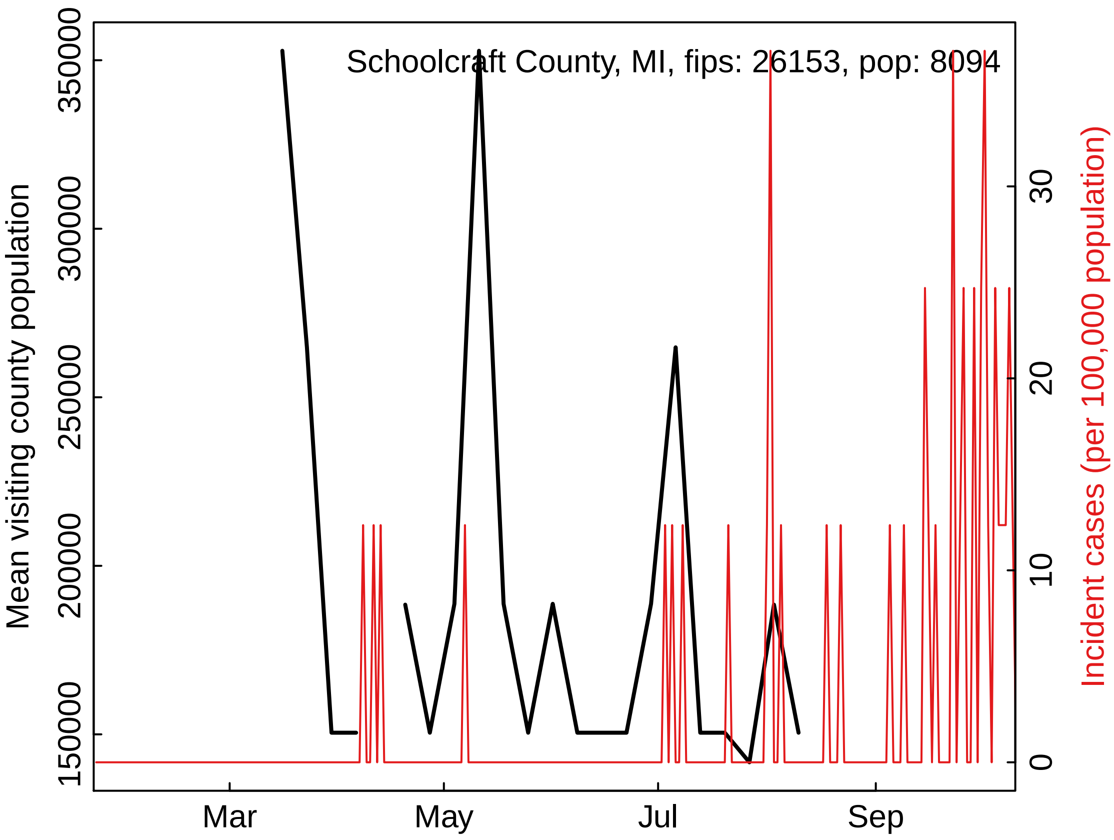

Schoolcraft County, MI, fips: 26153, pop: 8094
Mean visiting county population
150000
200000
250000
300000
350000
Incident cases (per 100,000 population)
0
10
20
30
Mar
May
Jul
Sep

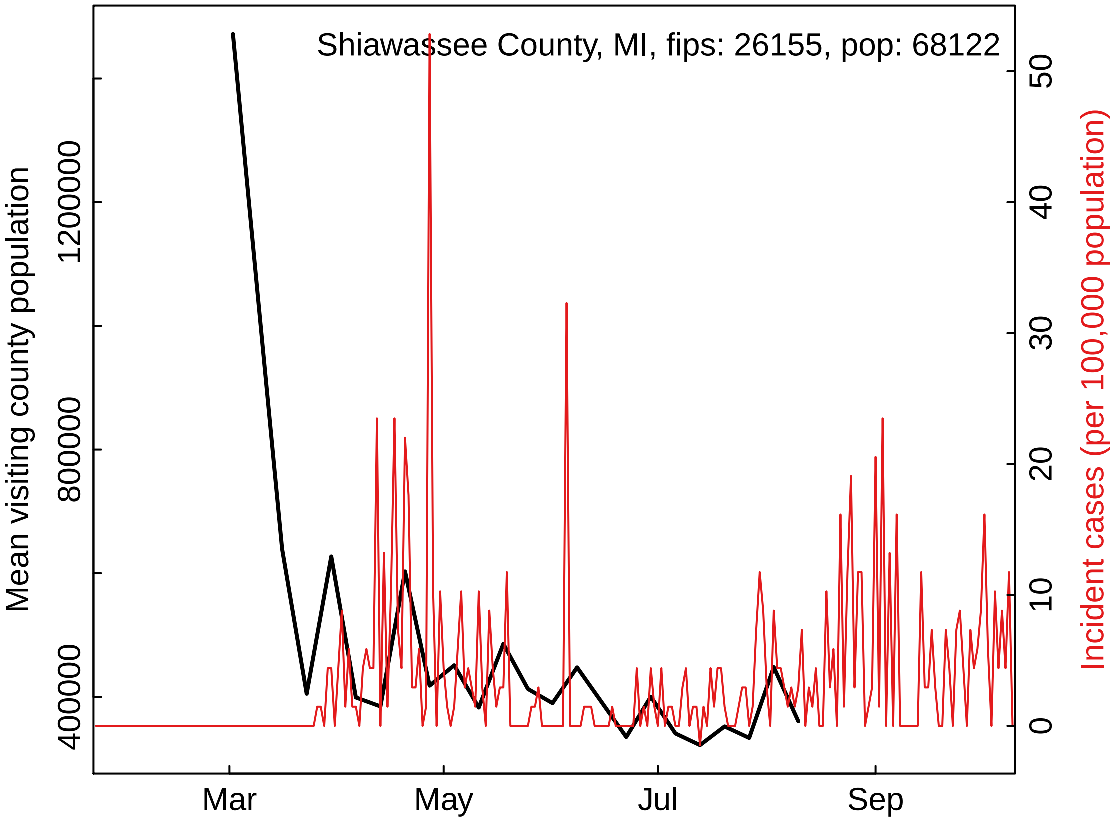

Shiawassee County, MI, fips: 26155, pop: 68122
Mean visiting county population
400000
800000
1200000
Incident cases (per 100,000 population)
0
10
20
30
40
50
Mar
May
Jul
Sep

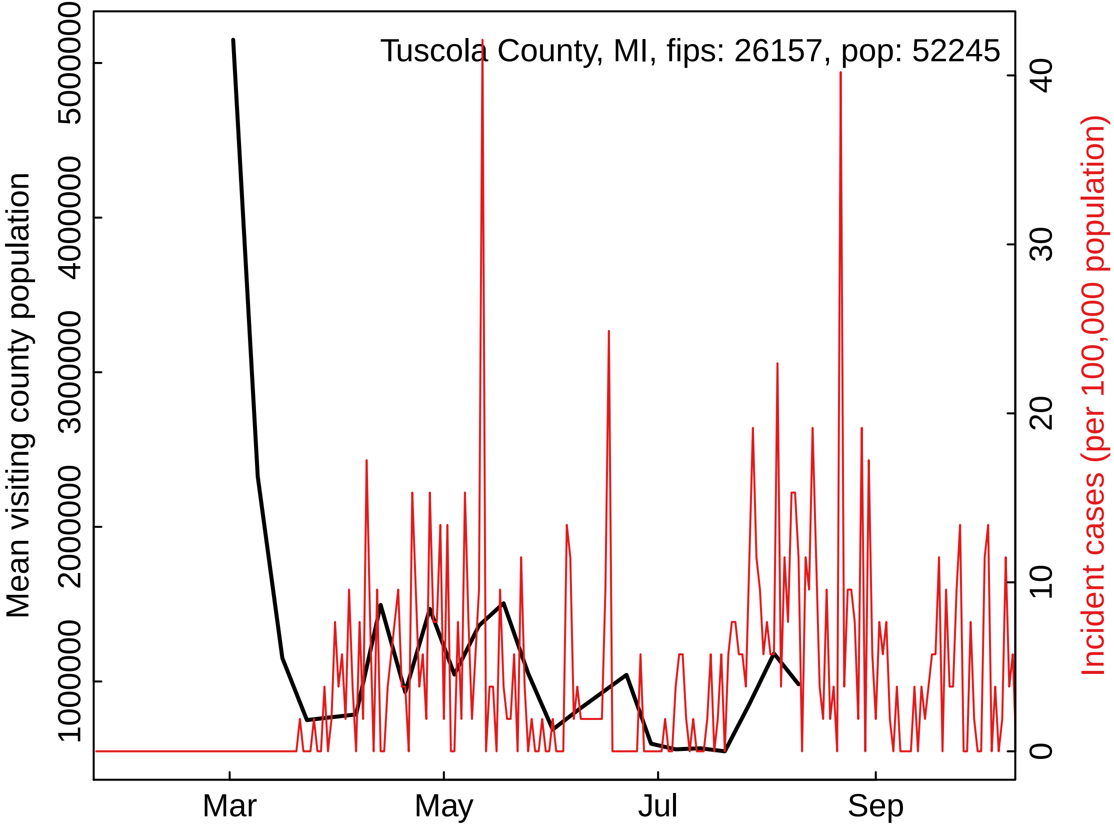

Tuscola County, MI, fips: 26157, pop: 52245
Mean visiting county population
1000000
2000000
3000000
4000000
5000000
Incident cases (per 100,000 population)
0
10
20
30
40
Mar
May
Jul
Sep

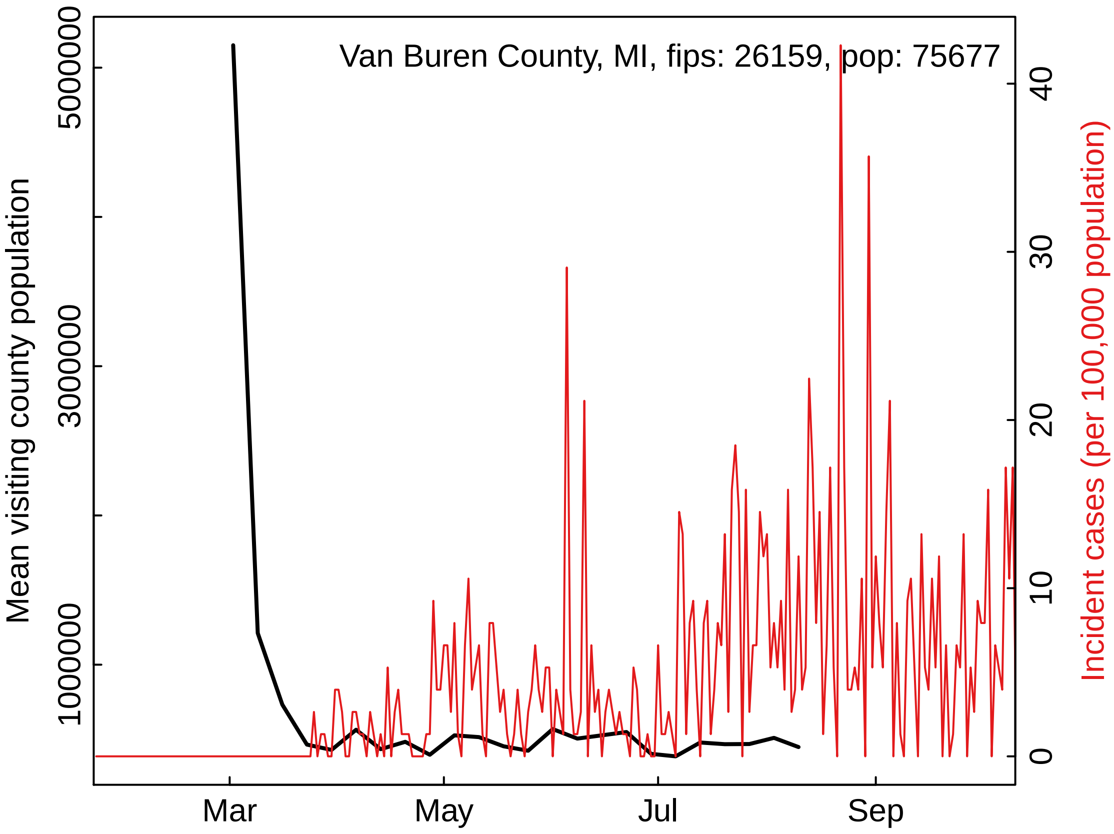
Van Buren County, MI, fips: 26159, pop: 75677
Mean visiting county population
Incident cases (per 100,000 population)
1000000
3000000
5000000
0
10
20
30
40
Mar
May
Jul
Sep

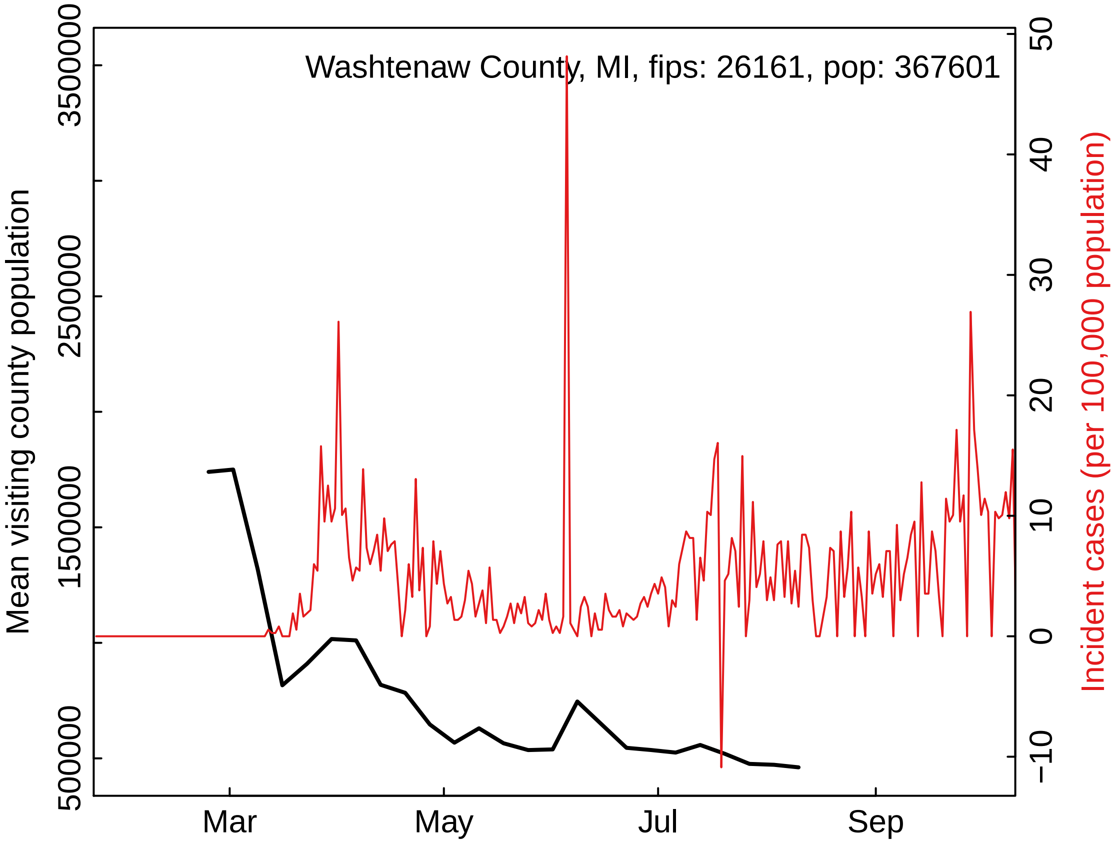

Washtenaw County, MI, fips: 26161, pop: 367601
Mean visiting county population
500000
1500000
2500000
3500000
Incident cases (per 100,000 population)
−10
0
10
20
30
40
50
Mar
May
Jul
Sep

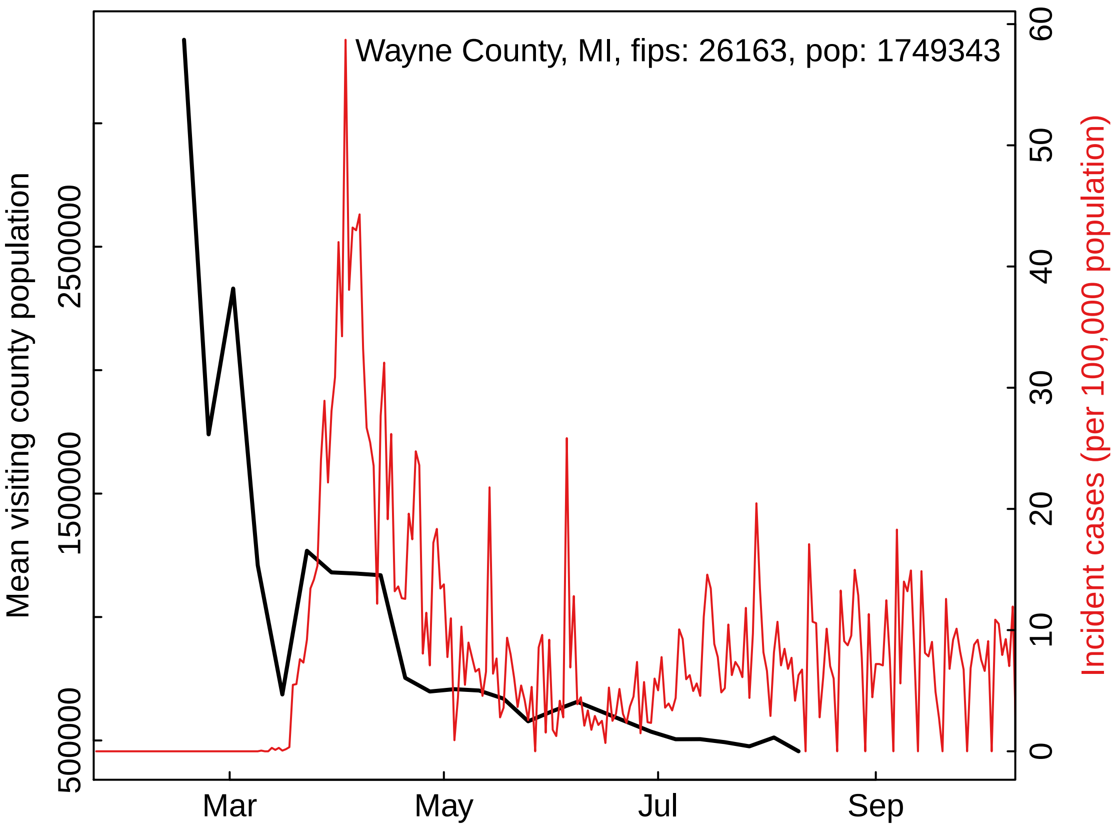

Wayne County, MI, fips: 26163, pop: 1749343
Mean visiting county population
500000
1500000
2500000
Mar
May
Jul
Sep
0
10
20
30
40
50
60
Incident cases (per 100,000 population)

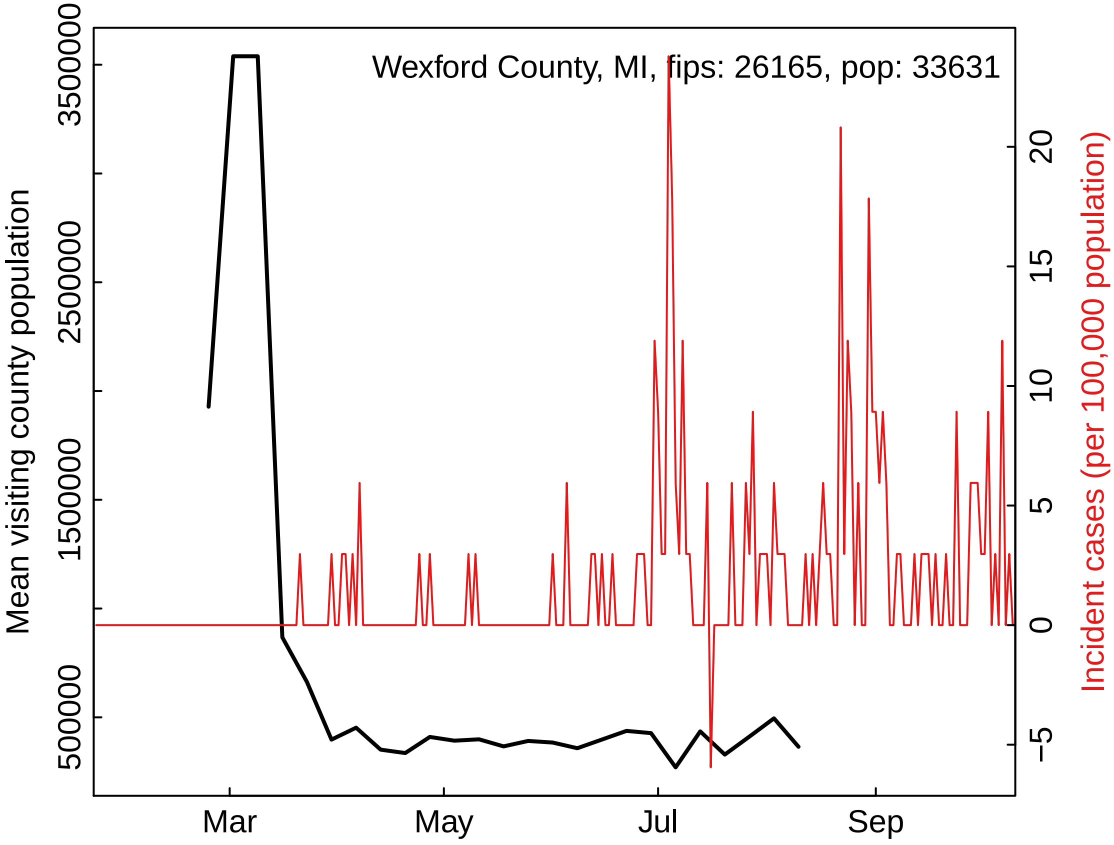

Wexford County, MI, fips: 26165, pop: 33631
Mean visiting county population
500000
1500000
2500000
3500000
Incident cases (per 100,000 population)
−5
0
5
10
15
20
Mar
May
Jul
Sep

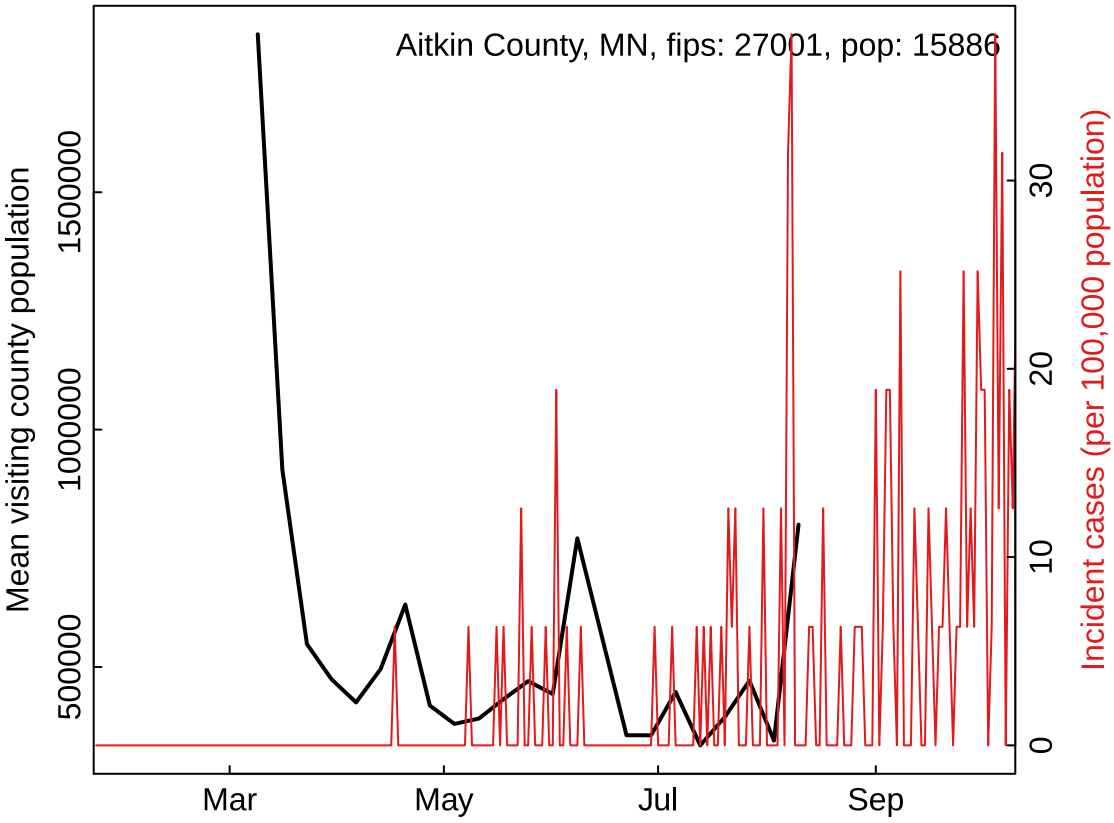

Aitkin County, MN, fips: 27001, pop: 15886
Mean visiting county population
500000
1000000
1500000
Incident cases (per 100,000 population)
0
10
20
30
Mar
May
Jul
Sep

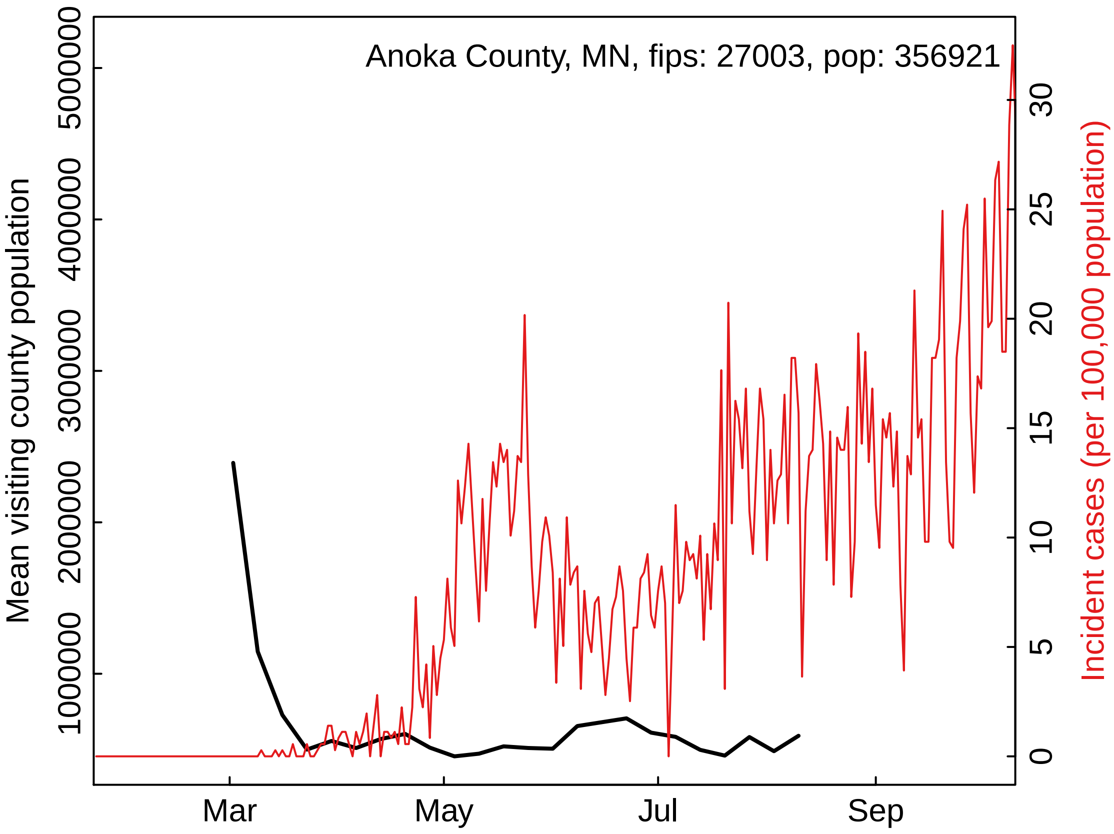

Anoka County, MN, fips: 27003, pop: 356921
Mean visiting county population
1000000
2000000
3000000
4000000
5000000
Incident cases (per 100,000 population)
0
5
10
15
20
25
30
Mar
May
Jul
Sep

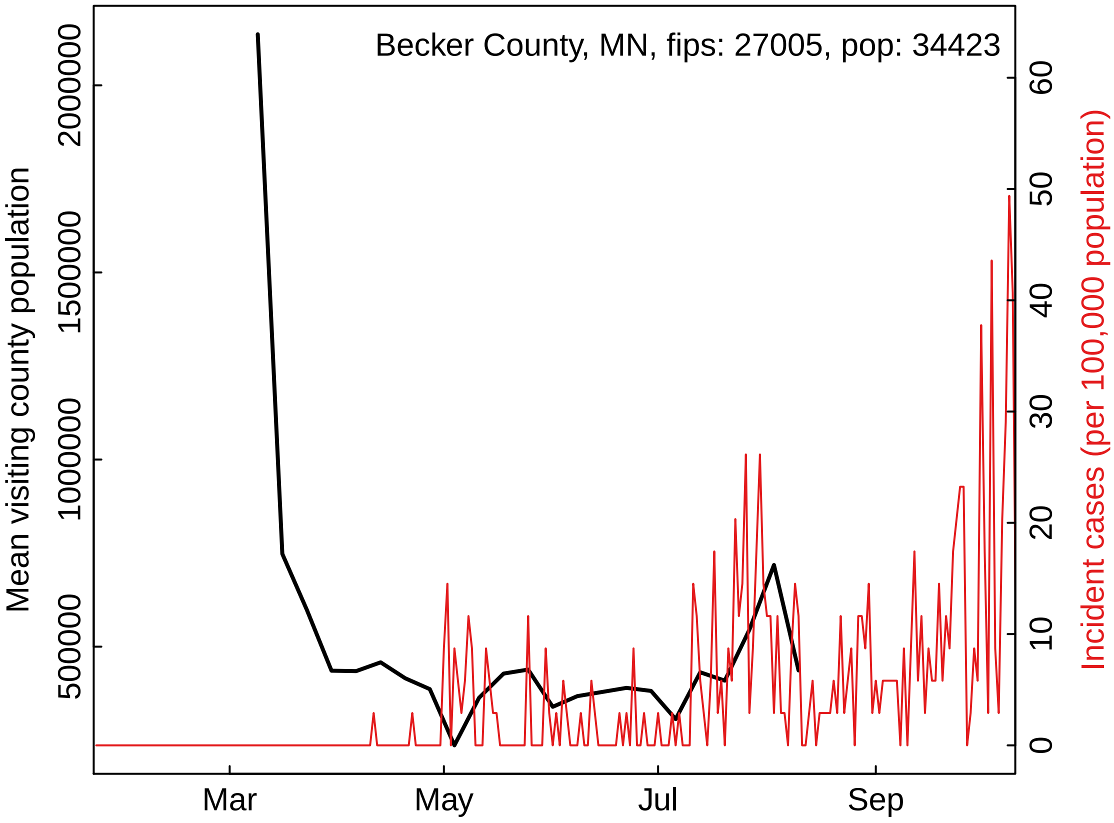
Becker County, MN, fips: 27005, pop: 34423
Mean visiting county population
500000
1000000
1500000
2000000
Incident cases (per 100,000 population)
0
10
20
30
40
50
60
Mar
May
Jul
Sep

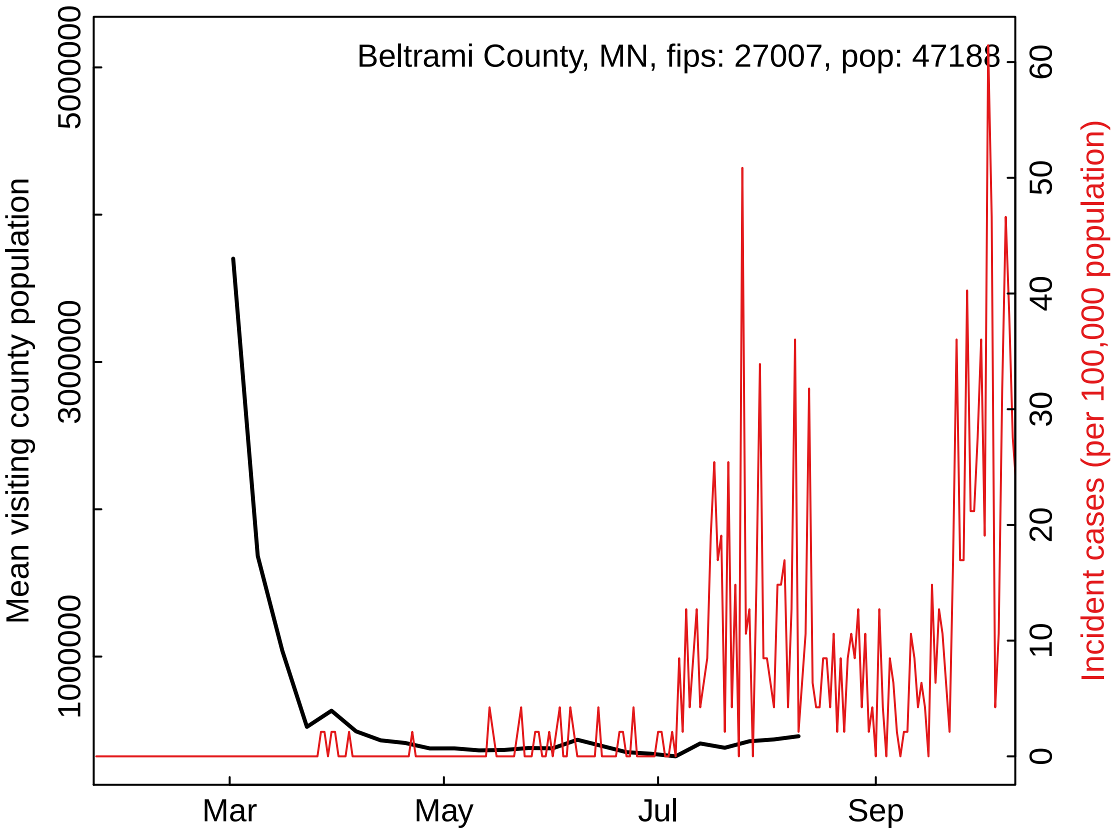
Beltrami County, MN, fips: 27007, pop: 47188
Mean visiting county population
1000000
3000000
5000000
Incident cases (per 100,000 population)
0
10
20
30
40
50
60
Mar
May
Jul
Sep

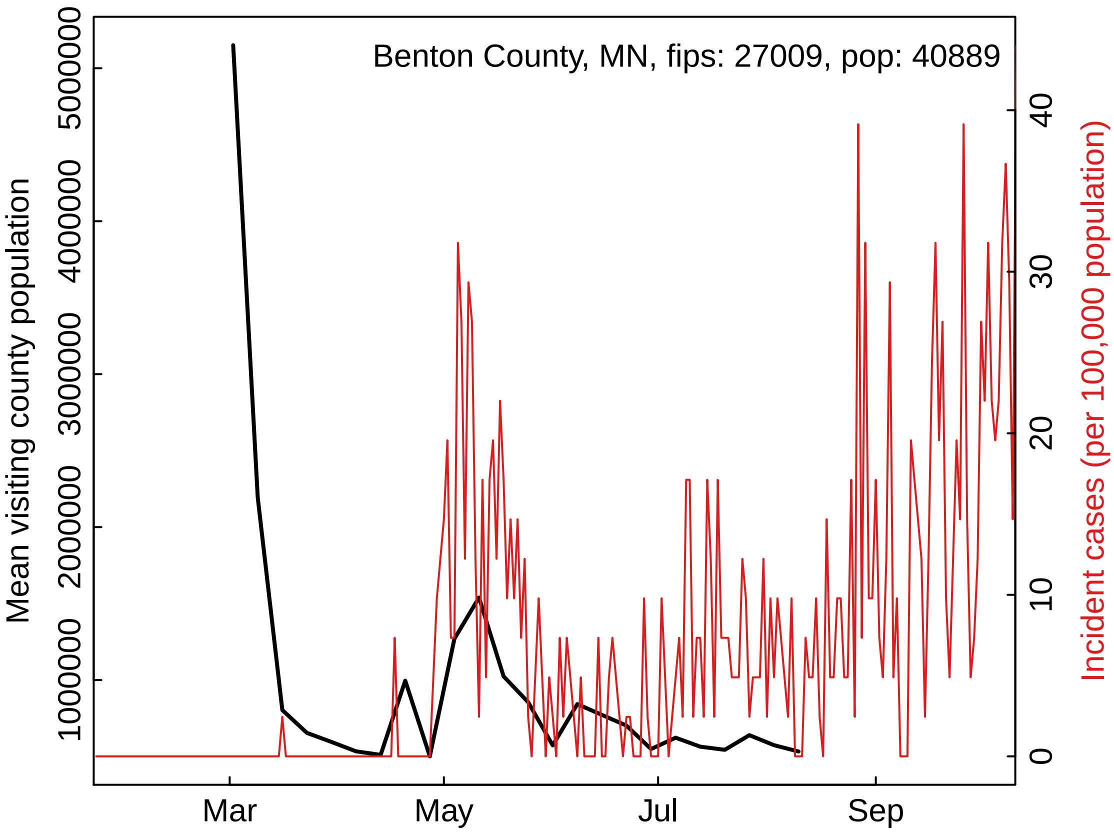

Benton County, MN, fips: 27009, pop: 40889
Mean visiting county population
Incident cases (per 100,000 population)
0
1000000
2000000
3000000
4000000
5000000
0
10
20
30
40
Mar
May
Jul
Sep

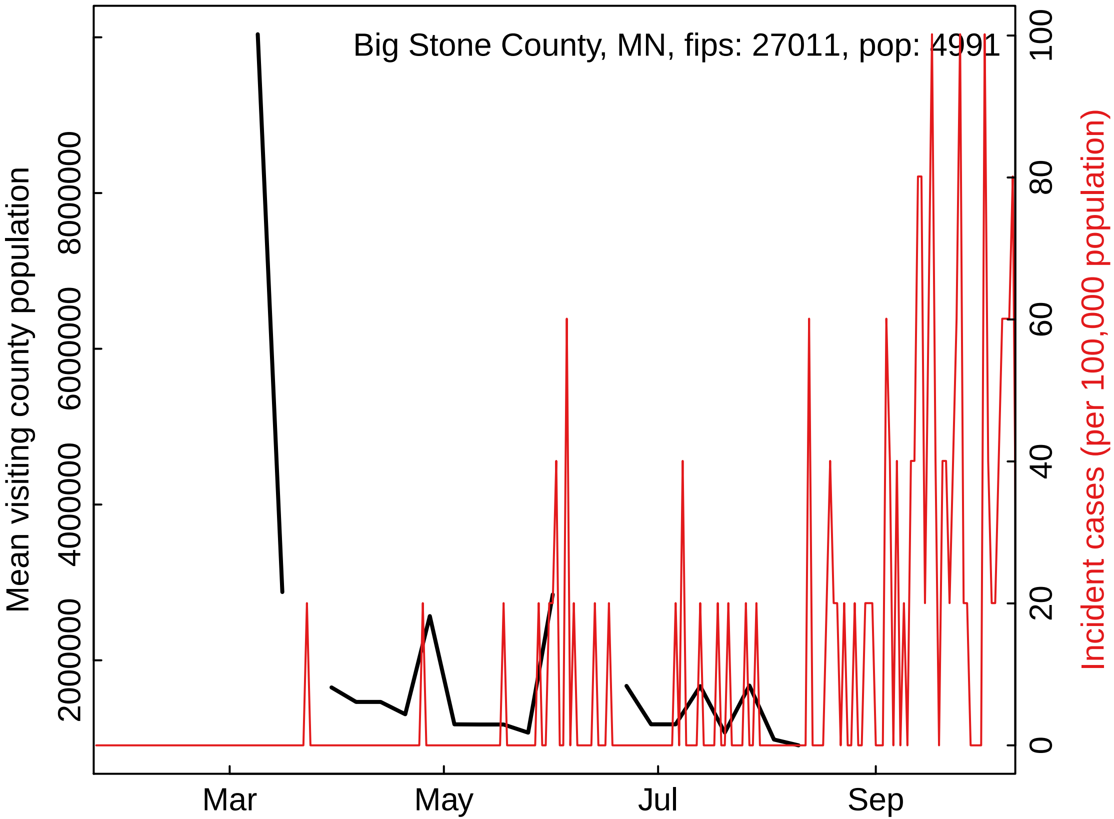
Big Stone County, MN, fips: 27011, pop: 4991
Mean visiting county population
20000000
40000000
60000000
80000000
Incident cases (per 100,000 population)
0
20
40
60
80
100
Mar
May
Jul
Sep

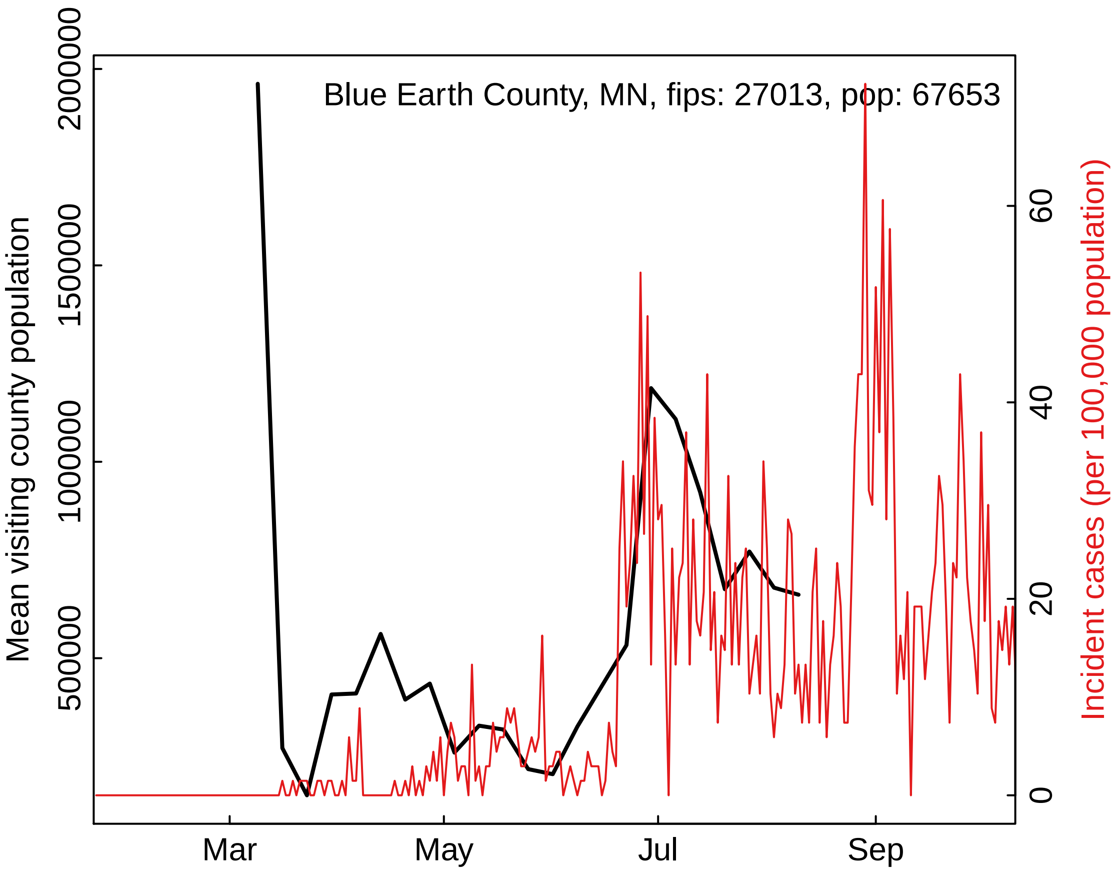

Blue Earth County, MN, fips: 27013, pop: 67653
Mean visiting county population
500000
1000000
1500000
2000000
Incident cases (per 100,000 population)
0
20
40
60
Mar
May
Jul
Sep

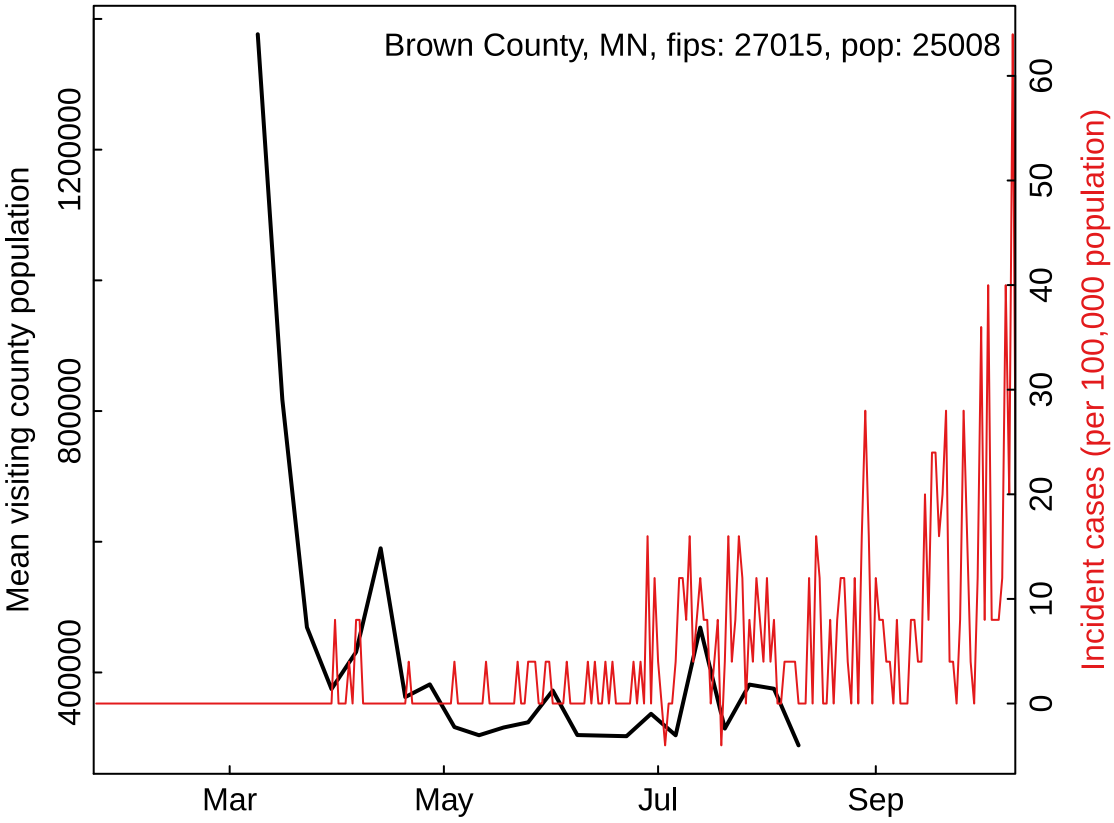

Brown County, MN, fips: 27015, pop: 25008
Mean visiting county population
400000
800000
1200000
Incident cases (per 100,000 population)
0
10
20
30
40
50
60
Mar
May
Jul
Sep

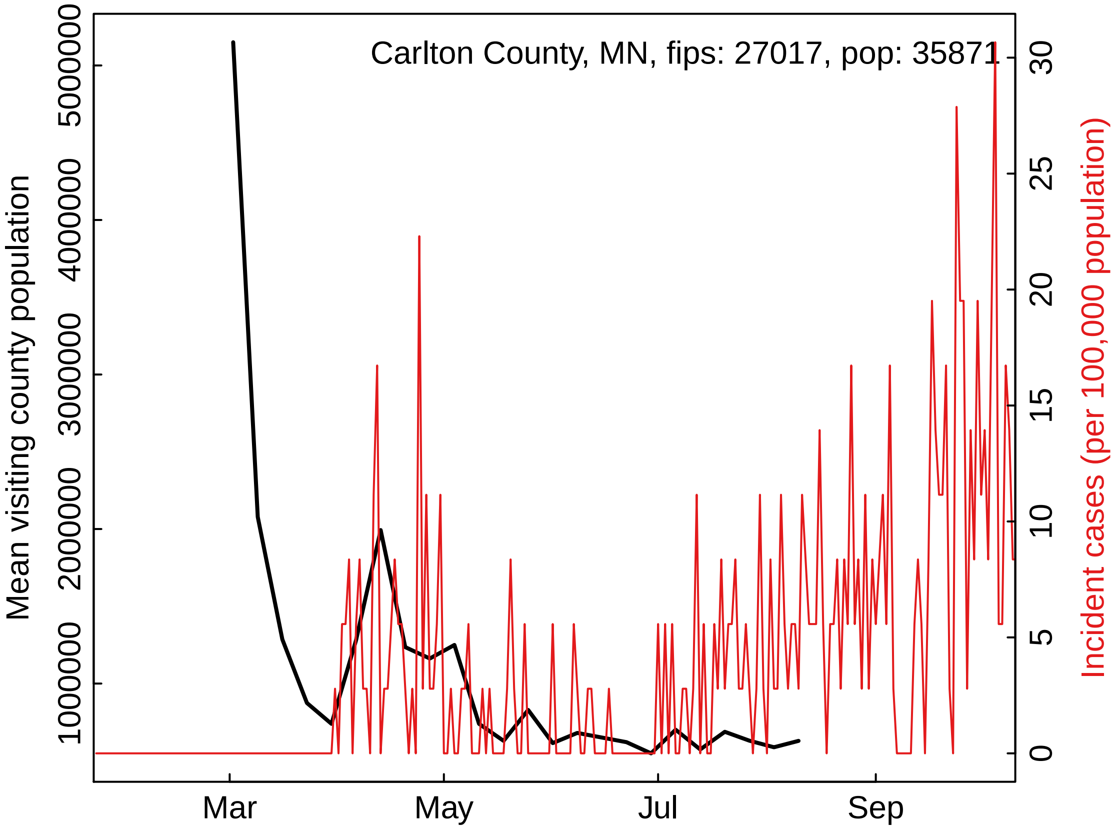

Carlton County, MN, fips: 27017, pop: 35871
Mean visiting county population
10000000
20000000
30000000
40000000
50000000
Incident cases (per 100,000 population)
0
5
10
15
20
25
30
Mar
May
Jul
Sep

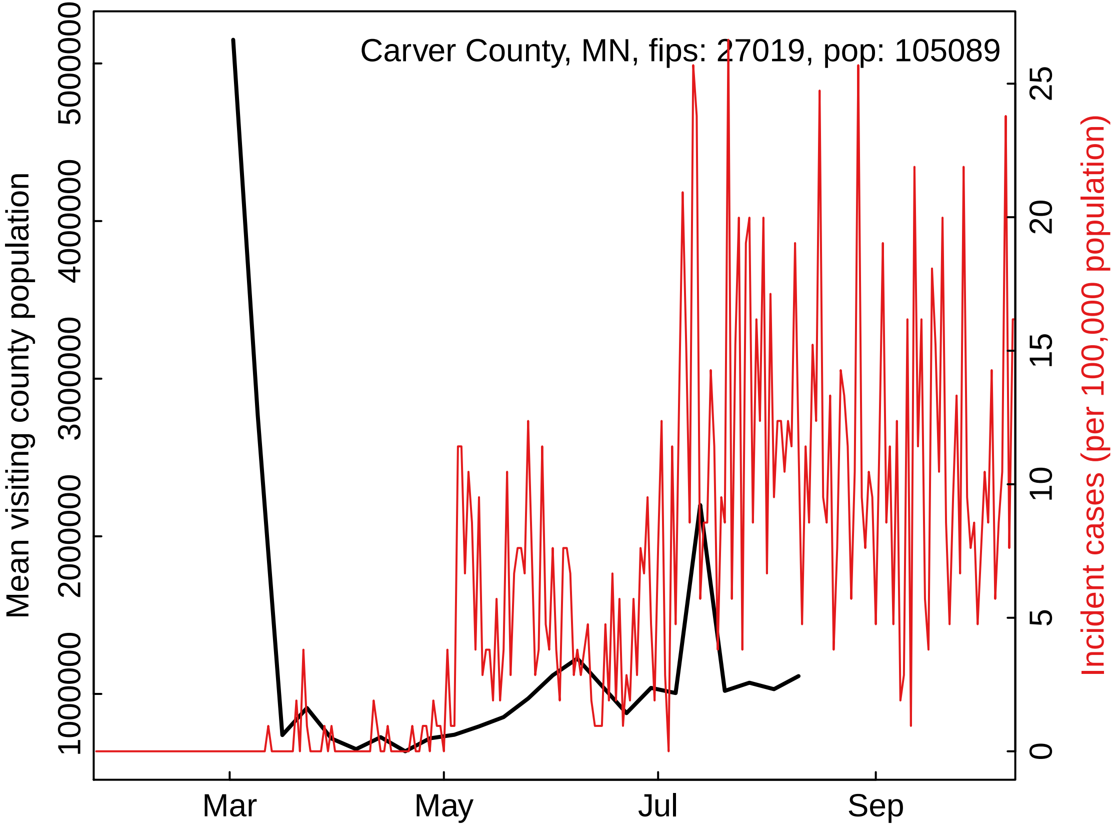

Carver County, MN, fips: 27019, pop: 105089
Mean visiting county population
1000000
2000000
3000000
4000000
5000000
Incident cases (per 100,000 population)
0
5
10
15
20
25
Mar
May
Jul
Sep

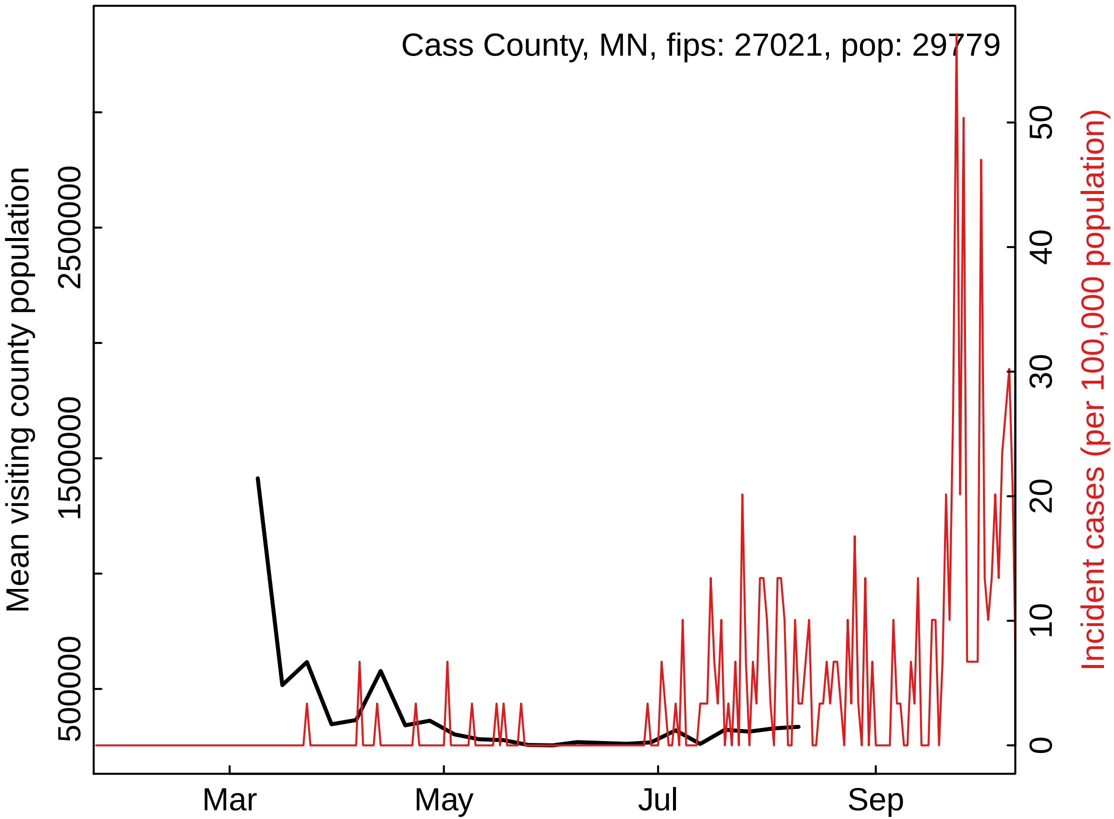

Cass County, MN, fips: 27021, pop: 29779
Mean visiting county population
500000
1500000
2500000
Mar
May
Jul
Sep
Incident cases (per 100,000 population)
0
10
20
30
40
50

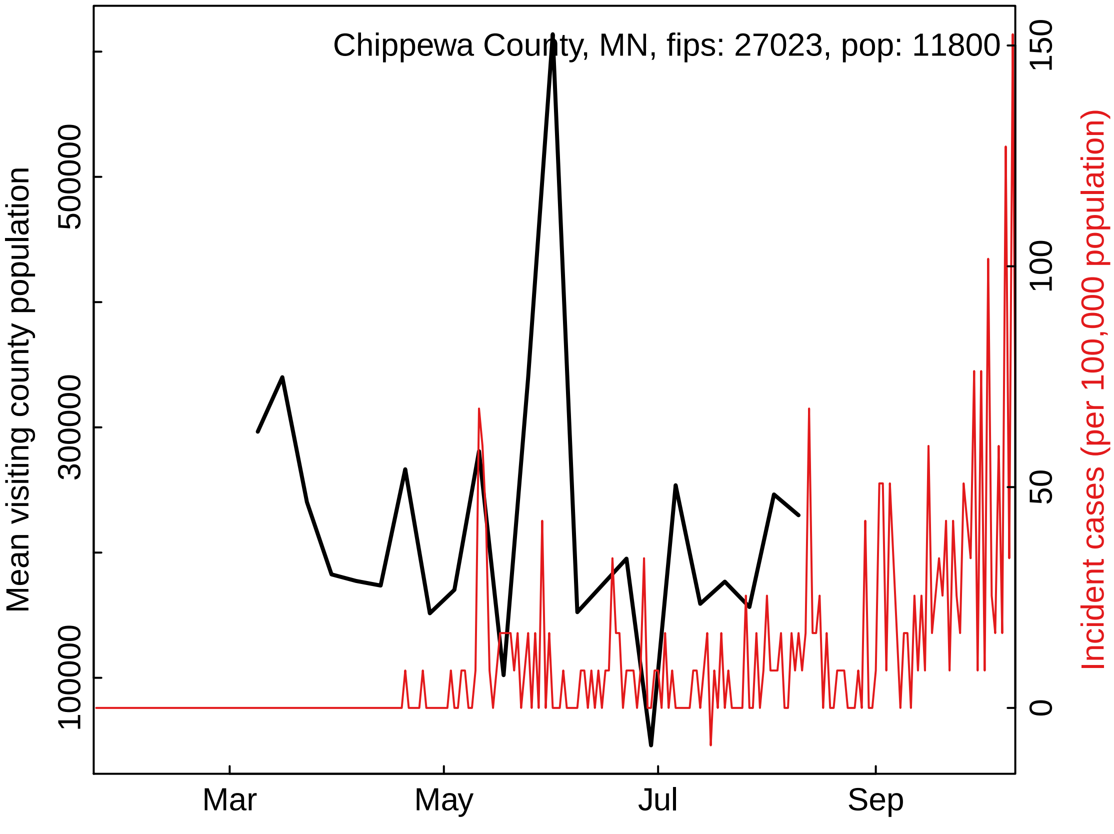
Chippewa County, MN, fips: 27023, pop: 11800
Mean visiting county population
100000
300000
500000
Incident cases (per 100,000 population)
0
50
100
150
Mar
May
Jul
Sep

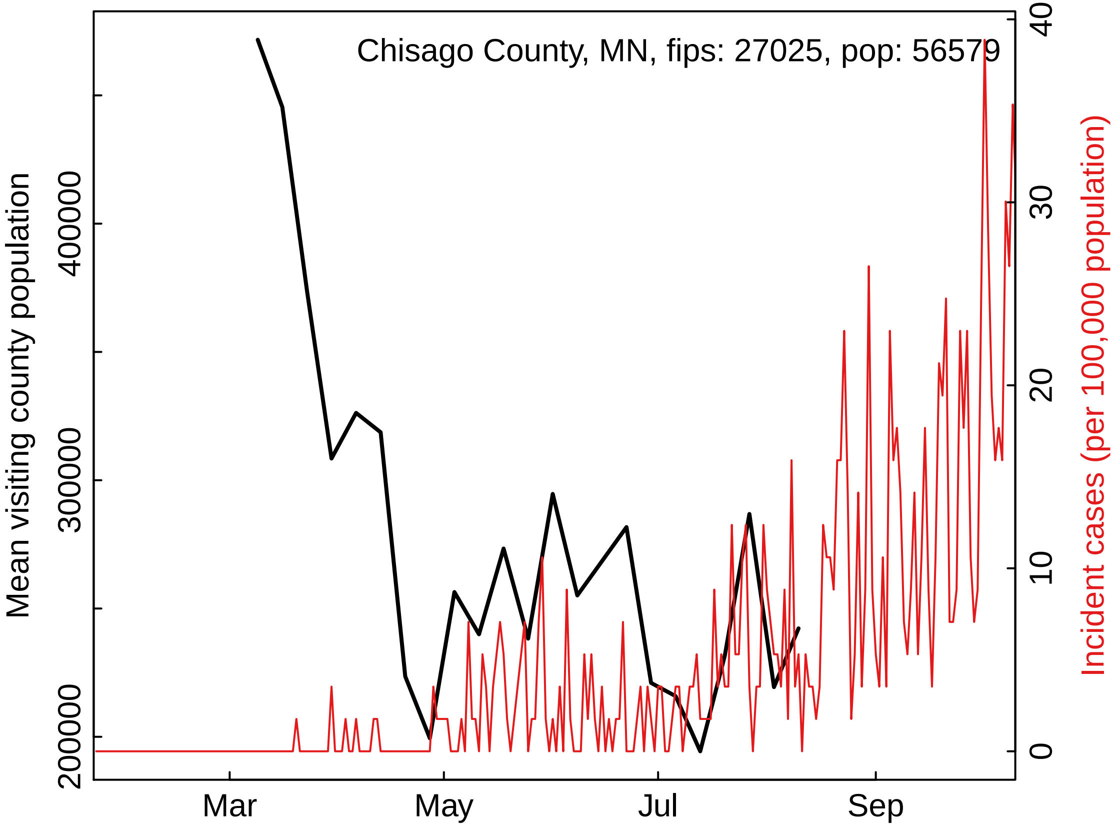

Chisago County, MN, fips: 27025, pop: 56579
Mean visiting county population
Incident cases (per 100,000 population)
200000
300000
400000
0
10
20
30
40
Mar
May
Jul
Sep

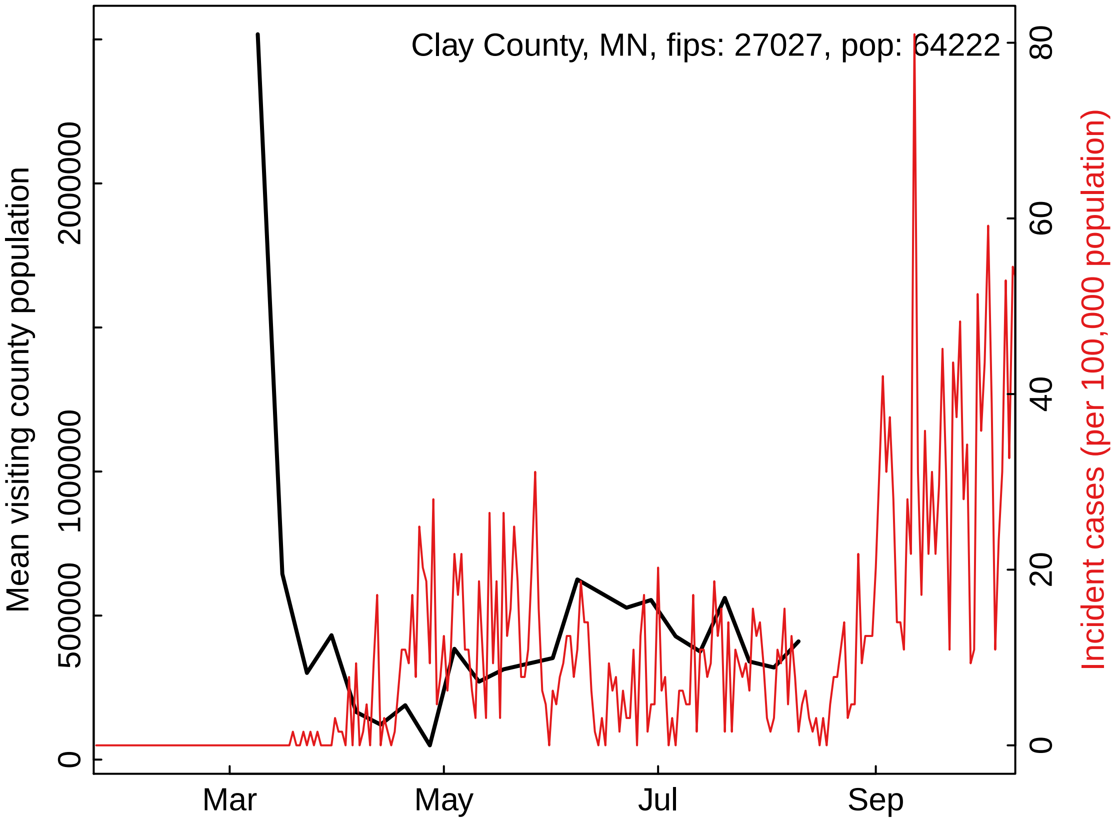

Clay County, MN, fips: 27027, pop: 64222
Mean visiting county population
Incident cases (per 100,000 population)
0
500000
1000000
2000000
0
20
40
60
80
Mar
May
Jul
Sep

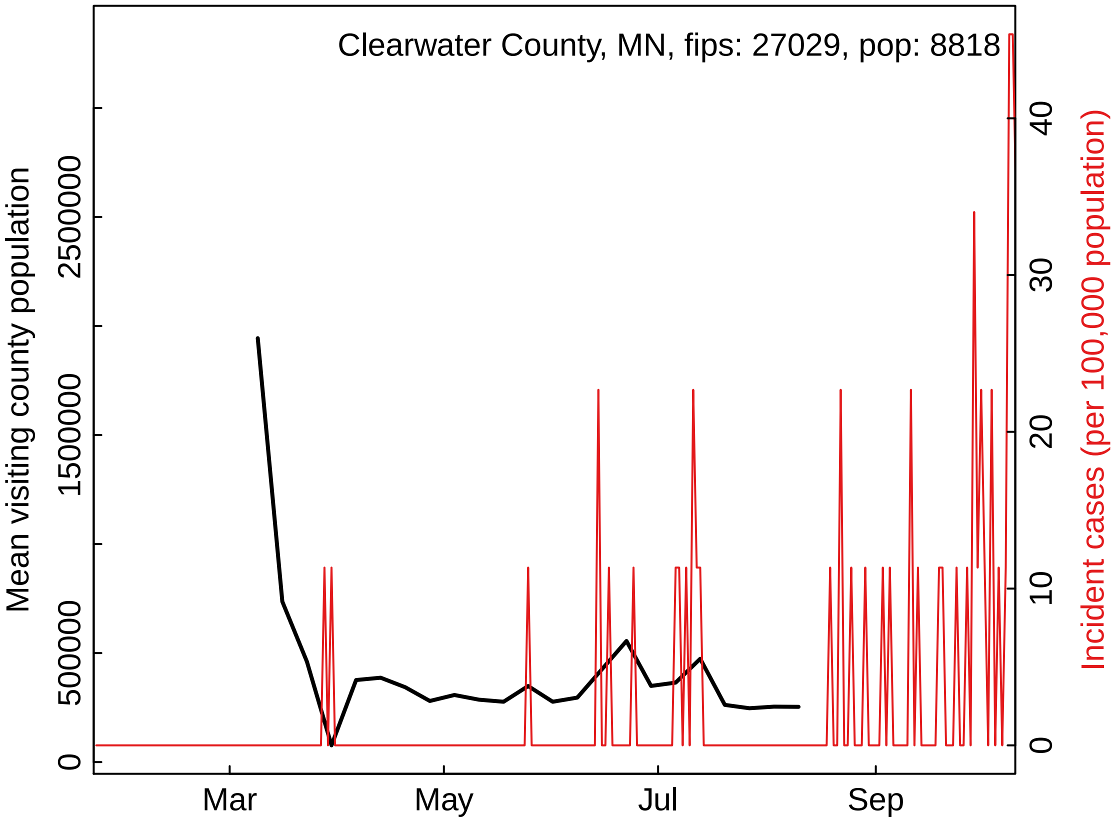

Clearwater County, MN, fips: 27029, pop: 8818
Mean visiting county population
0
500000
1500000
2500000
Incident cases (per 100,000 population)
0
10
20
30
40
Mar
May
Jul
Sep

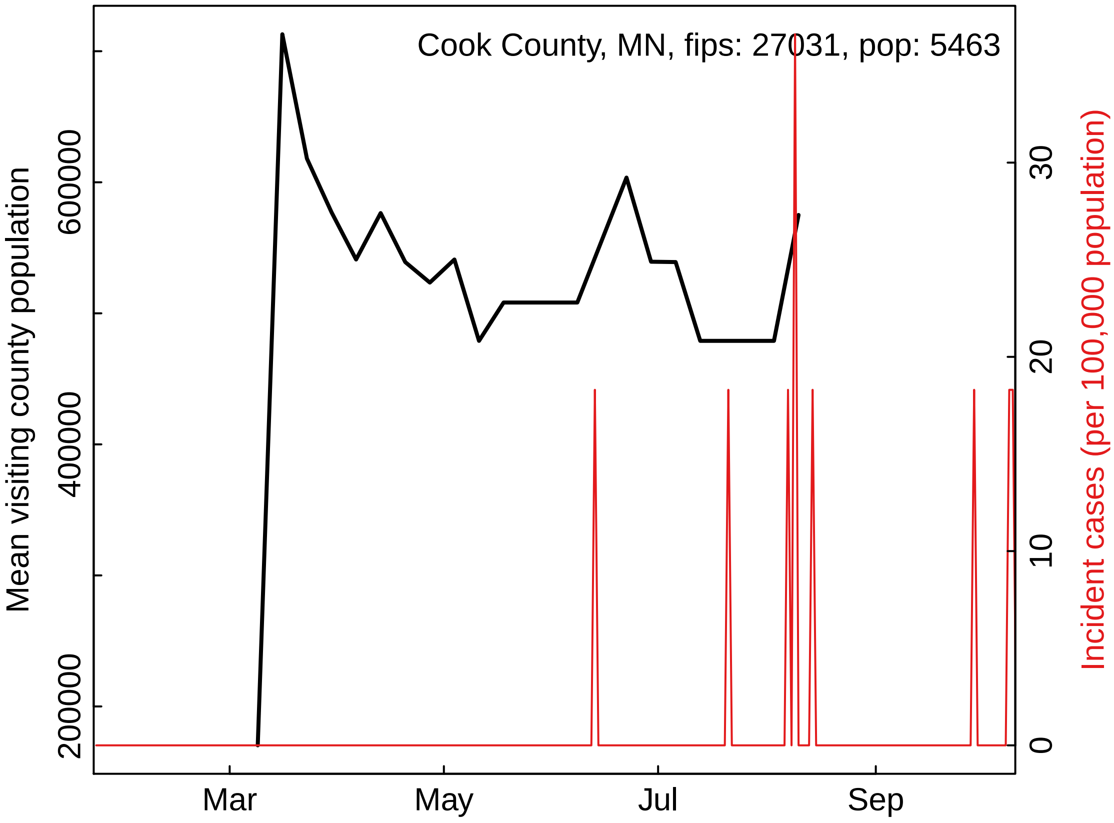

Cook County, MN, fips: 27031, pop: 5463
Mean visiting county population
200000
400000
600000
Incident cases (per 100,000 population)
0
10
20
30
Mar
May
Jul
Sep

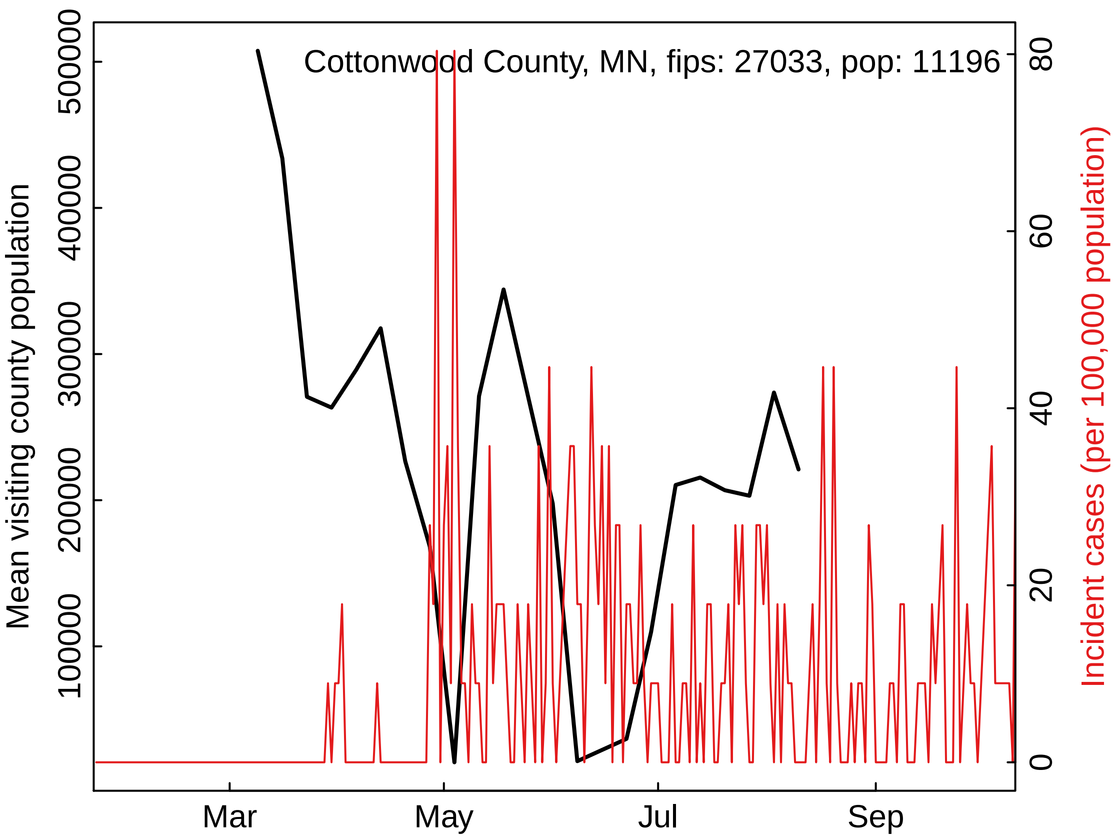
Cottonwood County, MN, fips: 27033, pop: 11196
Mean visiting county population
100000
200000
300000
400000
500000
Incident cases (per 100,000 population)
0
20
40
60
80
Mar
May
Jul
Sep

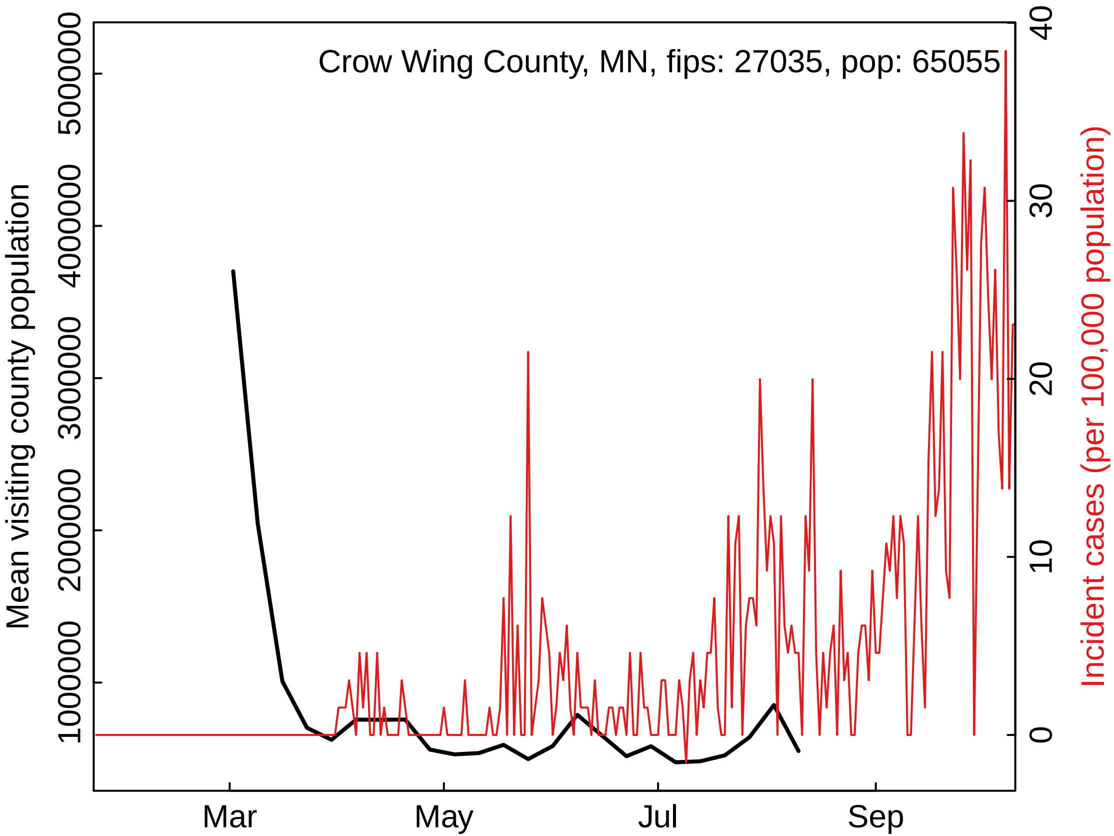

Crow Wing County, MN, fips: 27035, pop: 65055
Mean visiting county population
Incident cases (per 100,000 population)
1000000
2000000
3000000
4000000
5000000
0
10
20
30
40
Mar
May
Jul
Sep

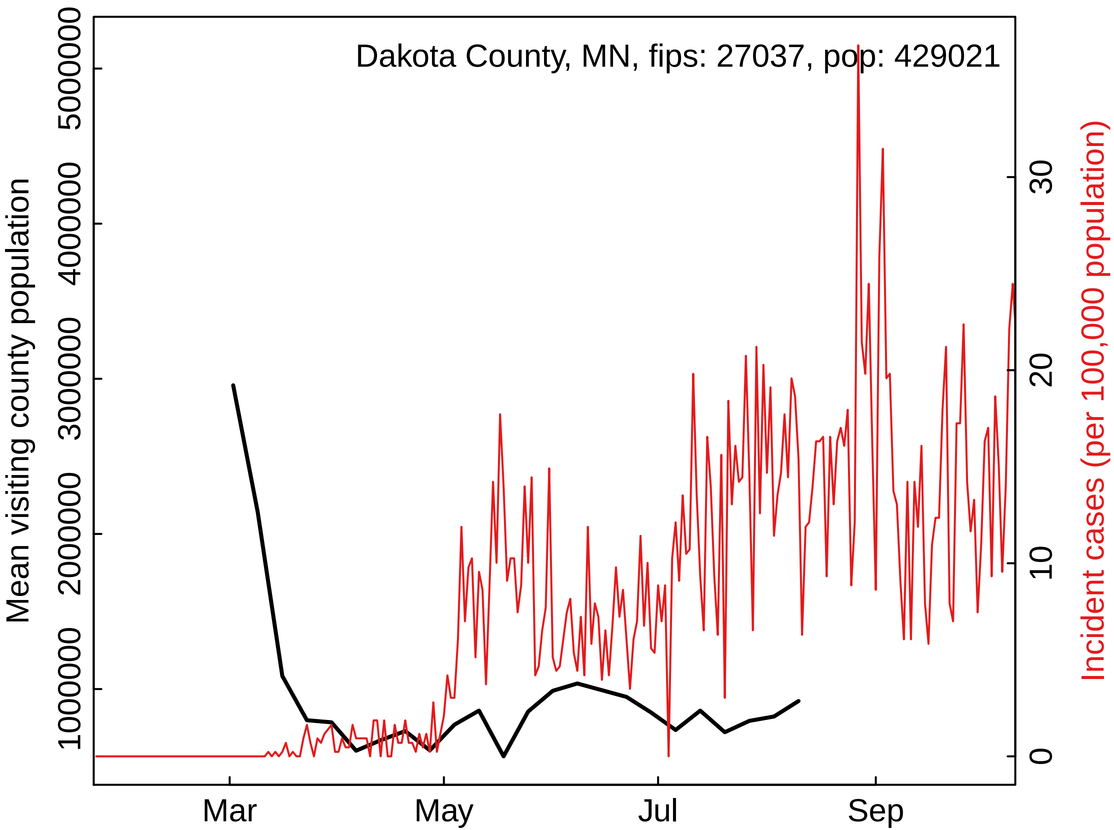

Dakota County, MN, fips: 27037, pop: 429021
Mean visiting county population
Incident cases (per 100,000 population)
10000000
20000000
30000000
40000000
50000000
0
10
20
30
Mar
May
Jul
Sep

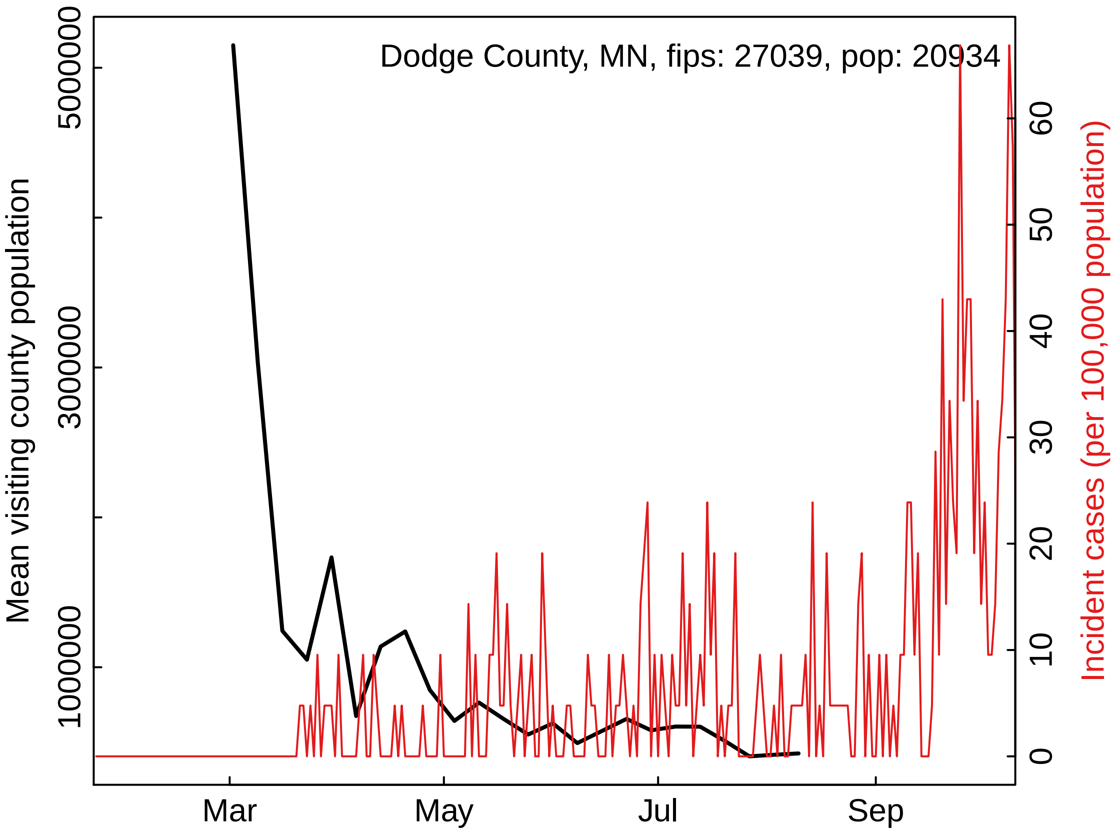
Dodge County, MN, fips: 27039, pop: 20934
Mean visiting county population
1000000
3000000
5000000
Incident cases (per 100,000 population)
0
10
20
30
40
50
60
Mar
May
Jul
Sep

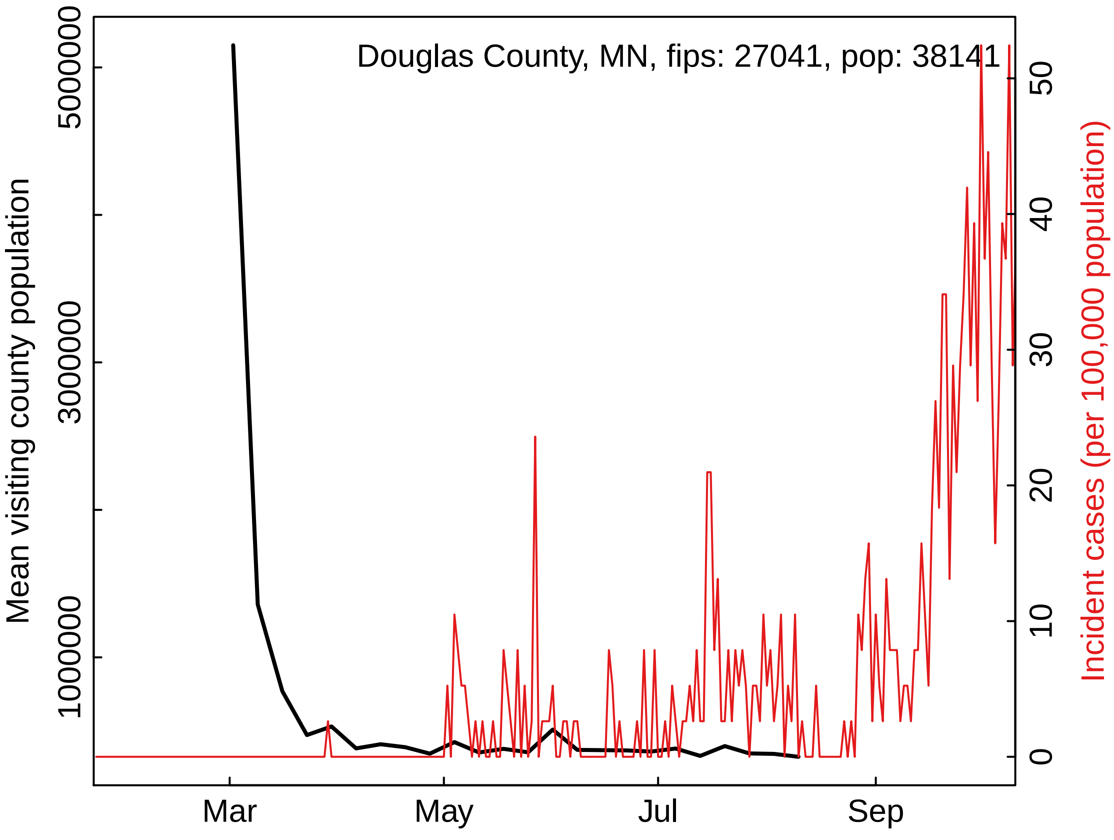

Douglas County, MN, fips: 27041, pop: 38141
Mean visiting county population
1000000
3000000
5000000
Incident cases (per 100,000 population)
0
10
20
30
40
50
Mar
May
Jul
Sep

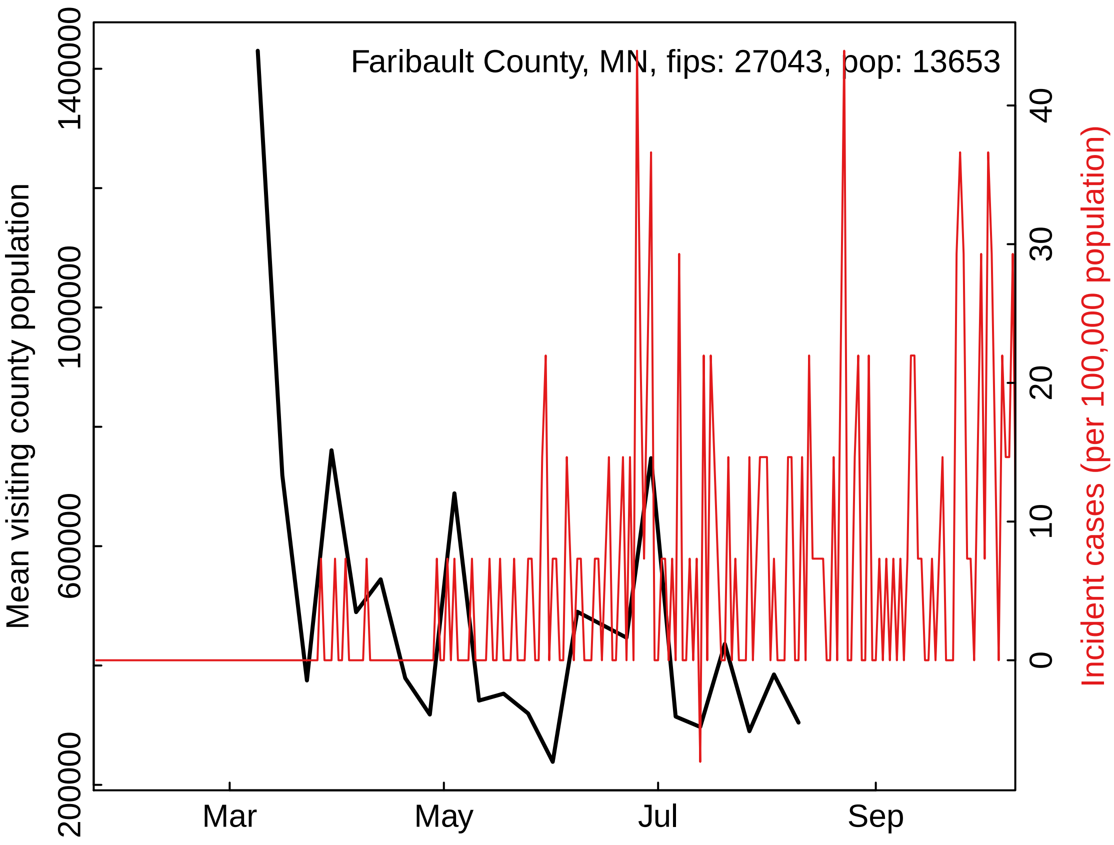

Faribault County, MN, fips: 27043, pop: 13653
Mean visiting county population
Incident cases (per 100,000 population)
2000000
6000000
10000000
14000000
0
10
20
30
40
Mar
May
Jul
Sep

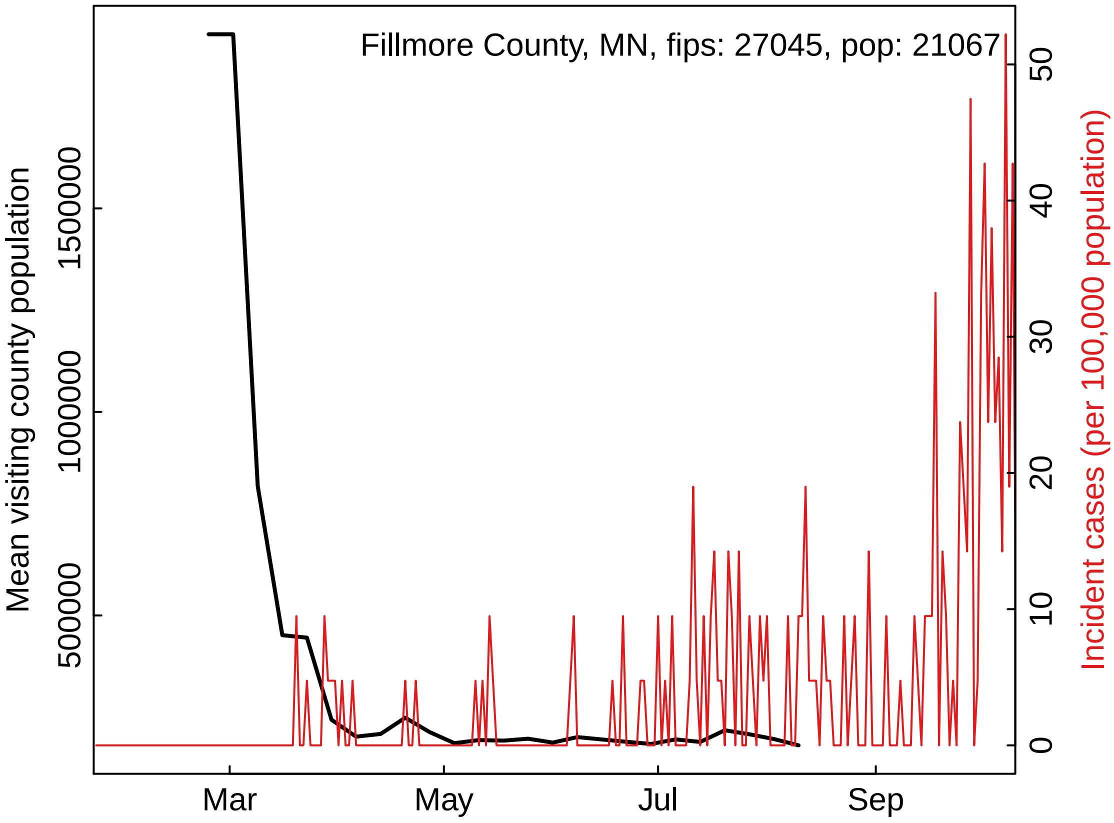
Fillmore County, MN, fips: 27045, pop: 21067
Mean visiting county population
500000
1000000
1500000
Incident cases (per 100,000 population)
0
10
20
30
40
50
Mar
May
Jul
Sep

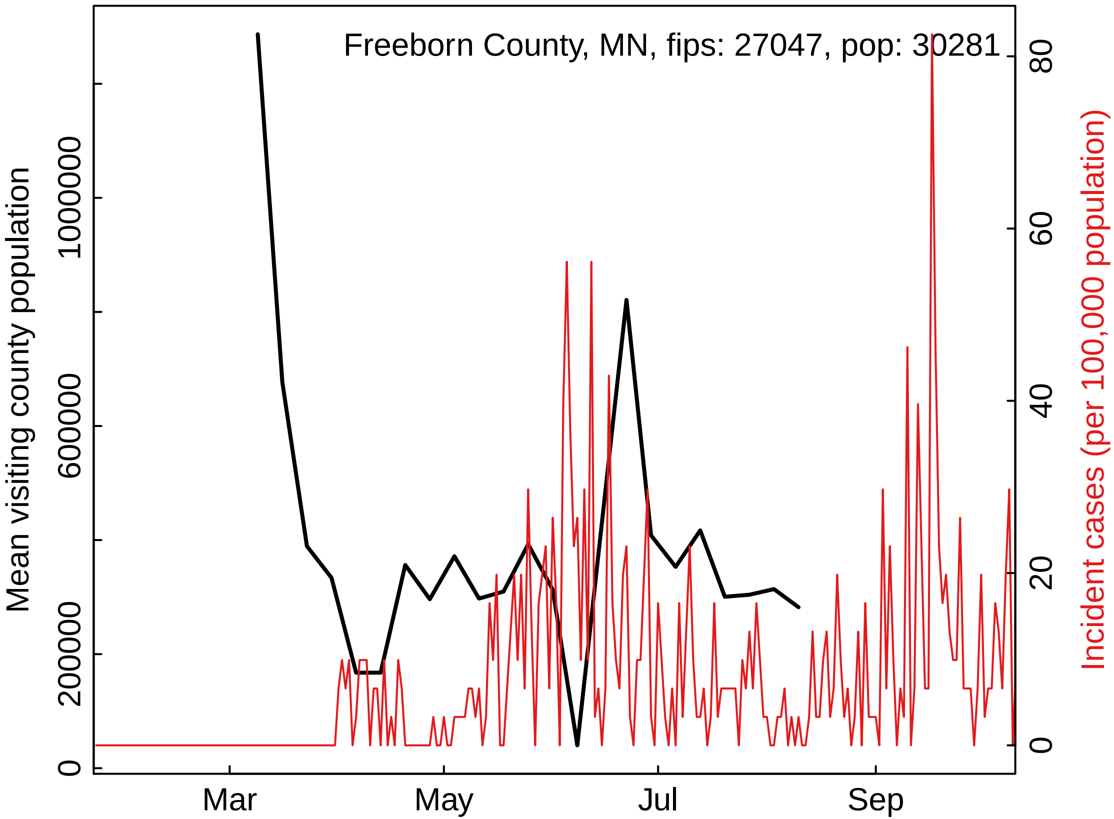

Freeborn County, MN, fips: 27047, pop: 30281
Mean visiting county population
Incident cases (per 100,000 population)
0
200000
600000
1000000
0
20
40
60
80
Mar
May
Jul
Sep

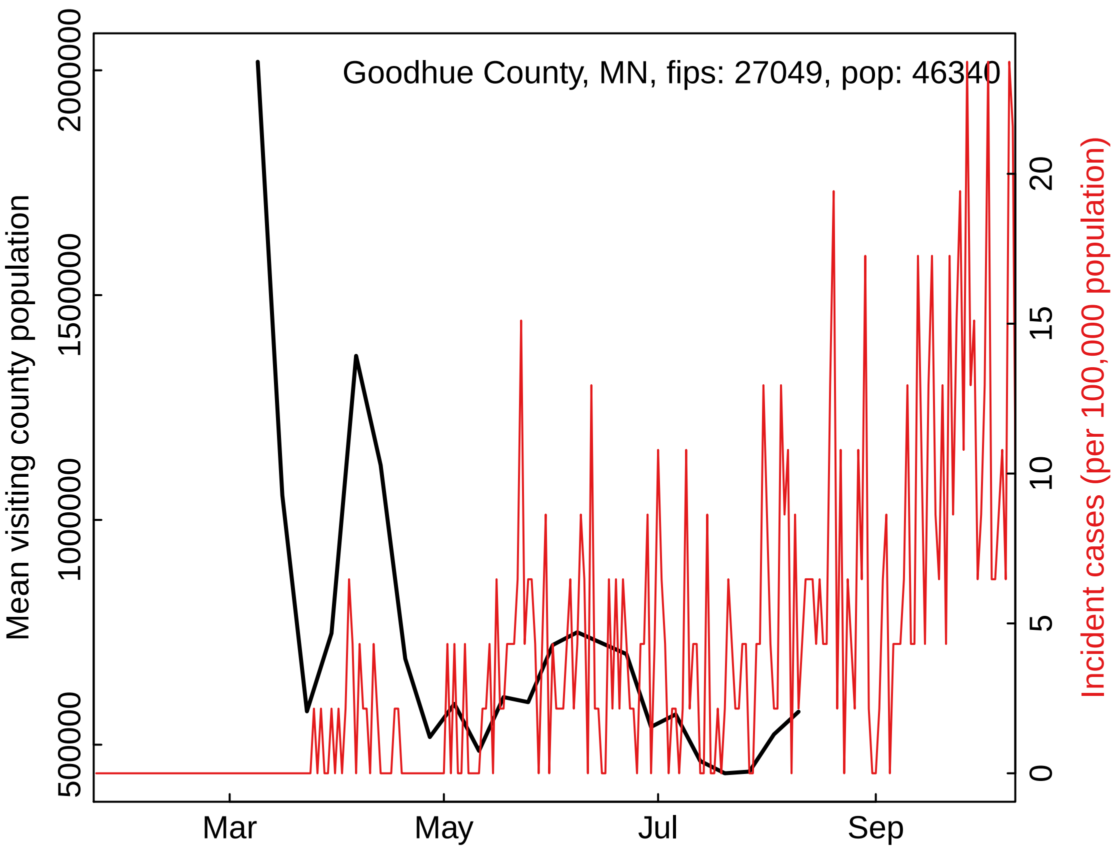
Goodhue County, MN, fips: 27049, pop: 46340
Mean visiting county population
500000
1000000
1500000
2000000
Incident cases (per 100,000 population)
0
5
10
15
20
Mar
May
Jul
Sep

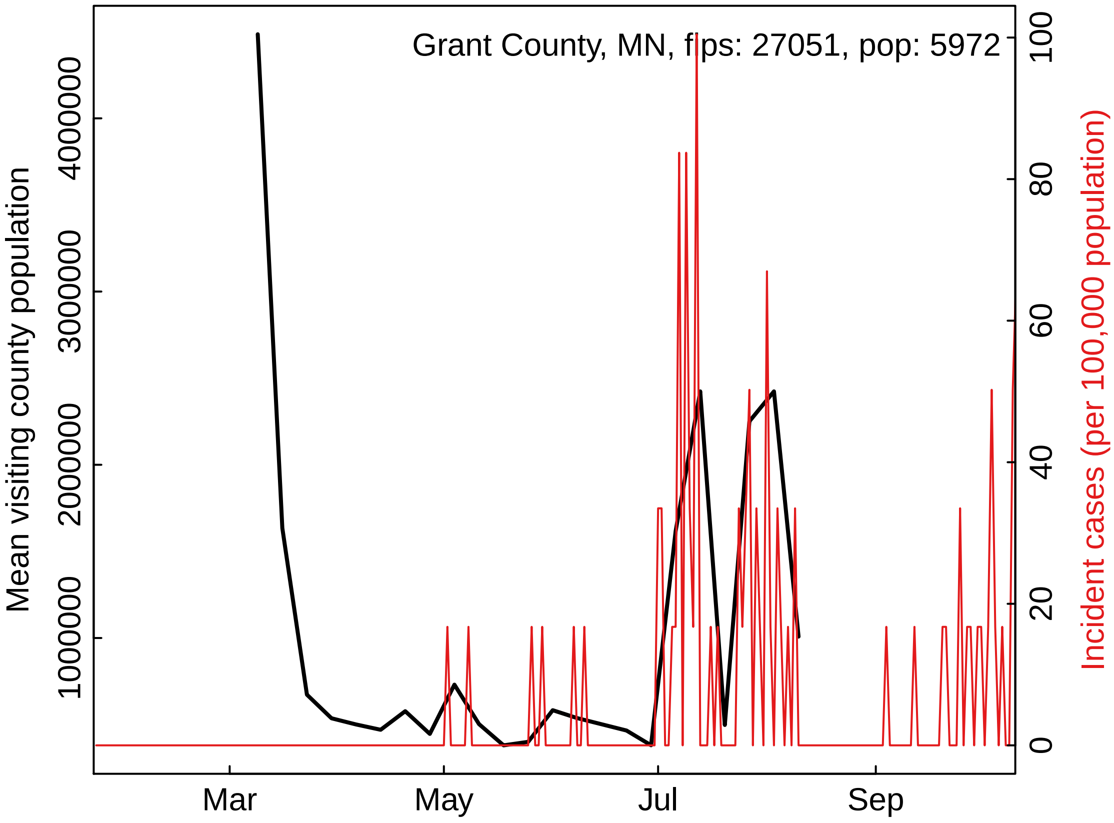

Grant County, MN, flips: 27051, pop: 5972
Mean visiting county population
1000000
2000000
3000000
4000000
Mar
May
Jul
Sep
0
20
40
60
80
100
Incident cases (per 100,000 population)

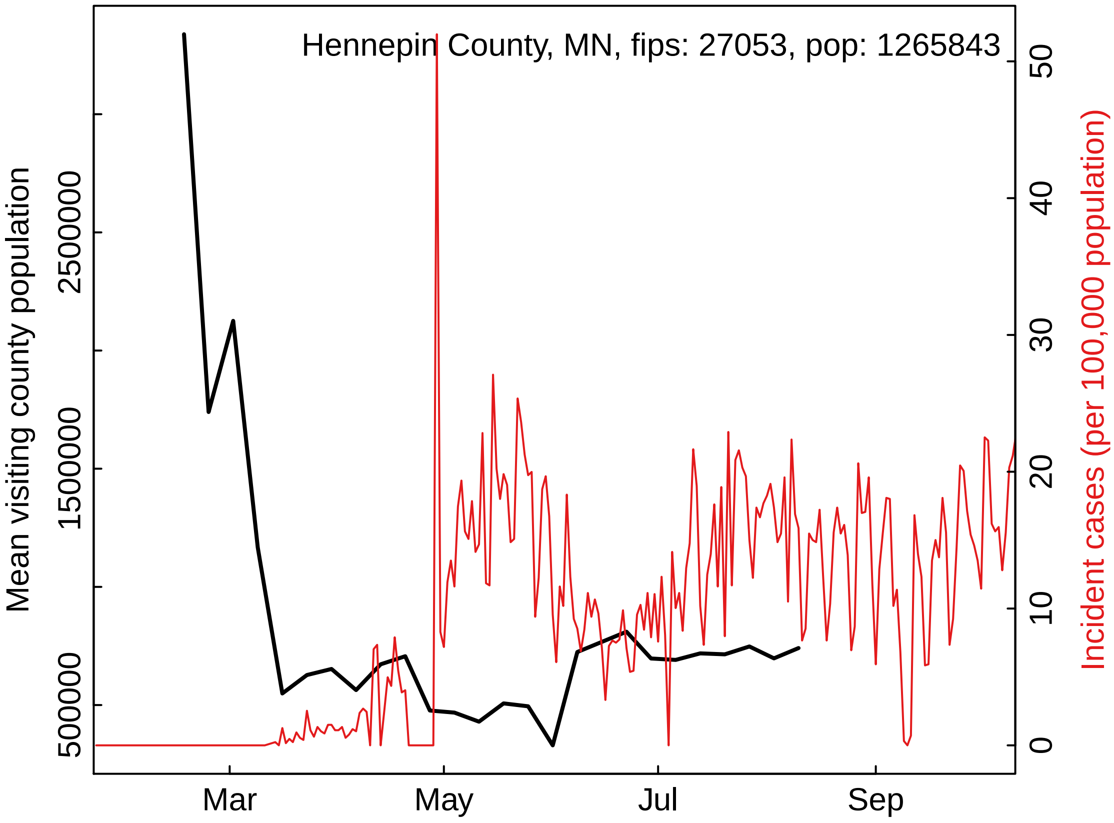

Hennepin County, MN, fips: 27053, pop: 1265843
Mean visiting county population
500000
1500000
2500000
Incident cases (per 100,000 population)
0
10
20
30
40
50
Mar
May
Jul
Sep

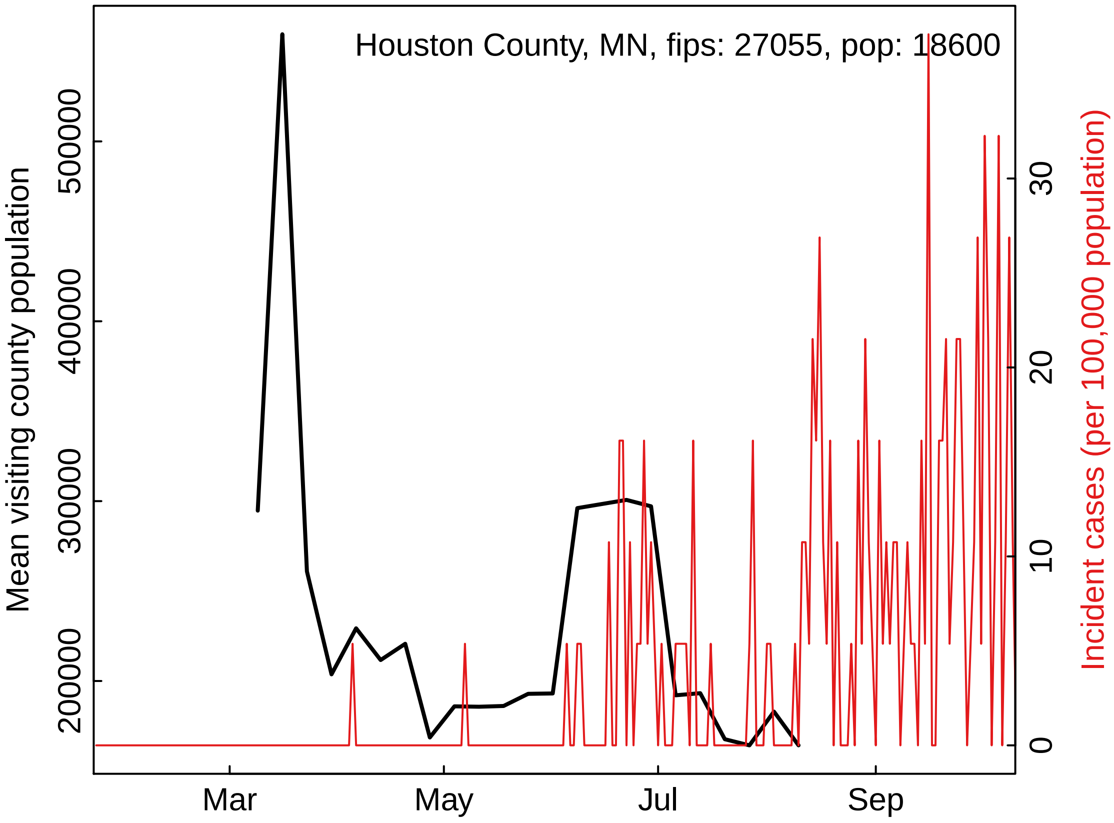
Houston County, MN, fips: 27055, pop: 18600
Mean visiting county population
200000
300000
400000
500000
Incident cases (per 100,000 population)
0
10
20
30
Mar
May
Jul
Sep

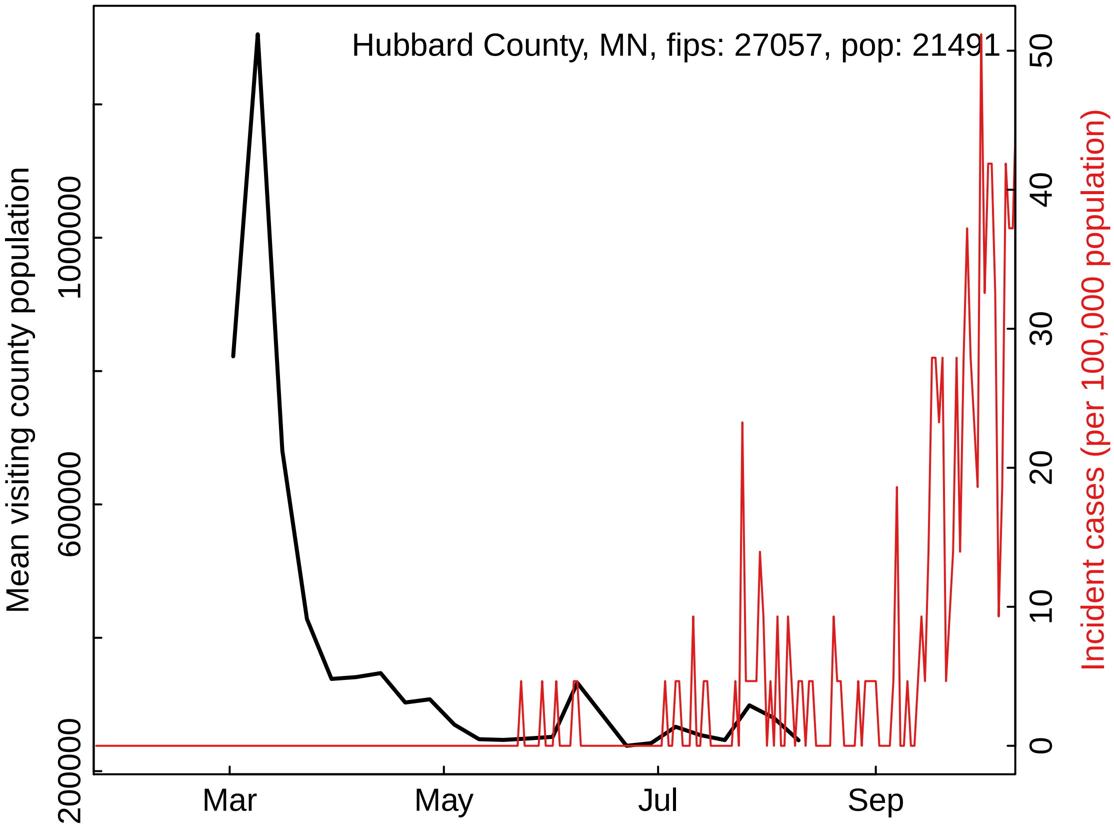

Hubbard County, MN, fips: 27057, pop: 21491
Mean visiting county population
200000
600000
1000000
Mar
May
Jul
Sep
0
10
20
30
40
50
Incident cases (per 100,000 population)

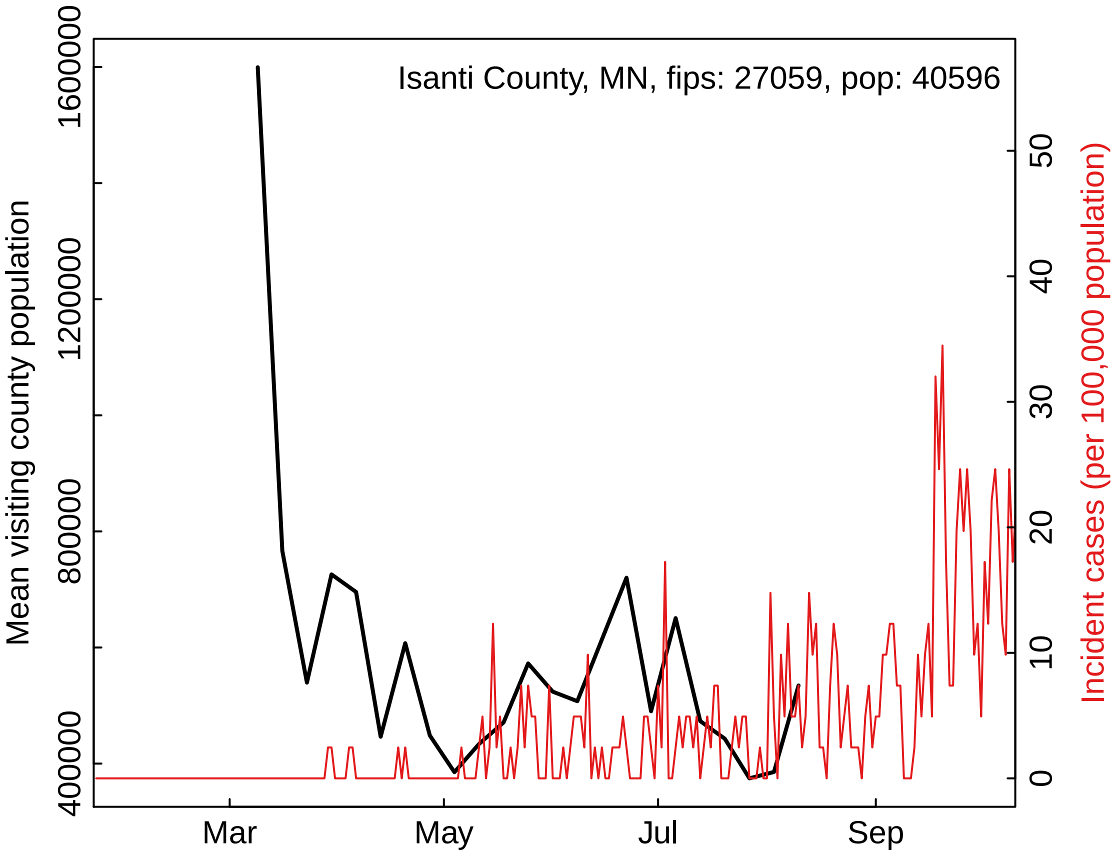

Isanti County, MN, fips: 27059, pop: 40596
Mean visiting county population
400000
800000
1200000
1600000
Mar
May
Jul
Sep
0
10
20
30
40
50
Incident cases (per 100,000 population)

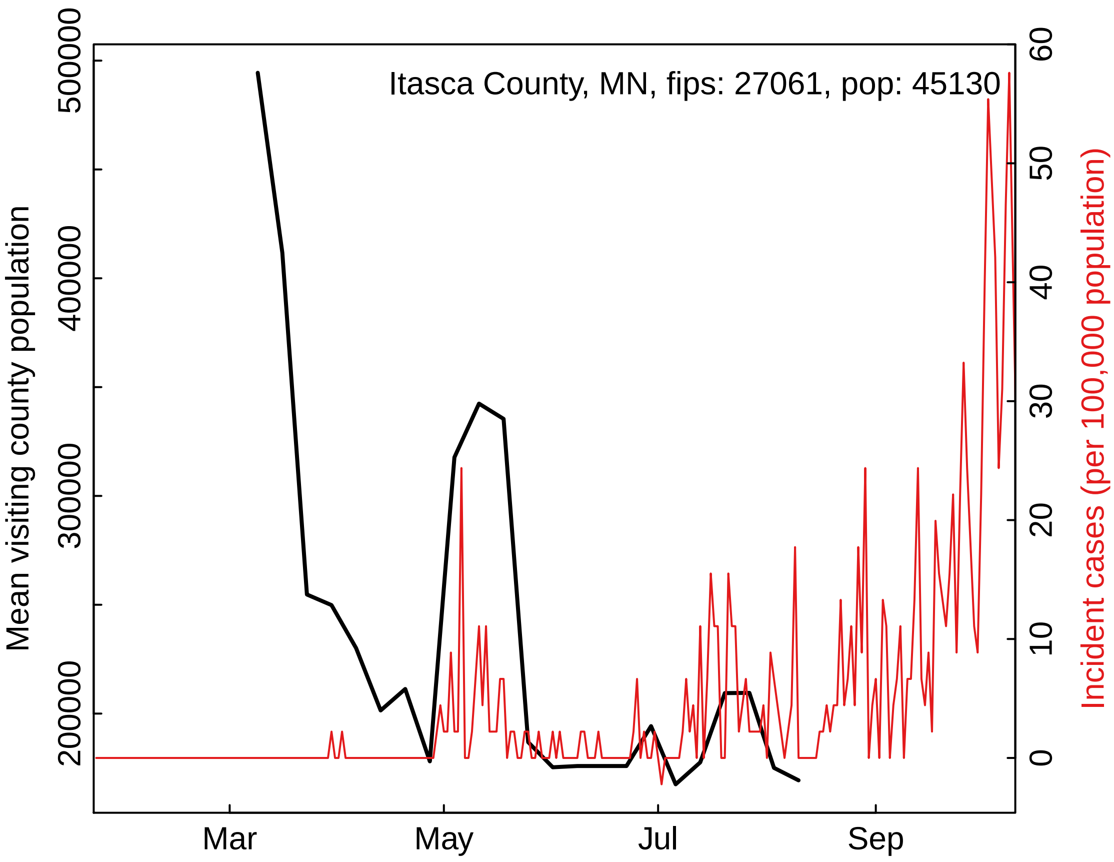

Itasca County, MN, fips: 27061, pop: 45130
Mean visiting county population
200000
300000
400000
500000
Incident cases (per 100,000 population)
0
10
20
30
40
50
60
Mar
May
Jul
Sep

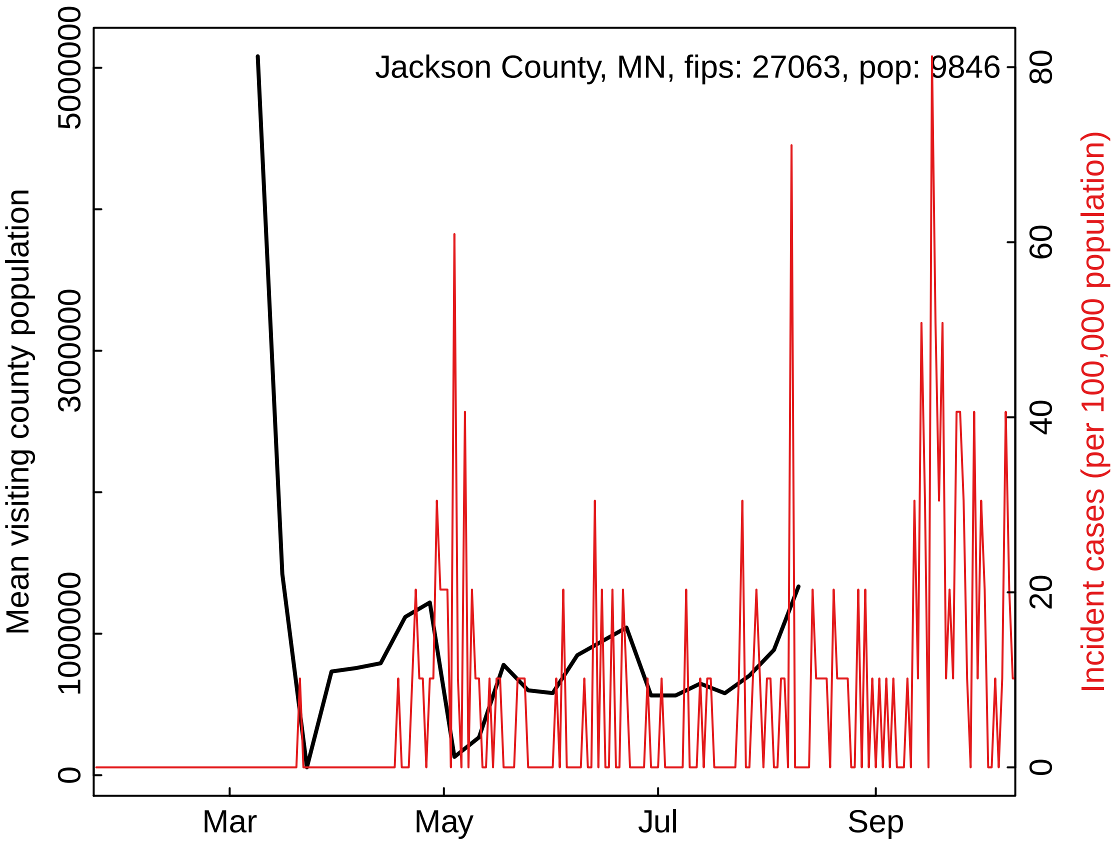

Jackson County, MN, fips: 27063, pop: 9846
Mean visiting county population
Incident cases (per 100,000 population)
0
1000000
3000000
5000000
0
20
40
60
80
Mar
May
Jul
Sep

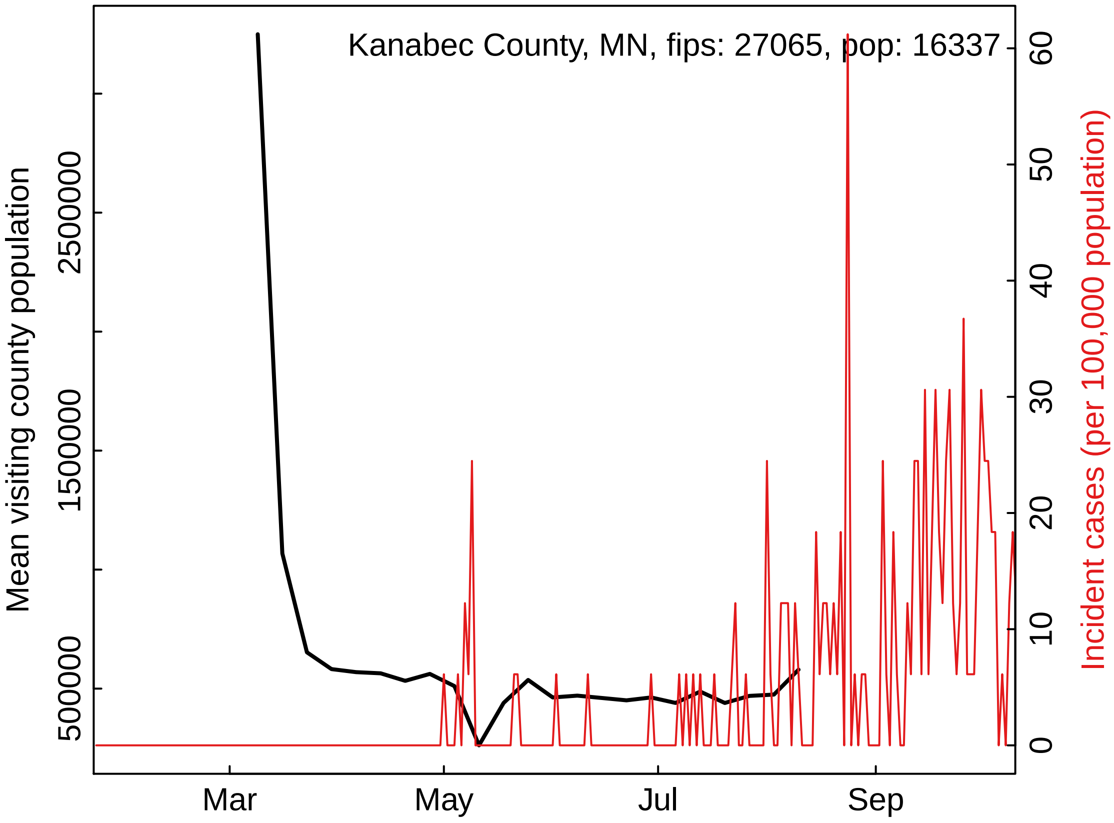

Kanabec County, MN, fips: 27065, pop: 16337
Mean visiting county population
500000
1500000
2500000
Mar
May
Jul
Sep
0
10
20
30
40
50
60
Incident cases (per 100,000 population)

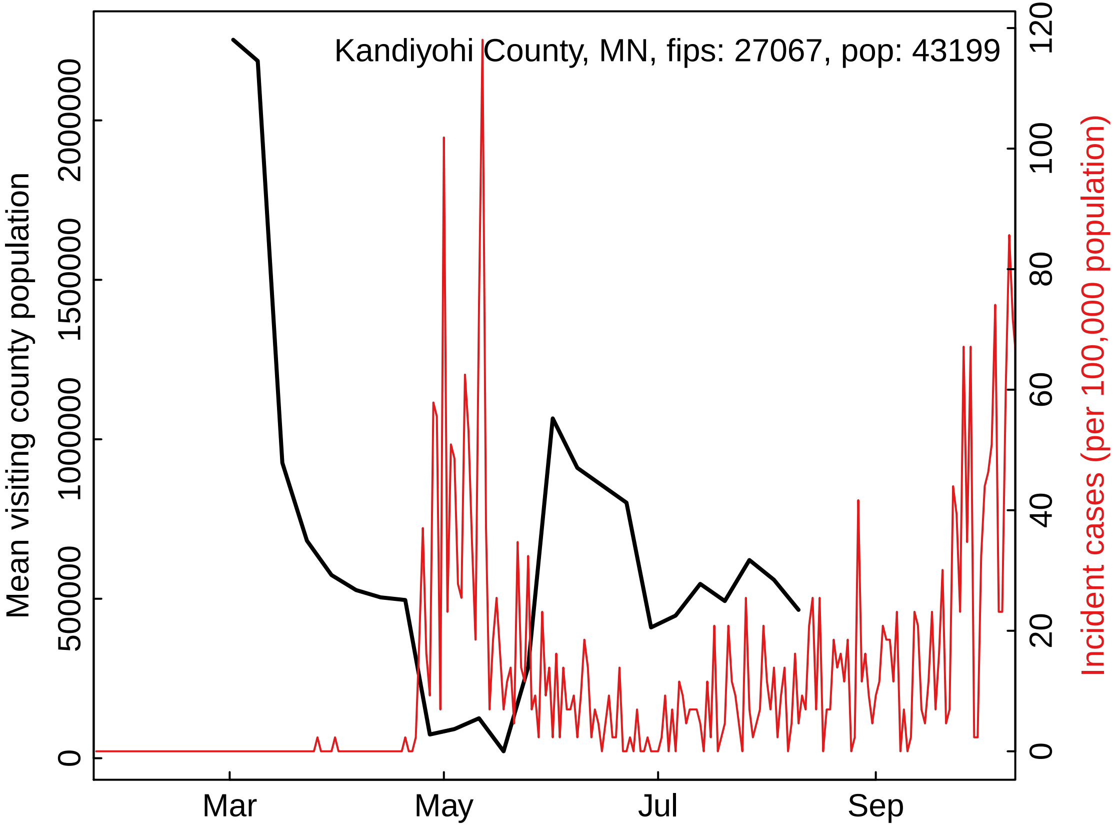

Kandiyohi County, MN, fips: 27067, pop: 43199
Mean visiting county population
0
500000
1000000
1500000
2000000
Incident cases (per 100,000 population)
0
20
40
60
80
100
120
Mar
May
Jul
Sep

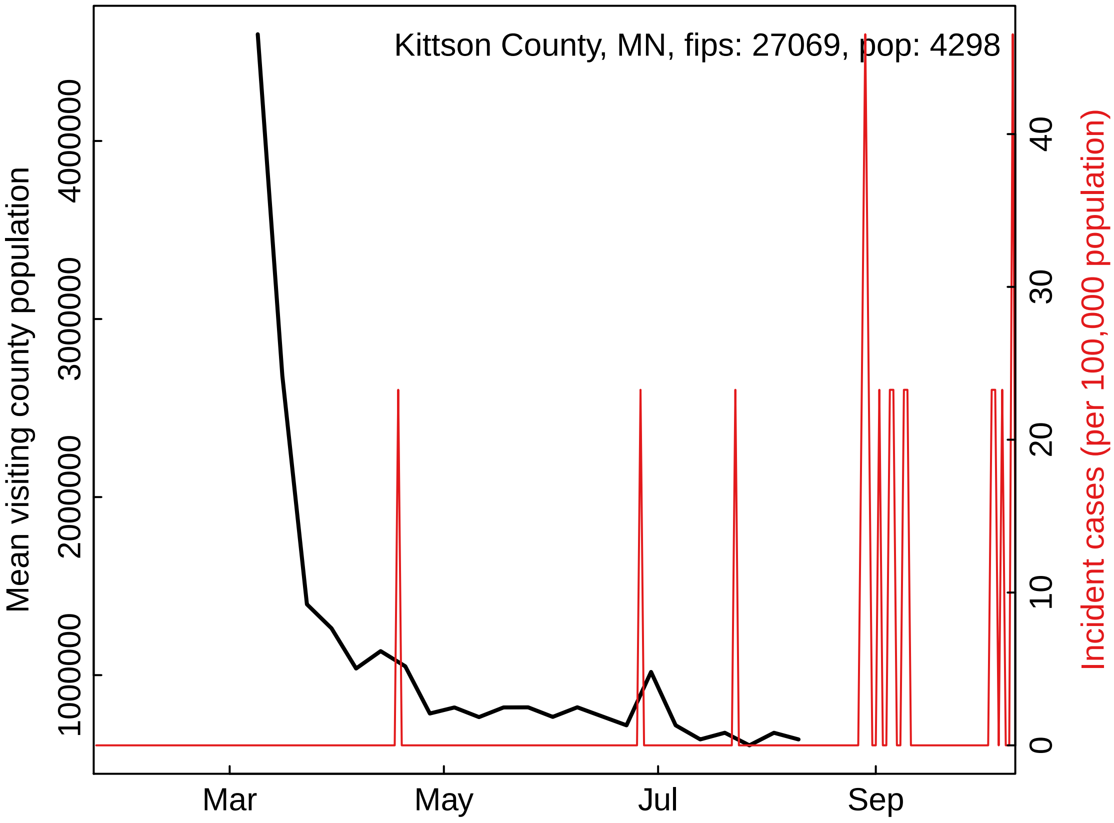

Kittson County, MN, fips: 27069, pop: 4298
Mean visiting county population
1000000
2000000
3000000
4000000
Incident cases (per 100,000 population)
0
10
20
30
40
Mar
May
Jul
Sep

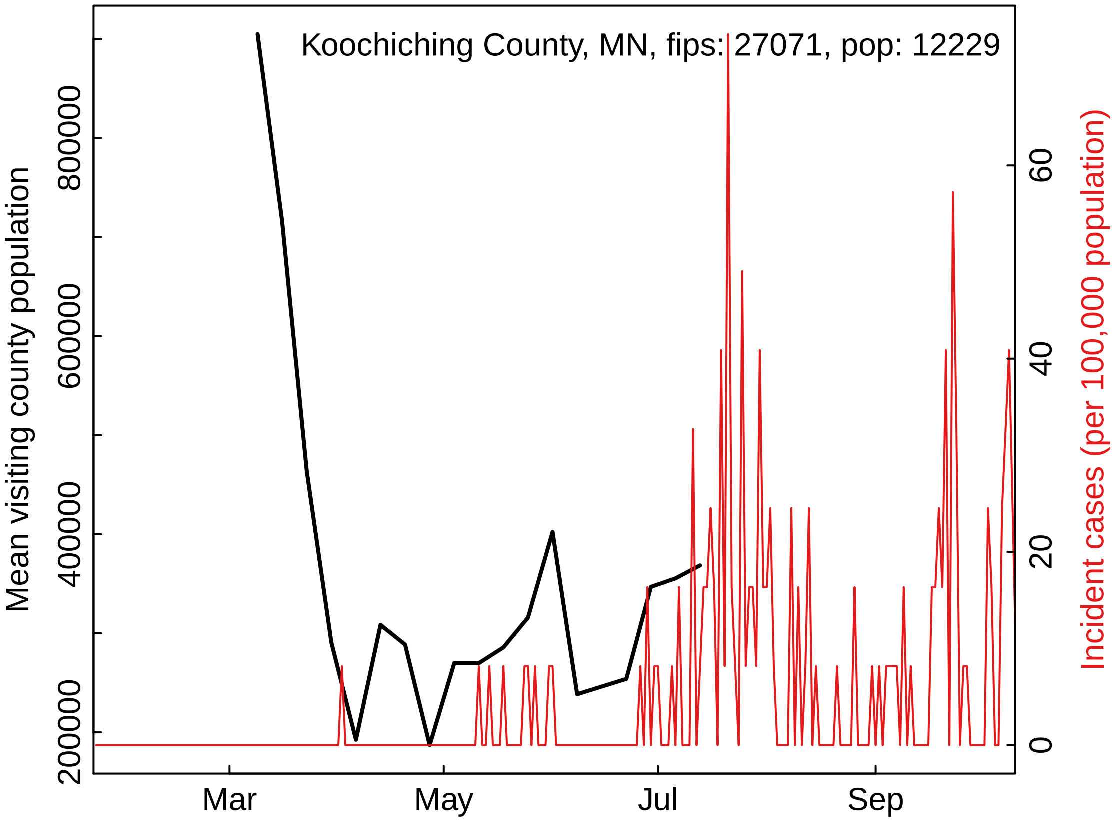
Koochiching County, MN, fips: 27071, pop: 12229
Mean visiting county population
200000
400000
600000
800000
Incident cases (per 100,000 population)
0
20
40
60
Mar
May
Jul
Sep

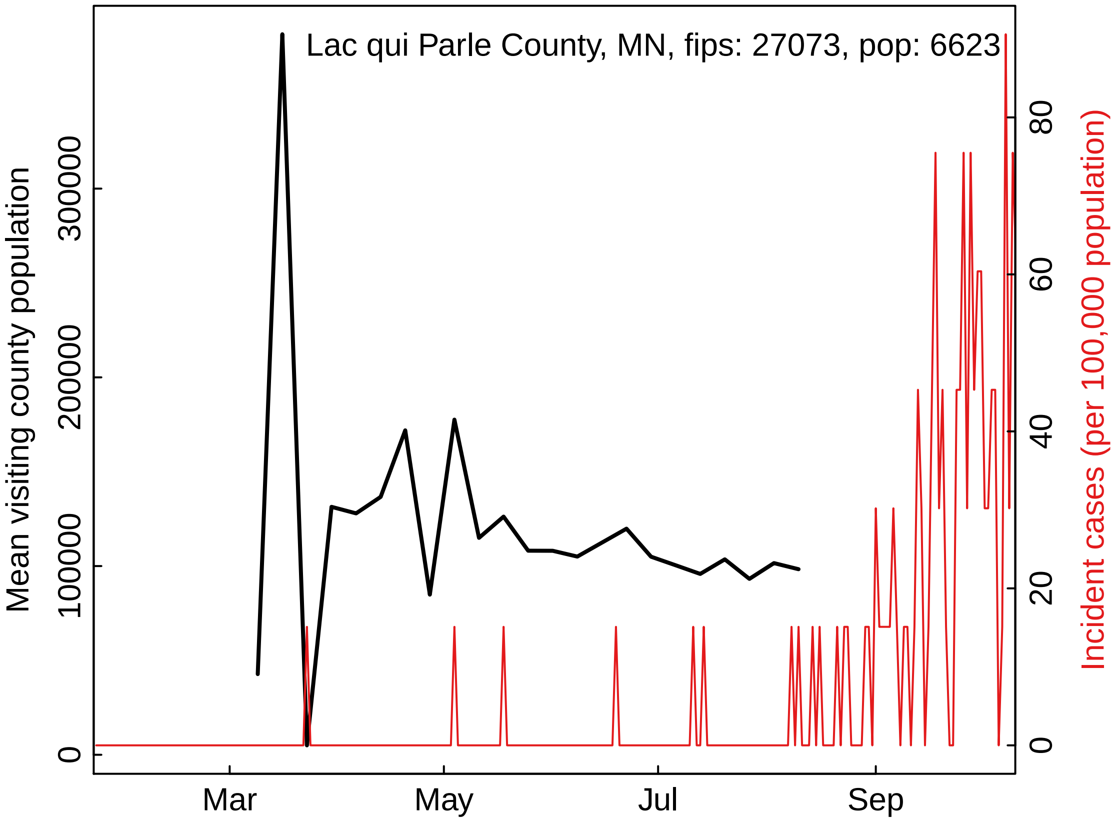

Lac qui Parle County, MN, fips: 27073, pop: 6623
Mean visiting county population
0
100000
200000
300000
Incident cases (per 100,000 population)
0
20
40
60
80
Mar
May
Jul
Sep

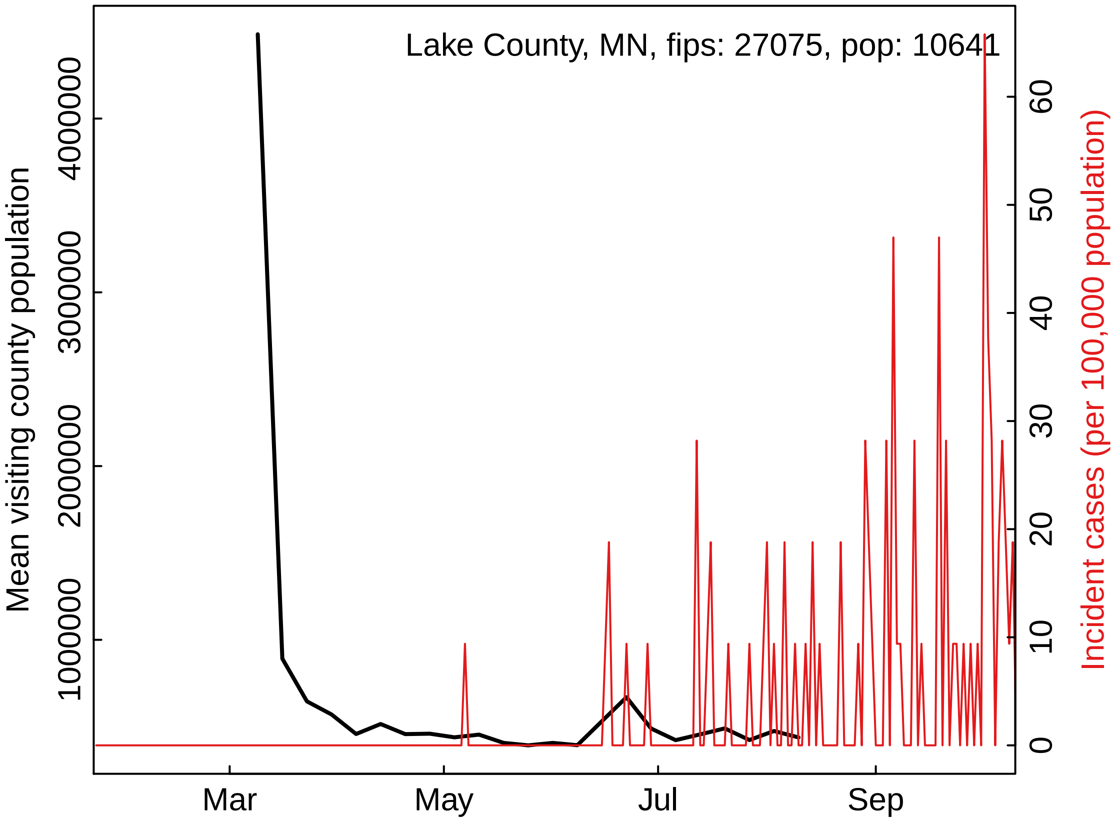

Lake County, MN, fips: 27075, pop: 10641
Mean visiting county population
1000000
2000000
3000000
4000000
Incident cases (per 100,000 population)
0
10
20
30
40
50
60
Mar
May
Jul
Sep

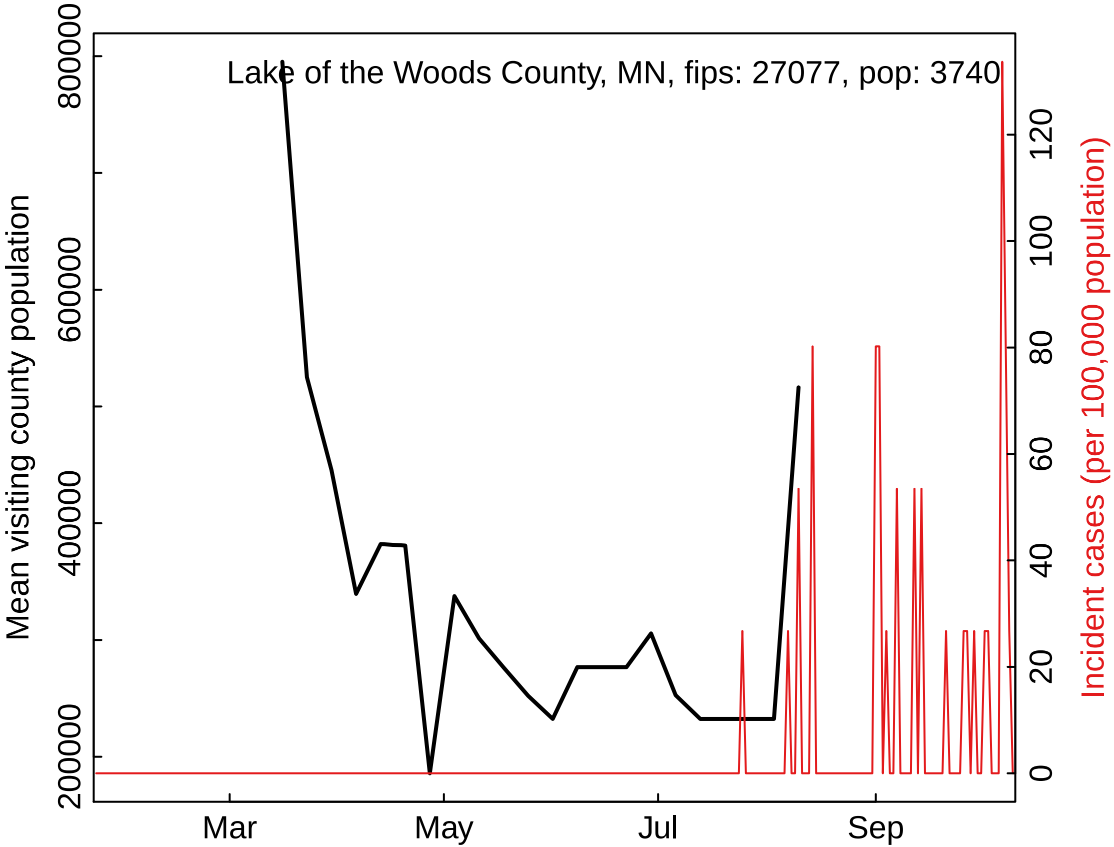

Lake of the Woods County, MN, fips: 27077, pop: 3740
Mean visiting county population
200000
400000
600000
800000
Mar
May
Jul
Sep
Incident cases (per 100,000 population)
0
20
40
60
80
100
120

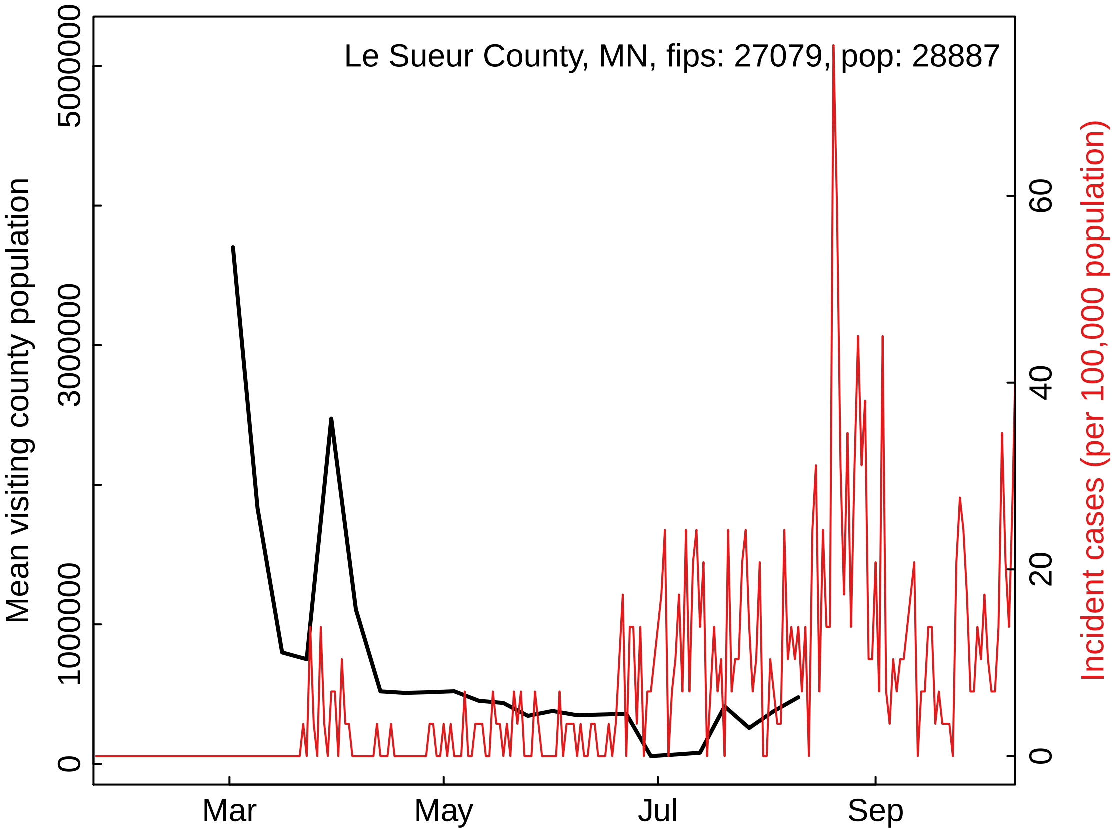
Le Sueur County, MN, fips: 27079, pop: 28887
Mean visiting county population
Incident cases (per 100,000 population)
0
1000000
3000000
5000000
0
20
40
60
Mar
May
Jul
Sep

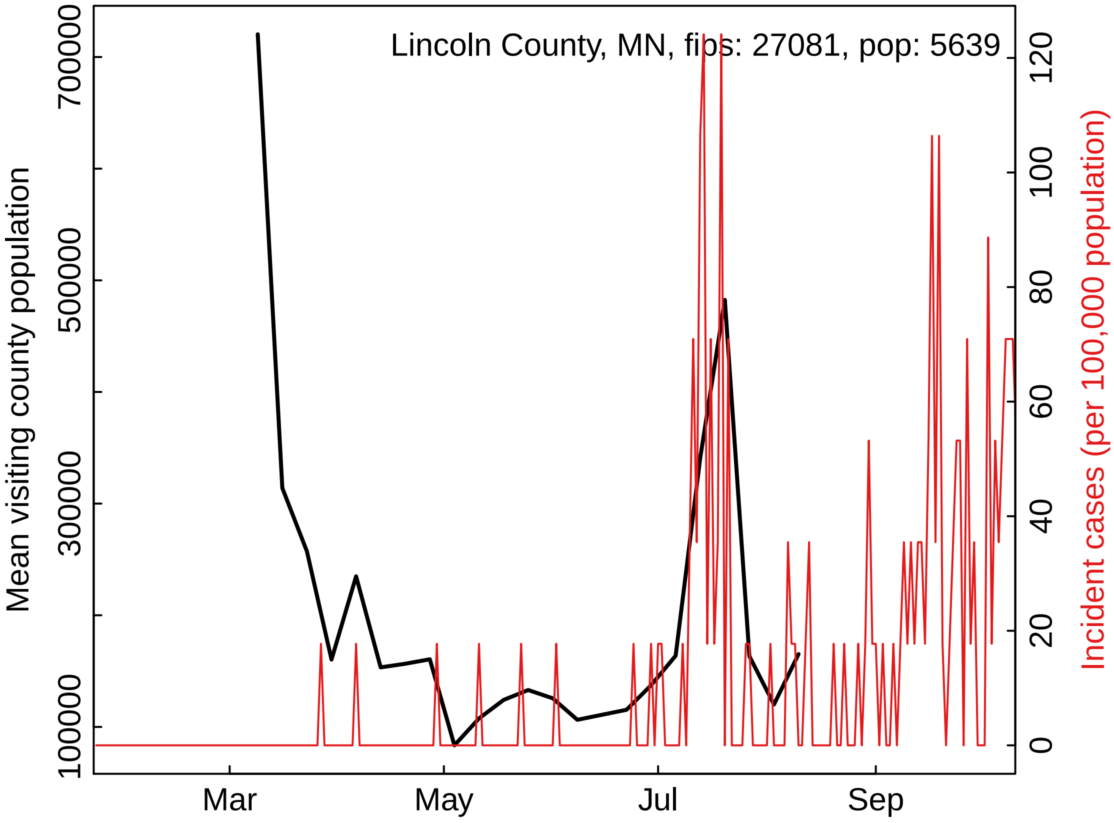

Lincoln County, MN, fips: 27081, pop: 5639
Mean visiting county population
100000
300000
500000
700000
Incident cases (per 100,000 population)
0
20
40
60
80
100
120
Mar
May
Jul
Sep

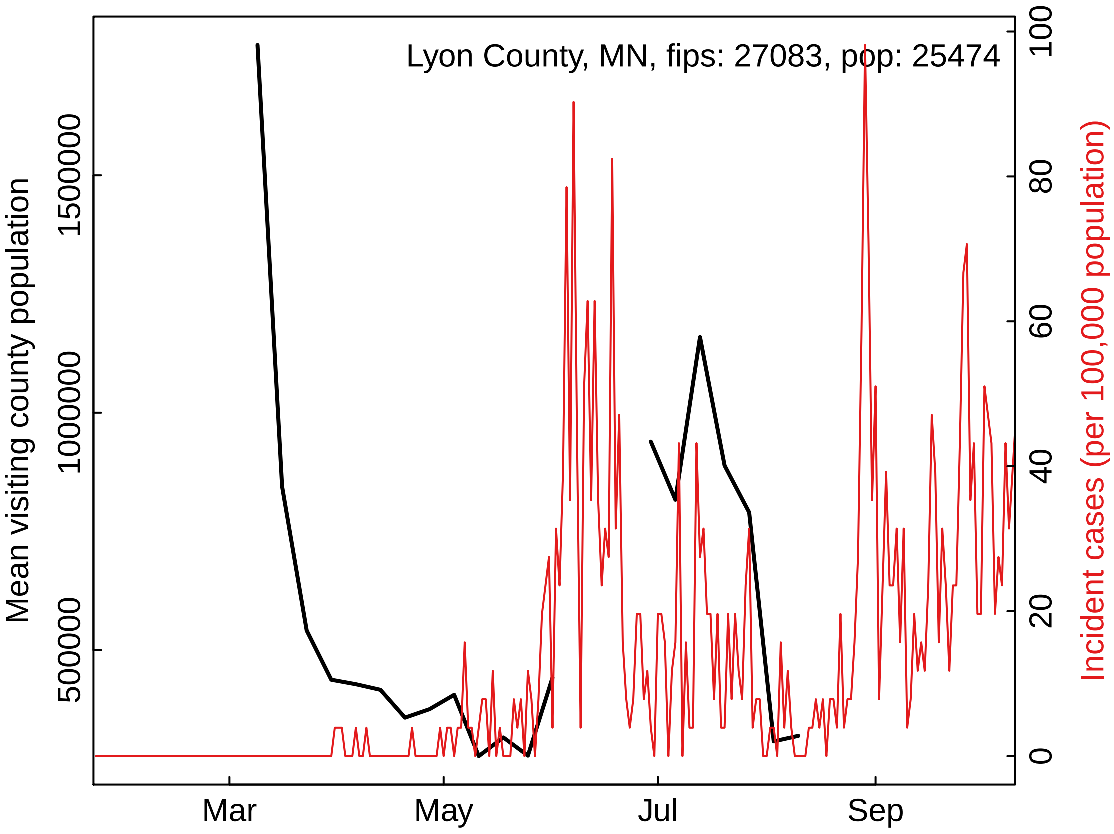

Lyon County, MN, fips: 27083, pop: 25474
Mean visiting county population
500000
1000000
1500000
Incident cases (per 100,000 population)
0
20
40
60
80
100
Mar
May
Jul
Sep

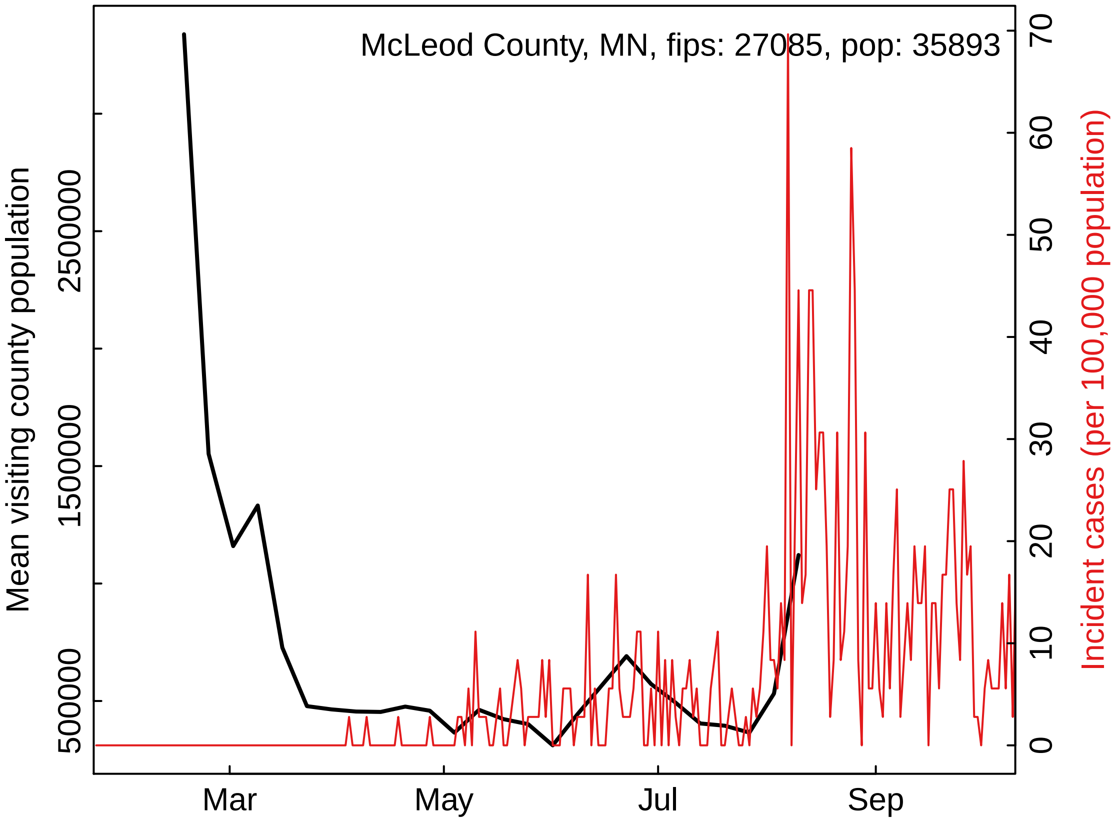
McLeod County, MN, fips: 27085, pop: 35893
Mean visiting county population
500000
1500000
2500000
Mar
May
Jul
Sep
0
10
20
30
40
50
60
70
Incident cases (per 100,000 population)

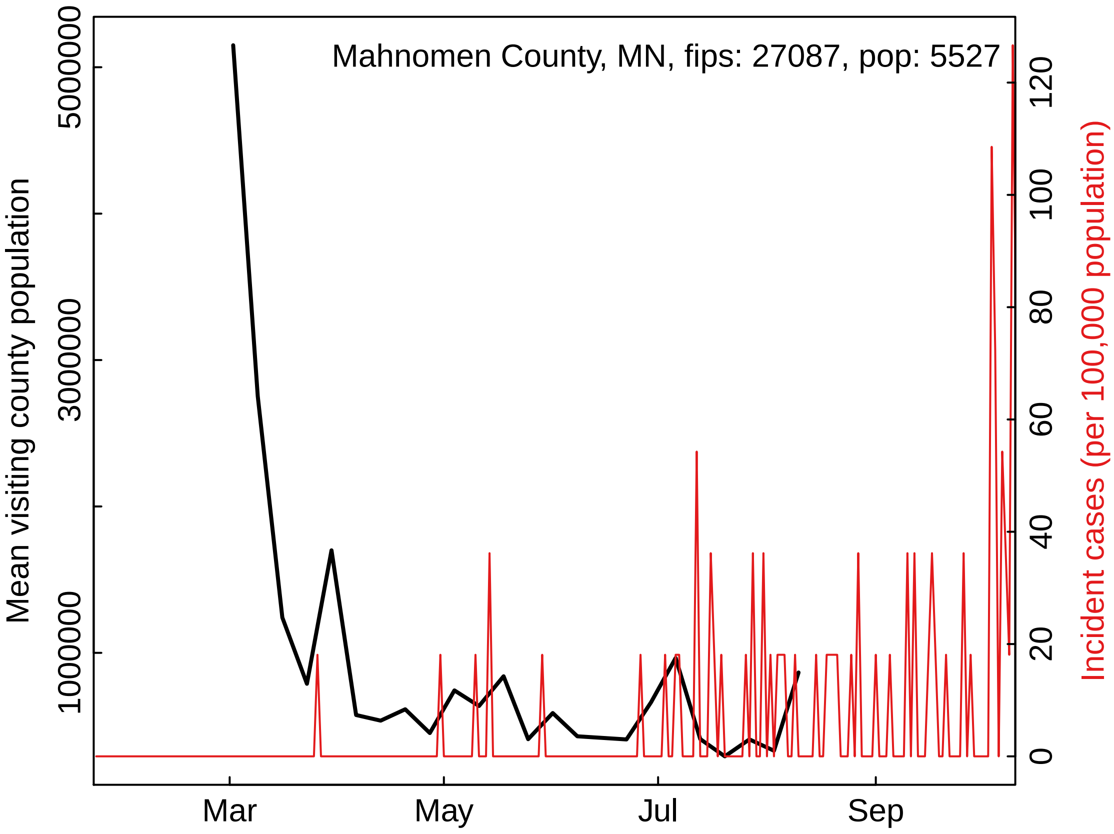
Mahnomen County, MN, fips: 27087, pop: 5527
Mean visiting county population
Incident cases (per 100,000 population)
1000000
3000000
5000000
0
20
40
60
80
100
120
Mar
May
Jul
Sep

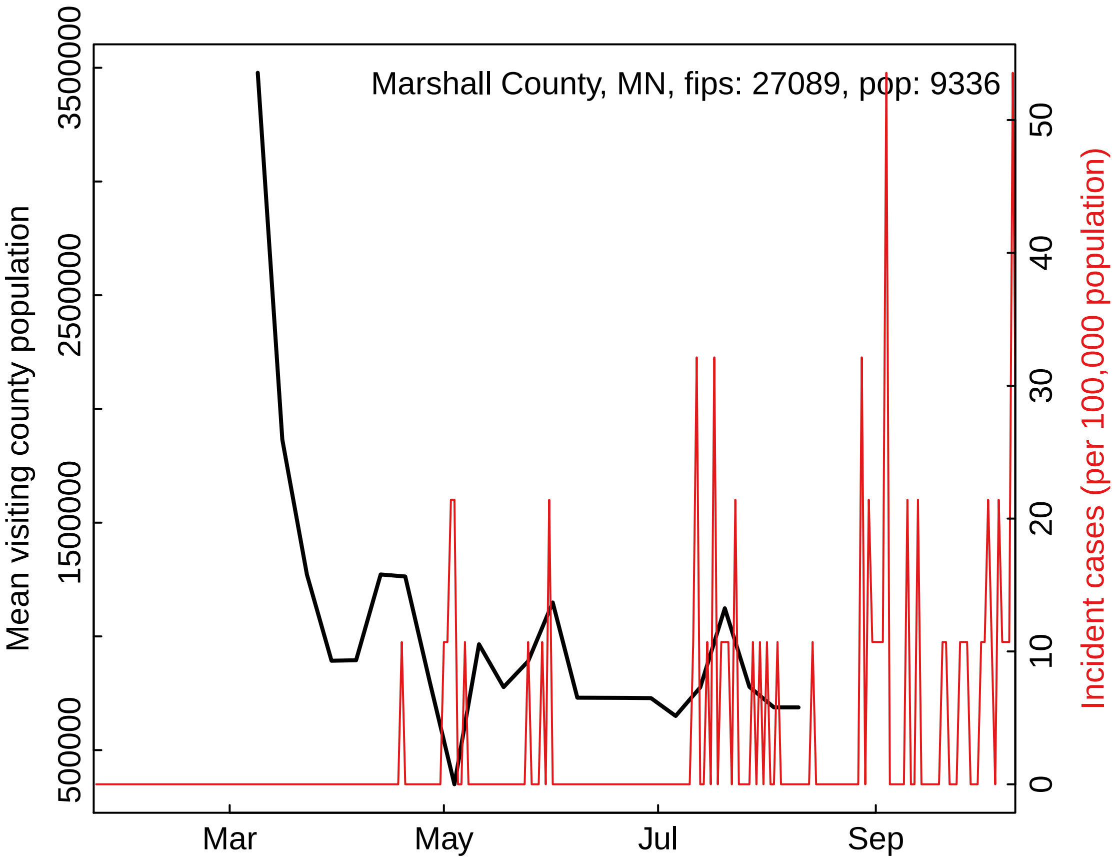

Marshall County, MN, fips: 27089, pop: 9336
Mean visiting county population
500000
1500000
2500000
3500000
Incident cases (per 100,000 population)
0
10
20
30
40
50
Mar
May
Jul
Sep

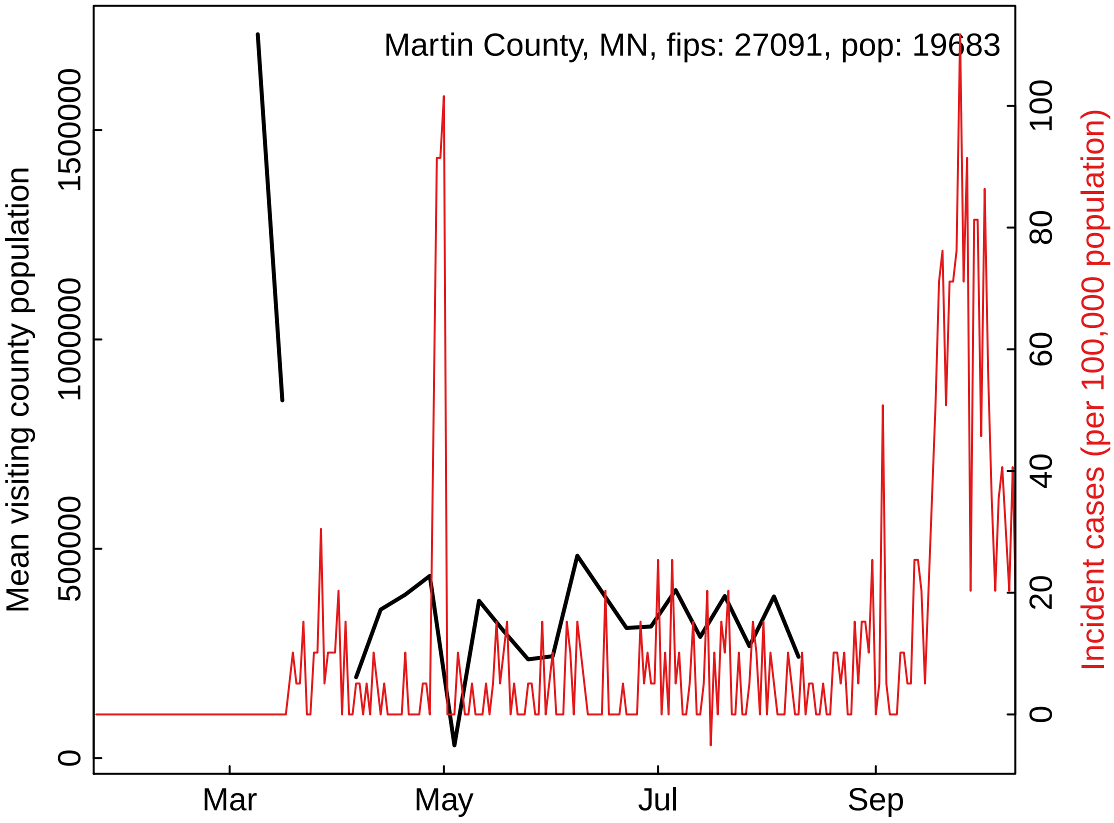

Martin County, MN, fips: 27091, pop: 19683
Mean visiting county population
0
500000
1000000
1500000
Incident cases (per 100,000 population)
0
20
40
60
80
100
Mar
May
Jul
Sep

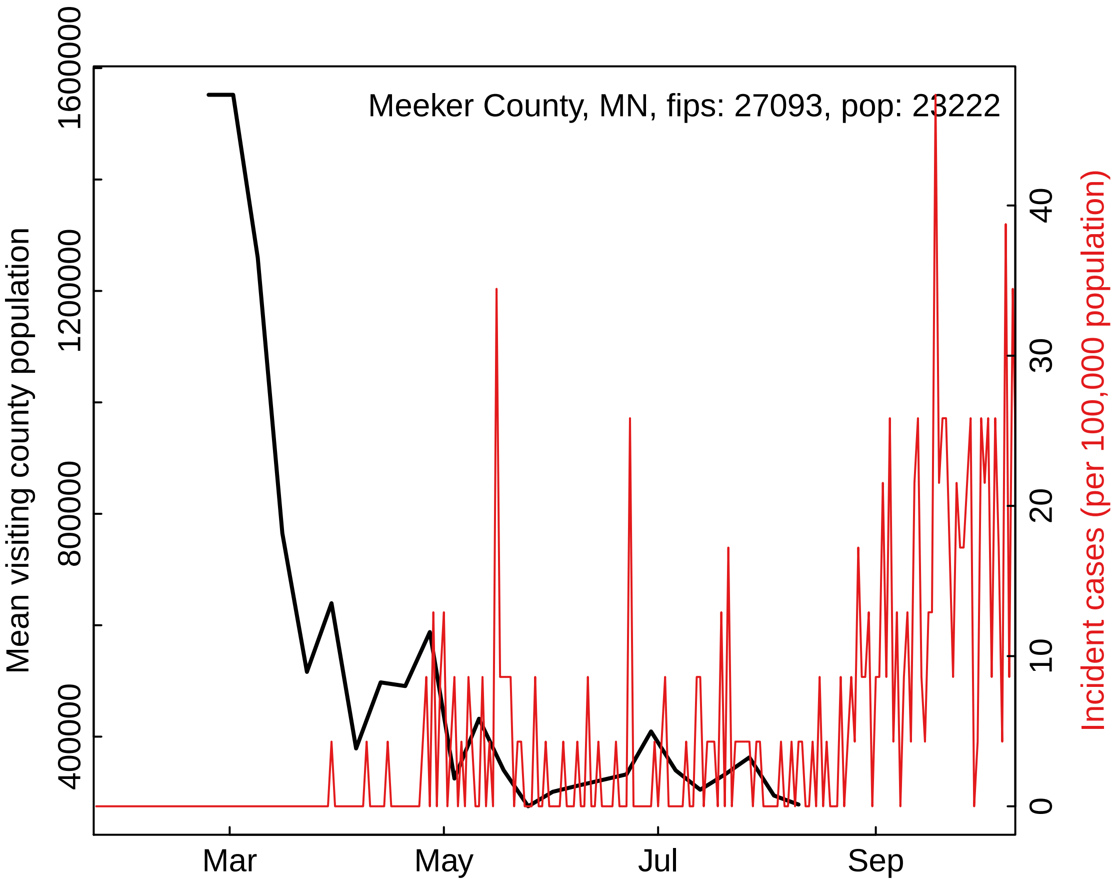

Meeker County, MN, fips: 27093, pop: 23222
Mean visiting county population
Incident cases (per 100,000 population)
400000
800000
1200000
1600000
0
10
20
30
40
Mar
May
Jul
Sep

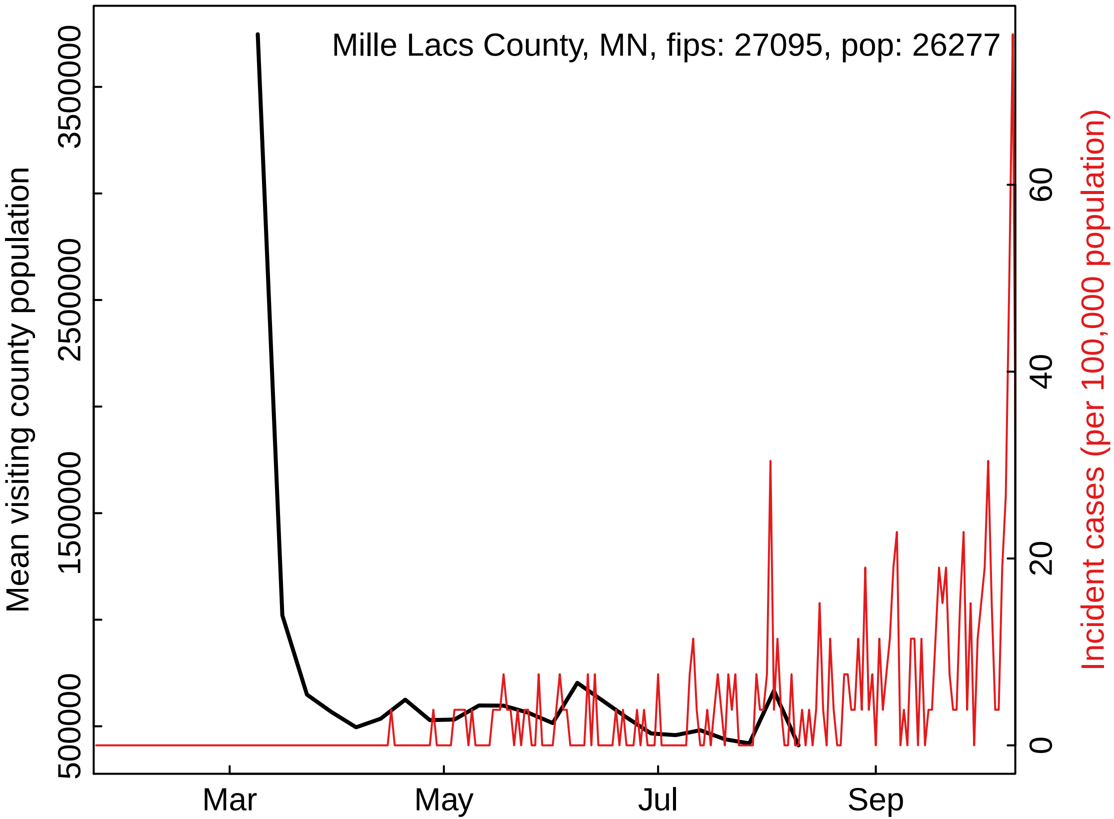

Mille Lacs County, MN, fips: 27095, pop: 26277
Mean visiting county population
500000
1500000
2500000
3500000
Incident cases (per 100,000 population)
0
20
40
60
Mar
May
Jul
Sep

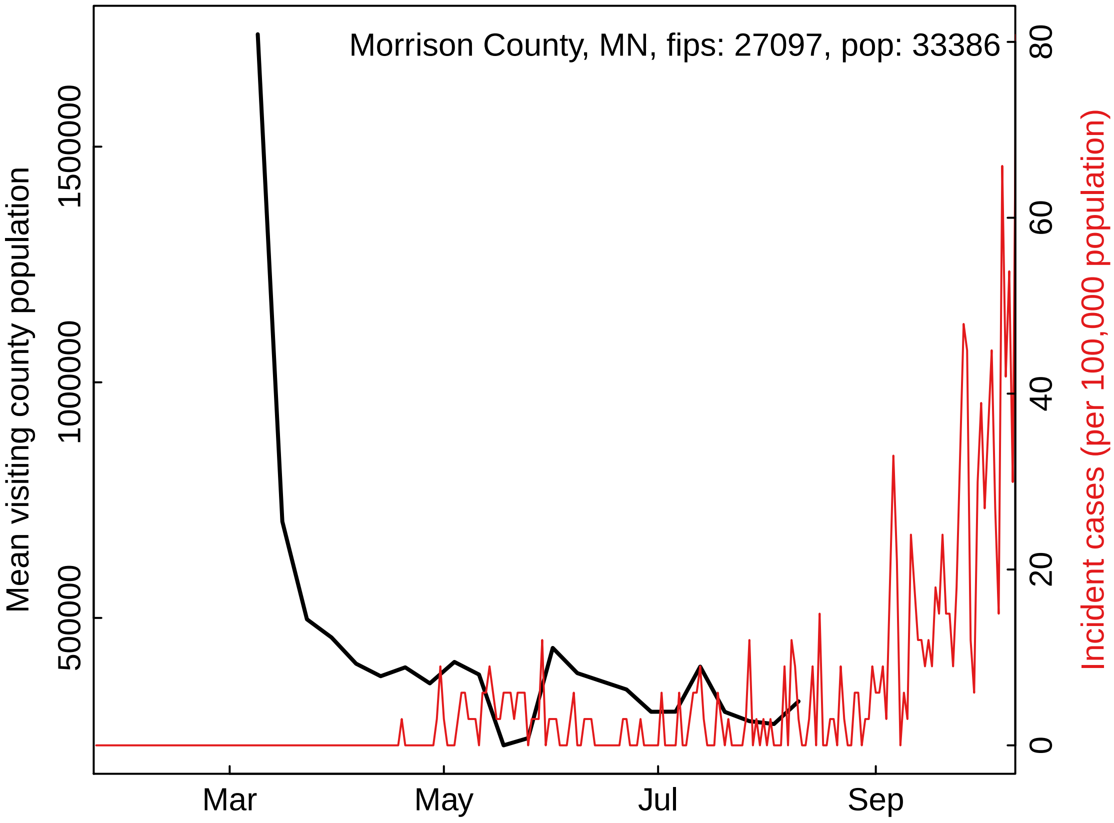

Morrison County, MN, fips: 27097, pop: 33386
Mean visiting county population
500000
1000000
1500000
Incident cases (per 100,000 population)
0
20
40
60
80
Mar
May
Jul
Sep

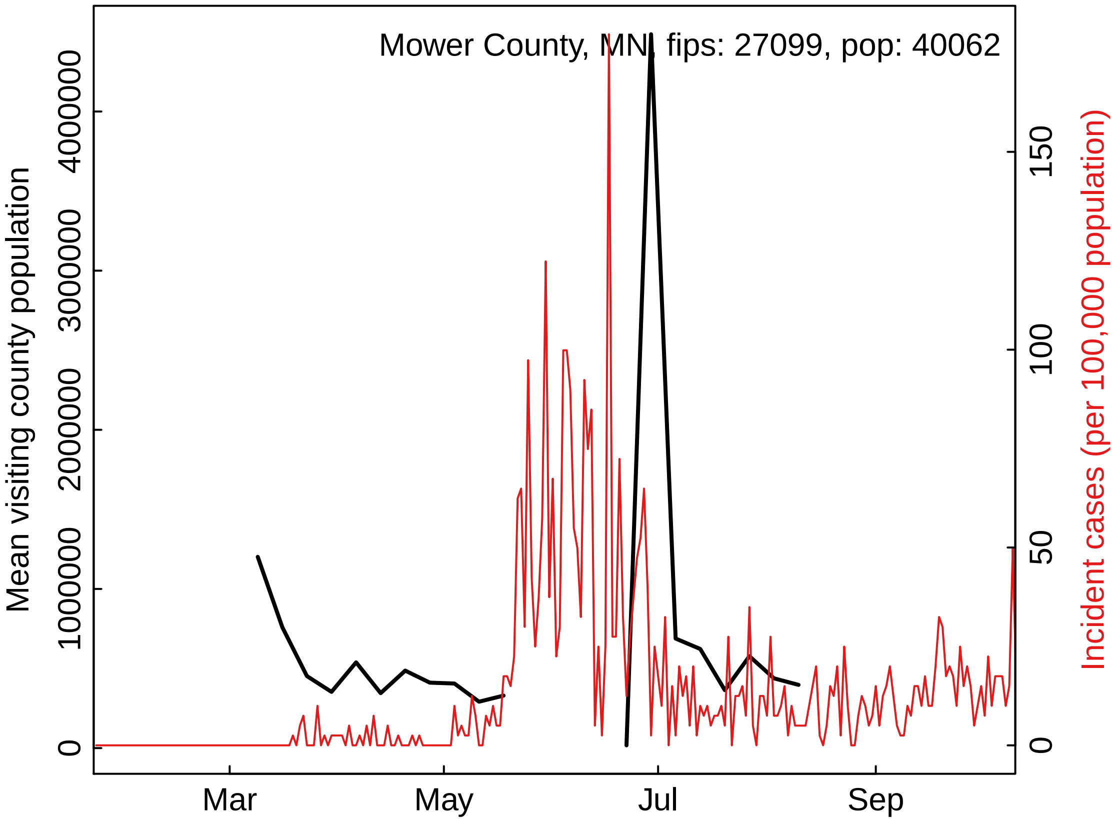
Mower County, MN, fips: 27099, pop: 40062
Mean visiting county population
0
1000000
2000000
3000000
4000000
Incident cases (per 100,000 population)
0
50
100
150
Mar
May
Jul
Sep

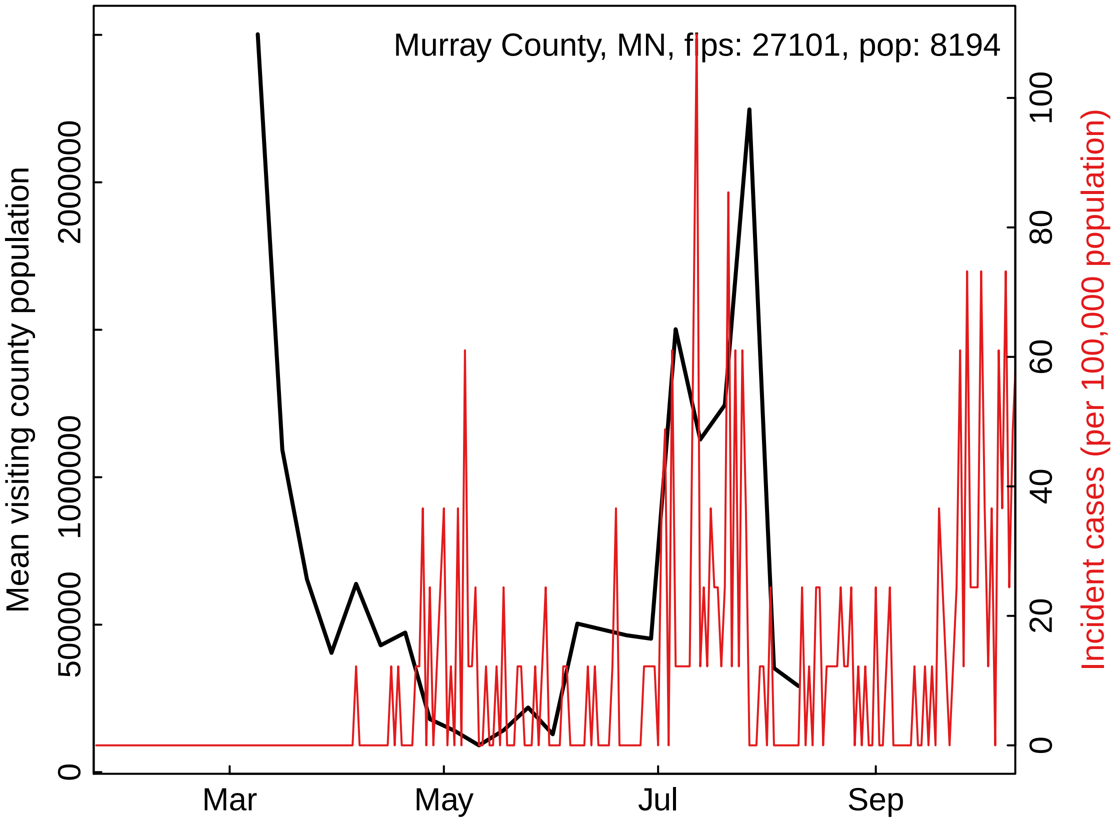

Murray County, MN, flips: 27101, pop: 8194
Mean visiting county population
Incident cases (per 100,000 population)
0
500000
1000000
2000000
0
20
40
60
80
100
Mar
May
Jul
Sep

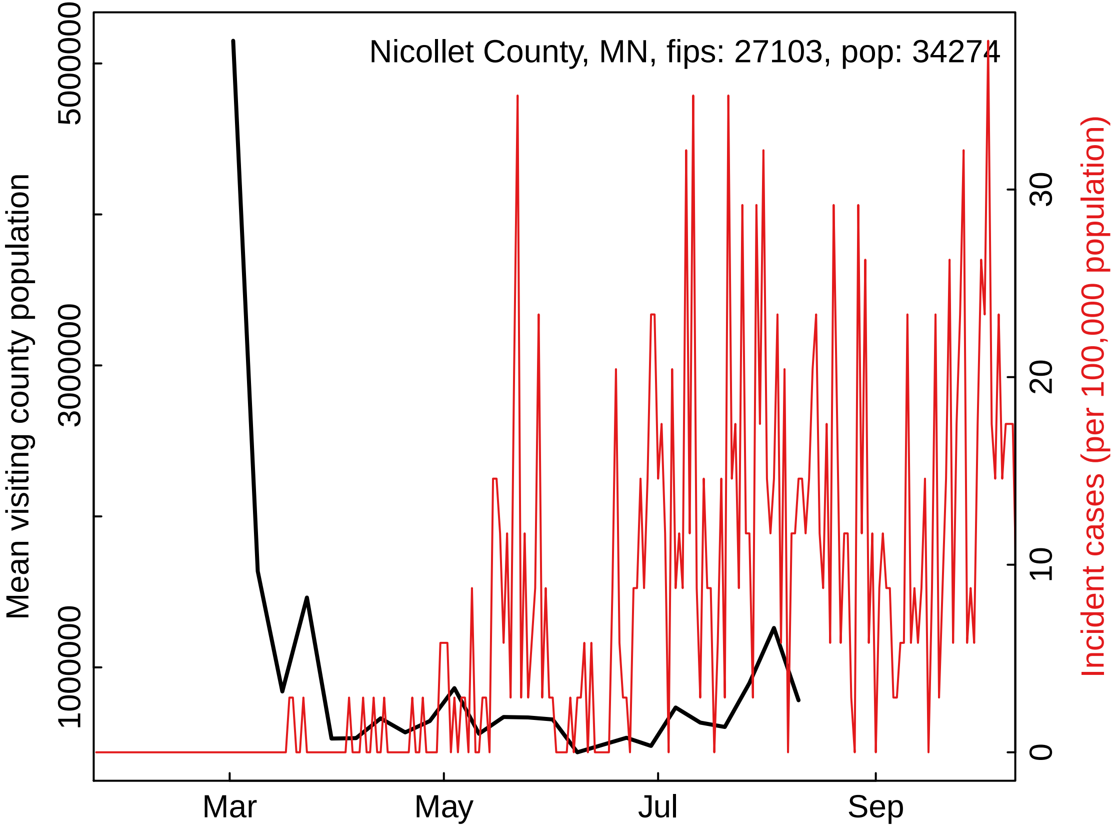

Nicollet County, MN, fips: 27103, pop: 34274
Mean visiting county population
1000000
3000000
5000000
Incident cases (per 100,000 population)
0
10
20
30
Mar
May
Jul
Sep

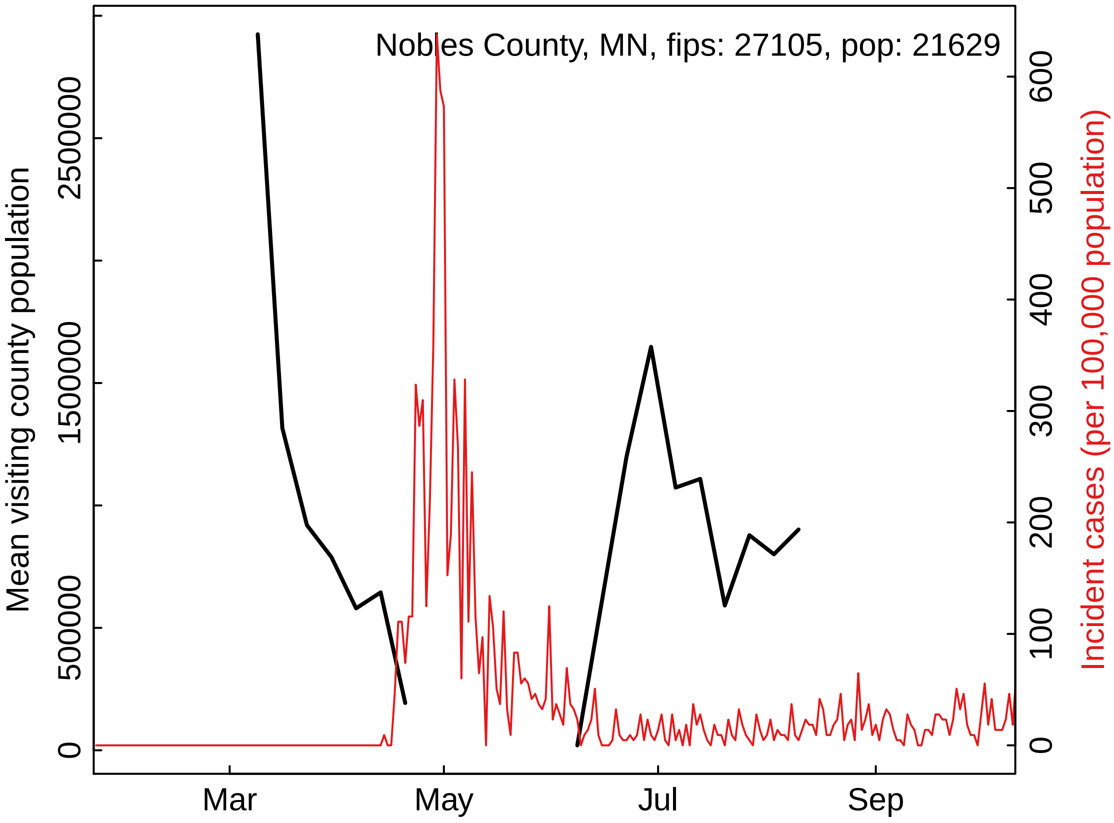

Nobles County, MN, fips: 27105, pop: 21629
Mean visiting county population
0
500000
1500000
2500000
Incident cases (per 100,000 population)
0
100
200
300
400
500
600
Mar
May
Jul
Sep

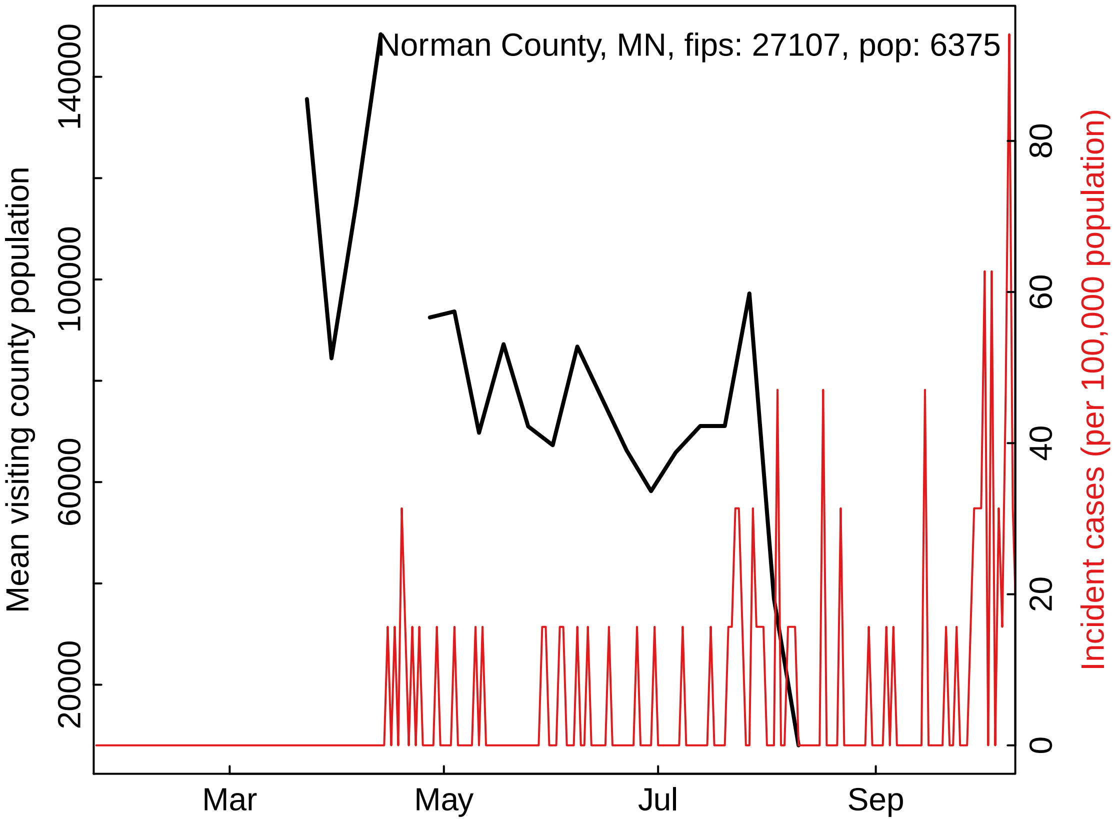

Norman County, MN, fips: 27107, pop: 6375
Mean visiting county population
20000
60000
100000
140000
Incident cases (per 100,000 population)
0
20
40
60
80
Mar
May
Jul
Sep

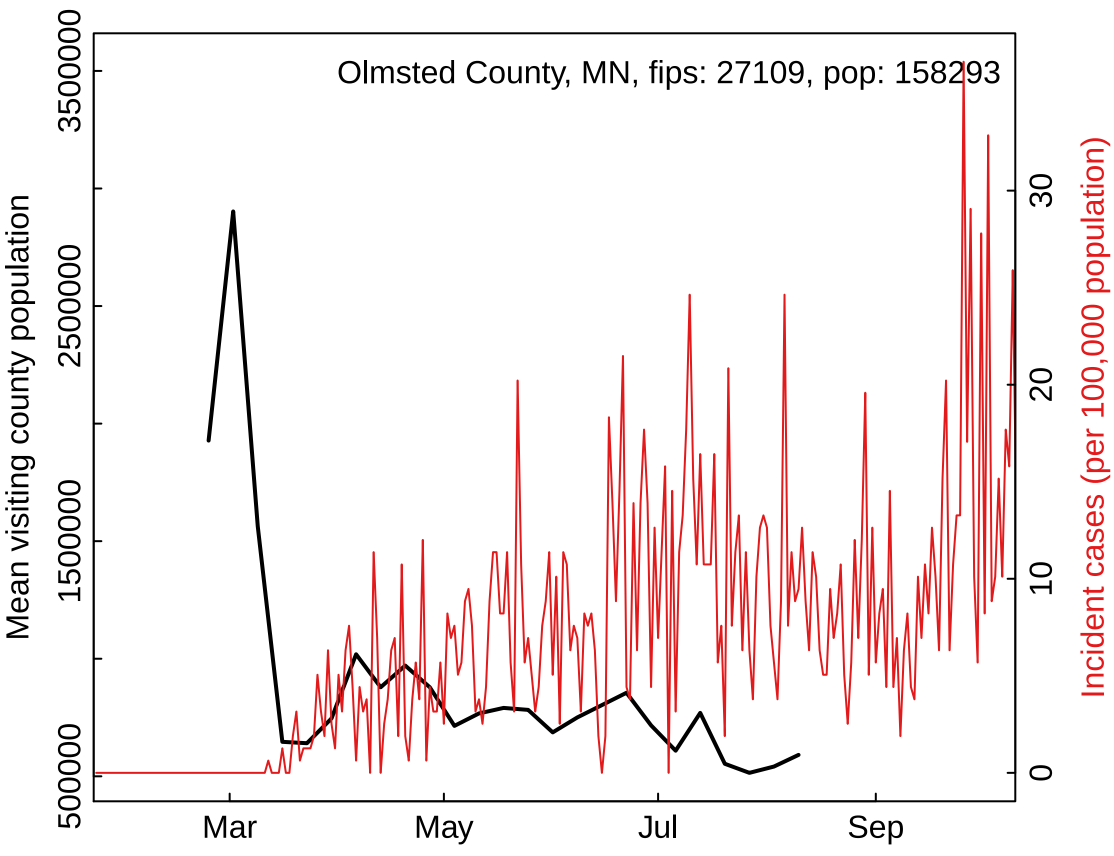

Olmsted County, MN, fips: 27109, pop: 158293
Mean visiting county population
500000
1500000
2500000
3500000
Mar
May
Jul
Sep
Incident cases (per 100,000 population)
0
10
20
30

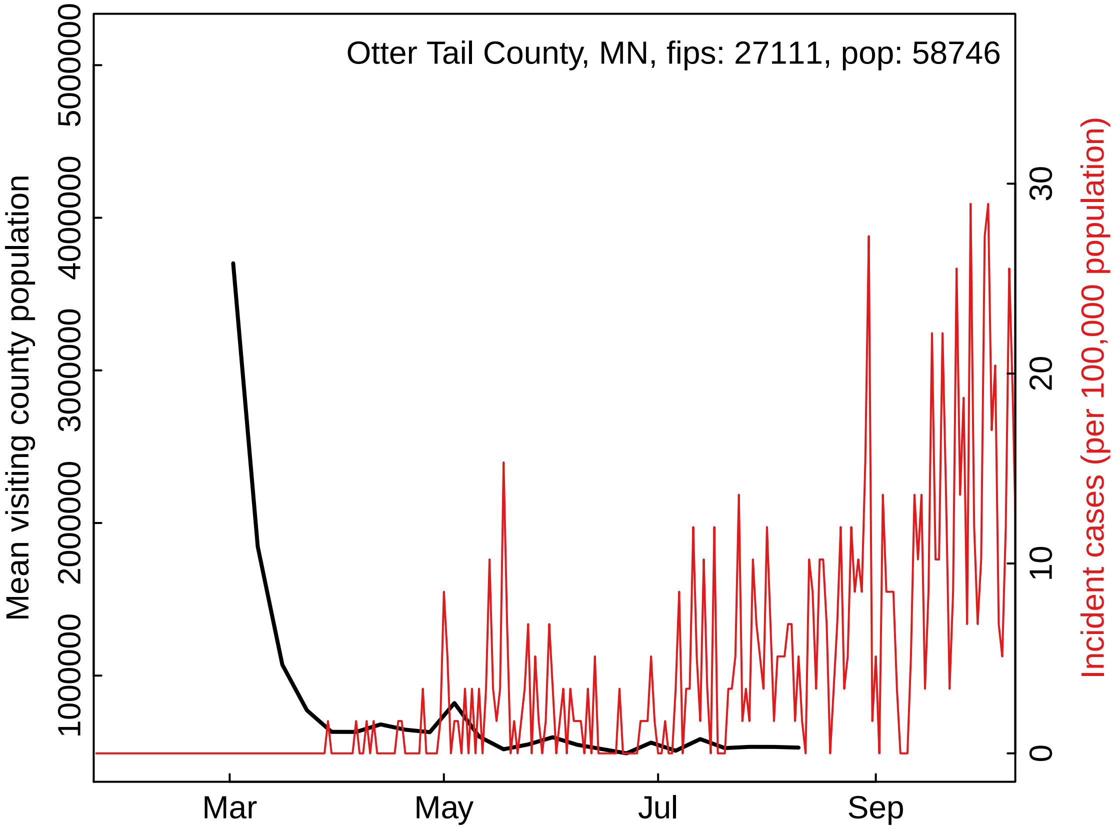

Otter Tail County, MN, fips: 27111, pop: 58746
Mean visiting county population
Incident cases (per 100,000 population)
0
1000000
2000000
3000000
4000000
5000000
0
10
20
30
Mar
May
Jul
Sep

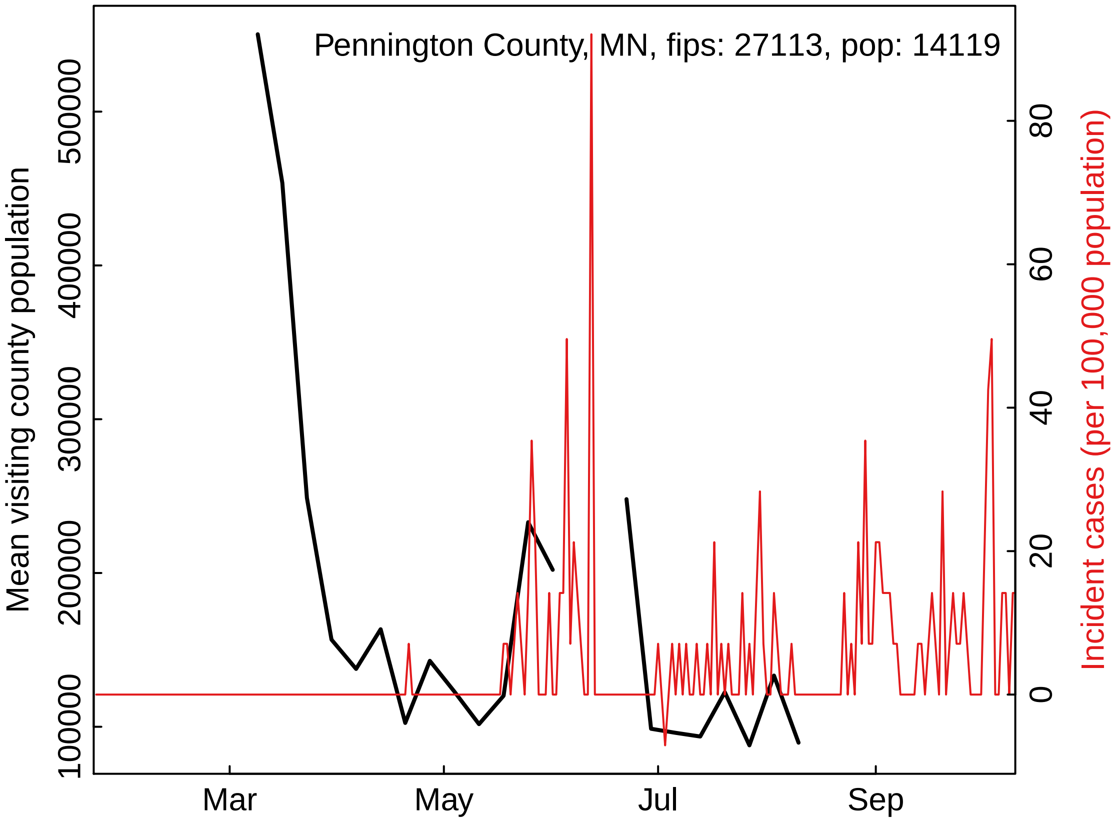

Pennington County, MN, fips: 27113, pop: 14119
Mean visiting county population
100000
200000
300000
400000
500000
Incident cases (per 100,000 population)
0
20
40
60
80
Mar
May
Jul
Sep

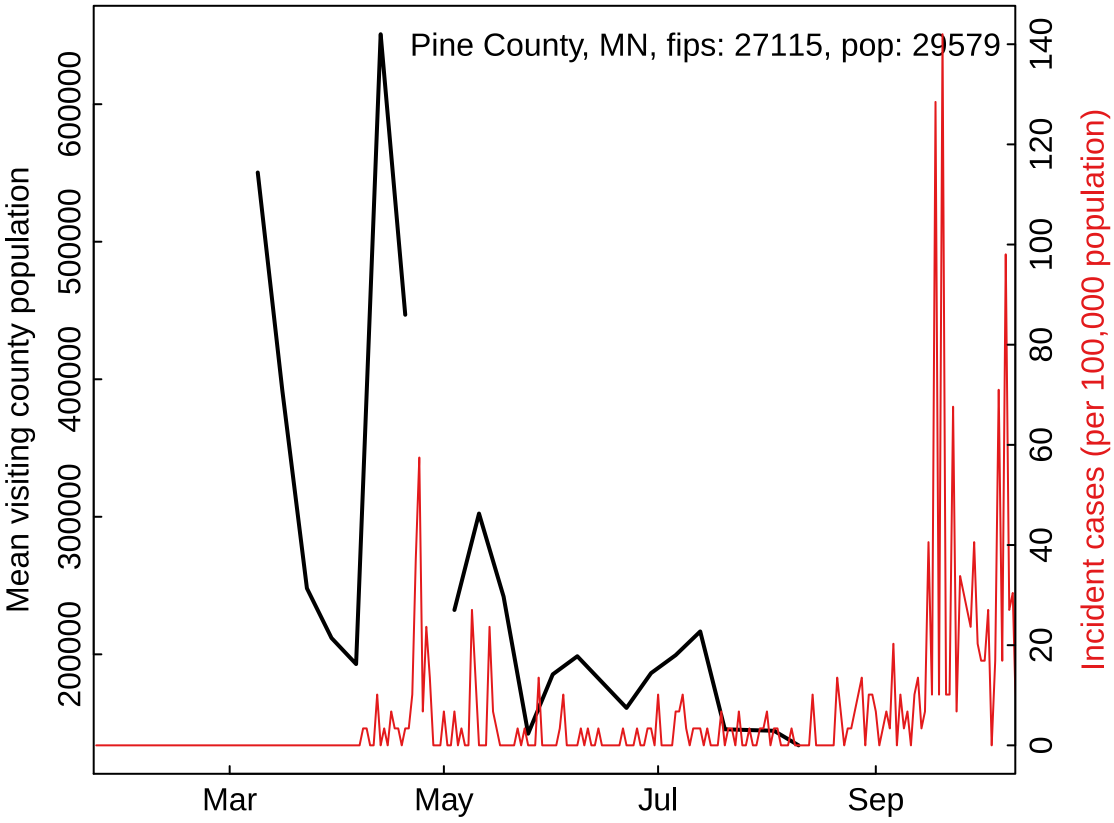

Pine County, MN, fips: 27115, pop: 29579
Mean visiting county population
200000
300000
400000
500000
600000
Incident cases (per 100,000 population)
0
20
40
60
80
100
120
140
Mar
May
Jul
Sep

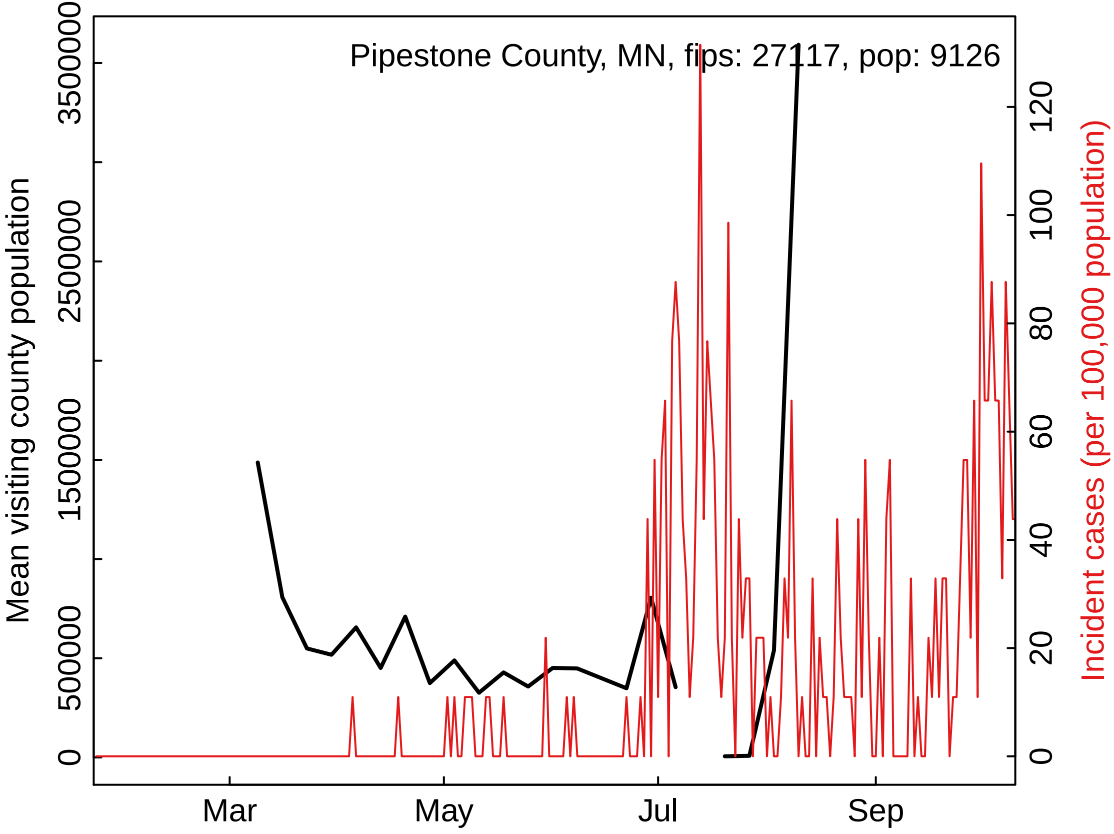
Pipestone County, MN, fips: 27117, pop: 9126
Mean visiting county population
Incident cases (per 100,000 population)
0
500000
1500000
2500000
3500000
0
20
40
60
80
100
120
Mar
May
Jul
Sep

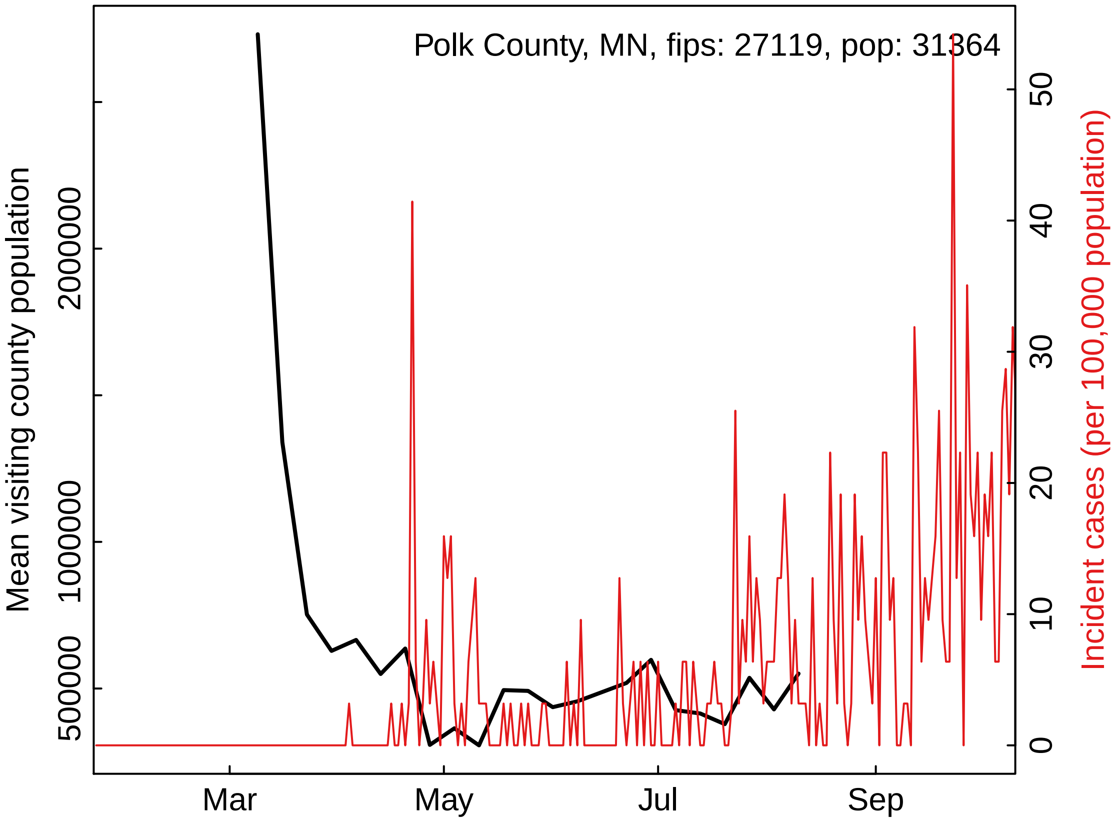

Polk County, MN, fips: 27119, pop: 31364
Mean visiting county population
500000
1000000
2000000
Mar
May
Jul
Sep
0
10
20
30
40
50
Incident cases (per 100,000 population)

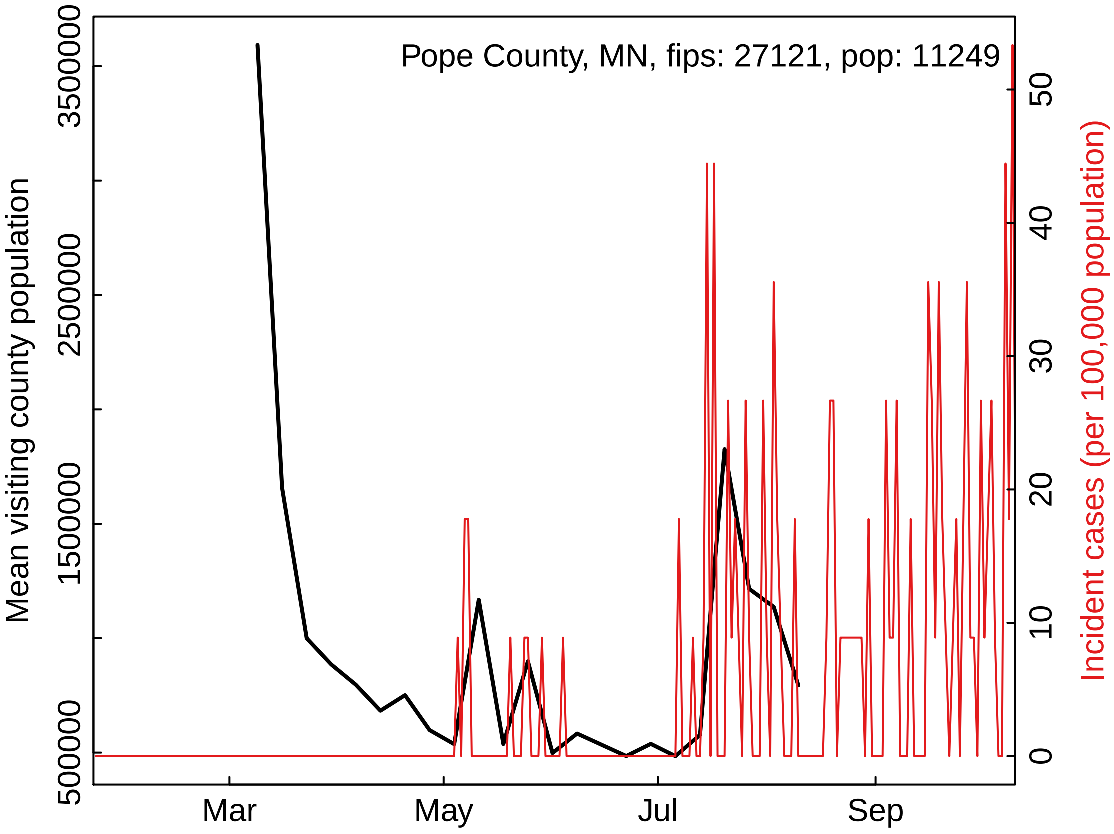

Pope County, MN, fips: 27121, pop: 11249
Mean visiting county population
Incident cases (per 100,000 population)
500000
1500000
2500000
3500000
0
10
20
30
40
50
Mar
May
Jul
Sep

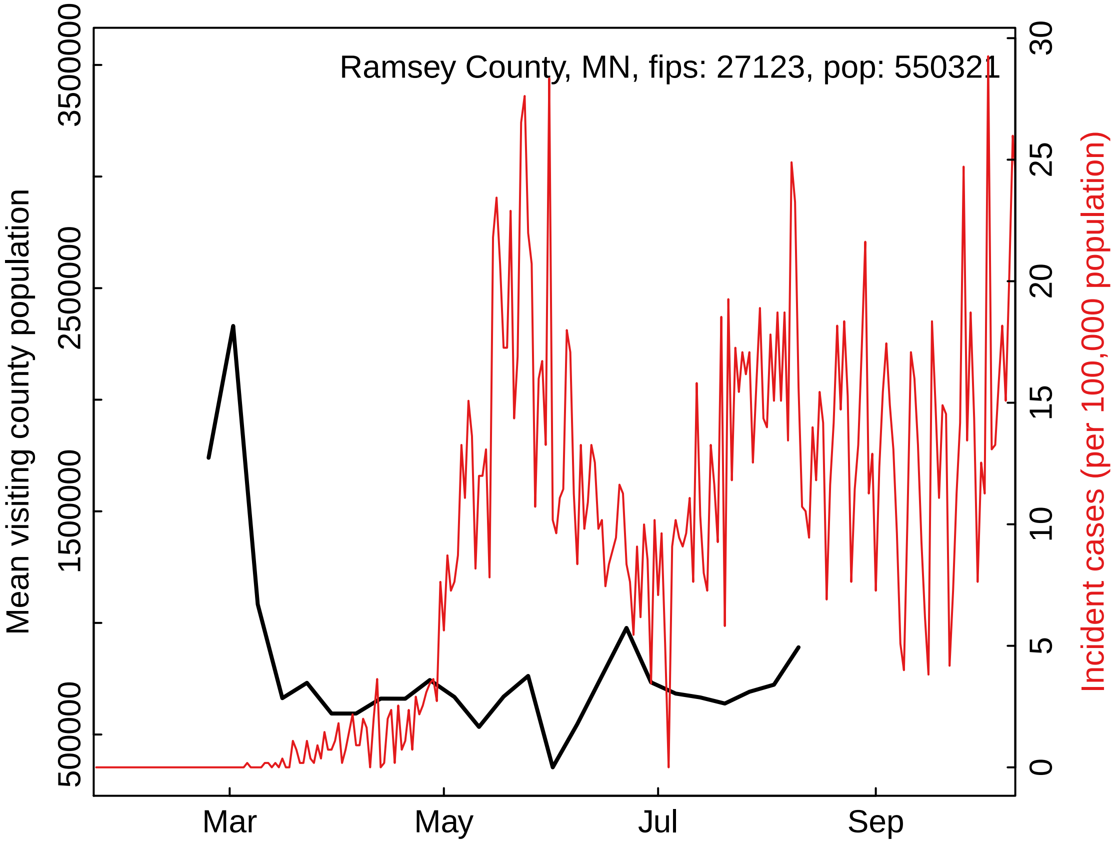

Ramsey County, MN, fips: 27123, pop: 550321
Mean visiting county population
500000
1500000
2500000
3500000
Incident cases (per 100,000 population)
0
5
10
15
20
25
30
Mar
May
Jul
Sep

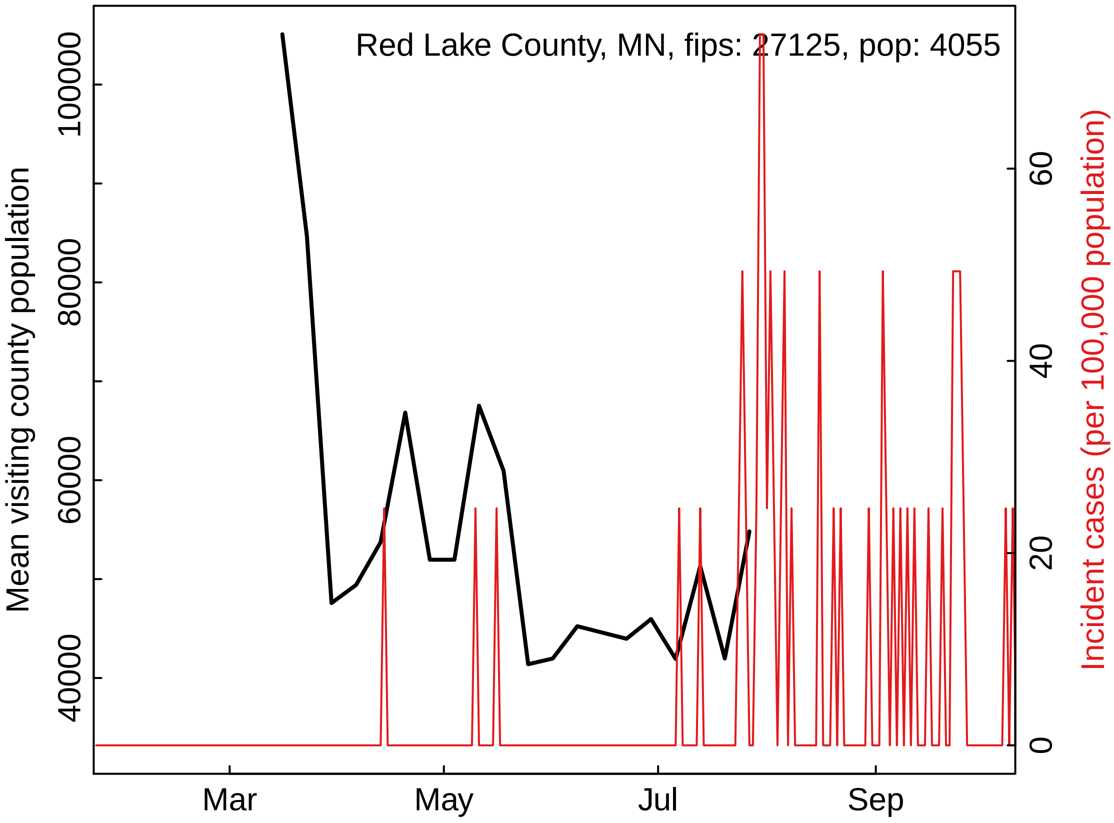

Red Lake County, MN, fips: 27125, pop: 4055
Mean visiting county population
40000
60000
80000
100000
Incident cases (per 100,000 population)
0
20
40
60
Mar
May
Jul
Sep

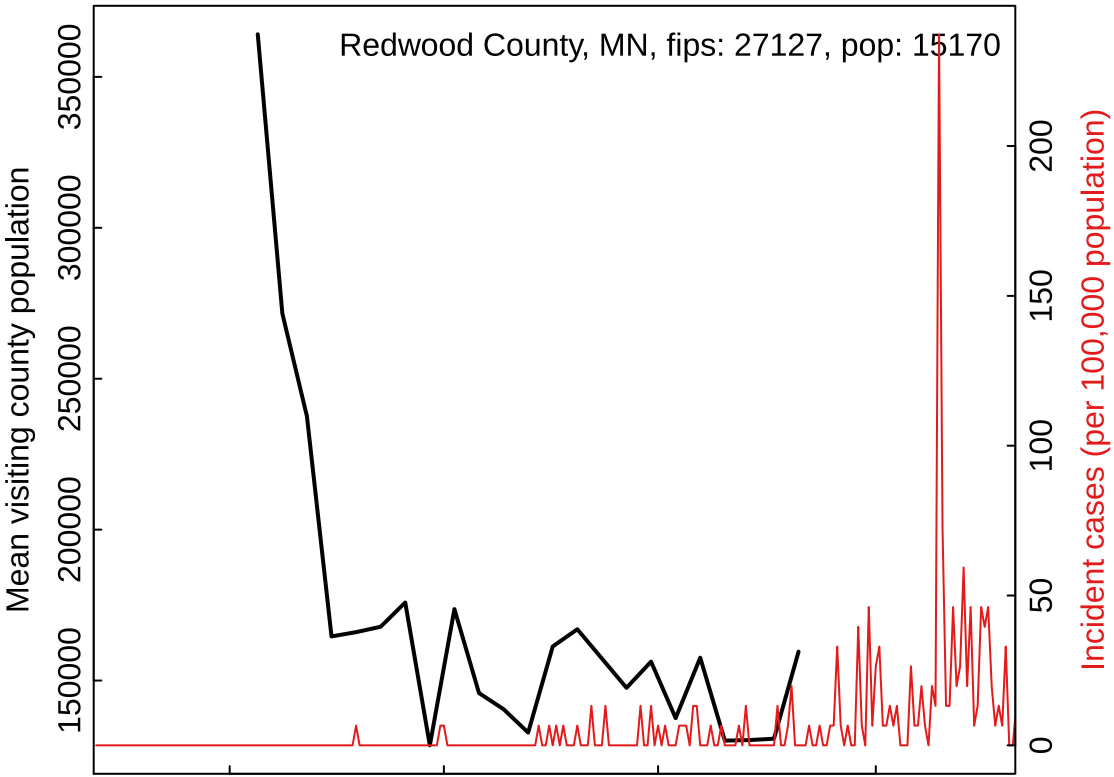

Redwood County, MN, fips: 27127, pop: 15170
Mean visiting county population
350000
300000
250000
200000
150000
Incident cases (per 100,000 population)
200
150
100
50
0
Mar
May
Jul
Sep

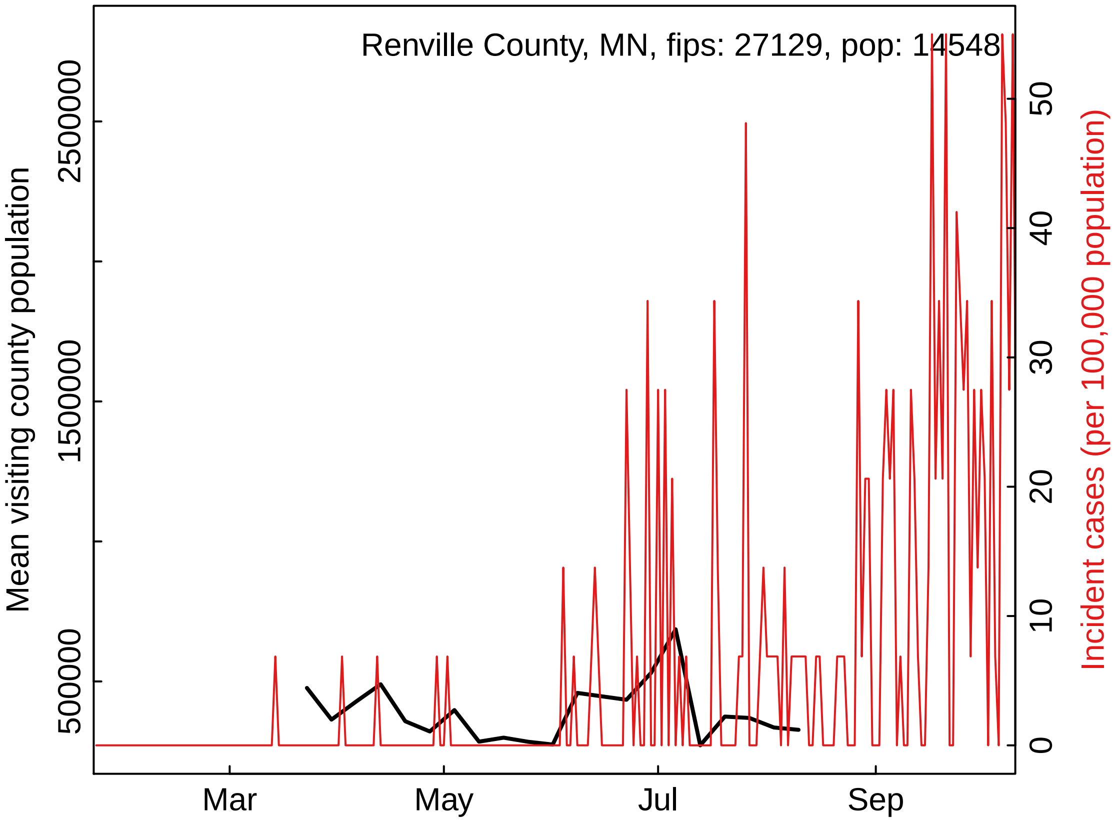

Renville County, MN, fips: 27129, pop: 14548
Mean visiting county population
500000
1500000
2500000
Incident cases (per 100,000 population)
0
10
20
30
40
50
Mar
May
Jul
Sep

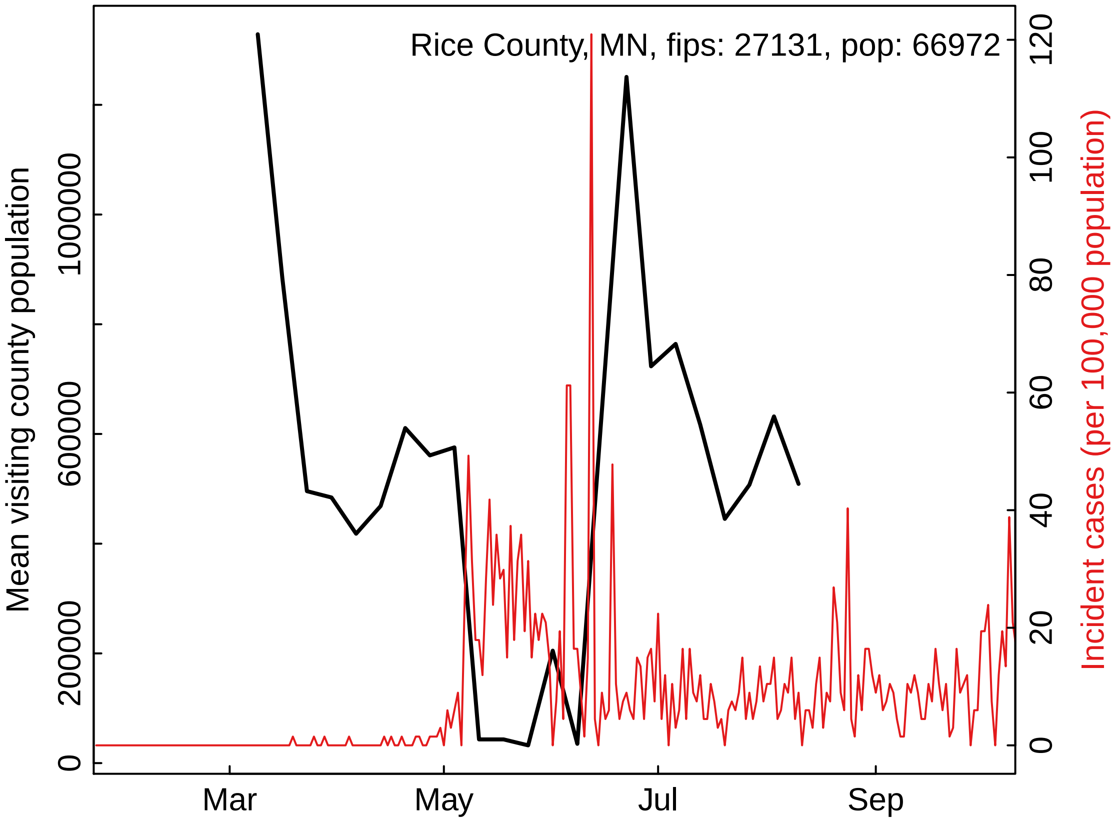

Rice County, MN, fips: 27131, pop: 66972
Mean visiting county population
0
200000
600000
1000000
Incident cases (per 100,000 population)
0
20
40
60
80
100
120
Mar
May
Jul
Sep

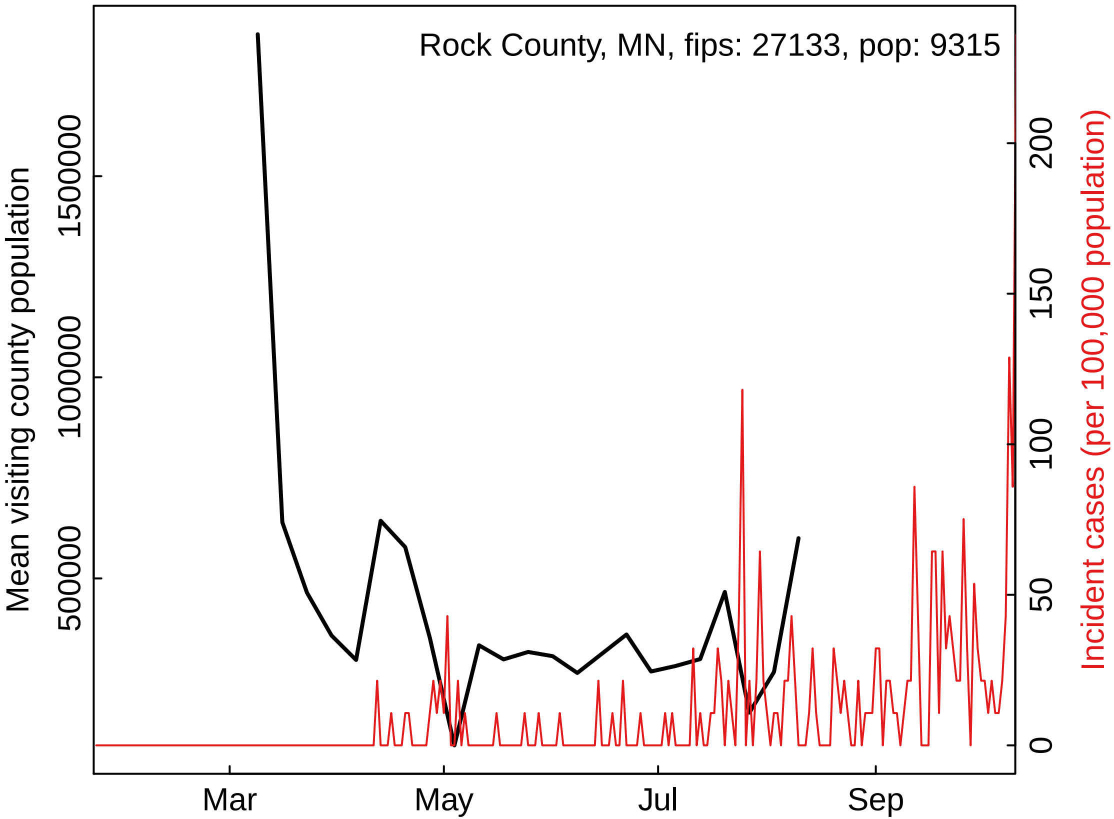
Rock County, MN, fips: 27133, pop: 9315
Mean visiting county population
500000
1000000
1500000
Incident cases (per 100,000 population)
0
50
100
150
200
Mar
May
Jul
Sep

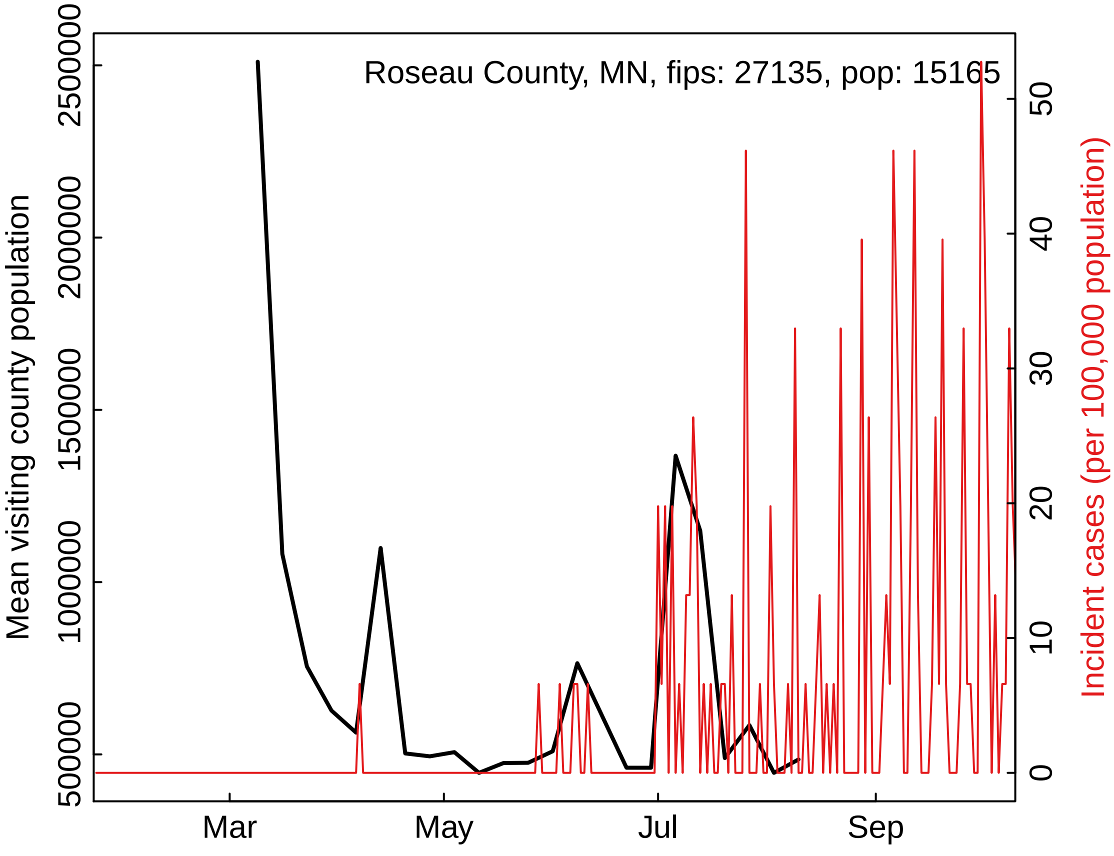
Roseau County, MN, fips: 27135, pop: 15165
Mean visiting county population
500000
1000000
1500000
2000000
2500000
Incident cases (per 100,000 population)
0
10
20
30
40
50
Mar
May
Jul
Sep

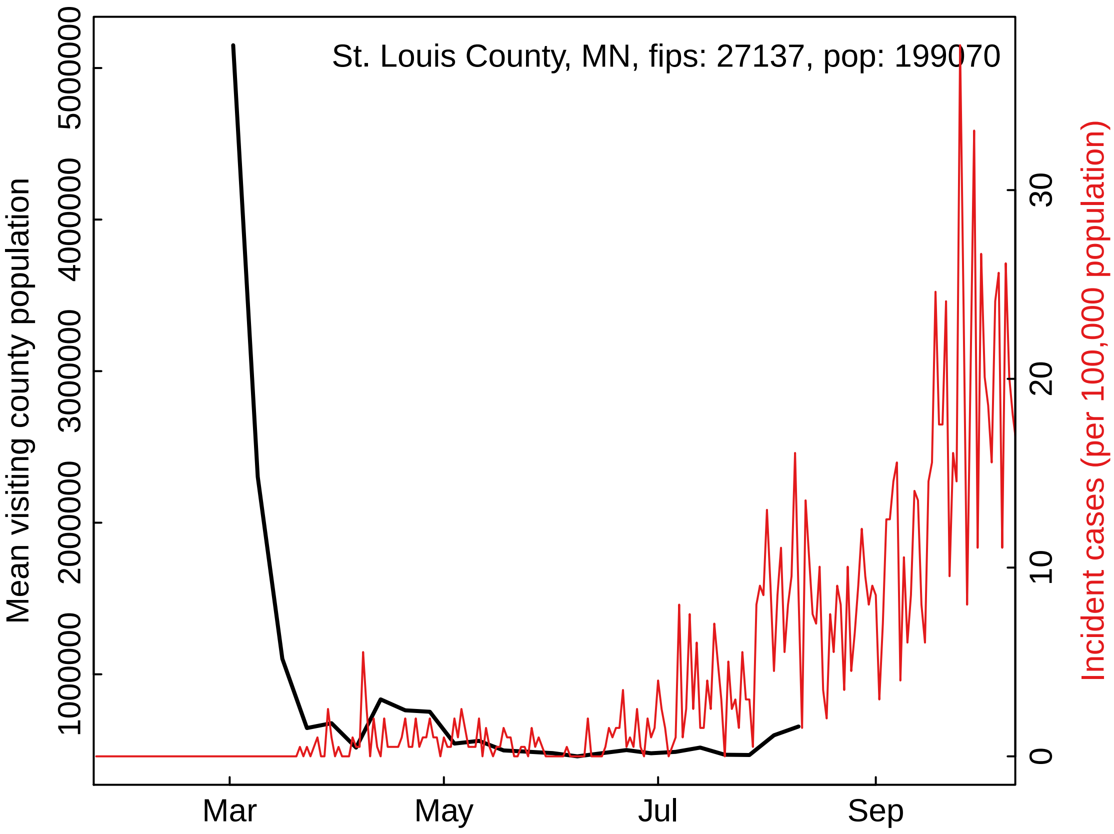
St. Louis County, MN, fips: 27137, pop: 199070
Mean visiting county population
Incident cases (per 100,000 population)
0
1000000
2000000
3000000
4000000
5000000
0
10
20
30
Mar
May
Jul
Sep

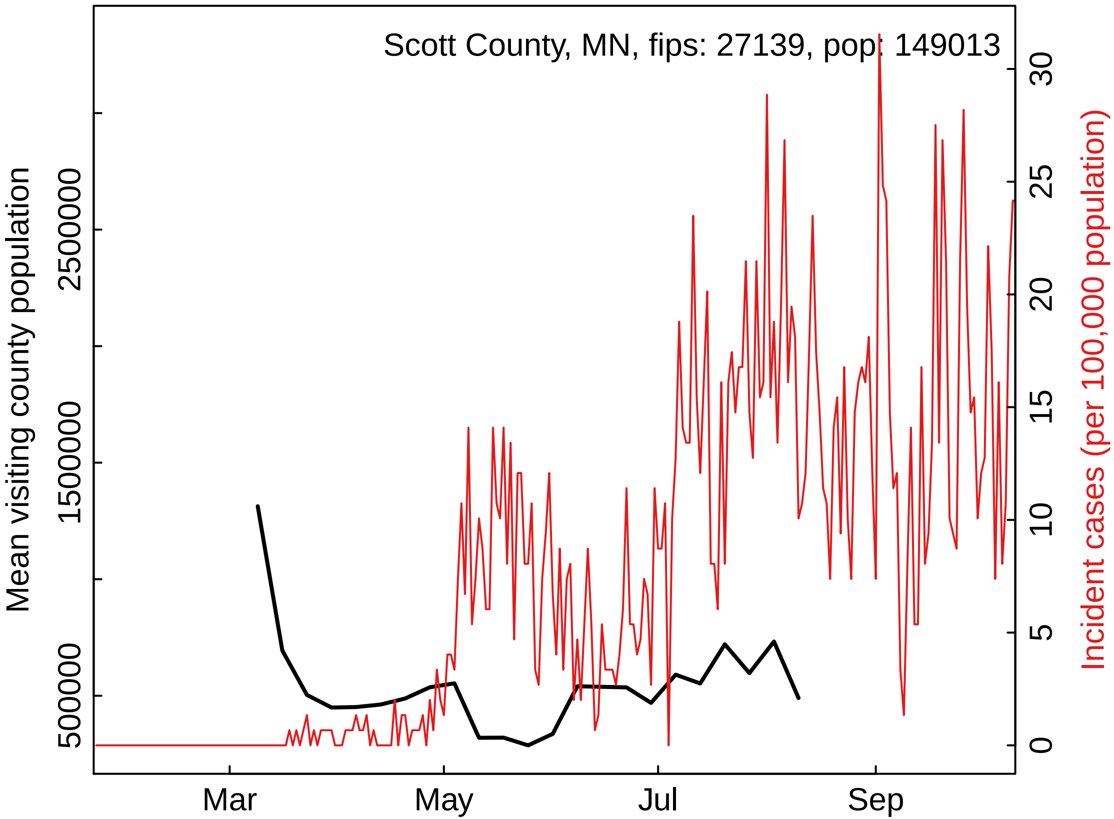
Scott County, MN, fips: 27139, pop: 149013
Mean visiting county population
500000
1500000
2500000
Incident cases (per 100,000 population)
0
5
10
15
20
25
30
Mar
May
Jul
Sep

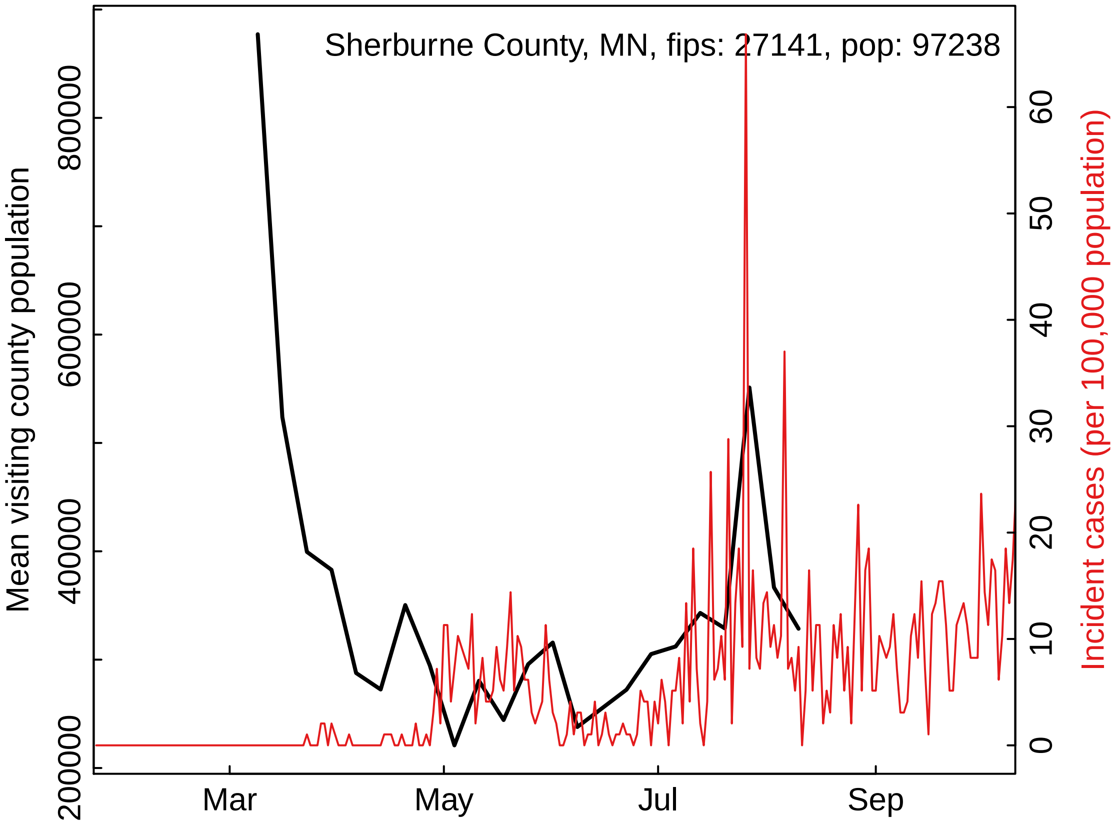

Sherburne County, MN, fips: 27141, pop: 97238
Mean visiting county population
200000
400000
600000
800000
Incident cases (per 100,000 population)
0
10
20
30
40
50
60
Mar
May
Jul
Sep

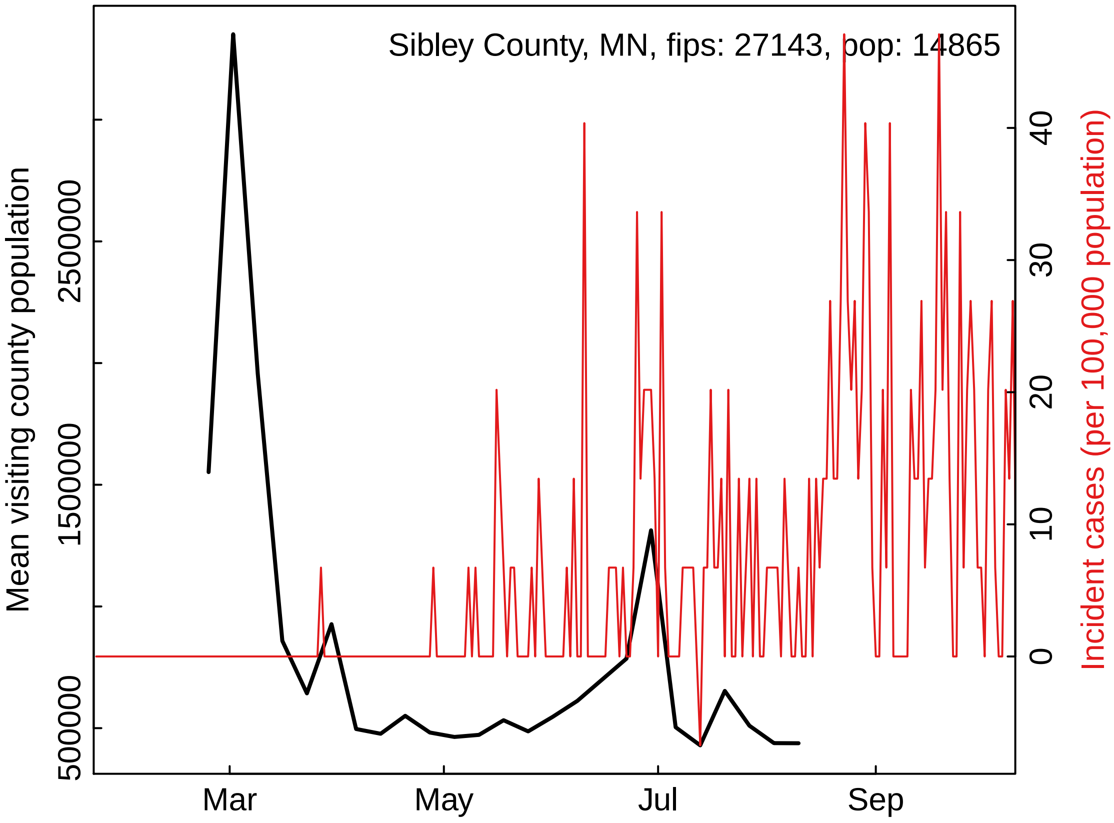

Sibley County, MN, fips: 27143, pop: 14865
Mean visiting county population
500000
1500000
2500000
Mar
May
Jul
Sep
0
10
20
30
40
Incident cases (per 100,000 population)

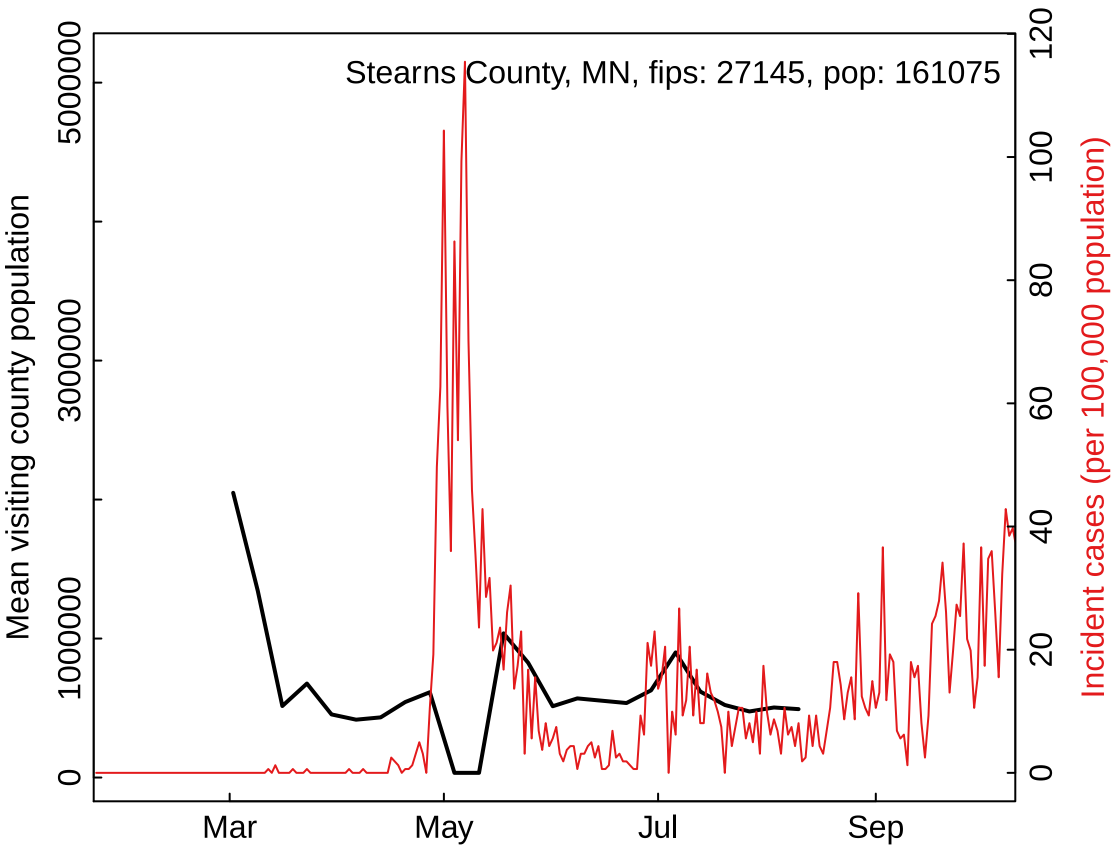

Stearns County, MN, fips: 27145, pop: 161075
Mean visiting county population
0
1000000
3000000
5000000
Incident cases (per 100,000 population)
0
20
40
60
80
100
120
Mar
May
Jul
Sep

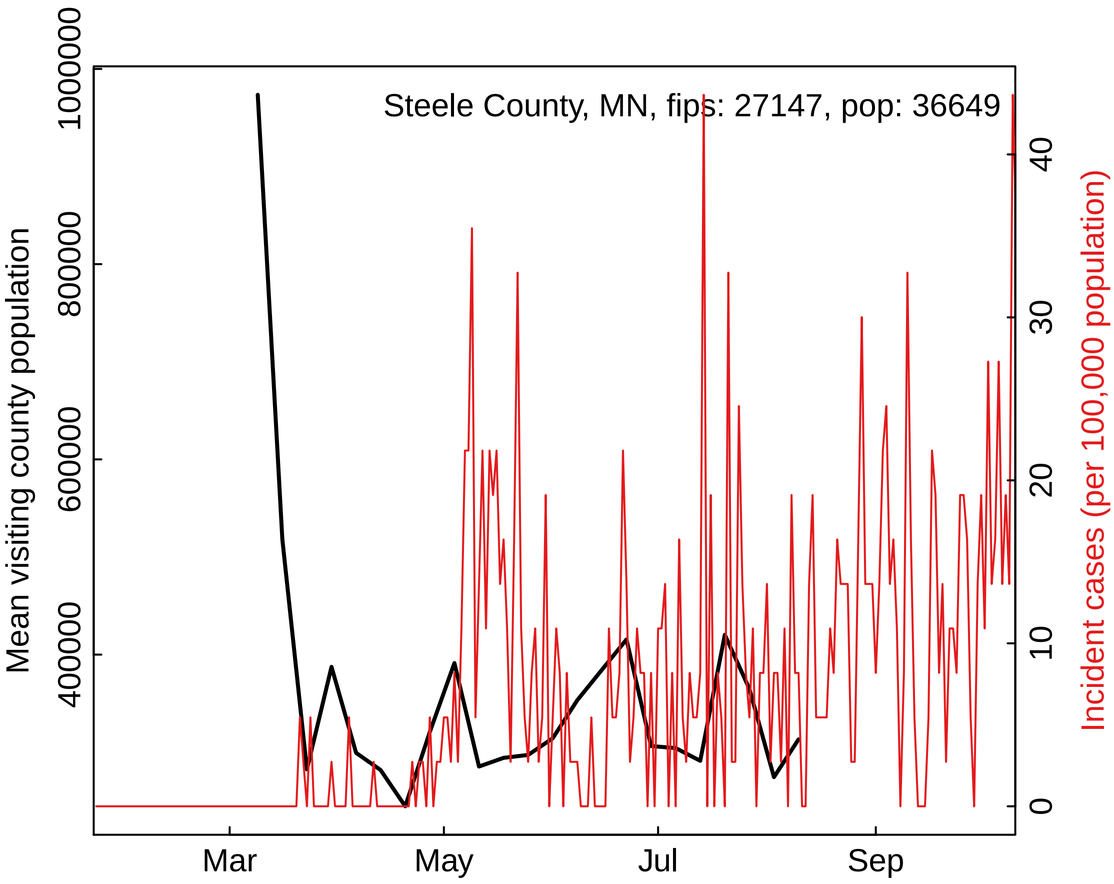
Steele County, MN, fips: 27147, pop: 36649
Mean visiting county population
400000
600000
800000
1000000
Incident cases (per 100,000 population)
0
10
20
30
40
Mar
May
Jul
Sep

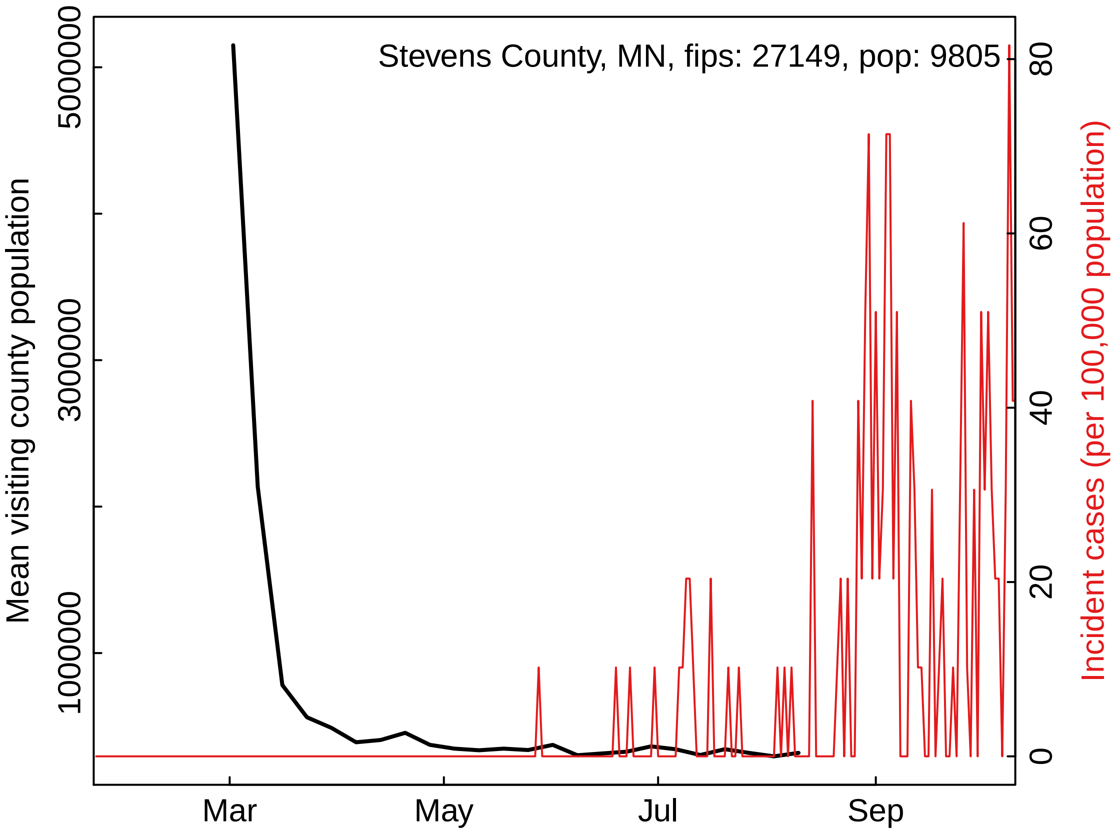

Stevens County, MN, fips: 27149, pop: 9805
Mean visiting county population
Incident cases (per 100,000 population)
1000000
3000000
5000000
0
20
40
60
80
Mar
May
Jul
Sep

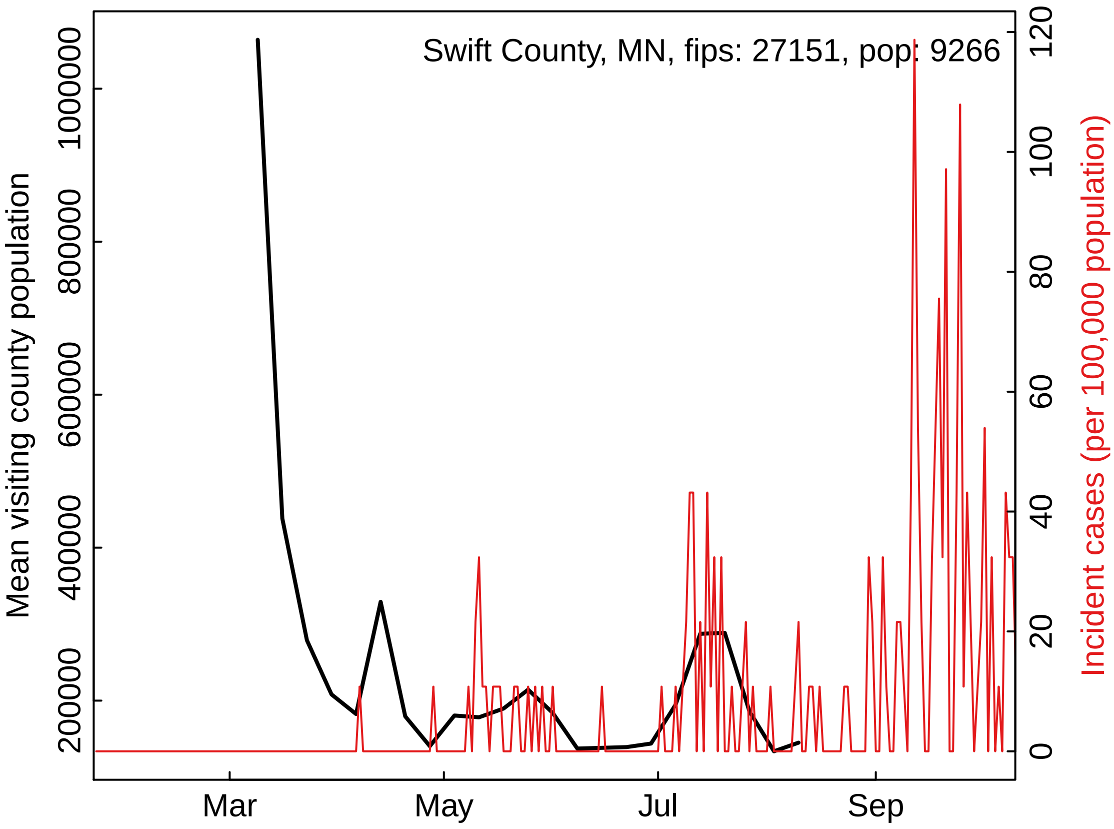
Swift County, MN, fips: 27151, pop: 9266
Mean visiting county population
200000
400000
600000
800000
1000000
Incident cases (per 100,000 population)
0
20
40
60
80
100
120
Mar
May
Jul
Sep

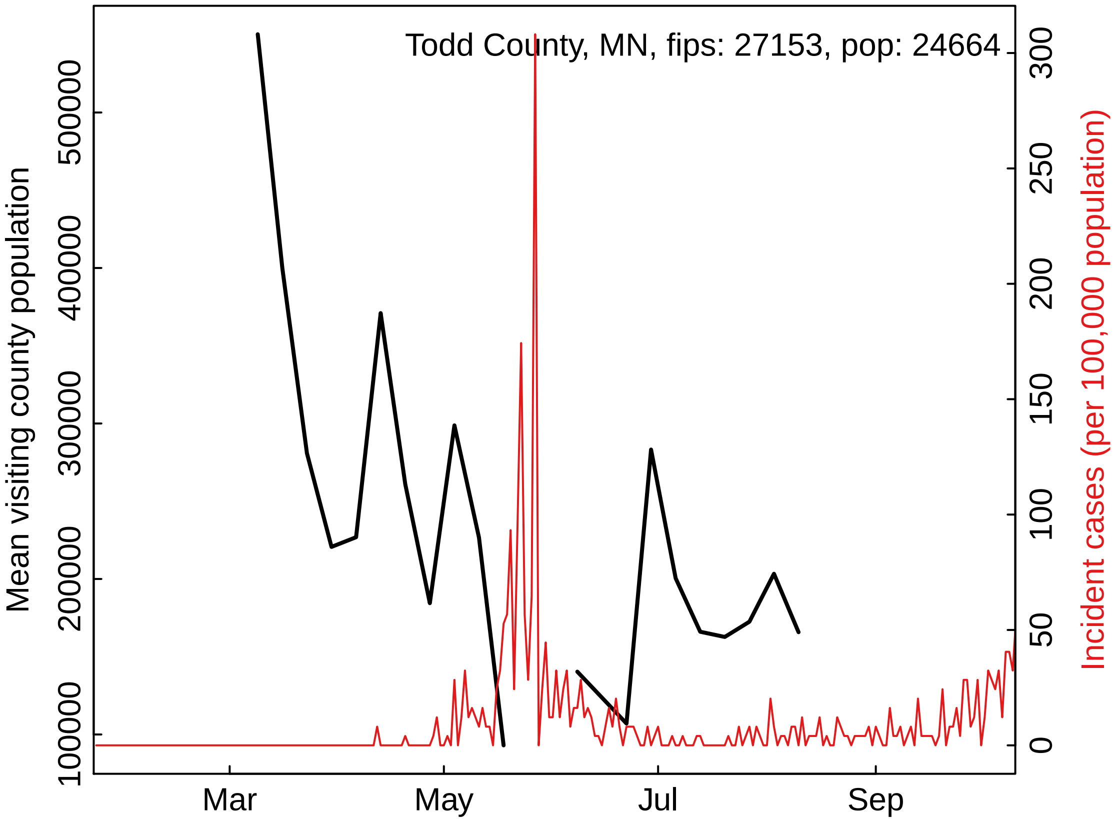

Todd County, MN, fips: 27153, pop: 24664
Mean visiting county population
Incident cases (per 100,000 population)
100000
200000
300000
400000
500000
0
50
100
150
200
250
300
Mar
May
Jul
Sep

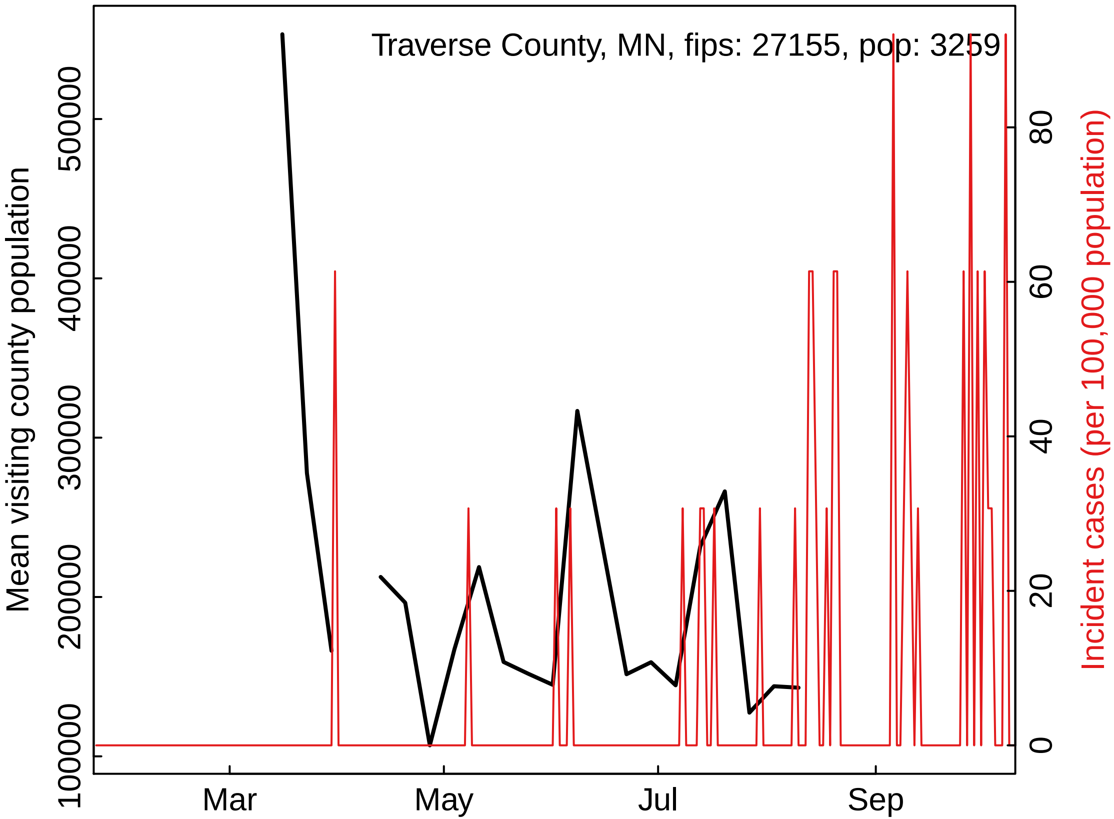
Traverse County, MN, fips: 27155, pop: 3259
Mean visiting county population
100000
200000
300000
400000
500000
Incident cases (per 100,000 population)
0
20
40
60
80
Mar
May
Jul
Sep

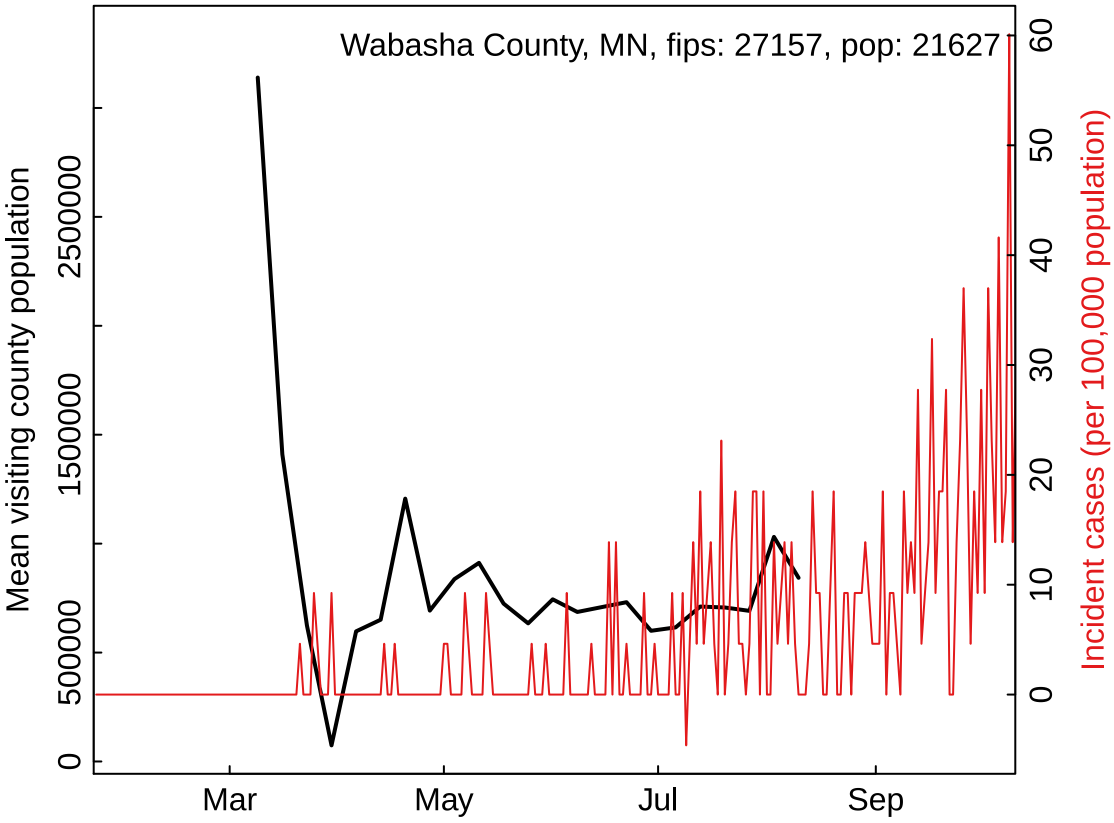

Wabasha County, MN, fips: 27157, pop: 21627
Mean visiting county population
0
500000
1500000
2500000
Incident cases (per 100,000 population)
0
10
20
30
40
50
60
Mar
May
Jul
Sep

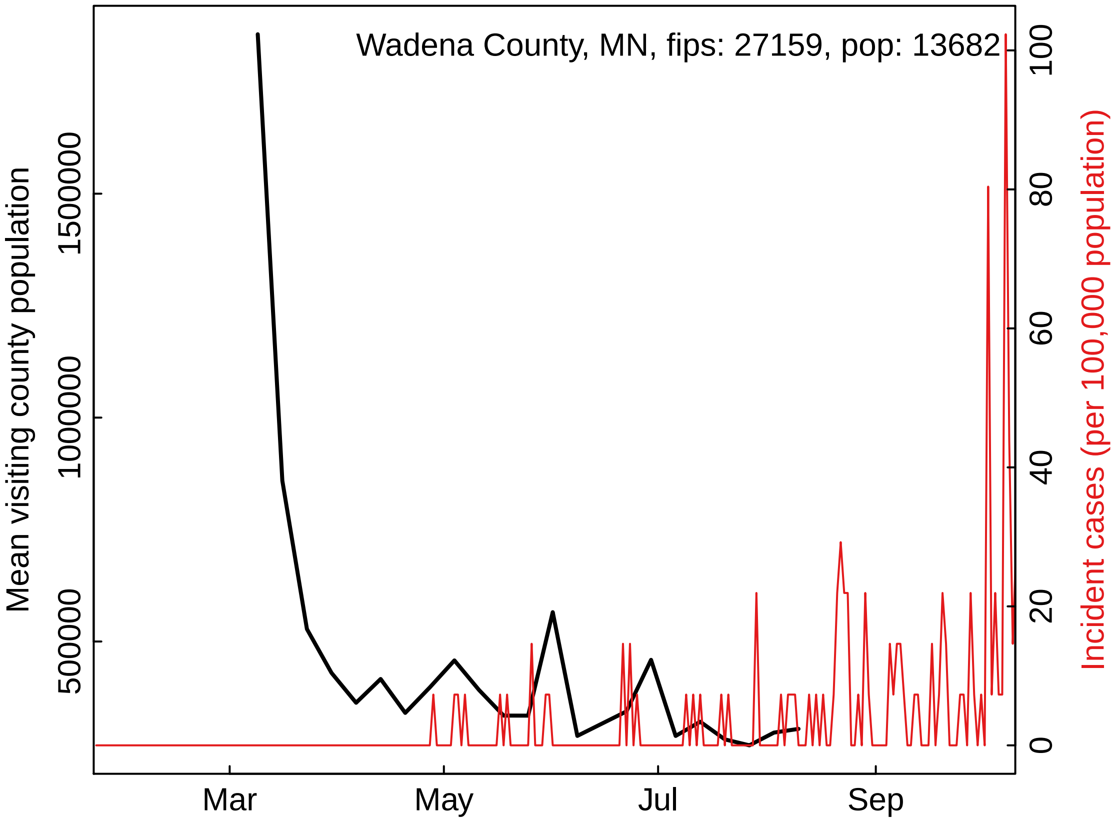

Wadena County, MN, fips: 27159, pop: 13682
Mean visiting county population
500000
1000000
1500000
Mar
May
Jul
Sep
0
20
40
60
80
100
Incident cases (per 100,000 population)

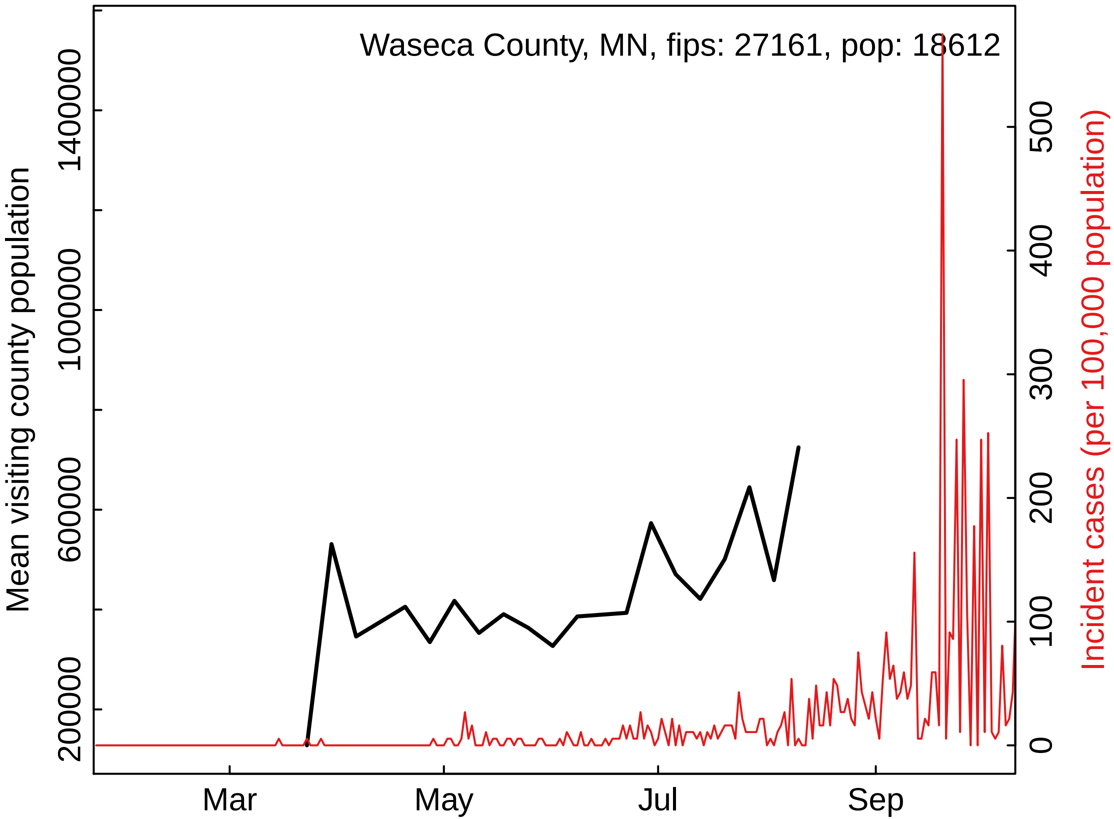

Waseca County, MN, fips: 27161, pop: 18612
Mean visiting county population
200000
600000
1000000
1400000
Incident cases (per 100,000 population)
0
100
200
300
400
500
Mar
May
Jul
Sep

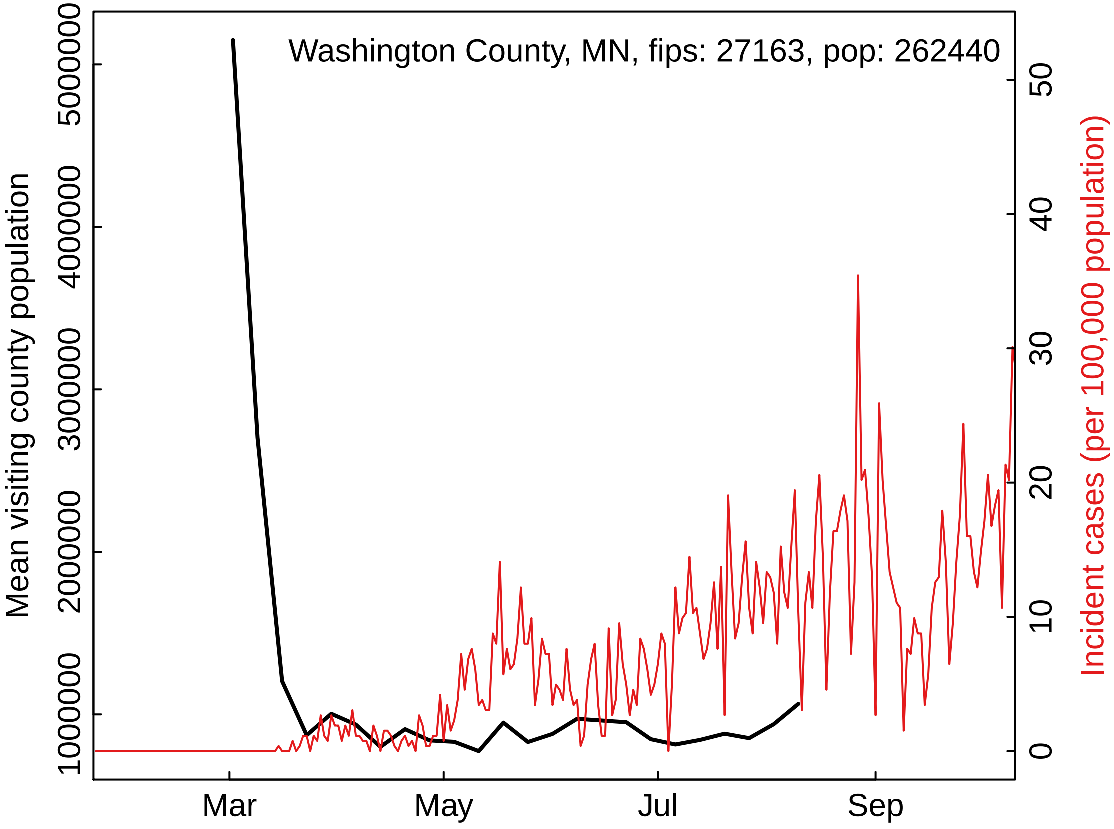

Washington County, MN, fips: 27163, pop: 262440
Mean visiting county population
Incident cases (per 100,000 population)
1000000
2000000
3000000
4000000
5000000
0
10
20
30
40
50
Mar
May
Jul
Sep

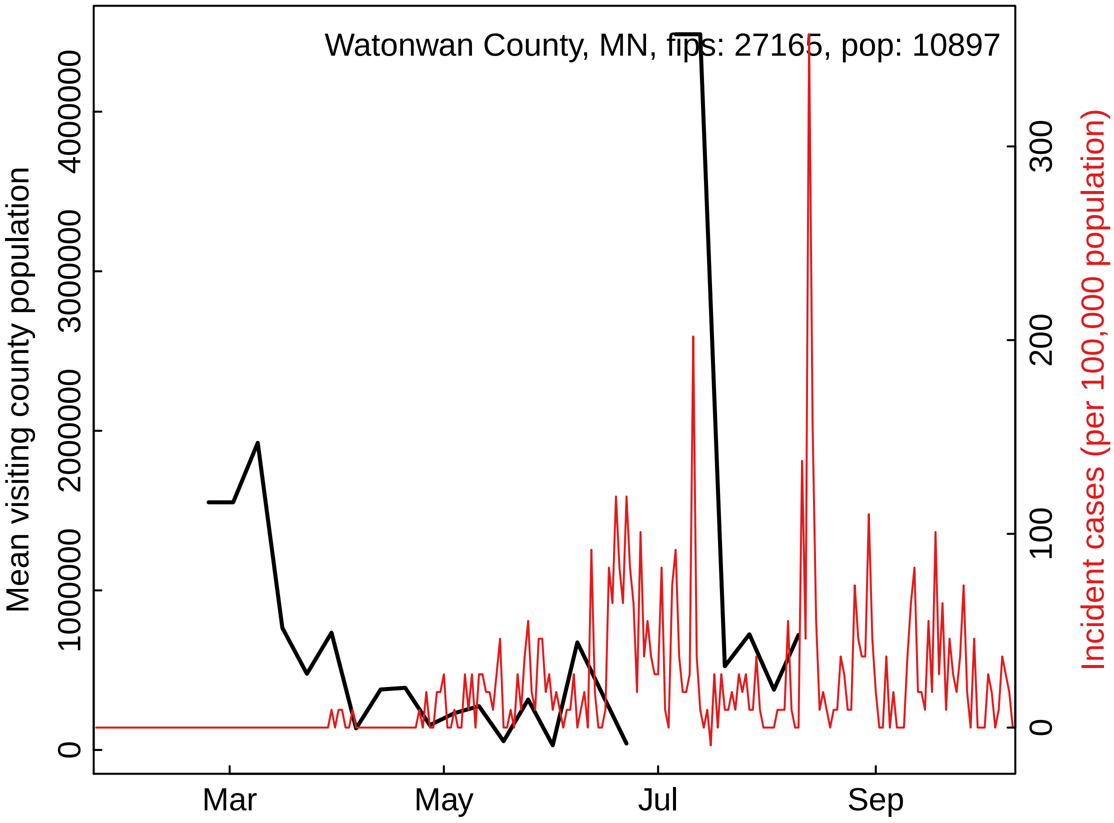

Watonwan County, MN, fips: 27165, pop: 10897
Mean visiting county population
0
1000000
2000000
3000000
4000000
Incident cases (per 100,000 population)
0
100
200
300
Mar
May
Jul
Sep

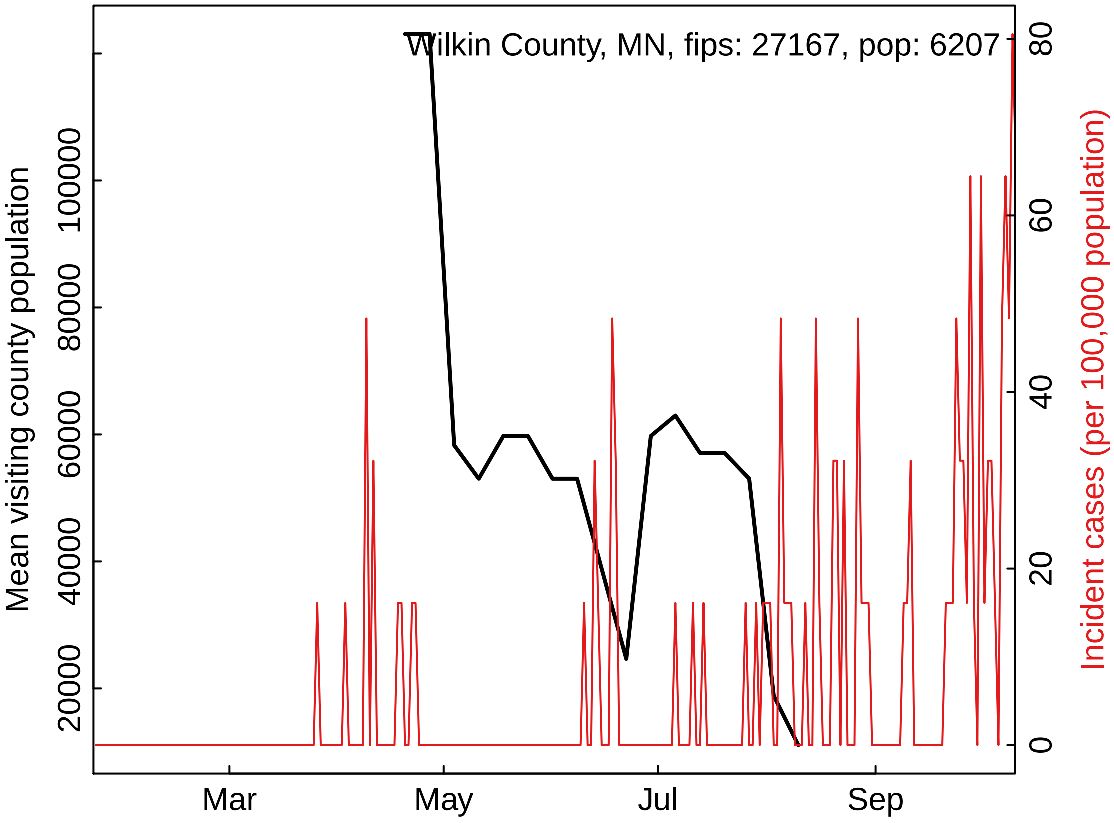
Wilkin County, MN, fips: 27167, pop: 6207
Mean visiting county population
20000
40000
60000
80000
100000
Incident cases (per 100,000 population)
0
20
40
60
80
Mar
May
Jul
Sep

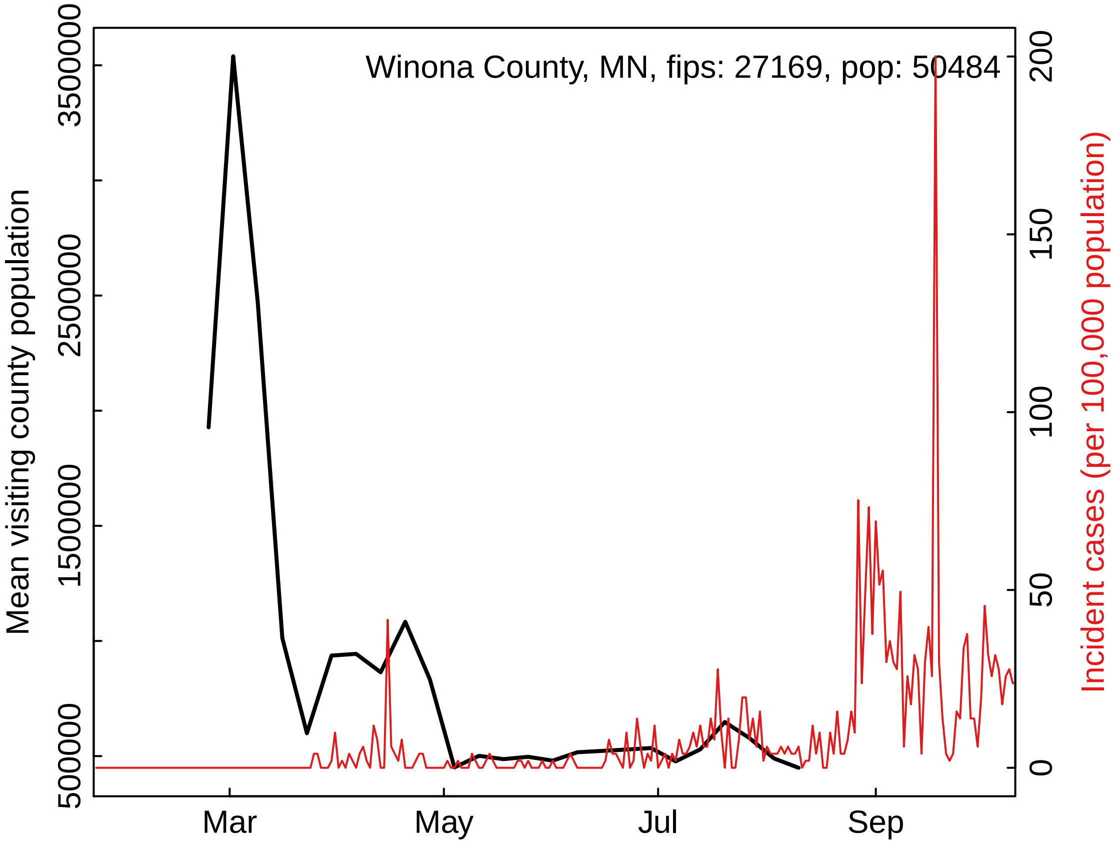

Winona County, MN, fips: 27169, pop: 50484
Mean visiting county population
500000
1500000
2500000
3500000
Incident cases (per 100,000 population)
0
50
100
150
200
Mar
May
Jul
Sep

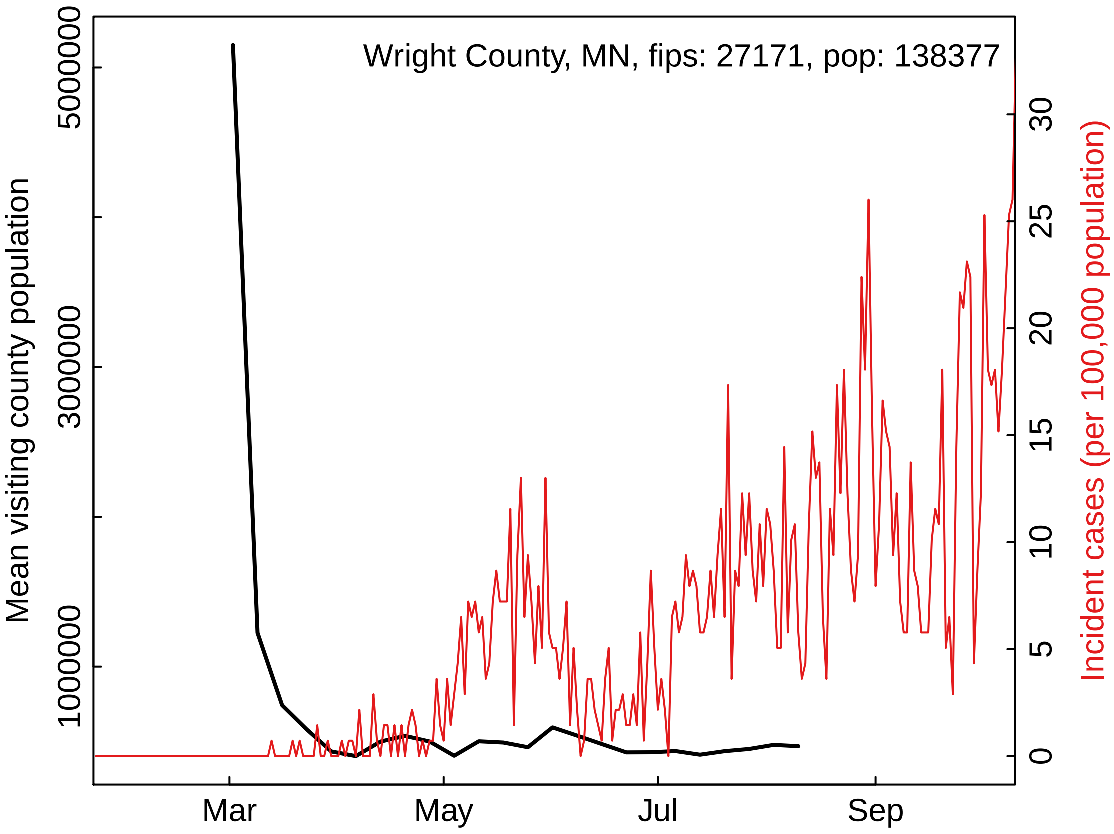

Wright County, MN, fips: 27171, pop: 138377
Mean visiting county population
1000000
3000000
5000000
Incident cases (per 100,000 population)
0
5
10
15
20
25
30
Mar
May
Jul
Sep

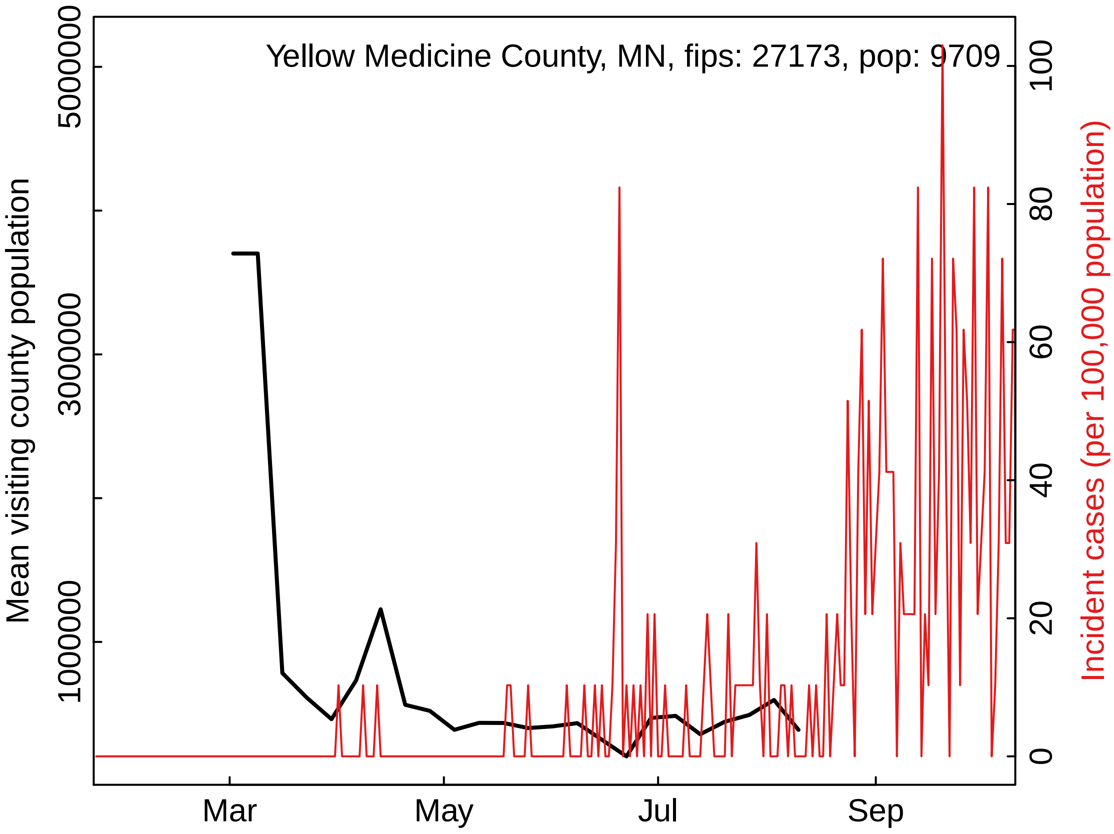
Yellow Medicine County, MN, fips: 27173, pop: 9709
Mean visiting county population
Incident cases (per 100,000 population)
5000000
3000000
1000000
100
80
60
40
20
0
Mar
May
Jul
Sep

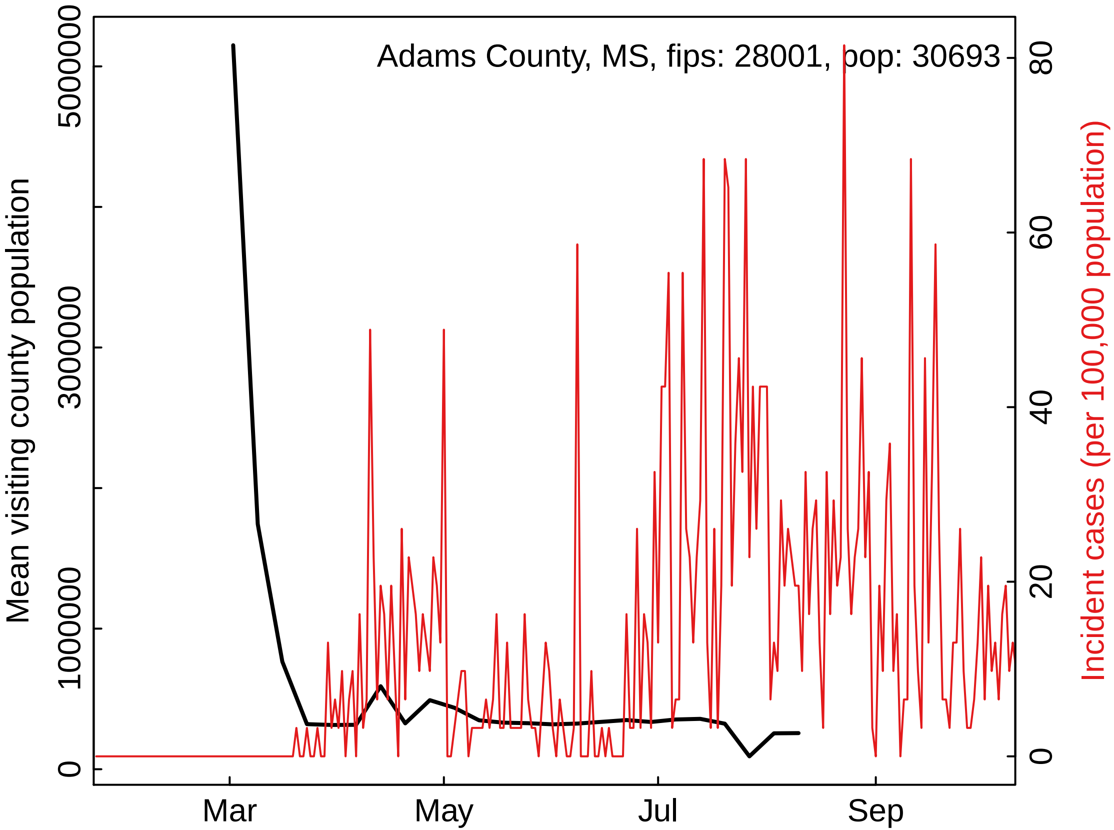

Adams County, MS, fips: 28001, pop: 30693
Mean visiting county population
0
1000000
3000000
5000000
Incident cases (per 100,000 population)
0
20
40
60
80
Mar
May
Jul
Sep

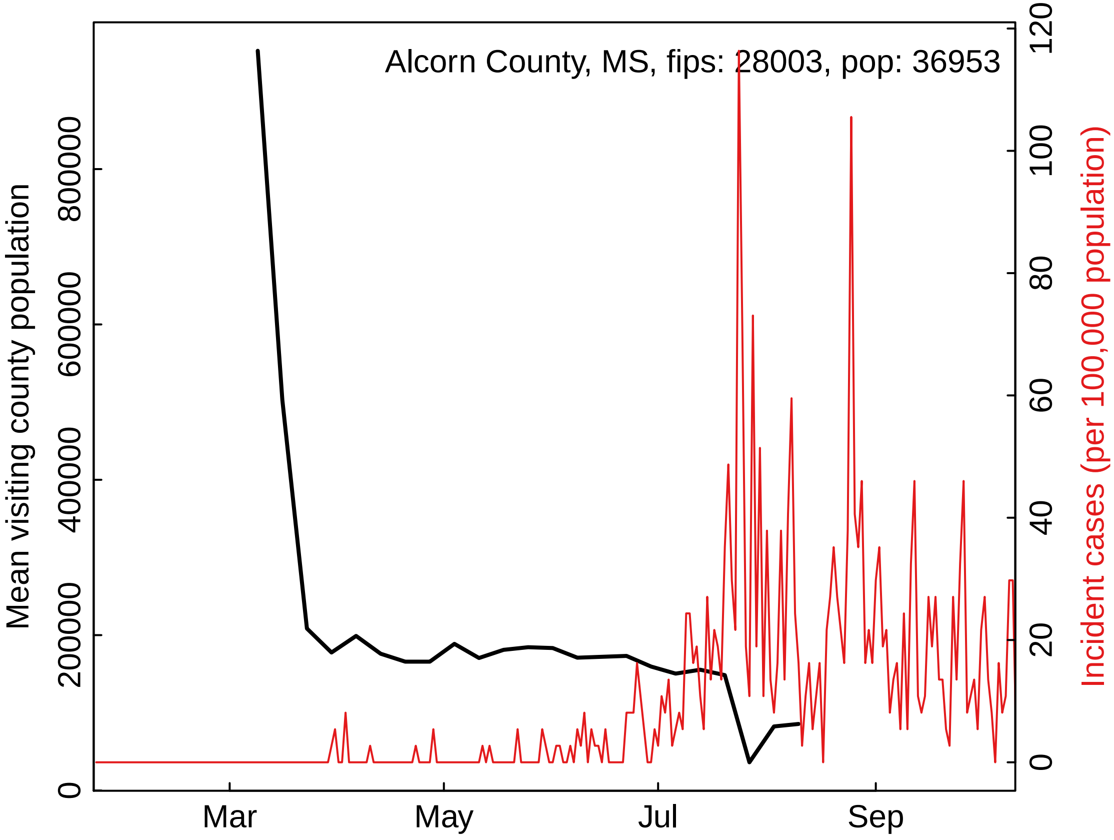

Alcorn County, MS, fips: 28003, pop: 36953
Mean visiting county population
0
200000
400000
600000
800000
Mar
May
Jul
Sep
Incident cases (per 100,000 population)
0
20
40
60
80
100
120

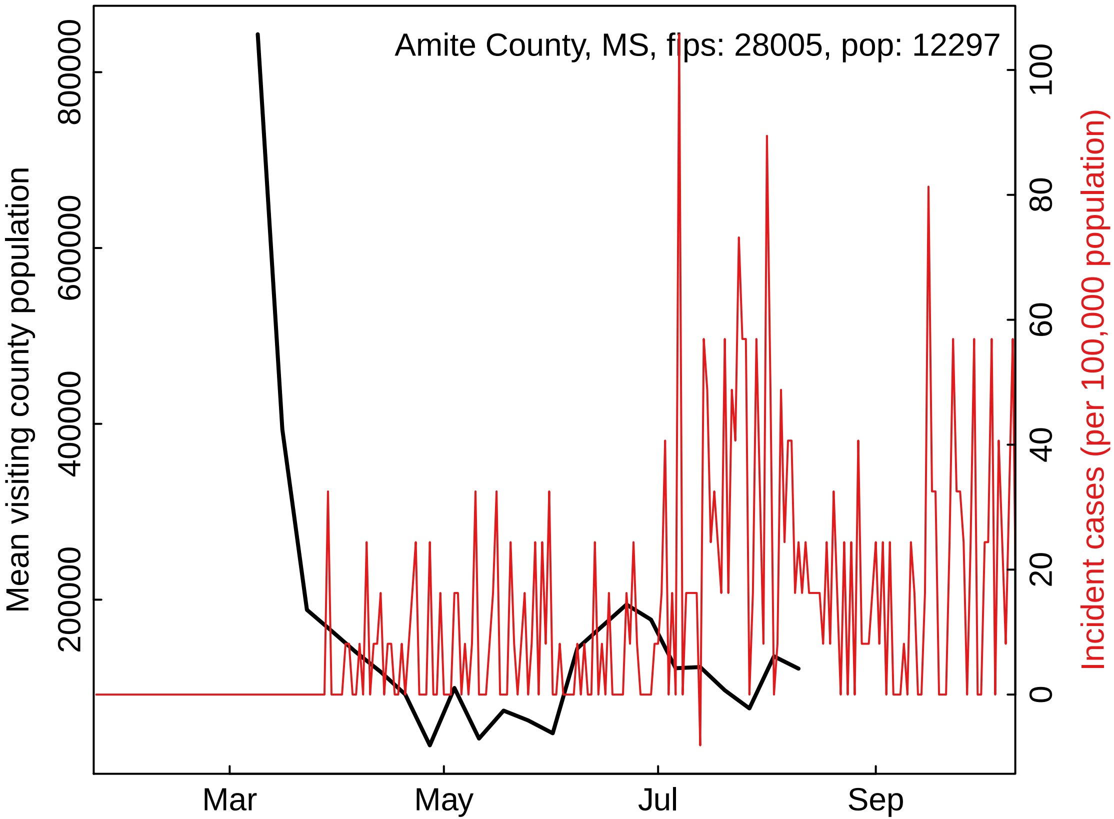
Amite County, MS, flips: 28005, pop: 12297
Mean visiting county population
200000
400000
600000
800000
Incident cases (per 100,000 population)
0
20
40
60
80
100
Mar
May
Jul
Sep

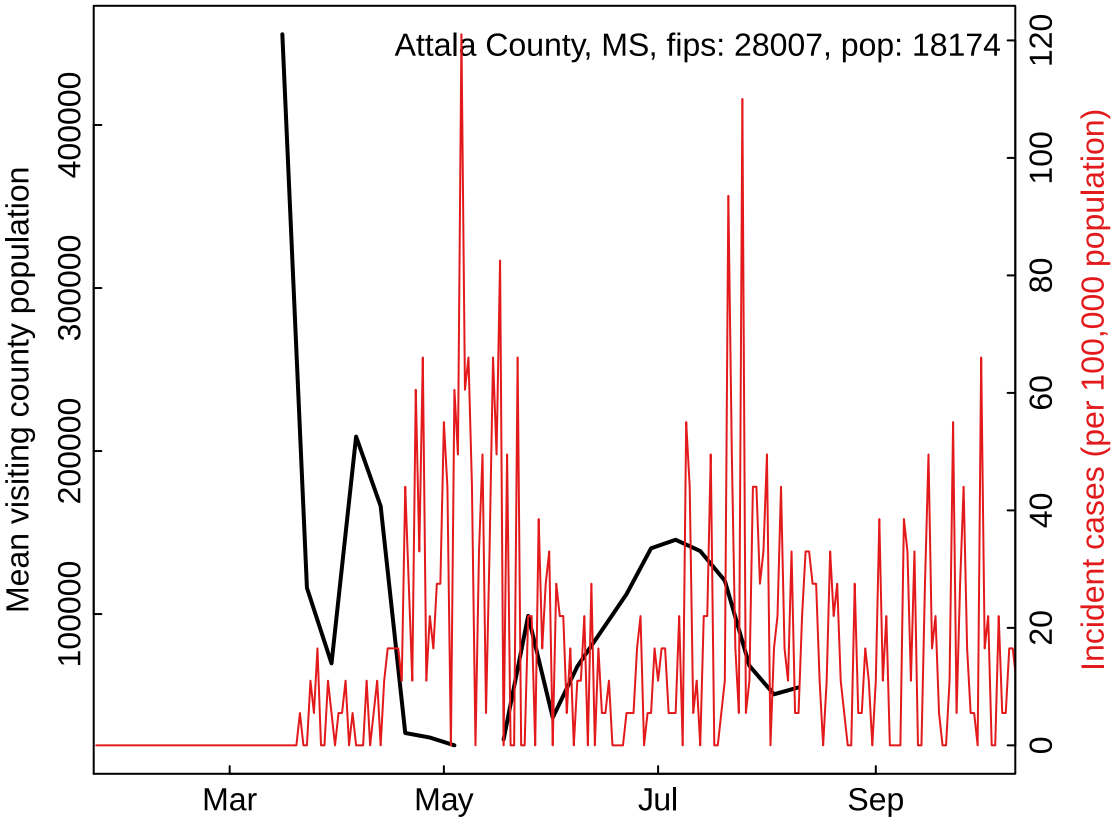
Attala County, MS, fips: 28007, pop: 18174
Mean visiting county population
100000
200000
300000
400000
Incident cases (per 100,000 population)
0
20
40
60
80
100
120
Mar
May
Jul
Sep

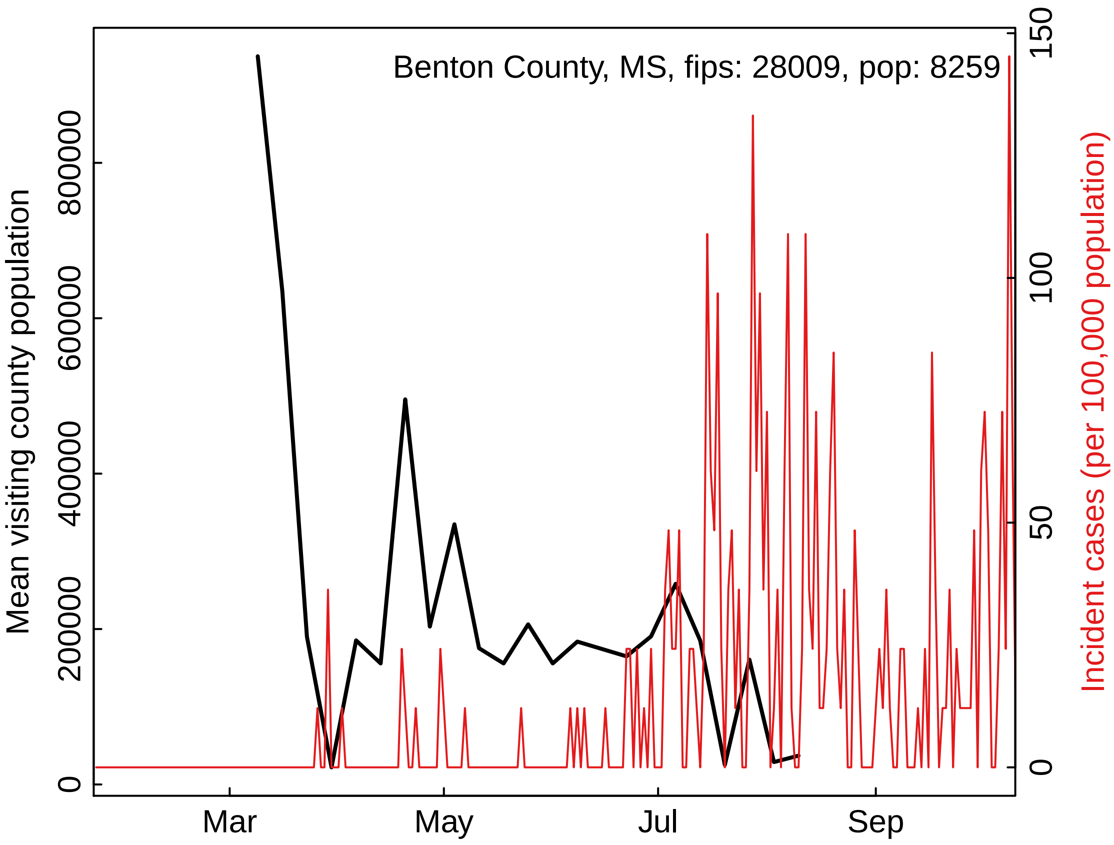

Benton County, MS, fips: 28009, pop: 8259
Mean visiting county population
0
200000
400000
600000
800000
Incident cases (per 100,000 population)
0
50
100
150
Mar
May
Jul
Sep

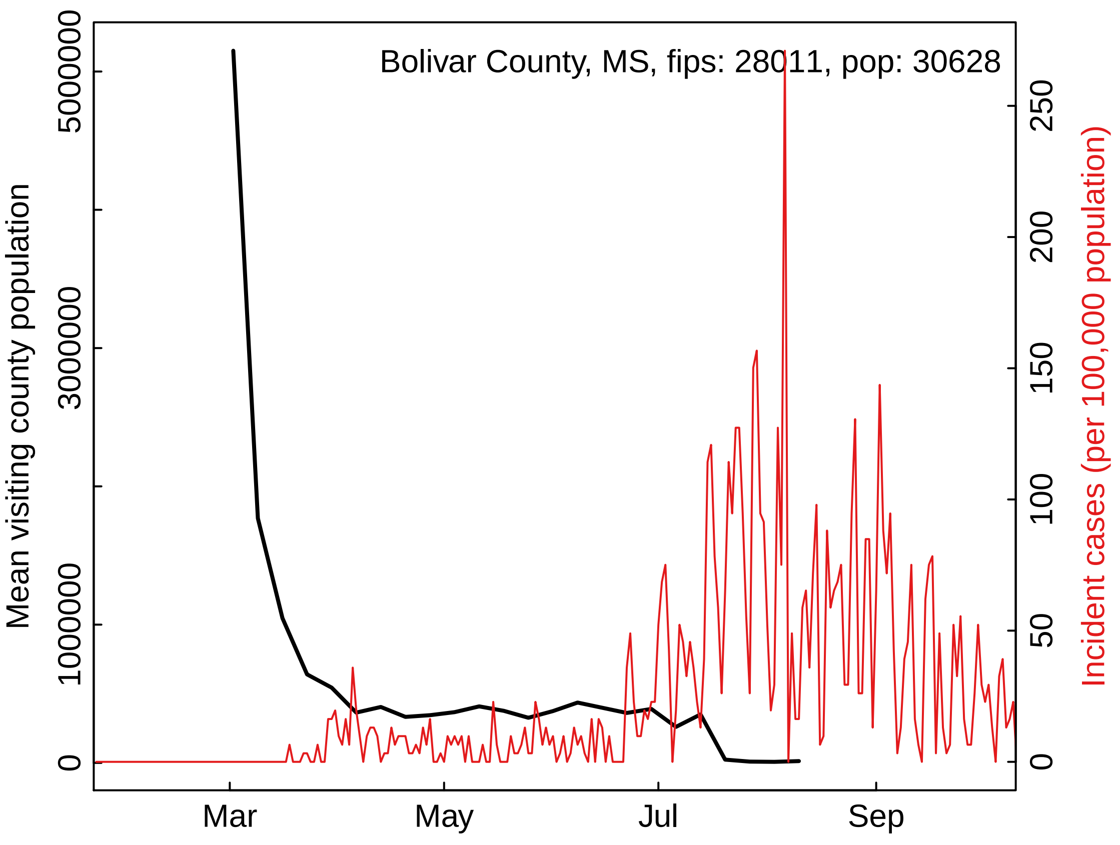

Bolivar County, MS, fips: 28011, pop: 30628
Mean visiting county population
0
1000000
3000000
5000000
Incident cases (per 100,000 population)
0
50
100
150
200
250
Mar
May
Jul
Sep

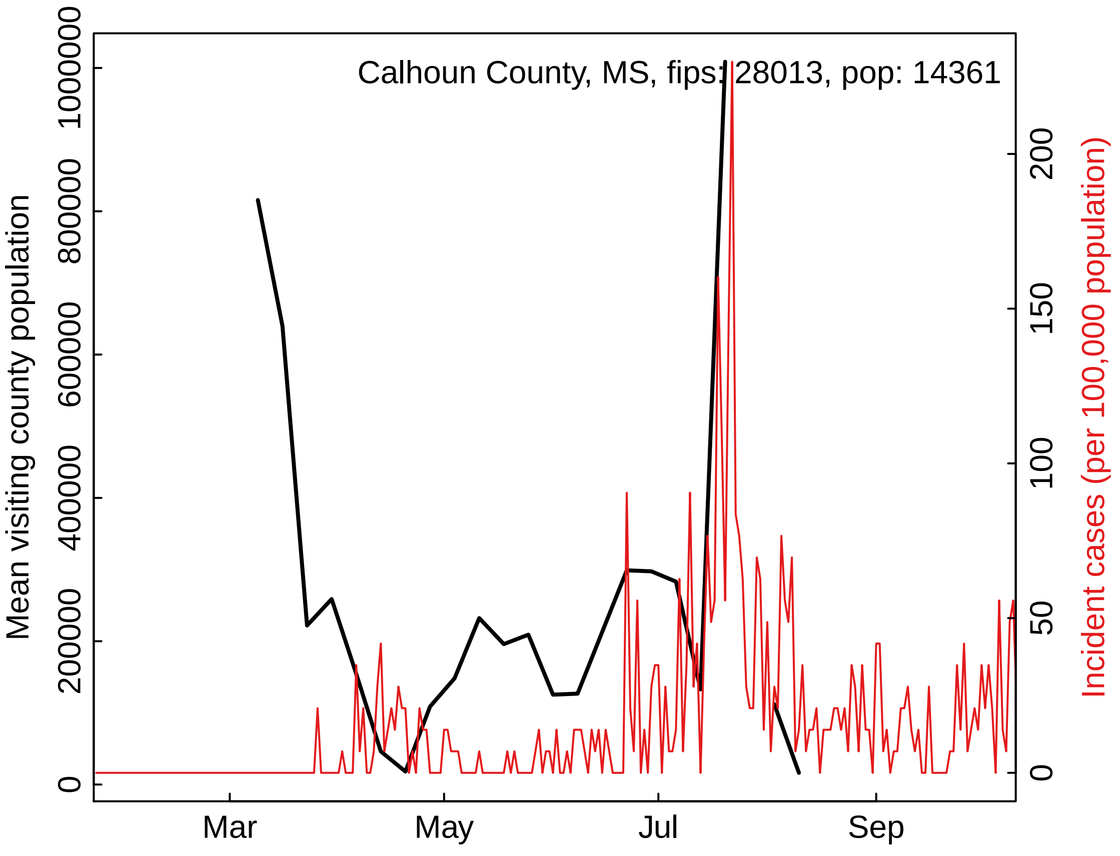

Calhoun County, MS, fips: 28013, pop: 14361
Mean visiting county population
Incident cases (per 100,000 population)
0
200000
400000
600000
800000
1000000
0
50
100
150
200
Mar
May
Jul
Sep

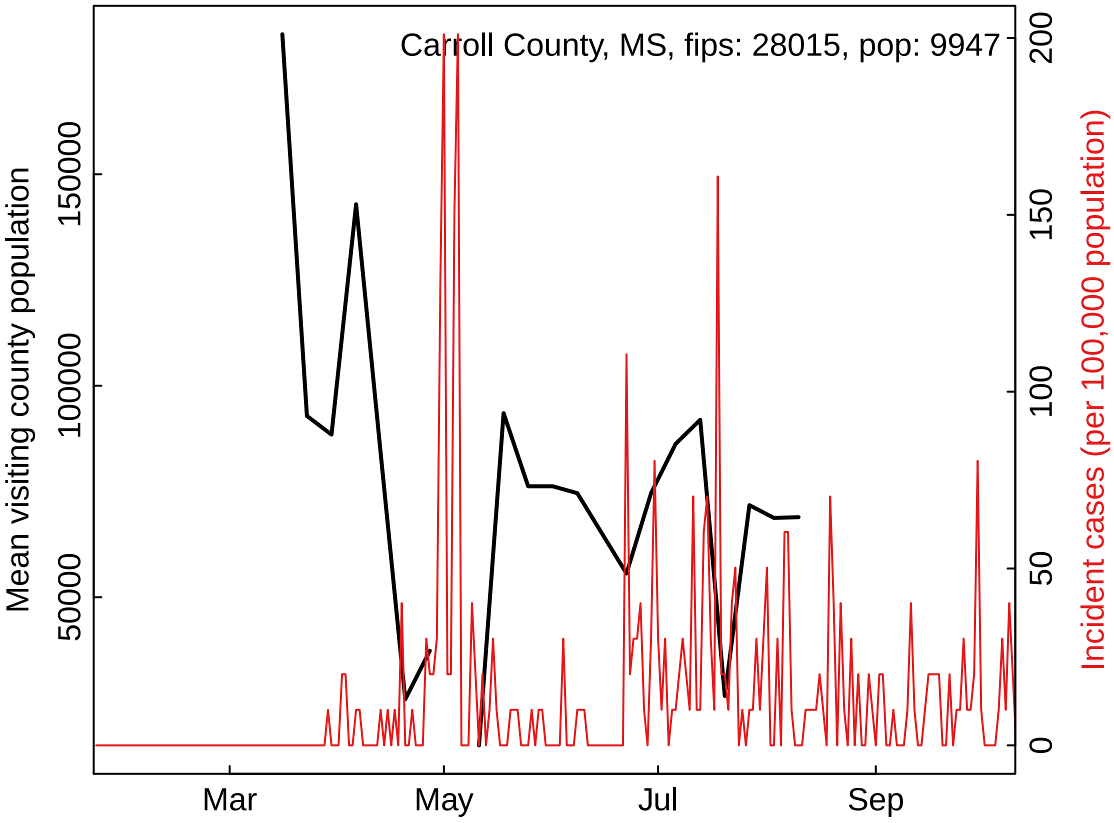

Carroll County, MS, fips: 28015, pop: 9947
Mean visiting county population
50000
100000
150000
Incident cases (per 100,000 population)
0
50
100
150
200
Mar
May
Jul
Sep

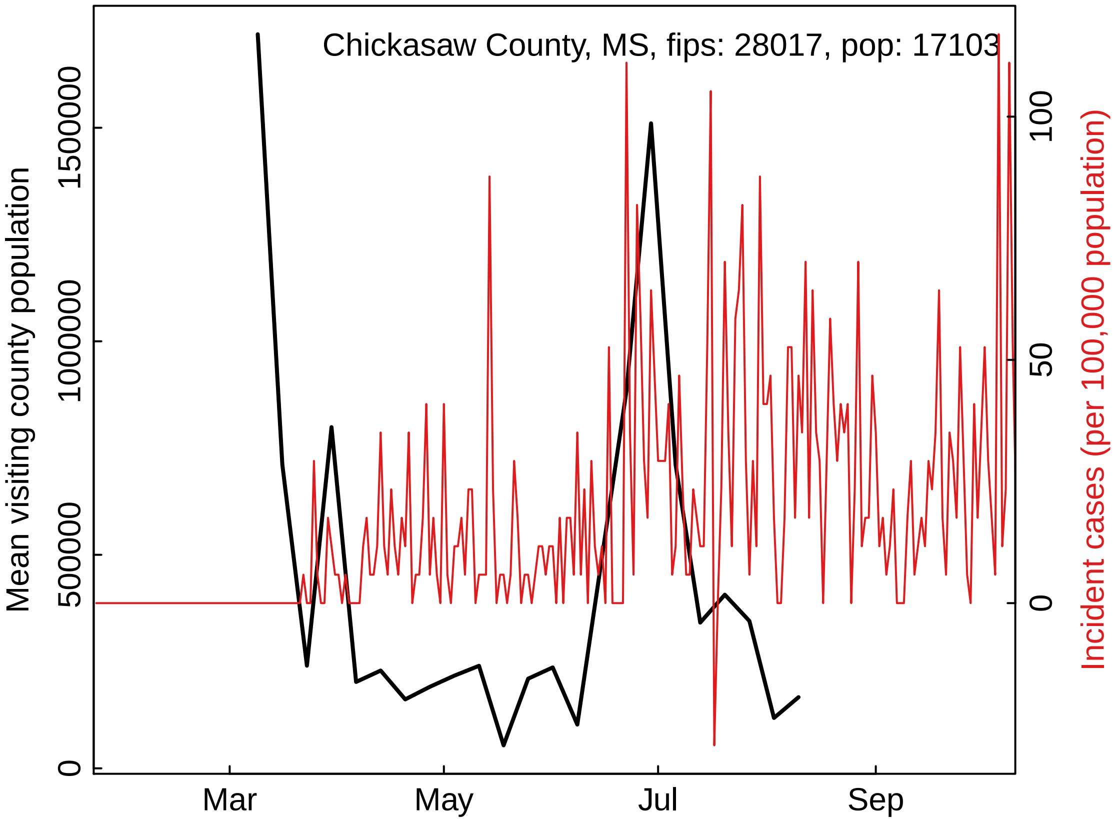
Chickasaw County, MS, fips: 28017, pop: 17103
Mean visiting county population
0
500000
1000000
1500000
Mar
May
Jul
Sep
0
50
100
Incident cases (per 100,000 population)

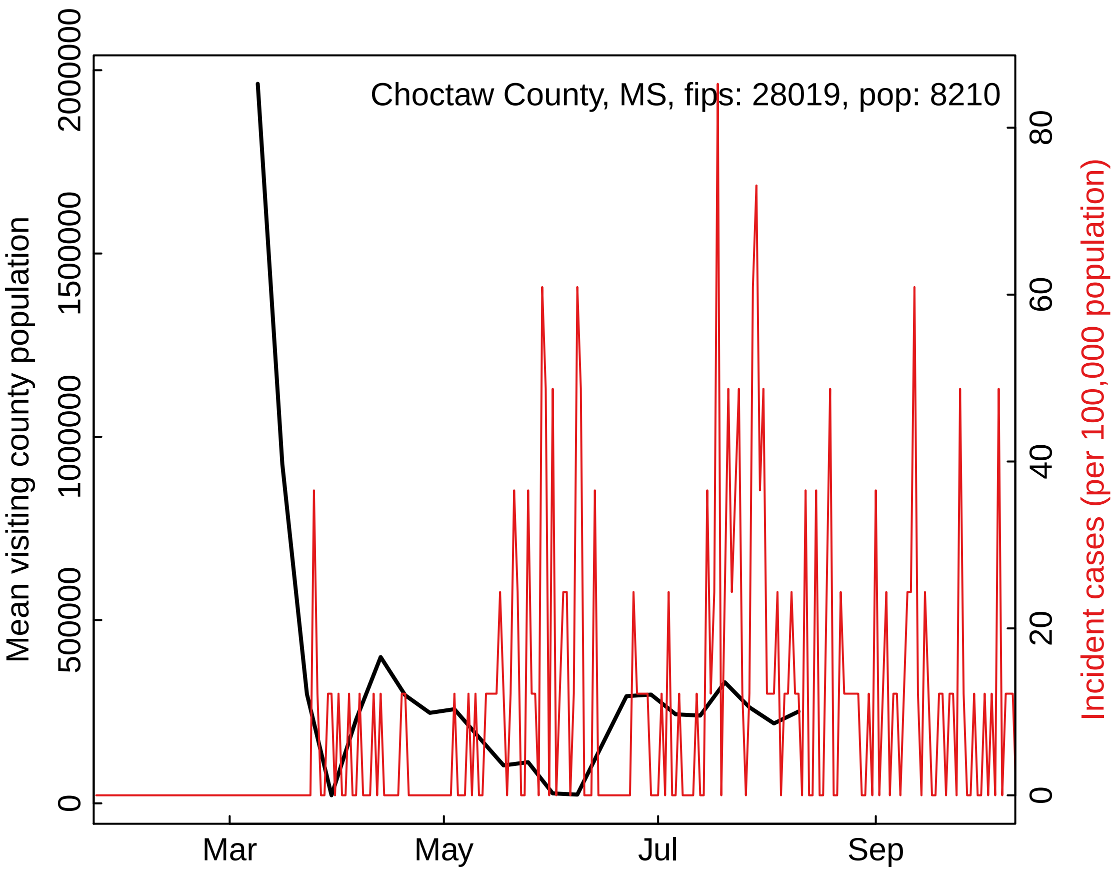

Choctaw County, MS, fips: 28019, pop: 8210
Mean visiting county population
Incident cases (per 100,000 population)
0
500000
1000000
1500000
2000000
0
20
40
60
80
Mar
May
Jul
Sep

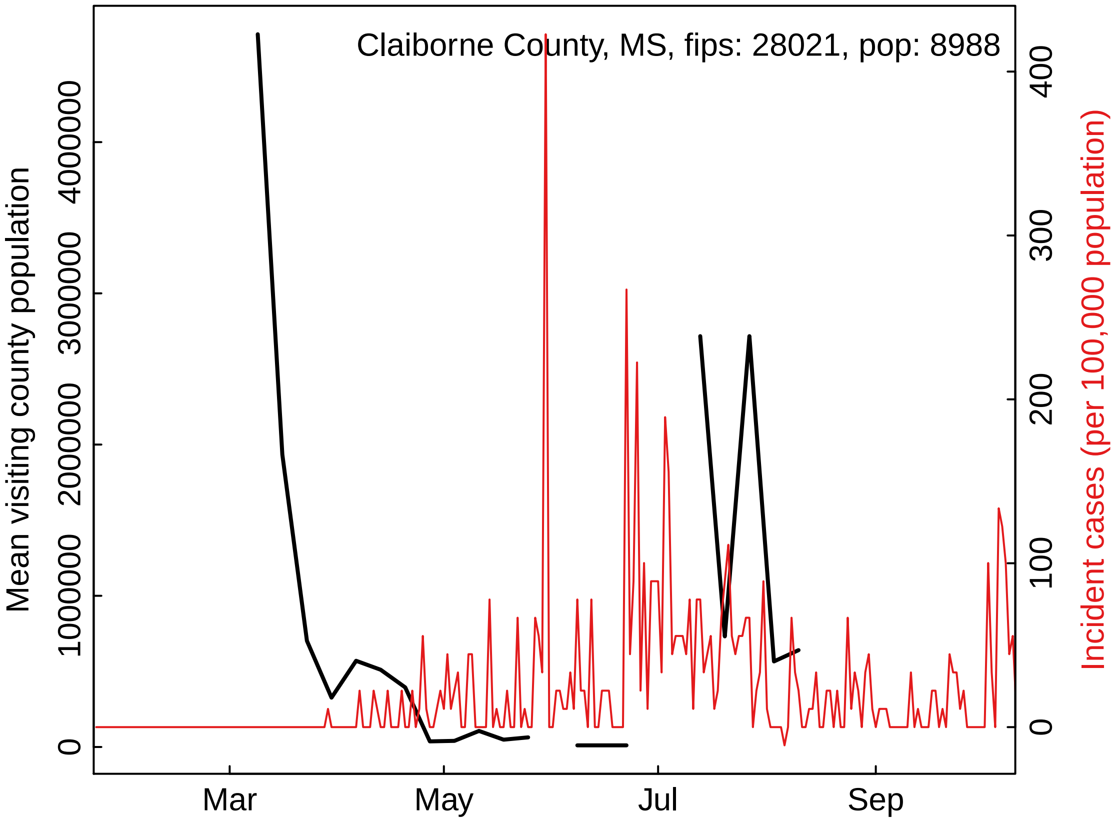

Claiborne County, MS, fips: 28021, pop: 8988
Mean visiting county population
0
1000000
2000000
3000000
4000000
Incident cases (per 100,000 population)
0
100
200
300
400
Mar
May
Jul
Sep

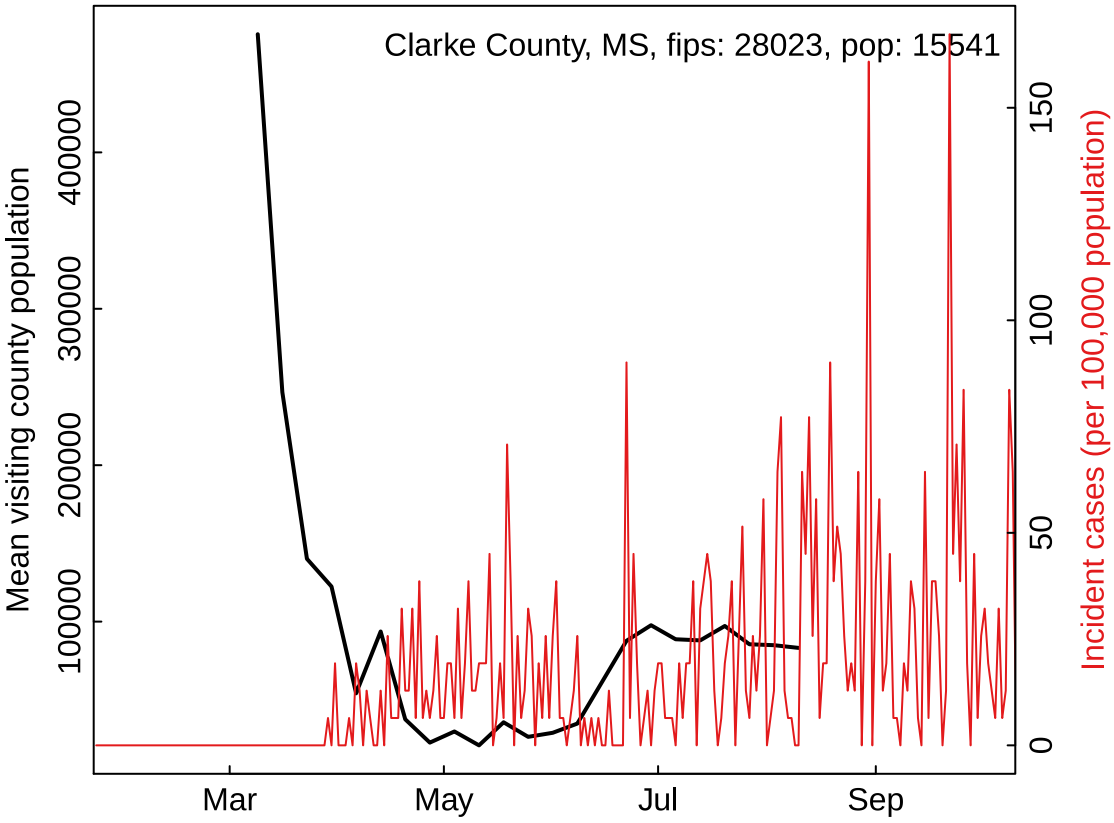

Clarke County, MS, fips: 28023, pop: 15541
Mean visiting county population
100000
200000
300000
400000
Incident cases (per 100,000 population)
0
50
100
150
Mar
May
Jul
Sep

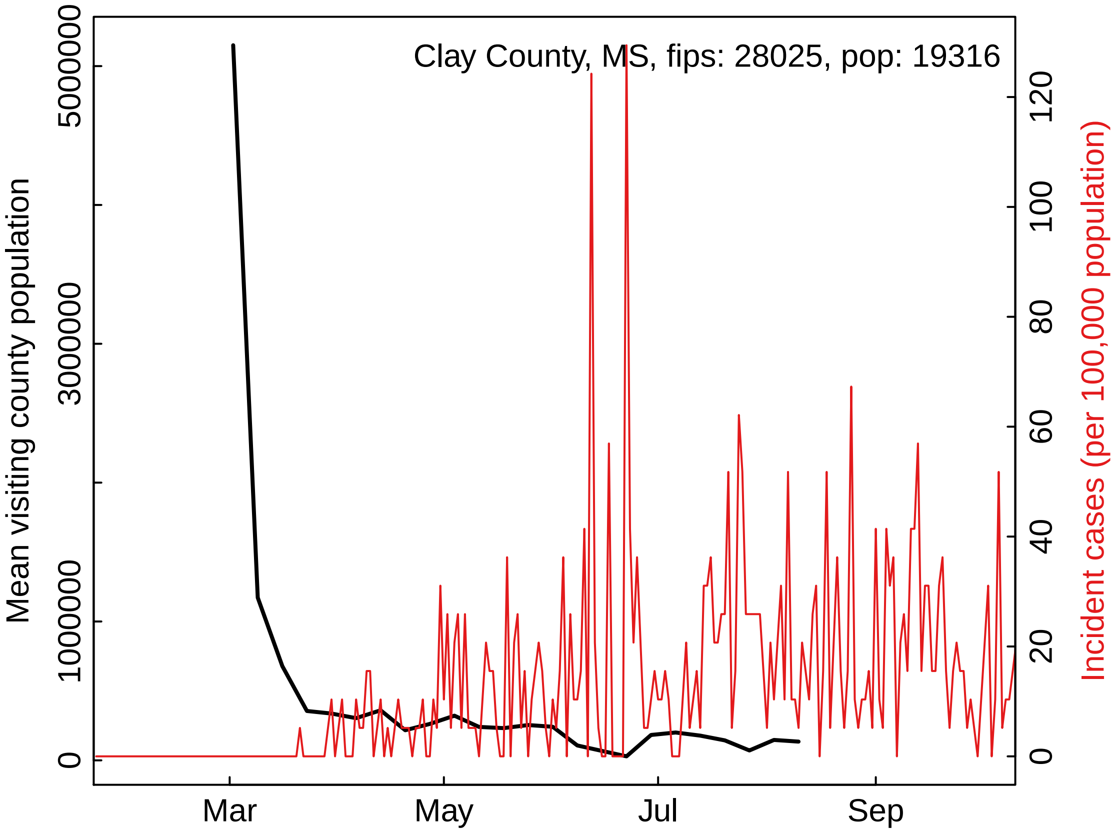

Clay County, MS, fips: 28025, pop: 19316
Mean visiting county population
Incident cases (per 100,000 population)
0
1000000
3000000
5000000
0
20
40
60
80
100
120
Mar
May
Jul
Sep

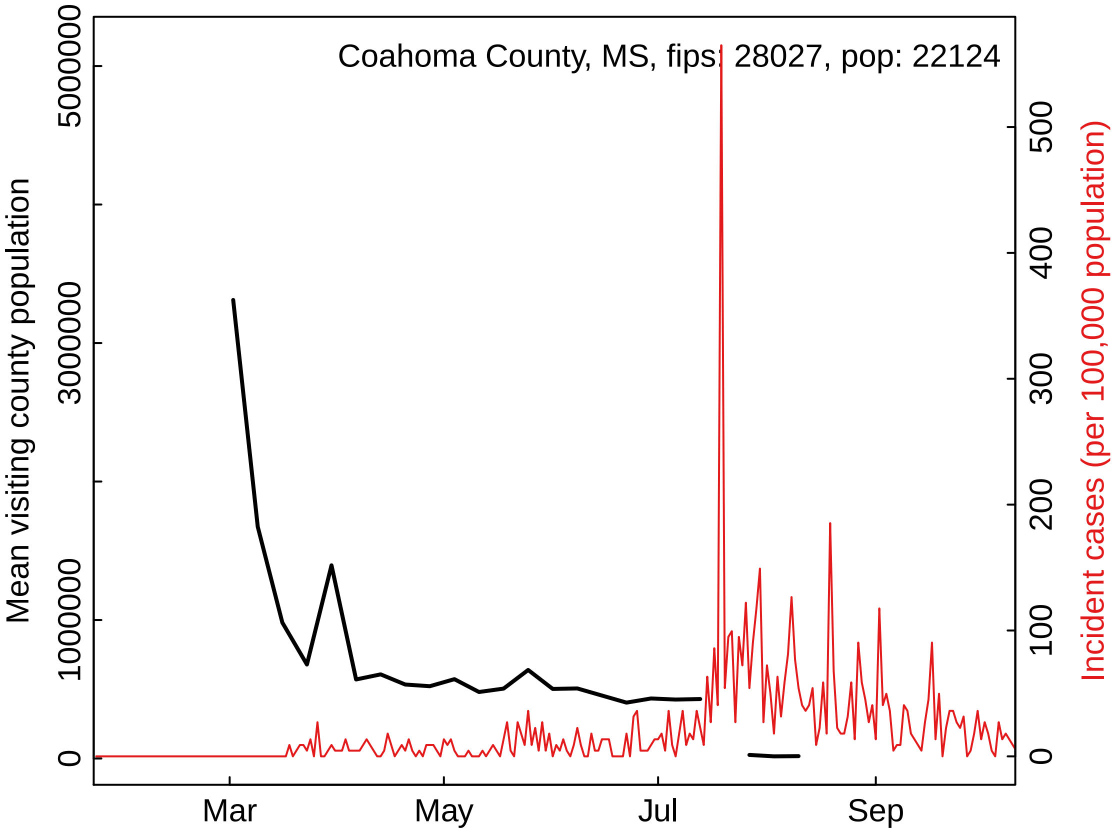

Coahoma County, MS, fips: 28027, pop: 22124
Mean visiting county population
0
1000000
3000000
5000000
Incident cases (per 100,000 population)
0
100
200
300
400
500
Mar
May
Jul
Sep

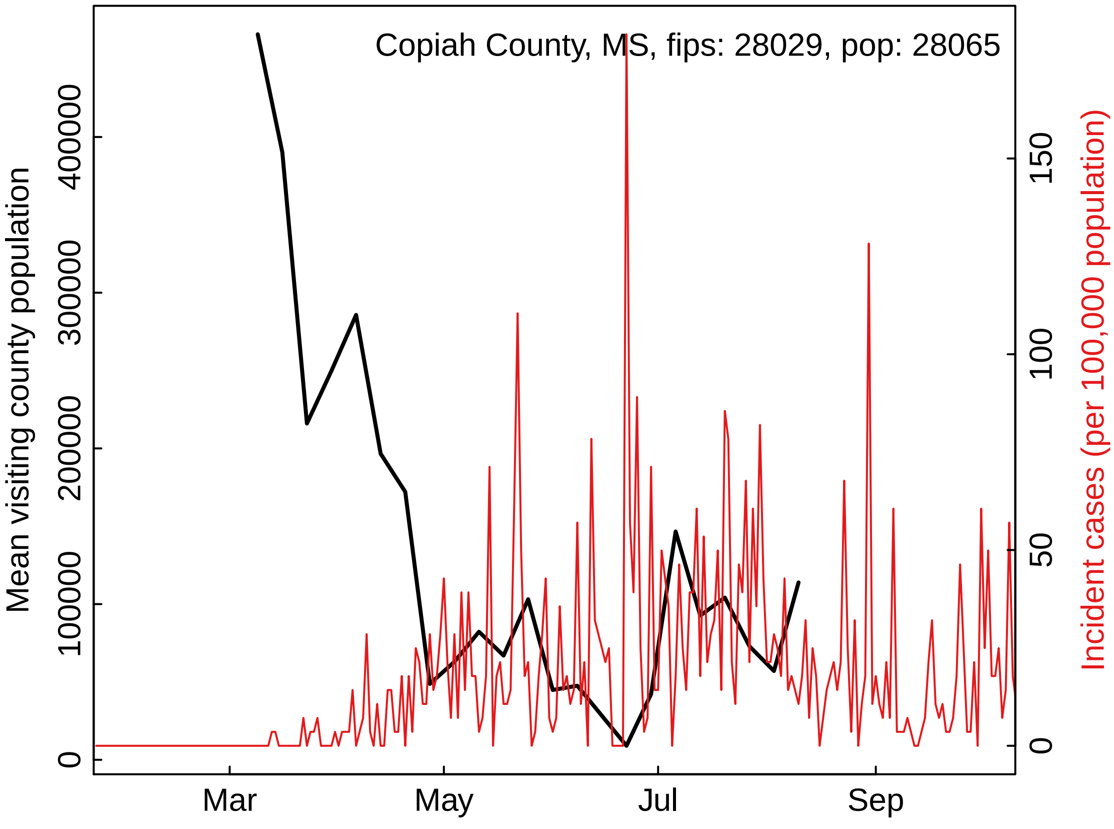

Copiah County, MS, fips: 28029, pop: 28065
Mean visiting county population
0
100000
200000
300000
400000
Incident cases (per 100,000 population)
0
50
100
150
Mar
May
Jul
Sep

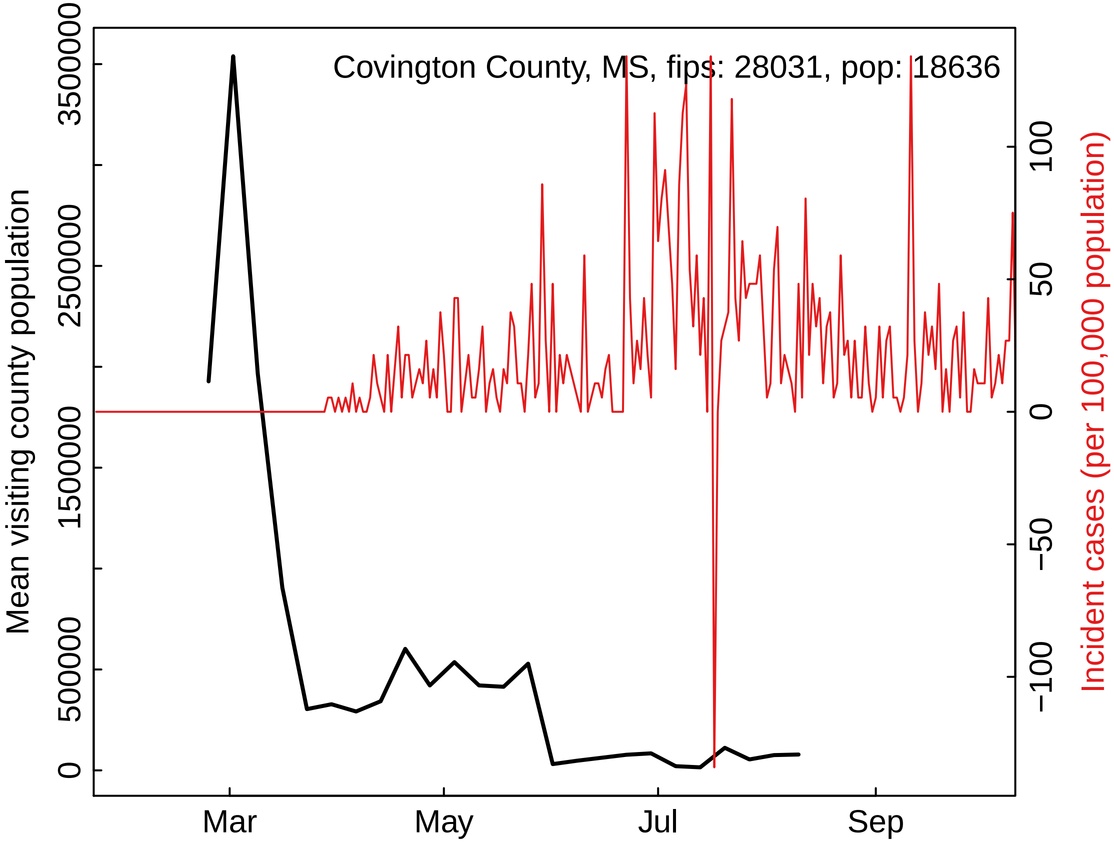

Covington County, MS, fips: 28031, pop: 18636
Mean visiting county population
0
500000
1500000
2500000
3500000
Incident cases (per 100,000 population)
−100
−50
0
50
100
Mar
May
Jul
Sep

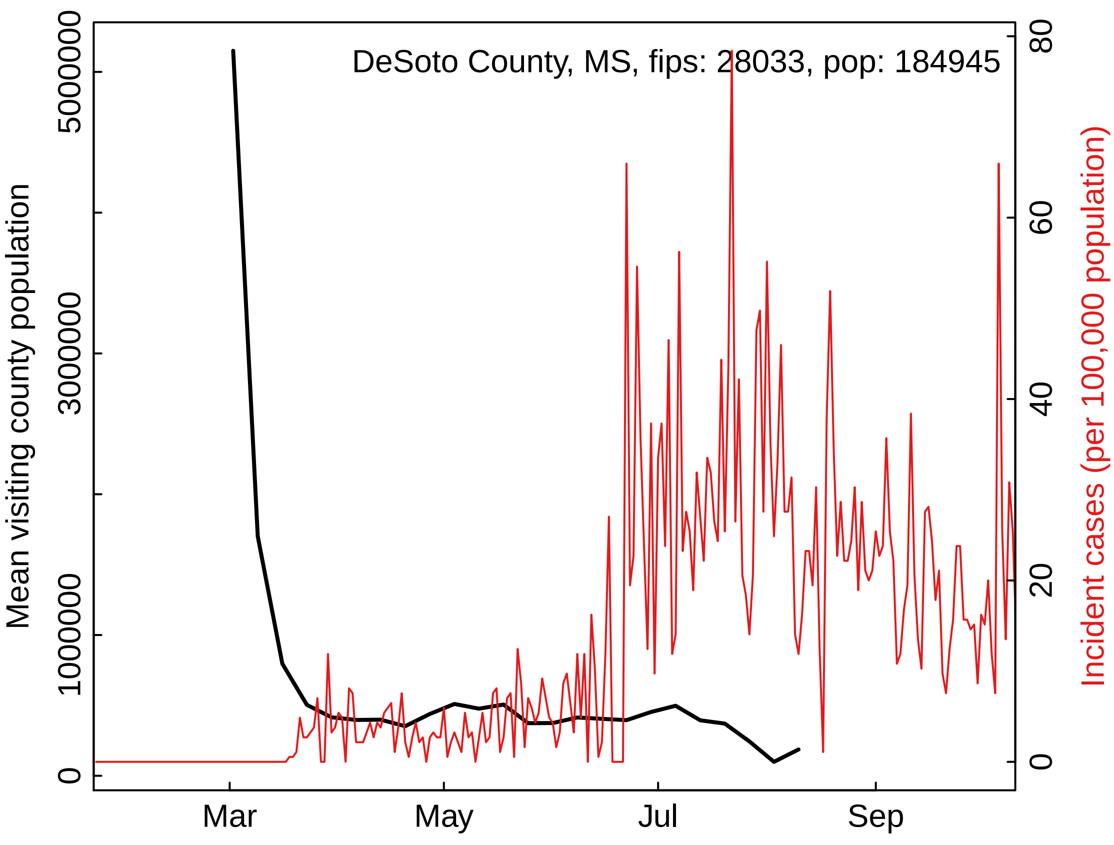
DeSoto County, MS, fips: 28033, pop: 184945
Mean visiting county population
0
1000000
3000000
5000000
Incident cases (per 100,000 population)
0
20
40
60
80
Mar
May
Jul
Sep

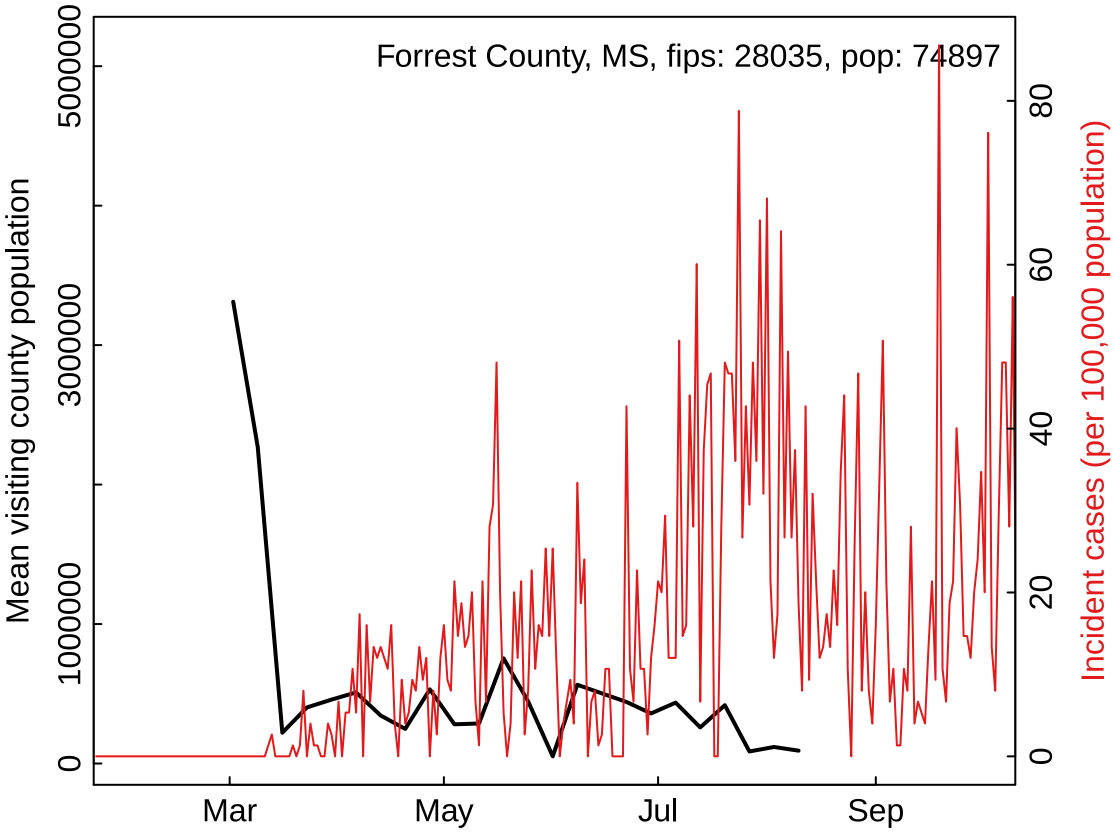

Forrest County, MS, fips: 28035, pop: 74897
Mean visiting county population
0
1000000
3000000
5000000
Incident cases (per 100,000 population)
0
20
40
60
80
Mar
May
Jul
Sep

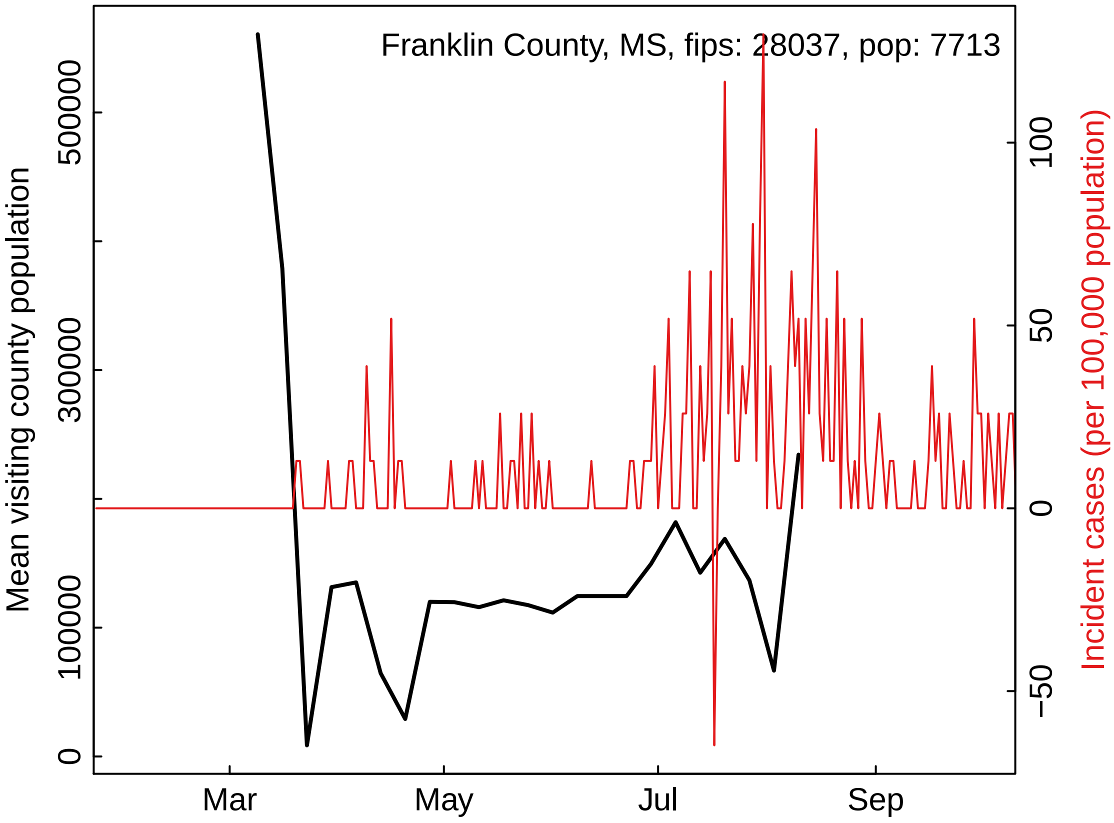

Franklin County, MS, fips: 28037, pop: 7713
Mean visiting county population
0
100000
300000
500000
Incident cases (per 100,000 population)
−50
0
50
100
Mar
May
Jul
Sep

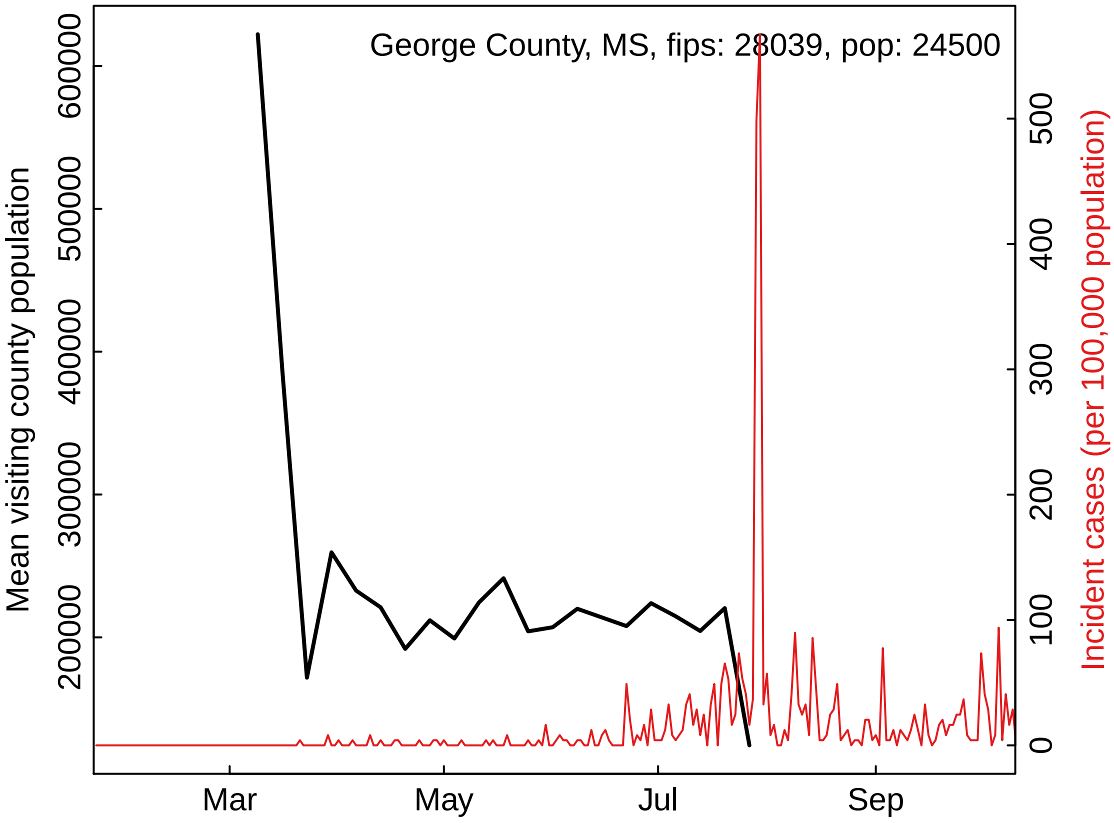
George County, MS, fips: 28039, pop: 24500
Mean visiting county population
200000
300000
400000
500000
600000
Incident cases (per 100,000 population)
0
100
200
300
400
500
Mar
May
Jul
Sep

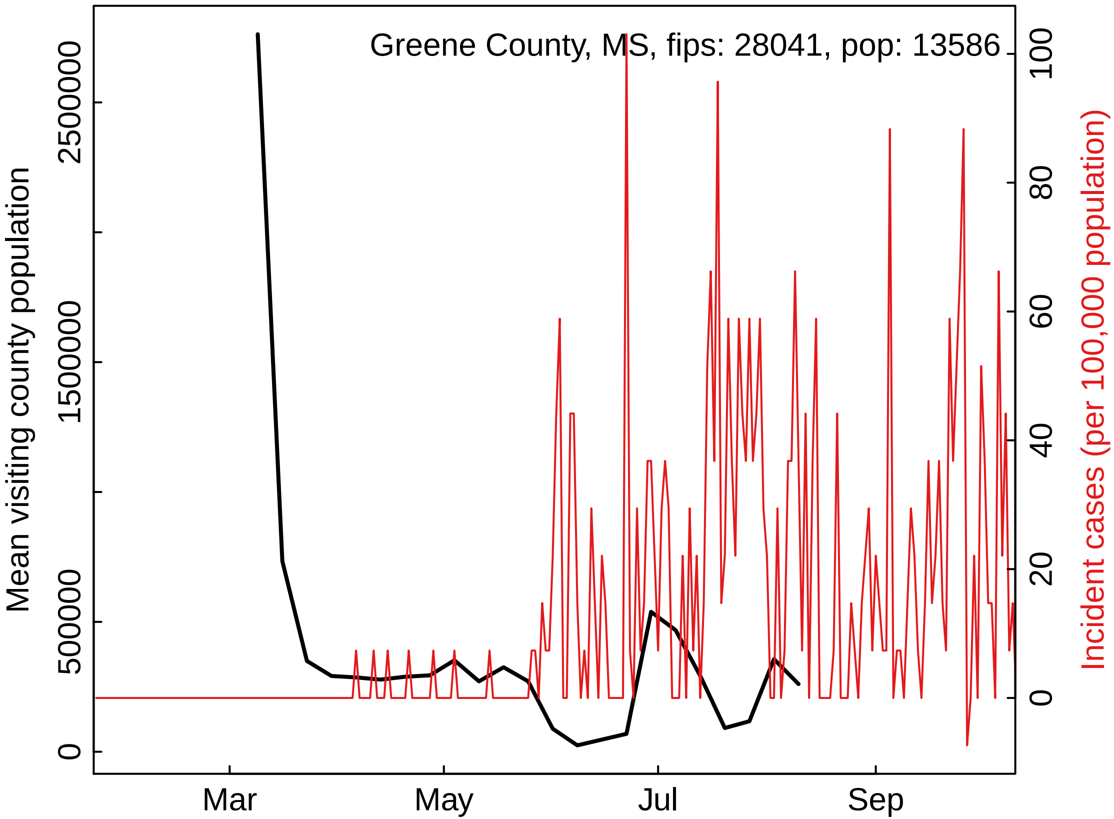

Greene County, MS, fips: 28041, pop: 13586
Mean visiting county population
0
500000
1500000
2500000
Mar
May
Jul
Sep
0
20
40
60
80
100
Incident cases (per 100,000 population)

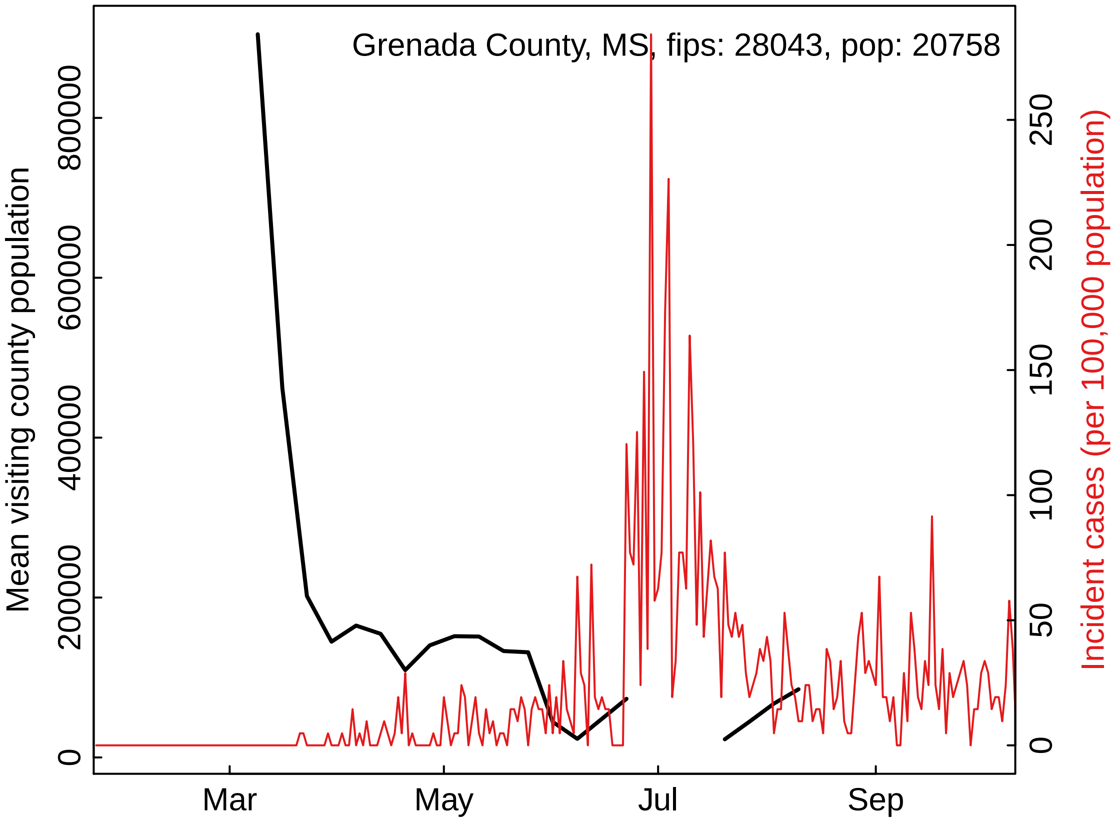

Grenada County, MS, fips: 28043, pop: 20758
Mean visiting county population
Incident cases (per 100,000 population)
0
200000
400000
600000
800000
0
50
100
150
200
250
Mar
May
Jul
Sep

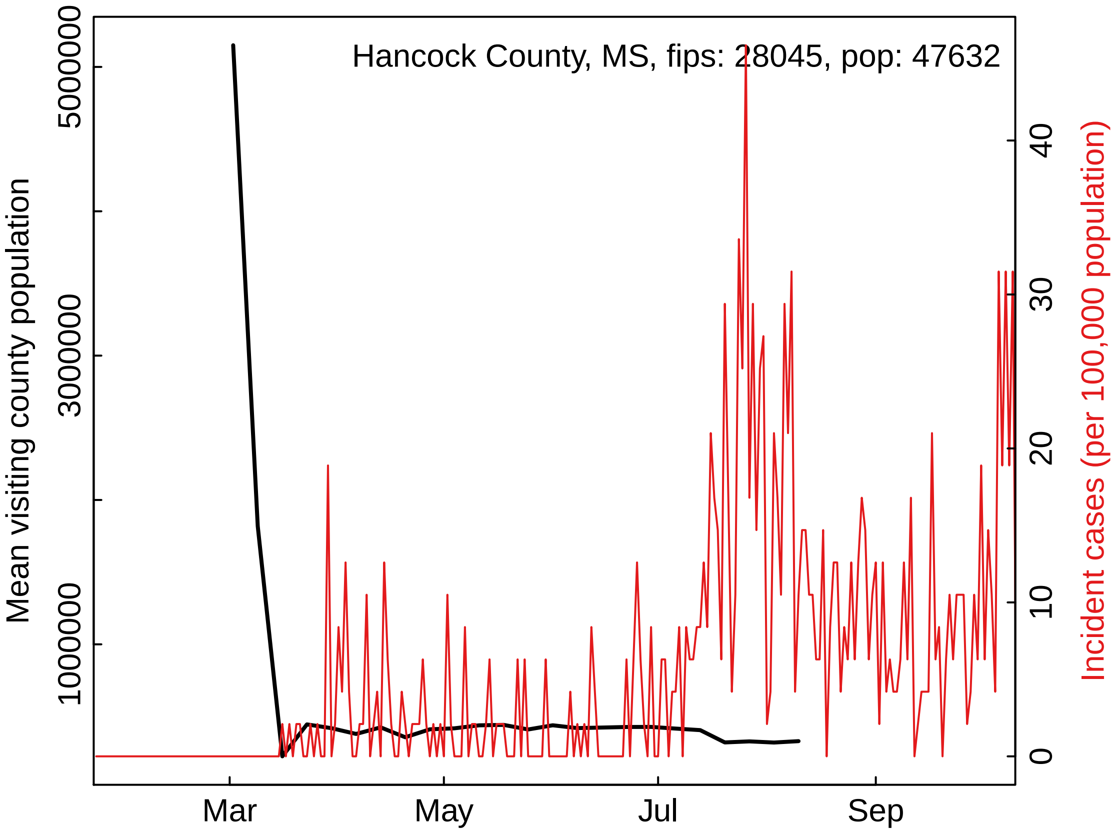

Hancock County, MS, fips: 28045, pop: 47632
Mean visiting county population
Incident cases (per 100,000 population)
1000000
3000000
5000000
0
10
20
30
40
Mar
May
Jul
Sep

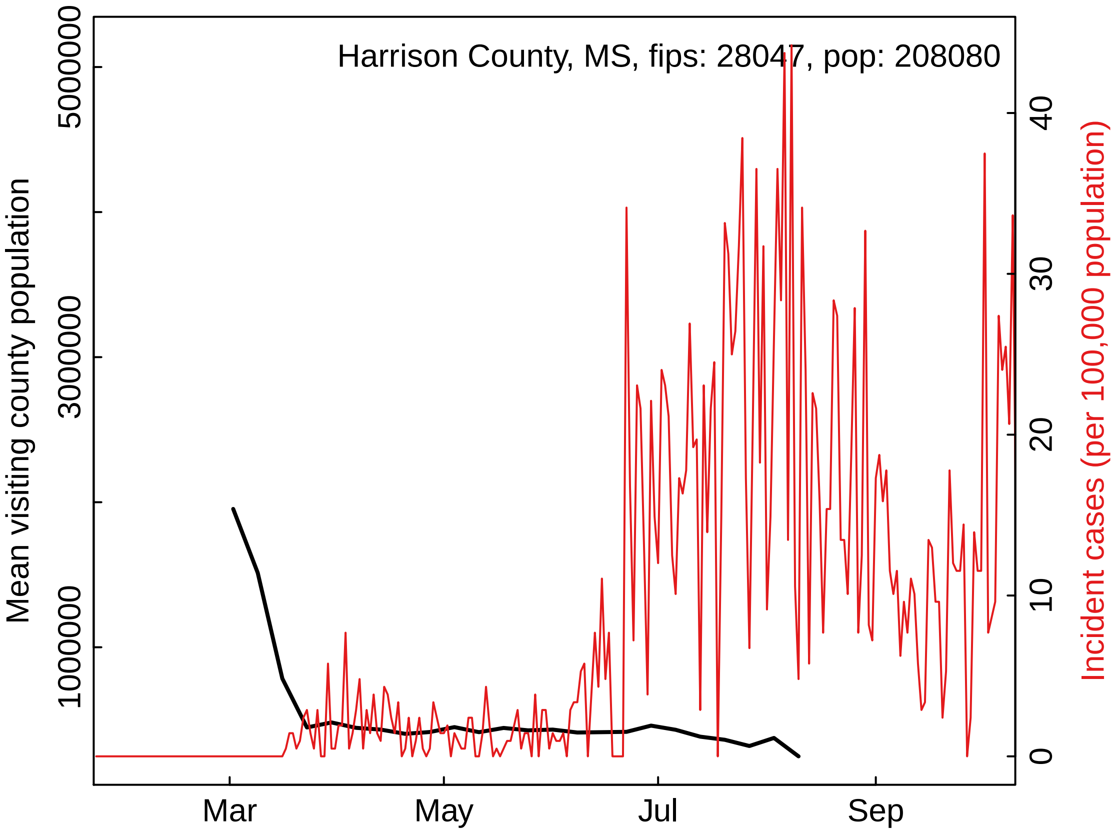

Harrison County, MS, fips: 28047, pop: 208080
Mean visiting county population
1000000
3000000
5000000
Incident cases (per 100,000 population)
0
10
20
30
40
Mar
May
Jul
Sep

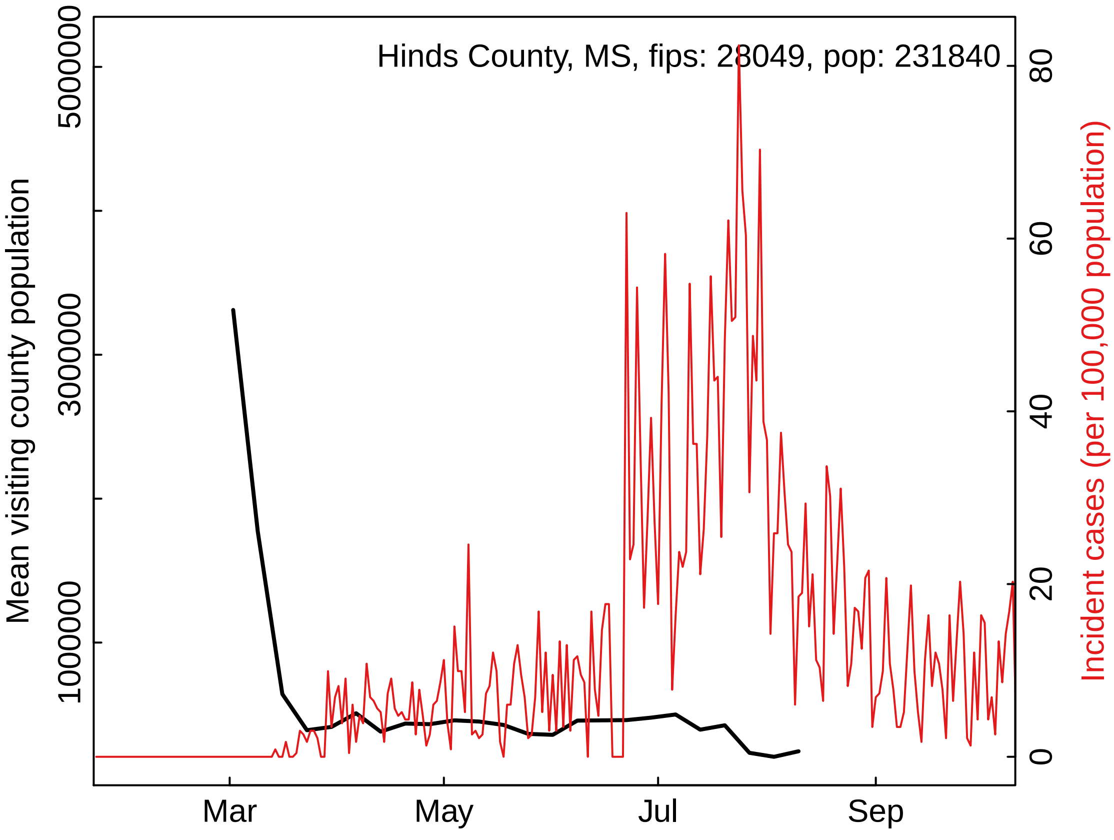

Hinds County, MS, fips: 28049, pop: 231840
Mean visiting county population
Incident cases (per 100,000 population)
1000000
3000000
5000000
0
20
40
60
80
Mar
May
Jul
Sep

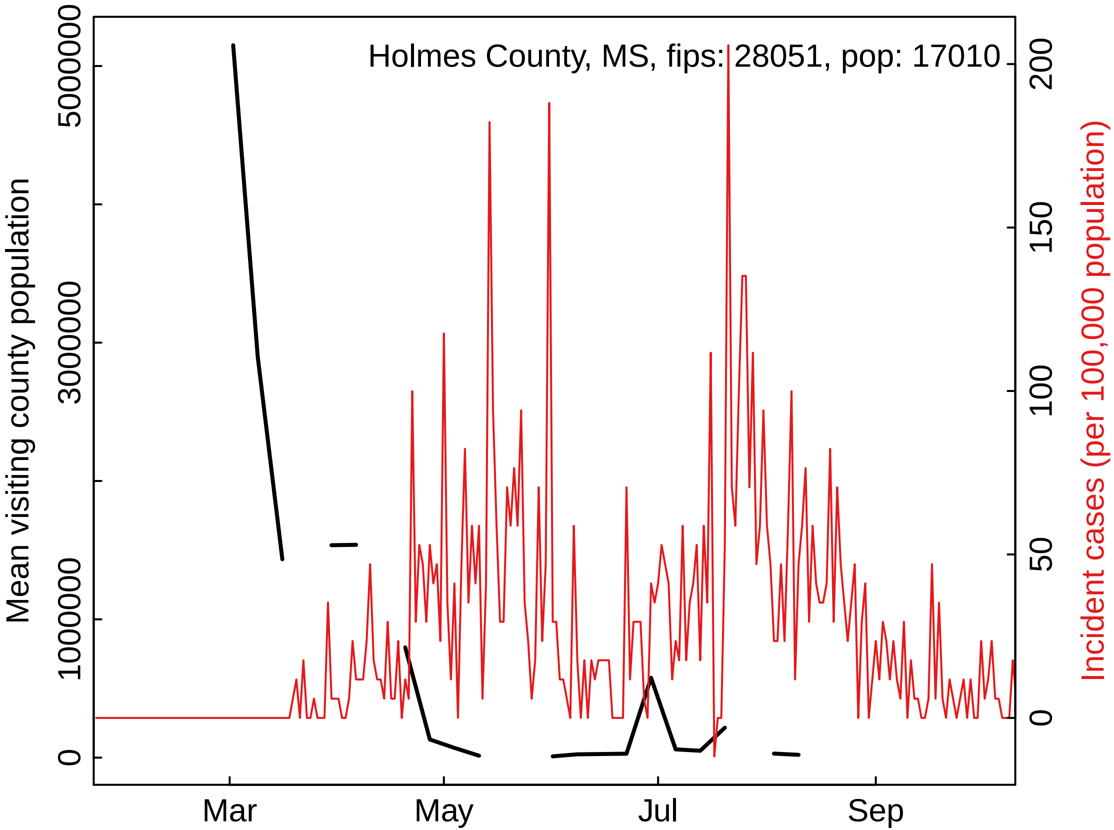
Holmes County, MS, fips: 28051, pop: 17010
Mean visiting county population
0
1000000
3000000
5000000
Incident cases (per 100,000 population)
0
50
100
150
200
Mar
May
Jul
Sep

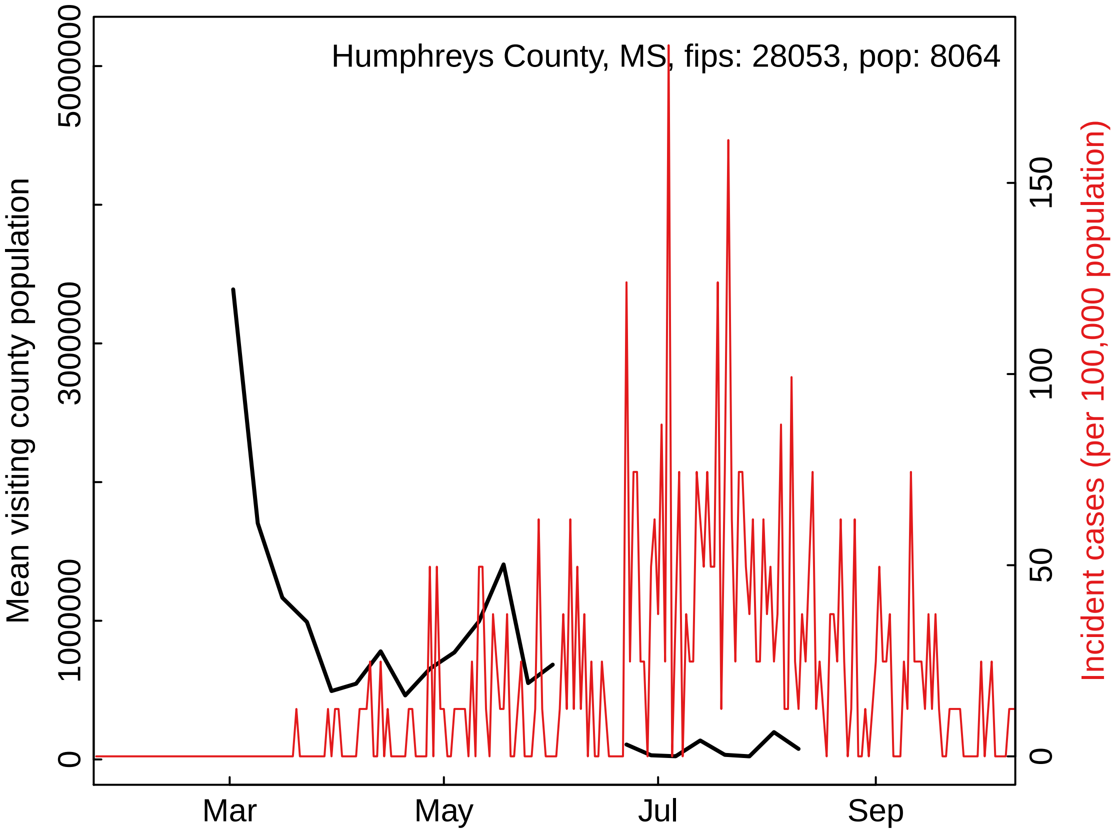
Humphreys County, MS, fips: 28053, pop: 8064
Mean visiting county population
0
1000000
3000000
5000000
Incident cases (per 100,000 population)
0
50
100
150
Mar
May
Jul
Sep

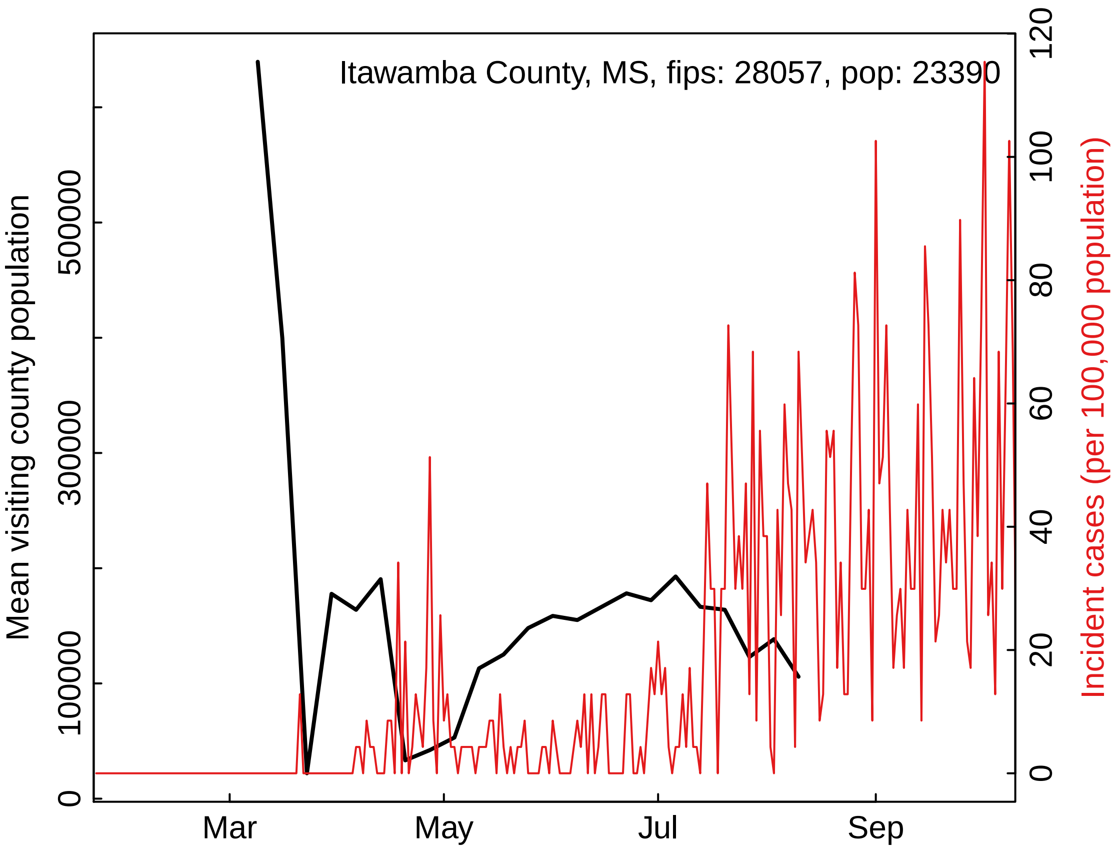
Itawamba County, MS, fips: 28057, pop: 23390
Mean visiting county population
0
100000
300000
500000
Incident cases (per 100,000 population)
0
20
40
60
80
100
120
Mar
May
Jul
Sep

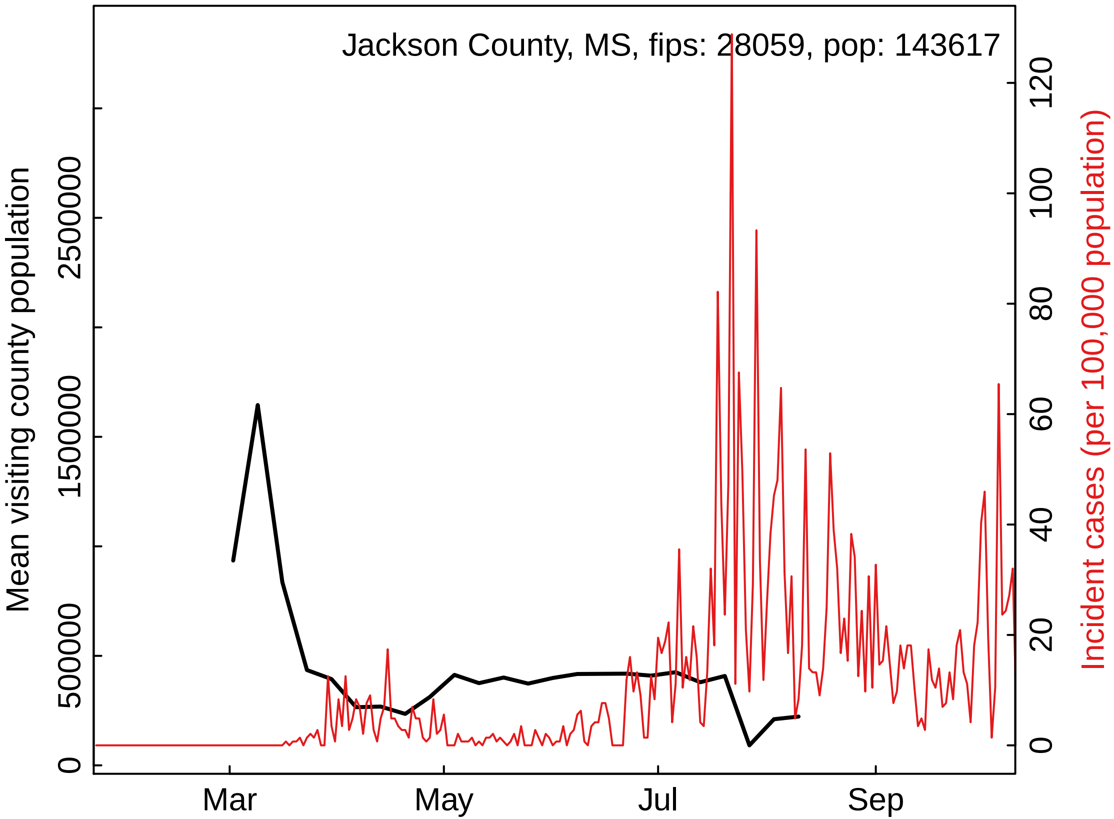

Jackson County, MS, fips: 28059, pop: 143617
Mean visiting county population
0
500000
1500000
2500000
Mar
May
Jul
Sep
Incident cases (per 100,000 population)
0
20
40
60
80
100
120

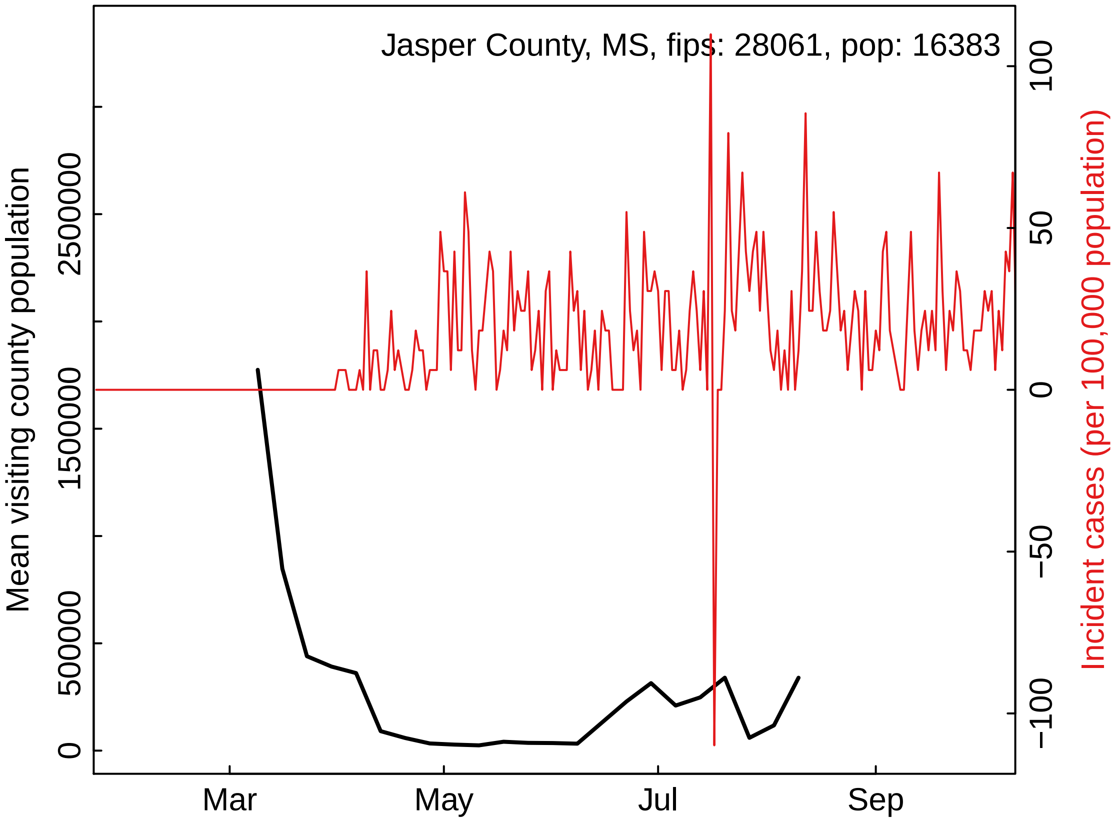

Jasper County, MS, fips: 28061, pop: 16383
Mean visiting county population
0
500000
1500000
2500000
Incident cases (per 100,000 population)
−100
−50
0
50
100
Mar
May
Jul
Sep

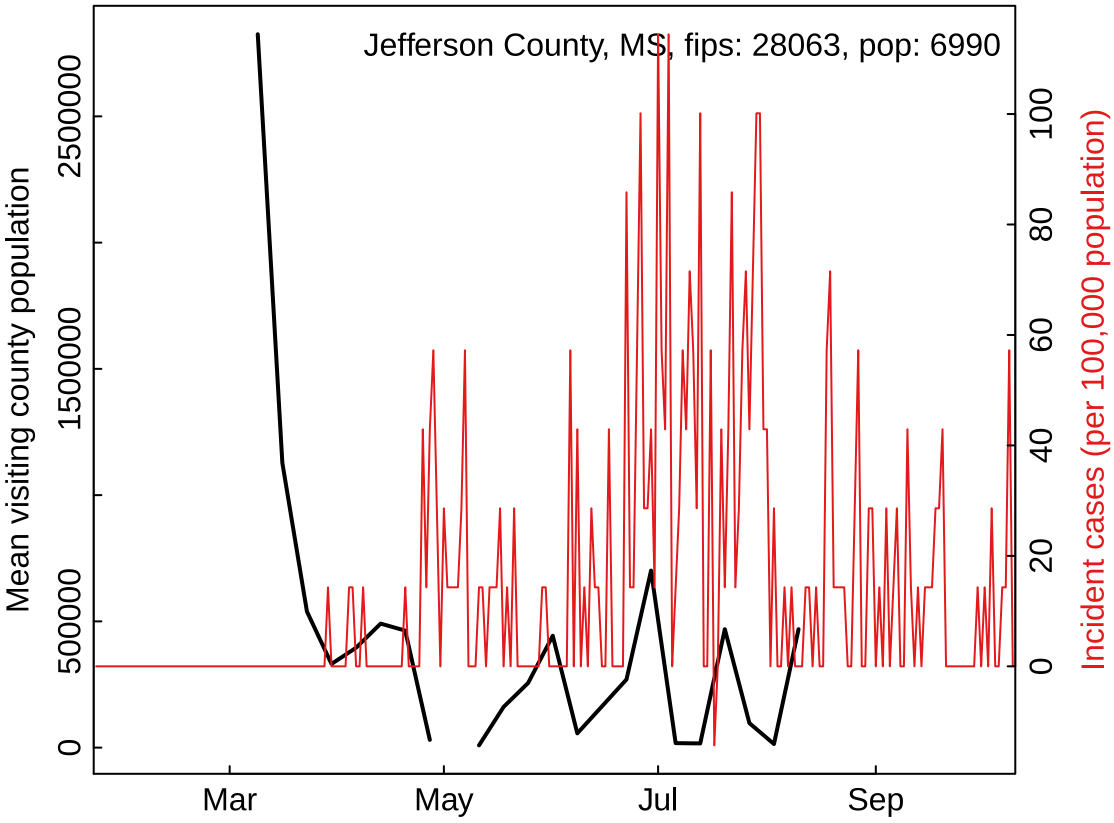

Jefferson County, MS, fips: 28063, pop: 6990
Mean visiting county population
Incident cases (per 100,000 population)
0
500000
1500000
2500000
0
20
40
60
80
100
Mar
May
Jul
Sep

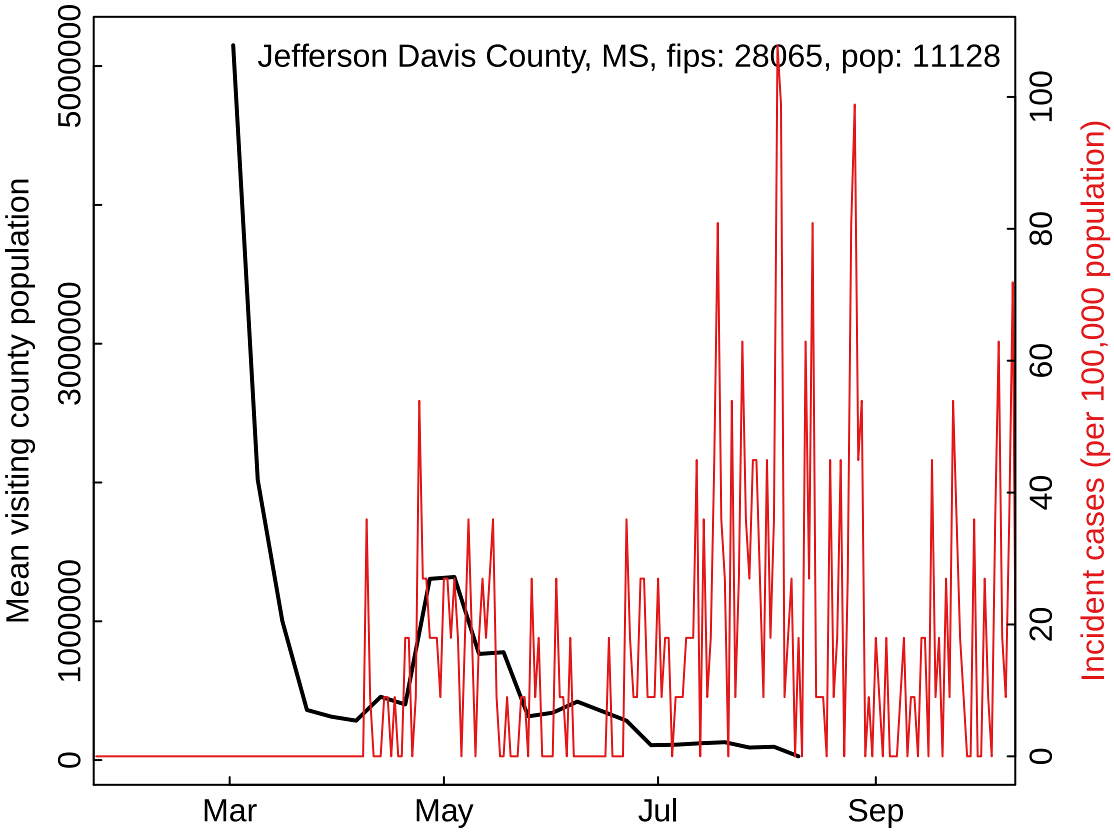

Jefferson Davis County, MS, fips: 28065, pop: 11128
Mean visiting county population
0
1000000
3000000
5000000
Incident cases (per 100,000 population)
0
20
40
60
80
100
Mar
May
Jul
Sep

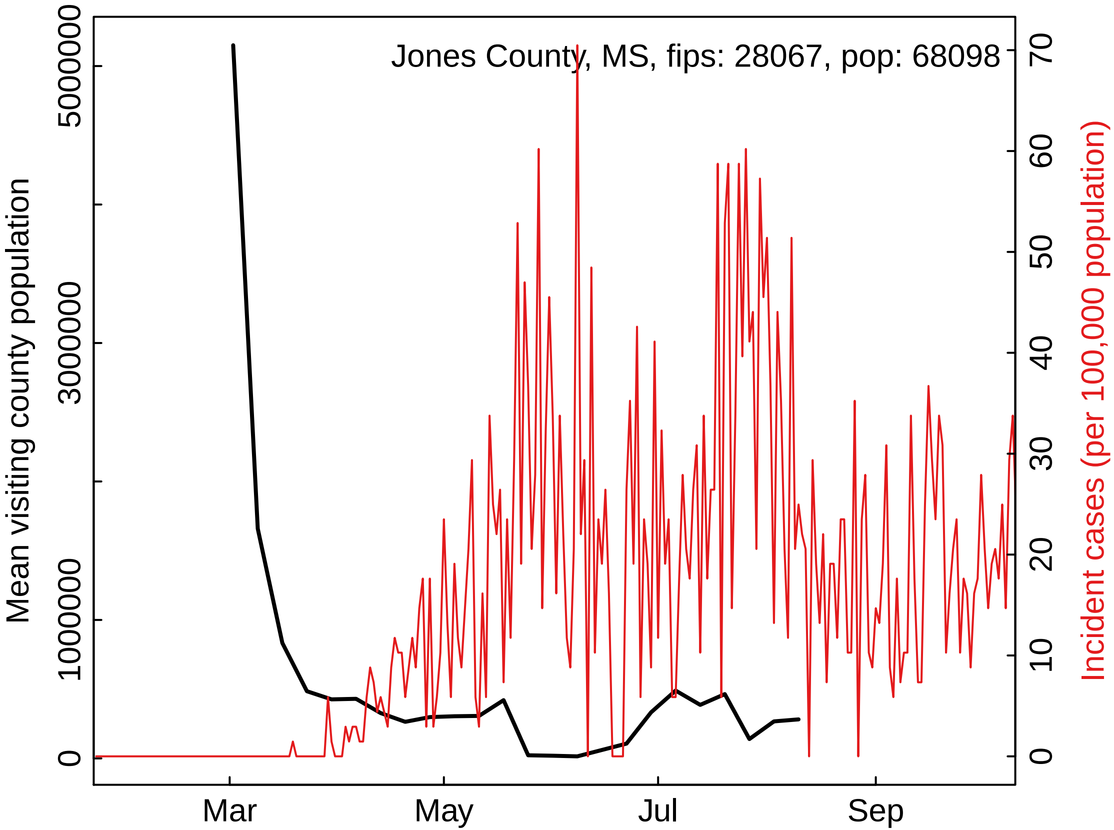

Jones County, MS, fips: 28067, pop: 68098
Mean visiting county population
0
1000000
3000000
5000000
Incident cases (per 100,000 population)
0
10
20
30
40
50
60
70
Mar
May
Jul
Sep

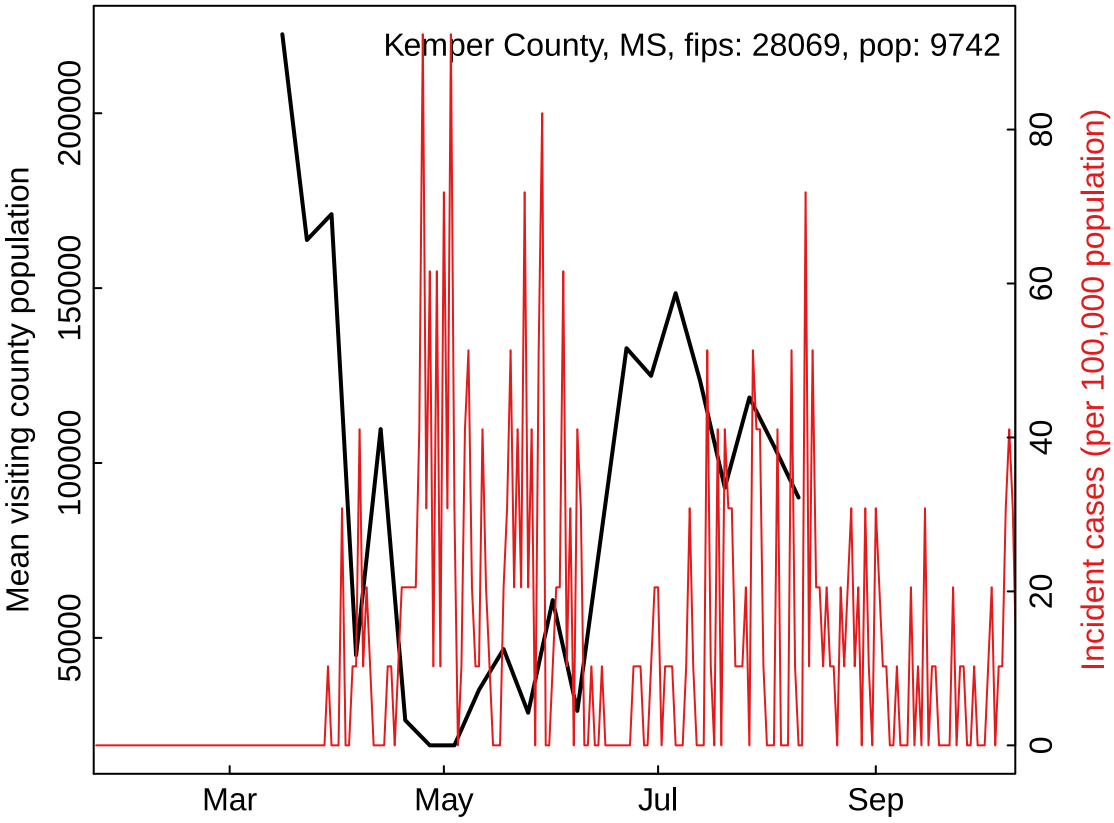

Kemper County, MS, fips: 28069, pop: 9742
Mean visiting county population
50000
100000
150000
200000
Incident cases (per 100,000 population)
0
20
40
60
80
Mar
May
Jul
Sep

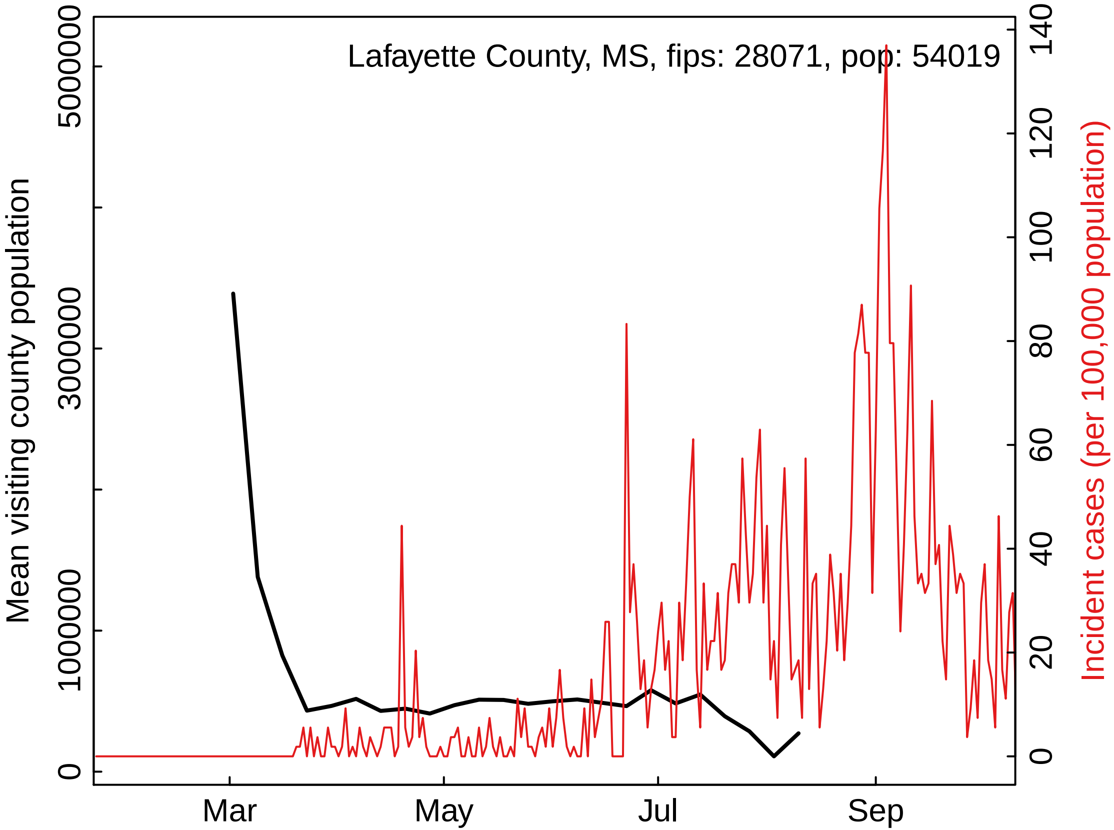

Lafayette County, MS, fips: 28071, pop: 54019
Mean visiting county population
0
1000000
3000000
5000000
Incident cases (per 100,000 population)
0
20
40
60
80
100
120
140
Mar
May
Jul
Sep

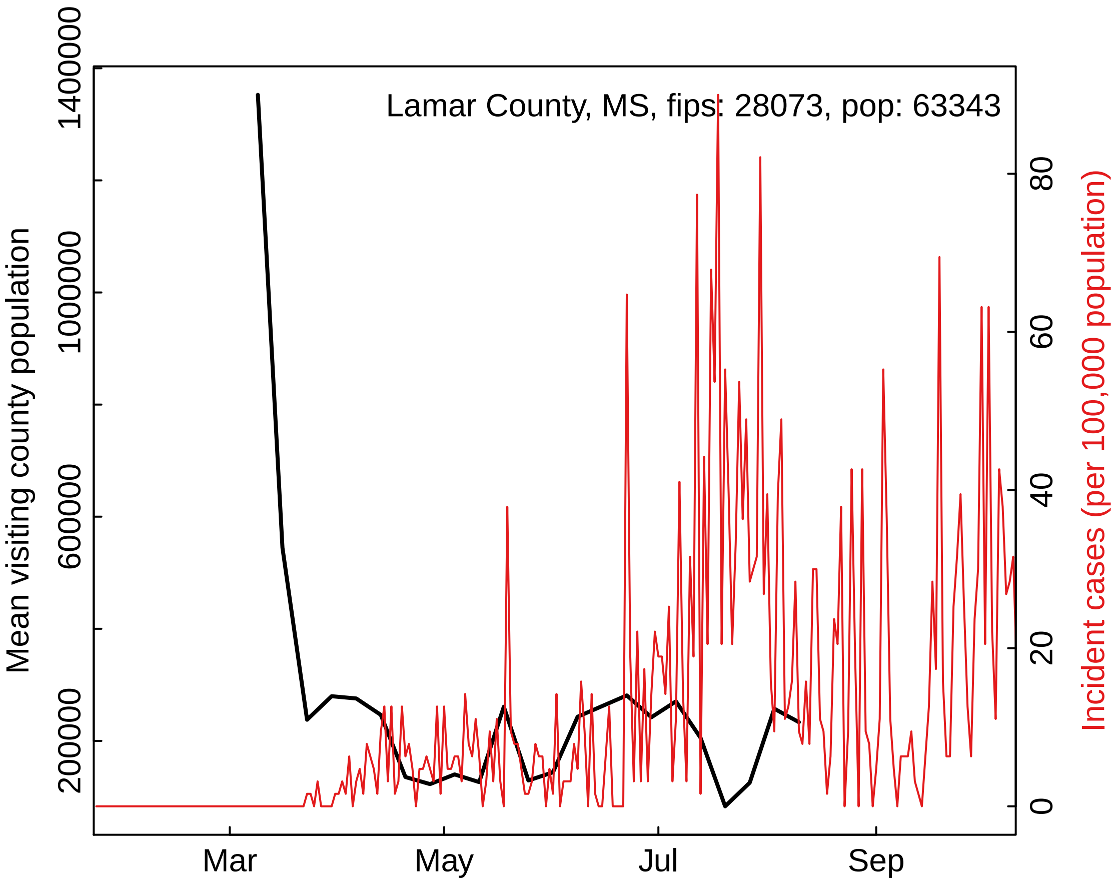

Lamar County, MS, fips: 28073, pop: 63343
Mean visiting county population
200000
600000
1000000
1400000
Incident cases (per 100,000 population)
0
20
40
60
80
Mar
May
Jul
Sep

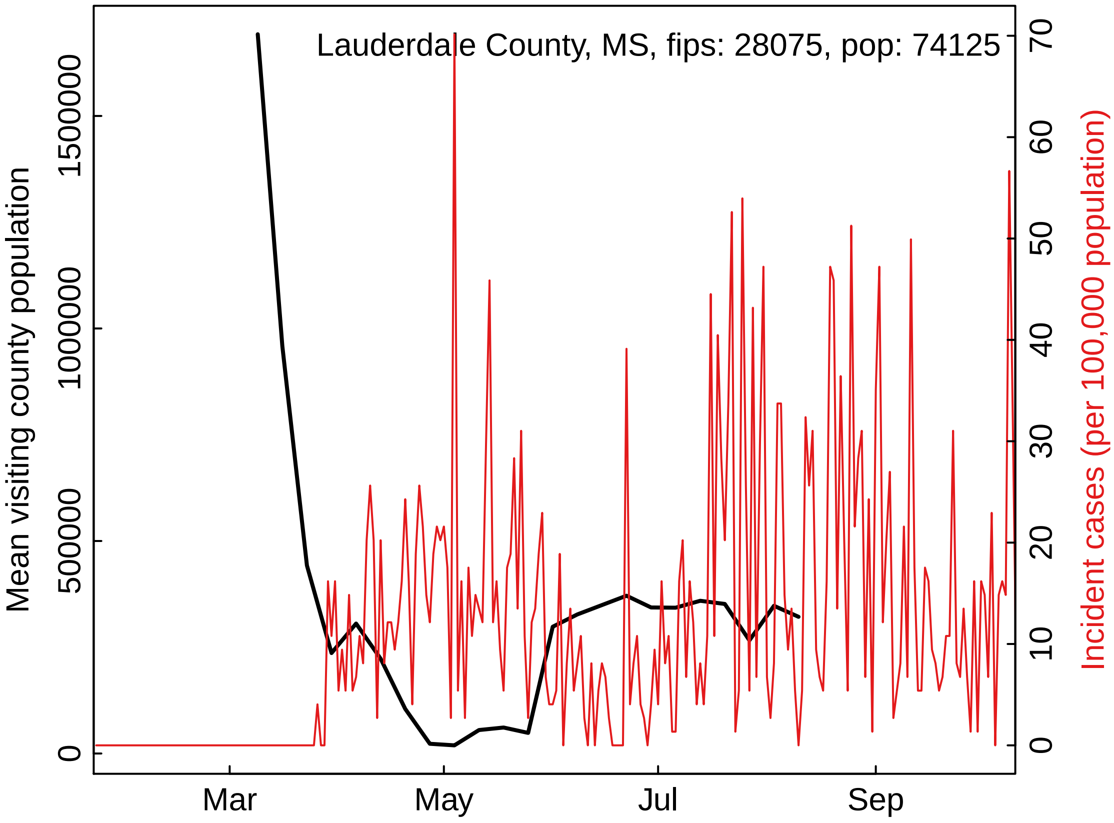

Lauderdale County, MS, fips: 28075, pop: 74125
Mean visiting county population
Incident cases (per 100,000 population)
0
500000
1000000
1500000
0
10
20
30
40
50
60
70
Mar
May
Jul
Sep

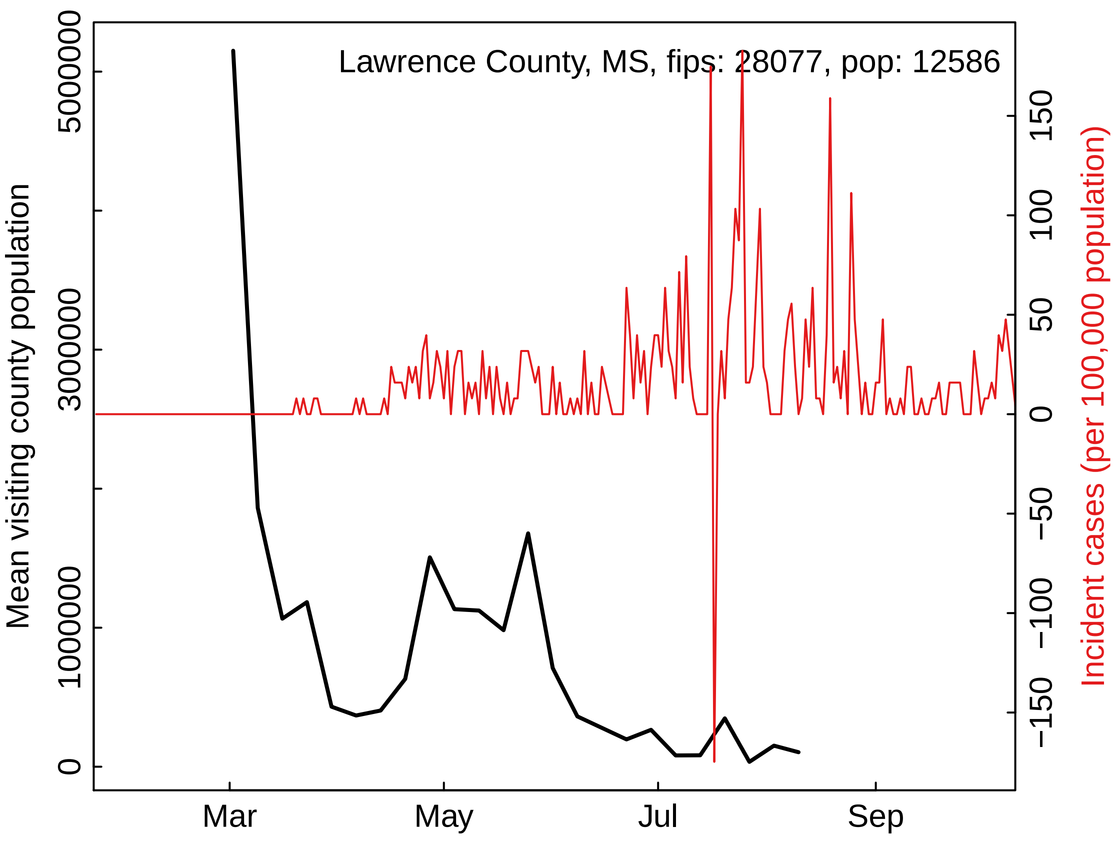

Lawrence County, MS, fips: 28077, pop: 12586
Mean visiting county population
0
1000000
3000000
5000000
Incident cases (per 100,000 population)
−150
−100
−50
0
50
100
150
Mar
May
Jul
Sep

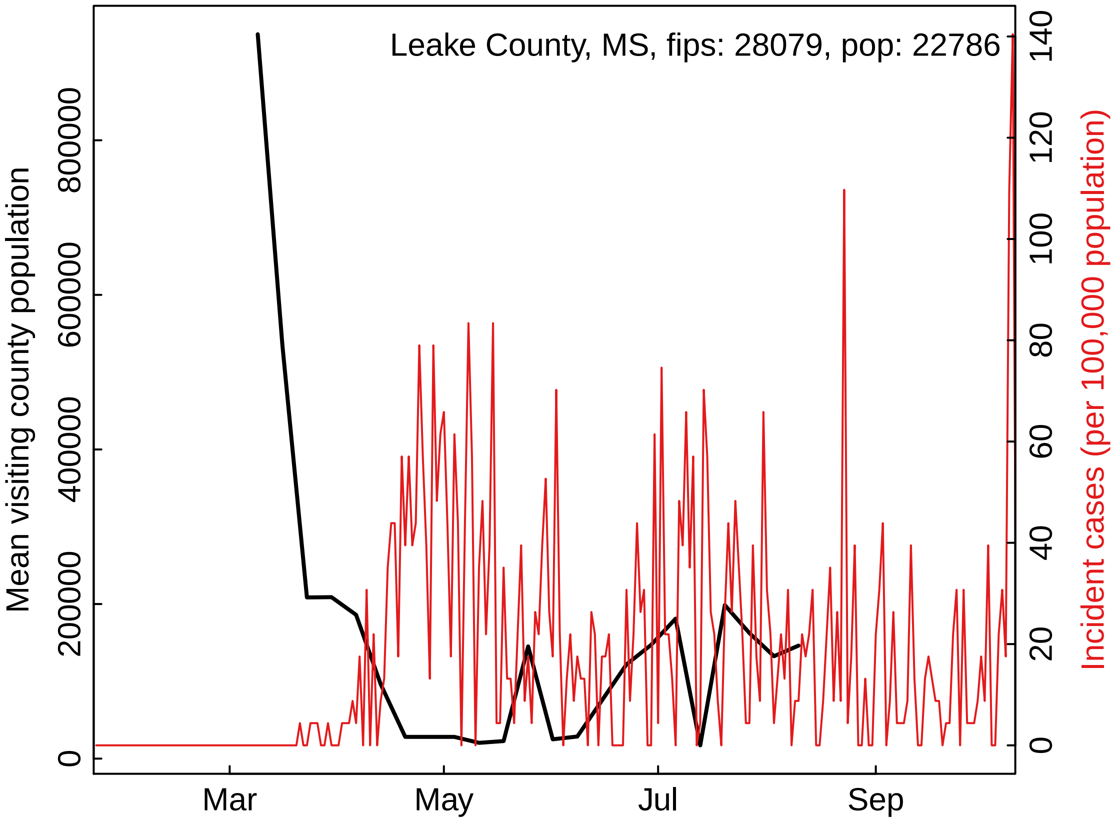

Leake County, MS, fips: 28079, pop: 22786
Mean visiting county population
0
200000
400000
600000
800000
Incident cases (per 100,000 population)
0
20
40
60
80
100
120
140
Mar
May
Jul
Sep

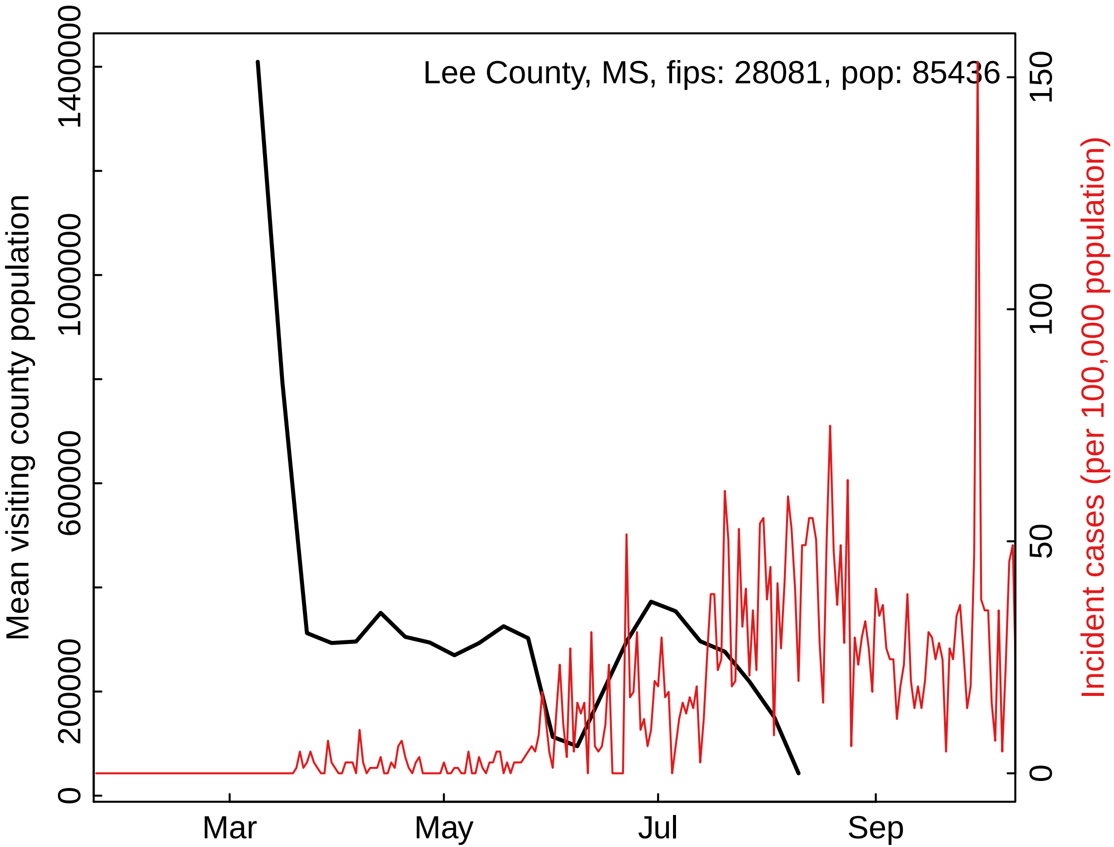

Lee County, MS, fips: 28081, pop: 85436
Mean visiting county population
Incident cases (per 100,000 population)
0
200000
600000
1000000
1400000
0
50
100
150
Mar
May
Jul
Sep

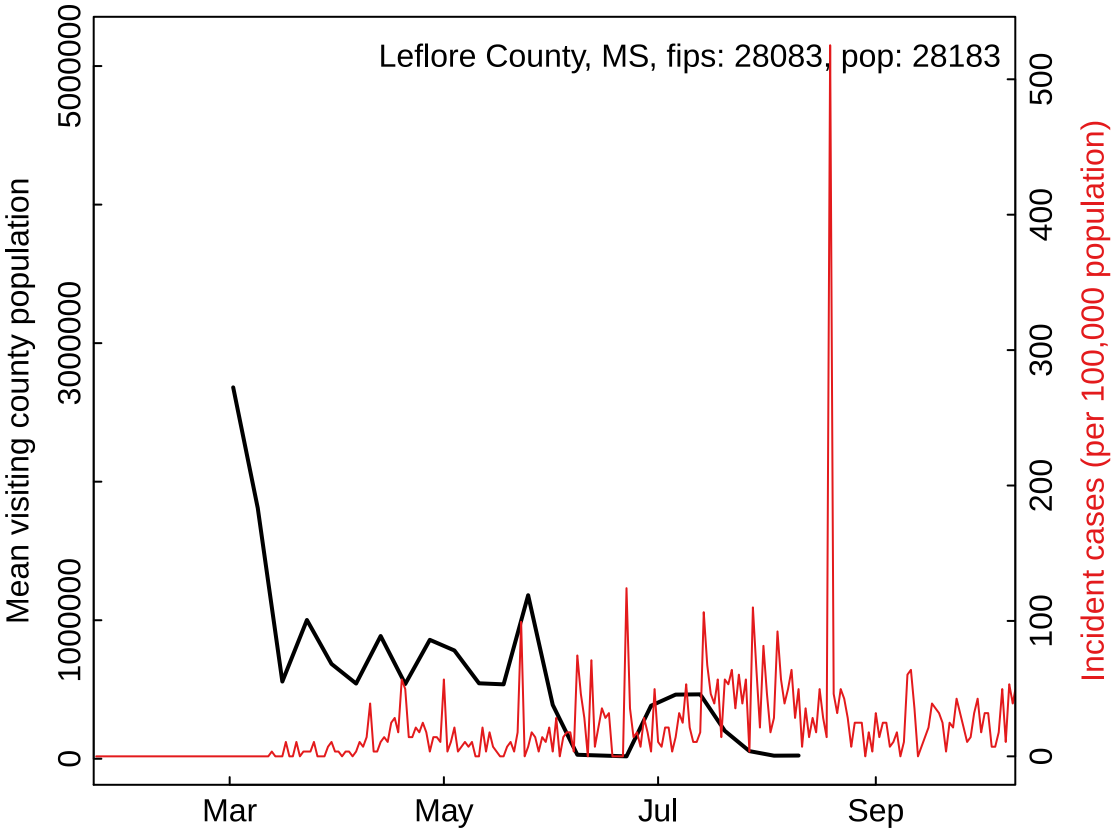

Leflore County, MS, fips: 28083, pop: 28183
Mean visiting county population
0
1000000
3000000
5000000
Incident cases (per 100,000 population)
0
100
200
300
400
500
Mar
May
Jul
Sep

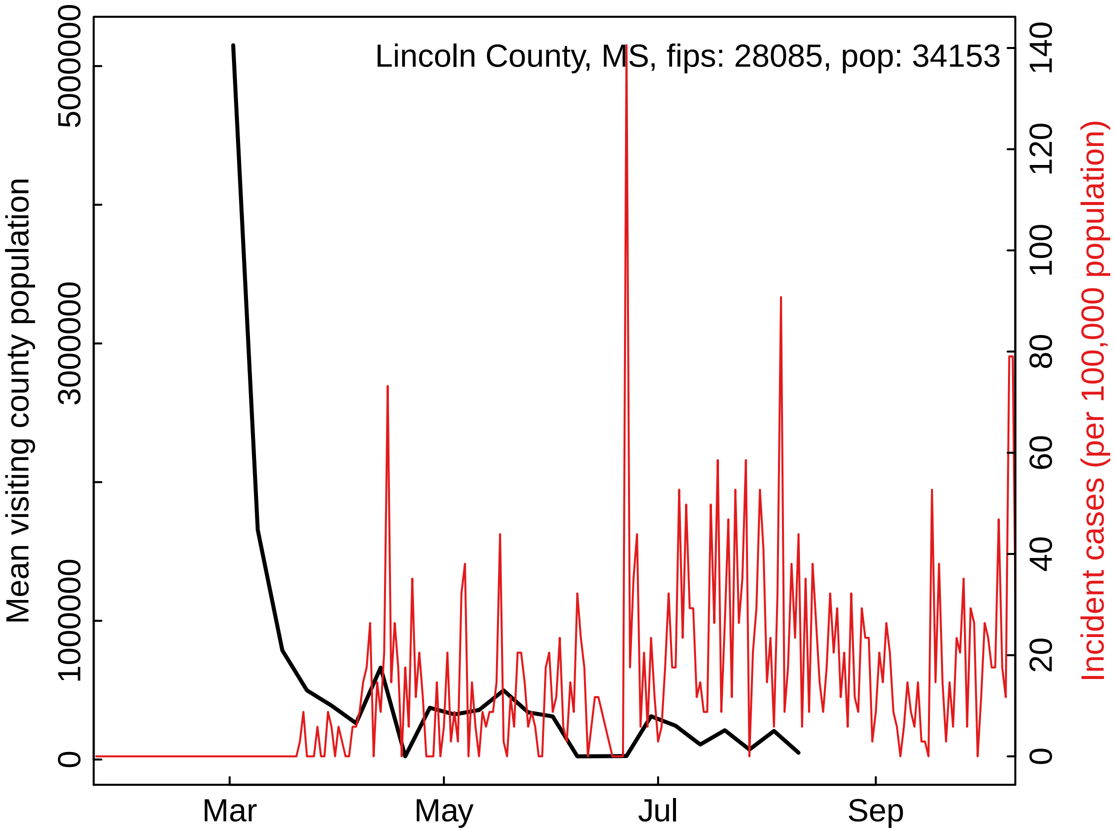

Lincoln County, MS, fips: 28085, pop: 34153
Mean visiting county population
0
1000000
3000000
5000000
Incident cases (per 100,000 population)
0
20
40
60
80
100
120
140
Mar
May
Jul
Sep

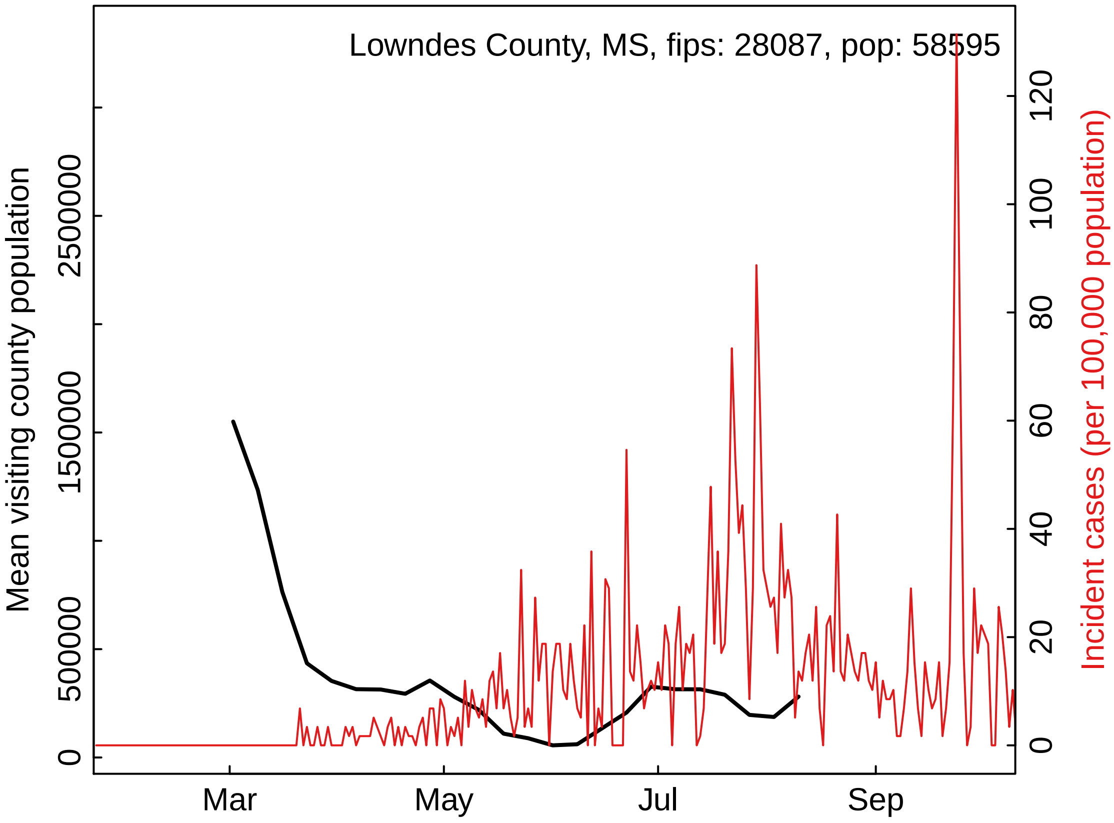

Lowndes County, MS, fips: 28087, pop: 58595
Mean visiting county population
0
500000
1500000
2500000
Incident cases (per 100,000 population)
0
20
40
60
80
100
120
Mar
May
Jul
Sep

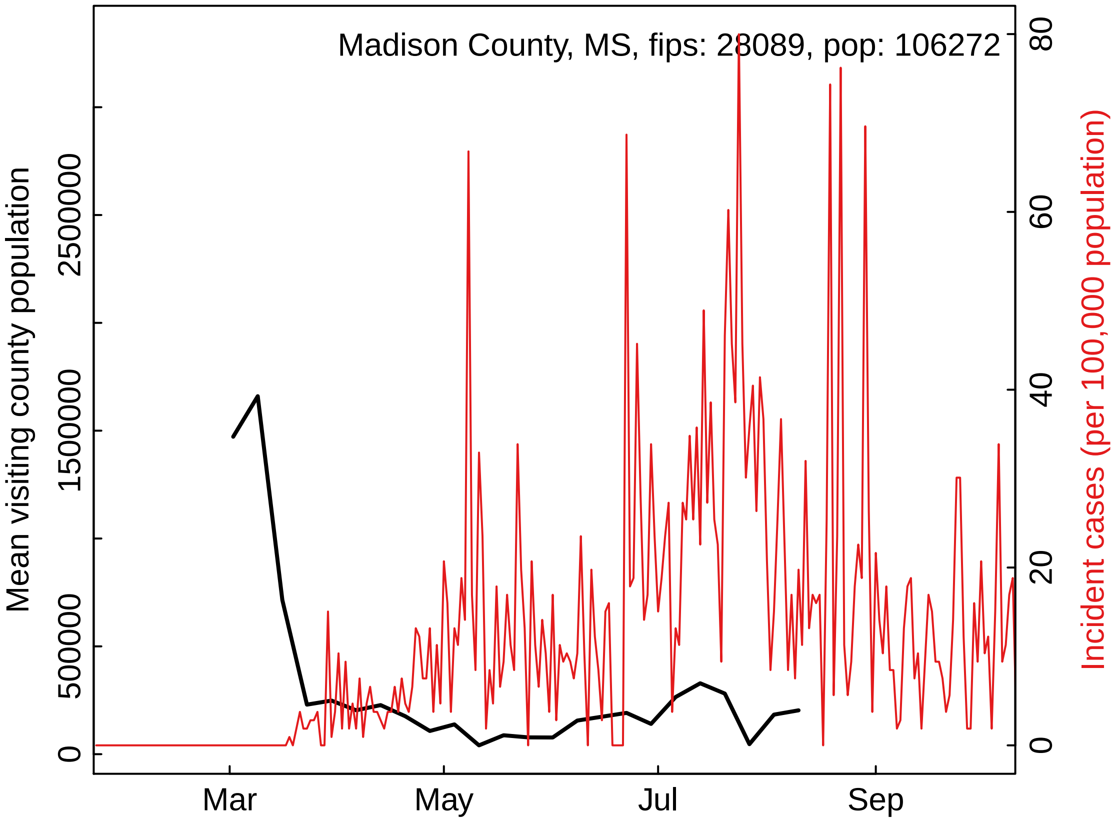

Madison County, MS, fips: 28089, pop: 106272
Mean visiting county population
0
500000
1500000
2500000
Incident cases (per 100,000 population)
0
20
40
60
80
Mar
May
Jul
Sep

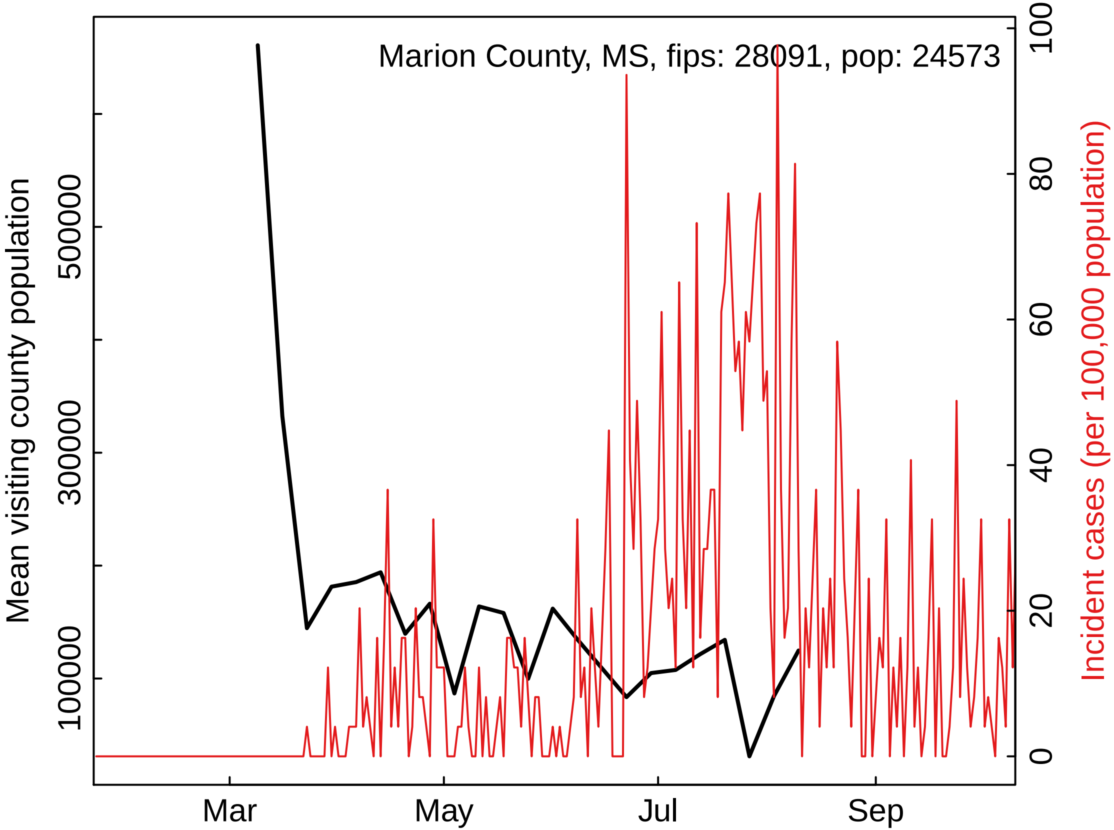
Marion County, MS, fips: 28091, pop: 24573
Mean visiting county population
100000
300000
500000
Mar
May
Jul
Sep
0
20
40
60
80
100
Incident cases (per 100,000 population)

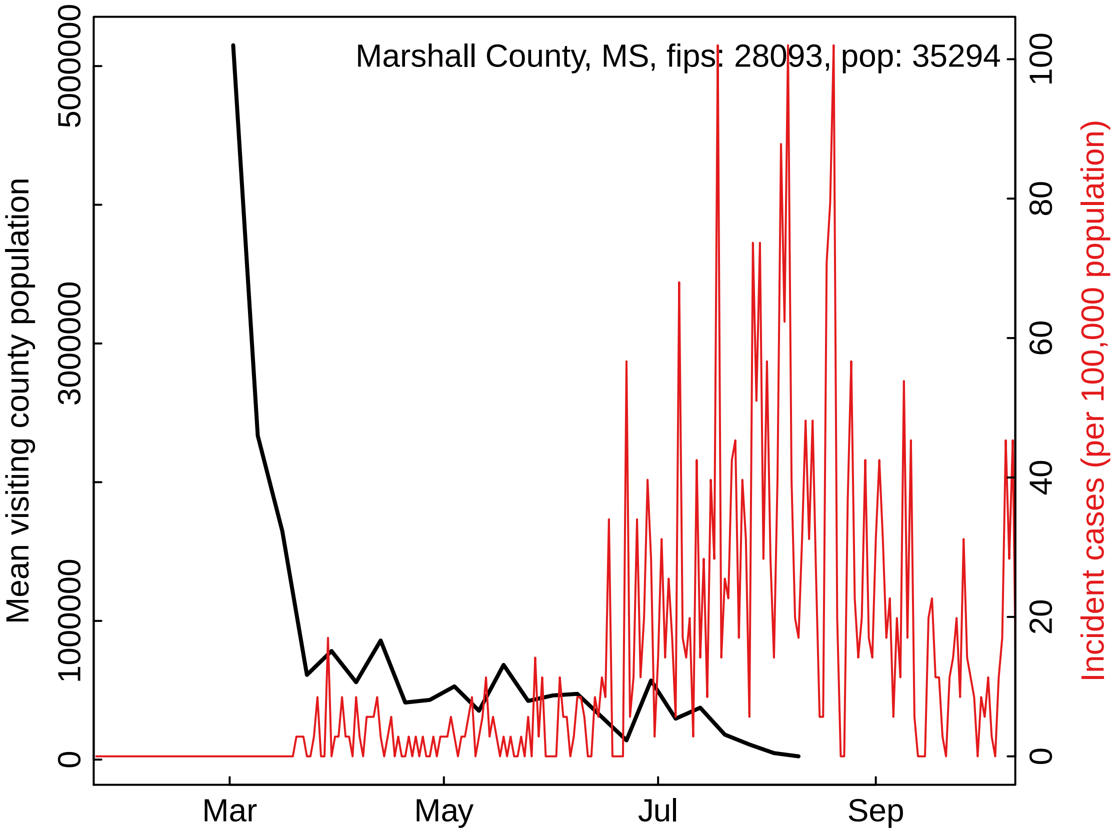
Marshall County, MS, fips: 28093, pop: 35294
Mean visiting county population
Incident cases (per 100,000 population)
0
1000000
3000000
5000000
0
20
40
60
80
100
Mar
May
Jul
Sep

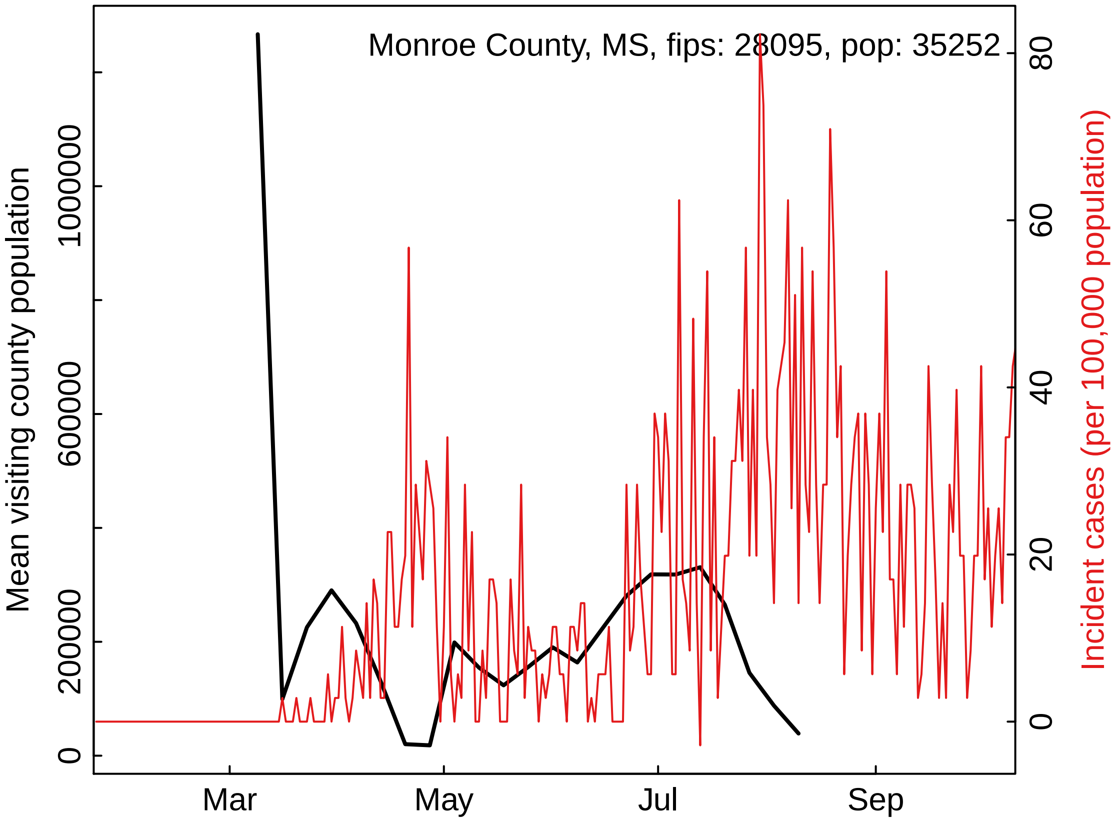

Monroe County, MS, fips: 28095, pop: 35252
Mean visiting county population
Incident cases (per 100,000 population)
0
200000
600000
1000000
0
20
40
60
80
Mar
May
Jul
Sep

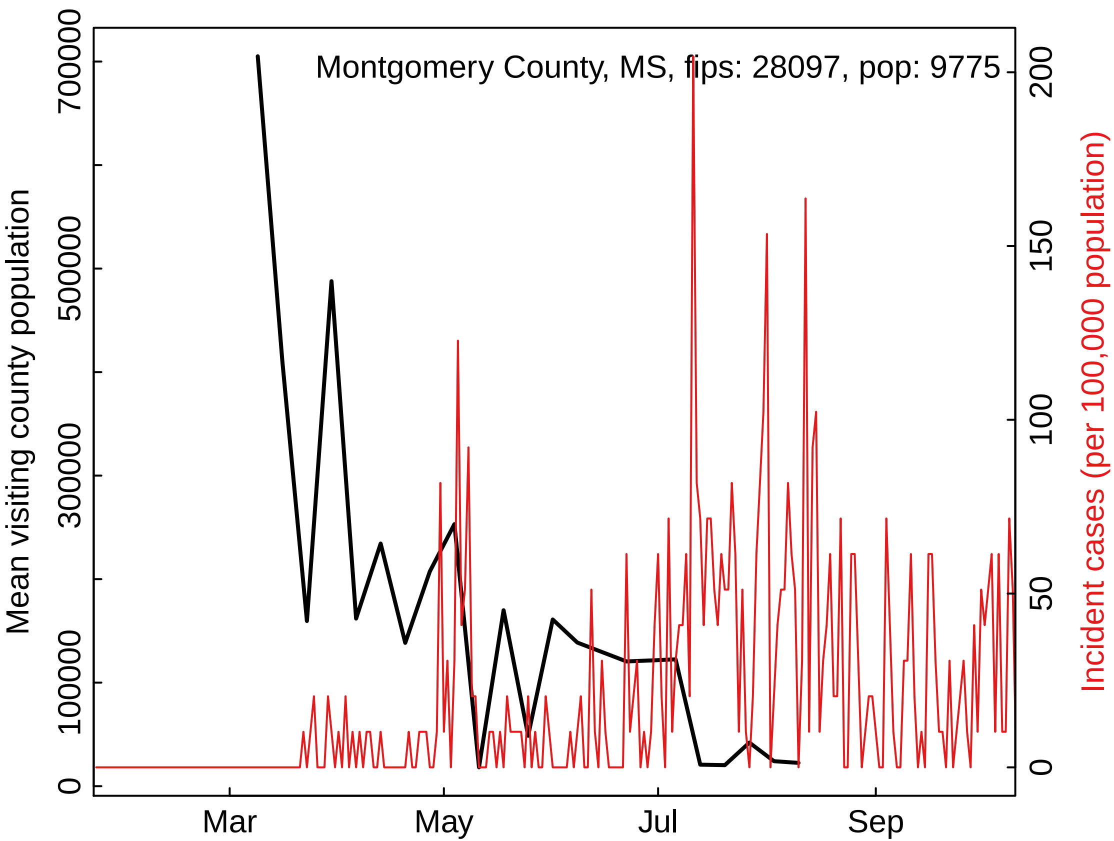

Montgomery County, MS, fips: 28097, pop: 9775
Mean visiting county population
Incident cases (per 100,000 population)
0
100000
300000
500000
700000
0
50
100
150
200
Mar
May
Jul
Sep

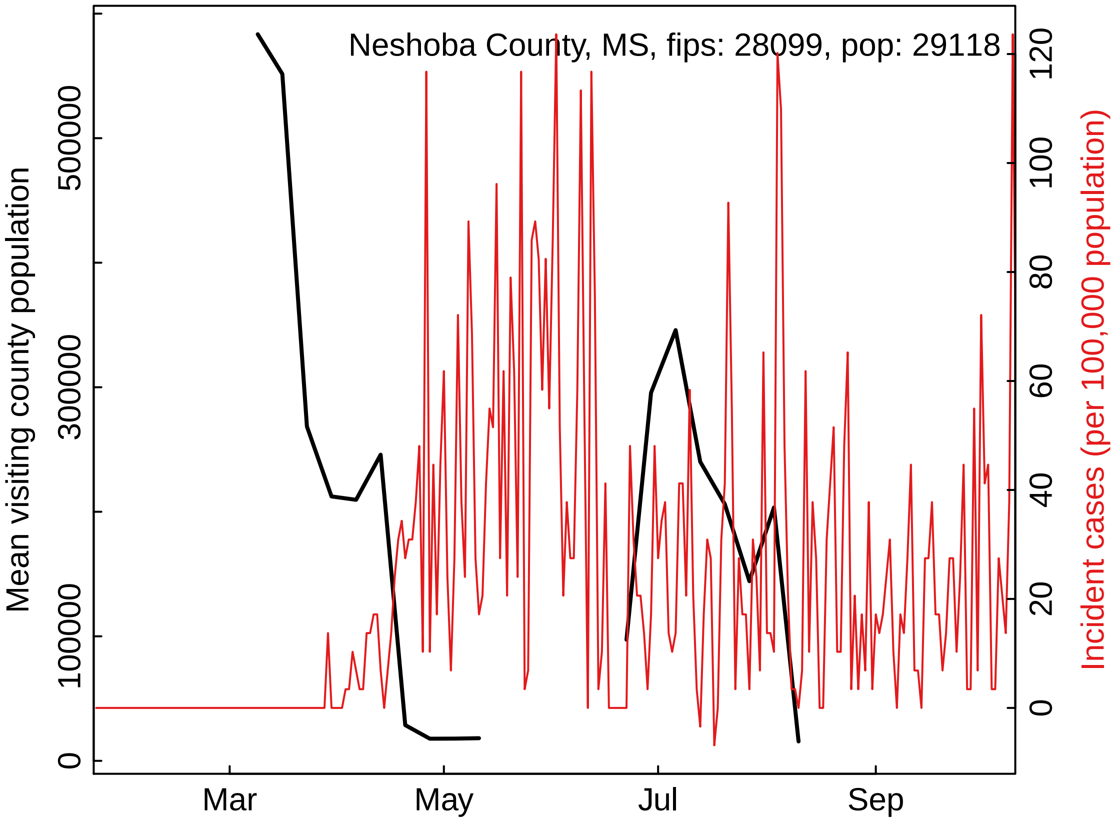
Neshoba County, MS, fips: 28099, pop: 29118
Mean visiting county population
0
100000
300000
500000
Incident cases (per 100,000 population)
0
20
40
60
80
100
120
Mar
May
Jul
Sep

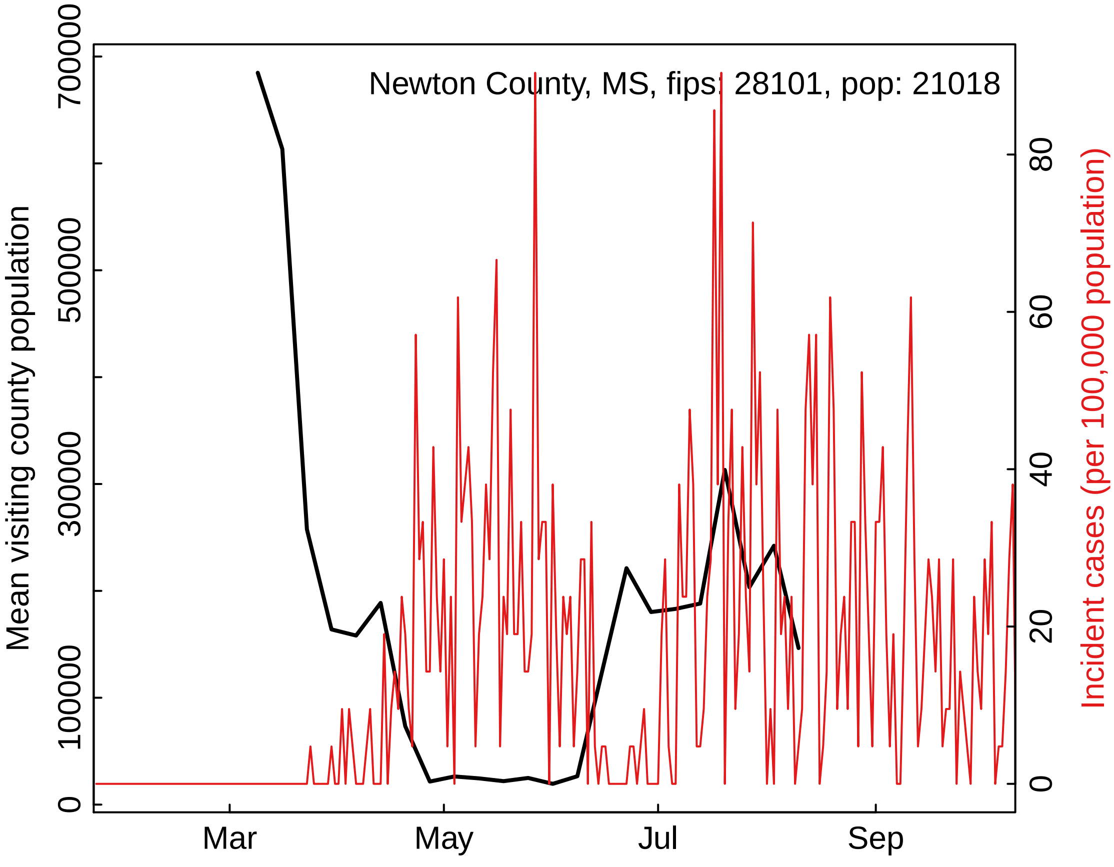

Newton County, MS, fips: 28101, pop: 21018
Mean visiting county population
0
100000
300000
500000
700000
Mar
May
Jul
Sep
0
20
40
60
80
Incident cases (per 100,000 population)

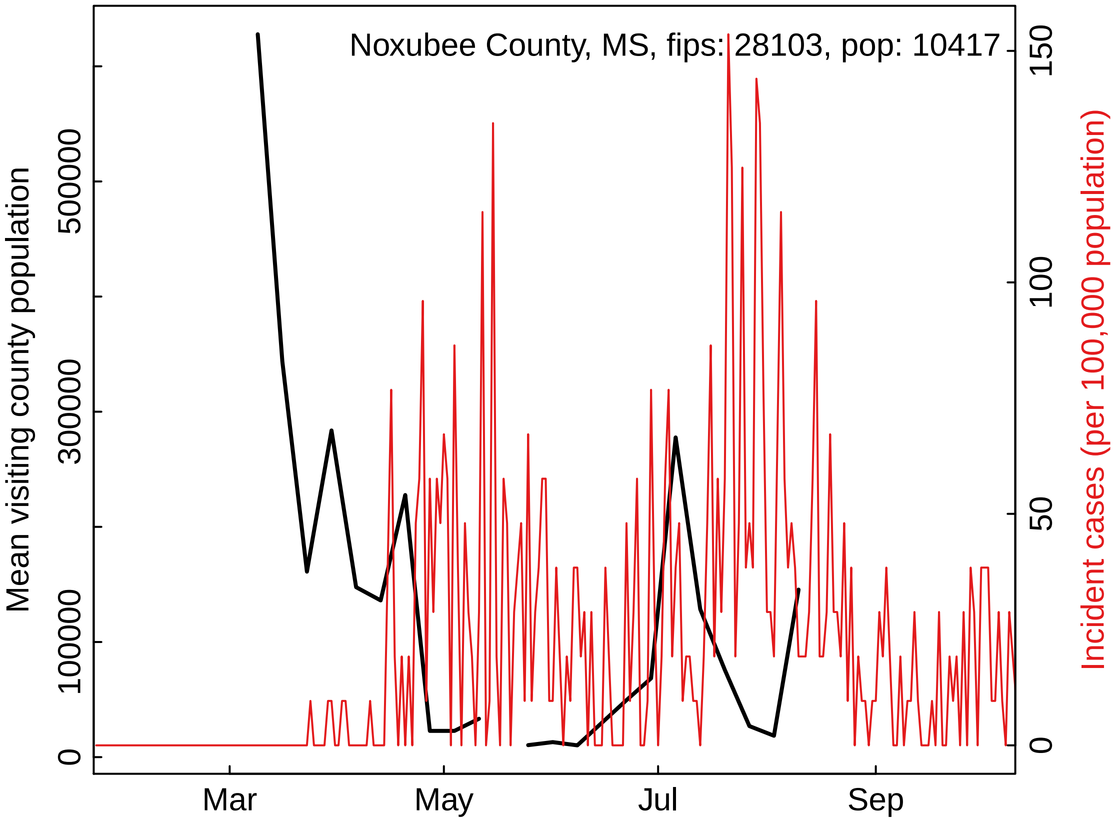

Noxubee County, MS, fips: 28103, pop: 10417
Mean visiting county population
0
100000
300000
500000
Mar
May
Jul
Sep
0
50
100
150
Incident cases (per 100,000 population)

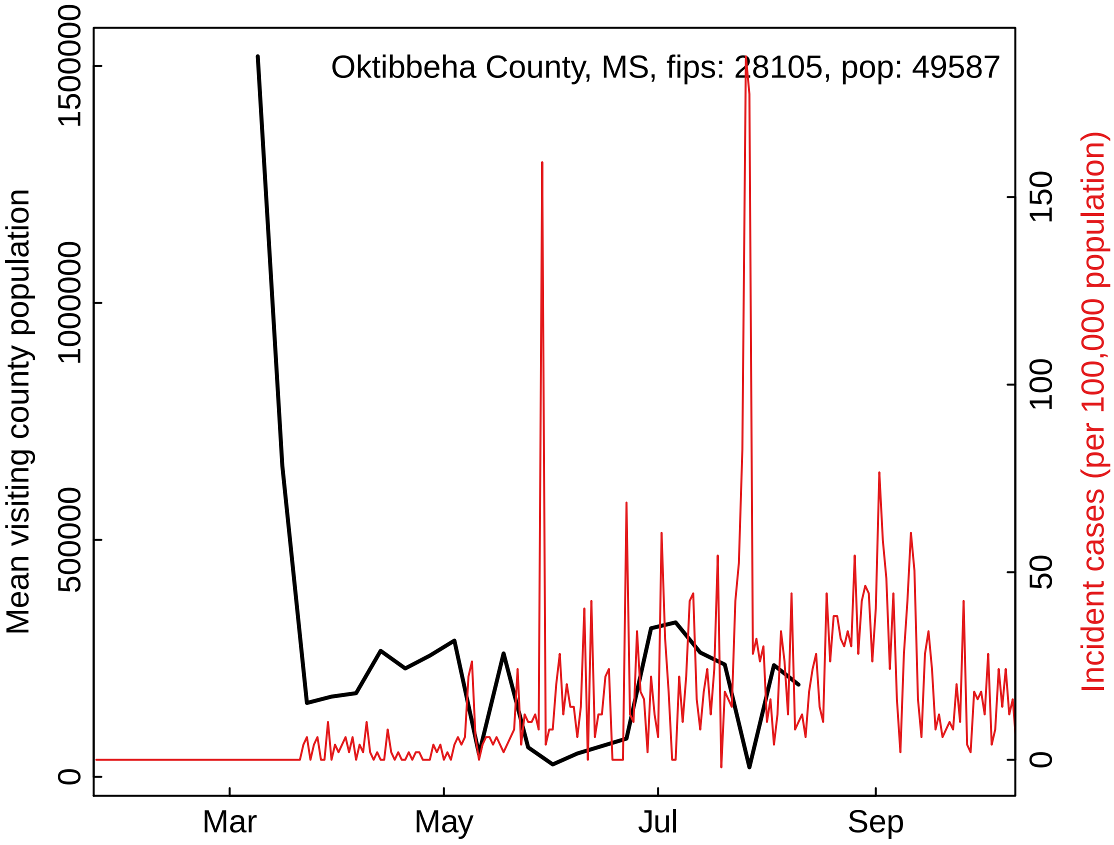

Oktibbeha County, MS, fips: 28105, pop: 49587
Mean visiting county population
0
500000
1000000
1500000
Incident cases (per 100,000 population)
0
50
100
150
Mar
May
Jul
Sep

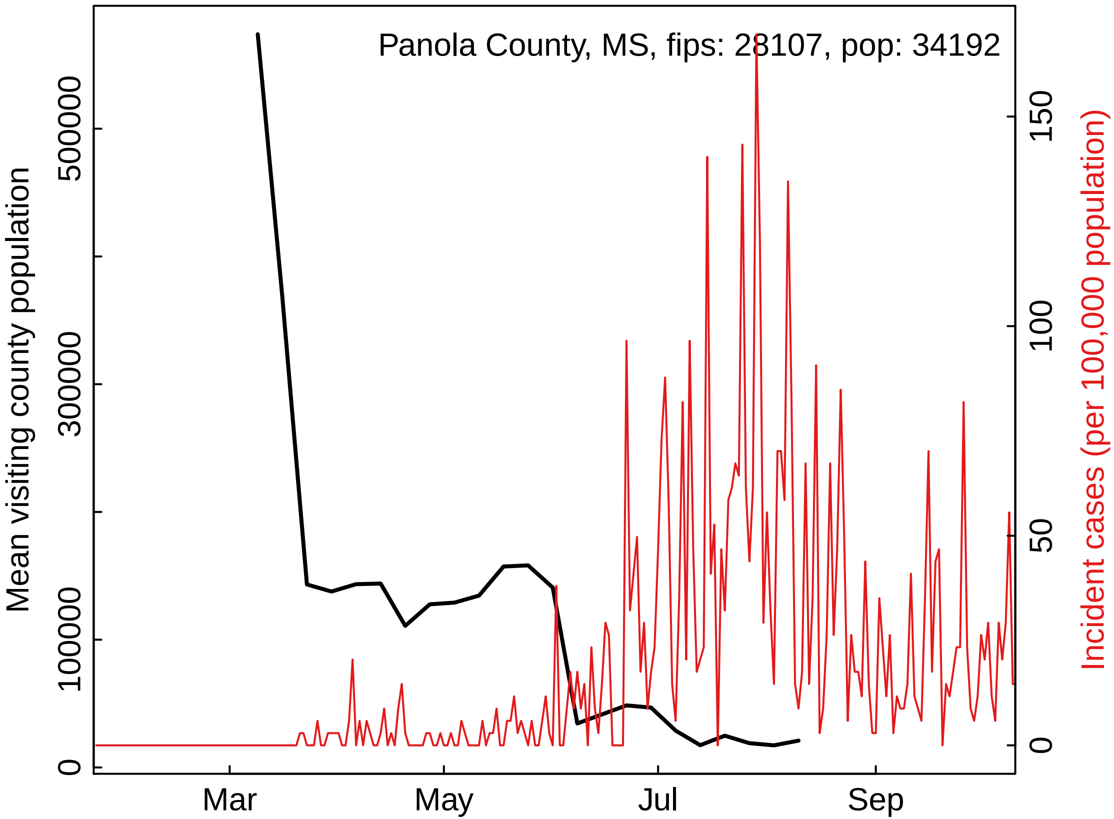

Panola County, MS, fips: 28107, pop: 34192
Mean visiting county population
Incident cases (per 100,000 population)
0
100000
300000
500000
0
50
100
150
Mar
May
Jul
Sep

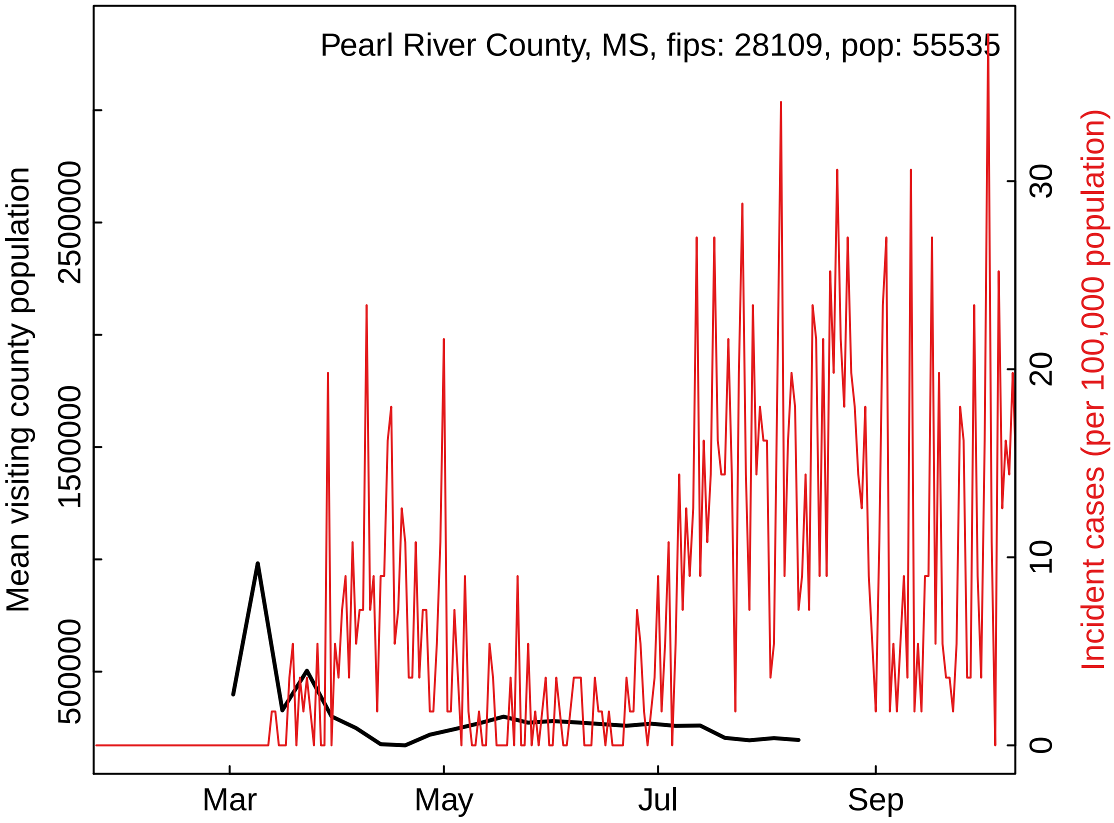

Pearl River County, MS, fips: 28109, pop: 55535
Mean visiting county population
500000
1500000
2500000
Incident cases (per 100,000 population)
0
10
20
30
Mar
May
Jul
Sep

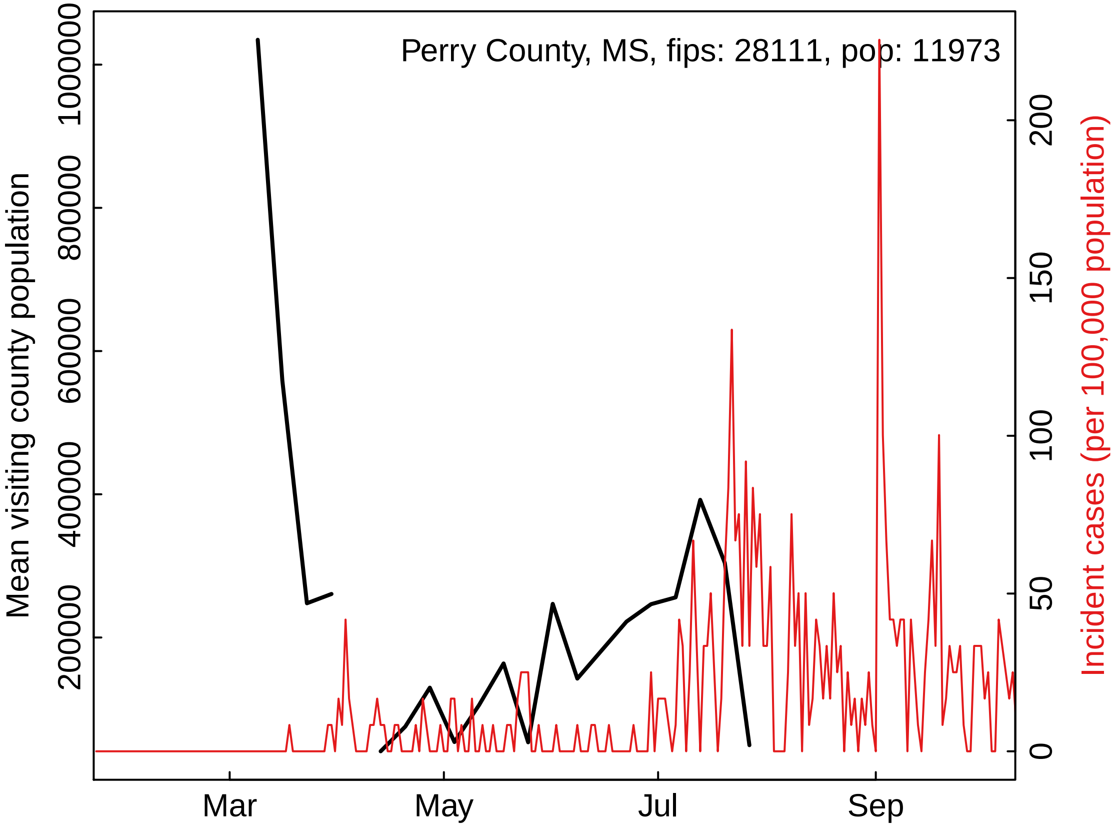

Perry County, MS, fips: 28111, pop: 11973
Mean visiting county population
200000
400000
600000
800000
1000000
Incident cases (per 100,000 population)
0
50
100
150
200
Mar
May
Jul
Sep

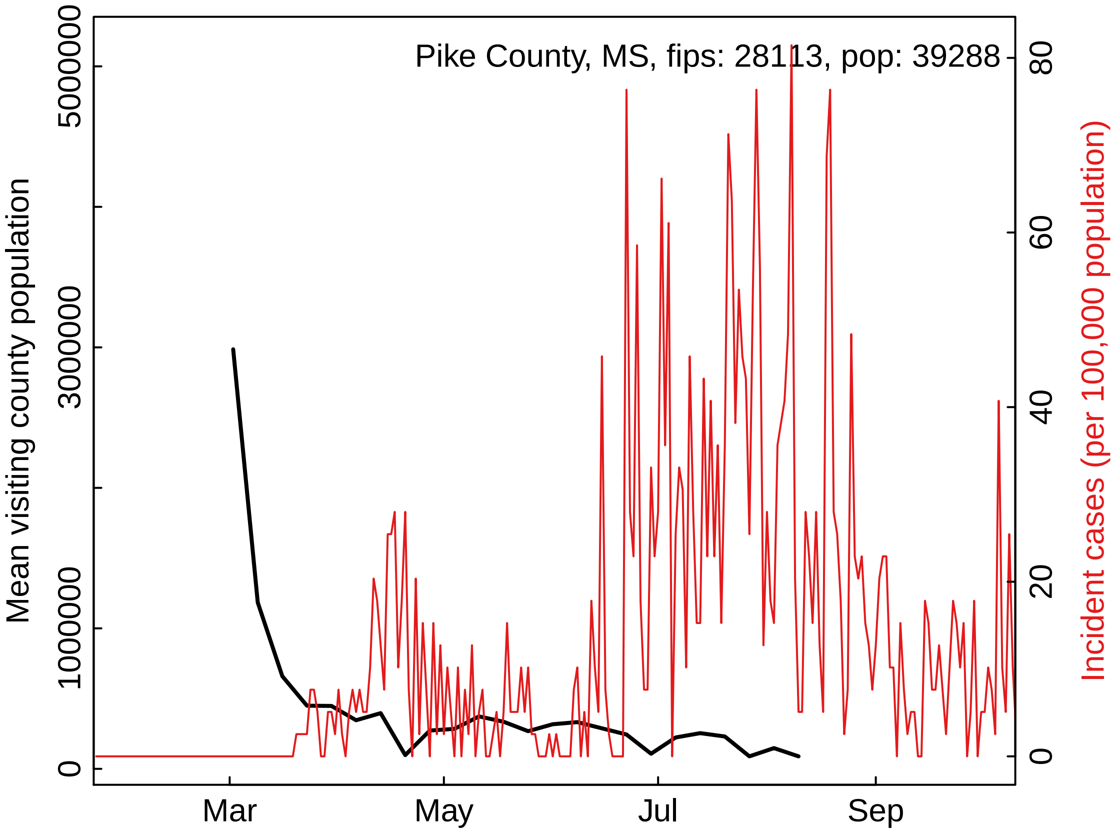

Pike County, MS, fips: 28113, pop: 39288
Mean visiting county population
0
1000000
3000000
5000000
Incident cases (per 100,000 population)
0
20
40
60
80
Mar
May
Jul
Sep

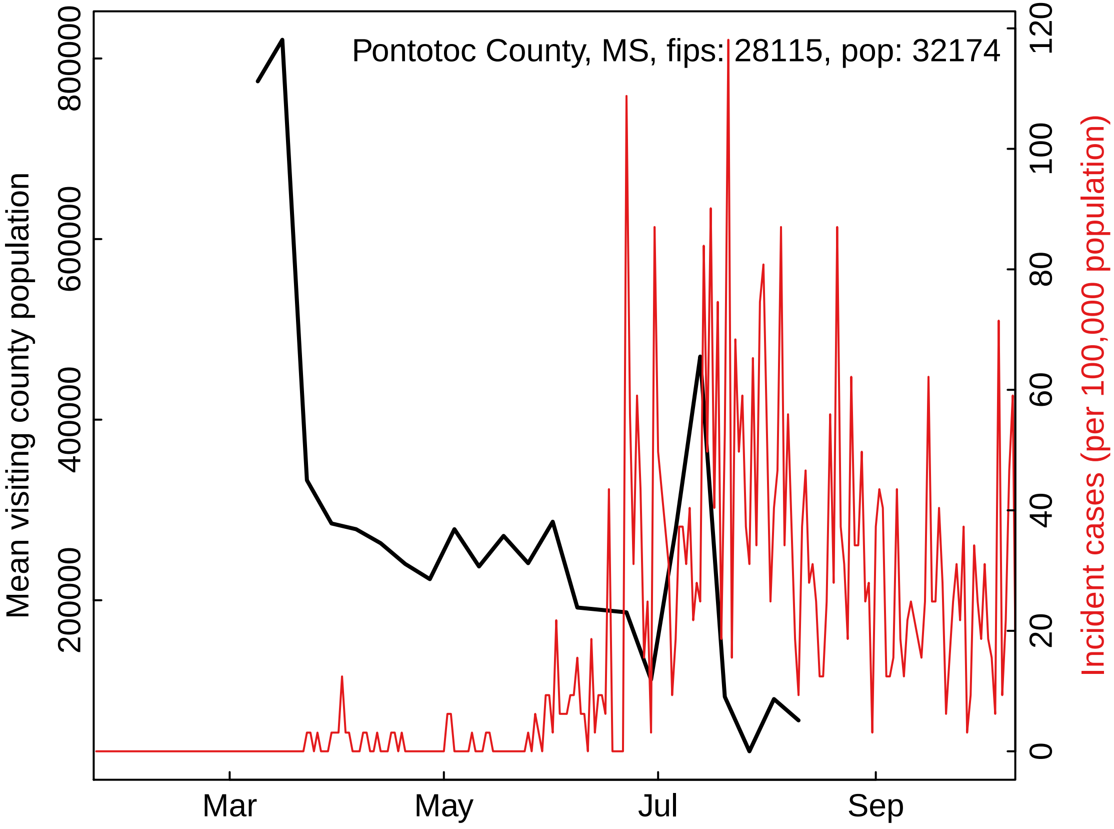
Pontotoc County, MS, fips: 28115, pop: 32174
Mean visiting county population
200000
400000
600000
800000
Incident cases (per 100,000 population)
0
20
40
60
80
100
120
Mar
May
Jul
Sep

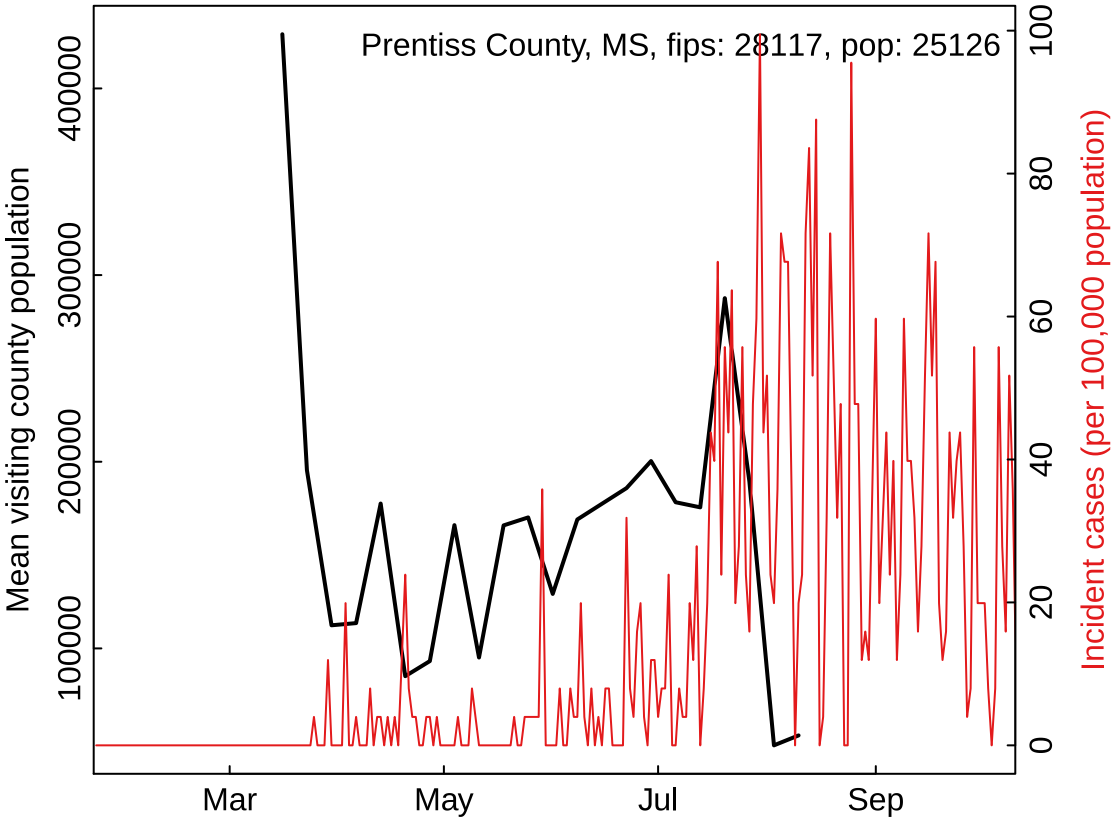

Prentiss County, MS, fips: 28117, pop: 25126
Mean visiting county population
100000
200000
300000
400000
Incident cases (per 100,000 population)
0
20
40
60
80
100
Mar
May
Jul
Sep

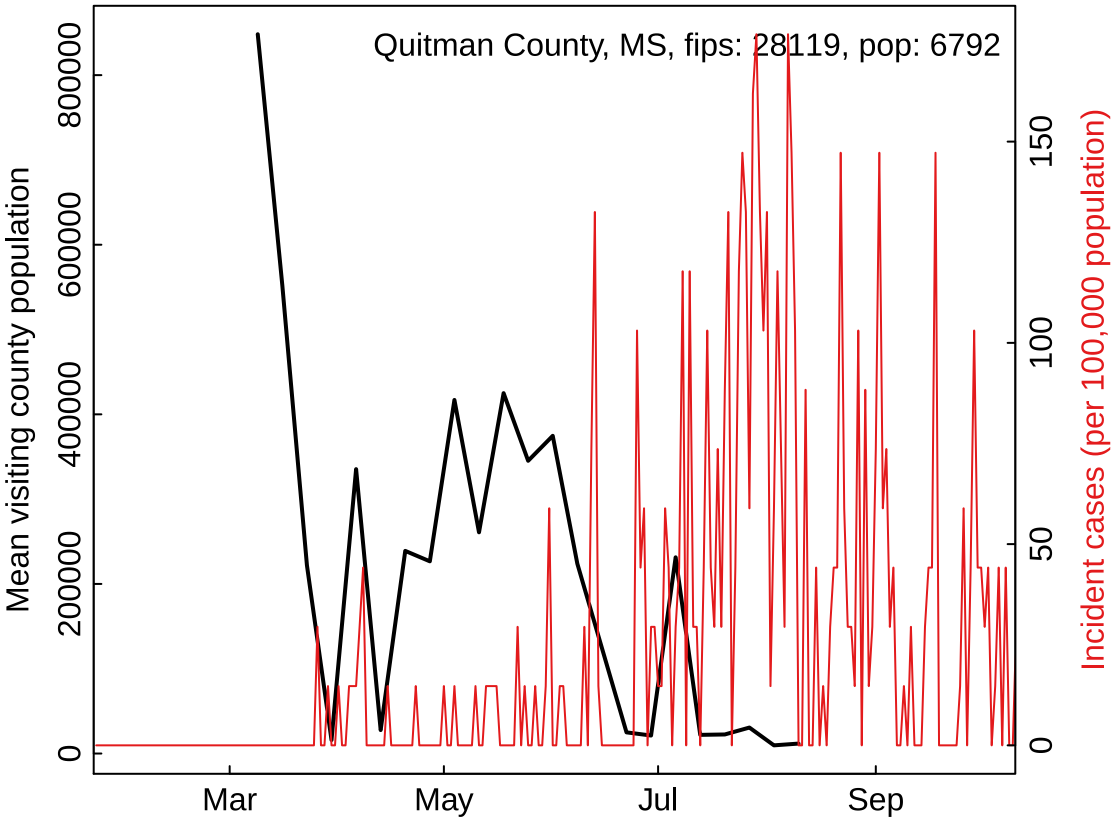

Quitman County, MS, fips: 28119, pop: 6792
Mean visiting county population
Incident cases (per 100,000 population)
0
200000
400000
600000
800000
0
50
100
150
Mar
May
Jul
Sep

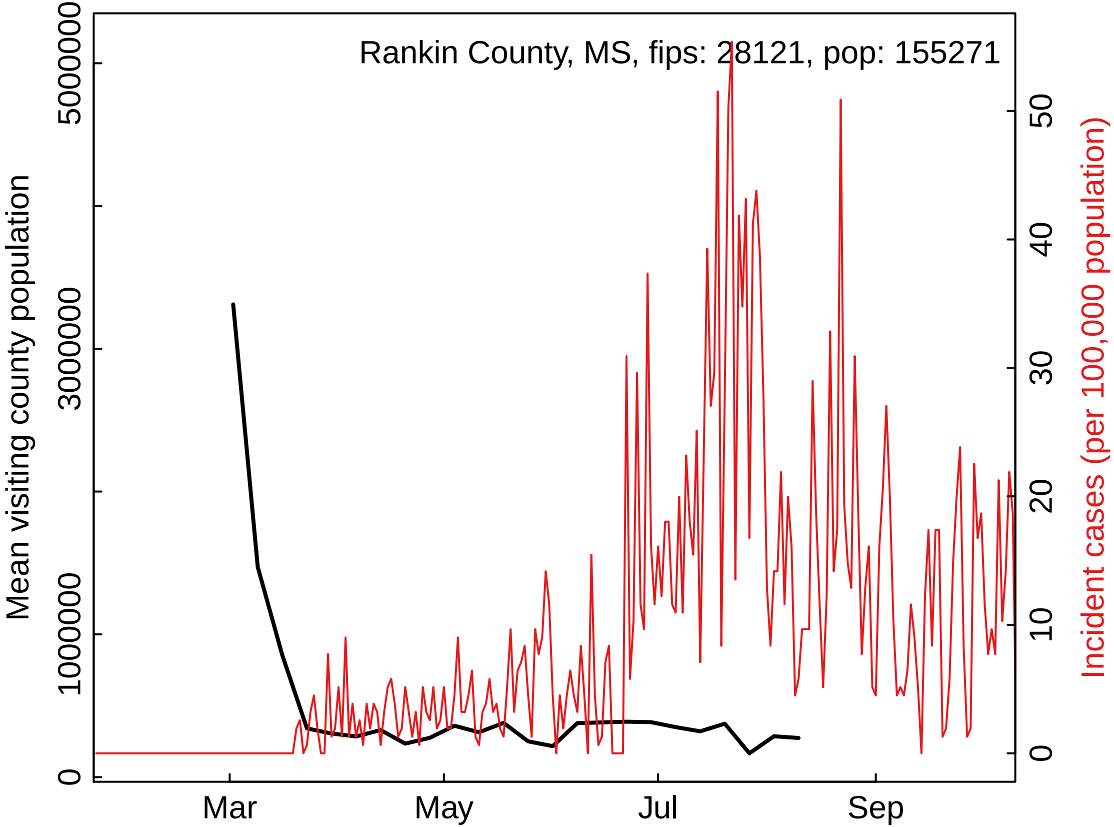

Rankin County, MS, fips: 28121, pop: 155271
Mean visiting county population
0
1000000
3000000
5000000
Incident cases (per 100,000 population)
0
10
20
30
40
50
Mar
May
Jul
Sep

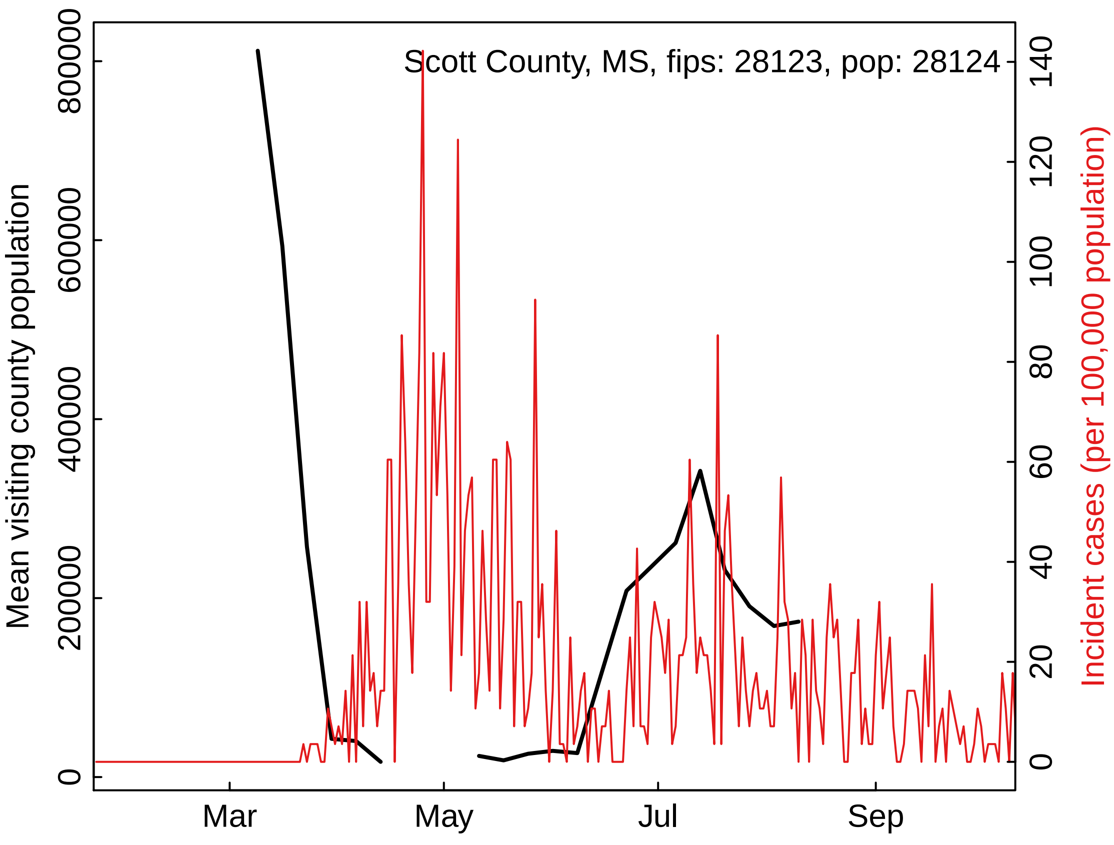

Scott County, MS, fips: 28123, pop: 28124
Mean visiting county population
0
200000
400000
600000
800000
Incident cases (per 100,000 population)
0
20
40
60
80
100
120
140
Mar
May
Jul
Sep

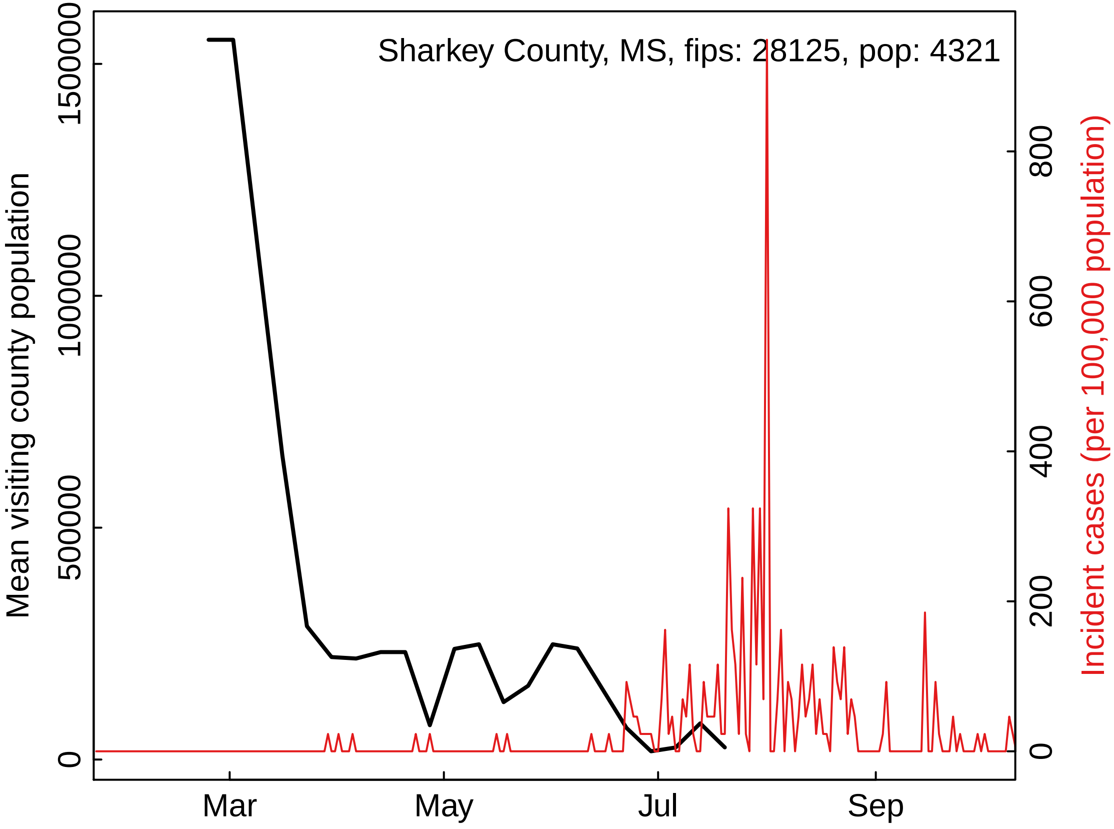

Sharkey County, MS, fips: 28125, pop: 4321
Mean visiting county population
0
500000
1000000
1500000
Incident cases (per 100,000 population)
0
200
400
600
800
Mar
May
Jul
Sep

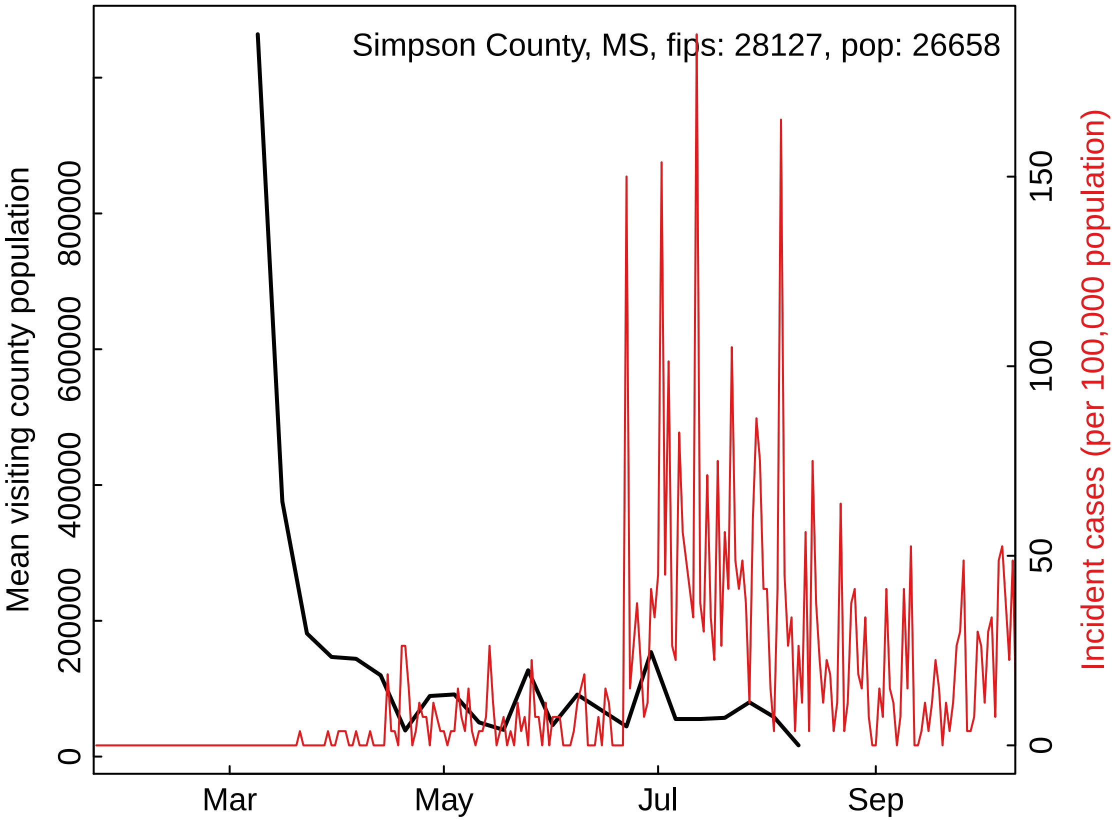

Simpson County, MS, fips: 28127, pop: 26658
Mean visiting county population
0
200000
400000
600000
800000
Incident cases (per 100,000 population)
0
50
100
150
Mar
May
Jul
Sep

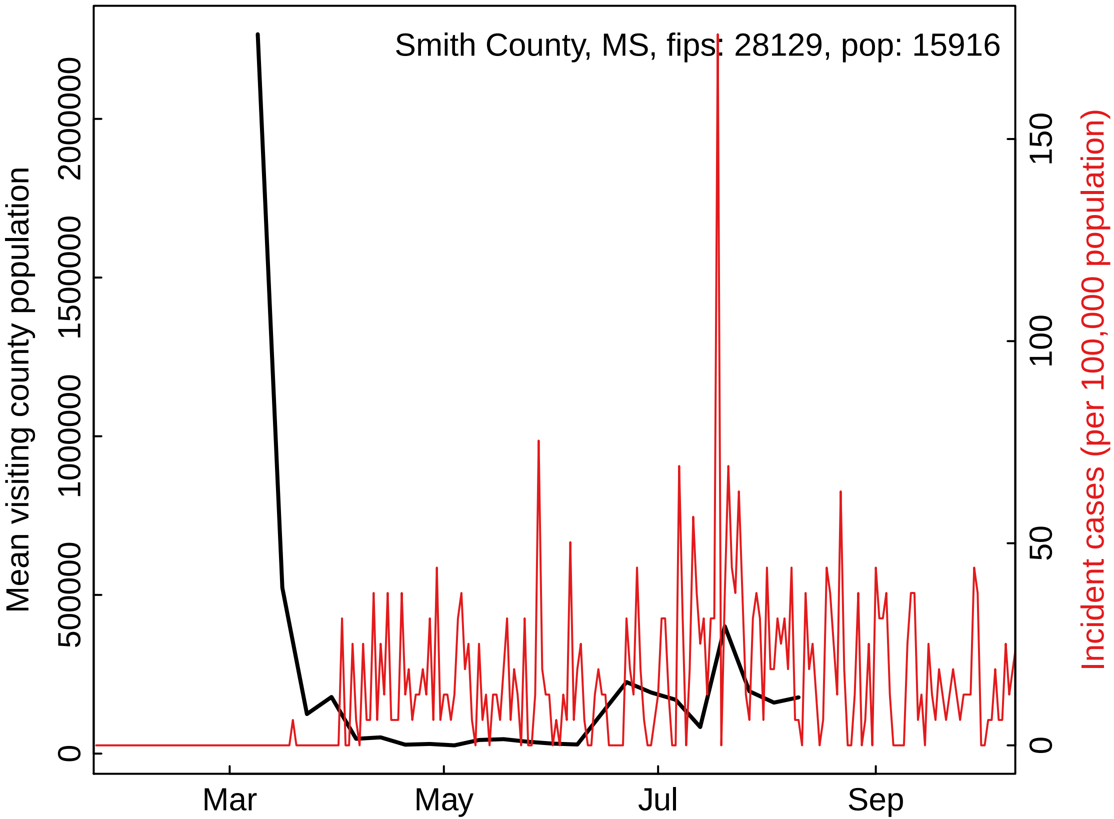

Smith County, MS, fips: 28129, pop: 15916
Mean visiting county population
Incident cases (per 100,000 population)
0
500000
1000000
1500000
2000000
0
50
100
150
Mar
May
Jul
Sep

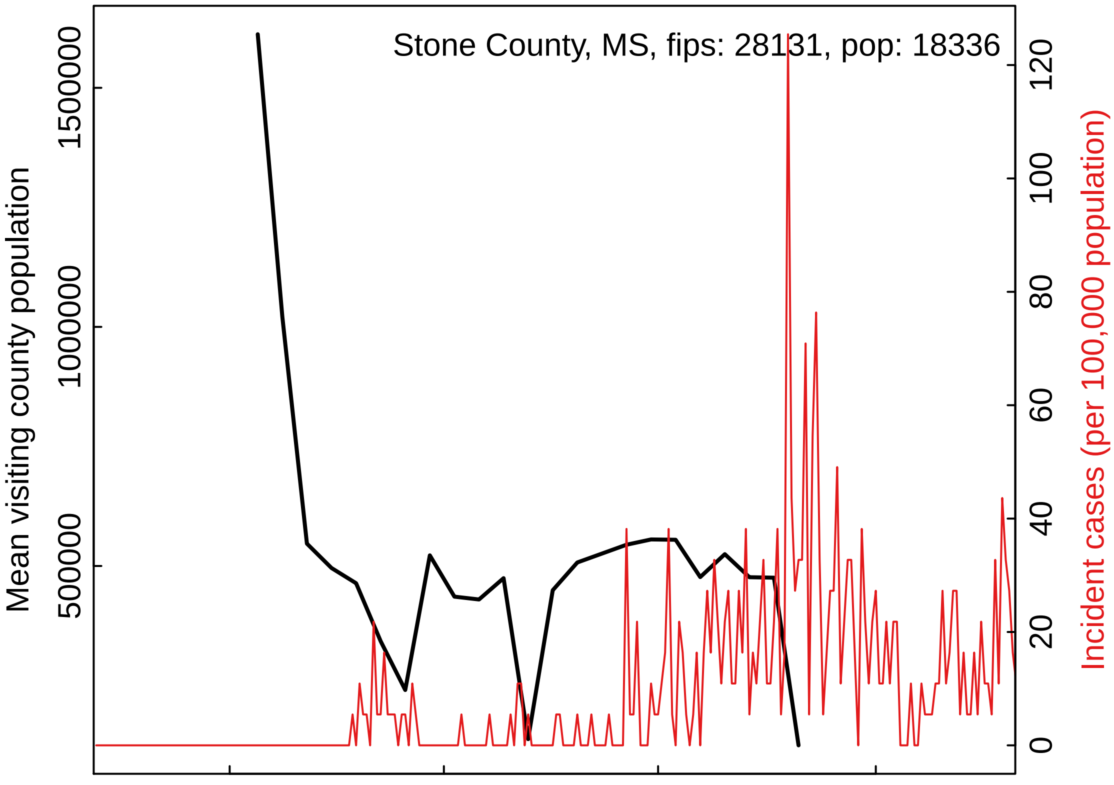

Stone County, MS, fips: 28131, pop: 18336
Mean visiting county population
500000
1000000
1500000
Incident cases (per 100,000 population)
0
20
40
60
80
100
120
Mar
May
Jul
Sep

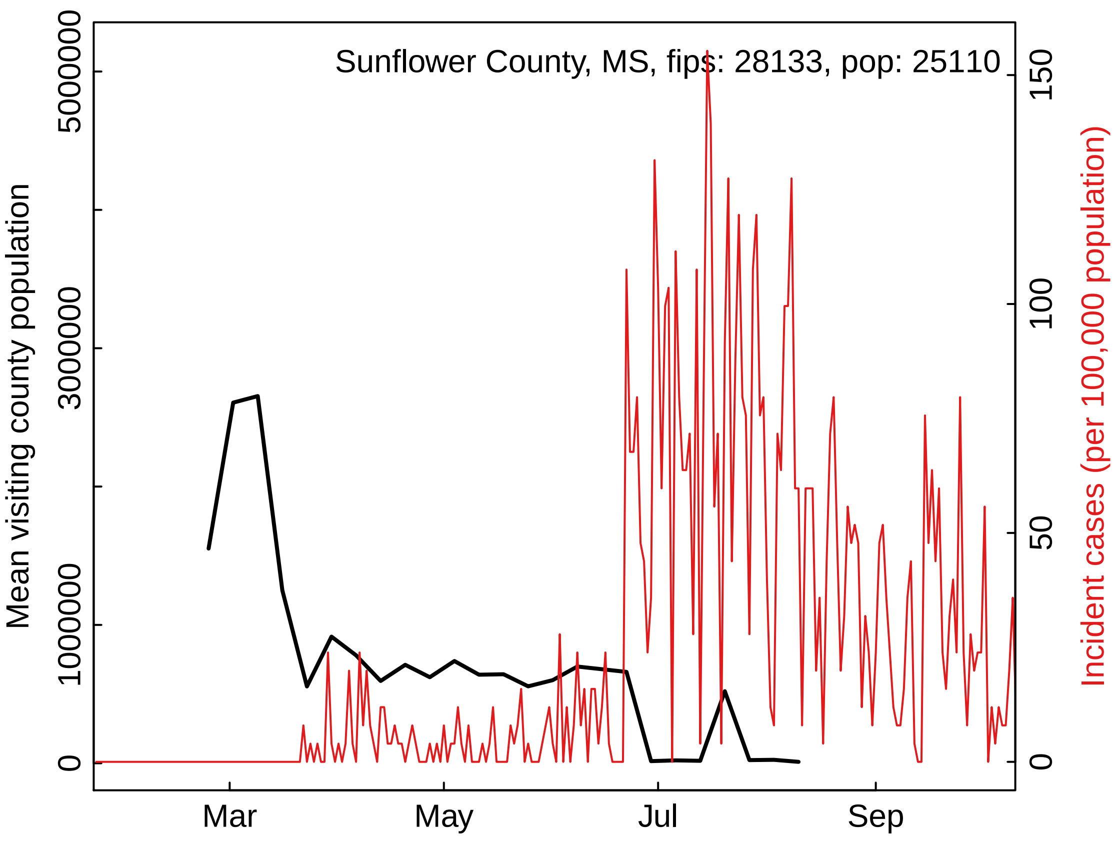

Sunflower County, MS, fips: 28133, pop: 25110
Mean visiting county population
0
1000000
3000000
5000000
Incident cases (per 100,000 population)
0
50
100
150
Mar
May
Jul
Sep

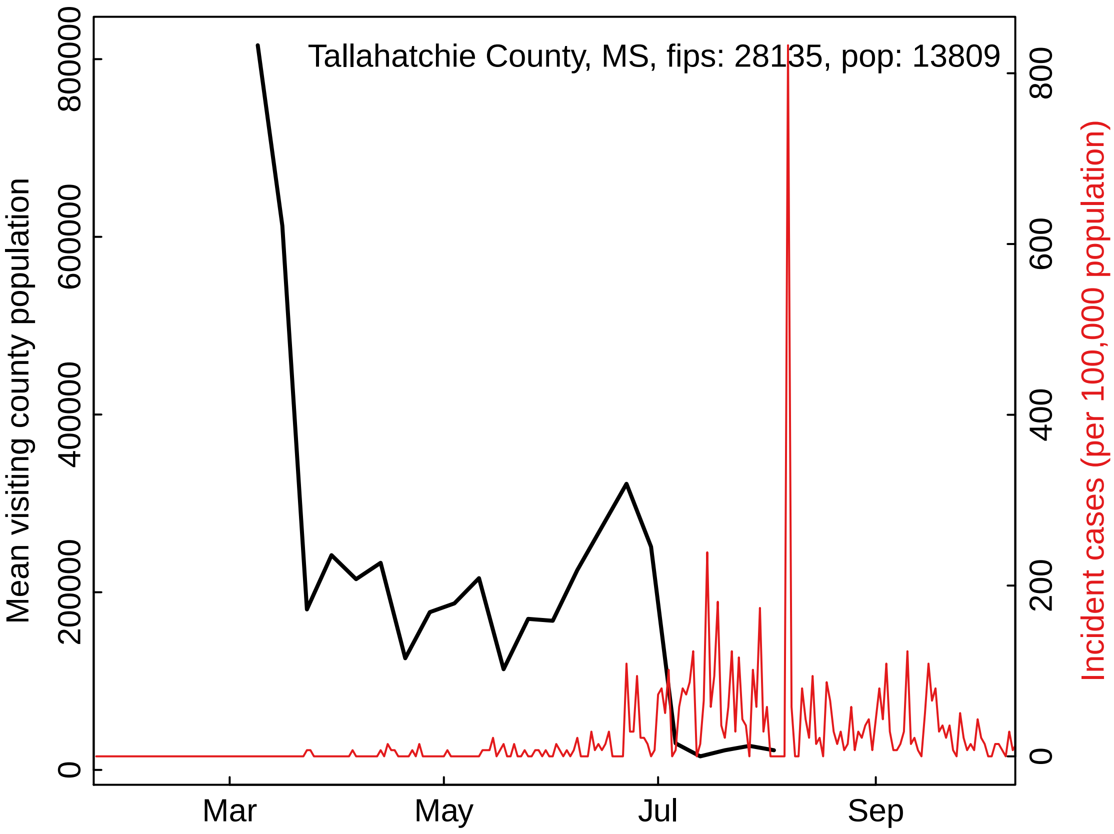

Tallahatchie County, MS, fips: 28135, pop: 13809
Mean visiting county population
Incident cases (per 100,000 population)
0
200000
400000
600000
800000
0
200
400
600
800
Mar
May
Jul
Sep

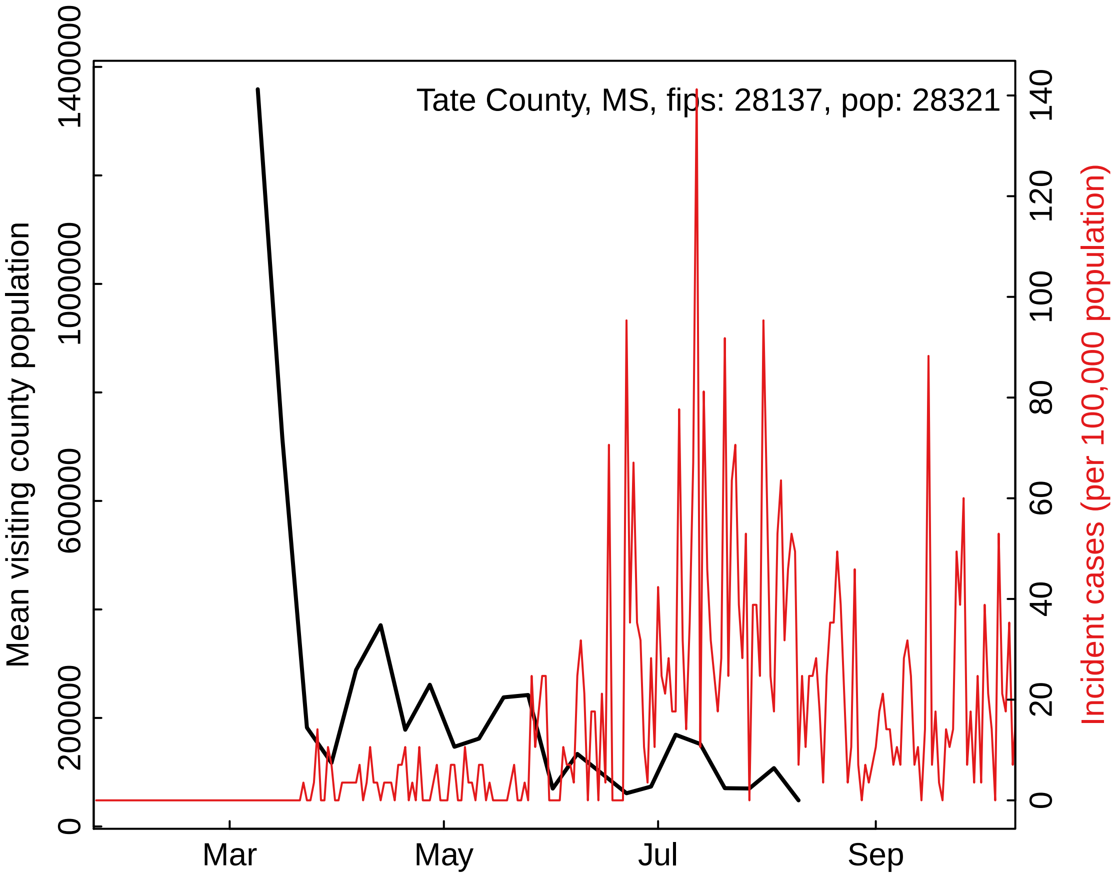

Tate County, MS, fips: 28137, pop: 28321
Mean visiting county population
Incident cases (per 100,000 population)
0
200000
600000
1000000
1400000
0
20
40
60
80
100
120
140
Mar
May
Jul
Sep

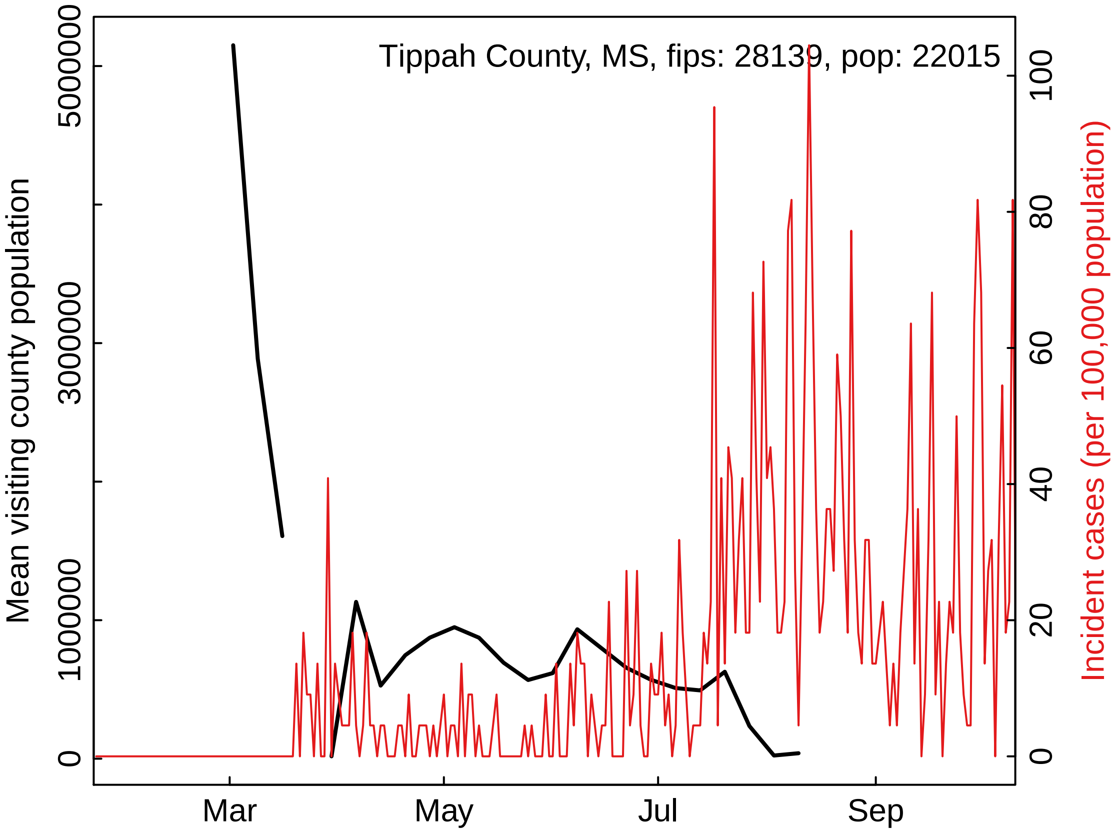

Tippah County, MS, fips: 28139, pop: 22015
Mean visiting county population
0
1000000
3000000
5000000
Incident cases (per 100,000 population)
0
20
40
60
80
100
Mar
May
Jul
Sep

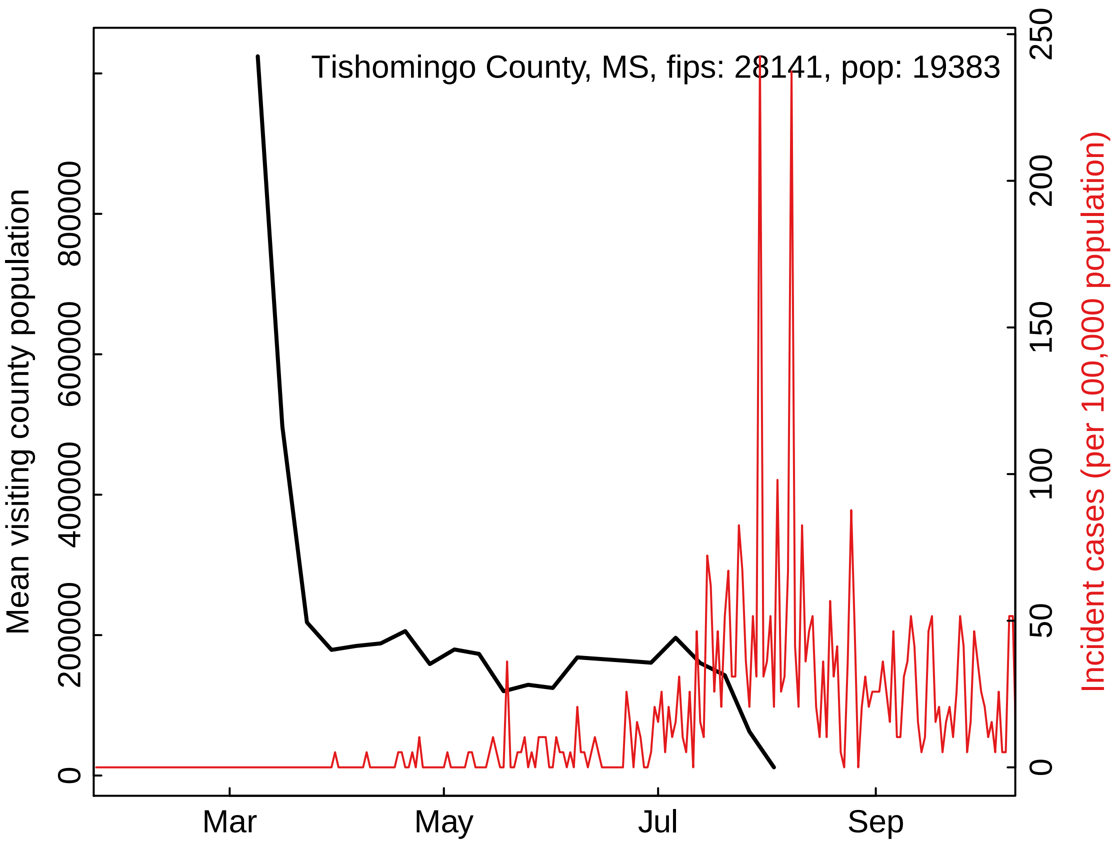

Tishomingo County, MS, fips: 28141, pop: 19383
Mean visiting county population
0
200000
400000
600000
800000
Incident cases (per 100,000 population)
0
50
100
150
200
250
Mar
May
Jul
Sep

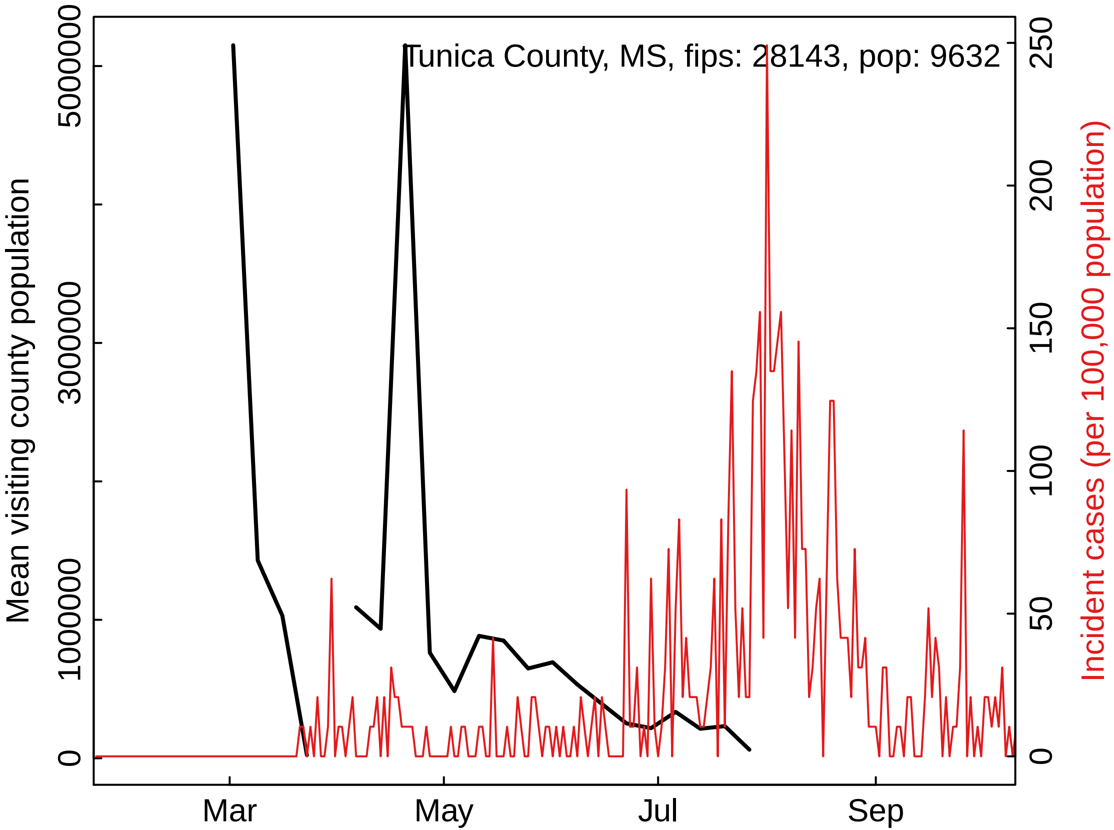

Tunica County, MS, fips: 28143, pop: 9632
Mean visiting county population
Incident cases (per 100,000 population)
0
1000000
3000000
5000000
0
50
100
150
200
250
Mar
May
Jul
Sep

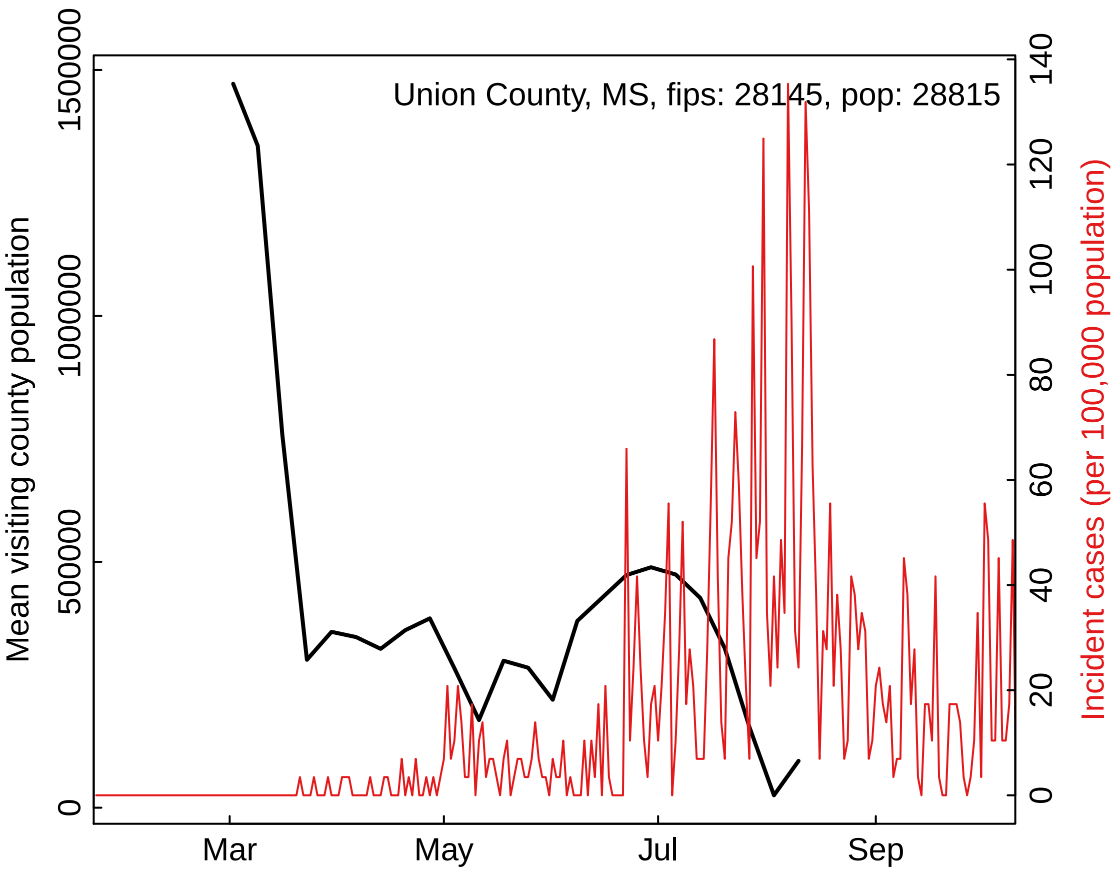

Union County, MS, fips: 28145, pop: 28815
Mean visiting county population
Incident cases (per 100,000 population)
0
500000
1000000
1500000
0
20
40
60
80
100
120
140
Mar
May
Jul
Sep

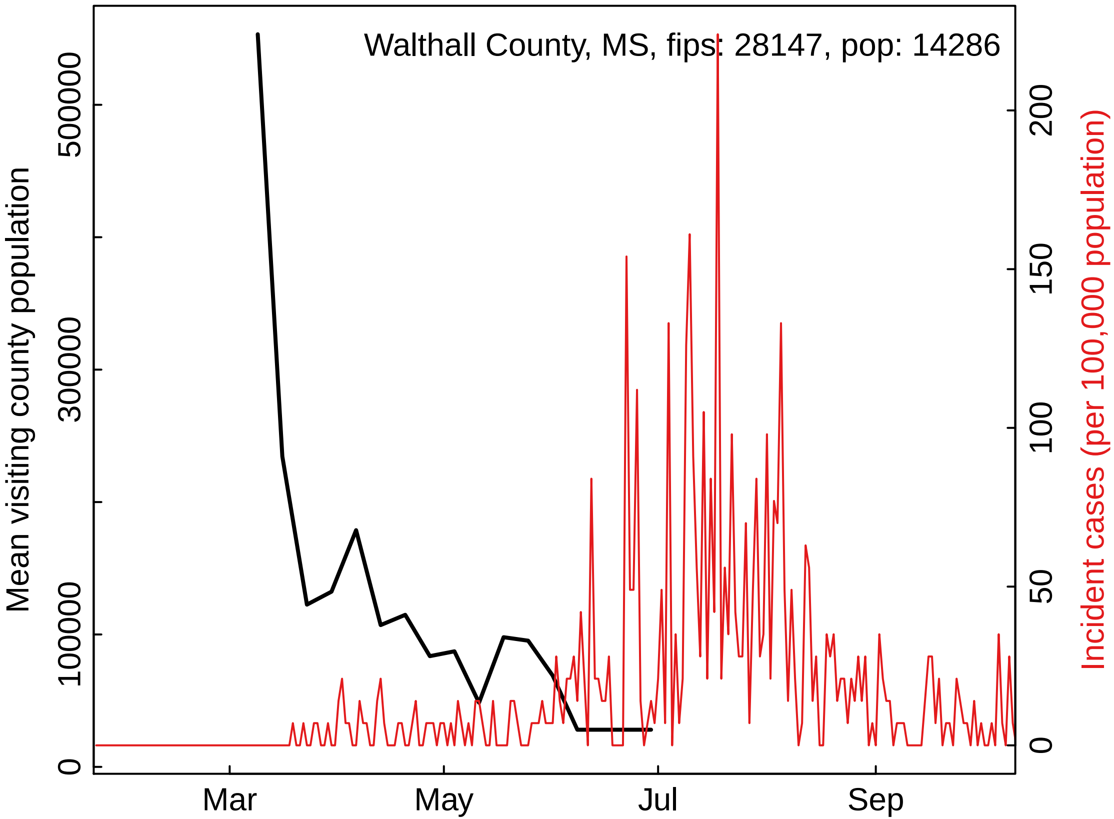

Walthall County, MS, fips: 28147, pop: 14286
Mean visiting county population
0
100000
300000
500000
Mar
May
Jul
Sep
0
50
100
150
200
Incident cases (per 100,000 population)

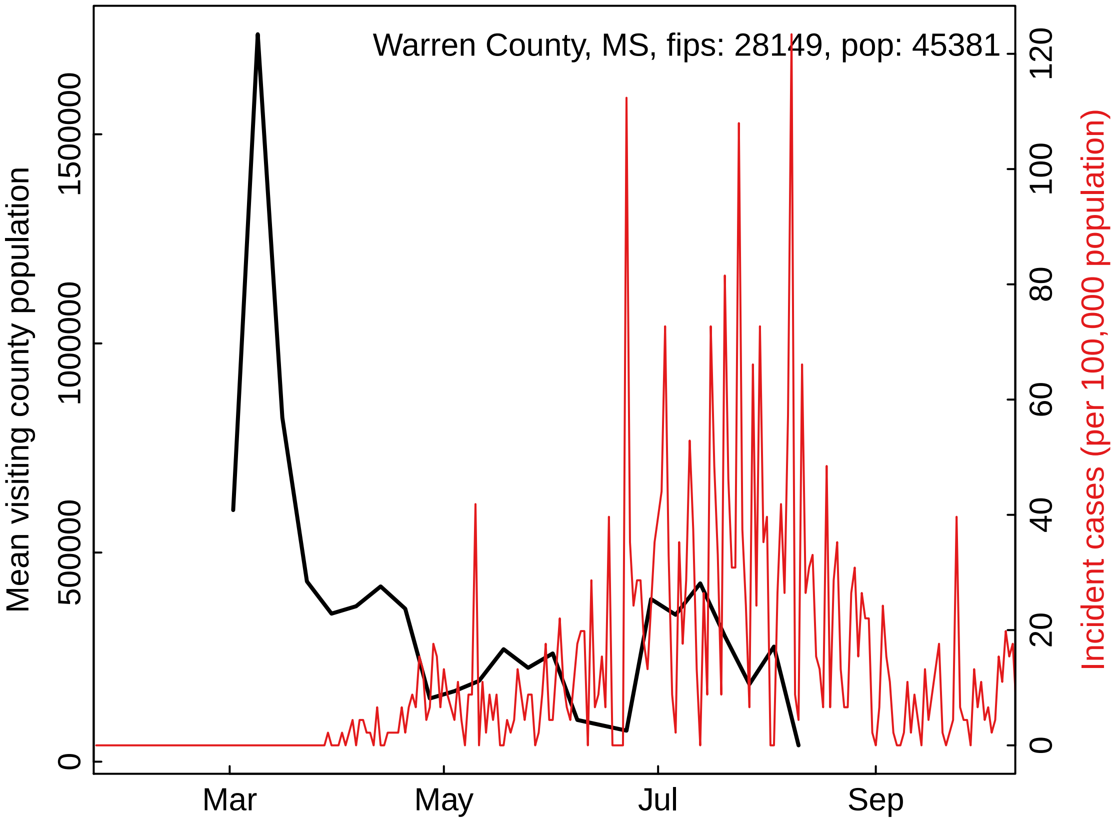

Warren County, MS, fips: 28149, pop: 45381
Mean visiting county population
0
5000000
10000000
15000000
Incident cases (per 100,000 population)
0
20
40
60
80
100
120
Mar
May
Jul
Sep

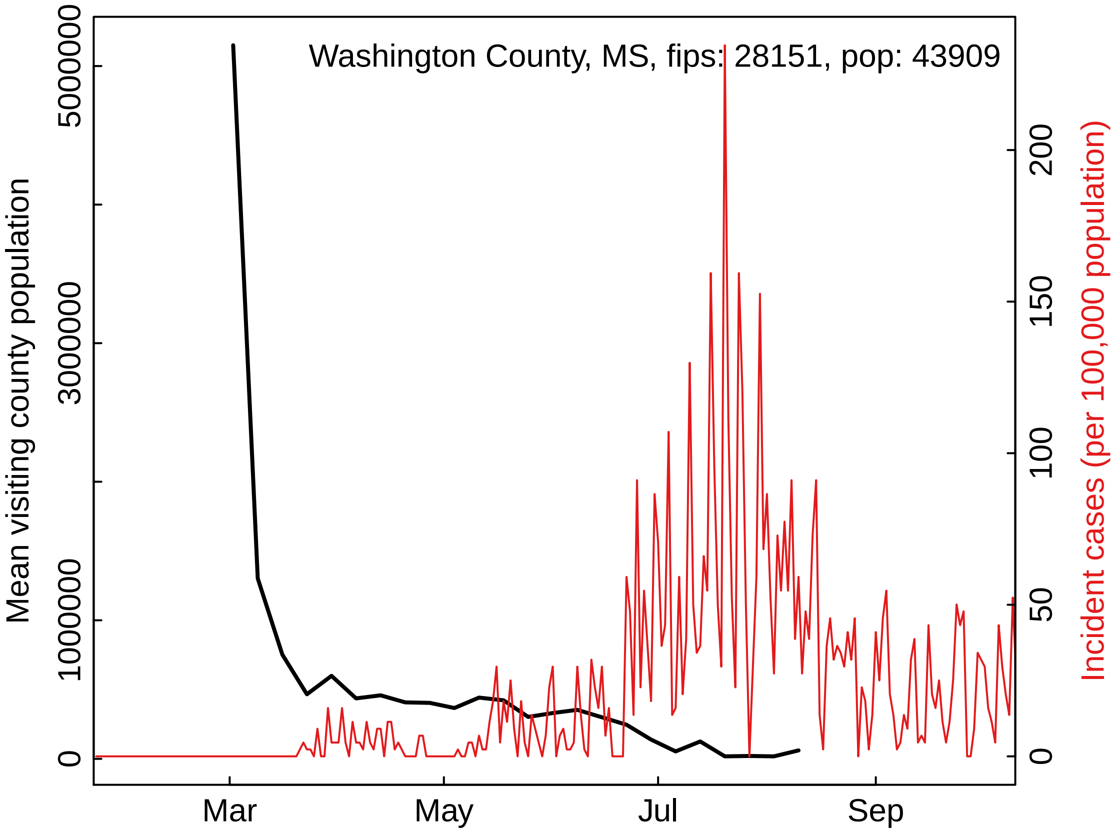

Washington County, MS, fips: 28151, pop: 43909
Mean visiting county population
Incident cases (per 100,000 population)
0
1000000
3000000
5000000
0
50
100
150
200
Mar
May
Jul
Sep

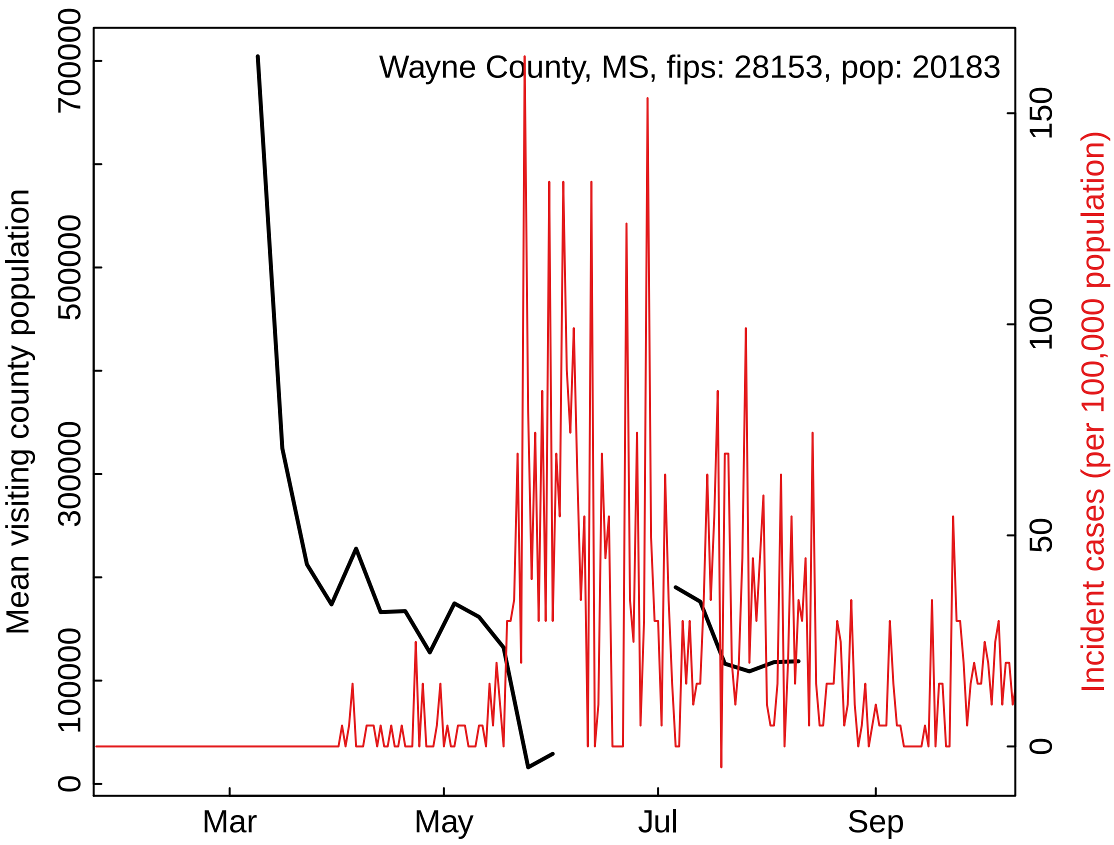

Wayne County, MS, fips: 28153, pop: 20183
Mean visiting county population
0
100000
300000
500000
700000
Incident cases (per 100,000 population)
0
50
100
150
Mar
May
Jul
Sep

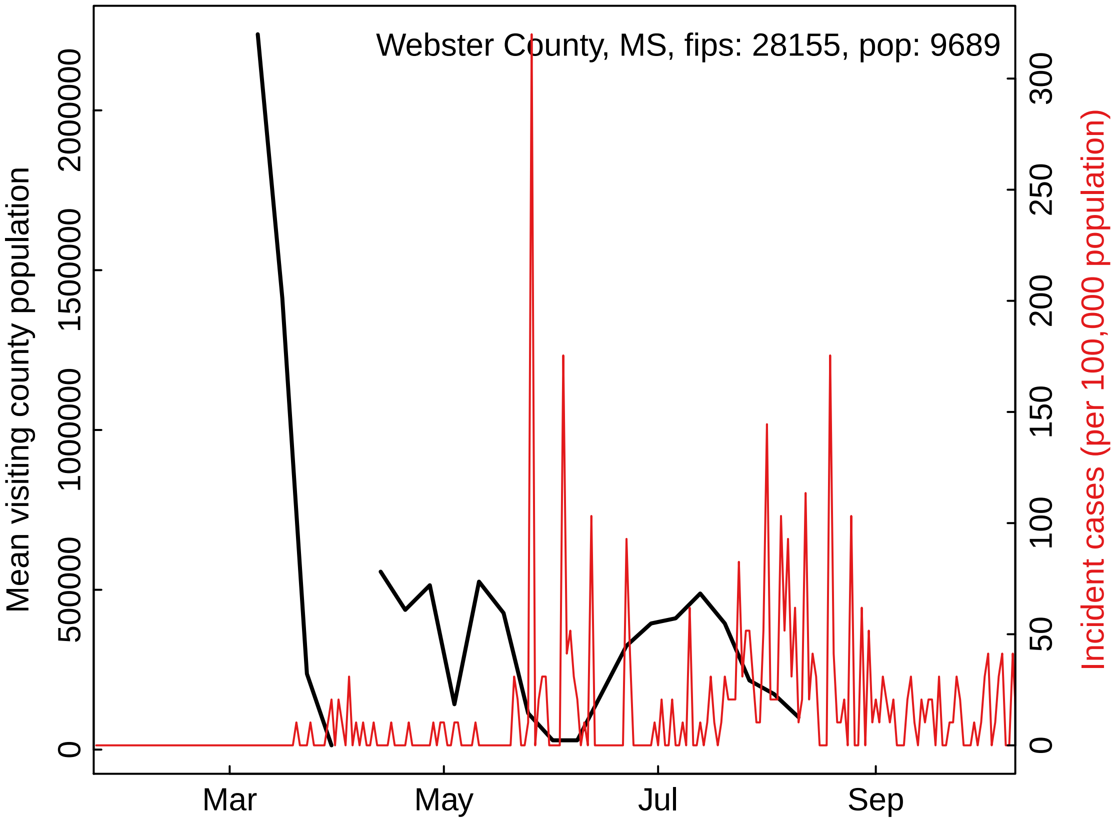
Webster County, MS, fips: 28155, pop: 9689
Mean visiting county population
0
500000
1000000
1500000
2000000
Incident cases (per 100,000 population)
0
50
100
150
200
250
300
Mar
May
Jul
Sep

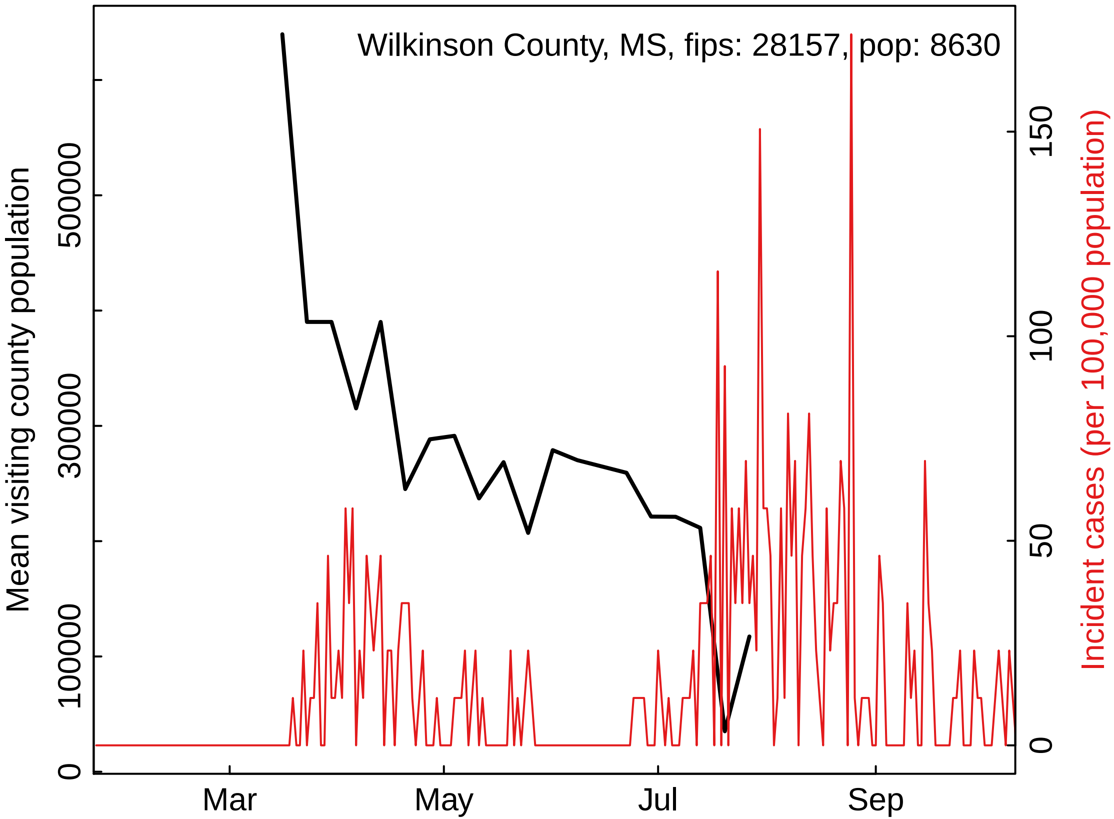

Wilkinson County, MS, fips: 28157, pop: 8630
Mean visiting county population
0
100000
300000
500000
Incident cases (per 100,000 population)
0
50
100
150
Mar
May
Jul
Sep

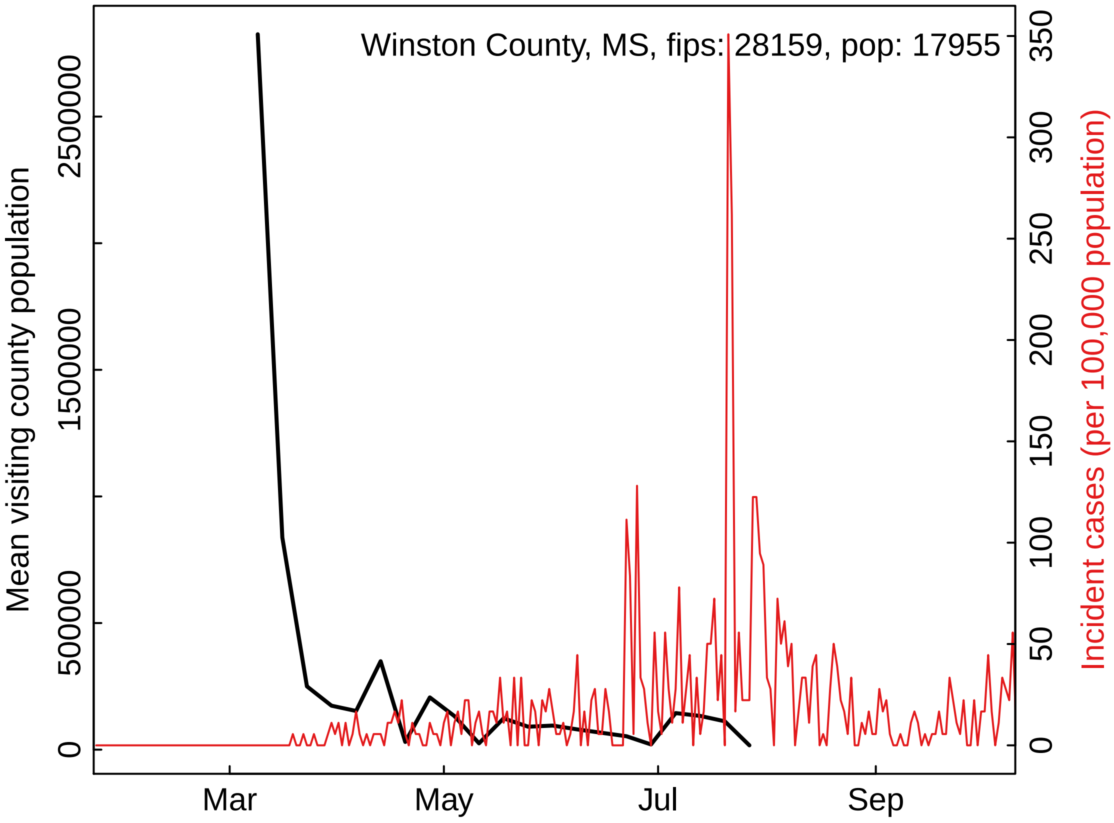

Winston County, MS, fips: 28159, pop: 17955
Mean visiting county population
0
500000
1500000
2500000
Incident cases (per 100,000 population)
0
50
100
150
200
250
300
350
Mar
May
Jul
Sep

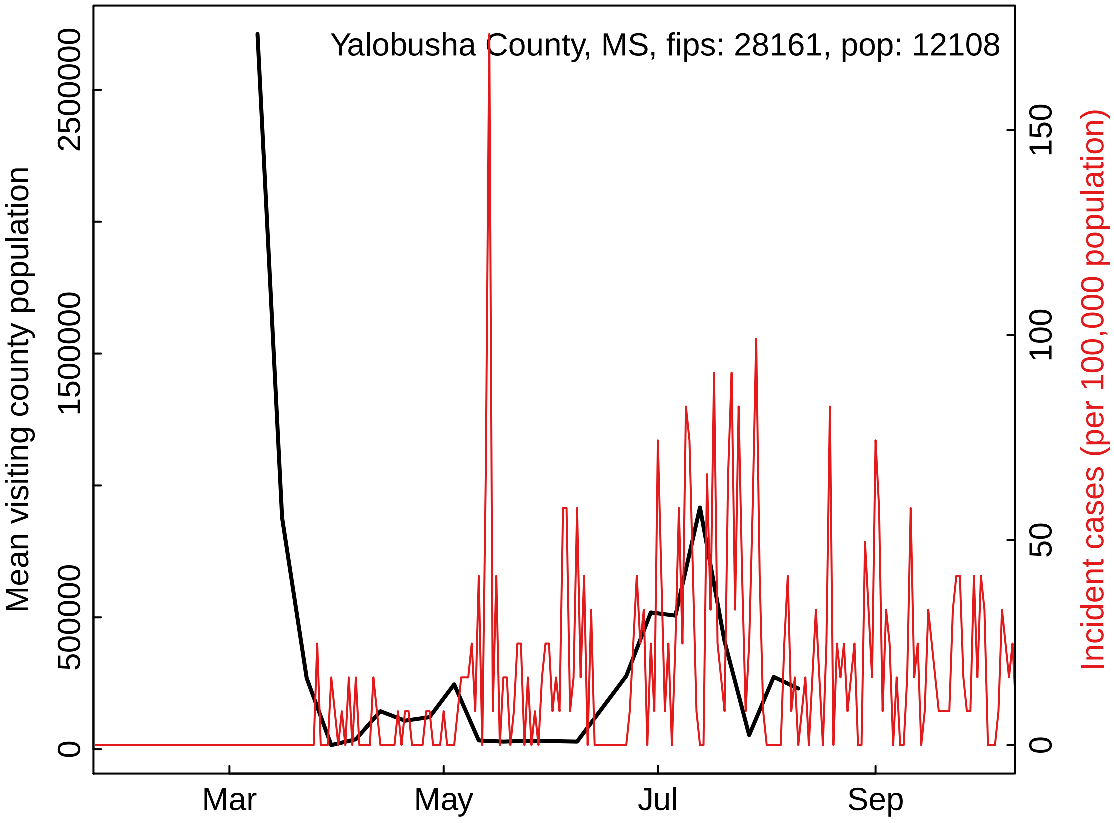

Yalobusha County, MS, fips: 28161, pop: 12108
Mean visiting county population
0
500000
1500000
2500000
Incident cases (per 100,000 population)
0
50
100
150
Mar
May
Jul
Sep

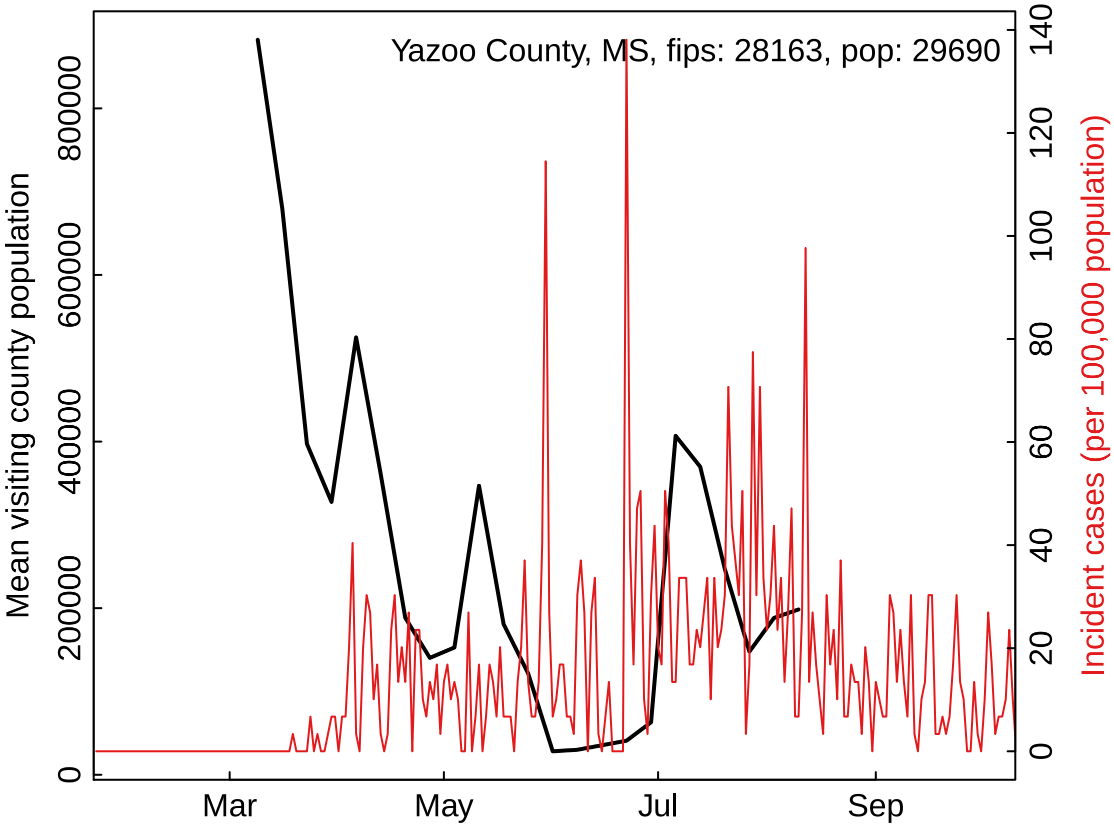

Yazoo County, MS, fips: 28163, pop: 29690
Mean visiting county population
Incident cases (per 100,000 population)
0
200000
400000
600000
800000
0
20
40
60
80
100
120
140
Mar
May
Jul
Sep

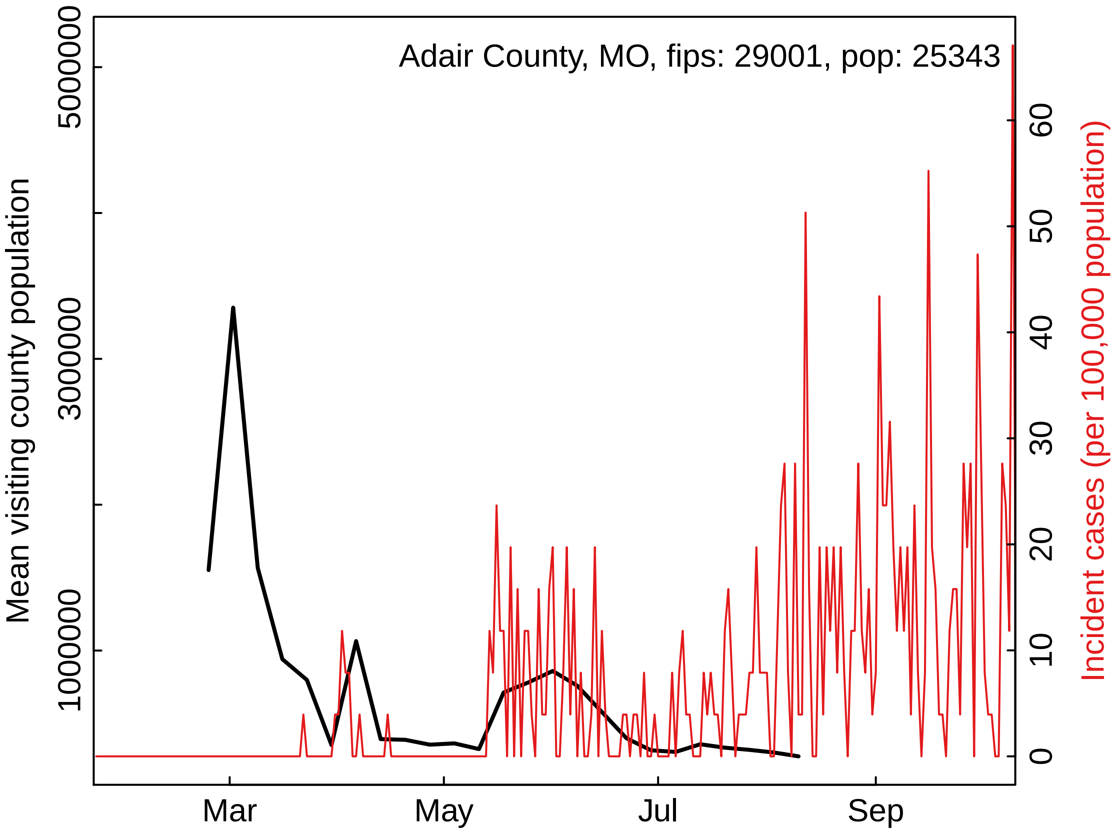

Adair County, MO, fips: 29001, pop: 25343
Mean visiting county population
1000000
3000000
5000000
Incident cases (per 100,000 population)
0
10
20
30
40
50
60
Mar
May
Jul
Sep

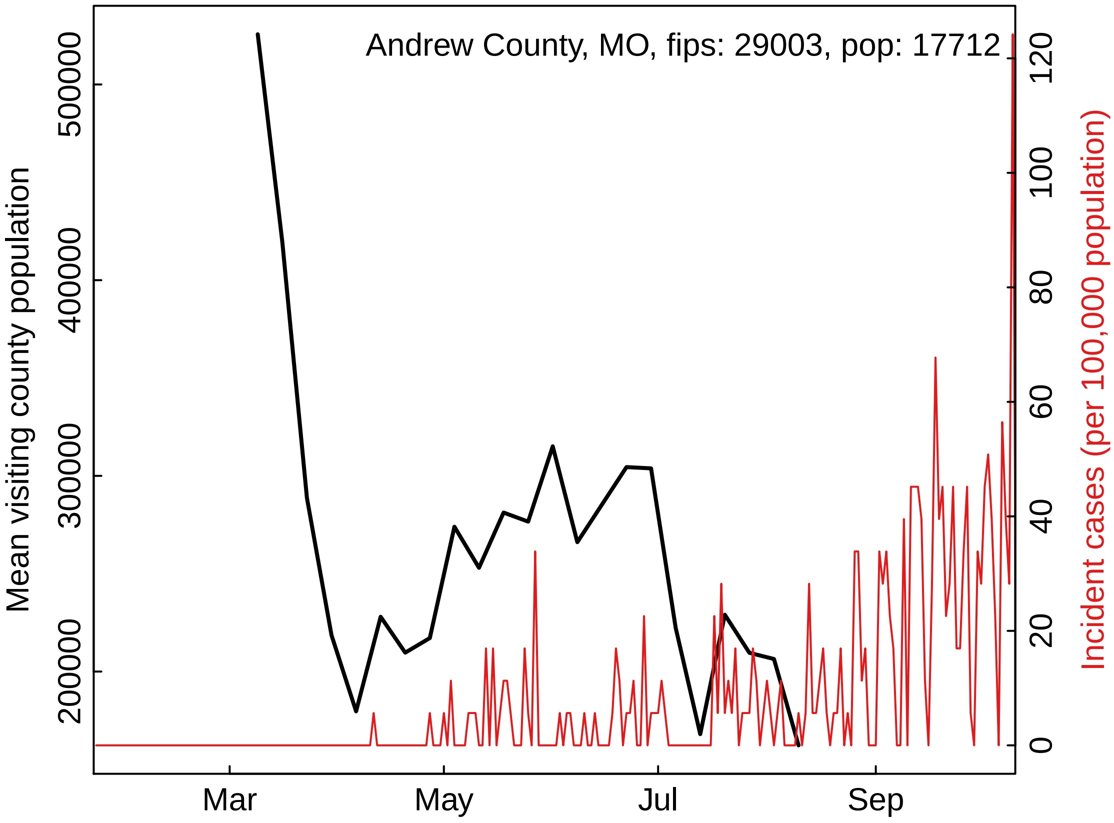

Andrew County, MO, fips: 29003, pop: 17712
Mean visiting county population
200000
300000
400000
500000
Incident cases (per 100,000 population)
0
20
40
60
80
100
120
Mar
May
Jul
Sep

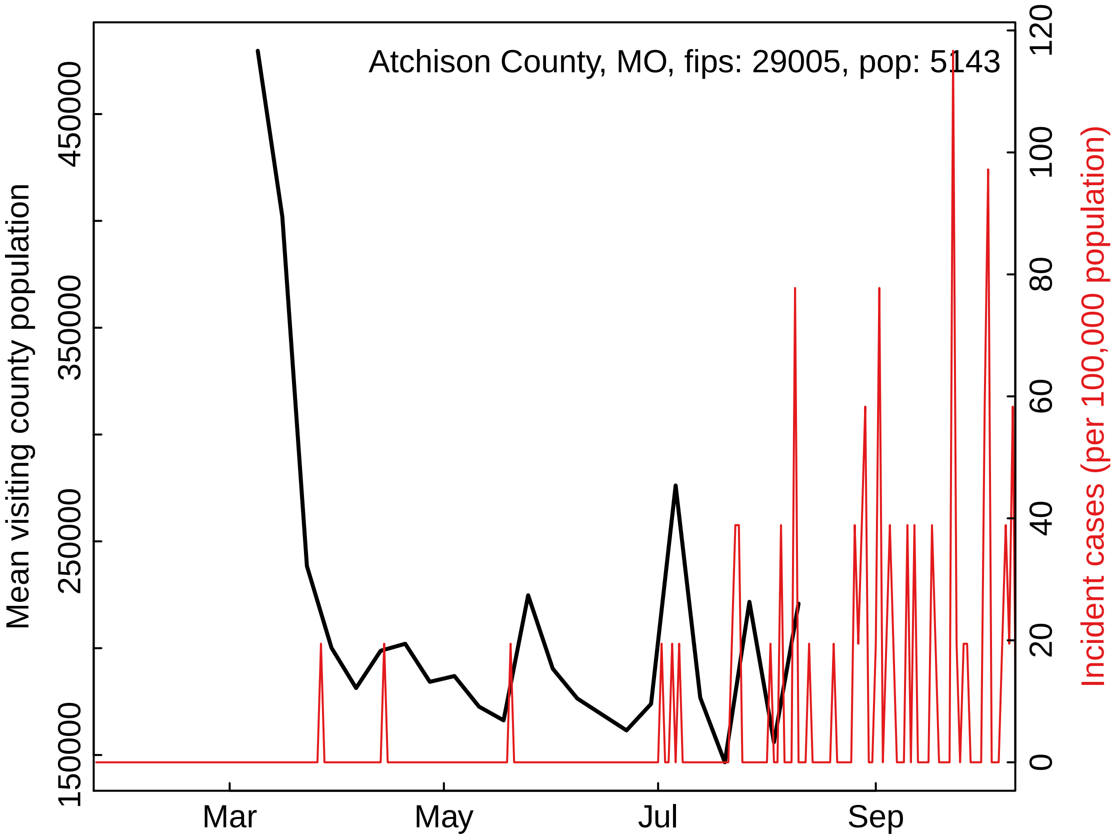

Atchison County, MO, fips: 29005, pop: 5143
Mean visiting county population
150000
250000
350000
450000
Incident cases (per 100,000 population)
0
20
40
60
80
100
120
Mar
May
Jul
Sep

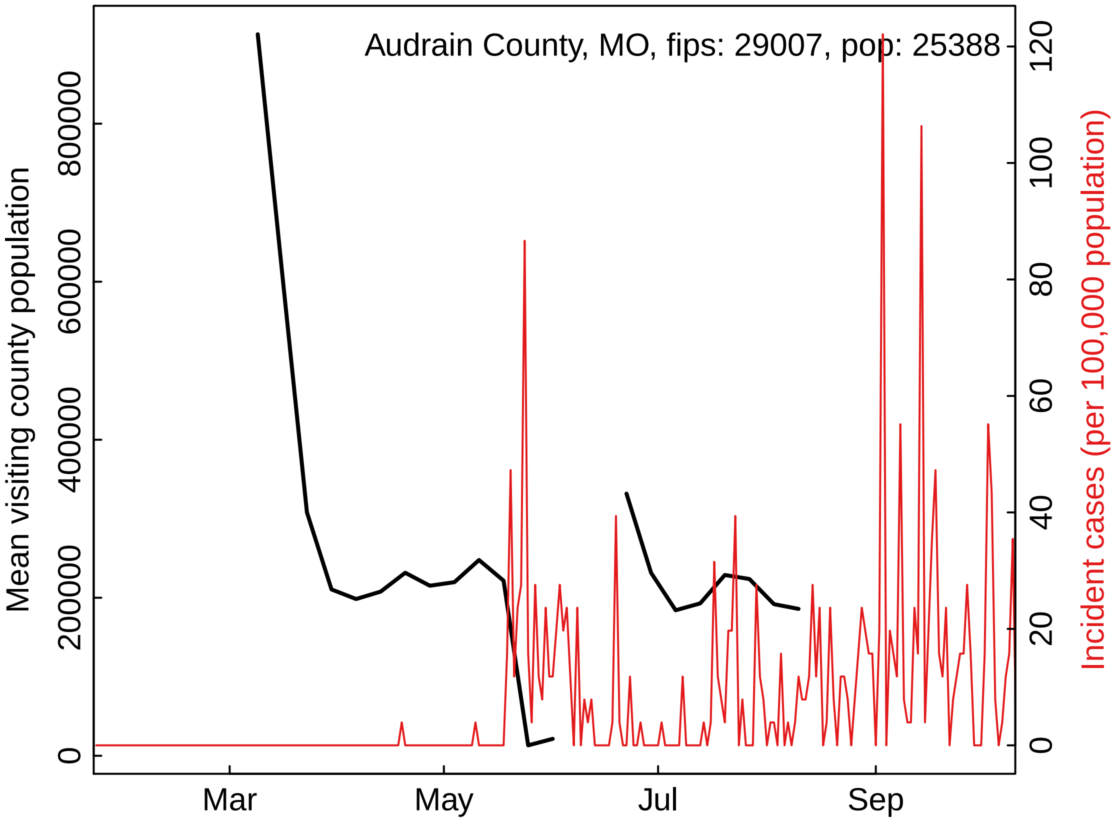

Audrain County, MO, fips: 29007, pop: 25388
Mean visiting county population
0
200000
400000
600000
800000
Mar
May
Jul
Sep
0
20
40
60
80
100
120
Incident cases (per 100,000 population)

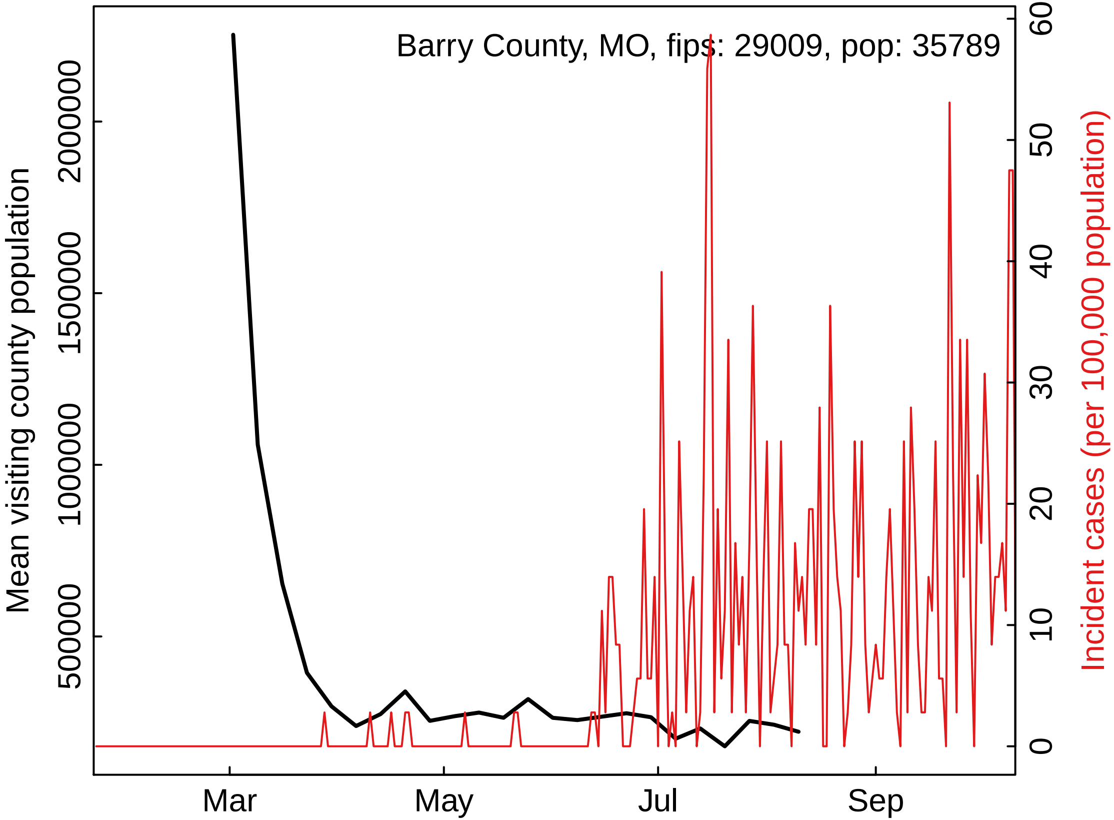

Barry County, MO, fips: 29009, pop: 35789
Mean visiting county population
500000
1000000
1500000
2000000
Incident cases (per 100,000 population)
0
10
20
30
40
50
60
Mar
May
Jul
Sep

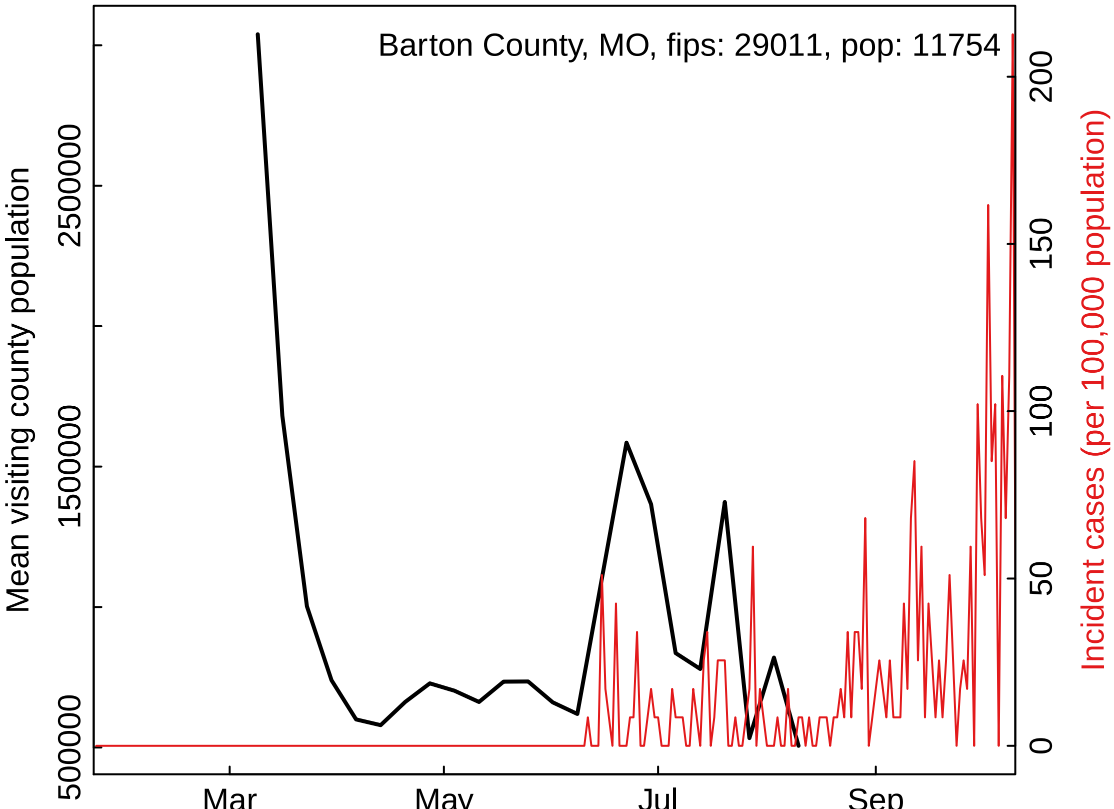

Barton County, MO, fips: 29011, pop: 11754
Mean visiting county population
500000
1500000
2500000
Incident cases (per 100,000 population)
0
50
100
150
200
Mar
May
Jul
Sep

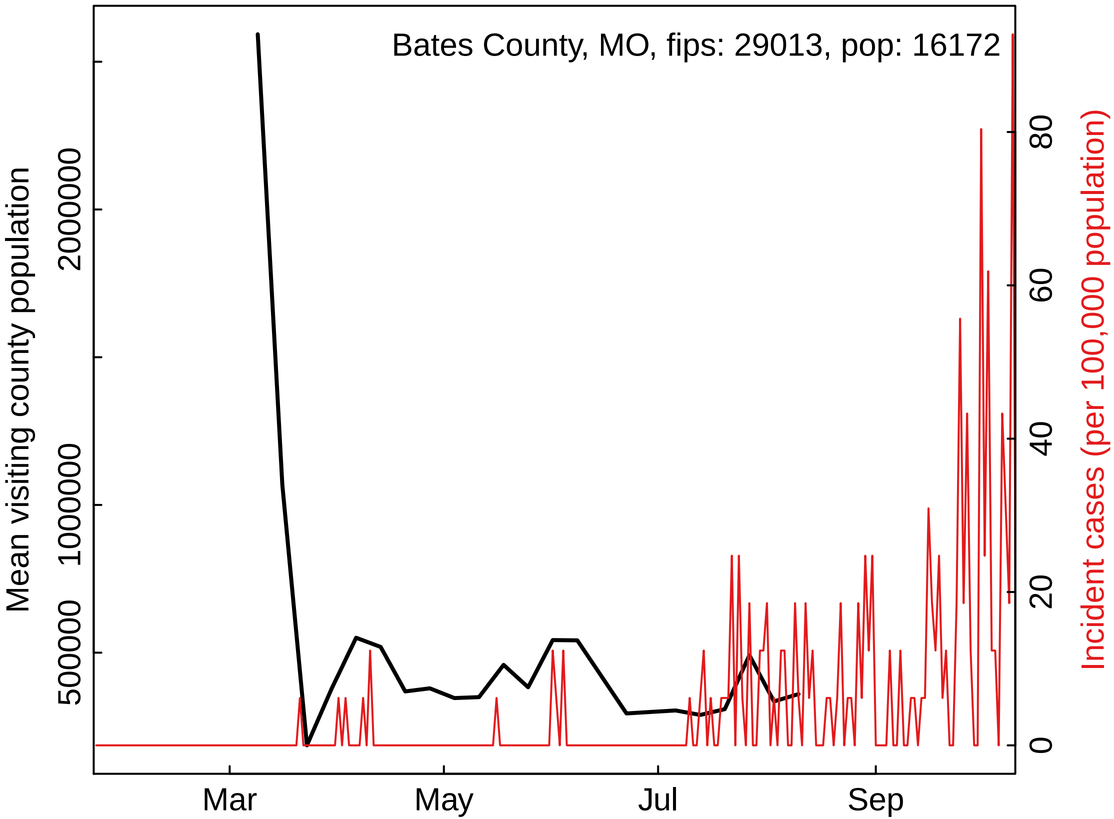

Bates County, MO, fips: 29013, pop: 16172
Mean visiting county population
500000
1000000
2000000
Incident cases (per 100,000 population)
0
20
40
60
80
Mar
May
Jul
Sep

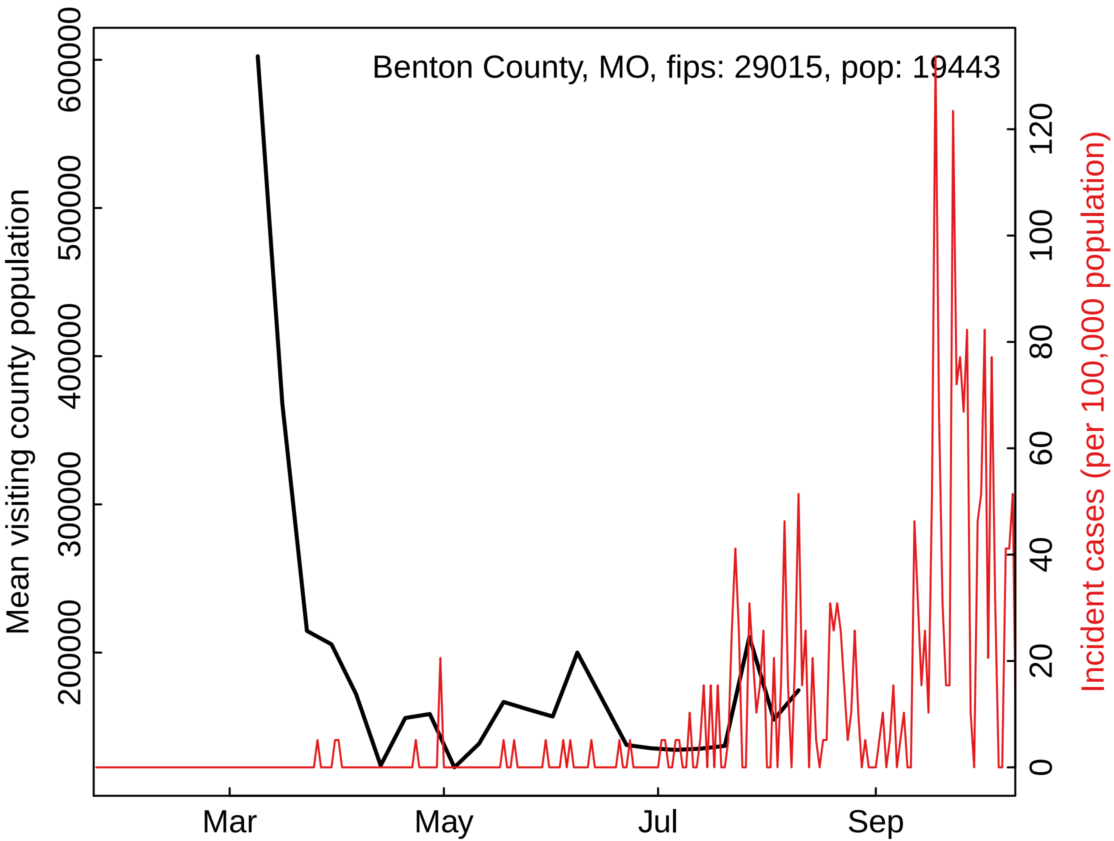

Benton County, MO, fips: 29015, pop: 19443
Mean visiting county population
200000
300000
400000
500000
600000
Incident cases (per 100,000 population)
0
20
40
60
80
100
120
Mar
May
Jul
Sep

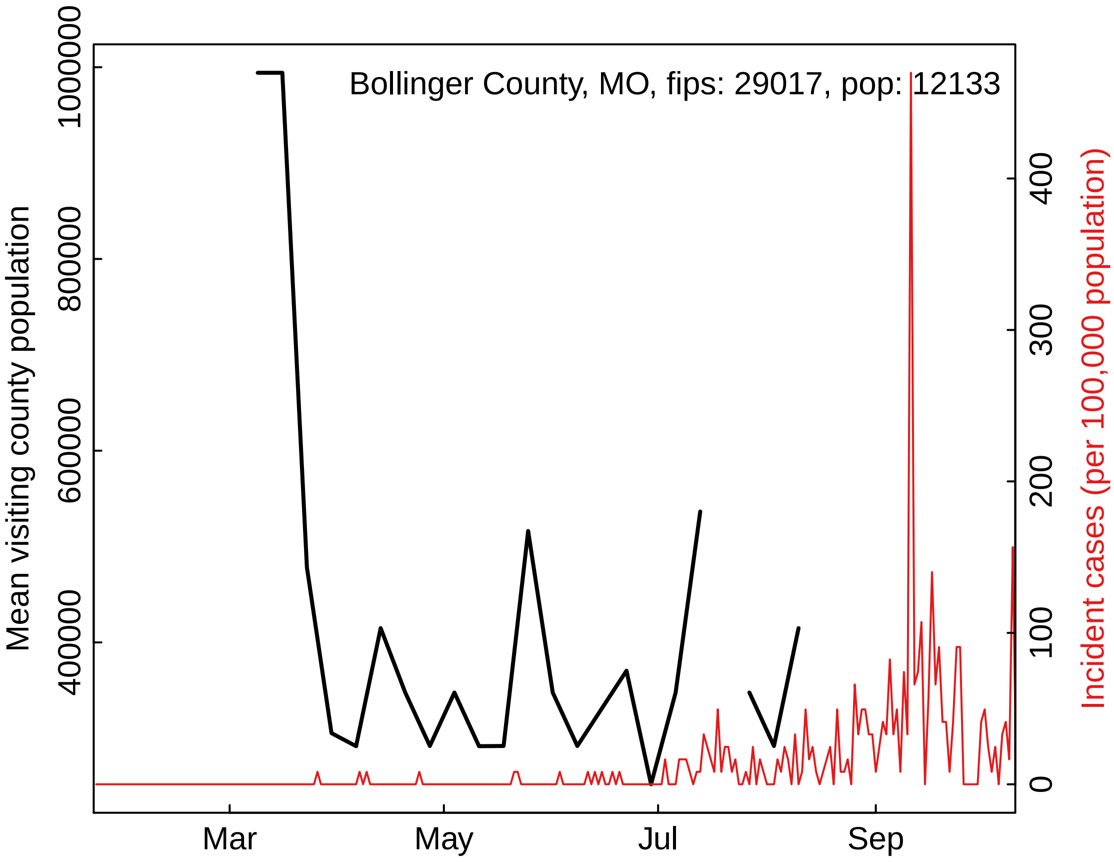

Bollinger County, MO, fips: 29017, pop: 12133
Mean visiting county population
400000
600000
800000
1000000
Incident cases (per 100,000 population)
0
100
200
300
400
Mar
May
Jul
Sep

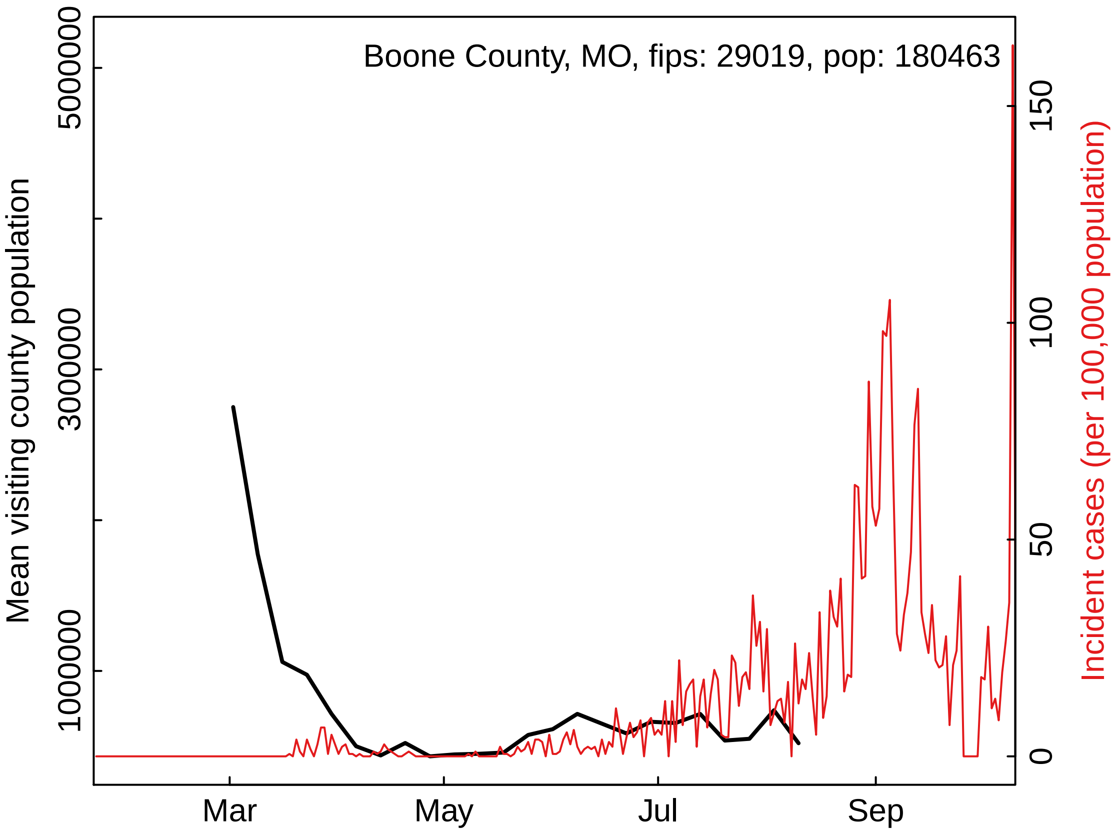

Boone County, MO, fips: 29019, pop: 180463
Mean visiting county population
1000000
3000000
5000000
Incident cases (per 100,000 population)
0
50
100
150
Mar
May
Jul
Sep

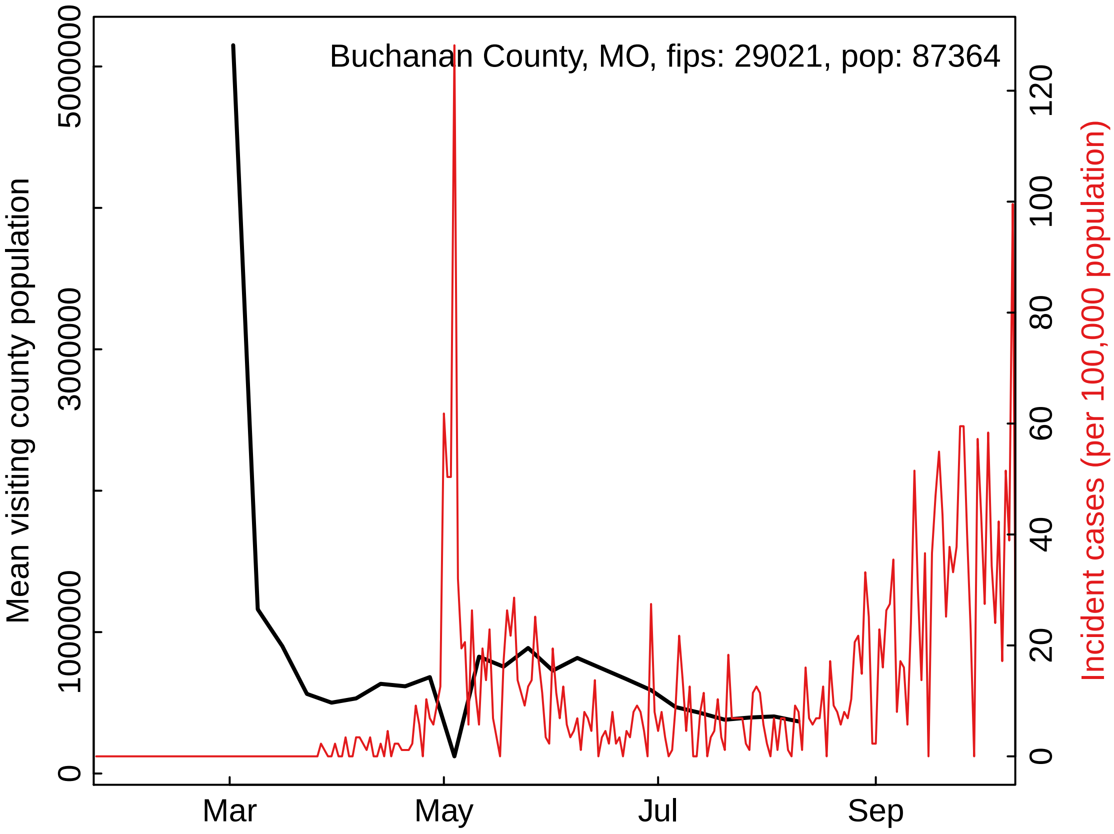

Buchanan County, MO, fips: 29021, pop: 87364
Mean visiting county population
0
1000000
3000000
5000000
Incident cases (per 100,000 population)
0
20
40
60
80
100
120
Mar
May
Jul
Sep

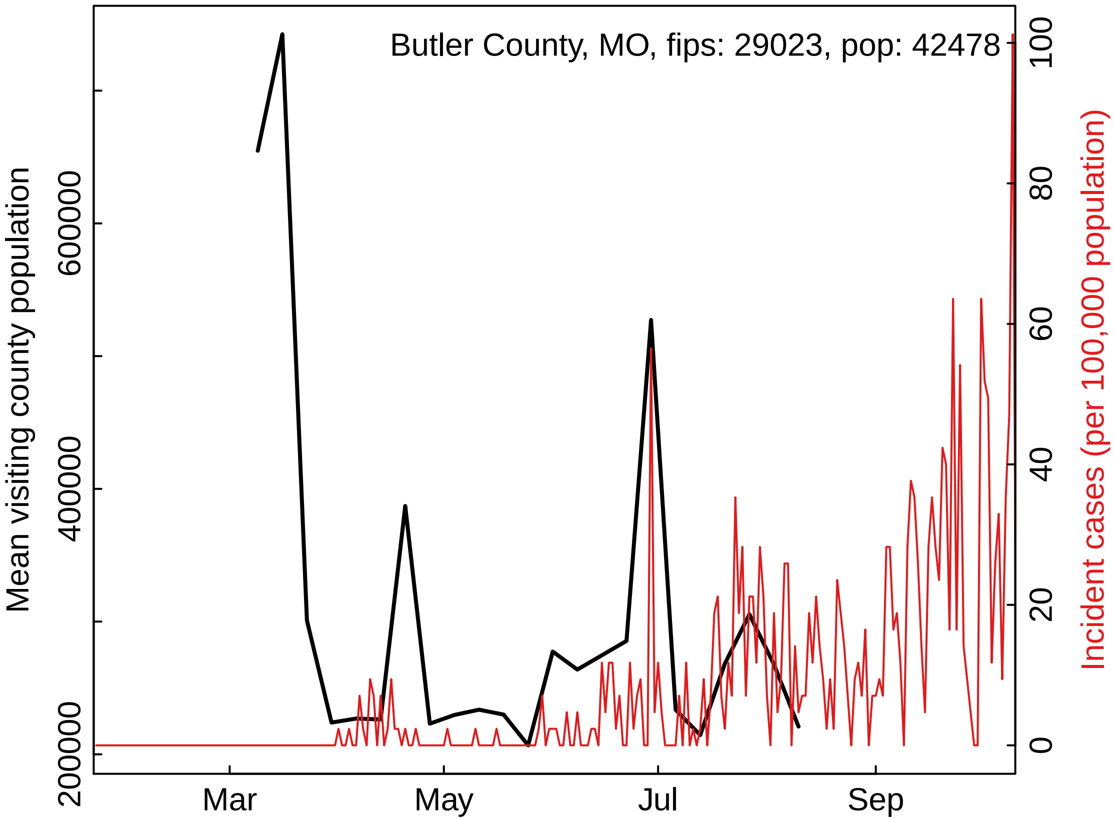

Butler County, MO, fips: 29023, pop: 42478
Mean visiting county population
200000
400000
600000
Mar
May
Jul
Sep
0
20
40
60
80
100
Incident cases (per 100,000 population)

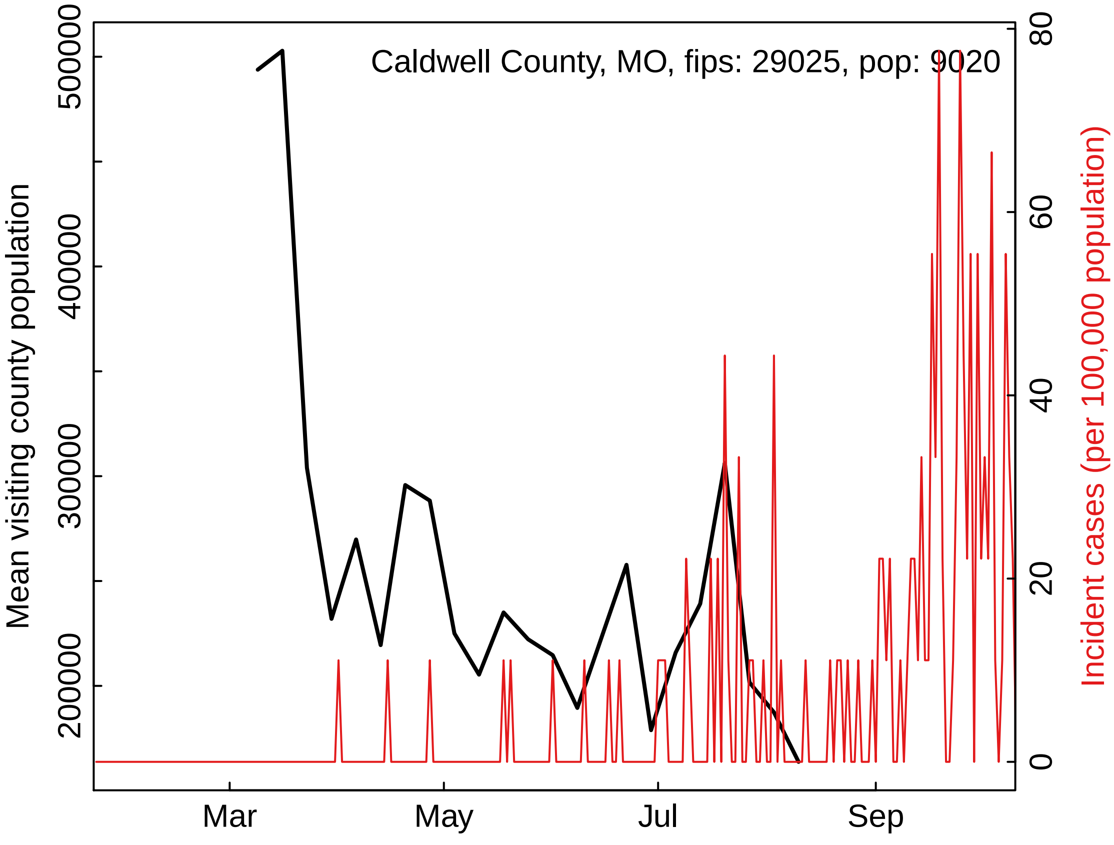

Caldwell County, MO, fips: 29025, pop: 9020
Mean visiting county population
200000
300000
400000
500000
Incident cases (per 100,000 population)
0
20
40
60
80
Mar
May
Jul
Sep

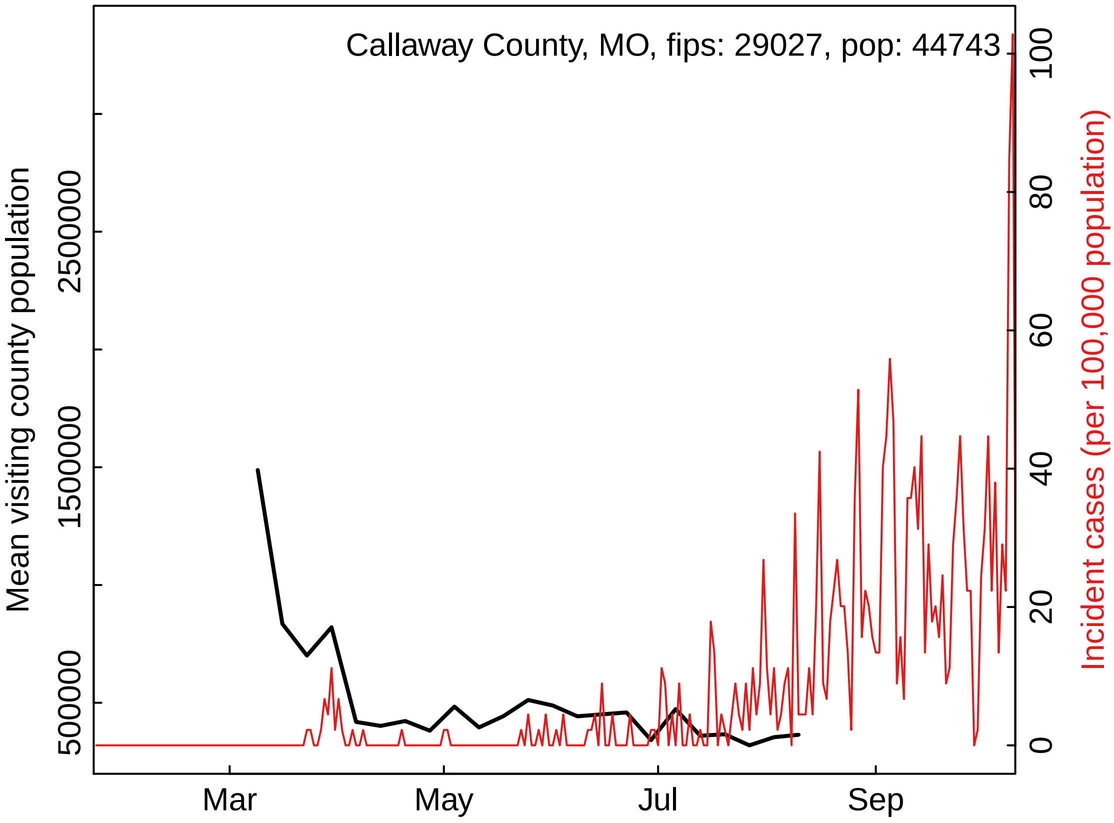

Callaway County, MO, fips: 29027, pop: 44743
Mean visiting county population
500000
1500000
2500000
Mar
May
Jul
Sep
0
20
40
60
80
100
Incident cases (per 100,000 population)

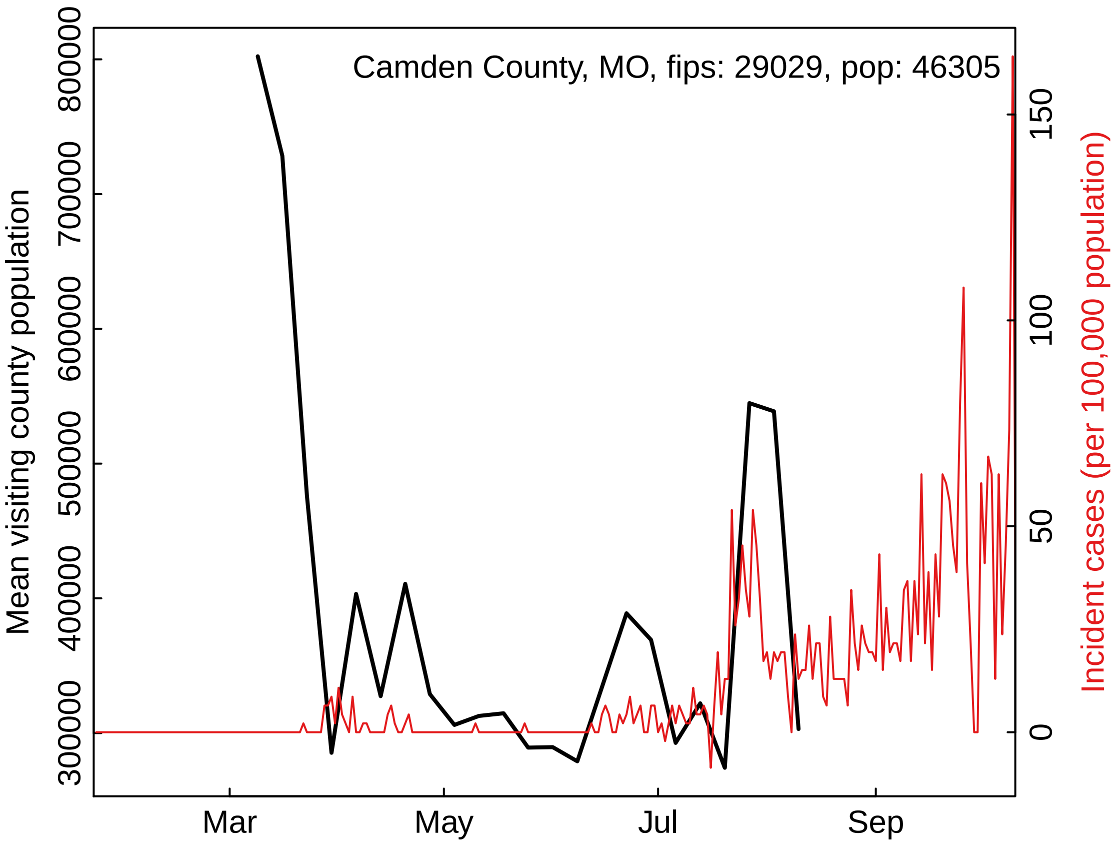

Camden County, MO, fips: 29029, pop: 46305
Mean visiting county population
300000
400000
500000
600000
700000
800000
Incident cases (per 100,000 population)
0
50
100
150
Mar
May
Jul
Sep

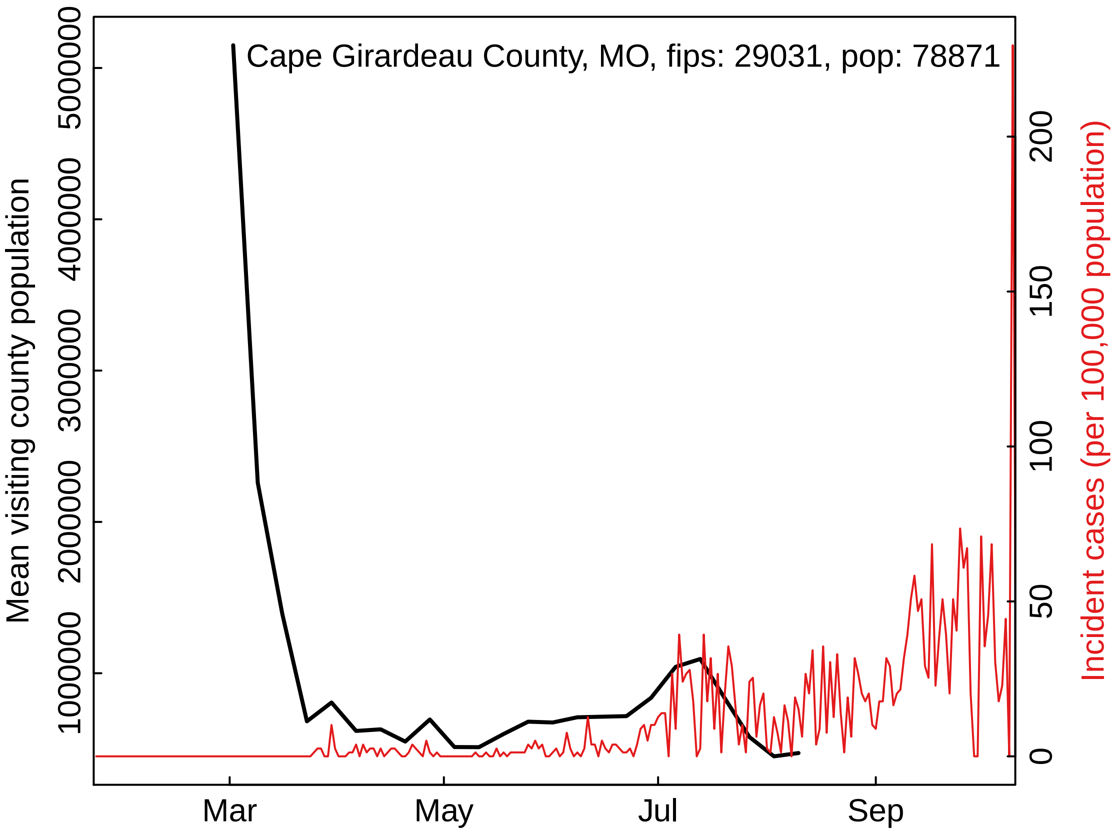

Cape Girardeau County, MO, fips: 29031, pop: 78871
Mean visiting county population
1000000
2000000
3000000
4000000
5000000
Incident cases (per 100,000 population)
0
50
100
150
200
Mar
May
Jul
Sep

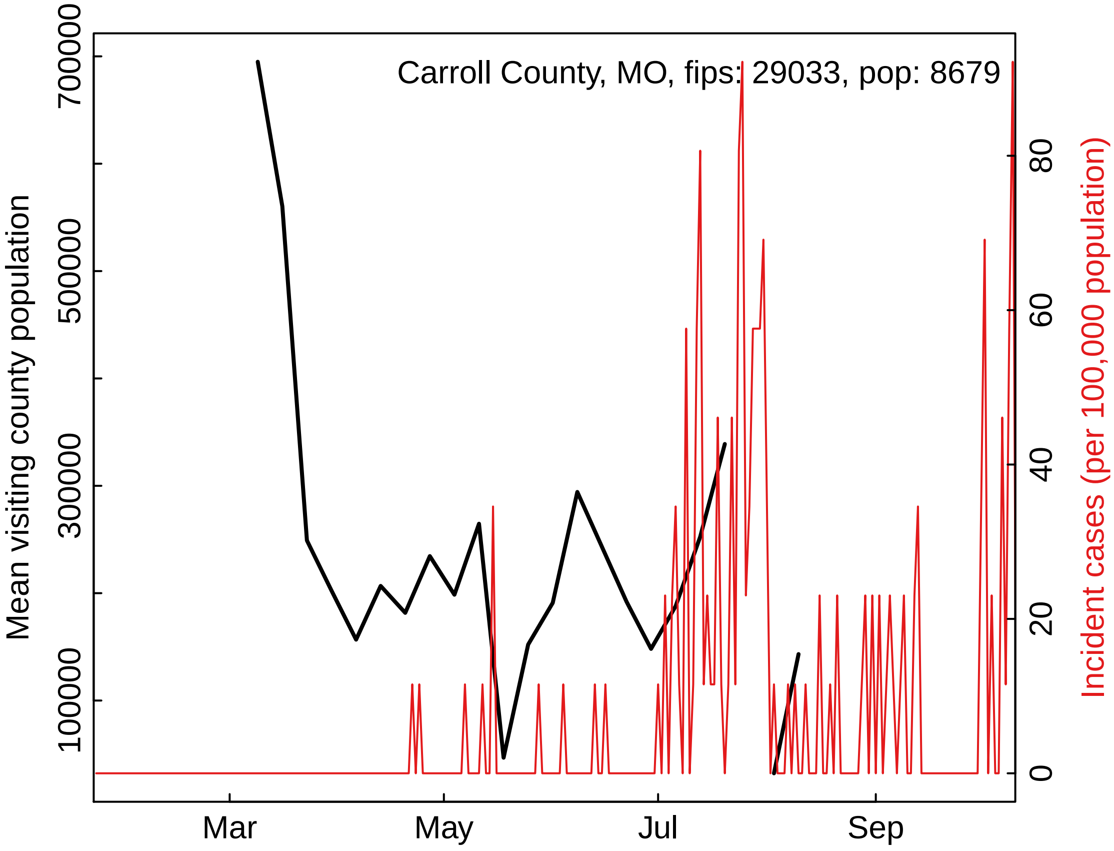
Carroll County, MO, fips: 29033, pop: 8679
Mean visiting county population
100000
300000
500000
700000
Incident cases (per 100,000 population)
0
20
40
60
80
Mar
May
Jul
Sep

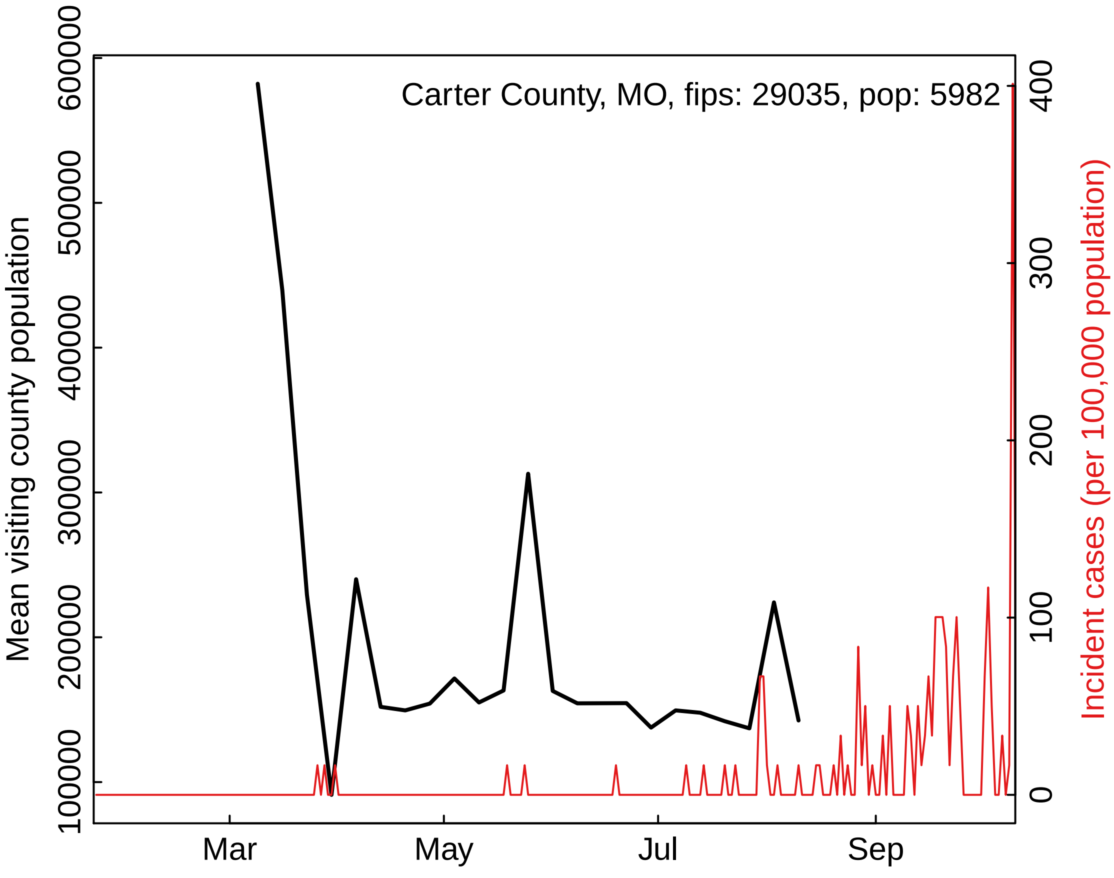

Carter County, MO, fips: 29035, pop: 5982
Mean visiting county population
100000
200000
300000
400000
500000
600000
Incident cases (per 100,000 population)
0
100
200
300
400
Mar
May
Jul
Sep

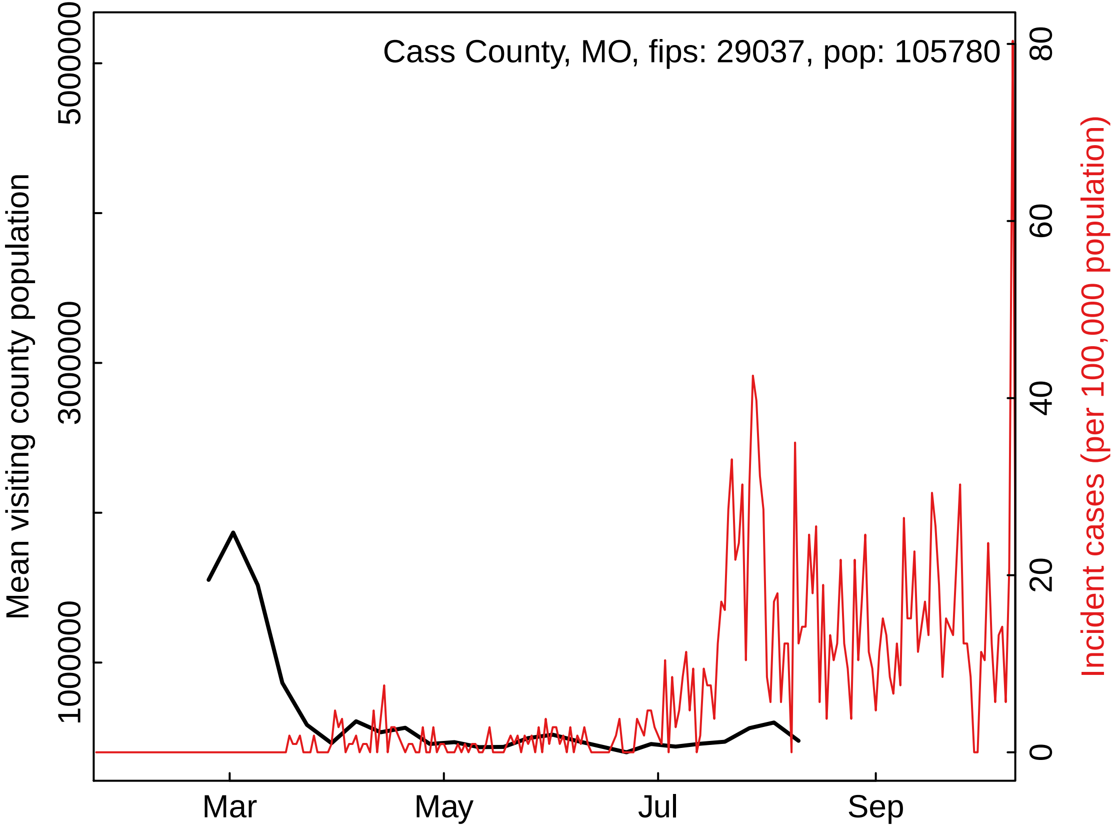

Cass County, MO, fips: 29037, pop: 105780
Mean visiting county population
1000000
3000000
5000000
Incident cases (per 100,000 population)
0
20
40
60
80
Mar
May
Jul
Sep

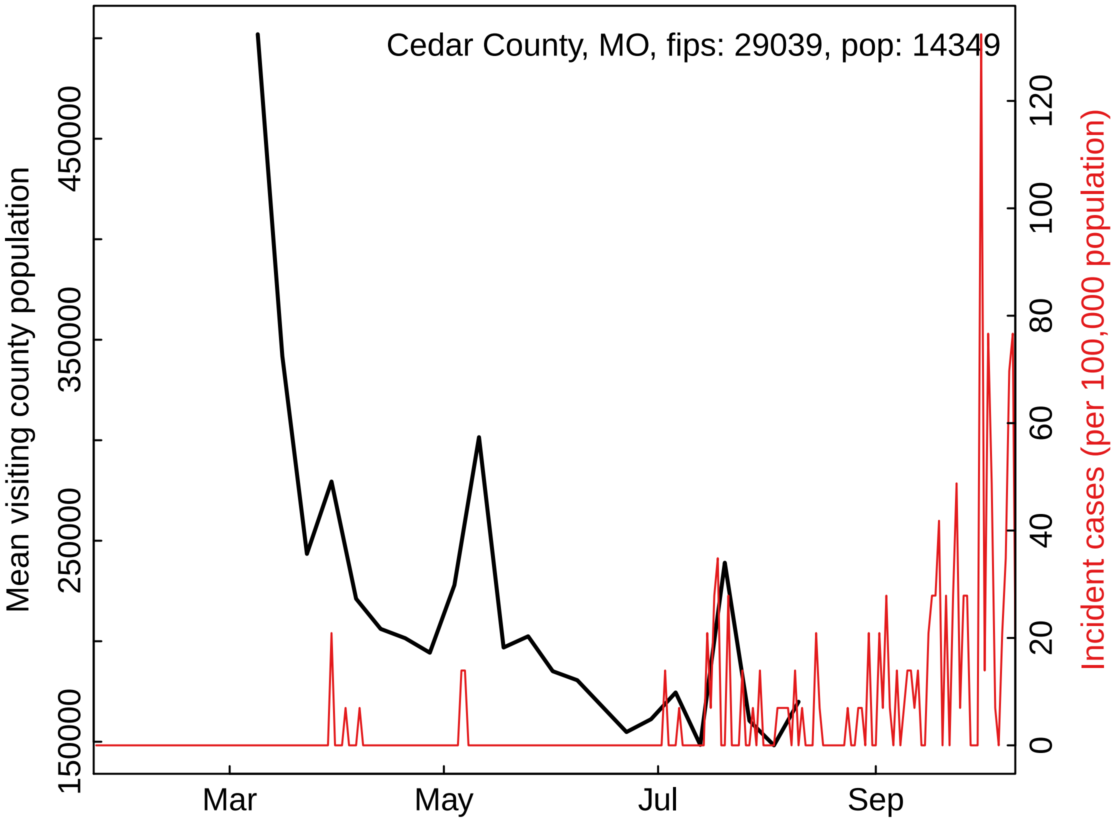
Cedar County, MO, fips: 29039, pop: 14349
Mean visiting county population
150000
250000
350000
450000
Incident cases (per 100,000 population)
0
20
40
60
80
100
120
Mar
May
Jul
Sep

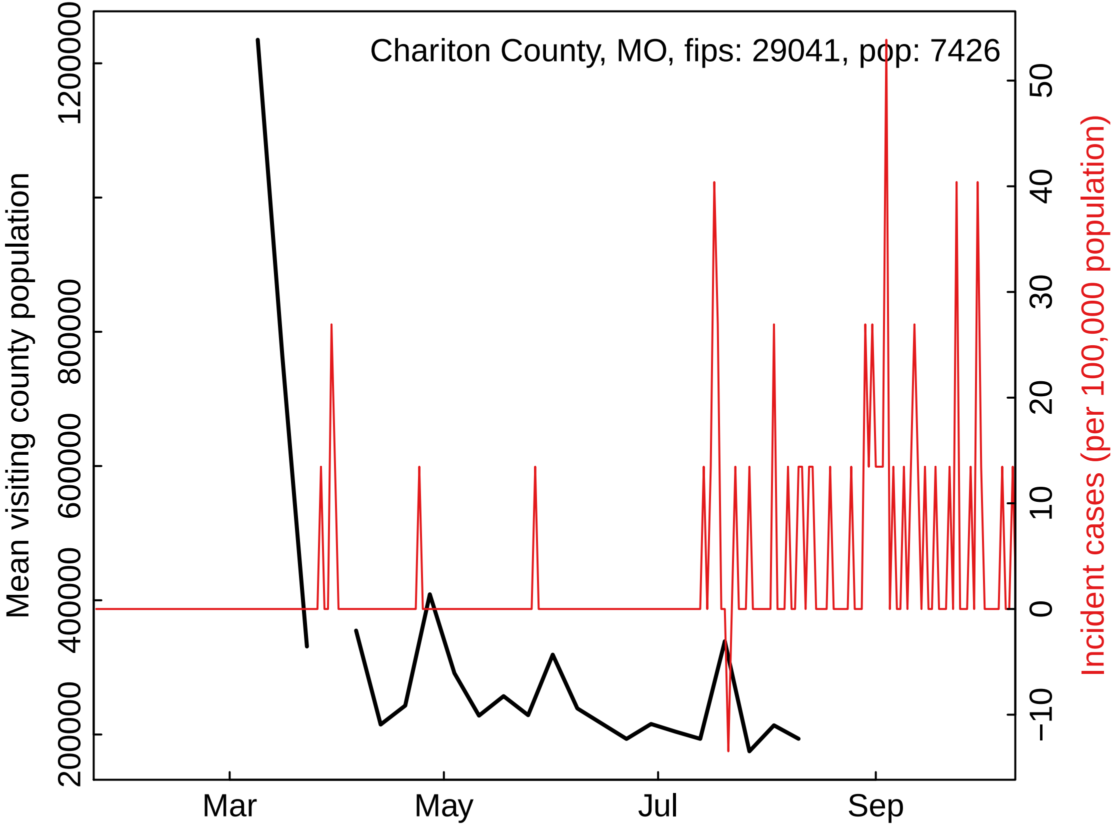

Chariton County, MO, fips: 29041, pop: 7426
Mean visiting county population
200000
400000
600000
800000
1200000
Incident cases (per 100,000 population)
−10
0
10
20
30
40
50
Mar
May
Jul
Sep

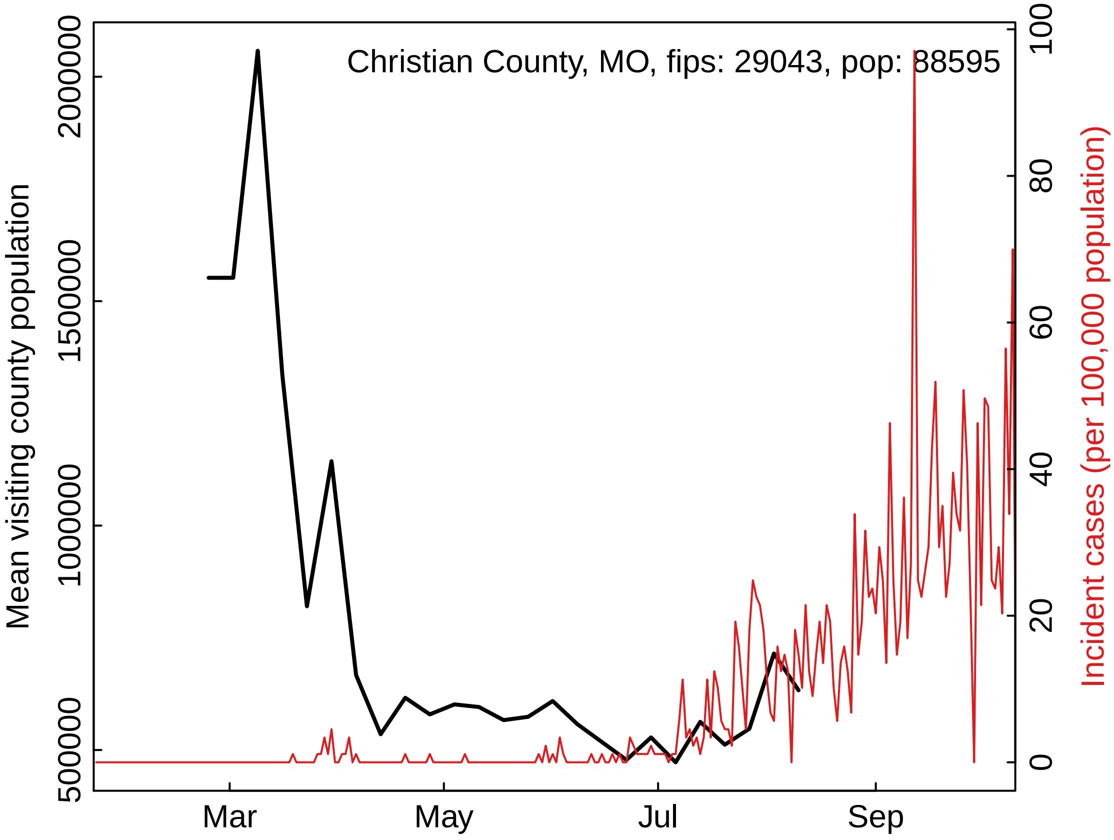

Christian County, MO, fips: 29043, pop: 88595
Mean visiting county population
500000
1000000
1500000
2000000
Incident cases (per 100,000 population)
0
20
40
60
80
100
Mar
May
Jul
Sep

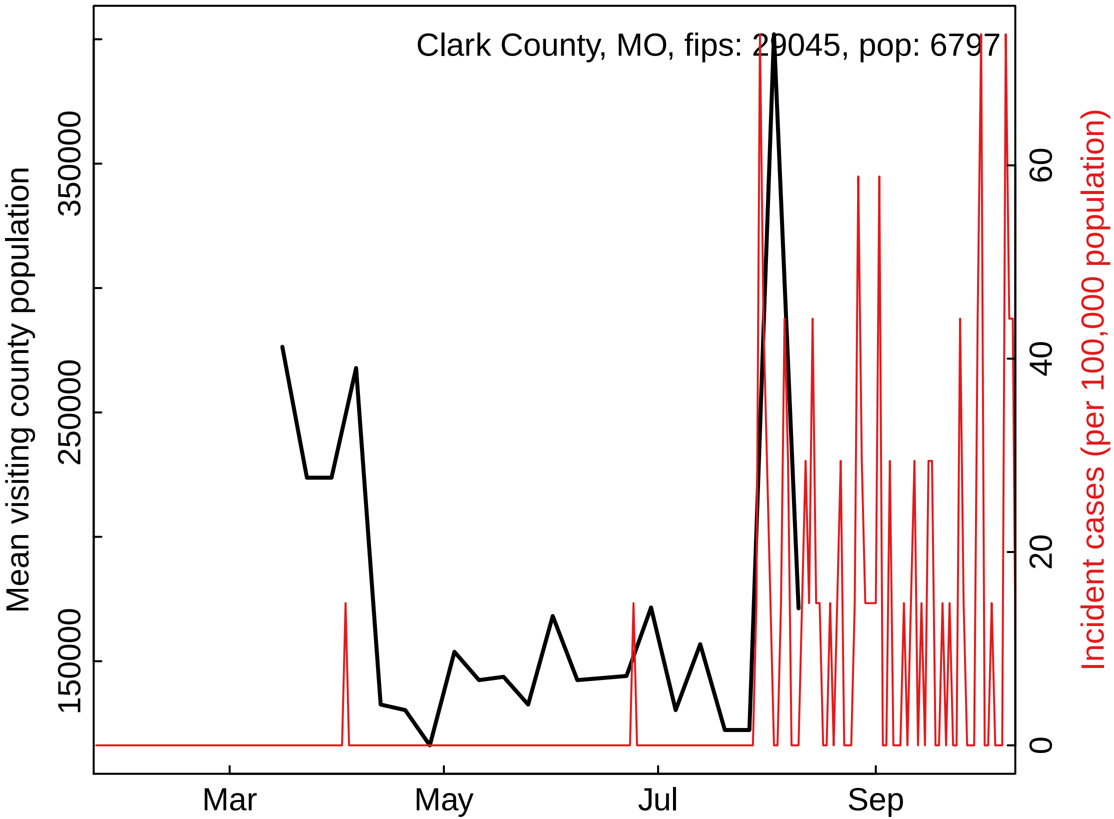

Clark County, MO, fips: 29045, pop: 6797
Mean visiting county population
150000
250000
350000
Incident cases (per 100,000 population)
0
20
40
60
Mar
May
Jul
Sep

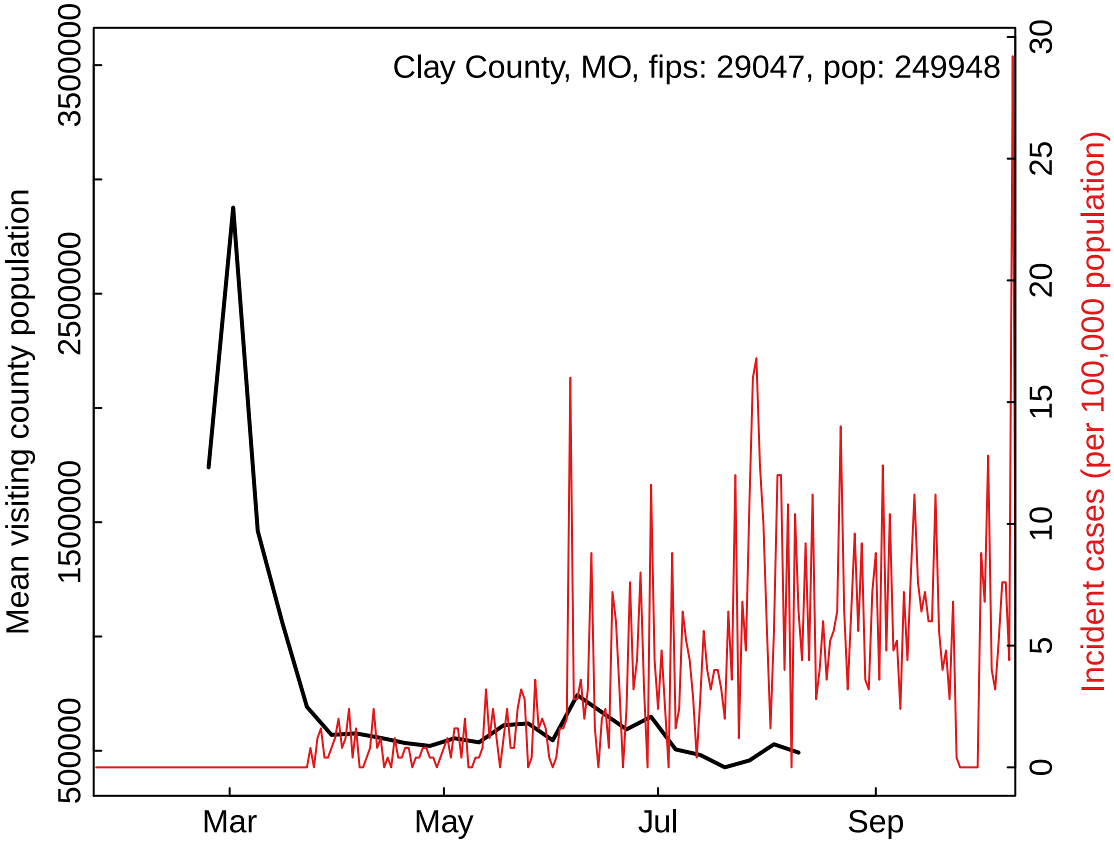
Clay County, MO, fips: 29047, pop: 249948
Mean visiting county population
500000
1500000
2500000
3500000
Incident cases (per 100,000 population)
0
5
10
15
20
25
30
Mar
May
Jul
Sep

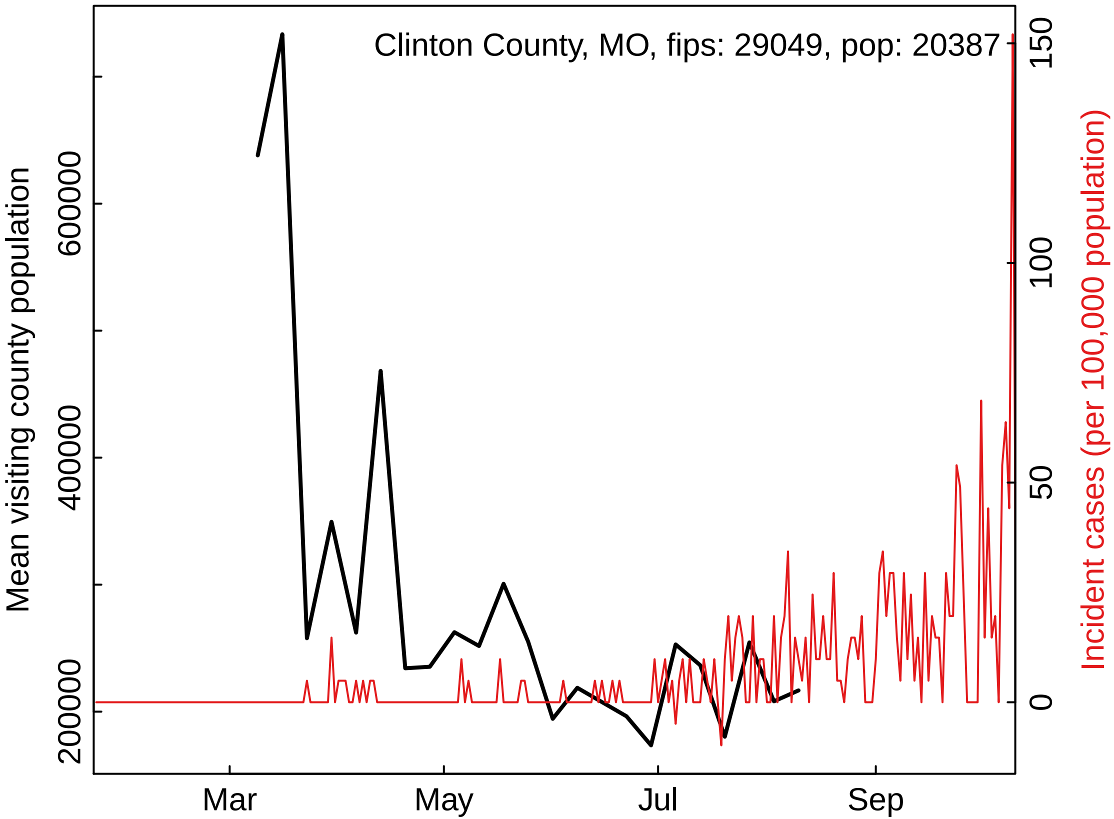

Clinton County, MO, fips: 29049, pop: 20387
Mean visiting county population
200000
400000
600000
Mar
May
Jul
Sep
Incident cases (per 100,000 population)
0
50
100
150

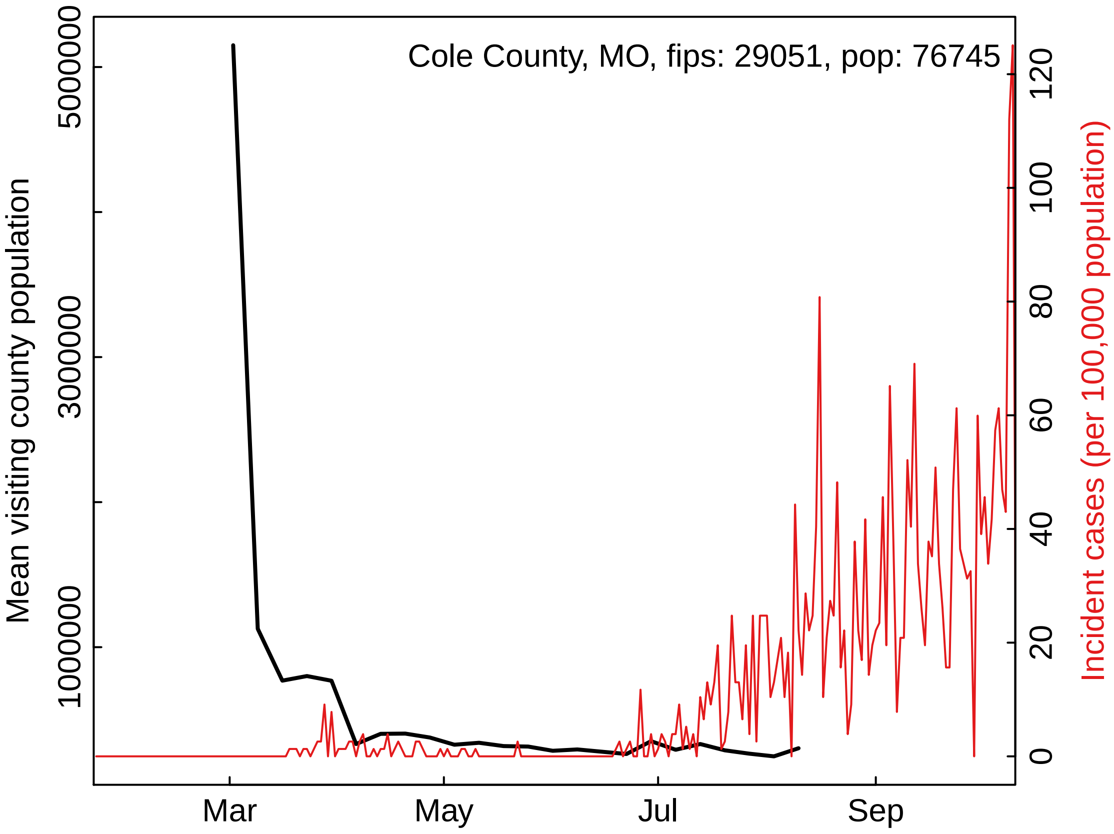

Cole County, MO, fips: 29051, pop: 76745
Mean visiting county population
10000000
30000000
50000000
0
20
40
60
80
100
120
Incident cases (per 100,000 population)
Mar
May
Jul
Sep

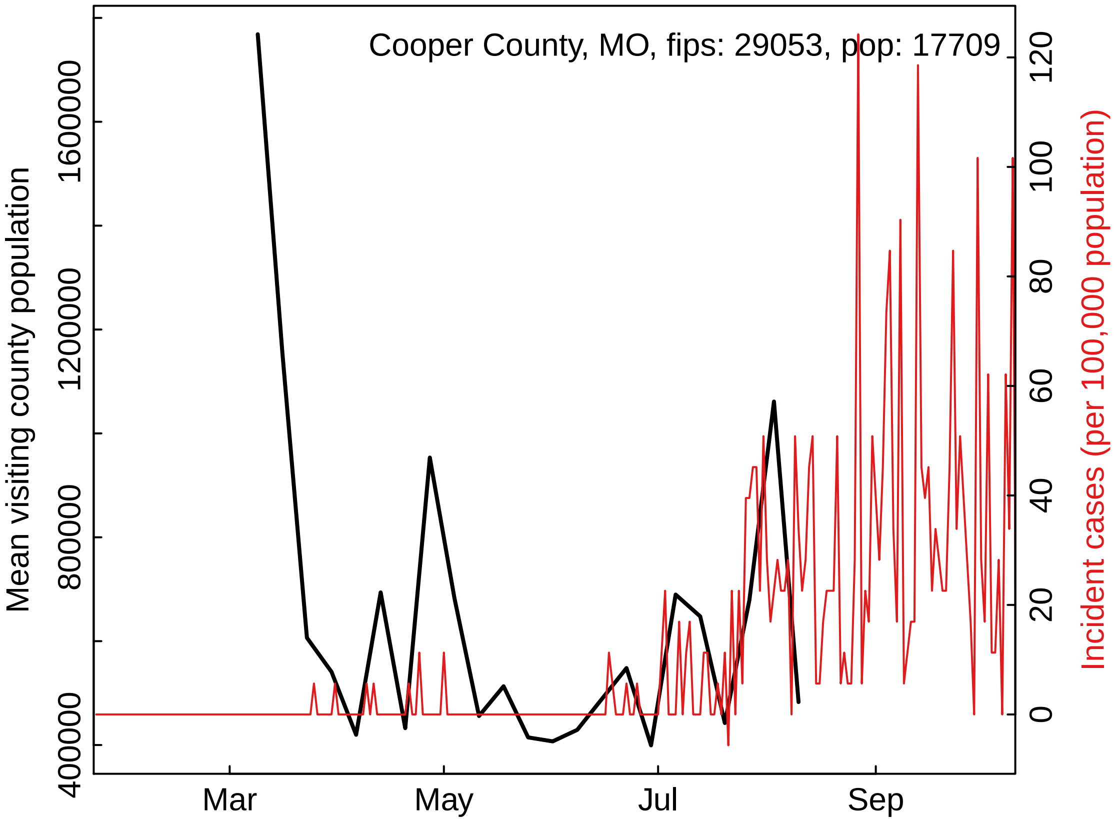
Cooper County, MO, fips: 29053, pop: 17709
Mean visiting county population
4000000
8000000
12000000
16000000
Incident cases (per 100,000 population)
0
20
40
60
80
100
120
Mar
May
Jul
Sep

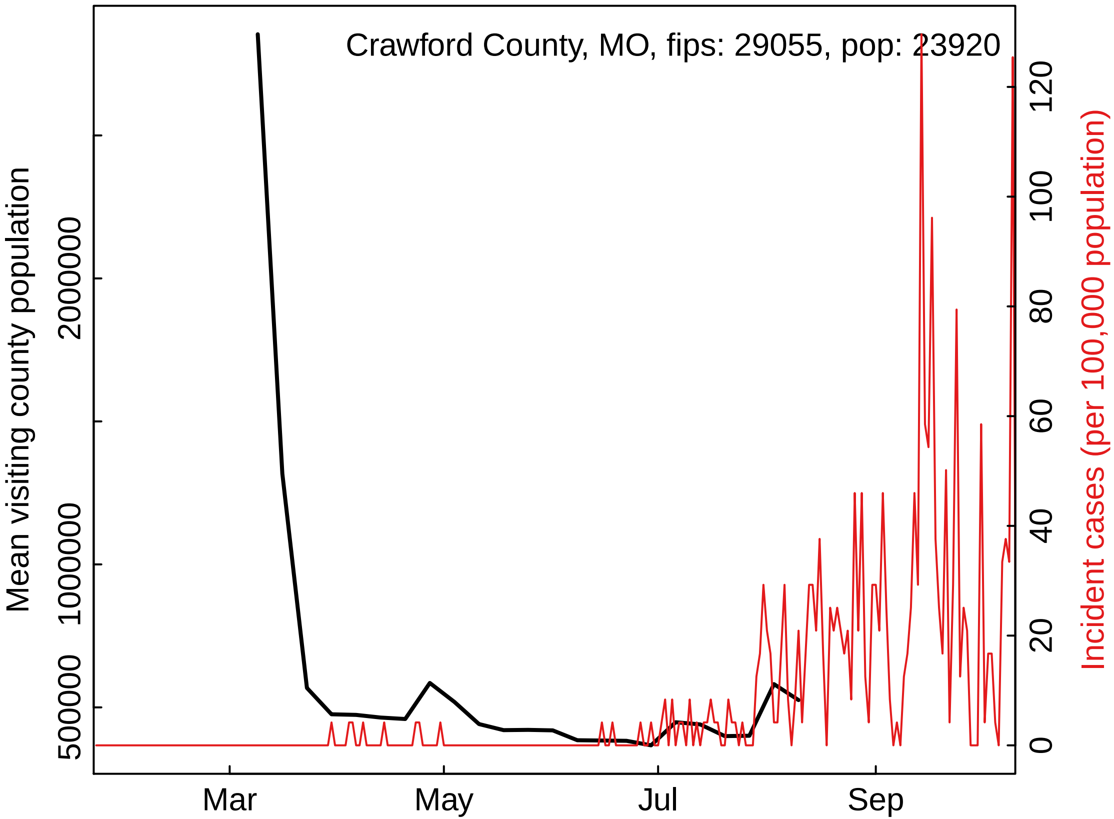

Crawford County, MO, fips: 29055, pop: 23920
Mean visiting county population
500000
1000000
2000000
Mar
May
Jul
Sep
0
20
40
60
80
100
120
Incident cases (per 100,000 population)

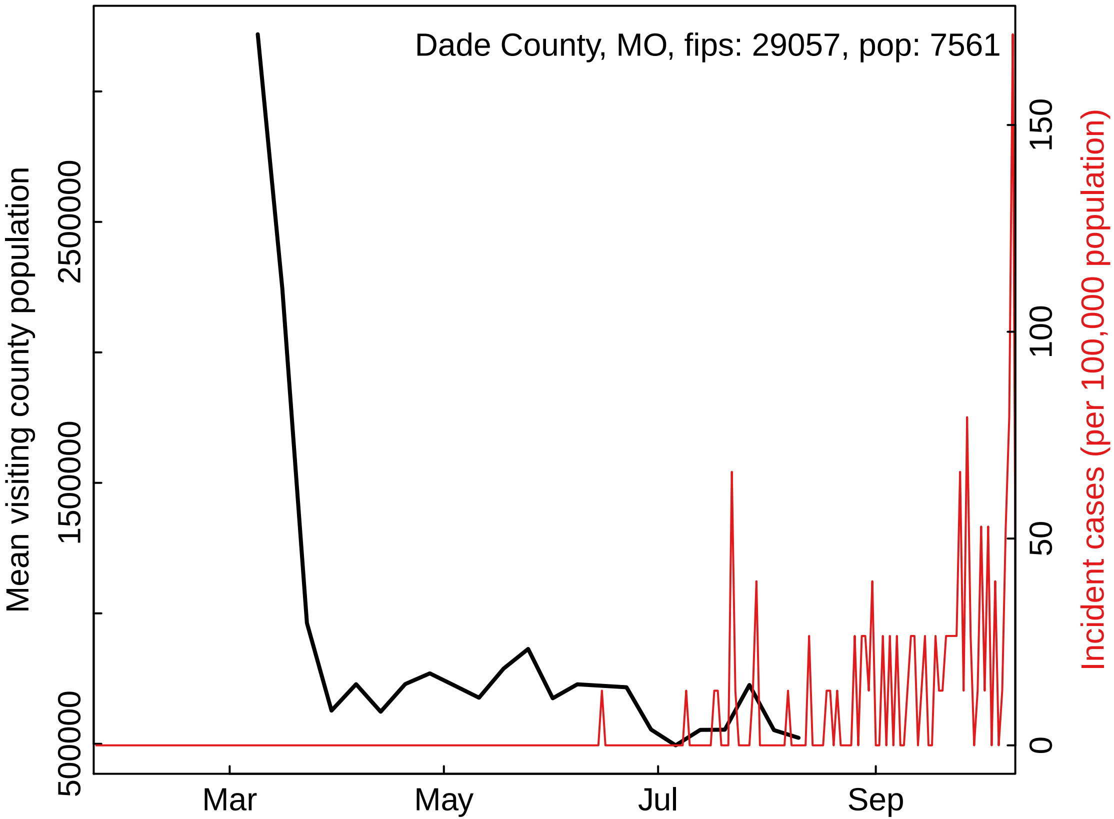

Dade County, MO, fips: 29057, pop: 7561
Mean visiting county population
500000
1500000
2500000
Incident cases (per 100,000 population)
0
50
100
150
Mar
May
Jul
Sep

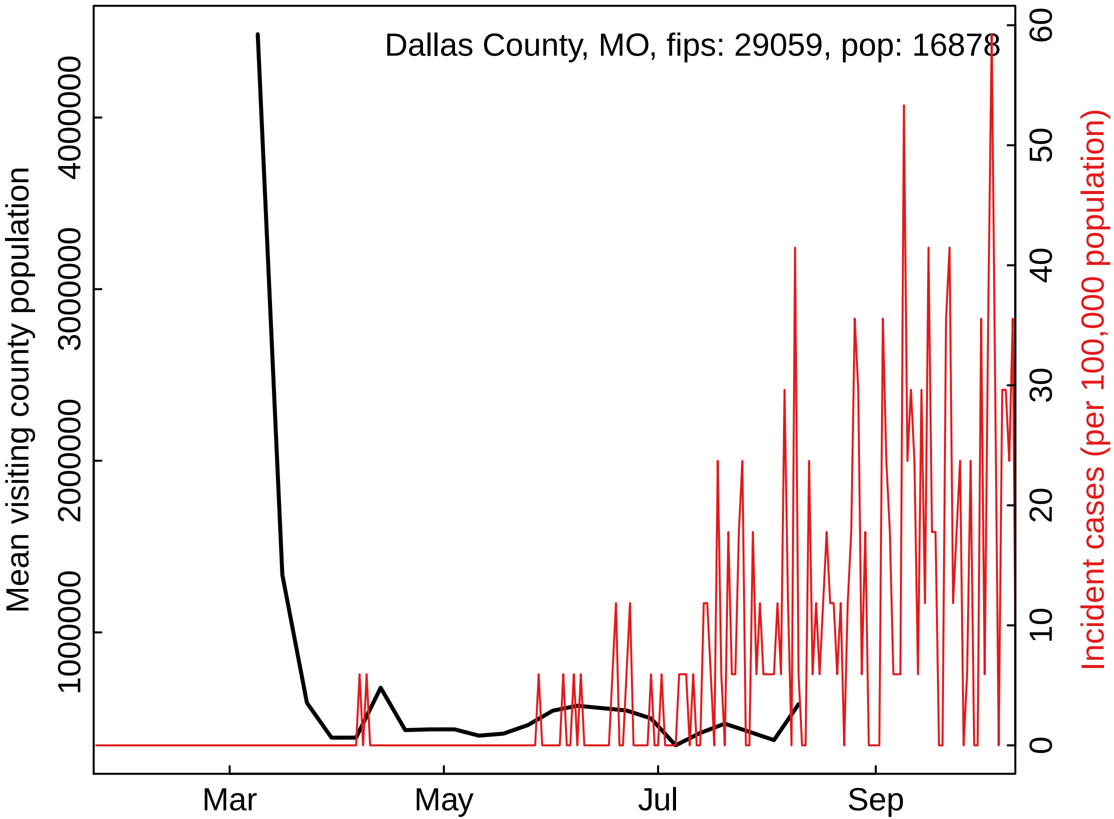

Dallas County, MO, fips: 29059, pop: 16878
Mean visiting county population
1000000
2000000
3000000
4000000
Incident cases (per 100,000 population)
0
10
20
30
40
50
60
Mar
May
Jul
Sep

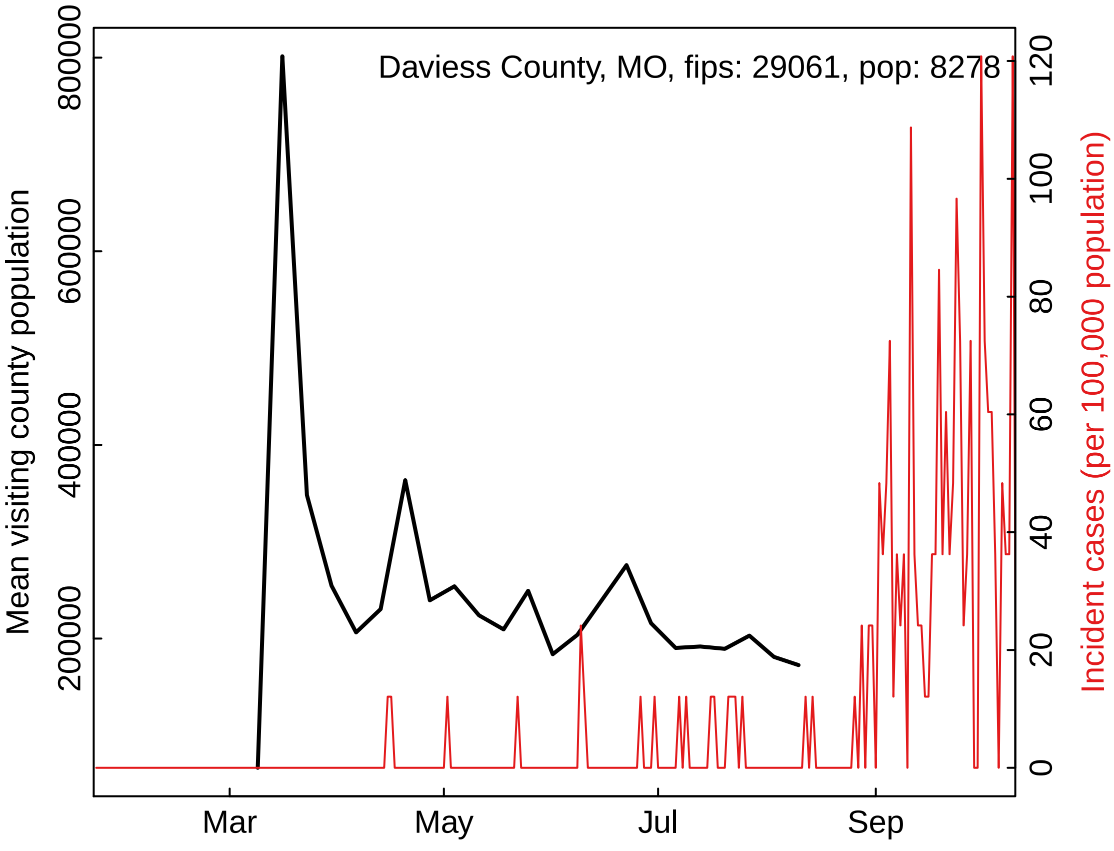
Daviess County, MO, fips: 29061, pop: 8278
Mean visiting county population
200000
400000
600000
800000
Incident cases (per 100,000 population)
0
20
40
60
80
100
120
Mar
May
Jul
Sep

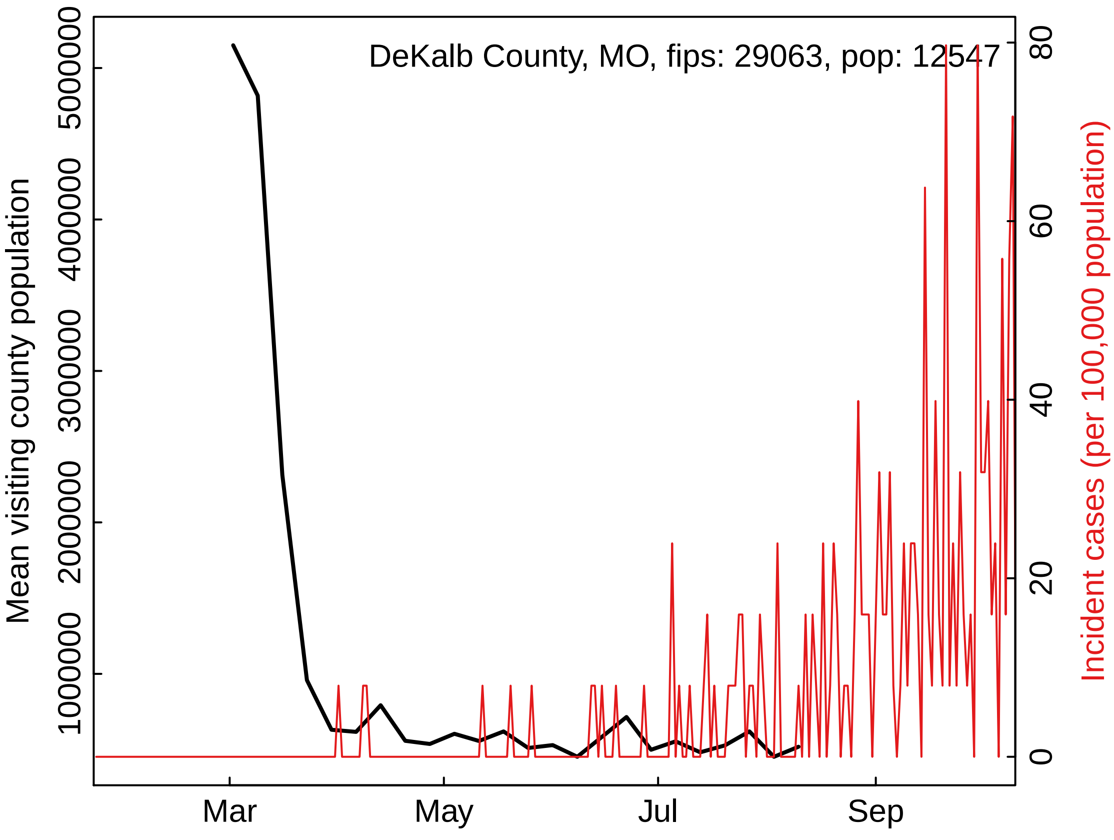
DeKalb County, MO, fips: 29063, pop: 12547
Mean visiting county population
1000000
2000000
3000000
4000000
5000000
Incident cases (per 100,000 population)
0
20
40
60
80
Mar
May
Jul
Sep

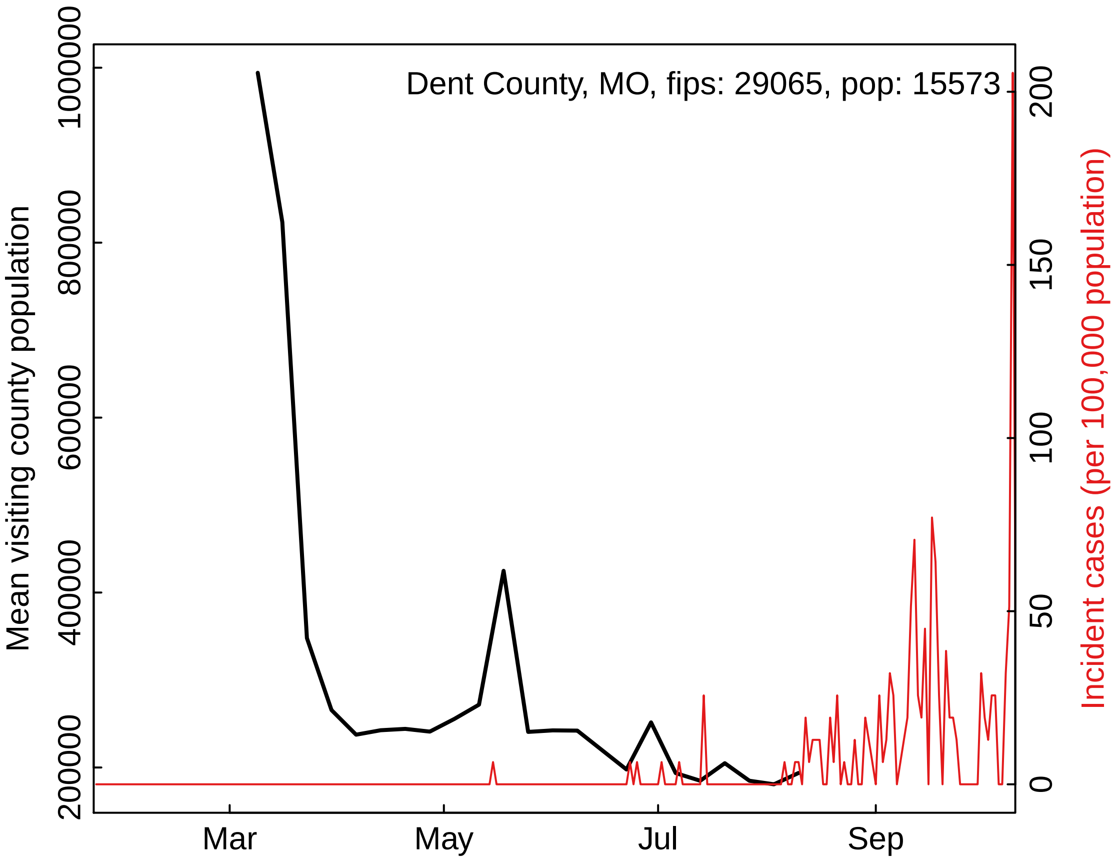

Dent County, MO, fips: 29065, pop: 15573
Mean visiting county population
200000
400000
600000
800000
1000000
Incident cases (per 100,000 population)
0
50
100
150
200
Mar
May
Jul
Sep

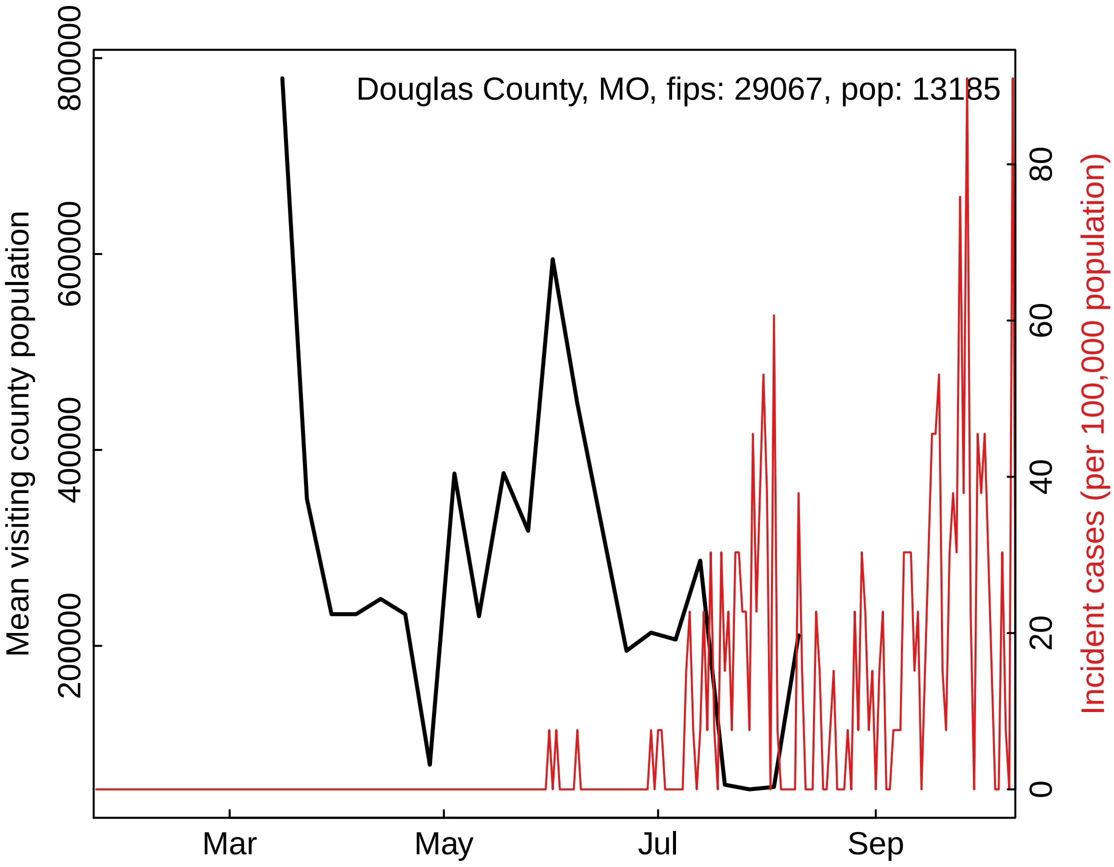
Douglas County, MO, fips: 29067, pop: 13185
Mean visiting county population
200000
400000
600000
800000
Incident cases (per 100,000 population)
0
20
40
60
80
Mar
May
Jul
Sep

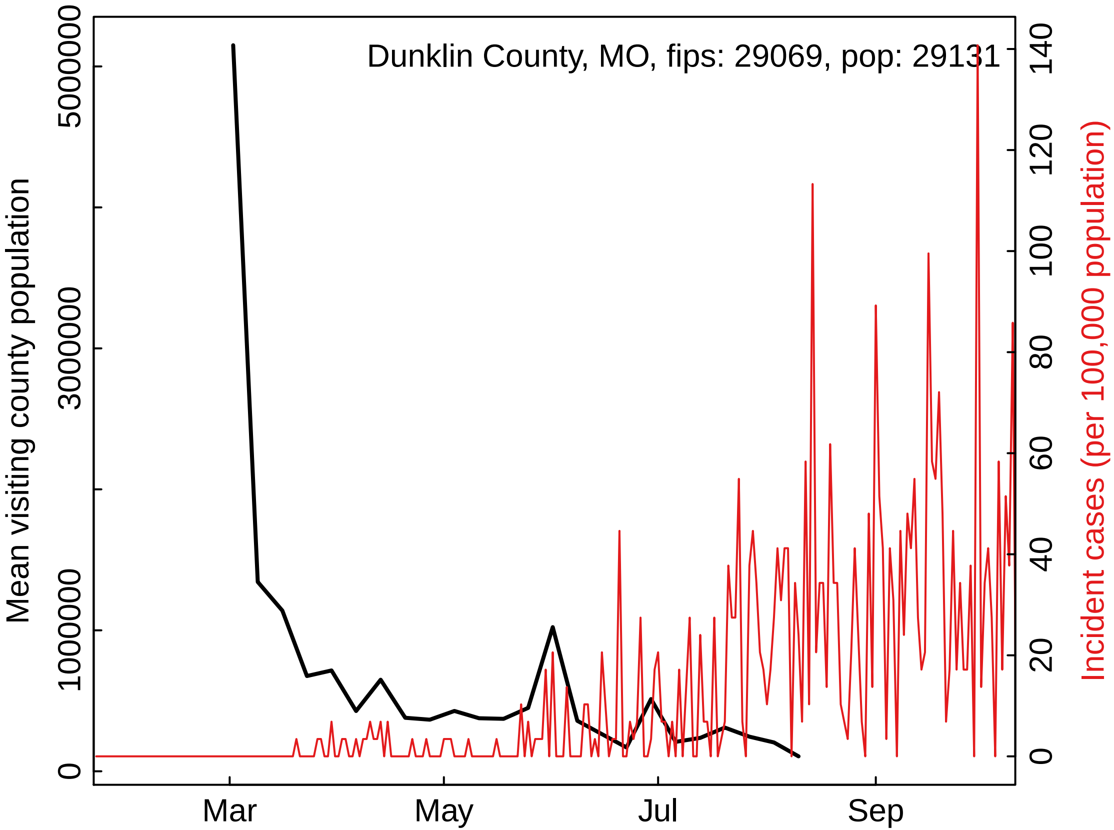

Dunklin County, MO, fips: 29069, pop: 29131
Mean visiting county population
0
1000000
3000000
5000000
Incident cases (per 100,000 population)
0
20
40
60
80
100
120
140
Mar
May
Jul
Sep

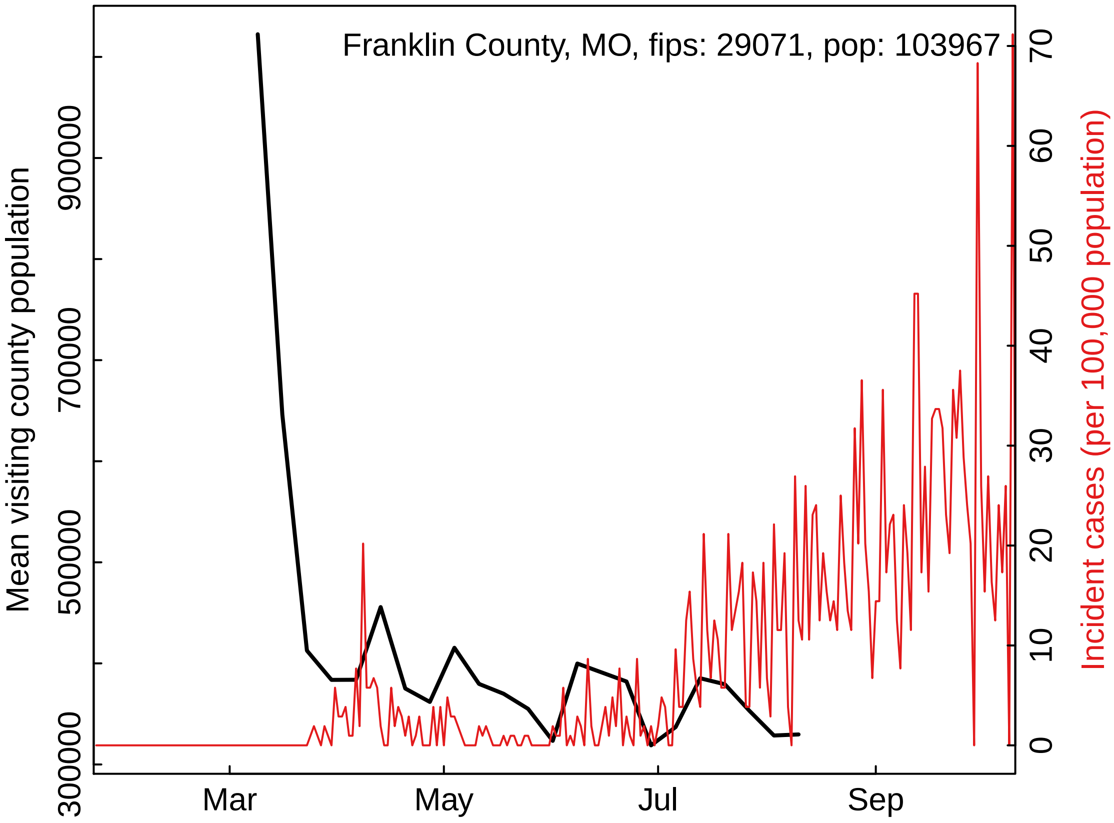

Franklin County, MO, fips: 29071, pop: 103967
Mean visiting county population
300000
500000
700000
900000
Incident cases (per 100,000 population)
0
10
20
30
40
50
60
70
Mar
May
Jul
Sep

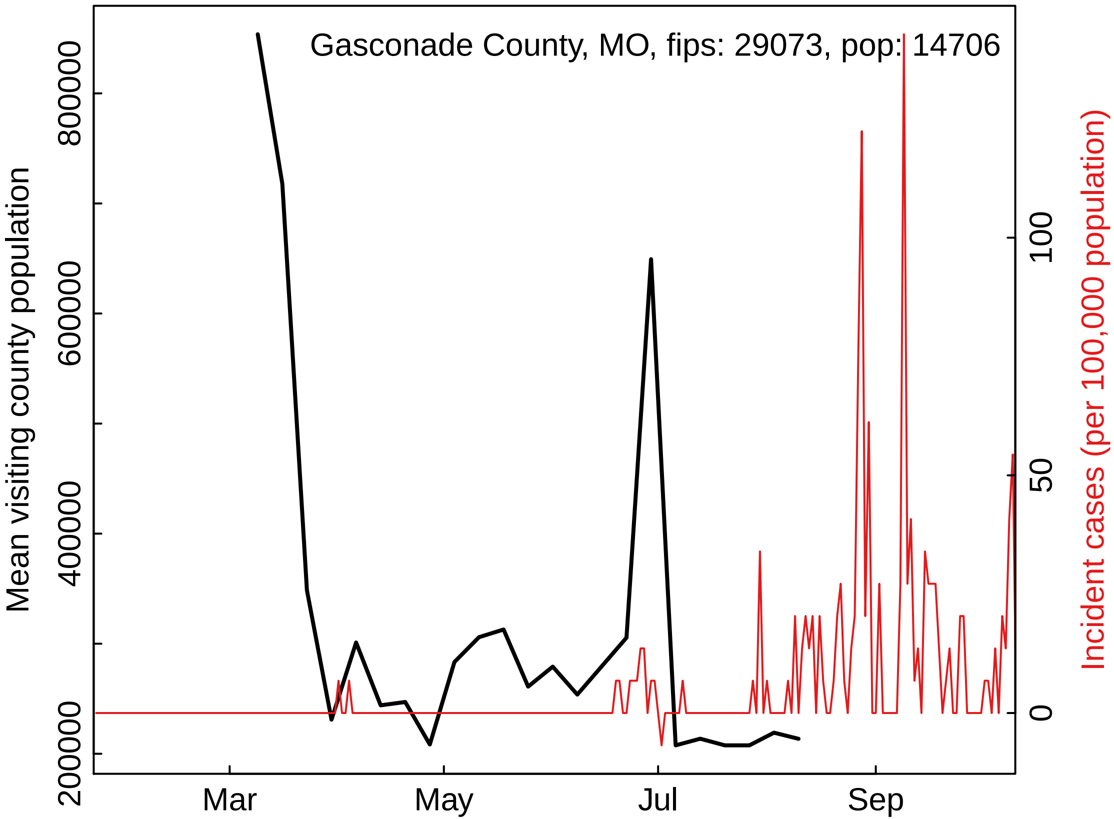

Gasconade County, MO, fips: 29073, pop: 14706
Mean visiting county population
200000
400000
600000
800000
Incident cases (per 100,000 population)
0
50
100
Mar
May
Jul
Sep

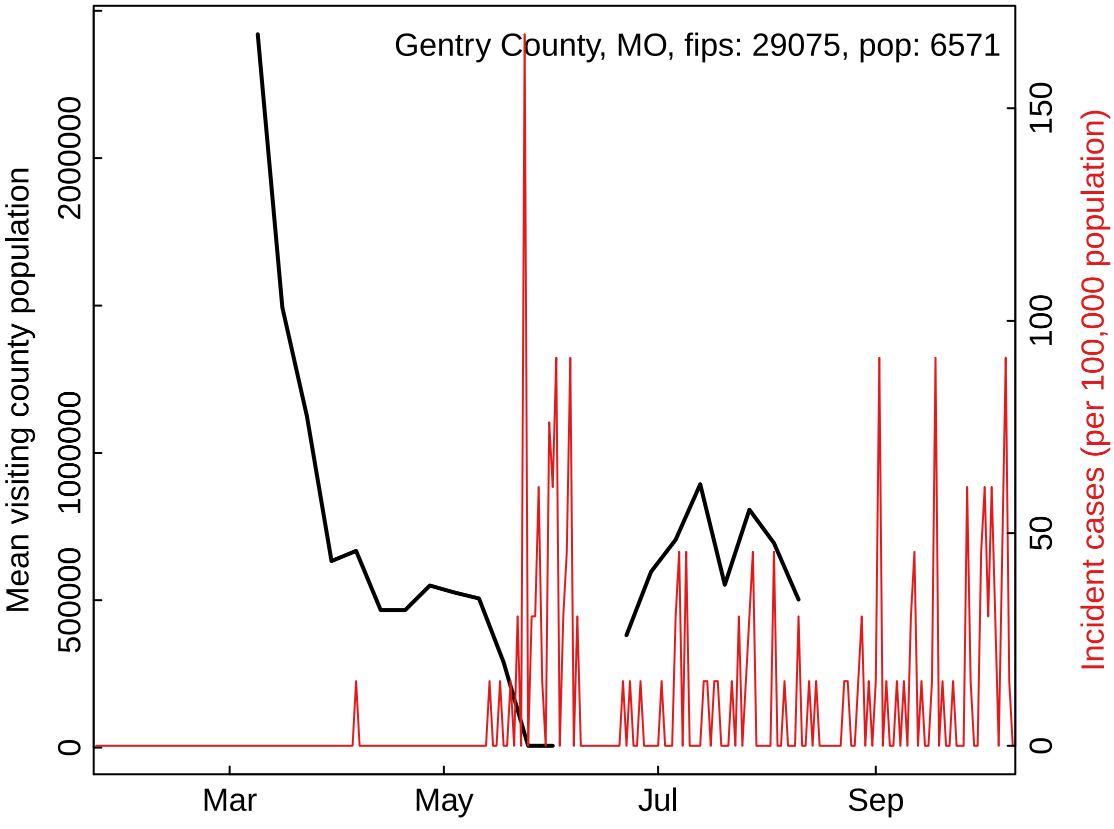

Gentry County, MO, fips: 29075, pop: 6571
Mean visiting county population
0
500000
1000000
2000000
Incident cases (per 100,000 population)
0
50
100
150
Mar
May
Jul
Sep

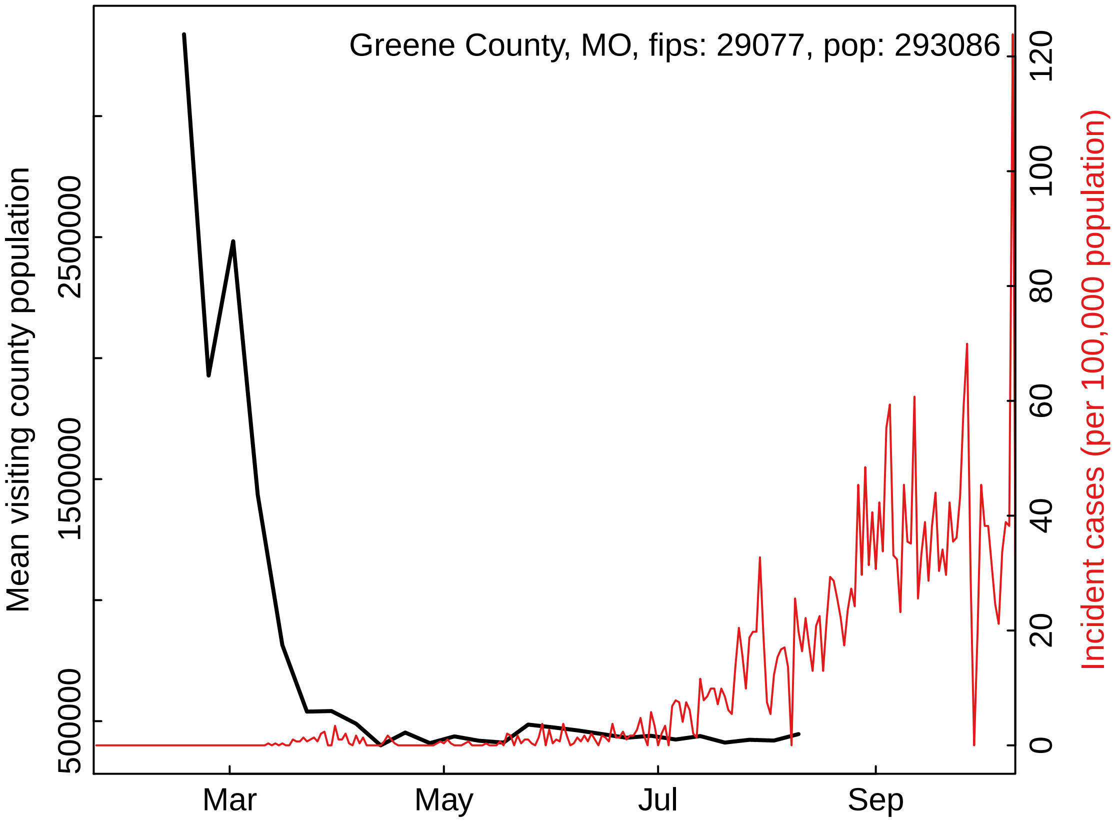

Greene County, MO, fips: 29077, pop: 293086
Mean visiting county population
500000
1500000
2500000
Mar
May
Jul
Sep
0
20
40
60
80
100
120
Incident cases (per 100,000 population)

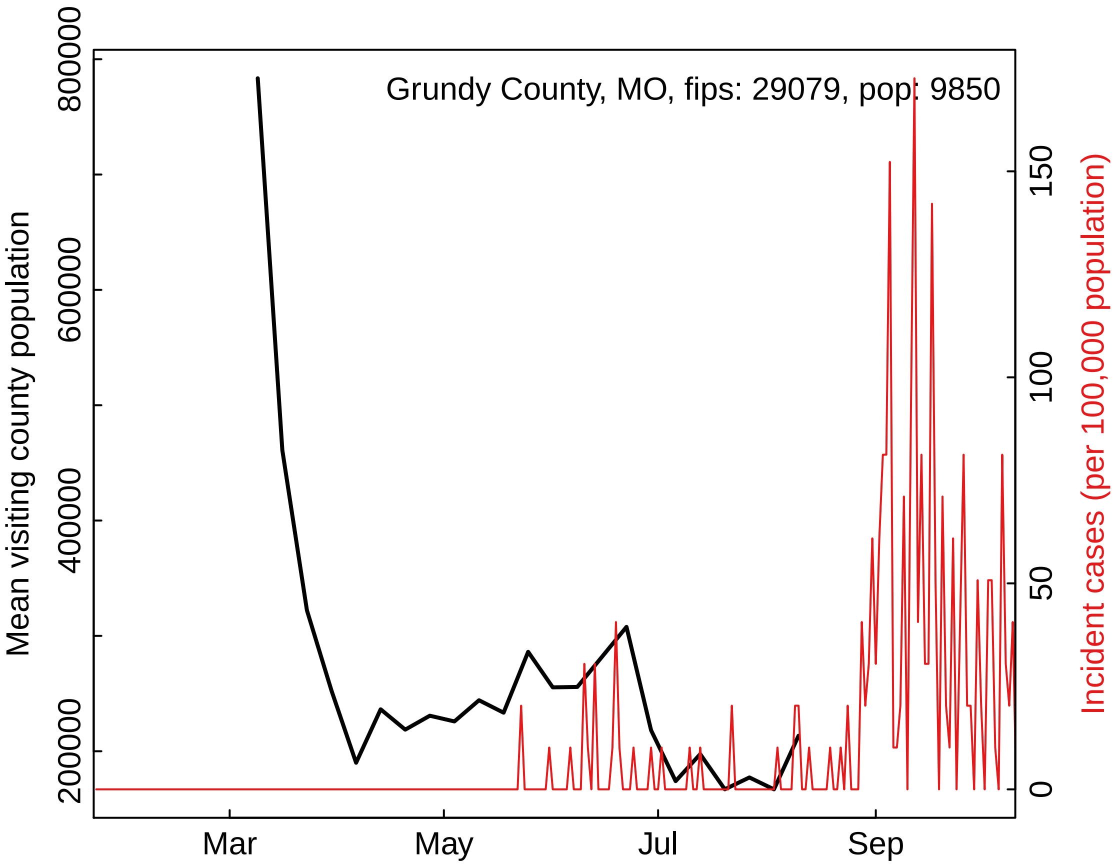

Grundy County, MO, fips: 29079, pop: 9850
Mean visiting county population
Incident cases (per 100,000 population)
8000000
6000000
4000000
2000000
150
100
50
0
Mar
May
Jul
Sep

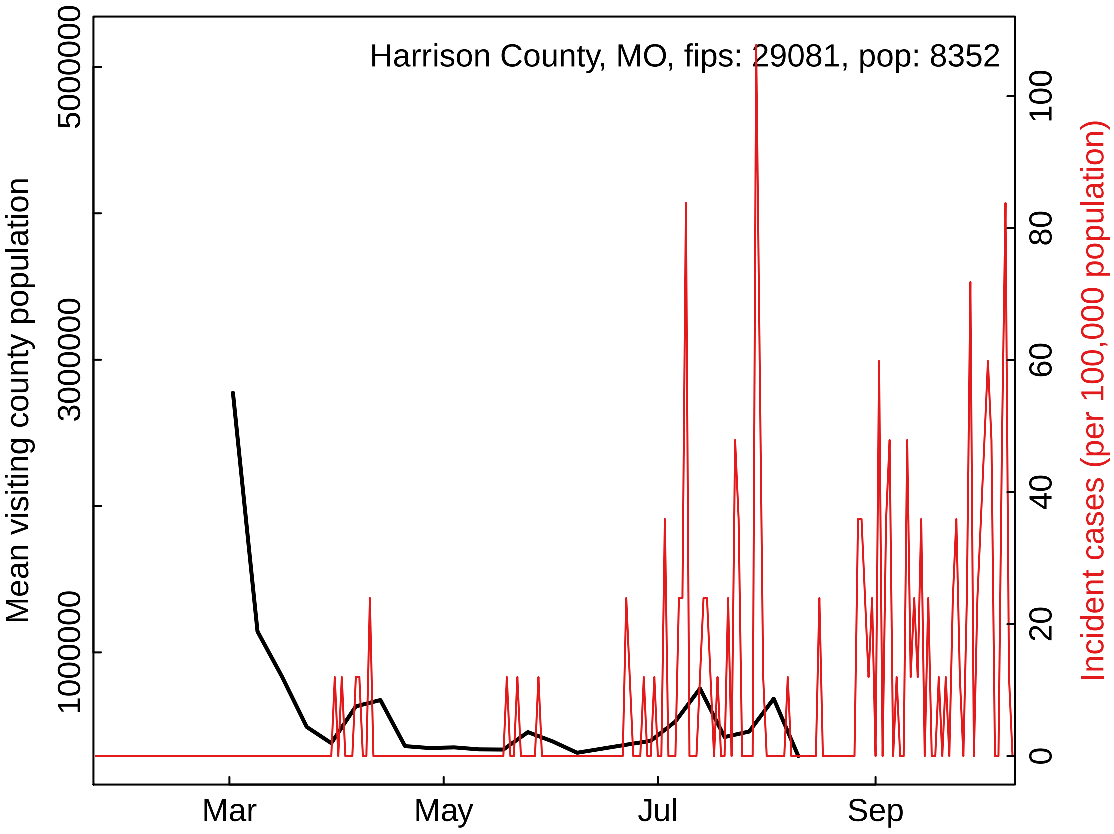

Harrison County, MO, fips: 29081, pop: 8352
Mean visiting county population
1000000
3000000
5000000
Incident cases (per 100,000 population)
0
20
40
60
80
100
Mar
May
Jul
Sep

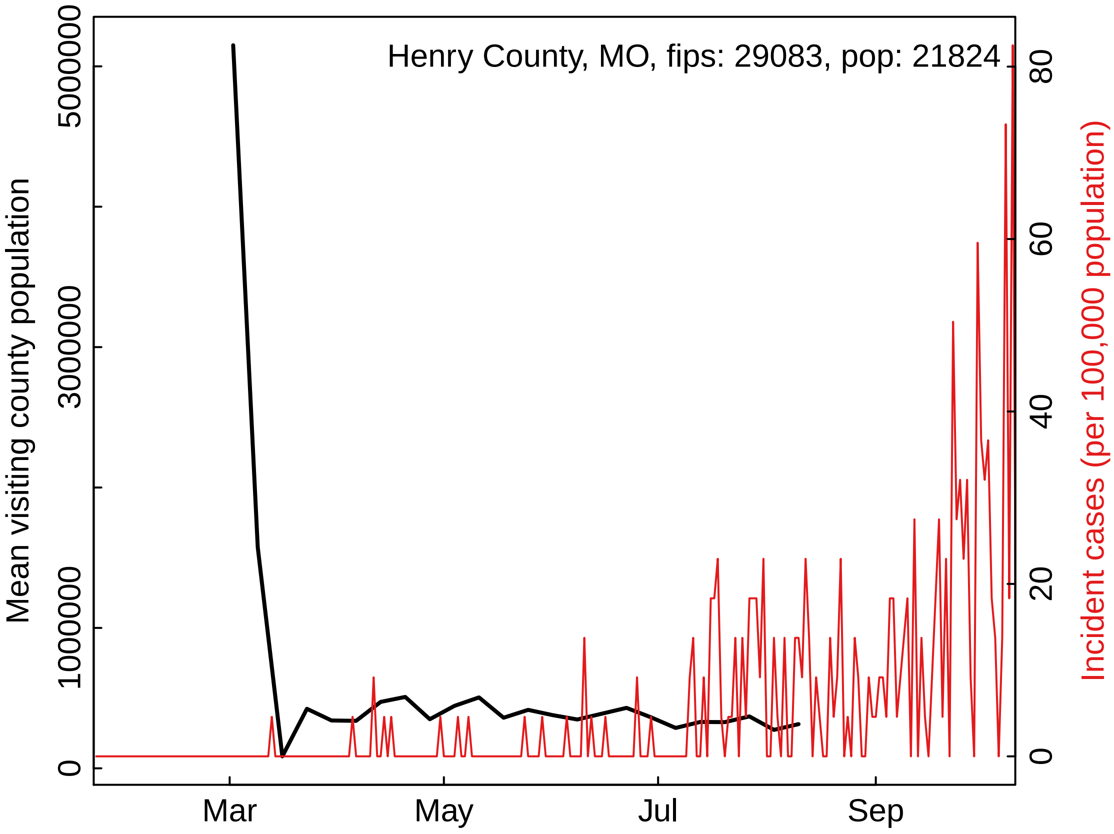

Henry County, MO, fips: 29083, pop: 21824
Mean visiting county population
0
1000000
3000000
5000000
Mar
May
Jul
Sep
0
20
40
60
80
Incident cases (per 100,000 population)

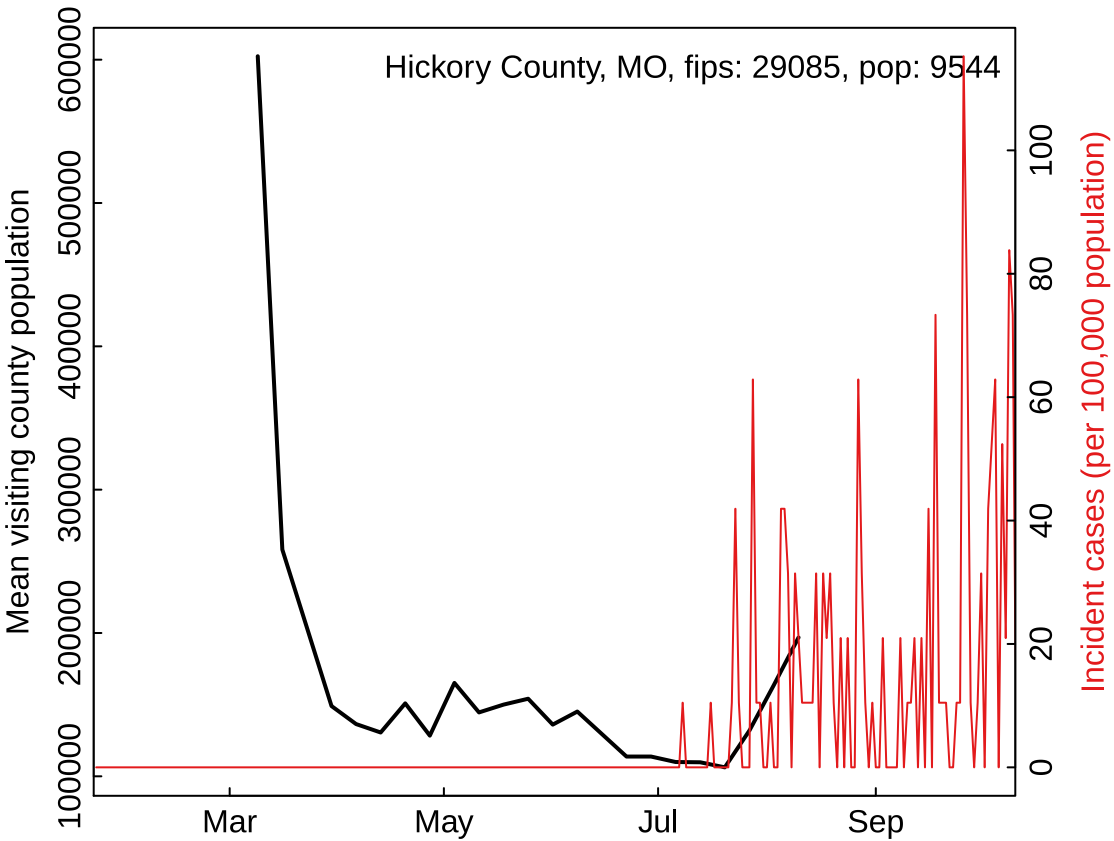

Hickory County, MO, fips: 29085, pop: 9544
Mean visiting county population
100000
200000
300000
400000
500000
600000
Mar
May
Jul
Sep
0
20
40
60
80
100
Incident cases (per 100,000 population)

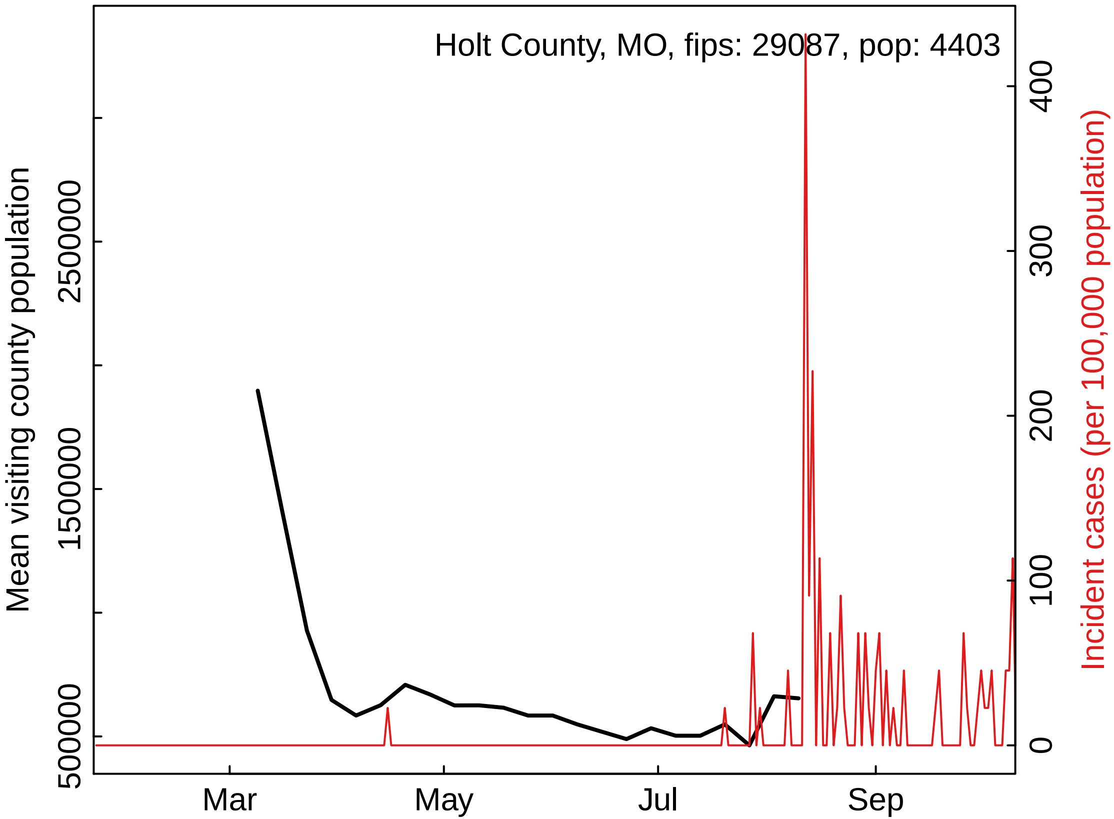

Holt County, MO, fips: 29087, pop: 4403
Mean visiting county population
500000
1500000
2500000
Mar
May
Jul
Sep
0
100
200
300
400
Incident cases (per 100,000 population)

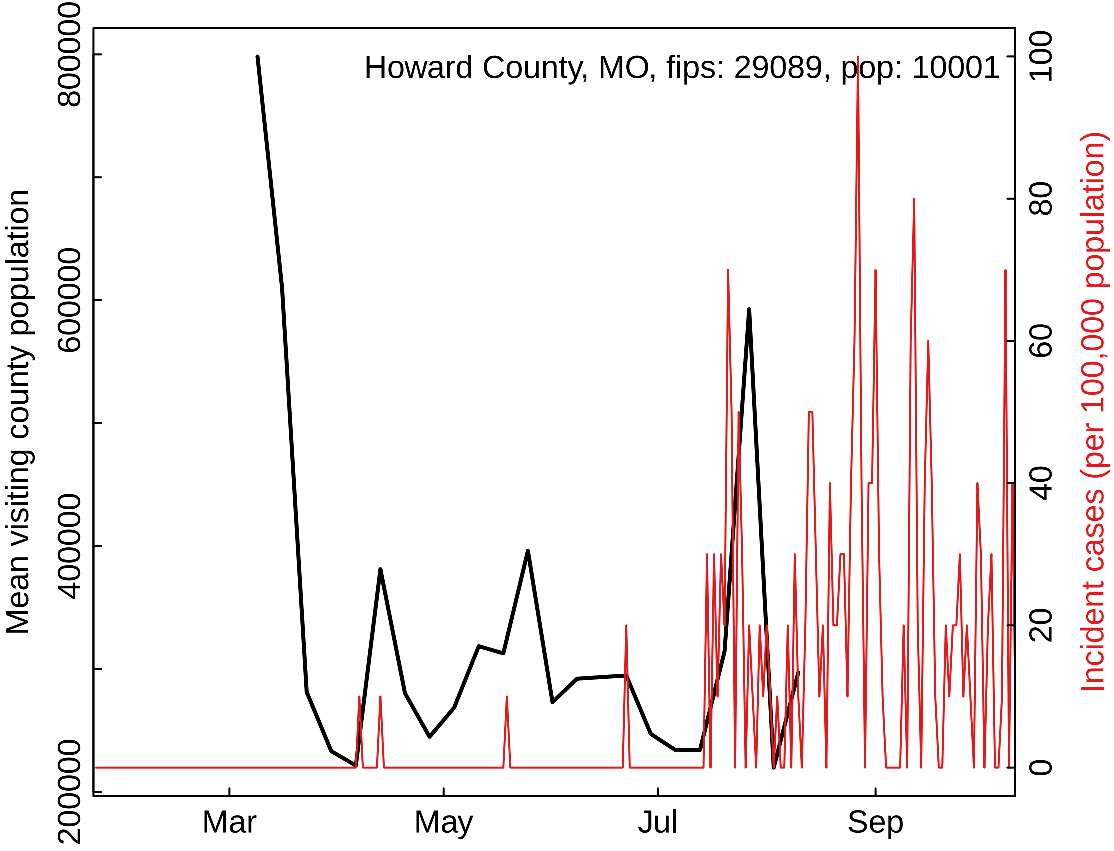

Howard County, MO, fips: 29089, pop: 10001
Mean visiting county population
200000
400000
600000
800000
Incident cases (per 100,000 population)
0
20
40
60
80
100
Mar
May
Jul
Sep

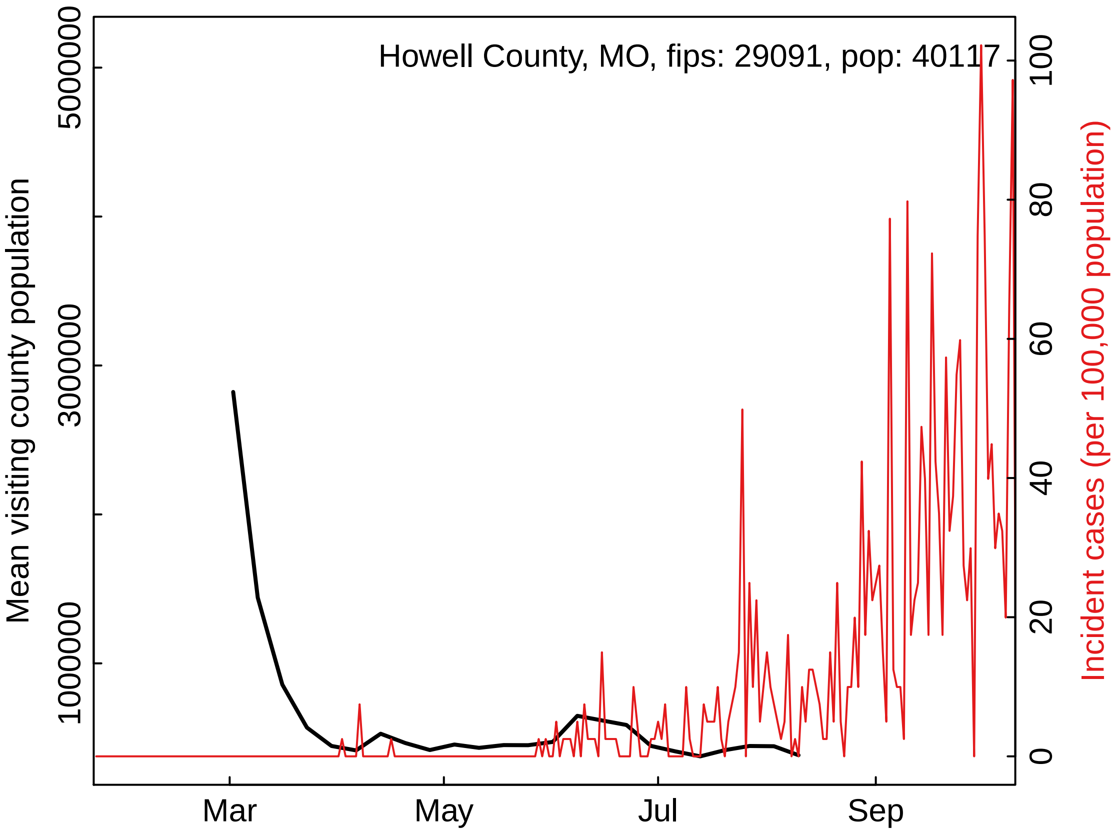

Howell County, MO, fips: 29091, pop: 40117
Mean visiting county population
1000000
3000000
5000000
Incident cases (per 100,000 population)
0
20
40
60
80
100
Mar
May
Jul
Sep

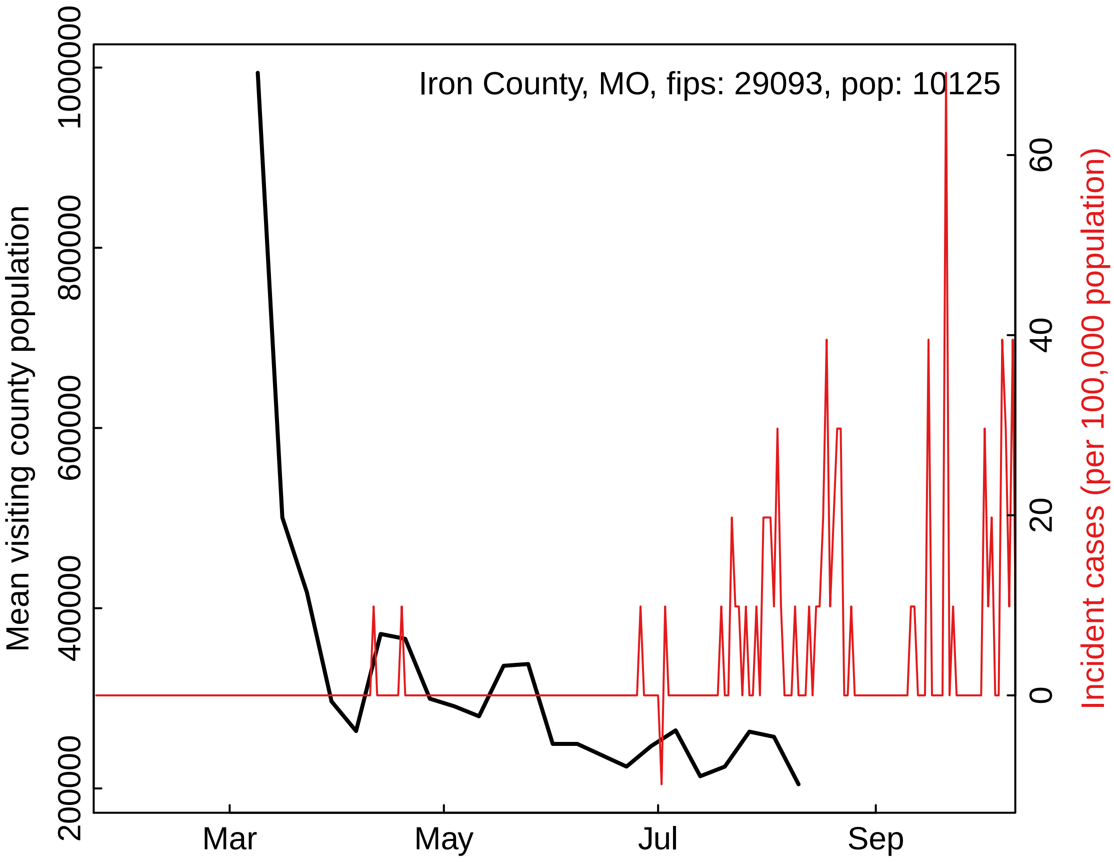
Iron County, MO, fips: 29093, pop: 10125
Mean visiting county population
200000
400000
600000
800000
1000000
Incident cases (per 100,000 population)
0
20
40
60
Mar
May
Jul
Sep

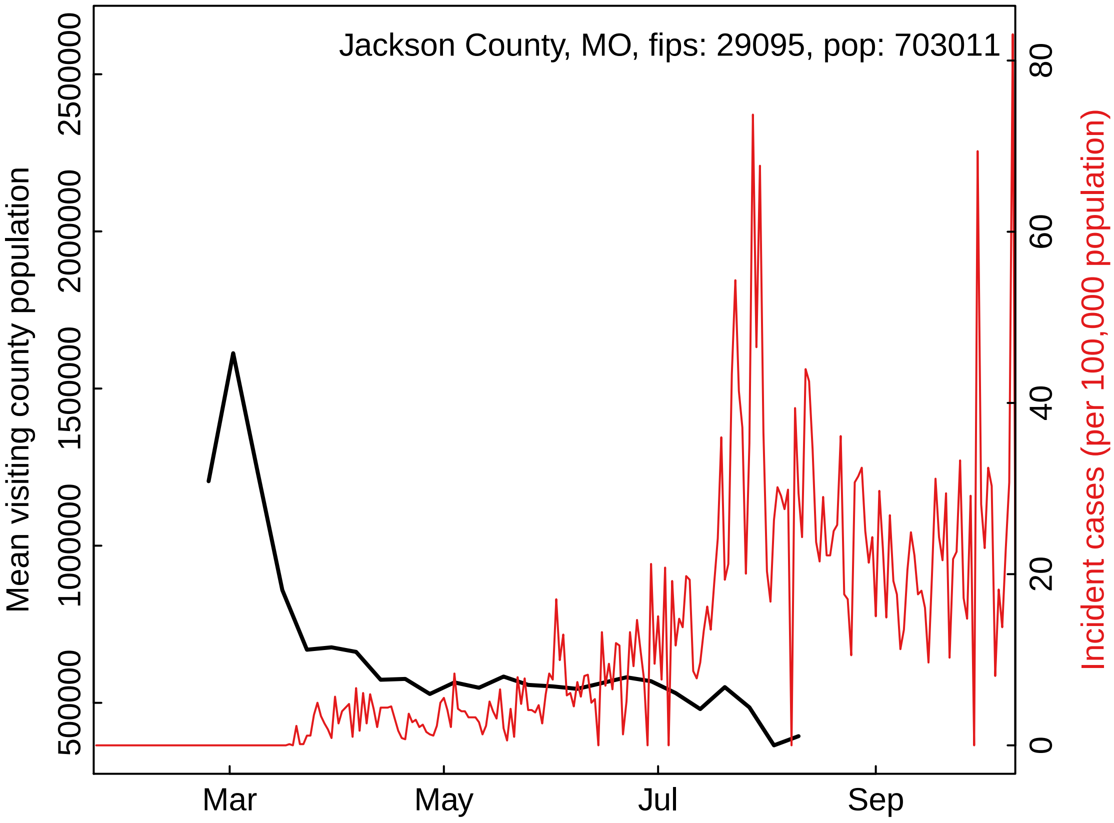

Jackson County, MO, fips: 29095, pop: 703011
Mean visiting county population
500000
1000000
1500000
2000000
2500000
Incident cases (per 100,000 population)
0
20
40
60
80
Mar
May
Jul
Sep

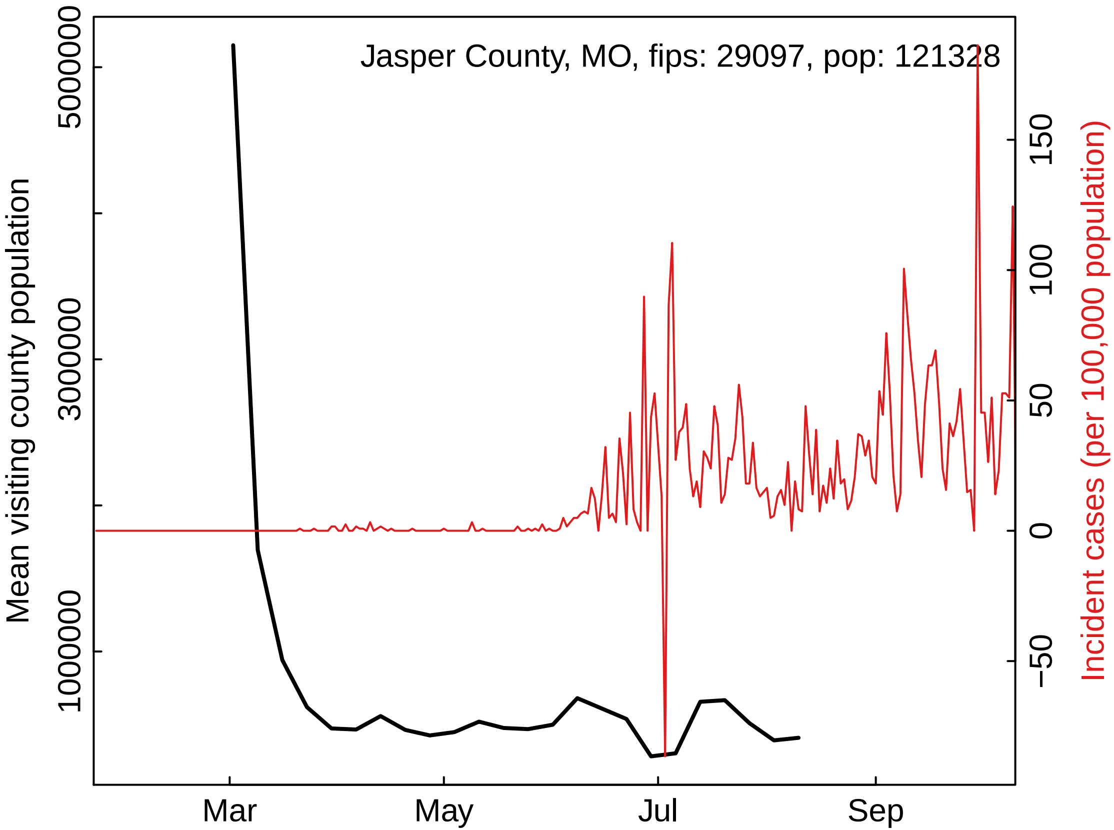
Jasper County, MO, fips: 29097, pop: 121328
Mean visiting county population
Incident cases (per 100,000 population)
1000000
3000000
5000000
−50
0
50
100
150
Mar
May
Jul
Sep

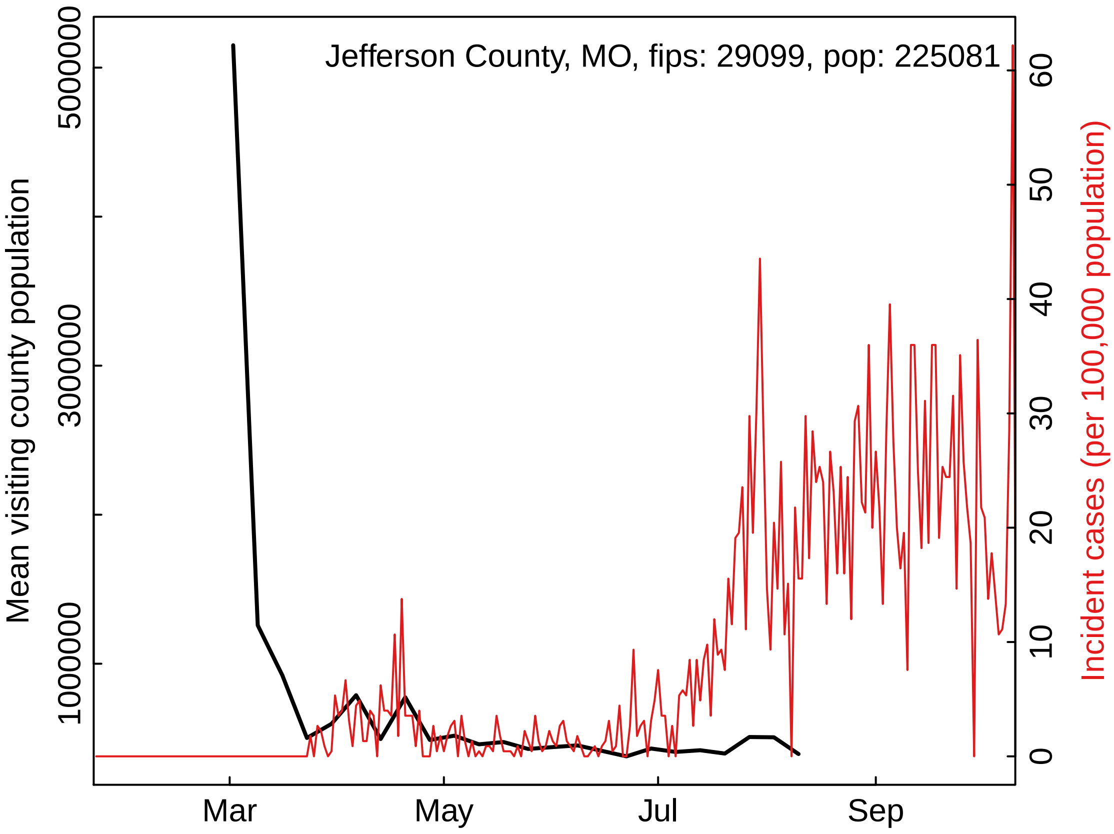

Jefferson County, MO, fips: 29099, pop: 225081
Mean visiting county population
Incident cases (per 100,000 population)
1000000
3000000
5000000
0
10
20
30
40
50
60
Mar
May
Jul
Sep

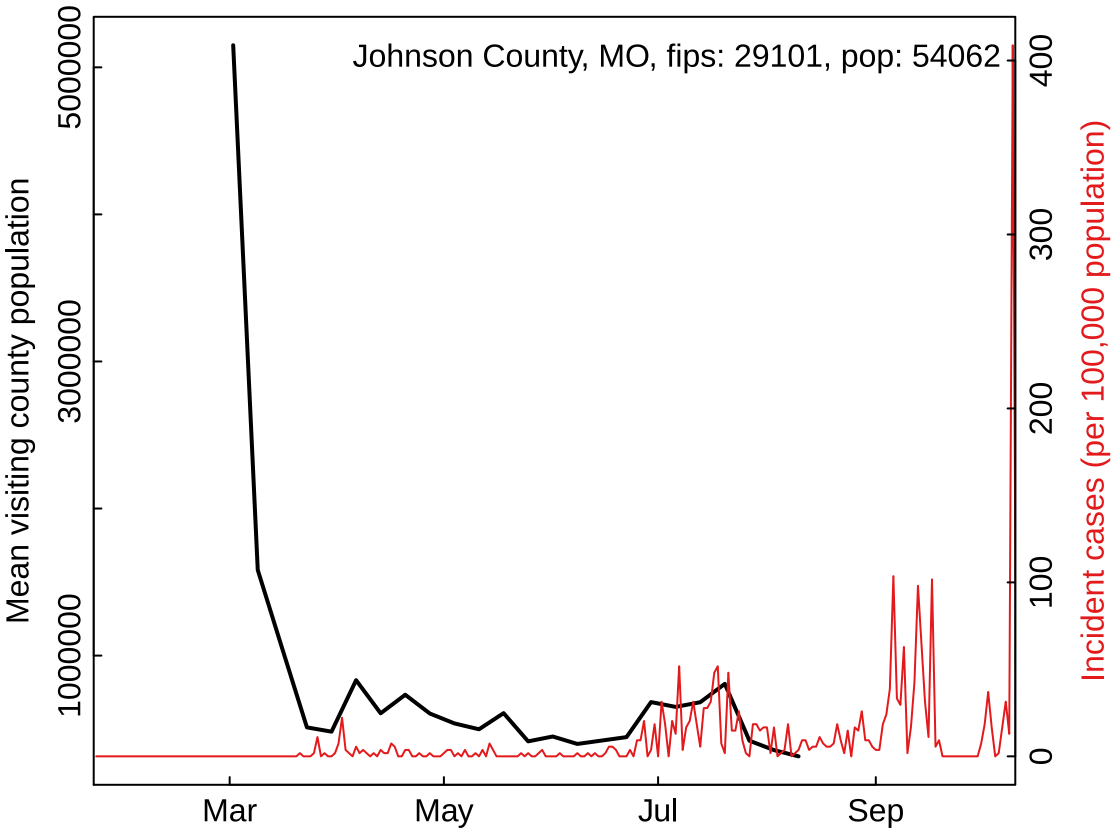
Johnson County, MO, fips: 29101, pop: 54062
Mean visiting county population
1000000
3000000
5000000
Incident cases (per 100,000 population)
0
100
200
300
400
Mar
May
Jul
Sep

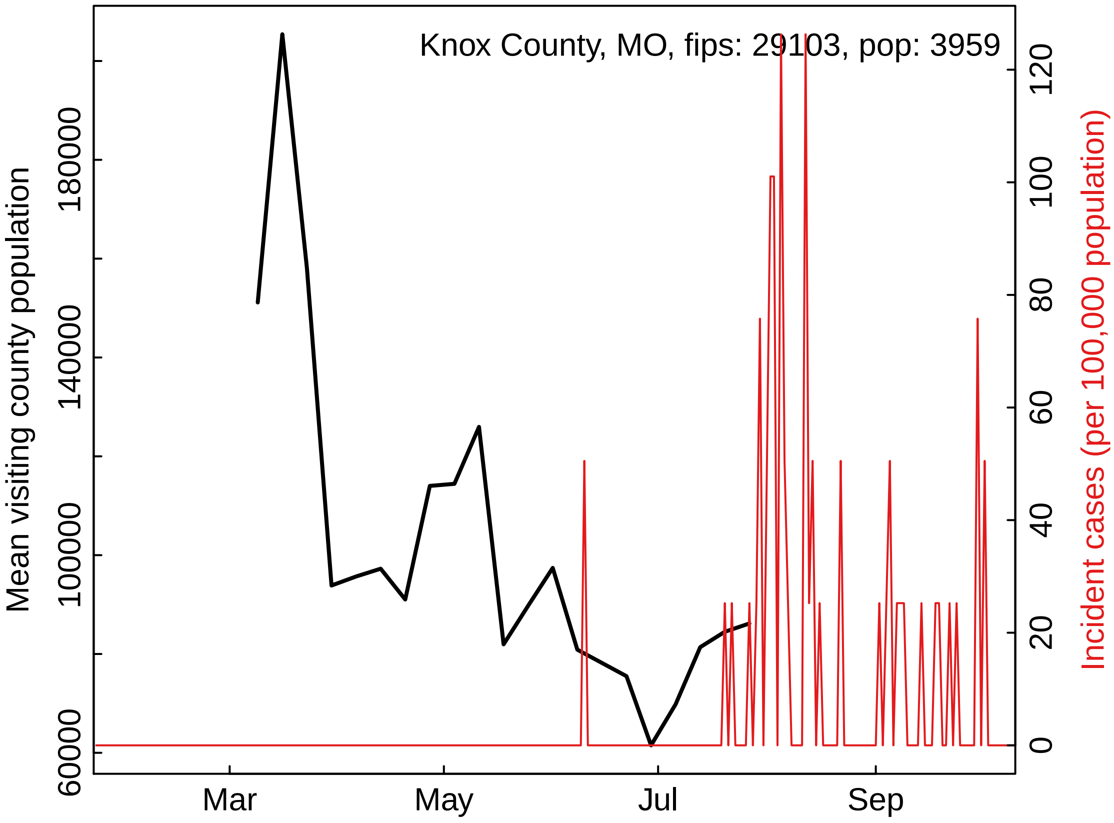
Knox County, MO, fips: 29103, pop: 3959
Mean visiting county population
Incident cases (per 100,000 population)
60000
100000
140000
180000
0
20
40
60
80
100
120
Mar
May
Jul
Sep

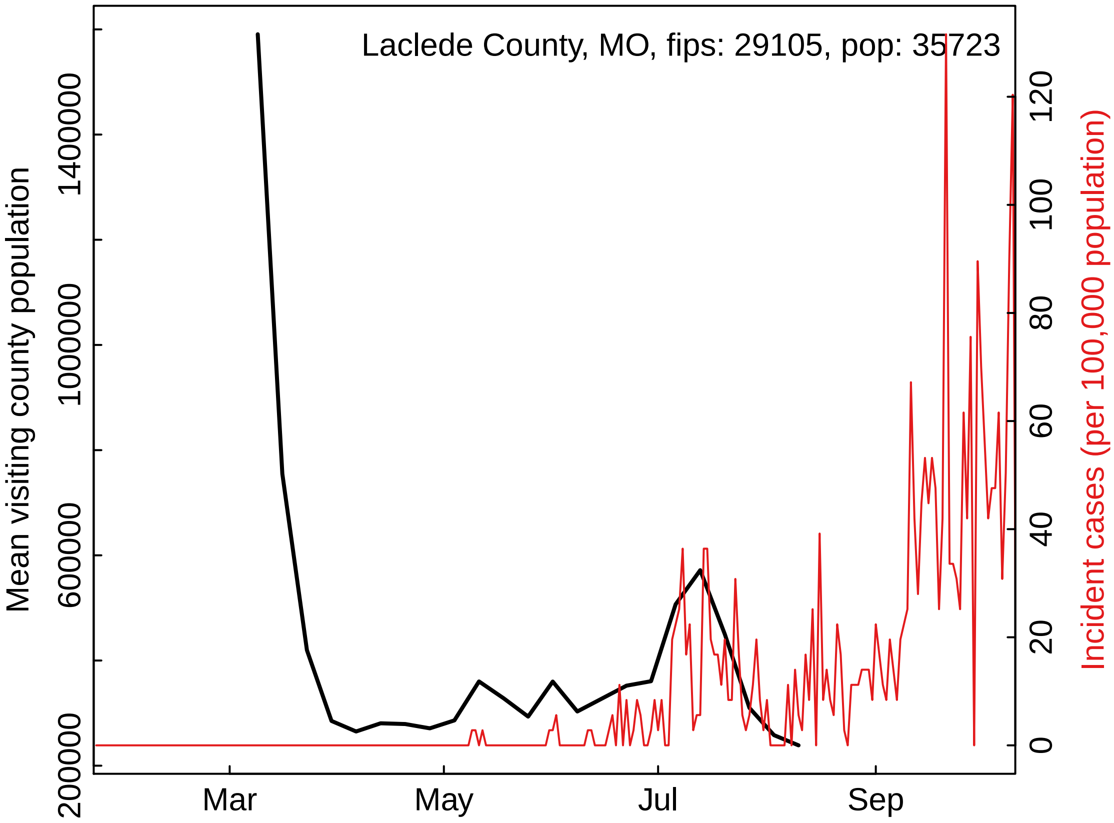

Laclede County, MO, fips: 29105, pop: 35723
Mean visiting county population
2000000
6000000
10000000
14000000
Mar
May
Jul
Sep
0
20
40
60
80
100
120
Incident cases (per 100,000 population)

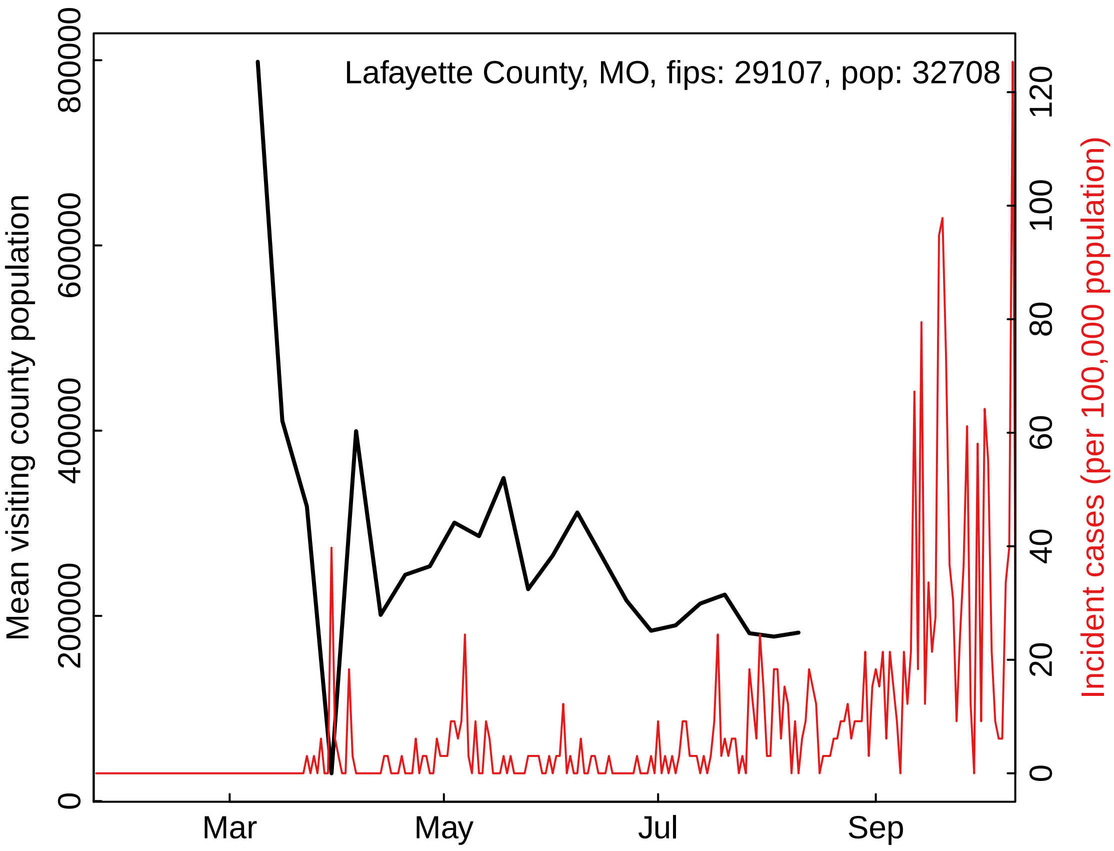
Lafayette County, MO, fips: 29107, pop: 32708
Mean visiting county population
0
200000
400000
600000
800000
Incident cases (per 100,000 population)
0
20
40
60
80
100
120
Mar
May
Jul
Sep

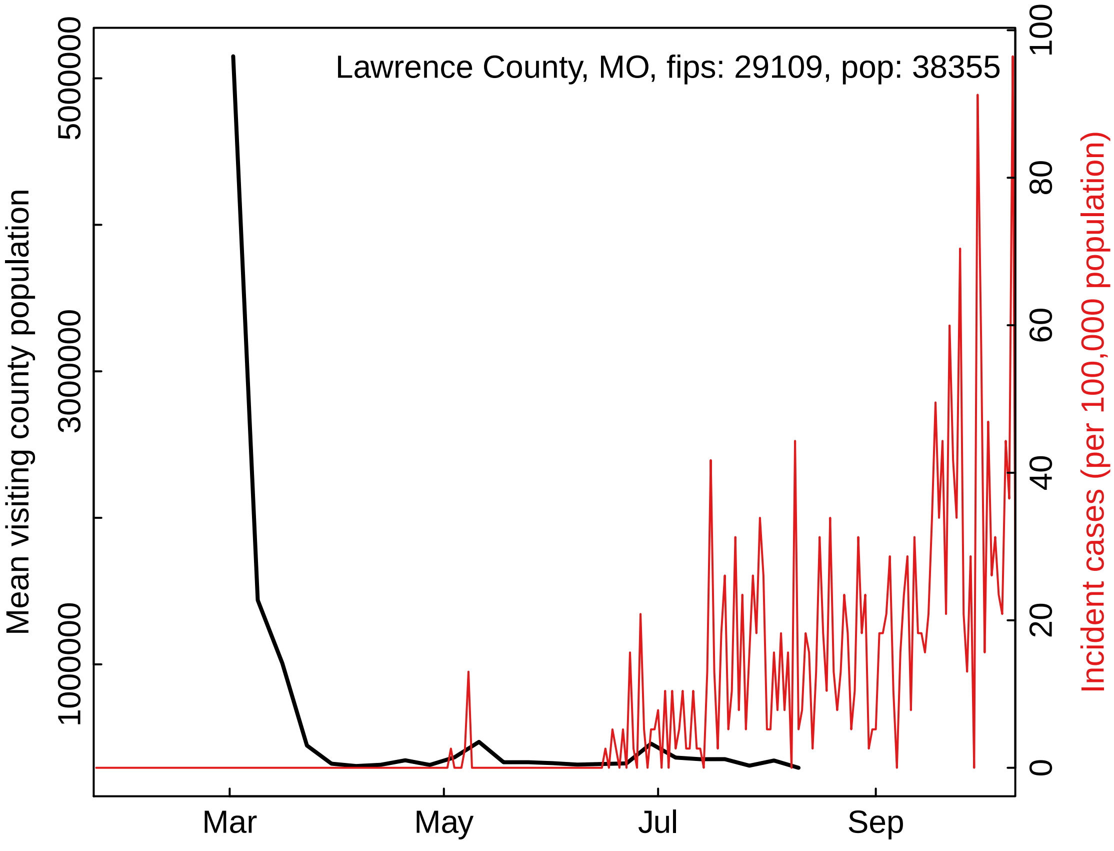

Lawrence County, MO, fips: 29109, pop: 38355
Mean visiting county population
Incident cases (per 100,000 population)
1000000
3000000
5000000
0
20
40
60
80
100
Mar
May
Jul
Sep

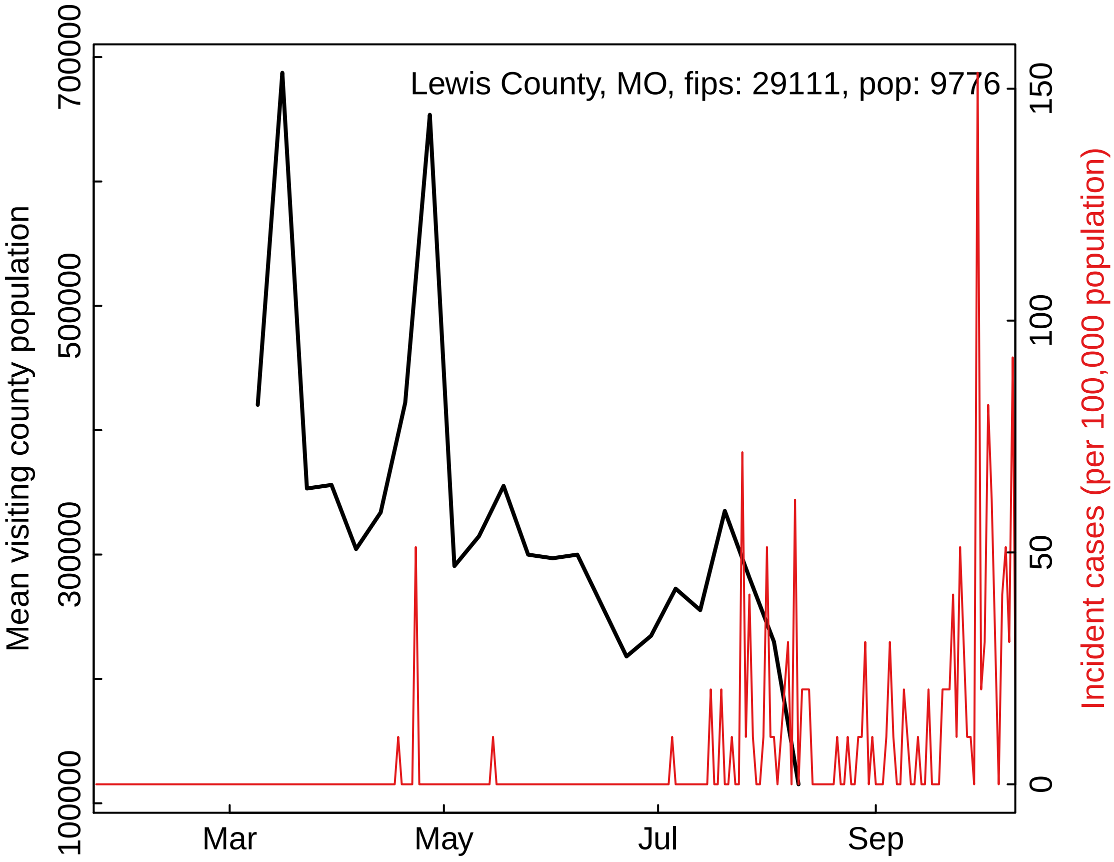
Lewis County, MO, fips: 29111, pop: 9776
Mean visiting county population
100000
300000
500000
700000
Incident cases (per 100,000 population)
0
50
100
150
Mar
May
Jul
Sep

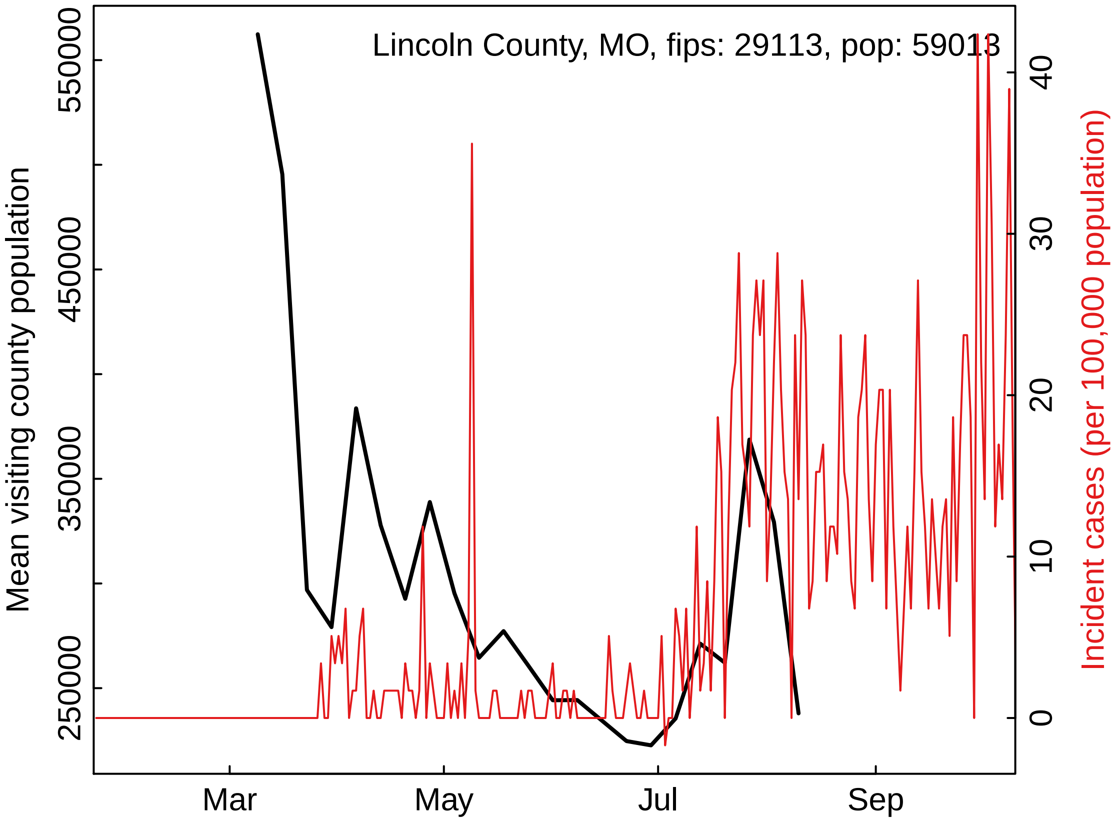

Lincoln County, MO, fips: 29113, pop: 59013
Mean visiting county population
250000
350000
450000
550000
Incident cases (per 100,000 population)
0
10
20
30
40
Mar
May
Jul
Sep

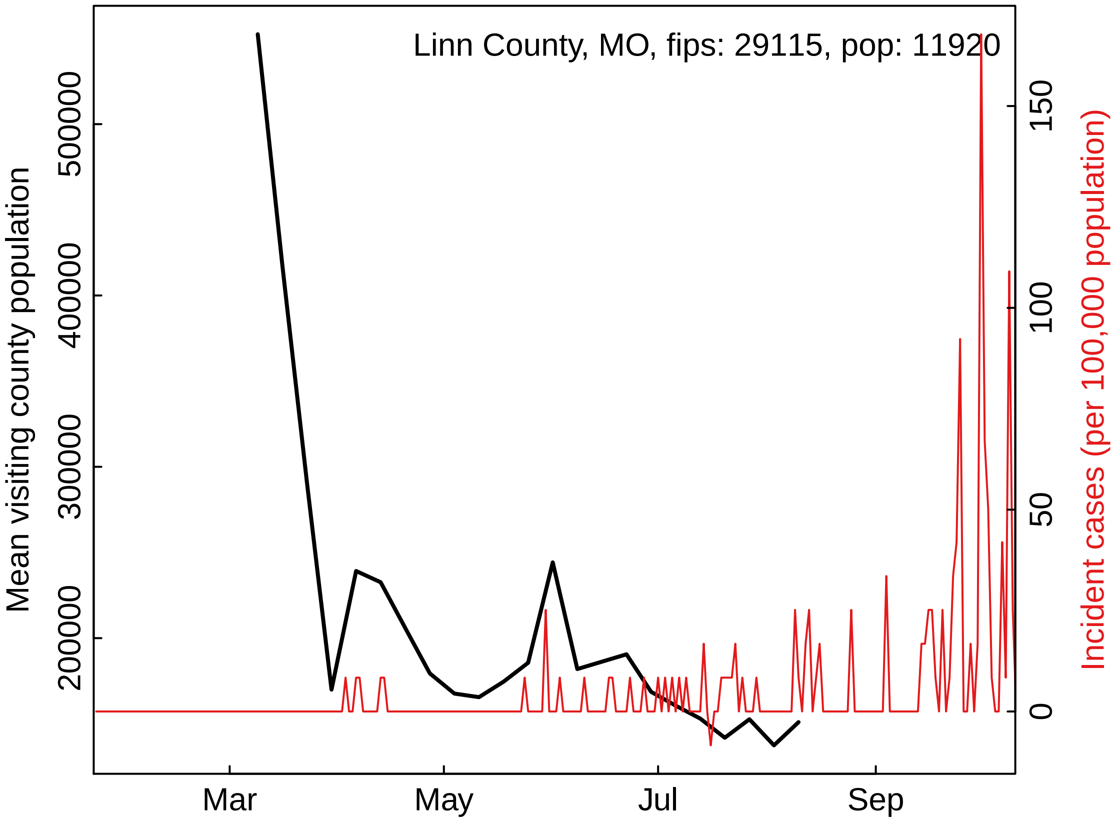

Linn County, MO, fips: 29115, pop: 11920
Mean visiting county population
200000
300000
400000
500000
Incident cases (per 100,000 population)
0
50
100
150
Mar
May
Jul
Sep

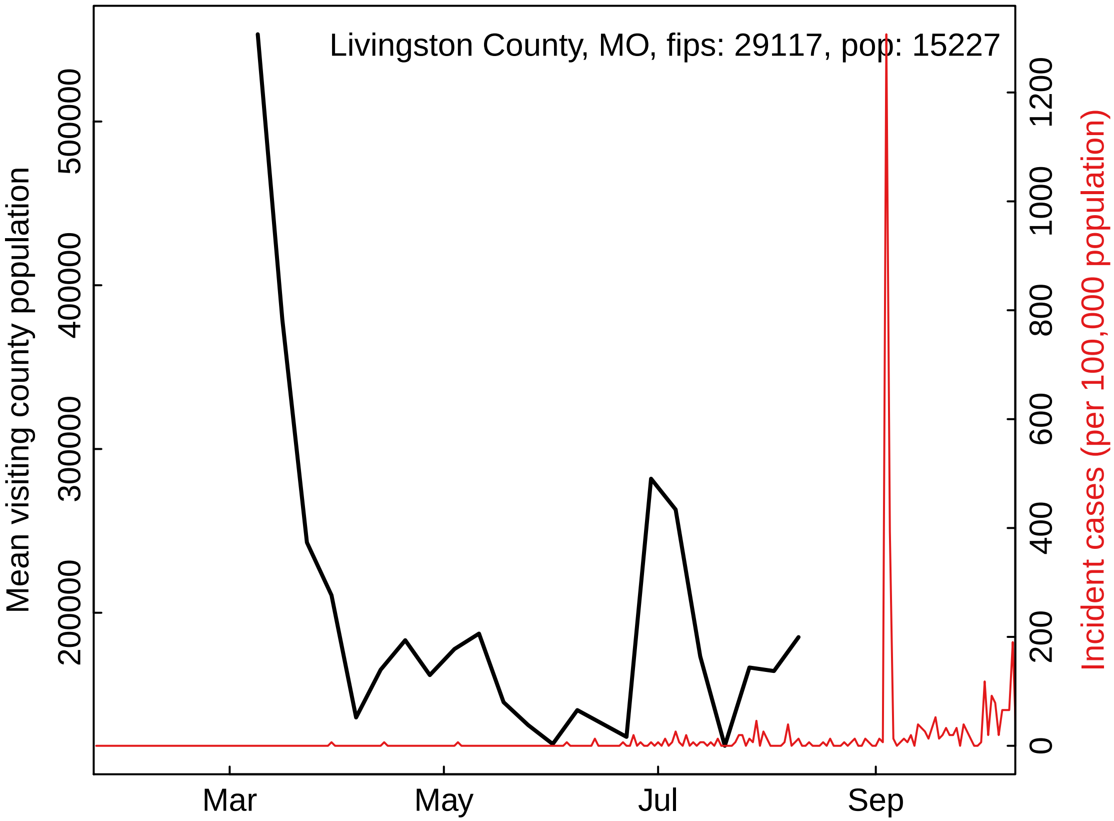

Livingston County, MO, fips: 29117, pop: 15227
Mean visiting county population
200000
300000
400000
500000
Mar
May
Jul
Sep
0
200
400
600
800
1000
1200
Incident cases (per 100,000 population)

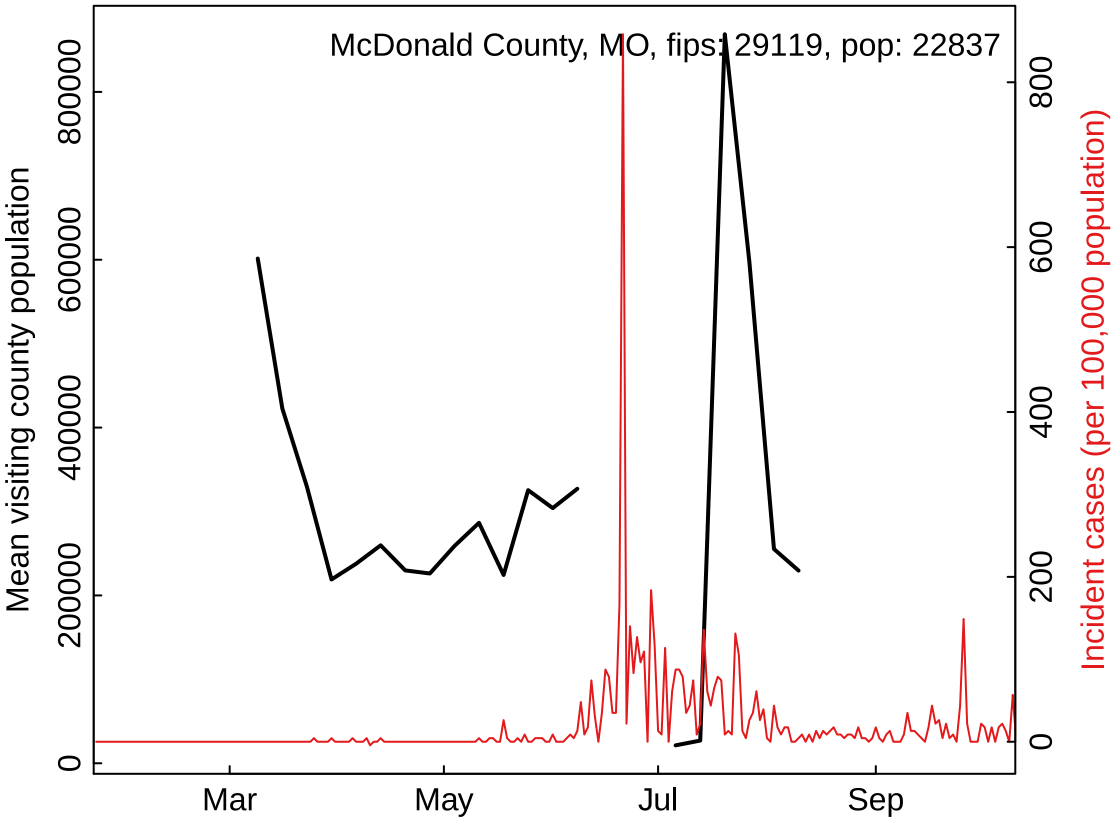
McDonald County, MO, fips: 29119, pop: 22837
Mean visiting county population
0
200000
400000
600000
800000
Incident cases (per 100,000 population)
0
200
400
600
800
Mar
May
Jul
Sep

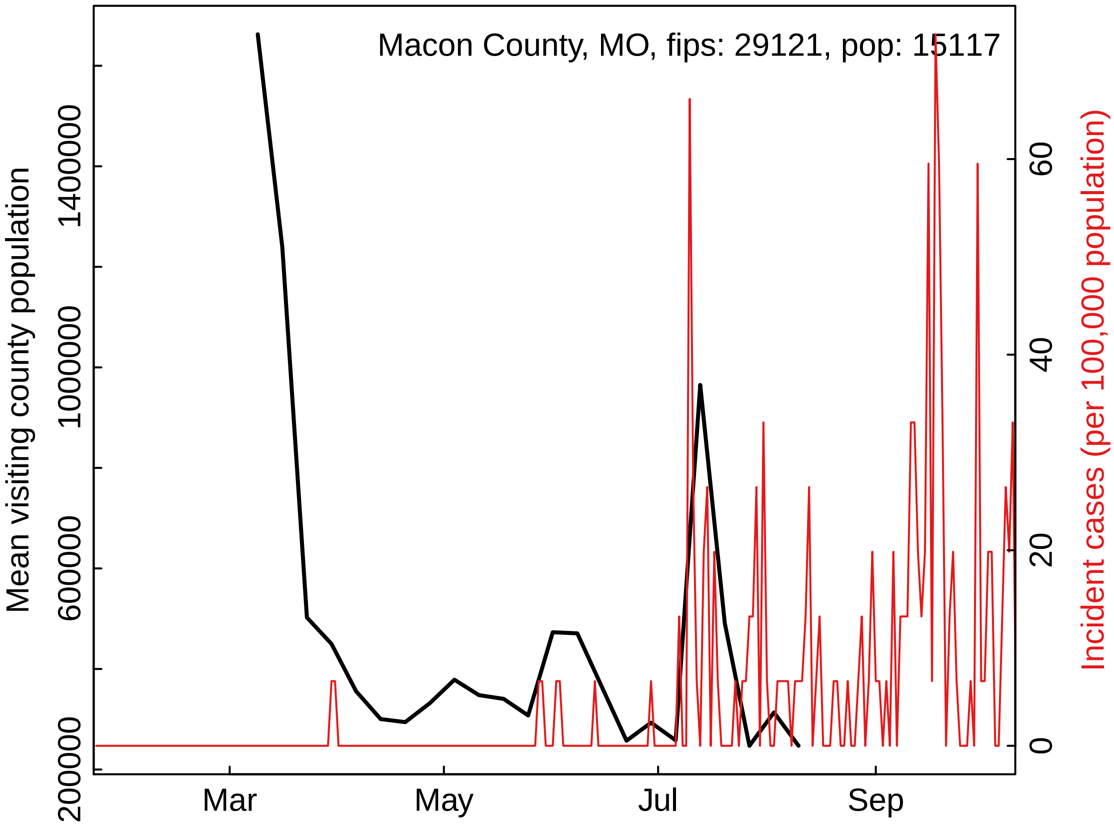

Macon County, MO, fips: 29121, pop: 15117
Mean visiting county population
200000
600000
1000000
1400000
Incident cases (per 100,000 population)
0
20
40
60
Mar
May
Jul
Sep

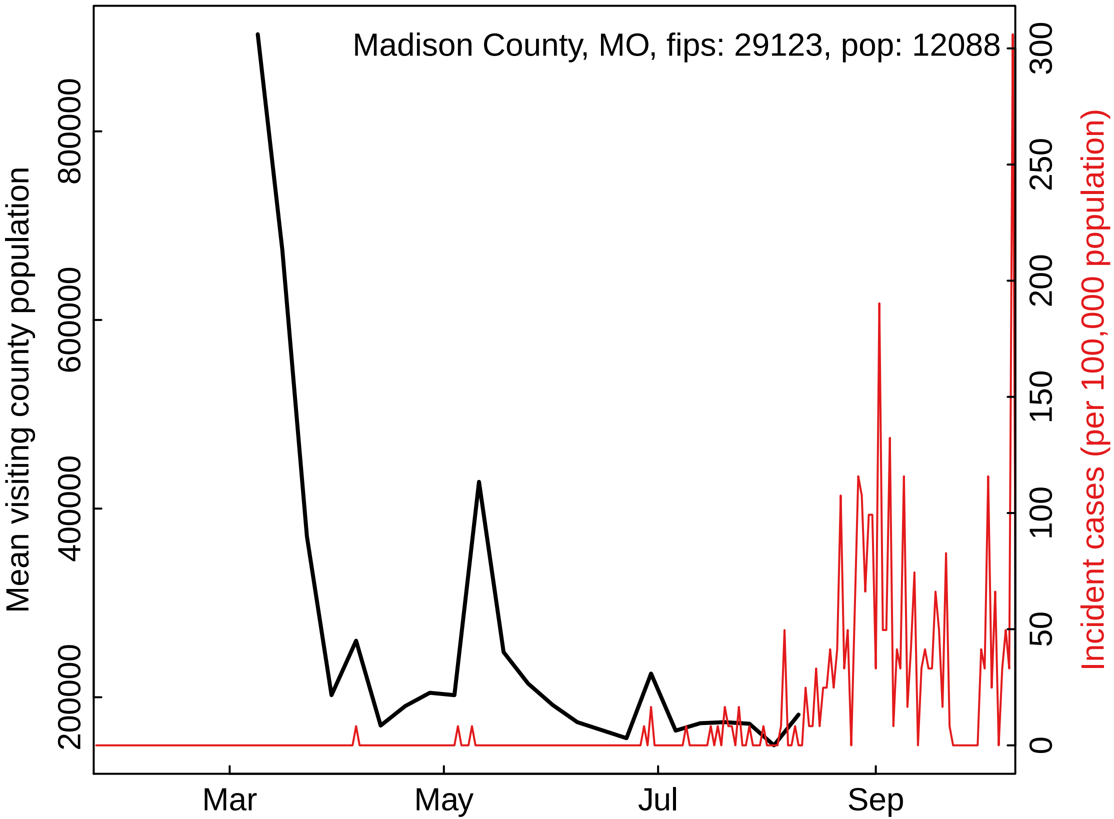

Madison County, MO, fips: 29123, pop: 12088
Mean visiting county population
200000
400000
600000
800000
Incident cases (per 100,000 population)
0
50
100
150
200
250
300
Mar
May
Jul
Sep

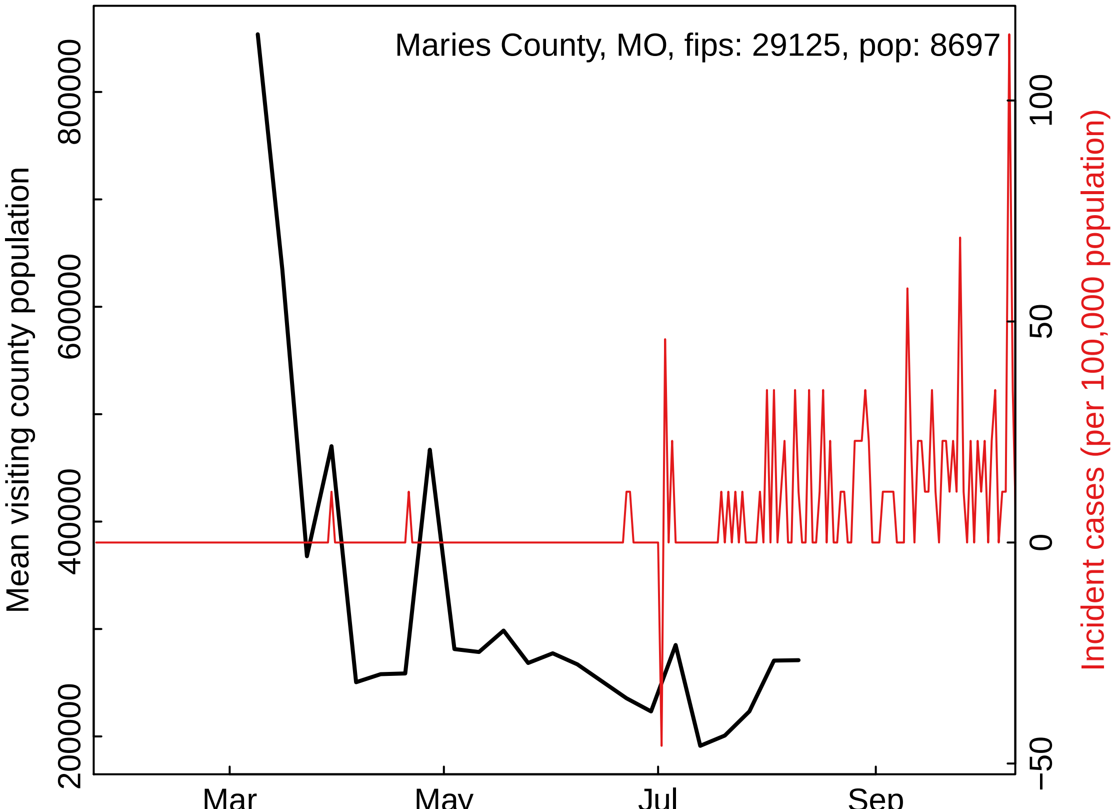

Maries County, MO, fips: 29125, pop: 8697
Mean visiting county population
200000
400000
600000
800000
Incident cases (per 100,000 population)
−50
0
50
100
Mar
May
Jul
Sep

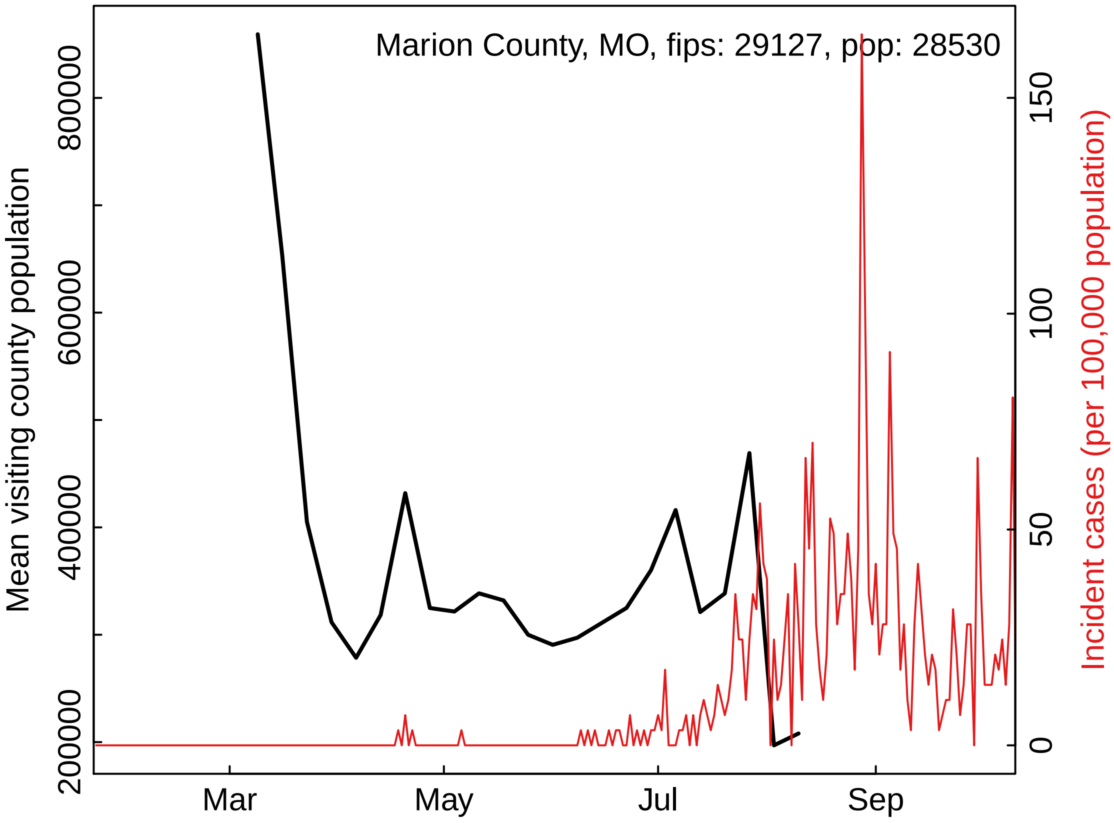
Marion County, MO, fips: 29127, pop: 28530
Mean visiting county population
200000
400000
600000
800000
Incident cases (per 100,000 population)
0
50
100
150
Mar
May
Jul
Sep

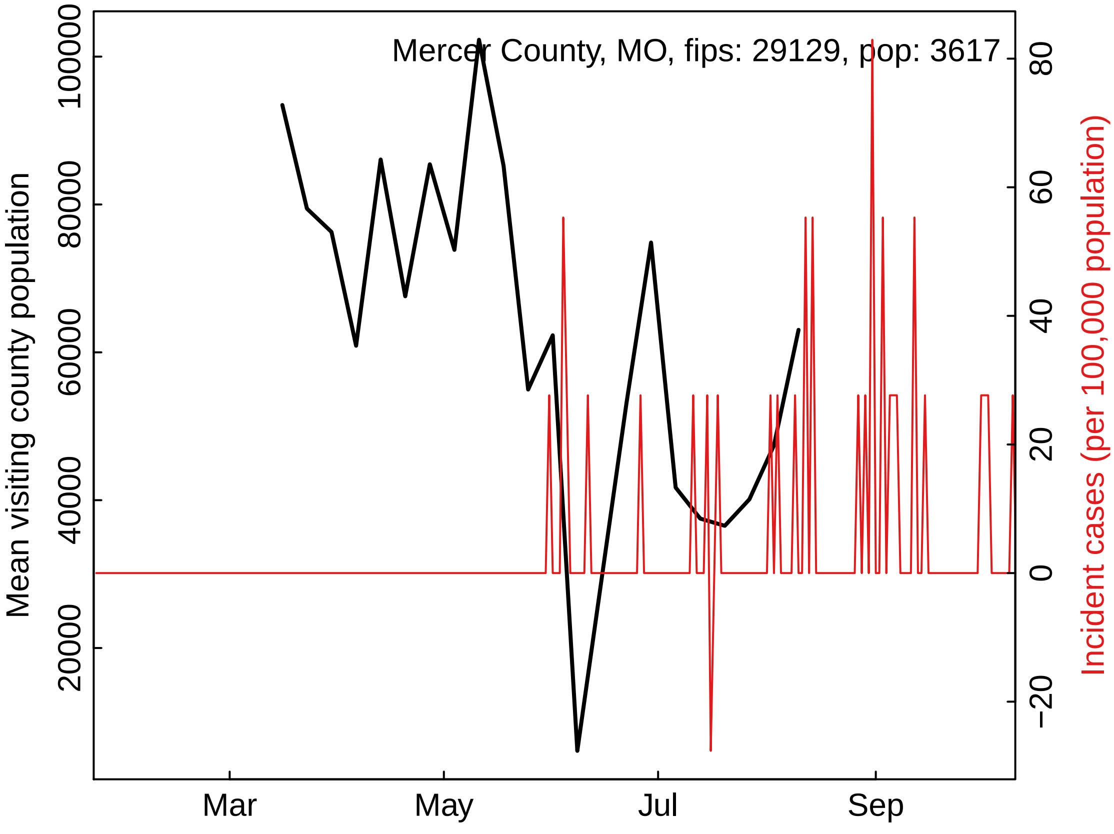

Mercer County, MO, fips: 29129, pop: 3617
Mean visiting county population
20000
40000
60000
80000
100000
Incident cases (per 100,000 population)
−20
0
20
40
60
80
Mar
May
Jul
Sep

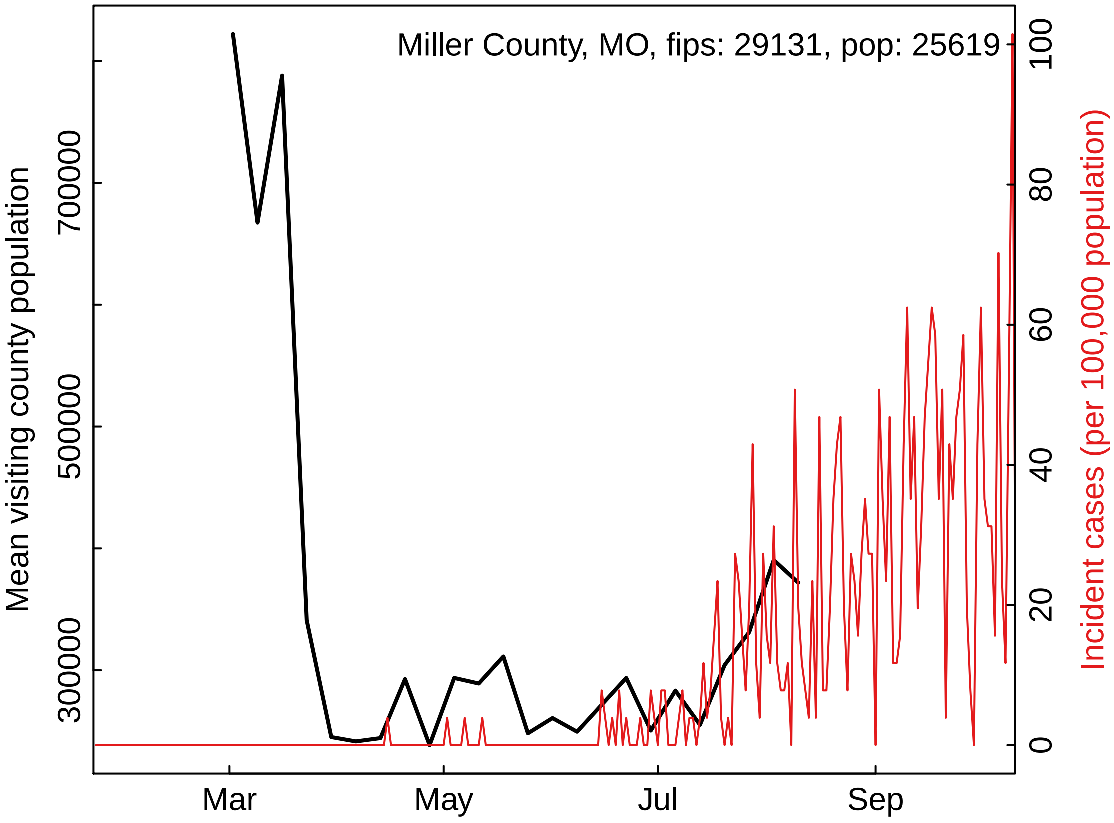
Miller County, MO, fips: 29131, pop: 25619
Mean visiting county population
300000
500000
700000
Mar
May
Jul
Sep
0
20
40
60
80
100
Incident cases (per 100,000 population)

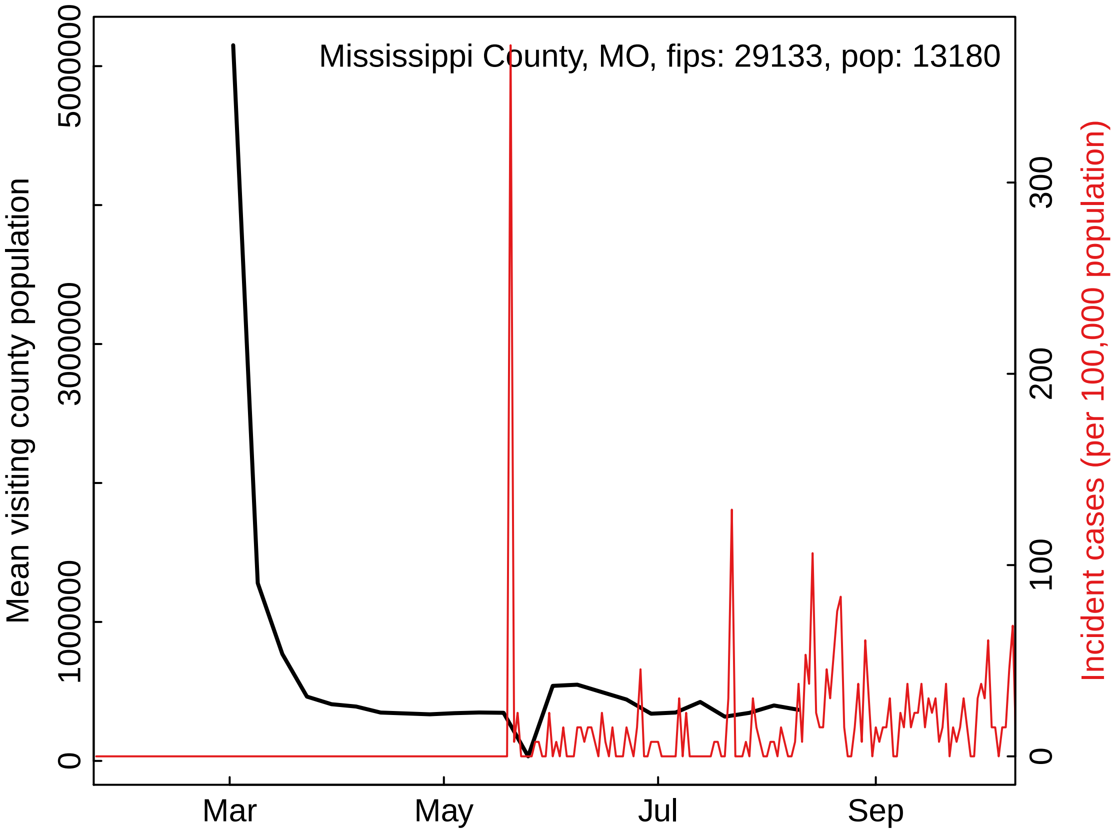

Mississippi County, MO, fips: 29133, pop: 13180
Mean visiting county population
0
1000000
3000000
5000000
Incident cases (per 100,000 population)
0
100
200
300
Mar
May
Jul
Sep

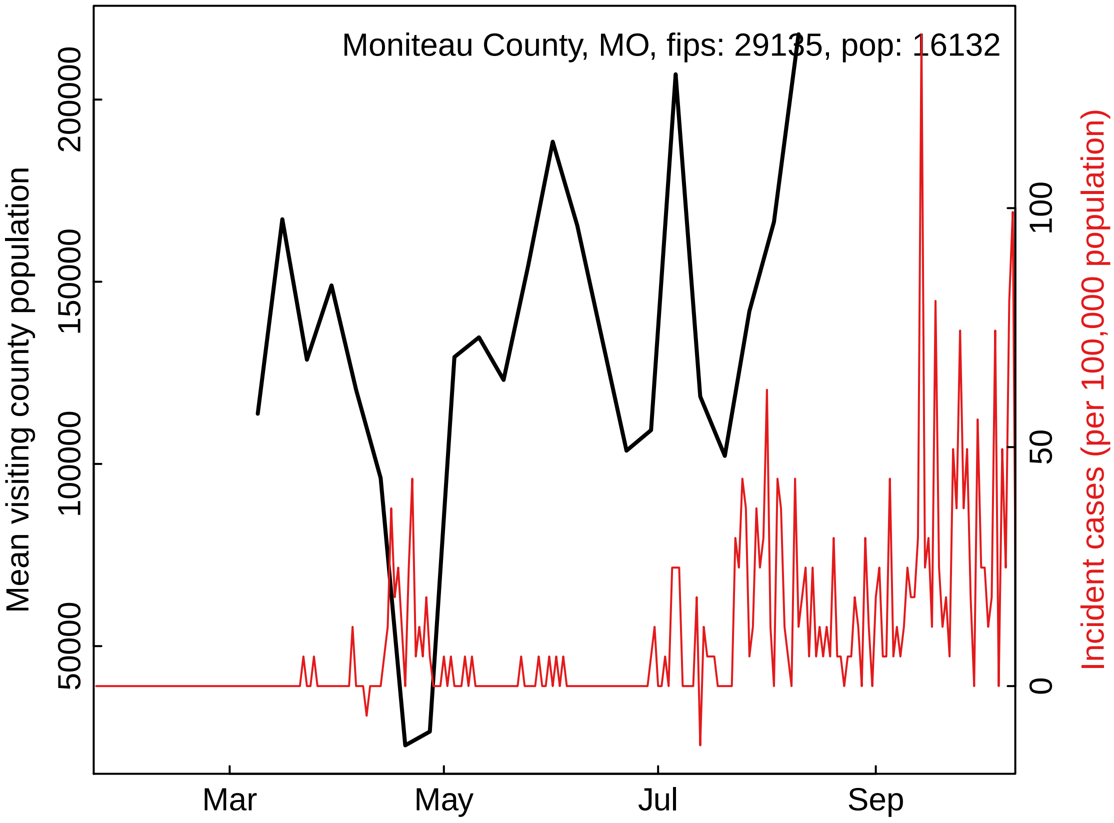

Moniteau County, MO, fips: 29135, pop: 16132
Mean visiting county population
50000
100000
150000
200000
Incident cases (per 100,000 population)
0
50
100
Mar
May
Jul
Sep

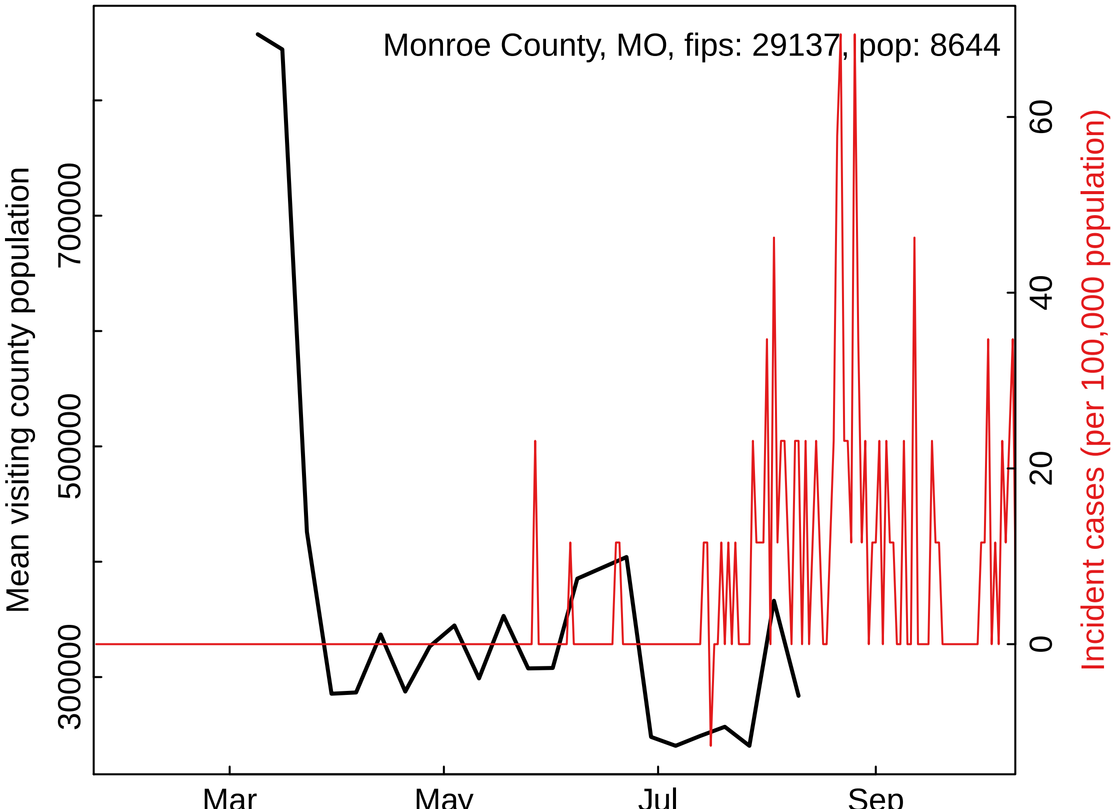

Monroe County, MO, fips: 29137, pop: 8644
Mean visiting county population
300000
500000
700000
Incident cases (per 100,000 population)
0
20
40
60
Mar
May
Jul
Sep

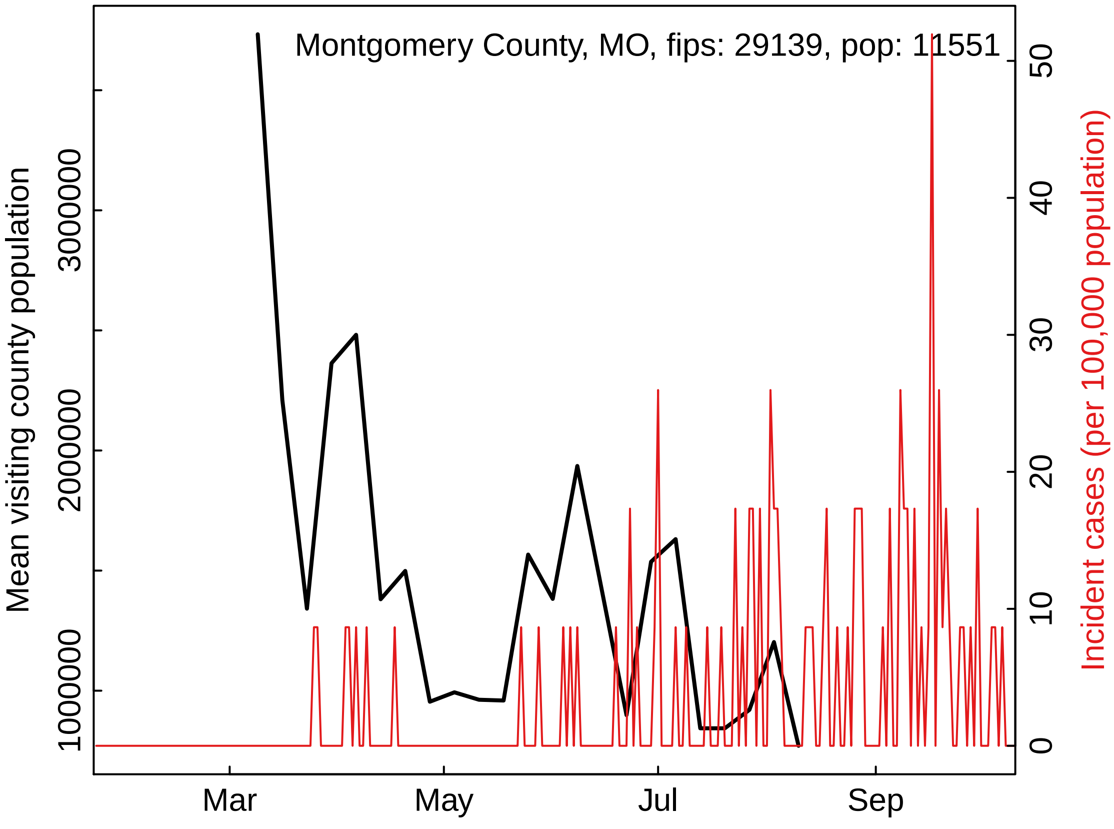
Montgomery County, MO, fips: 29139, pop: 11551
Mean visiting county population
1000000
2000000
3000000
Incident cases (per 100,000 population)
0
10
20
30
40
50
Mar
May
Jul
Sep

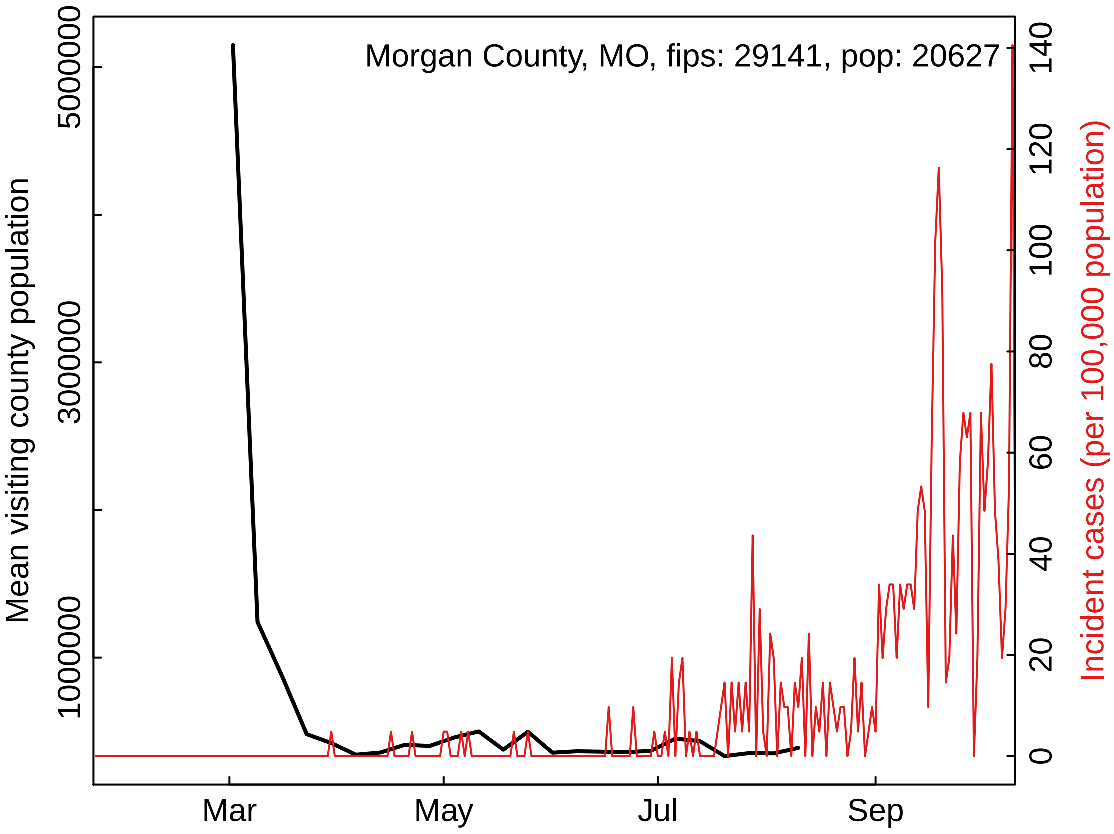
Morgan County, MO, fips: 29141, pop: 20627
Mean visiting county population
1000000
3000000
5000000
Incident cases (per 100,000 population)
0
20
40
60
80
100
120
140
Mar
May
Jul
Sep

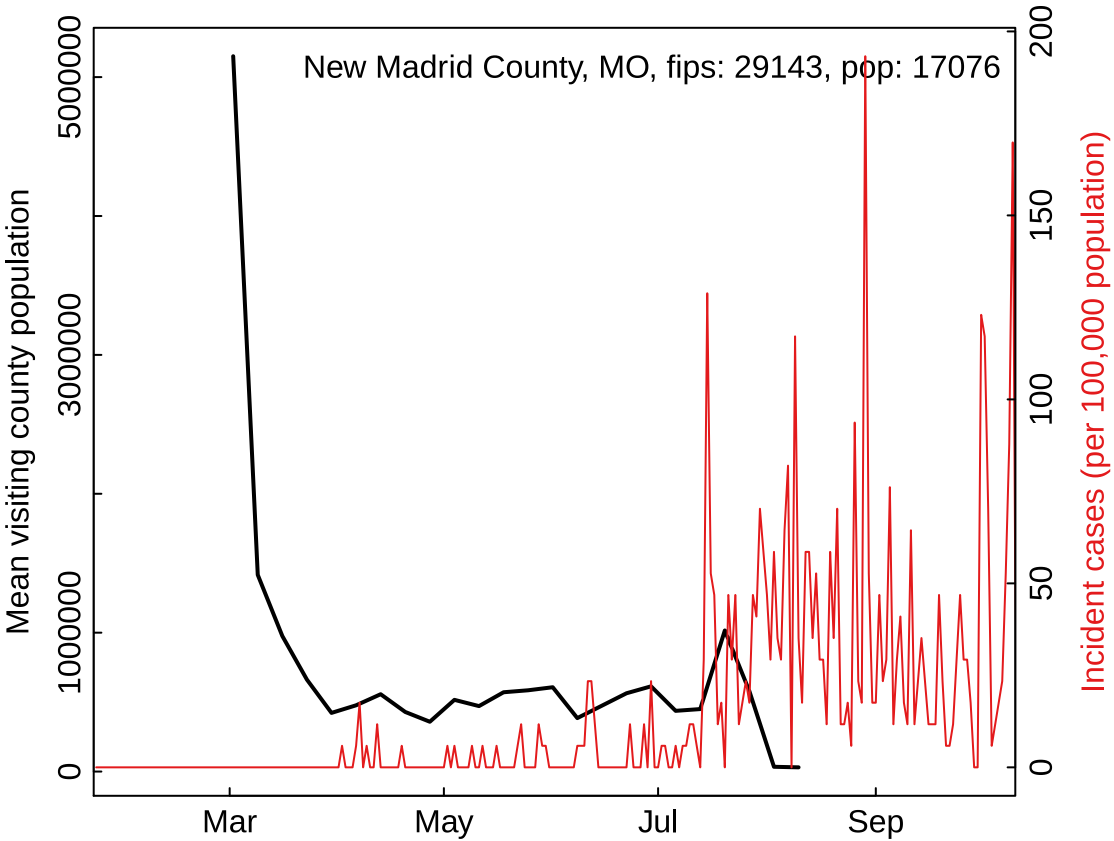

New Madrid County, MO, fips: 29143, pop: 17076
Mean visiting county population
Incident cases (per 100,000 population)
0
1000000
3000000
5000000
0
50
100
150
200
Mar
May
Jul
Sep

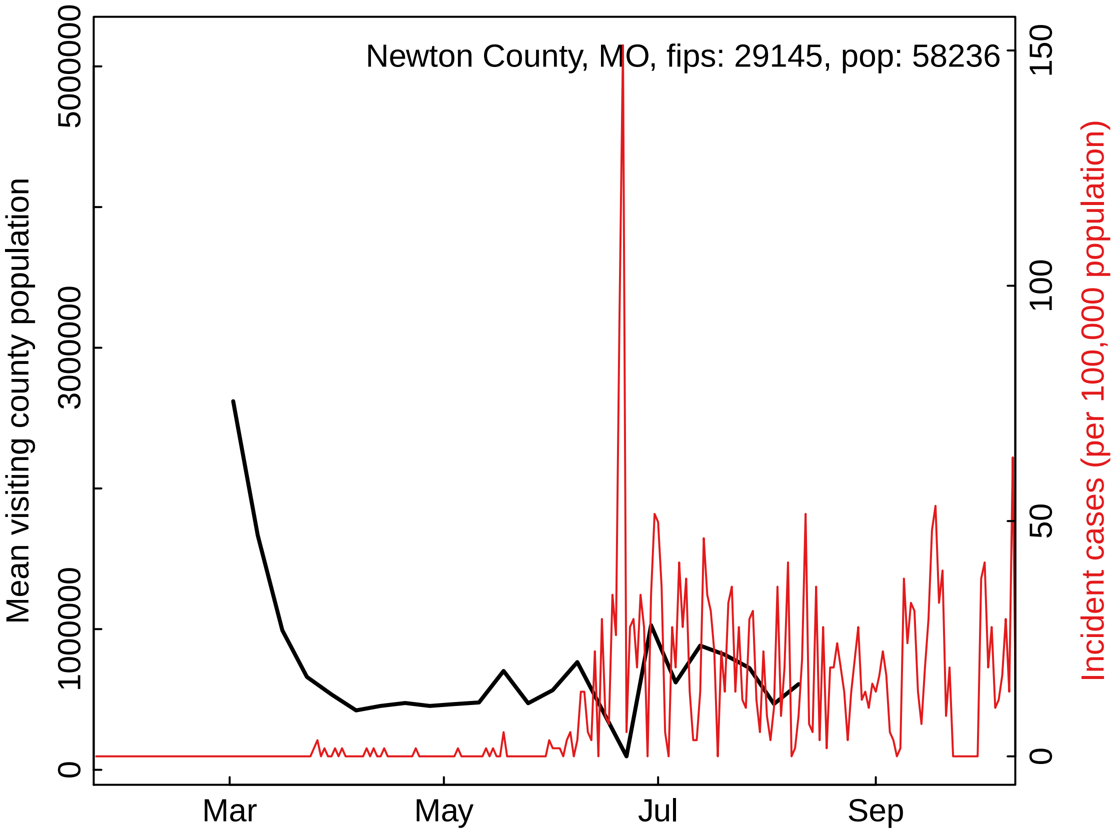

Newton County, MO, fips: 29145, pop: 58236
Mean visiting county population
0
1000000
3000000
5000000
Incident cases (per 100,000 population)
0
50
100
150
Mar
May
Jul
Sep

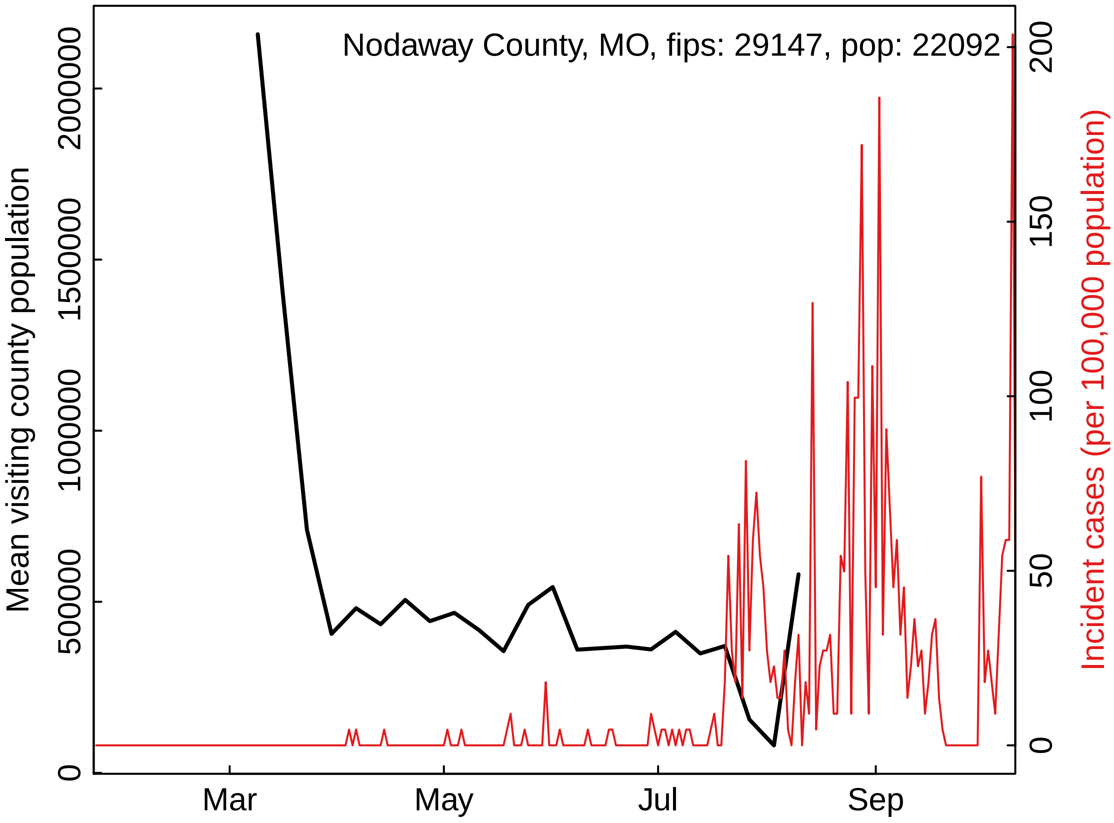

Nodaway County, MO, fips: 29147, pop: 22092
Mean visiting county population
0
500000
1000000
1500000
2000000
Incident cases (per 100,000 population)
0
50
100
150
200
Mar
May
Jul
Sep

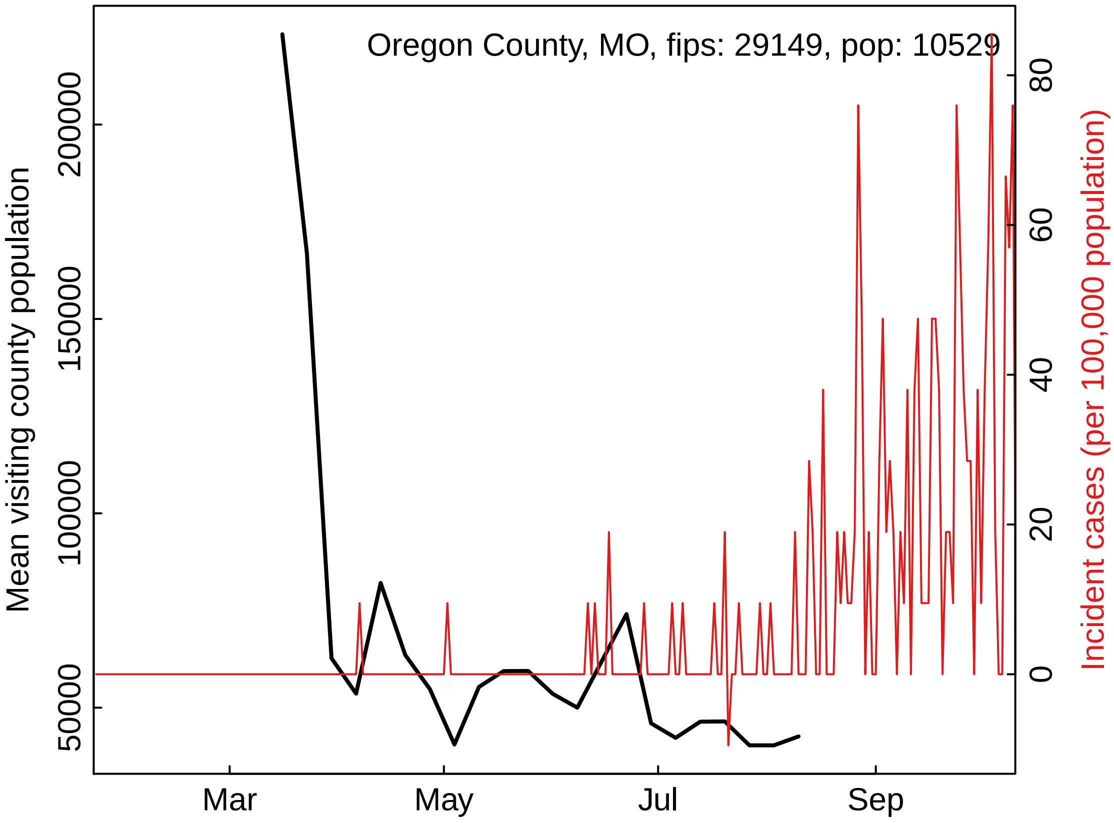

Oregon County, MO, fips: 29149, pop: 10529
Mean visiting county population
50000
100000
150000
200000
Incident cases (per 100,000 population)
0
20
40
60
80
Mar
May
Jul
Sep

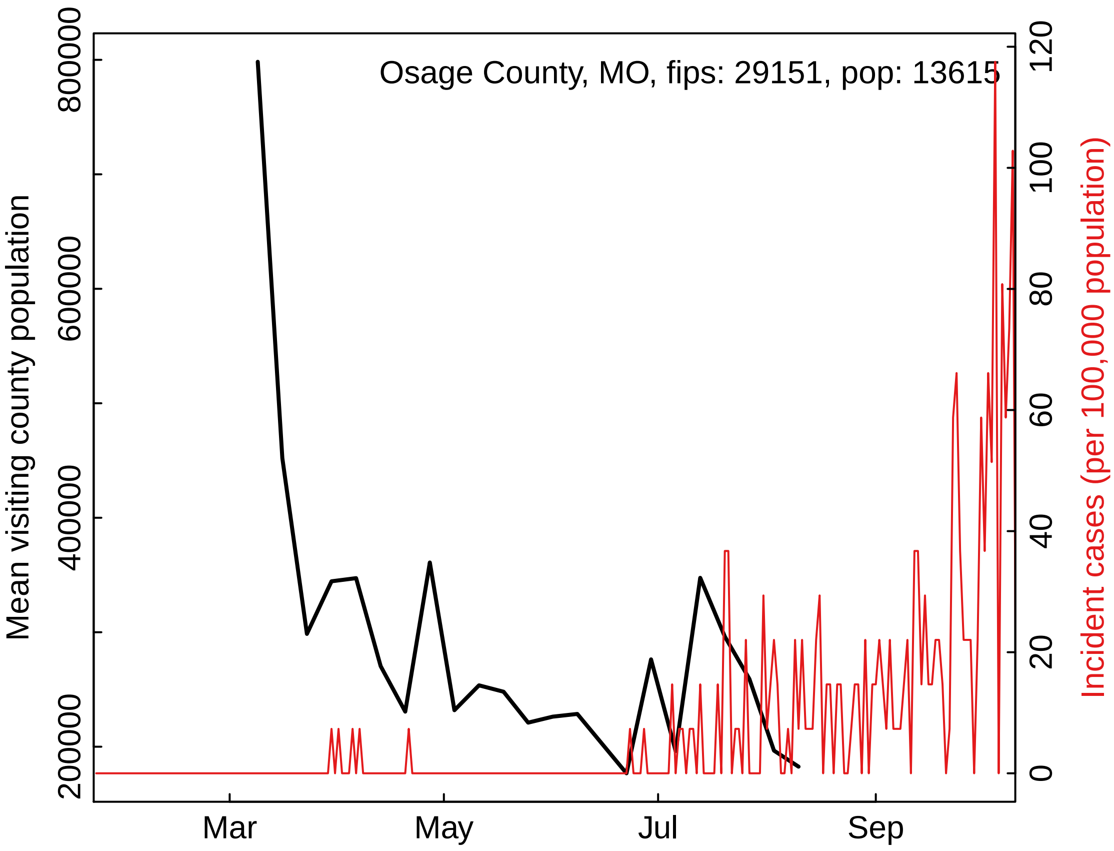

Osage County, MO, fips: 29151, pop: 13615
Mean visiting county population
Incident cases (per 100,000 population)
200000
400000
600000
800000
0
20
40
60
80
100
120
Mar
May
Jul
Sep

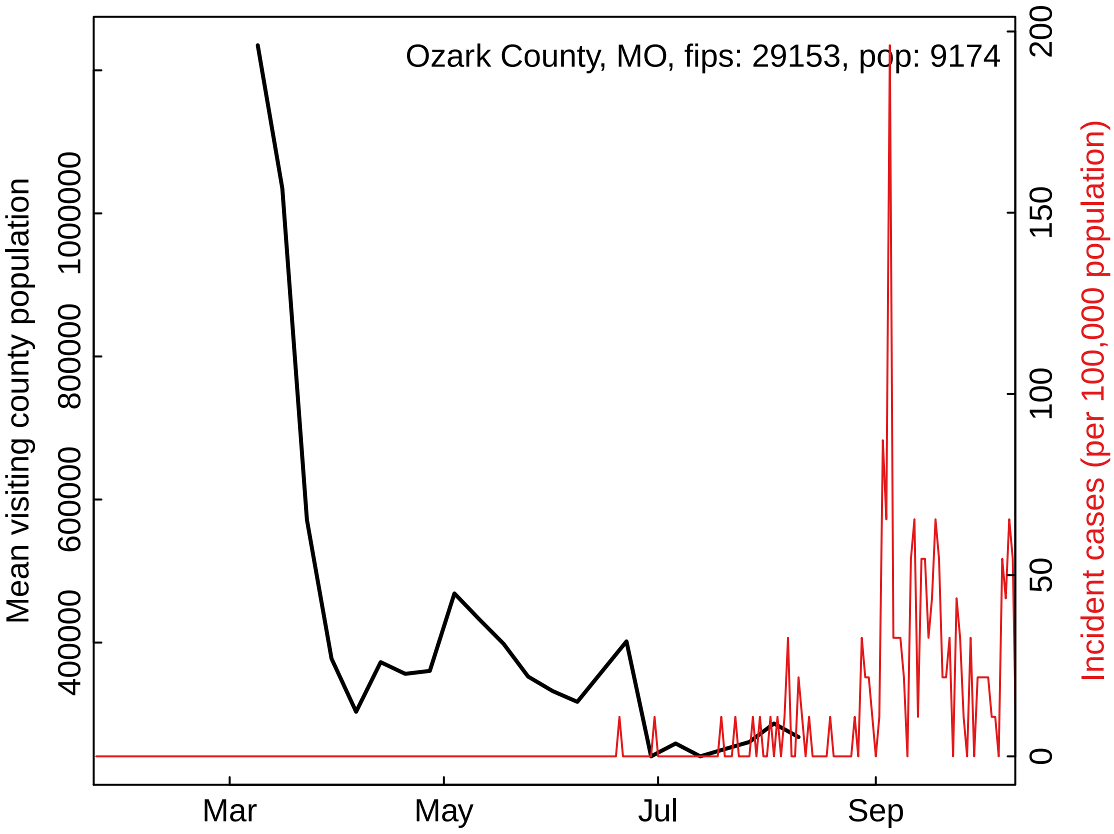

Ozark County, MO, fips: 29153, pop: 9174
Mean visiting county population
400000
600000
800000
1000000
Incident cases (per 100,000 population)
0
50
100
150
200
Mar
May
Jul
Sep

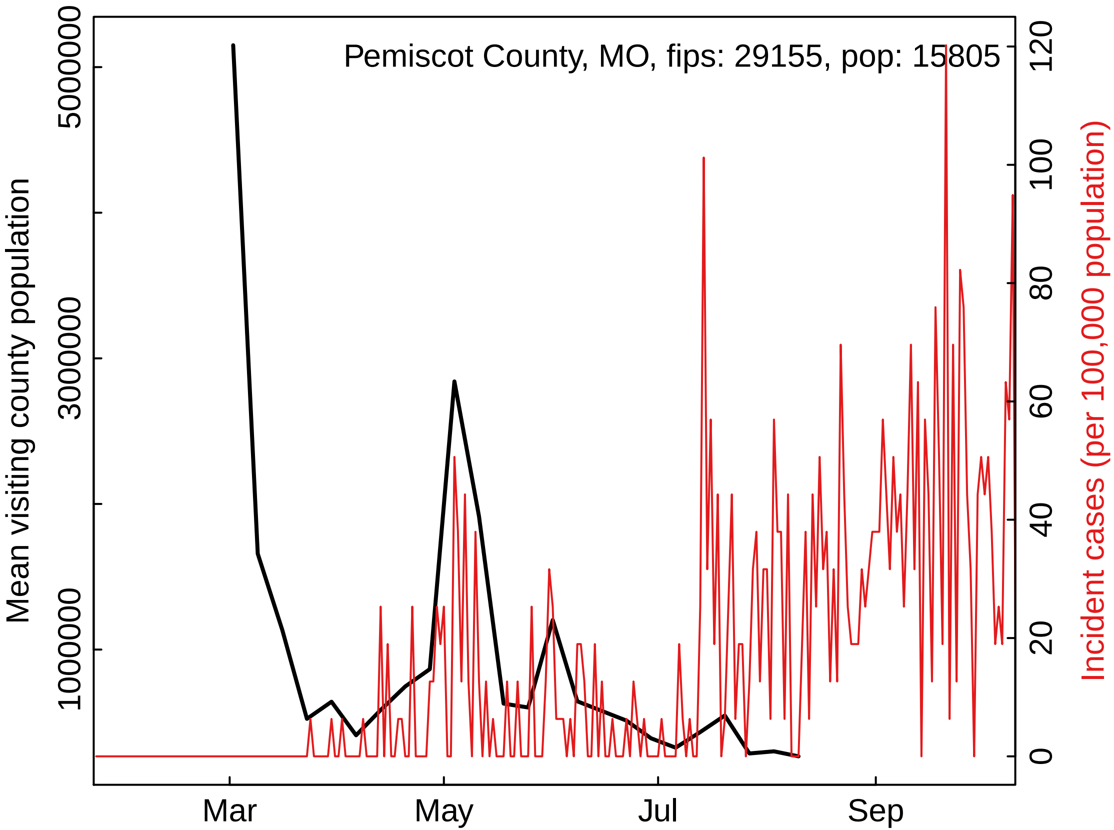
Pemiscot County, MO, fips: 29155, pop: 15805
Mean visiting county population
10000000
30000000
50000000
Incident cases (per 100,000 population)
0
20
40
60
80
100
120
Mar
May
Jul
Sep

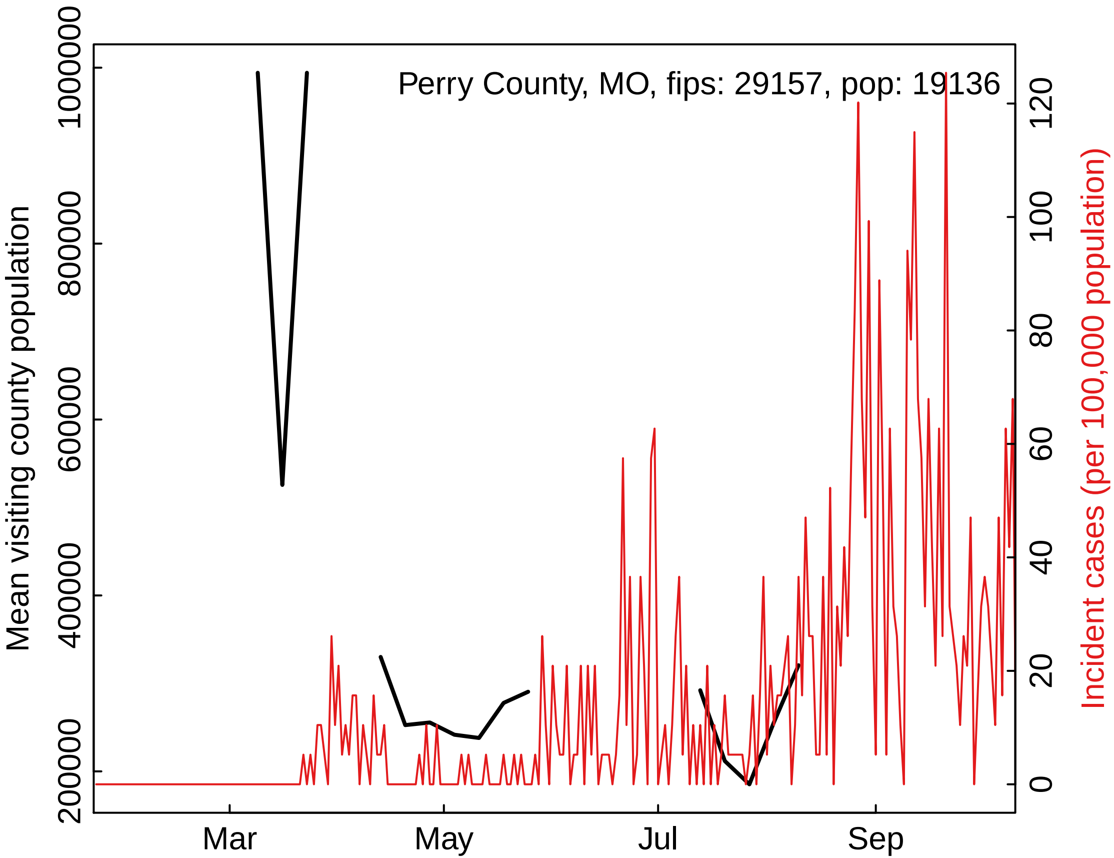

Perry County, MO, fips: 29157, pop: 19136
Mean visiting county population
200000
400000
600000
800000
1000000
Incident cases (per 100,000 population)
0
20
40
60
80
100
120
Mar
May
Jul
Sep

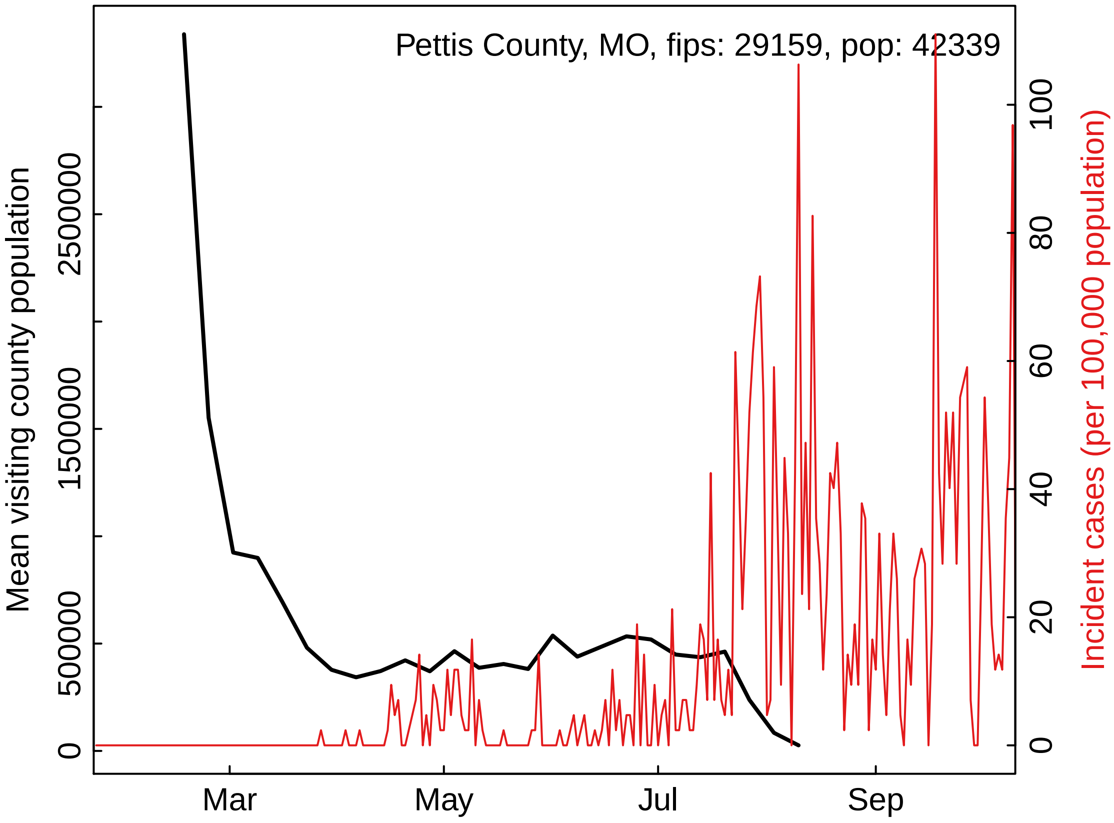

Pettis County, MO, fips: 29159, pop: 42339
Mean visiting county population
0
500000
1500000
2500000
Mar
May
Jul
Sep
0
20
40
60
80
100
Incident cases (per 100,000 population)

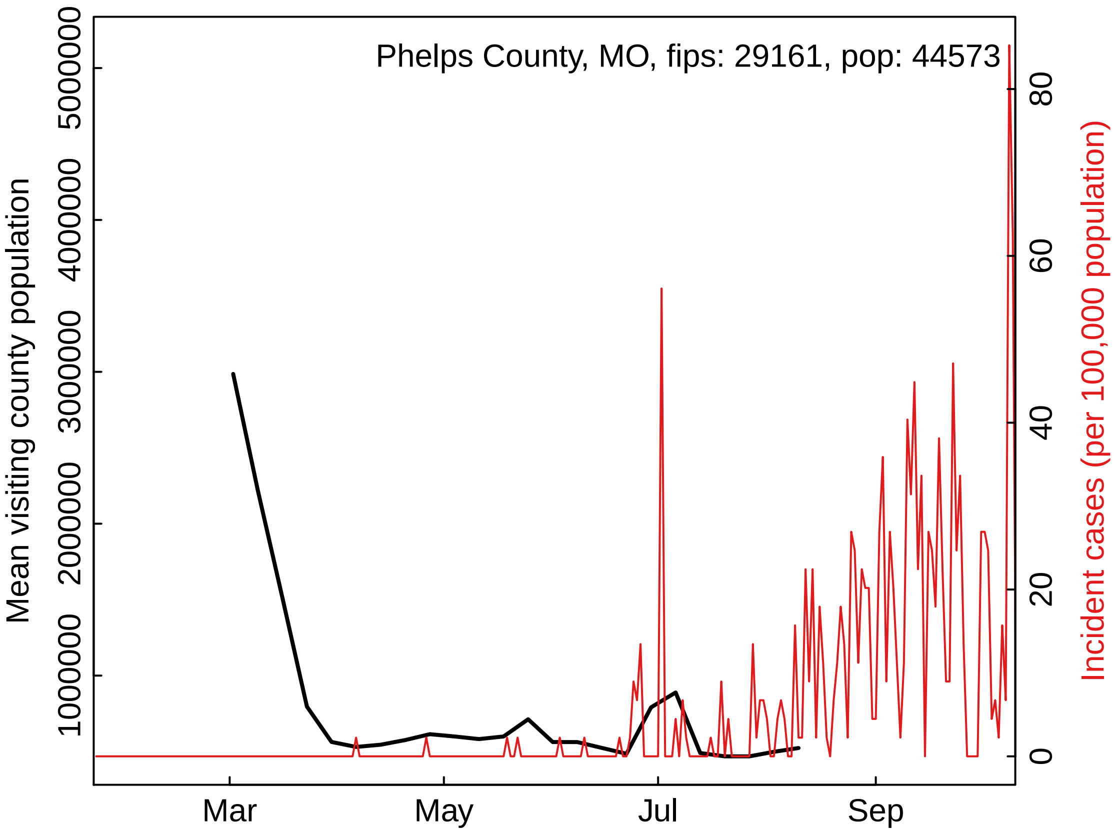

Phelps County, MO, fips: 29161, pop: 44573
Mean visiting county population
1000000
2000000
3000000
4000000
5000000
Incident cases (per 100,000 population)
0
20
40
60
80
Mar
May
Jul
Sep

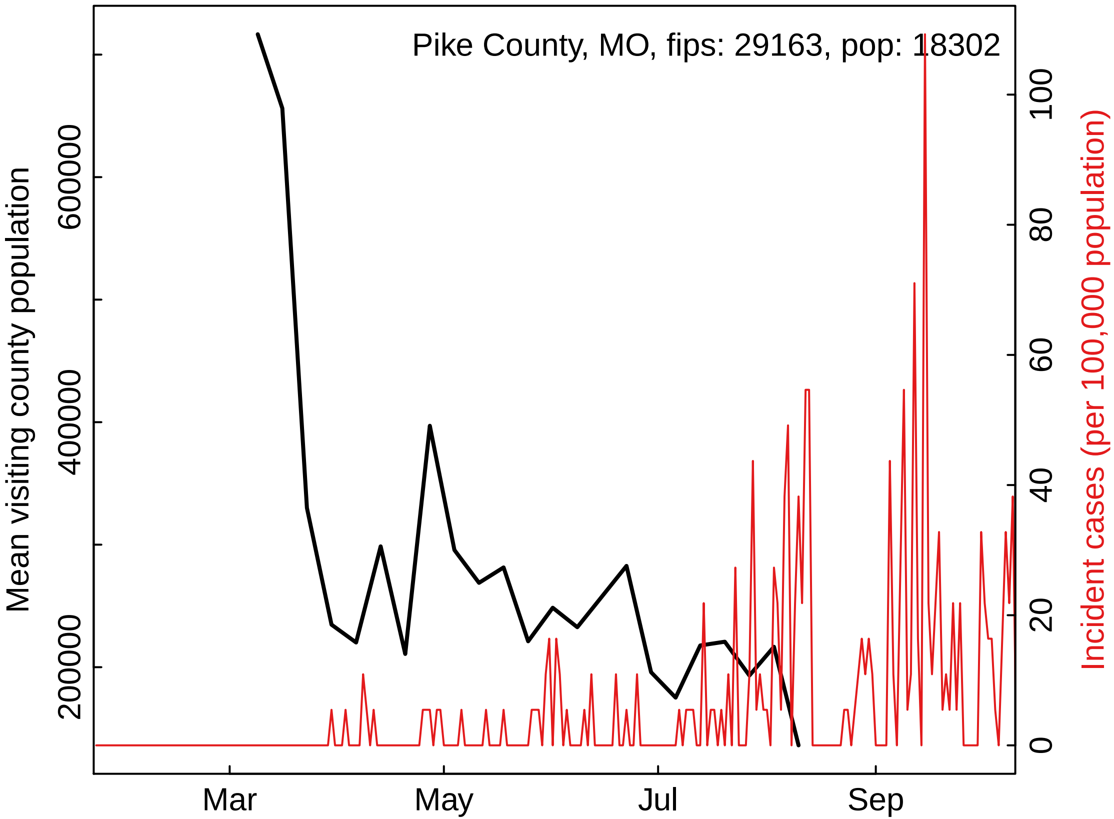

Pike County, MO, fips: 29163, pop: 18302
Mean visiting county population
200000
400000
600000
Incident cases (per 100,000 population)
0
20
40
60
80
100
Mar
May
Jul
Sep

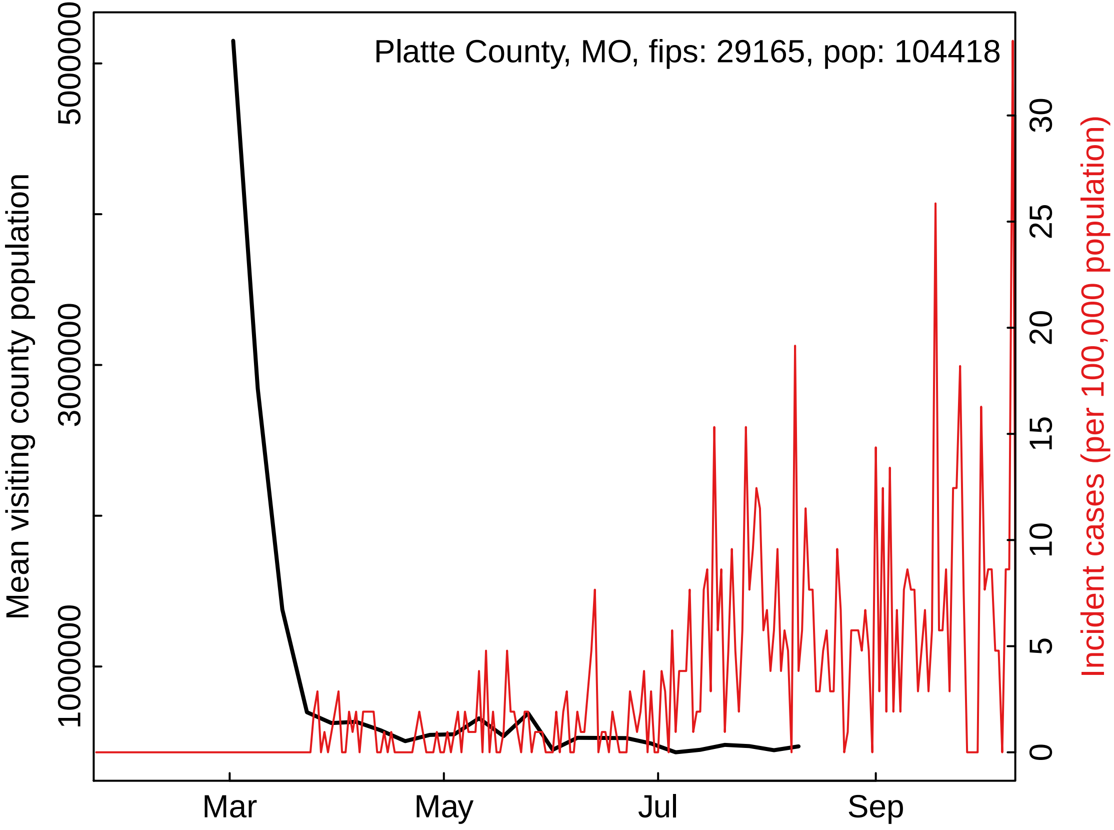
Platte County, MO, fips: 29165, pop: 104418
Mean visiting county population
1000000
3000000
5000000
Incident cases (per 100,000 population)
0
5
10
15
20
25
30
Mar
May
Jul
Sep

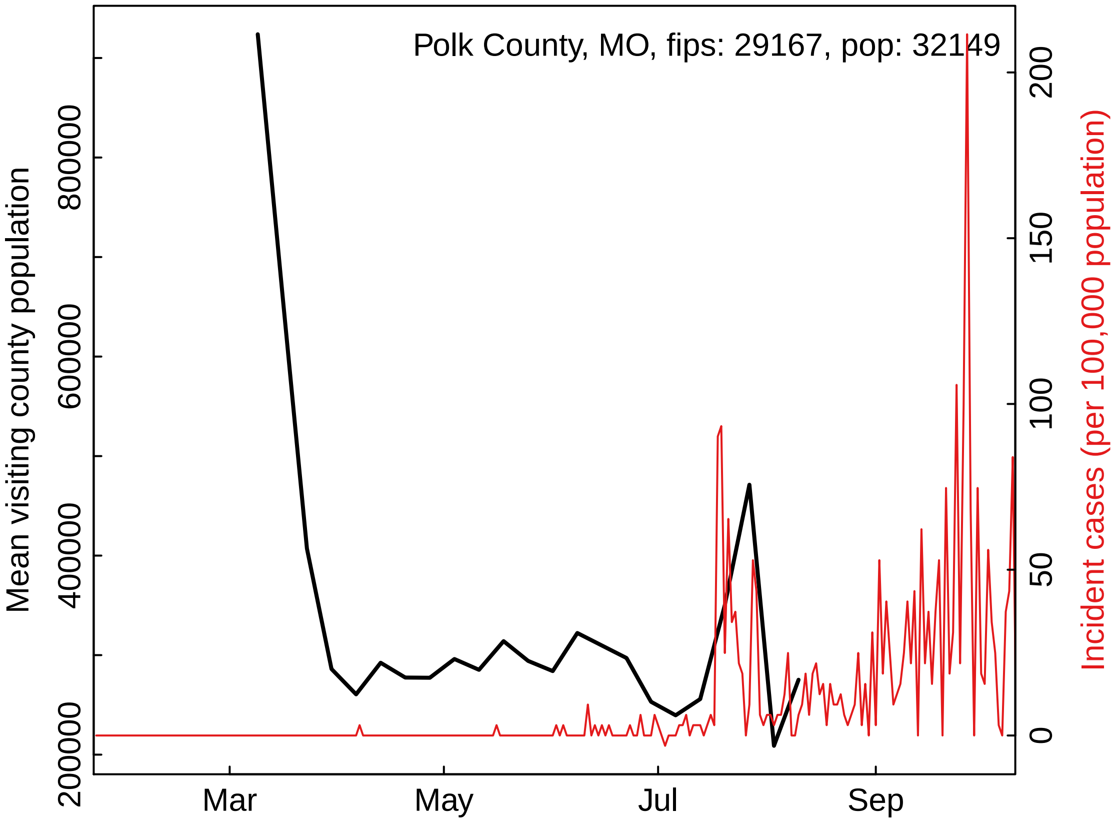
Polk County, MO, fips: 29167, pop: 32149
Mean visiting county population
200000
400000
600000
800000
Incident cases (per 100,000 population)
0
50
100
150
200
Mar
May
Jul
Sep

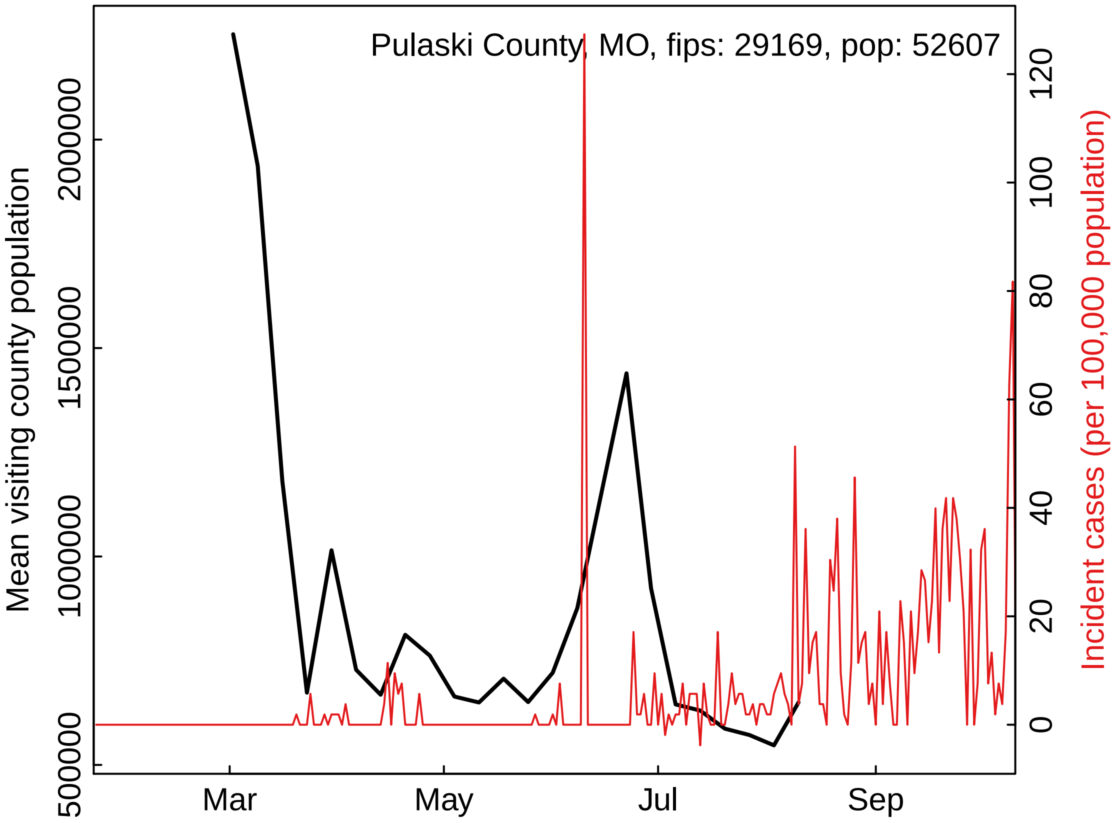
Pulaski County, MO, fips: 29169, pop: 52607
Mean visiting county population
5000000
10000000
15000000
20000000
Mar
May
Jul
Sep
Incident cases (per 100,000 population)
0
20
40
60
80
100
120

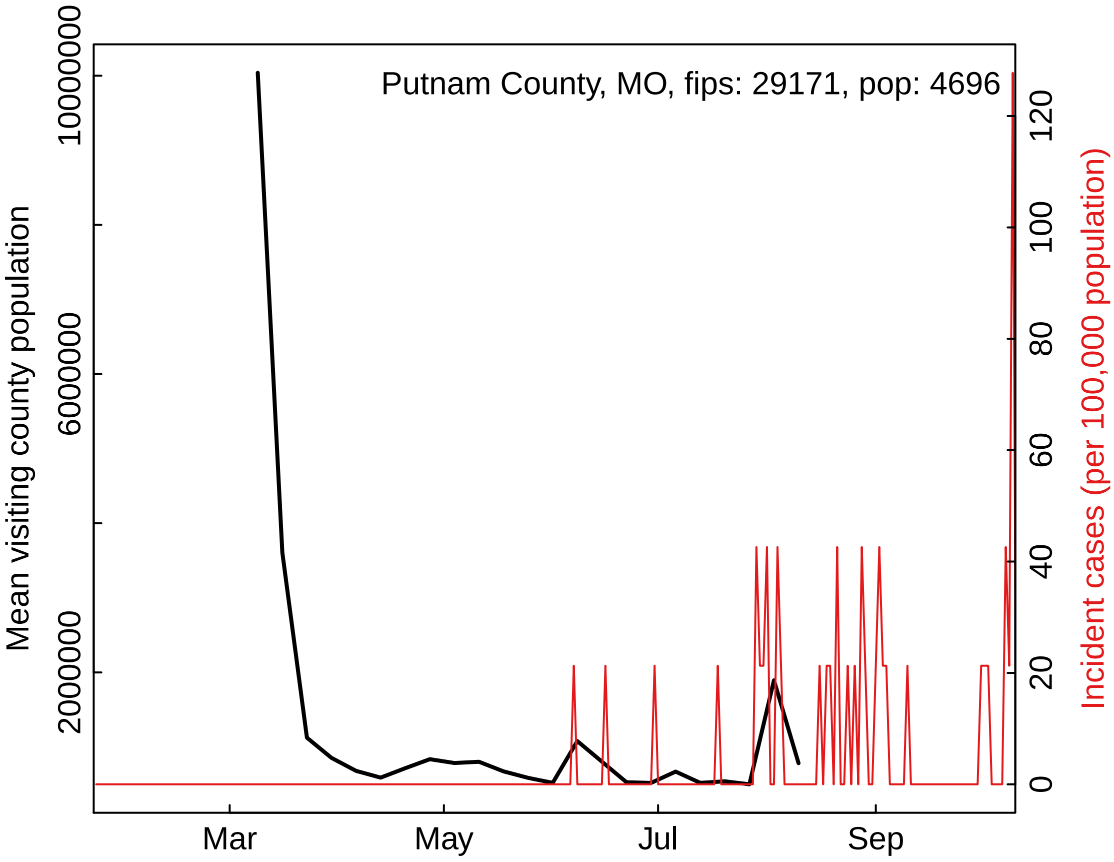

Putnam County, MO, fips: 29171, pop: 4696
Mean visiting county population
10000000
6000000
2000000
Incident cases (per 100,000 population)
0
20
40
60
80
100
120
Mar
May
Jul
Sep

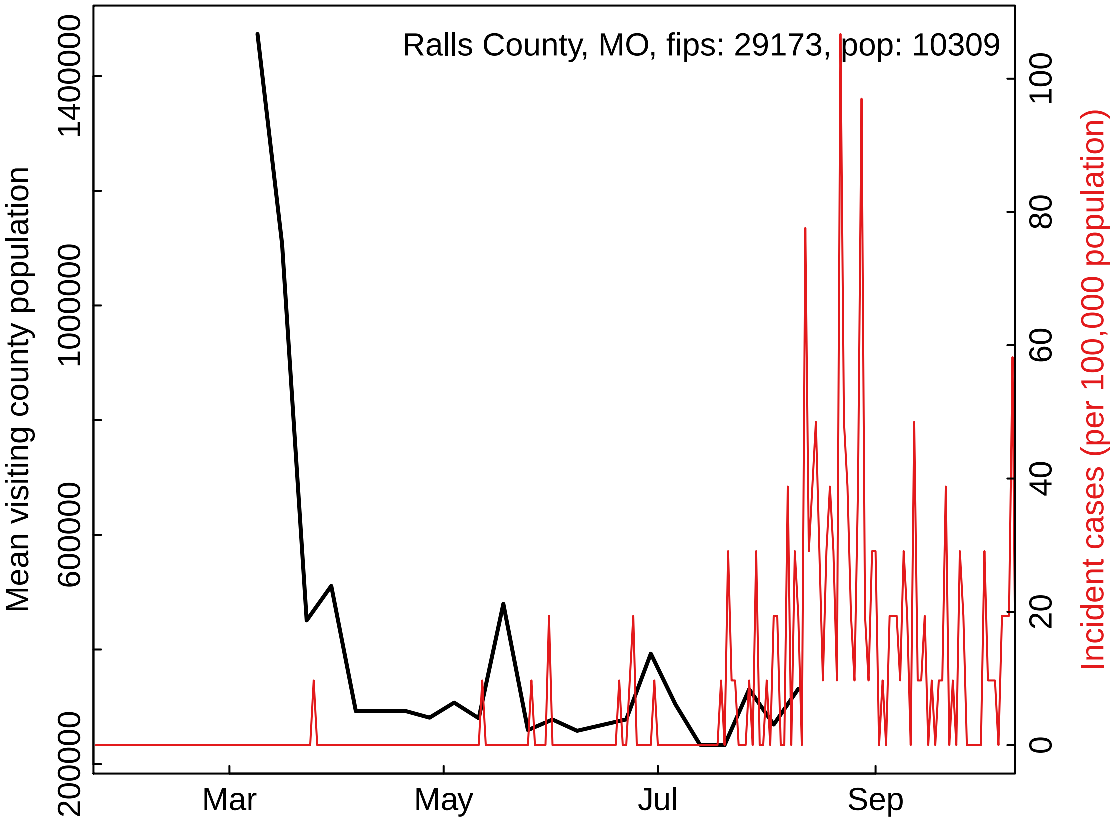
Ralls County, MO, fips: 29173, pop: 10309
Mean visiting county population
200000
600000
1000000
1400000
Incident cases (per 100,000 population)
0
20
40
60
80
100
Mar
May
Jul
Sep

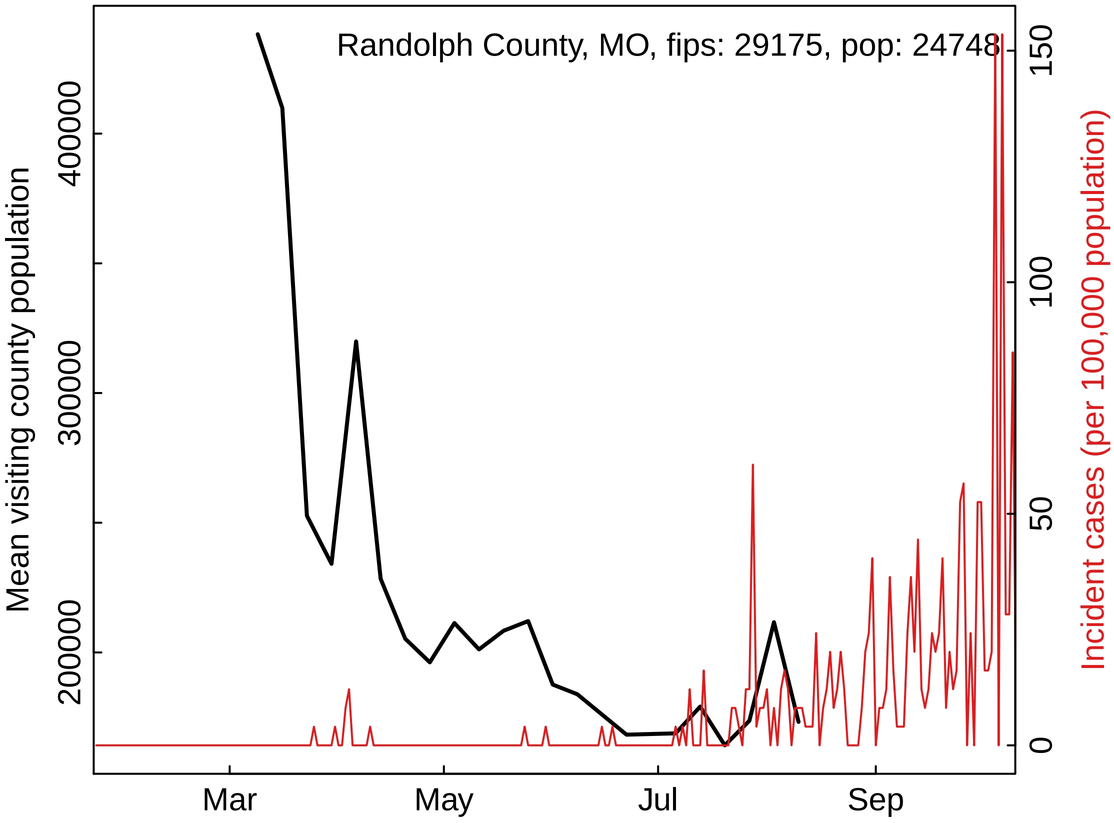
Randolph County, MO, fips: 29175, pop: 24748
Mean visiting county population
200000
300000
400000
Incident cases (per 100,000 population)
0
50
100
150
Mar
May
Jul
Sep

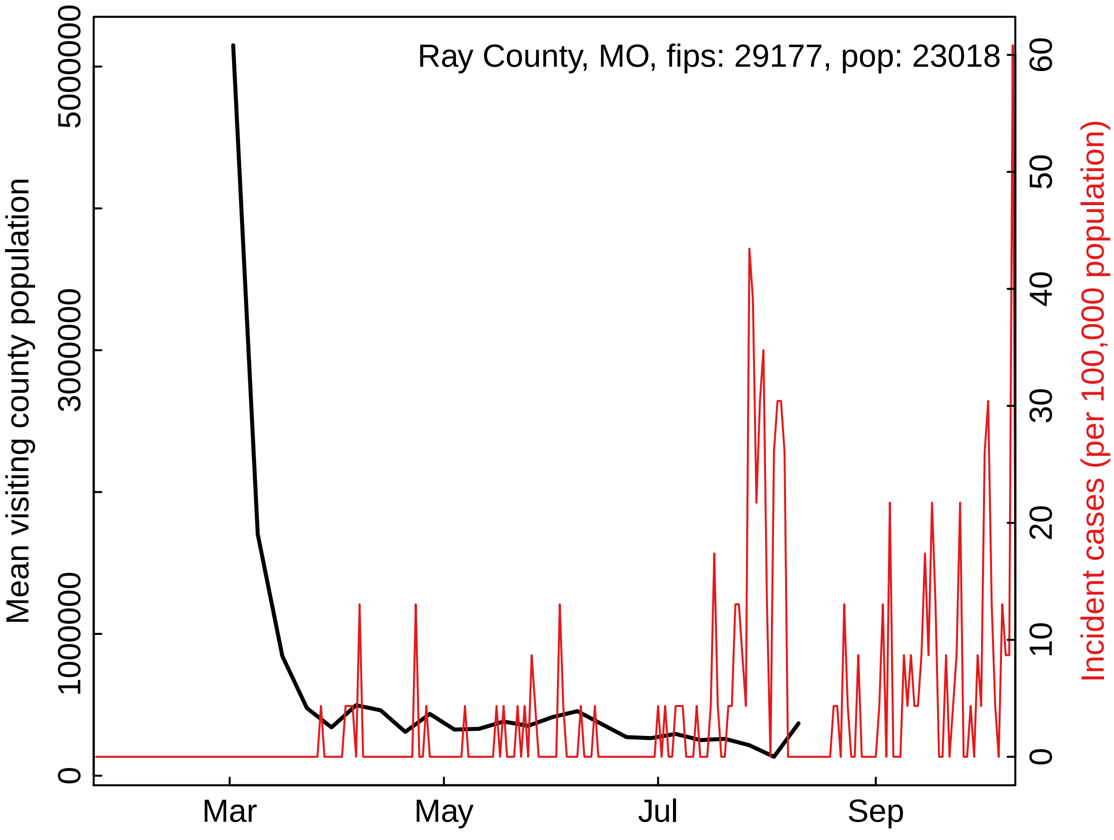

Ray County, MO, fips: 29177, pop: 23018
Mean visiting county population
0
1000000
3000000
5000000
Incident cases (per 100,000 population)
0
10
20
30
40
50
60
Mar
May
Jul
Sep

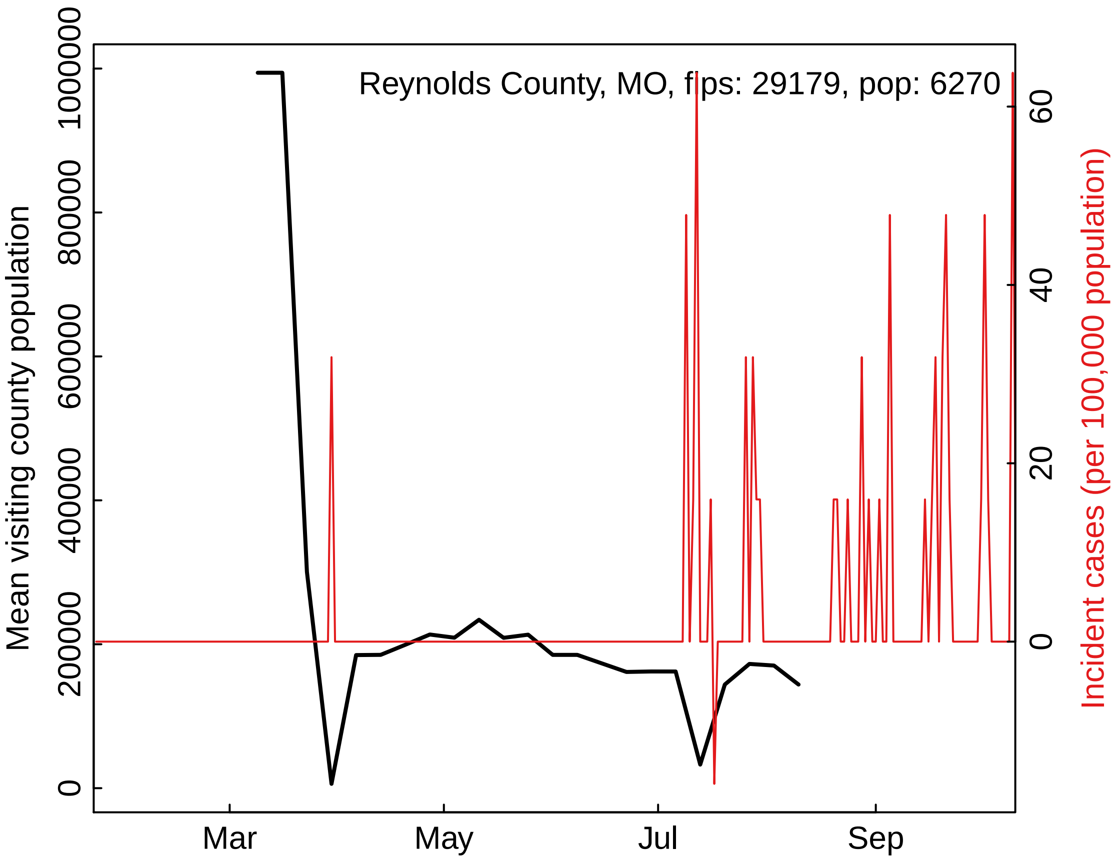

Reynolds County, MO, flips: 29179, pop: 6270
Mean visiting county population
0
200000
400000
600000
800000
1000000
Mar
May
Jul
Sep
0
20
40
60
Incident cases (per 100,000 population)

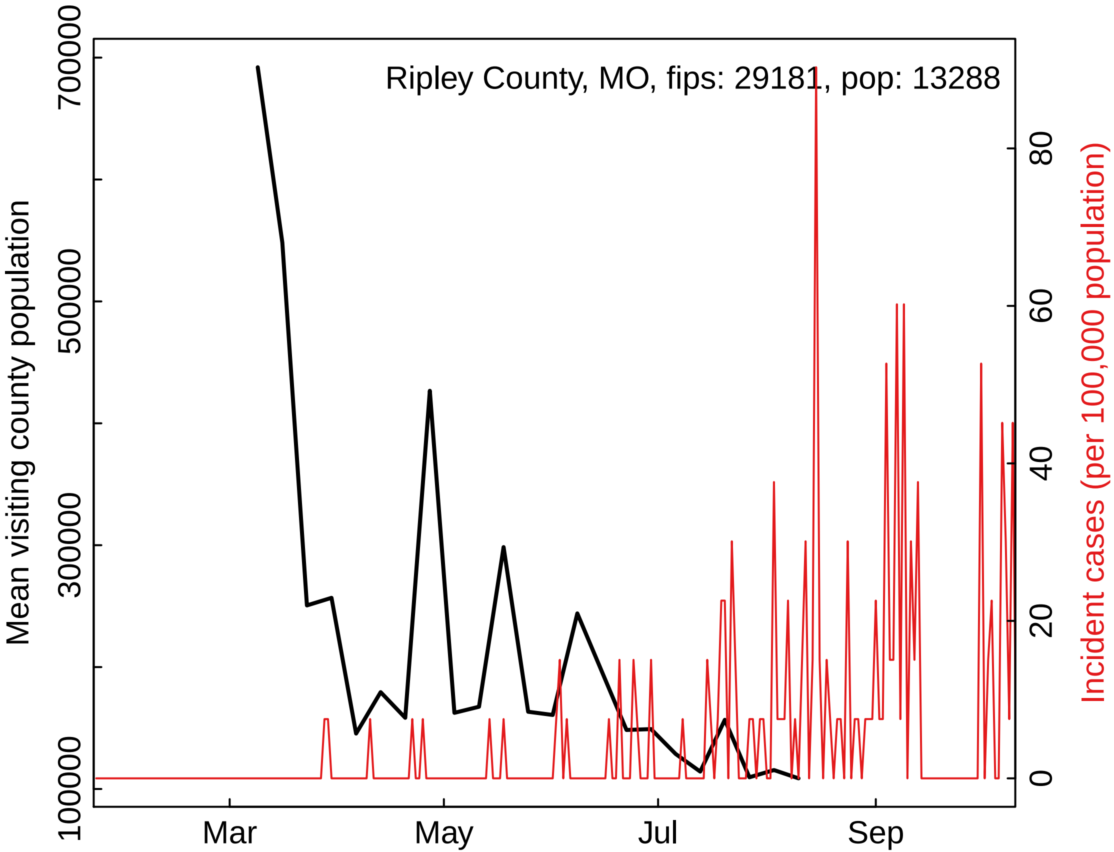

Ripley County, MO, fips: 29181, pop: 13288
Mean visiting county population
100000
300000
500000
700000
Mar
May
Jul
Sep
0
20
40
60
80
Incident cases (per 100,000 population)

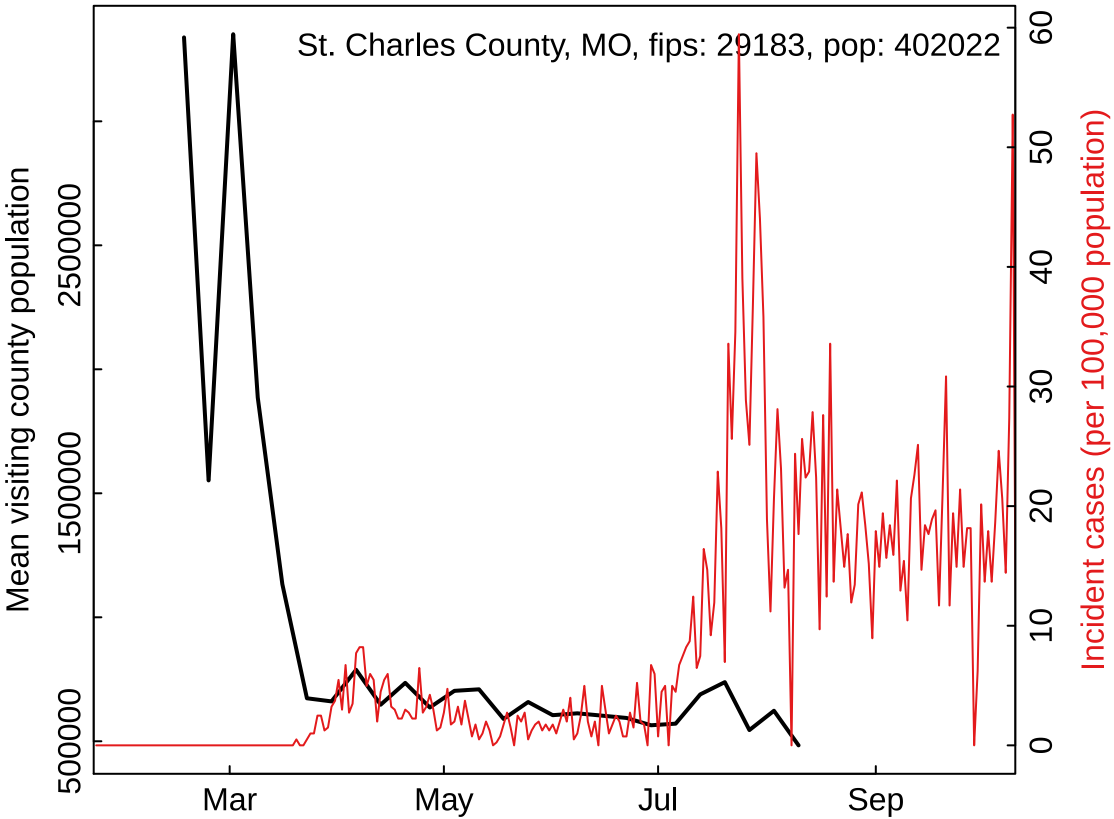
St. Charles County, MO, fips: 29183, pop: 402022
Mean visiting county population
500000
1500000
2500000
Incident cases (per 100,000 population)
0
10
20
30
40
50
60
Mar
May
Jul
Sep

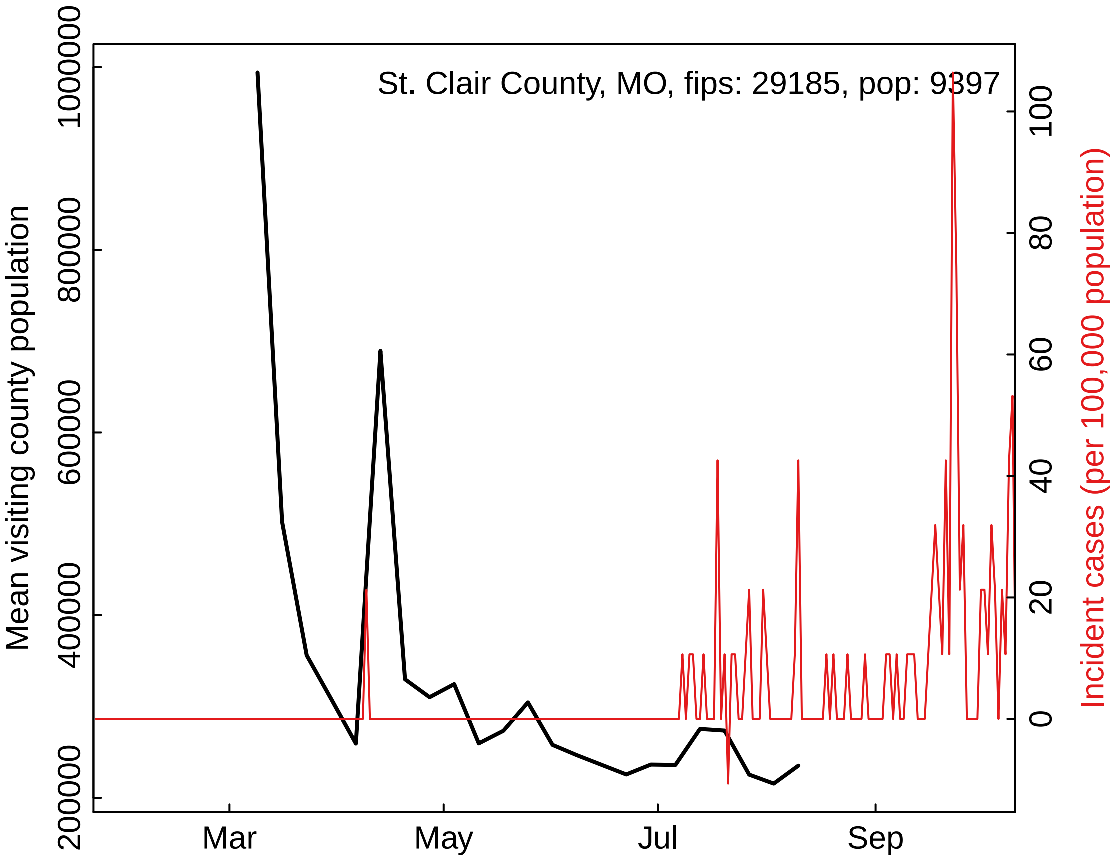
St. Clair County, MO, fips: 29185, pop: 9397
Mean visiting county population
200000
400000
600000
800000
1000000
Incident cases (per 100,000 population)
0
20
40
60
80
100
Mar
May
Jul
Sep

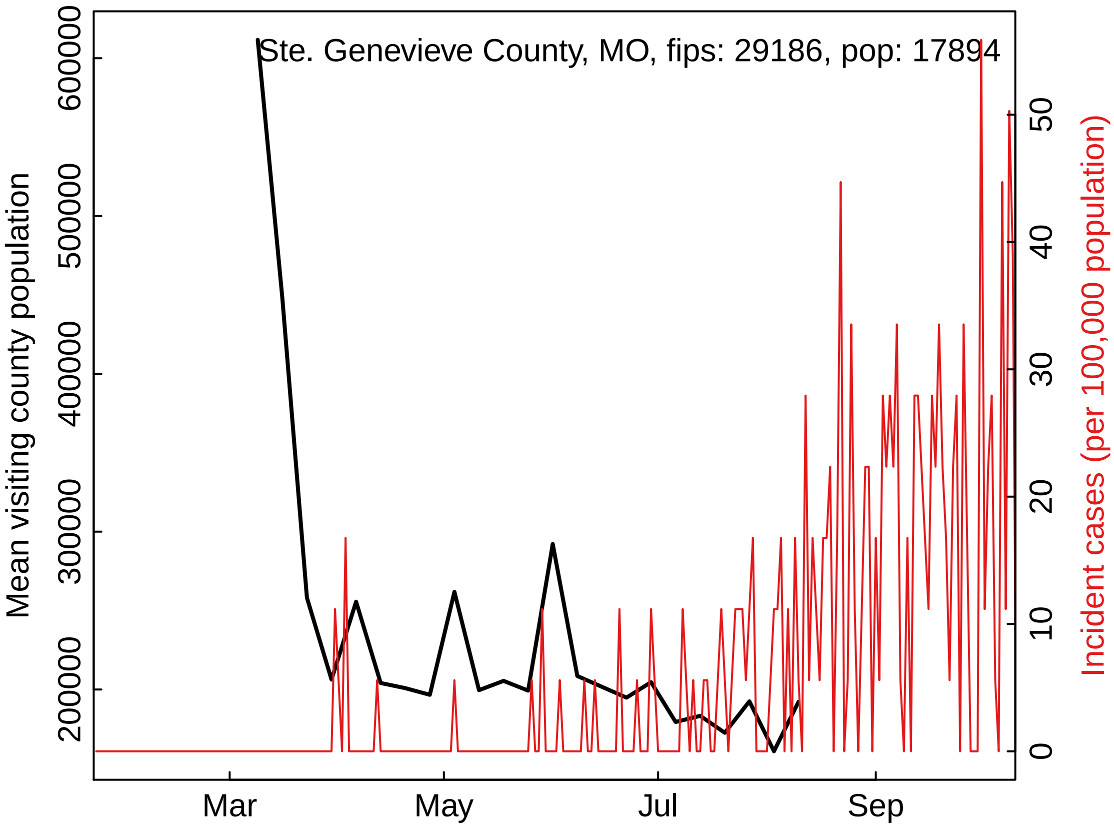

Ste. Genevieve County, MO, fips: 29186, pop: 17894
Mean visiting county population
200000
300000
400000
500000
600000
Incident cases (per 100,000 population)
0
10
20
30
40
50
Mar
May
Jul
Sep

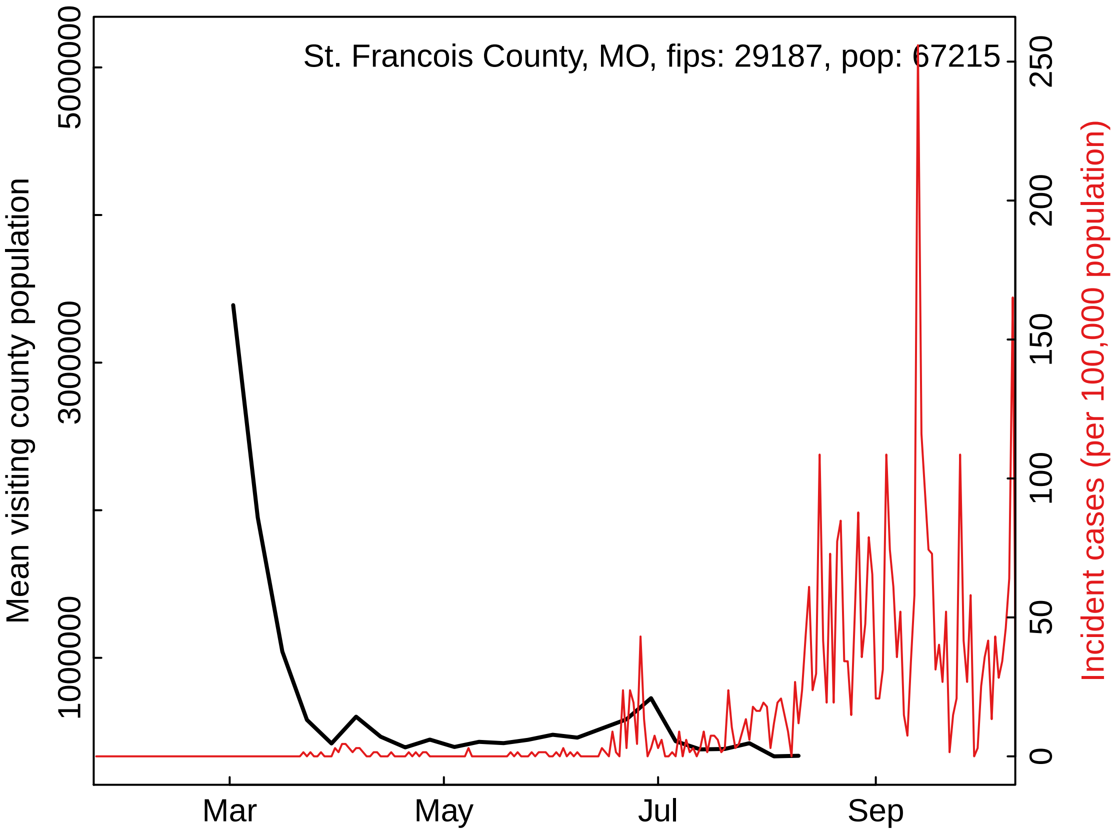

St. Francois County, MO, fips: 29187, pop: 67215
Mean visiting county population
1000000
3000000
5000000
Mar
May
Jul
Sep
0
50
100
150
200
250
Incident cases (per 100,000 population)

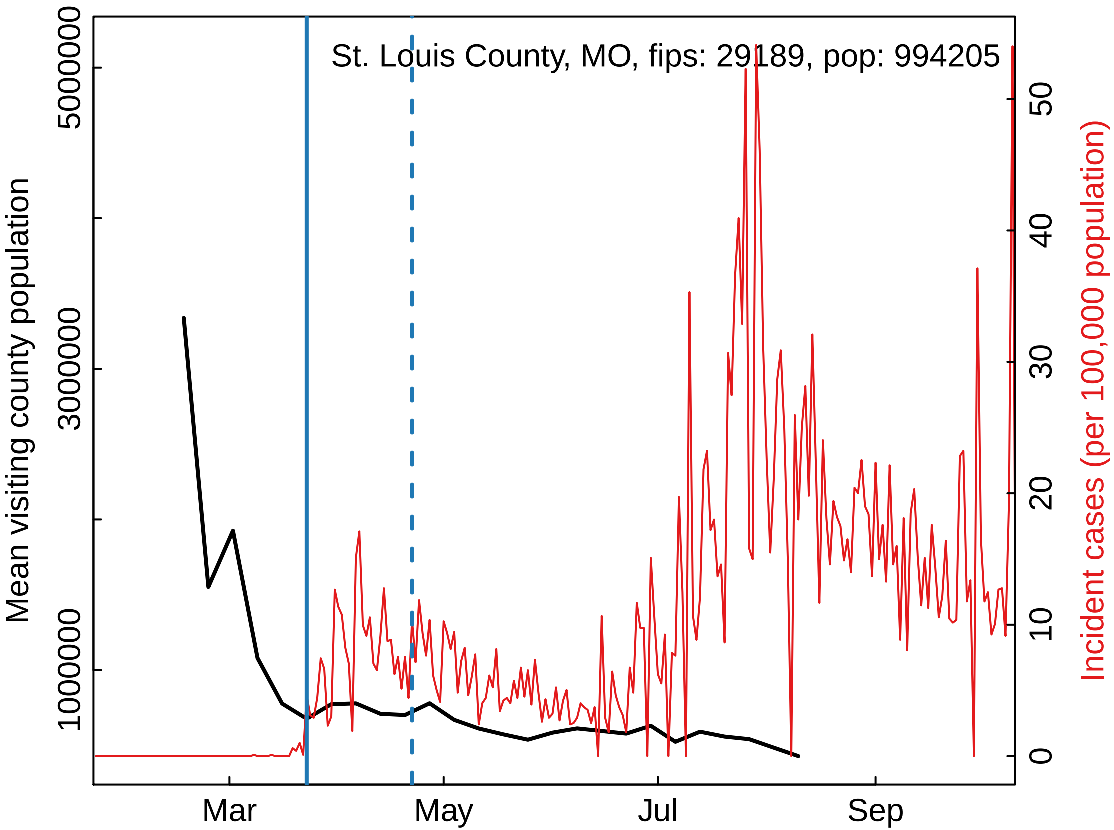
St. Louis County, MO, fips: 29189, pop: 994205
Mean visiting county population
1000000
3000000
5000000
Incident cases (per 100,000 population)
0
10
20
30
40
50
Mar
May
Jul
Sep

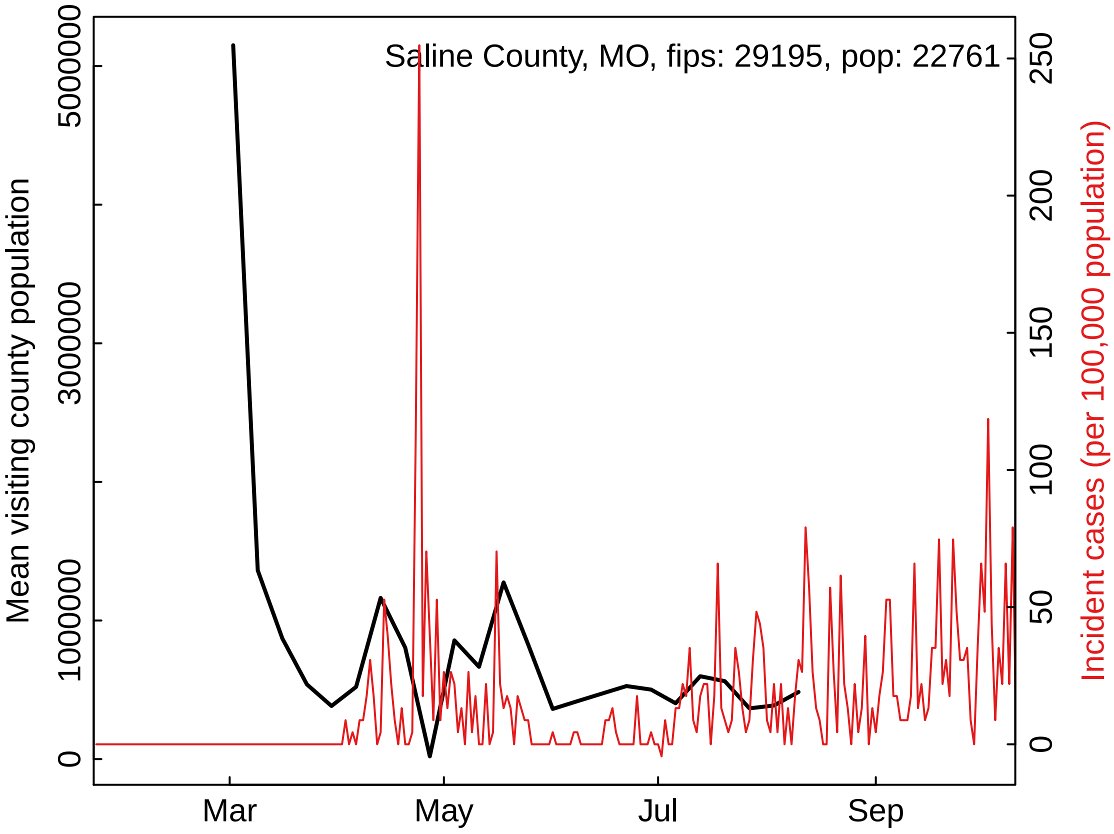

Saline County, MO, fips: 29195, pop: 22761
Mean visiting county population
0
1000000
3000000
5000000
Incident cases (per 100,000 population)
0
50
100
150
200
250
Mar
May
Jul
Sep

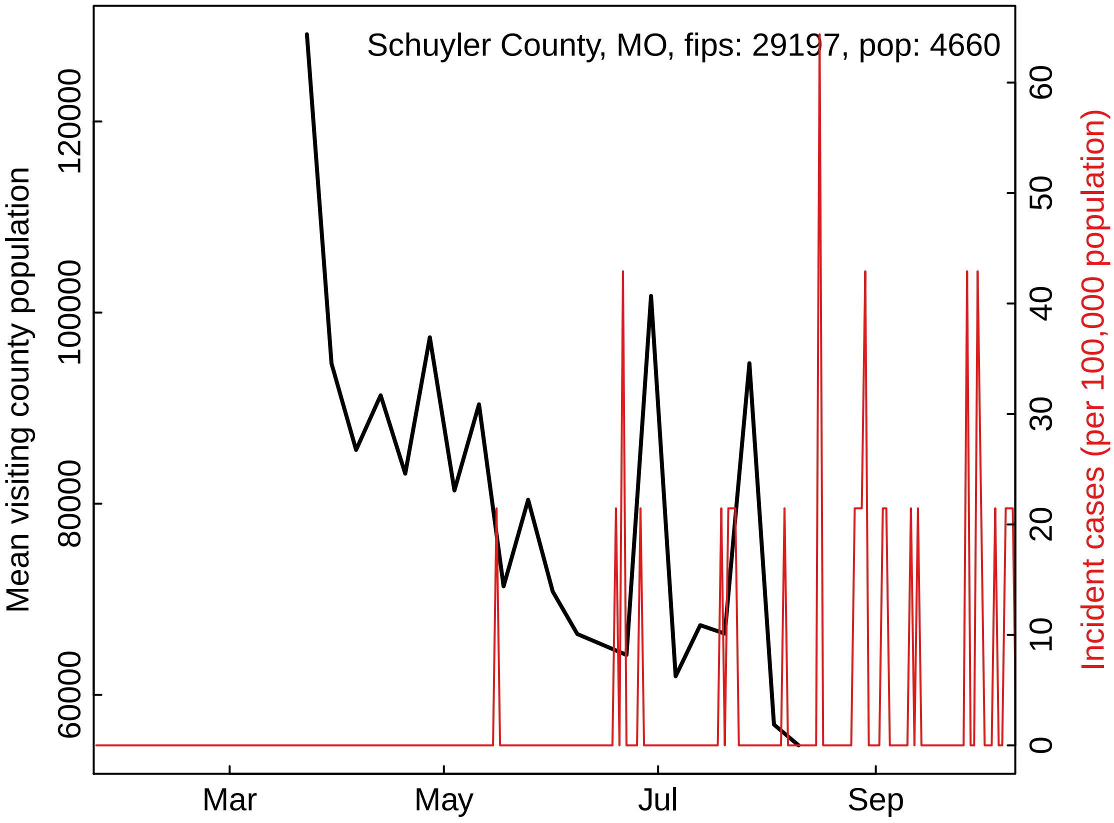

Schuyler County, MO, fips: 29197, pop: 4660
Mean visiting county population
60000
80000
100000
120000
Incident cases (per 100,000 population)
0
10
20
30
40
50
60
Mar
May
Jul
Sep

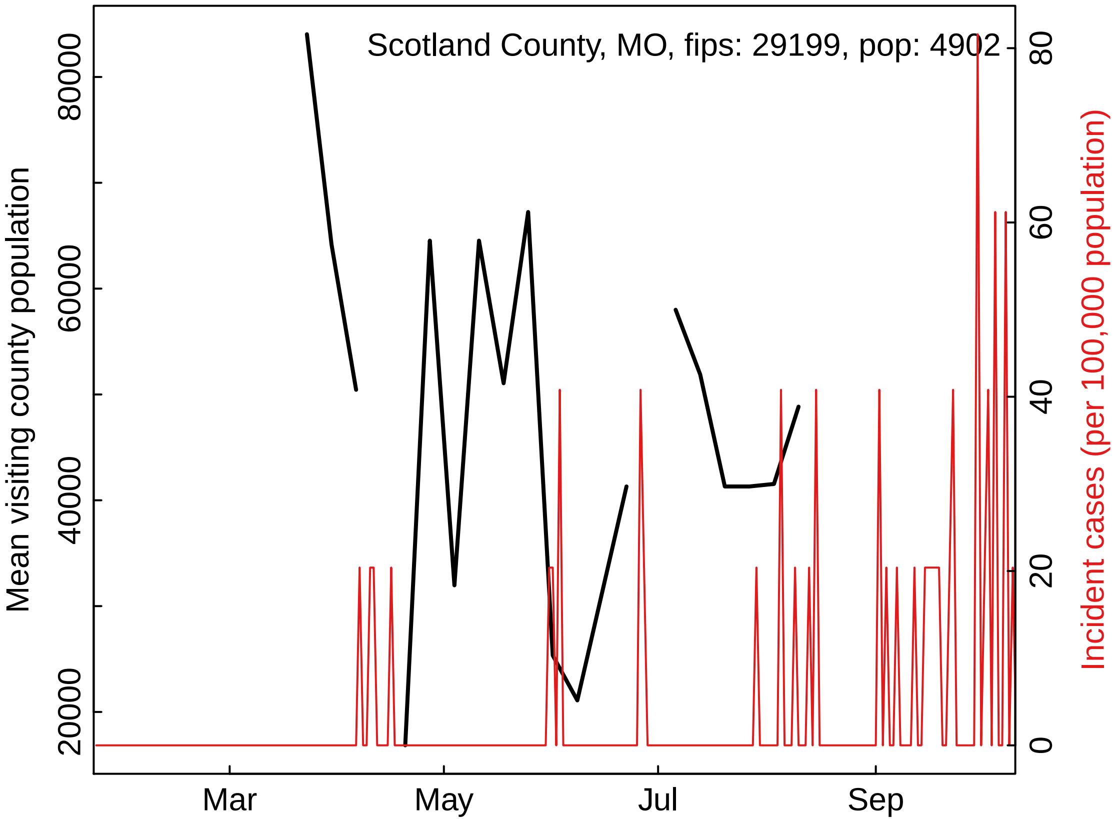

Scotland County, MO, fips: 29199, pop: 4902
Mean visiting county population
Incident cases (per 100,000 population)
20000
40000
60000
80000
0
20
40
60
80
Mar
May
Jul
Sep

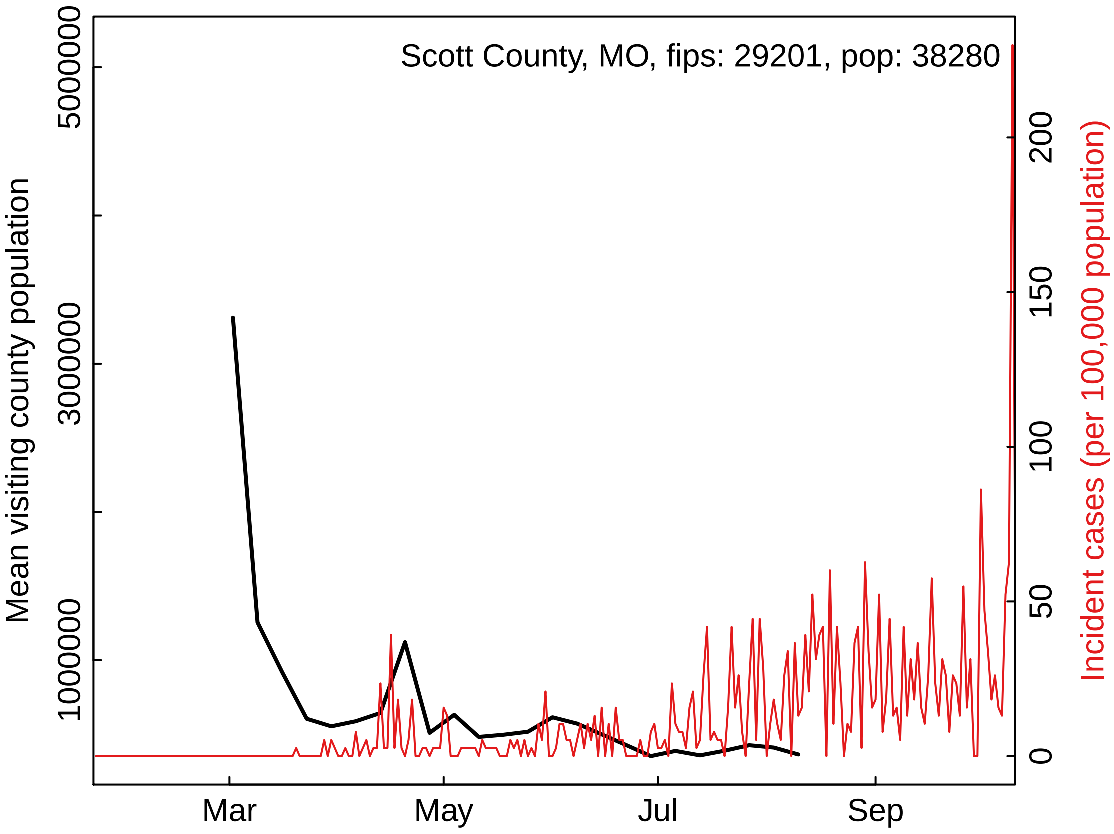

Scott County, MO, fips: 29201, pop: 38280
Mean visiting county population
1000000
3000000
5000000
Mar
May
Jul
Sep
0
50
100
150
200
Incident cases (per 100,000 population)

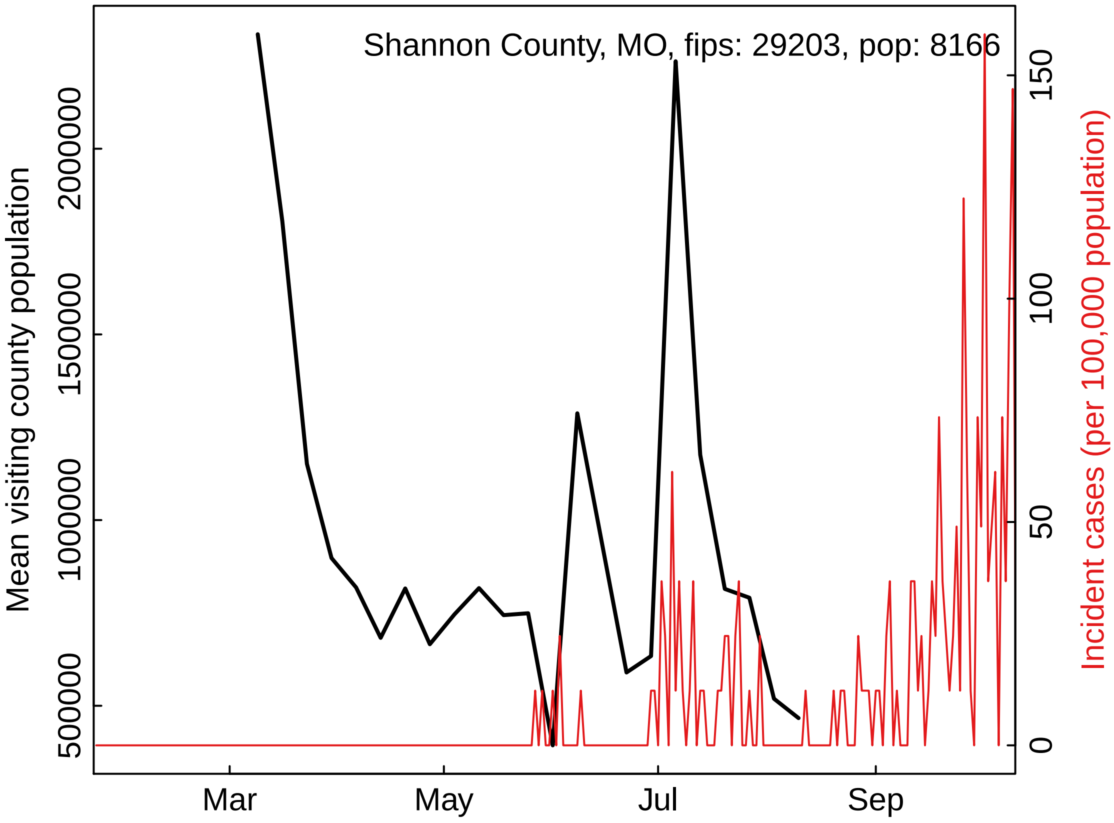

Shannon County, MO, fips: 29203, pop: 8166
Mean visiting county population
500000
1000000
1500000
2000000
Incident cases (per 100,000 population)
0
50
100
150
Mar
May
Jul
Sep

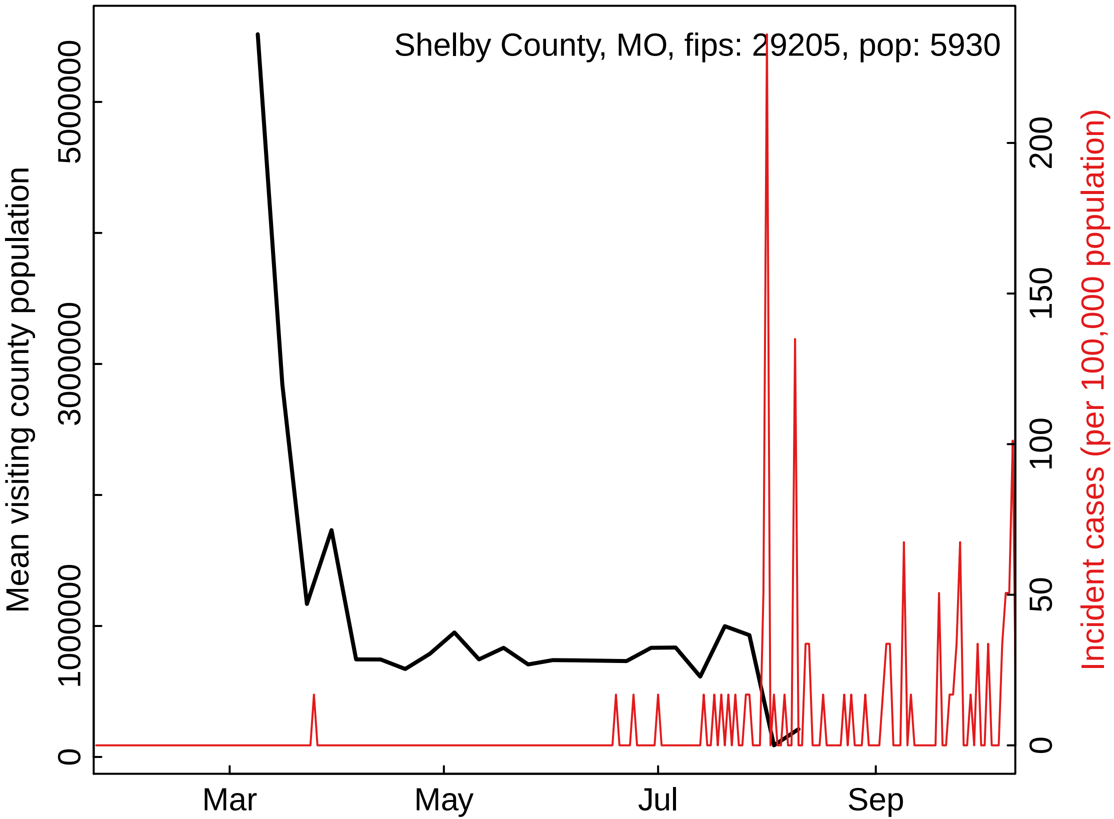

Shelby County, MO, fips: 29205, pop: 5930
Mean visiting county population
0
1000000
3000000
5000000
Incident cases (per 100,000 population)
0
50
100
150
200
Mar
May
Jul
Sep

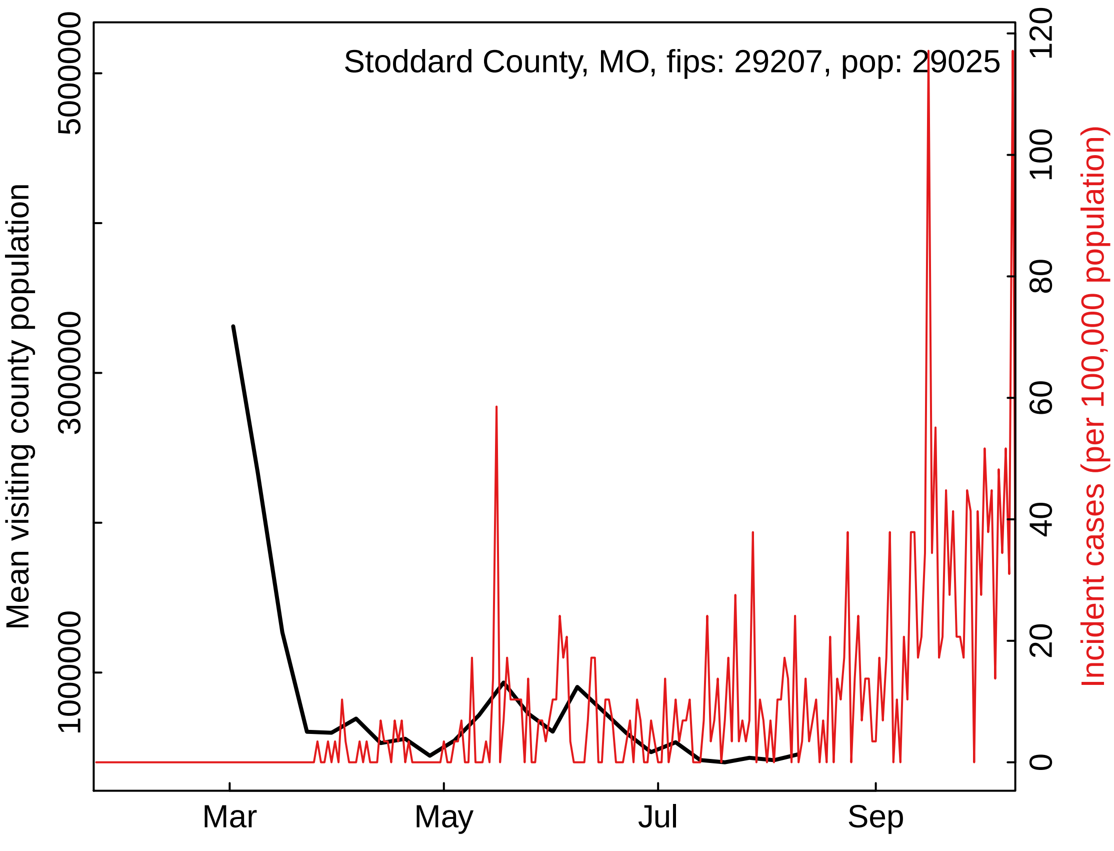
Stoddard County, MO, fips: 29207, pop: 29025
Mean visiting county population
1000000
3000000
5000000
Mar
May
Jul
Sep
0
20
40
60
80
100
120
Incident cases (per 100,000 population)

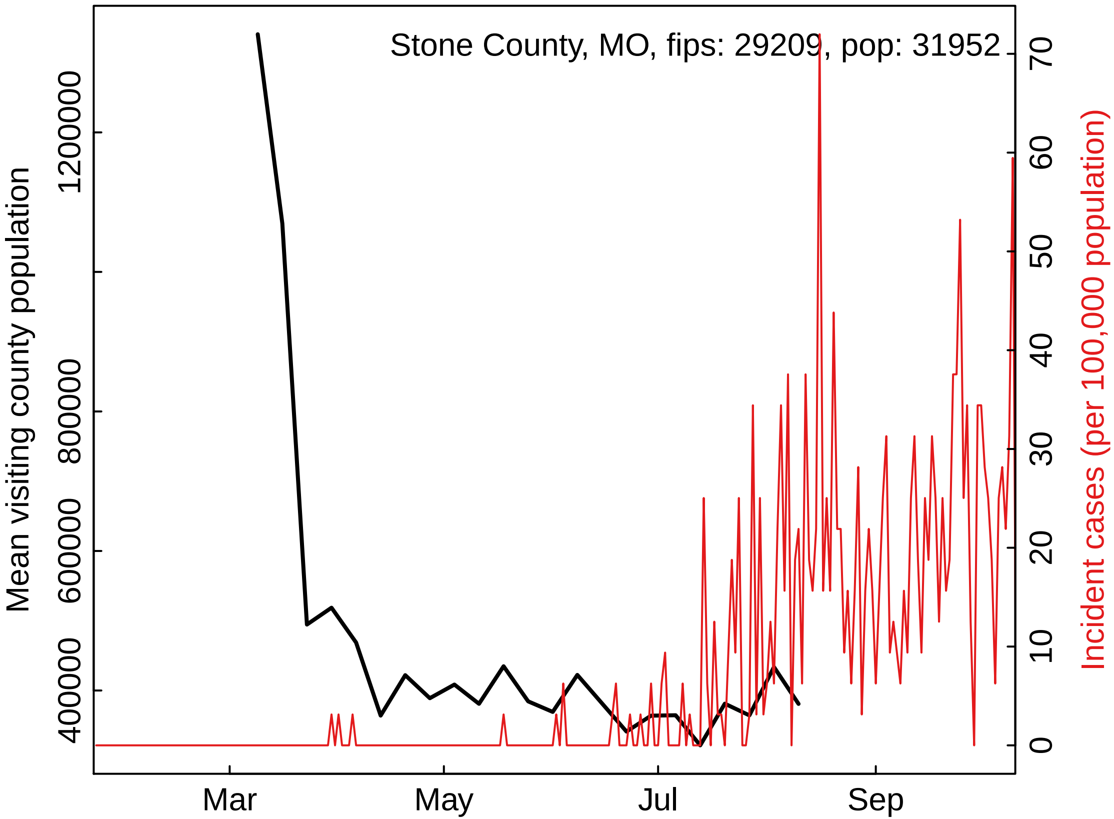

Stone County, MO, fips: 29209, pop: 31952
Mean visiting county population
400000
600000
800000
1200000
Incident cases (per 100,000 population)
0
10
20
30
40
50
60
70
Mar
May
Jul
Sep

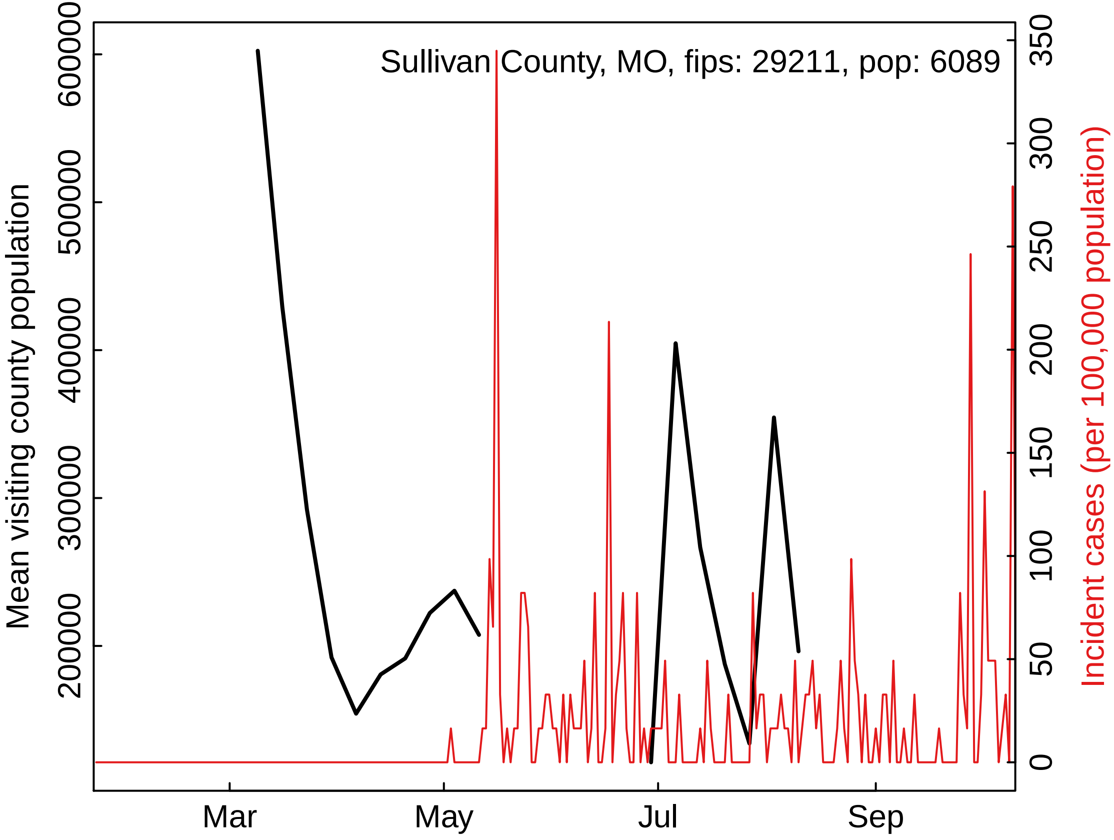
Sullivan County, MO, fips: 29211, pop: 6089
Mean visiting county population
Incident cases (per 100,000 population)
200000
300000
400000
500000
600000
0
50
100
150
200
250
300
350
Mar
May
Jul
Sep

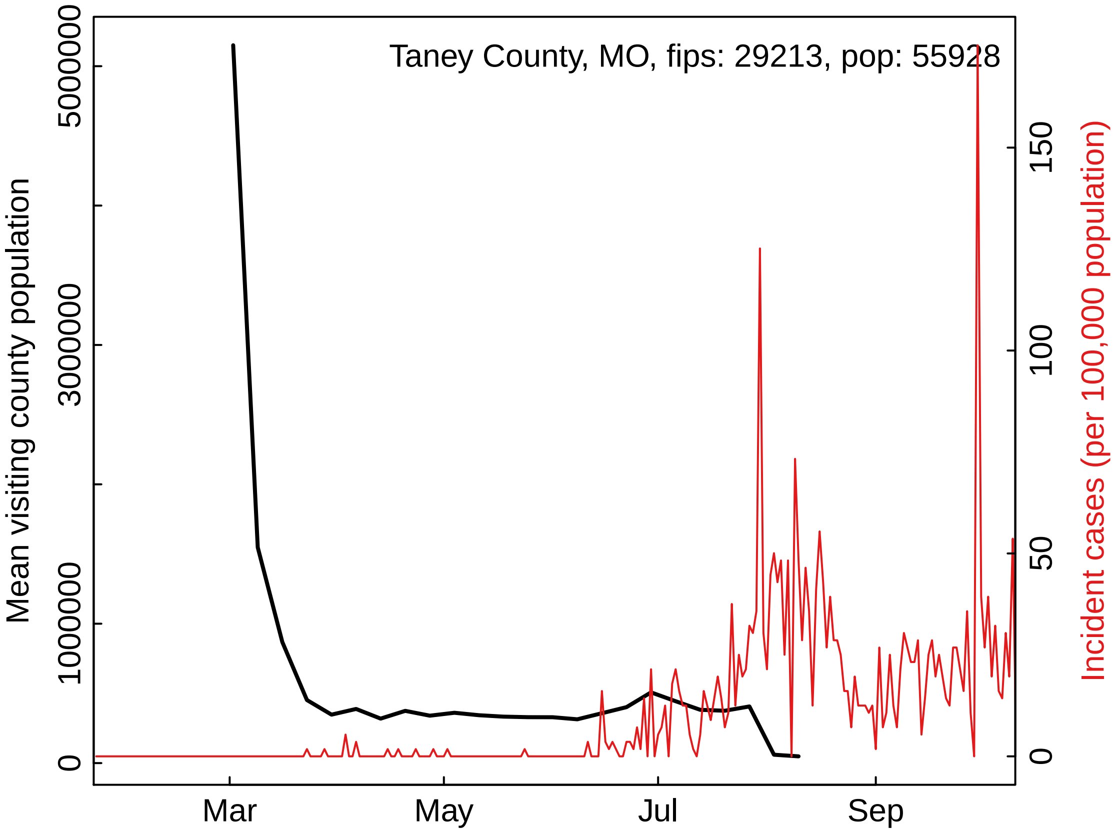
Taney County, MO, fips: 29213, pop: 55928
Mean visiting county population
0
1000000
3000000
5000000
Incident cases (per 100,000 population)
0
50
100
150
Mar
May
Jul
Sep

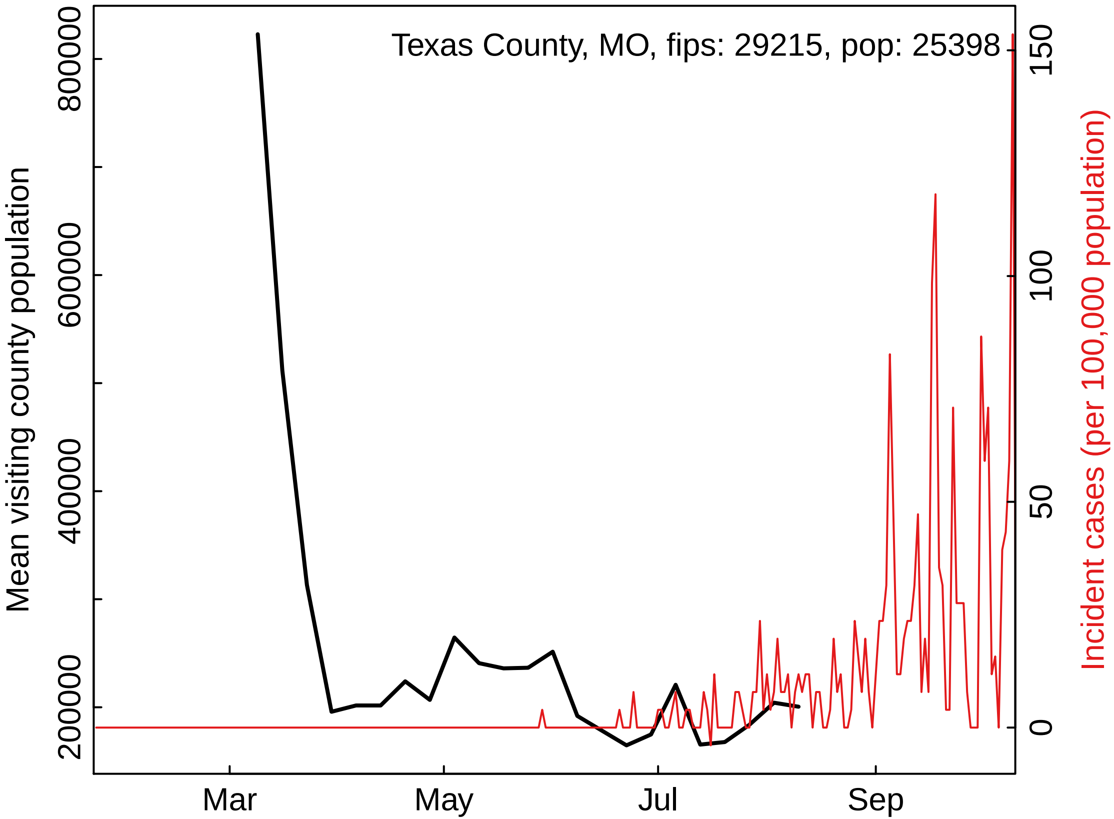
Texas County, MO, fips: 29215, pop: 25398
Mean visiting county population
200000
400000
600000
800000
Incident cases (per 100,000 population)
0
50
100
150
Mar
May
Jul
Sep

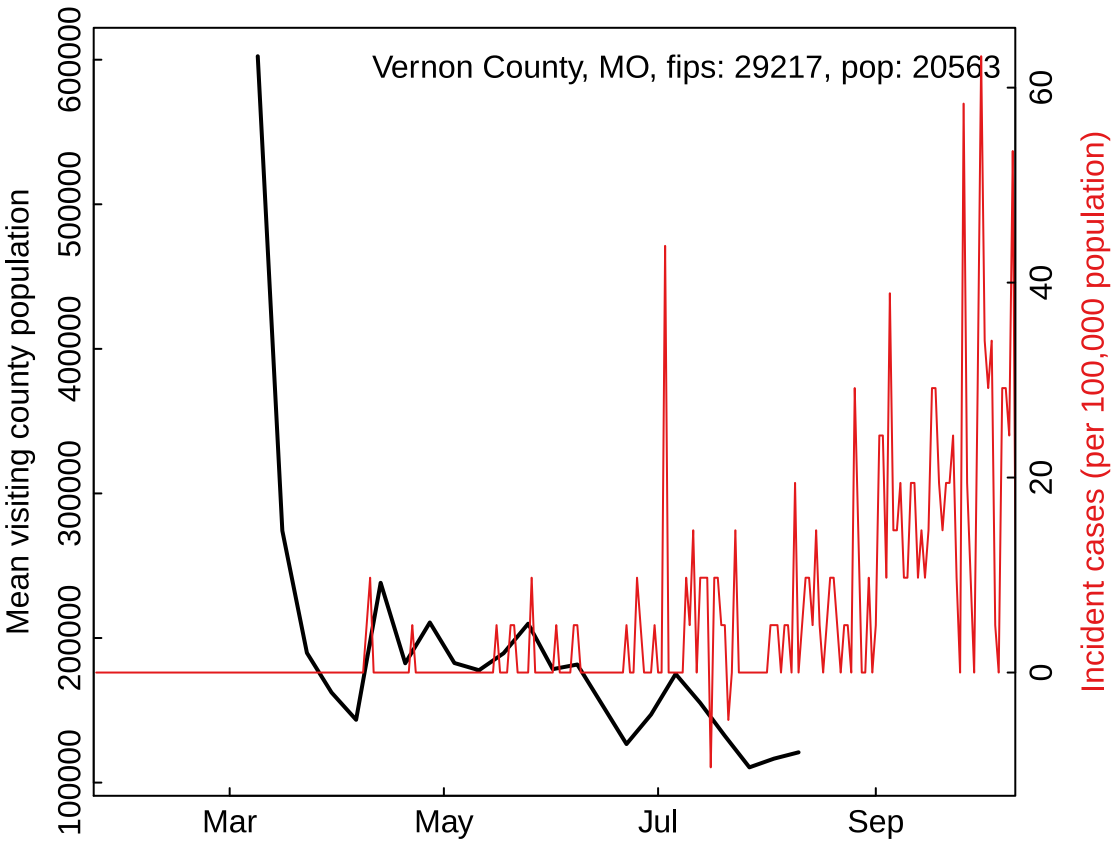

Vernon County, MO, fips: 29217, pop: 20563
Mean visiting county population
100000
200000
300000
400000
500000
600000
Mar
May
Jul
Sep
0
20
40
60
Incident cases (per 100,000 population)

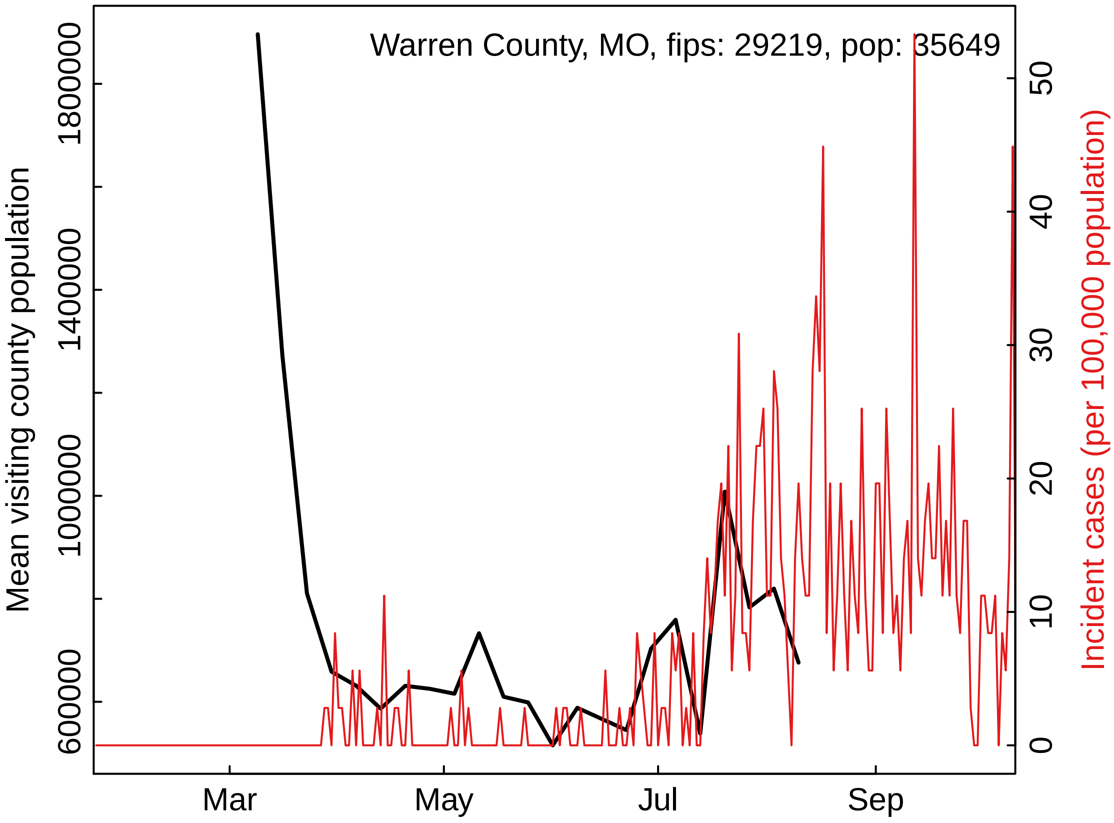

Warren County, MO, fips: 29219, pop: 35649
Mean visiting county population
600000
1000000
1400000
1800000
Incident cases (per 100,000 population)
0
10
20
30
40
50
Mar
May
Jul
Sep

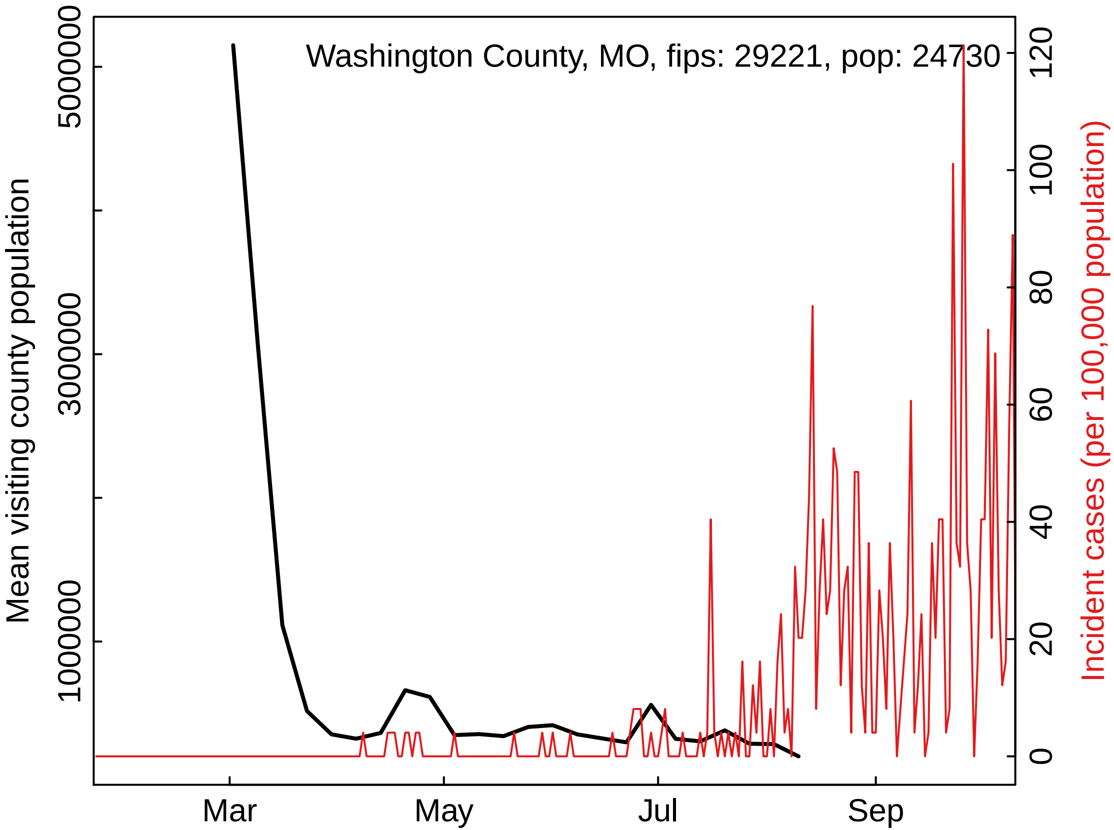

Washington County, MO, fips: 29221, pop: 24730
Mean visiting county population
Incident cases (per 100,000 population)
1000000
3000000
5000000
0
20
40
60
80
100
120
Mar
May
Jul
Sep

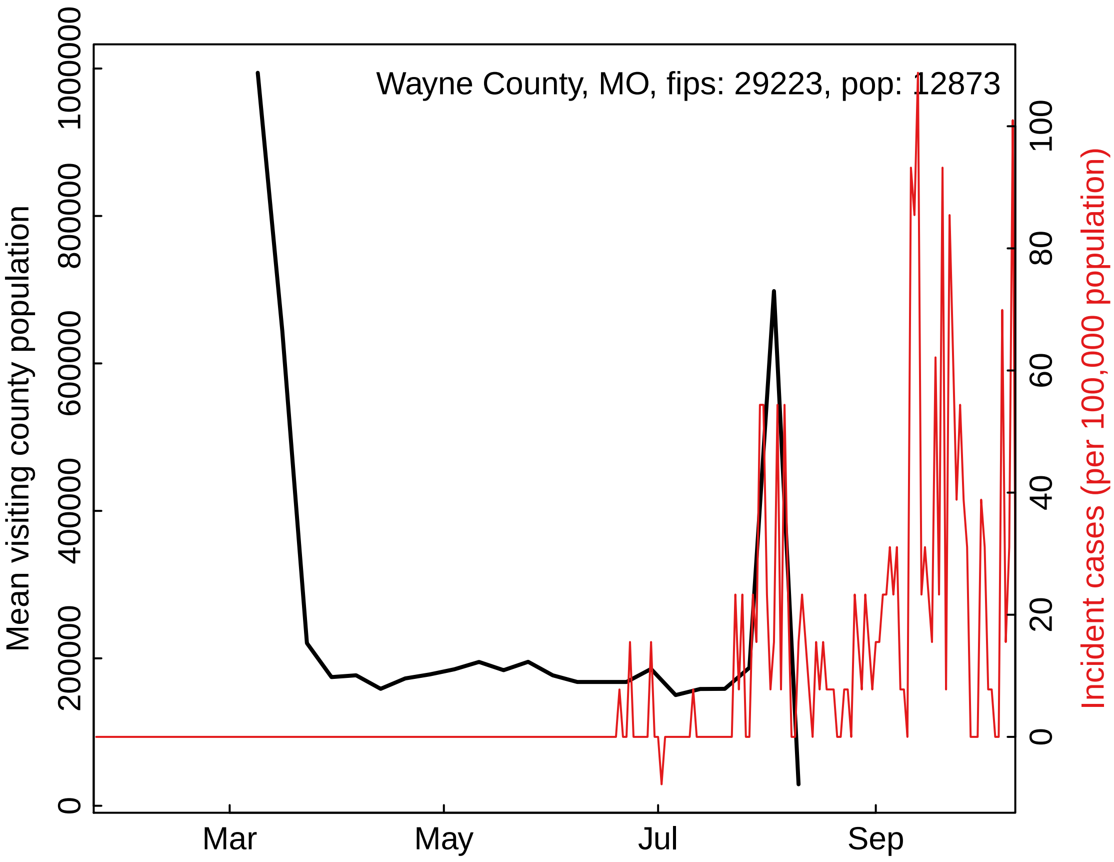
Wayne County, MO, fips: 29223, pop: 12873
Mean visiting county population
0
200000
400000
600000
800000
1000000
Mar
May
Jul
Sep
Incident cases (per 100,000 population)
0
20
40
60
80
100

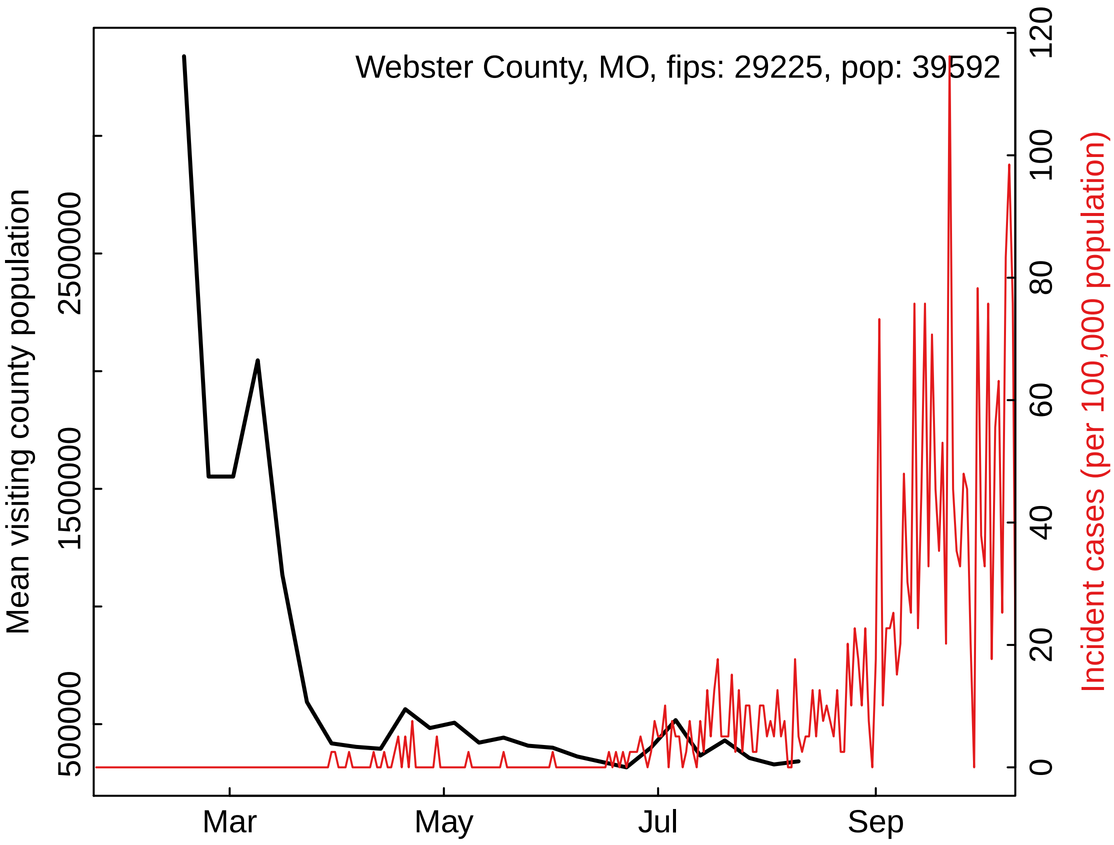

Webster County, MO, fips: 29225, pop: 39592
Mean visiting county population
500000
1500000
2500000
Mar
May
Jul
Sep
0
20
40
60
80
100
120
Incident cases (per 100,000 population)

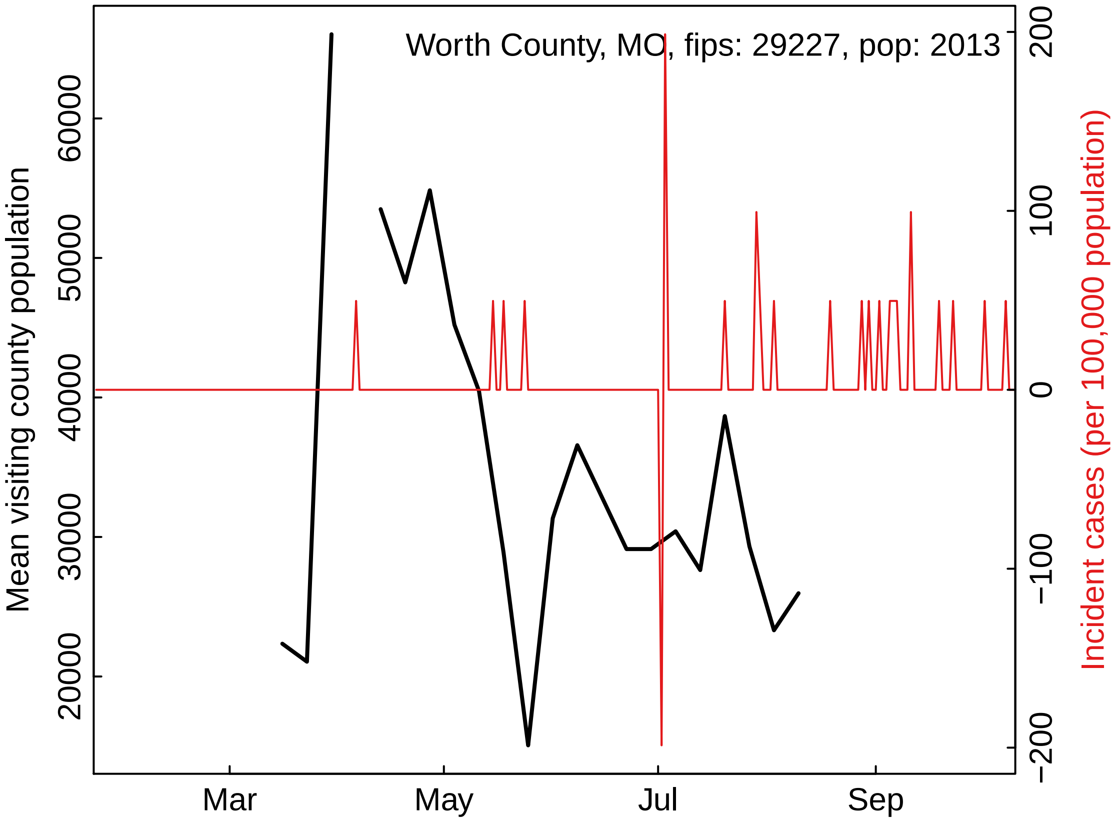

Worth County, MO, fips: 29227, pop: 2013
Mean visiting county population
20000
30000
40000
50000
60000
Incident cases (per 100,000 population)
−200
−100
0
100
200
Mar
May
Jul
Sep

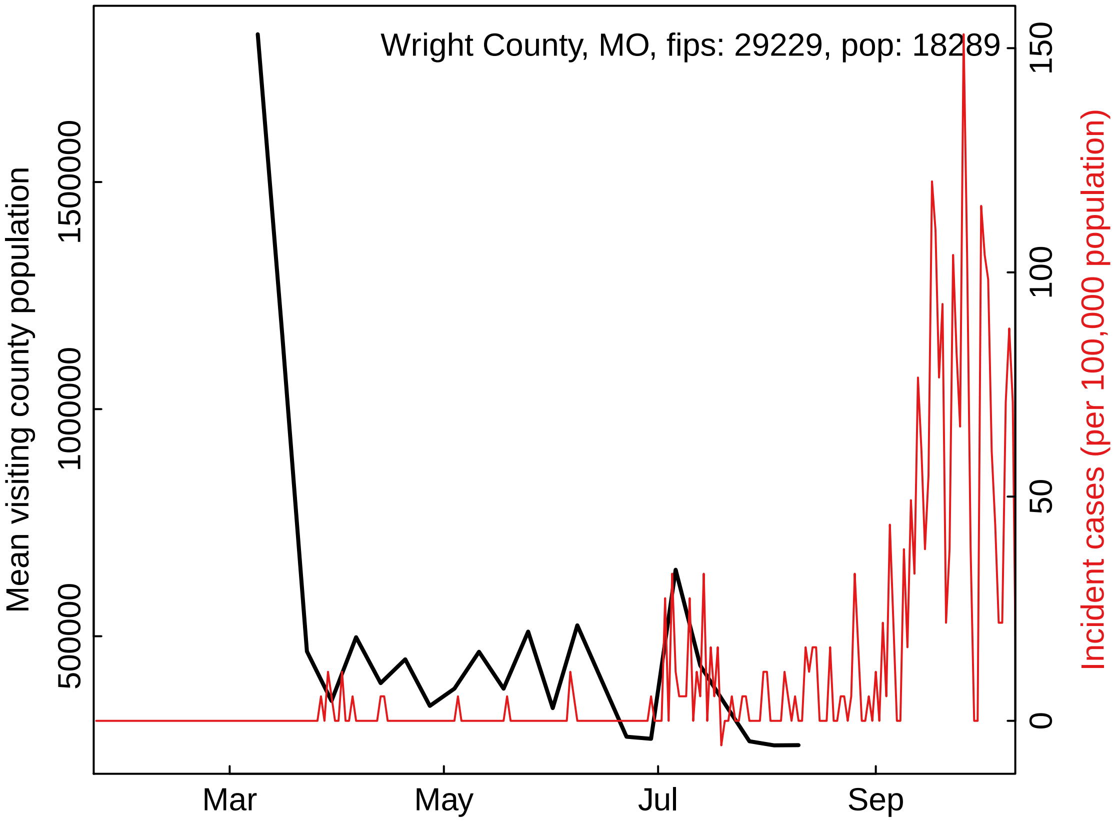

Wright County, MO, fips: 29229, pop: 18289
Mean visiting county population
500000
1000000
1500000
Incident cases (per 100,000 population)
0
50
100
150
Mar
May
Jul
Sep

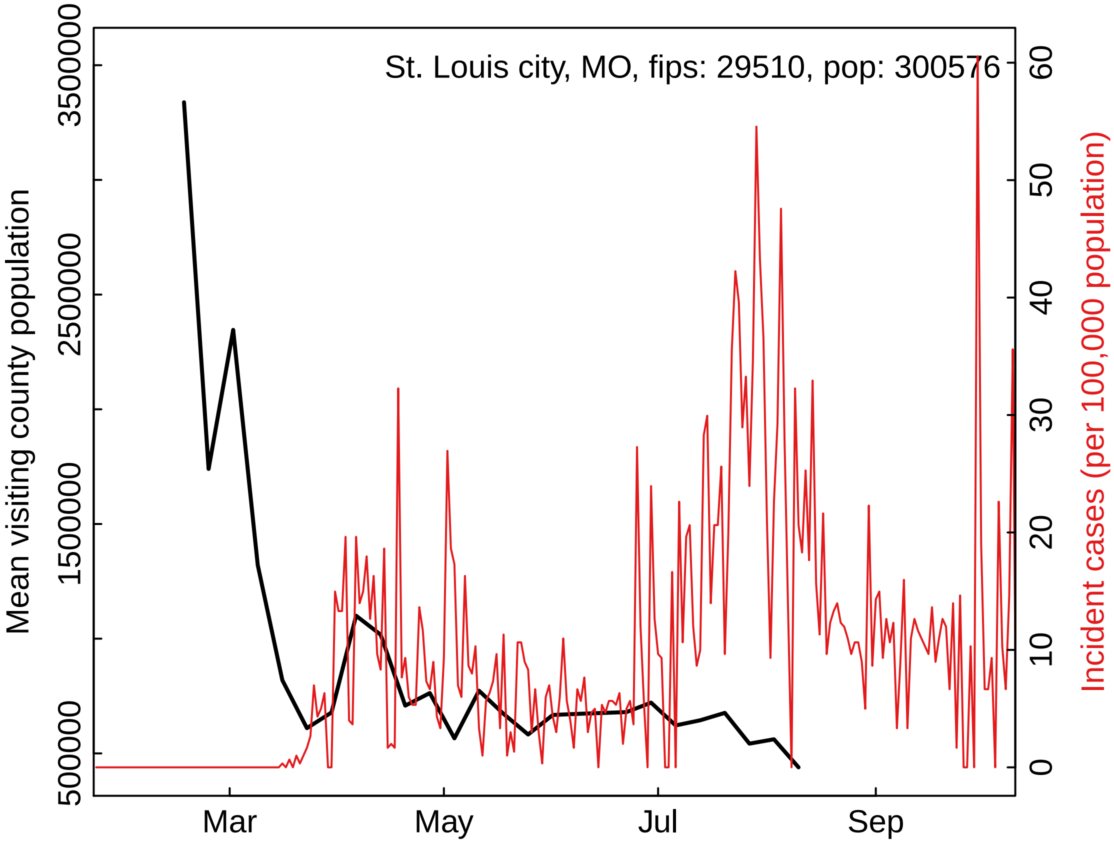
St. Louis city, MO, fips: 29510, pop: 300576
Mean visiting county population
Incident cases (per 100,000 population)
500000
1500000
2500000
3500000
0
10
20
30
40
50
60
Mar
May
Jul
Sep

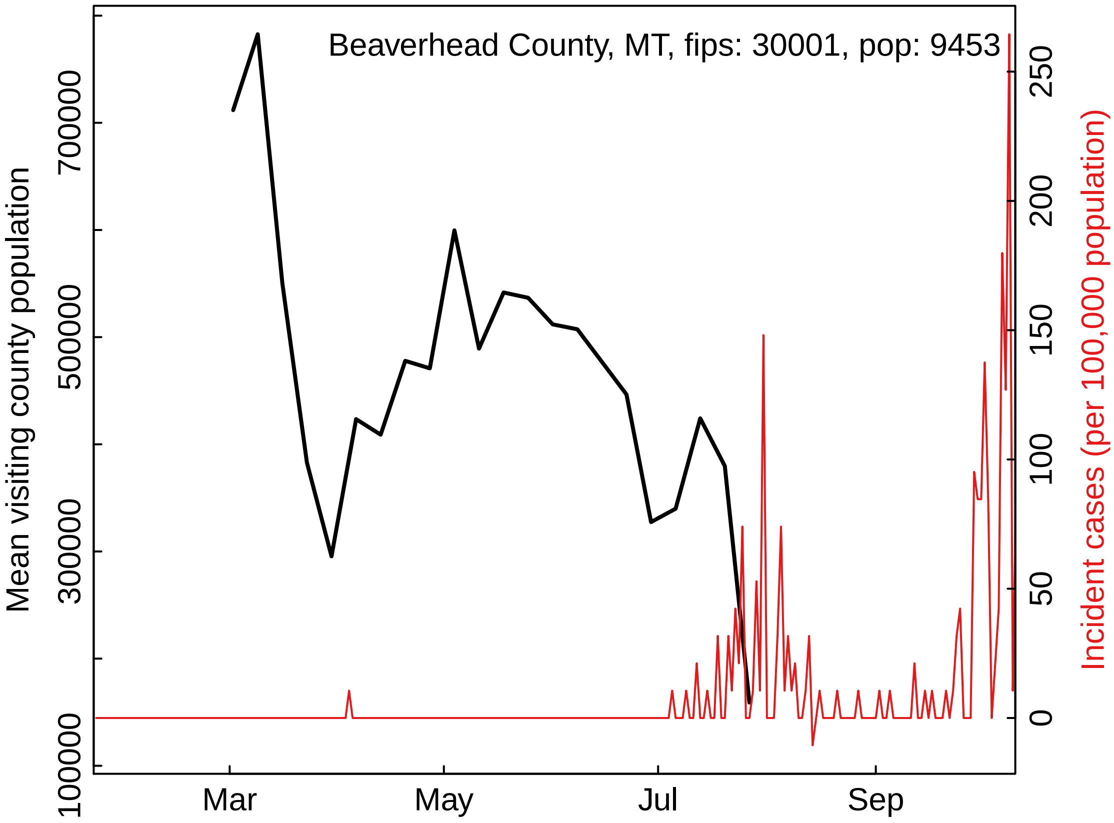

Beaverhead County, MT, fips: 30001, pop: 9453
Mean visiting county population
100000
300000
500000
700000
Mar
May
Jul
Sep
0
50
100
150
200
250
Incident cases (per 100,000 population)

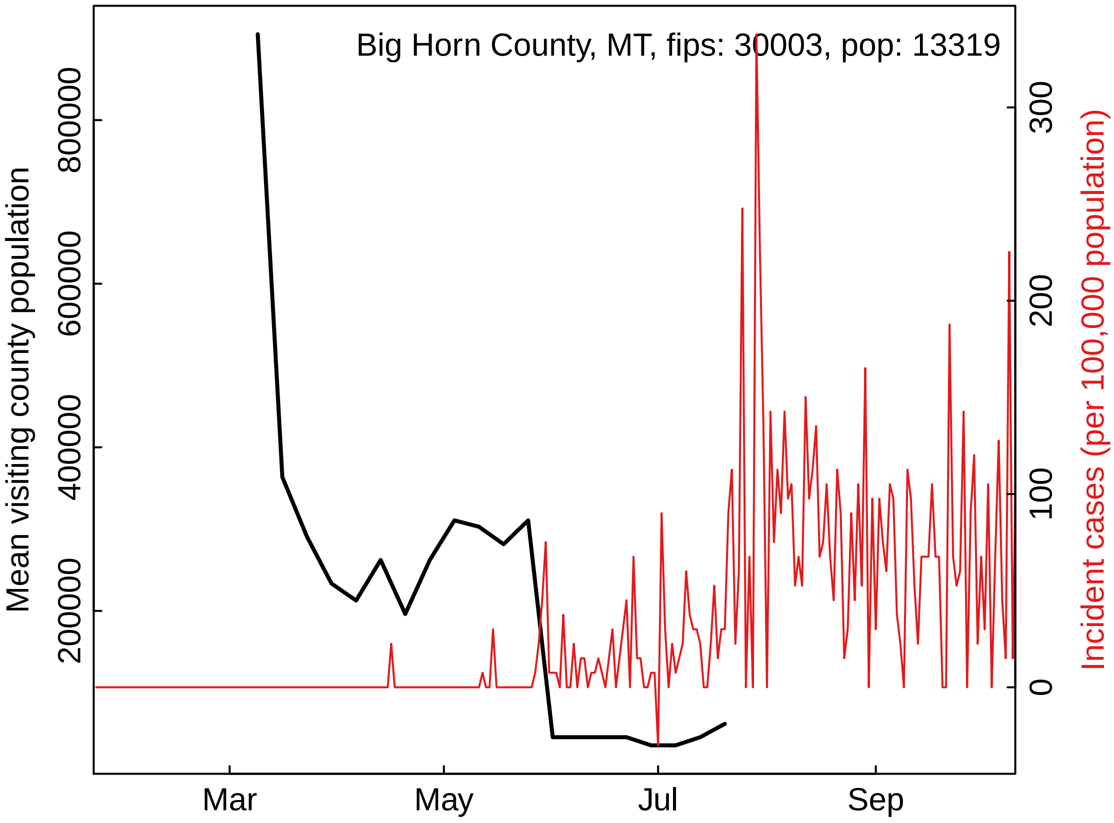

Big Horn County, MT, fips: 30003, pop: 13319
Mean visiting county population
200000
400000
600000
800000
Incident cases (per 100,000 population)
0
100
200
300
Mar
May
Jul
Sep

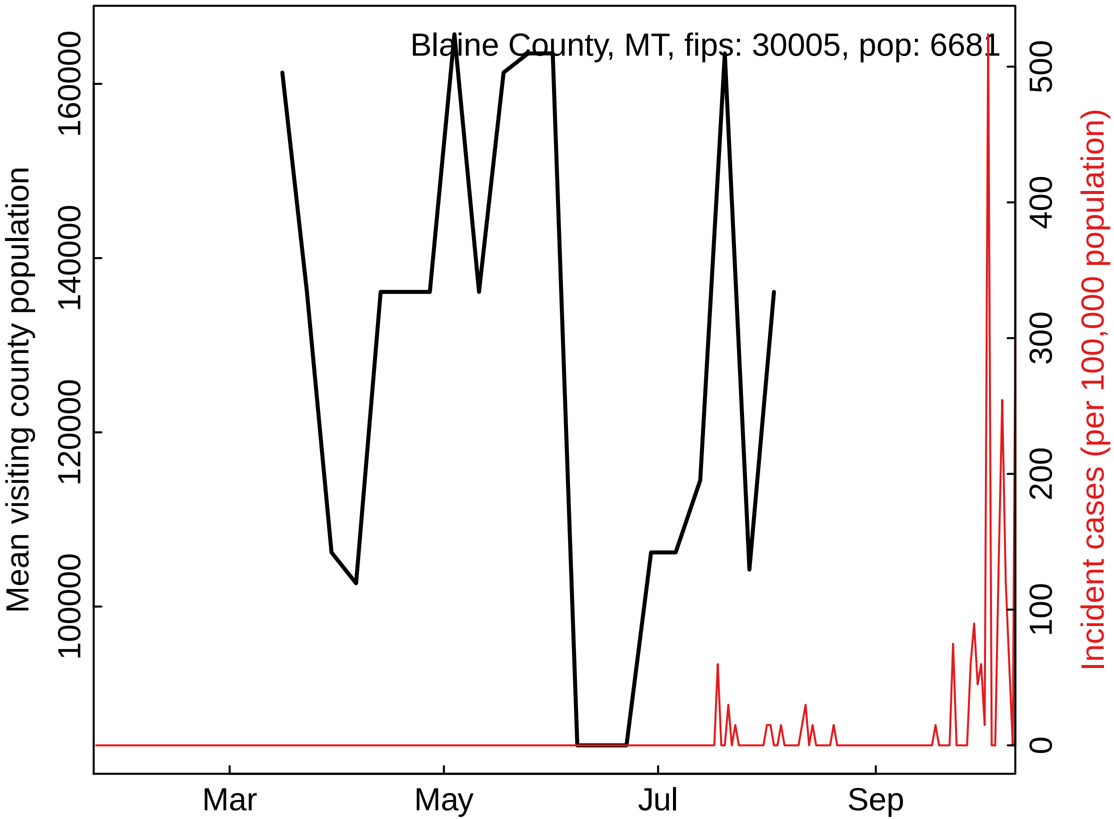

Blaine County, MT, fips: 30005, pop: 6681
Mean visiting county population
100000
120000
140000
160000
Incident cases (per 100,000 population)
0
100
200
300
400
500
Mar
May
Jul
Sep

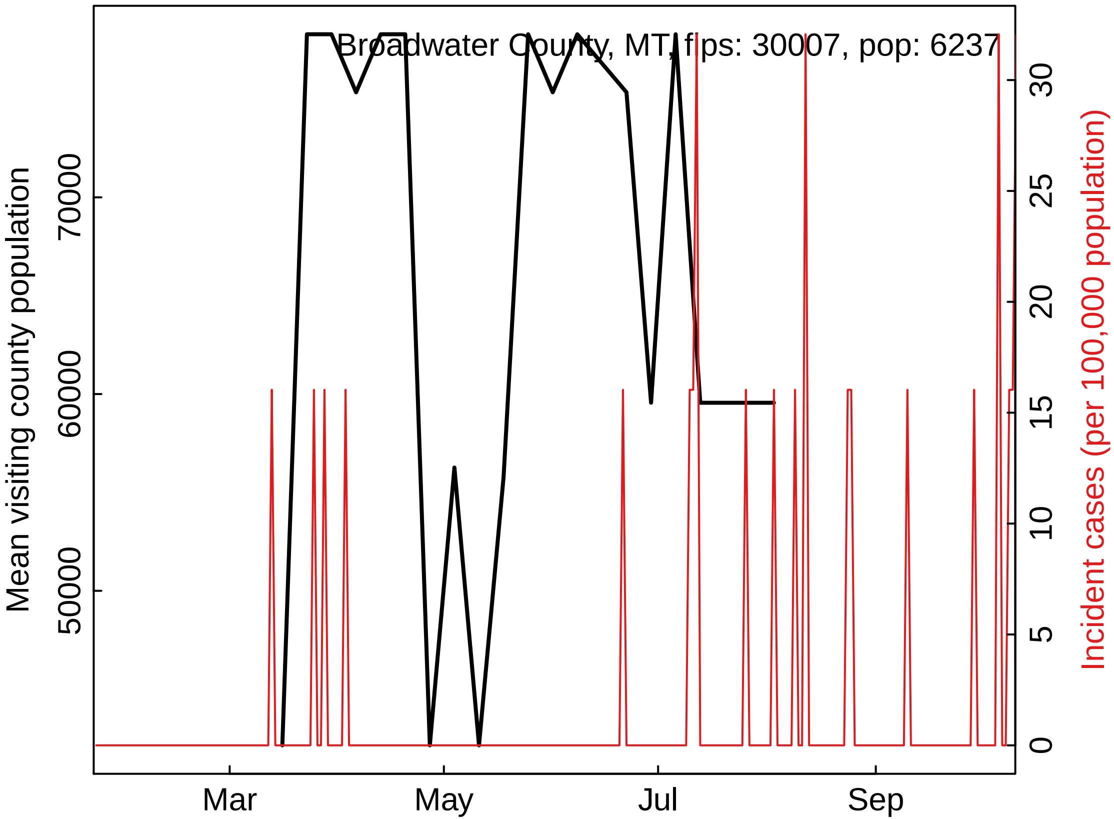

Broadwater County, MT, fips: 30007, pop: 6237
Mean visiting county population
50000
60000
70000
Mar
May
Jul
Sep
0
5
10
15
20
25
30
Incident cases (per 100,000 population)

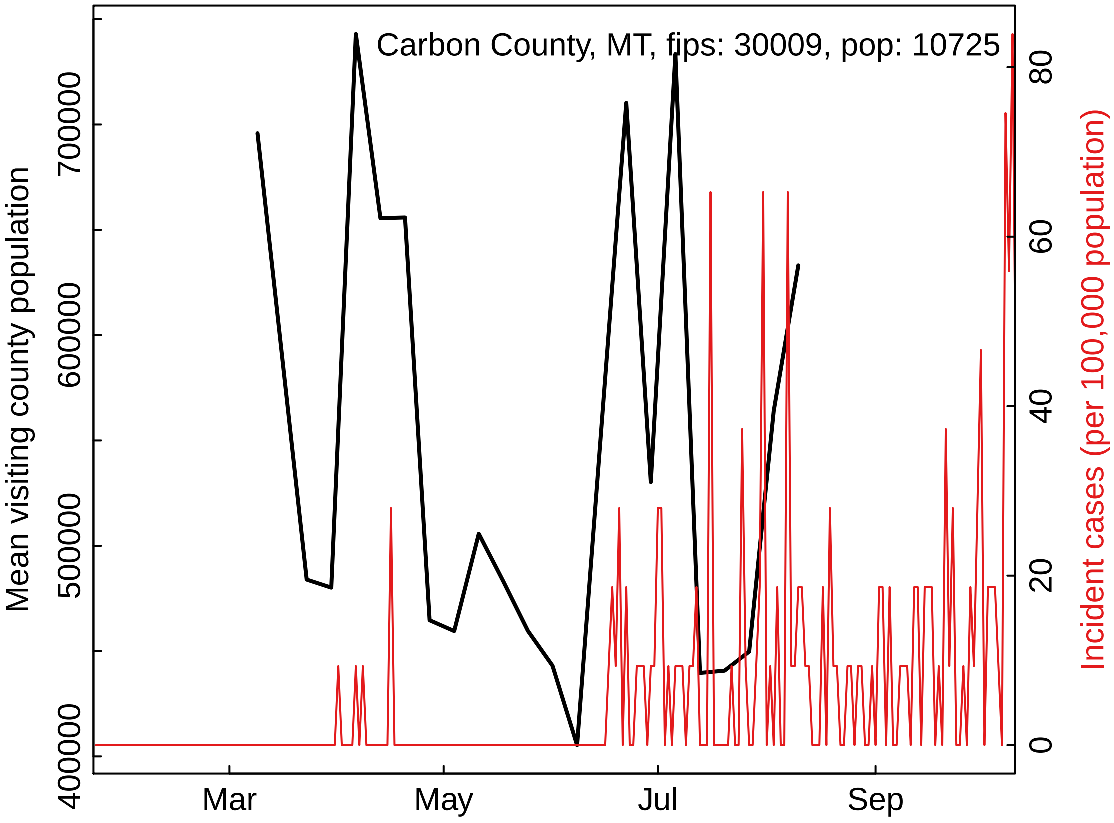

Carbon County, MT, fips: 30009, pop: 10725
Mean visiting county population
4000000
5000000
6000000
7000000
Mar
May
Jul
Sep
0
20
40
60
80
Incident cases (per 100,000 population)

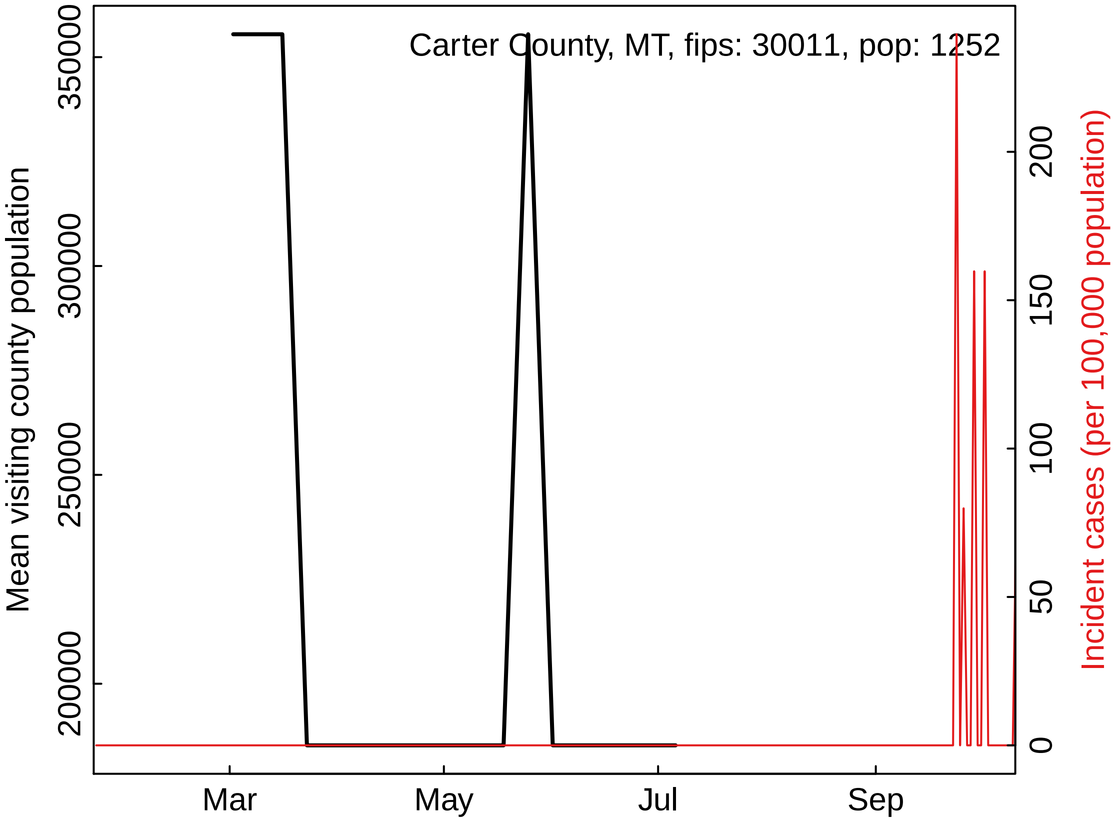

Carter County, MT, fips: 30011, pop: 1252
Mean visiting county population
200000
250000
300000
350000
Incident cases (per 100,000 population)
0
50
100
150
200
Mar
May
Jul
Sep

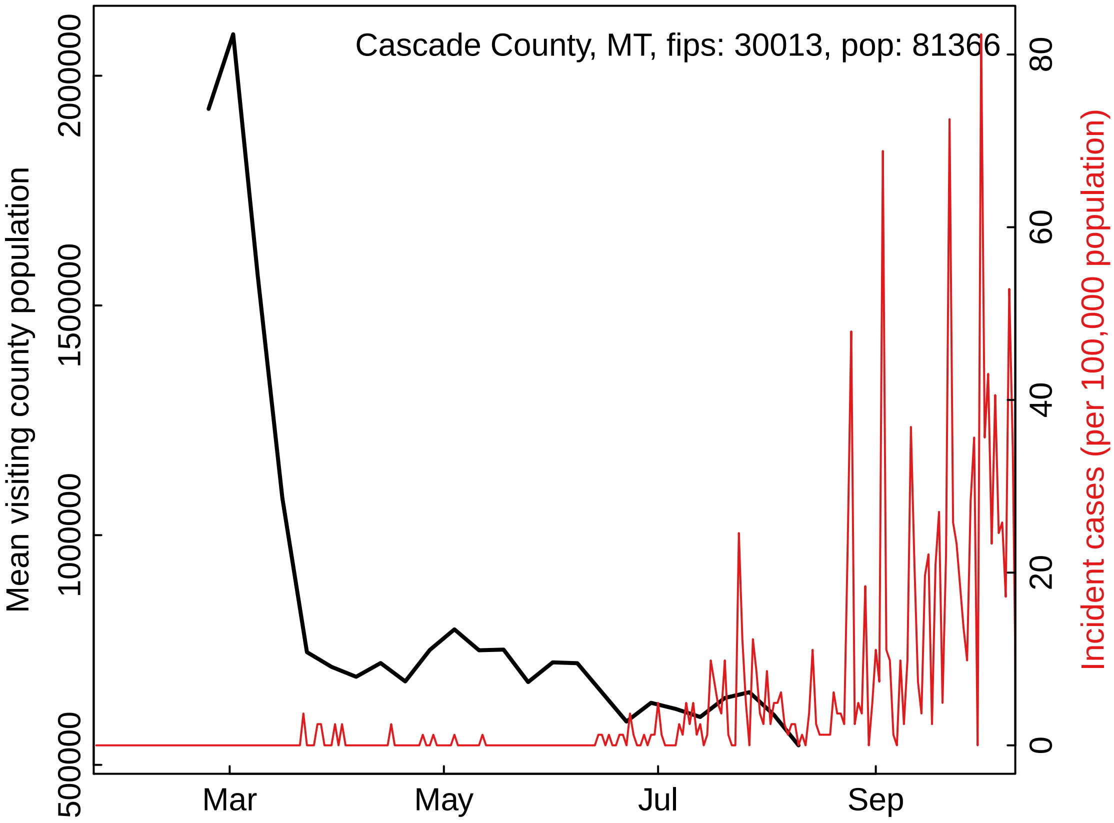
Cascade County, MT, fips: 30013, pop: 81366
Mean visiting county population
Incident cases (per 100,000 population)
5000000
10000000
15000000
20000000
0
20
40
60
80
Mar
May
Jul
Sep

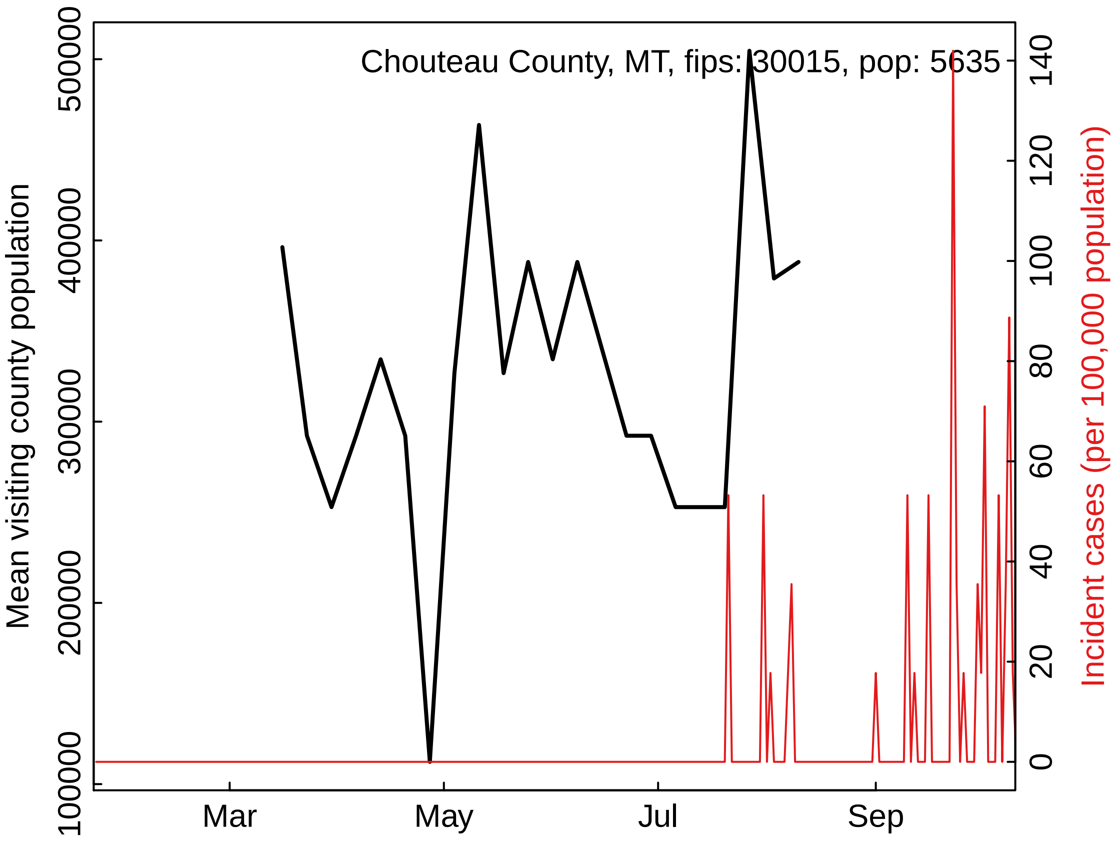
Chouteau County, MT, fips: 30015, pop: 5635
Mean visiting county population
Incident cases (per 100,000 population)
100000
200000
300000
400000
500000
0
20
40
60
80
100
120
140
Mar
May
Jul
Sep

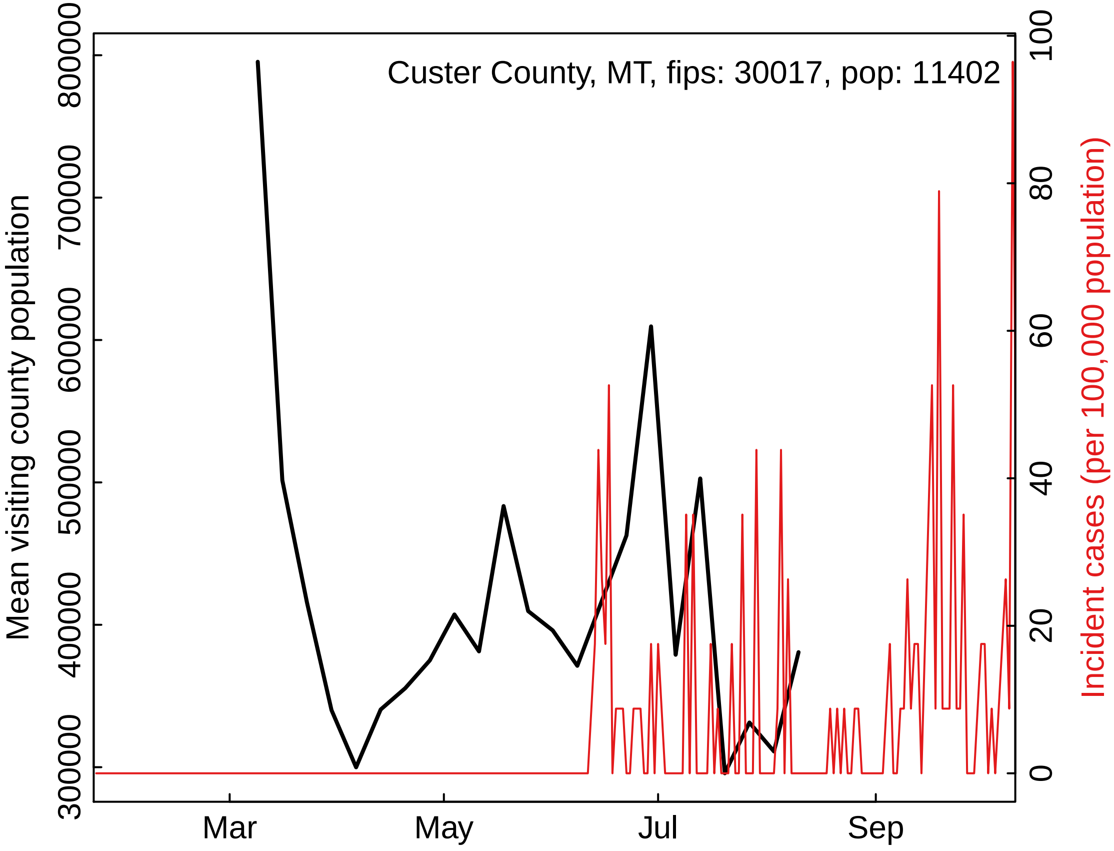
Custer County, MT, fips: 30017, pop: 11402
Mean visiting county population
300000
400000
500000
600000
700000
800000
Incident cases (per 100,000 population)
0
20
40
60
80
100
Mar
May
Jul
Sep

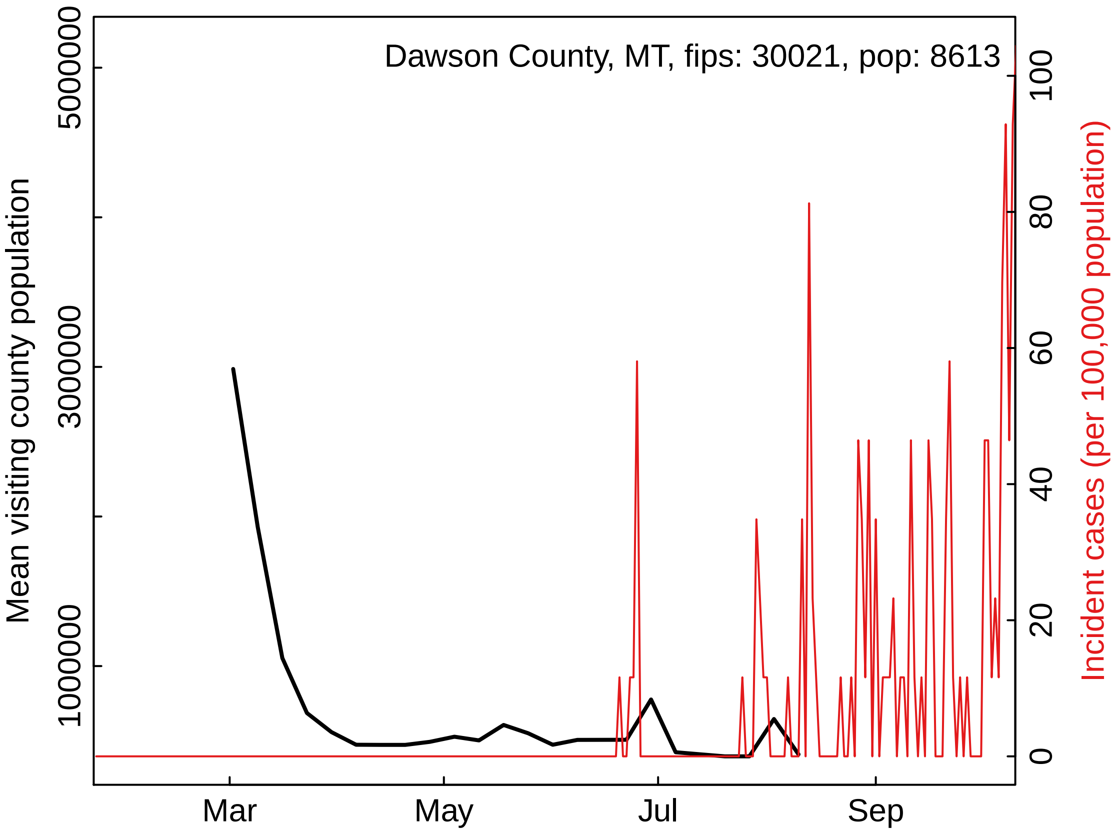
Dawson County, MT, fips: 30021, pop: 8613
Mean visiting county population
1000000
3000000
5000000
Incident cases (per 100,000 population)
0
20
40
60
80
100
Mar
May
Jul
Sep

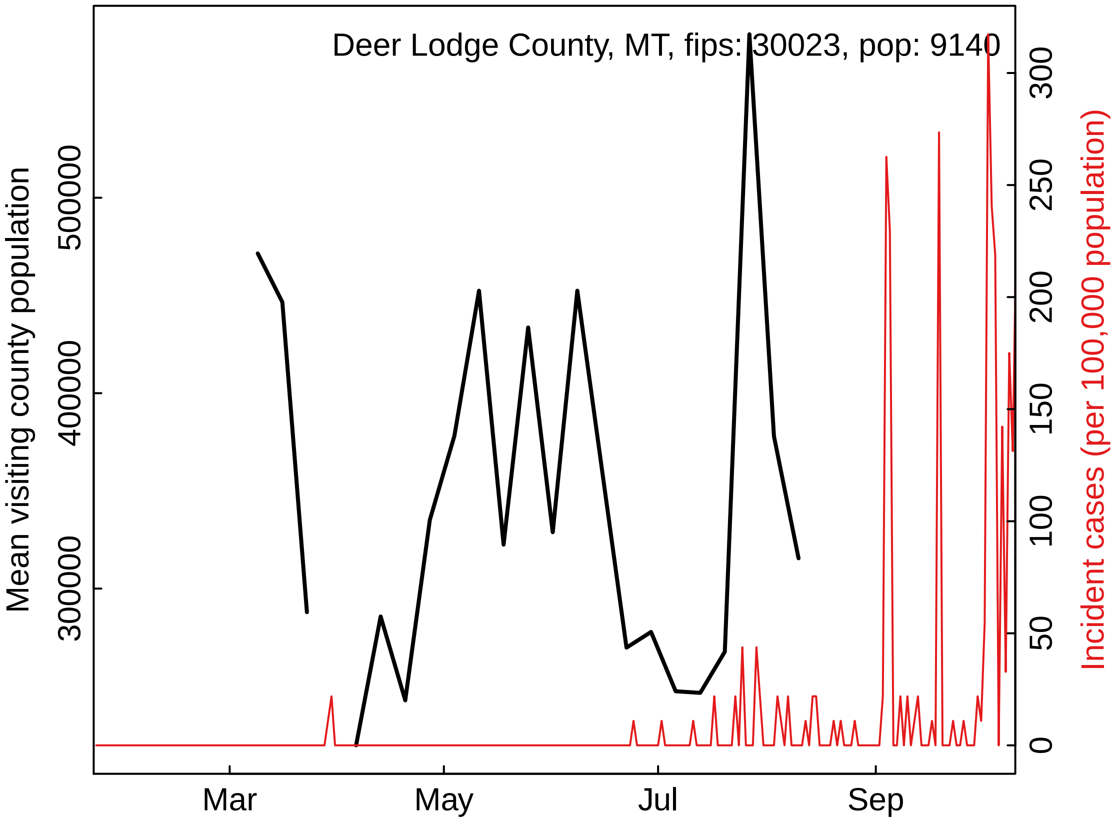
Deer Lodge County, MT, fips: 30023, pop: 9140
Mean visiting county population
300000
400000
500000
Incident cases (per 100,000 population)
0
50
100
150
200
250
300
Mar
May
Jul
Sep

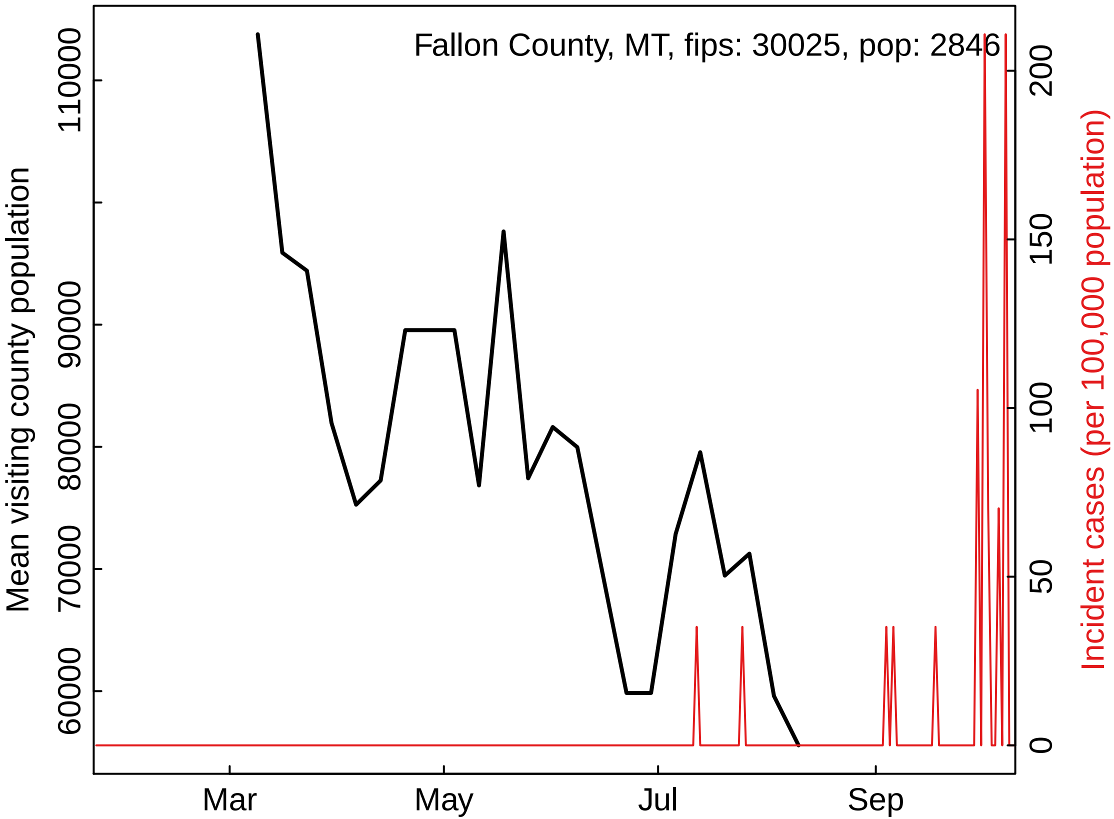
Fallon County, MT, fips: 30025, pop: 2846
Mean visiting county population
60000
70000
80000
90000
110000
Incident cases (per 100,000 population)
0
50
100
150
200
Mar
May
Jul
Sep

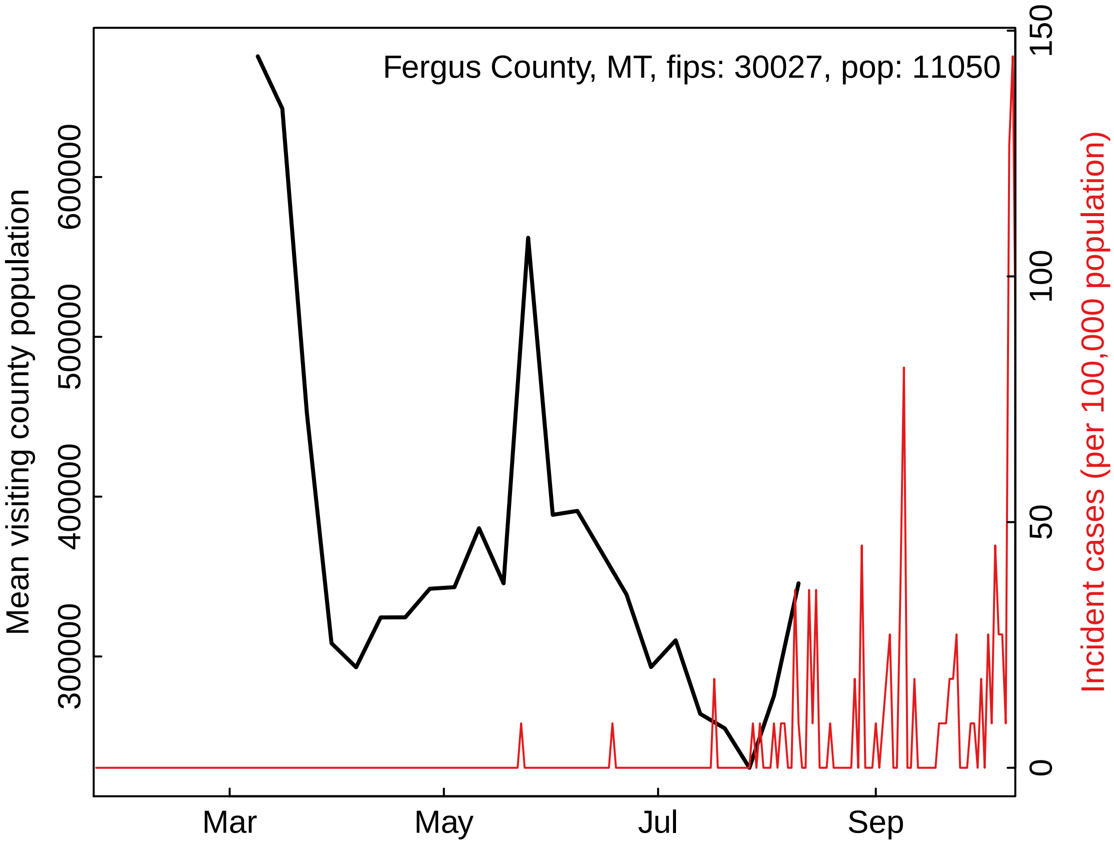

Fergus County, MT, fips: 30027, pop: 11050
Mean visiting county population
300000
400000
500000
600000
Incident cases (per 100,000 population)
0
50
100
150
Mar
May
Jul
Sep

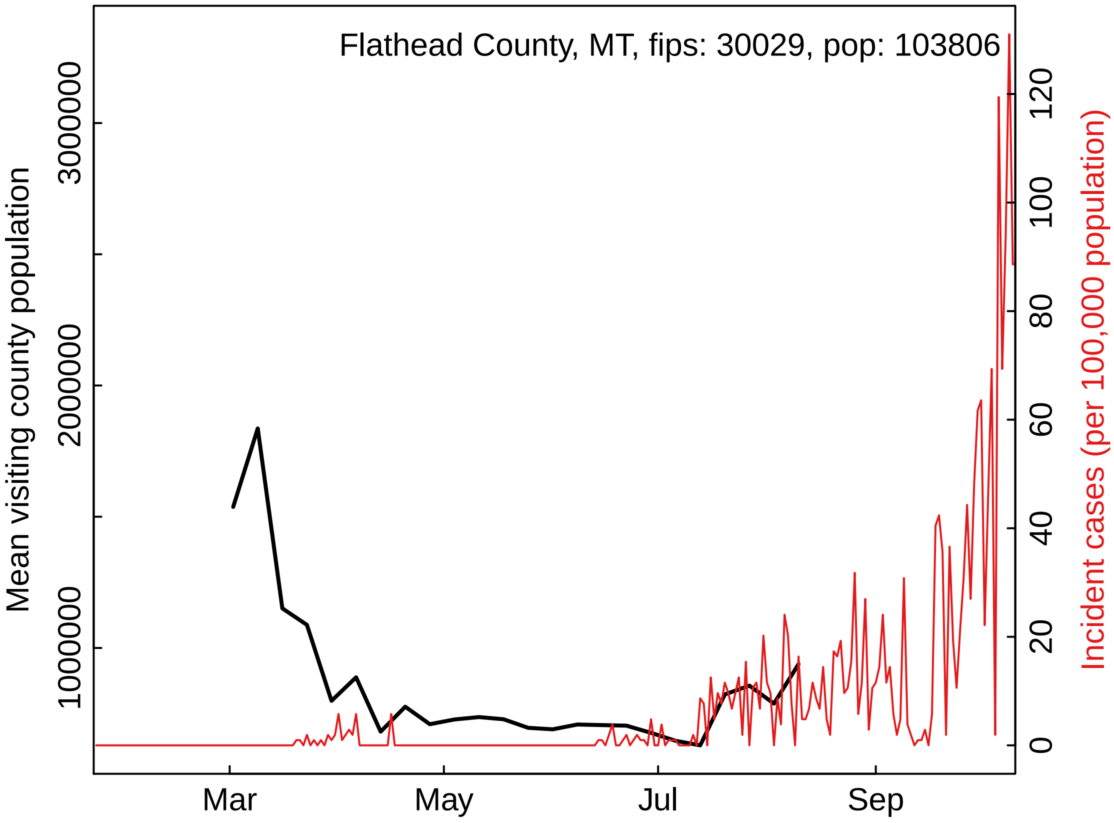

Flathead County, MT, fips: 30029, pop: 103806
Mean visiting county population
1000000
2000000
3000000
Incident cases (per 100,000 population)
0
20
40
60
80
100
120
Mar
May
Jul
Sep

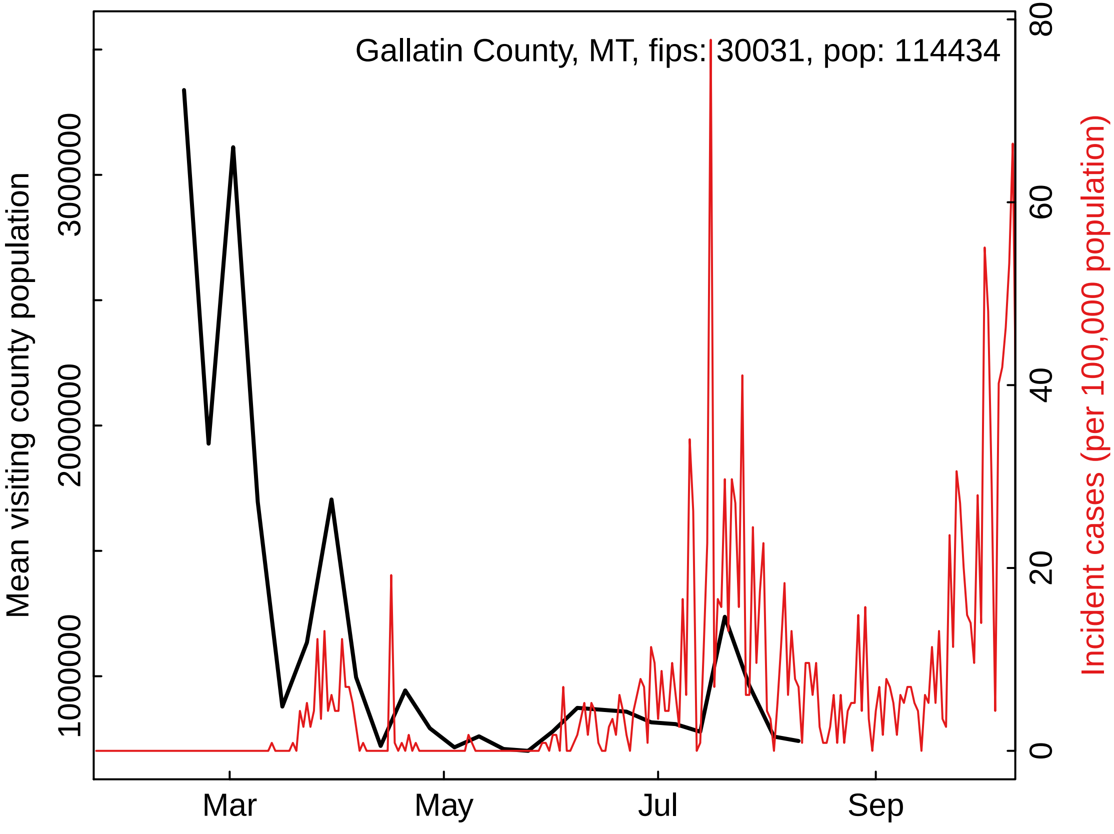

Gallatin County, MT, fips: 30031, pop: 114434
Mean visiting county population
10000000
20000000
30000000
Incident cases (per 100,000 population)
0
20
40
60
80
Mar
May
Jul
Sep

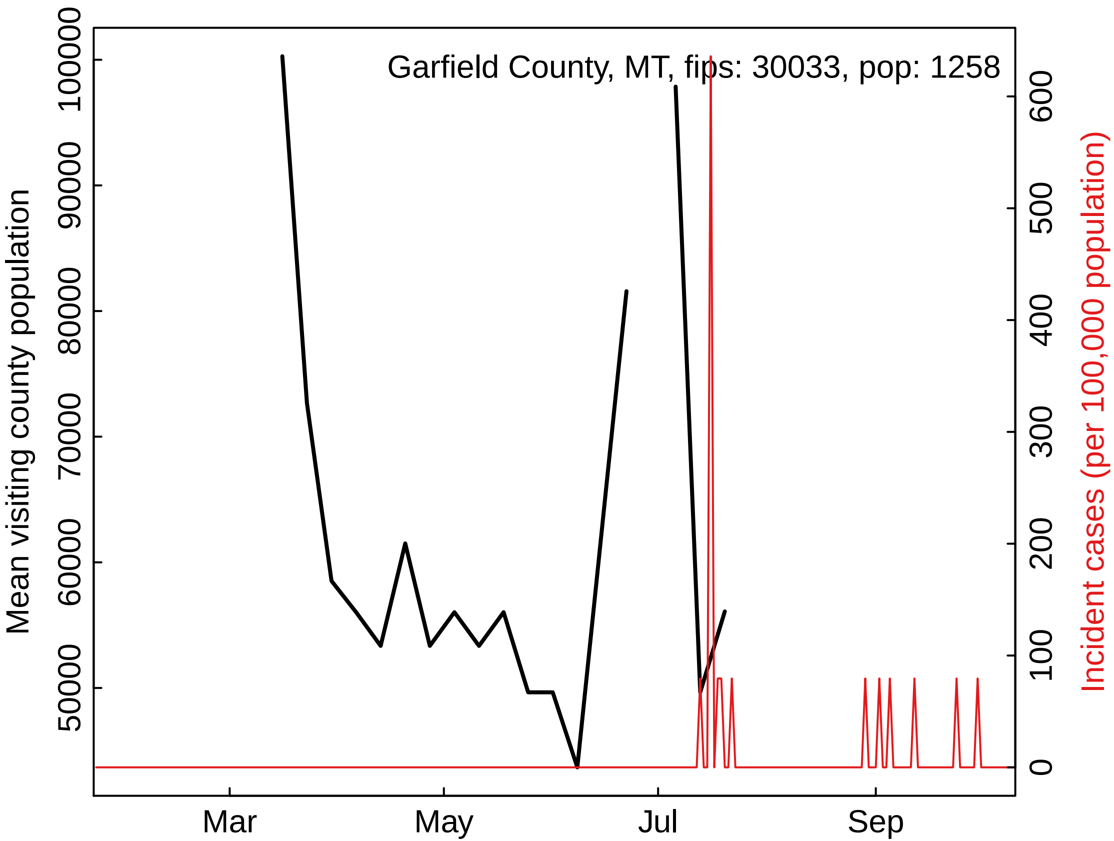

Garfield County, MT, fips: 30033, pop: 1258
Mean visiting county population
50000
60000
70000
80000
90000
100000
Incident cases (per 100,000 population)
0
100
200
300
400
500
600
Mar
May
Jul
Sep

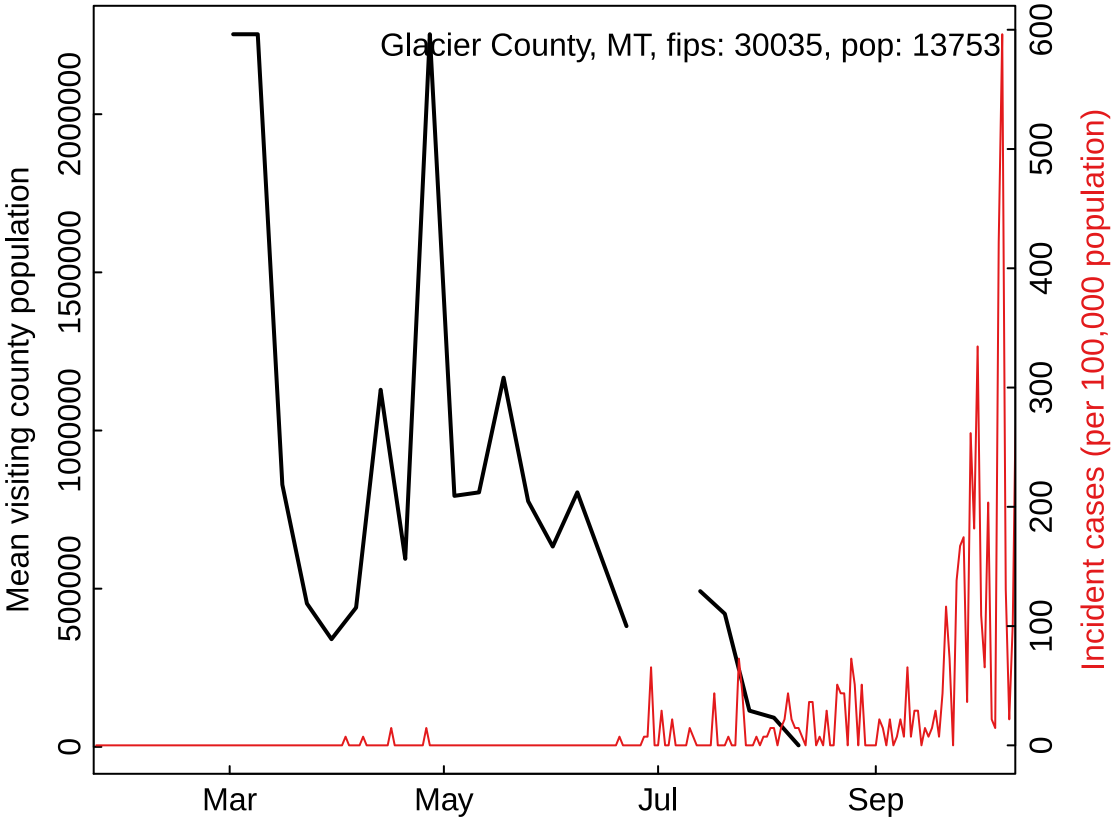
Glacier County, MT, fips: 30035, pop: 13753
Mean visiting county population
0
500000
1000000
1500000
2000000
Incident cases (per 100,000 population)
0
100
200
300
400
500
600
Mar
May
Jul
Sep

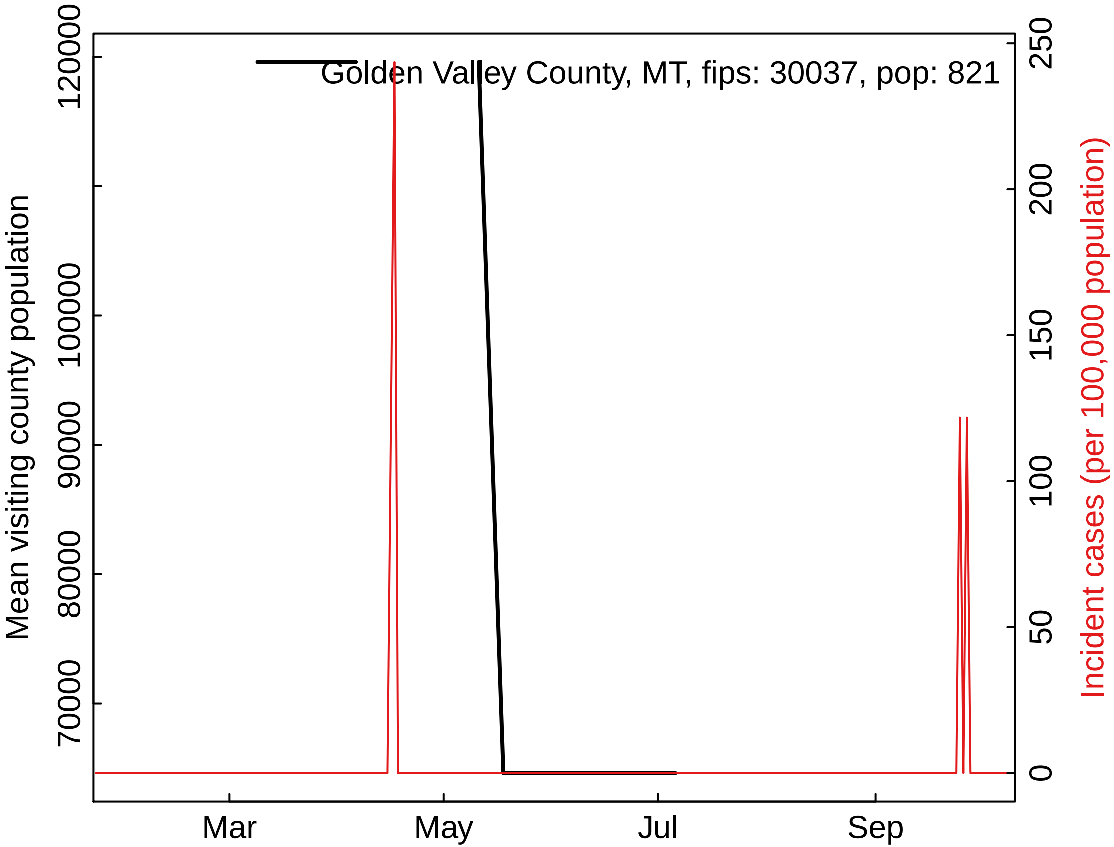

Golden Valley County, MT, fips: 30037, pop: 821
Mean visiting county population
70000
80000
90000
100000
120000
Incident cases (per 100,000 population)
0
50
100
150
200
250
Mar
May
Jul
Sep

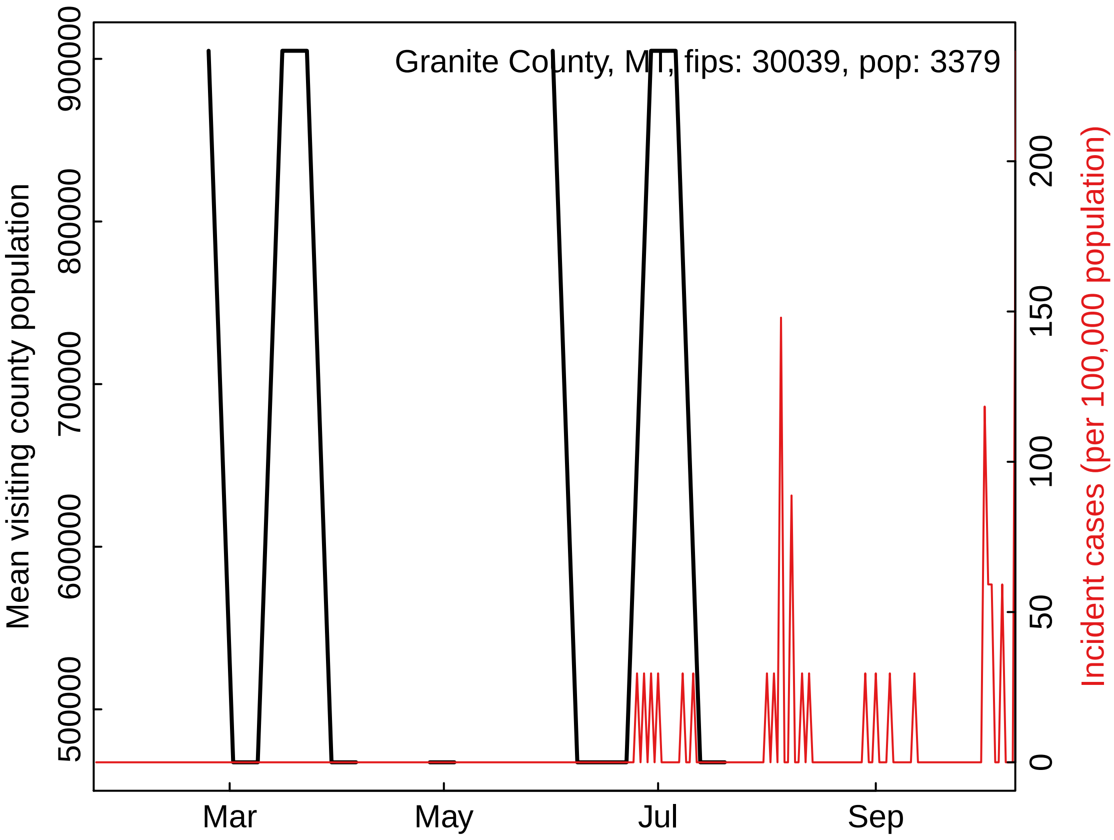

Granite County, MT, fips: 30039, pop: 3379
Mean visiting county population
500000
600000
700000
800000
900000
Incident cases (per 100,000 population)
0
50
100
150
200
Mar
May
Jul
Sep

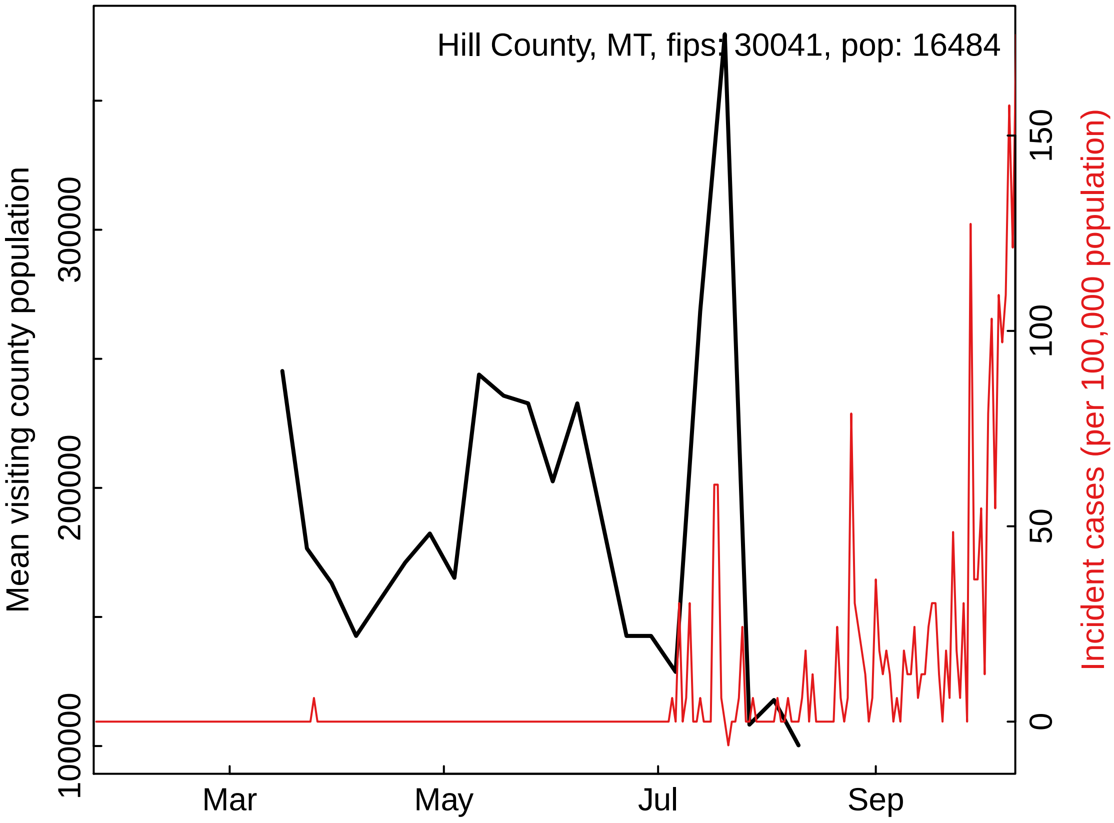
Hill County, MT, fips: 30041, pop: 16484
Mean visiting county population
Incident cases (per 100,000 population)
1000000
2000000
3000000
0
50
100
150
Mar
May
Jul
Sep

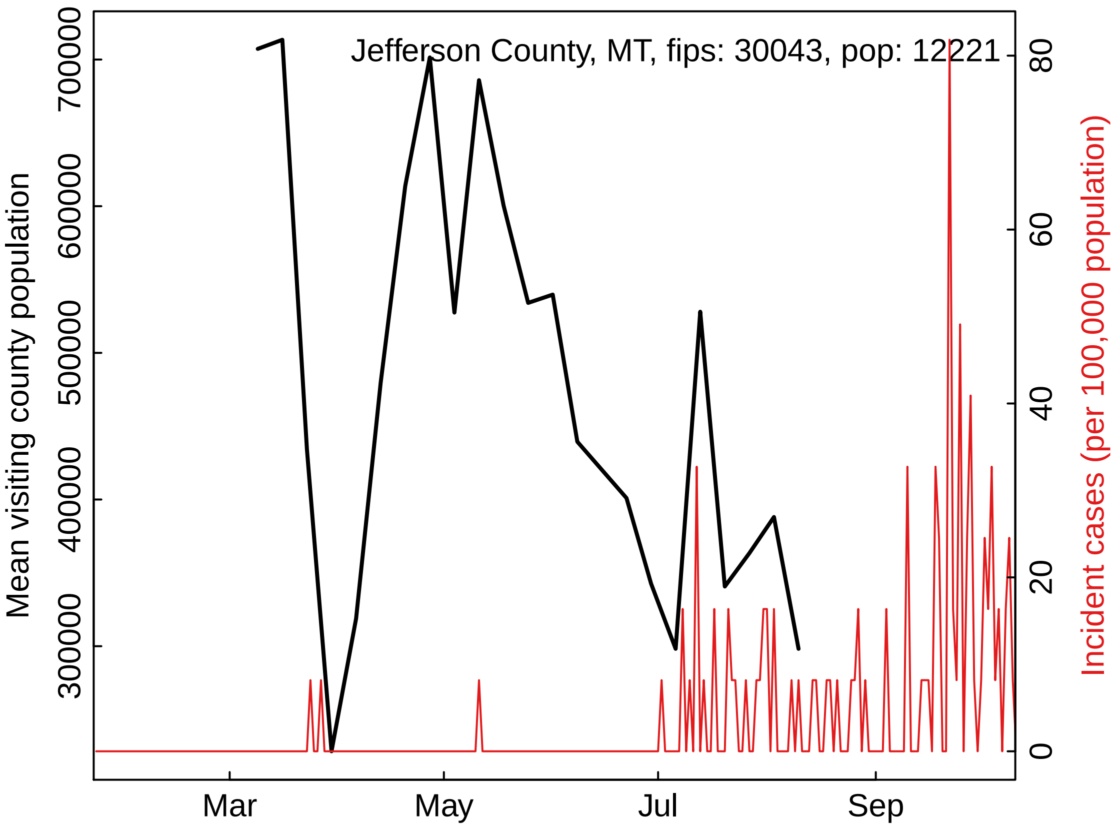

Jefferson County, MT, fips: 30043, pop: 12221
Mean visiting county population
300000
400000
500000
600000
700000
Incident cases (per 100,000 population)
0
20
40
60
80
Mar
May
Jul
Sep

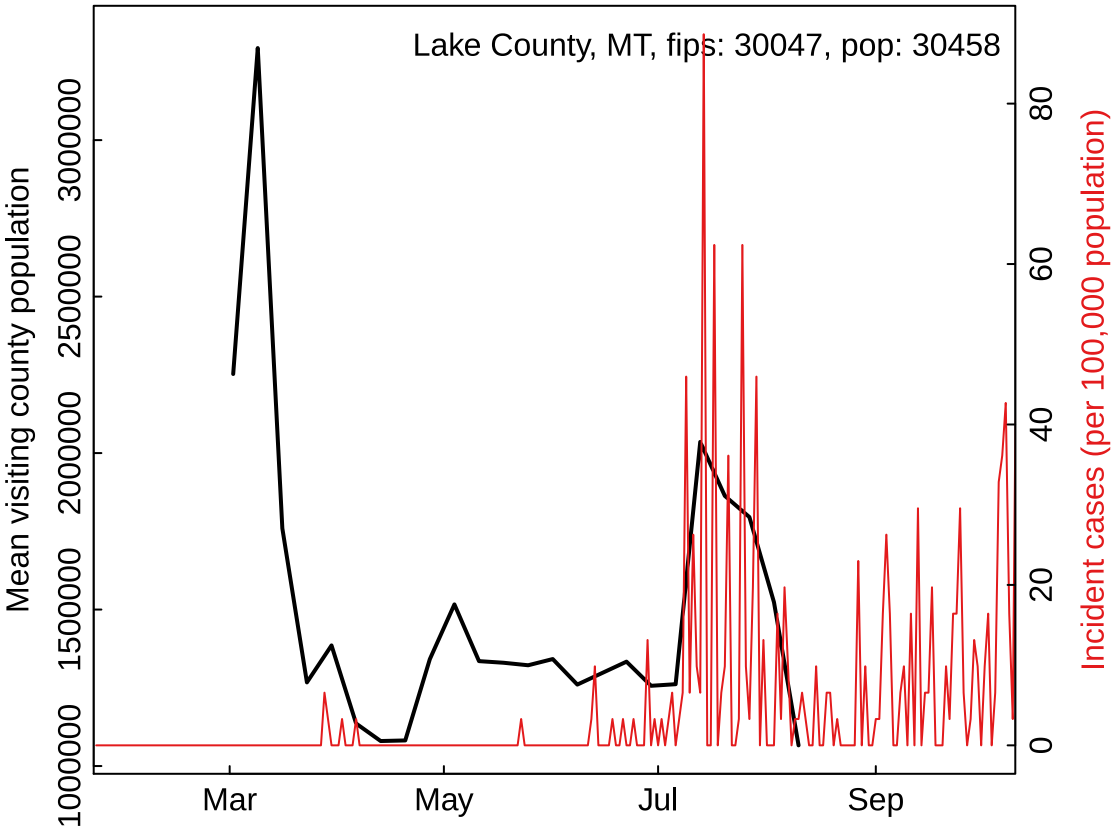

Lake County, MT, fips: 30047, pop: 30458
Mean visiting county population
10000000
1500000
2000000
2500000
3000000
Incident cases (per 100,000 population)
0
20
40
60
80
Mar
May
Jul
Sep

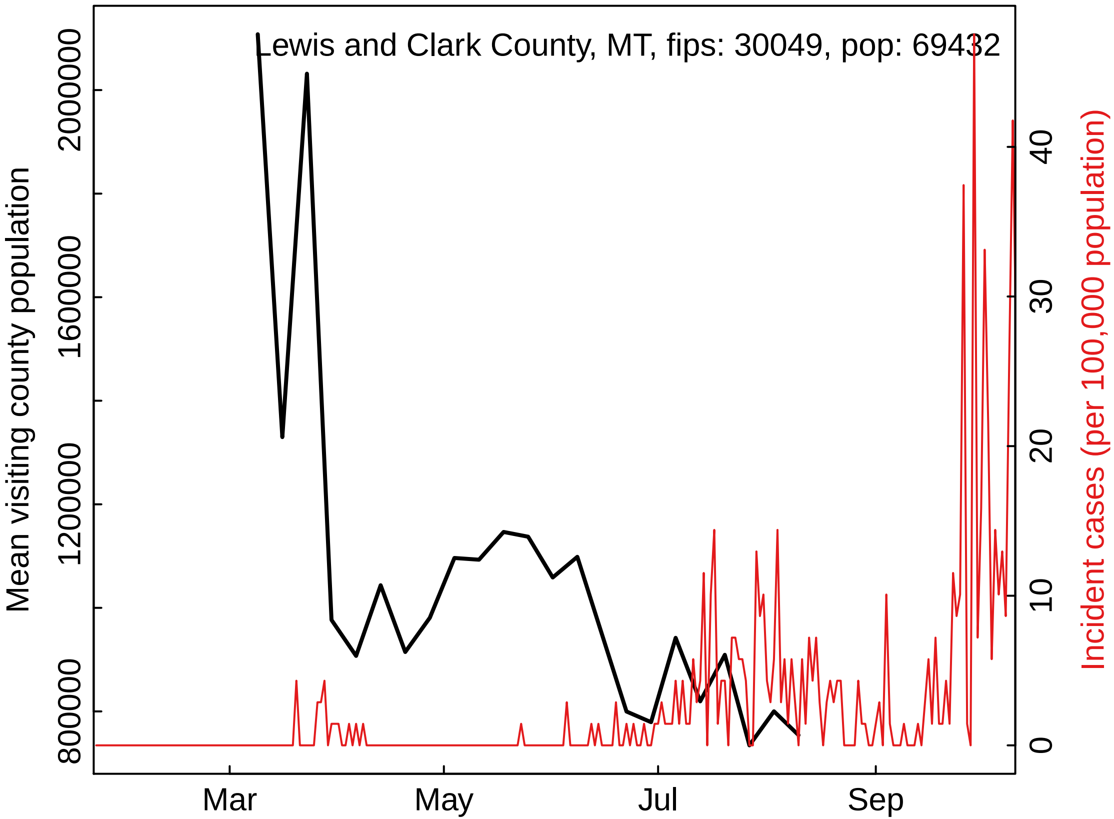

Lewis and Clark County, MT, fips: 30049, pop: 69432
Mean visiting county population
800000
1200000
1600000
2000000
Incident cases (per 100,000 population)
0
10
20
30
40
Mar
May
Jul
Sep

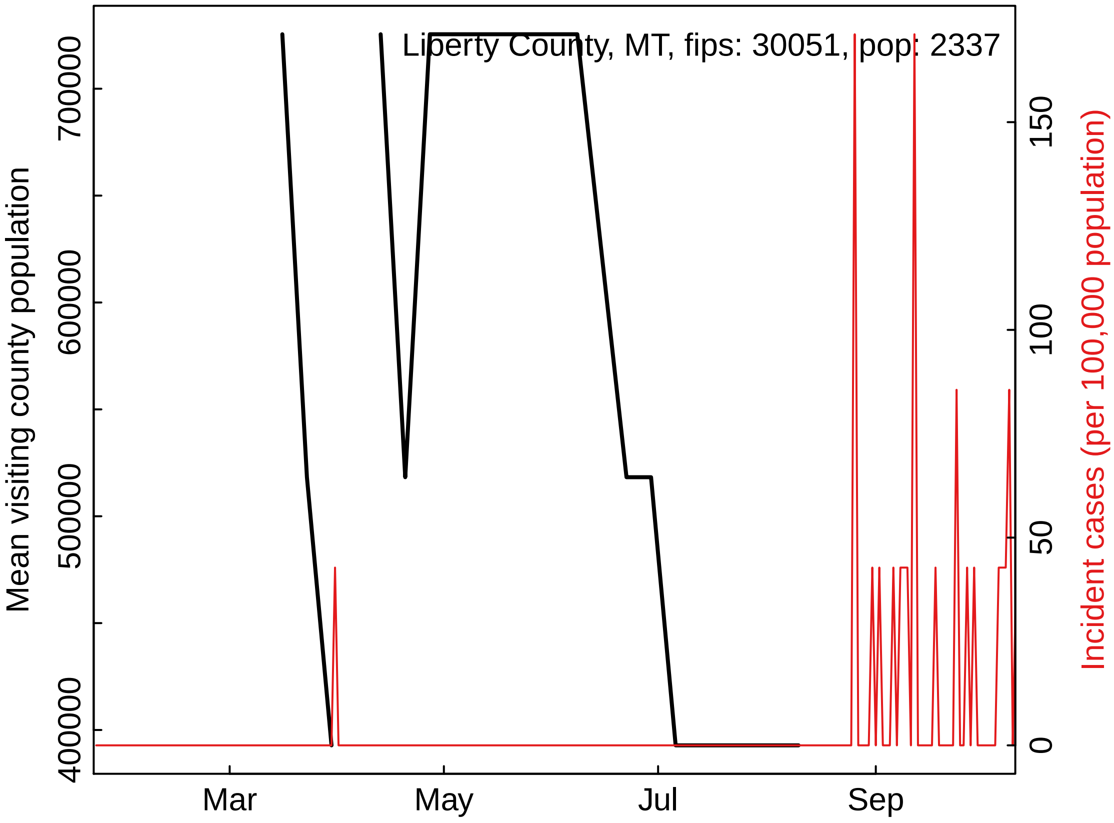

Liberty County, MT, fips: 30051, pop: 2337
Mean visiting county population
400000
500000
600000
700000
Incident cases (per 100,000 population)
0
50
100
150
Mar
May
Jul
Sep

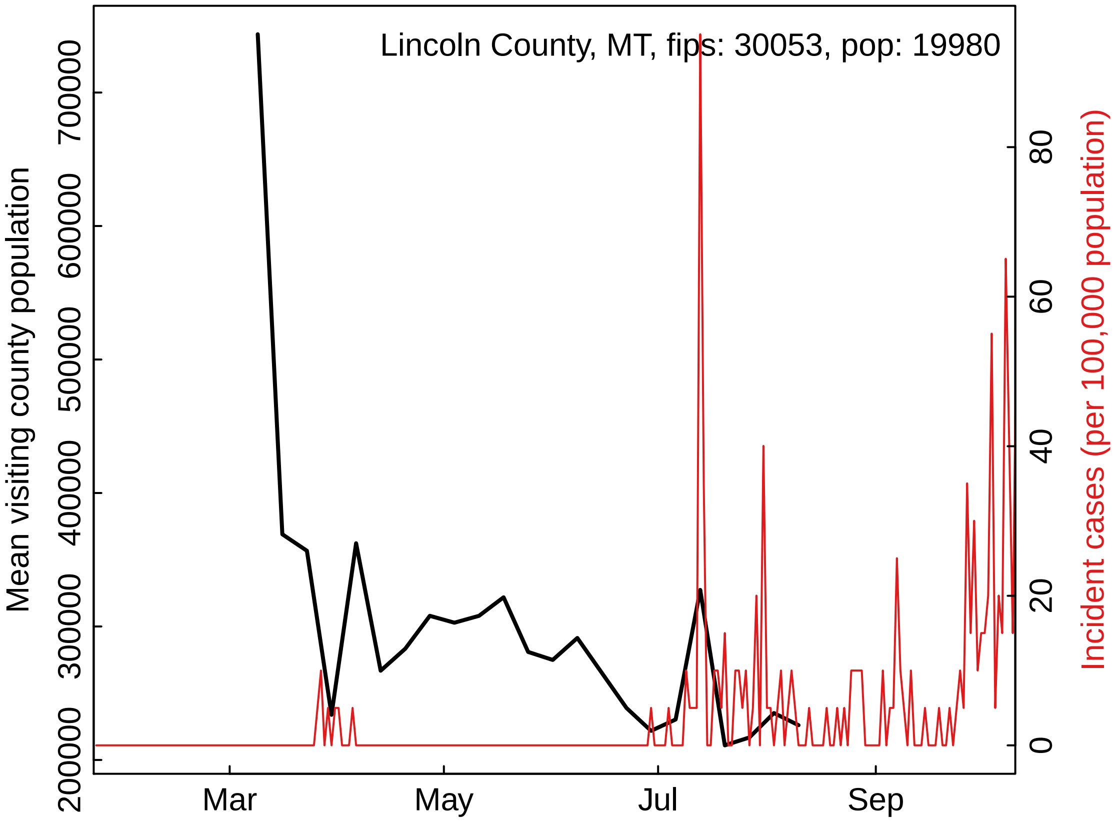

Lincoln County, MT, fips: 30053, pop: 19980
Mean visiting county population
200000
300000
400000
500000
600000
700000
Mar
May
Jul
Sep
0
20
40
60
80
Incident cases (per 100,000 population)

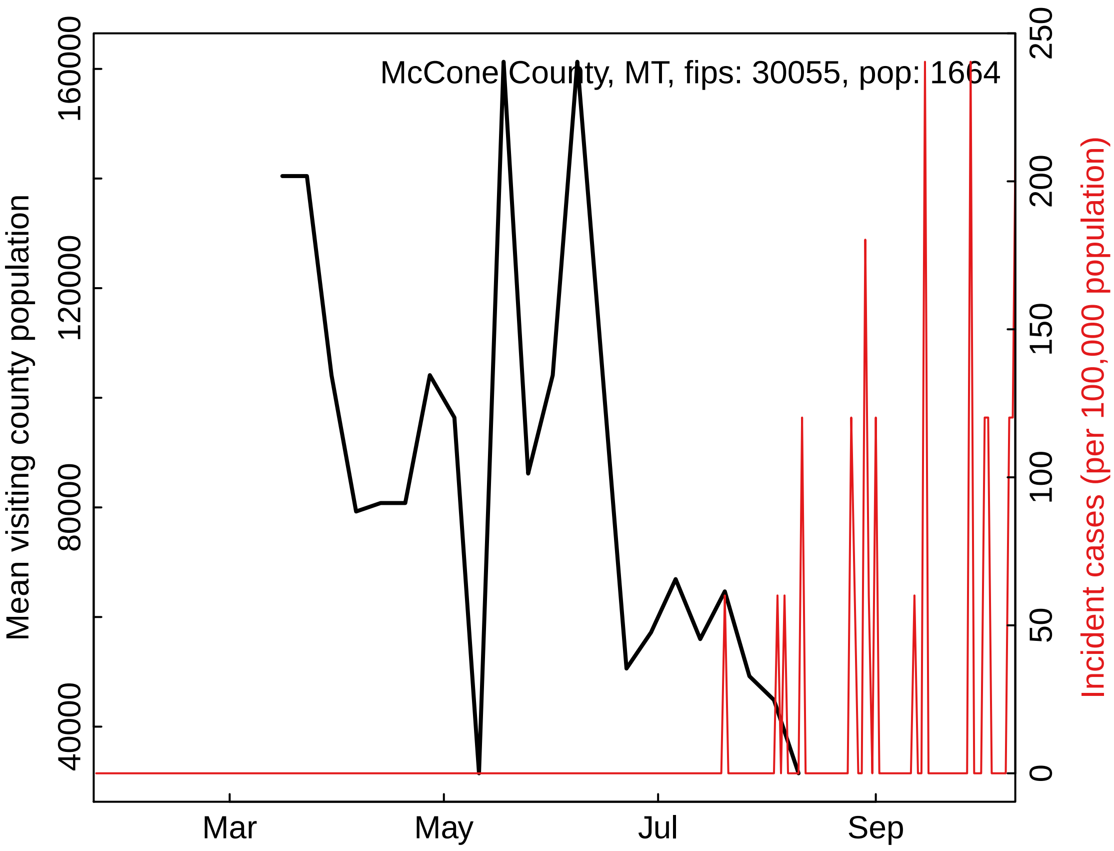
McCone County, MT, fips: 30055, pop: 1664
Mean visiting county population
40000
80000
120000
160000
Incident cases (per 100,000 population)
0
50
100
150
200
250
Mar
May
Jul
Sep

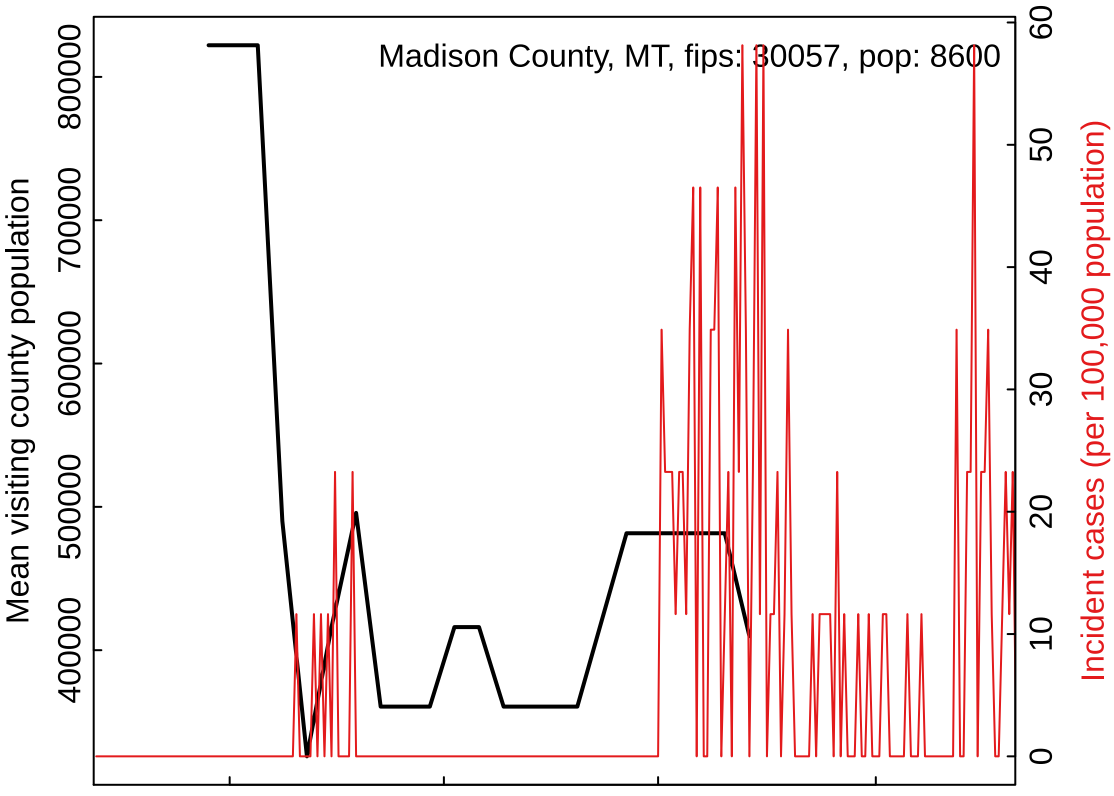
Madison County, MT, fips: 30057, pop: 8600
Mean visiting county population
400000
500000
600000
700000
800000
Incident cases (per 100,000 population)
0
10
20
30
40
50
60
Mar
May
Jul
Sep

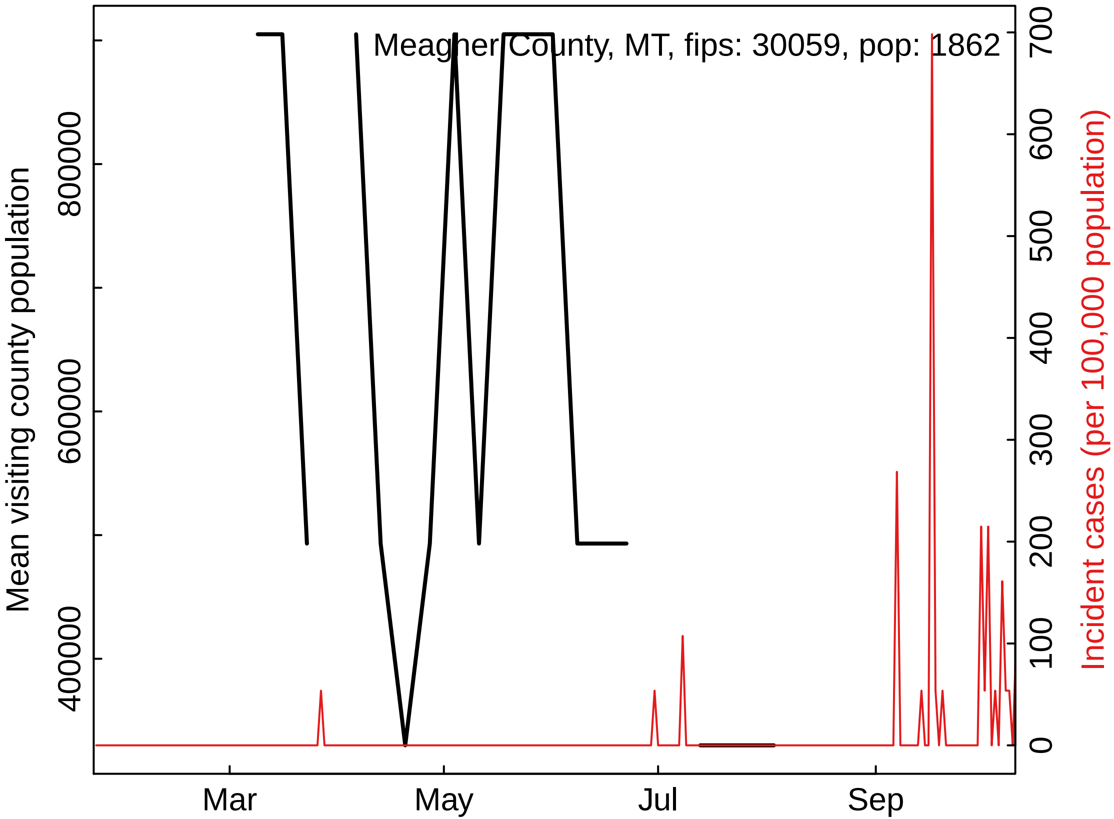

Meagher County, MT, fips: 30059, pop: 1862
Mean visiting county population
400000
600000
800000
Mar
May
Jul
Sep
0
100
200
300
400
500
600
700
Incident cases (per 100,000 population)

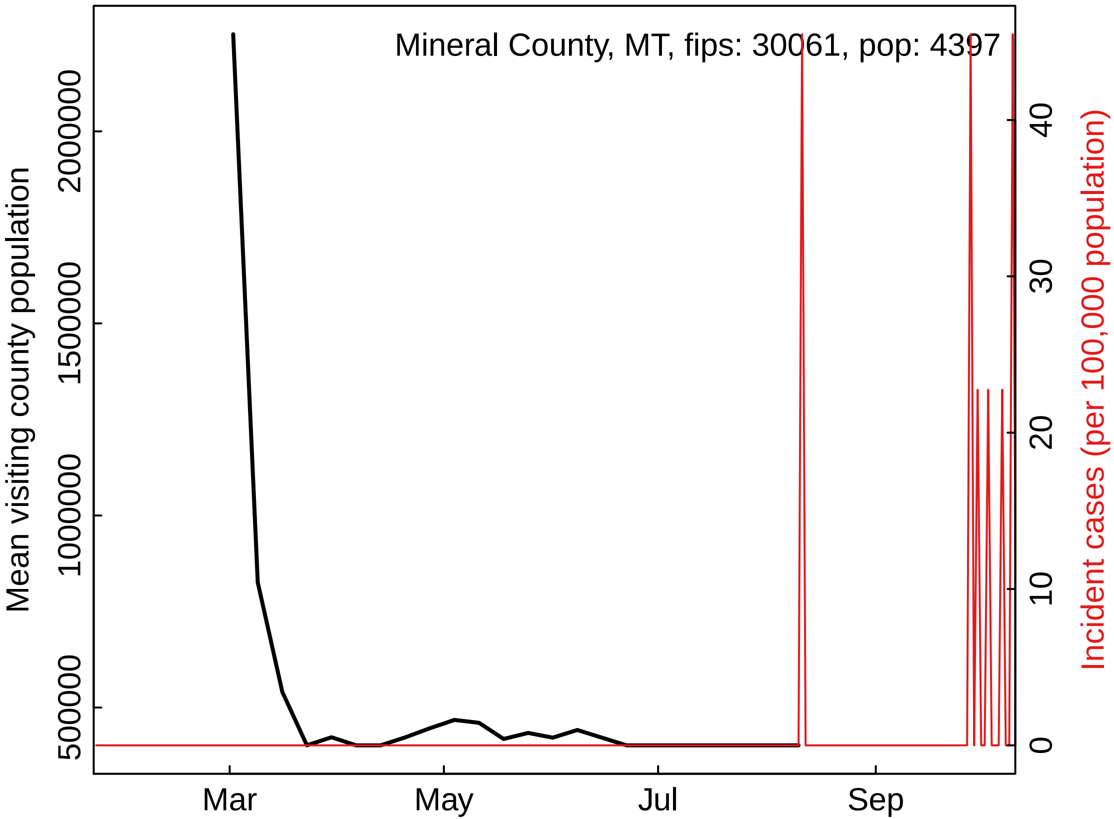

Mineral County, MT, fips: 30061, pop: 4397
Mean visiting county population
500000
1000000
1500000
2000000
Incident cases (per 100,000 population)
0
10
20
30
40
Mar
May
Jul
Sep

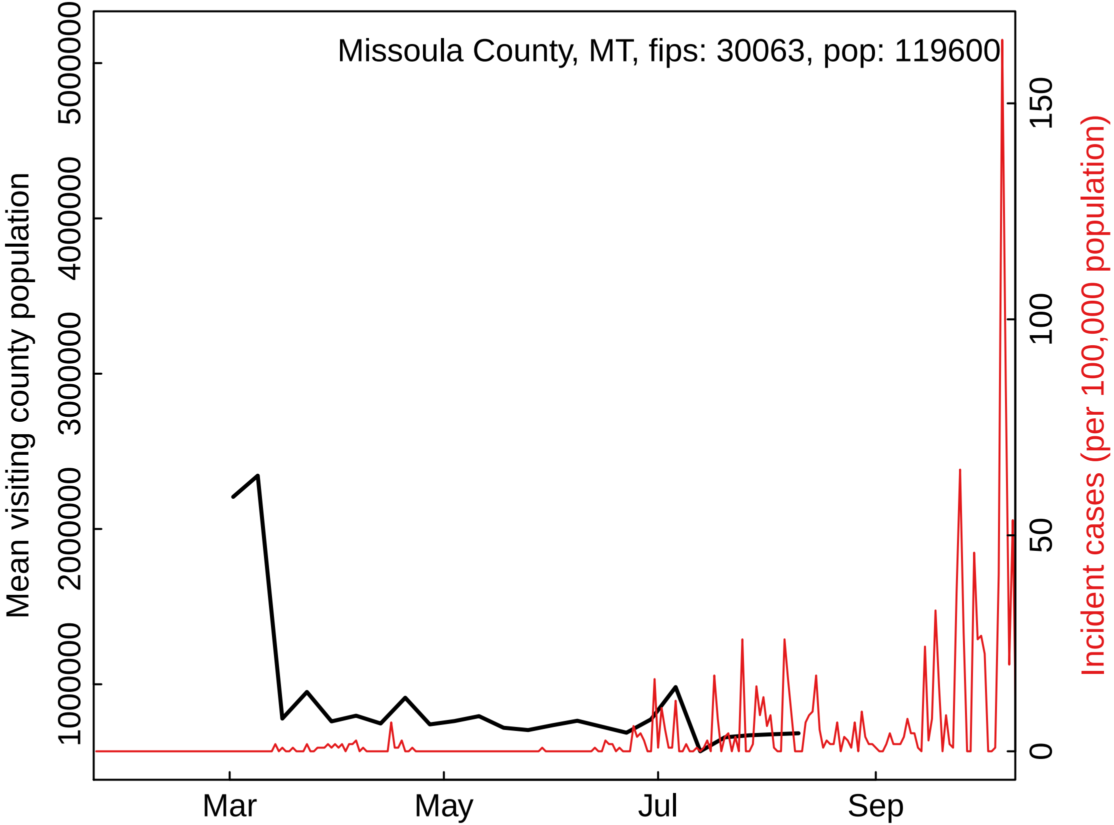
Missoula County, MT, fips: 30063, pop: 119600
Mean visiting county population
1000000
2000000
3000000
4000000
5000000
Incident cases (per 100,000 population)
0
50
100
150
Mar
May
Jul
Sep

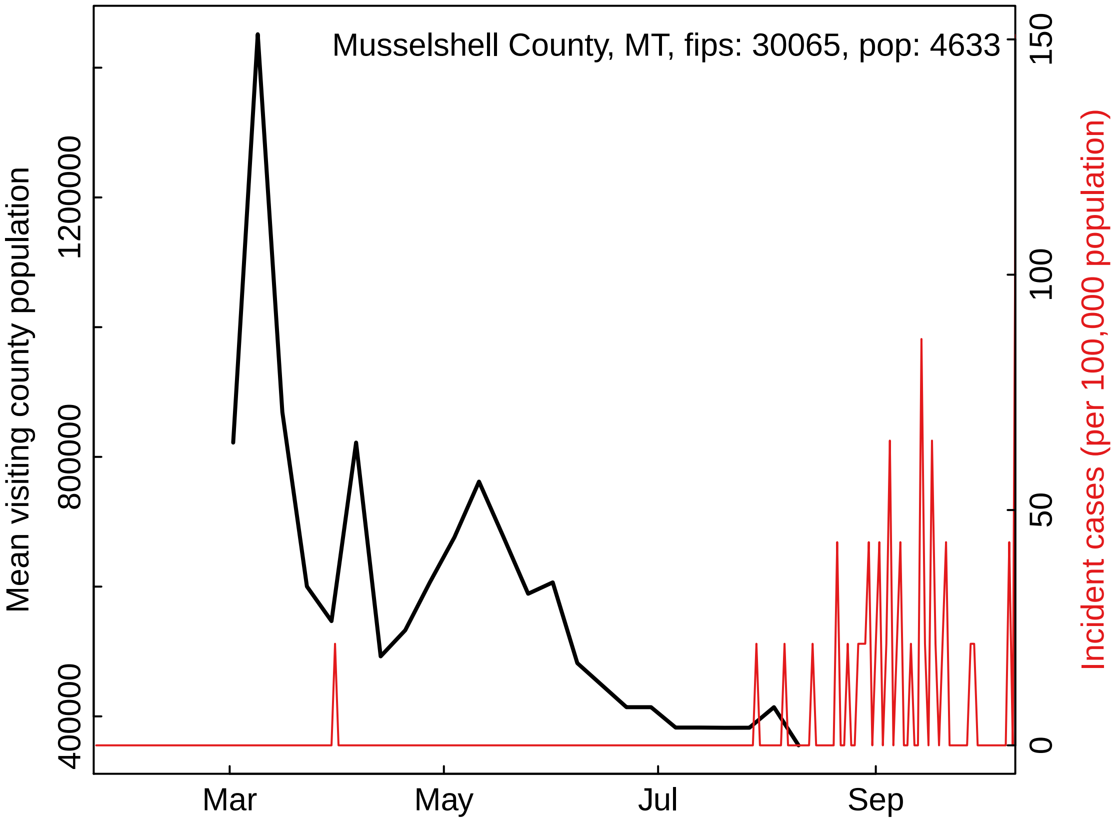

Musselshell County, MT, fips: 30065, pop: 4633
Mean visiting county population
400000
800000
1200000
Incident cases (per 100,000 population)
0
50
100
150
Mar
May
Jul
Sep

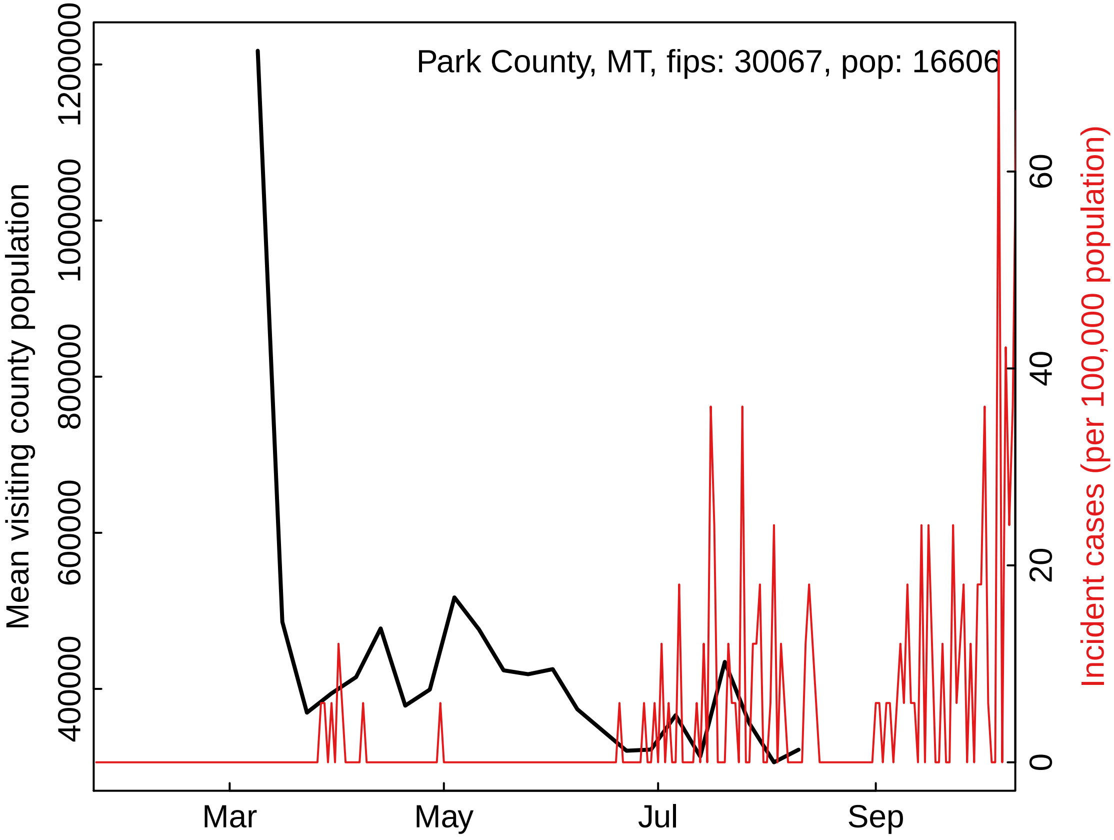

Park County, MT, fips: 30067, pop: 16606
Mean visiting county population
Incident cases (per 100,000 population)
400000
600000
800000
1000000
1200000
0
20
40
60
Mar
May
Jul
Sep

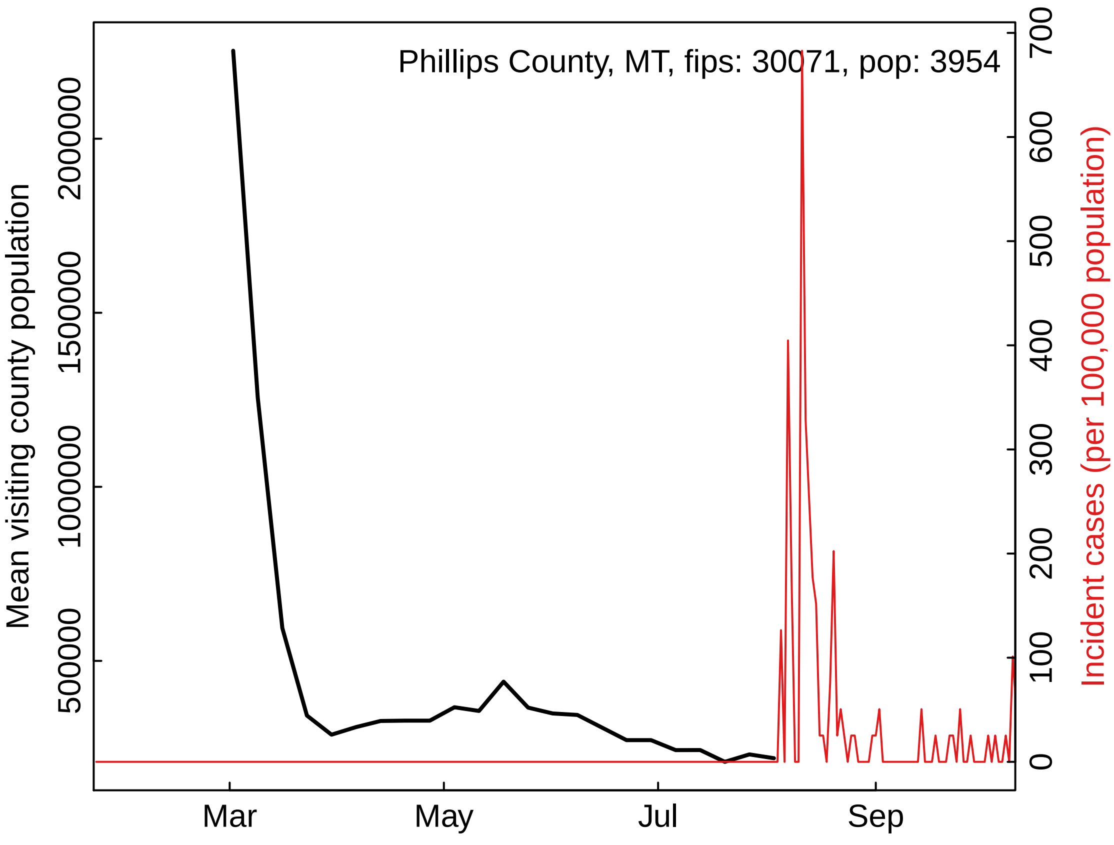
Phillips County, MT, fips: 30071, pop: 3954
Mean visiting county population
500000
1000000
1500000
2000000
Incident cases (per 100,000 population)
0
100
200
300
400
500
600
700
Mar
May
Jul
Sep

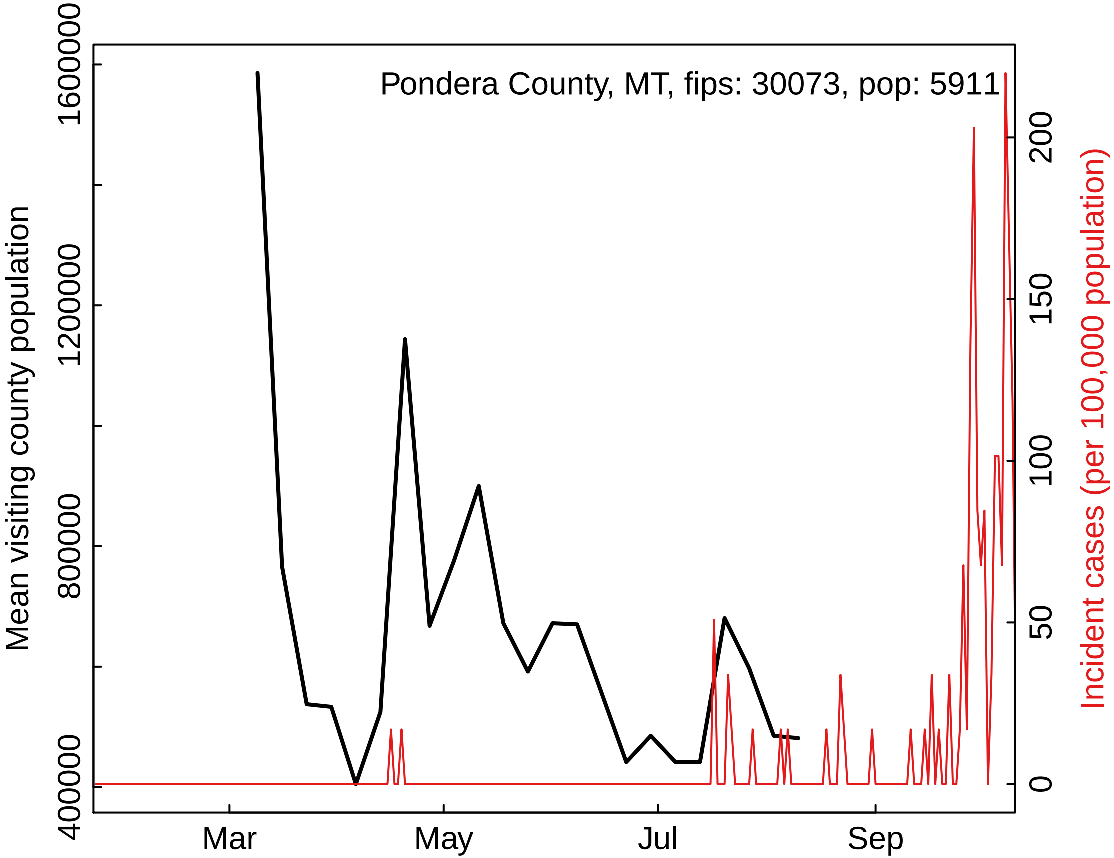
Pondera County, MT, fips: 30073, pop: 5911
Mean visiting county population
400000
800000
1200000
1600000
Incident cases (per 100,000 population)
0
50
100
150
200
Mar
May
Jul
Sep

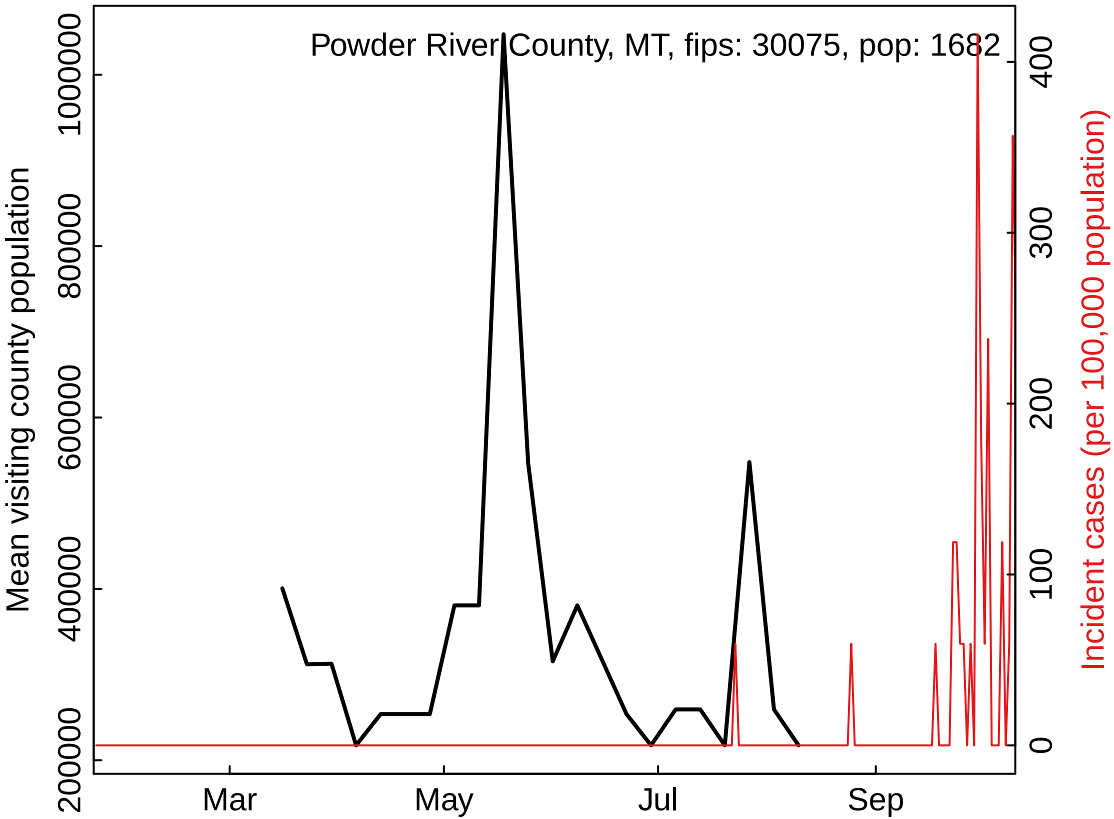
Powder River County, MT, fips: 30075, pop: 1682
Mean visiting county population
200000
400000
600000
800000
1000000
0
100
200
300
400
Incident cases (per 100,000 population)
Mar
May
Jul
Sep

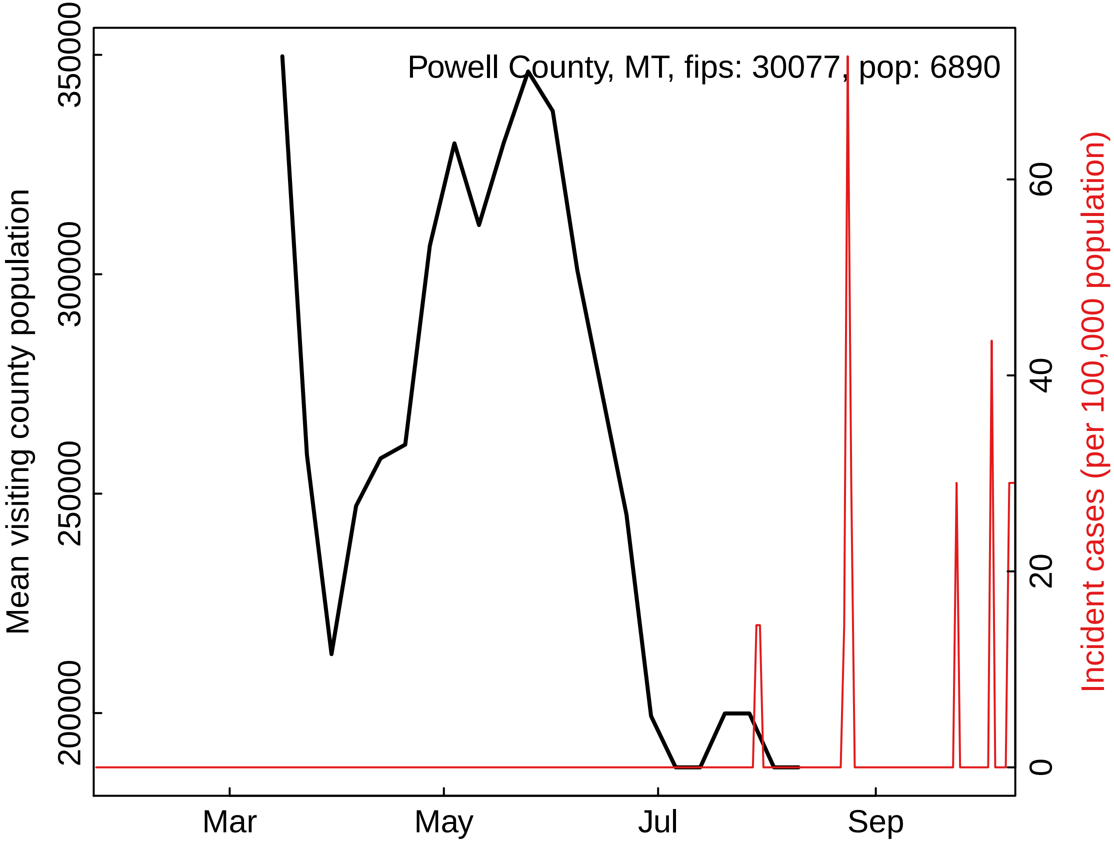
Powell County, MT, fips: 30077, pop: 6890
Mean visiting county population
200000
250000
300000
350000
Incident cases (per 100,000 population)
0
20
40
60
Mar
May
Jul
Sep

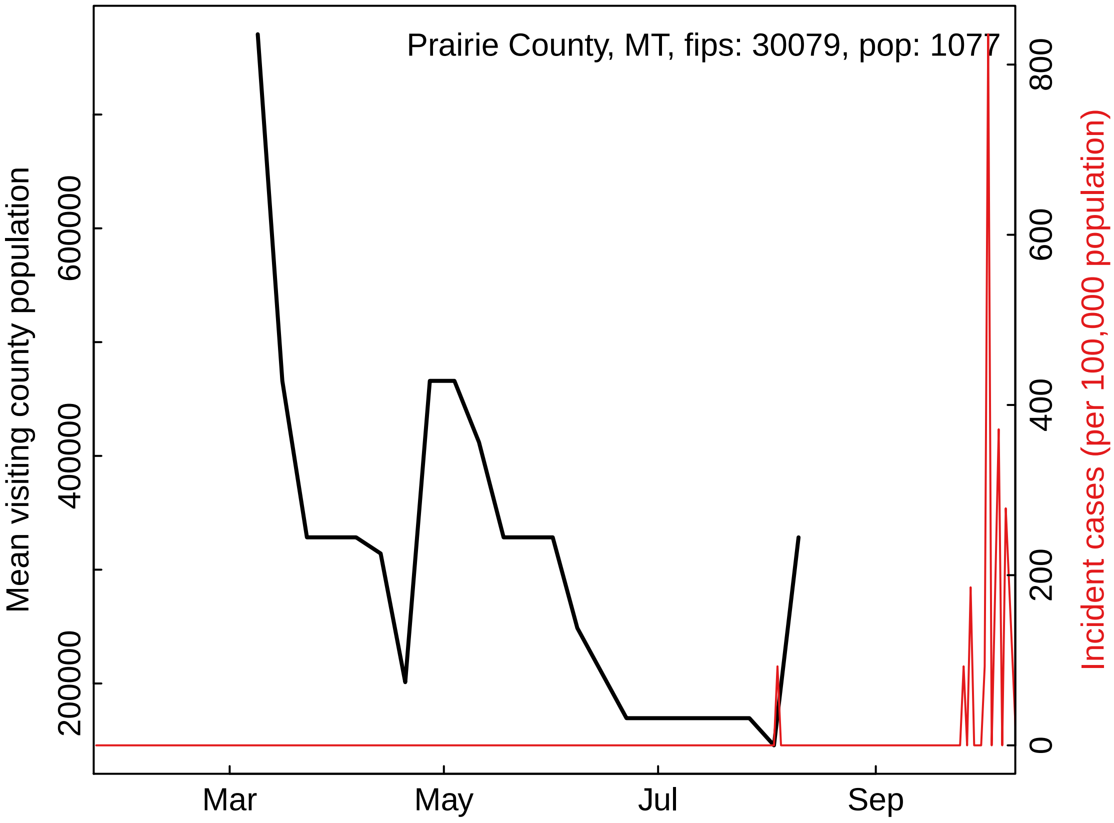

Prairie County, MT, fips: 30079, pop: 1077
Mean visiting county population
200000
400000
600000
Incident cases (per 100,000 population)
0
200
400
600
800
Mar
May
Jul
Sep

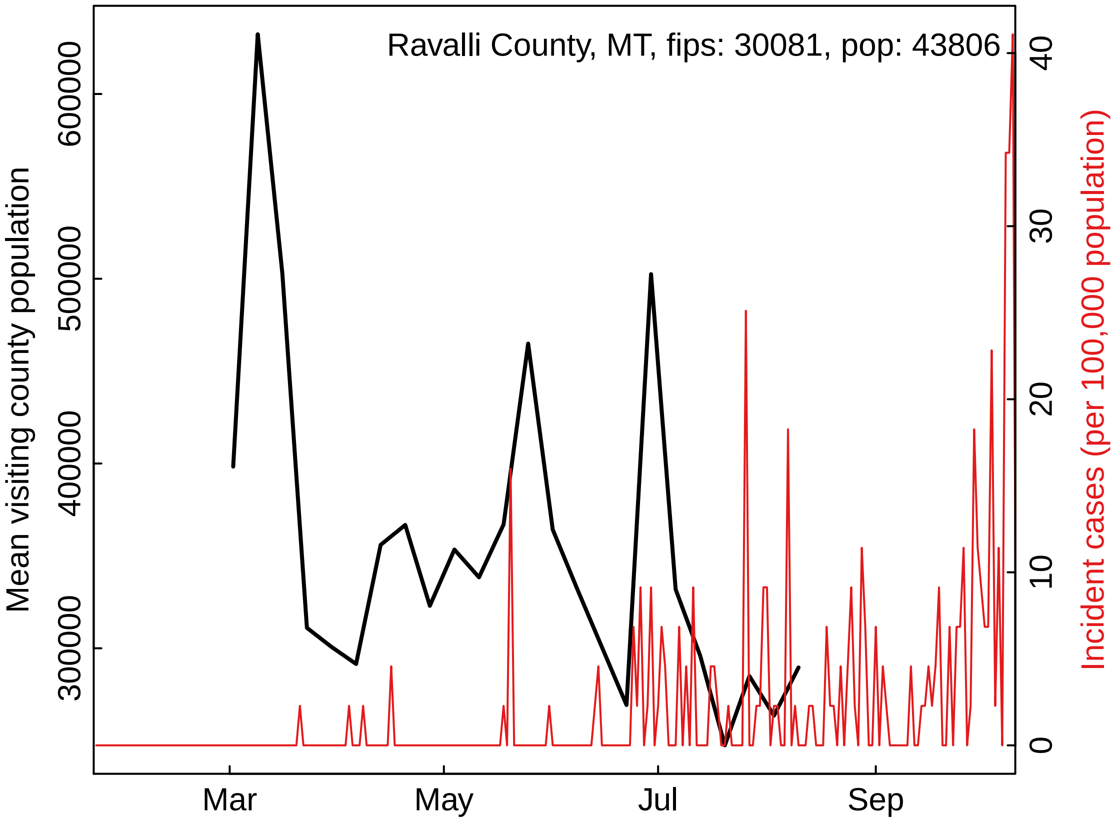
Ravalli County, MT, fips: 30081, pop: 43806
Mean visiting county population
300000
400000
500000
600000
Incident cases (per 100,000 population)
0
10
20
30
40
Mar
May
Jul
Sep

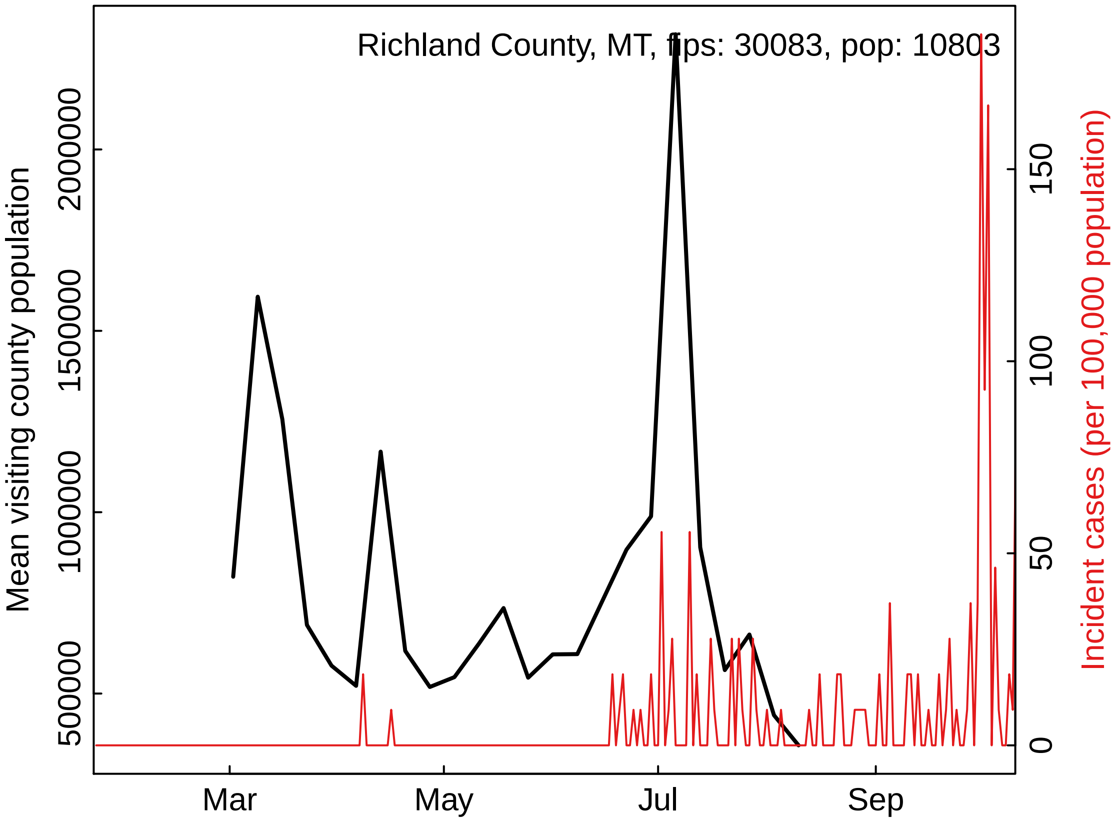
Richland County, MT, fips: 30083, pop: 10803
Mean visiting county population
500000
1000000
1500000
2000000
Incident cases (per 100,000 population)
0
50
100
150
Mar
May
Jul
Sep

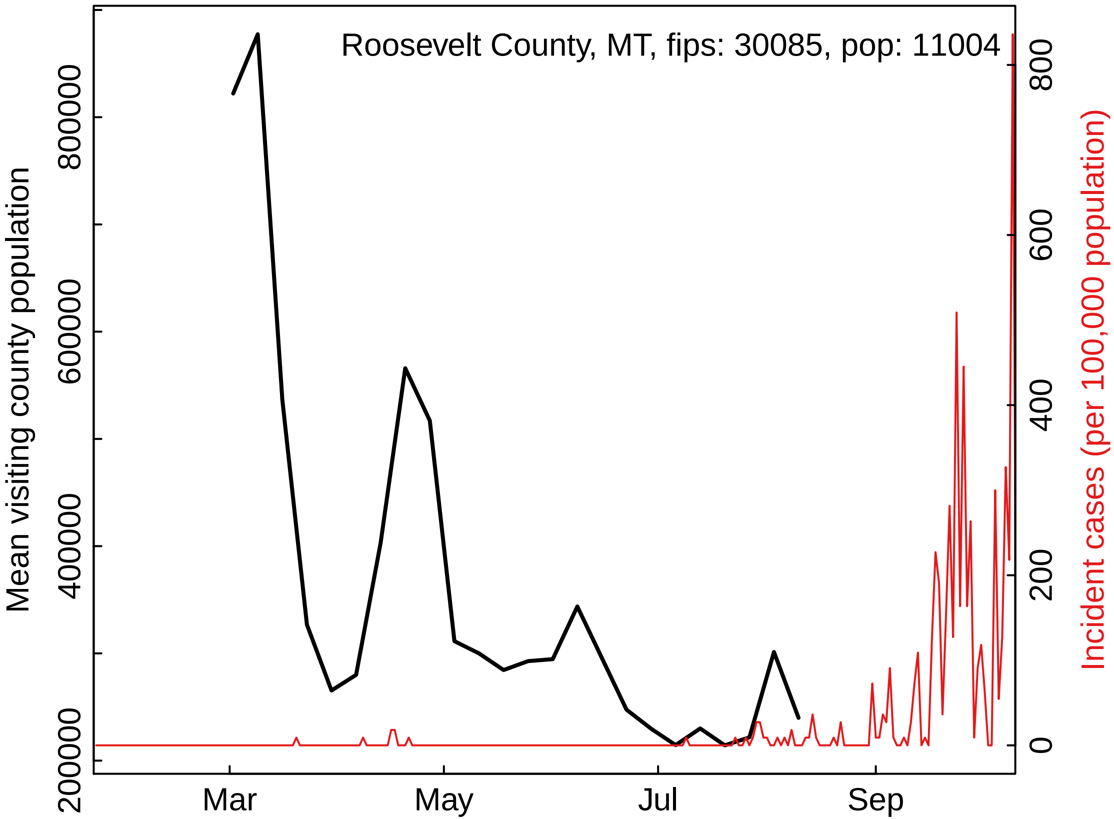
Roosevelt County, MT, fips: 30085, pop: 11004
Mean visiting county population
2000000
4000000
6000000
8000000
Incident cases (per 100,000 population)
0
200
400
600
800
Mar
May
Jul
Sep

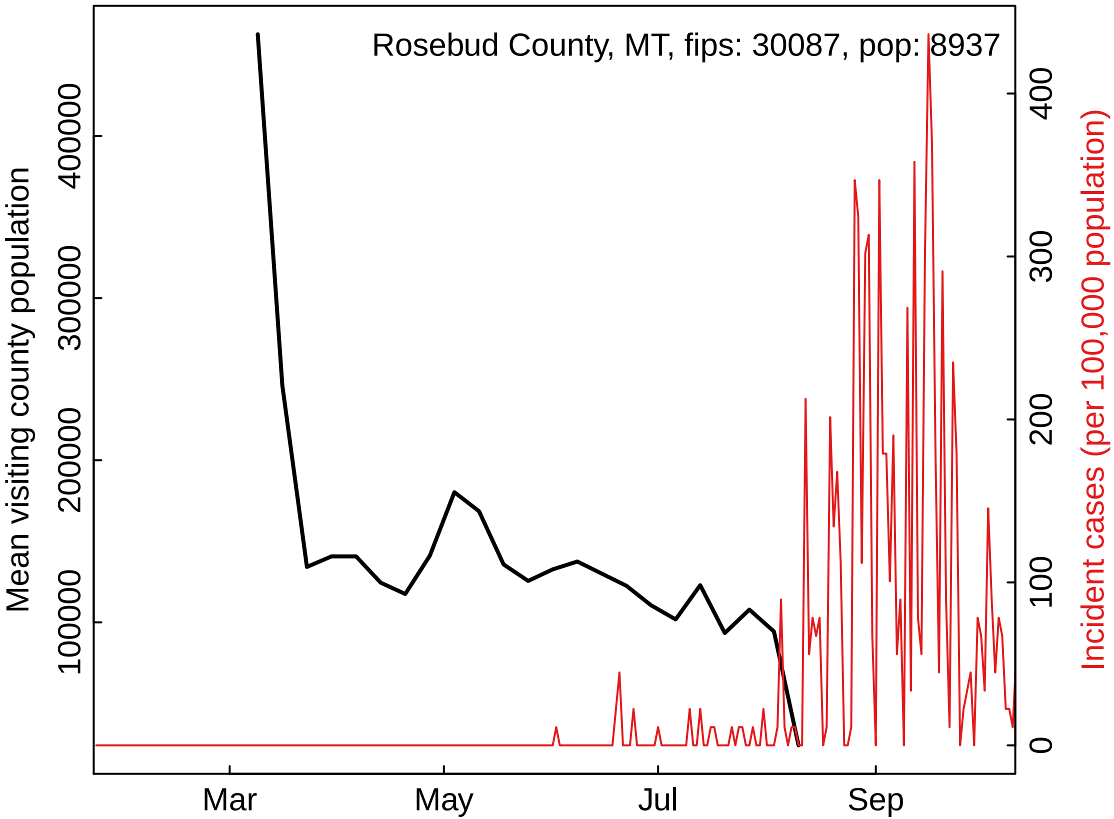

Rosebud County, MT, fips: 30087, pop: 8937
Mean visiting county population
100000
200000
300000
400000
Incident cases (per 100,000 population)
0
100
200
300
400
Mar
May
Jul
Sep

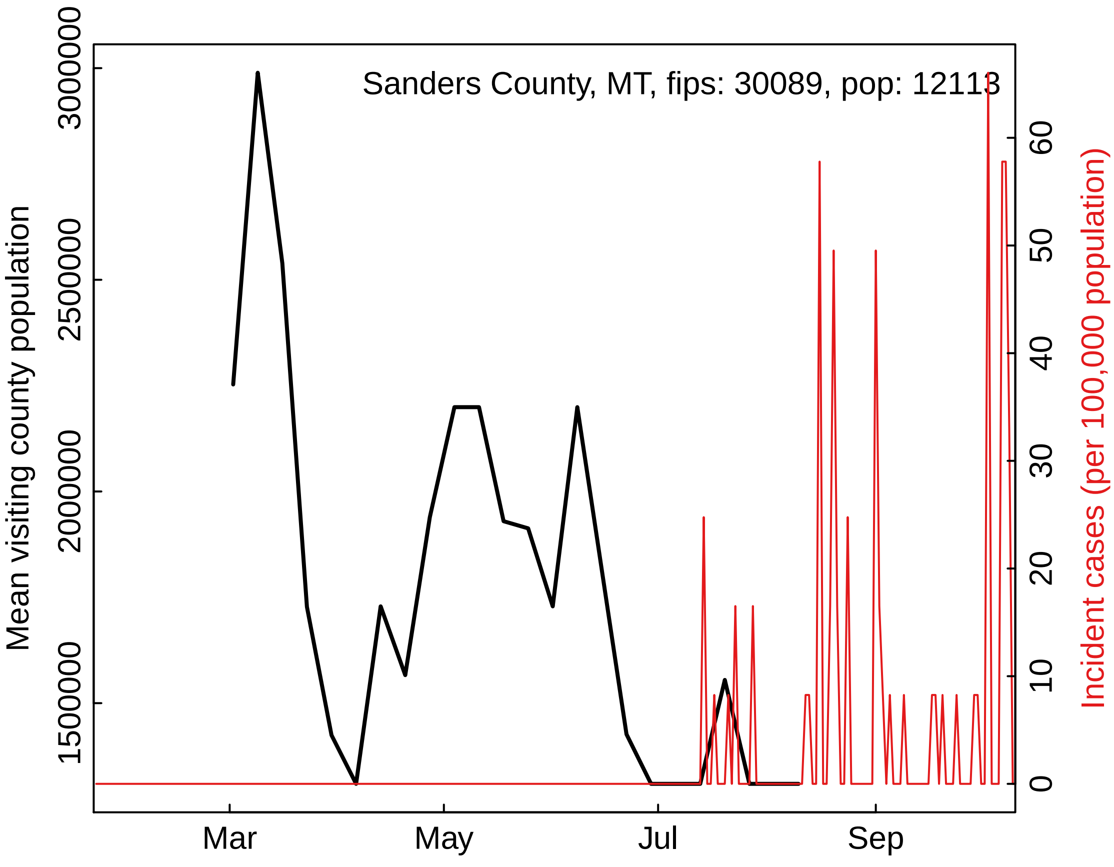

Sanders County, MT, fips: 30089, pop: 12113
Mean visiting county population
1500000
2000000
2500000
3000000
Incident cases (per 100,000 population)
0
10
20
30
40
50
60
Mar
May
Jul
Sep

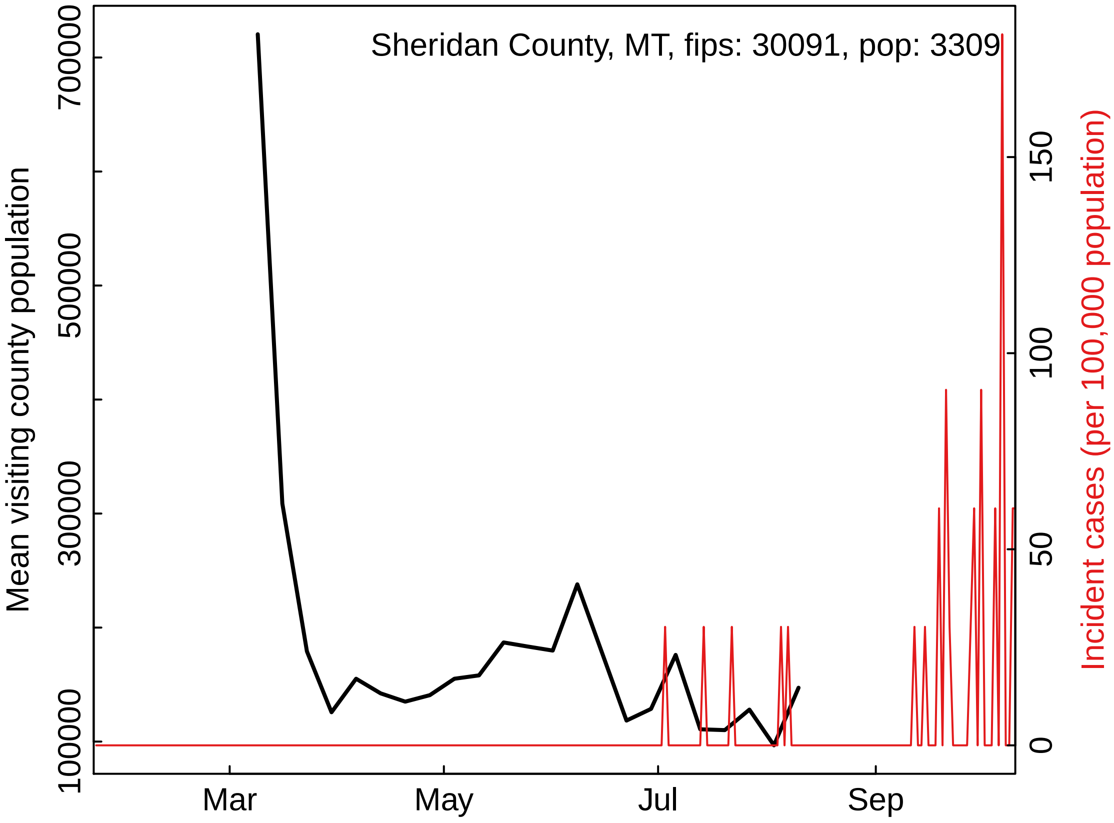

Sheridan County, MT, fips: 30091, pop: 3309
Mean visiting county population
100000
300000
500000
700000
Incident cases (per 100,000 population)
0
50
100
150
Mar
May
Jul
Sep

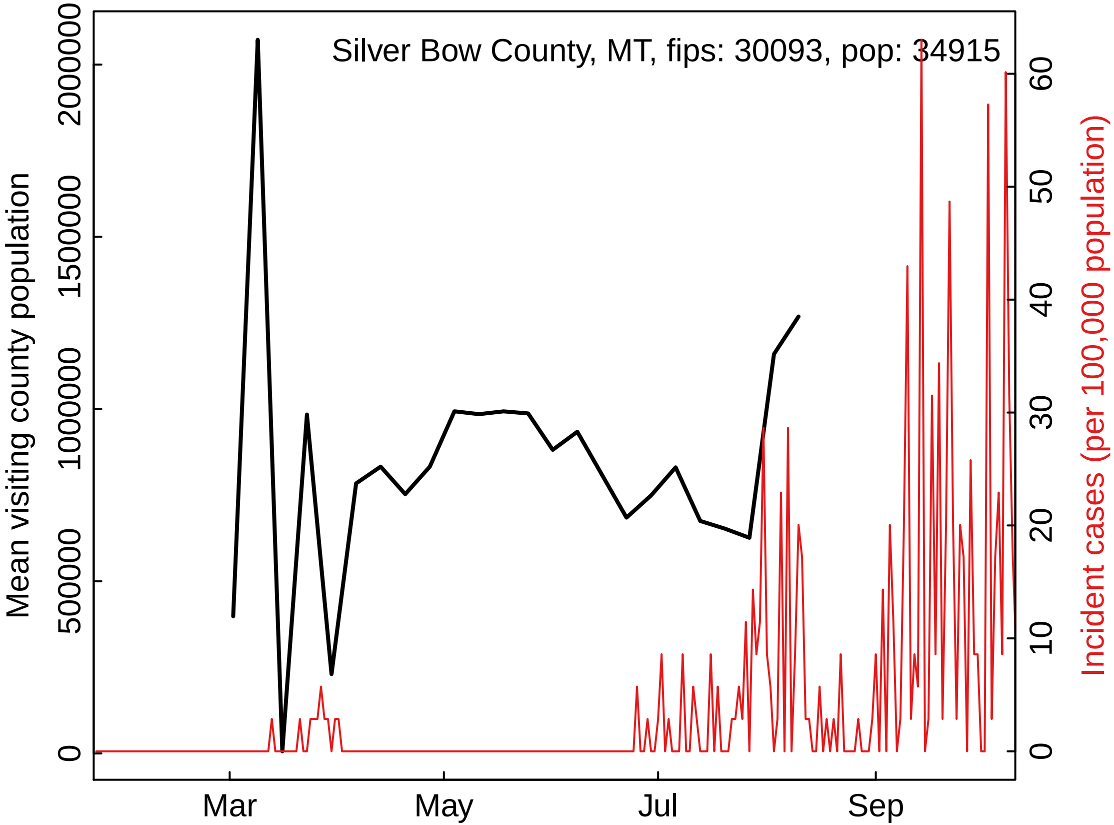

Silver Bow County, MT, fips: 30093, pop: 34915
Mean visiting county population
Incident cases (per 100,000 population)
0
500000
1000000
1500000
2000000
0
10
20
30
40
50
60
Mar
May
Jul
Sep

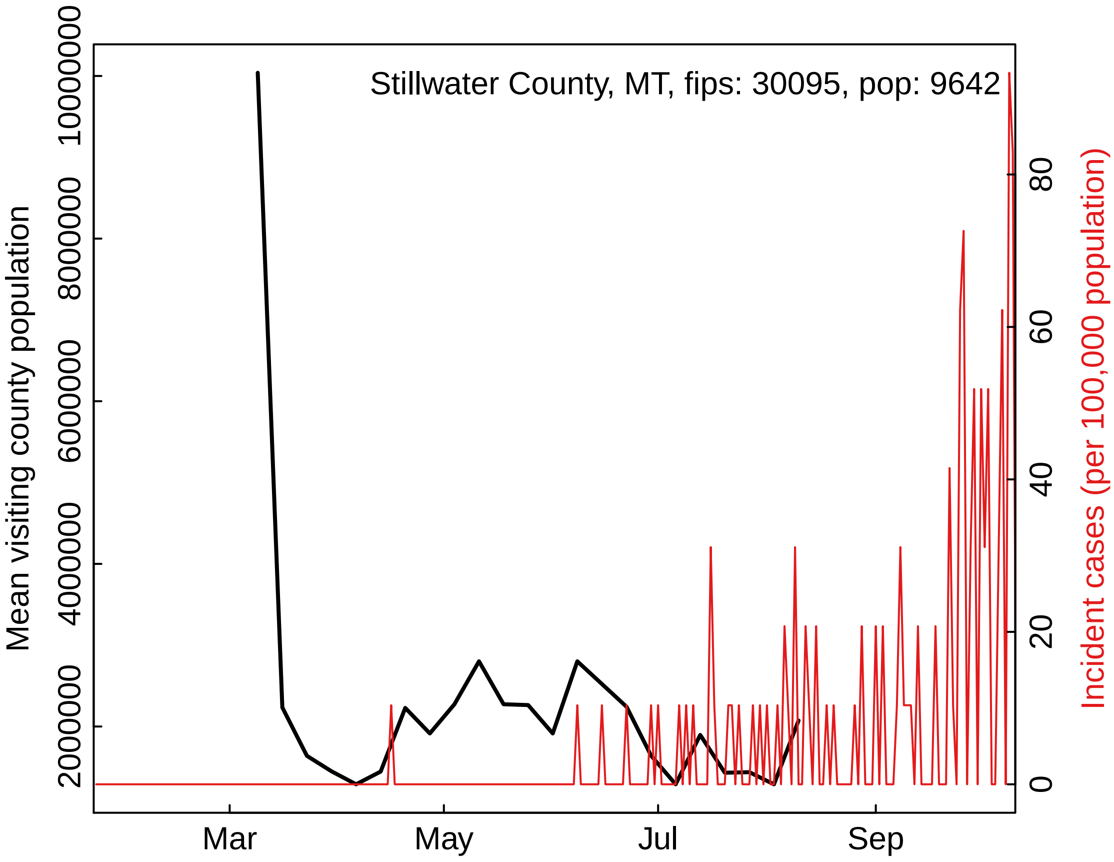

Stillwater County, MT, fips: 30095, pop: 9642
Mean visiting county population
2000000
4000000
6000000
8000000
10000000
Mar
May
Jul
Sep
0
20
40
60
80
Incident cases (per 100,000 population)

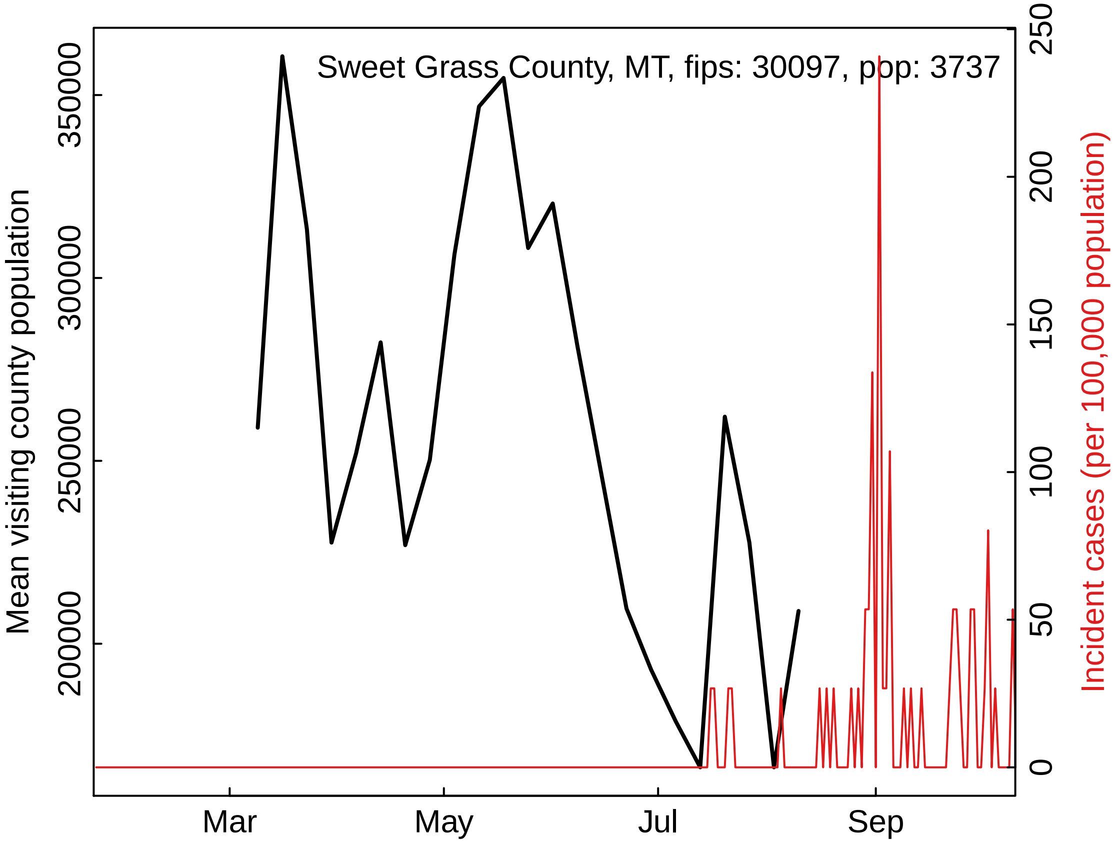

Sweet Grass County, MT, fips: 30097, pop: 3737
Mean visiting county population
200000
250000
300000
350000
Incident cases (per 100,000 population)
0
50
100
150
200
250
Mar
May
Jul
Sep

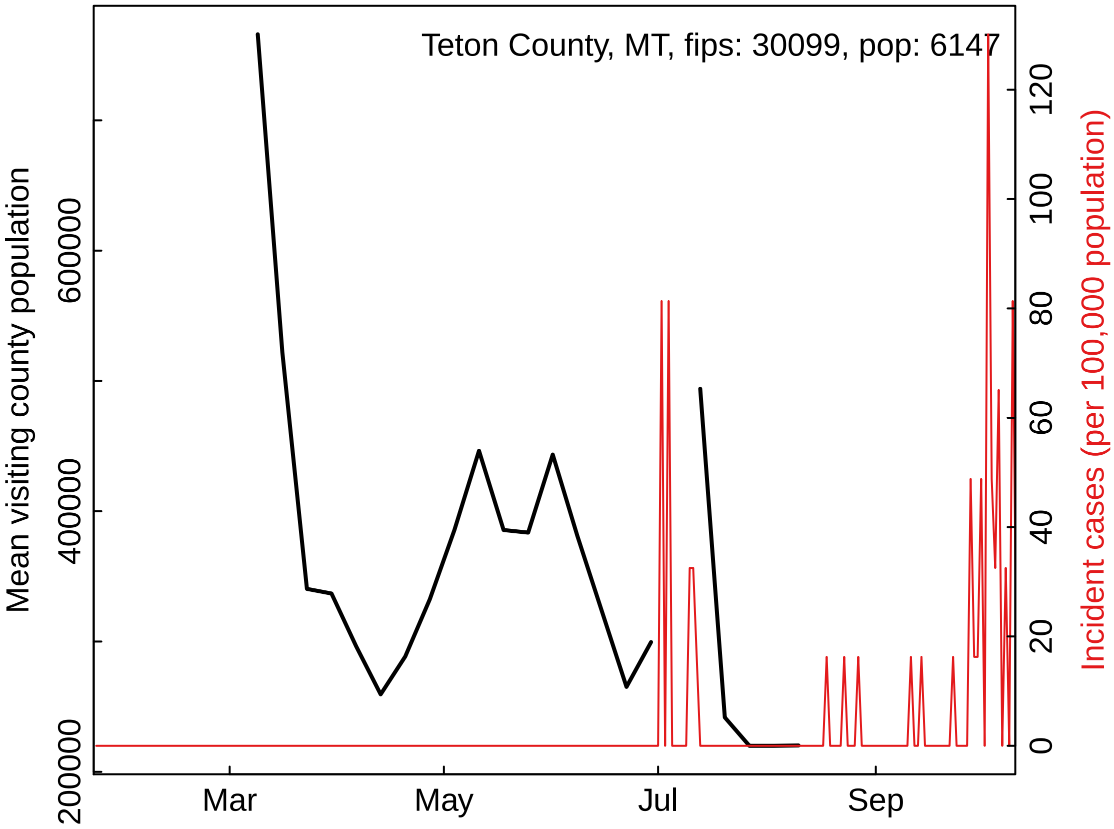

Teton County, MT, fips: 30099, pop: 6147
Mean visiting county population
200000
400000
600000
Incident cases (per 100,000 population)
0
20
40
60
80
100
120
Mar
May
Jul
Sep

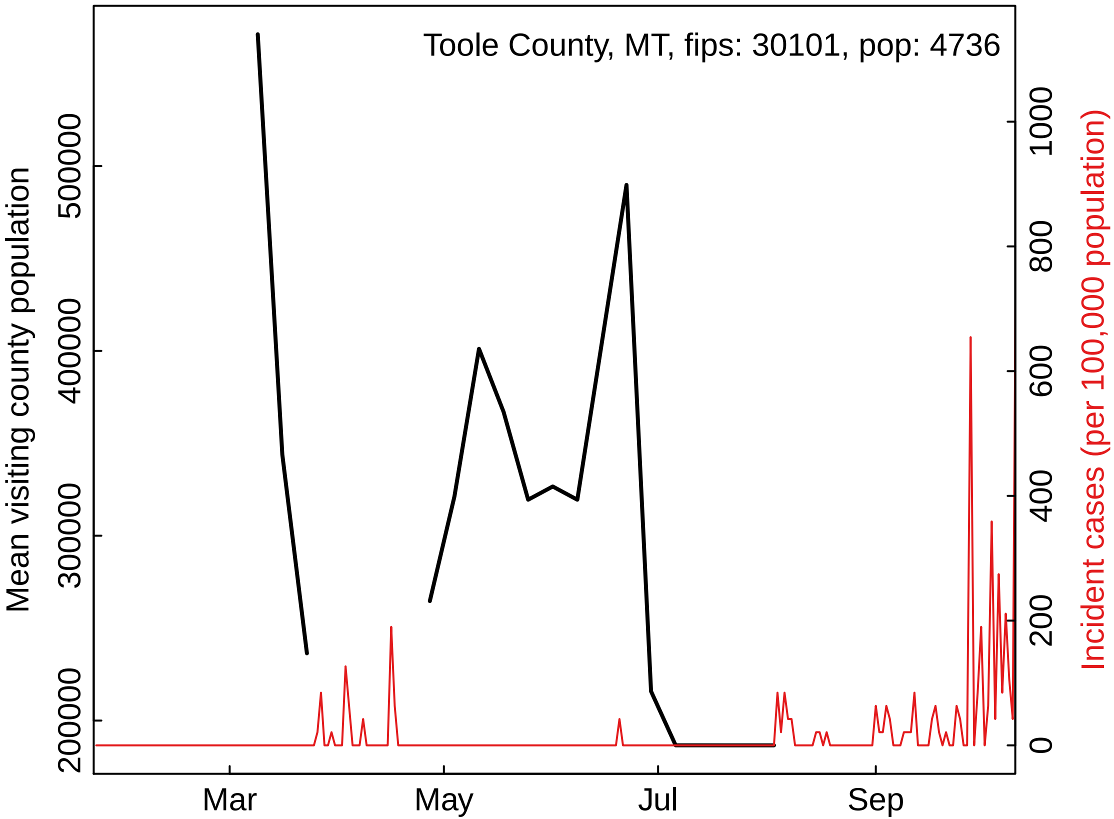

Toole County, MT, fips: 30101, pop: 4736
Mean visiting county population
200000
300000
400000
500000
Incident cases (per 100,000 population)
0
200
400
600
800
1000
Mar
May
Jul
Sep

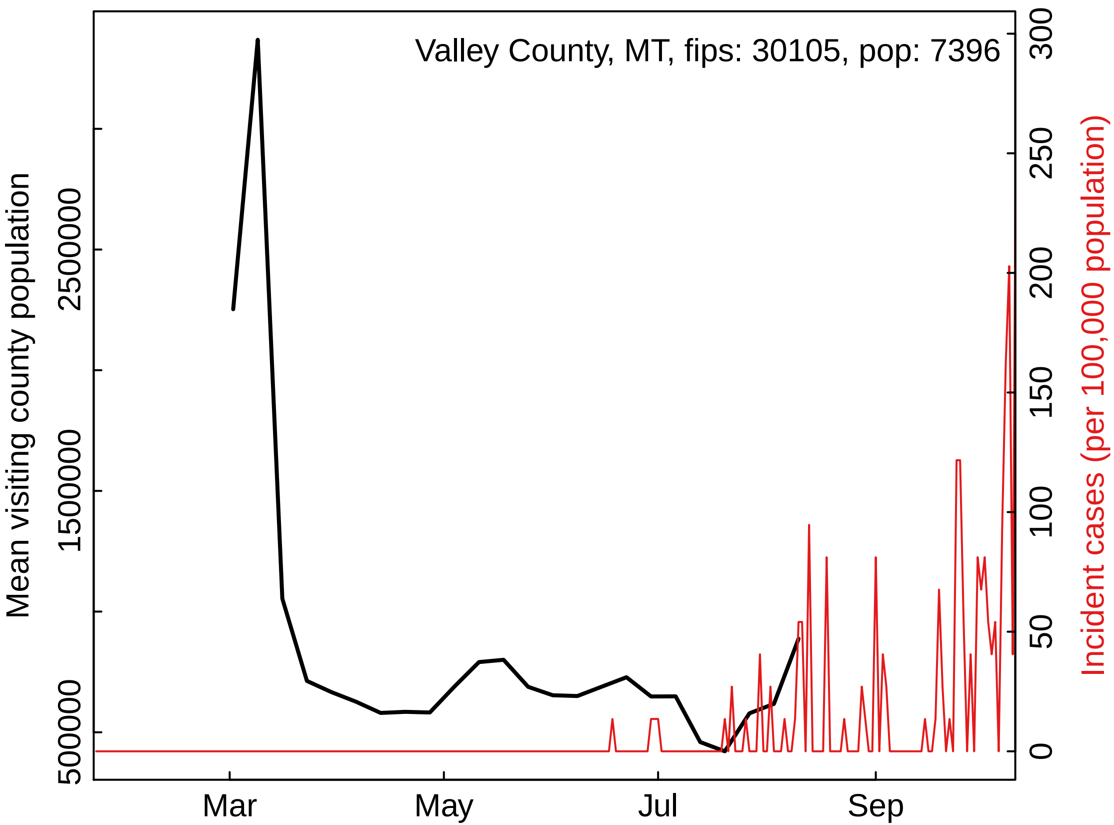
Valley County, MT, fips: 30105, pop: 7396
Mean visiting county population
500000
1500000
2500000
Mar
May
Jul
Sep
0
50
100
150
200
250
300
Incident cases (per 100,000 population)

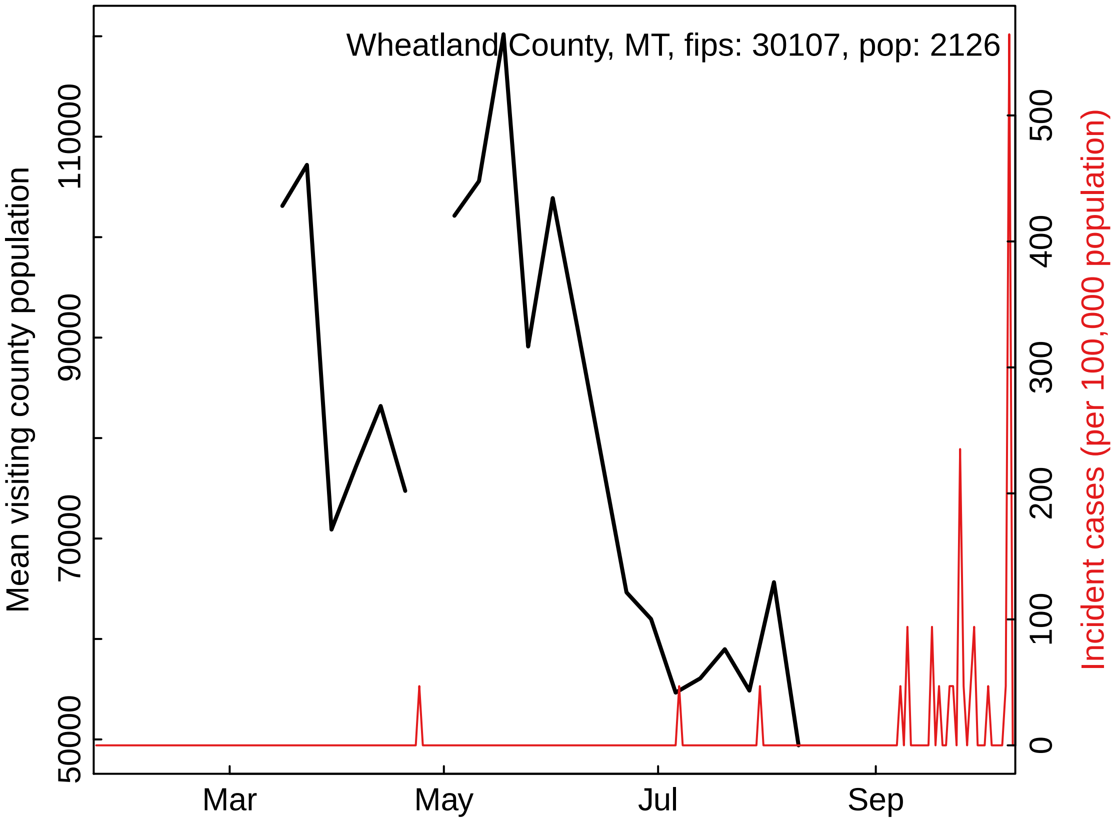

Wheatland County, MT, fips: 30107, pop: 2126
Mean visiting county population
50000
70000
90000
110000
Incident cases (per 100,000 population)
0
100
200
300
400
500
Mar
May
Jul
Sep

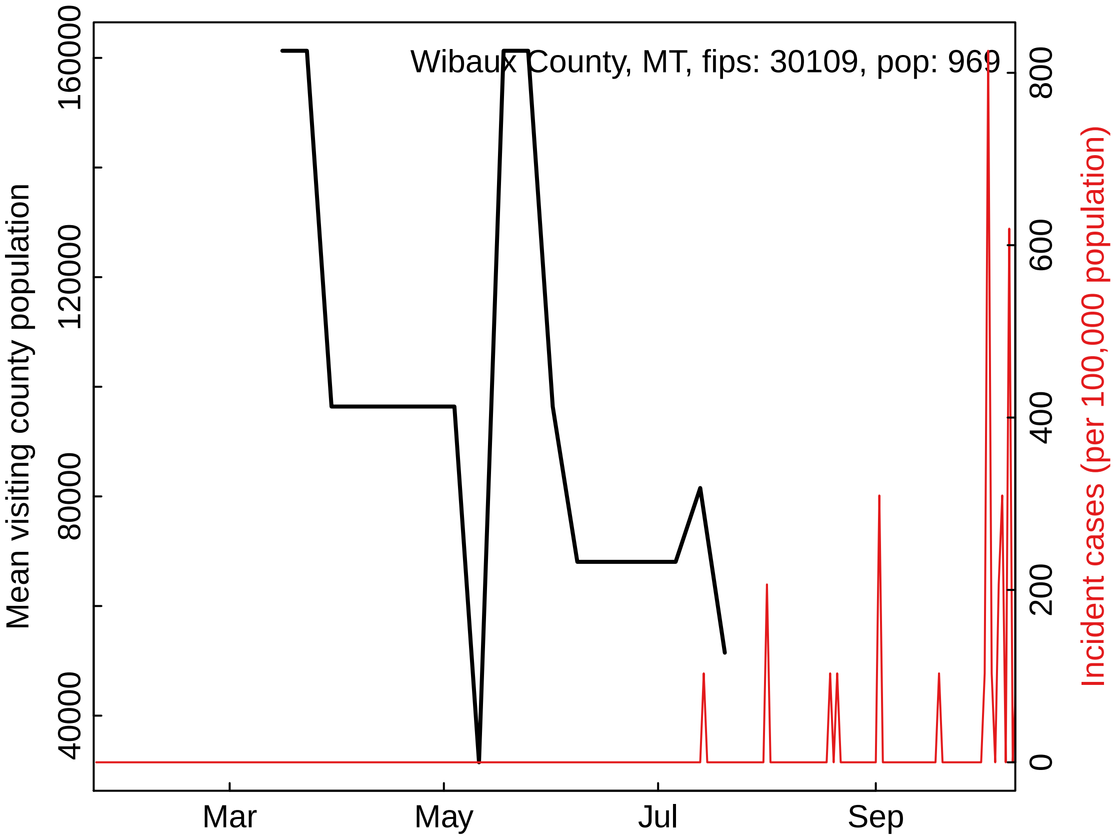

Wibaux County, MT, fips: 30109, pop: 969
Mean visiting county population
40000
80000
120000
160000
Incident cases (per 100,000 population)
0
200
400
600
800
Mar
May
Jul
Sep

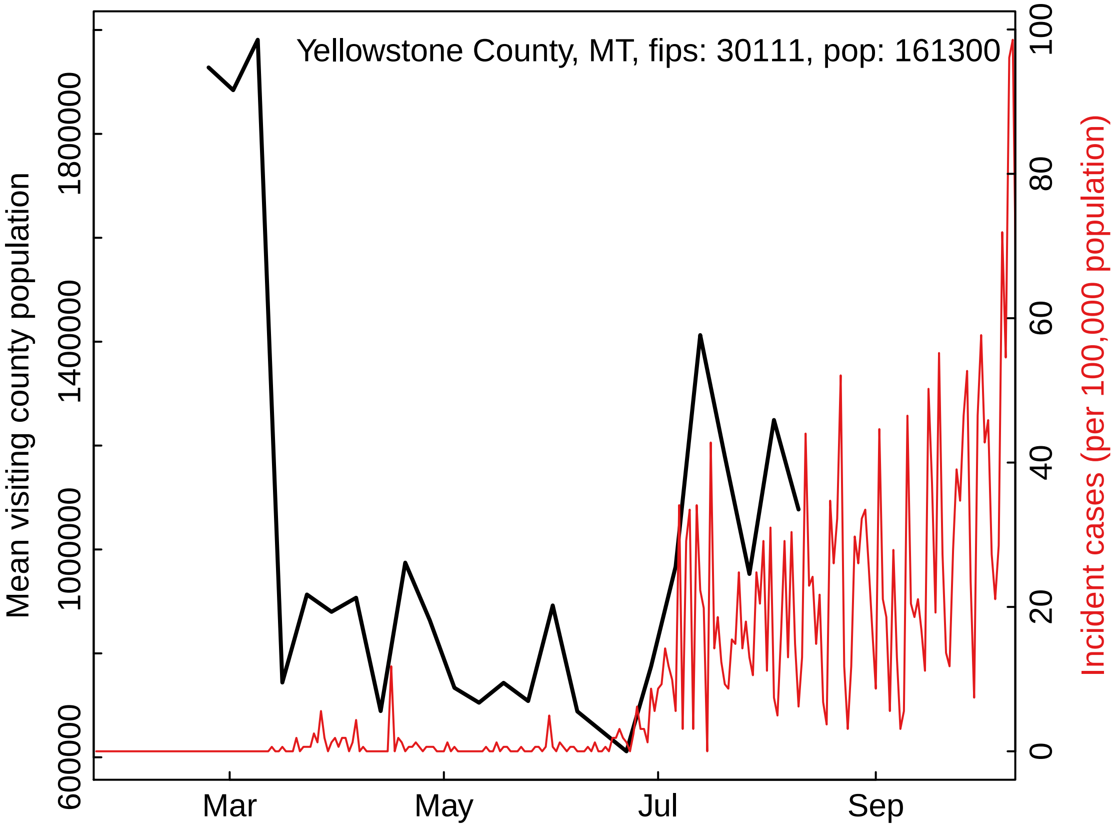

Yellowstone County, MT, fips: 30111, pop: 161300
Mean visiting county population
6000000
10000000
14000000
18000000
Incident cases (per 100,000 population)
0
20
40
60
80
100
Mar
May
Jul
Sep

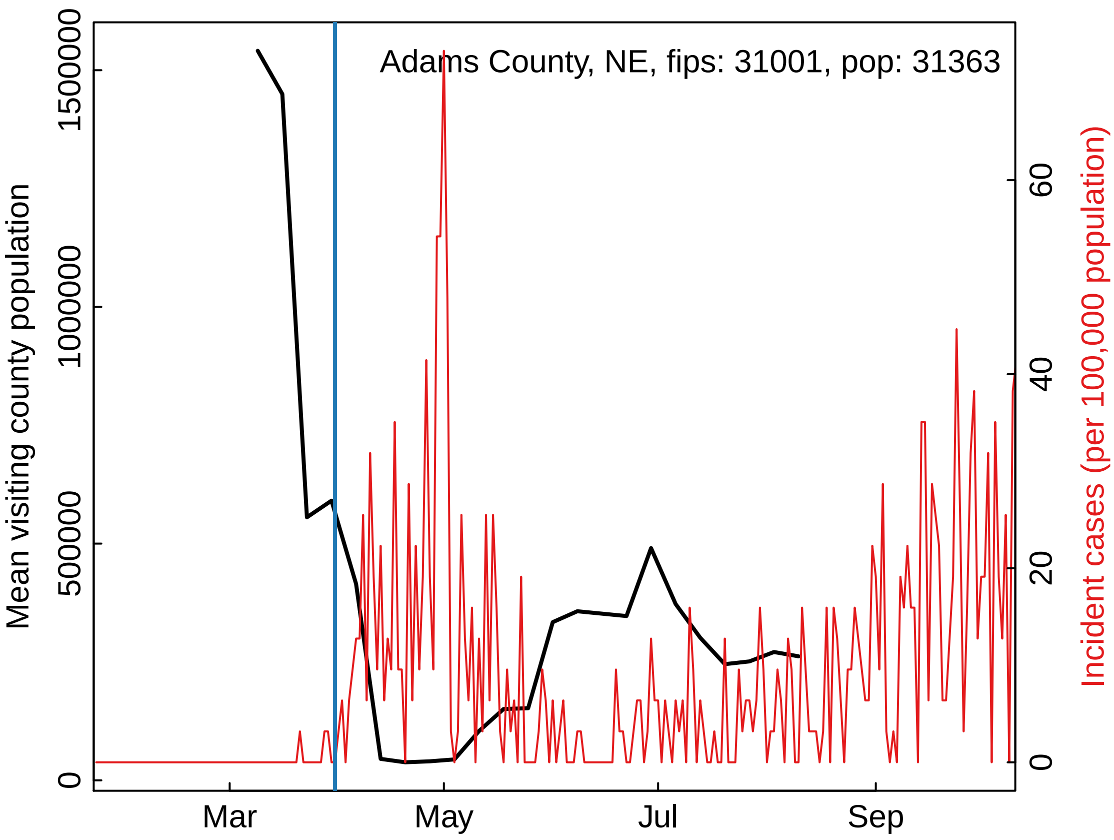

Adams County, NE, fips: 31001, pop: 31363
Mean visiting county population
Incident cases (per 100,000 population)
0
500000
1000000
1500000
0
20
40
60
Mar
May
Jul
Sep

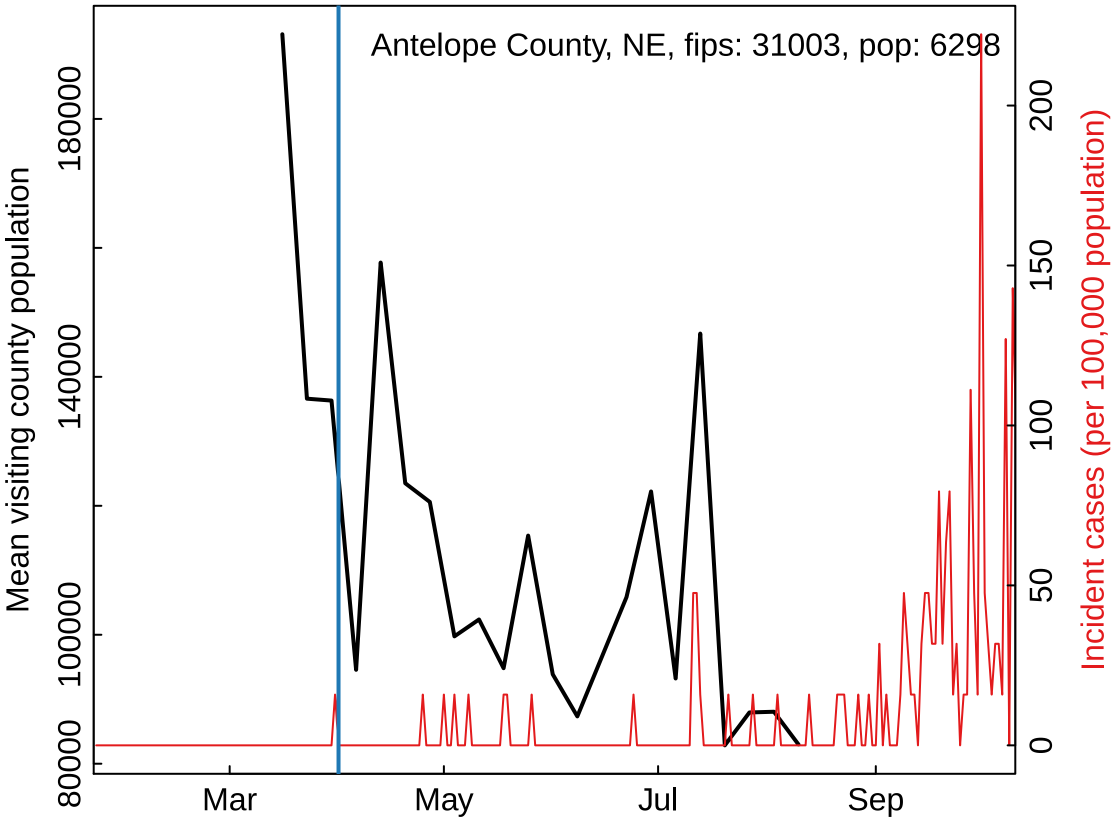

Antelope County, NE, fips: 31003, pop: 6298
Mean visiting county population
80000
100000
140000
180000
Mar
May
Jul
Sep
0
50
100
150
200
Incident cases (per 100,000 population)

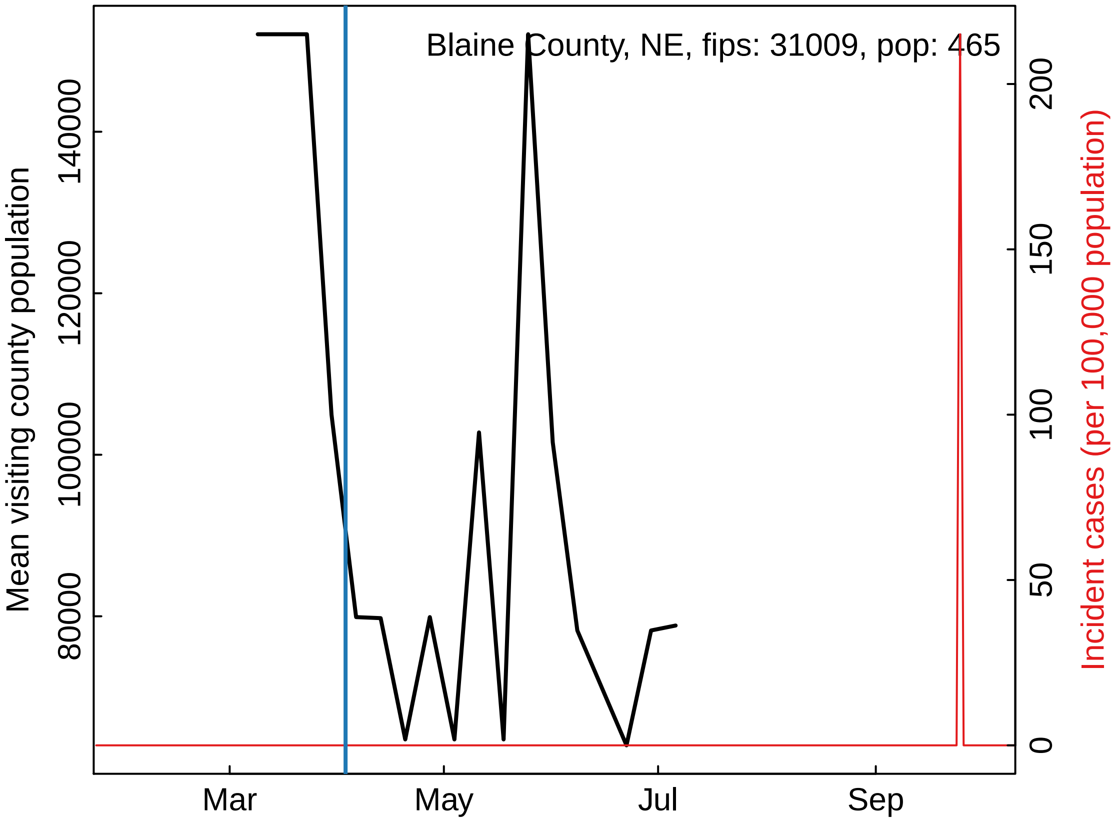

Blaine County, NE, fips: 31009, pop: 465
Mean visiting county population
80000
100000
120000
140000
Incident cases (per 100,000 population)
0
50
100
150
200
Mar
May
Jul
Sep

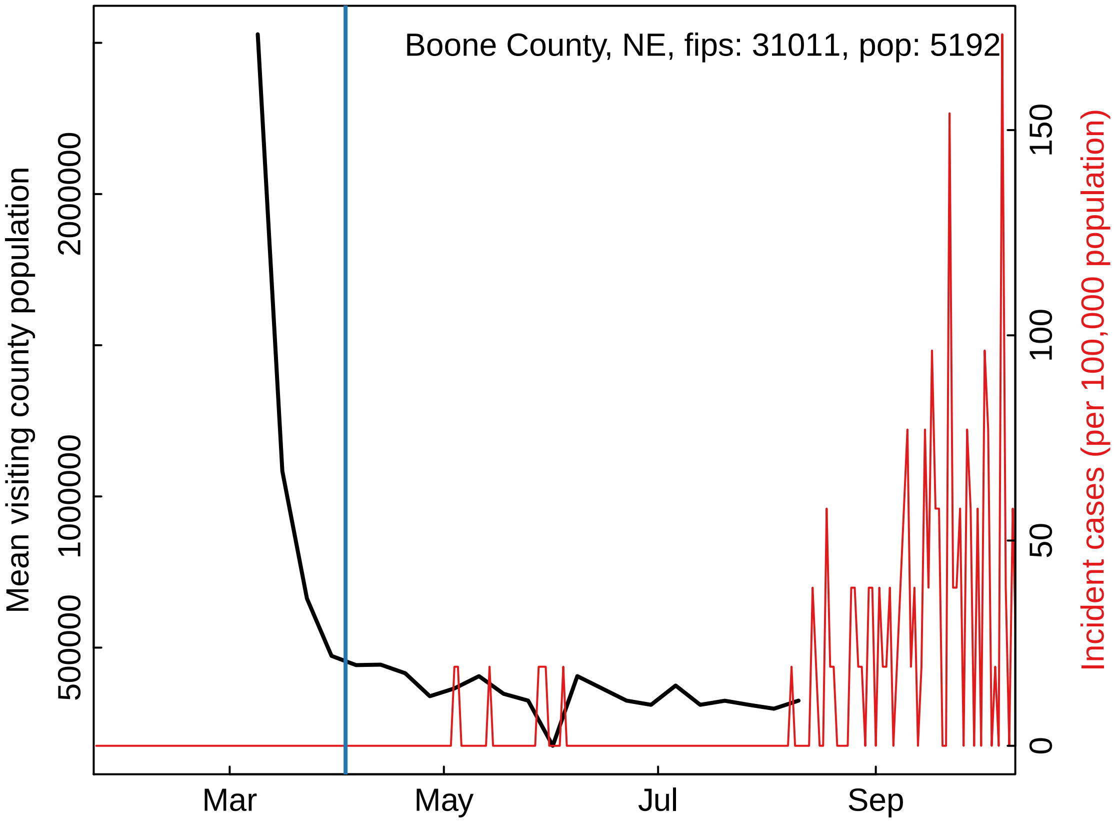

Boone County, NE, fips: 31011, pop: 5192
Mean visiting county population
500000
1000000
2000000
Mar
May
Jul
Sep
0
50
100
150
Incident cases (per 100,000 population)

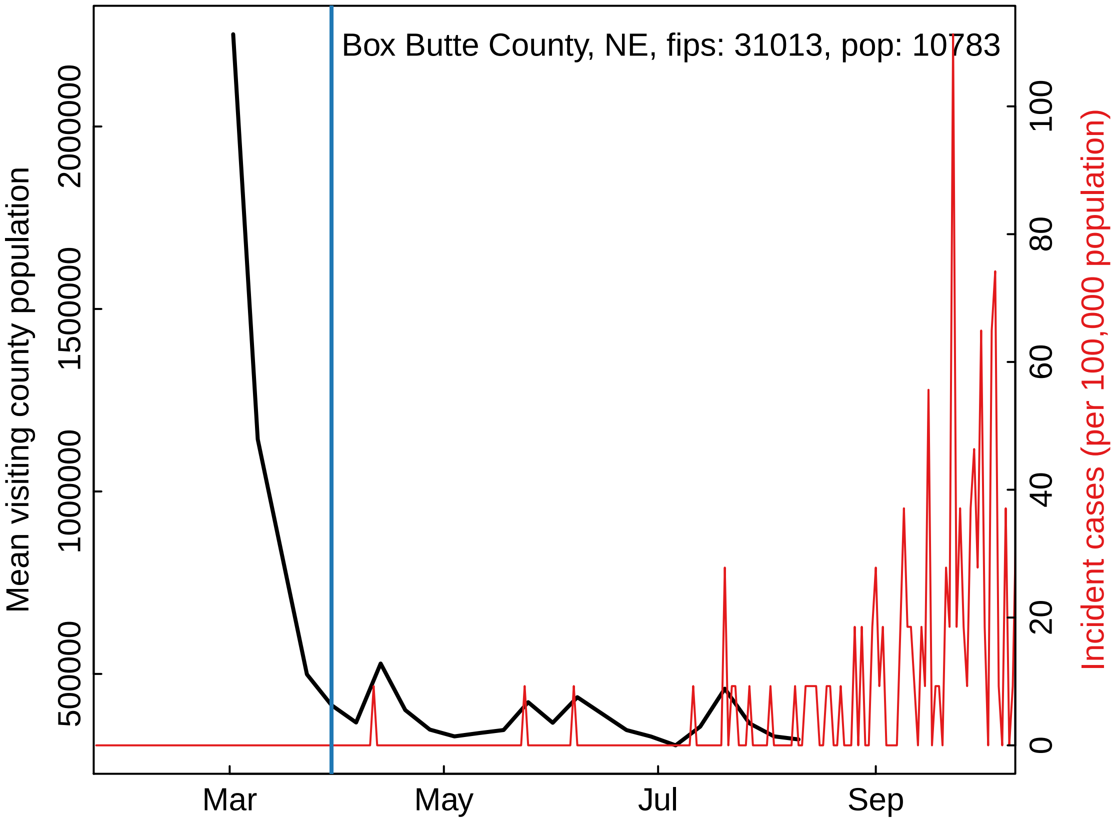

Box Butte County, NE, fips: 31013, pop: 10783
Mean visiting county population
500000
1000000
1500000
2000000
Incident cases (per 100,000 population)
0
20
40
60
80
100
Mar
May
Jul
Sep

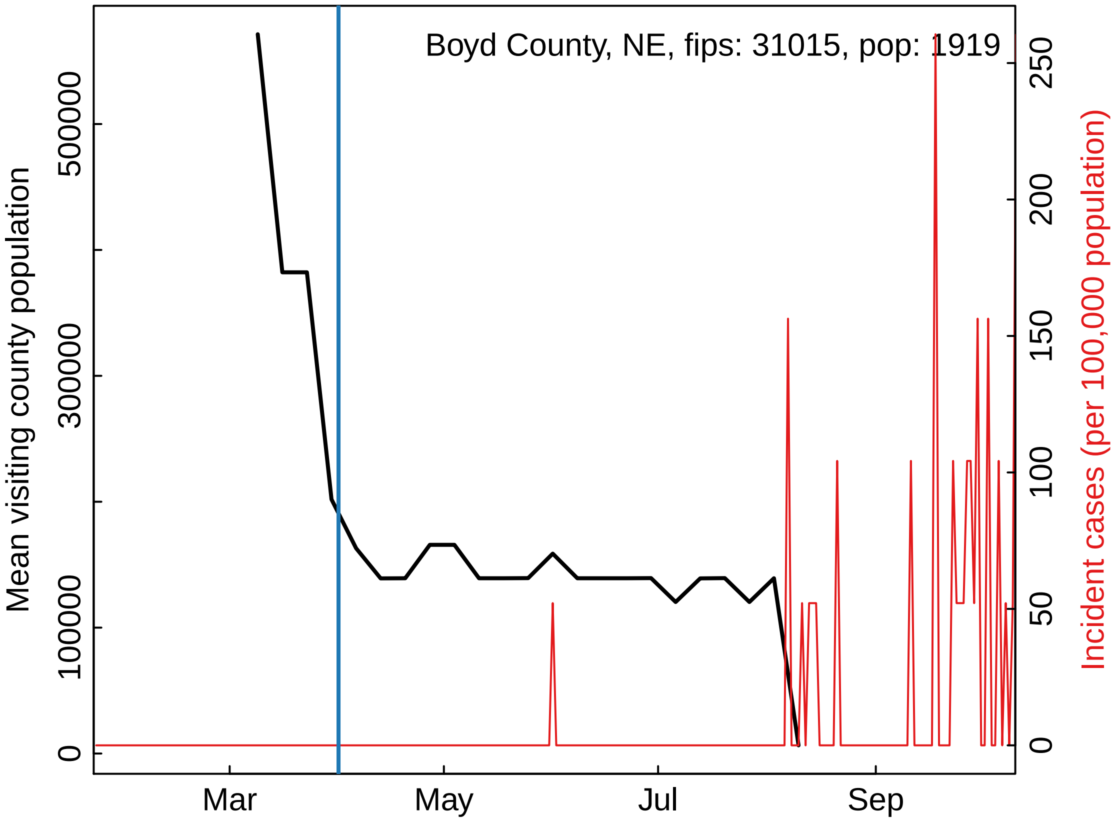

Boyd County, NE, fips: 31015, pop: 1919
Mean visiting county population
0
100000
300000
500000
Incident cases (per 100,000 population)
0
50
100
150
200
250
Mar
May
Jul
Sep

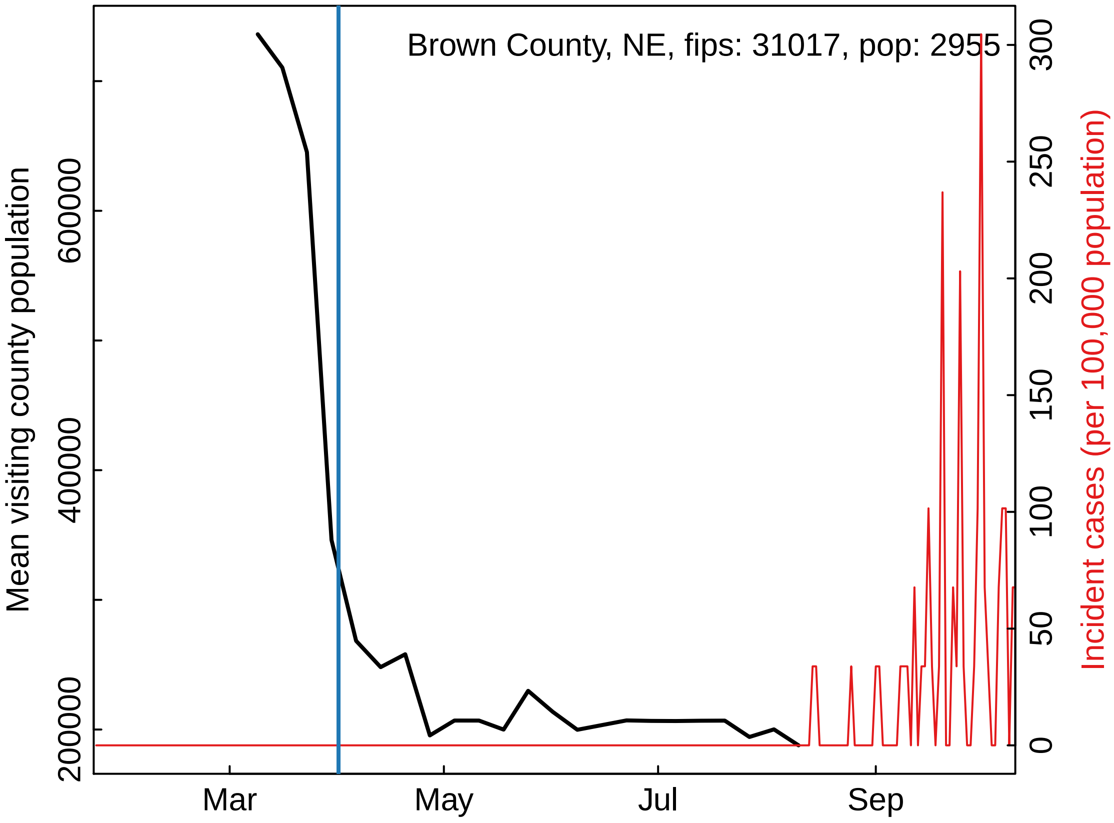

Brown County, NE, fips: 31017, pop: 2955
Mean visiting county population
200000
400000
600000
Incident cases (per 100,000 population)
0
50
100
150
200
250
300
Mar
May
Jul
Sep

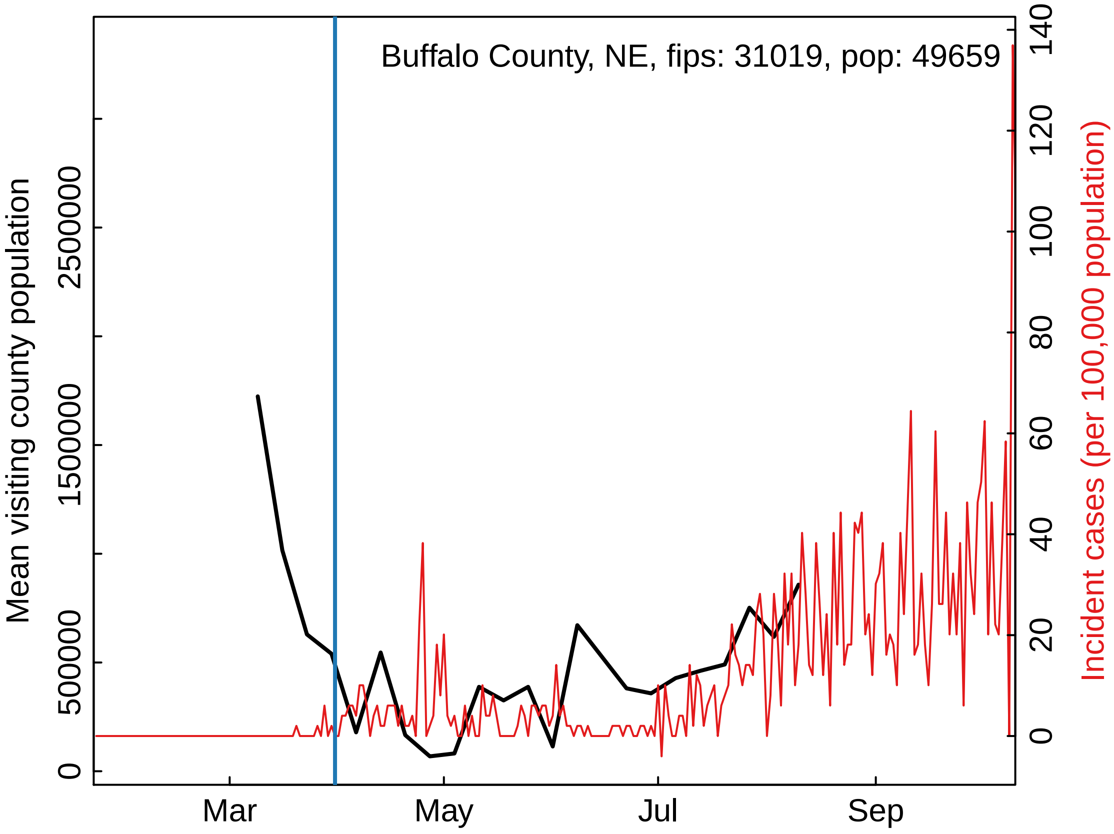
Buffalo County, NE, fips: 31019, pop: 49659
Mean visiting county population
0
500000
1500000
2500000
Incident cases (per 100,000 population)
0
20
40
60
80
100
120
140
Mar
May
Jul
Sep

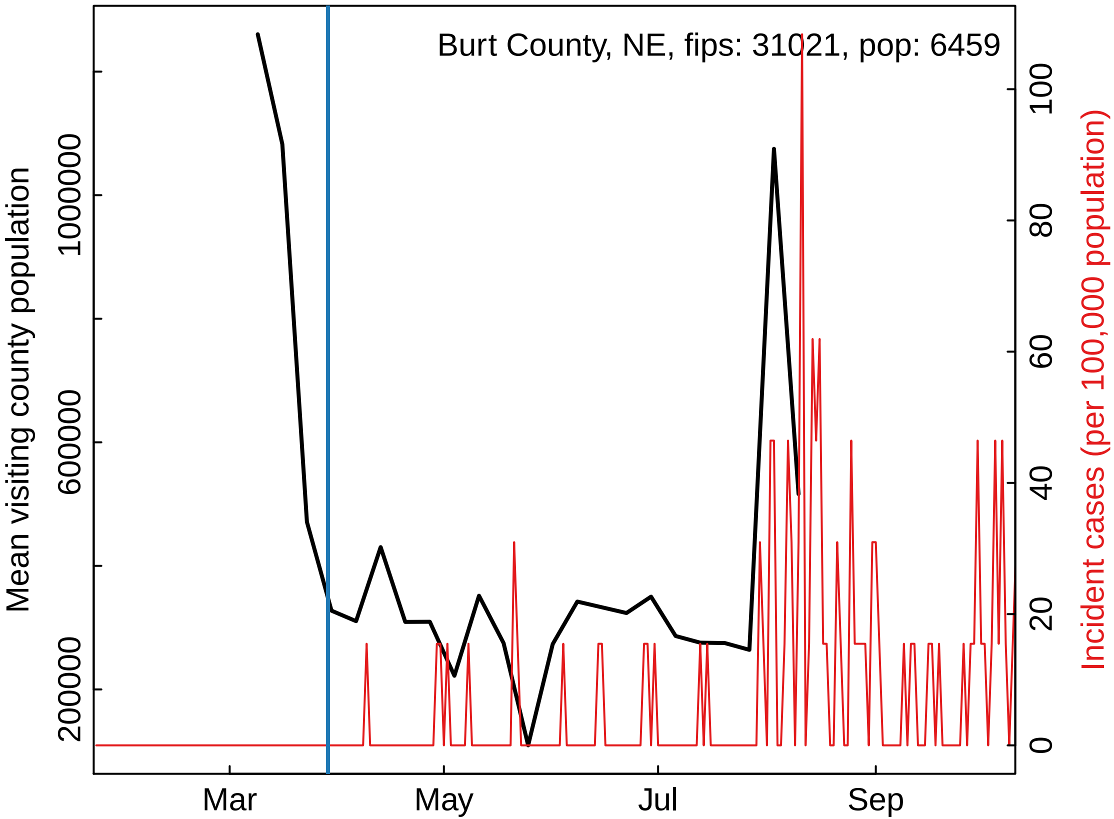

Burt County, NE, fips: 31021, pop: 6459
Mean visiting county population
200000
600000
1000000
Incident cases (per 100,000 population)
0
20
40
60
80
100
Mar
May
Jul
Sep

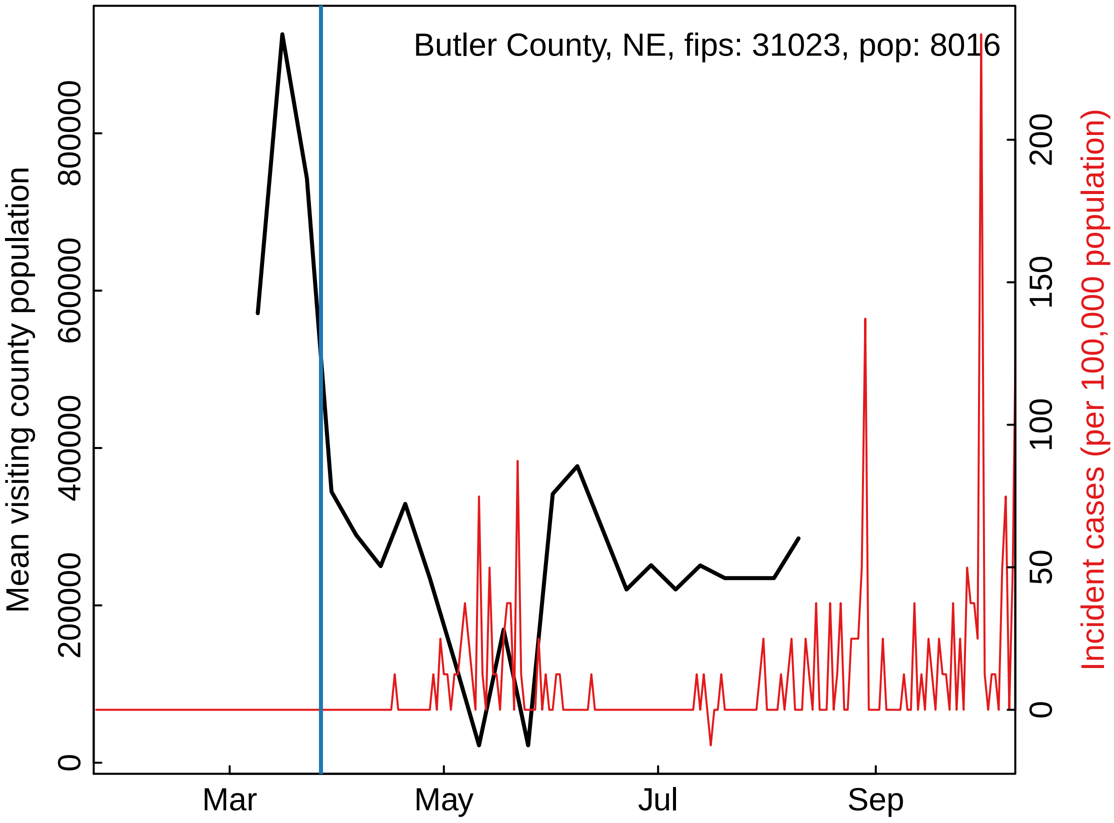

Butler County, NE, fips: 31023, pop: 8016
Mean visiting county population
0
200000
400000
600000
800000
Incident cases (per 100,000 population)
0
50
100
150
200
Mar
May
Jul
Sep

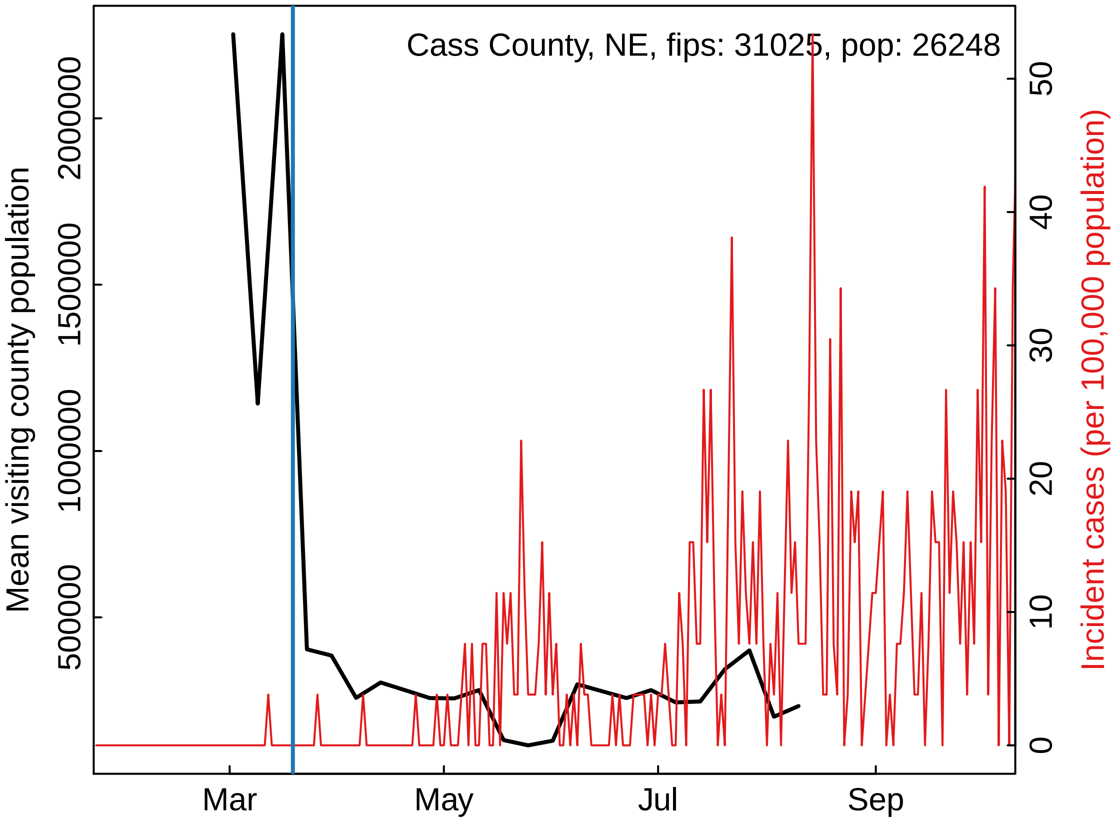

Cass County, NE, fips: 31025, pop: 26248
Mean visiting county population
500000
1000000
1500000
2000000
Incident cases (per 100,000 population)
0
10
20
30
40
50
Mar
May
Jul
Sep

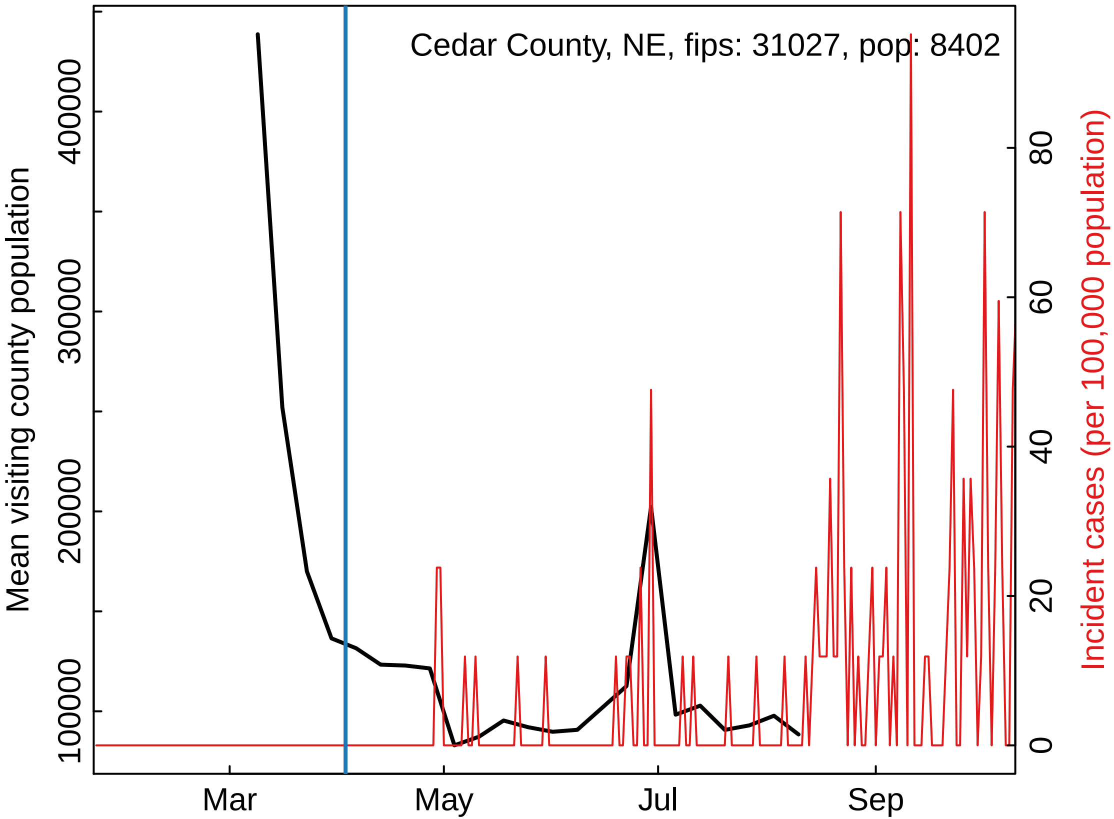

Cedar County, NE, fips: 31027, pop: 8402
Mean visiting county population
100000
200000
300000
400000
Incident cases (per 100,000 population)
0
20
40
60
80
Mar
May
Jul
Sep

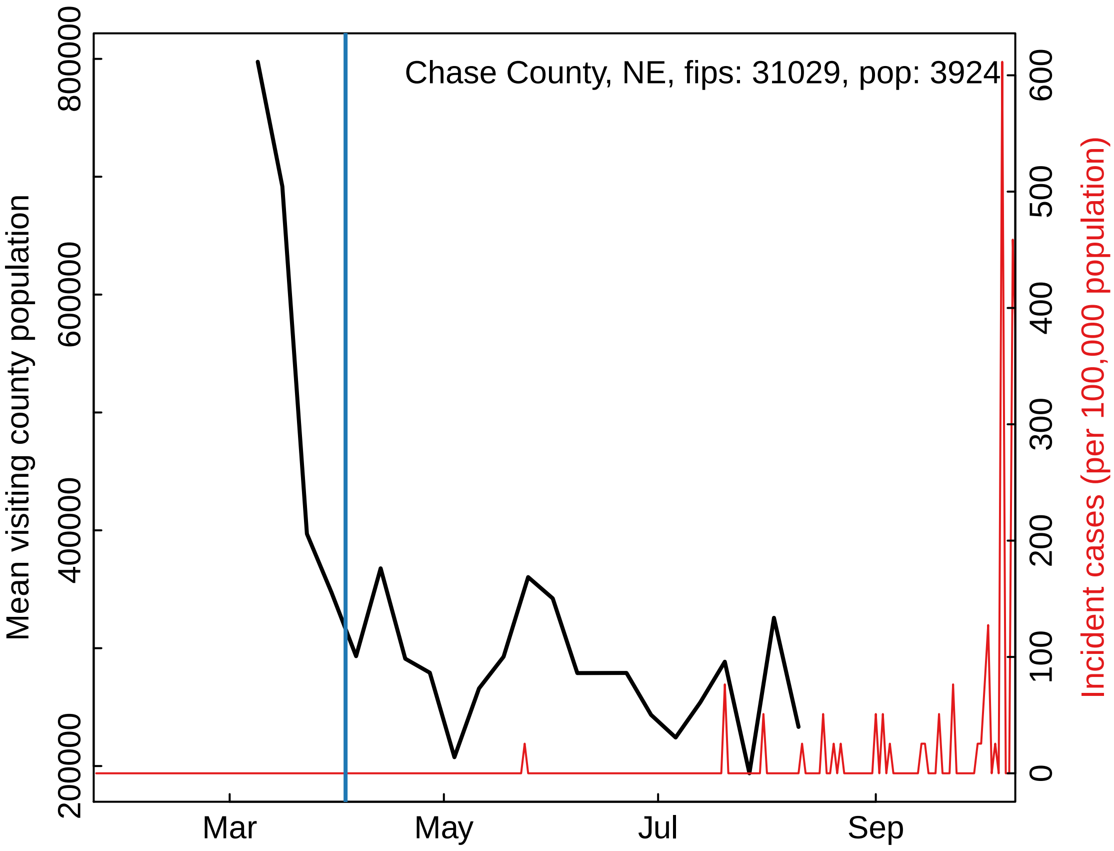

Chase County, NE, fips: 31029, pop: 3924
Mean visiting county population
200000
400000
600000
800000
Incident cases (per 100,000 population)
0
100
200
300
400
500
600
Mar
May
Jul
Sep

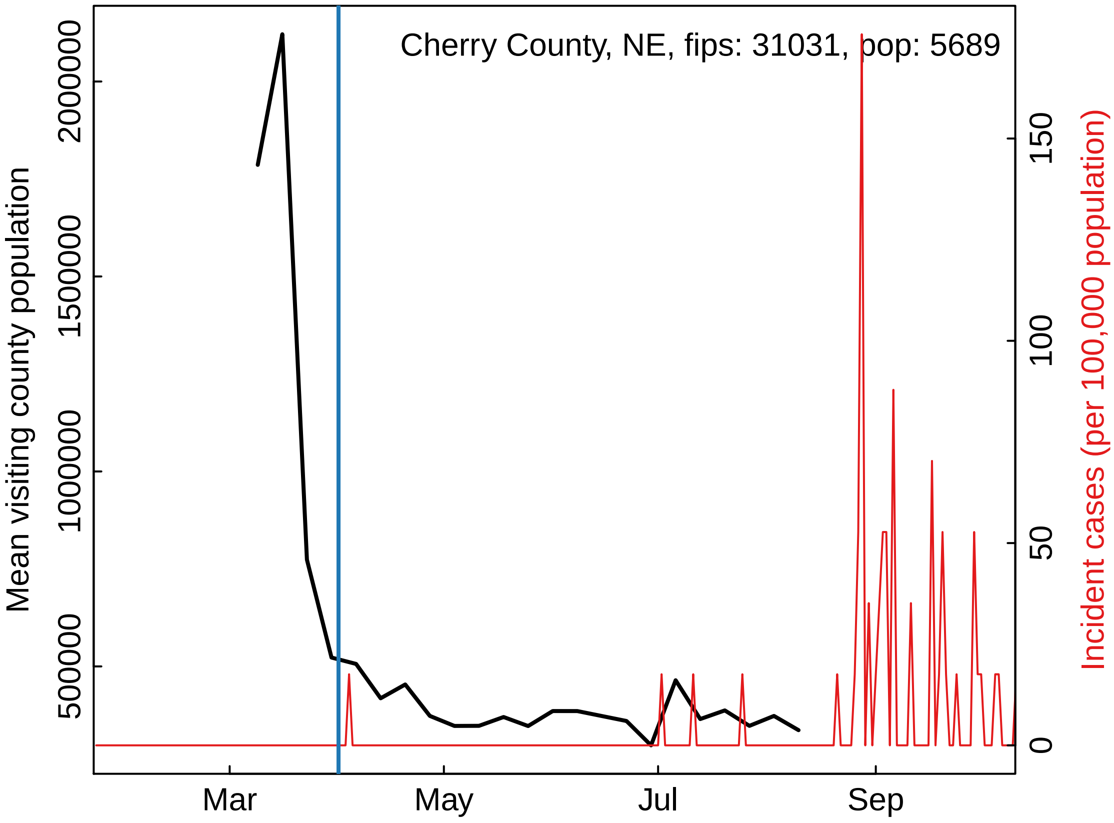

Cherry County, NE, fips: 31031, pop: 5689
Mean visiting county population
500000
1000000
1500000
2000000
Incident cases (per 100,000 population)
0
50
100
150
Mar
May
Jul
Sep

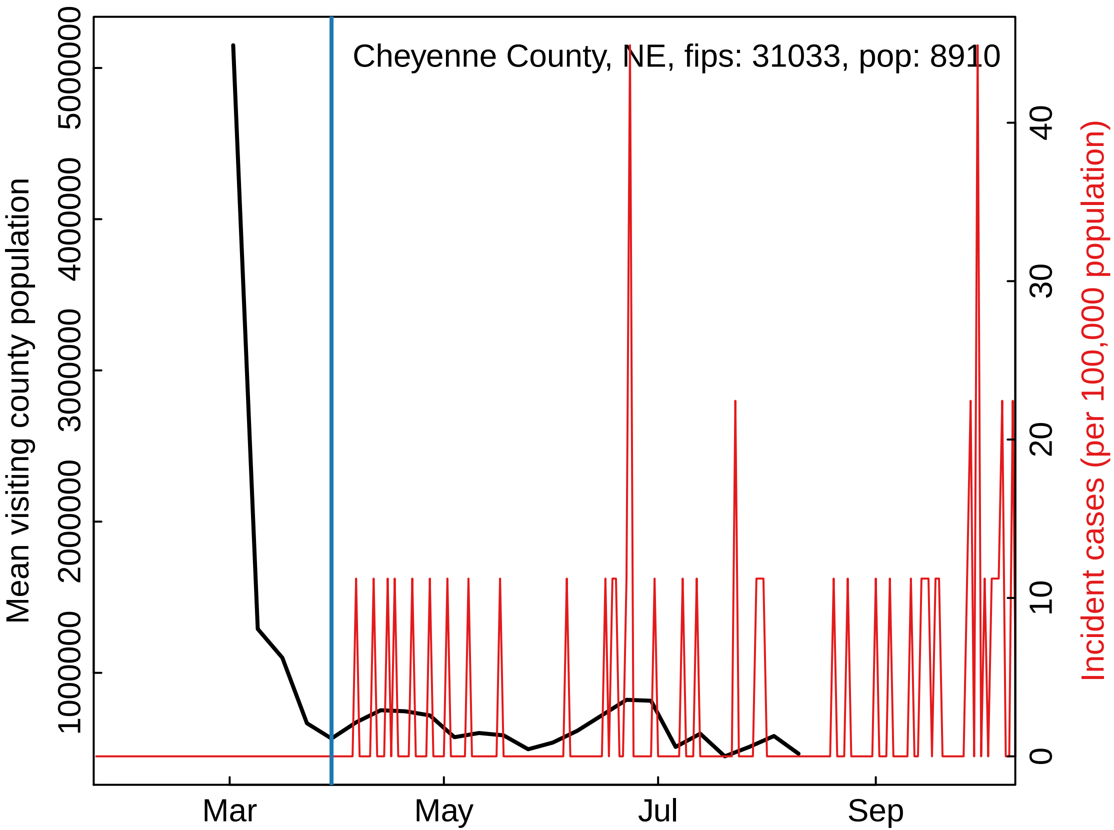

Cheyenne County, NE, fips: 31033, pop: 8910
Mean visiting county population
1000000
2000000
3000000
4000000
5000000
Incident cases (per 100,000 population)
0
10
20
30
40
Mar
May
Jul
Sep

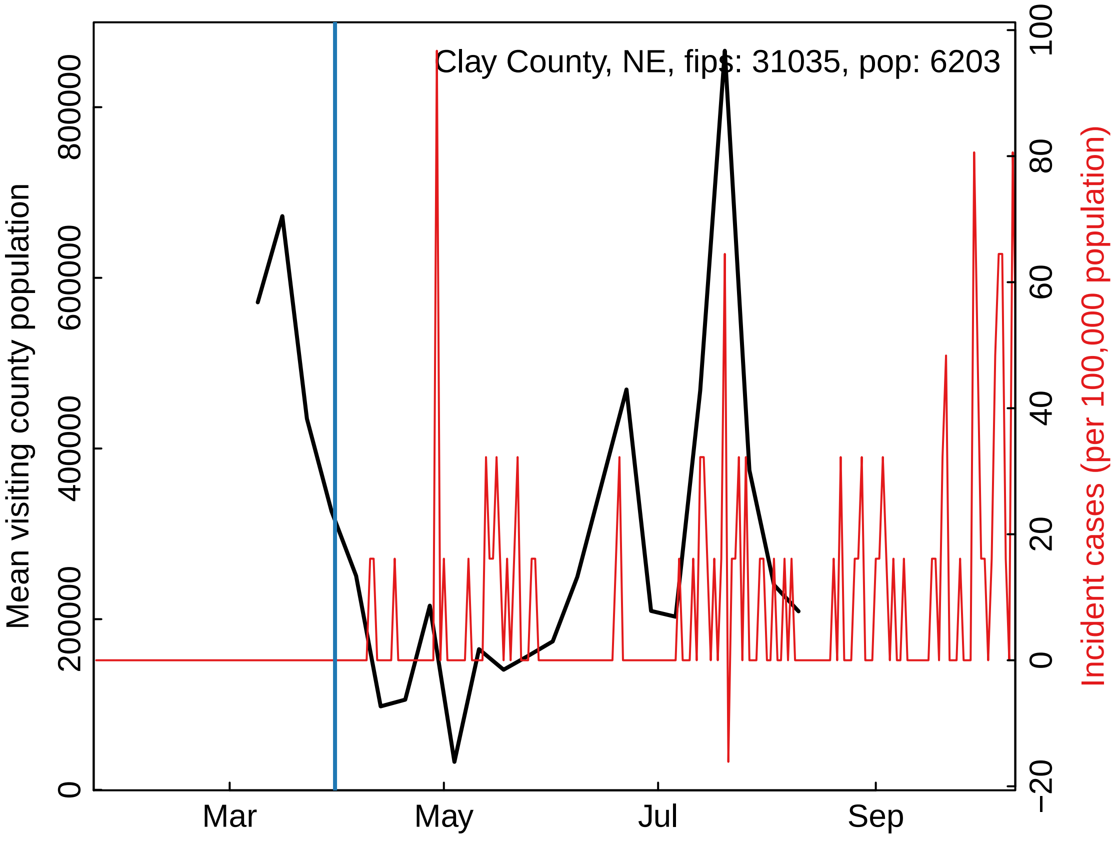
Clay County, NE, fips: 31035, pop: 6203
Mean visiting county population
0
200000
400000
600000
800000
Incident cases (per 100,000 population)
−20
0
20
40
60
80
100
Mar
May
Jul
Sep

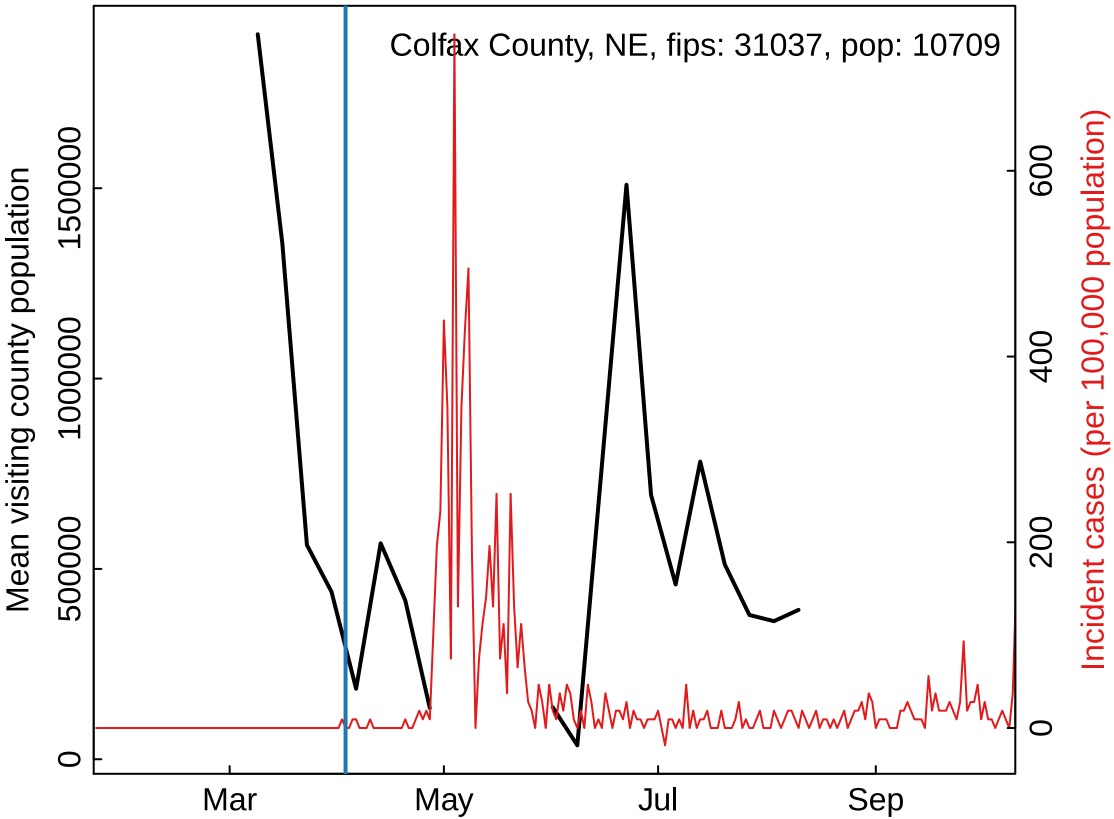

Colfax County, NE, fips: 31037, pop: 10709
Mean visiting county population
0
500000
1000000
1500000
Incident cases (per 100,000 population)
0
200
400
600
Mar
May
Jul
Sep

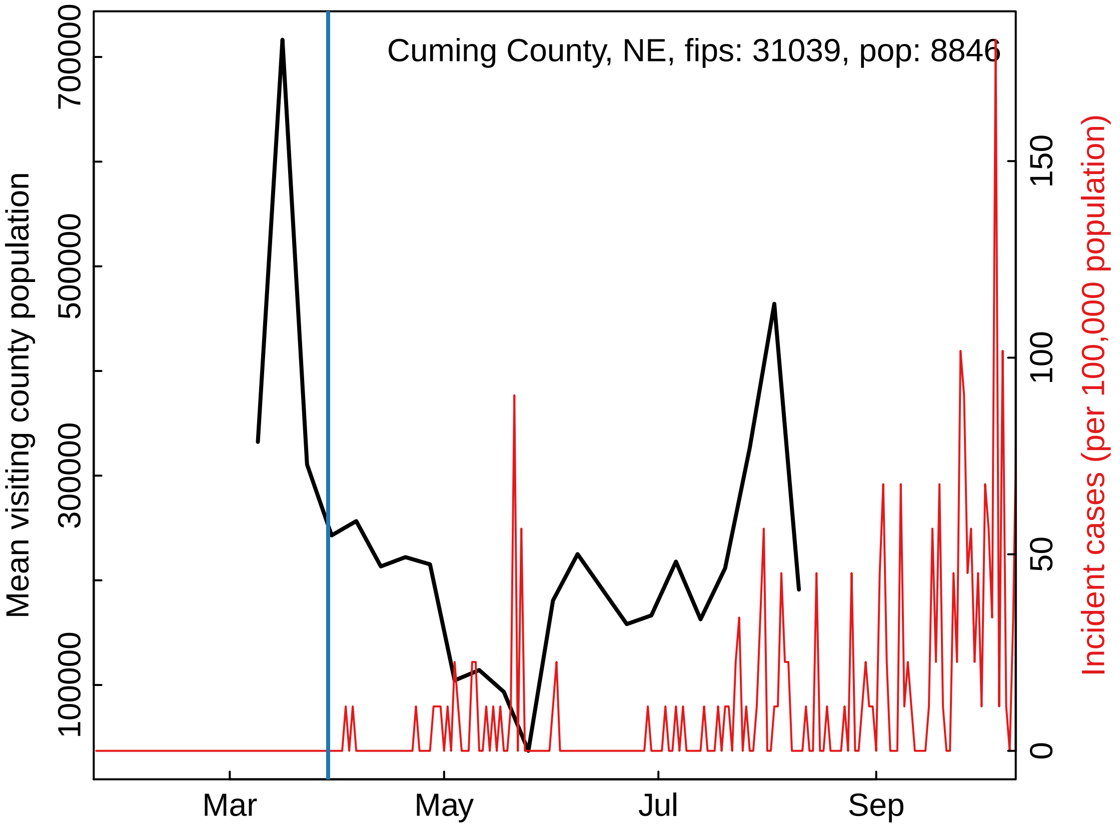

Cuming County, NE, fips: 31039, pop: 8846
Mean visiting county population
100000
300000
500000
700000
Incident cases (per 100,000 population)
0
50
100
150
Mar
May
Jul
Sep

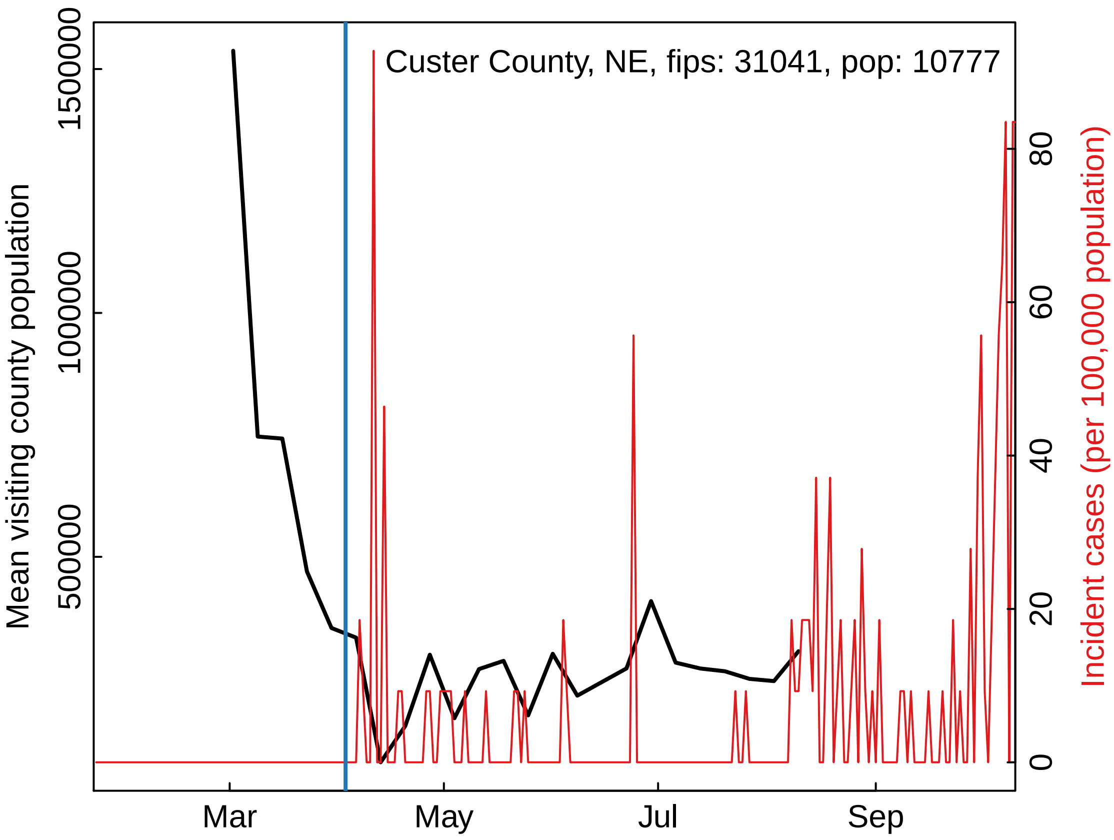

Custer County, NE, fips: 31041, pop: 10777
Mean visiting county population
500000
1000000
1500000
Incident cases (per 100,000 population)
0
20
40
60
80
Mar
May
Jul
Sep

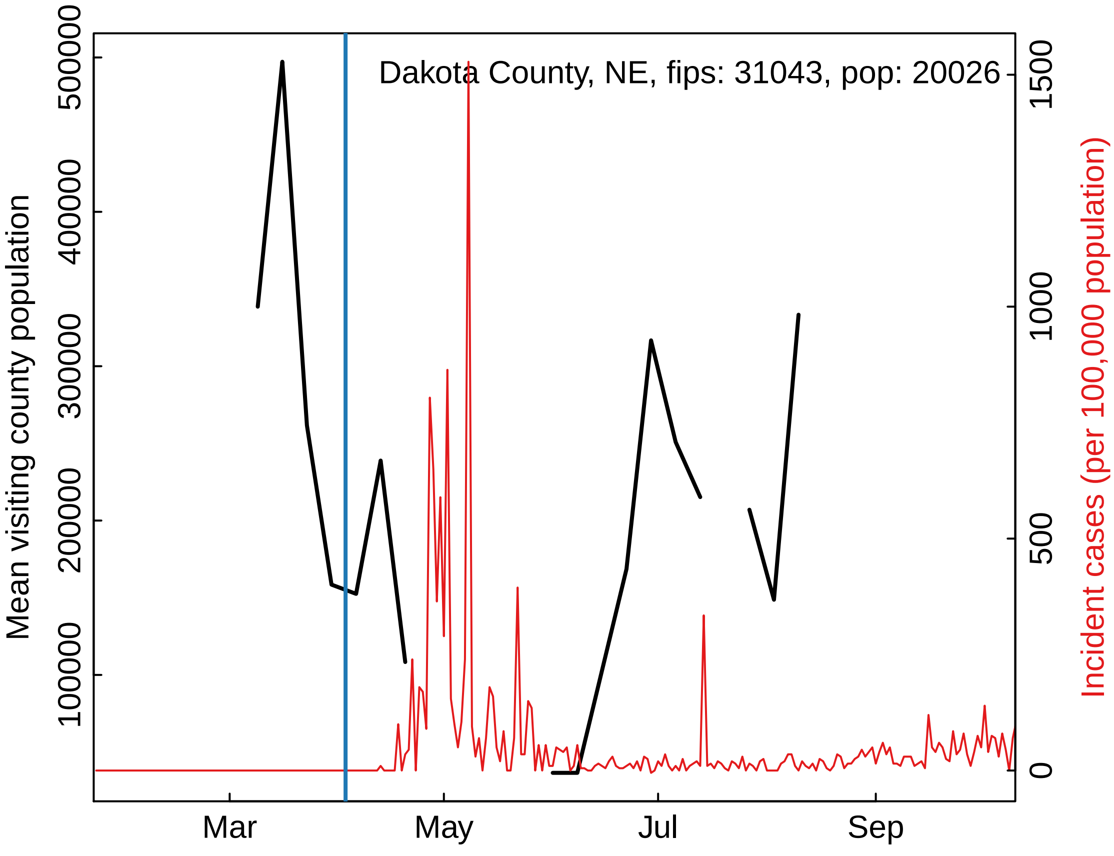

Dakota County, NE, fips: 31043, pop: 20026
Mean visiting county population
100000
200000
300000
400000
500000
Incident cases (per 100,000 population)
0
500
1000
1500
Mar
May
Jul
Sep

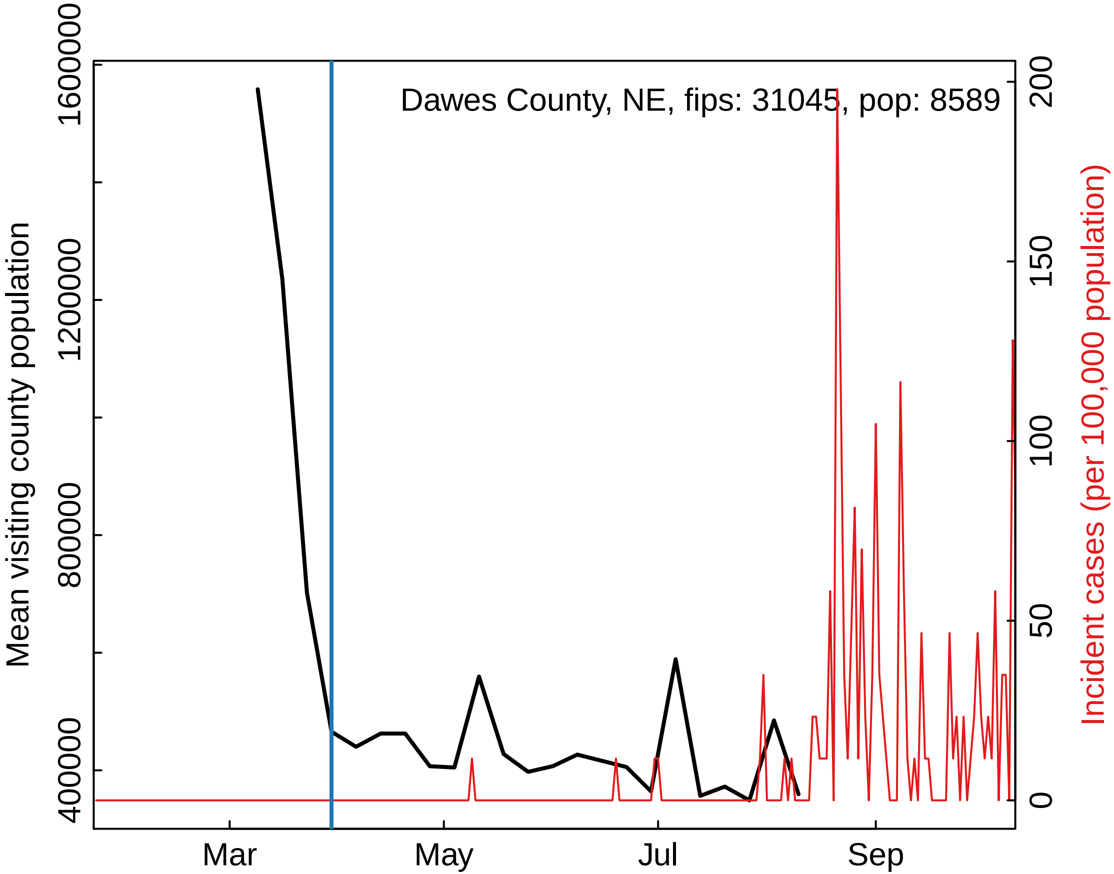

Dawes County, NE, fips: 31045, pop: 8589
Mean visiting county population
400000
800000
1200000
1600000
Incident cases (per 100,000 population)
0
50
100
150
200
Mar
May
Jul
Sep

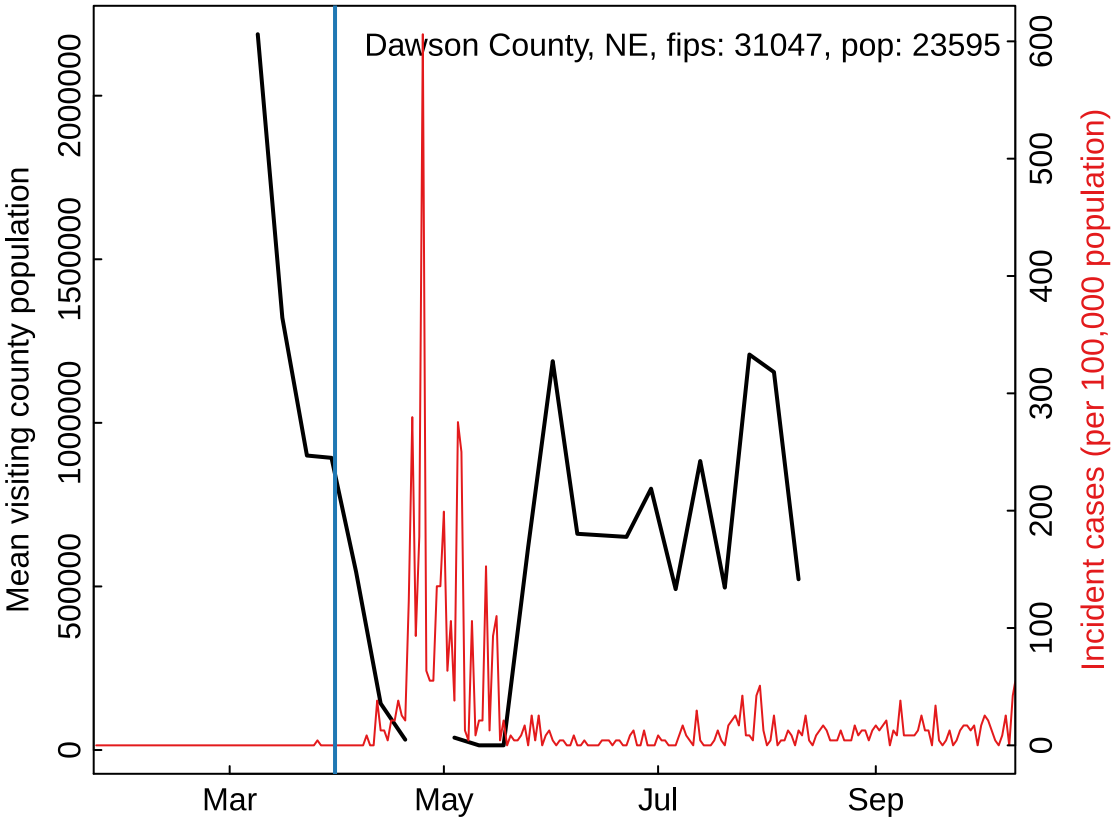

Dawson County, NE, fips: 31047, pop: 23595
Mean visiting county population
0
500000
1000000
1500000
2000000
Incident cases (per 100,000 population)
0
100
200
300
400
500
600
Mar
May
Jul
Sep

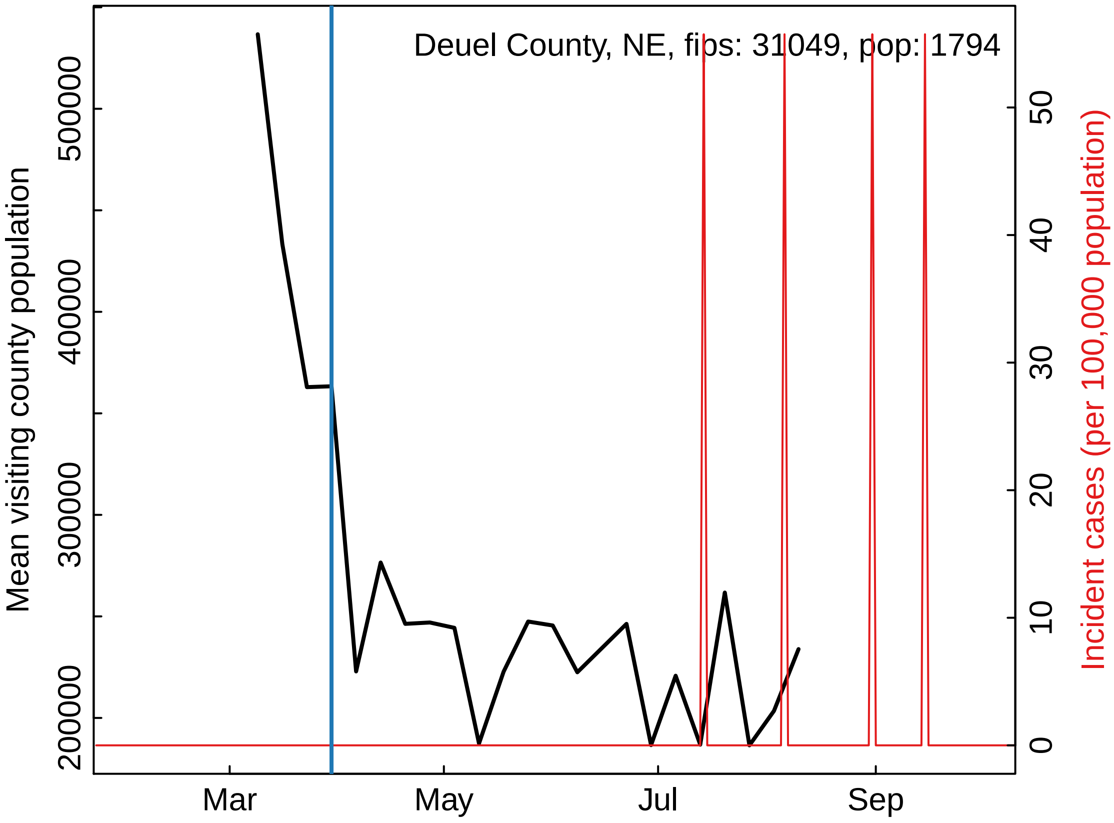

Deuel County, NE, fips: 31049, pop: 1794
Mean visiting county population
200000
300000
400000
500000
Incident cases (per 100,000 population)
0
10
20
30
40
50
Mar
May
Jul
Sep

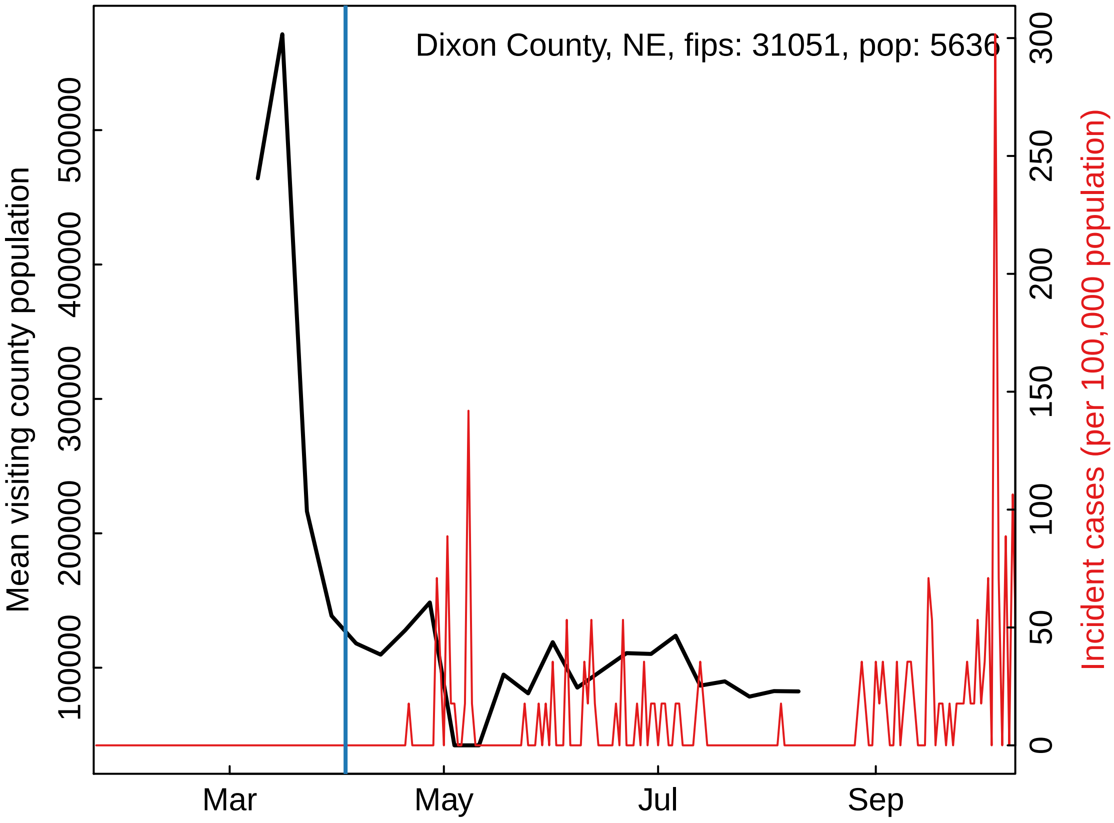

Dixon County, NE, fips: 31051, pop: 5636
Mean visiting county population
100000
200000
300000
400000
500000
Incident cases (per 100,000 population)
0
50
100
150
200
250
300
Mar
May
Jul
Sep

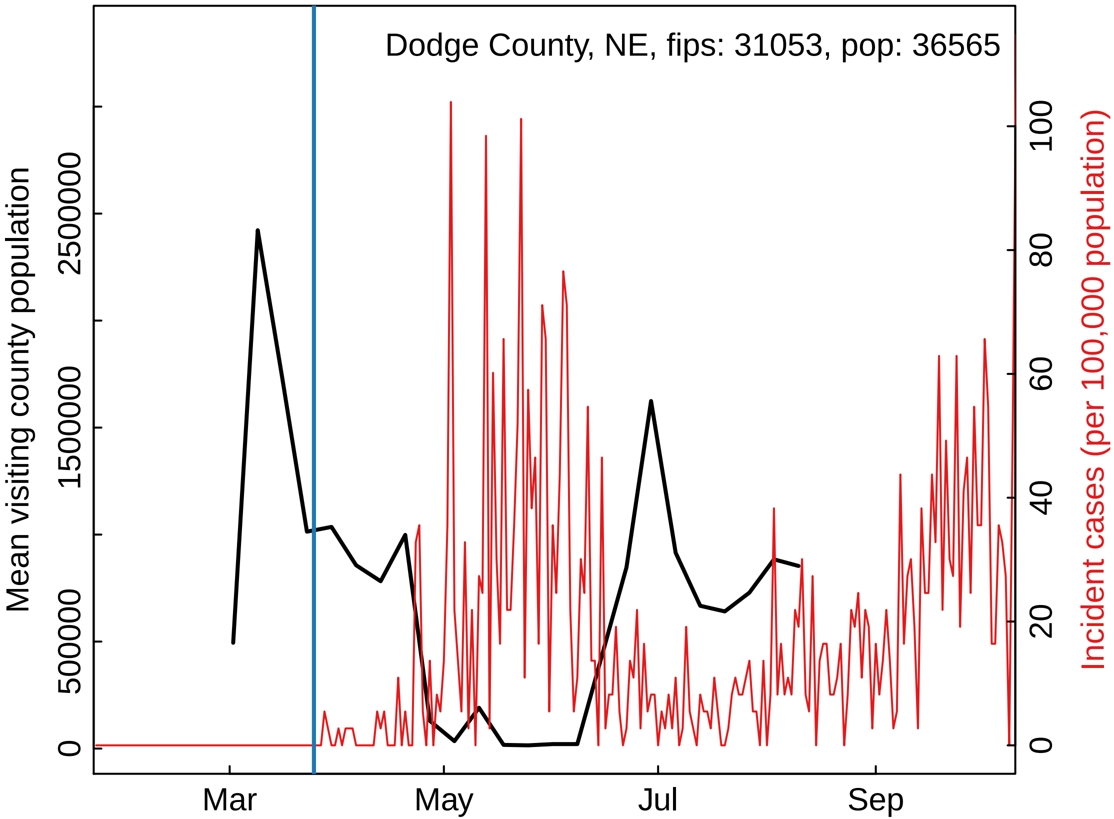

Dodge County, NE, fips: 31053, pop: 36565
Mean visiting county population
0
500000
1500000
2500000
Incident cases (per 100,000 population)
0
20
40
60
80
100
Mar
May
Jul
Sep

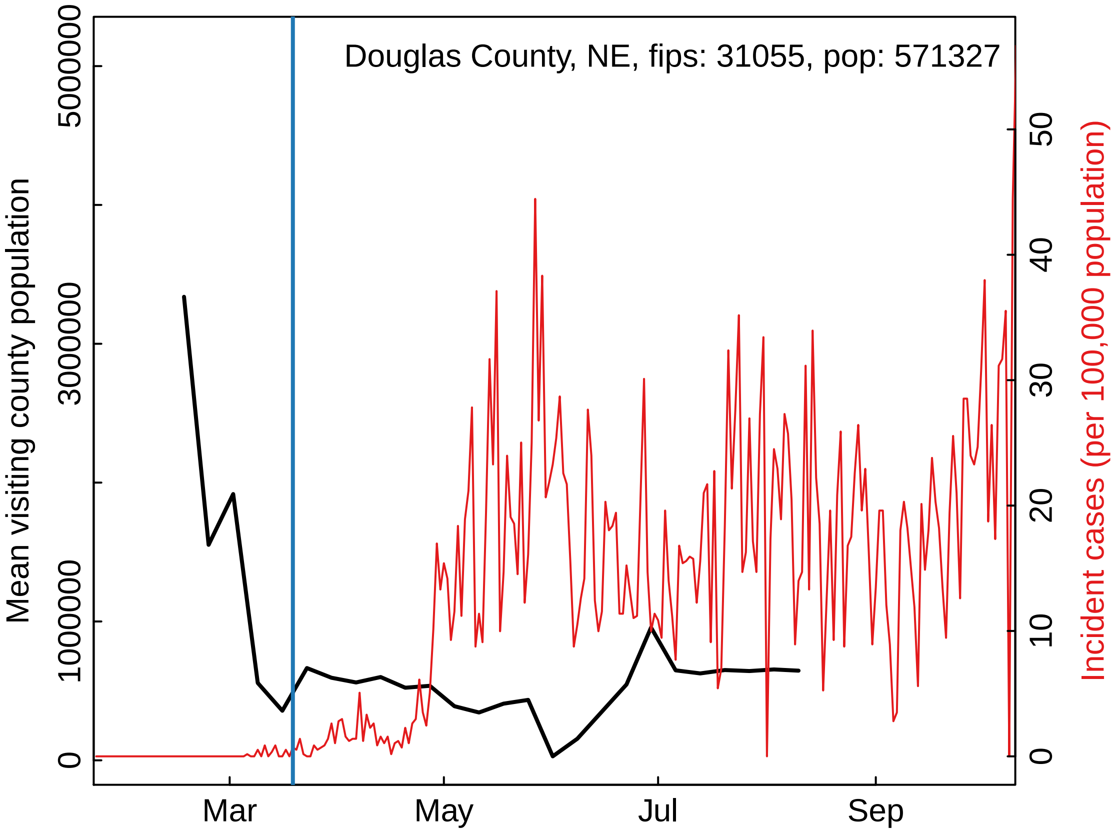

Douglas County, NE, fips: 31055, pop: 571327
Mean visiting county population
0
1000000
3000000
5000000
Incident cases (per 100,000 population)
0
10
20
30
40
50
Mar
May
Jul
Sep

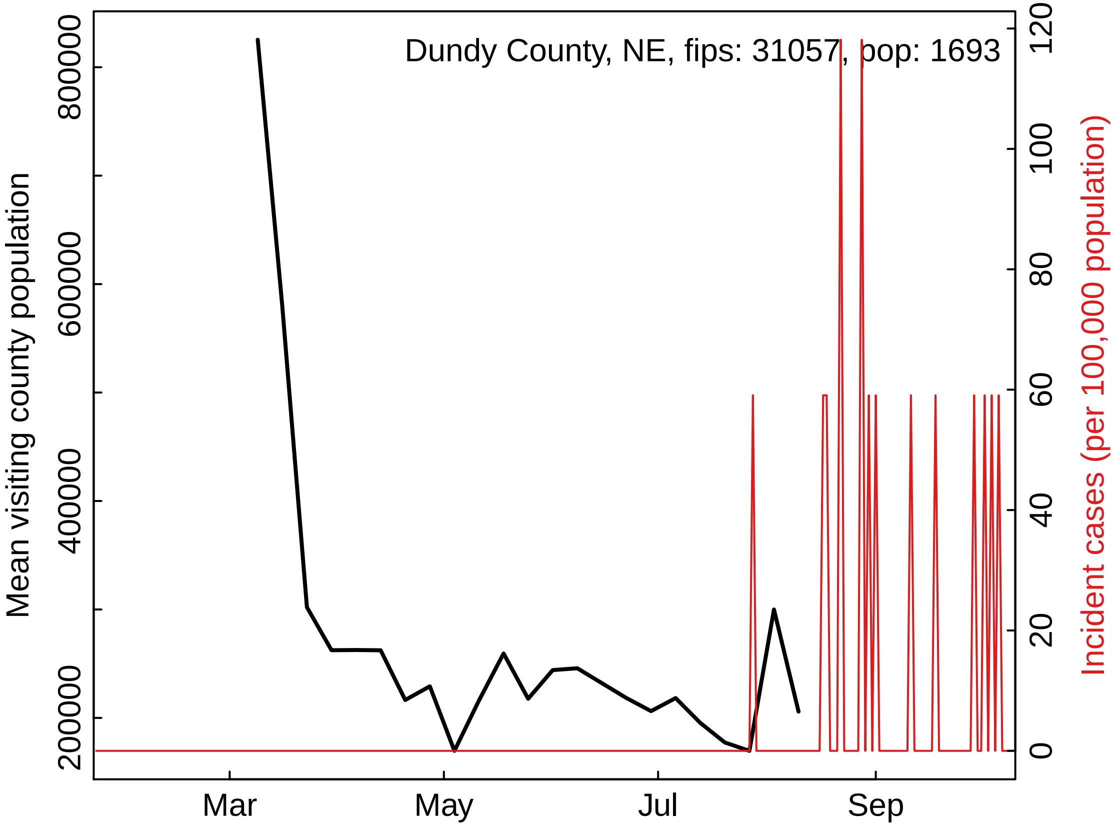

Dundy County, NE, fips: 31057, pop: 1693
Mean visiting county population
200000
400000
600000
800000
Incident cases (per 100,000 population)
0
20
40
60
80
100
120
Mar
May
Jul
Sep

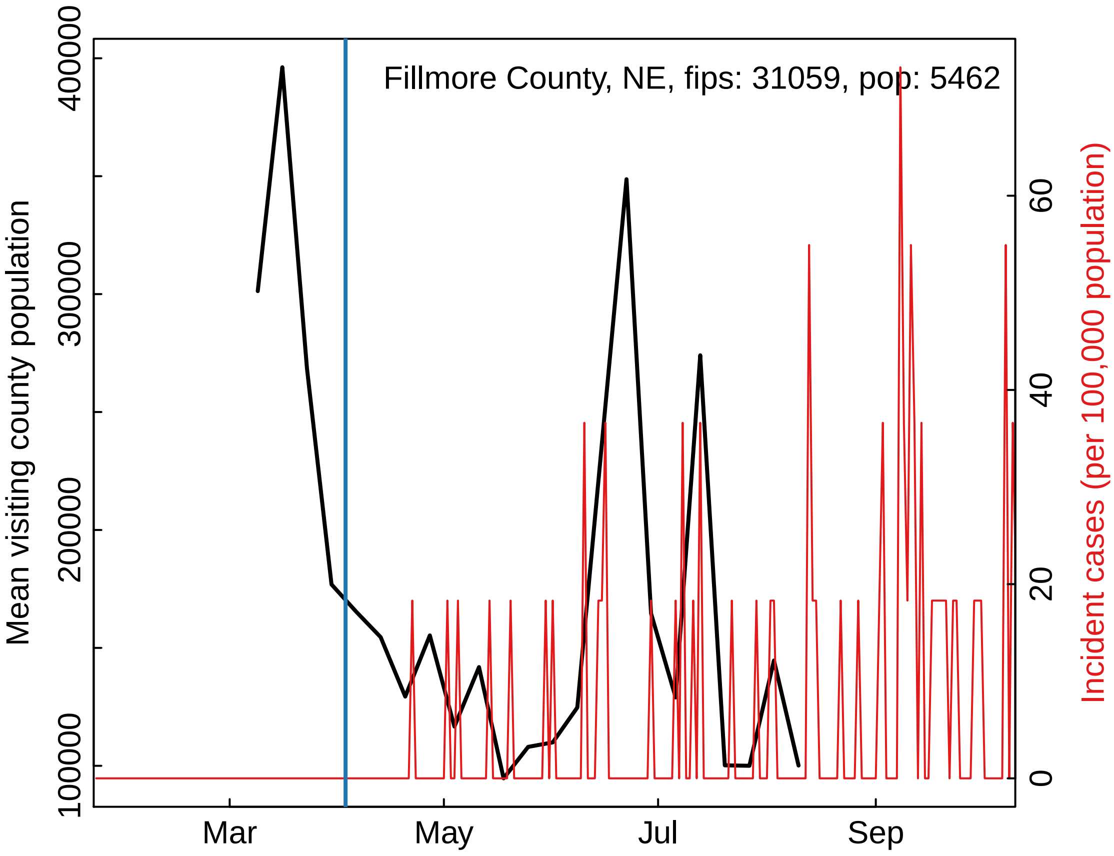

Fillmore County, NE, fips: 31059, pop: 5462
Mean visiting county population
100000
200000
300000
400000
Incident cases (per 100,000 population)
0
20
40
60
Mar
May
Jul
Sep

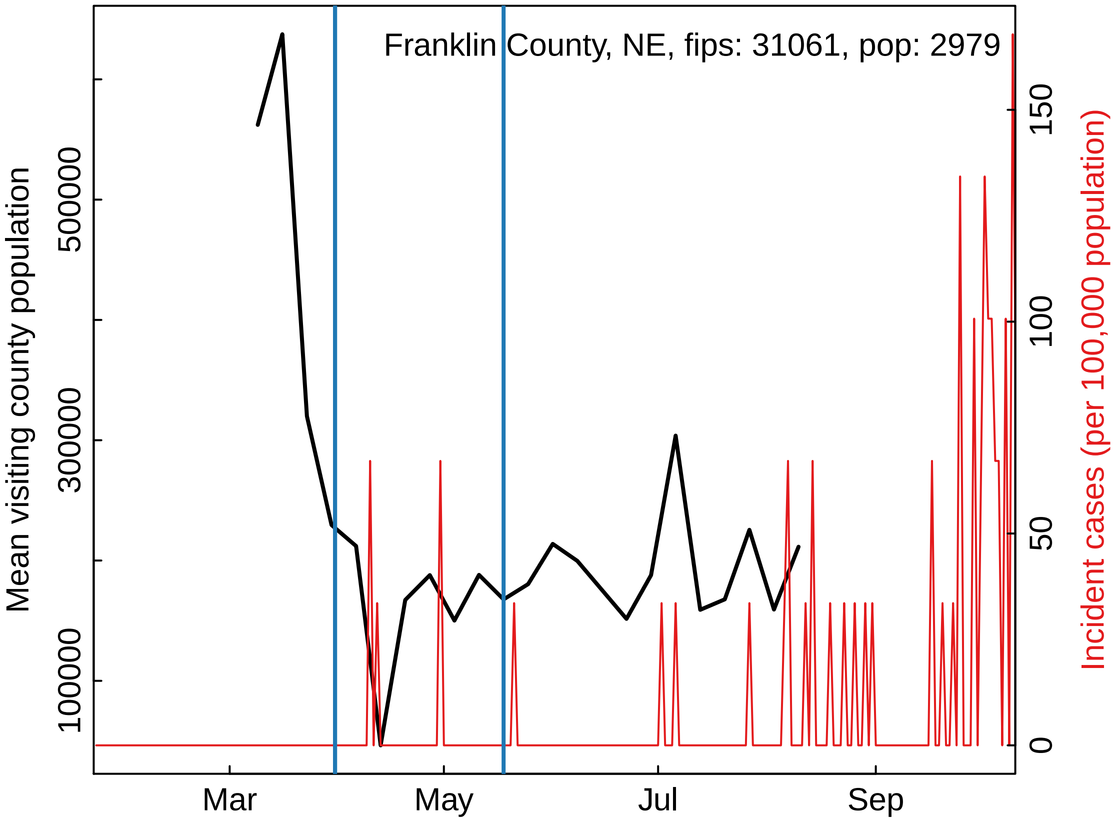
Franklin County, NE, fips: 31061, pop: 2979
Mean visiting county population
100000
300000
500000
Incident cases (per 100,000 population)
0
50
100
150
Mar
May
Jul
Sep

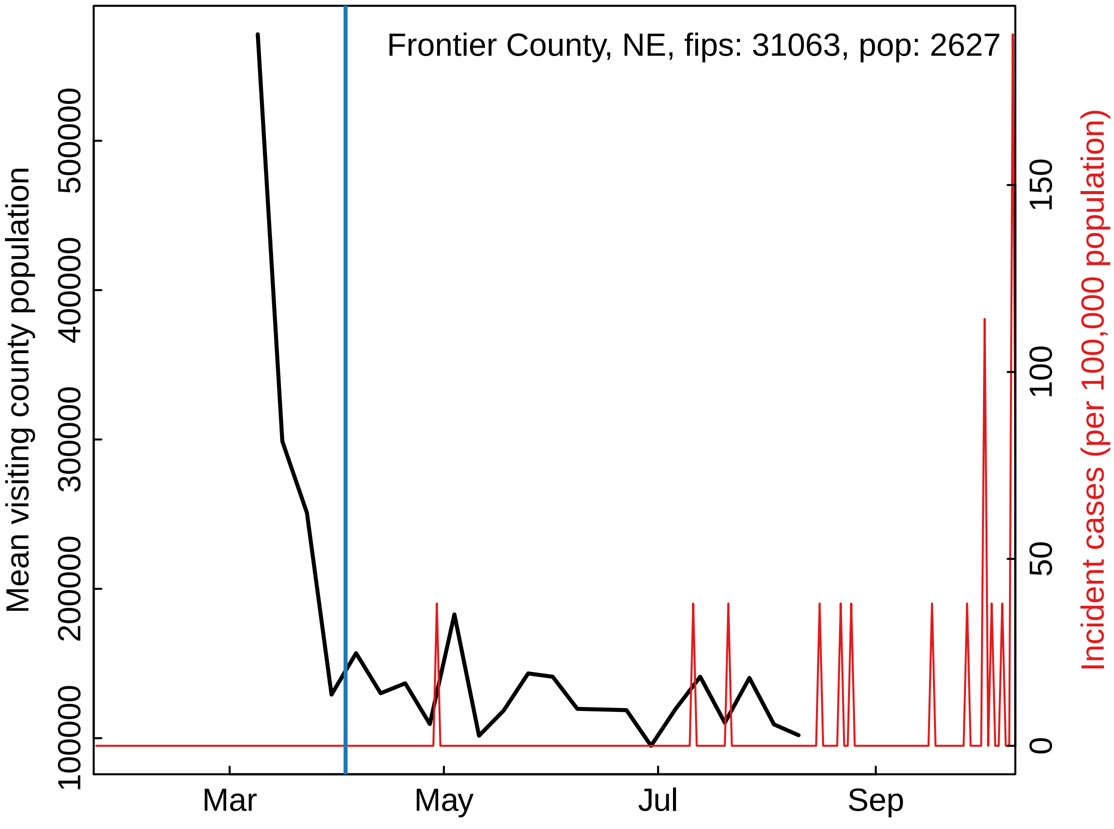

Frontier County, NE, fips: 31063, pop: 2627
Mean visiting county population
100000
200000
300000
400000
500000
Mar
May
Jul
Sep
0
50
100
150
Incident cases (per 100,000 population)

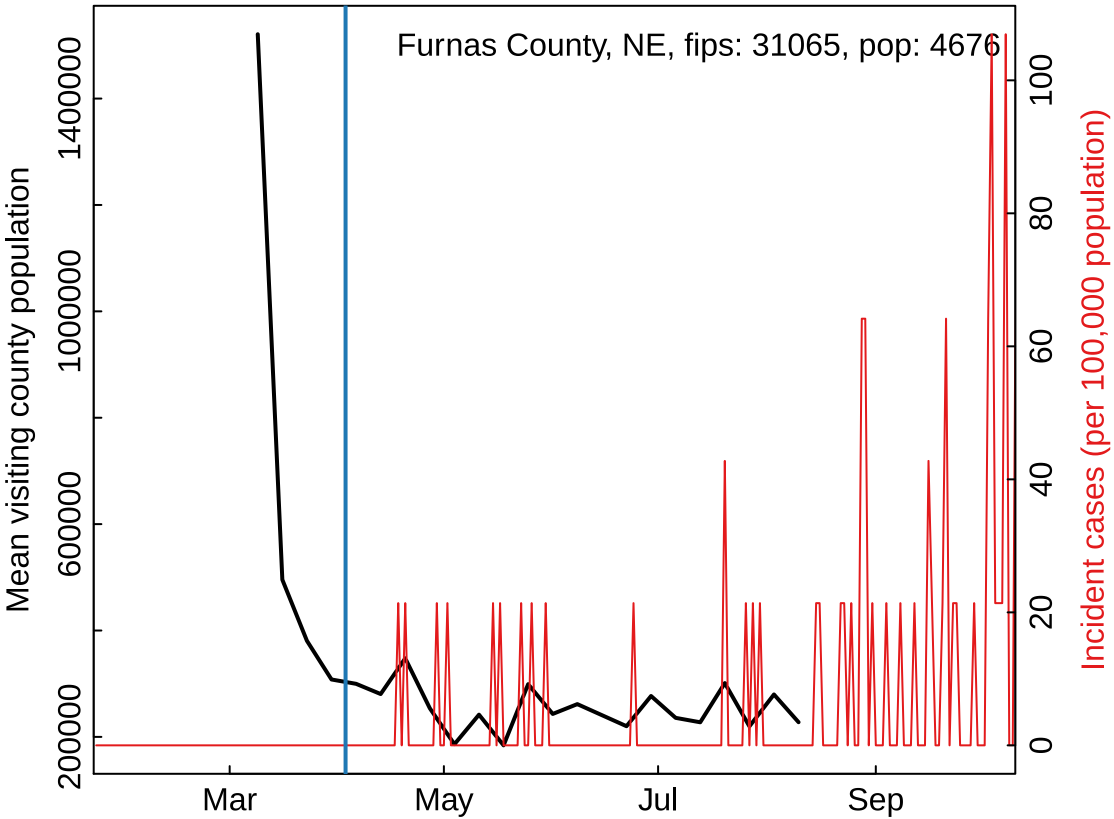

Furnas County, NE, fips: 31065, pop: 4676
Mean visiting county population
200000
600000
1000000
1400000
Incident cases (per 100,000 population)
0
20
40
60
80
100
Mar
May
Jul
Sep

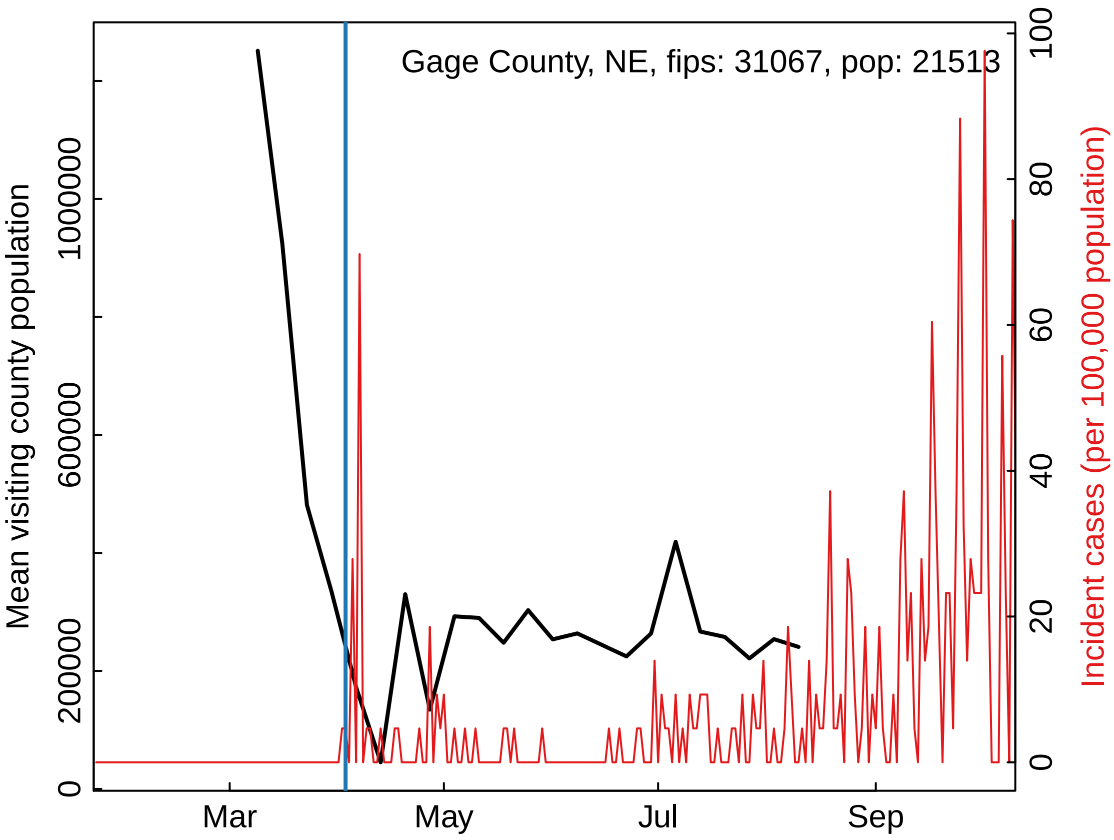

Gage County, NE, fips: 31067, pop: 21513
Mean visiting county population
0
200000
600000
1000000
Mar
May
Jul
Sep
Incident cases (per 100,000 population)
0
20
40
60
80
100

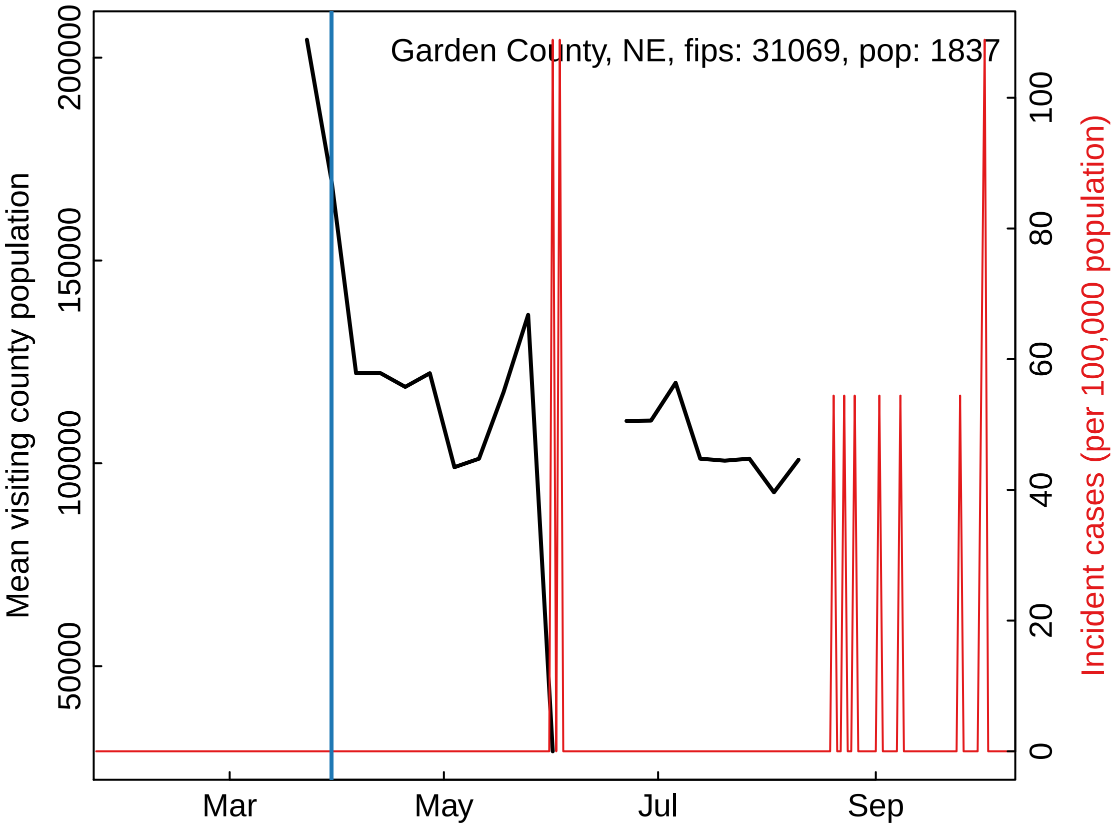

Garden County, NE, fips: 31069, pop: 1837
Mean visiting county population
50000
100000
150000
200000
Mar
May
Jul
Sep
0
20
40
60
80
100
Incident cases (per 100,000 population)

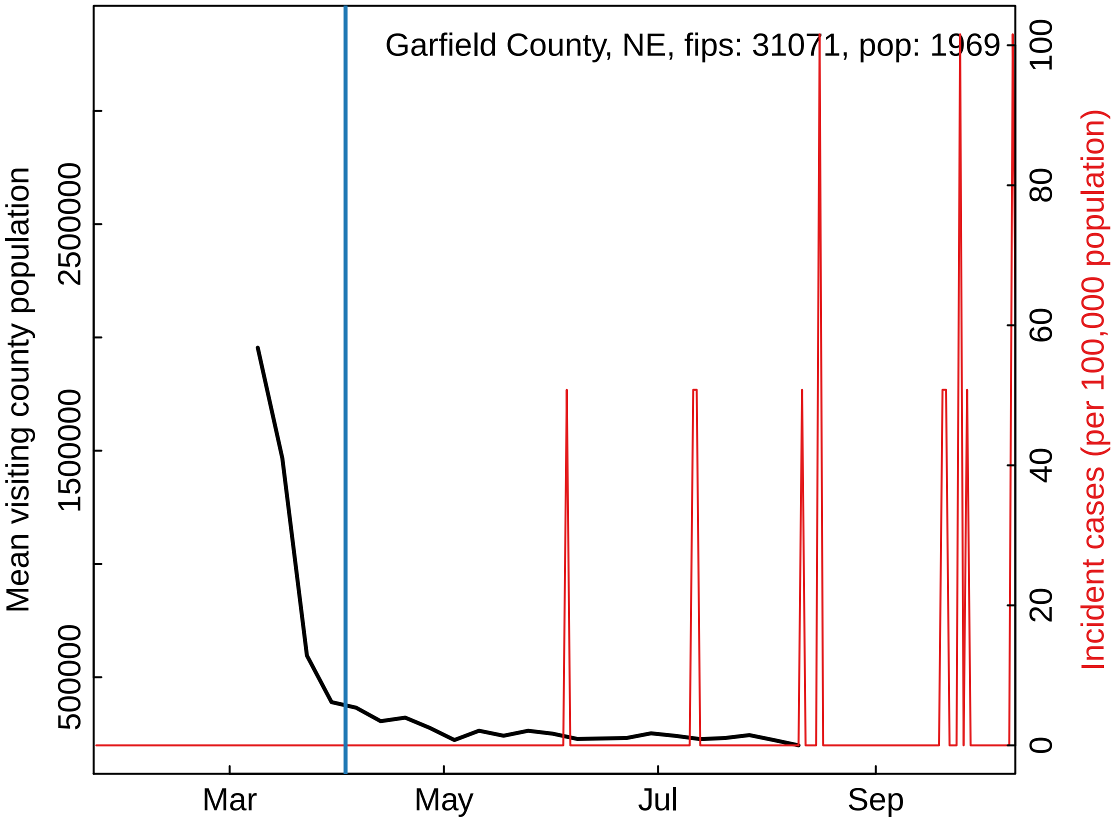

Garfield County, NE, fips: 31071, pop: 1969
Mean visiting county population
500000
1500000
2500000
Mar
May
Jul
Sep
0
20
40
60
80
100
Incident cases (per 100,000 population)

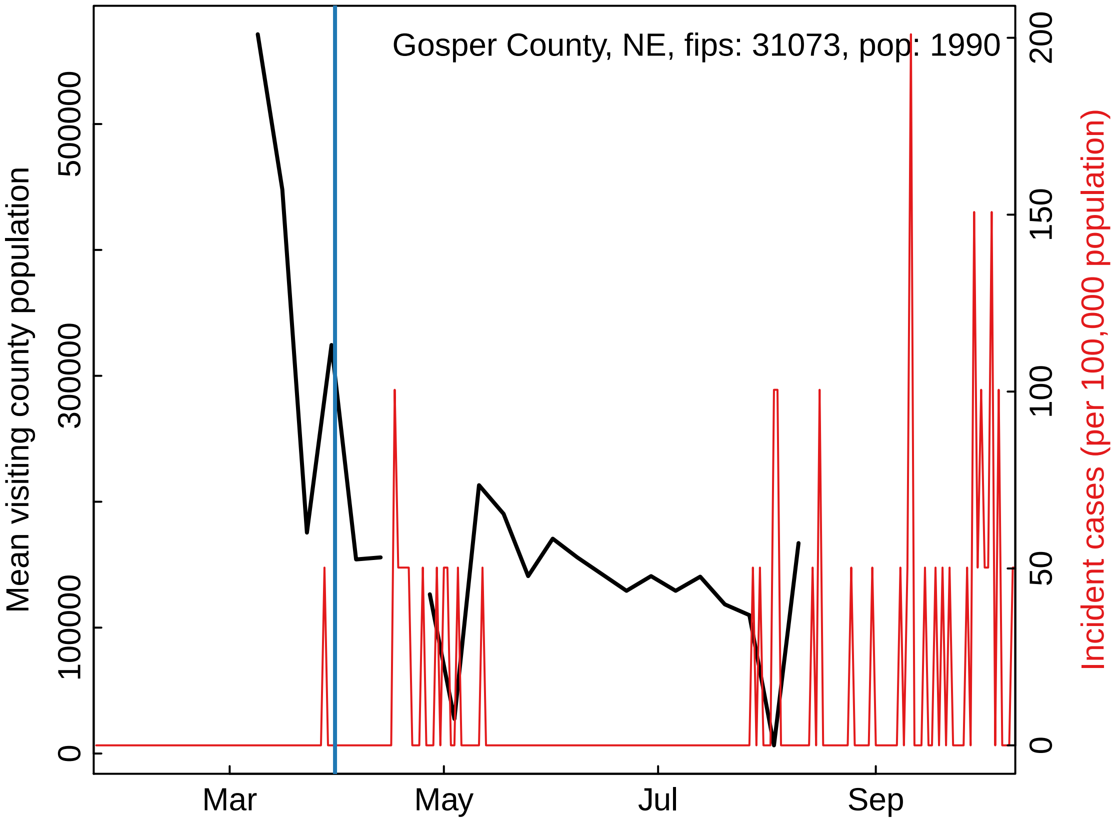
Gosper County, NE, fips: 31073, pop: 1990
Mean visiting county population
0
100000
300000
500000
Mar
May
Jul
Sep
Incident cases (per 100,000 population)
0
50
100
150
200

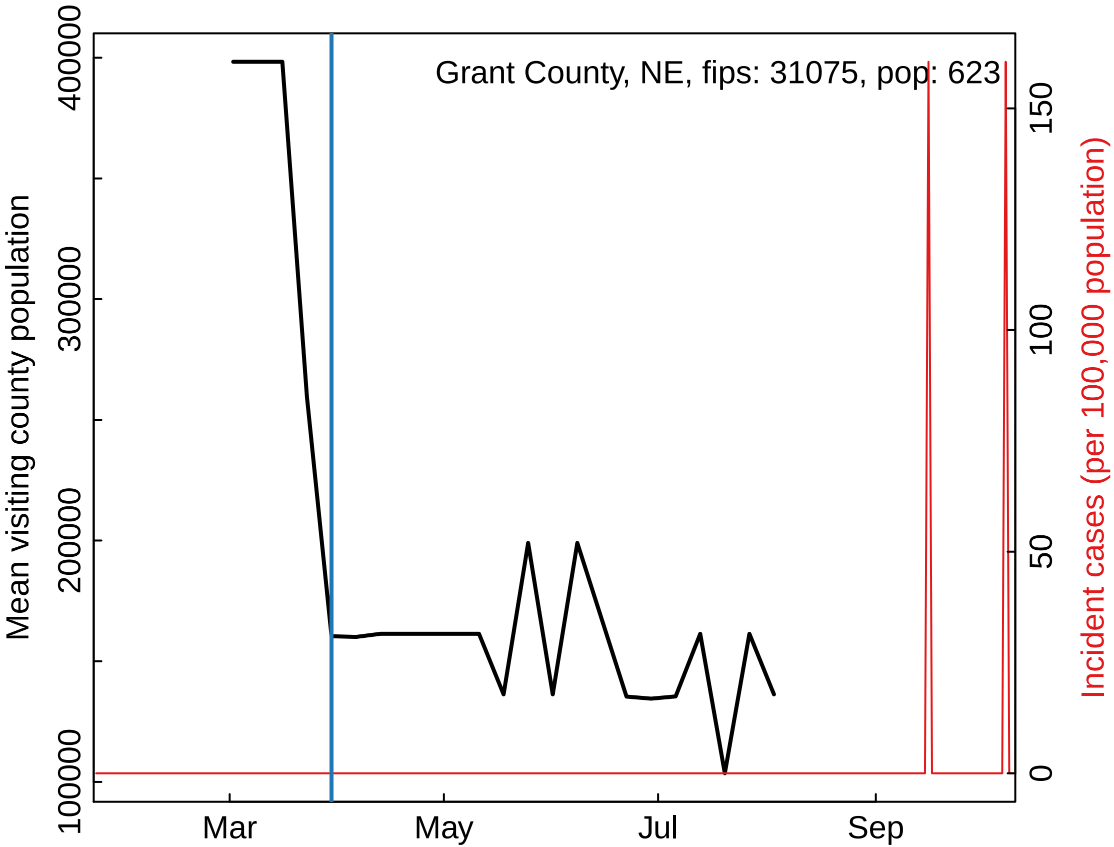
Grant County, NE, fips: 31075, pop: 623
Mean visiting county population
100000
200000
300000
400000
Mar
May
Jul
Sep
0
50
100
150
Incident cases (per 100,000 population)

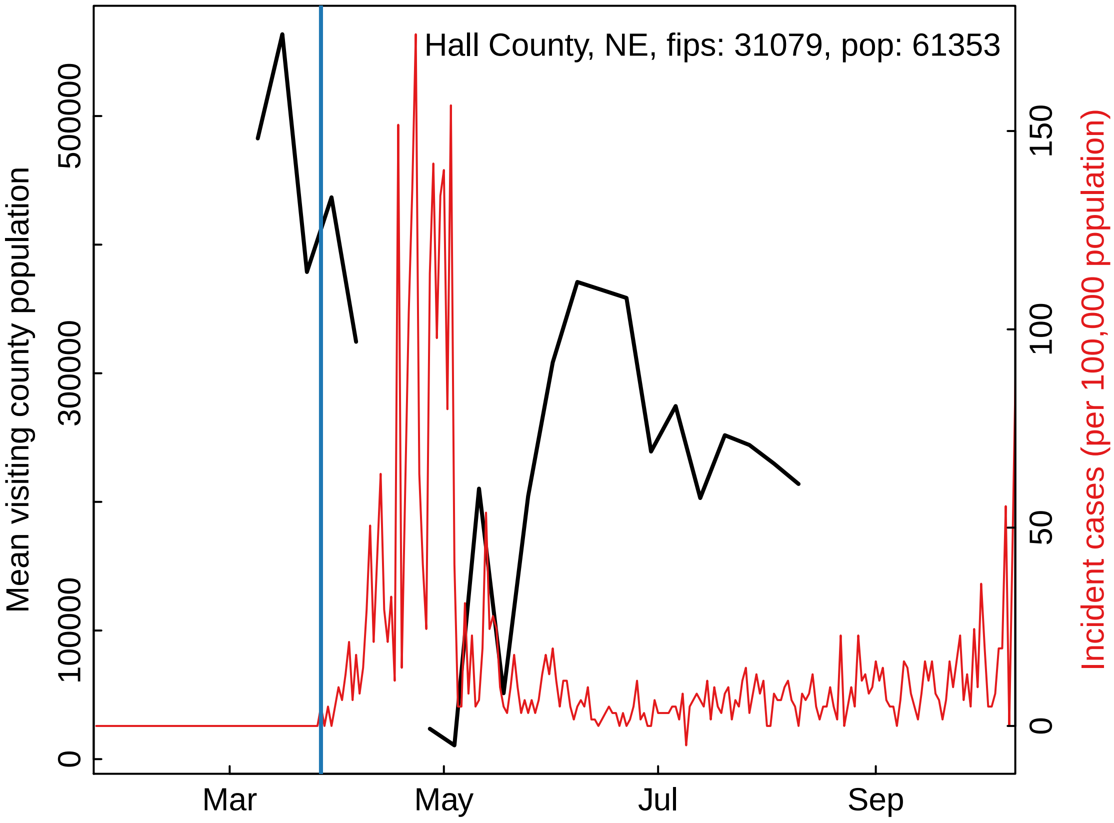
Hall County, NE, fips: 31079, pop: 61353
Mean visiting county population
Incident cases (per 100,000 population)
0
100000
300000
500000
0
50
100
150
Mar
May
Jul
Sep

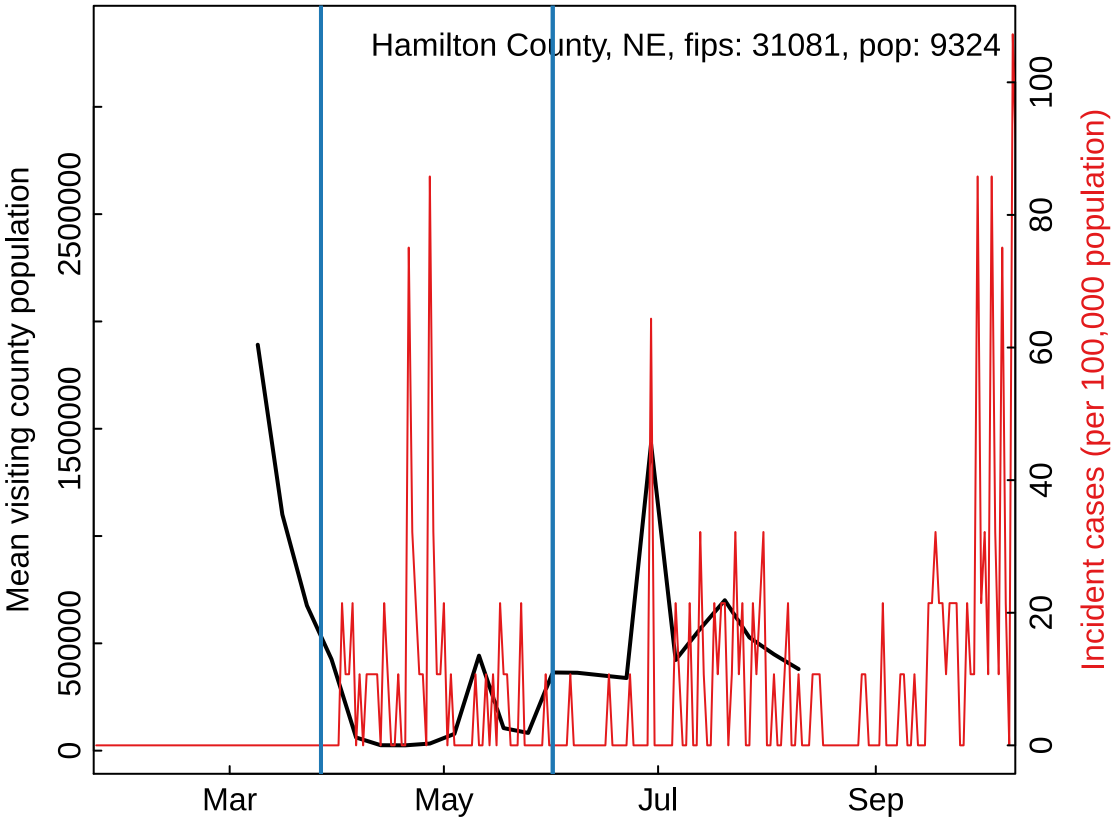

Hamilton County, NE, fips: 31081, pop: 9324
Mean visiting county population
0
500000
1500000
2500000
Incident cases (per 100,000 population)
0
20
40
60
80
100
Mar
May
Jul
Sep

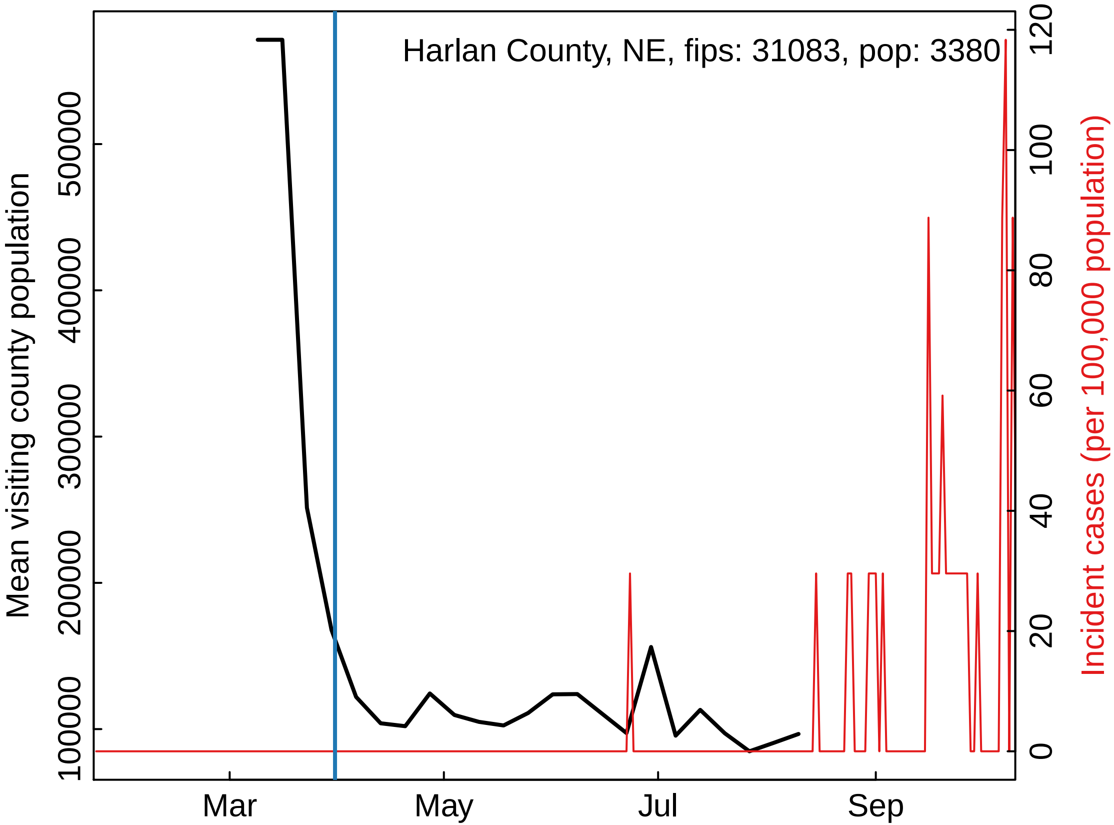

Harlan County, NE, fips: 31083, pop: 3380
Mean visiting county population
Incident cases (per 100,000 population)
100000
200000
300000
400000
500000
0
20
40
60
80
100
120
Mar
May
Jul
Sep

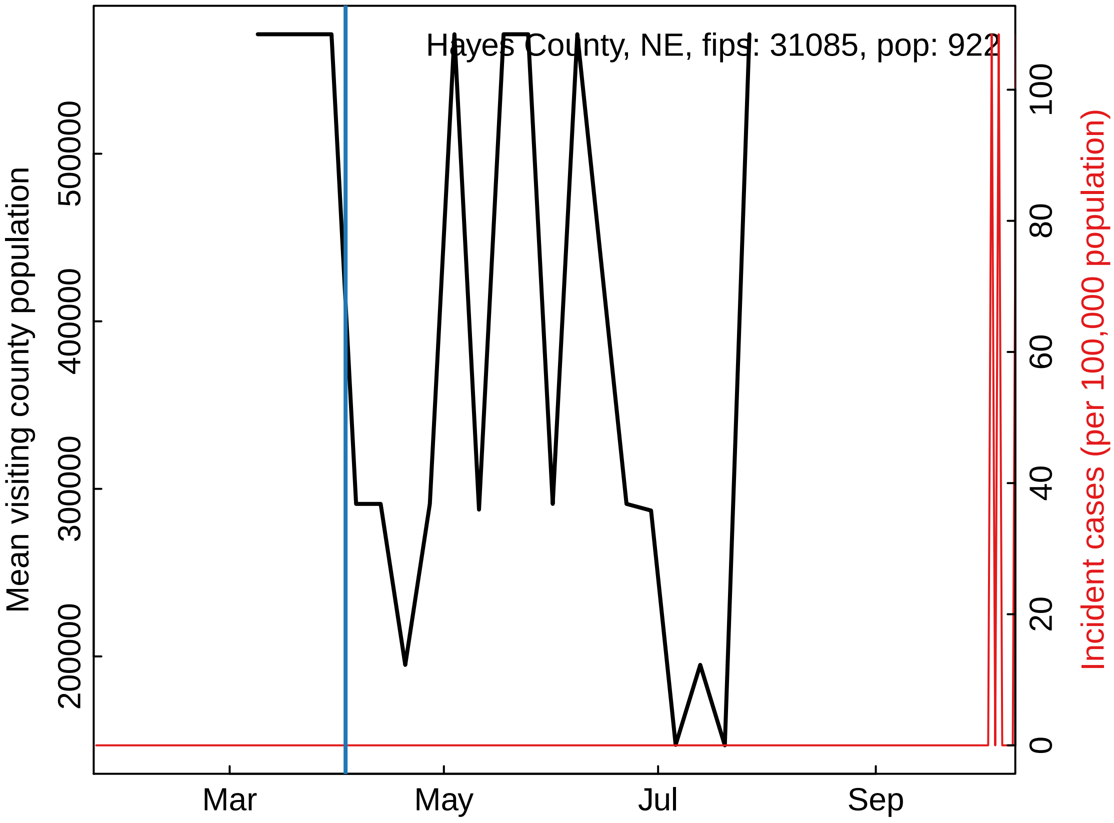

Hayes County, NE, fips: 31085, pop: 922
Mean visiting county population
200000
300000
400000
500000
Mar
May
Jul
Sep
0
20
40
60
80
100
Incident cases (per 100,000 population)

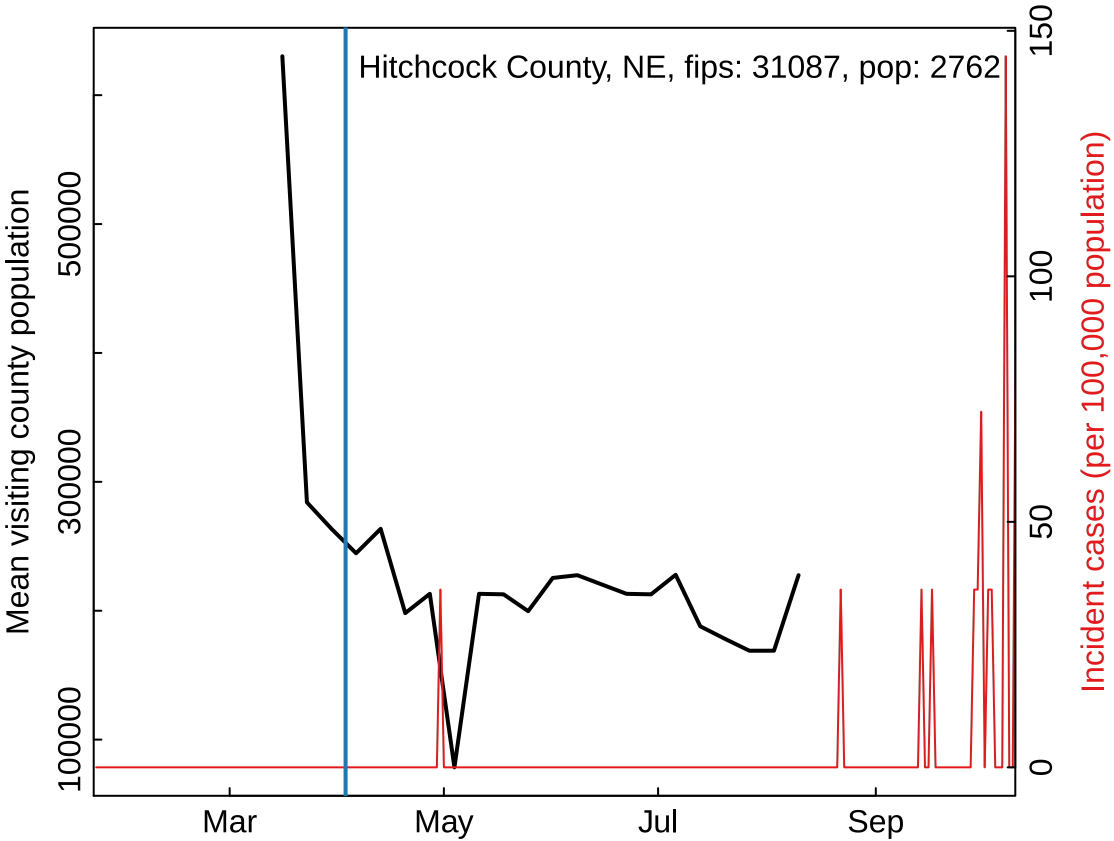

Hitchcock County, NE, fips: 31087, pop: 2762
Mean visiting county population
100000
300000
500000
Incident cases (per 100,000 population)
0
50
100
150
Mar
May
Jul
Sep

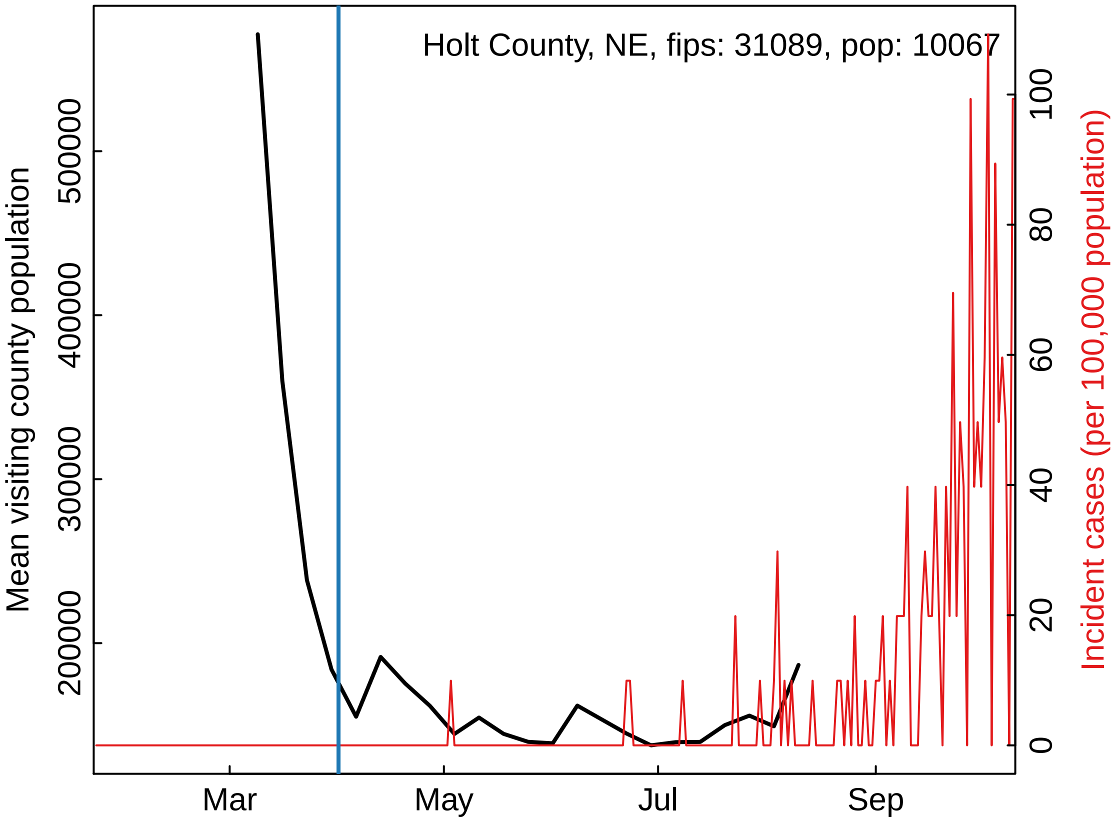

Holt County, NE, fips: 31089, pop: 10067
Mean visiting county population
200000
300000
400000
500000
Mar
May
Jul
Sep
0
20
40
60
80
100
Incident cases (per 100,000 population)

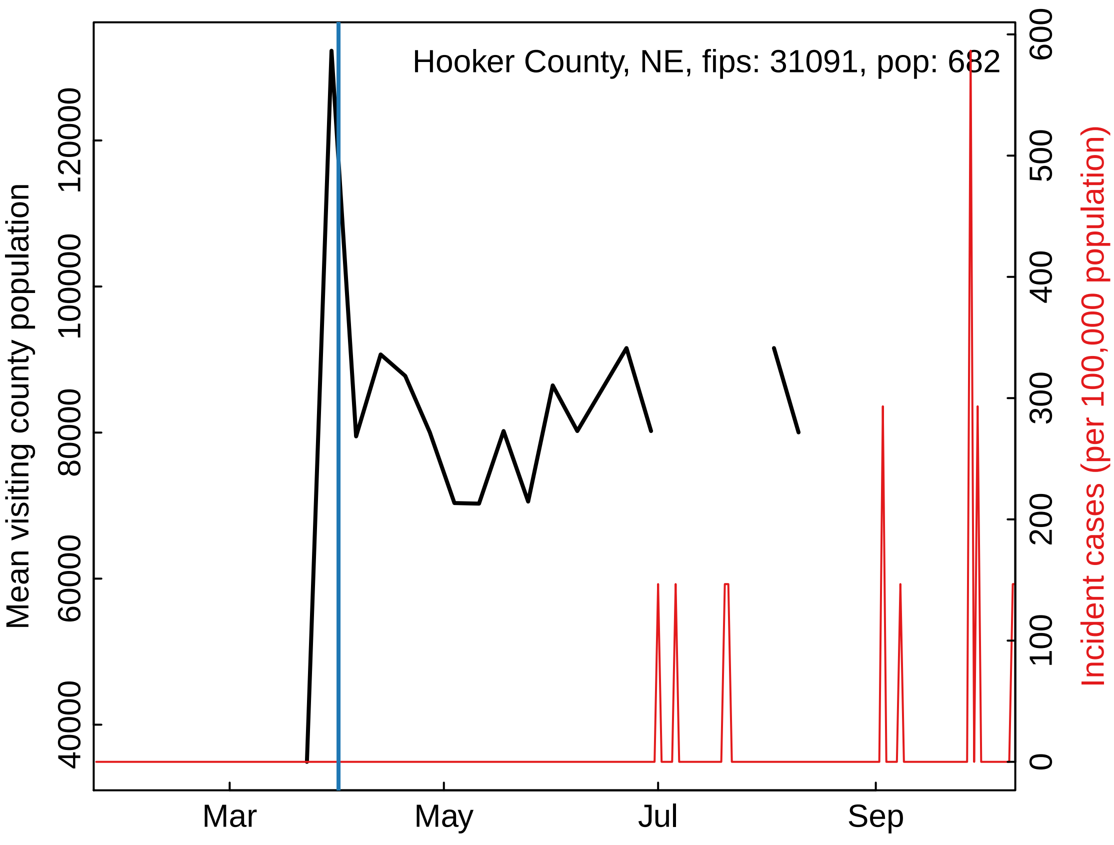
Hooker County, NE, fips: 31091, pop: 682
Mean visiting county population
40000
60000
80000
100000
120000
Incident cases (per 100,000 population)
0
100
200
300
400
500
600
Mar
May
Jul
Sep

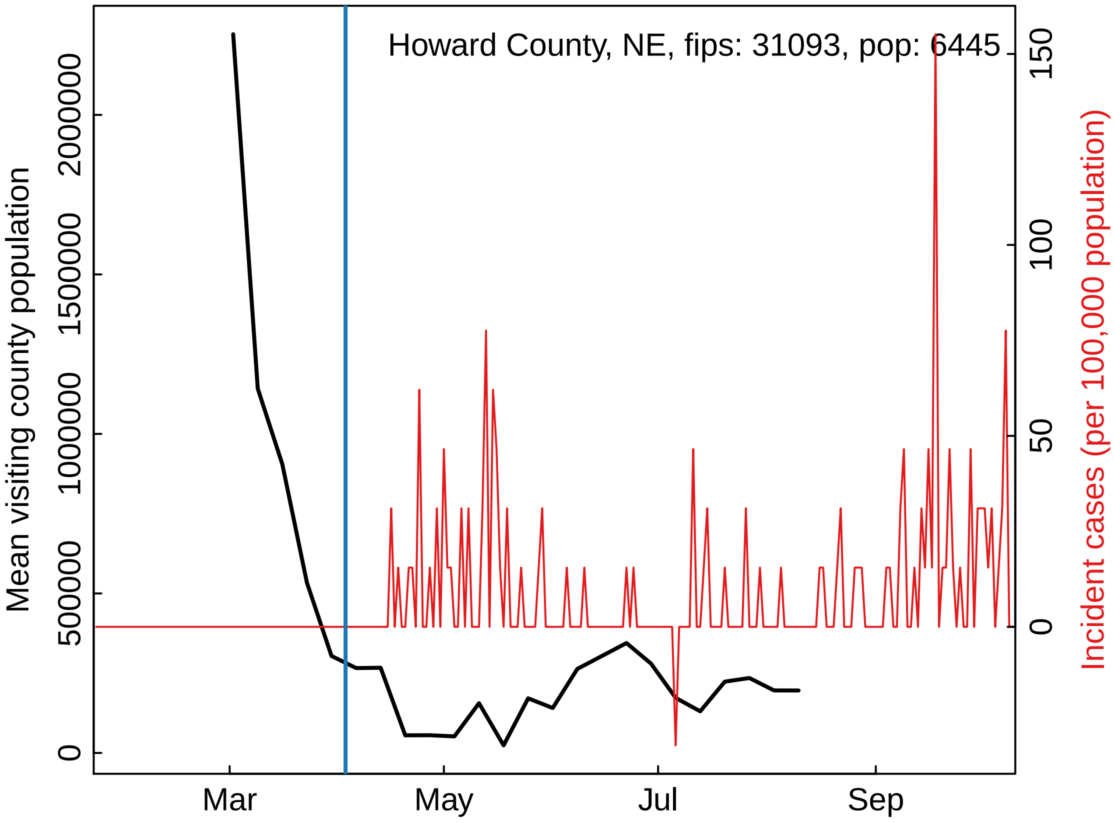

Howard County, NE, fips: 31093, pop: 6445
Mean visiting county population
0
500000
1000000
1500000
2000000
Incident cases (per 100,000 population)
0
50
100
150
Mar
May
Jul
Sep

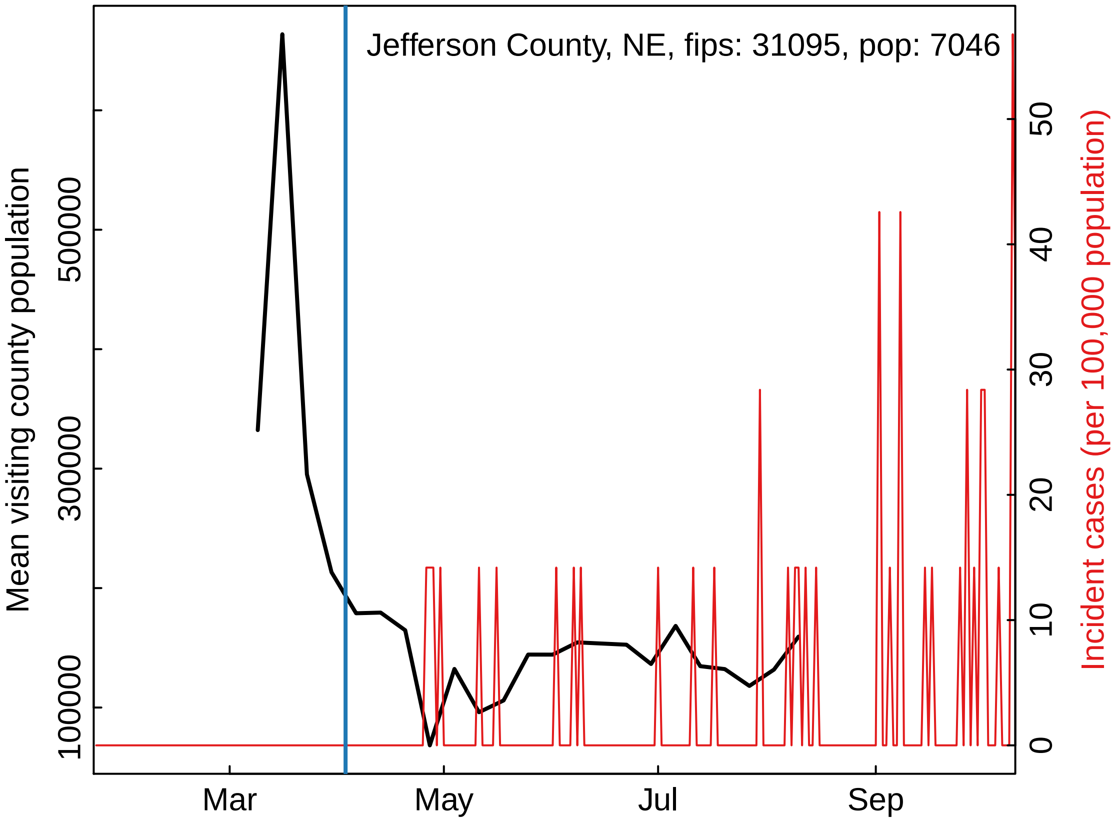

Jefferson County, NE, fips: 31095, pop: 7046
Mean visiting county population
100000
300000
500000
Mar
May
Jul
Sep
0
10
20
30
40
50
Incident cases (per 100,000 population)

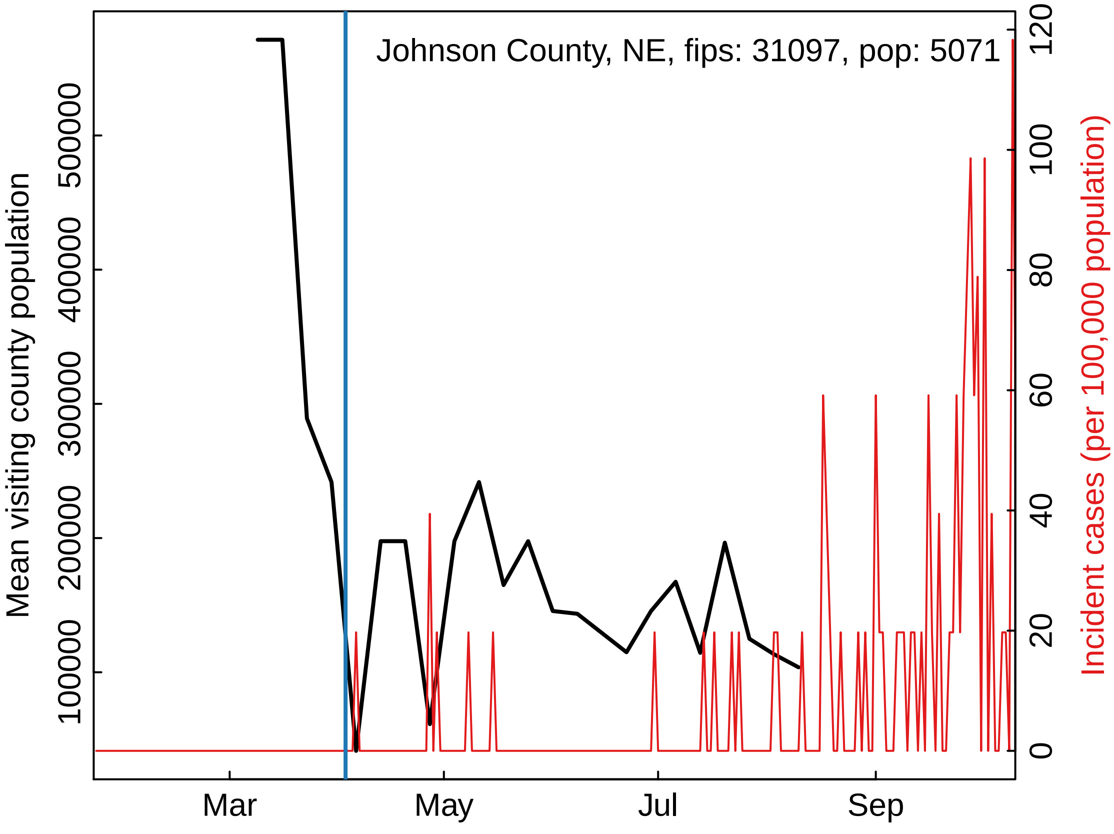

Johnson County, NE, fips: 31097, pop: 5071
Mean visiting county population
100000
200000
300000
400000
500000
Incident cases (per 100,000 population)
0
20
40
60
80
100
120
Mar
May
Jul
Sep

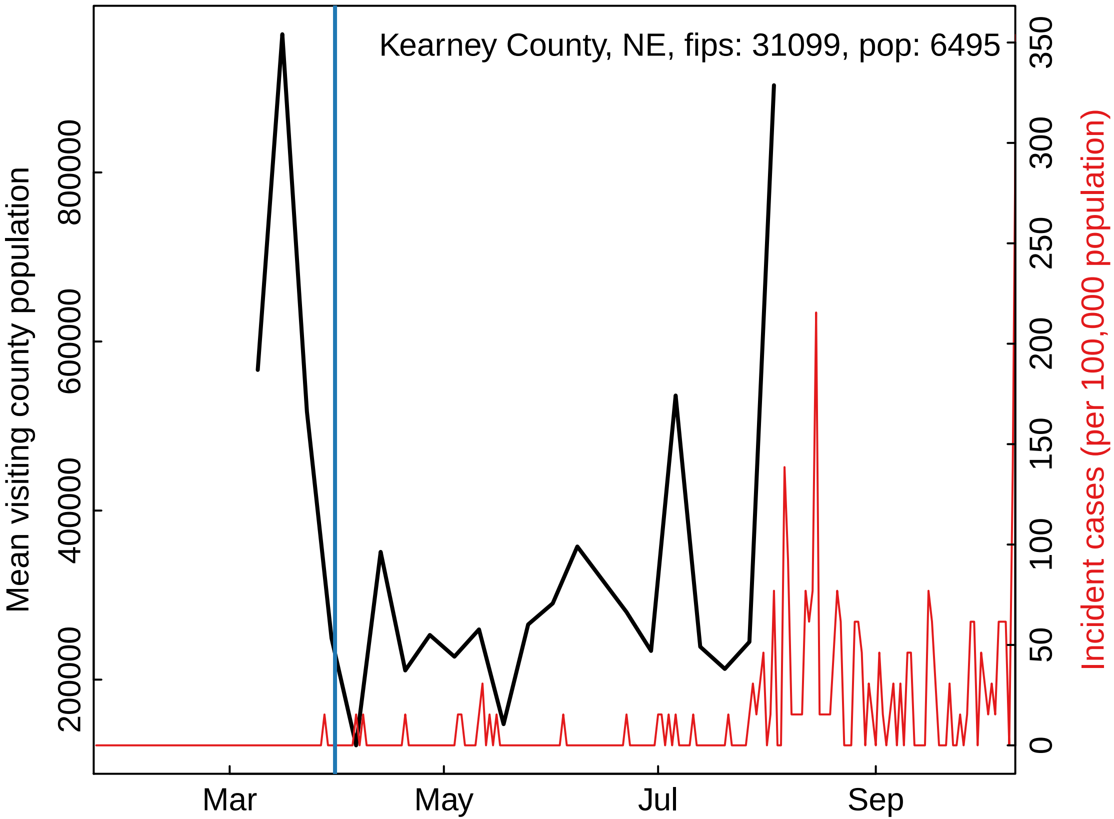

Kearney County, NE, fips: 31099, pop: 6495
Mean visiting county population
200000
400000
600000
800000
Incident cases (per 100,000 population)
0
50
100
150
200
250
300
350
Mar
May
Jul
Sep

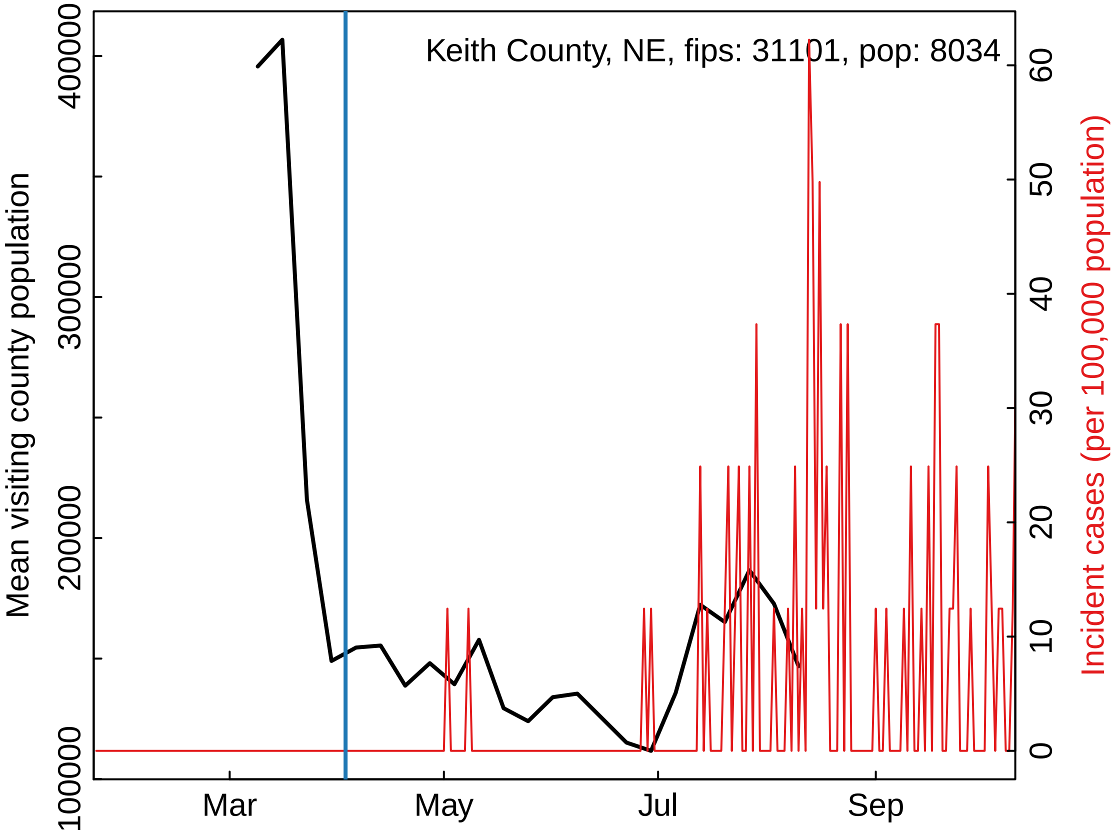

Keith County, NE, fips: 31101, pop: 8034
Mean visiting county population
100000
200000
300000
400000
Incident cases (per 100,000 population)
0
10
20
30
40
50
60
Mar
May
Jul
Sep

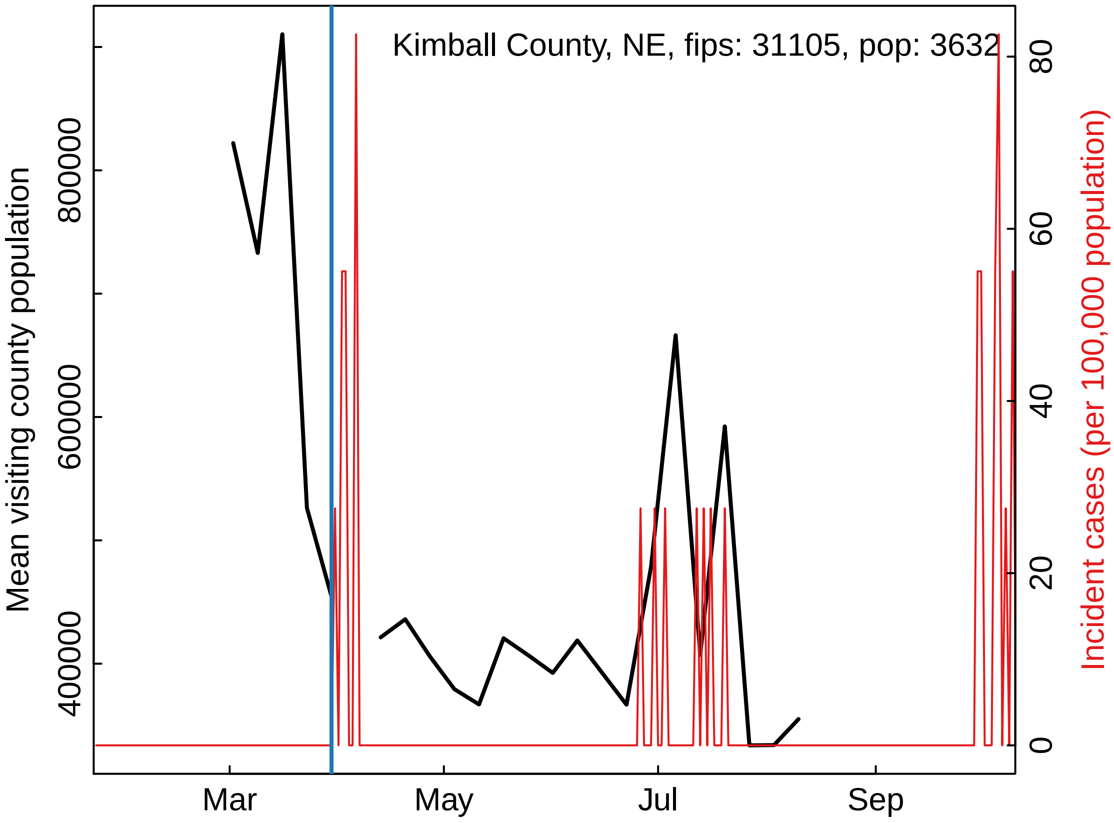
Kimball County, NE, fips: 31105, pop: 3632
Mean visiting county population
400000
600000
800000
Incident cases (per 100,000 population)
0
20
40
60
80
Mar
May
Jul
Sep

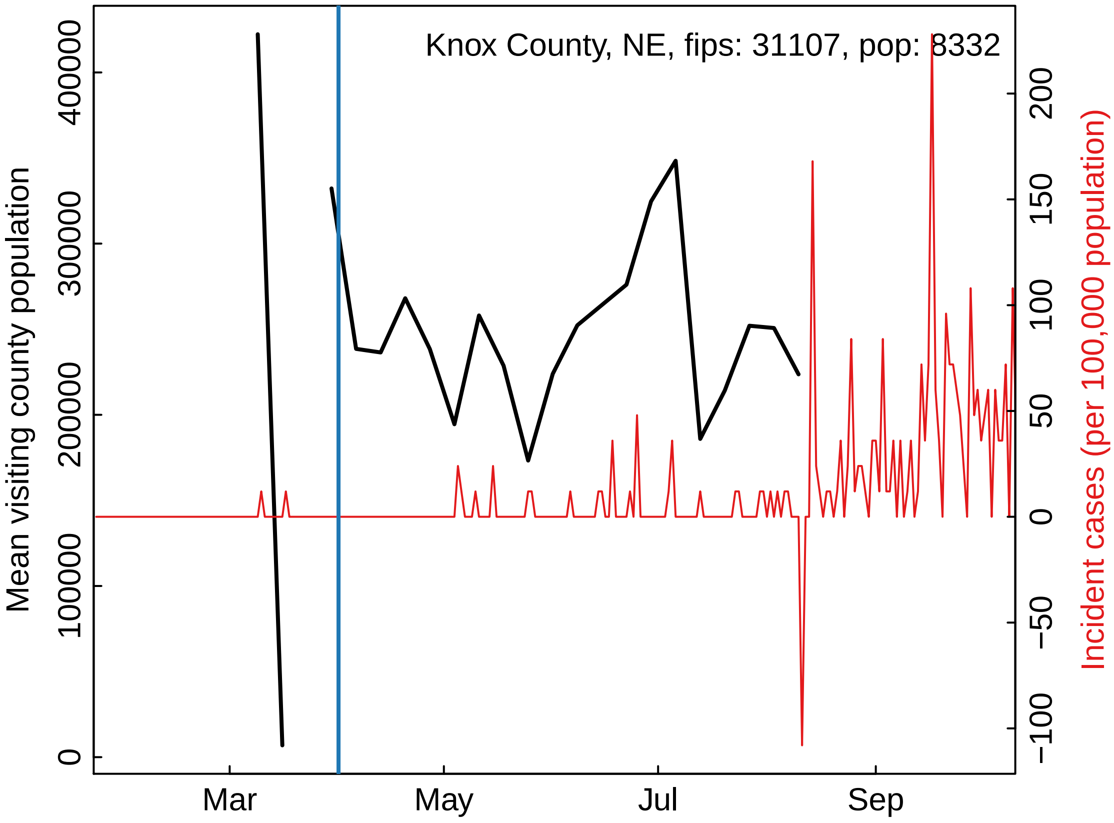

Knox County, NE, fips: 31107, pop: 8332
Mean visiting county population
0
100000
200000
300000
400000
Mar
May
Jul
Sep
Incident cases (per 100,000 population)
−100
−50
0
50
100
150
200

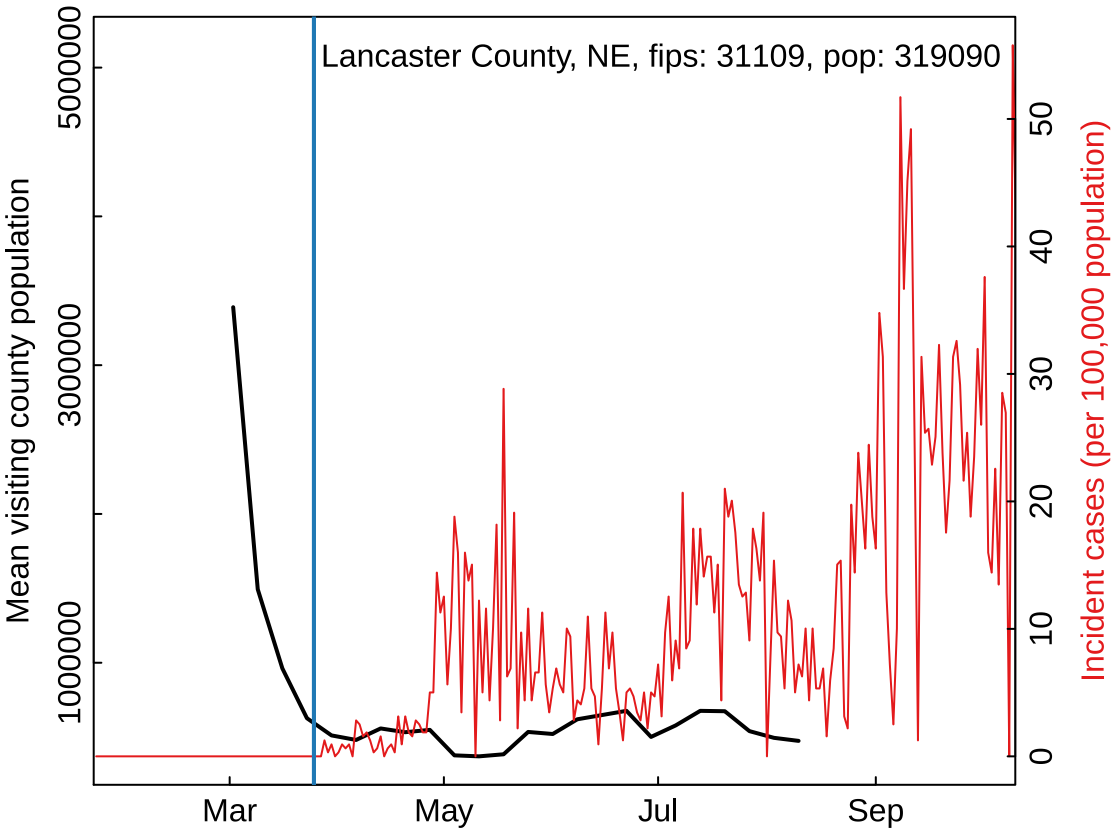
Lancaster County, NE, fips: 31109, pop: 319090
Mean visiting county population
1000000
3000000
5000000
Mar
May
Jul
Sep
0
10
20
30
40
50
Incident cases (per 100,000 population)

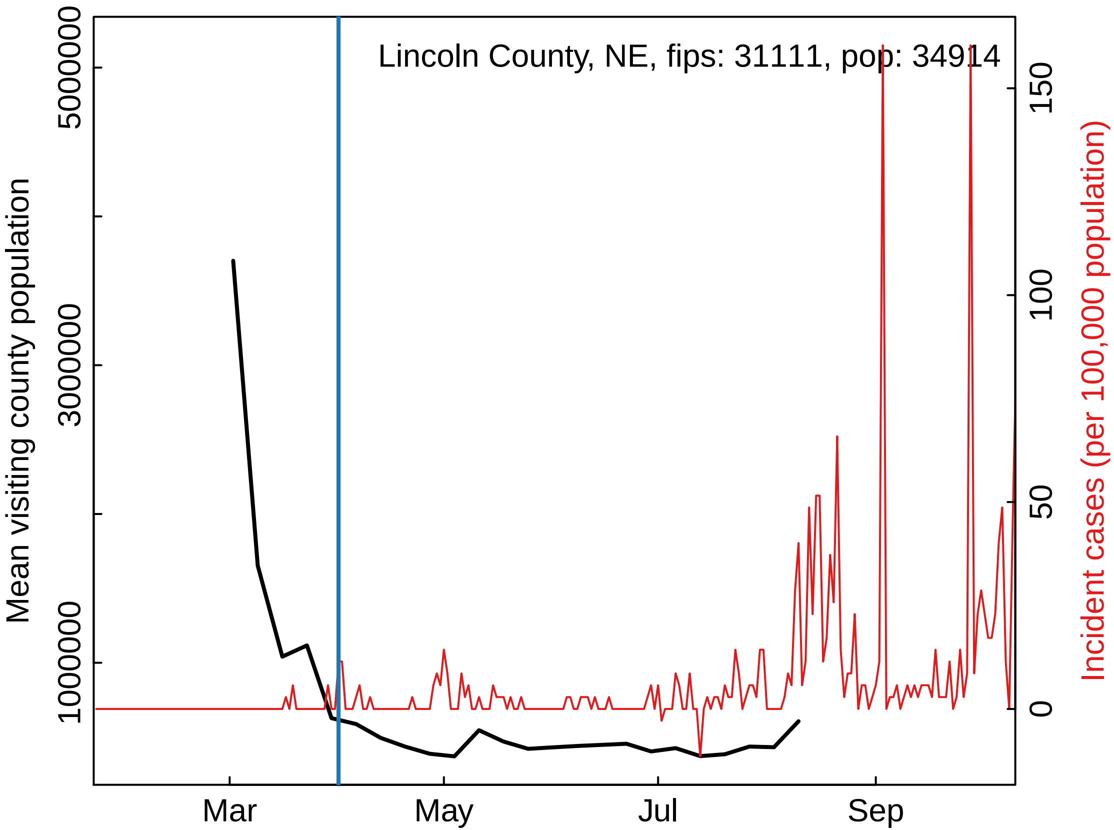

Lincoln County, NE, fips: 31111, pop: 34914
Mean visiting county population
1000000
3000000
5000000
Incident cases (per 100,000 population)
0
50
100
150
Mar
May
Jul
Sep

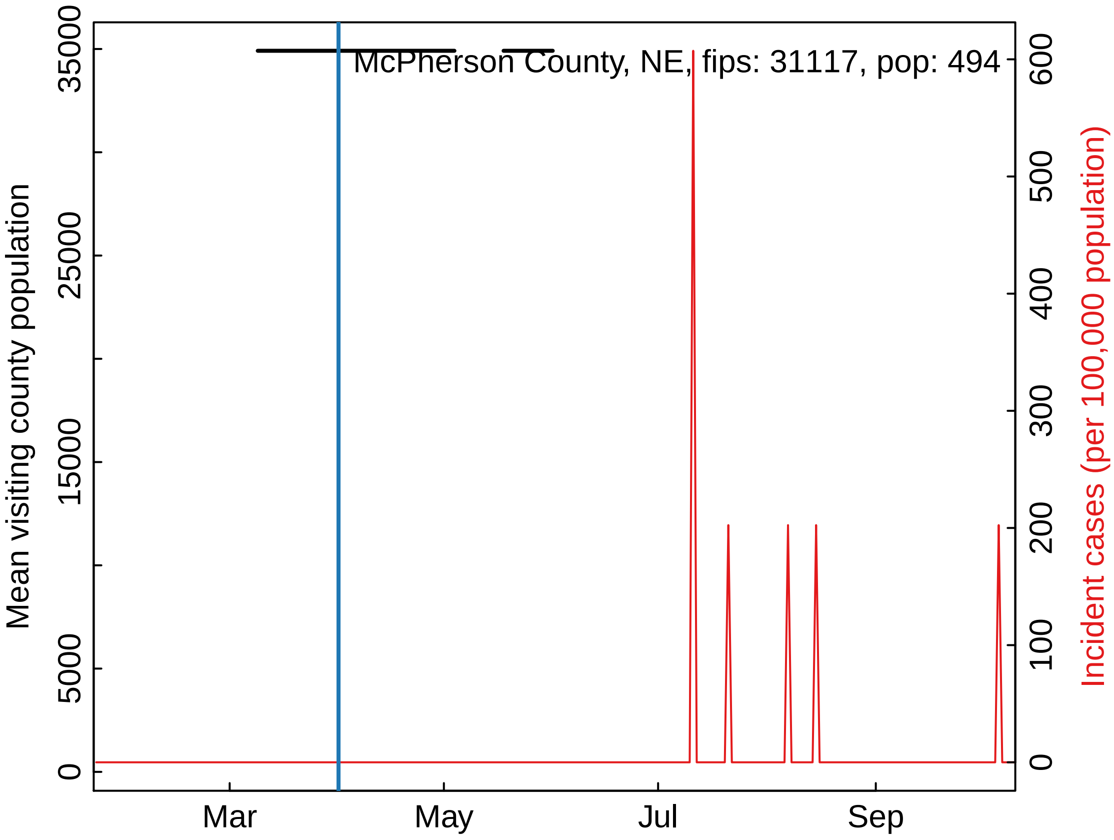
McPherson County, NE, fips: 31117, pop: 494
Mean visiting county population
Incident cases (per 100,000 population)
0
5000
15000
25000
35000
0
100
200
300
400
500
600
Mar
May
Jul
Sep

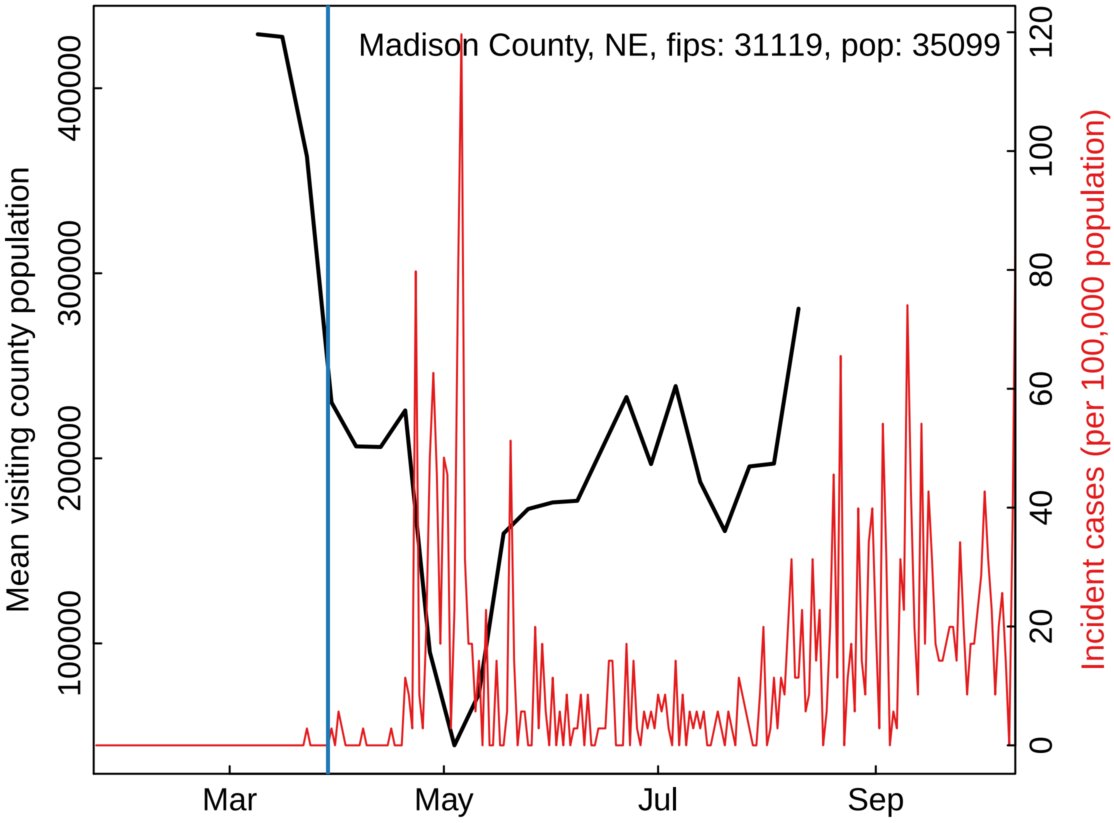

Madison County, NE, fips: 31119, pop: 35099
Mean visiting county population
100000
200000
300000
400000
Incident cases (per 100,000 population)
0
20
40
60
80
100
120
Mar
May
Jul
Sep

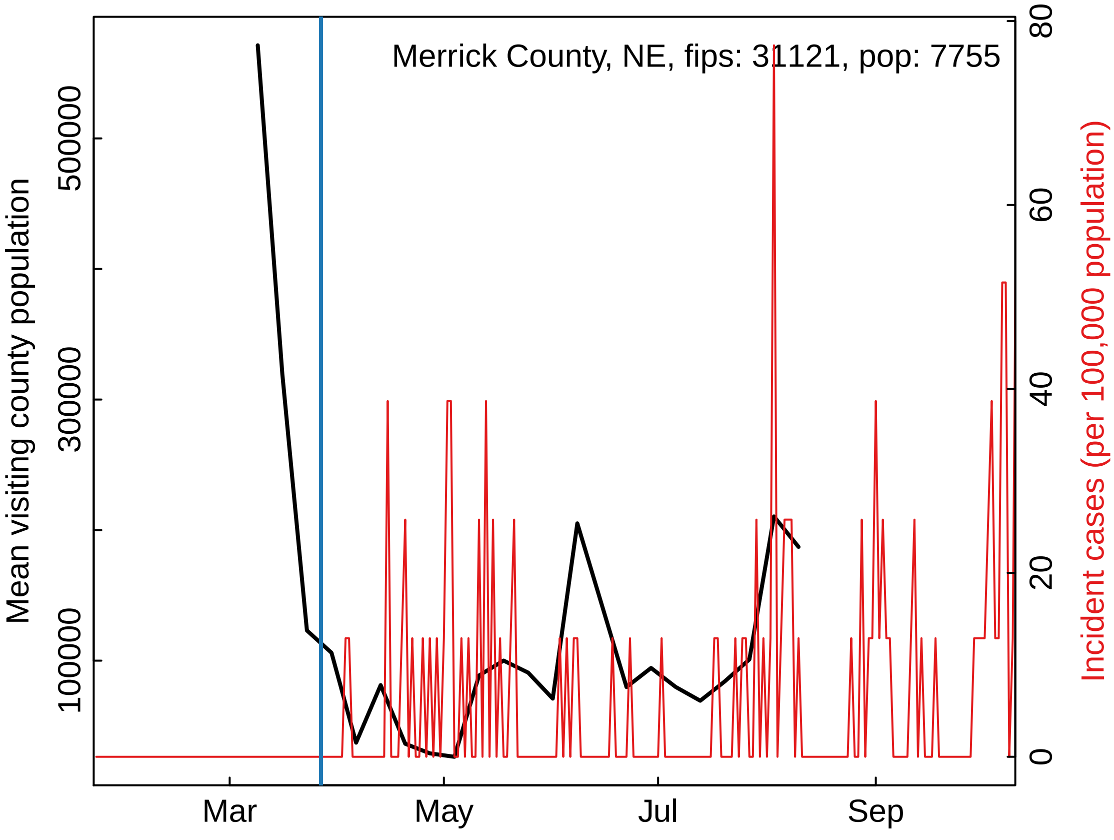

Merrick County, NE, fips: 31121, pop: 7755
Mean visiting county population
100000
300000
500000
Incident cases (per 100,000 population)
0
20
40
60
80
Mar
May
Jul
Sep

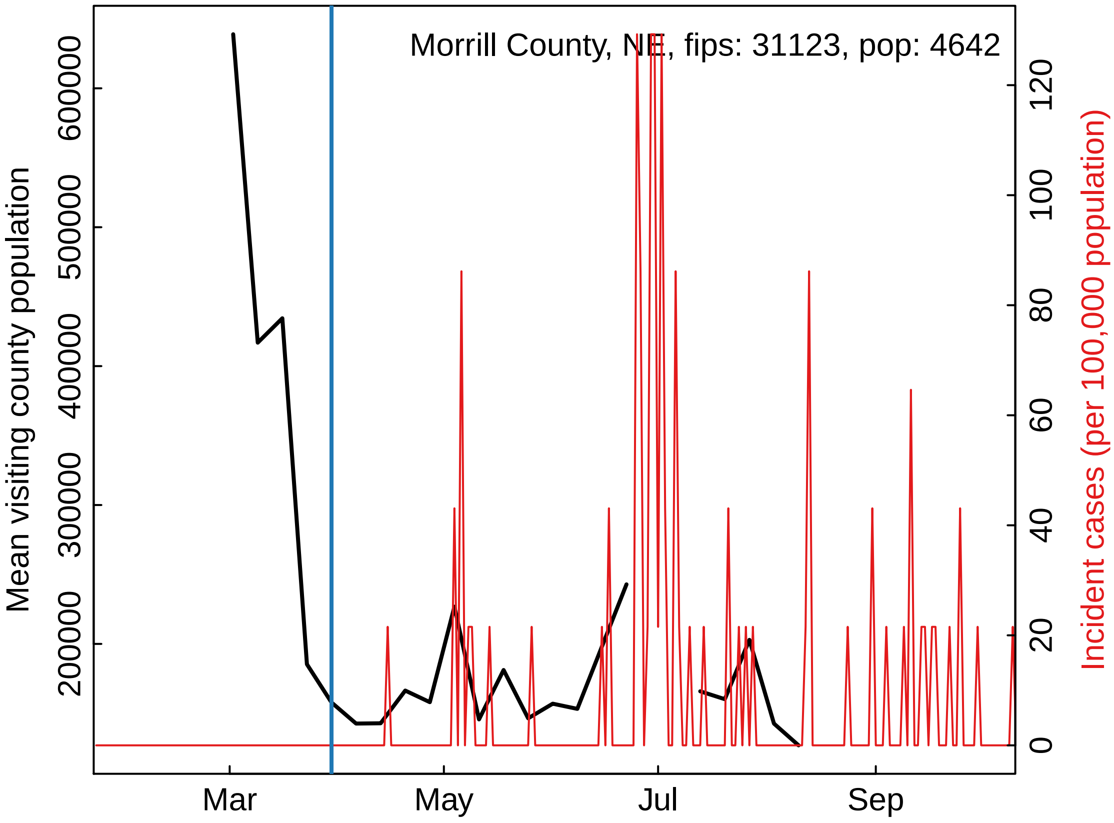

Morrill County, NE, fips: 31123, pop: 4642
Mean visiting county population
200000
300000
400000
500000
600000
Incident cases (per 100,000 population)
0
20
40
60
80
100
120
Mar
May
Jul
Sep

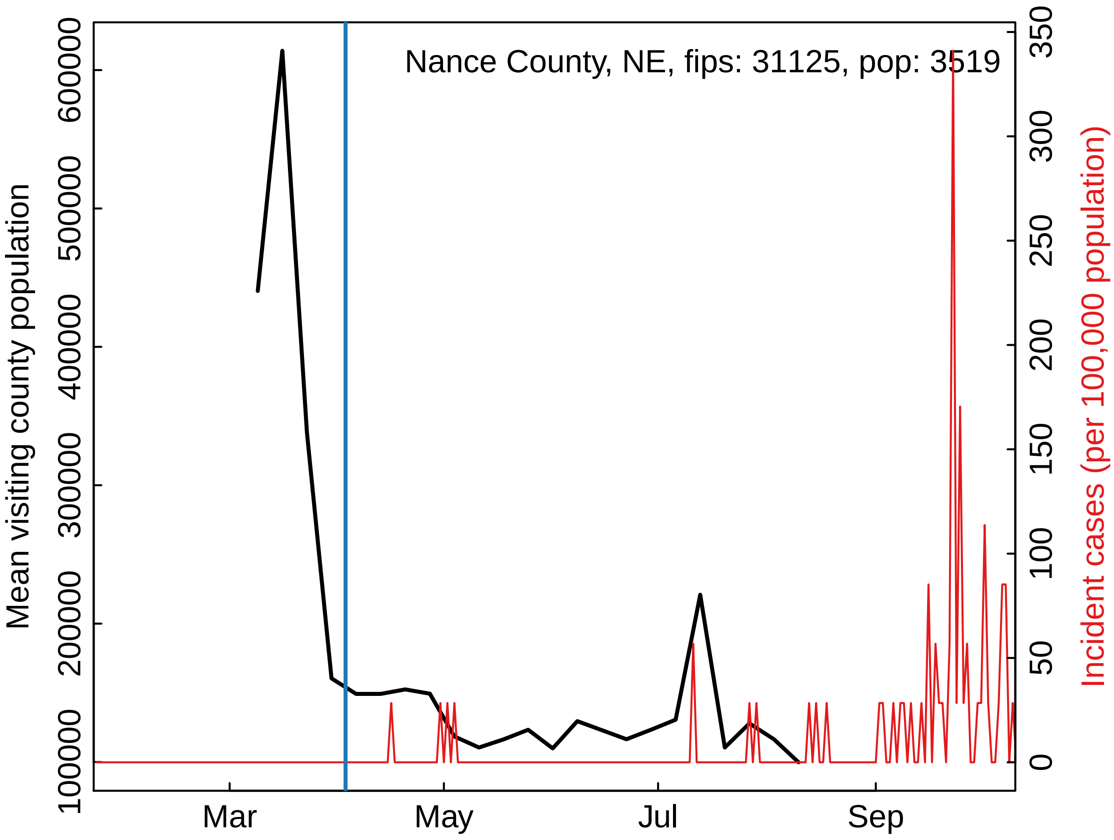

Nance County, NE, fips: 31125, pop: 3519
Mean visiting county population
100000
200000
300000
400000
500000
600000
Mar
May
Jul
Sep
0
50
100
150
200
250
300
350
Incident cases (per 100,000 population)

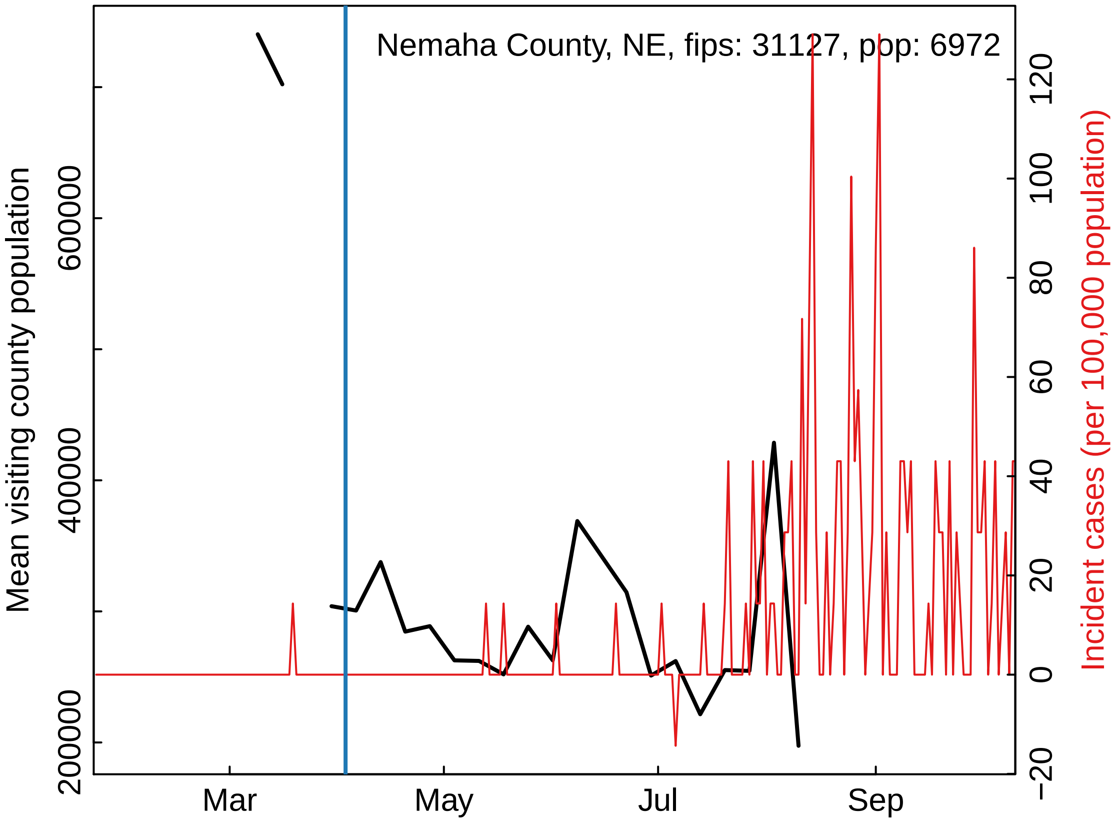

Nemaha County, NE, fips: 31127, pop: 6972
Mean visiting county population
200000
400000
600000
Mar
May
Jul
Sep
Incident cases (per 100,000 population)
−20
0
20
40
60
80
100
120

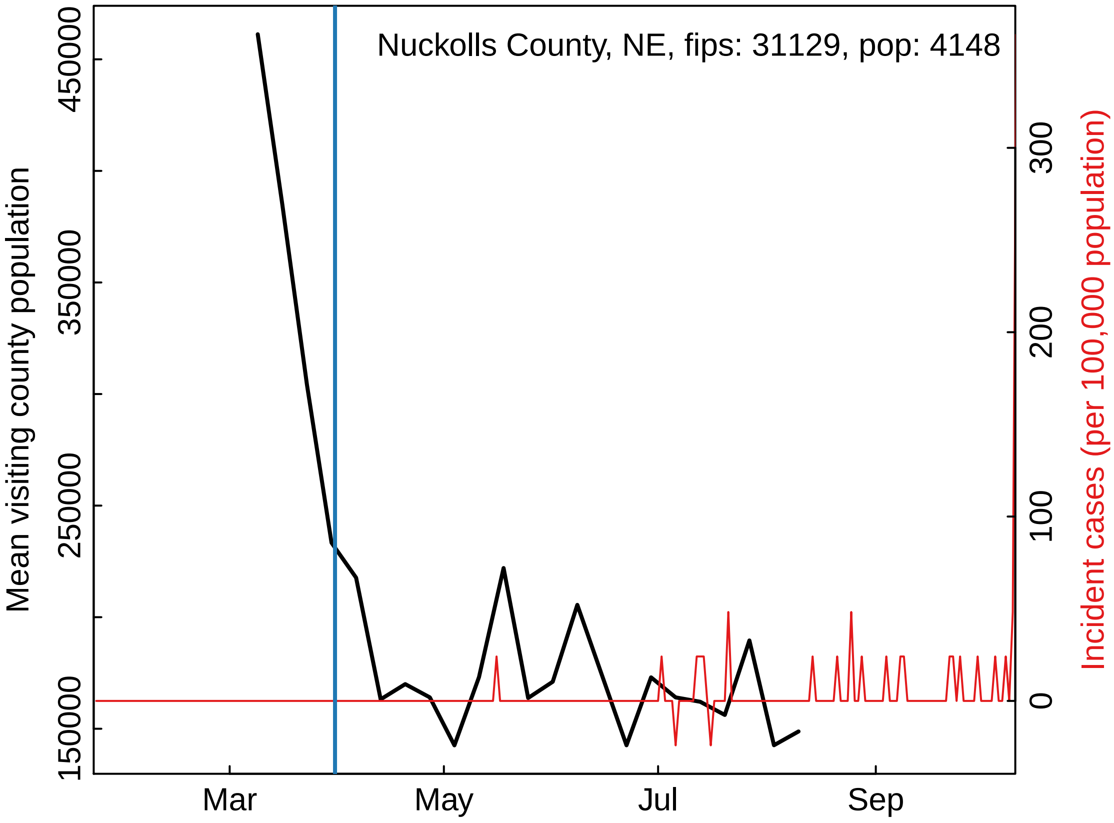
Nuckolls County, NE, fips: 31129, pop: 4148
Mean visiting county population
150000
250000
350000
450000
Incident cases (per 100,000 population)
0
100
200
300
Mar
May
Jul
Sep

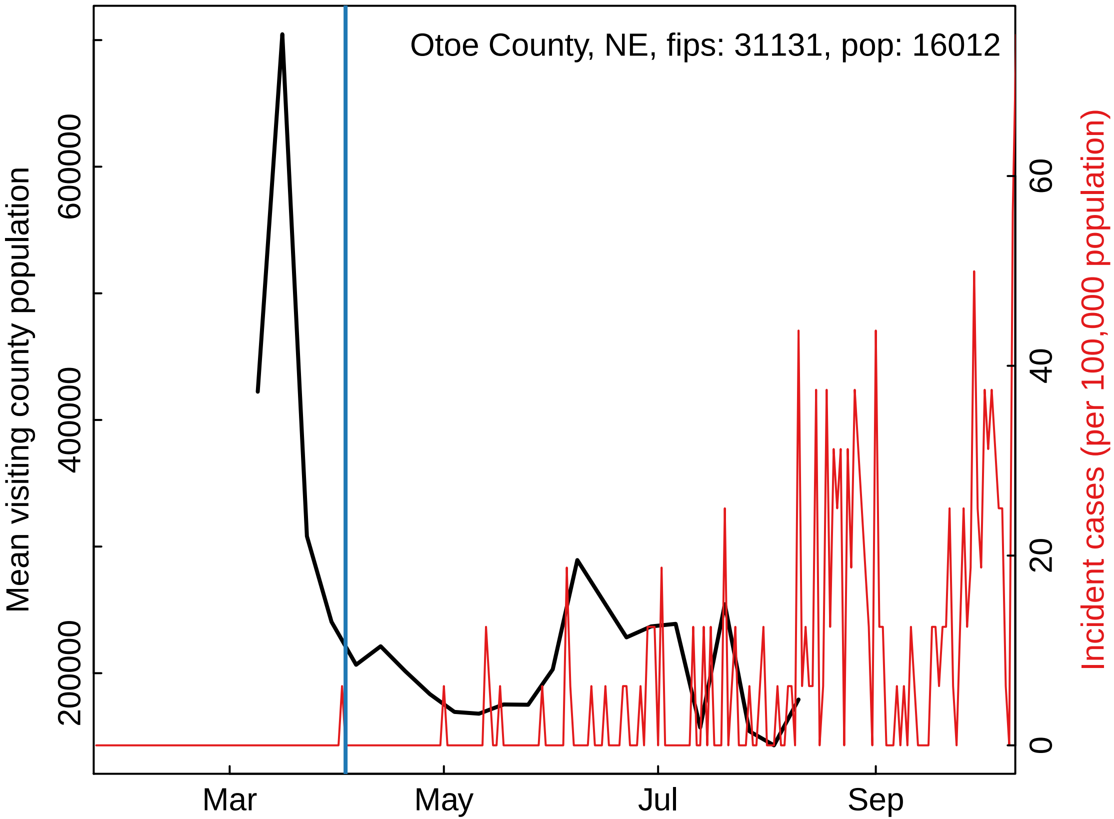
Otoe County, NE, fips: 31131, pop: 16012
Mean visiting county population
200000
400000
600000
Incident cases (per 100,000 population)
0
20
40
60
Mar
May
Jul
Sep

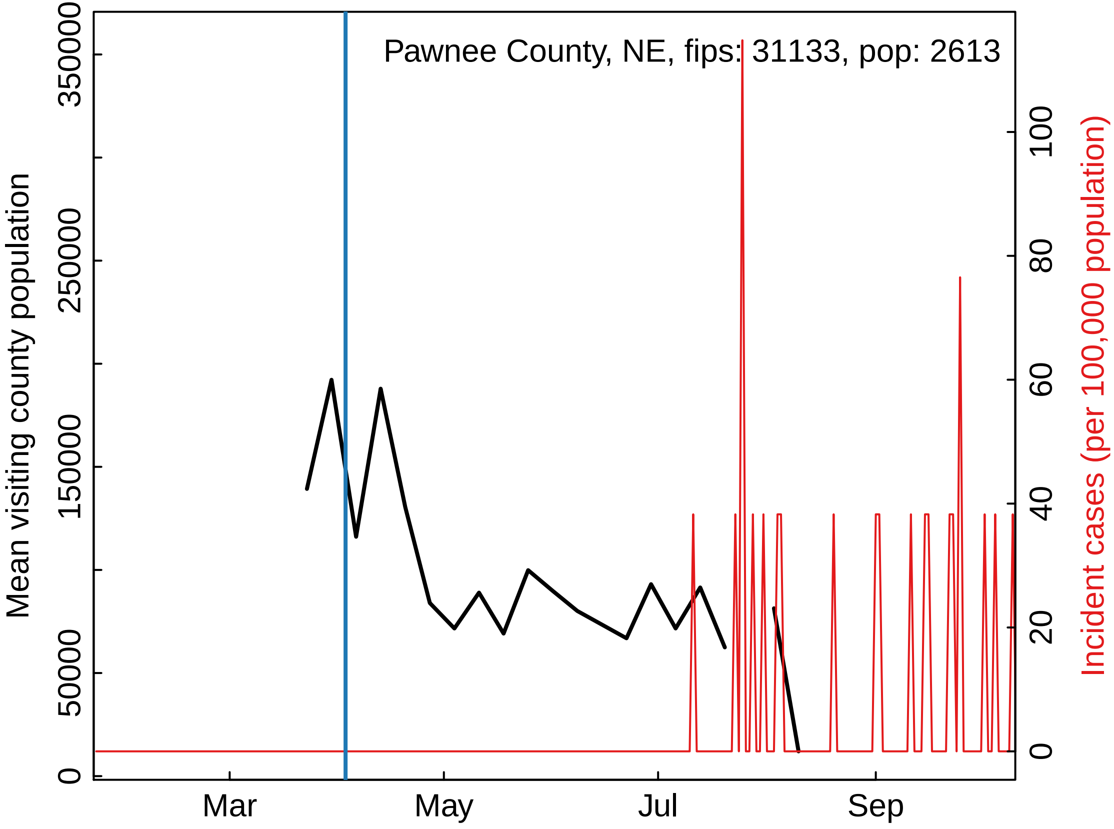

Pawnee County, NE, fips: 31133, pop: 2613
Mean visiting county population
Incident cases (per 100,000 population)
0
50000
150000
250000
350000
0
20
40
60
80
100
Mar
May
Jul
Sep

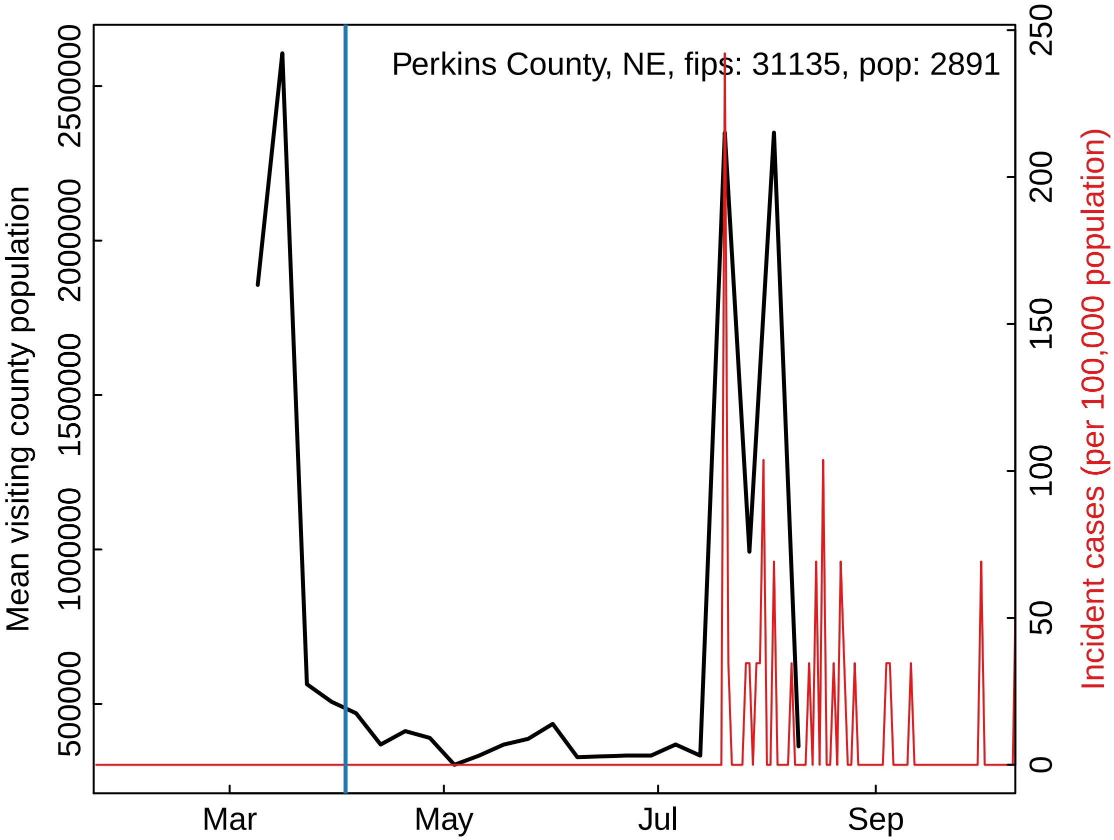

Perkins County, NE, fips: 31135, pop: 2891
Mean visiting county population
500000
1000000
1500000
2000000
2500000
Incident cases (per 100,000 population)
0
50
100
150
200
250
Mar
May
Jul
Sep

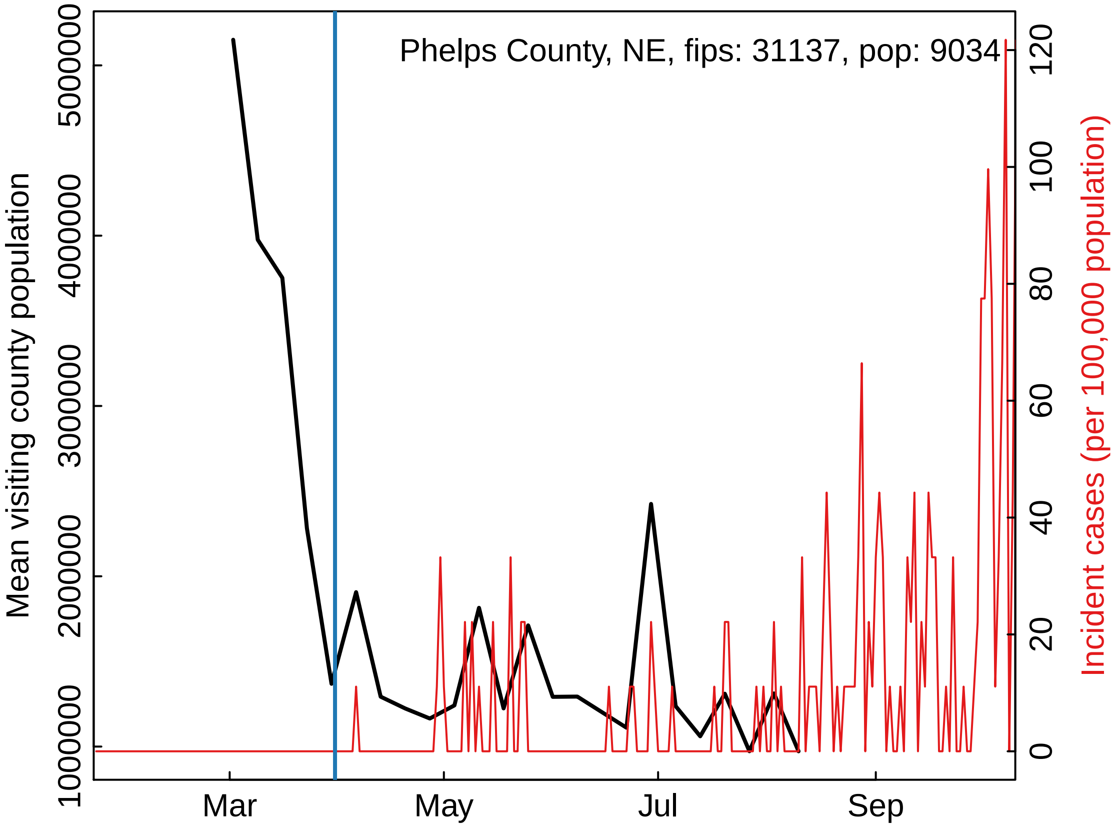
Phelps County, NE, fips: 31137, pop: 9034
Mean visiting county population
Incident cases (per 100,000 population)
1000000
2000000
3000000
4000000
5000000
0
20
40
60
80
100
120
Mar
May
Jul
Sep

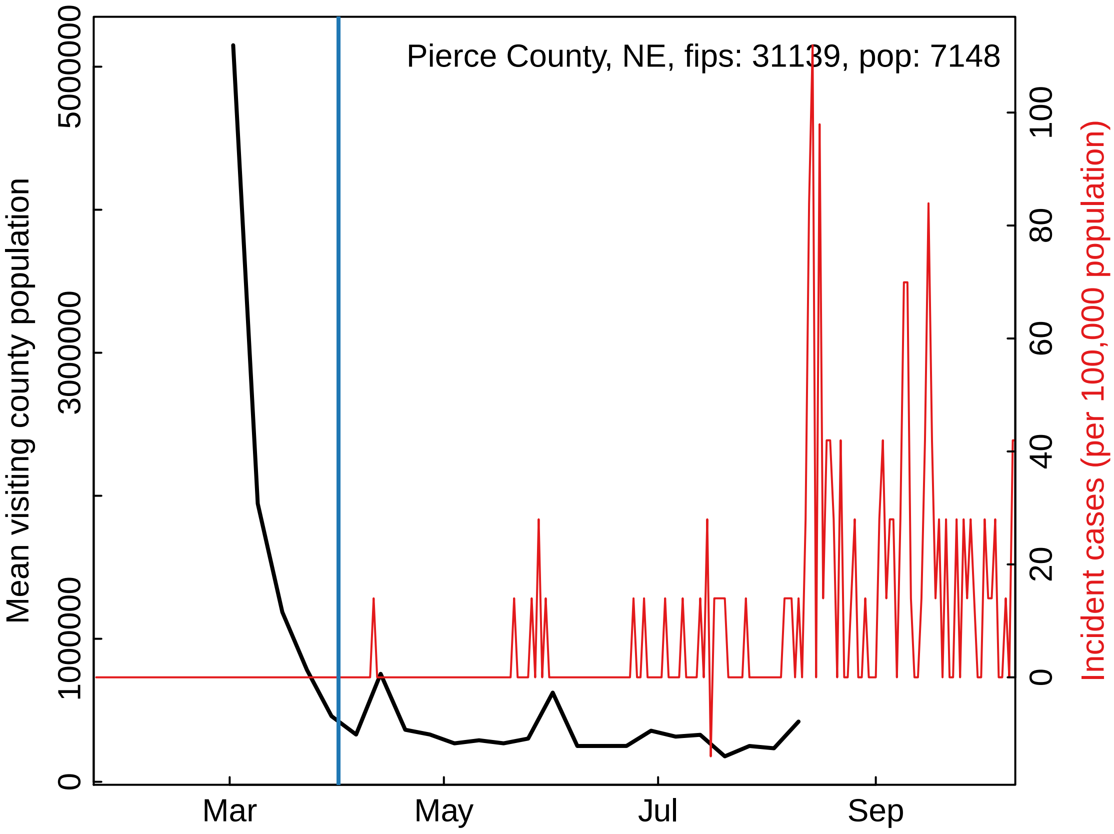

Pierce County, NE, fips: 31139, pop: 7148
Mean visiting county population
Incident cases (per 100,000 population)
0
1000000
3000000
5000000
0
20
40
60
80
100
Mar
May
Jul
Sep

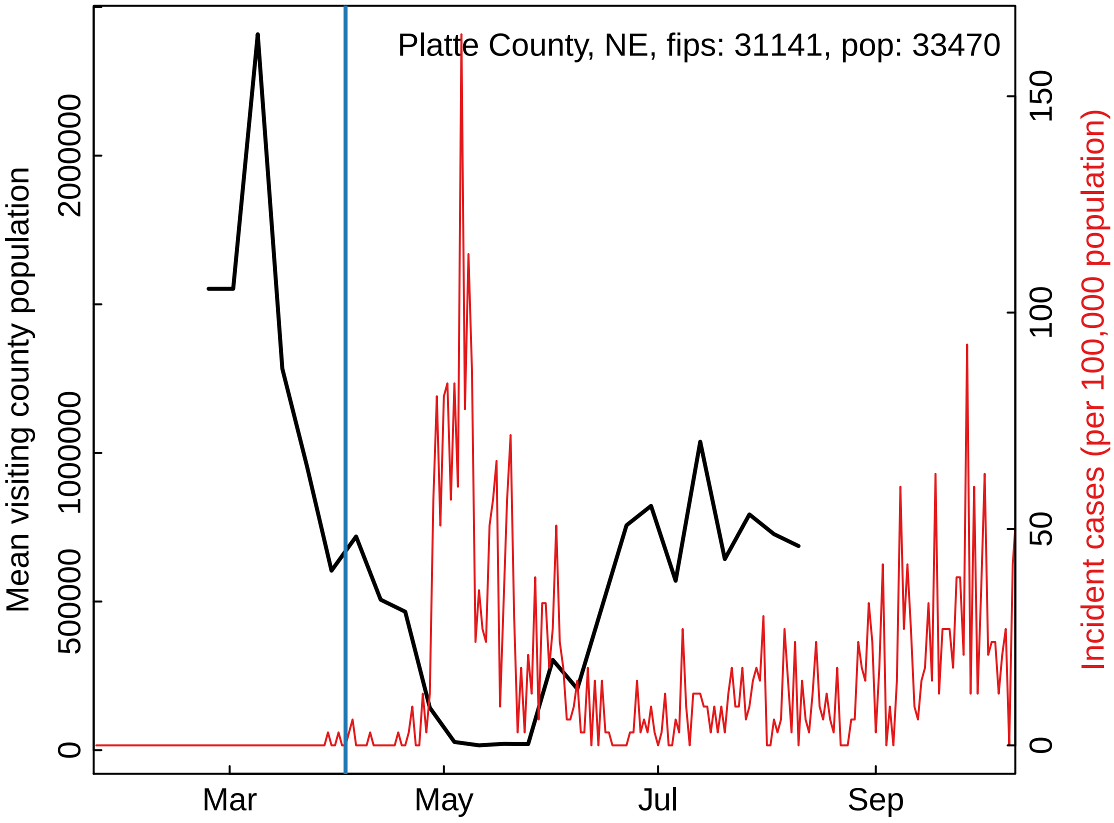
Platte County, NE, fips: 31141, pop: 33470
Mean visiting county population
Incident cases (per 100,000 population)
0
500000
1000000
2000000
0
50
100
150
Mar
May
Jul
Sep

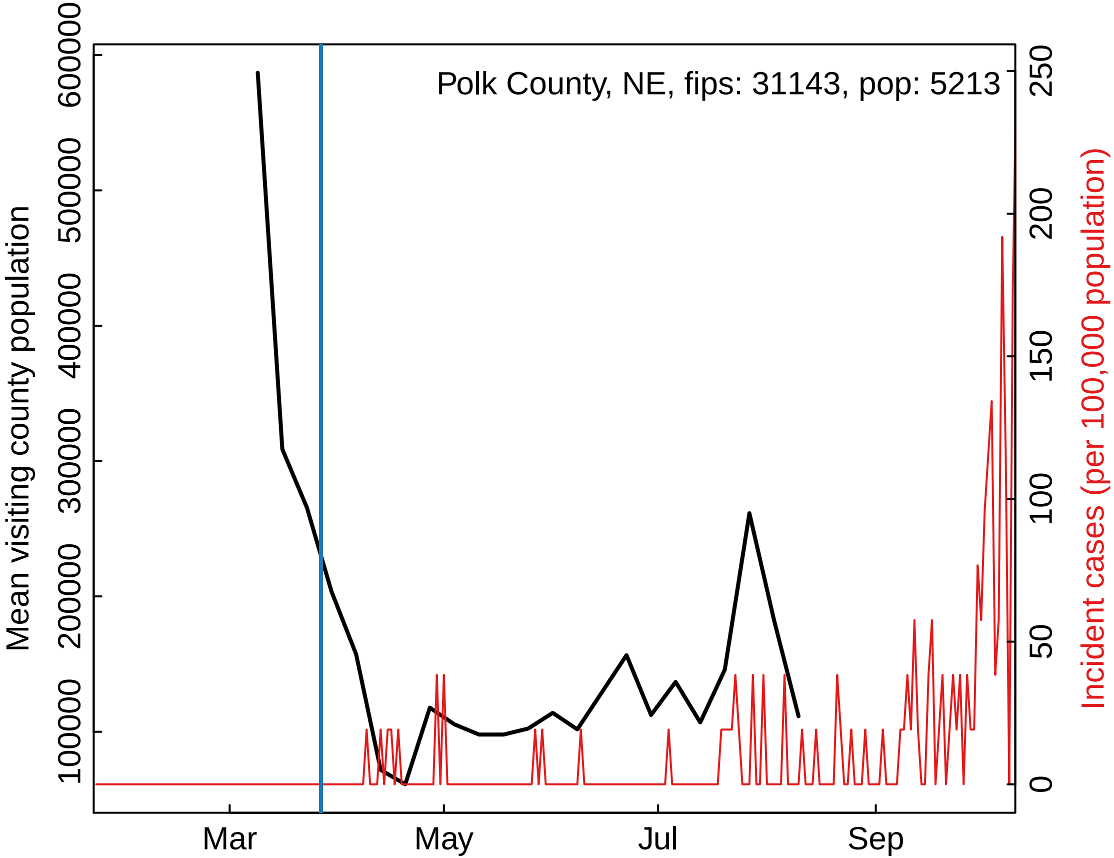

Polk County, NE, fips: 31143, pop: 5213
Mean visiting county population
100000
200000
300000
400000
500000
600000
Incident cases (per 100,000 population)
0
50
100
150
200
250
Mar
May
Jul
Sep

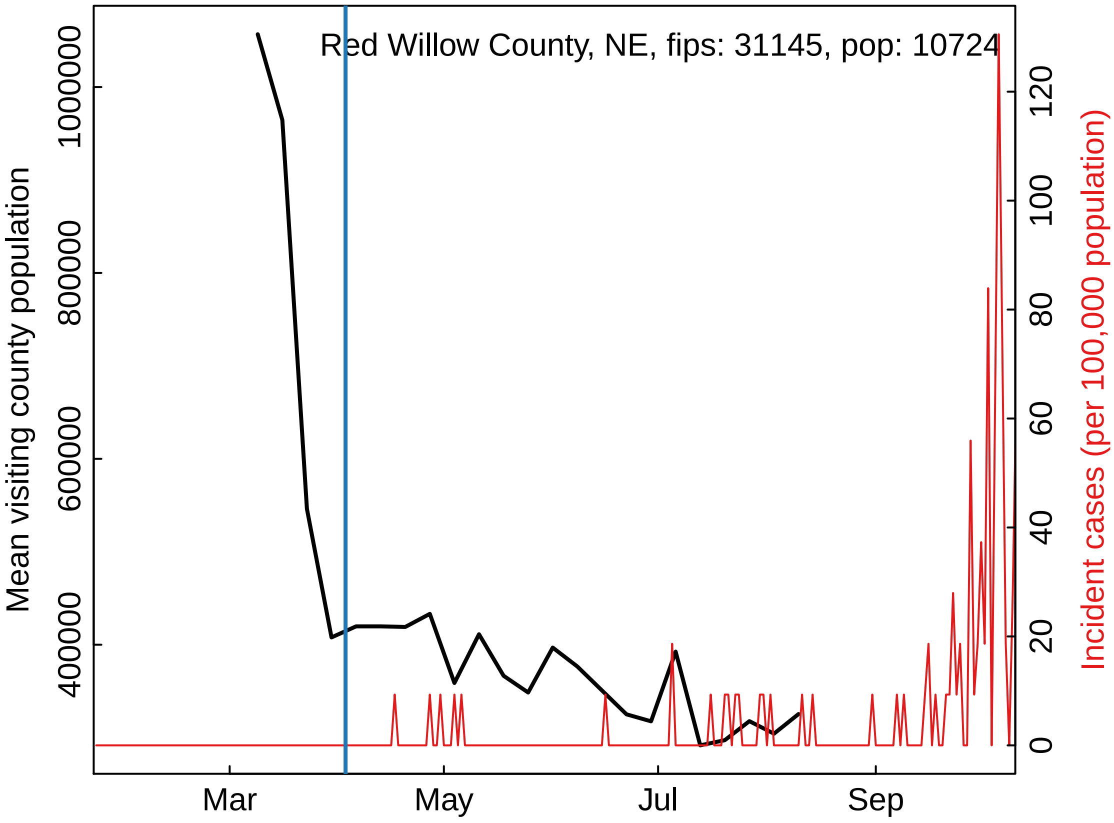

Red Willow County, NE, fips: 31145, pop: 10724
Mean visiting county population
400000
600000
800000
1000000
Incident cases (per 100,000 population)
0
20
40
60
80
100
120
Mar
May
Jul
Sep

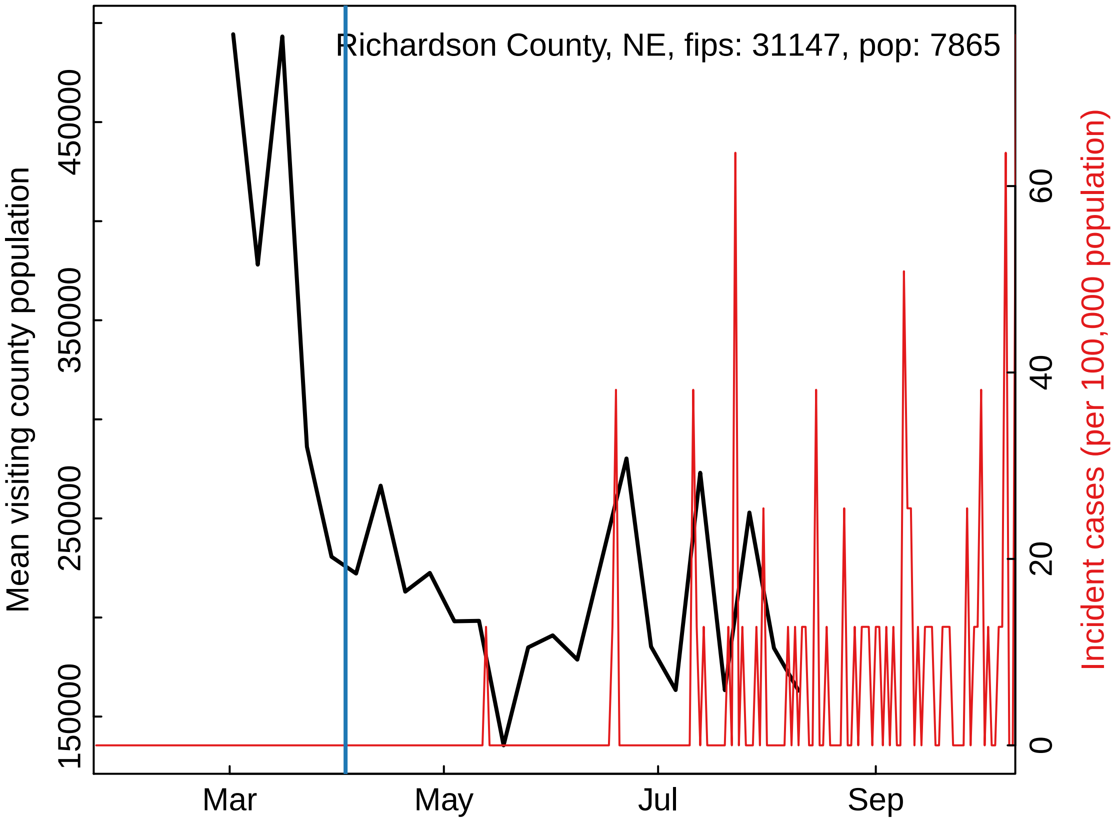
Richardson County, NE, fips: 31147, pop: 7865
Mean visiting county population
150000
250000
350000
450000
Incident cases (per 100,000 population)
0
20
40
60
Mar
May
Jul
Sep

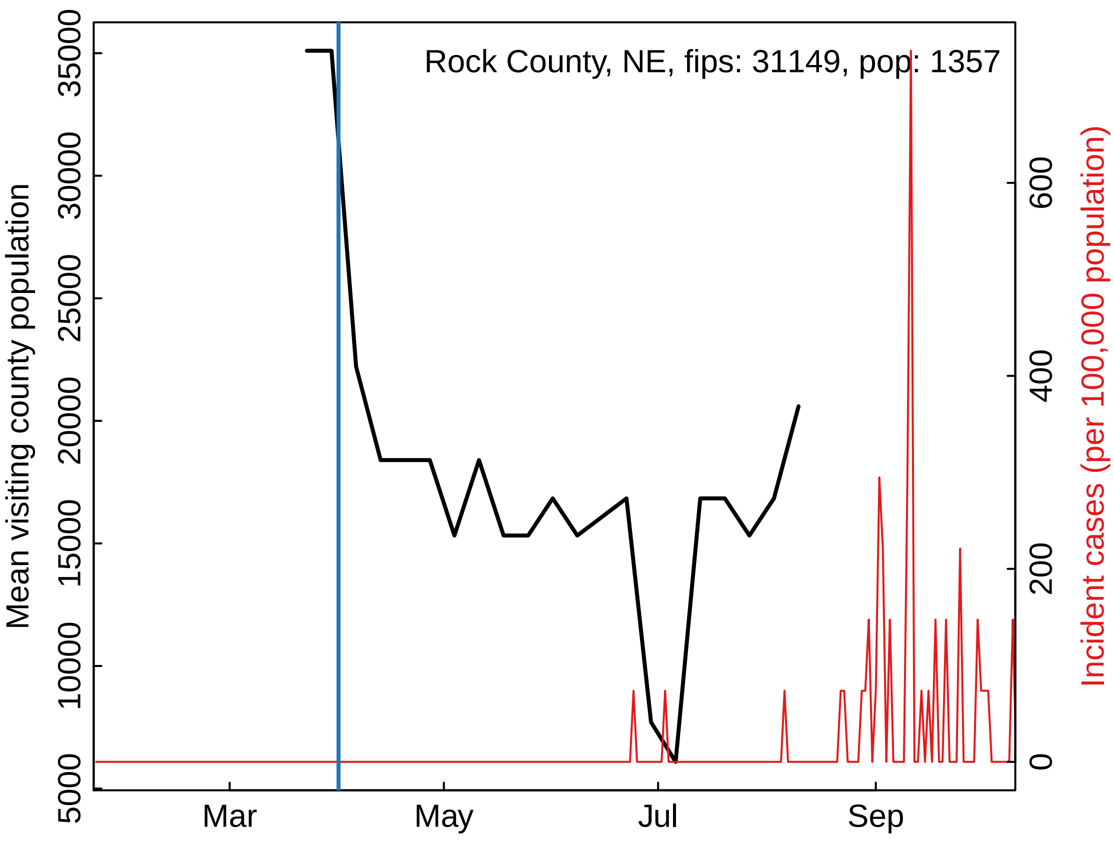

Rock County, NE, fips: 31149, pop: 1357
Mean visiting county population
5000
10000
15000
20000
25000
30000
35000
Mar
May
Jul
Sep
0
200
400
600
Incident cases (per 100,000 population)

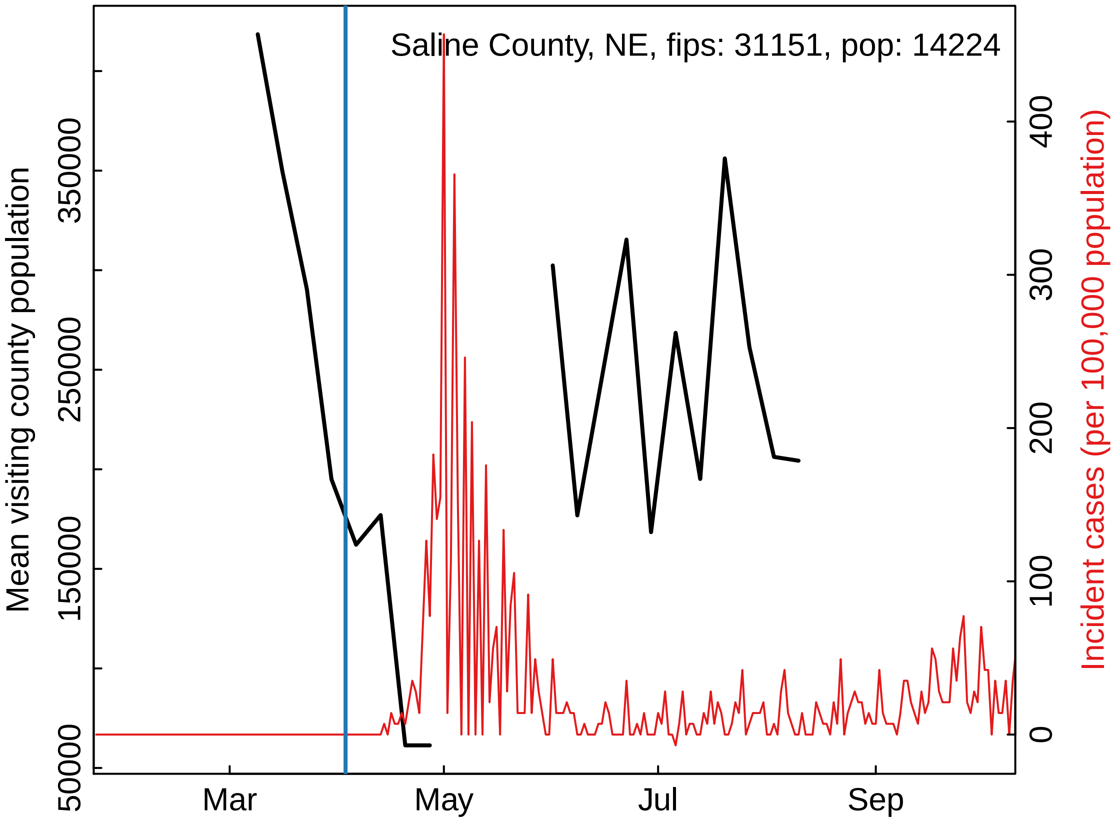

Saline County, NE, fips: 31151, pop: 14224
Mean visiting county population
50000
150000
250000
350000
Incident cases (per 100,000 population)
0
100
200
300
400
Mar
May
Jul
Sep

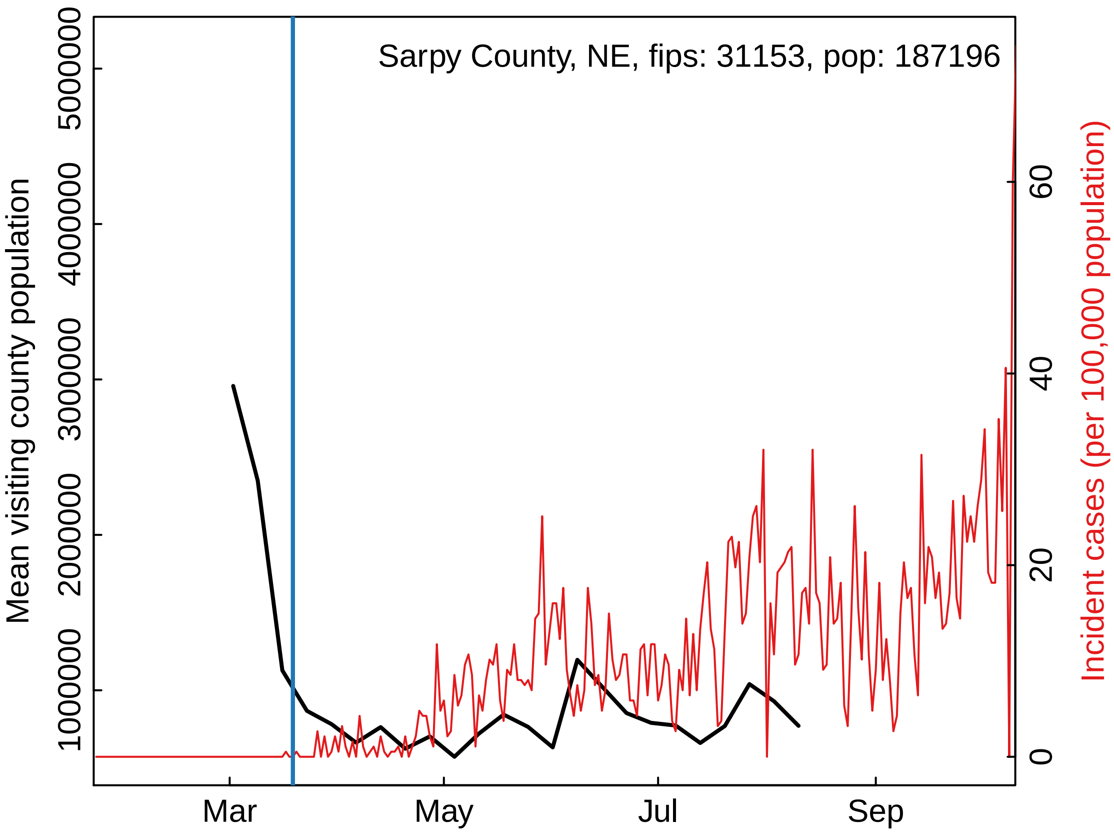

Sarpy County, NE, fips: 31153, pop: 187196
Mean visiting county population
1000000
2000000
3000000
4000000
5000000
Mar
May
Jul
Sep
0
20
40
60
Incident cases (per 100,000 population)

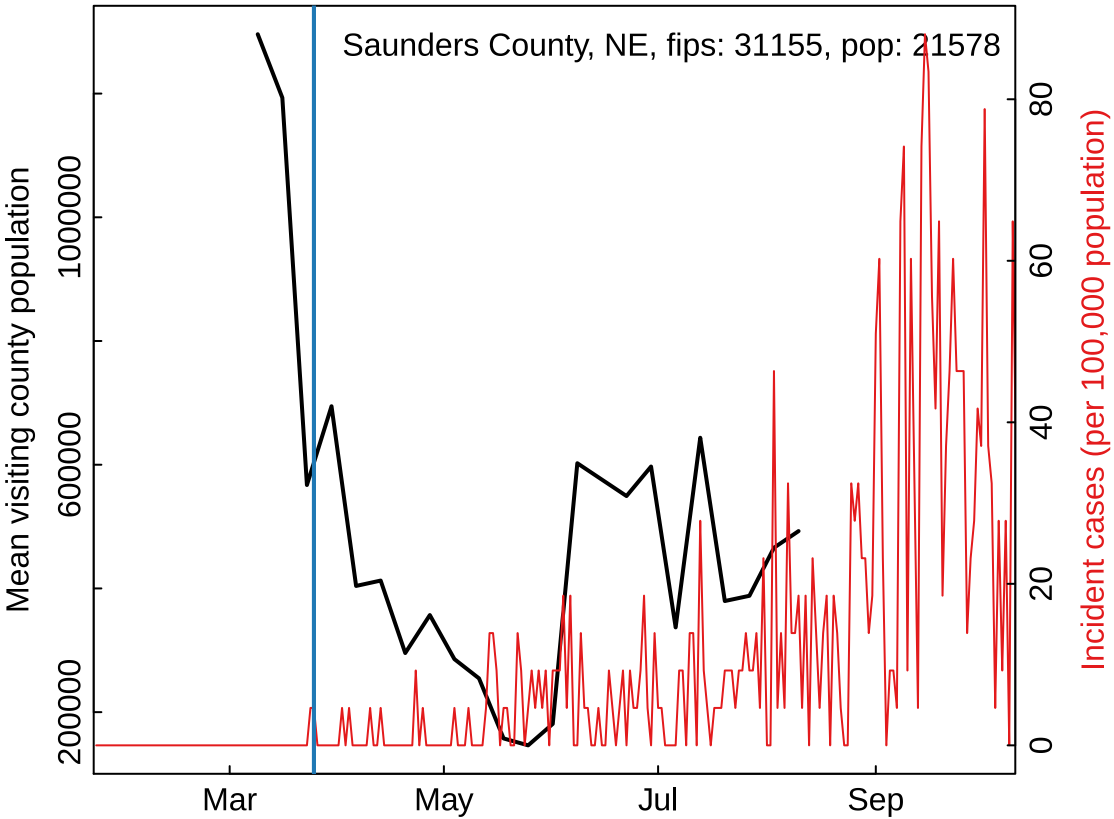
Saunders County, NE, fips: 31155, pop: 21578
Mean visiting county population
200000
600000
1000000
Incident cases (per 100,000 population)
0
20
40
60
80
Mar
May
Jul
Sep

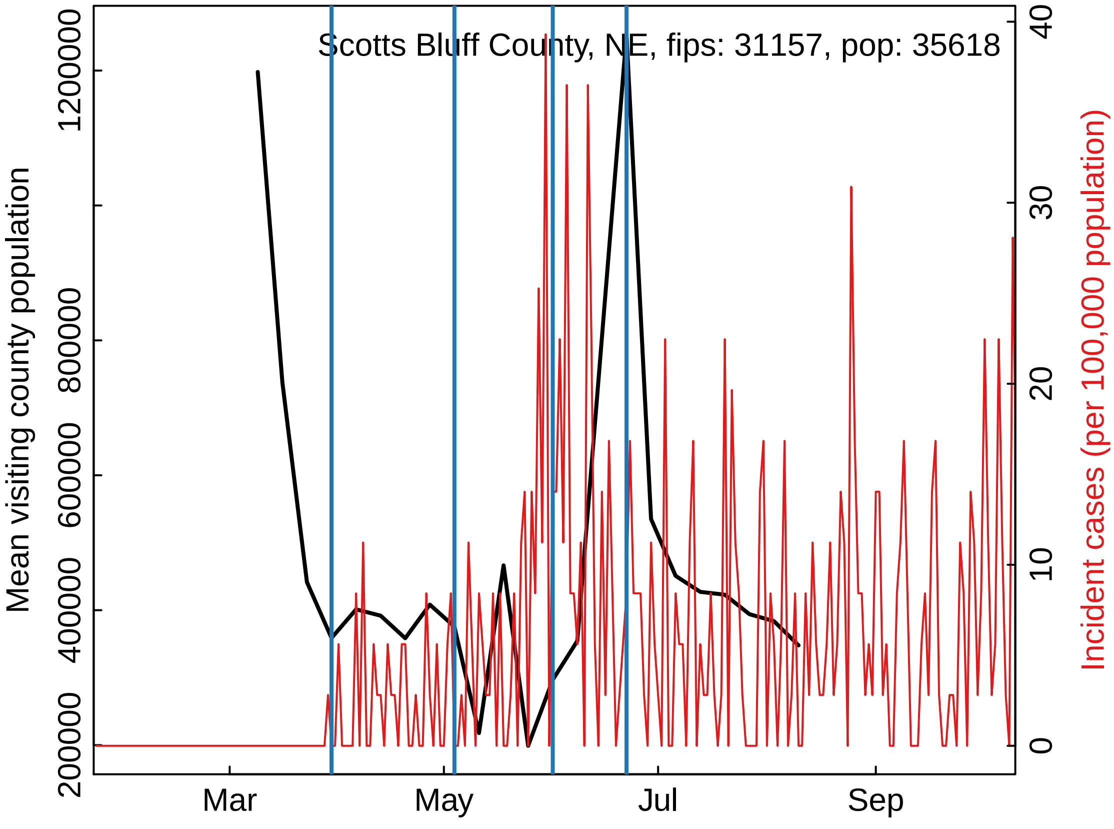

Scotts Bluff County, NE, fips: 31157, pop: 35618
Mean visiting county population
200000
400000
600000
800000
12000000
Incident cases (per 100,000 population)
0
10
20
30
40
Mar
May
Jul
Sep

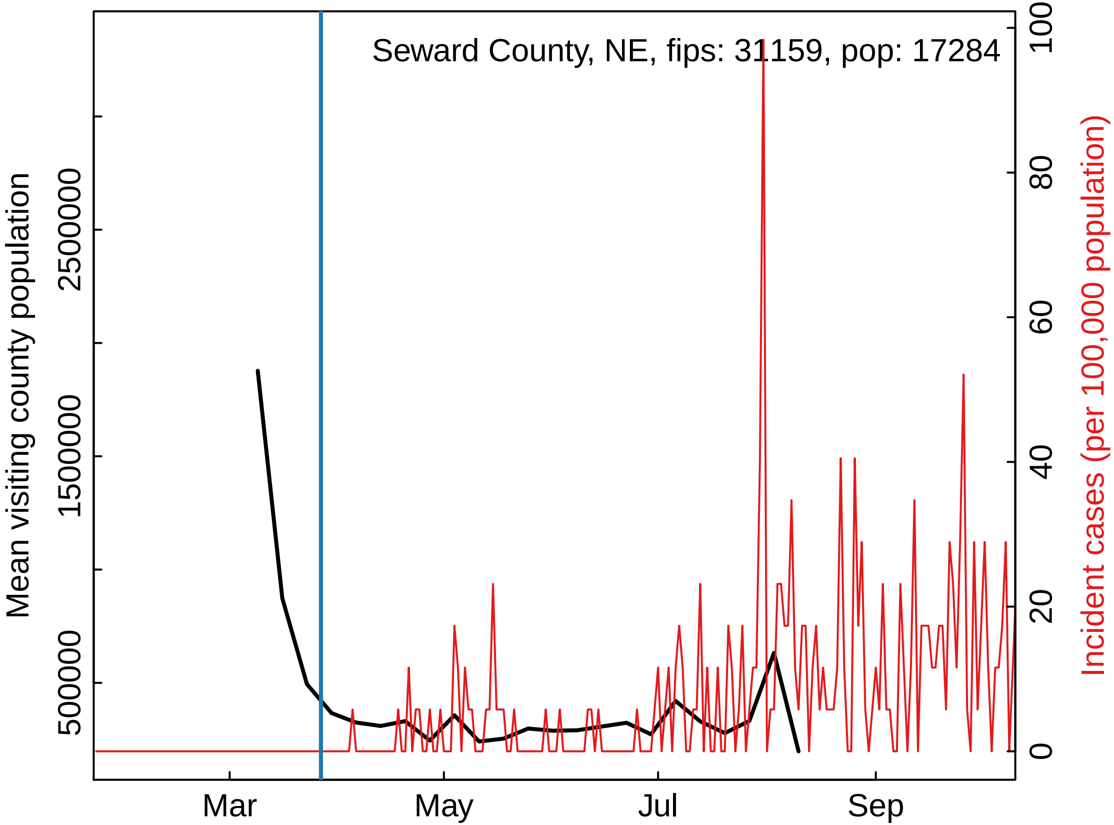

Seward County, NE, fips: 31159, pop: 17284
Mean visiting county population
500000
1500000
2500000
Mar
May
Jul
Sep
0
20
40
60
80
100
Incident cases (per 100,000 population)

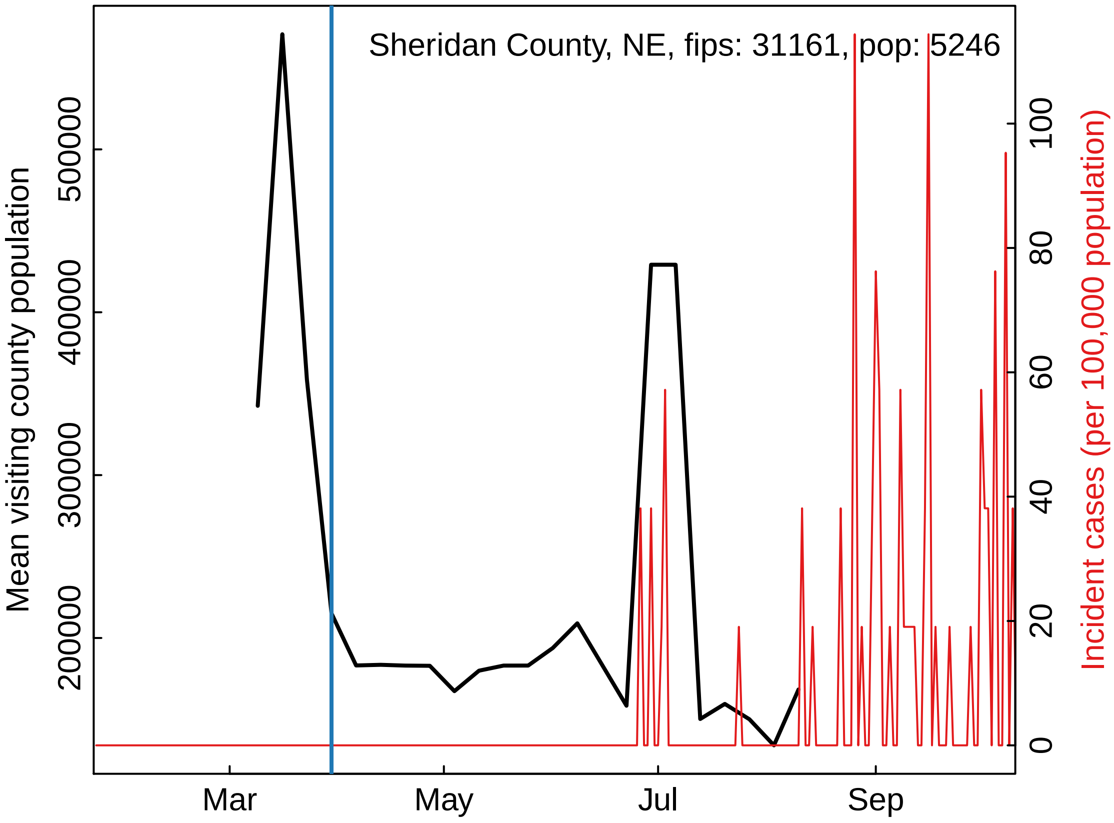

Sheridan County, NE, fips: 31161, pop: 5246
Mean visiting county population
200000
300000
400000
500000
Incident cases (per 100,000 population)
0
20
40
60
80
100
Mar
May
Jul
Sep

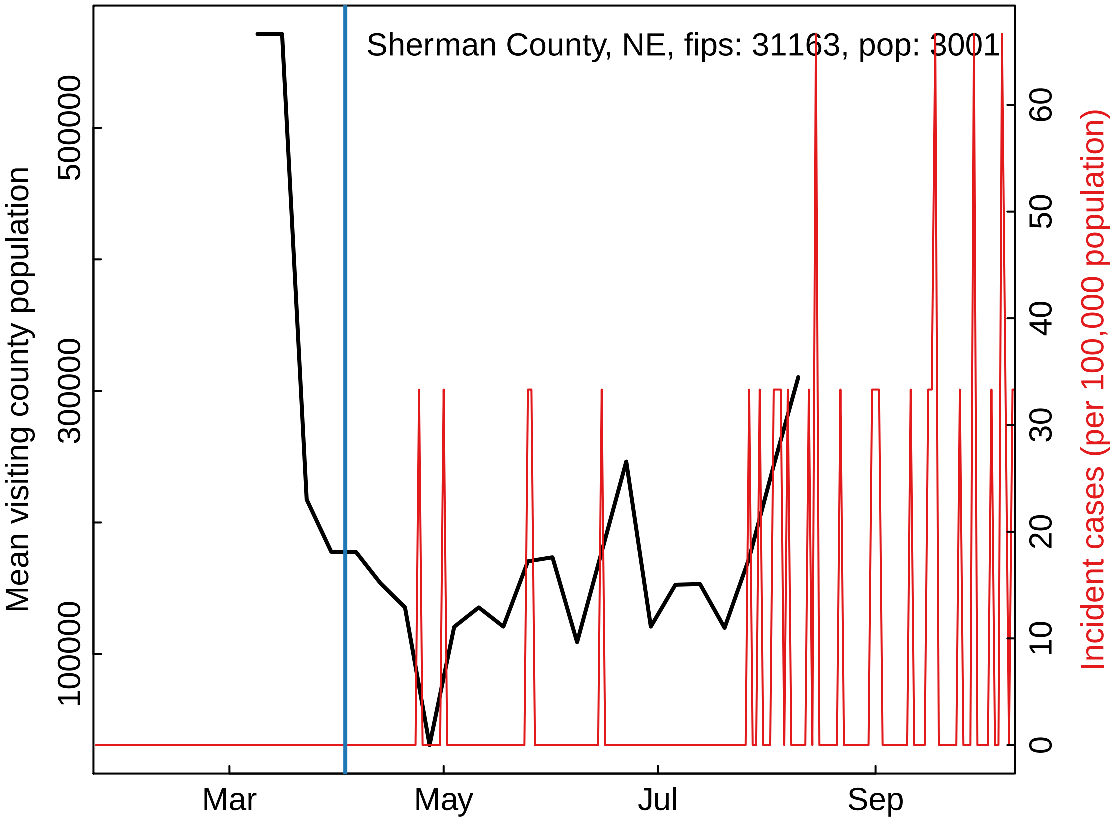
Sherman County, NE, fips: 31163, pop: 3001
Mean visiting county population
100000
300000
500000
Incident cases (per 100,000 population)
0
10
20
30
40
50
60
Mar
May
Jul
Sep

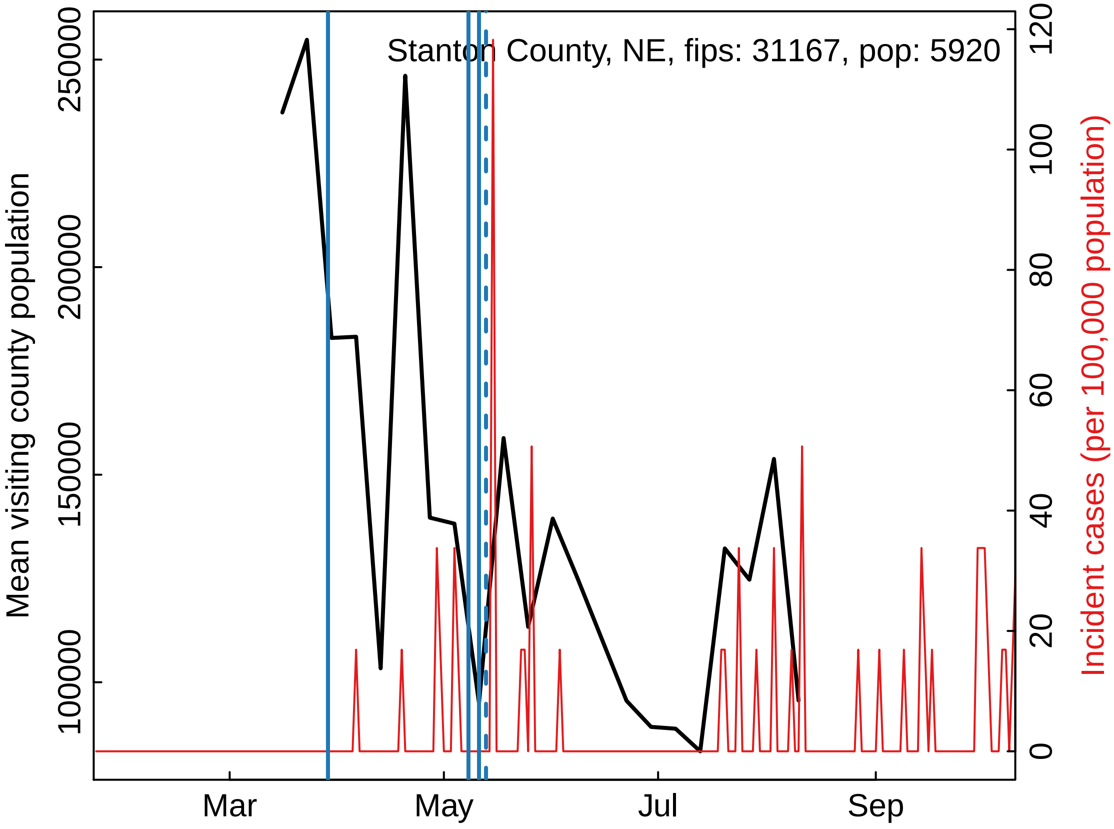

Stanton County, NE, fips: 31167, pop: 5920
Mean visiting county population
Incident cases (per 100,000 population)
100000
150000
200000
250000
0
20
40
60
80
100
120
Mar
May
Jul
Sep

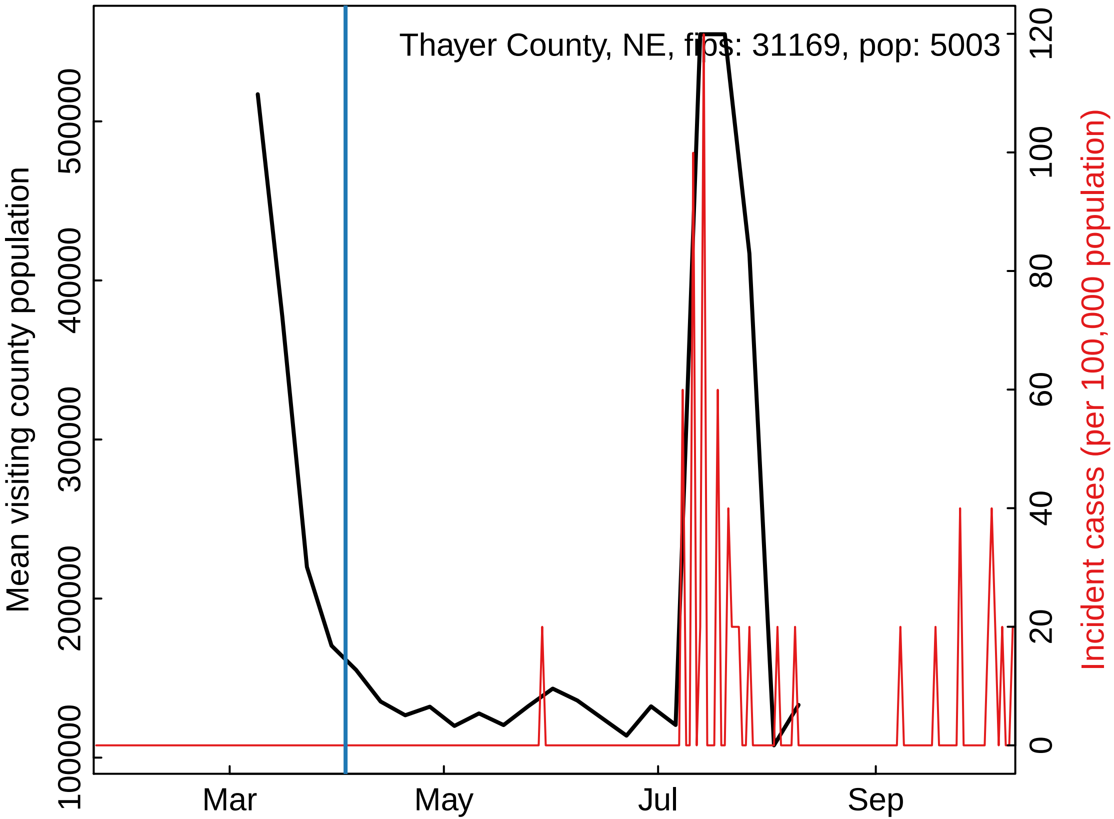
Thayer County, NE, fips: 31169, pop: 5003
Mean visiting county population
100000
200000
300000
400000
500000
Incident cases (per 100,000 population)
0
20
40
60
80
100
120
Mar
May
Jul
Sep

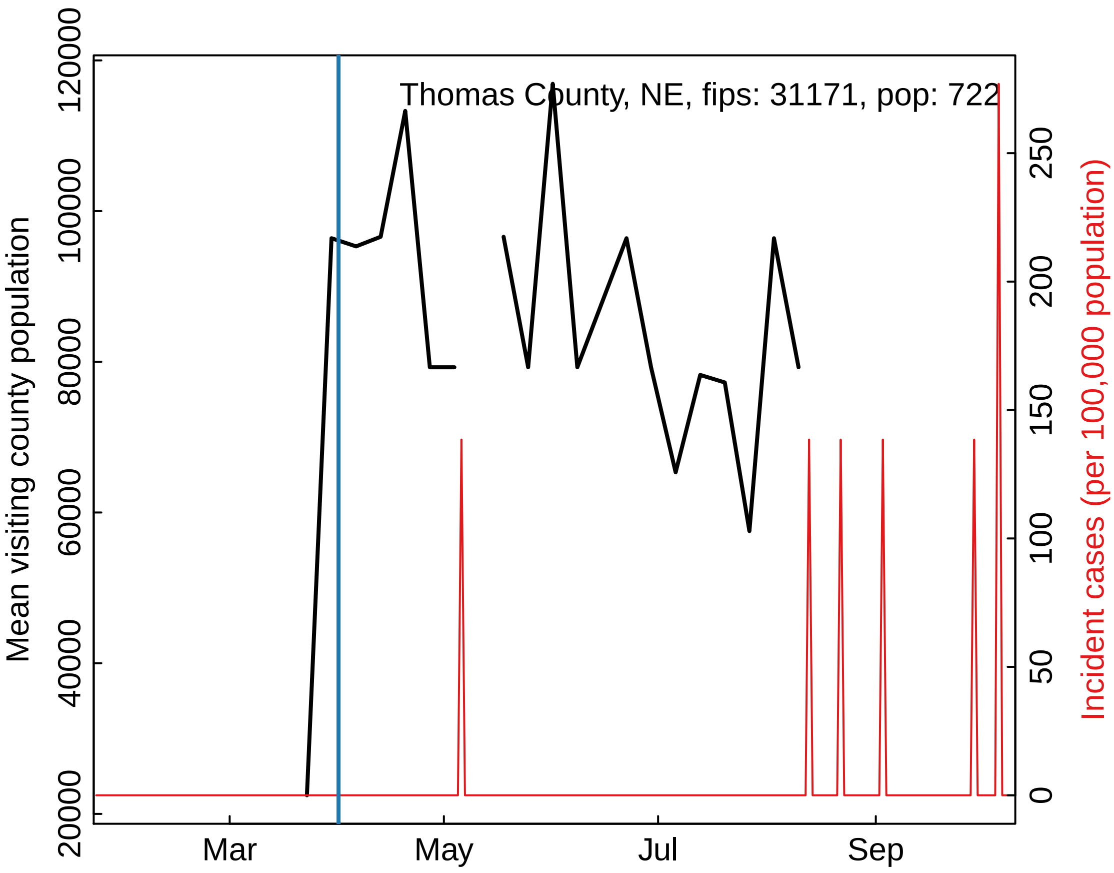

Thomas County, NE, fips: 31171, pop: 722
Mean visiting county population
20000
40000
60000
80000
100000
120000
Mar
May
Jul
Sep
0
50
100
150
200
250
Incident cases (per 100,000 population)

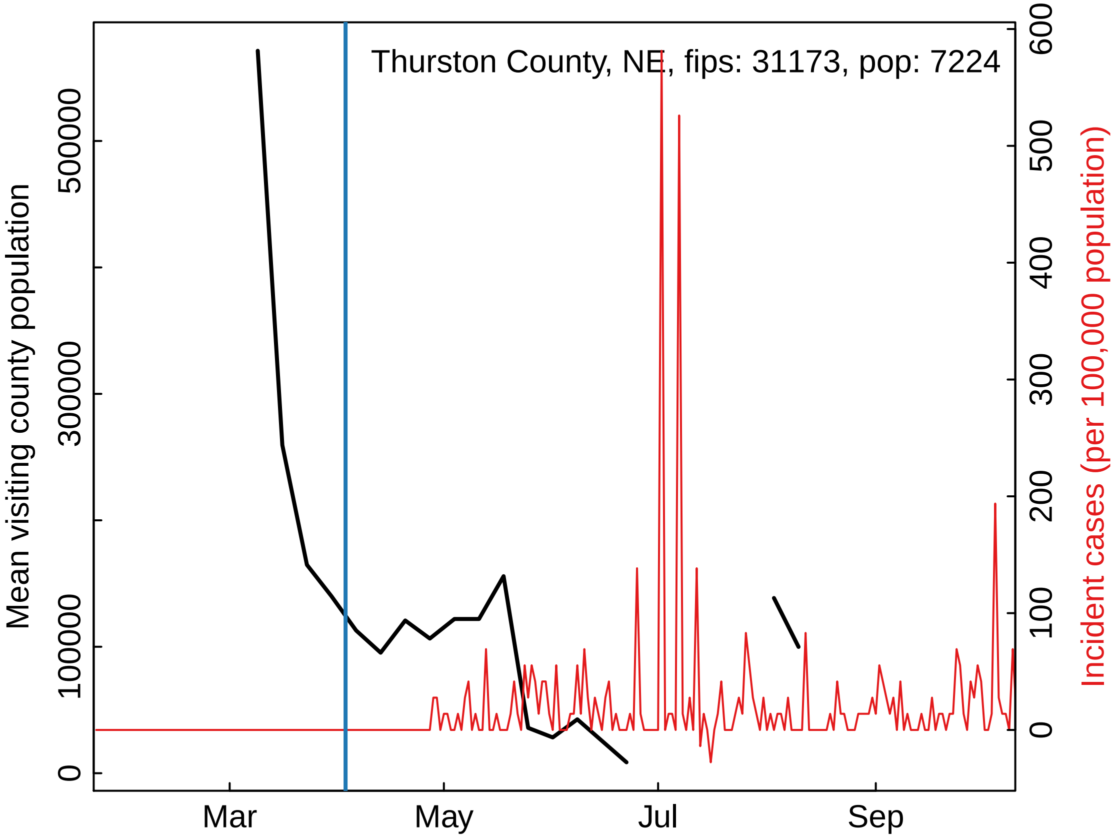

Thurston County, NE, fips: 31173, pop: 7224
Mean visiting county population
0
100000
300000
500000
Mar
May
Jul
Sep
0
100
200
300
400
500
600
Incident cases (per 100,000 population)

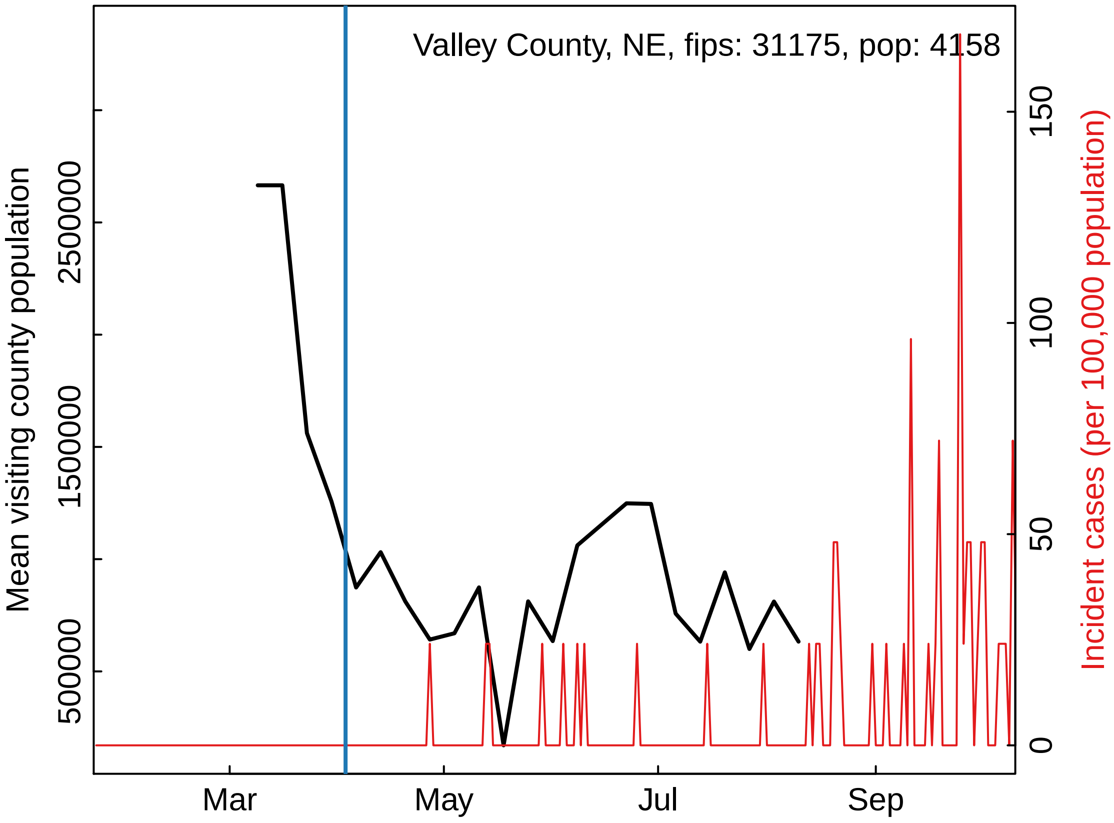

Valley County, NE, fips: 31175, pop: 4158
Mean visiting county population
500000
1500000
2500000
Mar
May
Jul
Sep
0
50
100
150
Incident cases (per 100,000 population)

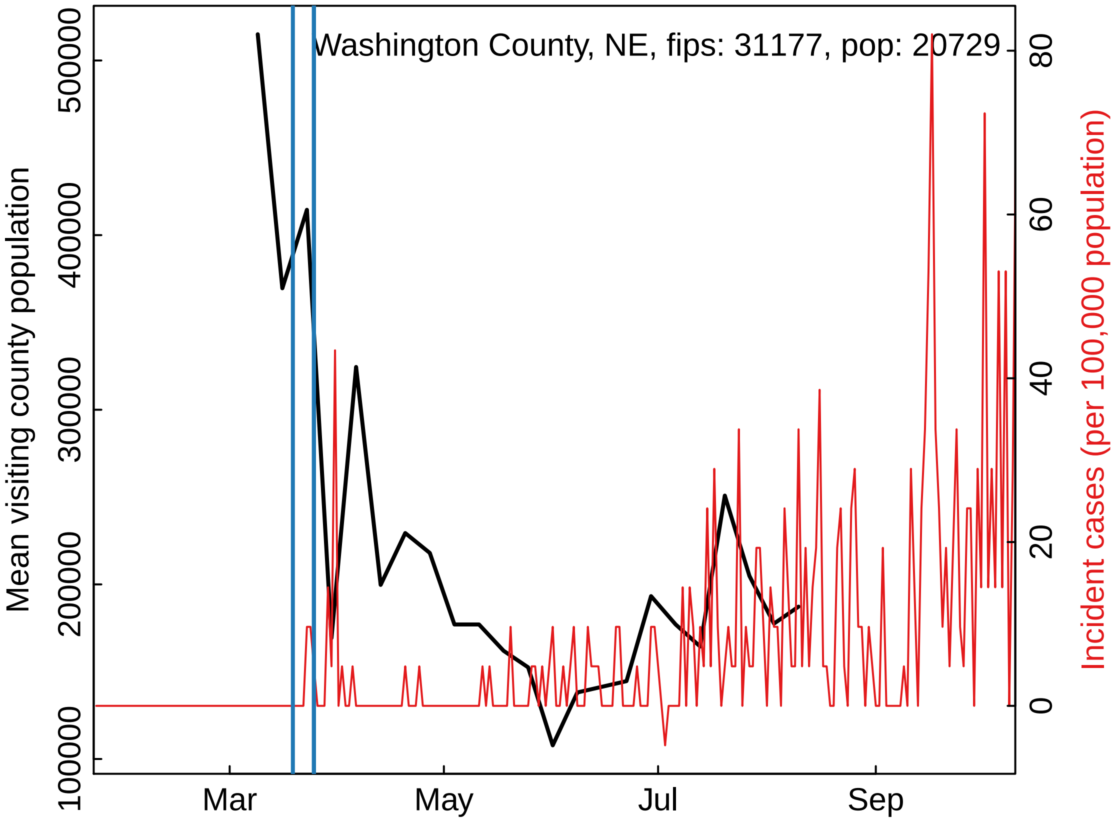

Washington County, NE, fips: 31177, pop: 20729
Mean visiting county population
100000
200000
300000
400000
500000
Incident cases (per 100,000 population)
0
20
40
60
80
Mar
May
Jul
Sep

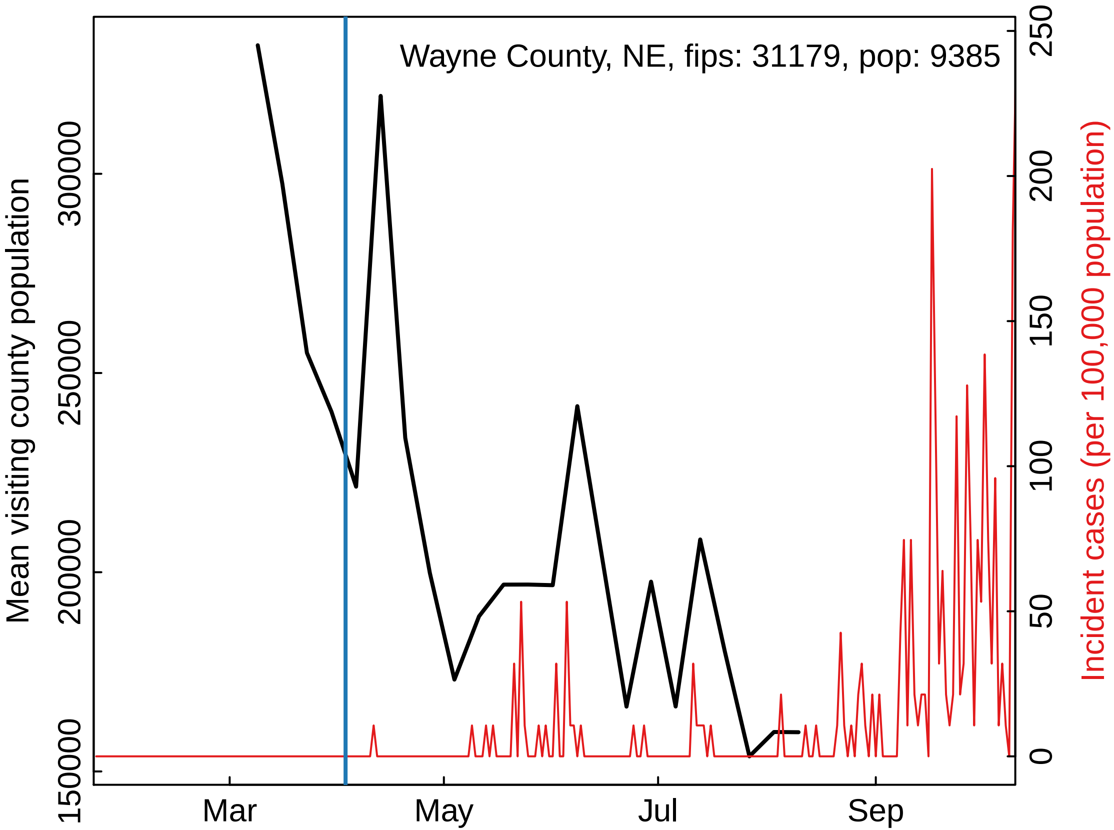

Wayne County, NE, fips: 31179, pop: 9385
Mean visiting county population
150000
200000
250000
300000
Mar
May
Jul
Sep
0
50
100
150
200
250
Incident cases (per 100,000 population)

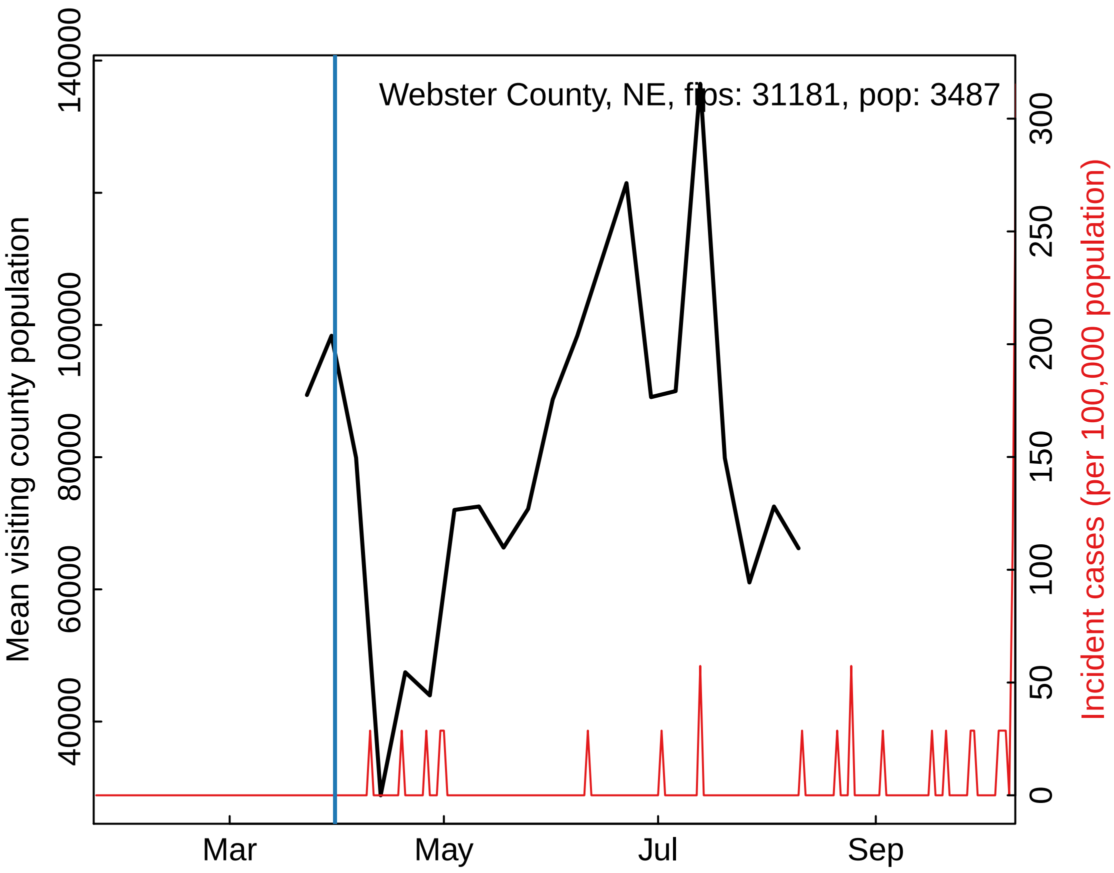

Webster County, NE, fips: 31181, pop: 3487
Mean visiting county population
40000
60000
80000
100000
140000
Incident cases (per 100,000 population)
0
50
100
150
200
250
300
Mar
May
Jul
Sep

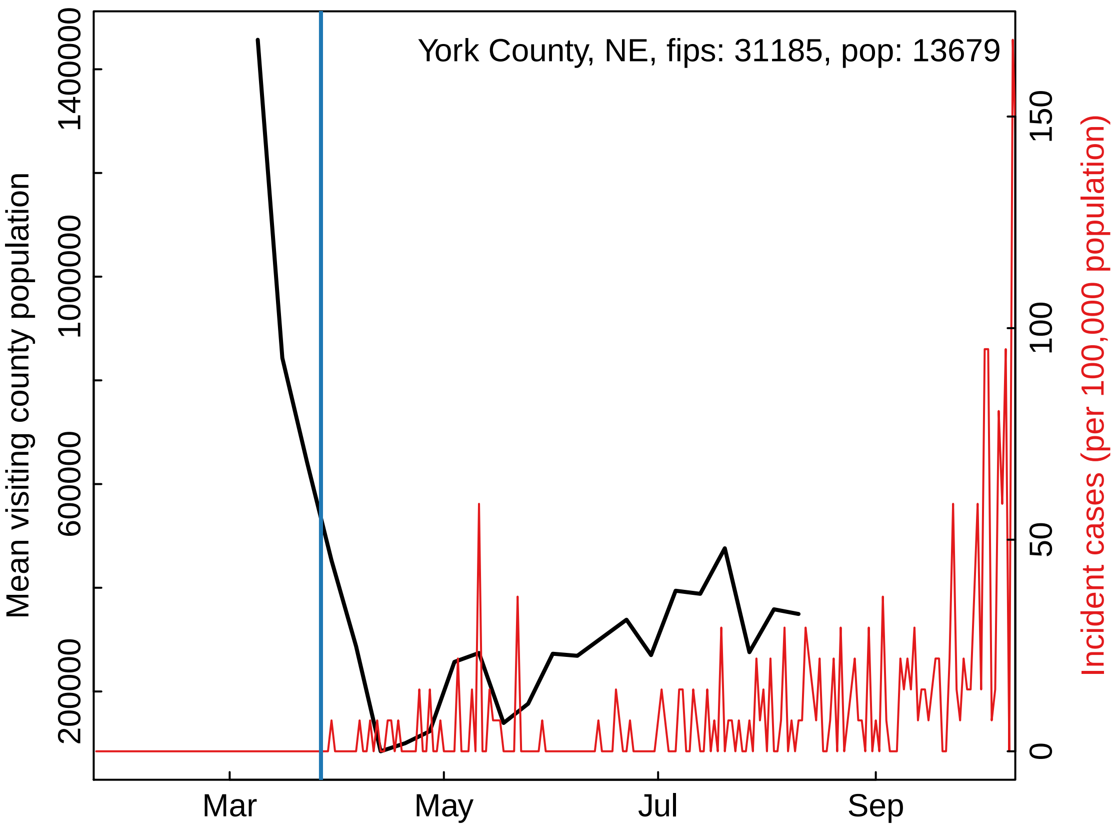

York County, NE, fips: 31185, pop: 13679
Mean visiting county population
200000
600000
1000000
1400000
Incident cases (per 100,000 population)
0
50
100
150
Mar
May
Jul
Sep

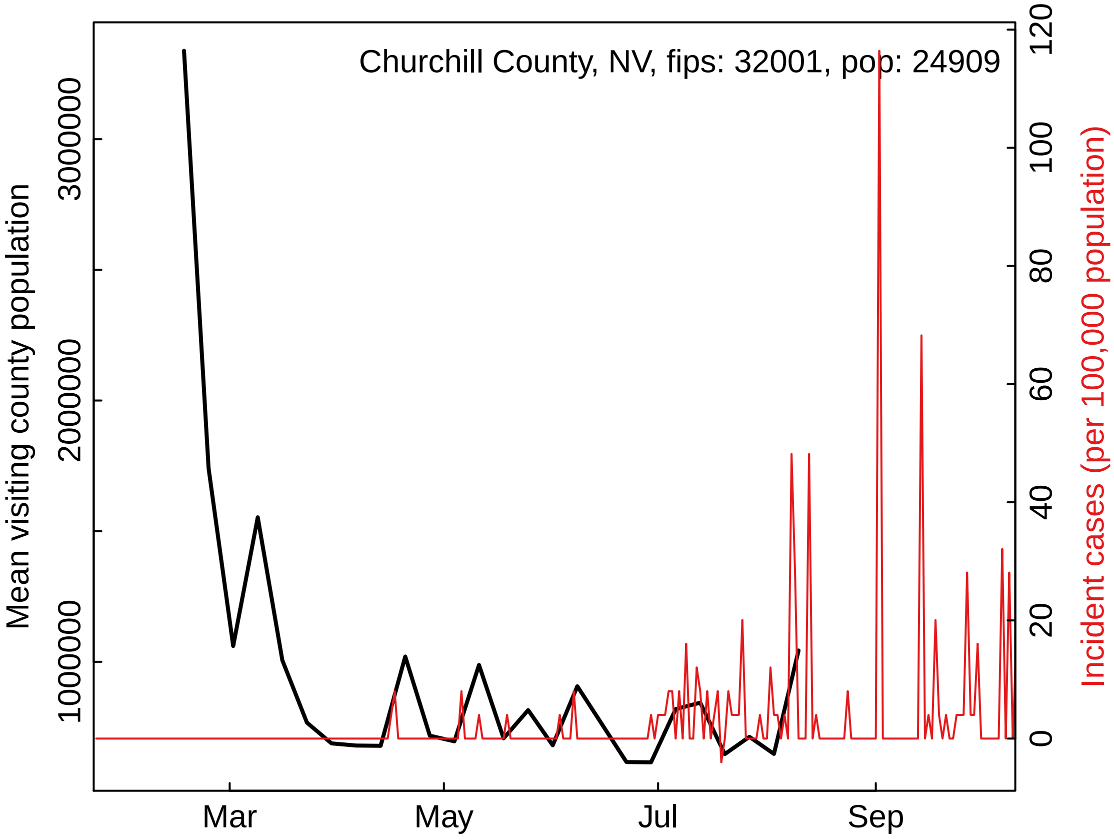

Churchill County, NV, fips: 32001, pop: 24909
Mean visiting county population
1000000
2000000
3000000
Incident cases (per 100,000 population)
0
20
40
60
80
100
120
Mar
May
Jul
Sep

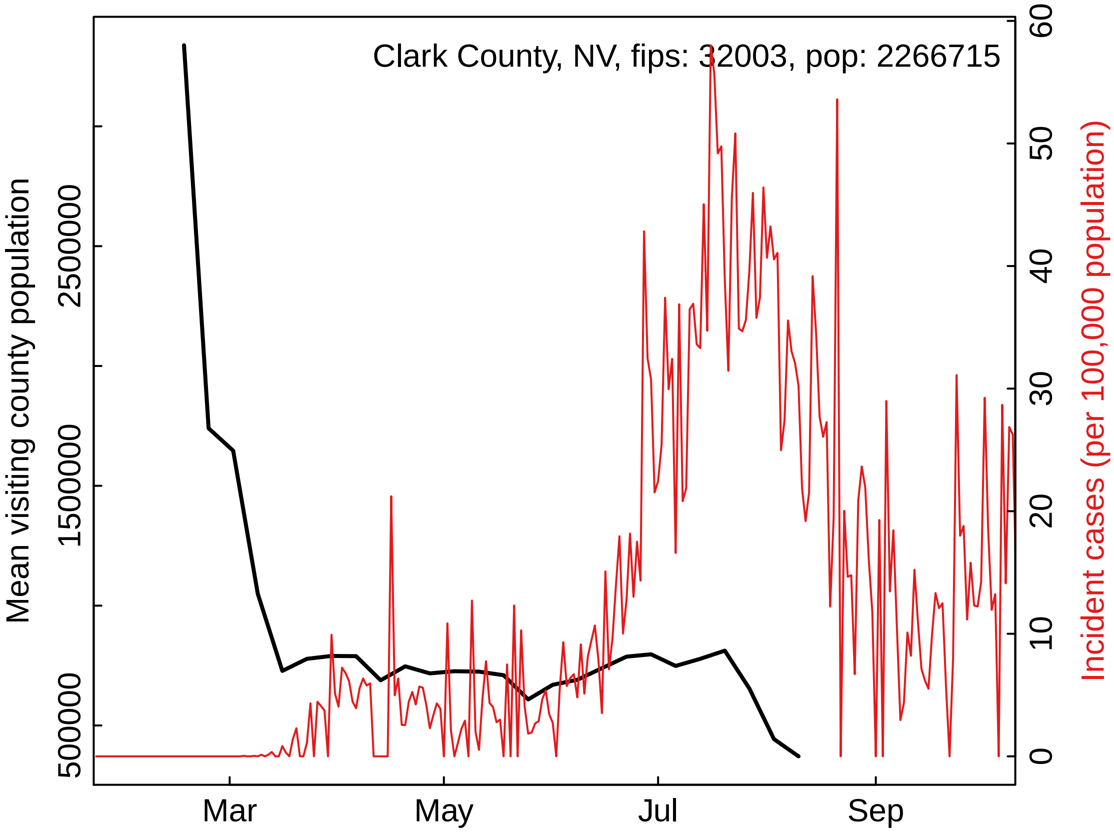

Clark County, NV, fips: 32003, pop: 2266715
Mean visiting county population
500000
1500000
2500000
Mar
May
Jul
Sep
0
10
20
30
40
50
60
Incident cases (per 100,000 population)

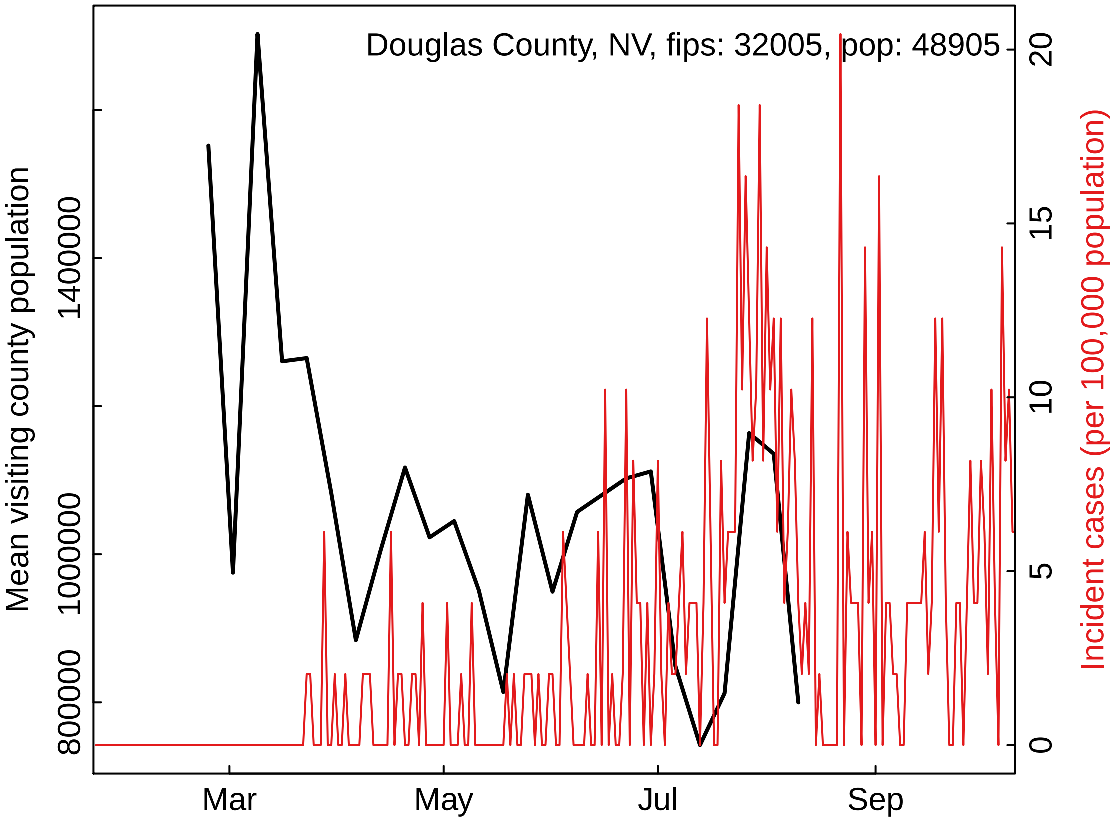

Douglas County, NV, fips: 32005, pop: 48905
Mean visiting county population
800000
1000000
1400000
Incident cases (per 100,000 population)
0
5
10
15
20
Mar
May
Jul
Sep

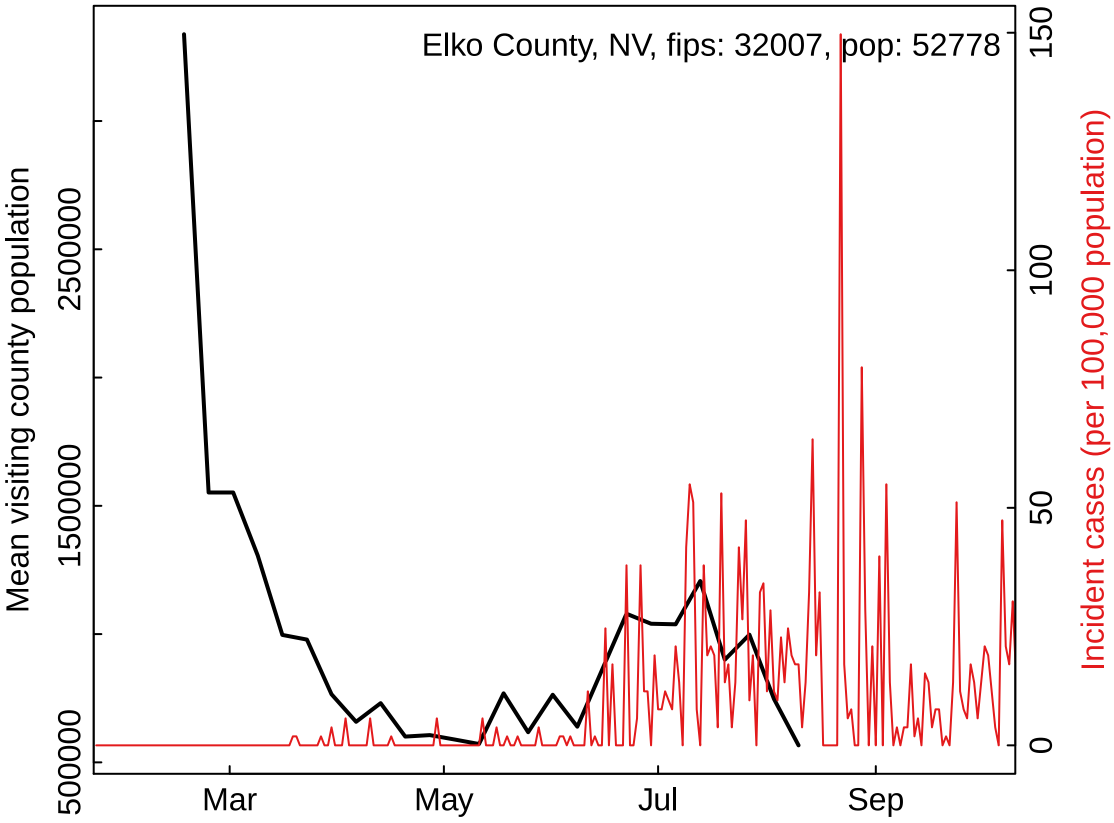
Elko County, NV, fips: 32007, pop: 52778
Mean visiting county population
500000
1500000
2500000
Incident cases (per 100,000 population)
0
50
100
150
Mar
May
Jul
Sep

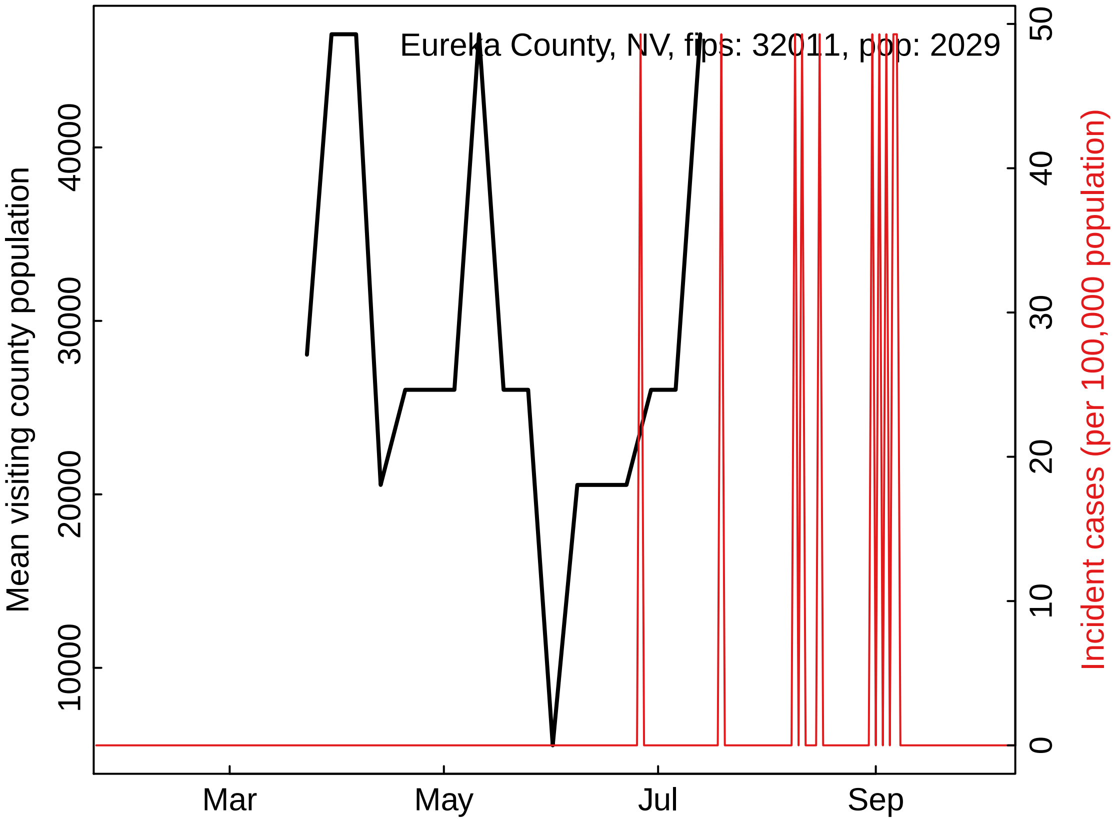

Eureka County, NV, fips: 32011, pop: 2029
Mean visiting county population
10000
20000
30000
40000
Incident cases (per 100,000 population)
0
10
20
30
40
50
Mar
May
Jul
Sep

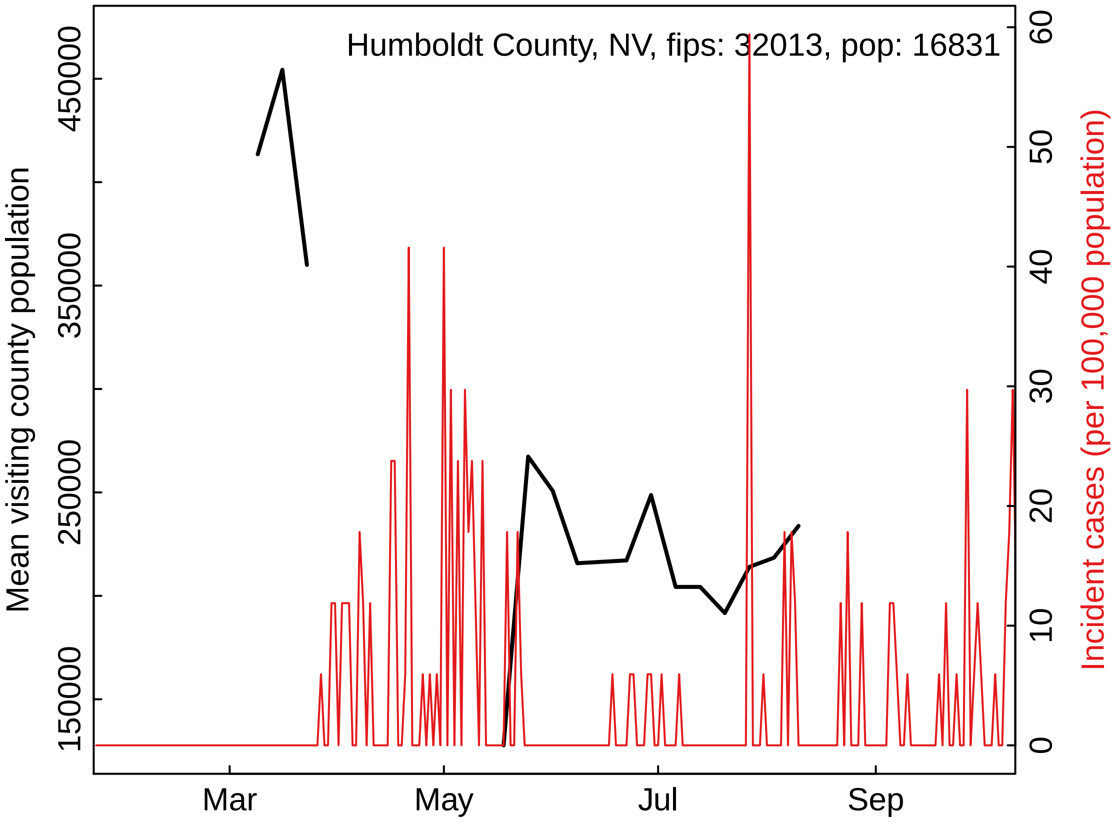

Humboldt County, NV, fips: 32013, pop: 16831
Mean visiting county population
150000
250000
350000
450000
Incident cases (per 100,000 population)
0
10
20
30
40
50
60
Mar
May
Jul
Sep

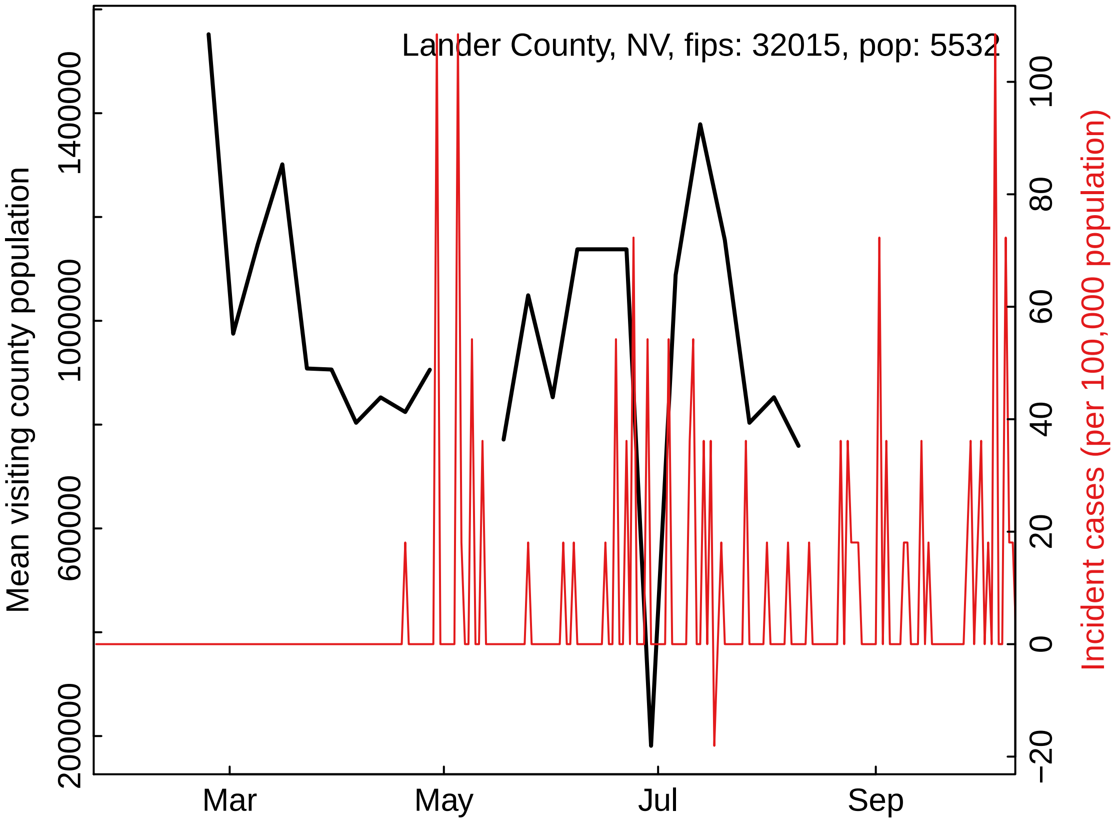

Lander County, NV, fips: 32015, pop: 5532
Mean visiting county population
200000
600000
1000000
1400000
Mar
May
Jul
Sep
Incident cases (per 100,000 population)
−20
0
20
40
60
80
100

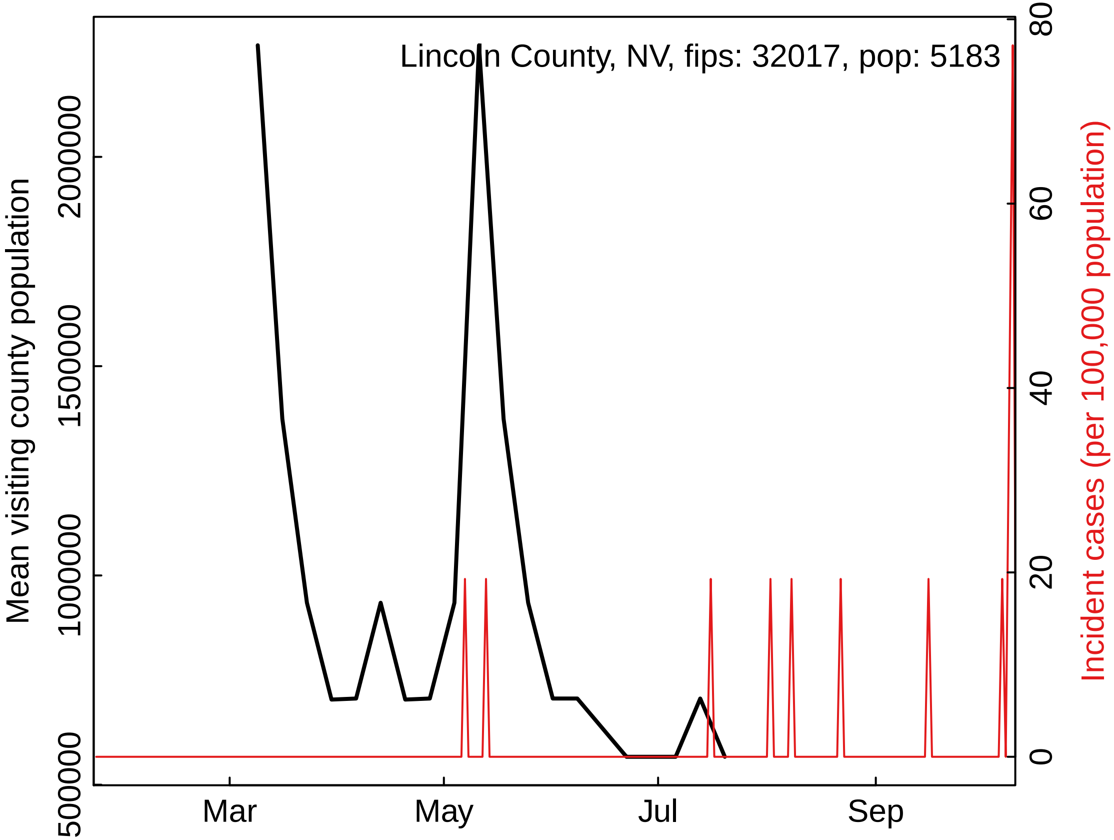

Lincoln County, NV, fips: 32017, pop: 5183
Mean visiting county population
500000
1000000
1500000
2000000
Mar
May
Jul
Sep
0
20
40
60
80
Incident cases (per 100,000 population)

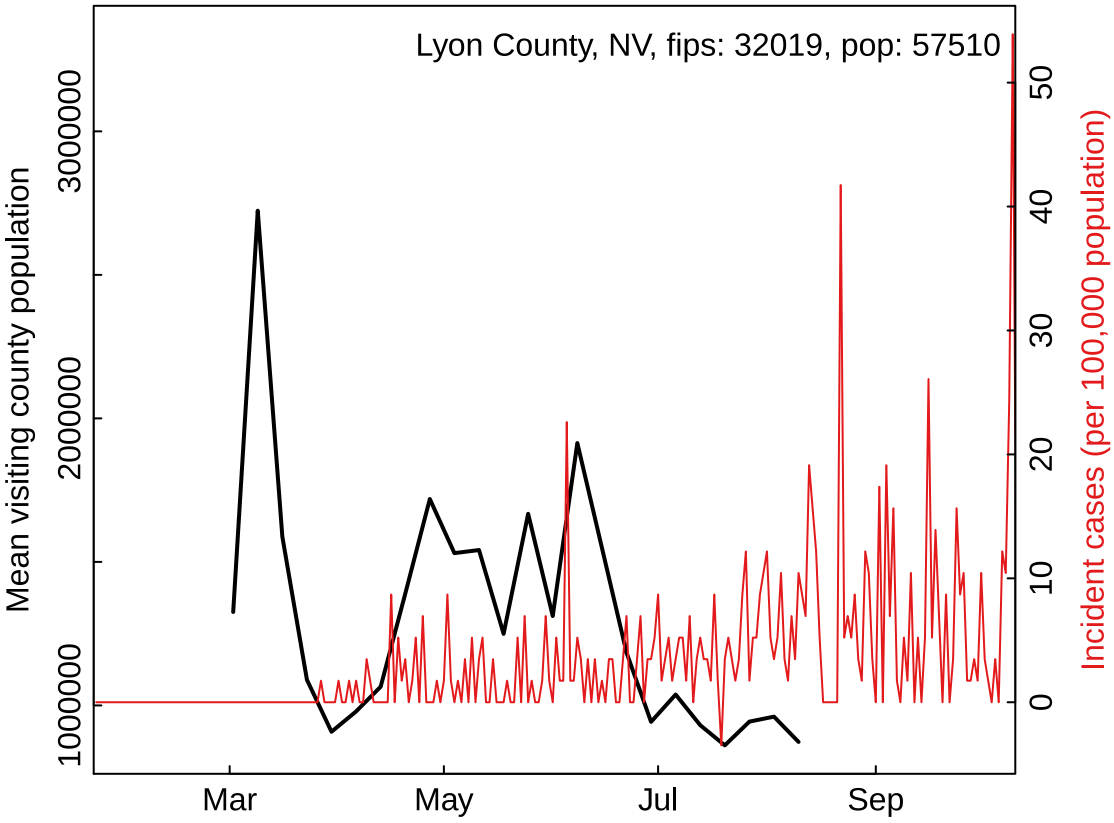

Lyon County, NV, fips: 32019, pop: 57510
Mean visiting county population
10000000
20000000
30000000
Incident cases (per 100,000 population)
0
10
20
30
40
50
Mar
May
Jul
Sep

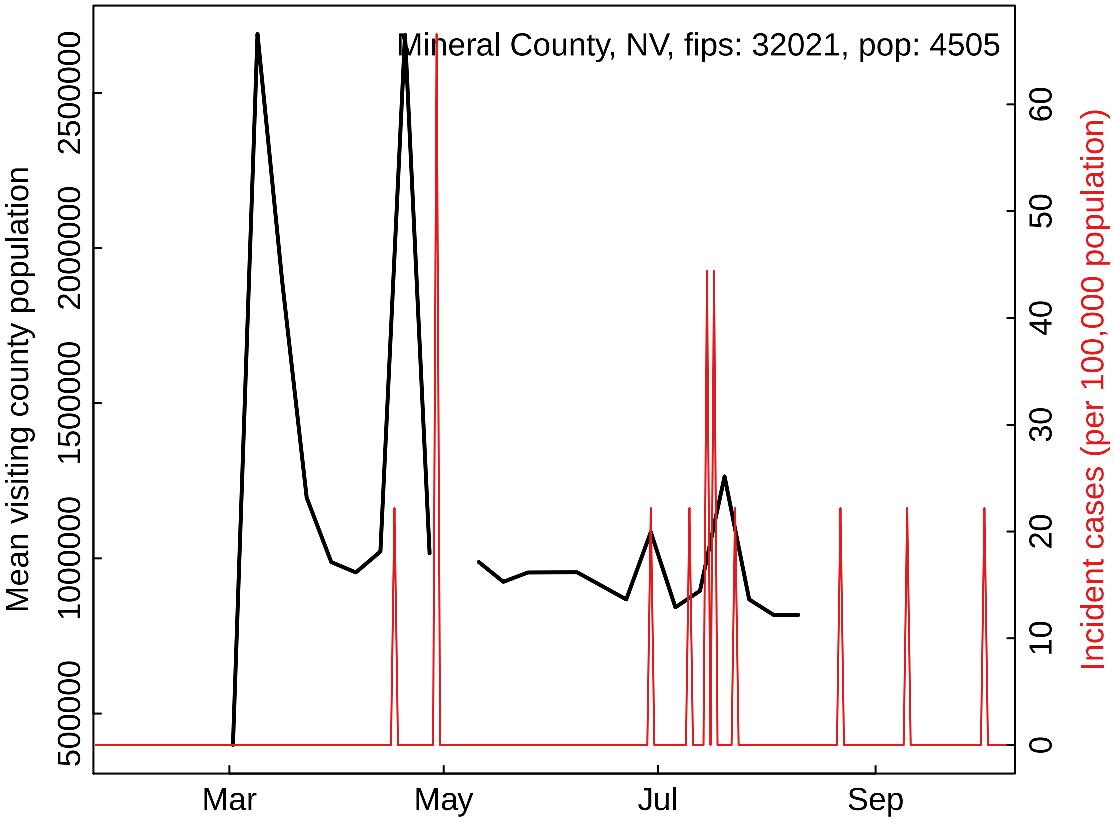
Mineral County, NV, fips: 32021, pop: 4505
Mean visiting county population
500000
1000000
1500000
2000000
2500000
Incident cases (per 100,000 population)
0
10
20
30
40
50
60
Mar
May
Jul
Sep

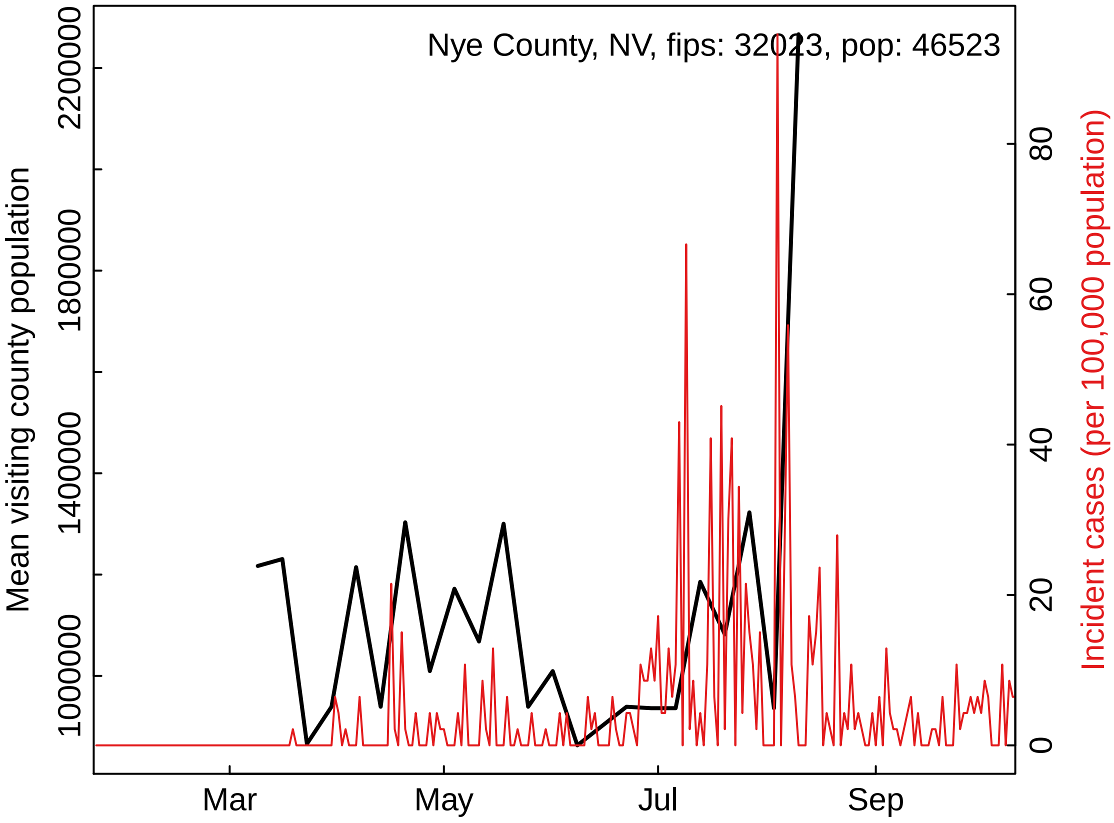

Nye County, NV, fips: 32023, pop: 46523
Mean visiting county population
1000000
1400000
1800000
2200000
Incident cases (per 100,000 population)
0
20
40
60
80
Mar
May
Jul
Sep

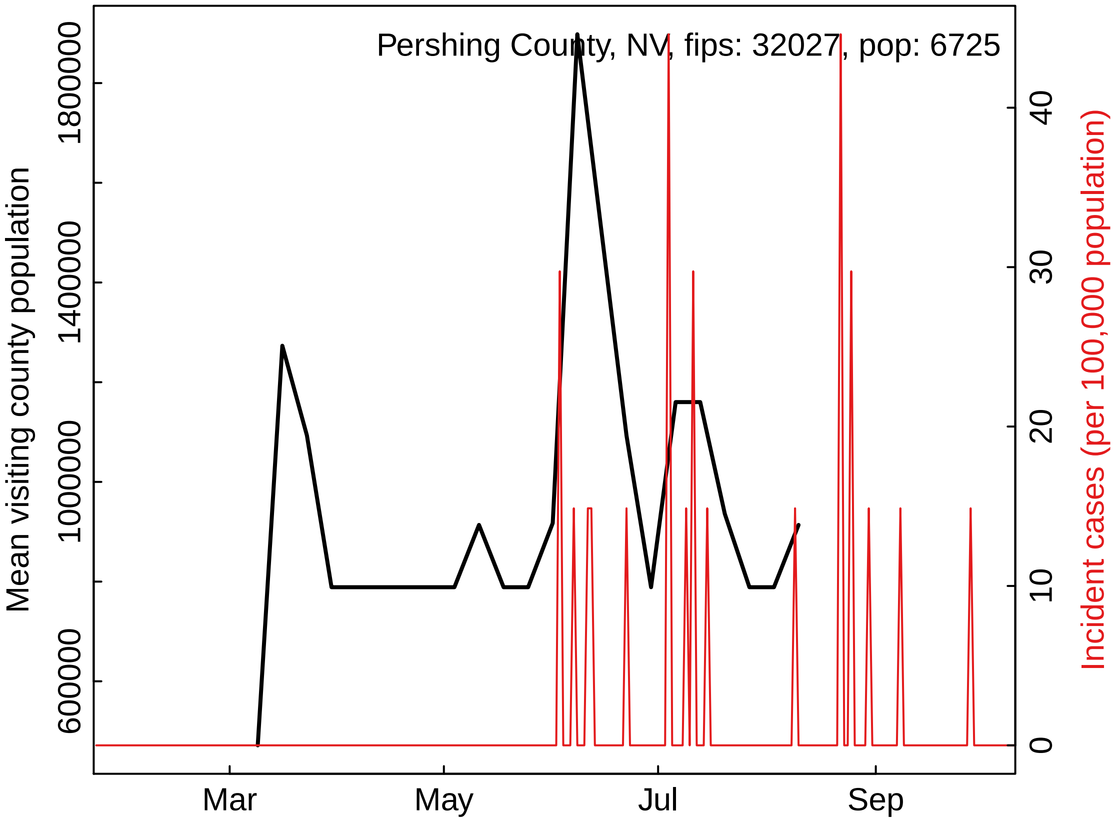

Pershing County, NV, fips: 32027, pop: 6725
Mean visiting county population
600000
1000000
1400000
1800000
Incident cases (per 100,000 population)
0
10
20
30
40
Mar
May
Jul
Sep

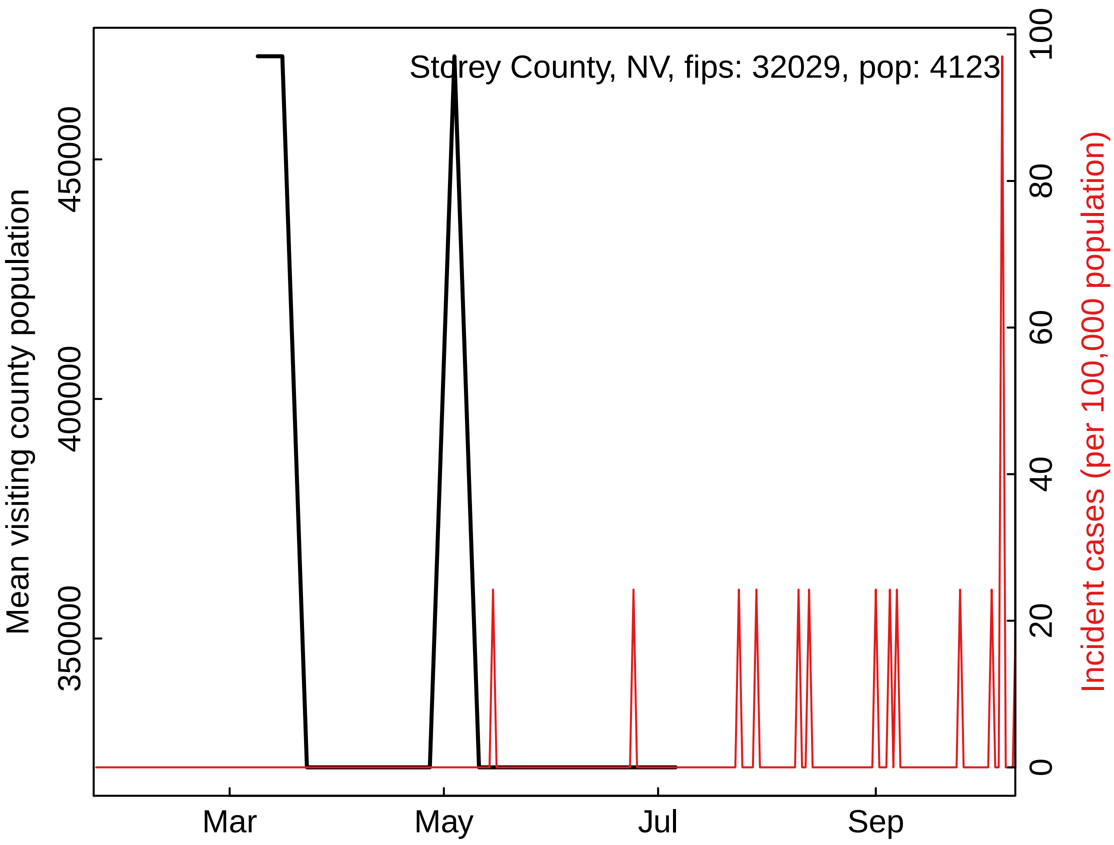

Storey County, NV, fips: 32029, pop: 4123
Mean visiting county population
350000
400000
450000
Mar
May
Jul
Sep
0
20
40
60
80
100
Incident cases (per 100,000 population)

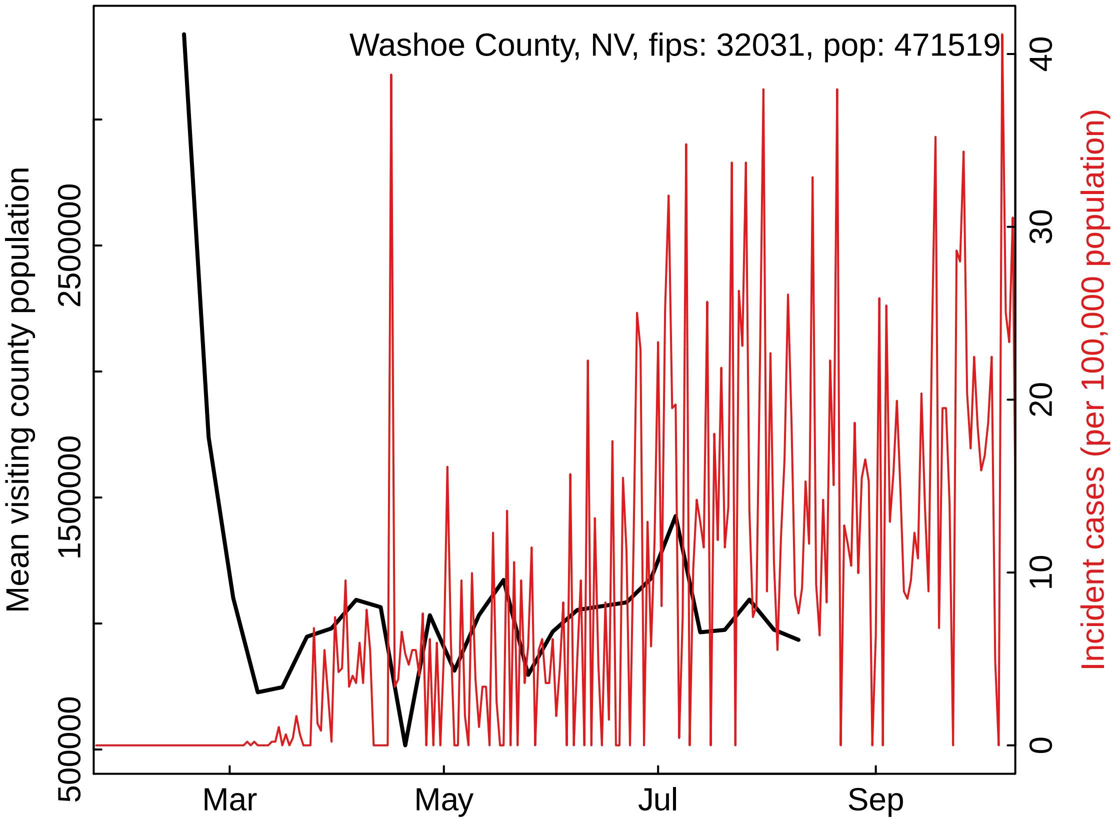

Washoe County, NV, fips: 32031, pop: 471519
Mean visiting county population
500000
1500000
2500000
Mar
May
Jul
Sep
0
10
20
30
40
Incident cases (per 100,000 population)

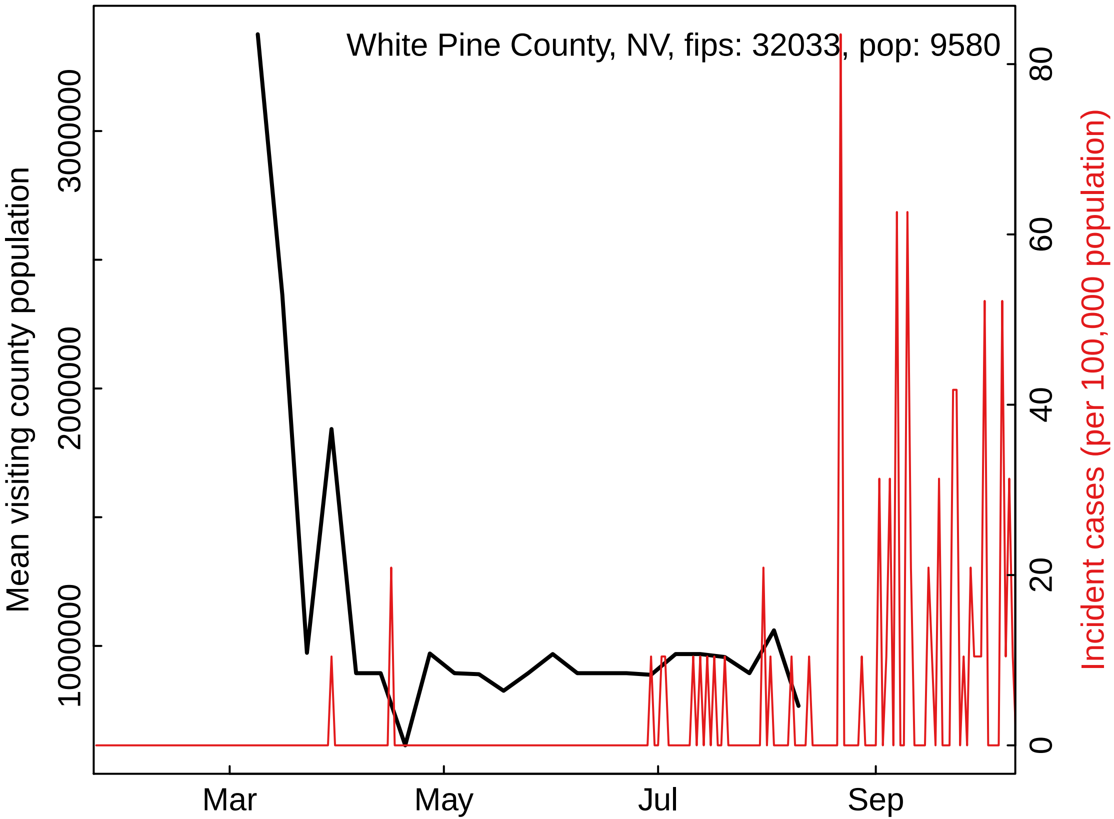
White Pine County, NV, fips: 32033, pop: 9580
Mean visiting county population
1000000
2000000
3000000
Incident cases (per 100,000 population)
0
20
40
60
80
Mar
May
Jul
Sep

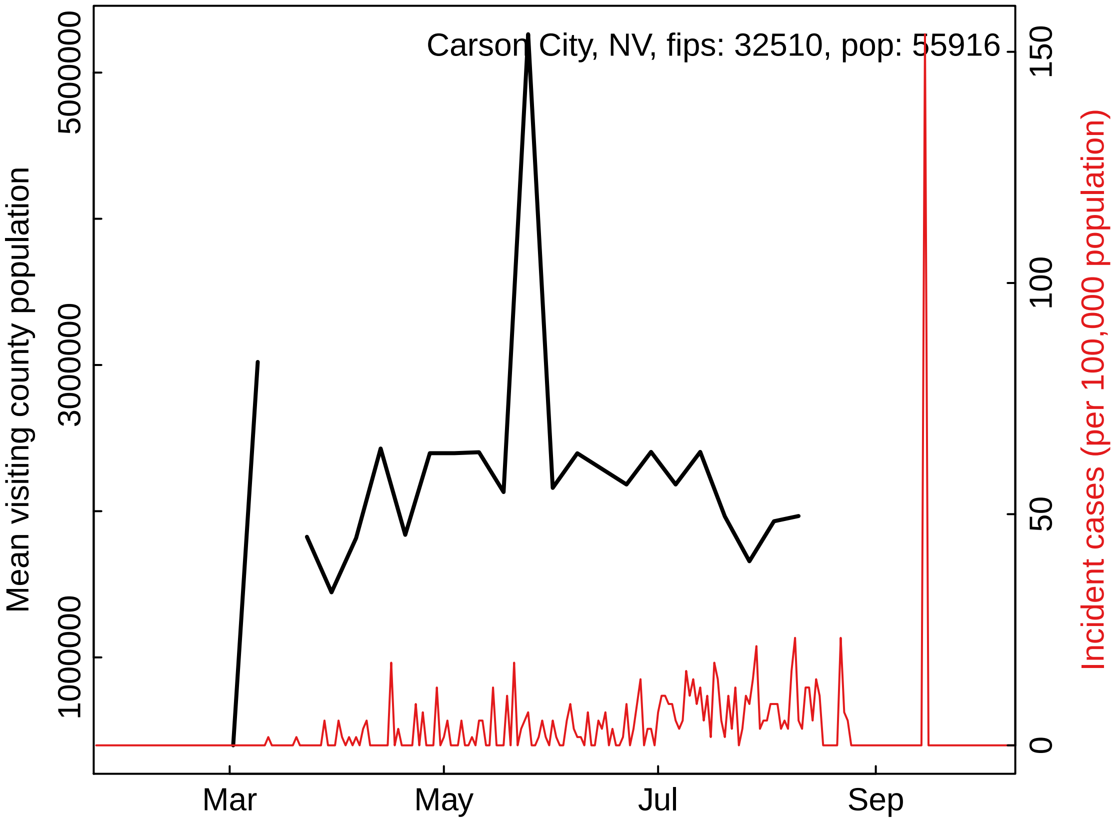

Carson City, NV, fips: 32510, pop: 55916
Mean visiting county population
1000000
3000000
5000000
Incident cases (per 100,000 population)
0
50
100
150
Mar
May
Jul
Sep

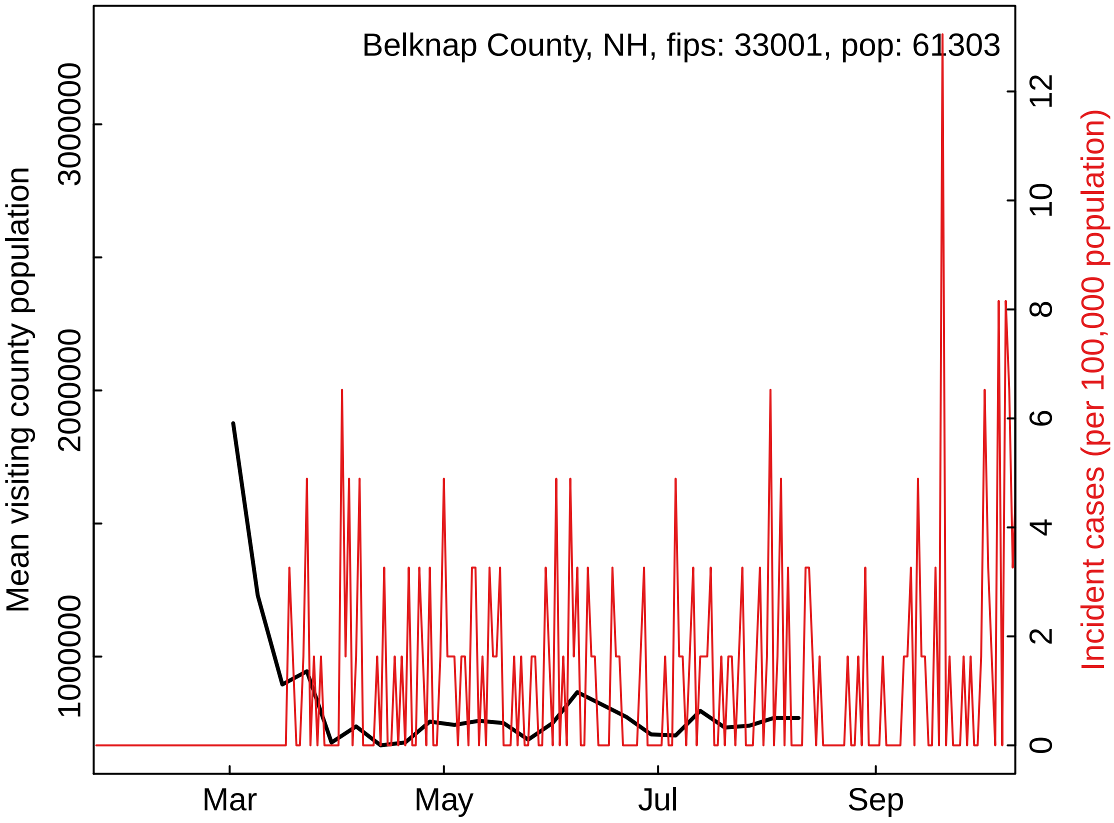

Belknap County, NH, fips: 33001, pop: 61303
Mean visiting county population
1000000
2000000
3000000
Incident cases (per 100,000 population)
0
2
4
6
8
10
12
Mar
May
Jul
Sep

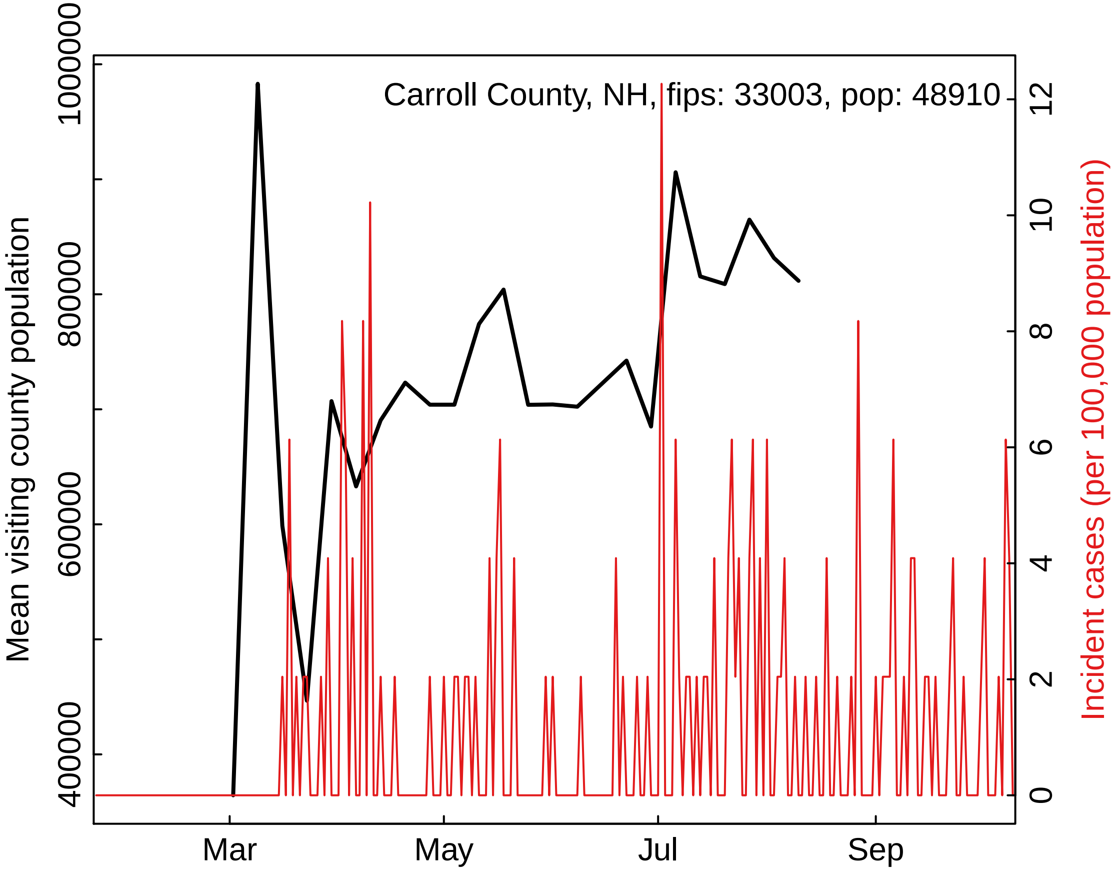

Carroll County, NH, fips: 33003, pop: 48910
Mean visiting county population
400000
600000
800000
1000000
Incident cases (per 100,000 population)
0
2
4
6
8
10
12
Mar
May
Jul
Sep

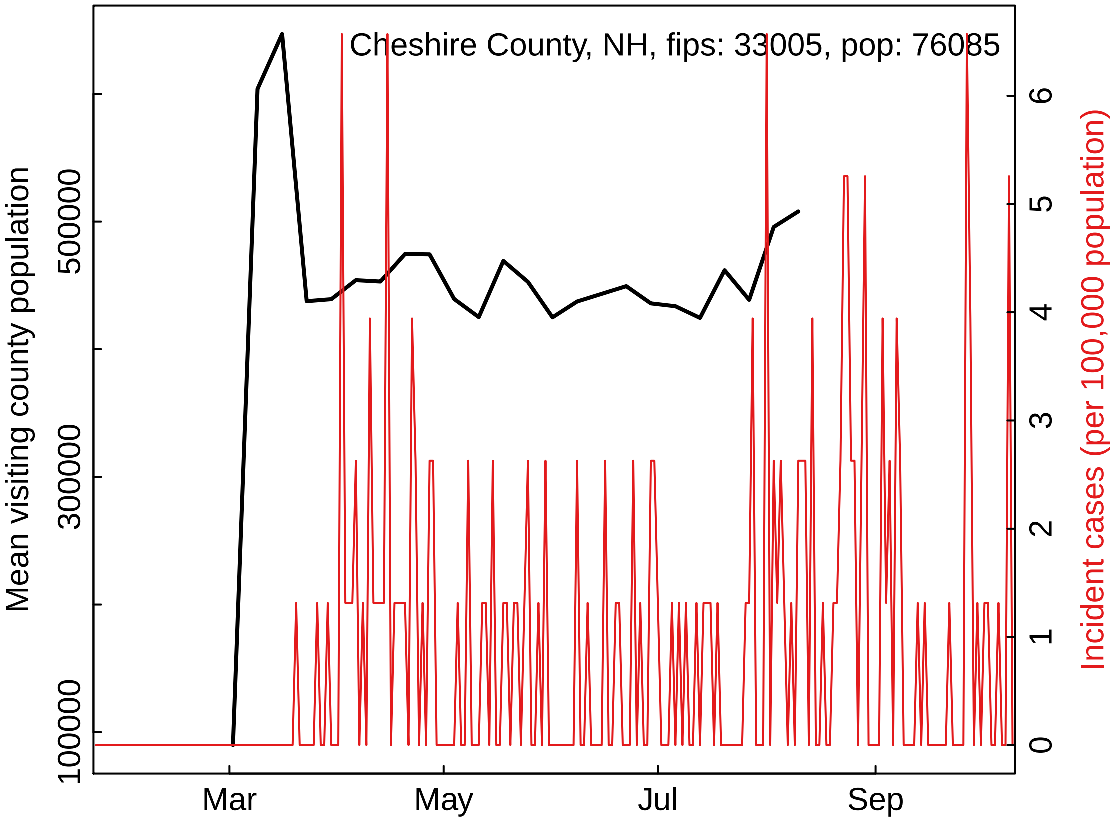

Cheshire County, NH, fips: 33005, pop: 76085
Mean visiting county population
100000
300000
500000
Mar
May
Jul
Sep
0
1
2
3
4
5
6
Incident cases (per 100,000 population)

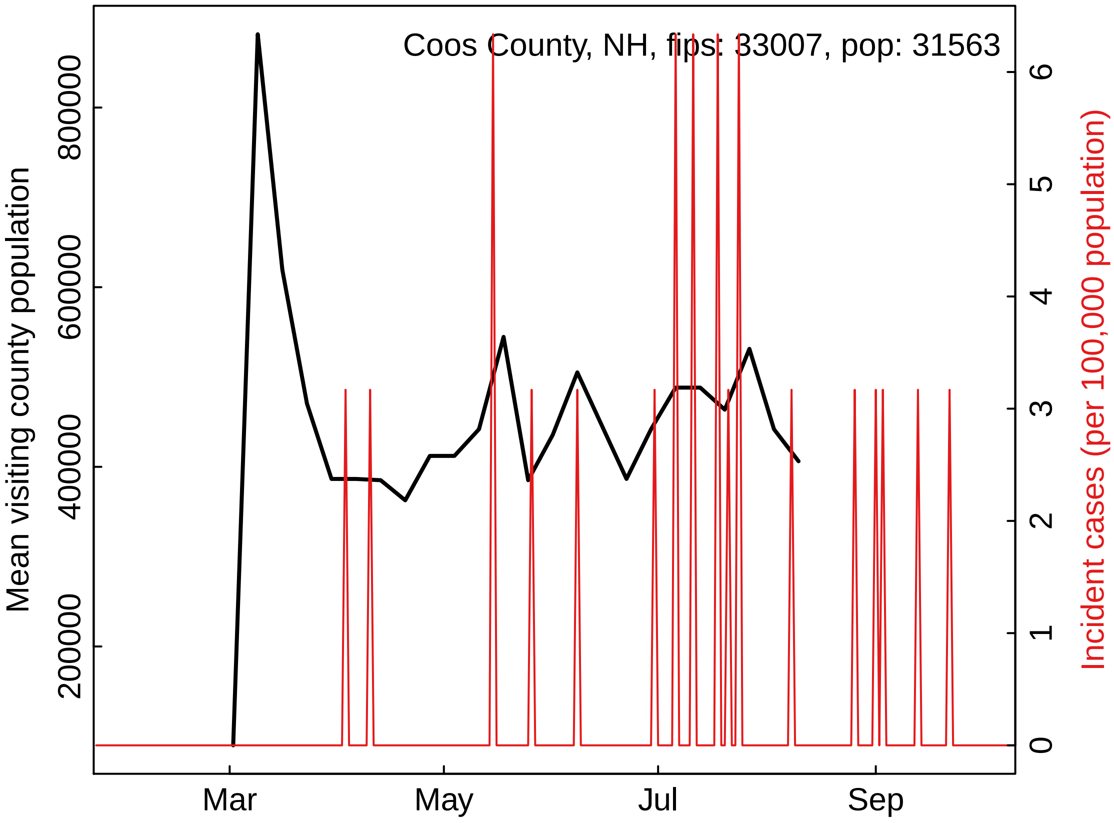

Coos County, NH, fips: 33007, pop: 31563
Mean visiting county population
200000
400000
600000
800000
Incident cases (per 100,000 population)
0
1
2
3
4
5
6
Mar
May
Jul
Sep

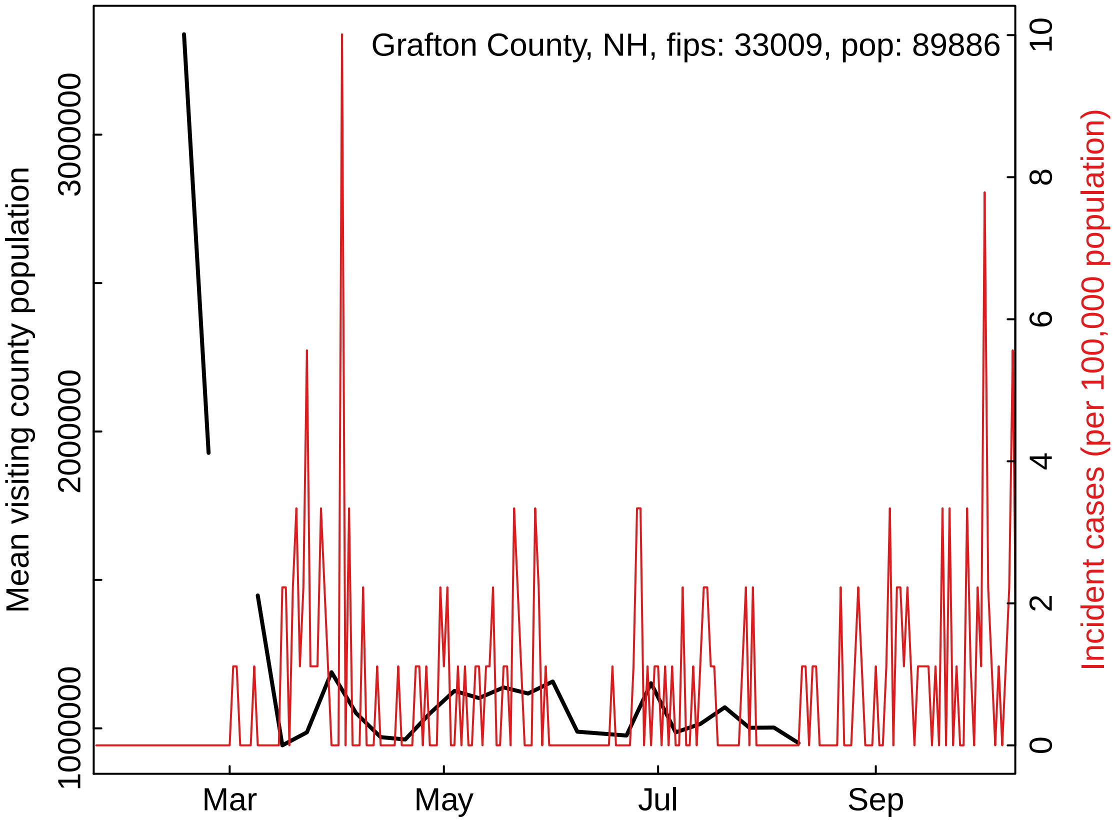
Grafton County, NH, fips: 33009, pop: 89886
Mean visiting county population
10000000
20000000
30000000
Incident cases (per 100,000 population)
0
2
4
6
8
10
Mar
May
Jul
Sep

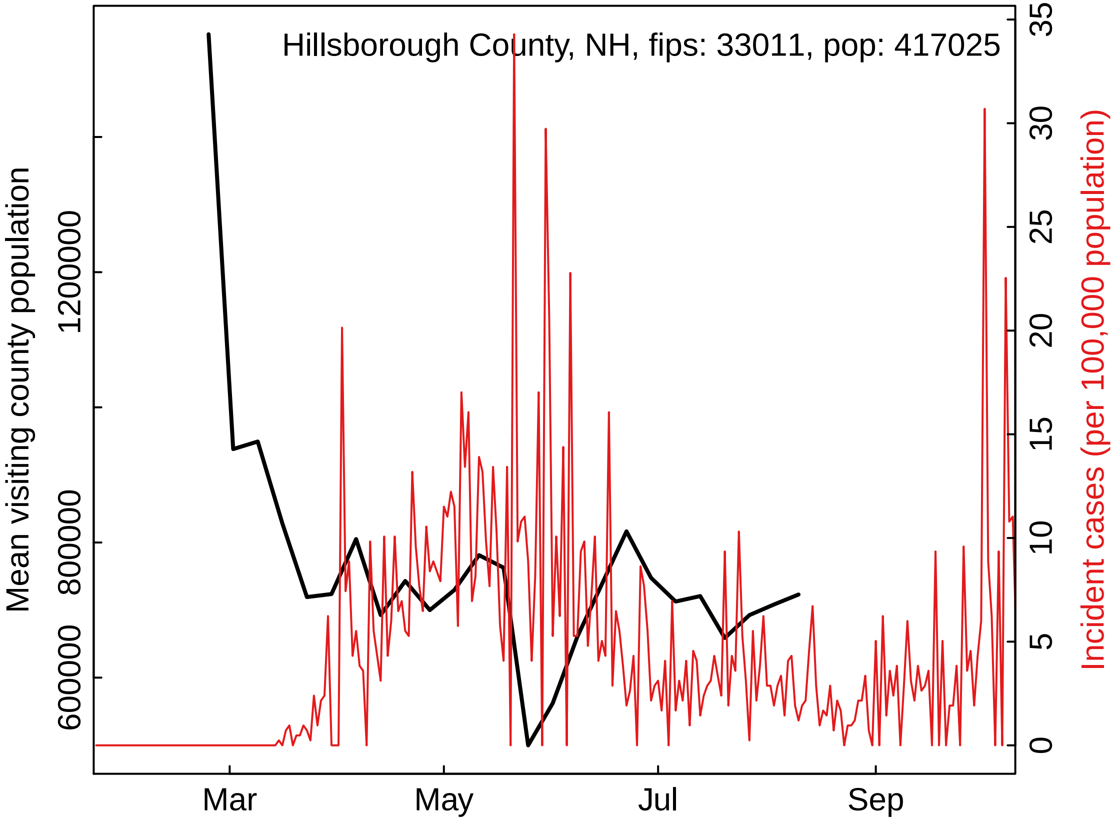

Hillsborough County, NH, fips: 33011, pop: 417025
Mean visiting county population
600000
800000
1200000
Incident cases (per 100,000 population)
0
5
10
15
20
25
30
35
Mar
May
Jul
Sep

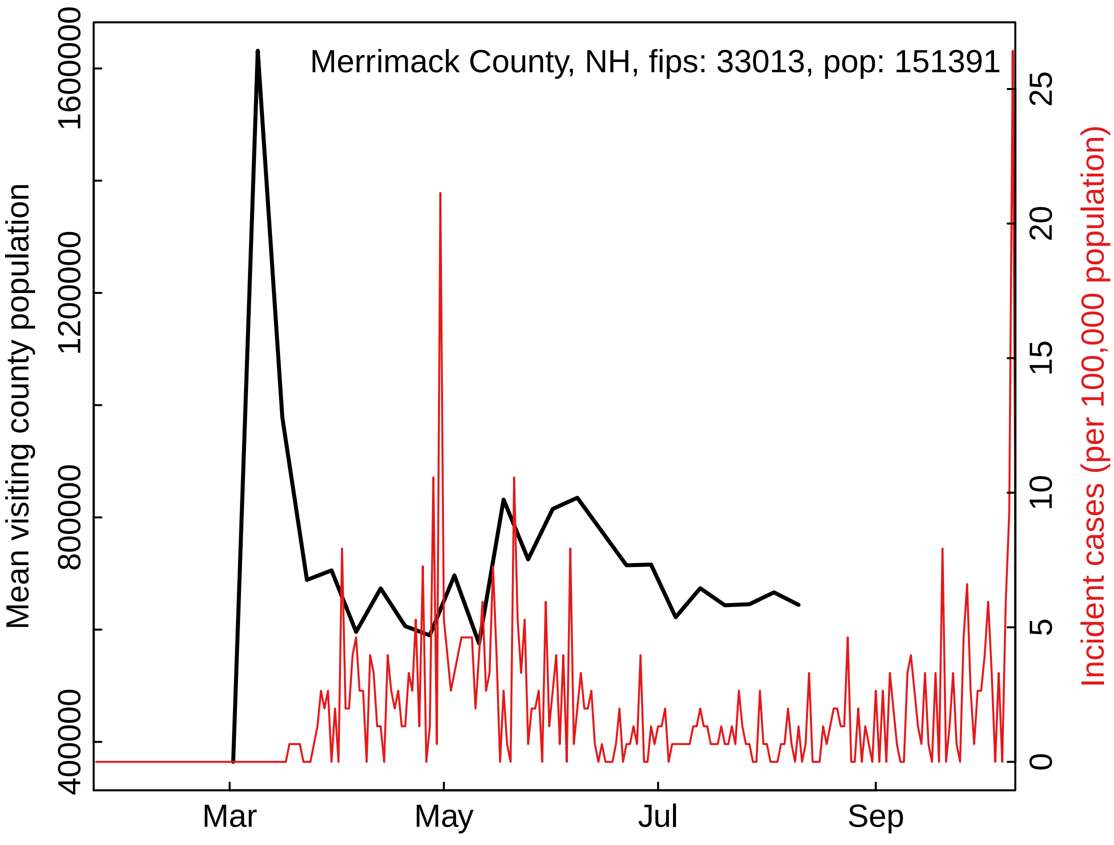

Merrimack County, NH, fips: 33013, pop: 151391
Mean visiting county population
400000
800000
1200000
1600000
Incident cases (per 100,000 population)
0
5
10
15
20
25
Mar
May
Jul
Sep

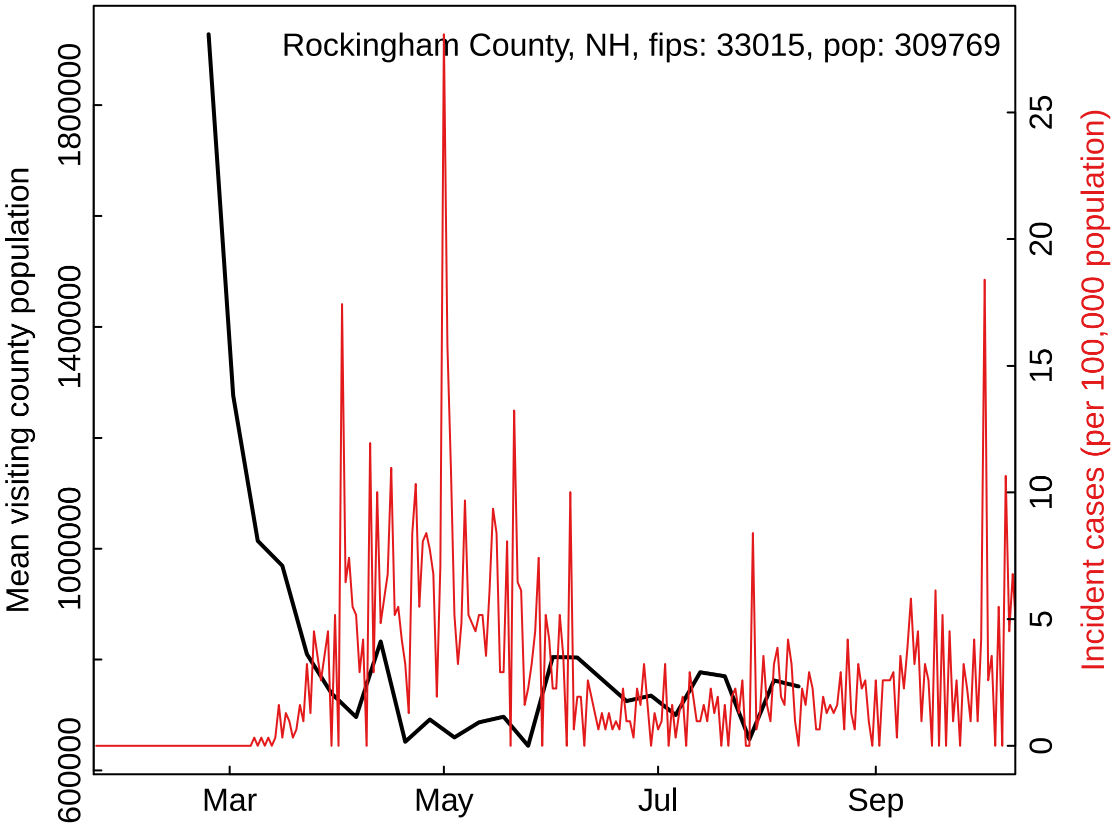
Rockingham County, NH, fips: 33015, pop: 309769
Mean visiting county population
600000
1000000
1400000
1800000
Mar
May
Jul
Sep
0
5
10
15
20
25
Incident cases (per 100,000 population)

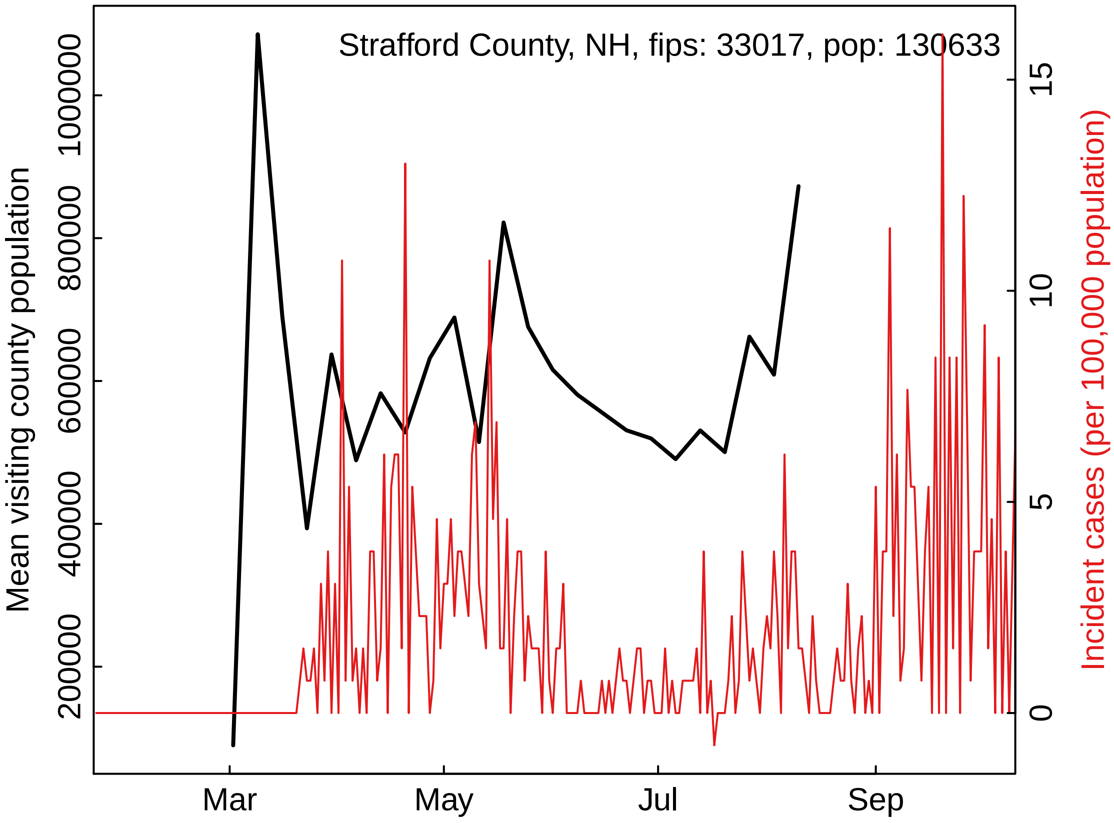

Strafford County, NH, fips: 33017, pop: 130633
Mean visiting county population
200000
400000
600000
800000
1000000
Incident cases (per 100,000 population)
0
5
10
15
Mar
May
Jul
Sep

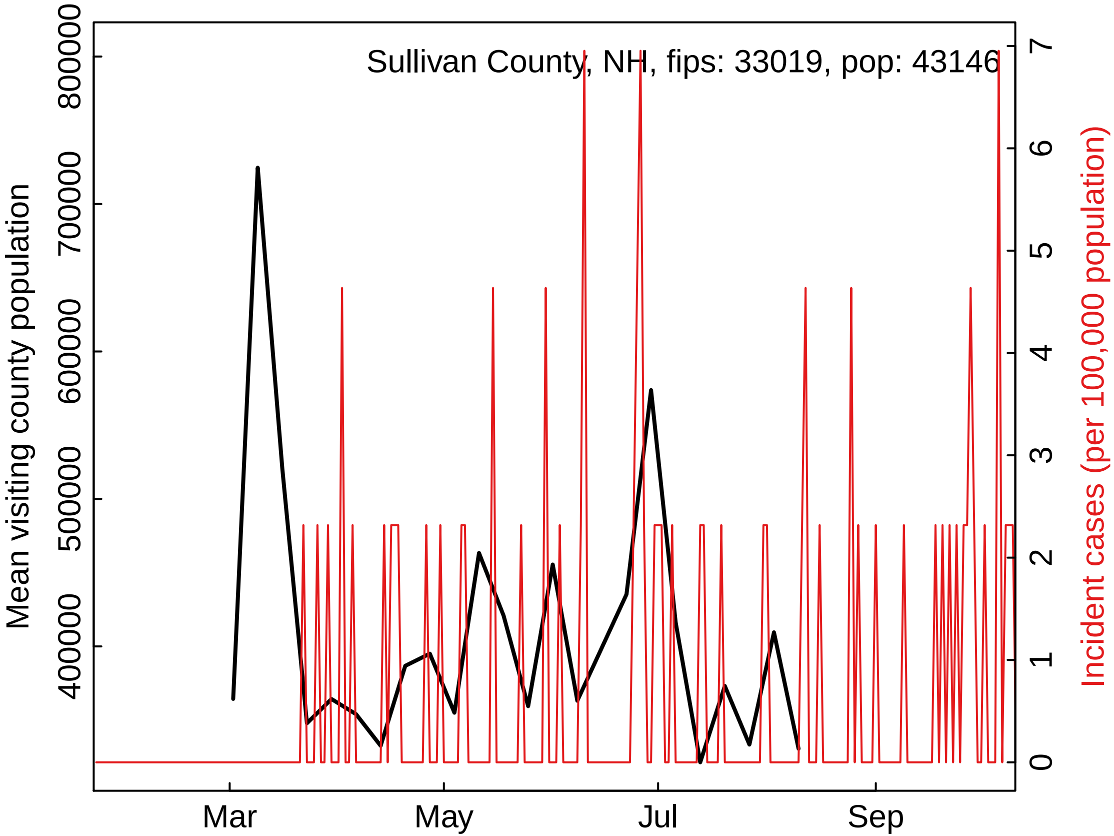

Sullivan County, NH, fips: 33019, pop: 43146
Mean visiting county population
400000
500000
600000
700000
800000
Incident cases (per 100,000 population)
0
1
2
3
4
5
6
7
Mar
May
Jul
Sep

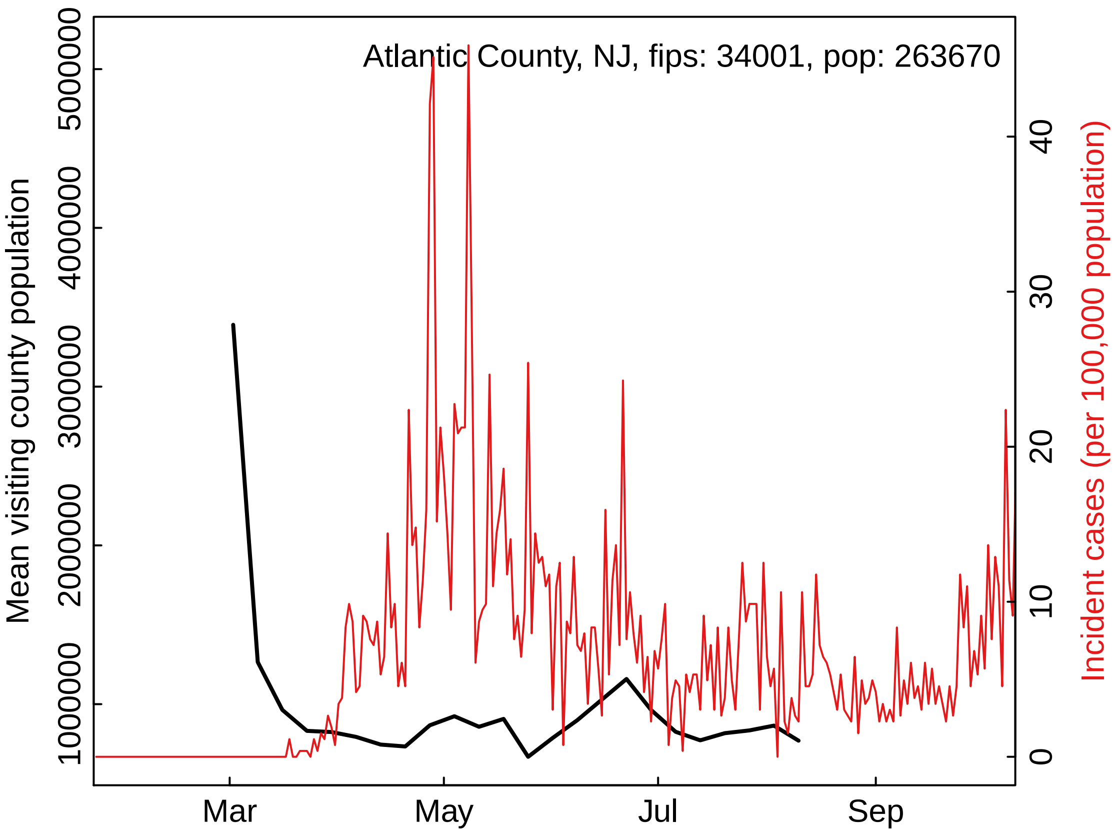

Atlantic County, NJ, fips: 34001, pop: 263670
Mean visiting county population
10000000
20000000
30000000
40000000
50000000
Incident cases (per 100,000 population)
0
10
20
30
40
Mar
May
Jul
Sep

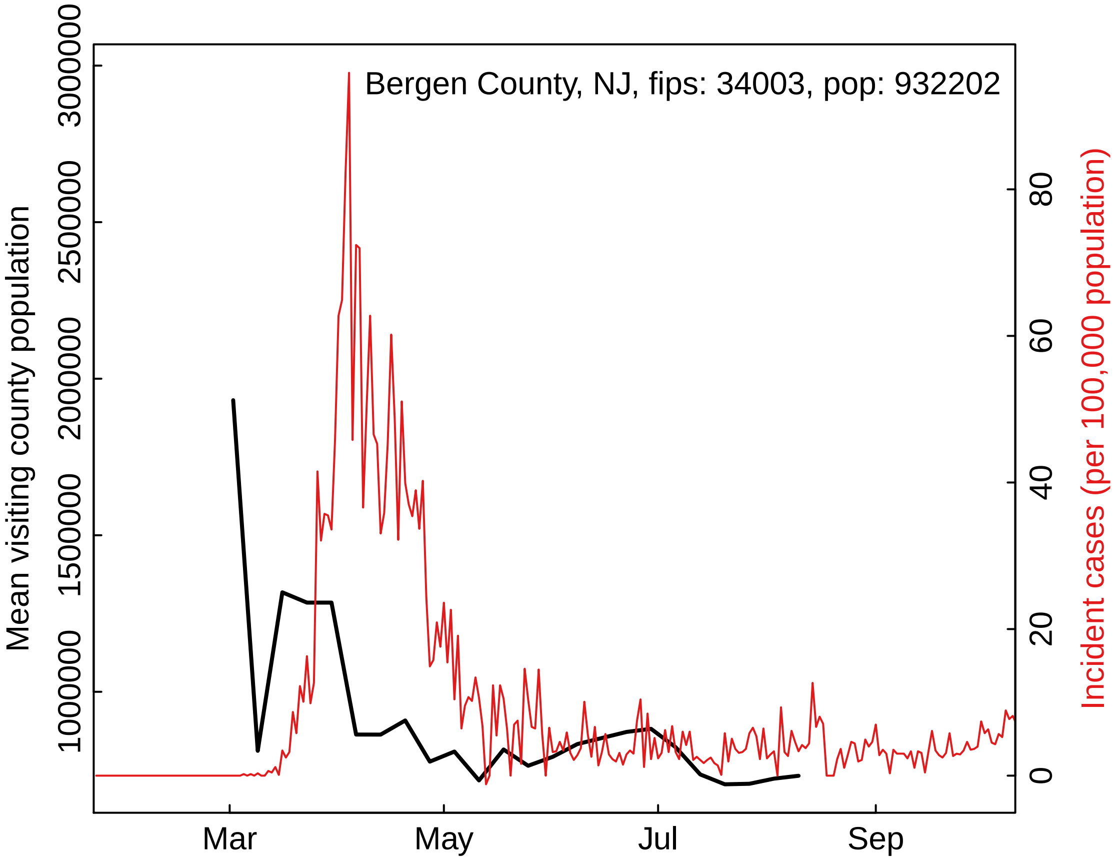

Bergen County, NJ, fips: 34003, pop: 932202
Mean visiting county population
10000000
1500000
2000000
2500000
3000000
Incident cases (per 100,000 population)
0
20
40
60
80
Mar
May
Jul
Sep

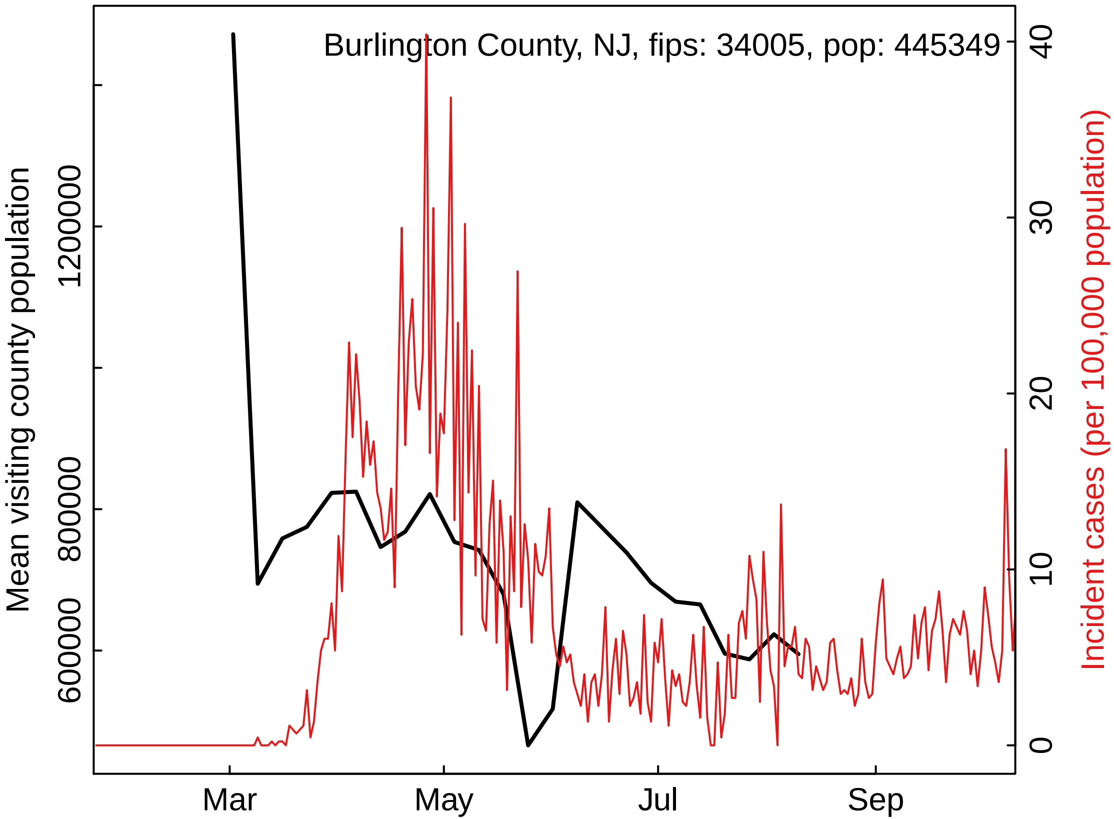

Burlington County, NJ, fips: 34005, pop: 445349
Mean visiting county population
600000
800000
1200000
Incident cases (per 100,000 population)
0
10
20
30
40
Mar
May
Jul
Sep

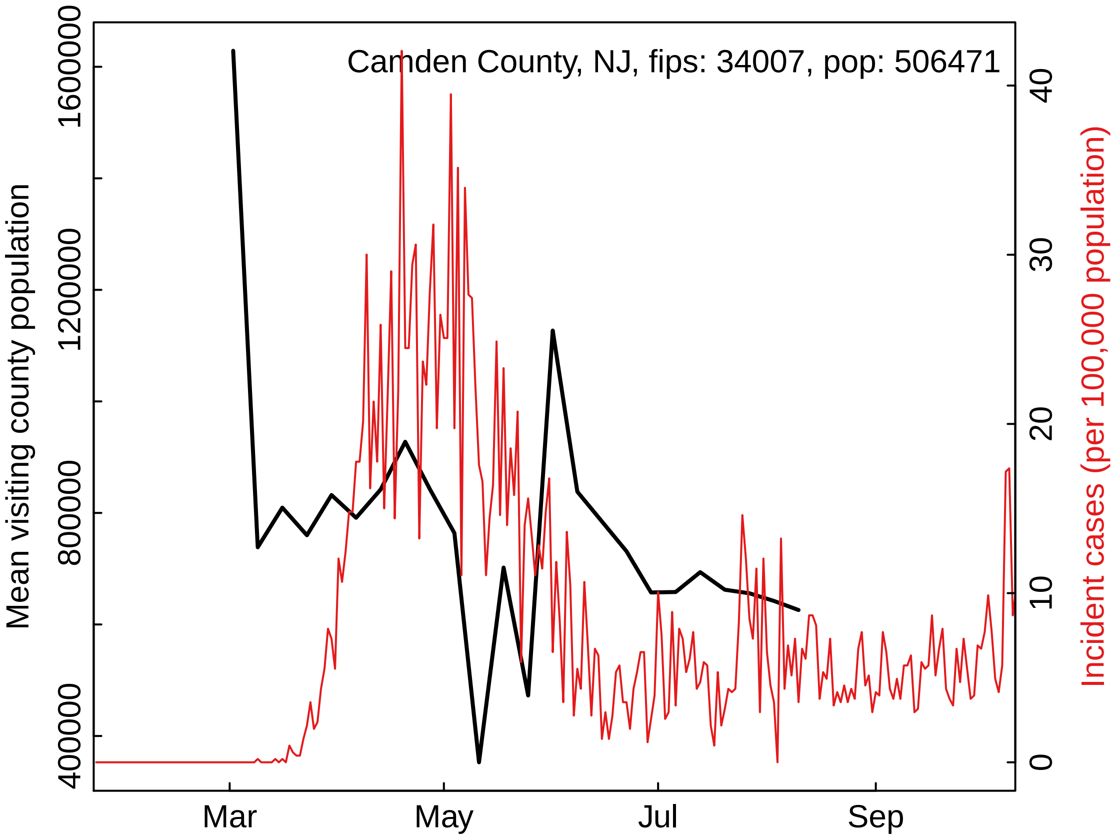

Camden County, NJ, fips: 34007, pop: 506471
Mean visiting county population
Incident cases (per 100,000 population)
400000
800000
1200000
1600000
0
10
20
30
40
Mar
May
Jul
Sep

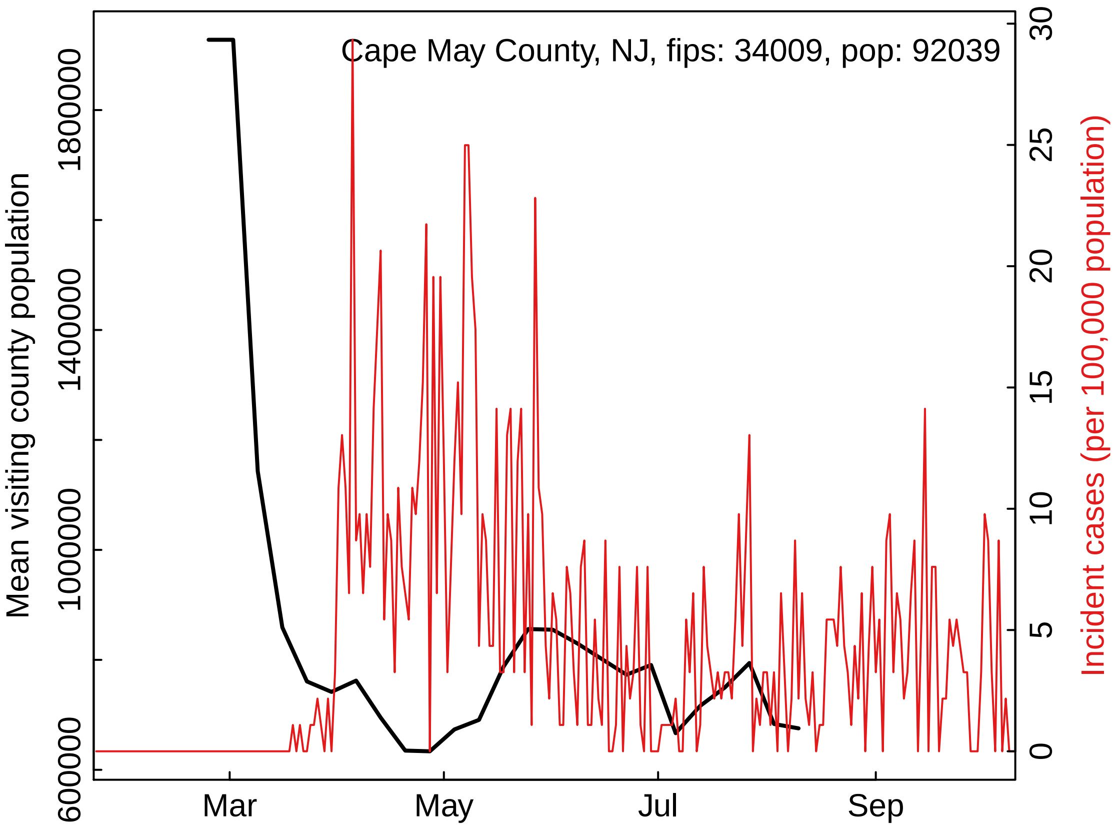
Cape May County, NJ, fips: 34009, pop: 92039
Mean visiting county population
600000
1000000
1400000
1800000
Incident cases (per 100,000 population)
0
5
10
15
20
25
30
Mar
May
Jul
Sep

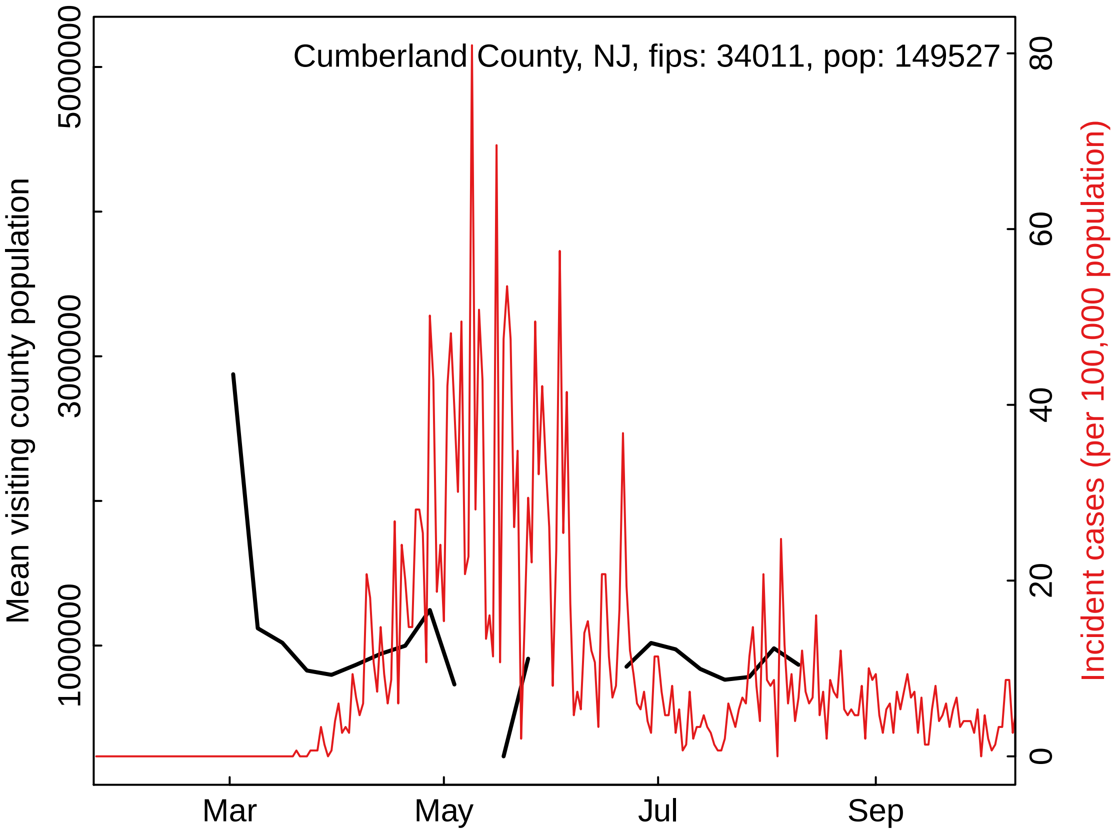

Cumberland County, NJ, fips: 34011, pop: 149527
Mean visiting county population
1000000
3000000
5000000
Incident cases (per 100,000 population)
0
20
40
60
80
Mar
May
Jul
Sep

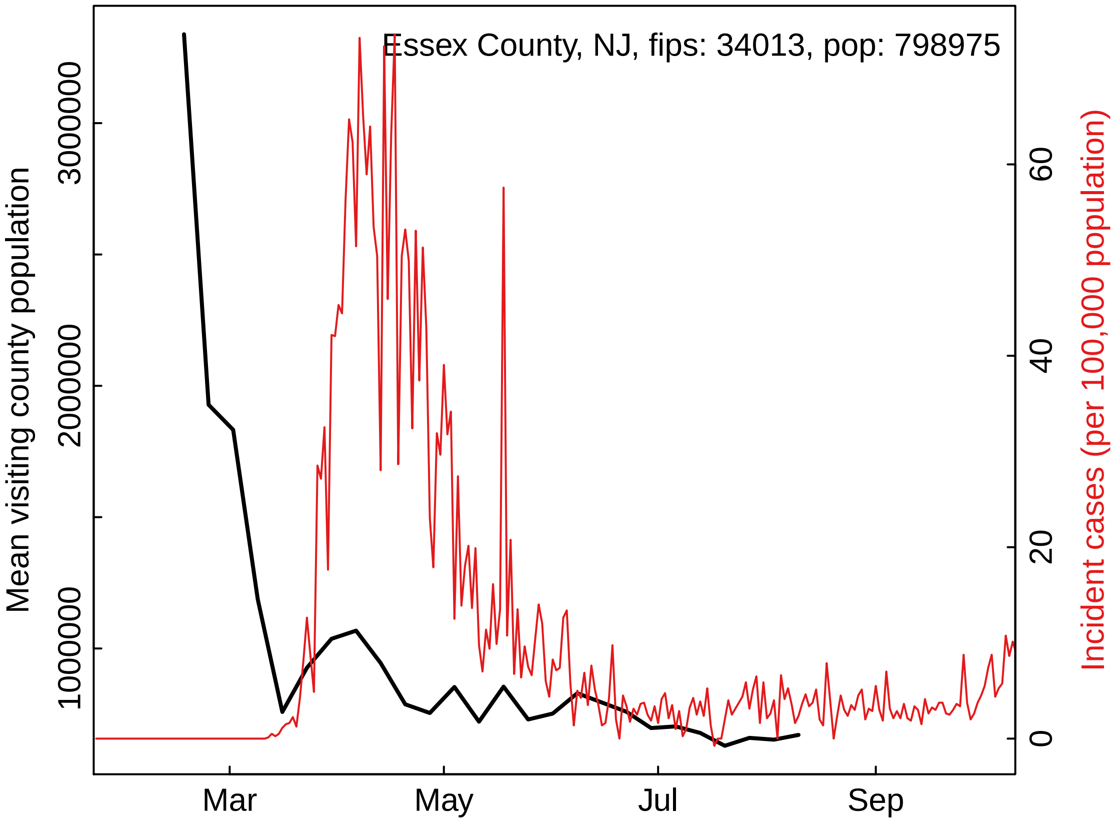

Essex County, NJ, fips: 34013, pop: 798975
Mean visiting county population
1000000
2000000
3000000
Incident cases (per 100,000 population)
0
20
40
60
Mar
May
Jul
Sep

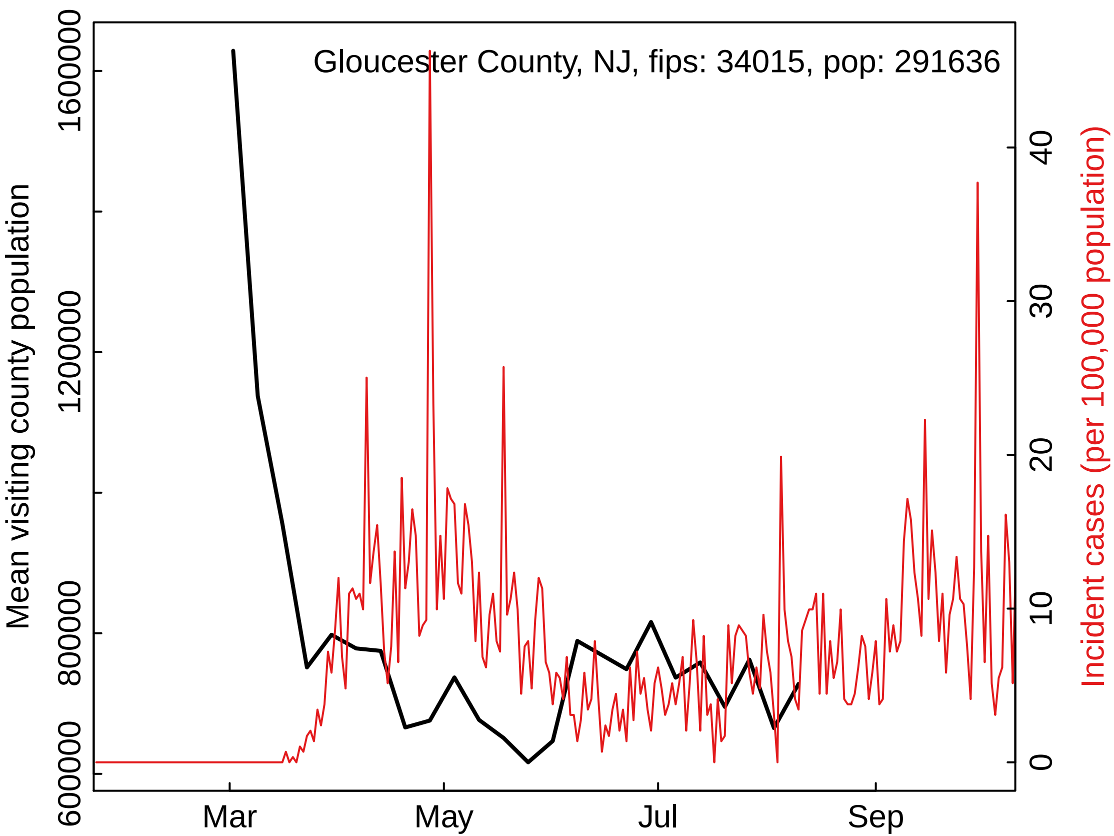

Gloucester County, NJ, fips: 34015, pop: 291636
Mean visiting county population
600000
800000
1200000
1600000
Incident cases (per 100,000 population)
0
10
20
30
40
Mar
May
Jul
Sep

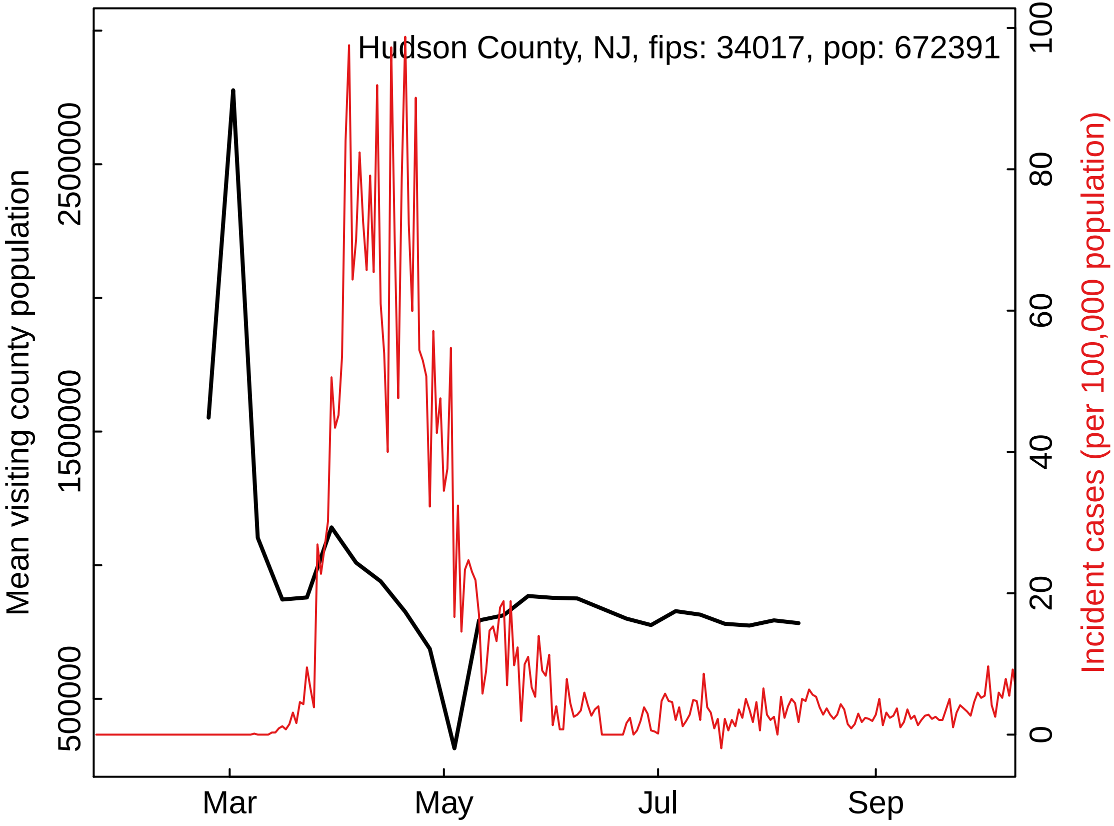

Hudson County, NJ, fips: 34017, pop: 672391
Mean visiting county population
500000
1500000
2500000
Incident cases (per 100,000 population)
0
20
40
60
80
100
Mar
May
Jul
Sep

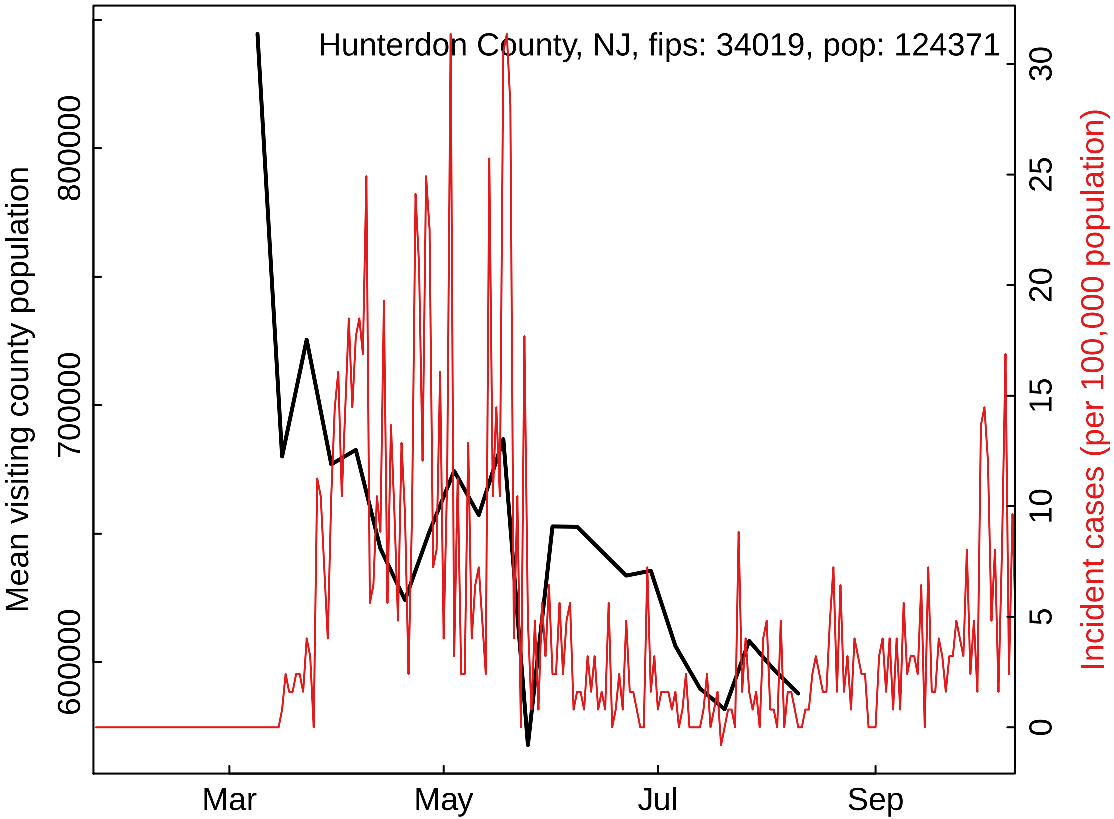

Hunterdon County, NJ, fips: 34019, pop: 124371
Mean visiting county population
600000
700000
800000
Incident cases (per 100,000 population)
0
5
10
15
20
25
30
Mar
May
Jul
Sep

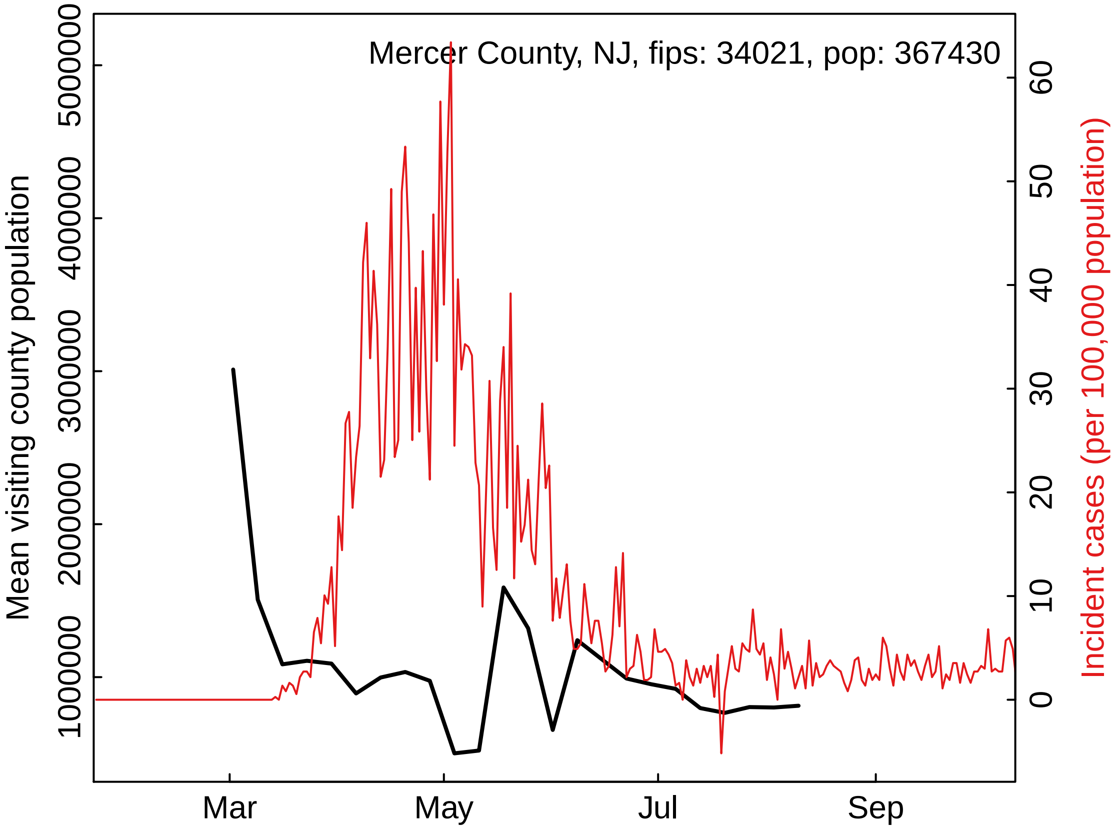

Mercer County, NJ, fips: 34021, pop: 367430
Mean visiting county population
1000000
2000000
3000000
4000000
5000000
Incident cases (per 100,000 population)
0
10
20
30
40
50
60
Mar
May
Jul
Sep

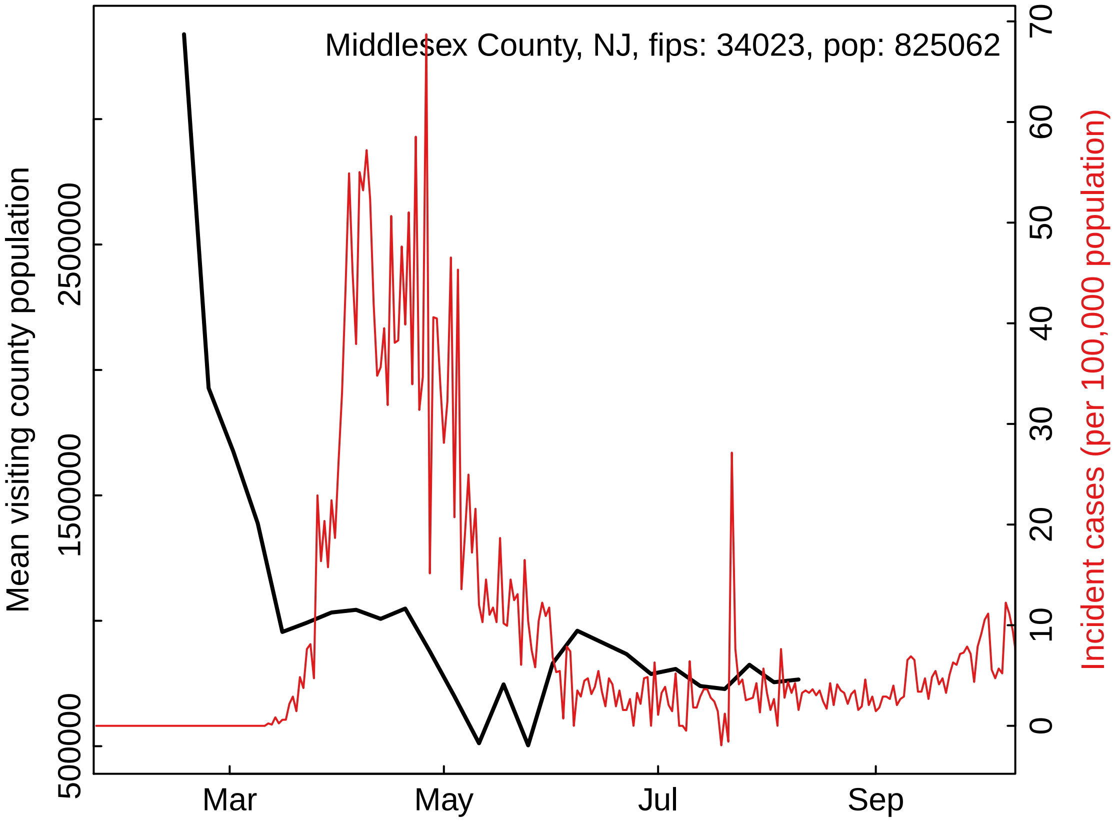
Middlesex County, NJ, fips: 34023, pop: 825062
Mean visiting county population
500000
1500000
2500000
Mar
May
Jul
Sep
0
10
20
30
40
50
60
70
Incident cases (per 100,000 population)

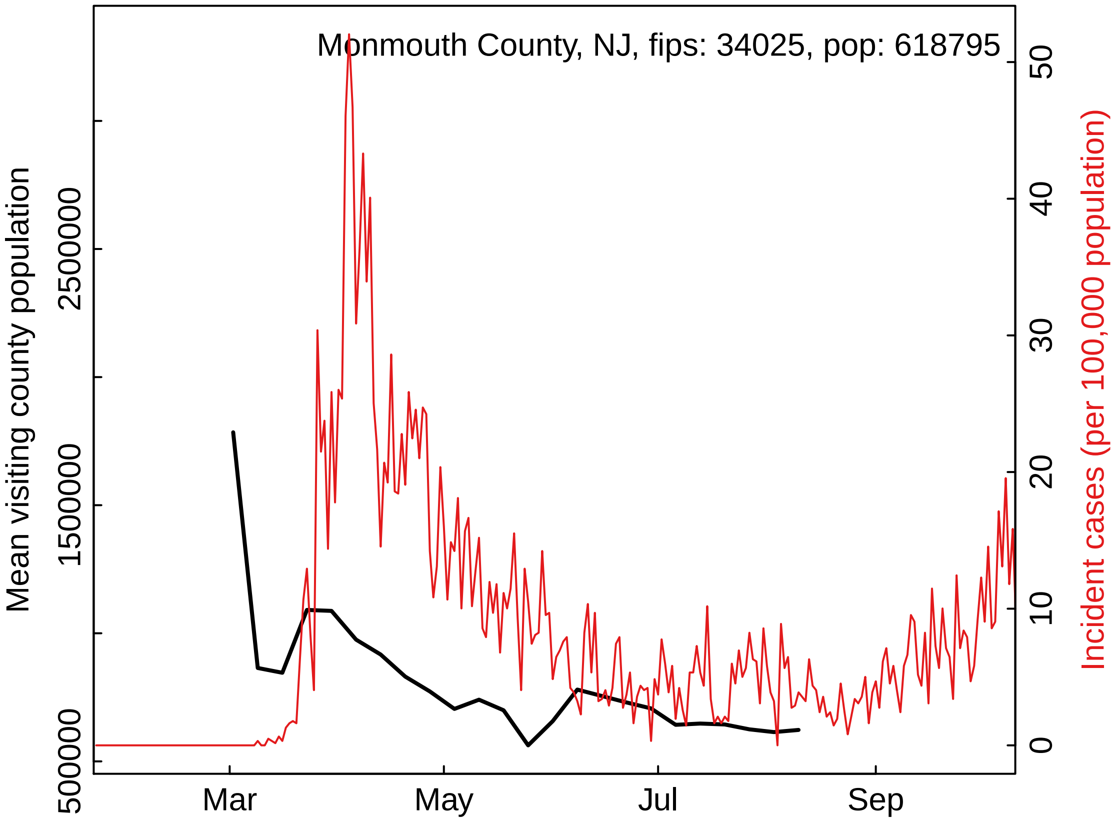

Monmouth County, NJ, fips: 34025, pop: 618795
Mean visiting county population
500000
1500000
2500000
Incident cases (per 100,000 population)
0
10
20
30
40
50
Mar
May
Jul
Sep

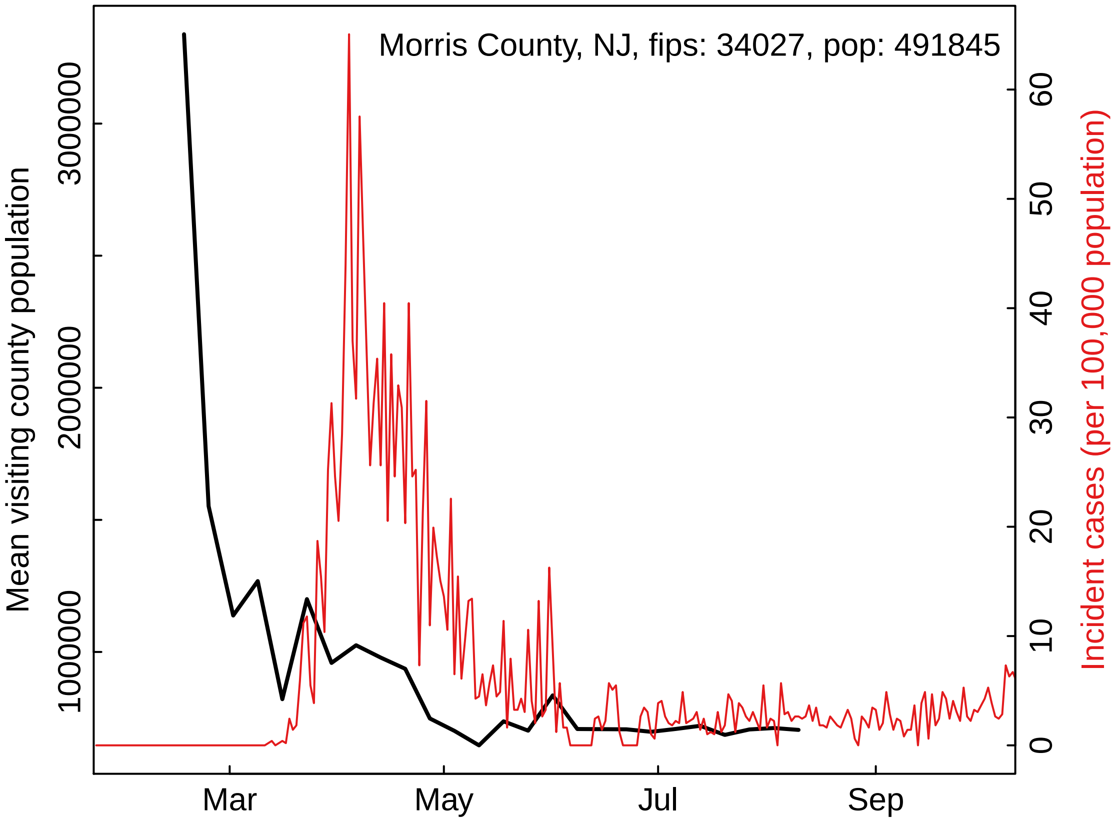

Morris County, NJ, fips: 34027, pop: 491845
Mean visiting county population
1000000
2000000
3000000
Incident cases (per 100,000 population)
0
10
20
30
40
50
60
Mar
May
Jul
Sep

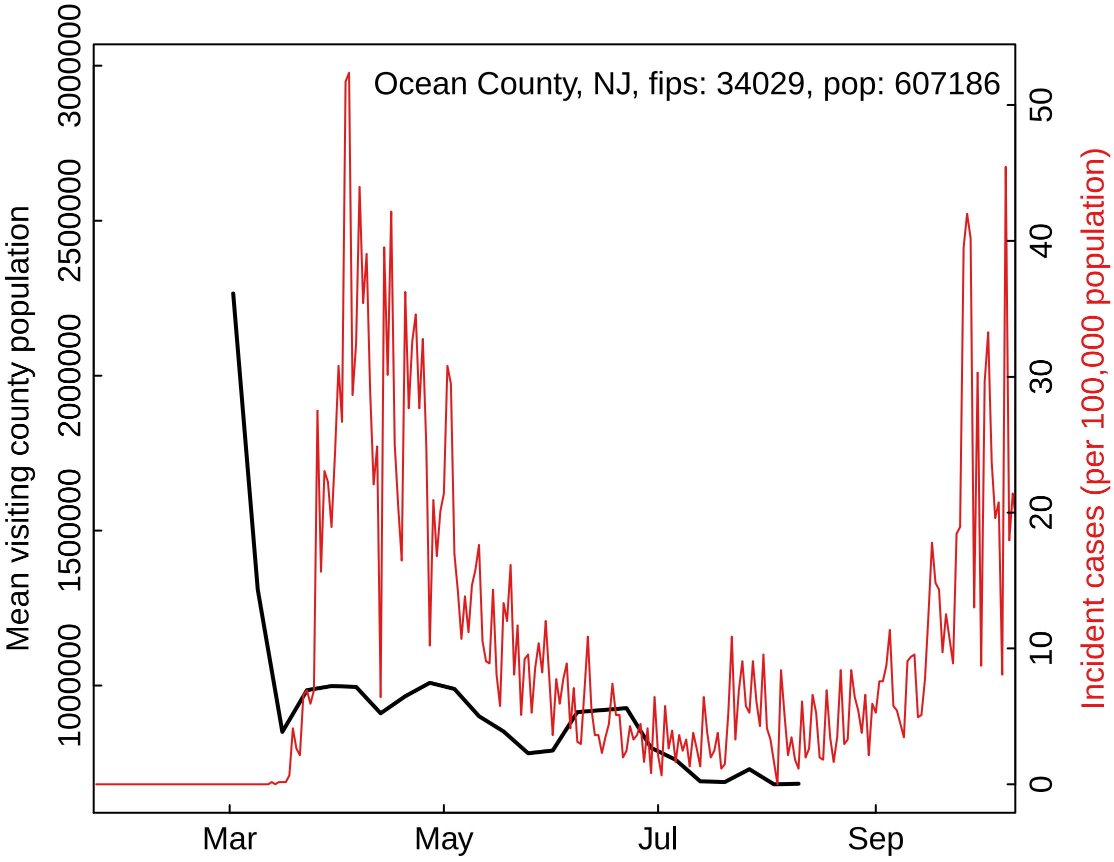
Ocean County, NJ, fips: 34029, pop: 607186
Mean visiting county population
1000000
1500000
2000000
2500000
3000000
Incident cases (per 100,000 population)
0
10
20
30
40
50
Mar
May
Jul
Sep

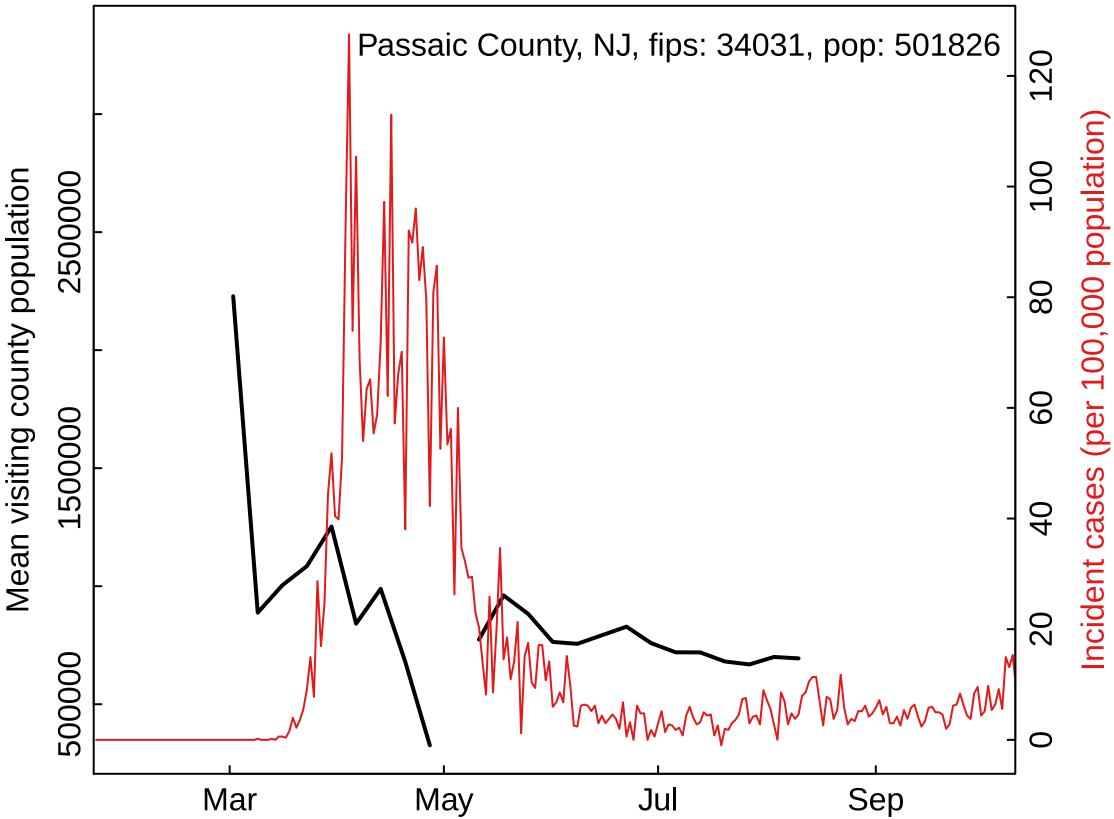

Passaic County, NJ, fips: 34031, pop: 501826
Mean visiting county population
500000
1500000
2500000
Incident cases (per 100,000 population)
0
20
40
60
80
100
120
Mar
May
Jul
Sep

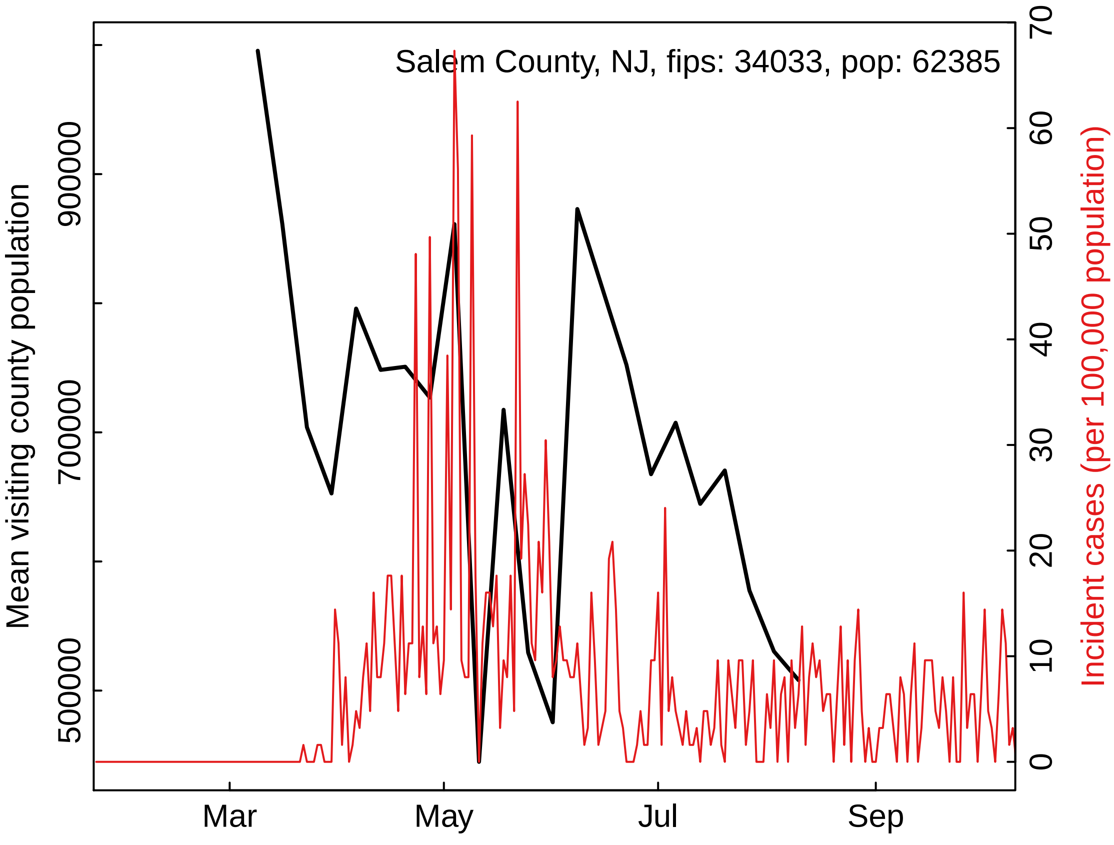

Salem County, NJ, fips: 34033, pop: 62385
Mean visiting county population
500000
700000
900000
Incident cases (per 100,000 population)
0
10
20
30
40
50
60
70
Mar
May
Jul
Sep

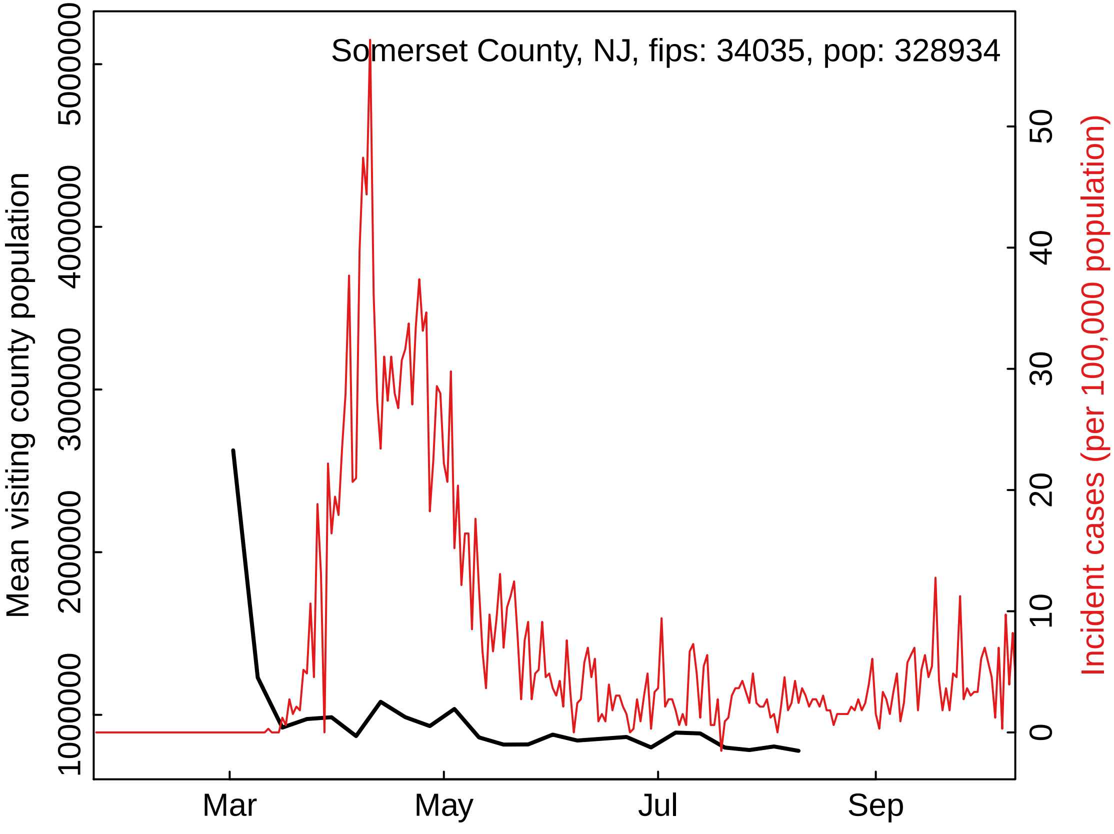

Somerset County, NJ, fips: 34035, pop: 328934
Mean visiting county population
Incident cases (per 100,000 population)
10000000
20000000
30000000
40000000
50000000
0
10
20
30
40
50
Mar
May
Jul
Sep

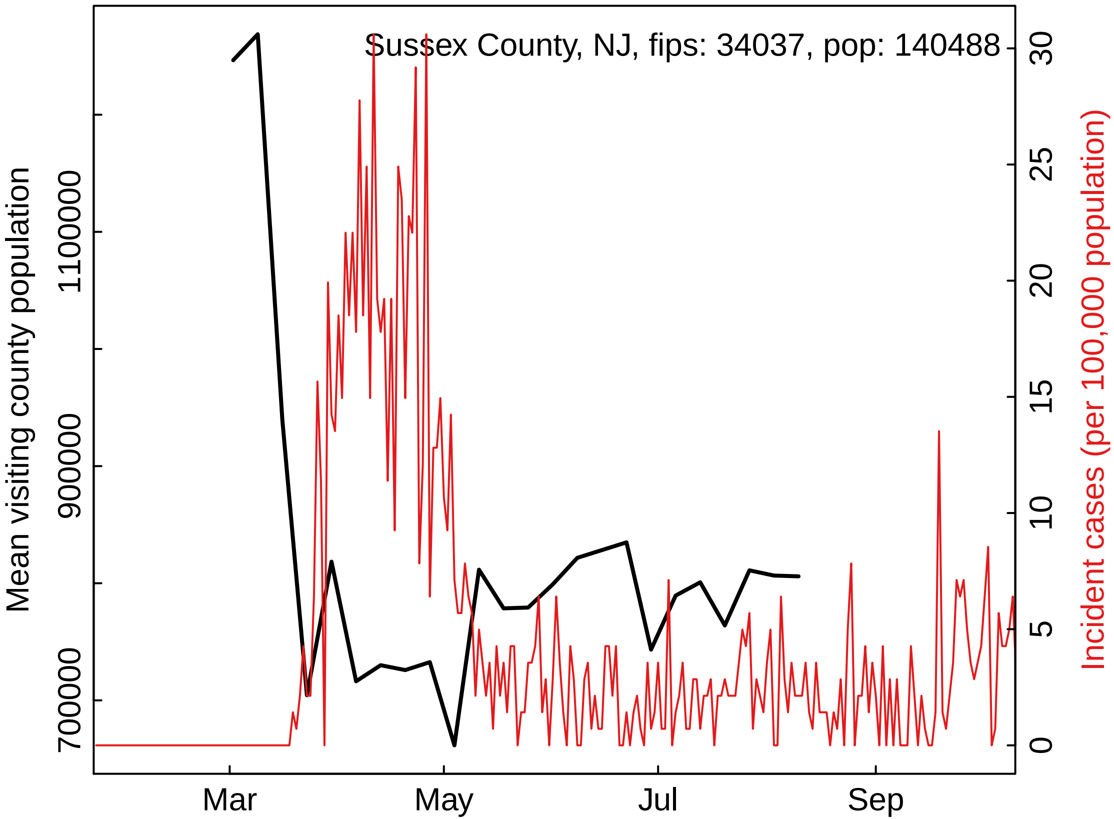
Sussex County, NJ, fips: 34037, pop: 140488
Mean visiting county population
700000
900000
1100000
Incident cases (per 100,000 population)
0
5
10
15
20
25
30
Mar
May
Jul
Sep

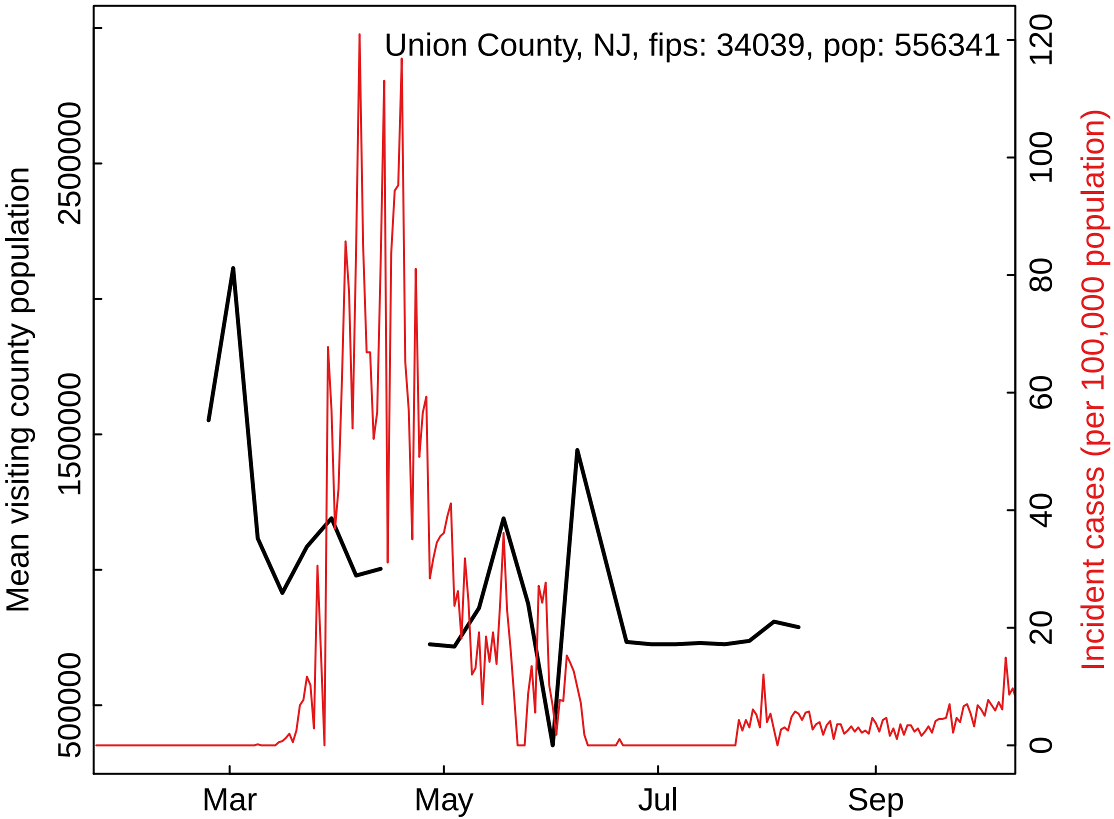

Union County, NJ, fips: 34039, pop: 556341
Mean visiting county population
500000
1500000
2500000
Incident cases (per 100,000 population)
0
20
40
60
80
100
120
Mar
May
Jul
Sep

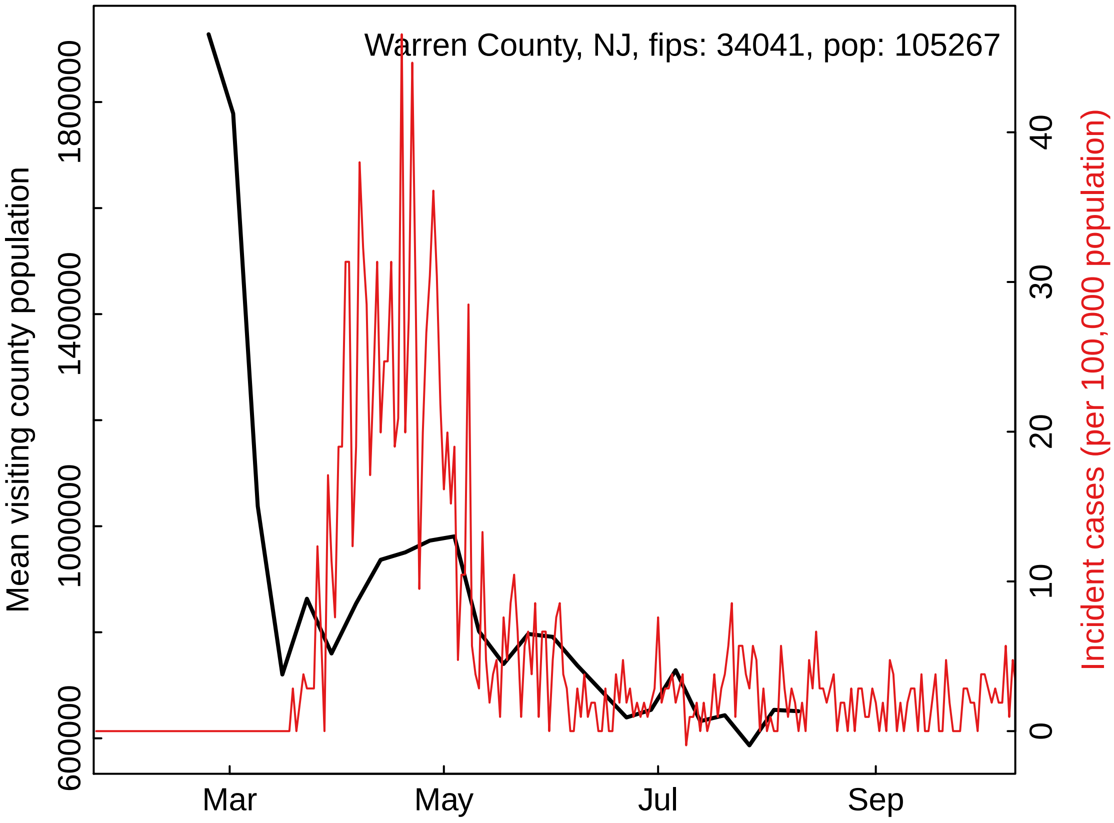
Warren County, NJ, fips: 34041, pop: 105267
Mean visiting county population
600000
1000000
1400000
1800000
Incident cases (per 100,000 population)
0
10
20
30
40
Mar
May
Jul
Sep

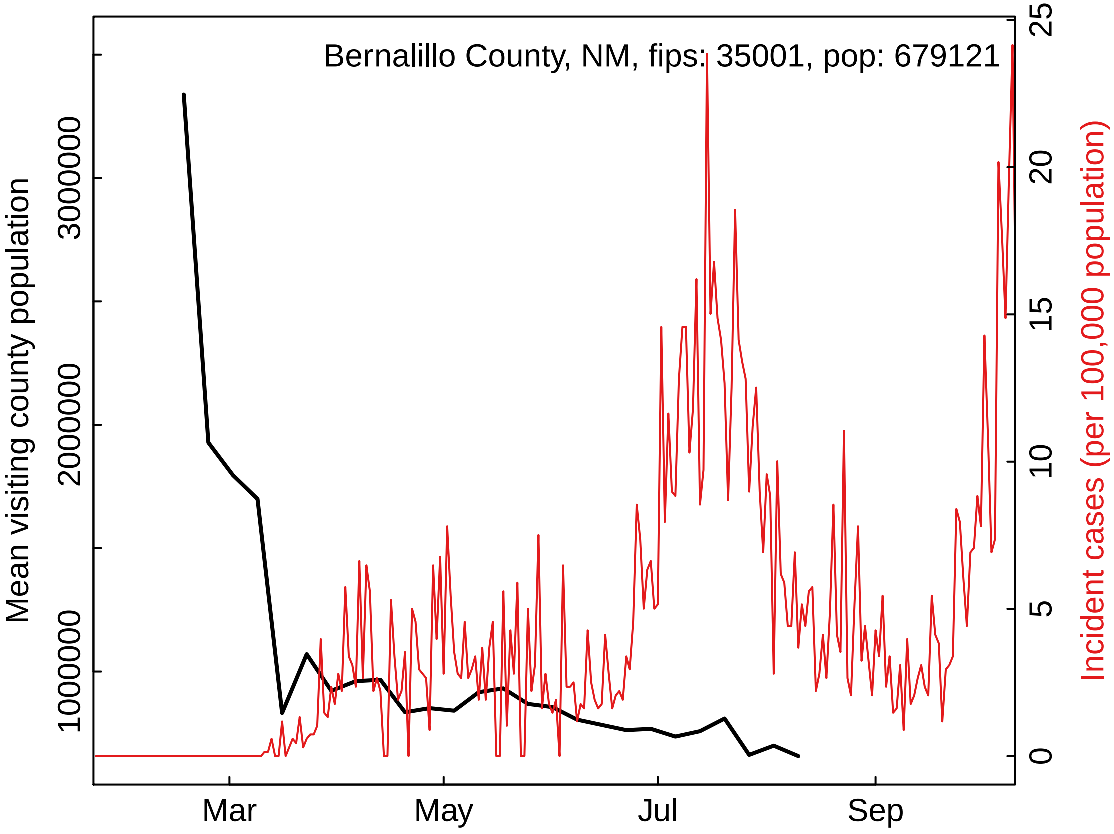

Bernalillo County, NM, fips: 35001, pop: 679121
Mean visiting county population
10000000
20000000
30000000
Incident cases (per 100,000 population)
0
5
10
15
20
25
Mar
May
Jul
Sep

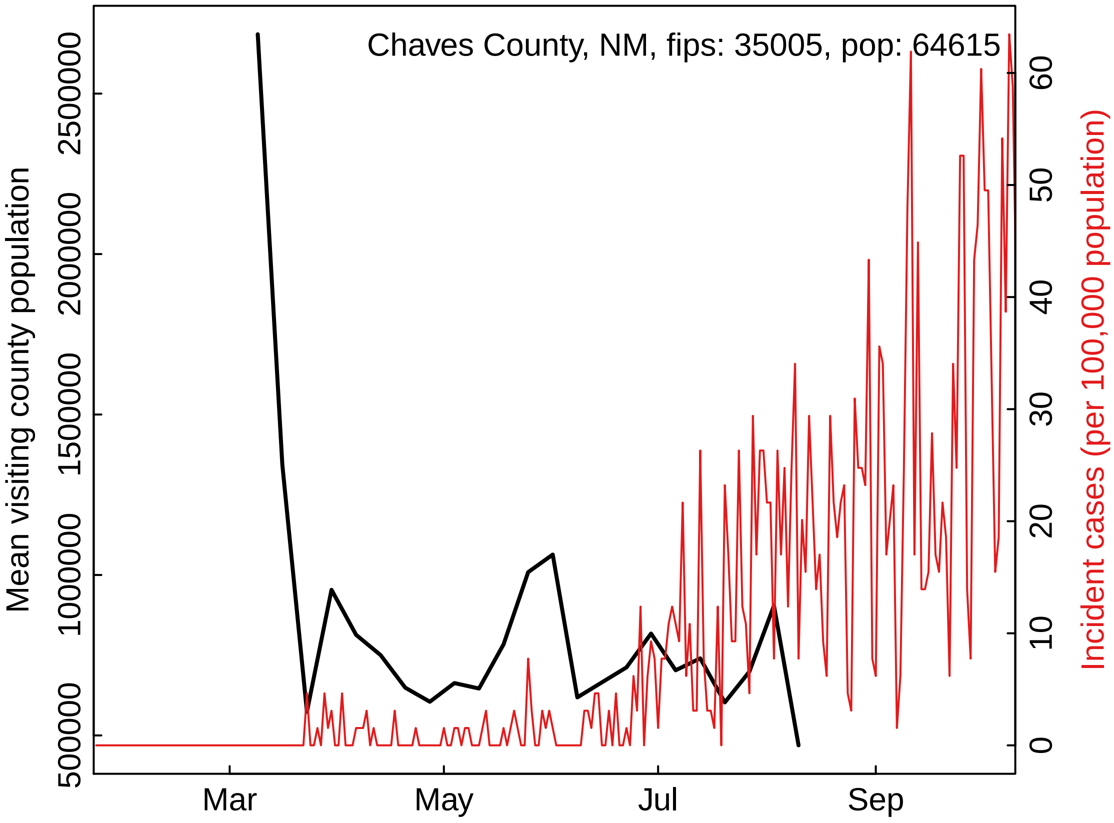
Chaves County, NM, fips: 35005, pop: 64615
Mean visiting county population
Incident cases (per 100,000 population)
500000
1000000
1500000
2000000
2500000
0
10
20
30
40
50
60
Mar
May
Jul
Sep

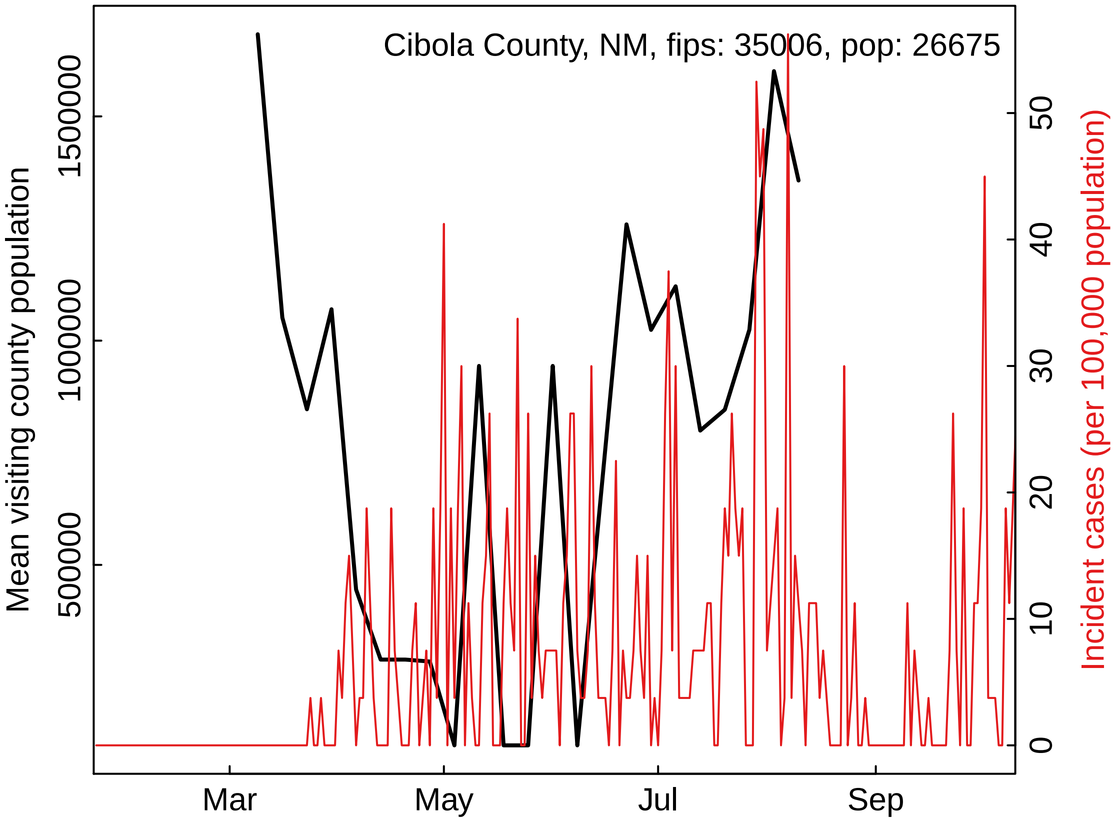

Cibola County, NM, fips: 35006, pop: 26675
Mean visiting county population
500000
1000000
1500000
Incident cases (per 100,000 population)
0
10
20
30
40
50
Mar
May
Jul
Sep

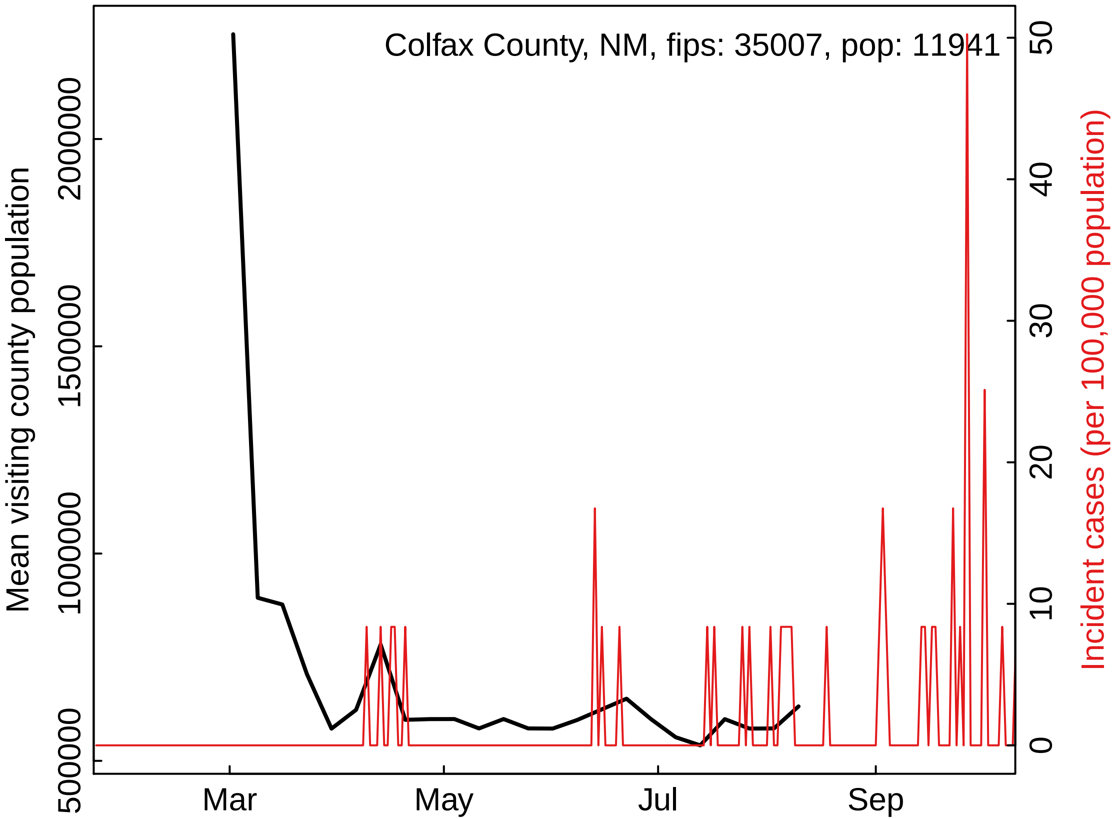

Colfax County, NM, fips: 35007, pop: 11941
Mean visiting county population
5000000
10000000
15000000
20000000
Mar
May
Jul
Sep
0
10
20
30
40
50
Incident cases (per 100,000 population)

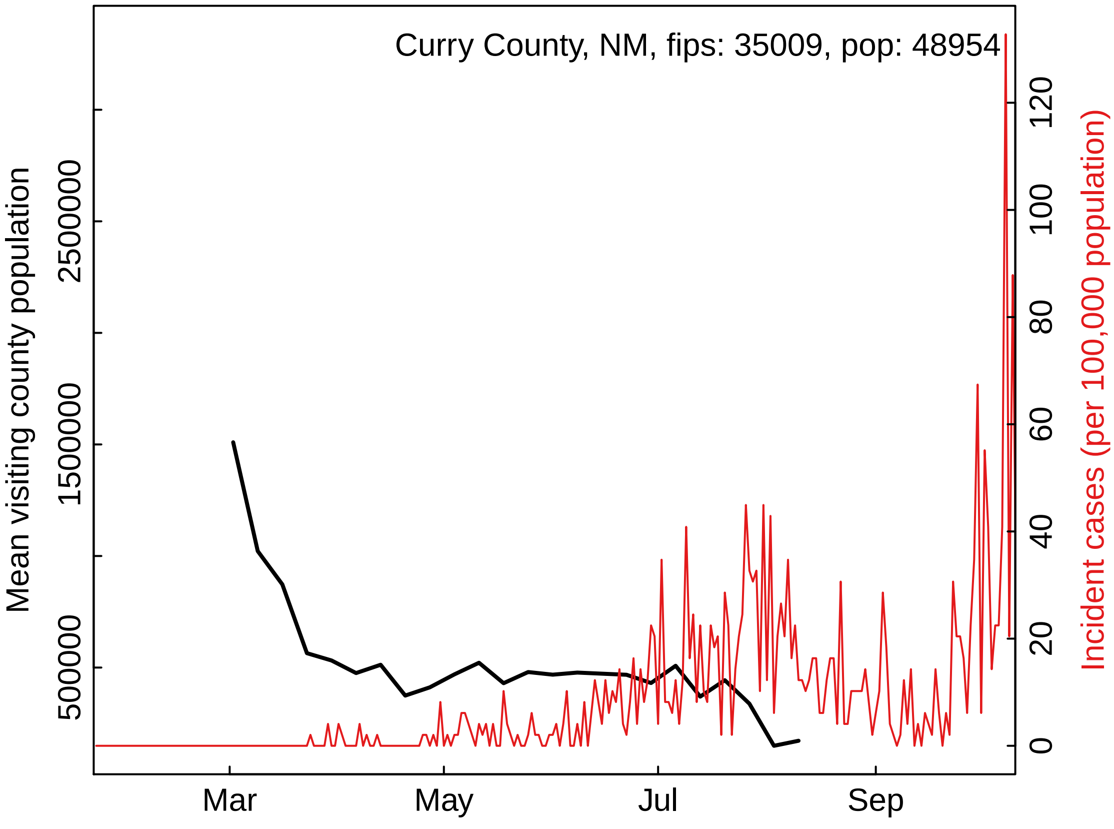

Curry County, NM, fips: 35009, pop: 48954
Mean visiting county population
500000
1500000
2500000
Incident cases (per 100,000 population)
0
20
40
60
80
100
120
Mar
May
Jul
Sep

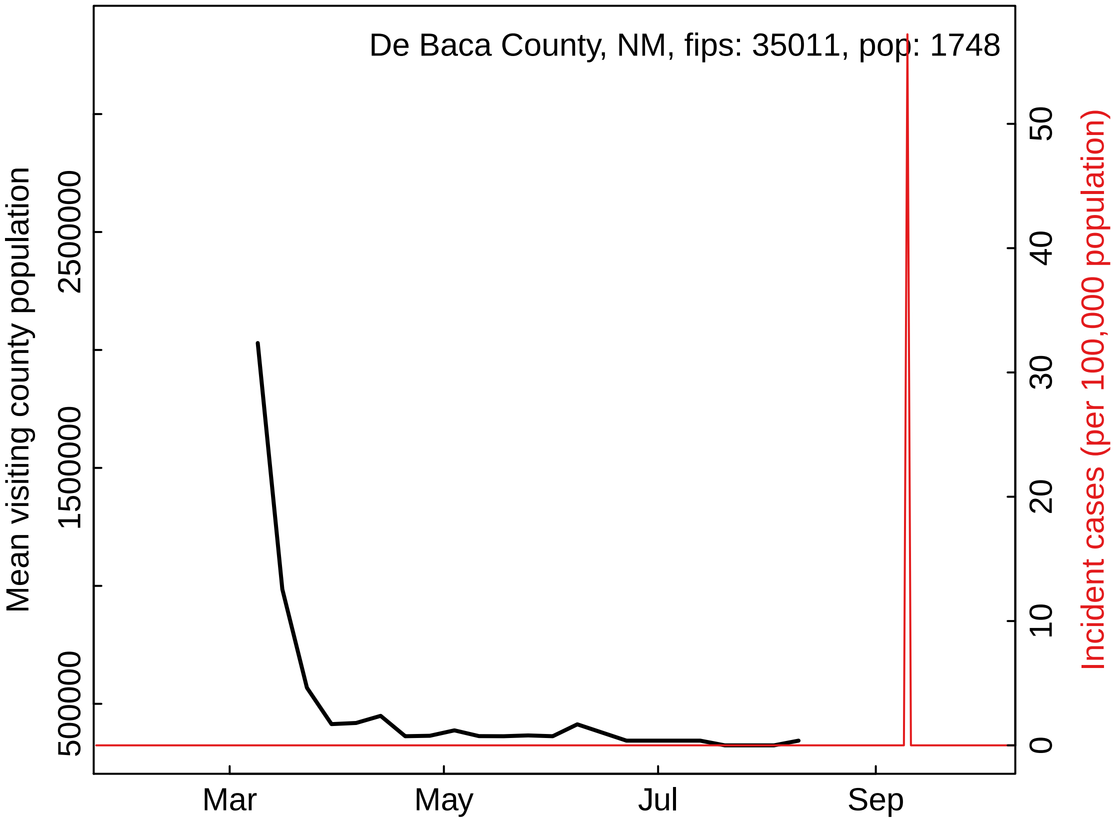

De Baca County, NM, fips: 35011, pop: 1748
Mean visiting county population
500000
1500000
2500000
Mar
May
Jul
Sep
0
10
20
30
40
50
Incident cases (per 100,000 population)

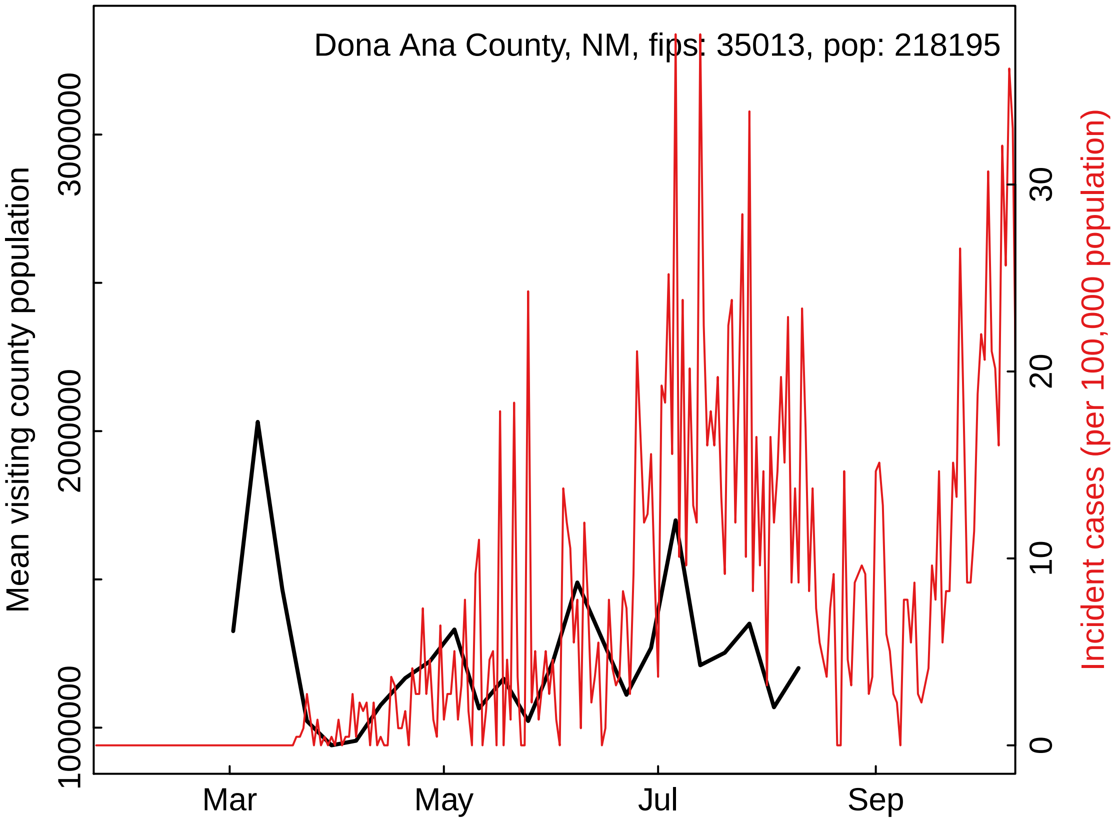
Dona Ana County, NM, fips: 35013, pop: 218195
Mean visiting county population
1000000
2000000
3000000
Incident cases (per 100,000 population)
0
10
20
30
Mar
May
Jul
Sep

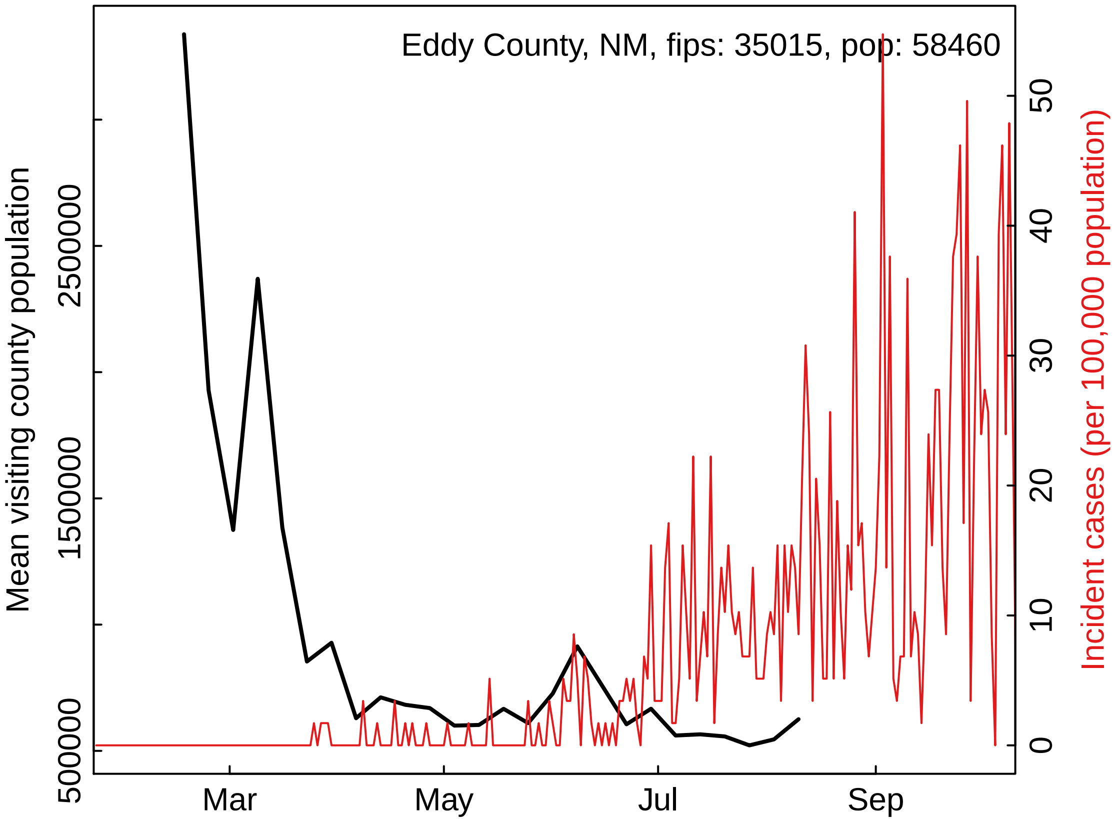
Eddy County, NM, fips: 35015, pop: 58460
Mean visiting county population
5000000
15000000
25000000
Mar
May
Jul
Sep
Incident cases (per 100,000 population)
0
10
20
30
40
50

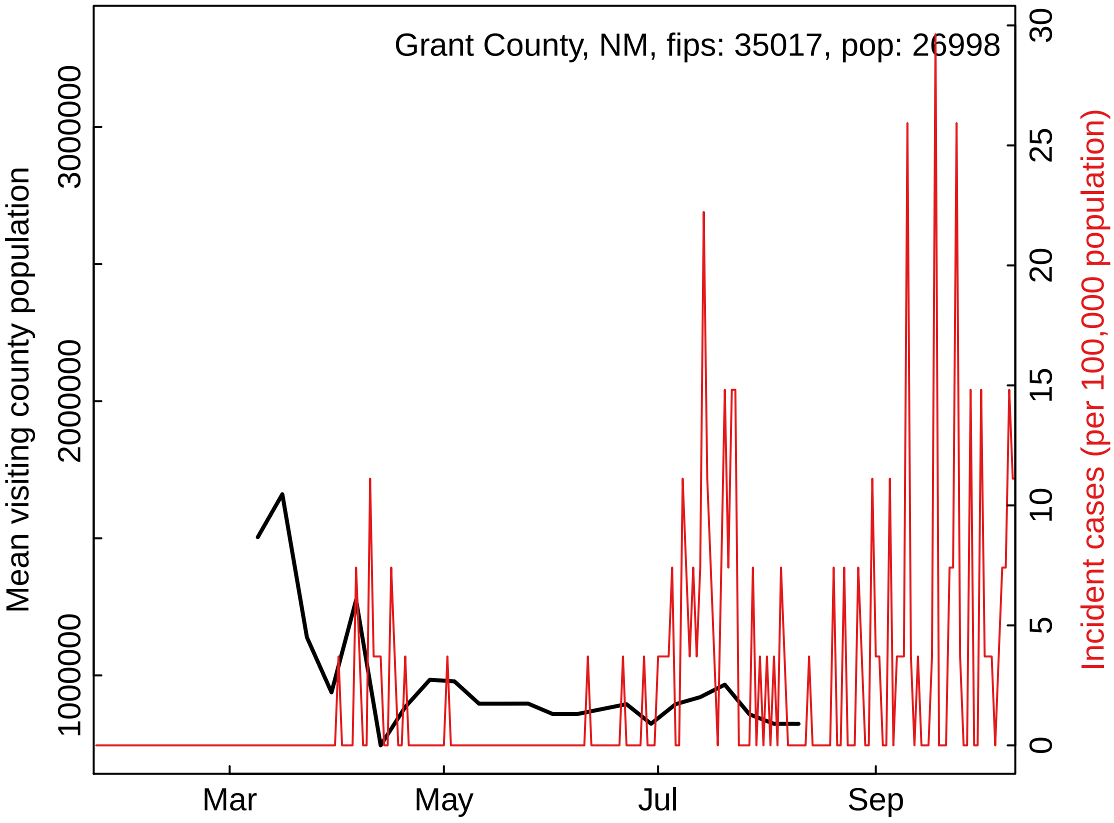
Grant County, NM, fips: 35017, pop: 26998
Mean visiting county population
1000000
2000000
3000000
Incident cases (per 100,000 population)
0
5
10
15
20
25
30
Mar
May
Jul
Sep

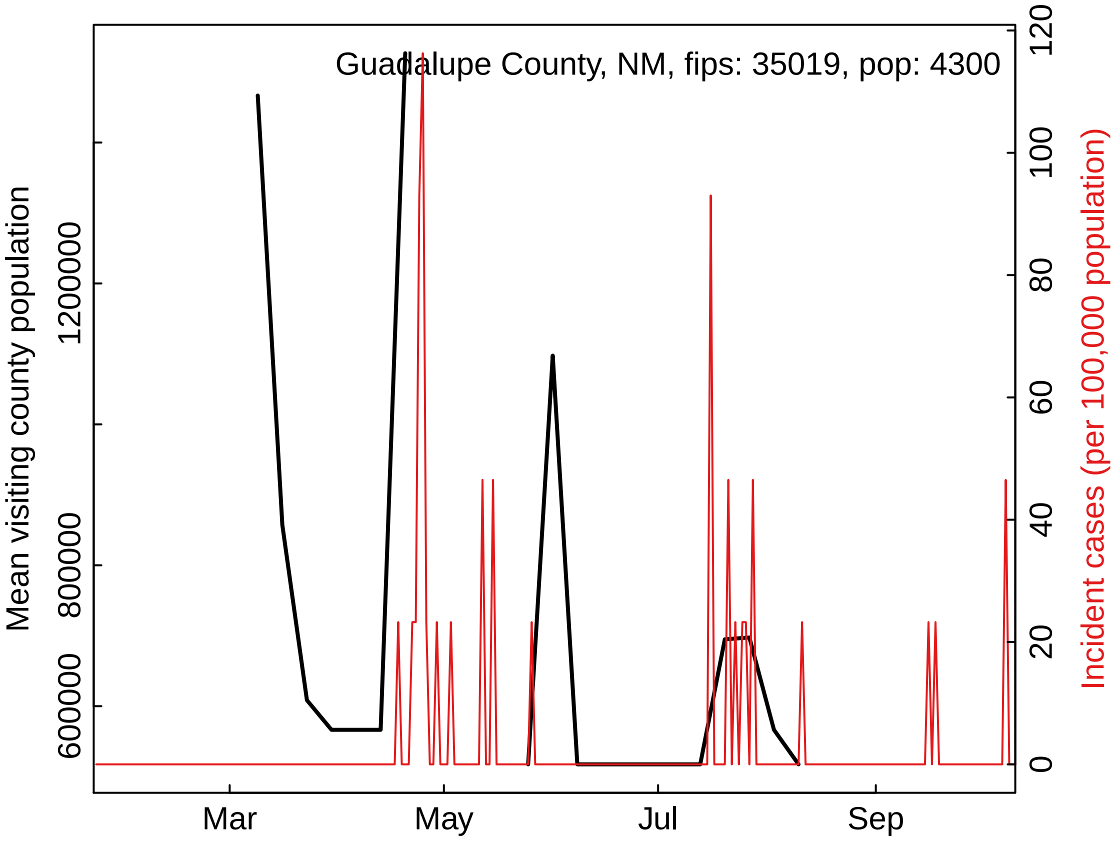

Guadalupe County, NM, fips: 35019, pop: 4300
Mean visiting county population
600000
800000
1200000
Incident cases (per 100,000 population)
0
20
40
60
80
100
120
Mar
May
Jul
Sep

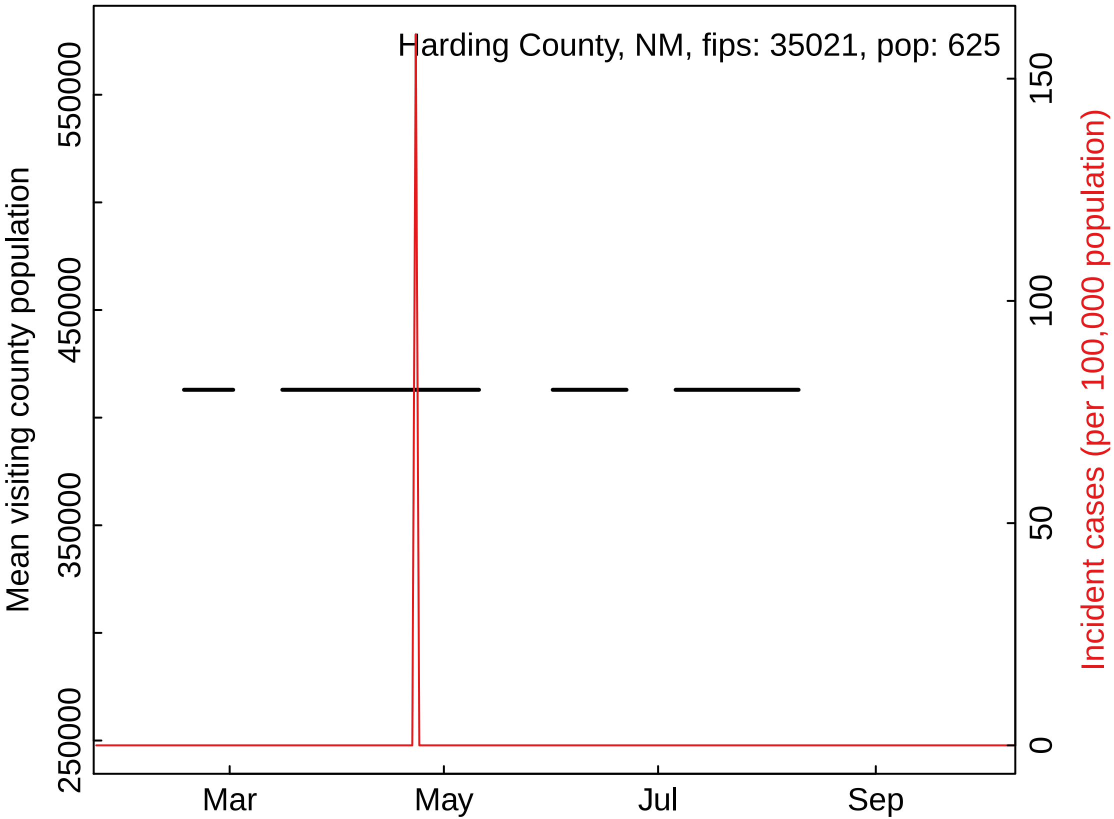

Harding County, NM, fips: 35021, pop: 625
Mean visiting county population
250000
350000
450000
550000
Incident cases (per 100,000 population)
0
50
100
150
Mar
May
Jul
Sep

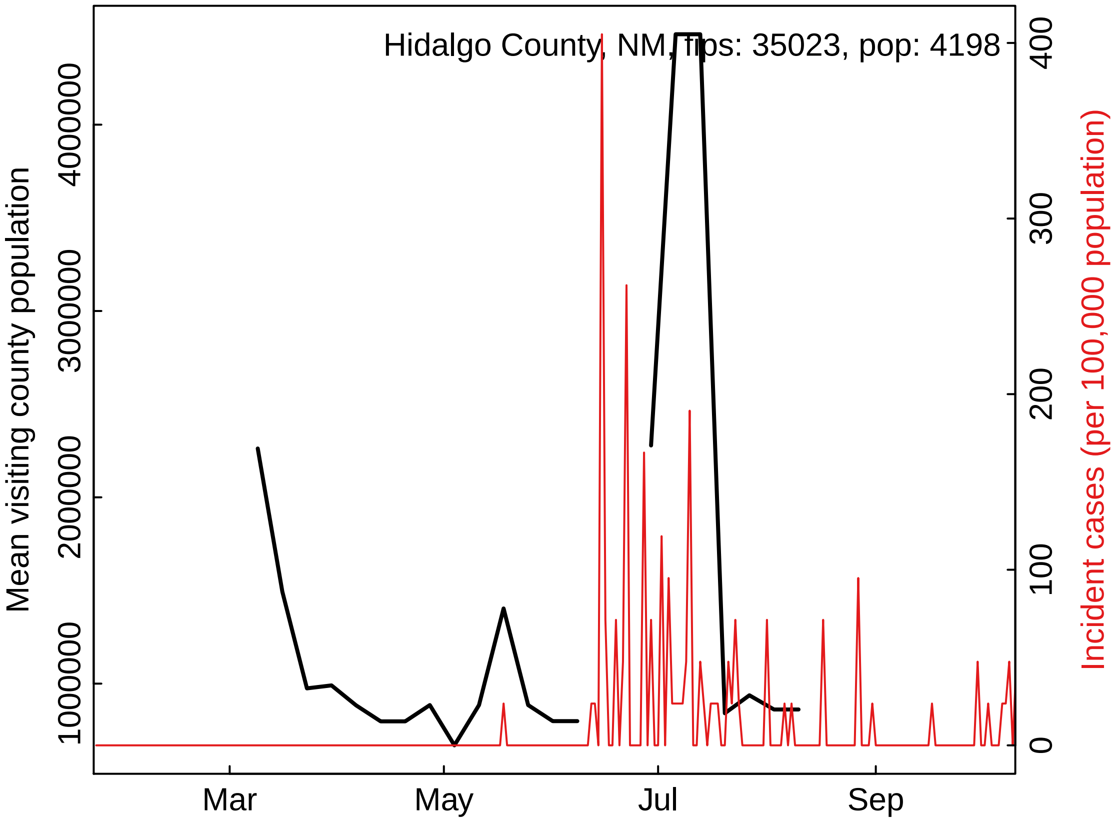
Hidalgo County, NM, fips: 35023, pop: 4198
Mean visiting county population
1000000
2000000
3000000
4000000
Incident cases (per 100,000 population)
0
100
200
300
400
Mar
May
Jul
Sep

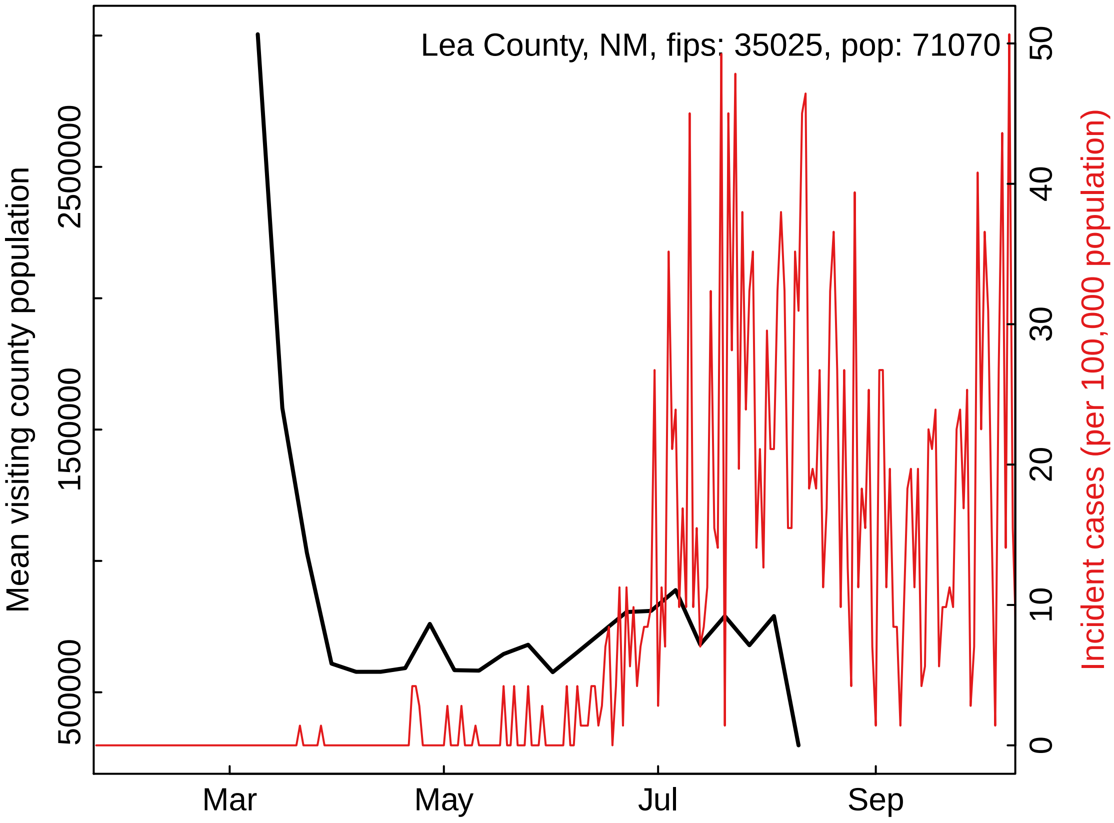

Lea County, NM, fips: 35025, pop: 71070
Mean visiting county population
500000
1500000
2500000
Mar
May
Jul
Sep
0
10
20
30
40
50
Incident cases (per 100,000 population)

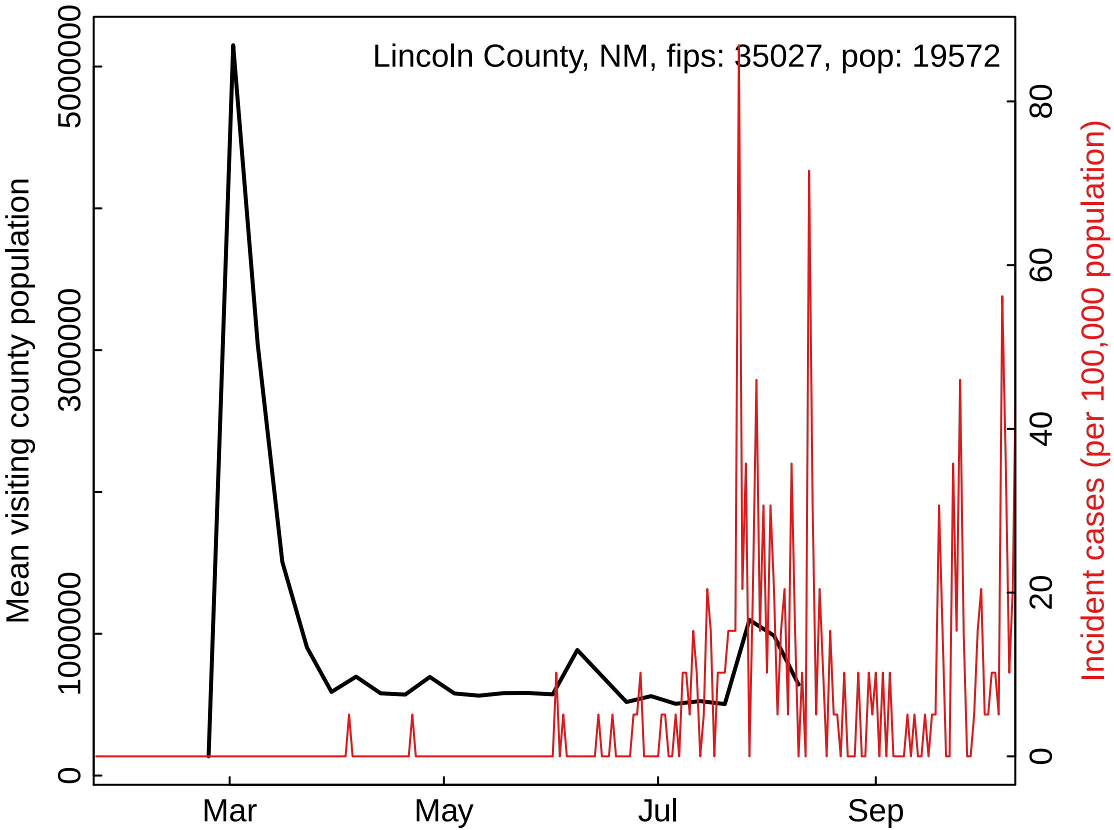

Lincoln County, NM, fips: 35027, pop: 19572
Mean visiting county population
0
1000000
3000000
5000000
Incident cases (per 100,000 population)
0
20
40
60
80
Mar
May
Jul
Sep

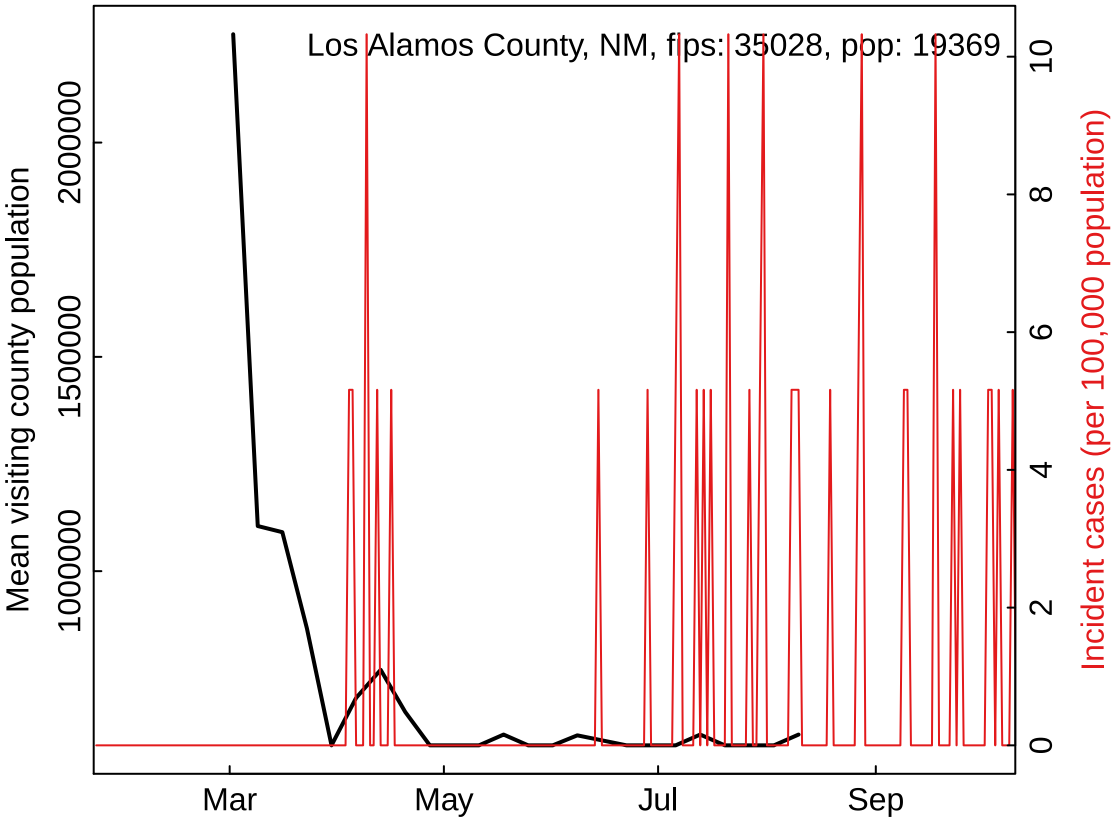

Los Alamos County, NM, fips: 35028, pop: 19369
Mean visiting county population
1000000
1500000
2000000
Incident cases (per 100,000 population)
0
2
4
6
8
10
Mar
May
Jul
Sep

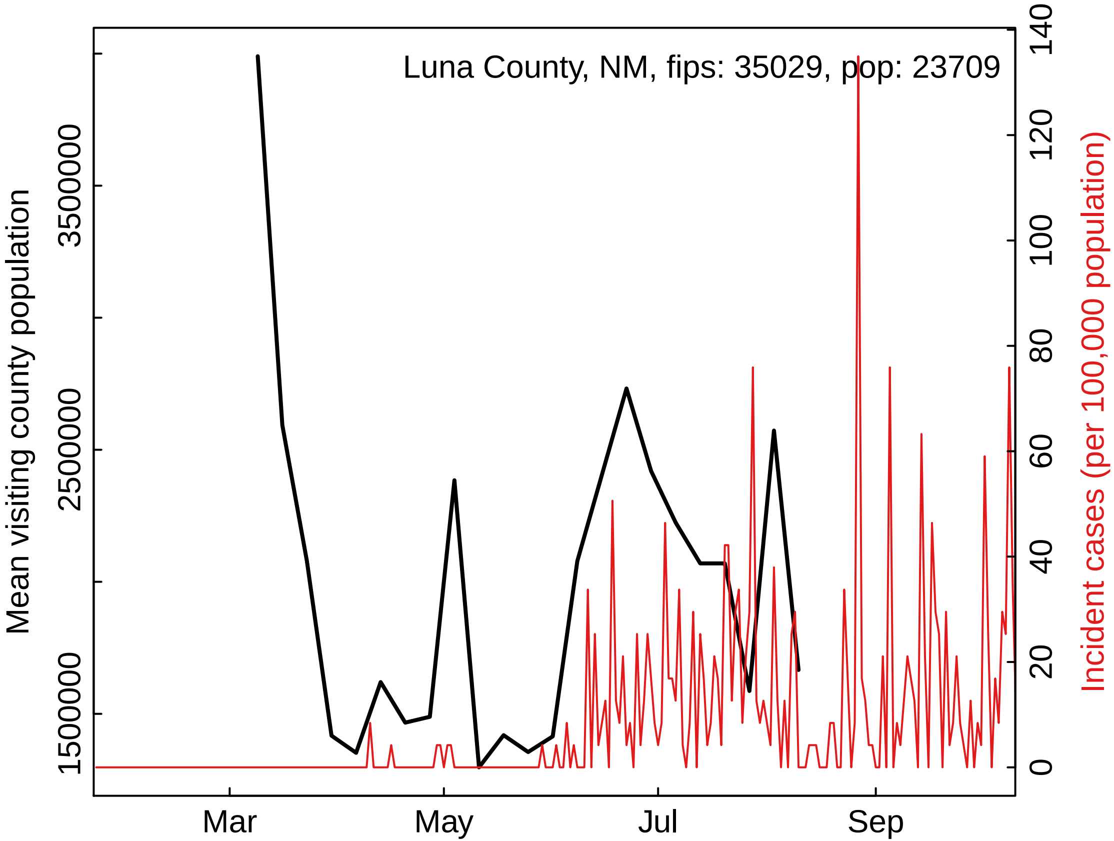

Luna County, NM, fips: 35029, pop: 23709
Mean visiting county population
1500000
2500000
3500000
Mar
May
Jul
Sep
0
20
40
60
80
100
120
140
Incident cases (per 100,000 population)

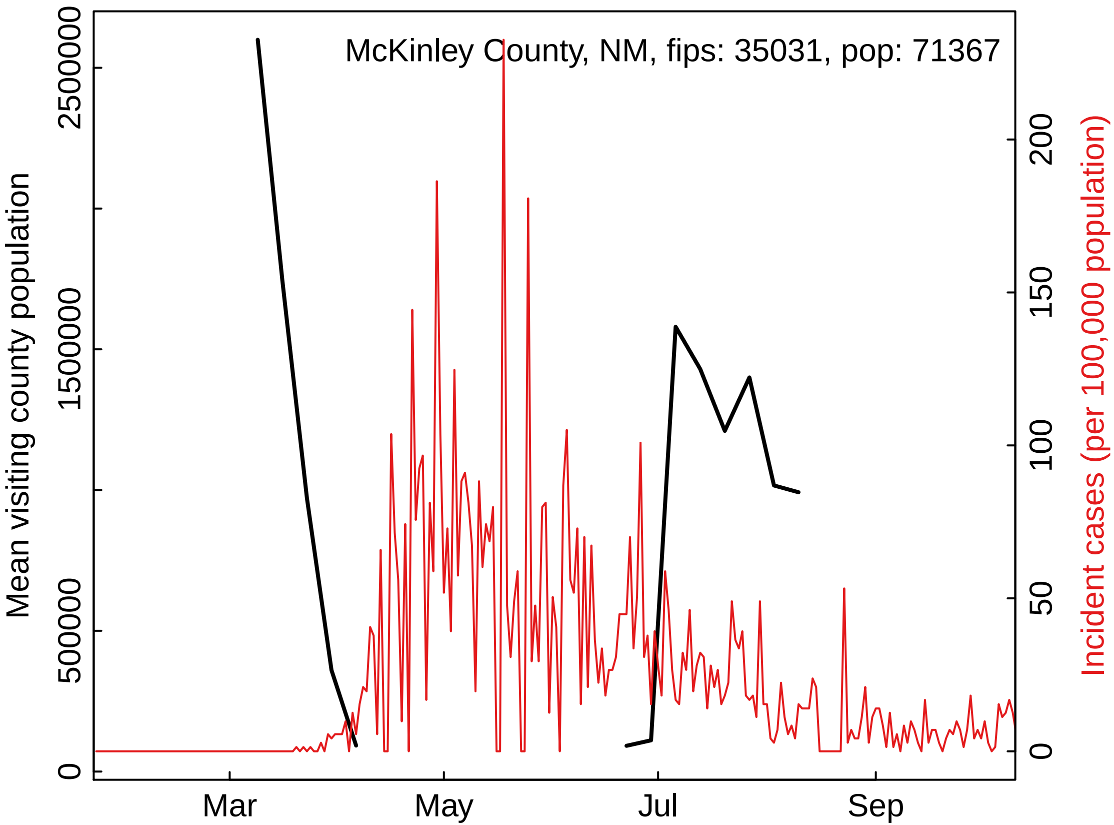

McKinley County, NM, fips: 35031, pop: 71367
Mean visiting county population
0
500000
1500000
2500000
Incident cases (per 100,000 population)
0
50
100
150
200
Mar
May
Jul
Sep

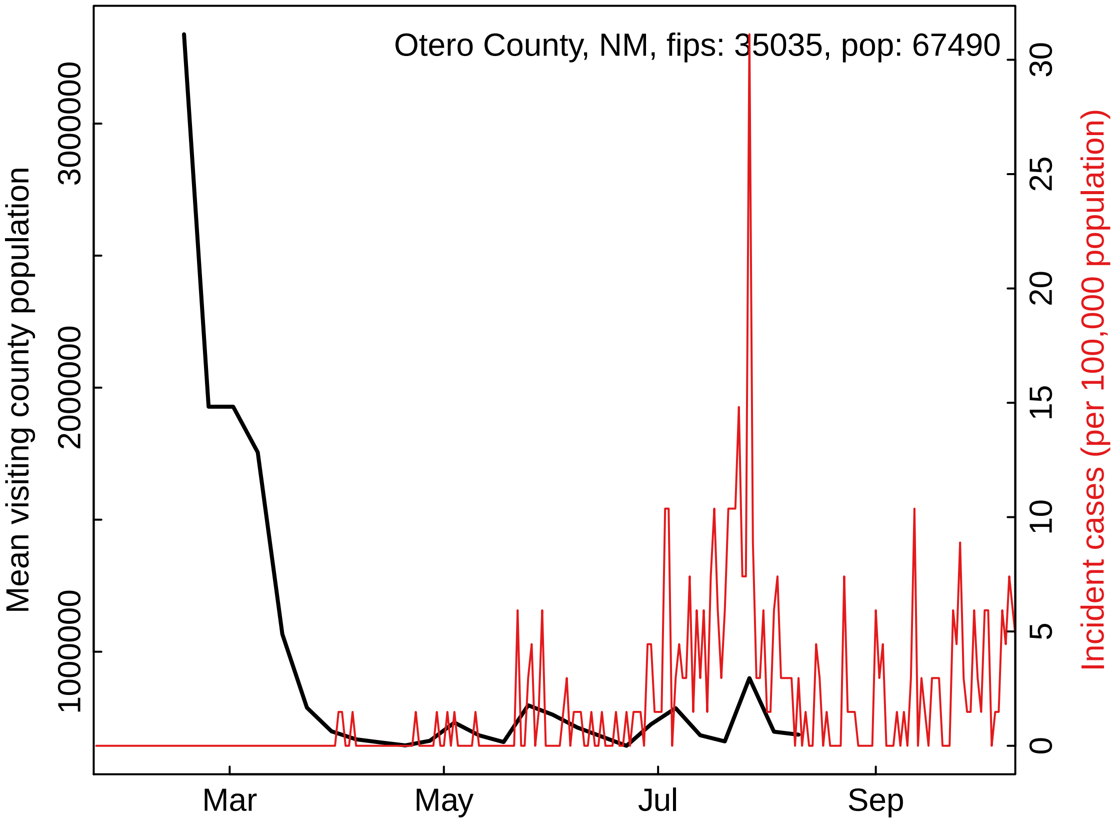

Otero County, NM, fips: 35035, pop: 67490
Mean visiting county population
10000000
2000000
3000000
Incident cases (per 100,000 population)
0
5
10
15
20
25
30
Mar
May
Jul
Sep

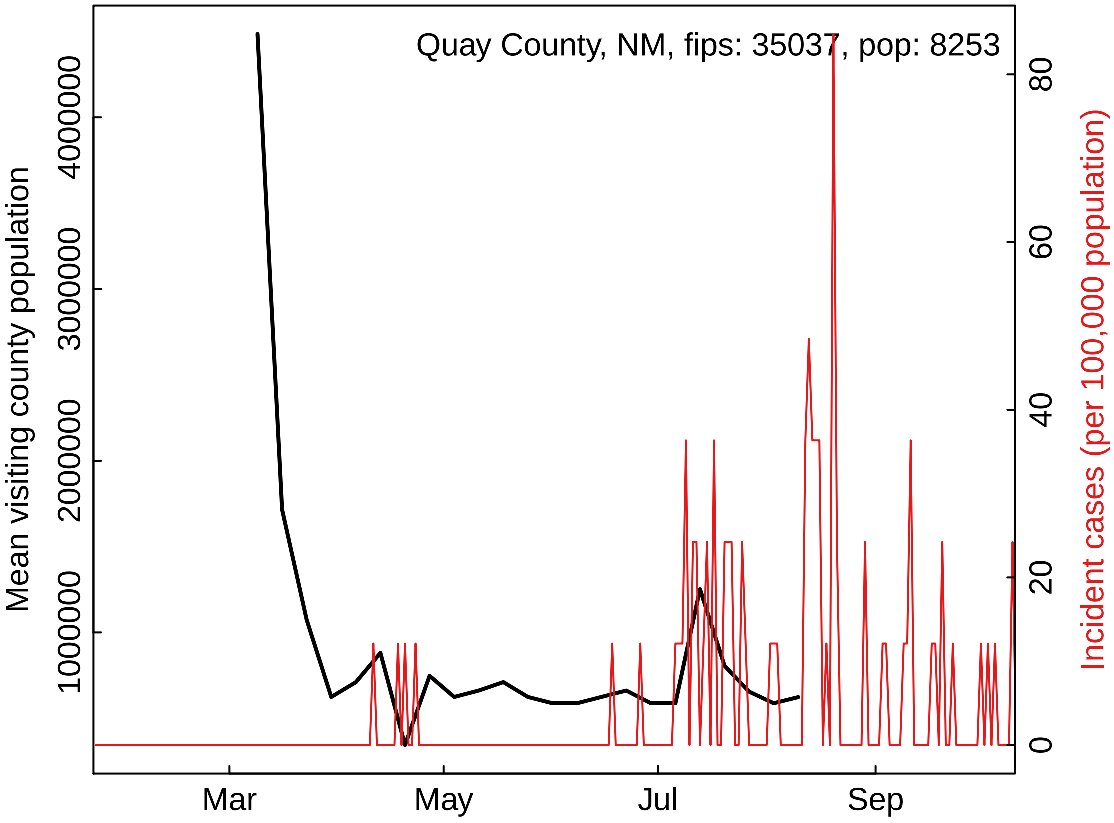

Quay County, NM, fips: 35037, pop: 8253
Mean visiting county population
10000000
20000000
30000000
40000000
Incident cases (per 100,000 population)
0
20
40
60
80
Mar
May
Jul
Sep

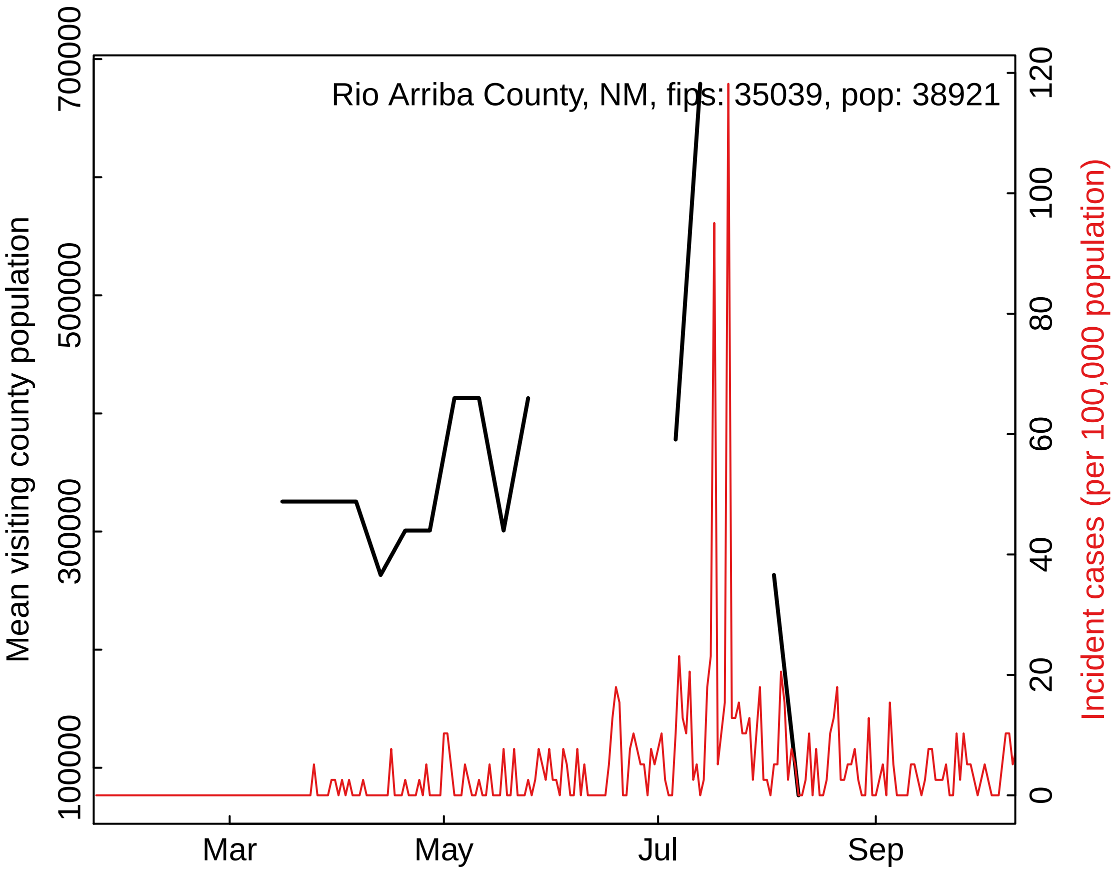
Rio Arriba County, NM, fips: 35039, pop: 38921
Mean visiting county population
100000
300000
500000
700000
Incident cases (per 100,000 population)
0
20
40
60
80
100
120
Mar
May
Jul
Sep

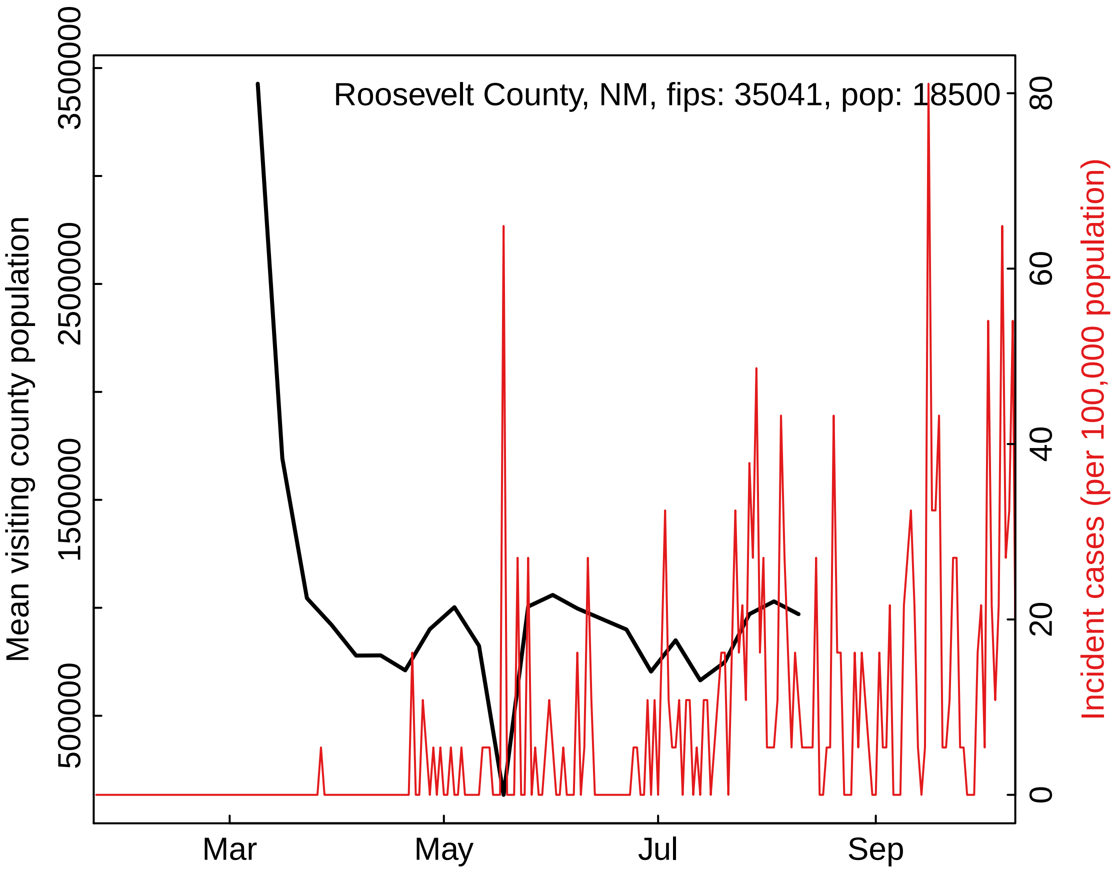
Roosevelt County, NM, fips: 35041, pop: 18500
Mean visiting county population
Incident cases (per 100,000 population)
500000
1500000
2500000
3500000
0
20
40
60
80
Mar
May
Jul
Sep

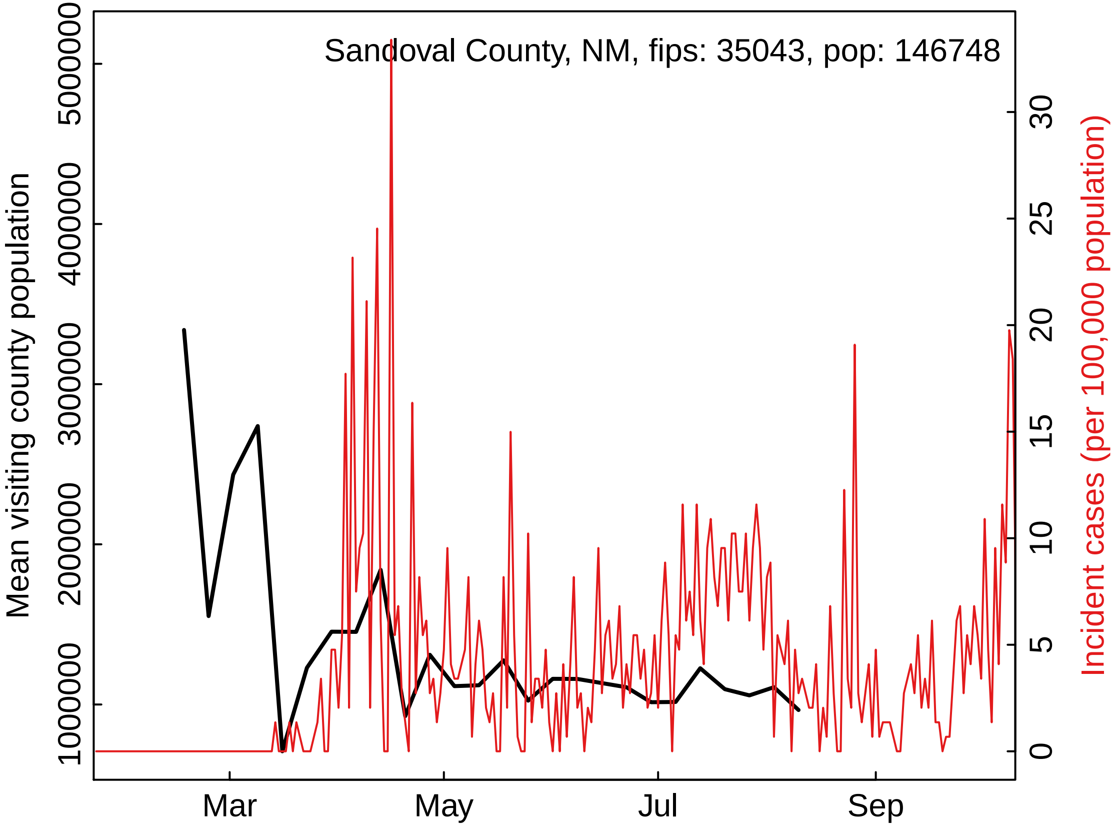

Sandoval County, NM, fips: 35043, pop: 146748
Mean visiting county population
10000000
20000000
30000000
40000000
50000000
Incident cases (per 100,000 population)
0
5
10
15
20
25
30
Mar
May
Jul
Sep

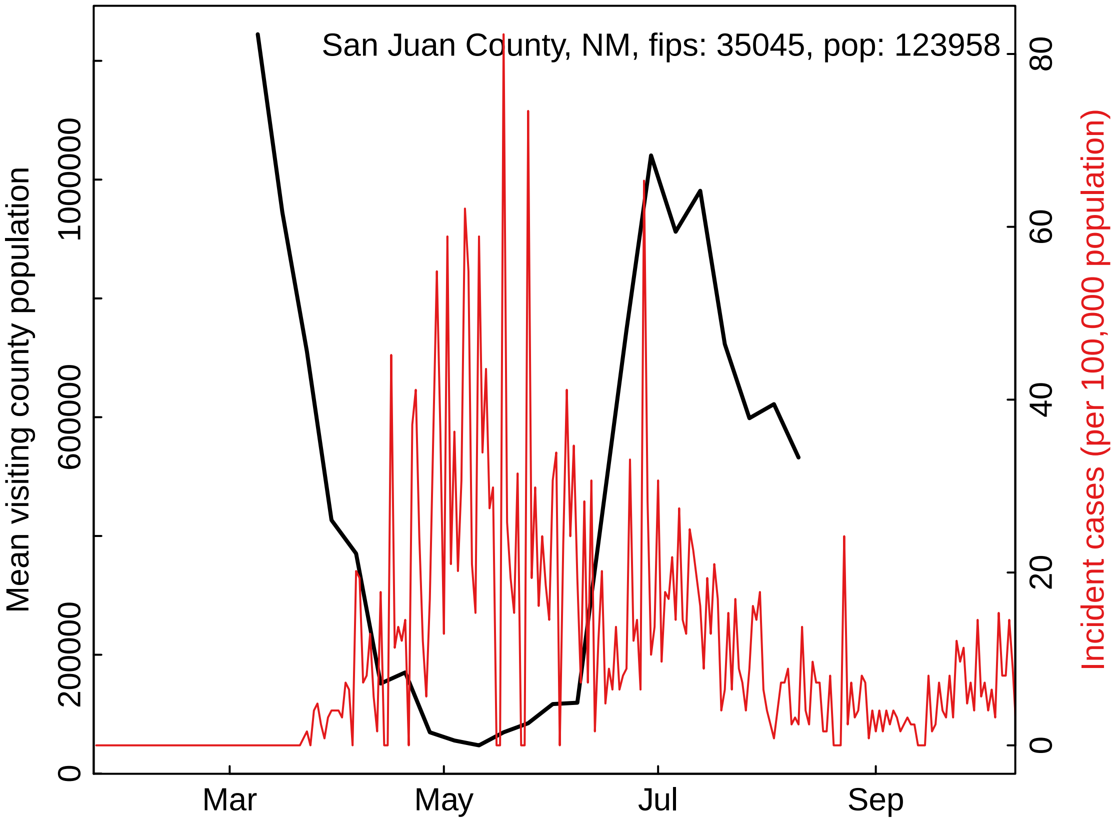

San Juan County, NM, fips: 35045, pop: 123958
Mean visiting county population
0
200000
600000
1000000
Incident cases (per 100,000 population)
0
20
40
60
80
Mar
May
Jul
Sep

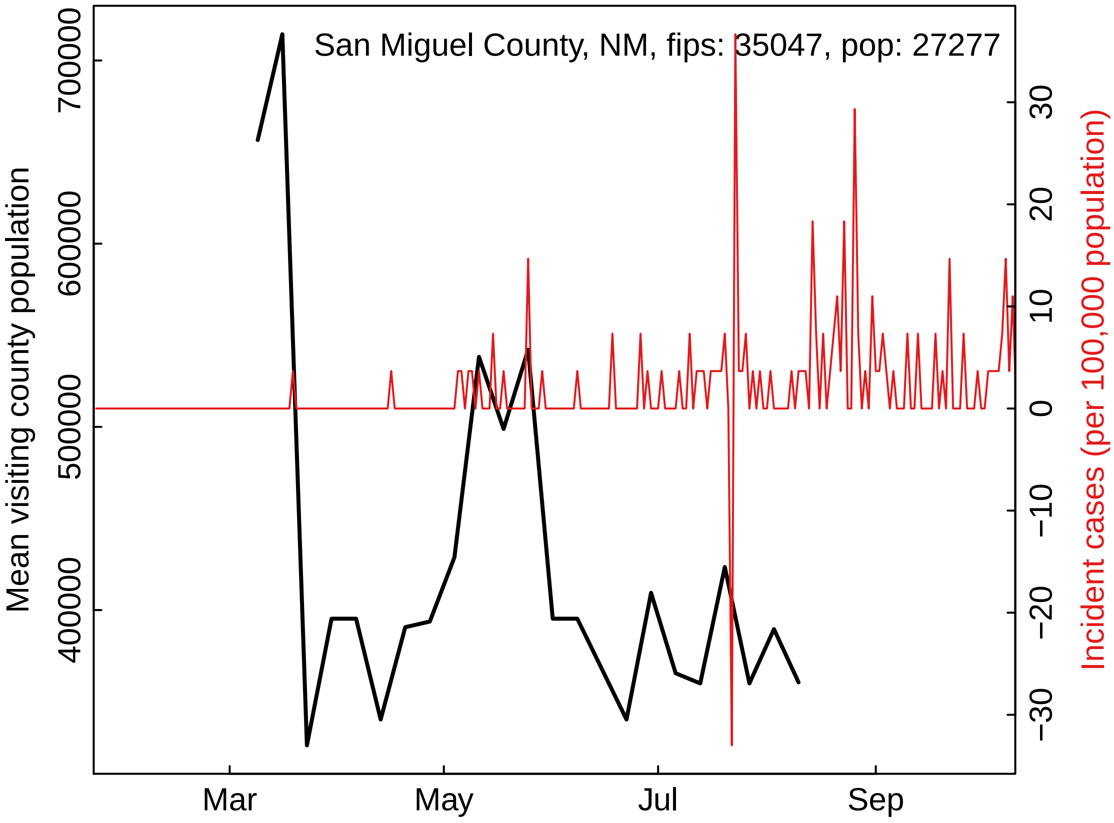
San Miguel County, NM, fips: 35047, pop: 27277
Mean visiting county population
400000
500000
600000
7000000
Incident cases (per 100,000 population)
−30
−20
−10
0
10
20
30
Mar
May
Jul
Sep

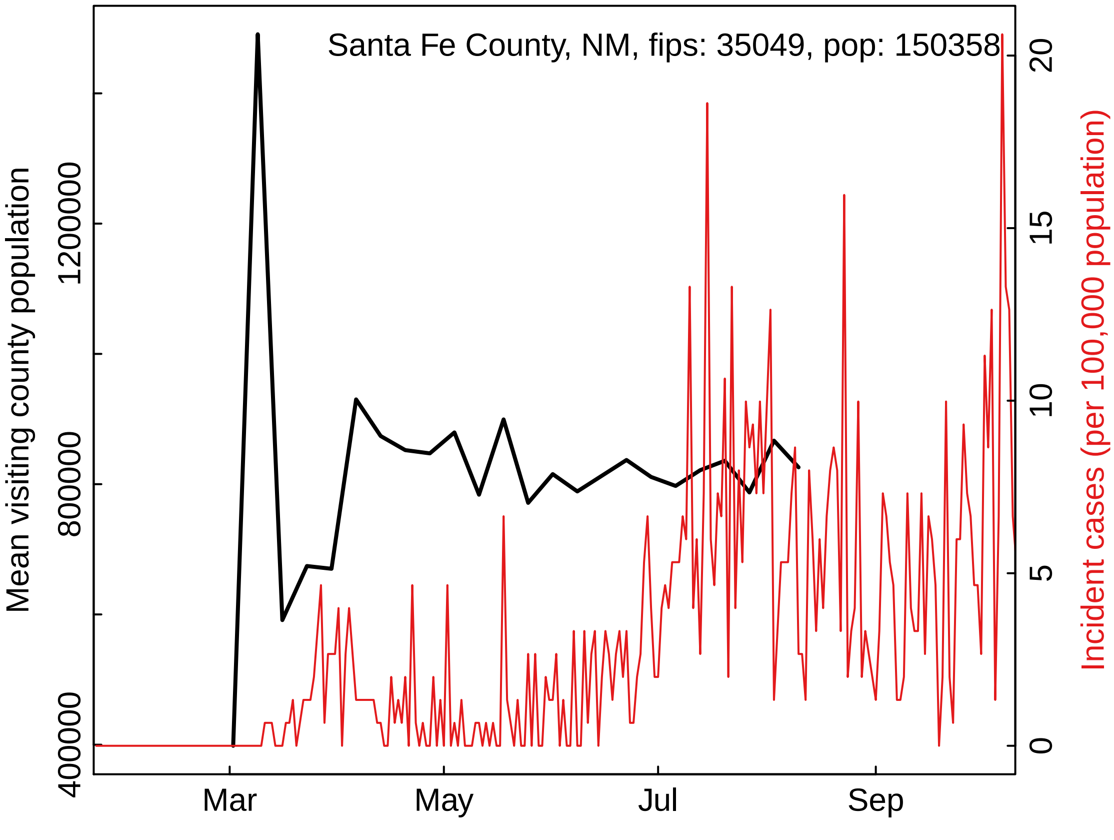

Santa Fe County, NM, fips: 35049, pop: 150358
Mean visiting county population
400000
800000
1200000
Incident cases (per 100,000 population)
0
5
10
15
20
Mar
May
Jul
Sep

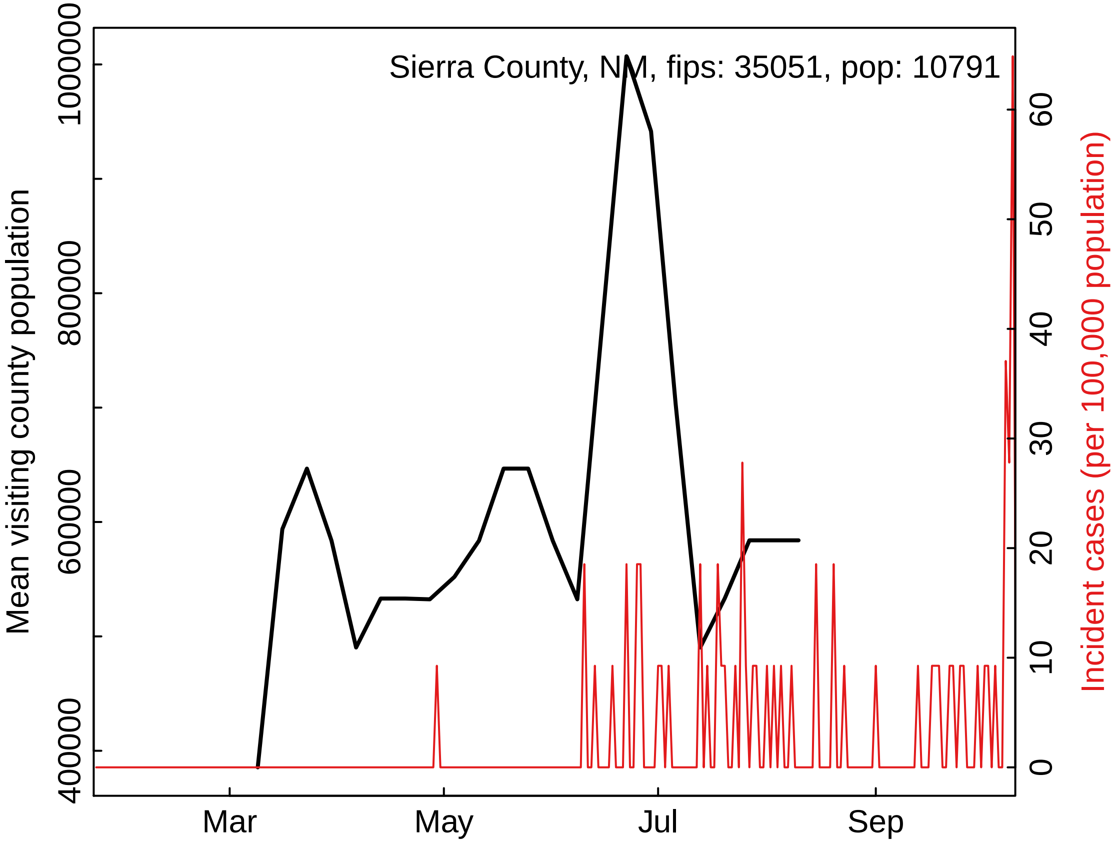
Sierra County, NM, fips: 35051, pop: 10791
Mean visiting county population
400000
600000
800000
1000000
Incident cases (per 100,000 population)
0
10
20
30
40
50
60
Mar
May
Jul
Sep

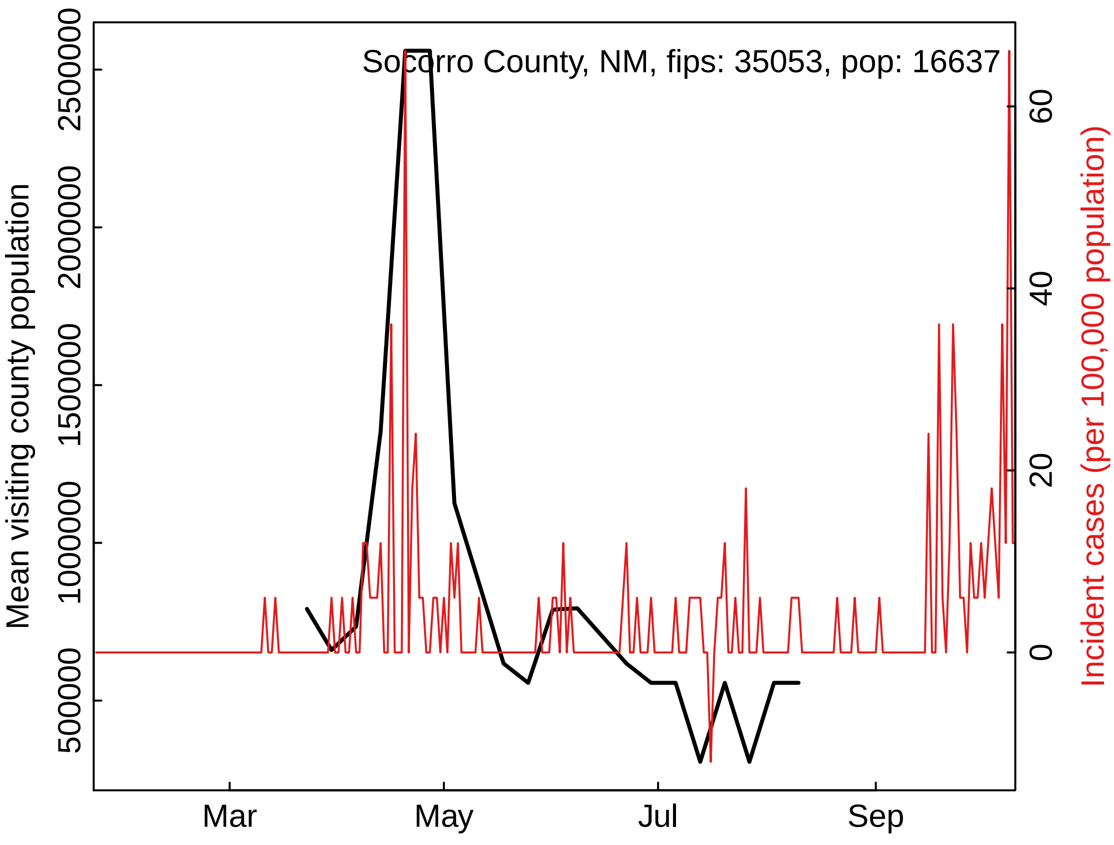

Socorro County, NM, fips: 35053, pop: 16637
Mean visiting county population
500000
1000000
1500000
2000000
2500000
Mar
May
Jul
Sep
0
20
40
60
Incident cases (per 100,000 population)

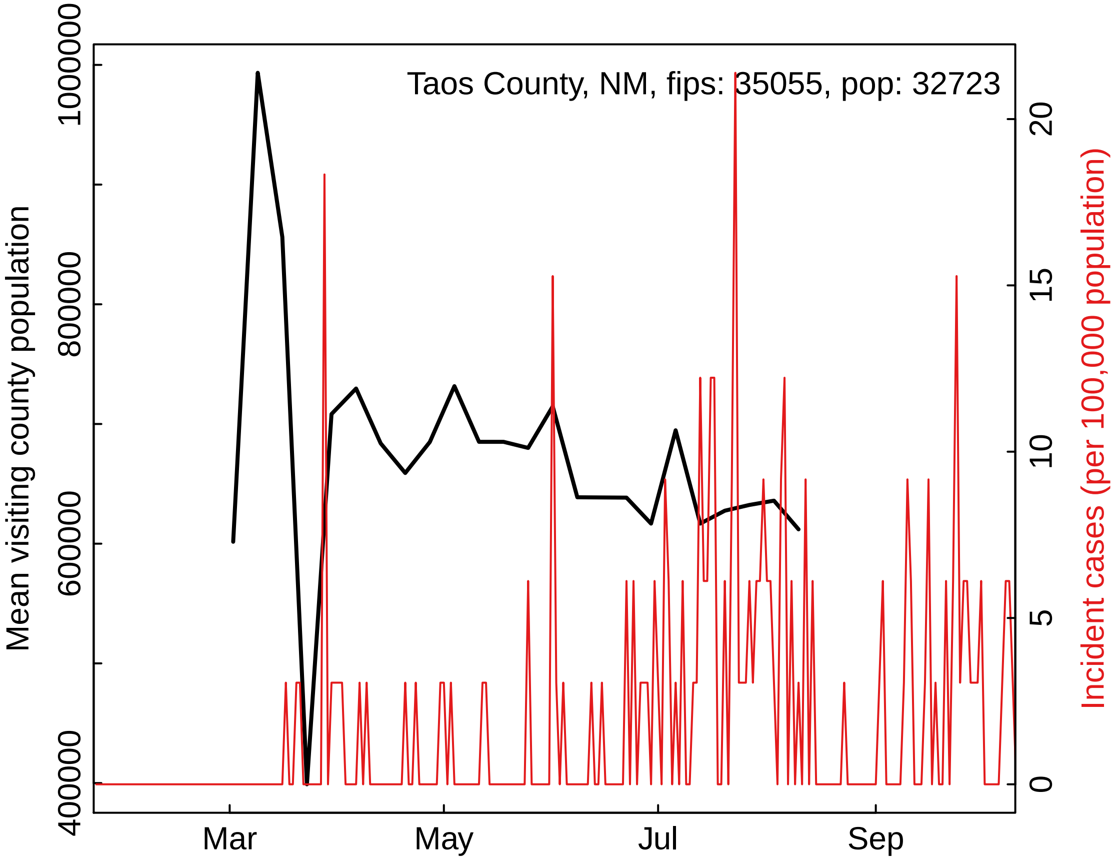

Taos County, NM, fips: 35055, pop: 32723
Mean visiting county population
Incident cases (per 100,000 population)
400000
600000
800000
1000000
0
5
10
15
20
Mar
May
Jul
Sep

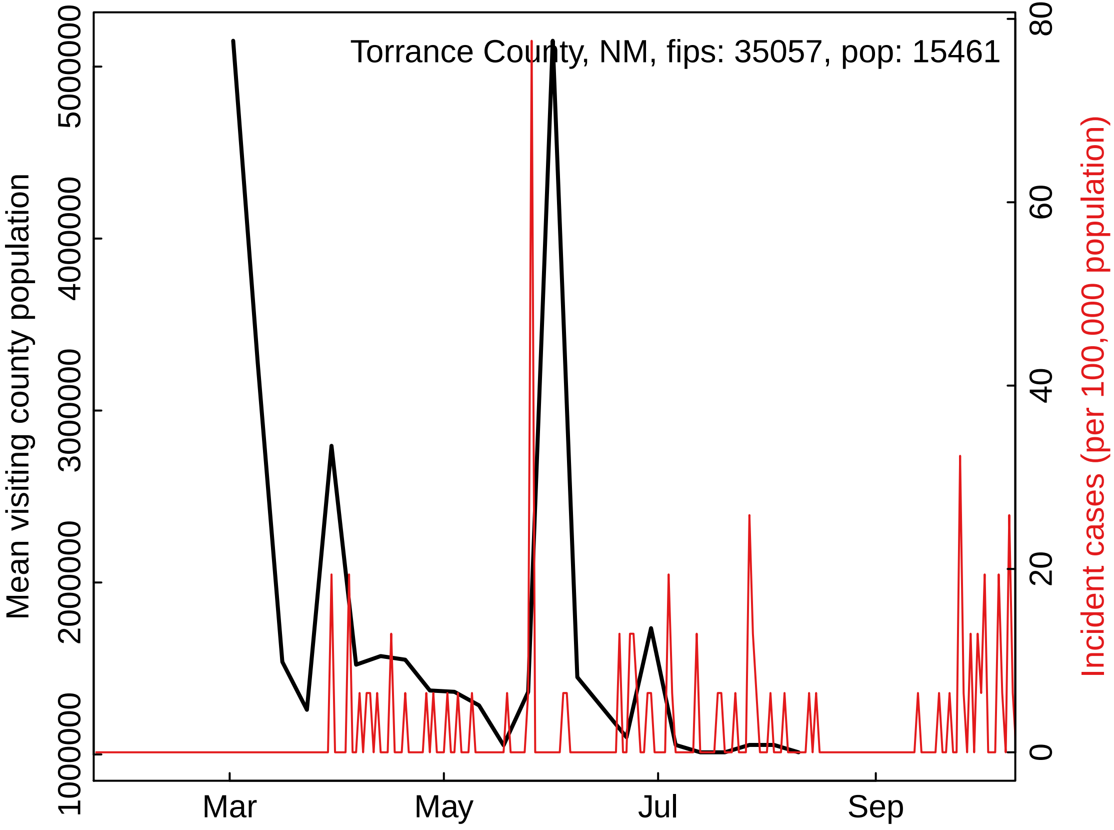

Torrance County, NM, fips: 35057, pop: 15461
Mean visiting county population
1000000
2000000
3000000
4000000
5000000
Incident cases (per 100,000 population)
0
20
40
60
80
Mar
May
Jul
Sep

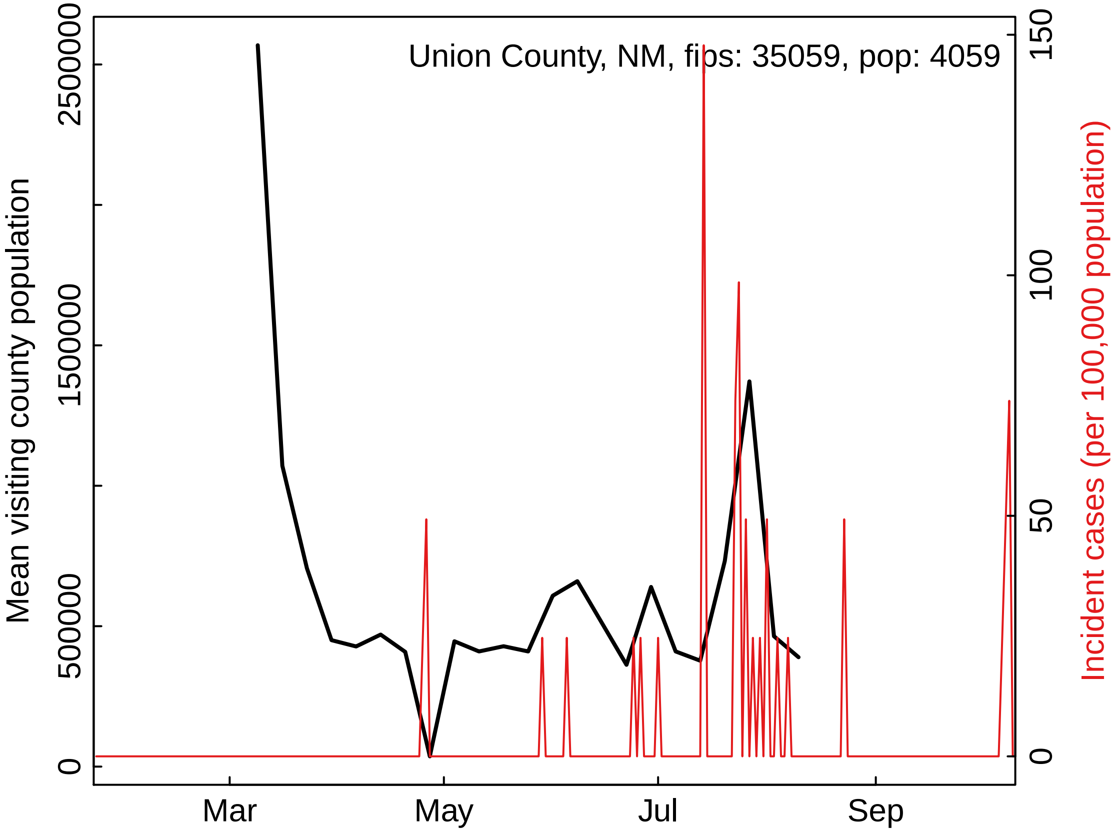

Union County, NM, fips: 35059, pop: 4059
Mean visiting county population
0
500000
1500000
2500000
Incident cases (per 100,000 population)
0
50
100
150
Mar
May
Jul
Sep

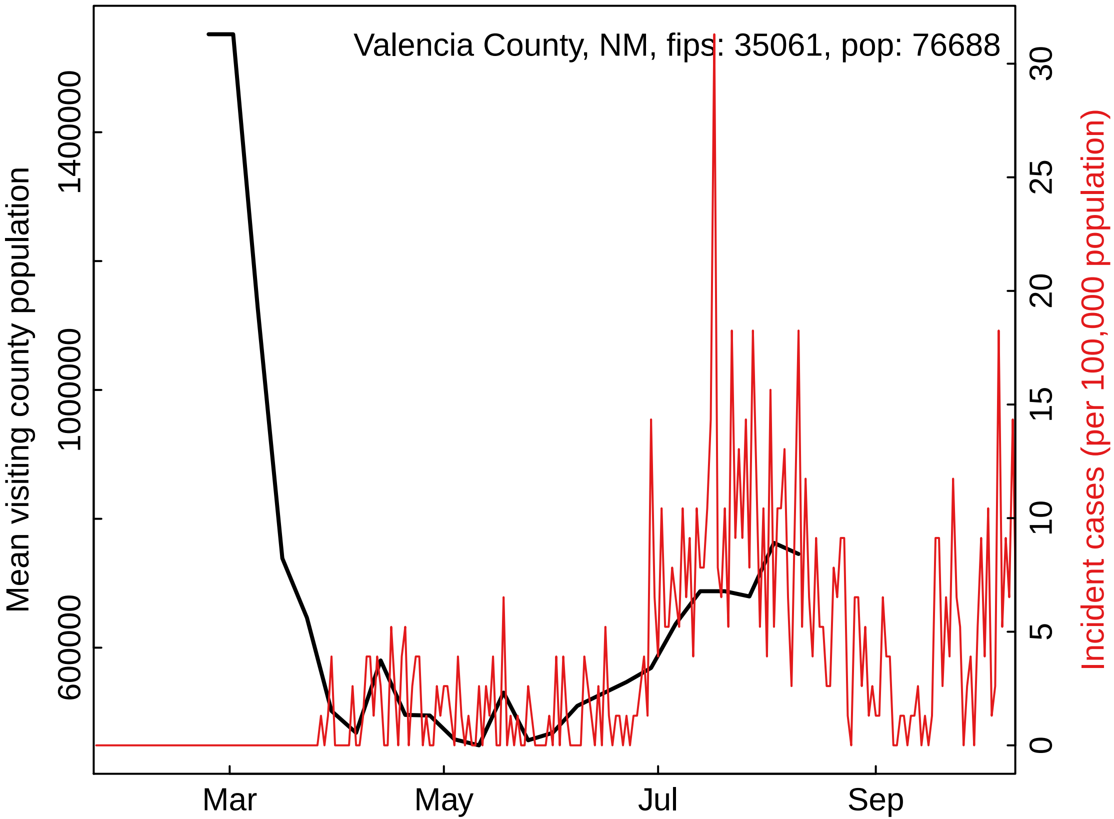

Valencia County, NM, fips: 35061, pop: 76688
Mean visiting county population
600000
1000000
1400000
Incident cases (per 100,000 population)
0
5
10
15
20
25
30
Mar
May
Jul
Sep

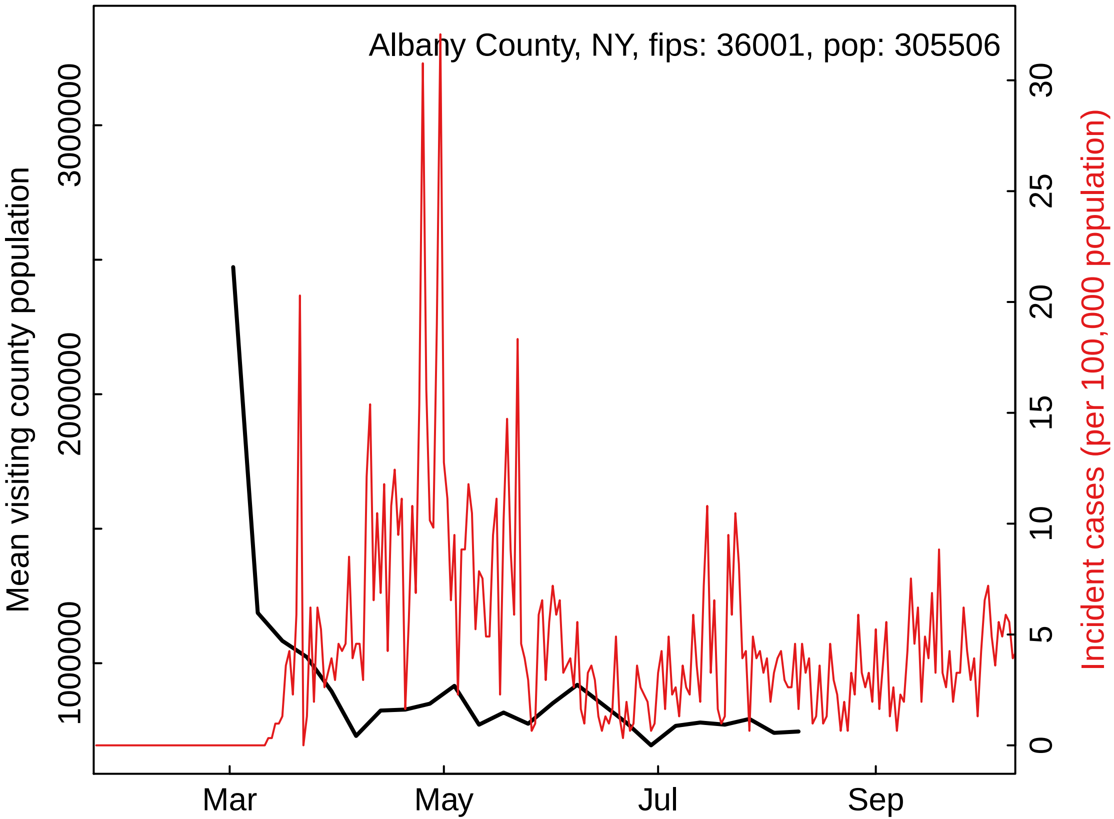

Albany County, NY, fips: 36001, pop: 305506
Mean visiting county population
1000000
2000000
3000000
Incident cases (per 100,000 population)
0
5
10
15
20
25
30
Mar
May
Jul
Sep

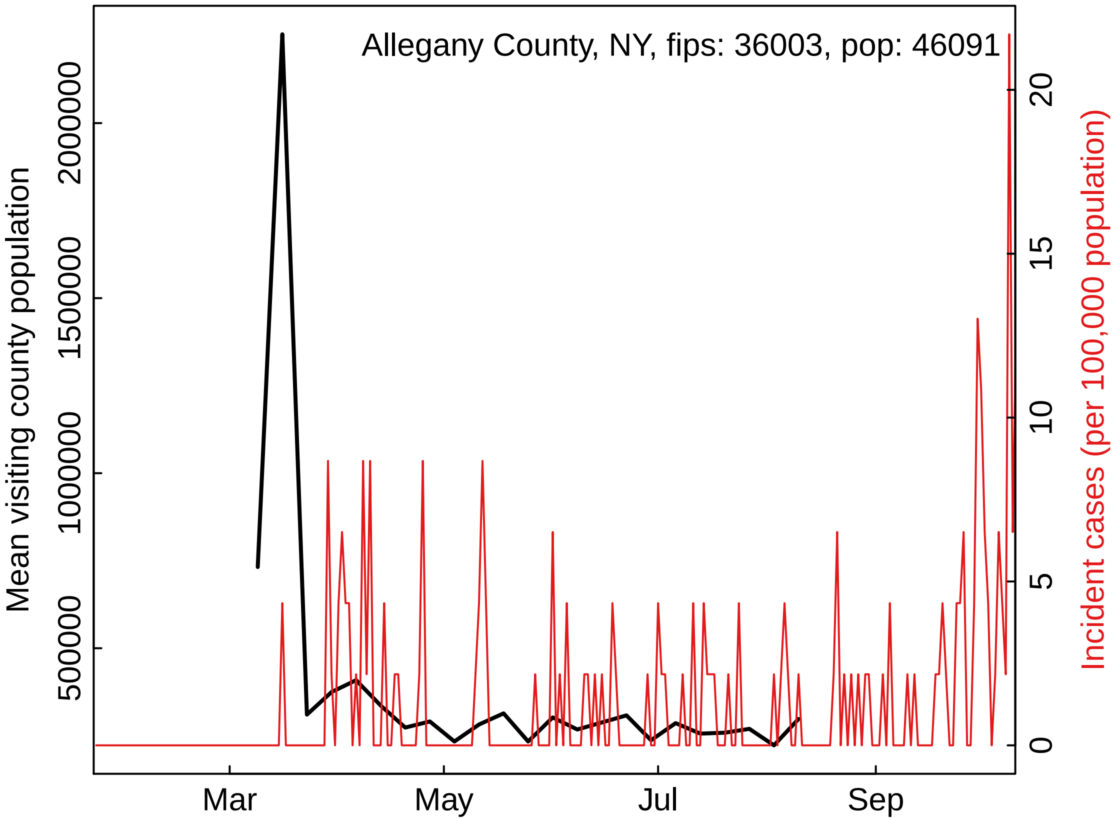

Allegany County, NY, fips: 36003, pop: 46091
Mean visiting county population
500000
1000000
1500000
2000000
Incident cases (per 100,000 population)
0
5
10
15
20
Mar
May
Jul
Sep

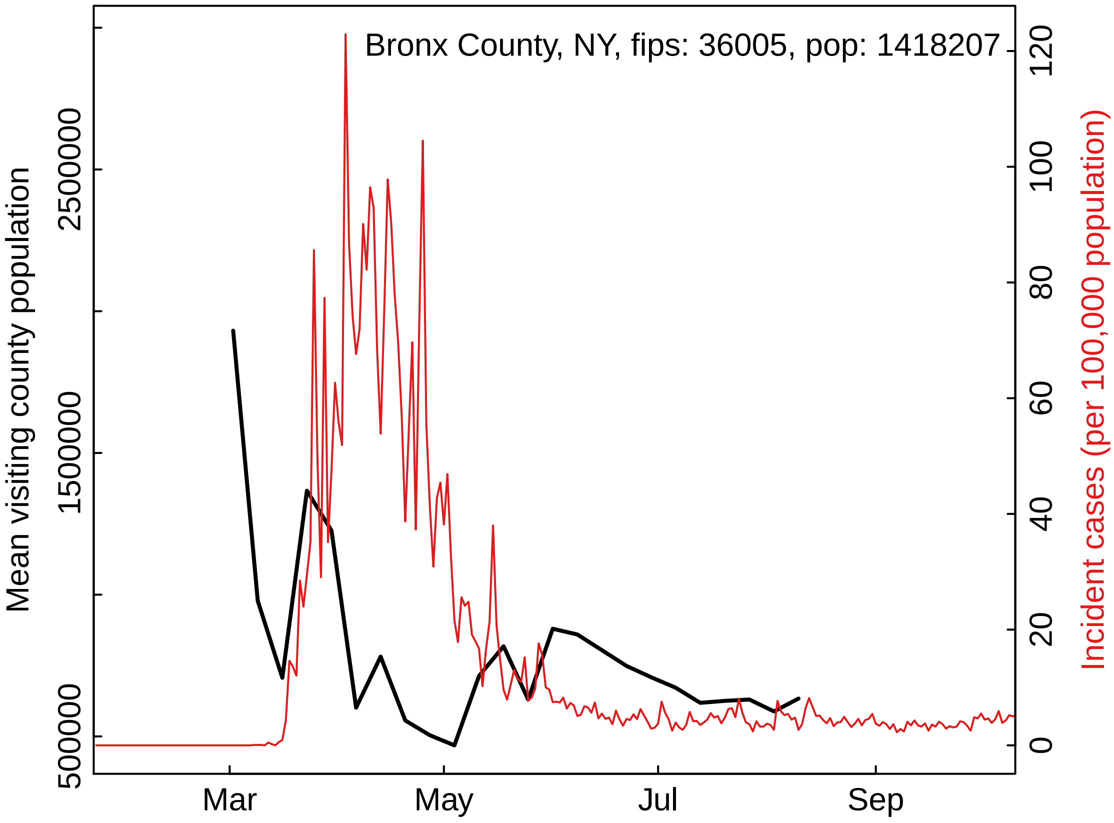

Bronx County, NY, fips: 36005, pop: 1418207
Mean visiting county population
500000
1500000
2500000
Mar
May
Jul
Sep
0
20
40
60
80
100
120
Incident cases (per 100,000 population)

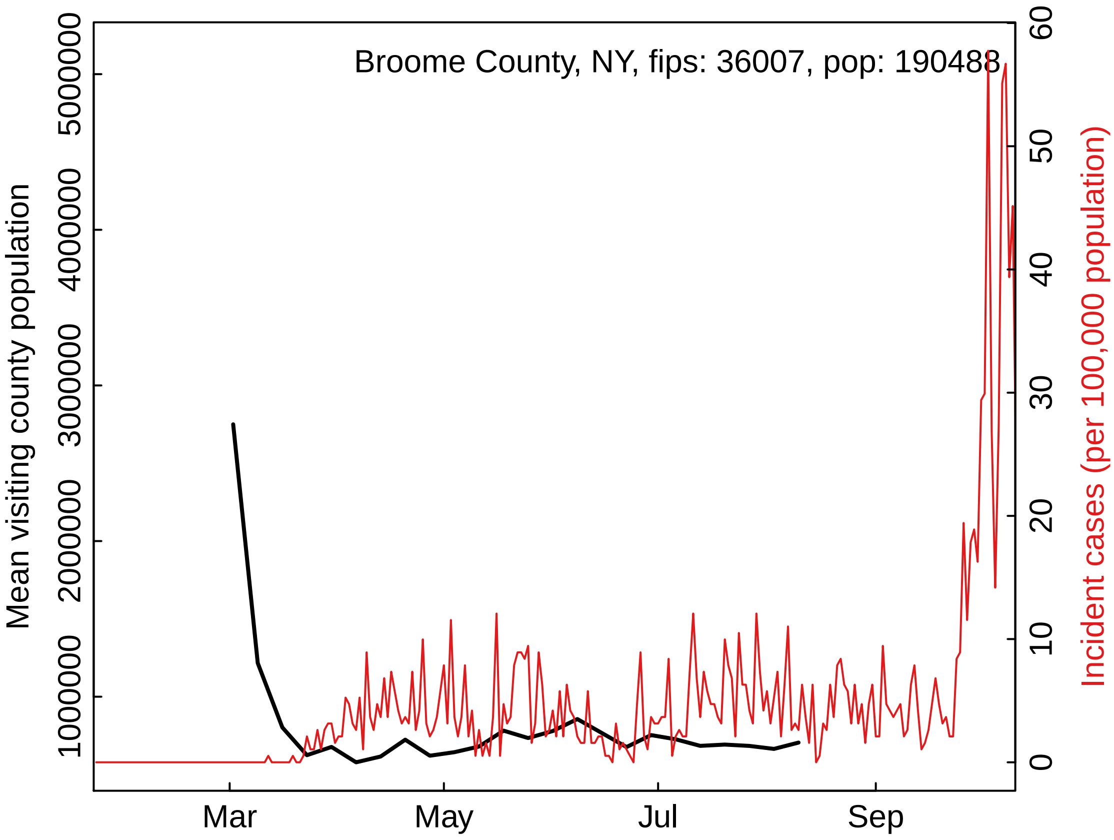

Broome County, NY, fips: 36007, pop: 190488
Mean visiting county population
1000000
2000000
3000000
4000000
5000000
Incident cases (per 100,000 population)
0
10
20
30
40
50
60
Mar
May
Jul
Sep

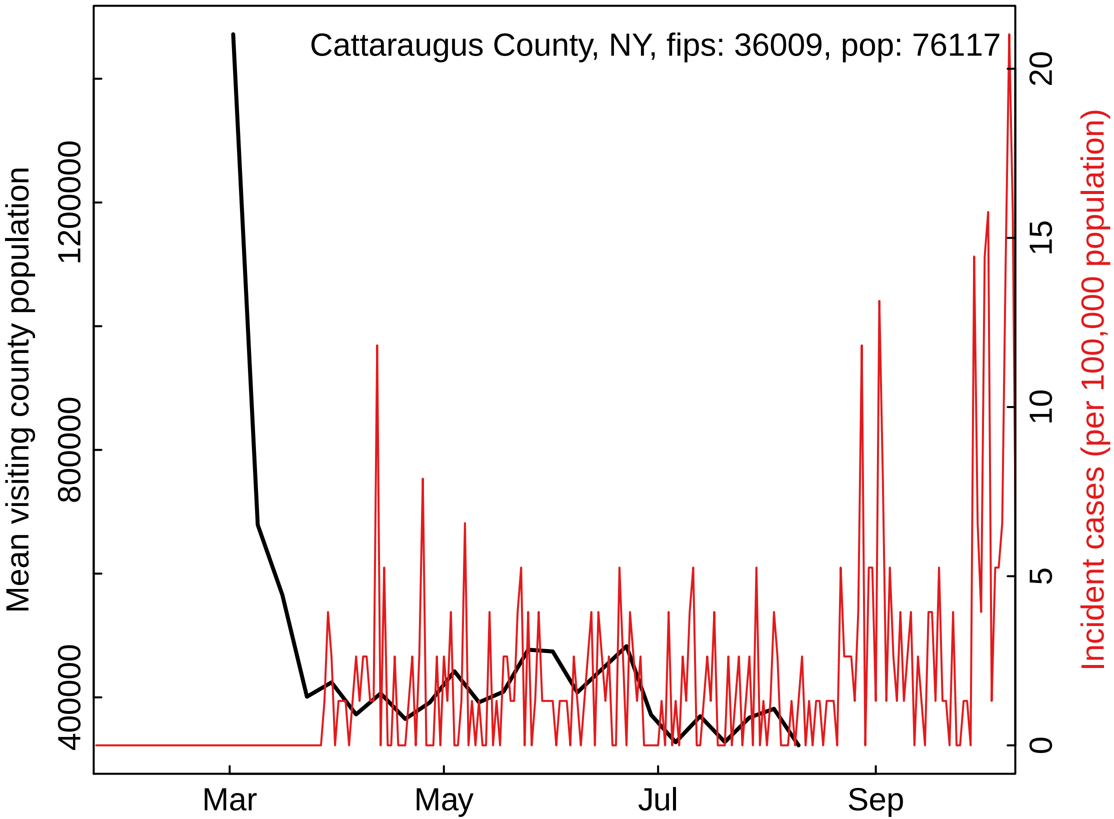
Cattaraugus County, NY, fips: 36009, pop: 76117
Mean visiting county population
400000
800000
1200000
Incident cases (per 100,000 population)
0
5
10
15
20
Mar
May
Jul
Sep

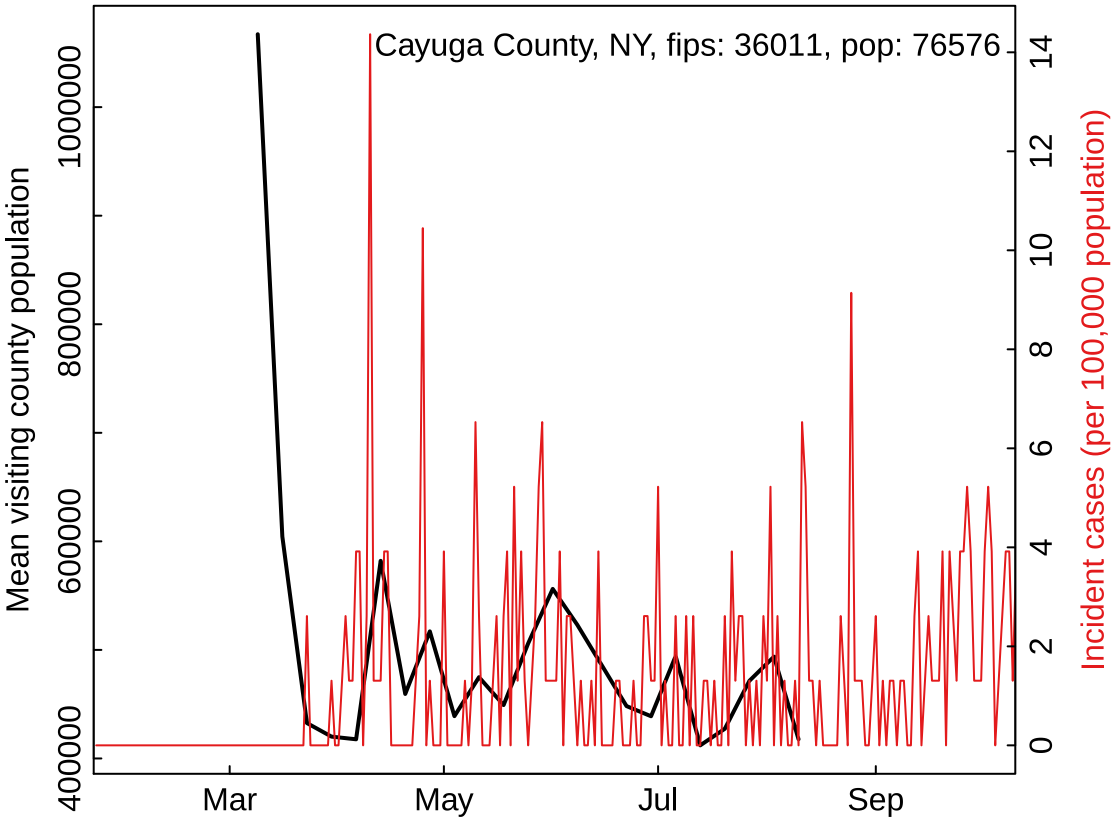

Cayuga County, NY, fips: 36011, pop: 76576
Mean visiting county population
400000
600000
800000
1000000
Mar
May
Jul
Sep
Incident cases (per 100,000 population)
0
2
4
6
8
10
12
14

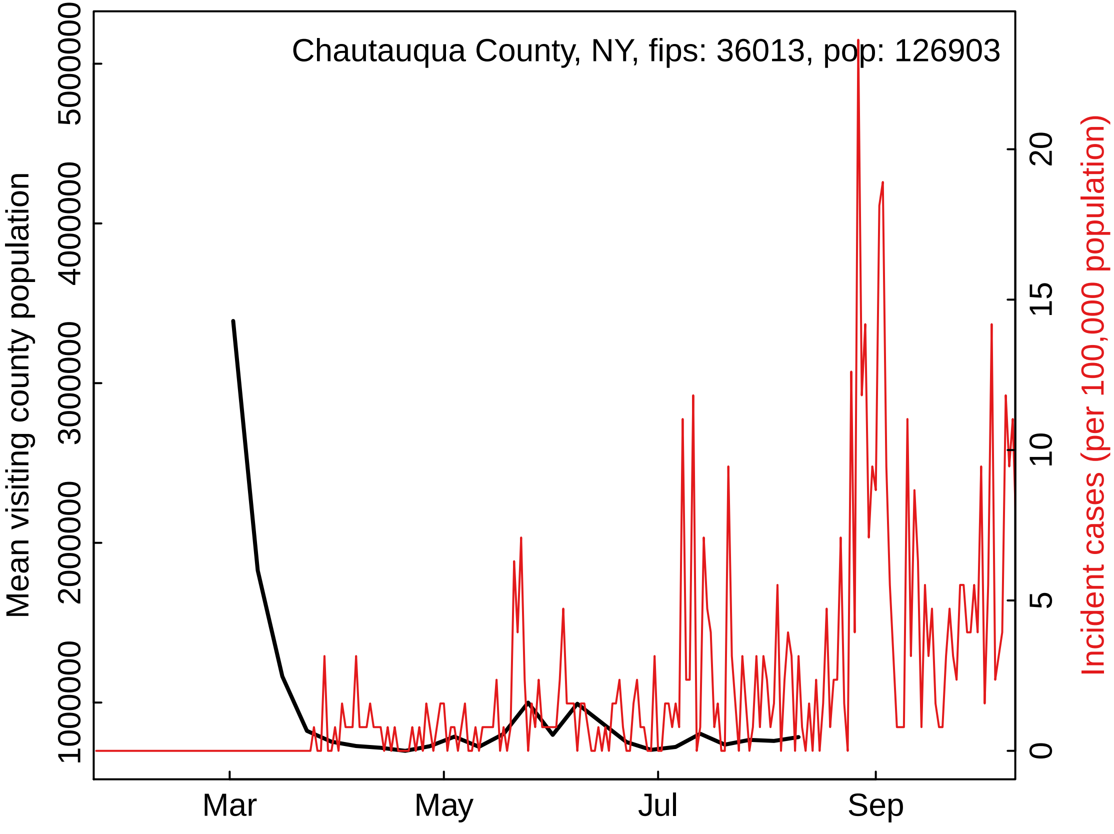

Chautauqua County, NY, fips: 36013, pop: 126903
Mean visiting county population
10000000
20000000
30000000
40000000
50000000
Incident cases (per 100,000 population)
0
5
10
15
20
Mar
May
Jul
Sep

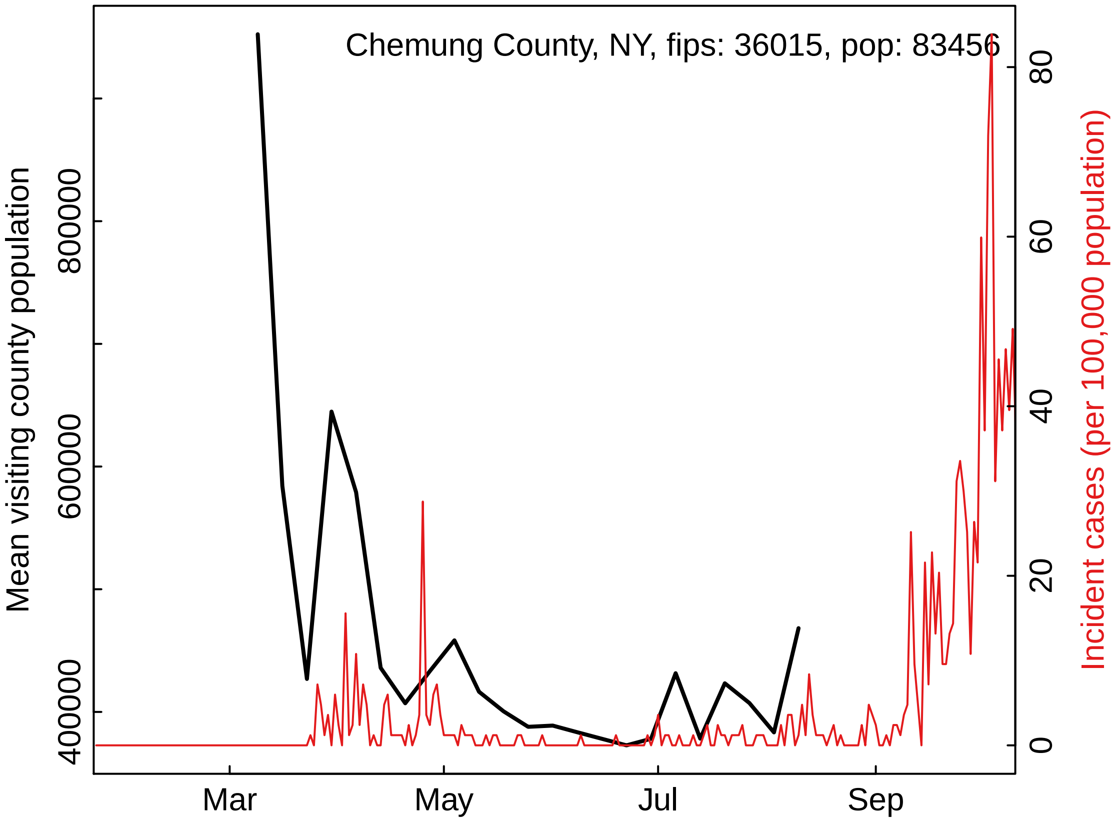

Chemung County, NY, fips: 36015, pop: 83456
Mean visiting county population
400000
600000
800000
Mar
May
Jul
Sep
0
20
40
60
80
Incident cases (per 100,000 population)

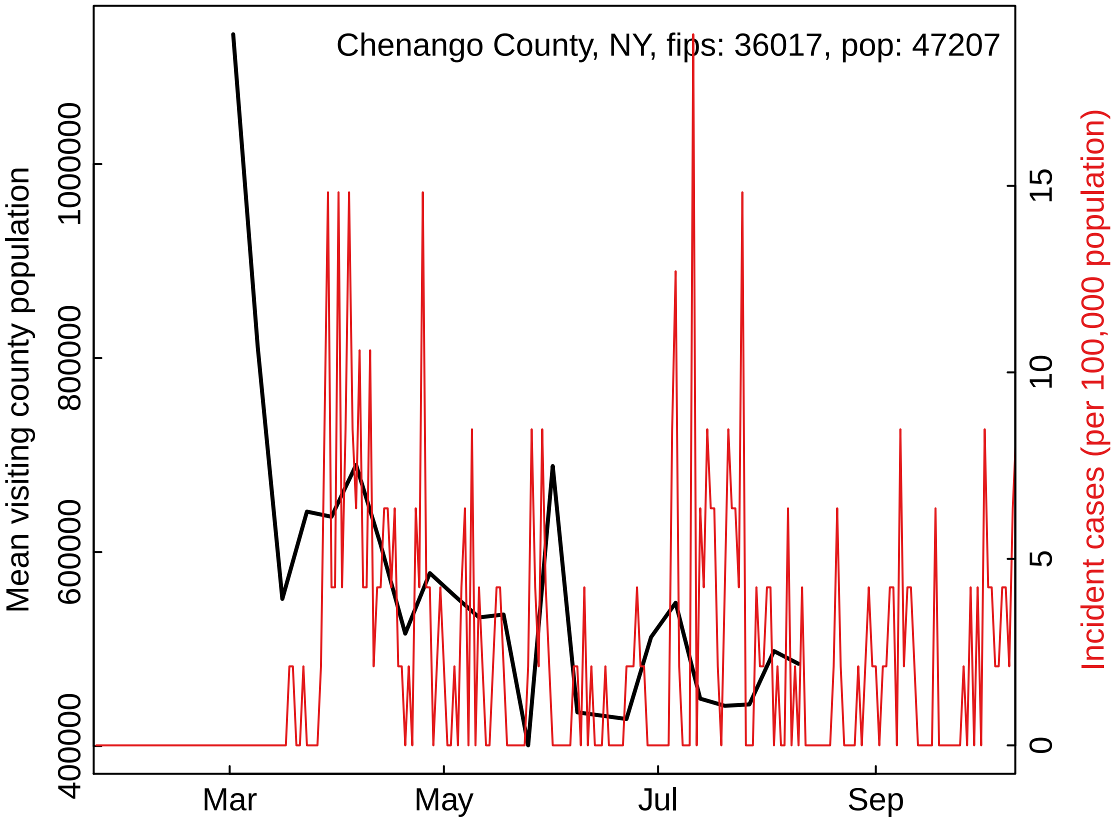

Chenango County, NY, fips: 36017, pop: 47207
Mean visiting county population
400000
600000
800000
1000000
Incident cases (per 100,000 population)
0
5
10
15
Mar
May
Jul
Sep

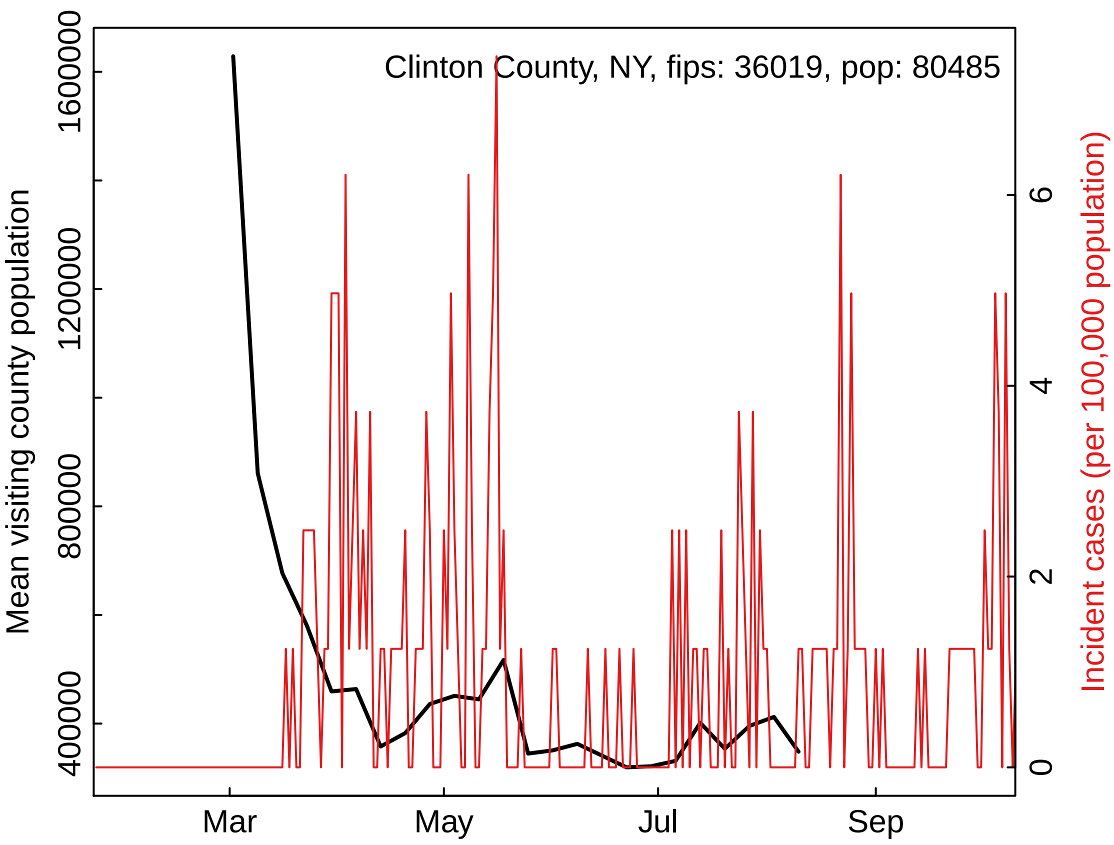

Clinton County, NY, fips: 36019, pop: 80485
Mean visiting county population
400000
800000
1200000
1600000
Mar
May
Jul
Sep
0
2
4
6
Incident cases (per 100,000 population)

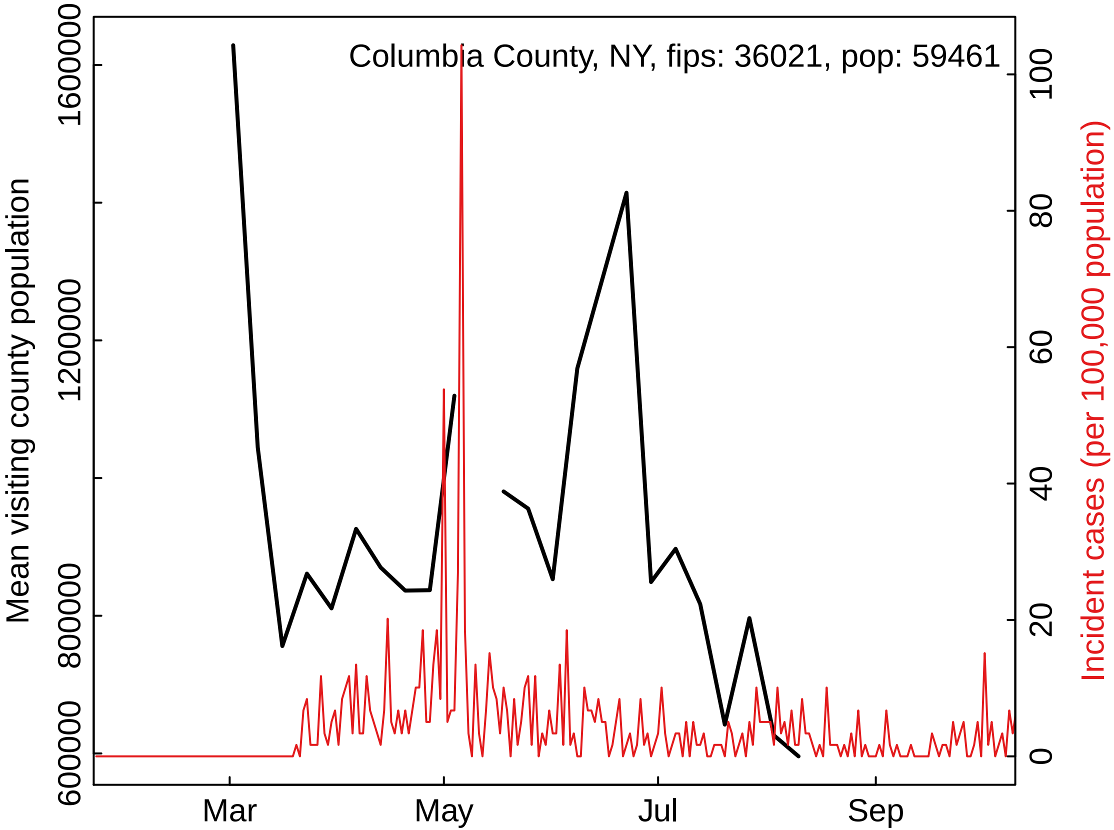

Columbia County, NY, fips: 36021, pop: 59461
Mean visiting county population
600000
800000
1200000
1600000
Incident cases (per 100,000 population)
0
20
40
60
80
100
Mar
May
Jul
Sep

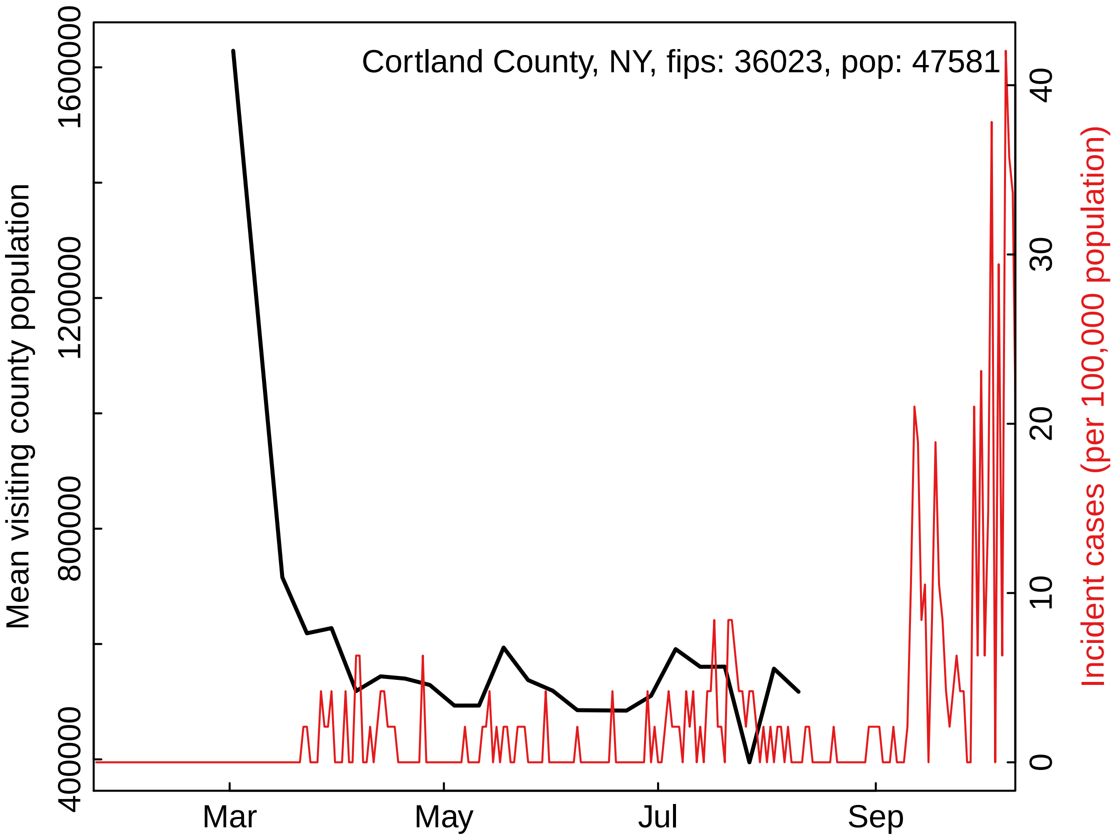

Cortland County, NY, fips: 36023, pop: 47581
Mean visiting county population
Incident cases (per 100,000 population)
400000
800000
1200000
1600000
0
10
20
30
40
Mar
May
Jul
Sep

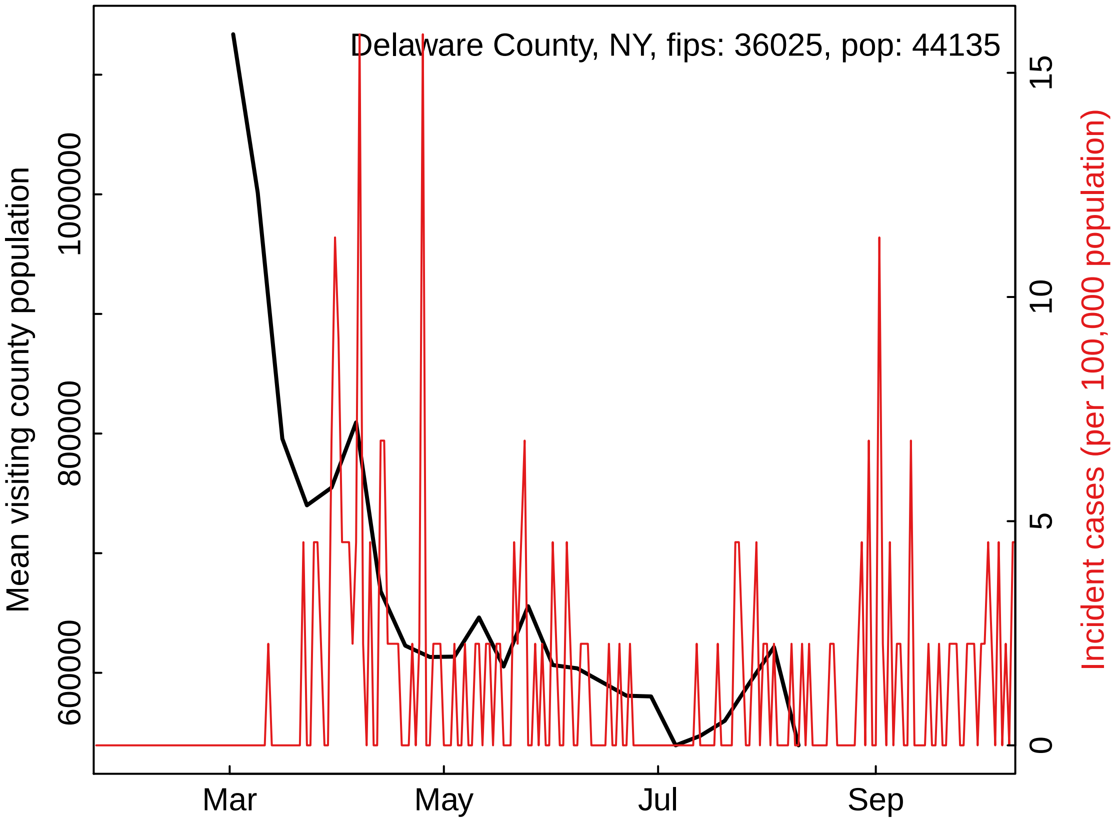
Delaware County, NY, fips: 36025, pop: 44135
Mean visiting county population
600000
800000
1000000
Incident cases (per 100,000 population)
0
5
10
15
Mar
May
Jul
Sep

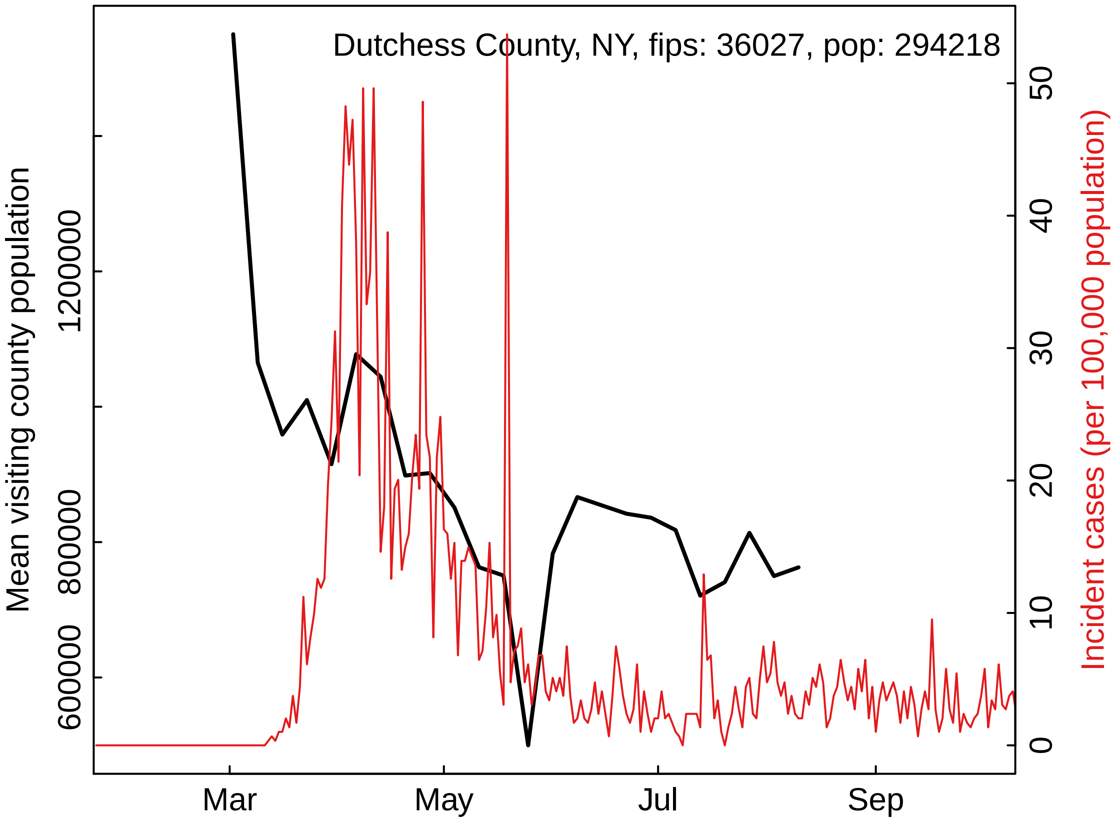

Dutchess County, NY, fips: 36027, pop: 294218
Mean visiting county population
600000
800000
1200000
Incident cases (per 100,000 population)
0
10
20
30
40
50
Mar
May
Jul
Sep

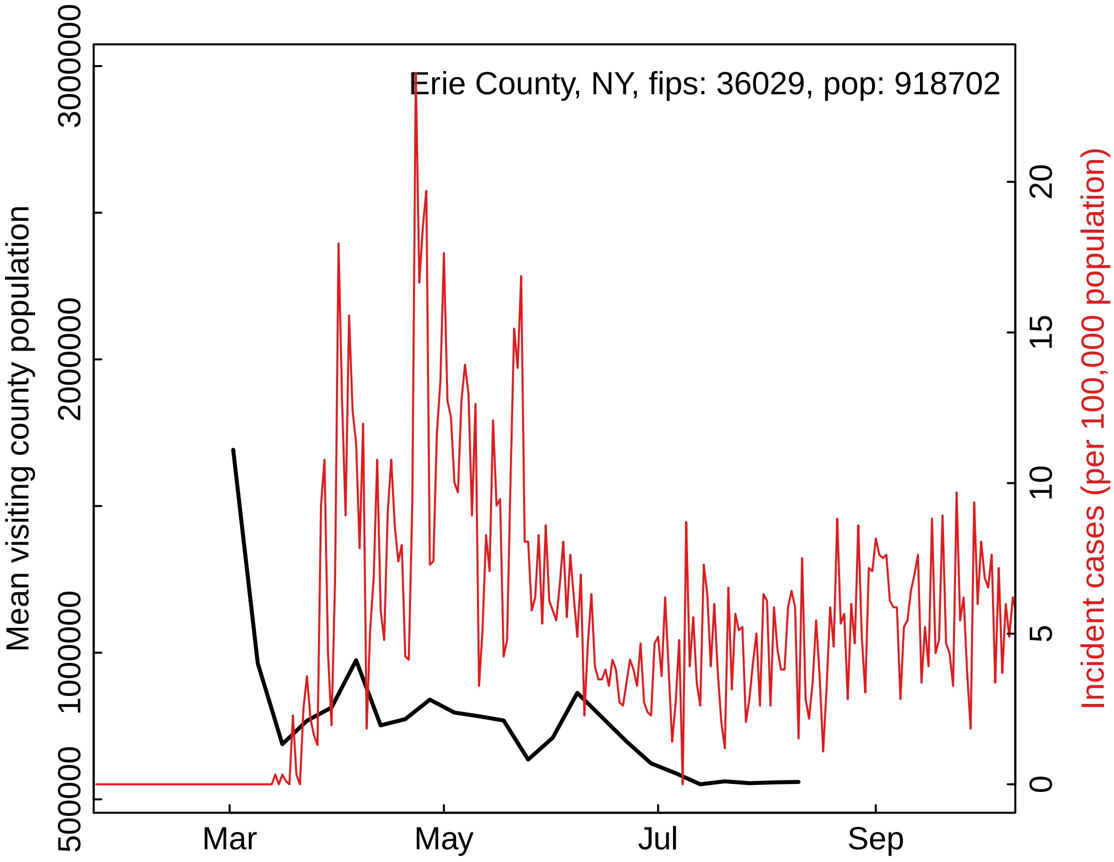

Erie County, NY, fips: 36029, pop: 918702
Mean visiting county population
Incident cases (per 100,000 population)
500000
1000000
2000000
3000000
0
5
10
15
20
Mar
May
Jul
Sep

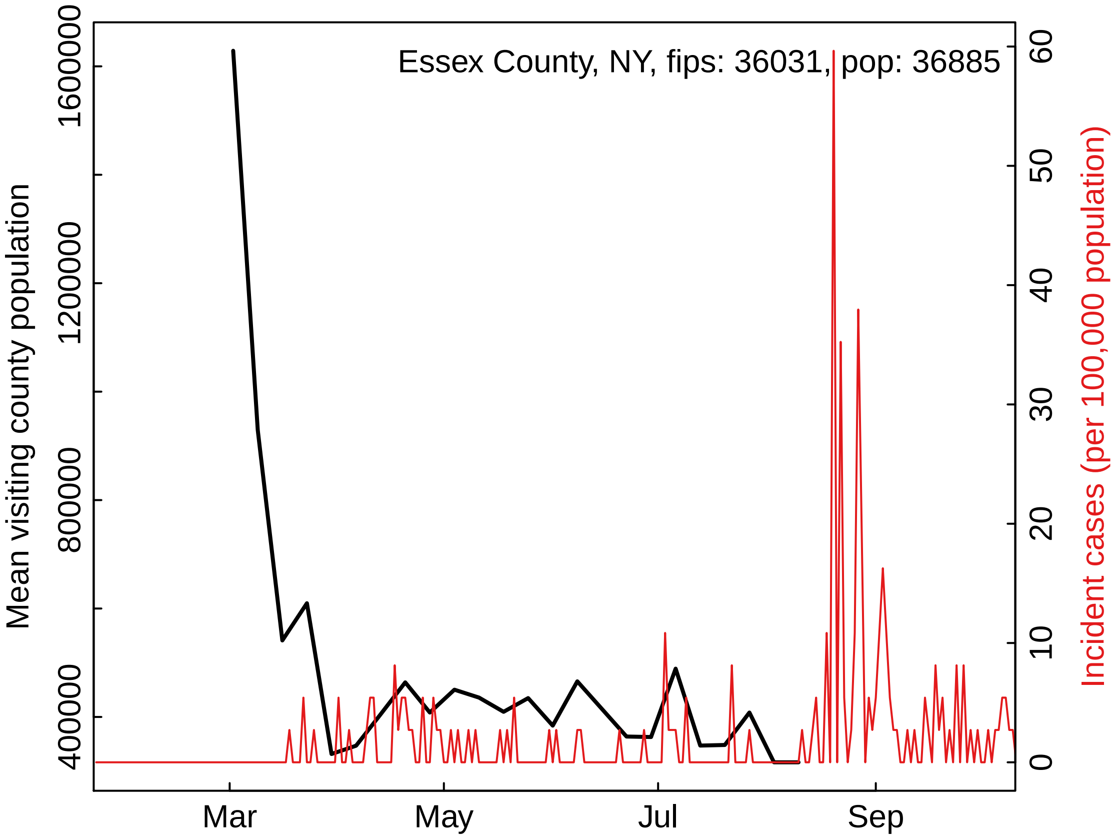

Essex County, NY, fips: 36031, pop: 36885
Mean visiting county population
400000
800000
1200000
1600000
Incident cases (per 100,000 population)
0
10
20
30
40
50
60
Mar
May
Jul
Sep

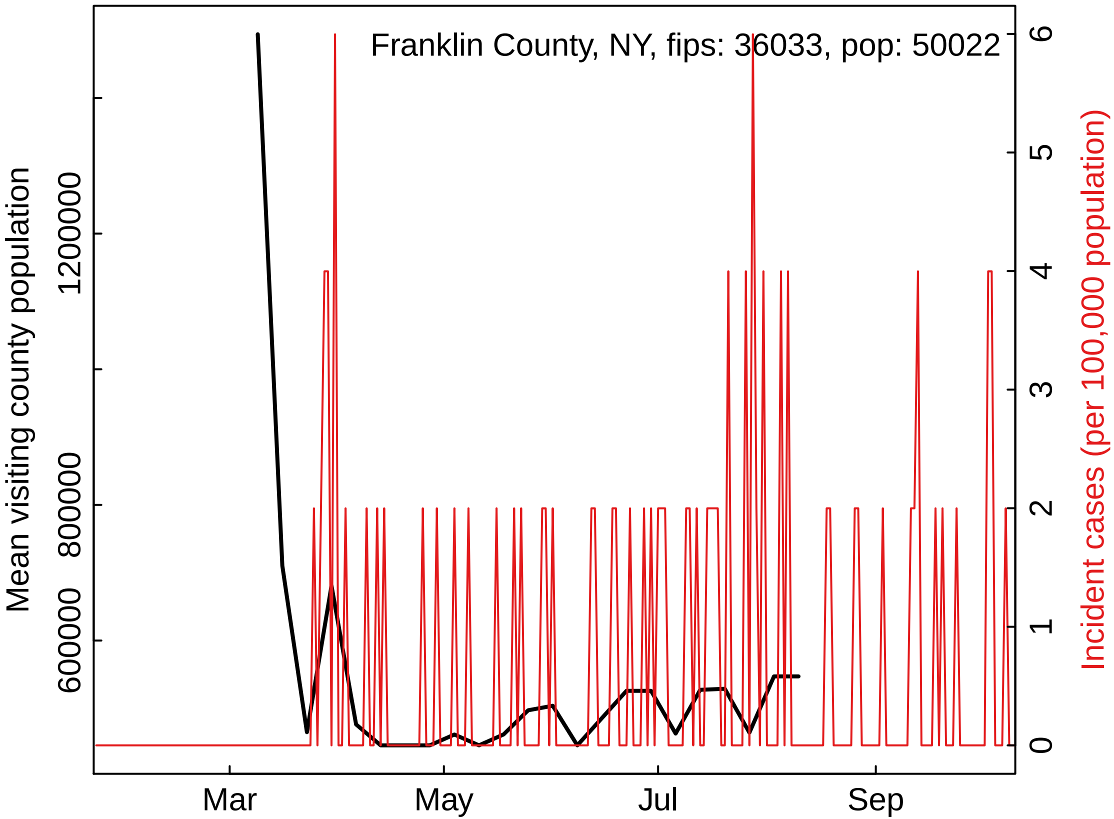

Franklin County, NY, fips: 36033, pop: 50022
Mean visiting county population
600000
800000
1200000
Incident cases (per 100,000 population)
0
1
2
3
4
5
6
Mar
May
Jul
Sep

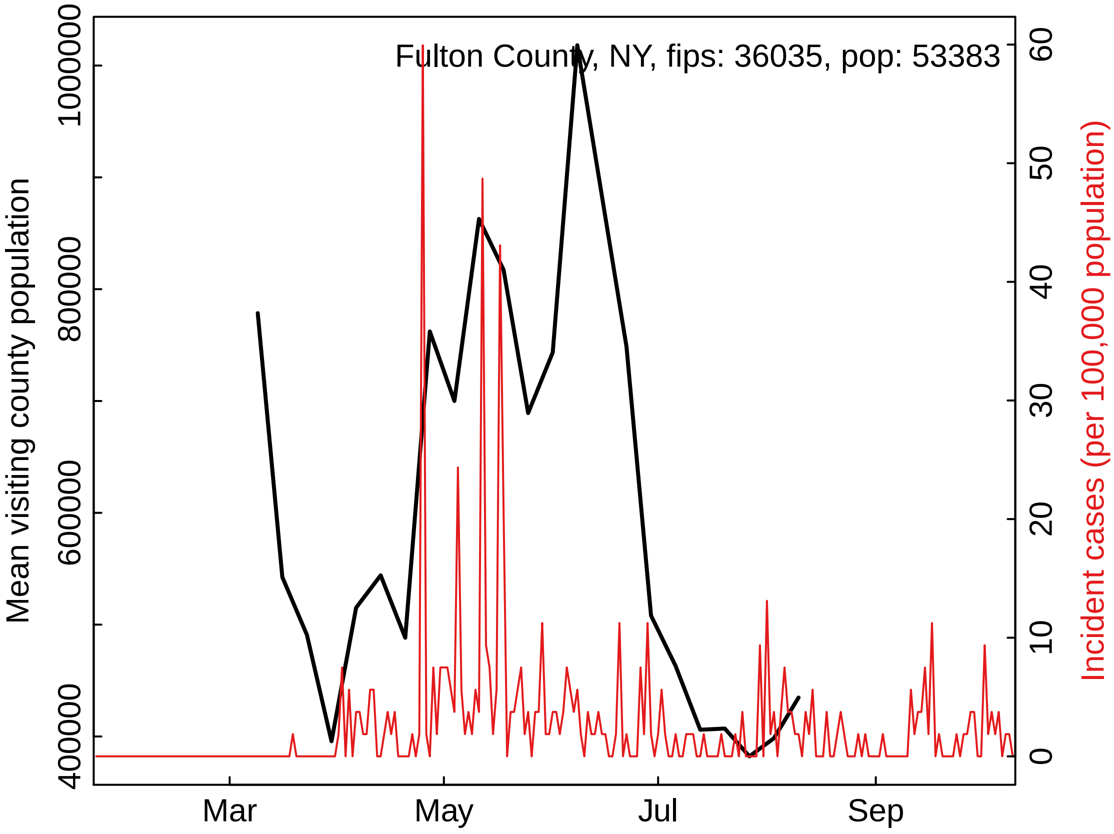

Fulton County, NY, fips: 36035, pop: 53383
Mean visiting county population
400000
600000
800000
1000000
Incident cases (per 100,000 population)
0
10
20
30
40
50
60
Mar
May
Jul
Sep

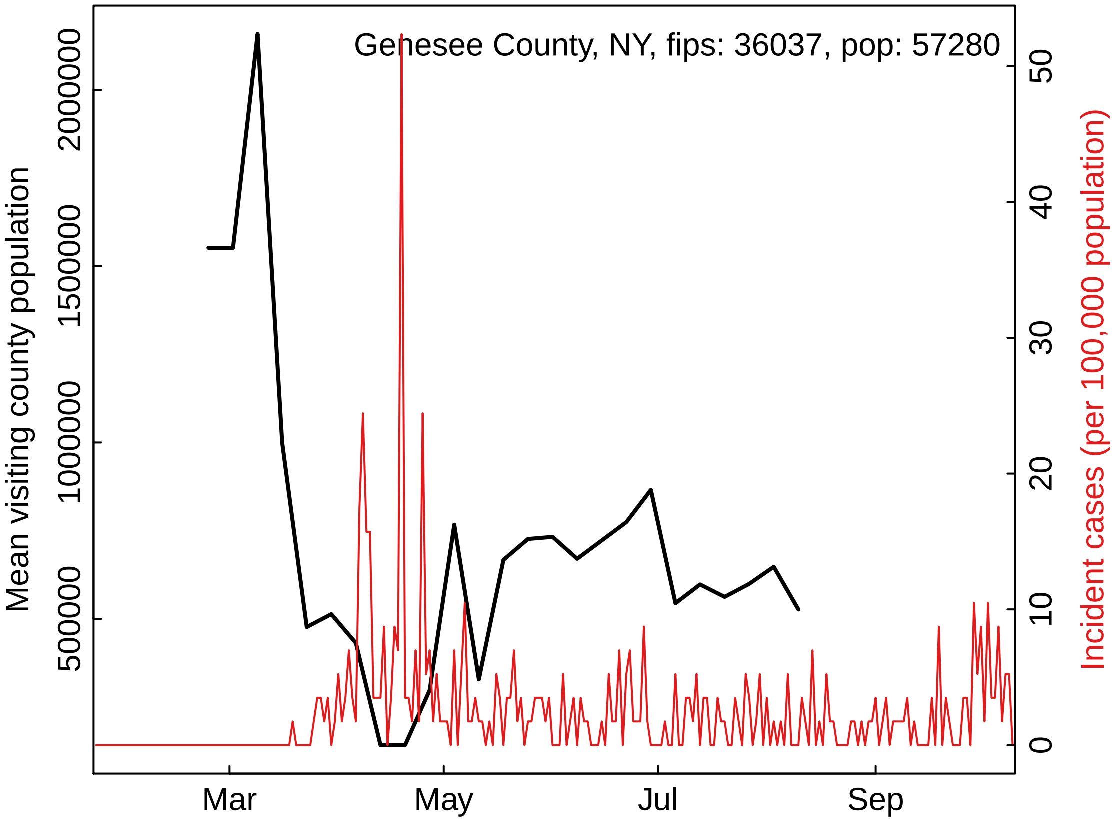

Genesee County, NY, fips: 36037, pop: 57280
Mean visiting county population
500000
1000000
1500000
2000000
Incident cases (per 100,000 population)
0
10
20
30
40
50
Mar
May
Jul
Sep

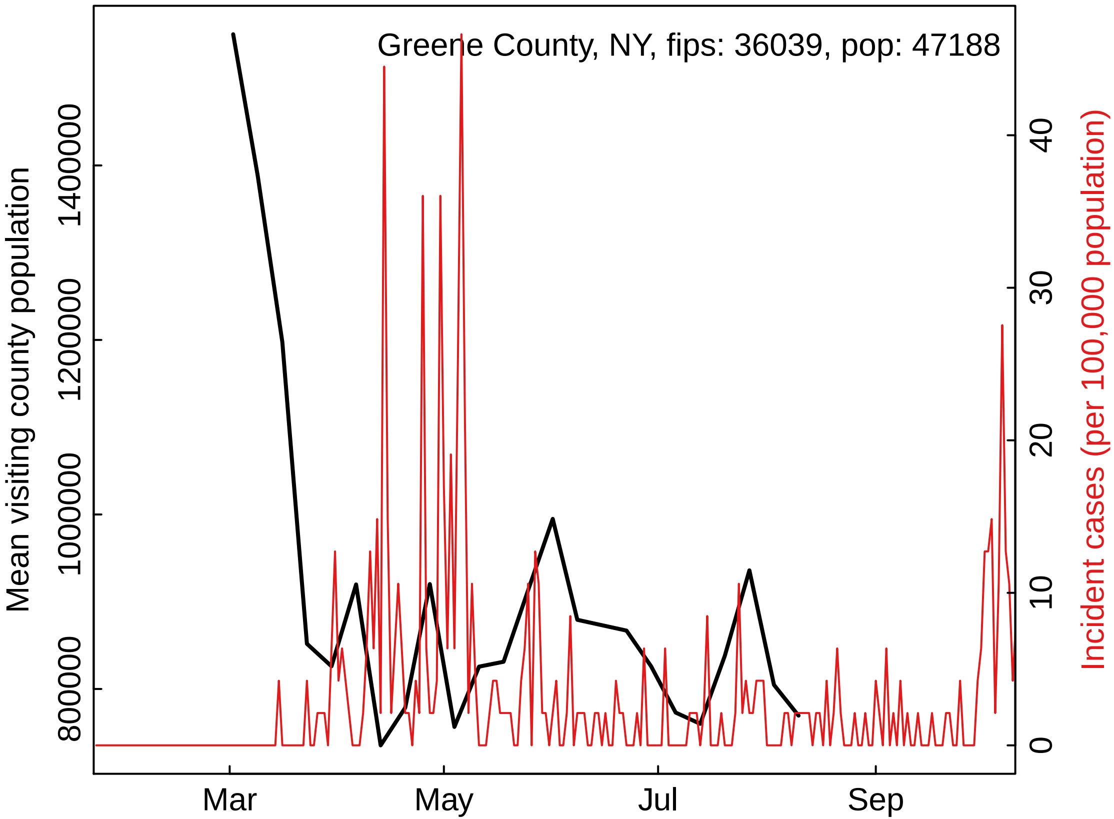

Greene County, NY, fips: 36039, pop: 47188
Mean visiting county population
800000
1000000
1200000
1400000
Mar
May
Jul
Sep
0
10
20
30
40
Incident cases (per 100,000 population)

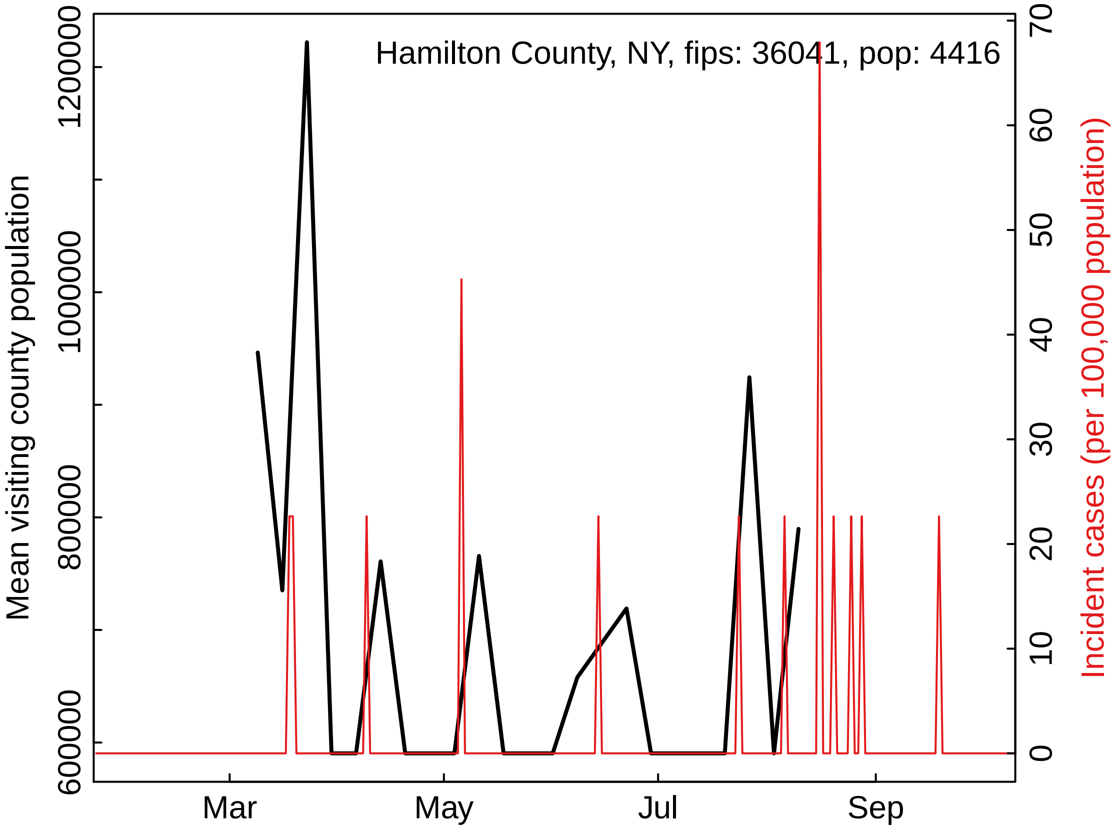

Hamilton County, NY, fips: 36041, pop: 4416
Mean visiting county population
Incident cases (per 100,000 population)
600000
800000
1000000
1200000
0
10
20
30
40
50
60
70
Mar
May
Jul
Sep

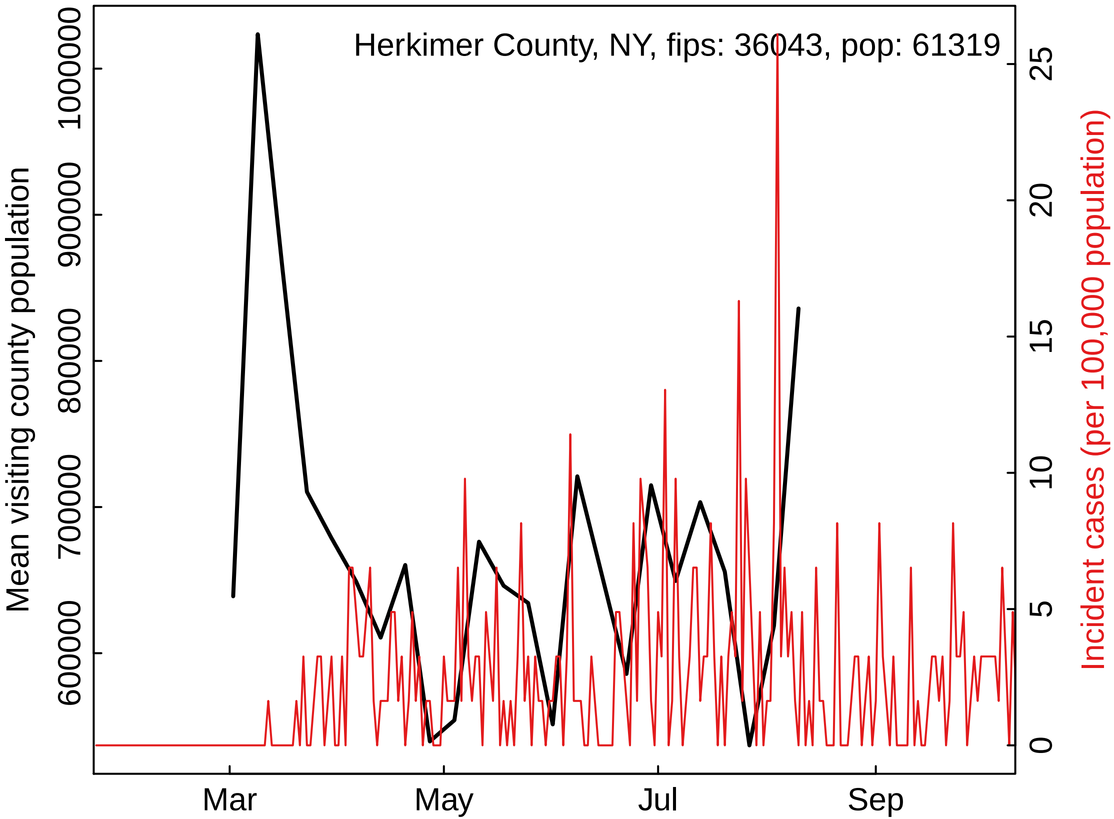

Herkimer County, NY, fips: 36043, pop: 61319
Mean visiting county population
600000
700000
800000
900000
1000000
Incident cases (per 100,000 population)
0
5
10
15
20
25
Mar
May
Jul
Sep

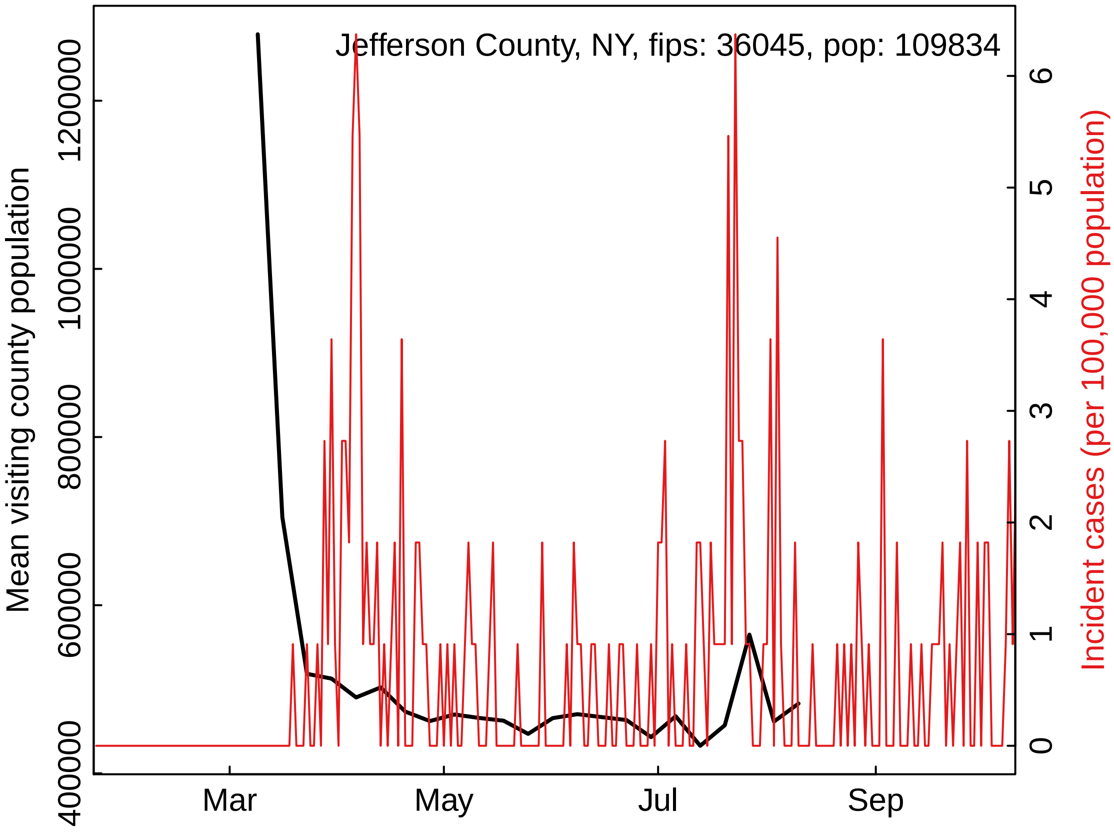

Jefferson County, NY, fips: 36045, pop: 109834
Mean visiting county population
400000
600000
800000
1000000
1200000
Incident cases (per 100,000 population)
0
1
2
3
4
5
6
Mar
May
Jul
Sep

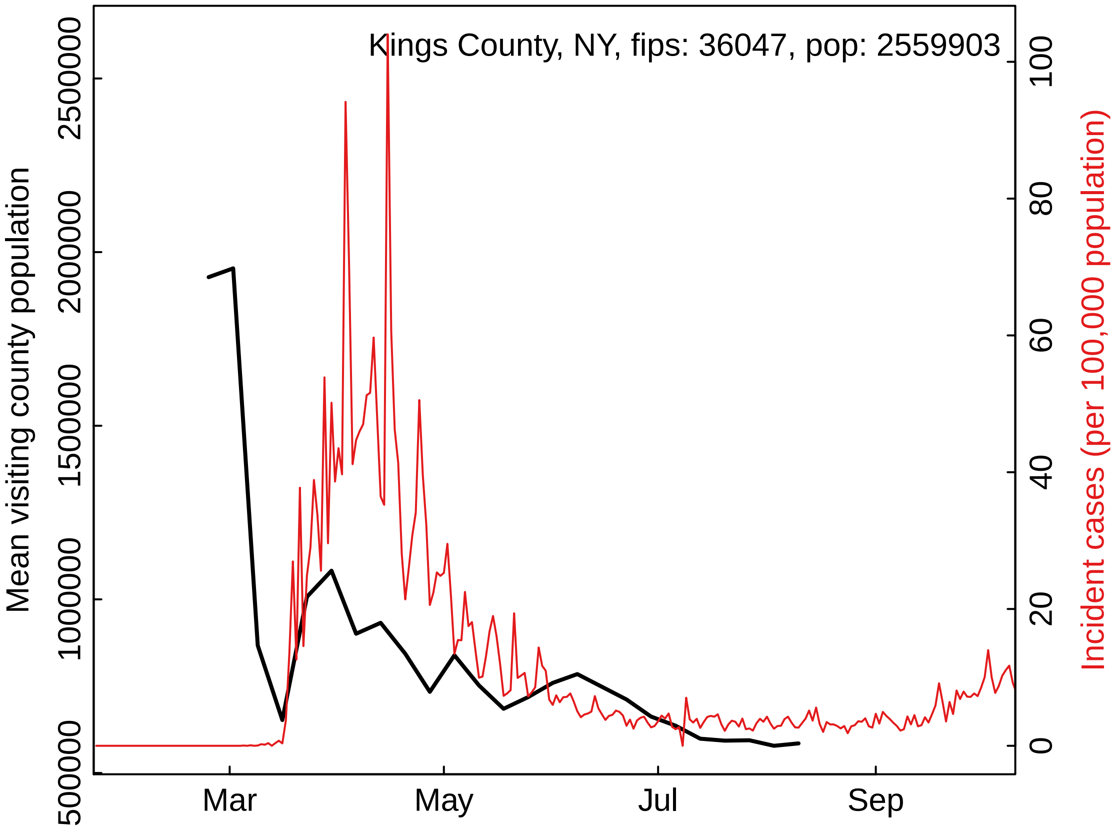

Kings County, NY, fips: 36047, pop: 2559903
Mean visiting county population
500000
1000000
1500000
2000000
2500000
Incident cases (per 100,000 population)
0
20
40
60
80
100
Mar
May
Jul
Sep

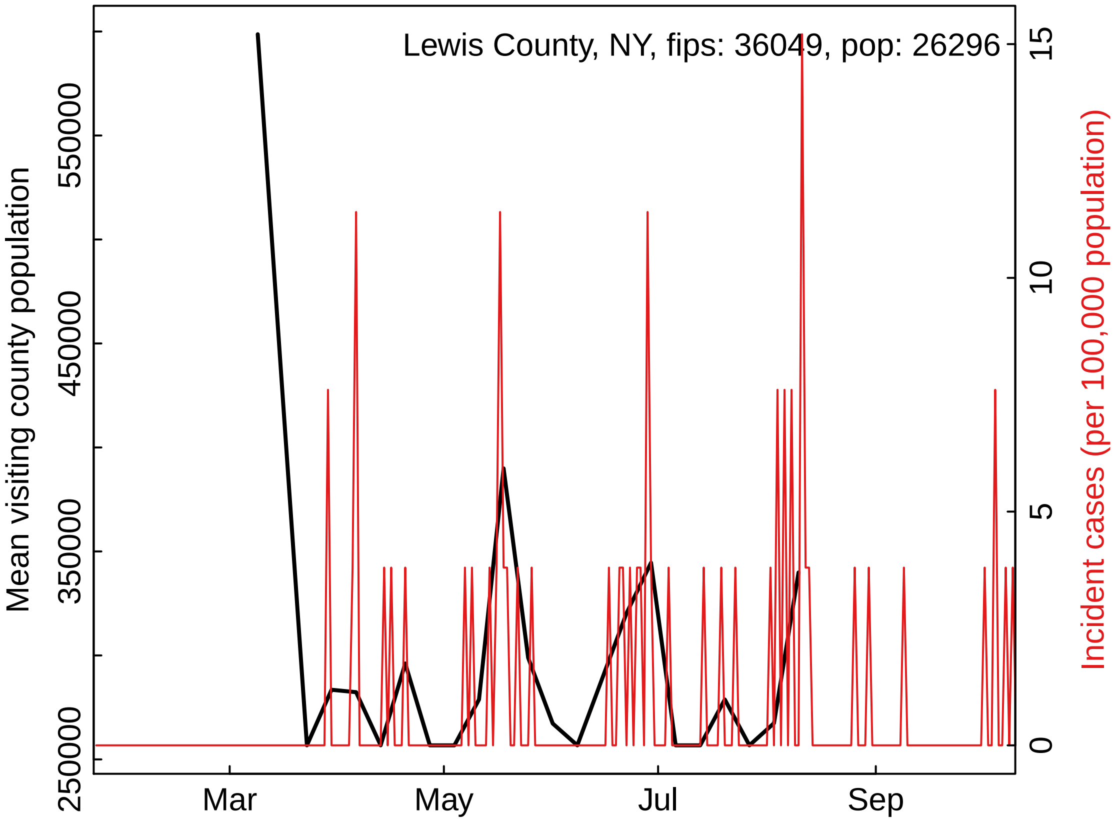

Lewis County, NY, fips: 36049, pop: 26296
Mean visiting county population
250000
350000
450000
550000
Incident cases (per 100,000 population)
0
5
10
15
Mar
May
Jul
Sep

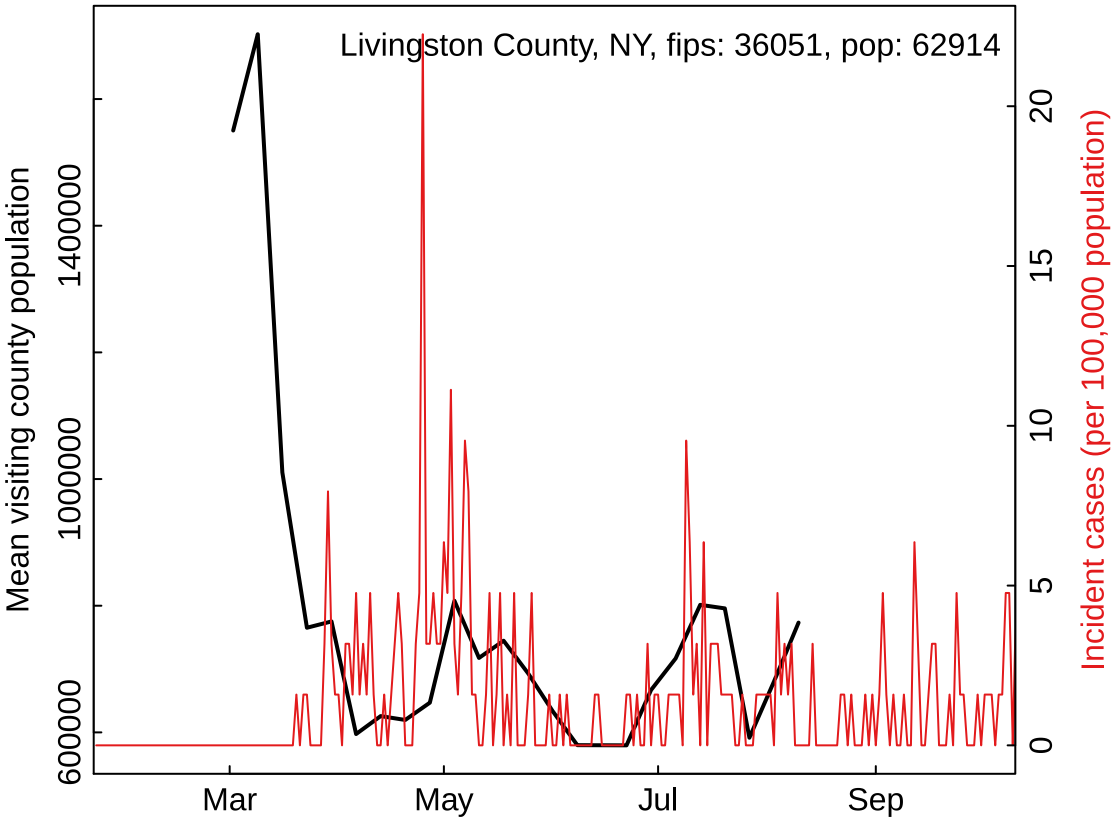

Livingston County, NY, fips: 36051, pop: 62914
Mean visiting county population
600000
1000000
1400000
Incident cases (per 100,000 population)
0
5
10
15
20
Mar
May
Jul
Sep

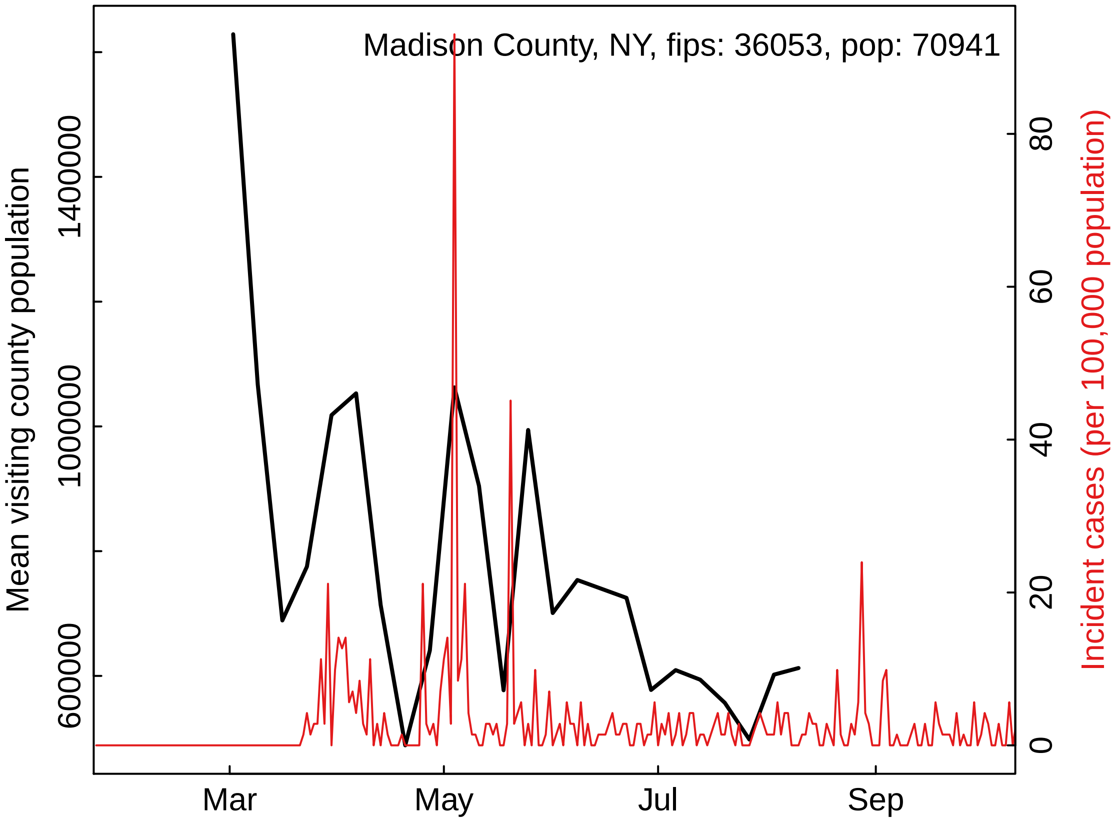

Madison County, NY, fips: 36053, pop: 70941
Mean visiting county population
600000
1000000
1400000
Incident cases (per 100,000 population)
0
20
40
60
80
Mar
May
Jul
Sep

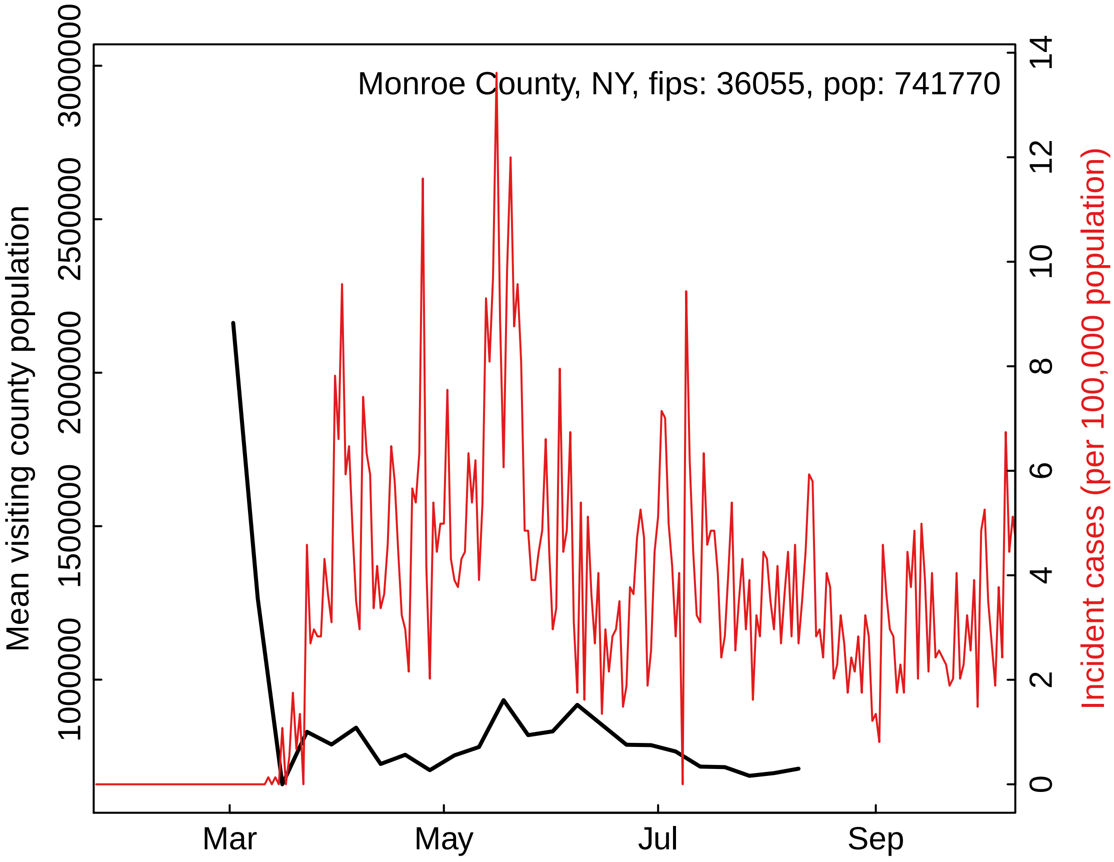

Monroe County, NY, fips: 36055, pop: 741770
Mean visiting county population
1000000
1500000
2000000
2500000
3000000
Incident cases (per 100,000 population)
0
2
4
6
8
10
12
14
Mar
May
Jul
Sep

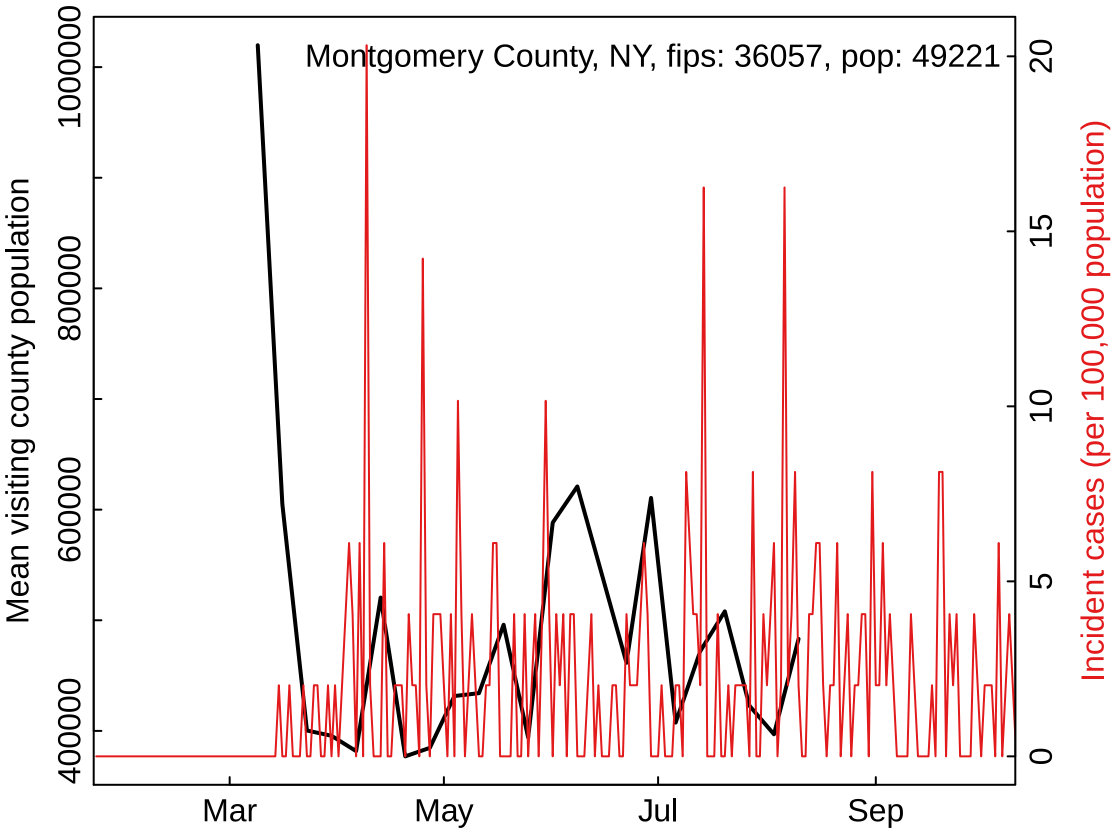

Montgomery County, NY, fips: 36057, pop: 49221
Mean visiting county population
400000
600000
800000
1000000
Incident cases (per 100,000 population)
0
5
10
15
20
Mar
May
Jul
Sep

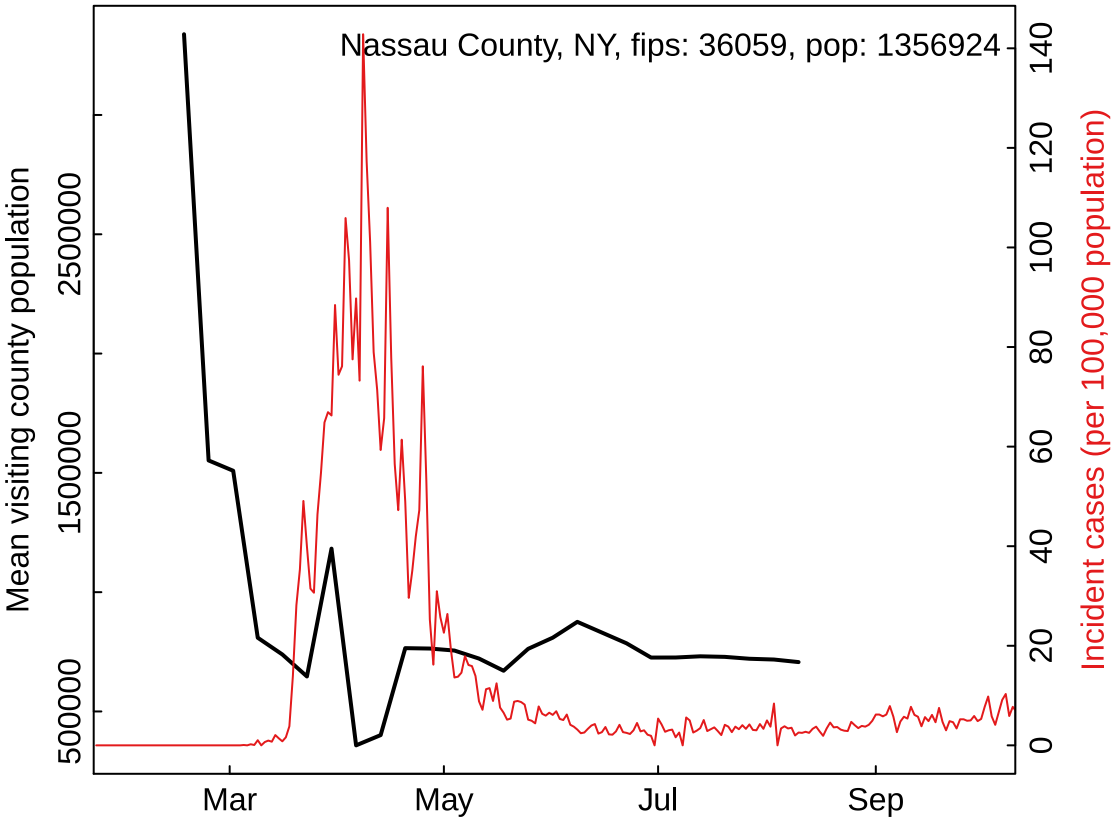

Nassau County, NY, fips: 36059, pop: 1356924
Mean visiting county population
500000
1500000
2500000
Incident cases (per 100,000 population)
0
20
40
60
80
100
120
140
Mar
May
Jul
Sep

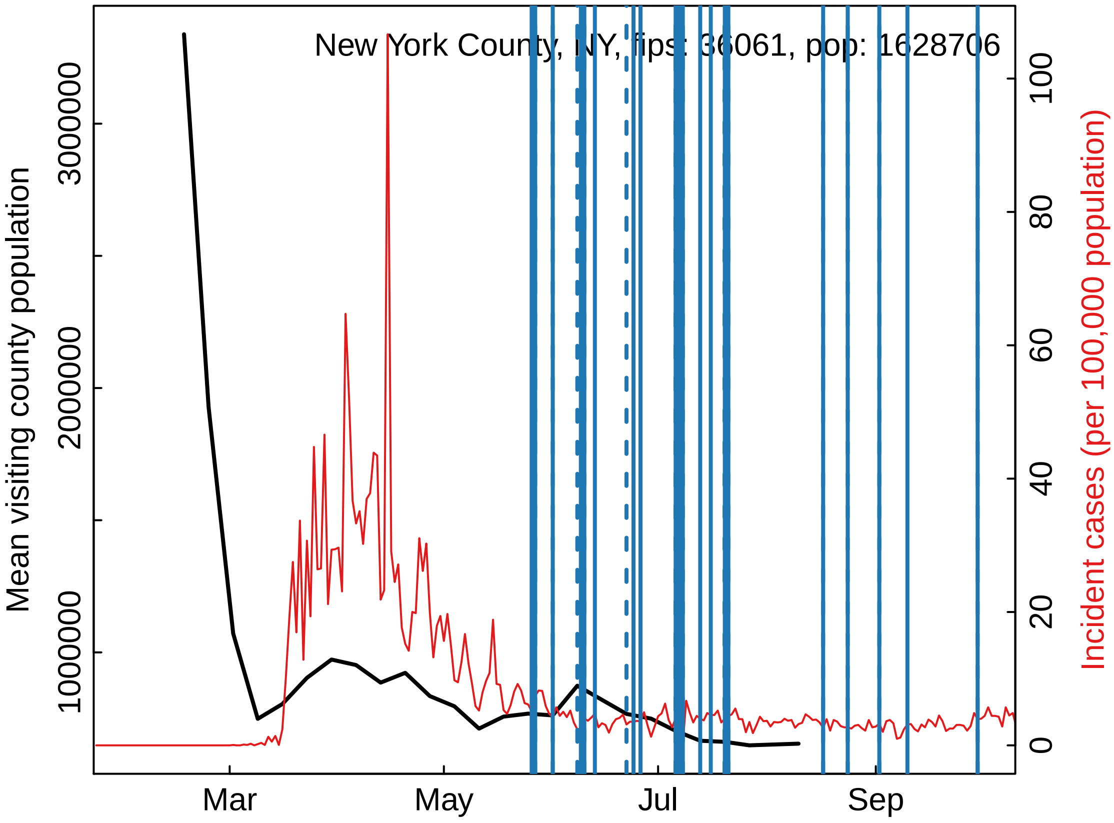
New York County, NY, fips: 36061, pop: 1628706
Mean visiting county population
1000000
2000000
3000000
Incident cases (per 100,000 population)
0
20
40
60
80
100
Mar
May
Jul
Sep

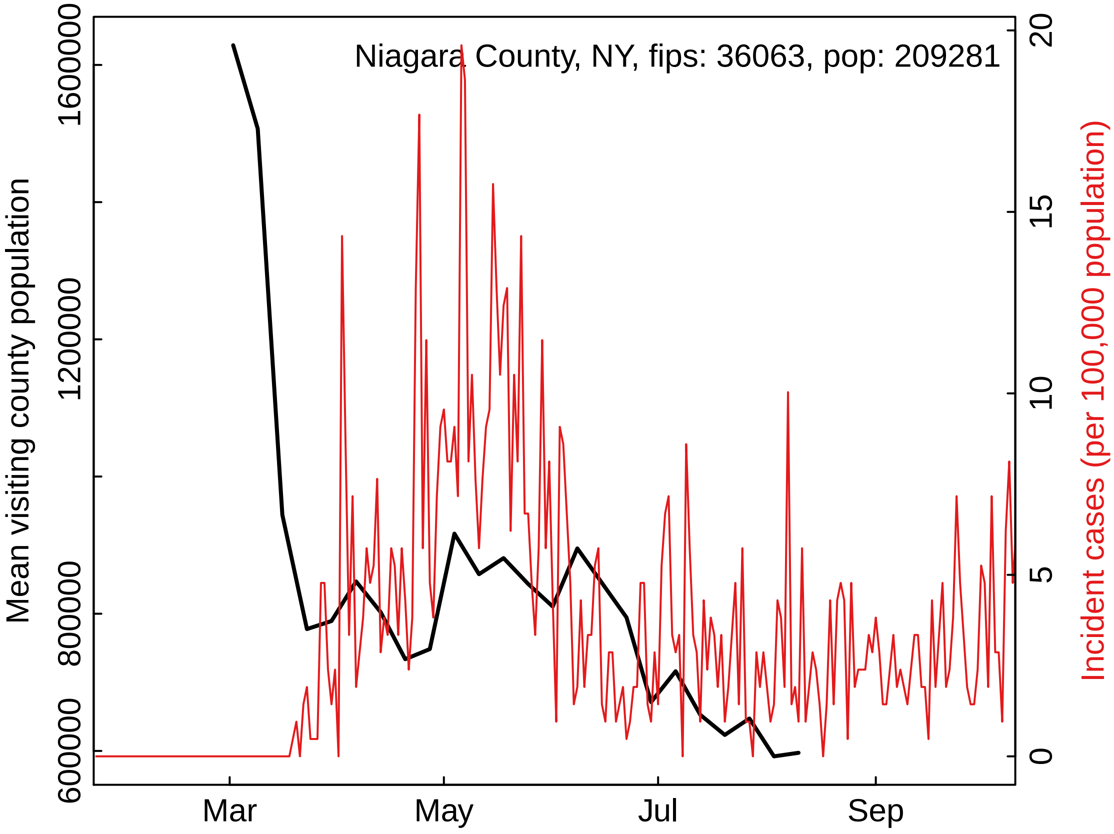

Niagara County, NY, fips: 36063, pop: 209281
Mean visiting county population
600000
800000
1200000
1600000
Incident cases (per 100,000 population)
0
5
10
15
20
Mar
May
Jul
Sep

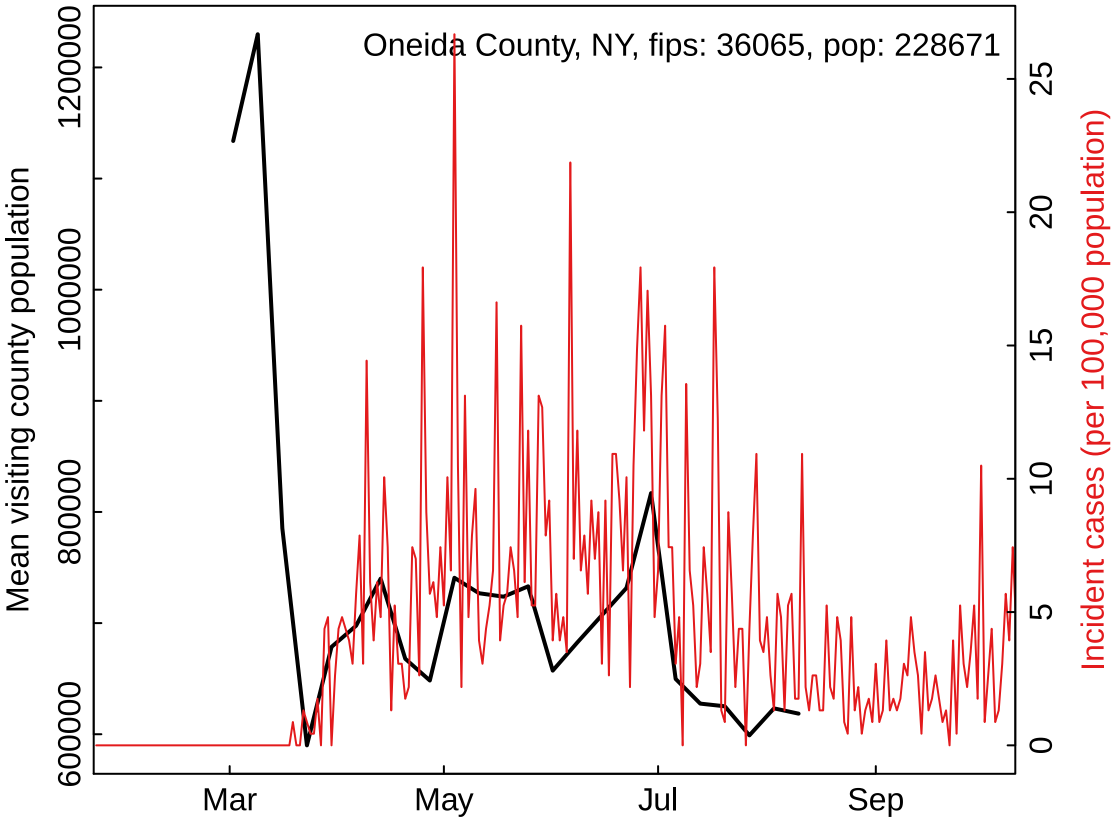

Oneida County, NY, fips: 36065, pop: 228671
Mean visiting county population
Incident cases (per 100,000 population)
600000
800000
1000000
1200000
0
5
10
15
20
25
Mar
May
Jul
Sep

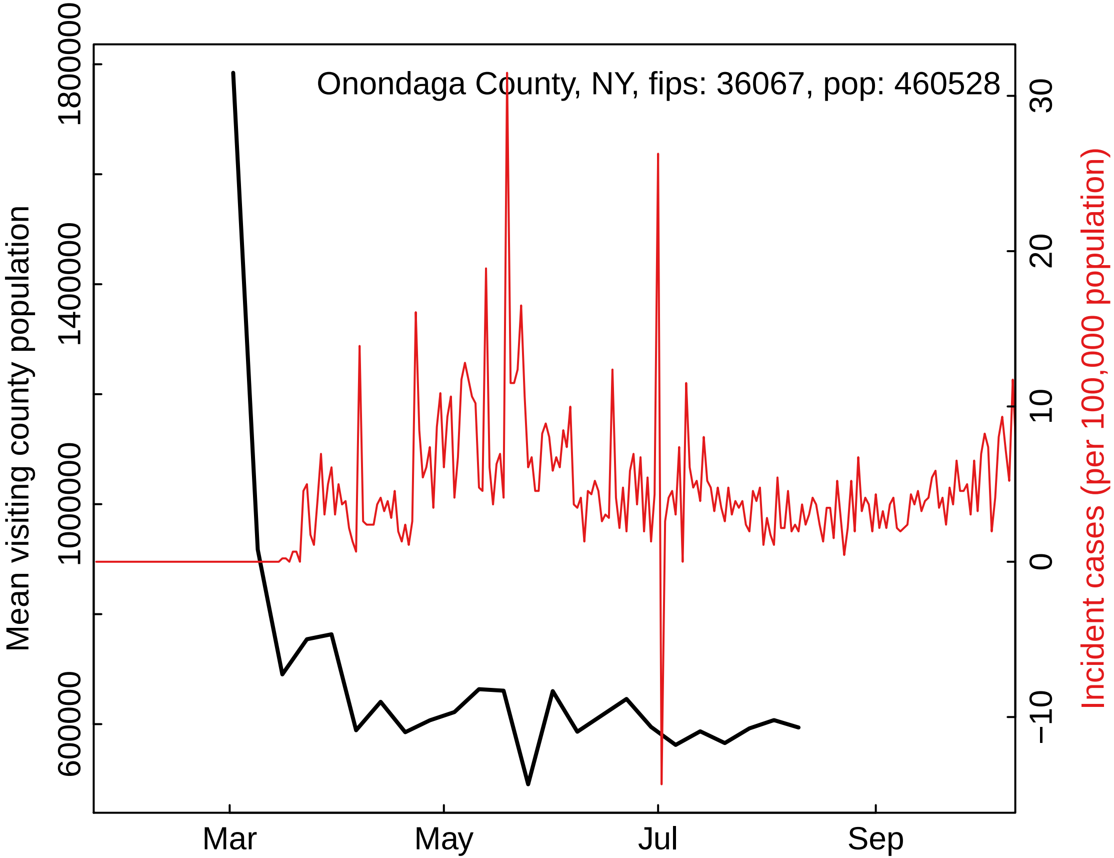

Onondaga County, NY, fips: 36067, pop: 460528
Mean visiting county population
600000
1000000
1400000
1800000
Incident cases (per 100,000 population)
−10
0
10
20
30
Mar
May
Jul
Sep

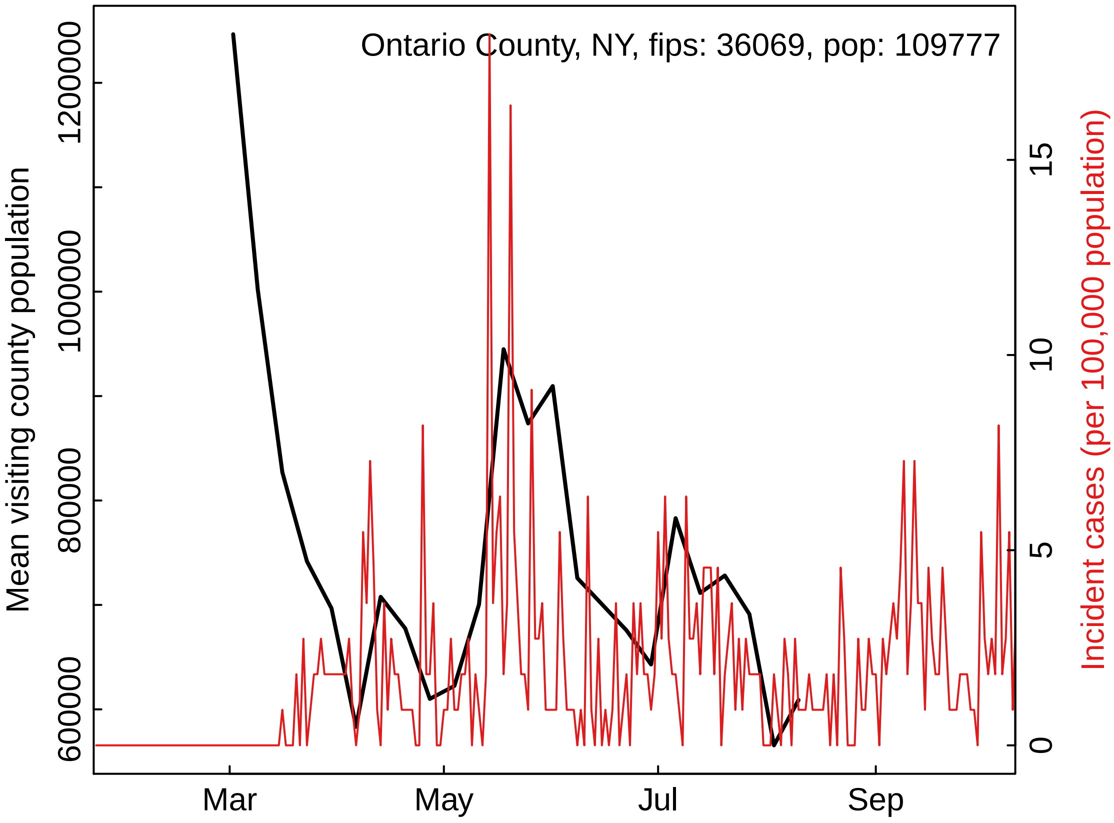

Ontario County, NY, fips: 36069, pop: 109777
Mean visiting county population
Incident cases (per 100,000 population)
600000
800000
1000000
1200000
0
5
10
15
Mar
May
Jul
Sep

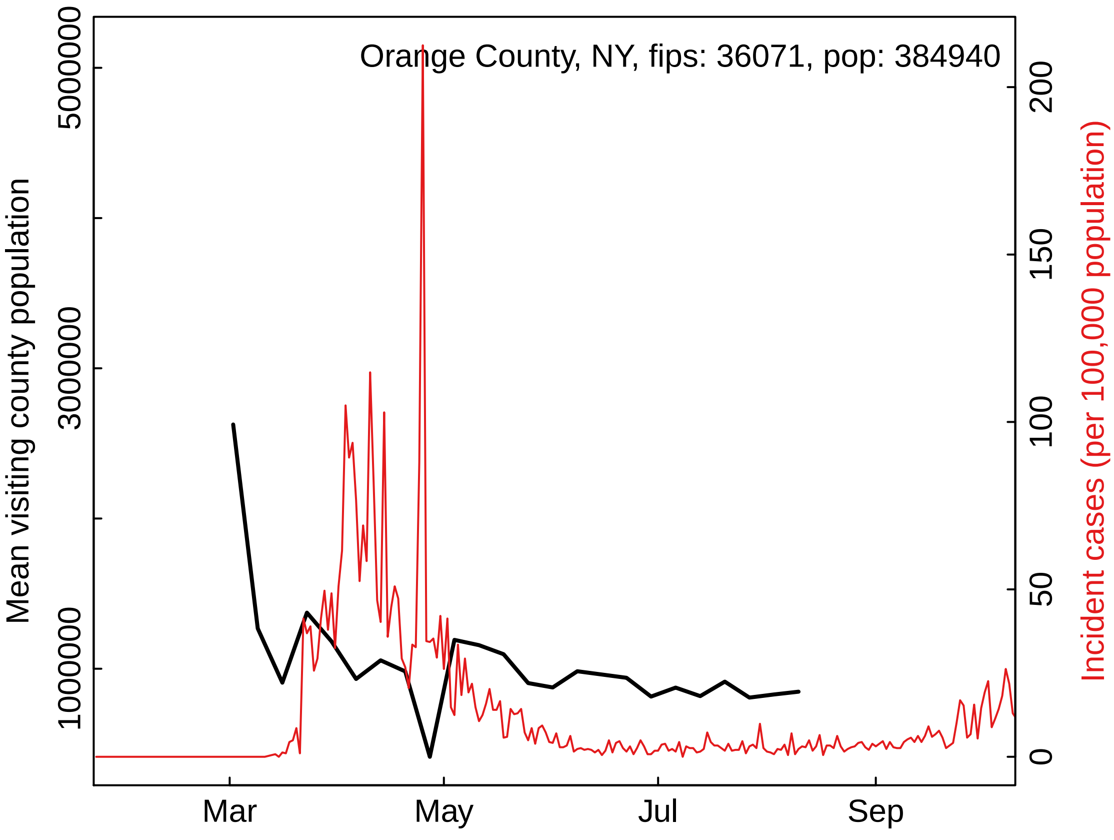
Orange County, NY, fips: 36071, pop: 384940
Mean visiting county population
1000000
3000000
5000000
Incident cases (per 100,000 population)
0
50
100
150
200
Mar
May
Jul
Sep

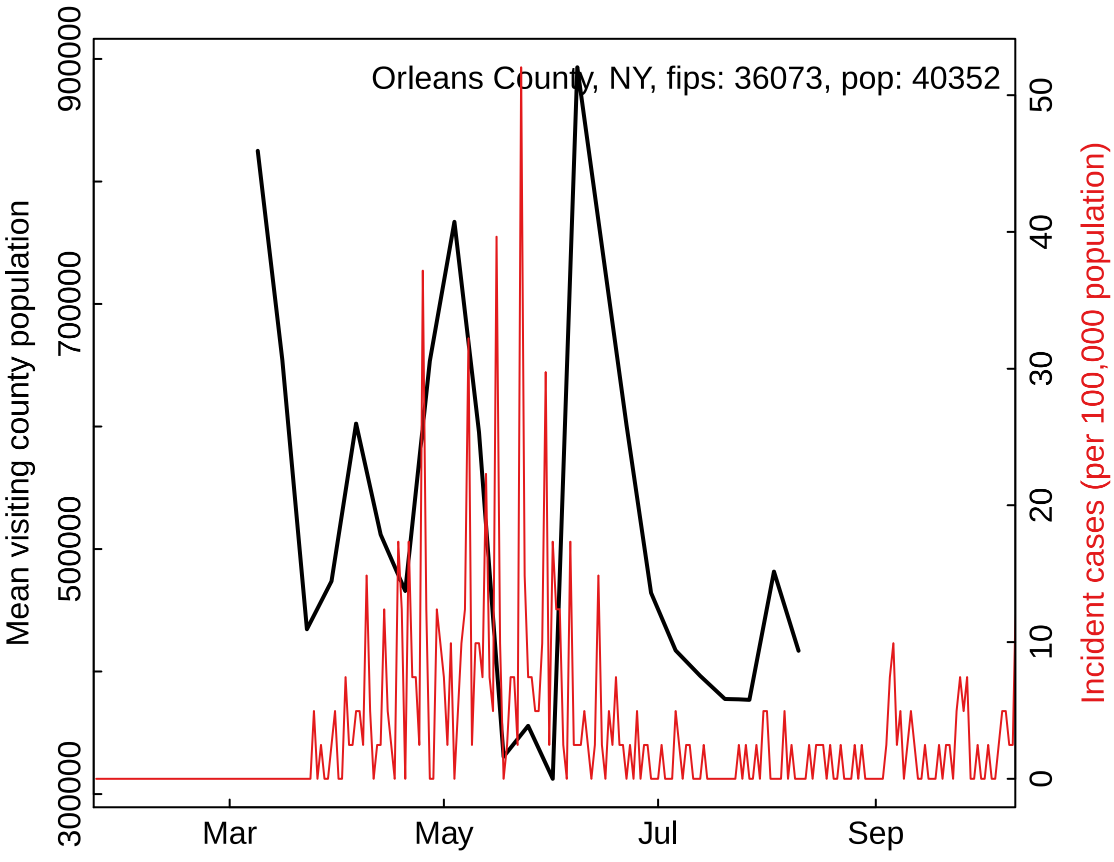

Orleans County, NY, fips: 36073, pop: 40352
Mean visiting county population
Incident cases (per 100,000 population)
3000000
5000000
7000000
9000000
0
10
20
30
40
50
Mar
May
Jul
Sep

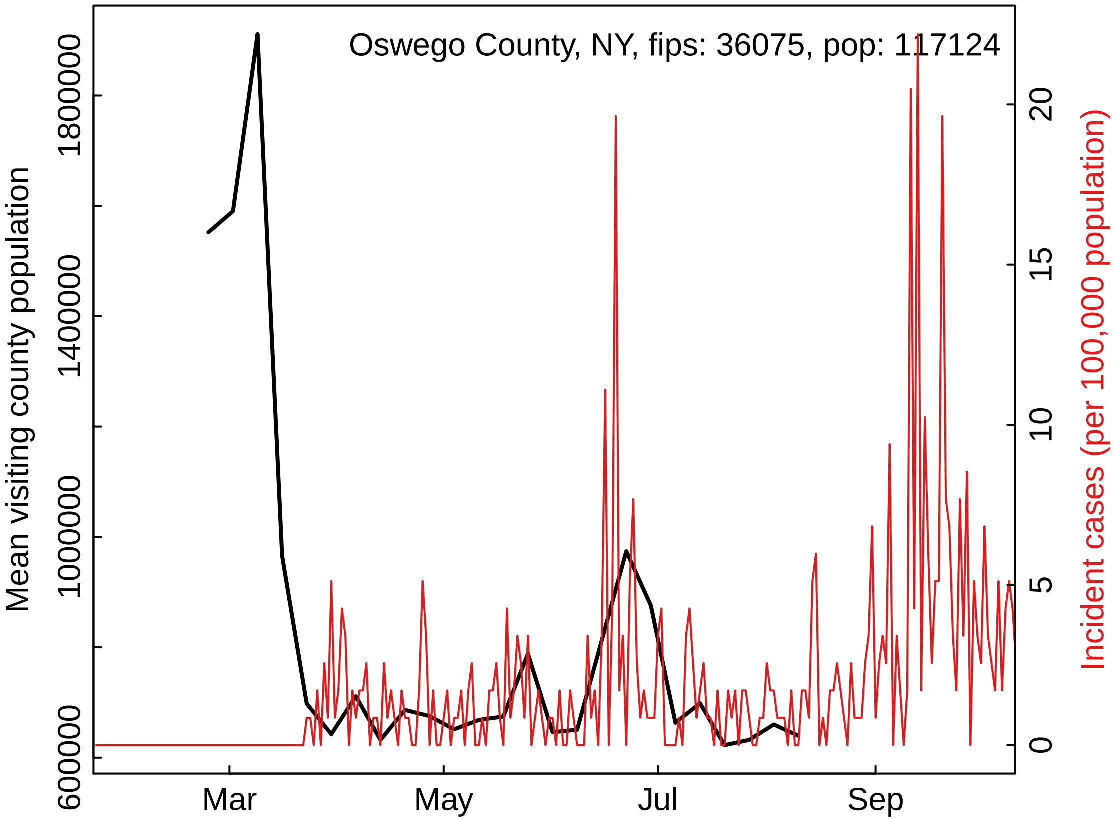

Oswego County, NY, fips: 36075, pop: 117124
Mean visiting county population
Incident cases (per 100,000 population)
600000
1000000
1400000
1800000
0
5
10
15
20
Mar
May
Jul
Sep

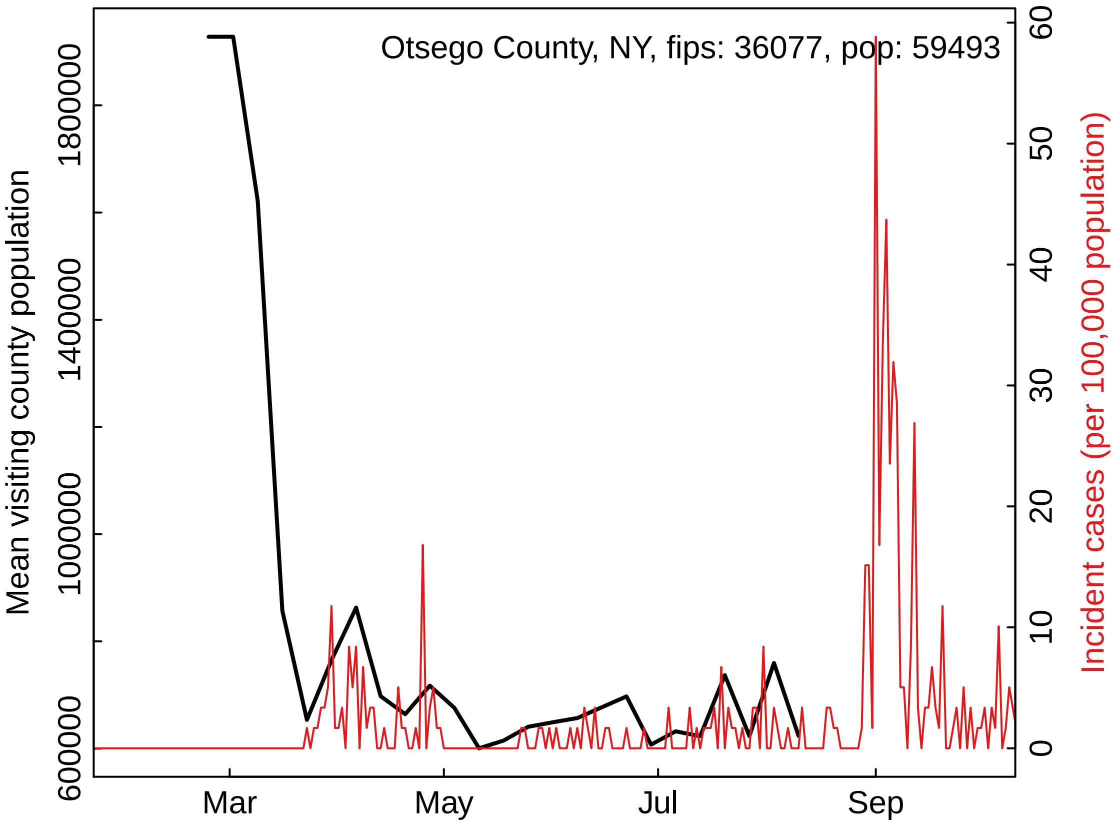

Otsego County, NY, fips: 36077, pop: 59493
Mean visiting county population
600000
1000000
1400000
1800000
Incident cases (per 100,000 population)
0
10
20
30
40
50
60
Mar
May
Jul
Sep

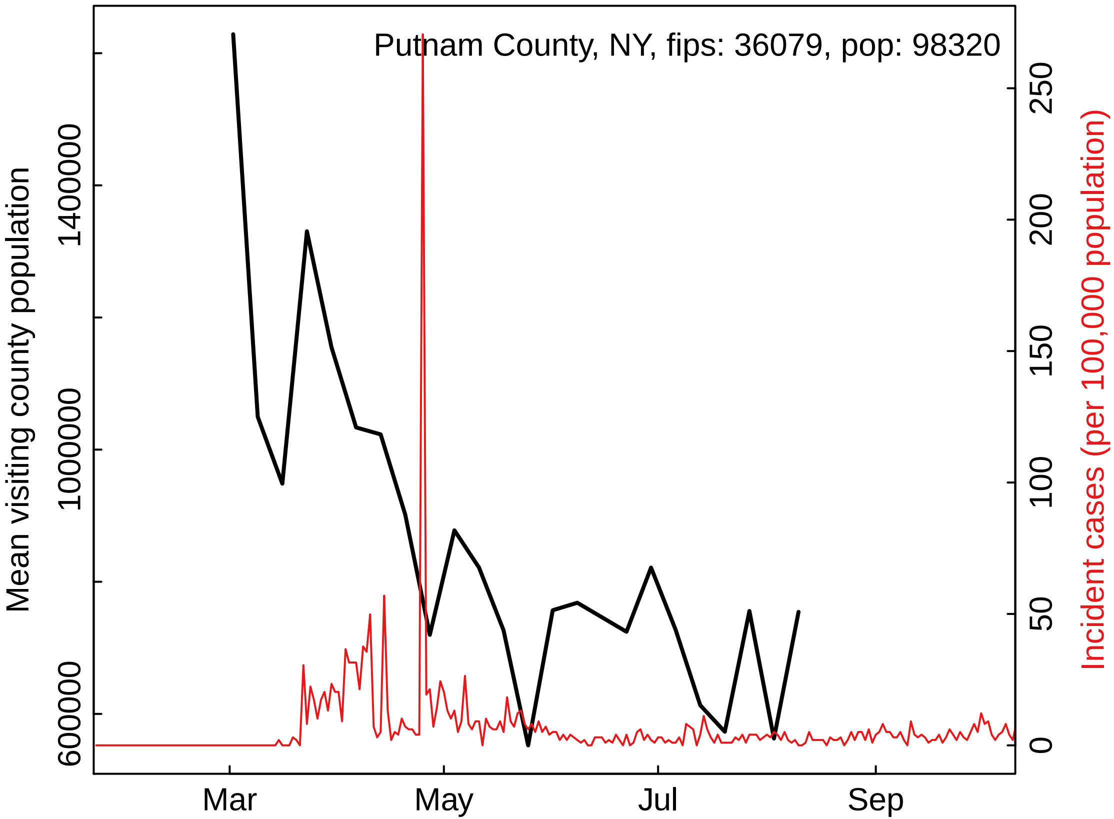

Putnam County, NY, fips: 36079, pop: 98320
Mean visiting county population
600000
1000000
1400000
Incident cases (per 100,000 population)
0
50
100
150
200
250
Mar
May
Jul
Sep

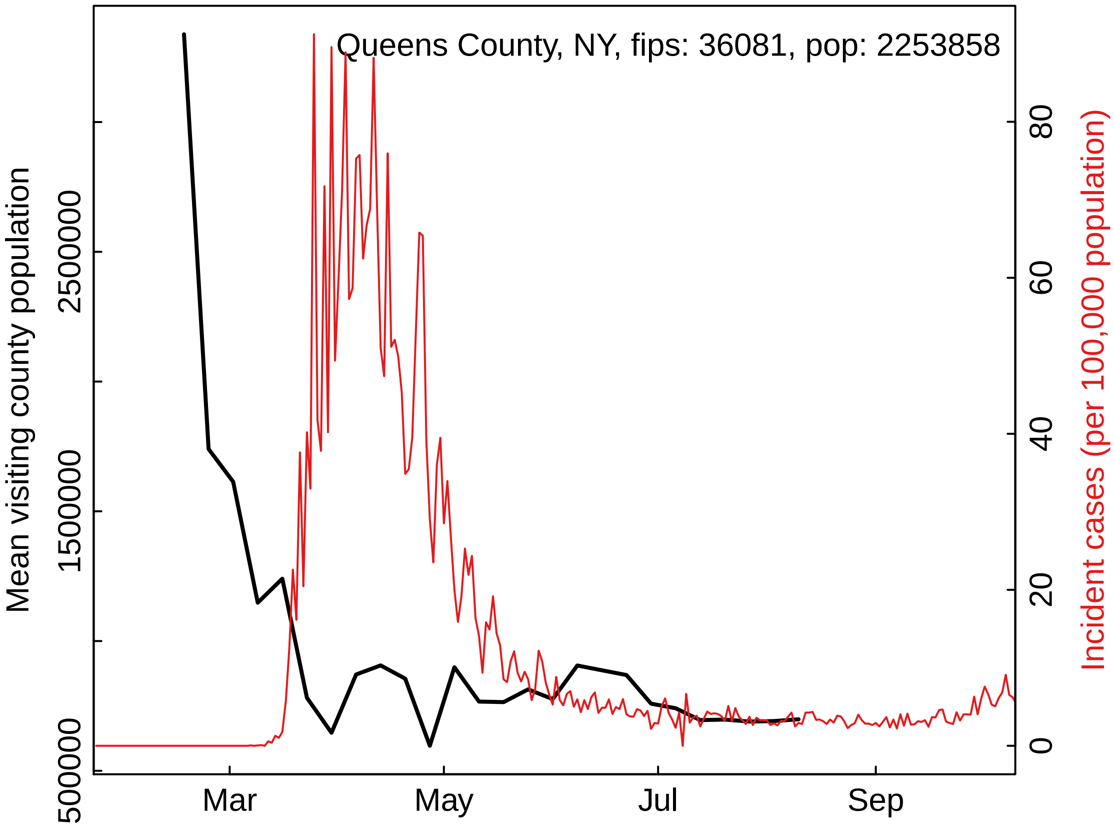
Queens County, NY, fips: 36081, pop: 2253858
Mean visiting county population
500000
1500000
2500000
Incident cases (per 100,000 population)
0
20
40
60
80
Mar
May
Jul
Sep

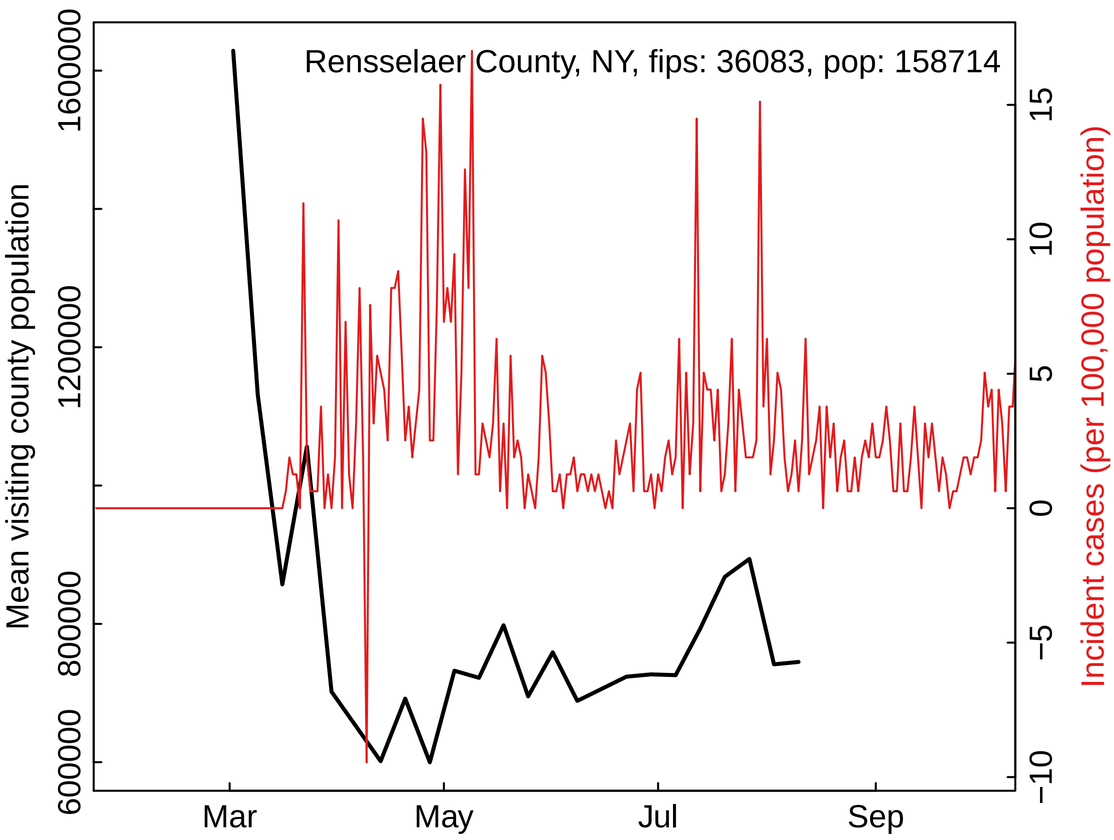

Rensselaer County, NY, fips: 36083, pop: 158714
Mean visiting county population
600000
800000
1200000
1600000
Mar
May
Jul
Sep
Incident cases (per 100,000 population)
−10
−5
0
5
10
15

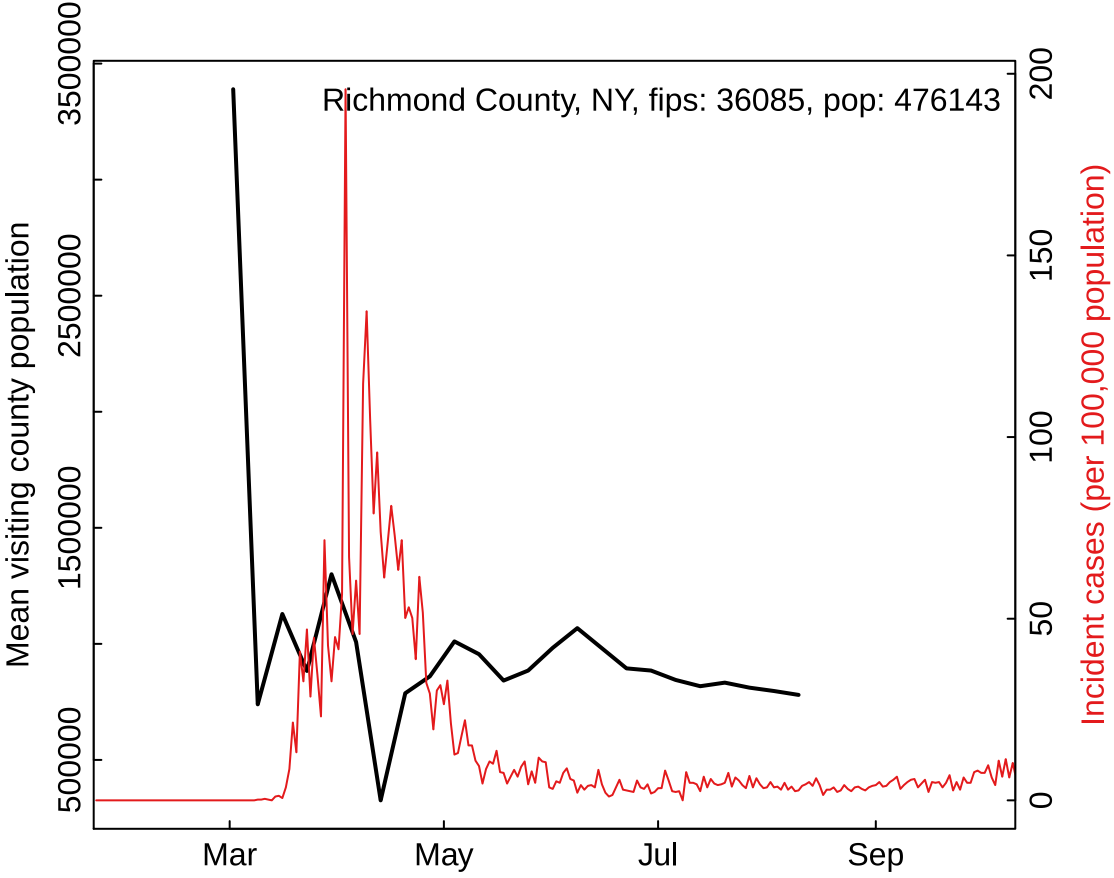

Richmond County, NY, fips: 36085, pop: 476143
Mean visiting county population
500000
1500000
2500000
3500000
Incident cases (per 100,000 population)
0
50
100
150
200
Mar
May
Jul
Sep

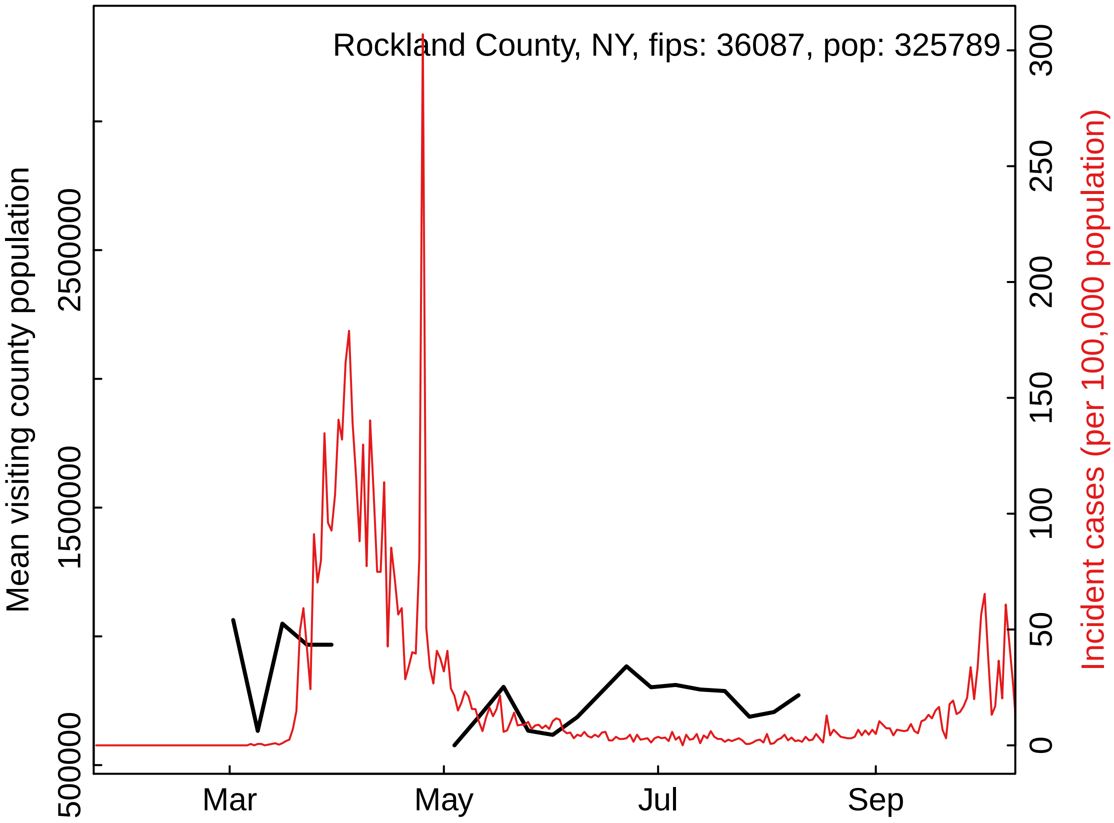

Rockland County, NY, fips: 36087, pop: 325789
Mean visiting county population
500000
1500000
2500000
Incident cases (per 100,000 population)
0
50
100
150
200
250
300
Mar
May
Jul
Sep

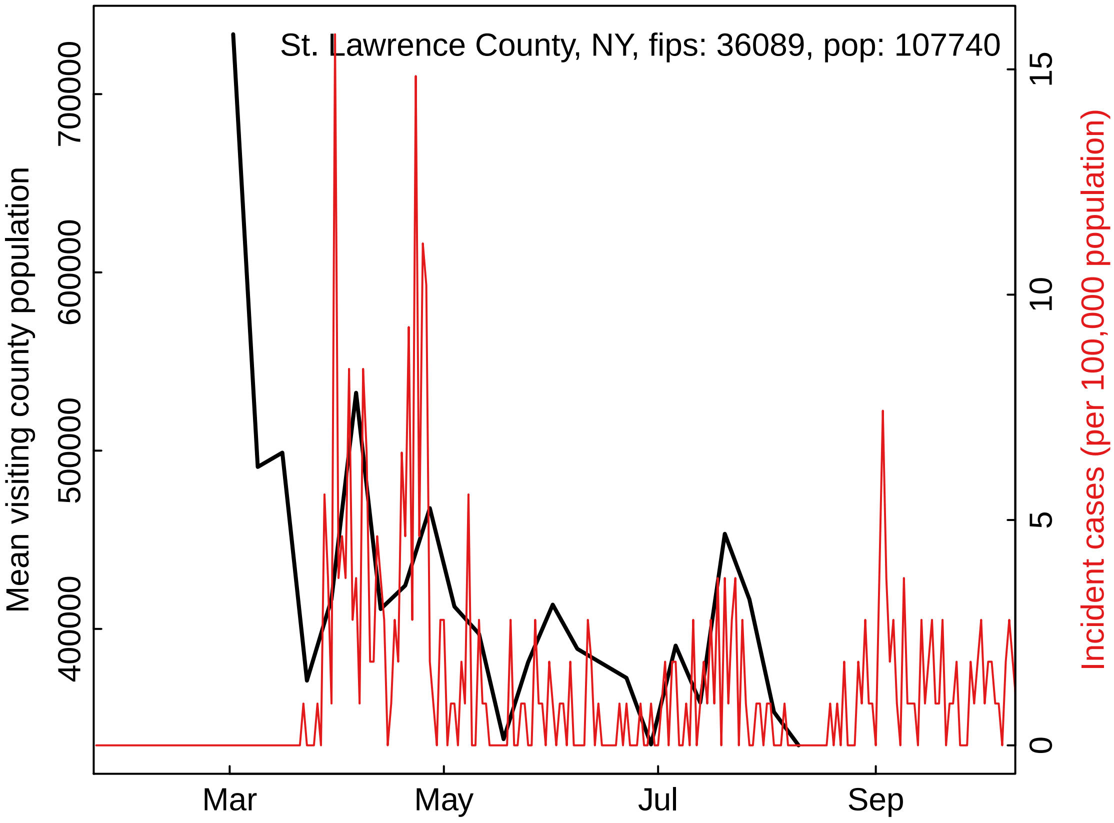
St. Lawrence County, NY, fips: 36089, pop: 107740
Mean visiting county population
400000
500000
600000
700000
Incident cases (per 100,000 population)
0
5
10
15
Mar
May
Jul
Sep

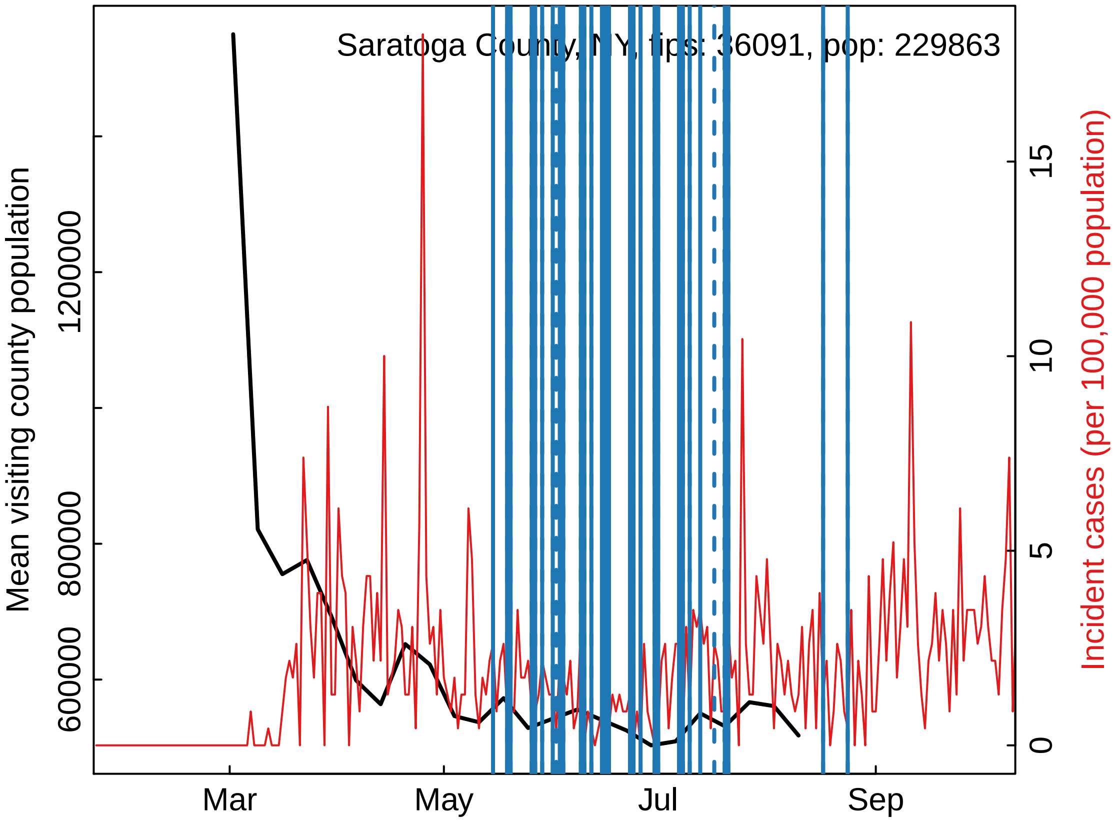

Saratoga County, NY, fips: 36091, pop: 229863
Mean visiting county population
600000
800000
1200000
Incident cases (per 100,000 population)
0
5
10
15
Mar
May
Jul
Sep

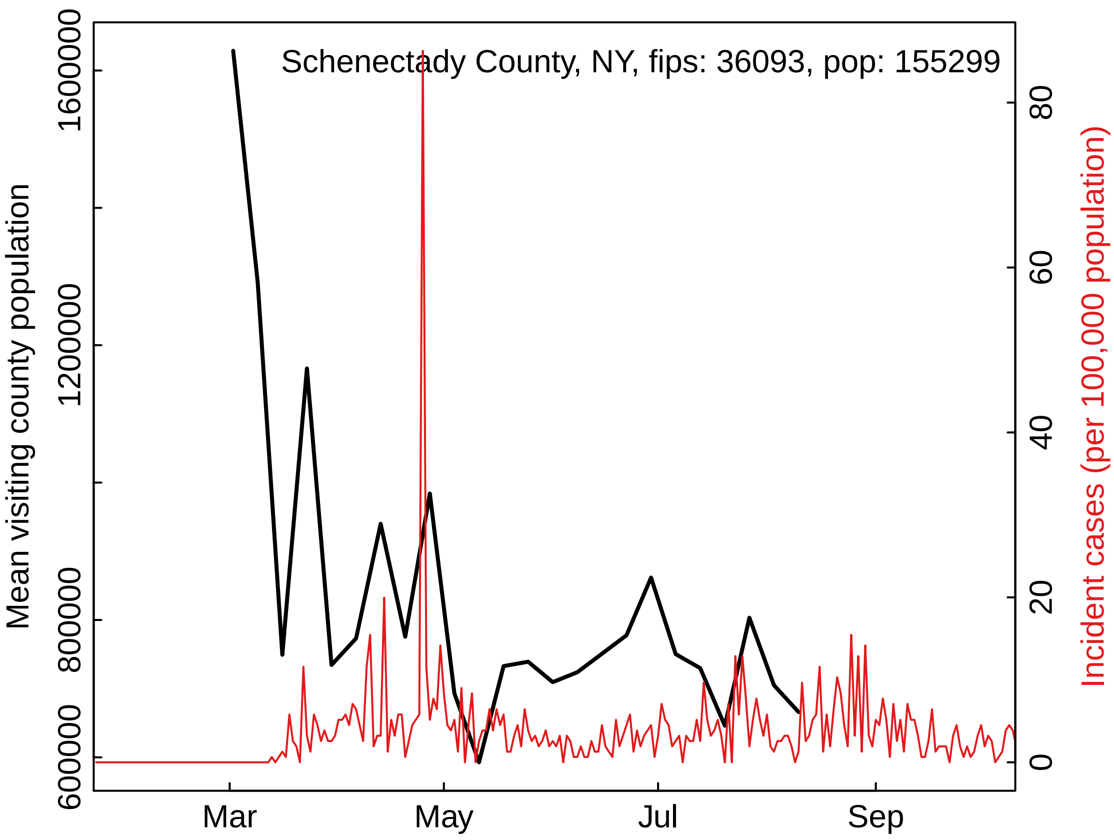

Schenectady County, NY, fips: 36093, pop: 155299
Mean visiting county population
Incident cases (per 100,000 population)
600000
800000
1200000
1600000
0
20
40
60
80
Mar
May
Jul
Sep

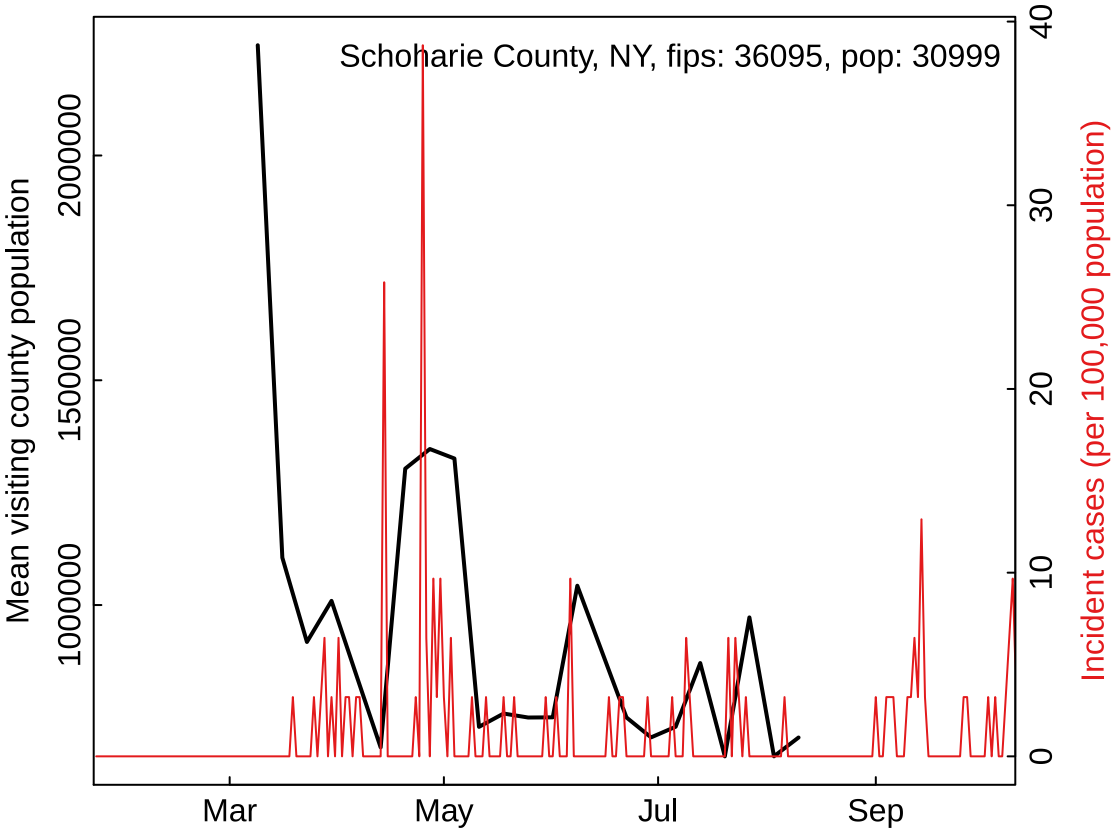

Schoharie County, NY, fips: 36095, pop: 30999
Mean visiting county population
1000000
1500000
2000000
Incident cases (per 100,000 population)
0
10
20
30
40
Mar
May
Jul
Sep

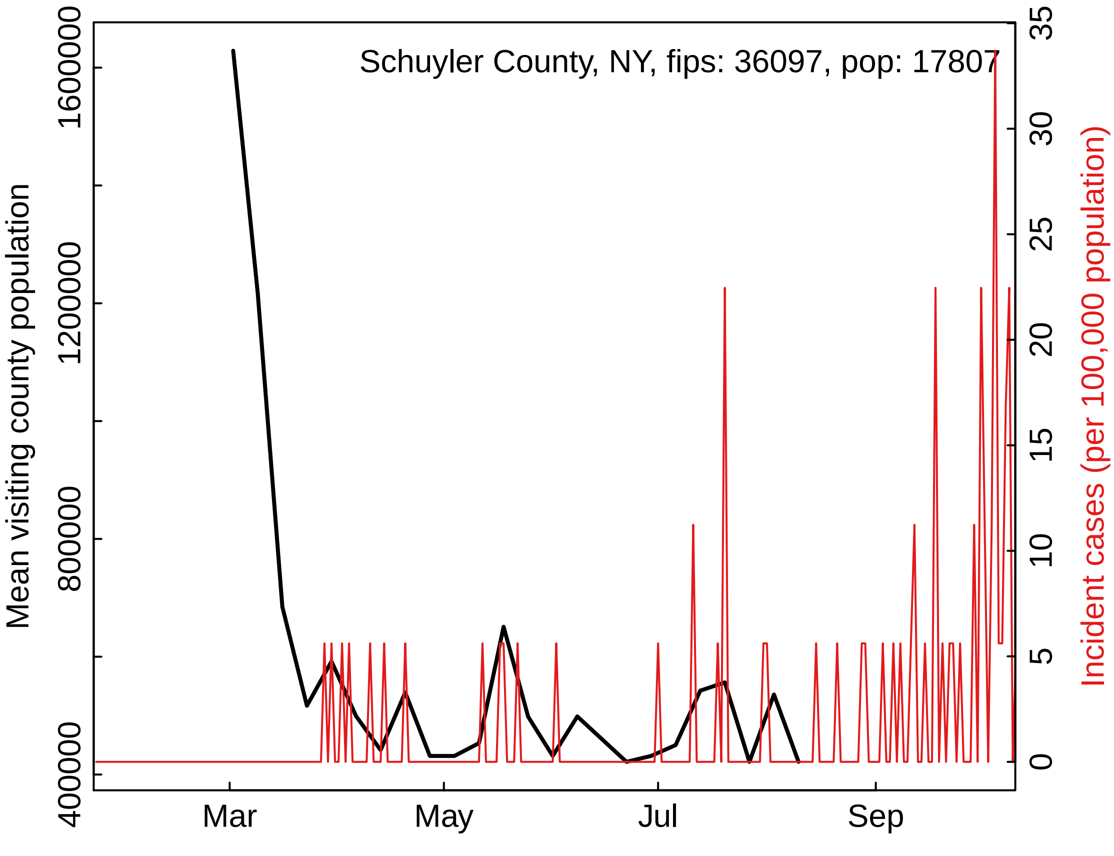
Schuyler County, NY, fips: 36097, pop: 17807
Mean visiting county population
400000
800000
1200000
1600000
Incident cases (per 100,000 population)
0
5
10
15
20
25
30
35
Mar
May
Jul
Sep

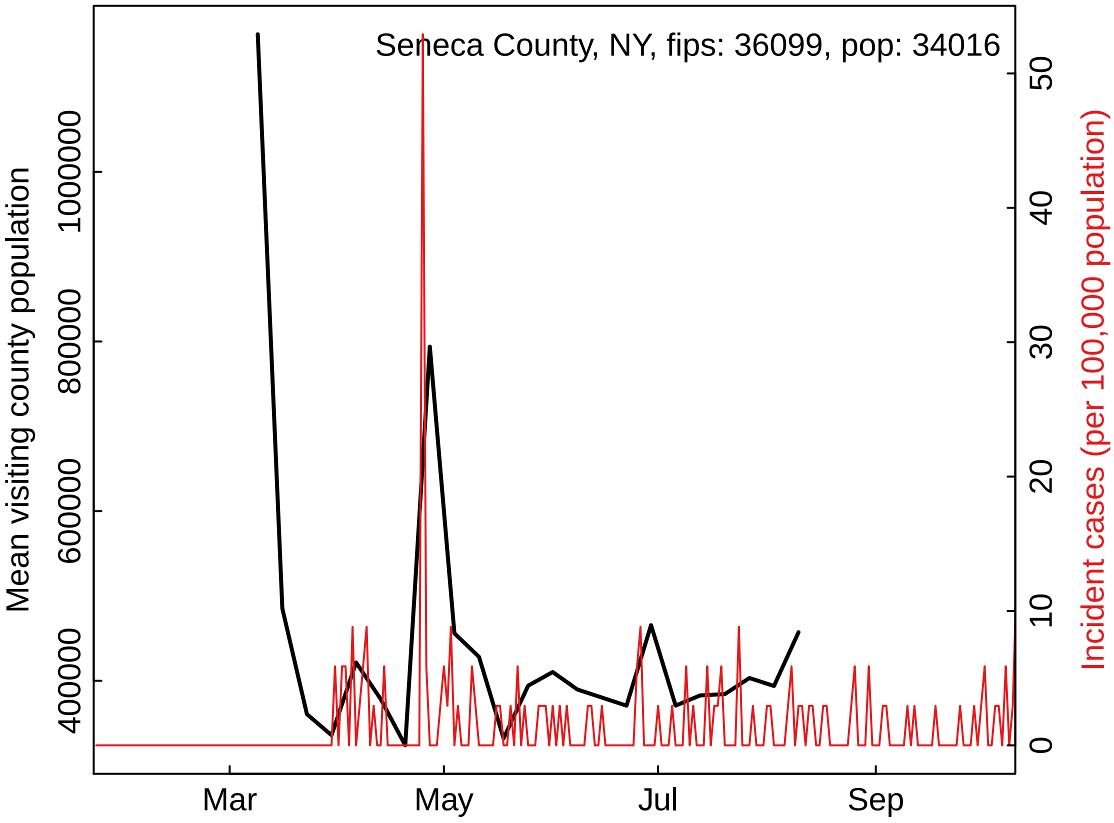

Seneca County, NY, fips: 36099, pop: 34016
Mean visiting county population
400000
600000
800000
1000000
Incident cases (per 100,000 population)
0
10
20
30
40
50
Mar
May
Jul
Sep

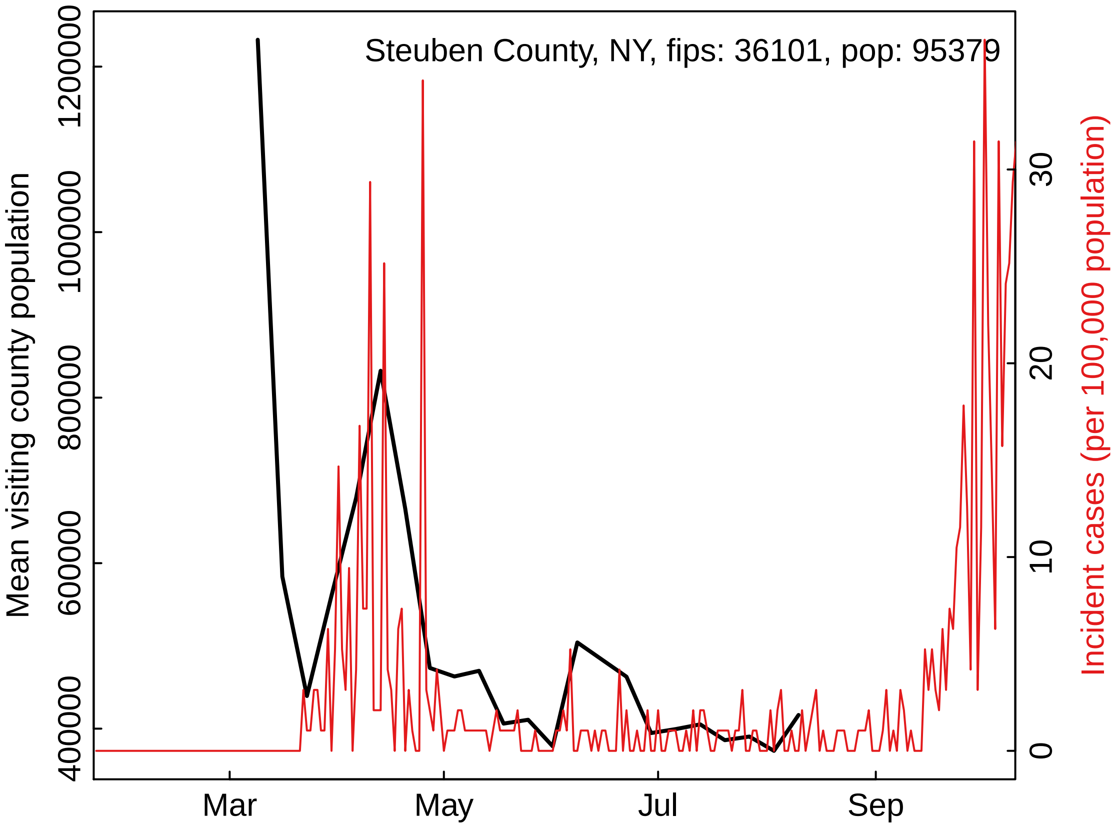

Steuben County, NY, fips: 36101, pop: 95379
Mean visiting county population
400000
600000
800000
1000000
1200000
Incident cases (per 100,000 population)
0
10
20
30
Mar
May
Jul
Sep

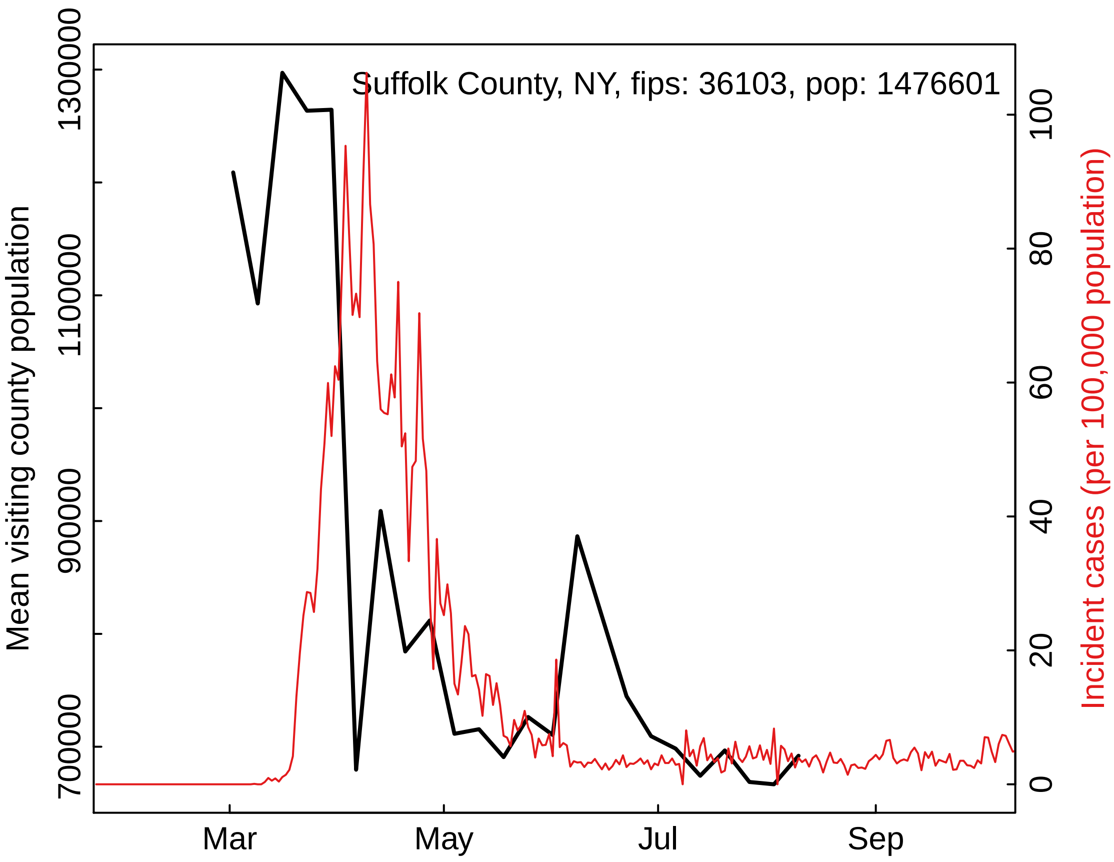
Suffolk County, NY, fips: 36103, pop: 1476601
Mean visiting county population
Incident cases (per 100,000 population)
700000
900000
1100000
1300000
0
20
40
60
80
100
Mar
May
Jul
Sep

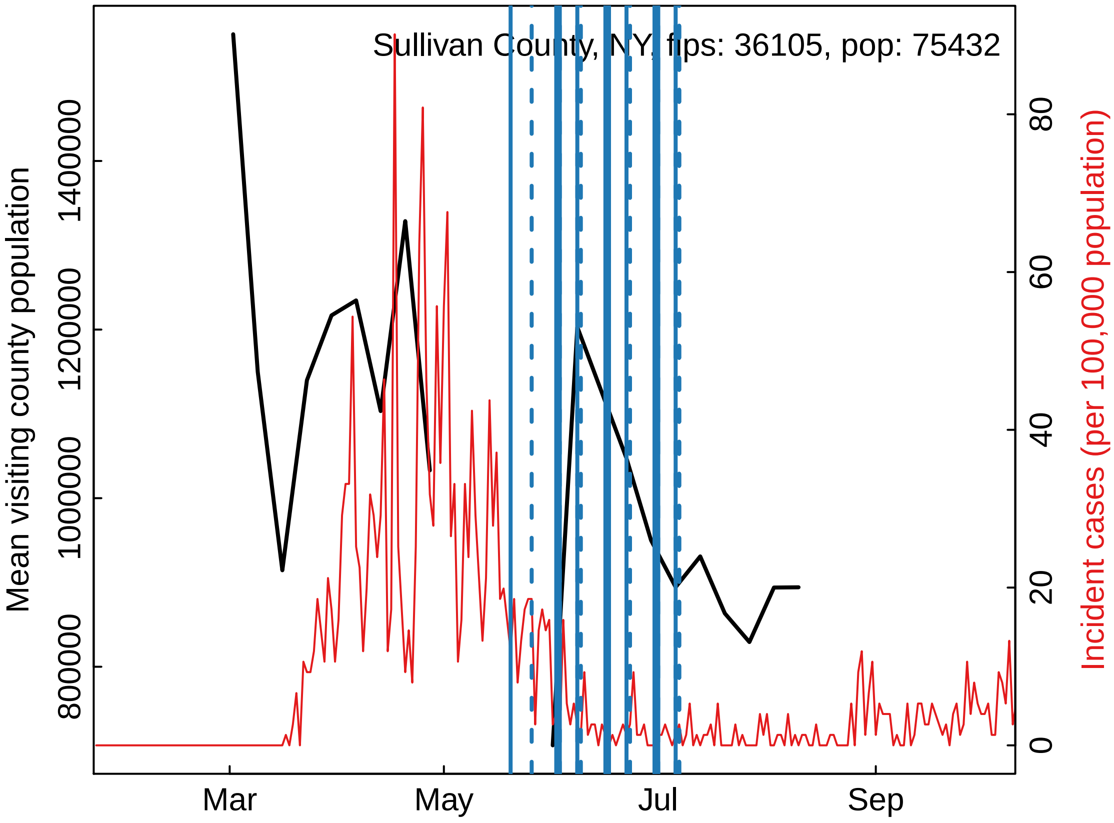

Sullivan County, NY, fips: 36105, pop: 75432
Mean visiting county population
800000
1000000
1200000
1400000
Incident cases (per 100,000 population)
0
20
40
60
80
Mar
May
Jul
Sep

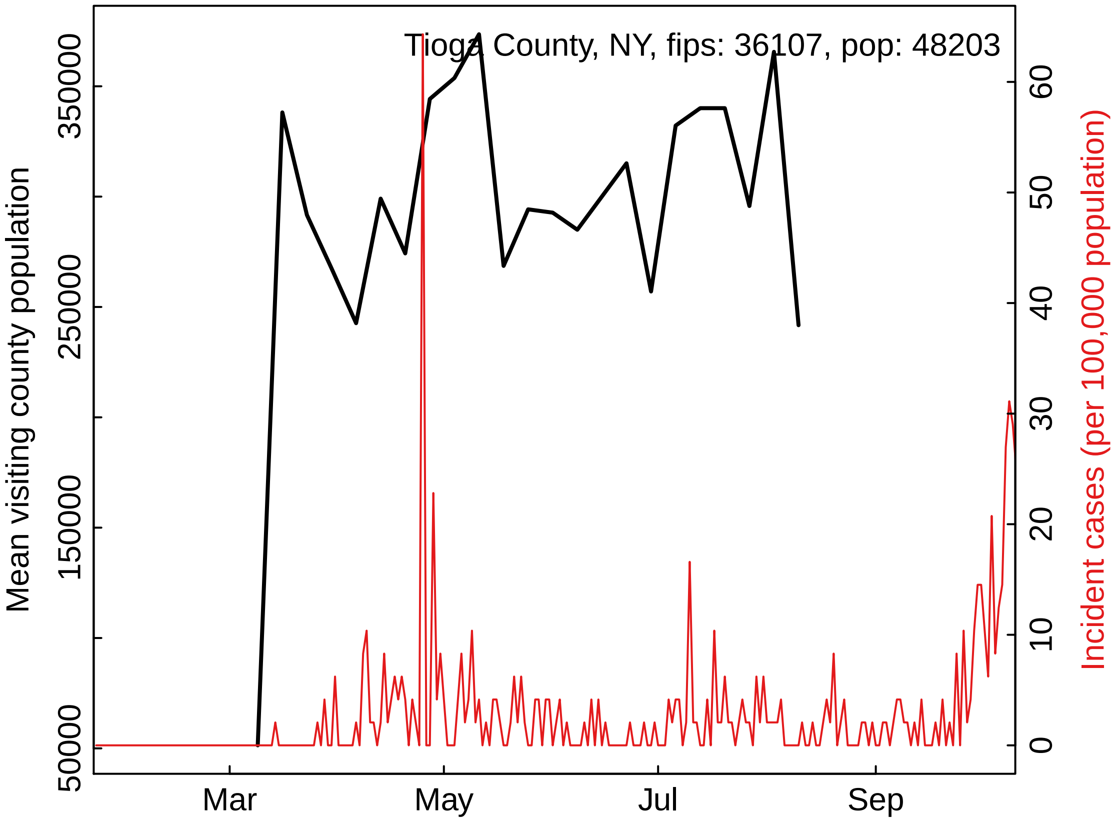

Tioga County, NY, fips: 36107, pop: 48203
Mean visiting county population
50000
150000
250000
350000
Incident cases (per 100,000 population)
0
10
20
30
40
50
60
Mar
May
Jul
Sep

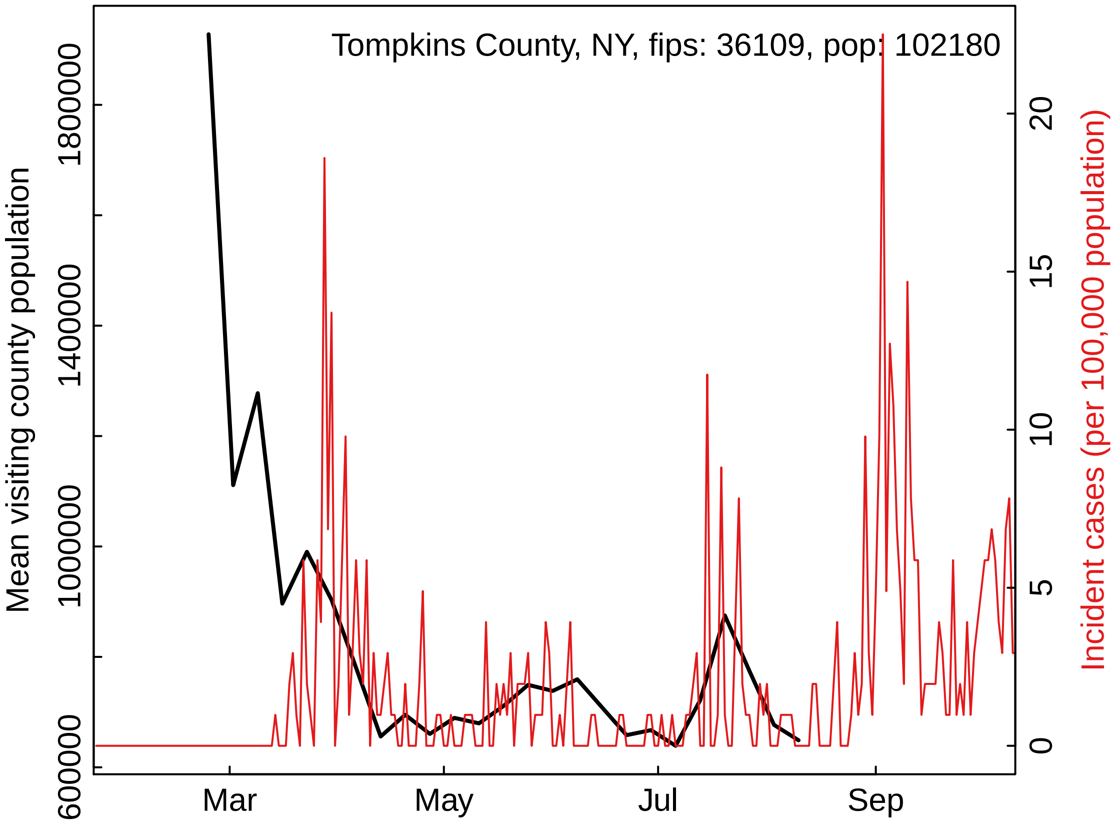

Tompkins County, NY, fips: 36109, pop: 102180
Mean visiting county population
Incident cases (per 100,000 population)
6000000
10000000
14000000
18000000
0
5
10
15
20
Mar
May
Jul
Sep

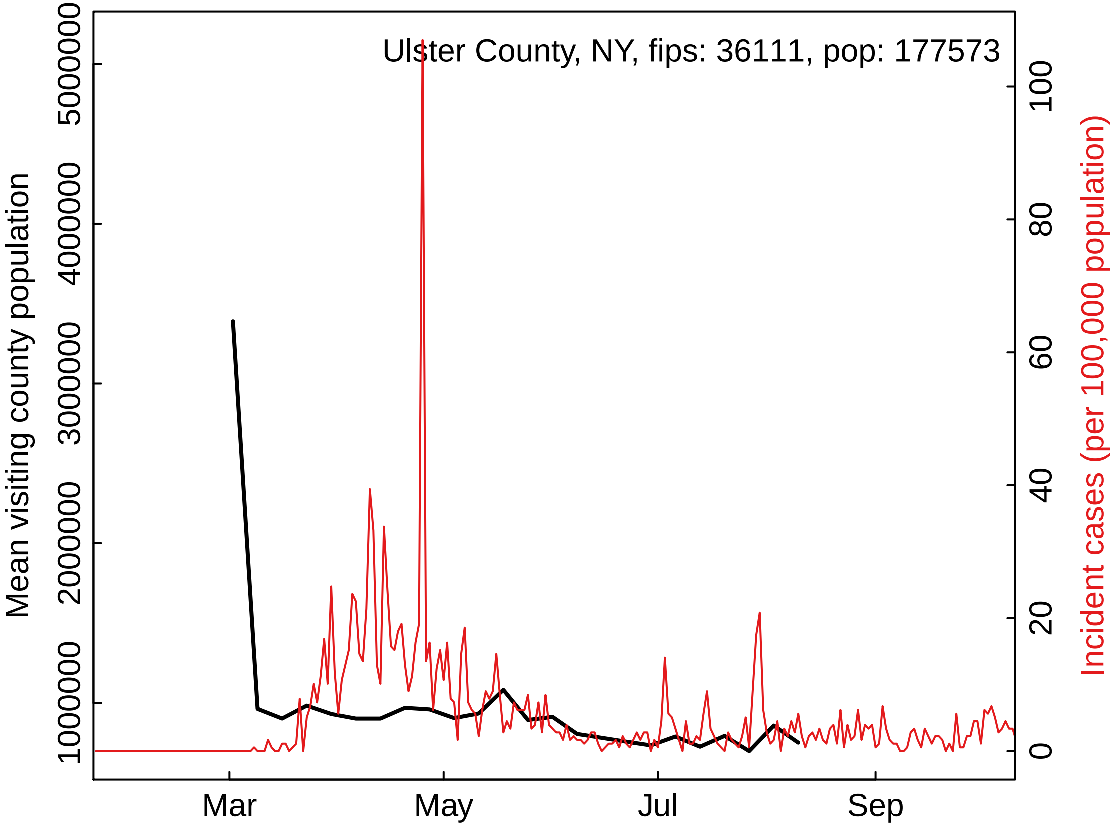

Ulster County, NY, fips: 36111, pop: 177573
Mean visiting county population
10000000
20000000
30000000
40000000
50000000
Incident cases (per 100,000 population)
0
20
40
60
80
100
Mar
May
Jul
Sep

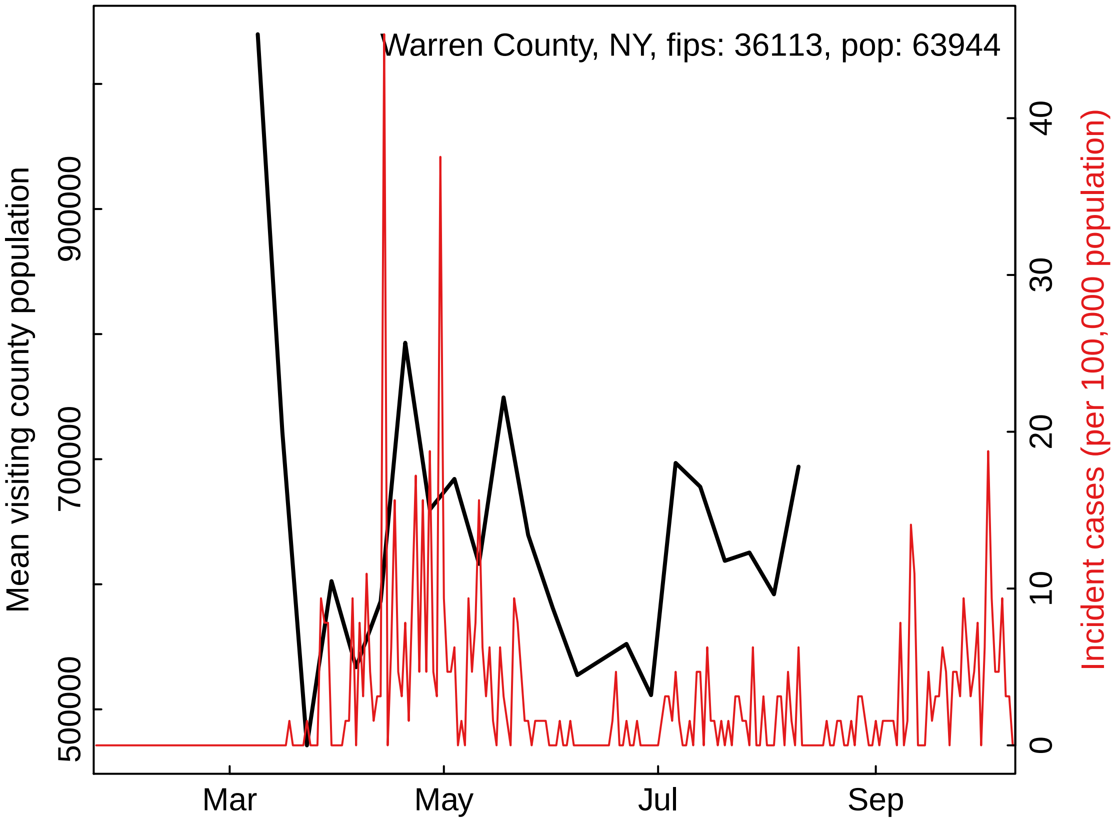
Warren County, NY, fips: 36113, pop: 63944
Mean visiting county population
500000
700000
900000
Incident cases (per 100,000 population)
0
10
20
30
40
Mar
May
Jul
Sep

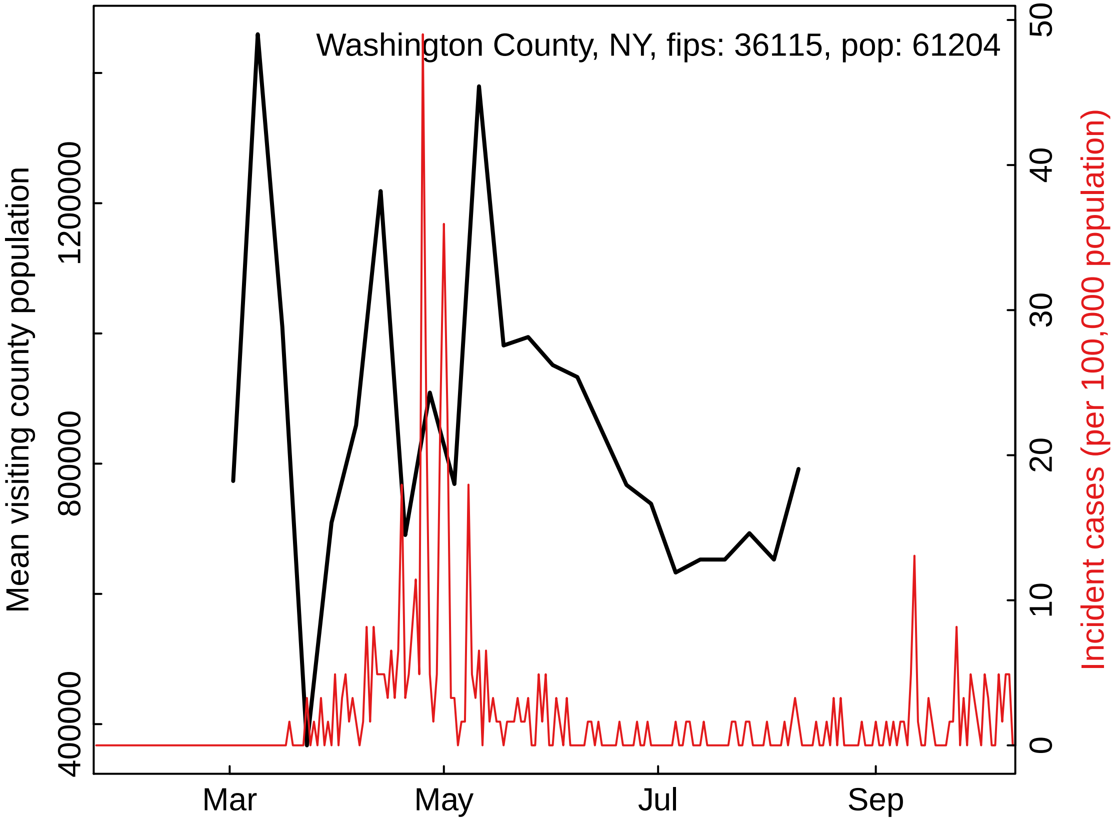
Washington County, NY, fips: 36115, pop: 61204
Mean visiting county population
400000
800000
1200000
Incident cases (per 100,000 population)
0
10
20
30
40
50
Mar
May
Jul
Sep

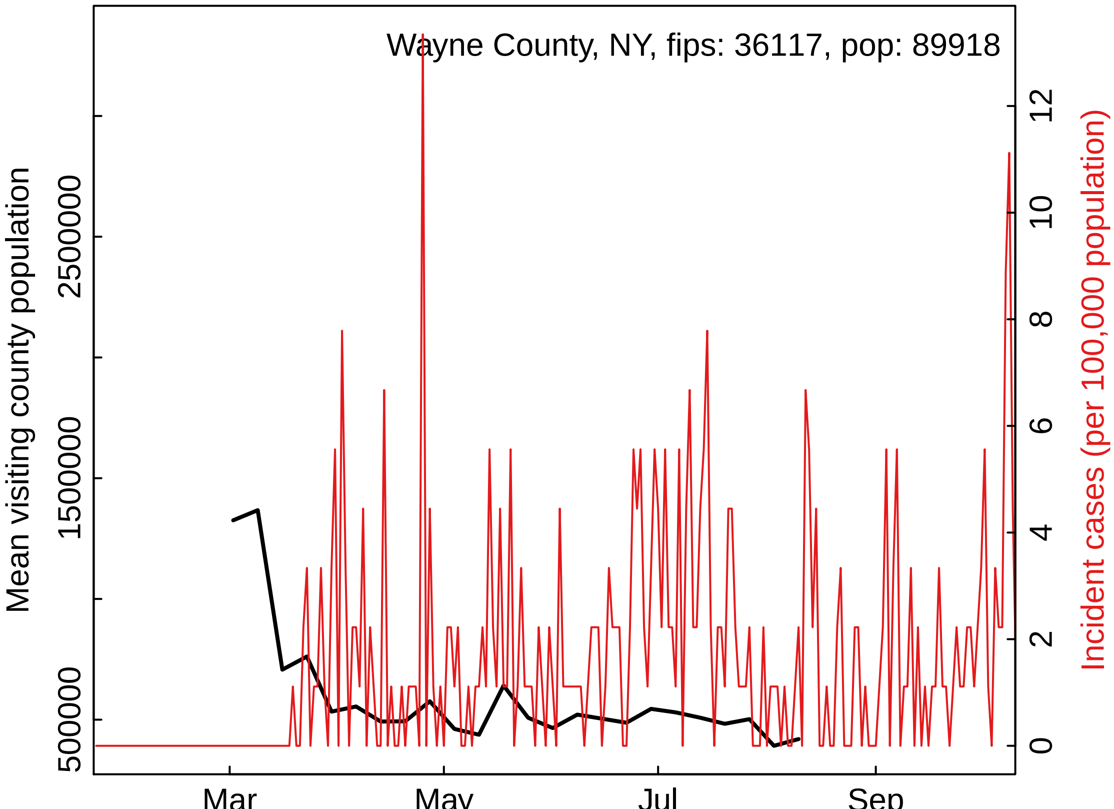

Wayne County, NY, fips: 36117, pop: 89918
Mean visiting county population
500000
1500000
2500000
Mar
May
Jul
Sep
0
2
4
6
8
10
12
Incident cases (per 100,000 population)

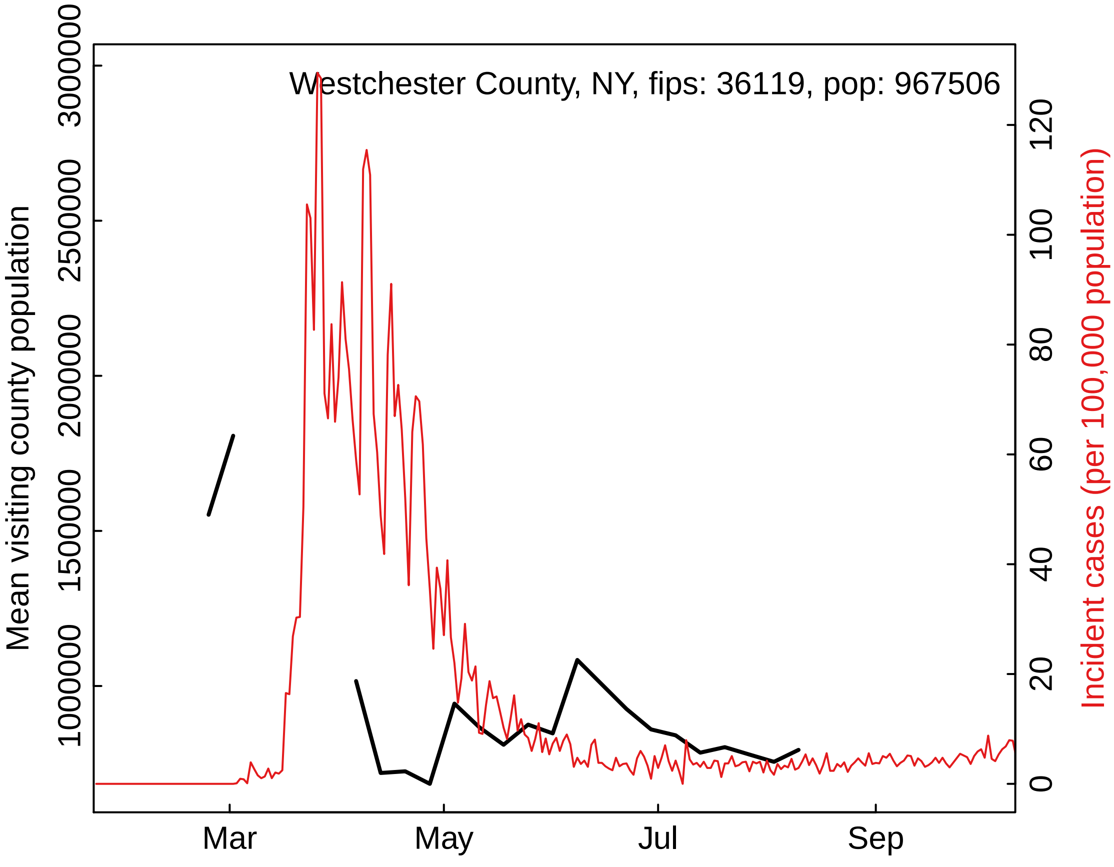

Westchester County, NY, fips: 36119, pop: 967506
Mean visiting county population
1000000
1500000
2000000
2500000
3000000
Mar
May
Jul
Sep
0
20
40
60
80
100
120
Incident cases (per 100,000 population)

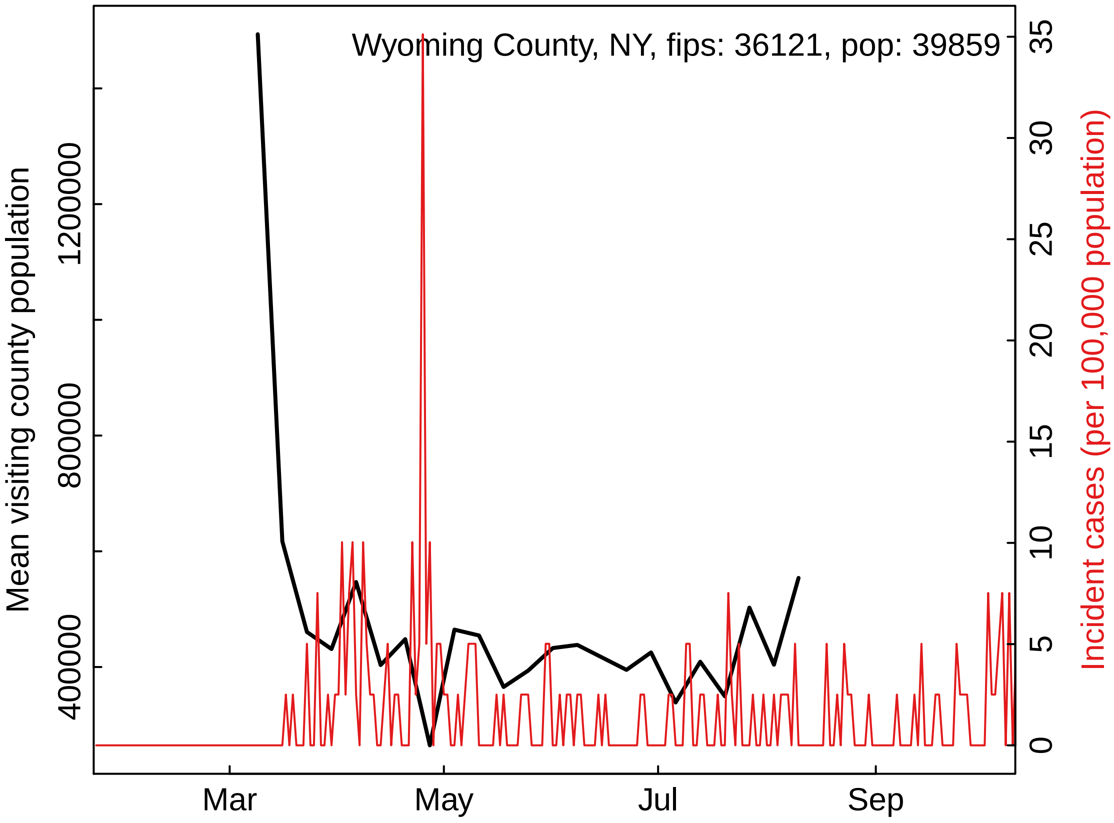

Wyoming County, NY, fips: 36121, pop: 39859
Mean visiting county population
400000
800000
1200000
Incident cases (per 100,000 population)
0
5
10
15
20
25
30
35
Mar
May
Jul
Sep

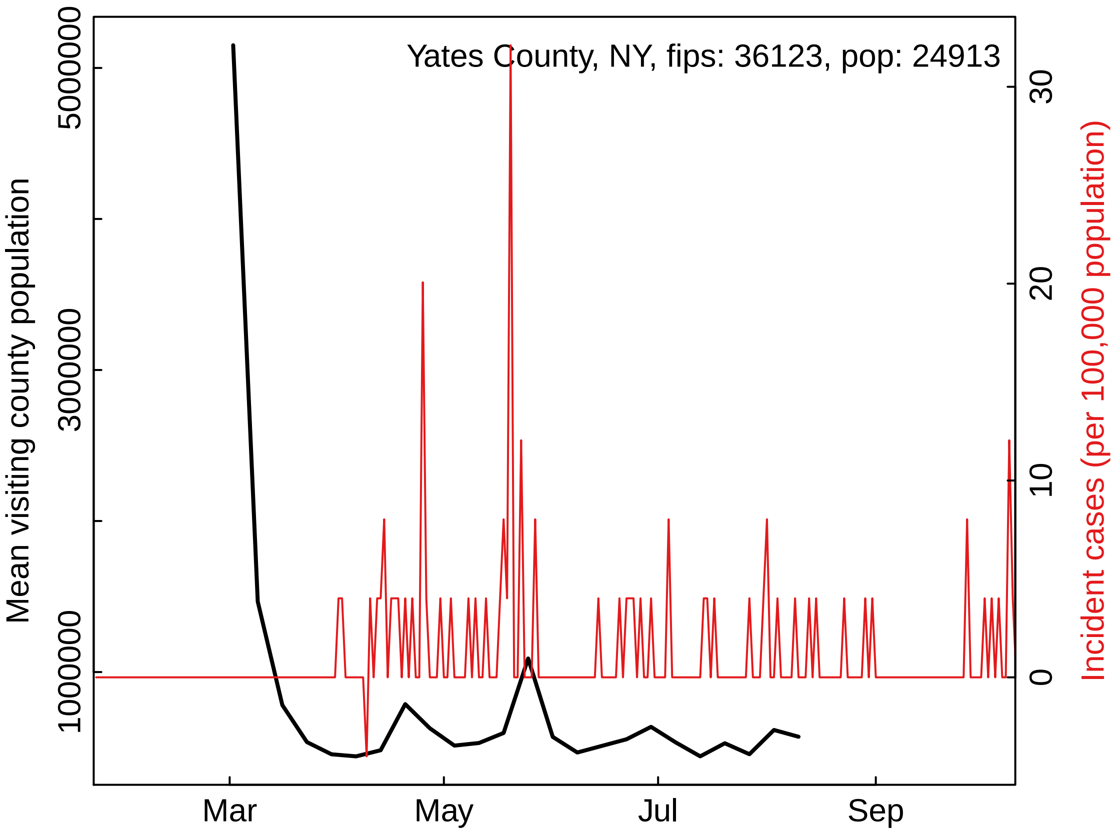

Yates County, NY, fips: 36123, pop: 24913
Mean visiting county population
1000000
3000000
5000000
Incident cases (per 100,000 population)
0
10
20
30
Mar
May
Jul
Sep

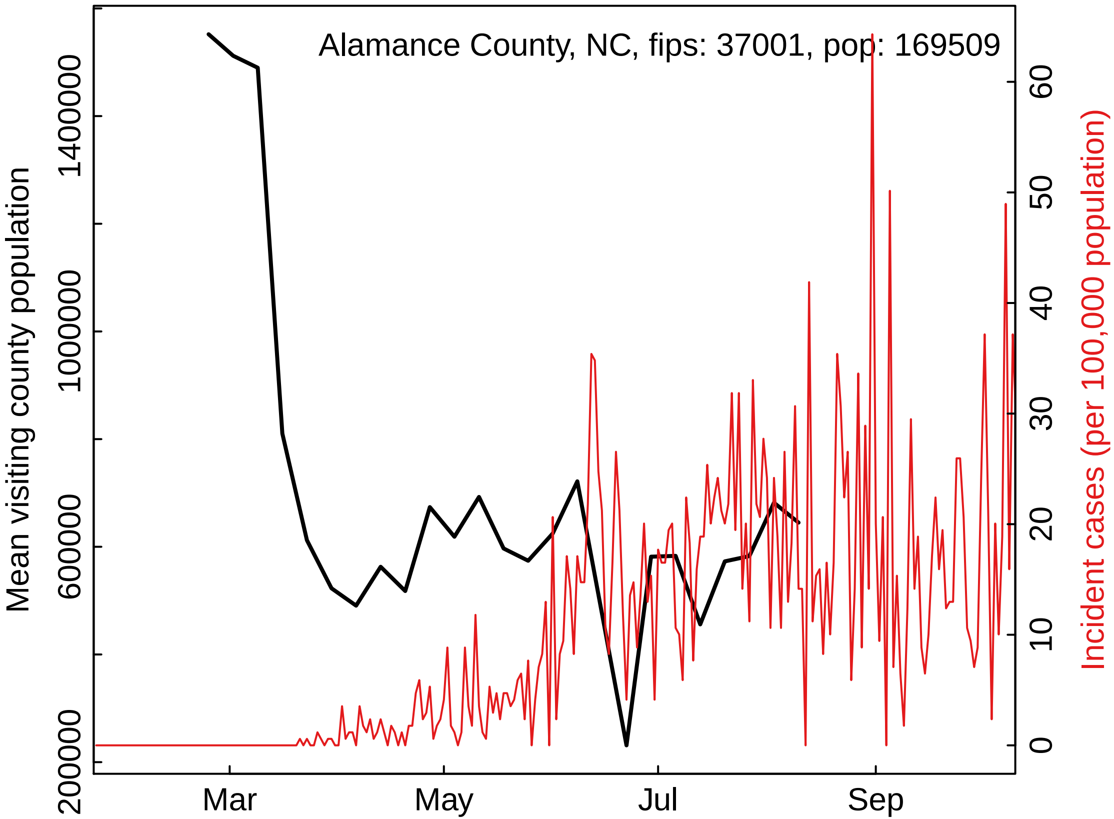
Alamance County, NC, fips: 37001, pop: 169509
Mean visiting county population
200000
600000
1000000
1400000
Mar
May
Jul
Sep
0
10
20
30
40
50
60
Incident cases (per 100,000 population)

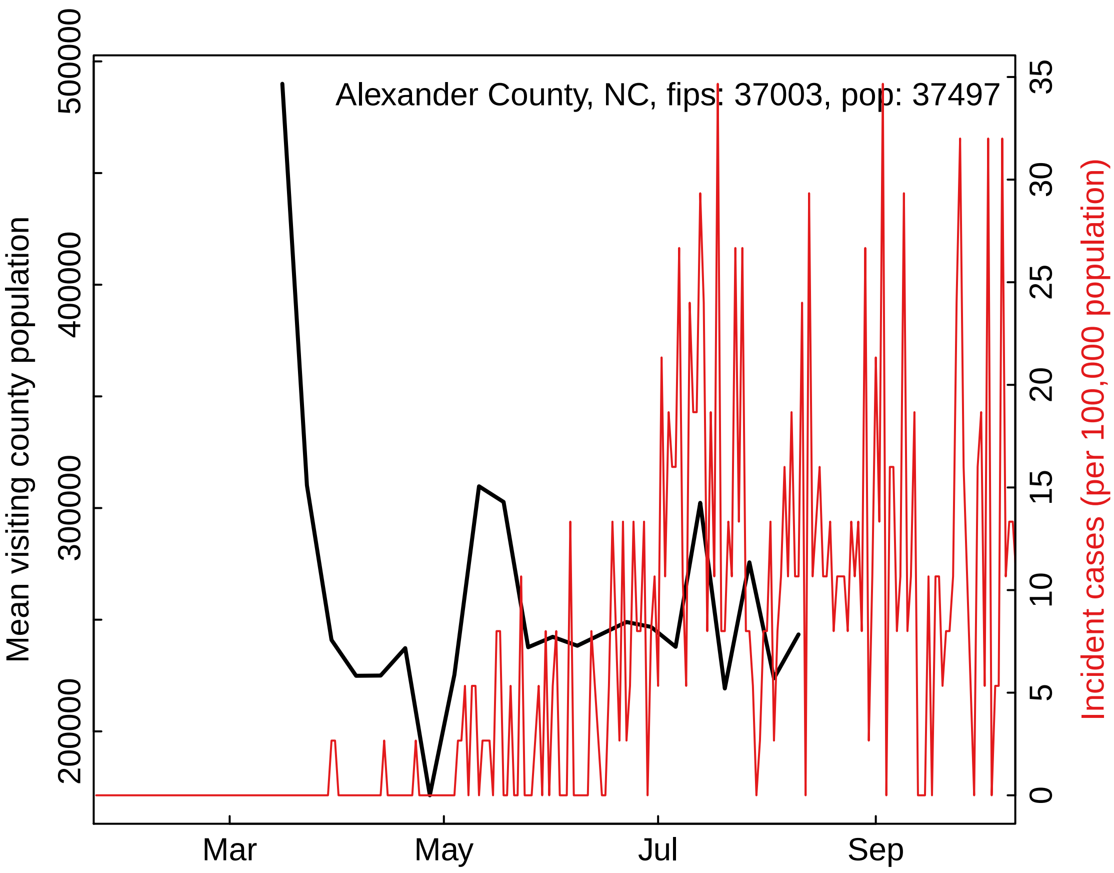

Alexander County, NC, fips: 37003, pop: 37497
Mean visiting county population
Incident cases (per 100,000 population)
200000
300000
400000
500000
0
5
10
15
20
25
30
35
Mar
May
Jul
Sep

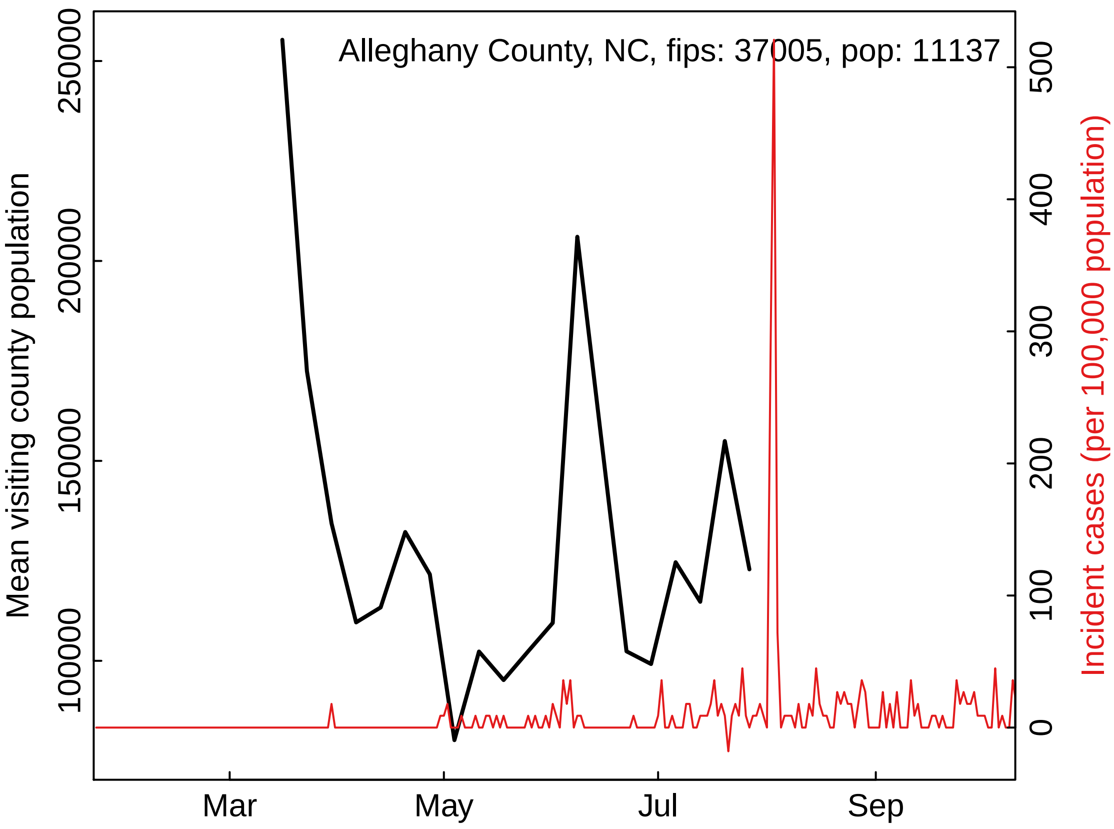
Alleghany County, NC, fips: 37005, pop: 11137
Mean visiting county population
100000
150000
200000
250000
Incident cases (per 100,000 population)
0
100
200
300
400
500
Mar
May
Jul
Sep

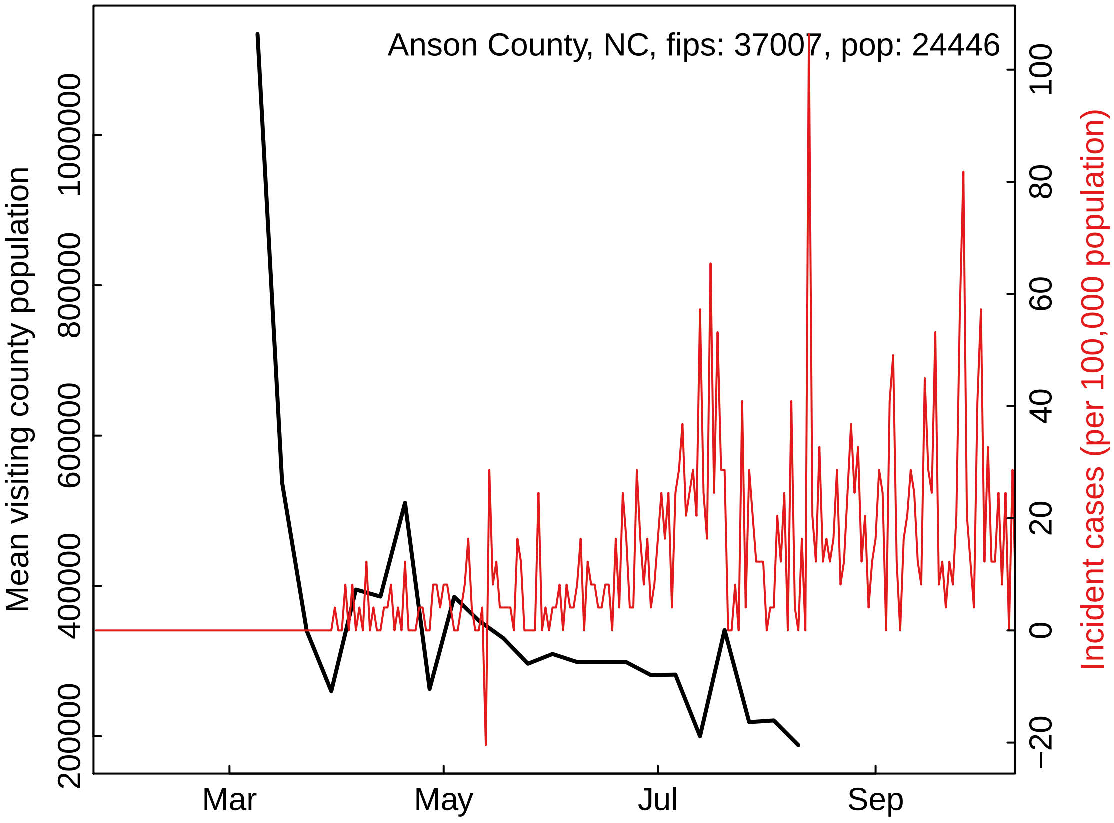

Anson County, NC, fips: 37007, pop: 24446
Mean visiting county population
Incident cases (per 100,000 population)
200000
400000
600000
800000
1000000
−20
0
20
40
60
80
100
Mar
May
Jul
Sep

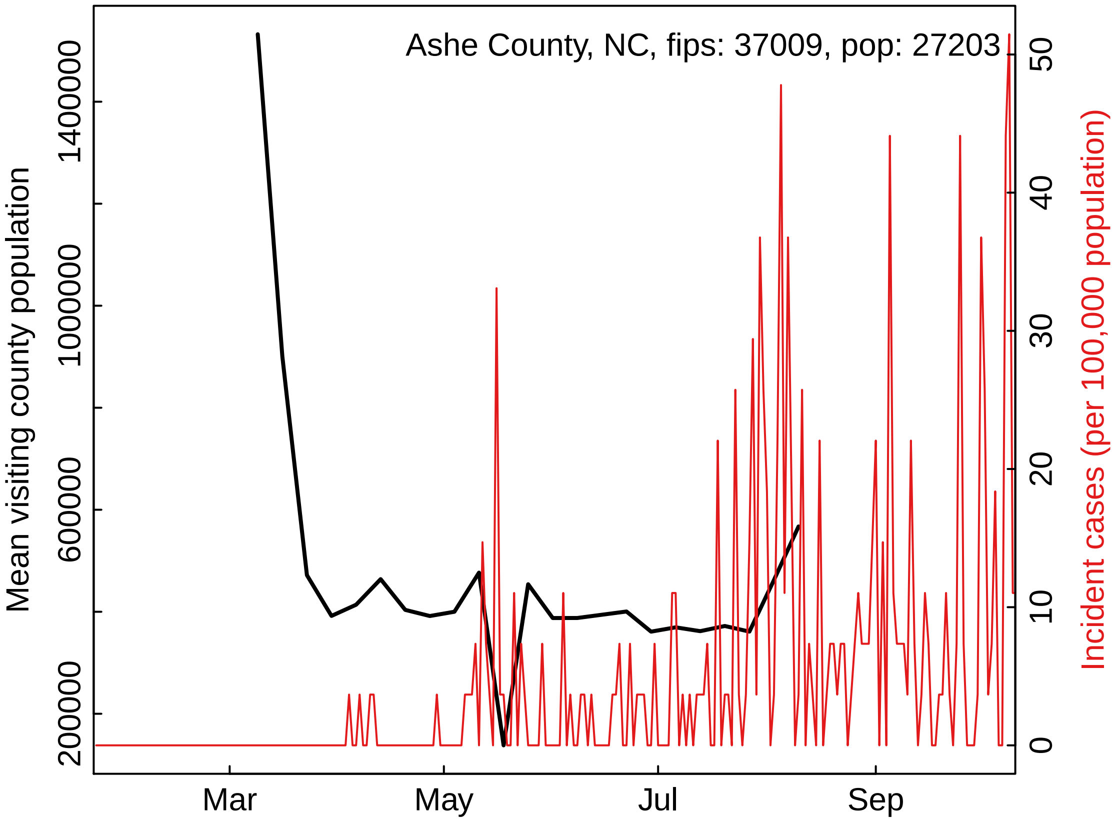

Ashe County, NC, fips: 37009, pop: 27203
Mean visiting county population
200000
600000
1000000
1400000
Incident cases (per 100,000 population)
0
10
20
30
40
50
Mar
May
Jul
Sep

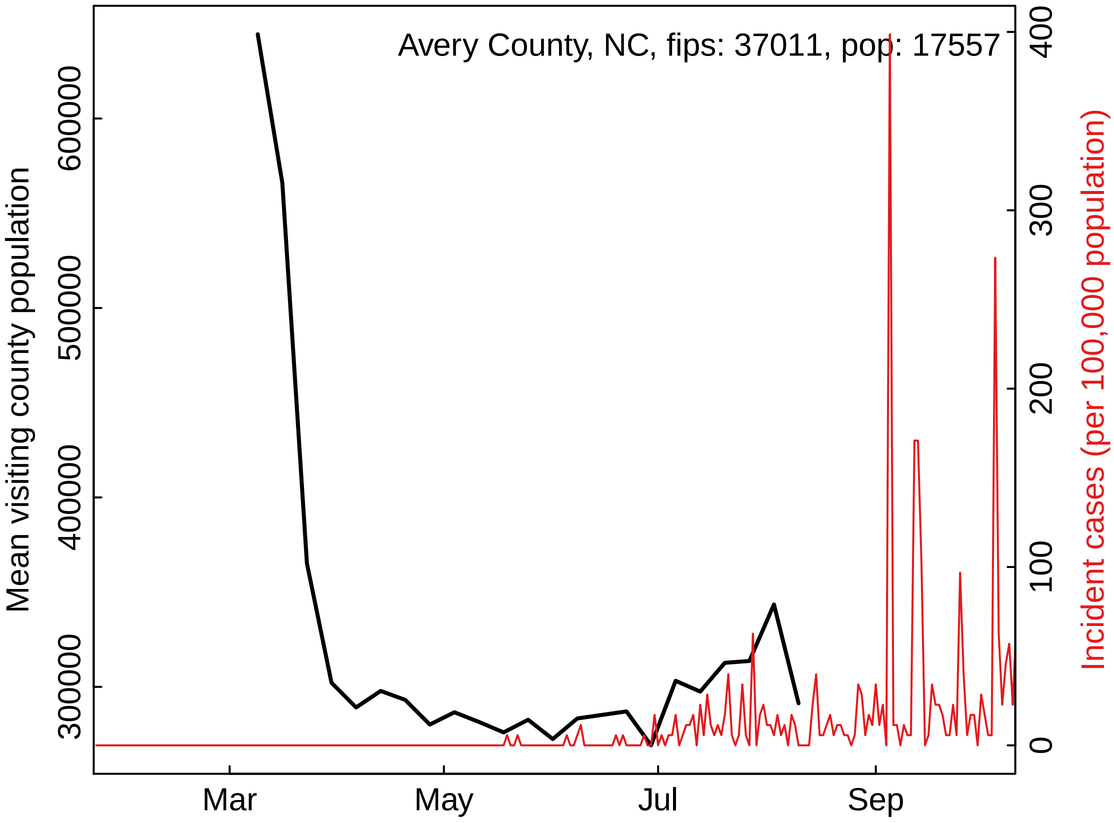
Avery County, NC, fips: 37011, pop: 17557
Mean visiting county population
300000
400000
500000
600000
Incident cases (per 100,000 population)
0
100
200
300
400
Mar
May
Jul
Sep

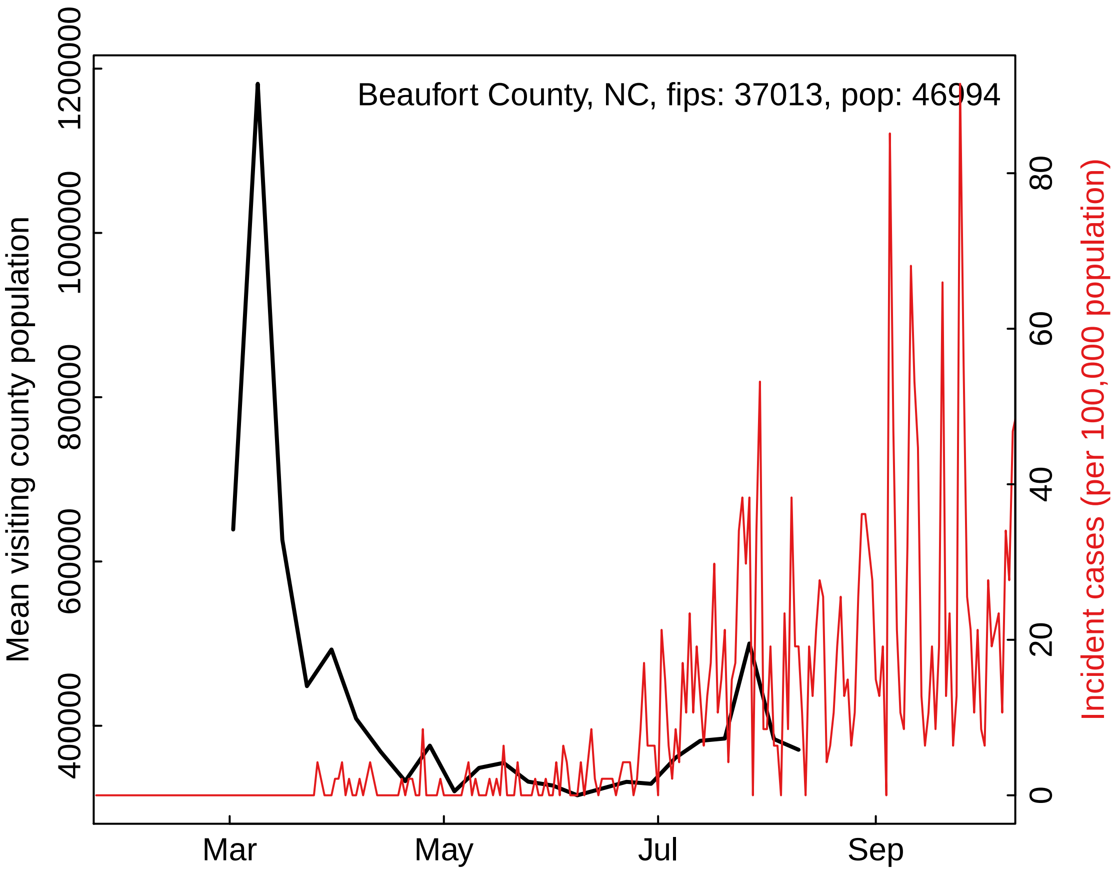
Beaufort County, NC, fips: 37013, pop: 46994
Mean visiting county population
400000
600000
800000
1000000
1200000
Incident cases (per 100,000 population)
0
20
40
60
80
Mar
May
Jul
Sep

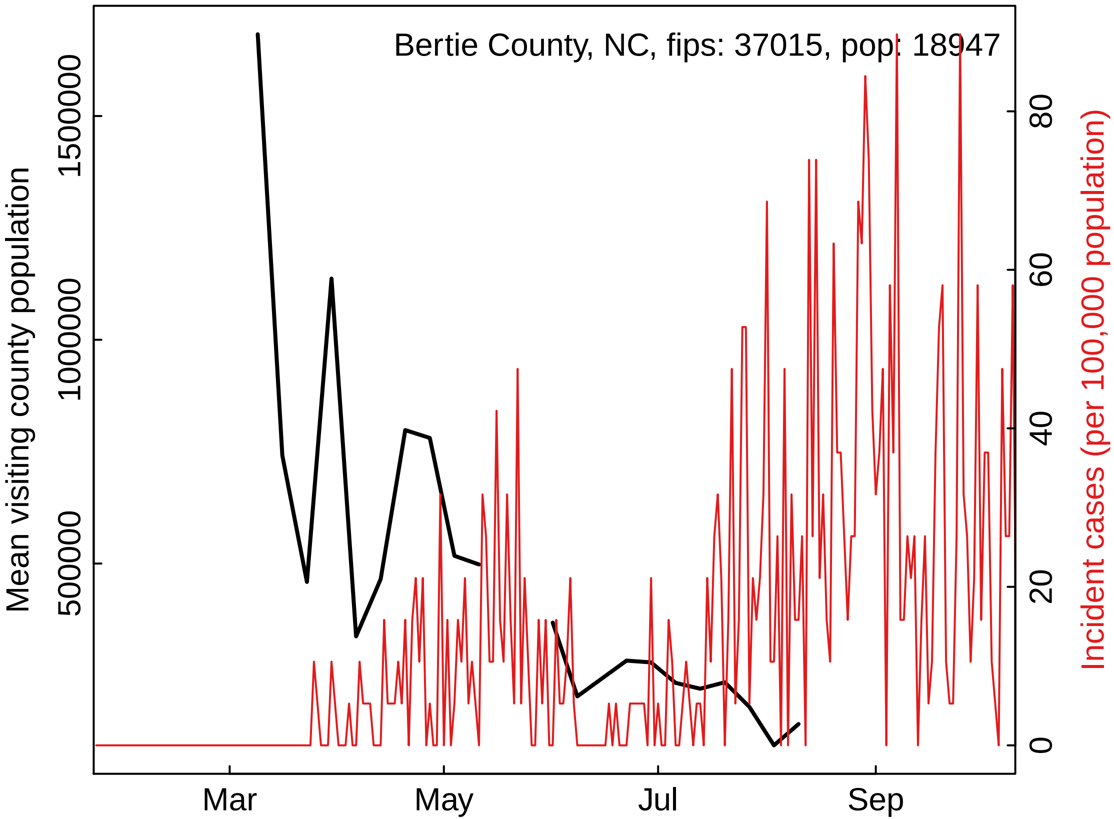
Bertie County, NC, fips: 37015, pop: 18947
Mean visiting county population
500000
1000000
1500000
Incident cases (per 100,000 population)
0
20
40
60
80
Mar
May
Jul
Sep

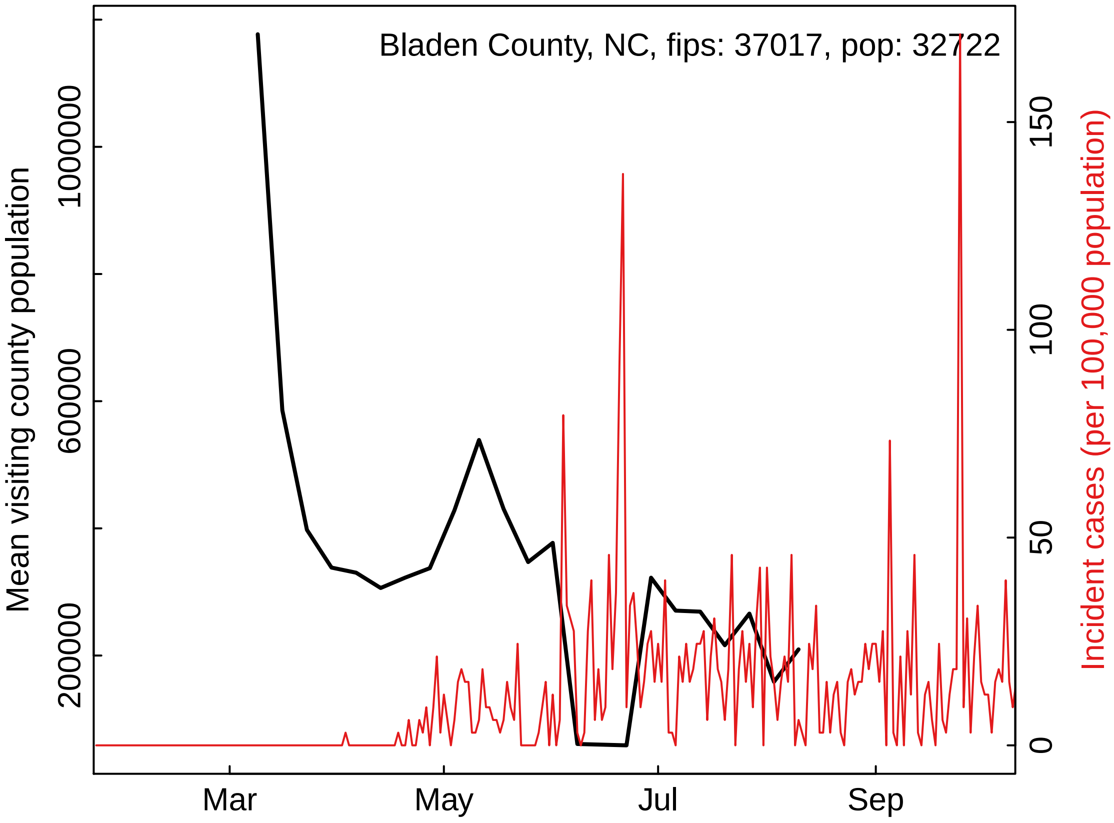

Bladen County, NC, fips: 37017, pop: 32722
Mean visiting county population
200000
600000
1000000
Incident cases (per 100,000 population)
0
50
100
150
Mar
May
Jul
Sep

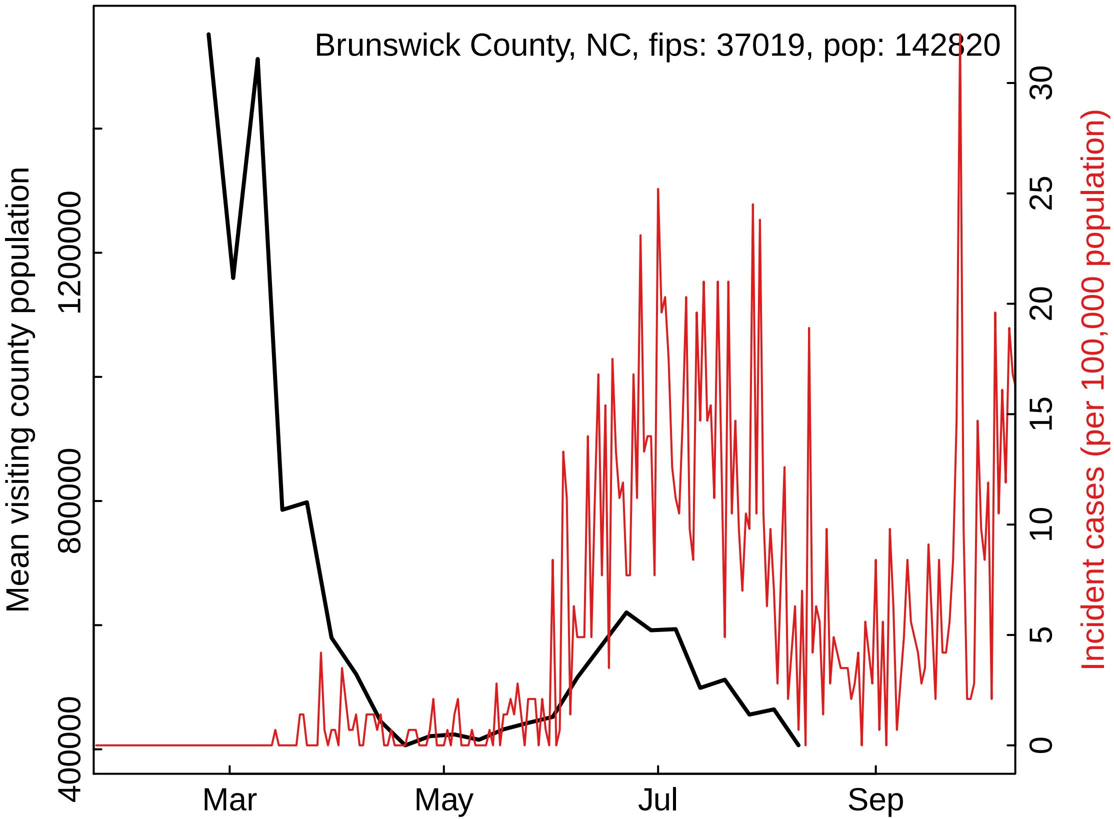

Brunswick County, NC, fips: 37019, pop: 142820
Mean visiting county population
400000
800000
1200000
Mar
May
Jul
Sep
0
5
10
15
20
25
30
Incident cases (per 100,000 population)

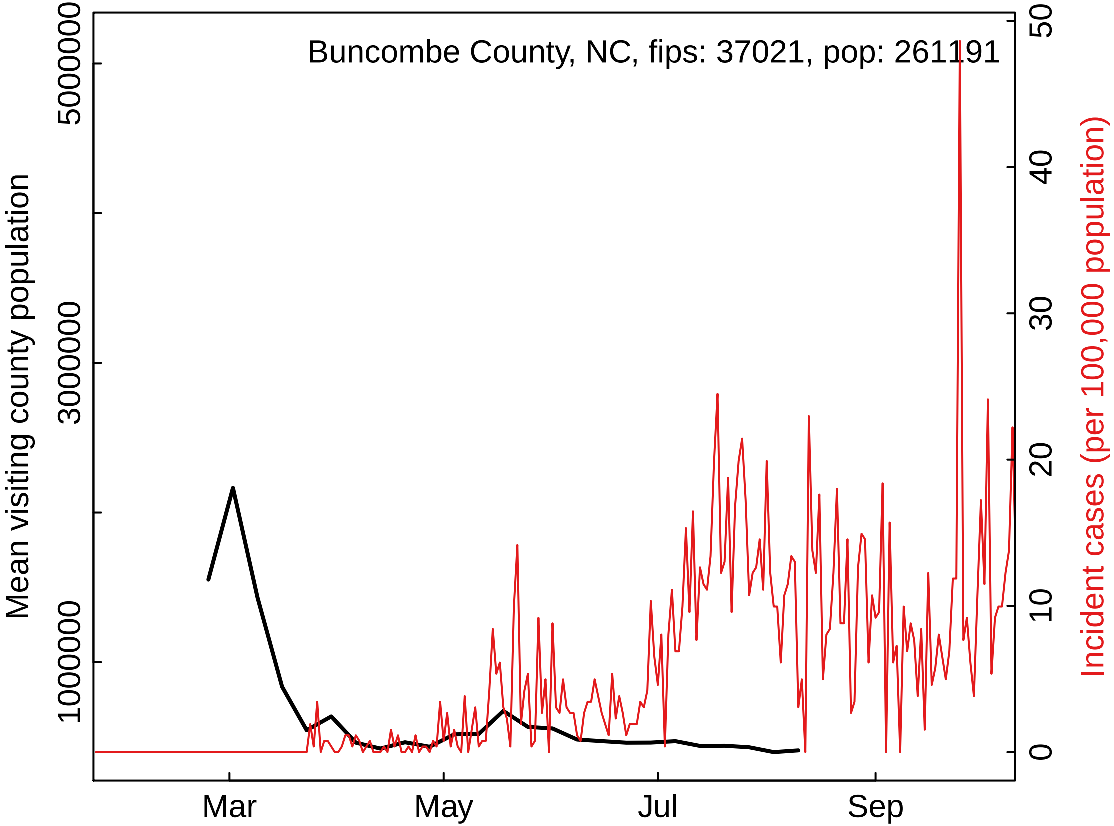

Buncombe County, NC, fips: 37021, pop: 261191
Mean visiting county population
1000000
3000000
5000000
Incident cases (per 100,000 population)
0
10
20
30
40
50
Mar
May
Jul
Sep

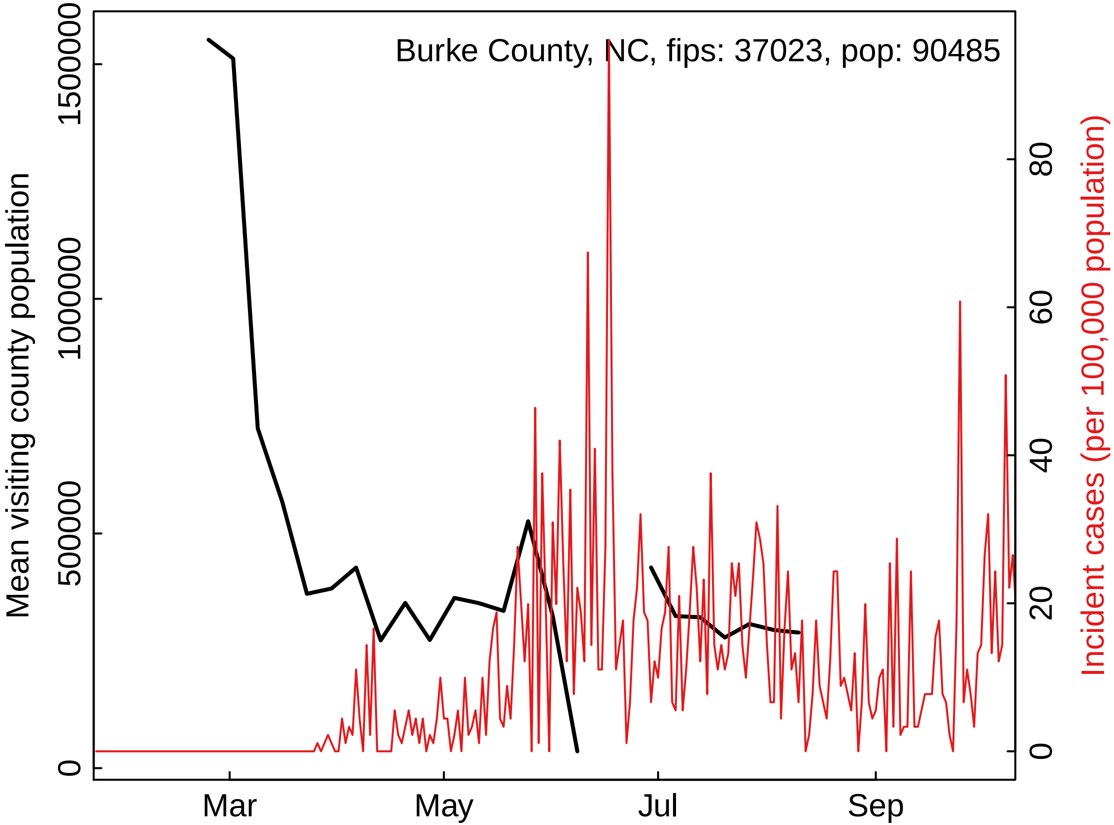

Burke County, NC, fips: 37023, pop: 90485
Mean visiting county population
0
500000
1000000
1500000
Incident cases (per 100,000 population)
0
20
40
60
80
Mar
May
Jul
Sep

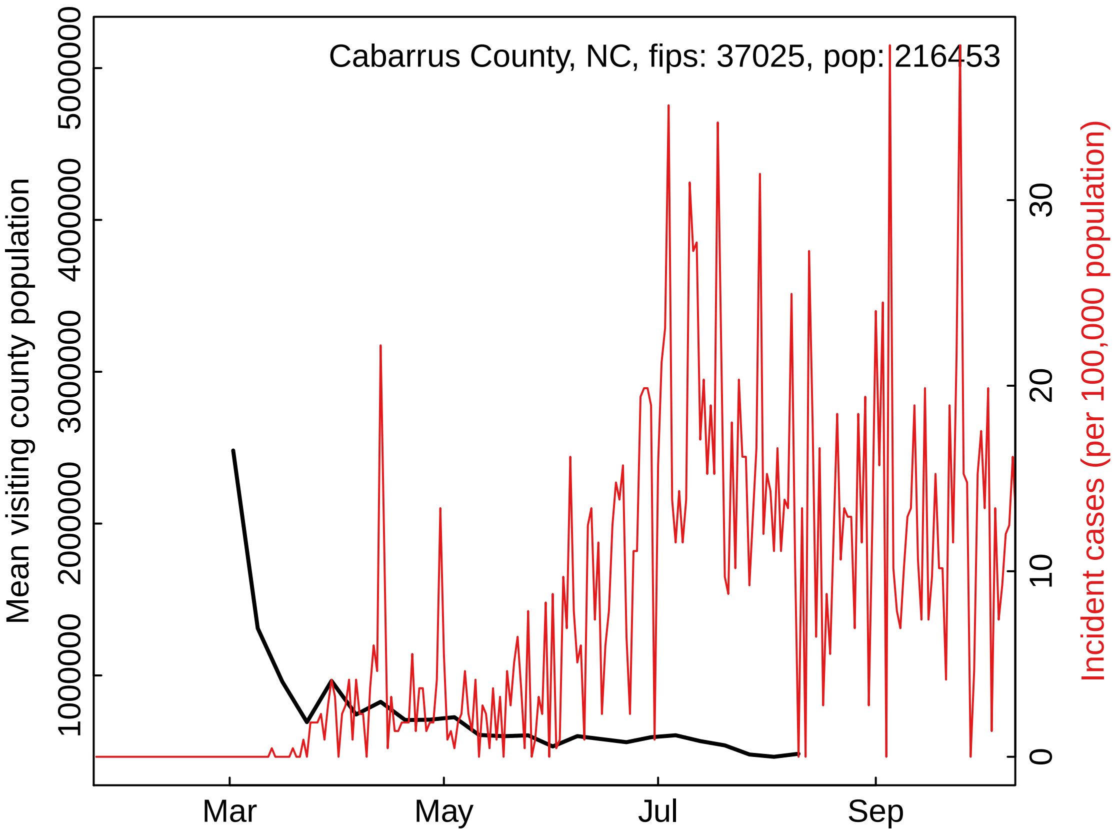

Cabarrus County, NC, fips: 37025, pop: 216453
Mean visiting county population
Incident cases (per 100,000 population)
1000000
2000000
3000000
4000000
5000000
0
10
20
30
Mar
May
Jul
Sep

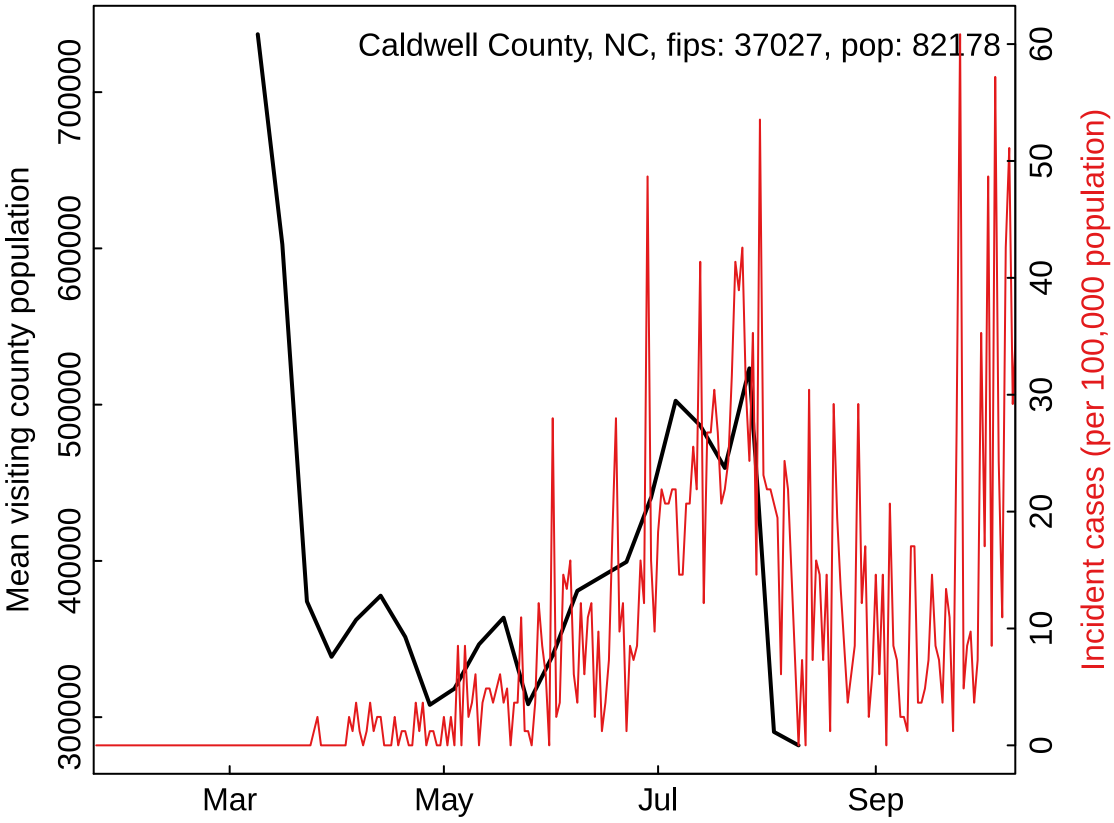
Caldwell County, NC, fips: 37027, pop: 82178
Mean visiting county population
300000
400000
500000
600000
700000
Incident cases (per 100,000 population)
0
10
20
30
40
50
60
Mar
May
Jul
Sep

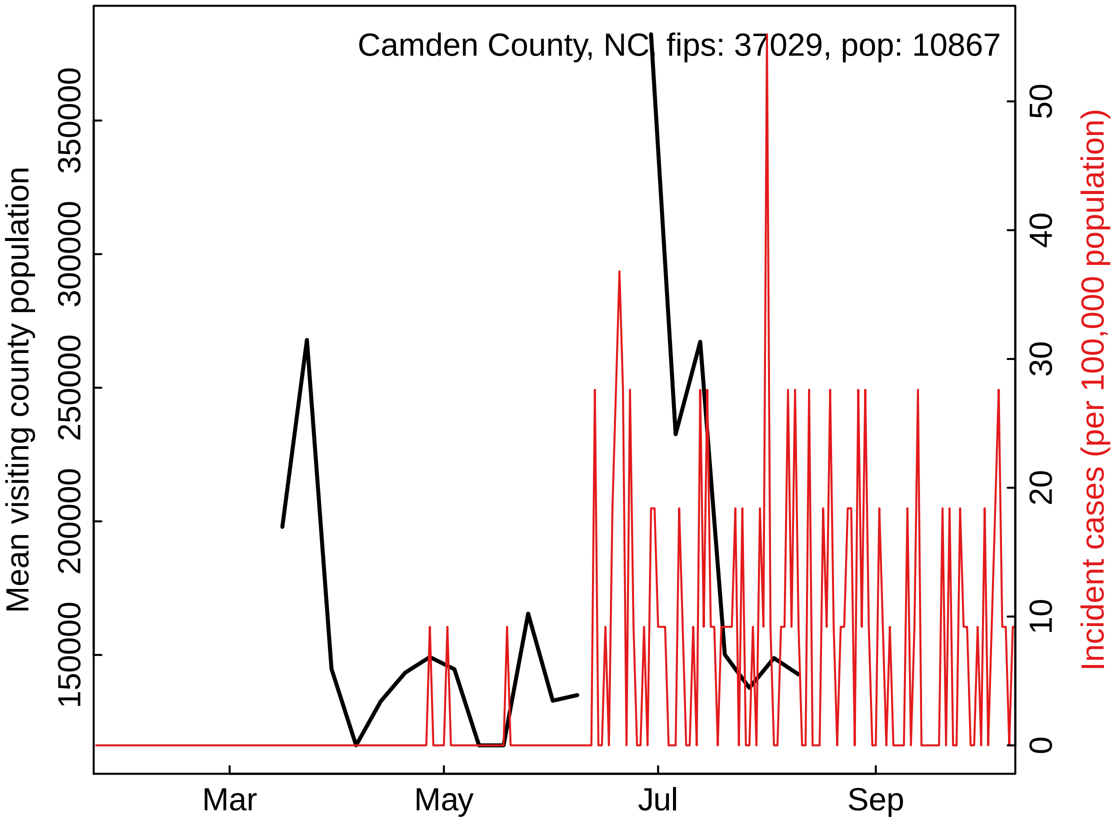

Camden County, NC, fips: 37029, pop: 10867
Mean visiting county population
150000
200000
250000
300000
350000
Incident cases (per 100,000 population)
0
10
20
30
40
50
Mar
May
Jul
Sep

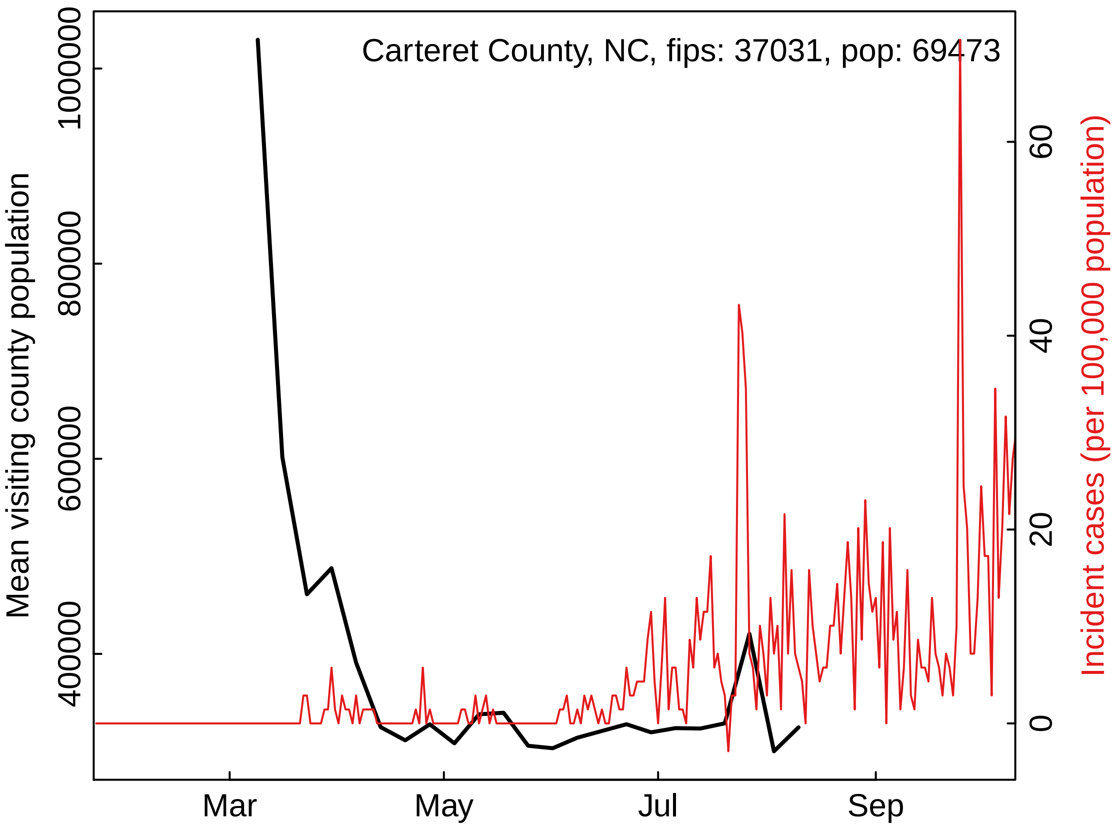

Carteret County, NC, fips: 37031, pop: 69473
Mean visiting county population
400000
600000
800000
1000000
Incident cases (per 100,000 population)
0
20
40
60
Mar
May
Jul
Sep

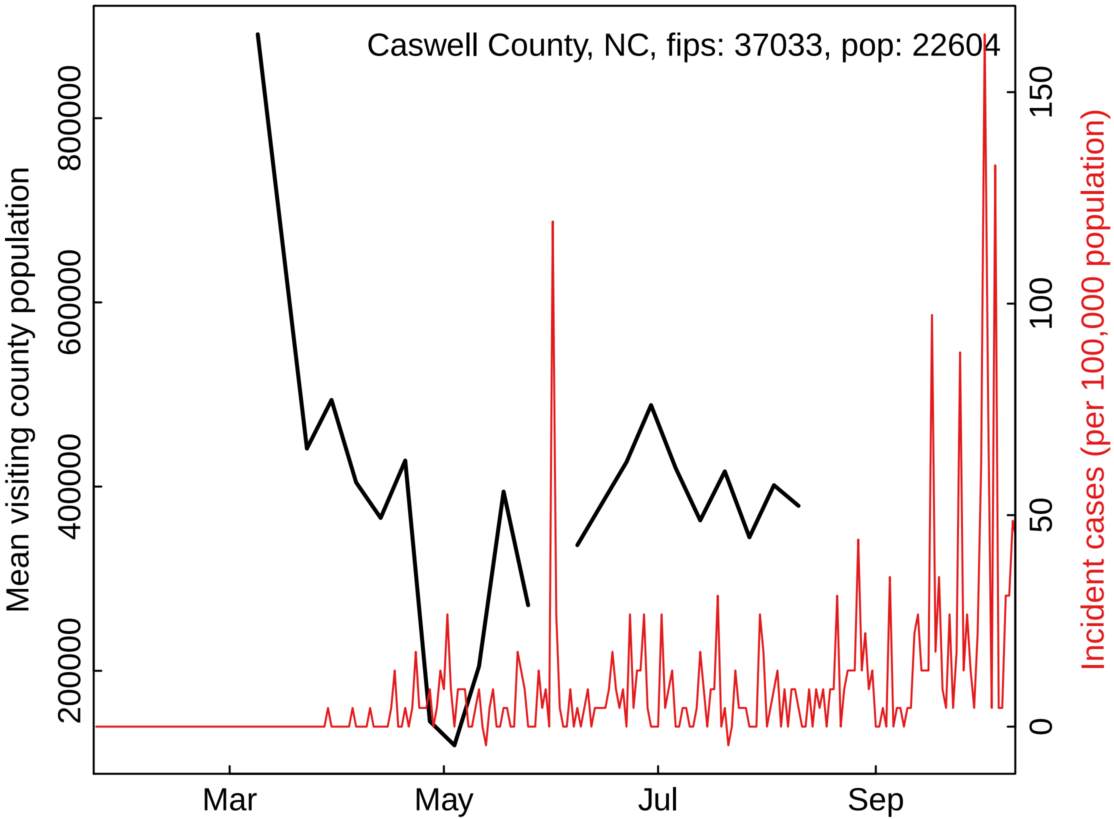

Caswell County, NC, fips: 37033, pop: 22604
Mean visiting county population
200000
400000
600000
800000
Incident cases (per 100,000 population)
0
50
100
150
Mar
May
Jul
Sep

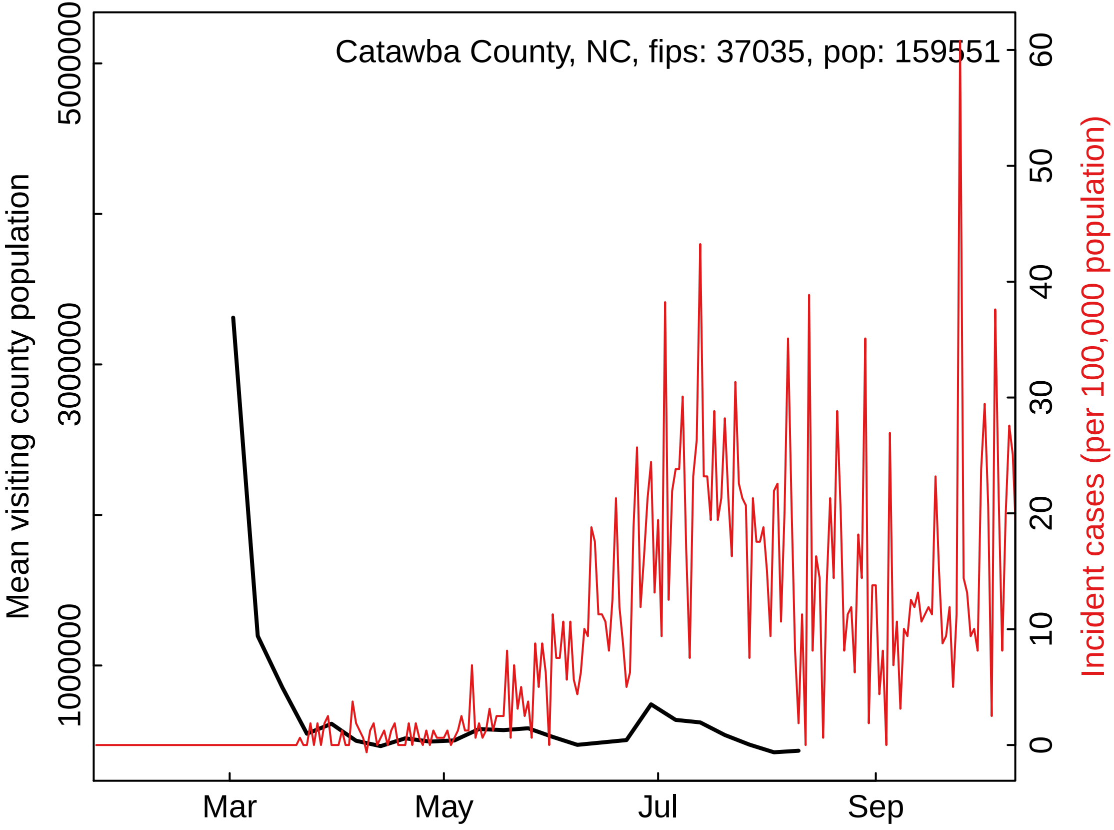

Catawba County, NC, fips: 37035, pop: 159551
Mean visiting county population
1000000
3000000
5000000
Incident cases (per 100,000 population)
0
10
20
30
40
50
60
Mar
May
Jul
Sep

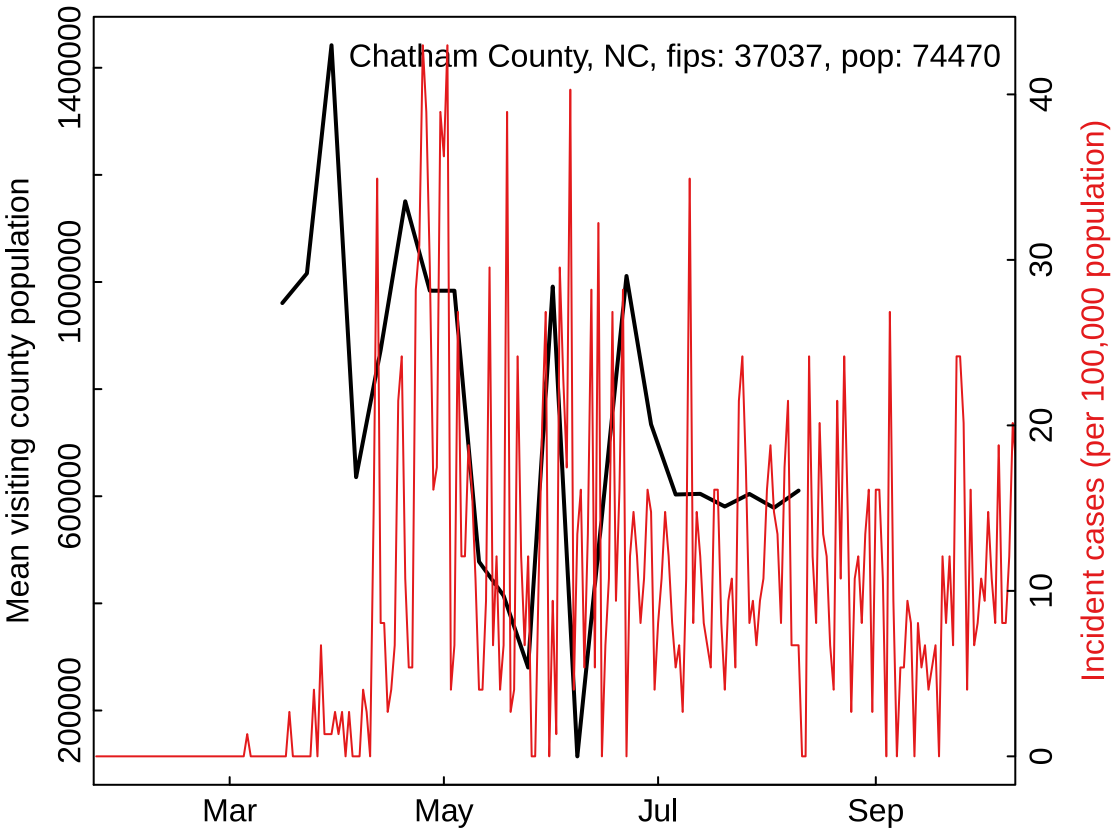

Chatham County, NC, fips: 37037, pop: 74470
Mean visiting county population
200000
600000
1000000
1400000
Incident cases (per 100,000 population)
0
10
20
30
40
Mar
May
Jul
Sep

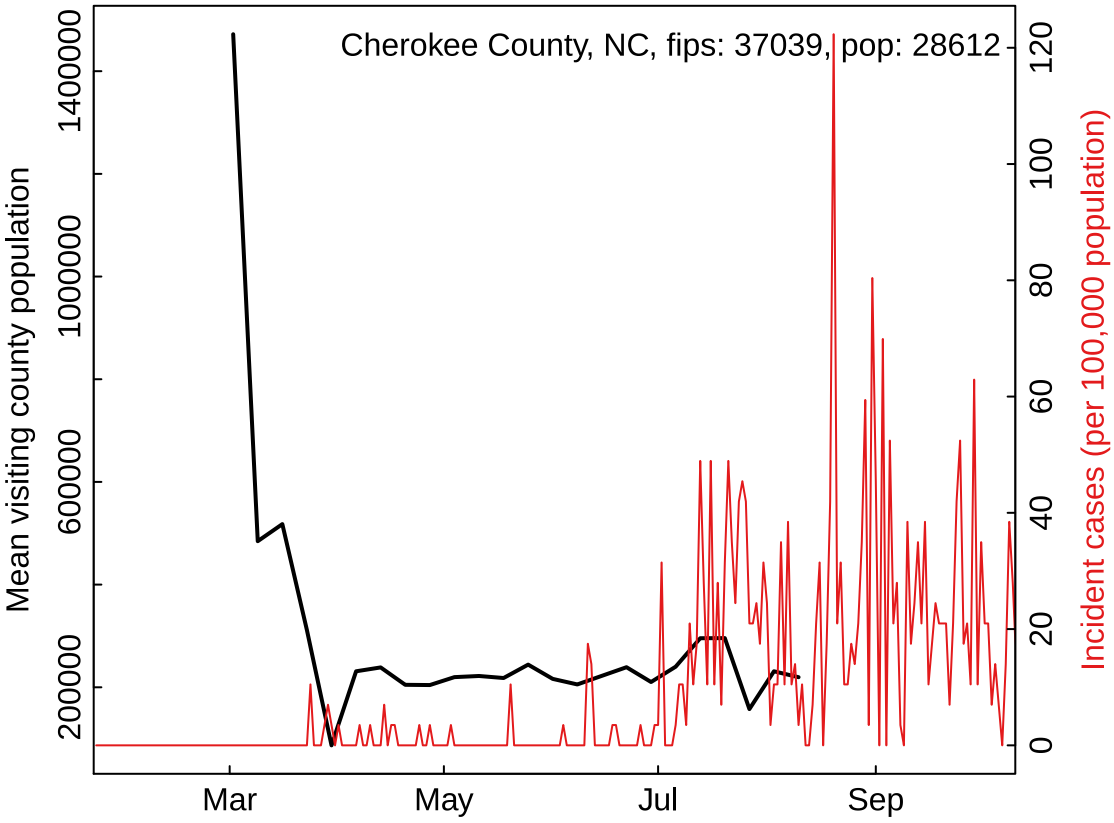
Cherokee County, NC, fips: 37039, pop: 28612
Mean visiting county population
200000
600000
1000000
1400000
Incident cases (per 100,000 population)
0
20
40
60
80
100
120
Mar
May
Jul
Sep

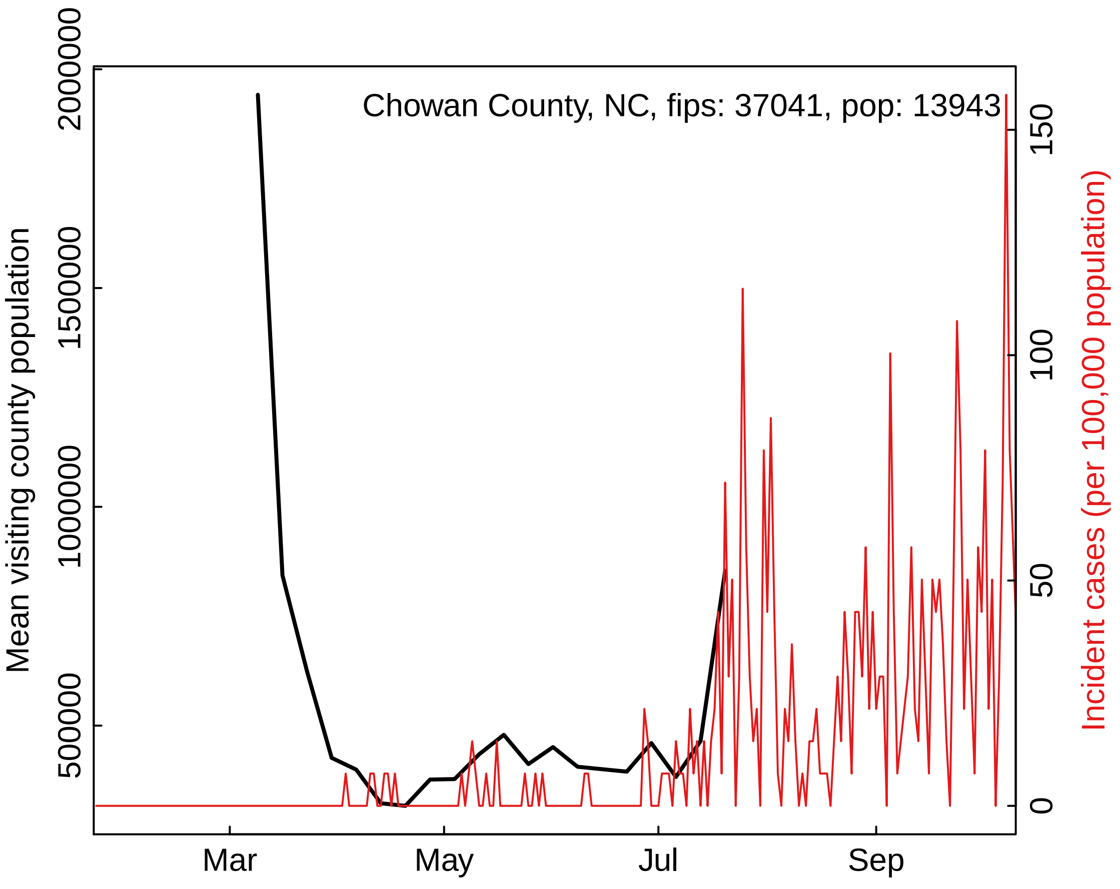

Chowan County, NC, fips: 37041, pop: 13943
Mean visiting county population
500000
1000000
1500000
2000000
Incident cases (per 100,000 population)
0
50
100
150
Mar
May
Jul
Sep

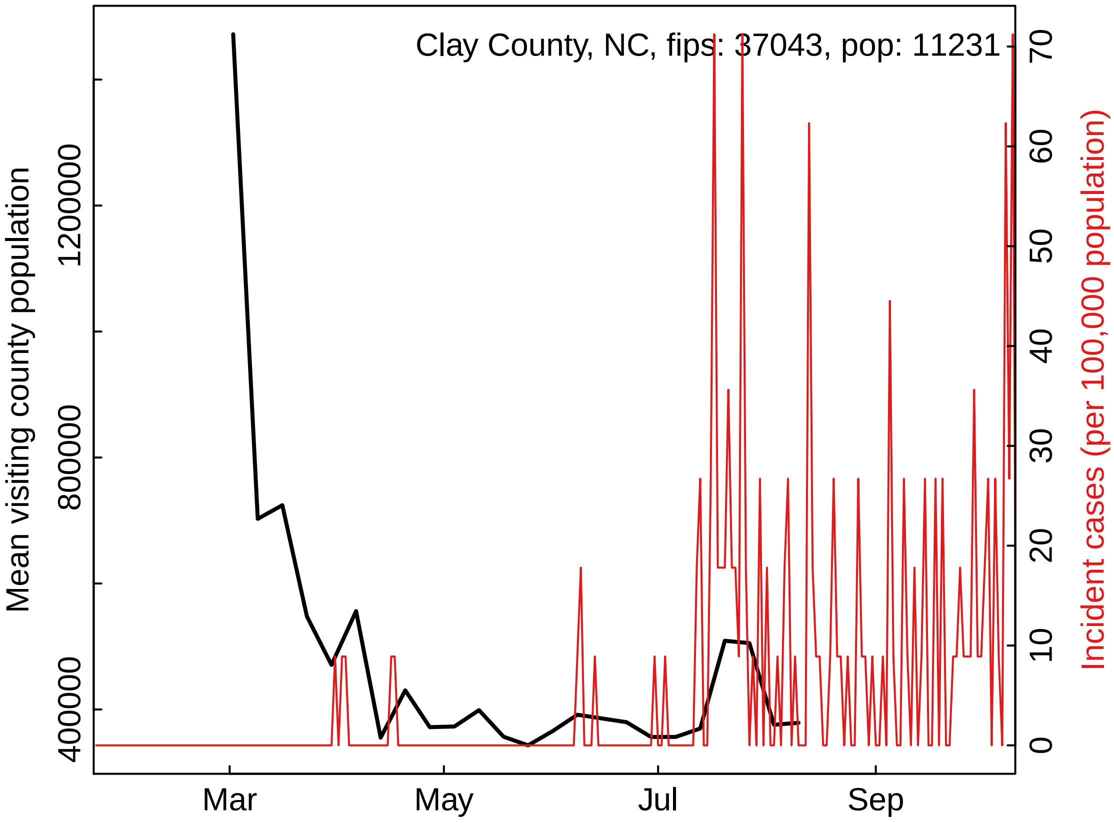

Clay County, NC, fips: 37043, pop: 11231
Mean visiting county population
400000
800000
1200000
Incident cases (per 100,000 population)
0
10
20
30
40
50
60
70
Mar
May
Jul
Sep

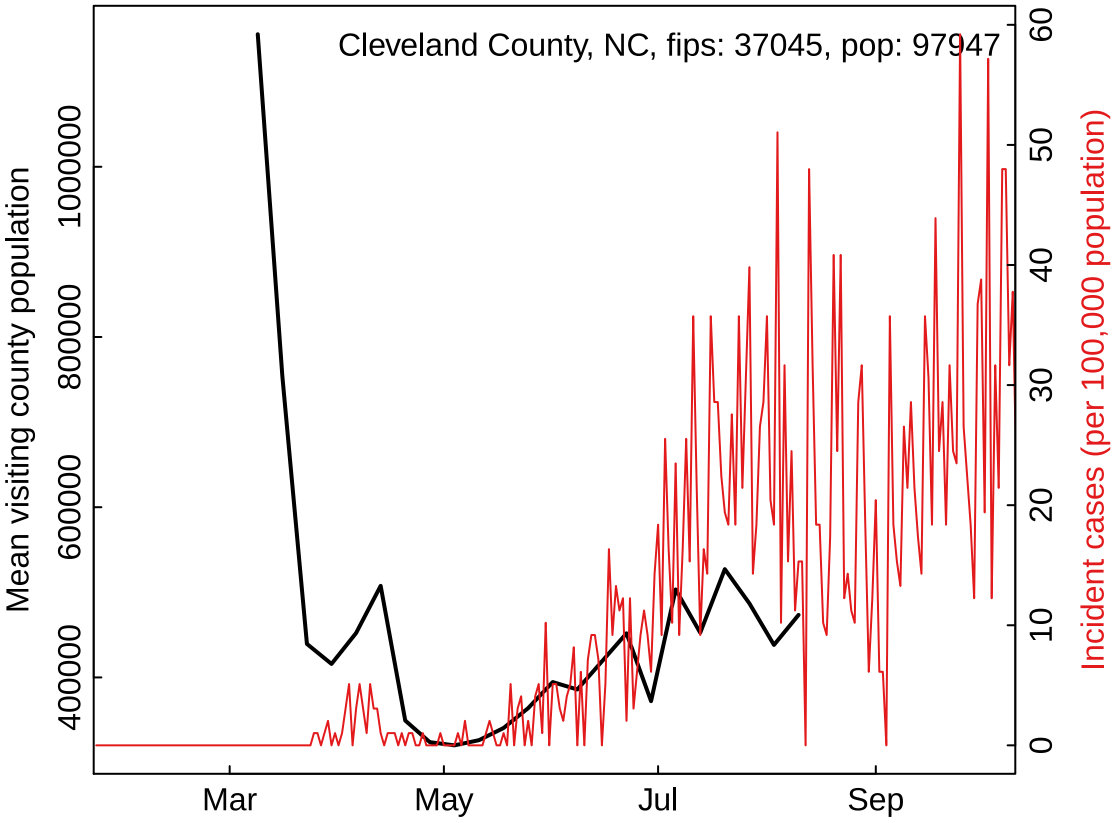

Cleveland County, NC, fips: 37045, pop: 97947
Mean visiting county population
400000
600000
800000
1000000
Incident cases (per 100,000 population)
0
10
20
30
40
50
60
Mar
May
Jul
Sep

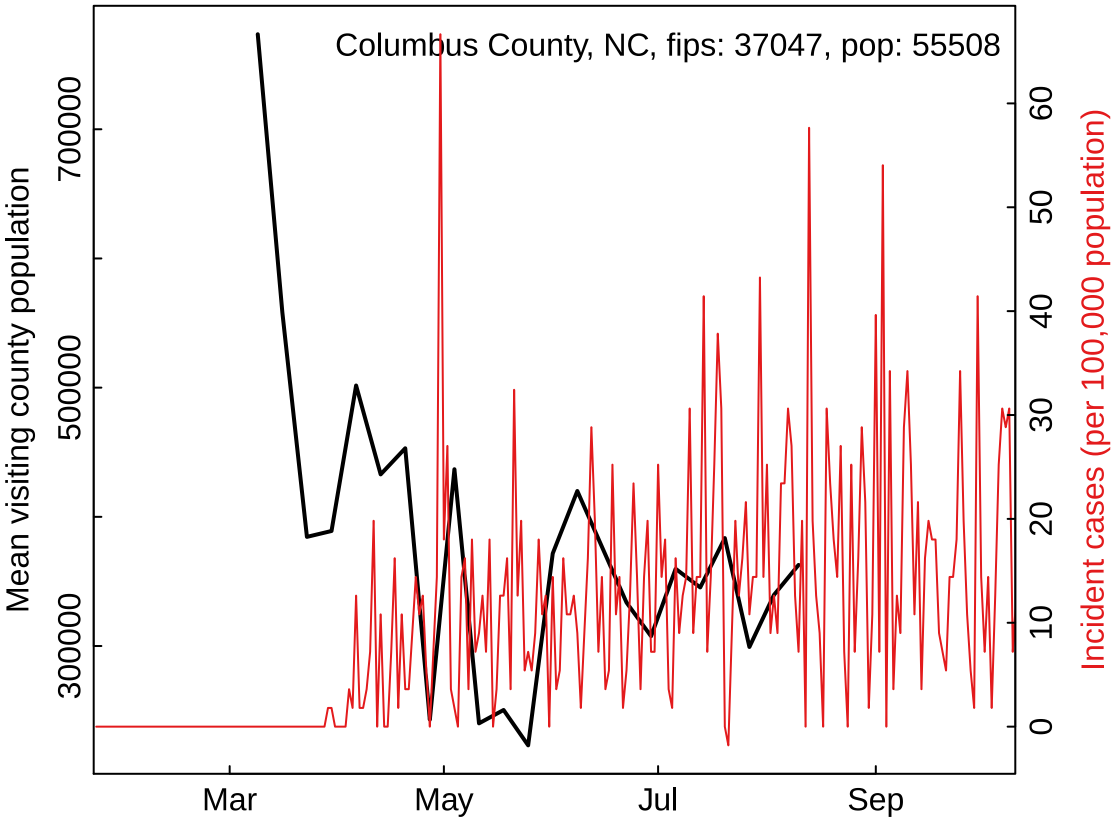

Columbus County, NC, fips: 37047, pop: 55508
Mean visiting county population
300000
500000
7000000
Incident cases (per 100,000 population)
0
10
20
30
40
50
60
Mar
May
Jul
Sep

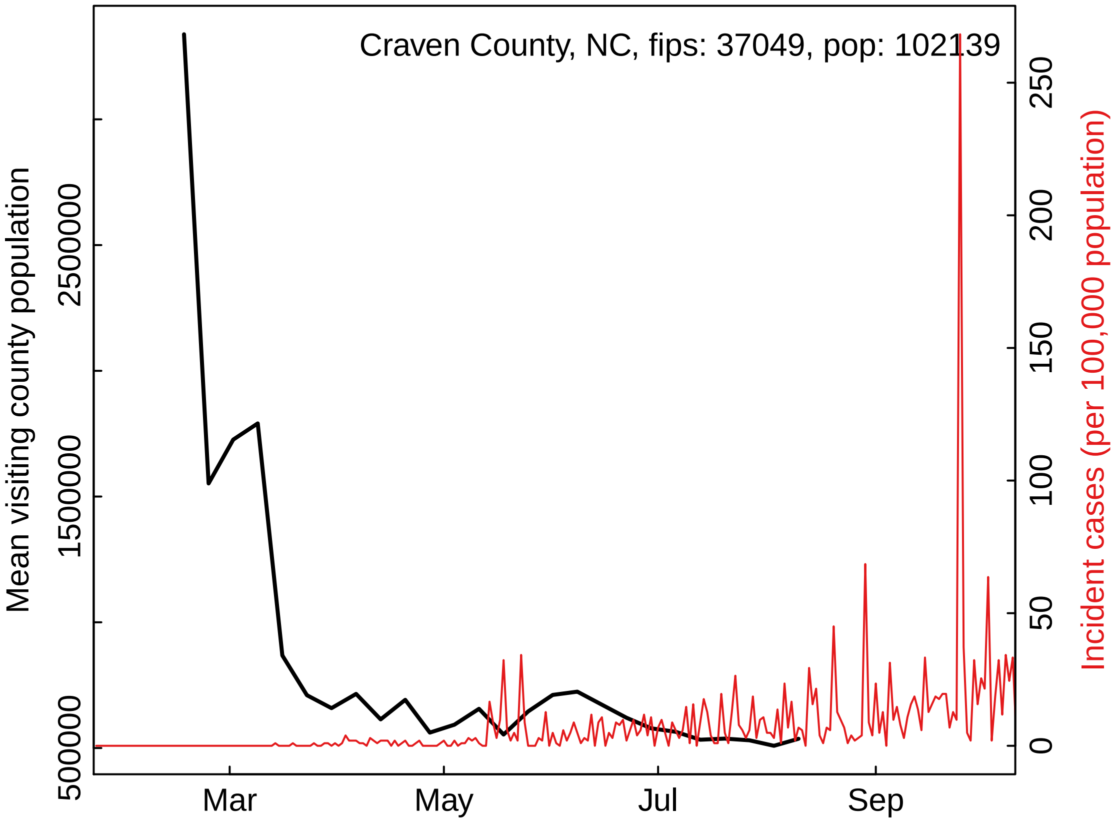

Craven County, NC, fips: 37049, pop: 102139
Mean visiting county population
500000
1500000
2500000
Incident cases (per 100,000 population)
0
50
100
150
200
250
Mar
May
Jul
Sep

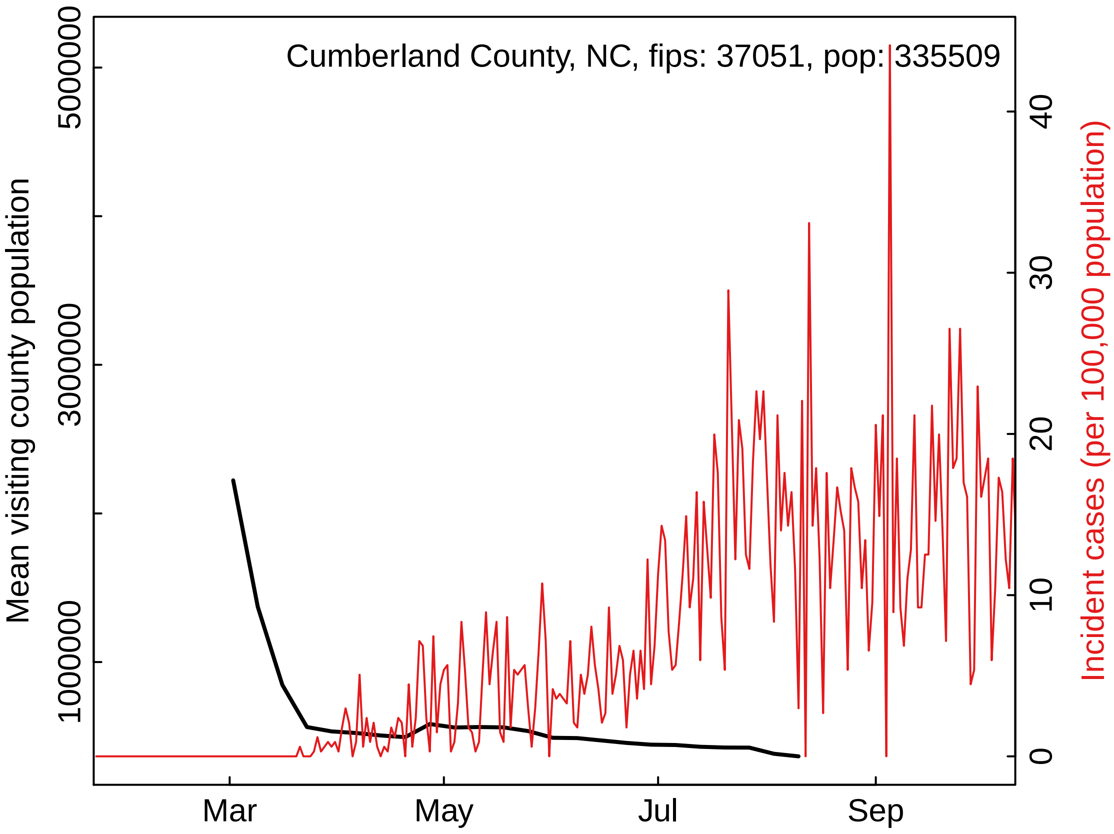
Cumberland County, NC, fips: 37051, pop: 335509
Mean visiting county population
1000000
3000000
5000000
Incident cases (per 100,000 population)
0
10
20
30
40
Mar
May
Jul
Sep

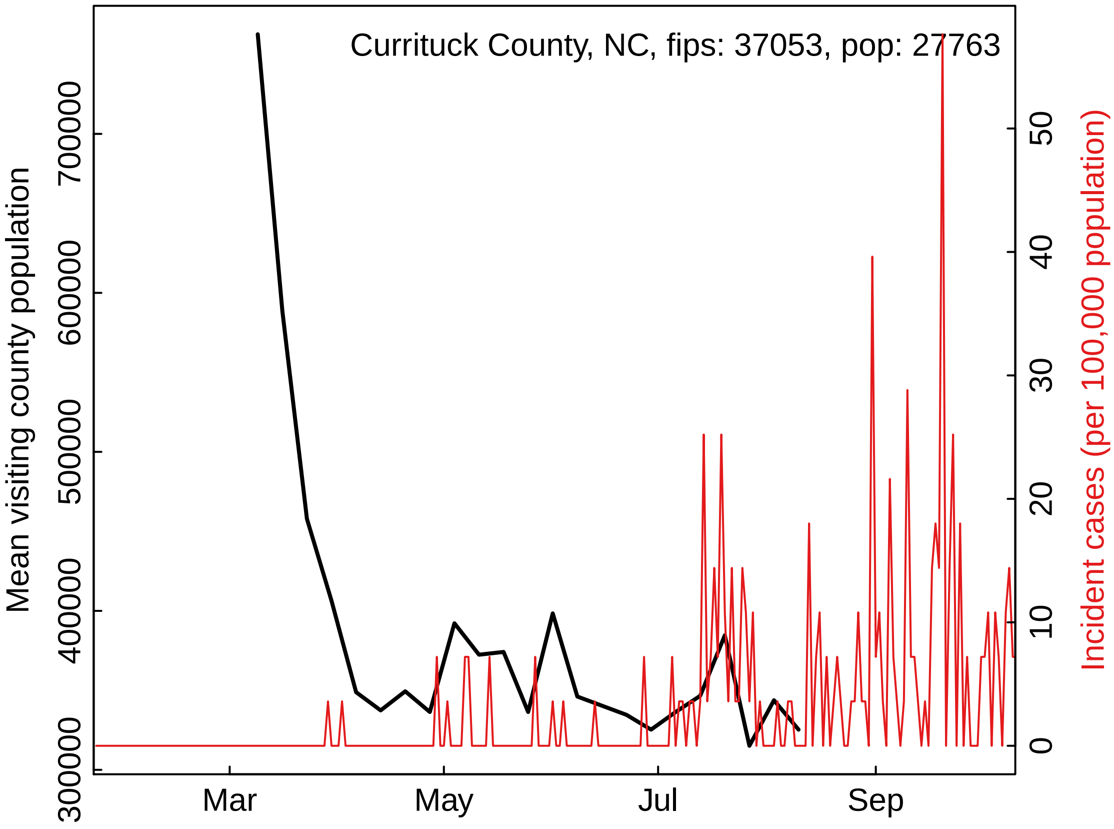
Currituck County, NC, fips: 37053, pop: 27763
Mean visiting county population
300000
400000
500000
600000
700000
Incident cases (per 100,000 population)
0
10
20
30
40
50
Mar
May
Jul
Sep

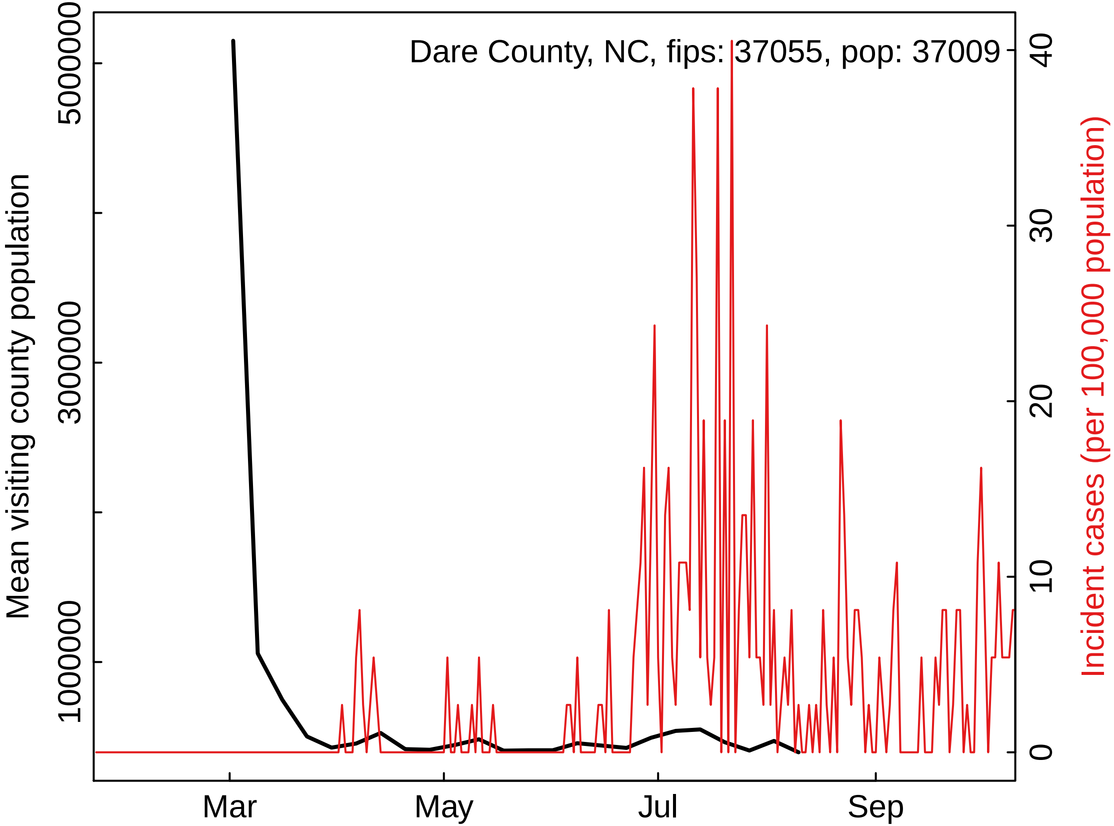

Dare County, NC, fips: 37055, pop: 37009
Mean visiting county population
1000000
3000000
5000000
Incident cases (per 100,000 population)
0
10
20
30
40
Mar
May
Jul
Sep

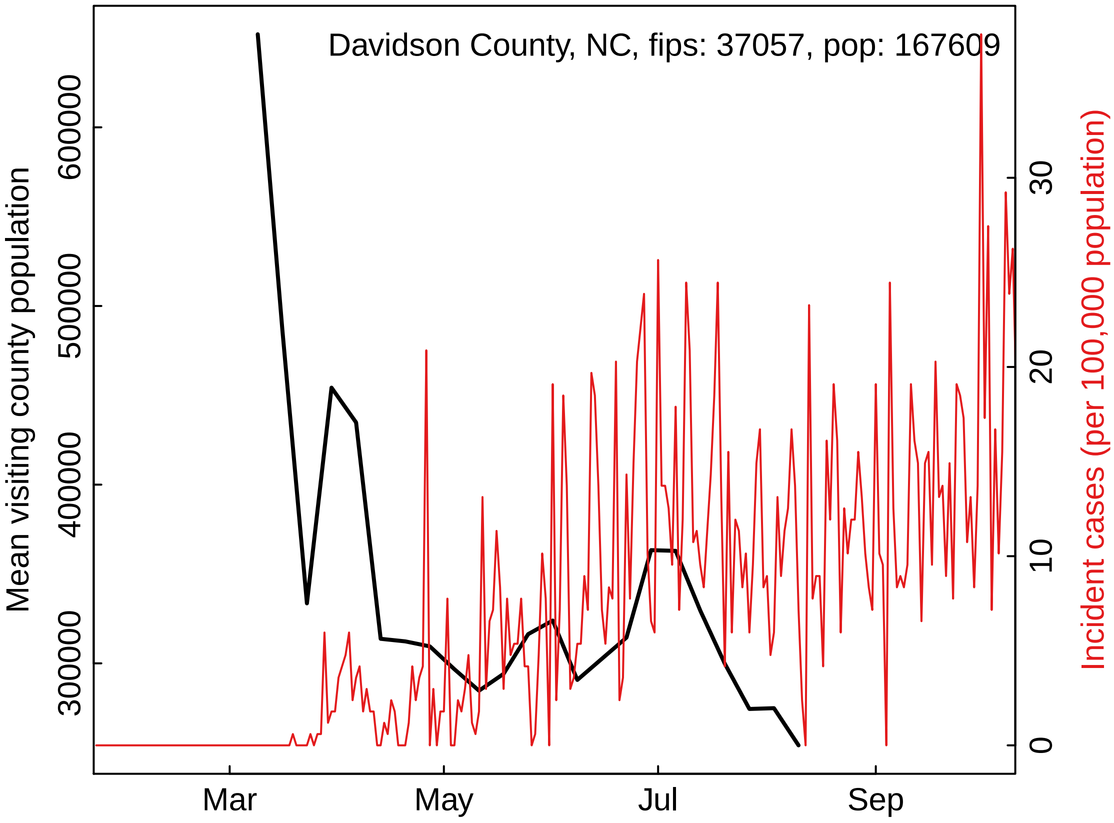

Davidson County, NC, fips: 37057, pop: 167609
Mean visiting county population
300000
400000
500000
600000
Incident cases (per 100,000 population)
0
10
20
30
Mar
May
Jul
Sep

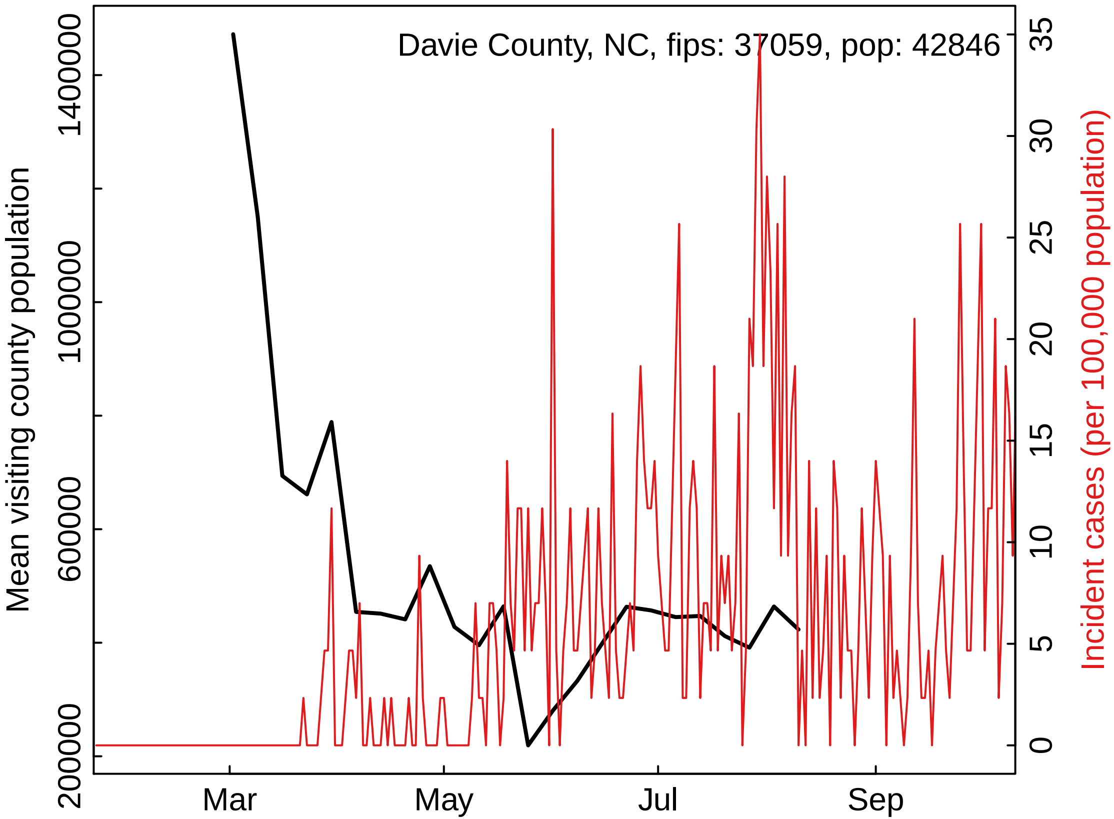
Davie County, NC, fips: 37059, pop: 42846
Mean visiting county population
2000000
6000000
10000000
14000000
Incident cases (per 100,000 population)
0
5
10
15
20
25
30
35
Mar
May
Jul
Sep

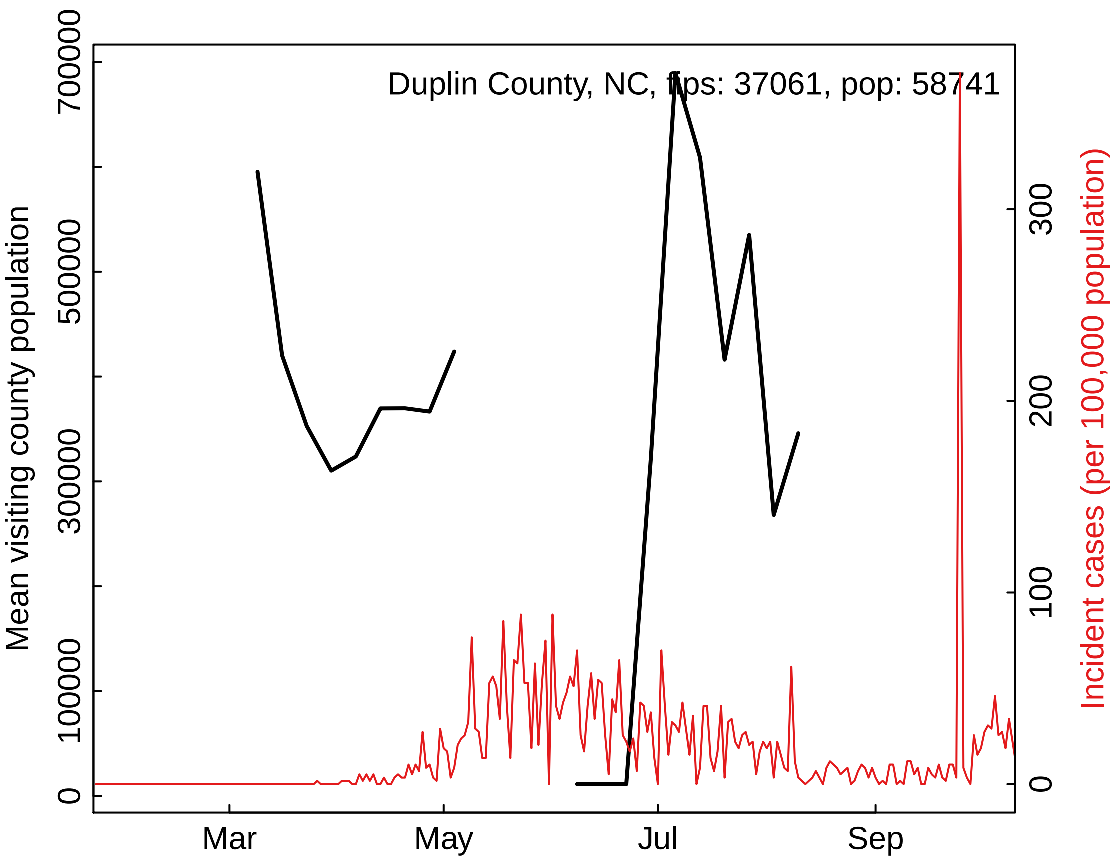
Duplin County, NC, fips: 37061, pop: 58741
Mean visiting county population
Incident cases (per 100,000 population)
0
100000
300000
500000
700000
0
100
200
300
Mar
May
Jul
Sep

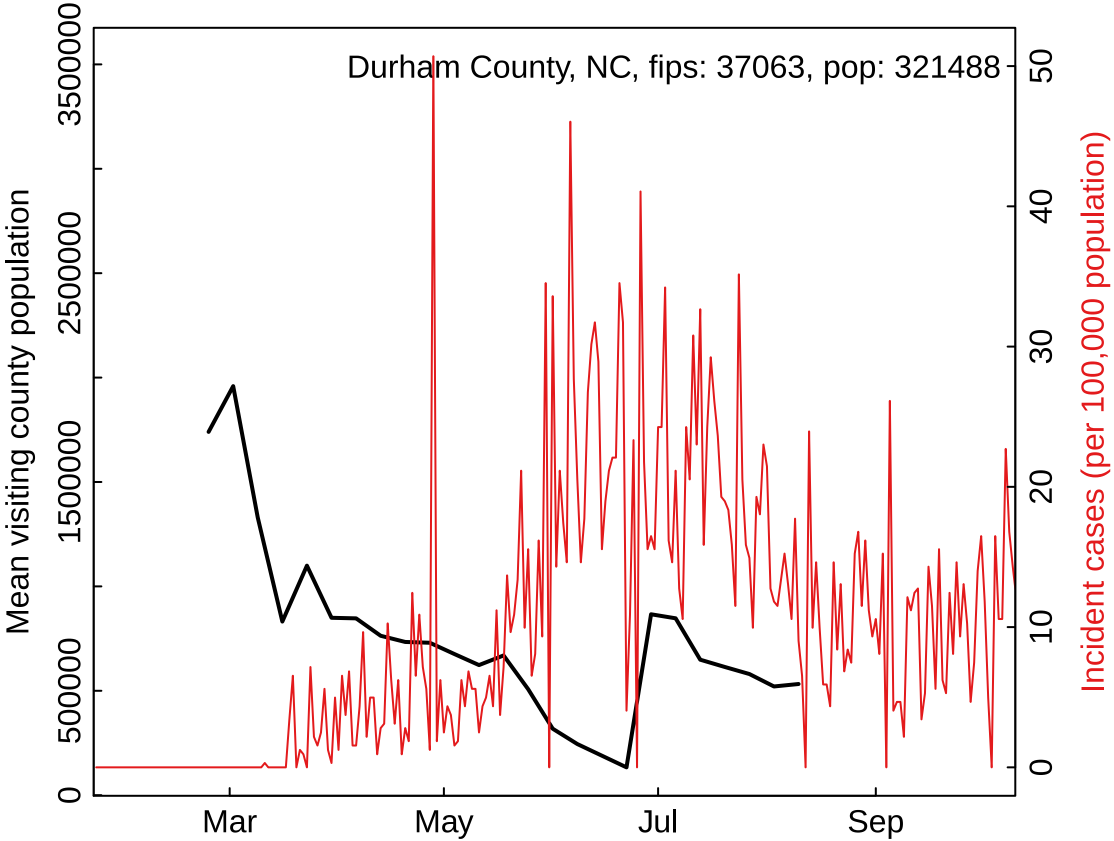

Durham County, NC, fips: 37063, pop: 321488
Mean visiting county population
Incident cases (per 100,000 population)
0
500000
1500000
2500000
3500000
0
10
20
30
40
50
Mar
May
Jul
Sep

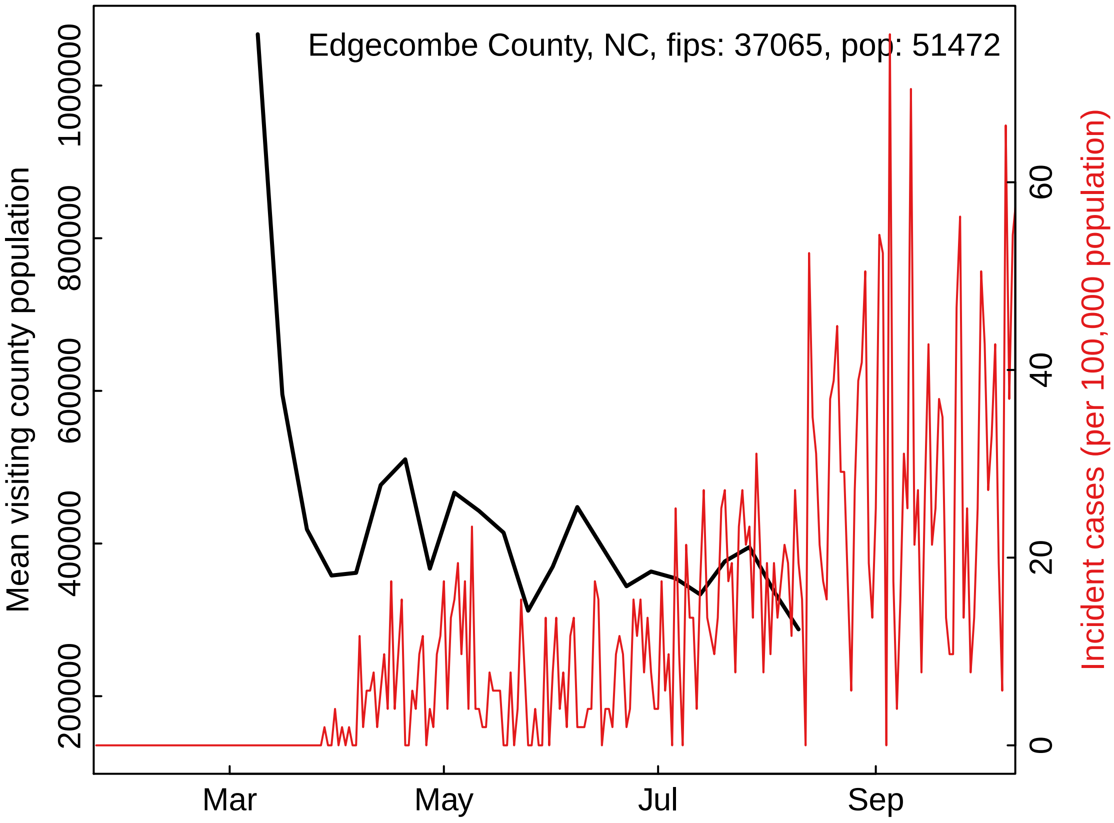

Edgecombe County, NC, fips: 37065, pop: 51472
Mean visiting county population
200000
400000
600000
800000
1000000
Incident cases (per 100,000 population)
0
20
40
60
Mar
May
Jul
Sep

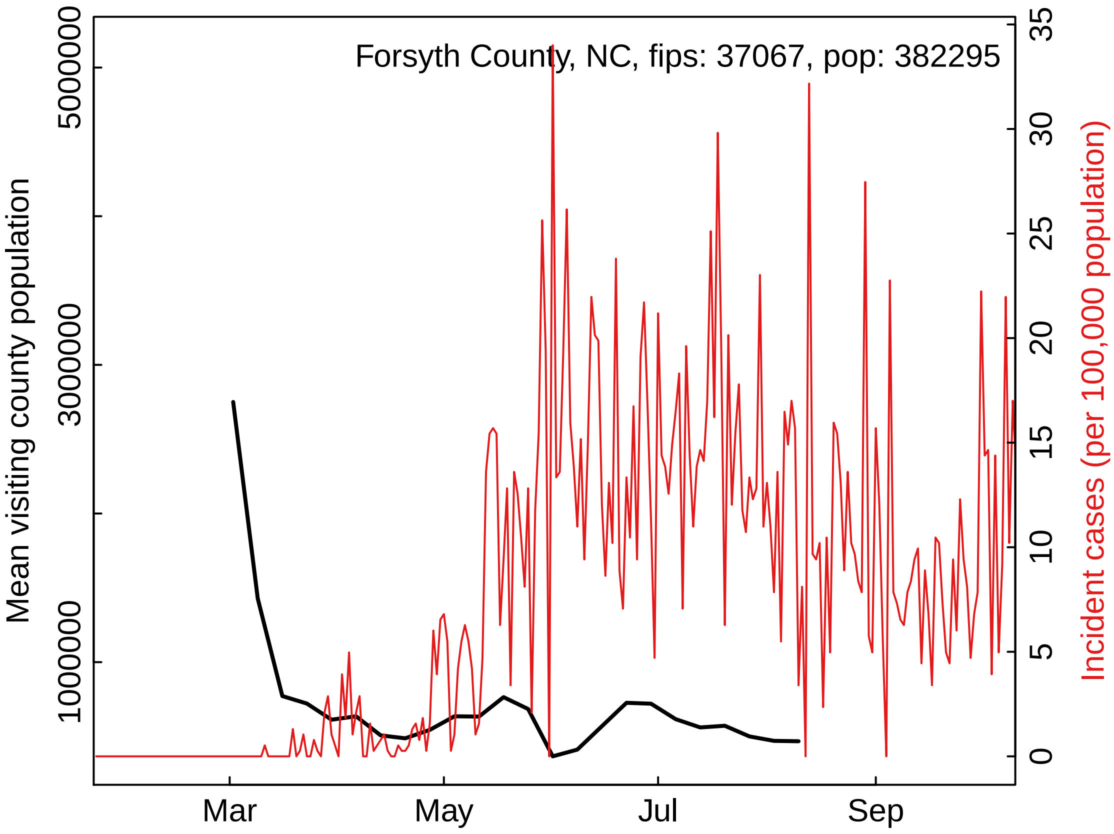

Forsyth County, NC, fips: 37067, pop: 382295
Mean visiting county population
1000000
3000000
5000000
Incident cases (per 100,000 population)
0
5
10
15
20
25
30
35
Mar
May
Jul
Sep

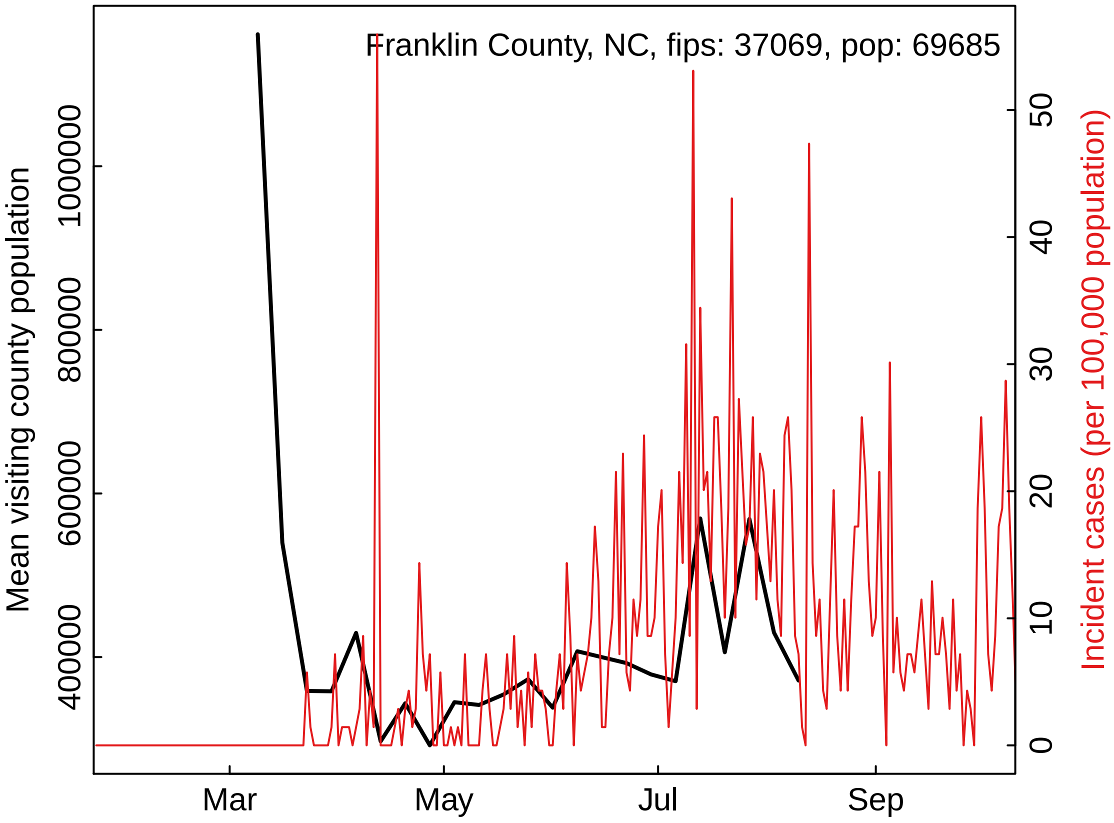
Franklin County, NC, fips: 37069, pop: 69685
Mean visiting county population
400000
600000
800000
1000000
Incident cases (per 100,000 population)
0
10
20
30
40
50
Mar
May
Jul
Sep

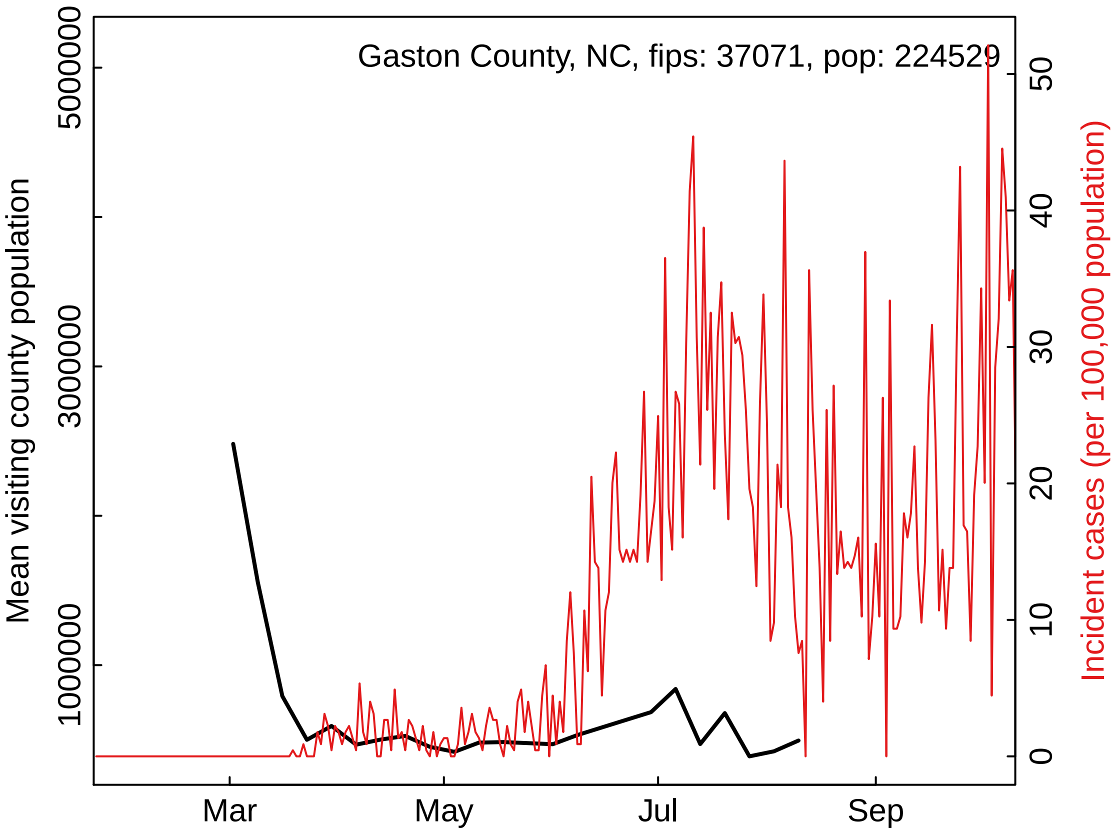

Gaston County, NC, fips: 37071, pop: 224529
Mean visiting county population
1000000
3000000
5000000
Incident cases (per 100,000 population)
0
10
20
30
40
50
Mar
May
Jul
Sep

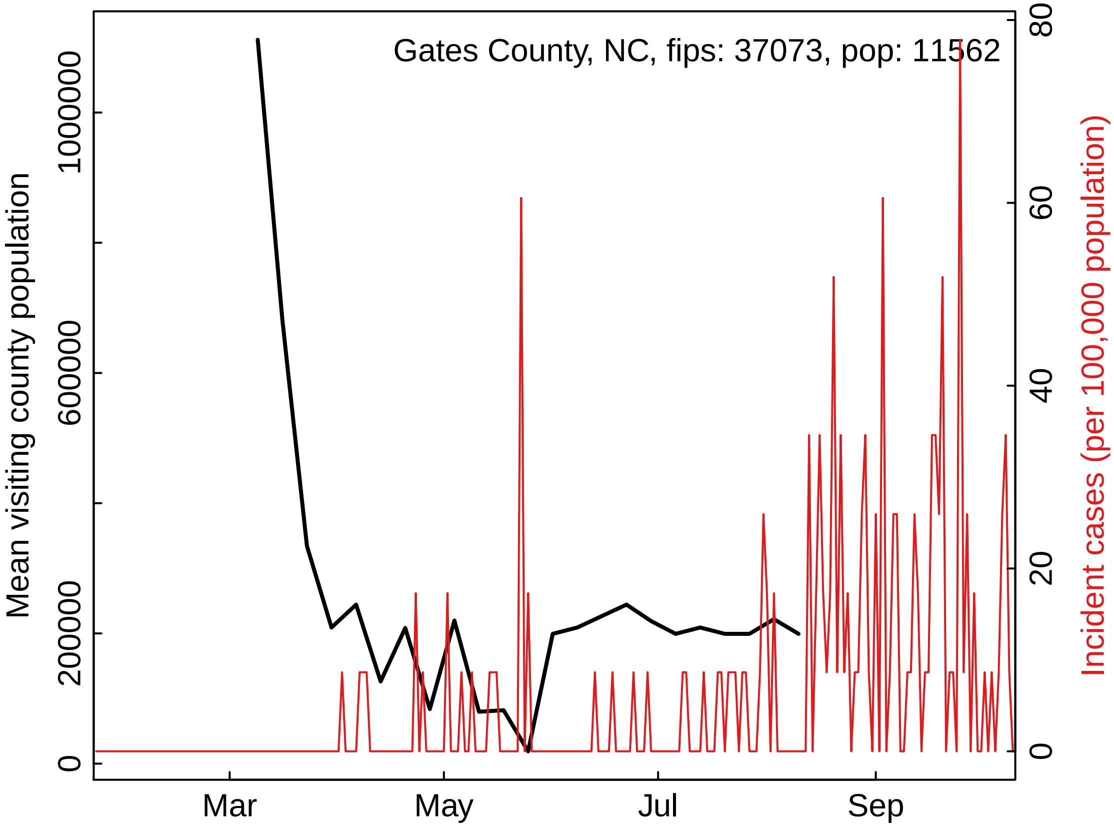

Gates County, NC, fips: 37073, pop: 11562
Mean visiting county population
0
200000
600000
1000000
Mar
May
Jul
Sep
Incident cases (per 100,000 population)
0
20
40
60
80

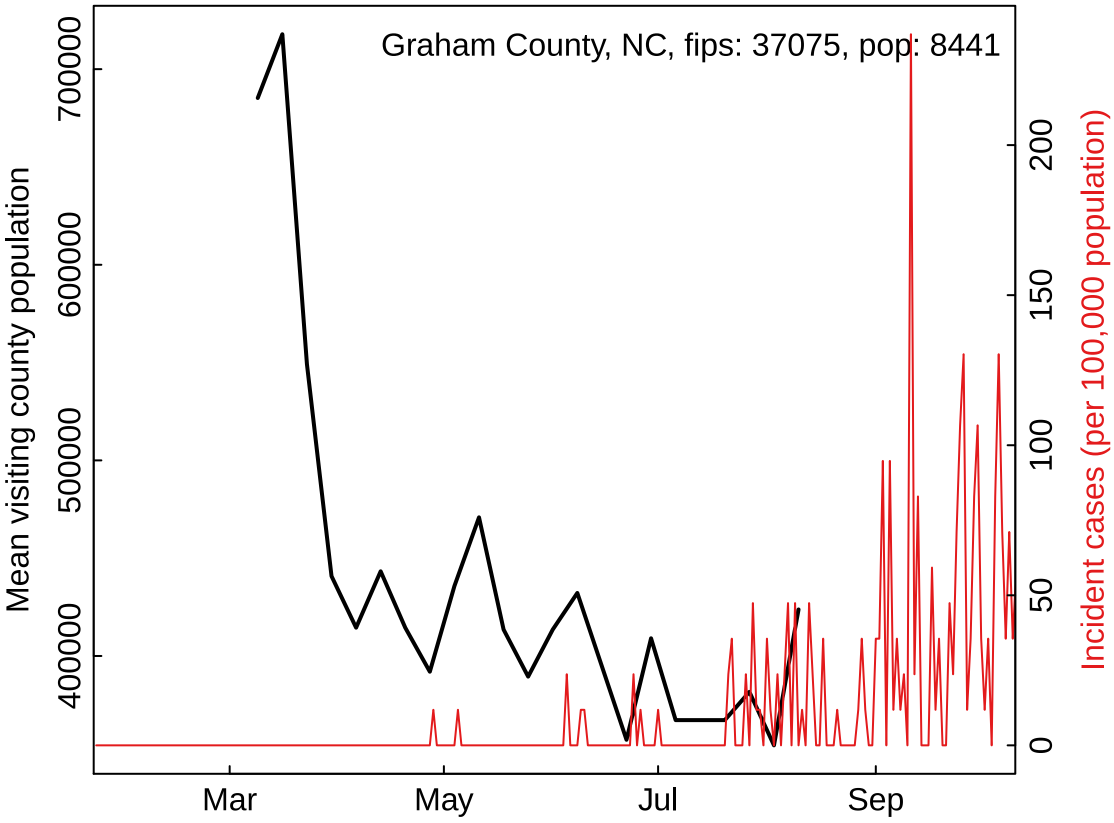

Graham County, NC, fips: 37075, pop: 8441
Mean visiting county population
400000
500000
600000
7000000
Incident cases (per 100,000 population)
0
50
100
150
200
Mar
May
Jul
Sep

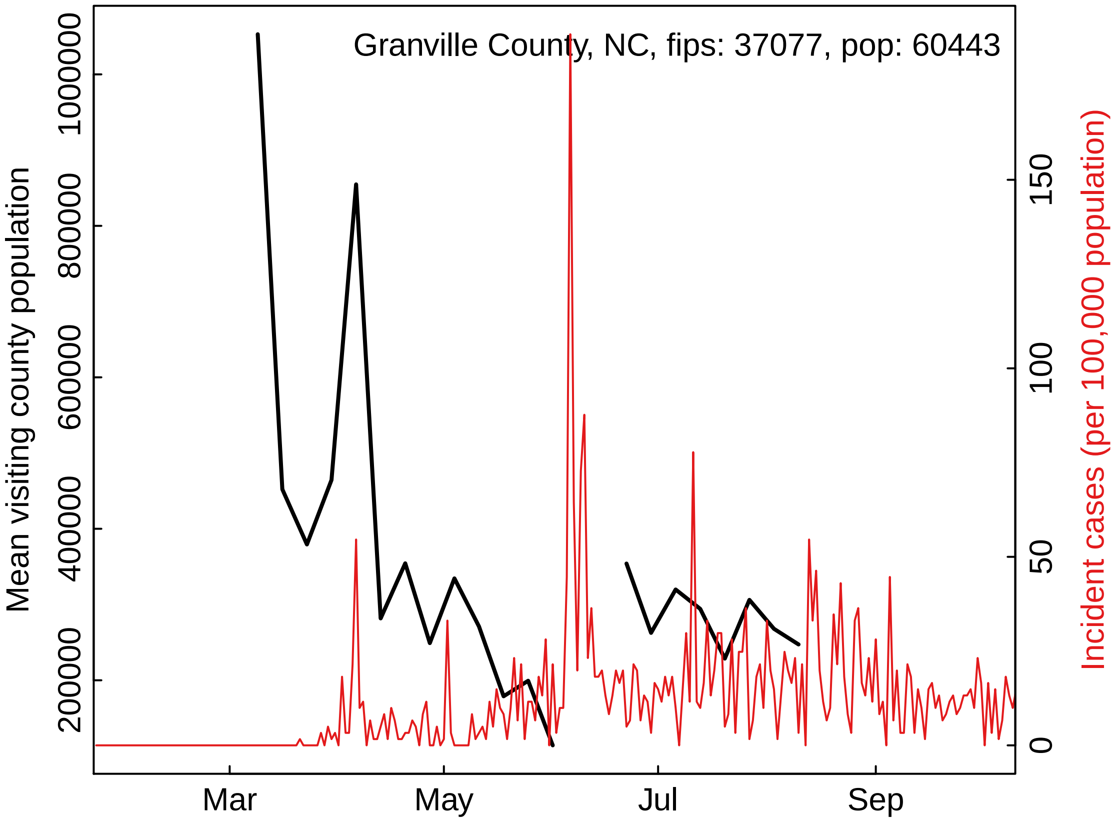

Granville County, NC, fips: 37077, pop: 60443
Mean visiting county population
200000
400000
600000
800000
1000000
Incident cases (per 100,000 population)
0
50
100
150
Mar
May
Jul
Sep

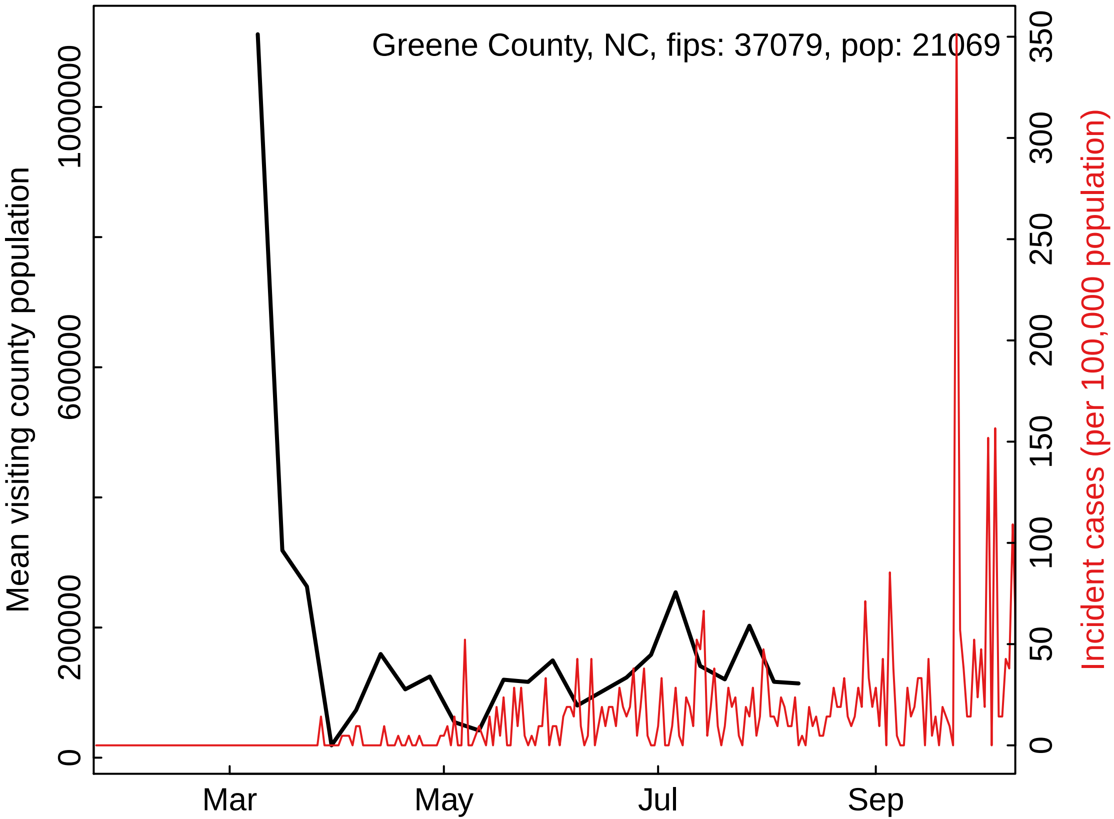

Greene County, NC, fips: 37079, pop: 21069
Mean visiting county population
0
200000
600000
1000000
Mar
May
Jul
Sep
0
50
100
150
200
250
300
350
Incident cases (per 100,000 population)

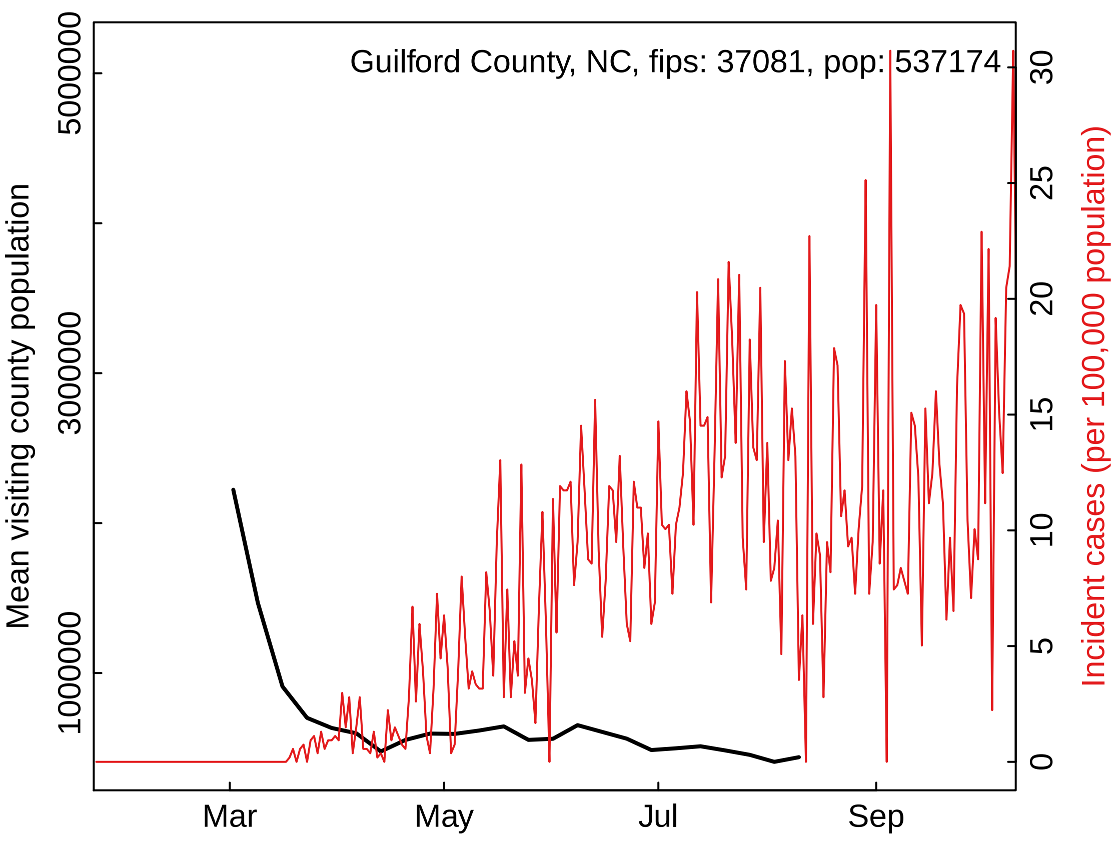

Guilford County, NC, fips: 37081, pop: 537174
Mean visiting county population
1000000
3000000
5000000
Incident cases (per 100,000 population)
0
5
10
15
20
25
30
Mar
May
Jul
Sep

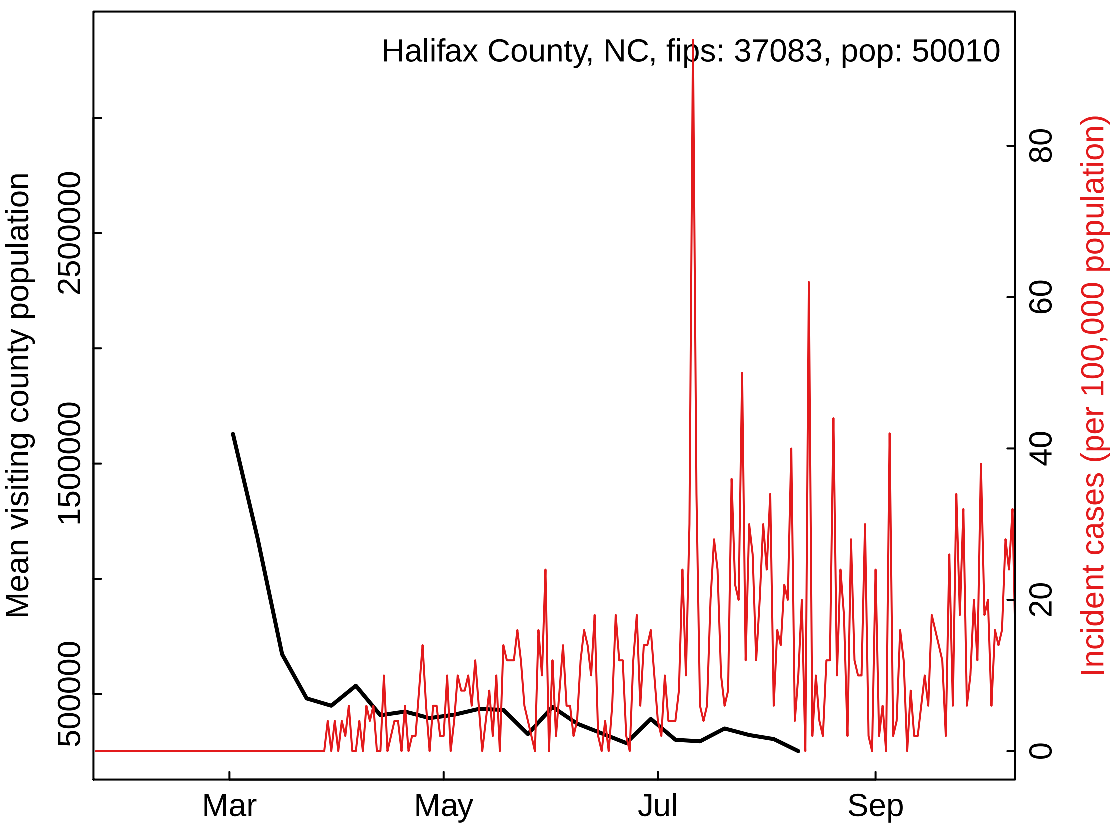
Halifax County, NC, fips: 37083, pop: 50010
Mean visiting county population
500000
1500000
2500000
Incident cases (per 100,000 population)
0
20
40
60
80
Mar
May
Jul
Sep

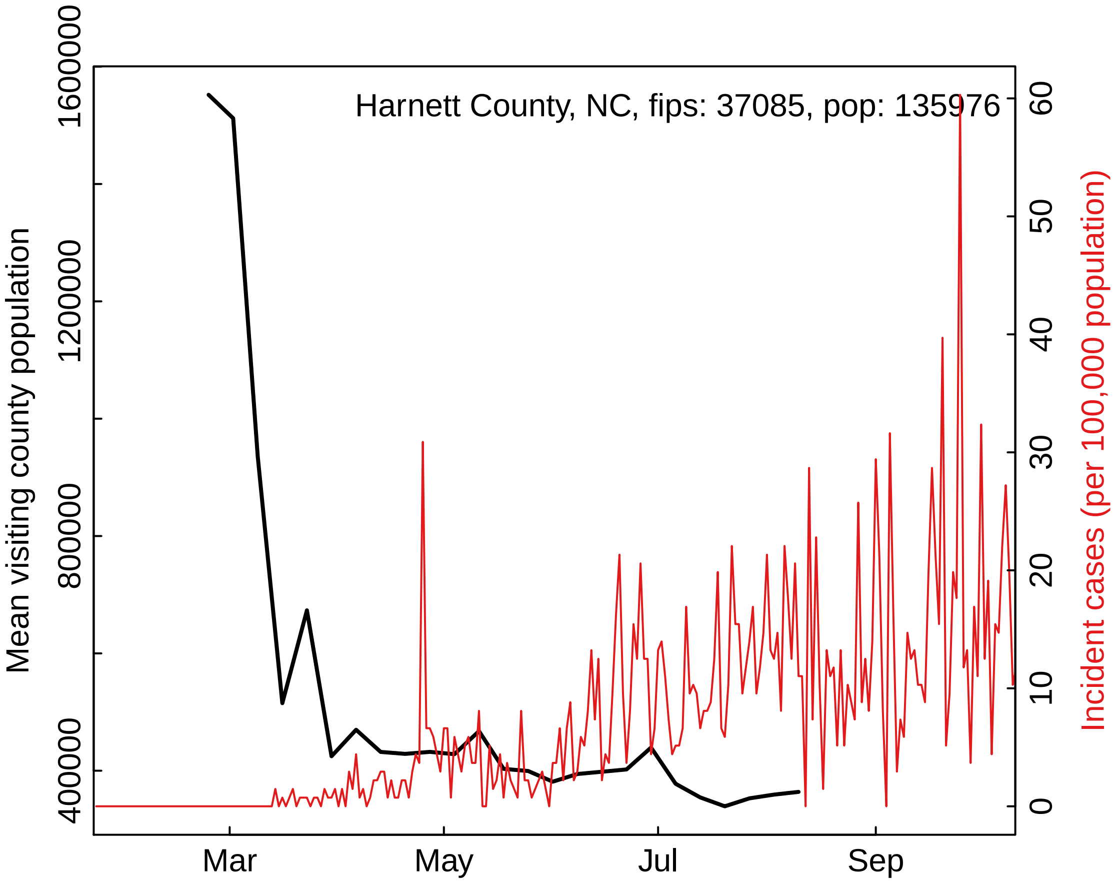

Harnett County, NC, fips: 37085, pop: 135976
Mean visiting county population
400000
800000
1200000
1600000
Mar
May
Jul
Sep
0
10
20
30
40
50
60
Incident cases (per 100,000 population)

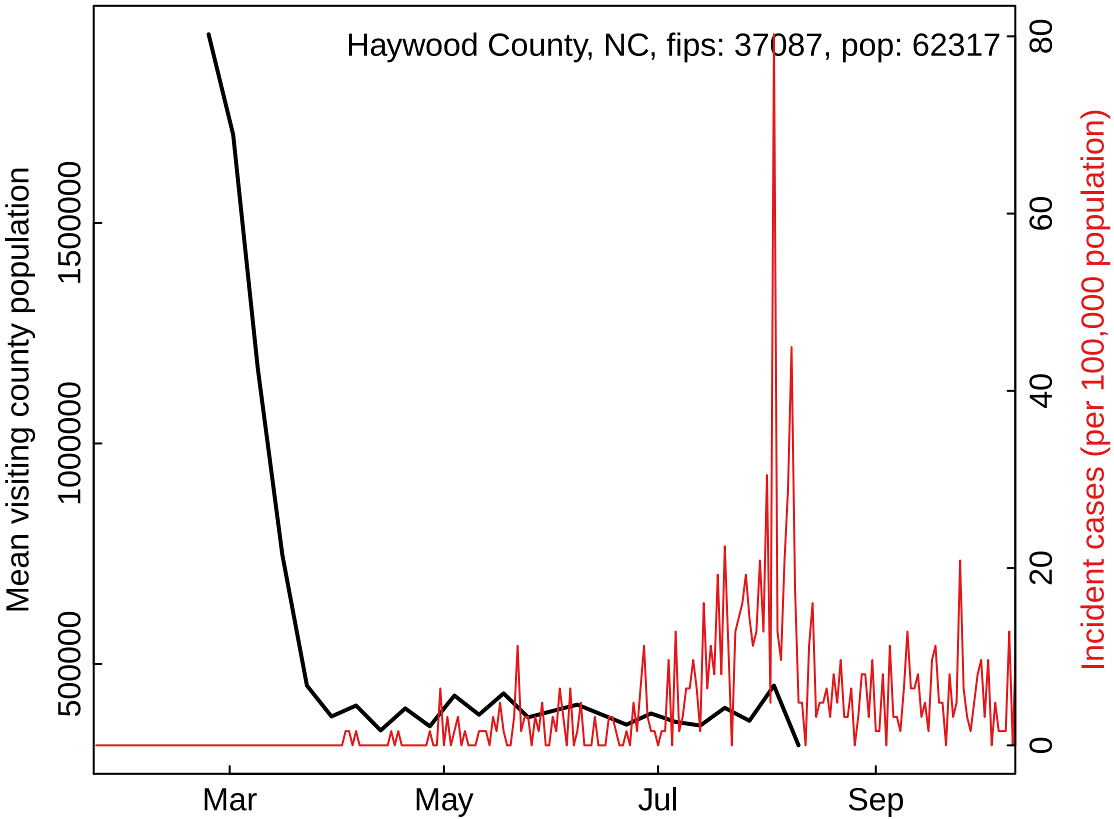

Haywood County, NC, fips: 37087, pop: 62317
Mean visiting county population
500000
1000000
1500000
Incident cases (per 100,000 population)
0
20
40
60
80
Mar
May
Jul
Sep

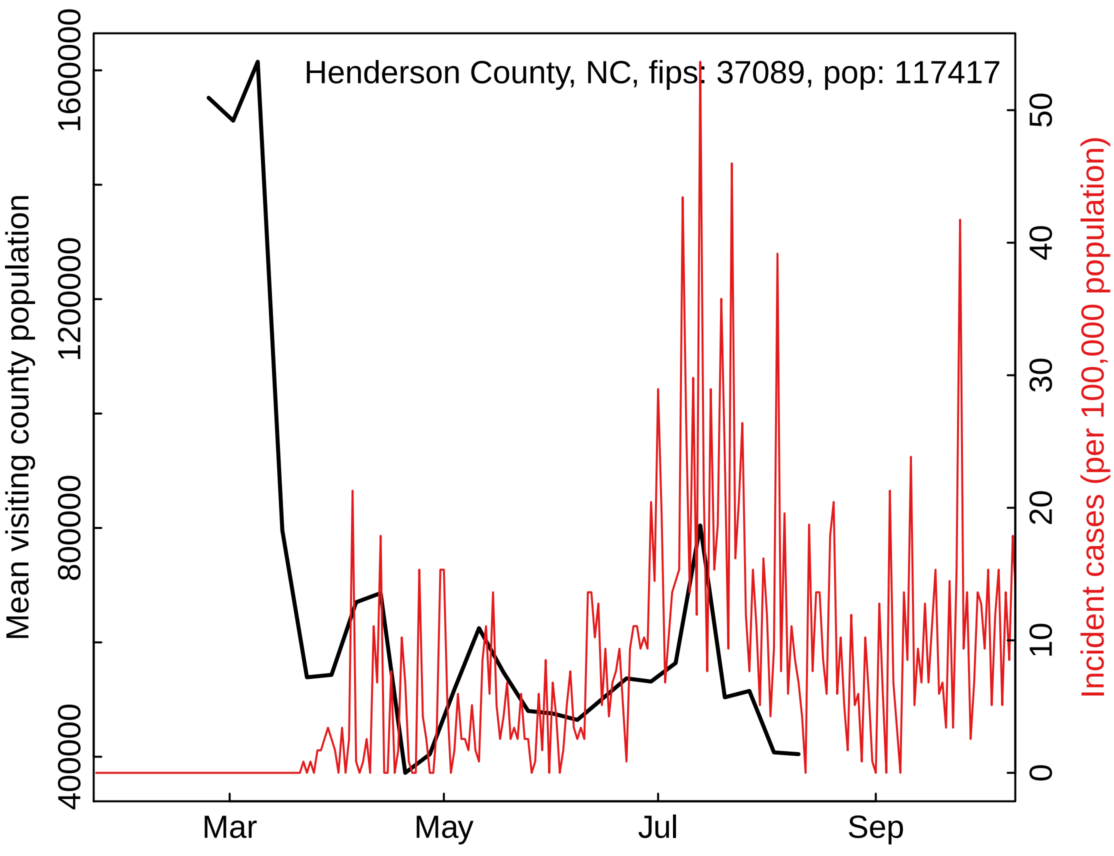

Henderson County, NC, fips: 37089, pop: 117417
Mean visiting county population
400000
800000
1200000
1600000
Incident cases (per 100,000 population)
0
10
20
30
40
50
Mar
May
Jul
Sep

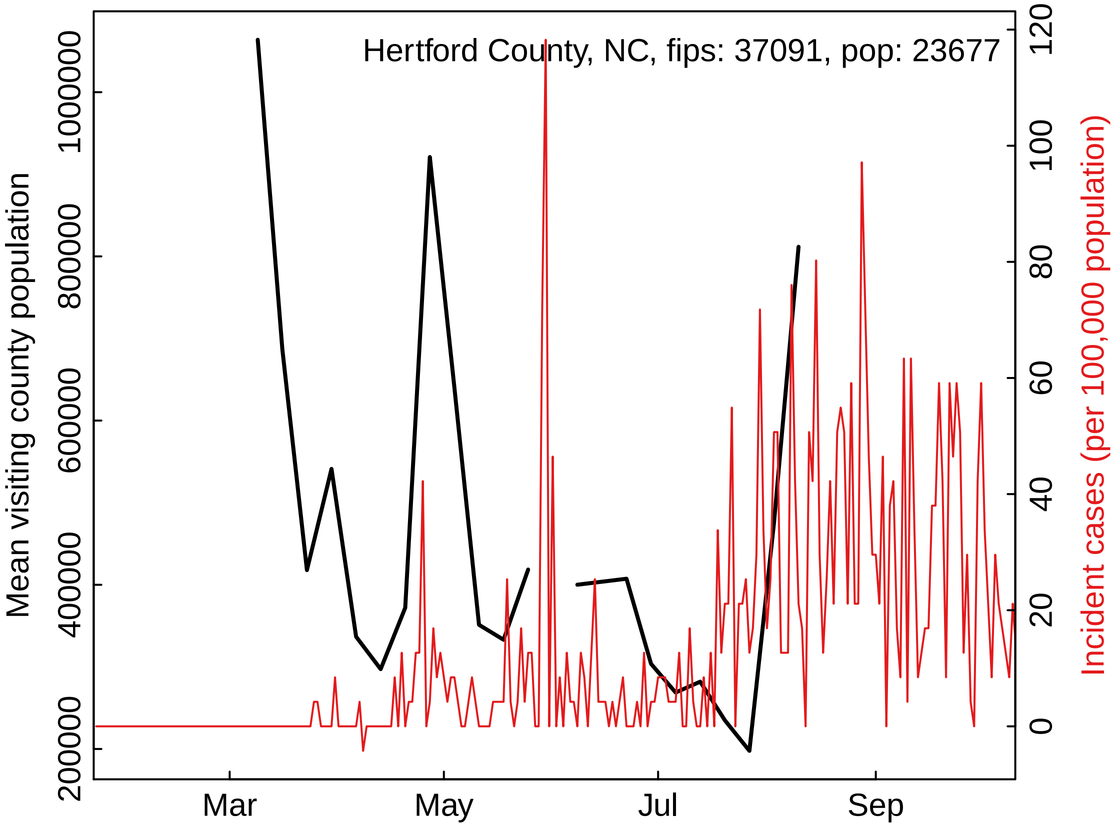
Hertford County, NC, fips: 37091, pop: 23677
Mean visiting county population
200000
400000
600000
800000
1000000
Incident cases (per 100,000 population)
0
20
40
60
80
100
120
Mar
May
Jul
Sep

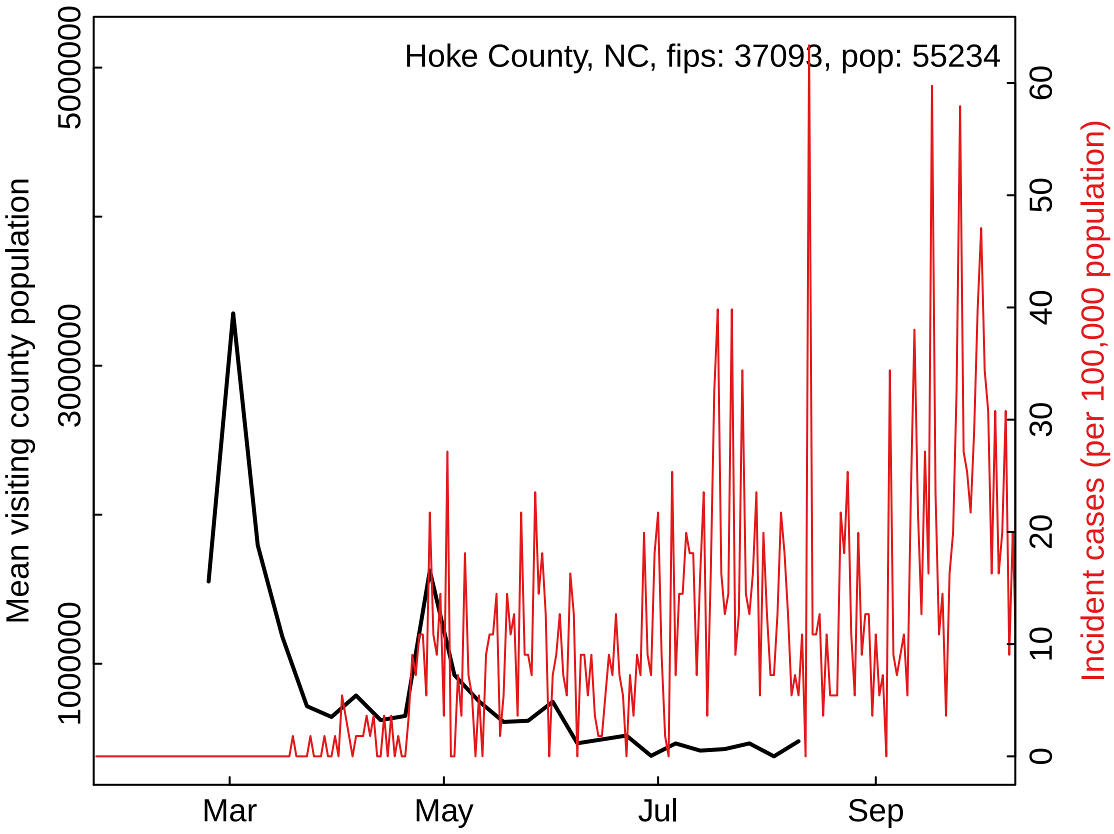
Hoke County, NC, fips: 37093, pop: 55234
Mean visiting county population
1000000
3000000
5000000
Incident cases (per 100,000 population)
0
10
20
30
40
50
60
Mar
May
Jul
Sep

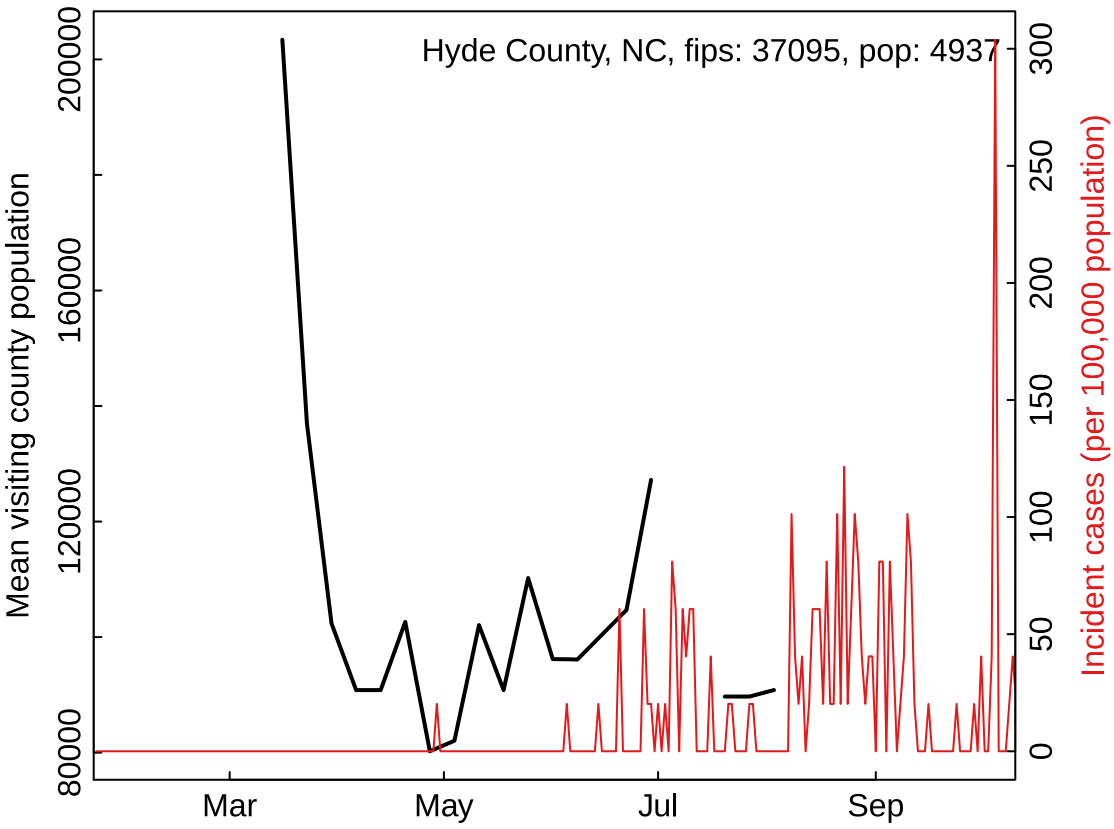
Hyde County, NC, fips: 37095, pop: 4937
Mean visiting county population
80000
120000
160000
200000
Mar
May
Jul
Sep
Incident cases (per 100,000 population)
0
50
100
150
200
250
300

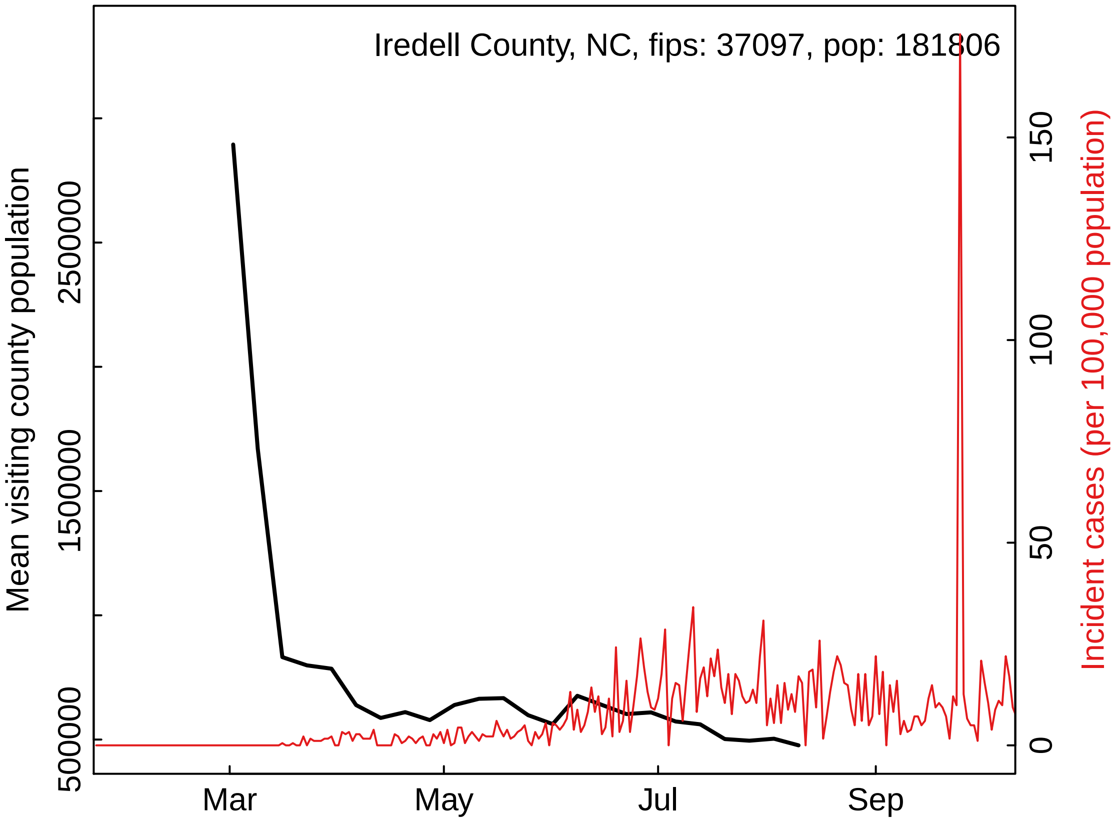

Iredell County, NC, fips: 37097, pop: 181806
Mean visiting county population
500000
1500000
2500000
Mar
May
Jul
Sep
0
50
100
150
Incident cases (per 100,000 population)

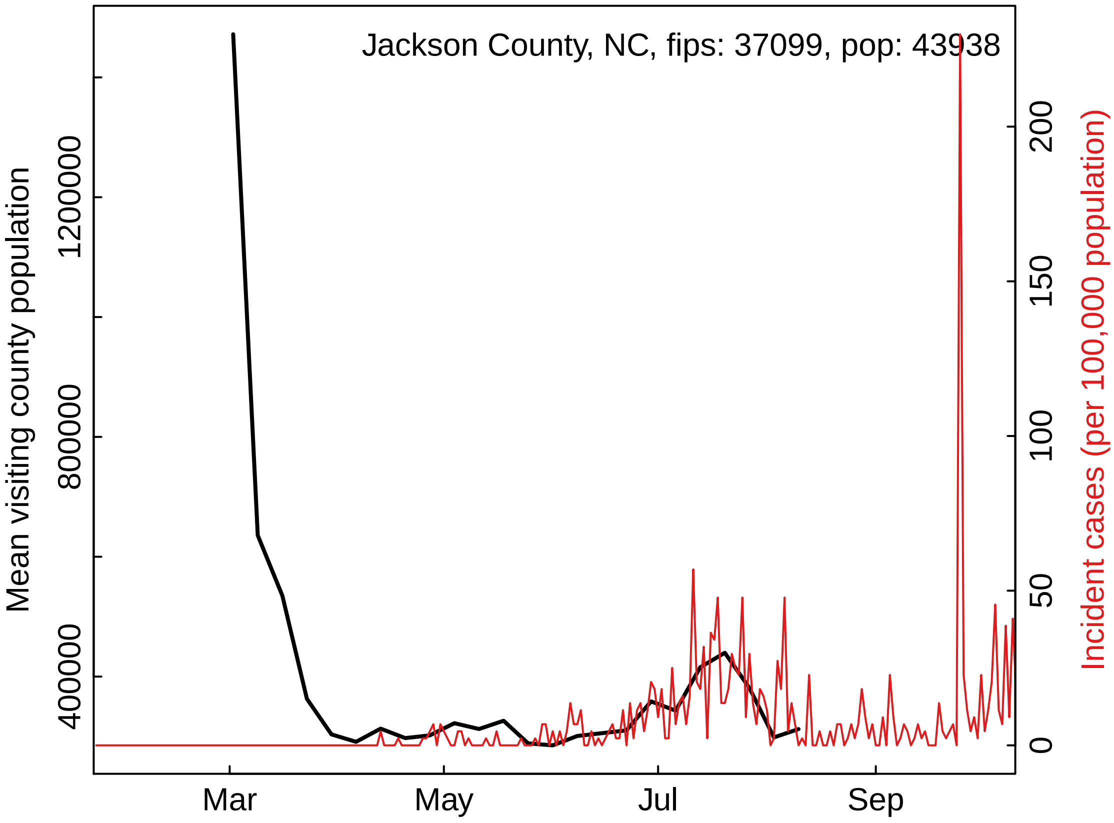

Jackson County, NC, fips: 37099, pop: 43938
Mean visiting county population
400000
800000
1200000
Mar
May
Jul
Sep
0
50
100
150
200
Incident cases (per 100,000 population)

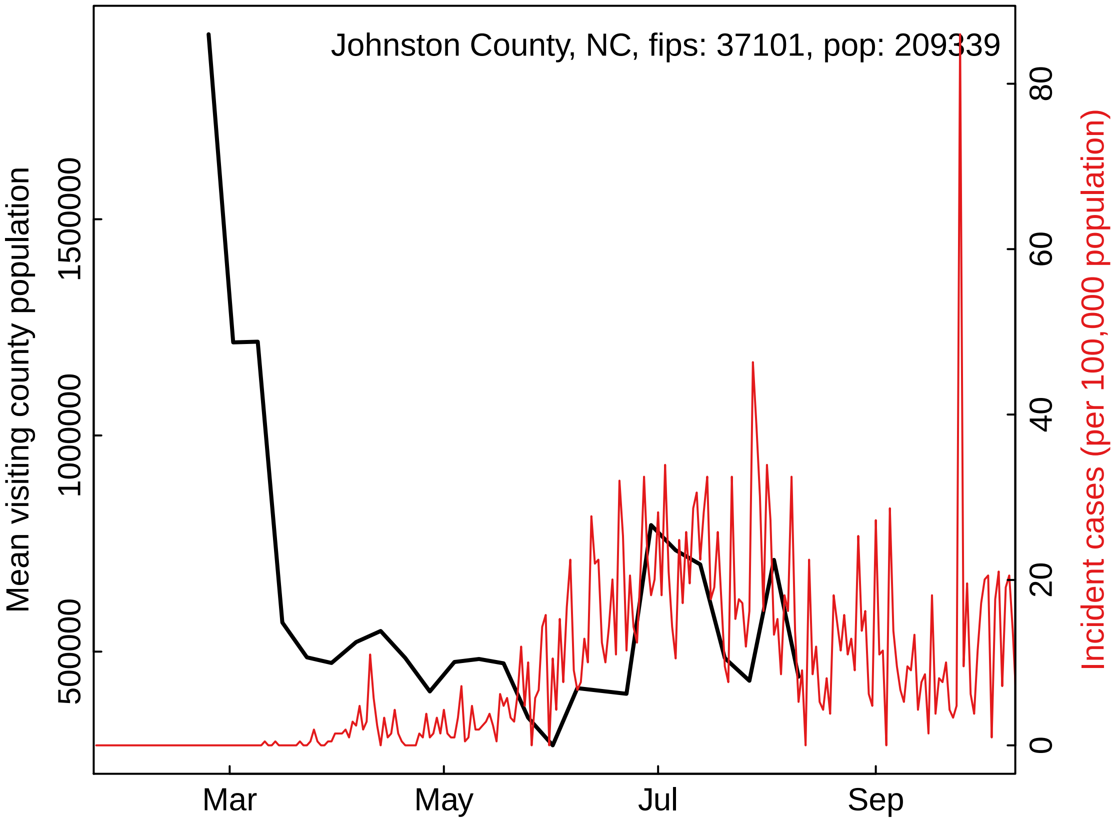

Johnston County, NC, fips: 37101, pop: 209339
Mean visiting county population
500000
1000000
1500000
Incident cases (per 100,000 population)
0
20
40
60
80
Mar
May
Jul
Sep

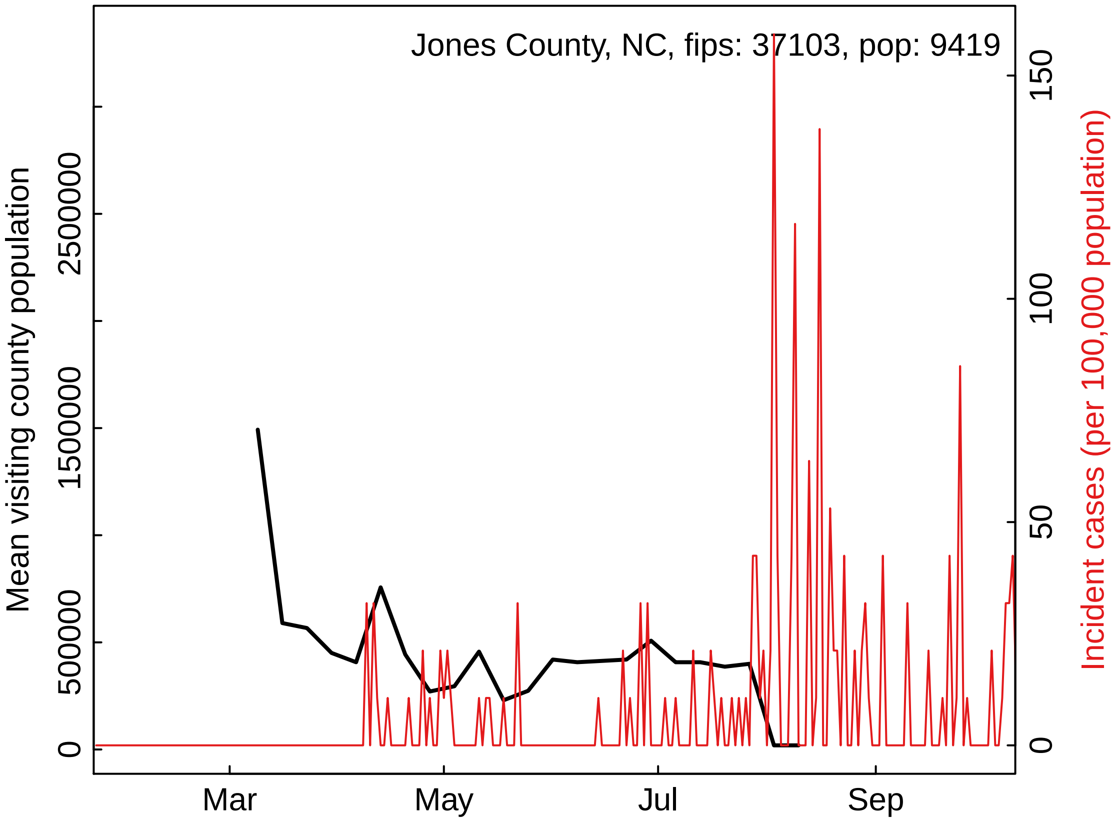

Jones County, NC, fips: 37103, pop: 9419
Mean visiting county population
0
500000
1500000
2500000
Incident cases (per 100,000 population)
0
50
100
150
Mar
May
Jul
Sep

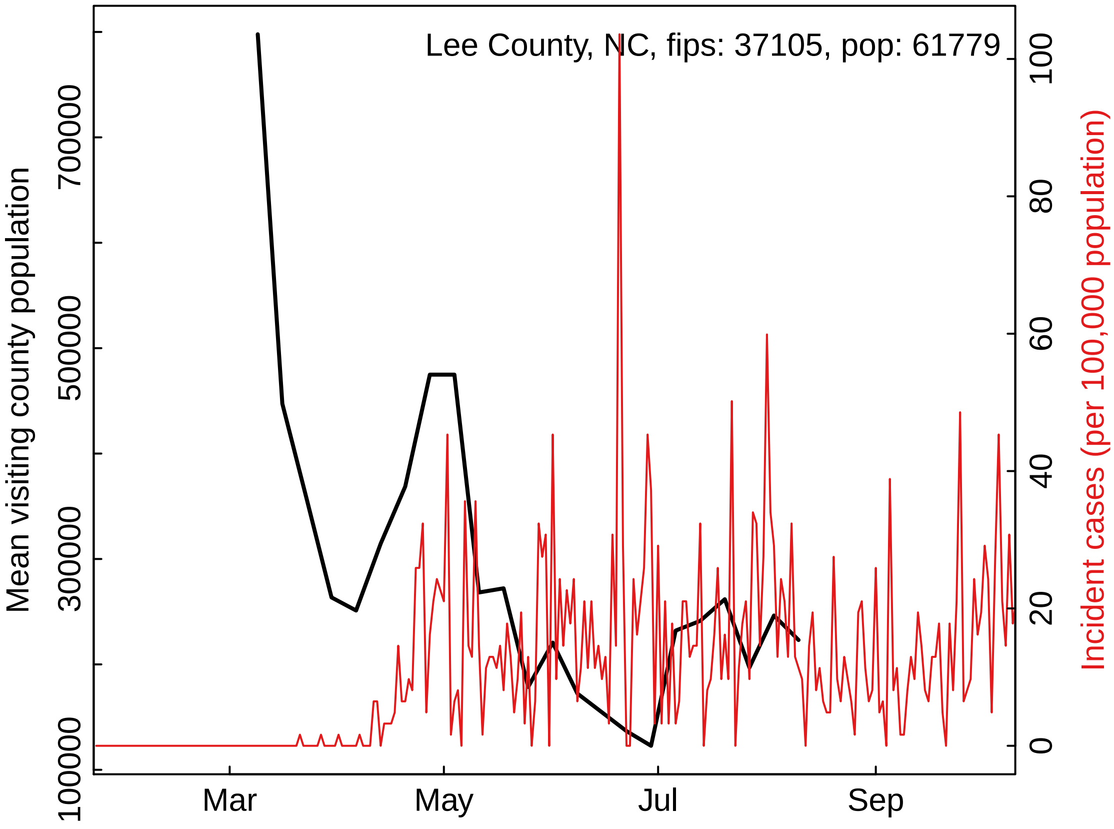

Lee County, NC, fips: 37105, pop: 61779
Mean visiting county population
1000000
3000000
5000000
7000000
Mar
May
Jul
Sep
0
20
40
60
80
100
Incident cases (per 100,000 population)

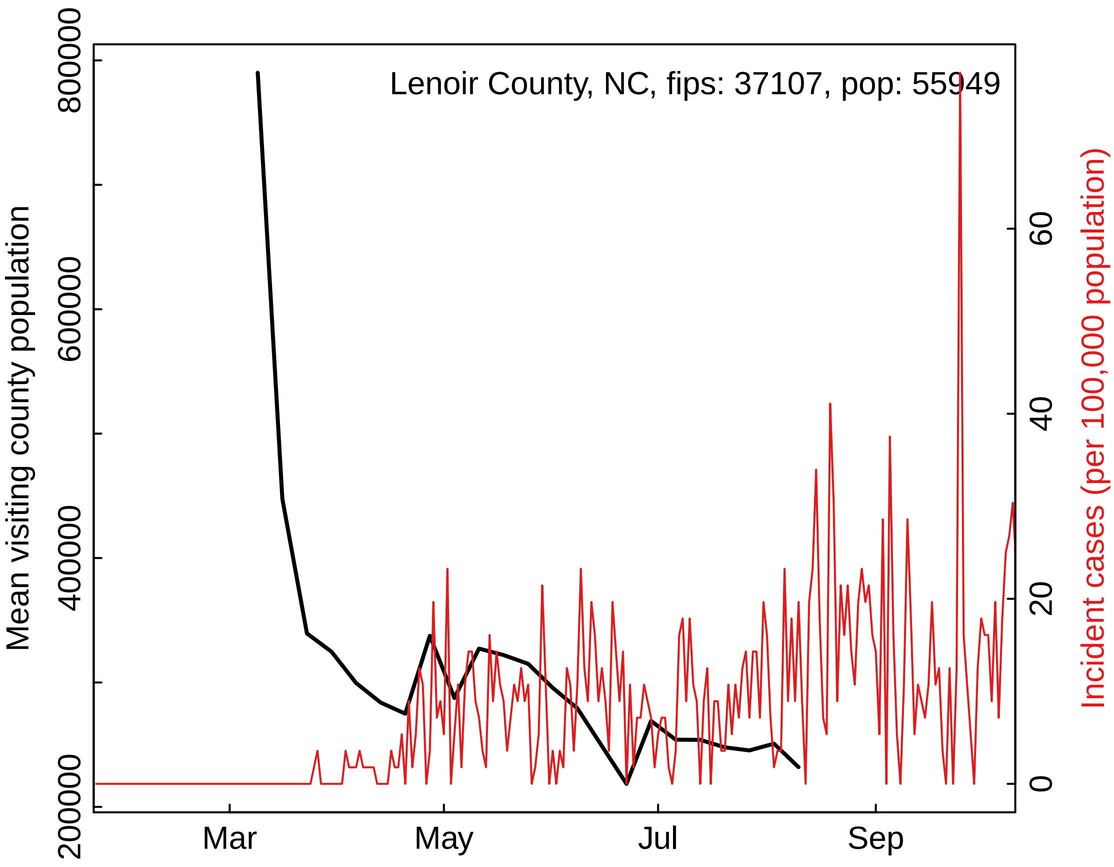

Lenoir County, NC, fips: 37107, pop: 55949
Mean visiting county population
200000
400000
600000
800000
Incident cases (per 100,000 population)
0
20
40
60
Mar
May
Jul
Sep

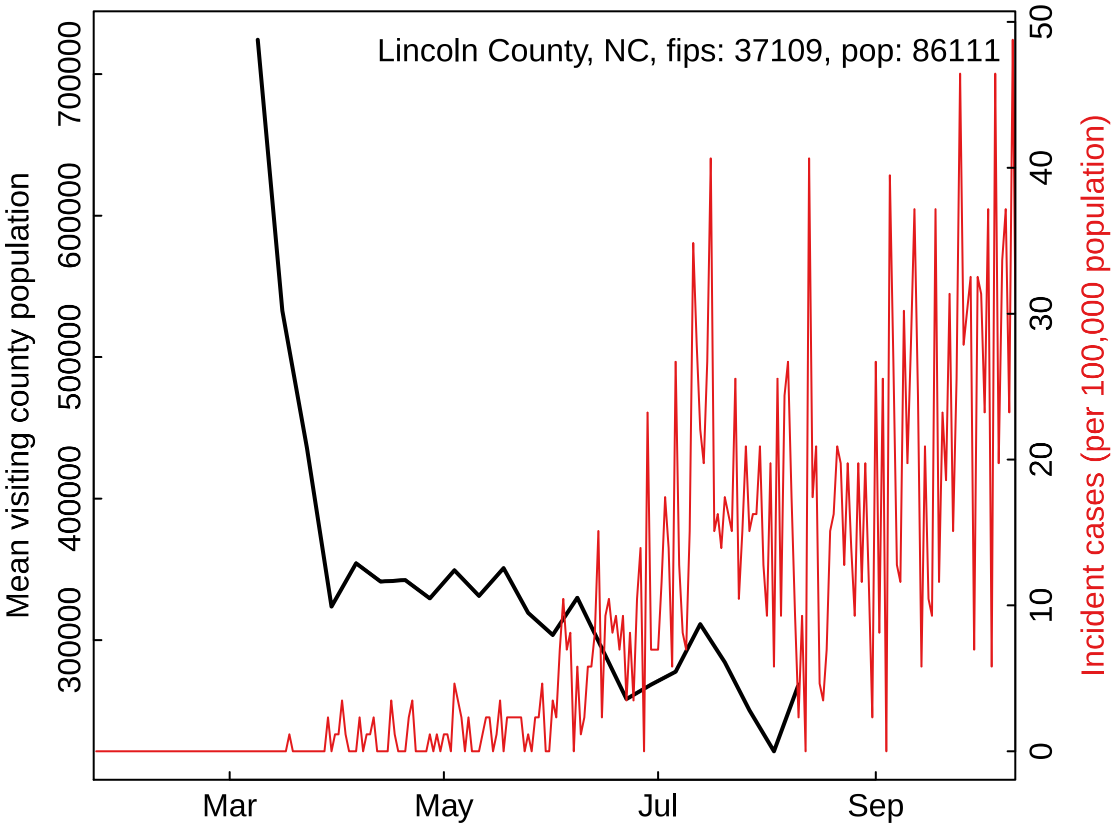
Lincoln County, NC, fips: 37109, pop: 86111
Mean visiting county population
300000
400000
500000
600000
700000
Incident cases (per 100,000 population)
0
10
20
30
40
50
Mar
May
Jul
Sep

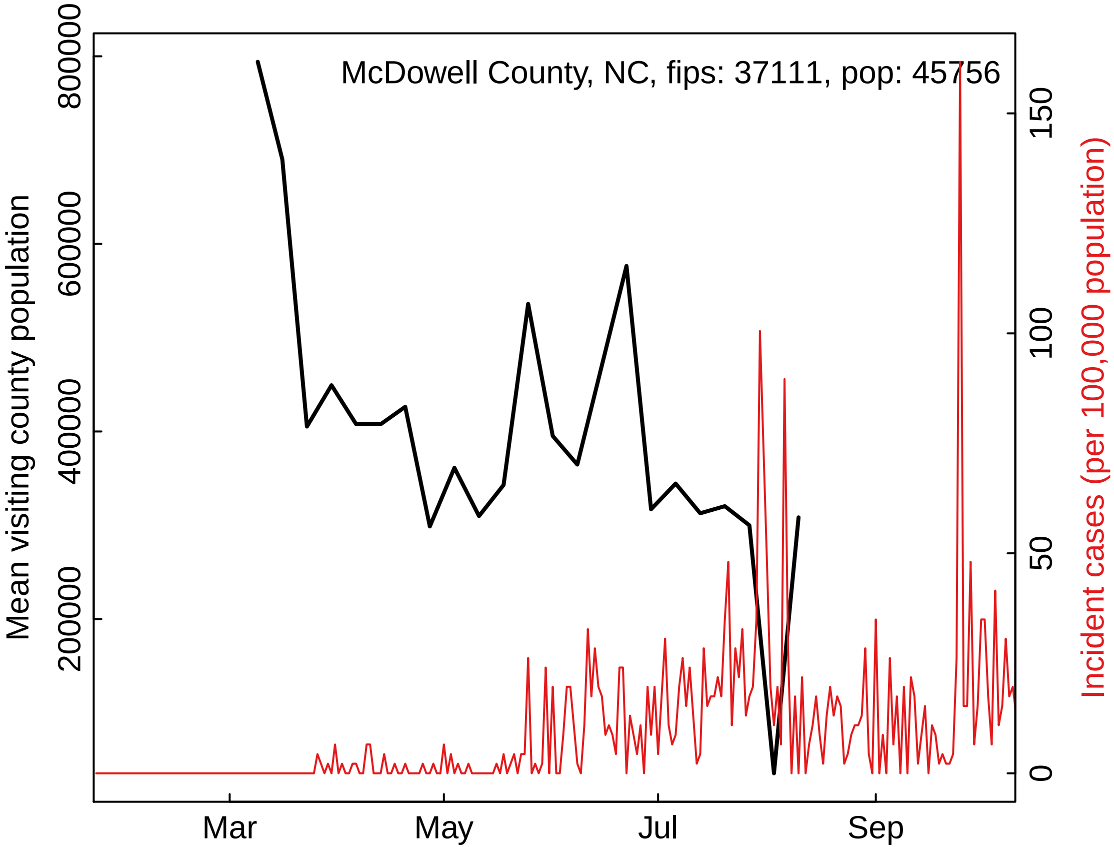

McDowell County, NC, fips: 37111, pop: 45756
Mean visiting county population
200000
400000
600000
800000
Incident cases (per 100,000 population)
0
50
100
150
Mar
May
Jul
Sep

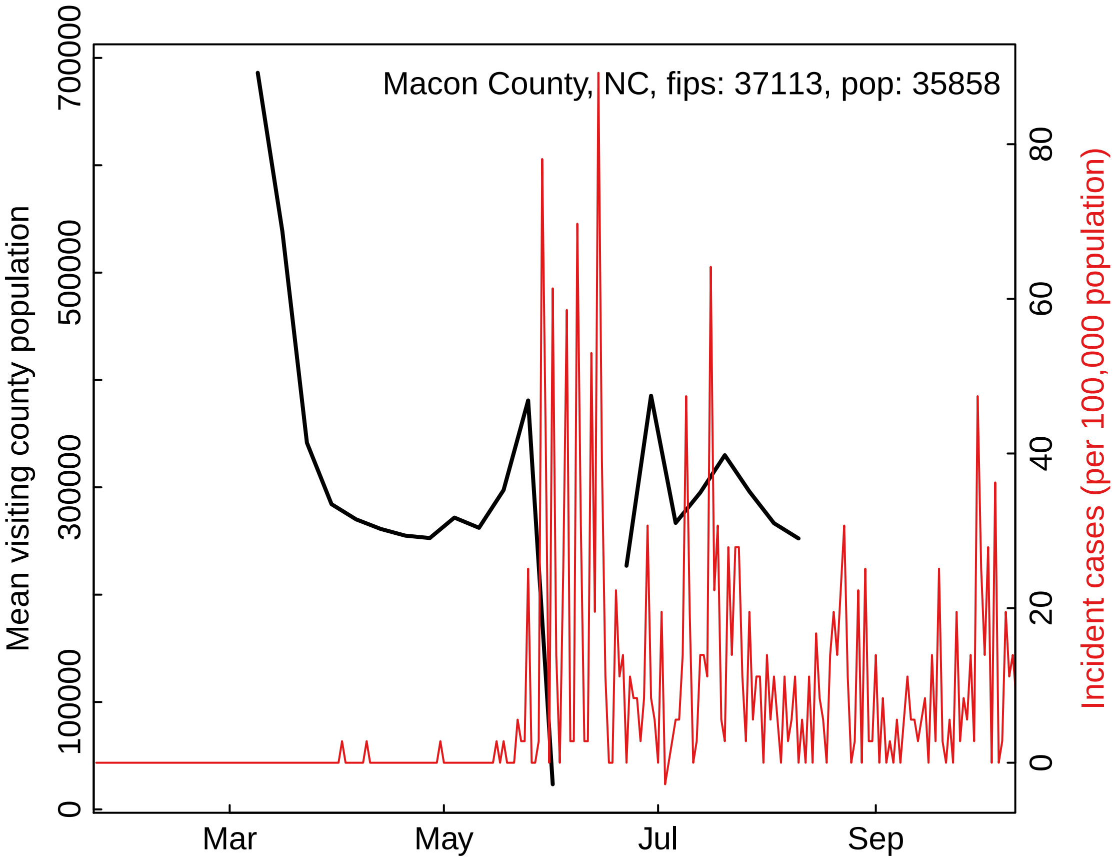

Macon County, NC, fips: 37113, pop: 35858
Mean visiting county population
0
100000
300000
500000
700000
Incident cases (per 100,000 population)
0
20
40
60
80
Mar
May
Jul
Sep

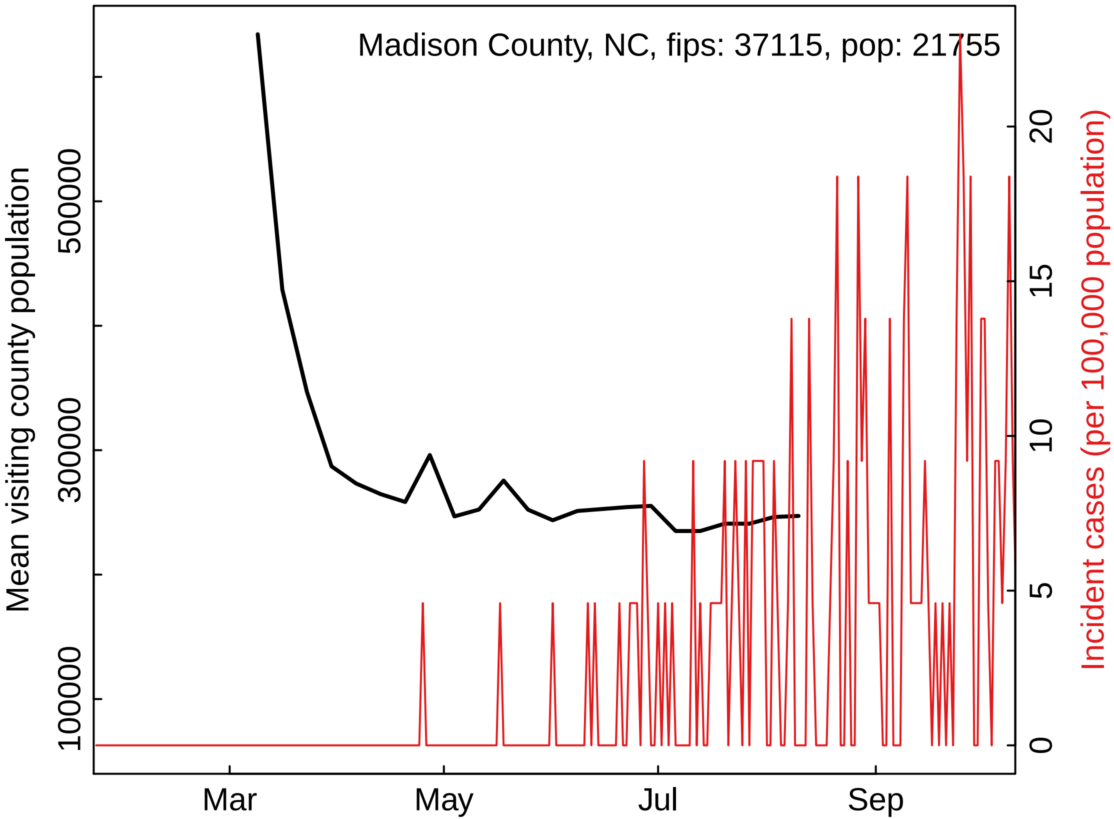

Madison County, NC, fips: 37115, pop: 21755
Mean visiting county population
100000
300000
500000
Mar
May
Jul
Sep
0
5
10
15
20
Incident cases (per 100,000 population)

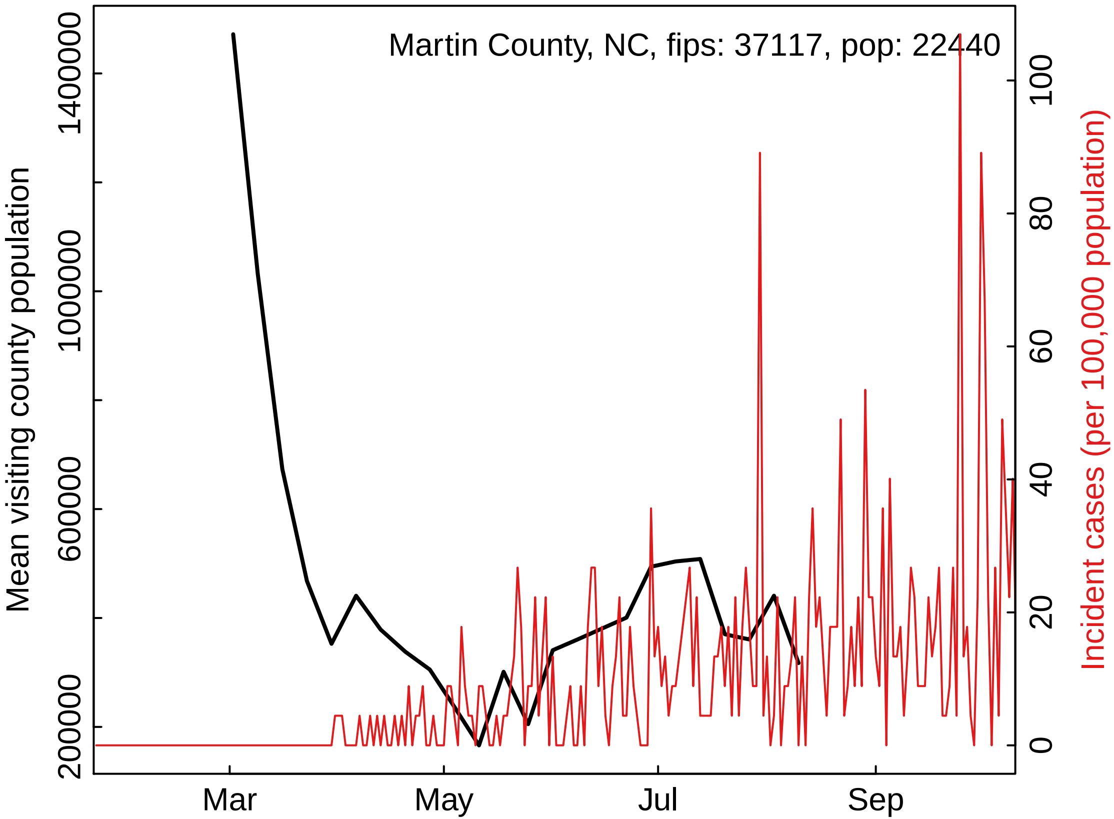

Martin County, NC, fips: 37117, pop: 22440
Mean visiting county population
200000
600000
1000000
1400000
Incident cases (per 100,000 population)
0
20
40
60
80
100
Mar
May
Jul
Sep

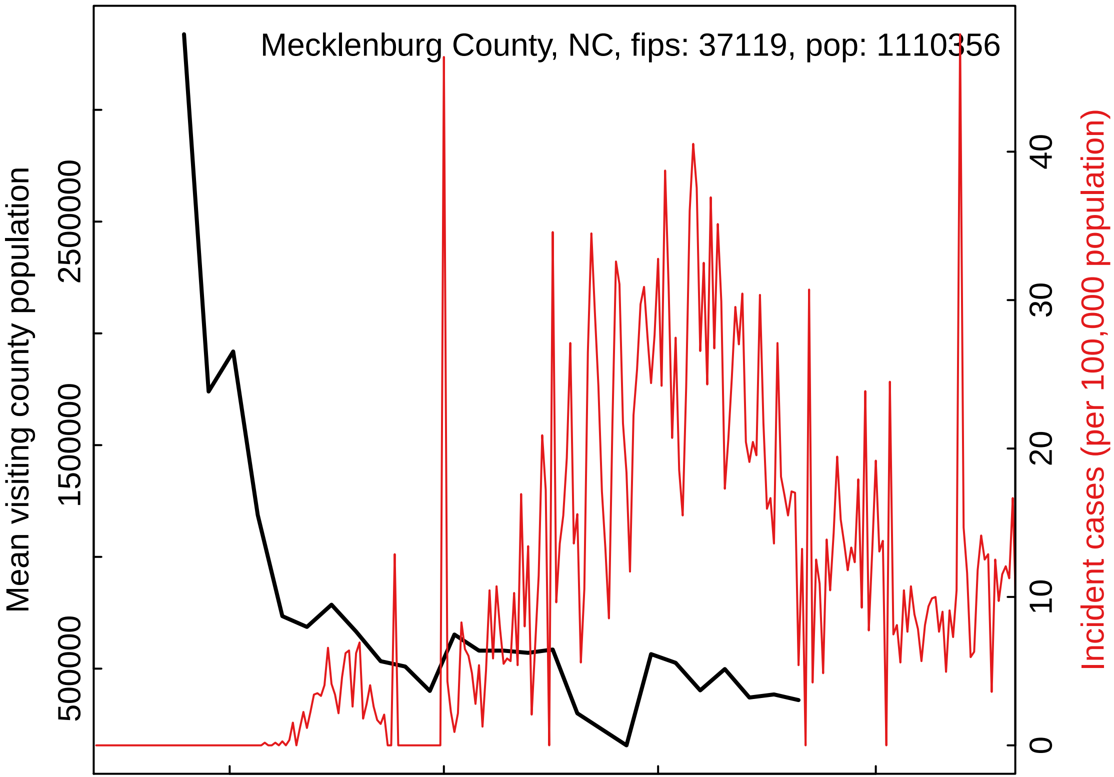

Mecklenburg County, NC, fips: 37119, pop: 1110356
Mean visiting county population
500000
1500000
2500000
Incident cases (per 100,000 population)
0
10
20
30
40
Mar
May
Jul
Sep

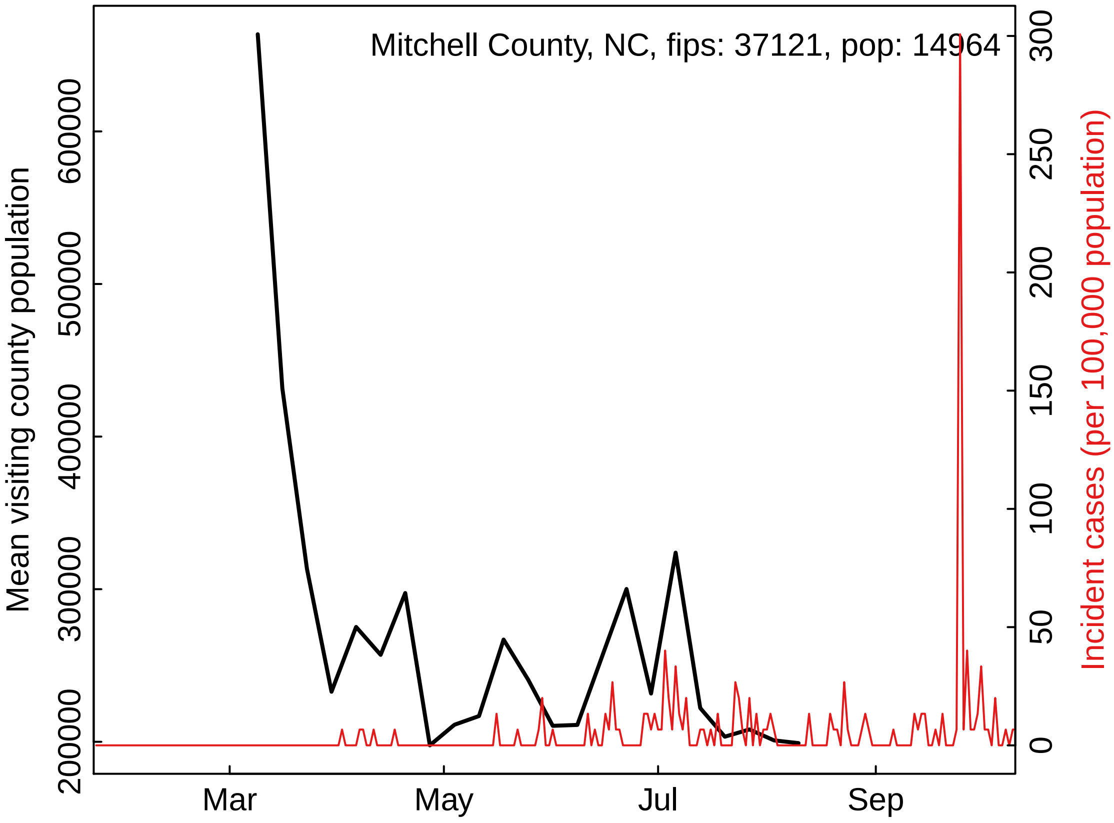
Mitchell County, NC, fips: 37121, pop: 14964
Mean visiting county population
200000
300000
400000
500000
600000
Mar
May
Jul
Sep
0
50
100
150
200
250
300
Incident cases (per 100,000 population)

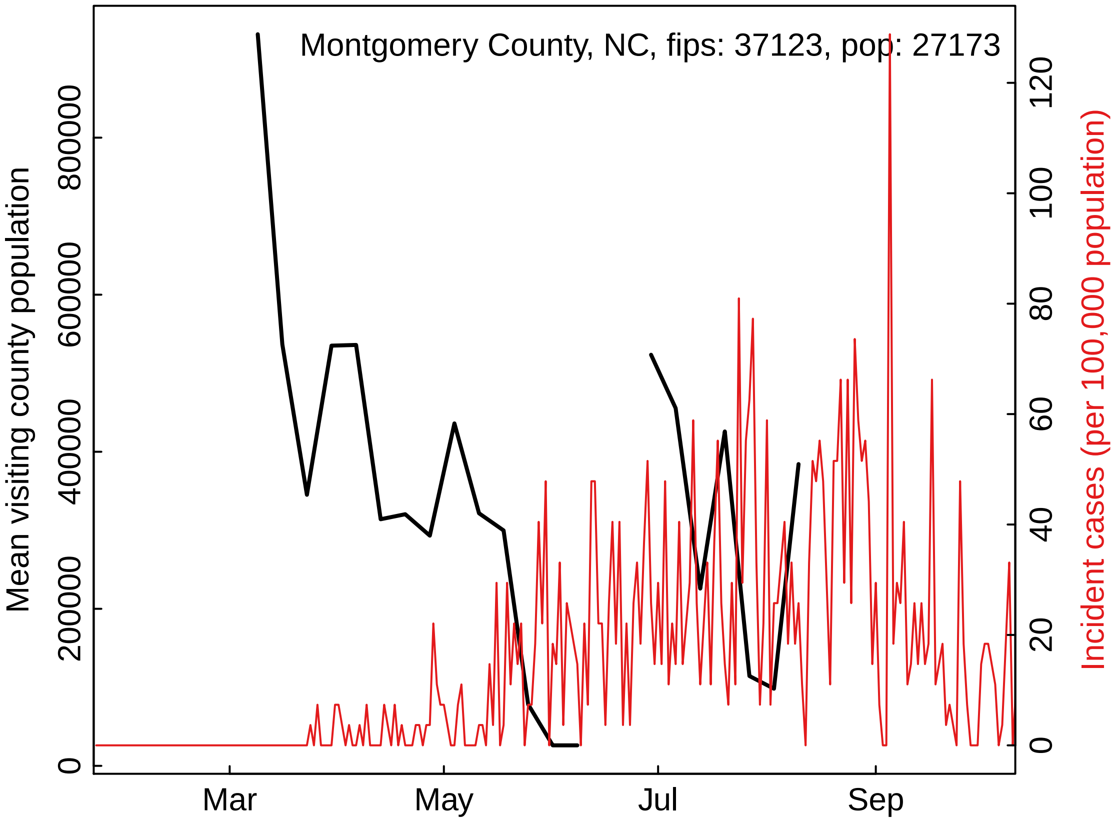

Montgomery County, NC, fips: 37123, pop: 27173
Mean visiting county population
0
200000
400000
600000
800000
Incident cases (per 100,000 population)
0
20
40
60
80
100
120
Mar
May
Jul
Sep

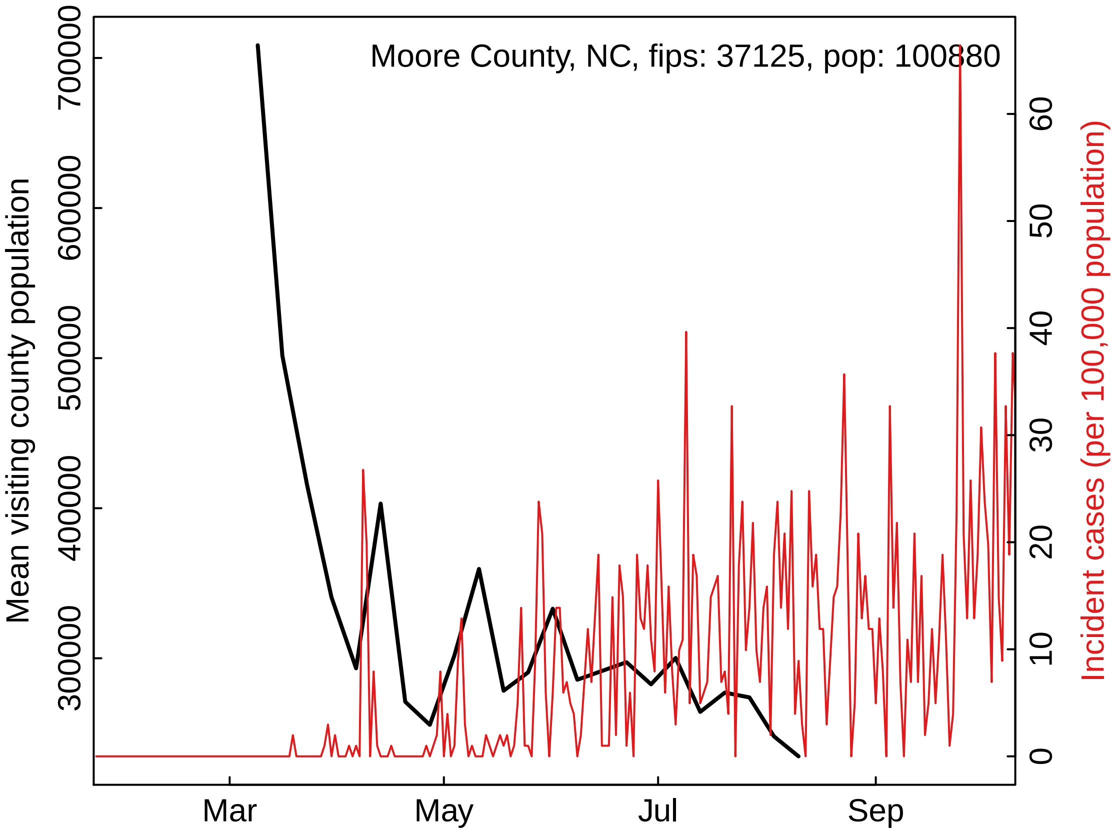

Moore County, NC, fips: 37125, pop: 100880
Mean visiting county population
300000
400000
500000
600000
700000
Incident cases (per 100,000 population)
0
10
20
30
40
50
60
Mar
May
Jul
Sep

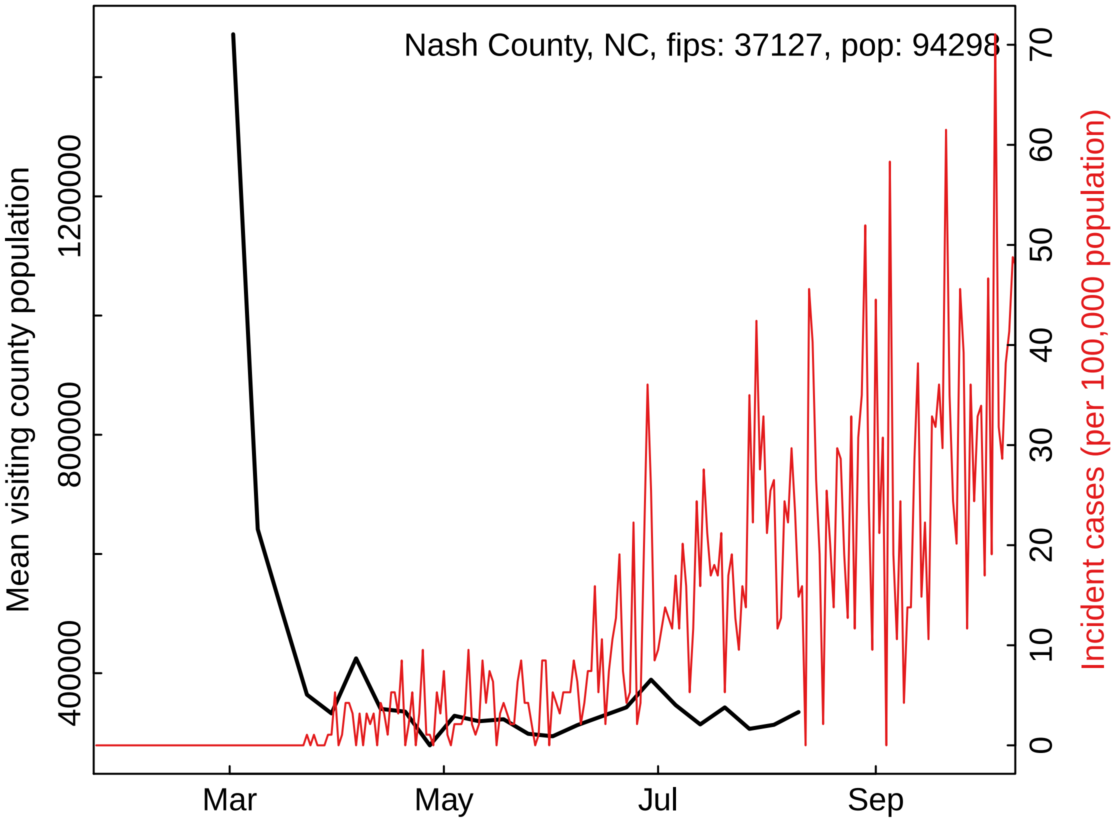

Nash County, NC, fips: 37127, pop: 94298
Mean visiting county population
400000
800000
1200000
Incident cases (per 100,000 population)
0
10
20
30
40
50
60
70
Mar
May
Jul
Sep

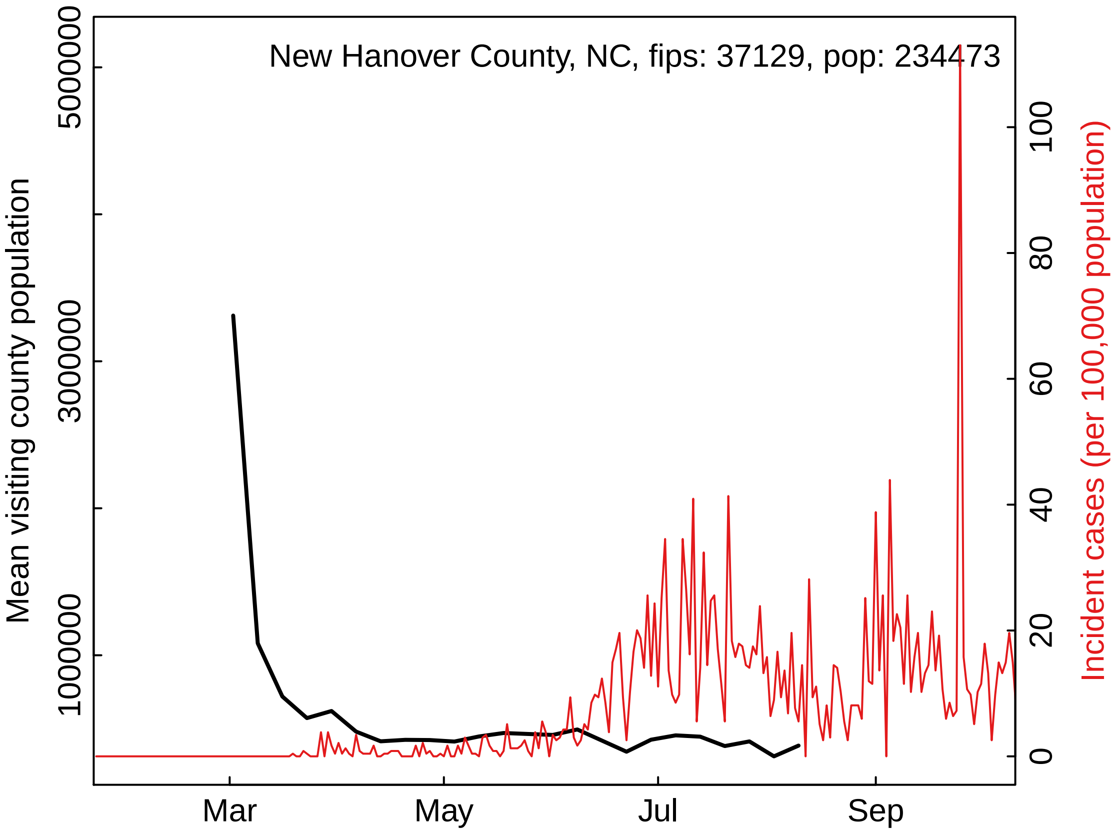

New Hanover County, NC, fips: 37129, pop: 234473
Mean visiting county population
1000000
3000000
5000000
Incident cases (per 100,000 population)
0
20
40
60
80
100
Mar
May
Jul
Sep

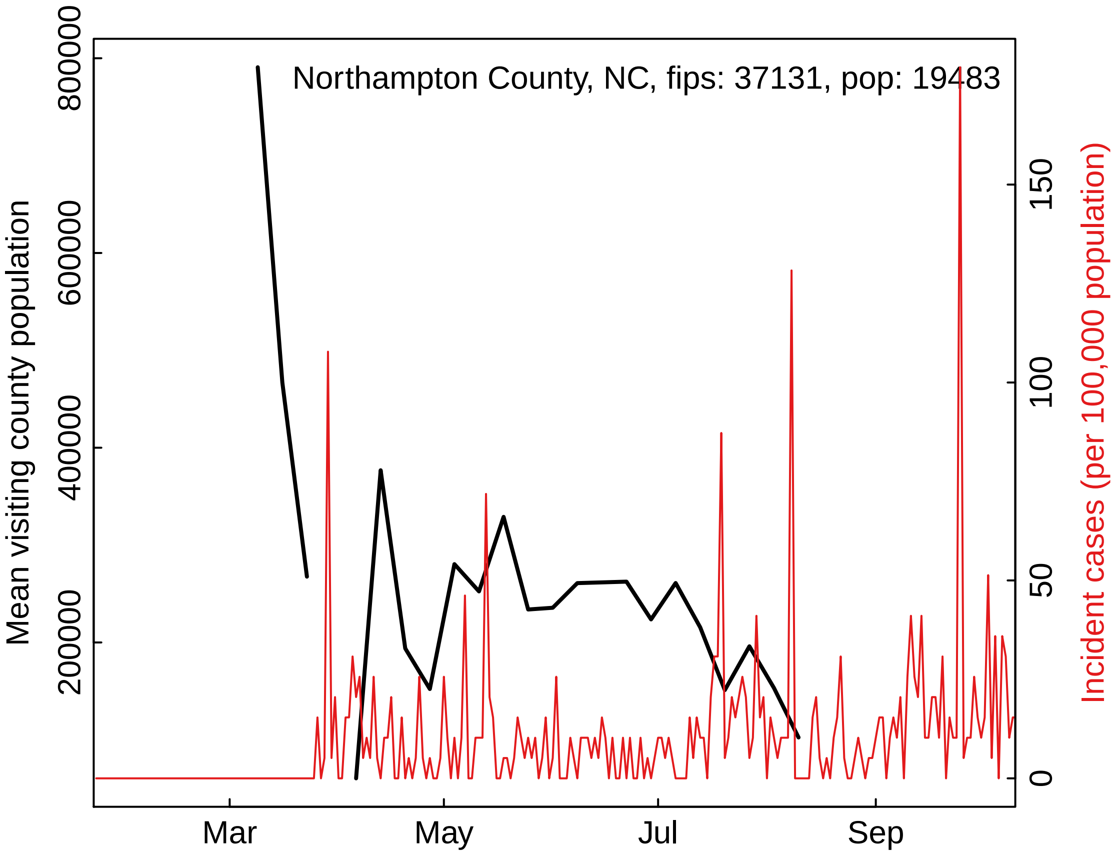

Northampton County, NC, fips: 37131, pop: 19483
Mean visiting county population
Incident cases (per 100,000 population)
200000
400000
600000
800000
0
50
100
150
Mar
May
Jul
Sep

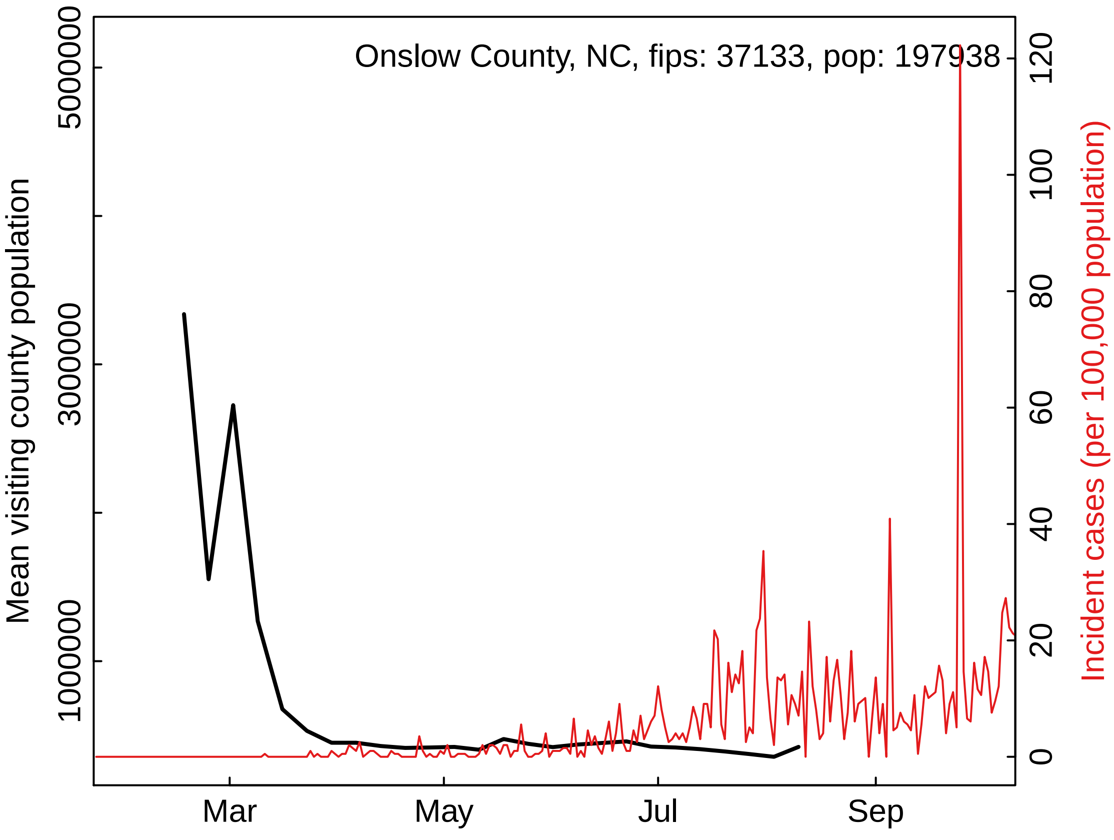
Onslow County, NC, fips: 37133, pop: 197938
Mean visiting county population
Incident cases (per 100,000 population)
1000000
3000000
5000000
0
20
40
60
80
100
120
Mar
May
Jul
Sep

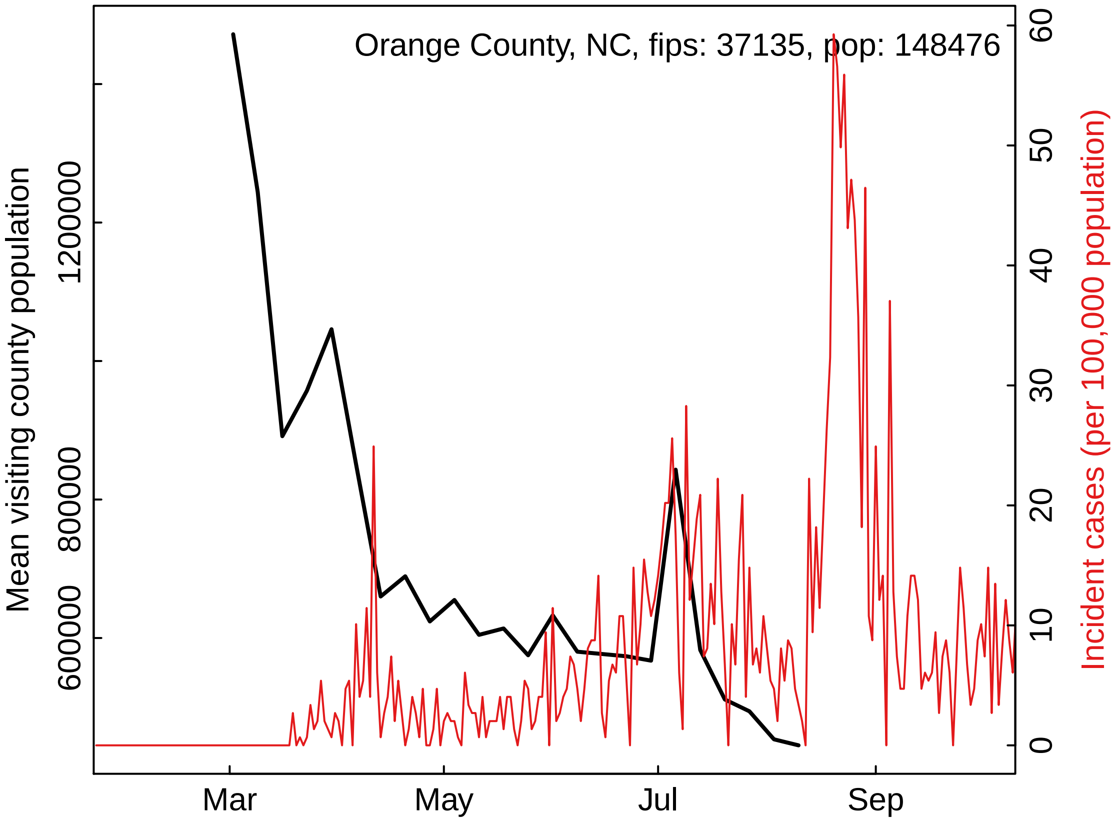
Orange County, NC, fips: 37135, pop: 148476
Mean visiting county population
600000
800000
1200000
Incident cases (per 100,000 population)
0
10
20
30
40
50
60
Mar
May
Jul
Sep

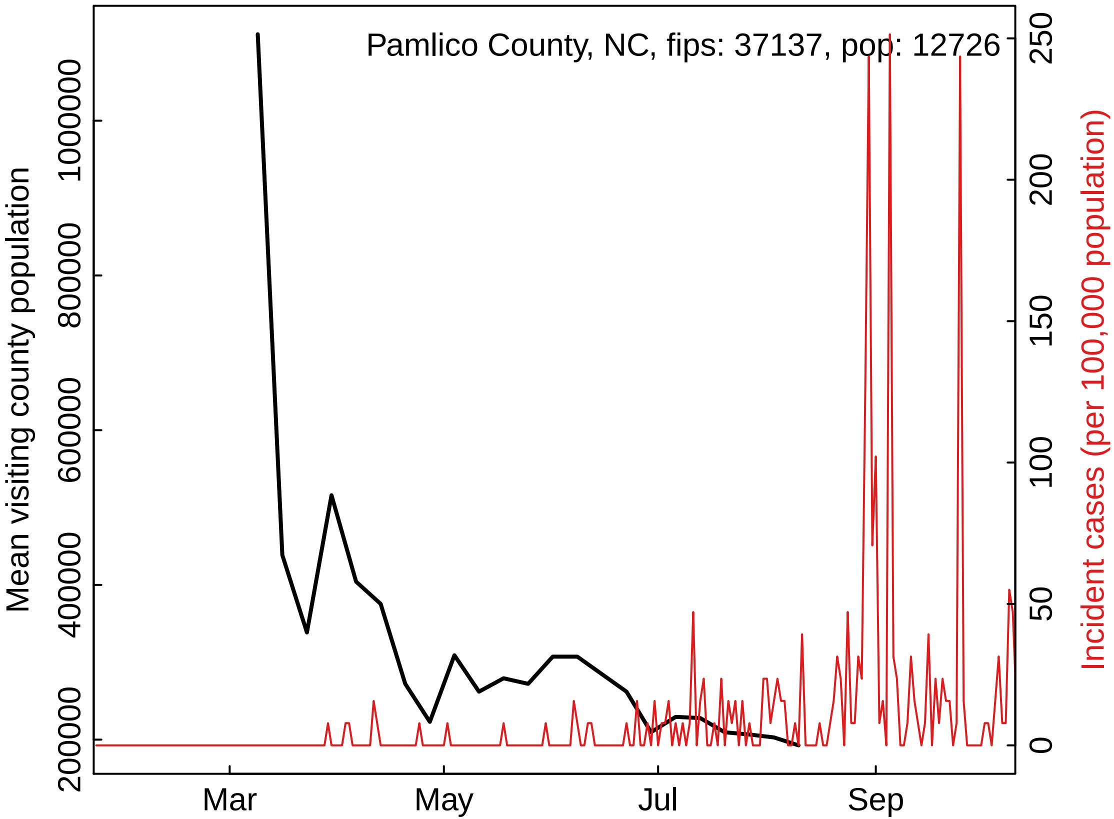

Pamlico County, NC, fips: 37137, pop: 12726
Mean visiting county population
200000
400000
600000
800000
1000000
Incident cases (per 100,000 population)
0
50
100
150
200
250
Mar
May
Jul
Sep

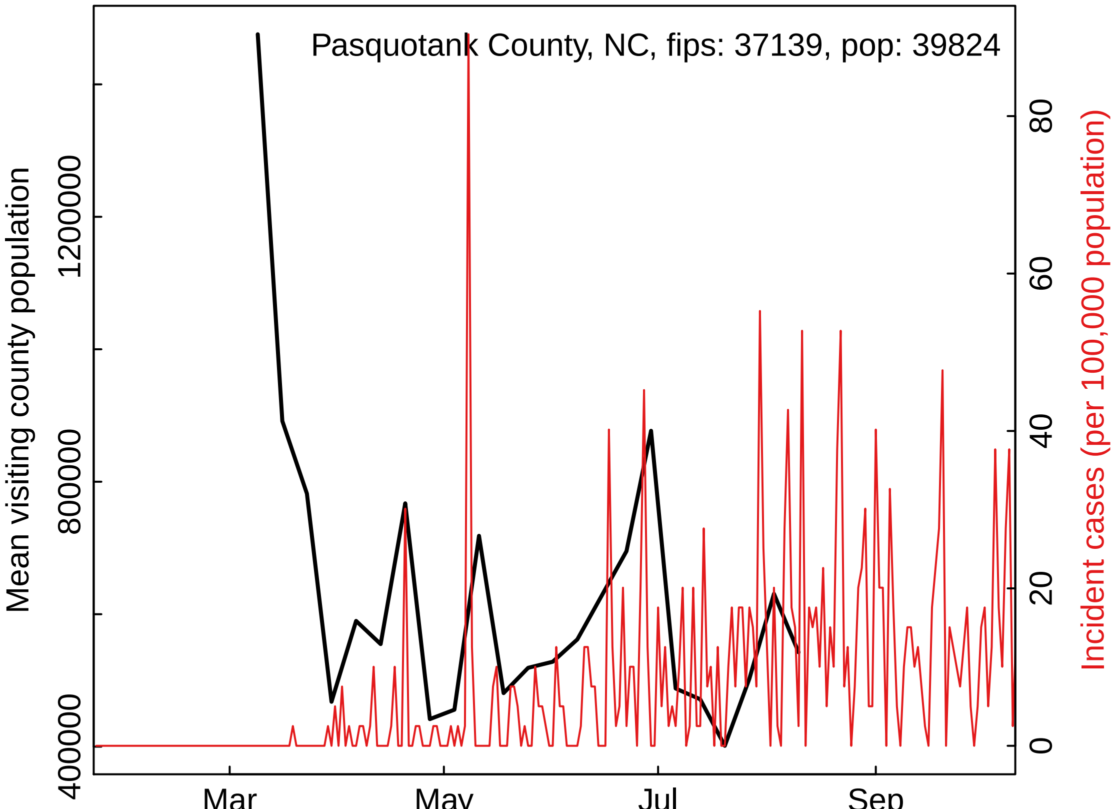

Pasquotank County, NC, fips: 37139, pop: 39824
Mean visiting county population
400000
800000
1200000
Mar
May
Jul
Sep
0
20
40
60
80
Incident cases (per 100,000 population)

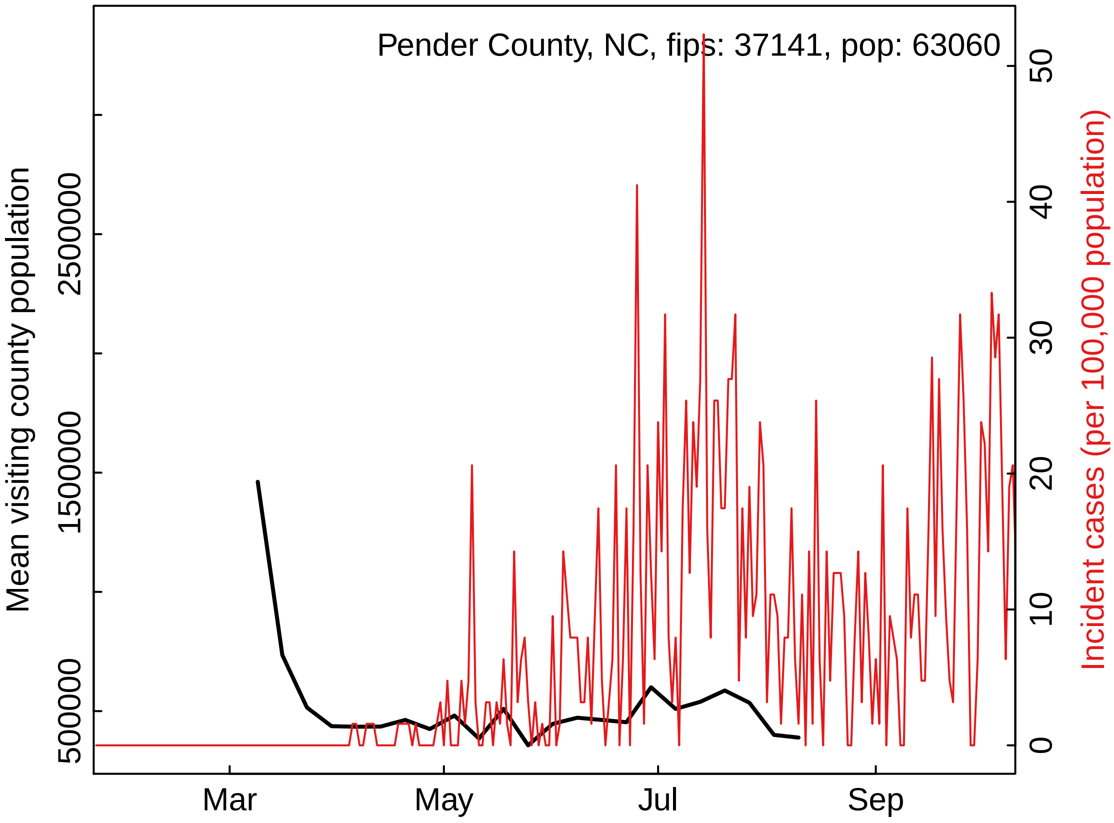

Pender County, NC, fips: 37141, pop: 63060
Mean visiting county population
500000
1500000
2500000
Incident cases (per 100,000 population)
0
10
20
30
40
50
Mar
May
Jul
Sep

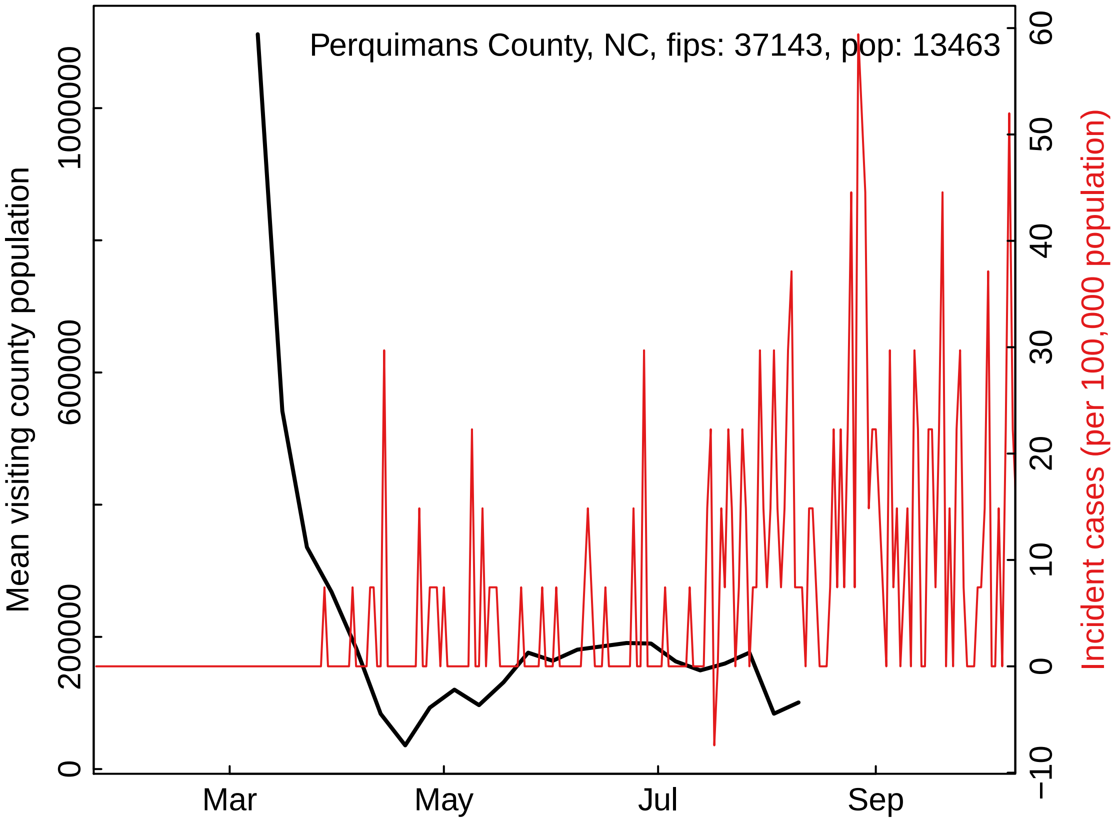
Perquimans County, NC, fips: 37143, pop: 13463
Mean visiting county population
0
200000
600000
1000000
Incident cases (per 100,000 population)
−10
0
10
20
30
40
50
60
Mar
May
Jul
Sep

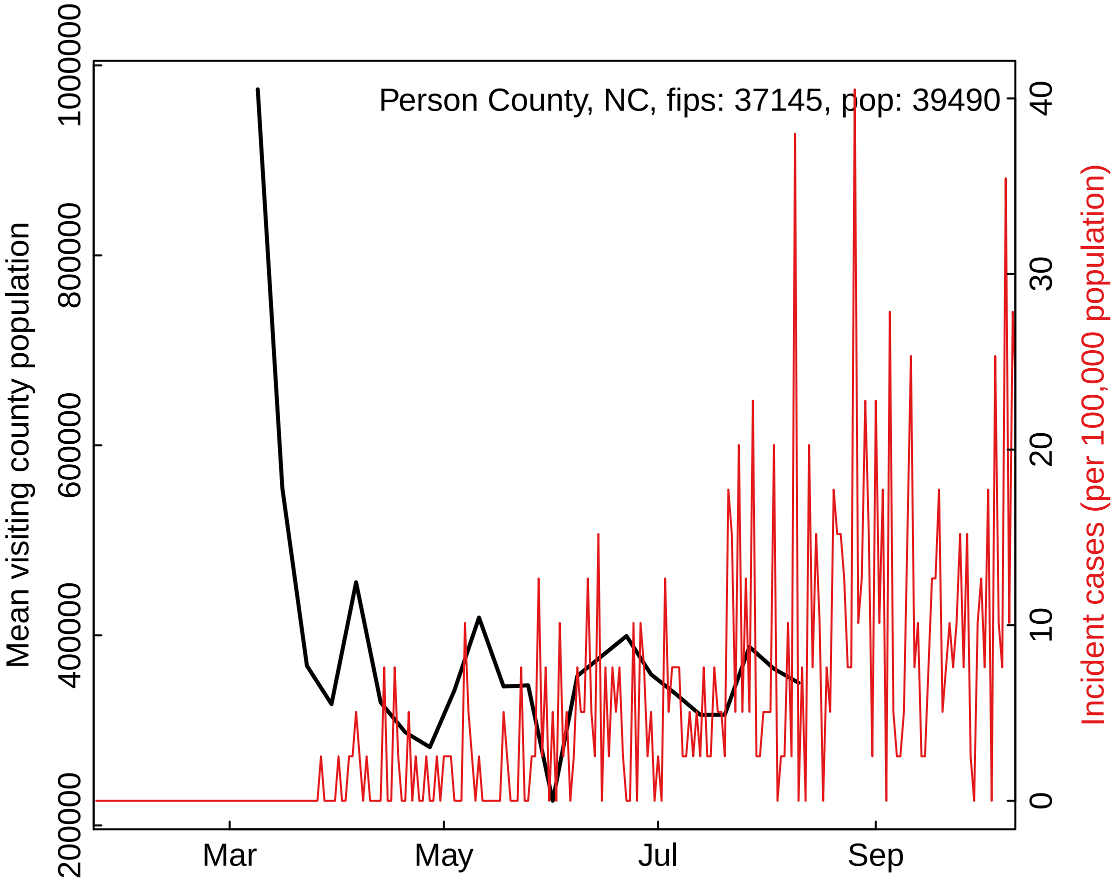
Person County, NC, fips: 37145, pop: 39490
Mean visiting county population
Incident cases (per 100,000 population)
200000
400000
600000
800000
1000000
0
10
20
30
40
Mar
May
Jul
Sep

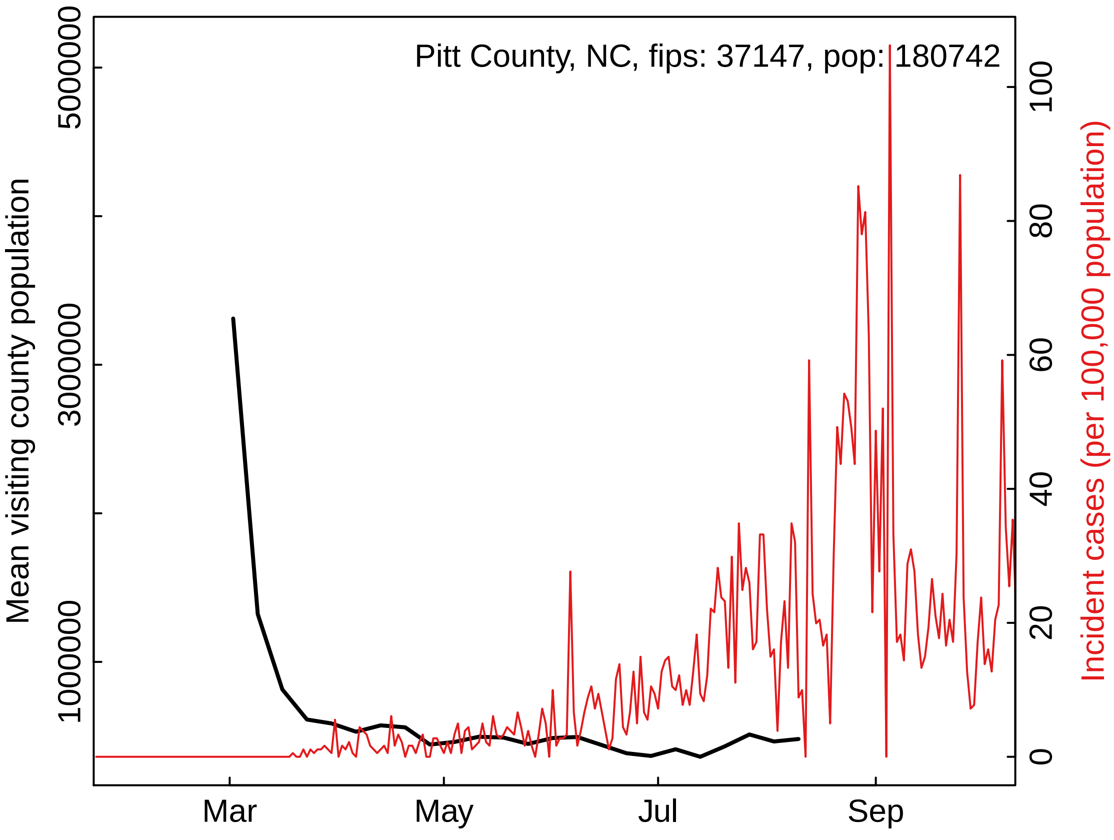
Pitt County, NC, fips: 37147, pop: 180742
Mean visiting county population
Incident cases (per 100,000 population)
1000000
3000000
5000000
0
20
40
60
80
100
Mar
May
Jul
Sep

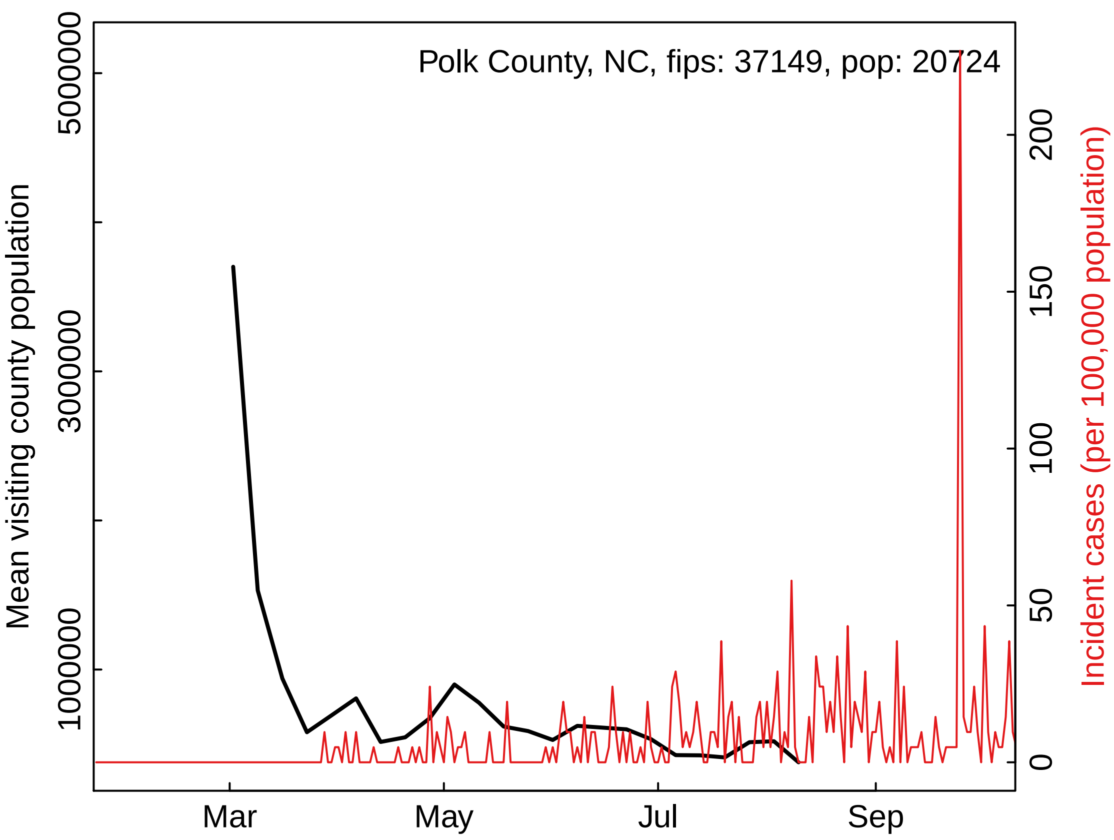

Polk County, NC, fips: 37149, pop: 20724
Mean visiting county population
10000000
30000000
50000000
Mar
May
Jul
Sep
0
50
100
150
200
Incident cases (per 100,000 population)

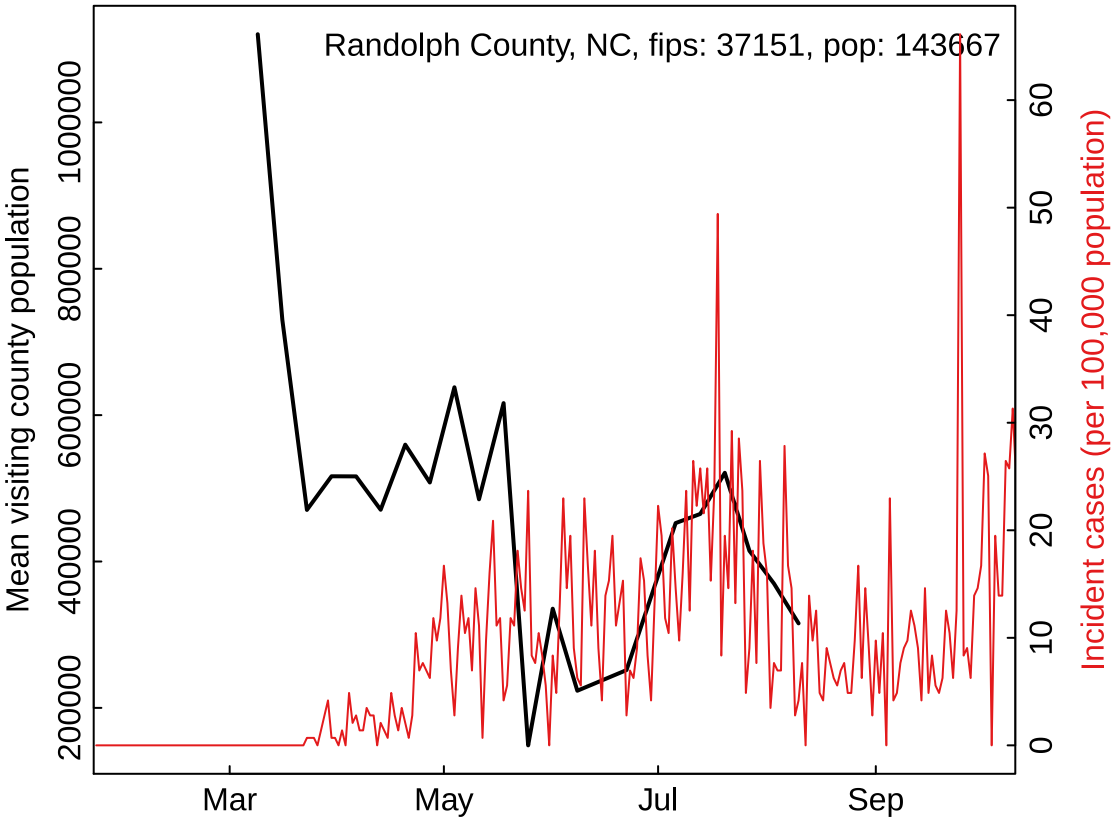

Randolph County, NC, fips: 37151, pop: 143667
Mean visiting county population
200000
400000
600000
800000
1000000
Incident cases (per 100,000 population)
0
10
20
30
40
50
60
Mar
May
Jul
Sep

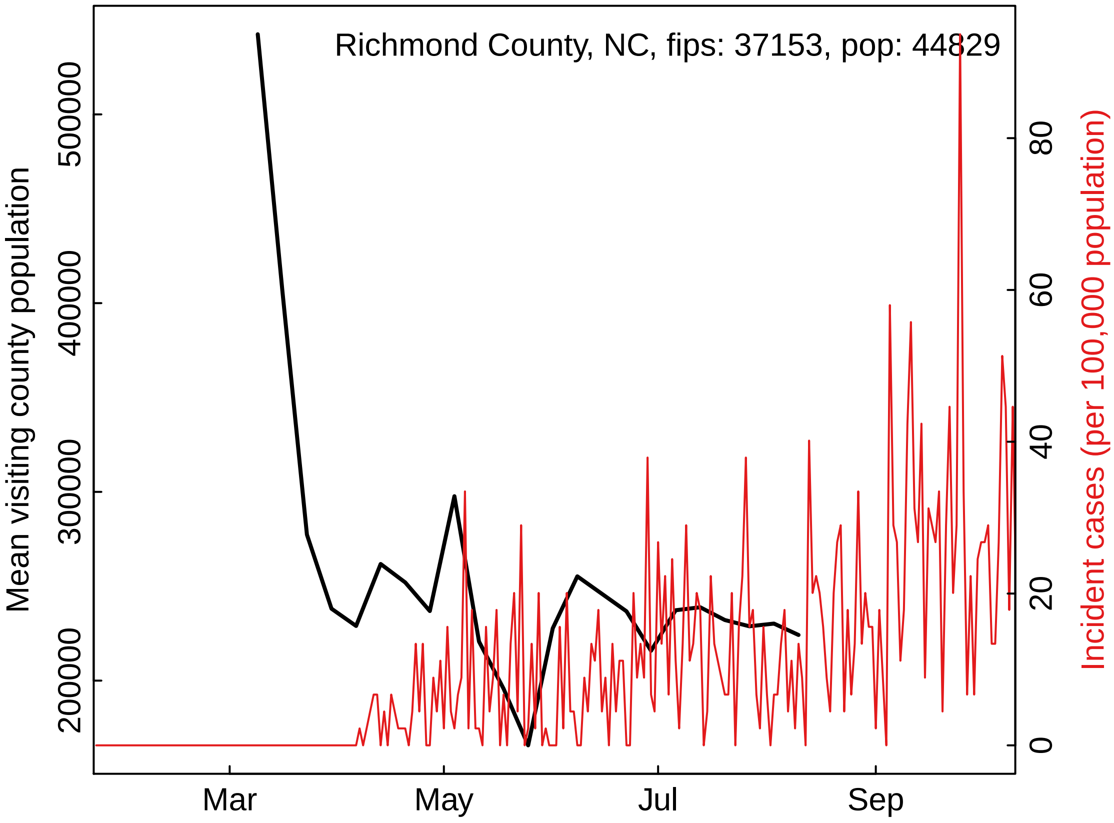

Richmond County, NC, fips: 37153, pop: 44829
Mean visiting county population
5000000
400000
300000
200000
Mar
May
Jul
Sep
0
20
40
60
80
Incident cases (per 100,000 population)

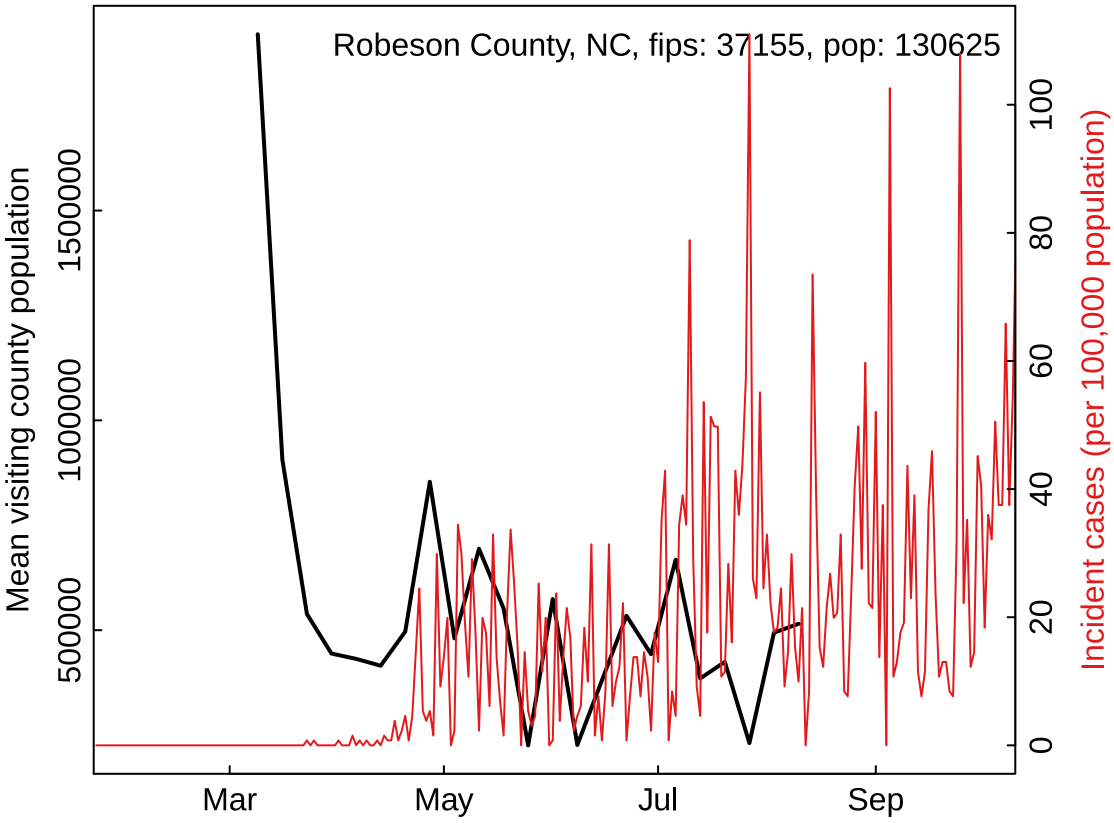
Robeson County, NC, fips: 37155, pop: 130625
Mean visiting county population
500000
1000000
1500000
Mar
May
Jul
Sep
0
20
40
60
80
100
Incident cases (per 100,000 population)

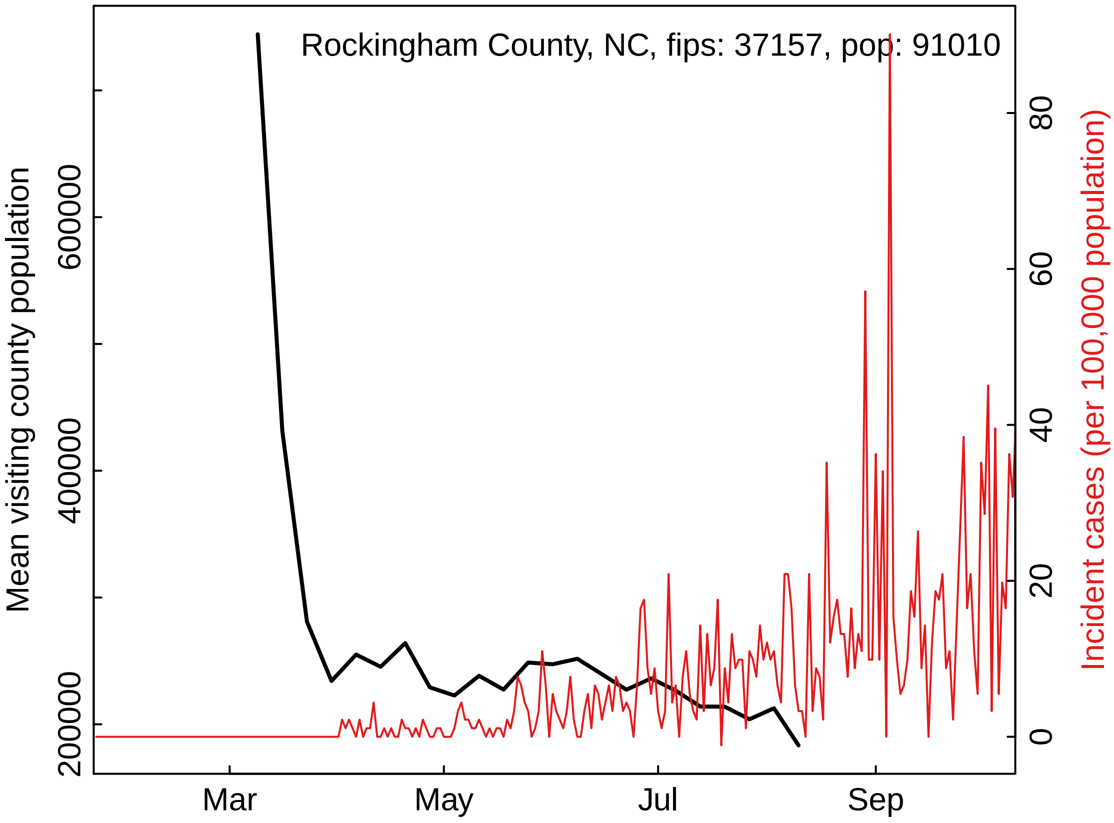

Rockingham County, NC, fips: 37157, pop: 91010
Mean visiting county population
200000
400000
600000
Incident cases (per 100,000 population)
0
20
40
60
80
Mar
May
Jul
Sep

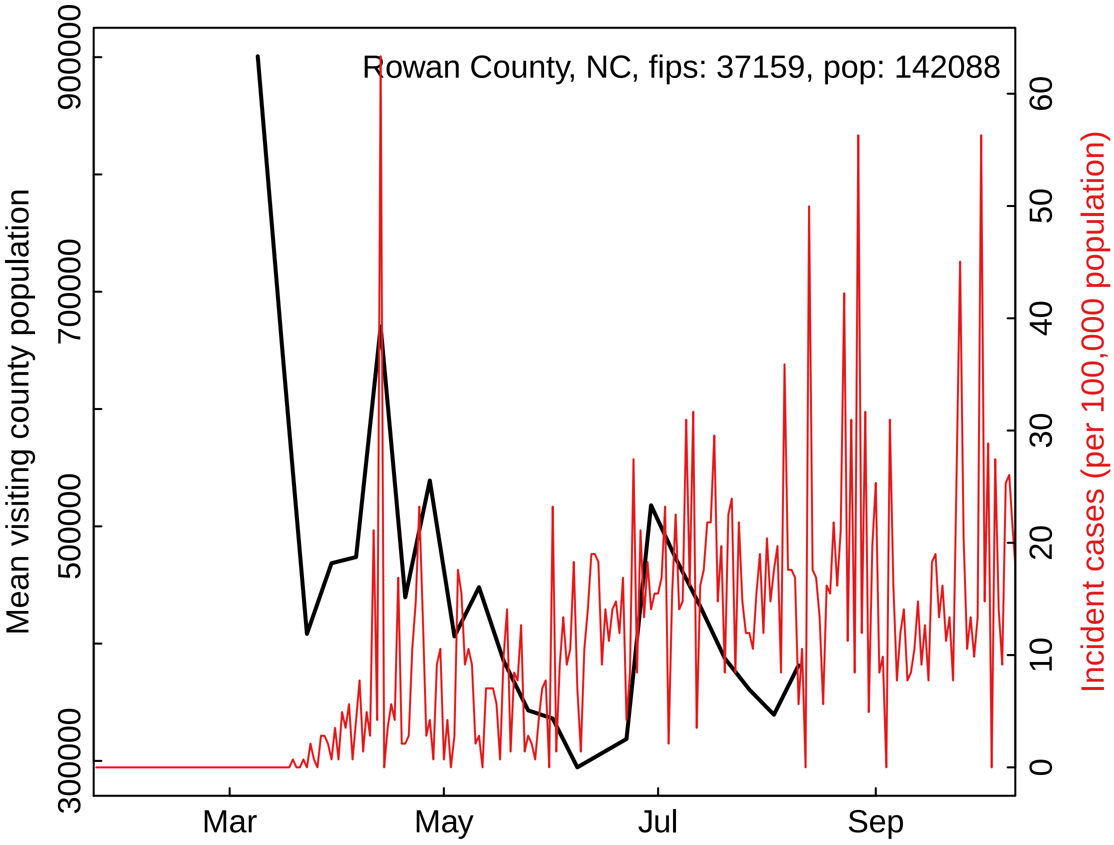

Rowan County, NC, fips: 37159, pop: 142088
Mean visiting county population
300000
500000
700000
900000
Incident cases (per 100,000 population)
0
10
20
30
40
50
60
Mar
May
Jul
Sep

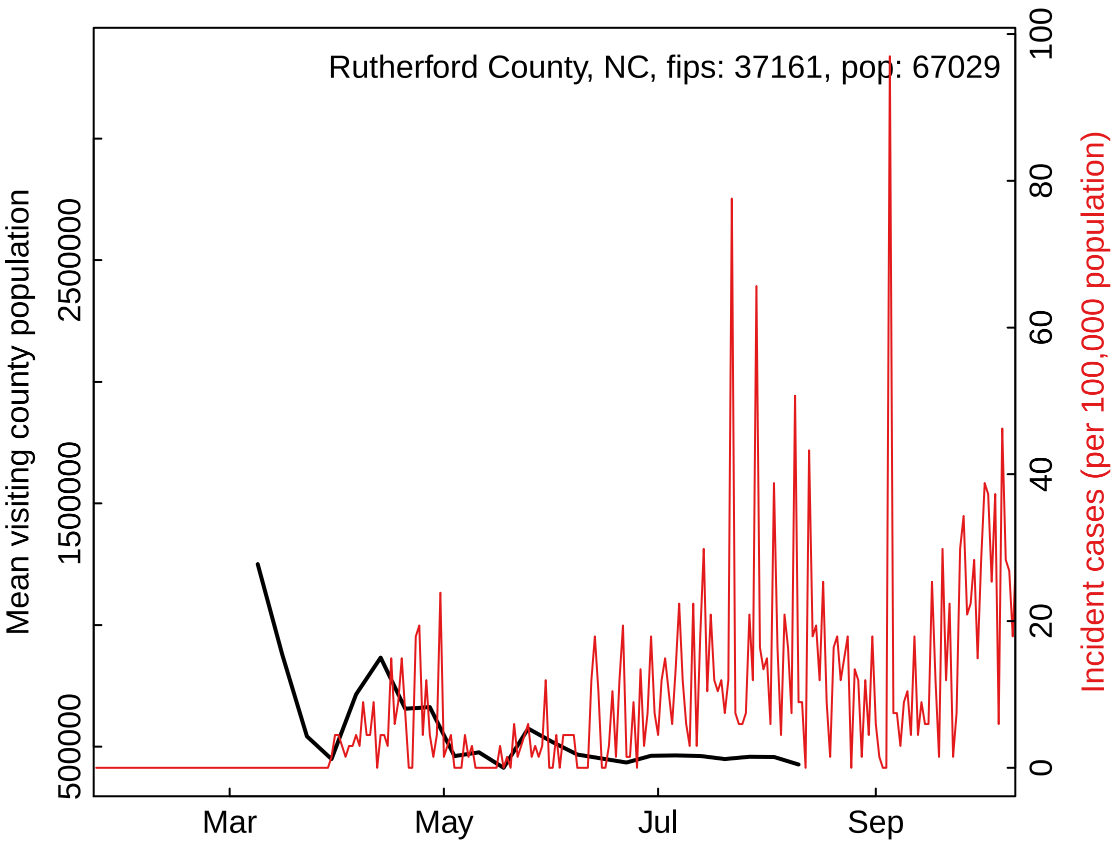

Rutherford County, NC, fips: 37161, pop: 67029
Mean visiting county population
500000
1500000
2500000
Mar
May
Jul
Sep
0
20
40
60
80
100
Incident cases (per 100,000 population)

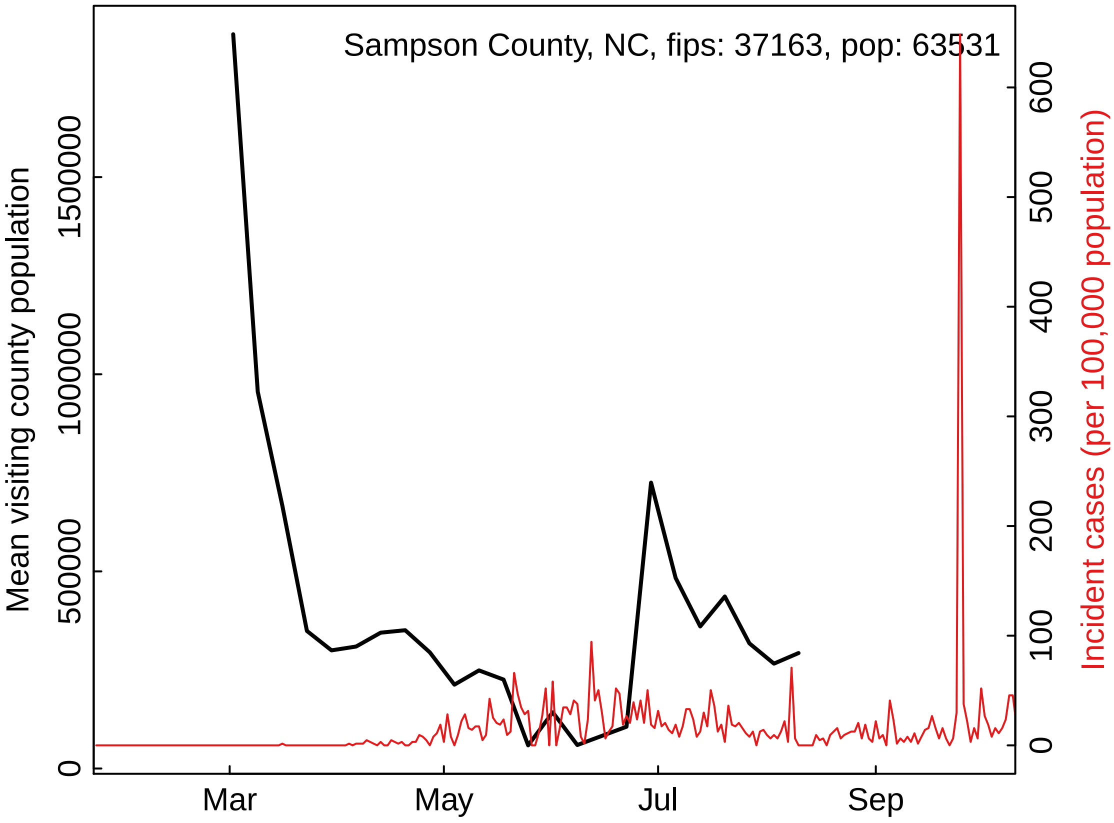
Sampson County, NC, fips: 37163, pop: 63531
Mean visiting county population
0
500000
1000000
1500000
Incident cases (per 100,000 population)
0
100
200
300
400
500
600
Mar
May
Jul
Sep

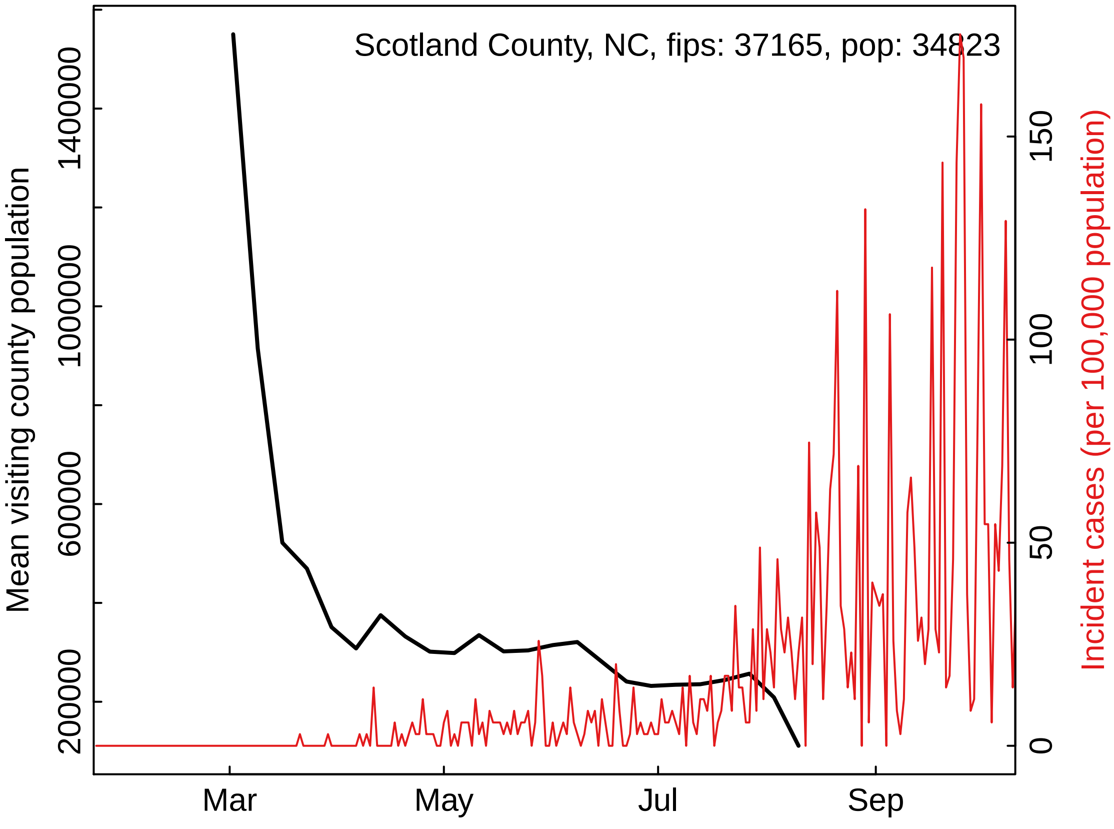
Scotland County, NC, fips: 37165, pop: 34823
Mean visiting county population
200000
600000
1000000
1400000
Mar
May
Jul
Sep
0
50
100
150
Incident cases (per 100,000 population)

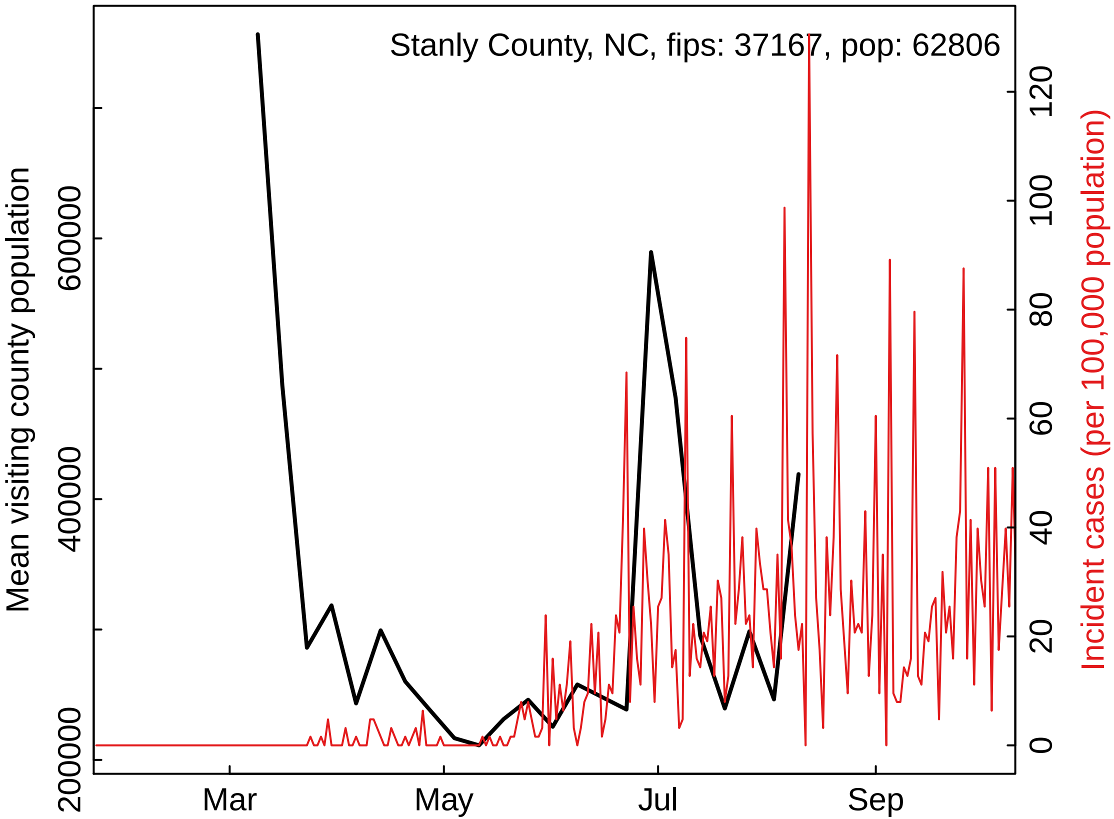

Stanly County, NC, fips: 37167, pop: 62806
Mean visiting county population
200000
400000
600000
Incident cases (per 100,000 population)
0
20
40
60
80
100
120
Mar
May
Jul
Sep

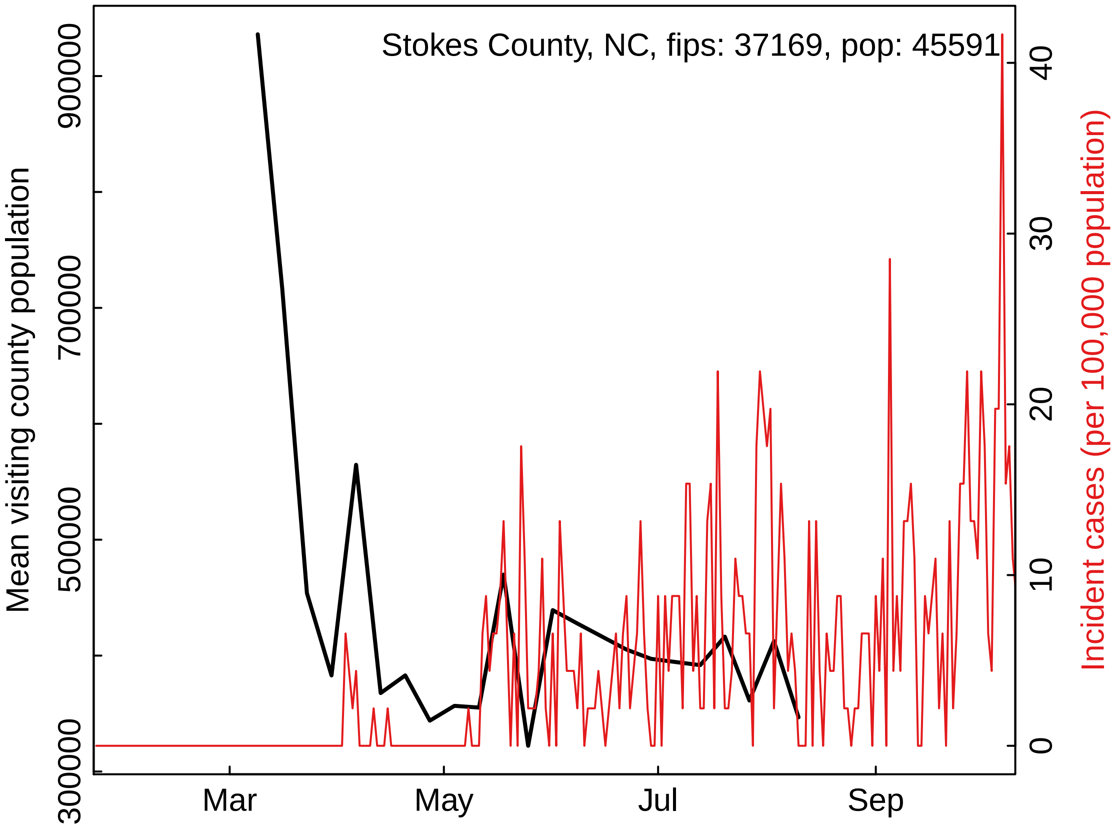

Stokes County, NC, fips: 37169, pop: 45591
Mean visiting county population
300000
500000
700000
900000
Mar
May
Jul
Sep
0
10
20
30
40
Incident cases (per 100,000 population)

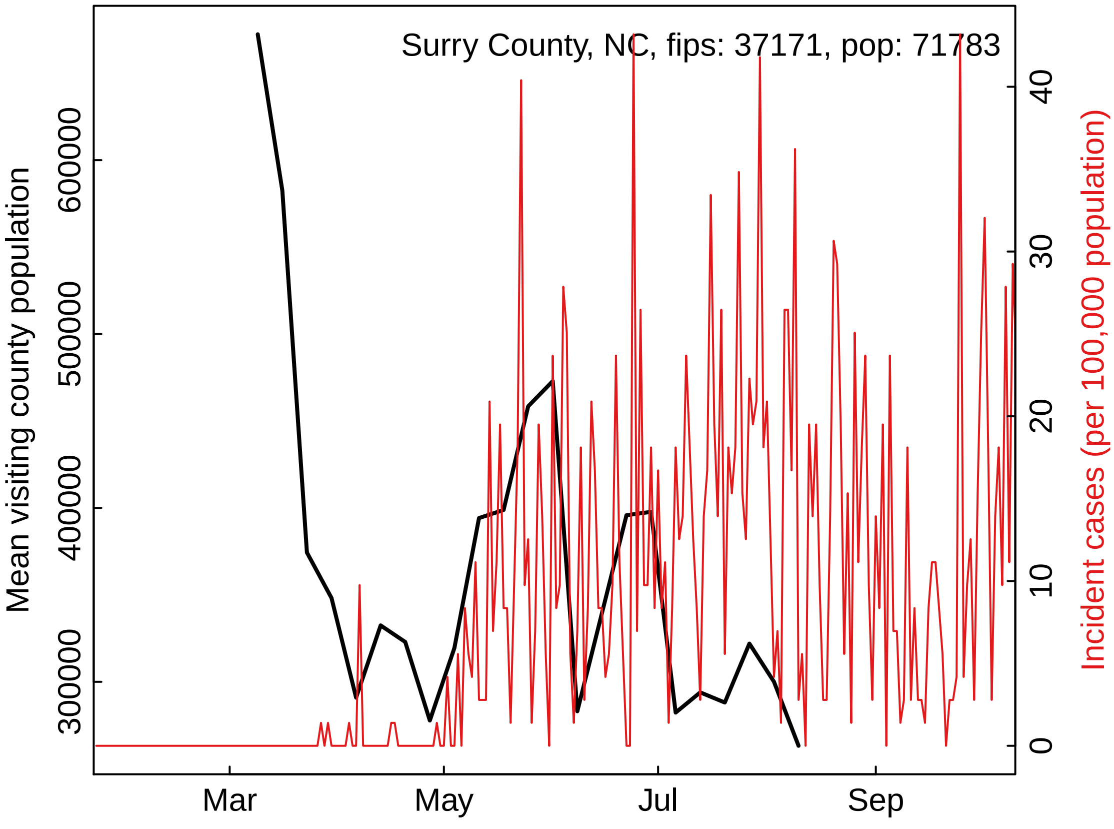
Surry County, NC, fips: 37171, pop: 71783
Mean visiting county population
300000
400000
500000
600000
Incident cases (per 100,000 population)
0
10
20
30
40
Mar
May
Jul
Sep

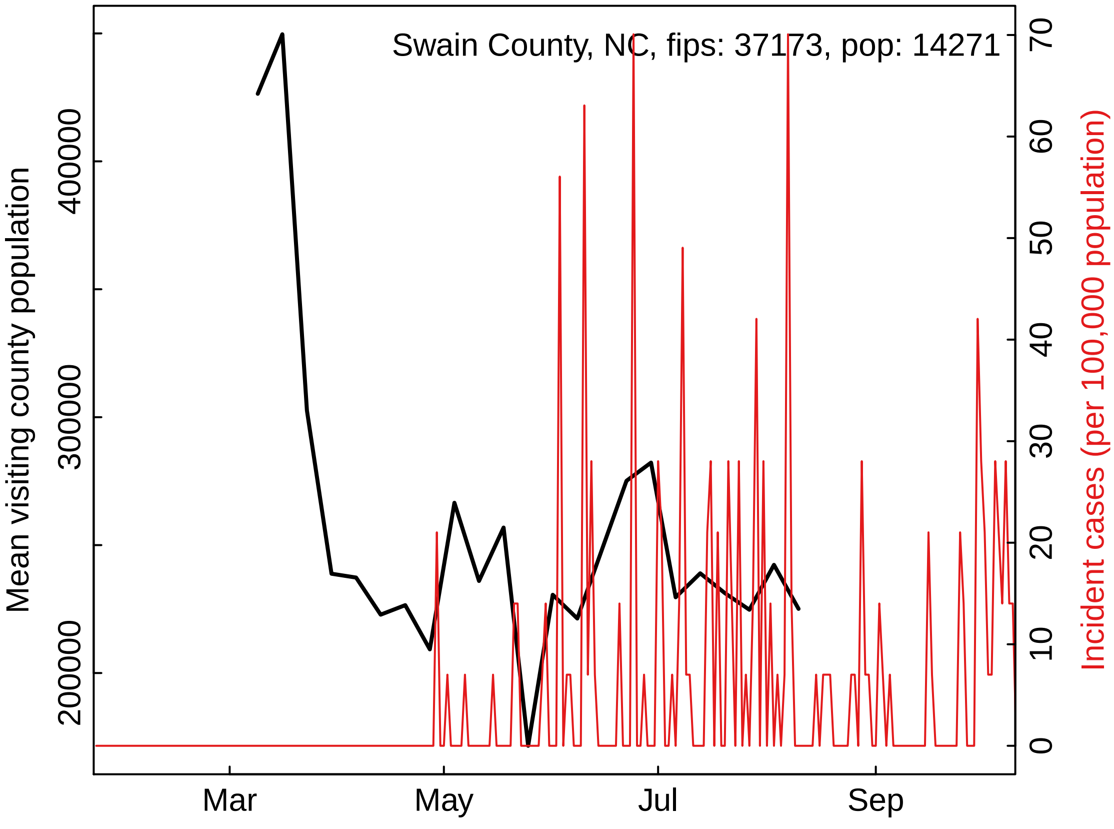

Swain County, NC, fips: 37173, pop: 14271
Mean visiting county population
200000
300000
400000
Incident cases (per 100,000 population)
0
10
20
30
40
50
60
70
Mar
May
Jul
Sep

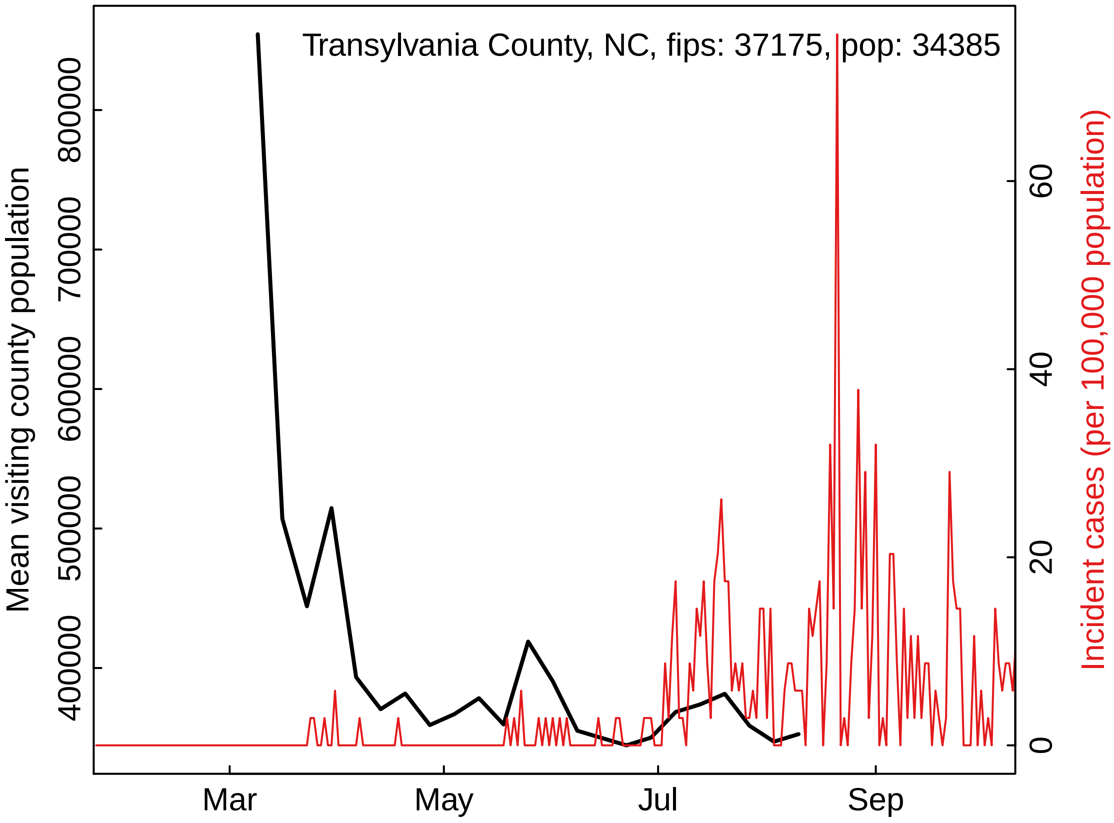

Transylvania County, NC, fips: 37175, pop: 34385
Mean visiting county population
400000
500000
600000
700000
800000
Incident cases (per 100,000 population)
0
20
40
60
Mar
May
Jul
Sep

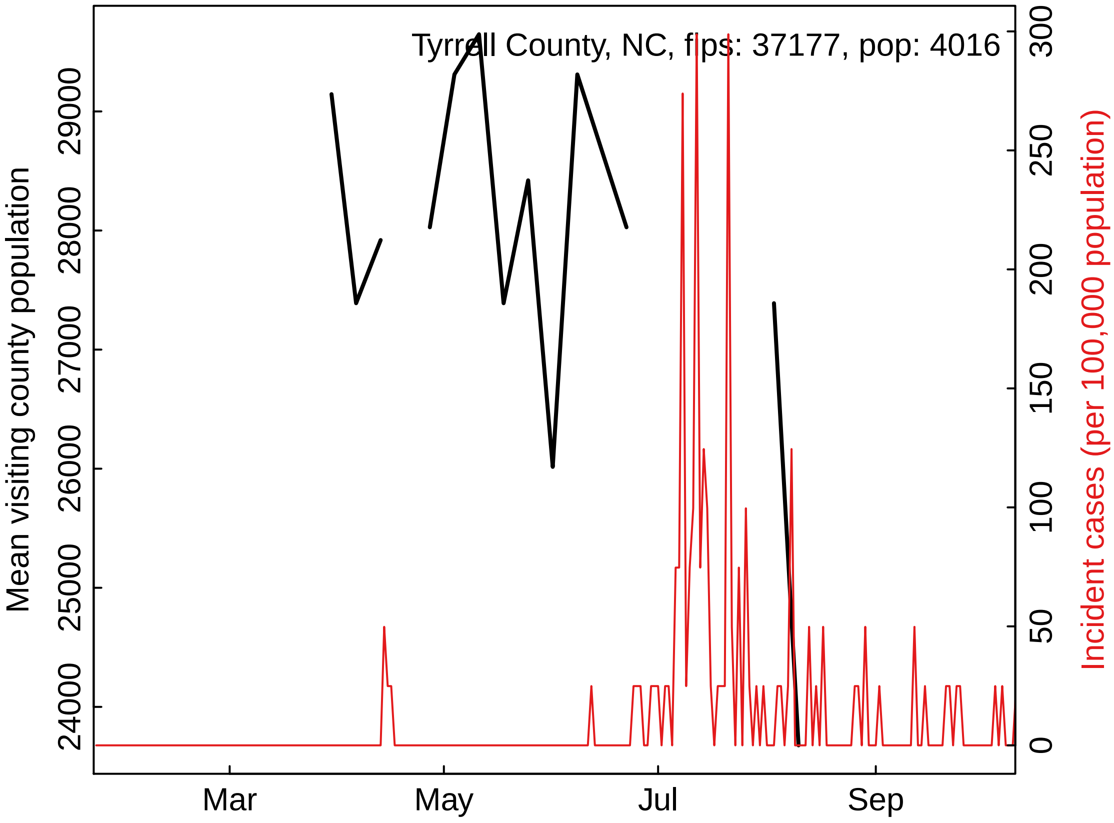

Tyrrell County, NC, fips: 37177, pop: 4016
Mean visiting county population
24000
25000
26000
27000
28000
29000
Incident cases (per 100,000 population)
0
50
100
150
200
250
300
Mar
May
Jul
Sep

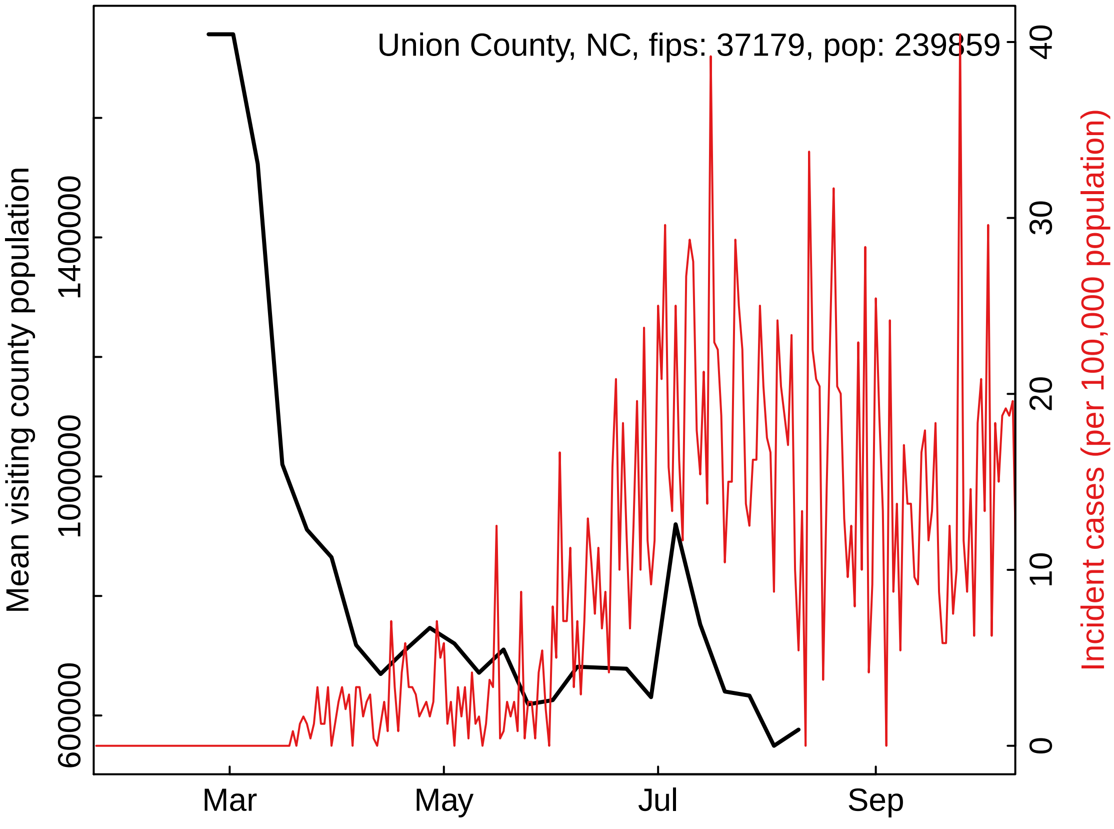

Union County, NC, fips: 37179, pop: 239859
Mean visiting county population
600000
1000000
1400000
Incident cases (per 100,000 population)
0
10
20
30
40
Mar
May
Jul
Sep

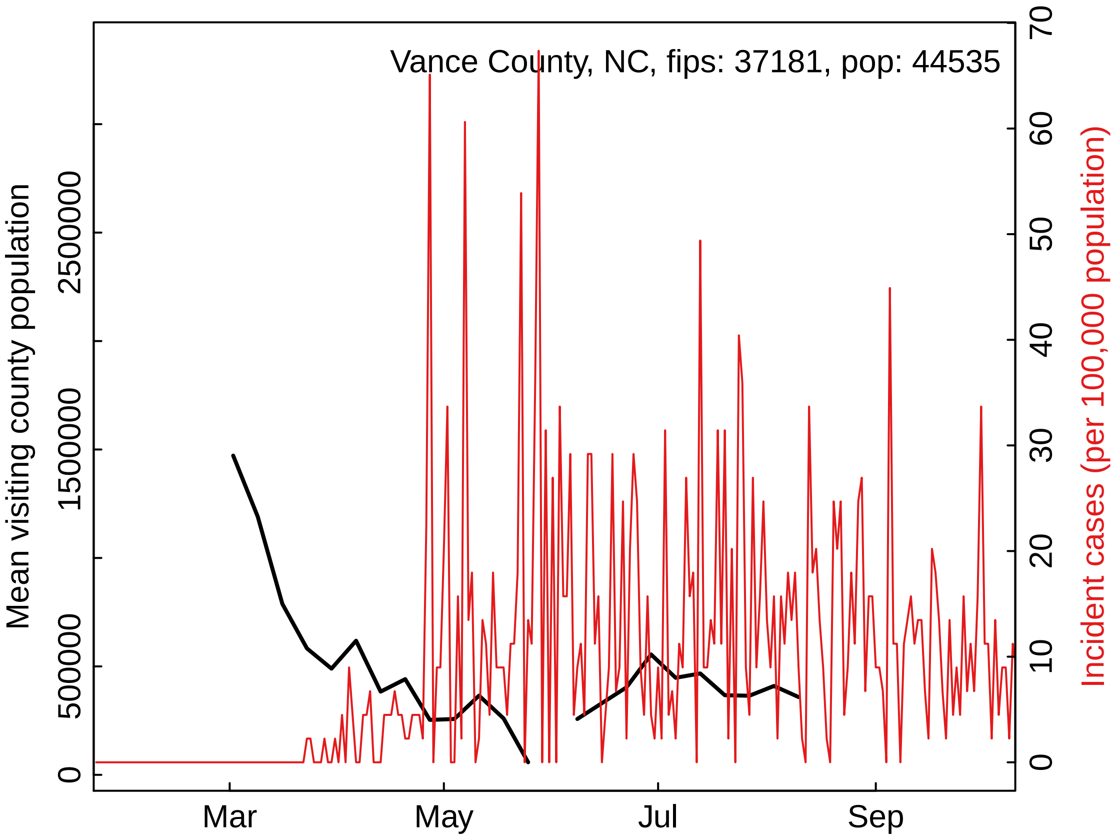

Vance County, NC, fips: 37181, pop: 44535
Mean visiting county population
0
500000
1500000
2500000
Incident cases (per 100,000 population)
0
10
20
30
40
50
60
70
Mar
May
Jul
Sep

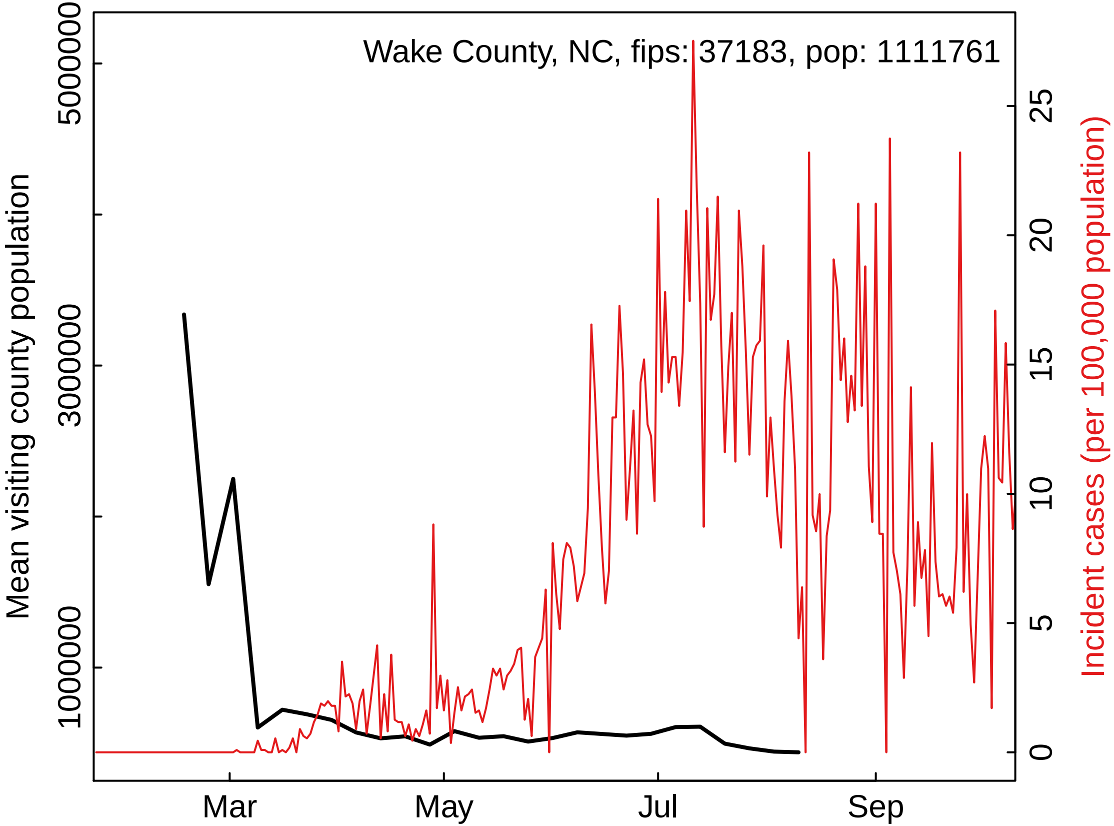

Wake County, NC, fips: 37183, pop: 1111761
Mean visiting county population
10000000
30000000
50000000
Incident cases (per 100,000 population)
0
5
10
15
20
25
Mar
May
Jul
Sep

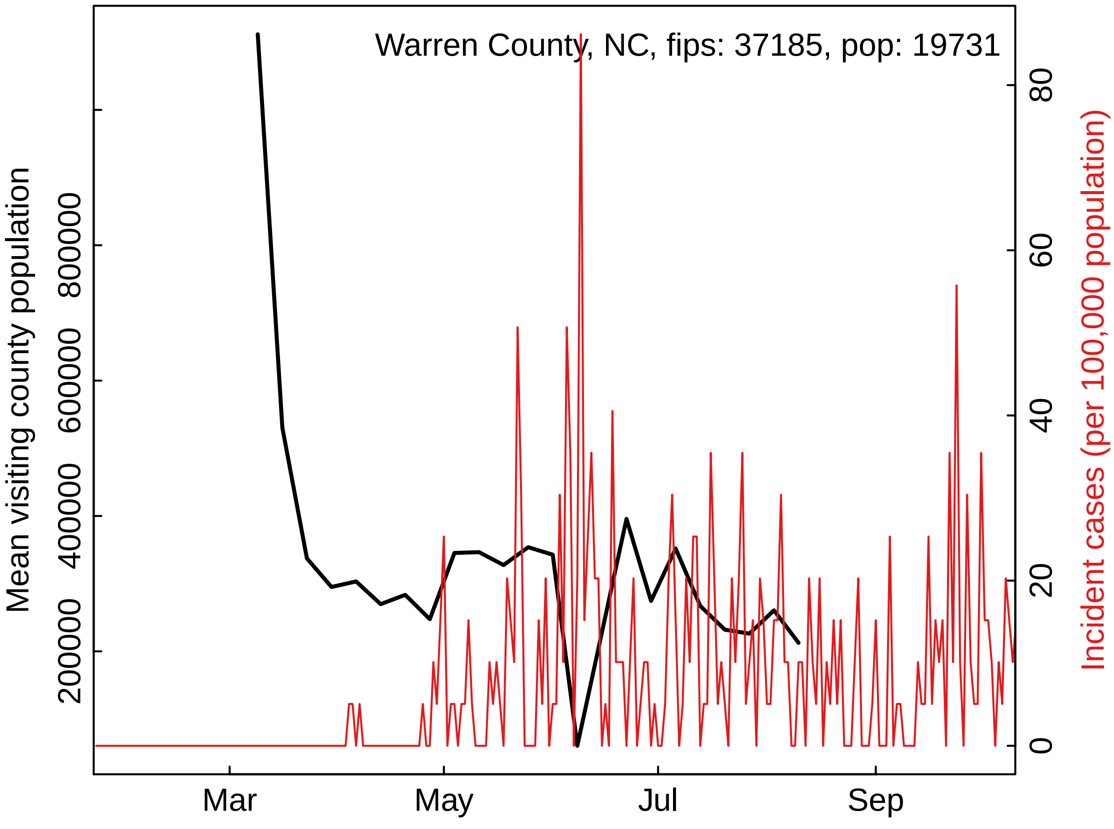

Warren County, NC, fips: 37185, pop: 19731
Mean visiting county population
200000
400000
600000
800000
Incident cases (per 100,000 population)
0
20
40
60
80
Mar
May
Jul
Sep

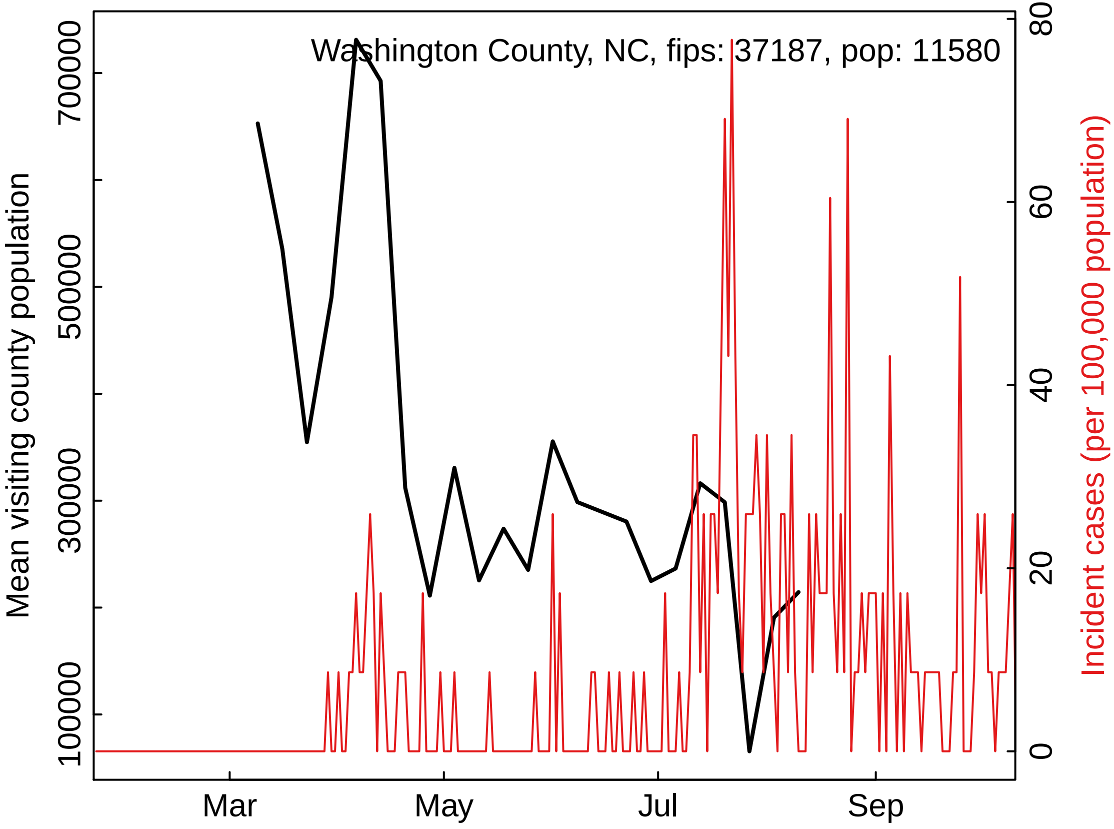

Washington County, NC, fips: 37187, pop: 11580
Mean visiting county population
100000
300000
500000
700000
Incident cases (per 100,000 population)
0
20
40
60
80
Mar
May
Jul
Sep

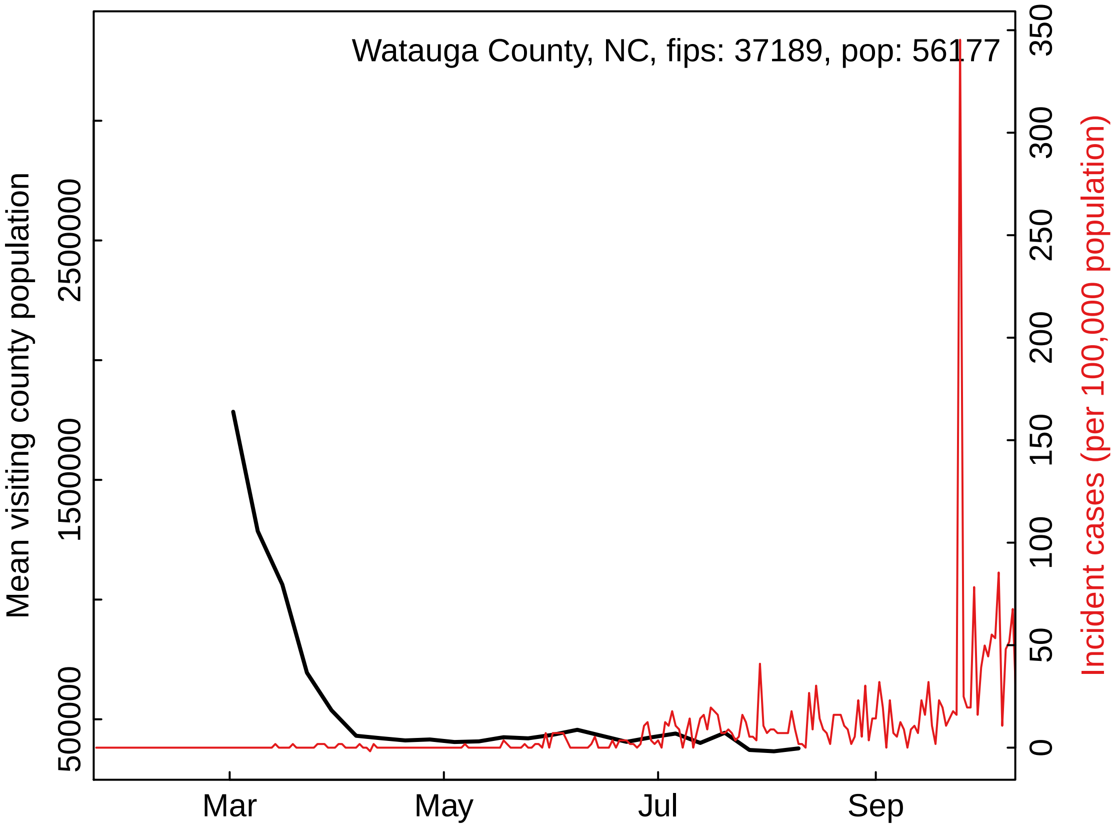

Watauga County, NC, fips: 37189, pop: 56177
Mean visiting county population
500000
1500000
2500000
Incident cases (per 100,000 population)
0
50
100
150
200
250
300
350
Mar
May
Jul
Sep

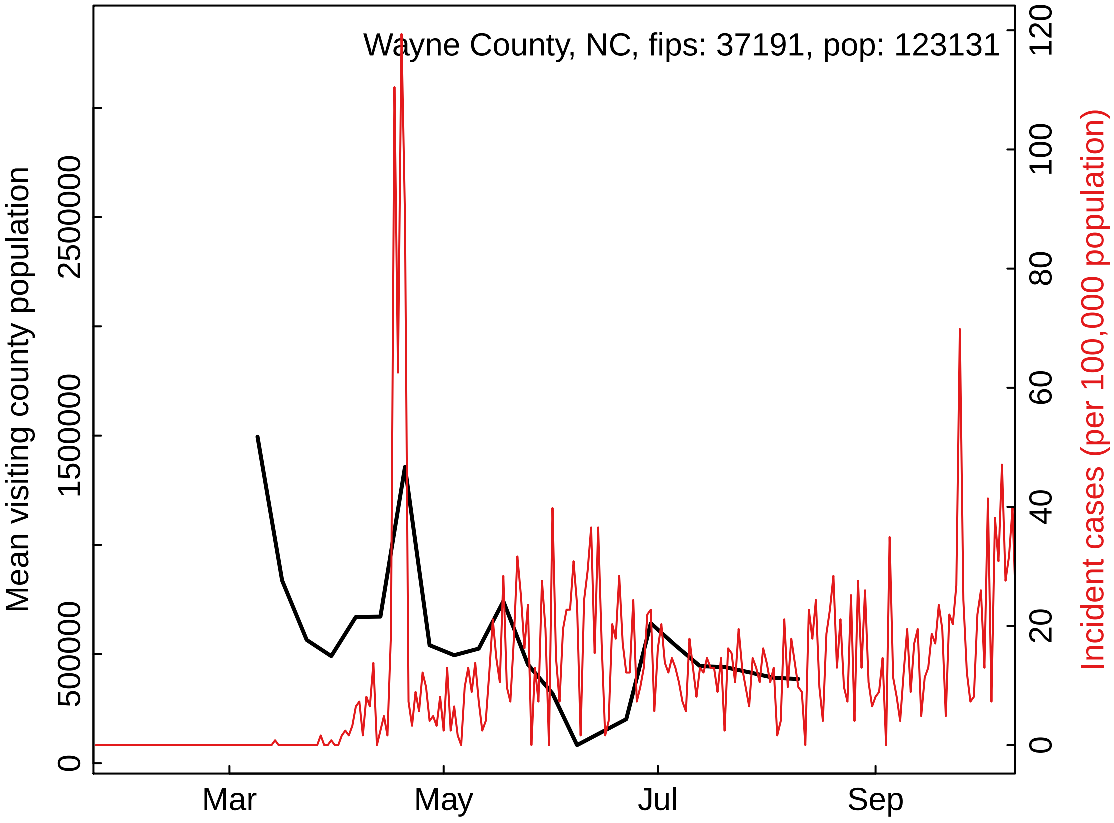

Wayne County, NC, fips: 37191, pop: 123131
Mean visiting county population
0
500000
1500000
2500000
Incident cases (per 100,000 population)
0
20
40
60
80
100
120
Mar
May
Jul
Sep

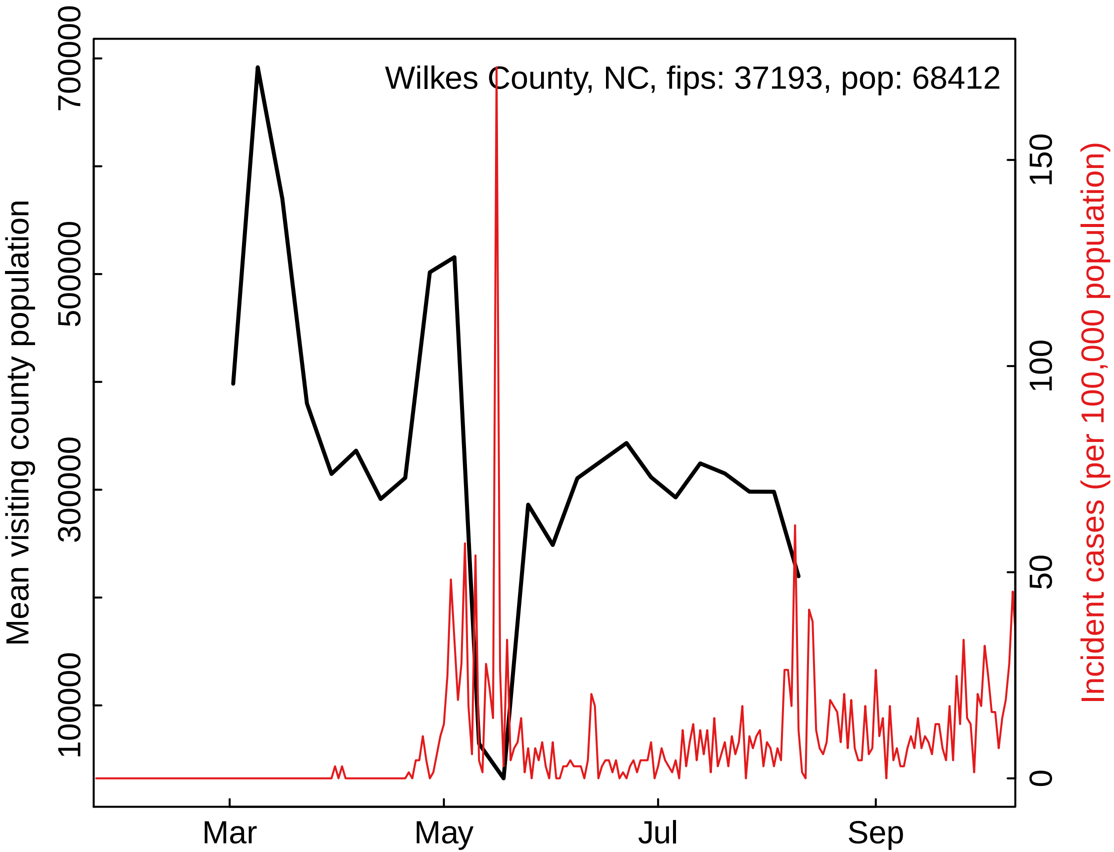

Wilkes County, NC, fips: 37193, pop: 68412
Mean visiting county population
100000
300000
500000
700000
Incident cases (per 100,000 population)
0
50
100
150
Mar
May
Jul
Sep

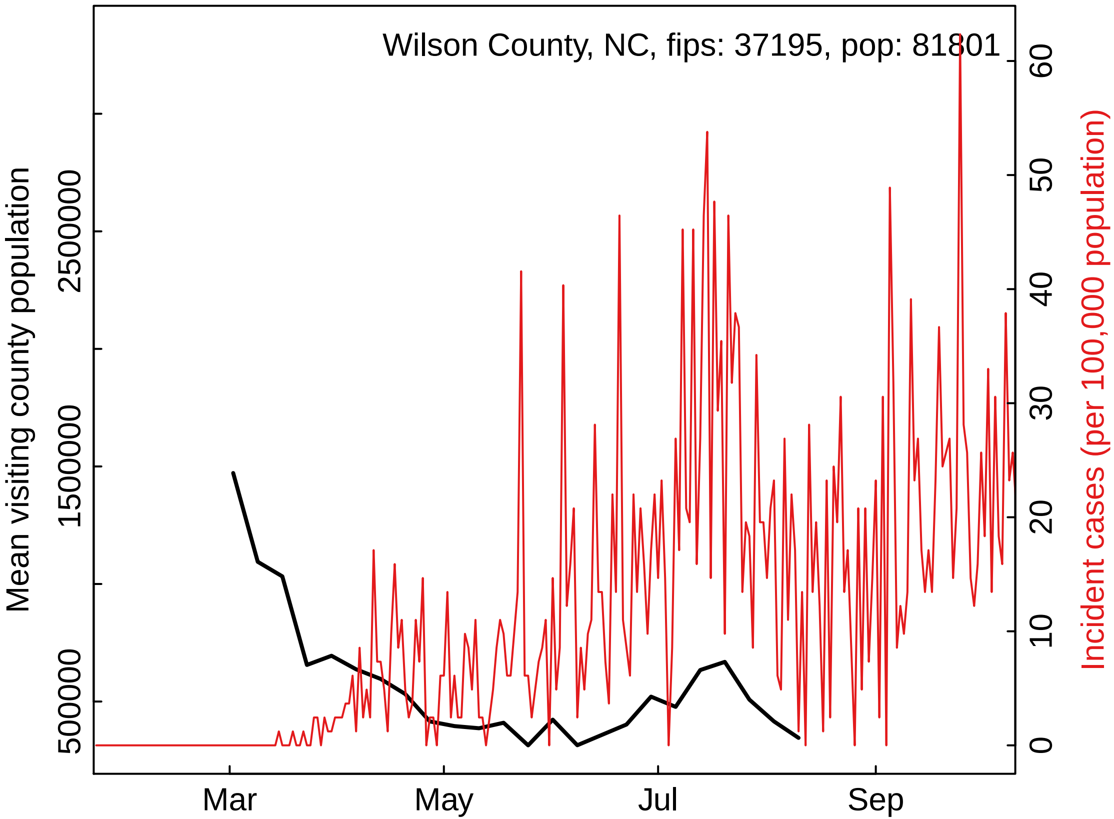
Wilson County, NC, fips: 37195, pop: 81801
Mean visiting county population
500000
1500000
2500000
Mar
May
Jul
Sep
0
10
20
30
40
50
60
Incident cases (per 100,000 population)

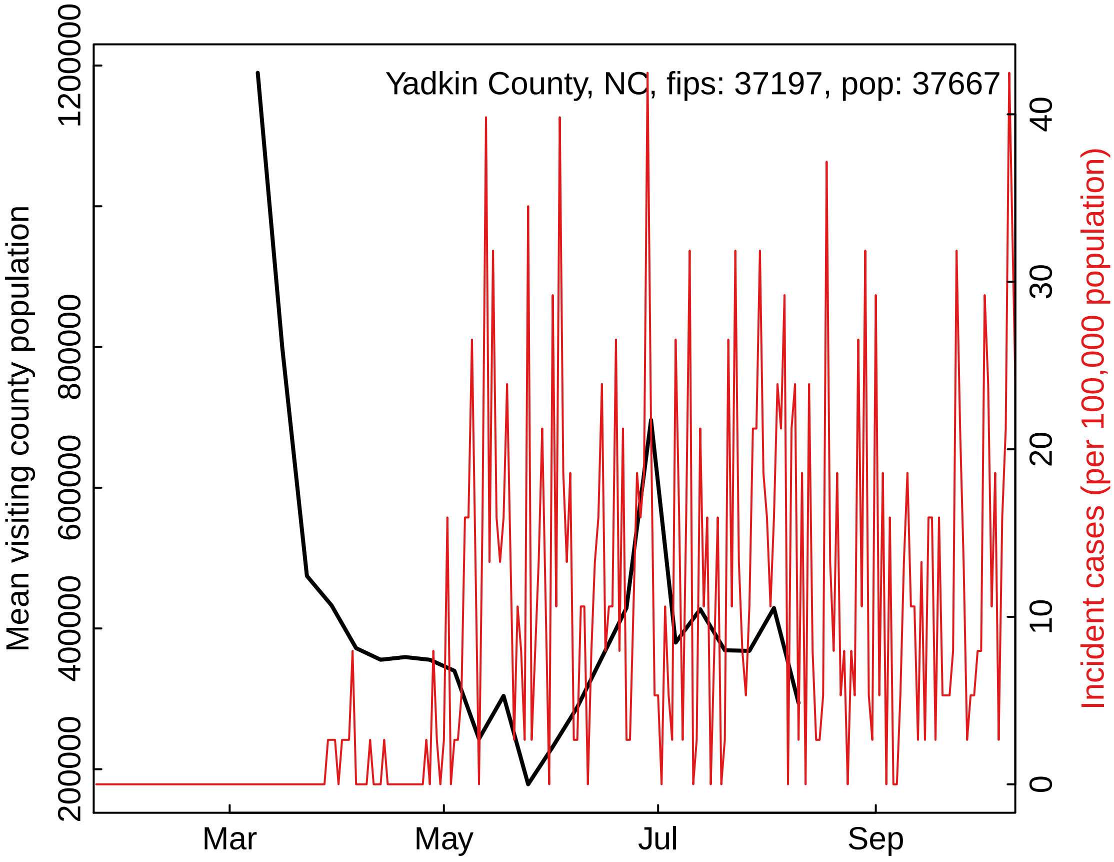

Yadkin County, NC, fips: 37197, pop: 37667
Mean visiting county population
Incident cases (per 100,000 population)
200000
400000
600000
800000
1200000
0
10
20
30
40
Mar
May
Jul
Sep

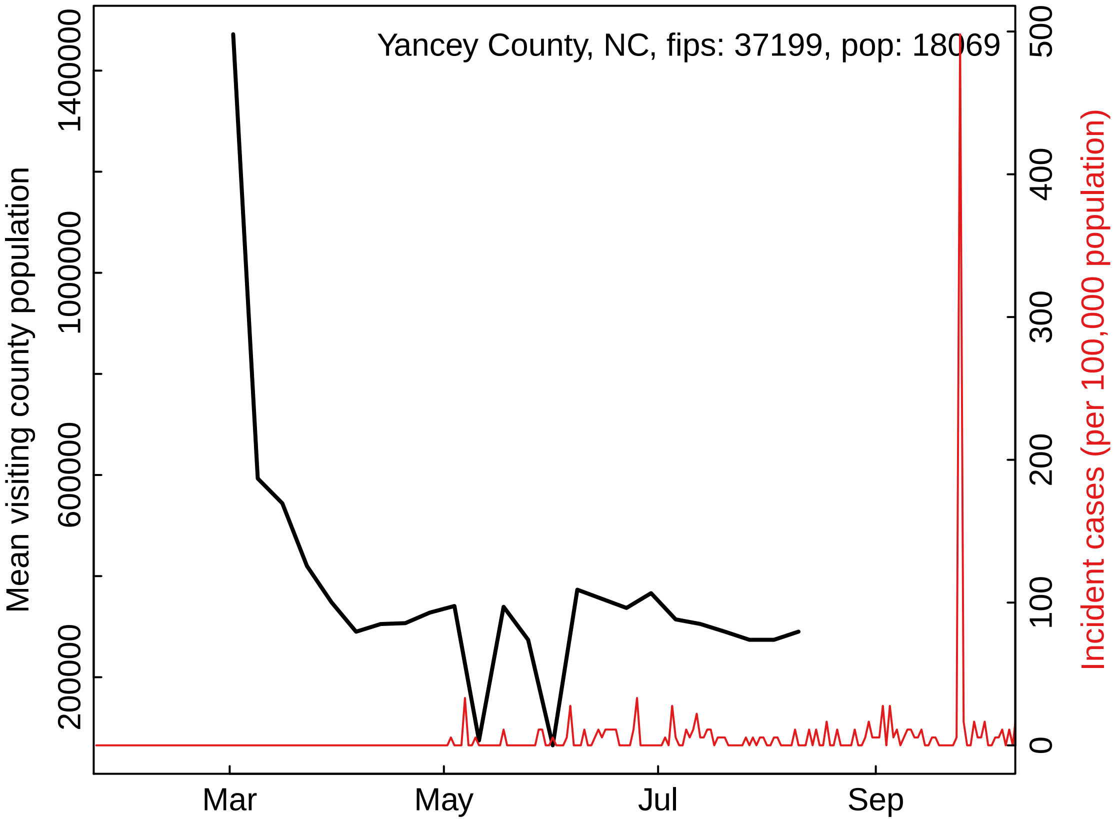

Yancey County, NC, fips: 37199, pop: 18069
Mean visiting county population
200000
600000
1000000
1400000
Incident cases (per 100,000 population)
0
100
200
300
400
500
Mar
May
Jul
Sep

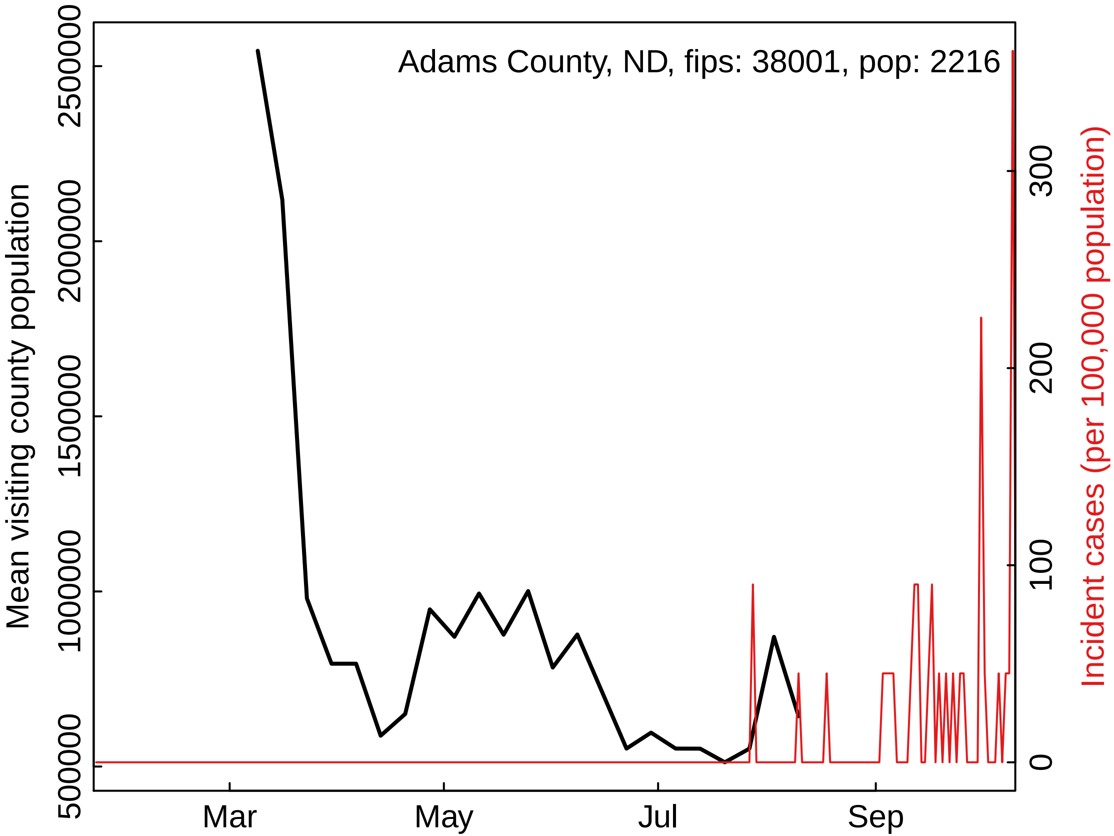

Adams County, ND, fips: 38001, pop: 2216
Mean visiting county population
500000
1000000
1500000
2000000
2500000
Incident cases (per 100,000 population)
0
100
200
300
Mar
May
Jul
Sep

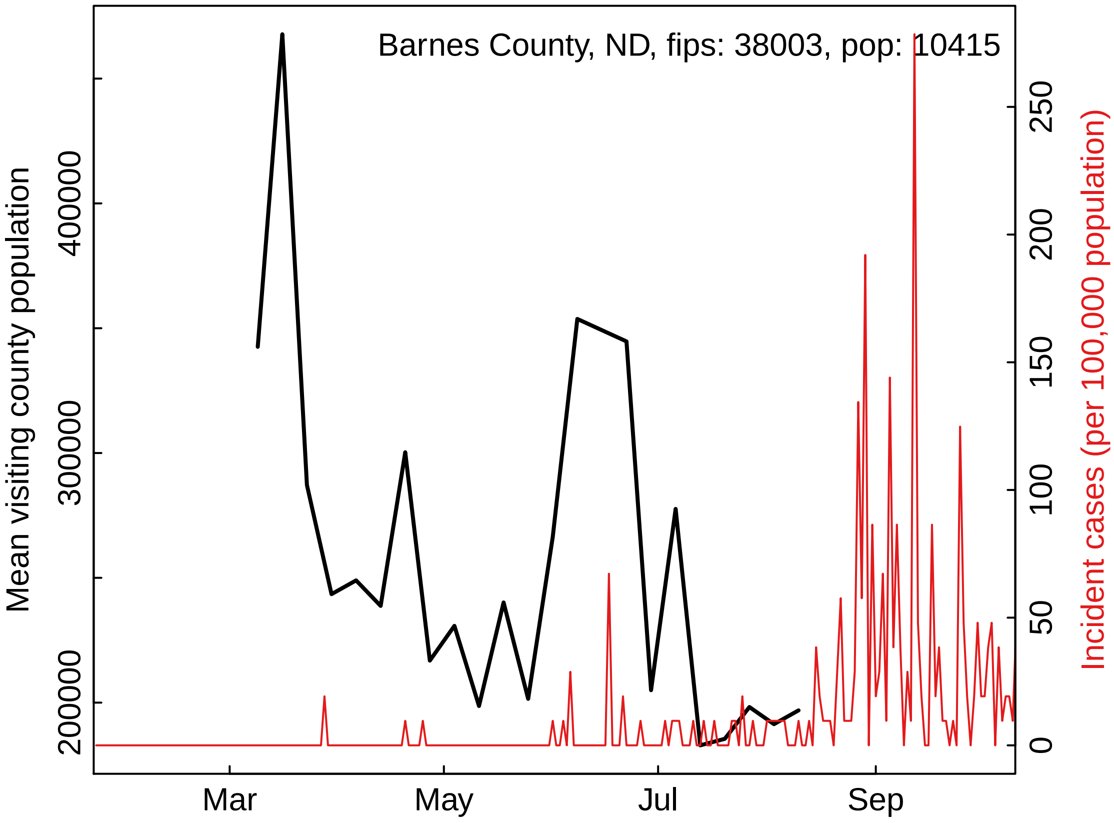

Barnes County, ND, fips: 38003, pop: 10415
Mean visiting county population
200000
300000
400000
Incident cases (per 100,000 population)
0
50
100
150
200
250
Mar
May
Jul
Sep

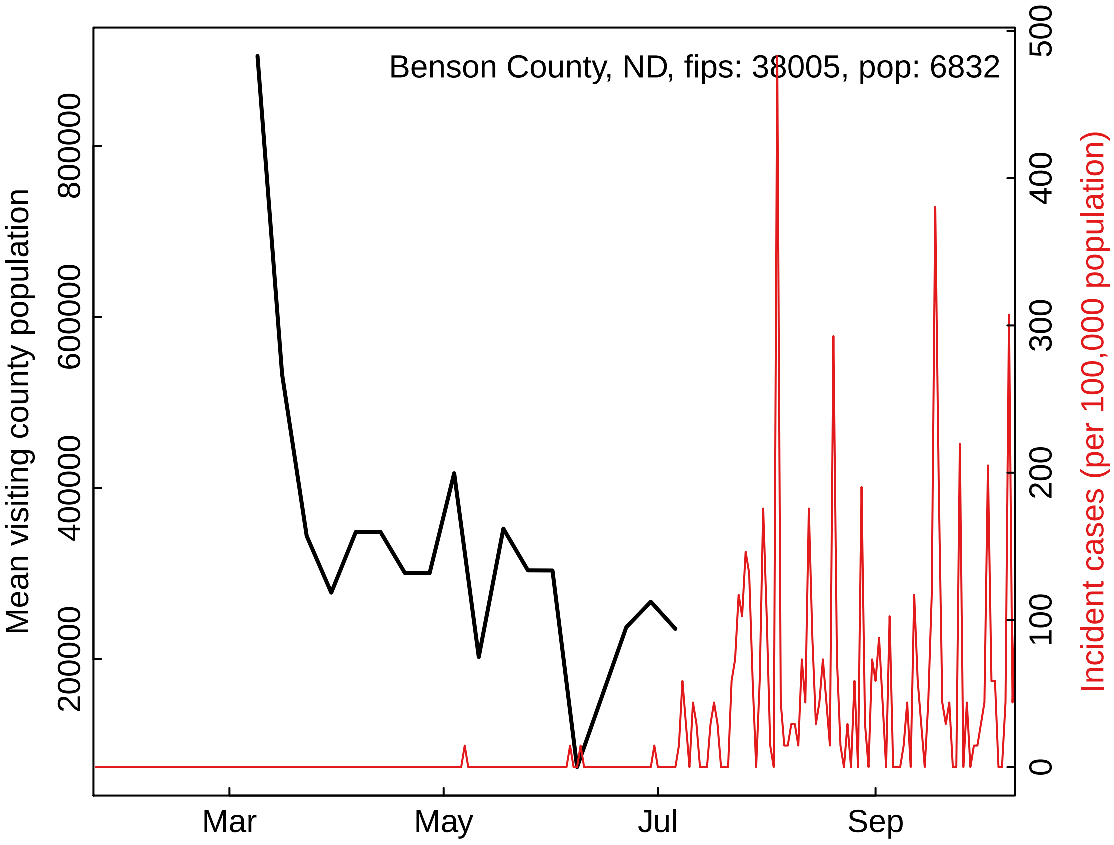

Benson County, ND, fips: 38005, pop: 6832
Mean visiting county population
200000
400000
600000
800000
Incident cases (per 100,000 population)
0
100
200
300
400
500
Mar
May
Jul
Sep

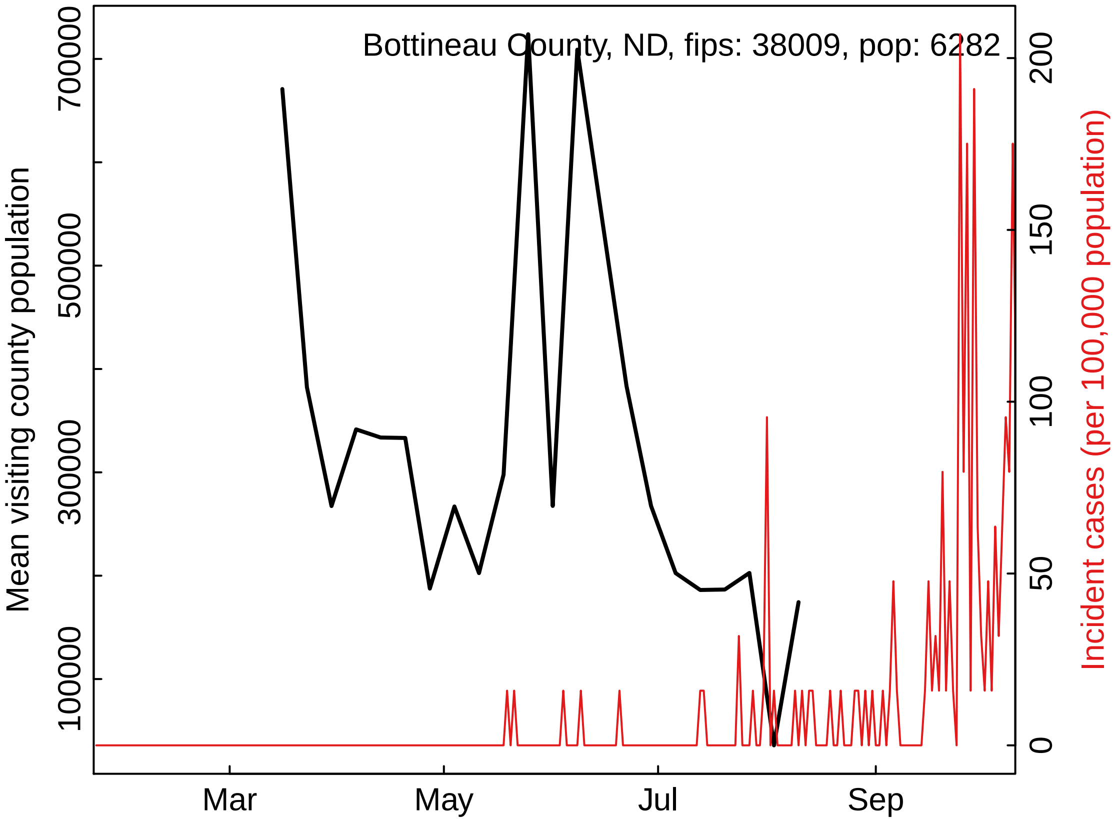

Bottineau County, ND, fips: 38009, pop: 6282
Mean visiting county population
100000
300000
500000
700000
Incident cases (per 100,000 population)
0
50
100
150
200
Mar
May
Jul
Sep

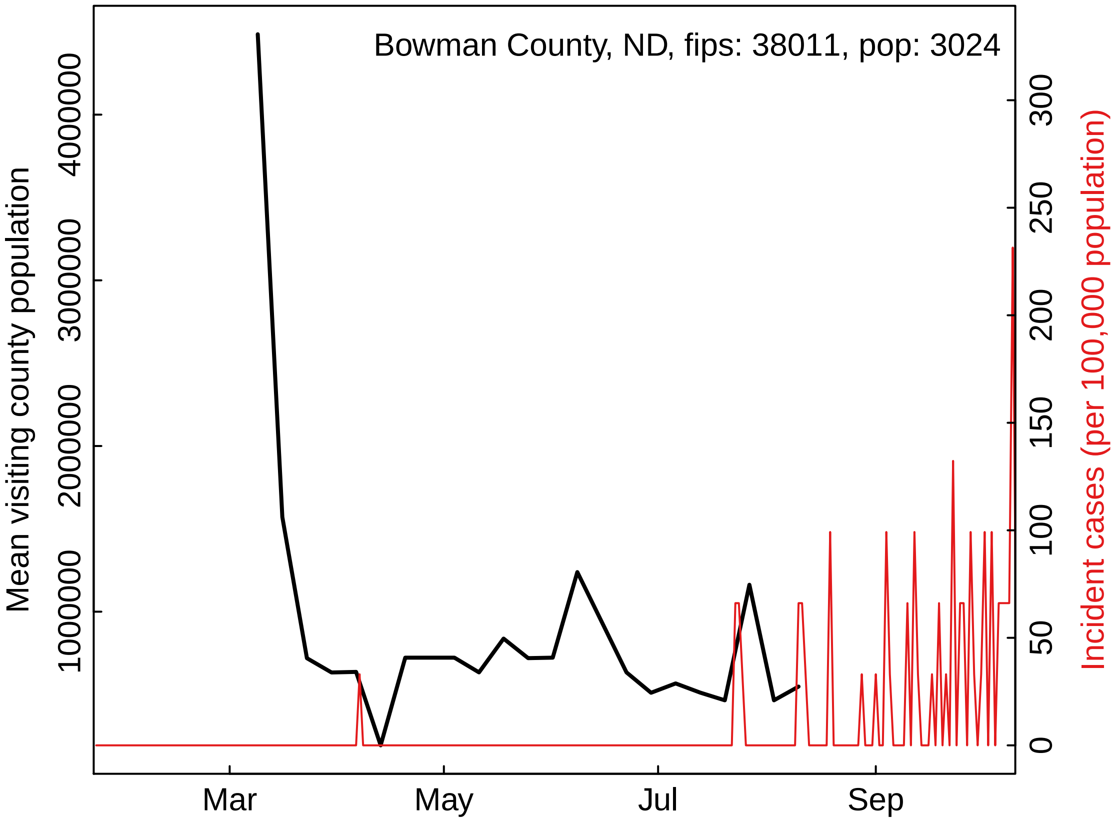
Bowman County, ND, fips: 38011, pop: 3024
Mean visiting county population
1000000
2000000
3000000
4000000
Incident cases (per 100,000 population)
0
50
100
150
200
250
300
Mar
May
Jul
Sep

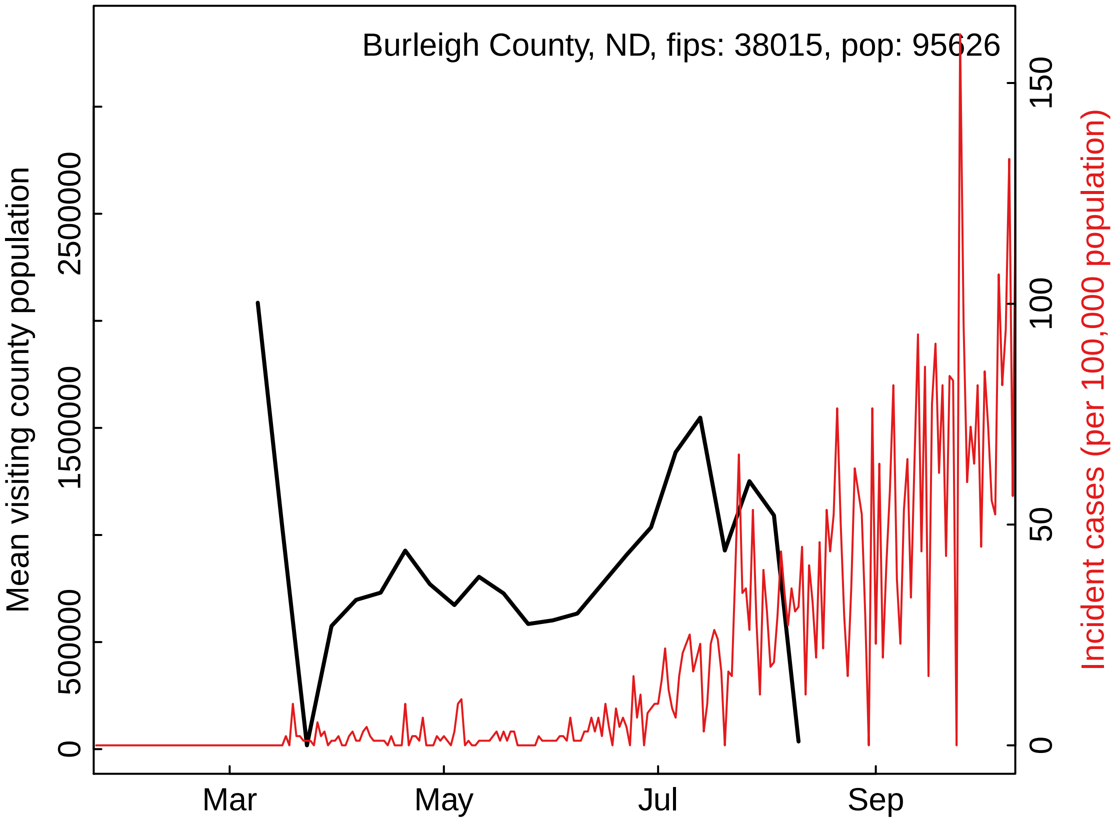

Burleigh County, ND, fips: 38015, pop: 95626
Mean visiting county population
0
500000
1500000
2500000
Incident cases (per 100,000 population)
0
50
100
150
Mar
May
Jul
Sep

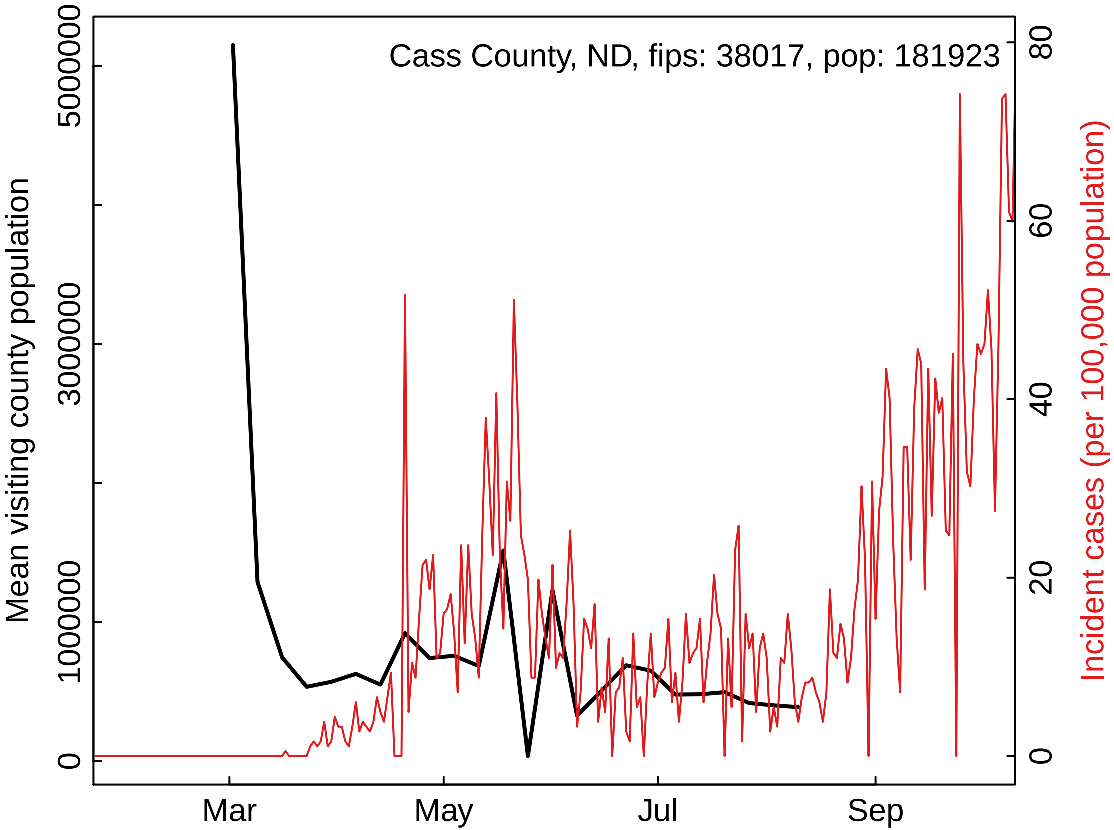
Cass County, ND, fips: 38017, pop: 181923
Mean visiting county population
Incident cases (per 100,000 population)
0
1000000
3000000
5000000
0
20
40
60
80
Mar
May
Jul
Sep

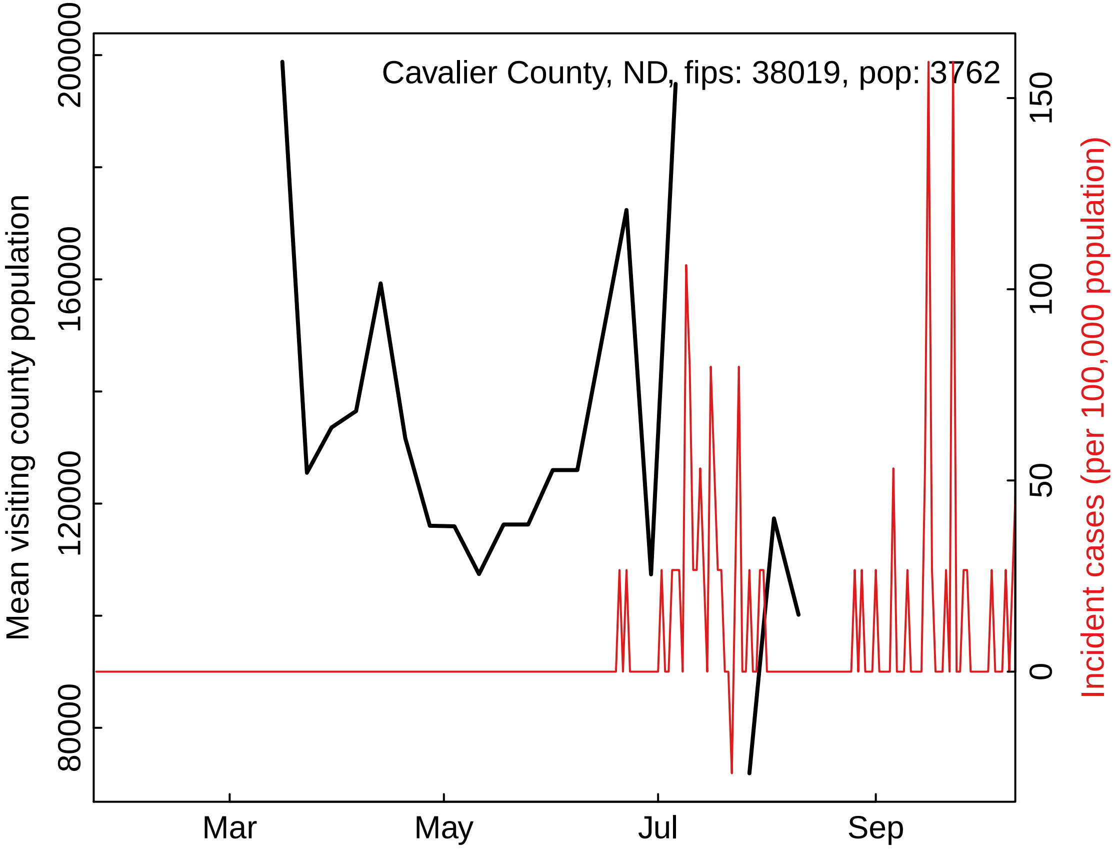

Cavalier County, ND, fips: 38019, pop: 3762
Mean visiting county population
80000
120000
160000
200000
Incident cases (per 100,000 population)
0
50
100
150
Mar
May
Jul
Sep

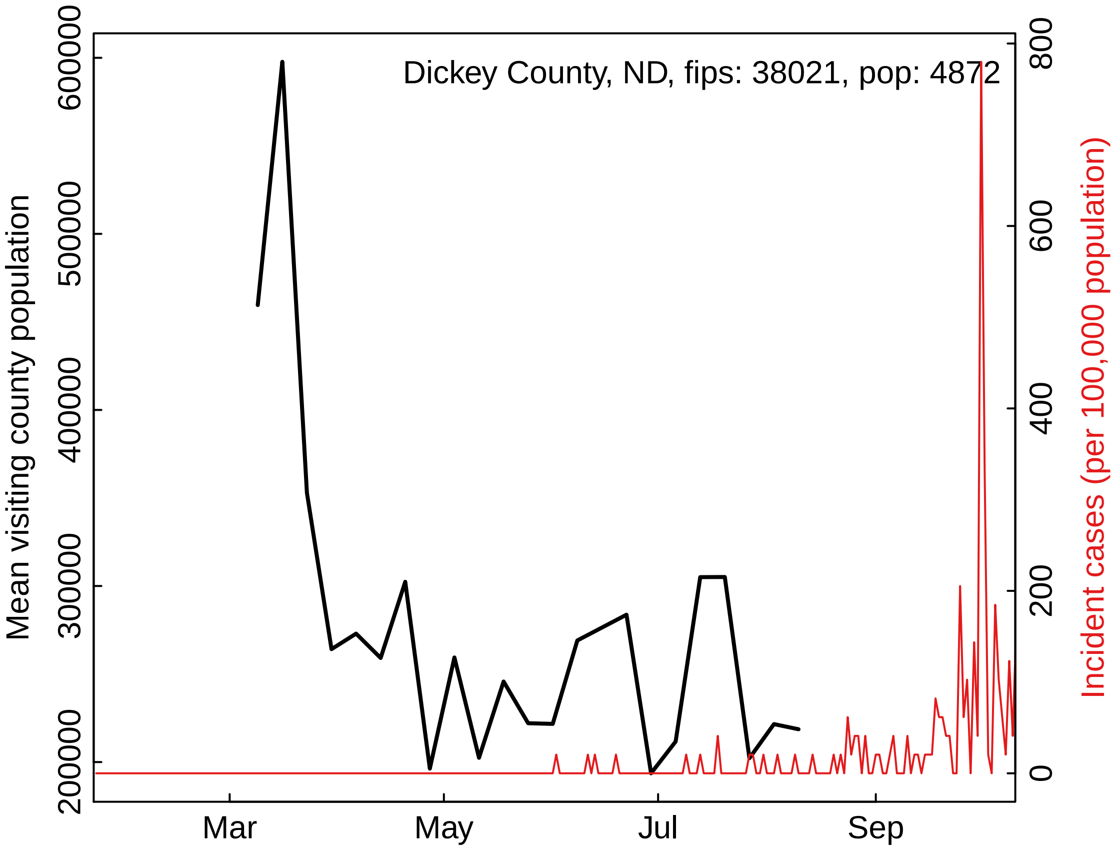
Dickey County, ND, fips: 38021, pop: 4872
Mean visiting county population
200000
300000
400000
500000
600000
Incident cases (per 100,000 population)
0
200
400
600
800
Mar
May
Jul
Sep

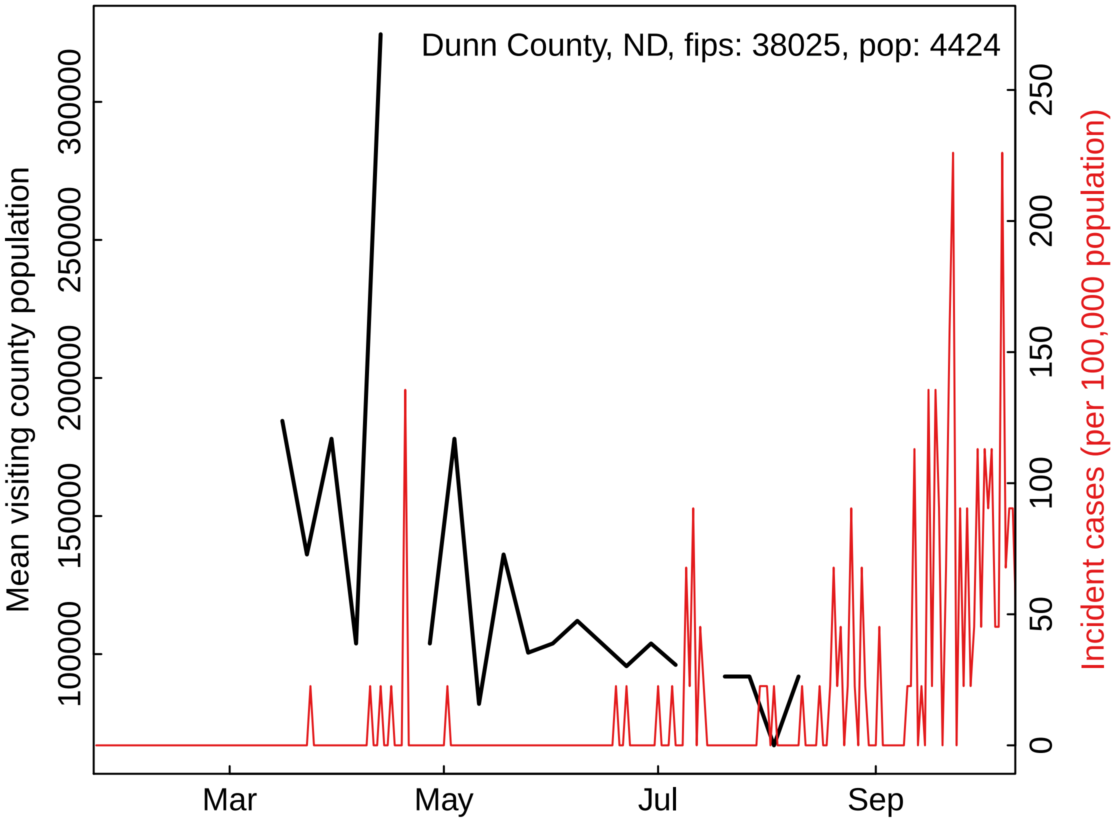
Dunn County, ND, fips: 38025, pop: 4424
Mean visiting county population
100000
150000
200000
250000
300000
Incident cases (per 100,000 population)
0
50
100
150
200
250
Mar
May
Jul
Sep

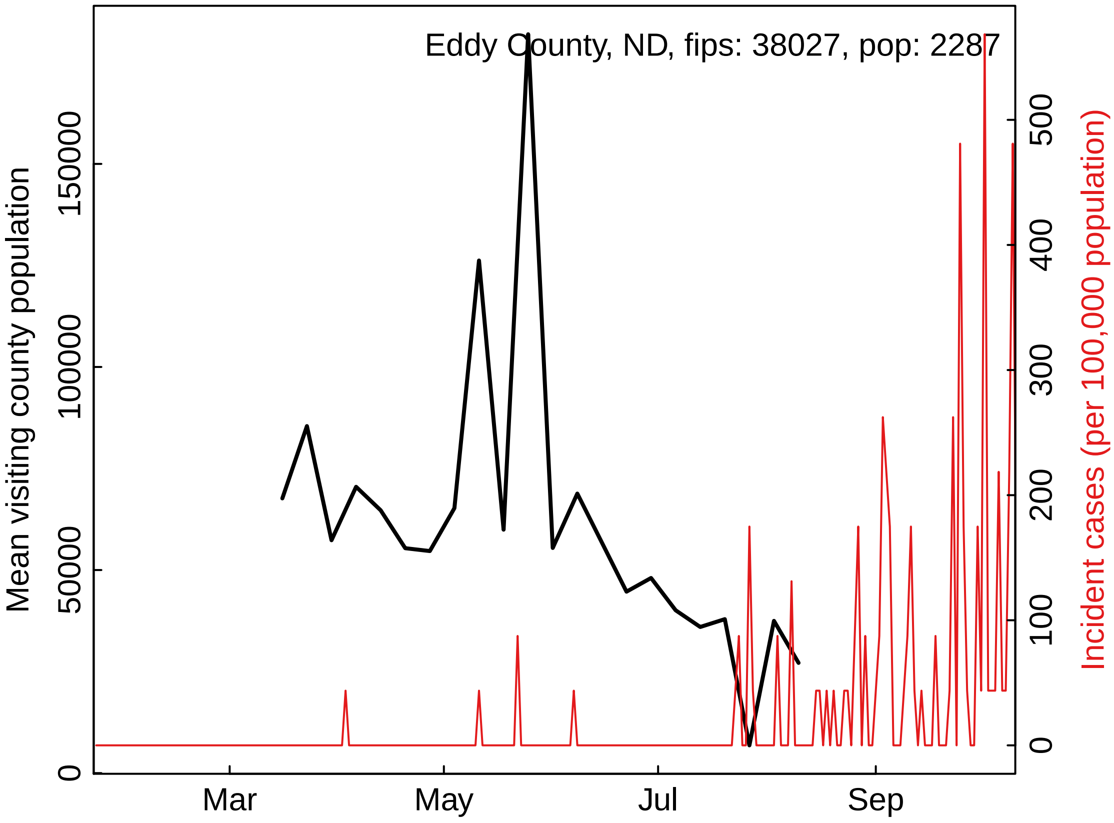

Eddy County, ND, fips: 38027, pop: 2287
Mean visiting county population
0
50000
100000
150000
Mar
May
Jul
Sep
0
100
200
300
400
500
Incident cases (per 100,000 population)

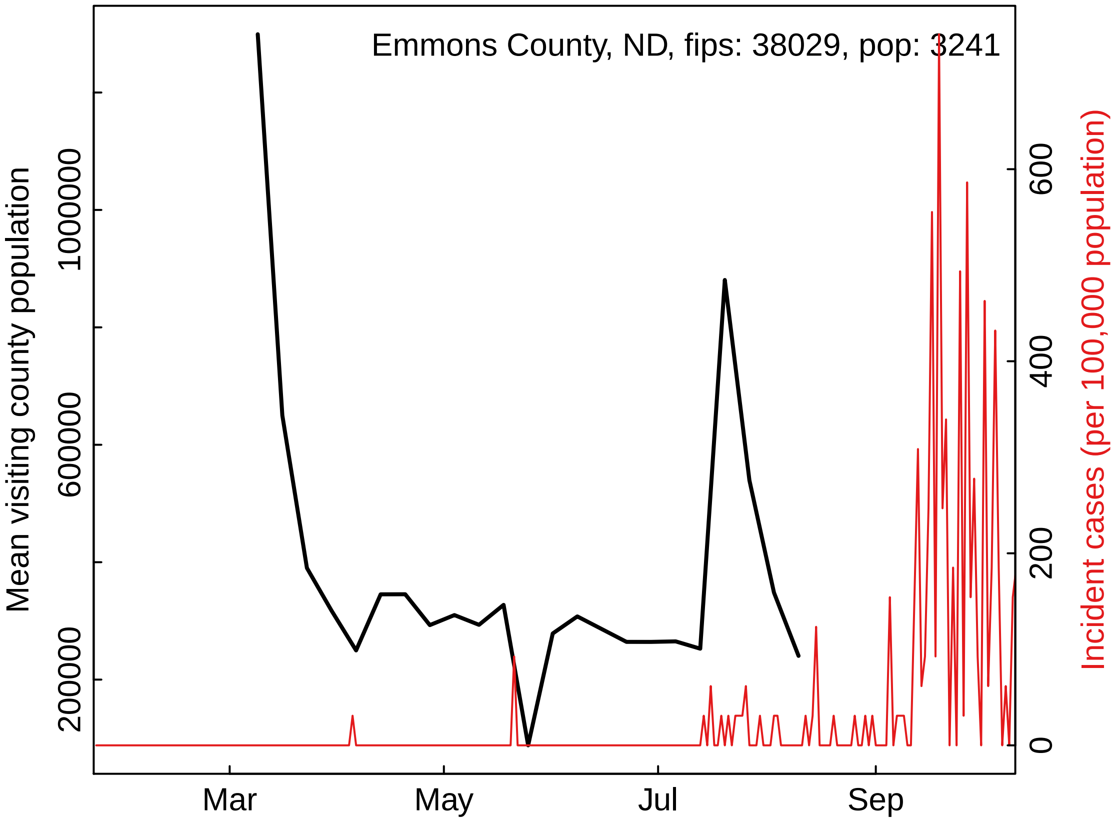

Emmons County, ND, fips: 38029, pop: 3241
Mean visiting county population
200000
600000
1000000
Incident cases (per 100,000 population)
0
200
400
600
Mar
May
Jul
Sep

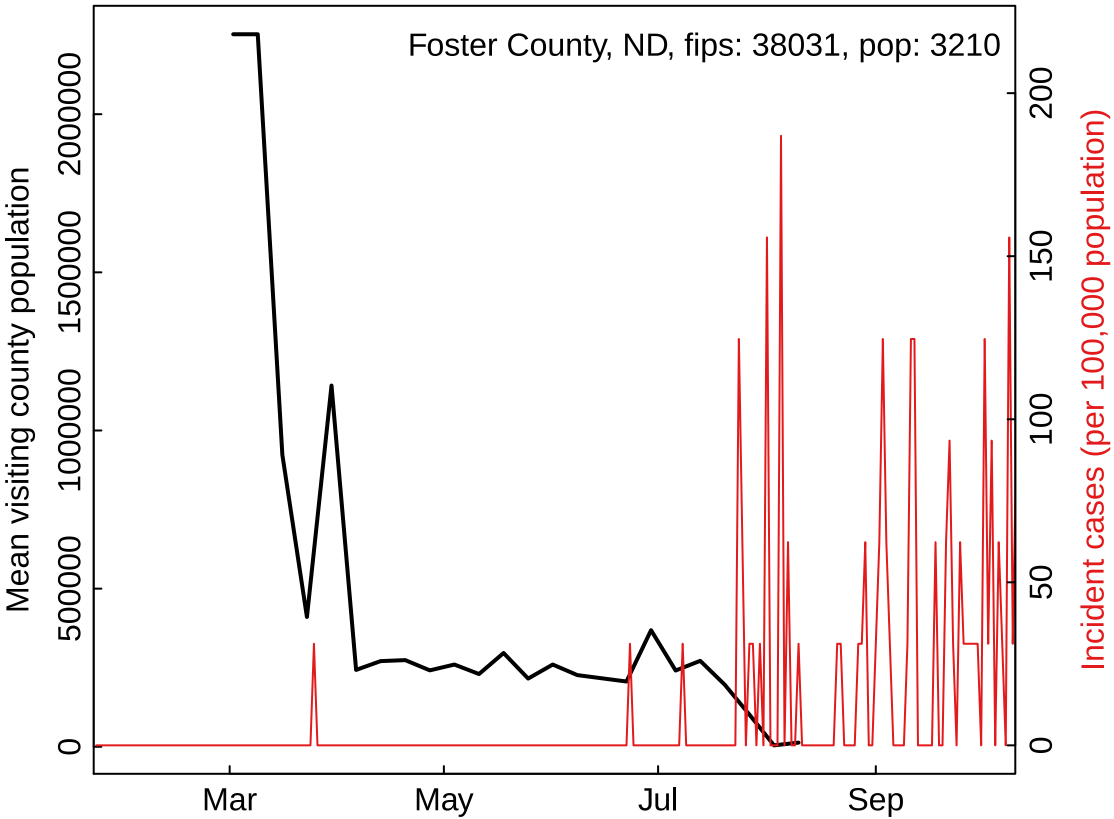
Foster County, ND, fips: 38031, pop: 3210
Mean visiting county population
0
500000
1000000
1500000
2000000
Incident cases (per 100,000 population)
0
50
100
150
200
Mar
May
Jul
Sep

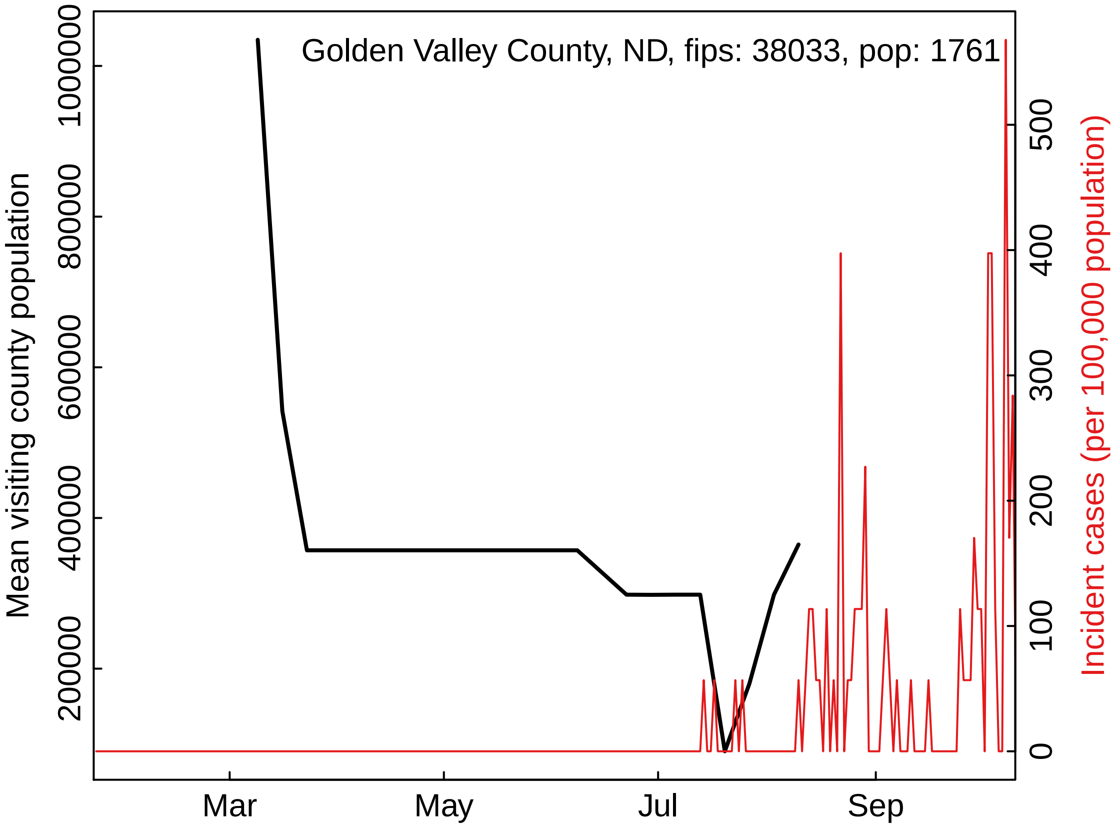

Golden Valley County, ND, fips: 38033, pop: 1761
Mean visiting county population
200000
400000
600000
800000
1000000
Incident cases (per 100,000 population)
0
100
200
300
400
500
Mar
May
Jul
Sep

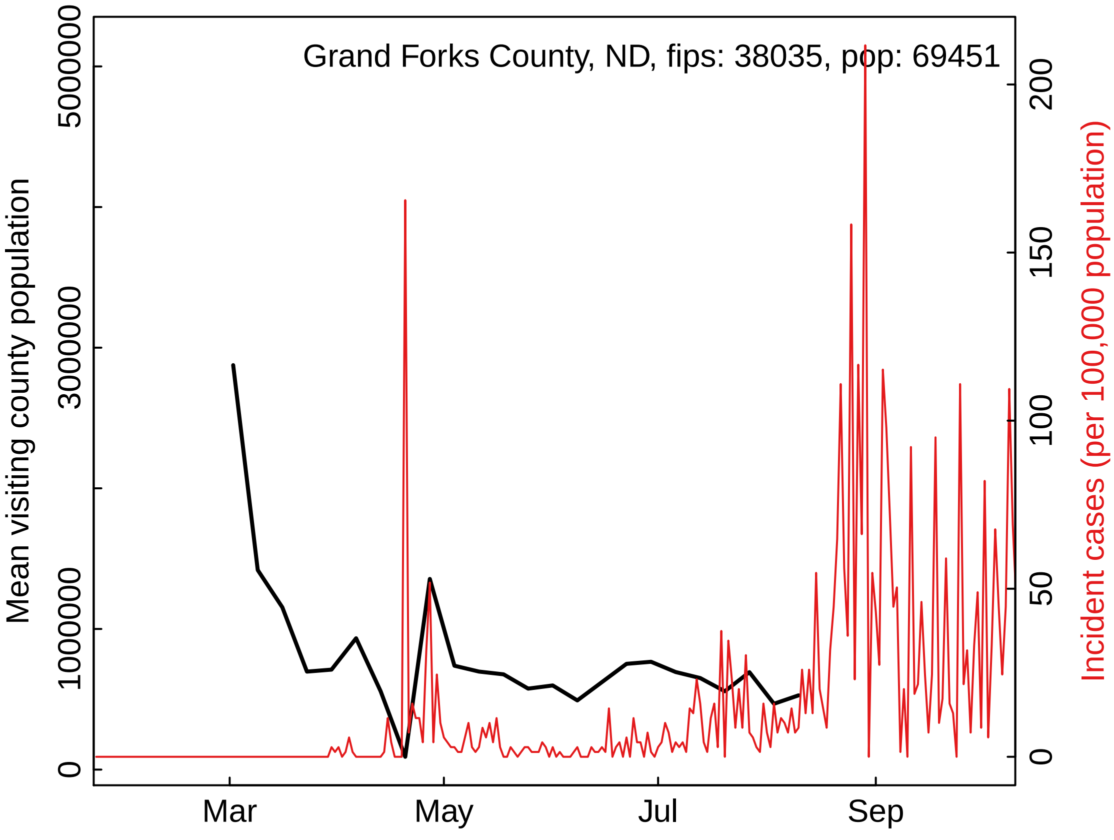

Grand Forks County, ND, fips: 38035, pop: 69451
Mean visiting county population
0
1000000
3000000
5000000
Incident cases (per 100,000 population)
0
50
100
150
200
Mar
May
Jul
Sep

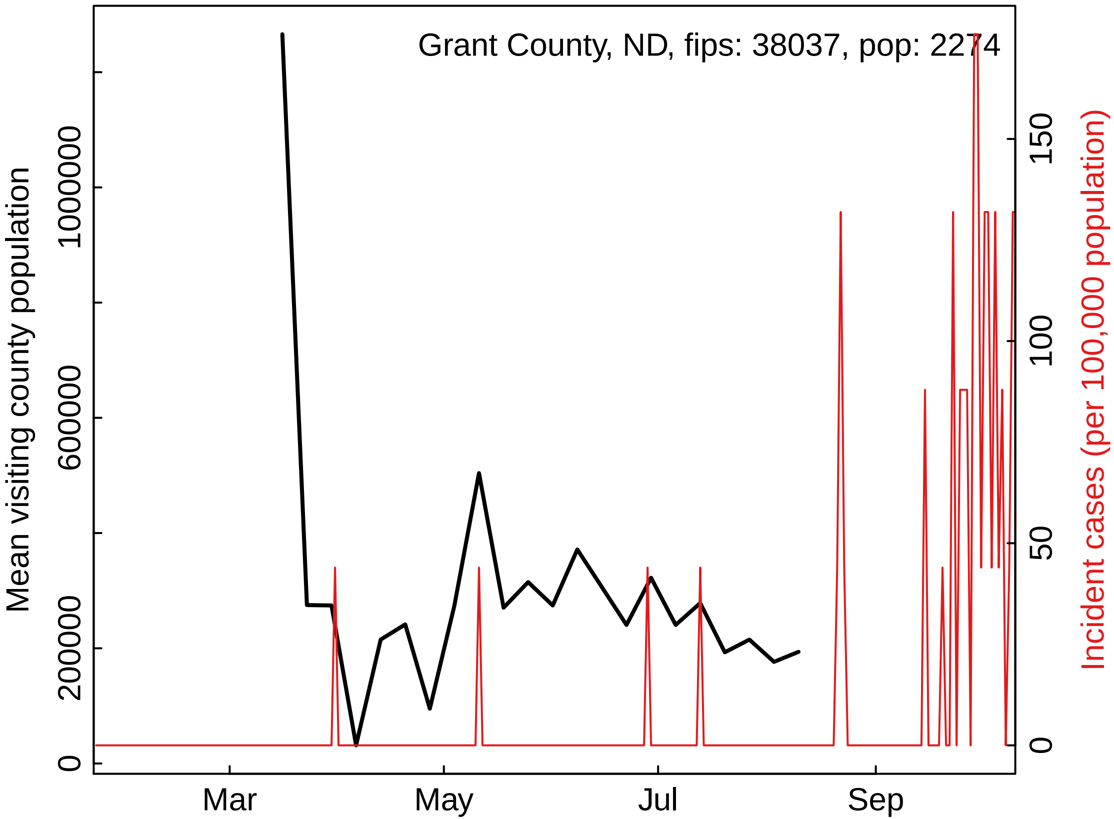

Grant County, ND, fips: 38037, pop: 2274
Mean visiting county population
0
200000
600000
1000000
Incident cases (per 100,000 population)
0
50
100
150
Mar
May
Jul
Sep

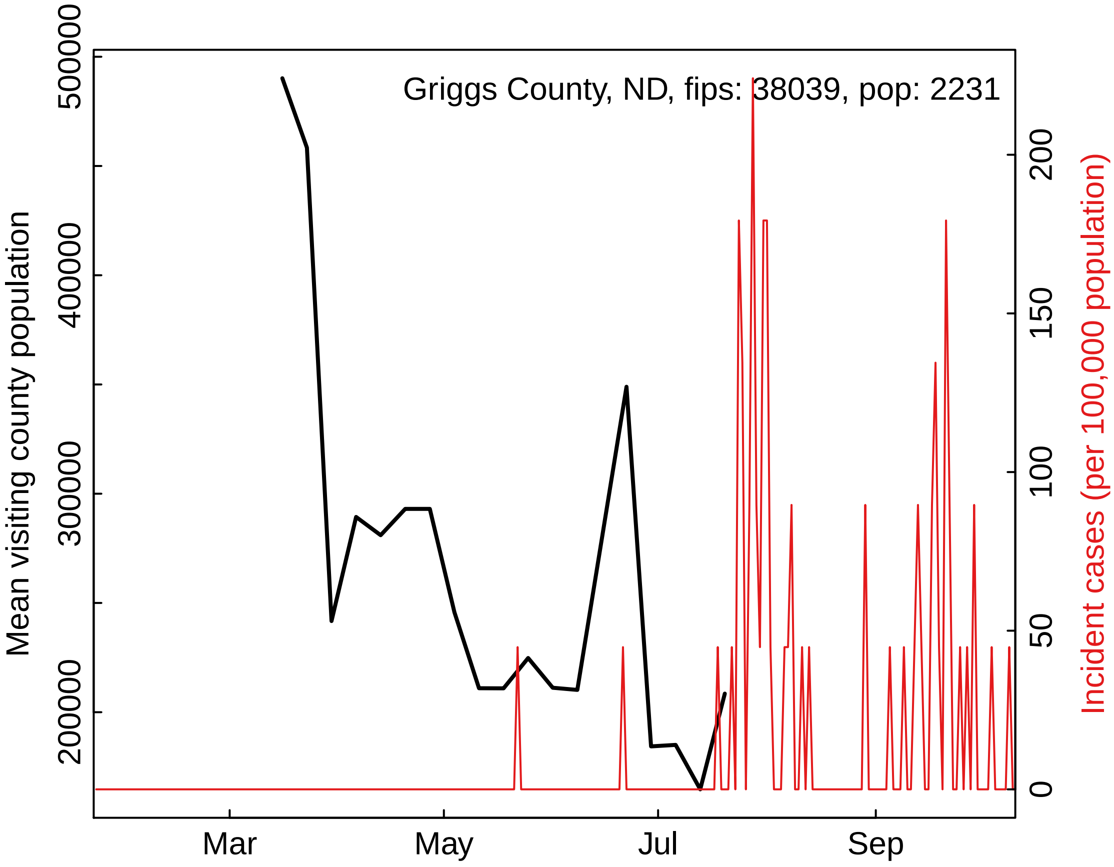

Griggs County, ND, fips: 38039, pop: 2231
Mean visiting county population
200000
300000
400000
500000
Incident cases (per 100,000 population)
0
50
100
150
200
Mar
May
Jul
Sep

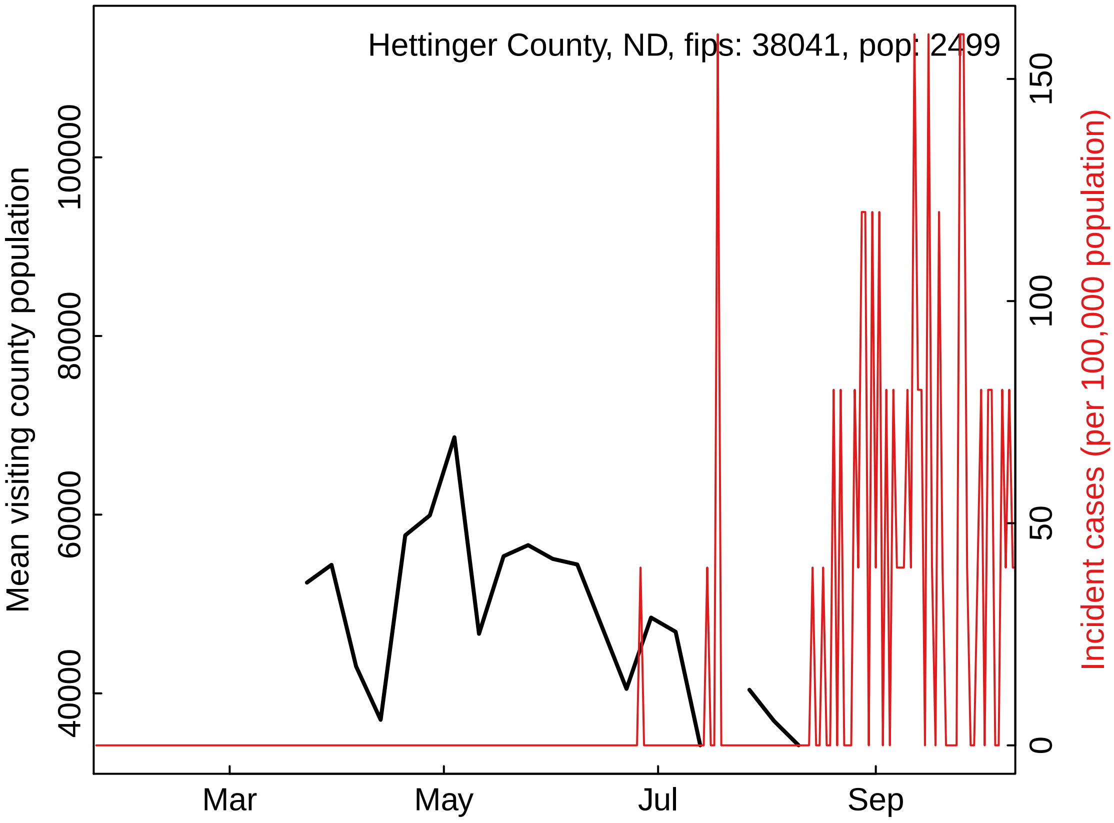

Hettinger County, ND, fips: 38041, pop: 2499
Mean visiting county population
40000
60000
80000
100000
Mar
May
Jul
Sep
0
50
100
150
Incident cases (per 100,000 population)

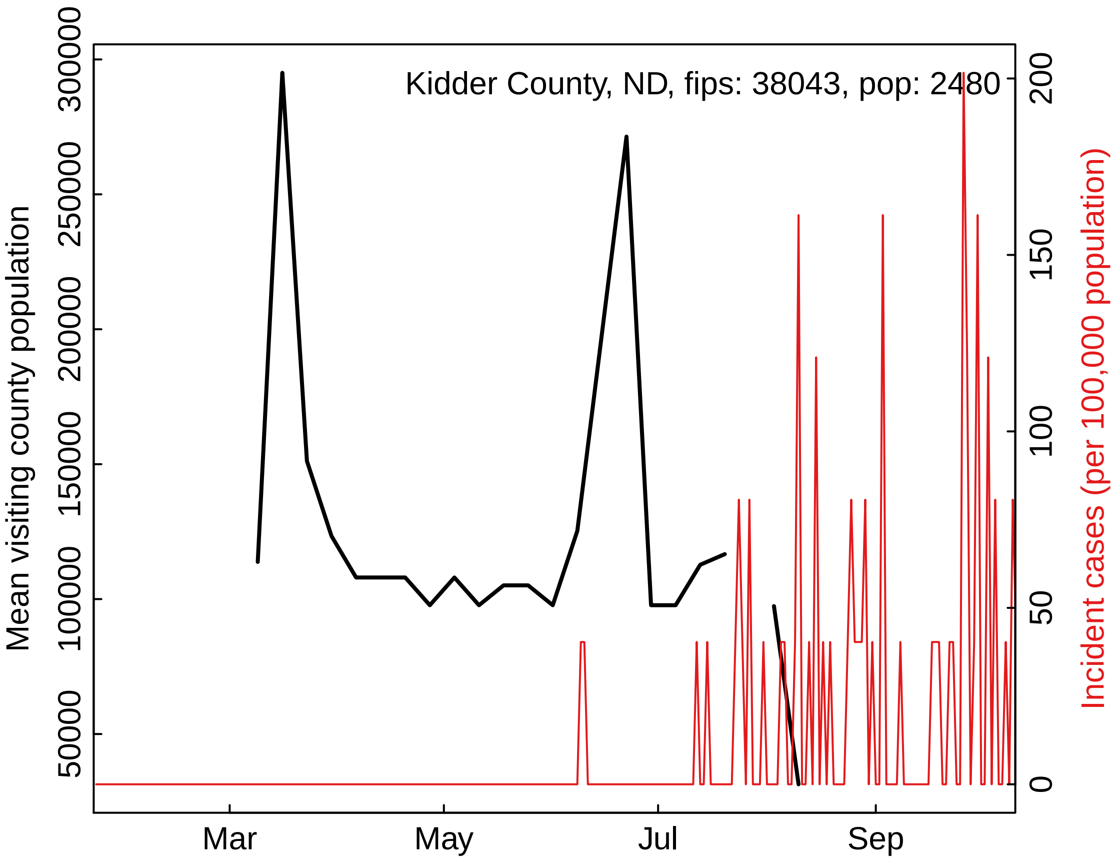

Kidder County, ND, fips: 38043, pop: 2480
Mean visiting county population
50000
100000
150000
200000
250000
300000
Incident cases (per 100,000 population)
0
50
100
150
200
Mar
May
Jul
Sep

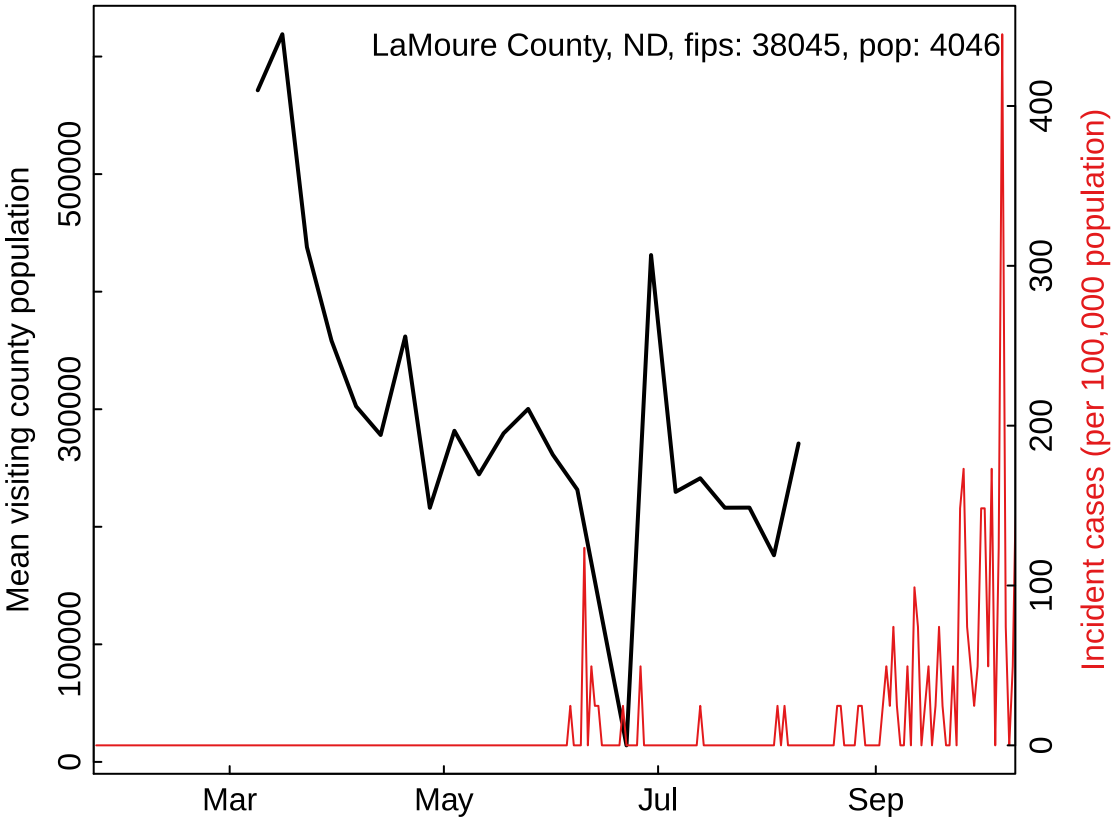

LaMoure County, ND, fips: 38045, pop: 4046
Mean visiting county population
0
100000
300000
500000
Mar
May
Jul
Sep
0
100
200
300
400
Incident cases (per 100,000 population)

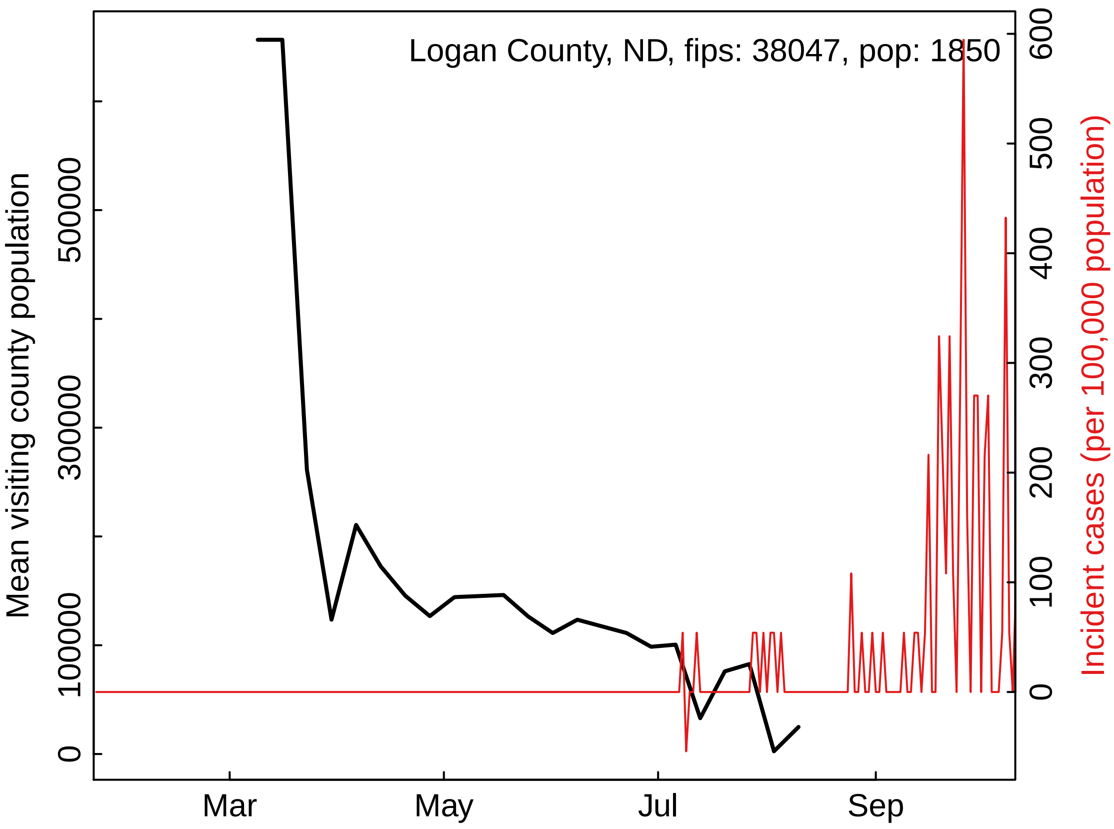

Logan County, ND, fips: 38047, pop: 1850
Mean visiting county population
0
100000
300000
500000
Mar
May
Jul
Sep
Incident cases (per 100,000 population)
0
100
200
300
400
500
600

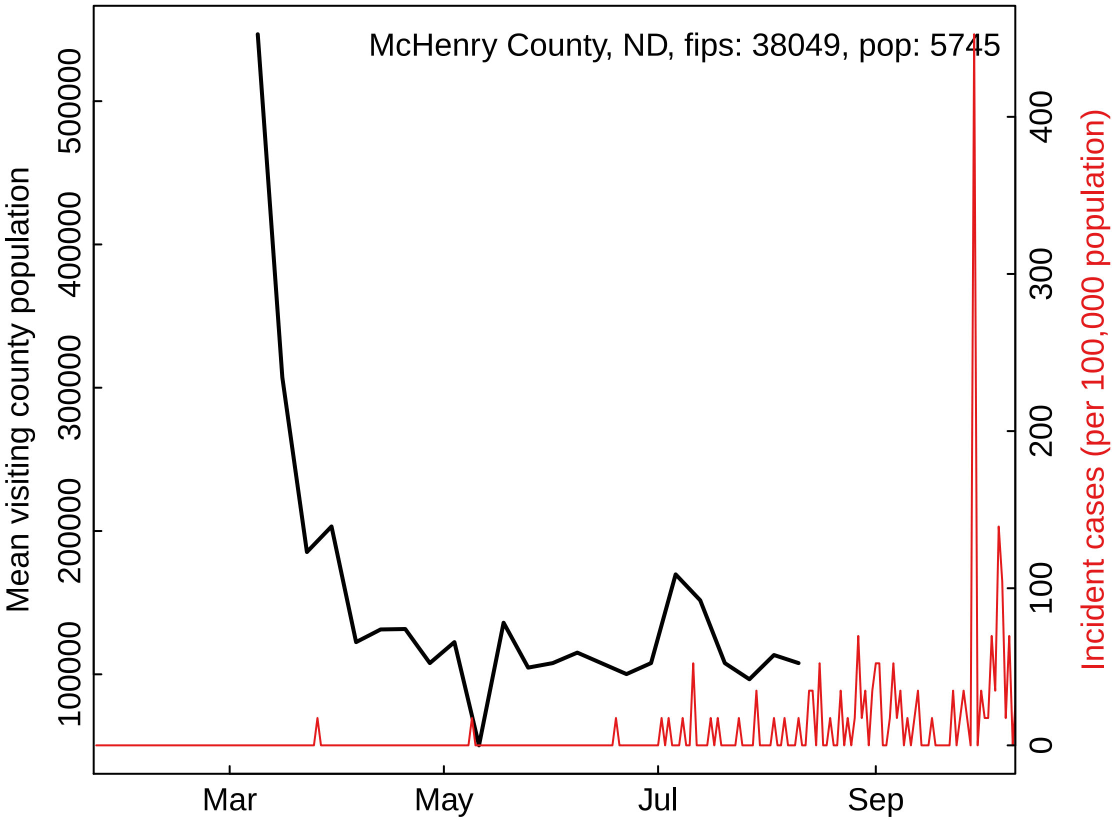

McHenry County, ND, fips: 38049, pop: 5745
Mean visiting county population
100000
200000
300000
400000
500000
Incident cases (per 100,000 population)
0
100
200
300
400
Mar
May
Jul
Sep

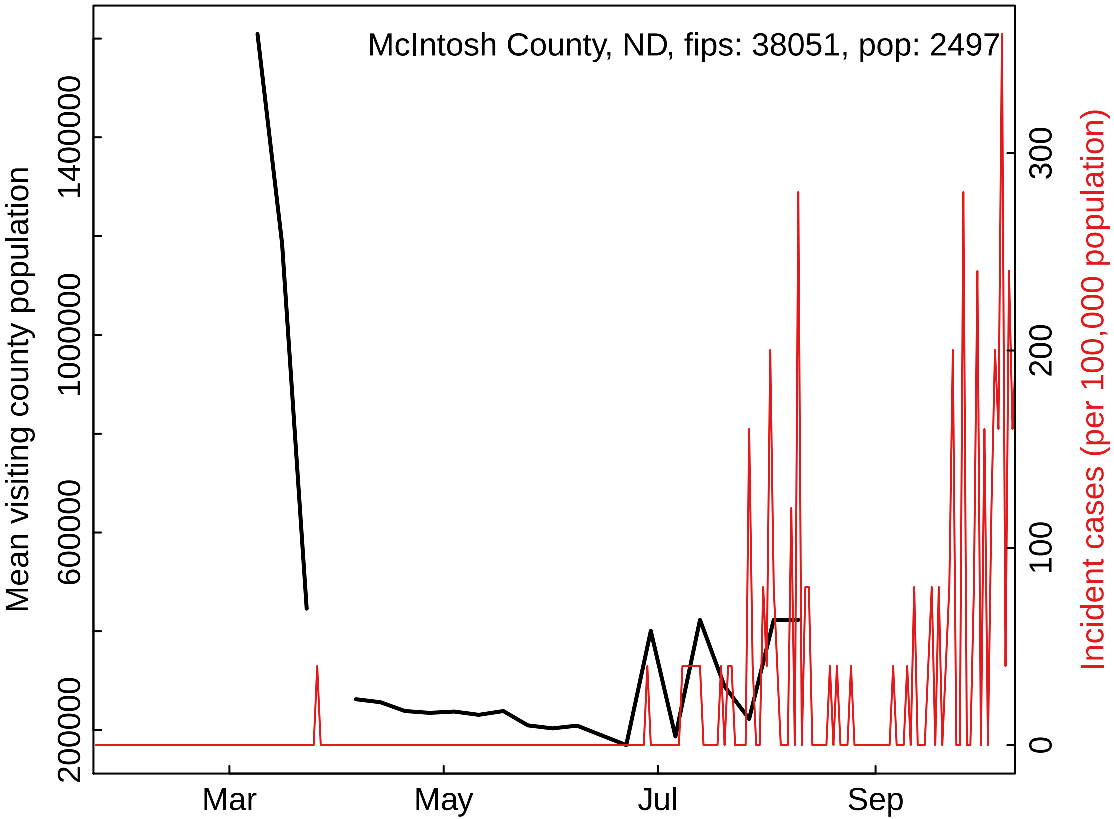

McIntosh County, ND, fips: 38051, pop: 2497
Mean visiting county population
Incident cases (per 100,000 population)
200000
600000
1000000
1400000
0
100
200
300
Mar
May
Jul
Sep

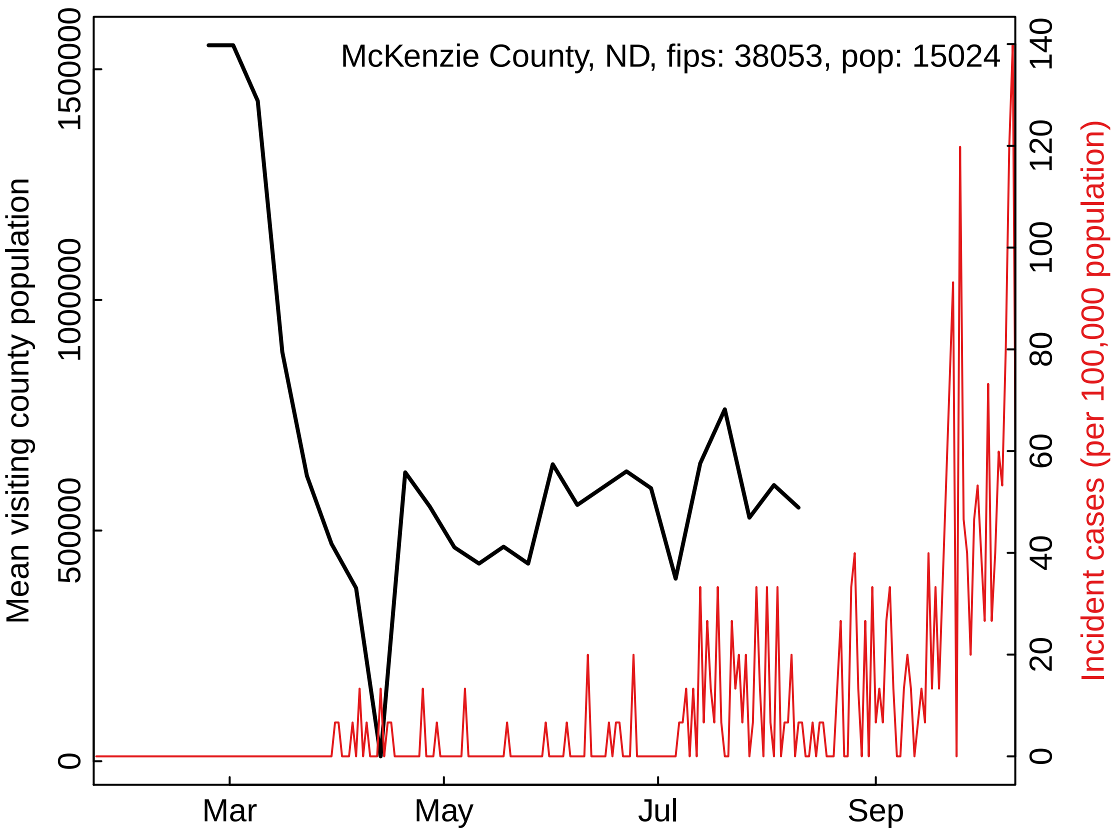

McKenzie County, ND, fips: 38053, pop: 15024
Mean visiting county population
0
500000
1000000
1500000
Incident cases (per 100,000 population)
0
20
40
60
80
100
120
140
Mar
May
Jul
Sep

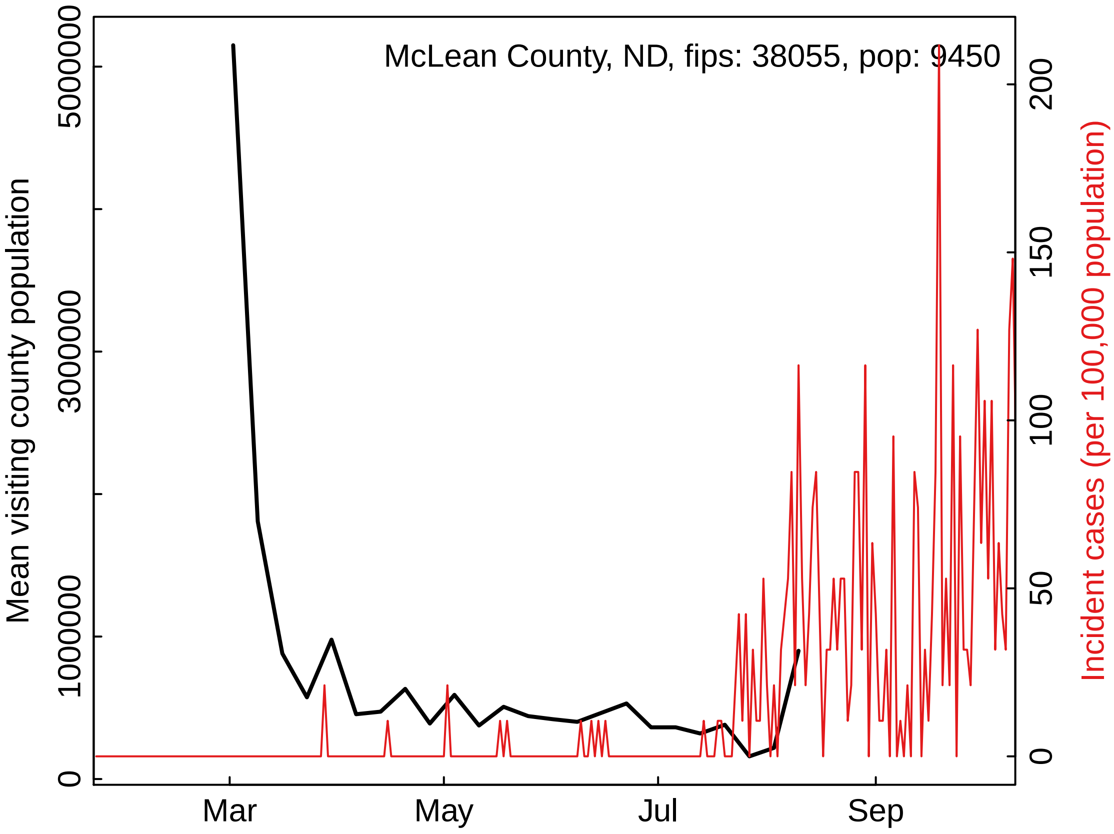

McLean County, ND, fips: 38055, pop: 9450
Mean visiting county population
Incident cases (per 100,000 population)
0
1000000
3000000
5000000
0
50
100
150
200
Mar
May
Jul
Sep

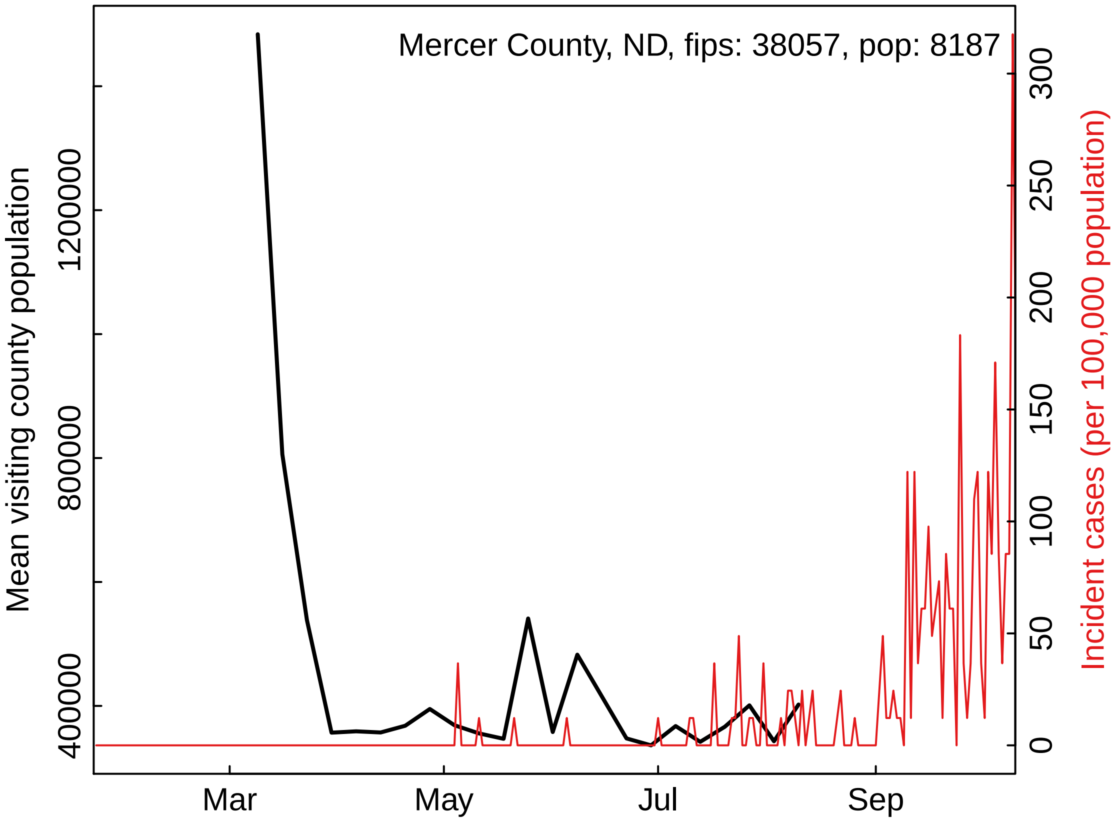

Mercer County, ND, fips: 38057, pop: 8187
Mean visiting county population
400000
800000
1200000
Incident cases (per 100,000 population)
0
50
100
150
200
250
300
Mar
May
Jul
Sep

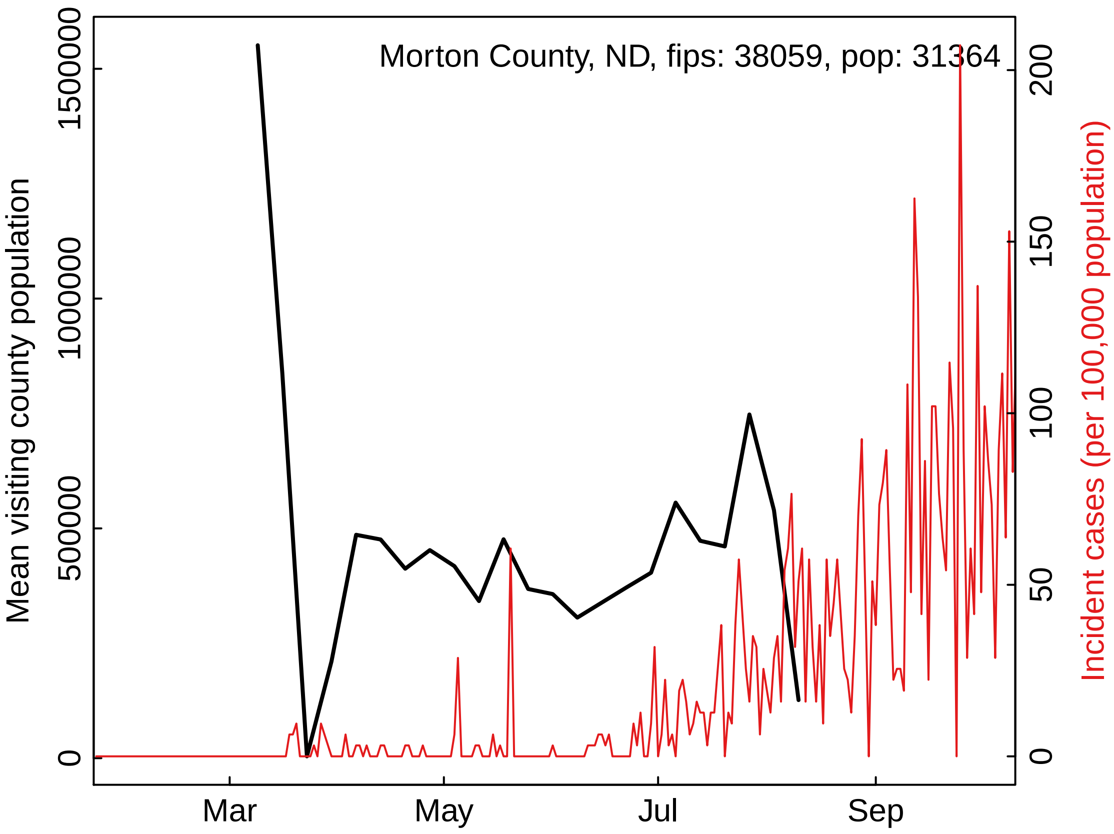

Morton County, ND, fips: 38059, pop: 31364
Mean visiting county population
0
500000
1000000
1500000
Incident cases (per 100,000 population)
0
50
100
150
200
Mar
May
Jul
Sep

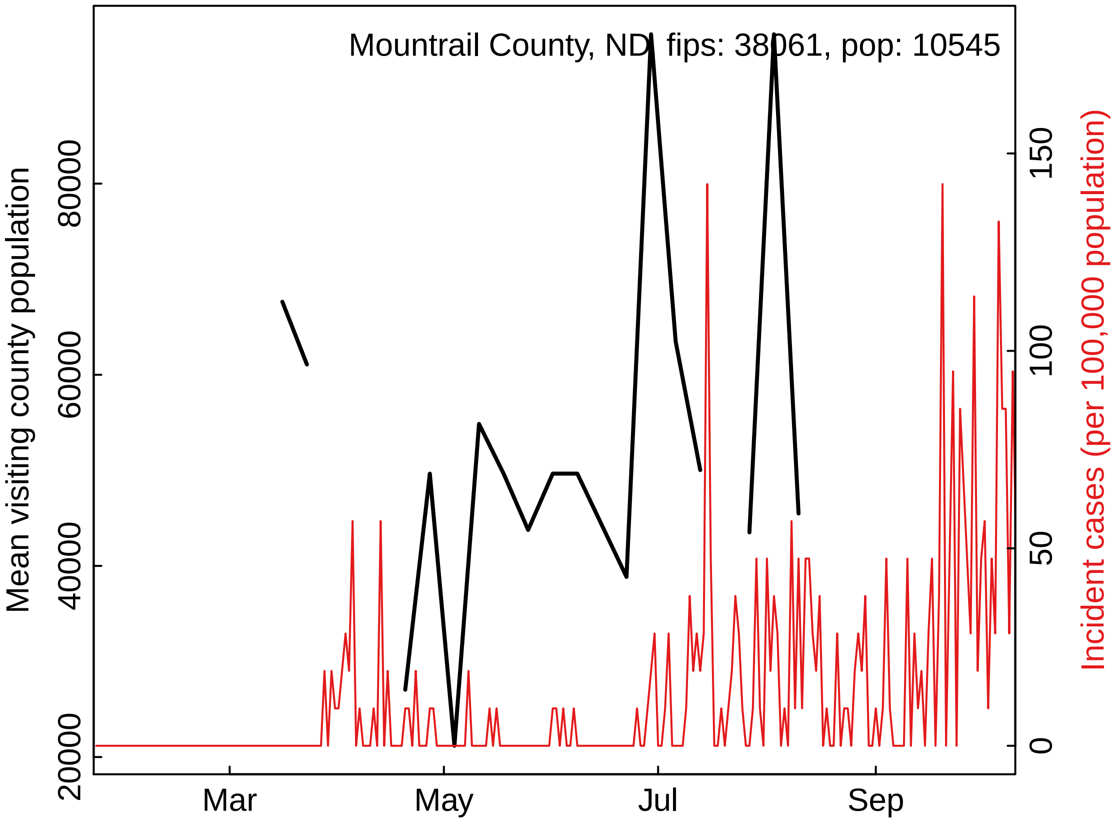

Mountrail County, ND fips: 38061, pop: 10545
Mean visiting county population
20000
40000
60000
80000
Mar
May
Jul
Sep
0
50
100
150
Incident cases (per 100,000 population)

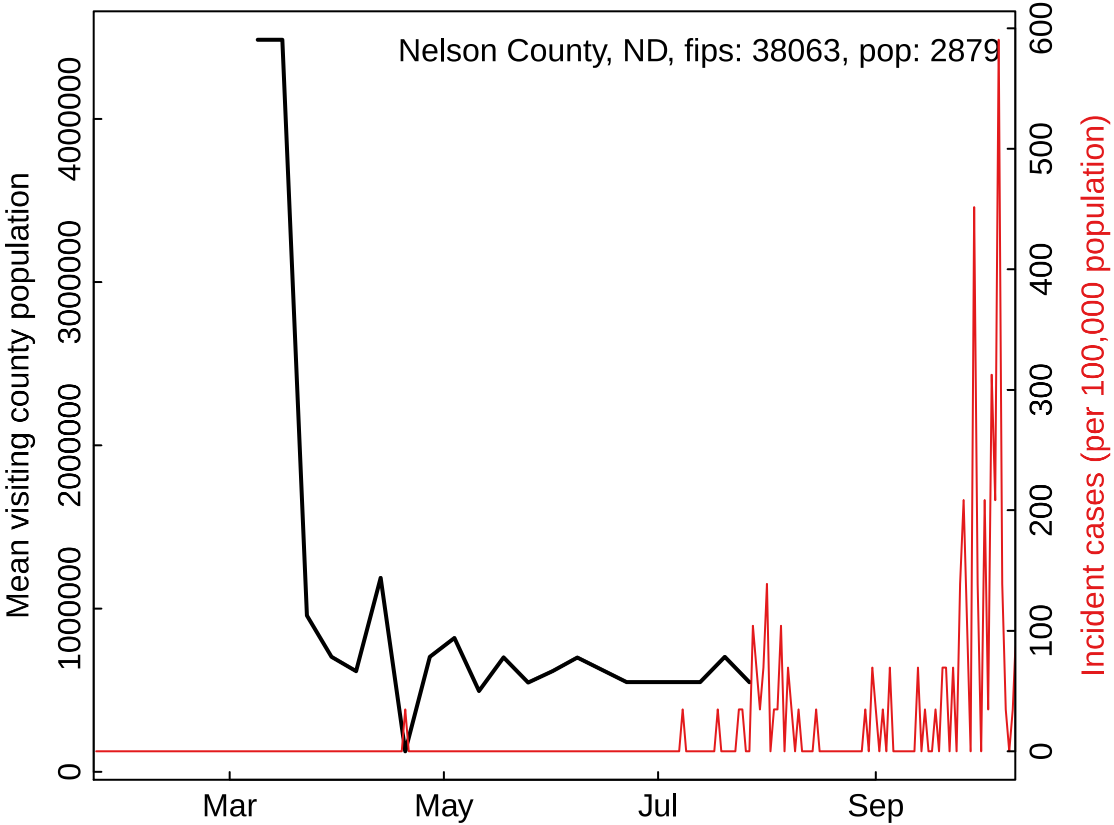

Nelson County, ND, fips: 38063, pop: 2879
Mean visiting county population
0
1000000
2000000
3000000
4000000
Incident cases (per 100,000 population)
0
100
200
300
400
500
600
Mar
May
Jul
Sep

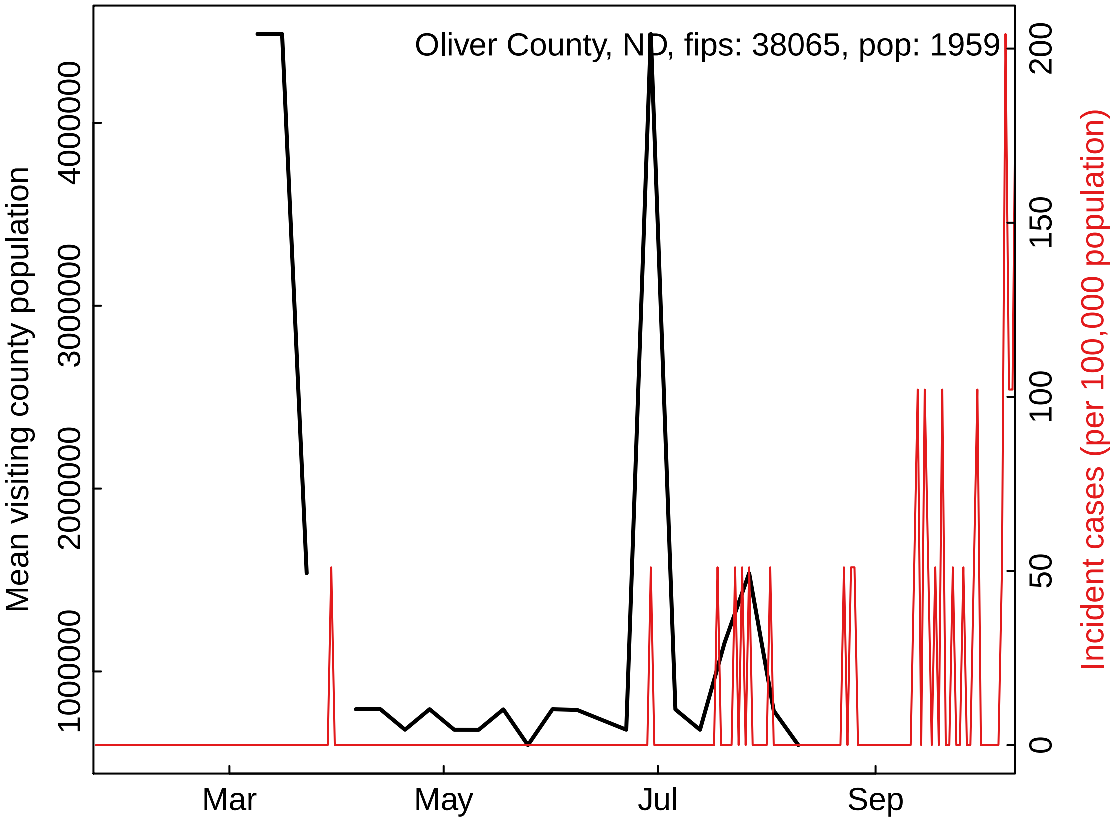
Oliver County, ND, fips: 38065, pop: 1959
Mean visiting county population
1000000
2000000
3000000
4000000
Incident cases (per 100,000 population)
0
50
100
150
200
Mar
May
Jul
Sep

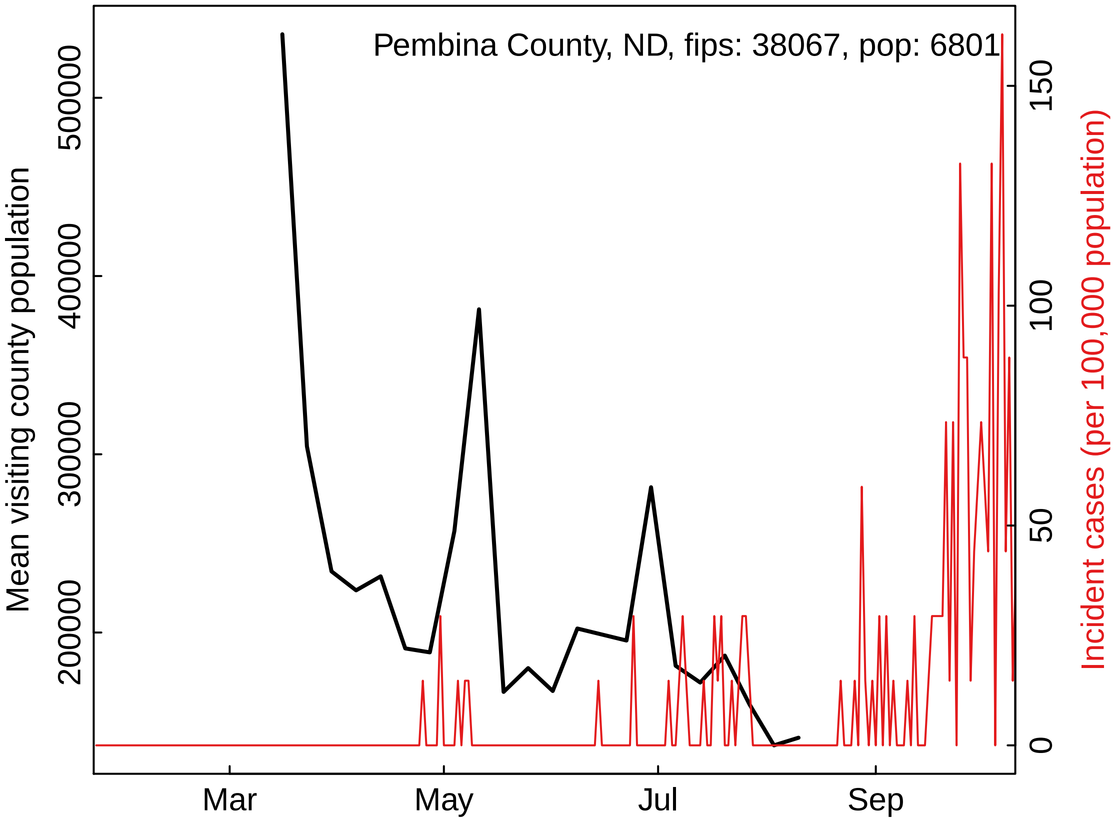

Pembina County, ND, fips: 38067, pop: 6801
Mean visiting county population
200000
300000
400000
500000
Incident cases (per 100,000 population)
0
50
100
150
Mar
May
Jul
Sep

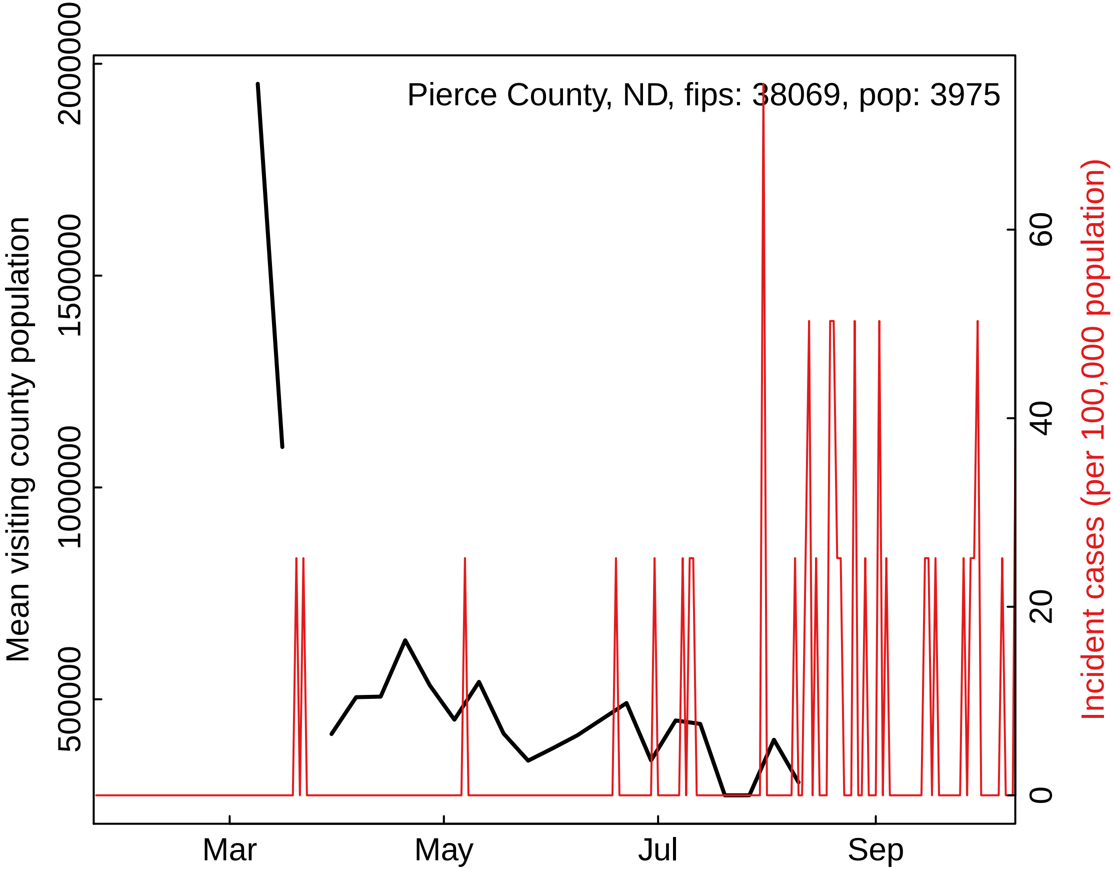
Pierce County, ND, fips: 38069, pop: 3975
Mean visiting county population
500000
1000000
1500000
2000000
Incident cases (per 100,000 population)
0
20
40
60
Mar
May
Jul
Sep

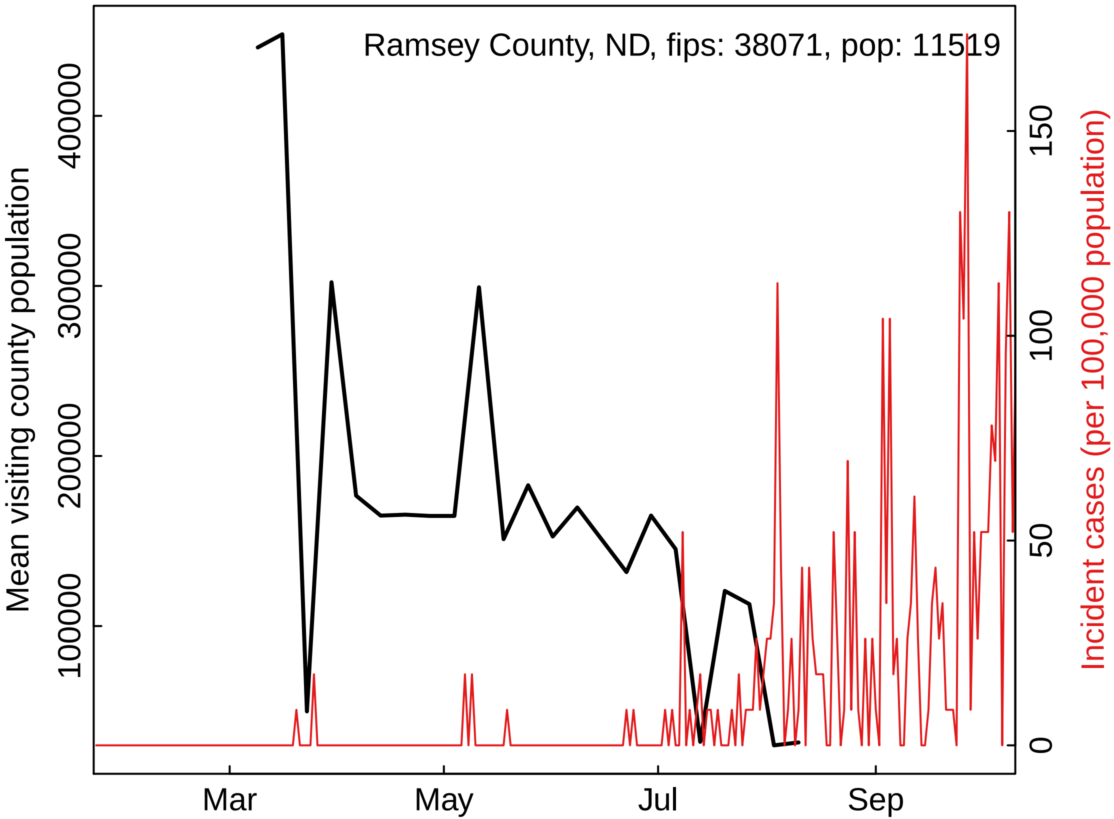

Ramsey County, ND, fips: 38071, pop: 11519
Mean visiting county population
100000
200000
300000
400000
Incident cases (per 100,000 population)
0
50
100
150
Mar
May
Jul
Sep

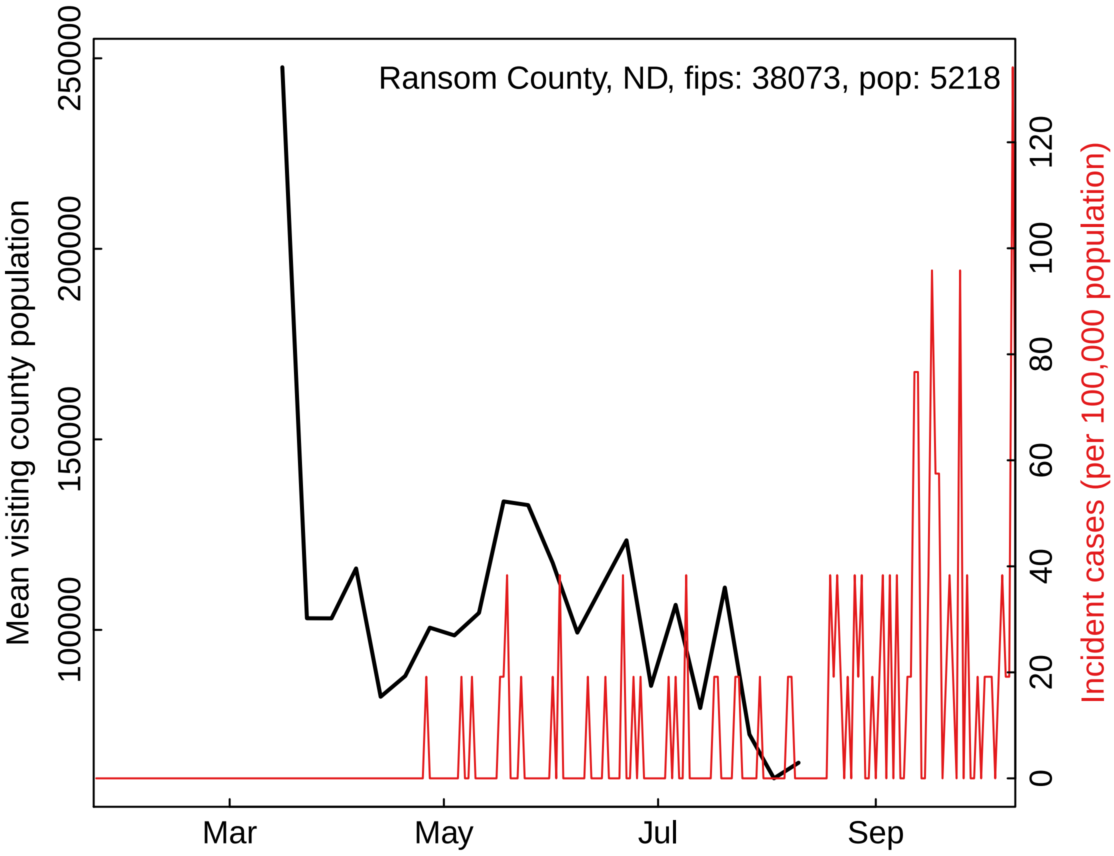
Ransom County, ND, fips: 38073, pop: 5218
Mean visiting county population
100000
150000
200000
250000
Incident cases (per 100,000 population)
0
20
40
60
80
100
120
Mar
May
Jul
Sep

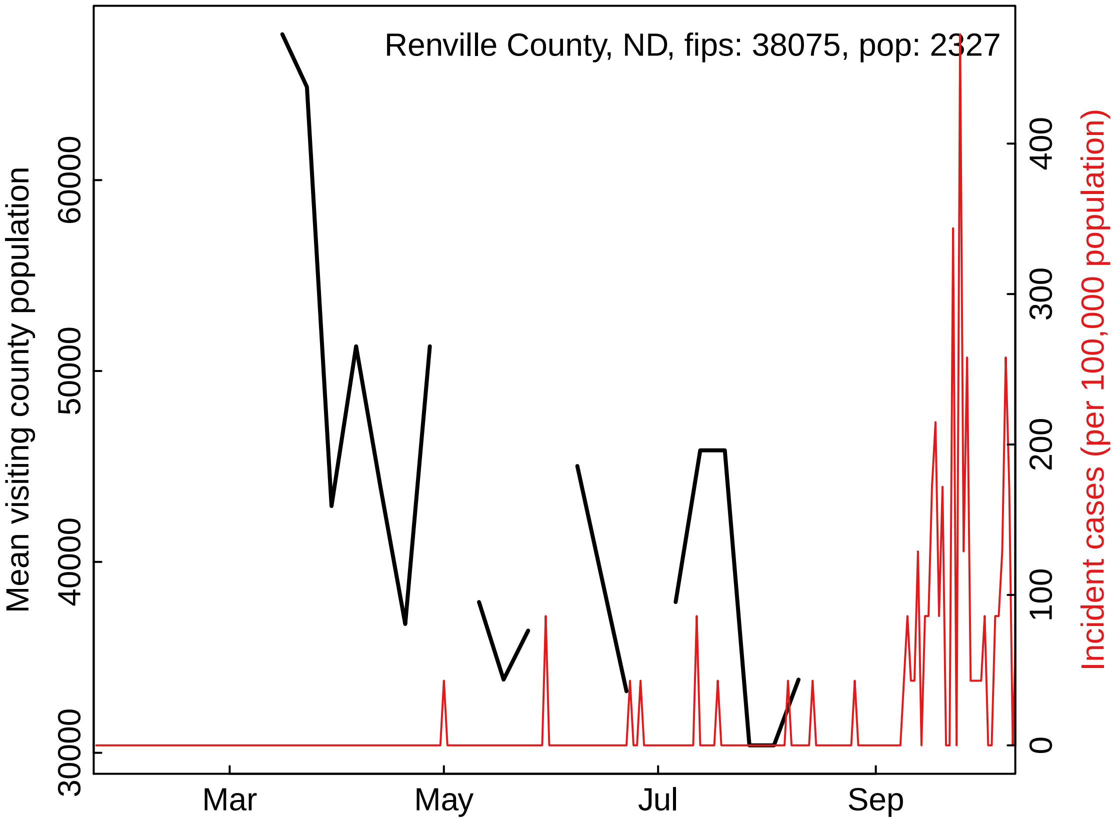

Renville County, ND, fips: 38075, pop: 2327
Mean visiting county population
30000
40000
50000
60000
Incident cases (per 100,000 population)
0
100
200
300
400
Mar
May
Jul
Sep

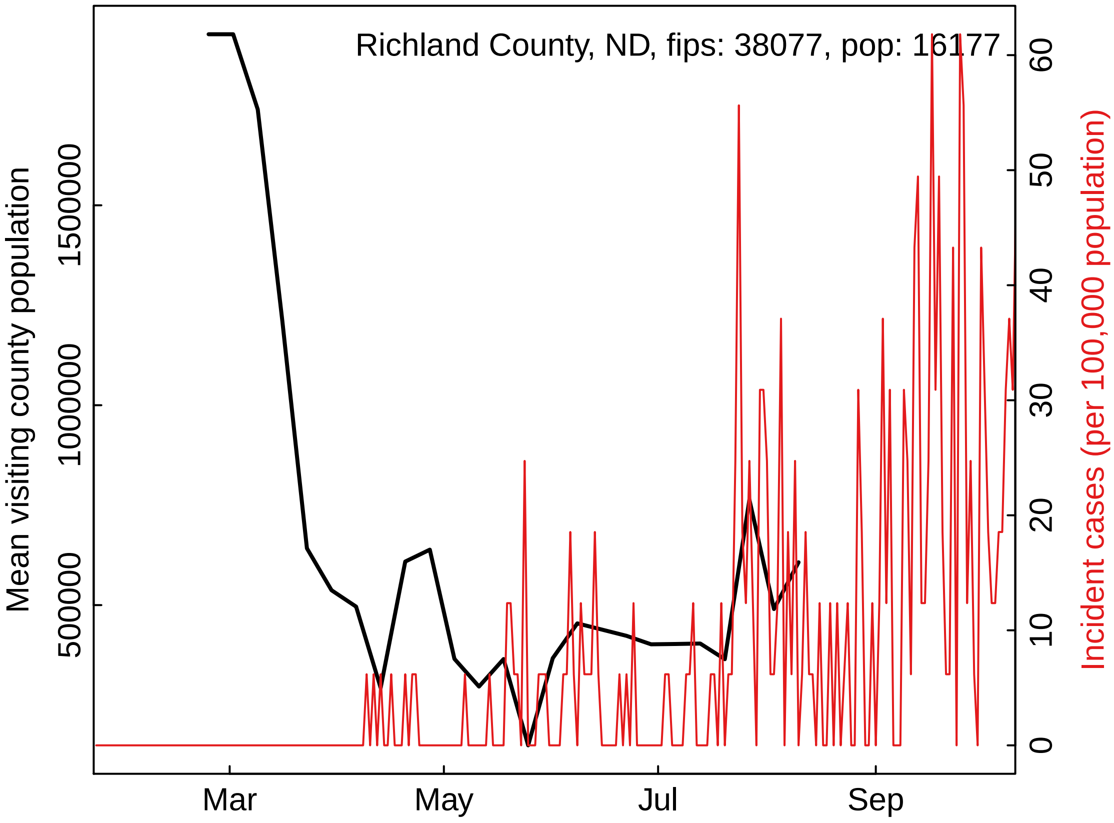

Richland County, ND, fips: 38077, pop: 16177
Mean visiting county population
500000
1000000
1500000
Mar
May
Jul
Sep
0
10
20
30
40
50
60
Incident cases (per 100,000 population)

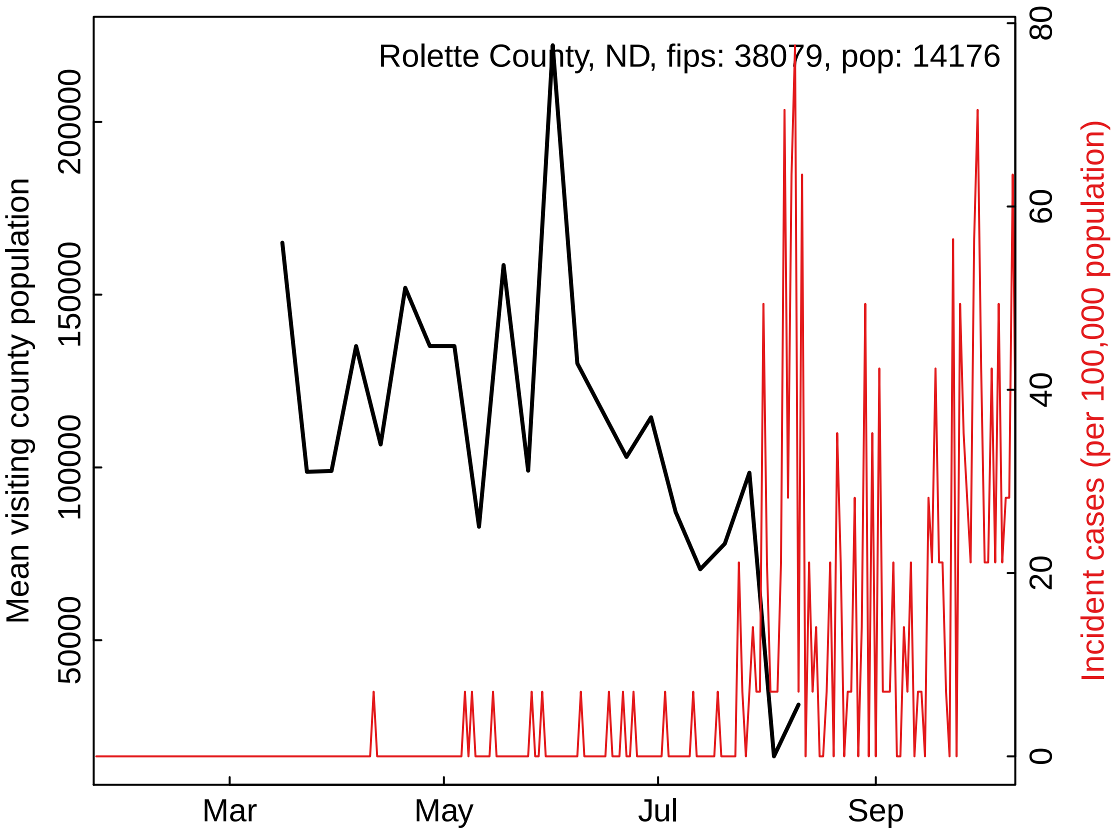
Rolette County, ND, fips: 38079, pop: 14176
Mean visiting county population
200000
150000
100000
50000
Mar
May
Jul
Sep
80
60
40
20
0
Incident cases (per 100,000 population)

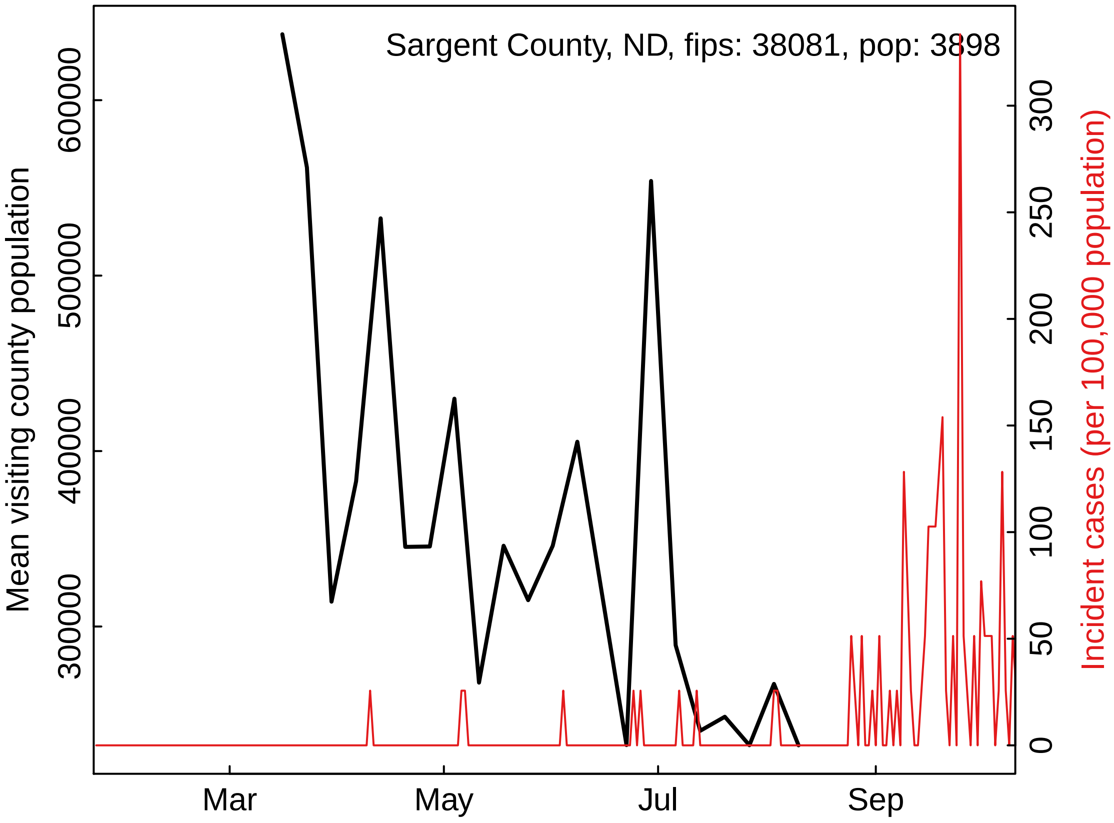
Sargent County, ND, fips: 38081, pop: 3898
Mean visiting county population
300000
400000
500000
600000
Incident cases (per 100,000 population)
0
50
100
150
200
250
300
Mar
May
Jul
Sep

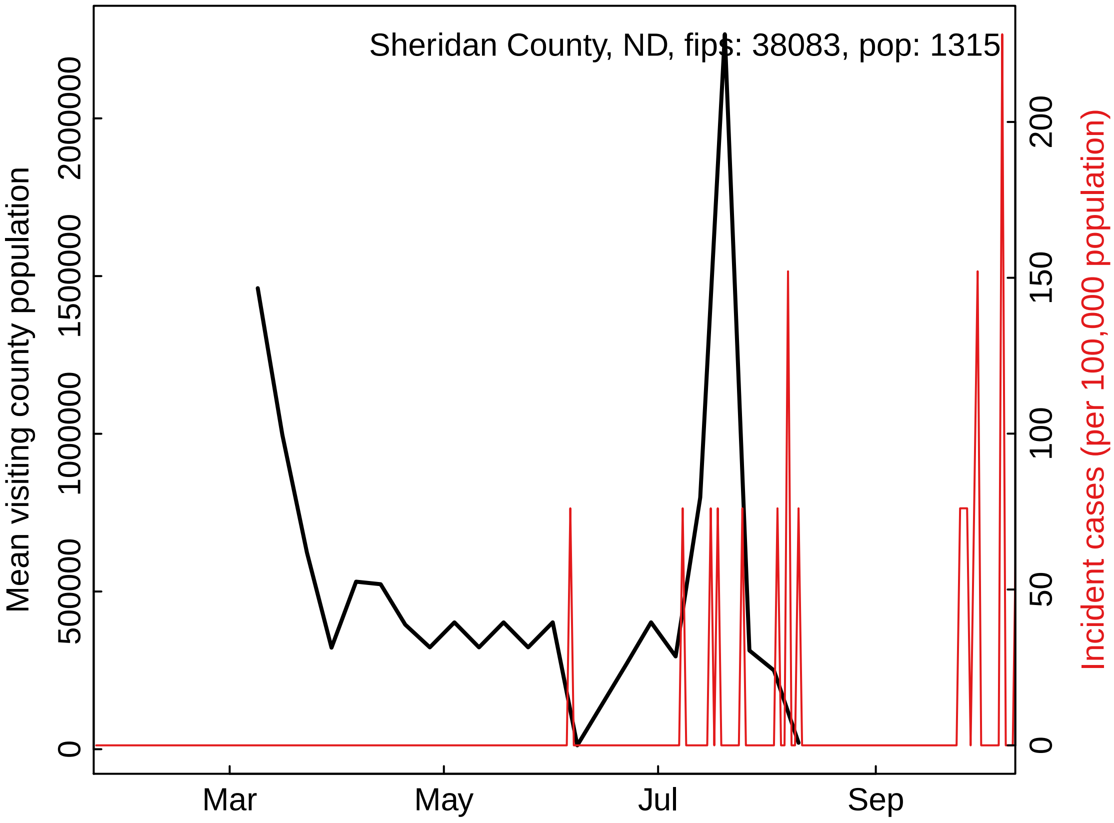

Sheridan County, ND, fips: 38083, pop: 1315
Mean visiting county population
0
500000
1000000
1500000
2000000
Incident cases (per 100,000 population)
0
50
100
150
200
Mar
May
Jul
Sep

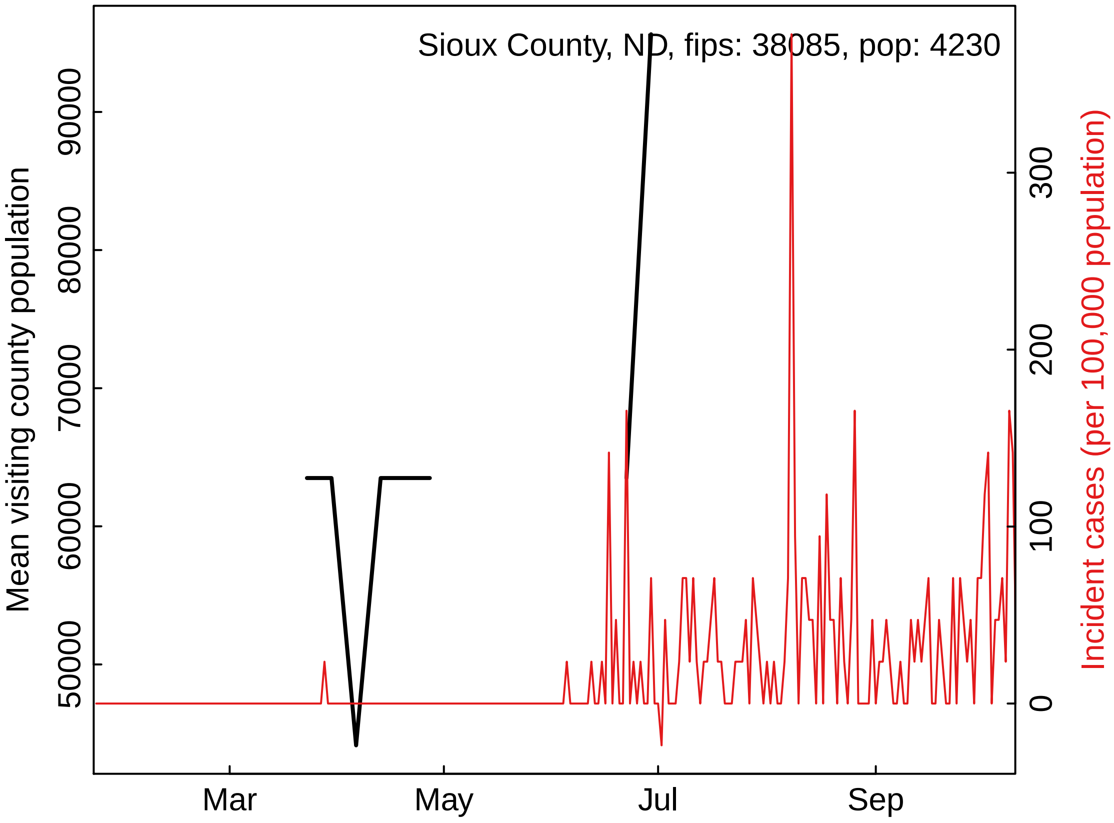
Sioux County, ND, fips: 38085, pop: 4230
Mean visiting county population
50000
60000
70000
80000
90000
Incident cases (per 100,000 population)
0
100
200
300
Mar
May
Jul
Sep

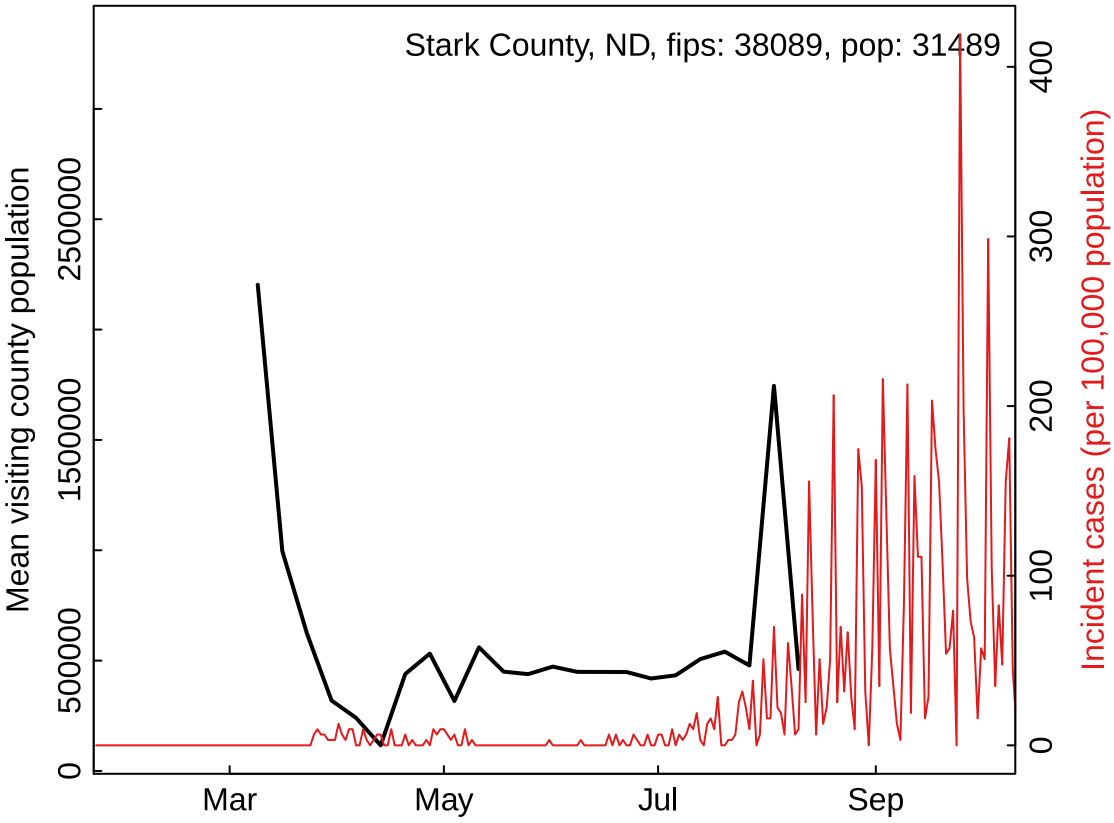

Stark County, ND, fips: 38089, pop: 31489
Mean visiting county population
0
500000
1500000
2500000
Incident cases (per 100,000 population)
0
100
200
300
400
Mar
May
Jul
Sep

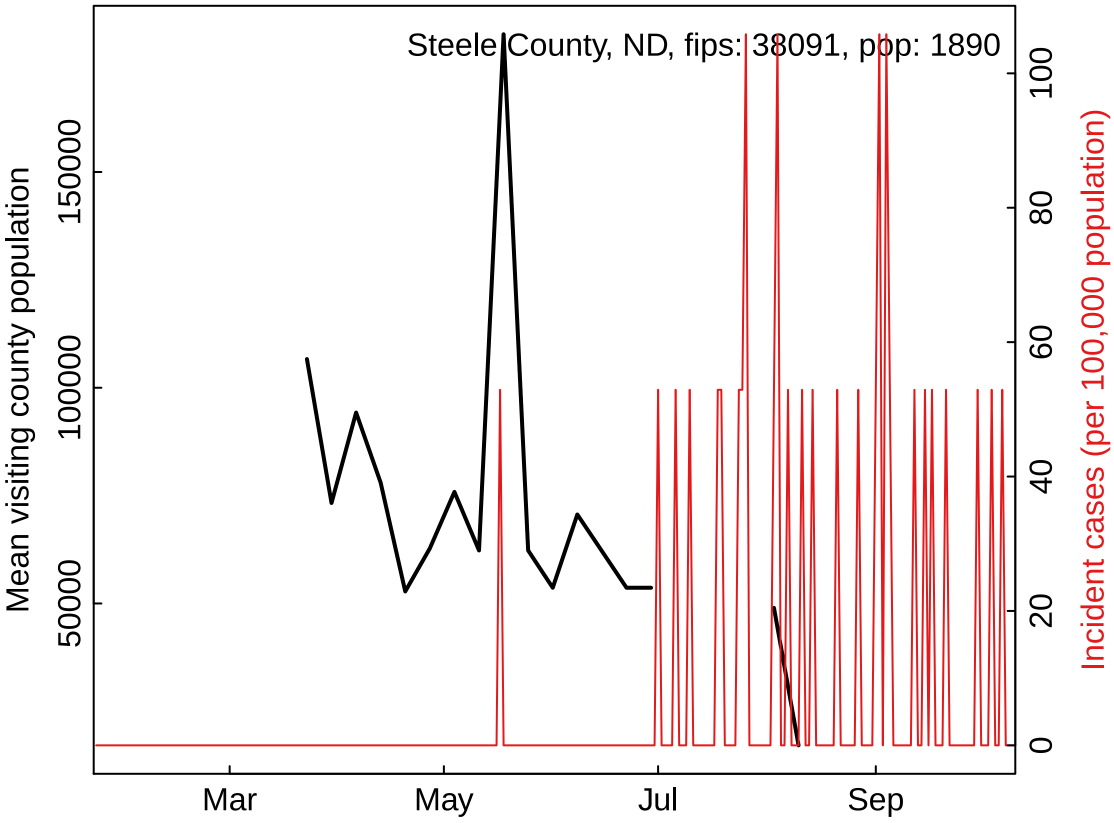

Steele County, ND, fips: 38091, pop: 1890
Mean visiting county population
50000
100000
150000
Incident cases (per 100,000 population)
0
20
40
60
80
100
Mar
May
Jul
Sep

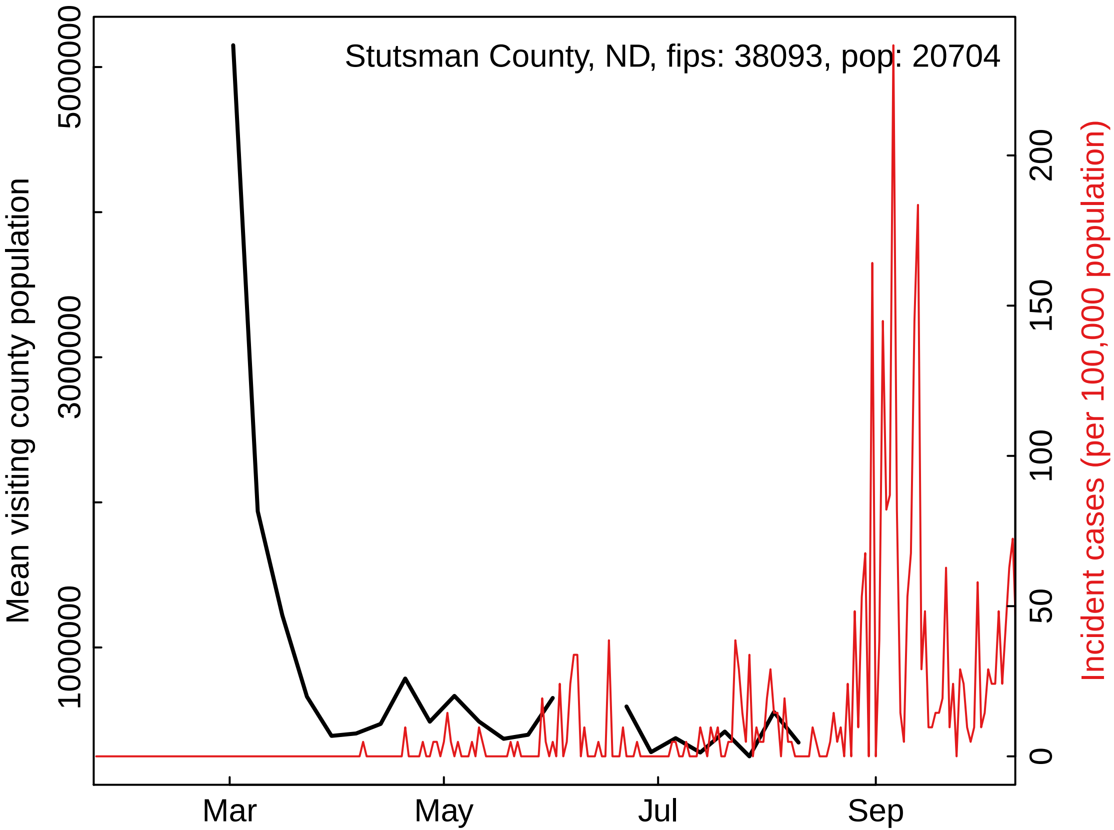

Stutsman County, ND, fips: 38093, pop: 20704
Mean visiting county population
1000000
3000000
5000000
Incident cases (per 100,000 population)
0
50
100
150
200
Mar
May
Jul
Sep

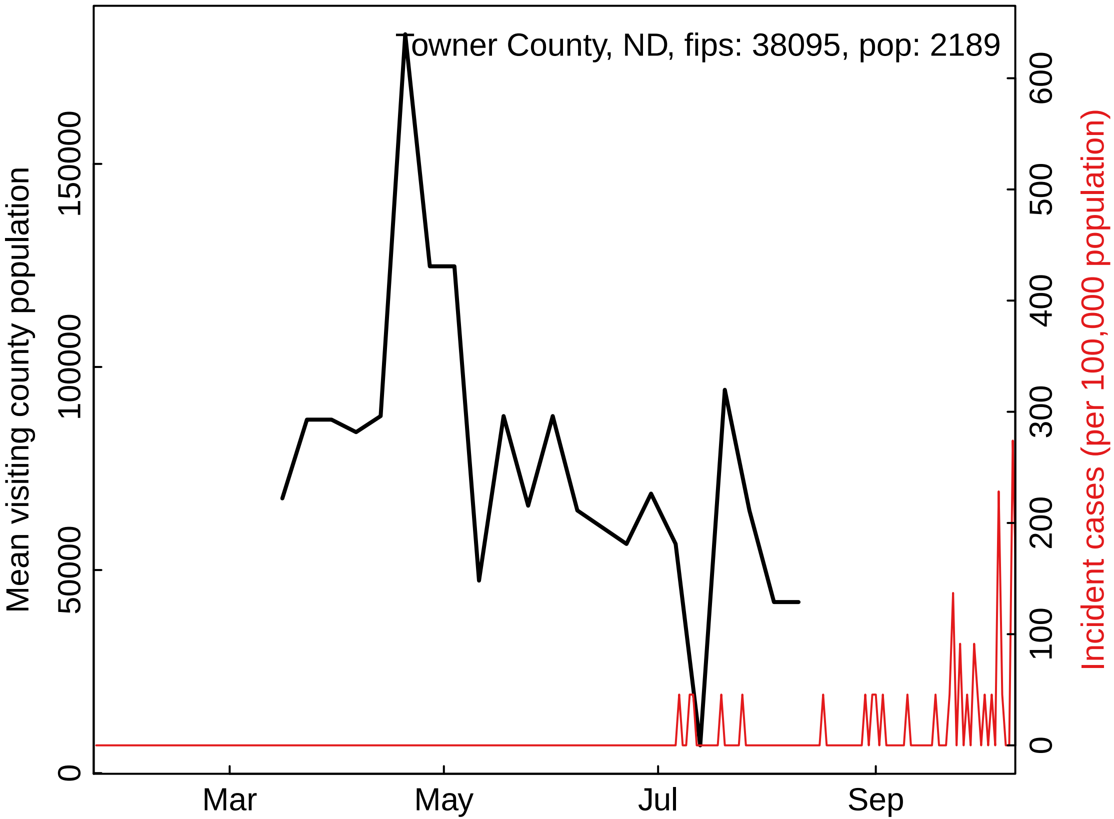

Towner County, ND, fips: 38095, pop: 2189
Mean visiting county population
0
50000
100000
150000
Mar
May
Jul
Sep
Incident cases (per 100,000 population)
0
100
200
300
400
500
600

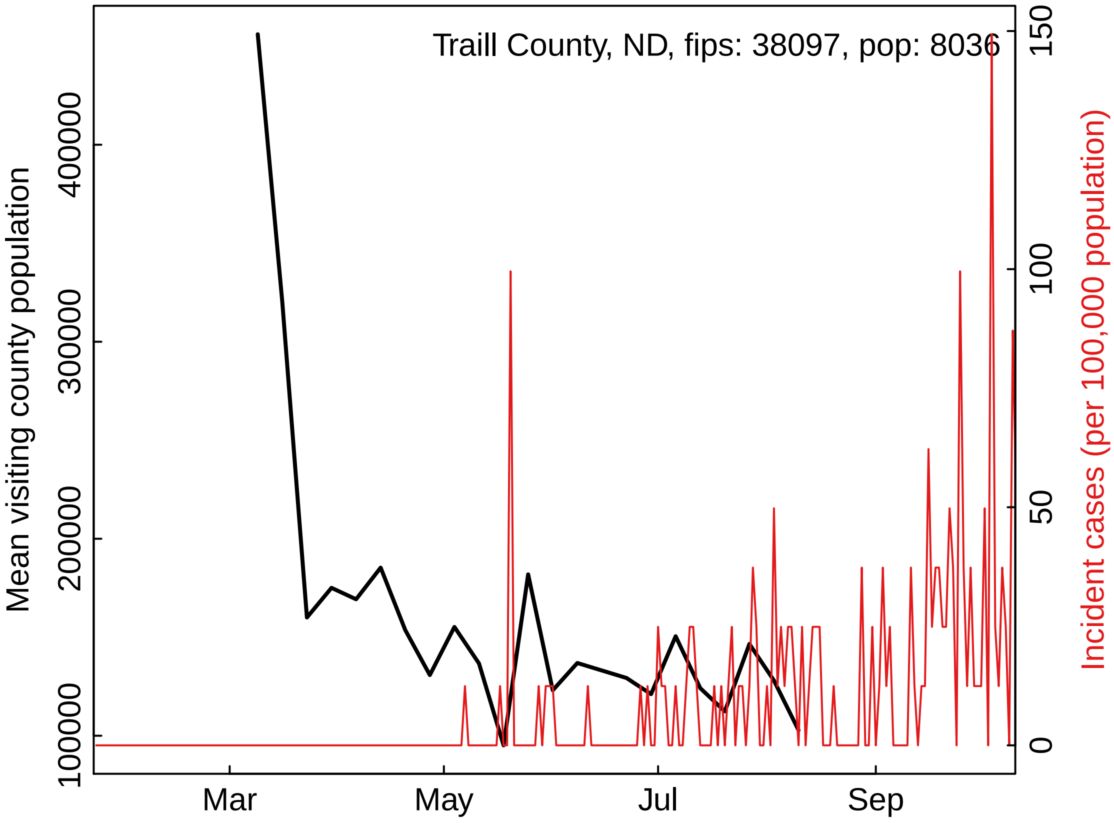

Traill County, ND, fips: 38097, pop: 8036
Mean visiting county population
100000
200000
300000
400000
Incident cases (per 100,000 population)
0
50
100
150
Mar
May
Jul
Sep

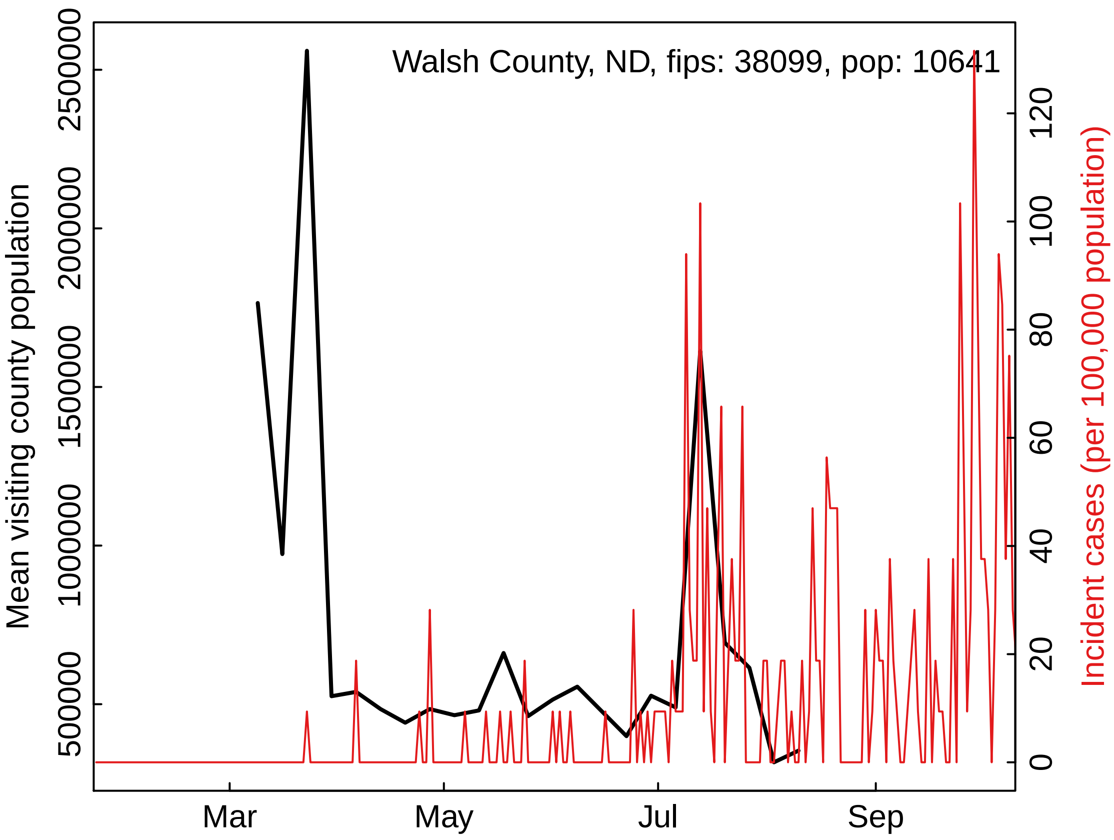

Walsh County, ND, fips: 38099, pop: 10641
Mean visiting county population
500000
1000000
1500000
2000000
2500000
Incident cases (per 100,000 population)
0
20
40
60
80
100
120
Mar
May
Jul
Sep

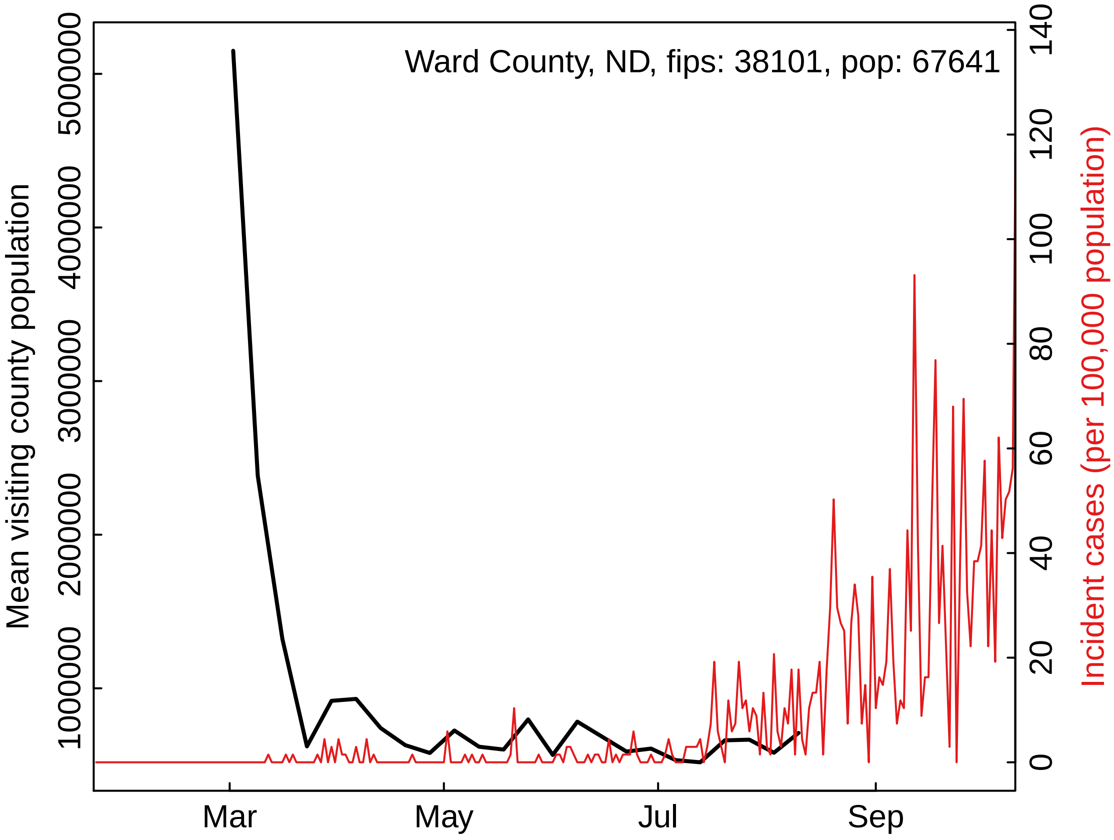

Ward County, ND, fips: 38101, pop: 67641
Mean visiting county population
1000000
2000000
3000000
4000000
5000000
Incident cases (per 100,000 population)
0
20
40
60
80
100
120
140
Mar
May
Jul
Sep

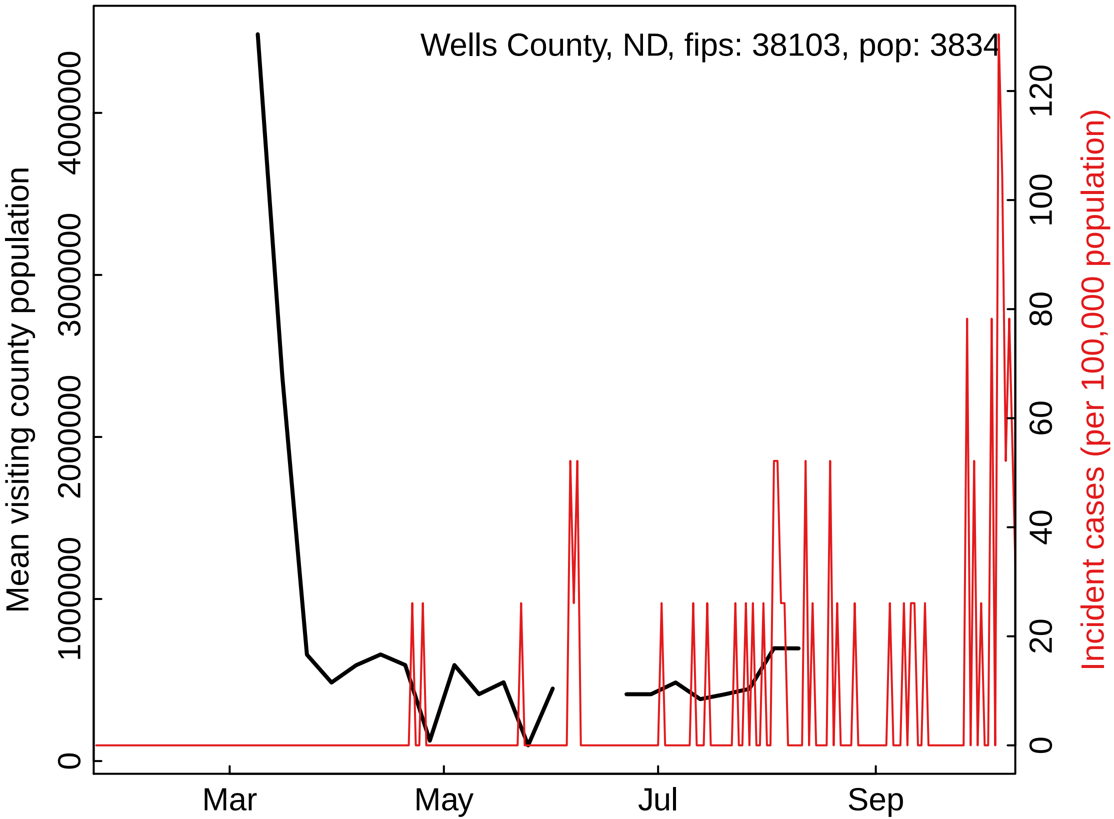

Wells County, ND, fips: 38103, pop: 3834
Mean visiting county population
0
1000000
2000000
3000000
4000000
Mar
May
Jul
Sep
0
20
40
60
80
100
120
Incident cases (per 100,000 population)

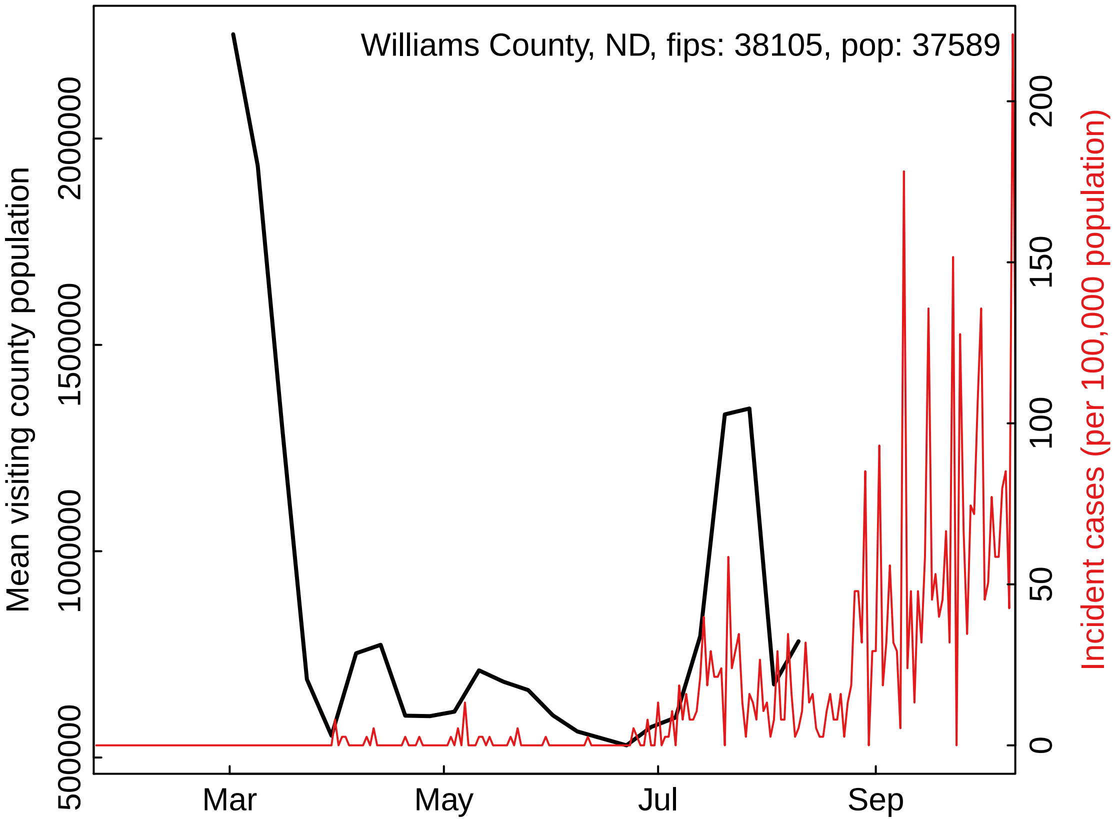

Williams County, ND, fips: 38105, pop: 37589
Mean visiting county population
5000000
10000000
15000000
20000000
Mar
May
Jul
Sep
0
50
100
150
200
Incident cases (per 100,000 population)

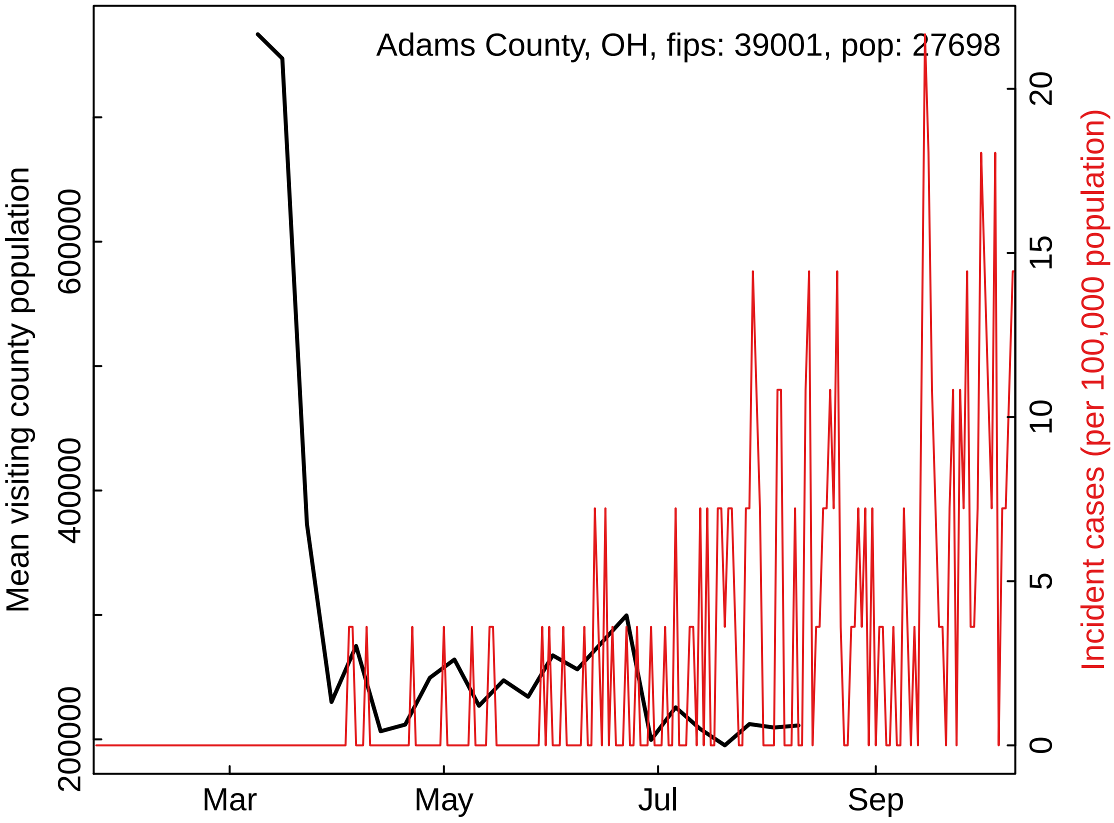

Adams County, OH, fips: 39001, pop: 27698
Mean visiting county population
200000
400000
600000
Incident cases (per 100,000 population)
0
5
10
15
20
Mar
May
Jul
Sep

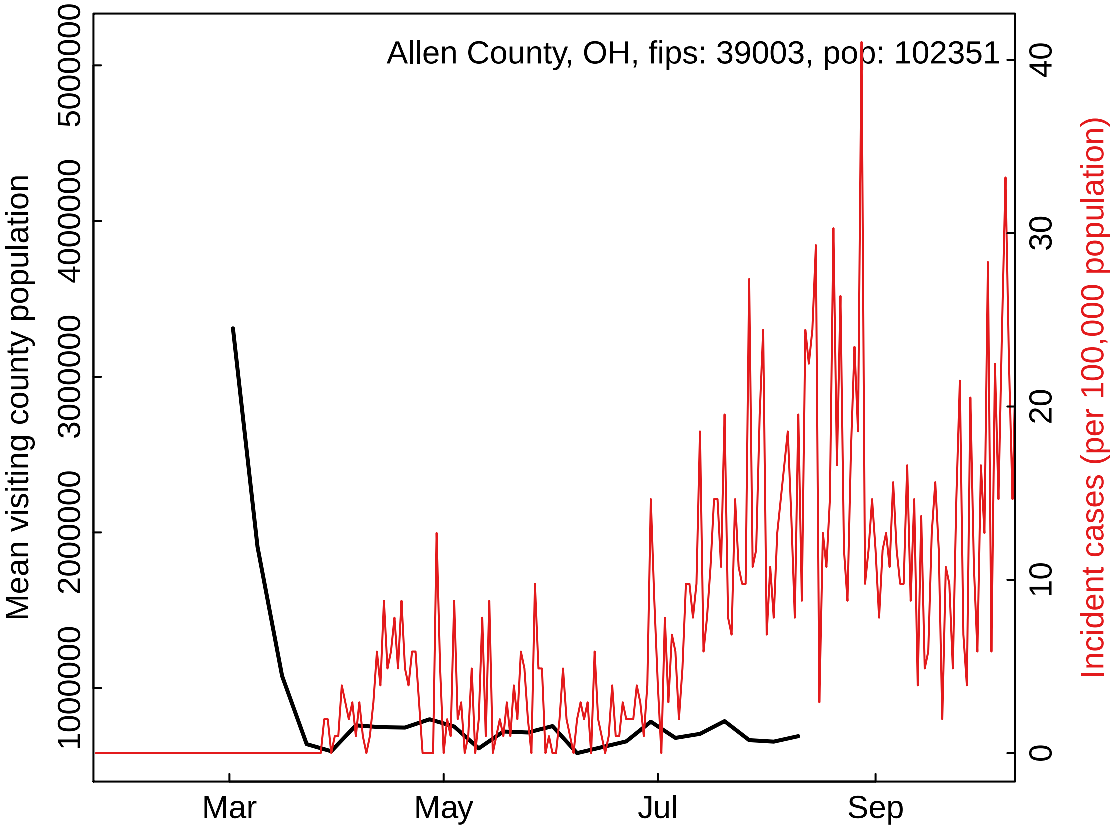

Allen County, OH, fips: 39003, pop: 102351
Mean visiting county population
10000000
20000000
30000000
40000000
50000000
Incident cases (per 100,000 population)
0
10
20
30
40
Mar
May
Jul
Sep

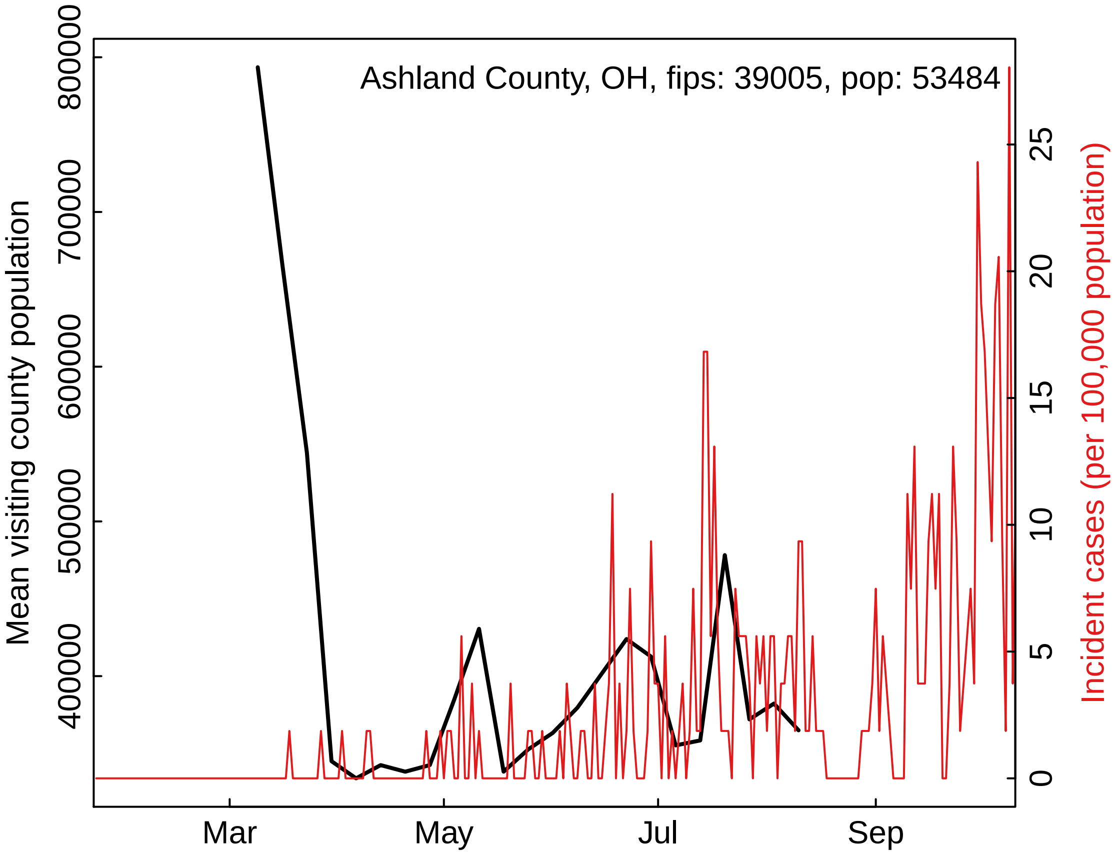

Ashland County, OH, fips: 39005, pop: 53484
Mean visiting county population
400000
500000
600000
700000
800000
Incident cases (per 100,000 population)
0
5
10
15
20
25
Mar
May
Jul
Sep

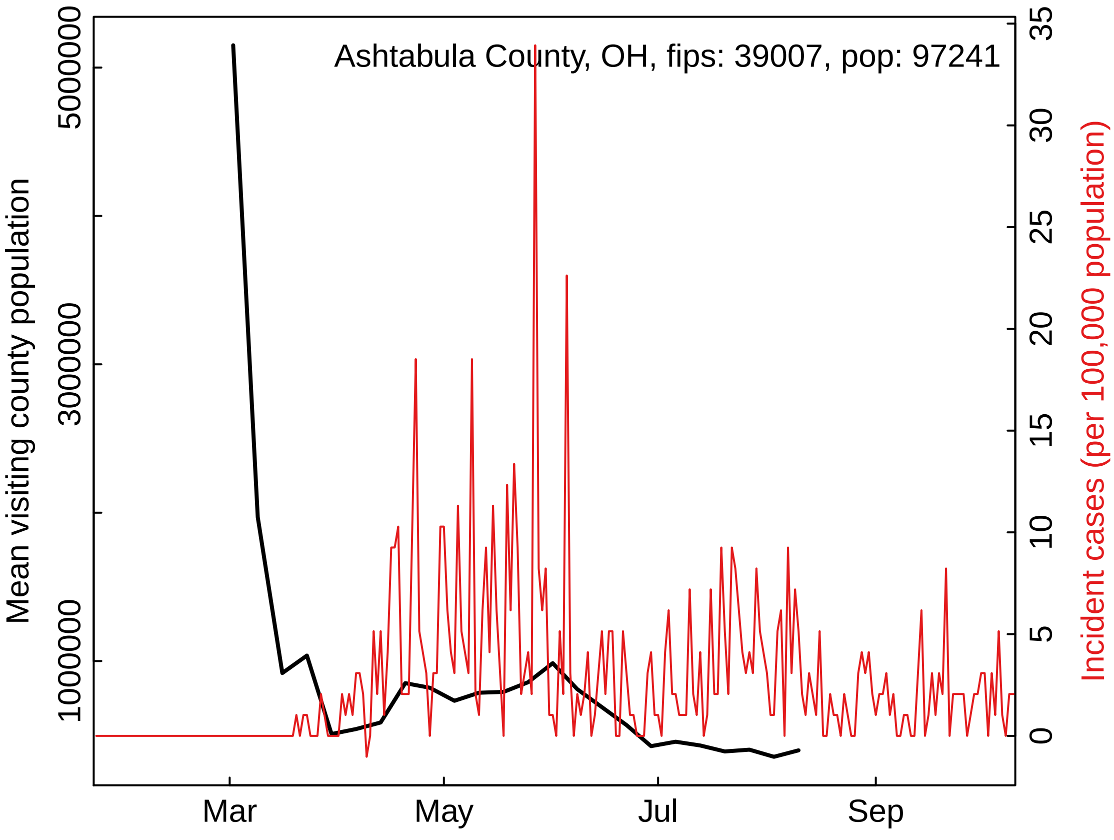

Ashtabula County, OH, fips: 39007, pop: 97241
Mean visiting county population
1000000
3000000
5000000
Incident cases (per 100,000 population)
0
5
10
15
20
25
30
35
Mar
May
Jul
Sep

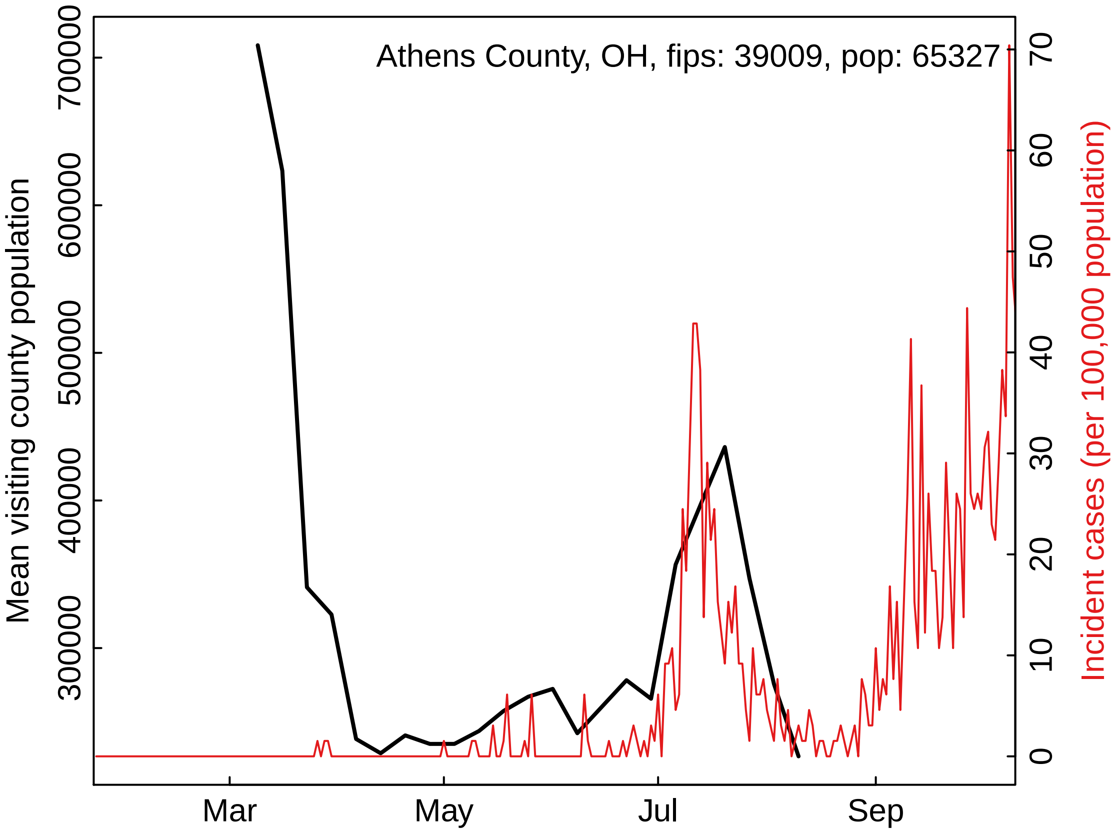

Athens County, OH, fips: 39009, pop: 65327
Mean visiting county population
300000
400000
500000
600000
700000
Incident cases (per 100,000 population)
0
10
20
30
40
50
60
70
Mar
May
Jul
Sep

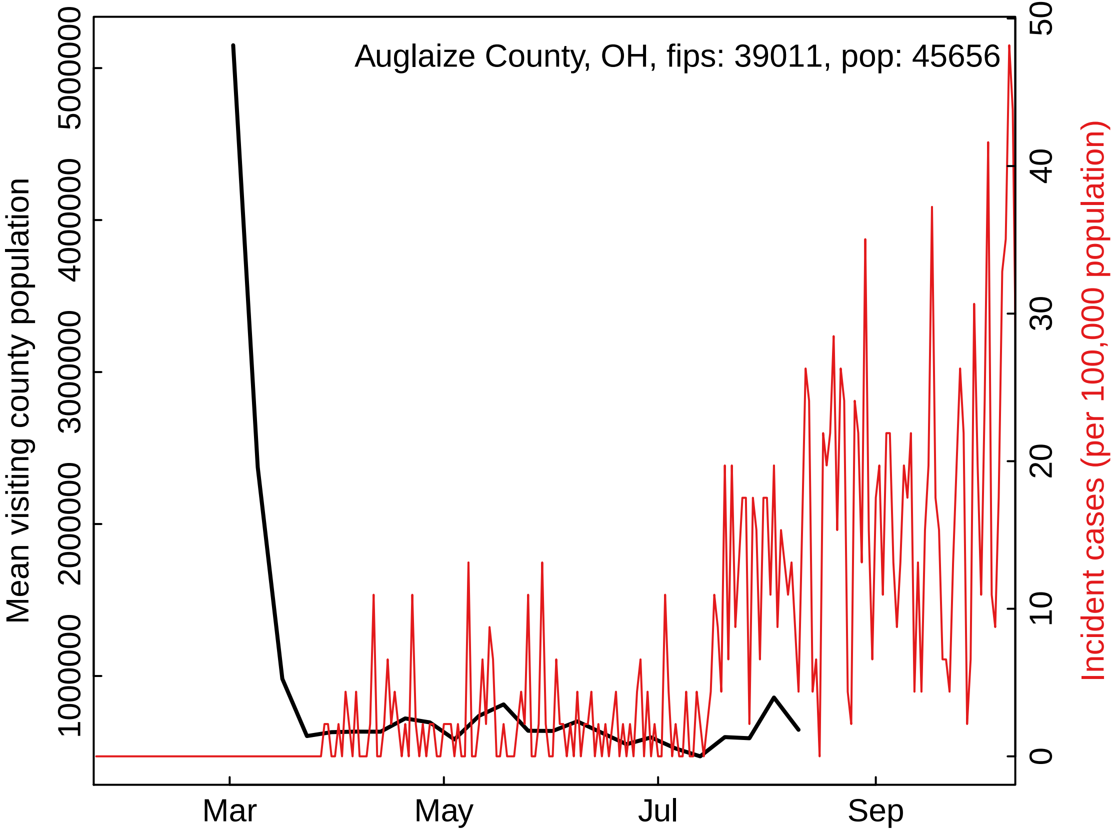

Auglaize County, OH, fips: 39011, pop: 45656
Mean visiting county population
Incident cases (per 100,000 population)
1000000
2000000
3000000
4000000
5000000
0
10
20
30
40
50
Mar
May
Jul
Sep

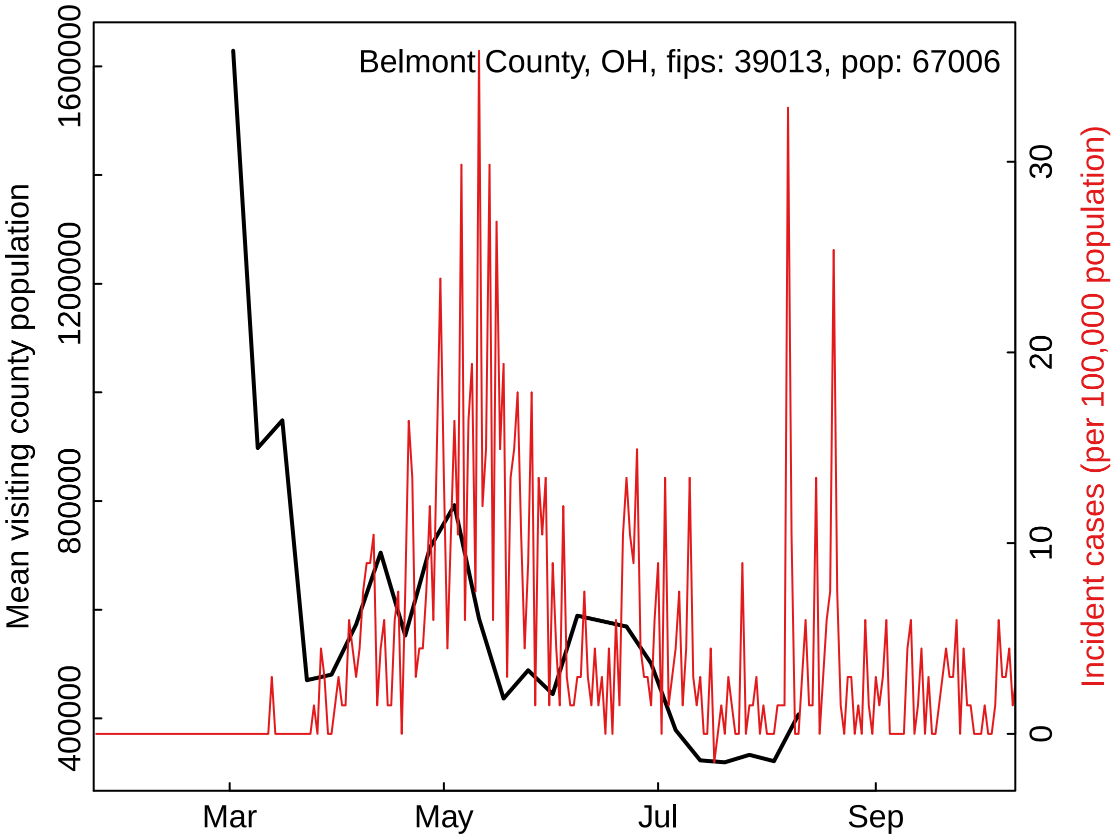
Belmont County, OH, fips: 39013, pop: 67006
Mean visiting county population
400000
800000
1200000
1600000
Incident cases (per 100,000 population)
0
10
20
30
Mar
May
Jul
Sep

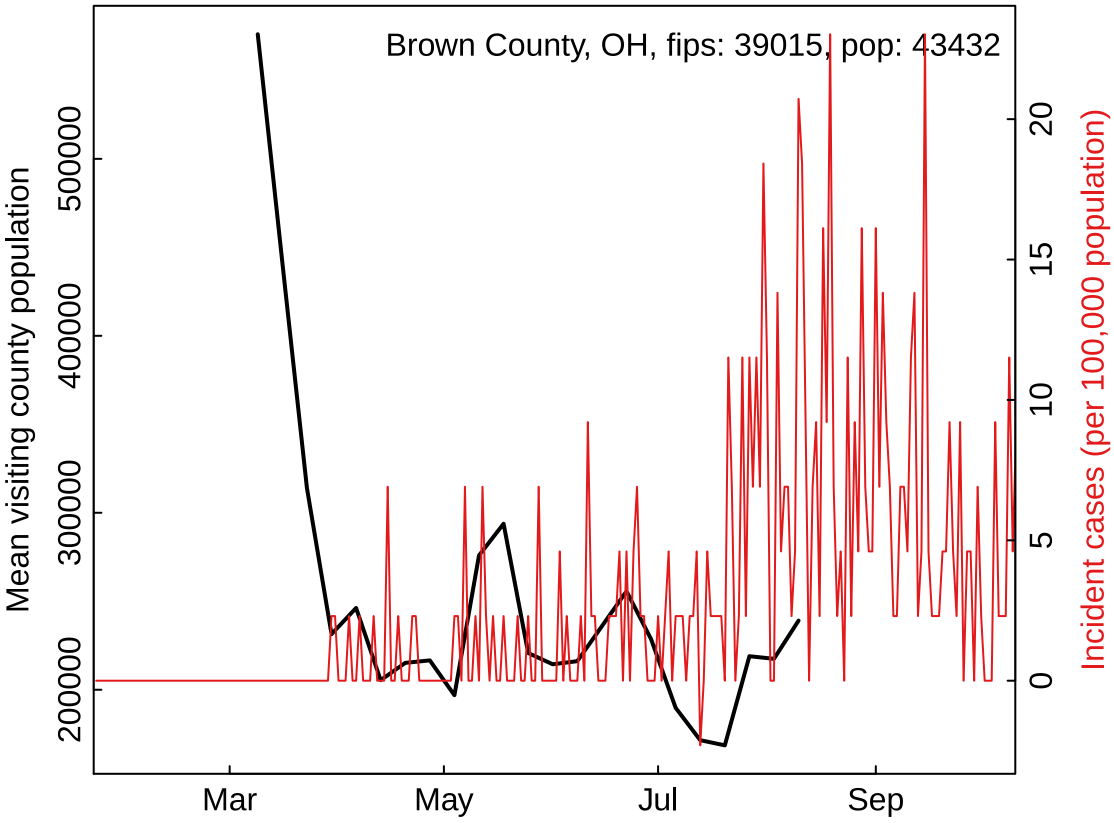
Brown County, OH, fips: 39015, pop: 43432
Mean visiting county population
200000
300000
400000
500000
Incident cases (per 100,000 population)
0
5
10
15
20
Mar
May
Jul
Sep

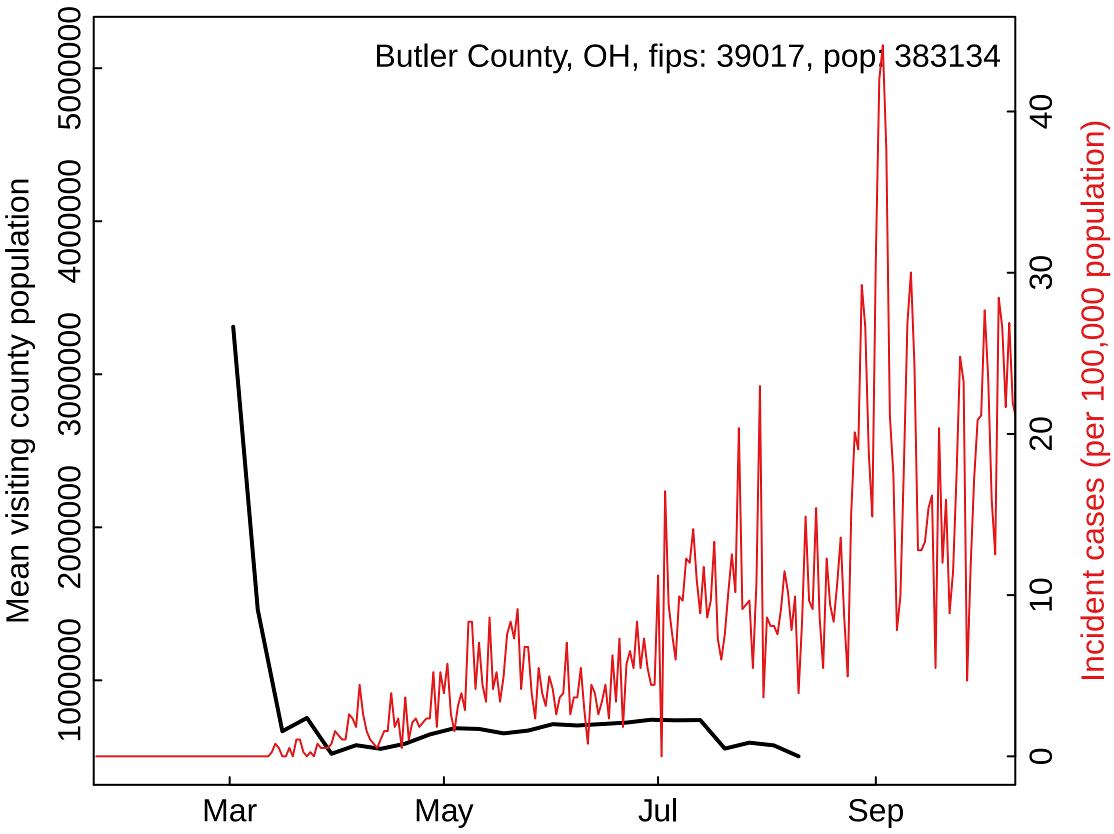
Butler County, OH, fips: 39017, pop: 383134
Mean visiting county population
Incident cases (per 100,000 population)
1000000
2000000
3000000
4000000
5000000
0
10
20
30
40
Mar
May
Jul
Sep

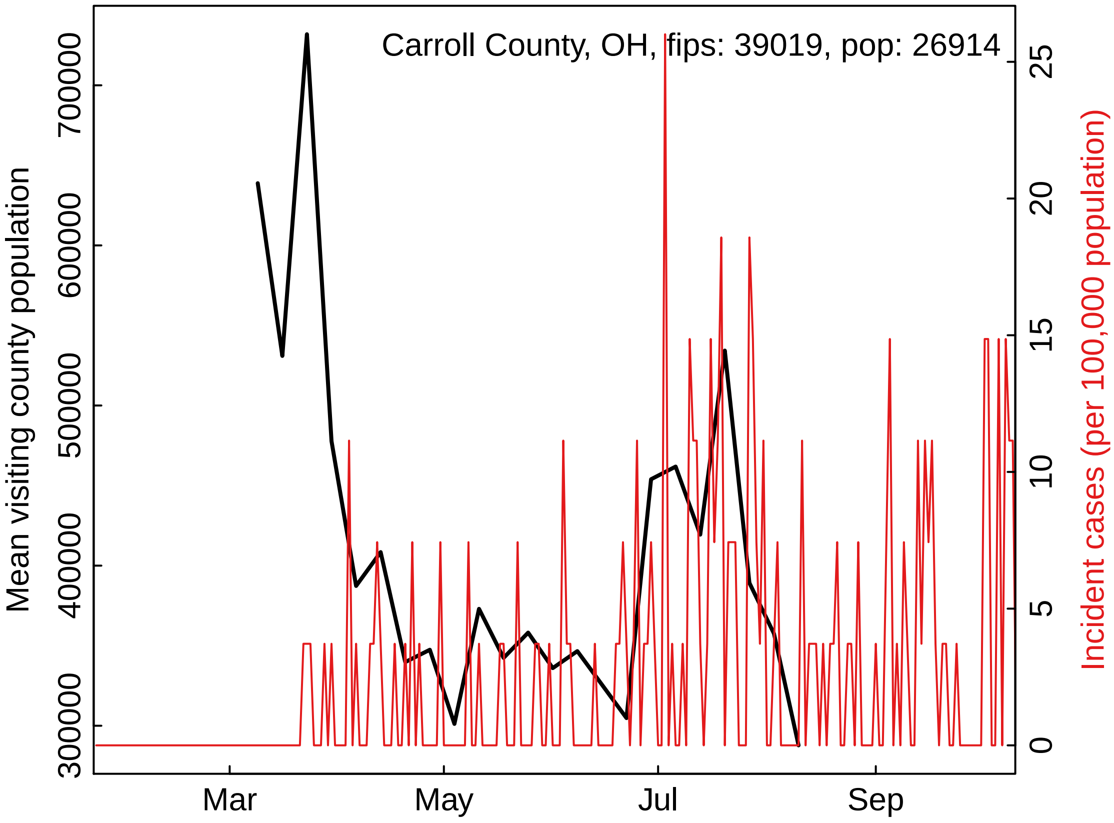

Carroll County, OH, fips: 39019, pop: 26914
Mean visiting county population
300000
400000
500000
600000
700000
Incident cases (per 100,000 population)
0
5
10
15
20
25
Mar
May
Jul
Sep

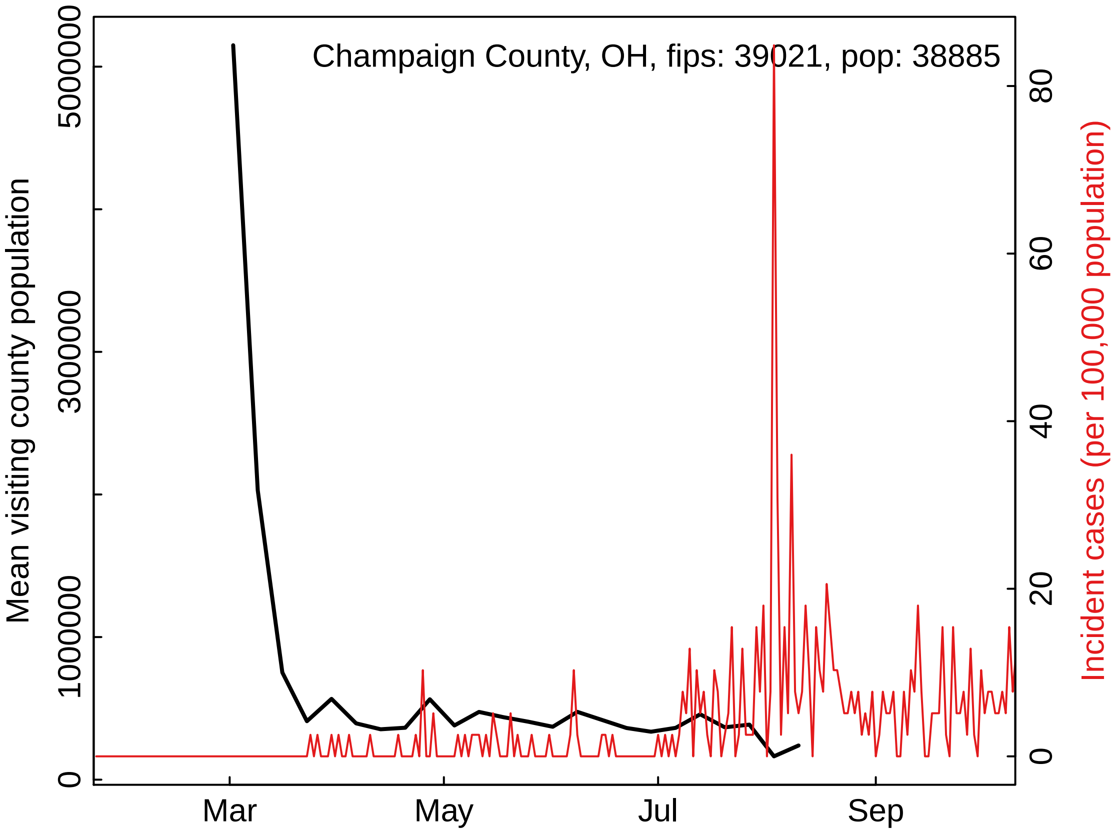
Champaign County, OH, fips: 39021, pop: 38885
Mean visiting county population
0
1000000
3000000
5000000
Incident cases (per 100,000 population)
0
20
40
60
80
Mar
May
Jul
Sep

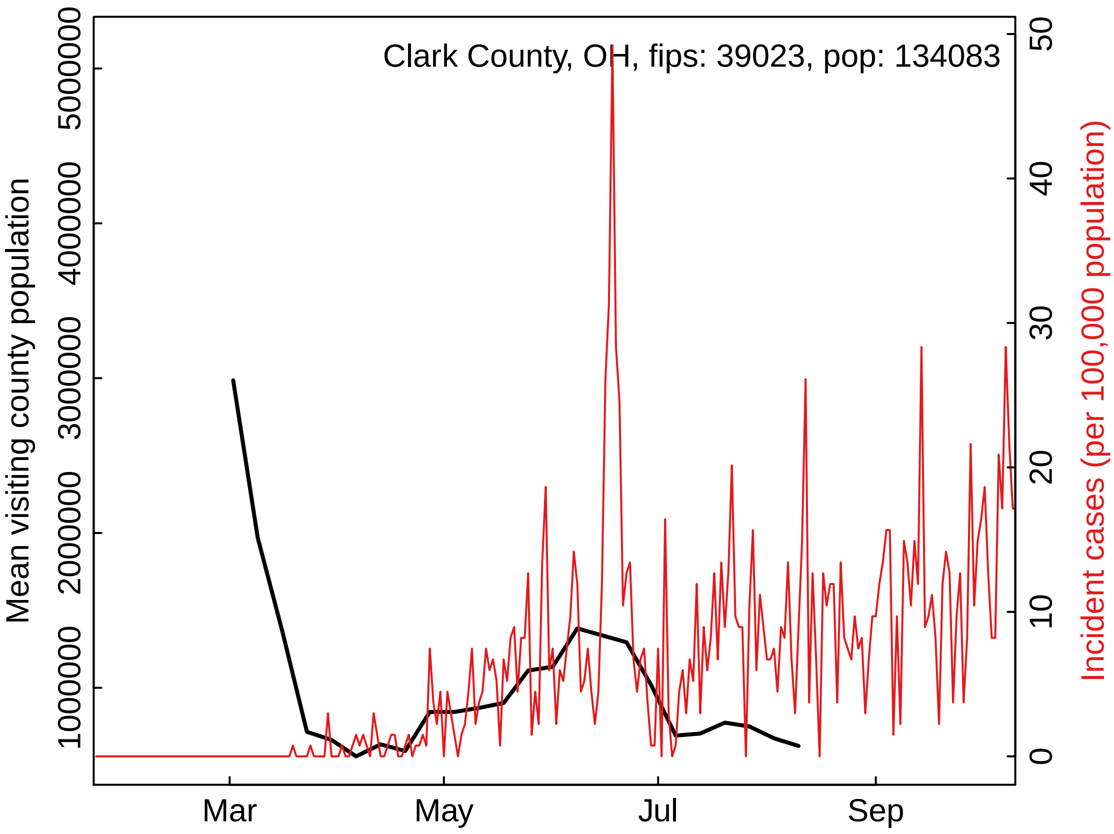
Clark County, OH, fips: 39023, pop: 134083
Mean visiting county population
Incident cases (per 100,000 population)
10000000
20000000
30000000
40000000
50000000
0
10
20
30
40
50
Mar
May
Jul
Sep

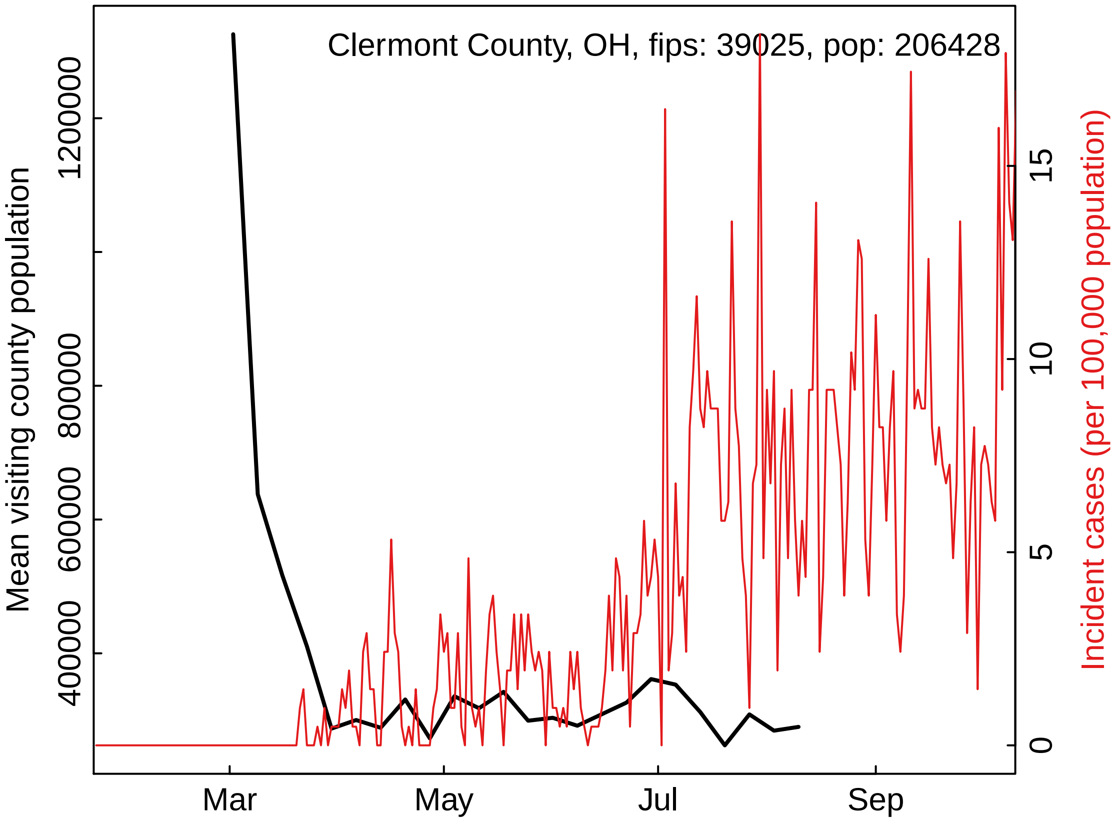
Clermont County, OH, fips: 39025, pop: 206428
Mean visiting county population
400000
600000
800000
1200000
Incident cases (per 100,000 population)
0
5
10
15
Mar
May
Jul
Sep

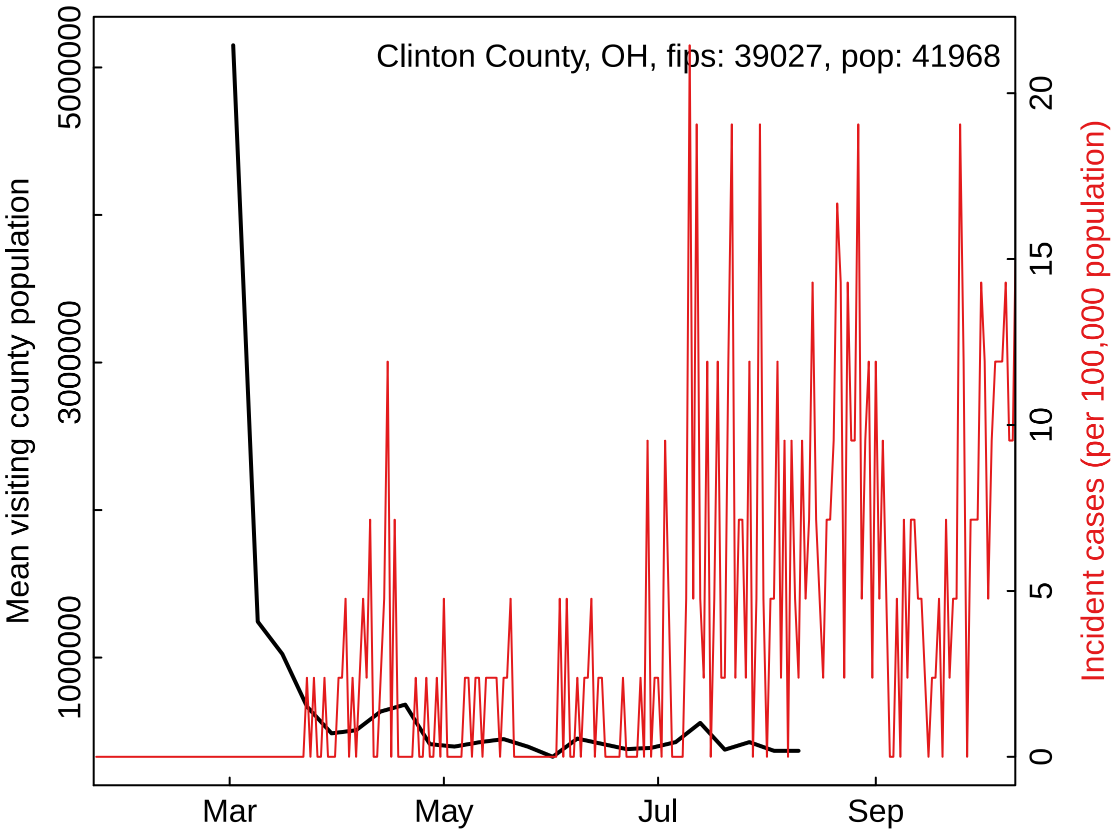
Clinton County, OH, fips: 39027, pop: 41968
Mean visiting county population
10000000
30000000
50000000
Incident cases (per 100,000 population)
0
5
10
15
20
Mar
May
Jul
Sep

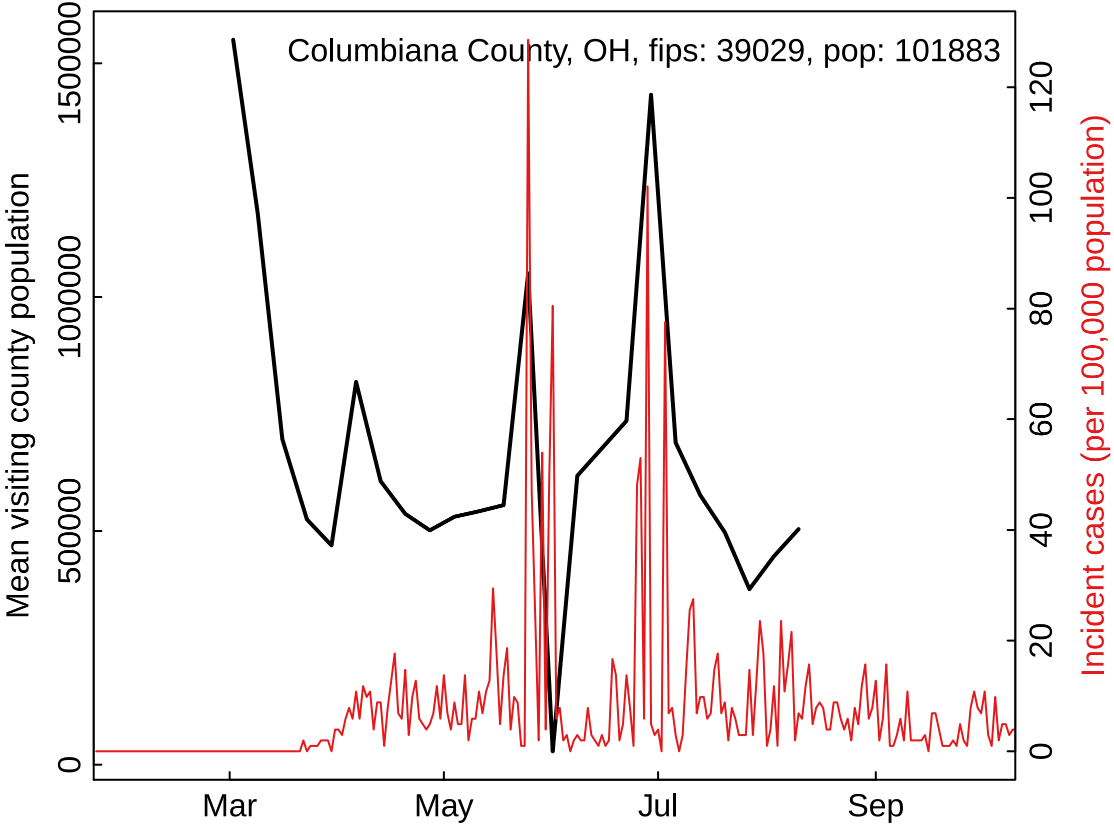
Columbiana County, OH, fips: 39029, pop: 101883
Mean visiting county population
Incident cases (per 100,000 population)
0
500000
1000000
1500000
0
20
40
60
80
100
120
Mar
May
Jul
Sep

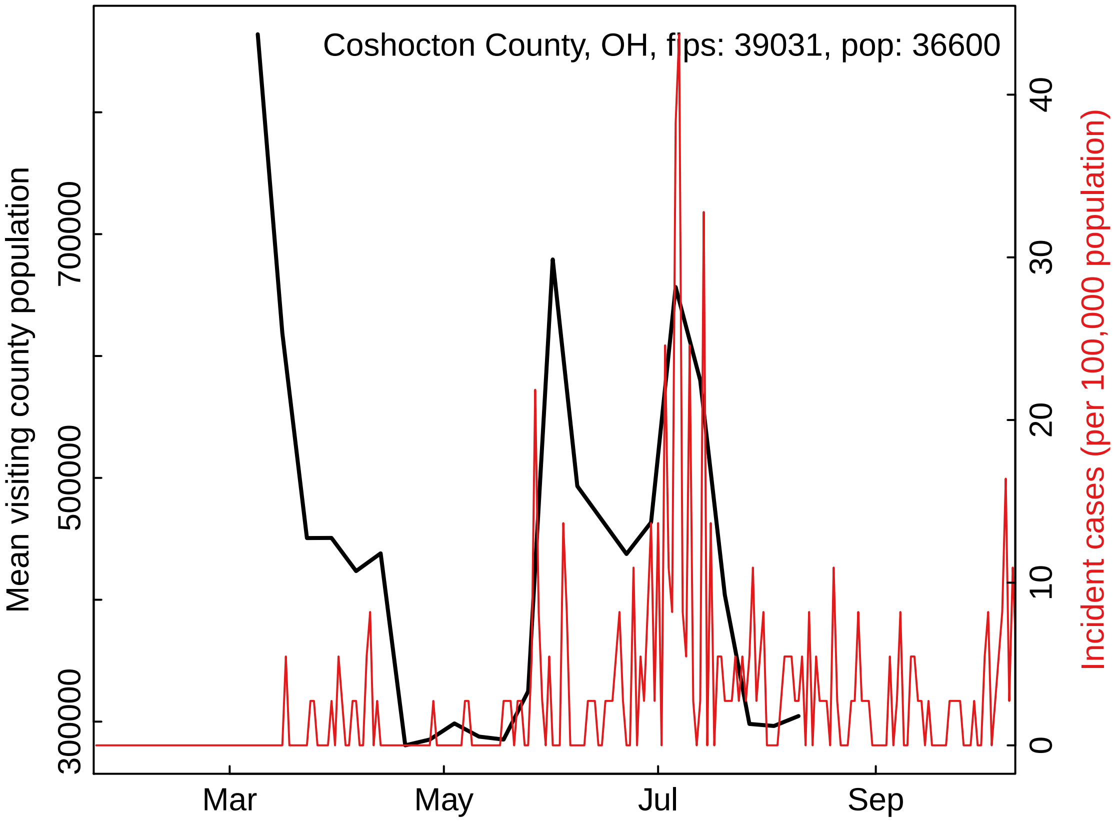

Coshocton County, OH, fips: 39031, pop: 36600
Mean visiting county population
300000
500000
700000
Incident cases (per 100,000 population)
0
10
20
30
40
Mar
May
Jul
Sep

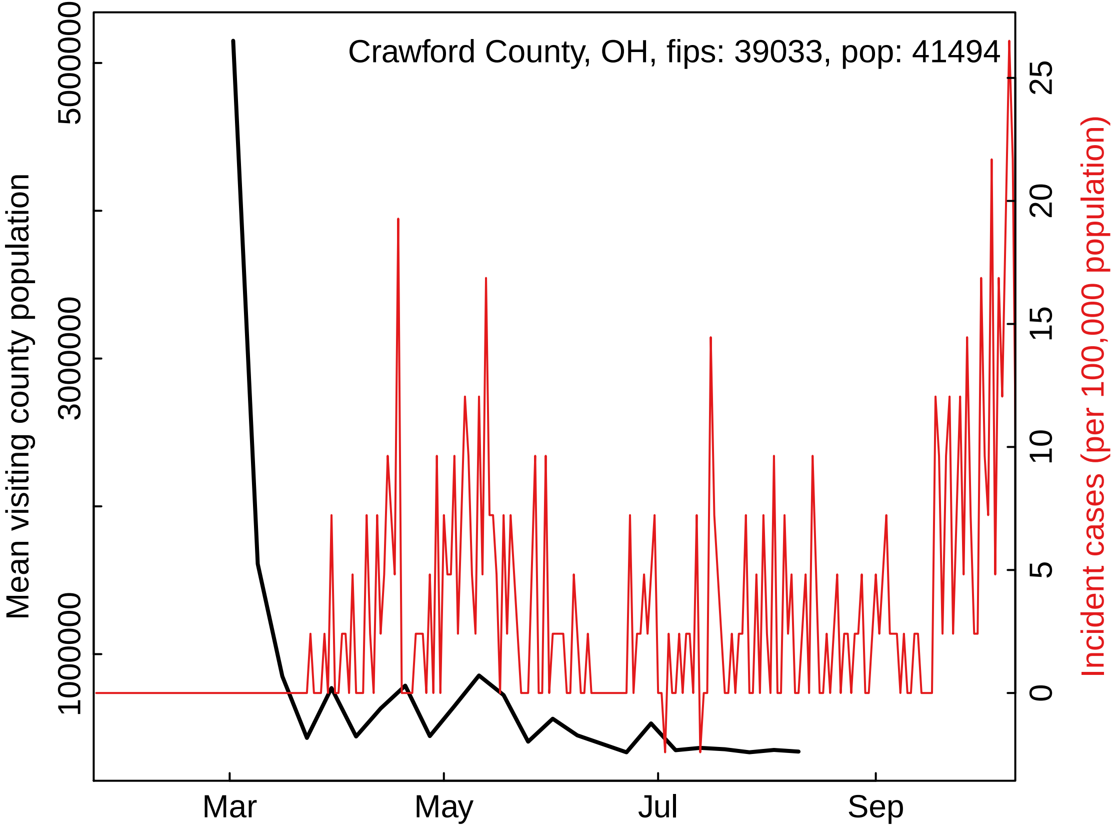

Crawford County, OH, fips: 39033, pop: 41494
Mean visiting county population
1000000
3000000
5000000
Mar
May
Jul
Sep
Incident cases (per 100,000 population)
0
5
10
15
20
25

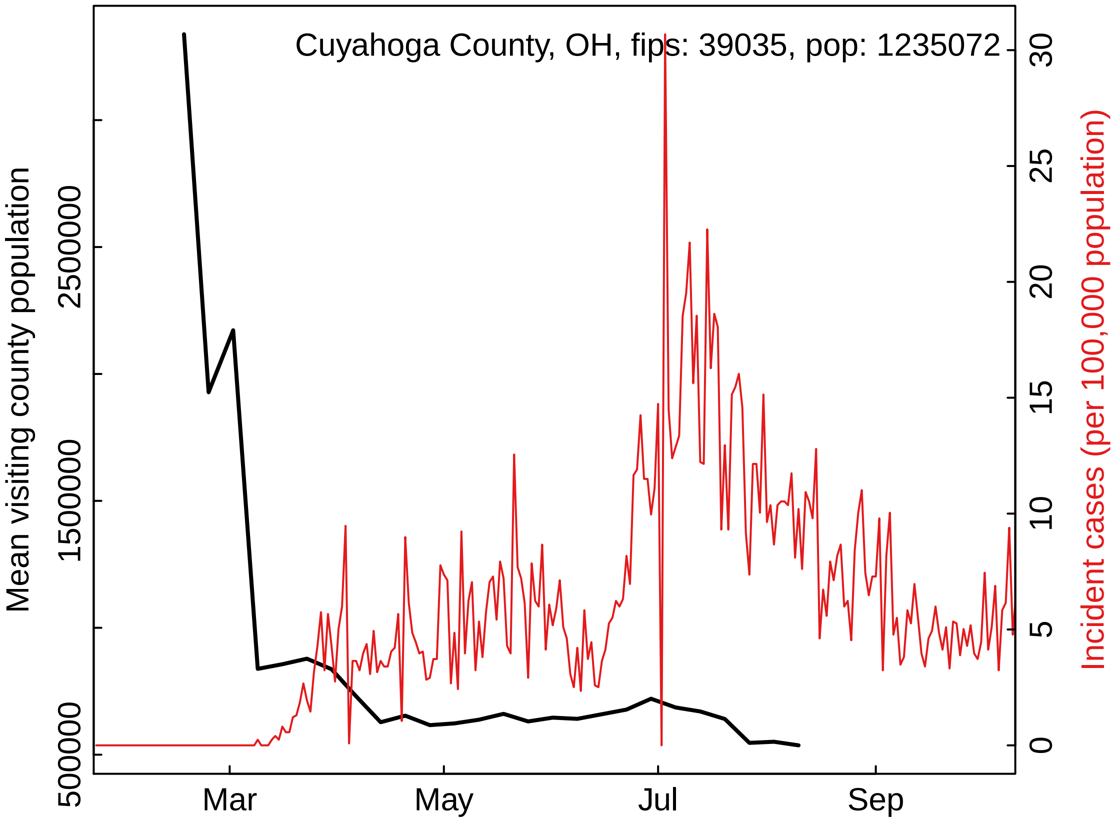

Cuyahoga County, OH, fips: 39035, pop: 1235072
Mean visiting county population
500000
1500000
2500000
Mar
May
Jul
Sep
0
5
10
15
20
25
30
Incident cases (per 100,000 population)

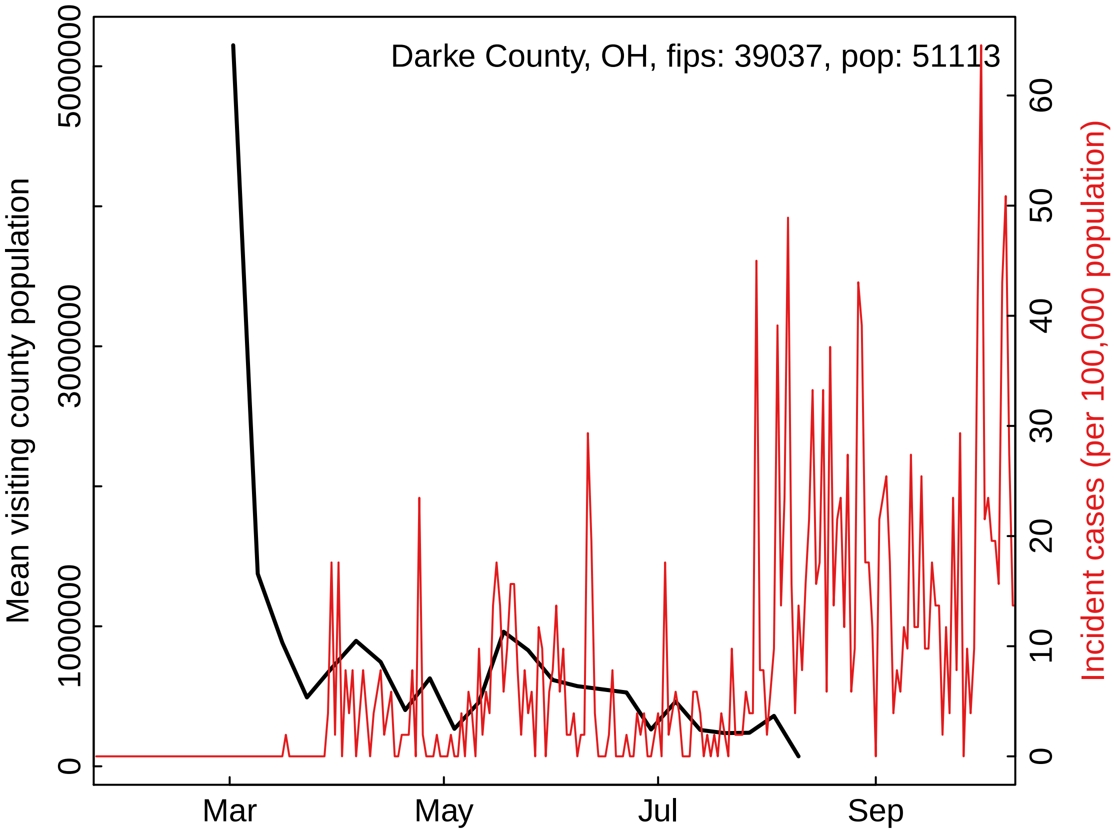

Darke County, OH, fips: 39037, pop: 51113
Mean visiting county population
0
1000000
3000000
5000000
Incident cases (per 100,000 population)
0
10
20
30
40
50
60
Mar
May
Jul
Sep

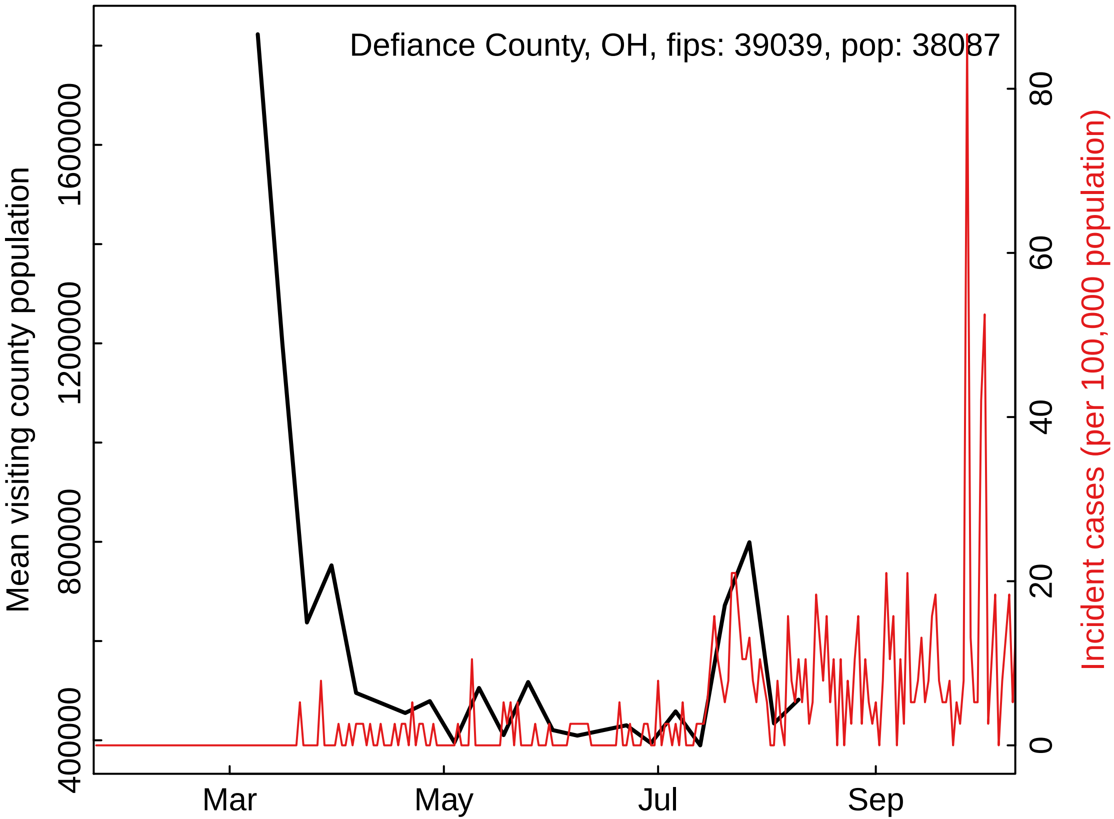

Defiance County, OH, fips: 39039, pop: 38087
Mean visiting county population
400000
800000
1200000
1600000
Incident cases (per 100,000 population)
0
20
40
60
80
Mar
May
Jul
Sep

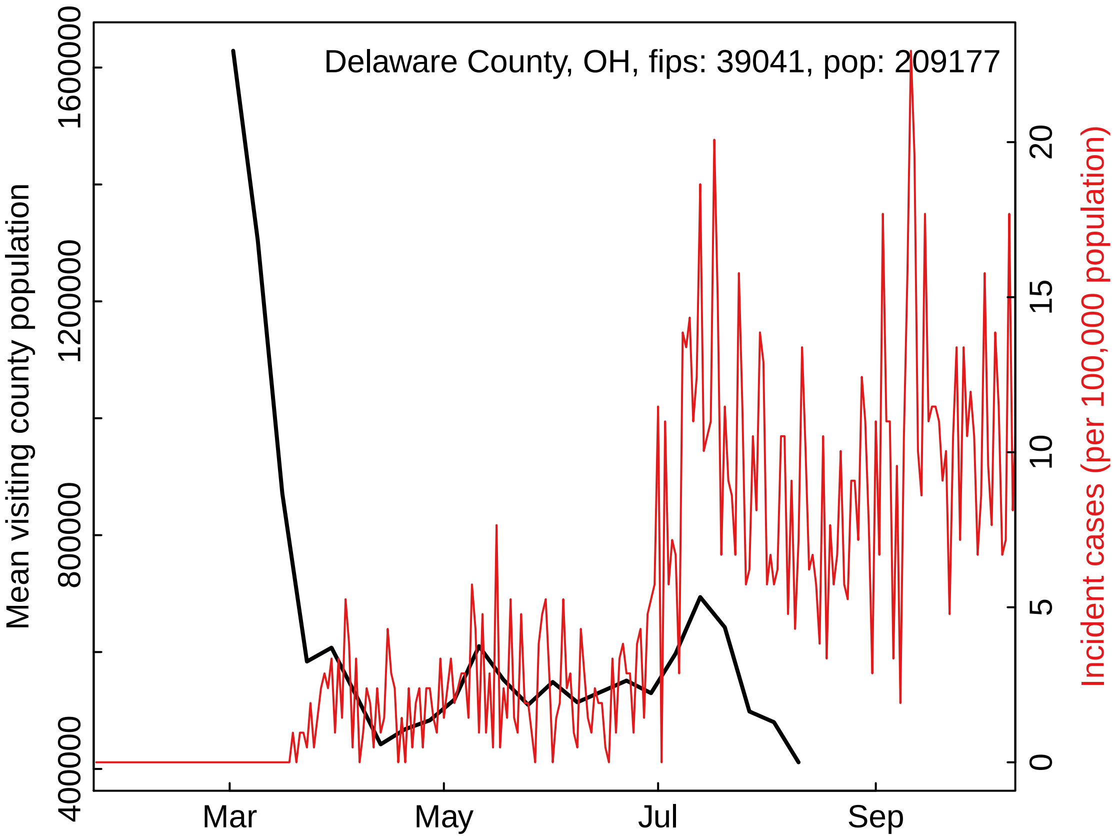

Delaware County, OH, fips: 39041, pop: 209177
Mean visiting county population
Incident cases (per 100,000 population)
4000000
800000
1200000
1600000
0
5
10
15
20
Mar
May
Jul
Sep

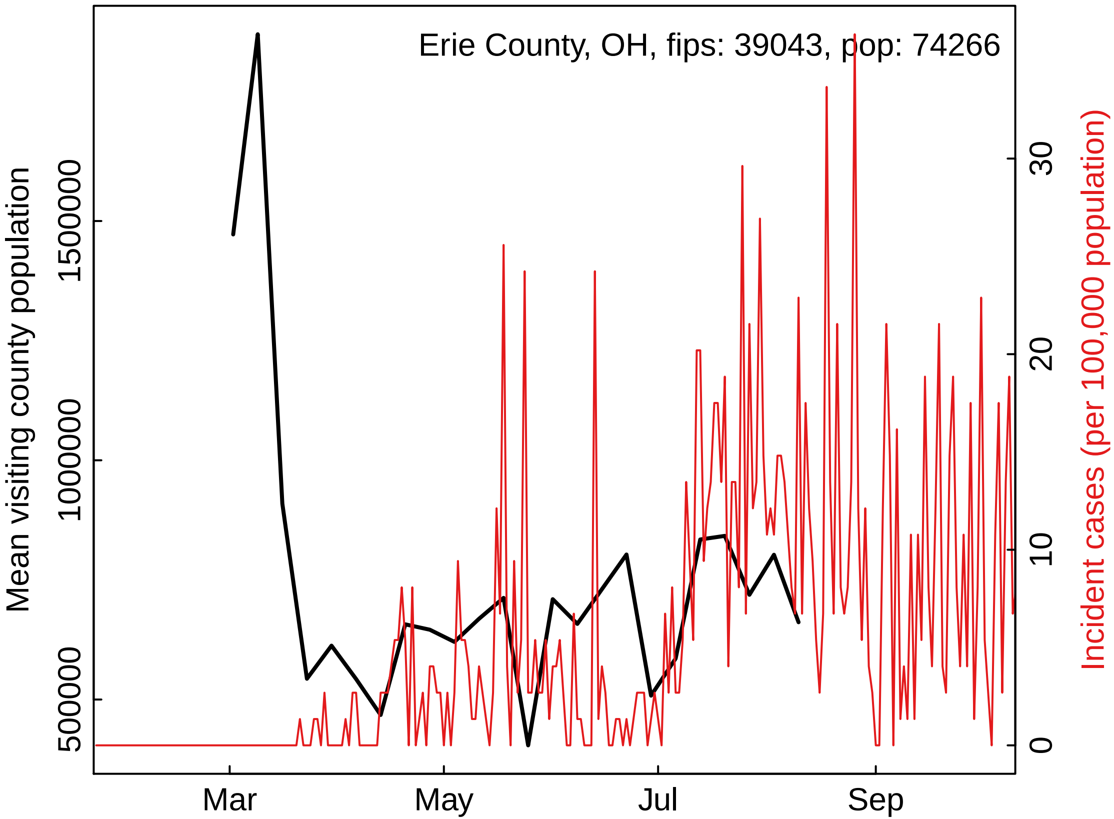
Erie County, OH, fips: 39043, pop: 74266
Mean visiting county population
500000
1000000
1500000
Incident cases (per 100,000 population)
0
10
20
30
Mar
May
Jul
Sep

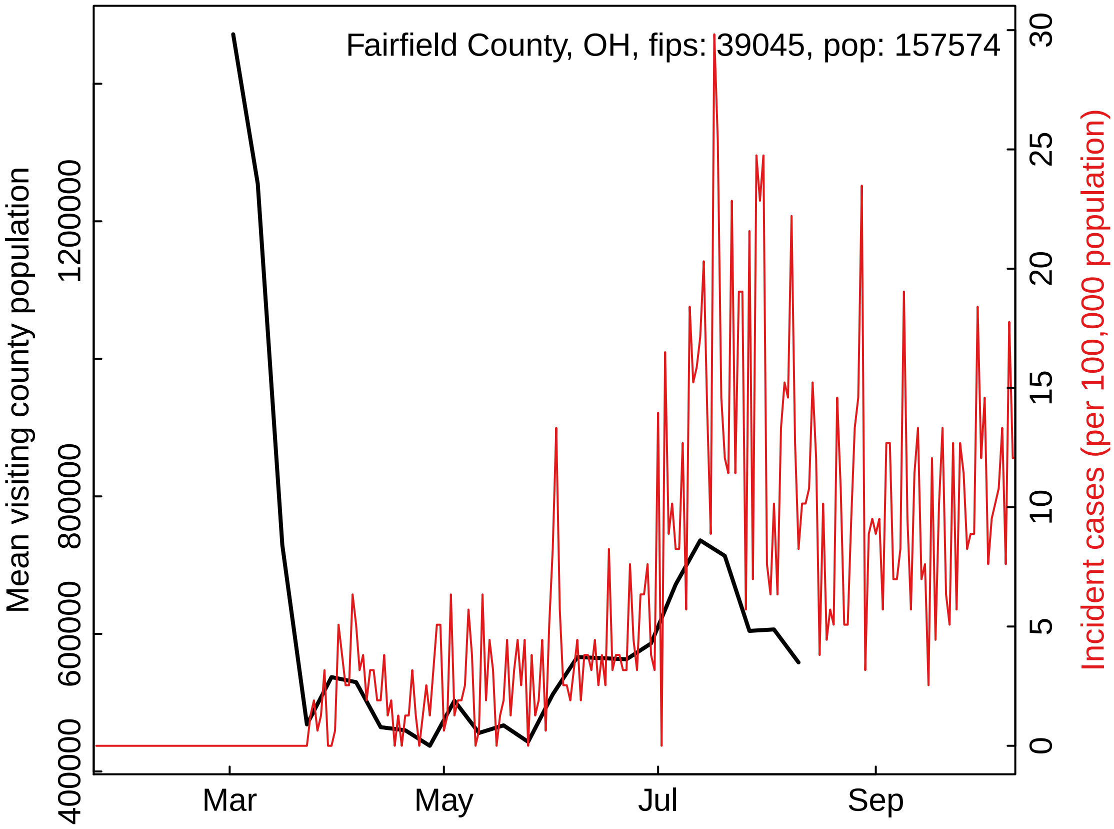

Fairfield County, OH, fips: 39045, pop: 157574
Mean visiting county population
400000
600000
800000
1200000
Incident cases (per 100,000 population)
0
5
10
15
20
25
30
Mar
May
Jul
Sep

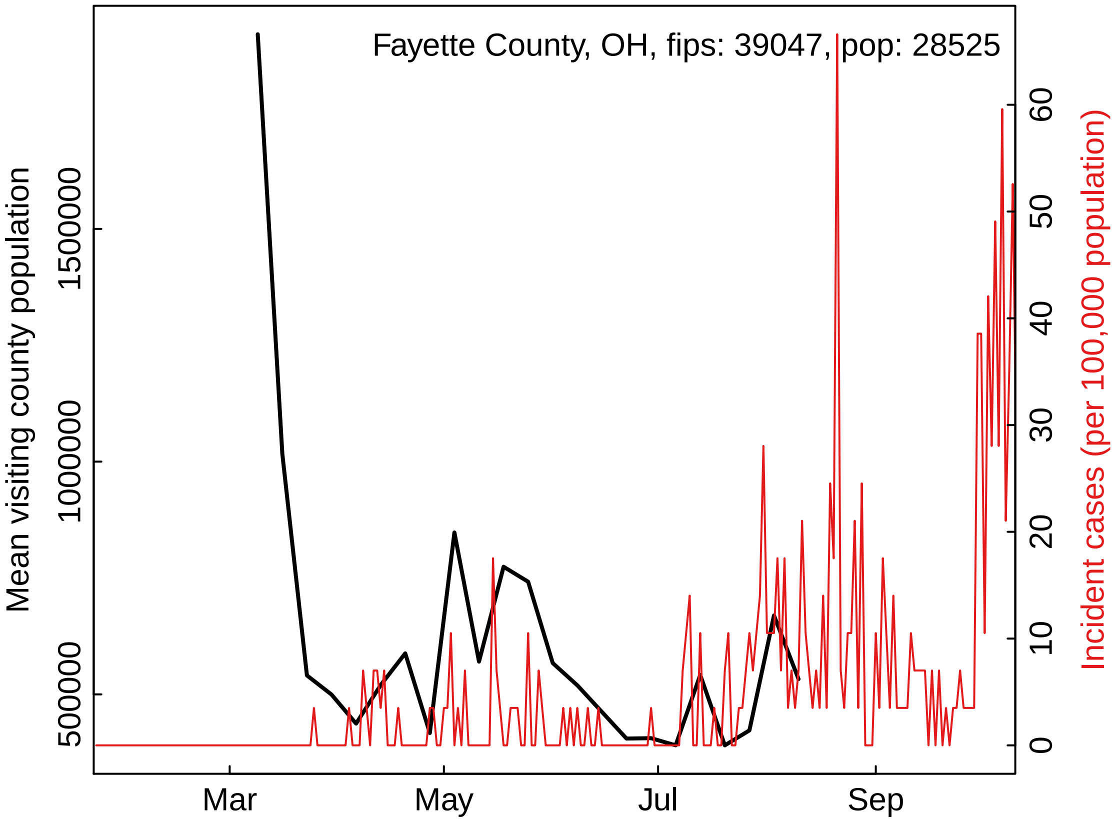

Fayette County, OH, fips: 39047, pop: 28525
Mean visiting county population
500000
1000000
1500000
Mar
May
Jul
Sep
0
10
20
30
40
50
60
Incident cases (per 100,000 population)

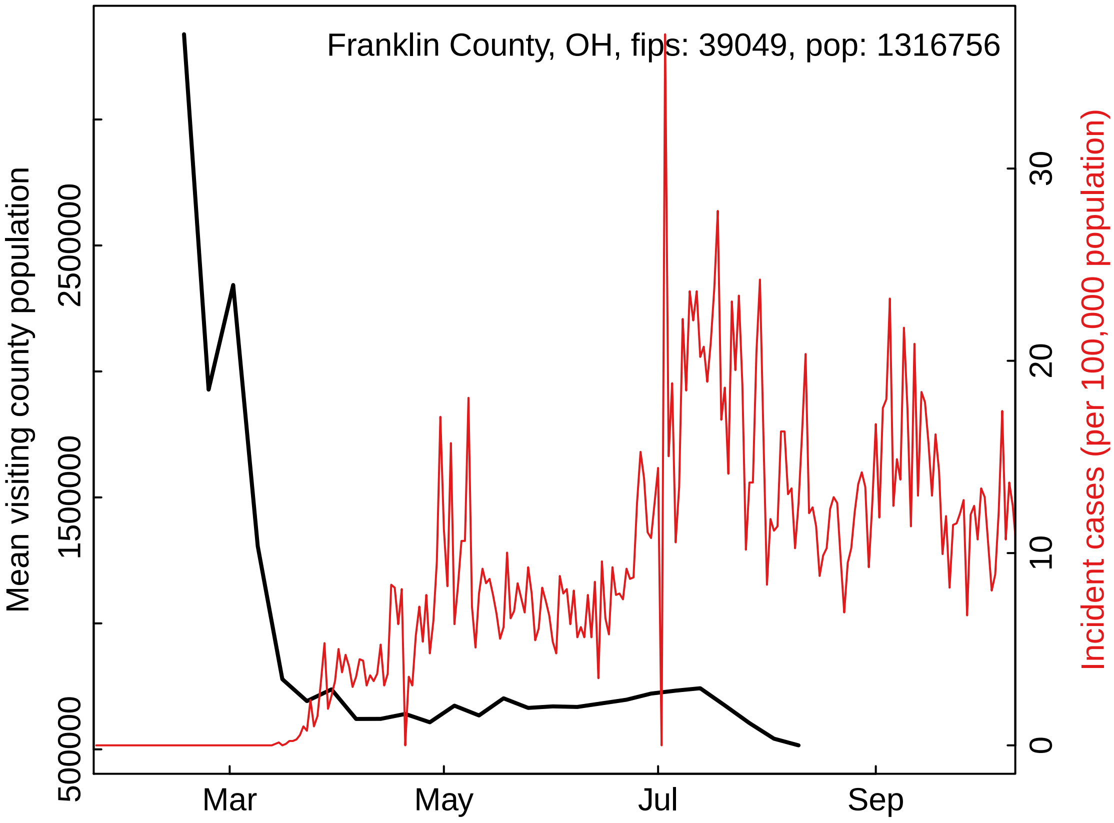

Franklin County, OH, fips: 39049, pop: 1316756
Mean visiting county population
500000
1500000
2500000
Mar
May
Jul
Sep
Incident cases (per 100,000 population)
0
10
20
30

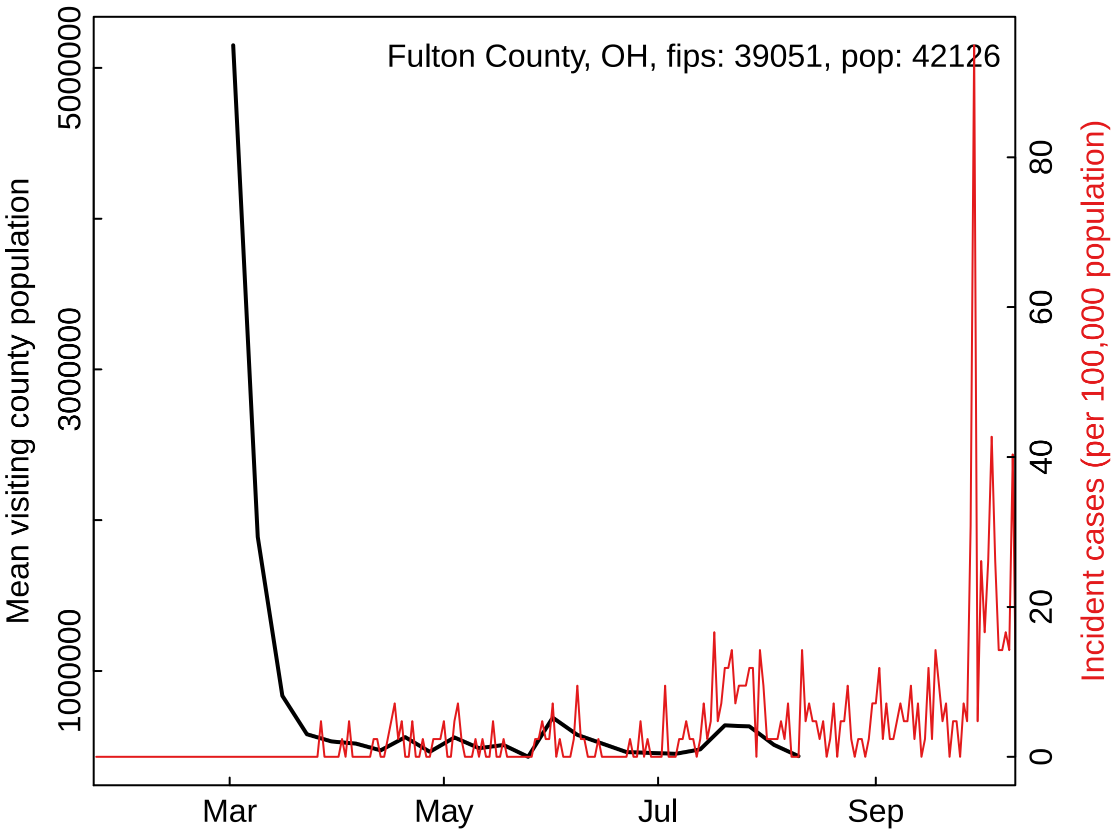
Fulton County, OH, fips: 39051, pop: 42126
Mean visiting county population
5000000
3000000
1000000
Incident cases (per 100,000 population)
80
60
40
20
0
Mar
May
Jul
Sep

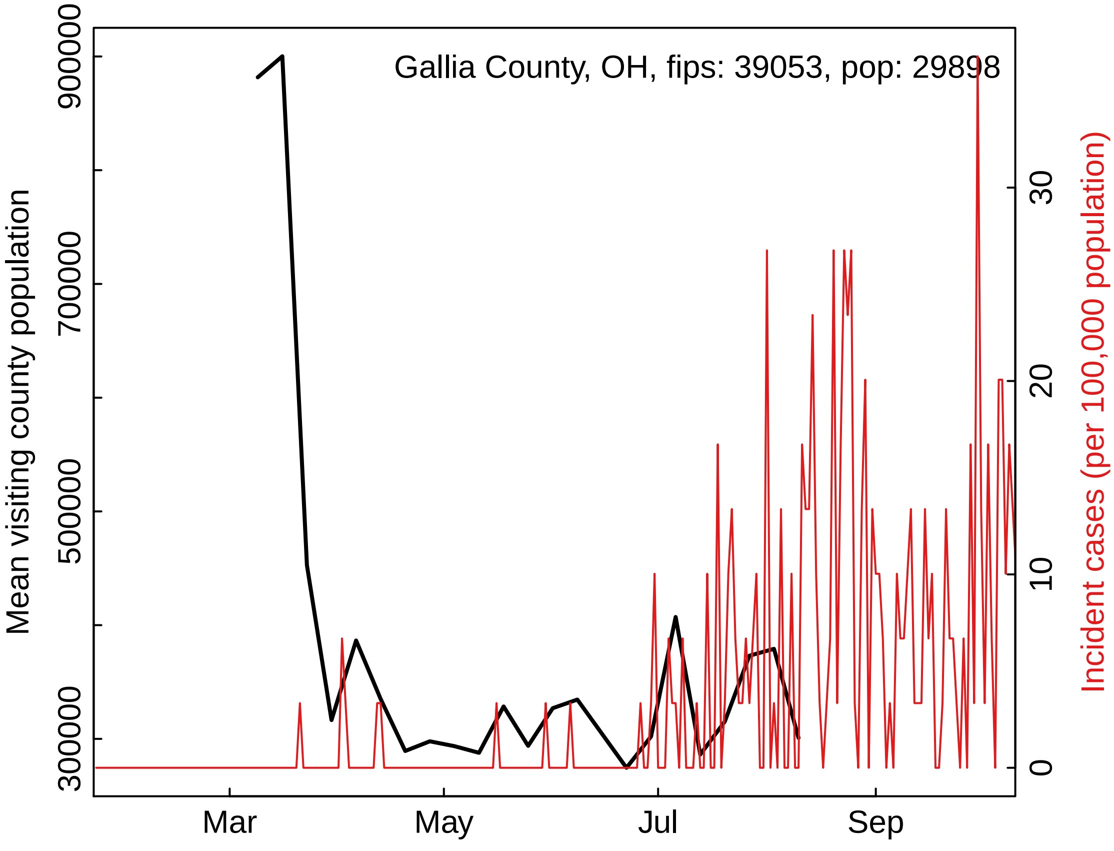

Gallia County, OH, fips: 39053, pop: 29898
Mean visiting county population
300000
500000
700000
900000
Mar
May
Jul
Sep
0
10
20
30
Incident cases (per 100,000 population)

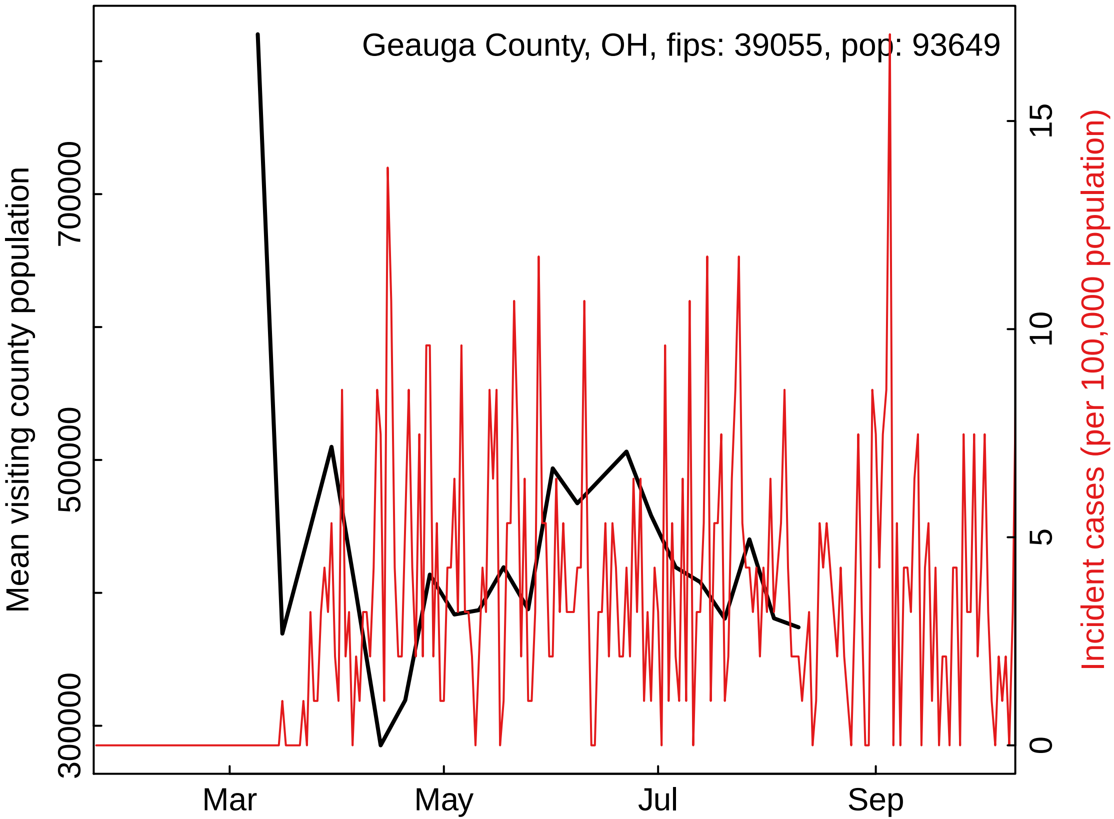

Geauga County, OH, fips: 39055, pop: 93649
Mean visiting county population
300000
500000
700000
Incident cases (per 100,000 population)
0
5
10
15
Mar
May
Jul
Sep

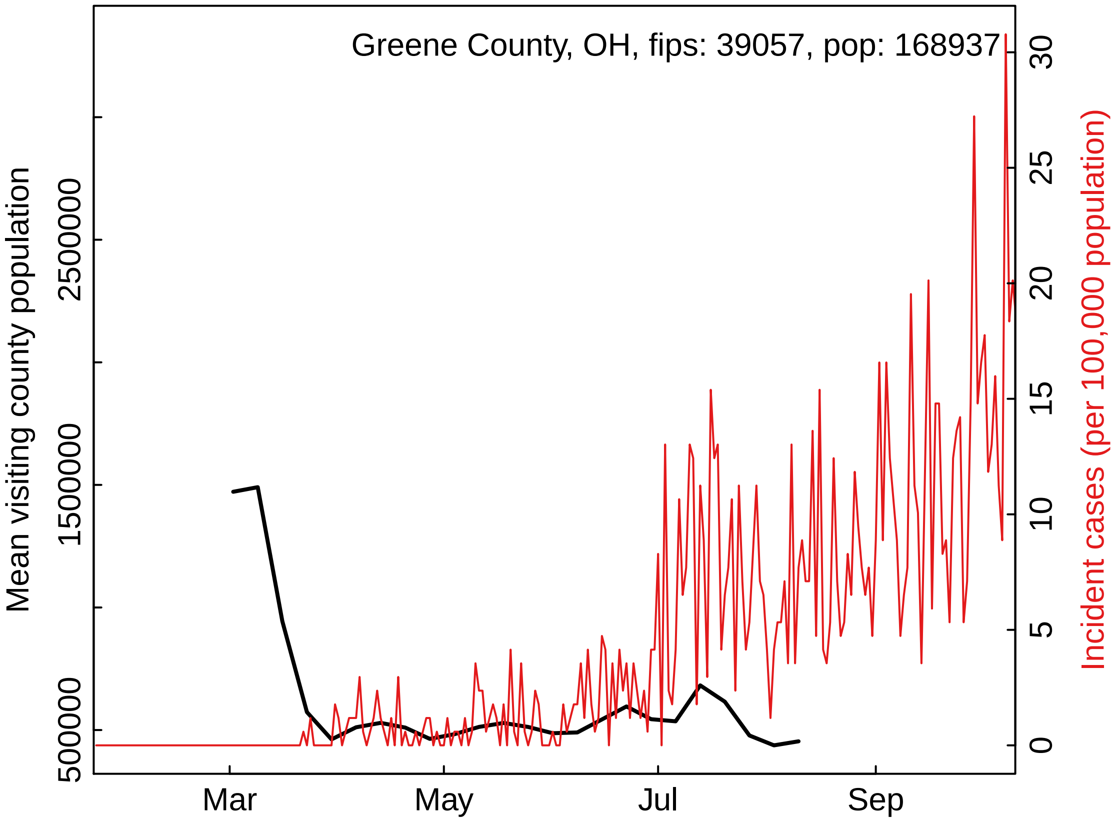

Greene County, OH, fips: 39057, pop: 168937
Mean visiting county population
500000
1500000
2500000
Incident cases (per 100,000 population)
0
5
10
15
20
25
30
Mar
May
Jul
Sep

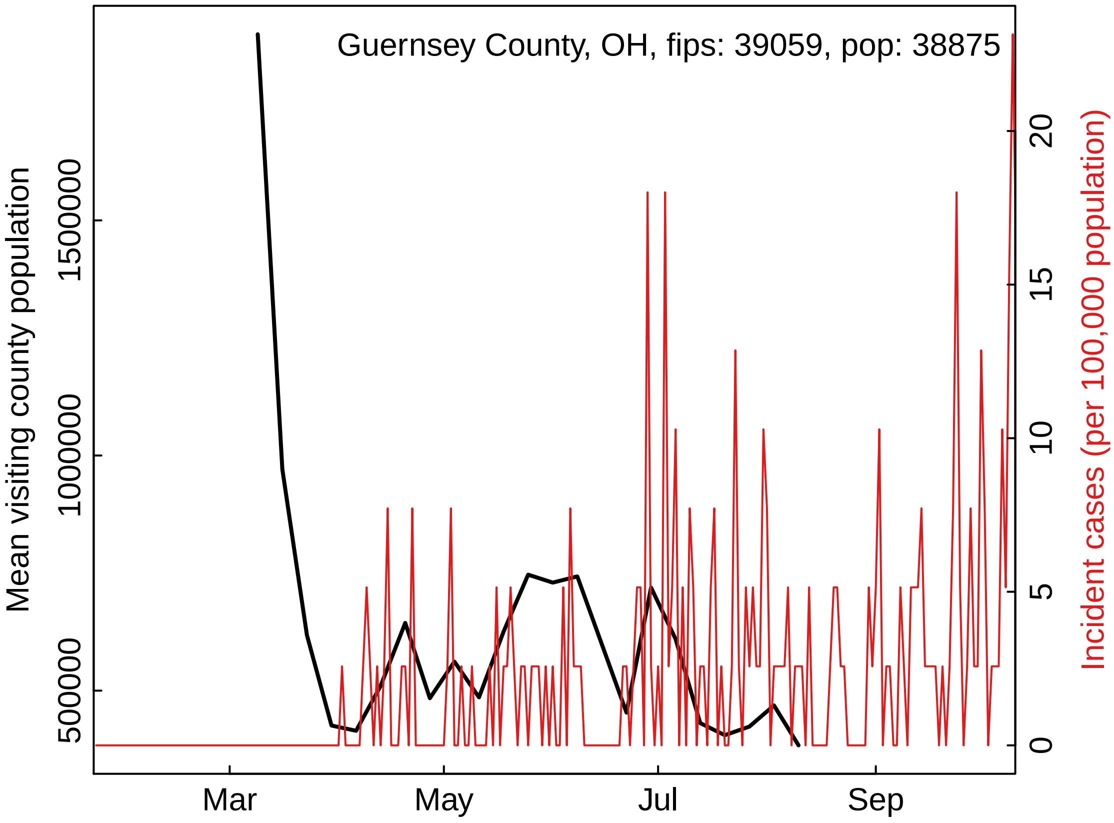

Guernsey County, OH, fips: 39059, pop: 38875
Mean visiting county population
500000
1000000
1500000
Mar
May
Jul
Sep
0
5
10
15
20
Incident cases (per 100,000 population)

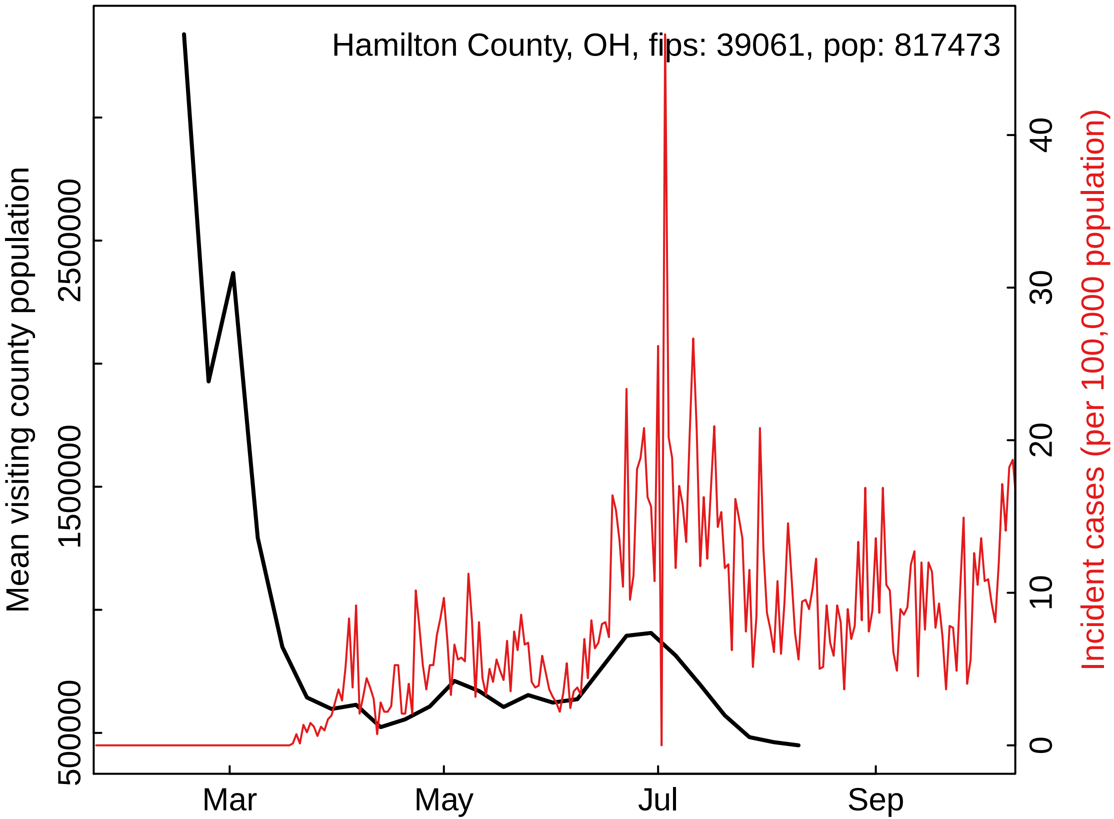

Hamilton County, OH, fips: 39061, pop: 817473
Mean visiting county population
500000
1500000
2500000
Incident cases (per 100,000 population)
0
10
20
30
40
Mar
May
Jul
Sep

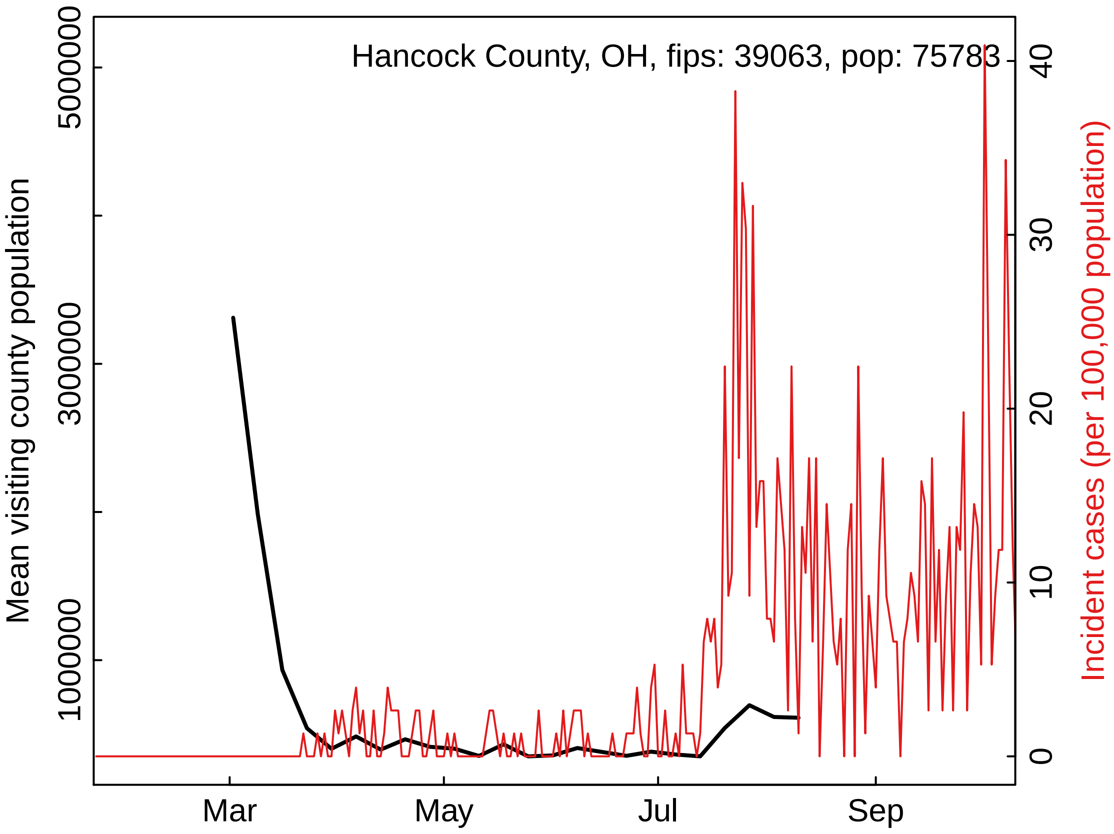

Hancock County, OH, fips: 39063, pop: 75783
Mean visiting county population
5000000
3000000
1000000
Incident cases (per 100,000 population)
0
10
20
30
40
Mar
May
Jul
Sep

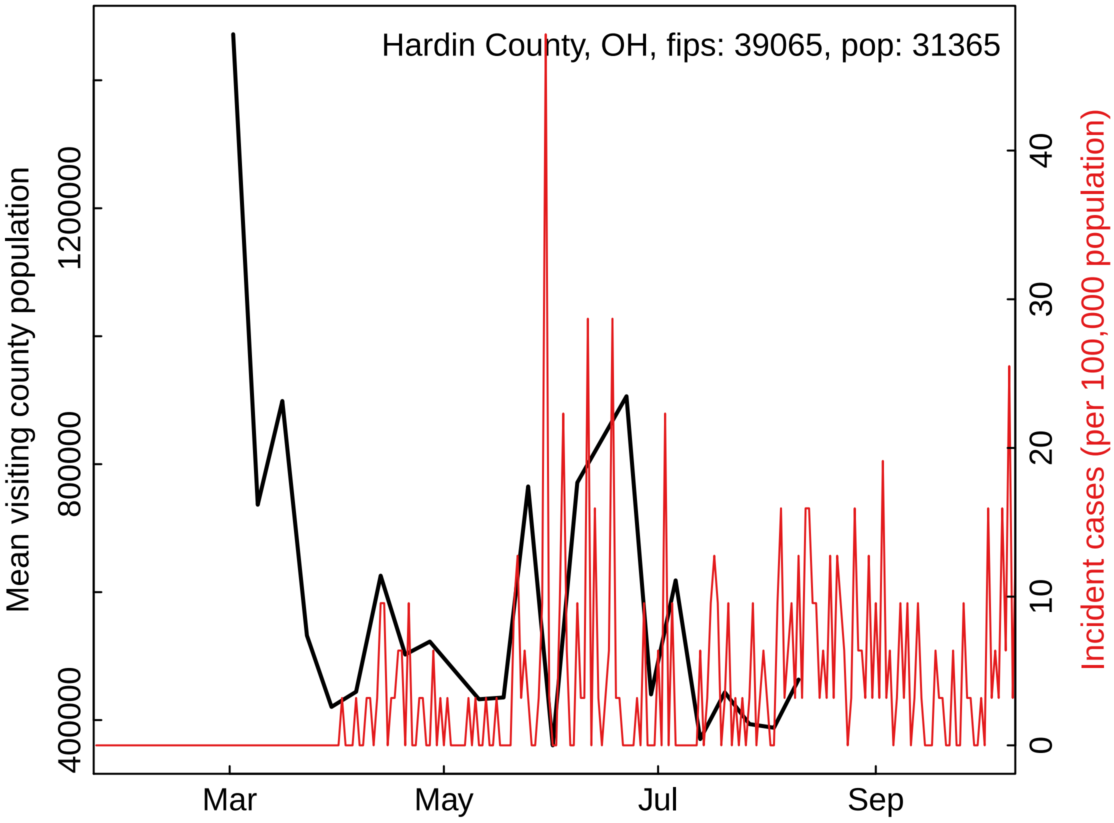

Hardin County, OH, fips: 39065, pop: 31365
Mean visiting county population
400000
800000
1200000
Mar
May
Jul
Sep
0
10
20
30
40
Incident cases (per 100,000 population)

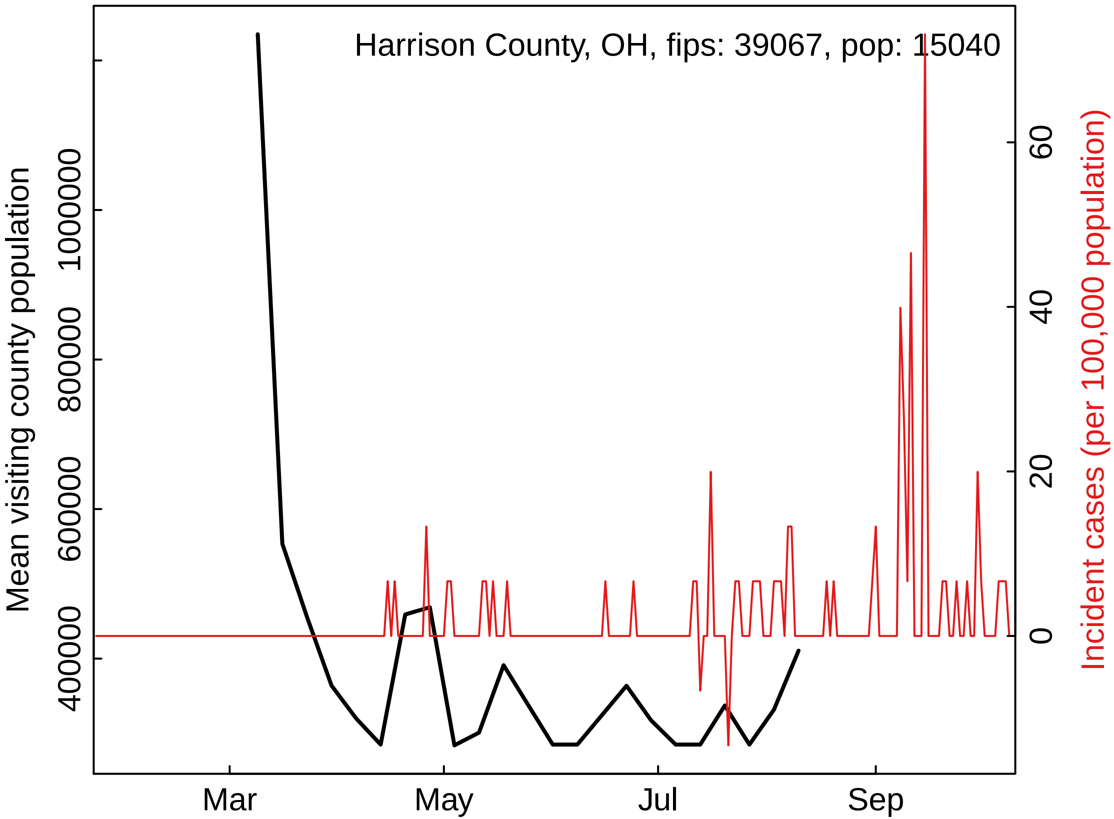

Harrison County, OH, fips: 39067, pop: 15040
Mean visiting county population
400000
600000
800000
1000000
Incident cases (per 100,000 population)
0
20
40
60
Mar
May
Jul
Sep

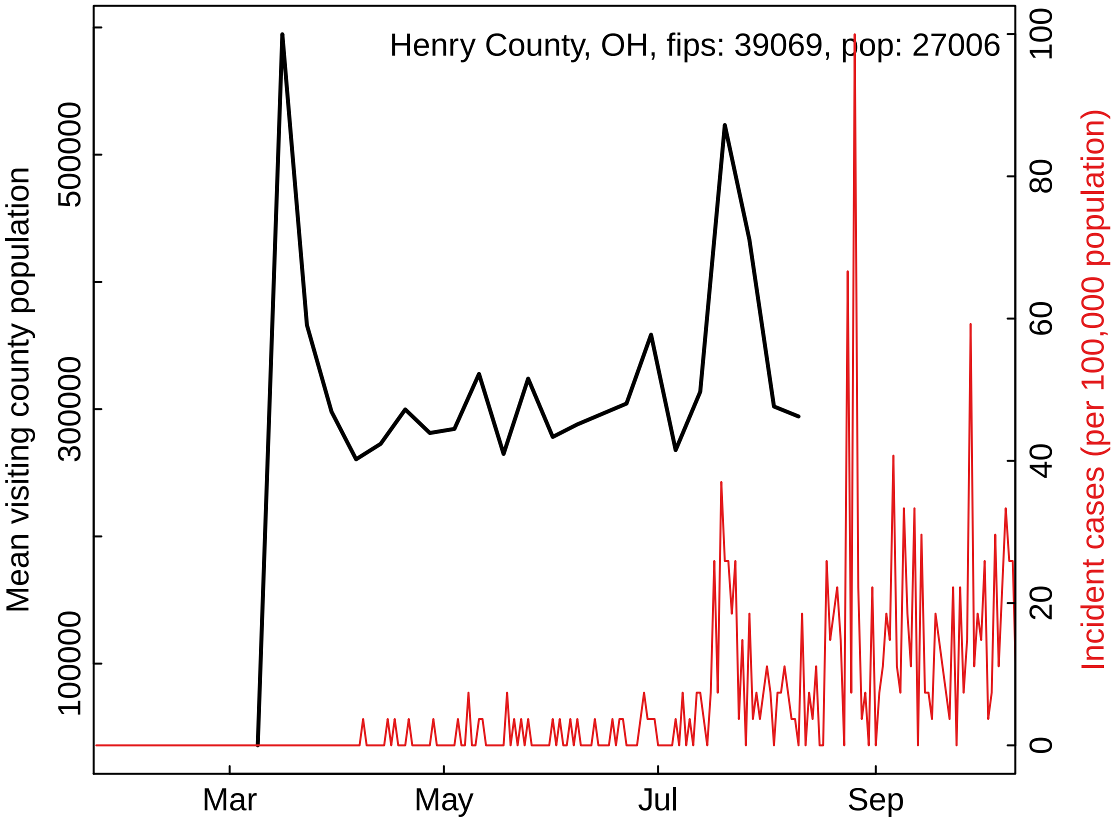

Henry County, OH, fips: 39069, pop: 27006
Mean visiting county population
100000
300000
500000
Incident cases (per 100,000 population)
0
20
40
60
80
100
Mar
May
Jul
Sep

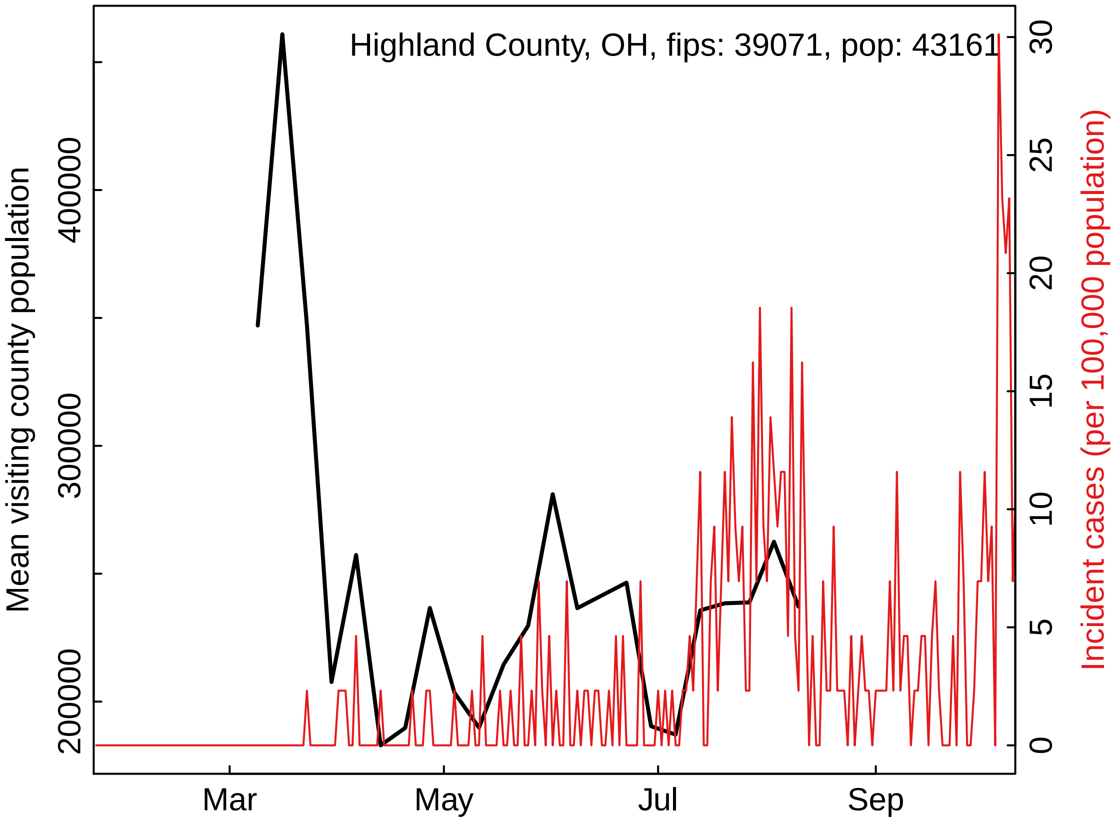
Highland County, OH, fips: 39071, pop: 43161
Mean visiting county population
200000
300000
400000
Incident cases (per 100,000 population)
0
5
10
15
20
25
30
Mar
May
Jul
Sep

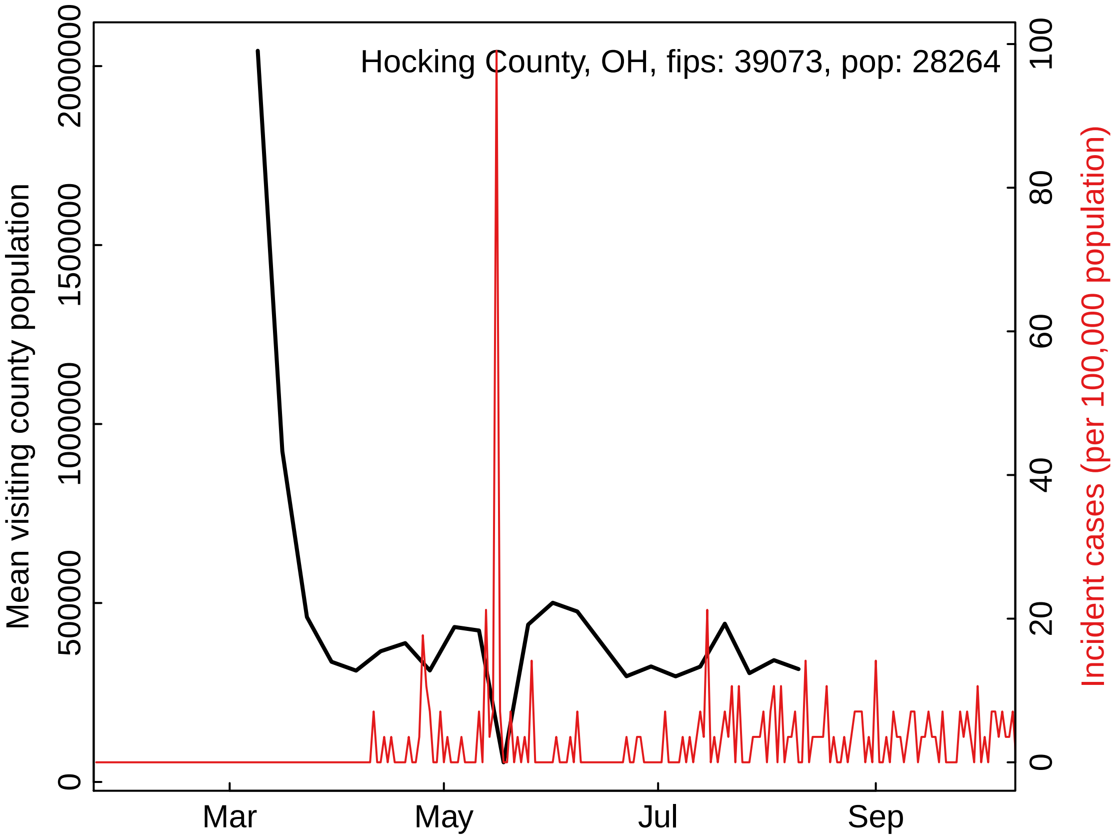

Hocking County, OH, fips: 39073, pop: 28264
Mean visiting county population
Incident cases (per 100,000 population)
0
500000
1000000
1500000
2000000
0
20
40
60
80
100
Mar
May
Jul
Sep

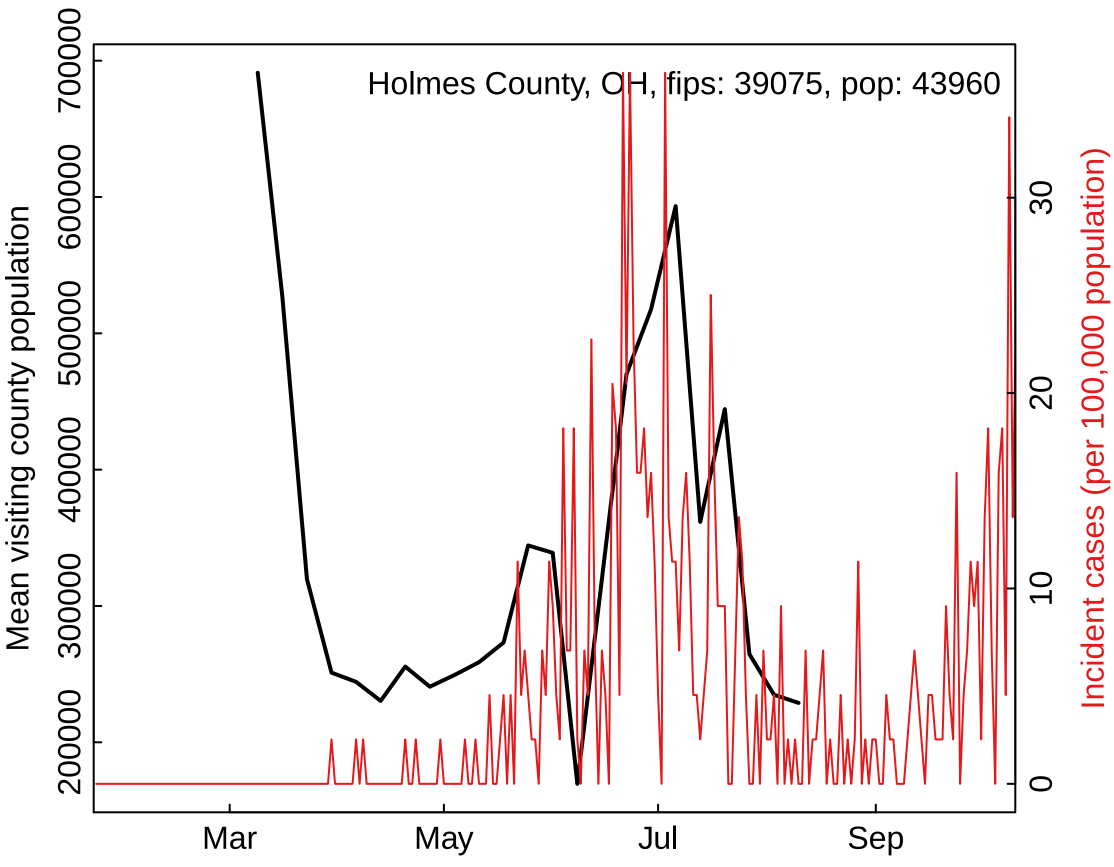

Holmes County, OH, fips: 39075, pop: 43960
Mean visiting county population
Incident cases (per 100,000 population)
200000
300000
400000
500000
600000
700000
0
10
20
30
Mar
May
Jul
Sep

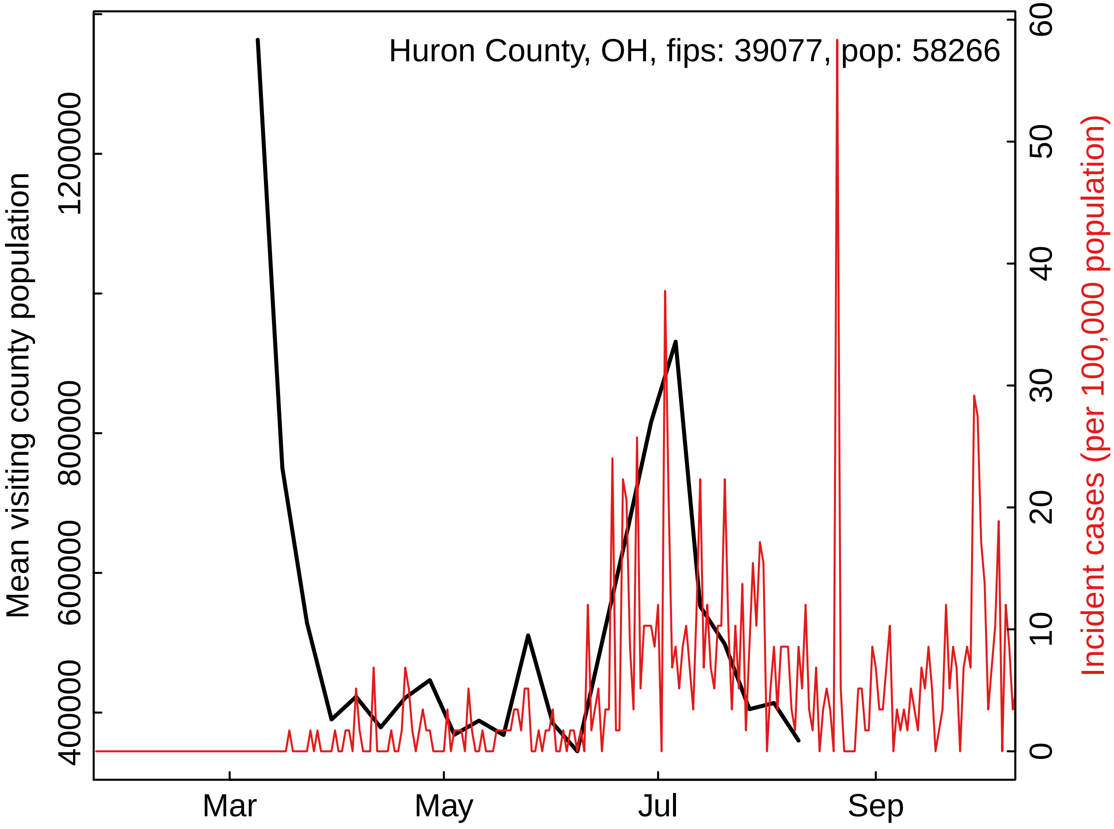
Huron County, OH, fips: 39077, pop: 58266
Mean visiting county population
400000
600000
800000
1200000
Incident cases (per 100,000 population)
0
10
20
30
40
50
60
Mar
May
Jul
Sep

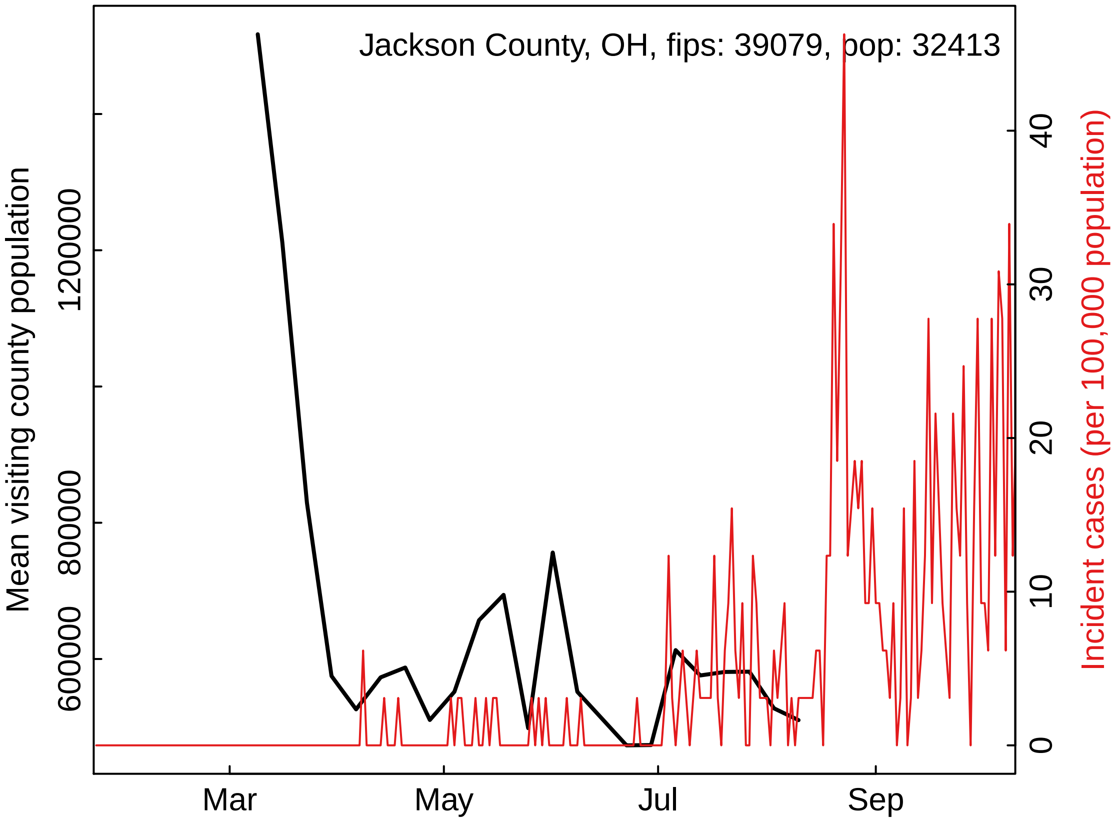

Jackson County, OH, fips: 39079, pop: 32413
Mean visiting county population
600000
800000
1200000
Incident cases (per 100,000 population)
0
10
20
30
40
Mar
May
Jul
Sep

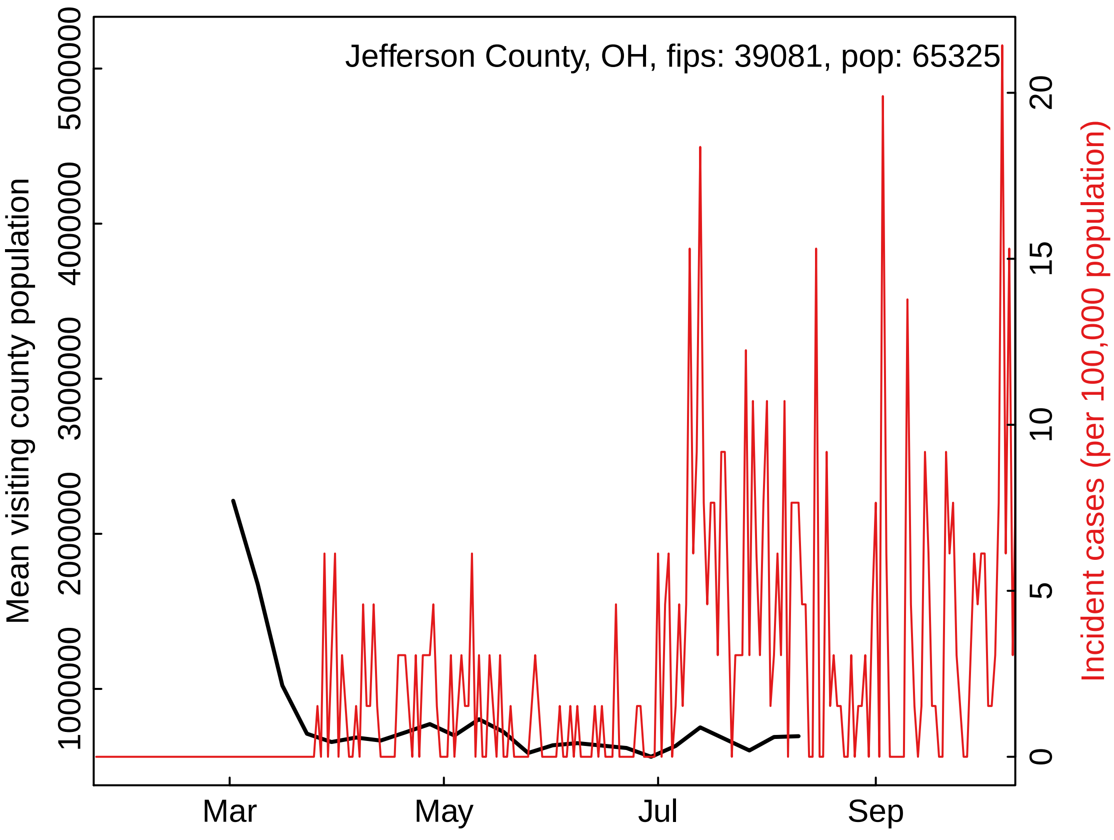

Jefferson County, OH, fips: 39081, pop: 65325
Mean visiting county population
10000000
20000000
30000000
40000000
50000000
Incident cases (per 100,000 population)
0
5
10
15
20
Mar
May
Jul
Sep

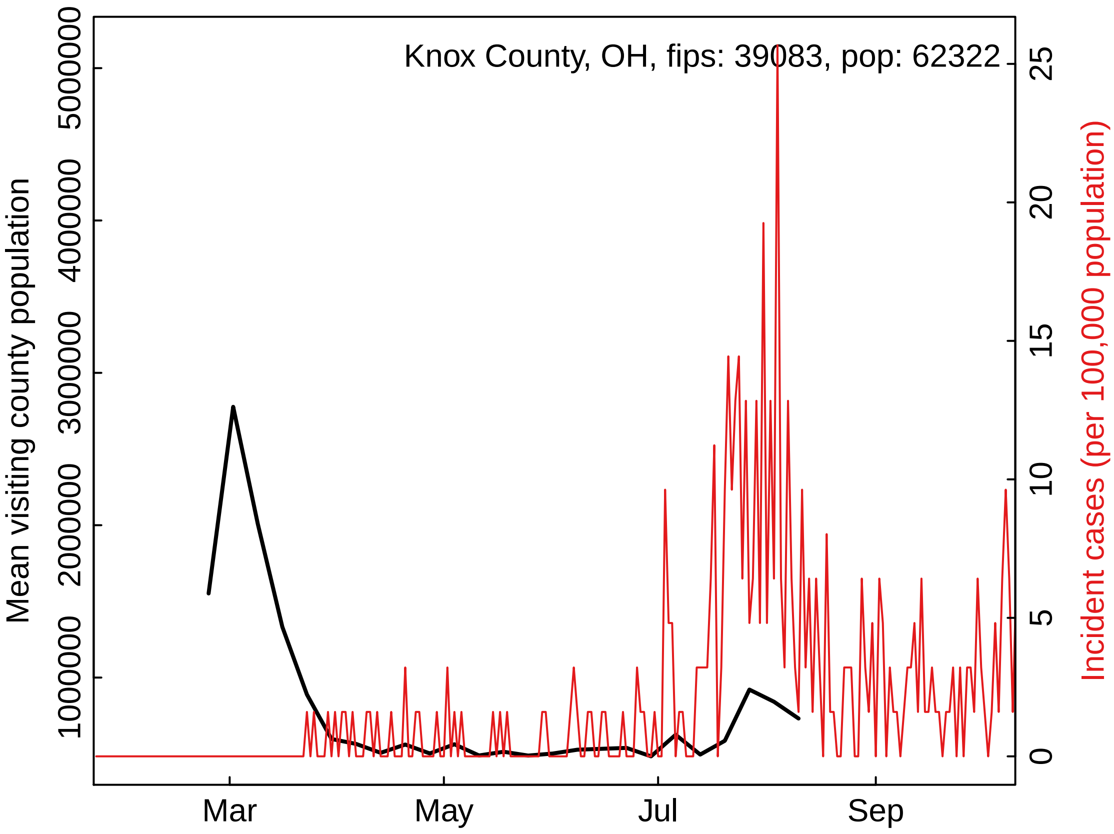

Knox County, OH, fips: 39083, pop: 62322
Mean visiting county population
1000000
2000000
3000000
4000000
5000000
Incident cases (per 100,000 population)
0
5
10
15
20
25
Mar
May
Jul
Sep

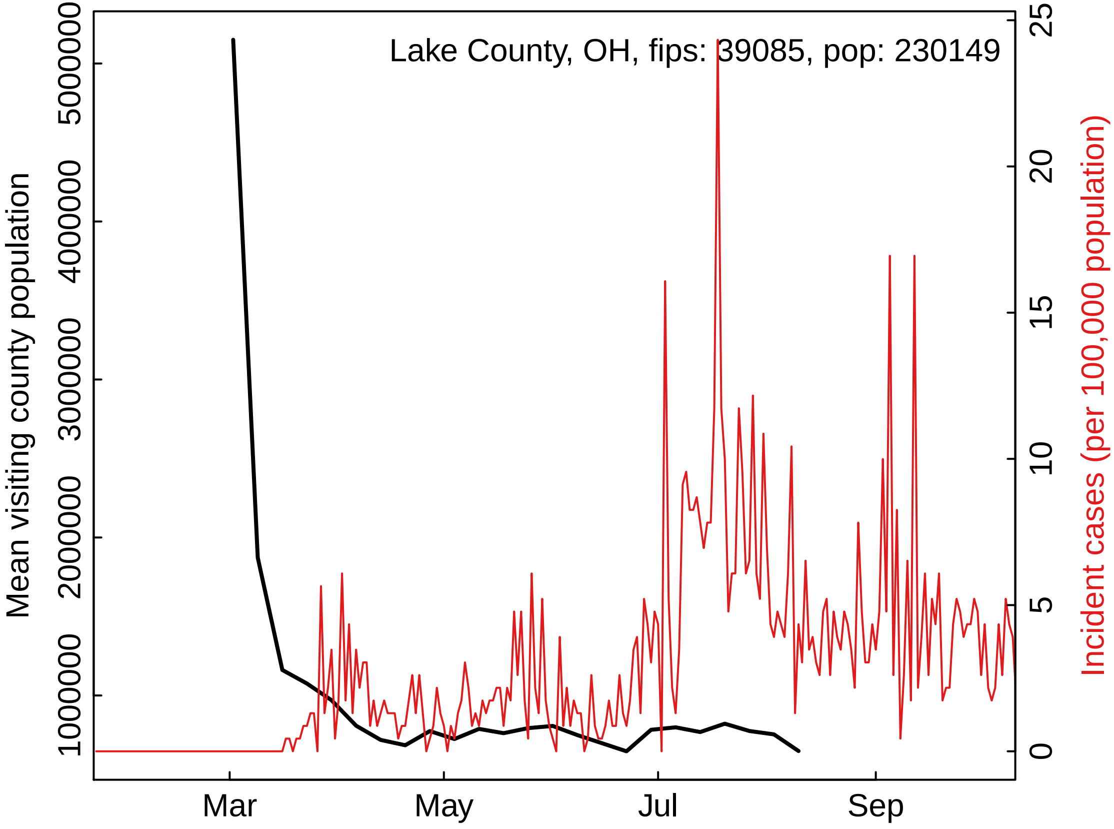

Lake County, OH, fips: 39085, pop: 230149
Mean visiting county population
10000000
20000000
30000000
40000000
50000000
Incident cases (per 100,000 population)
0
5
10
15
20
25
Mar
May
Jul
Sep

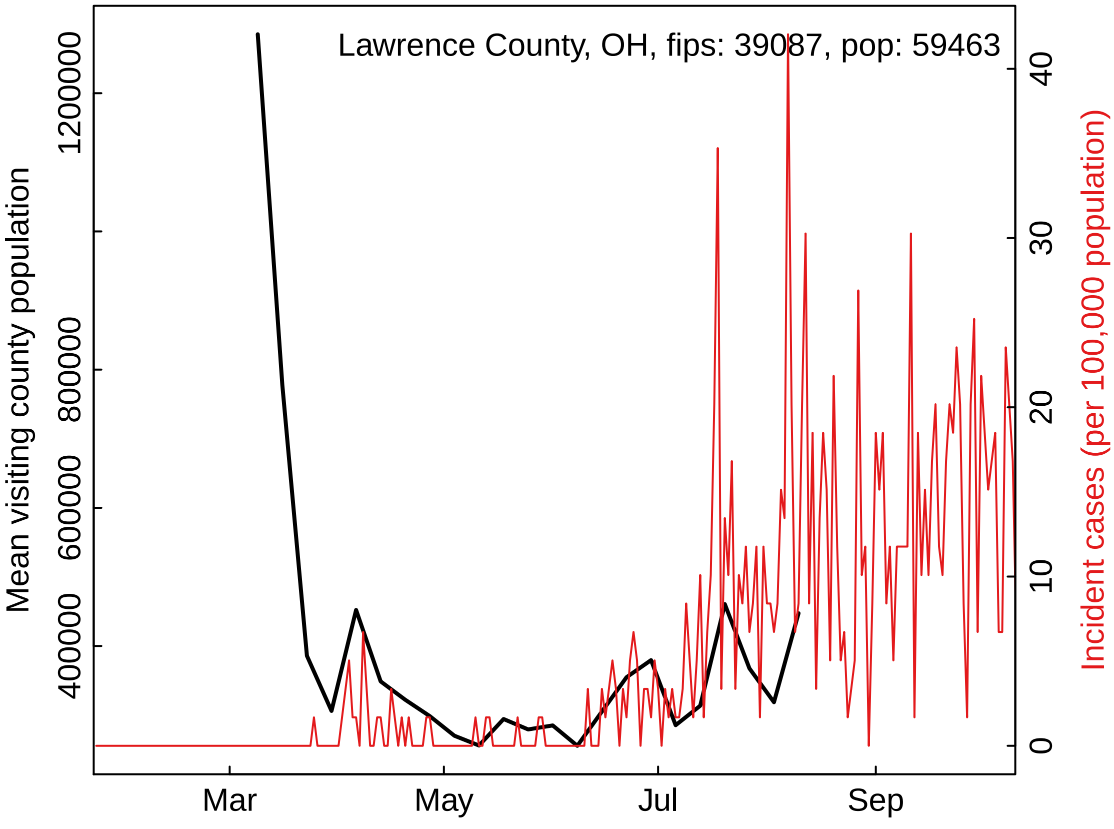

Lawrence County, OH, fips: 39087, pop: 59463
Mean visiting county population
400000
600000
800000
1200000
Incident cases (per 100,000 population)
0
10
20
30
40
Mar
May
Jul
Sep

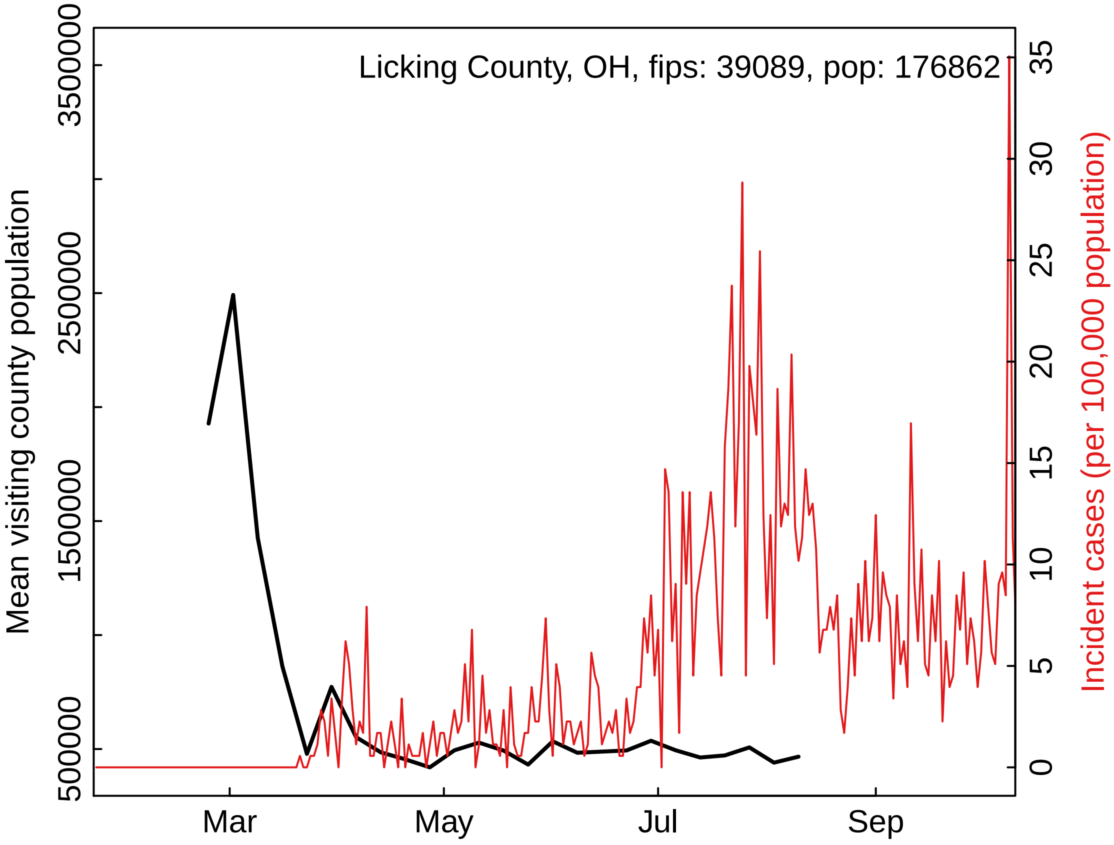
Licking County, OH, fips: 39089, pop: 176862
Mean visiting county population
500000
1500000
2500000
3500000
Incident cases (per 100,000 population)
0
5
10
15
20
25
30
35
Mar
May
Jul
Sep

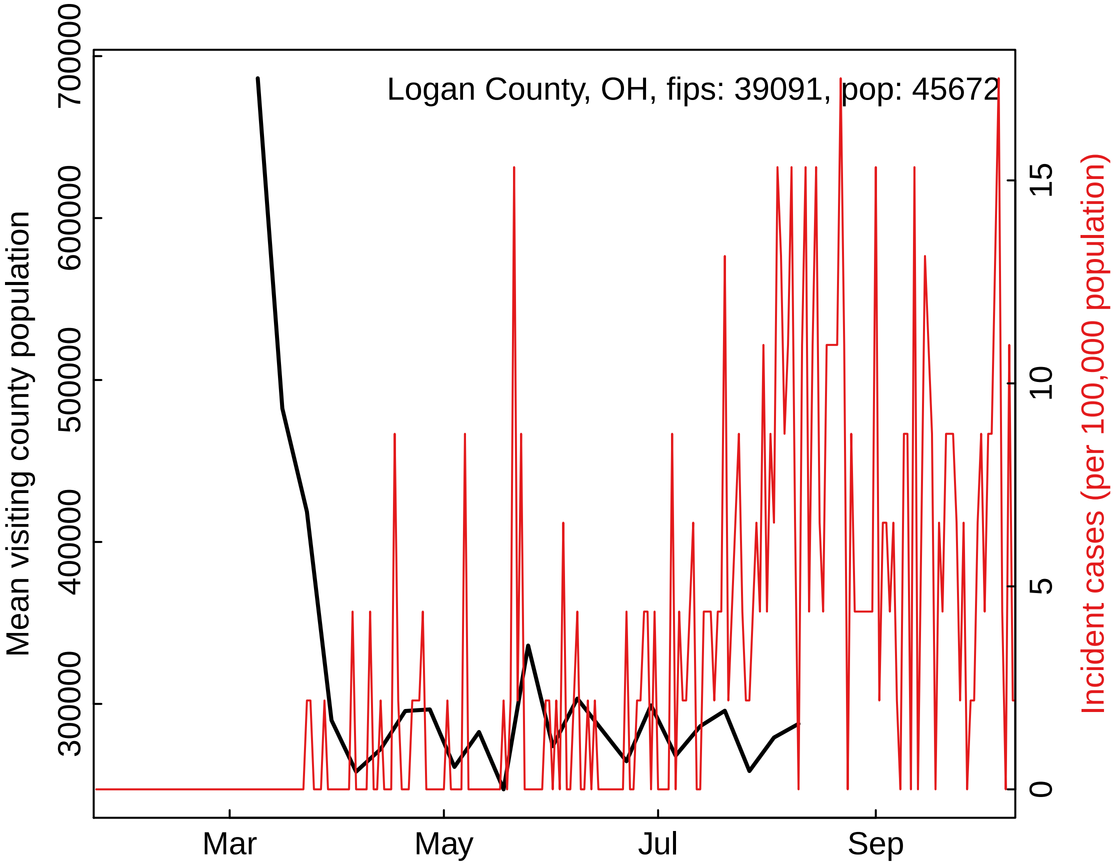

Logan County, OH, fips: 39091, pop: 45672
Mean visiting county population
Incident cases (per 100,000 population)
300000
400000
500000
600000
700000
0
5
10
15
Mar
May
Jul
Sep

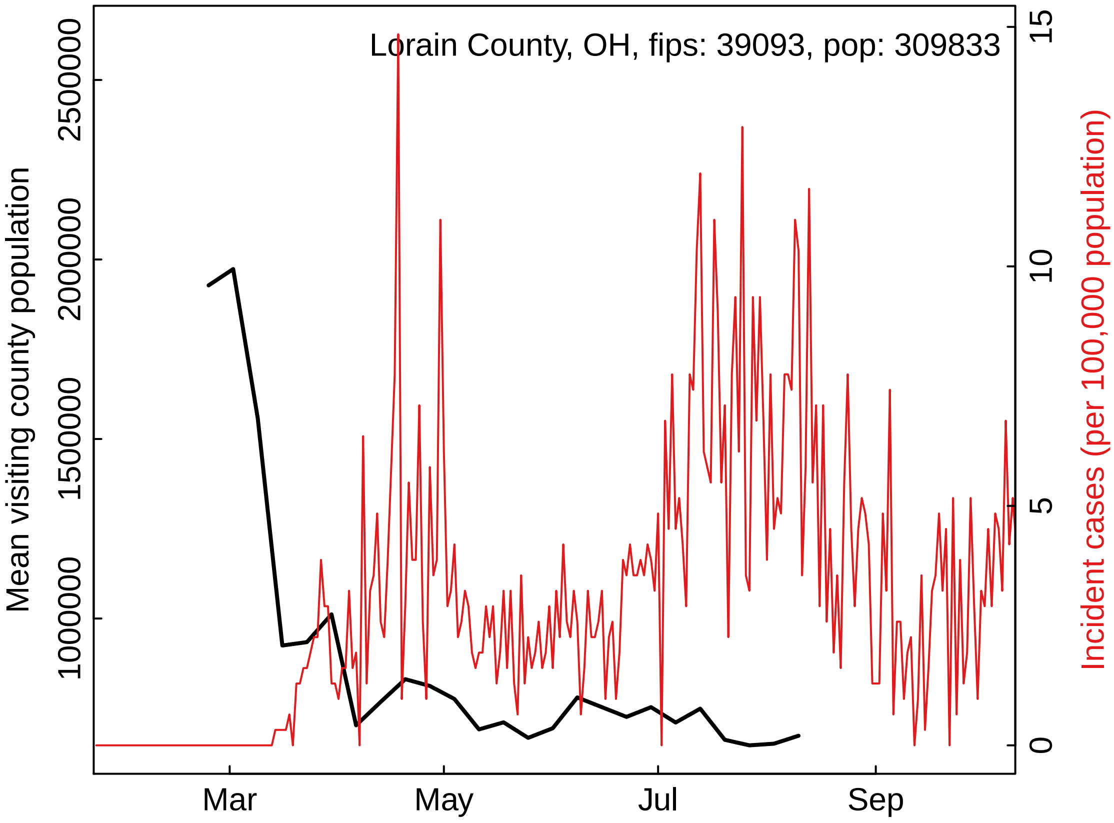
Lorain County, OH, fips: 39093, pop: 309833
Mean visiting county population
1000000
1500000
2000000
2500000
Incident cases (per 100,000 population)
0
5
10
15
Mar
May
Jul
Sep

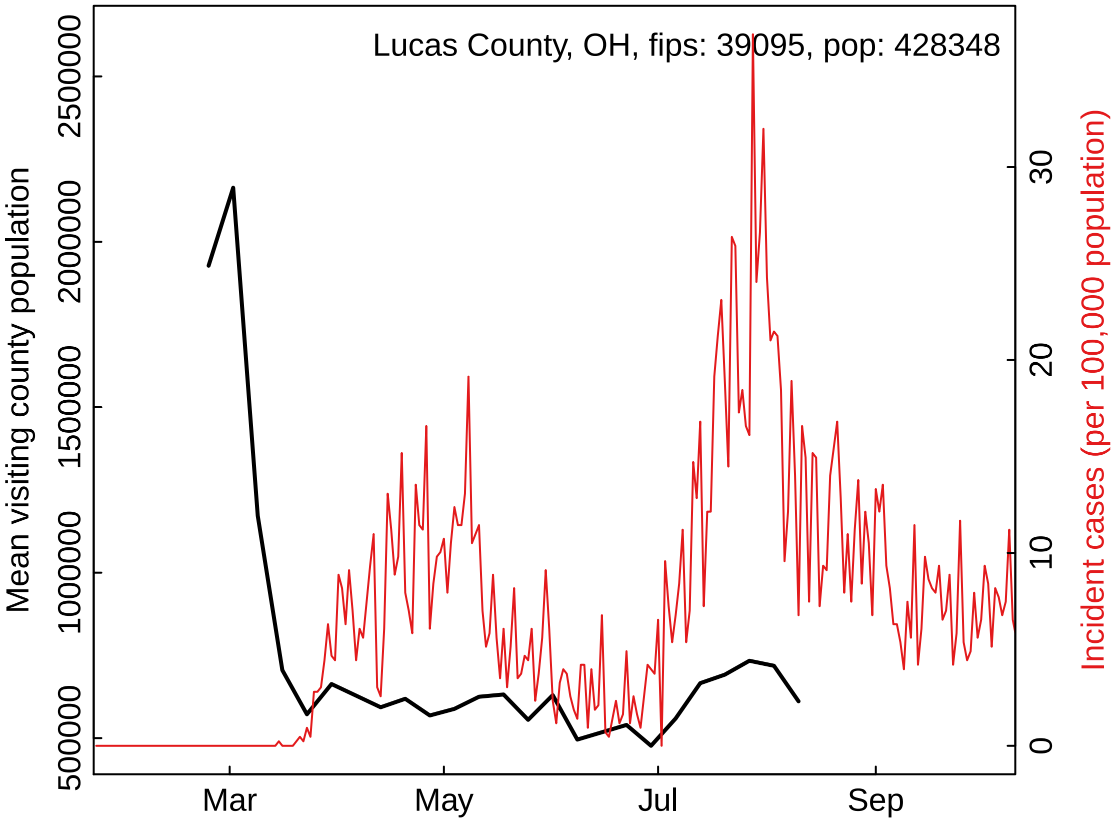
Lucas County, OH, fips: 39095, pop: 428348
Mean visiting county population
500000
1000000
1500000
2000000
2500000
Incident cases (per 100,000 population)
0
10
20
30
Mar
May
Jul
Sep

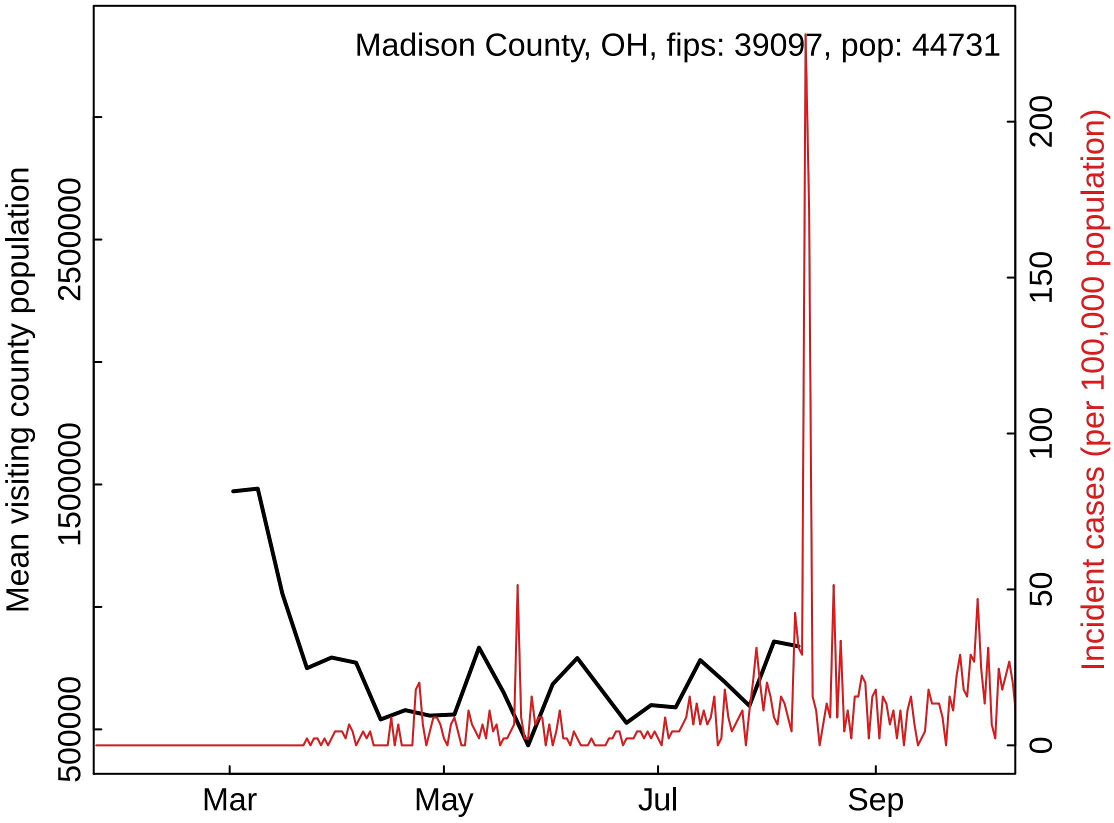
Madison County, OH, fips: 39097, pop: 44731
Mean visiting county population
500000
1500000
2500000
Mar
May
Jul
Sep
0
50
100
150
200
Incident cases (per 100,000 population)

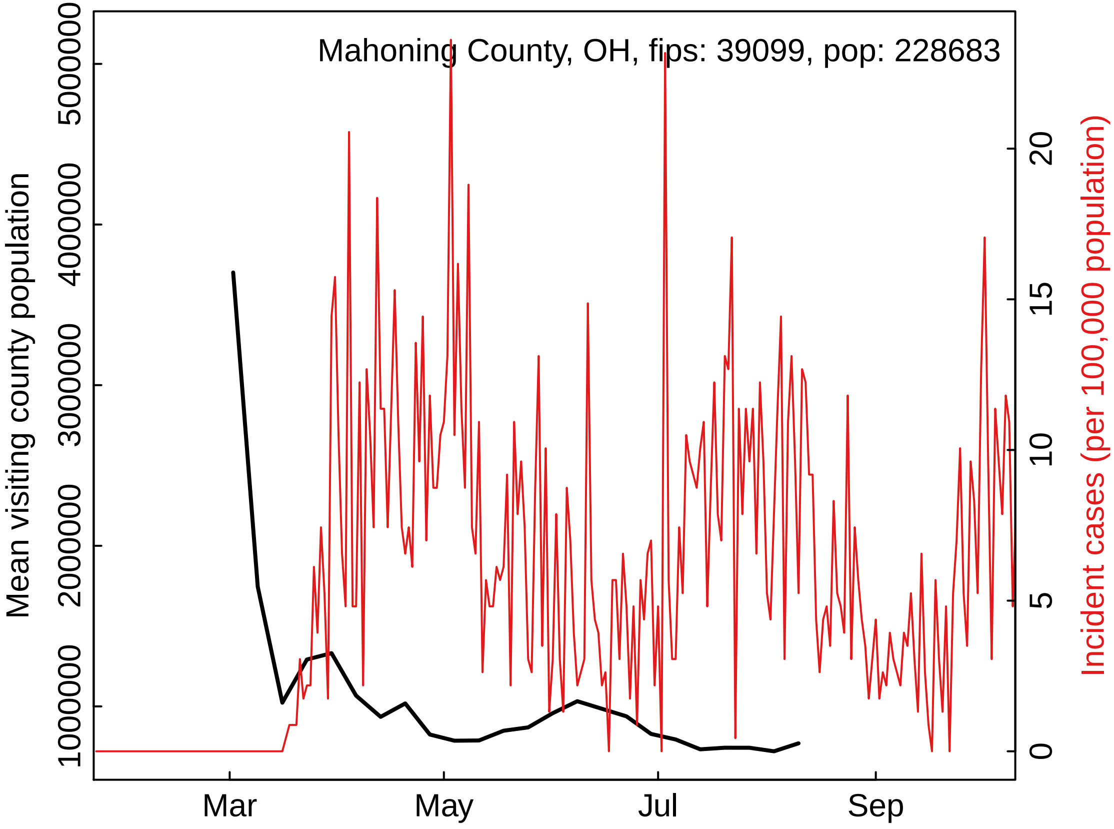

Mahoning County, OH, fips: 39099, pop: 228683
Mean visiting county population
10000000
20000000
30000000
40000000
50000000
Incident cases (per 100,000 population)
0
5
10
15
20
Mar
May
Jul
Sep

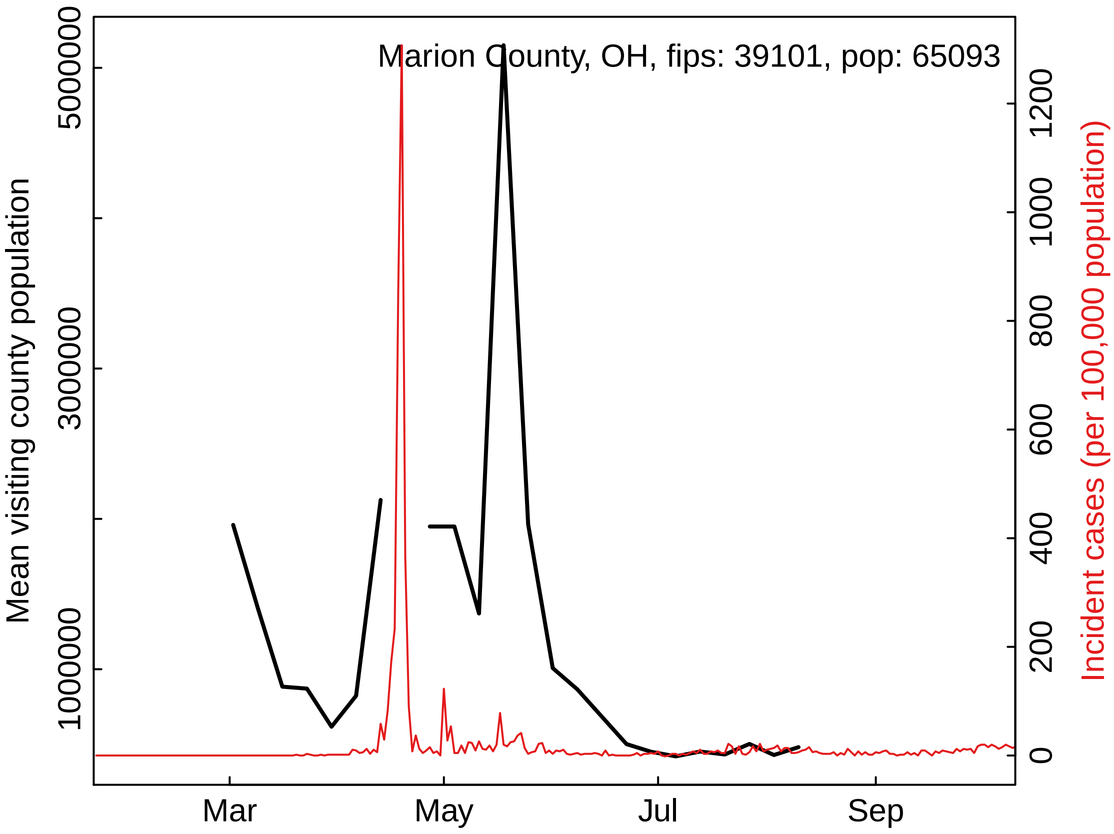

Marion County, OH, fips: 39101, pop: 65093
Mean visiting county population
1000000
3000000
5000000
Incident cases (per 100,000 population)
0
200
400
600
800
1000
1200
Mar
May
Jul
Sep

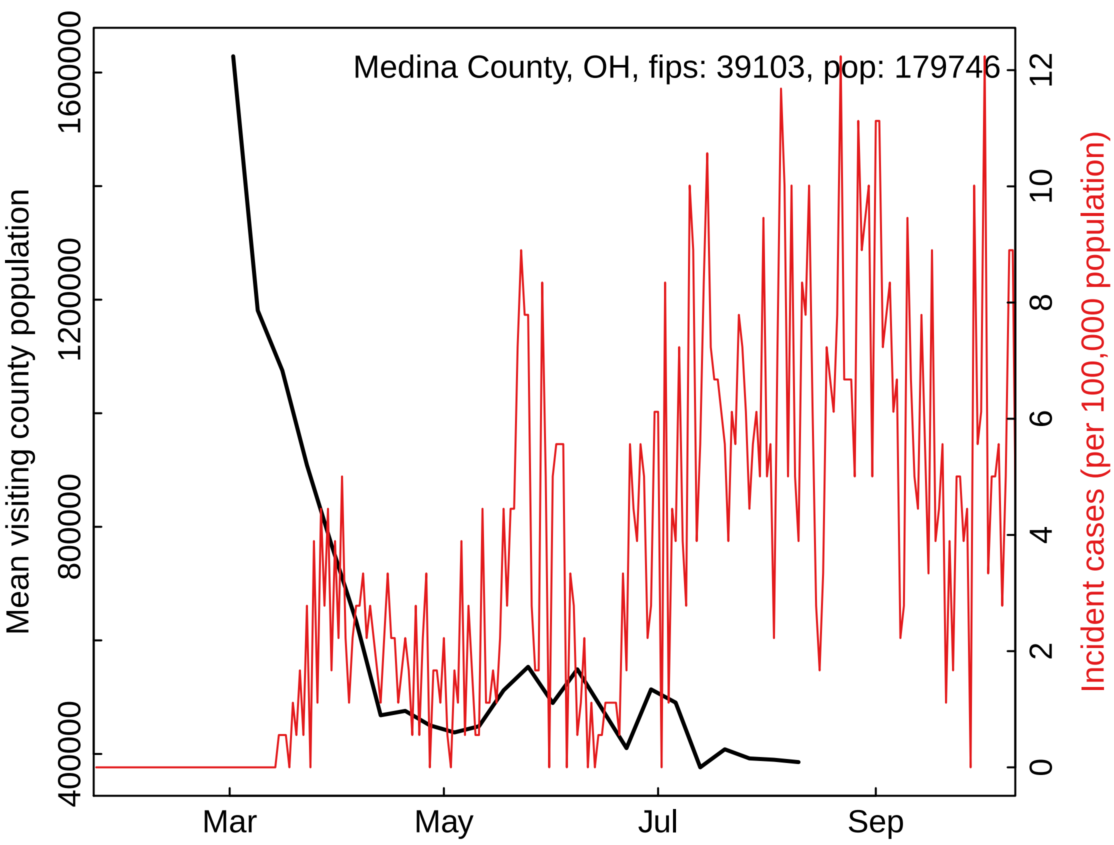

Medina County, OH, fips: 39103, pop: 179746
Mean visiting county population
Incident cases (per 100,000 population)
400000
800000
1200000
1600000
0
2
4
6
8
10
12
Mar
May
Jul
Sep

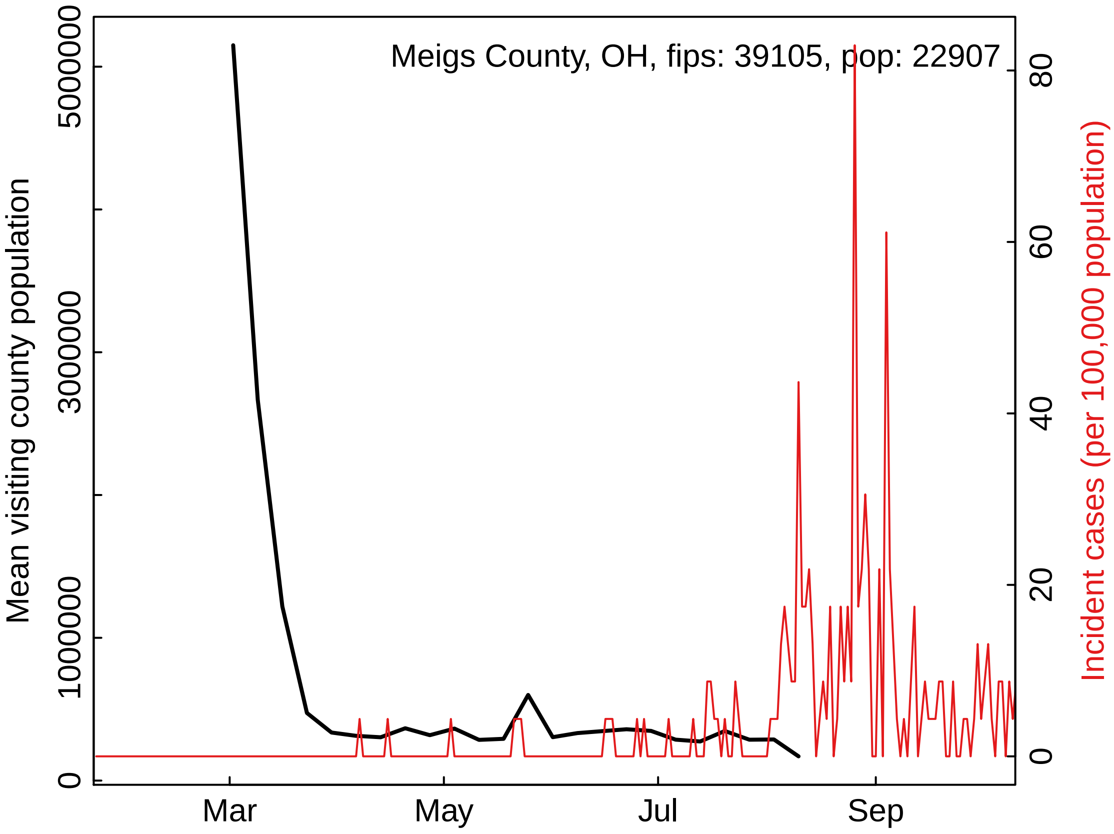

Meigs County, OH, fips: 39105, pop: 22907
Mean visiting county population
Incident cases (per 100,000 population)
0
1000000
3000000
5000000
0
20
40
60
80
Mar
May
Jul
Sep

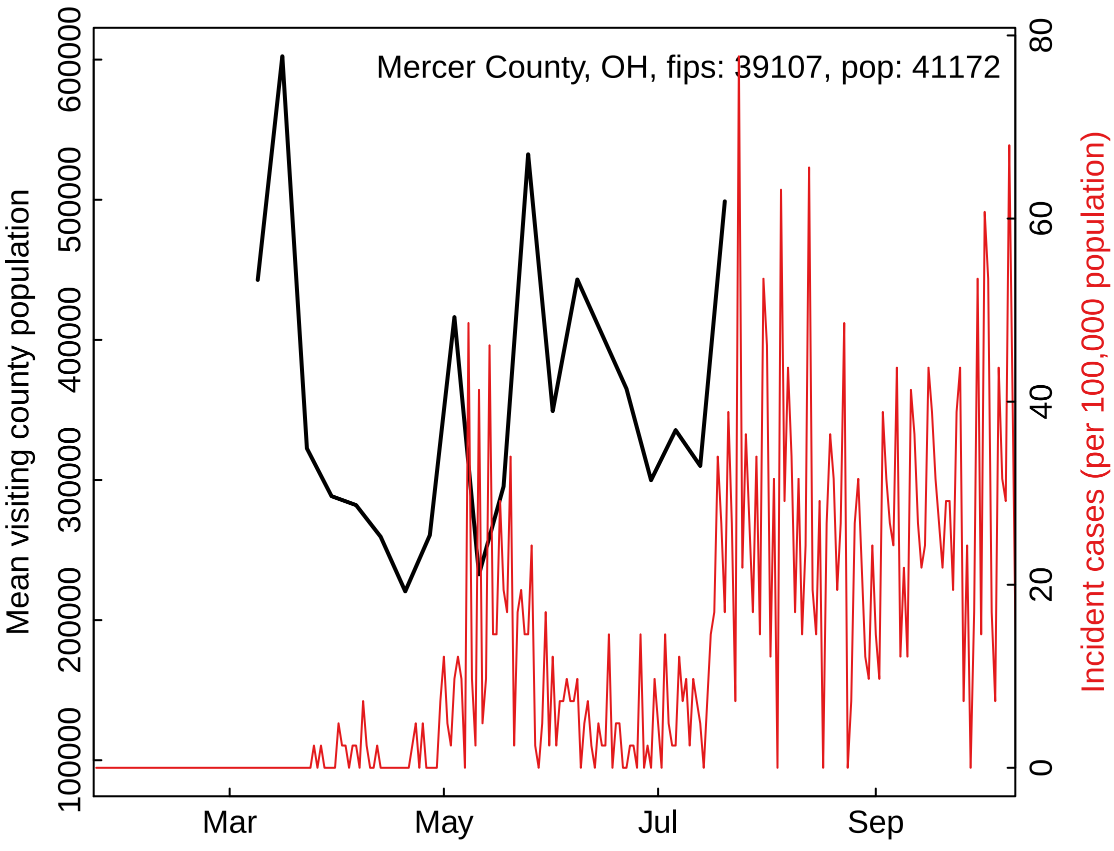

Mercer County, OH, fips: 39107, pop: 41172
Mean visiting county population
Incident cases (per 100,000 population)
100000
200000
300000
400000
500000
600000
0
20
40
60
80
Mar
May
Jul
Sep

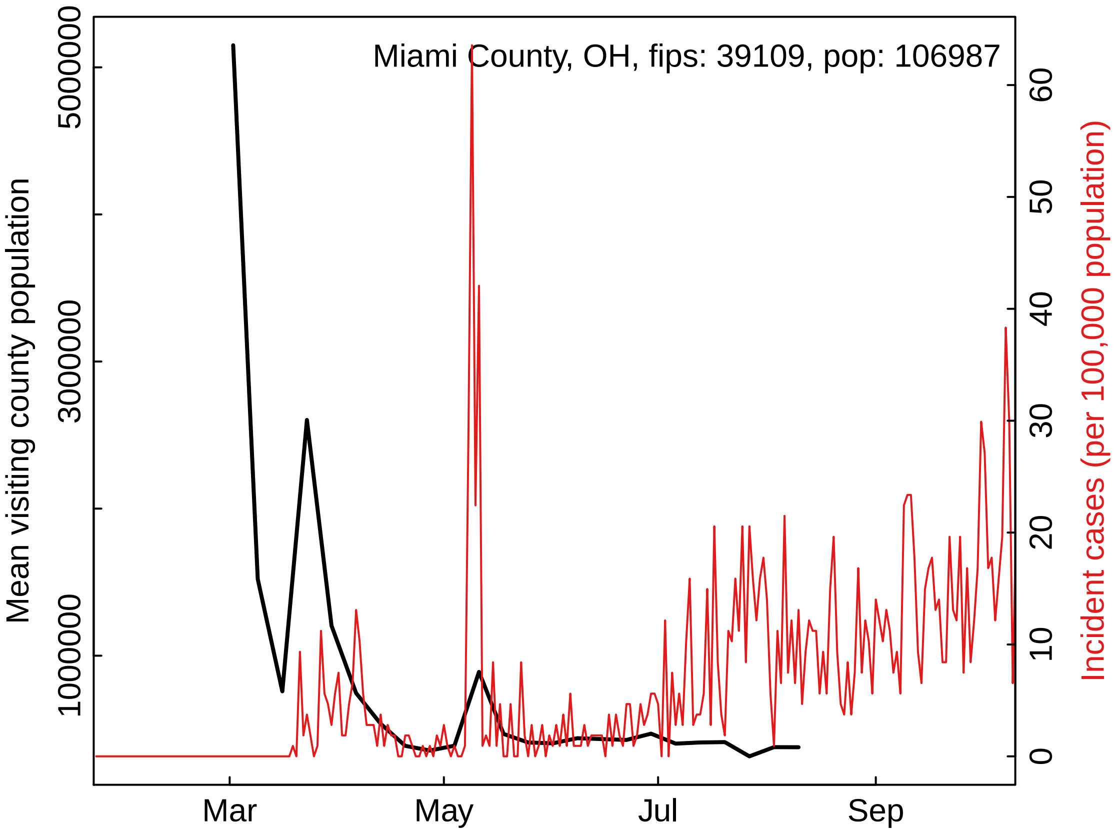

Miami County, OH, fips: 39109, pop: 106987
Mean visiting county population
1000000
3000000
5000000
Incident cases (per 100,000 population)
0
10
20
30
40
50
60
Mar
May
Jul
Sep

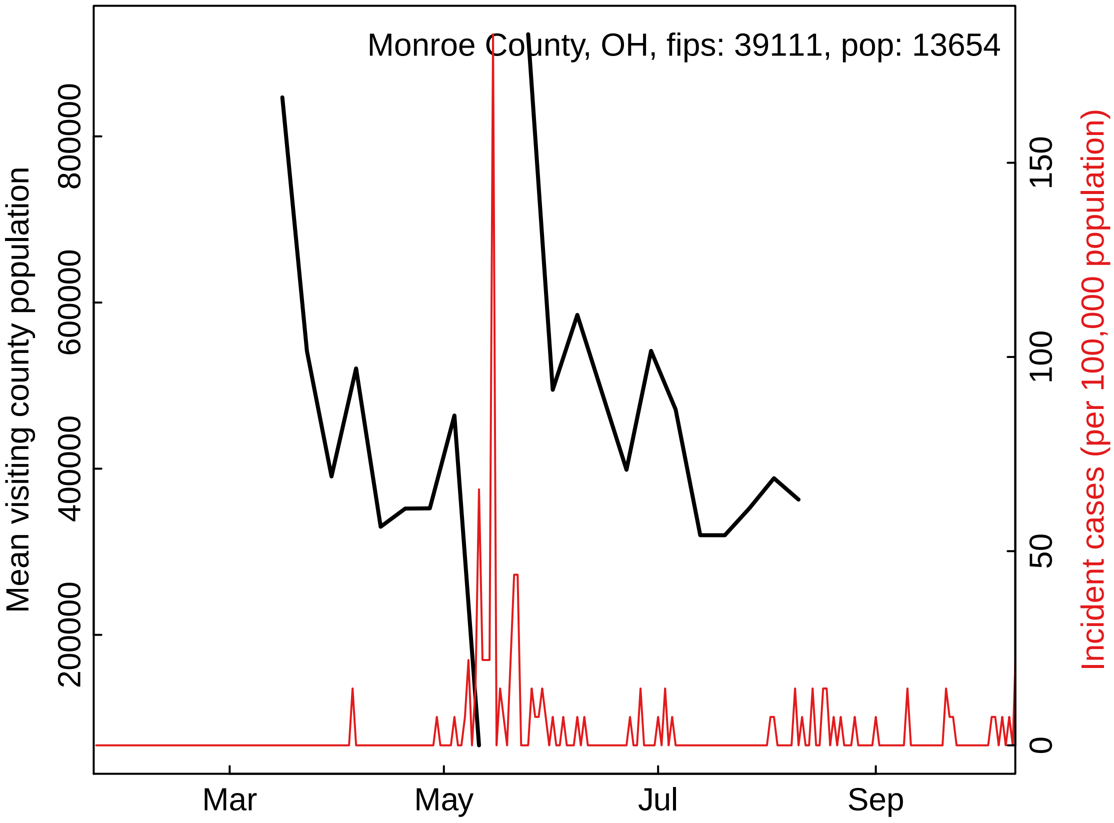

Monroe County, OH, fips: 39111, pop: 13654
Mean visiting county population
200000
400000
600000
800000
Incident cases (per 100,000 population)
0
50
100
150
Mar
May
Jul
Sep

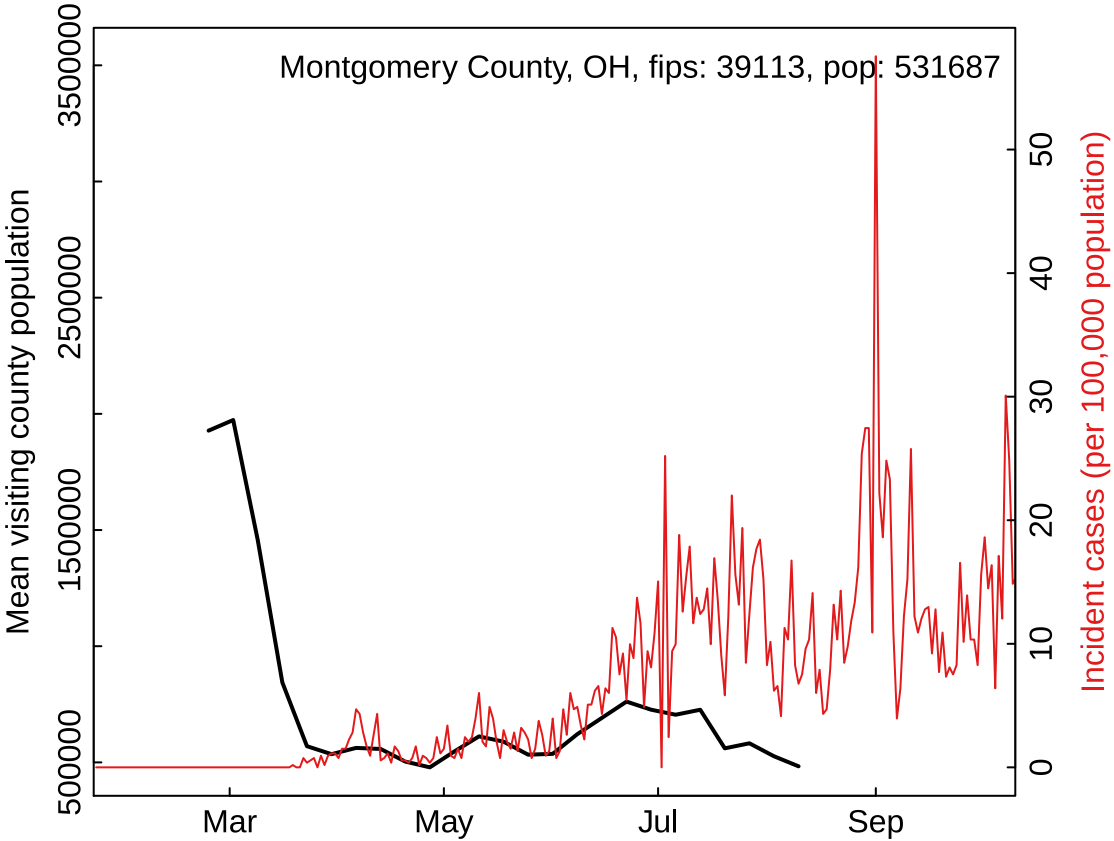
Montgomery County, OH, fips: 39113, pop: 531687
Mean visiting county population
500000
1500000
2500000
3500000
Incident cases (per 100,000 population)
0
10
20
30
40
50
Mar
May
Jul
Sep

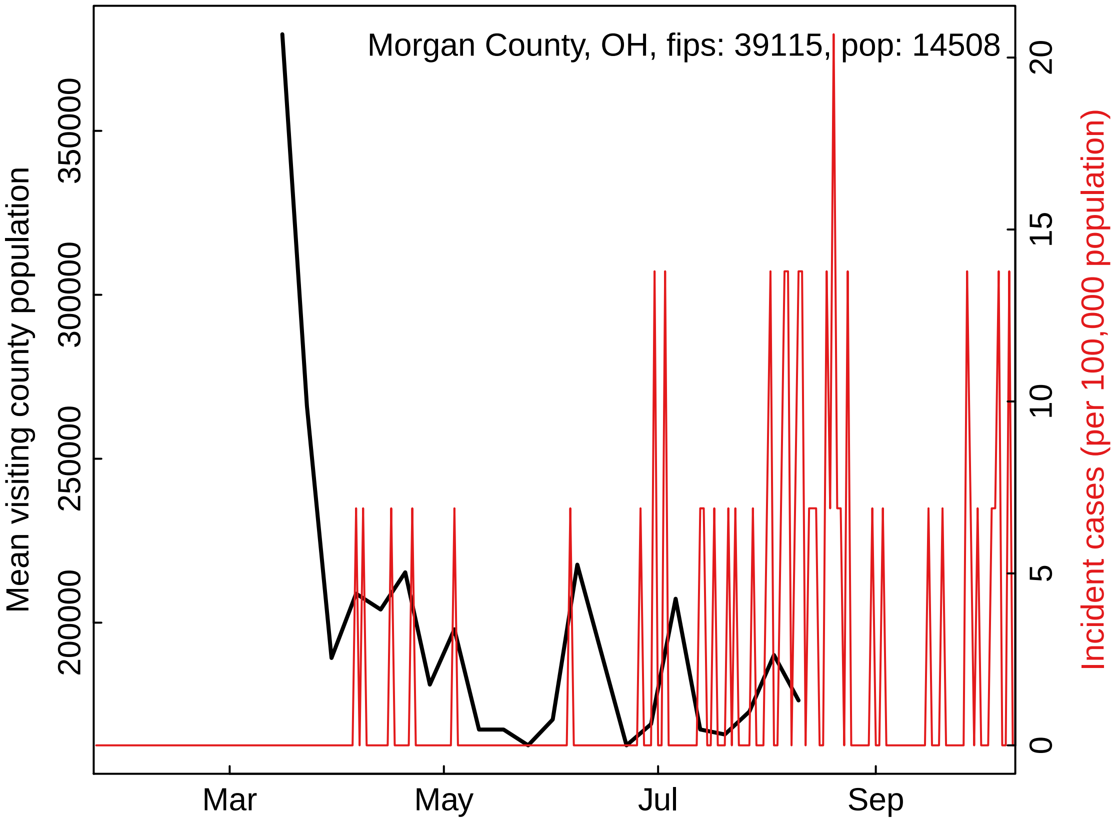

Morgan County, OH, fips: 39115, pop: 14508
Mean visiting county population
200000
250000
300000
350000
Incident cases (per 100,000 population)
0
5
10
15
20
Mar
May
Jul
Sep

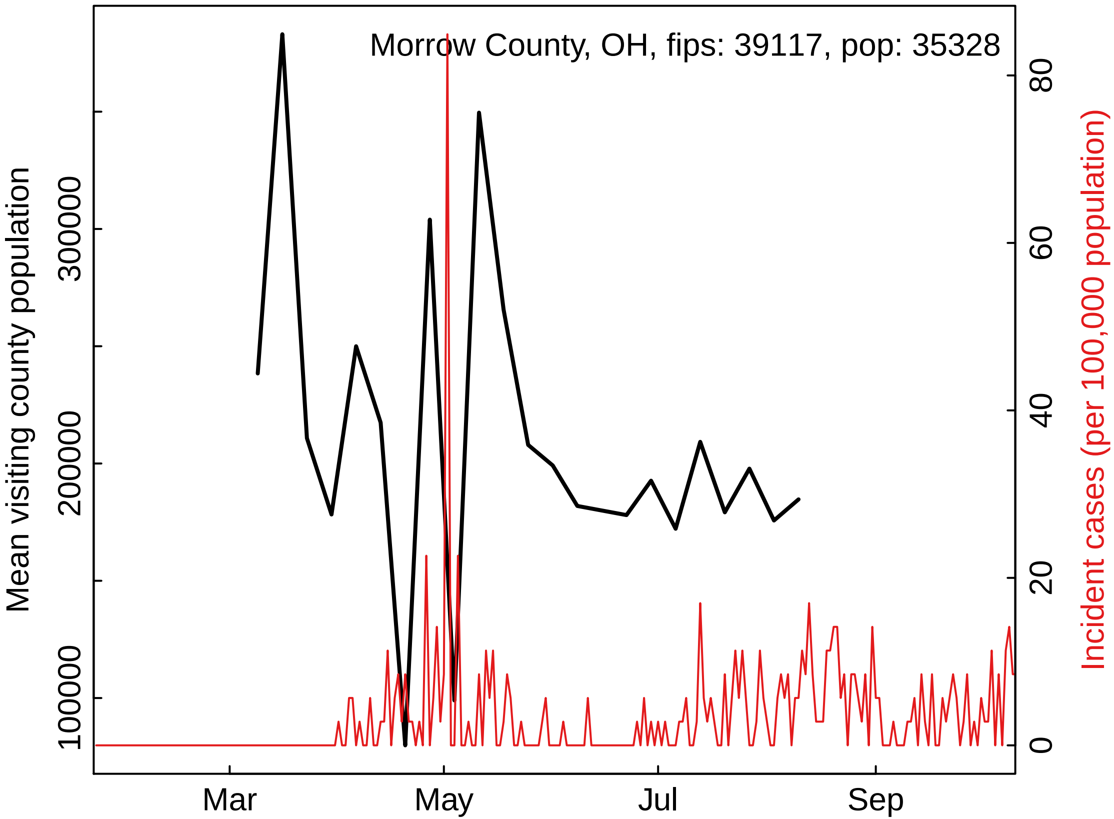
Morrow County, OH, fips: 39117, pop: 35328
Mean visiting county population
100000
200000
300000
Incident cases (per 100,000 population)
0
20
40
60
80
Mar
May
Jul
Sep

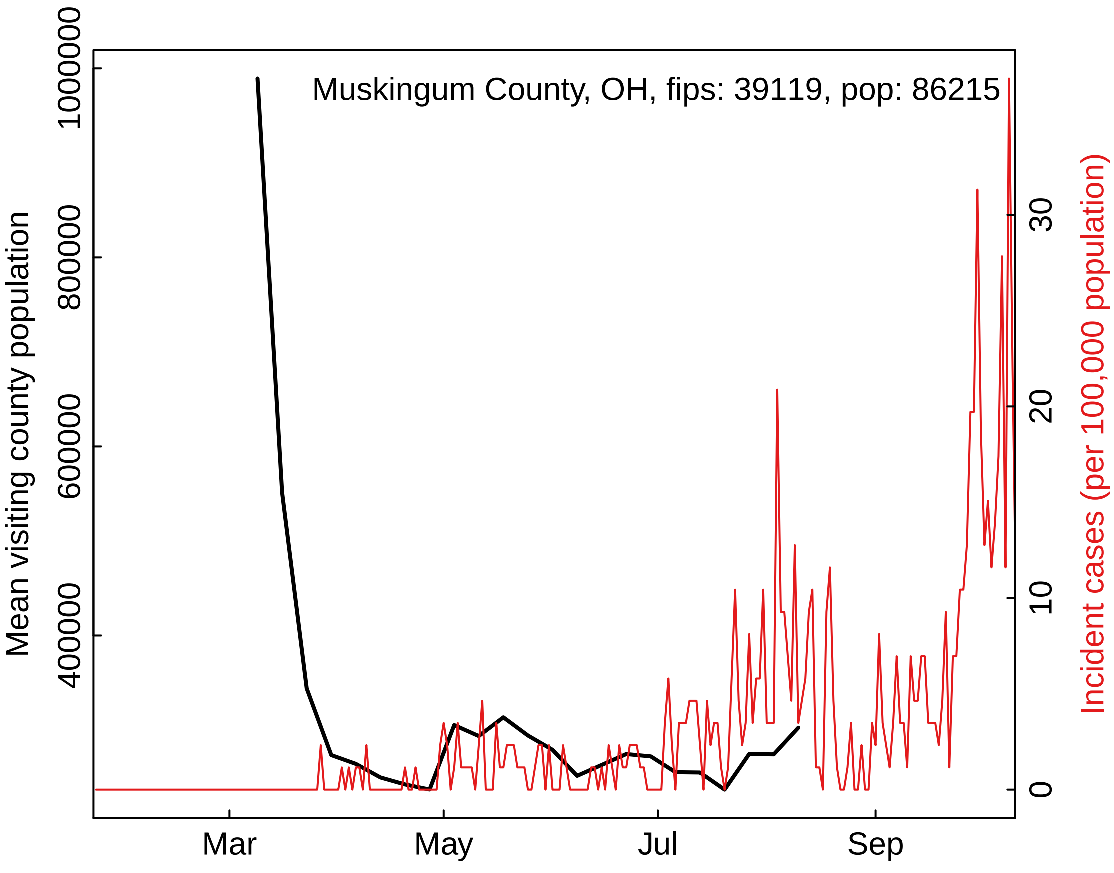
Muskingum County, OH, fips: 39119, pop: 86215
Mean visiting county population
400000
600000
800000
1000000
Incident cases (per 100,000 population)
0
10
20
30
Mar
May
Jul
Sep

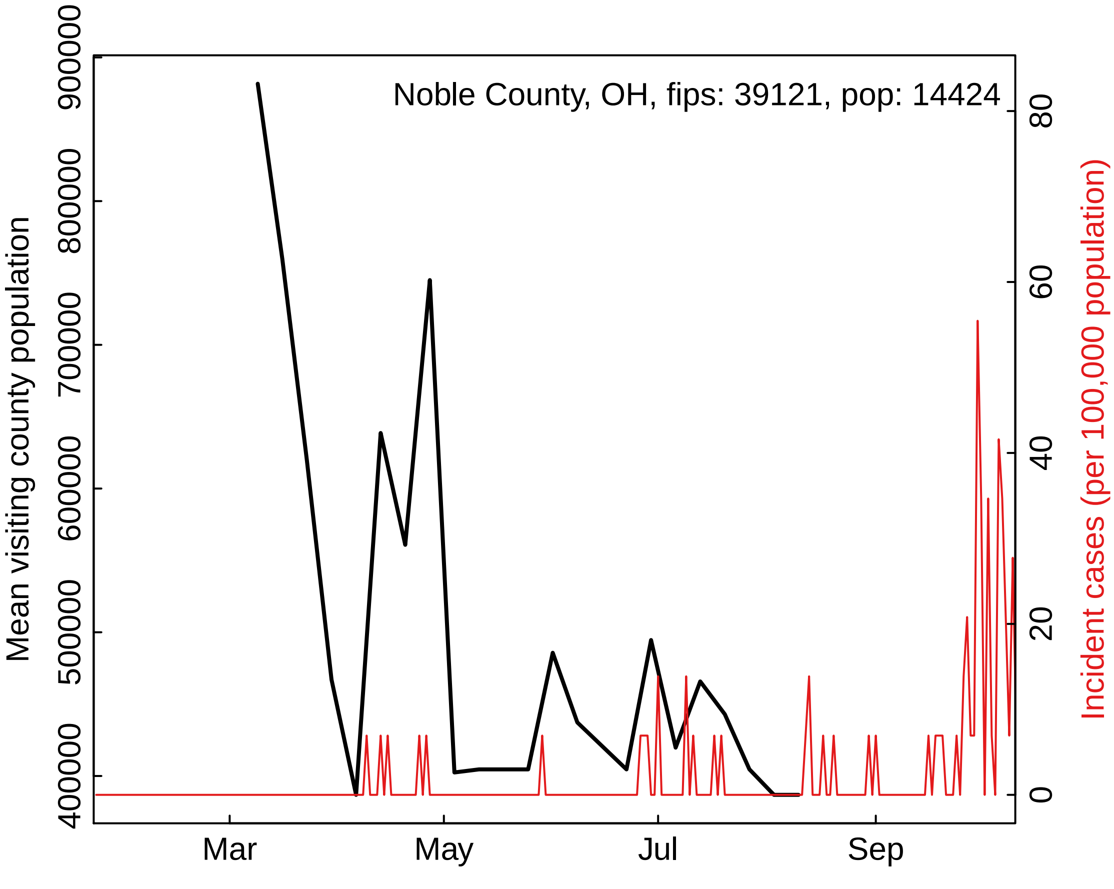

Noble County, OH, fips: 39121, pop: 14424
Mean visiting county population
400000
500000
600000
700000
800000
900000
Incident cases (per 100,000 population)
0
20
40
60
80
Mar
May
Jul
Sep

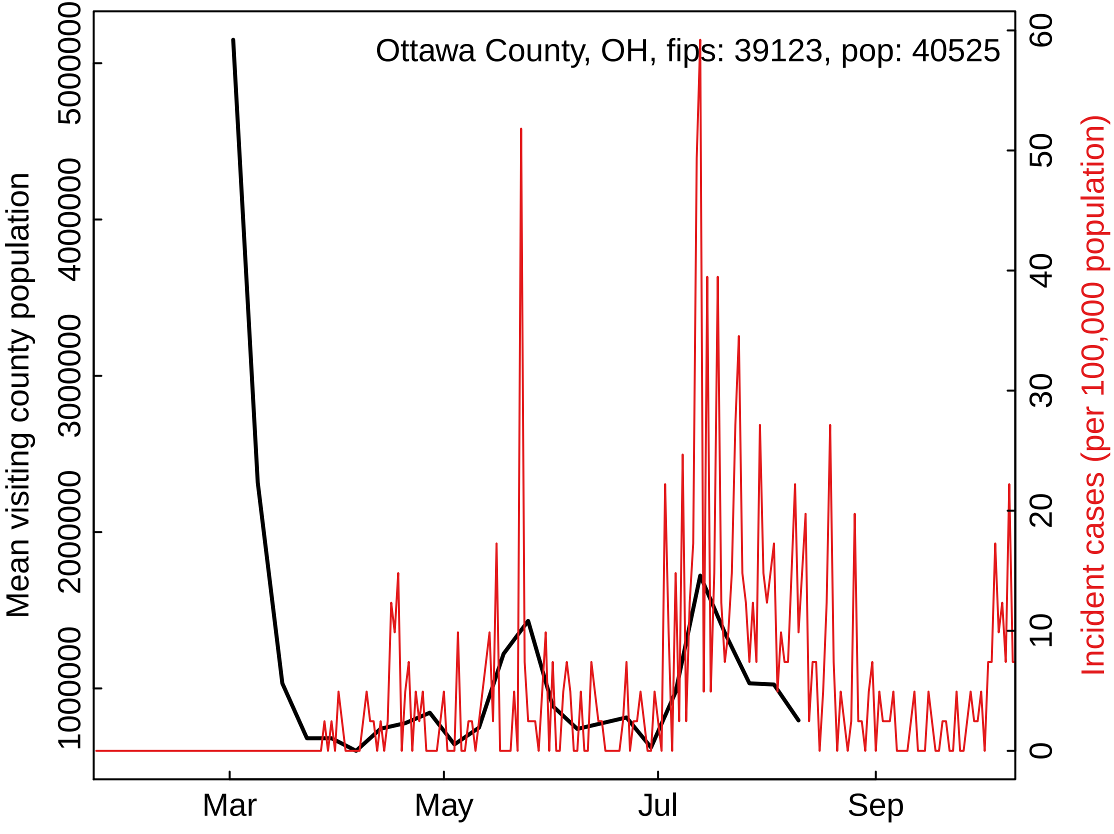

Ottawa County, OH, fips: 39123, pop: 40525
Mean visiting county population
10000000
20000000
30000000
40000000
50000000
Incident cases (per 100,000 population)
0
10
20
30
40
50
60
Mar
May
Jul
Sep

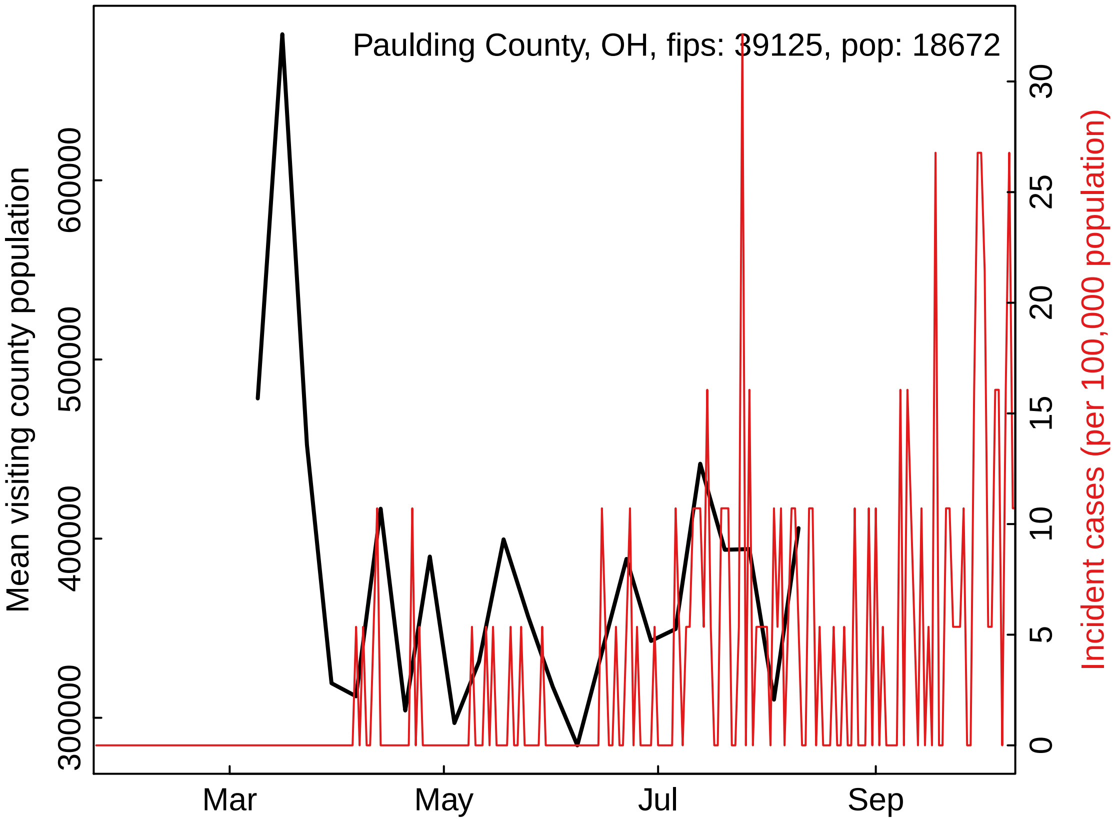

Paulding County, OH, fips: 39125, pop: 18672
Mean visiting county population
Incident cases (per 100,000 population)
300000
400000
500000
600000
0
5
10
15
20
25
30
Mar
May
Jul
Sep

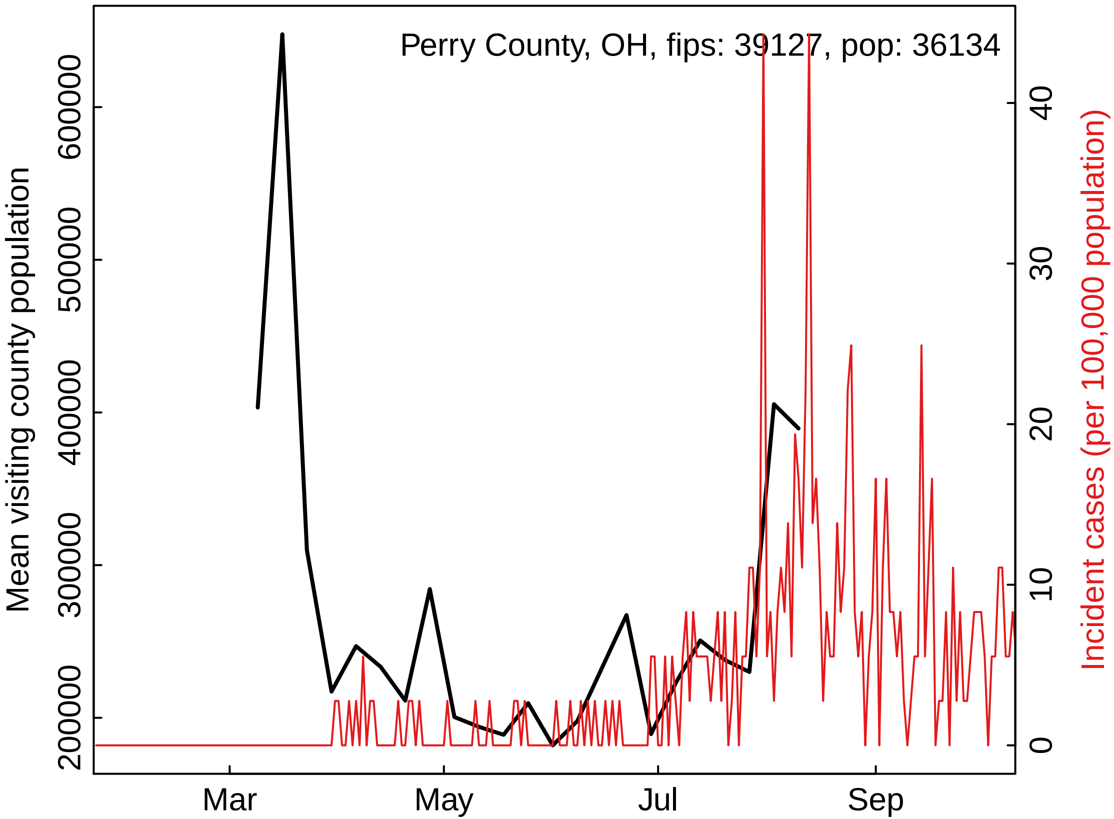
Perry County, OH, fips: 39127, pop: 36134
Mean visiting county population
200000
300000
400000
500000
600000
Incident cases (per 100,000 population)
0
10
20
30
40
Mar
May
Jul
Sep

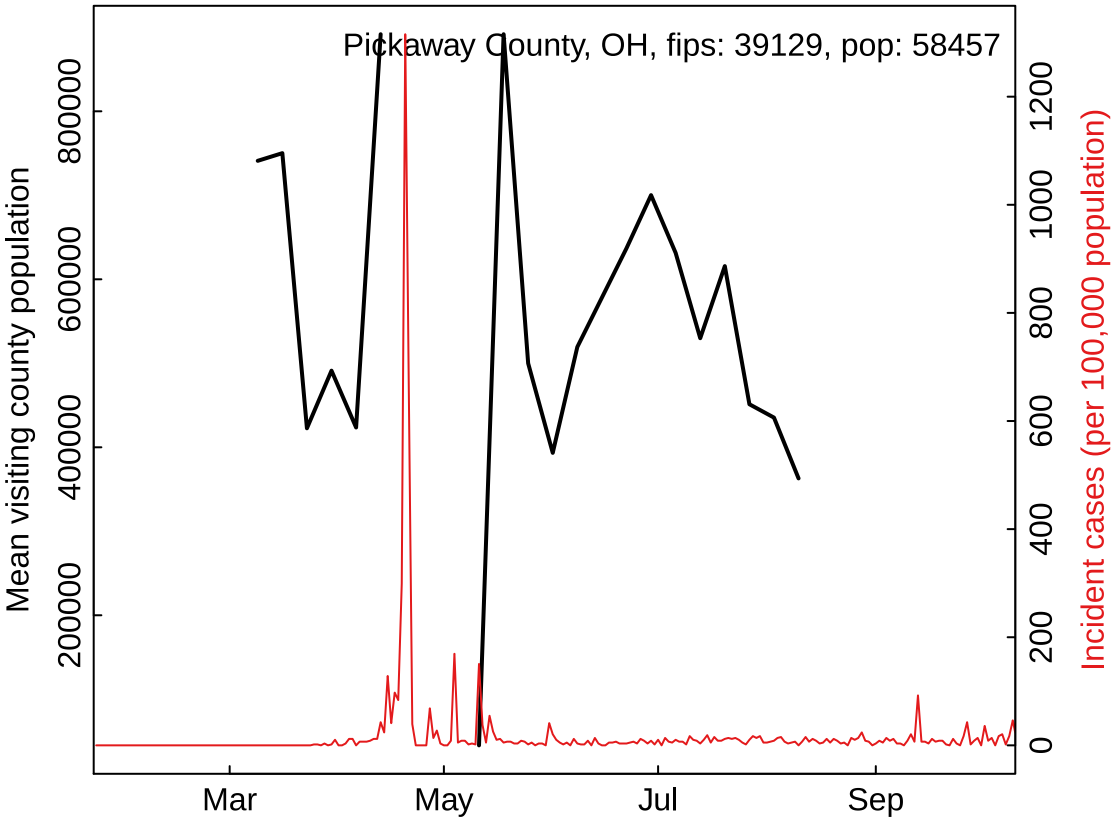

Pickaway County, OH, fips: 39129, pop: 58457
Mean visiting county population
200000
400000
600000
800000
Incident cases (per 100,000 population)
0
200
400
600
800
1000
1200
Mar
May
Jul
Sep

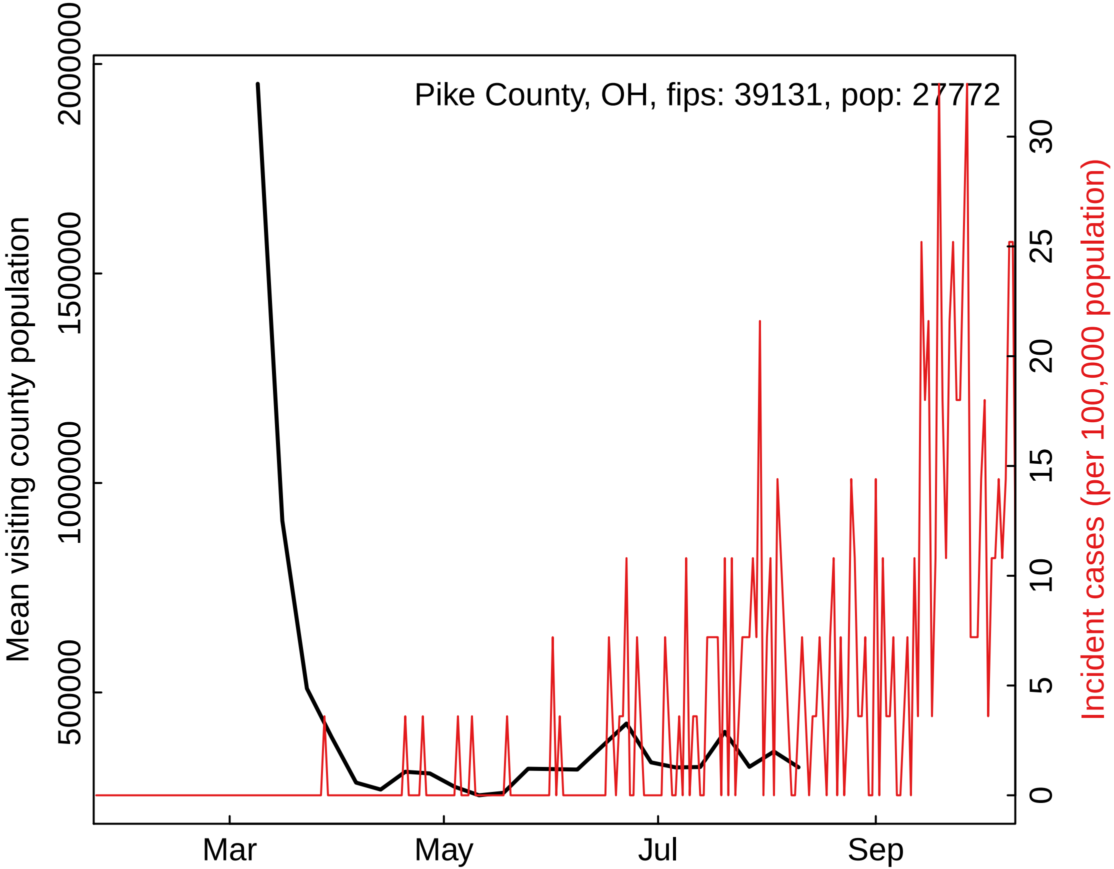

Pike County, OH, fips: 39131, pop: 27772
Mean visiting county population
Incident cases (per 100,000 population)
2000000
1500000
1000000
500000
0
5
10
15
20
25
30
Mar
May
Jul
Sep

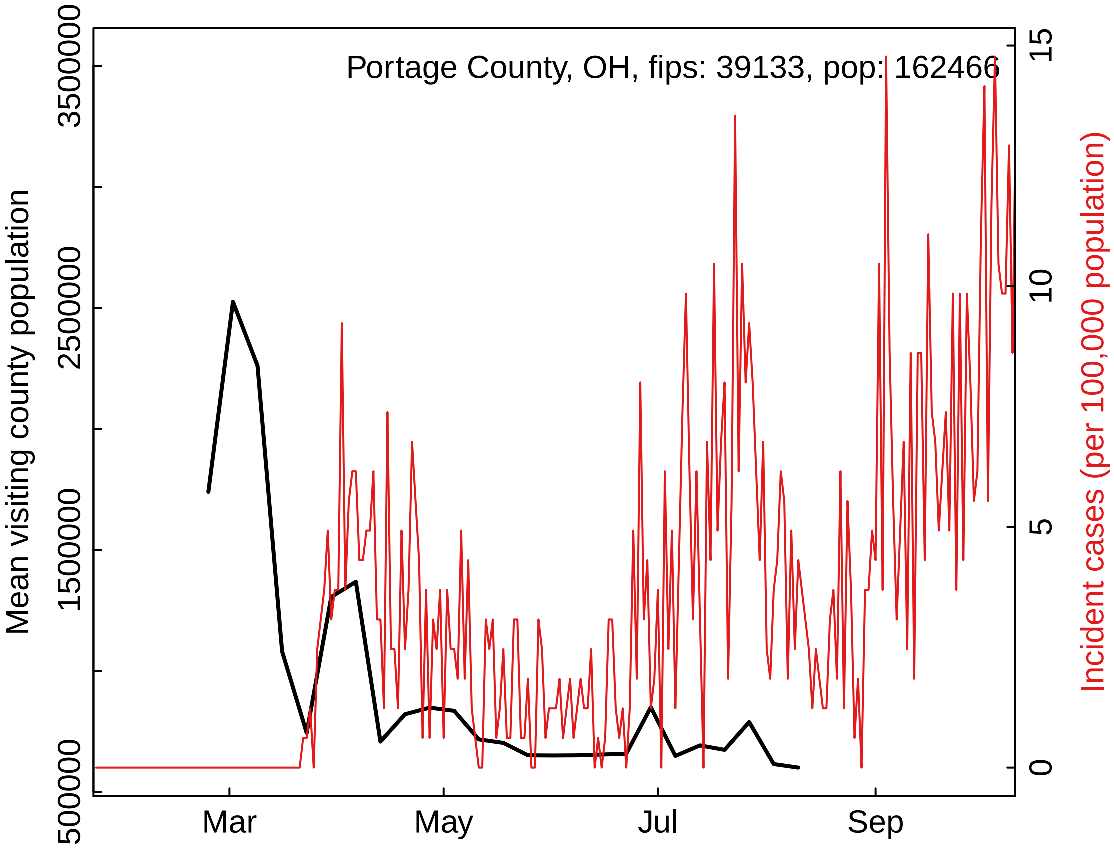

Portage County, OH, fips: 39133, pop: 162466
Mean visiting county population
500000
1500000
2500000
3500000
Incident cases (per 100,000 population)
0
5
10
15
Mar
May
Jul
Sep

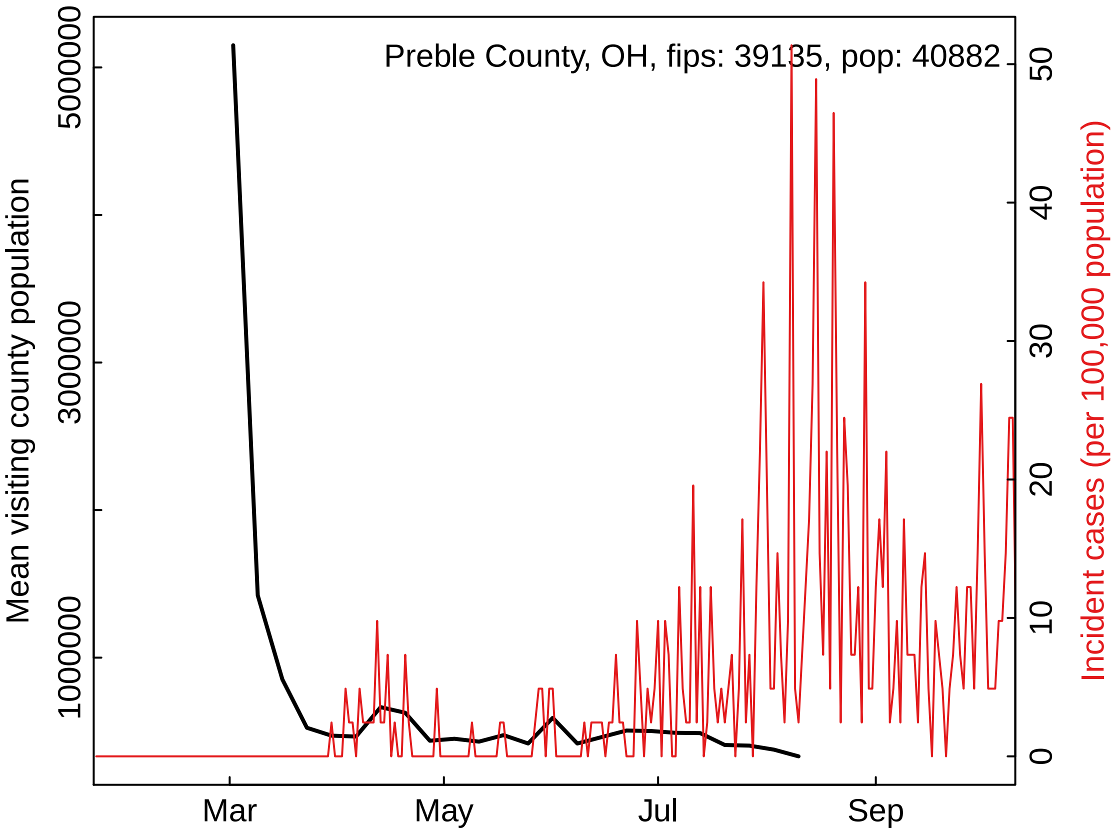

Preble County, OH, fips: 39135, pop: 40882
Mean visiting county population
1000000
3000000
5000000
Incident cases (per 100,000 population)
0
10
20
30
40
50
Mar
May
Jul
Sep

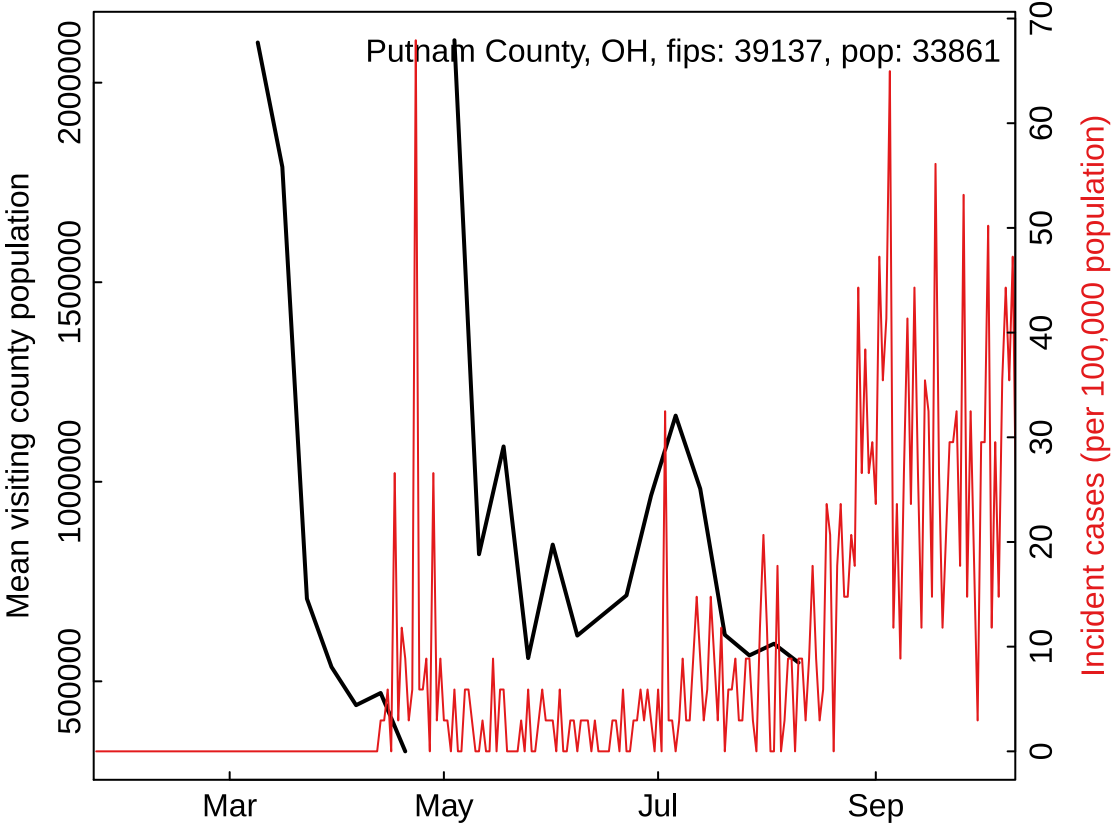

Putnam County, OH, fips: 39137, pop: 33861
Mean visiting county population
500000
1000000
1500000
2000000
Incident cases (per 100,000 population)
0
10
20
30
40
50
60
70
Mar
May
Jul
Sep

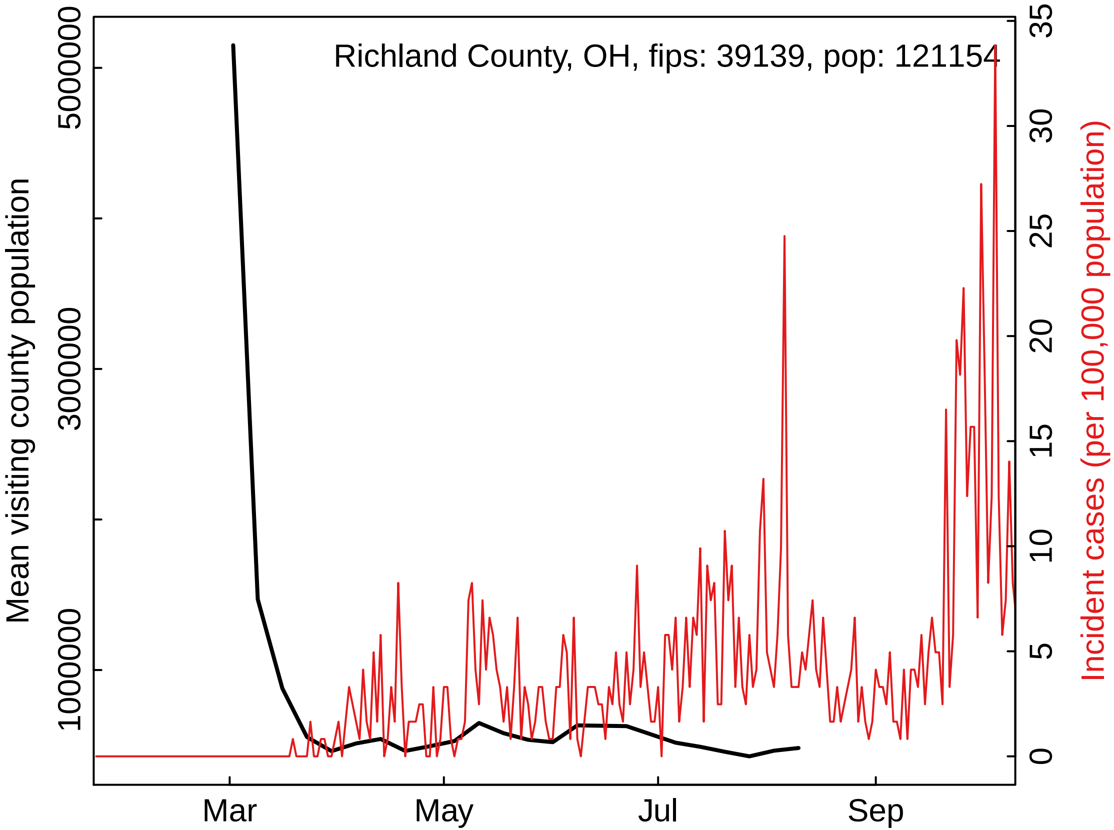
Richland County, OH, fips: 39139, pop: 121154
Mean visiting county population
10000000
30000000
50000000
Incident cases (per 100,000 population)
0
5
10
15
20
25
30
35
Mar
May
Jul
Sep

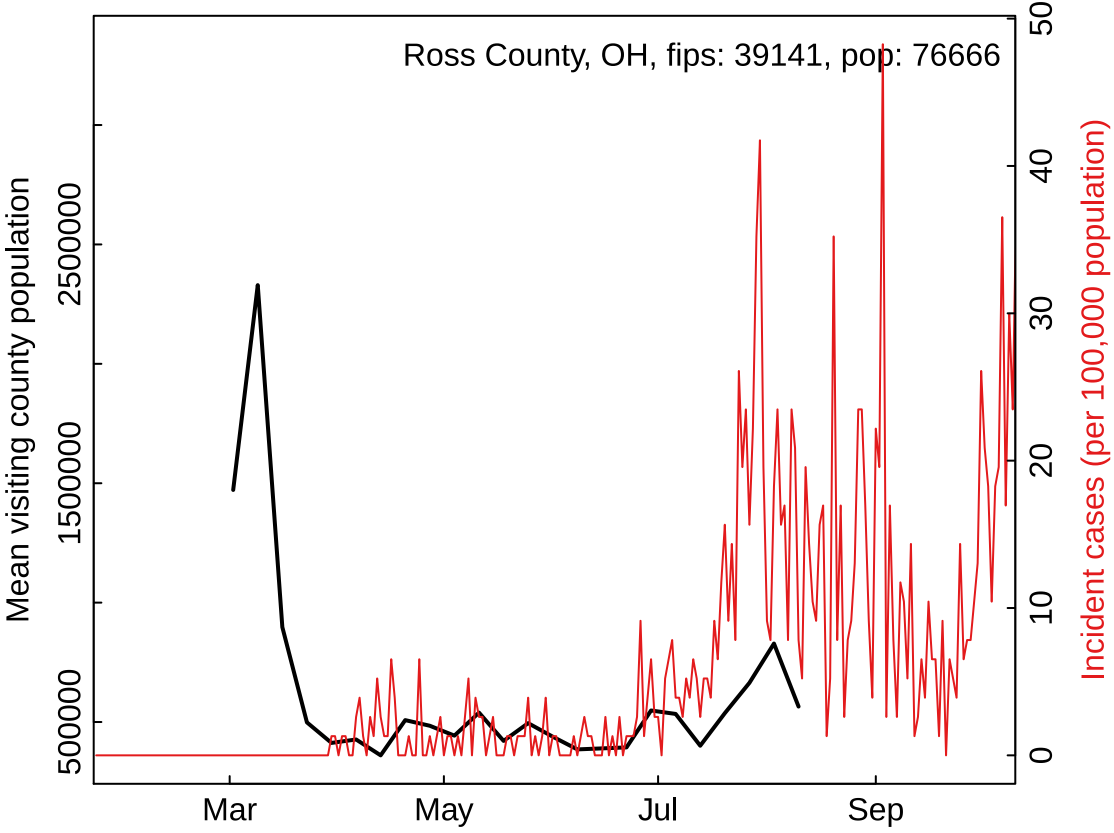

Ross County, OH, fips: 39141, pop: 76666
Mean visiting county population
500000
1500000
2500000
Incident cases (per 100,000 population)
0
10
20
30
40
50
Mar
May
Jul
Sep

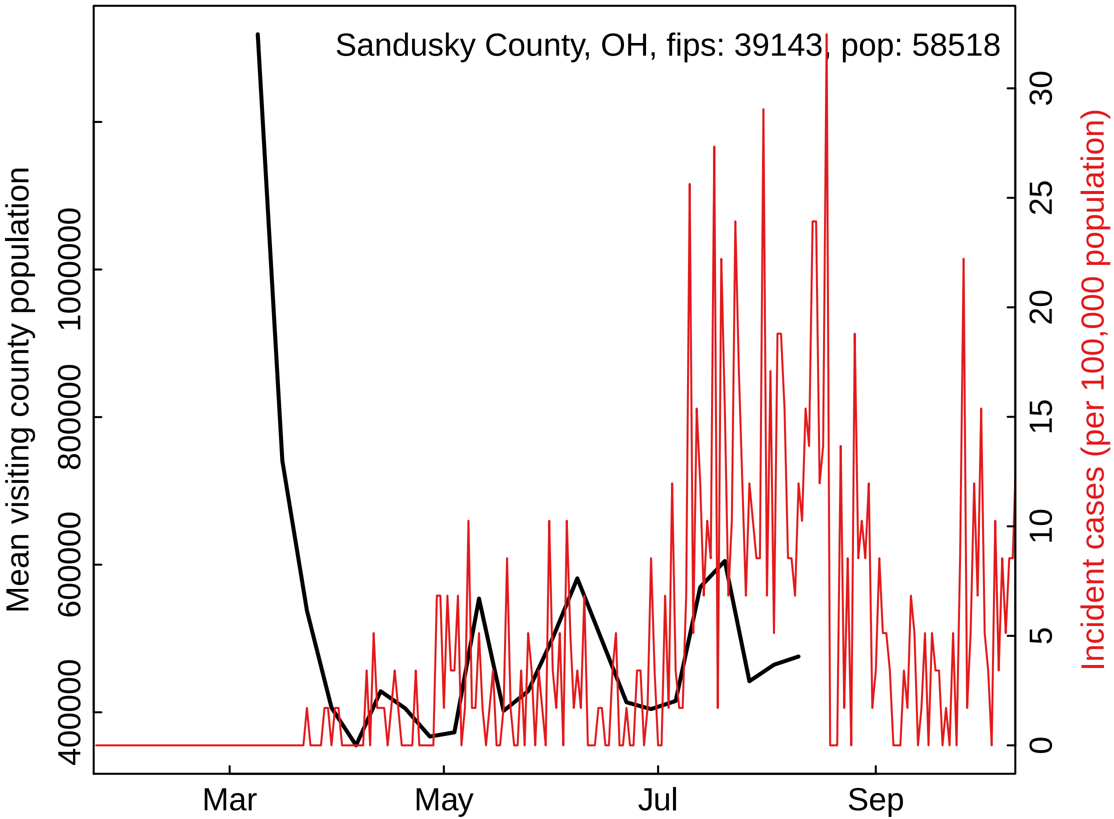

Sandusky County, OH, fips: 39143, pop: 58518
Mean visiting county population
400000
600000
800000
1000000
Incident cases (per 100,000 population)
0
5
10
15
20
25
30
Mar
May
Jul
Sep

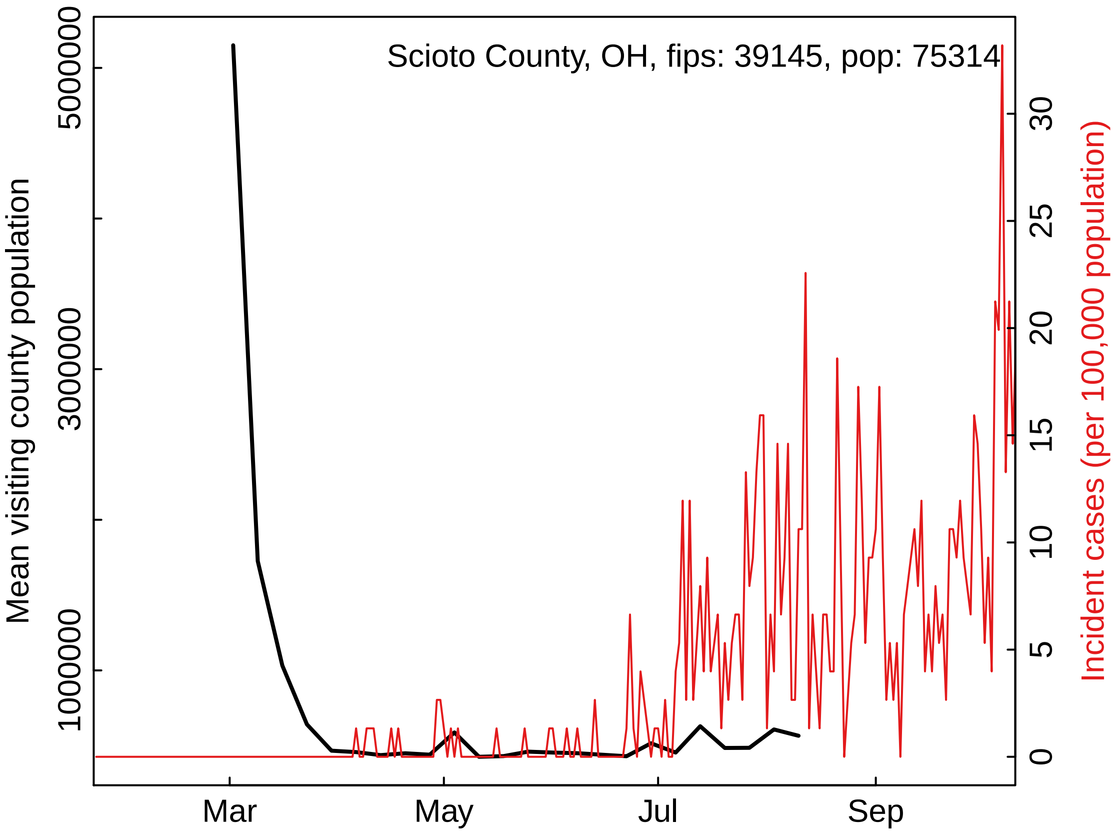

Scioto County, OH, fips: 39145, pop: 75314
Mean visiting county population
Incident cases (per 100,000 population)
10000000
30000000
50000000
0
5
10
15
20
25
30
Mar
May
Jul
Sep

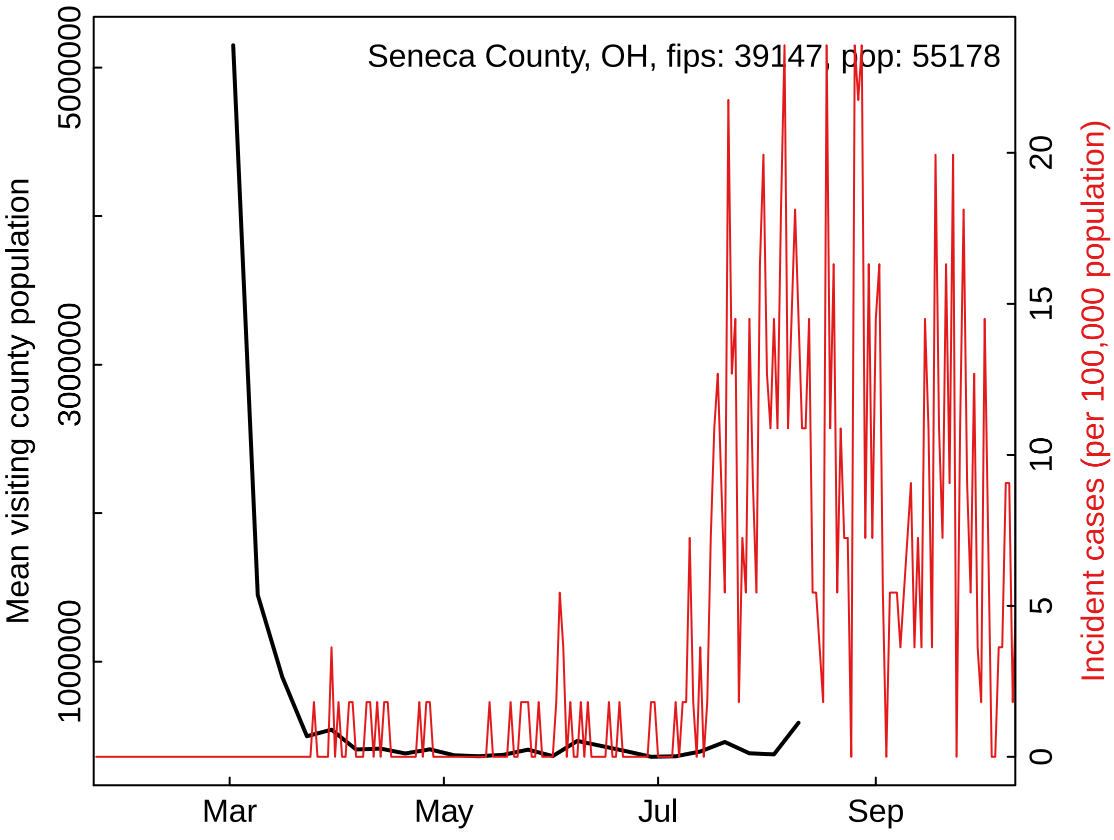

Seneca County, OH, fips: 39147, pop: 55178
Mean visiting county population
Incident cases (per 100,000 population)
1000000
3000000
5000000
0
5
10
15
20
Mar
May
Jul
Sep

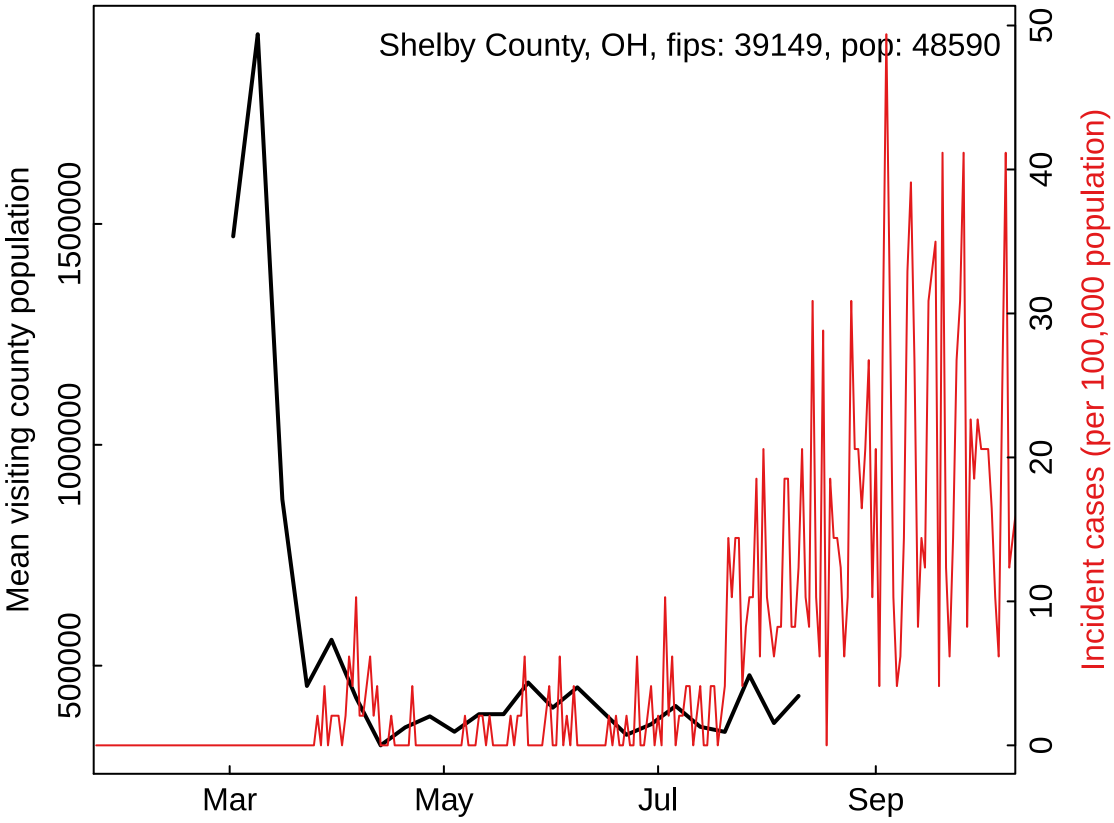

Shelby County, OH, fips: 39149, pop: 48590
Mean visiting county population
500000
1000000
1500000
Incident cases (per 100,000 population)
0
10
20
30
40
50
Mar
May
Jul
Sep

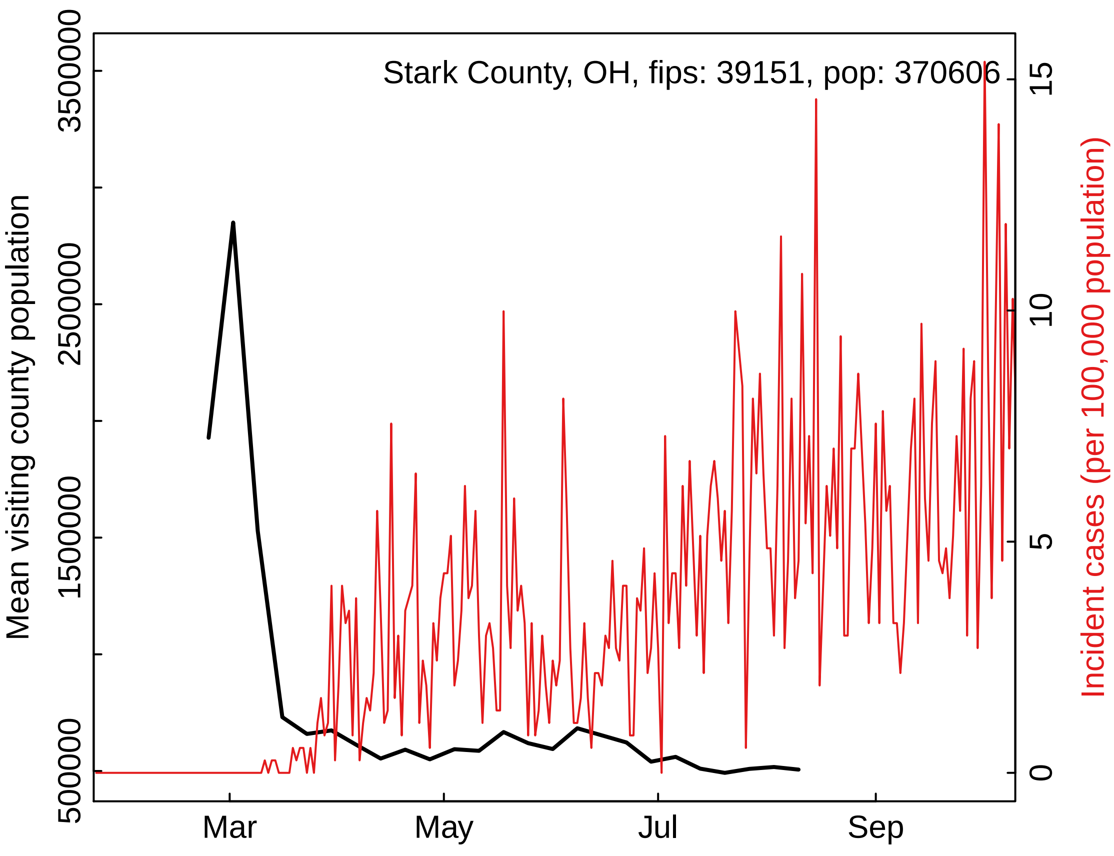

Stark County, OH, fips: 39151, pop: 370606
Mean visiting county population
Incident cases (per 100,000 population)
500000
1500000
2500000
3500000
0
5
10
15
Mar
May
Jul
Sep

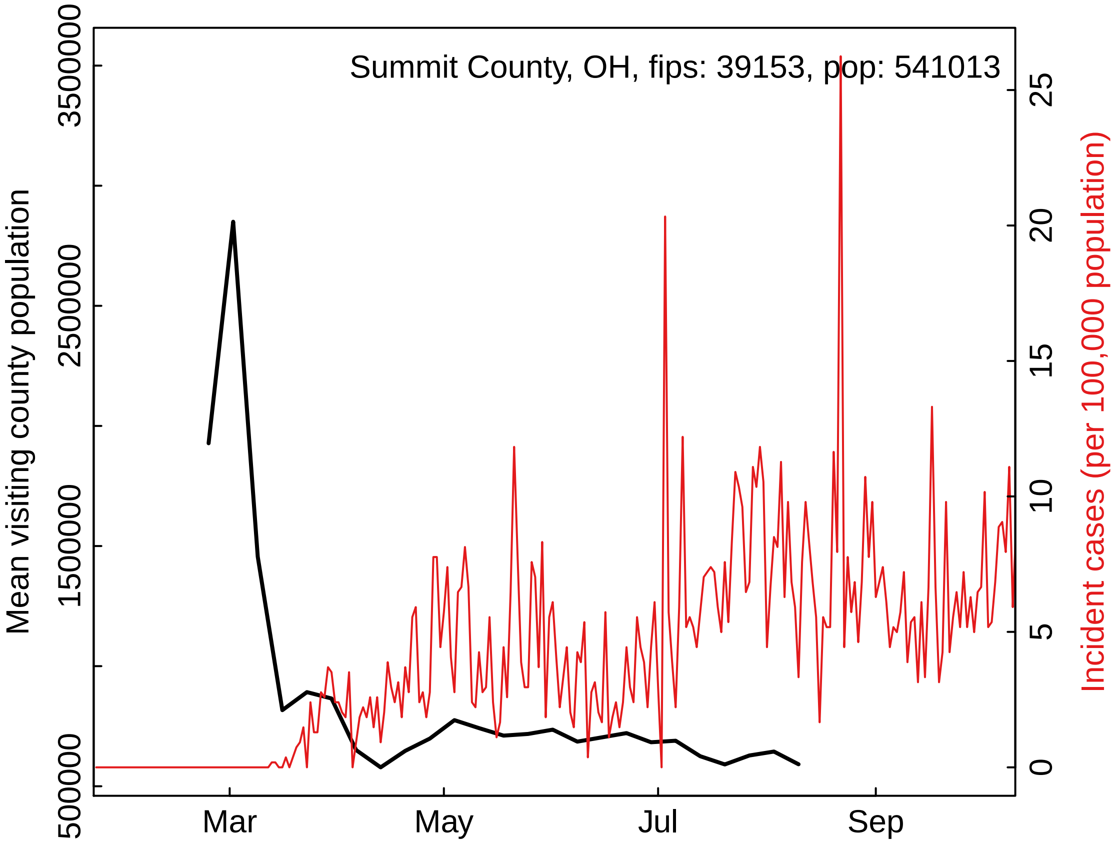
Summit County, OH, fips: 39153, pop: 541013
Mean visiting county population
500000
1500000
2500000
3500000
Incident cases (per 100,000 population)
0
5
10
15
20
25
Mar
May
Jul
Sep

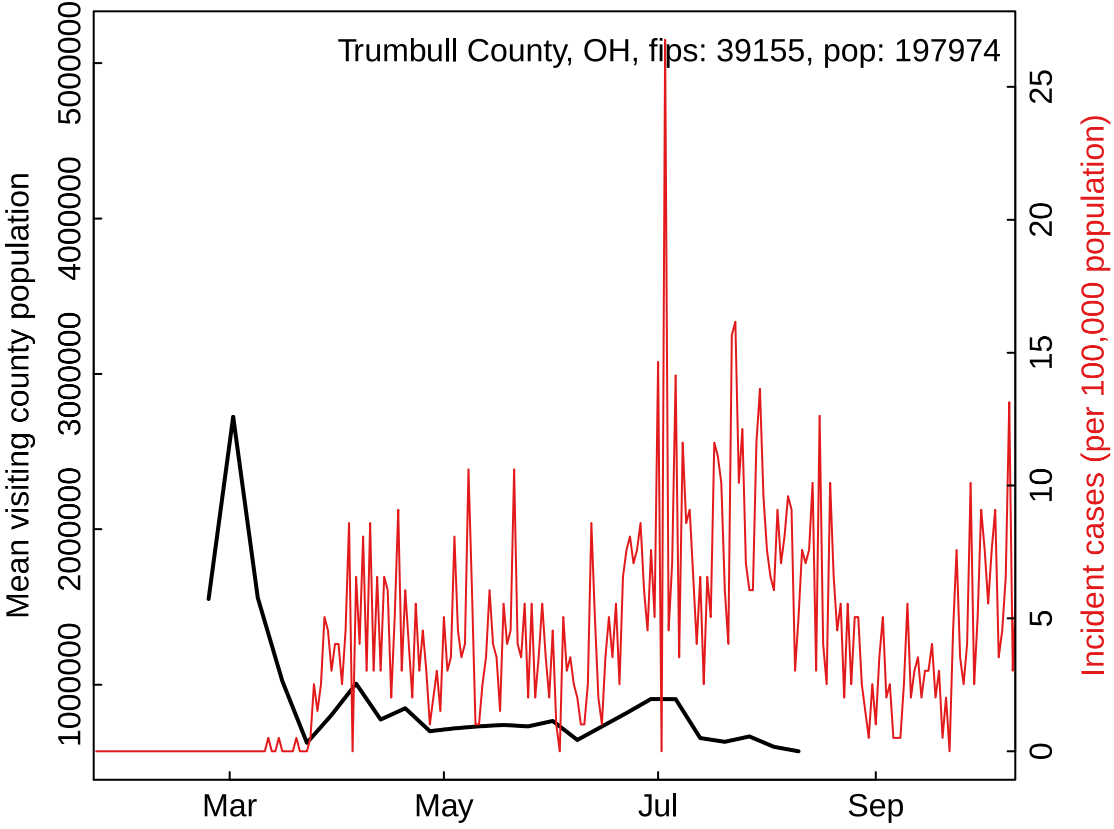

Trumbull County, OH, fips: 39155, pop: 197974
Mean visiting county population
10000000
20000000
30000000
40000000
50000000
Incident cases (per 100,000 population)
0
5
10
15
20
25
Mar
May
Jul
Sep

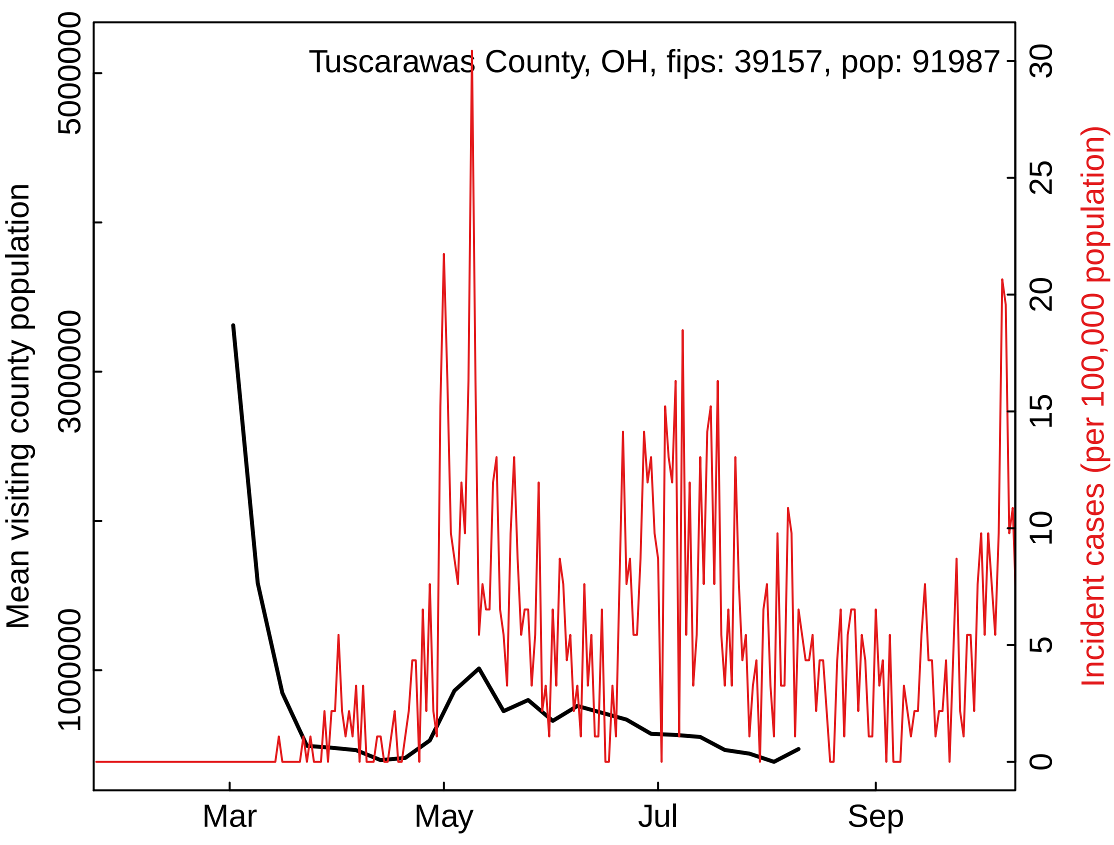

Tuscarawas County, OH, fips: 39157, pop: 91987
Mean visiting county population
Incident cases (per 100,000 population)
1000000
3000000
5000000
0
5
10
15
20
25
30
Mar
May
Jul
Sep

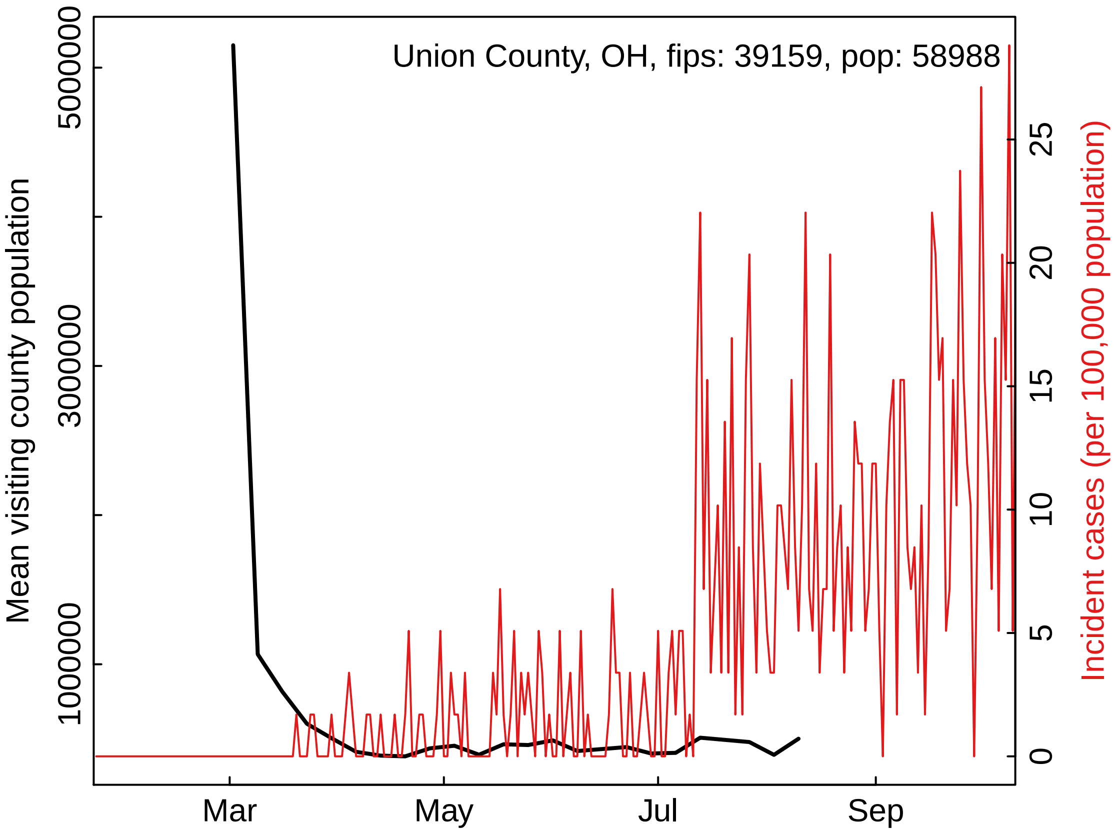

Union County, OH, fips: 39159, pop: 58988
Mean visiting county population
5000000
3000000
1000000
Incident cases (per 100,000 population)
0
5
10
15
20
25
Mar
May
Jul
Sep

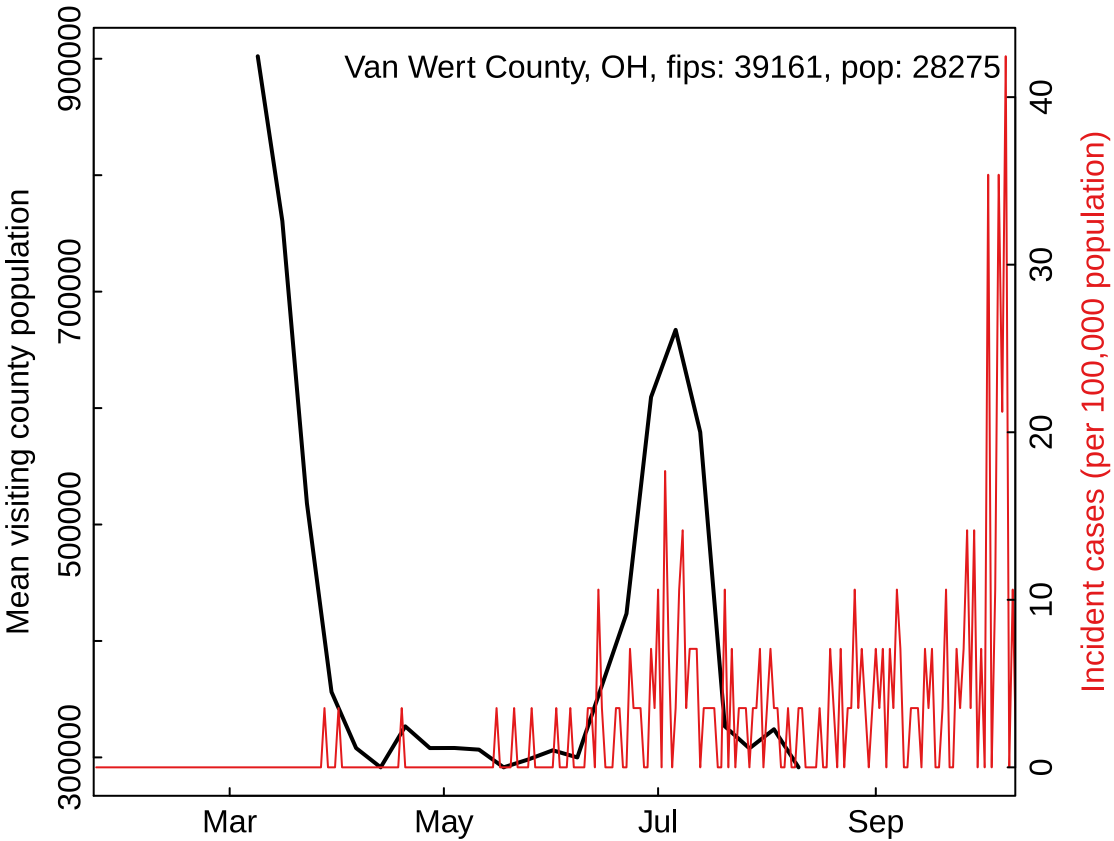
Van Wert County, OH, fips: 39161, pop: 28275
Mean visiting county population
300000
500000
700000
900000
Incident cases (per 100,000 population)
0
10
20
30
40
Mar
May
Jul
Sep

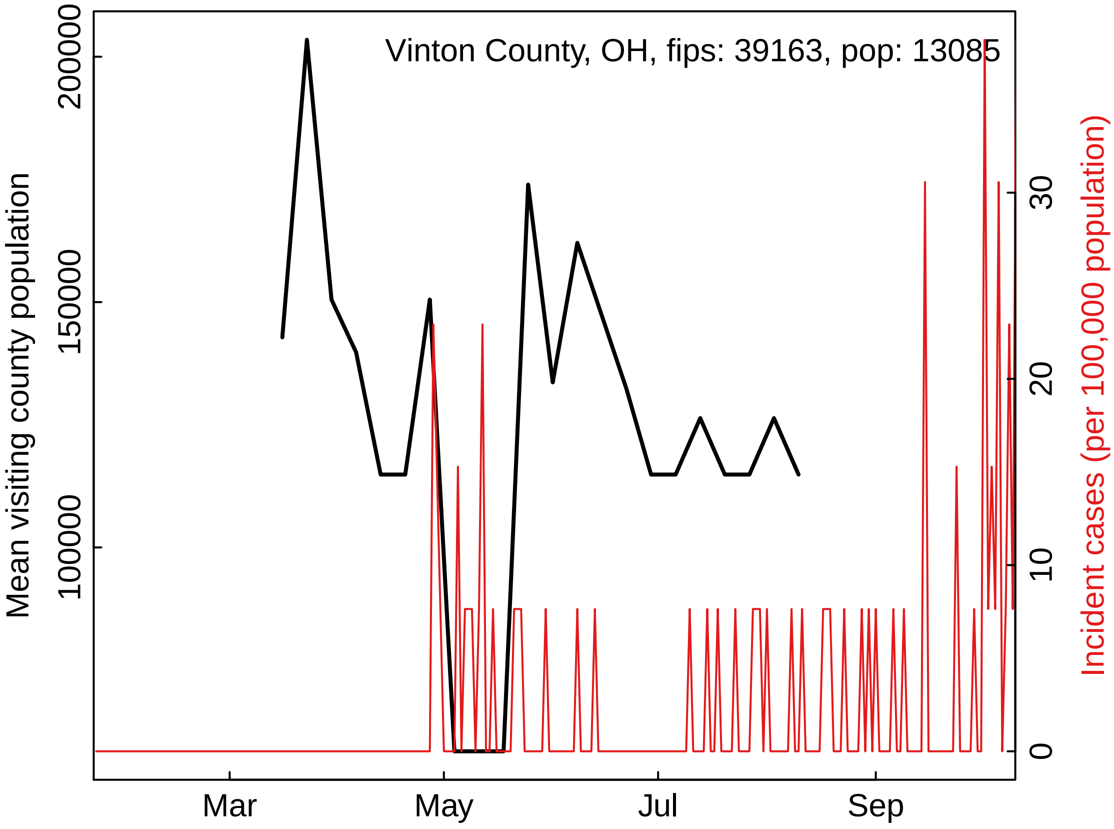

Vinton County, OH, fips: 39163, pop: 13085
Mean visiting county population
100000
150000
200000
Incident cases (per 100,000 population)
0
10
20
30
Mar
May
Jul
Sep

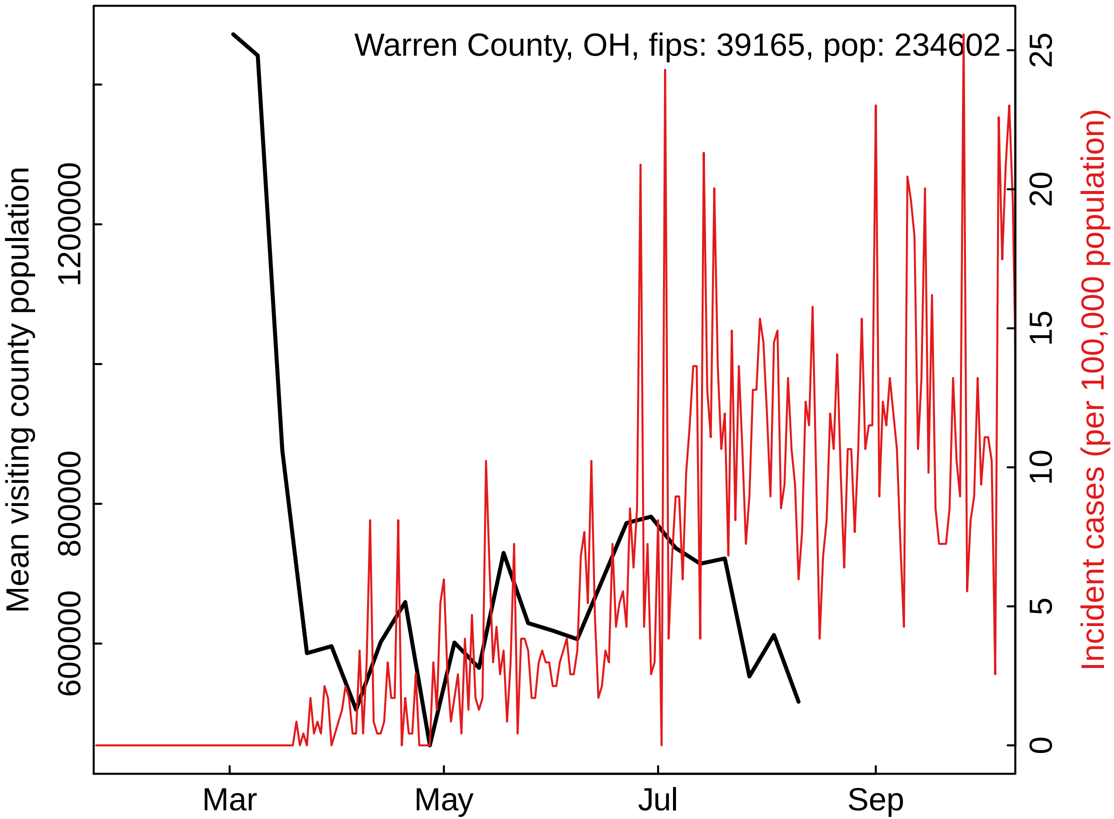

Warren County, OH, fips: 39165, pop: 234602
Mean visiting county population
600000
800000
1200000
Incident cases (per 100,000 population)
0
5
10
15
20
25
Mar
May
Jul
Sep

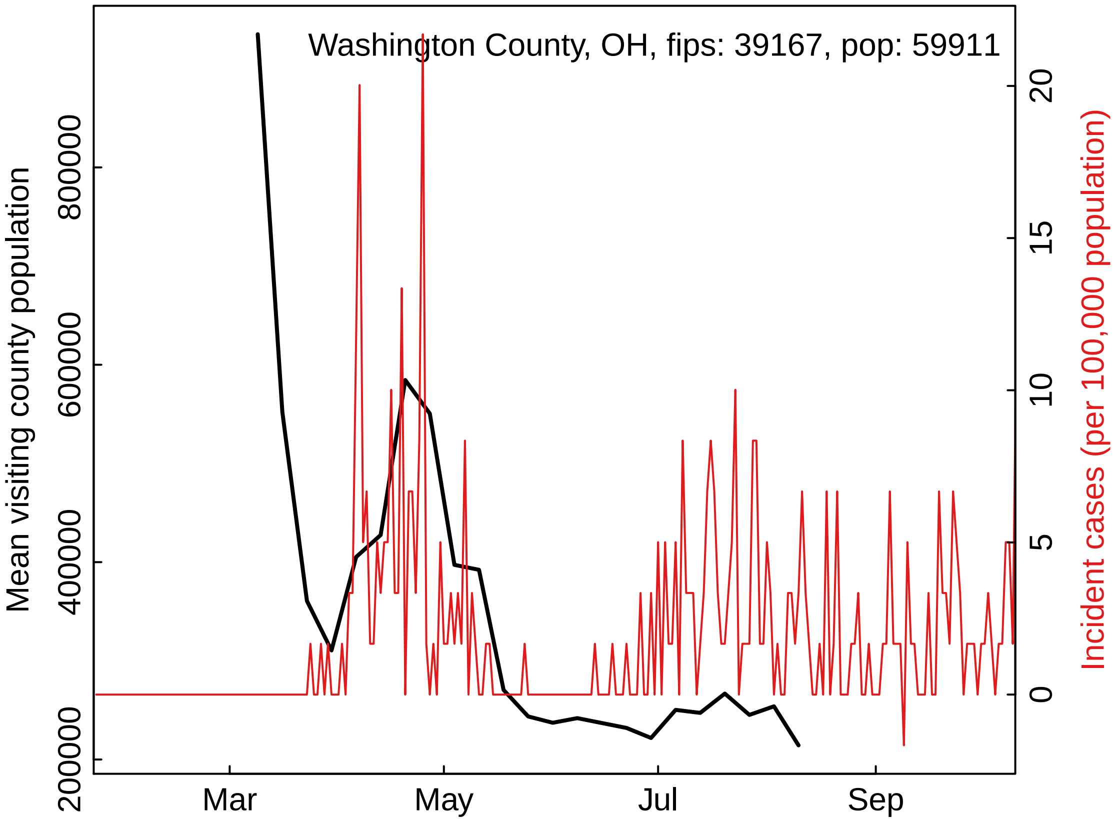
Washington County, OH, fips: 39167, pop: 59911
Mean visiting county population
200000
400000
600000
800000
Incident cases (per 100,000 population)
0
5
10
15
20
Mar
May
Jul
Sep

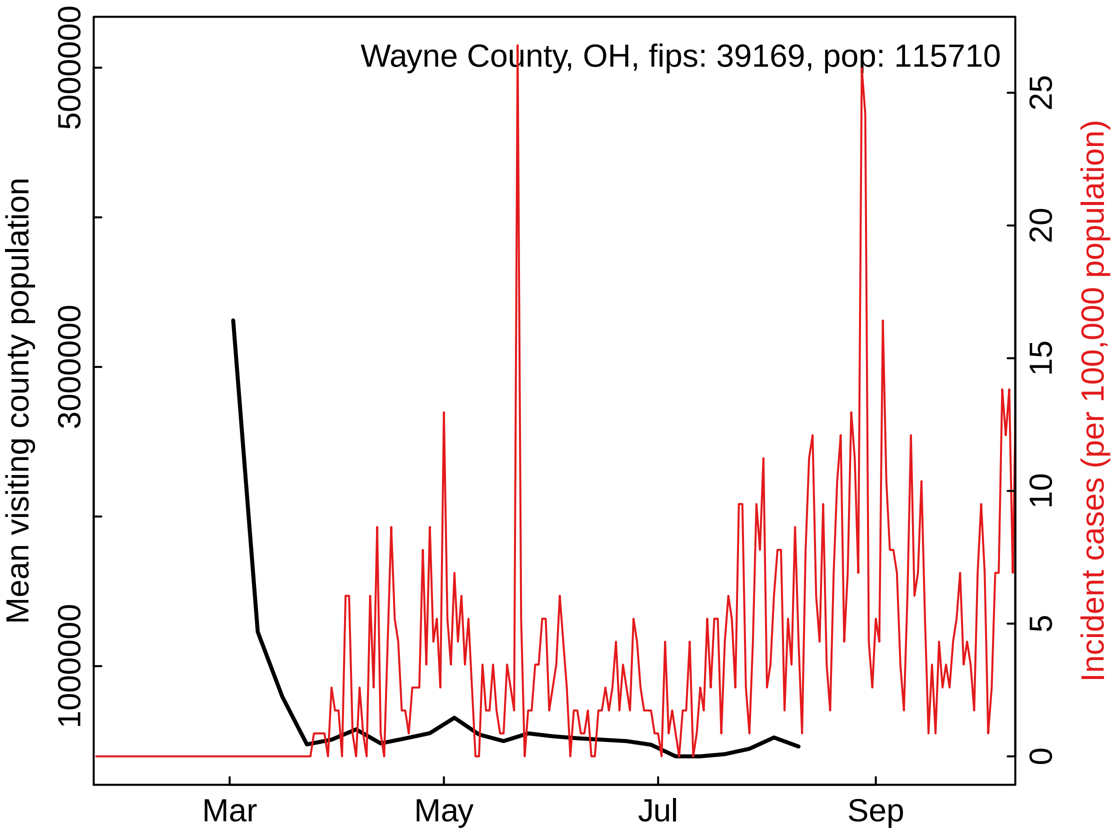
Wayne County, OH, fips: 39169, pop: 115710
Mean visiting county population
1000000
3000000
5000000
Incident cases (per 100,000 population)
0
5
10
15
20
25
Mar
May
Jul
Sep

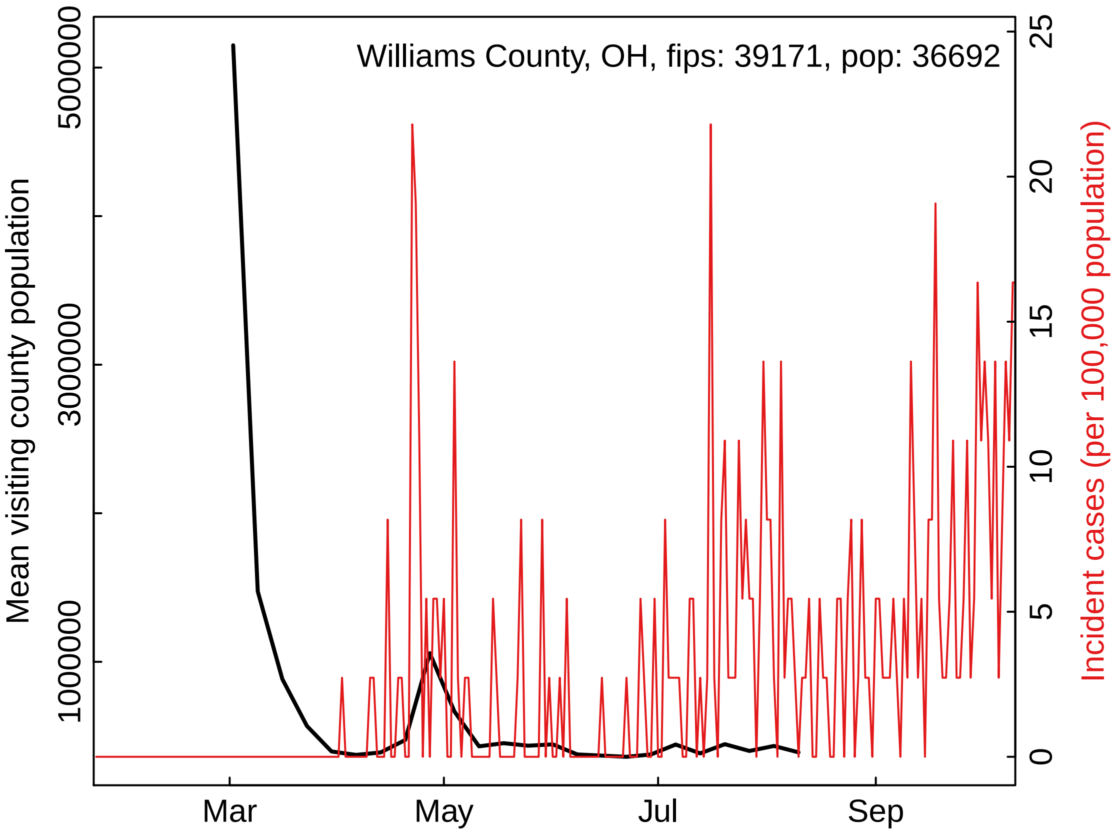

Williams County, OH, fips: 39171, pop: 36692
Mean visiting county population
Incident cases (per 100,000 population)
1000000
3000000
5000000
0
5
10
15
20
25
Mar
May
Jul
Sep

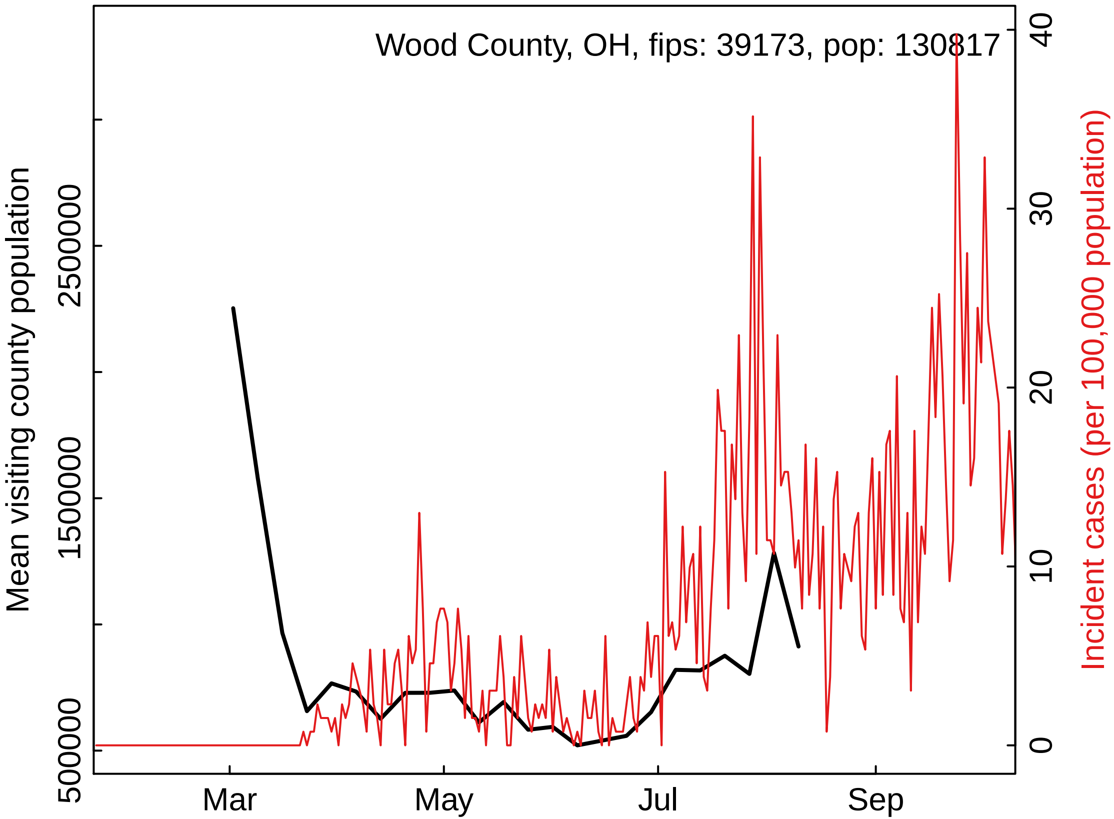
Wood County, OH, fips: 39173, pop: 130817
Mean visiting county population
500000
1500000
2500000
Incident cases (per 100,000 population)
0
10
20
30
40
Mar
May
Jul
Sep

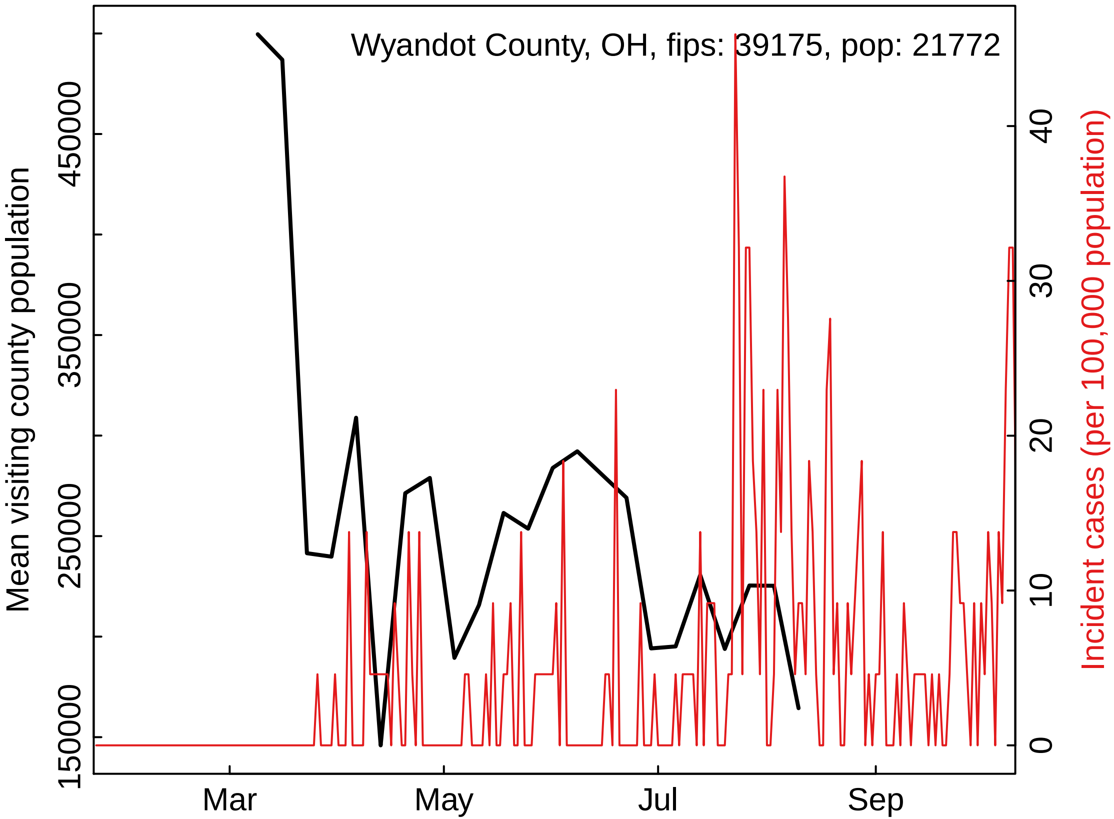
Wyandot County, OH, fips: 39175, pop: 21772
Mean visiting county population
150000
250000
350000
450000
Incident cases (per 100,000 population)
0
10
20
30
40
Mar
May
Jul
Sep

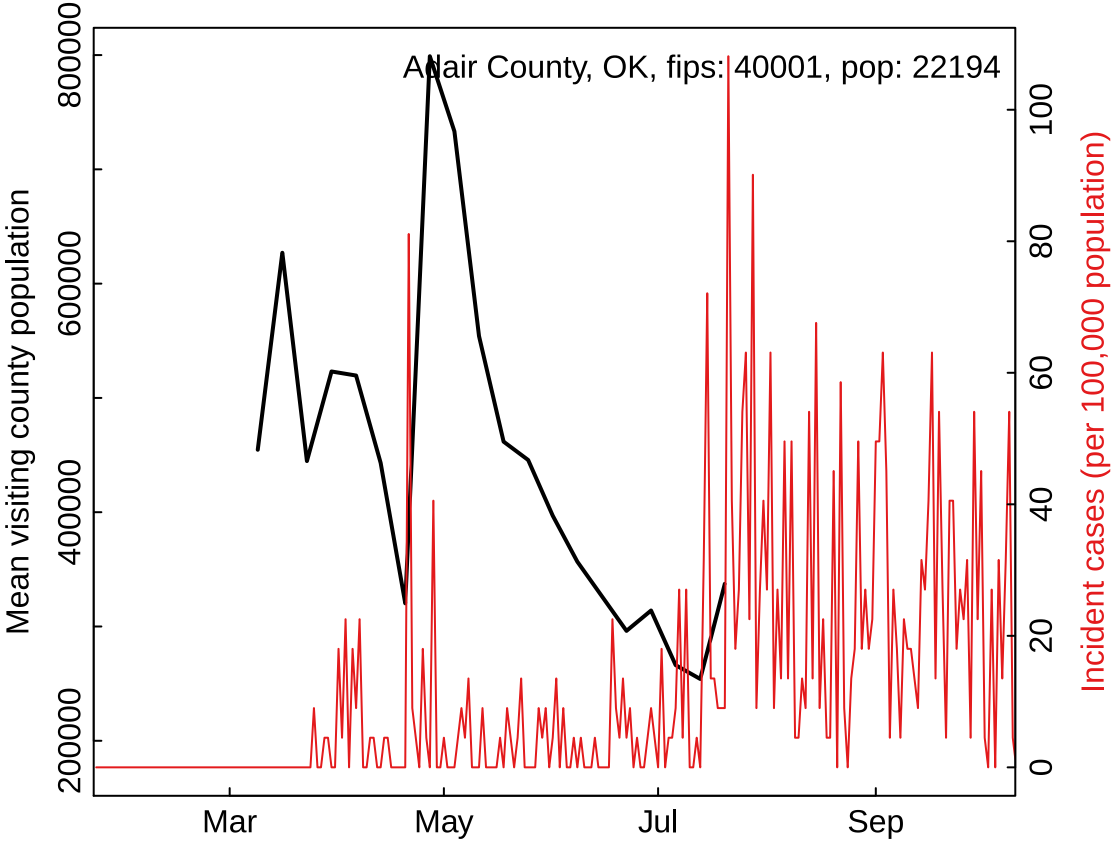

Adair County, OK, fips: 40001, pop: 22194
Mean visiting county population
200000
400000
600000
800000
Incident cases (per 100,000 population)
0
20
40
60
80
100
Mar
May
Jul
Sep

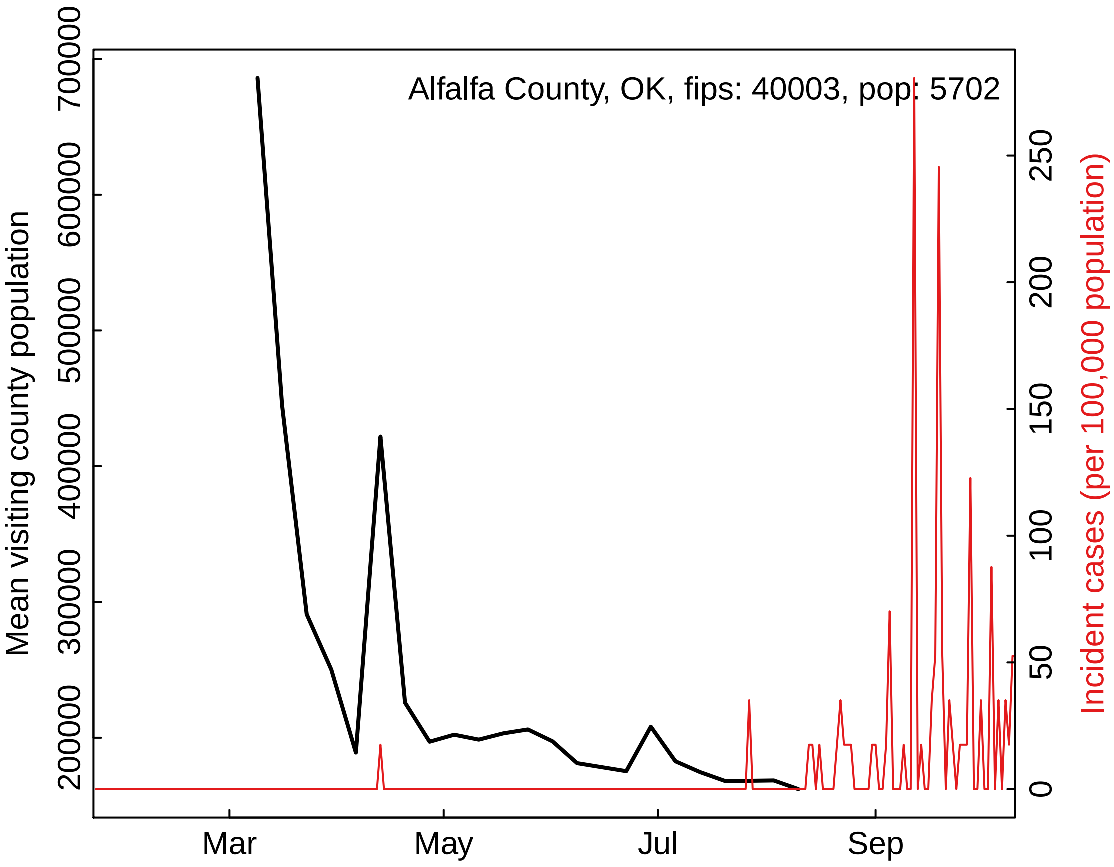

Alfalfa County, OK, fips: 40003, pop: 5702
Mean visiting county population
200000
300000
400000
500000
600000
700000
Incident cases (per 100,000 population)
0
50
100
150
200
250
Mar
May
Jul
Sep

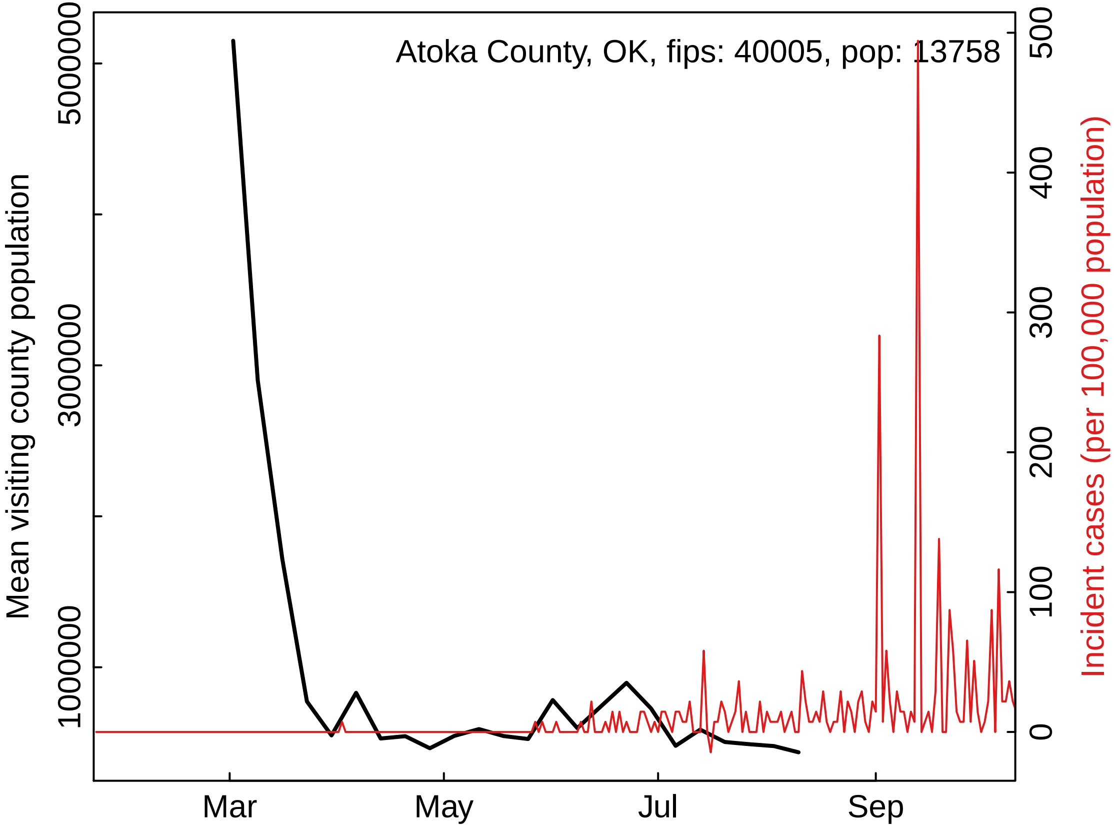

Atoka County, OK, fips: 40005, pop: 13758
Mean visiting county population
Incident cases (per 100,000 population)
1000000
3000000
5000000
0
100
200
300
400
500
Mar
May
Jul
Sep

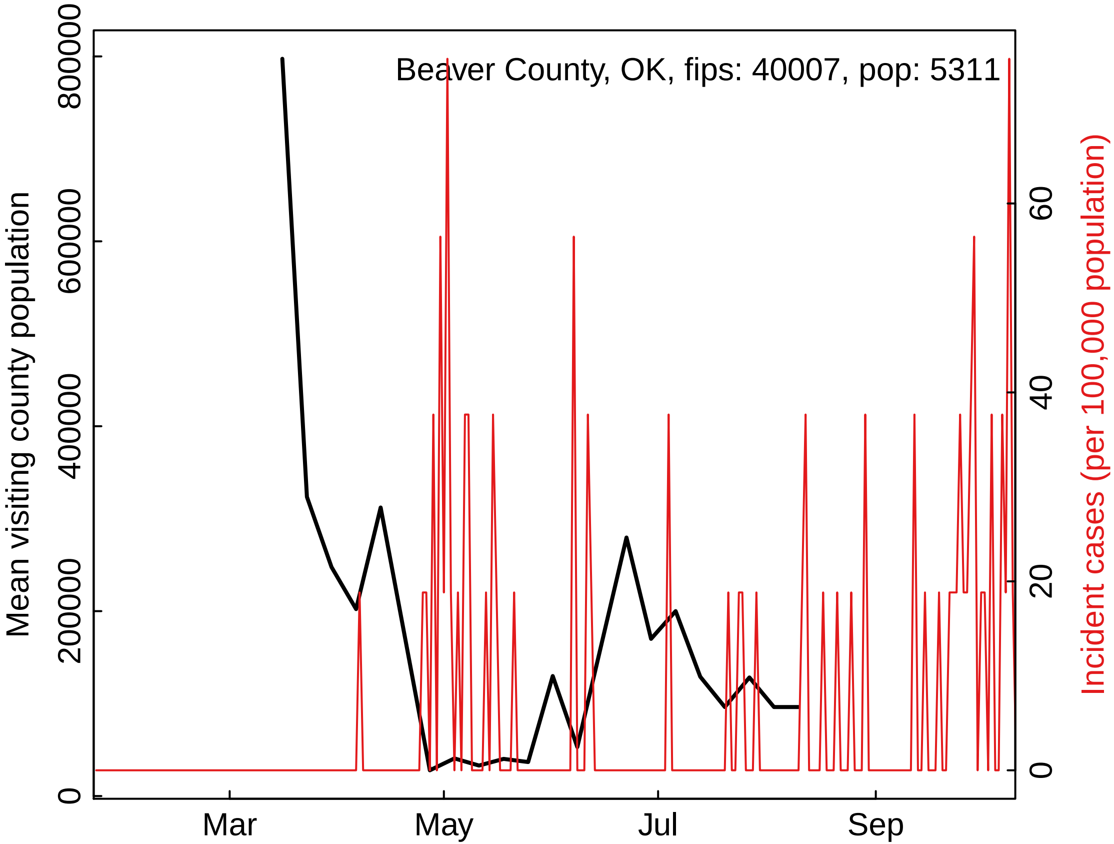

Beaver County, OK, fips: 40007, pop: 5311
Mean visiting county population
0
200000
400000
600000
800000
Incident cases (per 100,000 population)
0
20
40
60
Mar
May
Jul
Sep

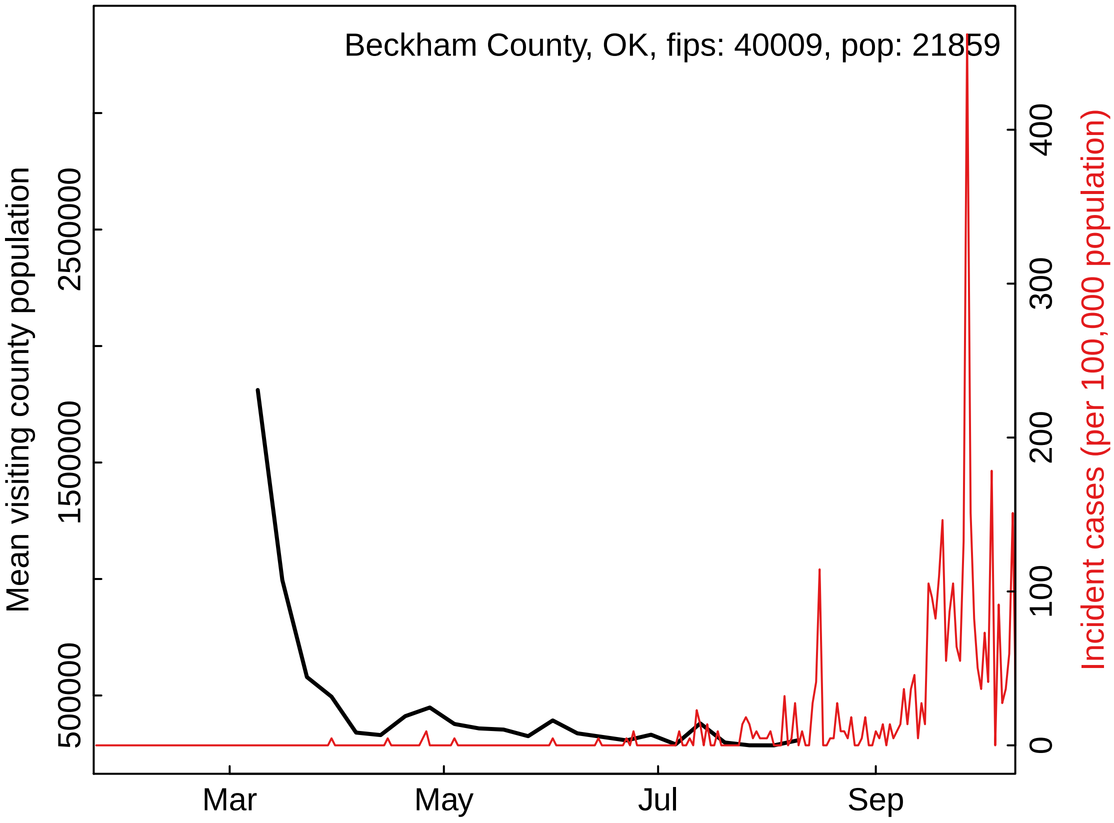

Beckham County, OK, fips: 40009, pop: 21859
Mean visiting county population
500000
1500000
2500000
Incident cases (per 100,000 population)
0
100
200
300
400
Mar
May
Jul
Sep

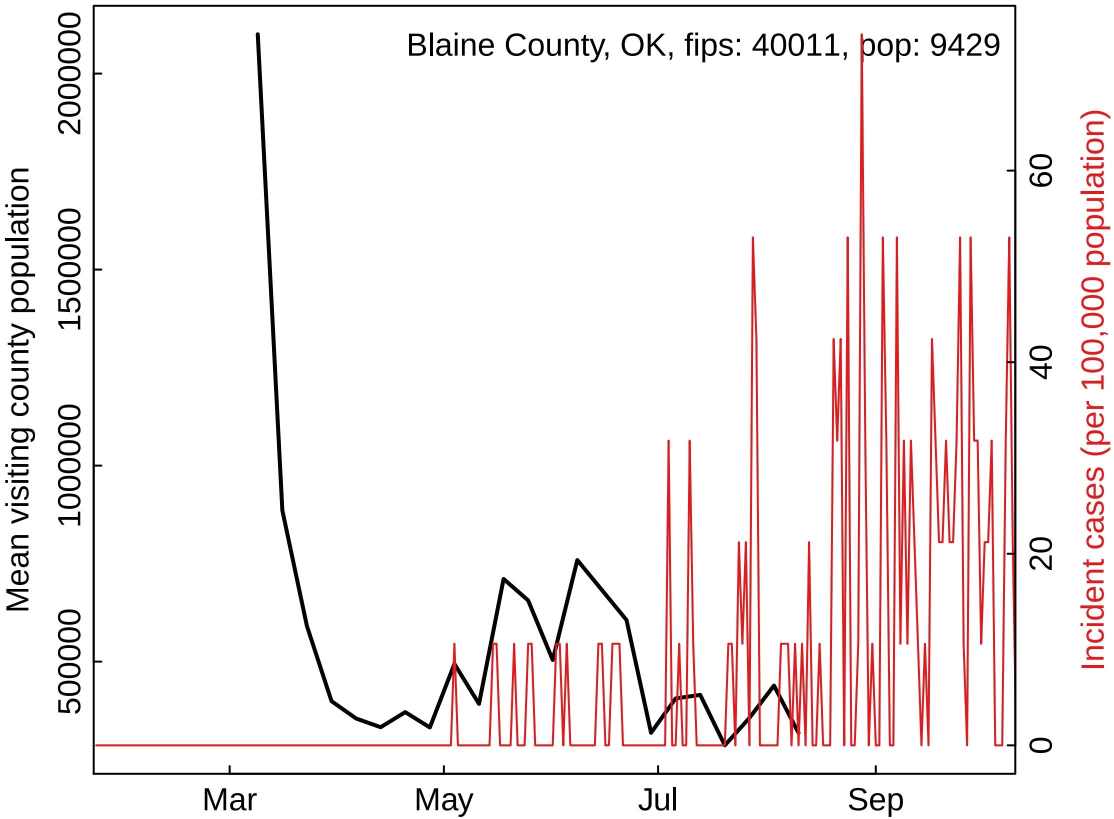
Blaine County, OK, fips: 40011, pop: 9429
Mean visiting county population
500000
1000000
1500000
2000000
Incident cases (per 100,000 population)
0
20
40
60
Mar
May
Jul
Sep

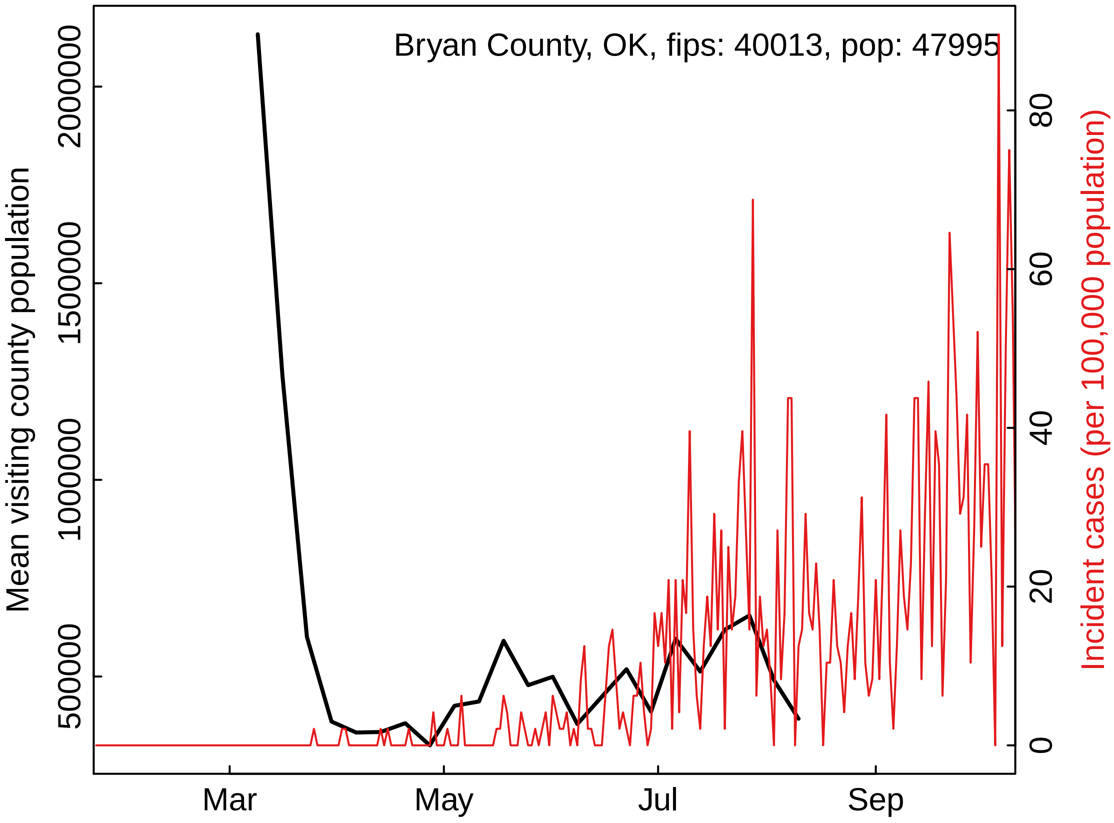

Bryan County, OK, fips: 40013, pop: 47995
Mean visiting county population
500000
1000000
1500000
2000000
Incident cases (per 100,000 population)
0
20
40
60
80
Mar
May
Jul
Sep

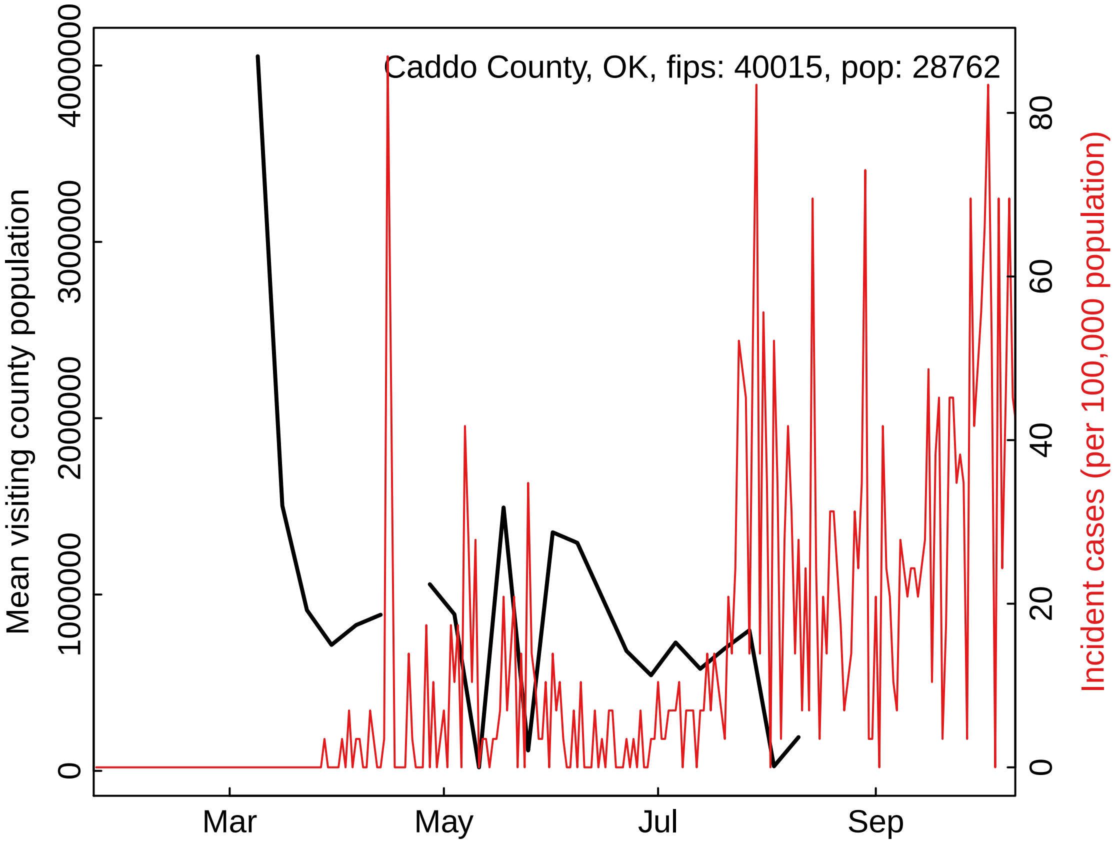
Caddo County, OK, fips: 40015, pop: 28762
Mean visiting county population
0
1000000
2000000
3000000
4000000
Incident cases (per 100,000 population)
0
20
40
60
80
Mar
May
Jul
Sep

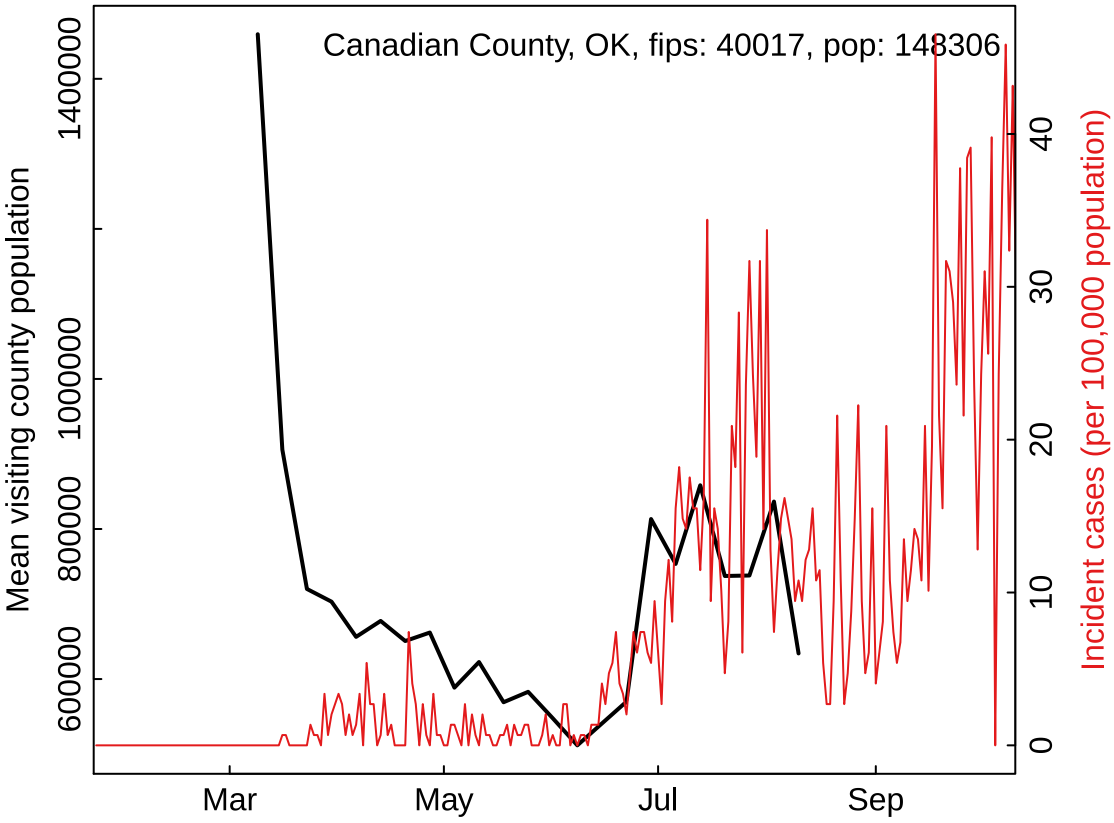

Canadian County, OK, fips: 40017, pop: 148306
Mean visiting county population
600000
800000
1000000
1400000
Incident cases (per 100,000 population)
0
10
20
30
40
Mar
May
Jul
Sep

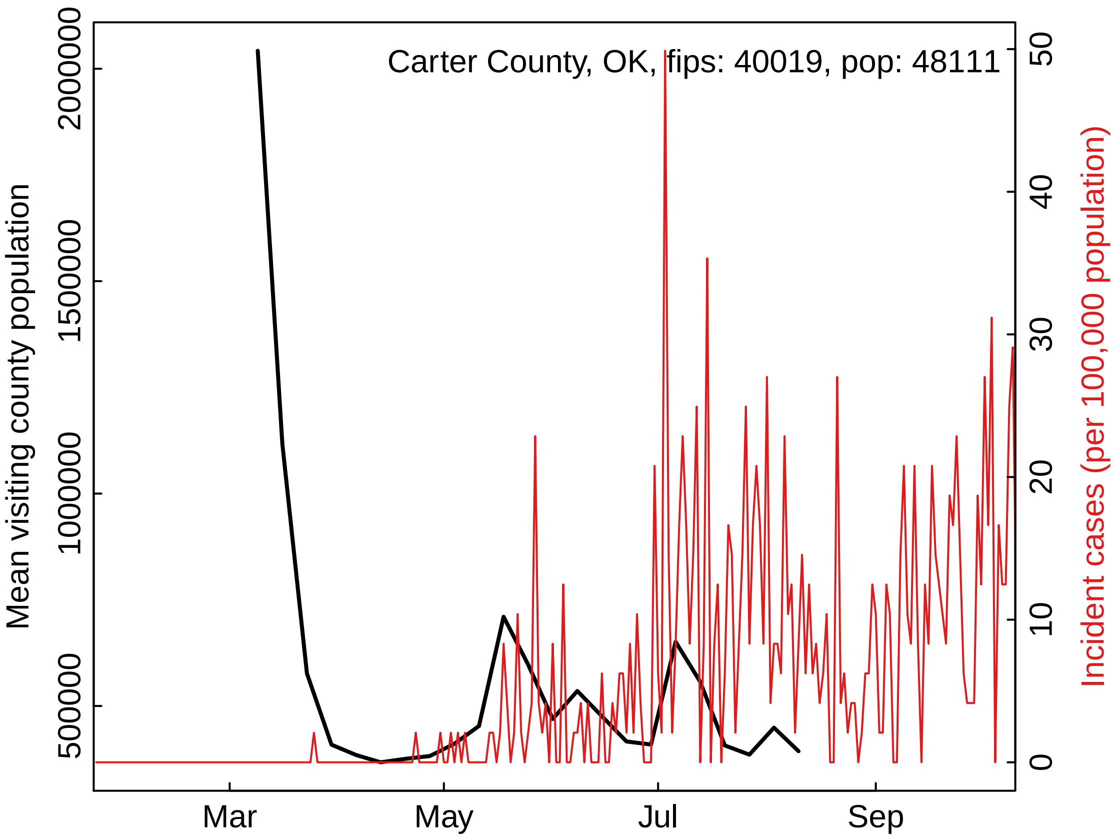

Carter County, OK, fips: 40019, pop: 48111
Mean visiting county population
500000
1000000
1500000
2000000
Incident cases (per 100,000 population)
0
10
20
30
40
50
Mar
May
Jul
Sep

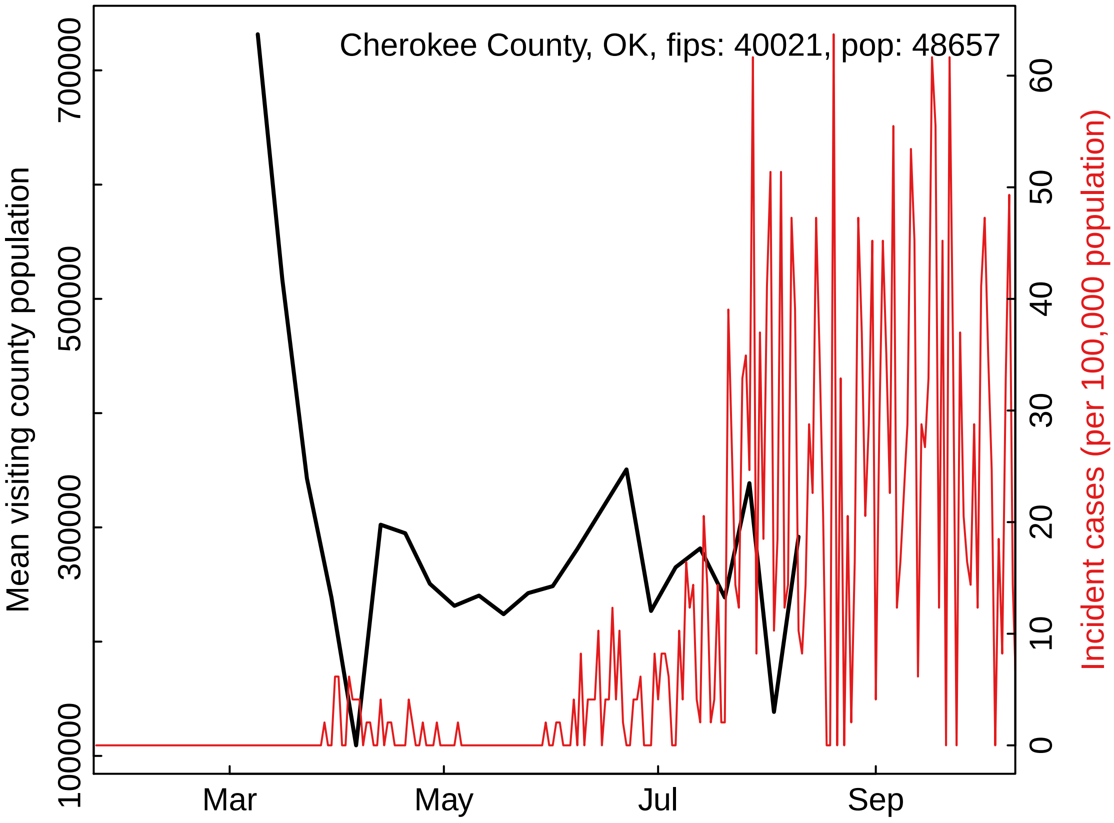
Cherokee County, OK, fips: 40021, pop: 48657
Mean visiting county population
Incident cases (per 100,000 population)
100000
300000
500000
700000
0
10
20
30
40
50
60
Mar
May
Jul
Sep

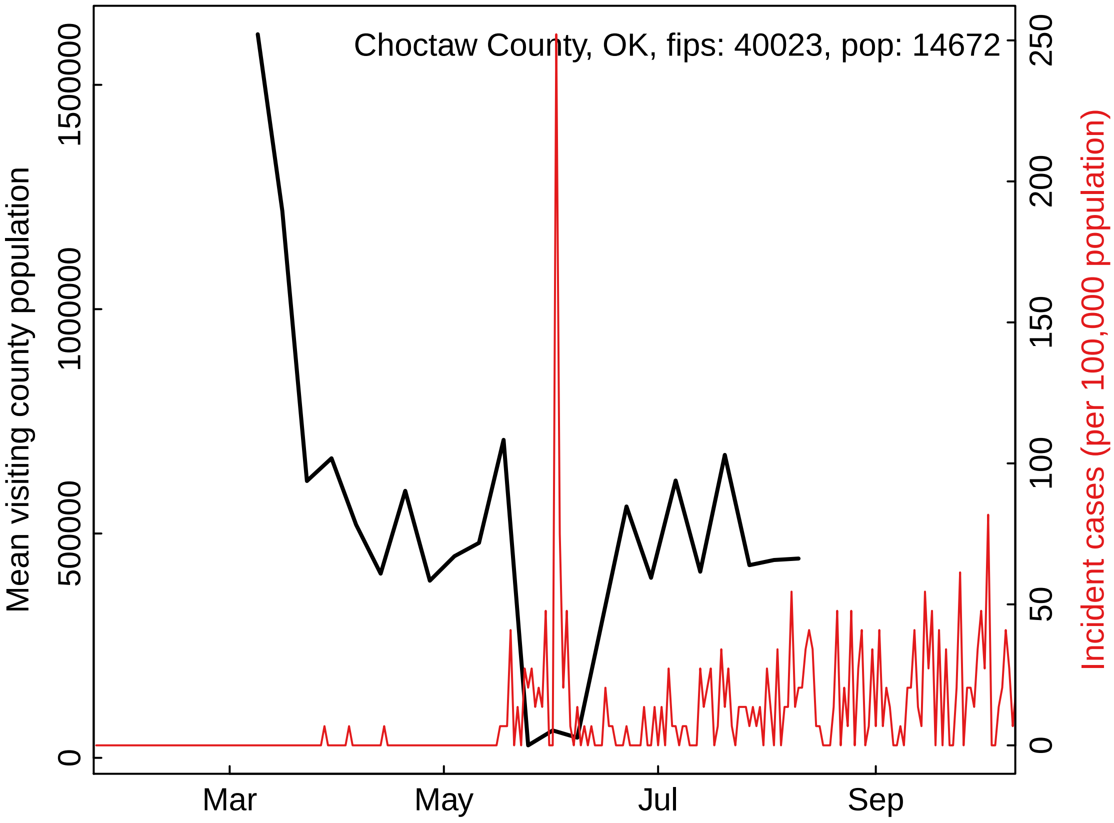
Choctaw County, OK, fips: 40023, pop: 14672
Mean visiting county population
0
500000
1000000
1500000
Incident cases (per 100,000 population)
0
50
100
150
200
250
Mar
May
Jul
Sep

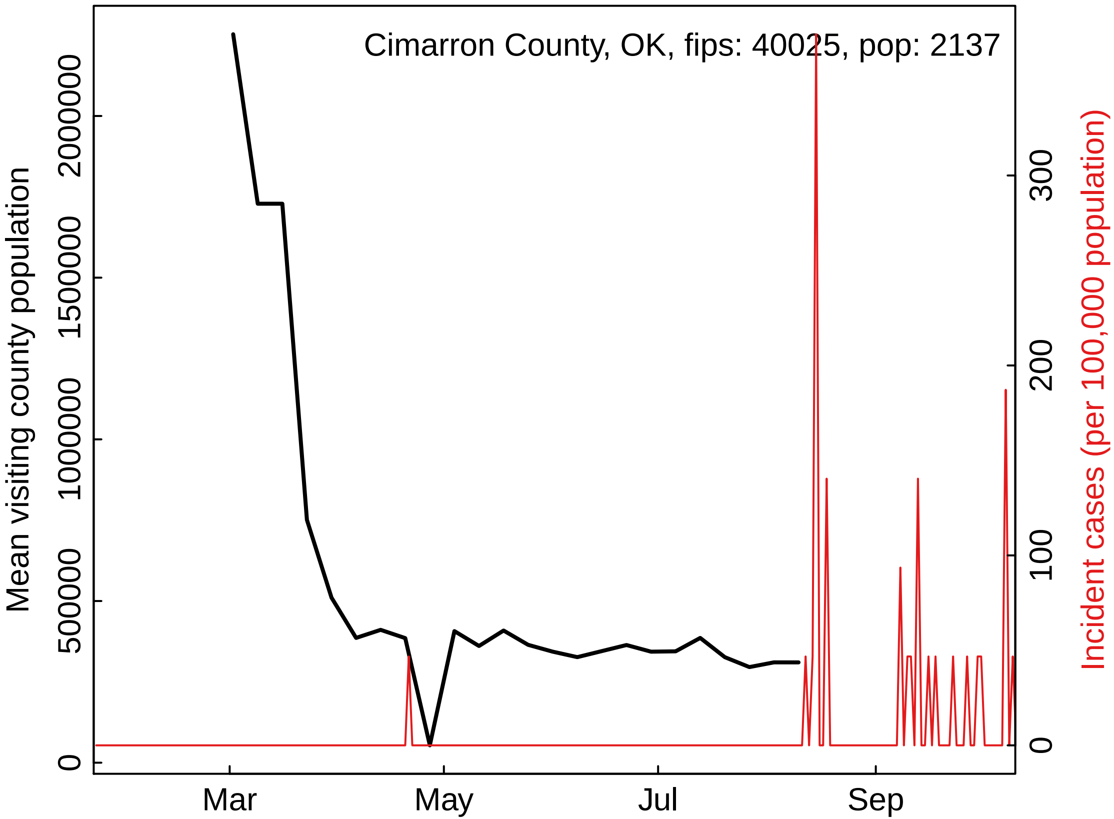

Cimarron County, OK, fips: 40025, pop: 2137
Mean visiting county population
0
500000
1000000
1500000
2000000
Incident cases (per 100,000 population)
0
100
200
300
Mar
May
Jul
Sep

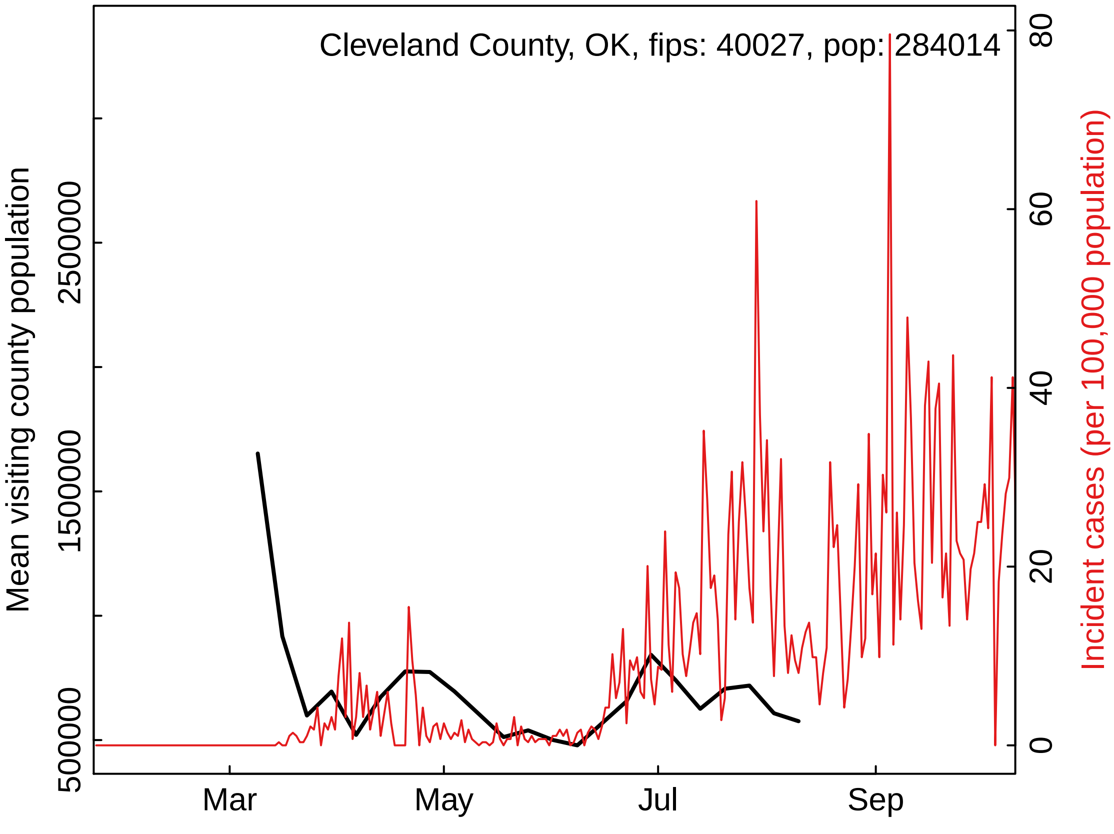
Cleveland County, OK, fips: 40027, pop: 284014
Mean visiting county population
500000
1500000
2500000
Incident cases (per 100,000 population)
0
20
40
60
80
Mar
May
Jul
Sep

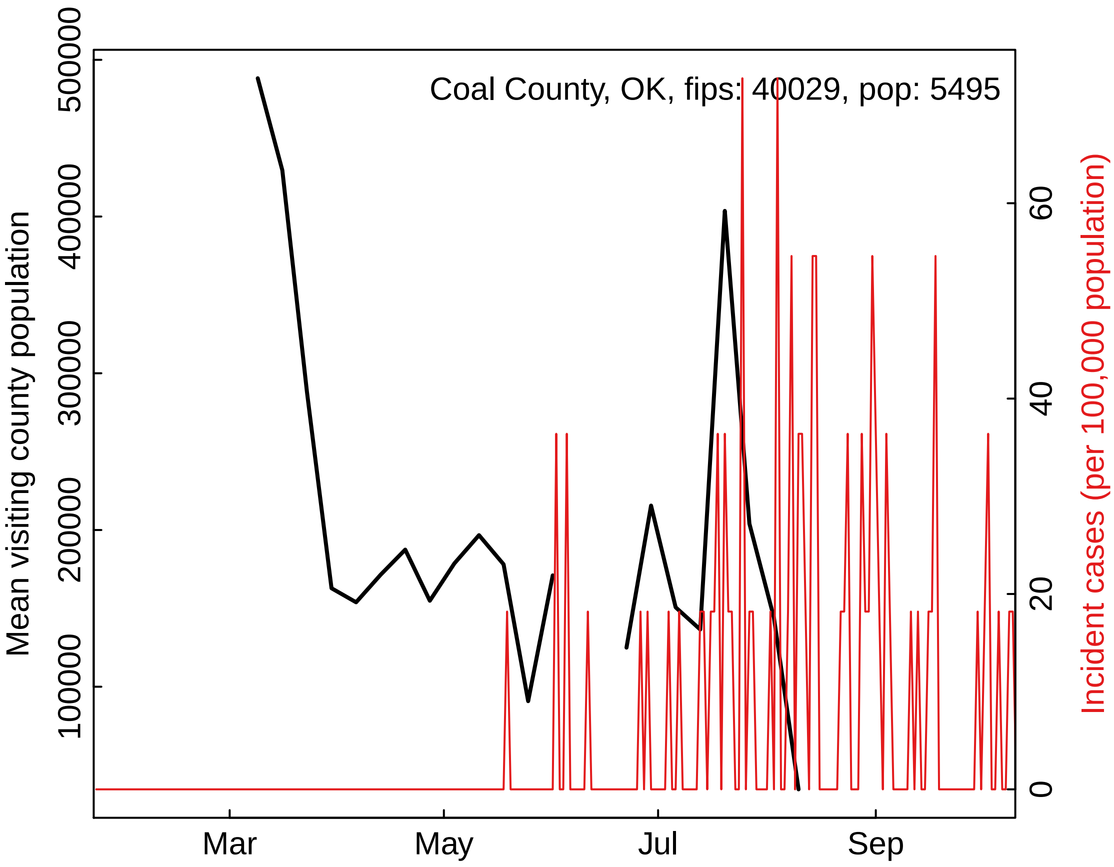

Coal County, OK, fips: 40029, pop: 5495
Mean visiting county population
100000
200000
300000
400000
500000
Incident cases (per 100,000 population)
0
20
40
60
Mar
May
Jul
Sep

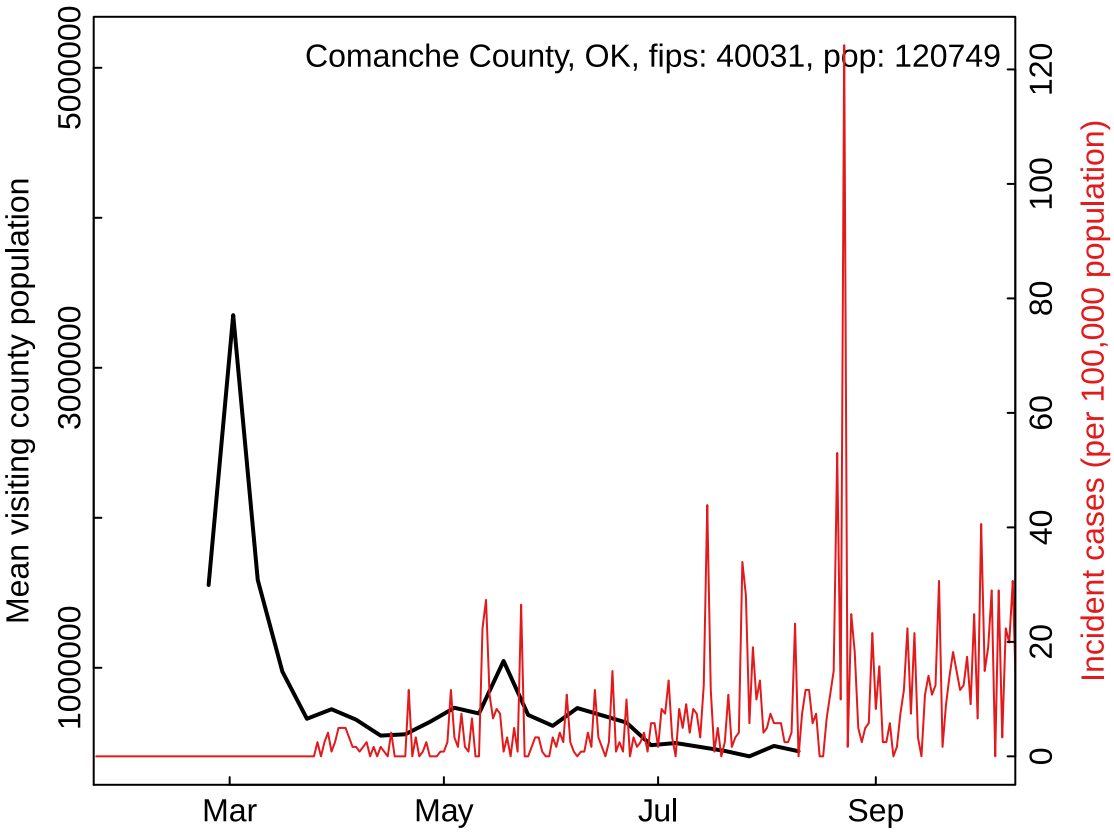

Comanche County, OK, fips: 40031, pop: 120749
Mean visiting county population
1000000
3000000
5000000
Incident cases (per 100,000 population)
0
20
40
60
80
100
120
Mar
May
Jul
Sep

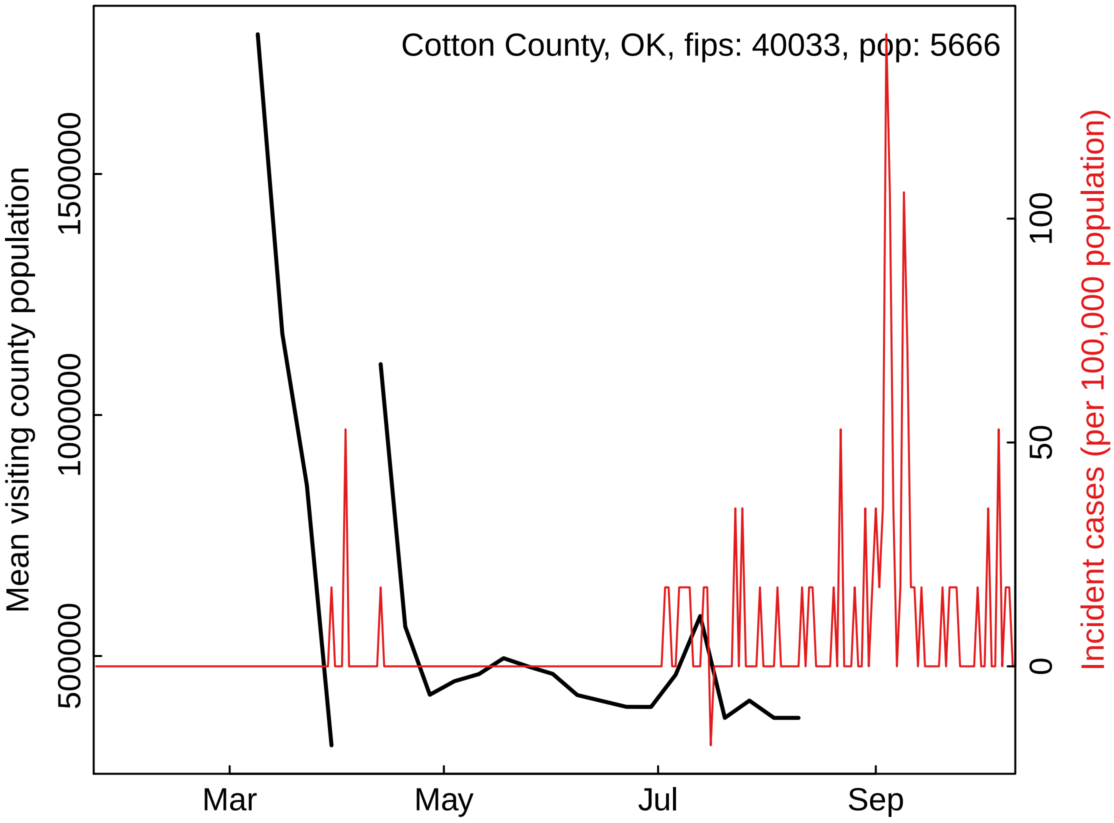

Cotton County, OK, fips: 40033, pop: 5666
Mean visiting county population
500000
1000000
1500000
Mar
May
Jul
Sep
0
50
100
Incident cases (per 100,000 population)

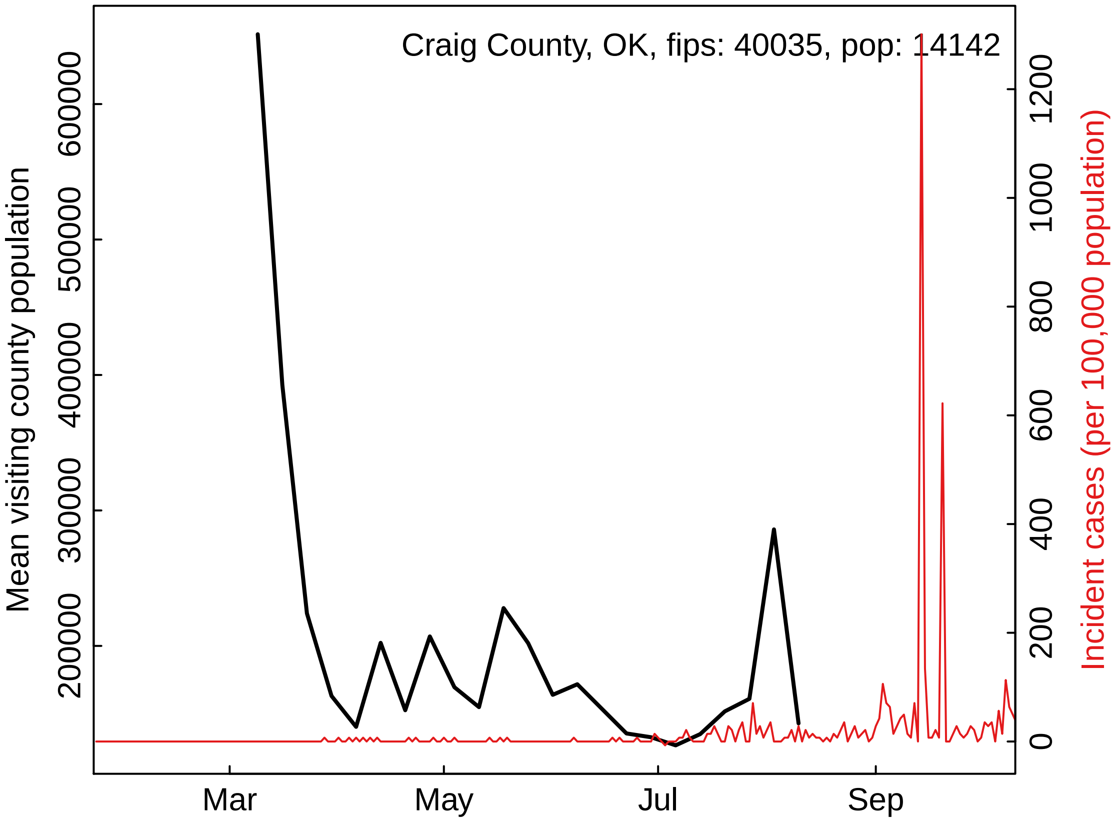
Craig County, OK, fips: 40035, pop: 14142
Mean visiting county population
200000
300000
400000
500000
600000
Incident cases (per 100,000 population)
0
200
400
600
800
1000
1200
Mar
May
Jul
Sep

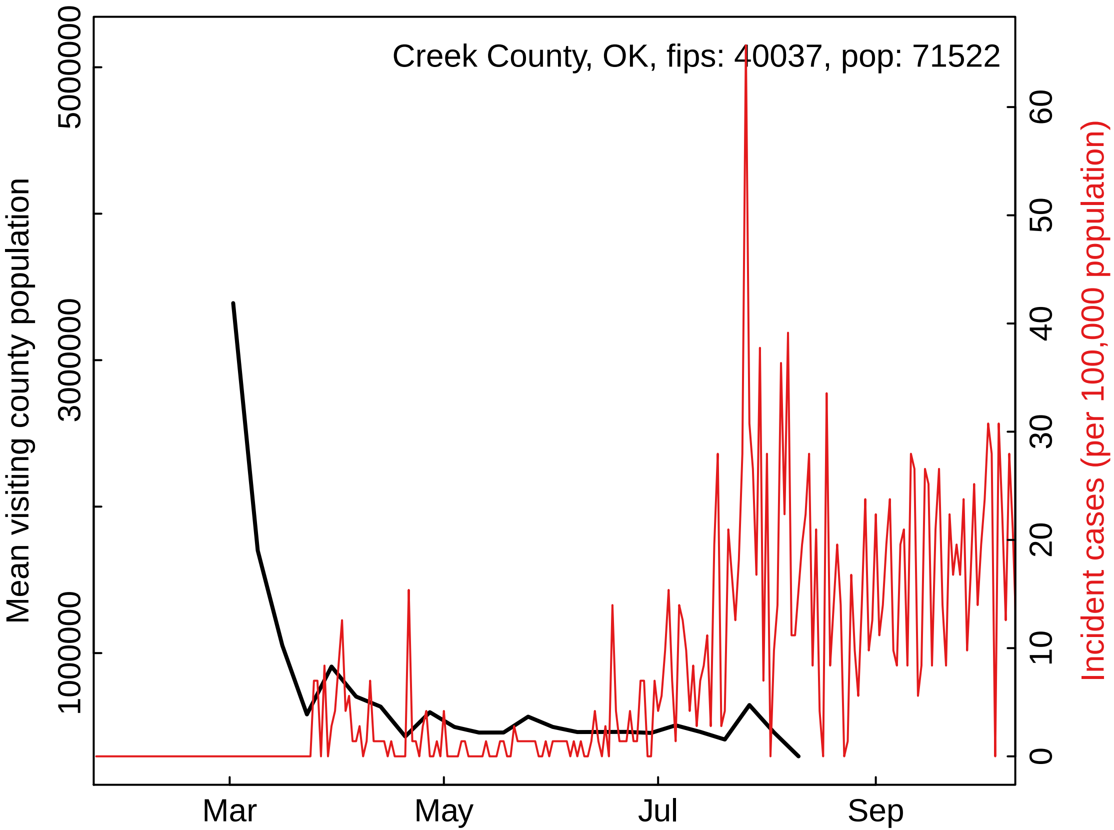

Creek County, OK, fips: 40037, pop: 71522
Mean visiting county population
1000000
3000000
5000000
Incident cases (per 100,000 population)
0
10
20
30
40
50
60
Mar
May
Jul
Sep

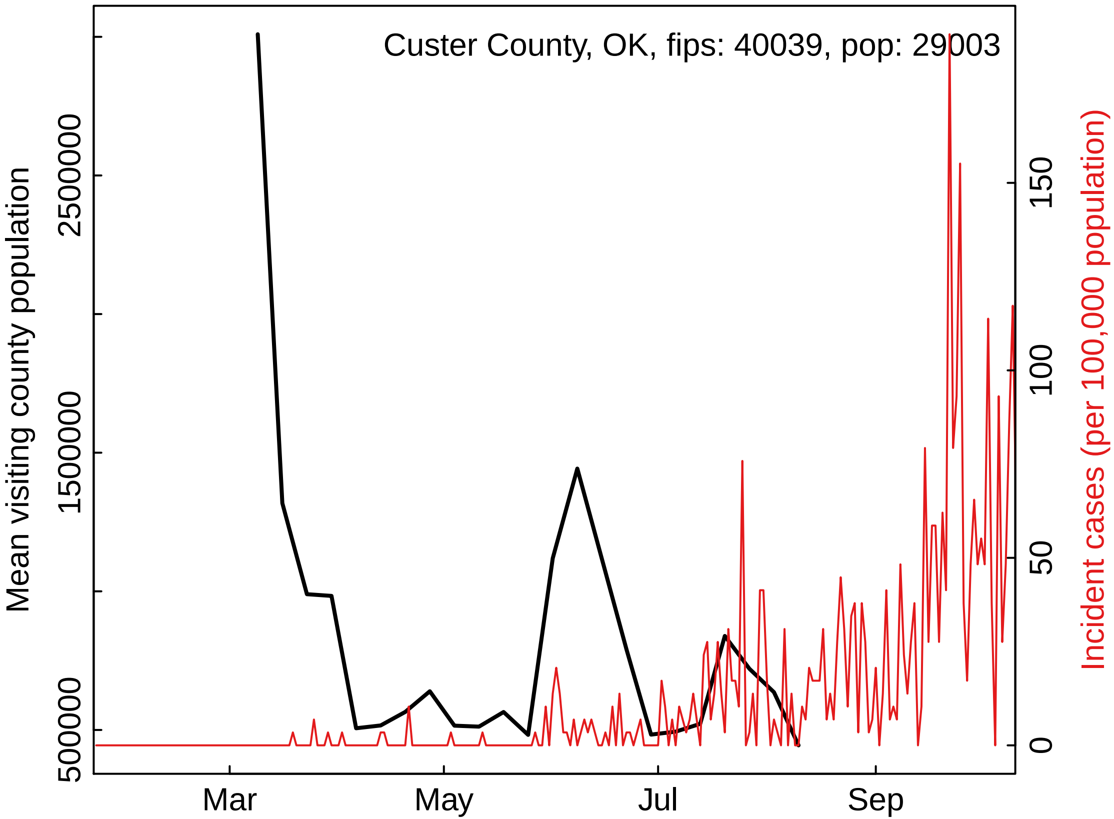

Custer County, OK, fips: 40039, pop: 29003
Mean visiting county population
500000
1500000
2500000
Incident cases (per 100,000 population)
0
50
100
150
Mar
May
Jul
Sep

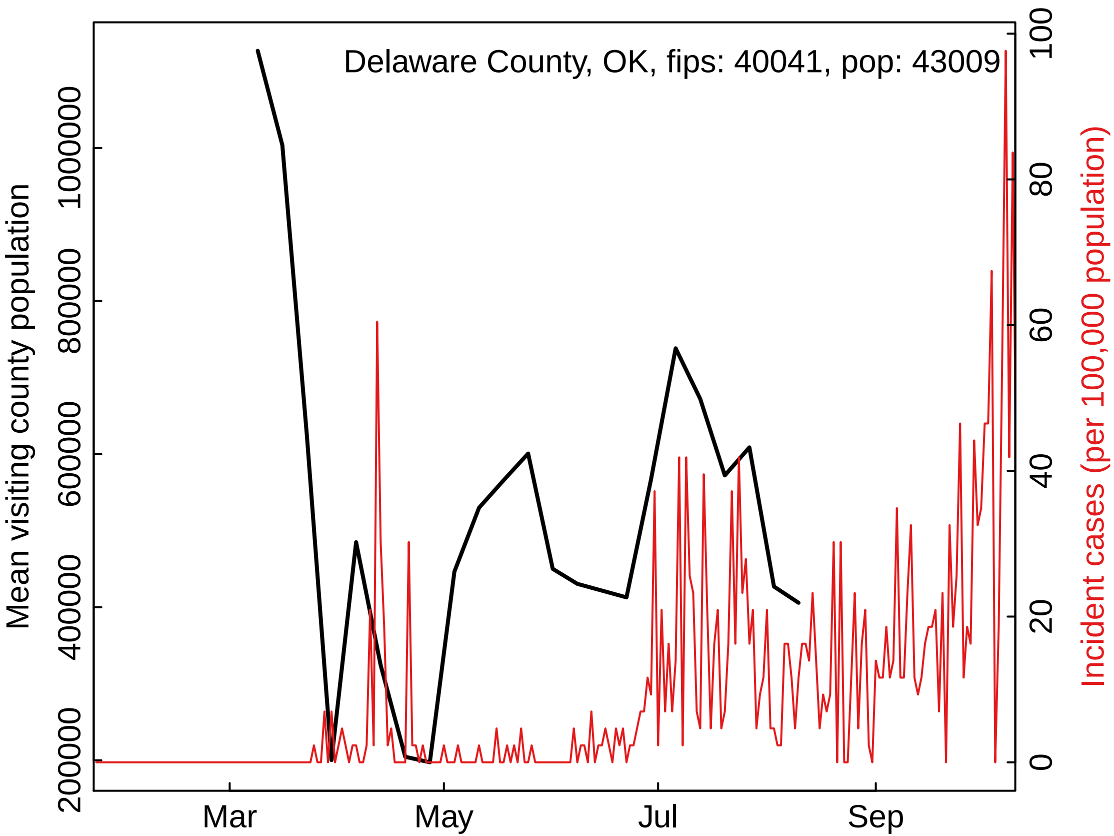

Delaware County, OK, fips: 40041, pop: 43009
Mean visiting county population
200000
400000
600000
800000
1000000
Mar
May
Jul
Sep
0
20
40
60
80
100
Incident cases (per 100,000 population)

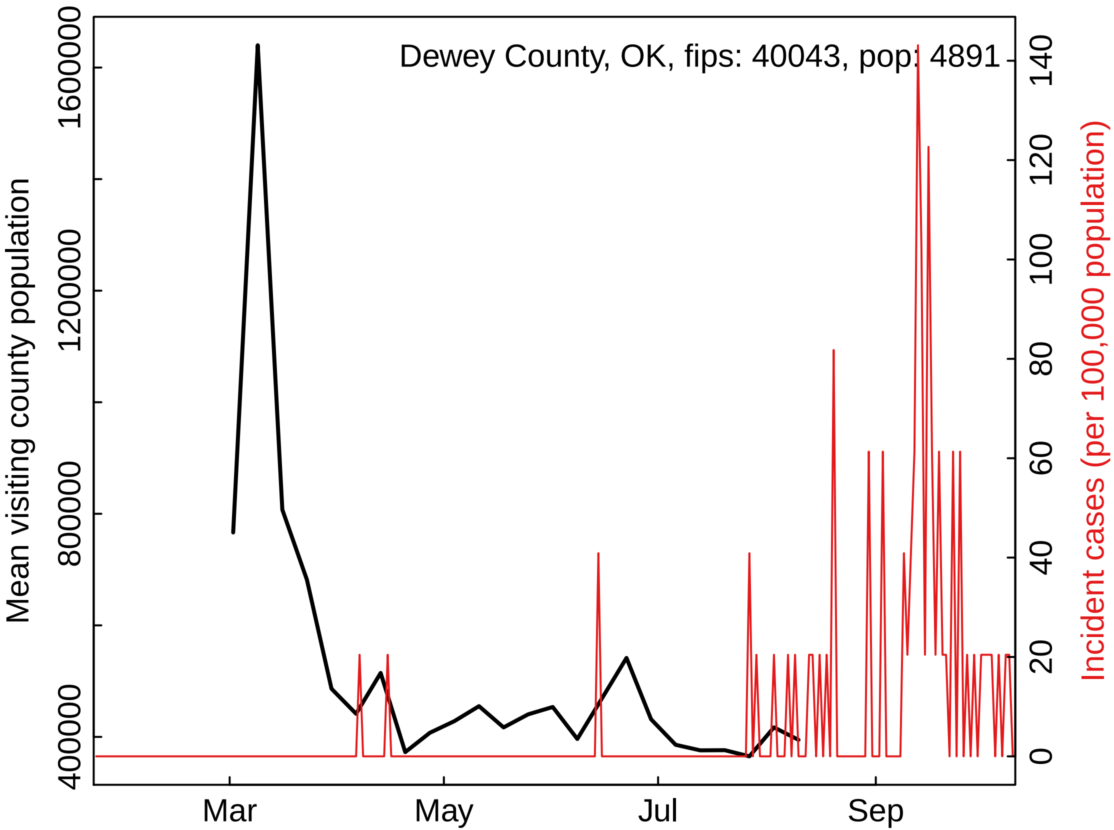
Dewey County, OK, fips: 40043, pop: 4891
Mean visiting county population
400000
800000
1200000
1600000
Mar
May
Jul
Sep
0
20
40
60
80
100
120
140
Incident cases (per 100,000 population)

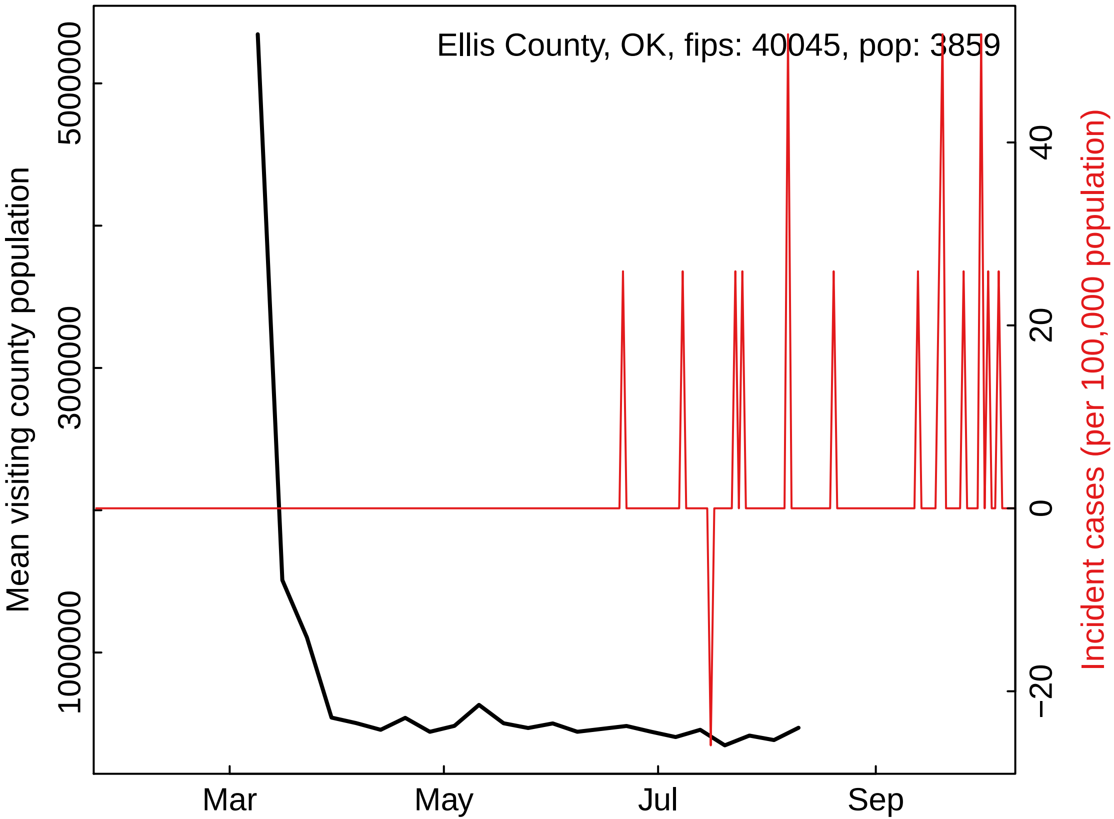

Ellis County, OK, fips: 40045, pop: 3859
Mean visiting county population
Incident cases (per 100,000 population)
1000000
3000000
5000000
−20
0
20
40
Mar
May
Jul
Sep

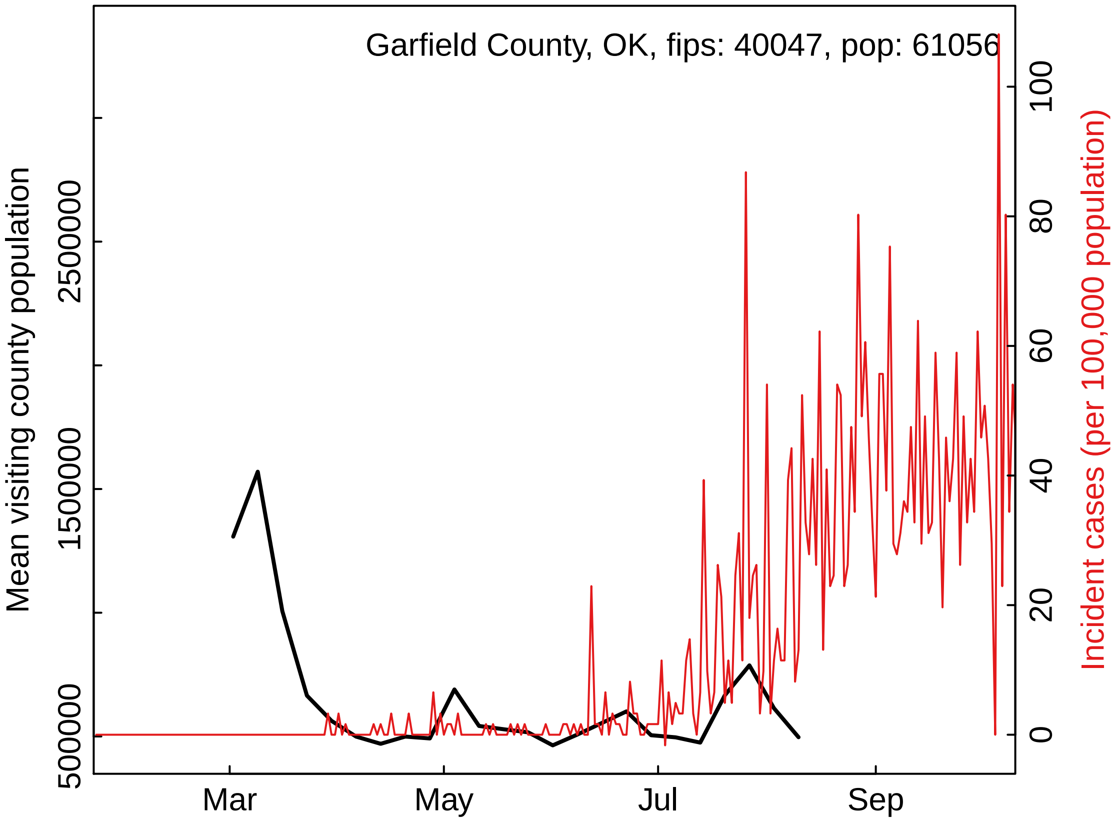

Garfield County, OK, fips: 40047, pop: 61056
Mean visiting county population
500000
1500000
2500000
Mar
May
Jul
Sep
0
20
40
60
80
100
Incident cases (per 100,000 population)

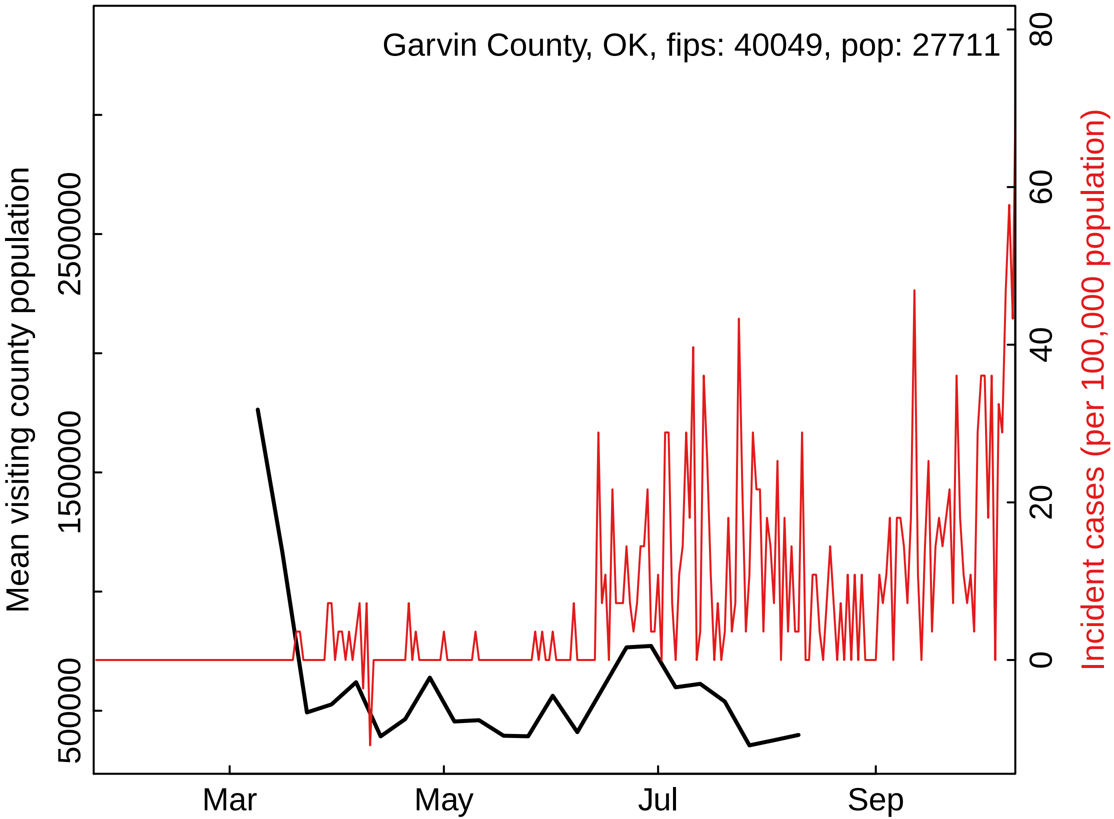

Garvin County, OK, fips: 40049, pop: 27711
Mean visiting county population
500000
1500000
2500000
Incident cases (per 100,000 population)
0
20
40
60
80
Mar
May
Jul
Sep

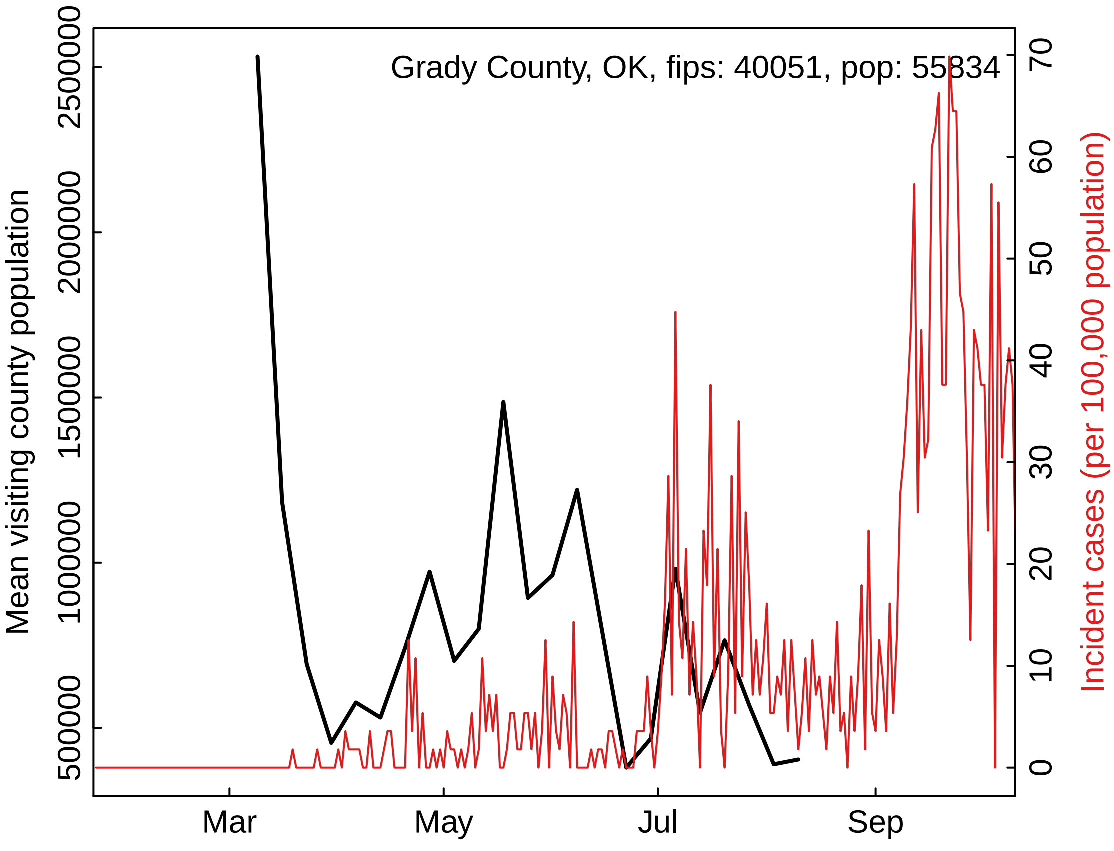

Grady County, OK, fips: 40051, pop: 55834
Mean visiting county population
Incident cases (per 100,000 population)
500000
1000000
1500000
2000000
2500000
0
10
20
30
40
50
60
70
Mar
May
Jul
Sep

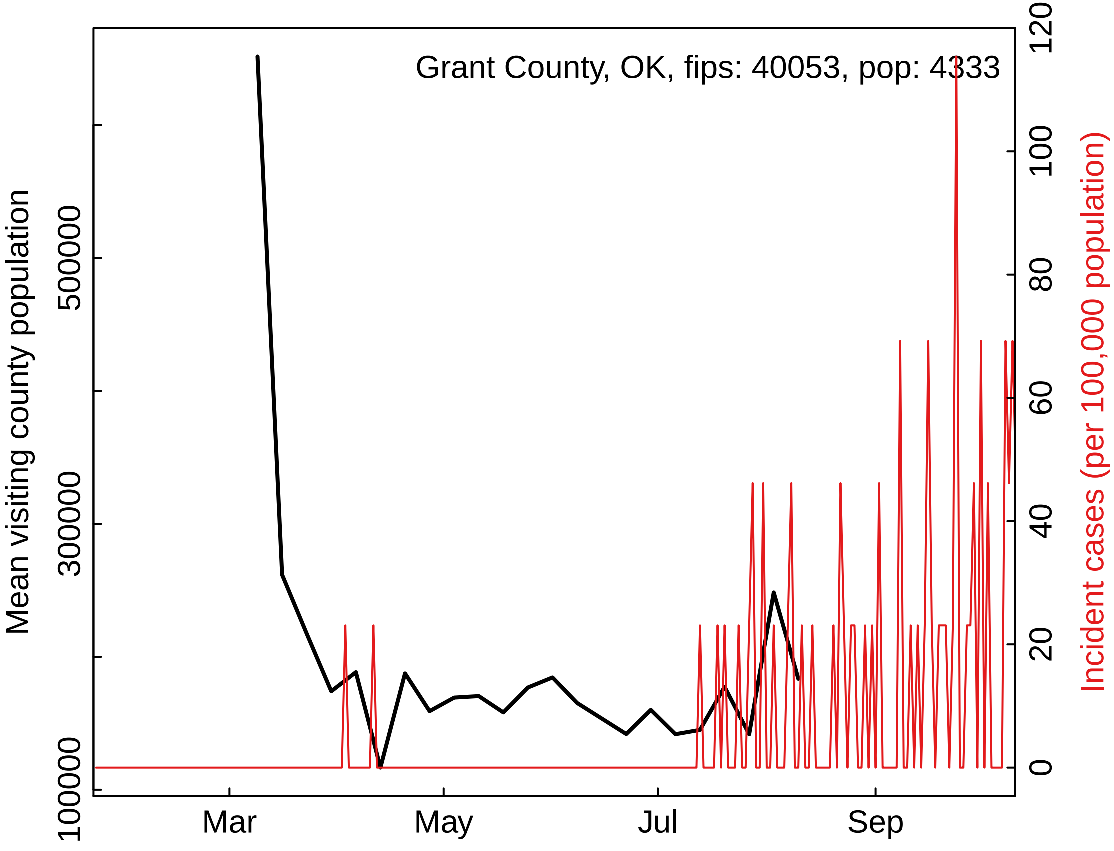

Grant County, OK, fips: 40053, pop: 4333
Mean visiting county population
100000
300000
500000
Mar
May
Jul
Sep
Incident cases (per 100,000 population)
0
20
40
60
80
100
120

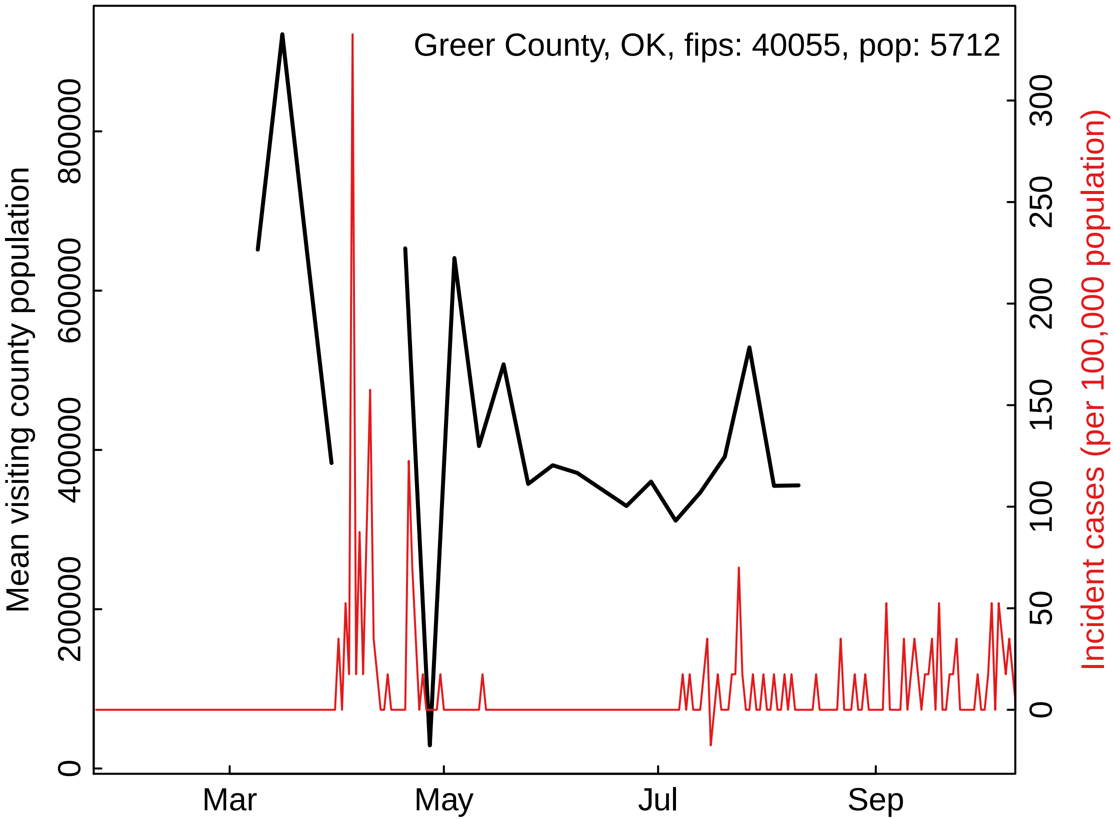

Greer County, OK, fips: 40055, pop: 5712
Mean visiting county population
0
200000
400000
600000
800000
Incident cases (per 100,000 population)
0
50
100
150
200
250
300
Mar
May
Jul
Sep

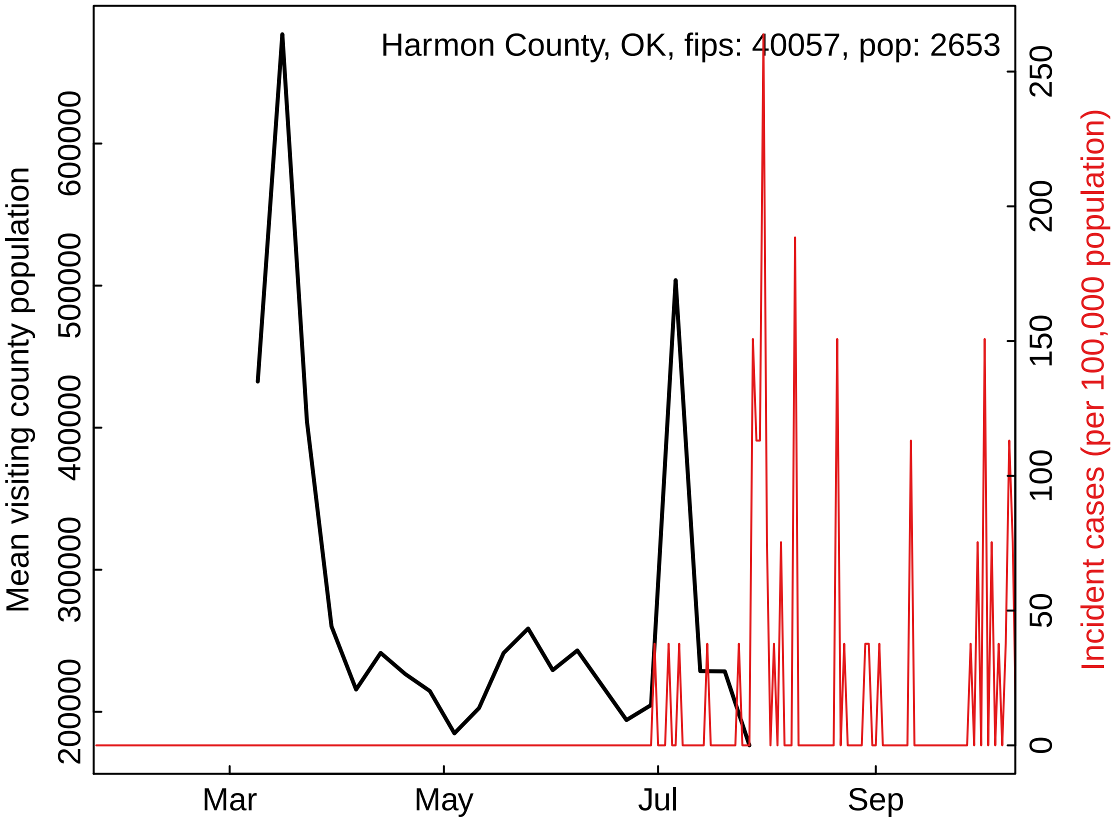
Harmon County, OK, fips: 40057, pop: 2653
Mean visiting county population
200000
300000
400000
500000
600000
Incident cases (per 100,000 population)
0
50
100
150
200
250
Mar
May
Jul
Sep

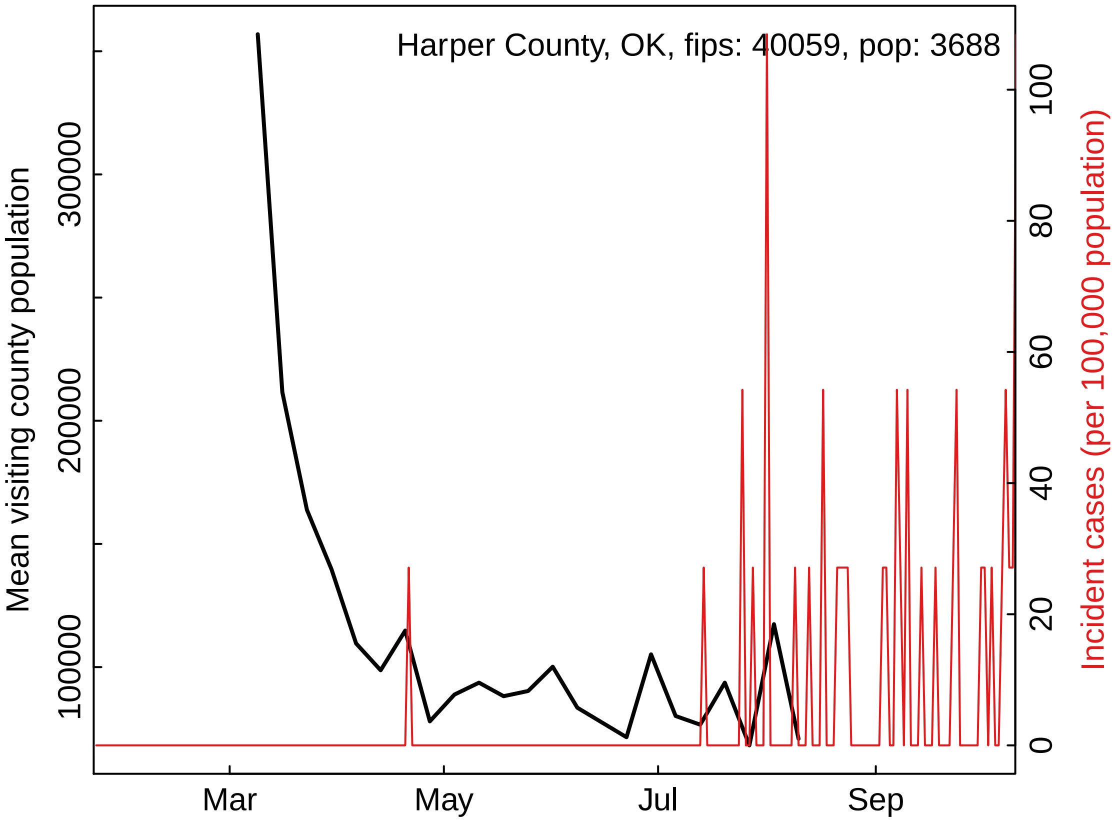

Harper County, OK, fips: 40059, pop: 3688
Mean visiting county population
100000
200000
300000
Mar
May
Jul
Sep
0
20
40
60
80
100
Incident cases (per 100,000 population)

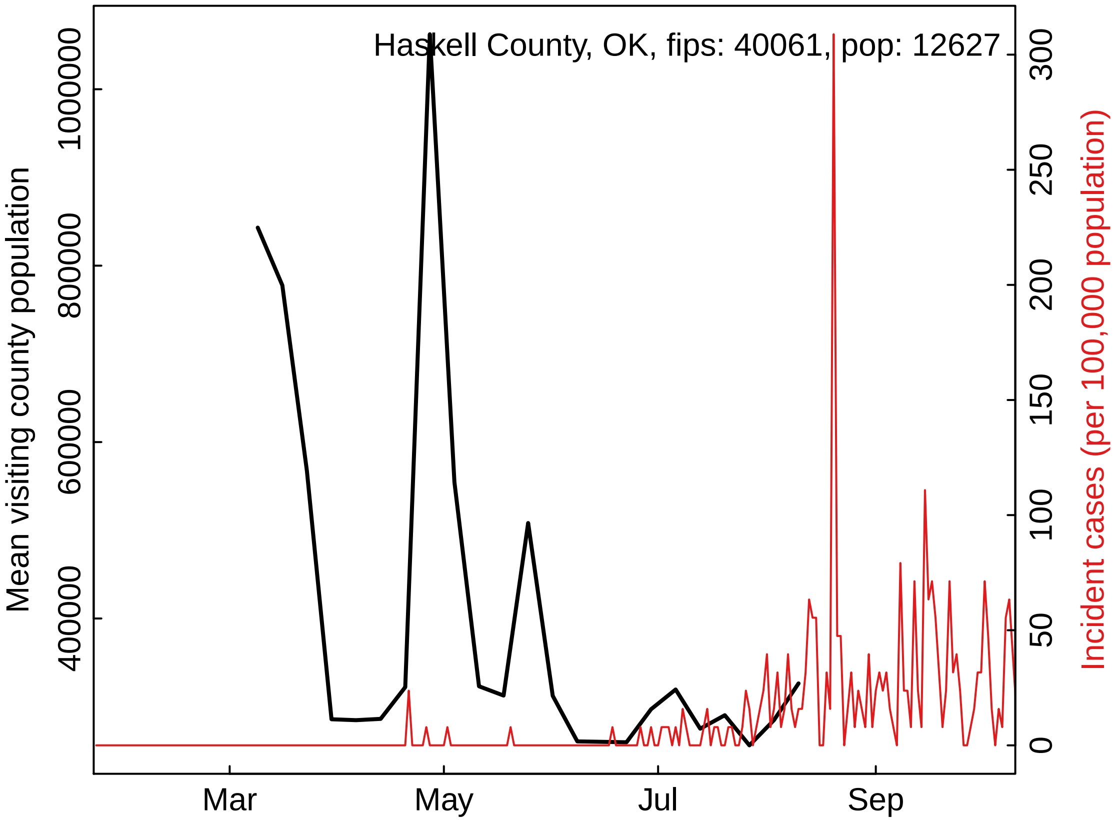

Haskell County, OK, fips: 40061, pop: 12627
Mean visiting county population
400000
600000
800000
10000000
Incident cases (per 100,000 population)
0
50
100
150
200
250
300
Mar
May
Jul
Sep

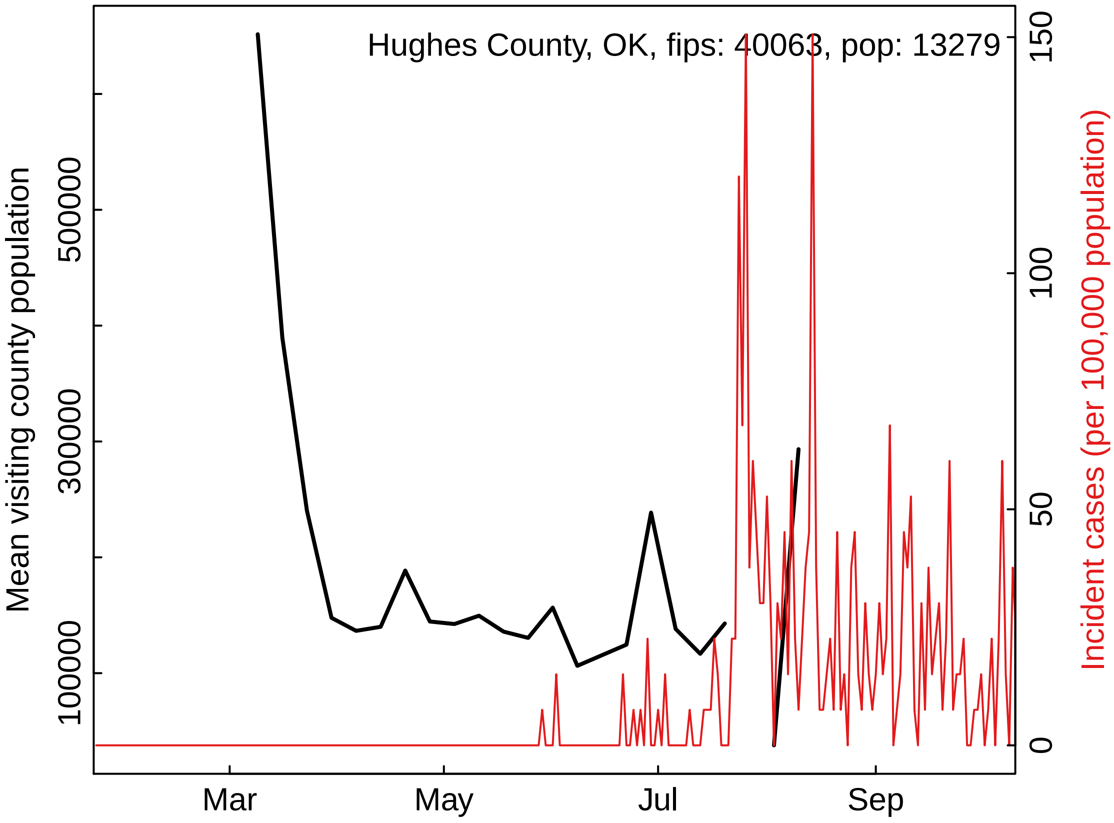

Hughes County, OK, fips: 40063, pop: 13279
Mean visiting county population
100000
300000
500000
Mar
May
Jul
Sep
0
50
100
150
Incident cases (per 100,000 population)

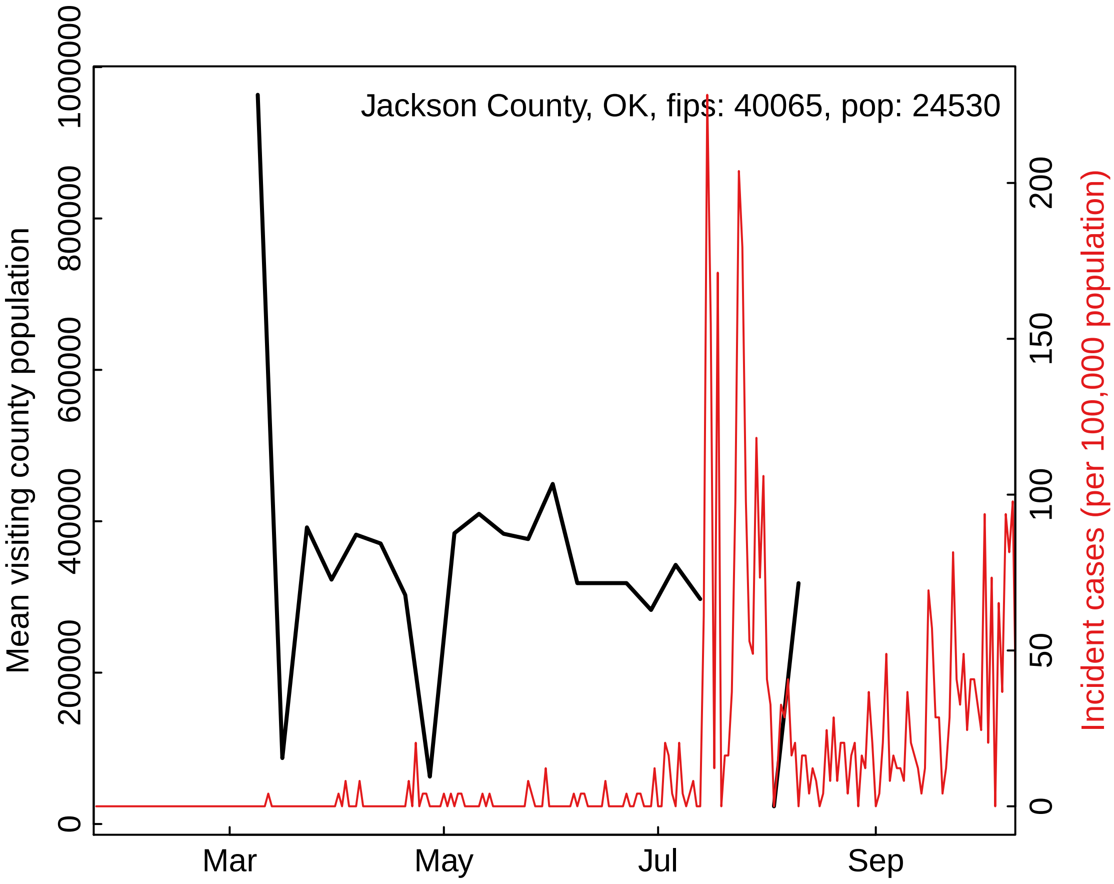

Jackson County, OK, fips: 40065, pop: 24530
Mean visiting county population
0
200000
400000
600000
800000
1000000
Incident cases (per 100,000 population)
0
50
100
150
200
Mar
May
Jul
Sep

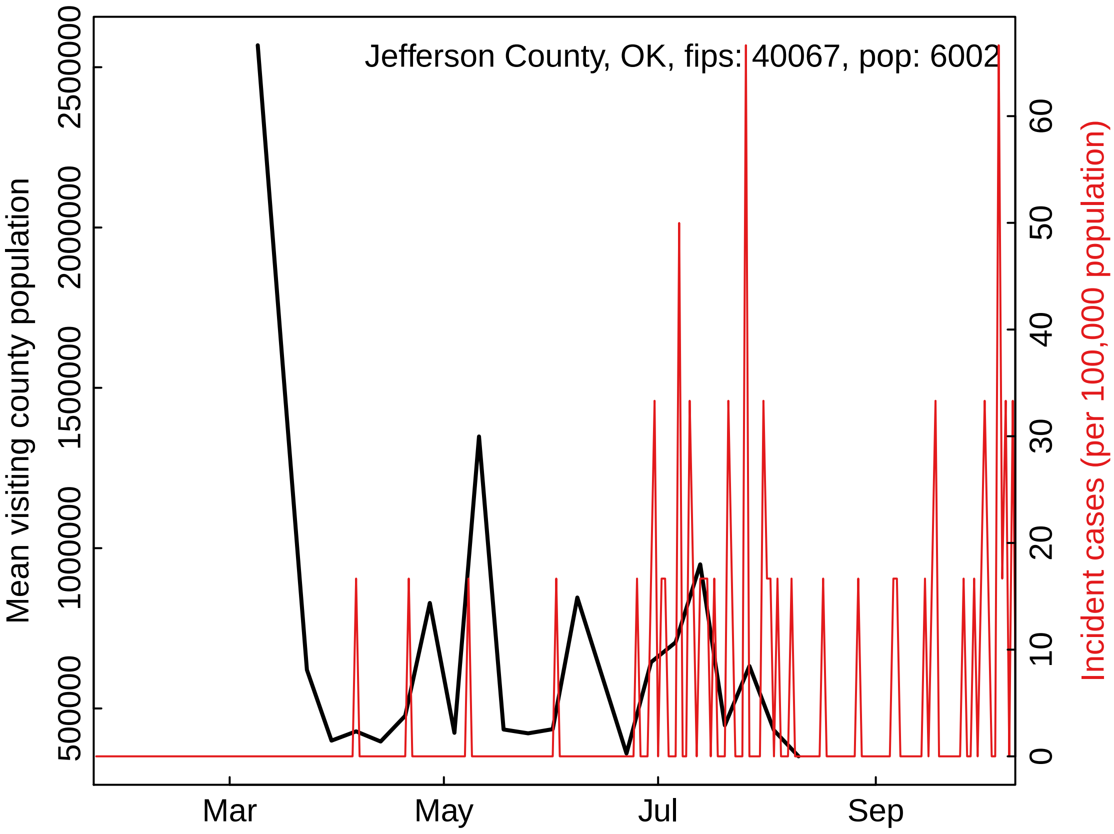

Jefferson County, OK, fips: 40067, pop: 6002
Mean visiting county population
500000
1000000
1500000
2000000
2500000
Incident cases (per 100,000 population)
0
10
20
30
40
50
60
Mar
May
Jul
Sep

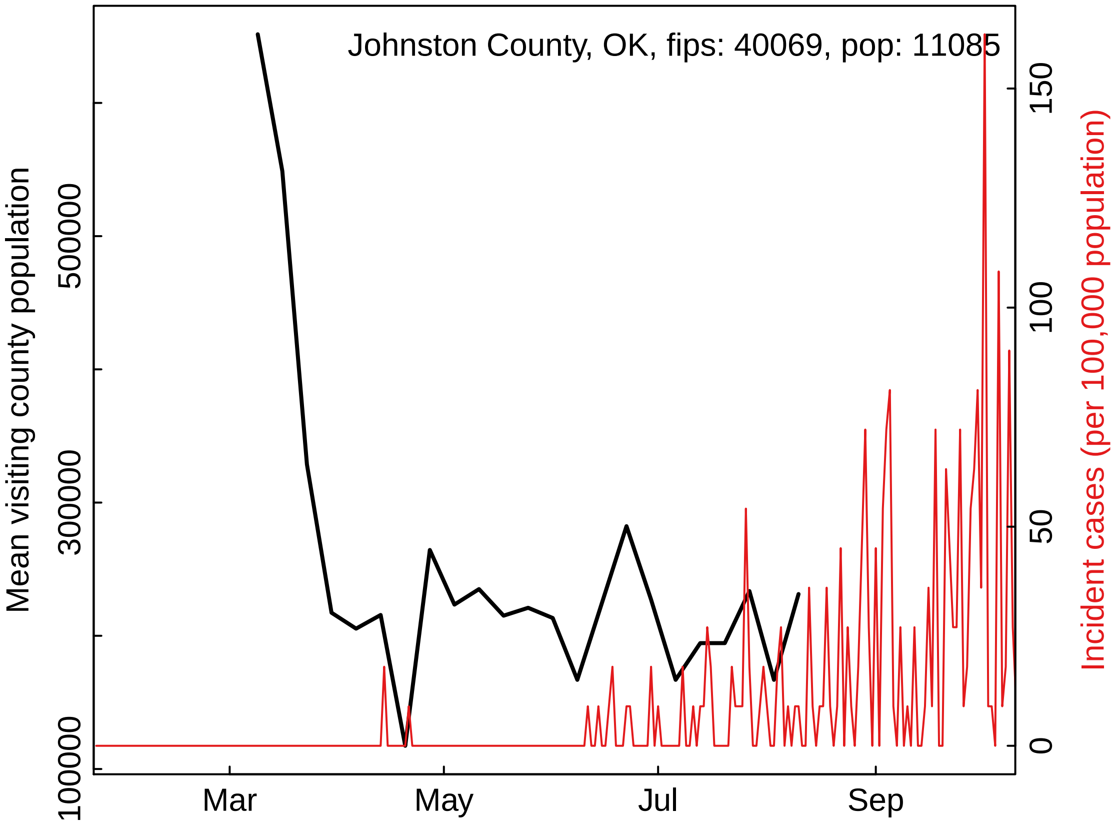

Johnston County, OK, fips: 40069, pop: 11085
Mean visiting county population
100000
300000
500000
Incident cases (per 100,000 population)
0
50
100
150
Mar
May
Jul
Sep

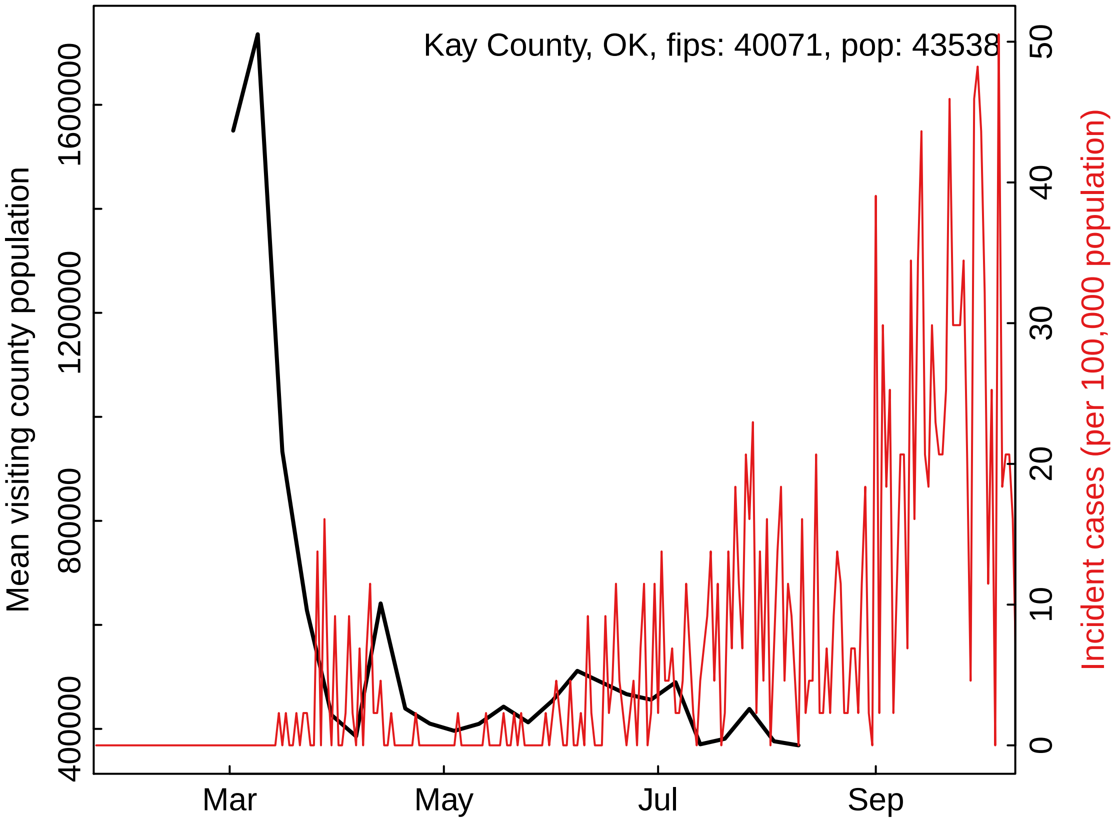

Kay County, OK, fips: 40071, pop: 43538
Mean visiting county population
Incident cases (per 100,000 population)
4000000
800000
1200000
1600000
0
10
20
30
40
50
Mar
May
Jul
Sep

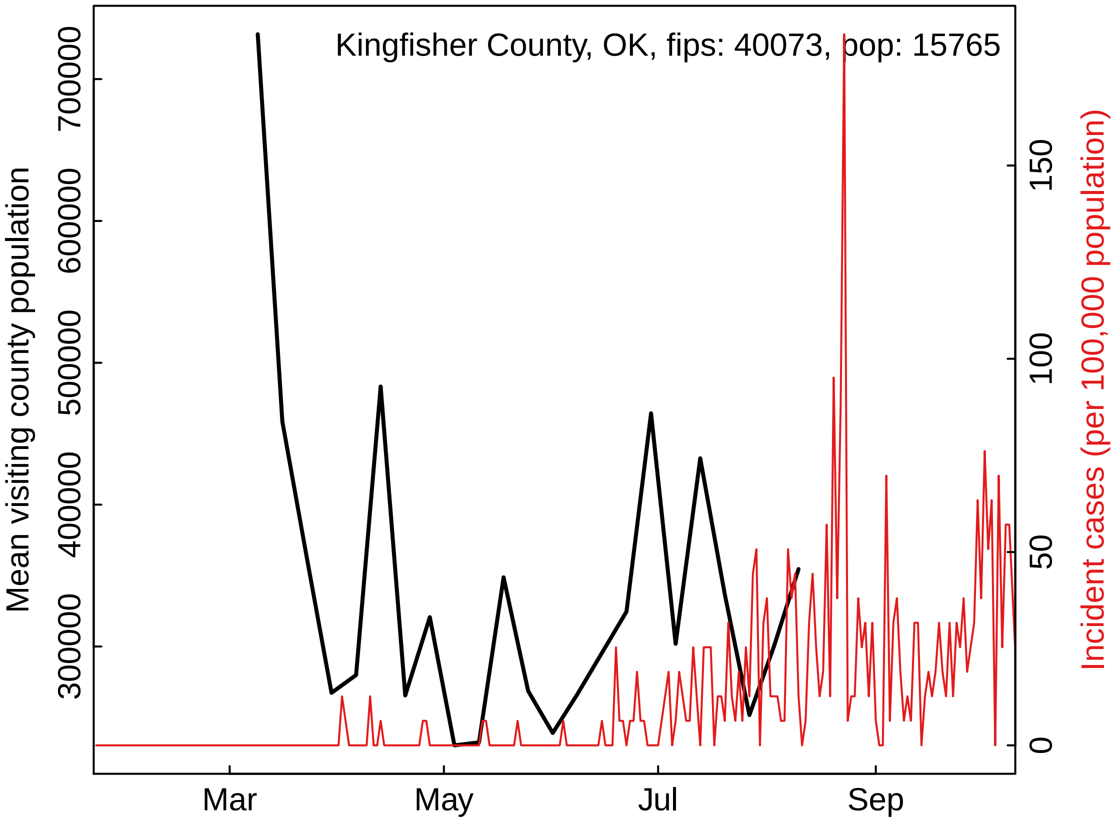
Kingfisher County, OK, fips: 40073, pop: 15765
Mean visiting county population
300000
400000
500000
600000
700000
Incident cases (per 100,000 population)
0
50
100
150
Mar
May
Jul
Sep

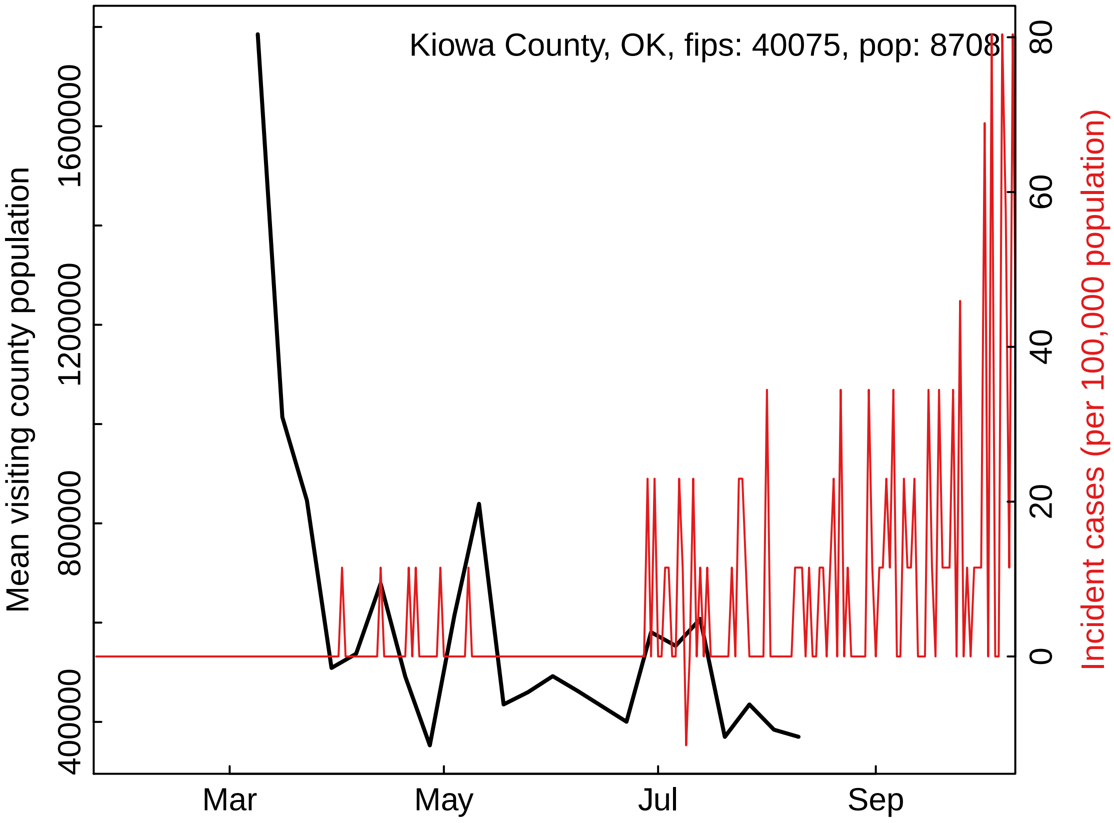

Kiowa County, OK, fips: 40075, pop: 8708
Mean visiting county population
400000
800000
1200000
1600000
Incident cases (per 100,000 population)
0
20
40
60
80
Mar
May
Jul
Sep

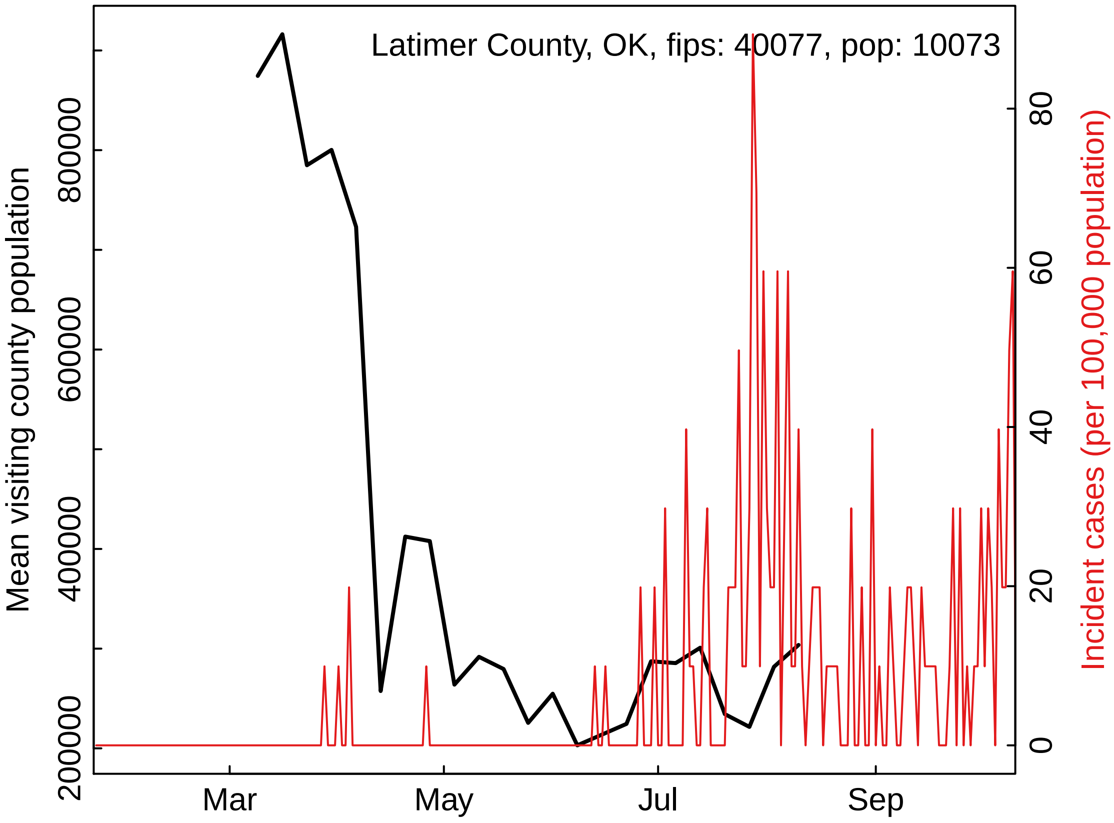

Latimer County, OK, fips: 40077, pop: 10073
Mean visiting county population
200000
400000
600000
800000
Incident cases (per 100,000 population)
0
20
40
60
80
Mar
May
Jul
Sep

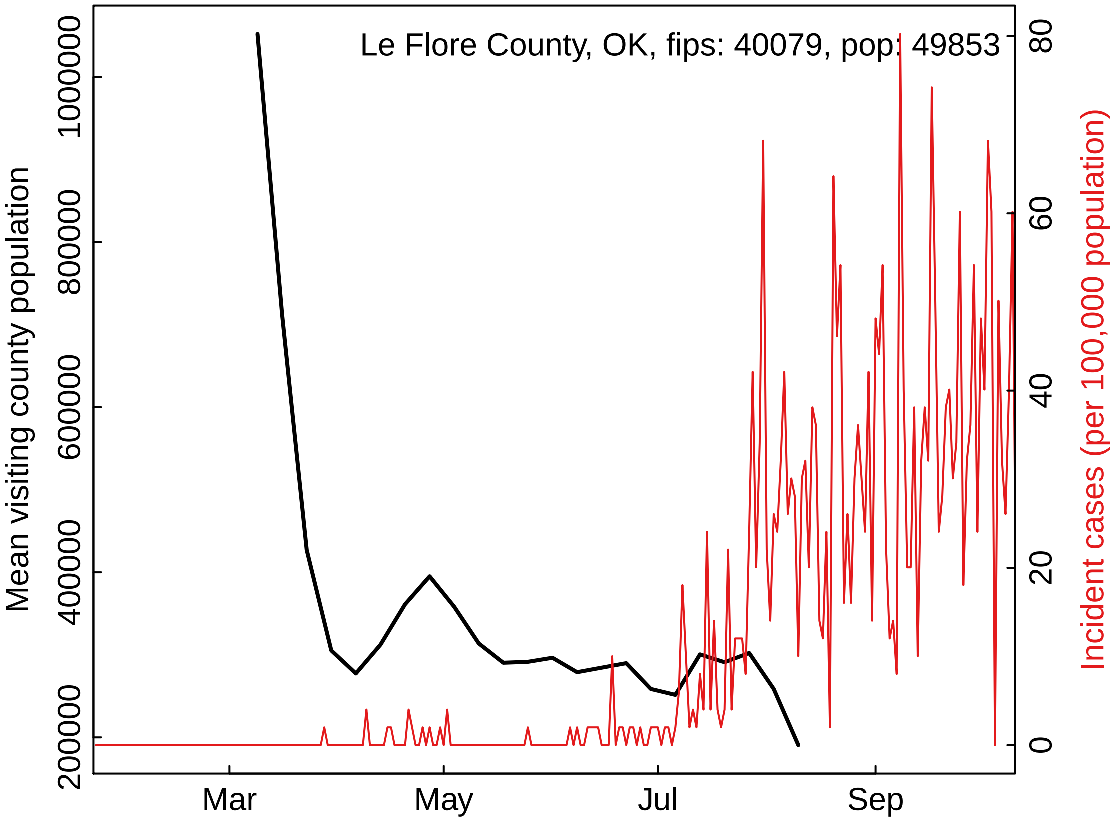

Le Flore County, OK, fips: 40079, pop: 49853
Mean visiting county population
200000
400000
600000
800000
1000000
Incident cases (per 100,000 population)
0
20
40
60
80
Mar
May
Jul
Sep

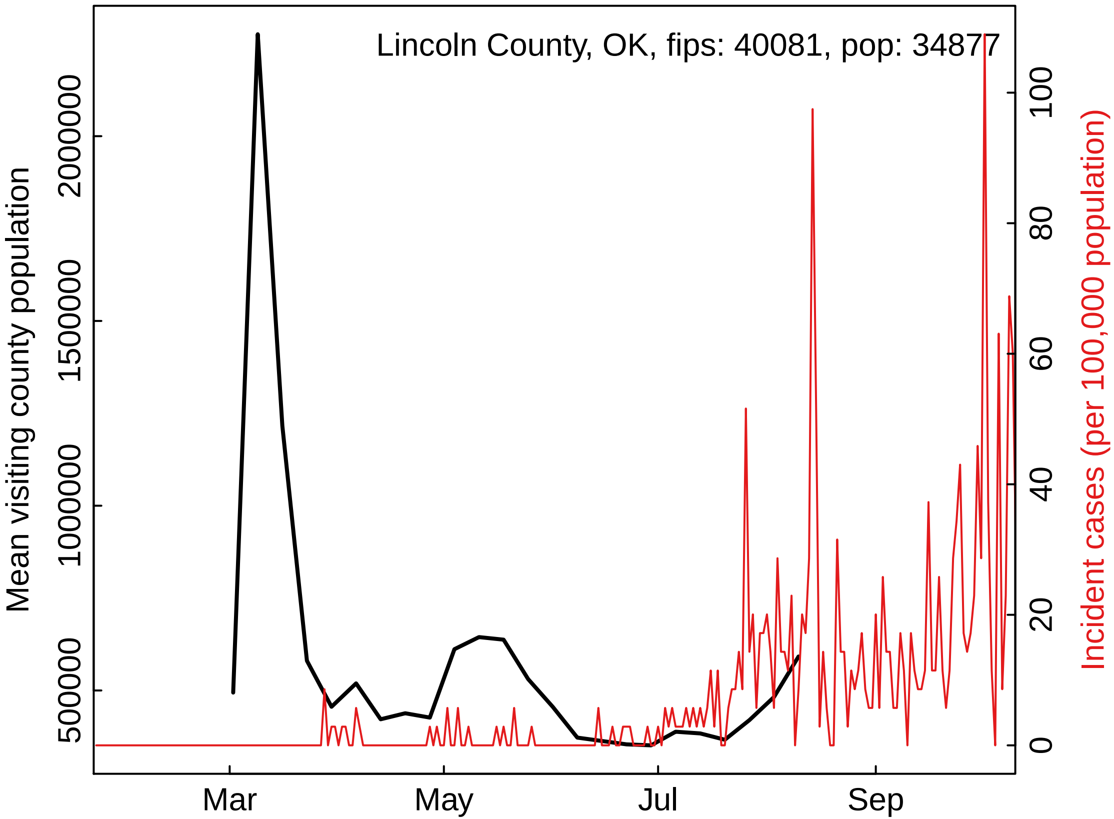

Lincoln County, OK, fips: 40081, pop: 34877
Mean visiting county population
500000
1000000
1500000
2000000
Incident cases (per 100,000 population)
0
20
40
60
80
100
Mar
May
Jul
Sep

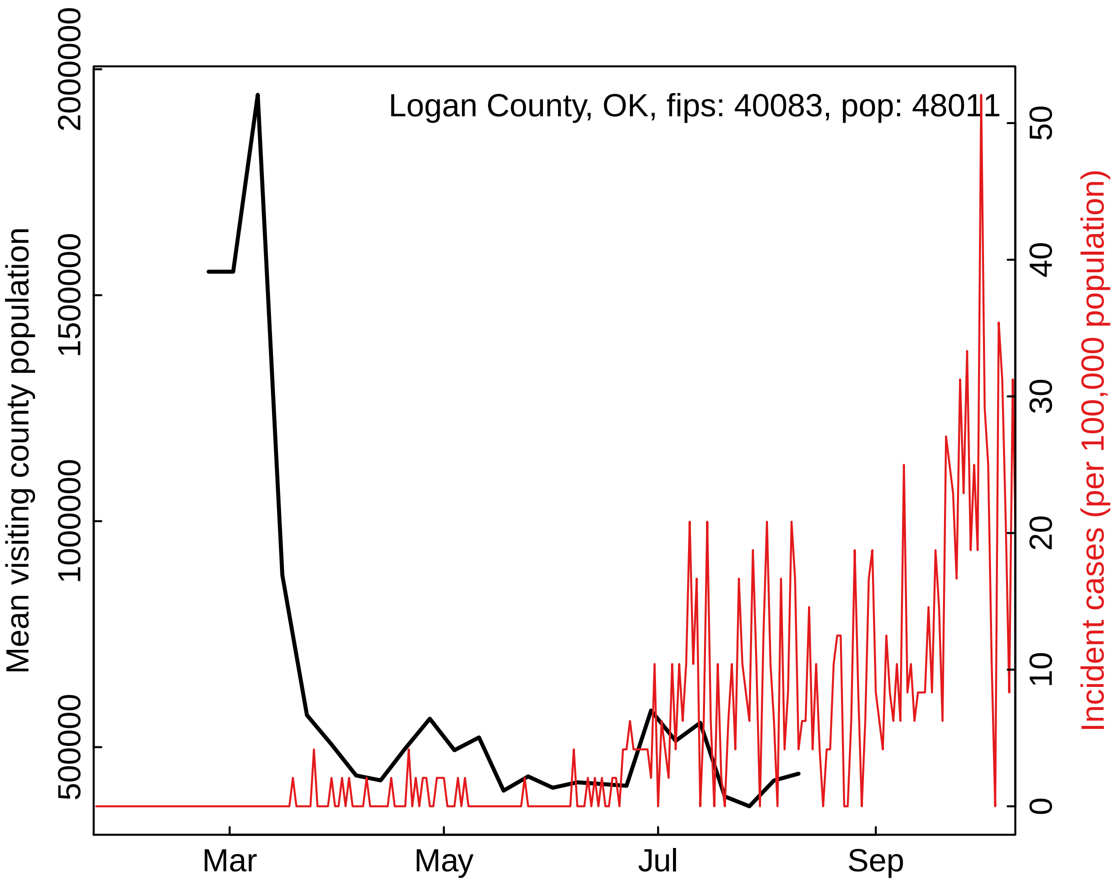

Logan County, OK, fips: 40083, pop: 48011
Mean visiting county population
500000
1000000
1500000
2000000
Incident cases (per 100,000 population)
0
10
20
30
40
50
Mar
May
Jul
Sep

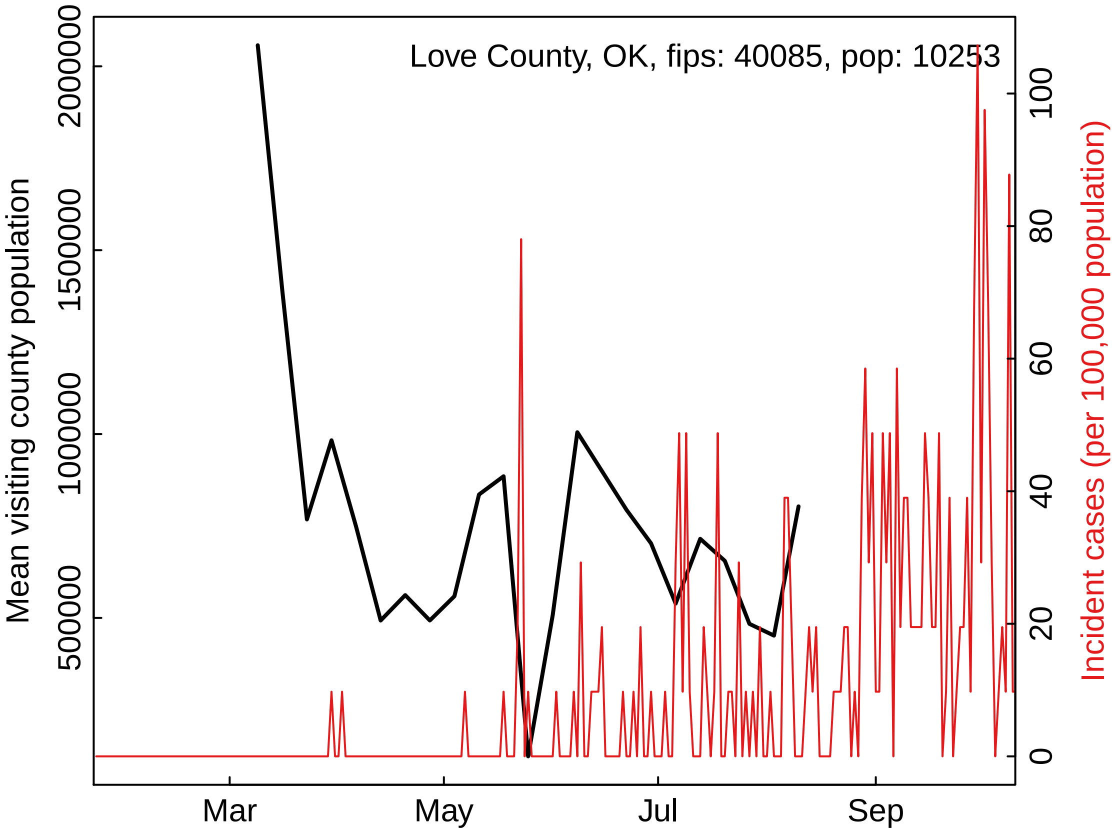

Love County, OK, fips: 40085, pop: 10253
Mean visiting county population
500000
1000000
1500000
2000000
Incident cases (per 100,000 population)
0
20
40
60
80
100
Mar
May
Jul
Sep

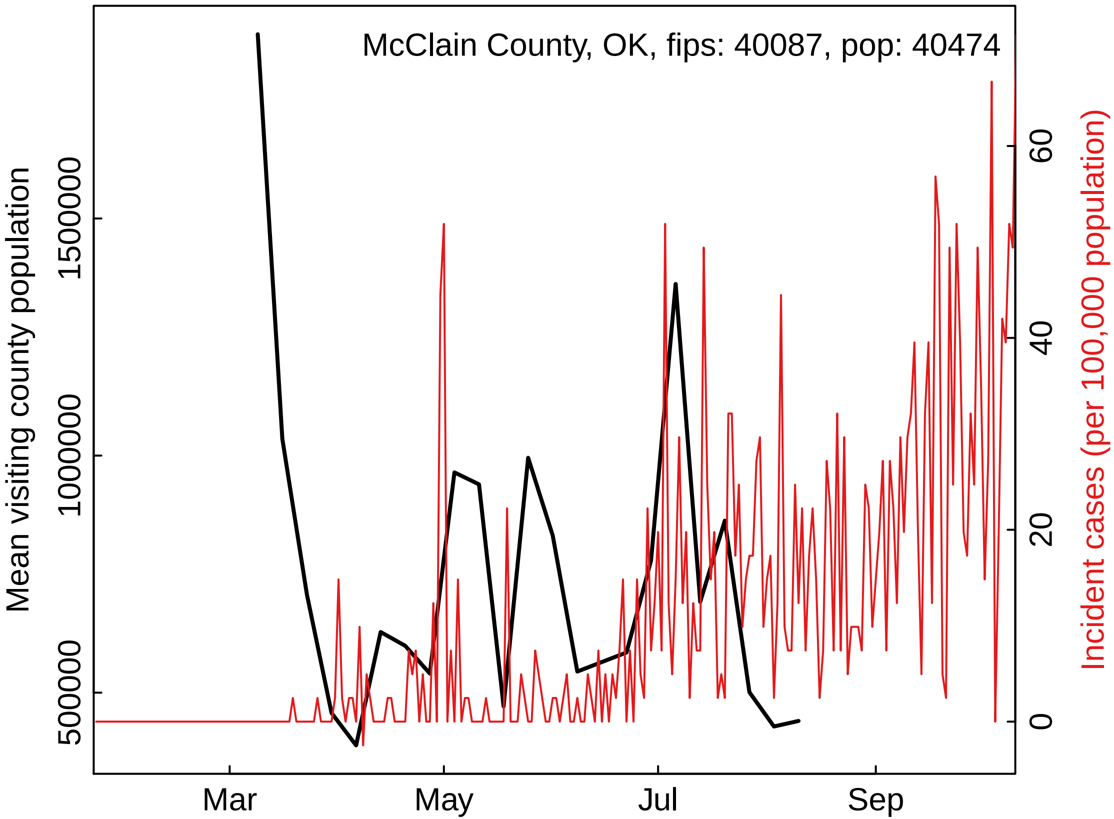
McClain County, OK, fips: 40087, pop: 40474
Mean visiting county population
500000
1000000
1500000
Incident cases (per 100,000 population)
0
20
40
60
Mar
May
Jul
Sep

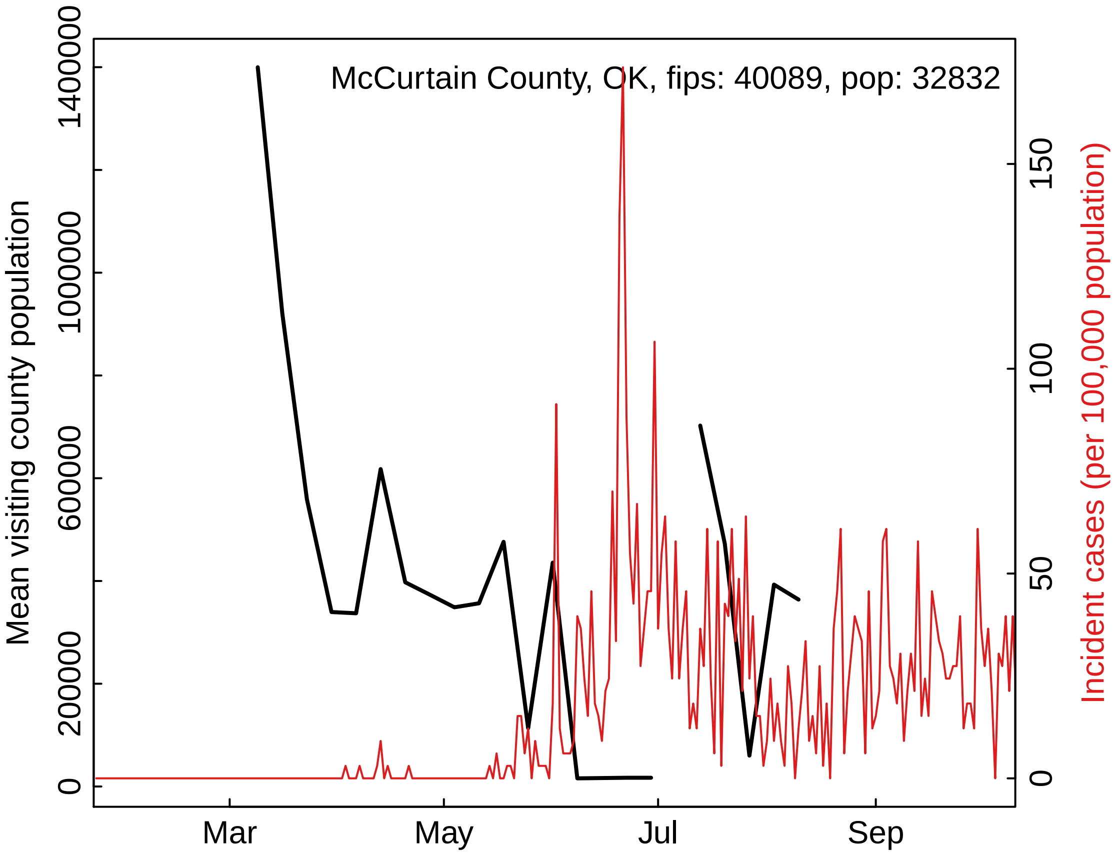
McCurtain County, OK, fips: 40089, pop: 32832
Mean visiting county population
0
200000
600000
1000000
1400000
Incident cases (per 100,000 population)
0
50
100
150
Mar
May
Jul
Sep

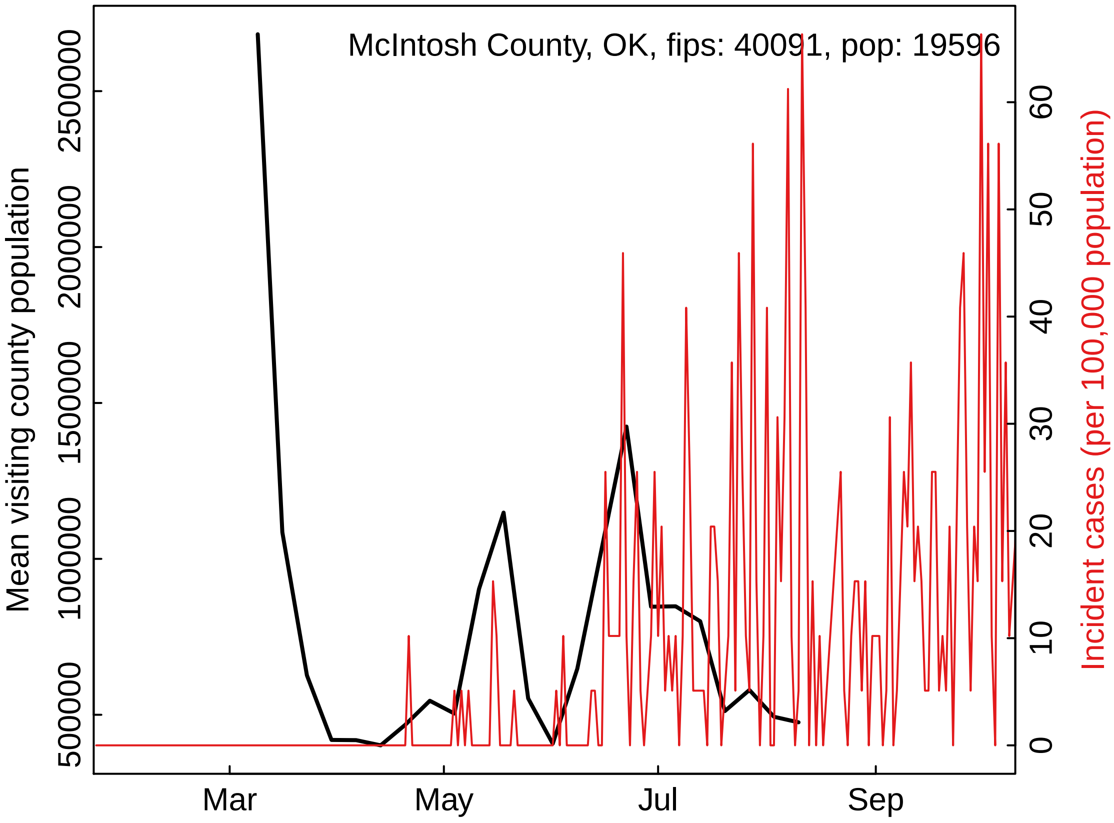

McIntosh County, OK, fips: 40091, pop: 19596
Mean visiting county population
500000
1000000
1500000
2000000
2500000
Incident cases (per 100,000 population)
0
10
20
30
40
50
60
Mar
May
Jul
Sep

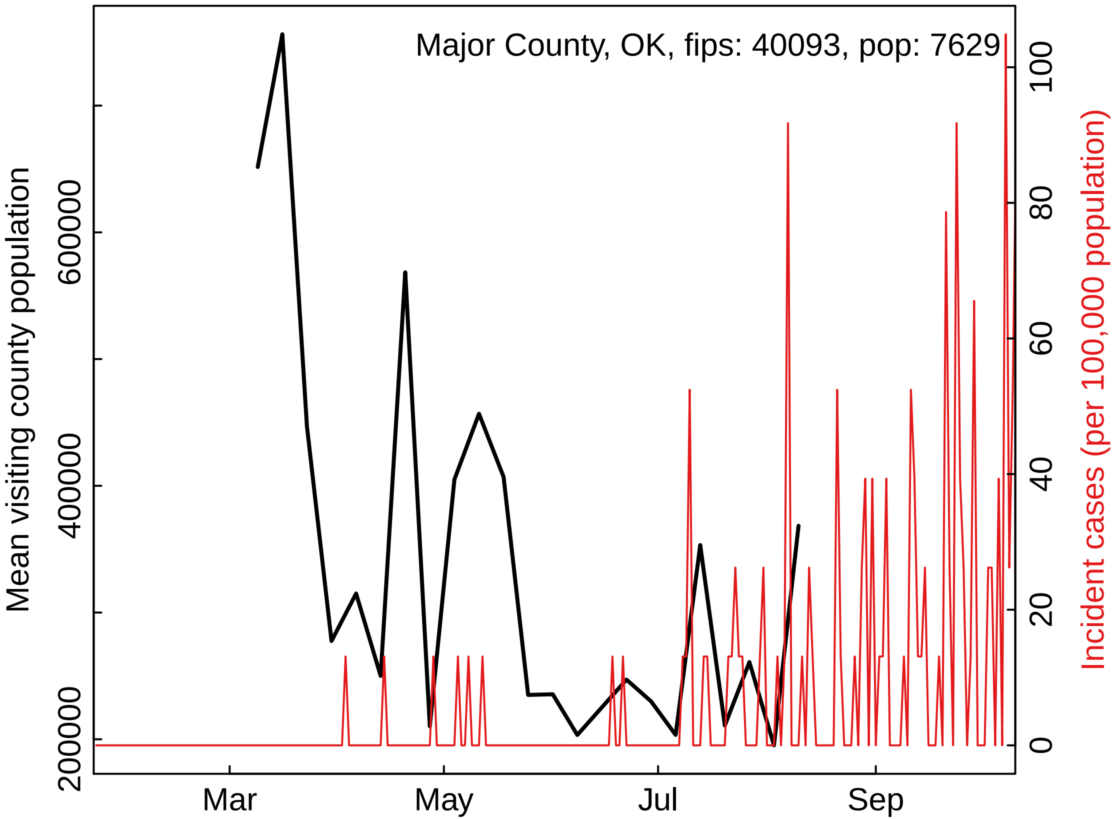

Major County, OK, fips: 40093, pop: 7629
Mean visiting county population
200000
400000
600000
Incident cases (per 100,000 population)
0
20
40
60
80
100
Mar
May
Jul
Sep

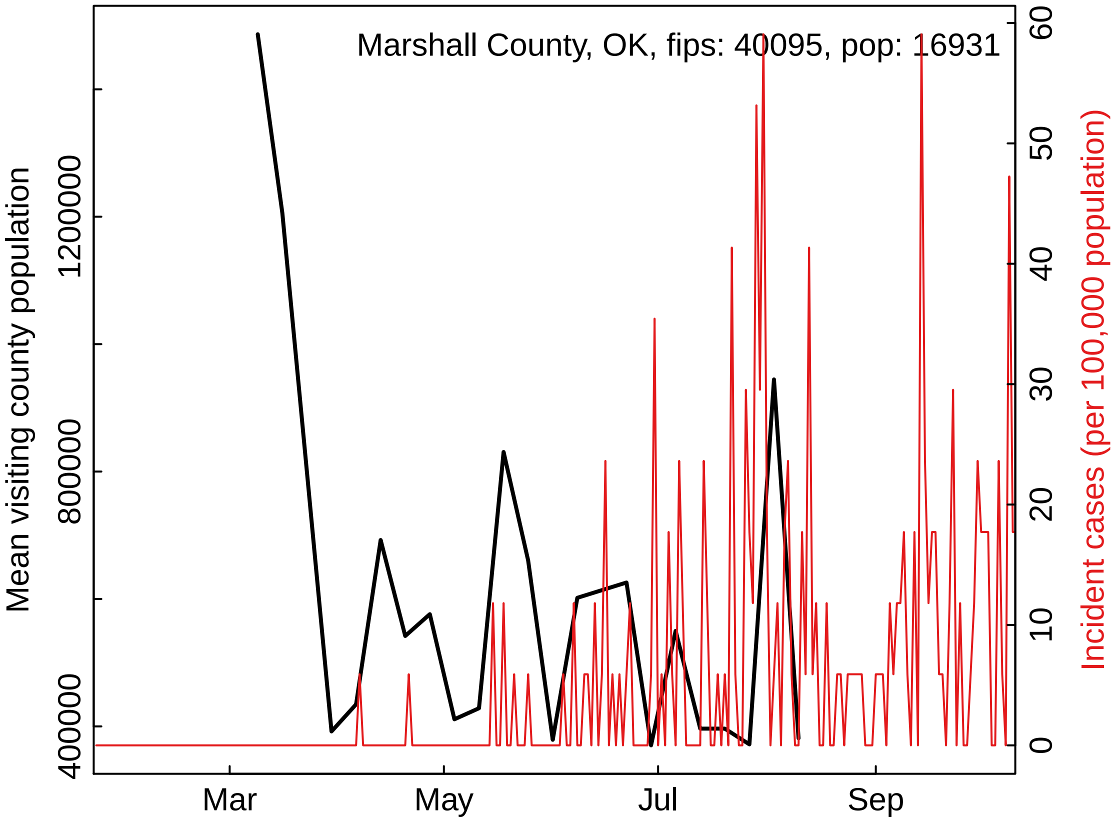

Marshall County, OK, fips: 40095, pop: 16931
Mean visiting county population
400000
800000
1200000
Incident cases (per 100,000 population)
0
10
20
30
40
50
60
Mar
May
Jul
Sep

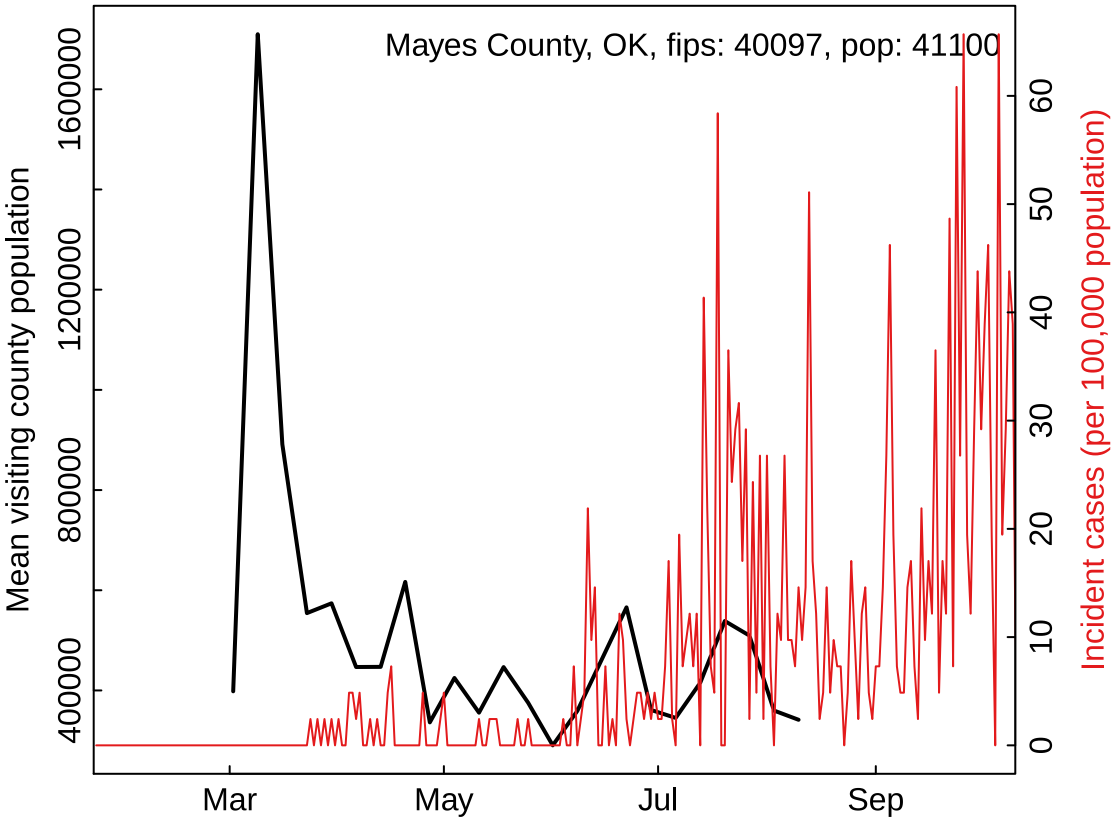

Mayes County, OK, fips: 40097, pop: 41100
Mean visiting county population
400000
800000
1200000
1600000
Mar
May
Jul
Sep
0
10
20
30
40
50
60
Incident cases (per 100,000 population)

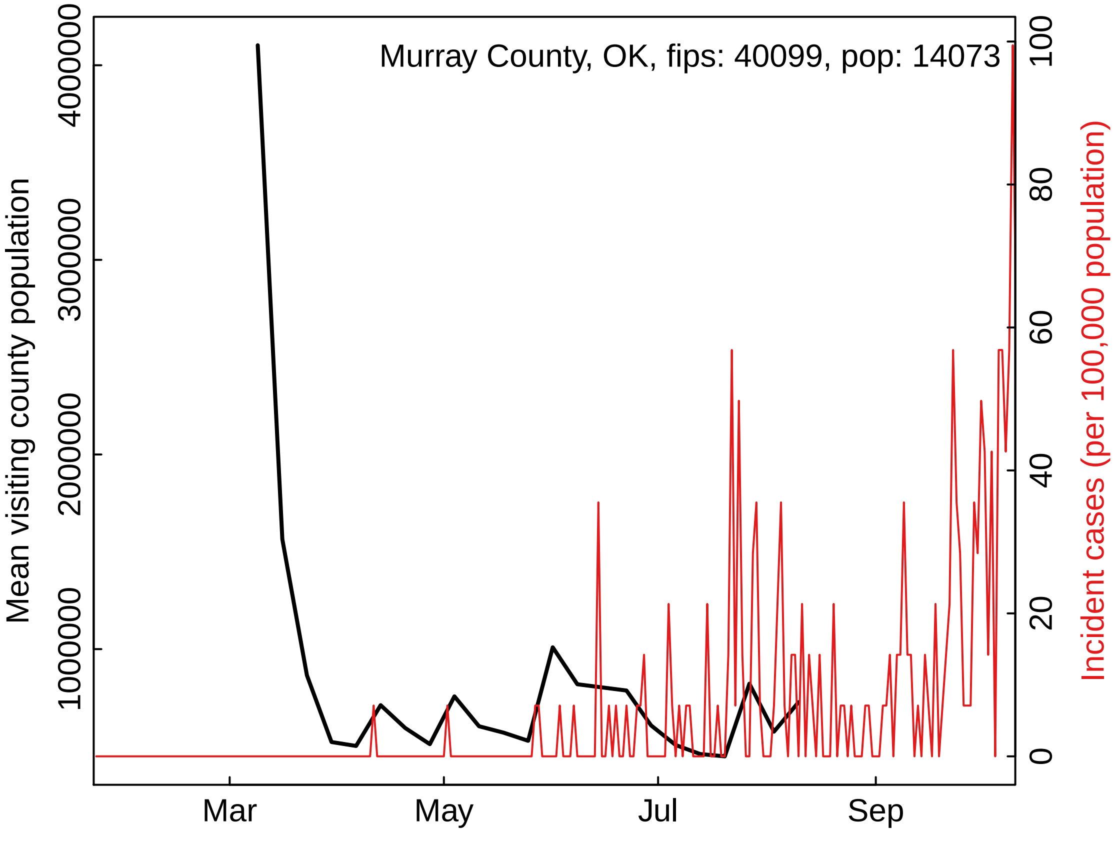

Murray County, OK, fips: 40099, pop: 14073
Mean visiting county population
10000000
20000000
30000000
40000000
Incident cases (per 100,000 population)
0
20
40
60
80
100
Mar
May
Jul
Sep

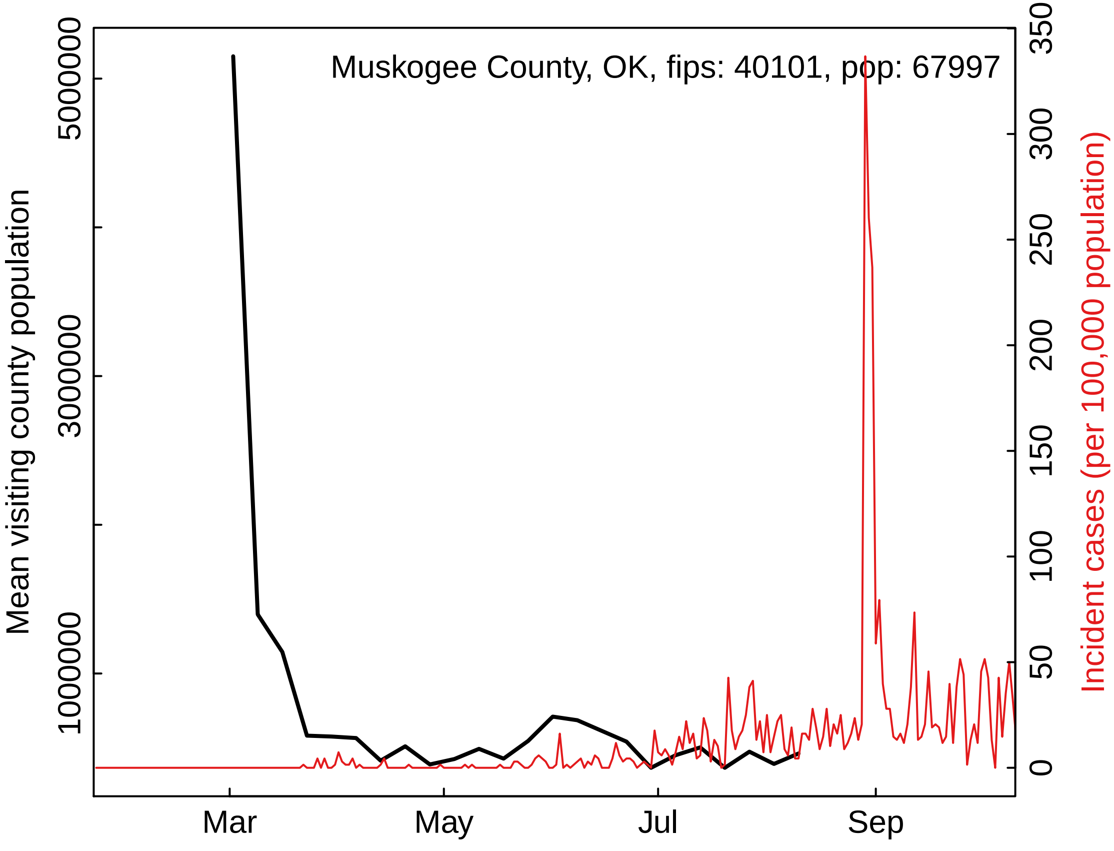

Muskogee County, OK, fips: 40101, pop: 67997
Mean visiting county population
1000000
3000000
5000000
Incident cases (per 100,000 population)
0
50
100
150
200
250
300
350
Mar
May
Jul
Sep

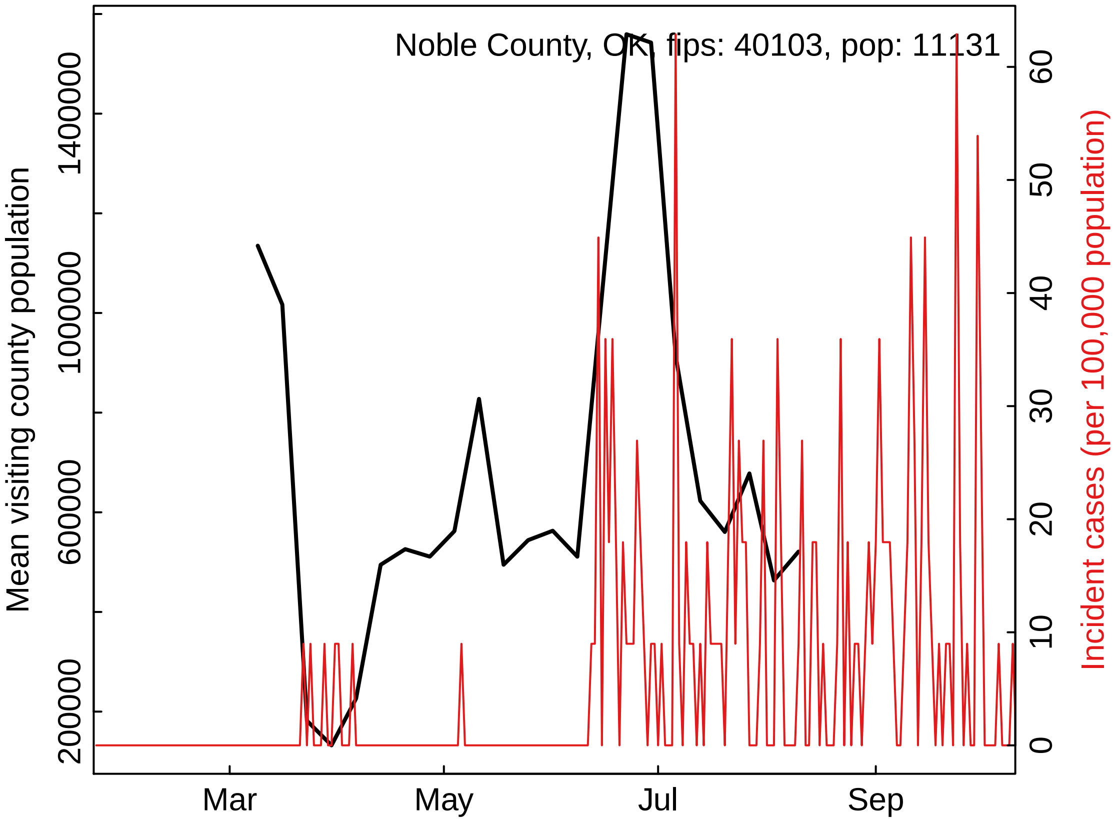

Noble County, OK, fips: 40103, pop: 11131
Mean visiting county population
200000
600000
1000000
1400000
Incident cases (per 100,000 population)
0
10
20
30
40
50
60
Mar
May
Jul
Sep

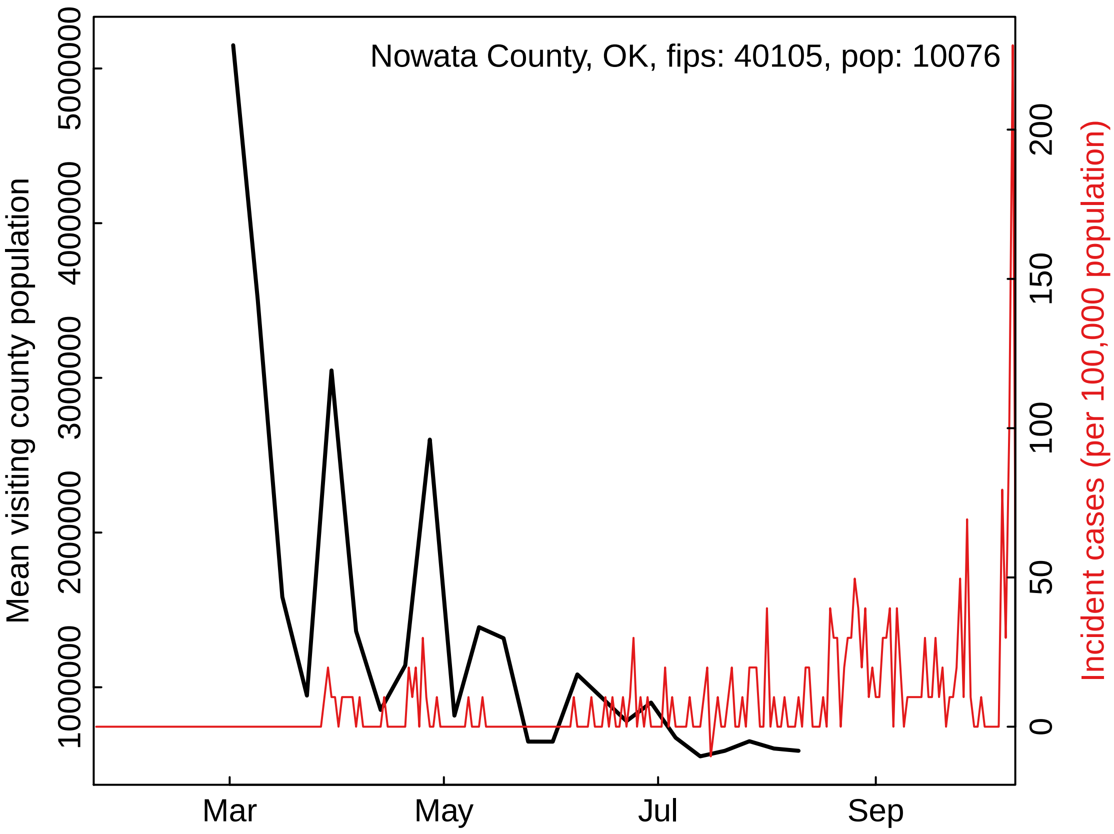

Nowata County, OK, fips: 40105, pop: 10076
Mean visiting county population
10000000
20000000
30000000
40000000
50000000
Incident cases (per 100,000 population)
0
50
100
150
200
Mar
May
Jul
Sep

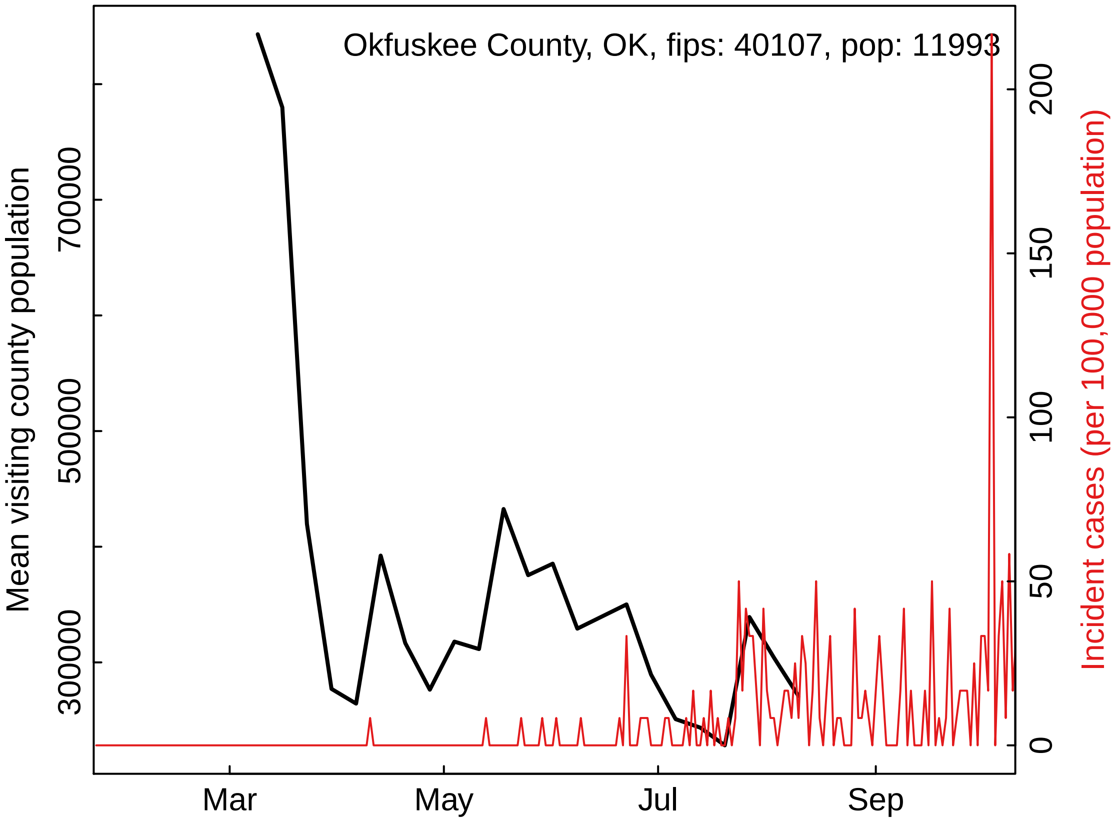

Okfuskee County, OK, fips: 40107, pop: 11993
Mean visiting county population
300000
500000
700000
Mar
May
Jul
Sep
0
50
100
150
200
Incident cases (per 100,000 population)

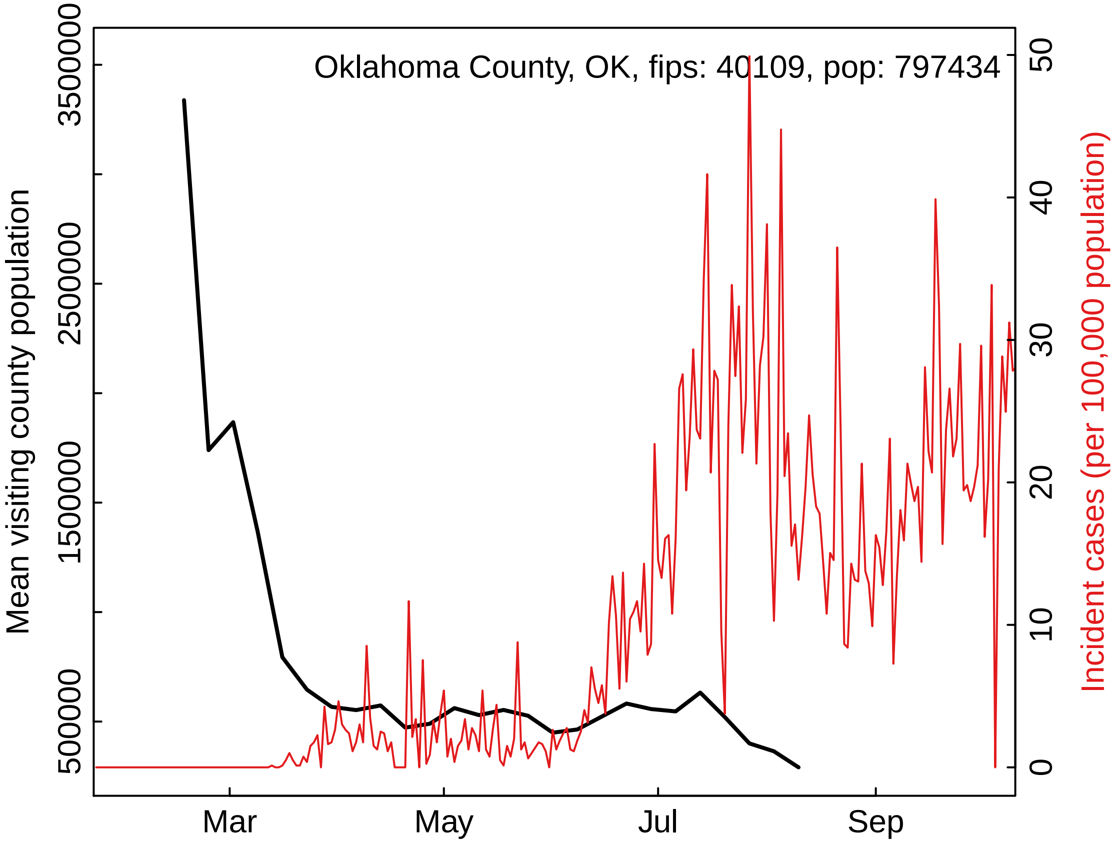
Oklahoma County, OK, fips: 40109, pop: 797434
Mean visiting county population
500000
1500000
2500000
3500000
Incident cases (per 100,000 population)
0
10
20
30
40
50
Mar
May
Jul
Sep

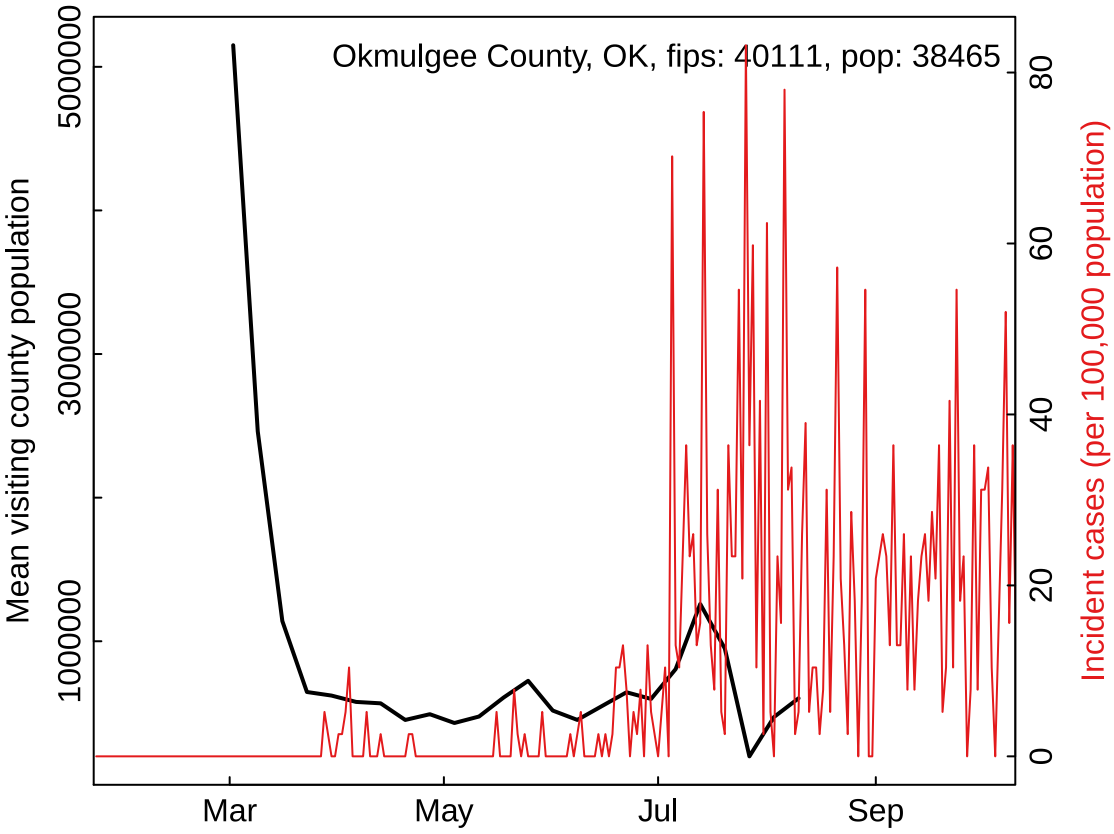

Okmulgee County, OK, fips: 40111, pop: 38465
Mean visiting county population
1000000
3000000
5000000
Mar
May
Jul
Sep
0
20
40
60
80
Incident cases (per 100,000 population)

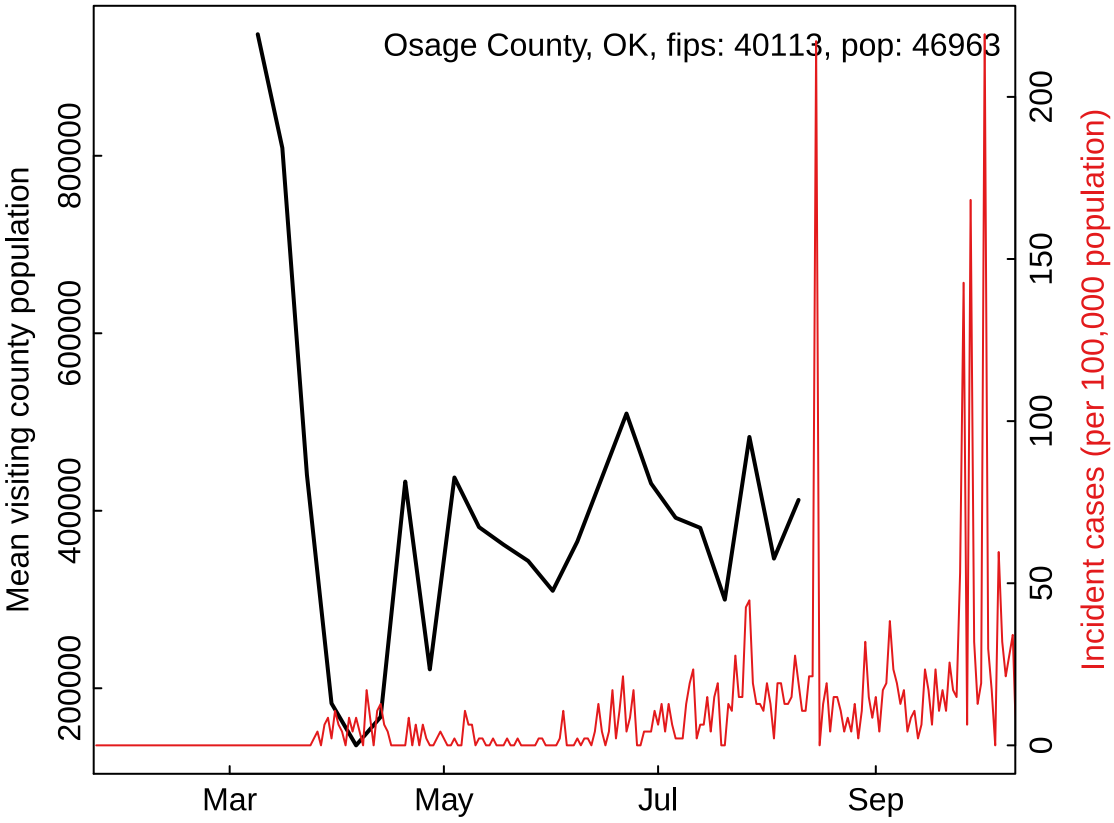

Osage County, OK, fips: 40113, pop: 46963
Mean visiting county population
200000
400000
600000
800000
Incident cases (per 100,000 population)
0
50
100
150
200
Mar
May
Jul
Sep

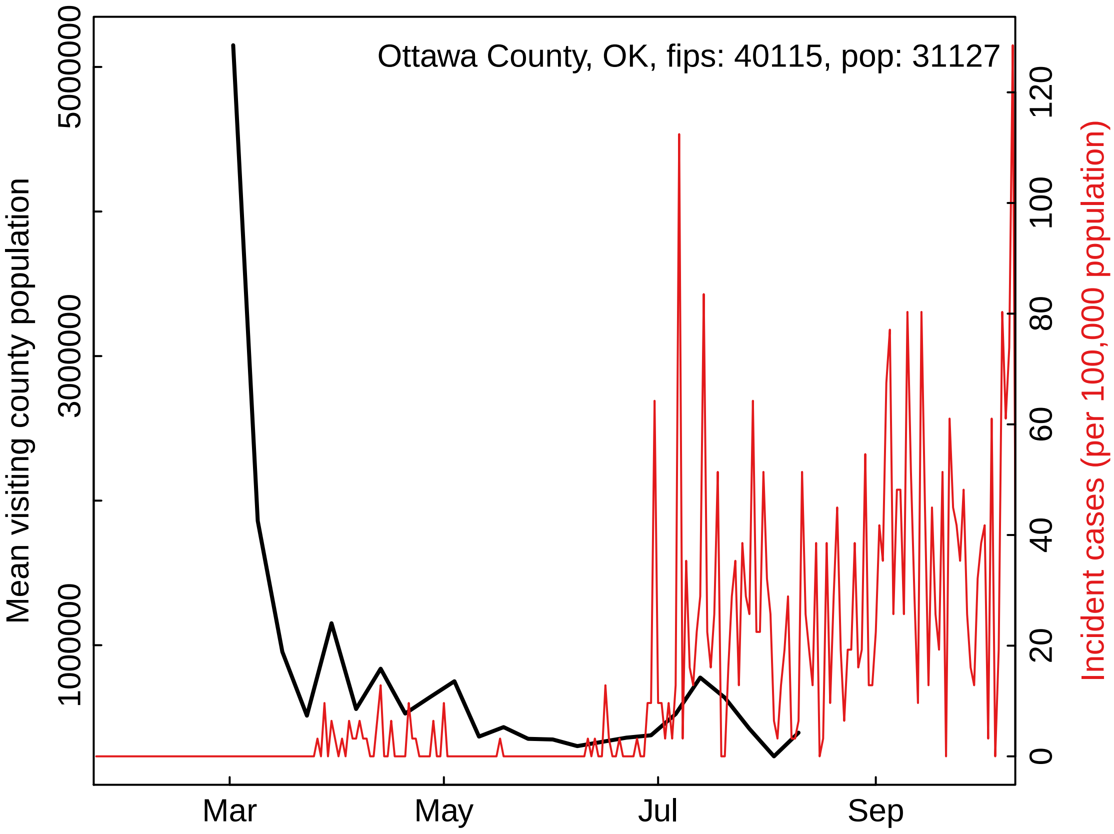

Ottawa County, OK, fips: 40115, pop: 31127
Mean visiting county population
1000000
3000000
5000000
Incident cases (per 100,000 population)
0
20
40
60
80
100
120
Mar
May
Jul
Sep

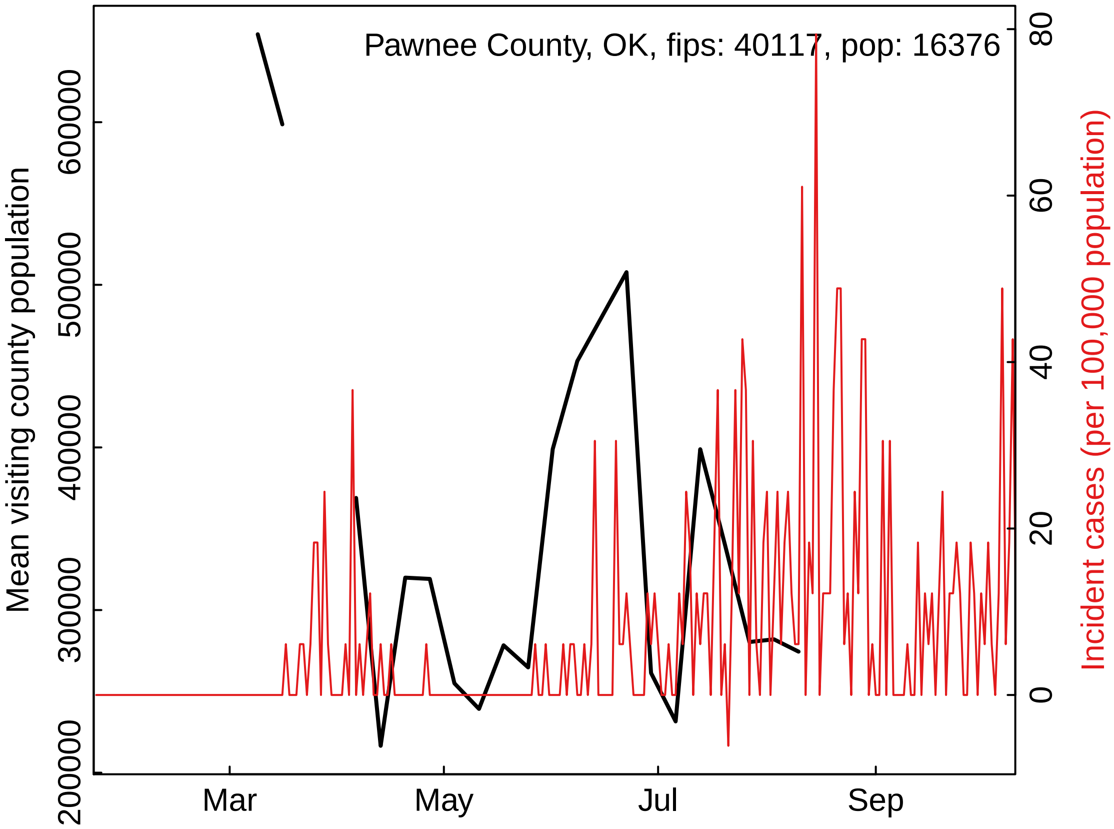

Pawnee County, OK, fips: 40117, pop: 16376
Mean visiting county population
200000
300000
400000
500000
600000
Mar
May
Jul
Sep
0
20
40
60
80
Incident cases (per 100,000 population)

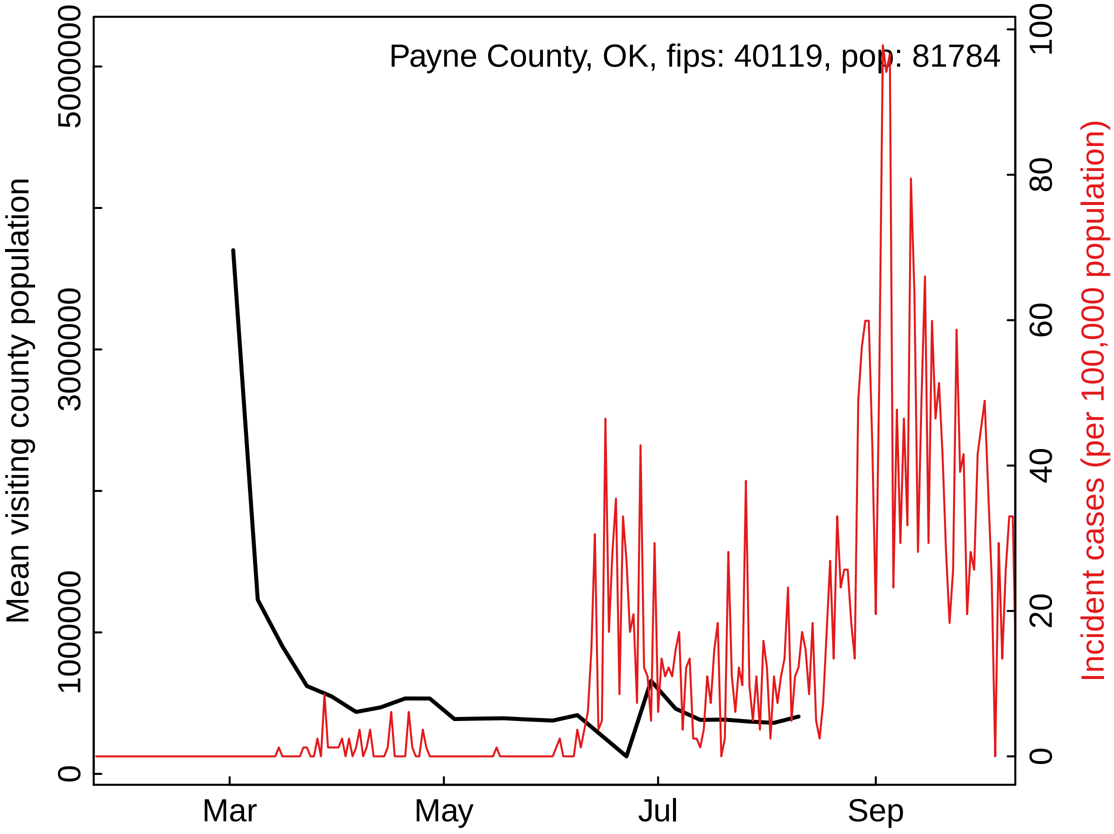

Payne County, OK, fips: 40119, pop: 81784
Mean visiting county population
0
1000000
3000000
5000000
Mar
May
Jul
Sep
Incident cases (per 100,000 population)
0
20
40
60
80
100

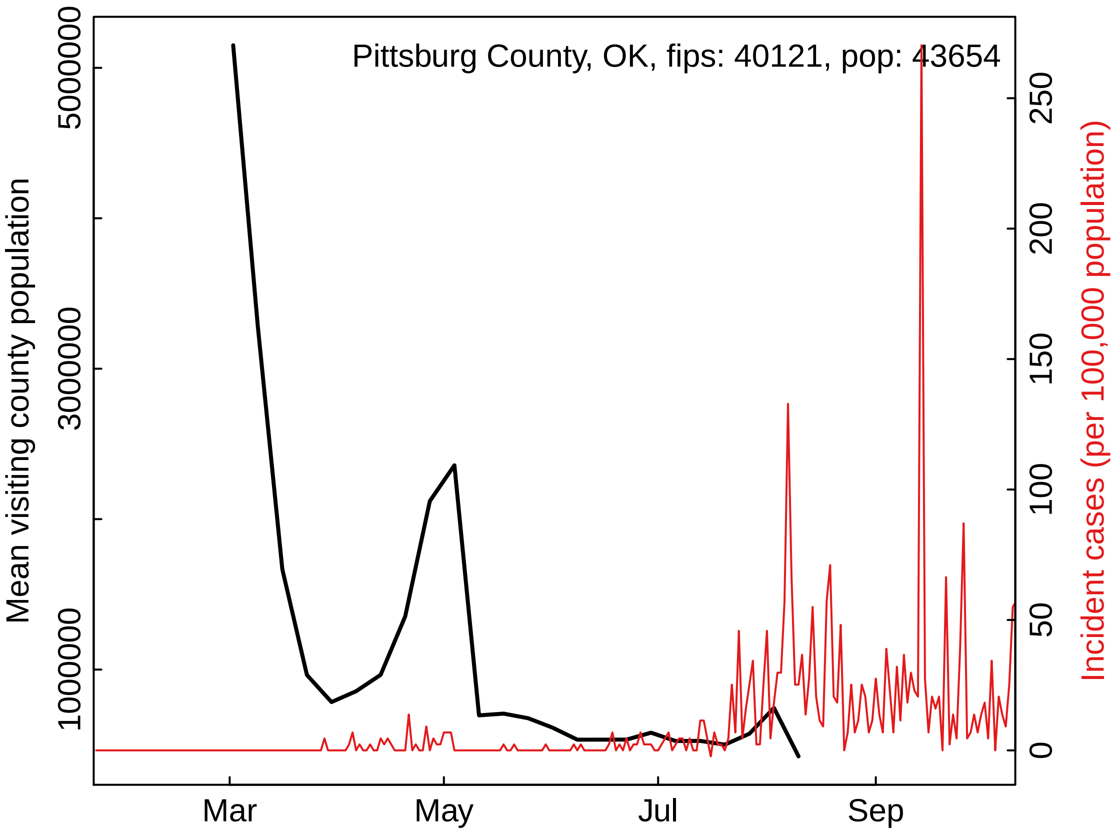

Pittsburg County, OK, fips: 40121, pop: 43654
Mean visiting county population
Incident cases (per 100,000 population)
1000000
3000000
5000000
0
50
100
150
200
250
Mar
May
Jul
Sep

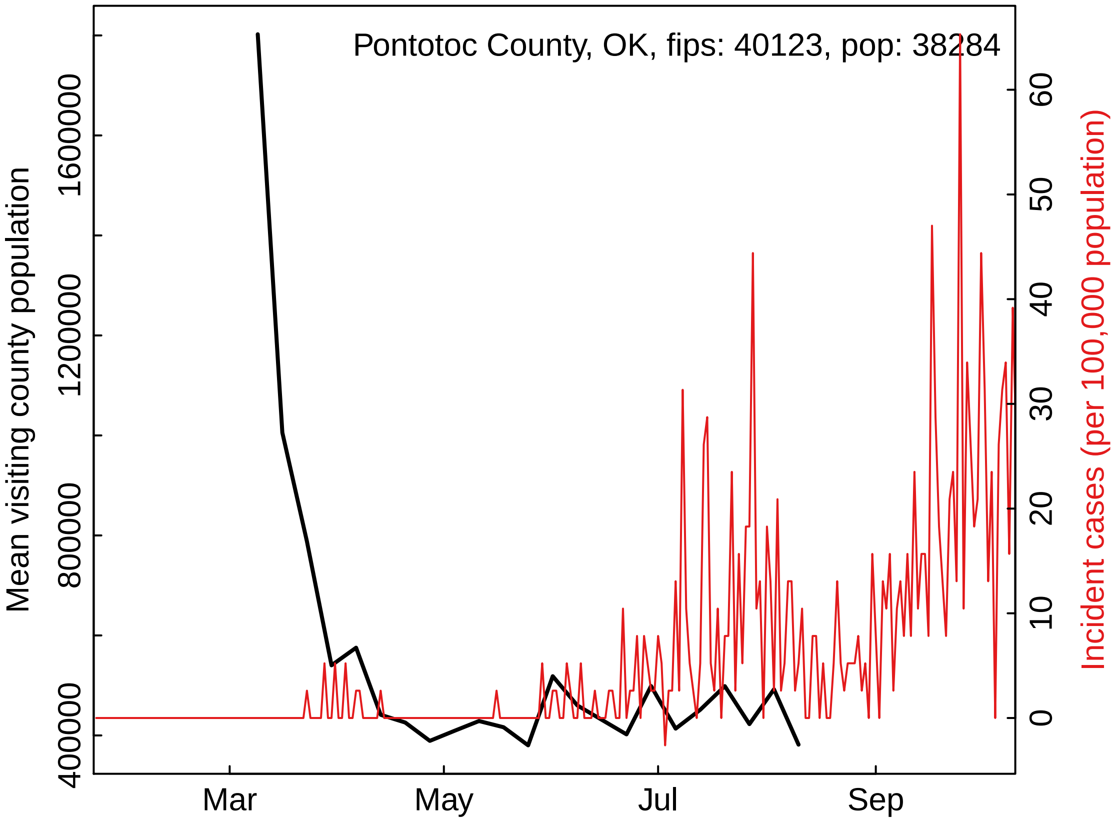

Pontotoc County, OK, fips: 40123, pop: 38284
Mean visiting county population
400000
800000
1200000
1600000
Incident cases (per 100,000 population)
0
10
20
30
40
50
60
Mar
May
Jul
Sep

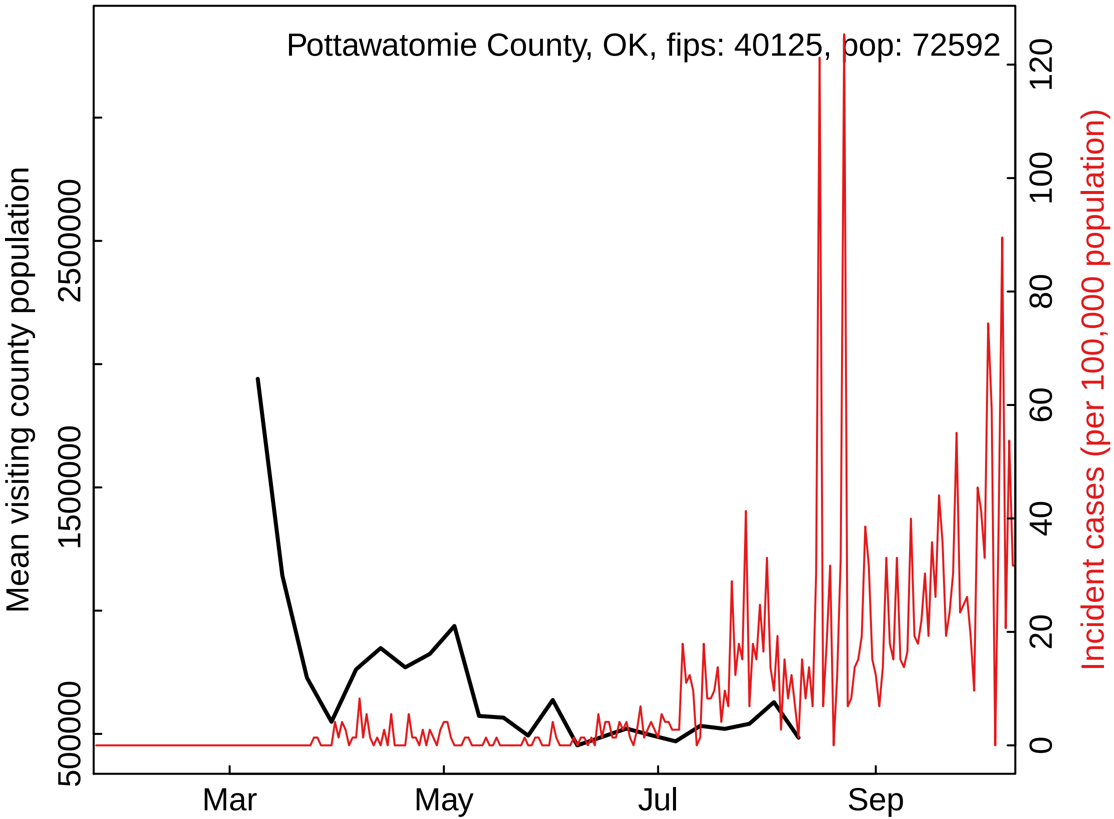

Pottawatomie County, OK, fips: 40125, pop: 72592
Mean visiting county population
500000
1500000
2500000
Mar
May
Jul
Sep
0
20
40
60
80
100
120
Incident cases (per 100,000 population)

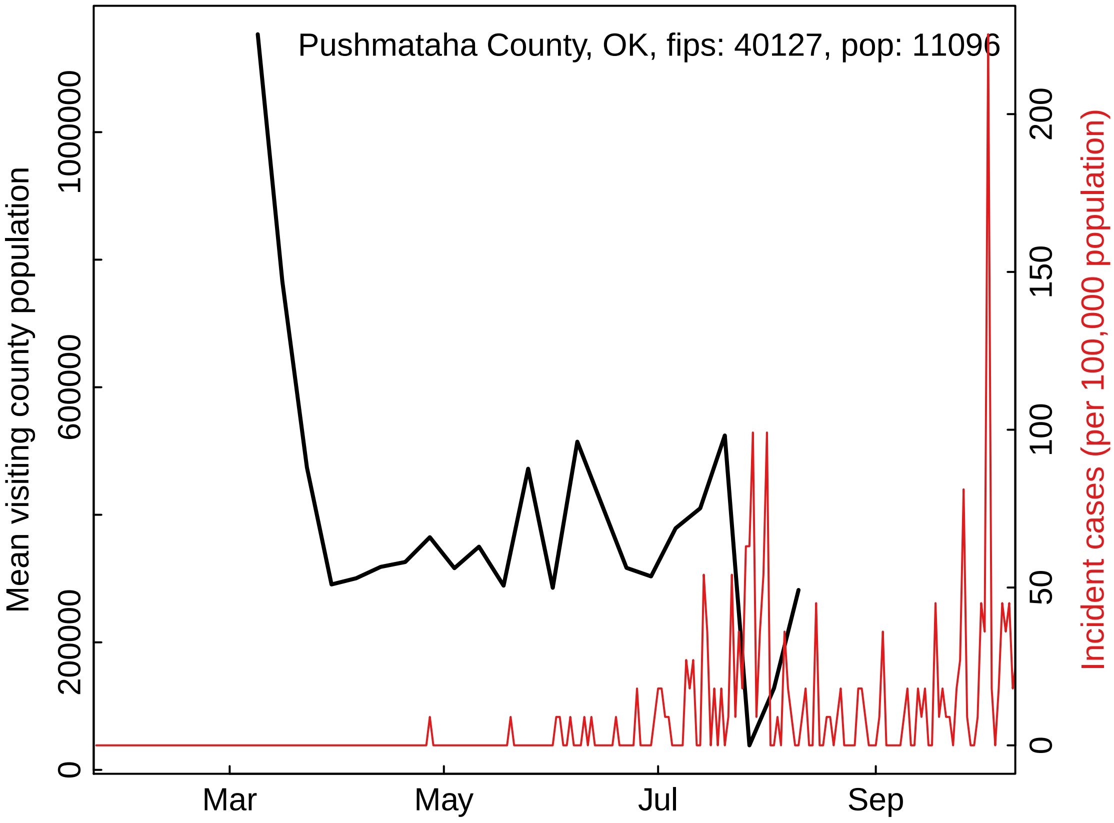
Pushmataha County, OK, fips: 40127, pop: 11096
Mean visiting county population
0
200000
600000
1000000
Incident cases (per 100,000 population)
0
50
100
150
200
Mar
May
Jul
Sep

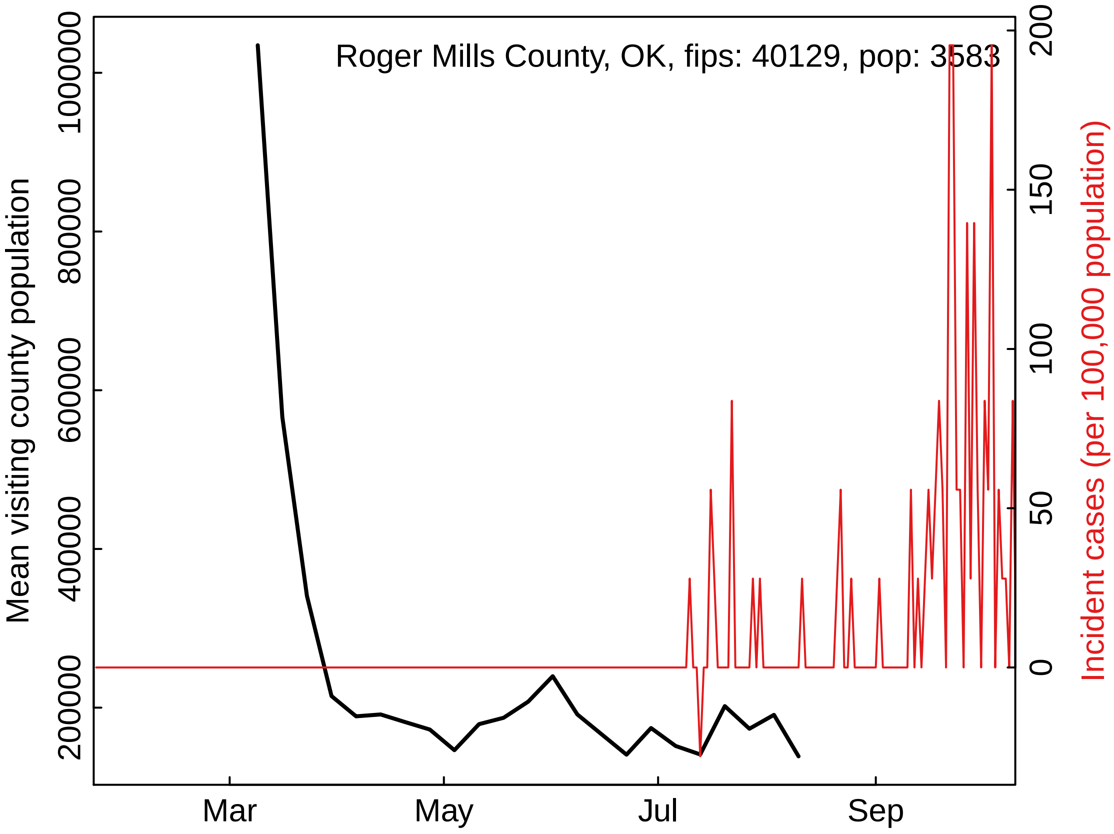
Roger Mills County, OK, fips: 40129, pop: 3583
Mean visiting county population
200000
400000
600000
800000
1000000
Incident cases (per 100,000 population)
0
50
100
150
200
Mar
May
Jul
Sep

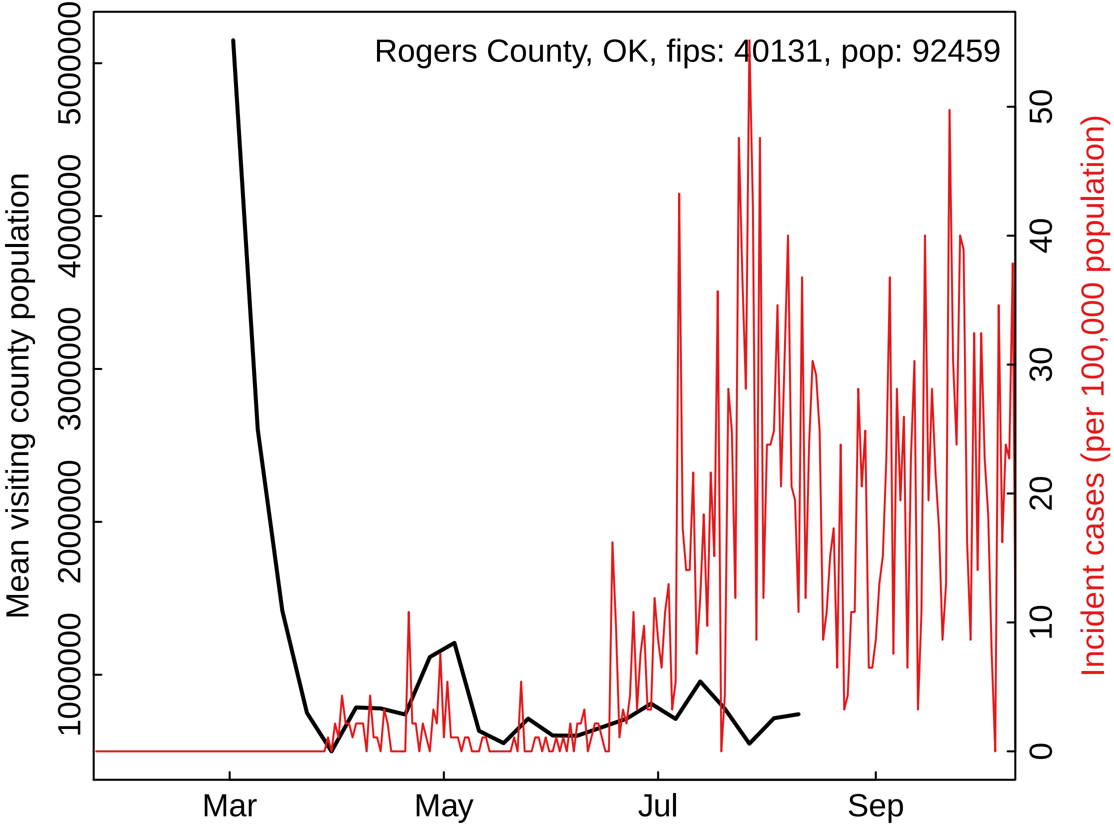

Rogers County, OK, fips: 40131, pop: 92459
Mean visiting county population
0
1000000
2000000
3000000
4000000
5000000
Incident cases (per 100,000 population)
0
10
20
30
40
50
Mar
May
Jul
Sep

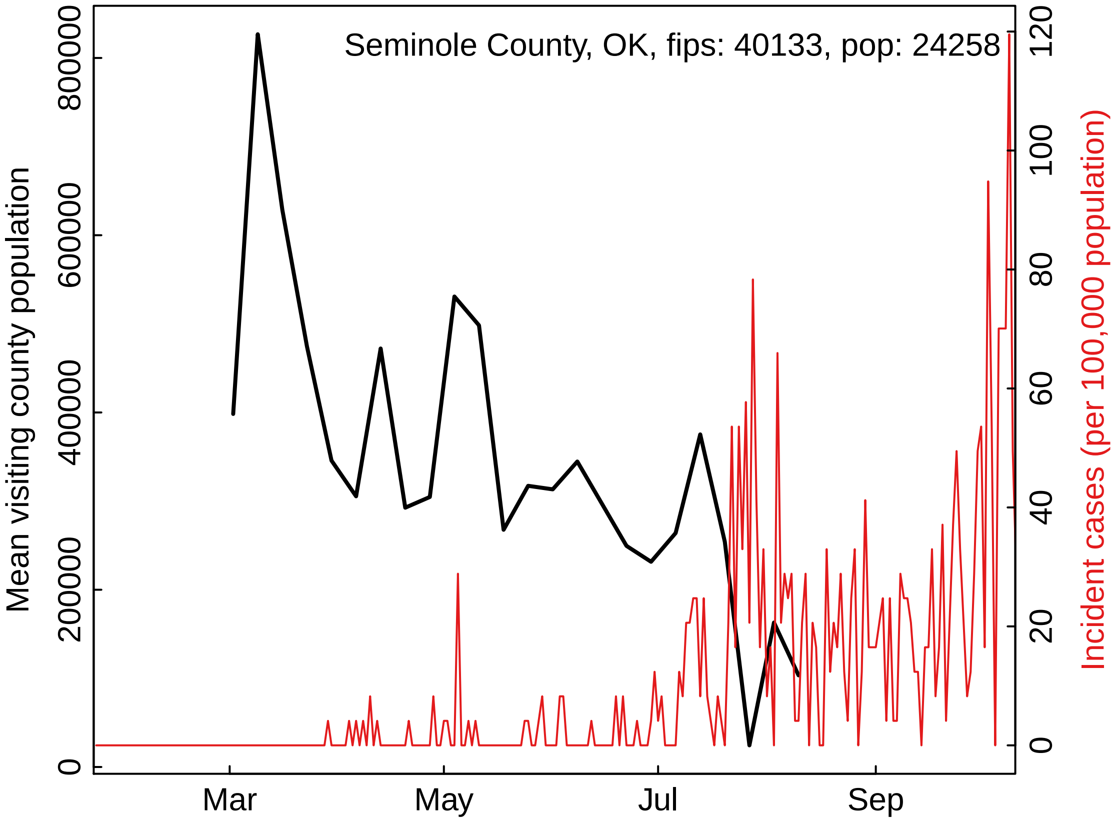

Seminole County, OK, fips: 40133, pop: 24258
Mean visiting county population
Incident cases (per 100,000 population)
0
200000
400000
600000
800000
0
20
40
60
80
100
120
Mar
May
Jul
Sep

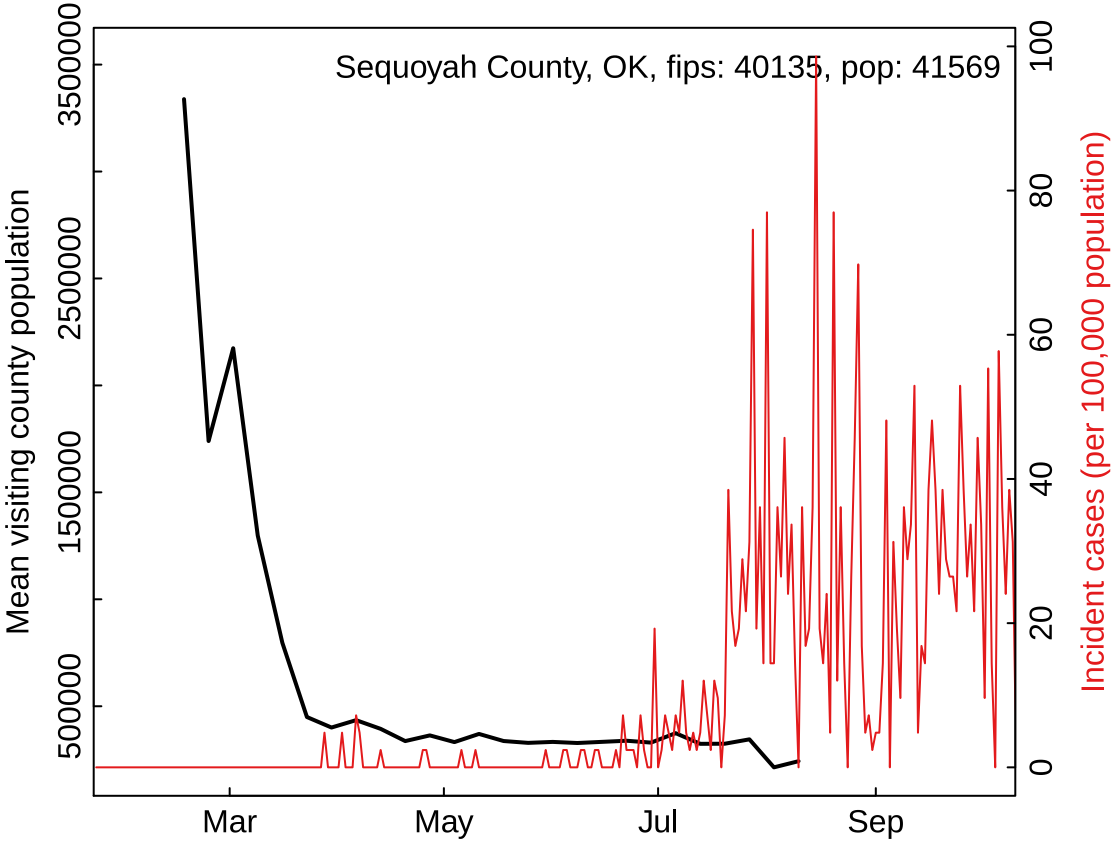
Sequoyah County, OK, fips: 40135, pop: 41569
Mean visiting county population
500000
1500000
2500000
3500000
Incident cases (per 100,000 population)
0
20
40
60
80
100
Mar
May
Jul
Sep

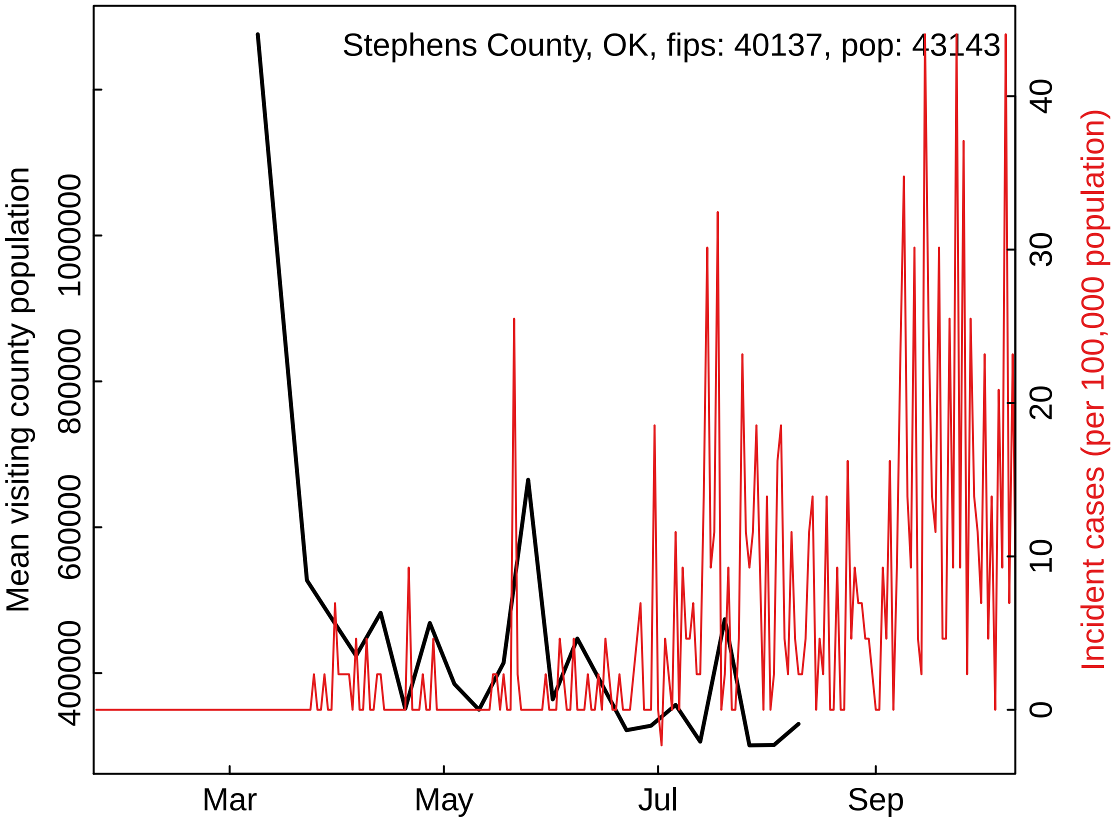

Stephens County, OK, fips: 40137, pop: 43143
Mean visiting county population
400000
600000
800000
1000000
Incident cases (per 100,000 population)
0
10
20
30
40
Mar
May
Jul
Sep

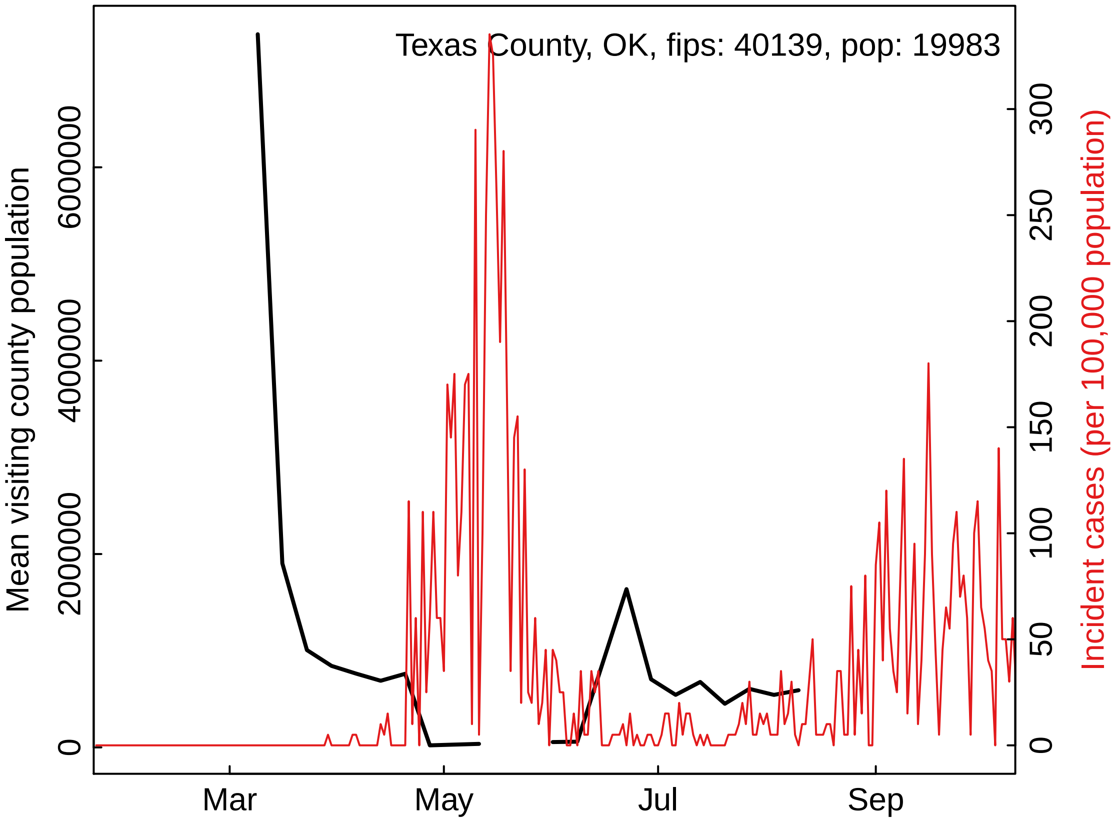

Texas County, OK, fips: 40139, pop: 19983
Mean visiting county population
0
2000000
4000000
6000000
Incident cases (per 100,000 population)
0
50
100
150
200
250
300
Mar
May
Jul
Sep

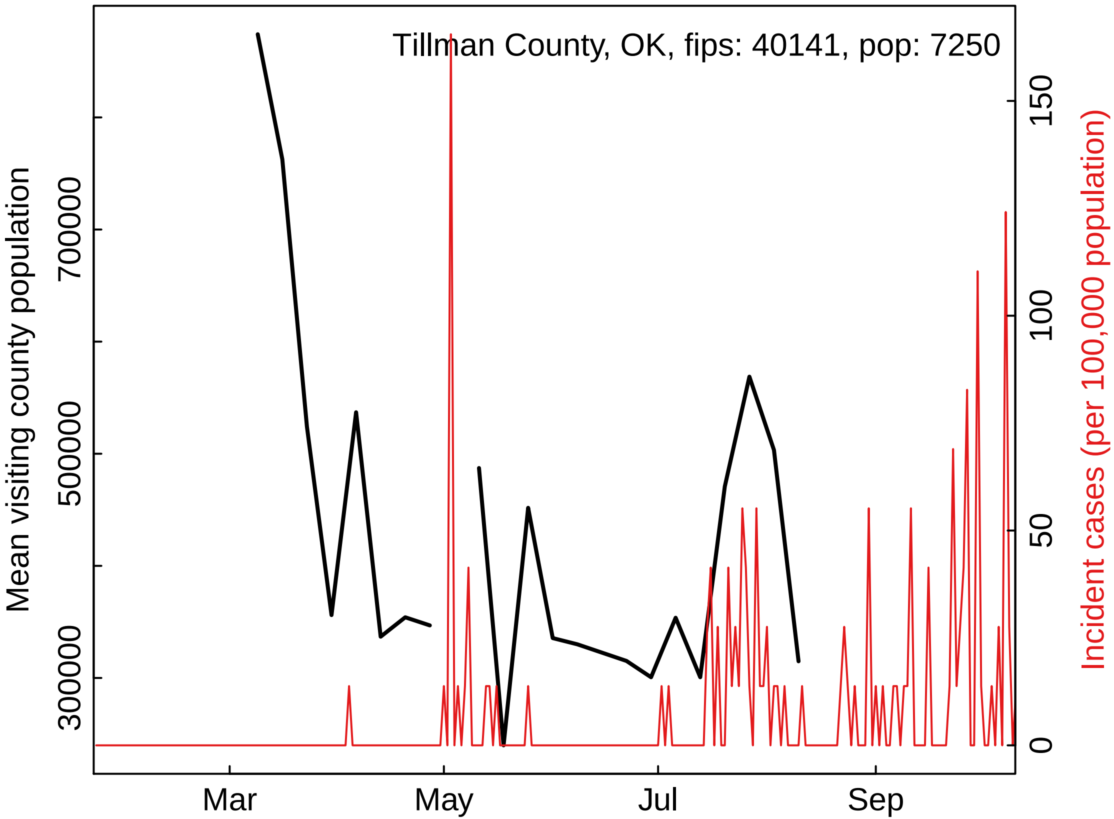

Tillman County, OK, fips: 40141, pop: 7250
Mean visiting county population
300000
500000
7000000
Incident cases (per 100,000 population)
0
50
100
150
Mar
May
Jul
Sep

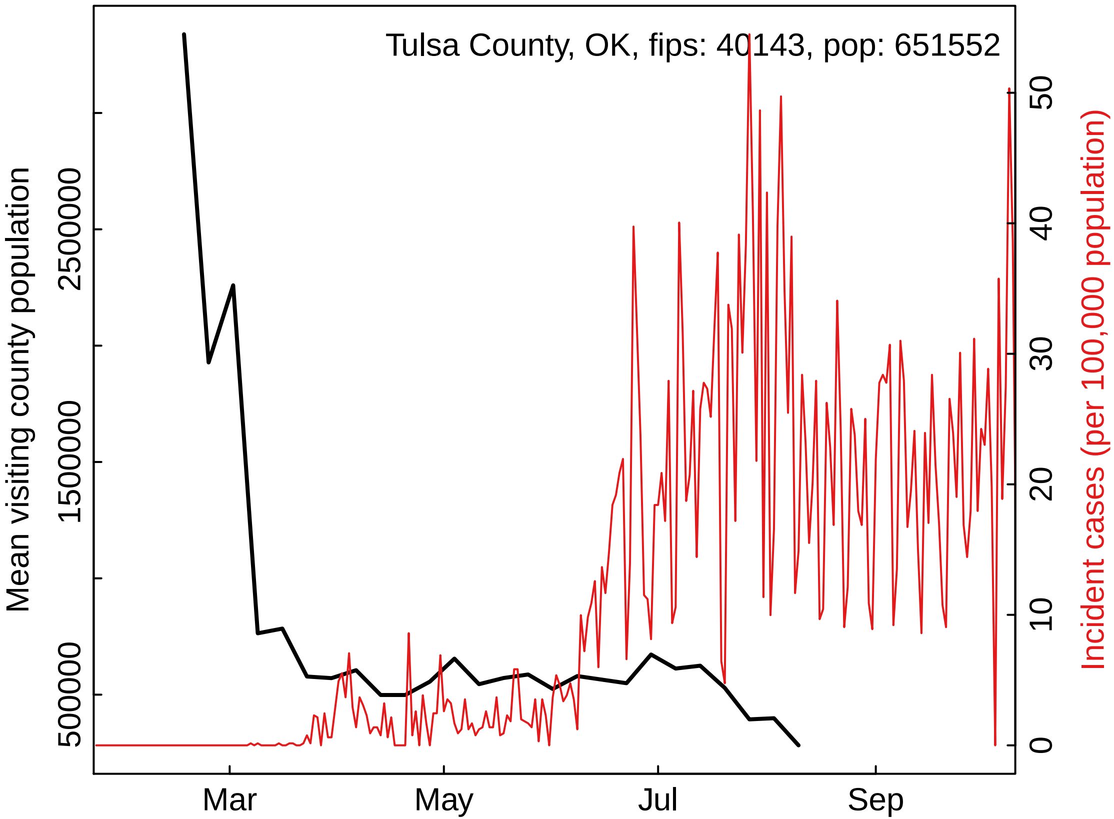

Tulsa County, OK, fips: 40143, pop: 651552
Mean visiting county population
500000
1500000
2500000
Mar
May
Jul
Sep
0
10
20
30
40
50
Incident cases (per 100,000 population)

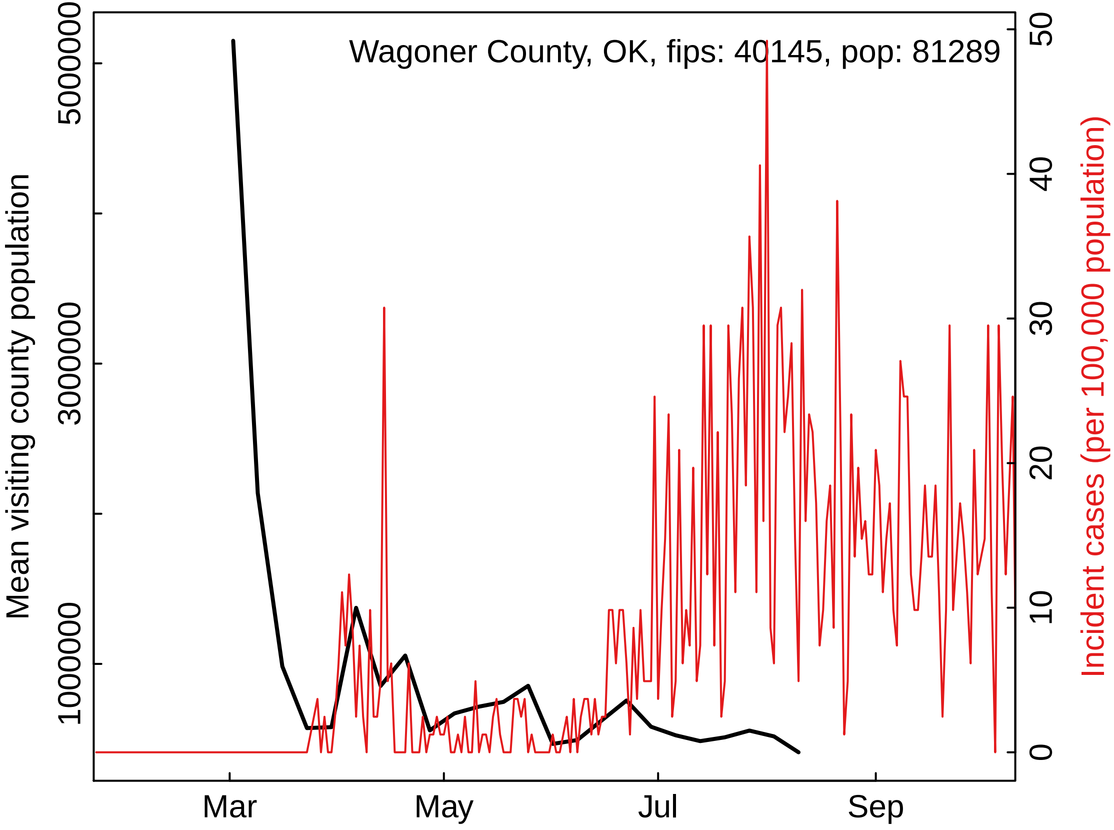

Wagoner County, OK, fips: 40145, pop: 81289
Mean visiting county population
1000000
3000000
5000000
Incident cases (per 100,000 population)
0
10
20
30
40
50
Mar
May
Jul
Sep

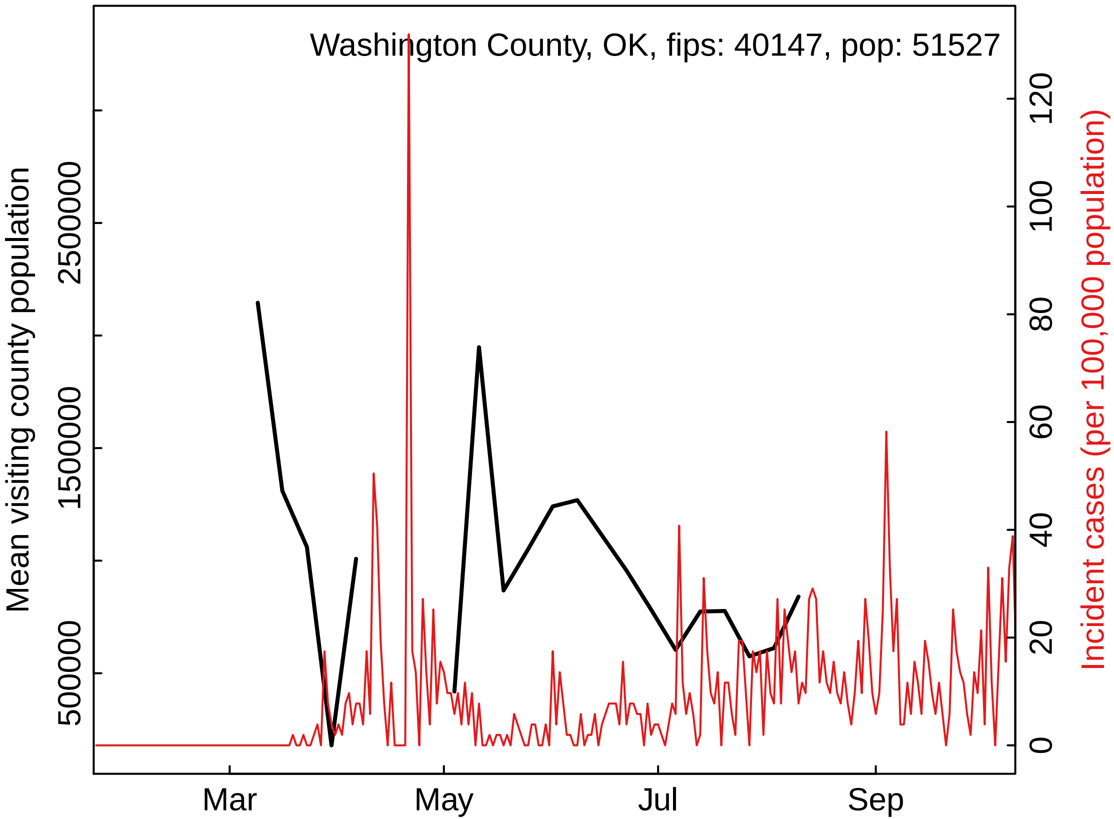

Washington County, OK, fips: 40147, pop: 51527
Mean visiting county population
500000
1500000
2500000
Mar
May
Jul
Sep
0
20
40
60
80
100
120
Incident cases (per 100,000 population)

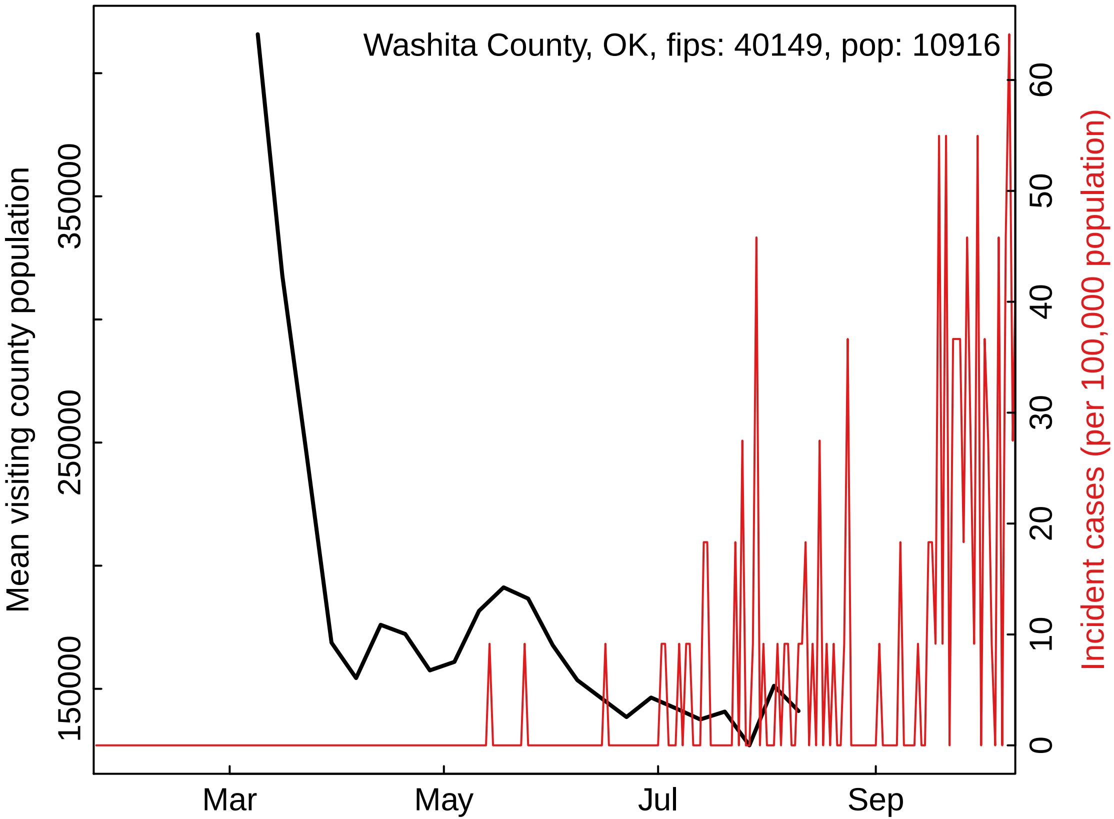
Washita County, OK, fips: 40149, pop: 10916
Mean visiting county population
150000
250000
350000
Incident cases (per 100,000 population)
0
10
20
30
40
50
60
Mar
May
Jul
Sep

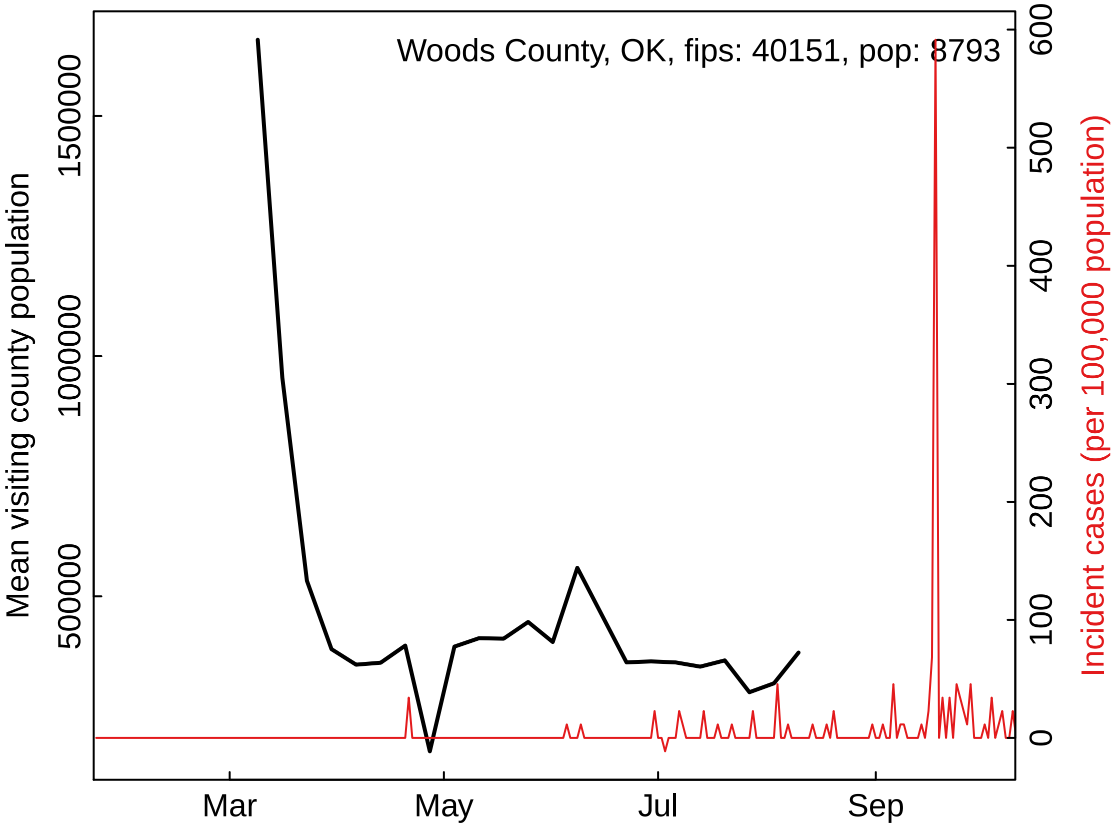

Woods County, OK, fips: 40151, pop: 8793
Mean visiting county population
5000000
10000000
15000000
Incident cases (per 100,000 population)
0
100
200
300
400
500
600
Mar
May
Jul
Sep

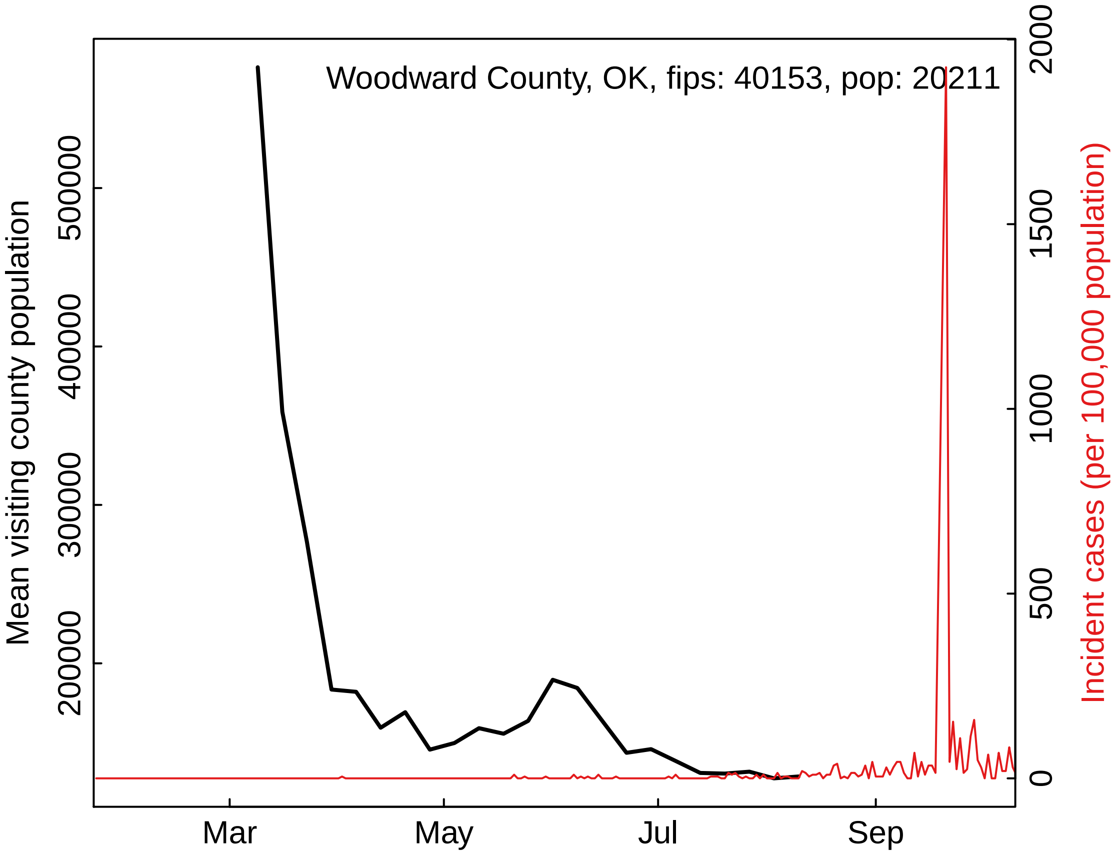
Woodward County, OK, fips: 40153, pop: 20211
Mean visiting county population
200000
300000
400000
500000
Incident cases (per 100,000 population)
0
500
1000
1500
2000
Mar
May
Jul
Sep

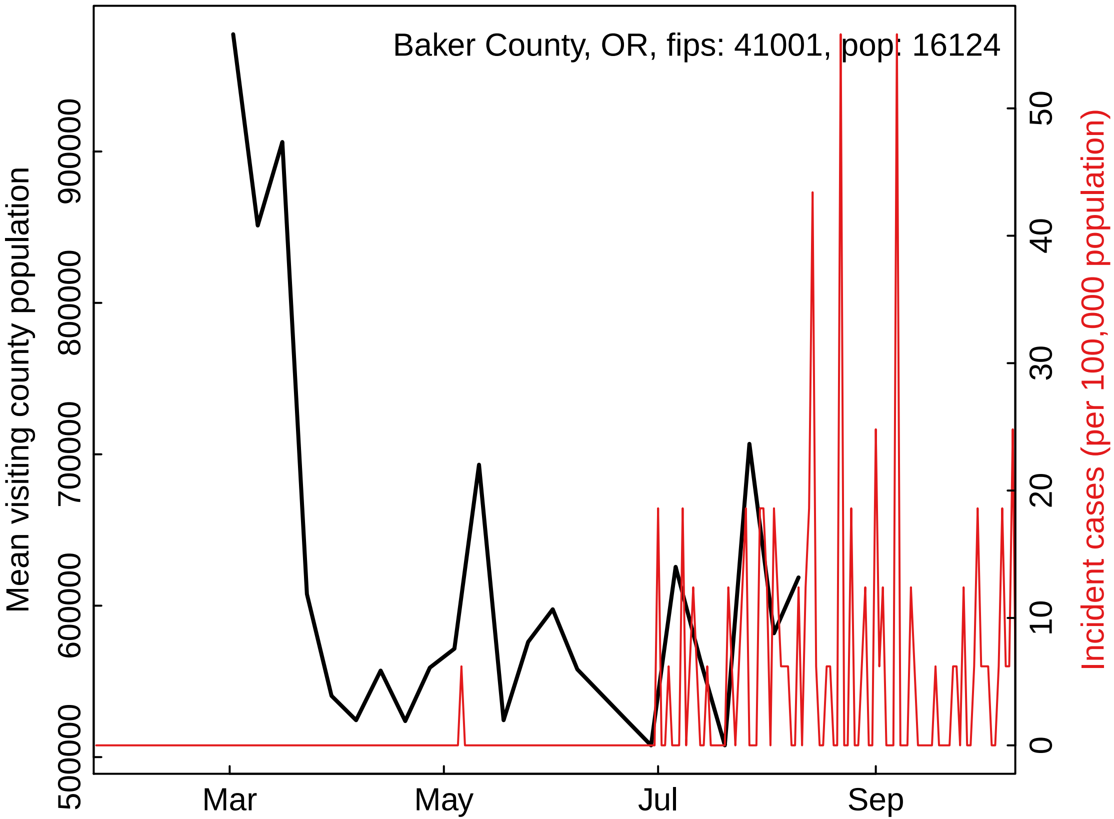

Baker County, OR, fips: 41001, pop: 16124
Mean visiting county population
Incident cases (per 100,000 population)
500000
600000
700000
800000
900000
0
10
20
30
40
50
Mar
May
Jul
Sep

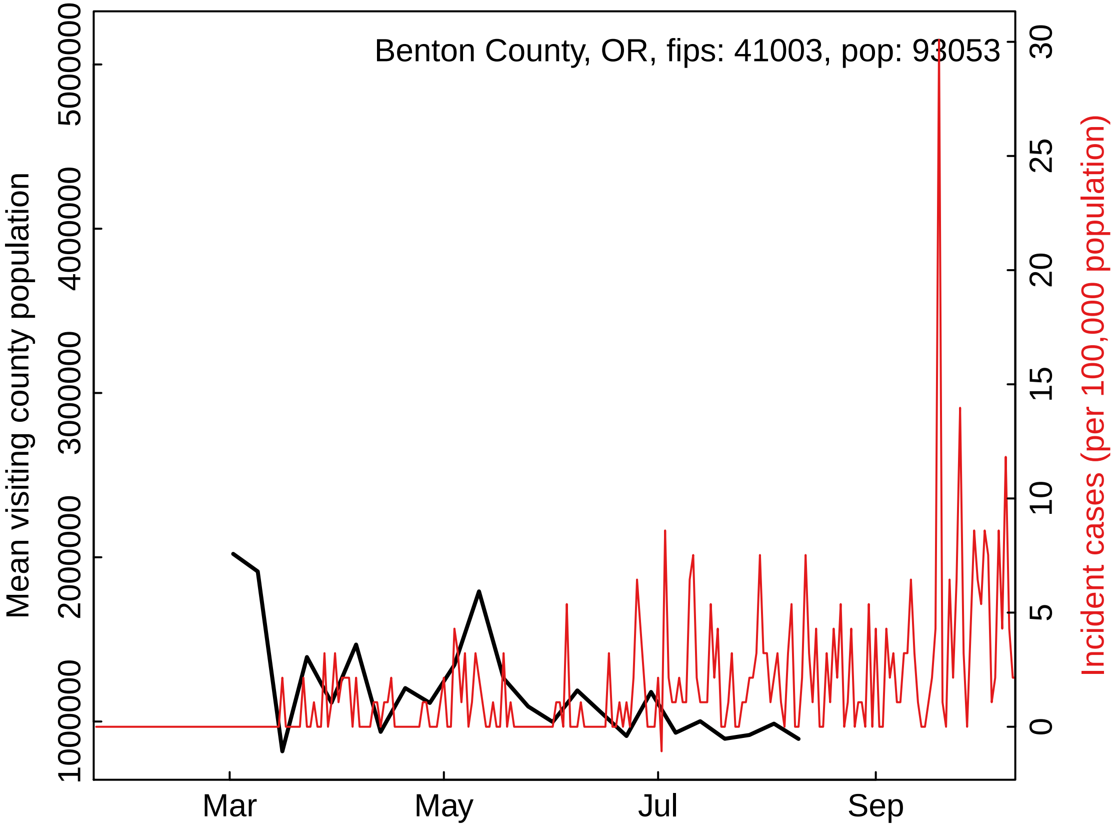
Benton County, OR, fips: 41003, pop: 93053
Mean visiting county population
Incident cases (per 100,000 population)
1000000
2000000
3000000
4000000
5000000
0
5
10
15
20
25
30
Mar
May
Jul
Sep

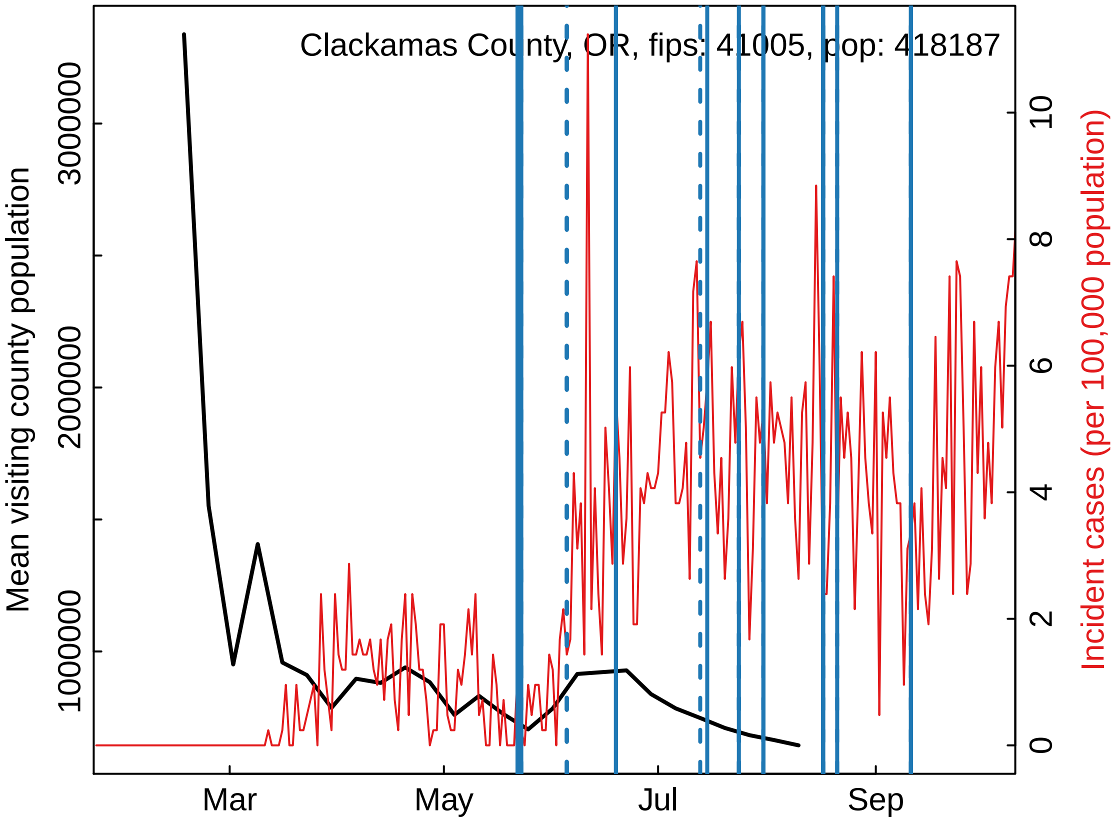
Clackamas County, OR, fips: 41005, pop: 418187
Mean visiting county population
30000000
20000000
10000000
Incident cases (per 100,000 population)
10
8
6
4
2
0
Mar
May
Jul
Sep

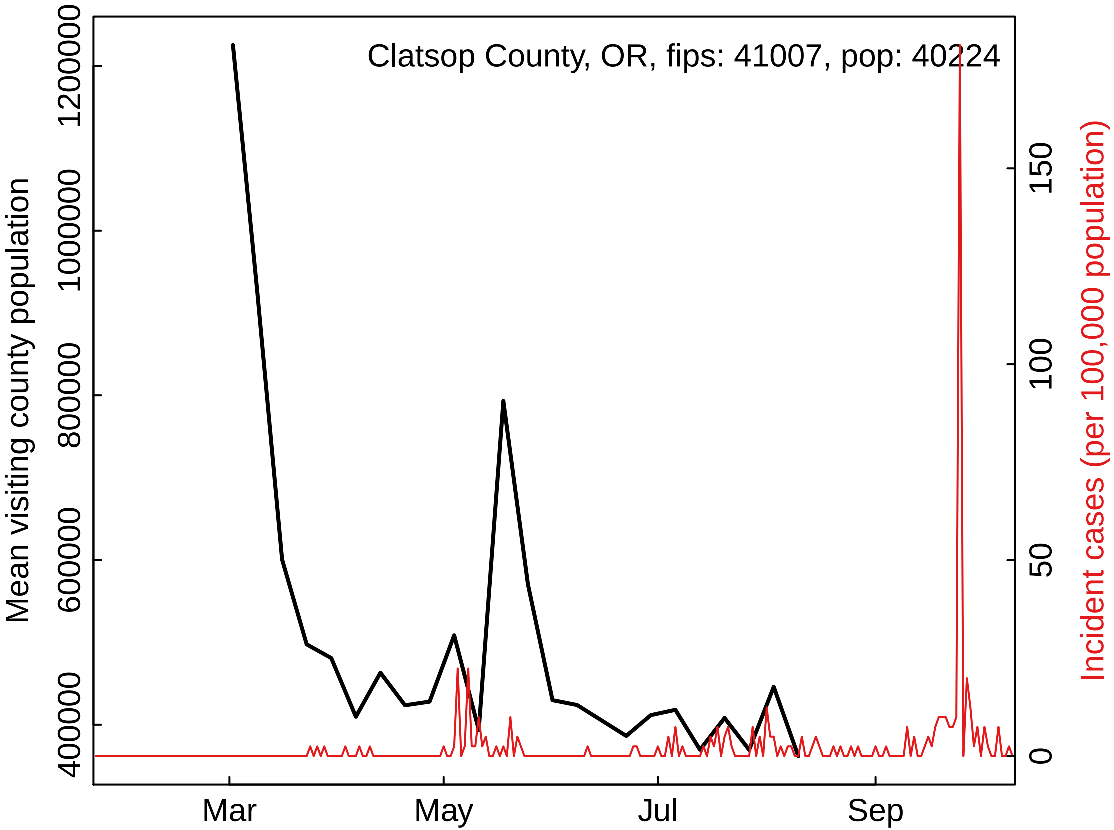

Clatsop County, OR, fips: 41007, pop: 40224
Mean visiting county population
400000
600000
800000
1000000
1200000
Incident cases (per 100,000 population)
0
50
100
150
Mar
May
Jul
Sep

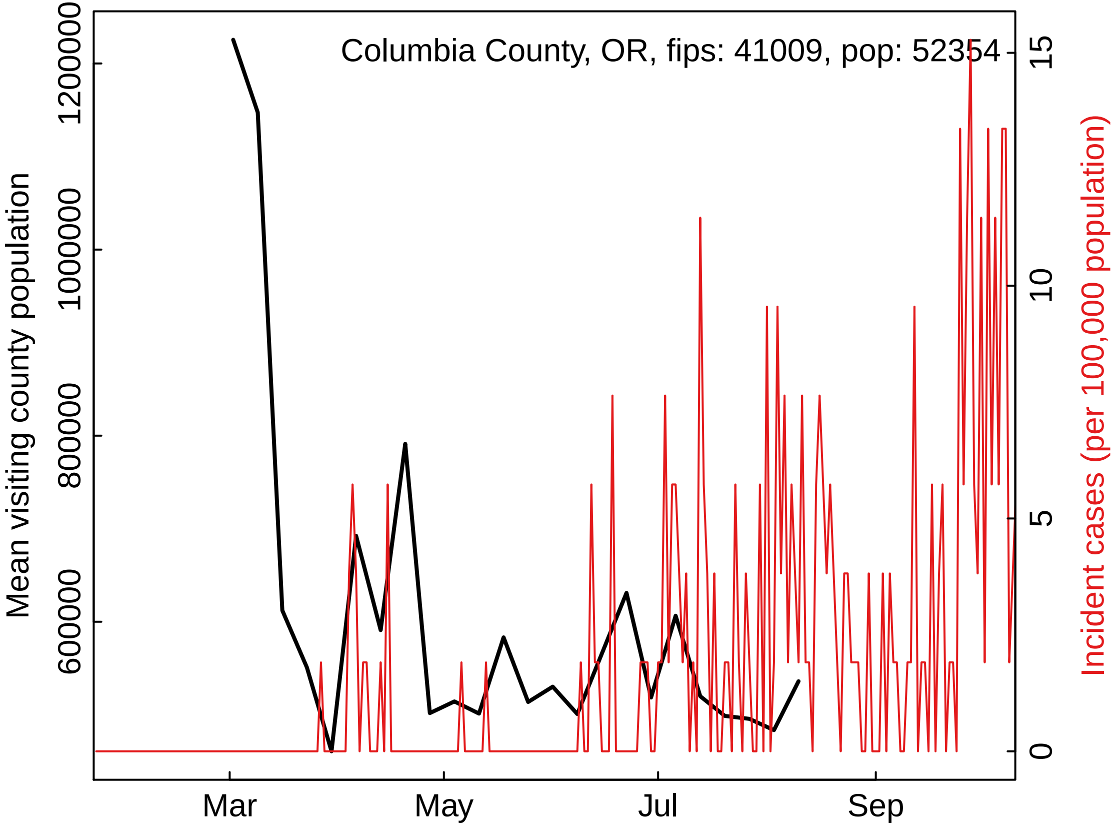

Columbia County, OR, fips: 41009, pop: 52354
Mean visiting county population
600000
800000
1000000
1200000
Incident cases (per 100,000 population)
0
5
10
15
Mar
May
Jul
Sep

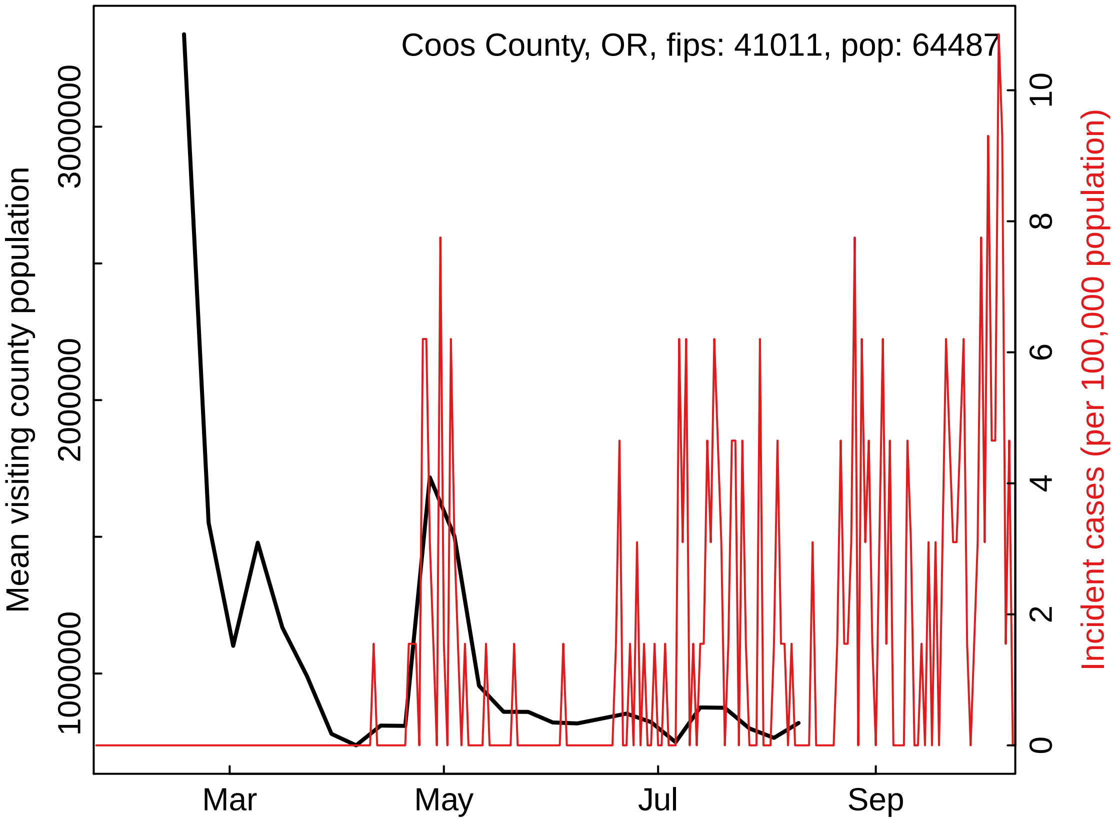

Coos County, OR, fips: 41011, pop: 64487
Mean visiting county population
Incident cases (per 100,000 population)
1000000
2000000
3000000
0
2
4
6
8
10
Mar
May
Jul
Sep

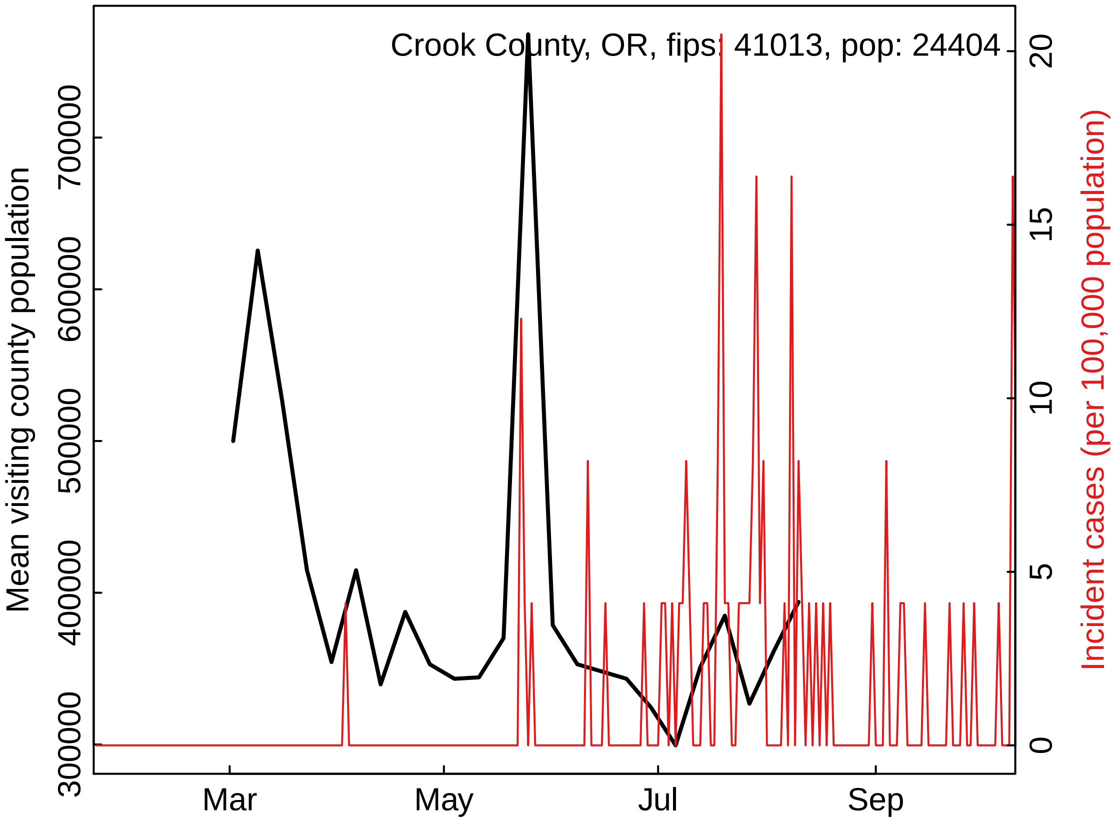

Crook County, OR, fips: 41013, pop: 24404
Mean visiting county population
300000
400000
500000
600000
700000
Incident cases (per 100,000 population)
0
5
10
15
20
Mar
May
Jul
Sep

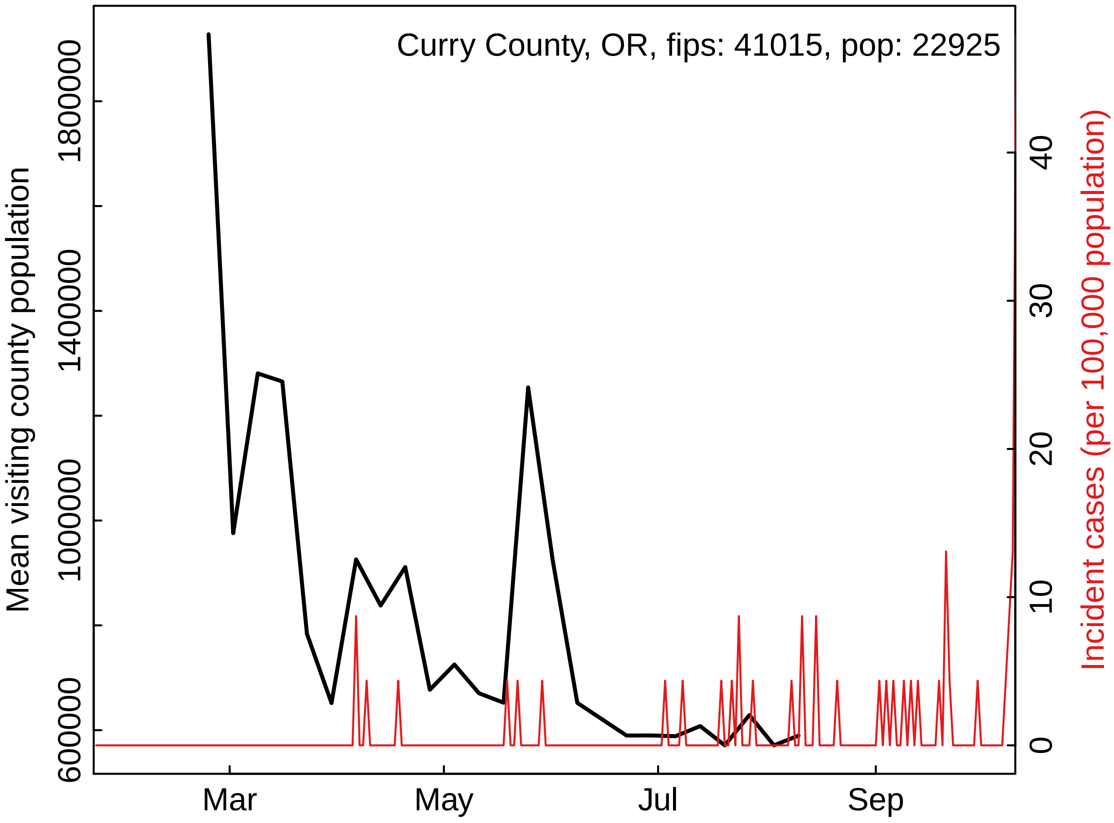

Curry County, OR, fips: 41015, pop: 22925
Mean visiting county population
600000
1000000
1400000
1800000
Incident cases (per 100,000 population)
0
10
20
30
40
Mar
May
Jul
Sep

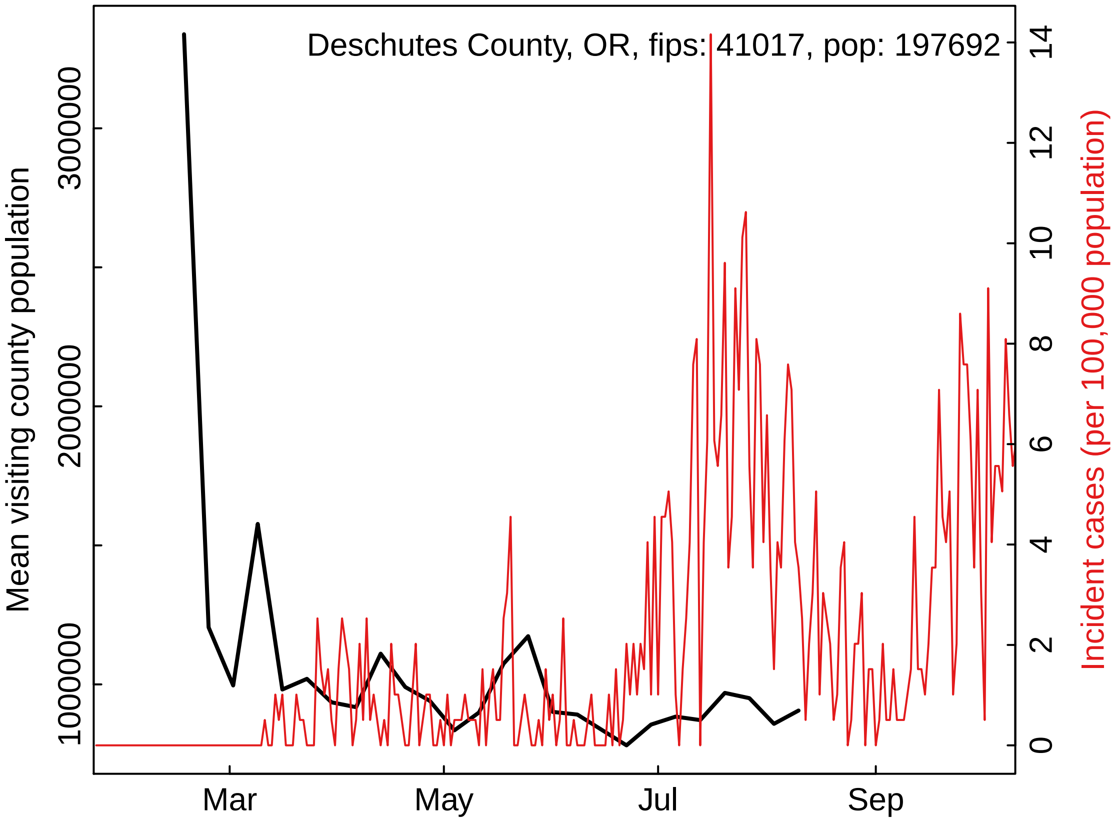

Deschutes County, OR, fips: 41017, pop: 197692
Mean visiting county population
10000000
20000000
30000000
Mar
May
Jul
Sep
0
2
4
6
8
10
12
14
Incident cases (per 100,000 population)

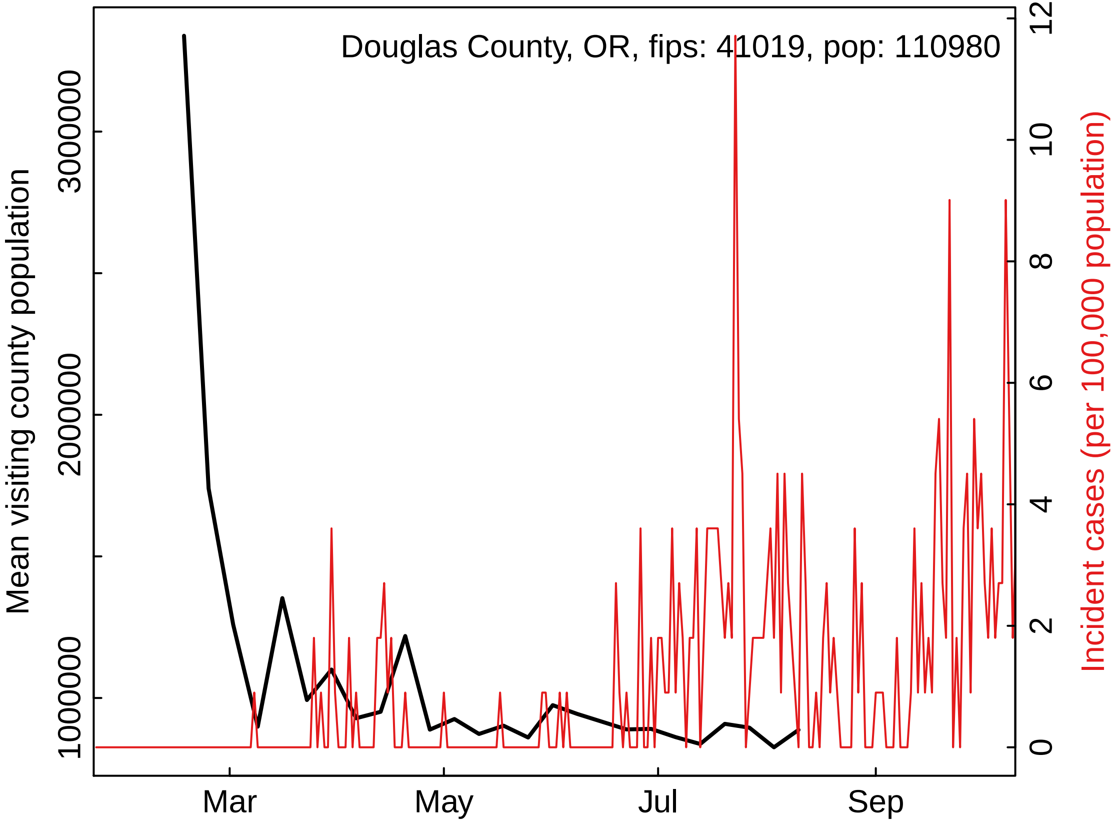

Douglas County, OR, fips: 41019, pop: 110980
Mean visiting county population
10000000
20000000
30000000
Incident cases (per 100,000 population)
0
2
4
6
8
10
12
Mar
May
Jul
Sep

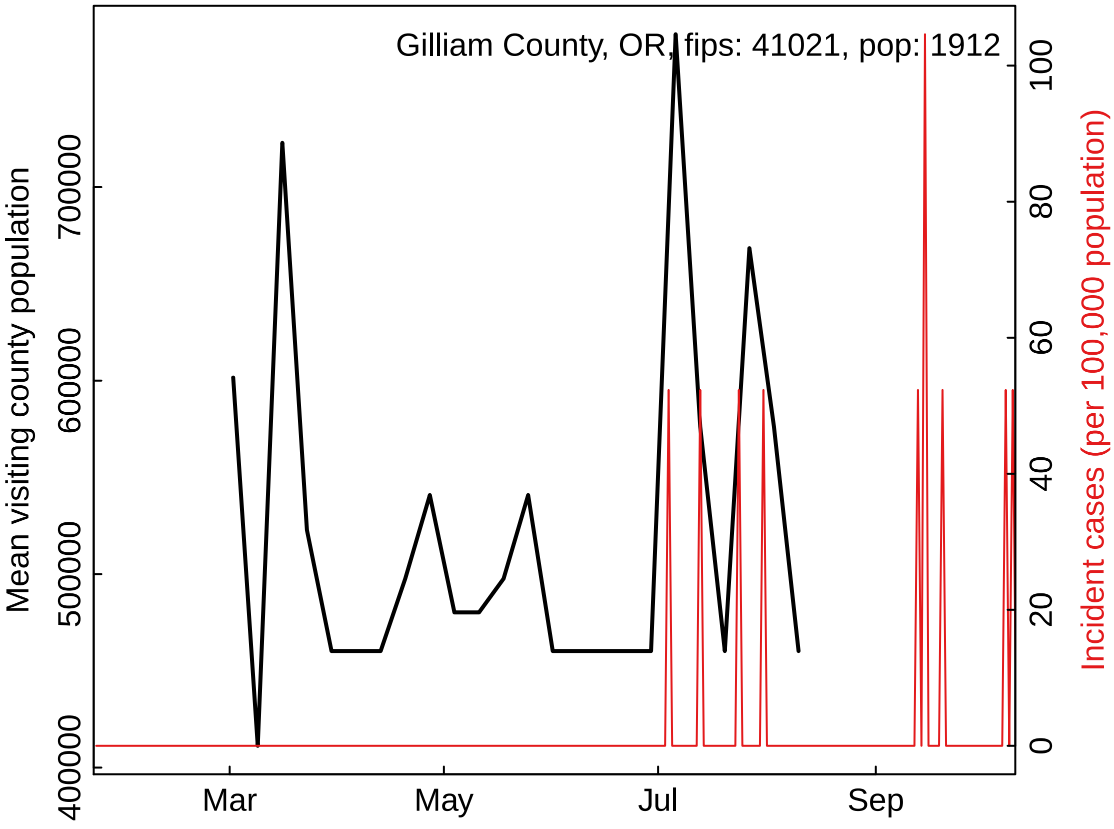

Gilliam County, OR, fips: 41021, pop: 1912
Mean visiting county population
400000
500000
600000
700000
Mar
May
Jul
Sep
0
20
40
60
80
100
Incident cases (per 100,000 population)

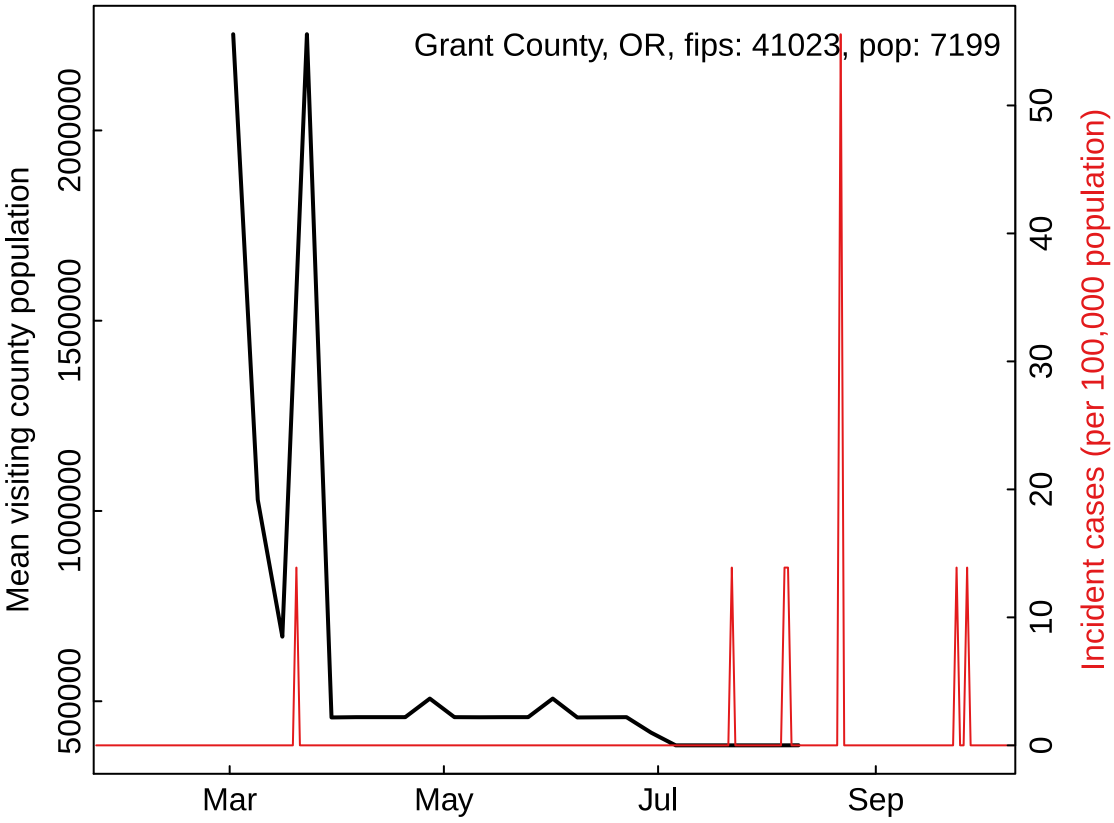
Grant County, OR, fips: 41023, pop: 7199
Mean visiting county population
500000
1000000
1500000
2000000
Incident cases (per 100,000 population)
0
10
20
30
40
50
Mar
May
Jul
Sep

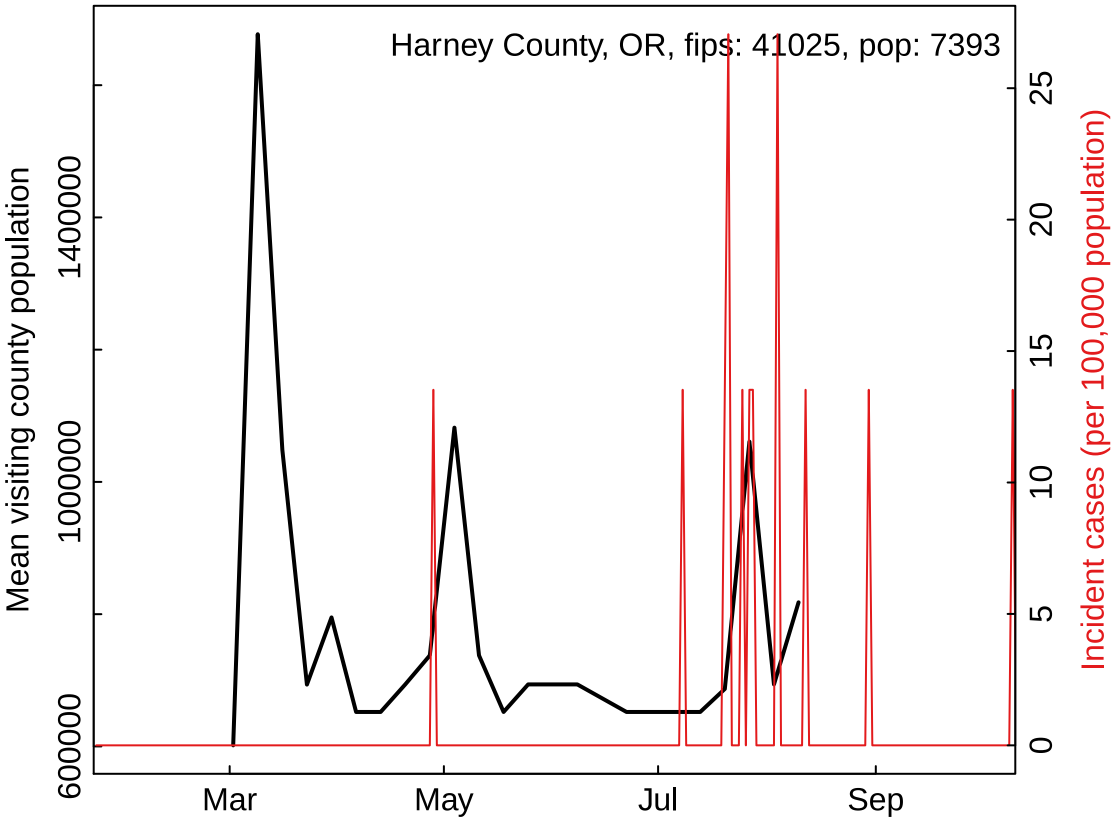
Harney County, OR, fips: 41025, pop: 7393
Mean visiting county population
600000
1000000
1400000
Incident cases (per 100,000 population)
0
5
10
15
20
25
Mar
May
Jul
Sep

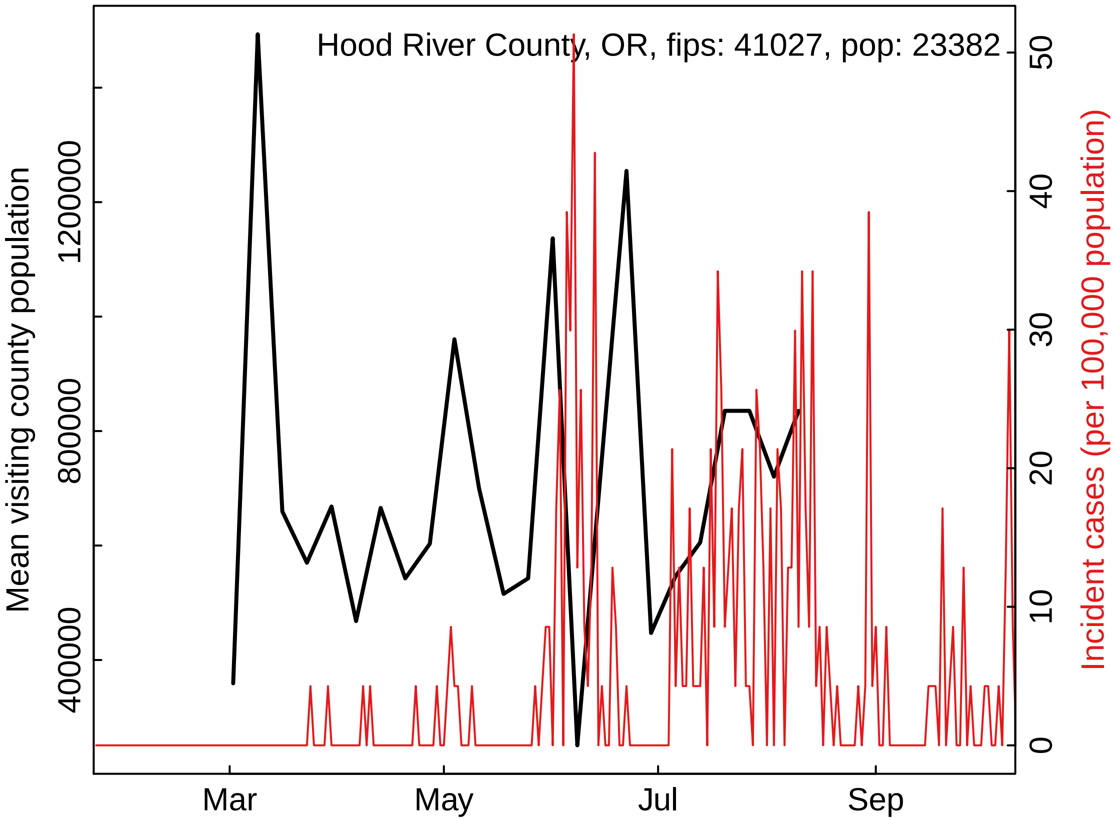

Hood River County, OR, fips: 41027, pop: 23382
Mean visiting county population
400000
800000
1200000
Incident cases (per 100,000 population)
0
10
20
30
40
50
Mar
May
Jul
Sep

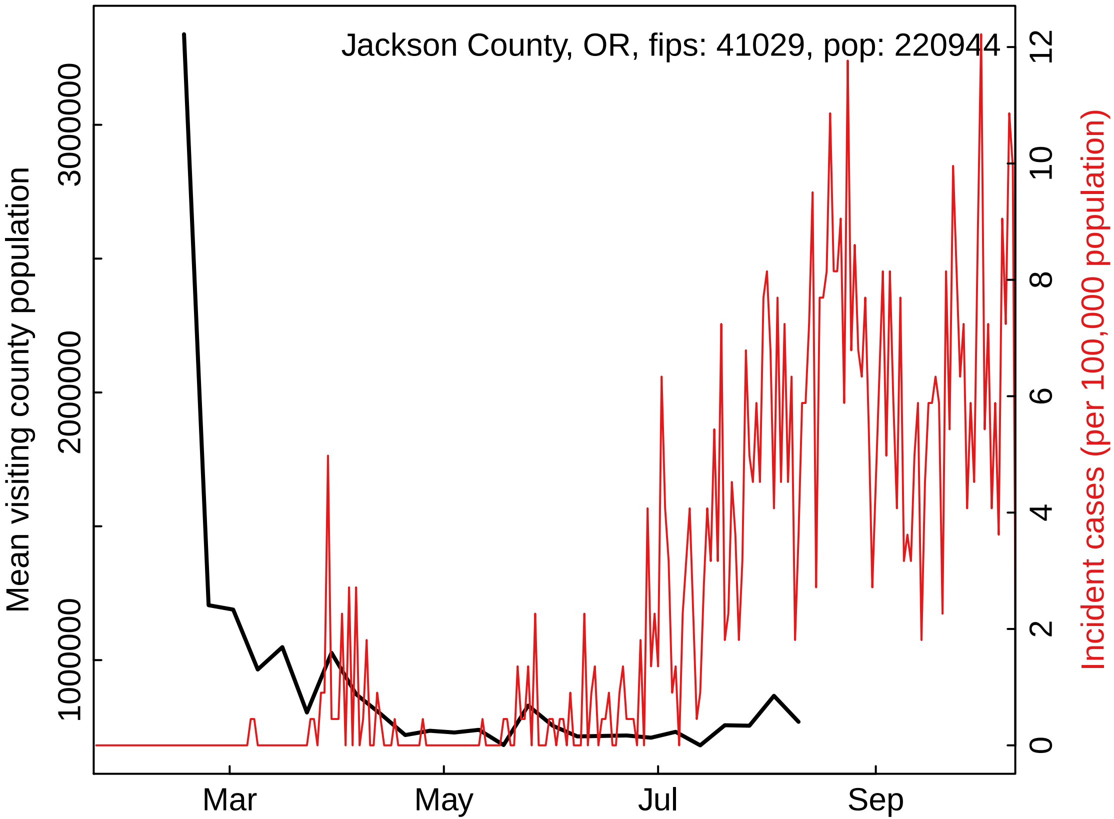

Jackson County, OR, fips: 41029, pop: 220944
Mean visiting county population
10000000
2000000
3000000
Incident cases (per 100,000 population)
0
2
4
6
8
10
12
Mar
May
Jul
Sep

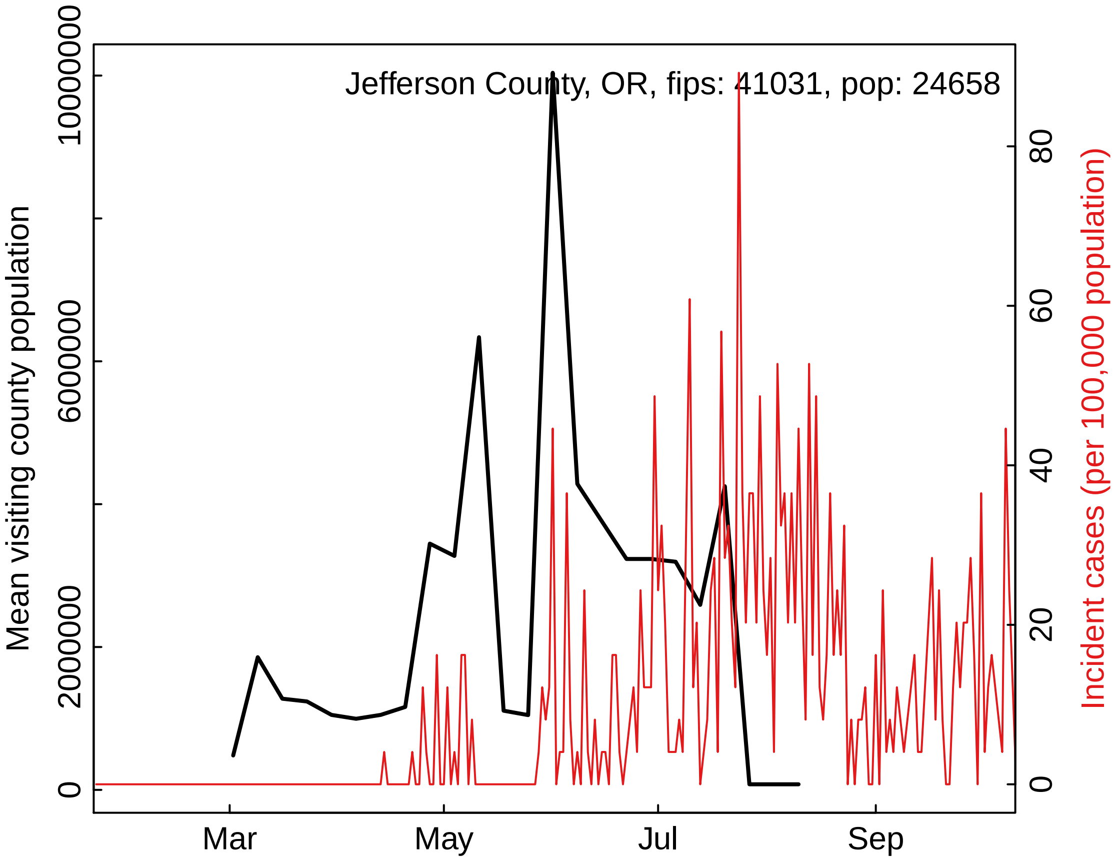

Jefferson County, OR, fips: 41031, pop: 24658
Mean visiting county population
0
2000000
6000000
10000000
Incident cases (per 100,000 population)
0
20
40
60
80
Mar
May
Jul
Sep

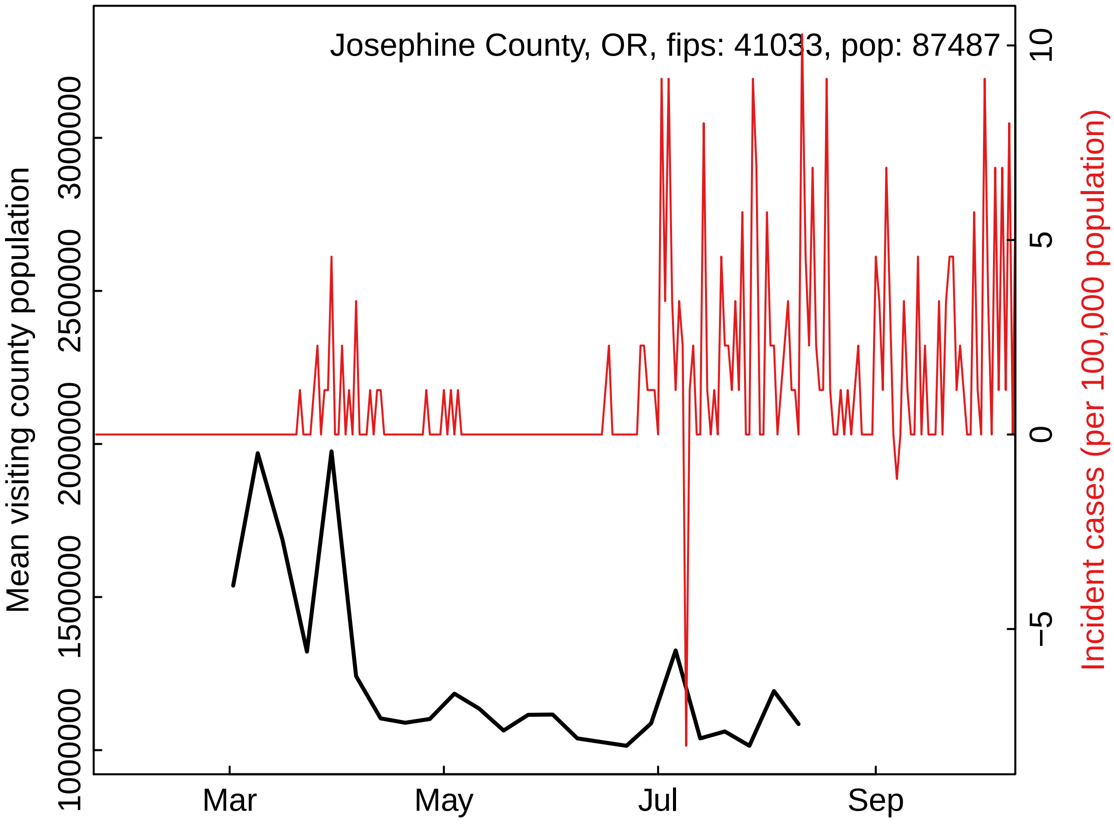

Josephine County, OR, fips: 41033, pop: 87487
Mean visiting county population
1000000
1500000
2000000
2500000
3000000
Mar
May
Jul
Sep
Incident cases (per 100,000 population)
−5
0
5
10

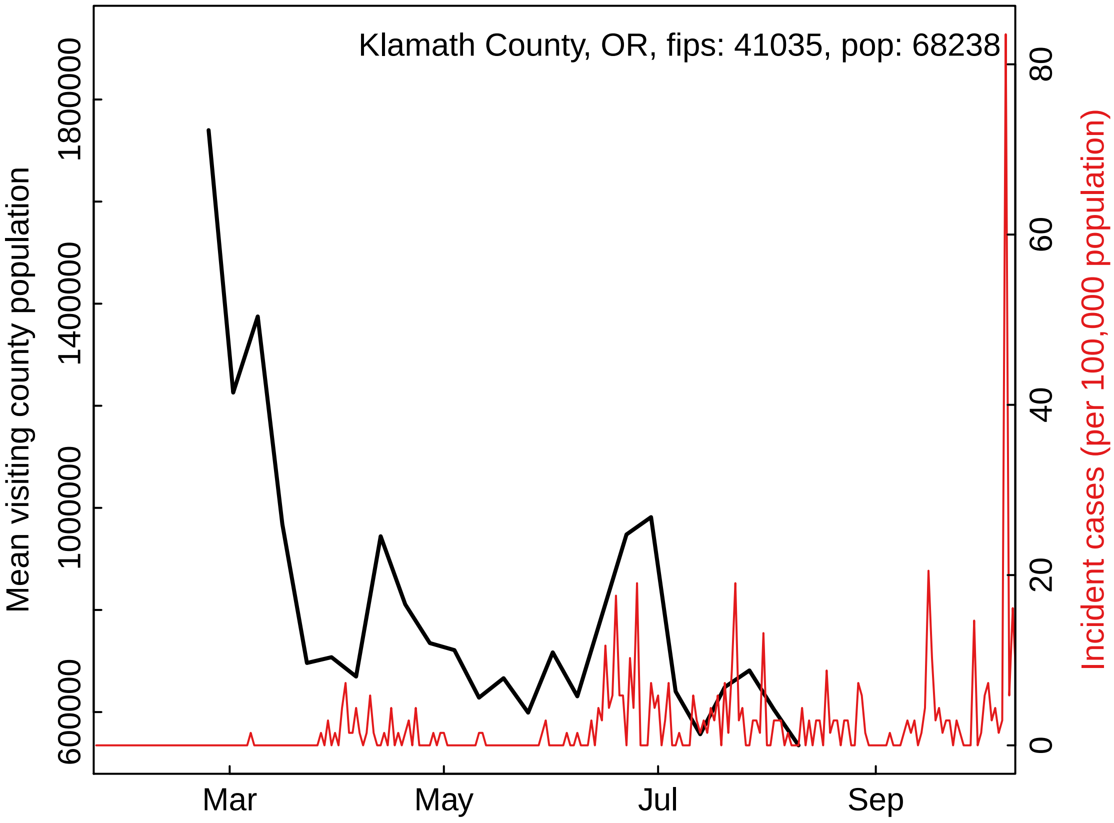

Klamath County, OR, fips: 41035, pop: 68238
Mean visiting county population
600000
1000000
1400000
1800000
Incident cases (per 100,000 population)
0
20
40
60
80
Mar
May
Jul
Sep

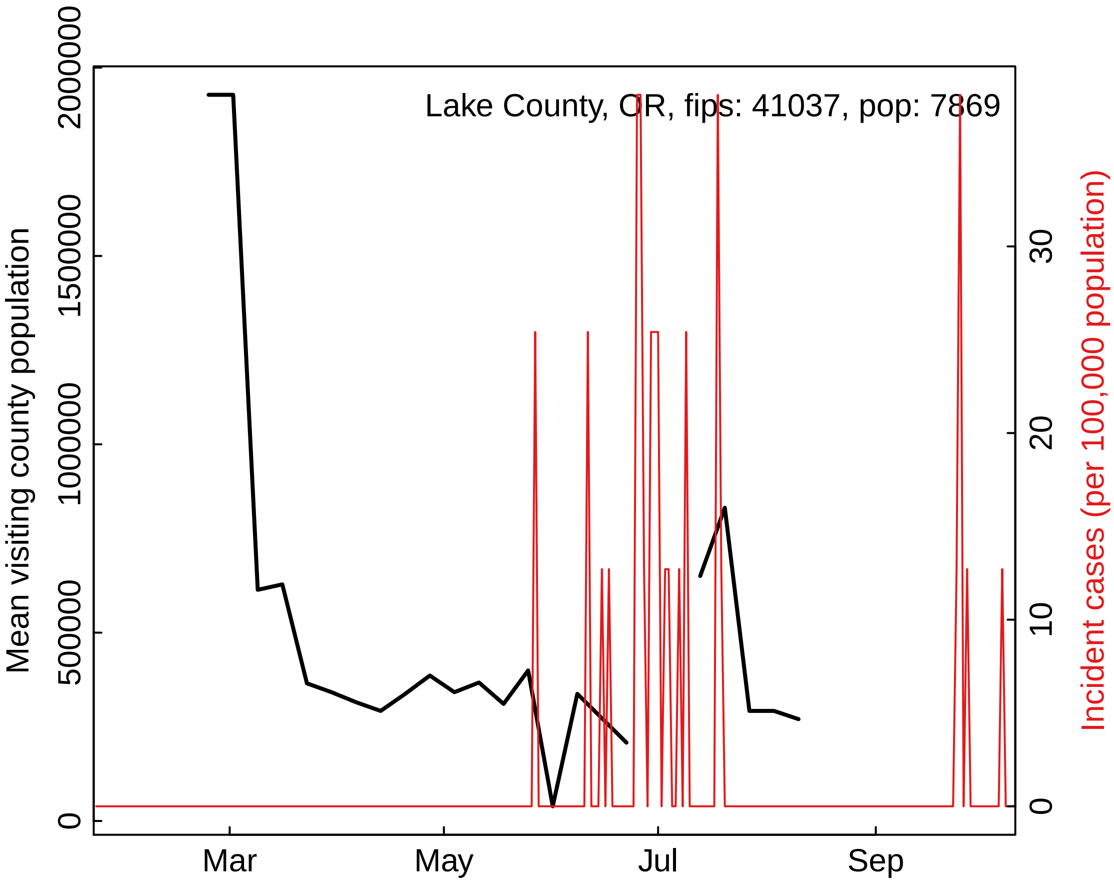

Lake County, OR, fips: 41037, pop: 7869
Mean visiting county population
0
500000
1000000
1500000
2000000
Incident cases (per 100,000 population)
0
10
20
30
Mar
May
Jul
Sep

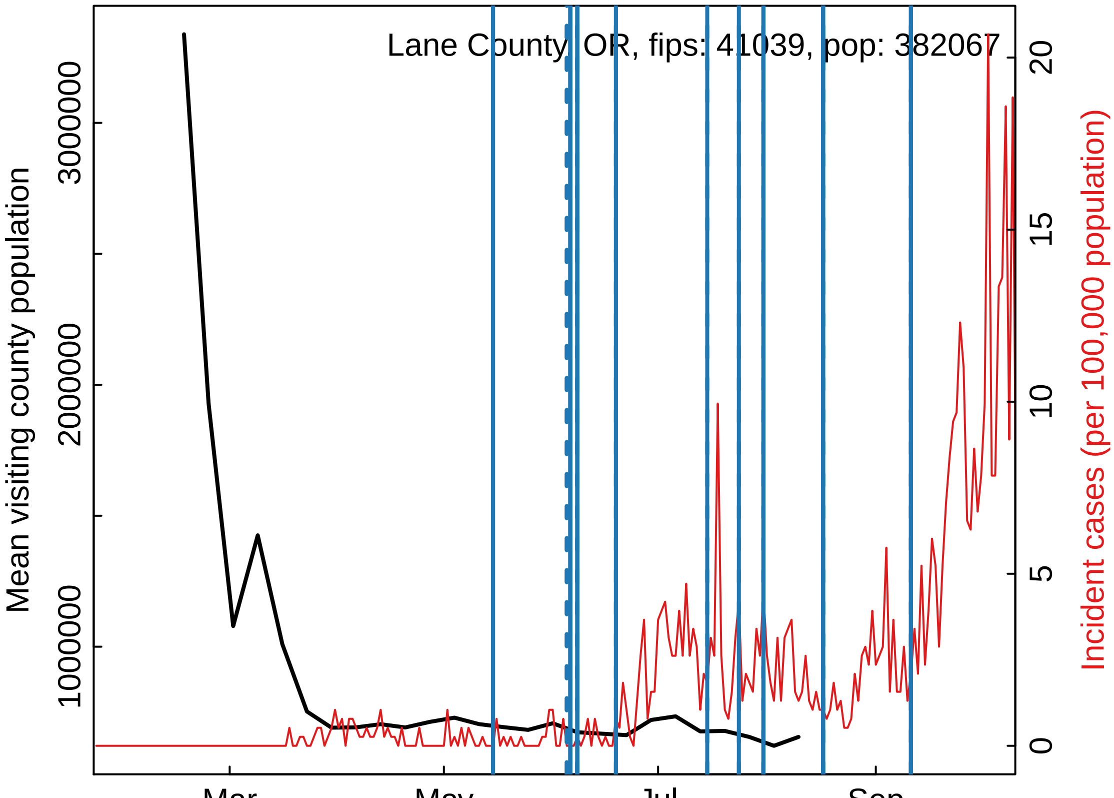

Lane County, OR, fips: 41039, pop: 382067
Mean visiting county population
10000000
20000000
30000000
Incident cases (per 100,000 population)
0
5
10
15
20
Mar
May
Jul
Sep

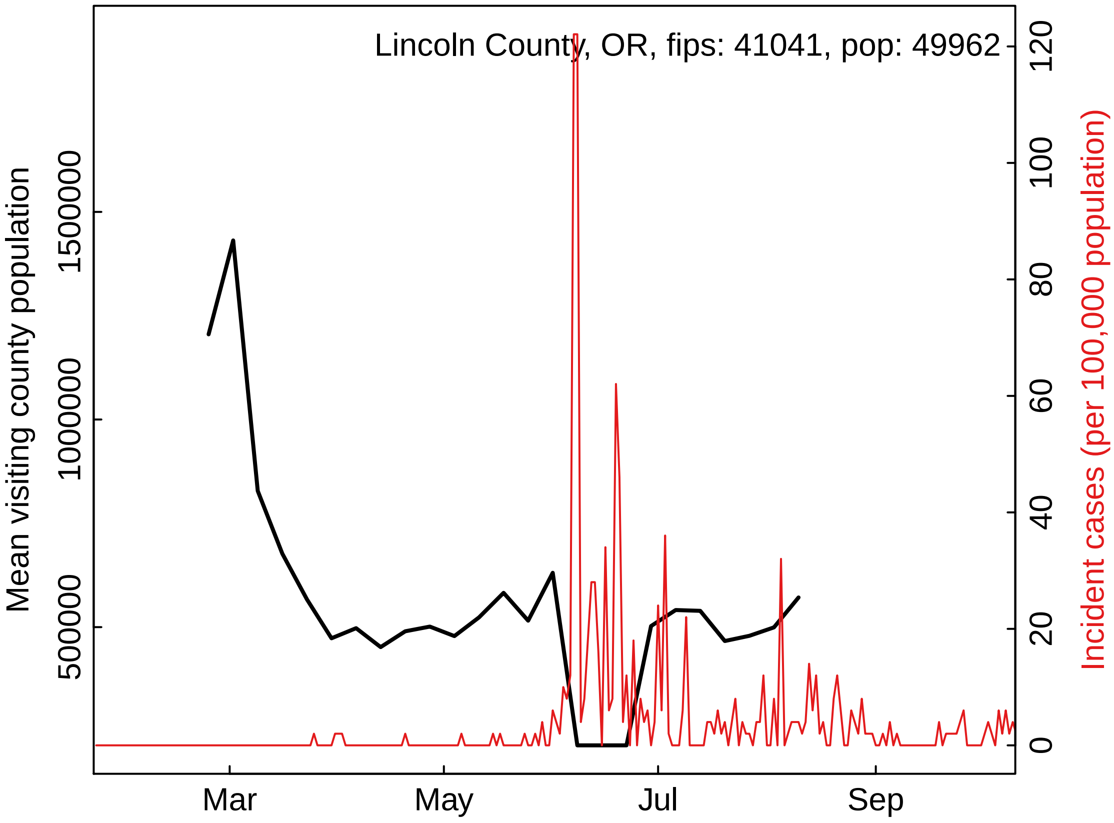
Lincoln County, OR, fips: 41041, pop: 49962
Mean visiting county population
Incident cases (per 100,000 population)
500000
1000000
1500000
0
20
40
60
80
100
120
Mar
May
Jul
Sep

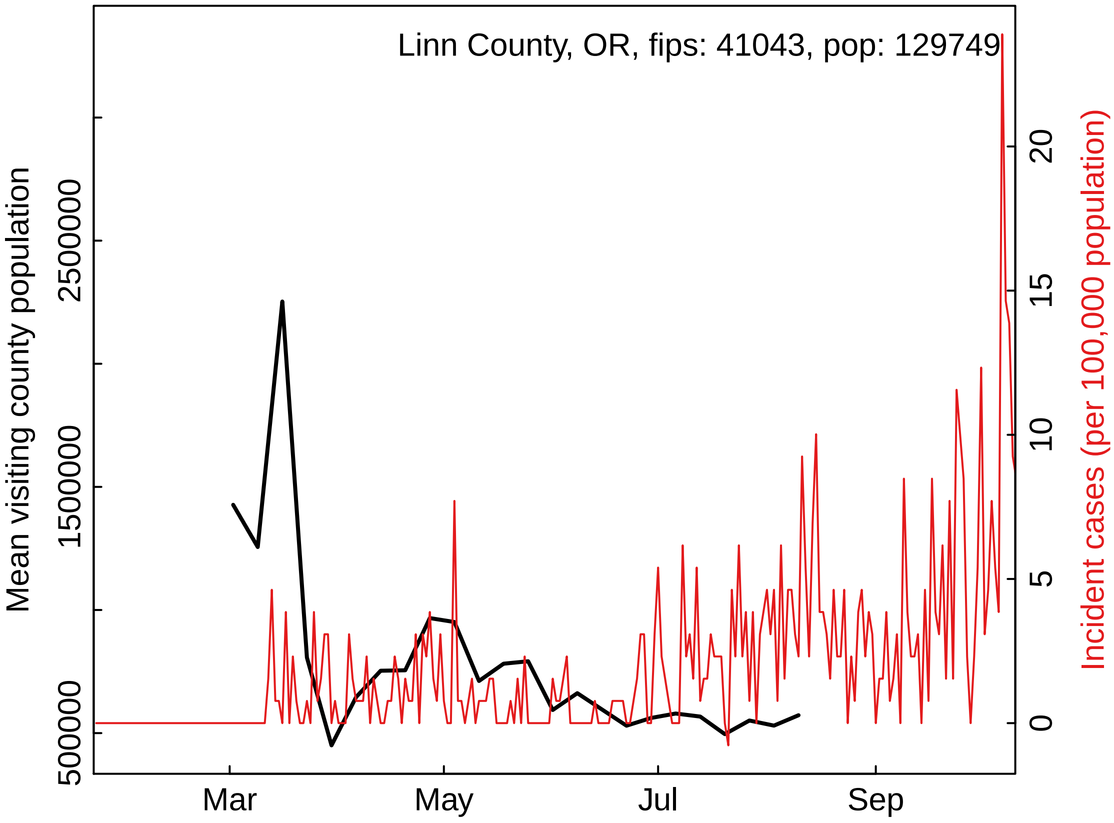

Linn County, OR, fips: 41043, pop: 129749
Mean visiting county population
500000
1500000
2500000
Incident cases (per 100,000 population)
0
5
10
15
20
Mar
May
Jul
Sep

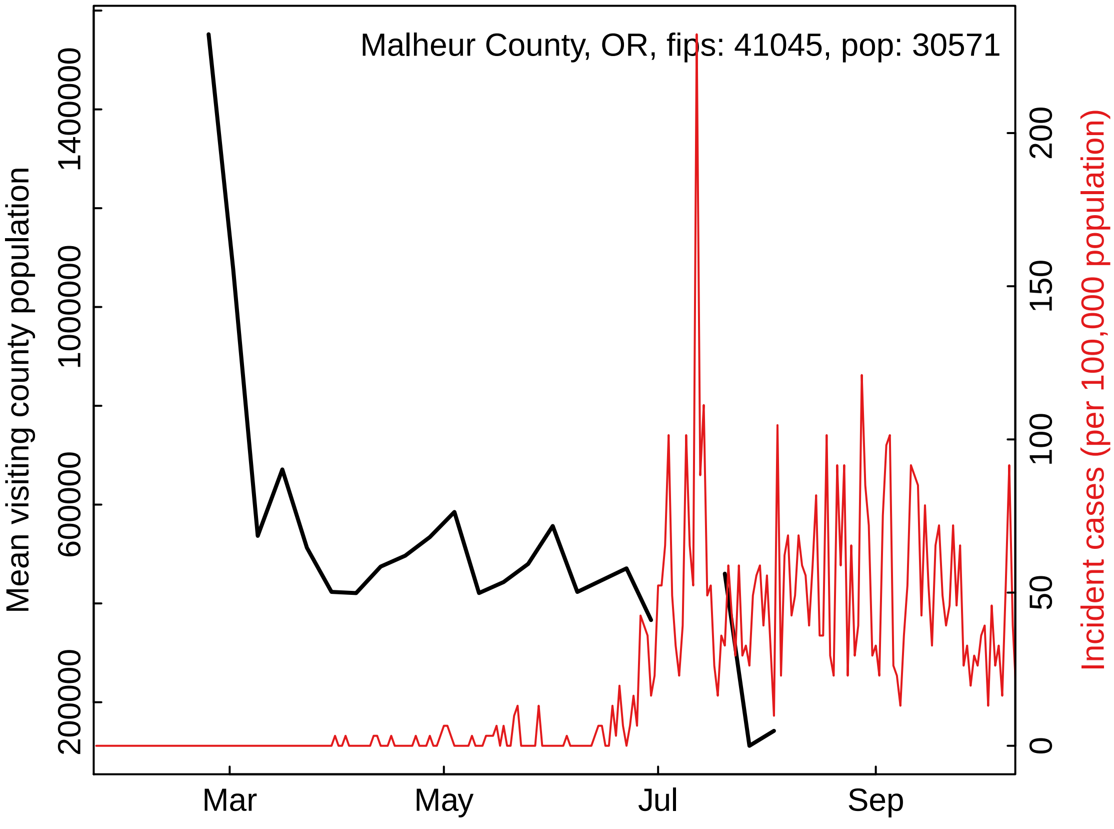

Malheur County, OR, fips: 41045, pop: 30571
Mean visiting county population
200000
600000
1000000
1400000
Incident cases (per 100,000 population)
0
50
100
150
200
Mar
May
Jul
Sep

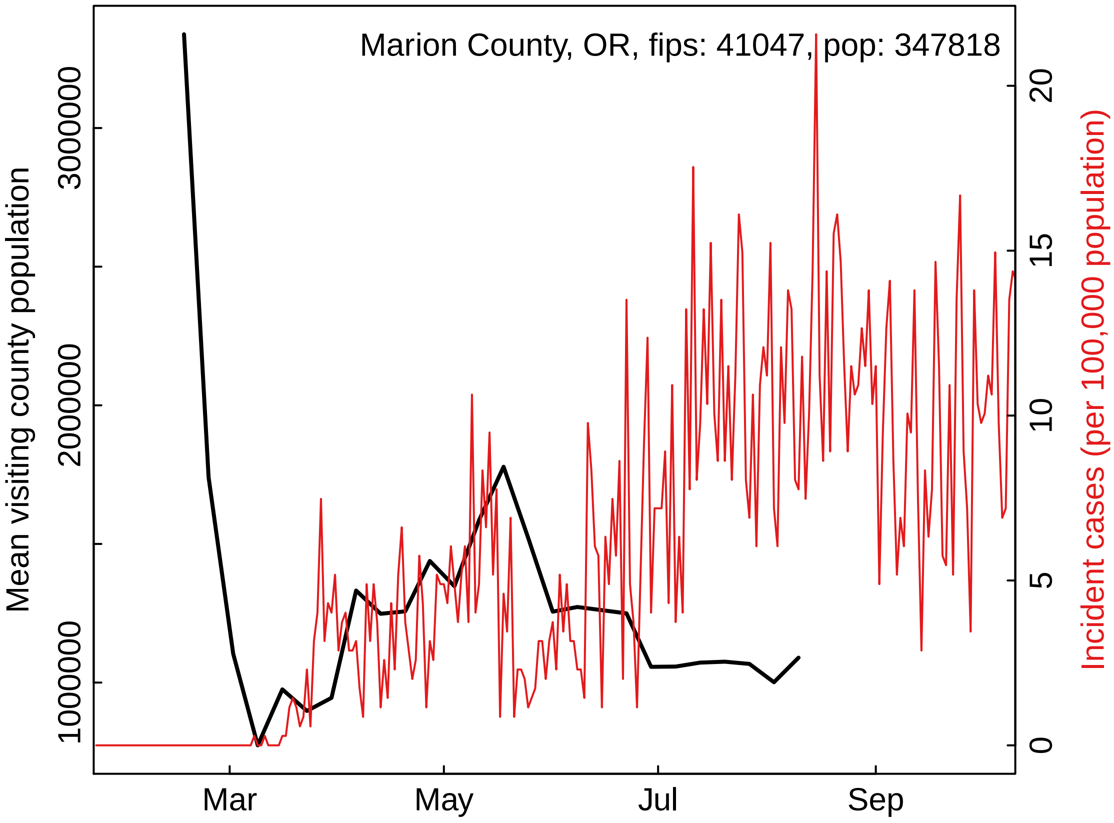

Marion County, OR, fips: 41047, pop: 347818
Mean visiting county population
Incident cases (per 100,000 population)
1000000
2000000
3000000
0
5
10
15
20
Mar
May
Jul
Sep

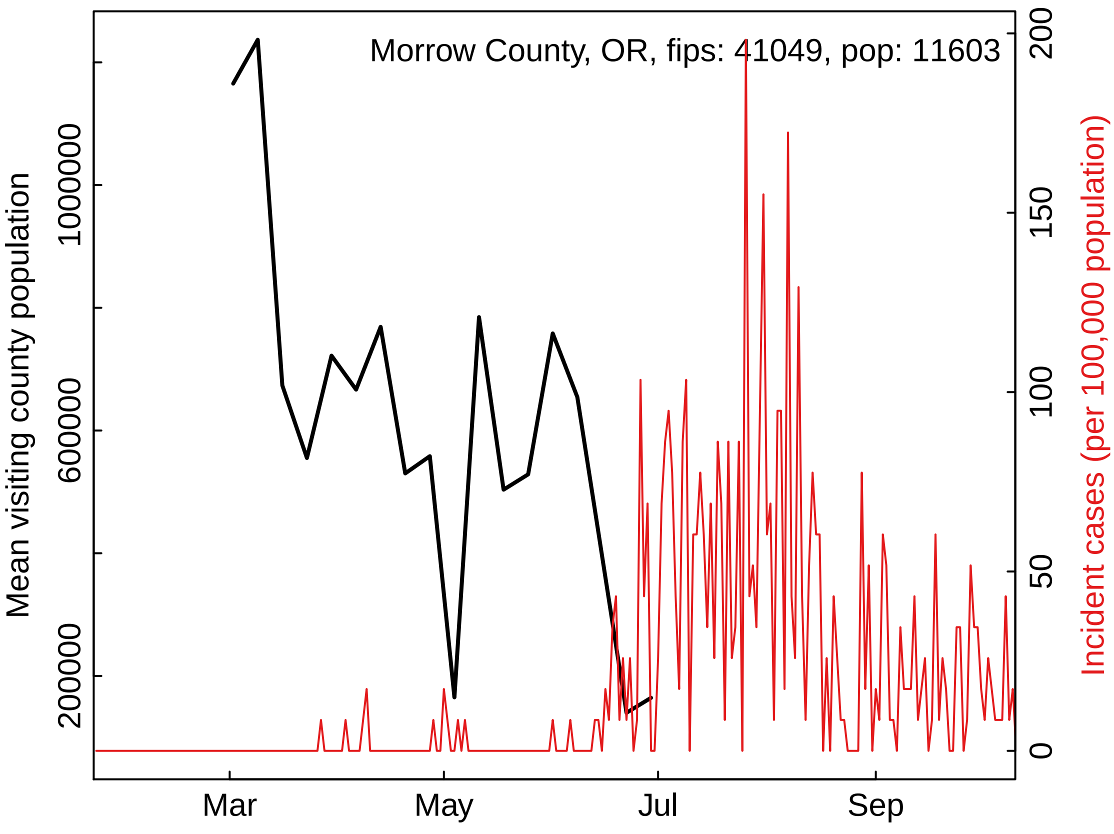

Morrow County, OR, fips: 41049, pop: 11603
Mean visiting county population
200000
600000
1000000
Incident cases (per 100,000 population)
0
50
100
150
200
Mar
May
Jul
Sep

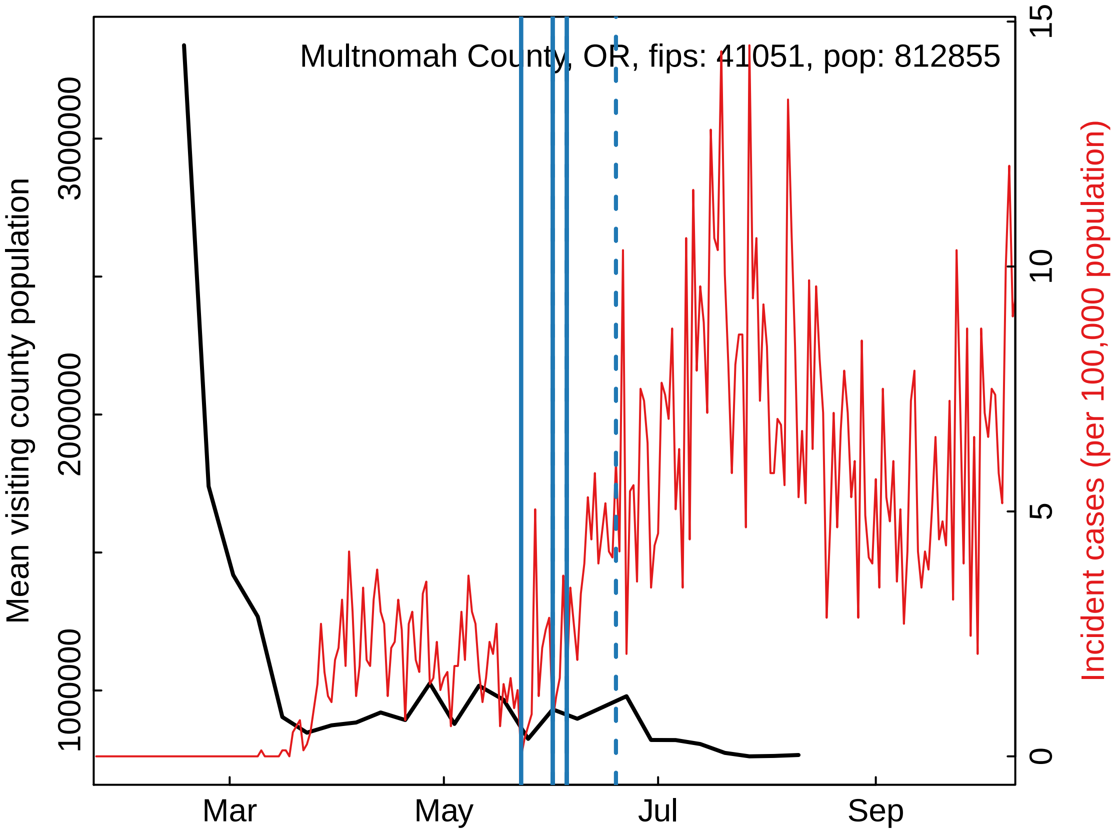

Multnomah County, OR, fips: 41051, pop: 812855
Mean visiting county population
1000000
2000000
3000000
Incident cases (per 100,000 population)
0
5
10
15
Mar
May
Jul
Sep

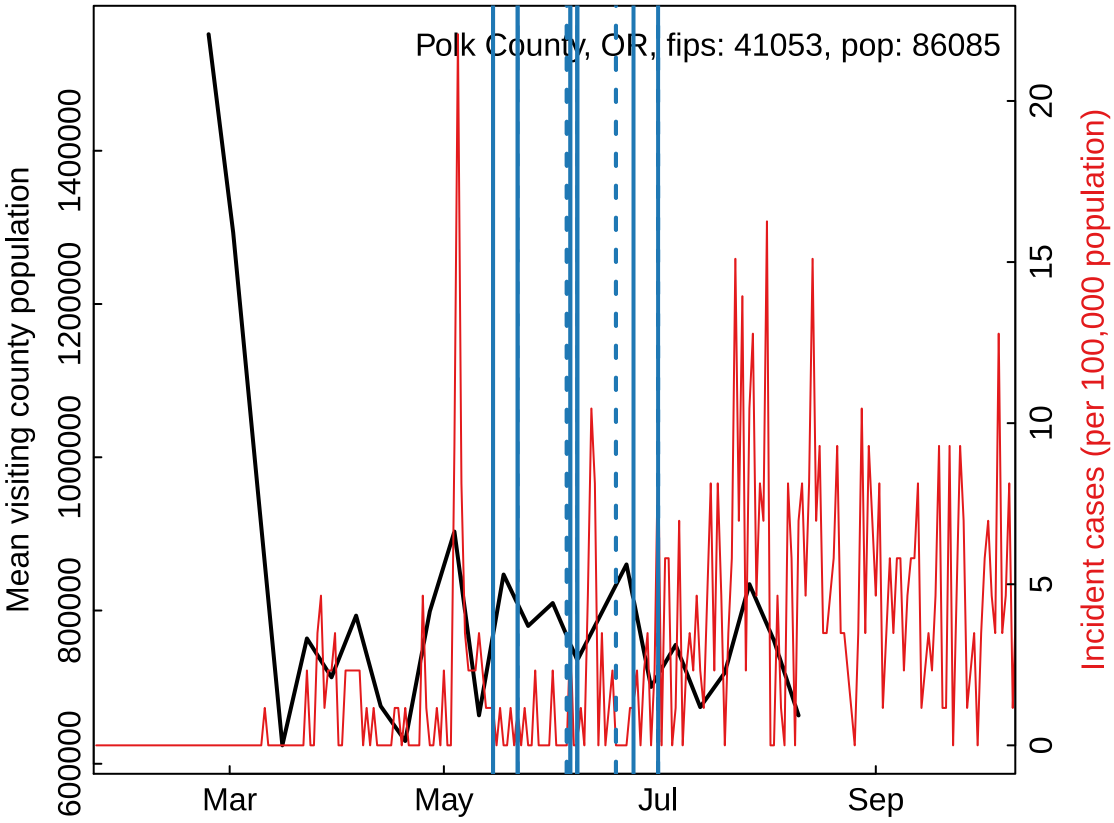
Polk County, OR, fips: 41053, pop: 86085
Mean visiting county population
600000
800000
1000000
1200000
1400000
Mar
May
Jul
Sep
0
5
10
15
20
Incident cases (per 100,000 population)

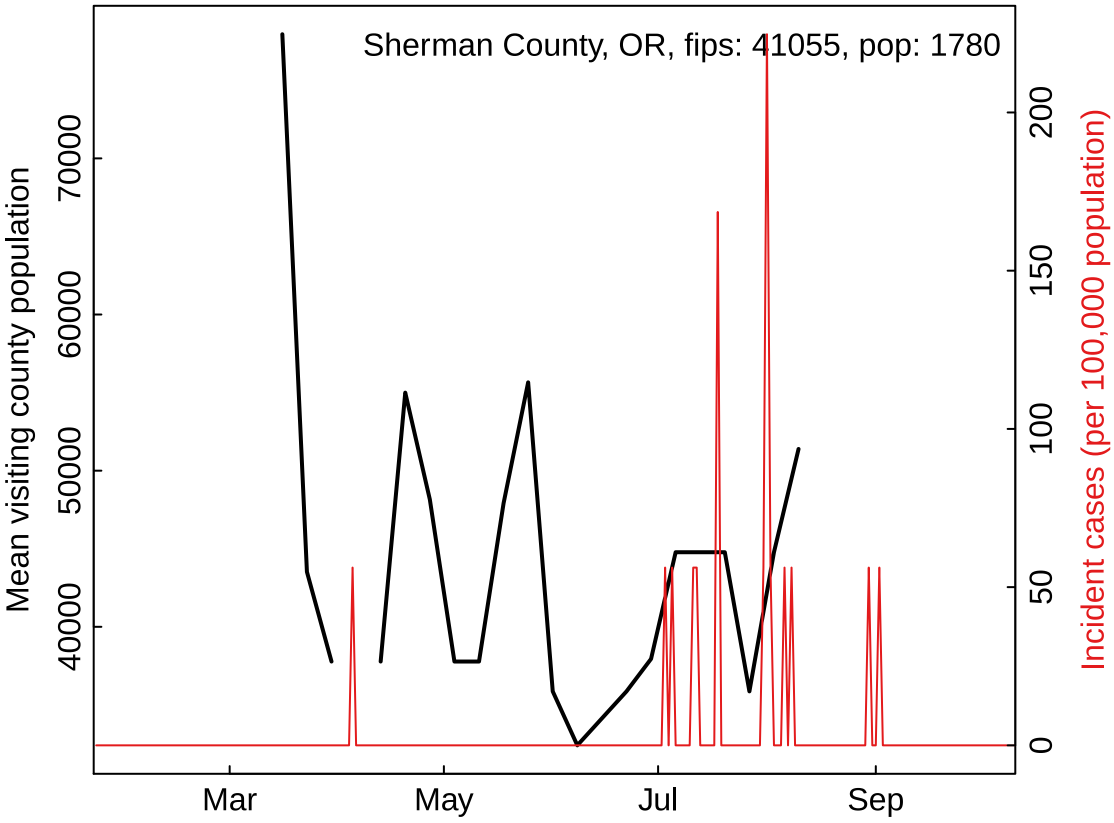
Sherman County, OR, fips: 41055, pop: 1780
Mean visiting county population
40000
50000
60000
70000
Incident cases (per 100,000 population)
0
50
100
150
200
Mar
May
Jul
Sep

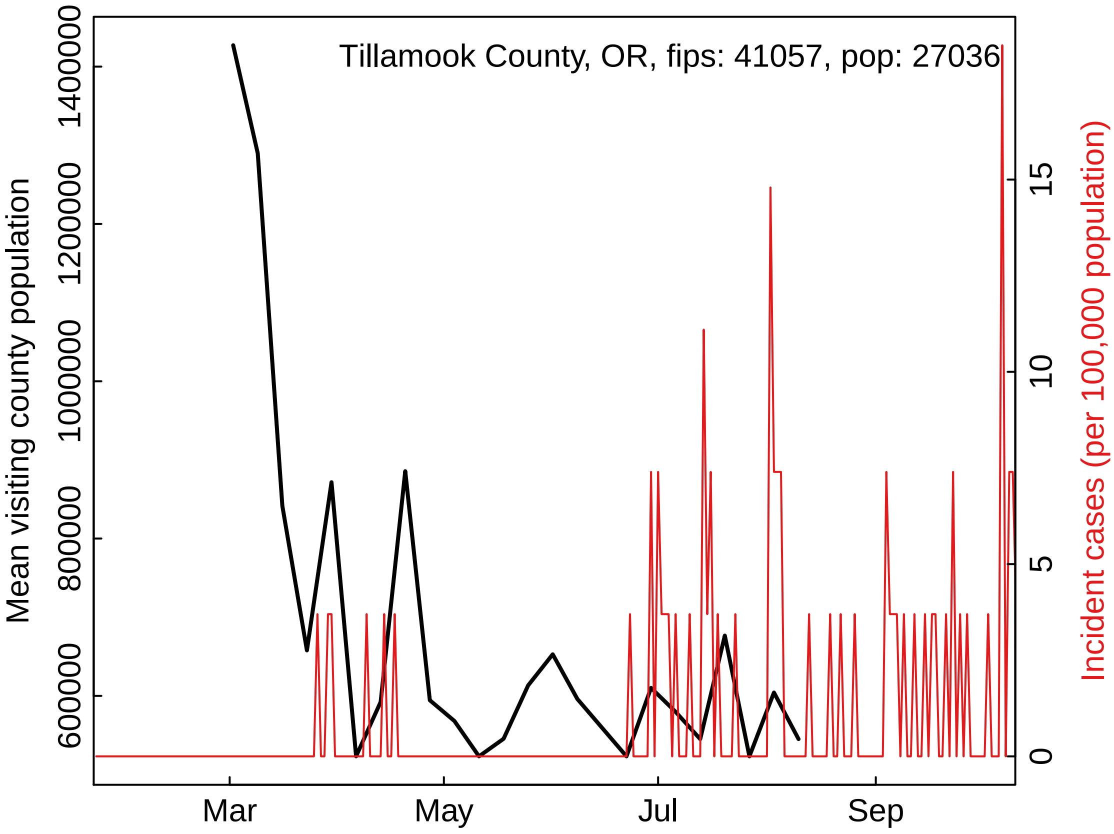

Tillamook County, OR, fips: 41057, pop: 27036
Mean visiting county population
600000
800000
1000000
1200000
1400000
Incident cases (per 100,000 population)
0
5
10
15
Mar
May
Jul
Sep

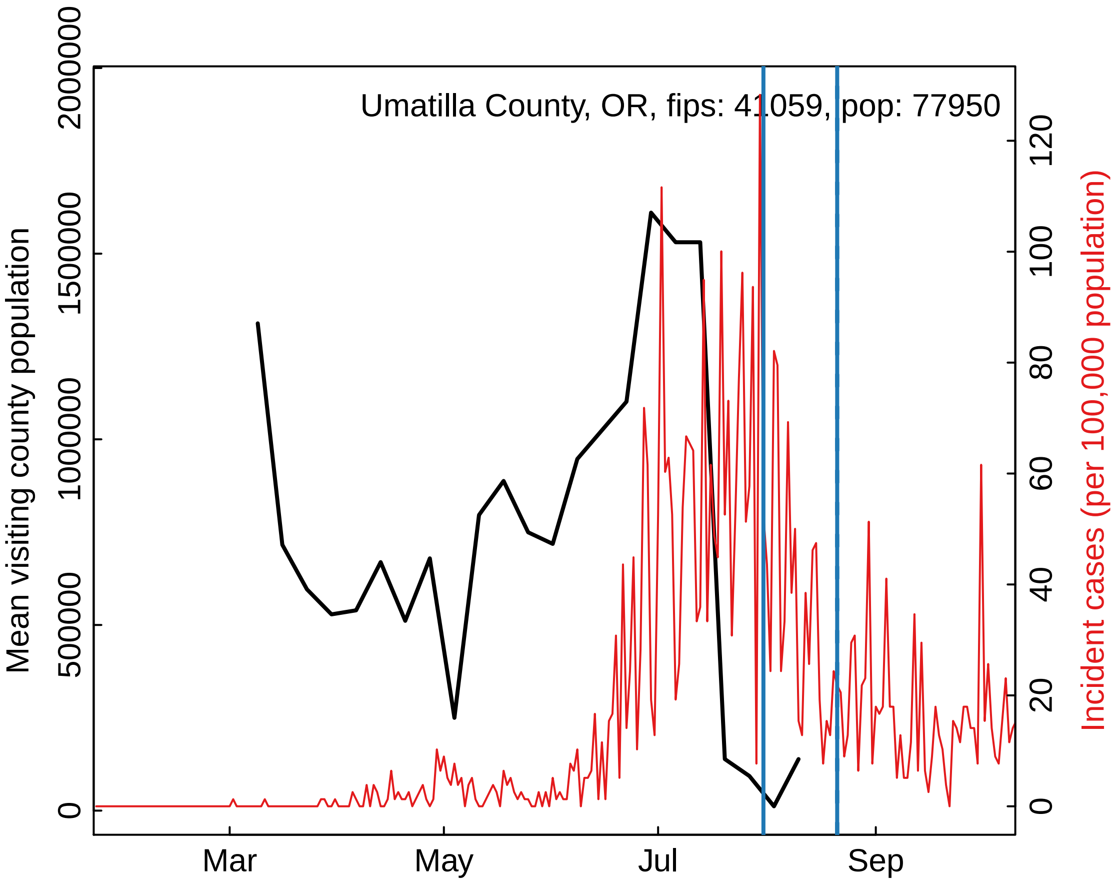

Umatilla County, OR, fips: 41059, pop: 77950
Mean visiting county population
Incident cases (per 100,000 population)
0
500000
1000000
1500000
2000000
0
20
40
60
80
100
120
Mar
May
Jul
Sep

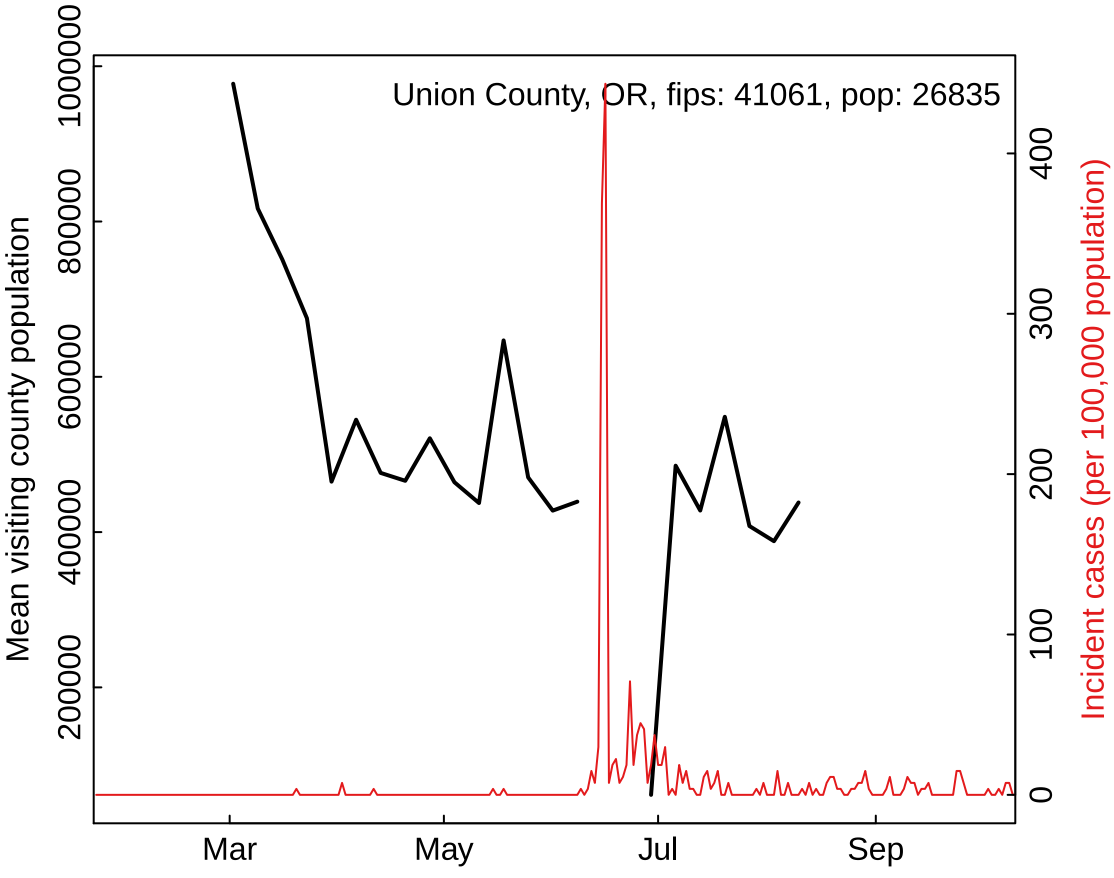

Union County, OR, fips: 41061, pop: 26835
Mean visiting county population
200000
400000
600000
800000
1000000
Incident cases (per 100,000 population)
0
100
200
300
400
Mar
May
Jul
Sep

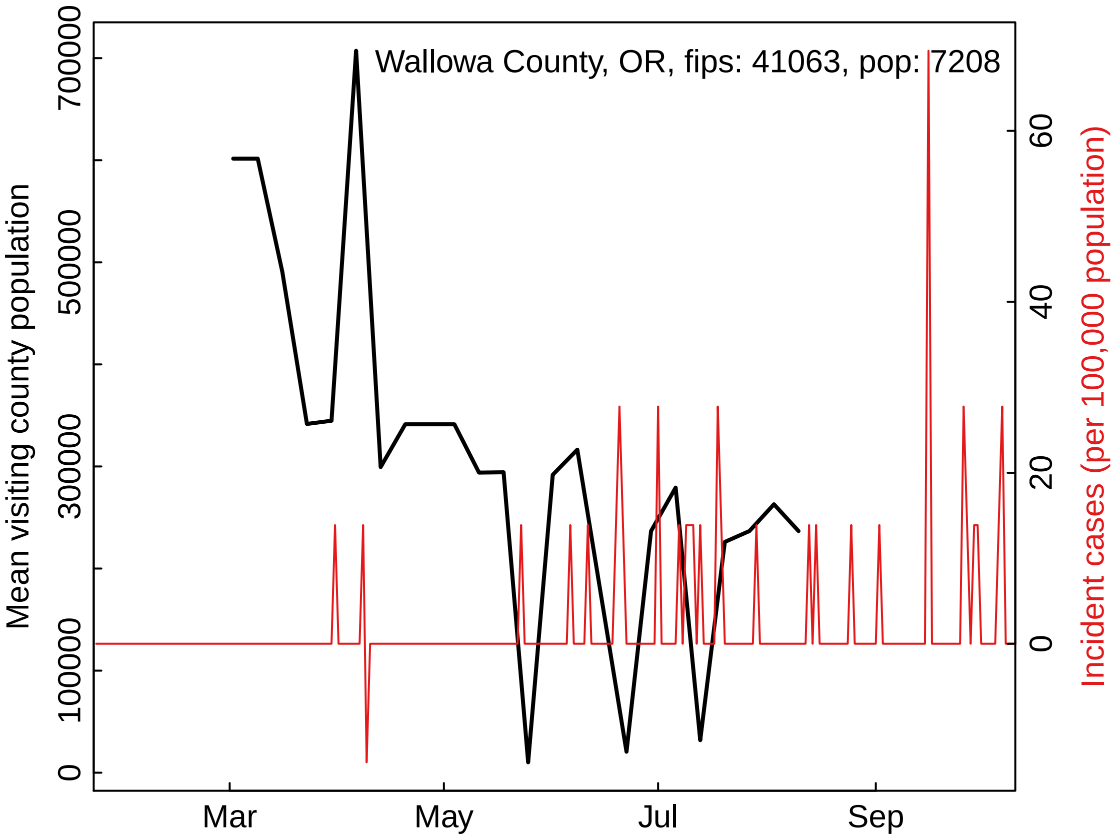
Wallowa County, OR, fips: 41063, pop: 7208
Mean visiting county population
0
100000
300000
500000
700000
Incident cases (per 100,000 population)
0
20
40
60
Mar
May
Jul
Sep

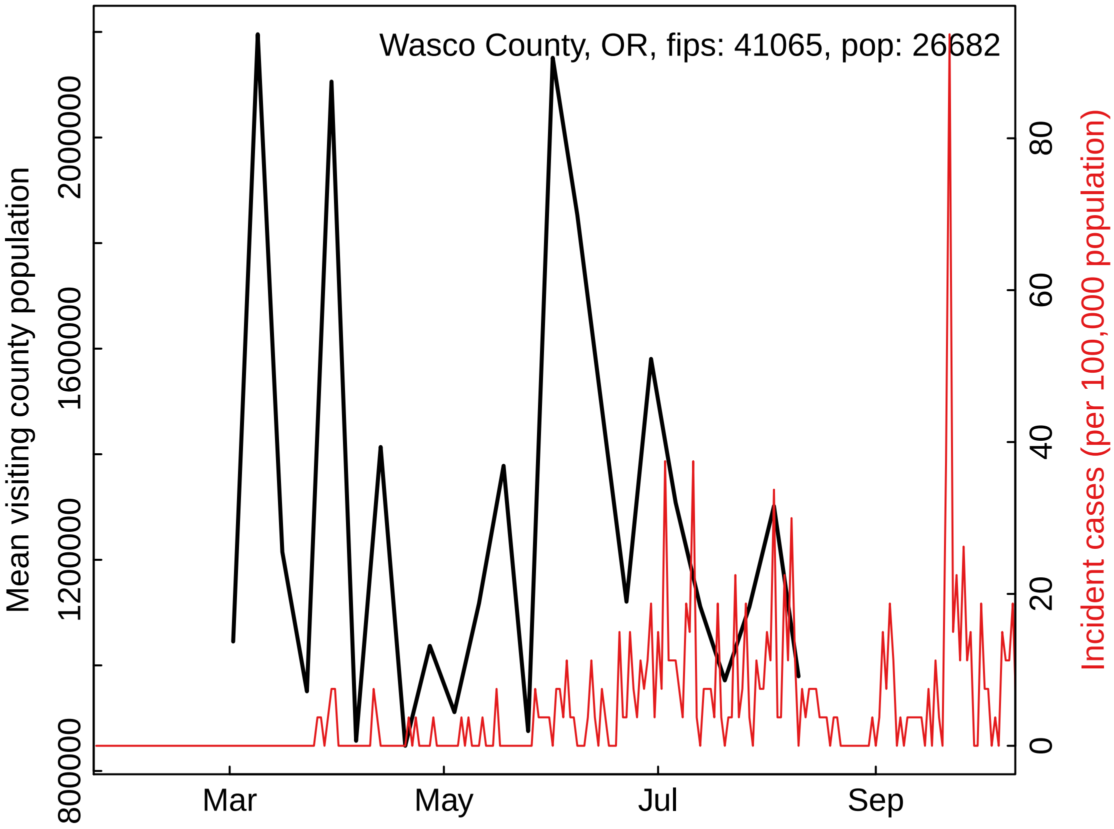

Wasco County, OR, fips: 41065, pop: 26682
Mean visiting county population
8000000
12000000
16000000
20000000
Incident cases (per 100,000 population)
0
20
40
60
80
Mar
May
Jul
Sep

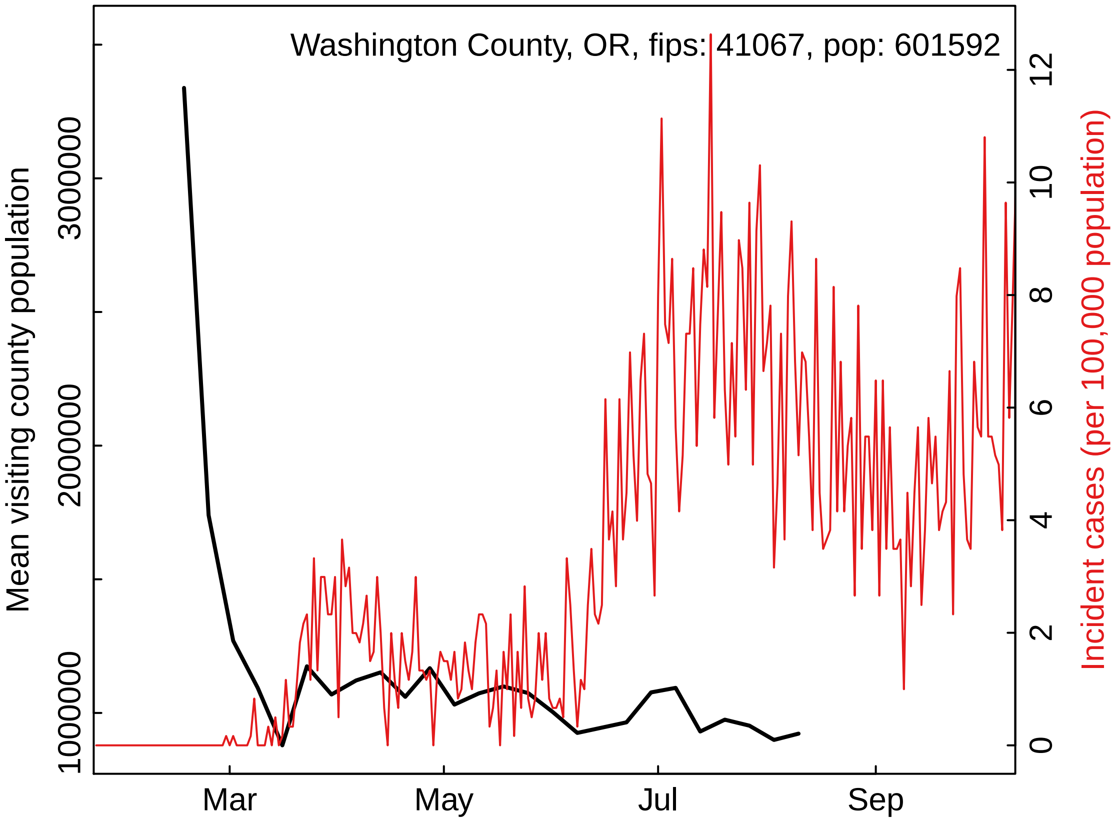

Washington County, OR, fips: 41067, pop: 601592
Mean visiting county population
Incident cases (per 100,000 population)
1000000
2000000
3000000
0
2
4
6
8
10
12
Mar
May
Jul
Sep

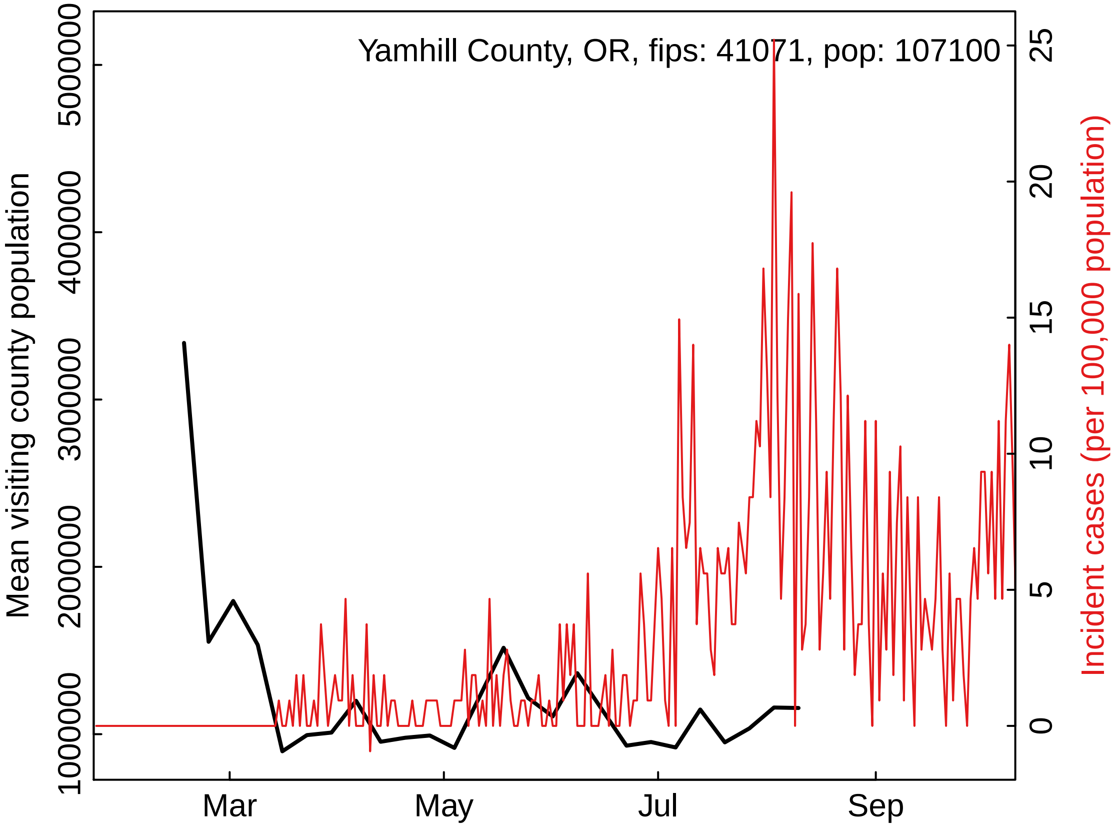

Yamhill County, OR, fips: 41071, pop: 107100
Mean visiting county population
10000000
20000000
30000000
40000000
50000000
Incident cases (per 100,000 population)
0
5
10
15
20
25
Mar
May
Jul
Sep

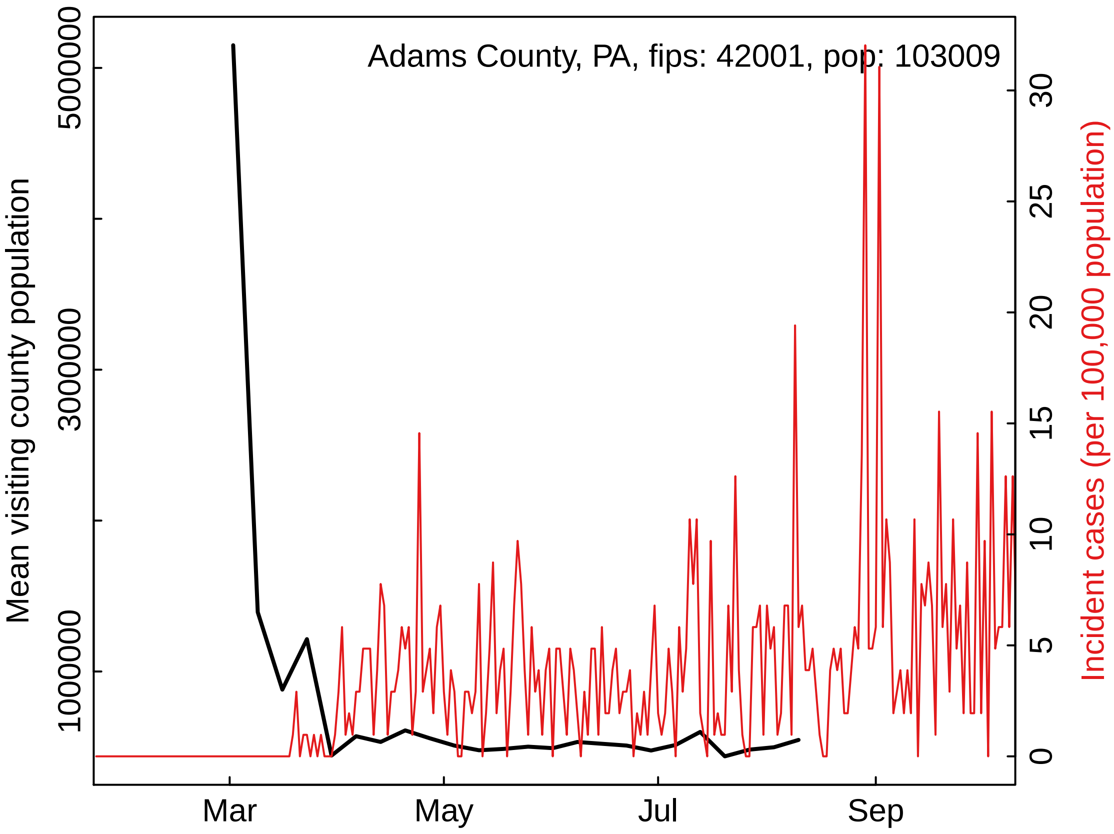

Adams County, PA, fips: 42001, pop: 103009
Mean visiting county population
10000000
30000000
50000000
Incident cases (per 100,000 population)
0
5
10
15
20
25
30
Mar
May
Jul
Sep

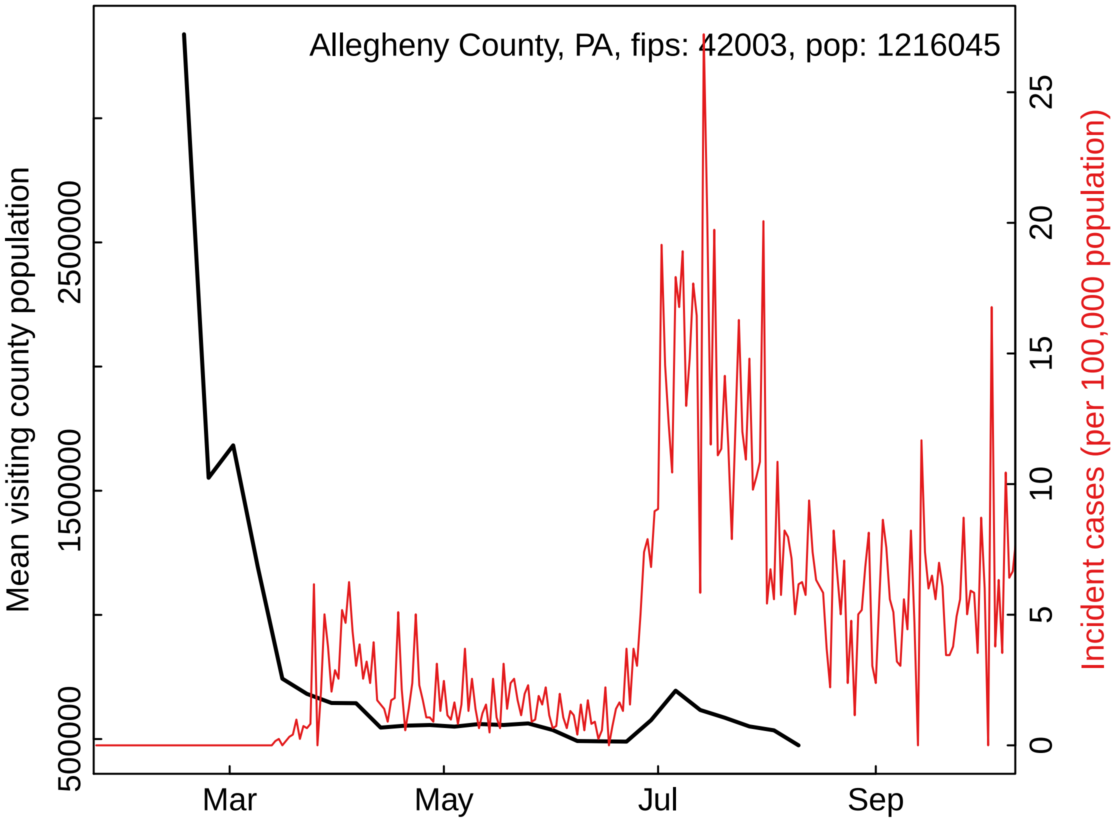

Allegheny County, PA, fips: 42003, pop: 1216045
Mean visiting county population
500000
1500000
2500000
Incident cases (per 100,000 population)
0
5
10
15
20
25
Mar
May
Jul
Sep

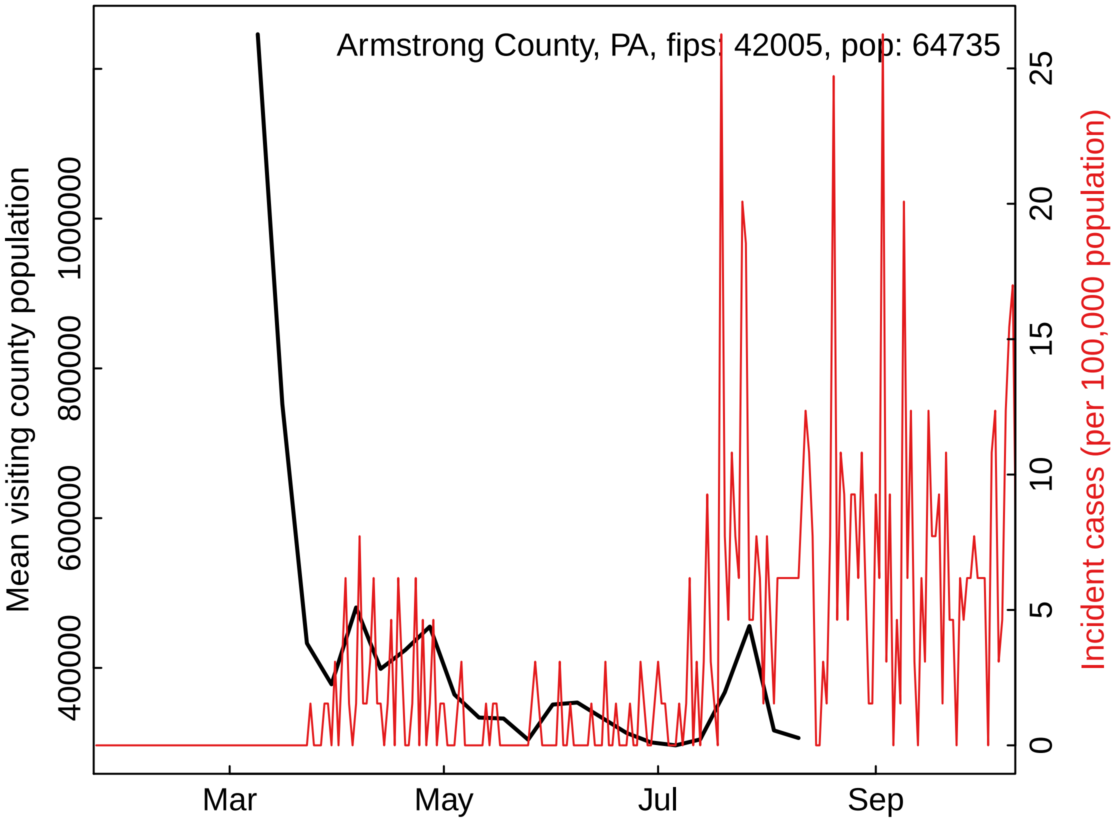
Armstrong County, PA, fips: 42005, pop: 64735
Mean visiting county population
400000
600000
800000
1000000
Incident cases (per 100,000 population)
0
5
10
15
20
25
Mar
May
Jul
Sep

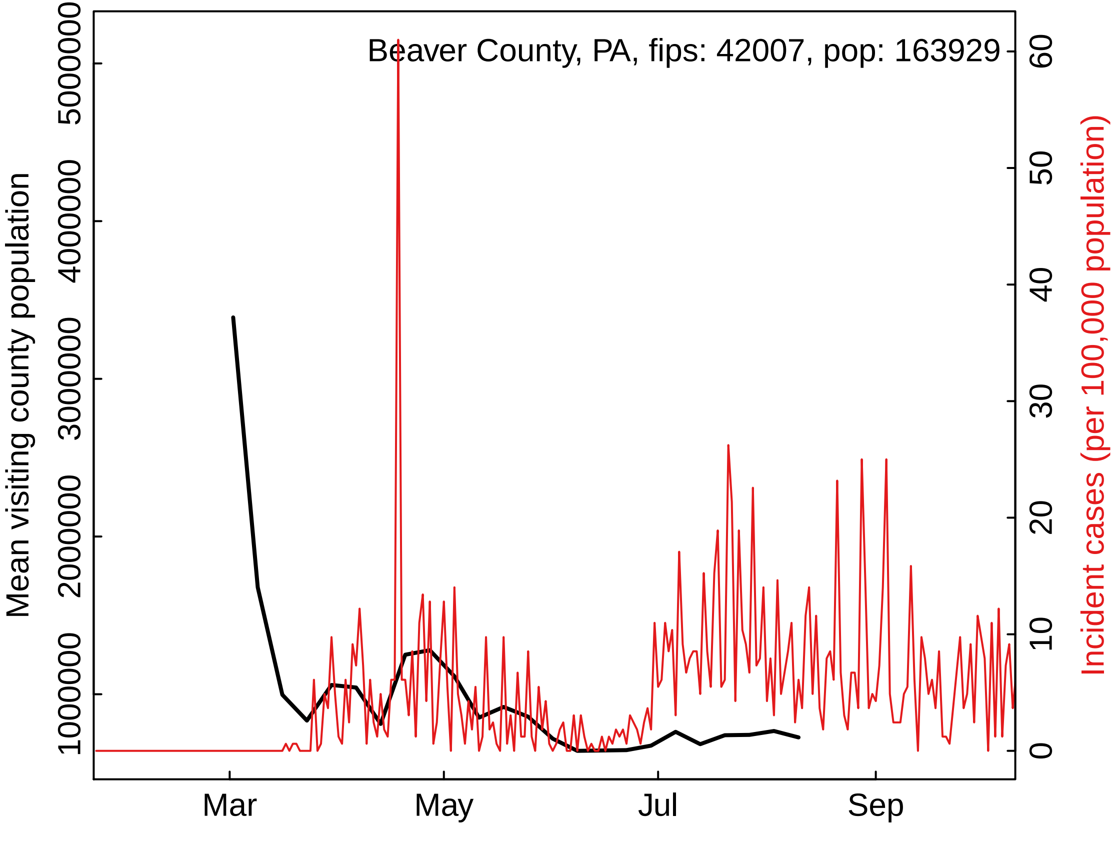
Beaver County, PA, fips: 42007, pop: 163929
Mean visiting county population
10000000
20000000
30000000
40000000
50000000
Incident cases (per 100,000 population)
0
10
20
30
40
50
60
Mar
May
Jul
Sep

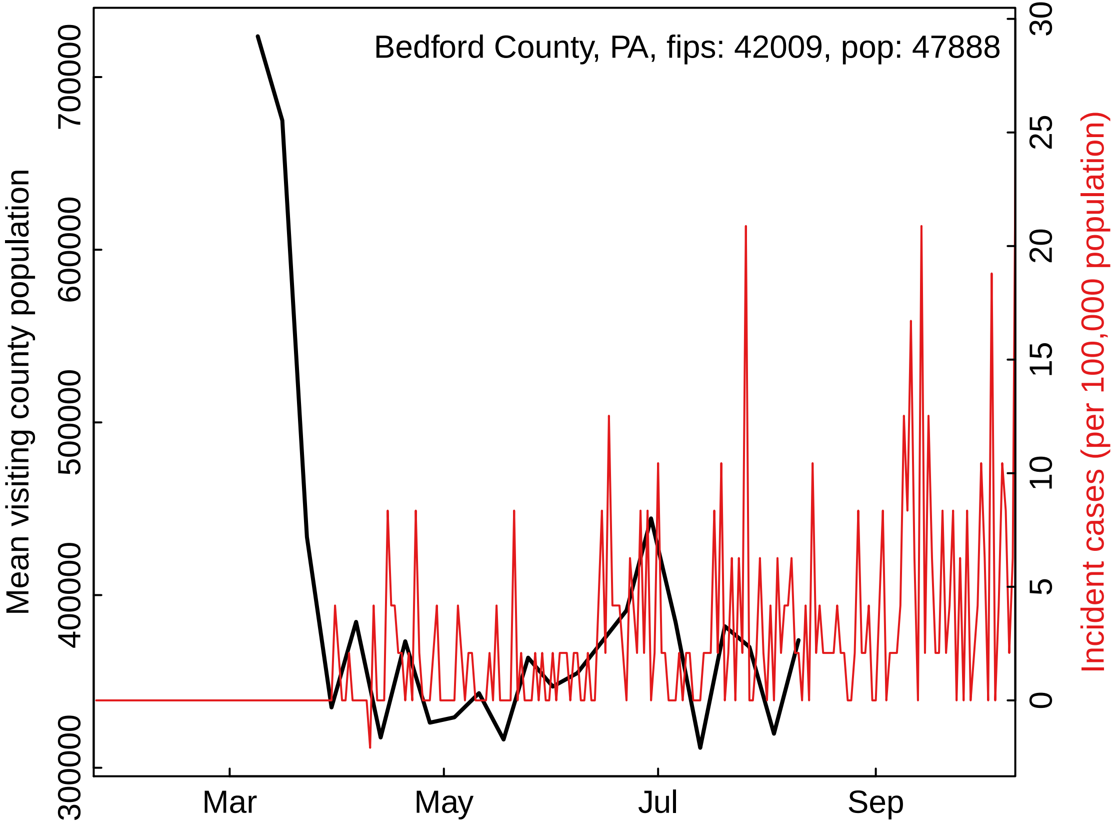

Bedford County, PA, fips: 42009, pop: 47888
Mean visiting county population
Incident cases (per 100,000 population)
3000000
4000000
5000000
6000000
7000000
0
5
10
15
20
25
30
Mar
May
Jul
Sep

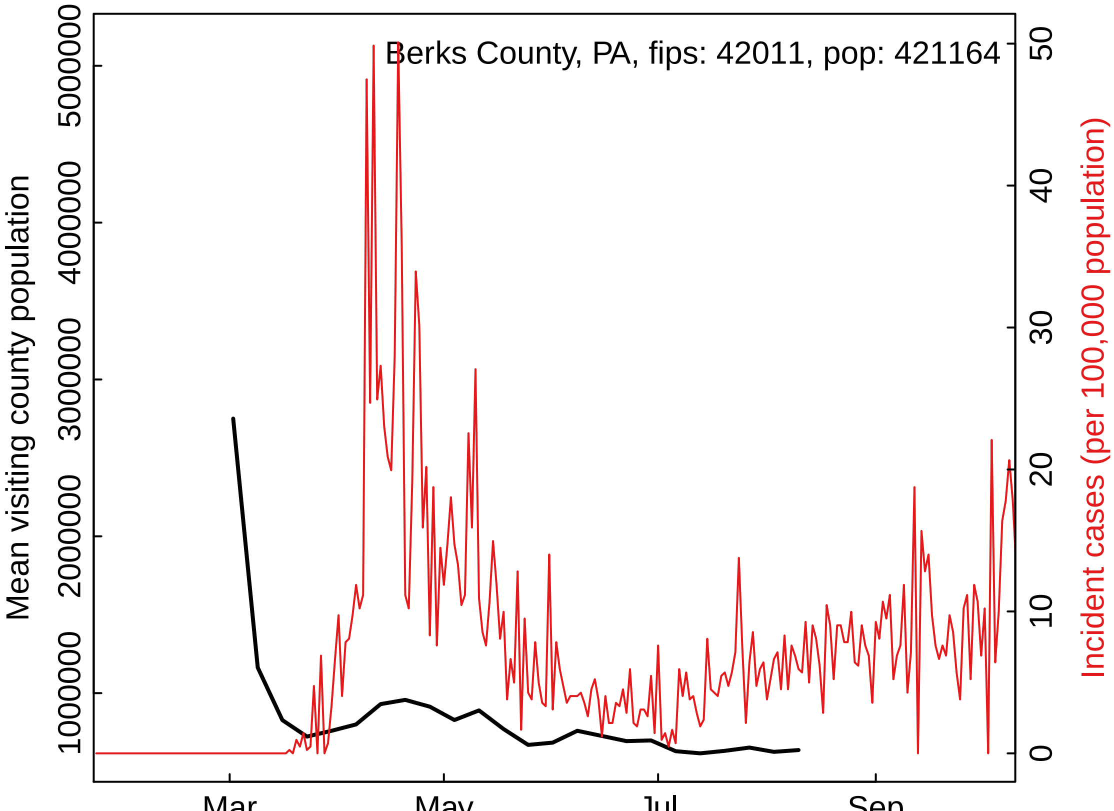

Berks County, PA, fips: 42011, pop: 421164
Mean visiting county population
1000000
2000000
3000000
4000000
5000000
Incident cases (per 100,000 population)
0
10
20
30
40
50
Mar
May
Jul
Sep

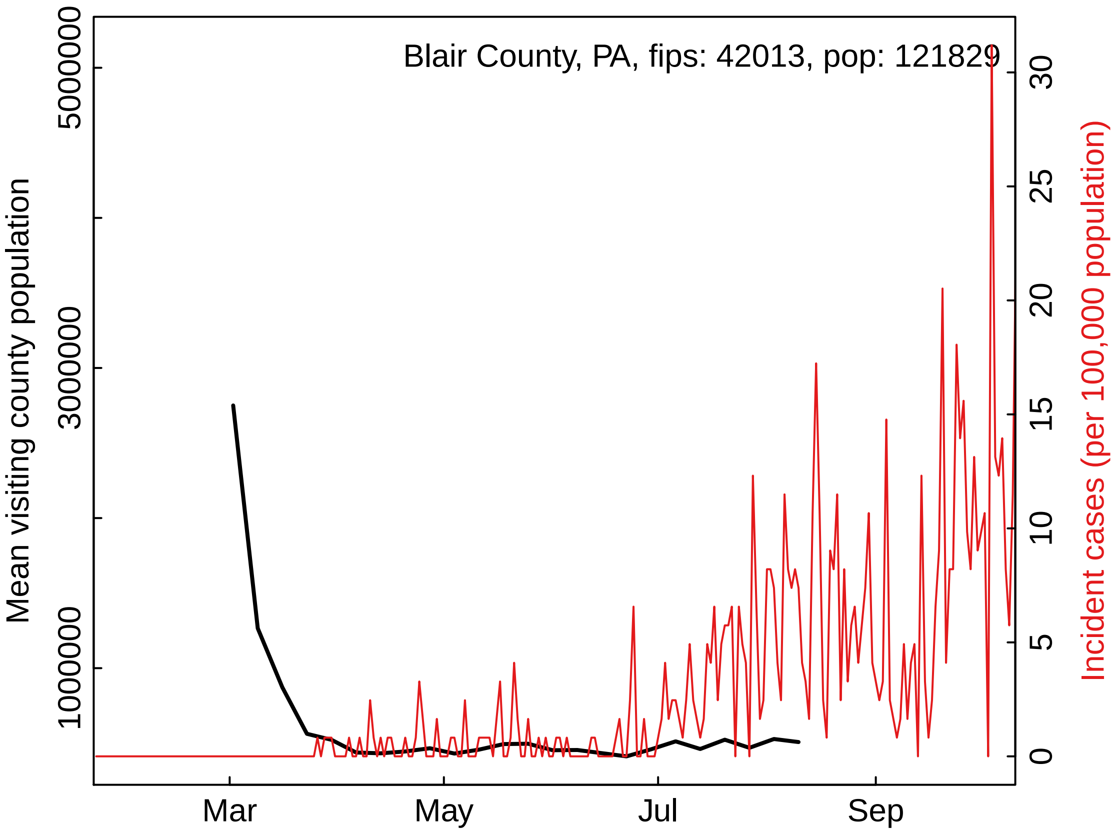

Blair County, PA, fips: 42013, pop: 121829
Mean visiting county population
1000000
3000000
5000000
Mar
May
Jul
Sep
0
5
10
15
20
25
30
Incident cases (per 100,000 population)

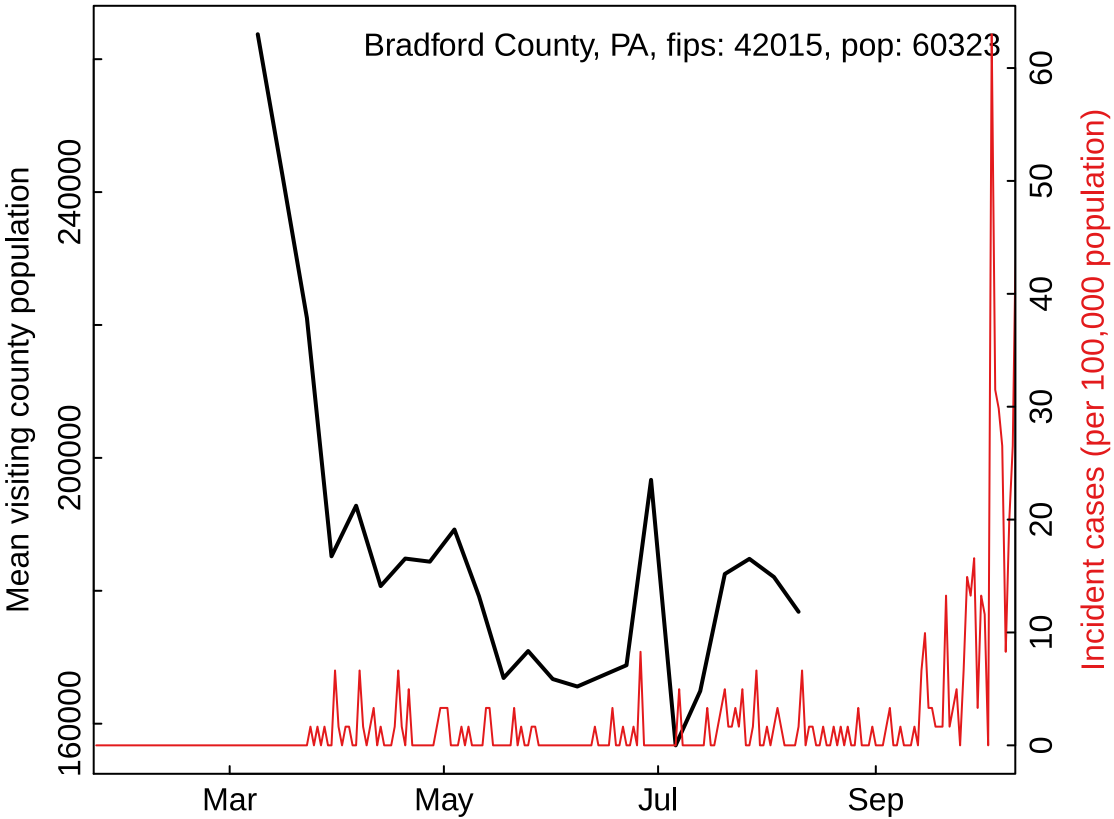

Bradford County, PA, fips: 42015, pop: 60323
Mean visiting county population
Incident cases (per 100,000 population)
160000
200000
240000
0
10
20
30
40
50
60
Mar
May
Jul
Sep

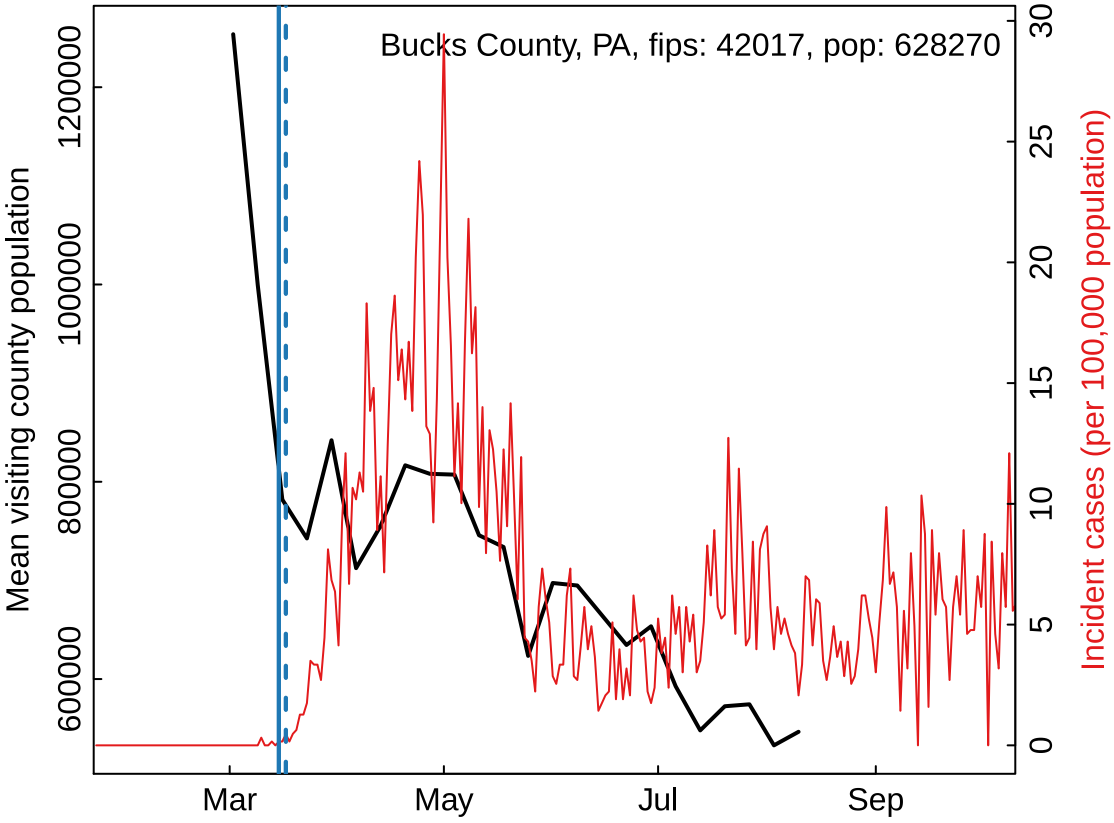

Bucks County, PA, fips: 42017, pop: 628270
Mean visiting county population
Incident cases (per 100,000 population)
600000
800000
1000000
1200000
0
5
10
15
20
25
30
Mar
May
Jul
Sep

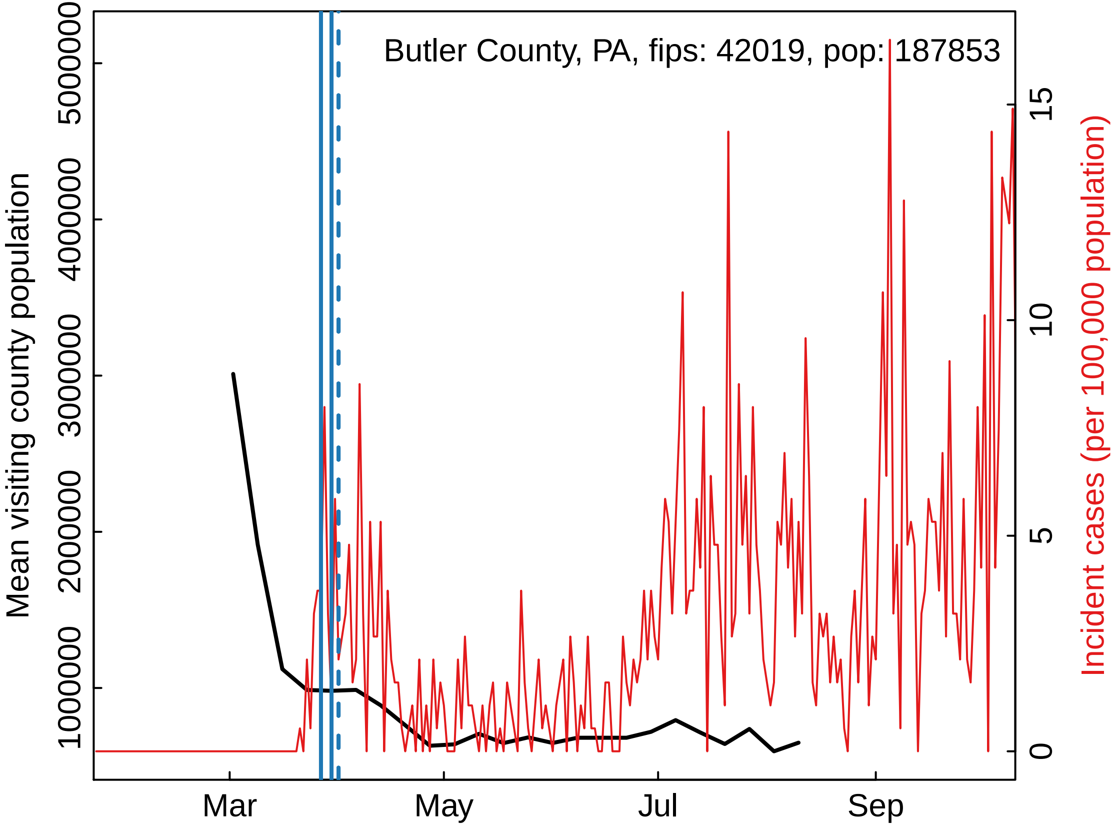

Butler County, PA, fips: 42019, pop: 187853
Mean visiting county population
10000000
20000000
30000000
40000000
50000000
Incident cases (per 100,000 population)
0
5
10
15
Mar
May
Jul
Sep

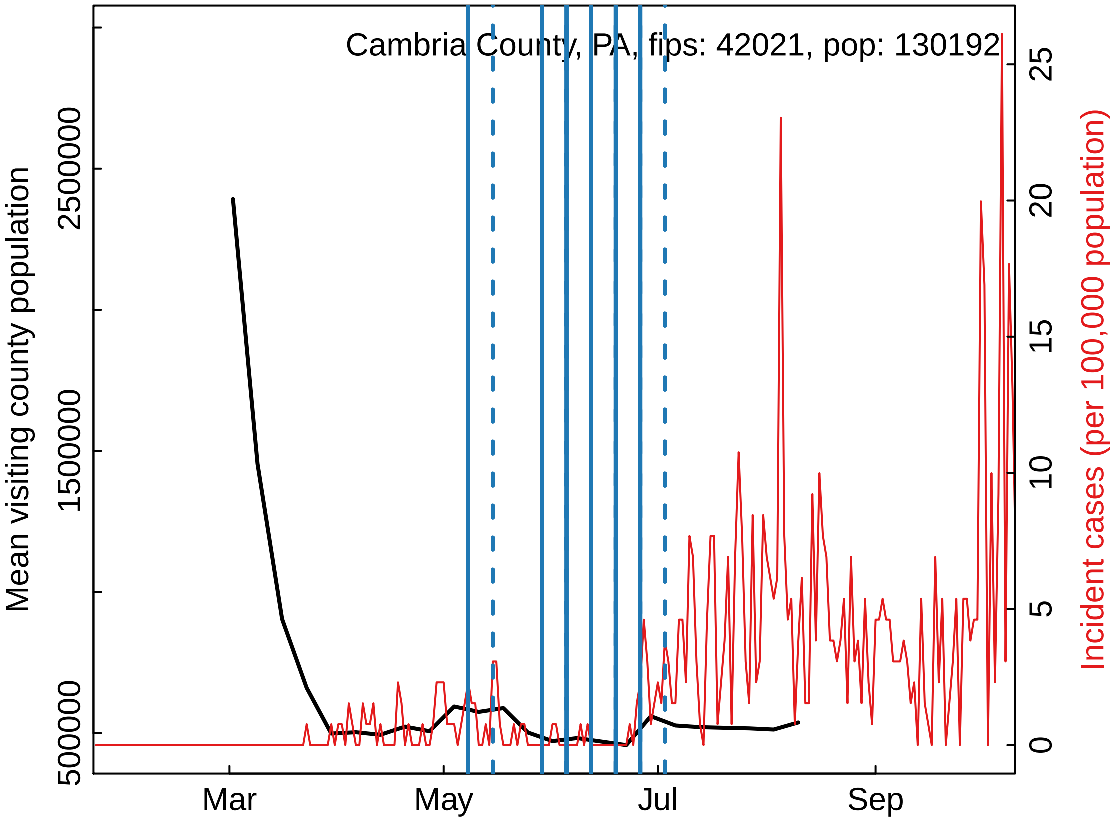
Cambria County, PA, fips: 42021, pop: 130192
Mean visiting county population
500000
1500000
2500000
Incident cases (per 100,000 population)
0
5
10
15
20
25
Mar
May
Jul
Sep

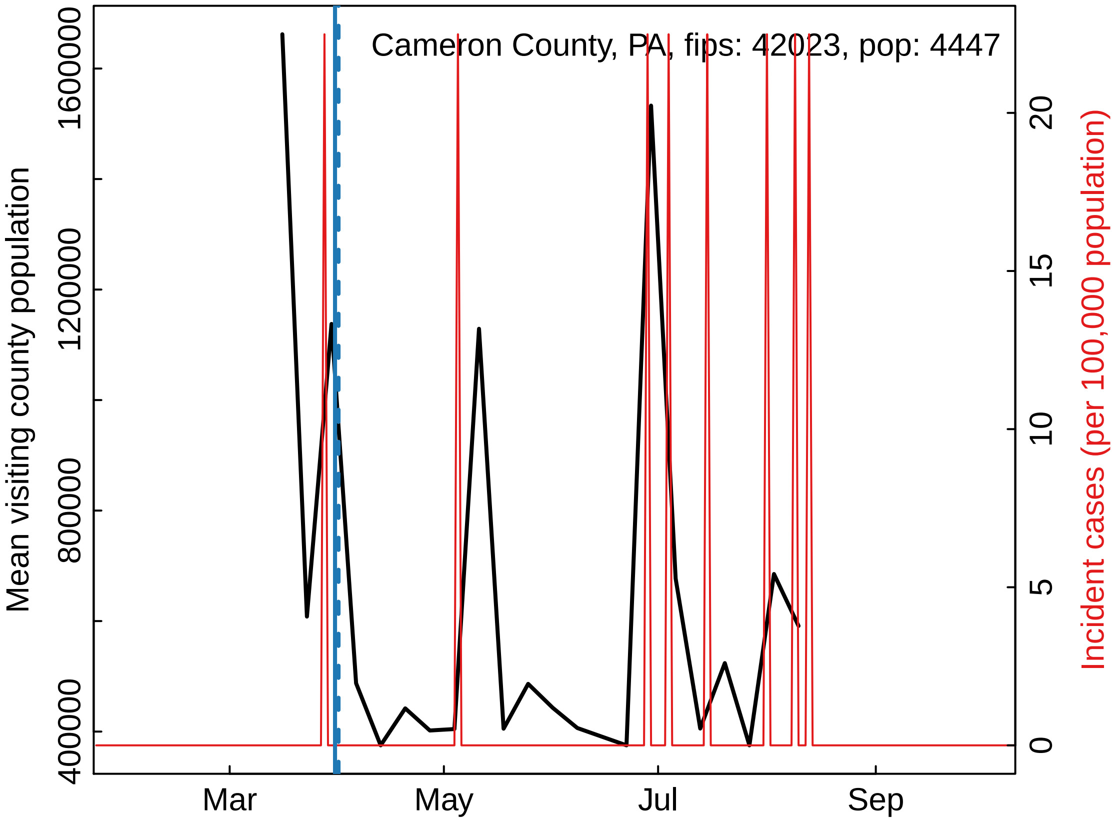
Cameron County, PA, fips: 42023, pop: 4447
Mean visiting county population
400000
800000
1200000
1600000
Incident cases (per 100,000 population)
0
5
10
15
20
Mar
May
Jul
Sep

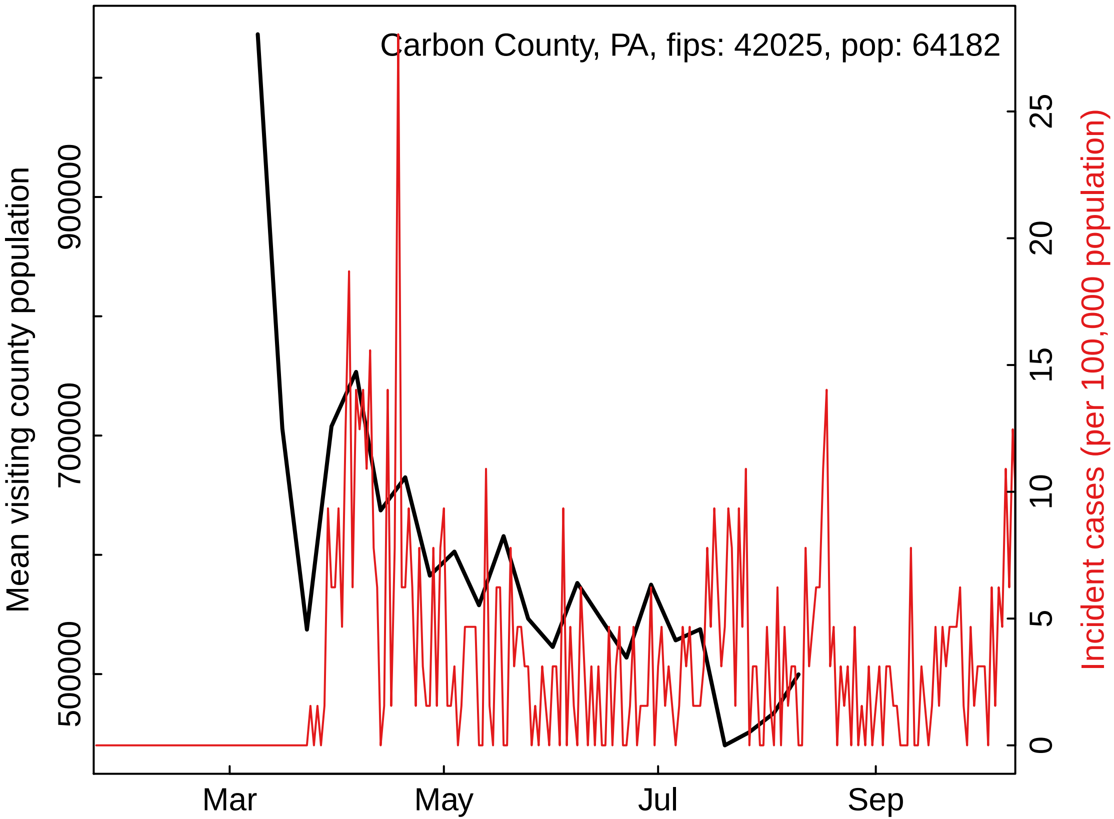

Carbon County, PA, fips: 42025, pop: 64182
Mean visiting county population
500000
700000
900000
Incident cases (per 100,000 population)
0
5
10
15
20
25
Mar
May
Jul
Sep

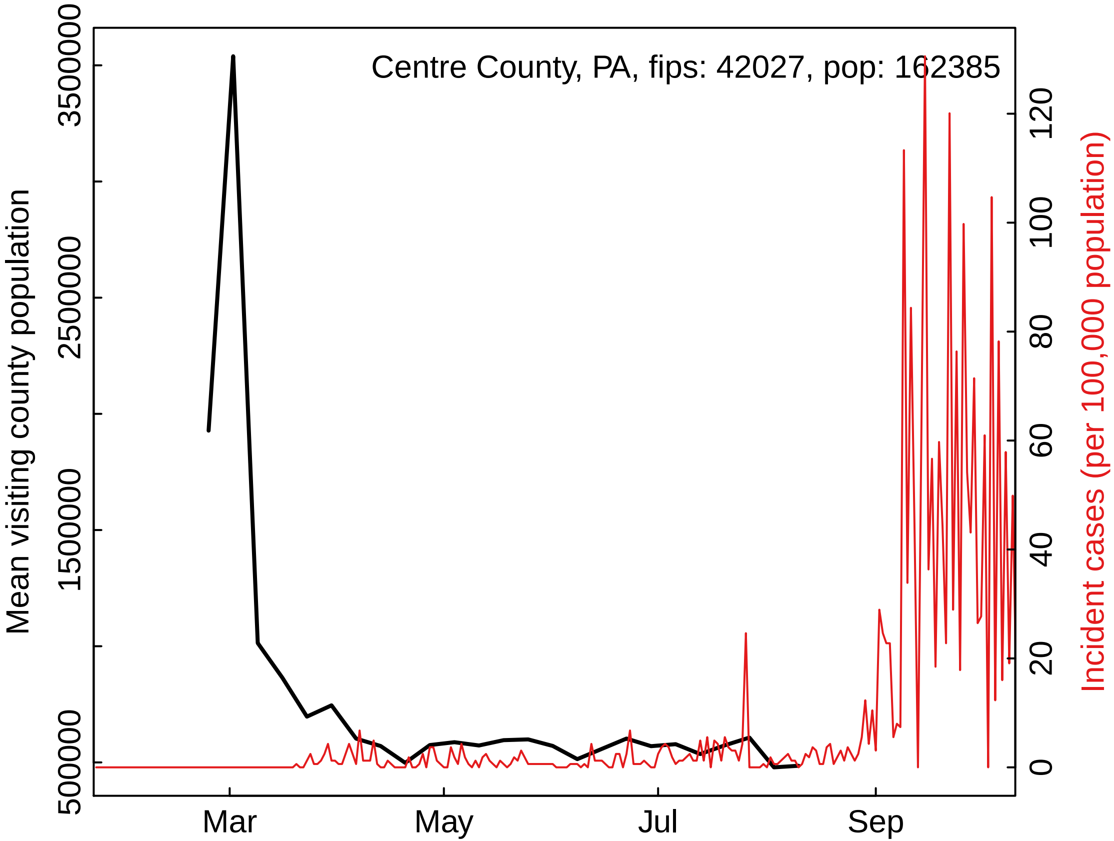

Centre County, PA, fips: 42027, pop: 162385
Mean visiting county population
500000
1500000
2500000
3500000
Incident cases (per 100,000 population)
0
20
40
60
80
100
120
Mar
May
Jul
Sep

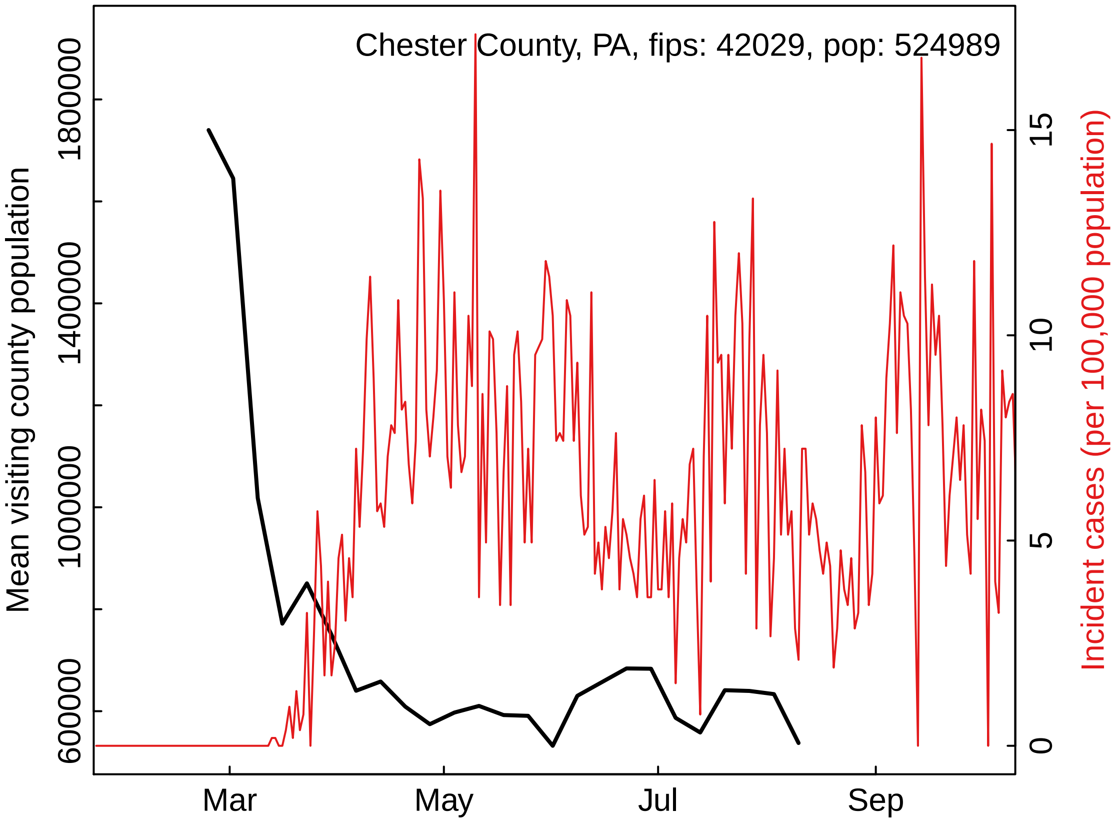

Chester County, PA, fips: 42029, pop: 524989
Mean visiting county population
Incident cases (per 100,000 population)
600000
1000000
1400000
1800000
0
5
10
15
Mar
May
Jul
Sep

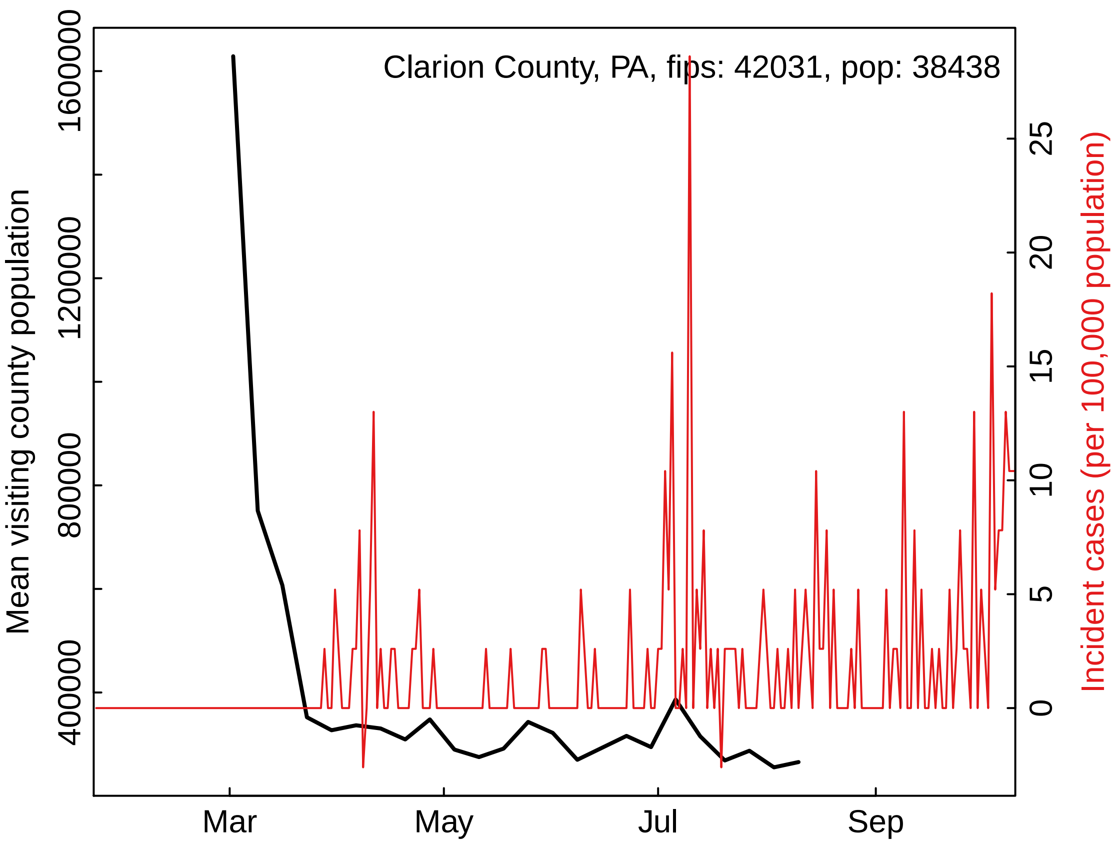

Clarion County, PA, fips: 42031, pop: 38438
Mean visiting county population
400000
800000
1200000
1600000
Incident cases (per 100,000 population)
0
5
10
15
20
25
Mar
May
Jul
Sep

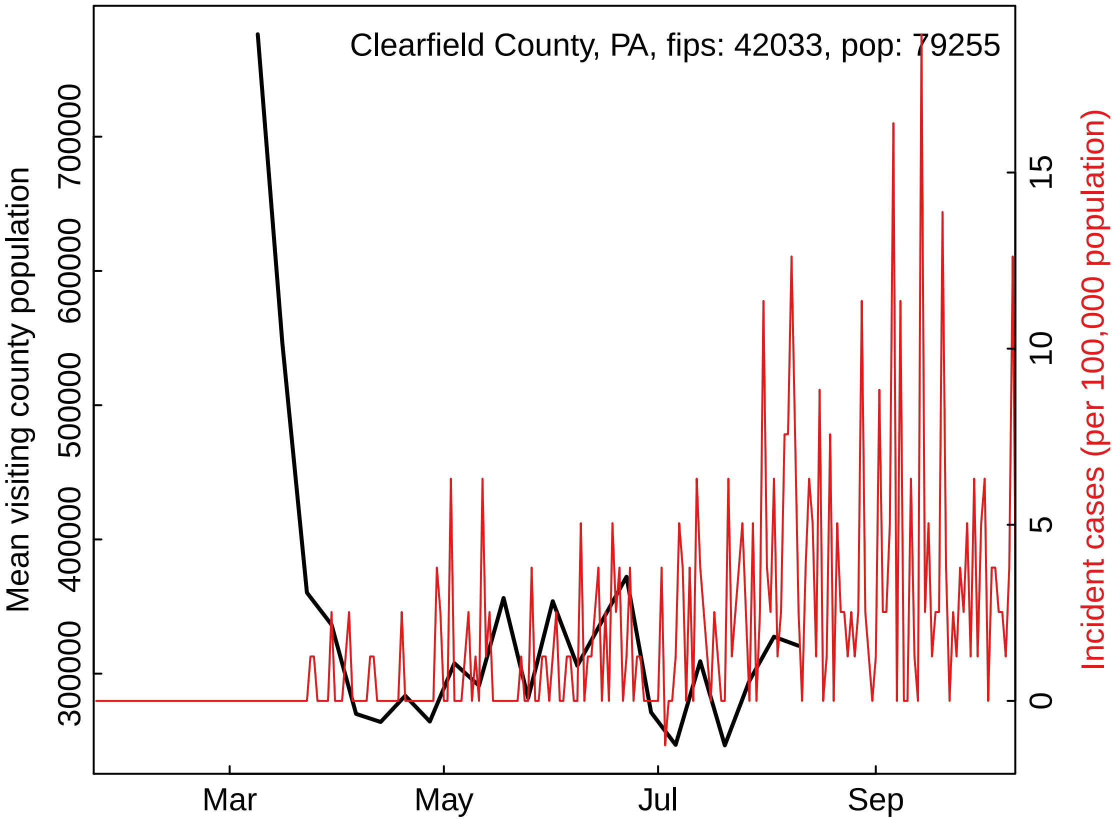

Clearfield County, PA, fips: 42033, pop: 79255
Mean visiting county population
300000
400000
500000
600000
700000
Incident cases (per 100,000 population)
0
5
10
15
Mar
May
Jul
Sep

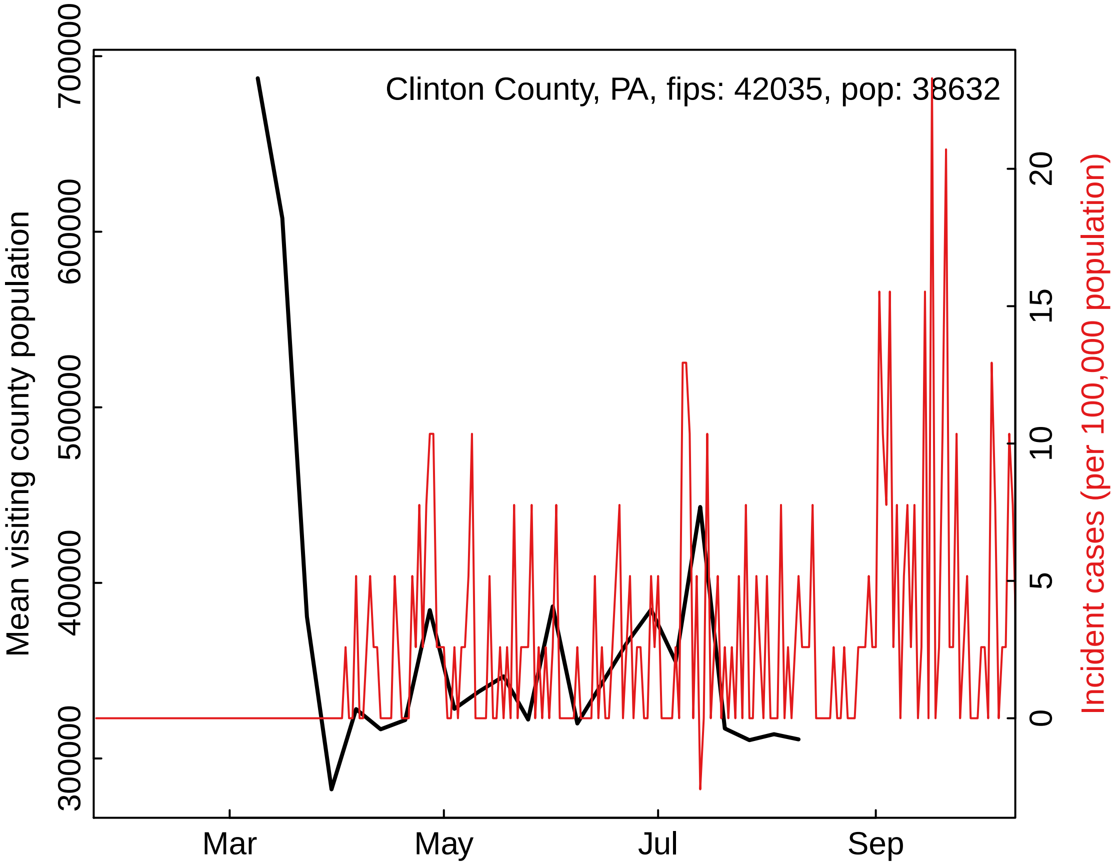
Clinton County, PA, fips: 42035, pop: 38632
Mean visiting county population
Incident cases (per 100,000 population)
300000
400000
500000
600000
700000
0
5
10
15
20
Mar
May
Jul
Sep

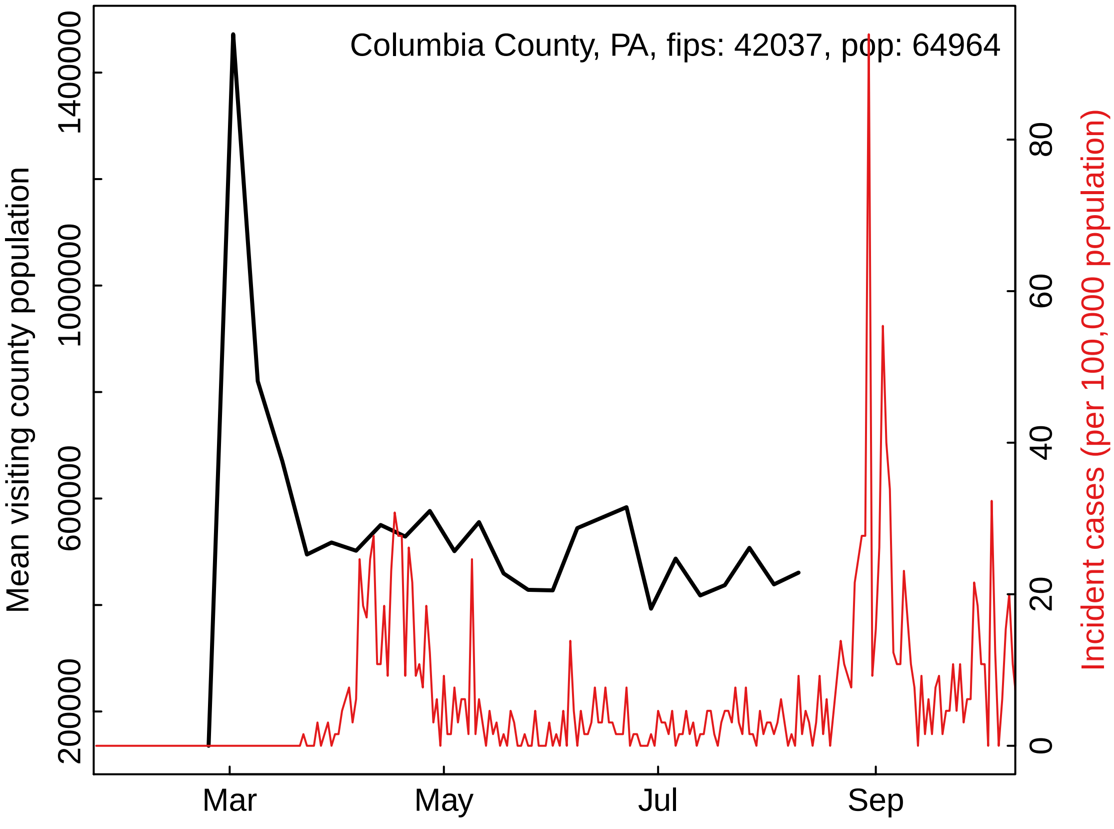

Columbia County, PA, fips: 42037, pop: 64964
Mean visiting county population
200000
600000
1000000
1400000
Incident cases (per 100,000 population)
0
20
40
60
80
Mar
May
Jul
Sep

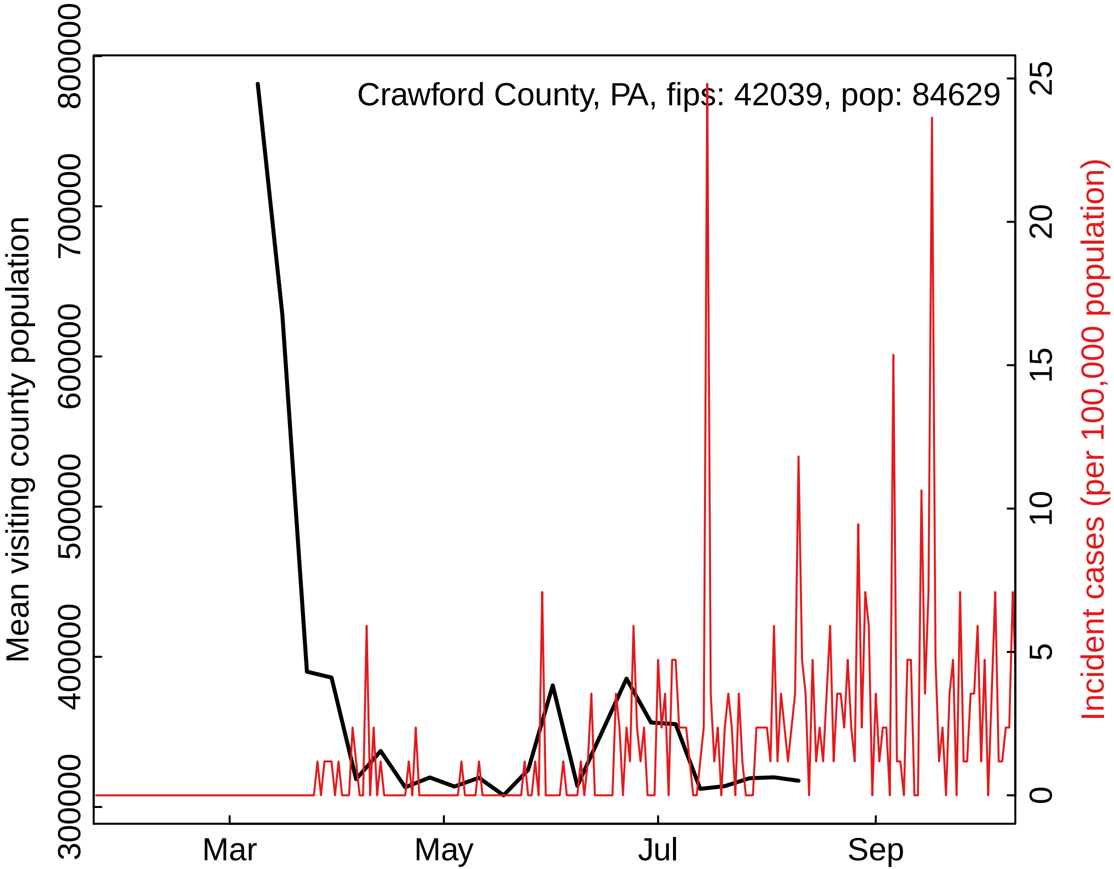

Crawford County, PA, fips: 42039, pop: 84629
Mean visiting county population
300000
400000
500000
600000
700000
800000
Mar
May
Jul
Sep
0
5
10
15
20
25
Incident cases (per 100,000 population)

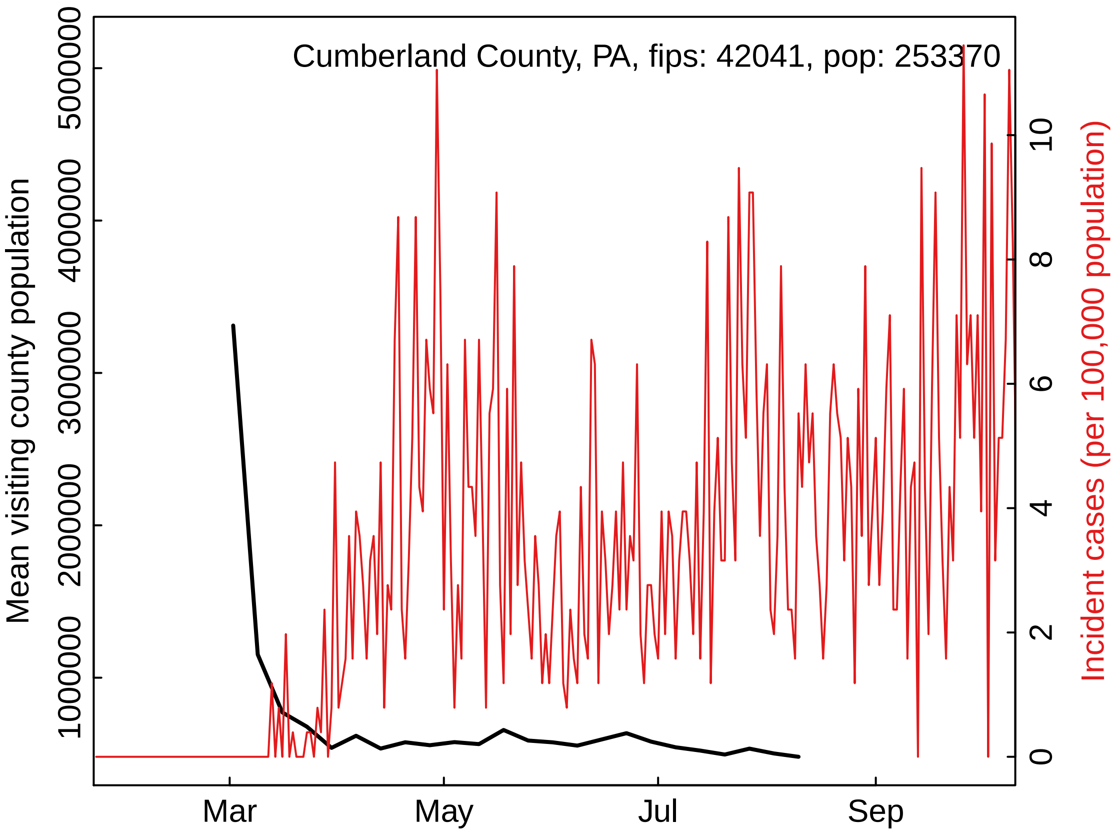

Cumberland County, PA, fips: 42041, pop: 253370
Mean visiting county population
Incident cases (per 100,000 population)
10000000
20000000
30000000
40000000
50000000
0
2
4
6
8
10
Mar
May
Jul
Sep

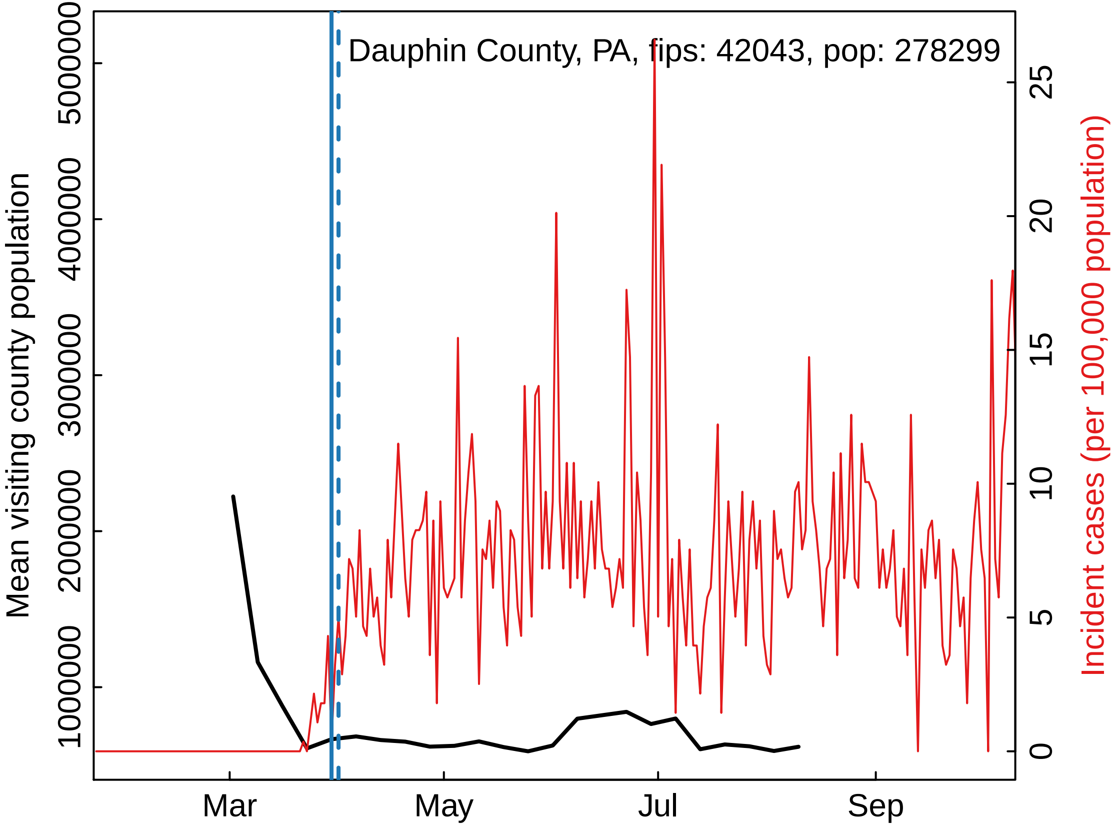

Dauphin County, PA, fips: 42043, pop: 278299
Mean visiting county population
10000000
20000000
30000000
40000000
50000000
Mar
May
Jul
Sep
0
5
10
15
20
25
Incident cases (per 100,000 population)

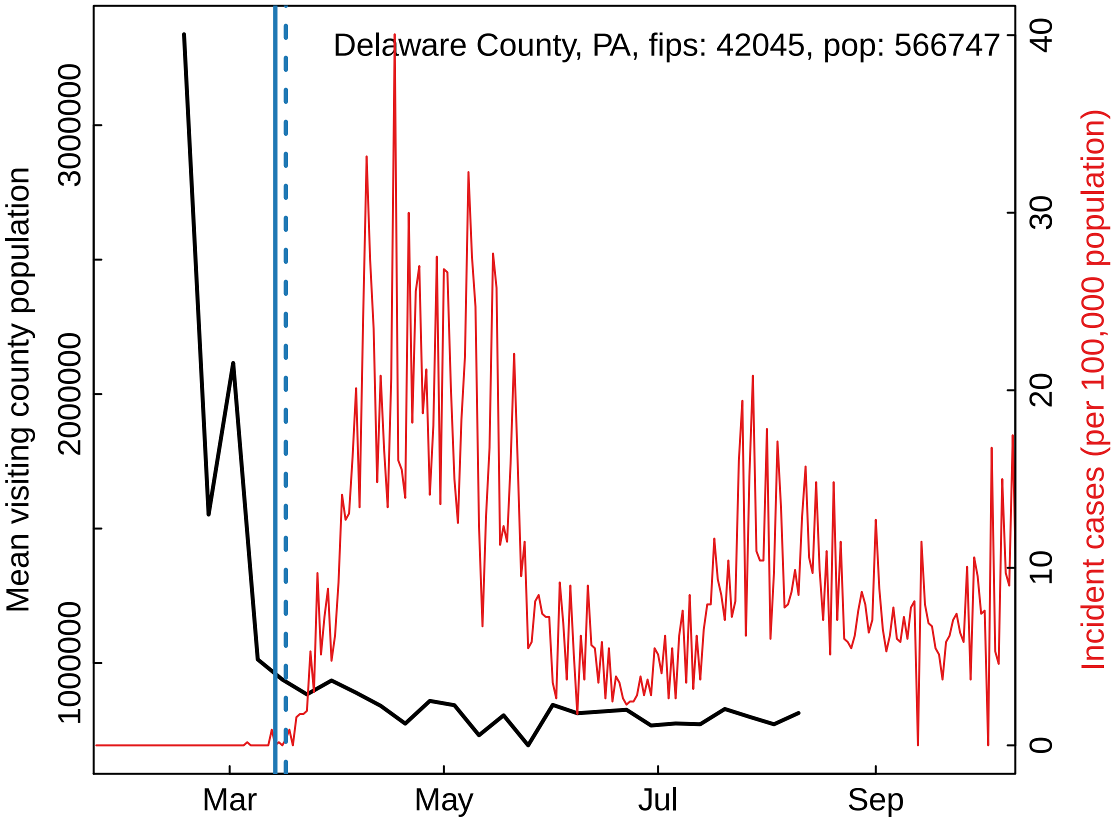

Delaware County, PA, fips: 42045, pop: 566747
Mean visiting county population
1000000
2000000
3000000
Incident cases (per 100,000 population)
0
10
20
30
40
Mar
May
Jul
Sep

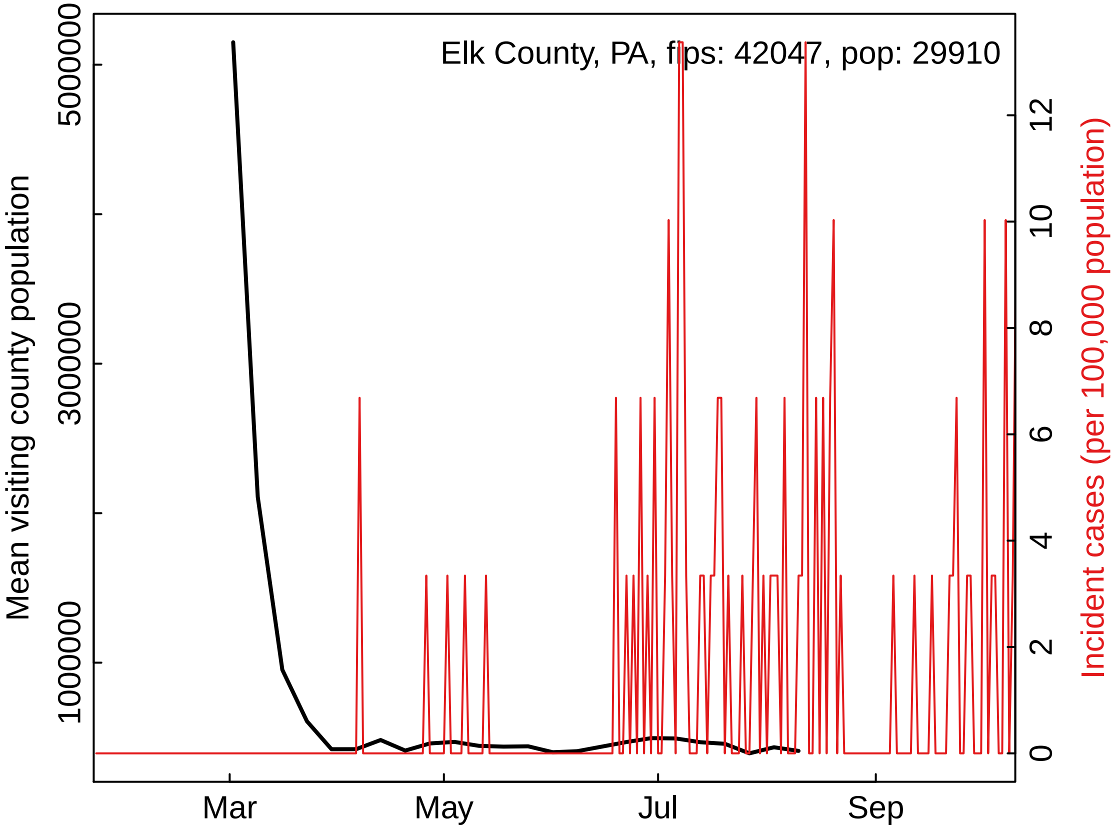

Elk County, PA, fips: 42047, pop: 29910
Mean visiting county population
Incident cases (per 100,000 population)
1000000
3000000
5000000
0
2
4
6
8
10
12
Mar
May
Jul
Sep

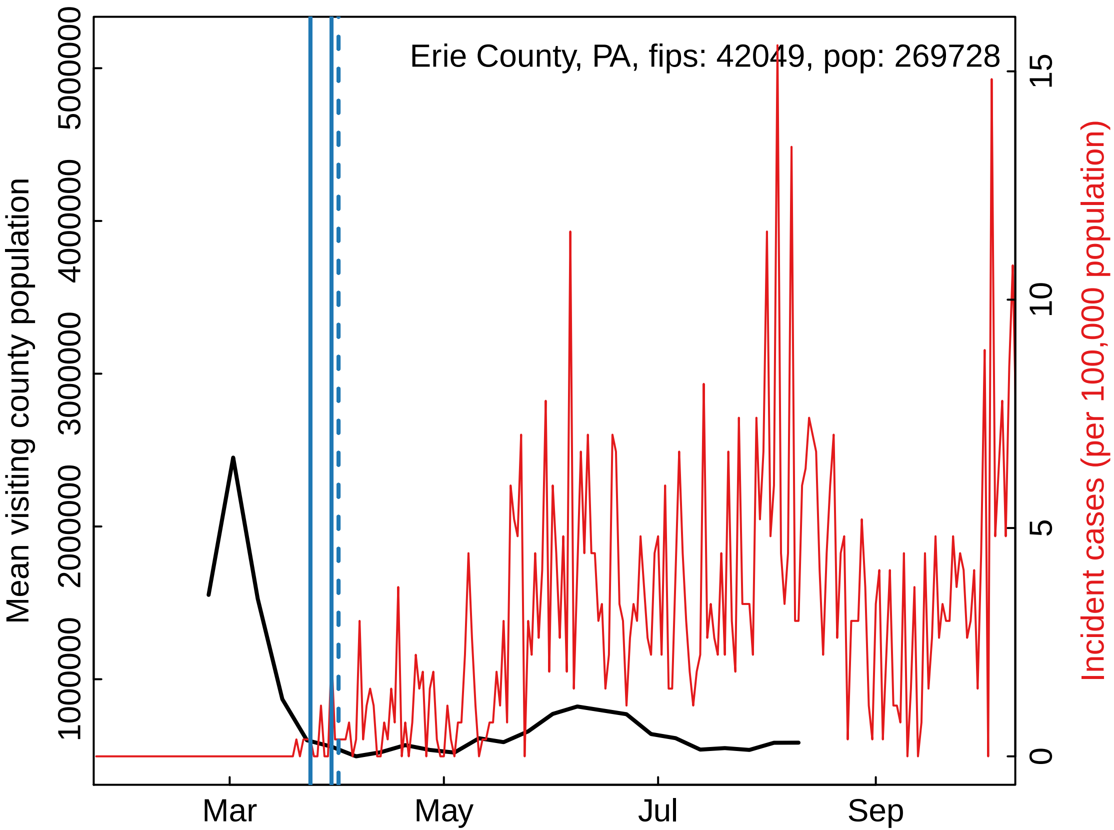

Erie County, PA, fips: 42049, pop: 269728
Mean visiting county population
1000000
2000000
3000000
4000000
5000000
Incident cases (per 100,000 population)
0
5
10
15
Mar
May
Jul
Sep

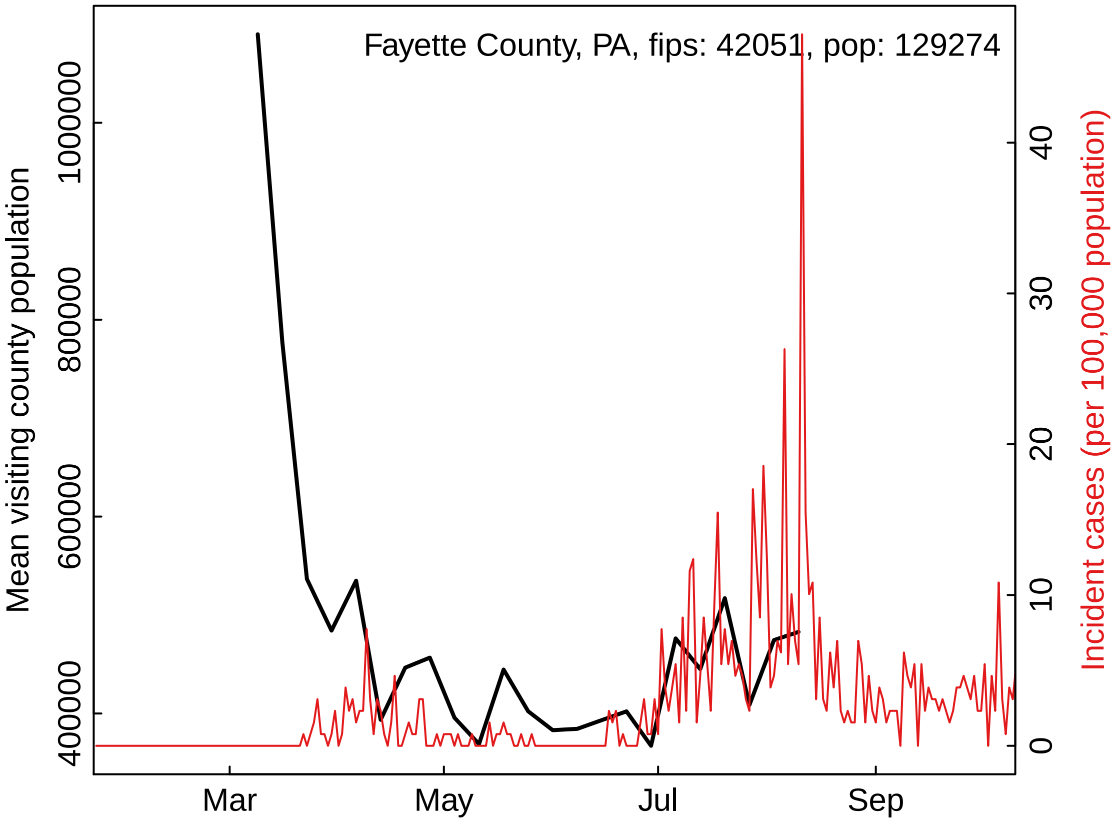

Fayette County, PA, fips: 42051, pop: 129274
Mean visiting county population
400000
600000
800000
1000000
Incident cases (per 100,000 population)
0
10
20
30
40
Mar
May
Jul
Sep

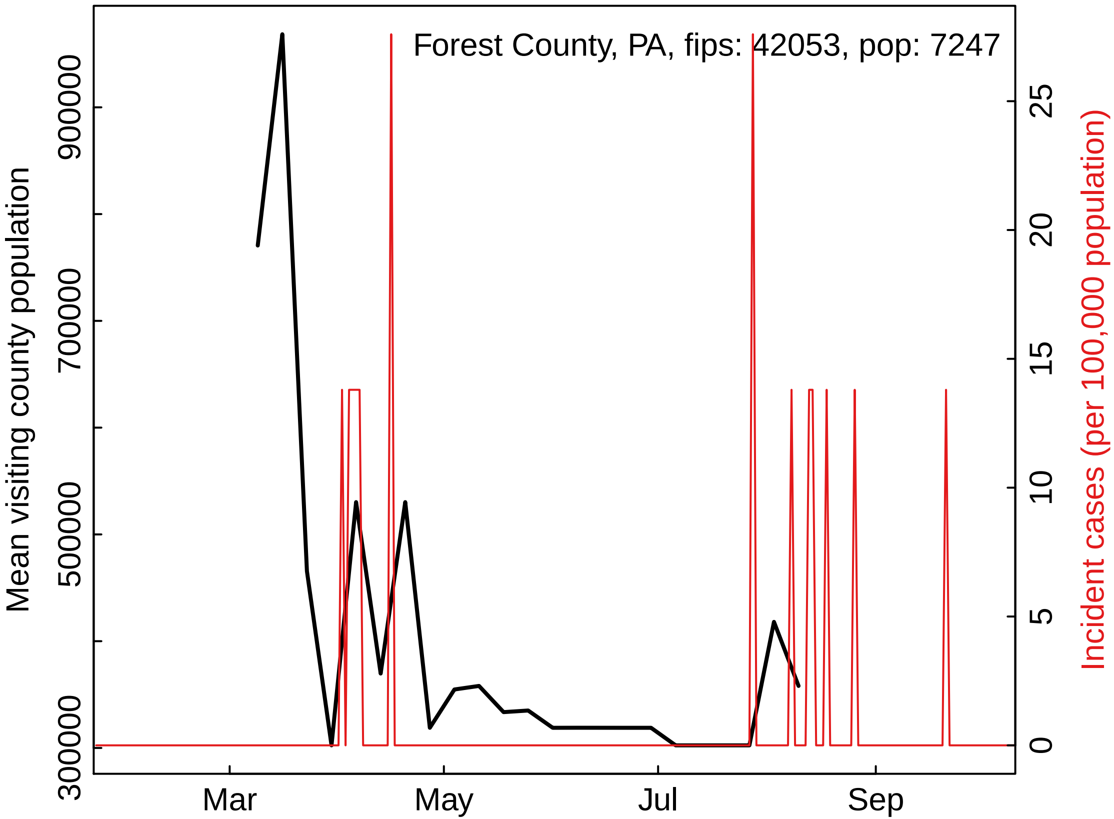
Forest County, PA, fips: 42053, pop: 7247
Mean visiting county population
300000
500000
700000
900000
Incident cases (per 100,000 population)
0
5
10
15
20
25
Mar
May
Jul
Sep

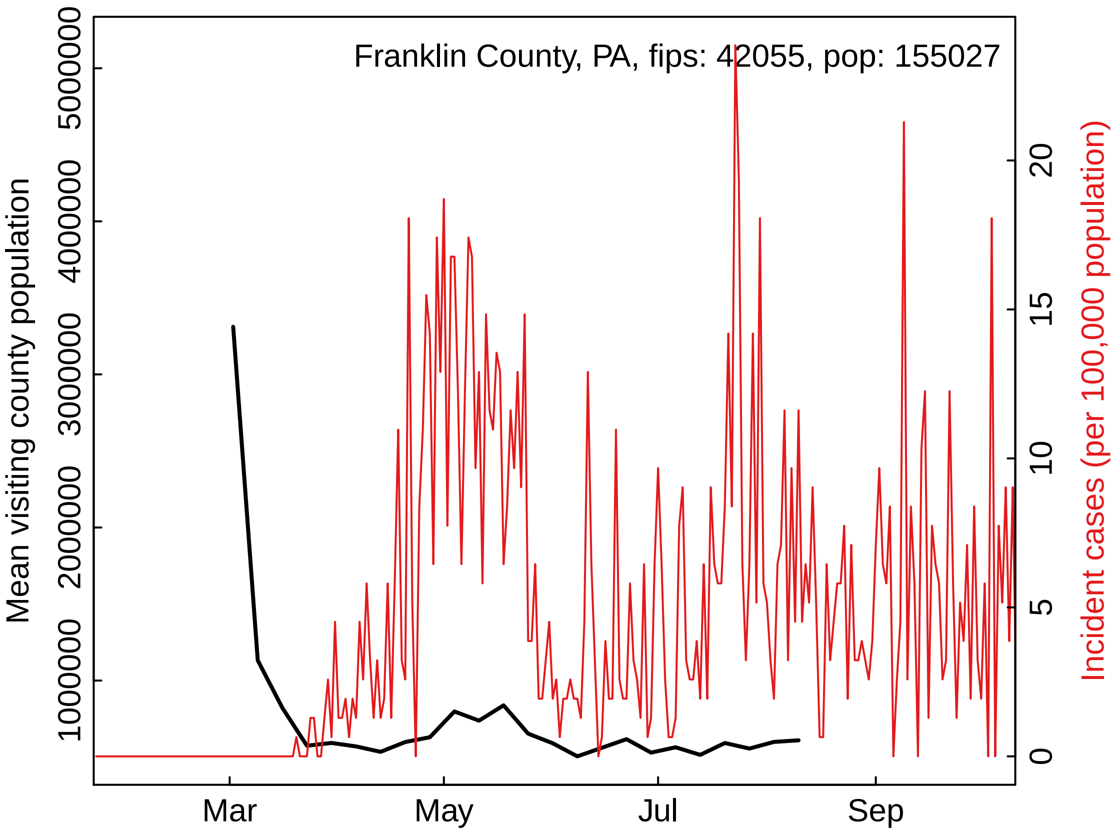

Franklin County, PA, fips: 42055, pop: 155027
Mean visiting county population
Incident cases (per 100,000 population)
1000000
2000000
3000000
4000000
5000000
0
5
10
15
20
Mar
May
Jul
Sep

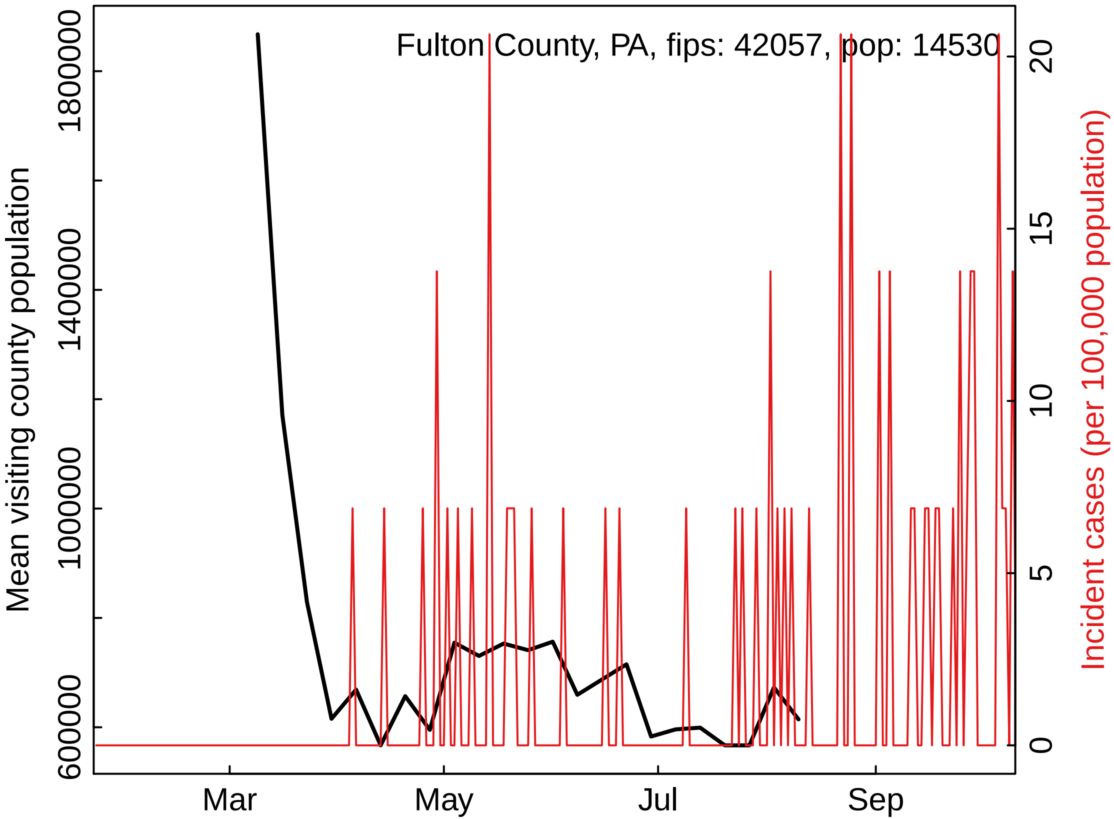

Fulton County, PA, fips: 42057, pop: 14530
Mean visiting county population
600000
1000000
1400000
1800000
Incident cases (per 100,000 population)
0
5
10
15
20
Mar
May
Jul
Sep

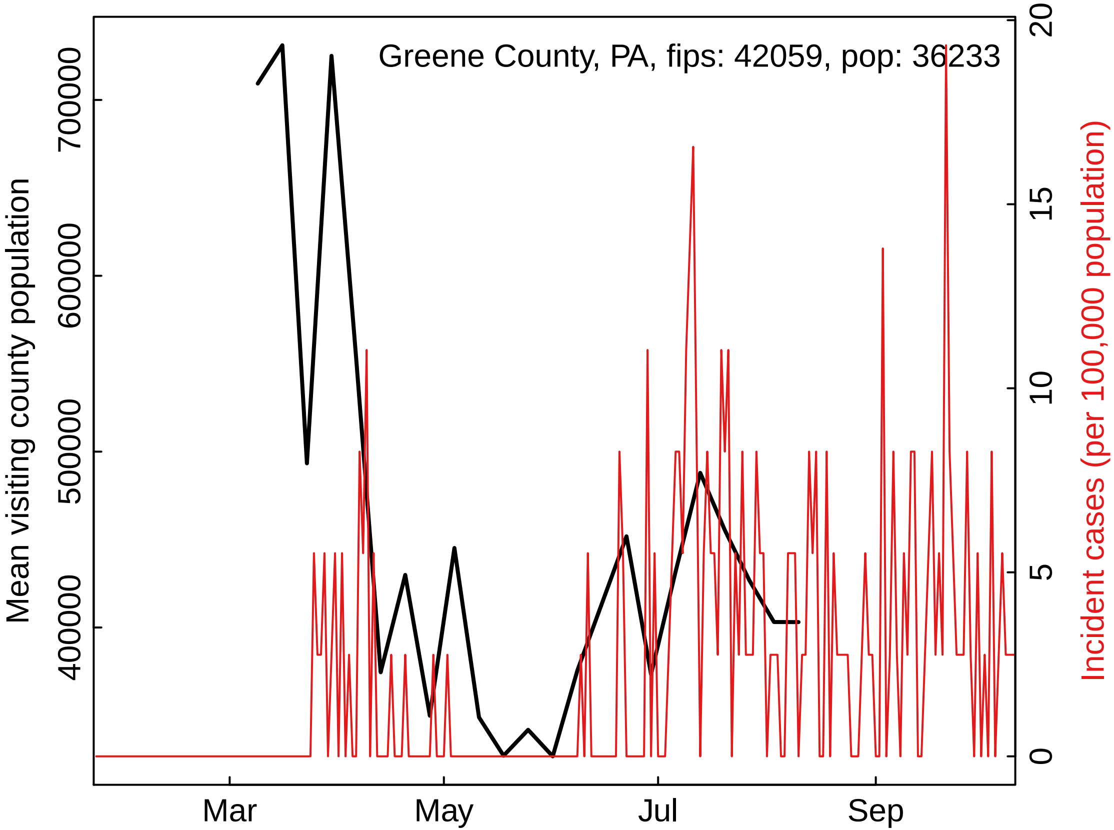

Greene County, PA, fips: 42059, pop: 36233
Mean visiting county population
400000
500000
600000
7000000
Incident cases (per 100,000 population)
0
5
10
15
20
Mar
May
Jul
Sep

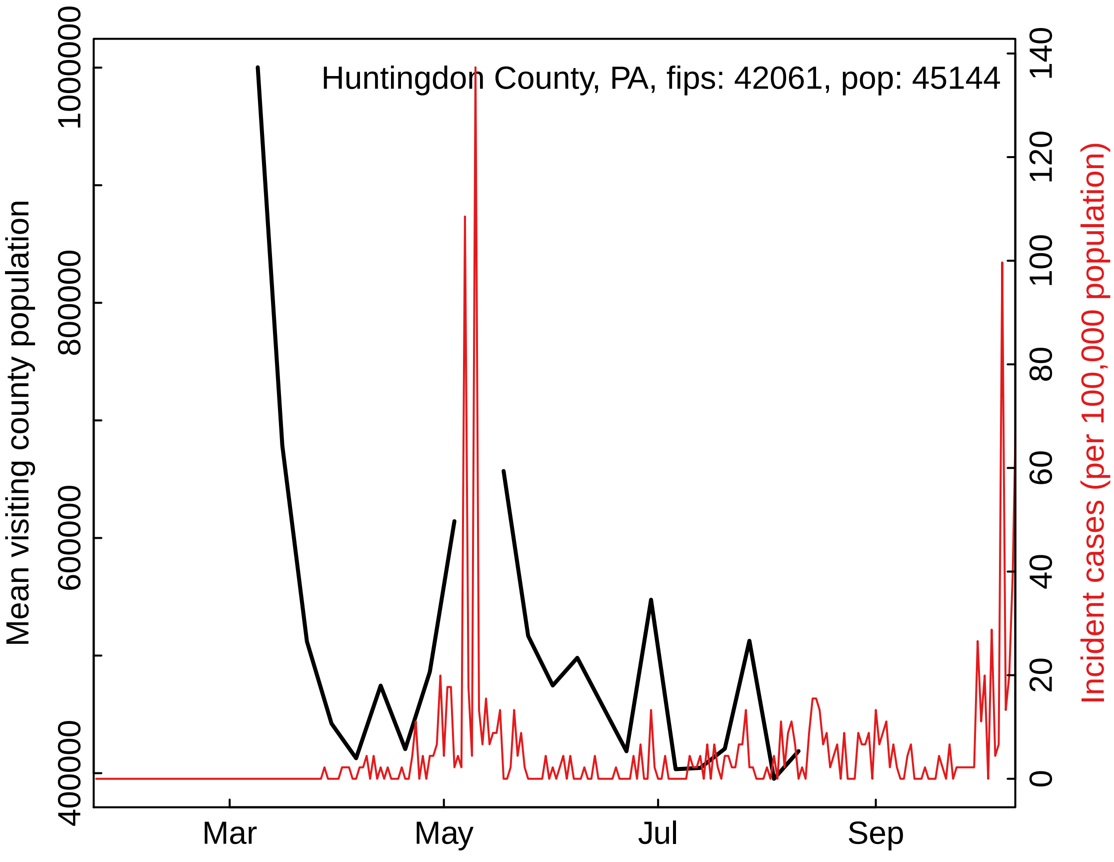

Huntingdon County, PA, fips: 42061, pop: 45144
Mean visiting county population
400000
600000
800000
1000000
Incident cases (per 100,000 population)
0
20
40
60
80
100
120
140
Mar
May
Jul
Sep

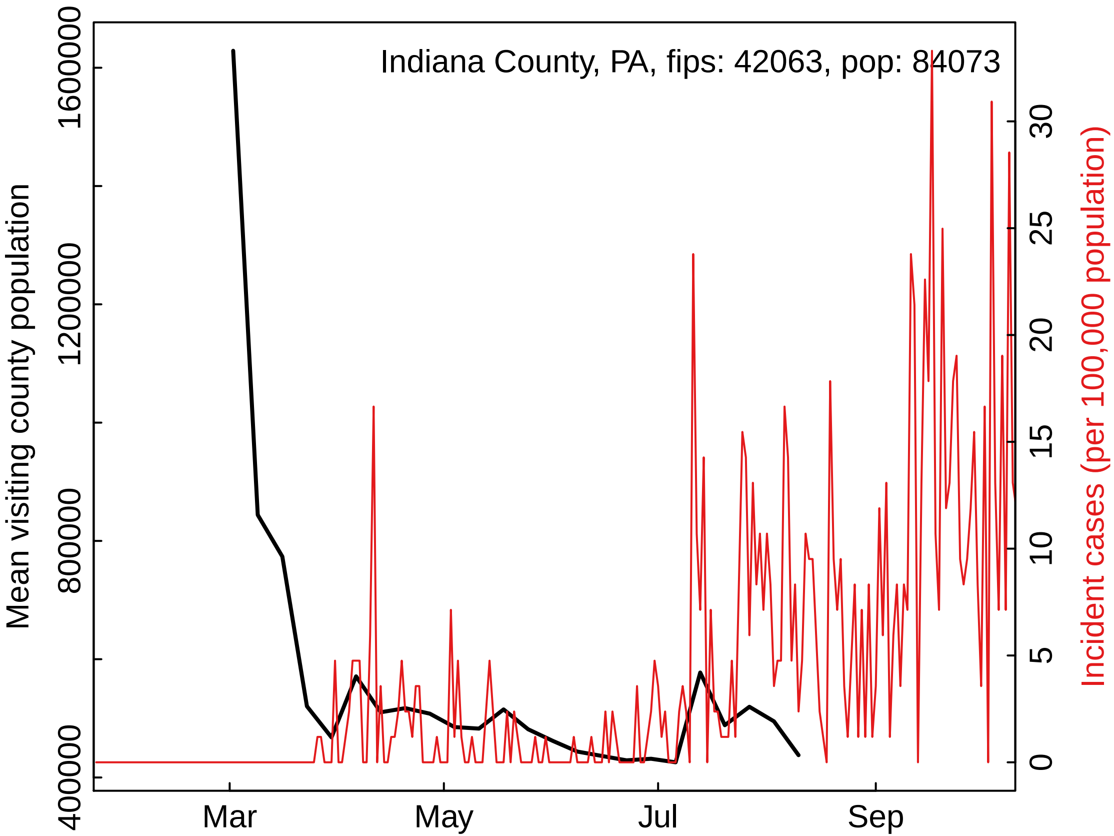
Indiana County, PA, fips: 42063, pop: 84073
Mean visiting county population
4000000
800000
1200000
1600000
Mar
May
Jul
Sep
0
5
10
15
20
25
30
Incident cases (per 100,000 population)

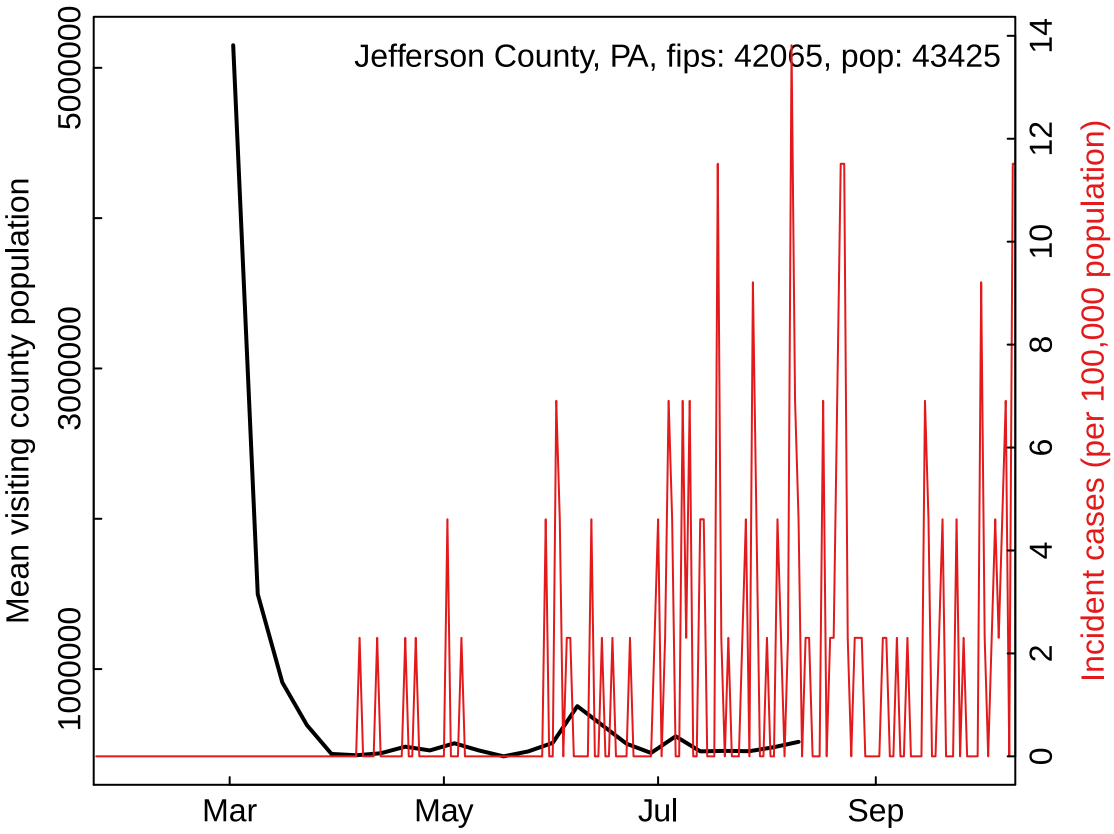

Jefferson County, PA, fips: 42065, pop: 43425
Mean visiting county population
Incident cases (per 100,000 population)
10000000
30000000
50000000
0
2
4
6
8
10
12
14
Mar
May
Jul
Sep

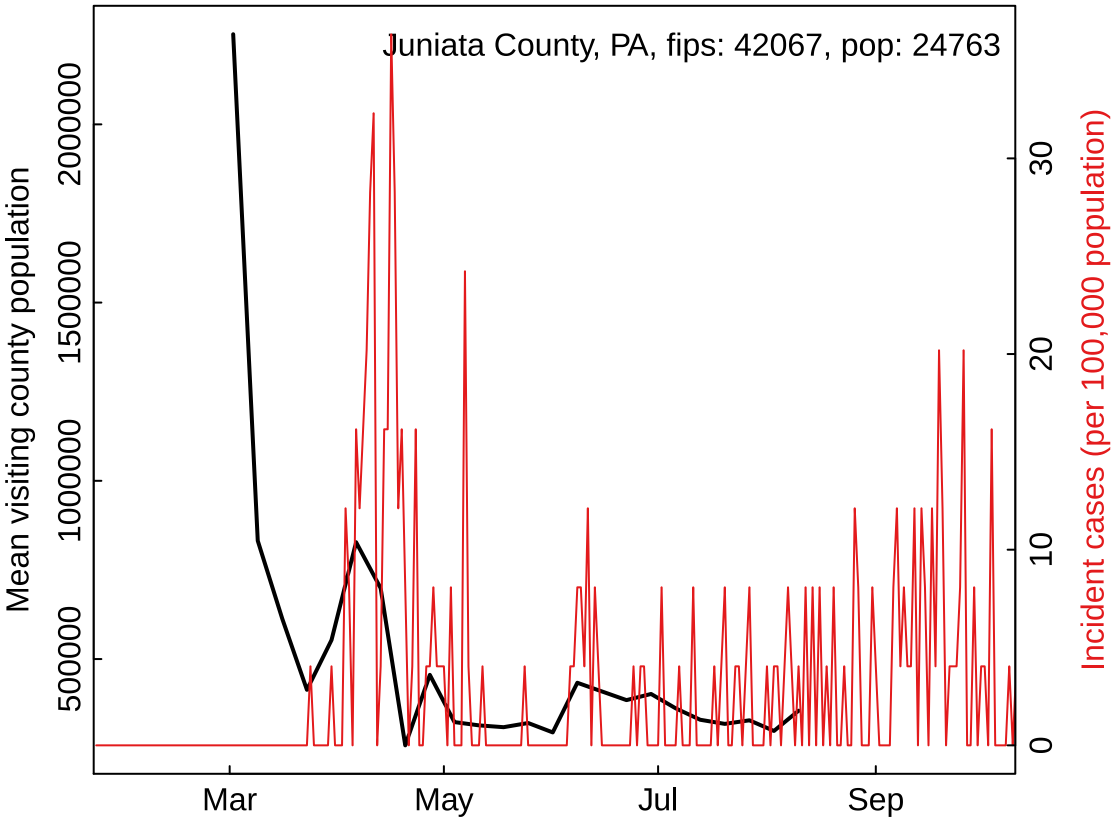

Juniata County, PA, fips: 42067, pop: 24763
Mean visiting county population
500000
1000000
1500000
2000000
Incident cases (per 100,000 population)
0
10
20
30
Mar
May
Jul
Sep

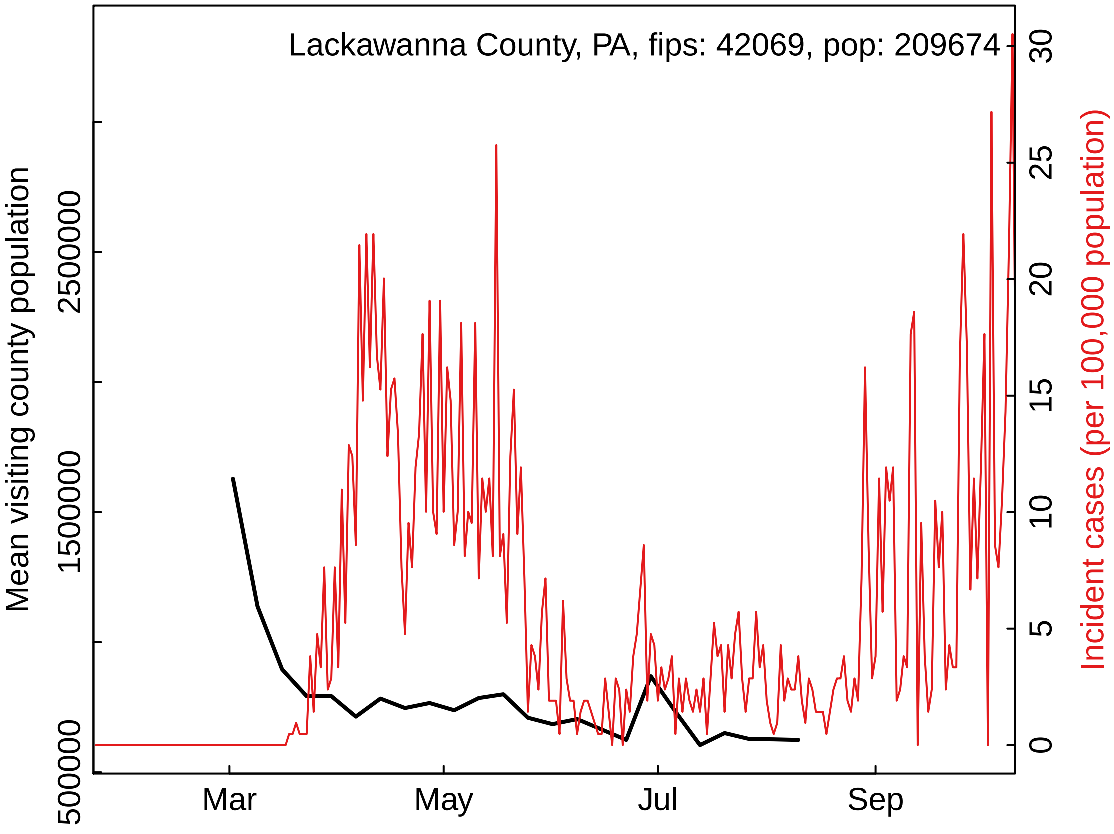

Lackawanna County, PA, fips: 42069, pop: 209674
Mean visiting county population
500000
1500000
2500000
Mar
May
Jul
Sep
Incident cases (per 100,000 population)
0
5
10
15
20
25
30

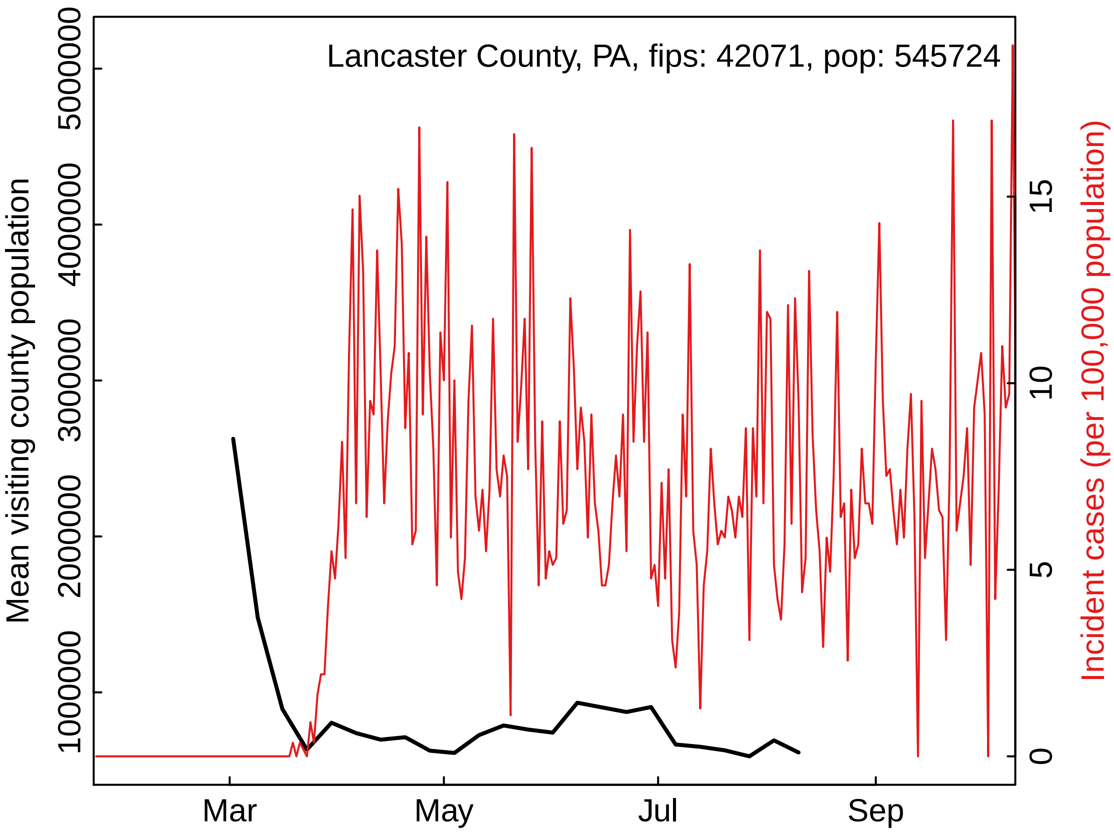
Lancaster County, PA, fips: 42071, pop: 545724
Mean visiting county population
Incident cases (per 100,000 population)
10000000
20000000
30000000
40000000
50000000
0
5
10
15
Mar
May
Jul
Sep

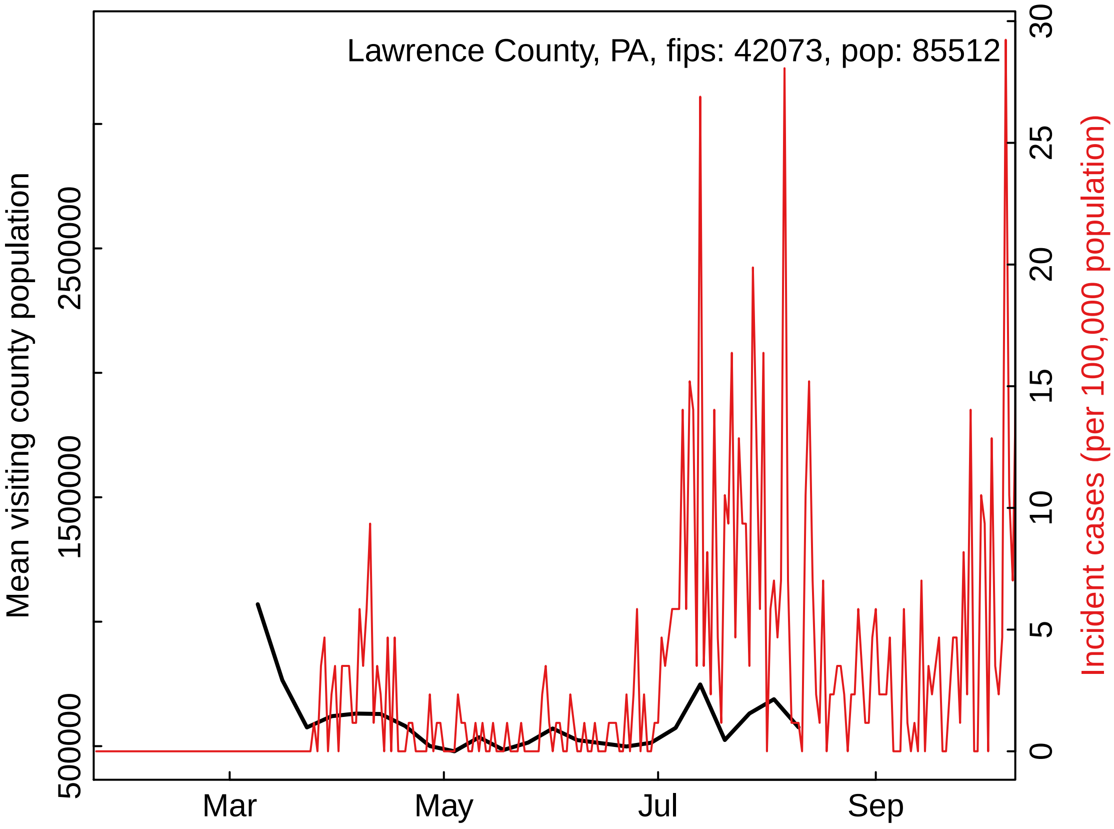

Lawrence County, PA, fips: 42073, pop: 85512
Mean visiting county population
500000
1500000
2500000
Incident cases (per 100,000 population)
0
5
10
15
20
25
30
Mar
May
Jul
Sep

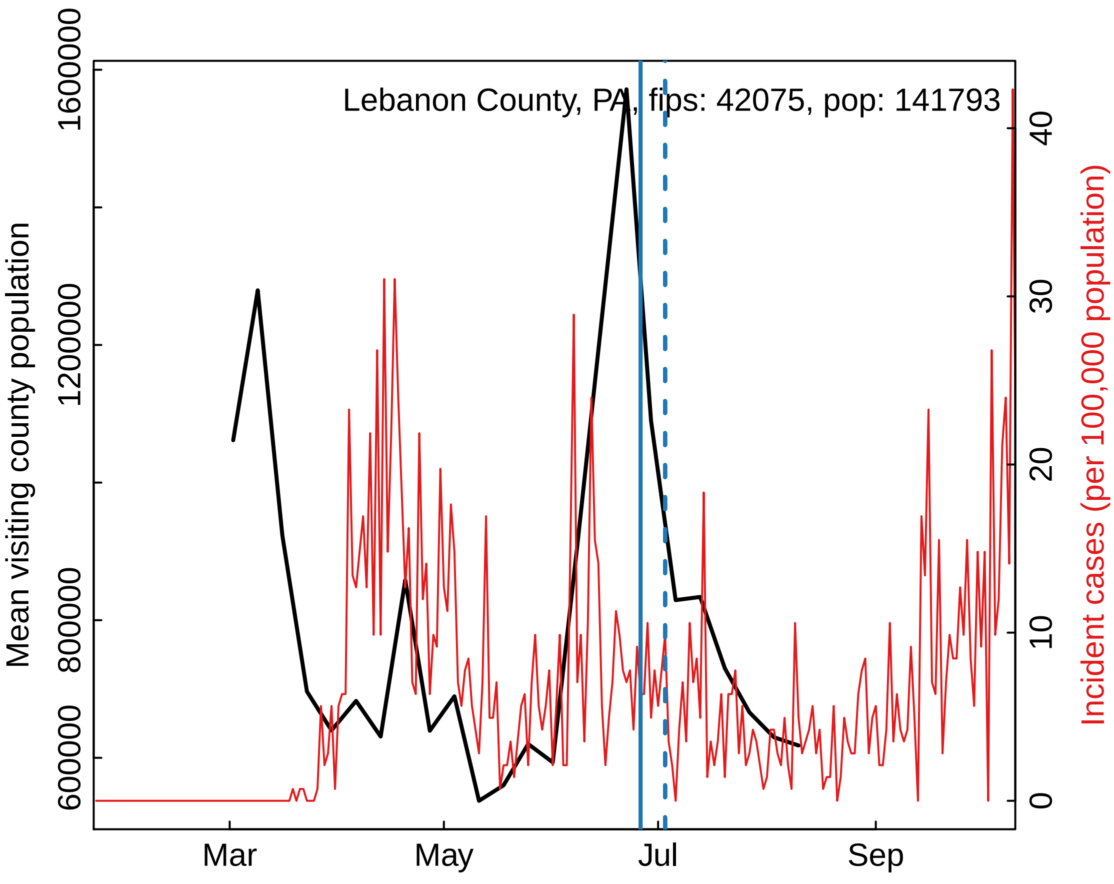

Lebanon County, PA, fips: 42075, pop: 141793
Mean visiting county population
600000
800000
1200000
1600000
Incident cases (per 100,000 population)
0
10
20
30
40
Mar
May
Jul
Sep

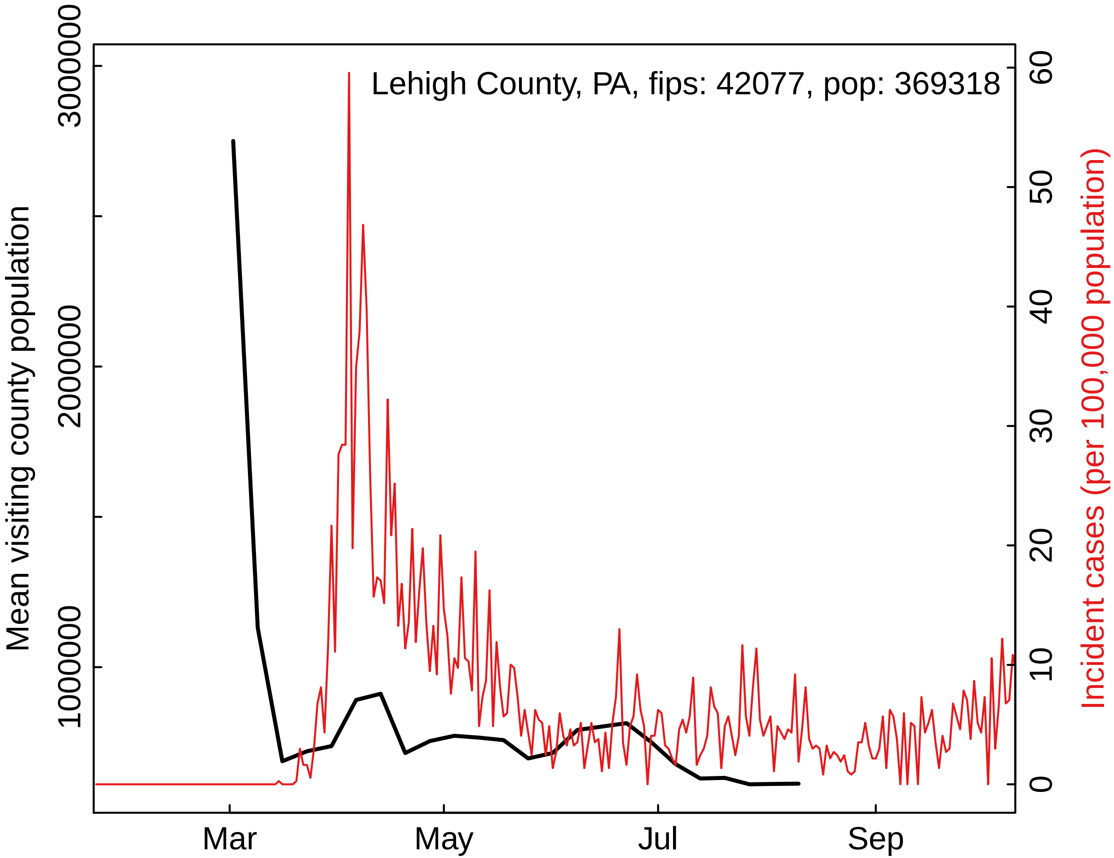

Lehigh County, PA, fips: 42077, pop: 369318
Mean visiting county population
1000000
2000000
3000000
Incident cases (per 100,000 population)
0
10
20
30
40
50
60
Mar
May
Jul
Sep

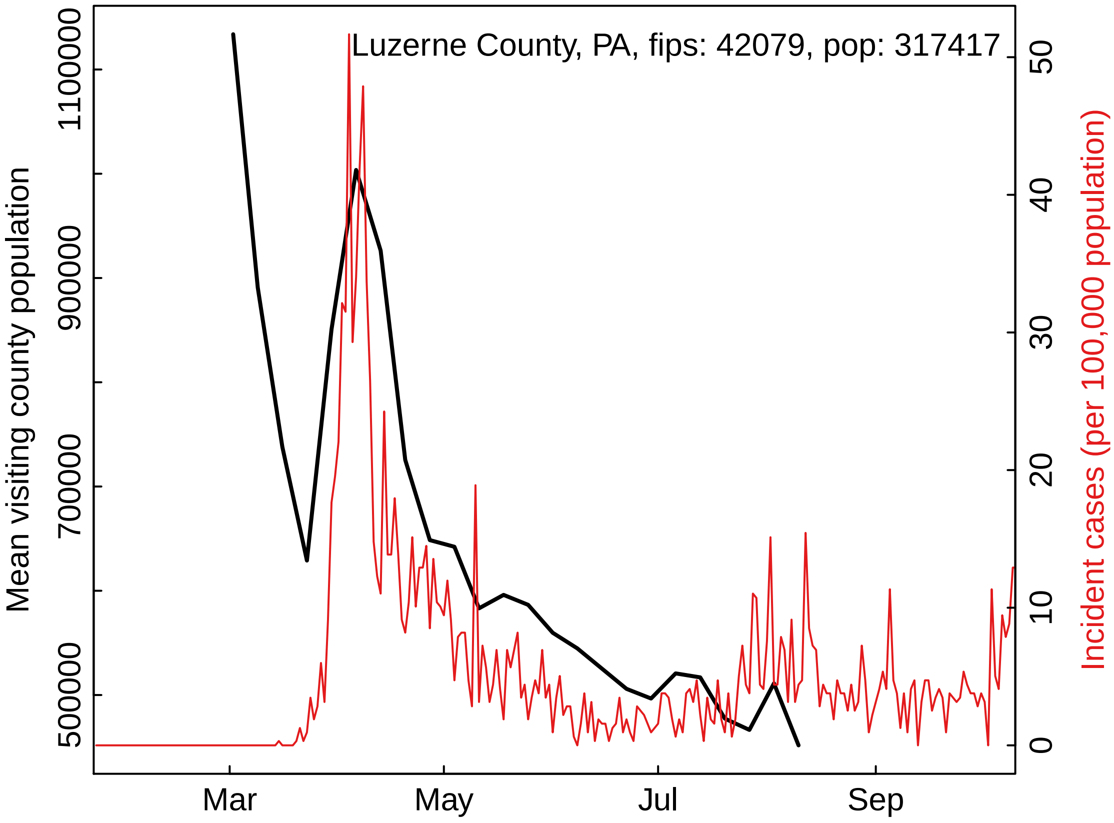
Luzerne County, PA, fips: 42079, pop: 317417
Mean visiting county population
500000
700000
900000
1100000
Incident cases (per 100,000 population)
0
10
20
30
40
50
Mar
May
Jul
Sep

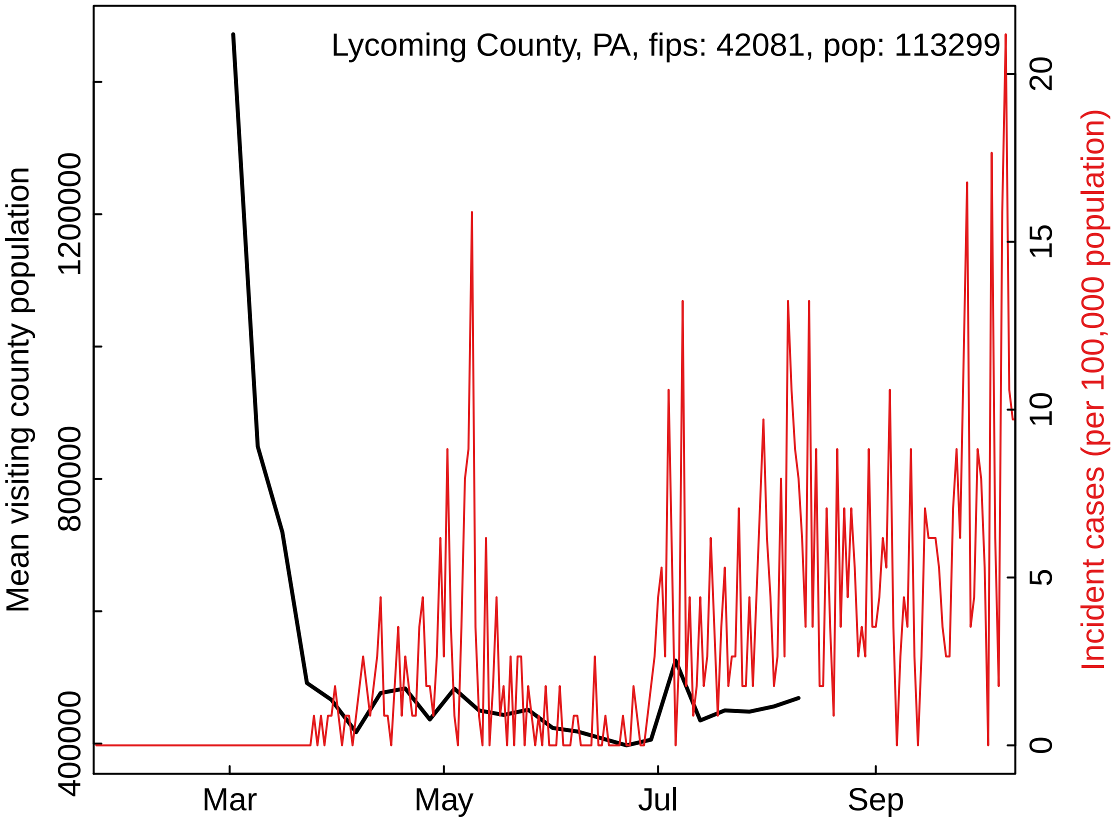

Lycoming County, PA, fips: 42081, pop: 113299
Mean visiting county population
400000
800000
1200000
Mar
May
Jul
Sep
0
5
10
15
20
Incident cases (per 100,000 population)

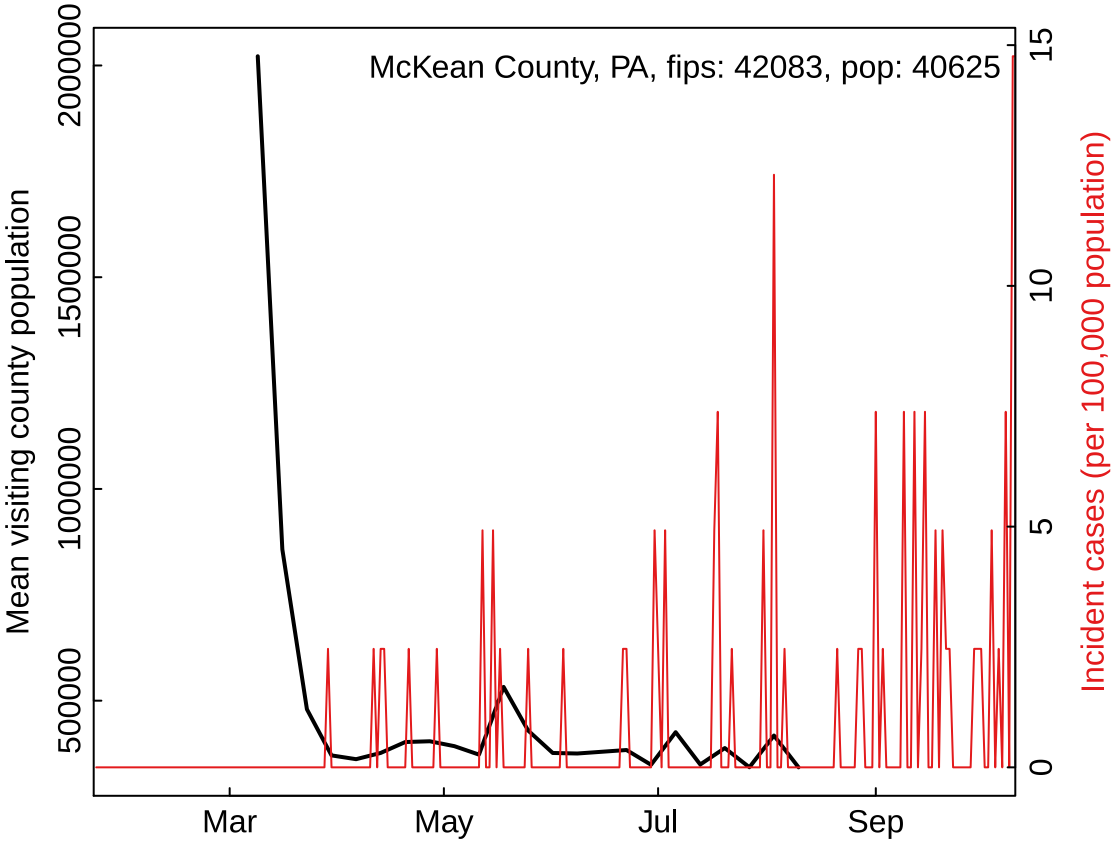

McKean County, PA, fips: 42083, pop: 40625
Mean visiting county population
500000
1000000
1500000
2000000
Incident cases (per 100,000 population)
0
5
10
15
Mar
May
Jul
Sep

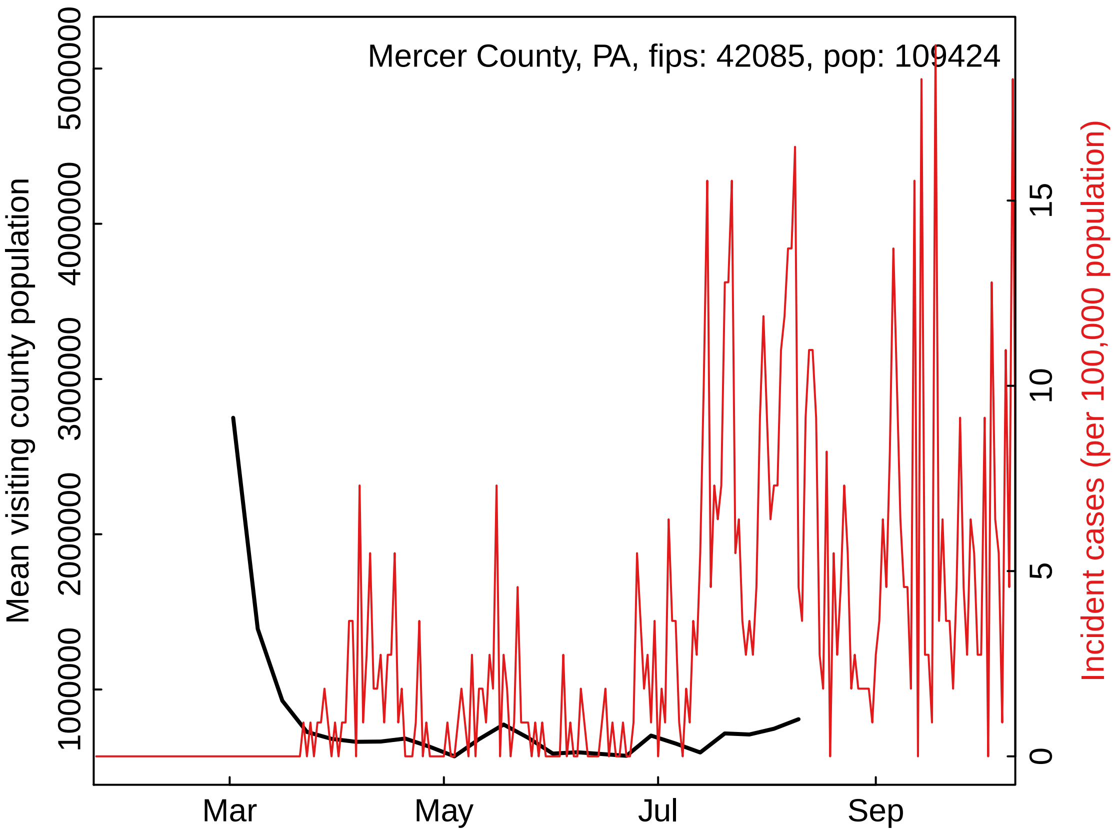

Mercer County, PA, fips: 42085, pop: 109424
Mean visiting county population
1000000
2000000
3000000
4000000
5000000
Incident cases (per 100,000 population)
0
5
10
15
Mar
May
Jul
Sep

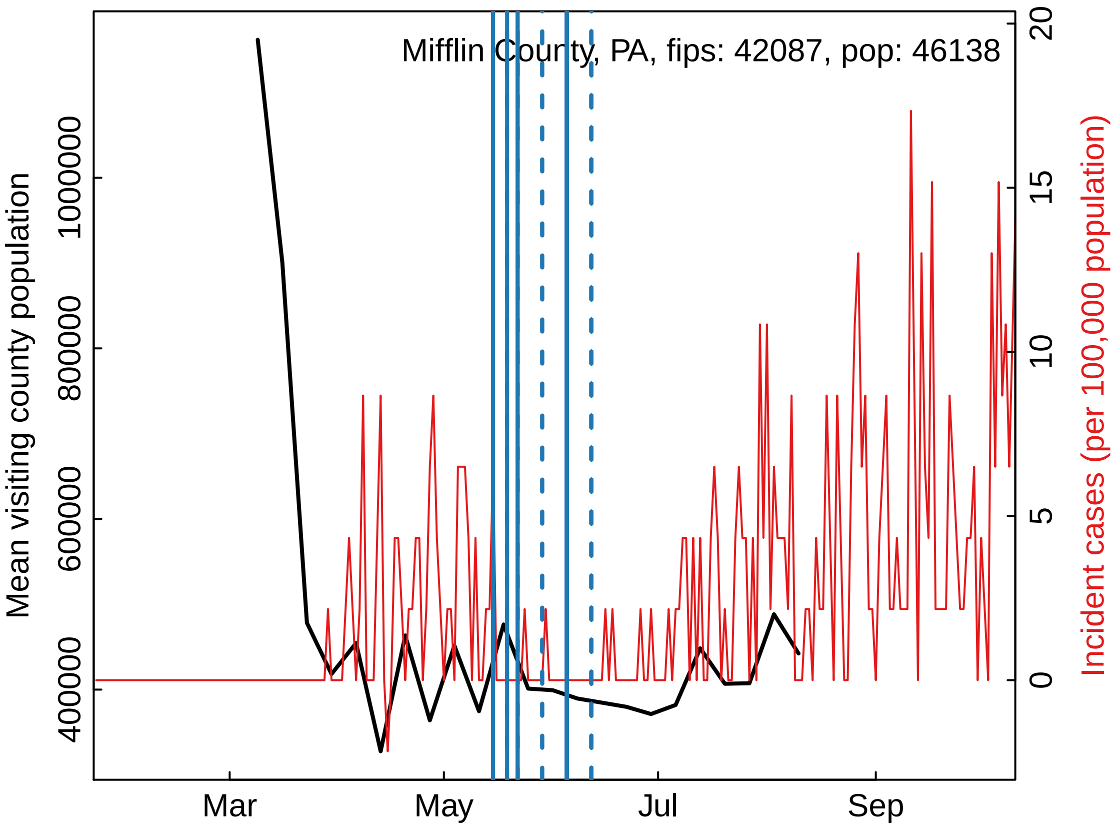

Mifflin County, PA, fips: 42087, pop: 46138
Mean visiting county population
400000
600000
800000
1000000
Incident cases (per 100,000 population)
0
5
10
15
20
Mar
May
Jul
Sep

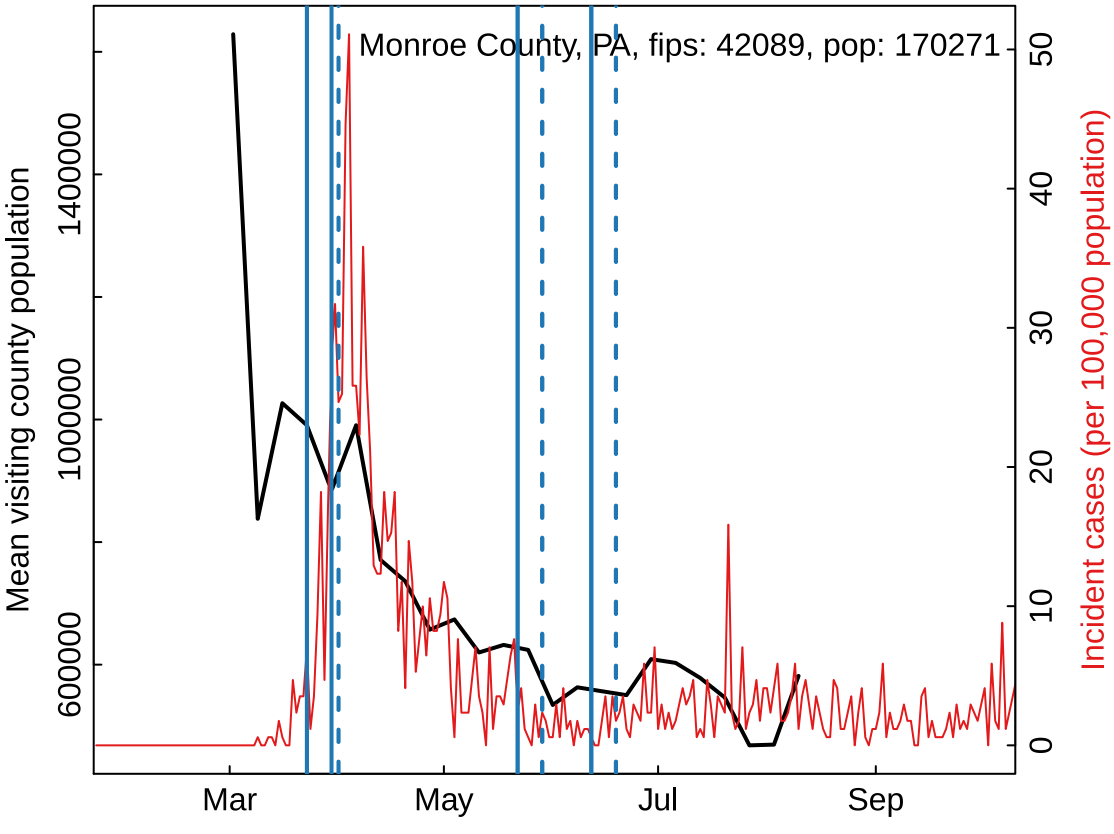

Monroe County, PA, fips: 42089, pop: 170271
Mean visiting county population
600000
1000000
1400000
Mar
May
Jul
Sep
0
10
20
30
40
50
Incident cases (per 100,000 population)

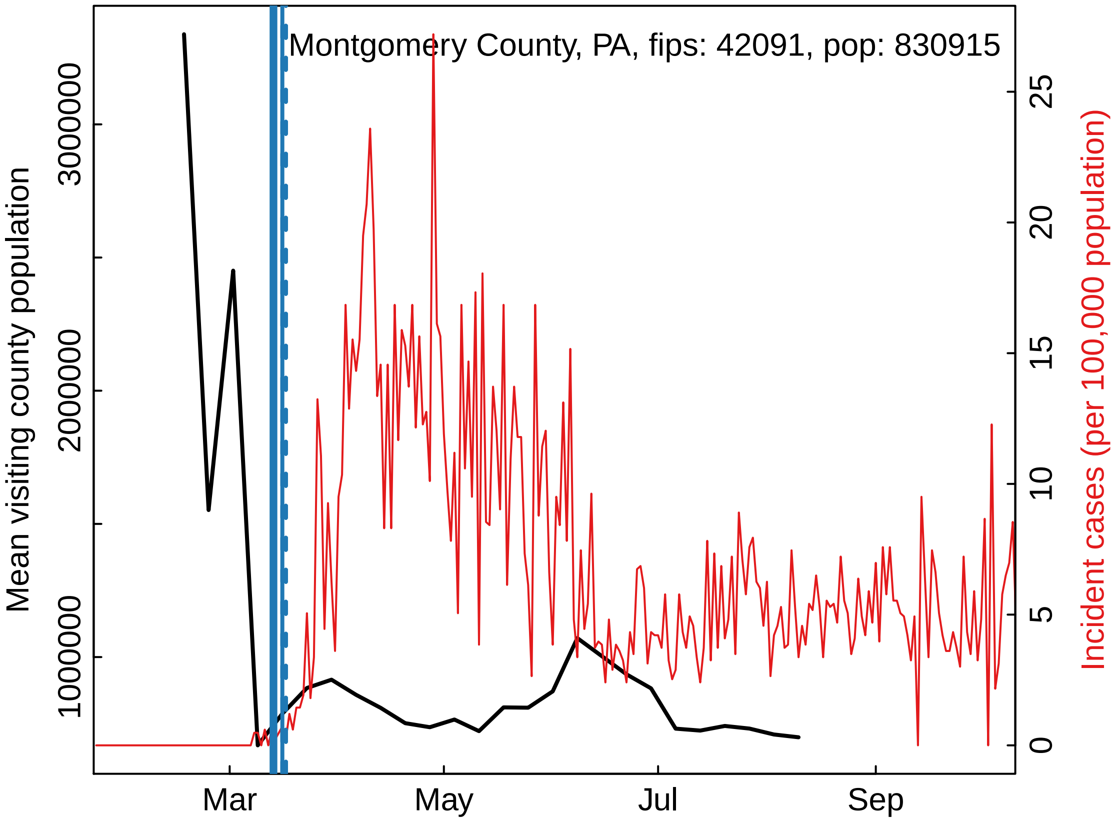

Montgomery County, PA, fips: 42091, pop: 830915
Mean visiting county population
1000000
2000000
3000000
Mar
May
Jul
Sep
Incident cases (per 100,000 population)
0
5
10
15
20
25

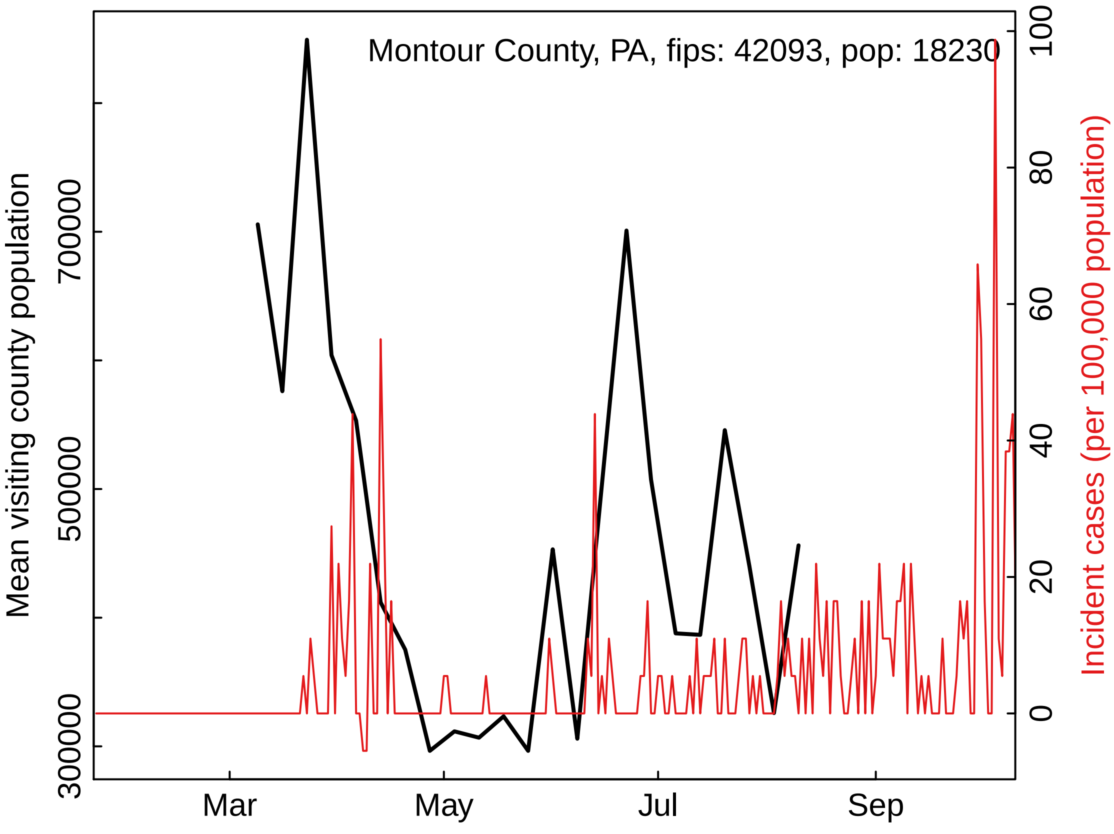

Montour County, PA, fips: 42093, pop: 18230
Mean visiting county population
300000
500000
700000
Incident cases (per 100,000 population)
0
20
40
60
80
100
Mar
May
Jul
Sep

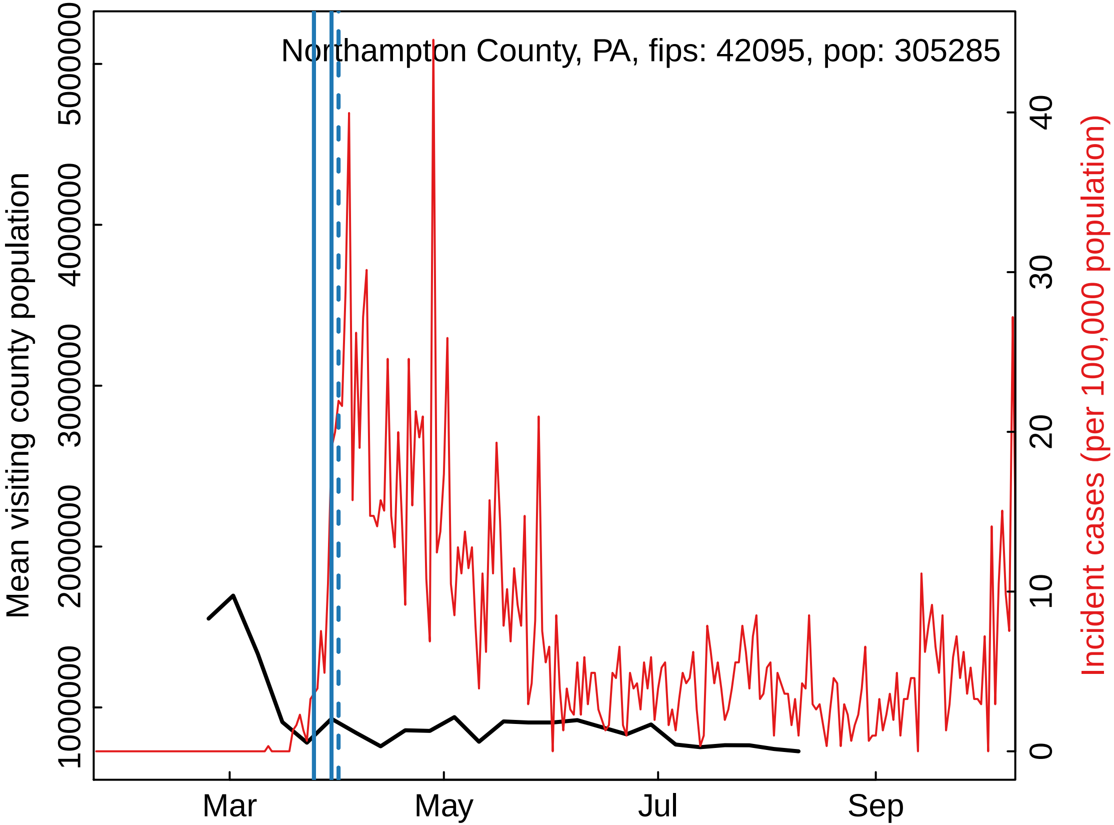

Northampton County, PA, fips: 42095, pop: 305285
Mean visiting county population
10000000
20000000
30000000
40000000
50000000
Mar
May
Jul
Sep
0
10
20
30
40
Incident cases (per 100,000 population)

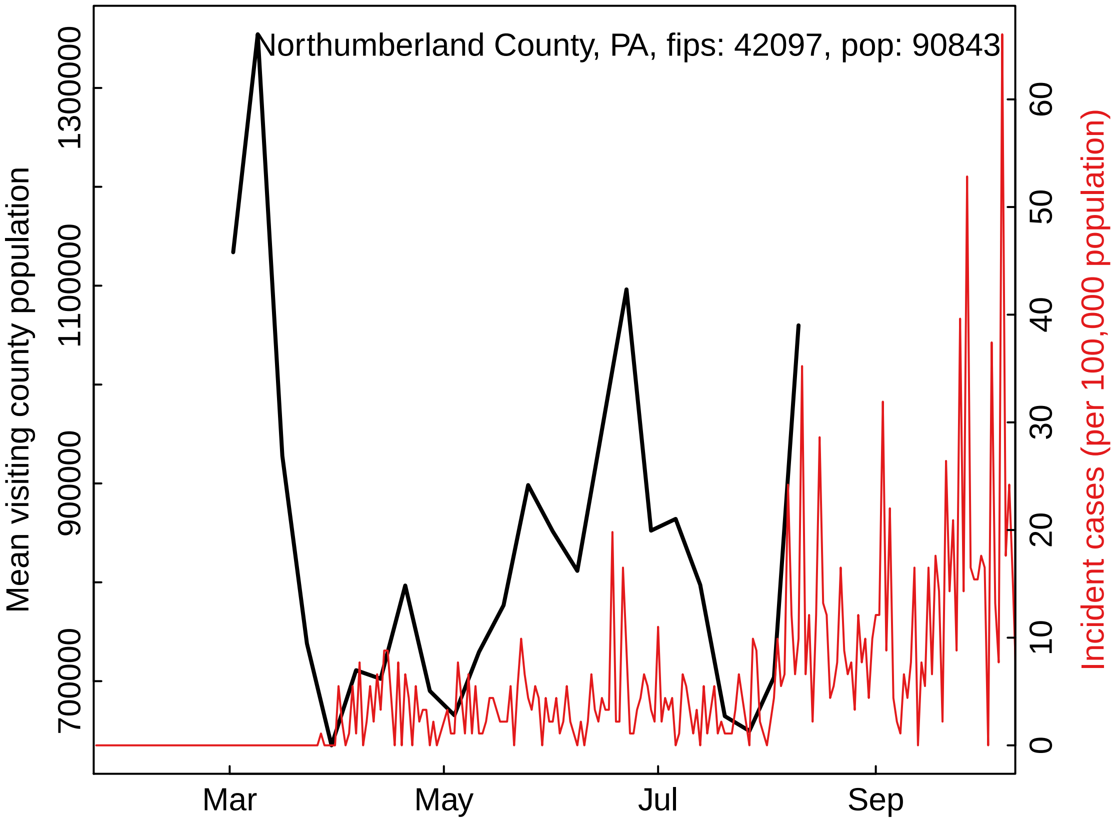

Northumberland County, PA, fips: 42097, pop: 90843
Mean visiting county population
700000
900000
1100000
1300000
Incident cases (per 100,000 population)
0
10
20
30
40
50
60
Mar
May
Jul
Sep

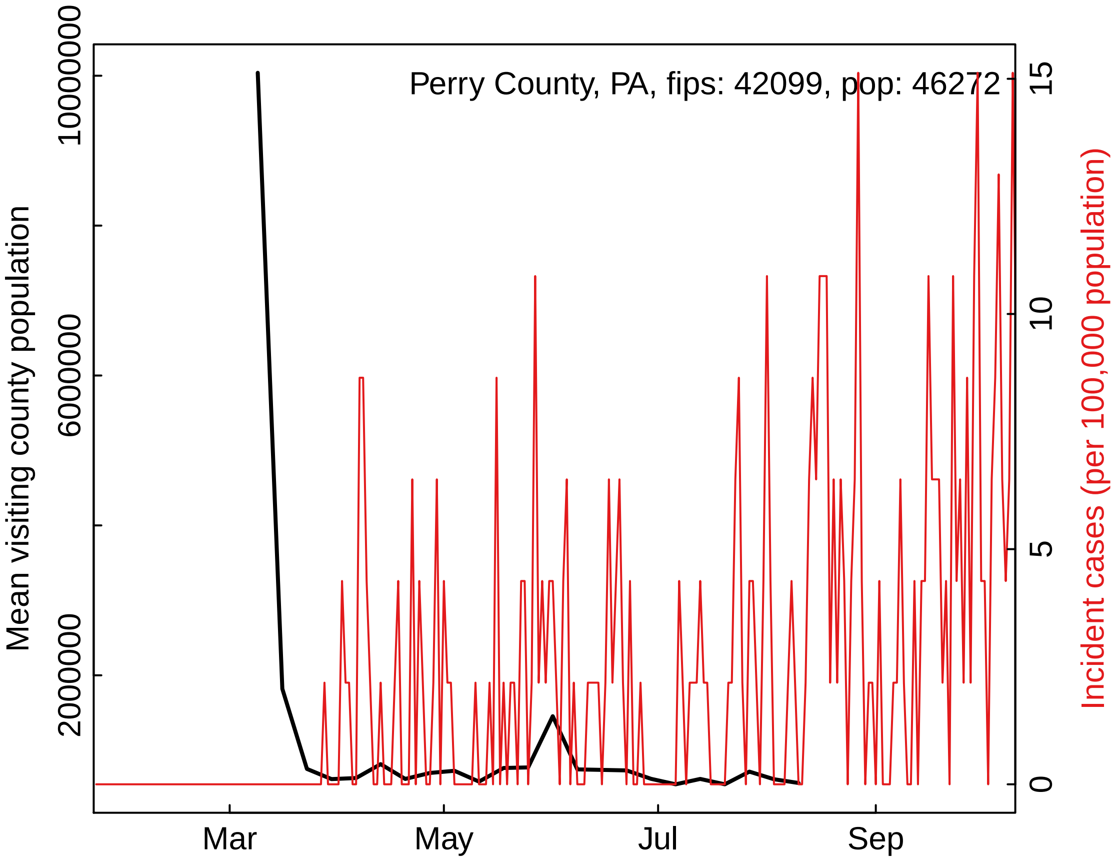
Perry County, PA, fips: 42099, pop: 46272
Mean visiting county population
2000000
6000000
10000000
Incident cases (per 100,000 population)
0
5
10
15
Mar
May
Jul
Sep

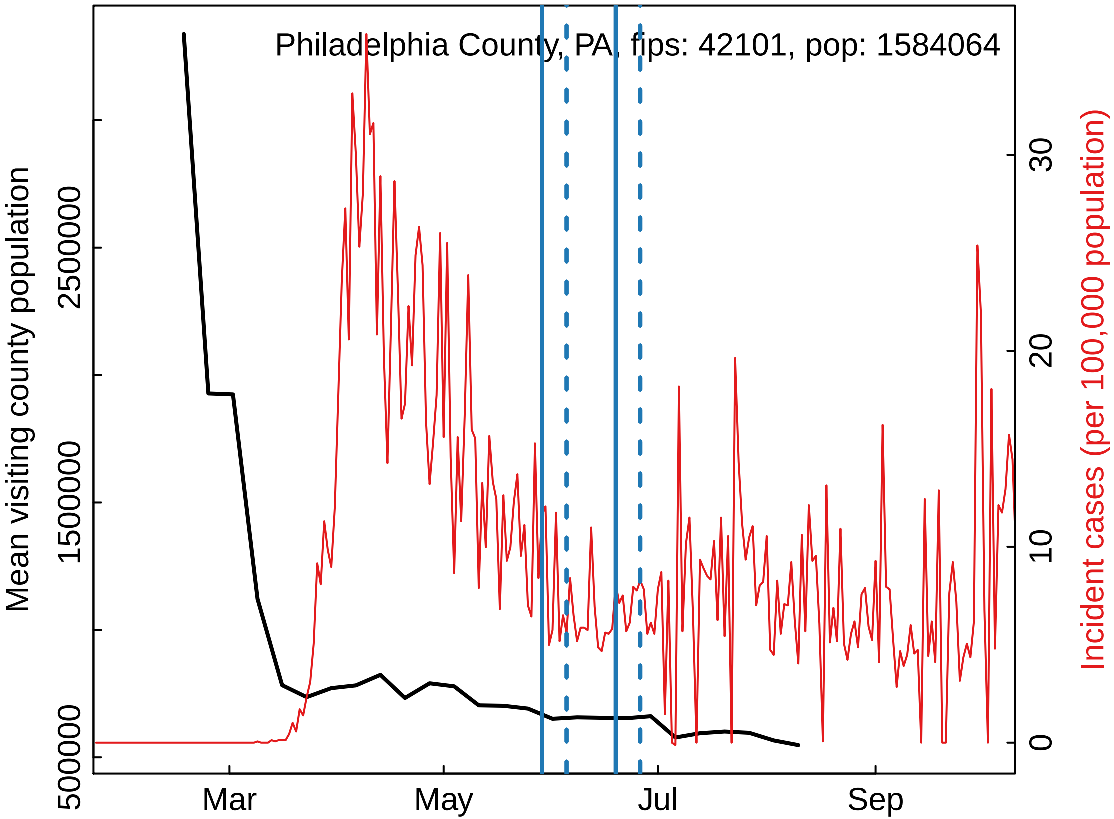
Philadelphia County, PA, fips: 42101, pop: 1584064
Mean visiting county population
Incident cases (per 100,000 population)
500000
1500000
2500000
0
10
20
30
Mar
May
Jul
Sep

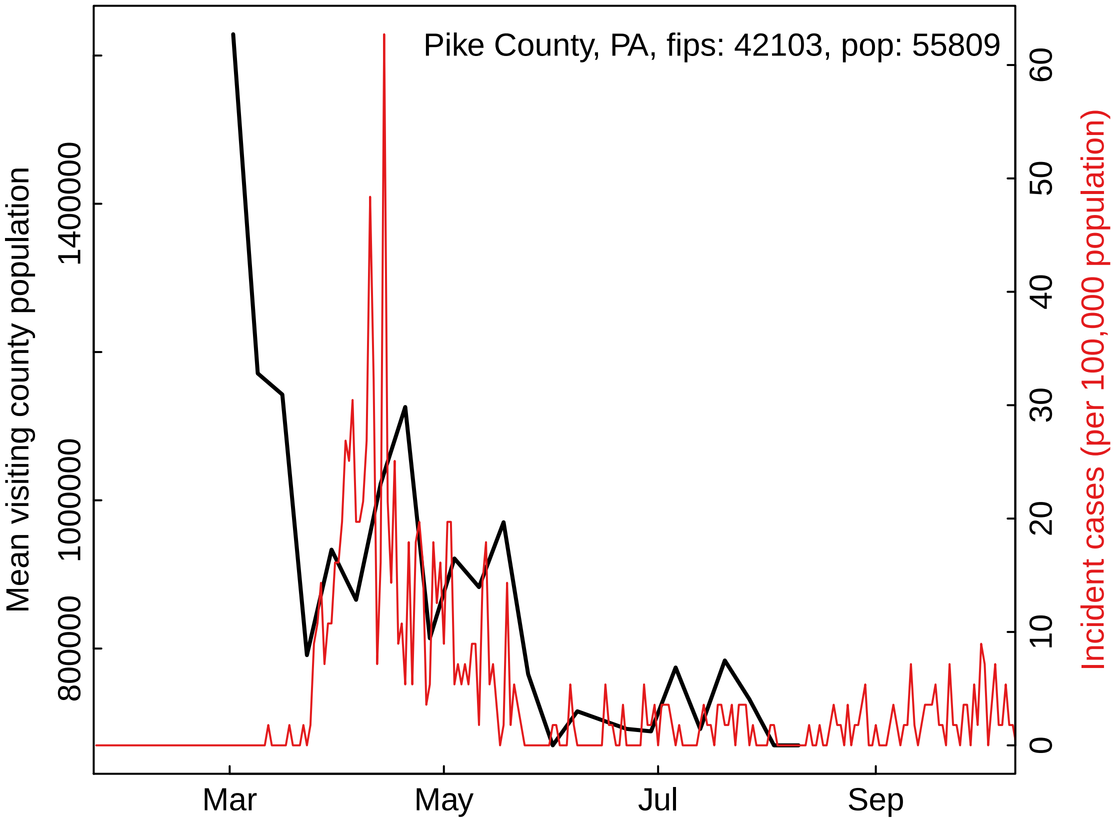

Pike County, PA, fips: 42103, pop: 55809
Mean visiting county population
800000
1000000
1400000
Mar
May
Jul
Sep
0
10
20
30
40
50
60
Incident cases (per 100,000 population)

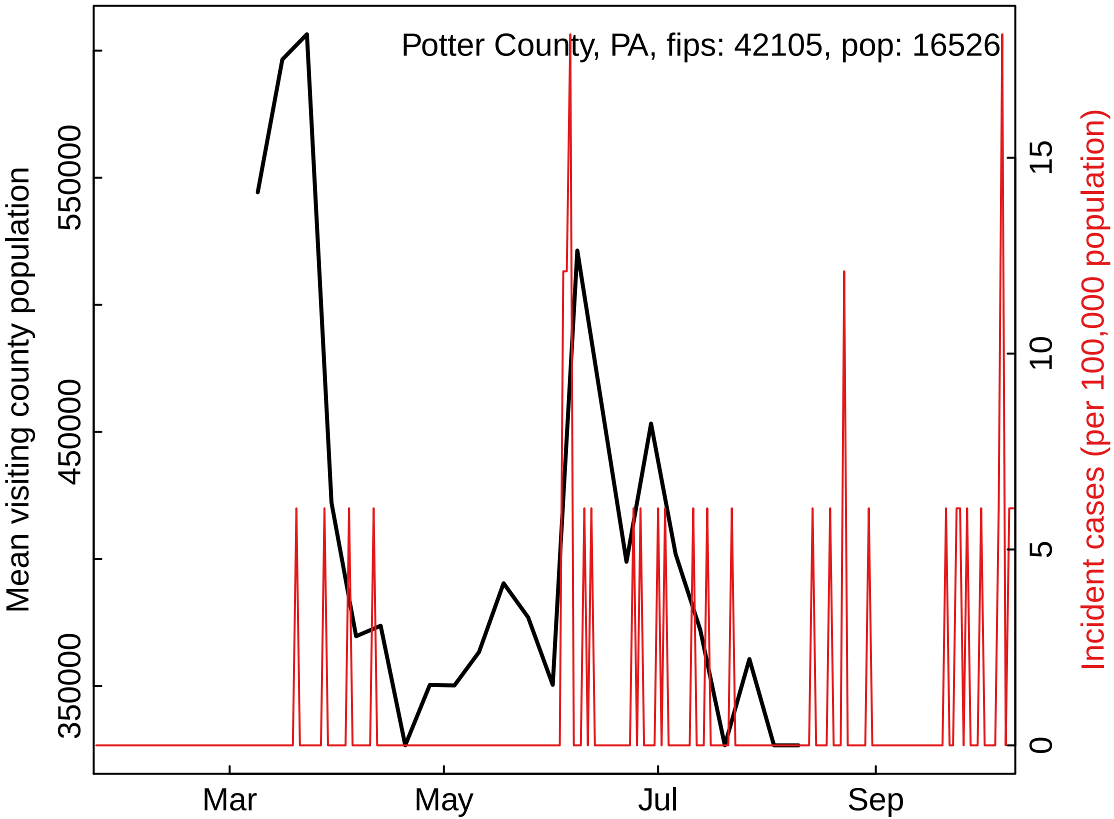
Potter County, PA, fips: 42105, pop: 16526
Mean visiting county population
350000
450000
550000
Incident cases (per 100,000 population)
0
5
10
15
Mar
May
Jul
Sep

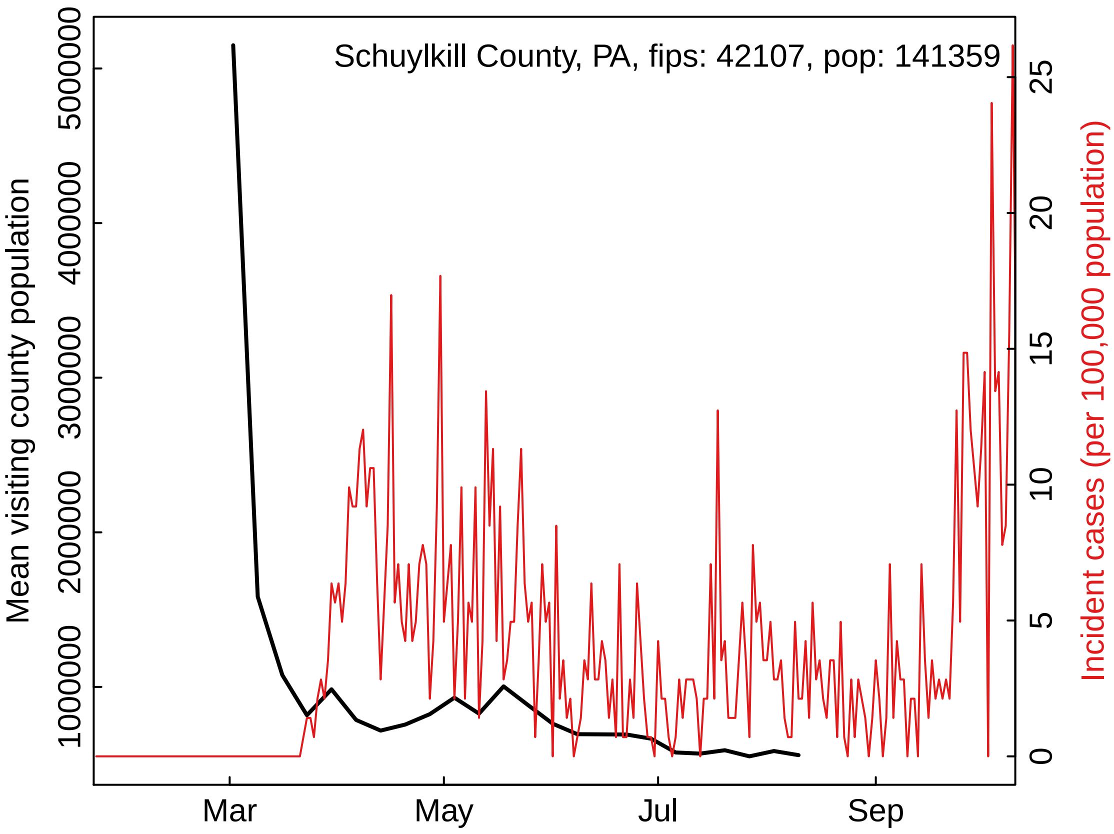
Schuylkill County, PA, fips: 42107, pop: 141359
Mean visiting county population
Incident cases (per 100,000 population)
10000000
20000000
30000000
40000000
50000000
0
5
10
15
20
25
Mar
May
Jul
Sep

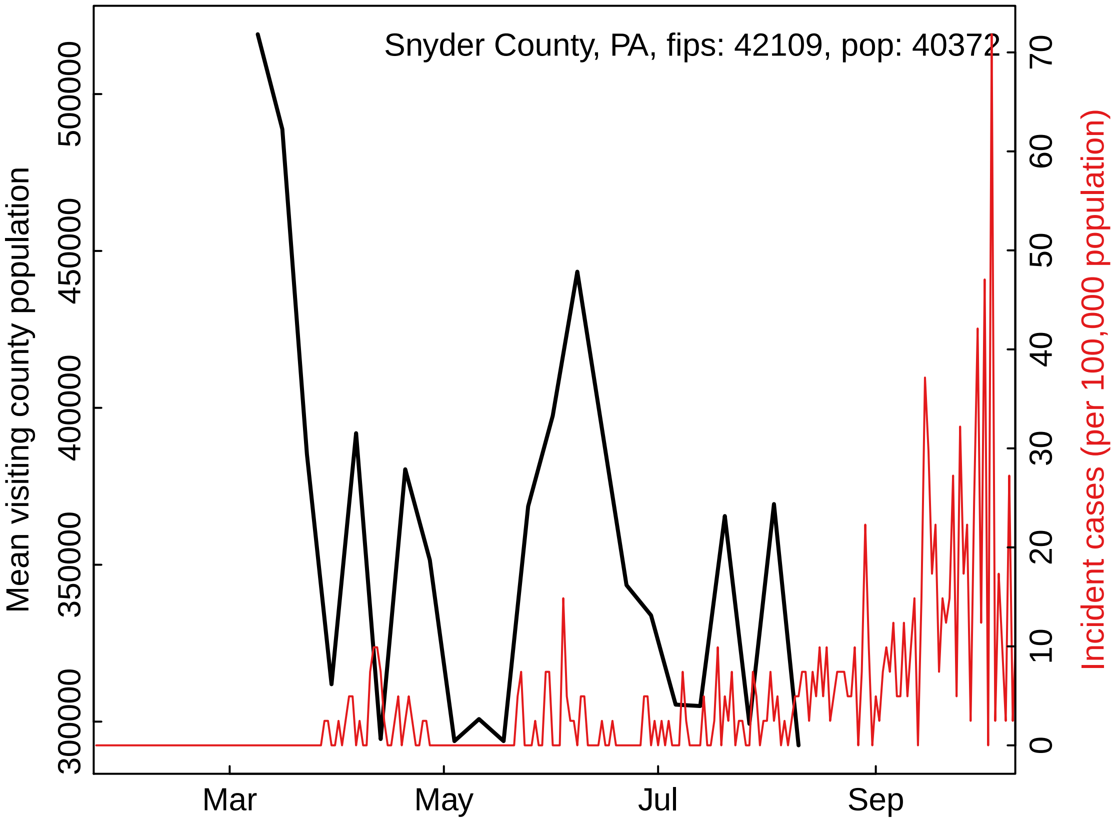
Snyder County, PA, fips: 42109, pop: 40372
Mean visiting county population
300000
350000
400000
450000
500000
Incident cases (per 100,000 population)
0
10
20
30
40
50
60
70
Mar
May
Jul
Sep

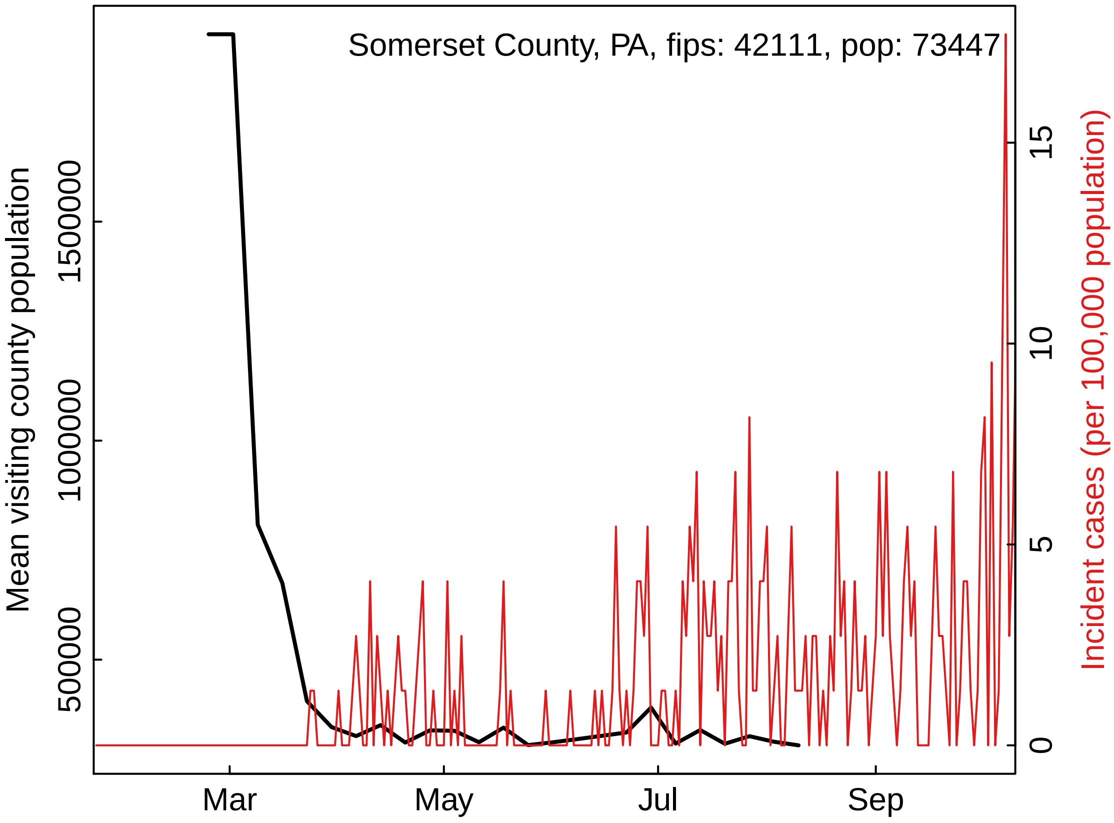

Somerset County, PA, fips: 42111, pop: 73447
Mean visiting county population
500000
1000000
1500000
Incident cases (per 100,000 population)
0
5
10
15
Mar
May
Jul
Sep

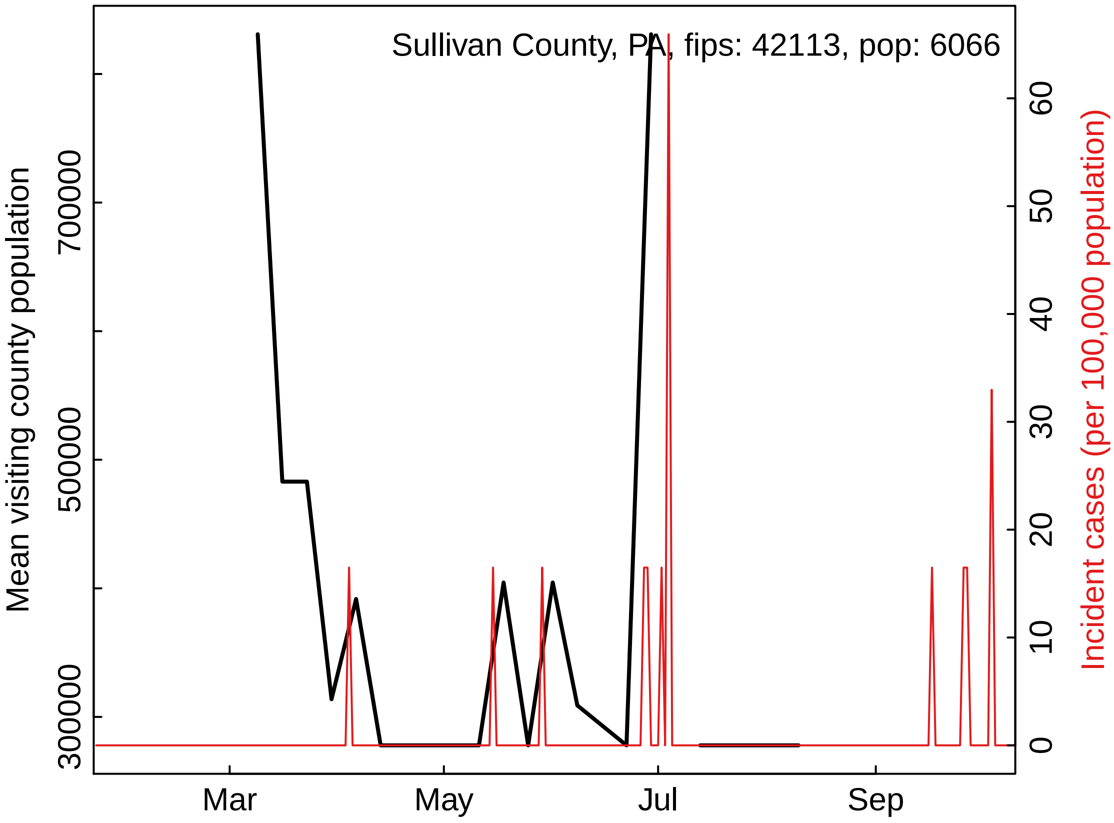

Sullivan County, PA, fips: 42113, pop: 6066
Mean visiting county population
300000
500000
700000
Incident cases (per 100,000 population)
0
10
20
30
40
50
60
Mar
May
Jul
Sep

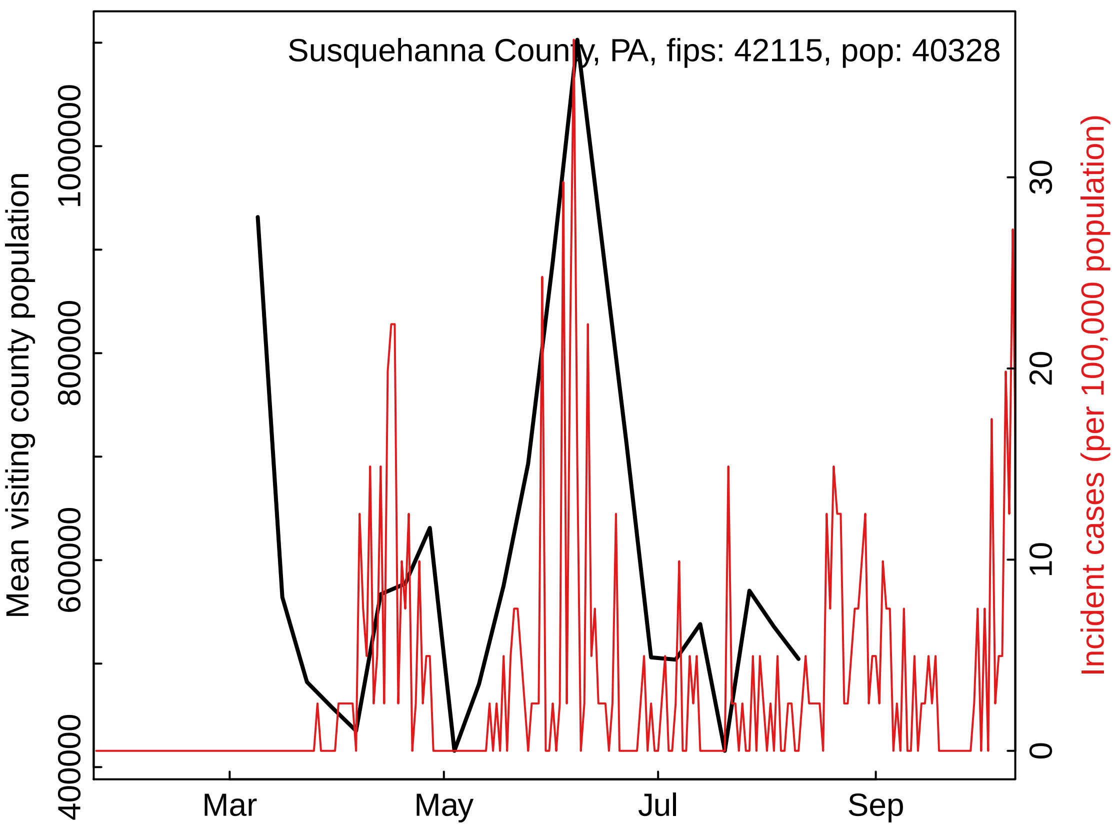
Susquehanna County, PA, fips: 42115, pop: 40328
Mean visiting county population
400000
600000
800000
1000000
Incident cases (per 100,000 population)
0
10
20
30
Mar
May
Jul
Sep

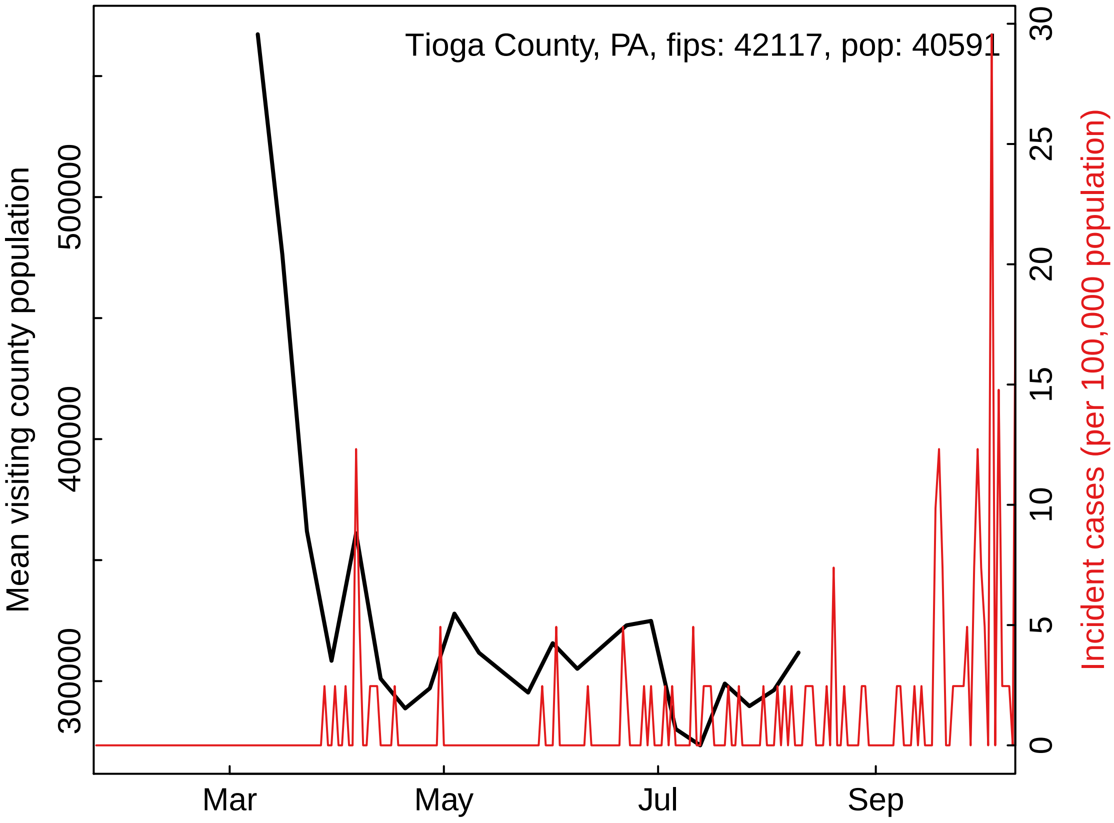

Tioga County, PA, fips: 42117, pop: 40591
Mean visiting county population
300000
400000
500000
Incident cases (per 100,000 population)
0
5
10
15
20
25
30
Mar
May
Jul
Sep

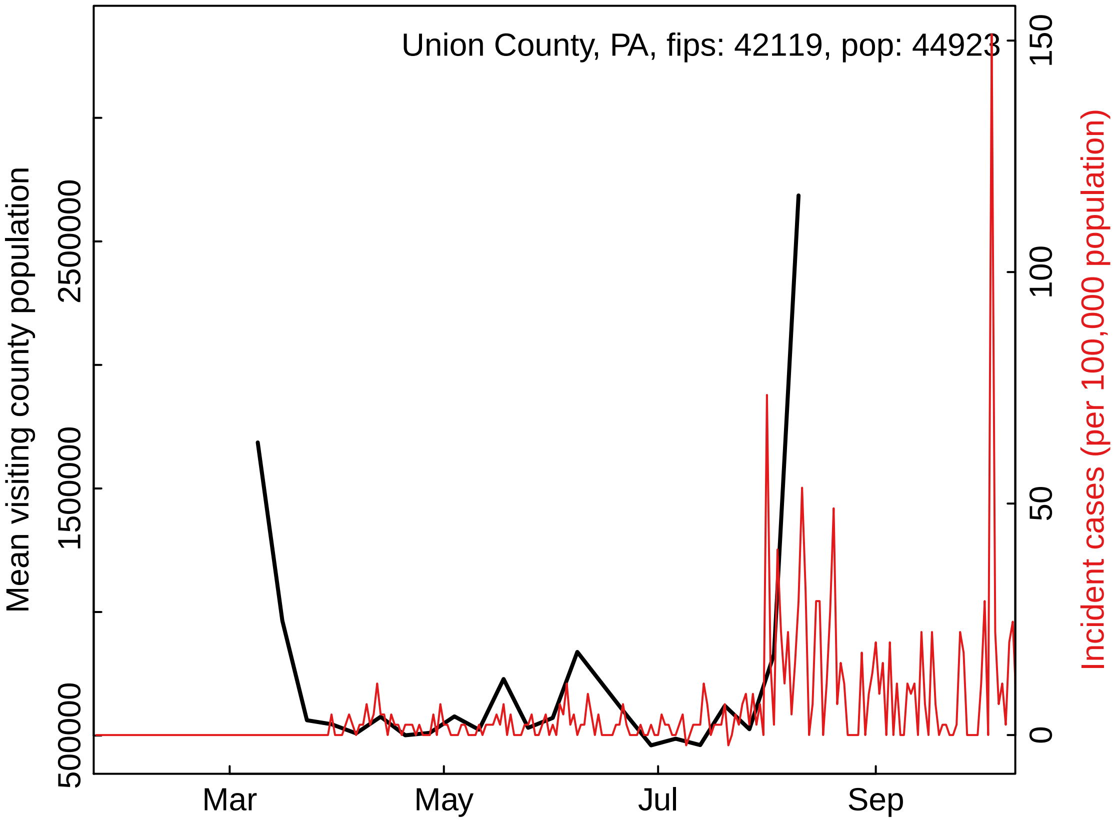

Union County, PA, fips: 42119, pop: 44923
Mean visiting county population
Incident cases (per 100,000 population)
500000
1500000
2500000
0
50
100
150
Mar
May
Jul
Sep

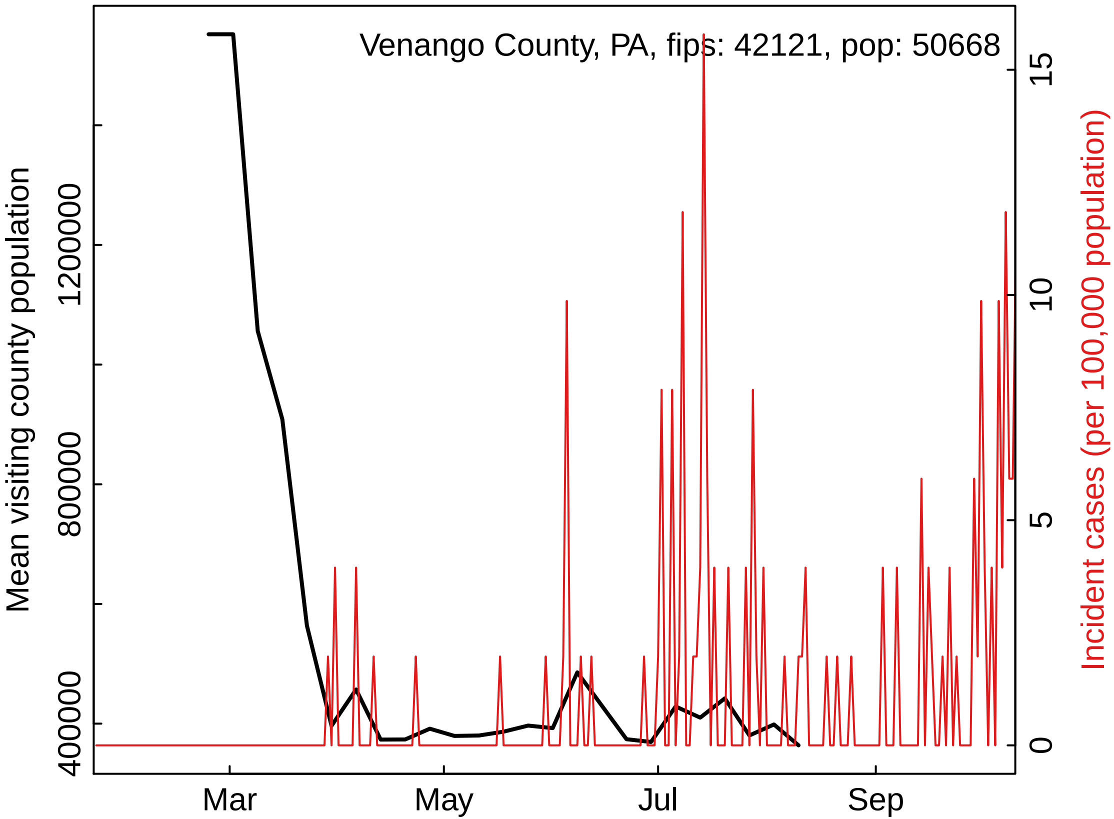

Venango County, PA, fips: 42121, pop: 50668
Mean visiting county population
400000
800000
1200000
Mar
May
Jul
Sep
0
5
10
15
Incident cases (per 100,000 population)

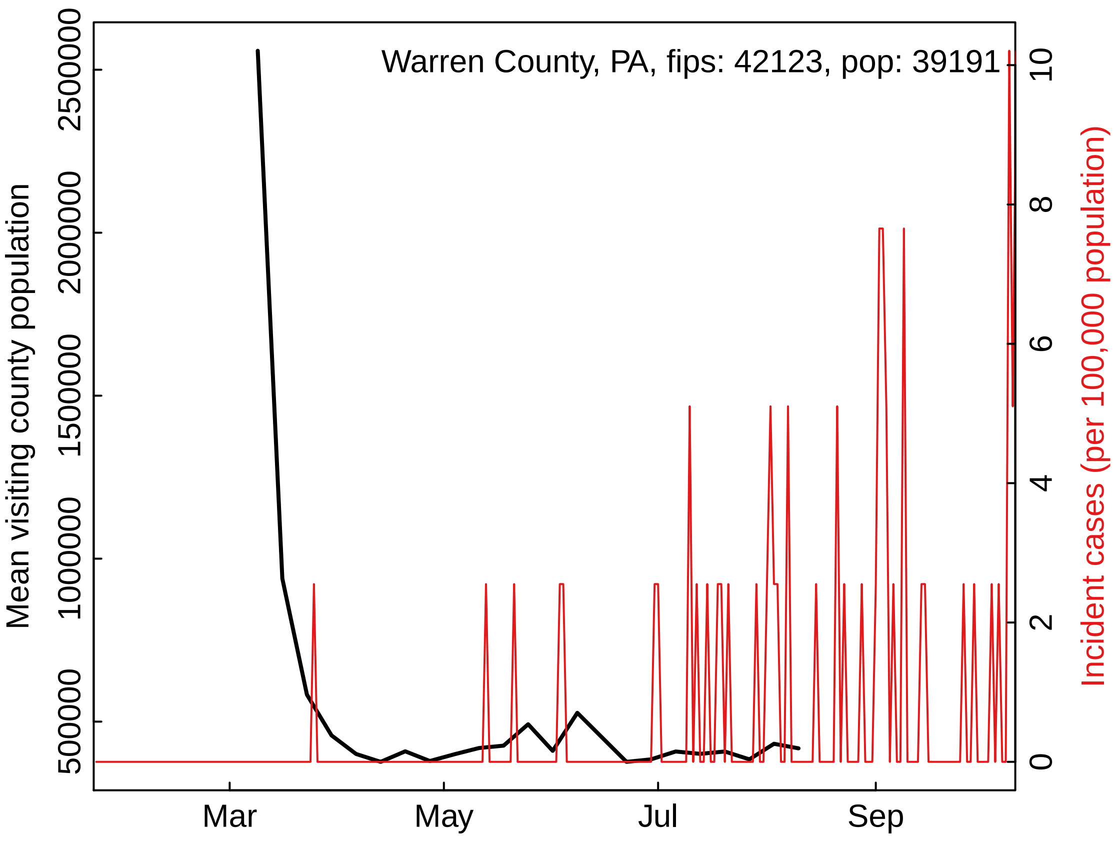

Warren County, PA, fips: 42123, pop: 39191
Mean visiting county population
500000
1000000
1500000
2000000
2500000
Incident cases (per 100,000 population)
0
2
4
6
8
10
Mar
May
Jul
Sep

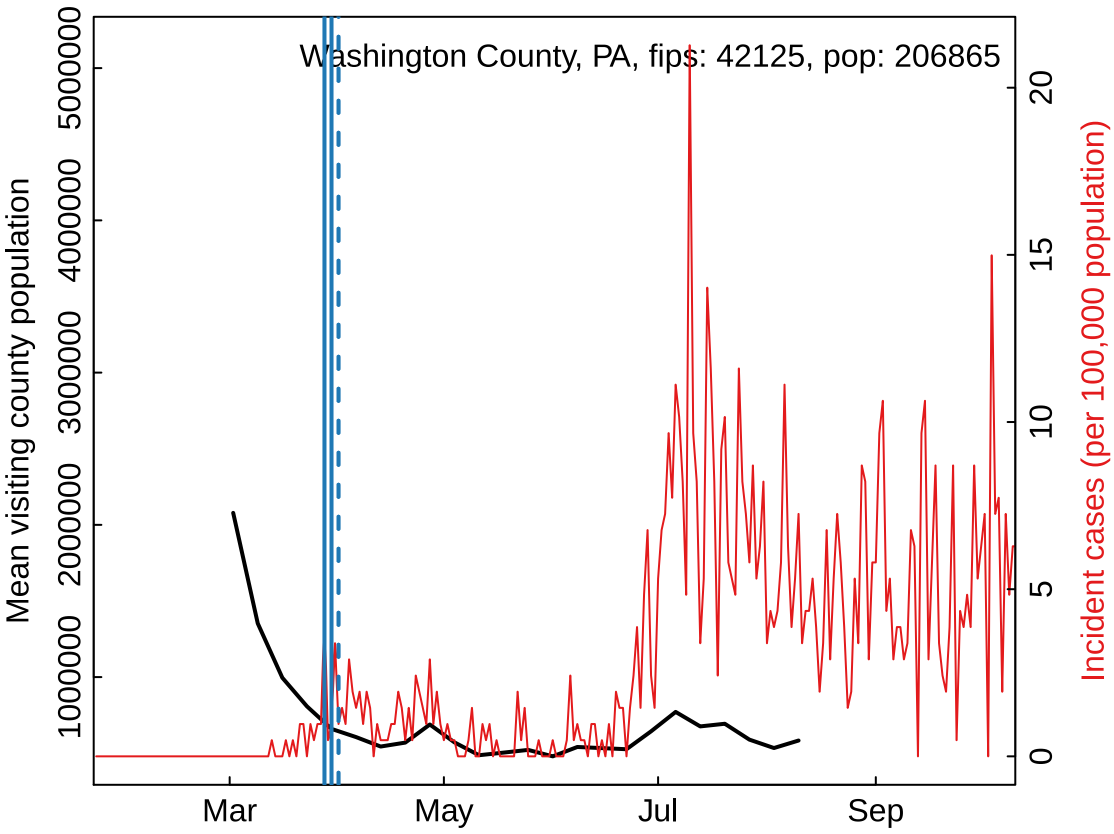

Washington County, PA, fips: 42125, pop: 206865
Mean visiting county population
10000000
20000000
30000000
40000000
50000000
Incident cases (per 100,000 population)
0
5
10
15
20
Mar
May
Jul
Sep

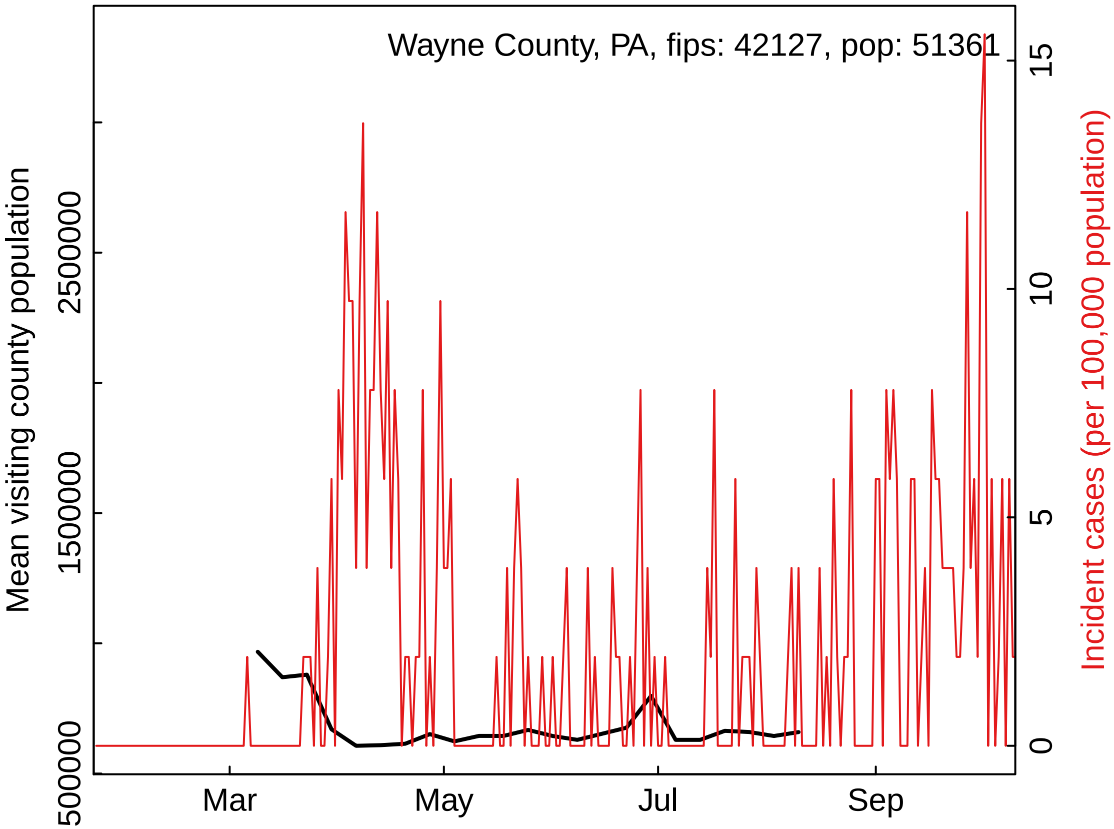

Wayne County, PA, fips: 42127, pop: 51361
Mean visiting county population
500000
1500000
2500000
Mar
May
Jul
Sep
Incident cases (per 100,000 population)
0
5
10
15

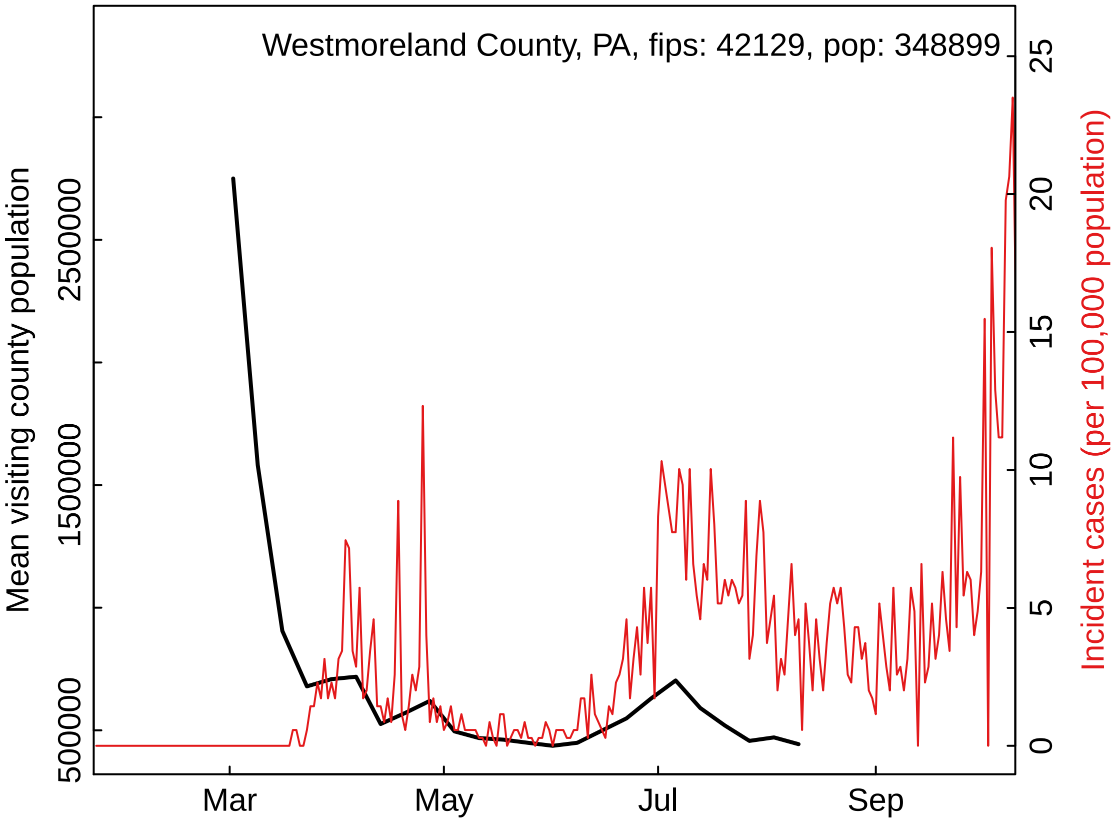

Westmoreland County, PA, fips: 42129, pop: 348899
Mean visiting county population
500000
1500000
2500000
Incident cases (per 100,000 population)
0
5
10
15
20
25
Mar
May
Jul
Sep

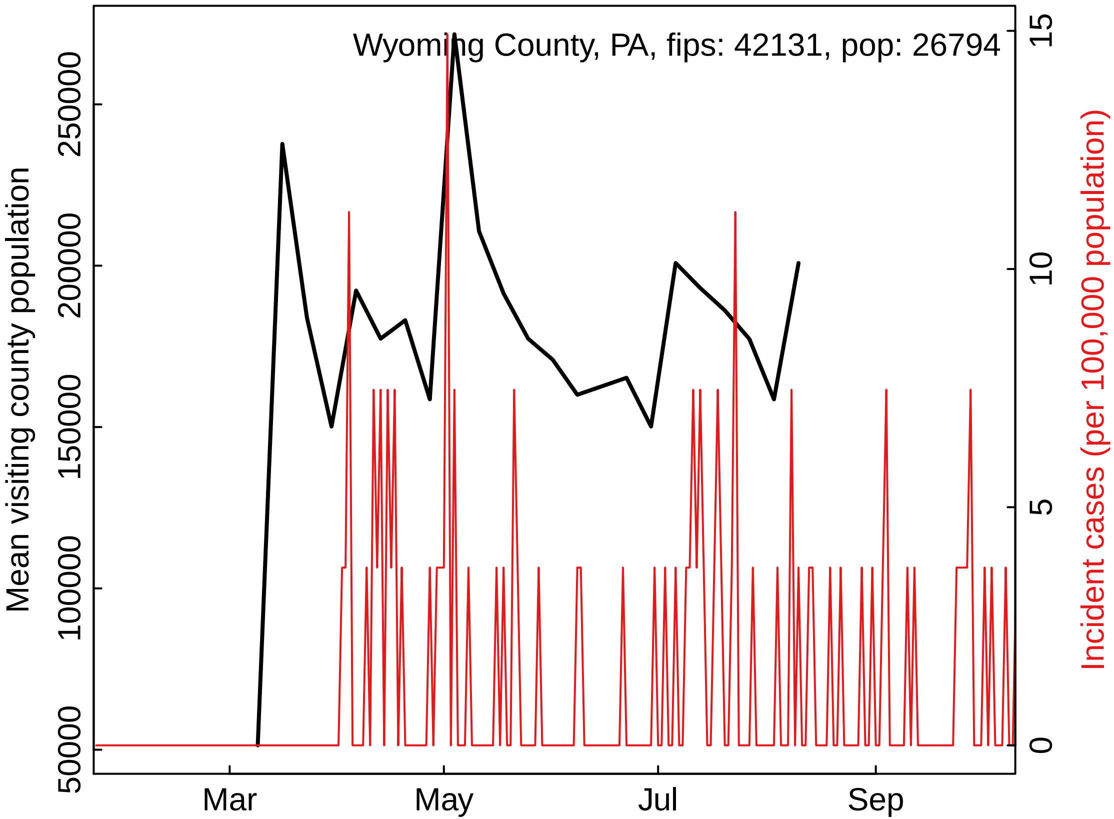

Wyoming County, PA, fips: 42131, pop: 26794
Mean visiting county population
50000
100000
150000
200000
250000
Incident cases (per 100,000 population)
0
5
10
15
Mar
May
Jul
Sep

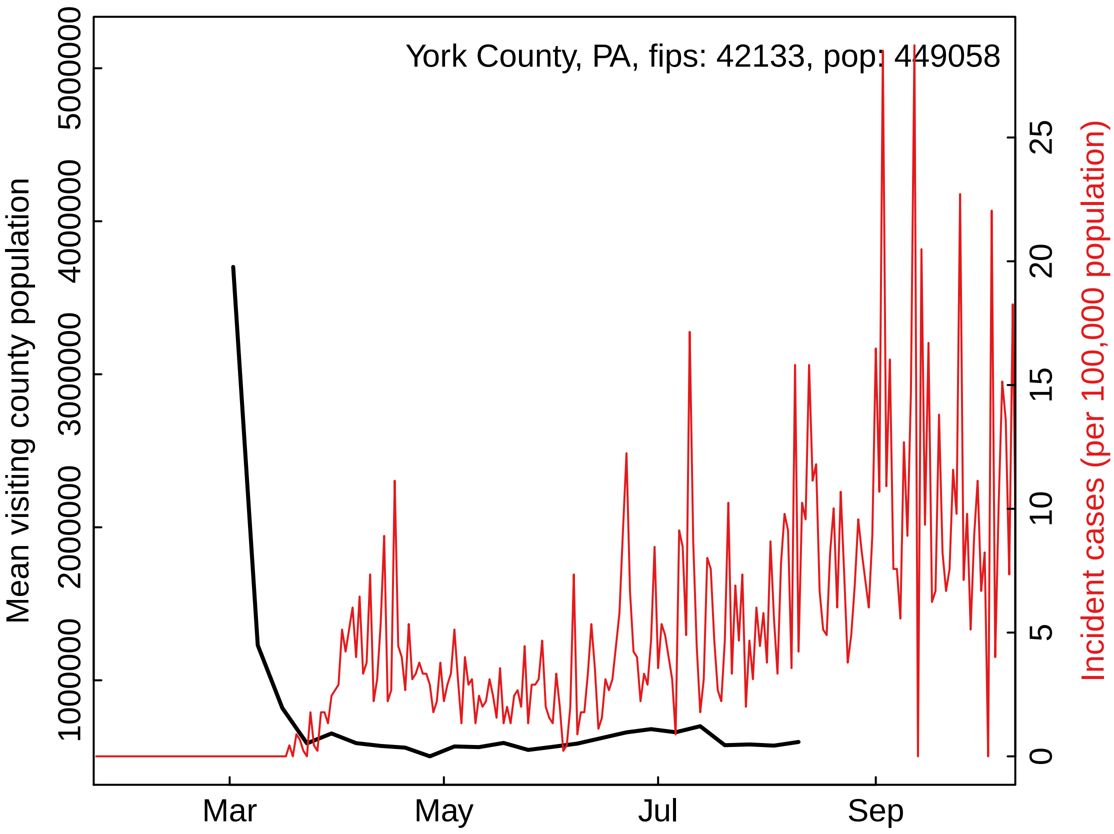
York County, PA, fips: 42133, pop: 449058
Mean visiting county population
1000000
2000000
3000000
4000000
5000000
Incident cases (per 100,000 population)
0
5
10
15
20
25
Mar
May
Jul
Sep

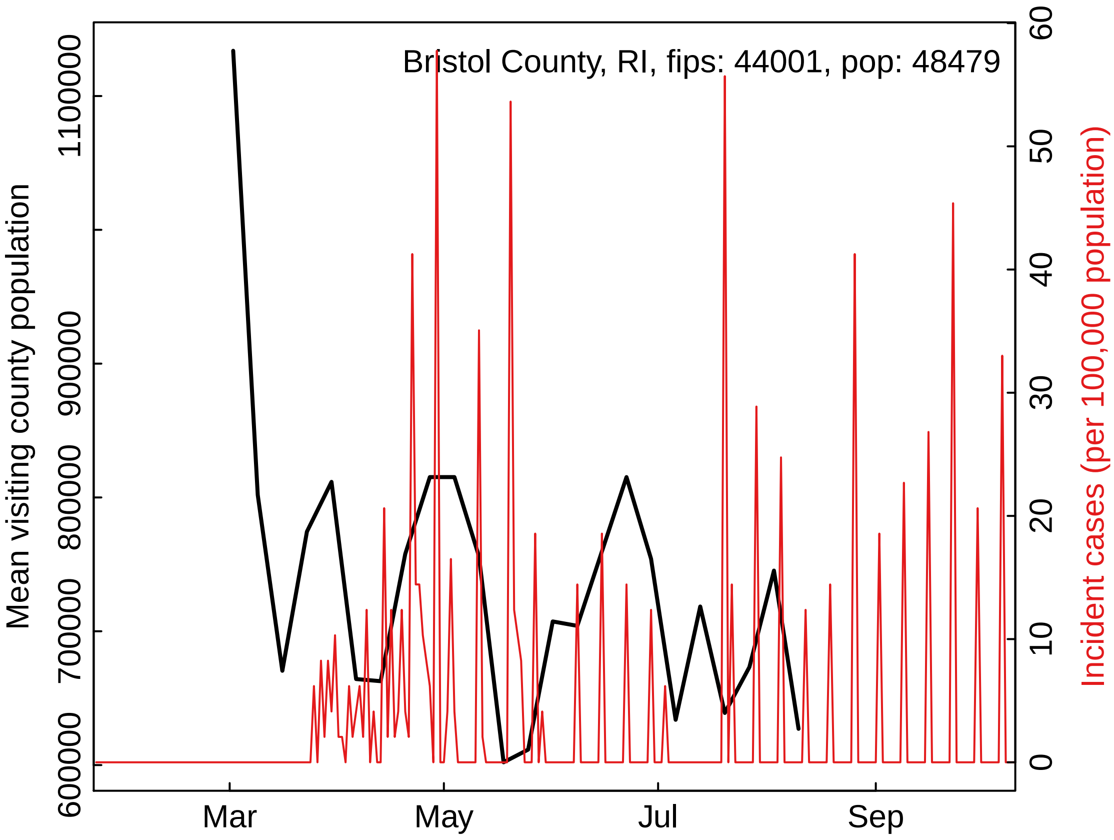

Bristol County, RI, fips: 44001, pop: 48479
Mean visiting county population
600000
700000
800000
900000
1100000
Incident cases (per 100,000 population)
0
10
20
30
40
50
60
Mar
May
Jul
Sep

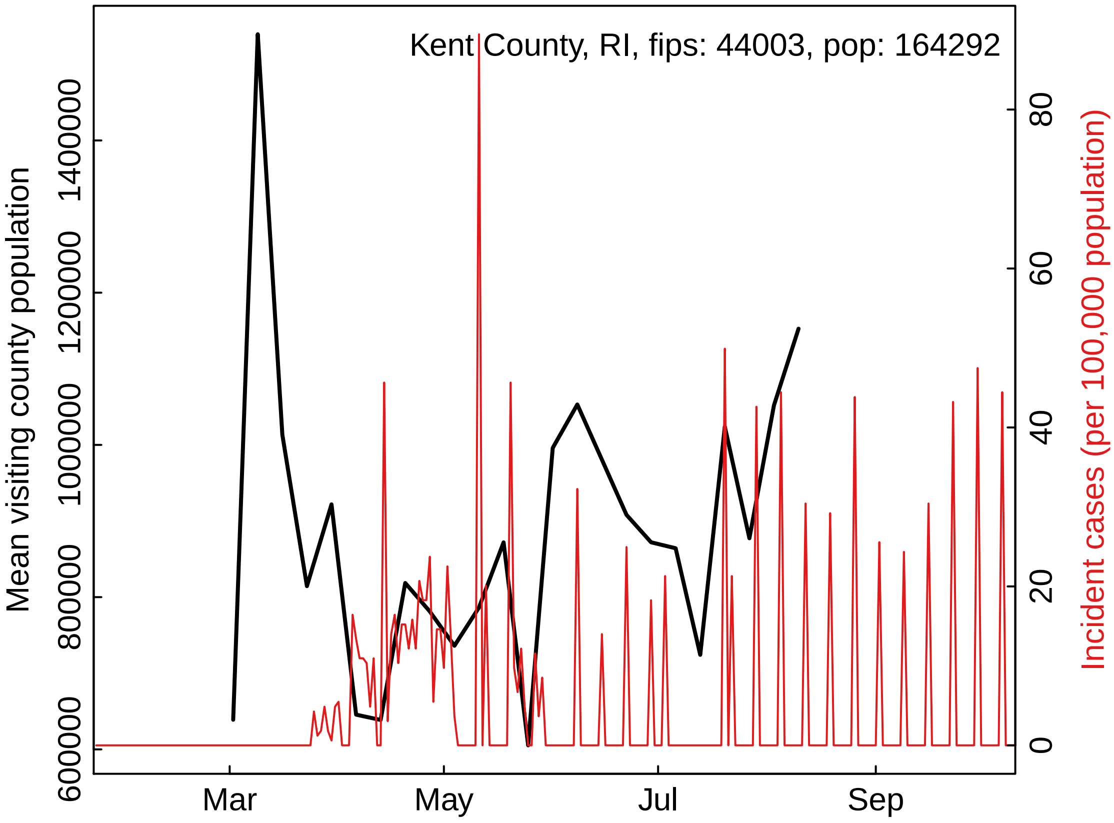
Kent County, RI, fips: 44003, pop: 164292
Mean visiting county population
600000
800000
1000000
1200000
1400000
Incident cases (per 100,000 population)
0
20
40
60
80
Mar
May
Jul
Sep

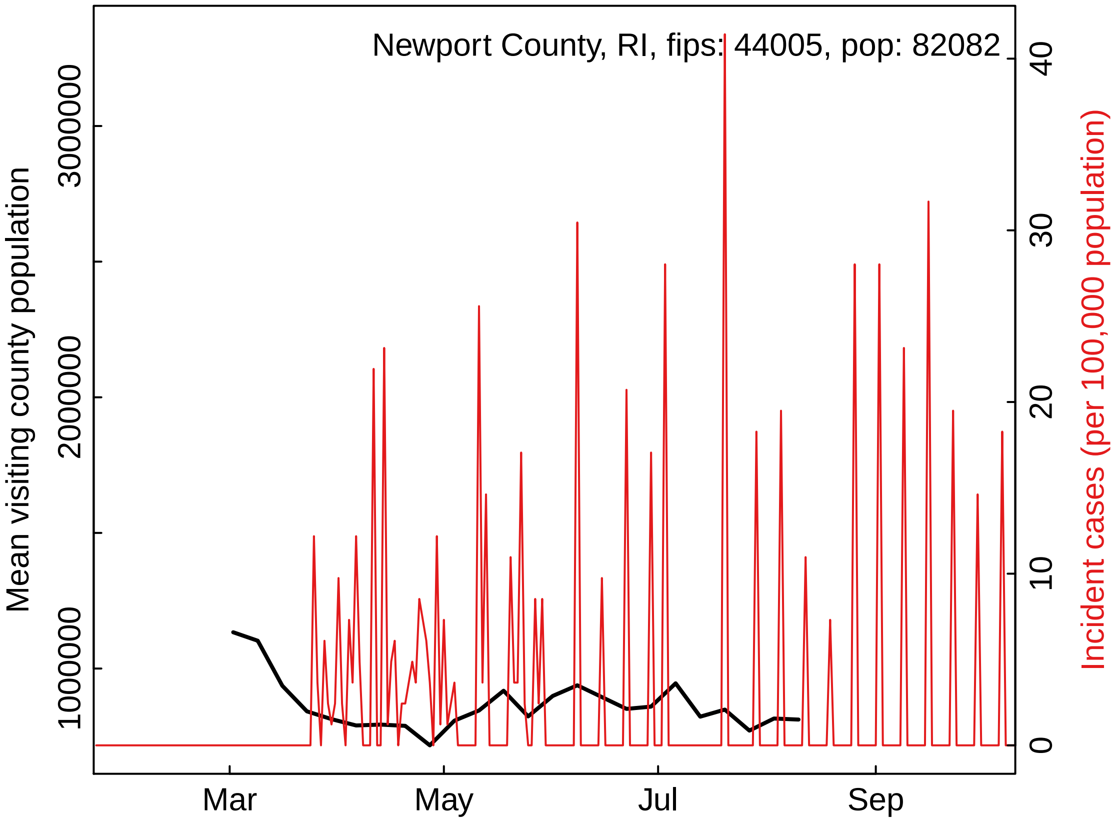

Newport County, RI, fips: 44005, pop: 82082
Mean visiting county population
1000000
2000000
3000000
Incident cases (per 100,000 population)
0
10
20
30
40
Mar
May
Jul
Sep

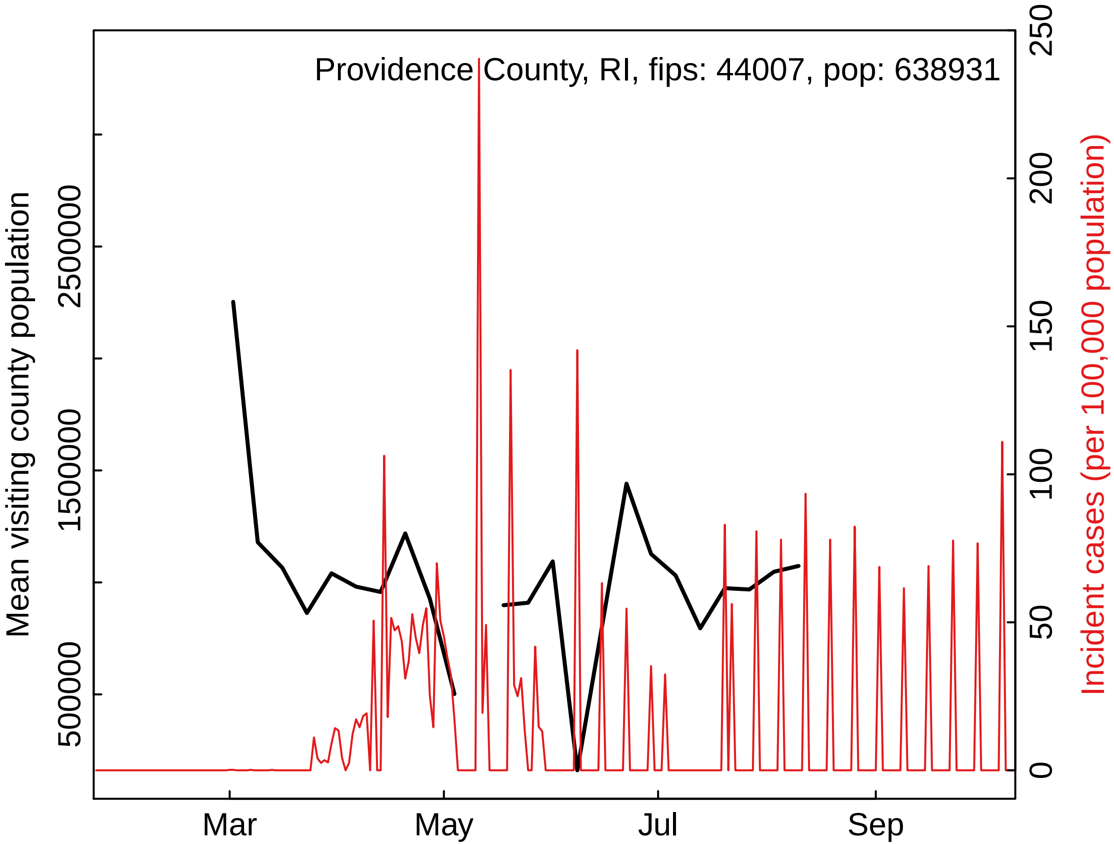

Providence County, RI, fips: 44007, pop: 638931
Mean visiting county population
500000
1500000
2500000
Incident cases (per 100,000 population)
0
50
100
150
200
250
Mar
May
Jul
Sep

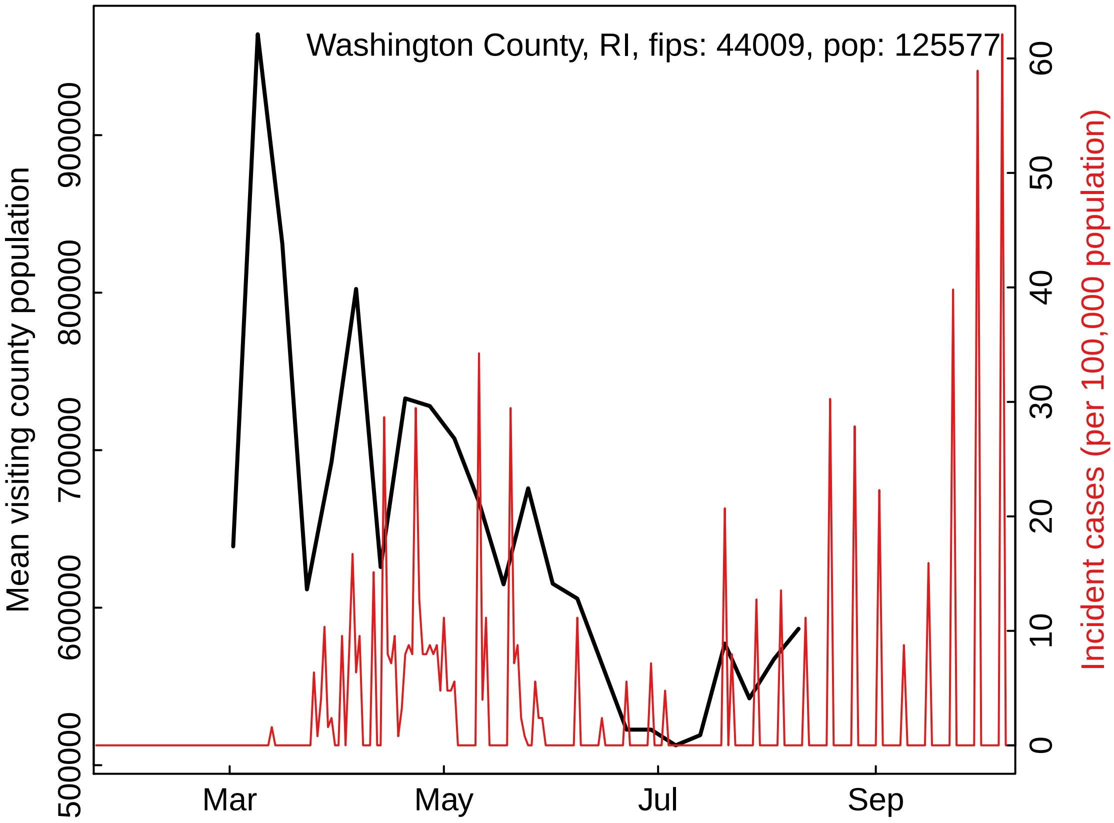

Washington County, RI, fips: 44009, pop: 125577
Mean visiting county population
500000
600000
700000
800000
900000
Incident cases (per 100,000 population)
0
10
20
30
40
50
60
Mar
May
Jul
Sep

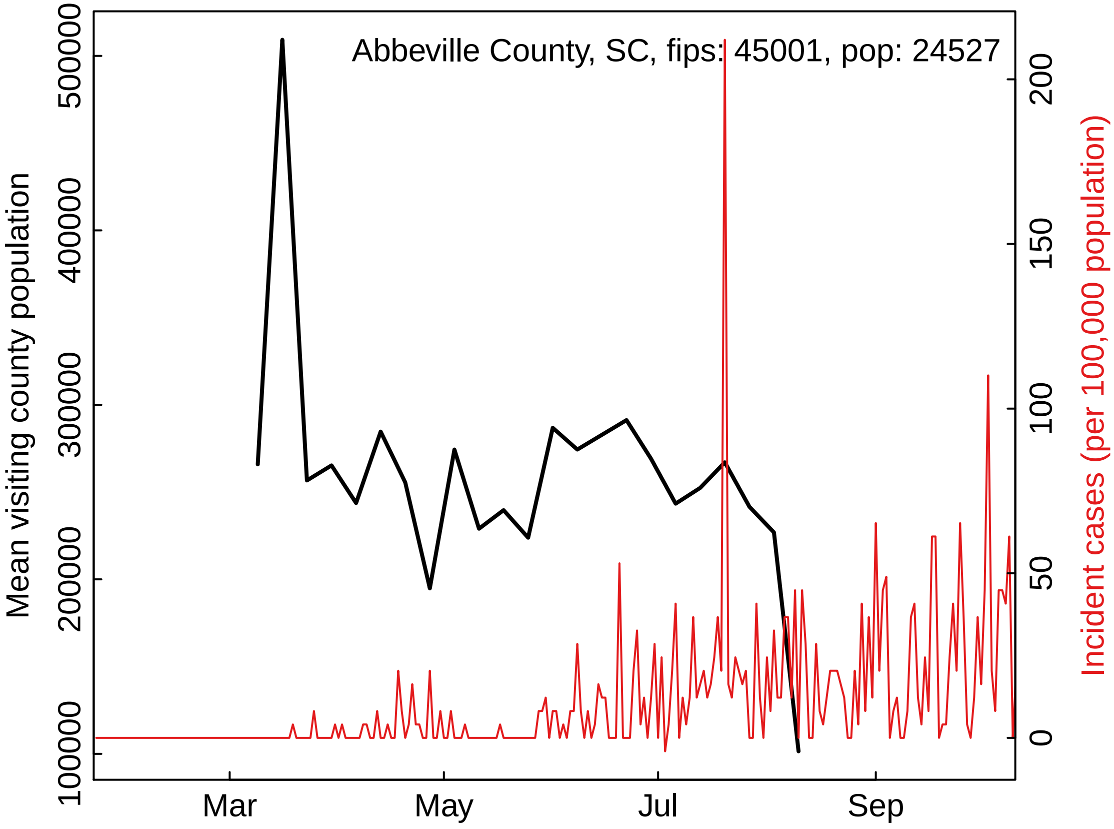

Abbeville County, SC, fips: 45001, pop: 24527
Mean visiting county population
100000
200000
300000
400000
500000
Incident cases (per 100,000 population)
0
50
100
150
200
Mar
May
Jul
Sep

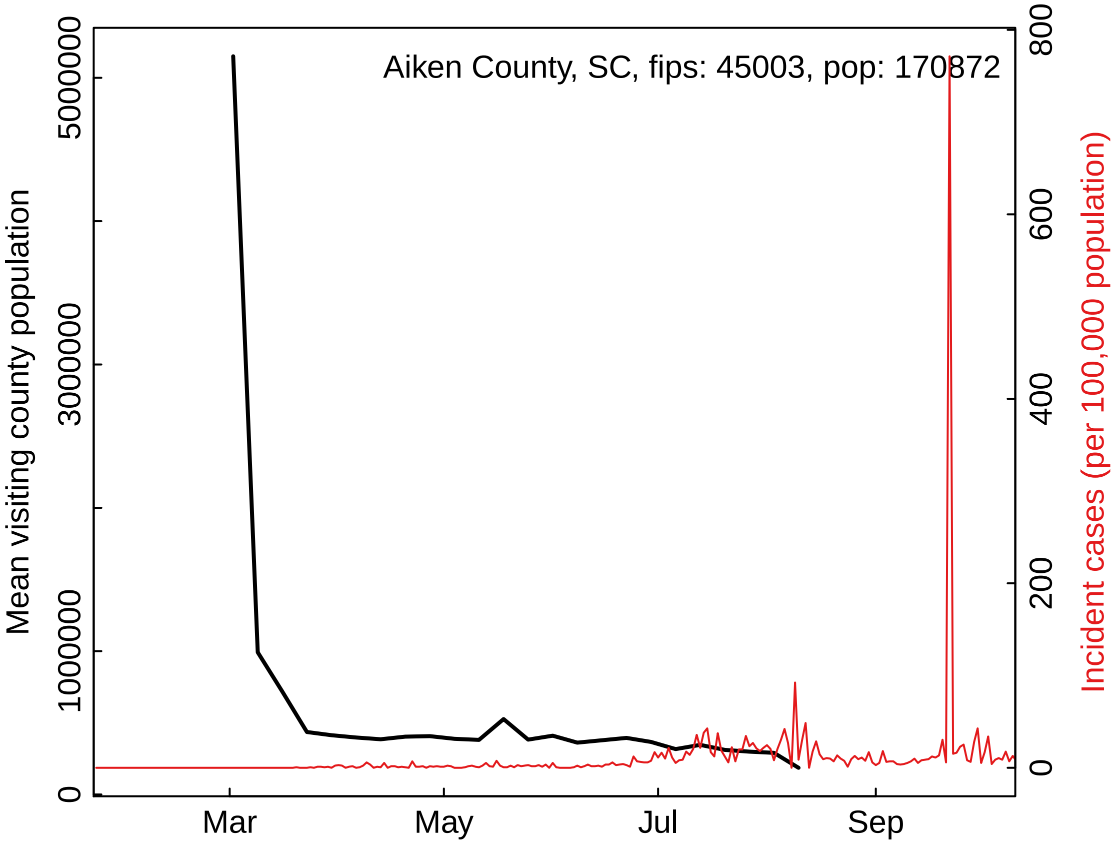

Aiken County, SC, fips: 45003, pop: 170872
Mean visiting county population
Incident cases (per 100,000 population)
0
1000000
3000000
5000000
0
200
400
600
800
Mar
May
Jul
Sep

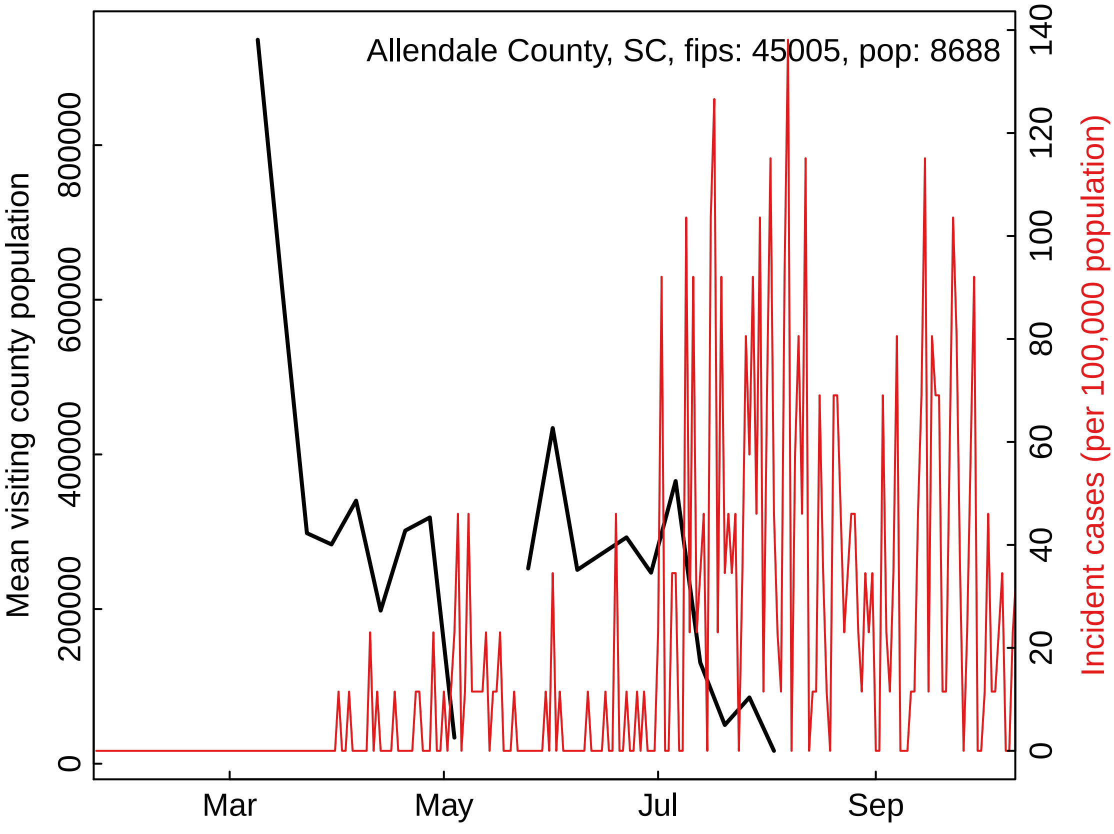

Allendale County, SC, fips: 45005, pop: 8688
Mean visiting county population
0
200000
400000
600000
800000
Incident cases (per 100,000 population)
0
20
40
60
80
100
120
140
Mar
May
Jul
Sep

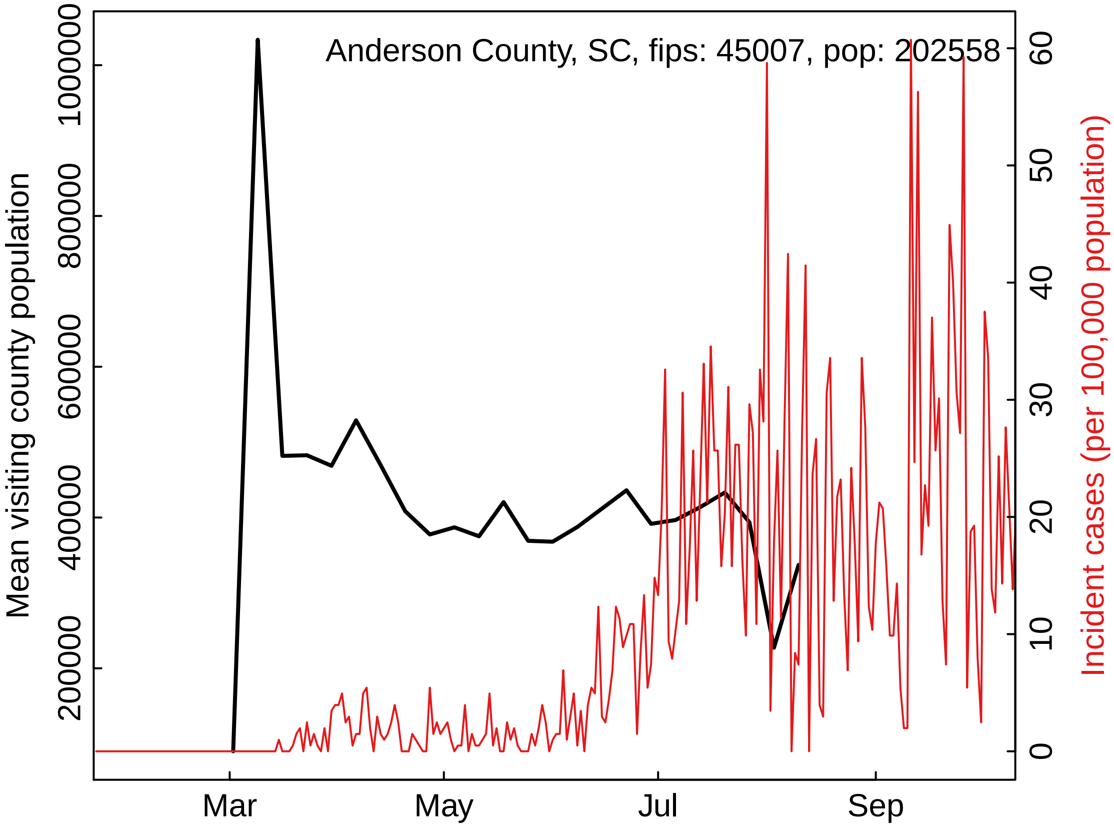

Anderson County, SC, fips: 45007, pop: 202558
Mean visiting county population
Incident cases (per 100,000 population)
200000
400000
600000
800000
1000000
0
10
20
30
40
50
60
Mar
May
Jul
Sep

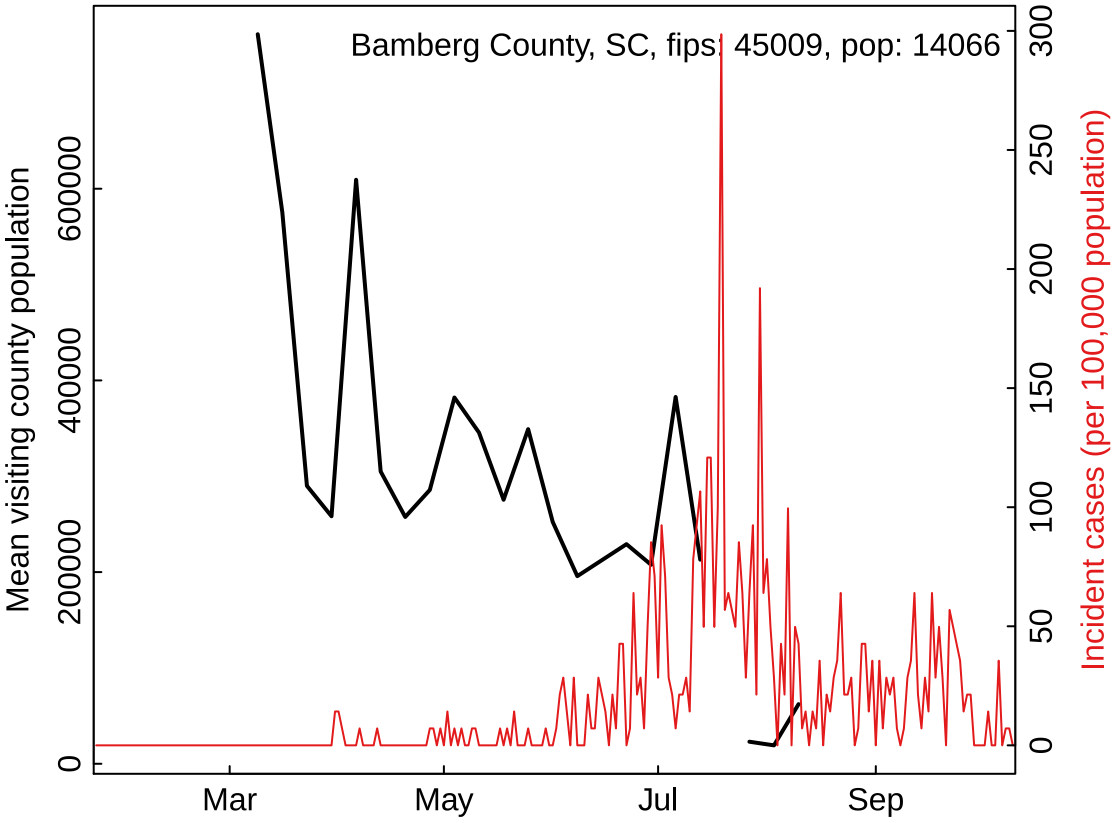

Bamberg County, SC, fips: 45009, pop: 14066
Mean visiting county population
0
200000
400000
600000
Incident cases (per 100,000 population)
0
50
100
150
200
250
300
Mar
May
Jul
Sep

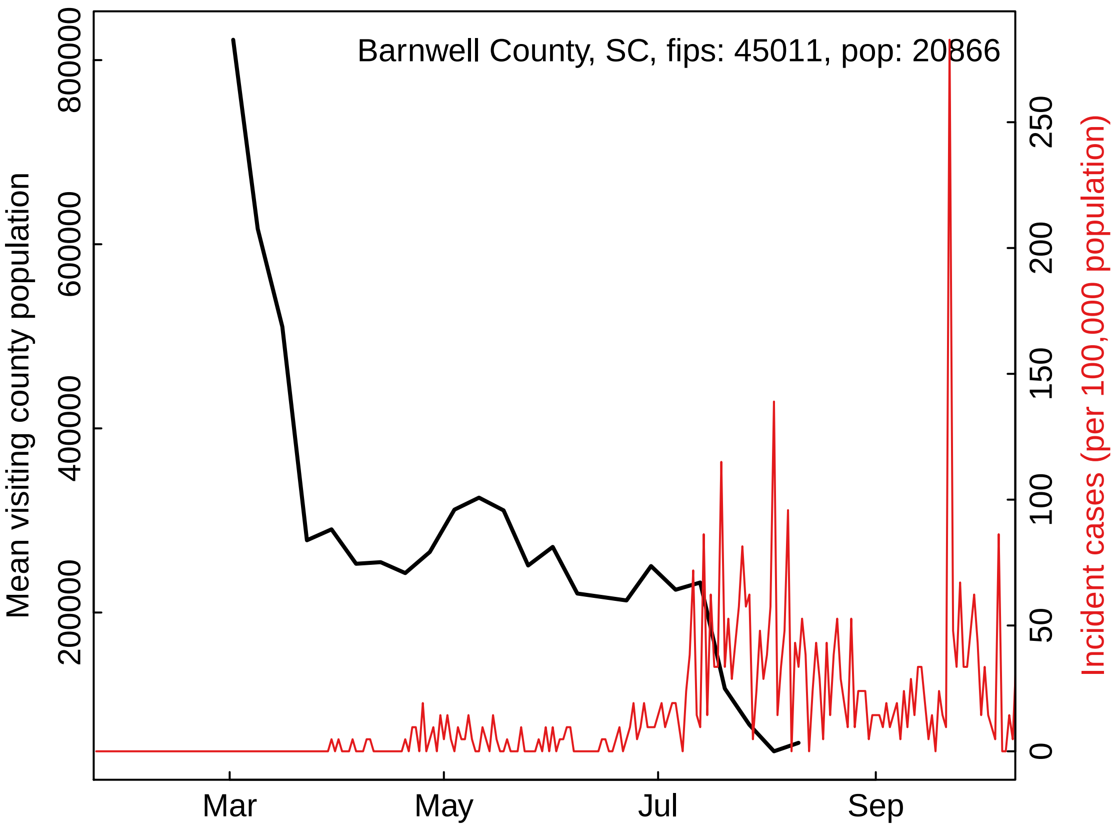

Barnwell County, SC, fips: 45011, pop: 20866
Mean visiting county population
200000
400000
600000
800000
Incident cases (per 100,000 population)
0
50
100
150
200
250
Mar
May
Jul
Sep

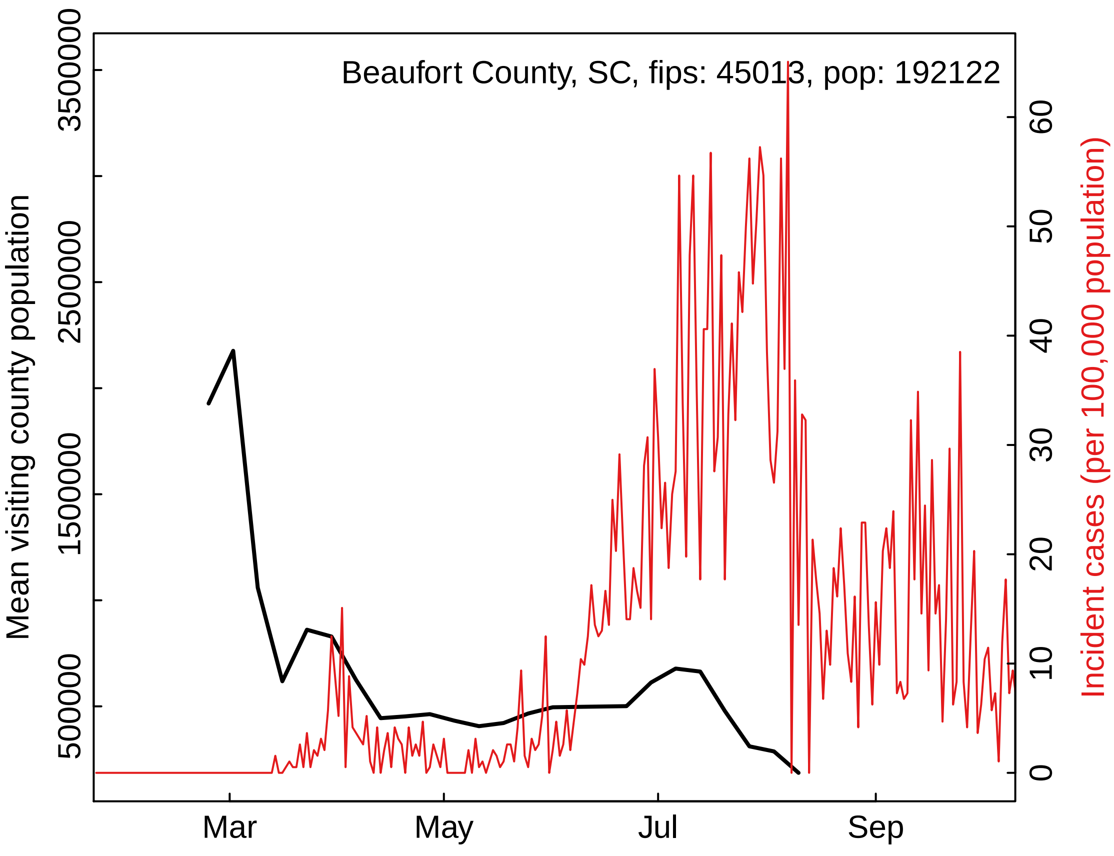

Beaufort County, SC, fips: 45013, pop: 192122
Mean visiting county population
500000
1500000
2500000
3500000
Incident cases (per 100,000 population)
0
10
20
30
40
50
60
Mar
May
Jul
Sep

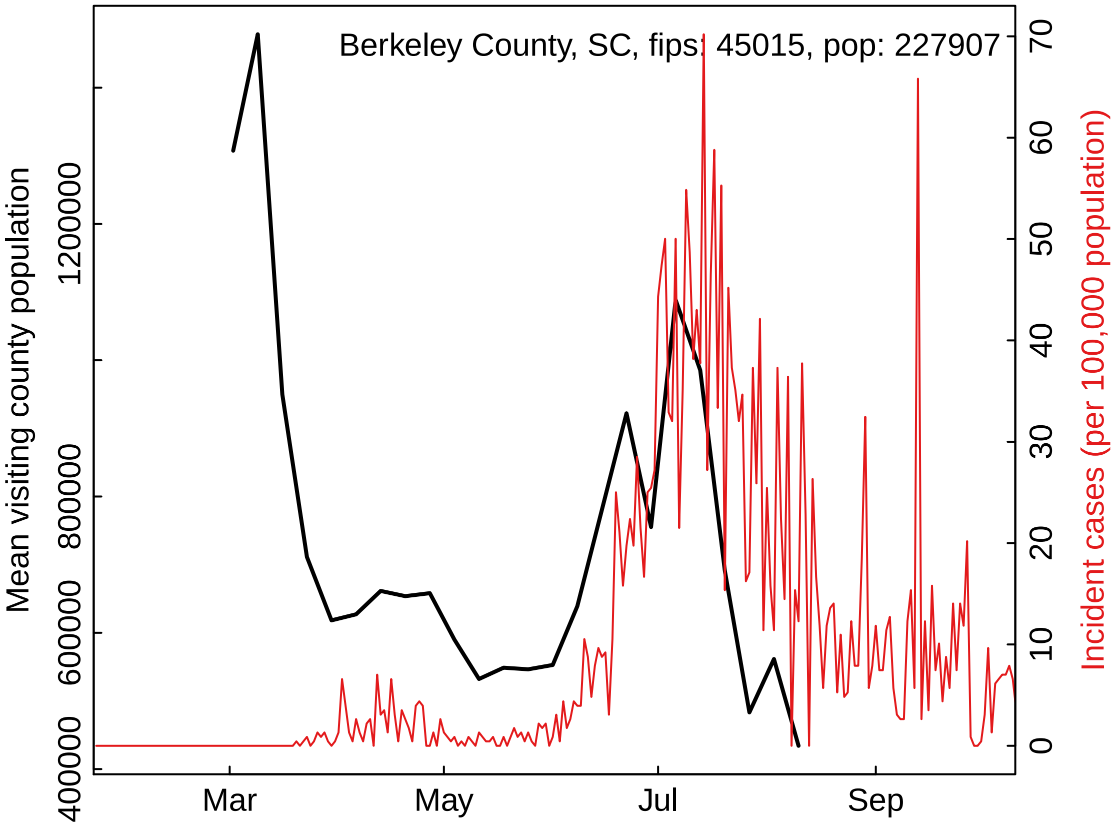

Berkeley County, SC, fips: 45015, pop: 227907
Mean visiting county population
400000
600000
800000
1200000
Mar
May
Jul
Sep
0
10
20
30
40
50
60
70
Incident cases (per 100,000 population)

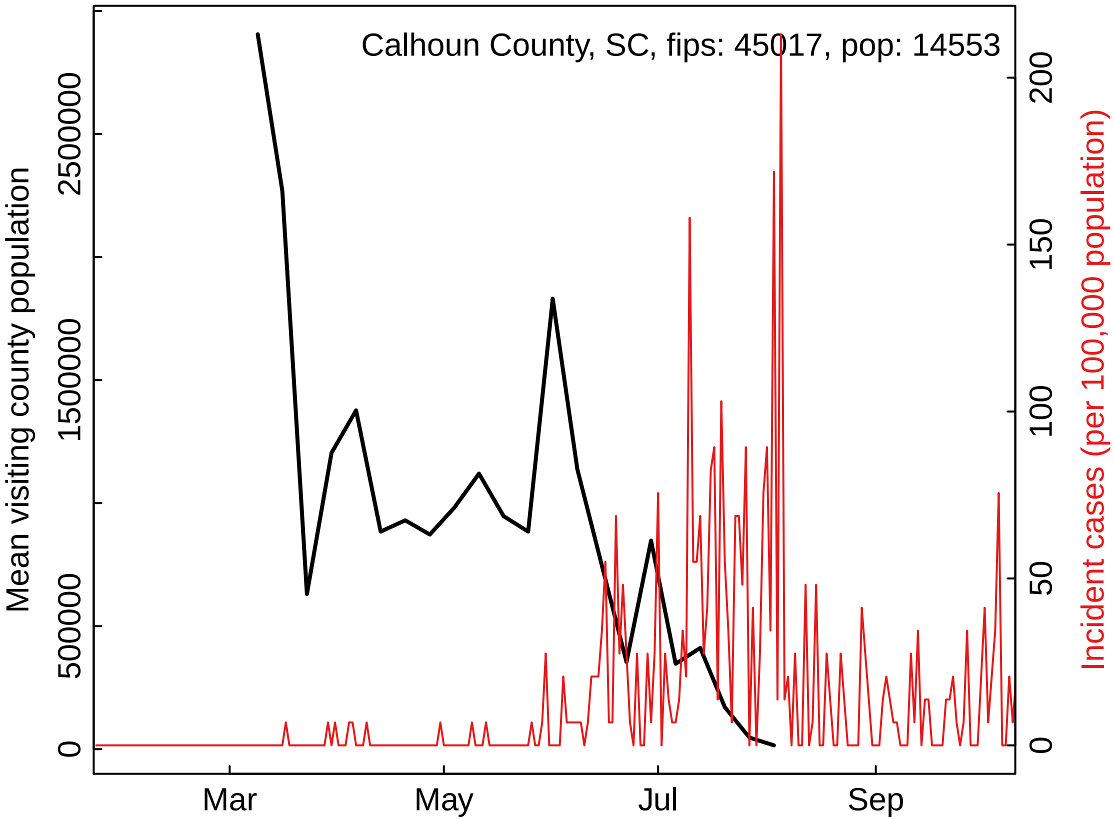

Calhoun County, SC, fips: 45017, pop: 14553
Mean visiting county population
0
500000
1500000
2500000
Incident cases (per 100,000 population)
0
50
100
150
200
Mar
May
Jul
Sep

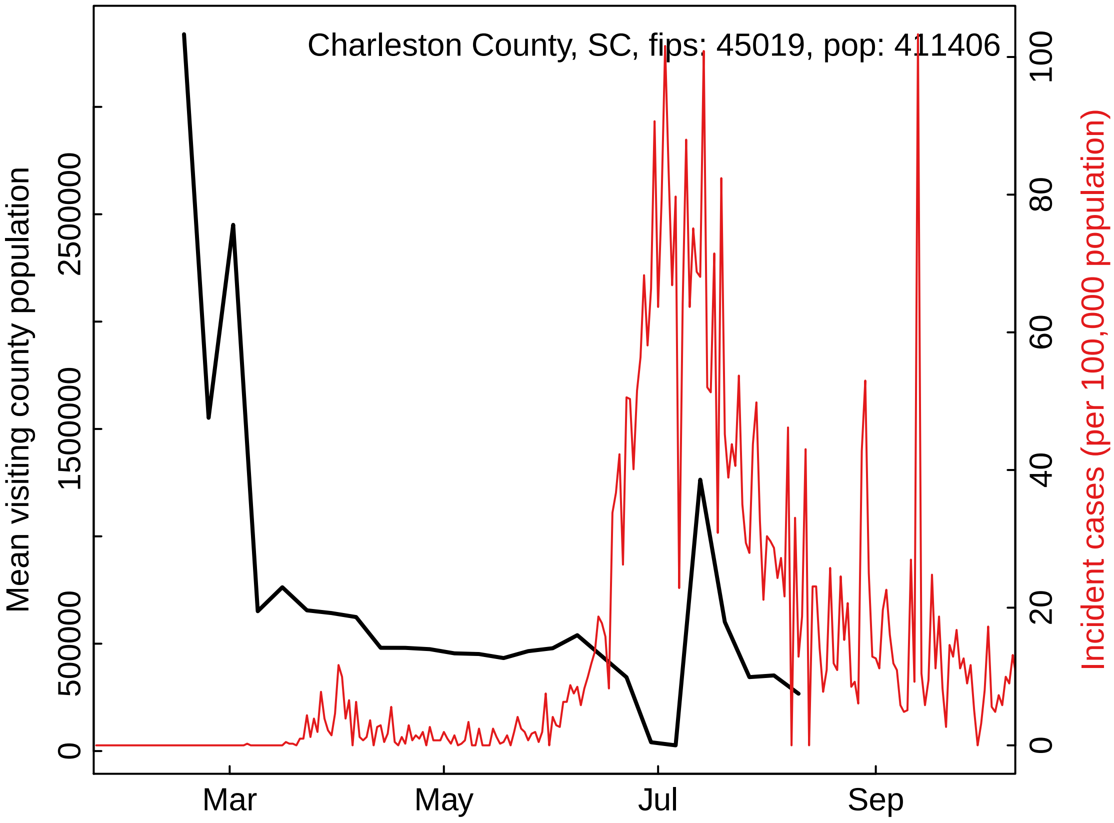

Charleston County, SC, fips: 45019, pop: 411406
Mean visiting county population
0
500000
1500000
2500000
Incident cases (per 100,000 population)
0
20
40
60
80
100
Mar
May
Jul
Sep

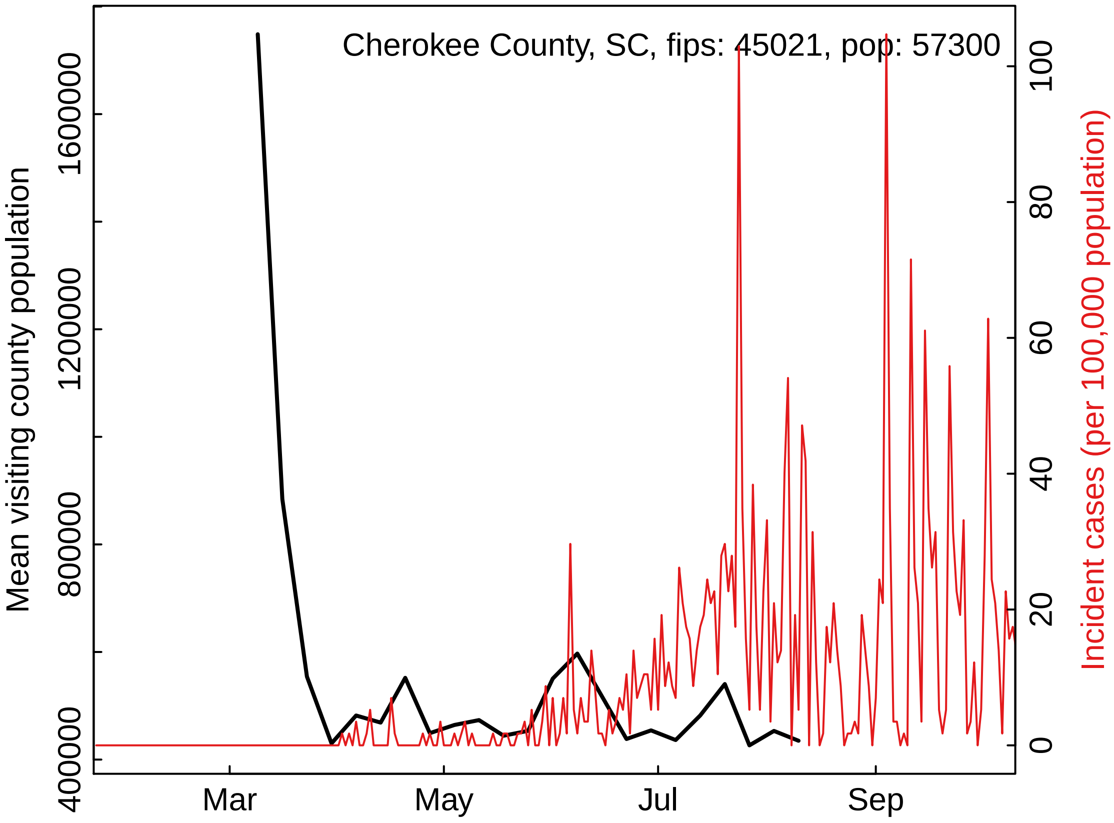

Cherokee County, SC, fips: 45021, pop: 57300
Mean visiting county population
4000000
8000000
12000000
16000000
Mar
May
Jul
Sep
0
20
40
60
80
100
Incident cases (per 100,000 population)

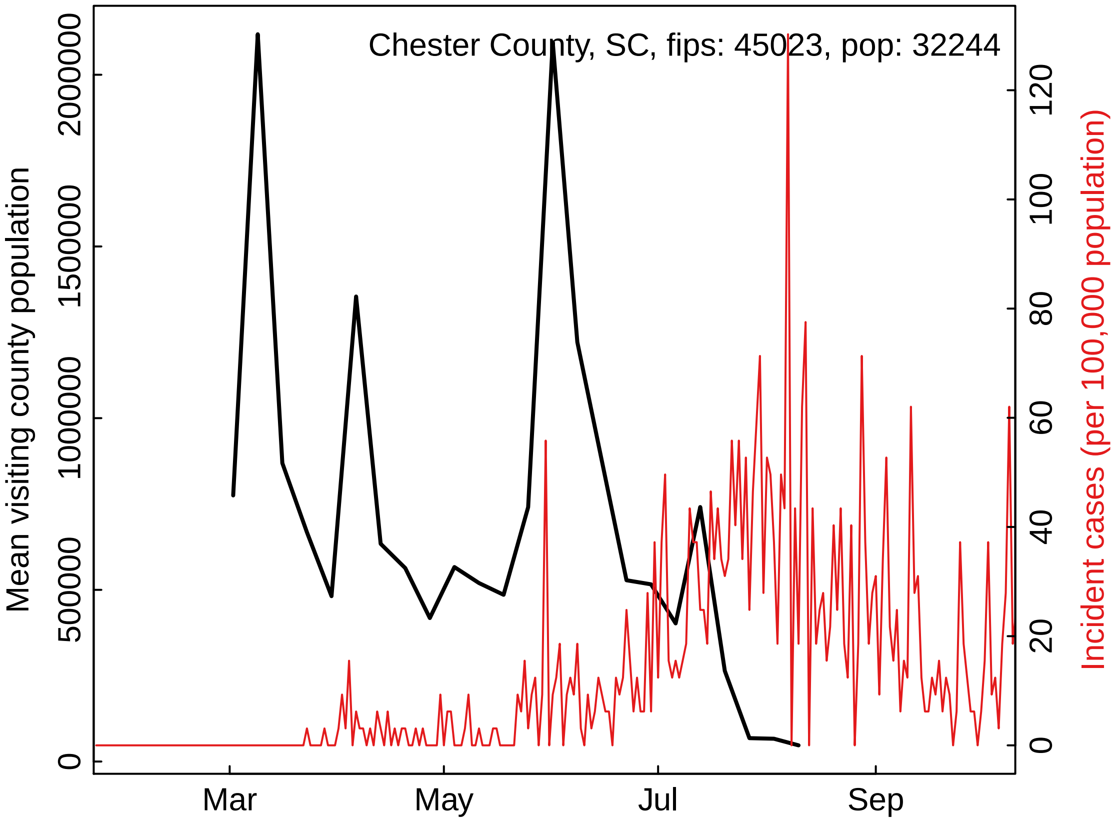

Chester County, SC, fips: 45023, pop: 32244
Mean visiting county population
0
500000
1000000
1500000
2000000
Incident cases (per 100,000 population)
0
20
40
60
80
100
120
Mar
May
Jul
Sep

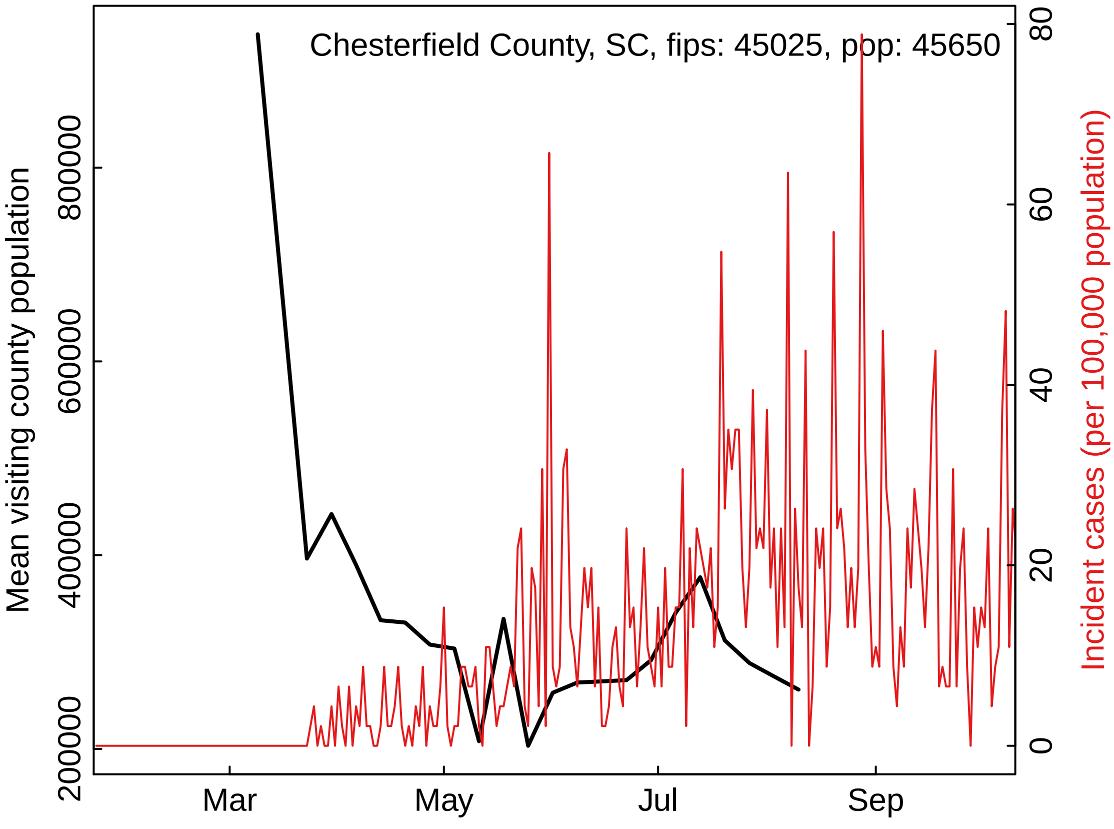

Chesterfield County, SC, fips: 45025, pop: 45650
Mean visiting county population
200000
400000
600000
800000
Incident cases (per 100,000 population)
0
20
40
60
80
Mar
May
Jul
Sep

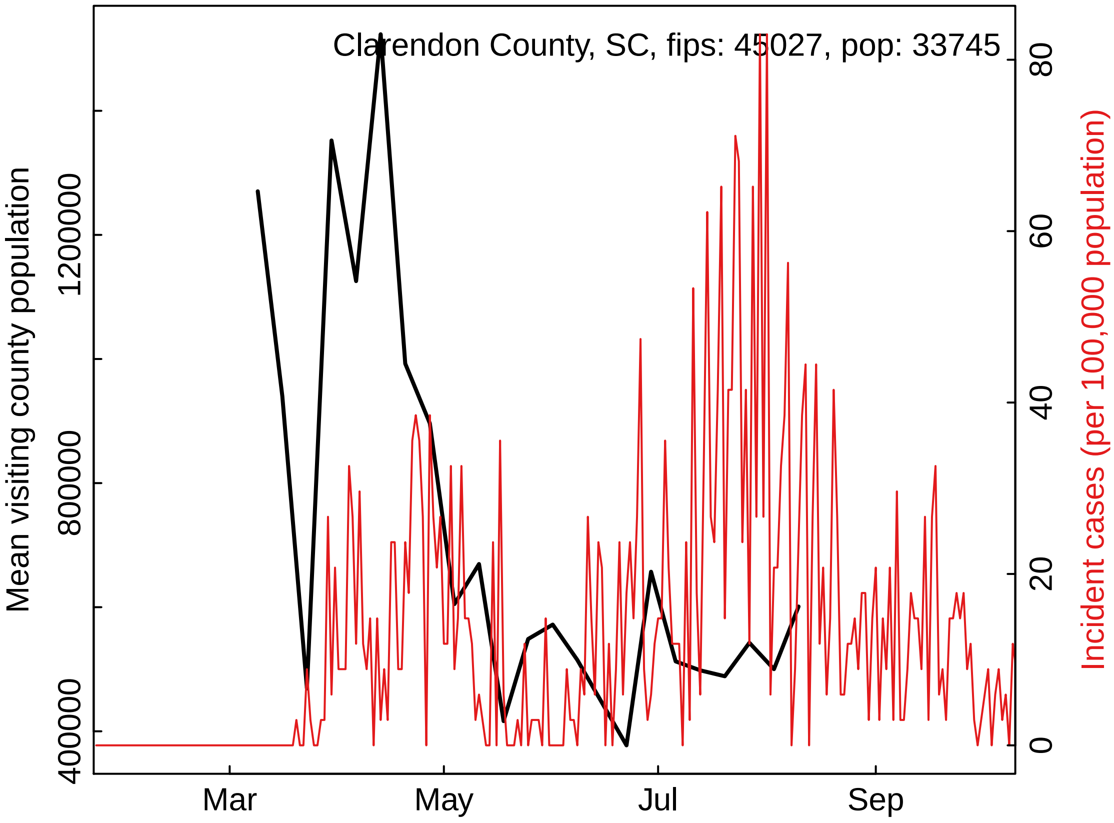

Clarendon County, SC, fips: 45027, pop: 33745
Mean visiting county population
400000
800000
1200000
Incident cases (per 100,000 population)
0
20
40
60
80
Mar
May
Jul
Sep

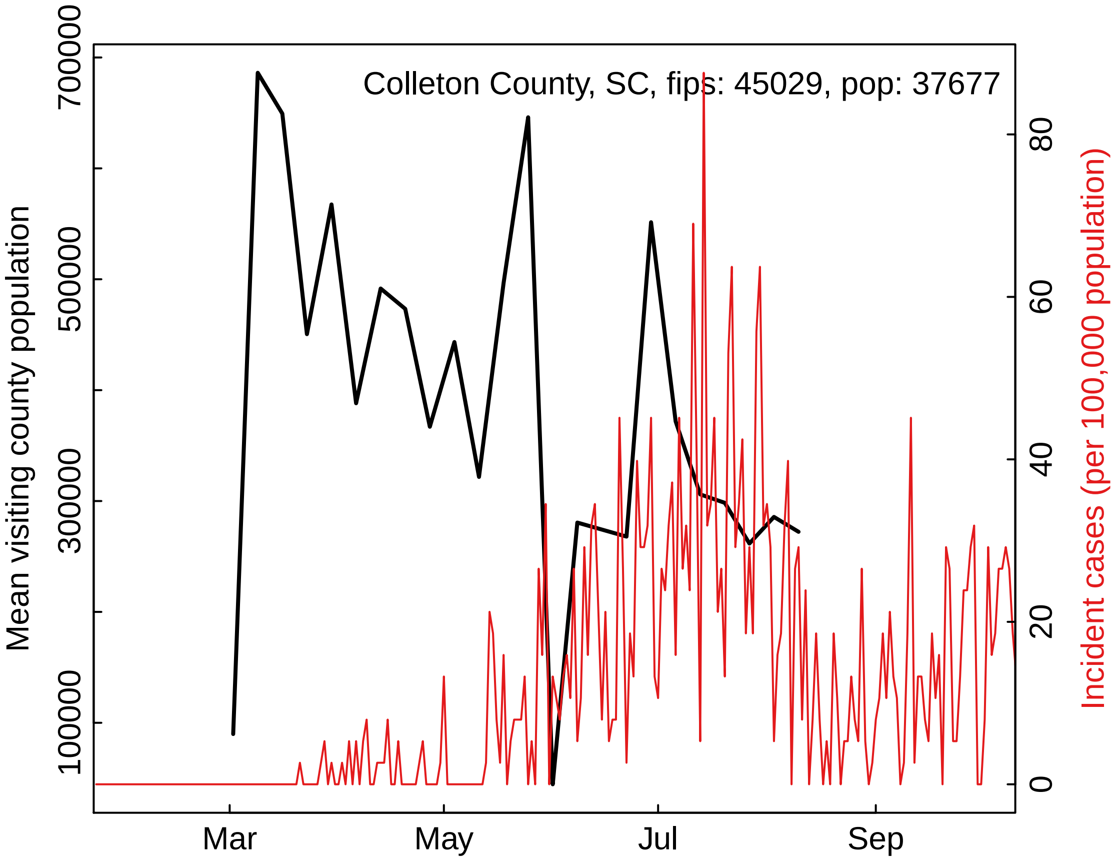

Colleton County, SC, fips: 45029, pop: 37677
Mean visiting county population
100000
300000
500000
700000
Incident cases (per 100,000 population)
0
20
40
60
80
Mar
May
Jul
Sep

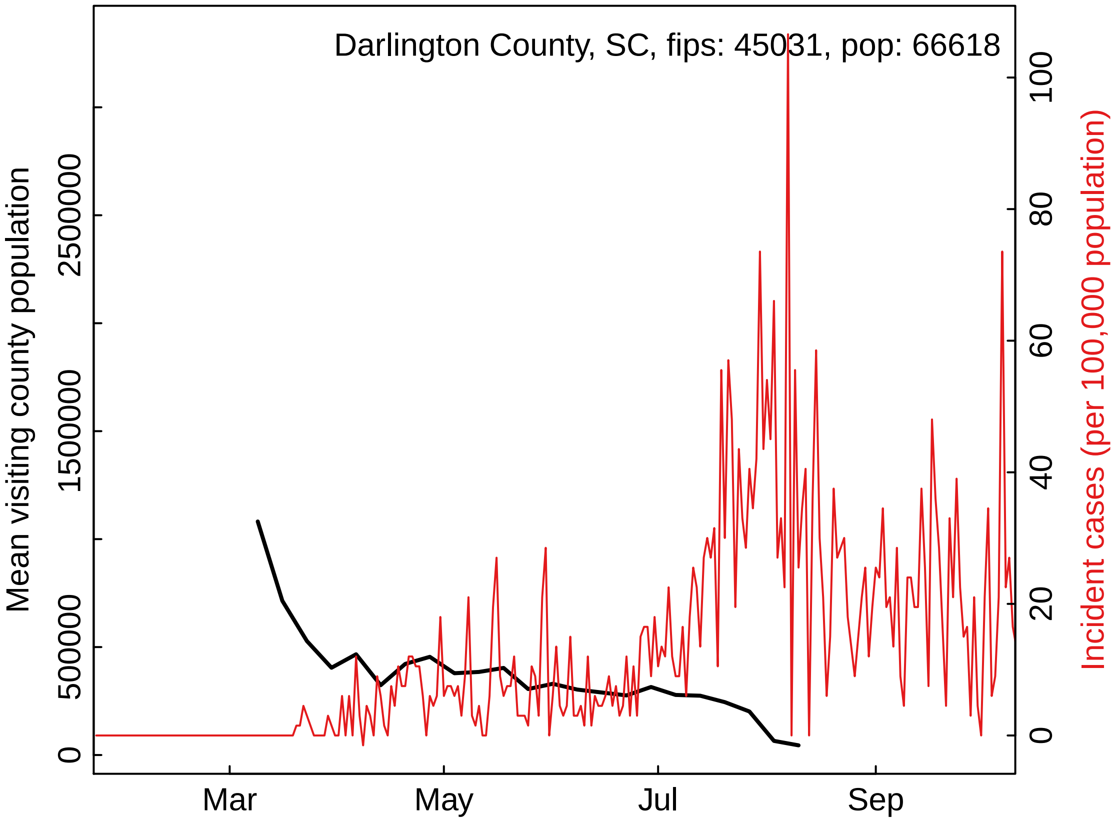
Darlington County, SC, fips: 45031, pop: 66618
Mean visiting county population
0
500000
1500000
2500000
Incident cases (per 100,000 population)
0
20
40
60
80
100
Mar
May
Jul
Sep

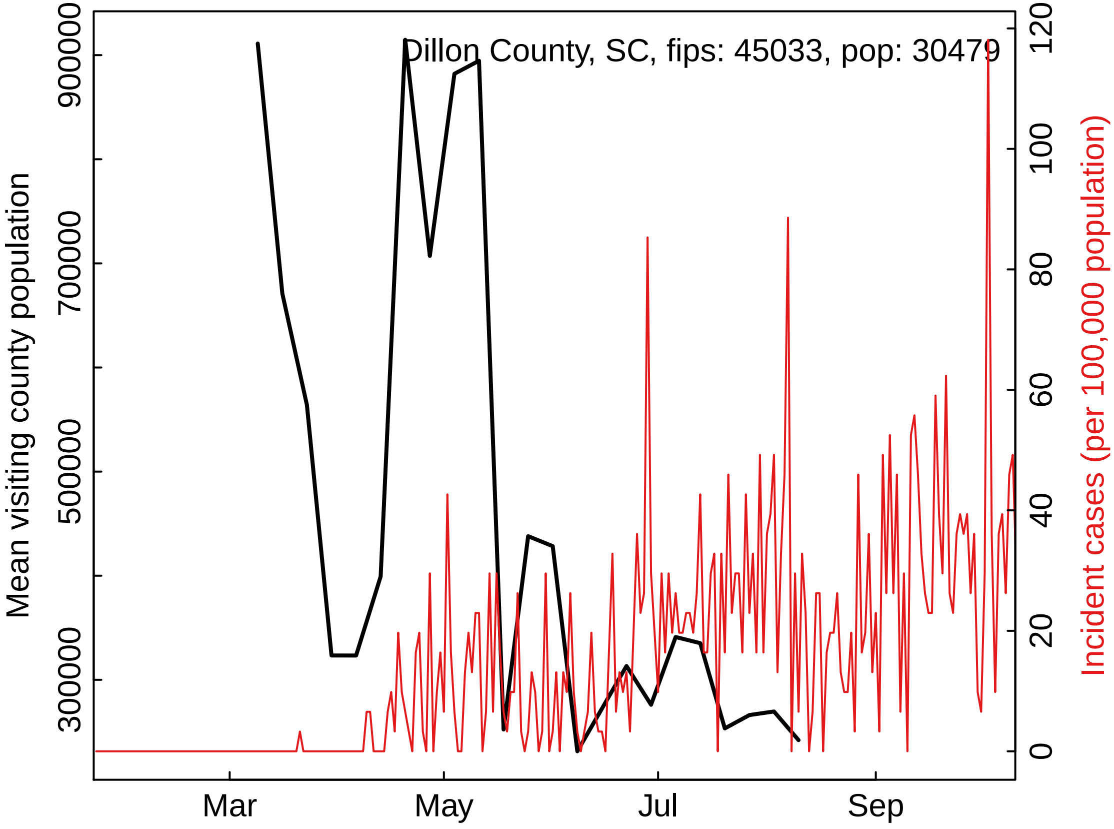

Dillon County, SC, fips: 45033, pop: 30479
Mean visiting county population
300000
500000
700000
900000
Incident cases (per 100,000 population)
0
20
40
60
80
100
120
Mar
May
Jul
Sep

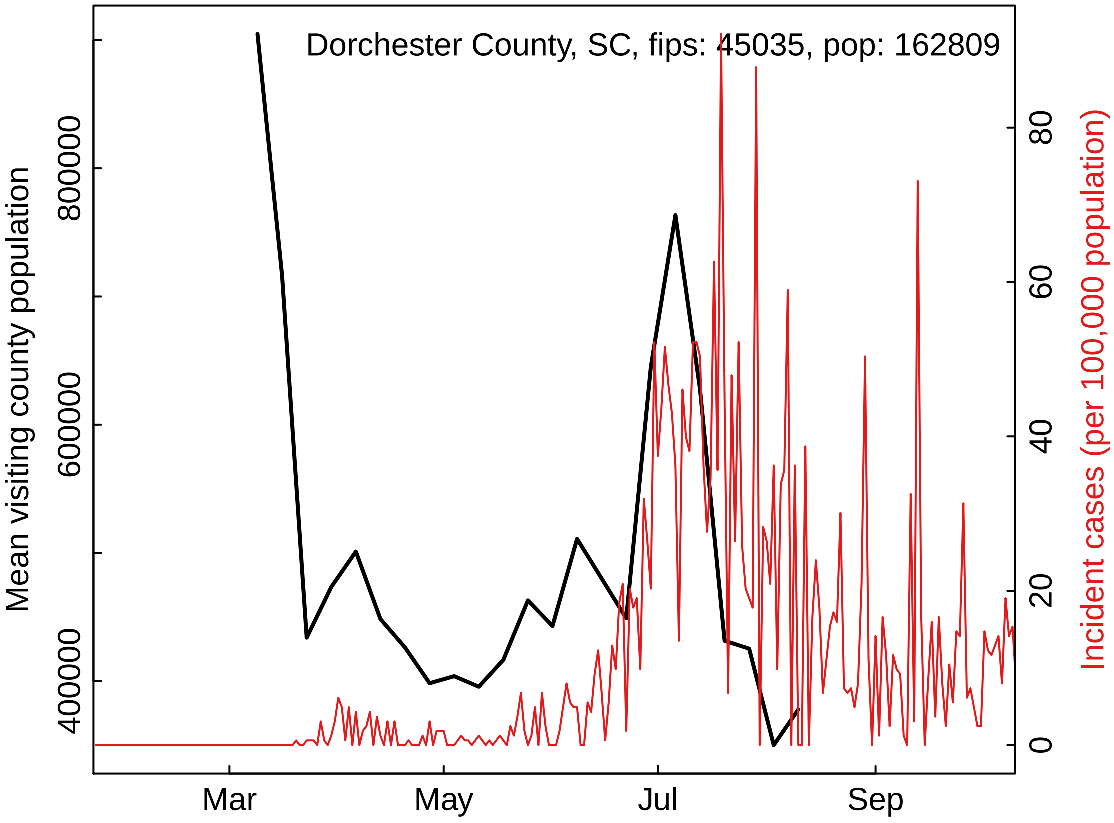
Dorchester County, SC, fips: 45035, pop: 162809
Mean visiting county population
400000
600000
800000
Incident cases (per 100,000 population)
0
20
40
60
80
Mar
May
Jul
Sep

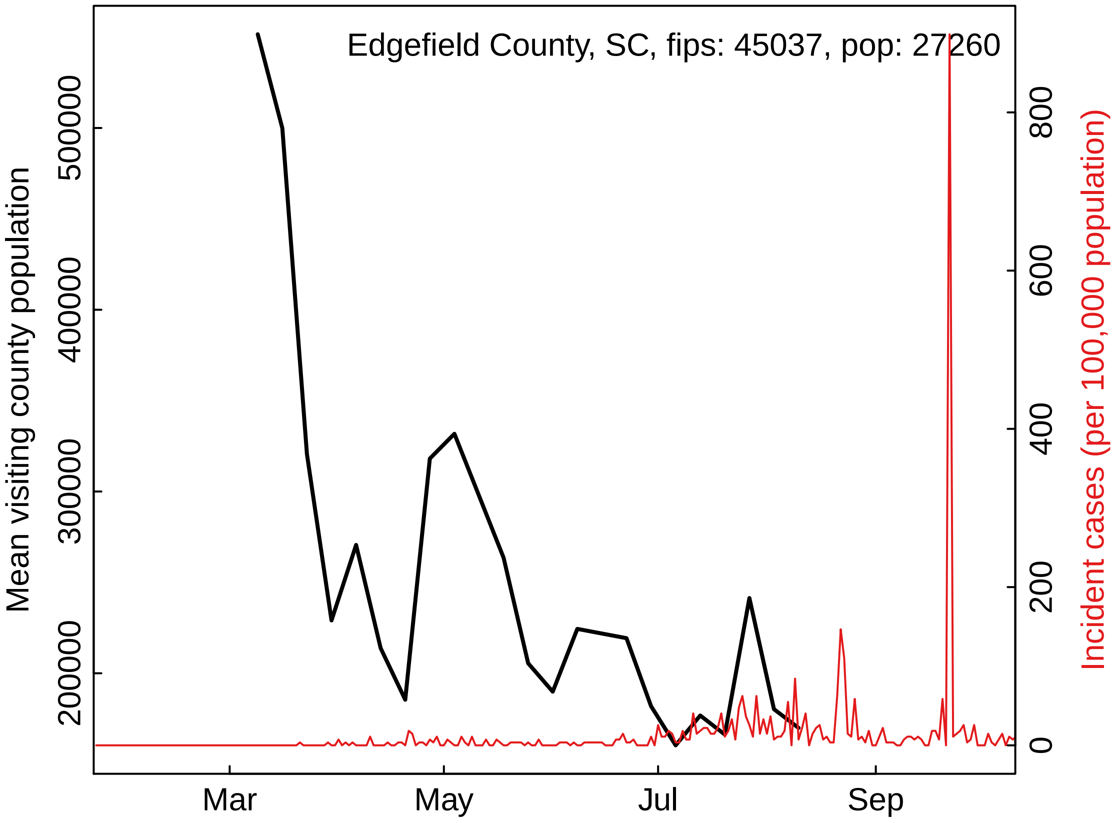

Edgefield County, SC, fips: 45037, pop: 27260
Mean visiting county population
200000
300000
400000
500000
Incident cases (per 100,000 population)
0
200
400
600
800
Mar
May
Jul
Sep

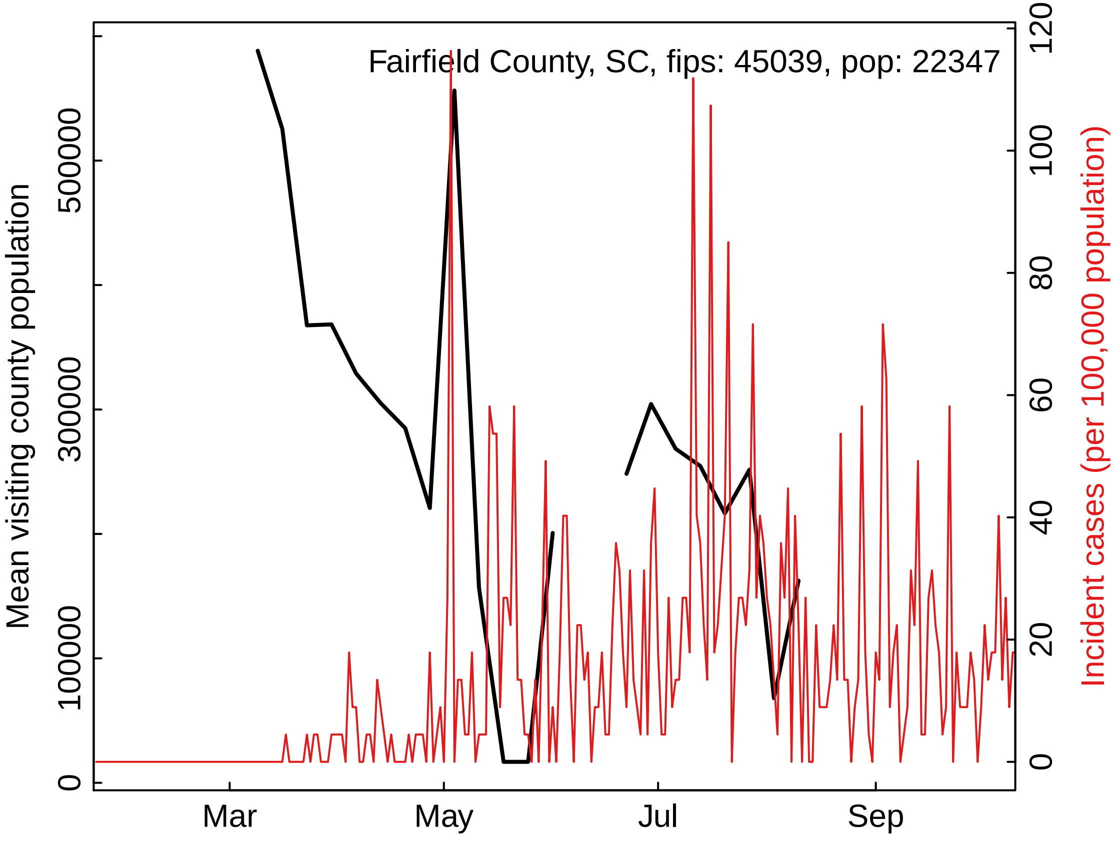

Fairfield County, SC, fips: 45039, pop: 22347
Mean visiting county population
0
100000
300000
500000
Mar
May
Jul
Sep
0
20
40
60
80
100
120
Incident cases (per 100,000 population)

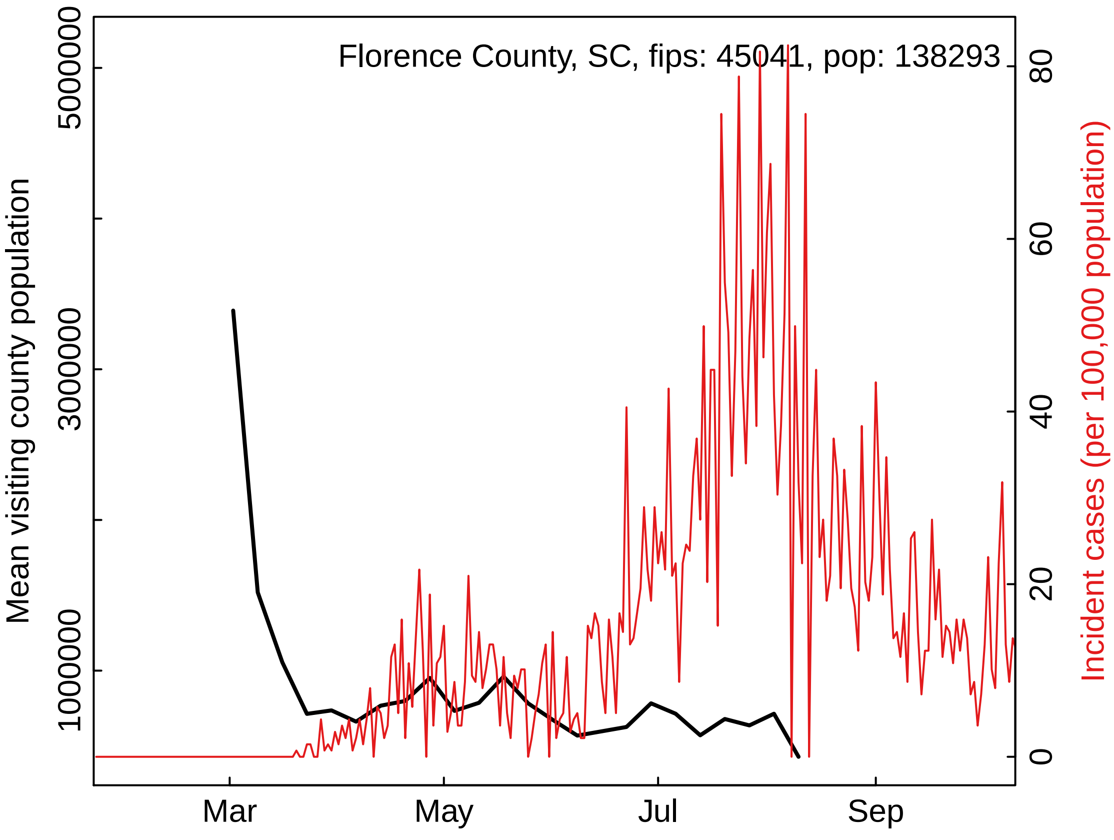

Florence County, SC, fips: 45041, pop: 138293
Mean visiting county population
1000000
3000000
5000000
Incident cases (per 100,000 population)
0
20
40
60
80
Mar
May
Jul
Sep

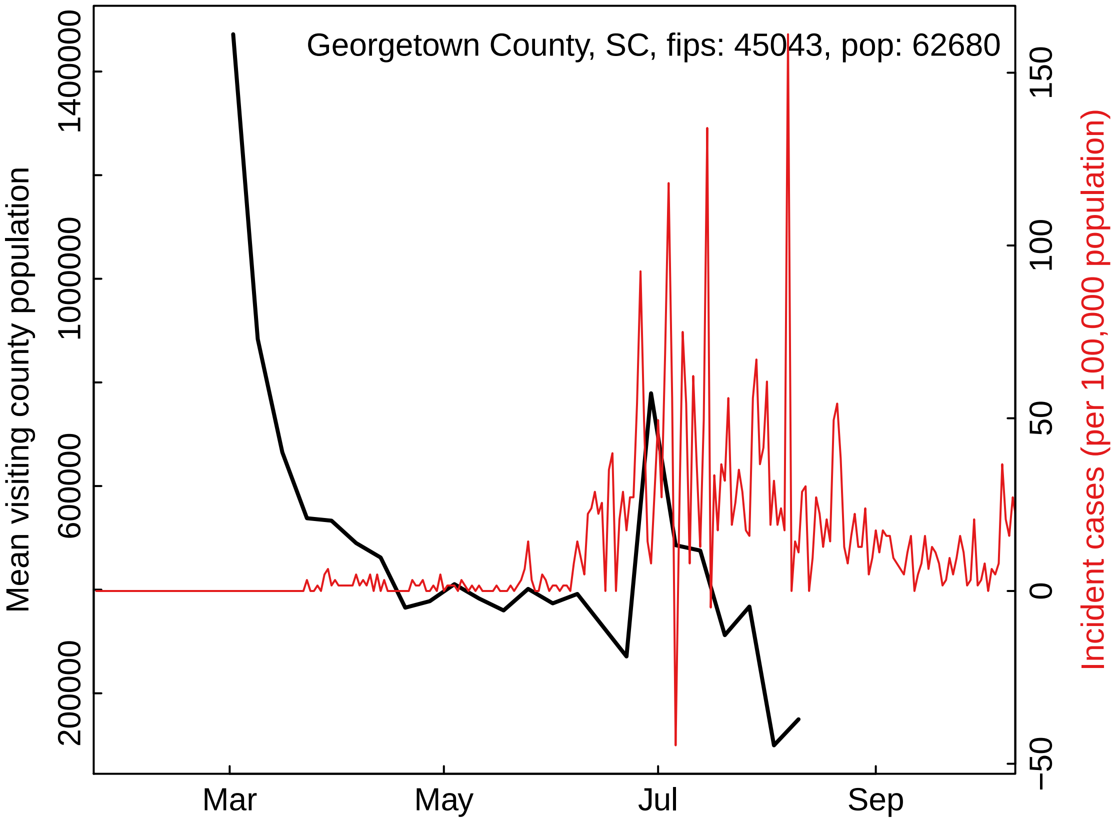

Georgetown County, SC, fips: 45043, pop: 62680
Mean visiting county population
200000
600000
1000000
1400000
Incident cases (per 100,000 population)
−50
0
50
100
150
Mar
May
Jul
Sep

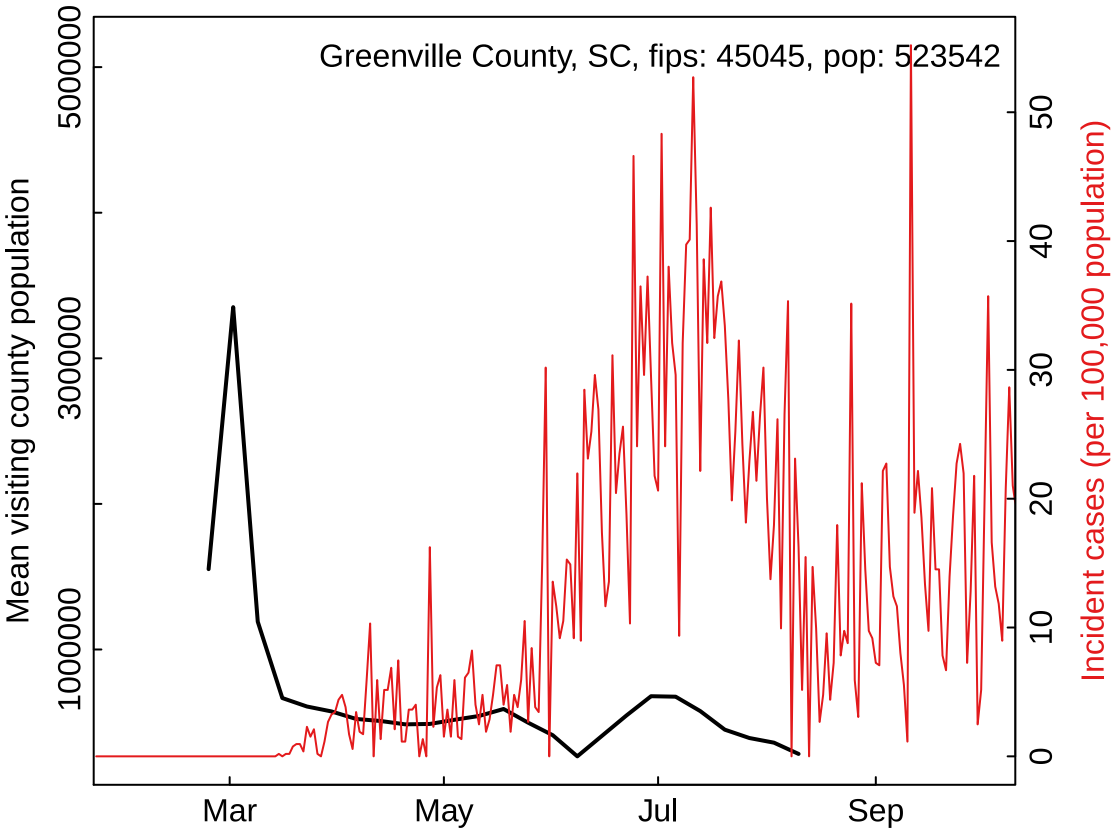

Greenville County, SC, fips: 45045, pop: 523542
Mean visiting county population
1000000
3000000
5000000
Incident cases (per 100,000 population)
0
10
20
30
40
50
Mar
May
Jul
Sep

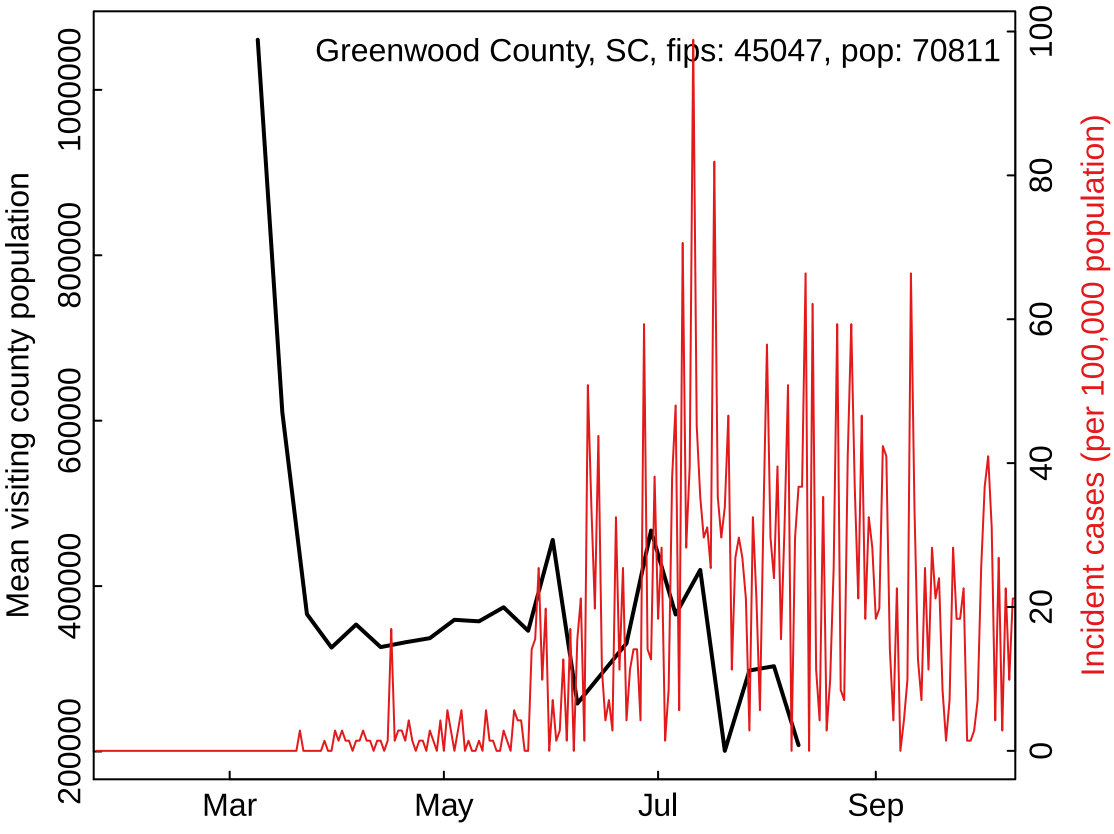

Greenwood County, SC, fips: 45047, pop: 70811
Mean visiting county population
Incident cases (per 100,000 population)
200000
400000
600000
800000
1000000
0
20
40
60
80
100
Mar
May
Jul
Sep

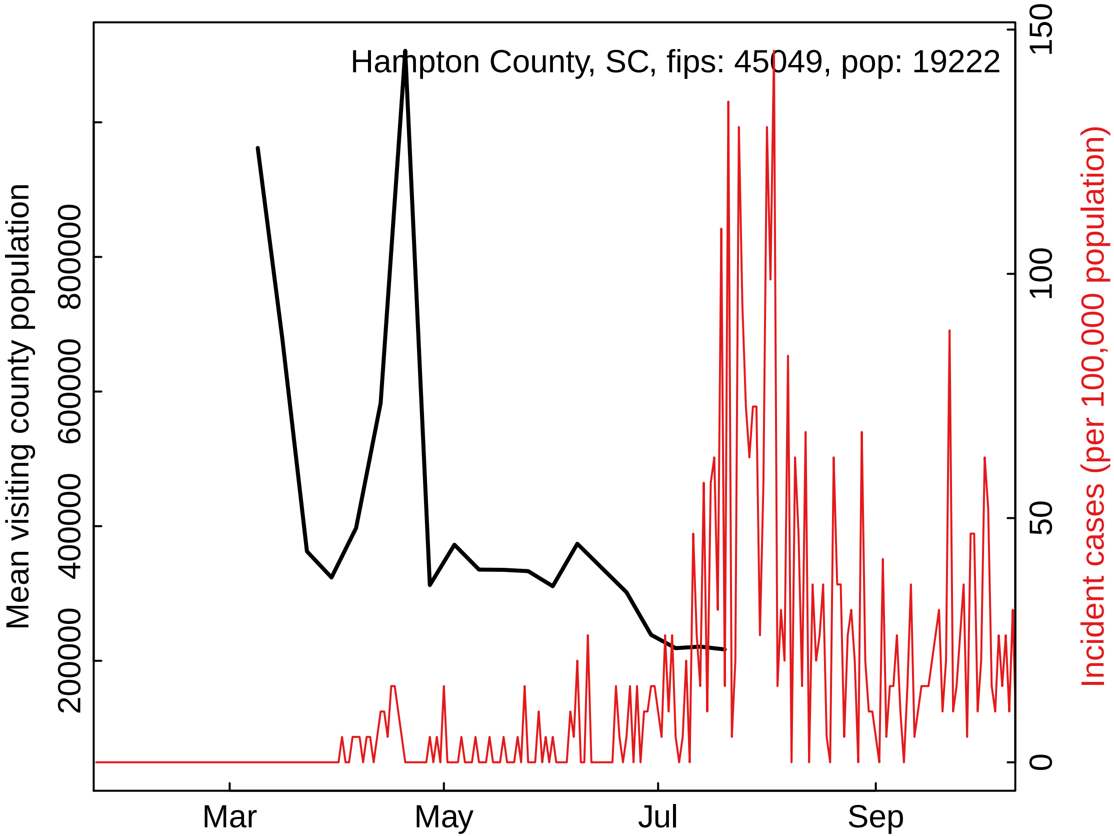

Hampton County, SC, fips: 45049, pop: 19222
Mean visiting county population
200000
400000
600000
800000
Incident cases (per 100,000 population)
0
50
100
150
Mar
May
Jul
Sep

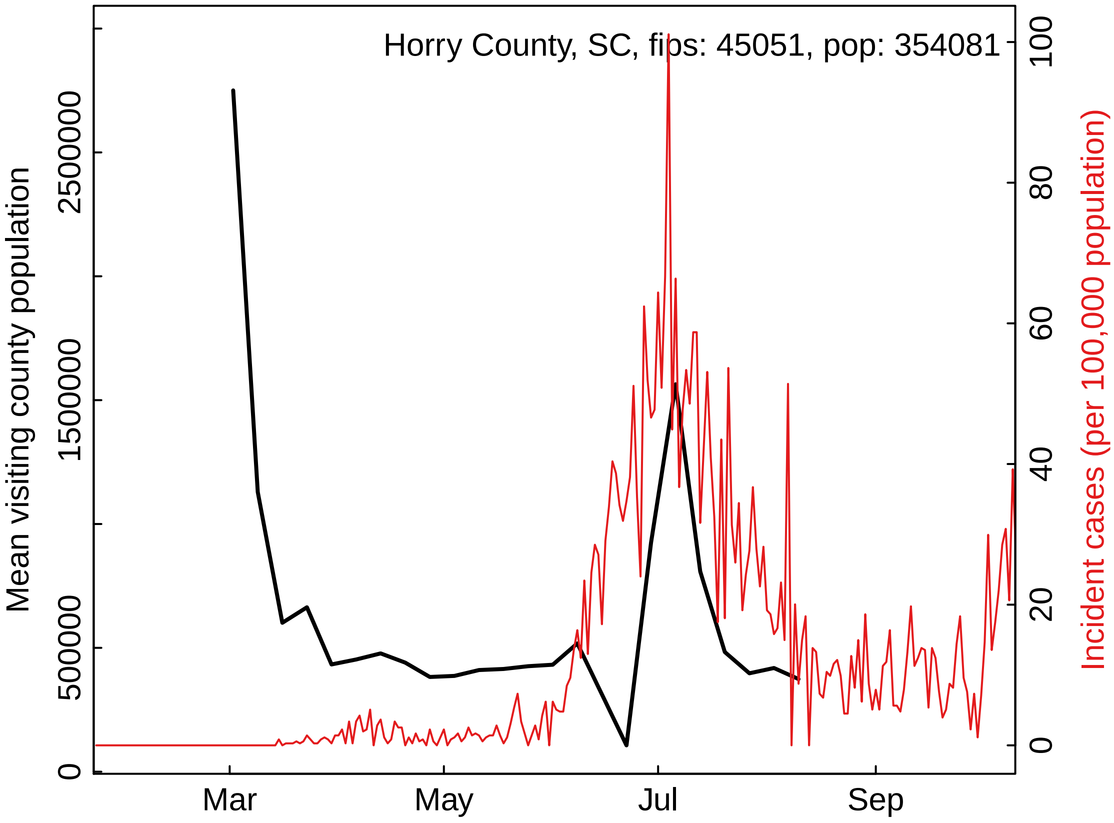
Horry County, SC, fips: 45051, pop: 354081
Mean visiting county population
Incident cases (per 100,000 population)
0
500000
1500000
2500000
0
20
40
60
80
100
Mar
May
Jul
Sep

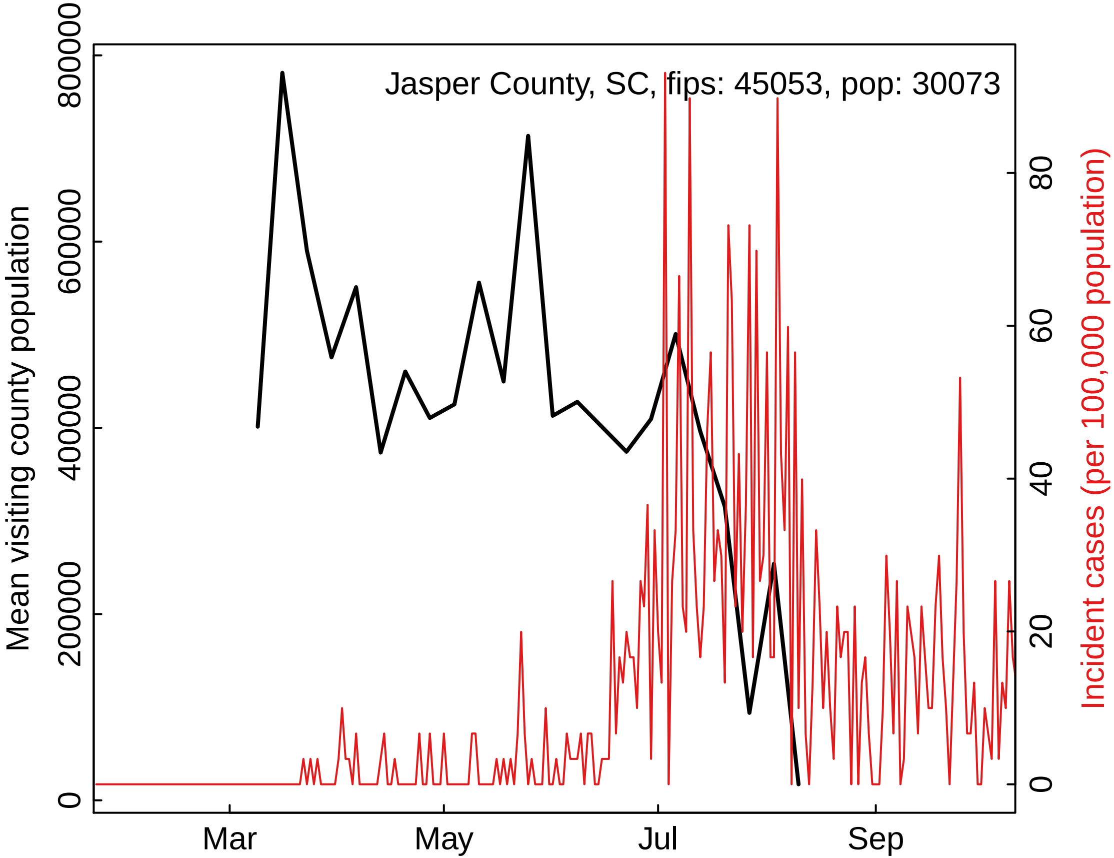
Jasper County, SC, fips: 45053, pop: 30073
Mean visiting county population
0
200000
400000
600000
800000
Incident cases (per 100,000 population)
0
20
40
60
80
Mar
May
Jul
Sep

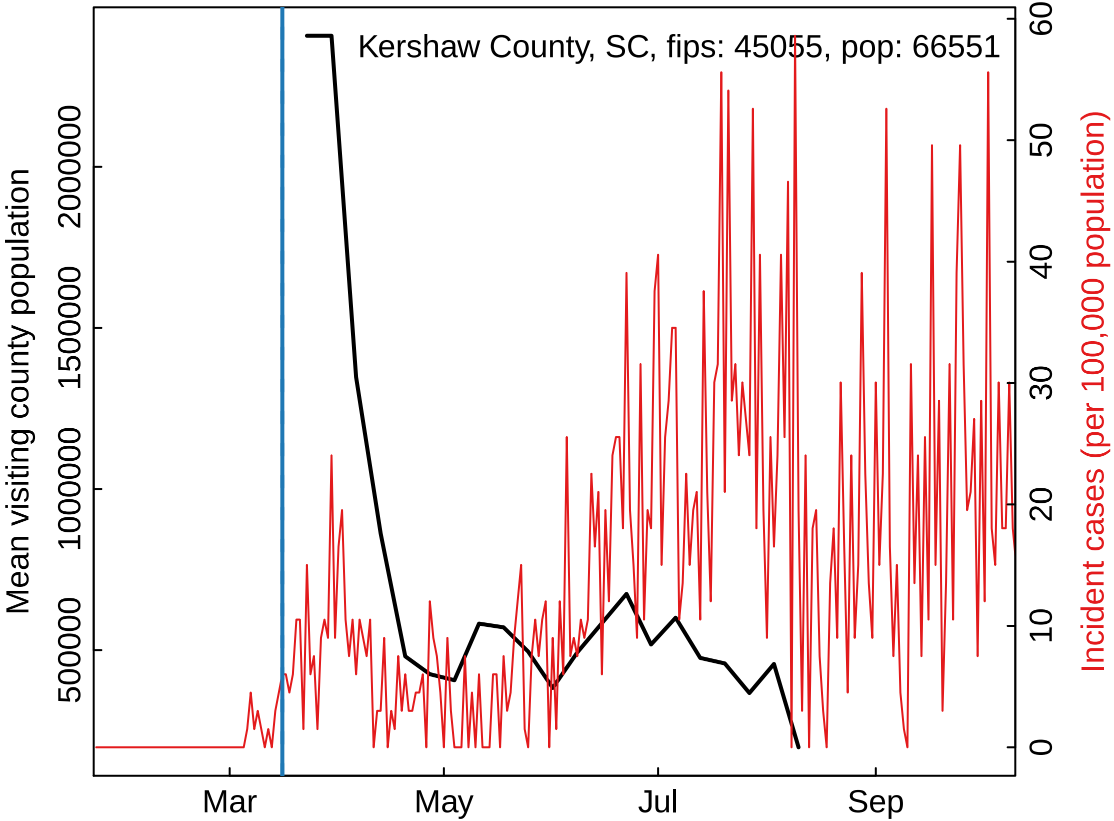

Kershaw County, SC, fips: 45055, pop: 66551
Mean visiting county population
500000
1000000
1500000
2000000
Incident cases (per 100,000 population)
0
10
20
30
40
50
60
Mar
May
Jul
Sep

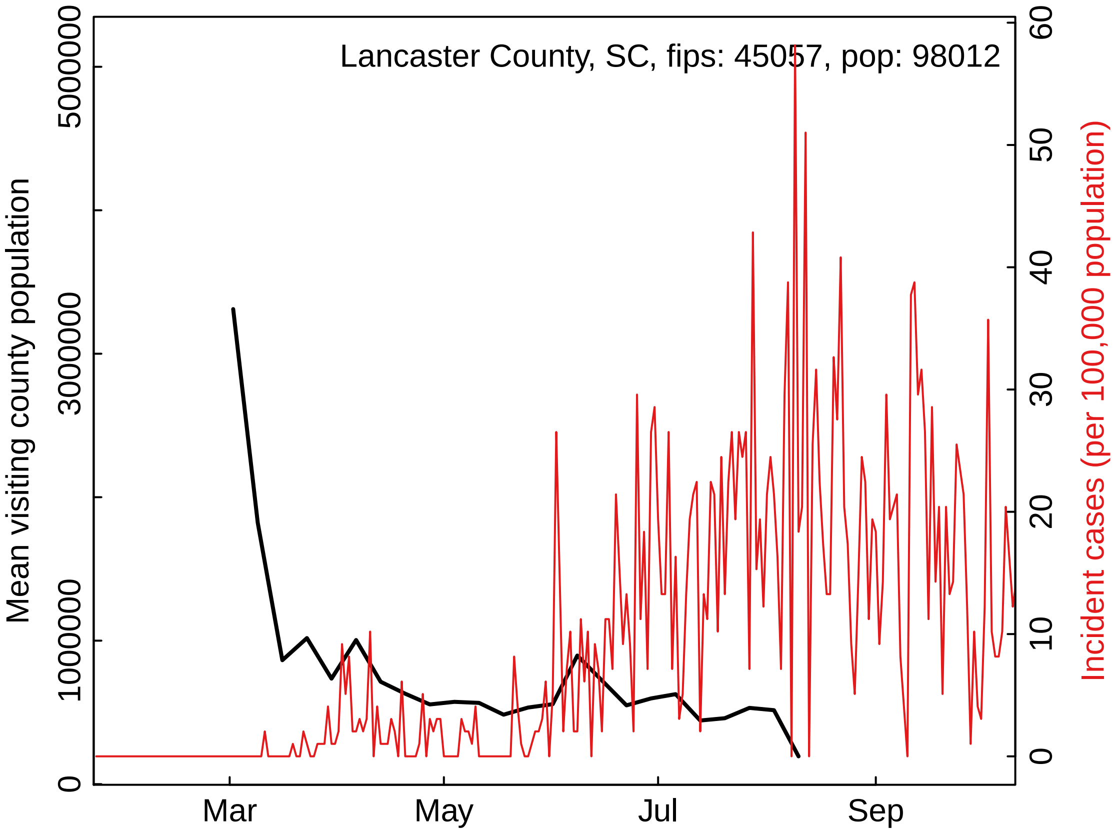

Lancaster County, SC, fips: 45057, pop: 98012
Mean visiting county population
0
1000000
3000000
5000000
Incident cases (per 100,000 population)
0
10
20
30
40
50
60
Mar
May
Jul
Sep

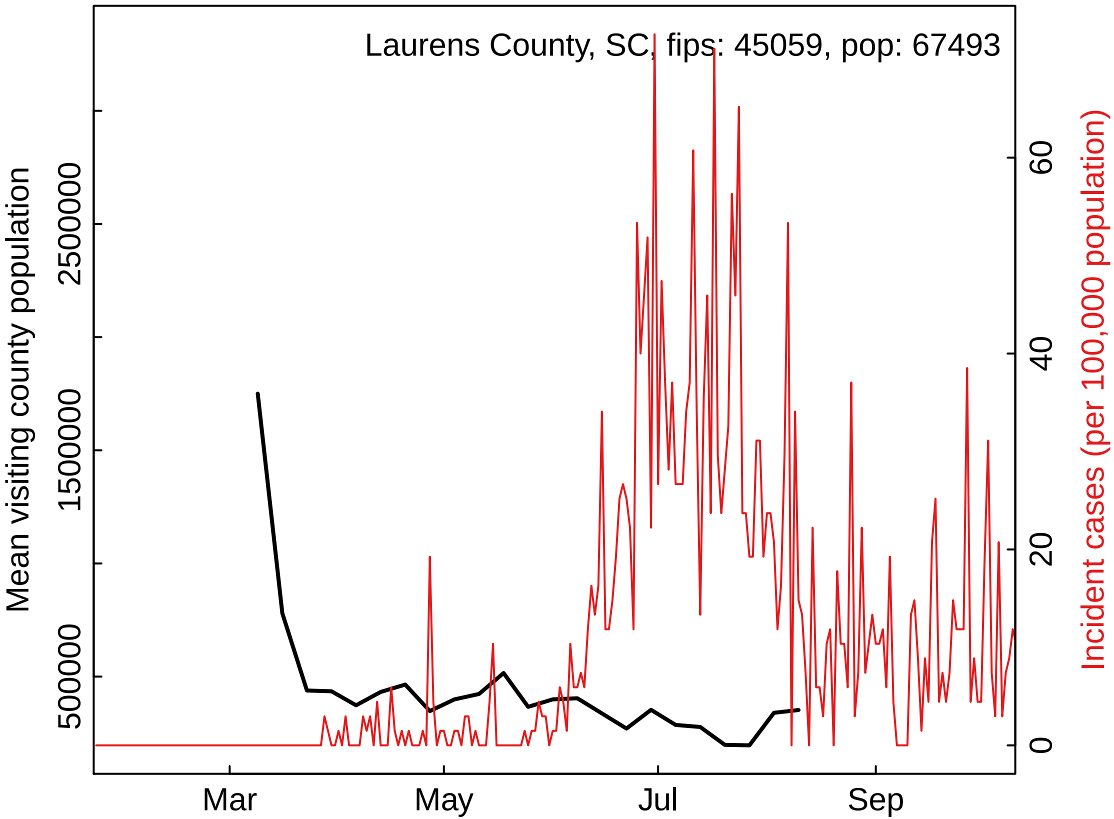
Laurens County, SC, fips: 45059, pop: 67493
Mean visiting county population
500000
1500000
2500000
Mar
May
Jul
Sep
0
20
40
60
Incident cases (per 100,000 population)

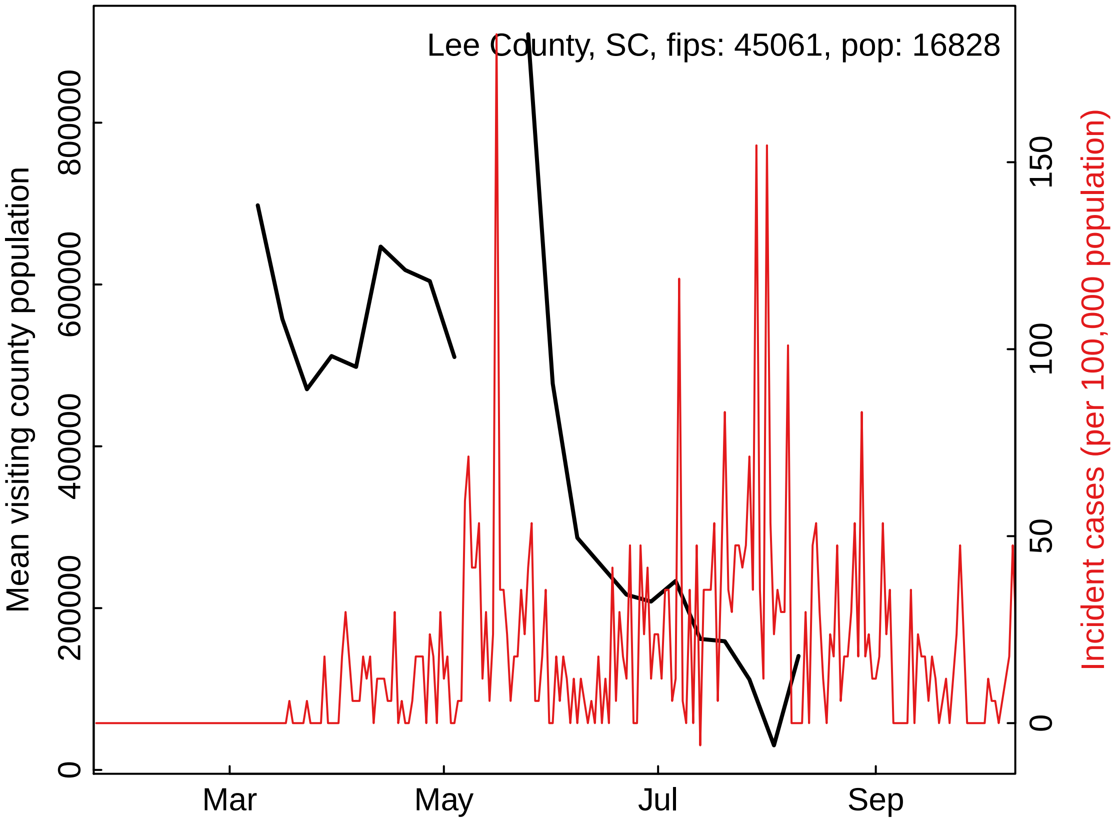
Lee County, SC, fips: 45061, pop: 16828
Mean visiting county population
0
200000
400000
600000
800000
Incident cases (per 100,000 population)
0
50
100
150
Mar
May
Jul
Sep

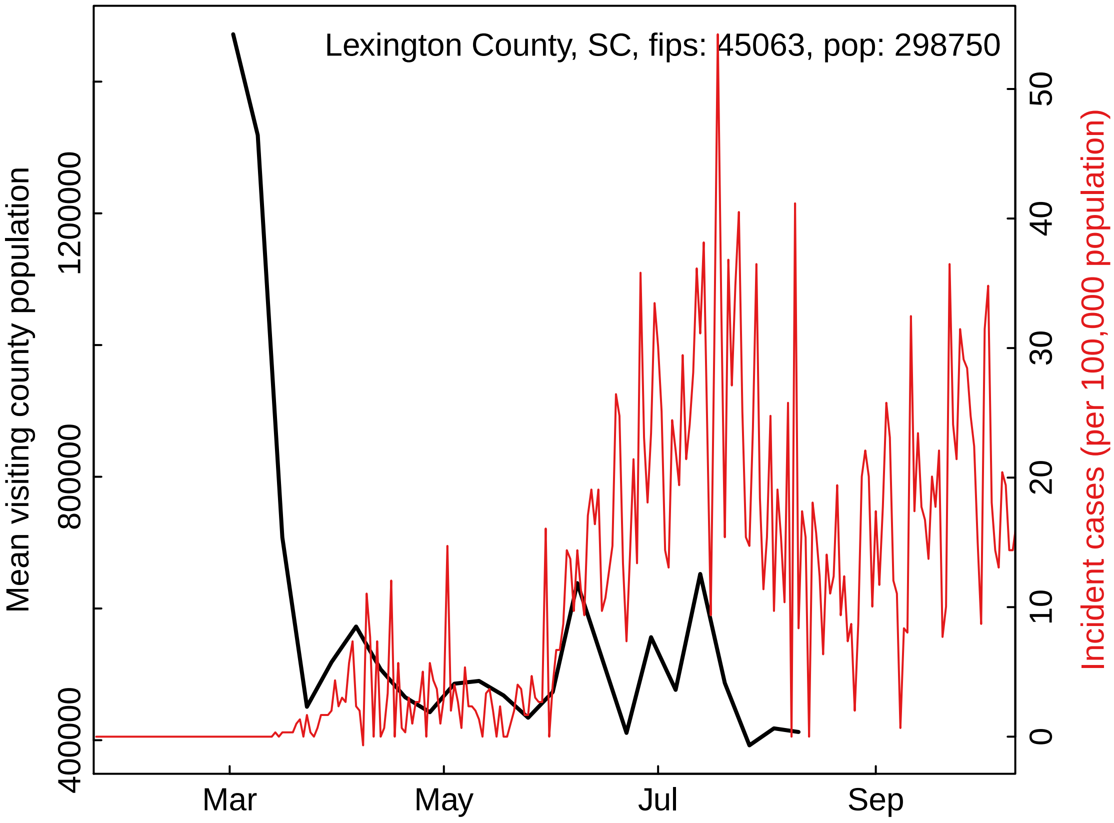

Lexington County, SC, fips: 45063, pop: 298750
Mean visiting county population
400000
800000
1200000
Incident cases (per 100,000 population)
0
10
20
30
40
50
Mar
May
Jul
Sep

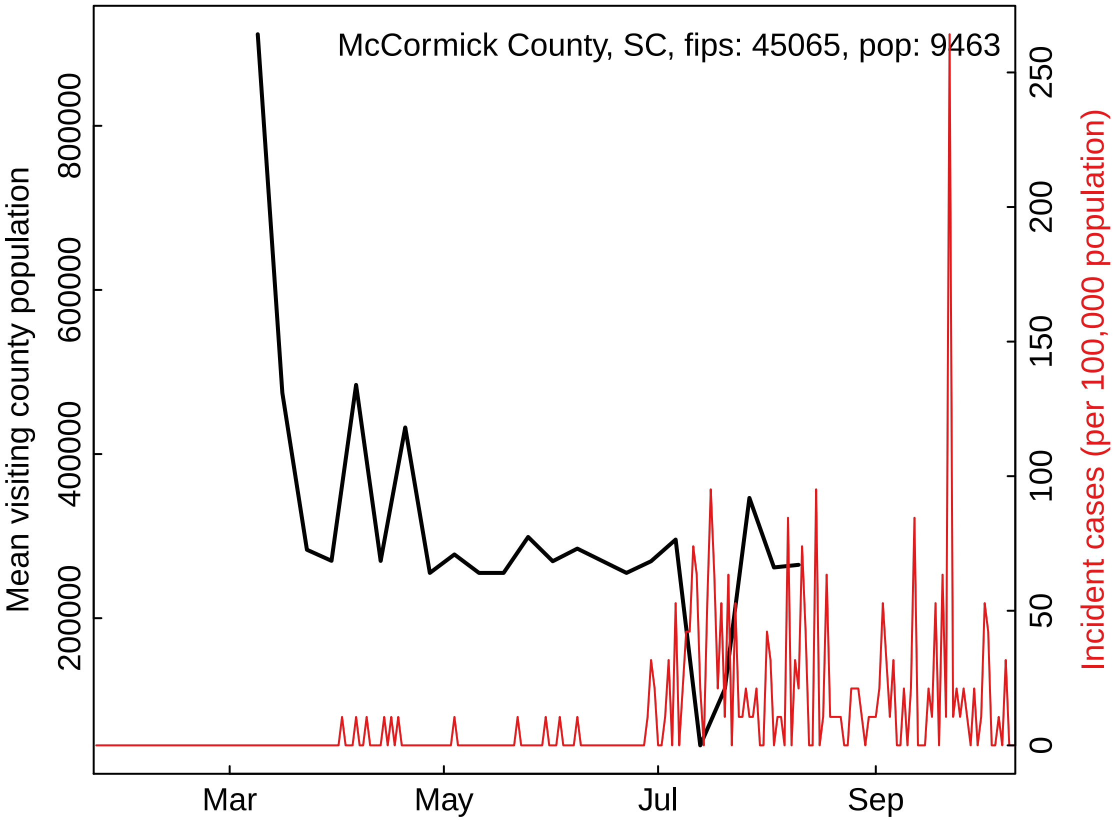
McCormick County, SC, fips: 45065, pop: 9463
Mean visiting county population
200000
400000
600000
800000
Incident cases (per 100,000 population)
0
50
100
150
200
250
Mar
May
Jul
Sep

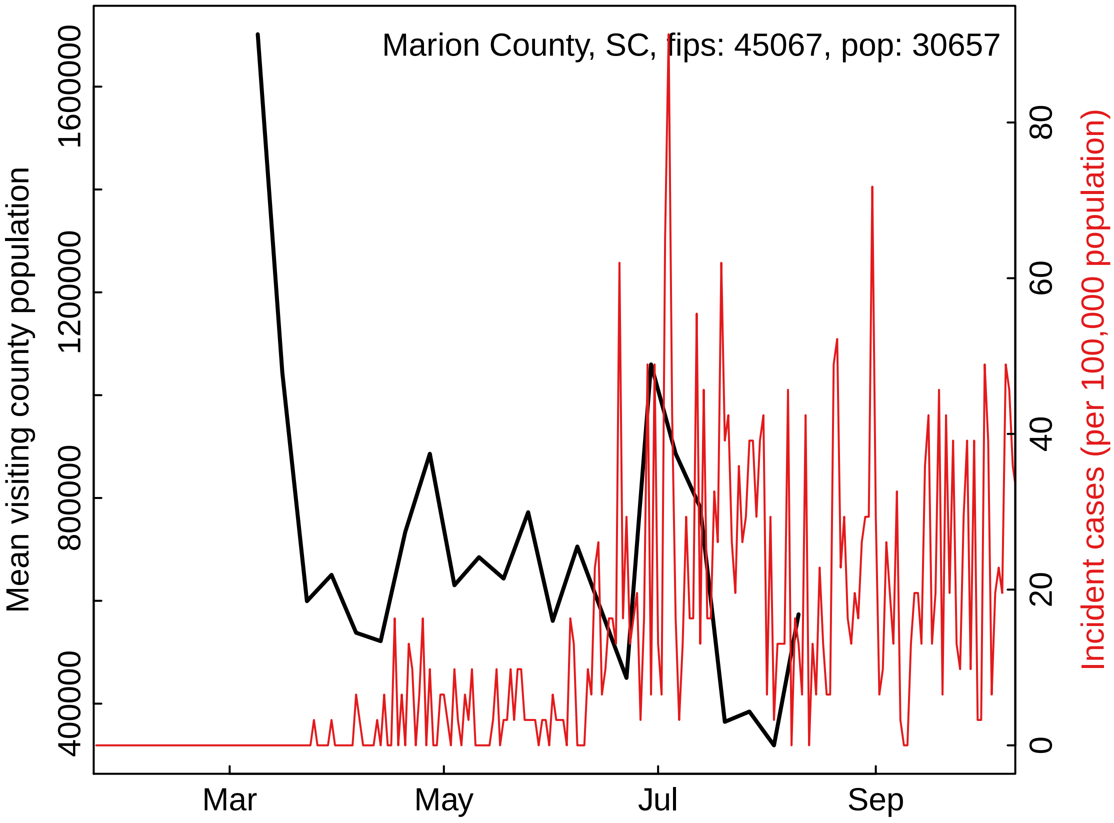

Marion County, SC, fips: 45067, pop: 30657
Mean visiting county population
400000
800000
1200000
1600000
Incident cases (per 100,000 population)
0
20
40
60
80
Mar
May
Jul
Sep

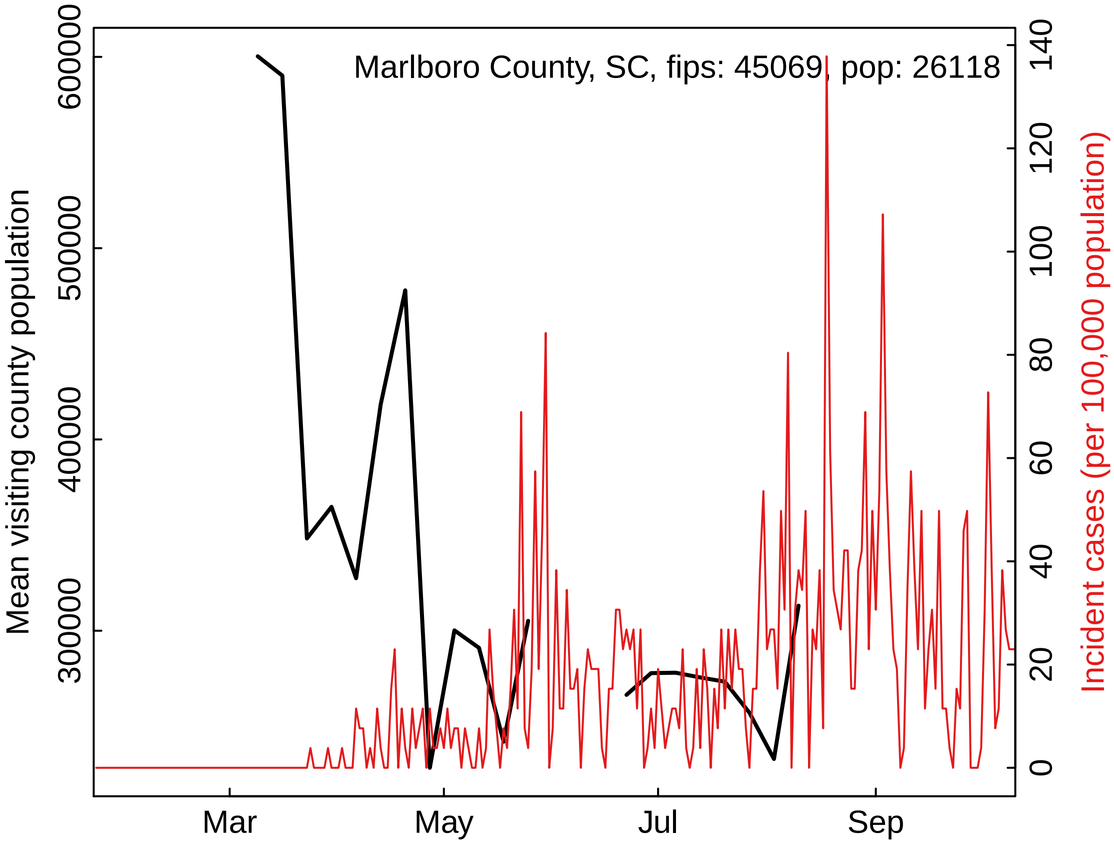
Marlboro County, SC, fips: 45069, pop: 26118
Mean visiting county population
300000
400000
500000
600000
Incident cases (per 100,000 population)
0
20
40
60
80
100
120
140
Mar
May
Jul
Sep

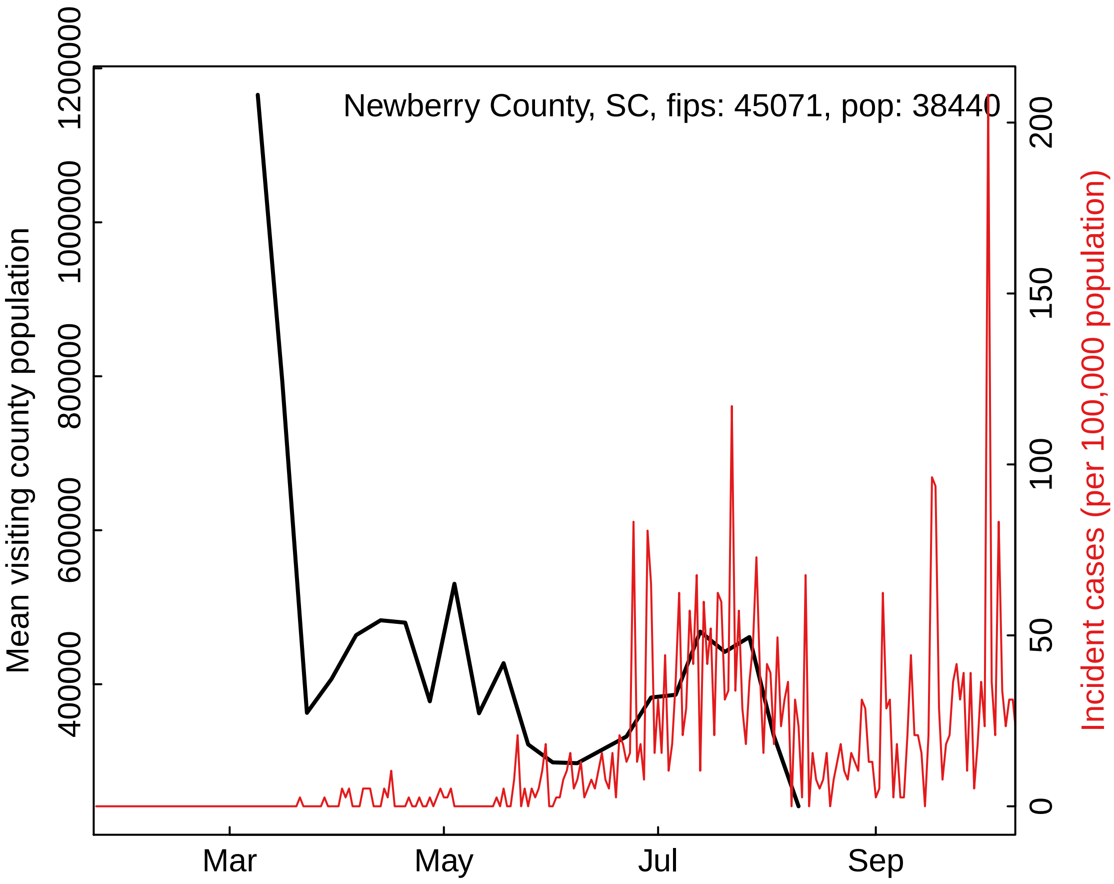

Newberry County, SC, fips: 45071, pop: 38440
Mean visiting county population
400000
600000
800000
1000000
1200000
Incident cases (per 100,000 population)
0
50
100
150
200
Mar
May
Jul
Sep

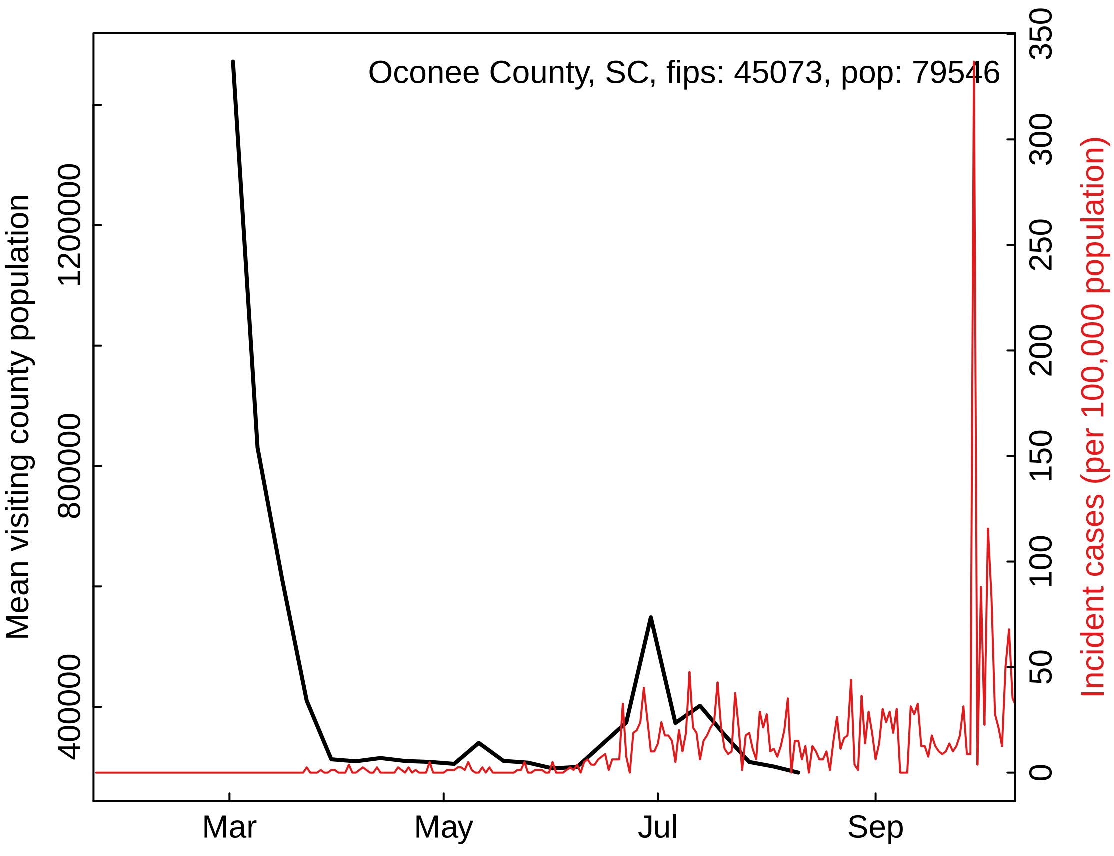

Oconee County, SC, fips: 45073, pop: 79546
Mean visiting county population
400000
800000
1200000
Mar
May
Jul
Sep
0
50
100
150
200
250
300
350
Incident cases (per 100,000 population)

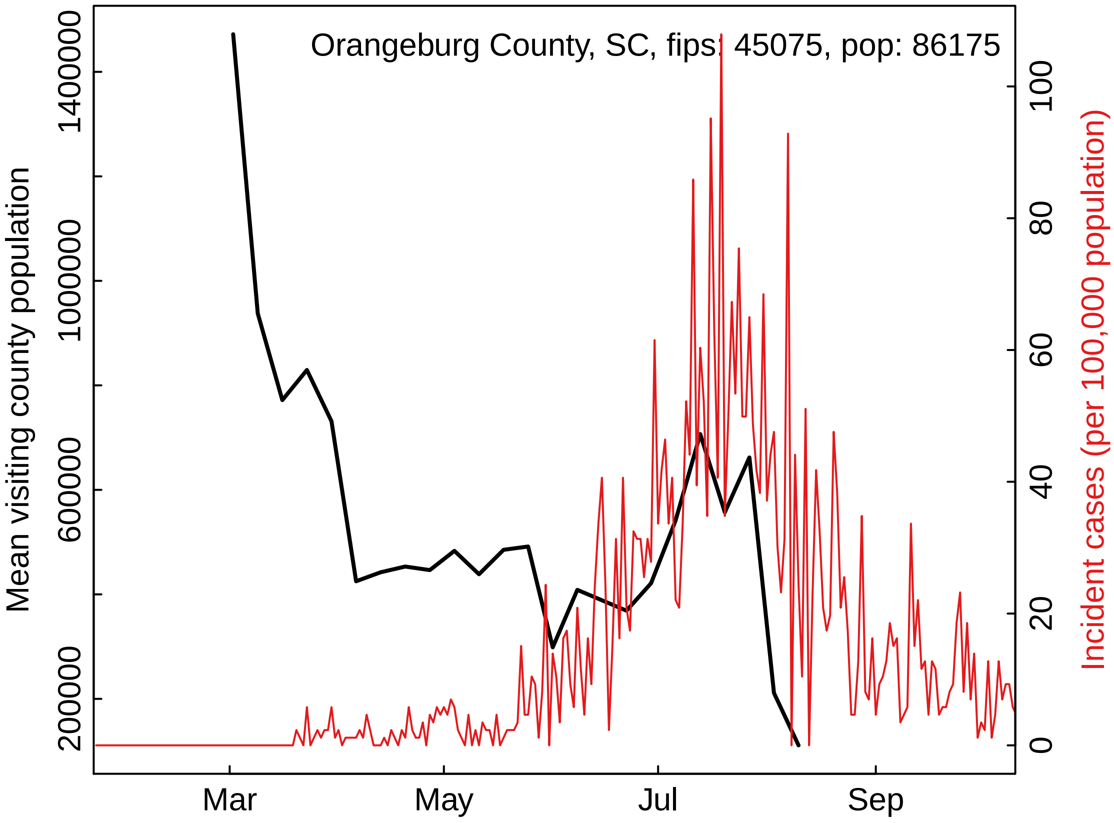

Orangeburg County, SC, fips: 45075, pop: 86175
Mean visiting county population
Incident cases (per 100,000 population)
200000
600000
1000000
1400000
0
20
40
60
80
100
Mar
May
Jul
Sep

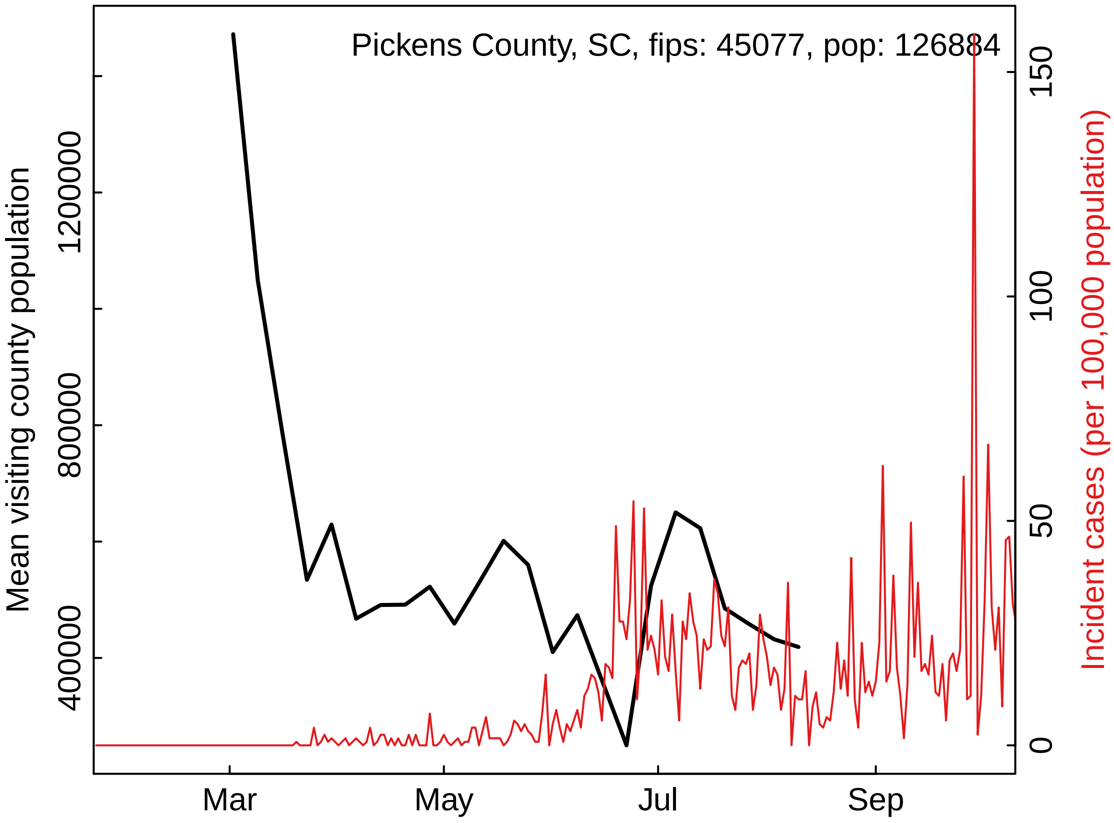

Pickens County, SC, fips: 45077, pop: 126884
Mean visiting county population
400000
800000
1200000
Incident cases (per 100,000 population)
0
50
100
150
Mar
May
Jul
Sep

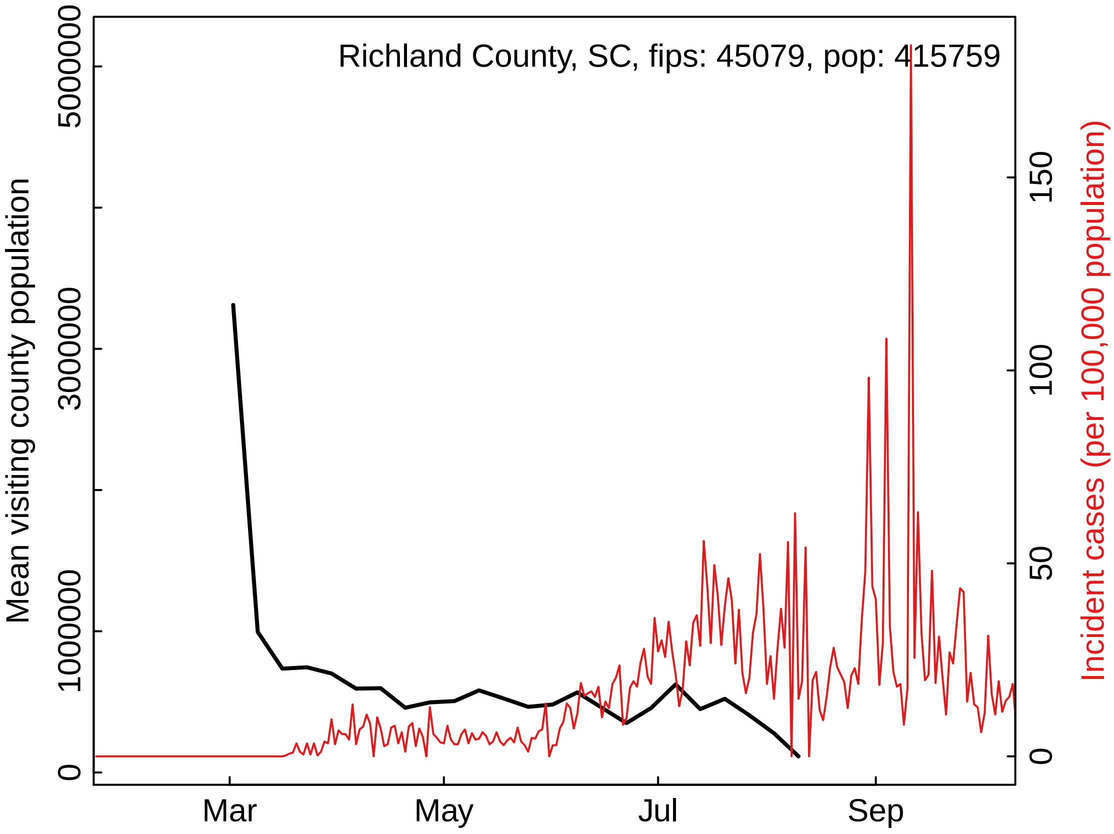

Richland County, SC, fips: 45079, pop: 415759
Mean visiting county population
0
1000000
3000000
5000000
Incident cases (per 100,000 population)
0
50
100
150
Mar
May
Jul
Sep

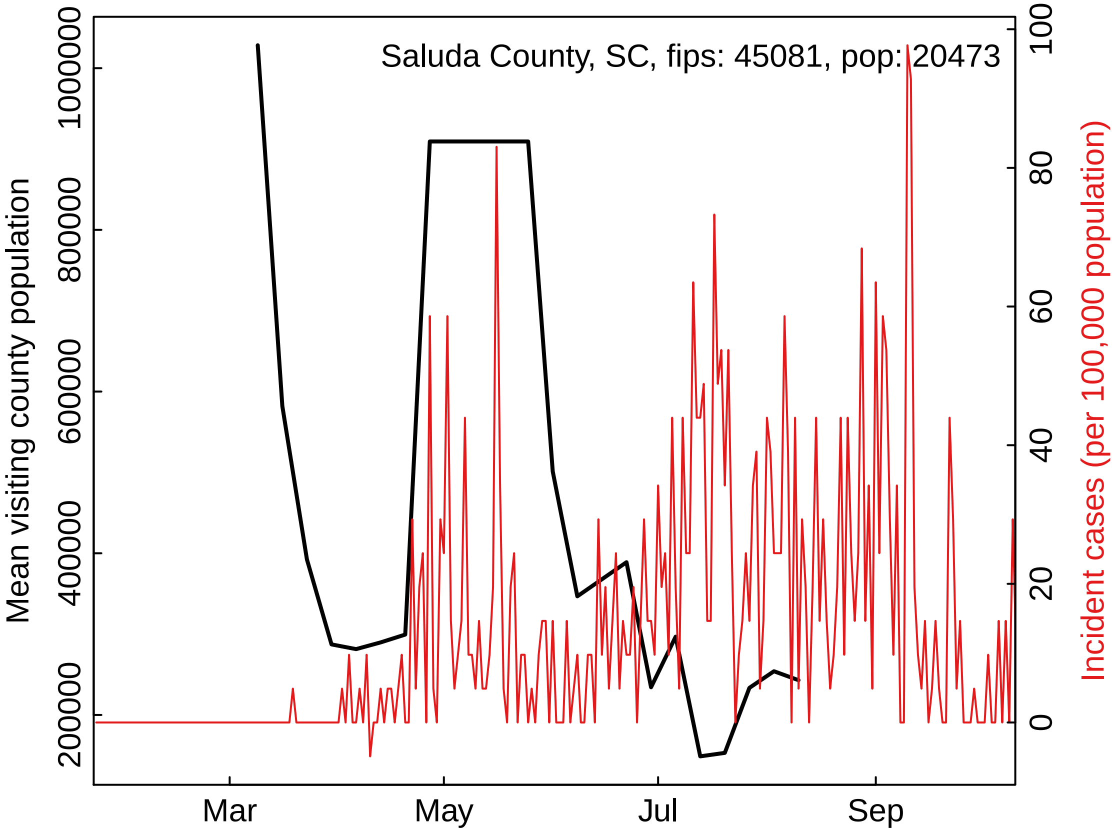

Saluda County, SC, fips: 45081, pop: 20473
Mean visiting county population
200000
400000
600000
800000
1000000
Incident cases (per 100,000 population)
0
20
40
60
80
100
Mar
May
Jul
Sep

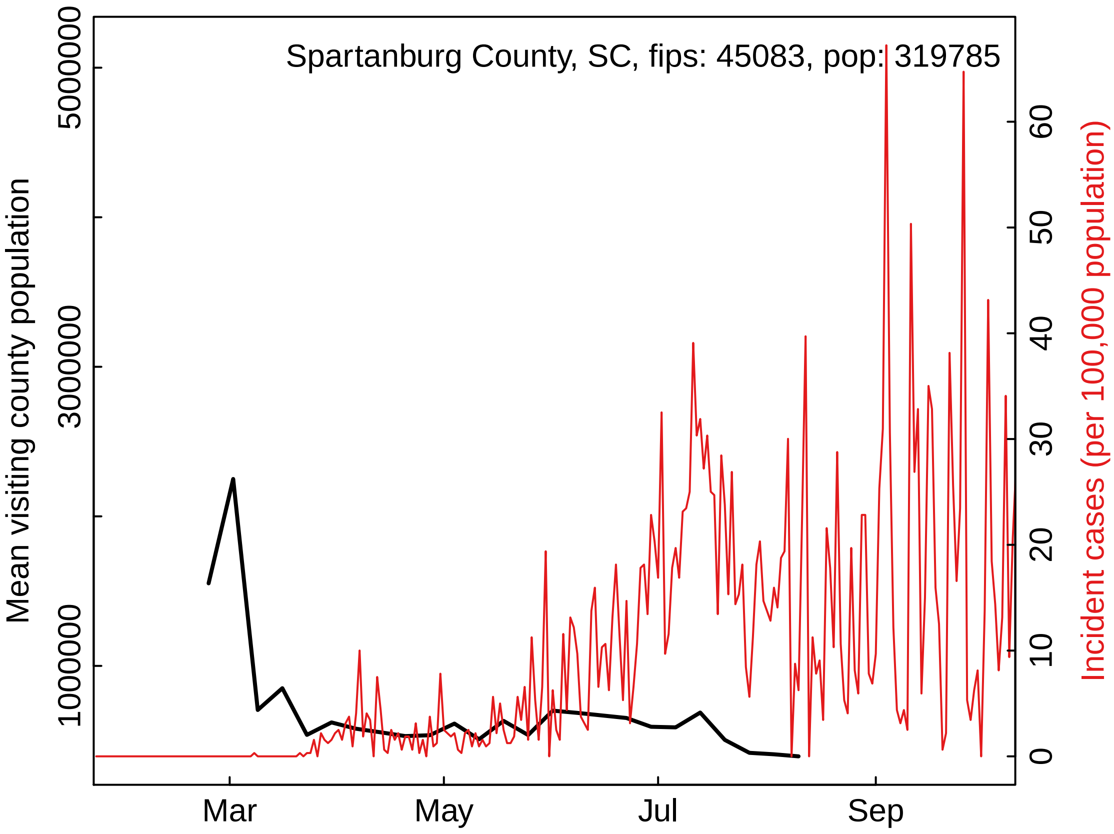

Spartanburg County, SC, fips: 45083, pop: 319785
Mean visiting county population
1000000
3000000
5000000
Incident cases (per 100,000 population)
0
10
20
30
40
50
60
Mar
May
Jul
Sep

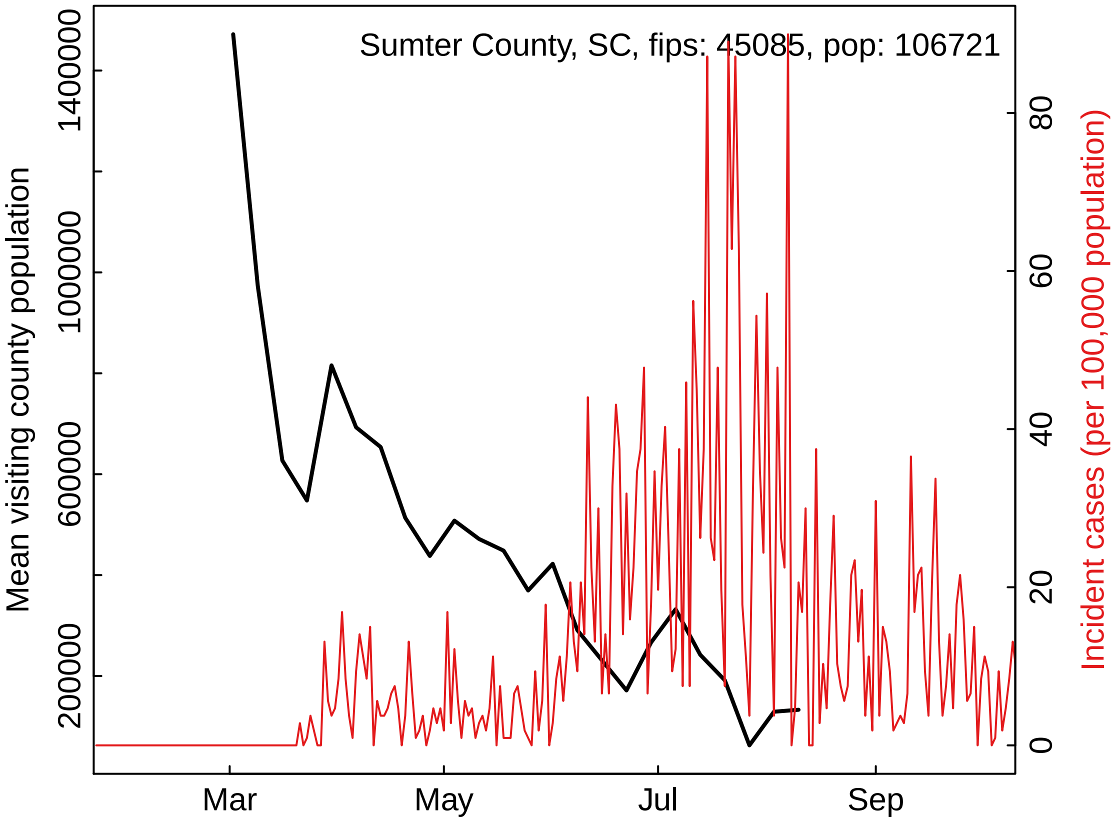

Sumter County, SC, fips: 45085, pop: 106721
Mean visiting county population
200000
600000
1000000
1400000
Incident cases (per 100,000 population)
0
20
40
60
80
Mar
May
Jul
Sep

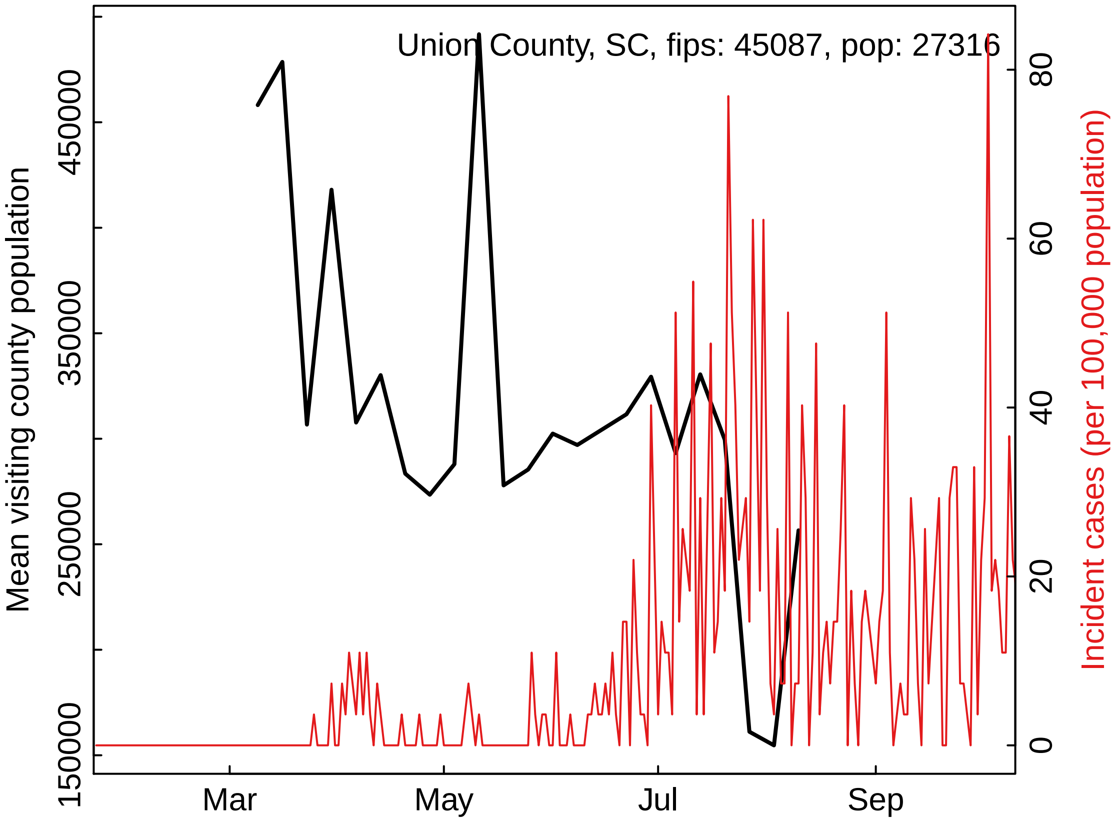

Union County, SC, fips: 45087, pop: 27316
Mean visiting county population
Incident cases (per 100,000 population)
150000
250000
350000
450000
0
20
40
60
80
Mar
May
Jul
Sep

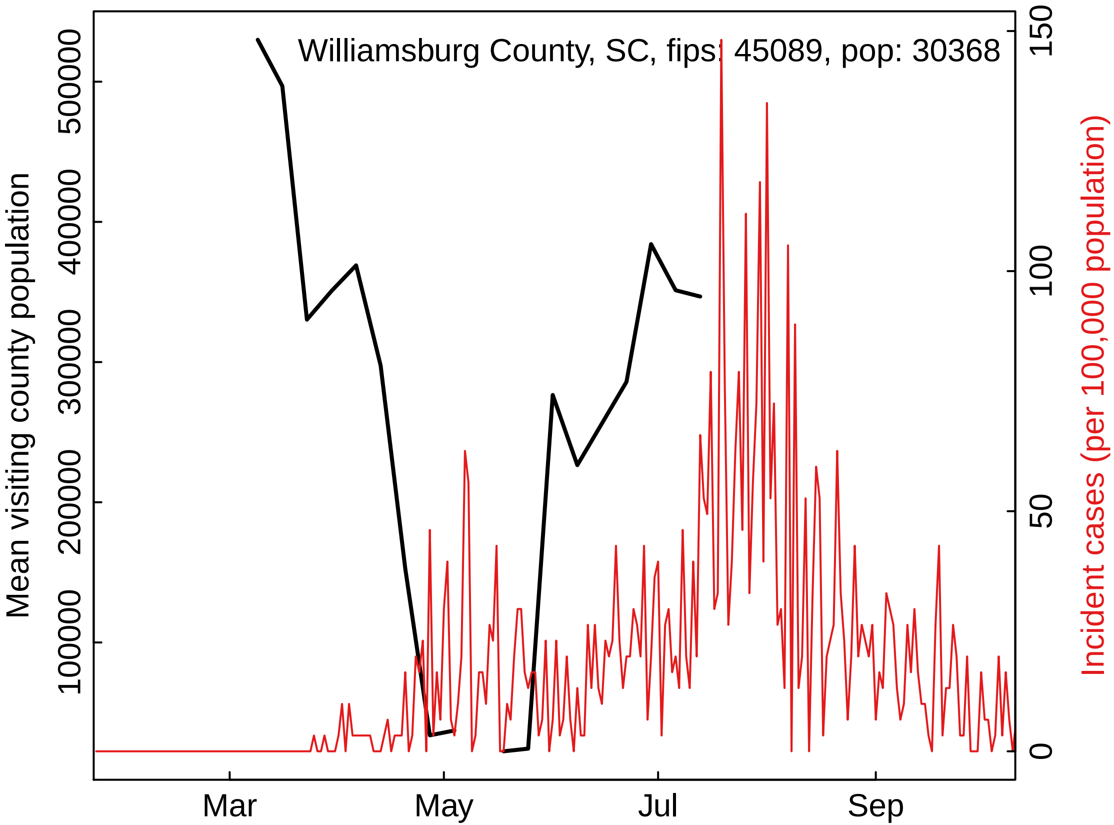

Williamsburg County, SC, fips: 45089, pop: 30368
Mean visiting county population
100000
200000
300000
400000
500000
Incident cases (per 100,000 population)
0
50
100
150
Mar
May
Jul
Sep

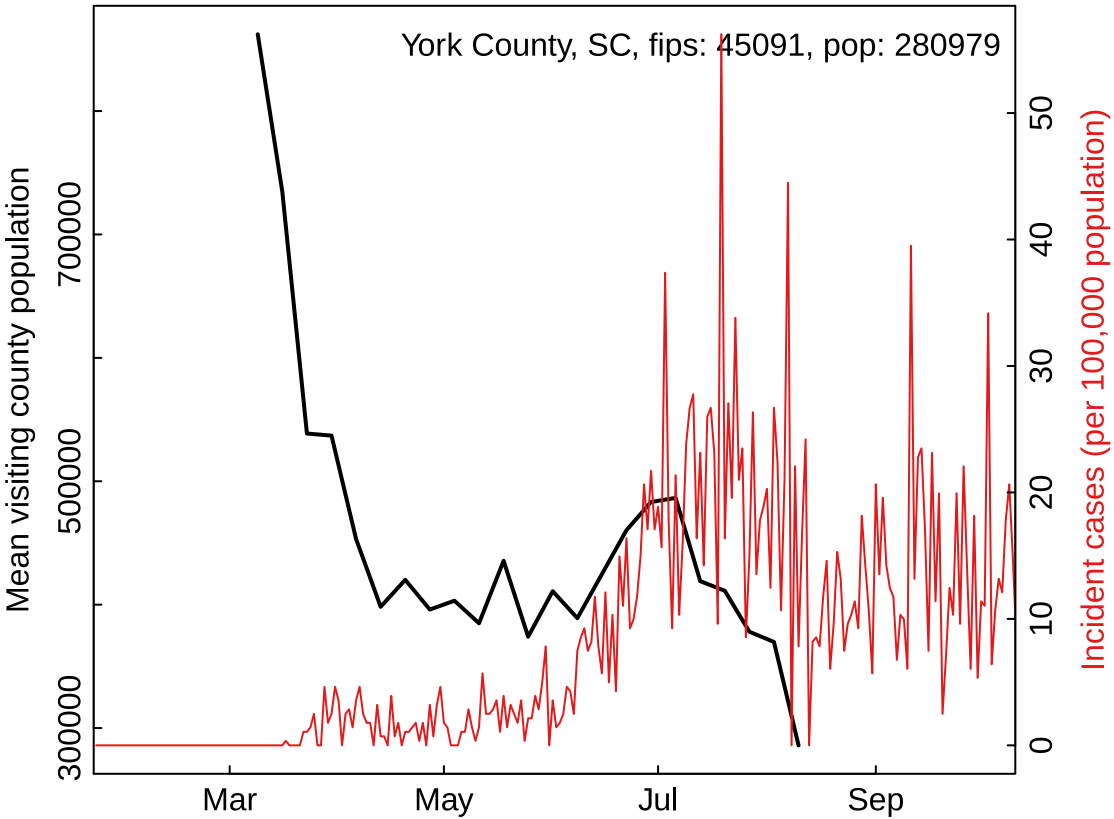

York County, SC, fips: 45091, pop: 280979
Mean visiting county population
300000
500000
700000
Incident cases (per 100,000 population)
0
10
20
30
40
50
Mar
May
Jul
Sep

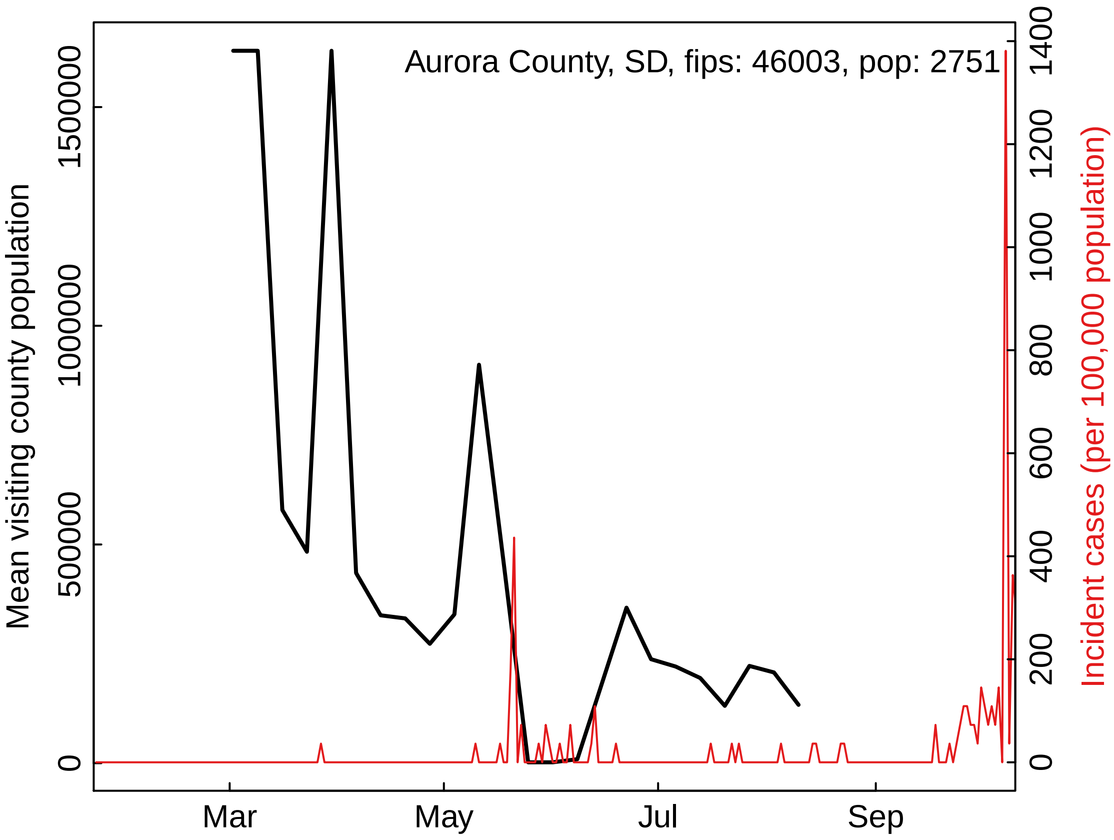

Aurora County, SD, fips: 46003, pop: 2751
Mean visiting county population
0
500000
1000000
1500000
Incident cases (per 100,000 population)
0
200
400
600
800
1000
1200
1400
Mar
May
Jul
Sep

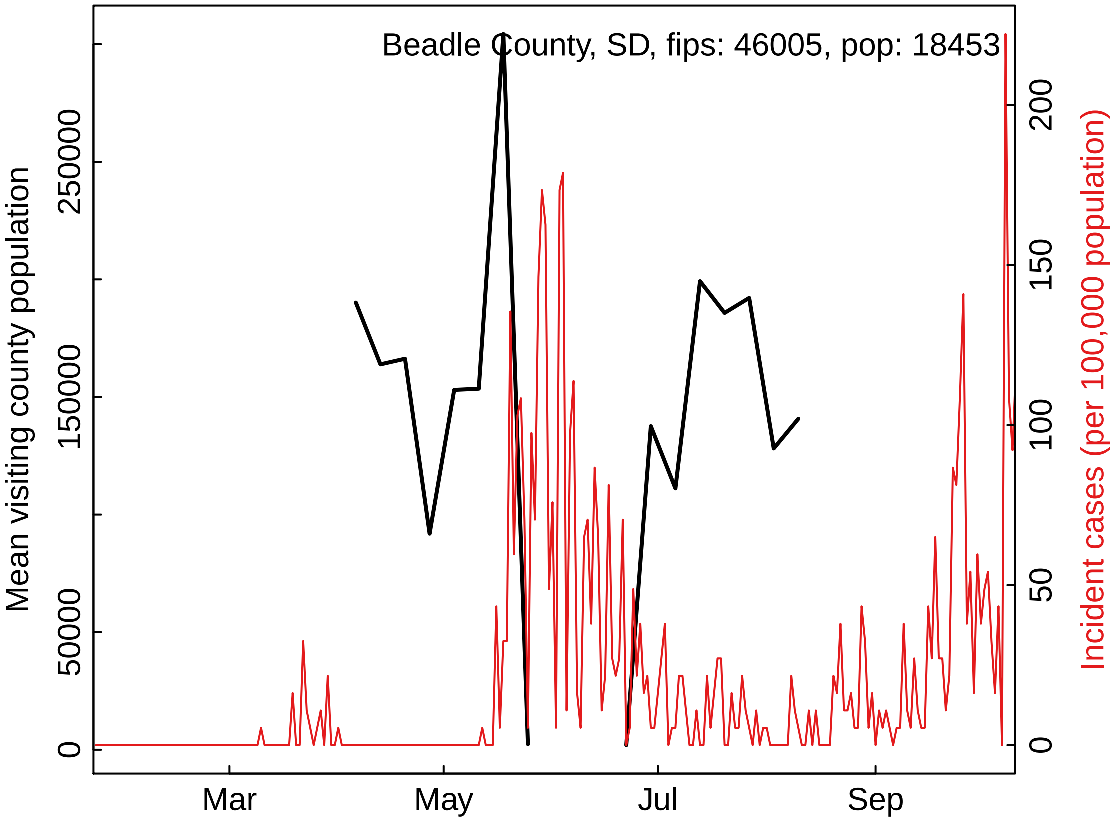

Beadle County, SD, fips: 46005, pop: 18453
Mean visiting county population
0
50000
150000
250000
Incident cases (per 100,000 population)
0
50
100
150
200
Mar
May
Jul
Sep

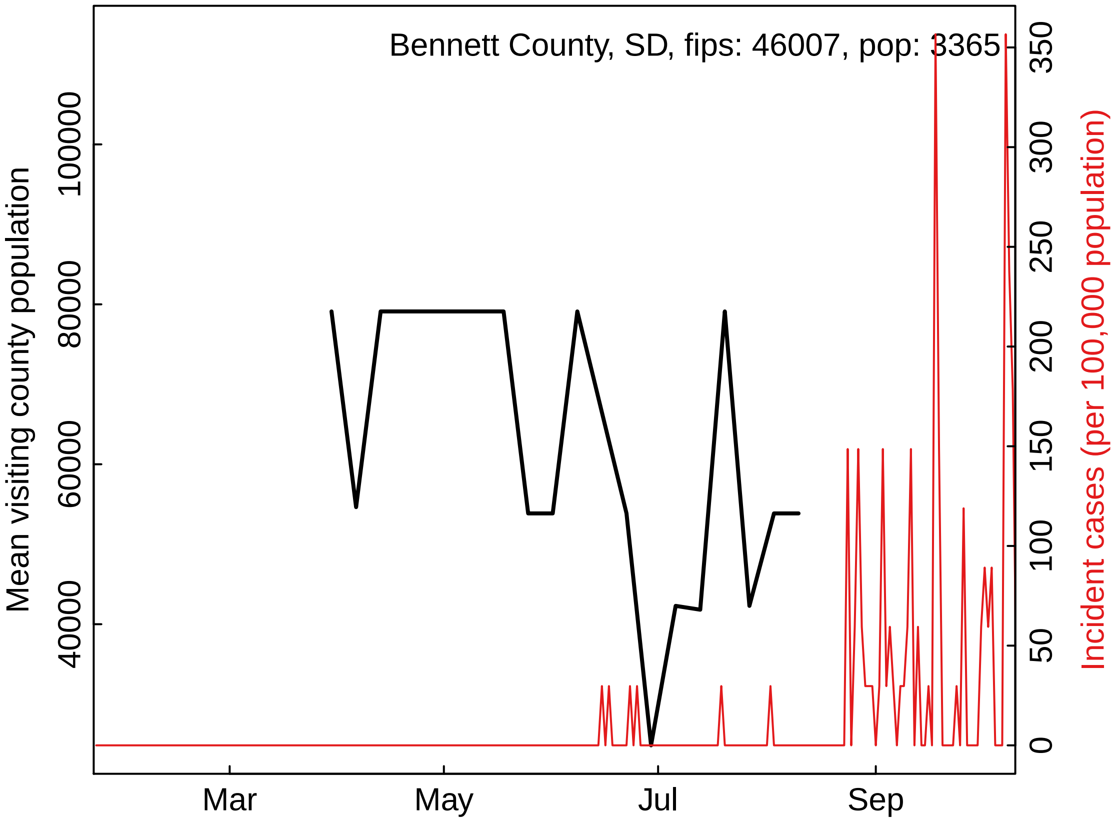

Bennett County, SD, fips: 46007, pop: 3365
Mean visiting county population
40000
60000
80000
100000
Incident cases (per 100,000 population)
0
50
100
150
200
250
300
350
Mar
May
Jul
Sep

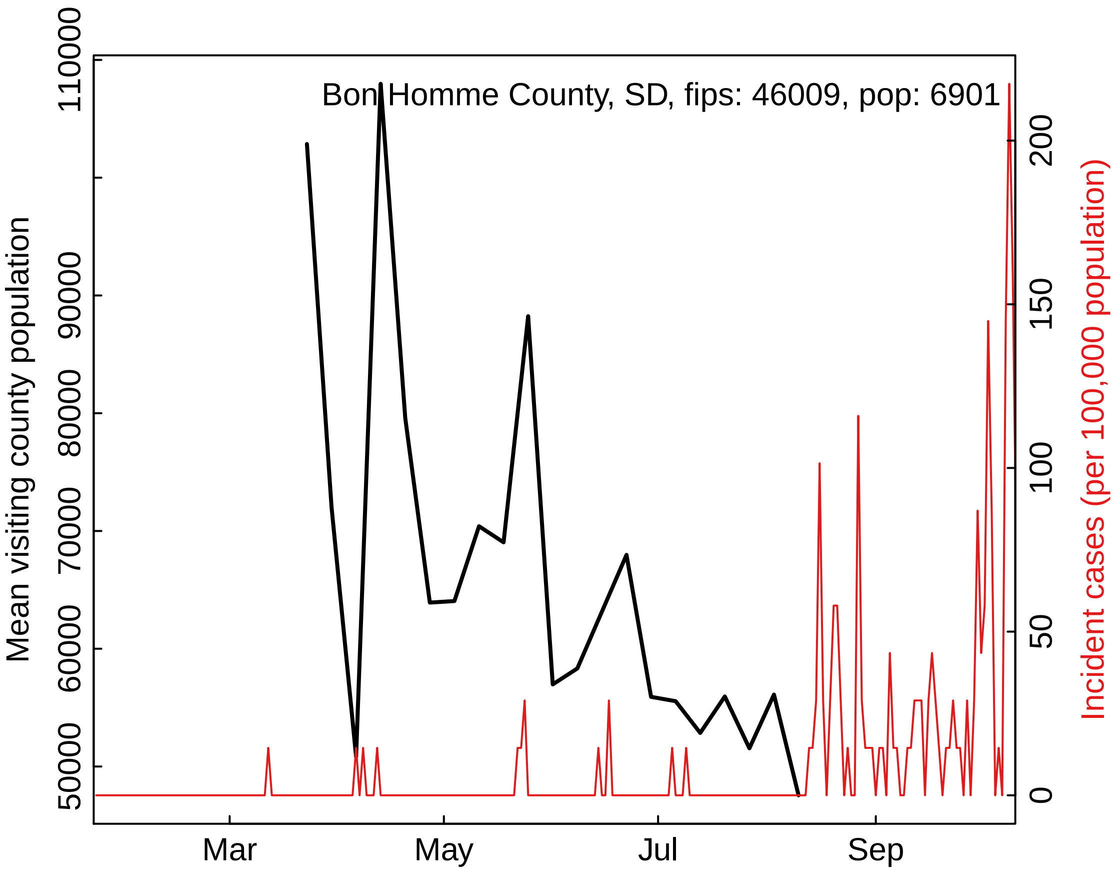

Bon Homme County, SD, fips: 46009, pop: 6901
Mean visiting county population
50000
60000
70000
80000
90000
110000
Incident cases (per 100,000 population)
0
50
100
150
200
Mar
May
Jul
Sep

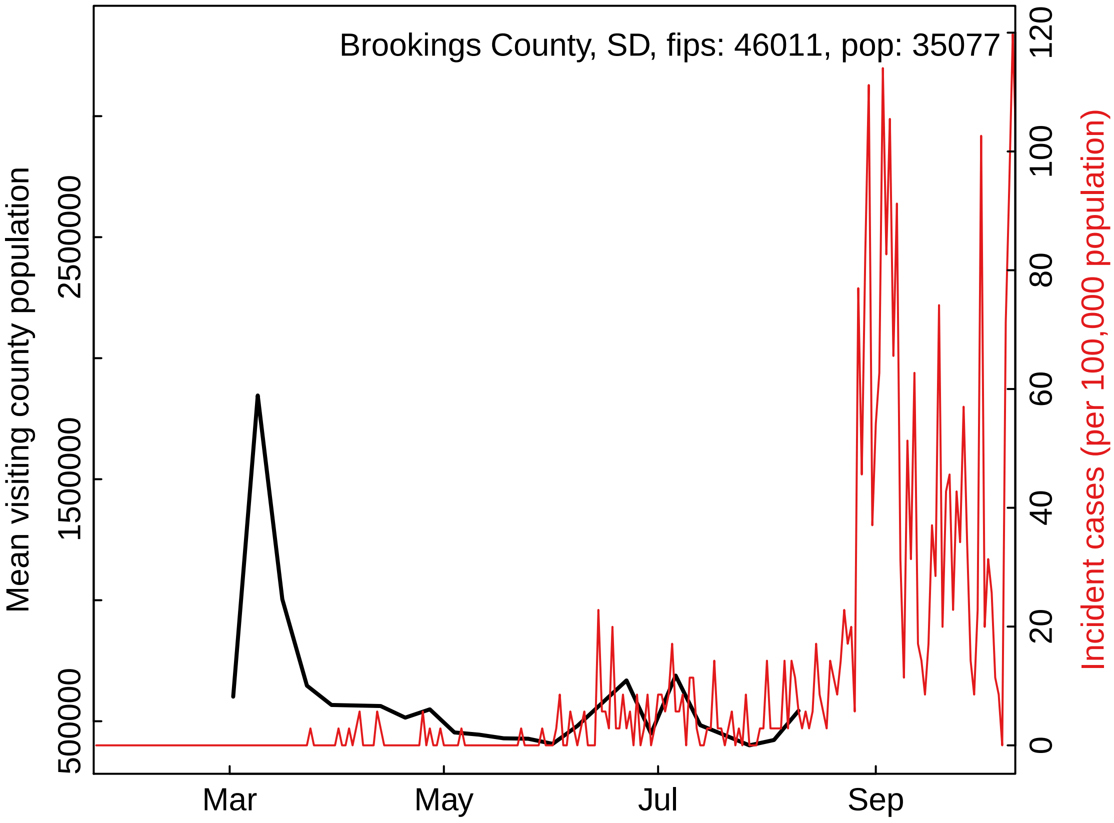

Brookings County, SD, fips: 46011, pop: 35077
Mean visiting county population
5000000
1500000
2500000
Incident cases (per 100,000 population)
0
20
40
60
80
100
120
Mar
May
Jul
Sep

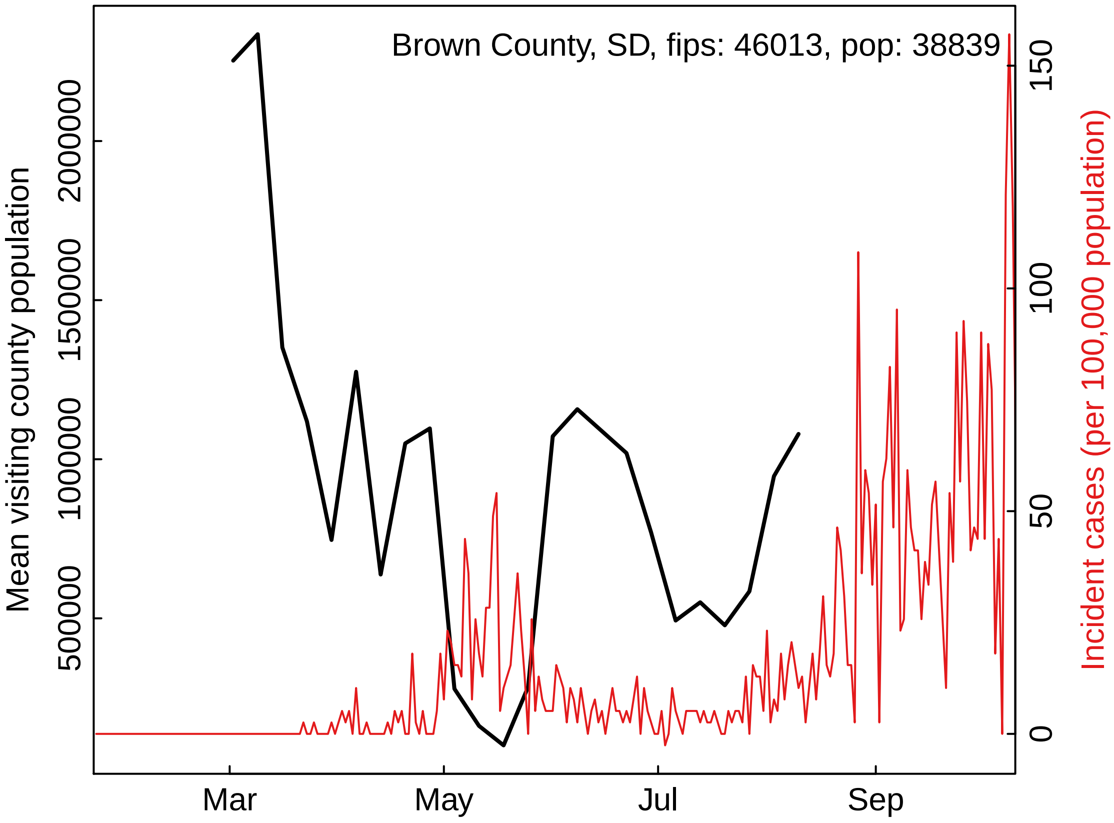
Brown County, SD, fips: 46013, pop: 38839
Mean visiting county population
500000
1000000
1500000
2000000
Incident cases (per 100,000 population)
0
50
100
150
Mar
May
Jul
Sep

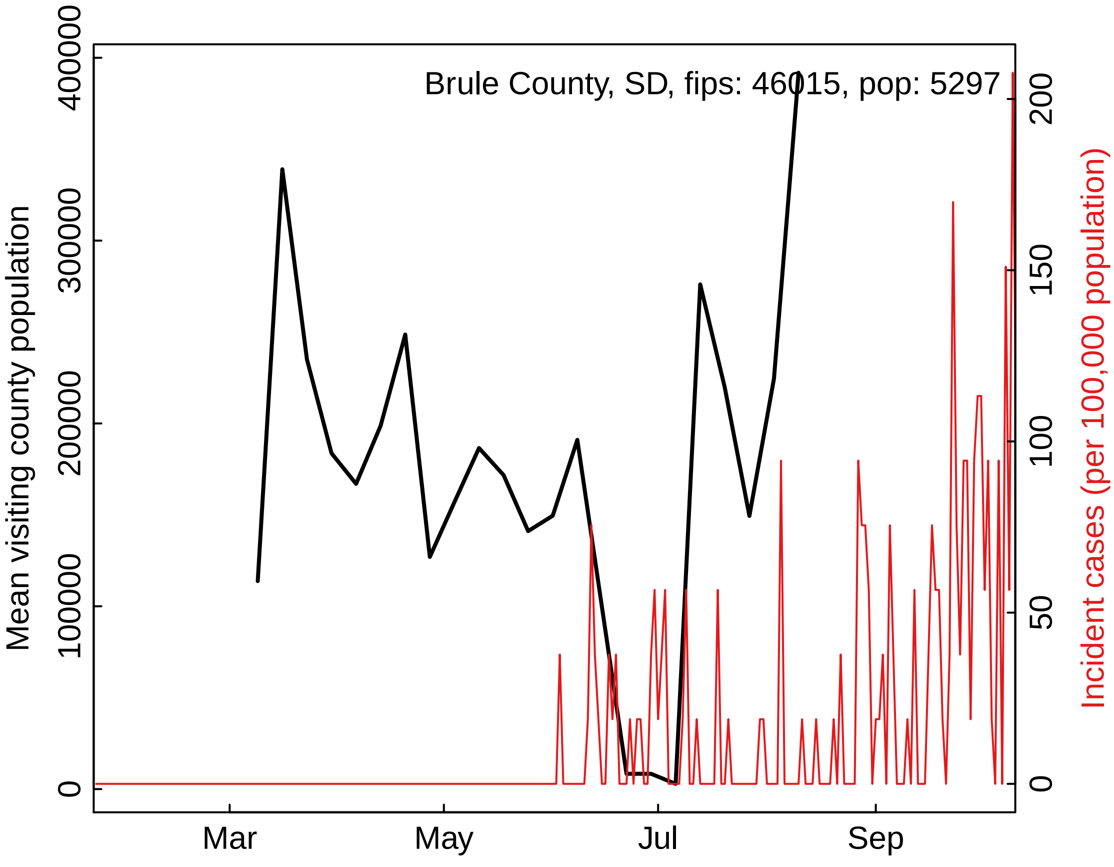

Brule County, SD, fips: 46015, pop: 5297
Mean visiting county population
0
100000
200000
300000
400000
Incident cases (per 100,000 population)
0
50
100
150
200
Mar
May
Jul
Sep

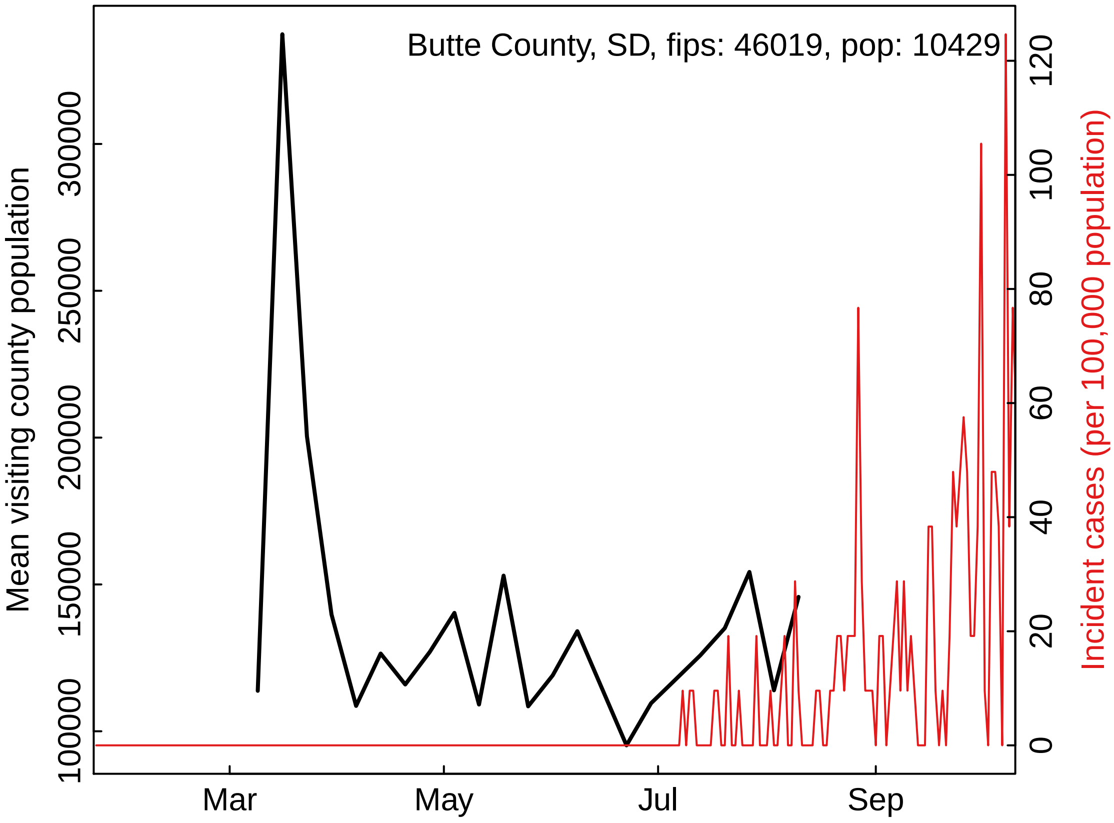
Butte County, SD, fips: 46019, pop: 10429
Mean visiting county population
100000
150000
200000
250000
300000
Mar
May
Jul
Sep
0
20
40
60
80
100
120
Incident cases (per 100,000 population)

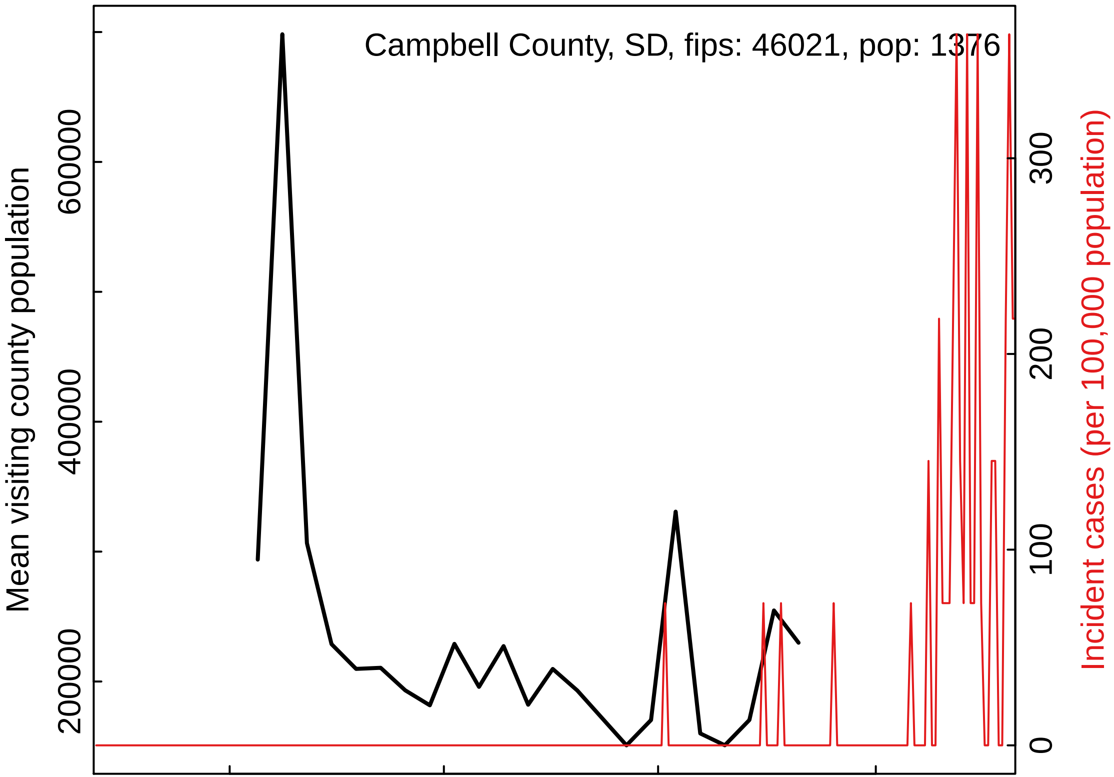
Campbell County, SD, fips: 46021, pop: 1376
Mean visiting county population
200000
400000
600000
Incident cases (per 100,000 population)
0
100
200
300
Mar
May
Jul
Sep

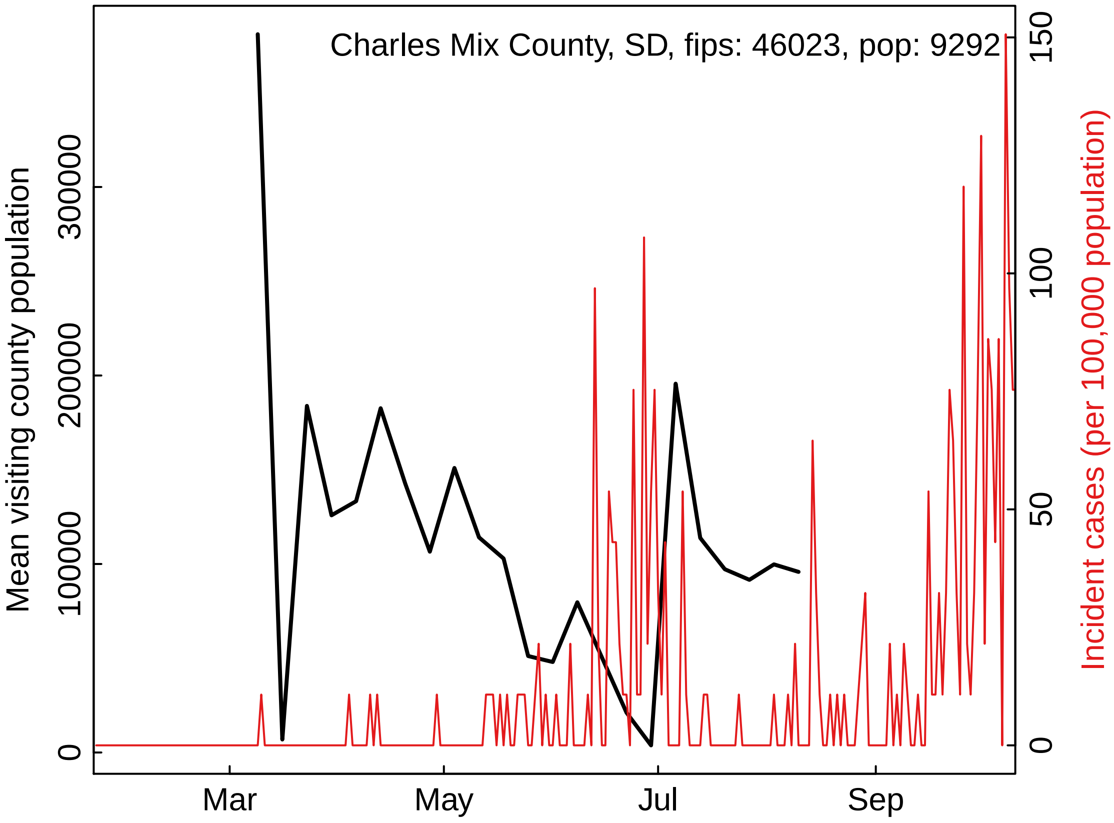

Charles Mix County, SD, fips: 46023, pop: 9292
Mean visiting county population
Incident cases (per 100,000 population)
0
100000
200000
300000
0
50
100
150
Mar
May
Jul
Sep

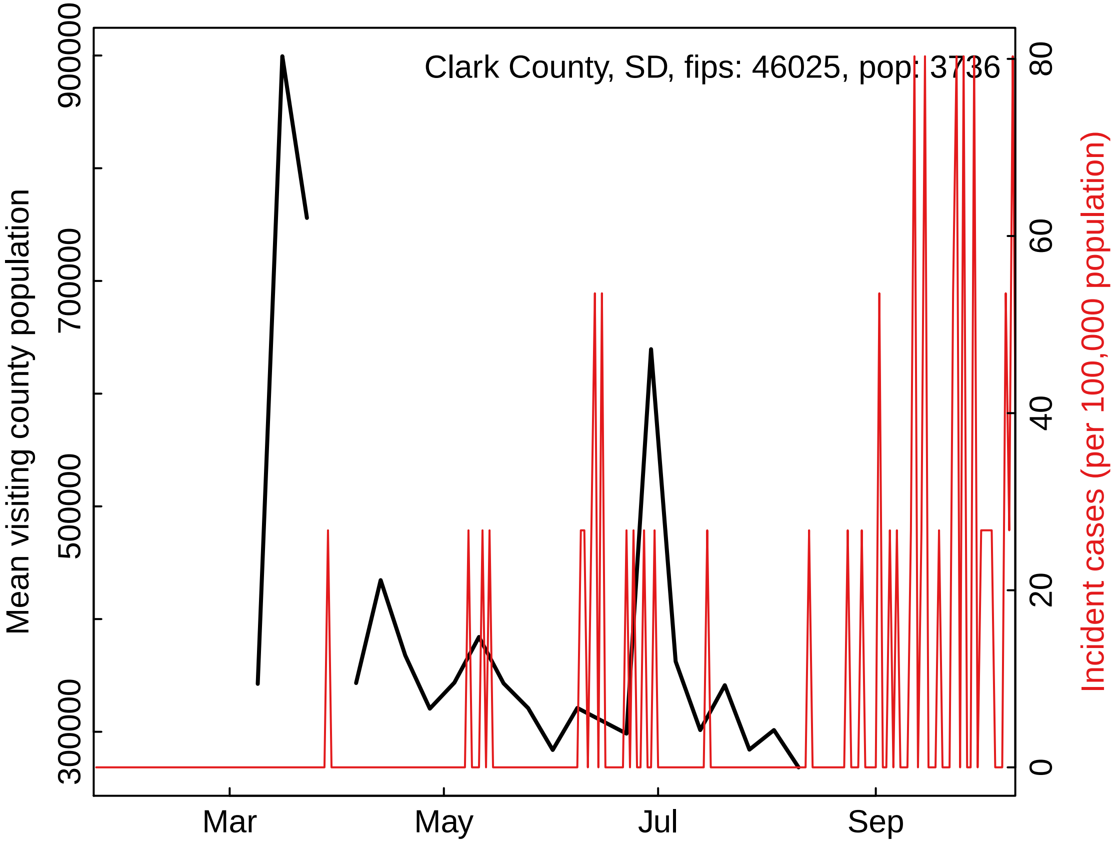

Clark County, SD, fips: 46025, pop: 3736
Mean visiting county population
Incident cases (per 100,000 population)
300000
500000
700000
900000
0
20
40
60
80
Mar
May
Jul
Sep

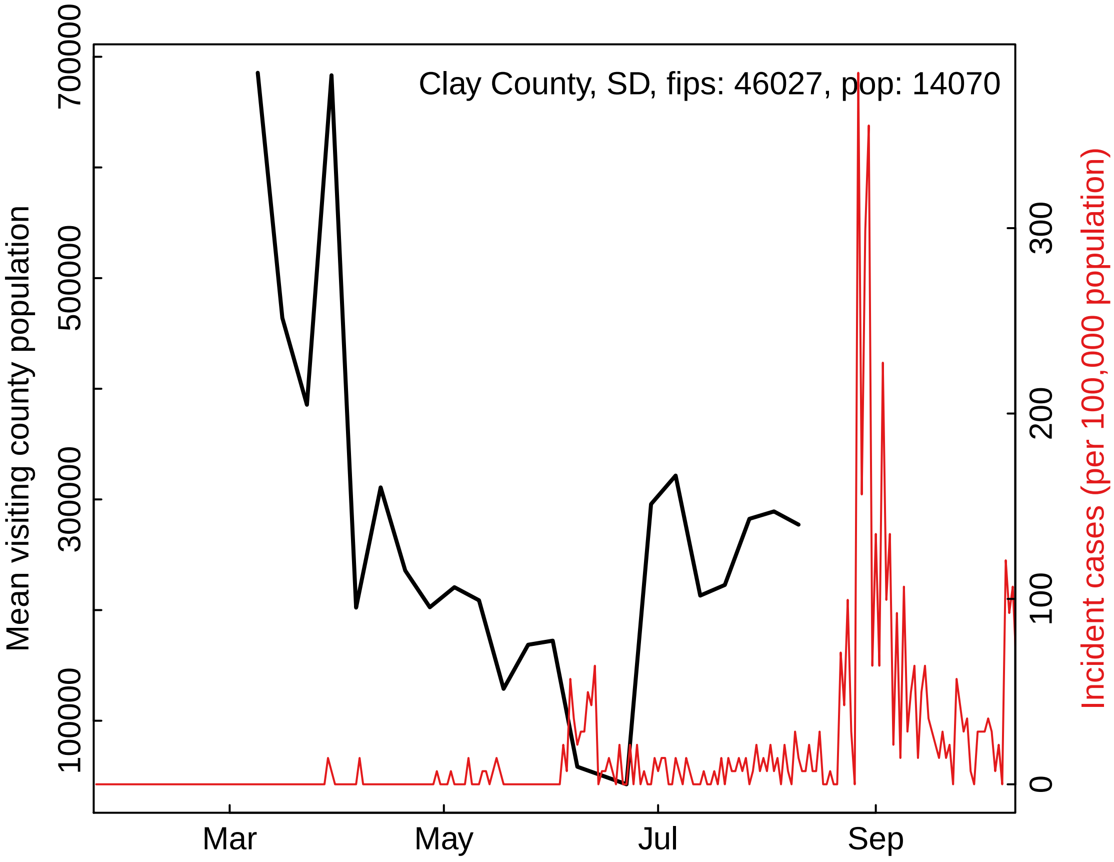

Clay County, SD, fips: 46027, pop: 14070
Mean visiting county population
100000
300000
500000
700000
Incident cases (per 100,000 population)
0
100
200
300
Mar
May
Jul
Sep

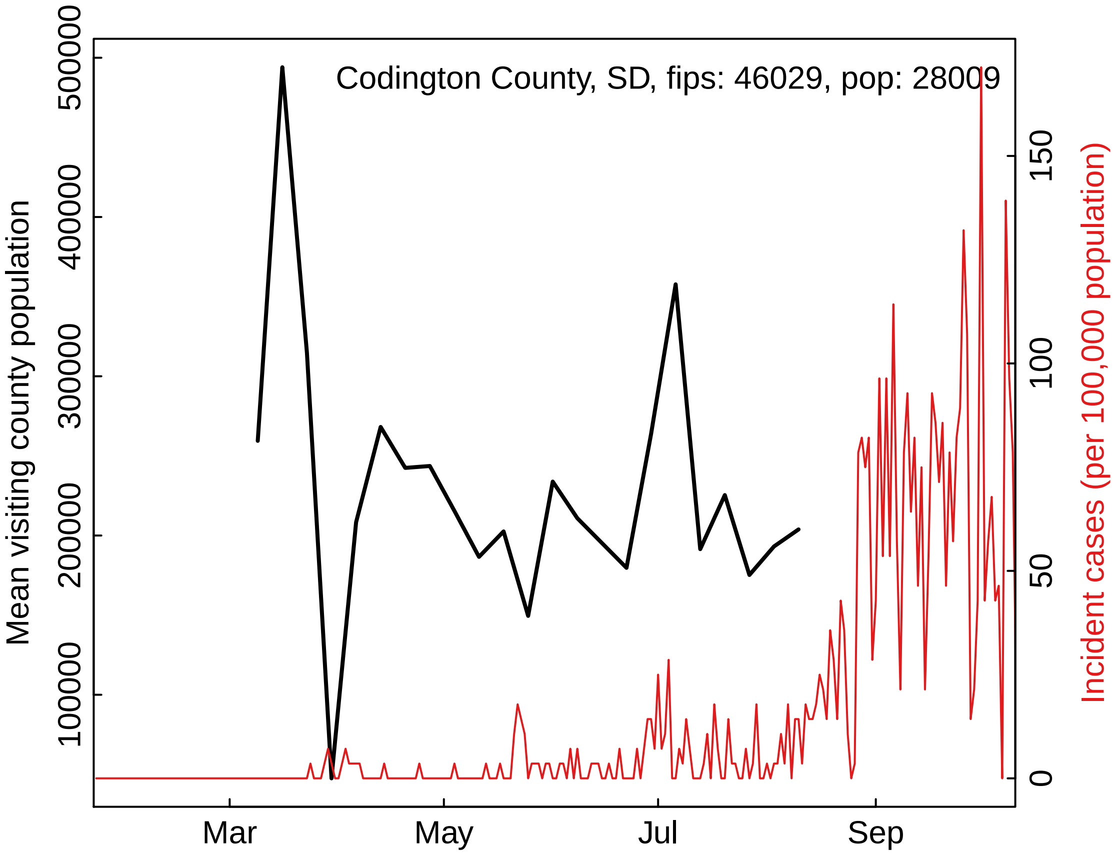
Codington County, SD, fips: 46029, pop: 28009
Mean visiting county population
100000
200000
300000
400000
500000
Incident cases (per 100,000 population)
0
50
100
150
Mar
May
Jul
Sep

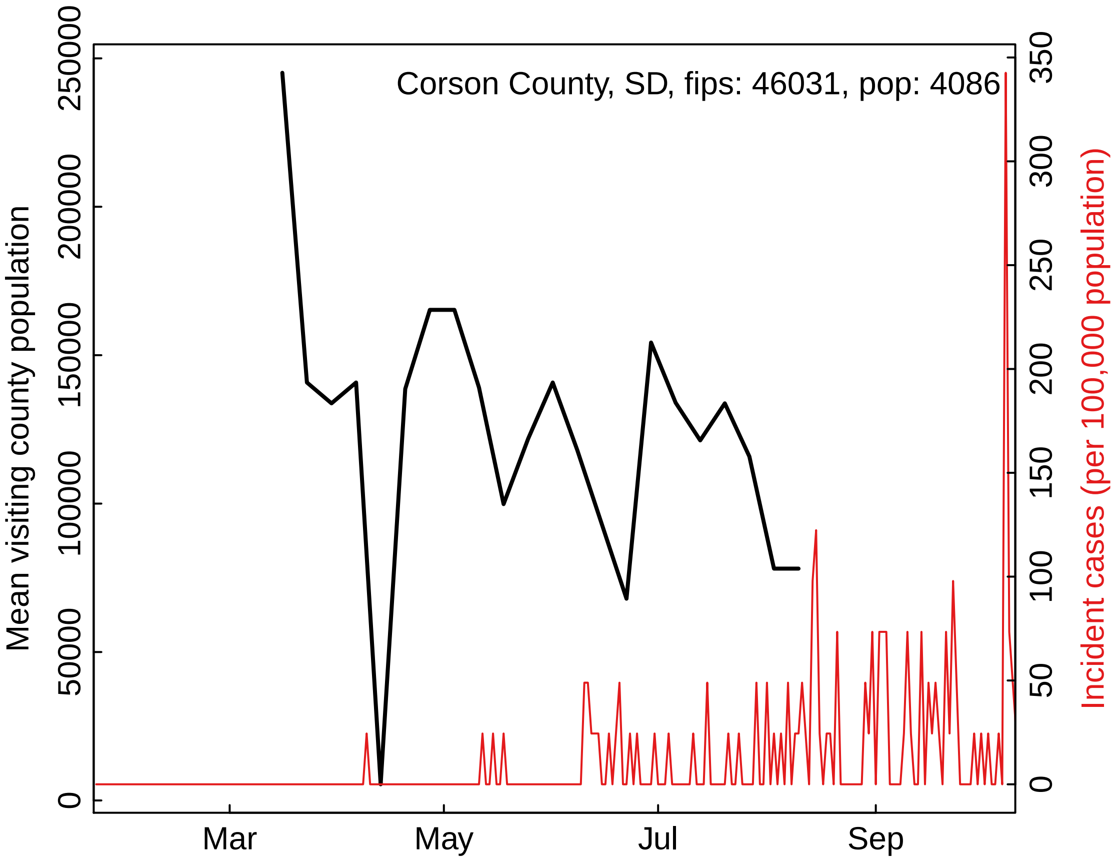

Corson County, SD, fips: 46031, pop: 4086
Mean visiting county population
0
50000
100000
150000
200000
250000
Incident cases (per 100,000 population)
0
50
100
150
200
250
300
350
Mar
May
Jul
Sep

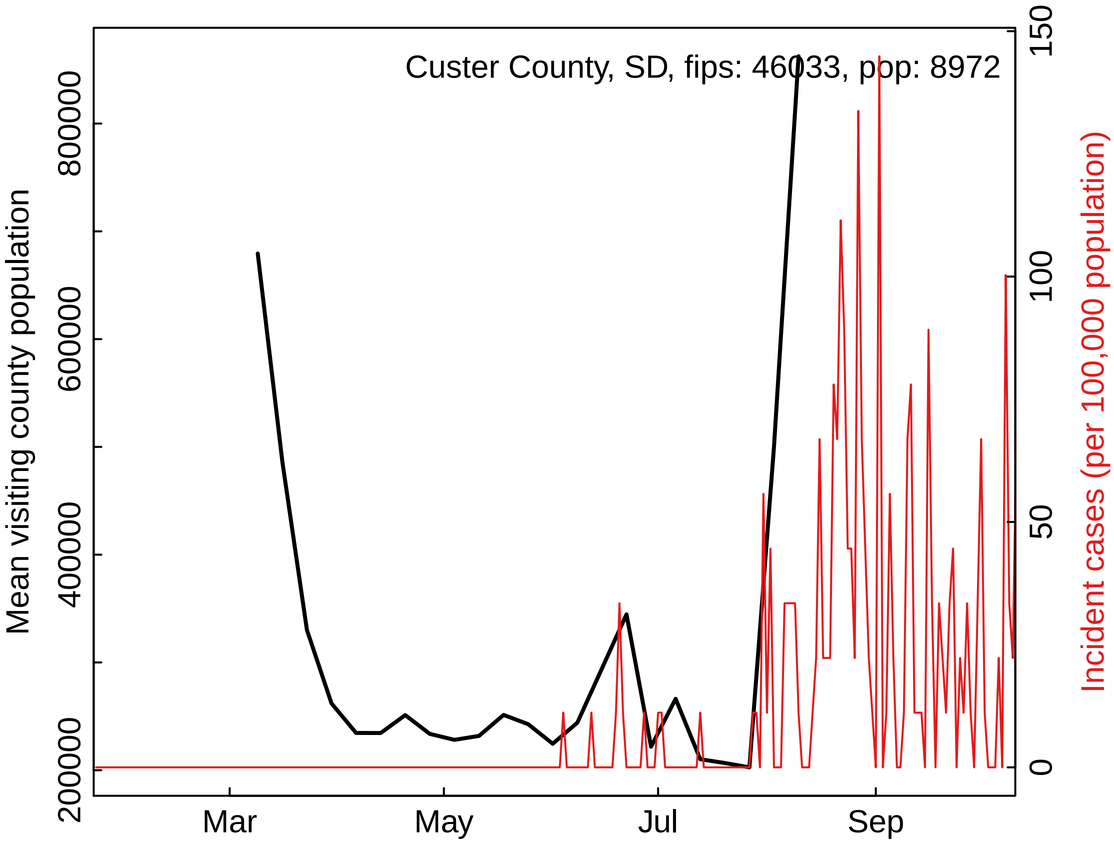

Custer County, SD, fips: 46033, pop: 8972
Mean visiting county population
200000
400000
600000
800000
Incident cases (per 100,000 population)
0
50
100
150
Mar
May
Jul
Sep

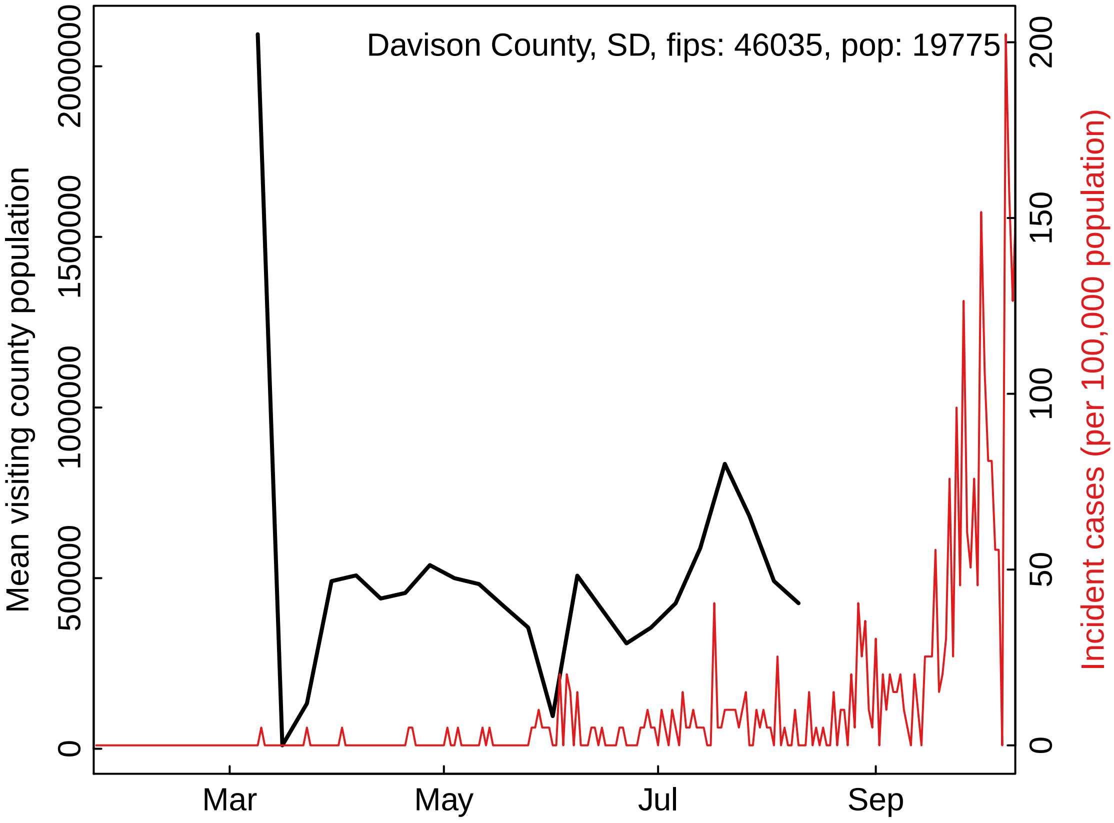

Davison County, SD, fips: 46035, pop: 19775
Mean visiting county population
0
500000
1000000
1500000
2000000
Incident cases (per 100,000 population)
0
50
100
150
200
Mar
May
Jul
Sep

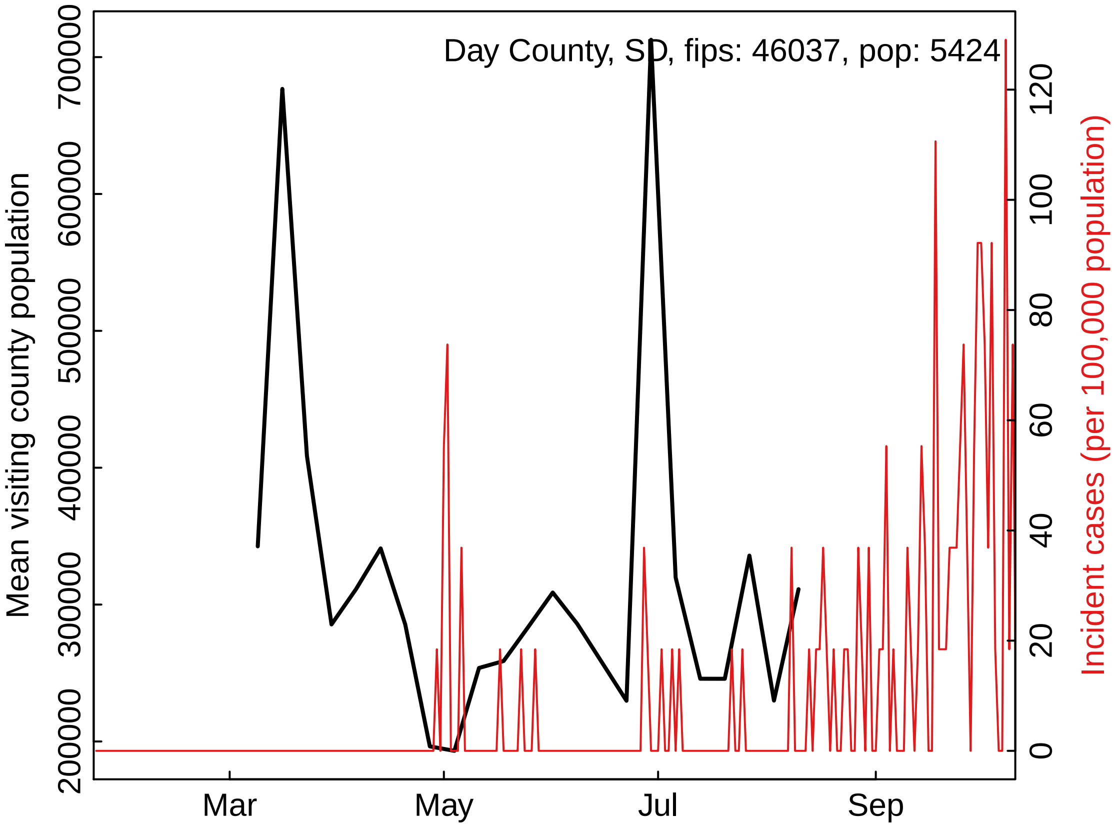

Day County, SD, fips: 46037, pop: 5424
Mean visiting county population
200000
300000
400000
500000
600000
700000
Incident cases (per 100,000 population)
0
20
40
60
80
100
120
Mar
May
Jul
Sep

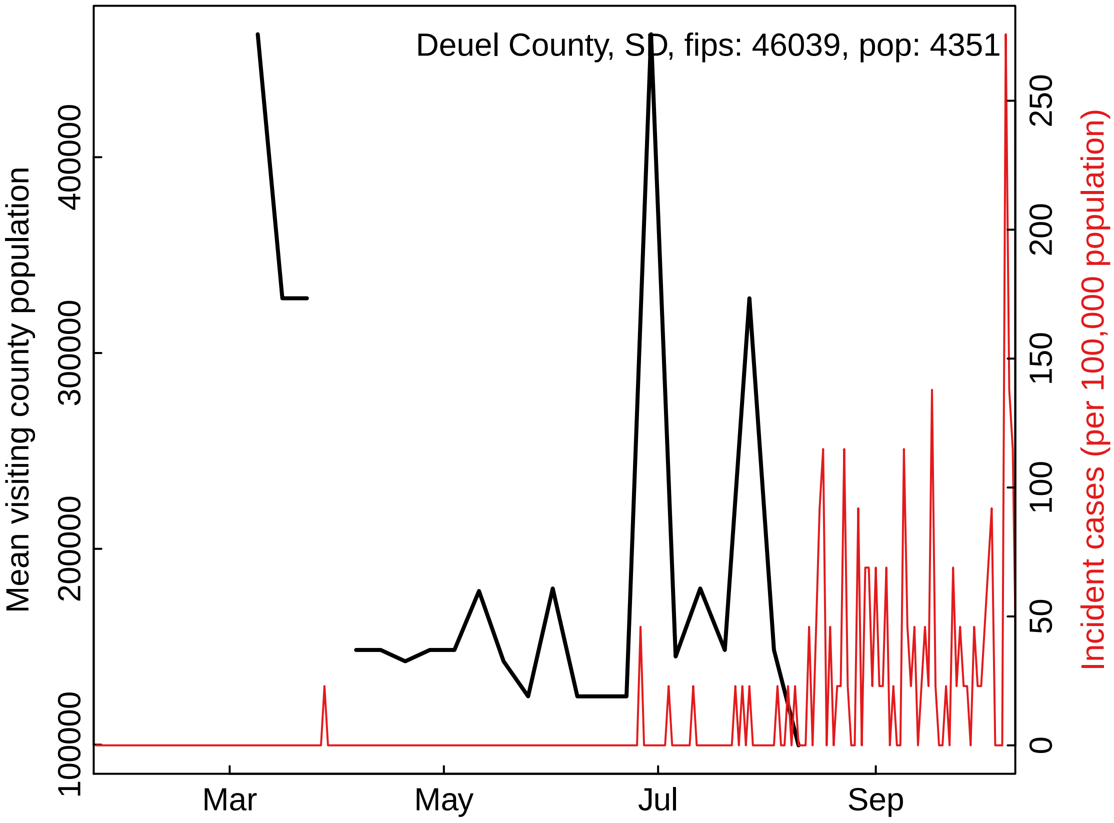

Deuel County, SD, fips: 46039, pop: 4351
Mean visiting county population
100000
200000
300000
400000
Incident cases (per 100,000 population)
0
50
100
150
200
250
Mar
May
Jul
Sep

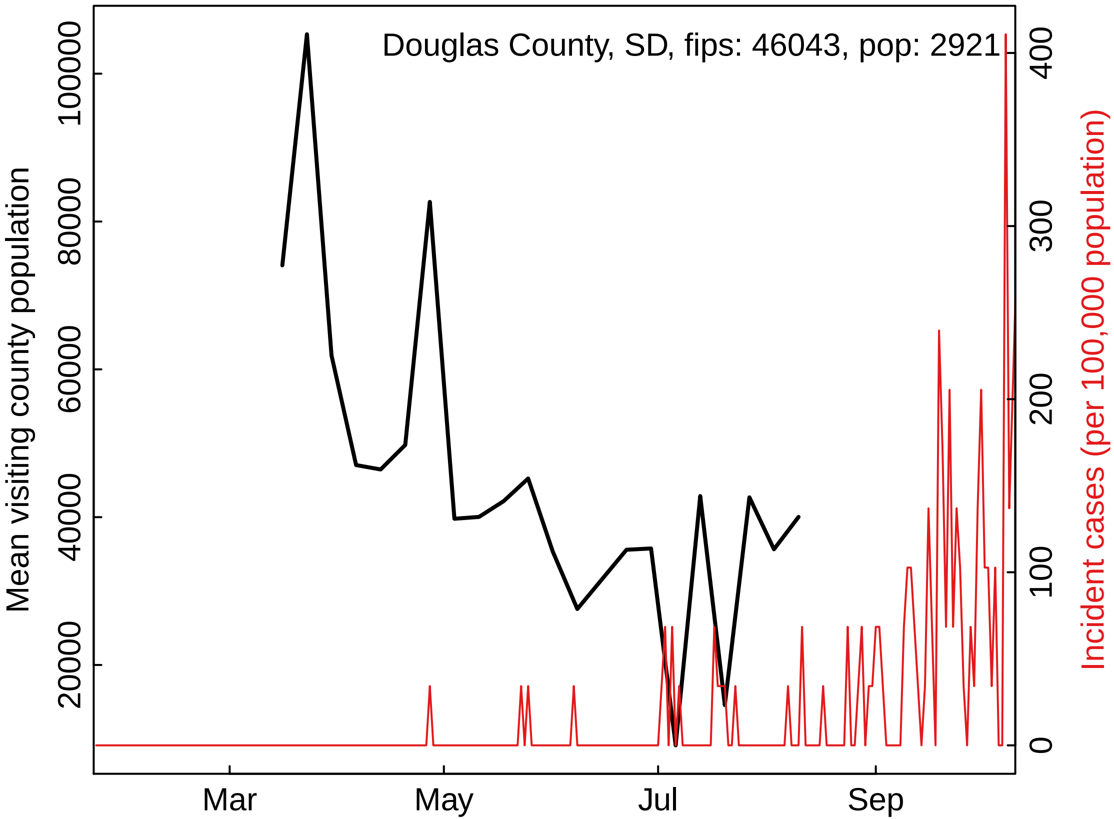

Douglas County, SD, fips: 46043, pop: 2921
Mean visiting county population
20000
40000
60000
80000
100000
Incident cases (per 100,000 population)
0
100
200
300
400
Mar
May
Jul
Sep

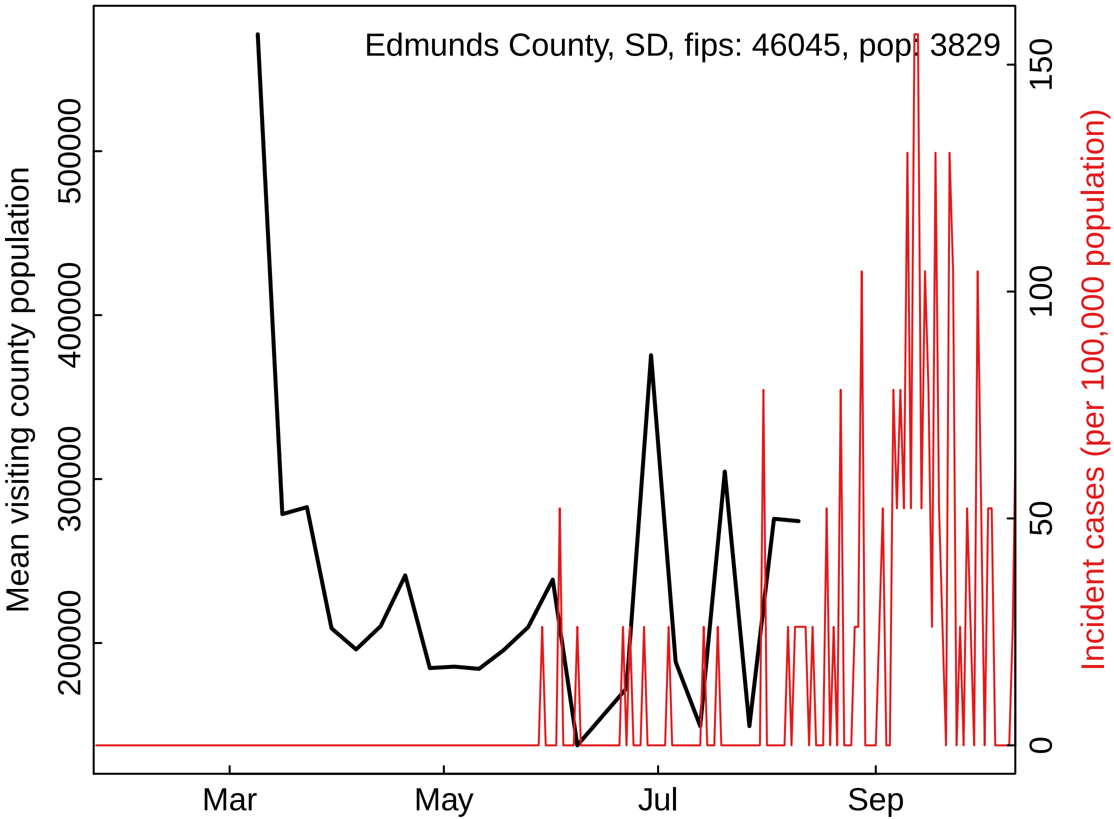
Edmunds County, SD, fips: 46045, pop: 3829
Mean visiting county population
200000
300000
400000
500000
Incident cases (per 100,000 population)
0
50
100
150
Mar
May
Jul
Sep

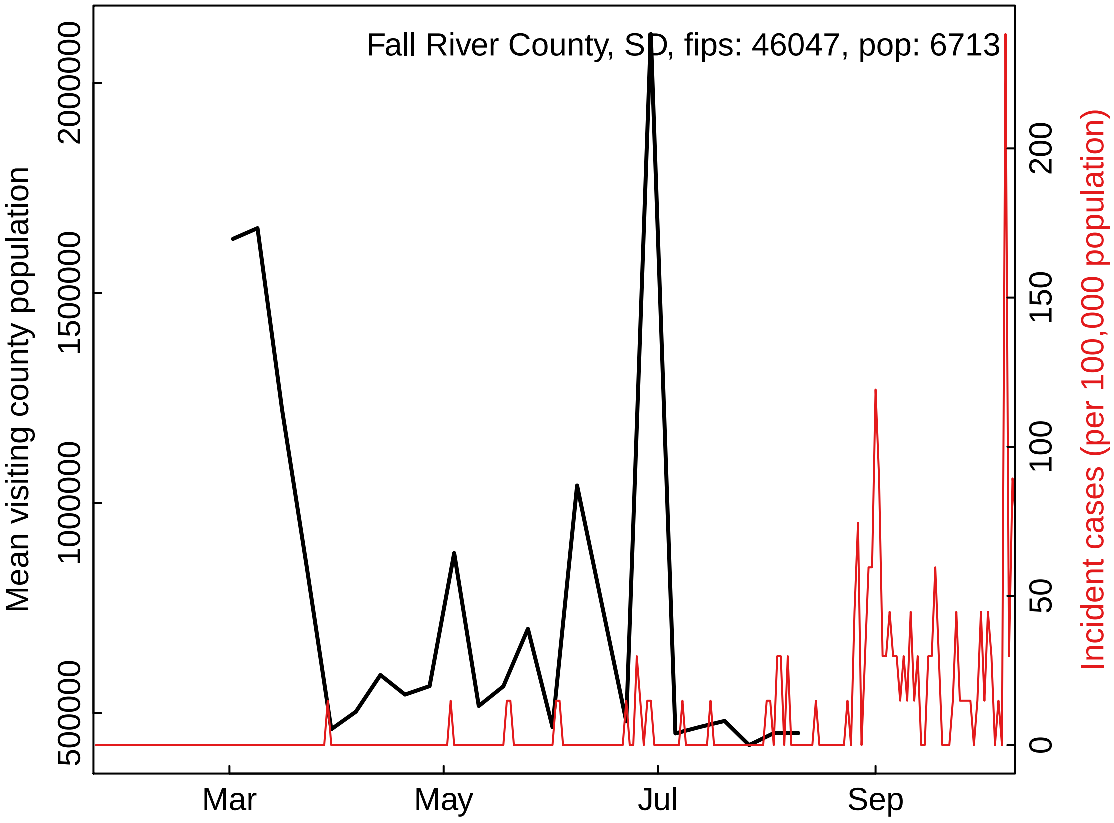

Fall River County, SD, fips: 46047, pop: 6713
Mean visiting county population
500000
1000000
1500000
2000000
Incident cases (per 100,000 population)
0
50
100
150
200
Mar
May
Jul
Sep

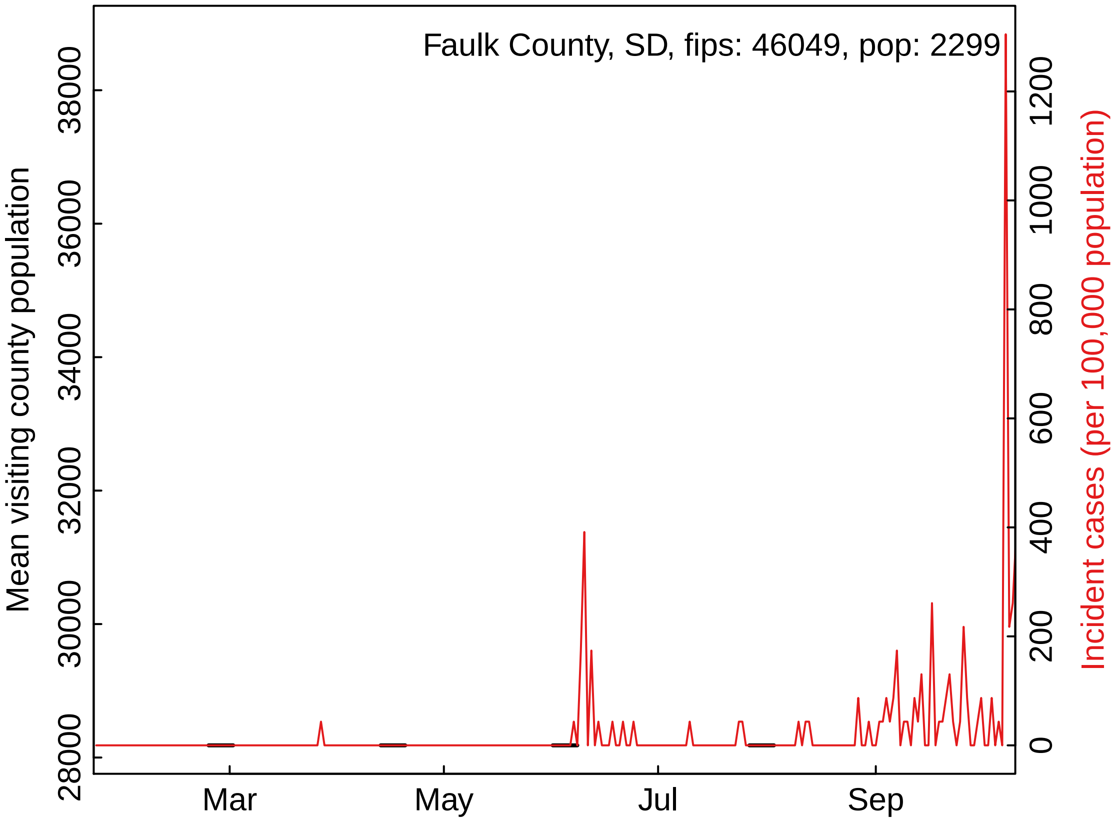

Faulk County, SD, fips: 46049, pop: 2299
Mean visiting county population
28000
30000
32000
34000
36000
38000
Incident cases (per 100,000 population)
0
200
400
600
800
1000
1200
Mar
May
Jul
Sep

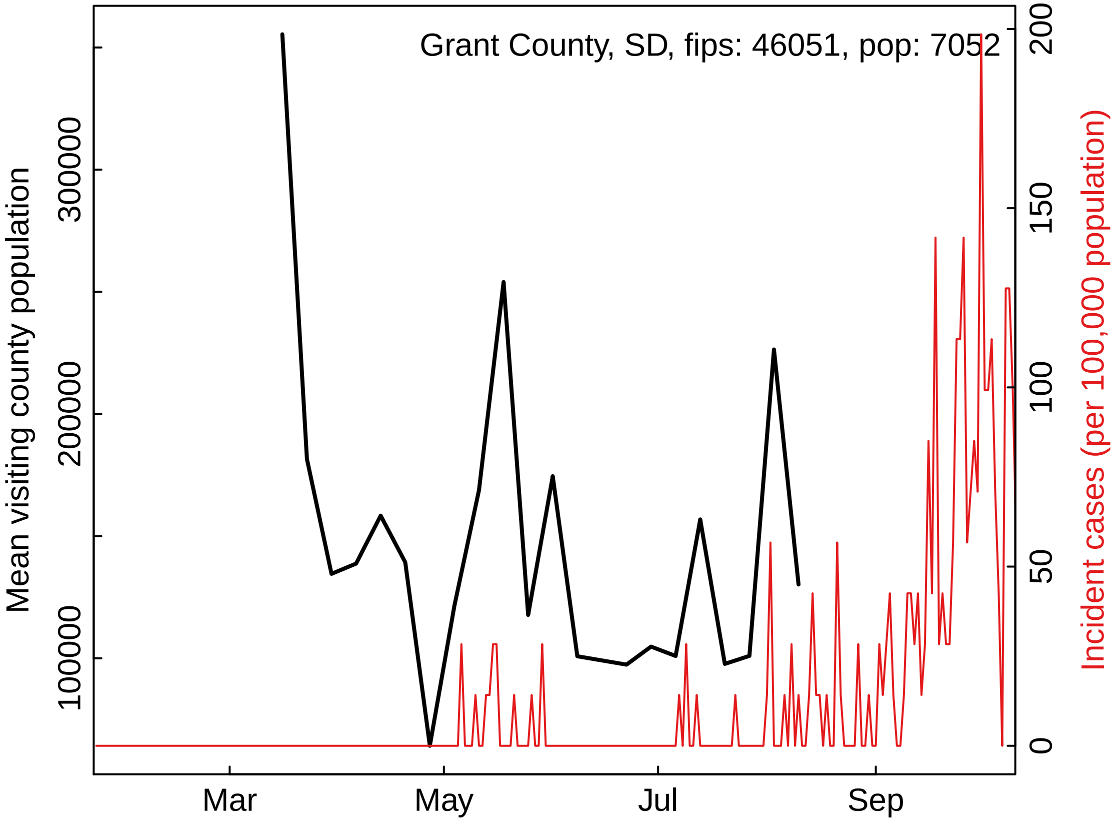
Grant County, SD, fips: 46051, pop: 7052
Mean visiting county population
Incident cases (per 100,000 population)
100000
200000
300000
0
50
100
150
200
Mar
May
Jul
Sep

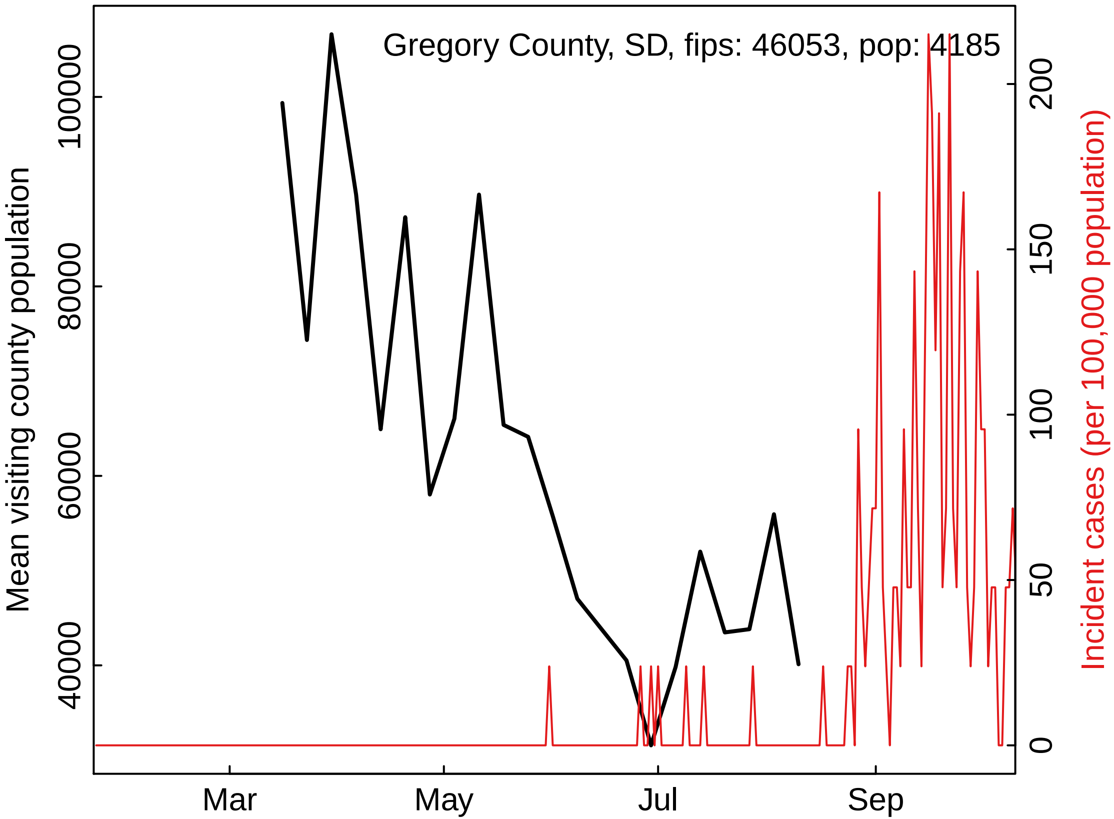

Gregory County, SD, fips: 46053, pop: 4185
Mean visiting county population
40000
60000
80000
100000
Incident cases (per 100,000 population)
0
50
100
150
200
Mar
May
Jul
Sep

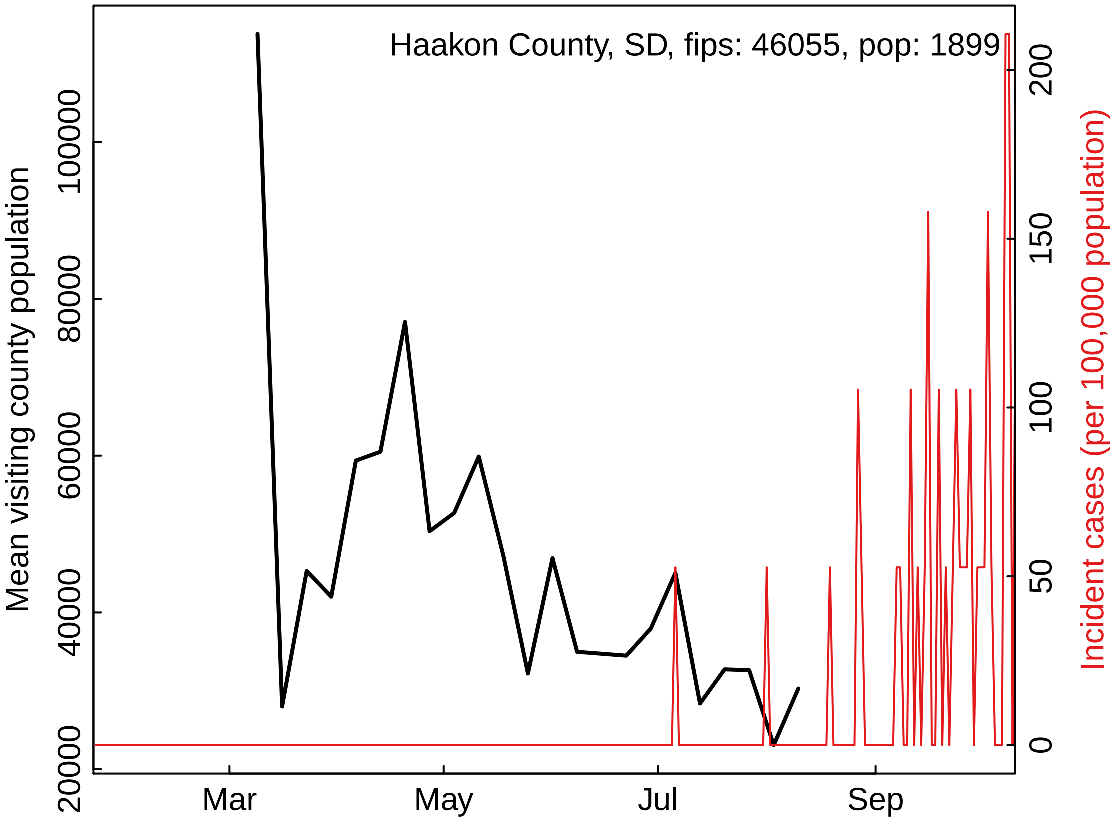

Haakon County, SD, fips: 46055, pop: 1899
Mean visiting county population
Incident cases (per 100,000 population)
20000
40000
60000
80000
100000
0
50
100
150
200
Mar
May
Jul
Sep

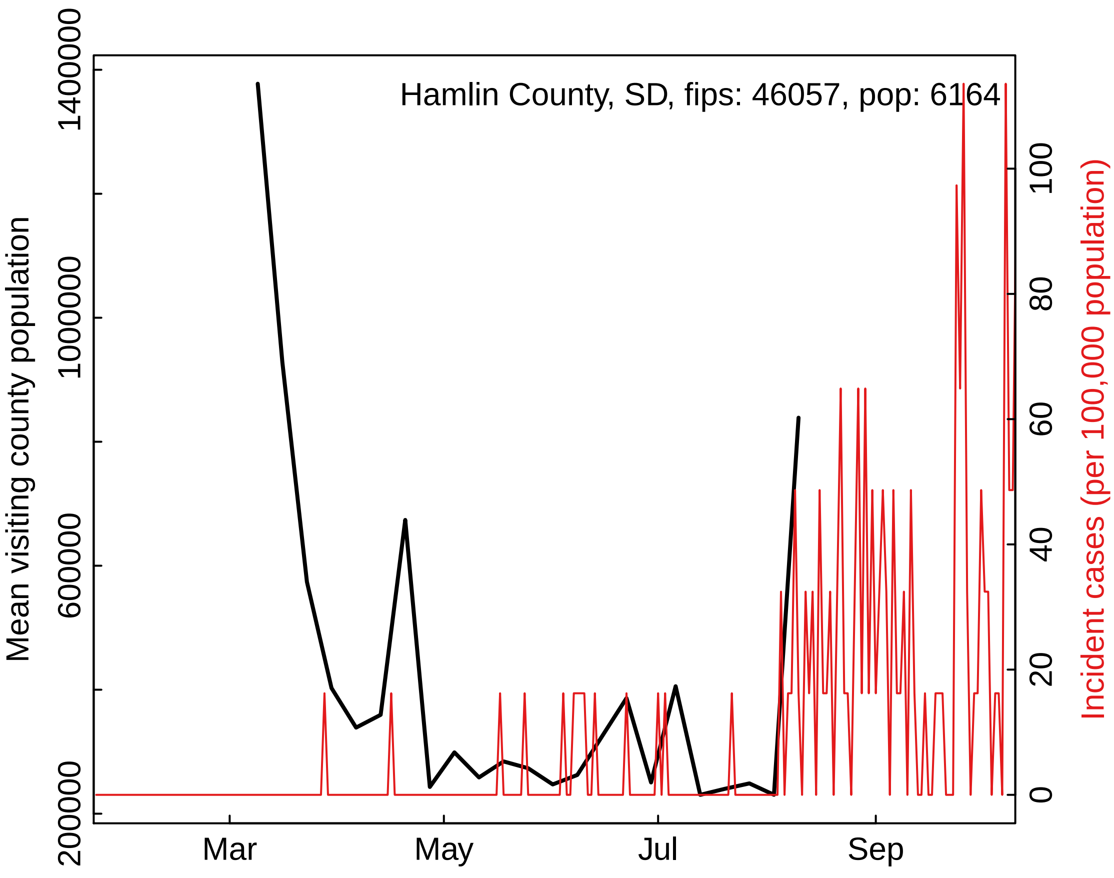

Hamlin County, SD, fips: 46057, pop: 6164
Mean visiting county population
200000
600000
1000000
1400000
Incident cases (per 100,000 population)
0
20
40
60
80
100
Mar
May
Jul
Sep

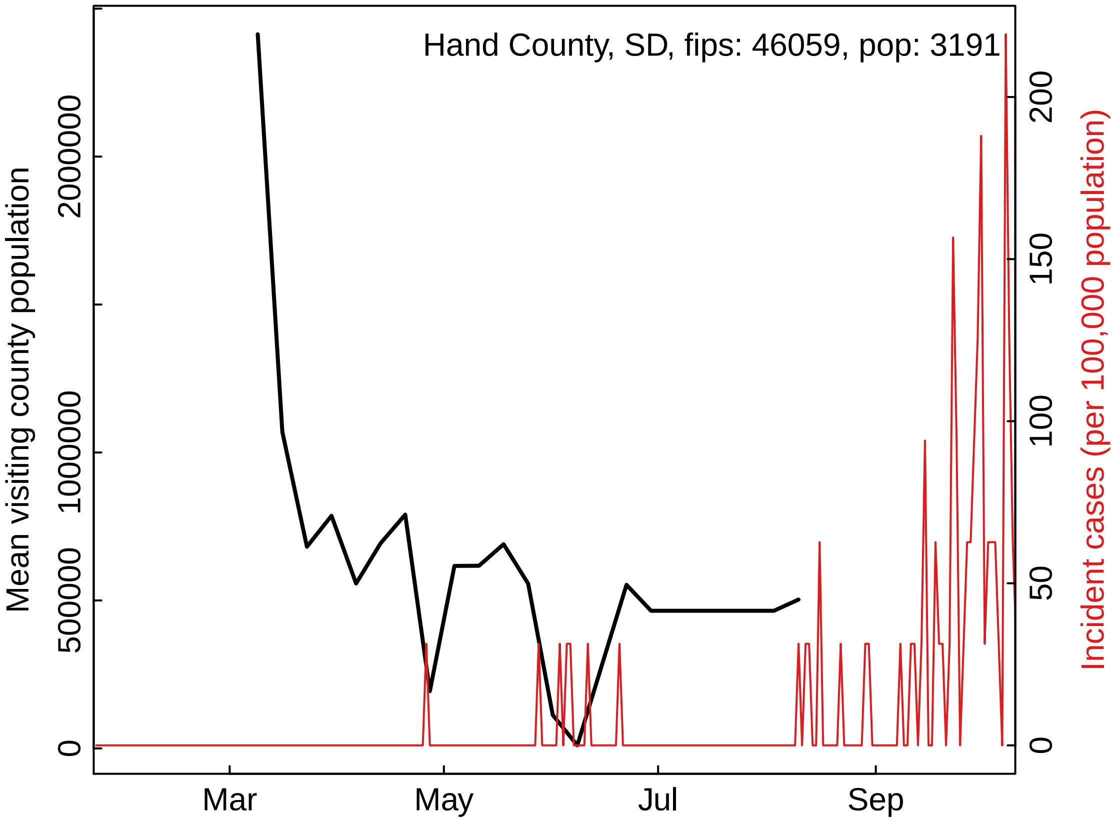

Hand County, SD, fips: 46059, pop: 3191
Mean visiting county population
0
500000
1000000
2000000
Incident cases (per 100,000 population)
0
50
100
150
200
Mar
May
Jul
Sep

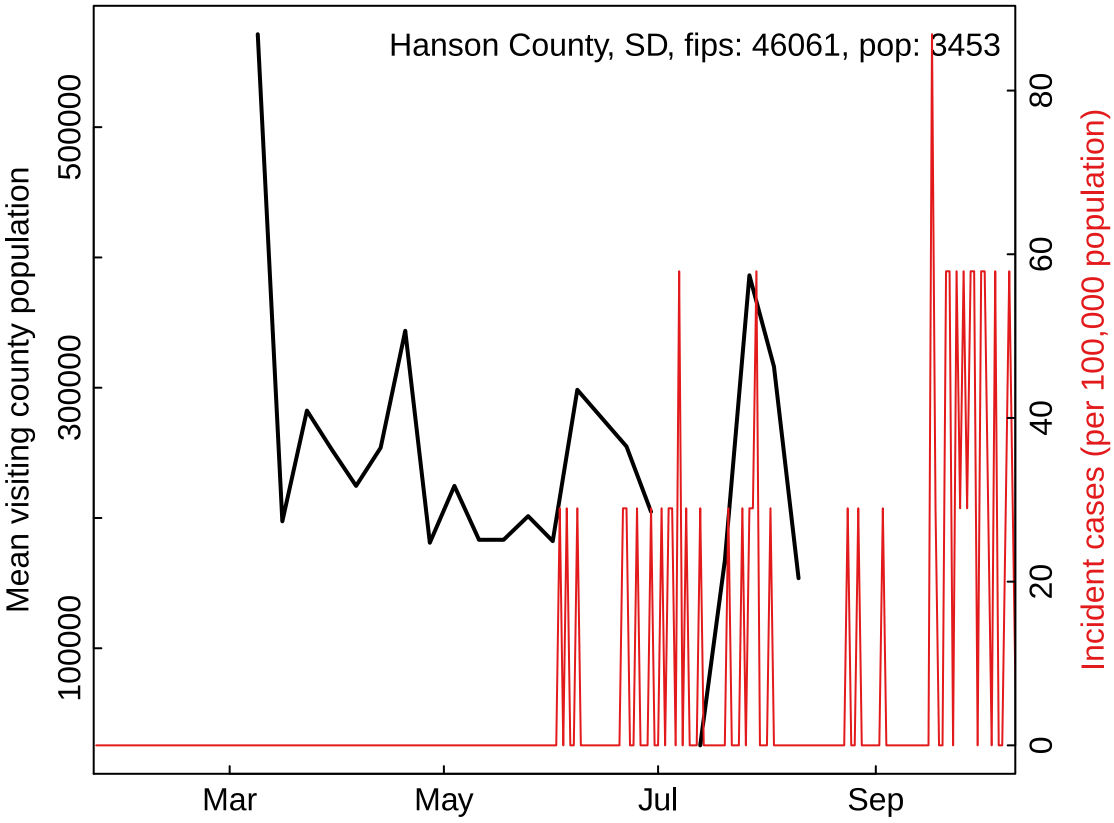

Hanson County, SD, fips: 46061, pop: 3453
Mean visiting county population
100000
300000
500000
Incident cases (per 100,000 population)
0
20
40
60
80
Mar
May
Jul
Sep

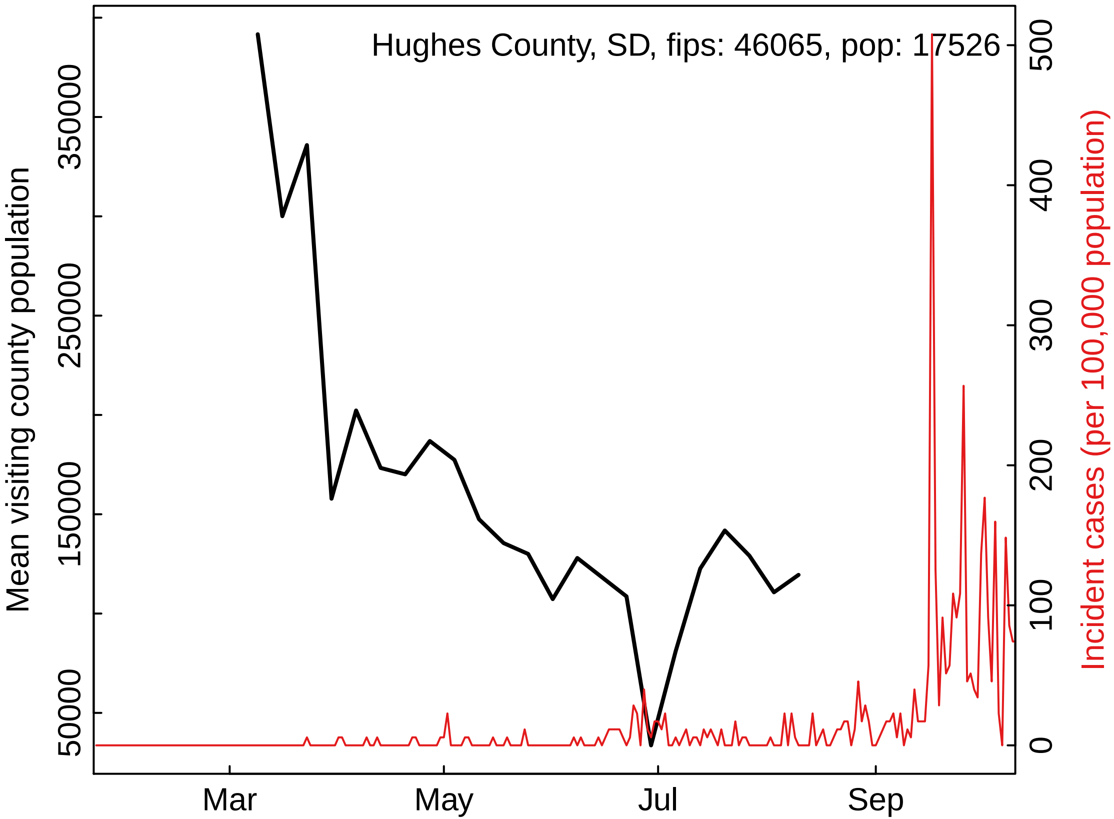
Hughes County, SD, fips: 46065, pop: 17526
Mean visiting county population
50000
150000
250000
350000
Incident cases (per 100,000 population)
0
100
200
300
400
500
Mar
May
Jul
Sep

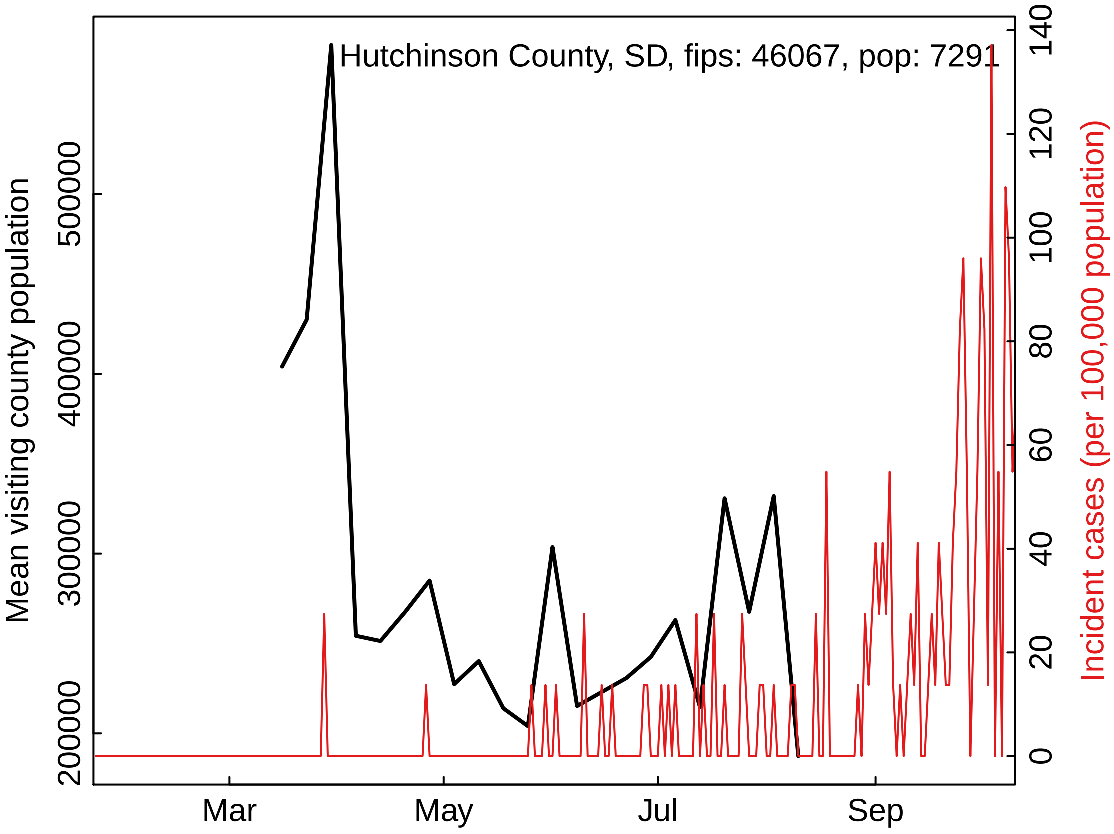

Hutchinson County, SD, fips: 46067, pop: 7291
Mean visiting county population
200000
300000
400000
500000
Incident cases (per 100,000 population)
0
20
40
60
80
100
120
140
Mar
May
Jul
Sep

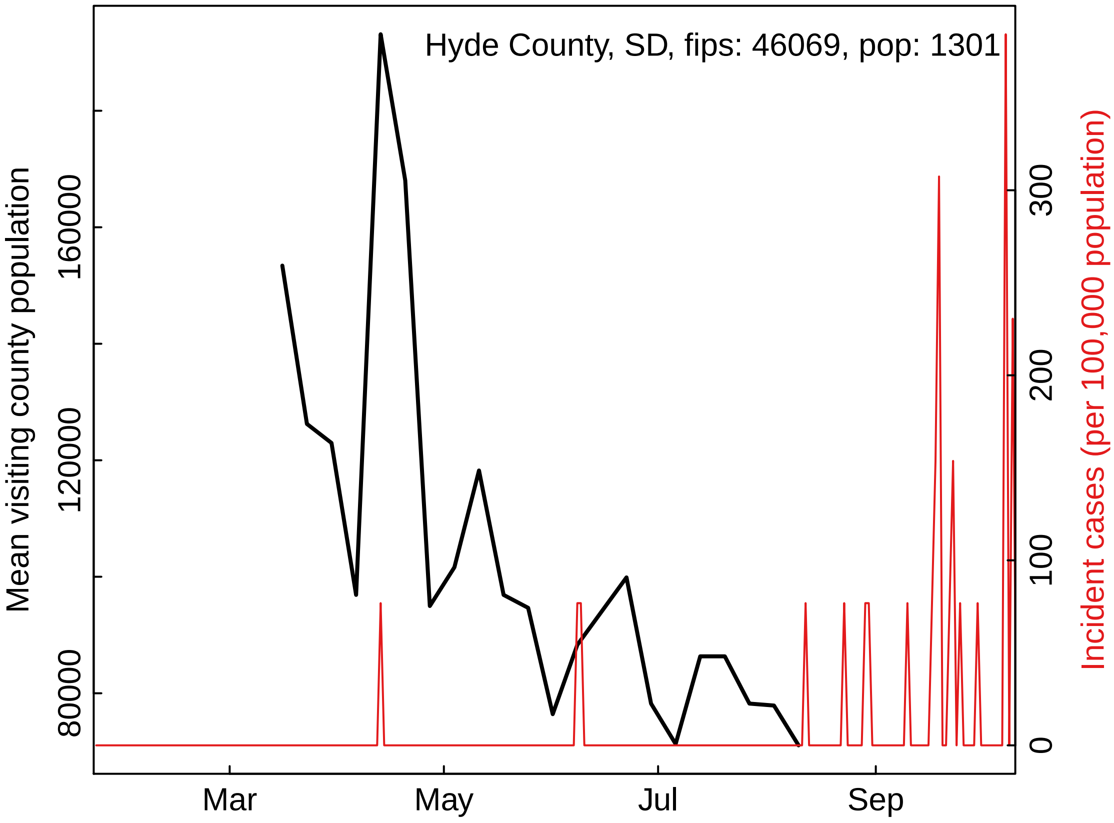

Hyde County, SD, fips: 46069, pop: 1301
Mean visiting county population
80000
120000
160000
Mar
May
Jul
Sep
Incident cases (per 100,000 population)
0
100
200
300

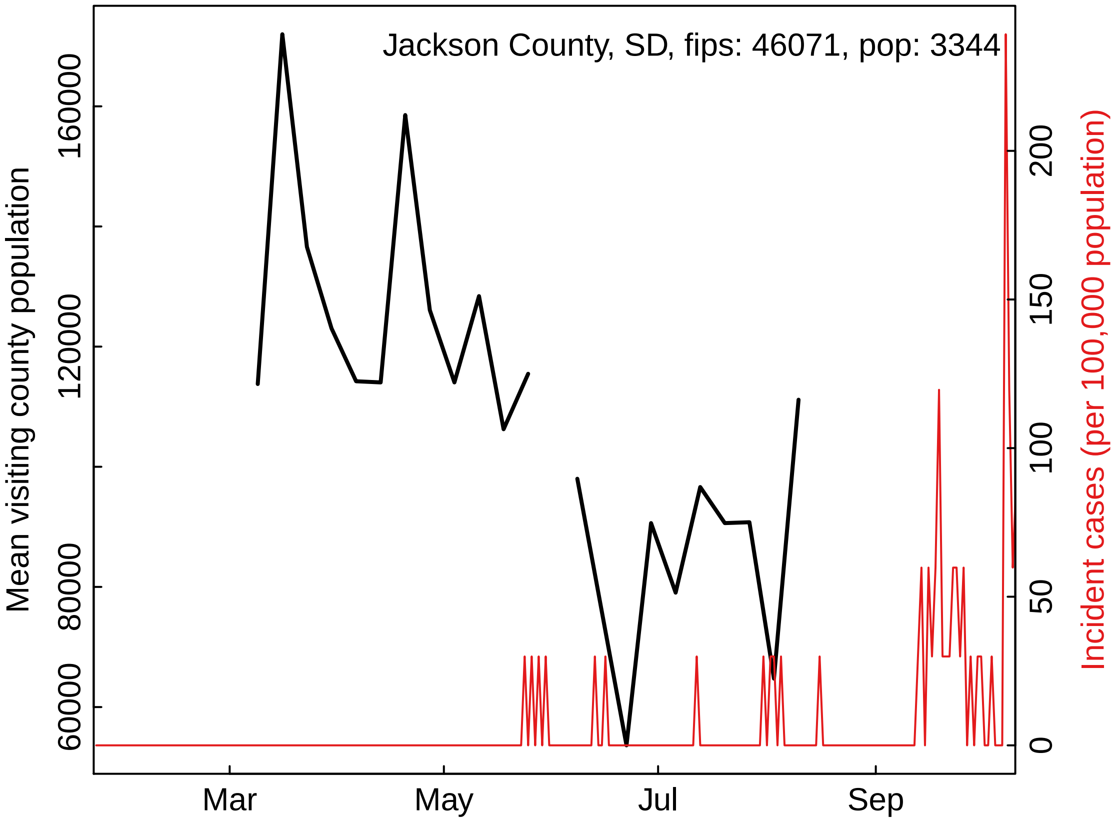

Jackson County, SD, fips: 46071, pop: 3344
Mean visiting county population
Incident cases (per 100,000 population)
1600000
1200000
80000
60000
200
150
100
50
0
Mar
May
Jul
Sep

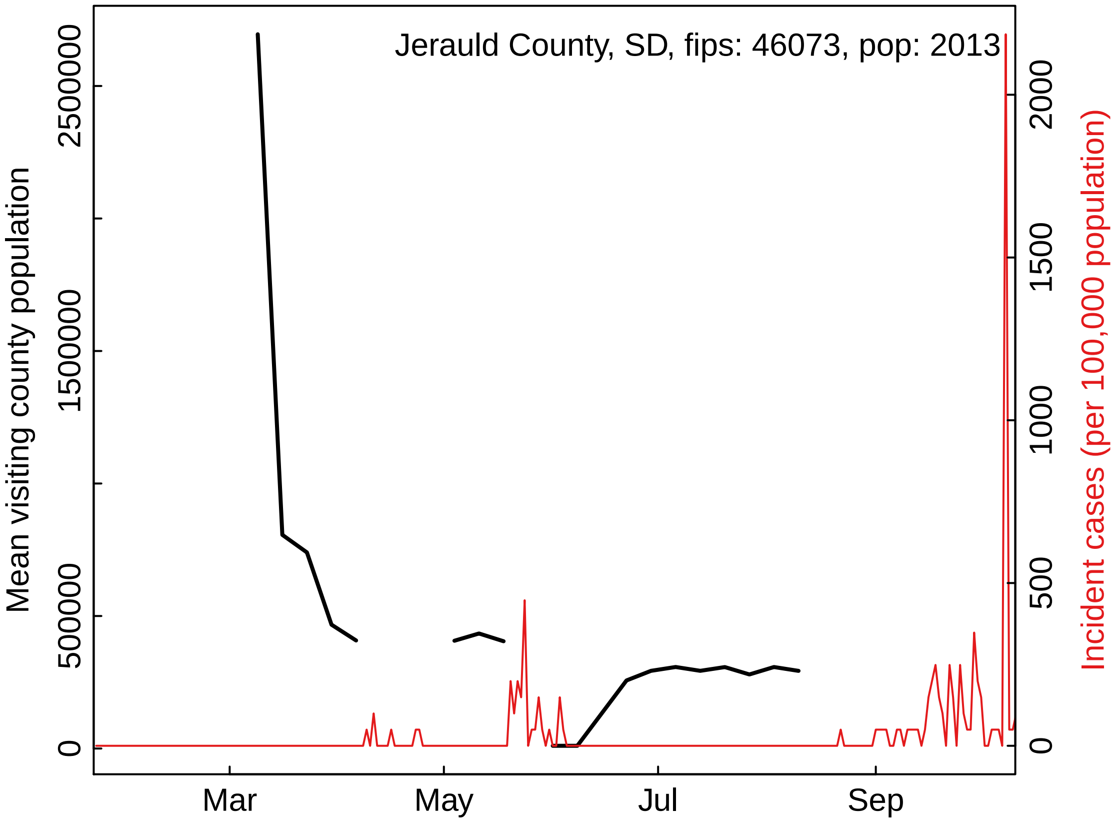

Jerauld County, SD, fips: 46073, pop: 2013
Mean visiting county population
0
500000
1500000
2500000
Mar
May
Jul
Sep
0
500
1000
1500
2000
Incident cases (per 100,000 population)

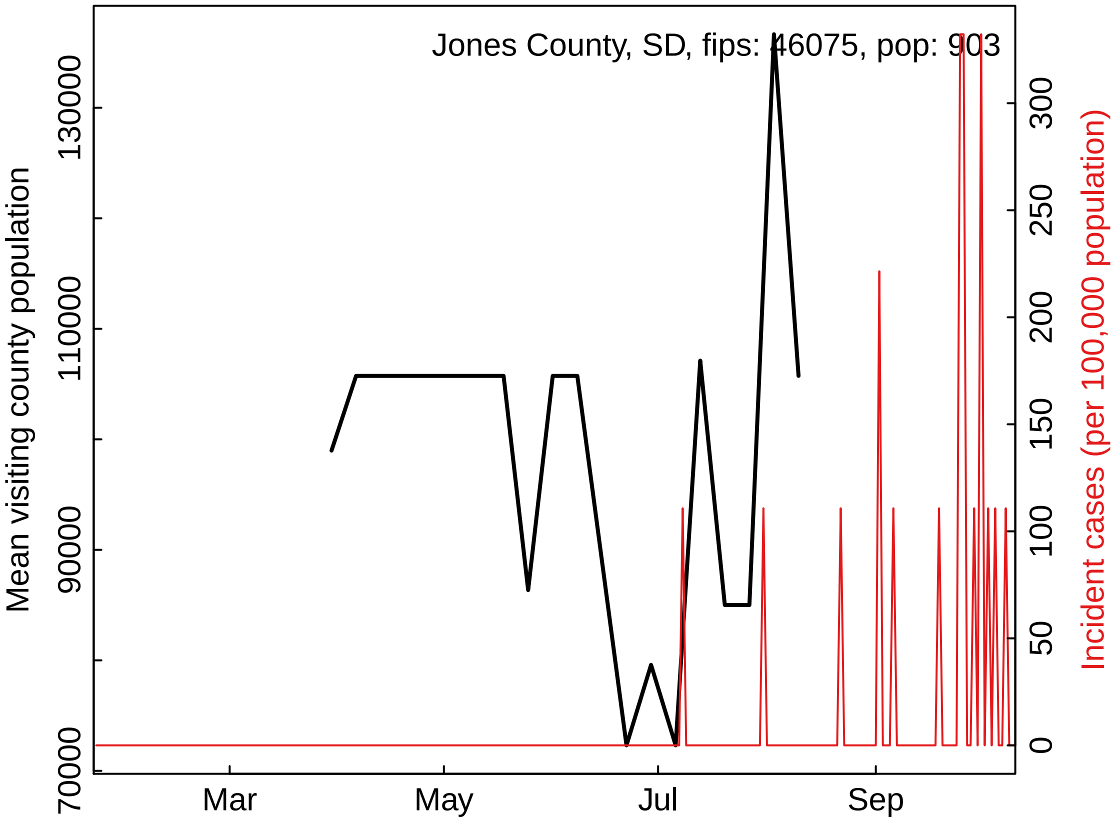

Jones County, SD, fips: 46075, pop: 903
Mean visiting county population
70000
90000
110000
130000
Mar
May
Jul
Sep
0
50
100
150
200
250
300
Incident cases (per 100,000 population)

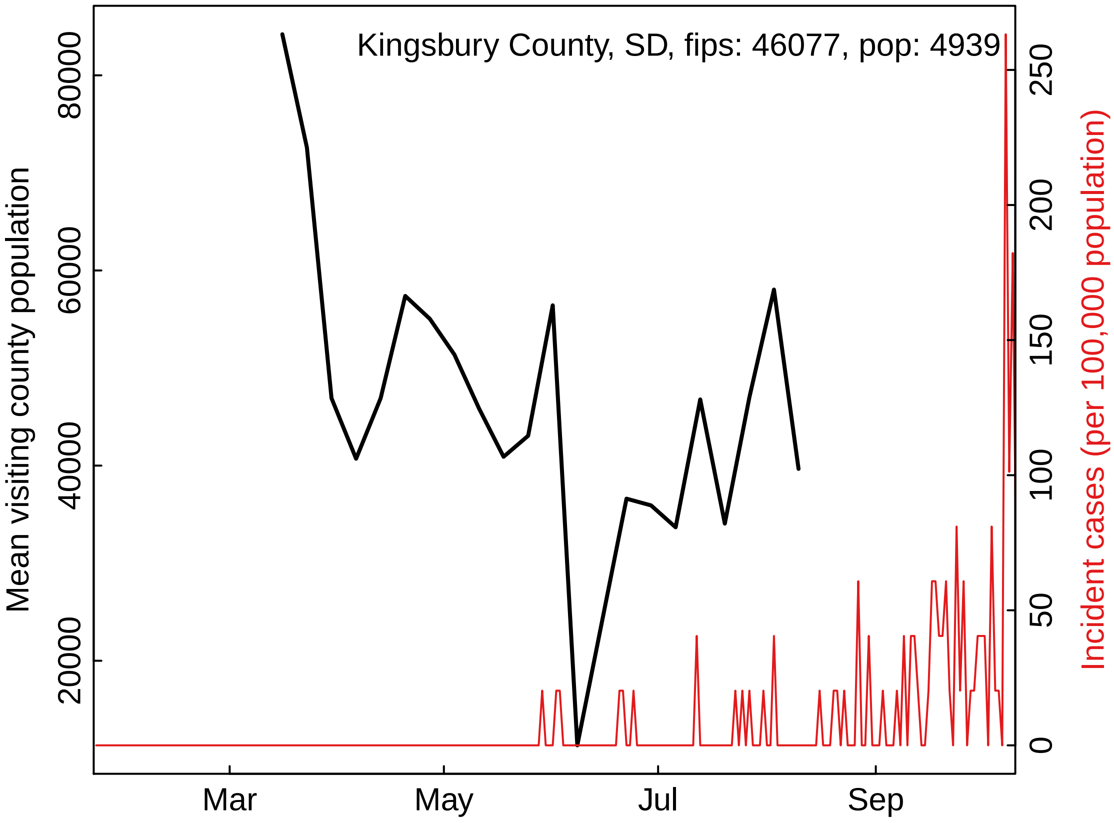

Kingsbury County, SD, fips: 46077, pop: 4939
Mean visiting county population
20000
40000
60000
80000
Incident cases (per 100,000 population)
0
50
100
150
200
250
Mar
May
Jul
Sep

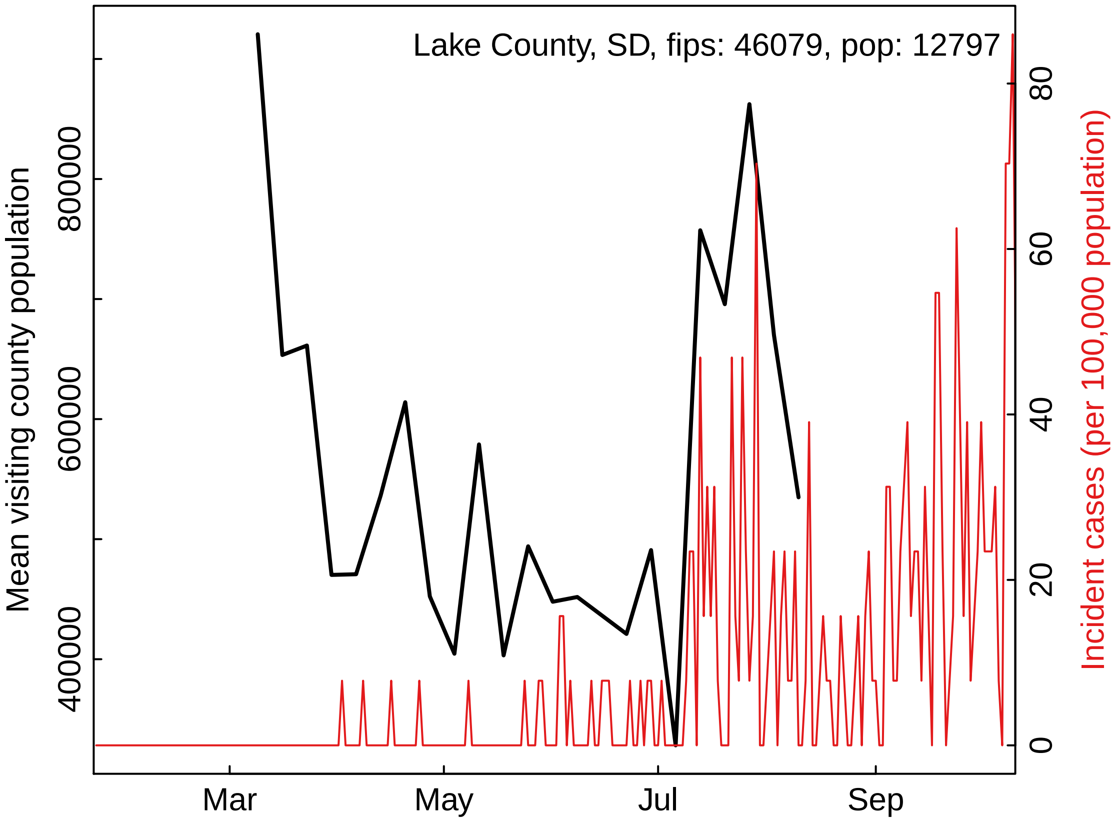
Lake County, SD, fips: 46079, pop: 12797
Mean visiting county population
400000
600000
800000
Incident cases (per 100,000 population)
0
20
40
60
80
Mar
May
Jul
Sep

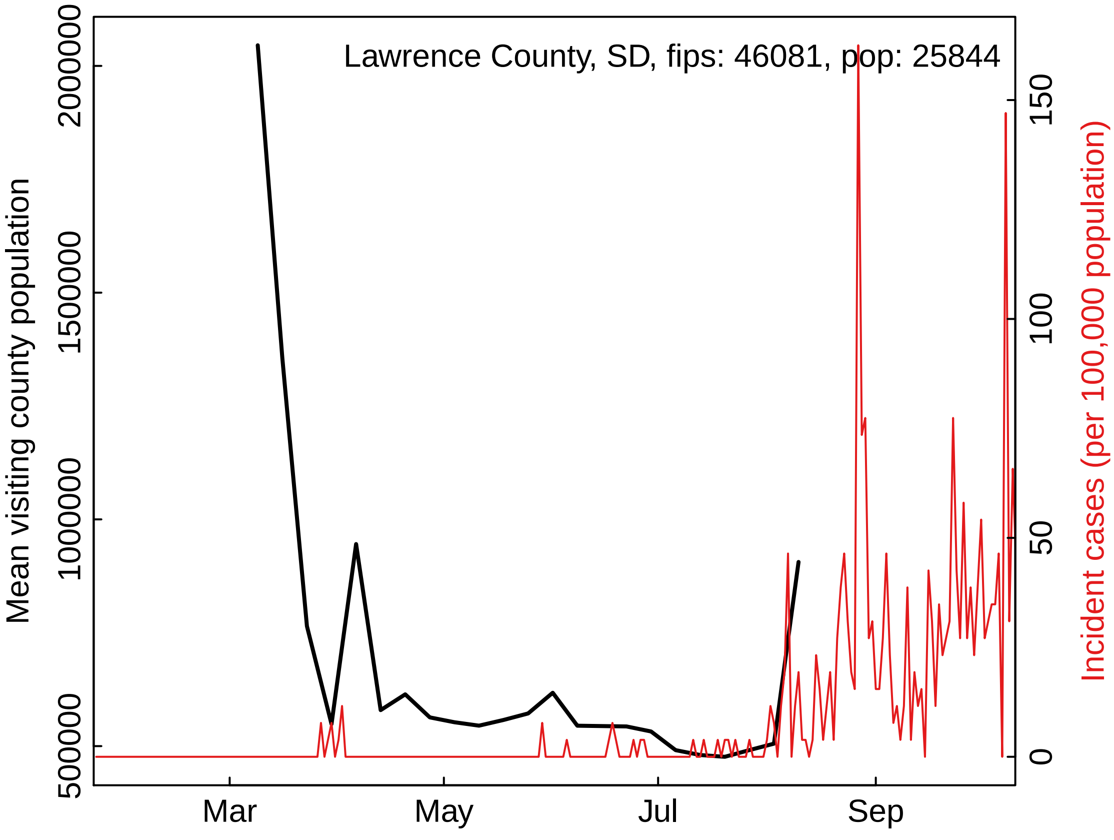

Lawrence County, SD, fips: 46081, pop: 25844
Mean visiting county population
Incident cases (per 100,000 population)
2000000
1500000
1000000
500000
150
100
50
0
Mar
May
Jul
Sep

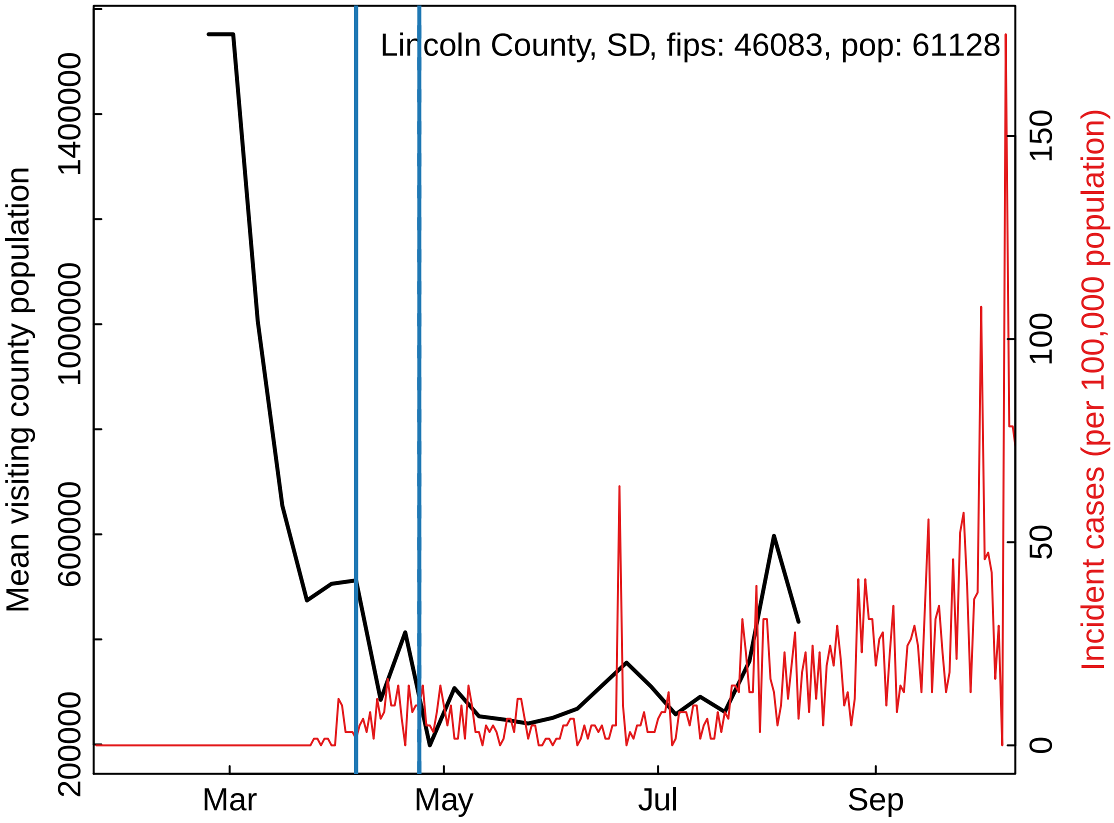

Lincoln County, SD, fips: 46083, pop: 61128
Mean visiting county population
200000
600000
1000000
1400000
Mar
May
Jul
Sep
Incident cases (per 100,000 population)
0
50
100
150

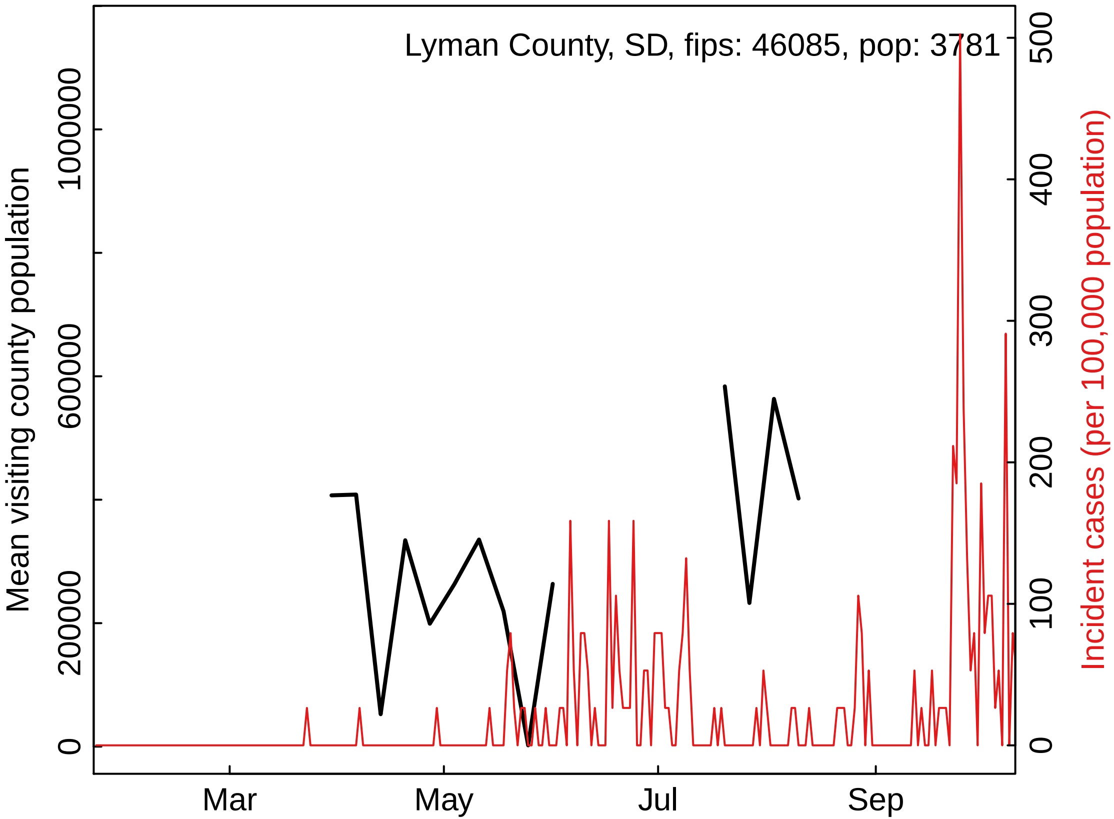

Lyman County, SD, fips: 46085, pop: 3781
Mean visiting county population
0
200000
600000
1000000
Incident cases (per 100,000 population)
0
100
200
300
400
500
Mar
May
Jul
Sep

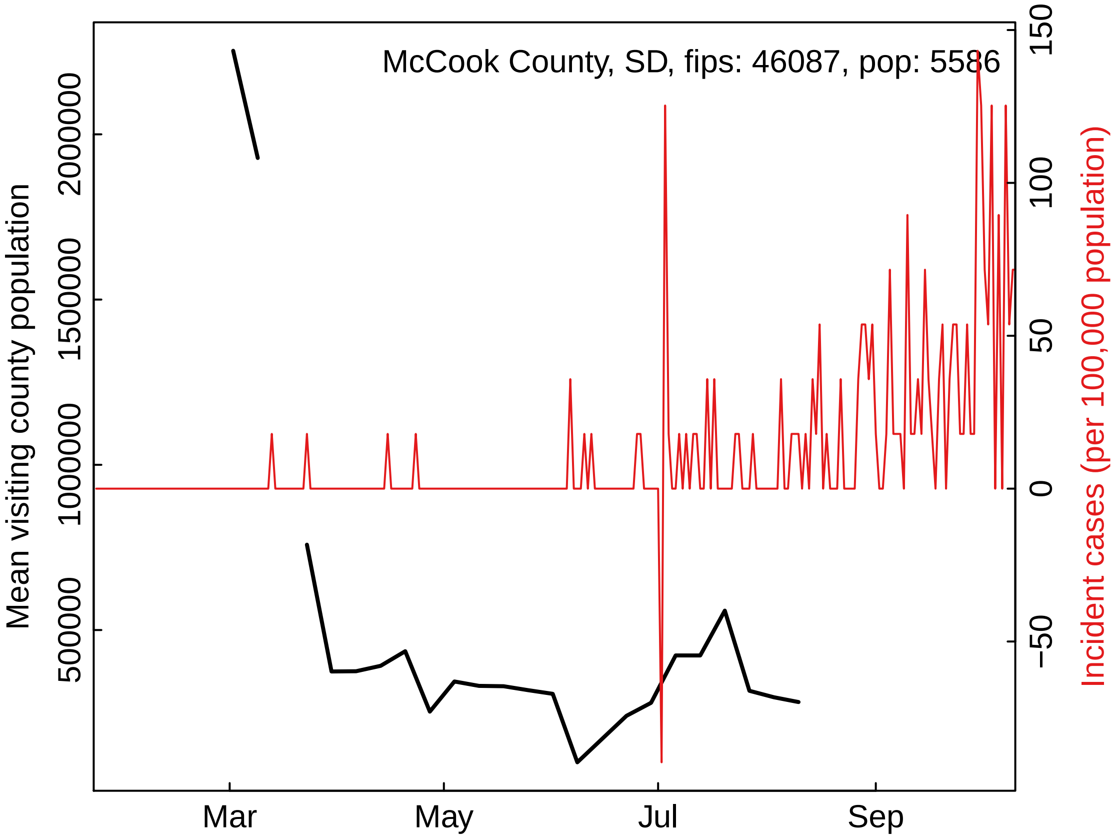

McCook County, SD, fips: 46087, pop: 5586
Mean visiting county population
500000
1000000
1500000
2000000
Incident cases (per 100,000 population)
−50
0
50
100
150
Mar
May
Jul
Sep

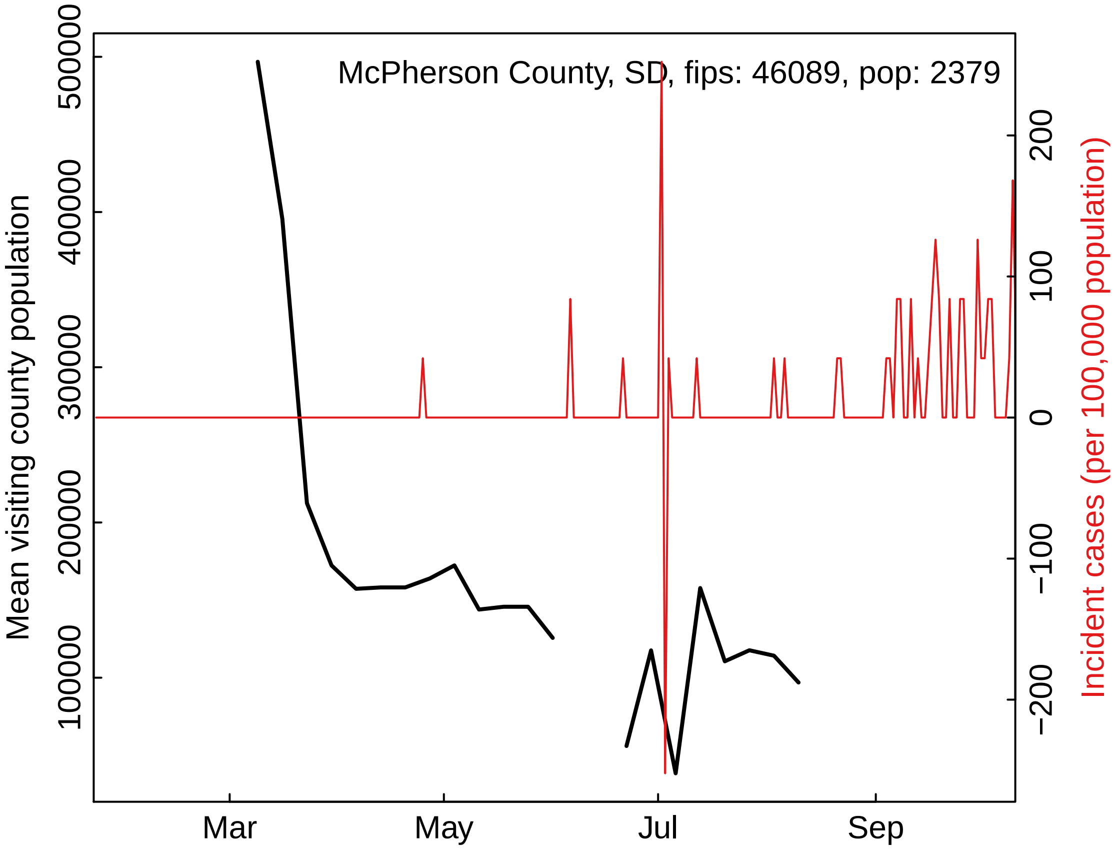
McPherson County, SD, fips: 46089, pop: 2379
Mean visiting county population
100000
200000
300000
400000
500000
Incident cases (per 100,000 population)
−200
−100
0
100
200
Mar
May
Jul
Sep

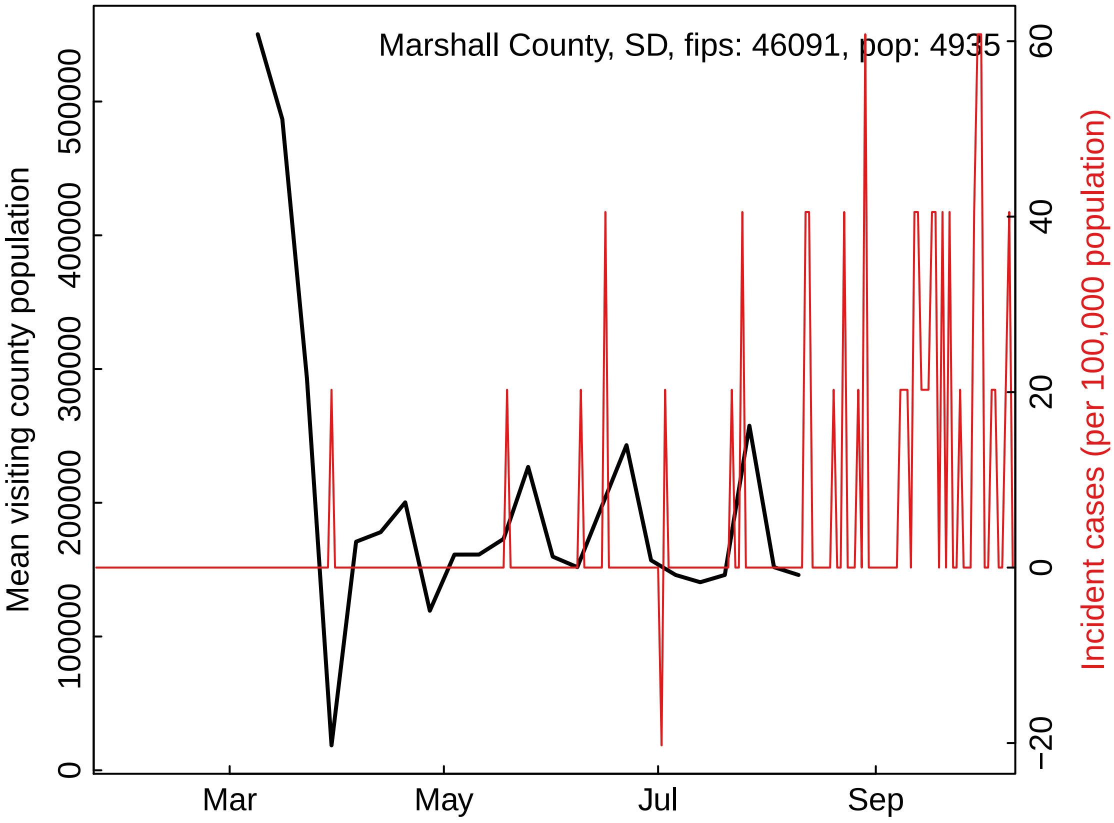

Marshall County, SD, fips: 46091, pop: 4935
Mean visiting county population
0
100000
200000
300000
400000
500000
Incident cases (per 100,000 population)
−20
0
20
40
60
Mar
May
Jul
Sep

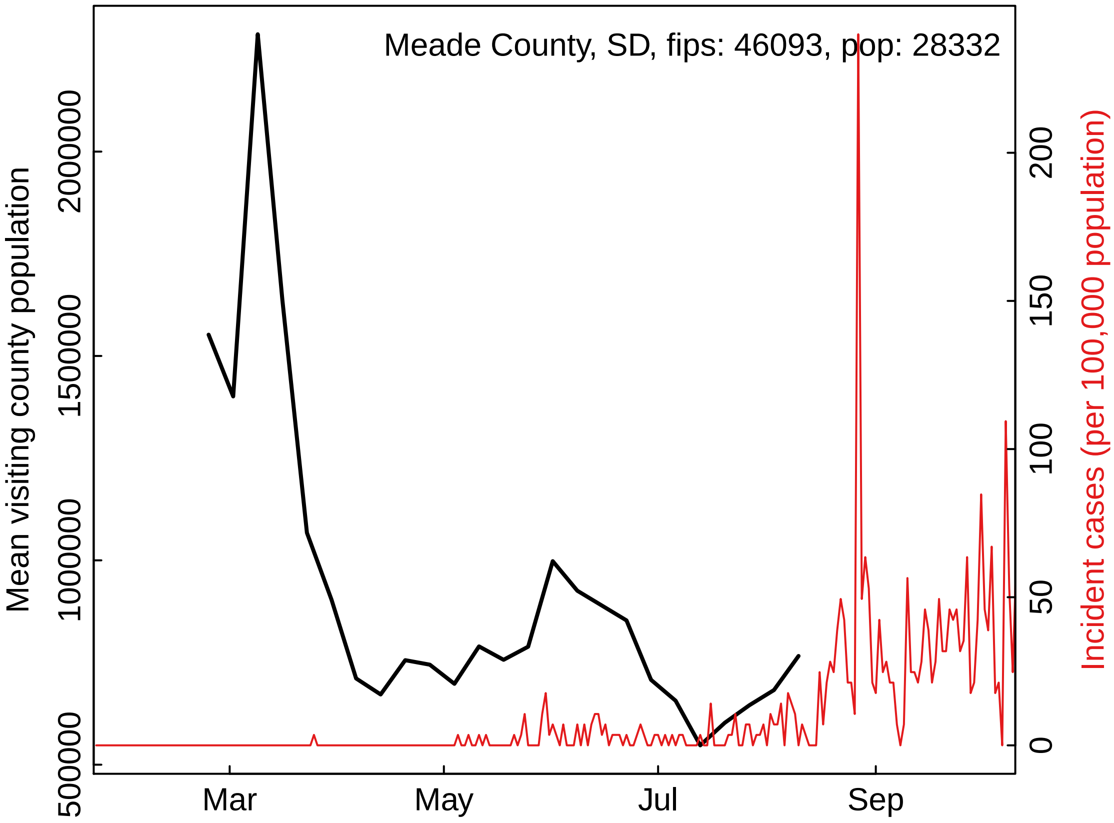

Meade County, SD, fips: 46093, pop: 28332
Mean visiting county population
500000
1000000
1500000
2000000
Mar
May
Jul
Sep
0
50
100
150
200
Incident cases (per 100,000 population)

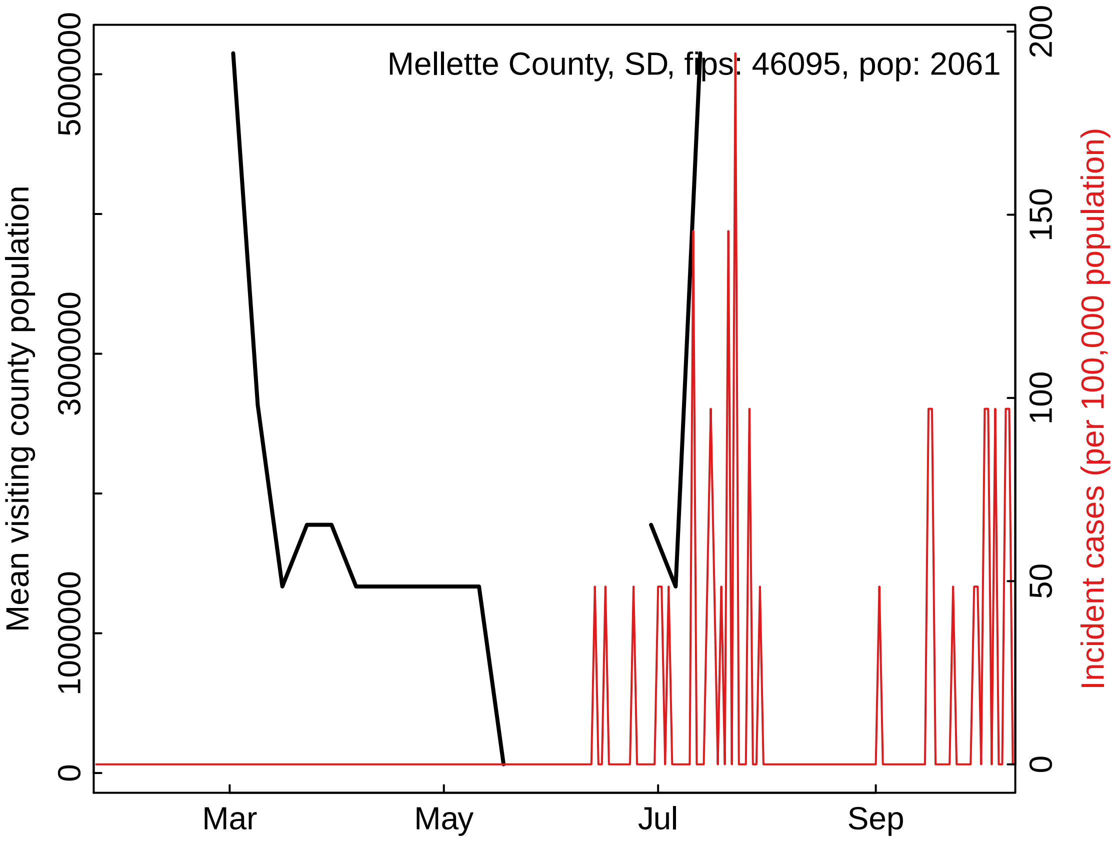

Mellette County, SD, fips: 46095, pop: 2061
Mean visiting county population
0
1000000
3000000
5000000
Incident cases (per 100,000 population)
0
50
100
150
200
Mar
May
Jul
Sep

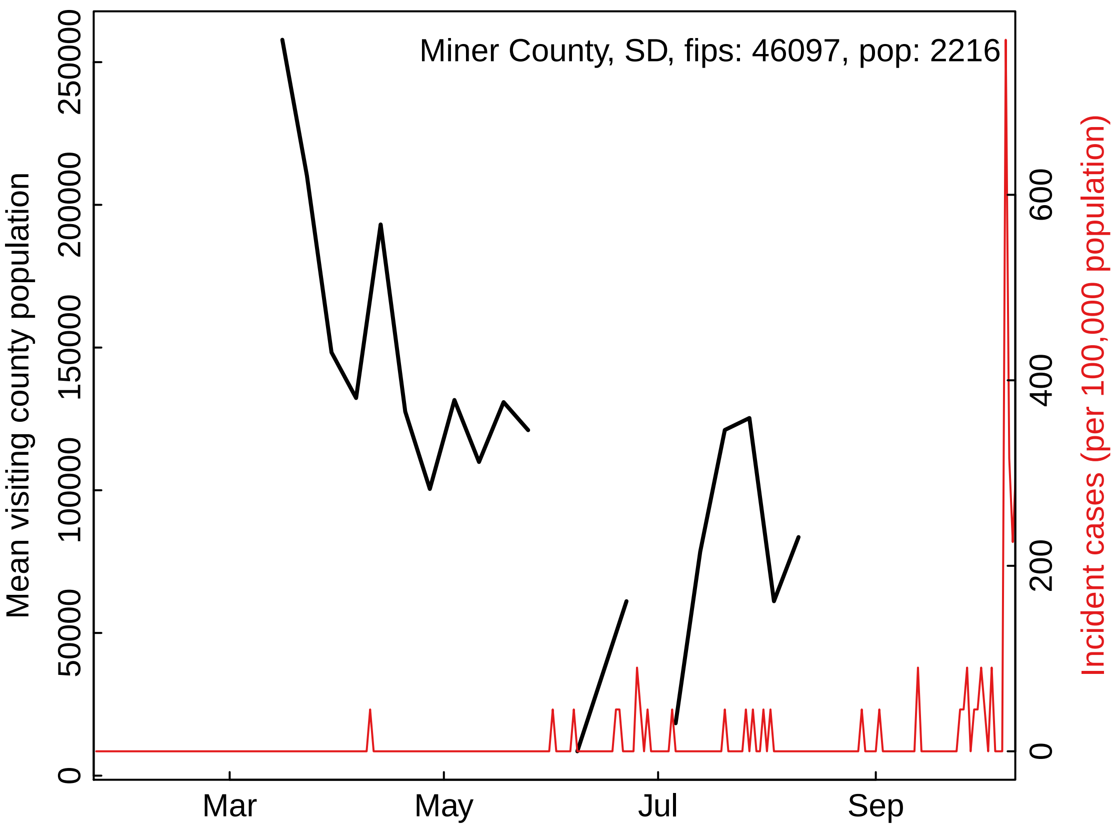
Miner County, SD, fips: 46097, pop: 2216
Mean visiting county population
0
50000
100000
150000
200000
250000
Incident cases (per 100,000 population)
0
200
400
600
Mar
May
Jul
Sep

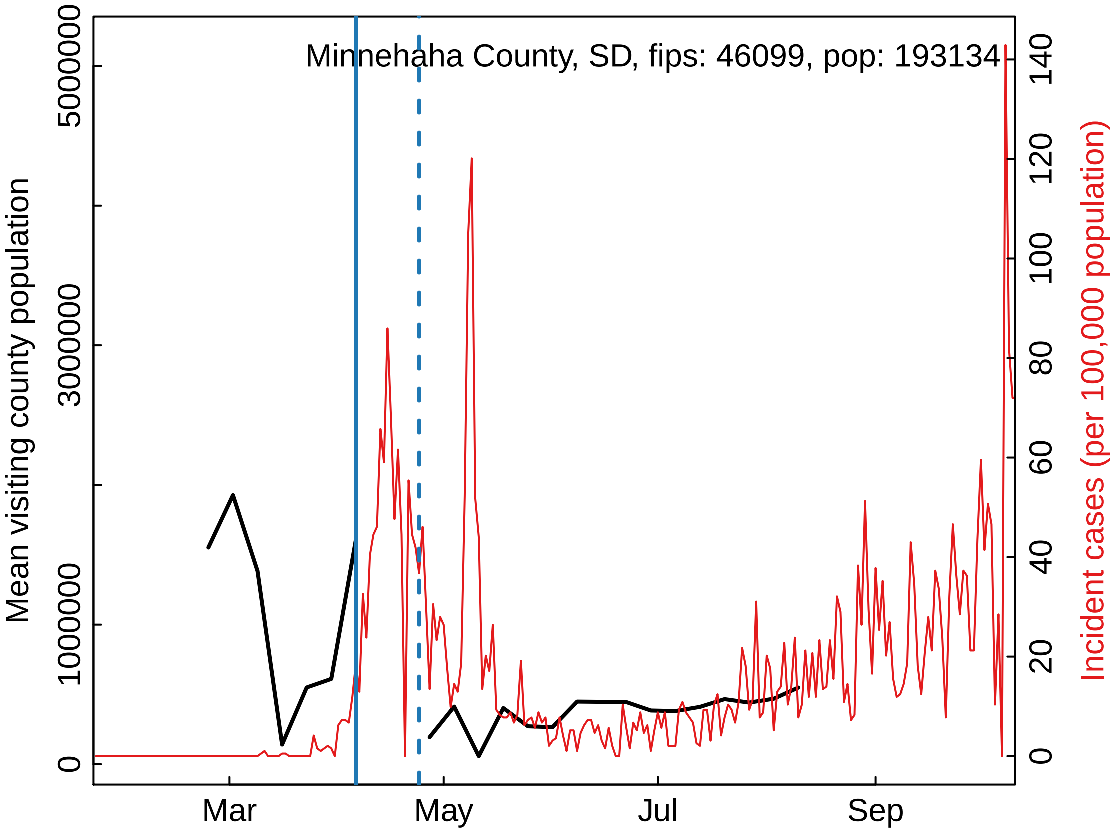
Minnehaha County, SD, fips: 46099, pop: 193134
Mean visiting county population
0
1000000
3000000
5000000
Incident cases (per 100,000 population)
0
20
40
60
80
100
120
140
Mar
May
Jul
Sep

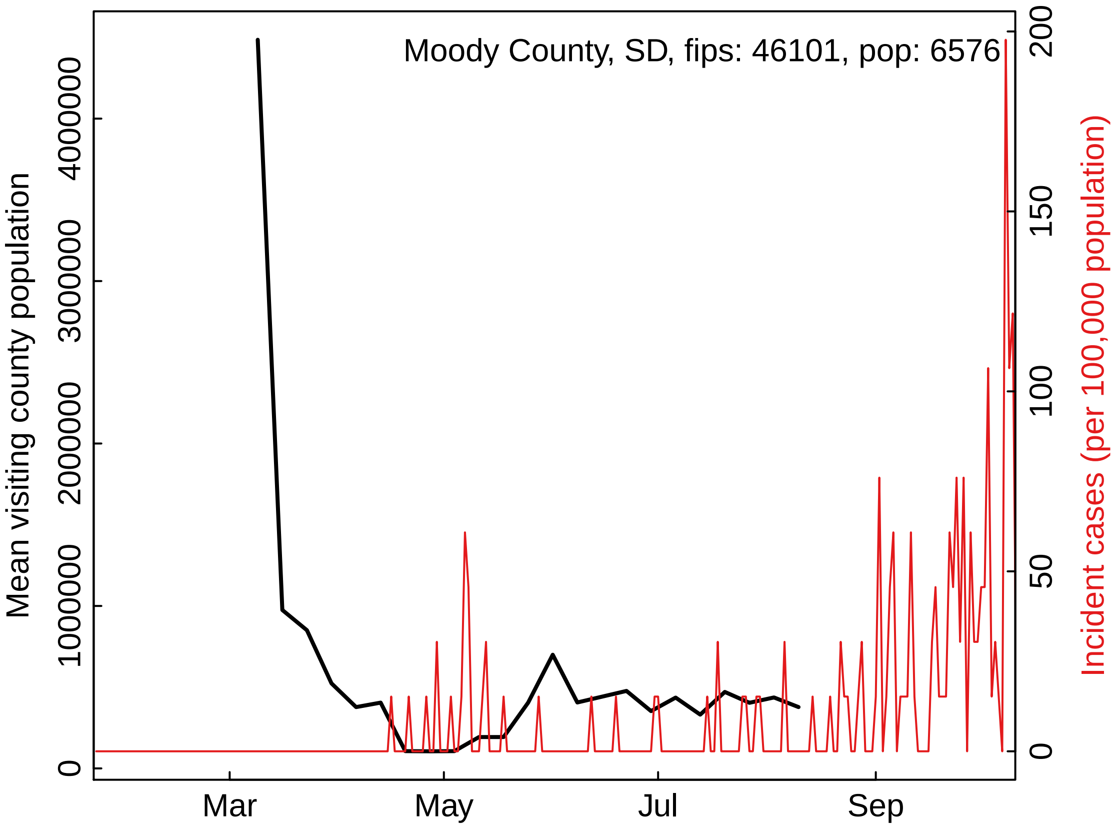

Moody County, SD, fips: 46101, pop: 6576
Mean visiting county population
0
1000000
2000000
3000000
4000000
Mar
May
Jul
Sep
0
50
100
150
200
Incident cases (per 100,000 population)

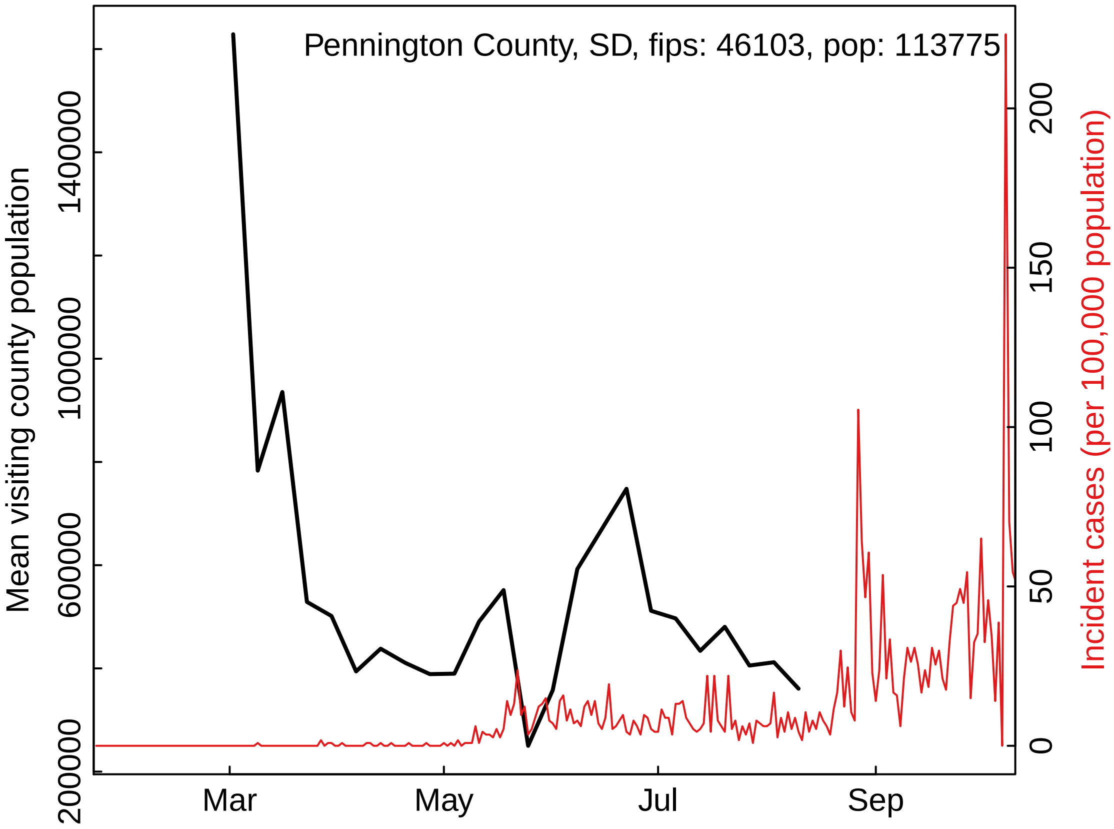

Pennington County, SD, fips: 46103, pop: 113775
Mean visiting county population
200000
600000
1000000
1400000
Mar
May
Jul
Sep
0
50
100
150
200
Incident cases (per 100,000 population)

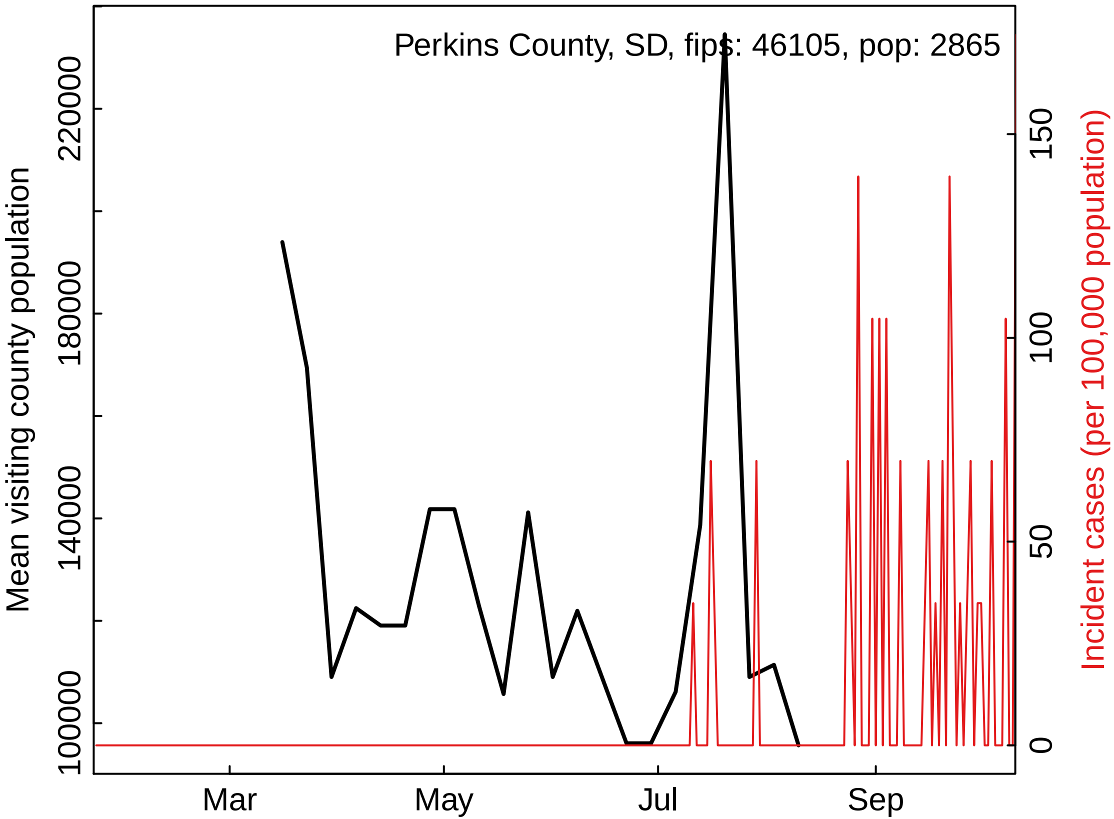

Perkins County, SD, fips: 46105, pop: 2865
Mean visiting county population
100000
140000
180000
220000
Incident cases (per 100,000 population)
0
50
100
150
Mar
May
Jul
Sep

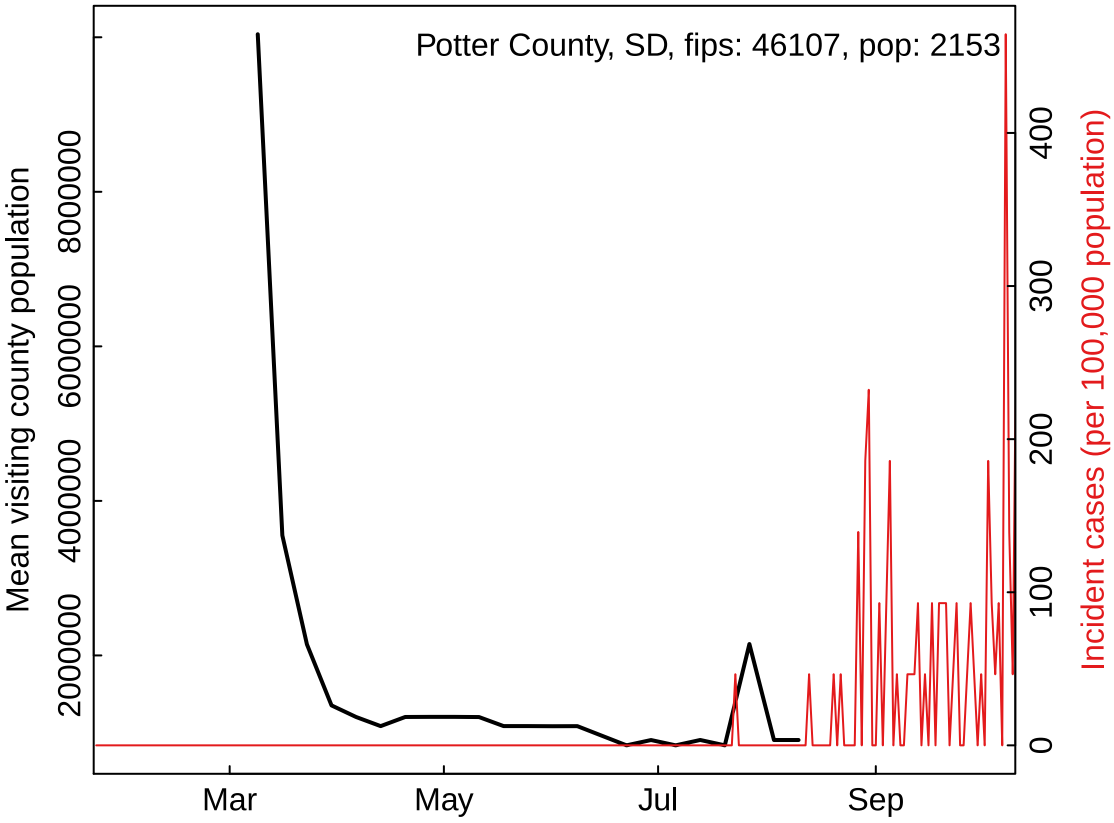

Potter County, SD, fips: 46107, pop: 2153
Mean visiting county population
2000000
4000000
6000000
8000000
Incident cases (per 100,000 population)
0
100
200
300
400
Mar
May
Jul
Sep

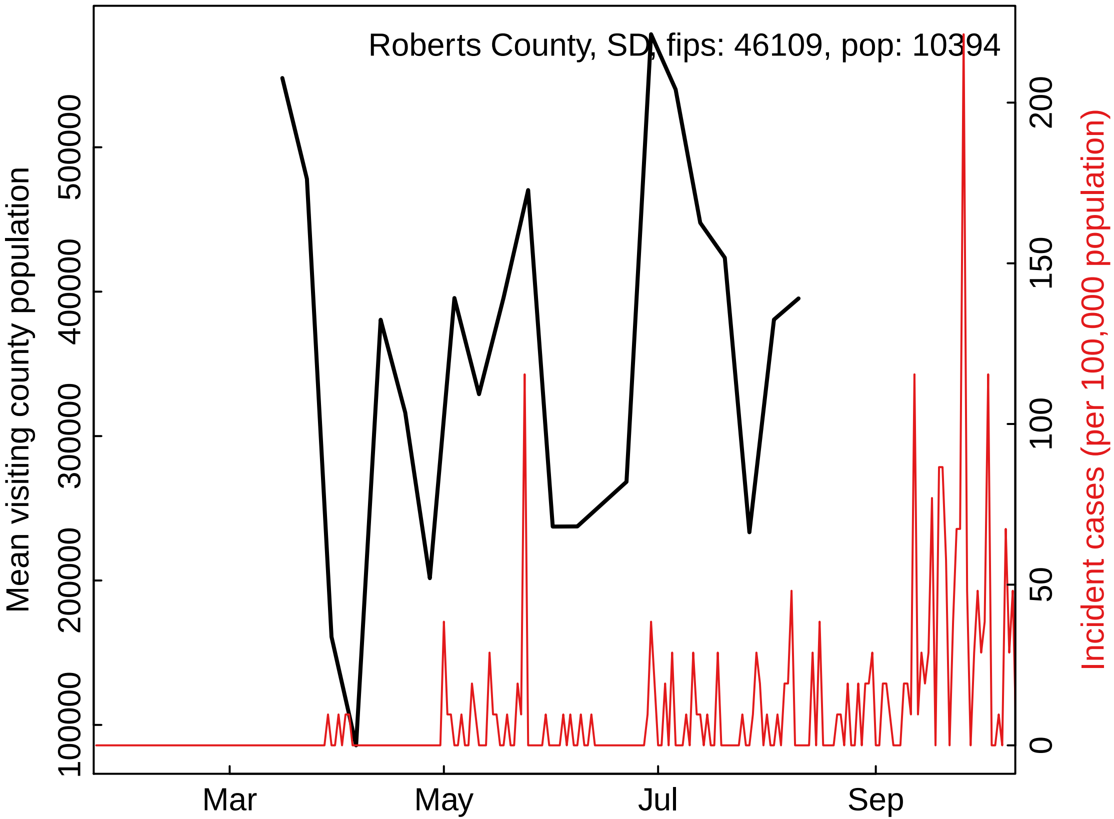

Roberts County, SD, fips: 46109, pop: 10394
Mean visiting county population
100000
200000
300000
400000
500000
Incident cases (per 100,000 population)
0
50
100
150
200
Mar
May
Jul
Sep

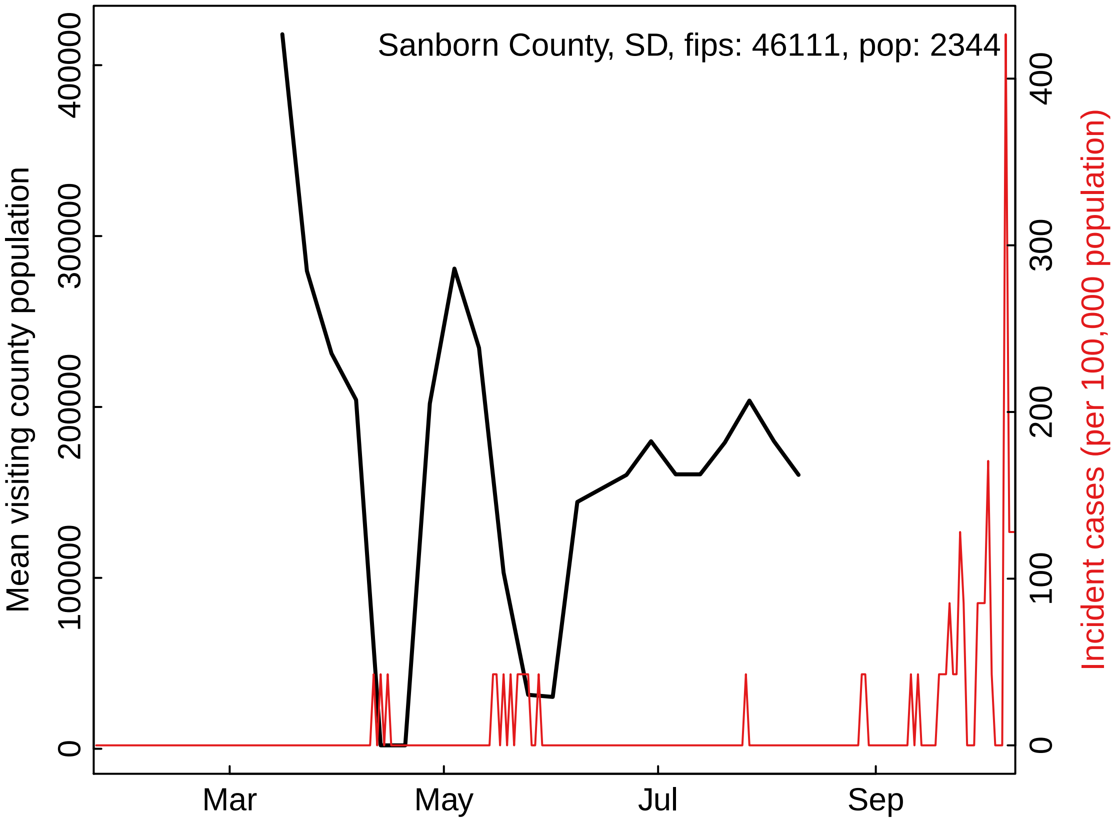

Sanborn County, SD, fips: 46111, pop: 2344
Mean visiting county population
Incident cases (per 100,000 population)
0
100000
200000
300000
400000
0
100
200
300
400
Mar
May
Jul
Sep

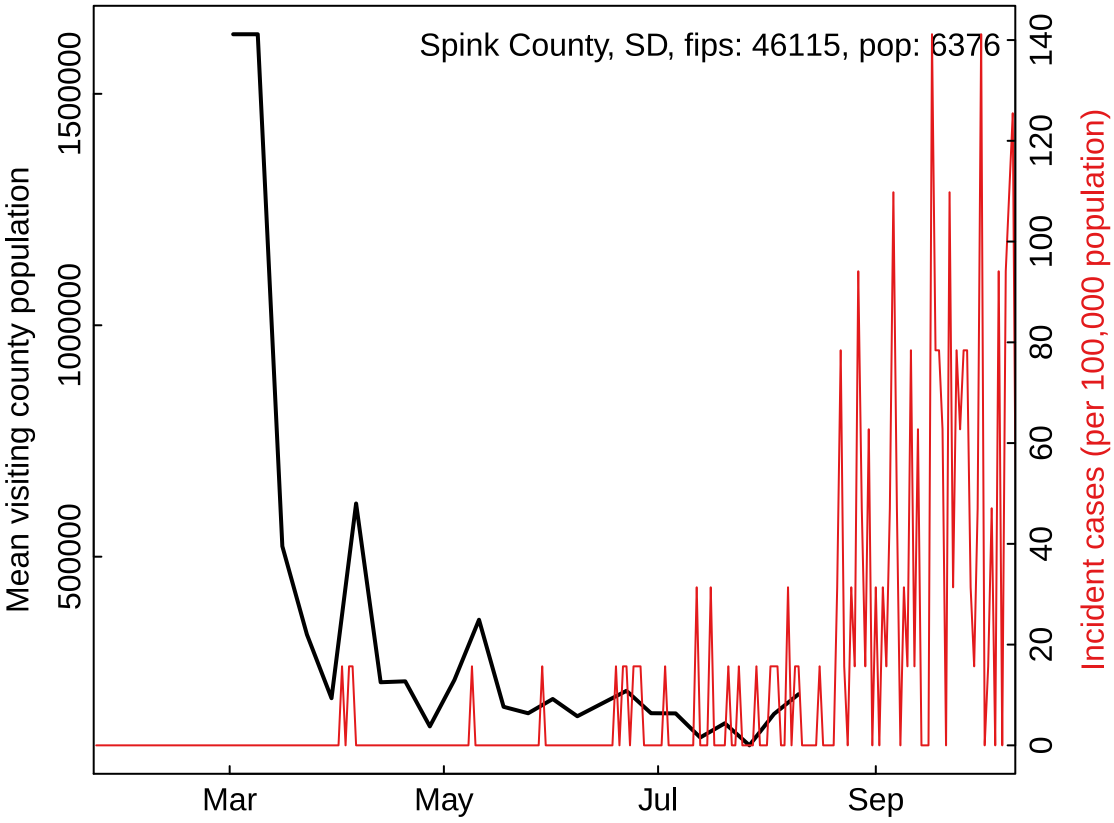
Spink County, SD, fips: 46115, pop: 6376
Mean visiting county population
500000
1000000
1500000
Incident cases (per 100,000 population)
0
20
40
60
80
100
120
140
Mar
May
Jul
Sep

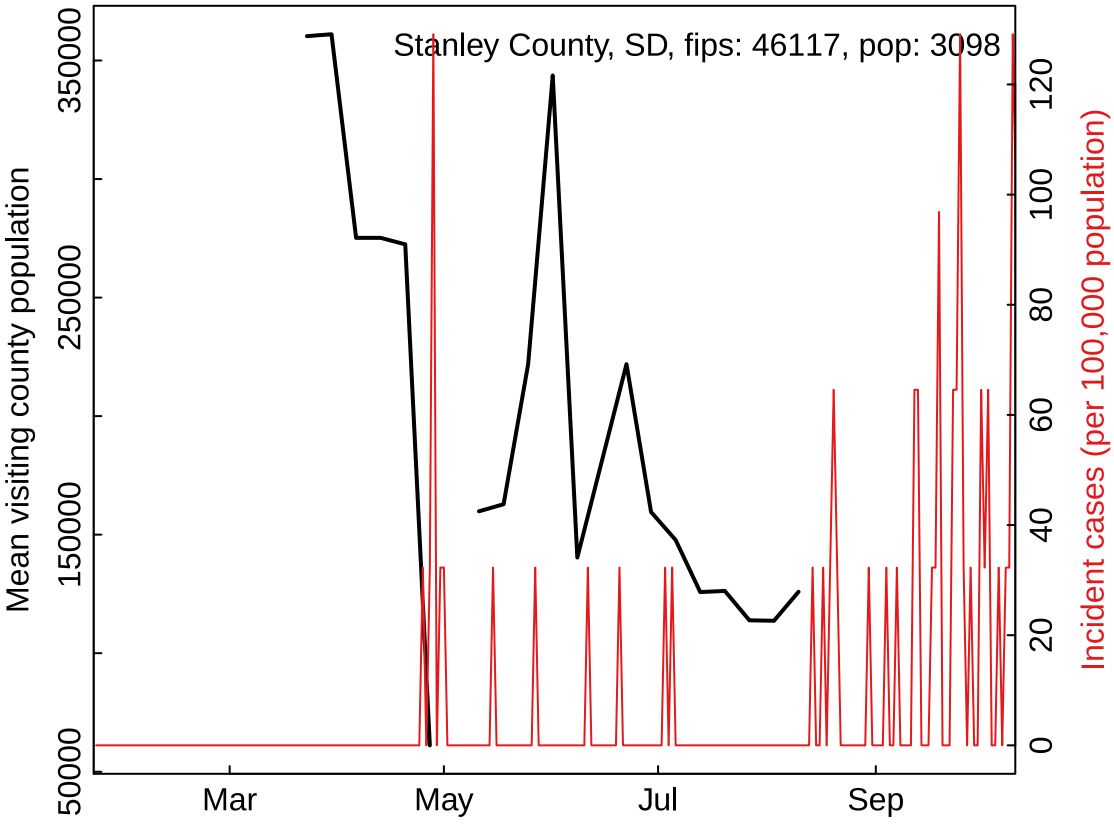

Stanley County, SD, fips: 46117, pop: 3098
Mean visiting county population
50000
150000
250000
350000
Incident cases (per 100,000 population)
0
20
40
60
80
100
120
Mar
May
Jul
Sep

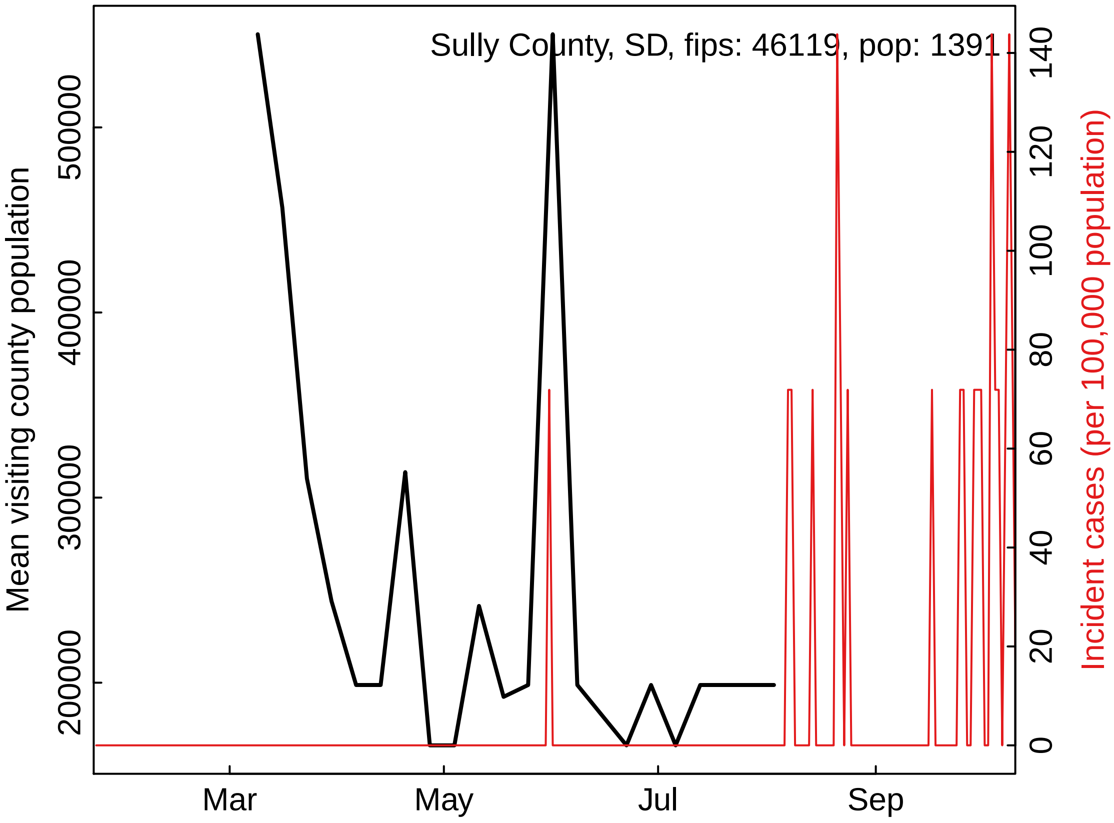

Sully County, SD, fips: 46119, pop: 1391
Mean visiting county population
200000
300000
400000
500000
Incident cases (per 100,000 population)
0
20
40
60
80
100
120
140
Mar
May
Jul
Sep

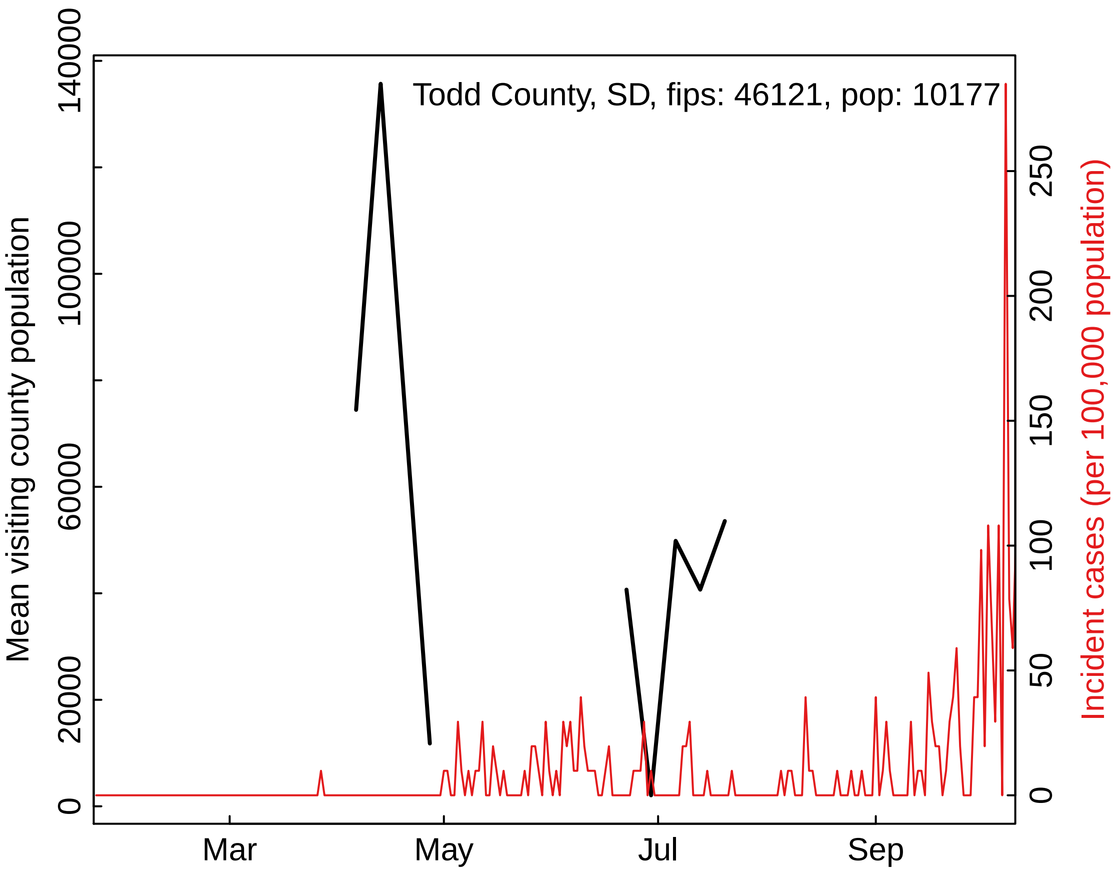
Todd County, SD, fips: 46121, pop: 10177
Mean visiting county population
Incident cases (per 100,000 population)
0
20000
60000
100000
140000
0
50
100
150
200
250
Mar
May
Jul
Sep

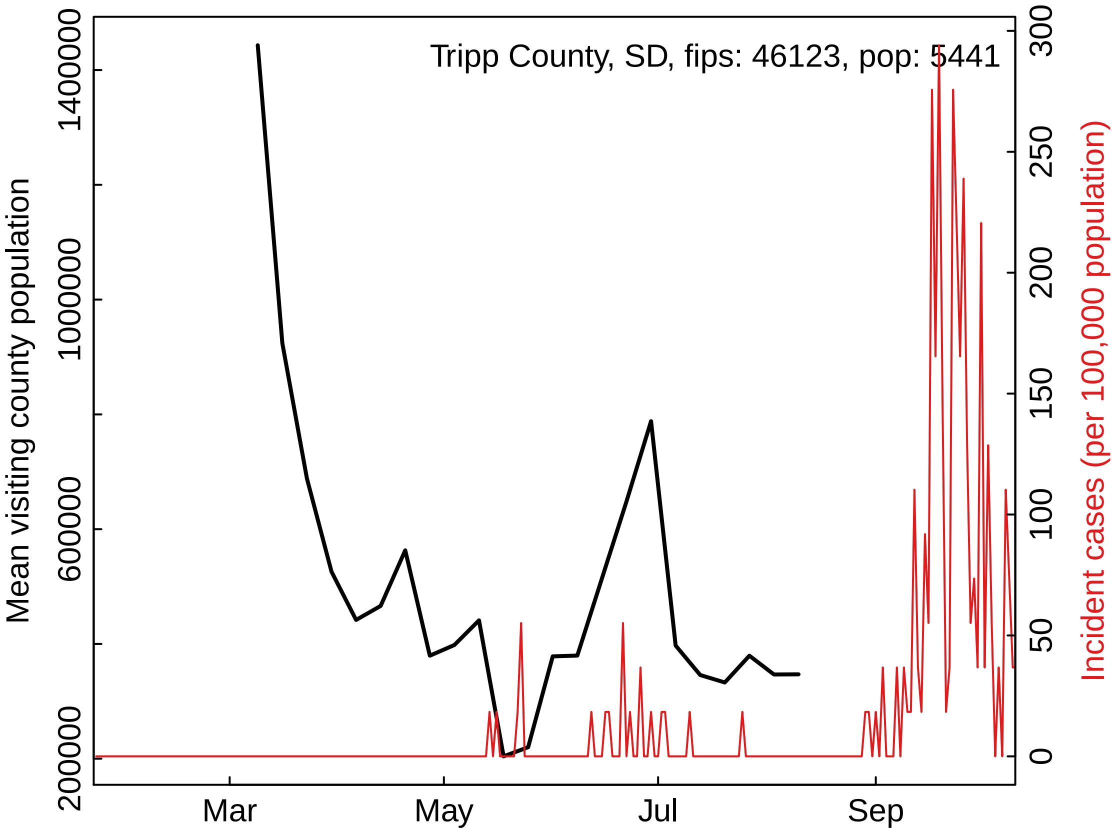

Tripp County, SD, fips: 46123, pop: 5441
Mean visiting county population
200000
600000
1000000
1400000
Incident cases (per 100,000 population)
0
50
100
150
200
250
300
Mar
May
Jul
Sep

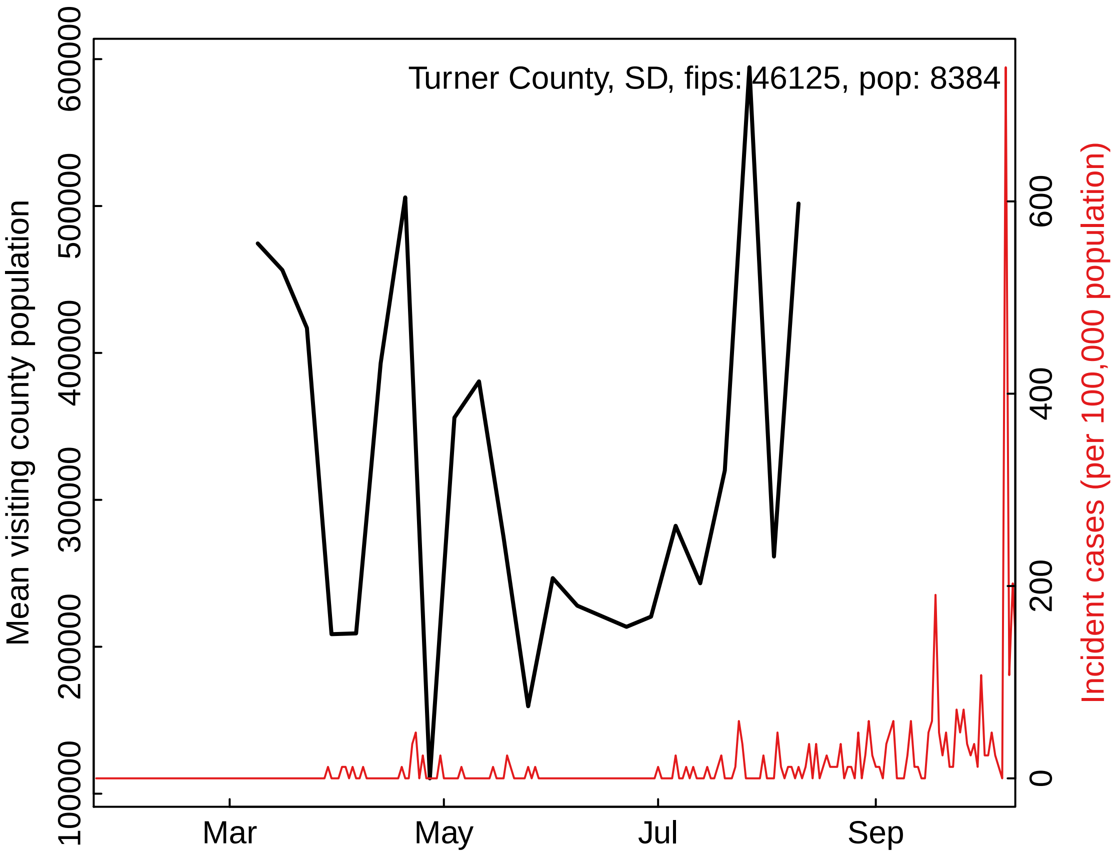

Turner County, SD, fips: 46125, pop: 8384
Mean visiting county population
100000
200000
300000
400000
500000
600000
Mar
May
Jul
Sep
0
200
400
600
Incident cases (per 100,000 population)

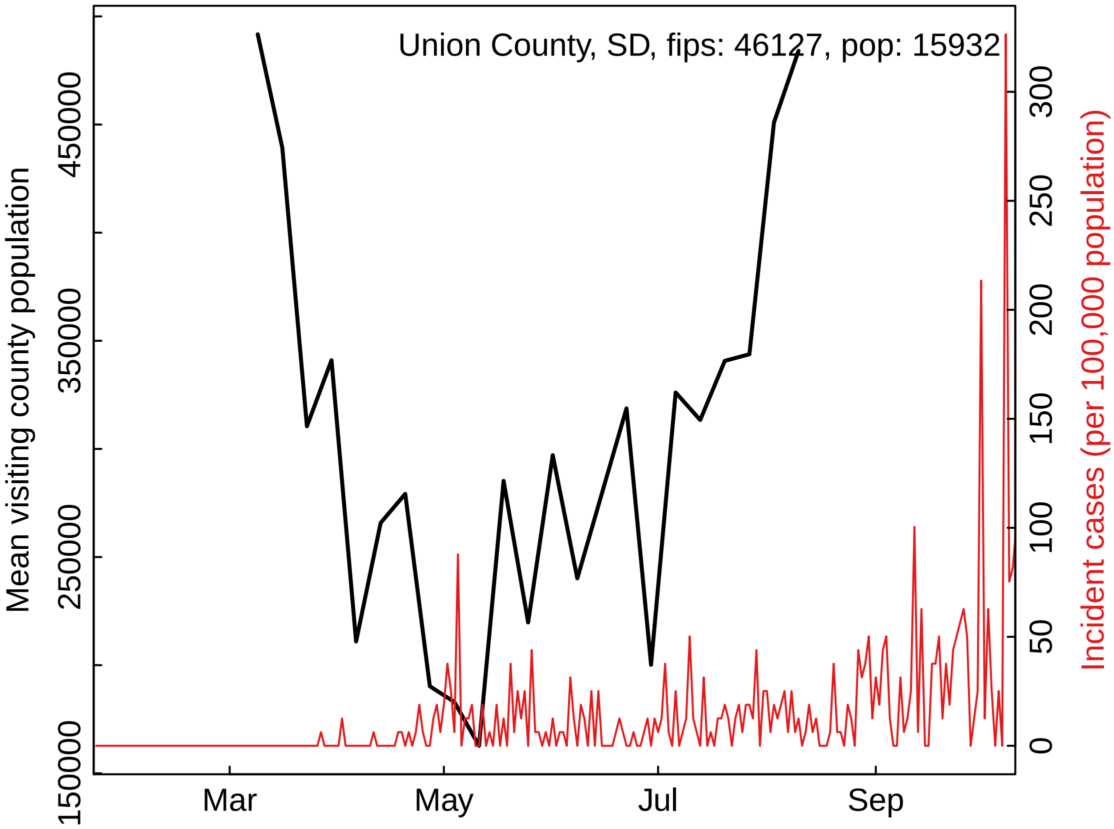

Union County, SD, fips: 46127, pop: 15932
Mean visiting county population
150000
250000
350000
450000
Incident cases (per 100,000 population)
0
50
100
150
200
250
300
Mar
May
Jul
Sep

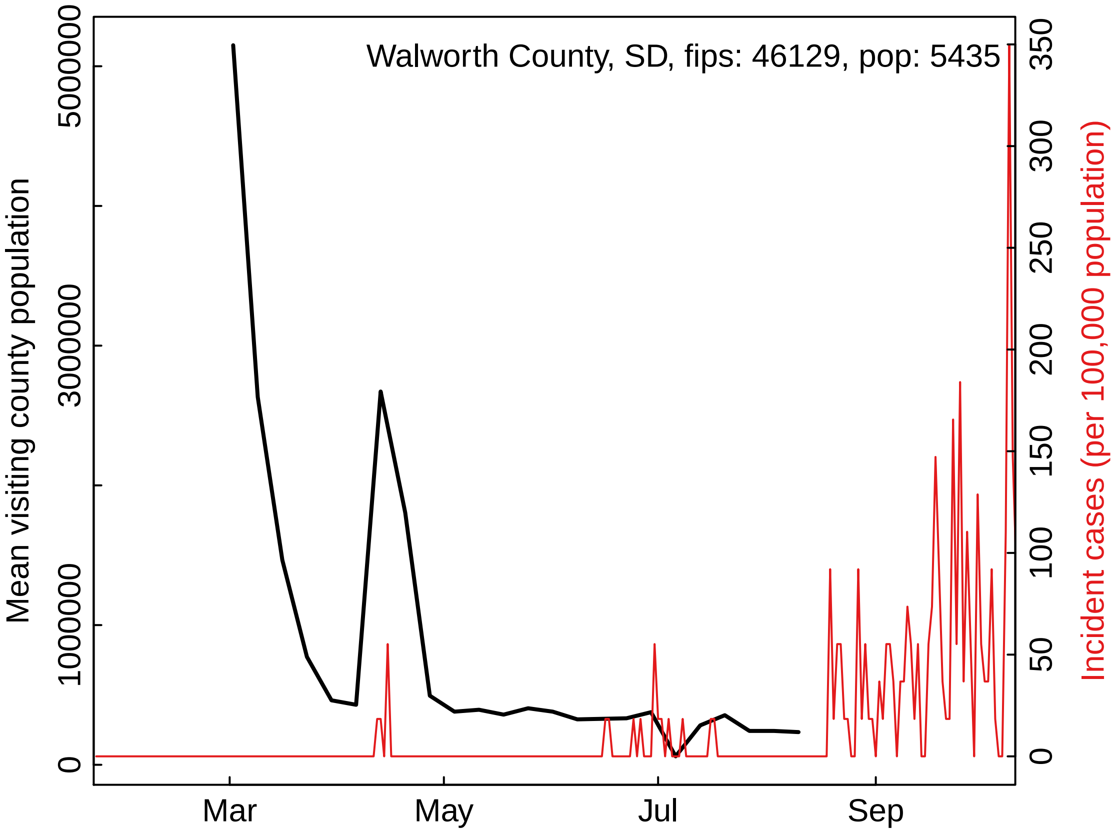
Walworth County, SD, fips: 46129, pop: 5435
Mean visiting county population
0
1000000
3000000
5000000
Incident cases (per 100,000 population)
0
50
100
150
200
250
300
350
Mar
May
Jul
Sep

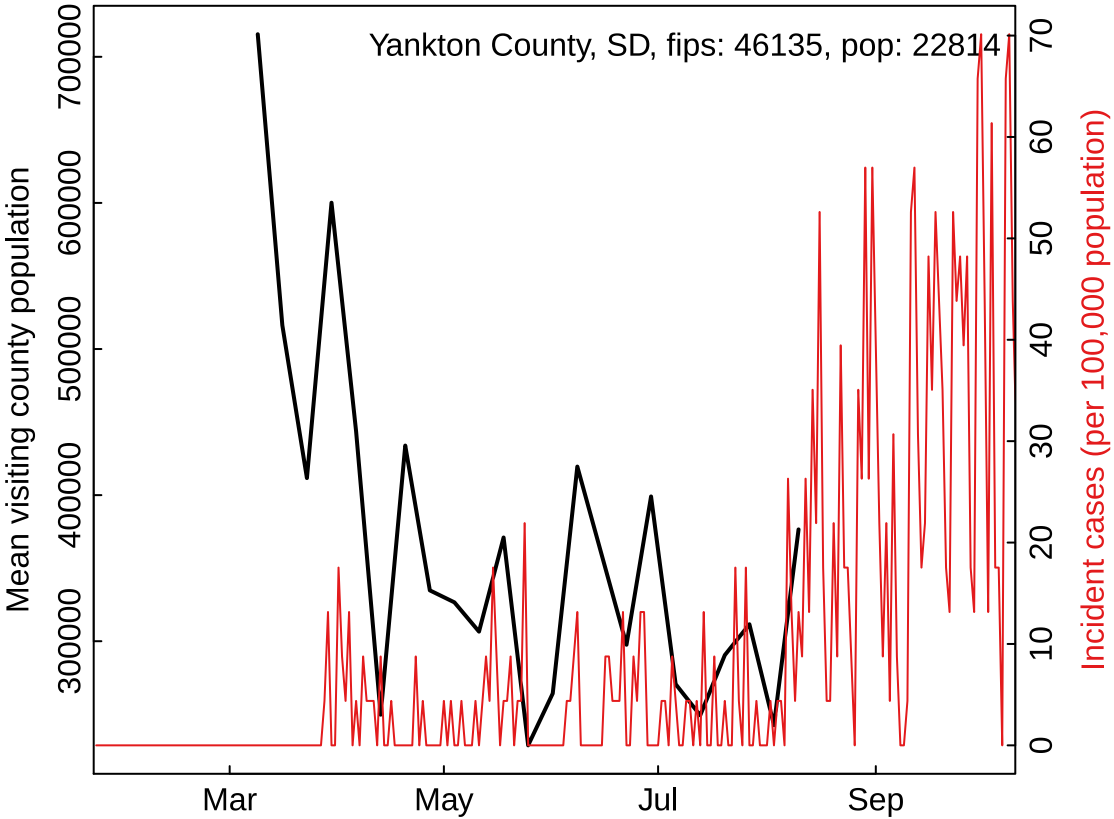

Yankton County, SD, fips: 46135, pop: 22814
Mean visiting county population
Incident cases (per 100,000 population)
300000
400000
500000
600000
700000
0
10
20
30
40
50
60
70
Mar
May
Jul
Sep

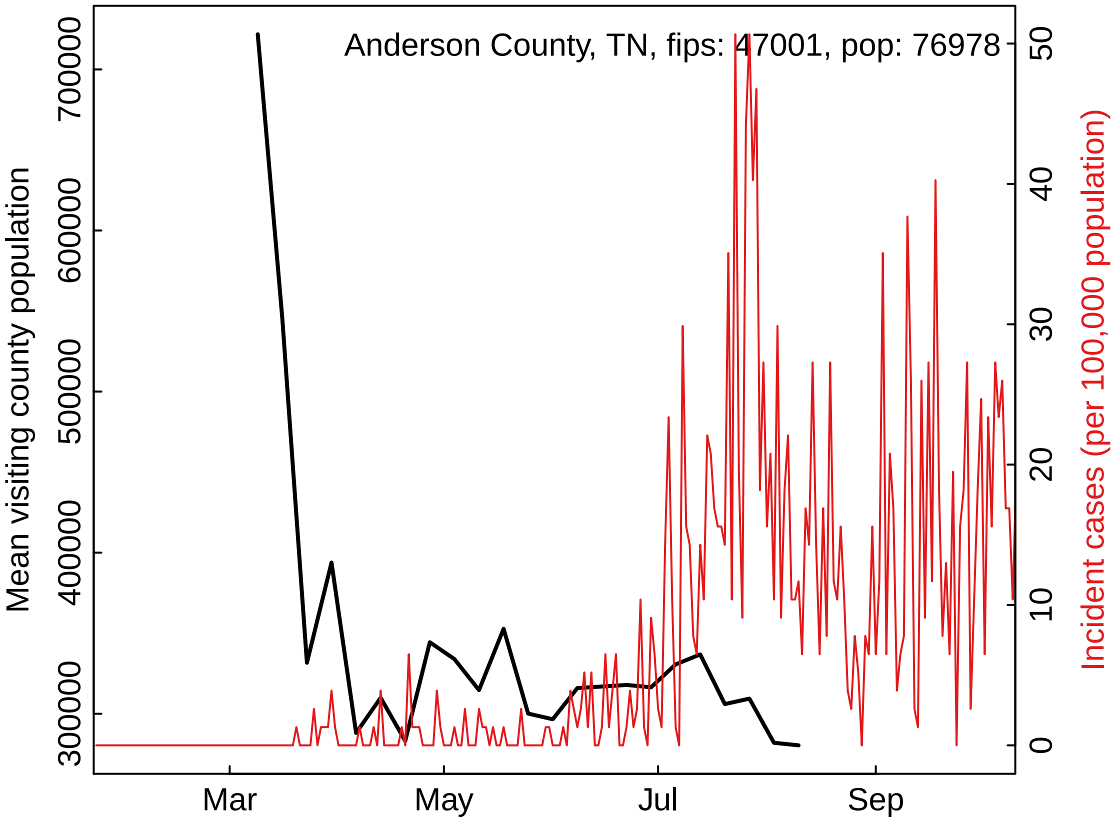

Anderson County, TN, fips: 47001, pop: 76978
Mean visiting county population
300000
400000
500000
600000
700000
Mar
May
Jul
Sep
Incident cases (per 100,000 population)
0
10
20
30
40
50

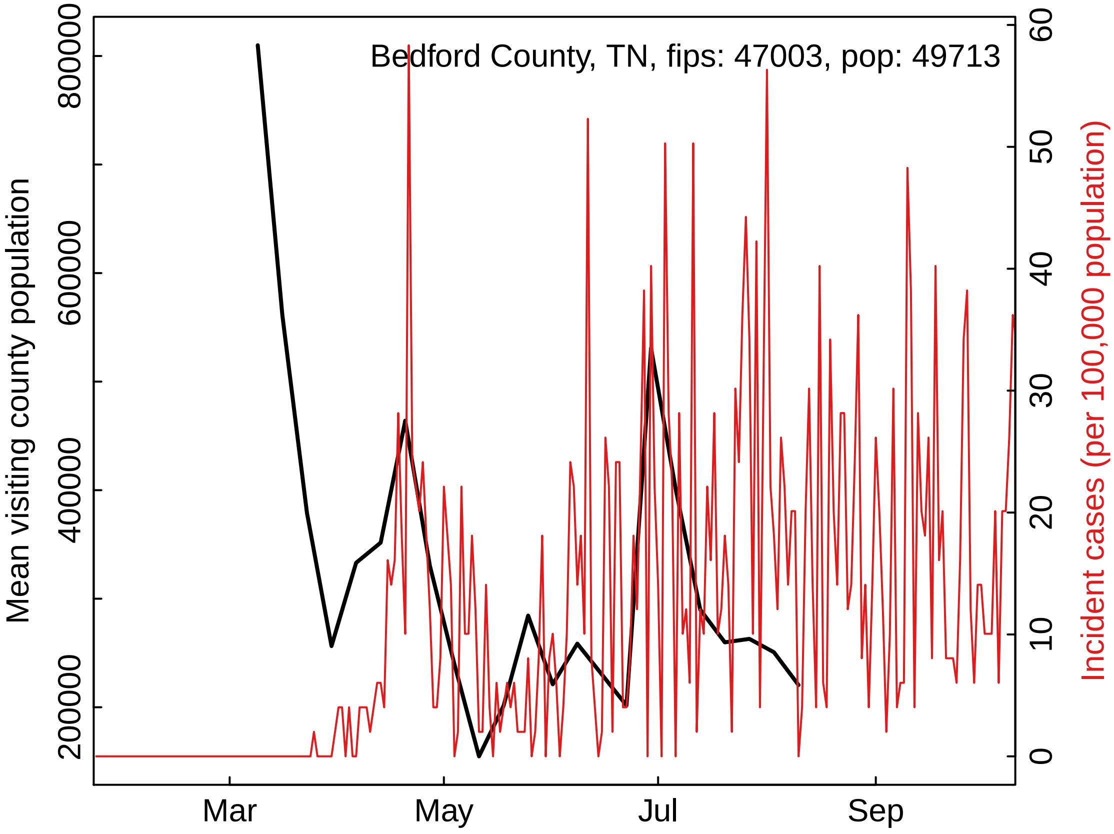

Bedford County, TN, fips: 47003, pop: 49713
Mean visiting county population
Incident cases (per 100,000 population)
200000
400000
600000
800000
0
10
20
30
40
50
60
Mar
May
Jul
Sep

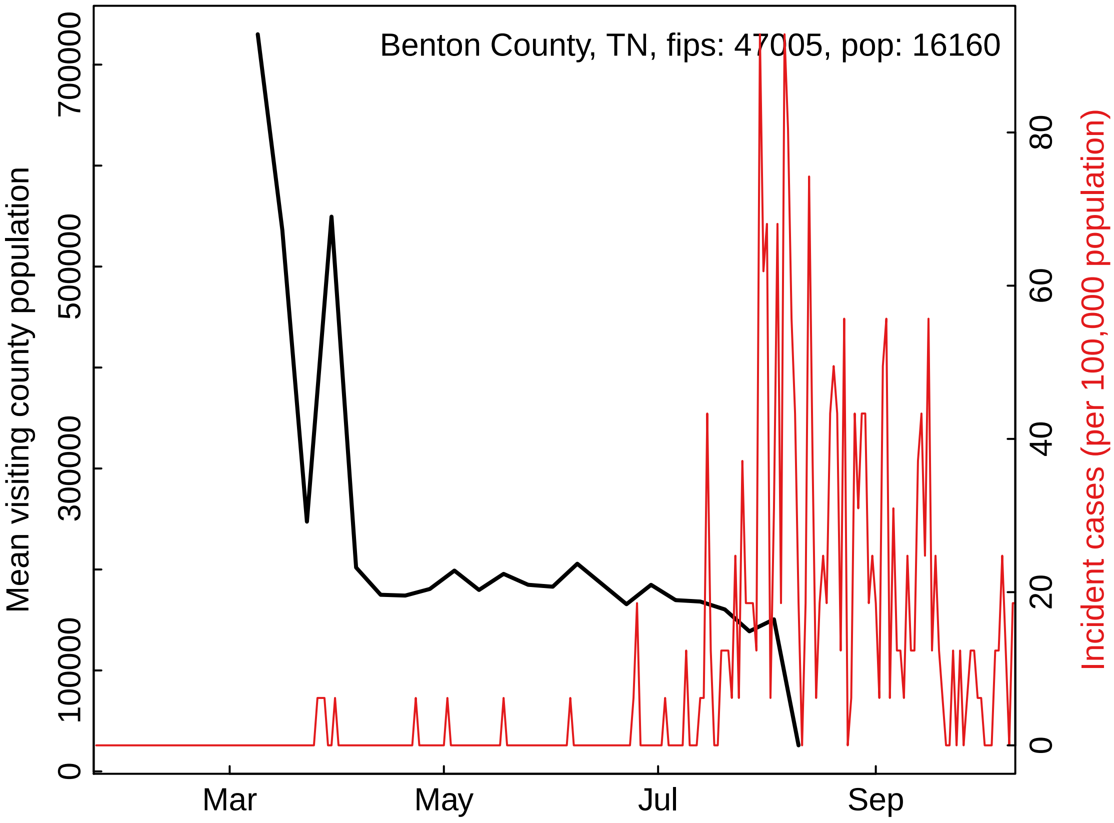

Benton County, TN, fips: 47005, pop: 16160
Mean visiting county population
0
100000
300000
500000
700000
Mar
May
Jul
Sep
0
20
40
60
80
Incident cases (per 100,000 population)

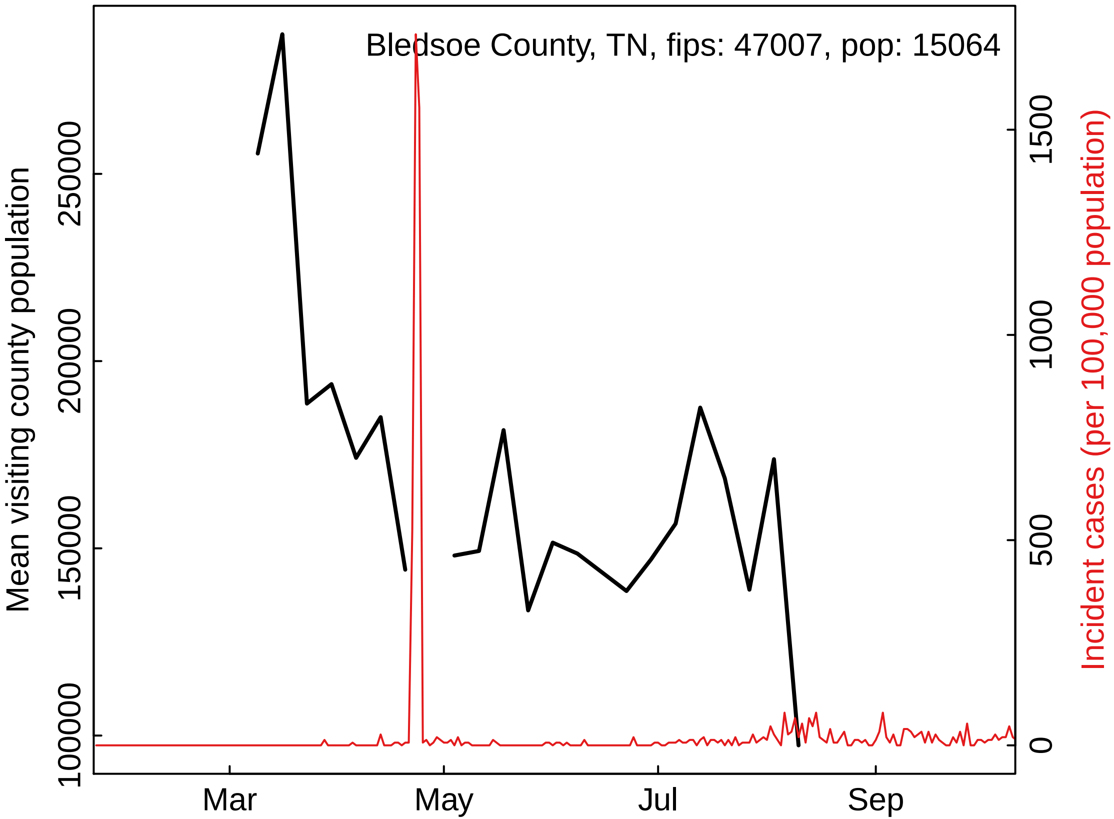

Bledsoe County, TN, fips: 47007, pop: 15064
Mean visiting county population
100000
150000
200000
250000
Incident cases (per 100,000 population)
0
500
1000
1500
Mar
May
Jul
Sep

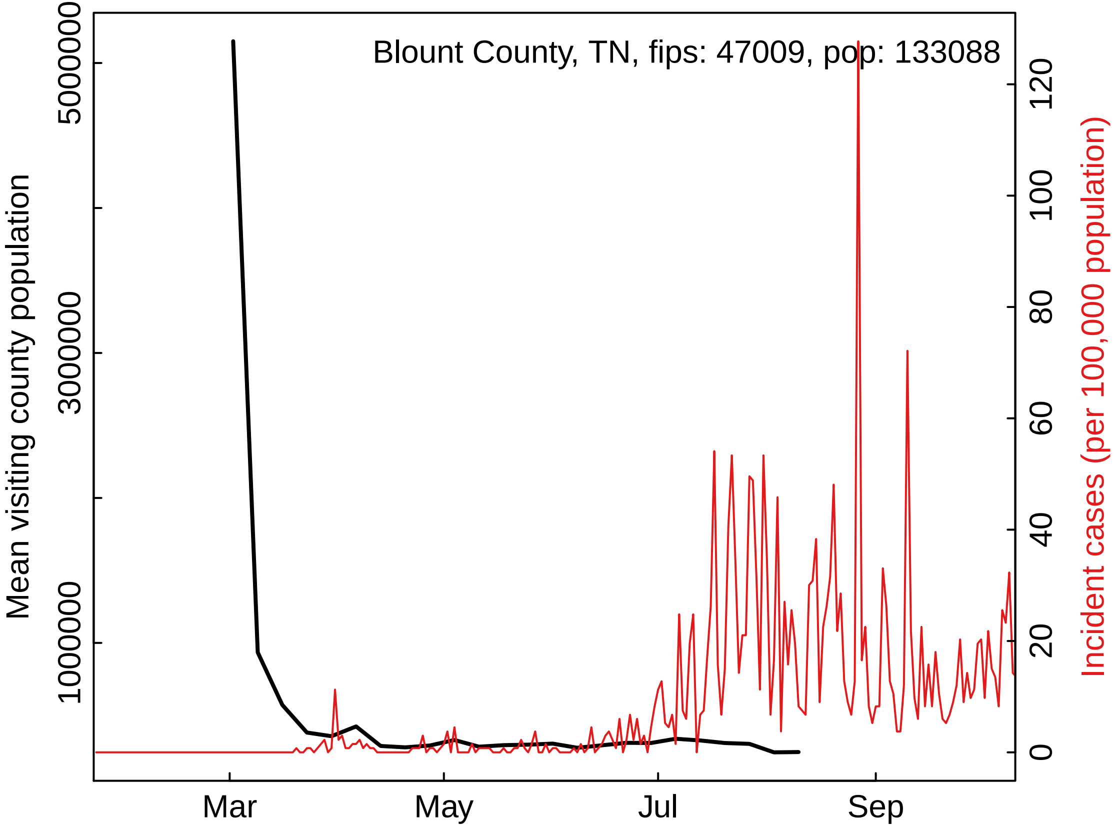

Blount County, TN, fips: 47009, pop: 133088
Mean visiting county population
1000000
3000000
5000000
Incident cases (per 100,000 population)
0
20
40
60
80
100
120
Mar
May
Jul
Sep

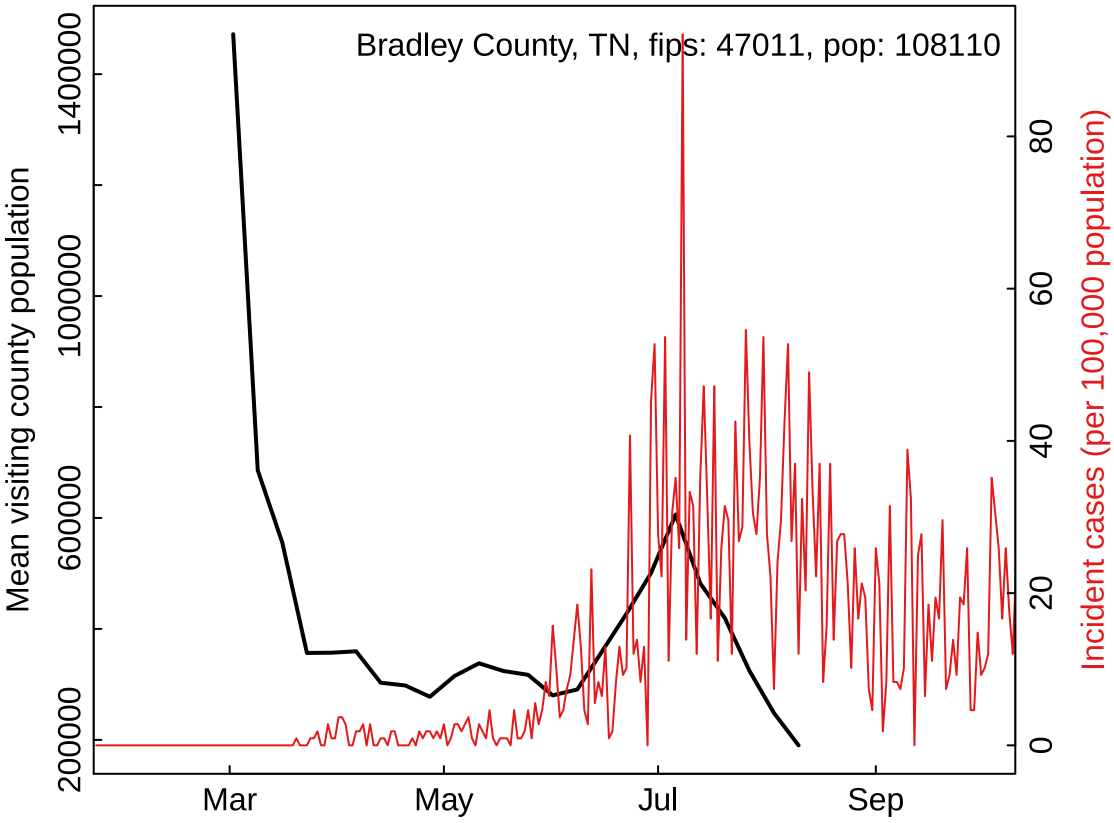

Bradley County, TN, fips: 47011, pop: 108110
Mean visiting county population
200000
600000
1000000
1400000
Incident cases (per 100,000 population)
0
20
40
60
80
Mar
May
Jul
Sep

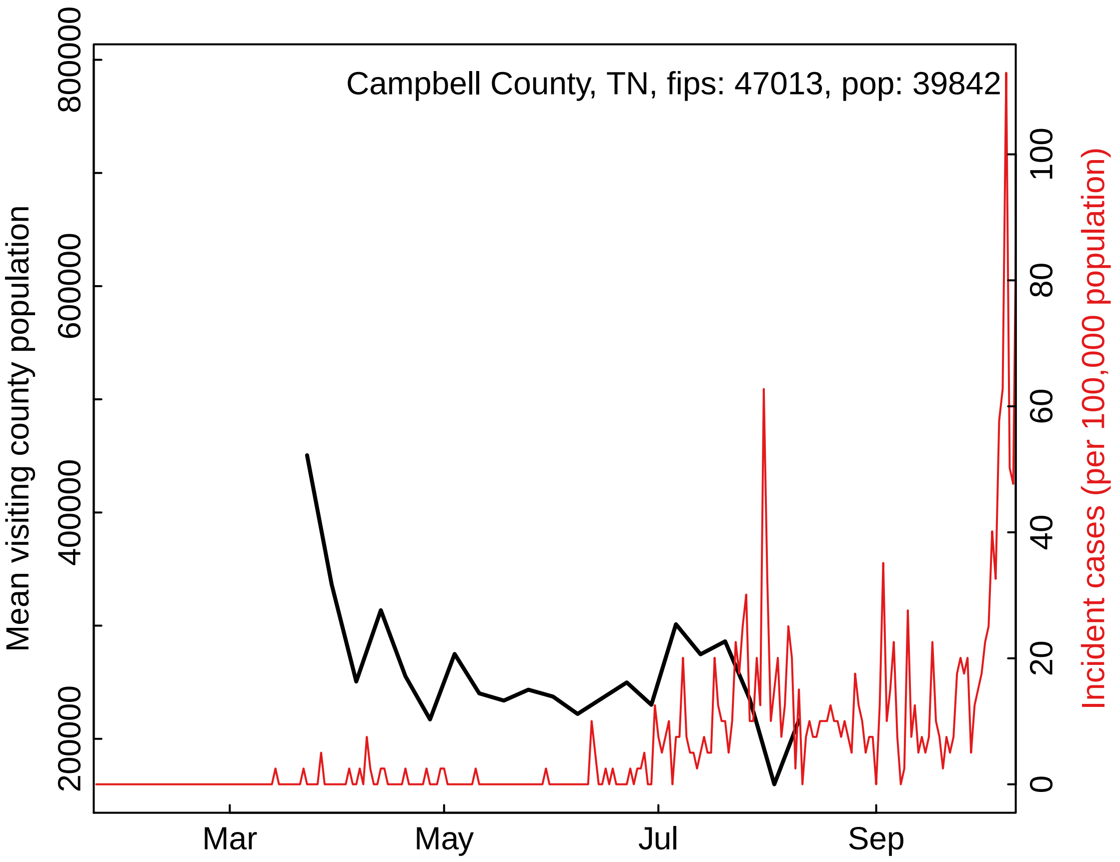

Campbell County, TN, fips: 47013, pop: 39842
Mean visiting county population
200000
400000
600000
800000
Incident cases (per 100,000 population)
0
20
40
60
80
100
Mar
May
Jul
Sep

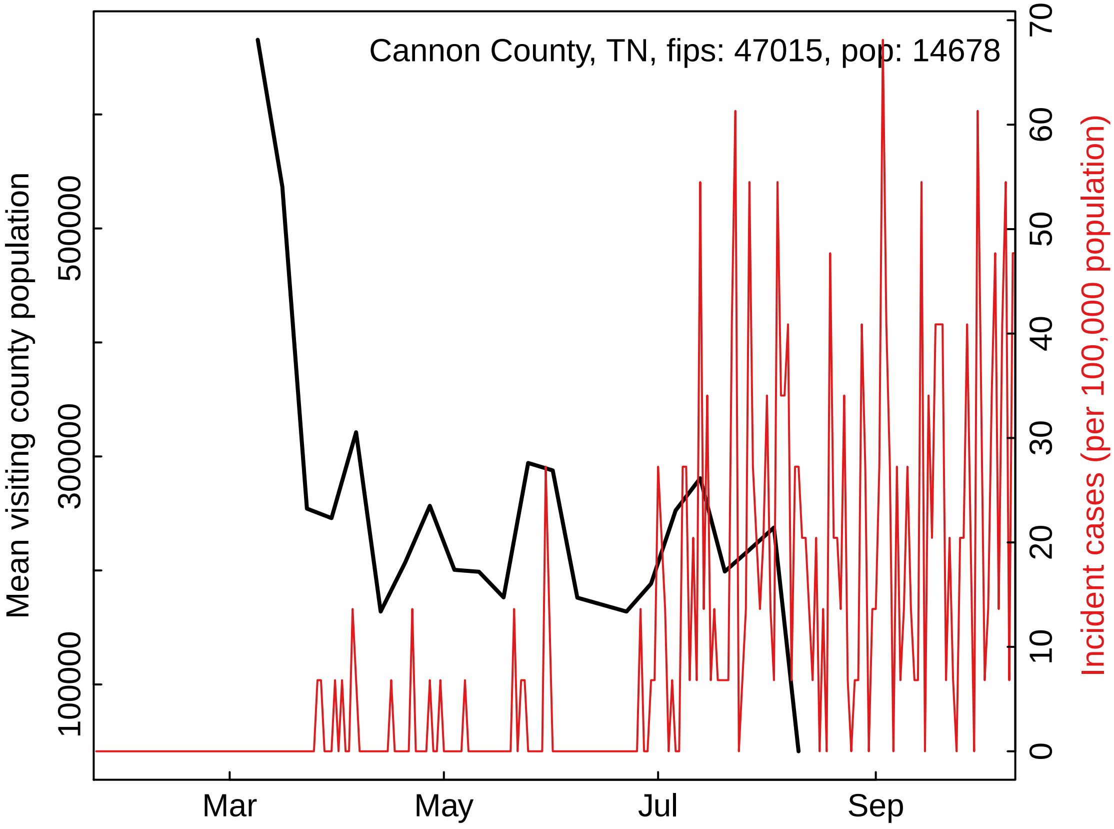

Cannon County, TN, fips: 47015, pop: 14678
Mean visiting county population
100000
300000
500000
Incident cases (per 100,000 population)
0
10
20
30
40
50
60
70
Mar
May
Jul
Sep

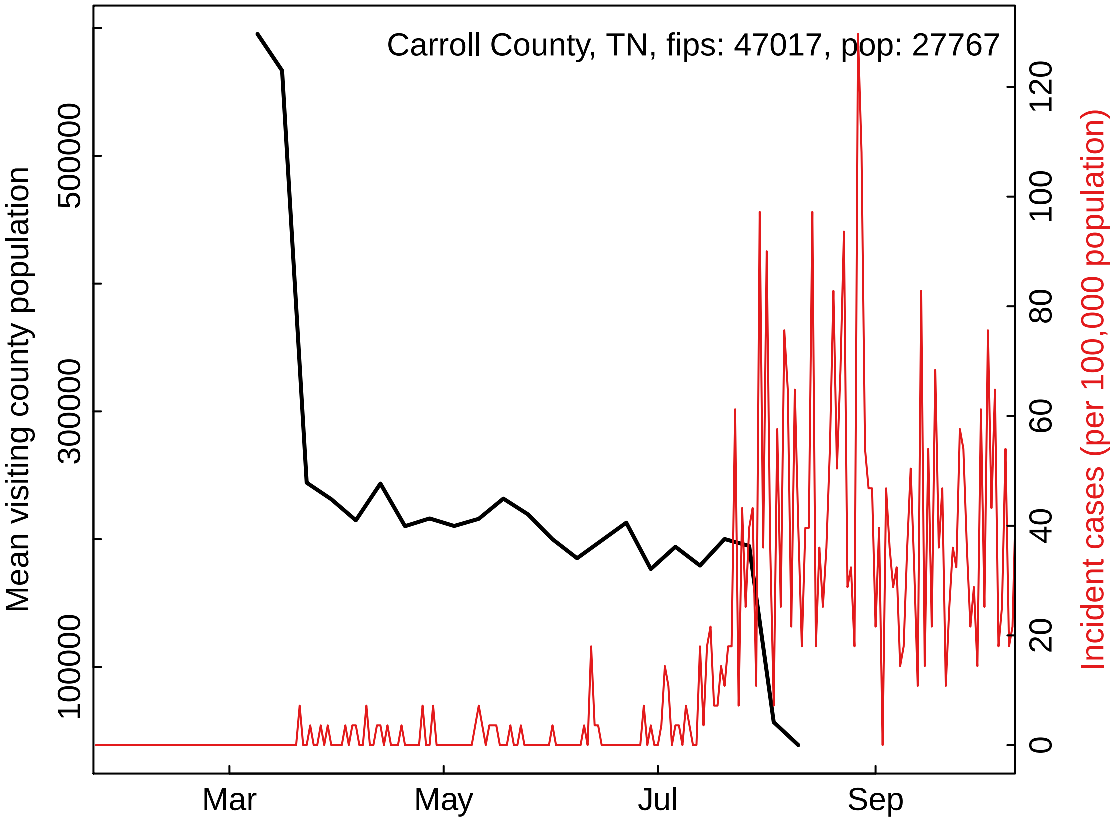

Carroll County, TN, fips: 47017, pop: 27767
Mean visiting county population
100000
300000
500000
Incident cases (per 100,000 population)
0
20
40
60
80
100
120
Mar
May
Jul
Sep

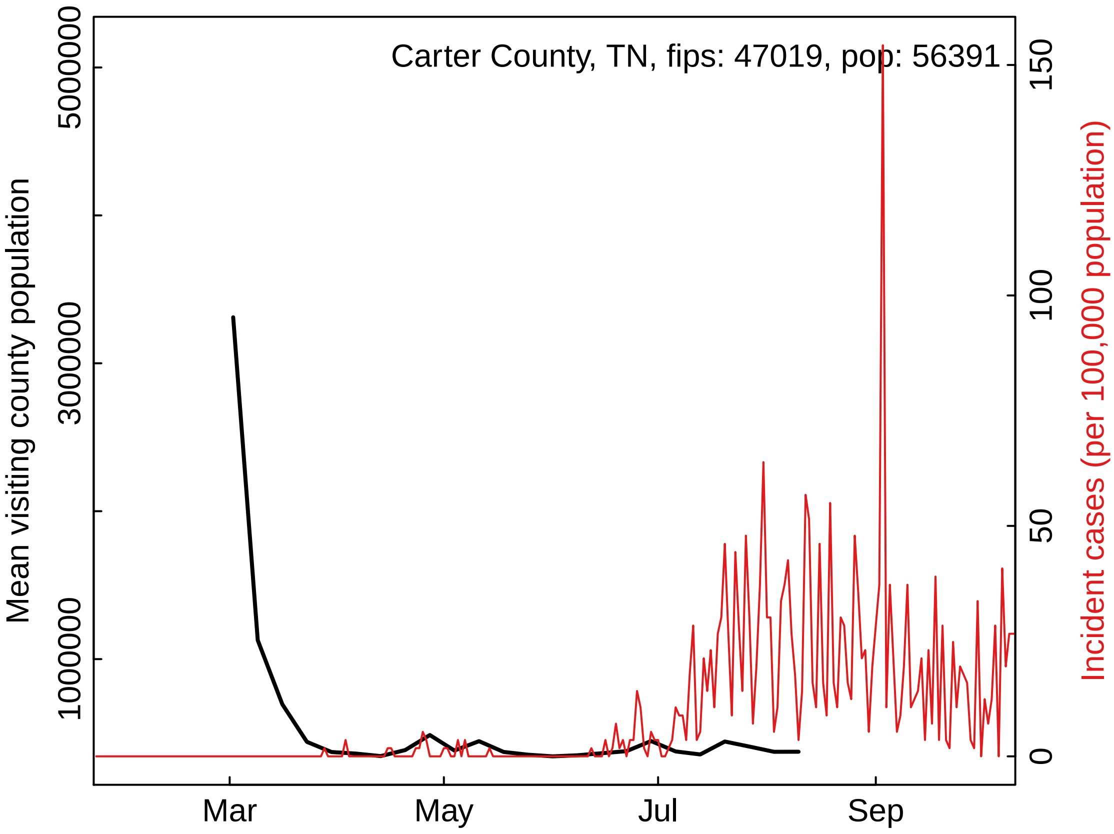

Carter County, TN, fips: 47019, pop: 56391
Mean visiting county population
1000000
3000000
5000000
Incident cases (per 100,000 population)
0
50
100
150
Mar
May
Jul
Sep

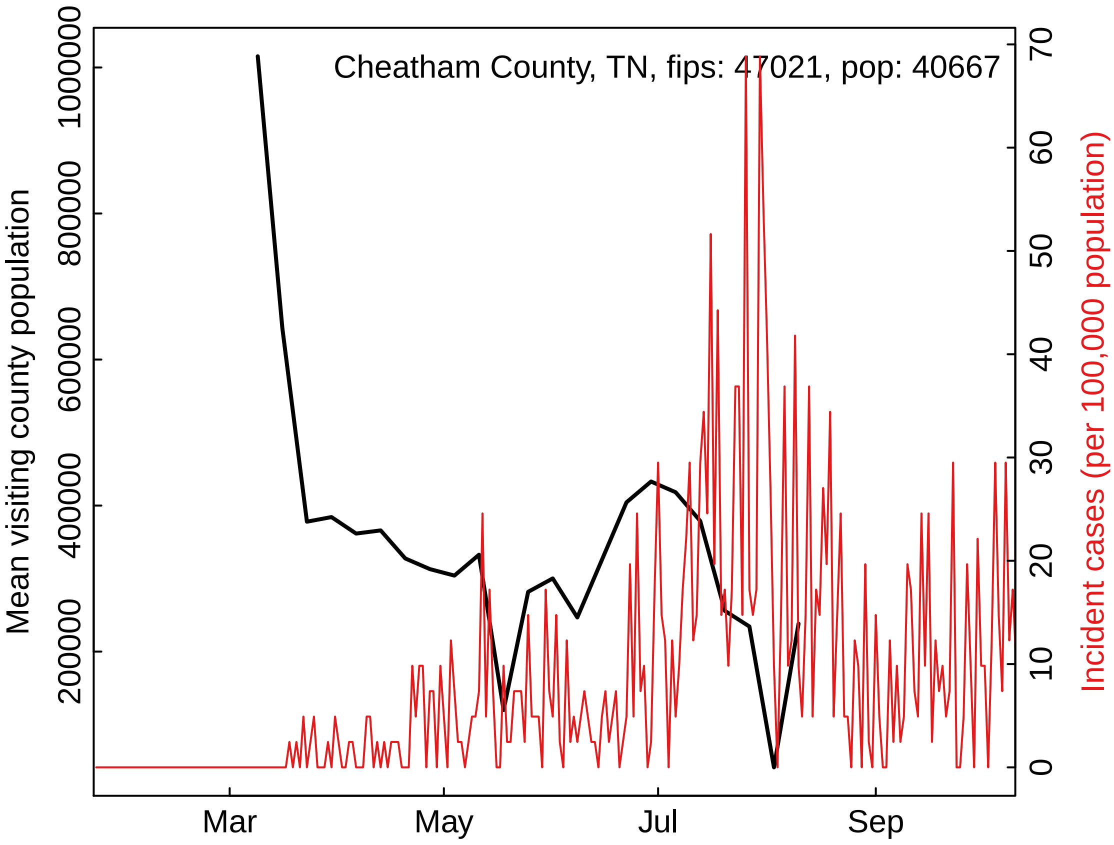

Cheatham County, TN, fips: 47021, pop: 40667
Mean visiting county population
200000
400000
600000
800000
1000000
Incident cases (per 100,000 population)
0
10
20
30
40
50
60
70
Mar
May
Jul
Sep

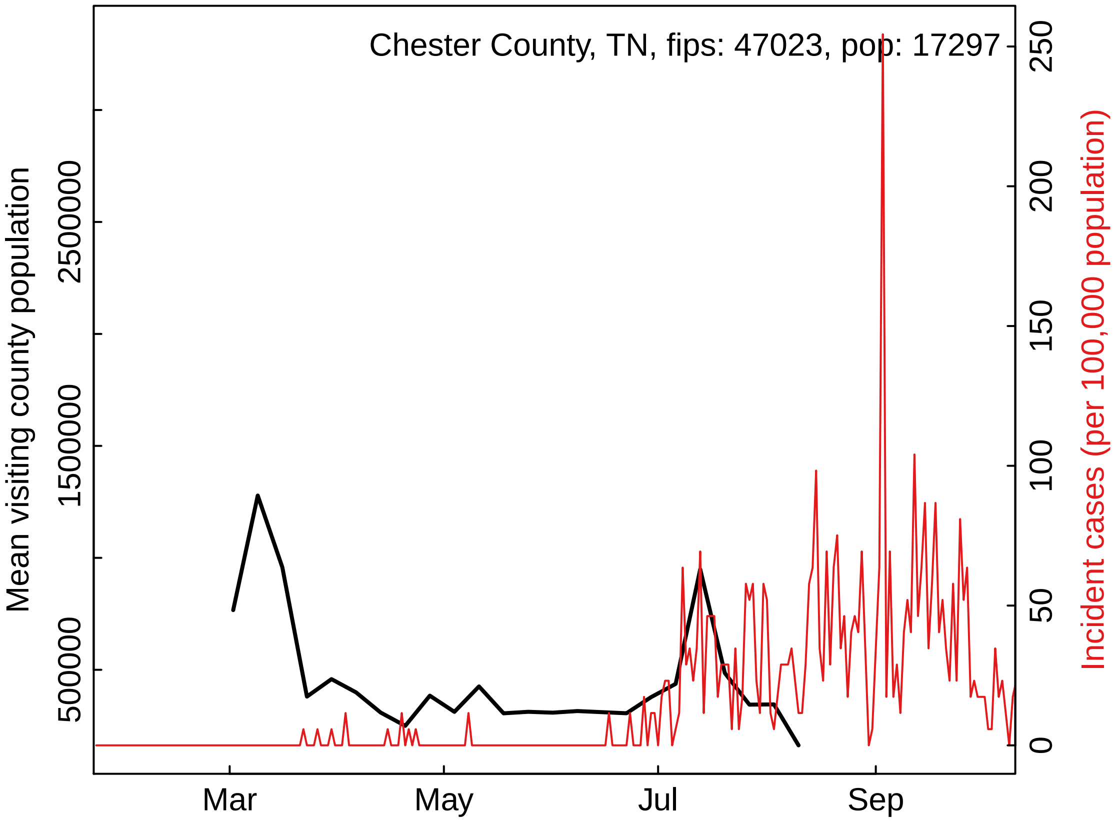
Chester County, TN, fips: 47023, pop: 17297
Mean visiting county population
500000
1500000
2500000
Incident cases (per 100,000 population)
0
50
100
150
200
250
Mar
May
Jul
Sep

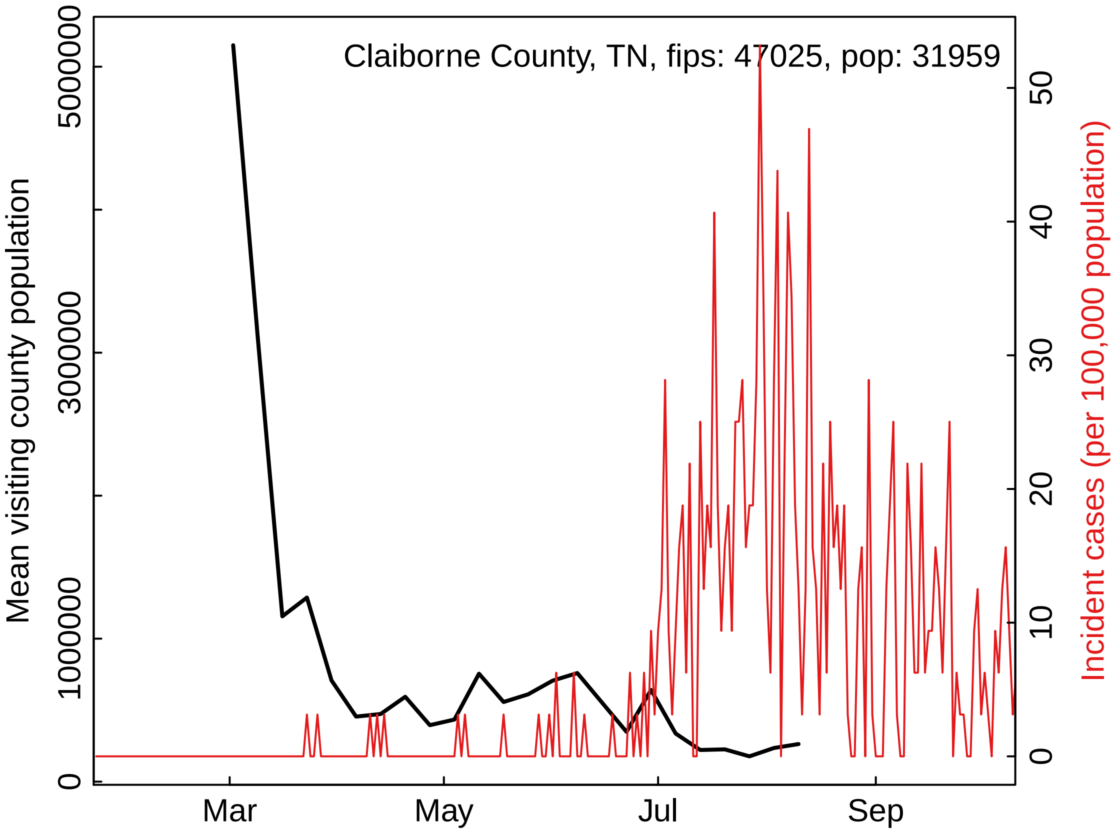

Claiborne County, TN, fips: 47025, pop: 31959
Mean visiting county population
0
1000000
3000000
5000000
Incident cases (per 100,000 population)
0
10
20
30
40
50
Mar
May
Jul
Sep

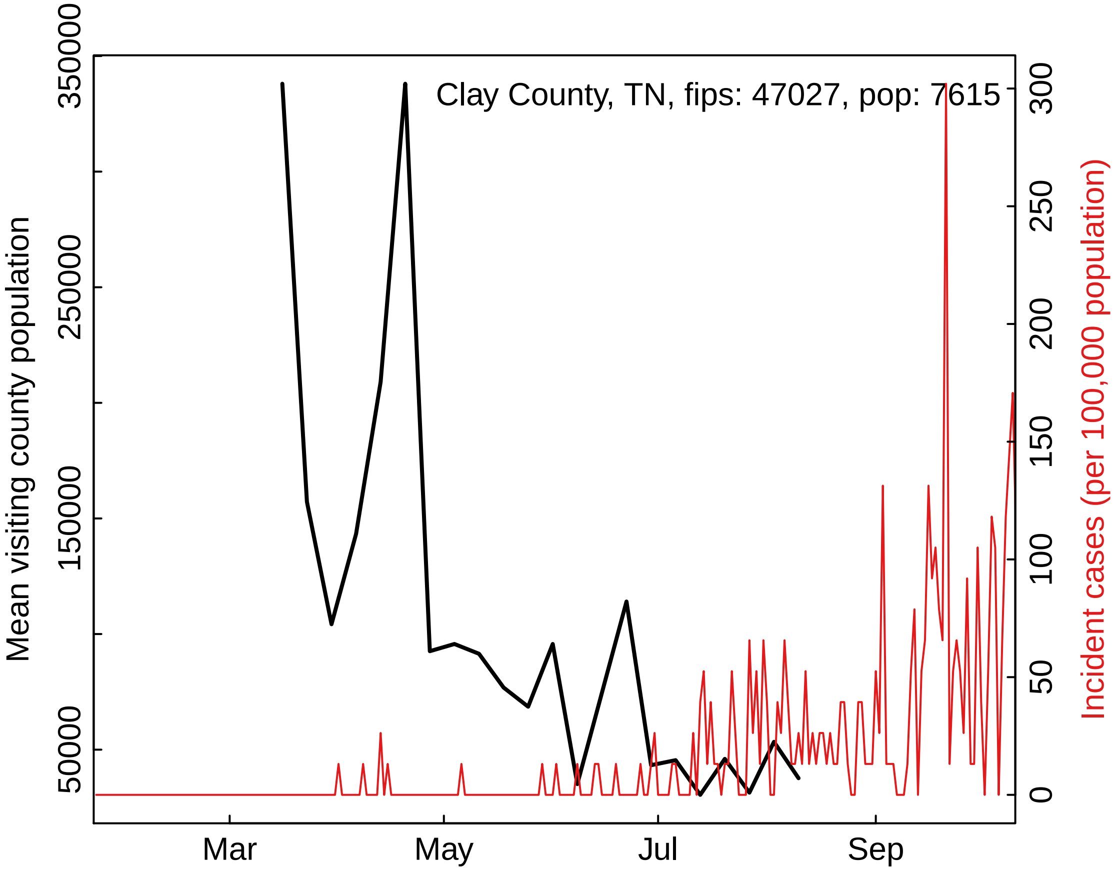

Clay County, TN, fips: 47027, pop: 7615
Mean visiting county population
Incident cases (per 100,000 population)
50000
150000
250000
350000
0
50
100
150
200
250
300
Mar
May
Jul
Sep

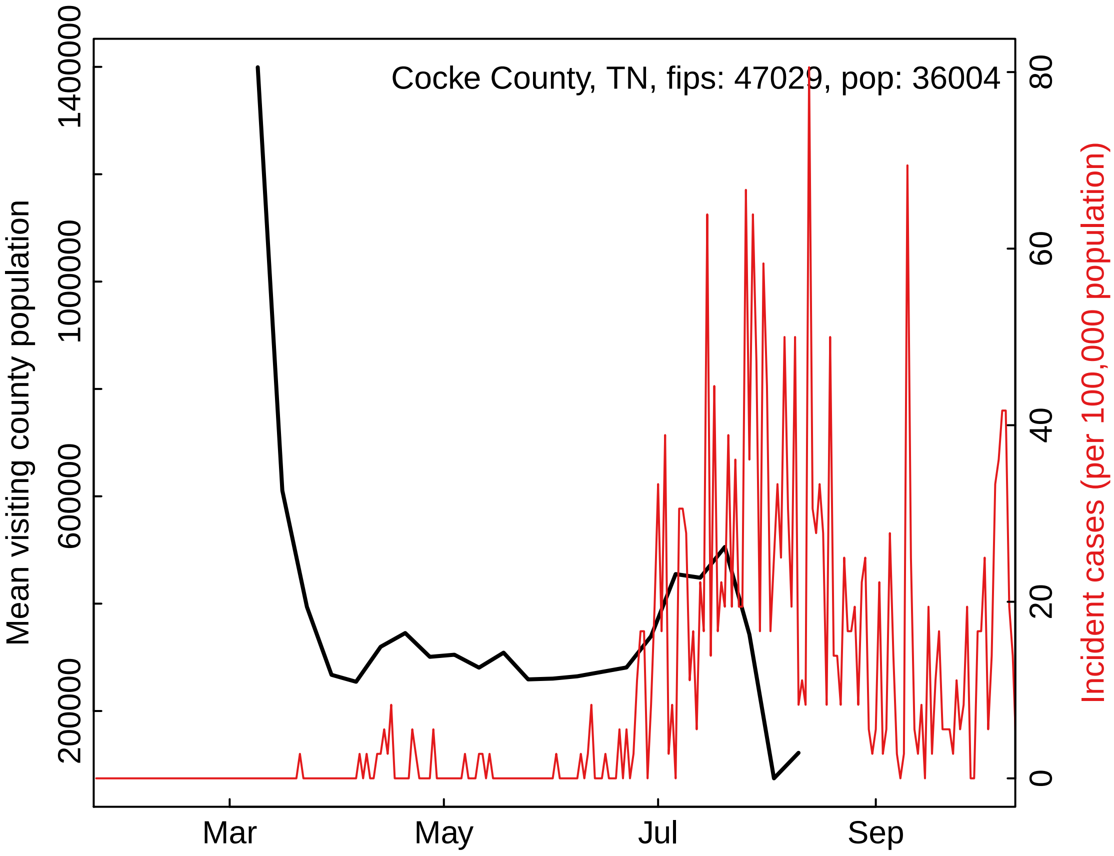
Cocke County, TN, fips: 47029, pop: 36004
Mean visiting county population
200000
600000
1000000
1400000
Incident cases (per 100,000 population)
0
20
40
60
80
Mar
May
Jul
Sep

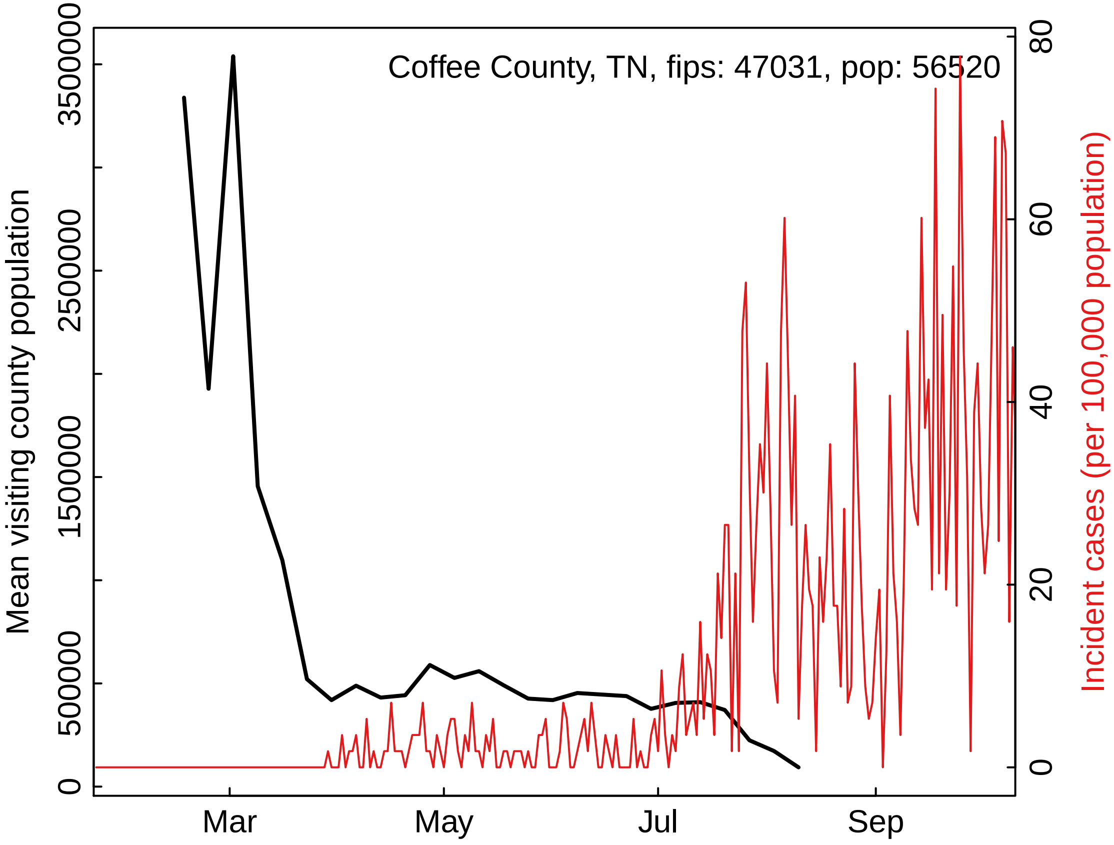

Coffee County, TN, fips: 47031, pop: 56520
Mean visiting county population
0
500000
1500000
2500000
3500000
Incident cases (per 100,000 population)
0
20
40
60
80
Mar
May
Jul
Sep

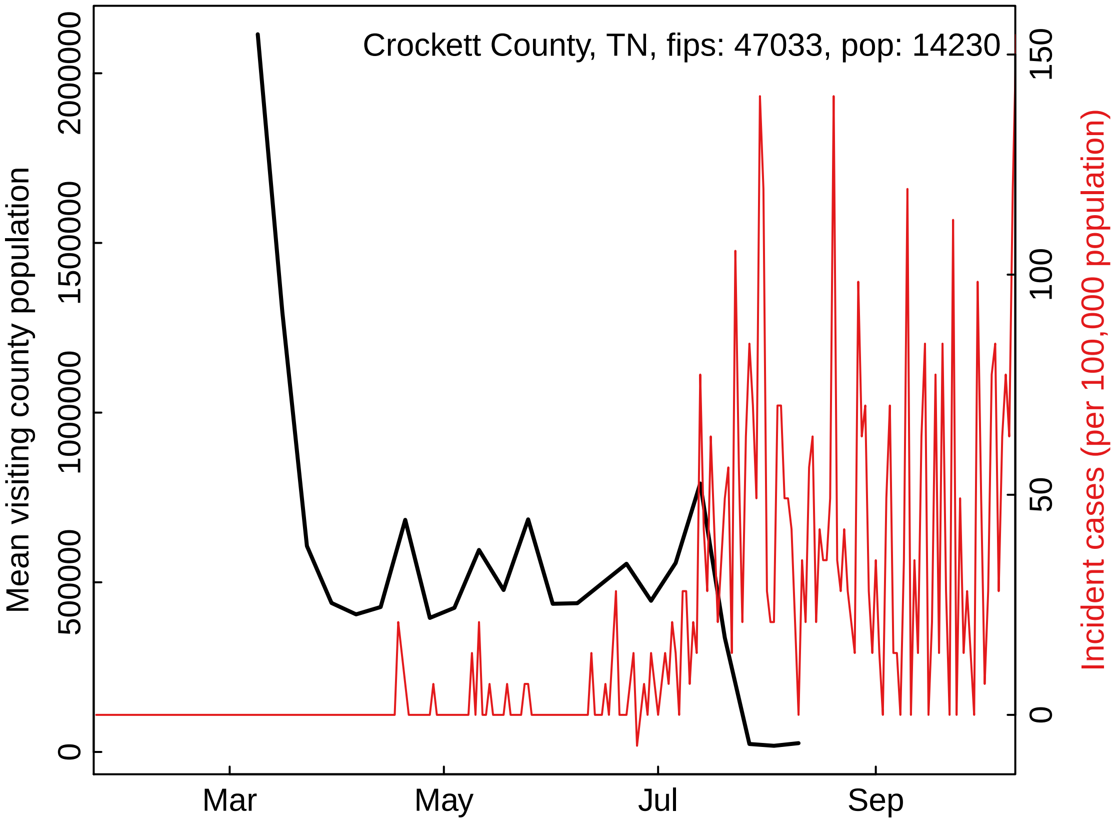

Crockett County, TN, fips: 47033, pop: 14230
Mean visiting county population
0
500000
1000000
1500000
2000000
Incident cases (per 100,000 population)
0
50
100
150
Mar
May
Jul
Sep

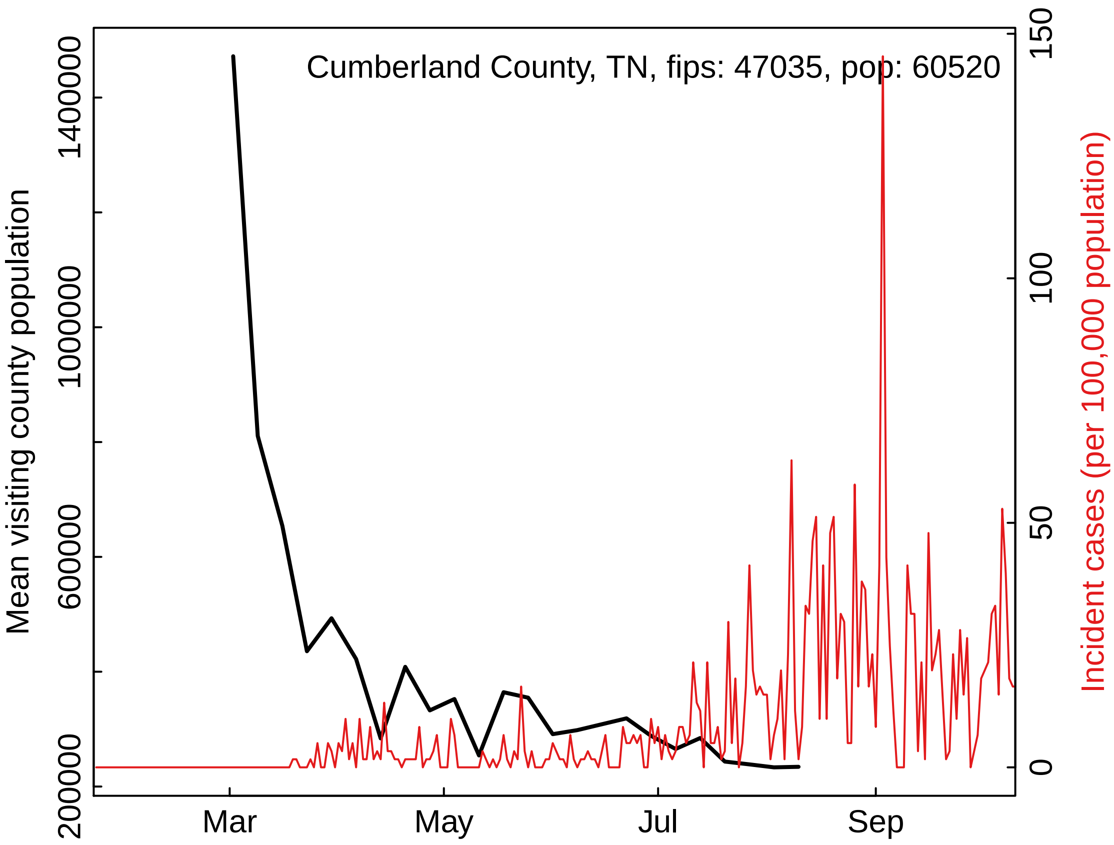

Cumberland County, TN, fips: 47035, pop: 60520
Mean visiting county population
200000
600000
1000000
1400000
Mar
May
Jul
Sep
0
50
100
150
Incident cases (per 100,000 population)

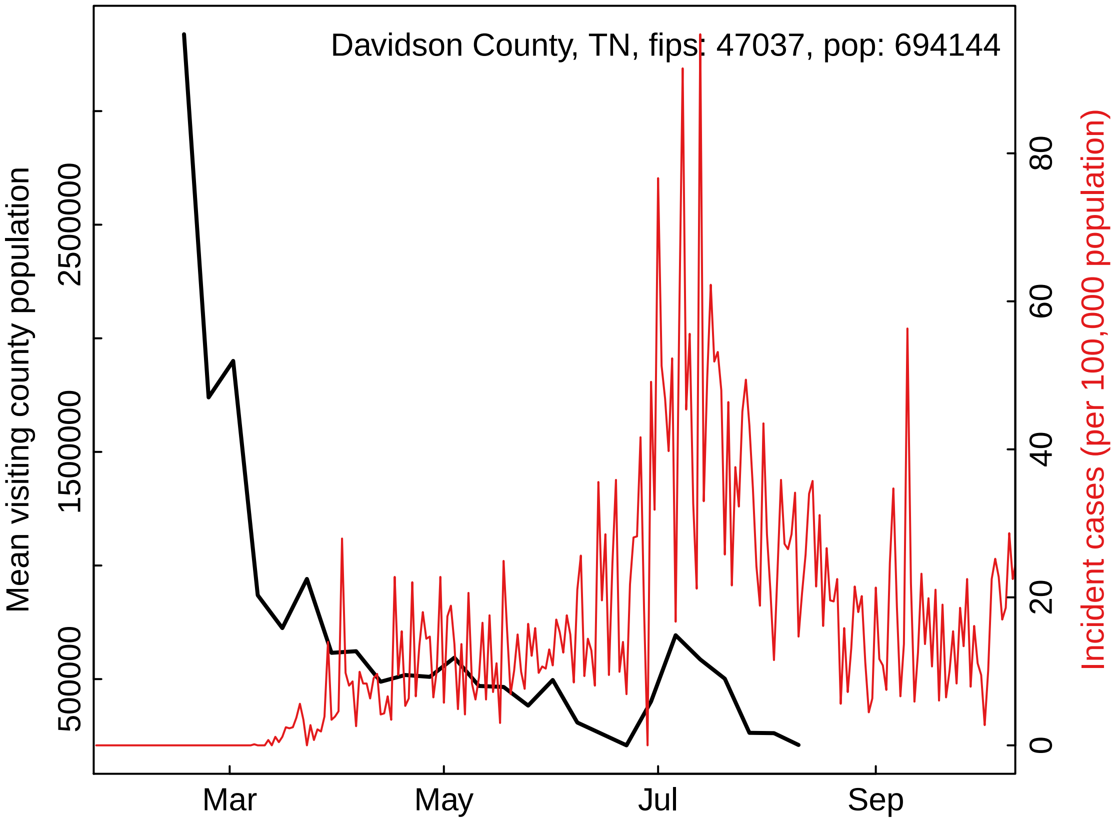
Davidson County, TN, fips: 47037, pop: 694144
Mean visiting county population
500000
1500000
2500000
Incident cases (per 100,000 population)
0
20
40
60
80
Mar
May
Jul
Sep

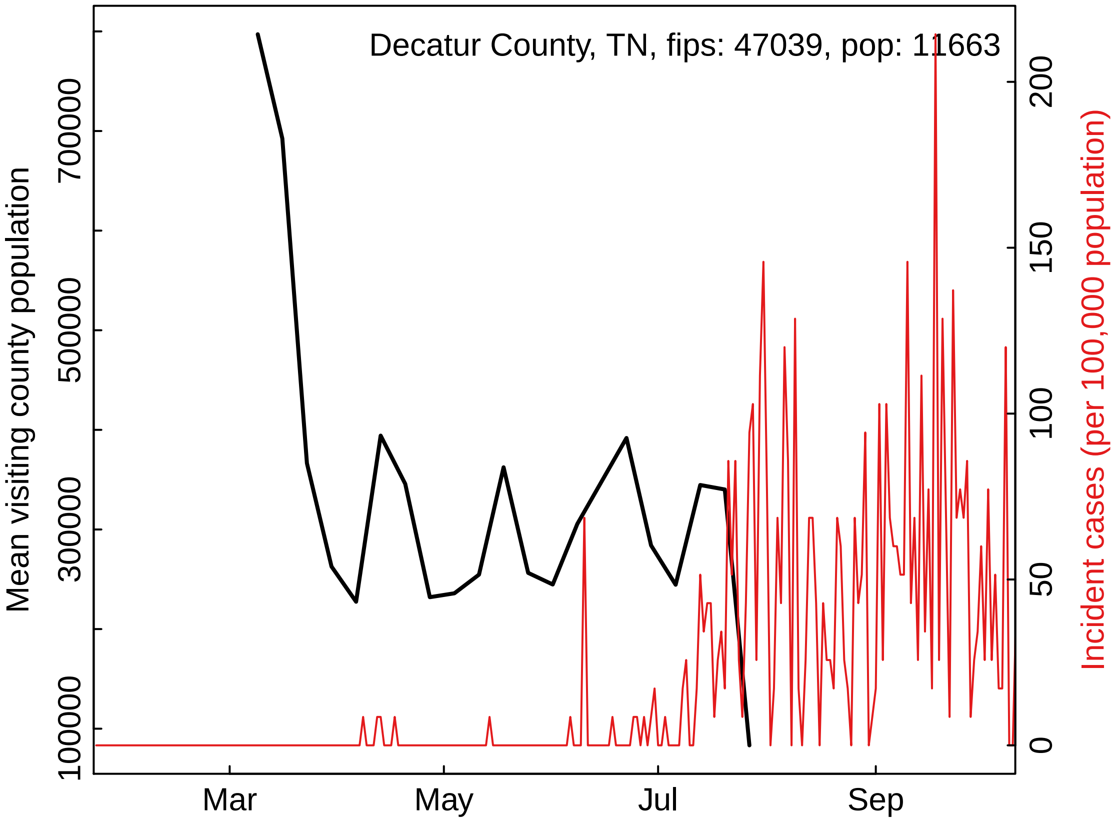

Decatur County, TN, fips: 47039, pop: 11663
Mean visiting county population
100000
300000
500000
700000
Incident cases (per 100,000 population)
0
50
100
150
200
Mar
May
Jul
Sep

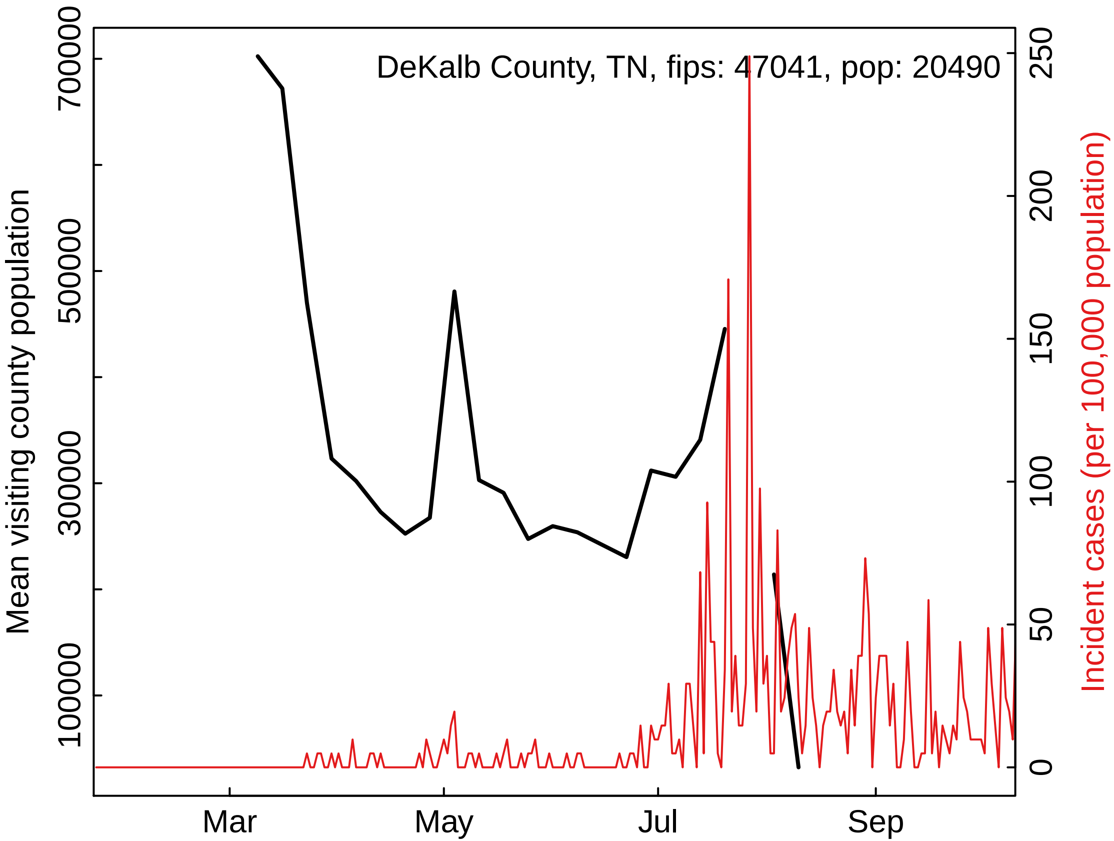
DeKalb County, TN, fips: 47041, pop: 20490
Mean visiting county population
100000
300000
500000
700000
Incident cases (per 100,000 population)
0
50
100
150
200
250
Mar
May
Jul
Sep

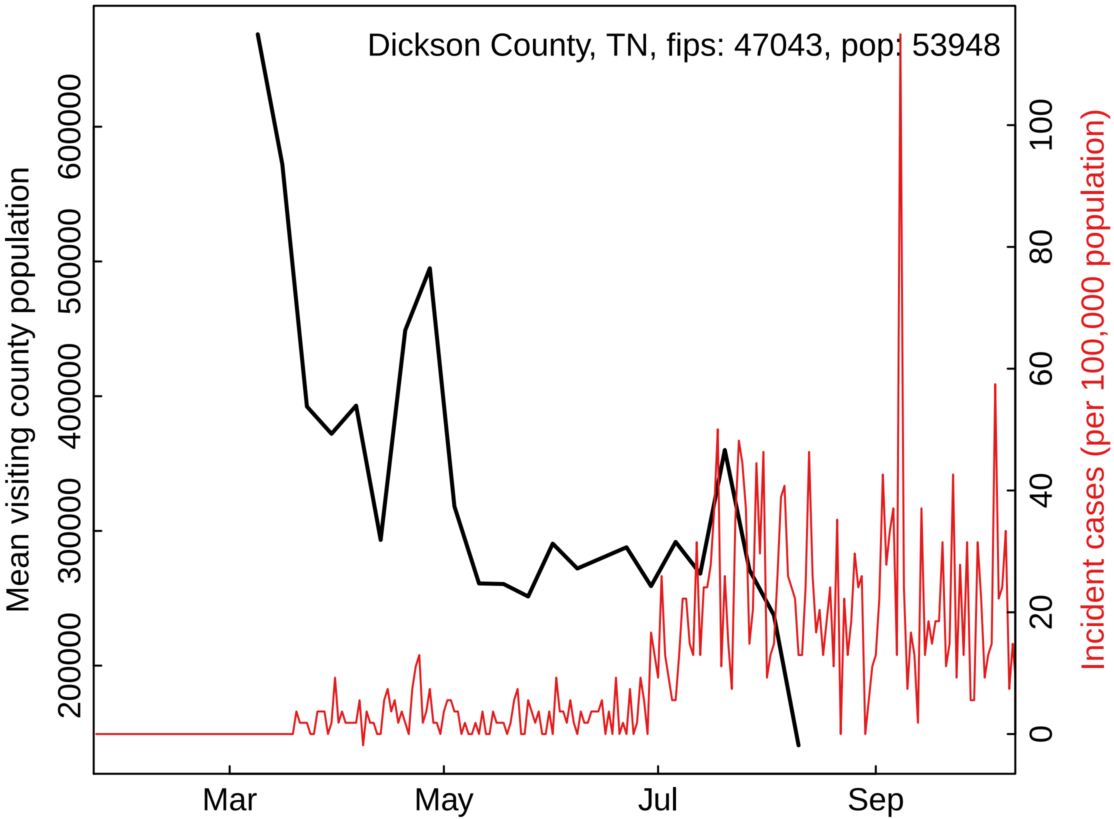

Dickson County, TN, fips: 47043, pop: 53948
Mean visiting county population
200000
300000
400000
500000
600000
Incident cases (per 100,000 population)
0
20
40
60
80
100
Mar
May
Jul
Sep

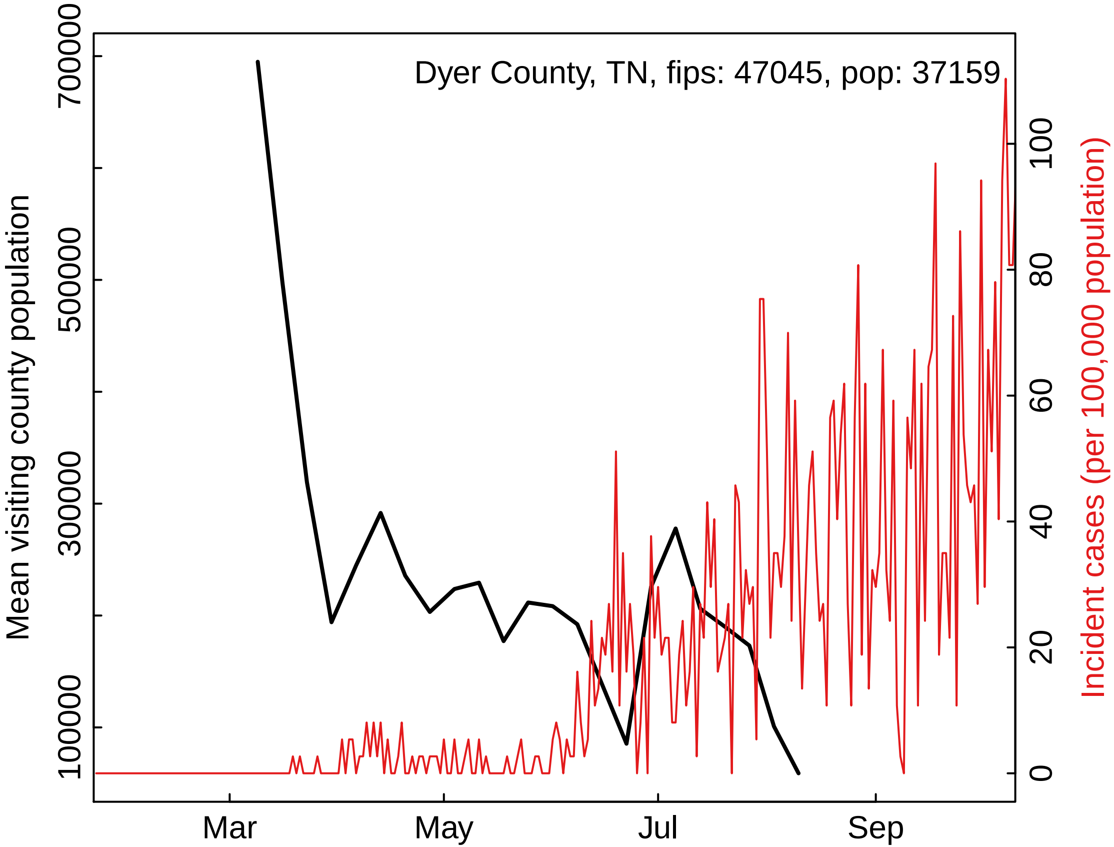

Dyer County, TN, fips: 47045, pop: 37159
Mean visiting county population
100000
300000
500000
700000
Incident cases (per 100,000 population)
0
20
40
60
80
100
Mar
May
Jul
Sep

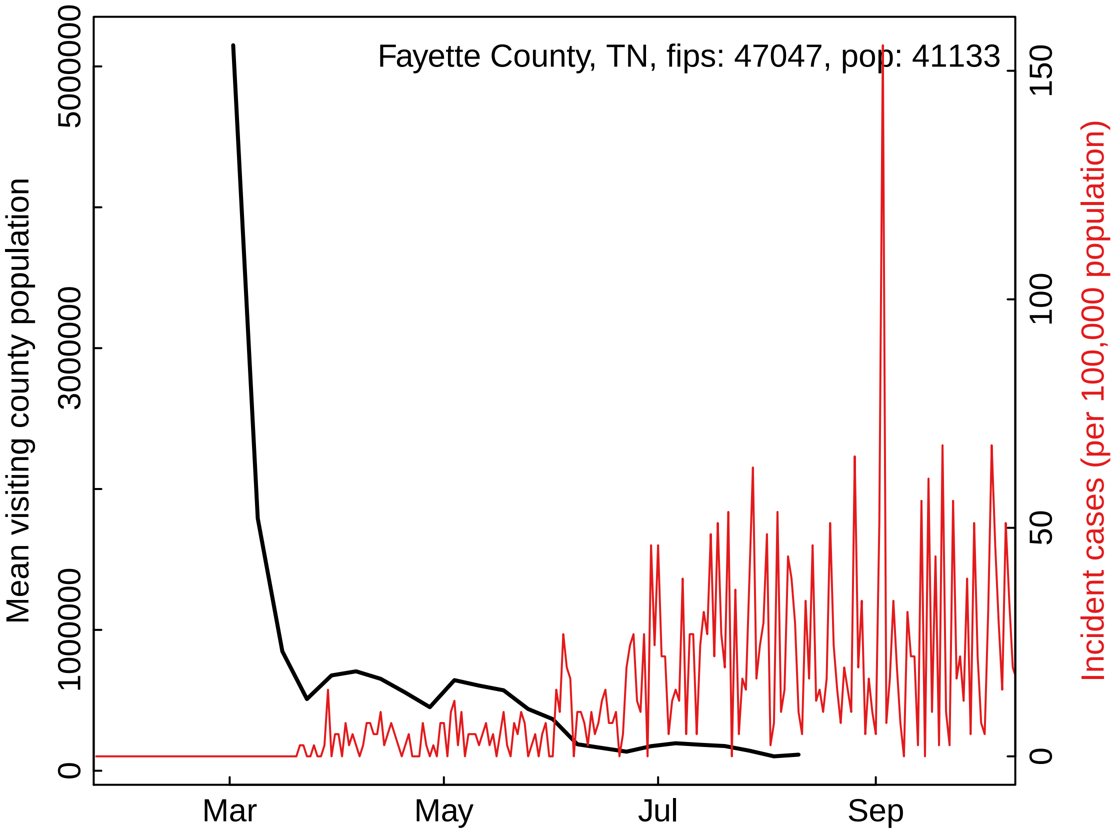

Fayette County, TN, fips: 47047, pop: 41133
Mean visiting county population
0
1000000
3000000
5000000
Mar
May
Jul
Sep
Incident cases (per 100,000 population)
0
50
100
150

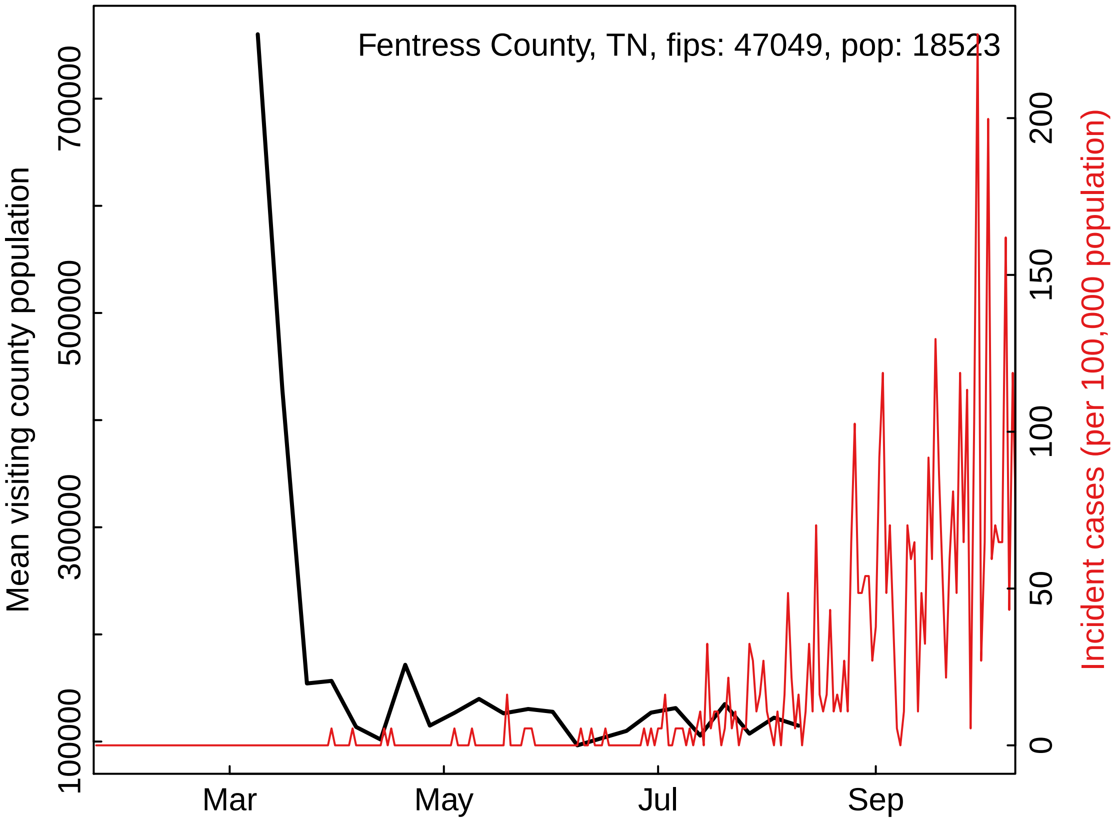
Fentress County, TN, fips: 47049, pop: 18523
Mean visiting county population
100000
300000
500000
700000
Incident cases (per 100,000 population)
0
50
100
150
200
Mar
May
Jul
Sep

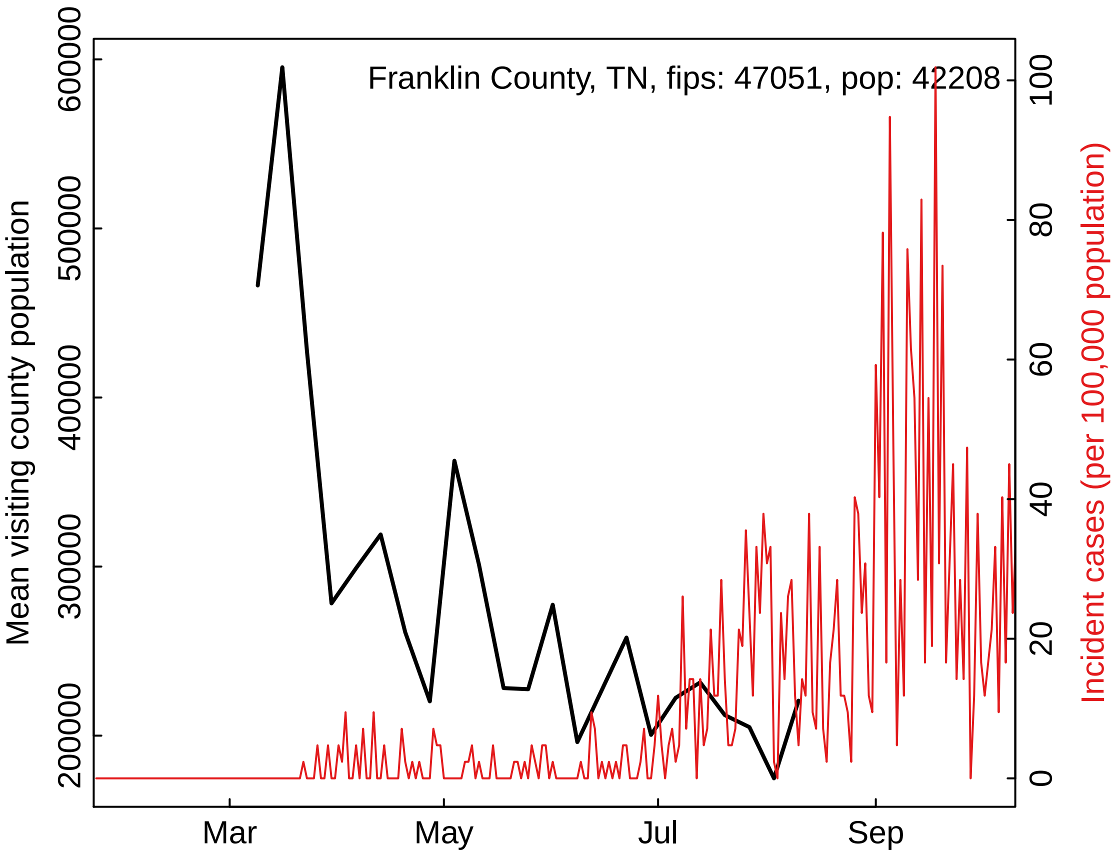

Franklin County, TN, fips: 47051, pop: 42208
Mean visiting county population
Incident cases (per 100,000 population)
200000
300000
400000
500000
600000
0
20
40
60
80
100
Mar
May
Jul
Sep

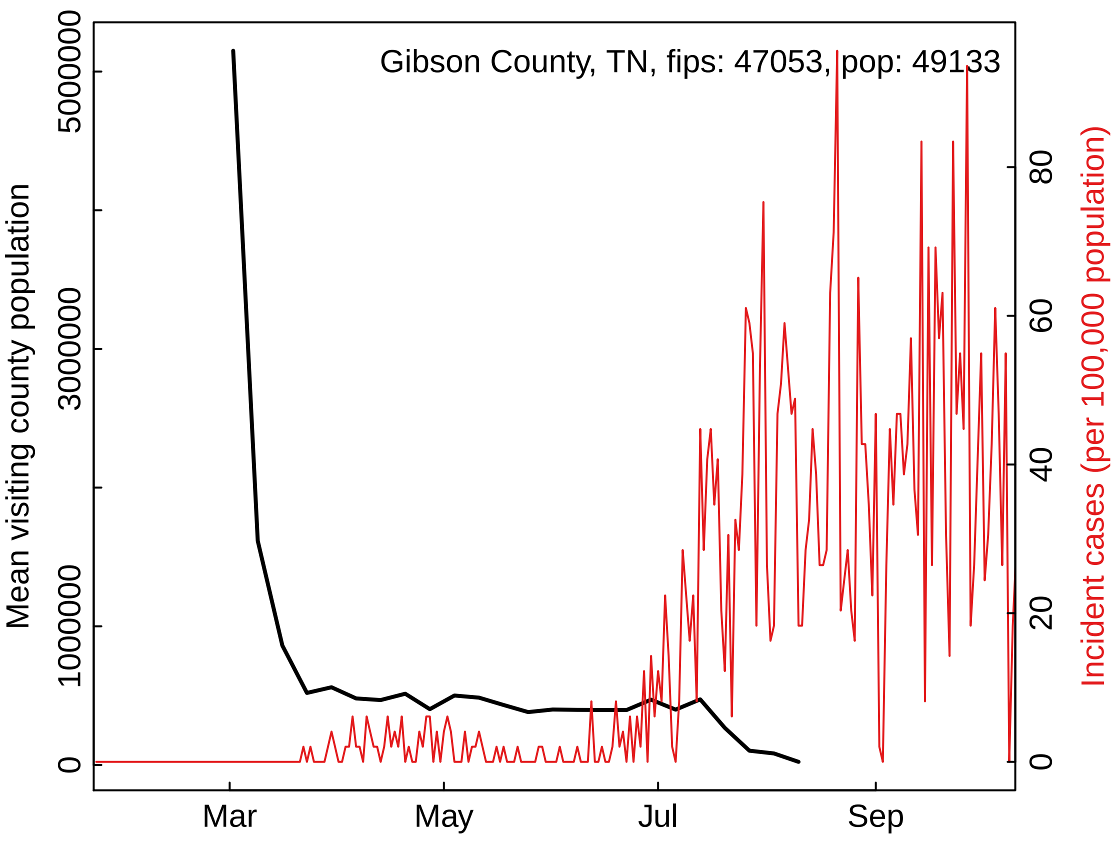
Gibson County, TN, fips: 47053, pop: 49133
Mean visiting county population
0
1000000
3000000
5000000
Incident cases (per 100,000 population)
0
20
40
60
80
Mar
May
Jul
Sep

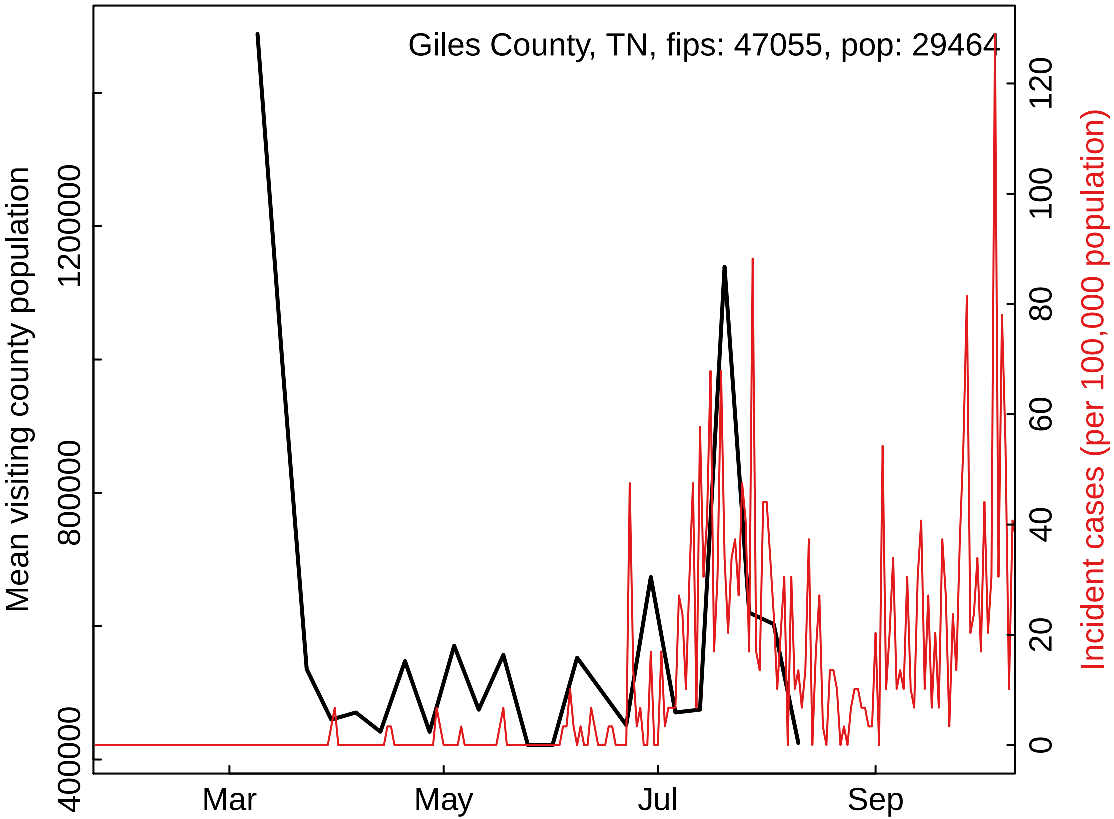

Giles County, TN, fips: 47055, pop: 29464
Mean visiting county population
400000
800000
1200000
Incident cases (per 100,000 population)
0
20
40
60
80
100
120
Mar
May
Jul
Sep

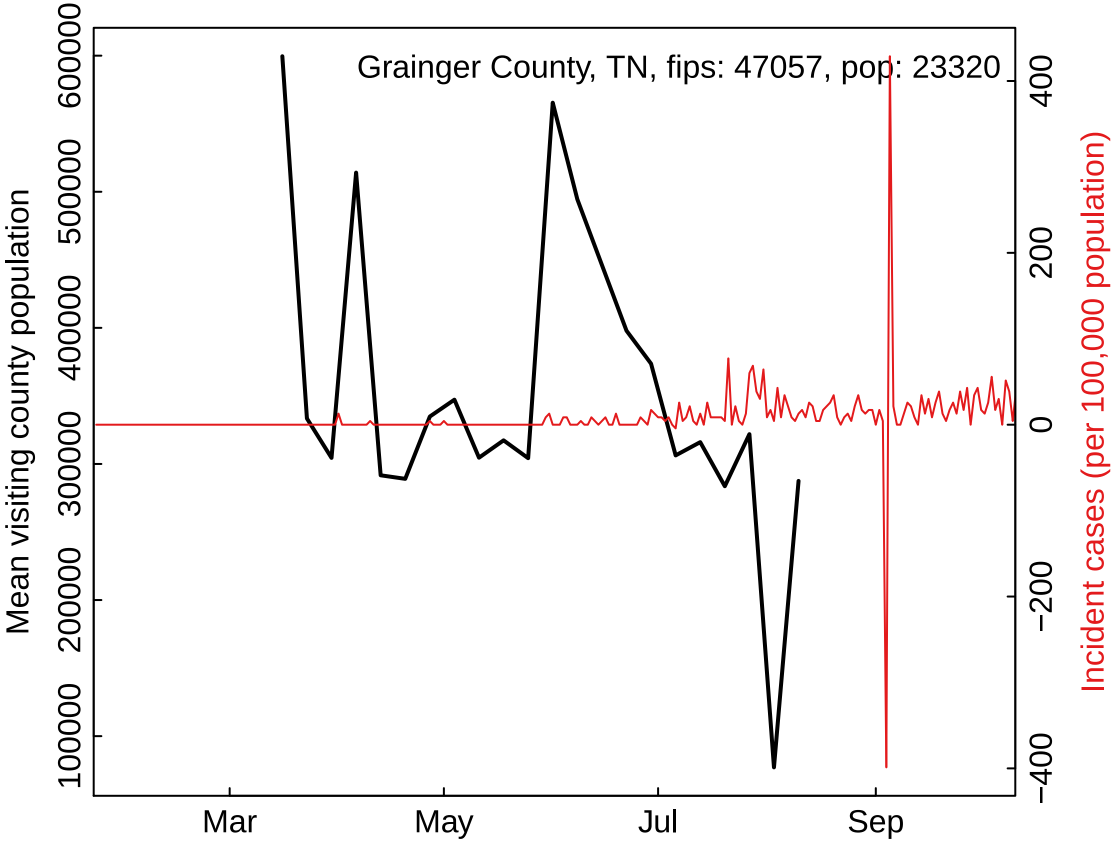

Grainger County, TN, fips: 47057, pop: 23320
Mean visiting county population
100000
200000
300000
400000
500000
600000
Incident cases (per 100,000 population)
−400
−200
0
200
400
Mar
May
Jul
Sep

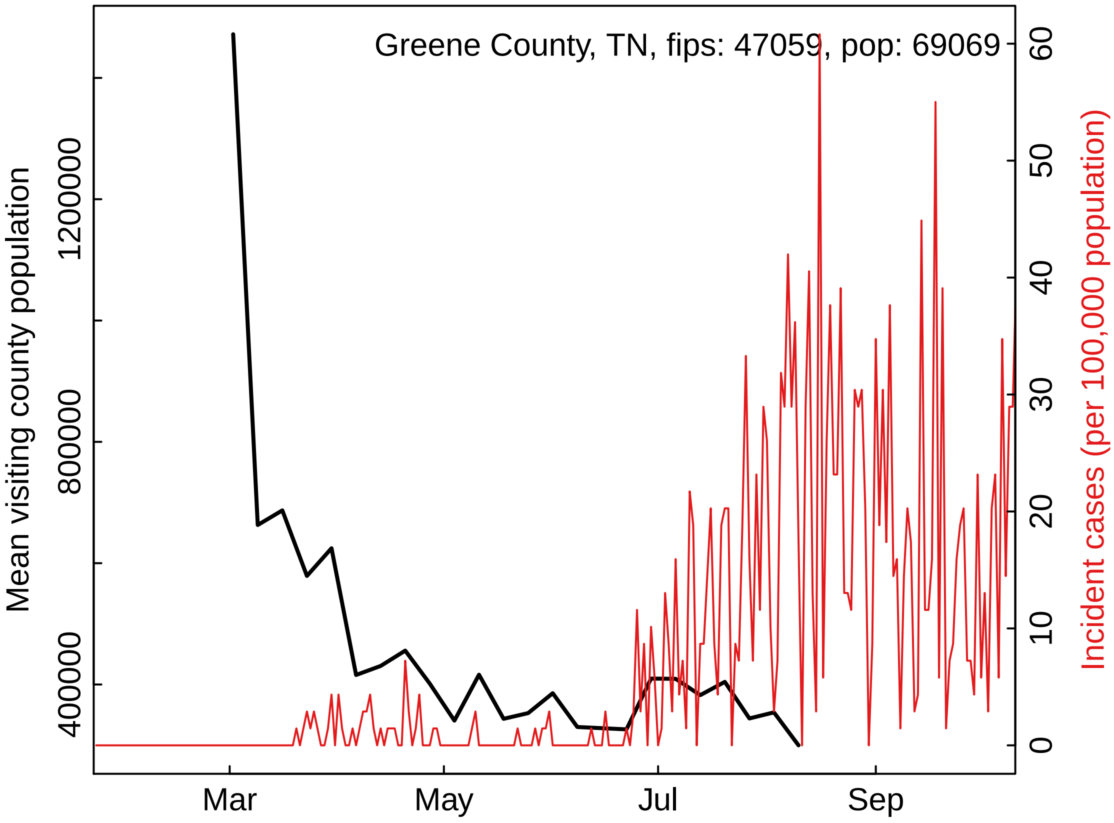

Greene County, TN, fips: 47059, pop: 69069
Mean visiting county population
400000
800000
1200000
Incident cases (per 100,000 population)
0
10
20
30
40
50
60
Mar
May
Jul
Sep

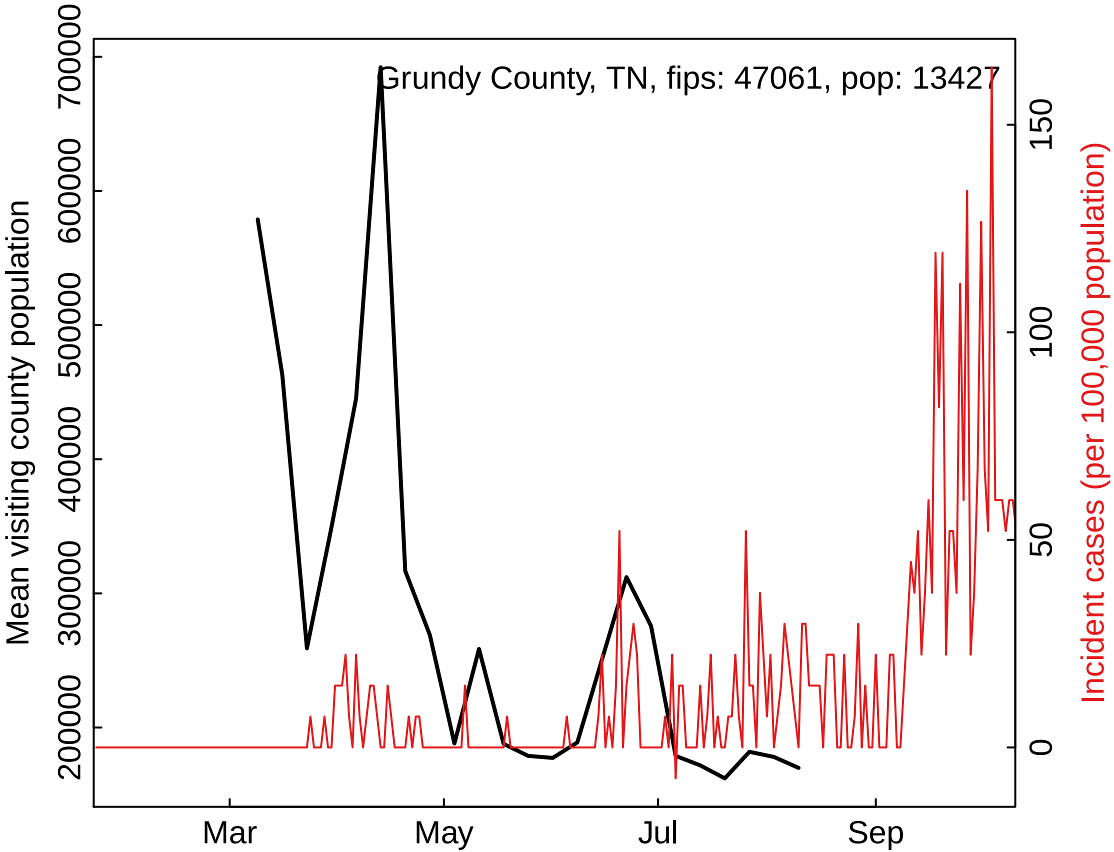

Grundy County, TN, fips: 47061, pop: 13427
Mean visiting county population
200000
300000
400000
500000
600000
700000
Incident cases (per 100,000 population)
0
50
100
150
Mar
May
Jul
Sep

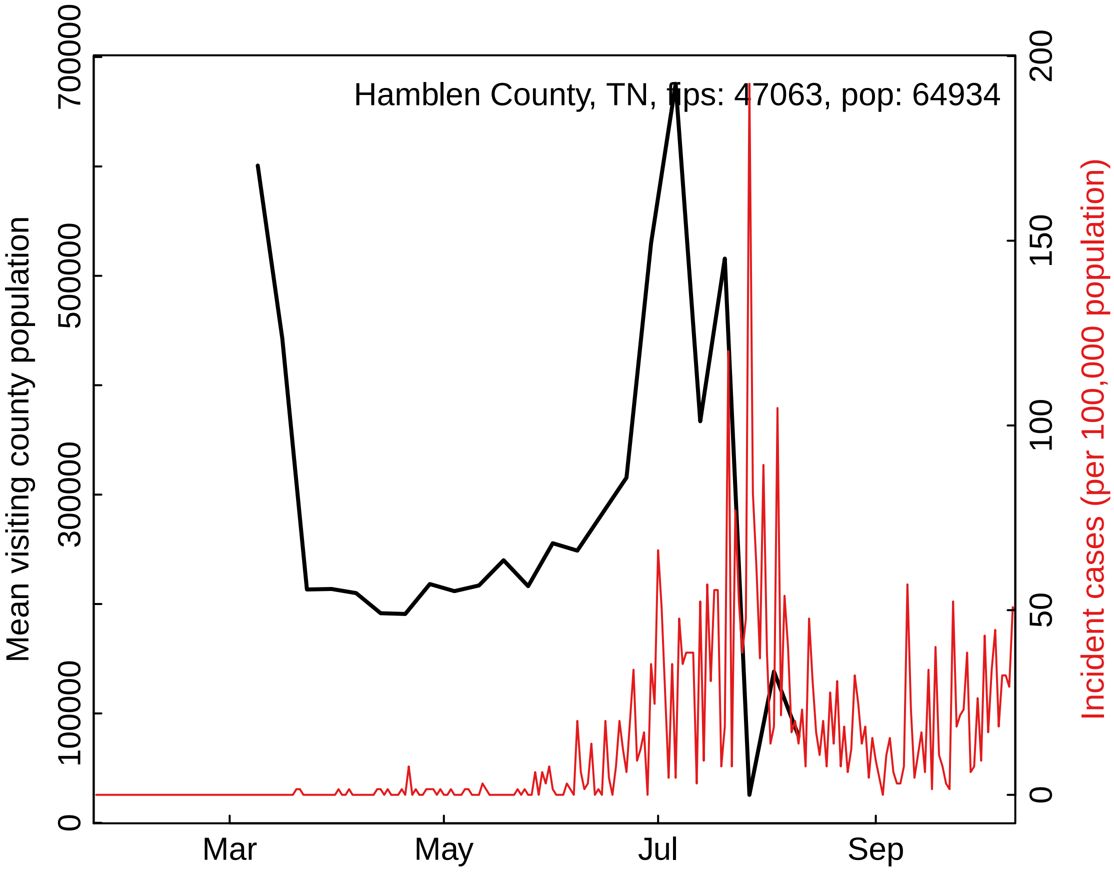
Hamblen County, TN, fips: 47063, pop: 64934
Mean visiting county population
Incident cases (per 100,000 population)
0
100000
300000
500000
700000
0
50
100
150
200
Mar
May
Jul
Sep

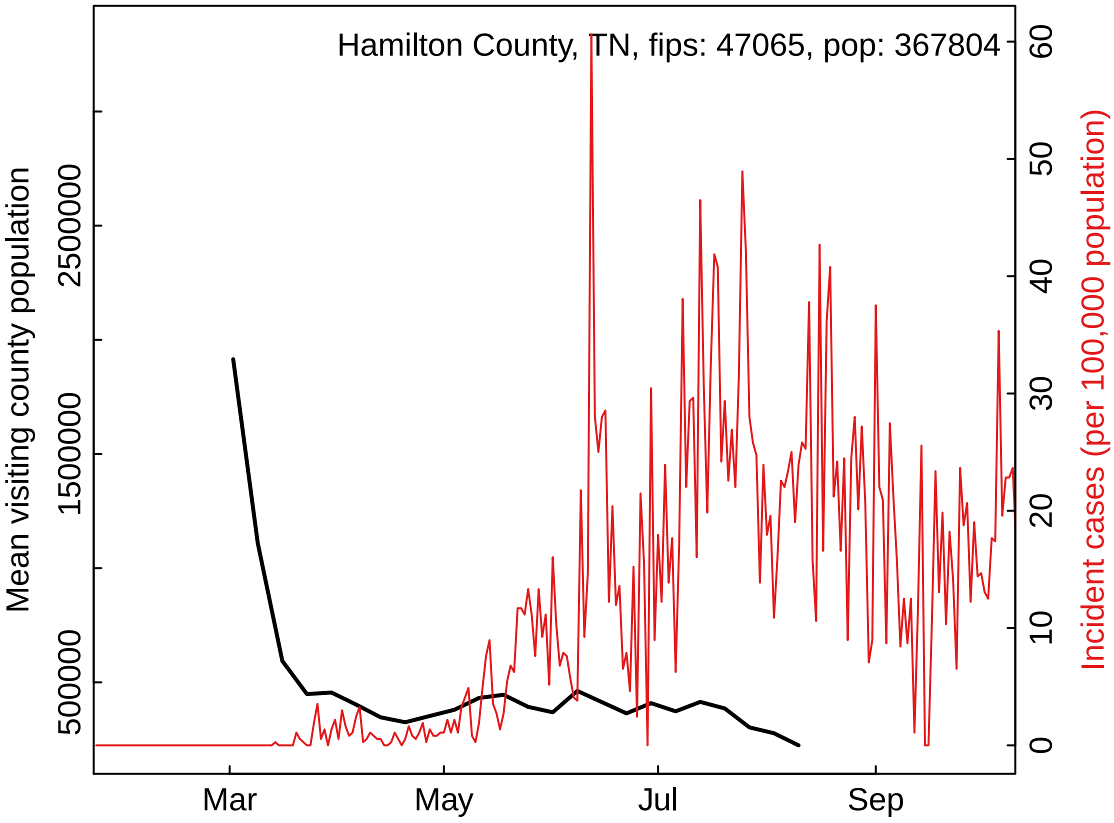

Hamilton County, TN, fips: 47065, pop: 367804
Mean visiting county population
500000
1500000
2500000
Incident cases (per 100,000 population)
0
10
20
30
40
50
60
Mar
May
Jul
Sep

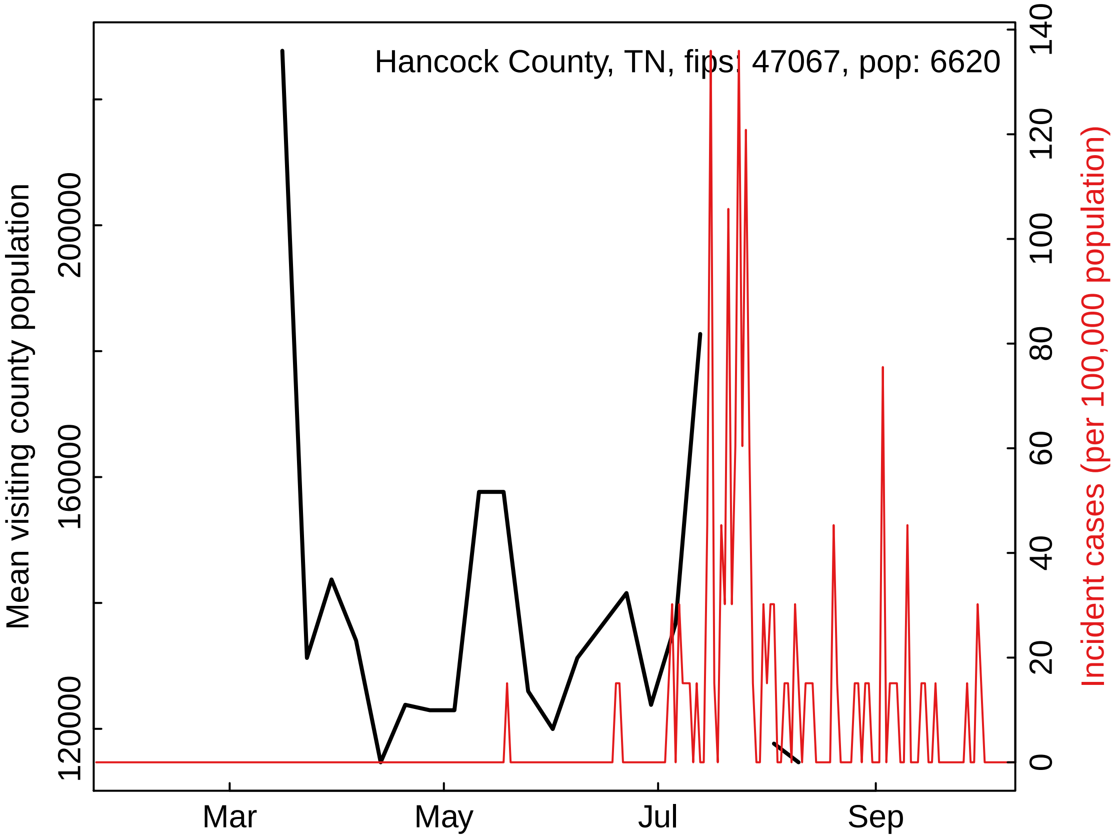
Hancock County, TN, fips: 47067, pop: 6620
Mean visiting county population
120000
160000
200000
Incident cases (per 100,000 population)
0
20
40
60
80
100
120
140
Mar
May
Jul
Sep

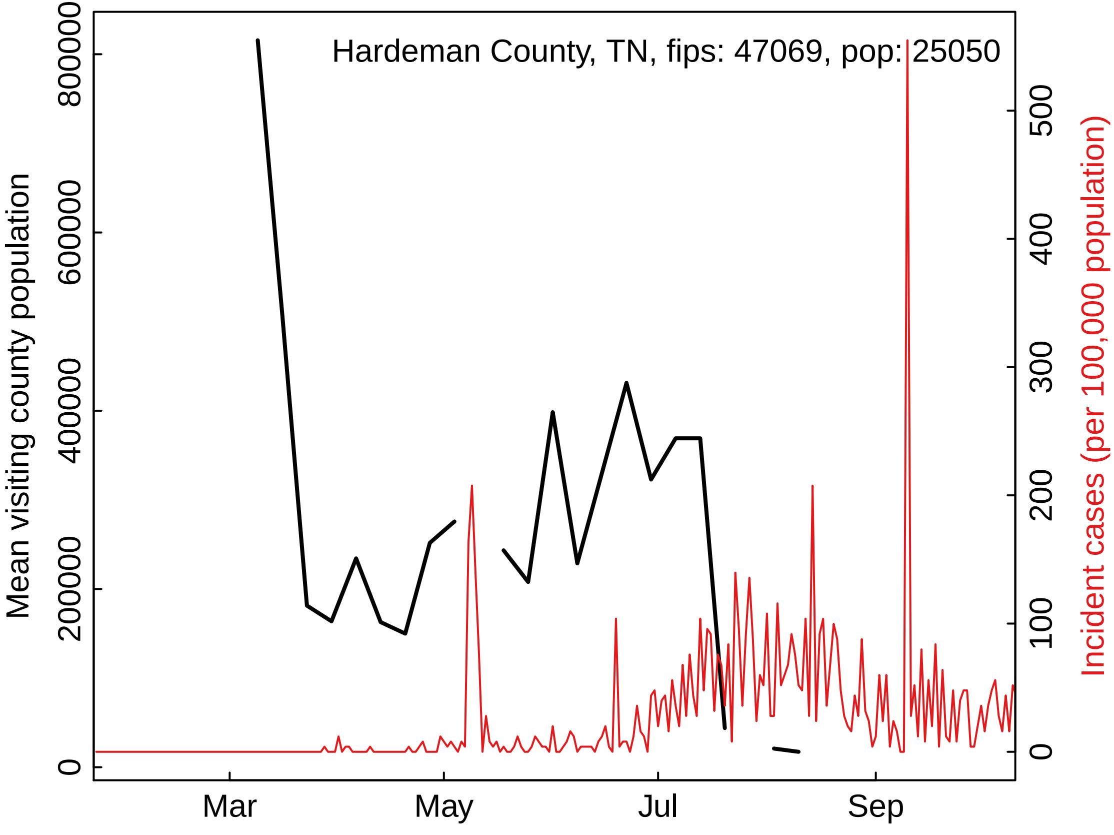

Hardeman County, TN, fips: 47069, pop: 25050
Mean visiting county population
0
200000
400000
600000
800000
Incident cases (per 100,000 population)
0
100
200
300
400
500
Mar
May
Jul
Sep

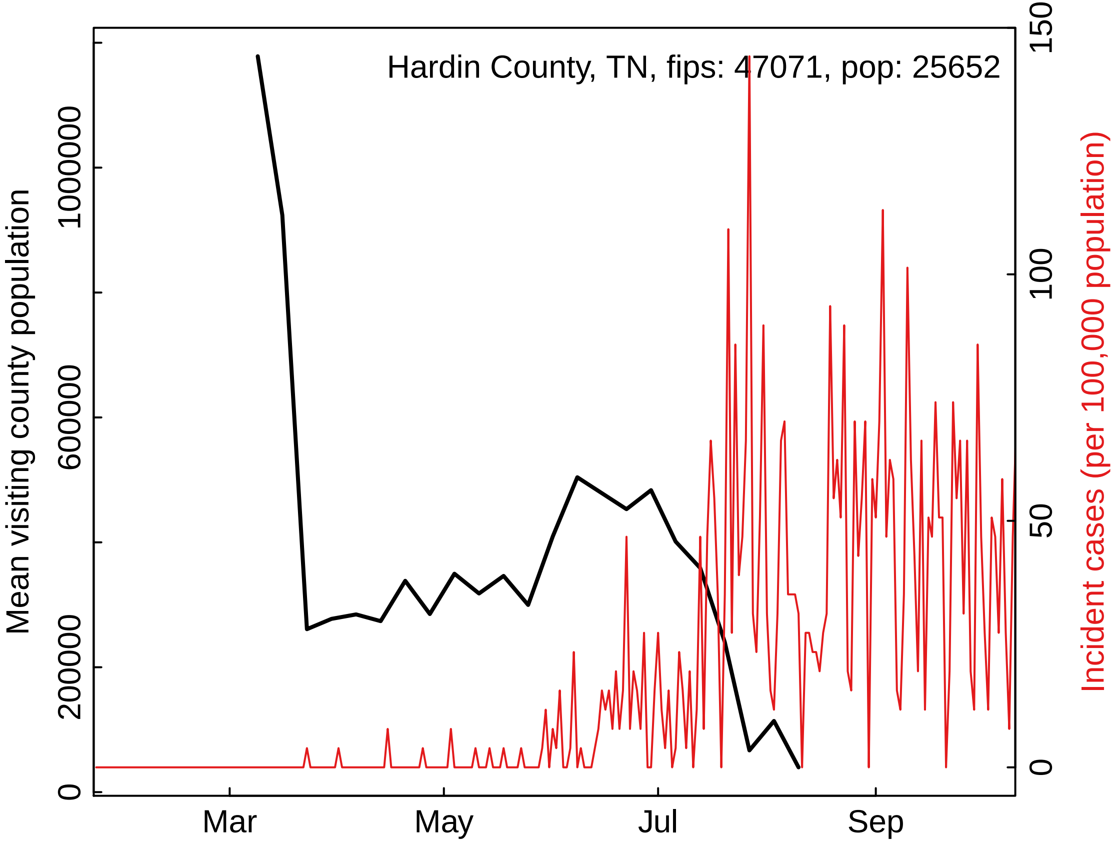

Hardin County, TN, fips: 47071, pop: 25652
Mean visiting county population
Incident cases (per 100,000 population)
0
200000
600000
1000000
0
50
100
150
Mar
May
Jul
Sep

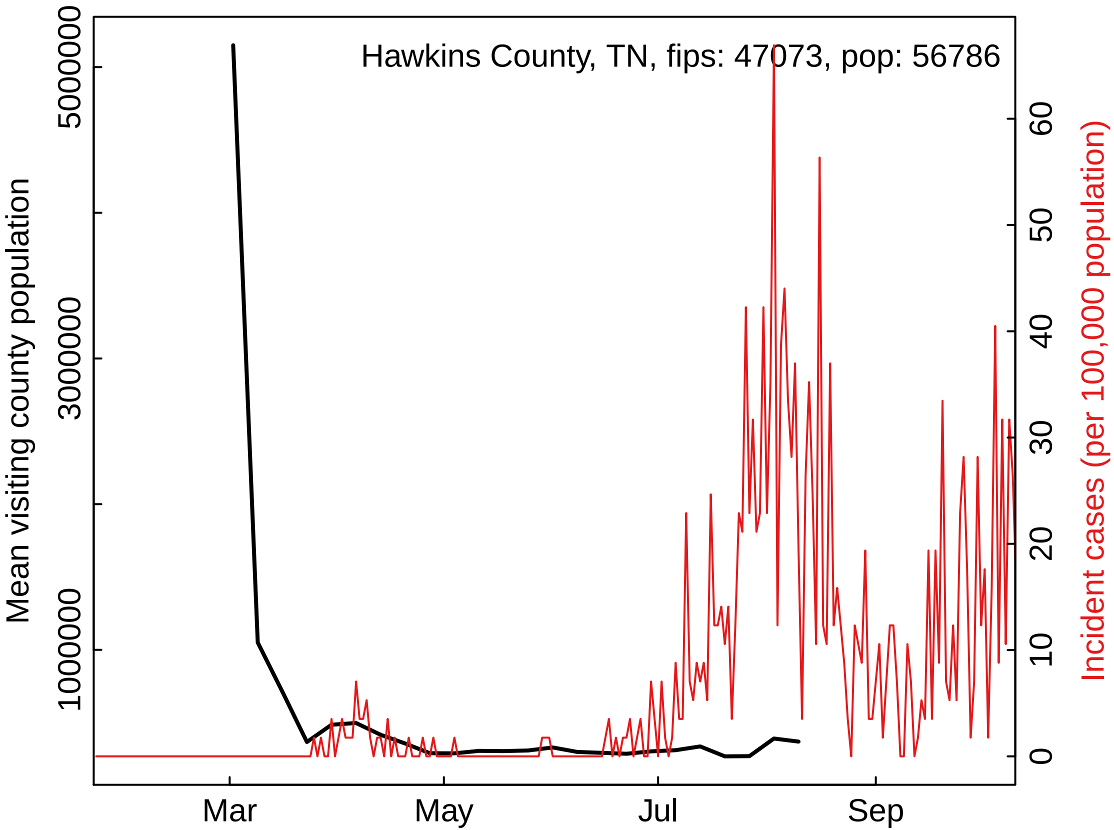

Hawkins County, TN, fips: 47073, pop: 56786
Mean visiting county population
1000000
3000000
5000000
Incident cases (per 100,000 population)
0
10
20
30
40
50
60
Mar
May
Jul
Sep

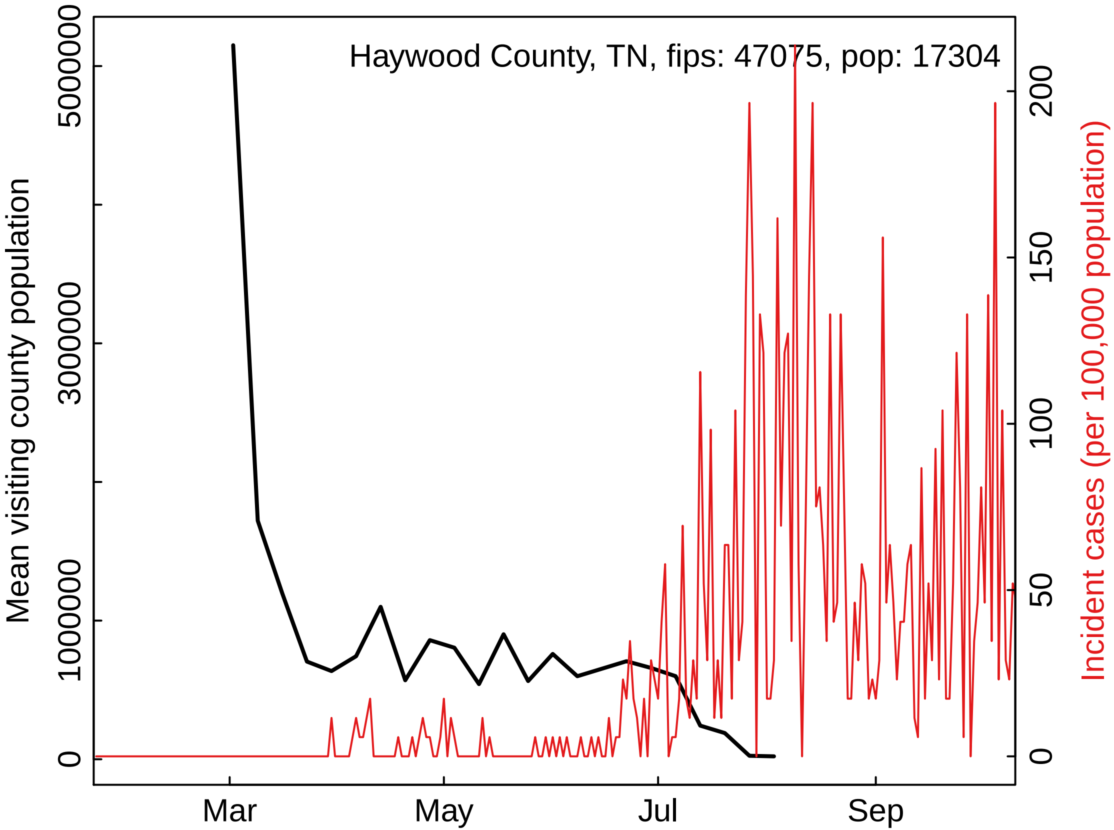

Haywood County, TN, fips: 47075, pop: 17304
Mean visiting county population
0
1000000
3000000
5000000
Incident cases (per 100,000 population)
0
50
100
150
200
Mar
May
Jul
Sep

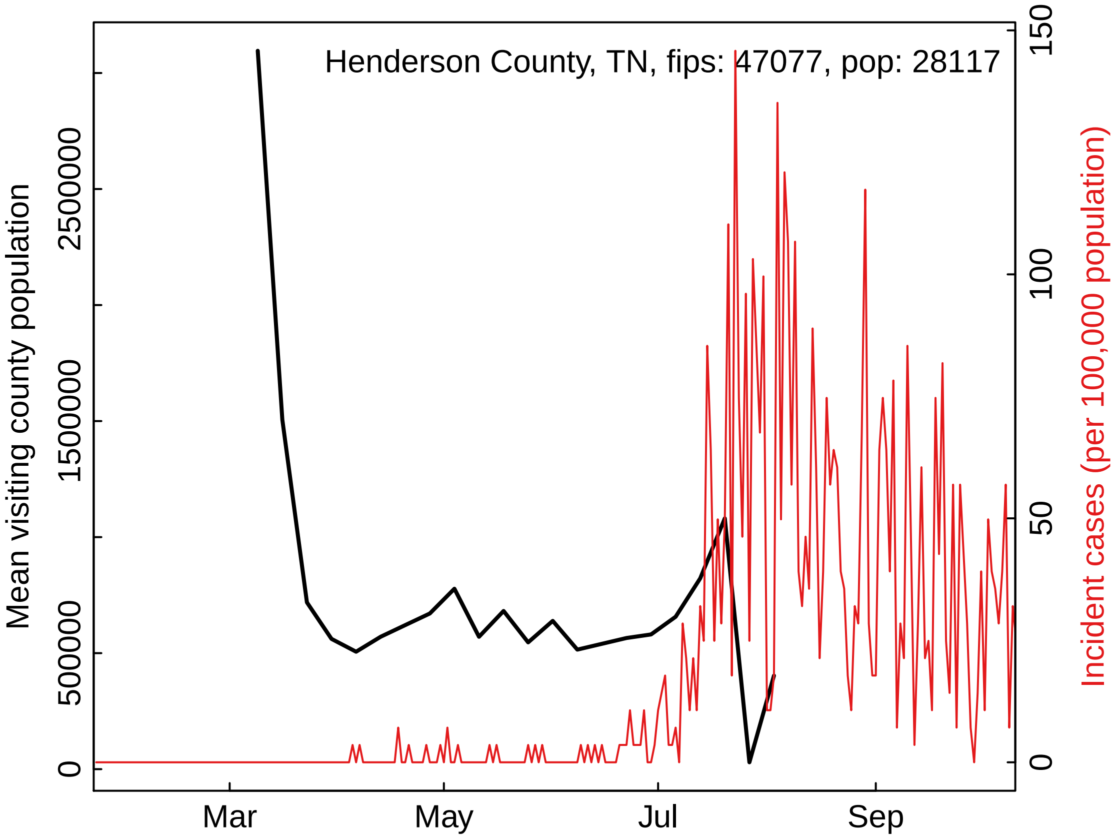
Henderson County, TN, fips: 47077, pop: 28117
Mean visiting county population
0
500000
1500000
2500000
Incident cases (per 100,000 population)
0
50
100
150
Mar
May
Jul
Sep

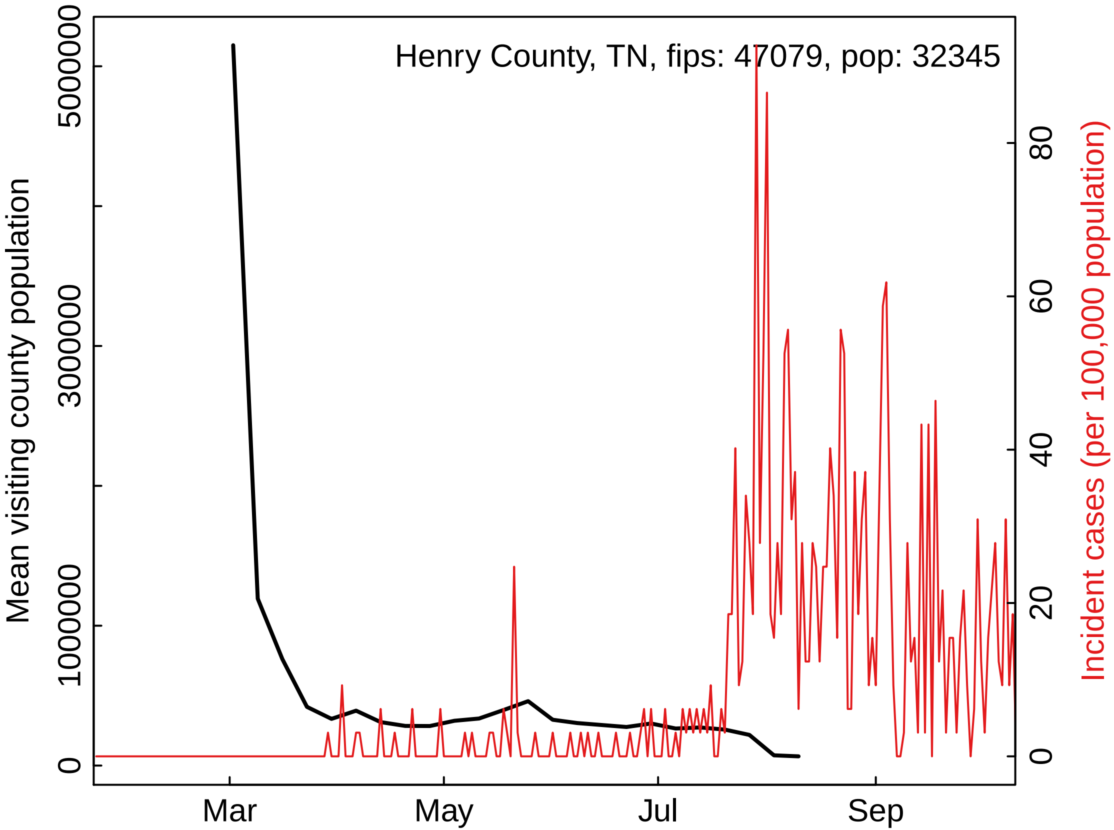
Henry County, TN, fips: 47079, pop: 32345
Mean visiting county population
0
1000000
3000000
5000000
Incident cases (per 100,000 population)
0
20
40
60
80
Mar
May
Jul
Sep

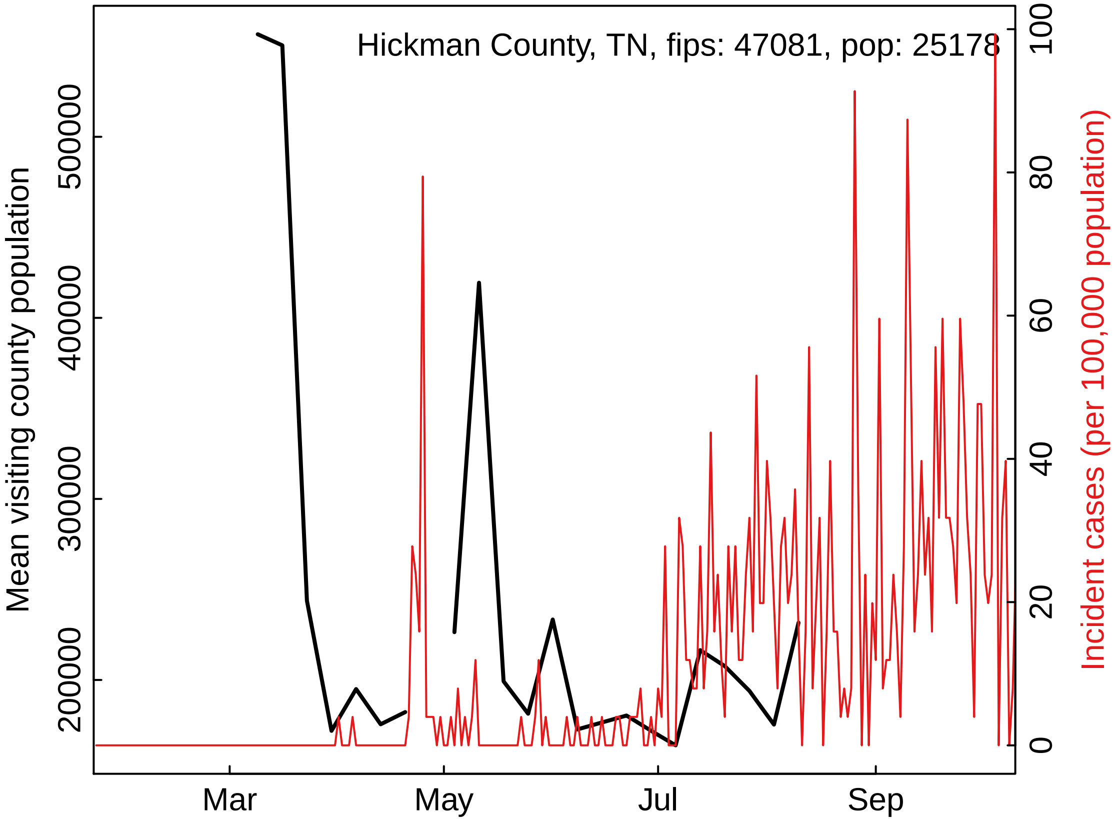

Hickman County, TN, fips: 47081, pop: 25178
Mean visiting county population
200000
300000
400000
5000000
Incident cases (per 100,000 population)
0
20
40
60
80
100
Mar
May
Jul
Sep

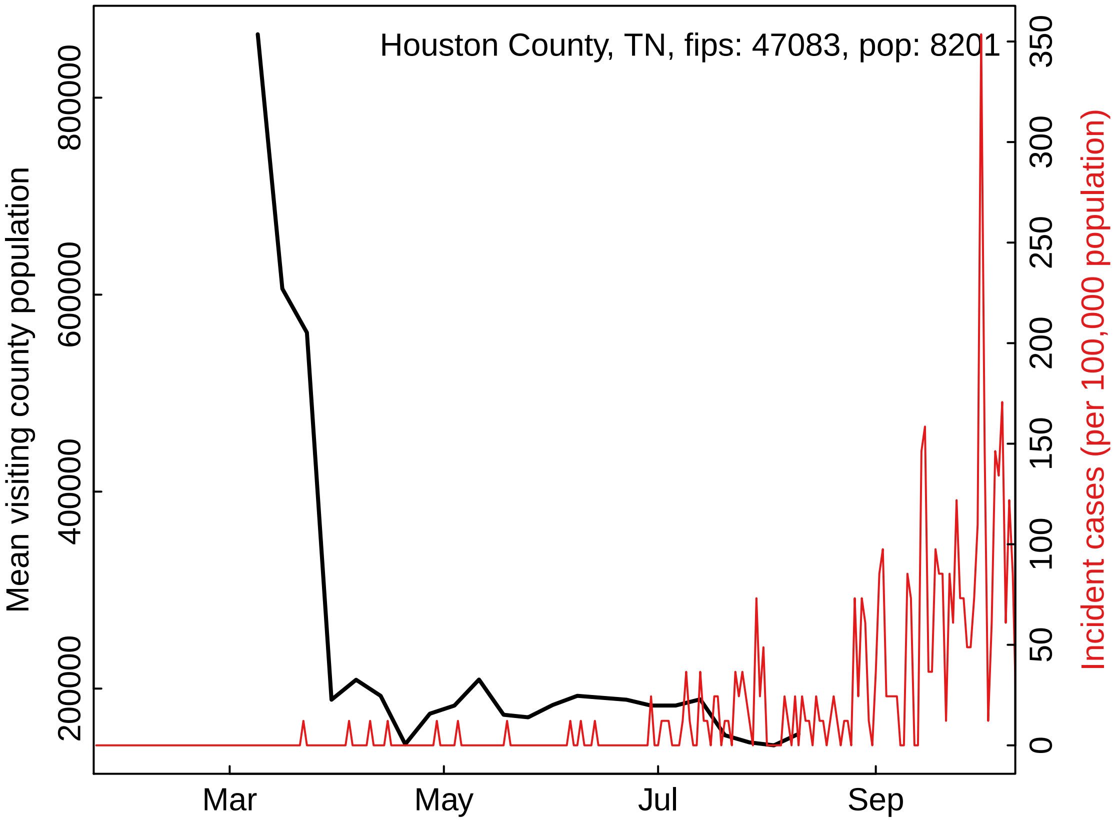

Houston County, TN, fips: 47083, pop: 8201
Mean visiting county population
200000
400000
600000
800000
Incident cases (per 100,000 population)
0
50
100
150
200
250
300
350
Mar
May
Jul
Sep

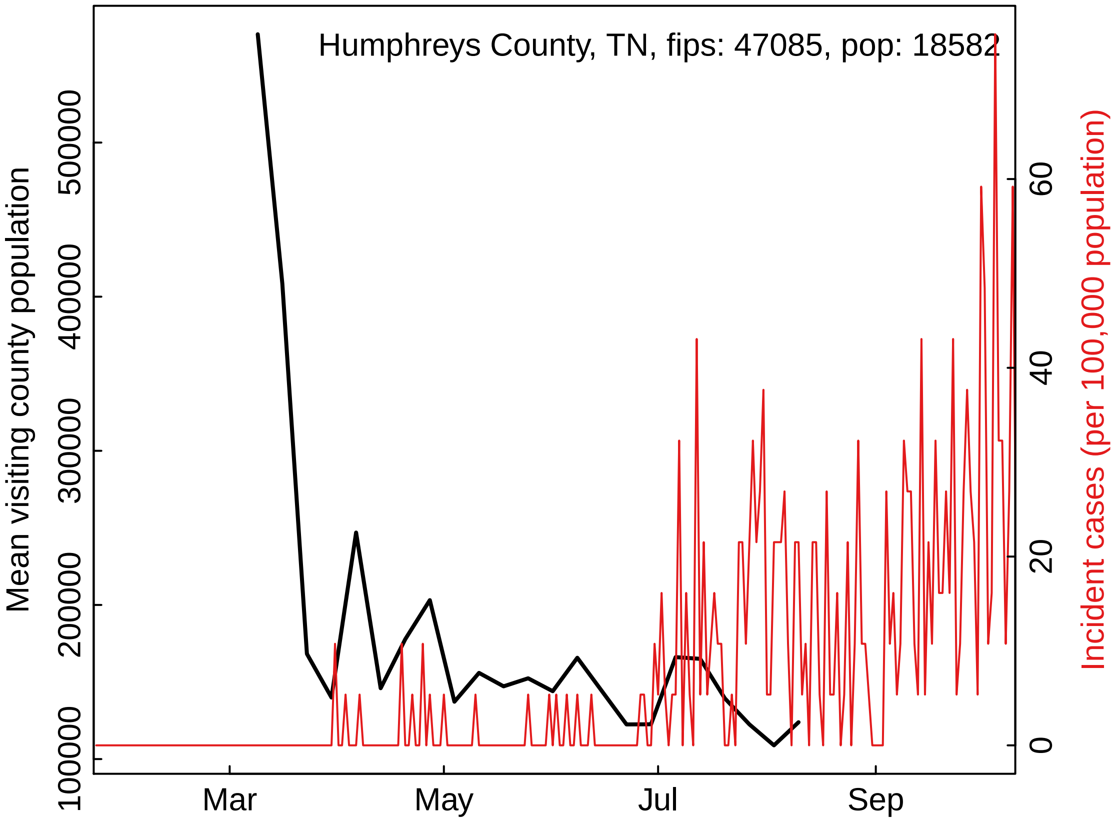

Humphreys County, TN, fips: 47085, pop: 18582
Mean visiting county population
100000
200000
300000
400000
500000
Incident cases (per 100,000 population)
0
20
40
60
Mar
May
Jul
Sep

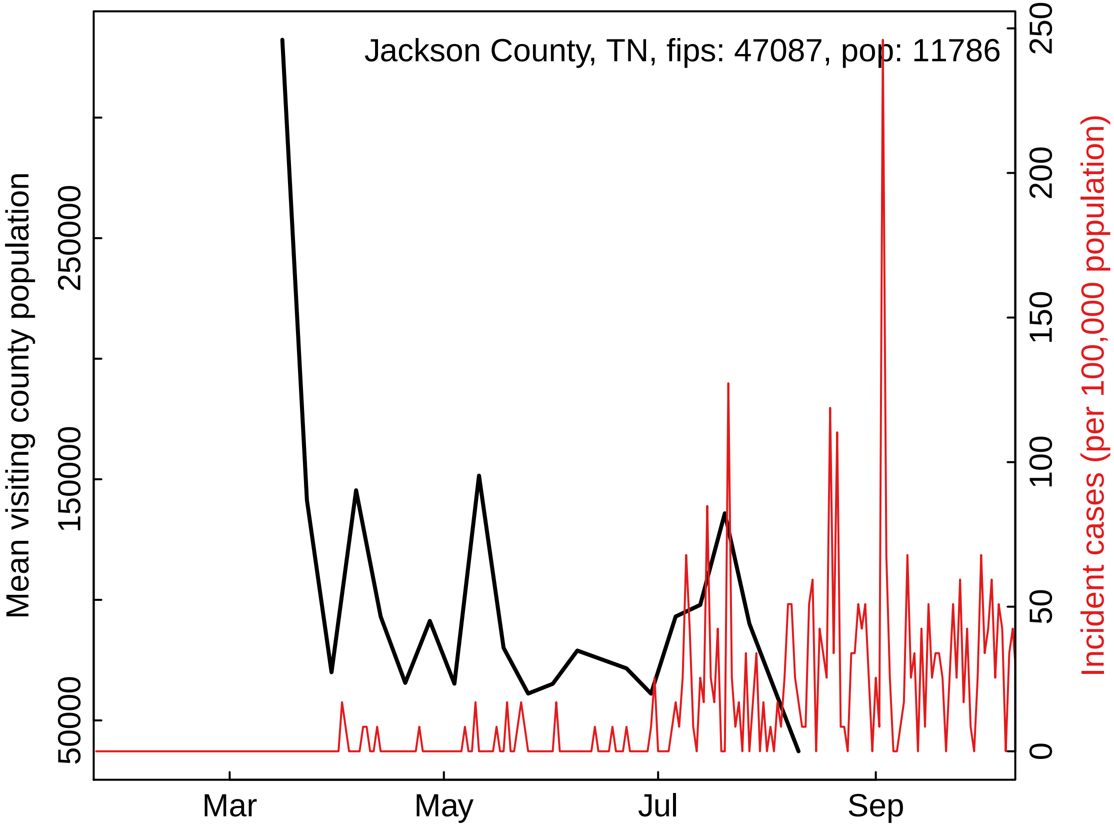
Jackson County, TN, fips: 47087, pop: 11786
Mean visiting county population
50000
150000
250000
Mar
May
Jul
Sep
Incident cases (per 100,000 population)
0
50
100
150
200
250

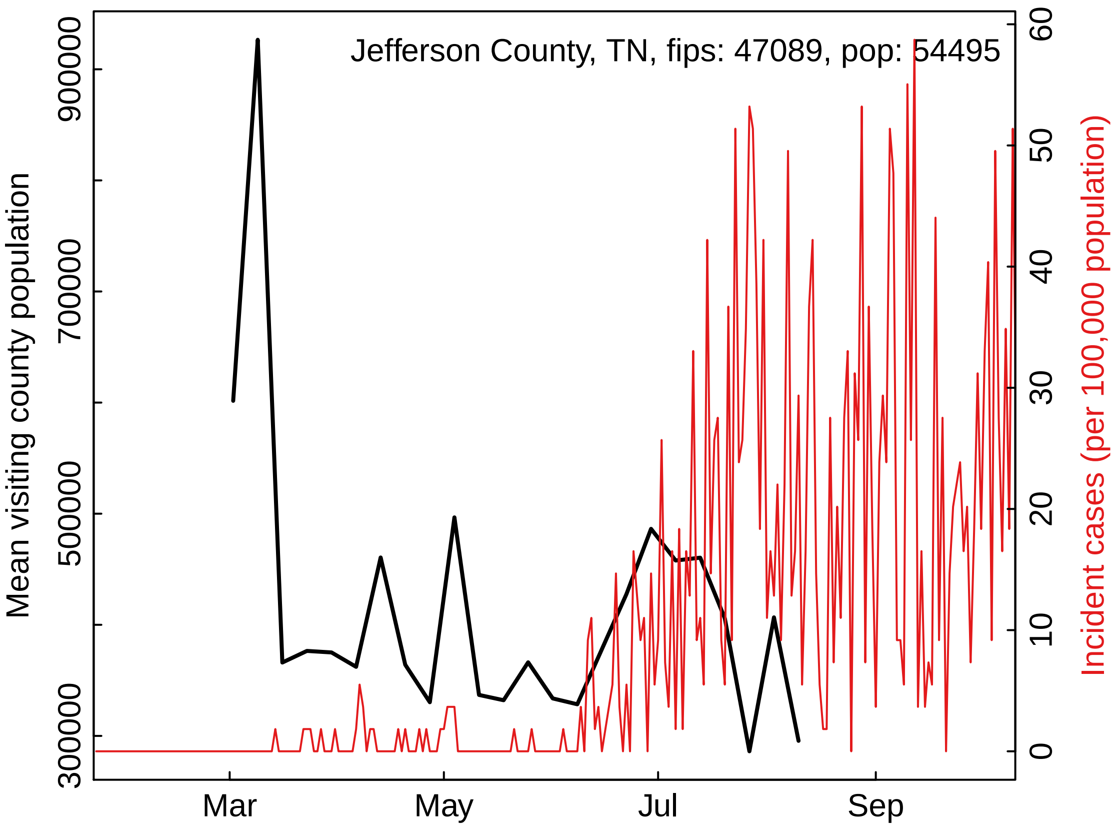
Jefferson County, TN, fips: 47089, pop: 54495
Mean visiting county population
300000
500000
700000
900000
Incident cases (per 100,000 population)
0
10
20
30
40
50
60
Mar
May
Jul
Sep

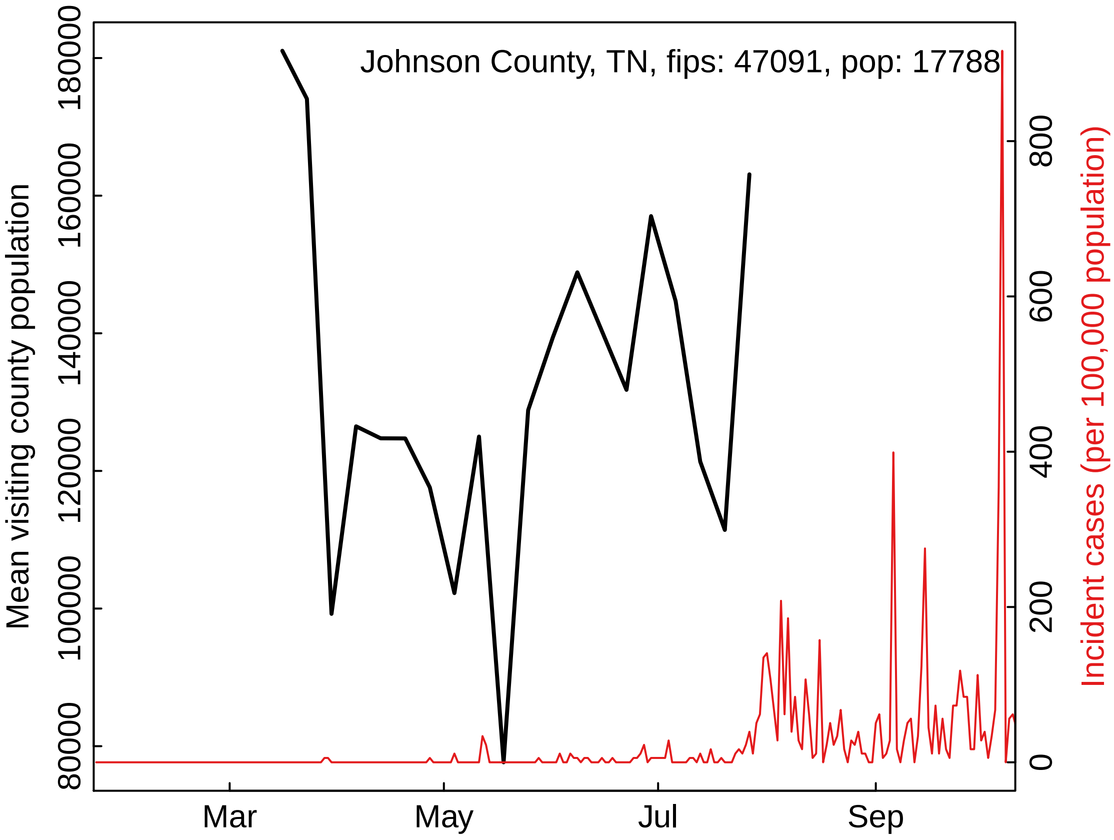

Johnson County, TN, fips: 47091, pop: 17788
Mean visiting county population
80000
100000
120000
140000
160000
180000
Mar
May
Jul
Sep
0
200
400
600
800
Incident cases (per 100,000 population)

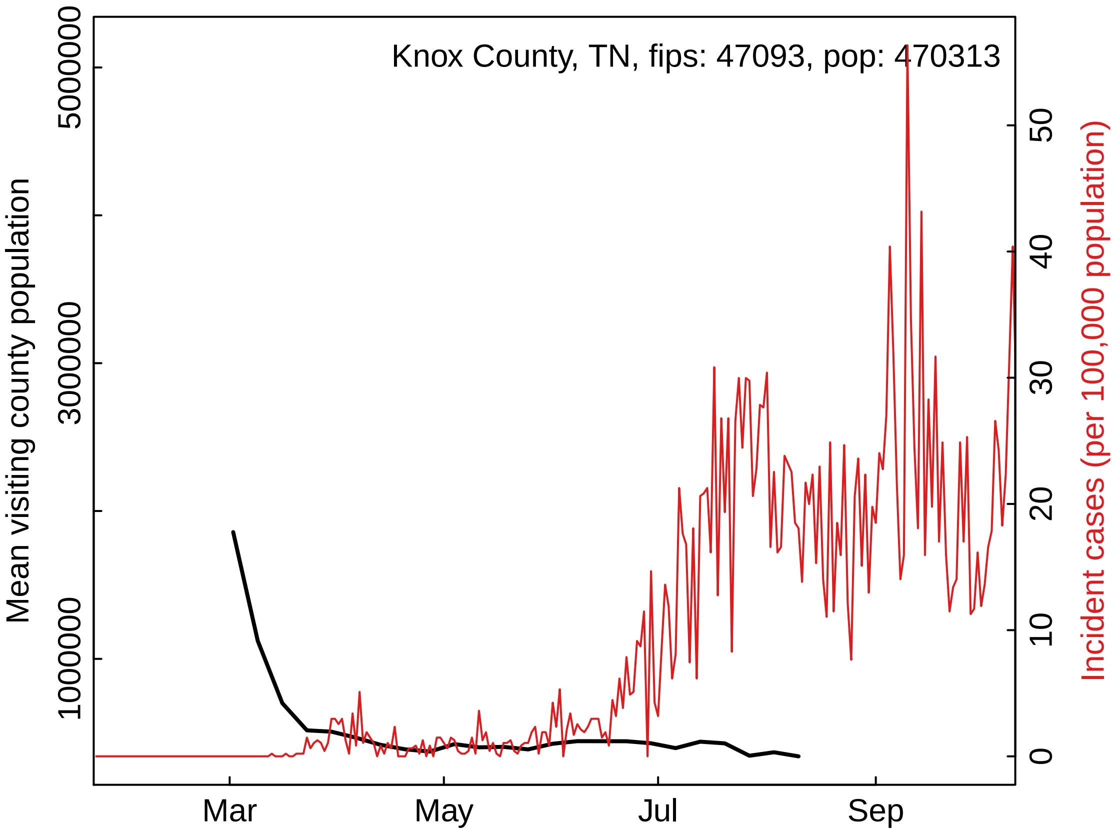
Knox County, TN, fips: 47093, pop: 470313
Mean visiting county population
1000000
3000000
5000000
Incident cases (per 100,000 population)
0
10
20
30
40
50
Mar
May
Jul
Sep

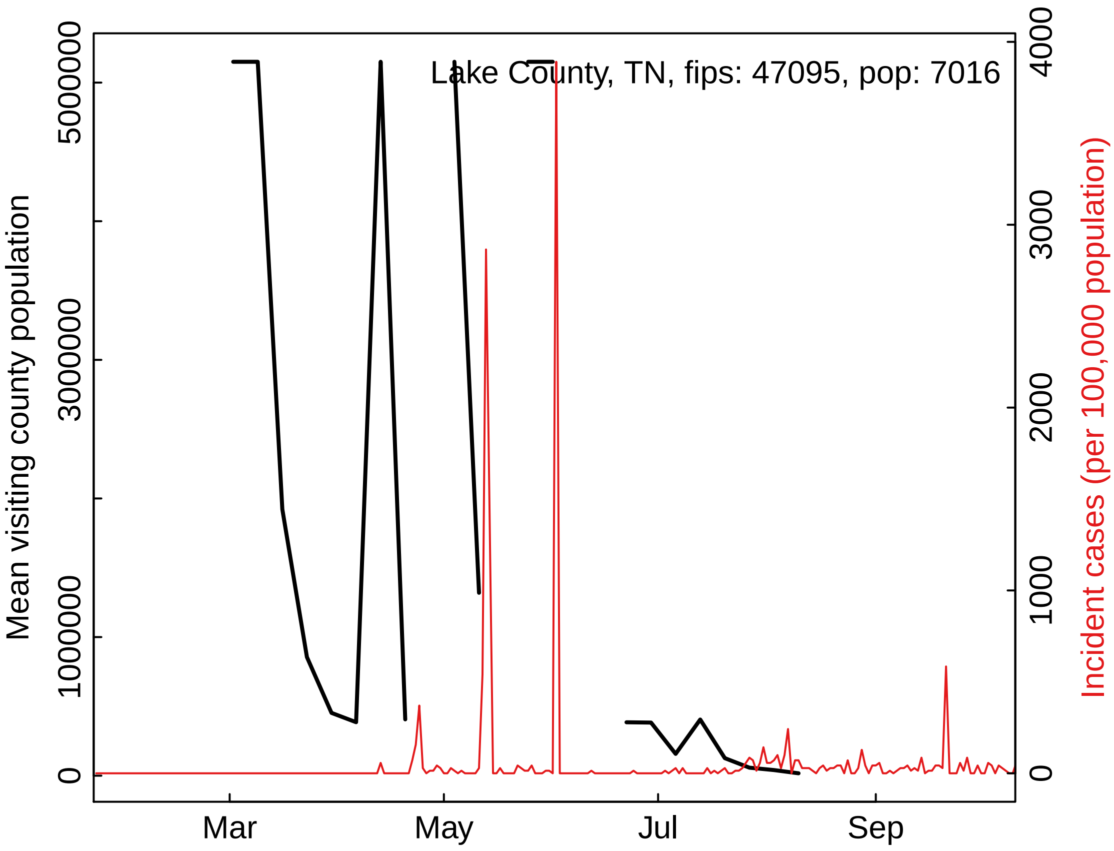

Lake County, TN, fips: 47095, pop: 7016
Mean visiting county population
Incident cases (per 100,000 population)
0
1000000
3000000
5000000
0
1000
2000
3000
4000
Mar
May
Jul
Sep

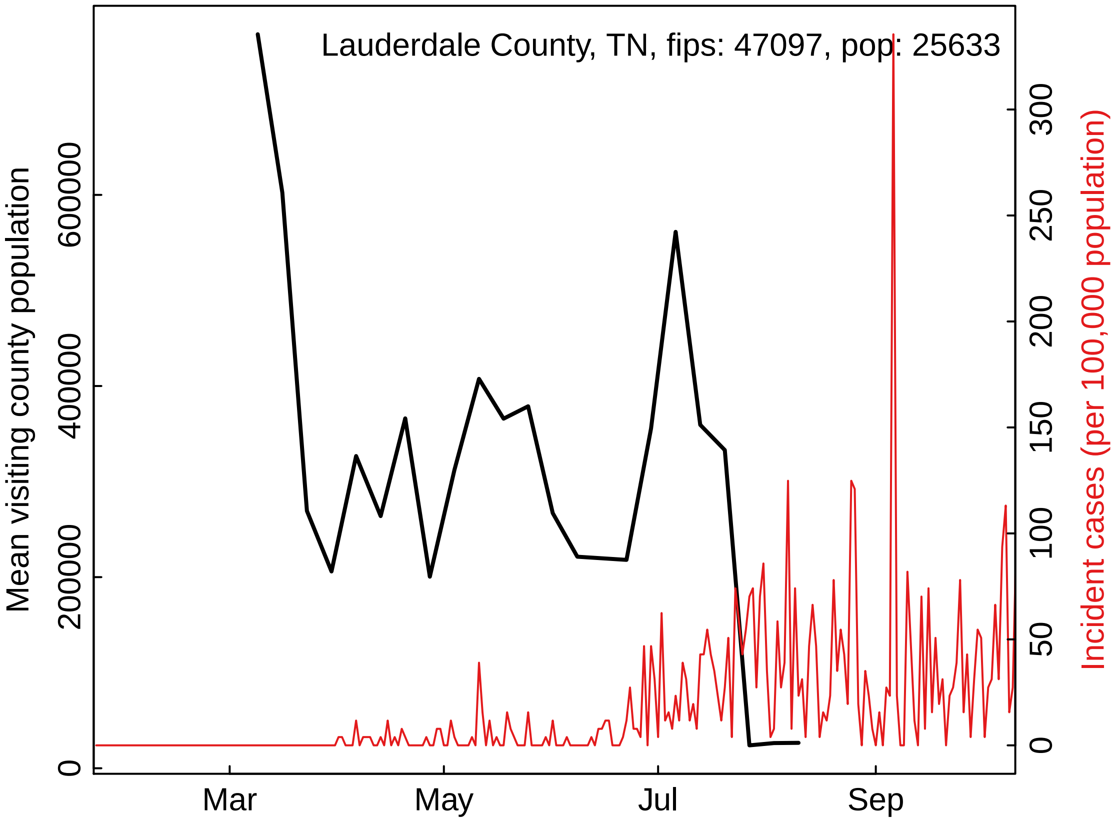

Lauderdale County, TN, fips: 47097, pop: 25633
Mean visiting county population
0
200000
400000
600000
Incident cases (per 100,000 population)
0
50
100
150
200
250
300
Mar
May
Jul
Sep

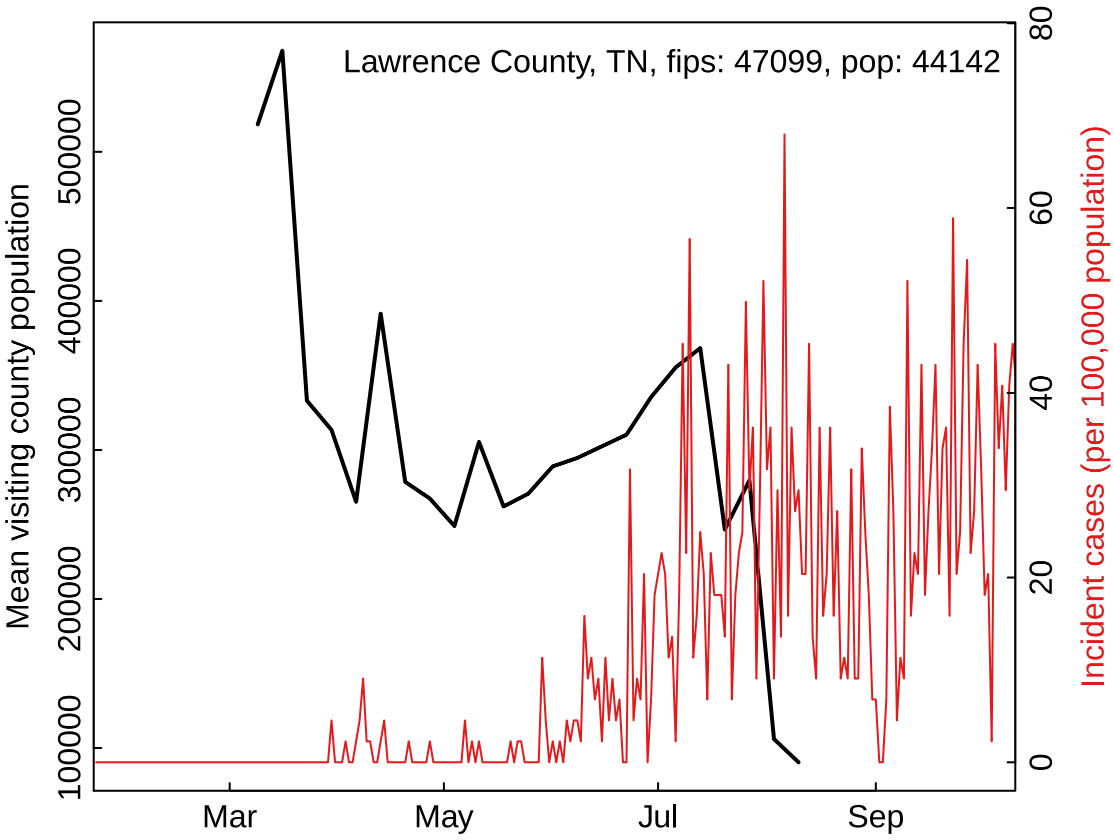

Lawrence County, TN, fips: 47099, pop: 44142
Mean visiting county population
100000
200000
300000
400000
500000
Incident cases (per 100,000 population)
0
20
40
60
80
Mar
May
Jul
Sep

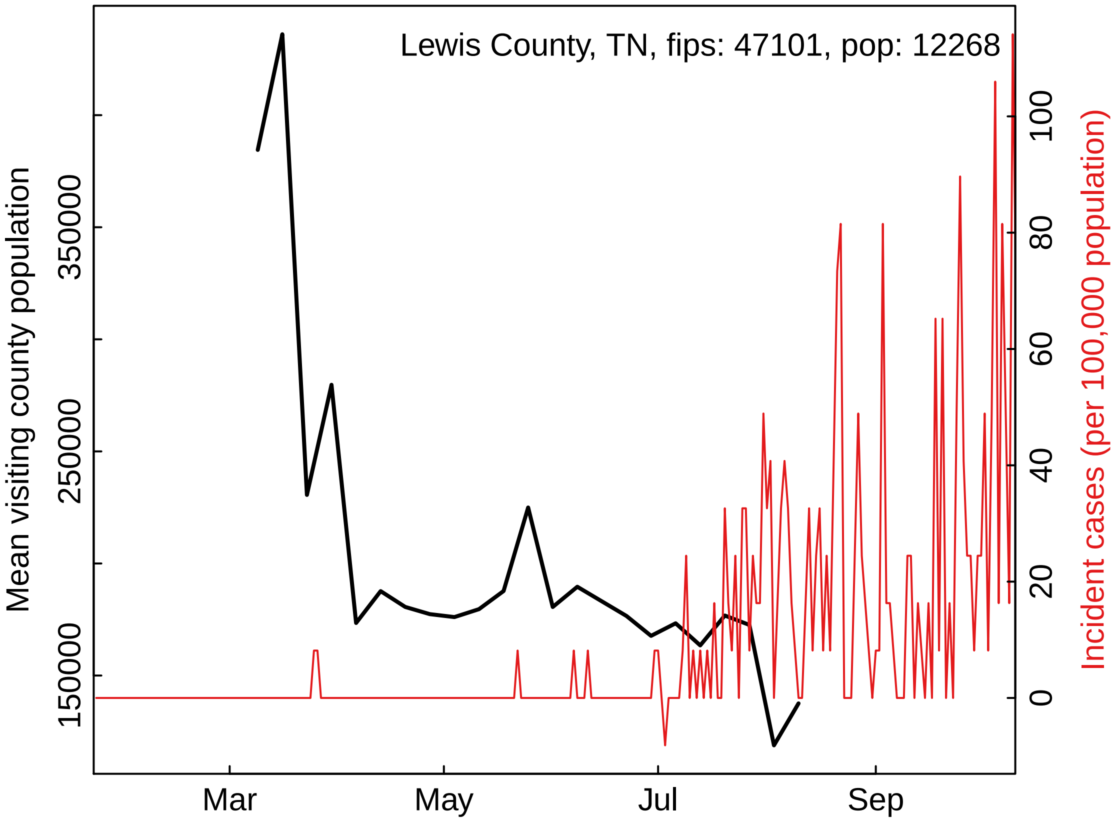

Lewis County, TN, fips: 47101, pop: 12268
Mean visiting county population
150000
250000
350000
Incident cases (per 100,000 population)
0
20
40
60
80
100
Mar
May
Jul
Sep

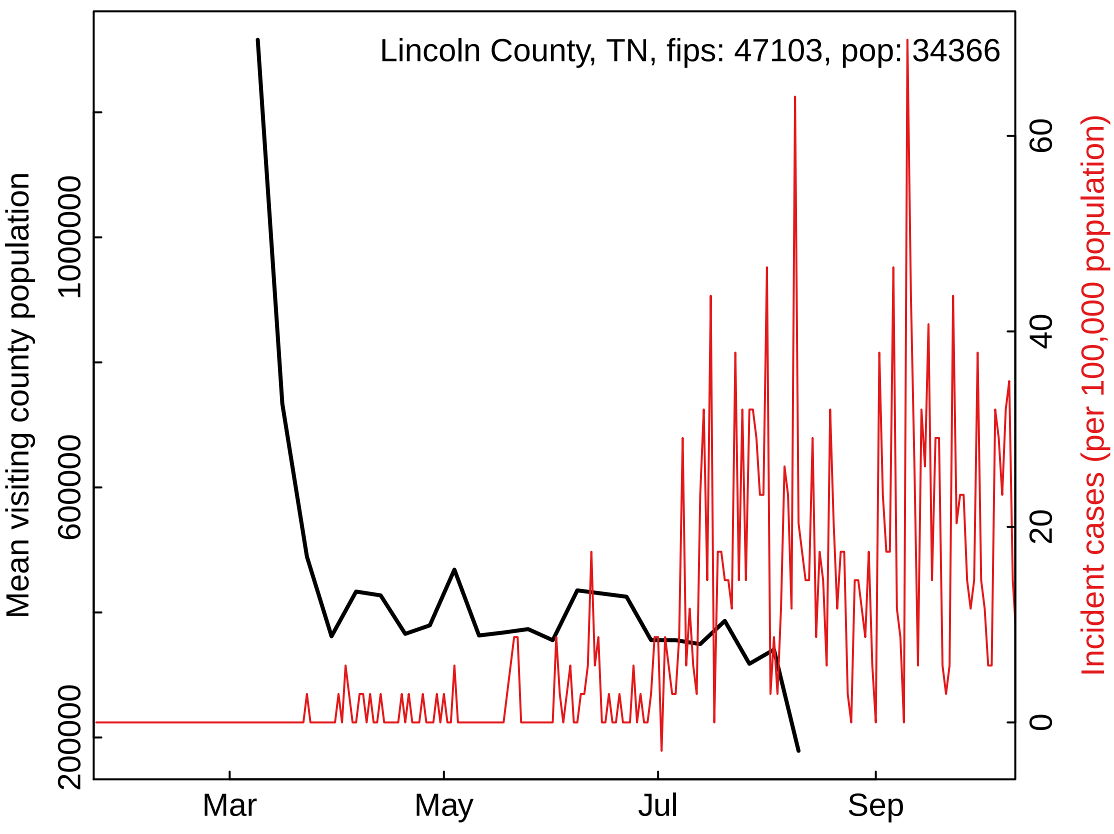

Lincoln County, TN, fips: 47103, pop: 34366
Mean visiting county population
200000
600000
1000000
Incident cases (per 100,000 population)
0
20
40
60
Mar
May
Jul
Sep

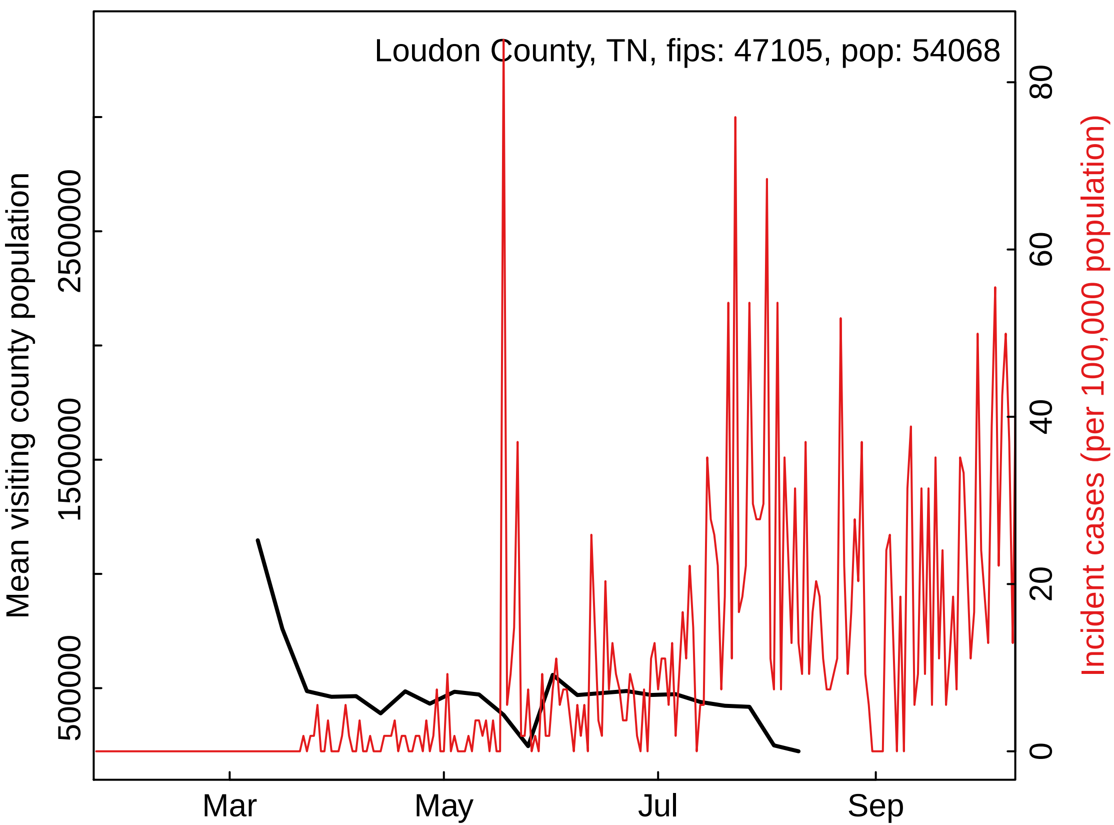

Loudon County, TN, fips: 47105, pop: 54068
Mean visiting county population
500000
1500000
2500000
Mar
May
Jul
Sep
0
20
40
60
80
Incident cases (per 100,000 population)

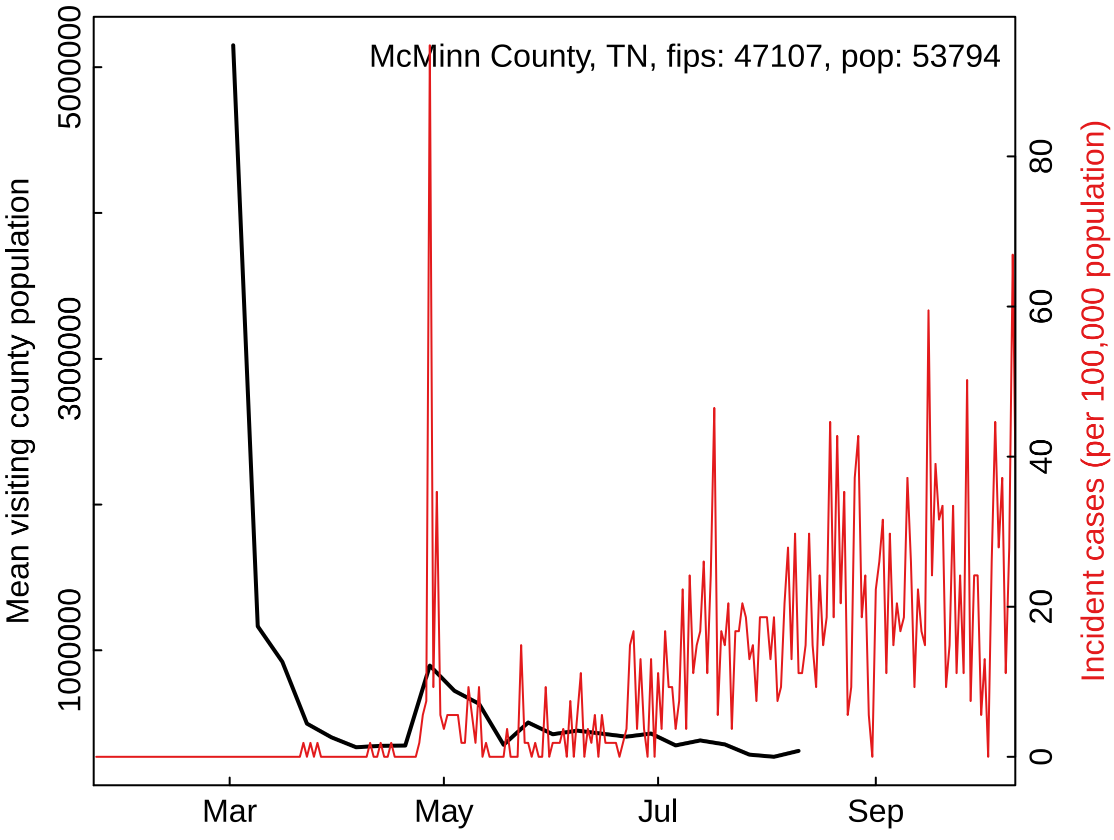

McMinn County, TN, fips: 47107, pop: 53794
Mean visiting county population
1000000
3000000
5000000
Incident cases (per 100,000 population)
0
20
40
60
80
Mar
May
Jul
Sep

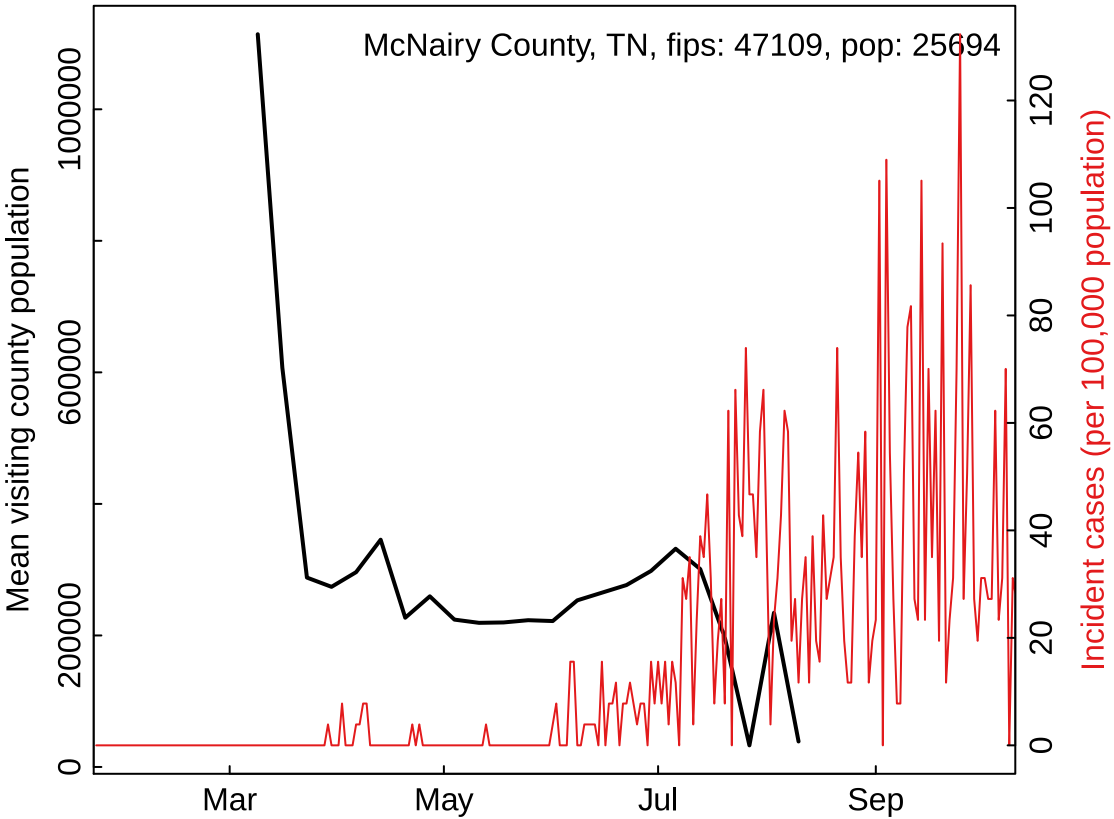

McNairy County, TN, fips: 47109, pop: 25694
Mean visiting county population
0
200000
600000
1000000
Incident cases (per 100,000 population)
0
20
40
60
80
100
120
Mar
May
Jul
Sep

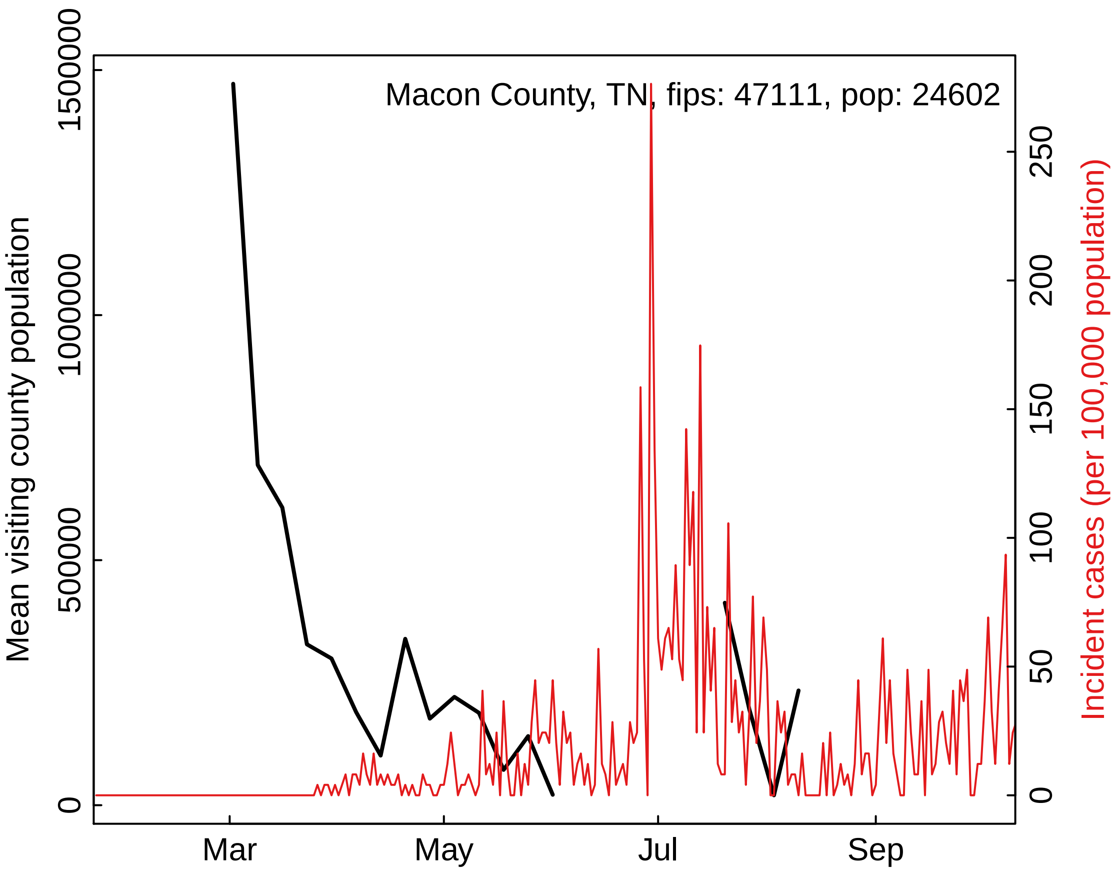

Macon County, TN, fips: 47111, pop: 24602
Mean visiting county population
0
500000
1000000
1500000
Incident cases (per 100,000 population)
0
50
100
150
200
250
Mar
May
Jul
Sep

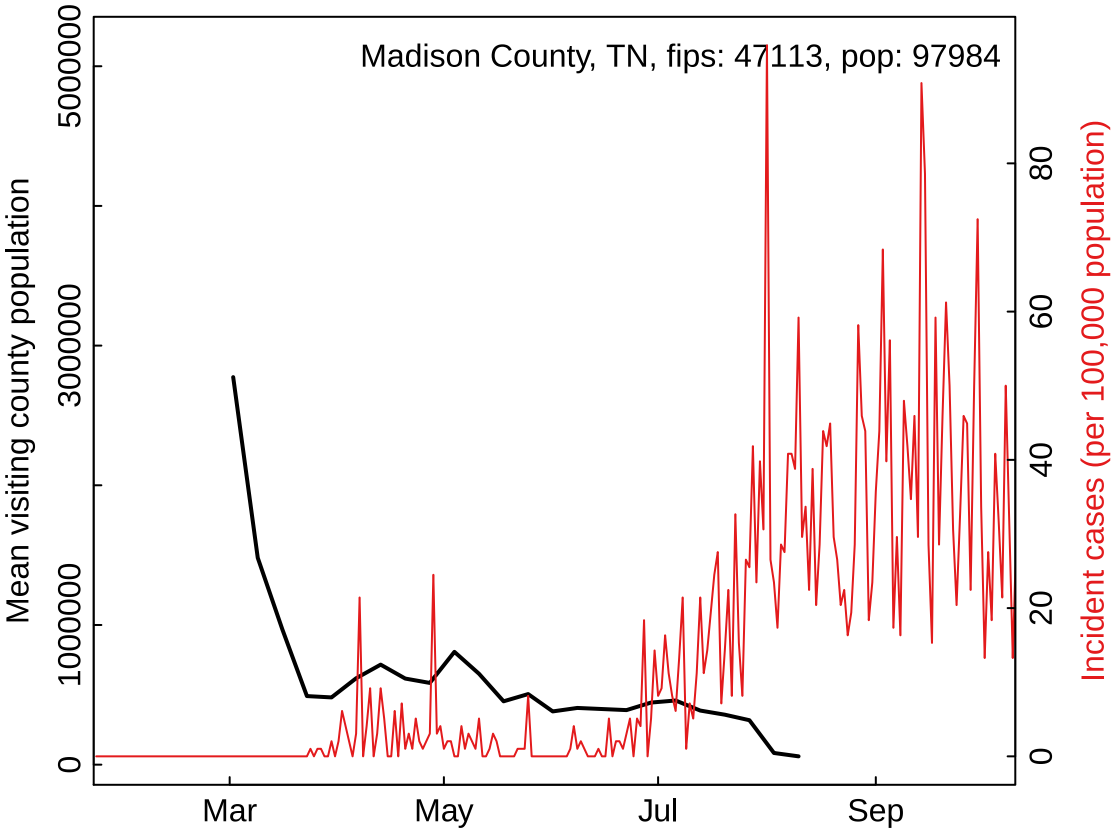

Madison County, TN, fips: 47113, pop: 97984
Mean visiting county population
0
1000000
3000000
5000000
Incident cases (per 100,000 population)
0
20
40
60
80
Mar
May
Jul
Sep

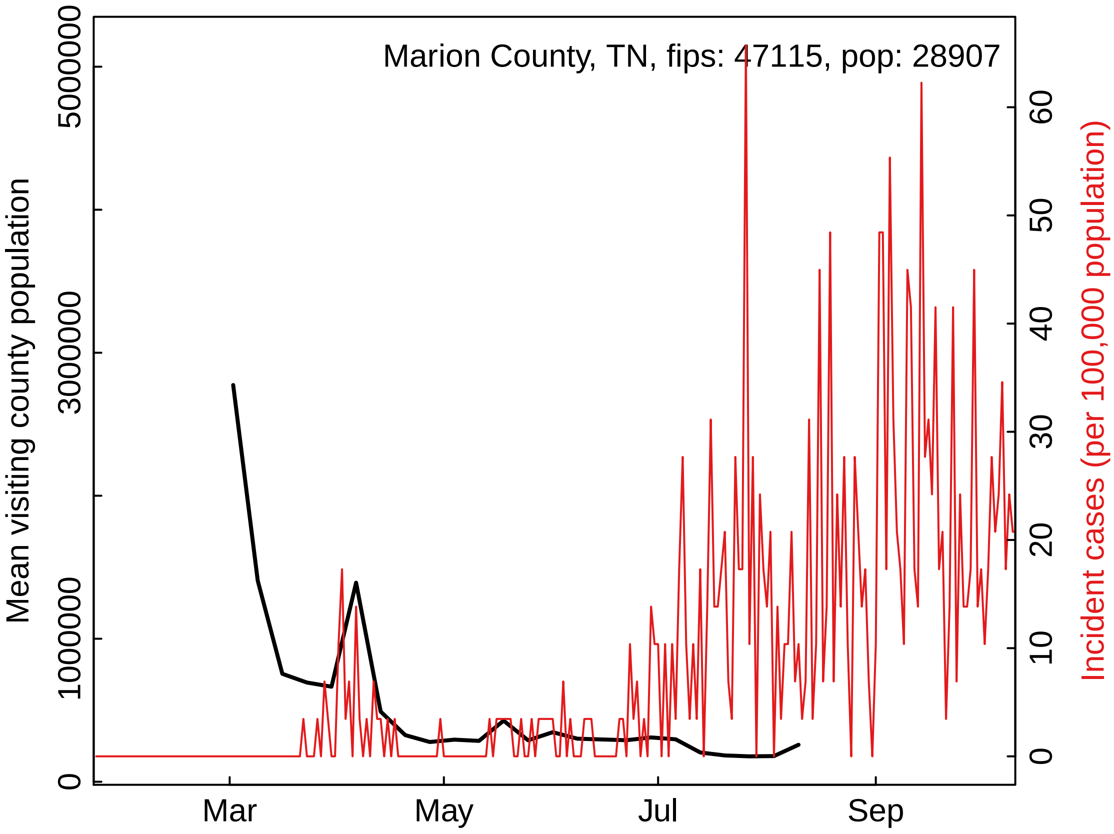

Marion County, TN, fips: 47115, pop: 28907
Mean visiting county population
0
1000000
3000000
5000000
Incident cases (per 100,000 population)
0
10
20
30
40
50
60
Mar
May
Jul
Sep

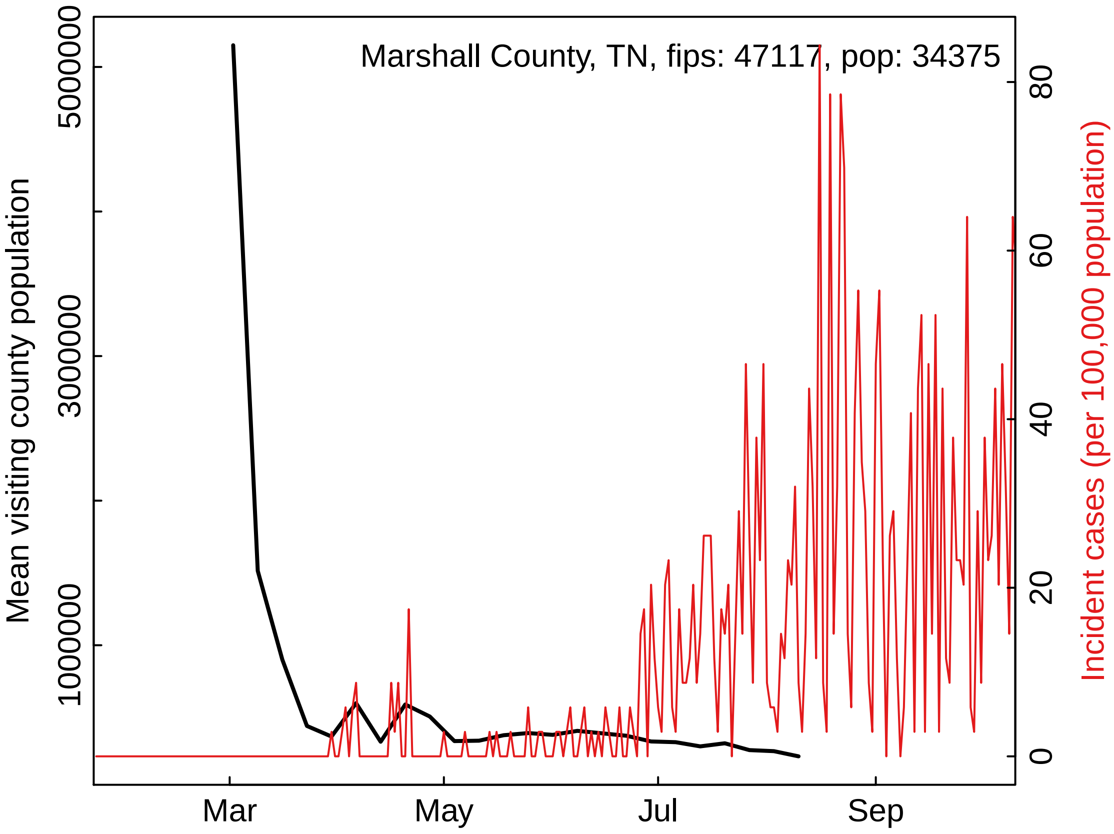

Marshall County, TN, fips: 47117, pop: 34375
Mean visiting county population
10000000
30000000
50000000
Incident cases (per 100,000 population)
0
20
40
60
80
Mar
May
Jul
Sep

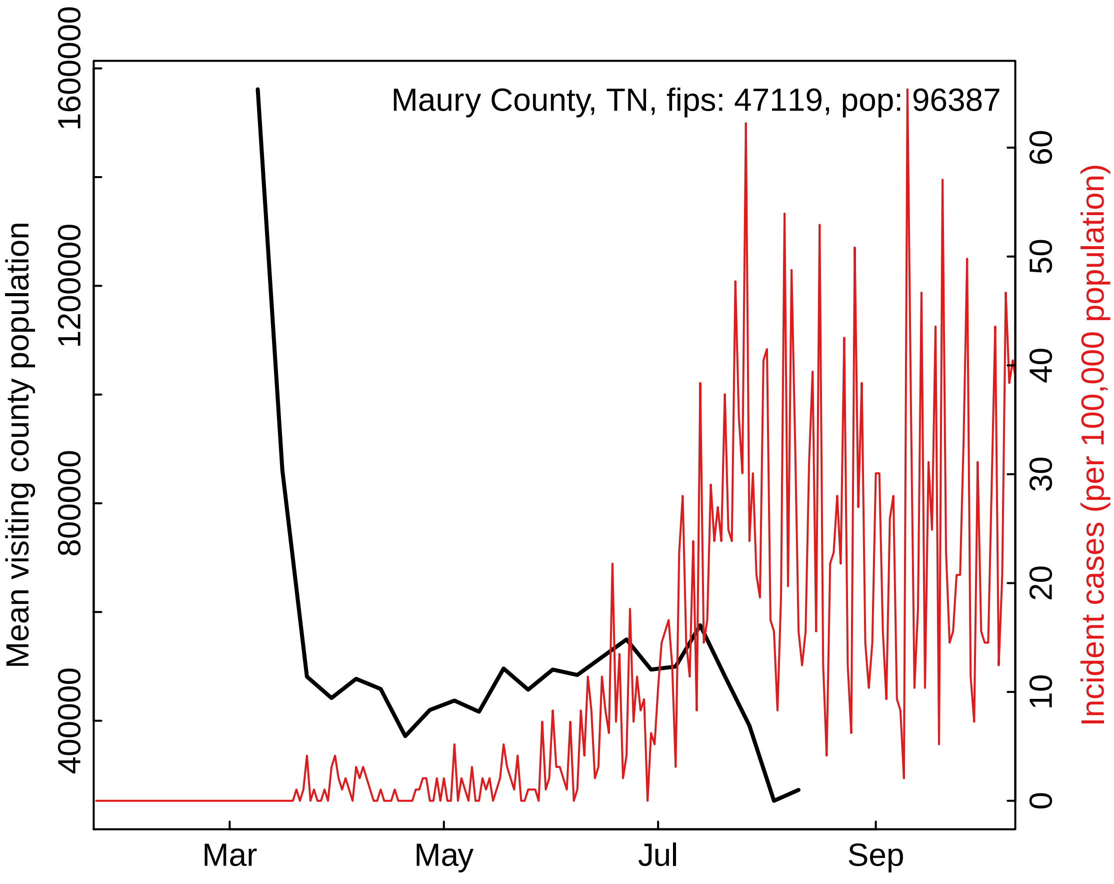

Maury County, TN, fips: 47119, pop: 96387
Mean visiting county population
400000
800000
1200000
1600000
Incident cases (per 100,000 population)
0
10
20
30
40
50
60
Mar
May
Jul
Sep

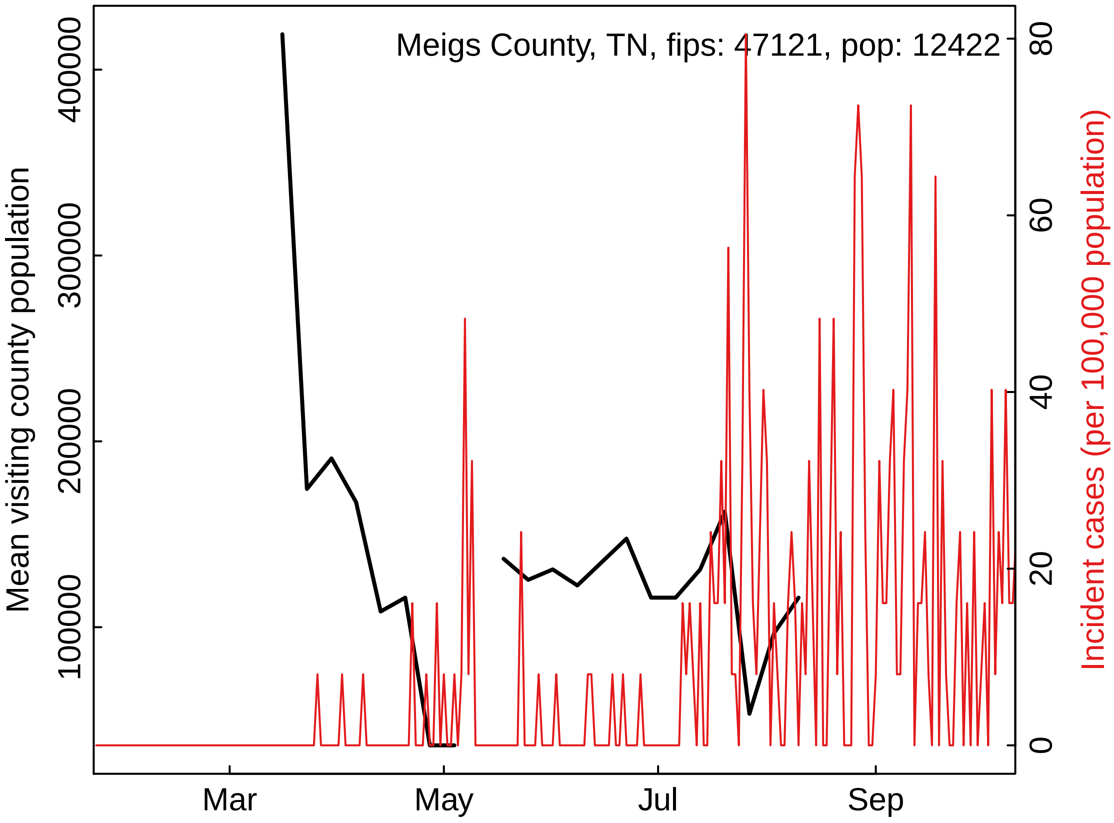

Meigs County, TN, fips: 47121, pop: 12422
Mean visiting county population
100000
200000
300000
400000
Incident cases (per 100,000 population)
0
20
40
60
80
Mar
May
Jul
Sep

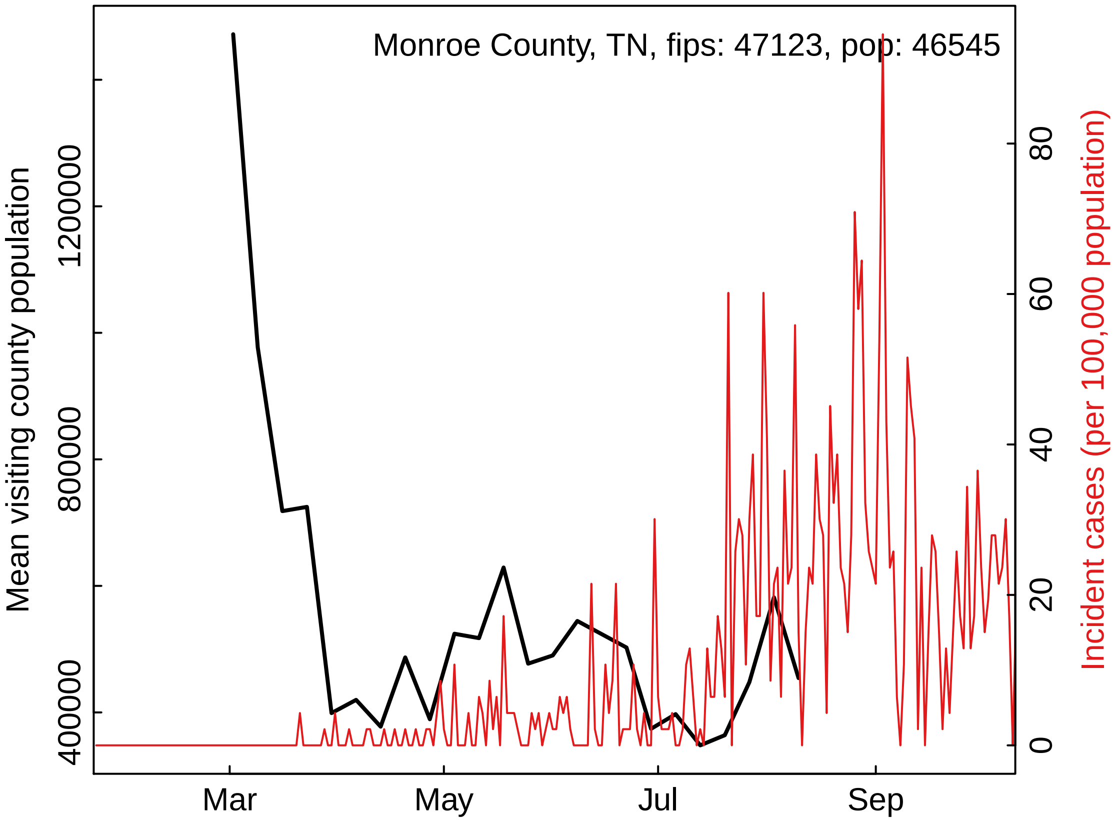

Monroe County, TN, fips: 47123, pop: 46545
Mean visiting county population
400000
800000
1200000
Mar
May
Jul
Sep
0
20
40
60
80
Incident cases (per 100,000 population)

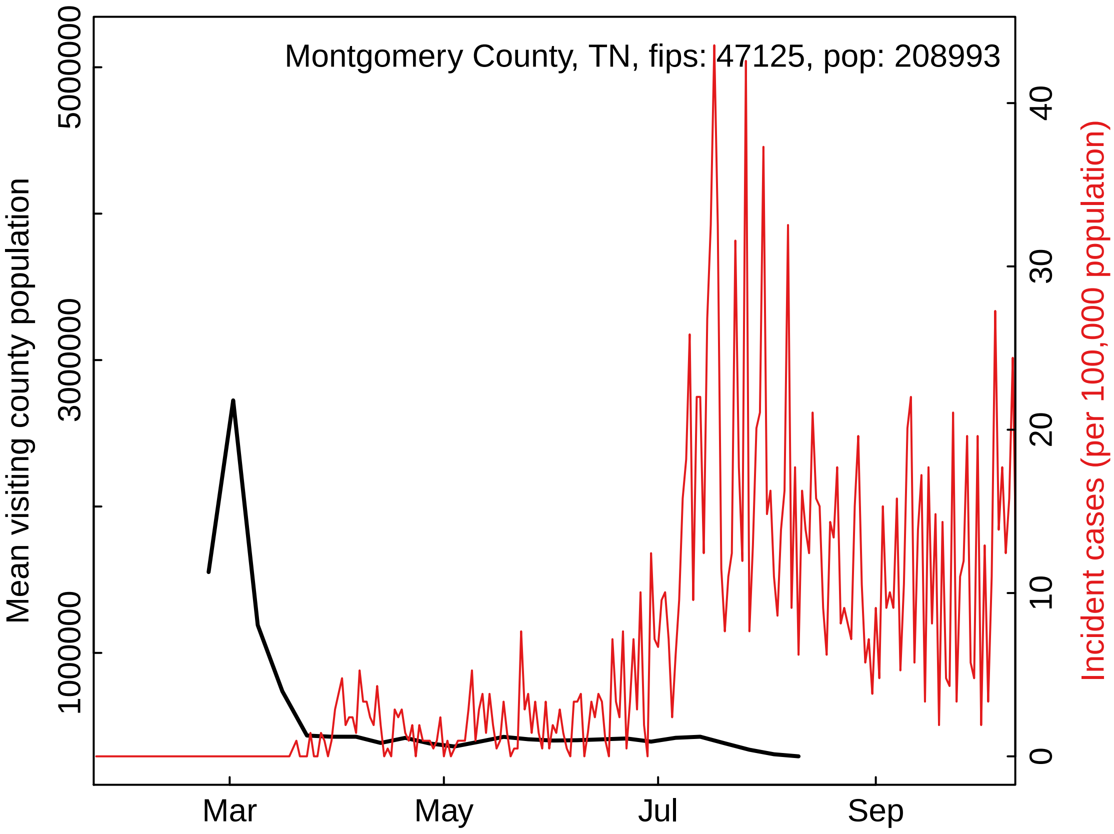

Montgomery County, TN, fips: 47125, pop: 208993
Mean visiting county population
1000000
3000000
5000000
Incident cases (per 100,000 population)
0
10
20
30
40
Mar
May
Jul
Sep

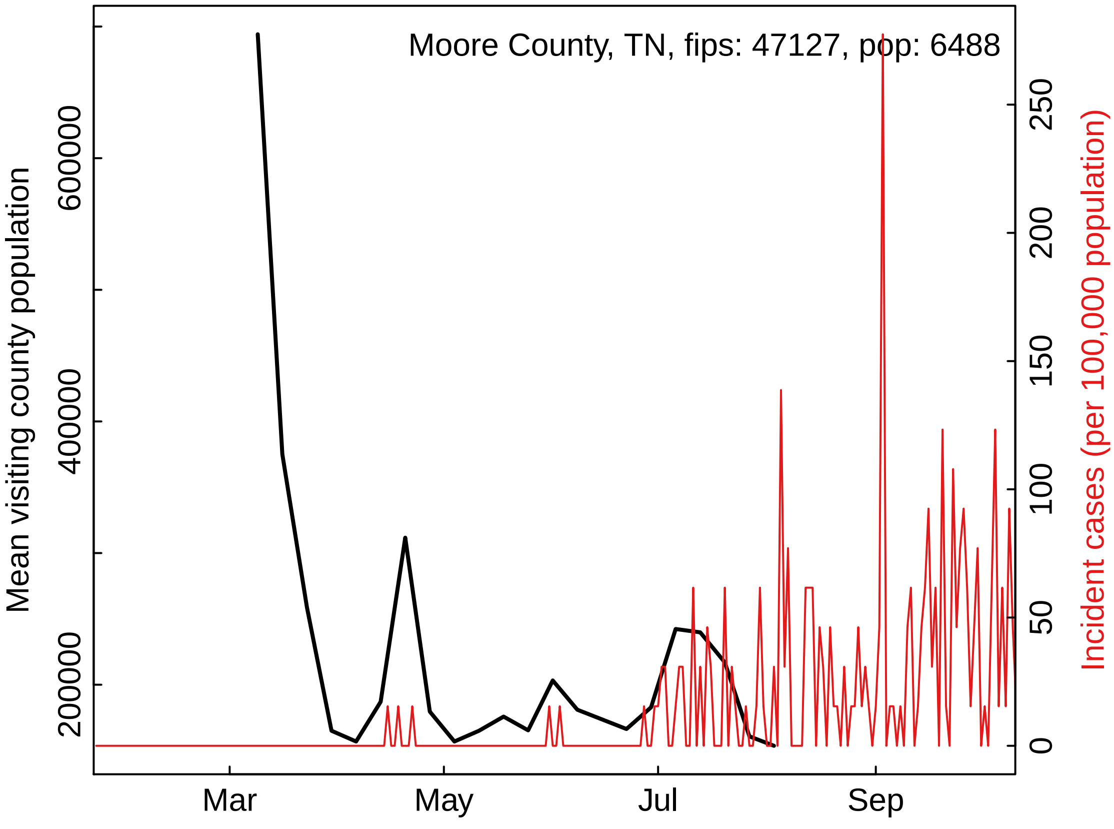

Moore County, TN, fips: 47127, pop: 6488
Mean visiting county population
200000
400000
600000
Incident cases (per 100,000 population)
0
50
100
150
200
250
Mar
May
Jul
Sep

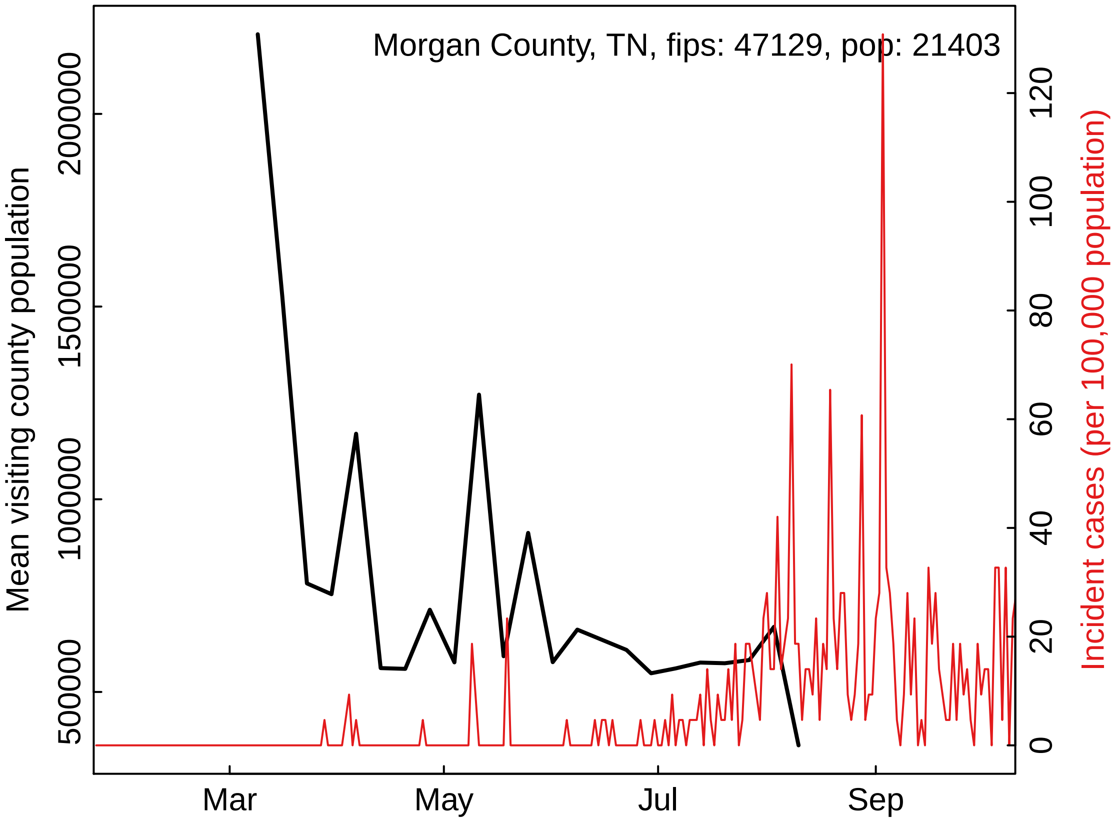

Morgan County, TN, fips: 47129, pop: 21403
Mean visiting county population
Incident cases (per 100,000 population)
500000
1000000
1500000
2000000
0
20
40
60
80
100
120
Mar
May
Jul
Sep

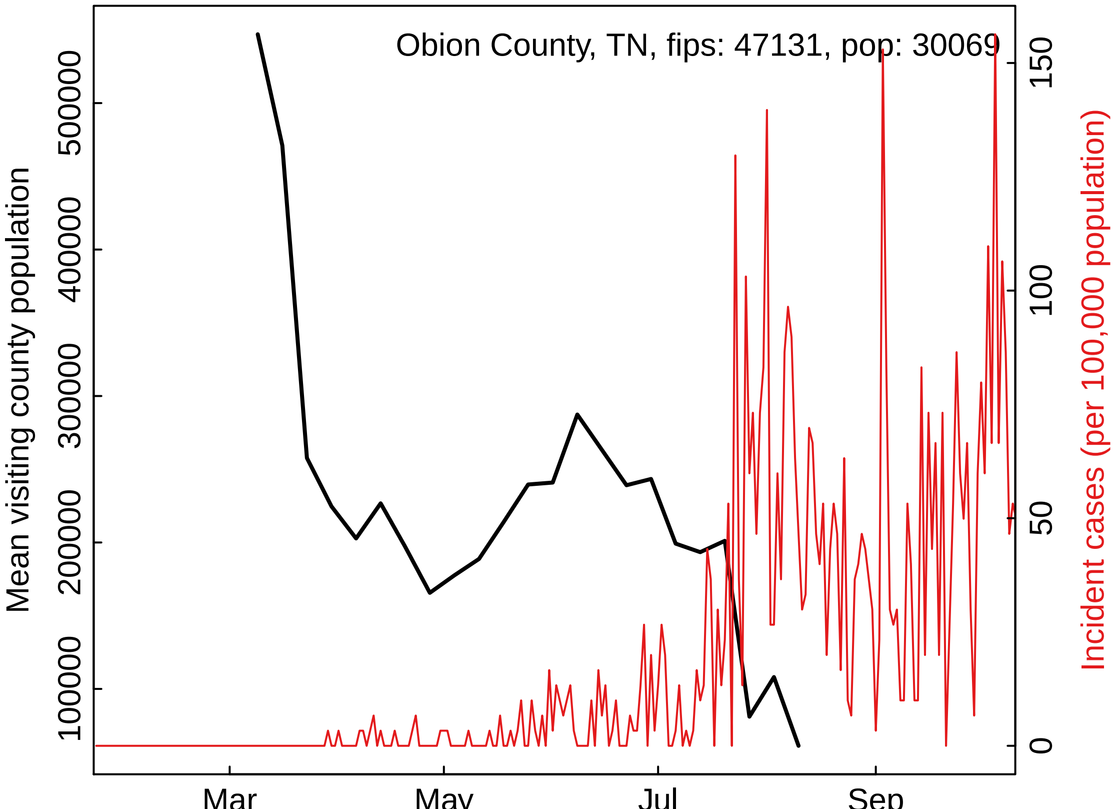

Obion County, TN, fips: 47131, pop: 30069
Mean visiting county population
100000
200000
300000
400000
500000
Incident cases (per 100,000 population)
0
50
100
150
Mar
May
Jul
Sep

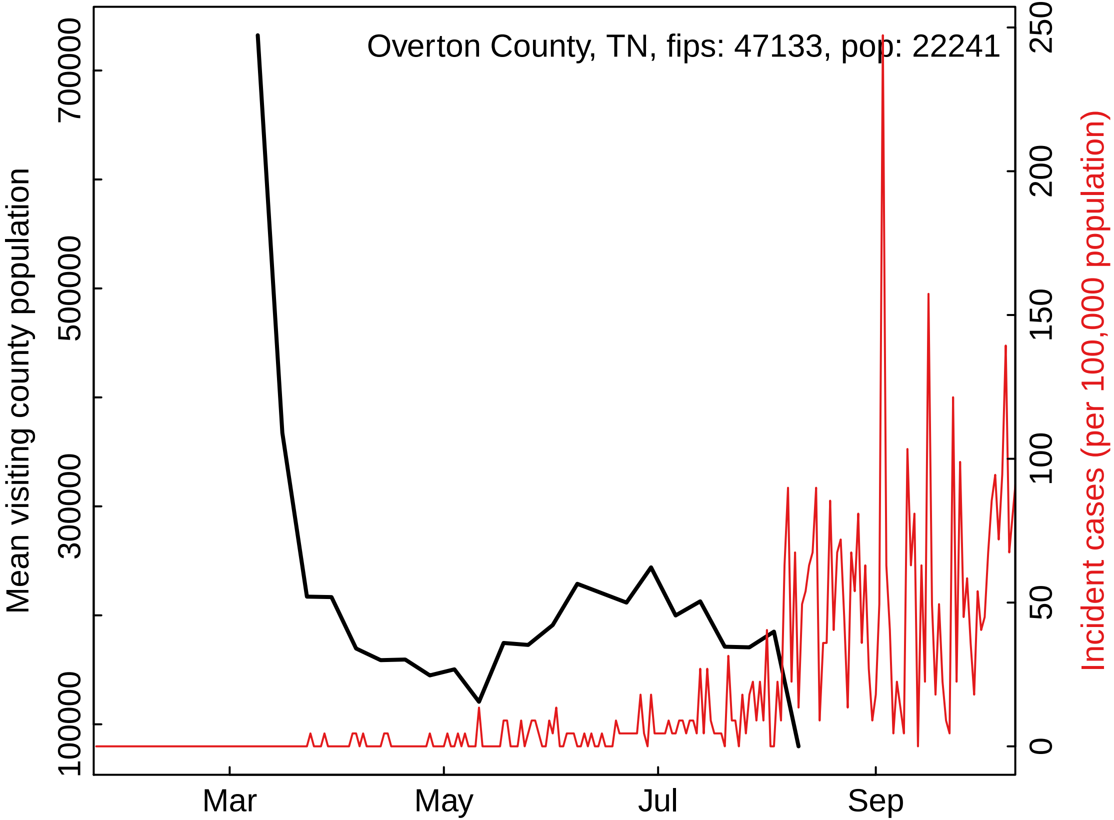

Overton County, TN, fips: 47133, pop: 22241
Mean visiting county population
100000
300000
500000
700000
Incident cases (per 100,000 population)
0
50
100
150
200
250
Mar
May
Jul
Sep

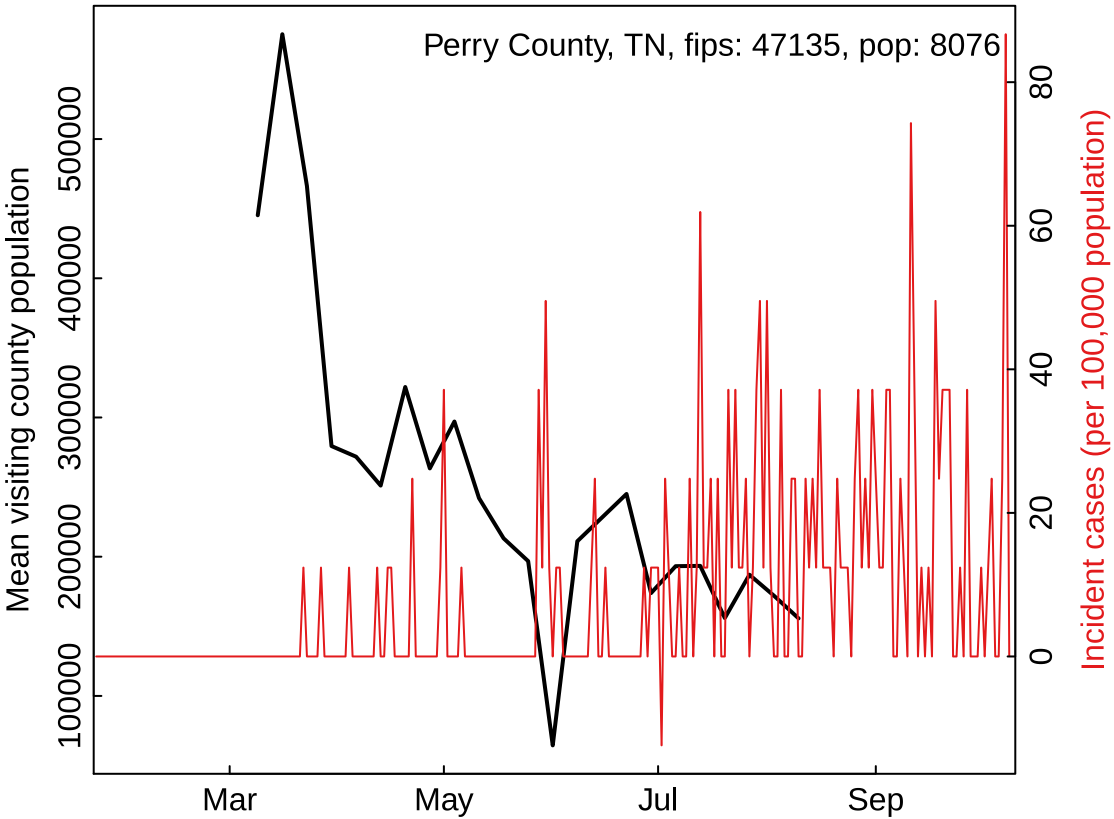
Perry County, TN, fips: 47135, pop: 8076
Mean visiting county population
100000
200000
300000
400000
500000
Incident cases (per 100,000 population)
0
20
40
60
80
Mar
May
Jul
Sep

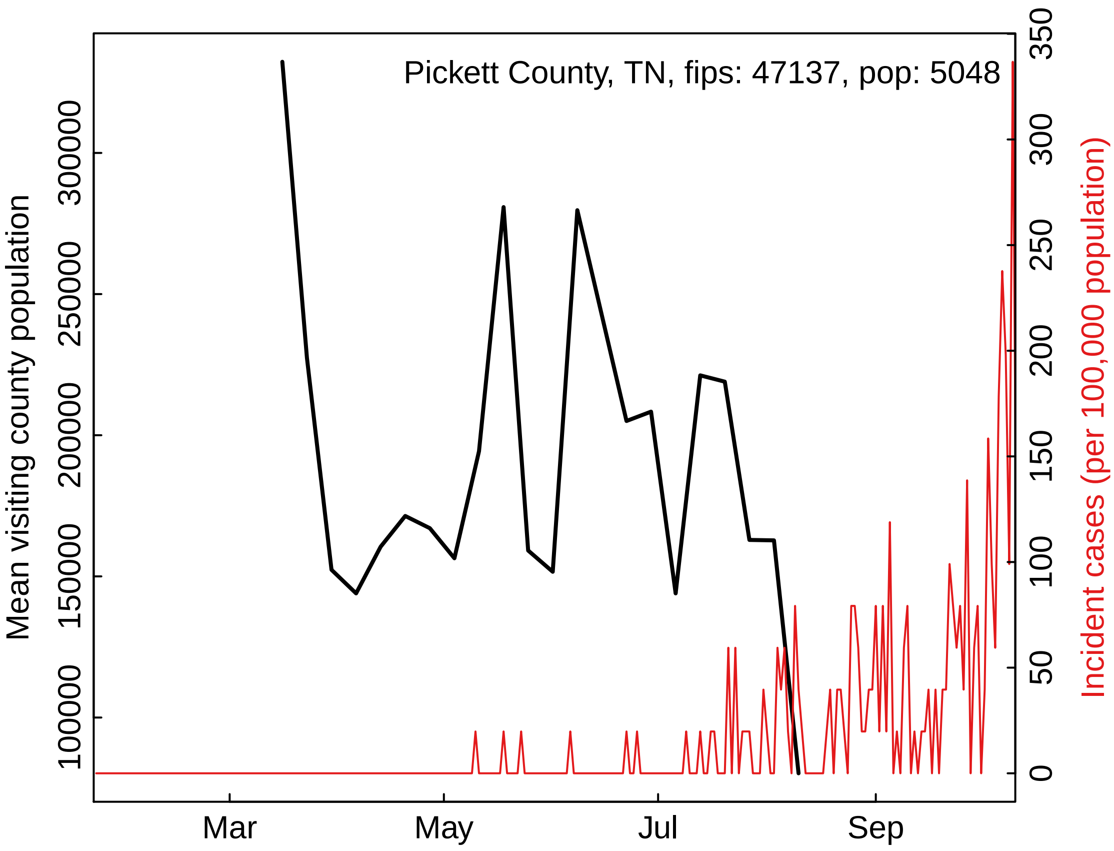

Pickett County, TN, fips: 47137, pop: 5048
Mean visiting county population
100000
150000
200000
250000
300000
Mar
May
Jul
Sep
0
50
100
150
200
250
300
350
Incident cases (per 100,000 population)

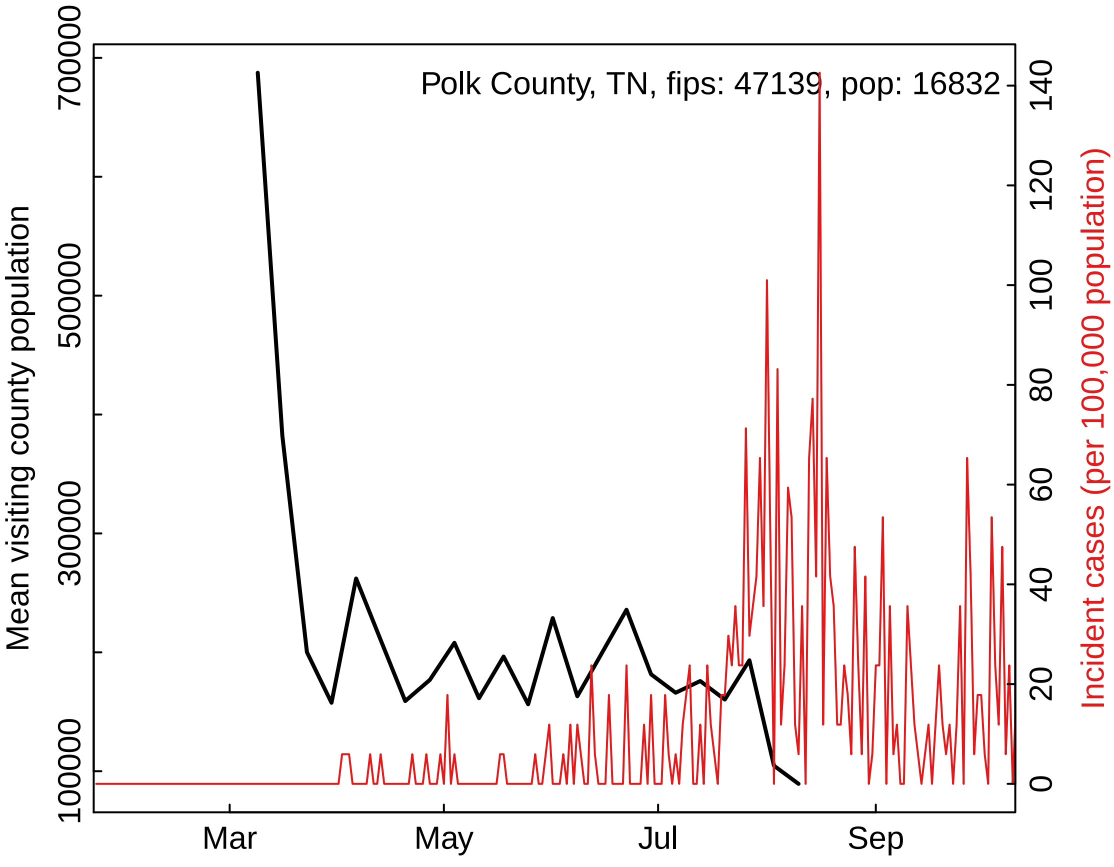

Polk County, TN, fips: 47139, pop: 16832
Mean visiting county population
100000
300000
500000
700000
Incident cases (per 100,000 population)
0
20
40
60
80
100
120
140
Mar
May
Jul
Sep

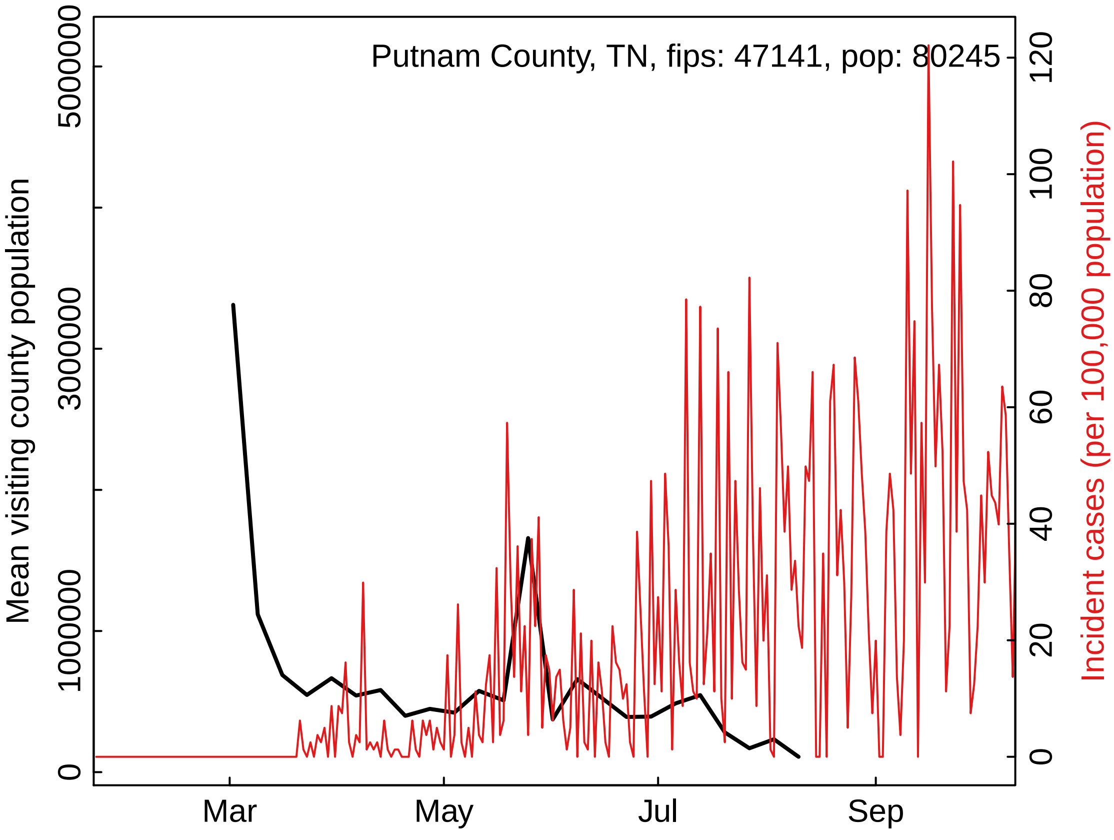

Putnam County, TN, fips: 47141, pop: 80245
Mean visiting county population
0
1000000
3000000
5000000
Incident cases (per 100,000 population)
0
20
40
60
80
100
120
Mar
May
Jul
Sep

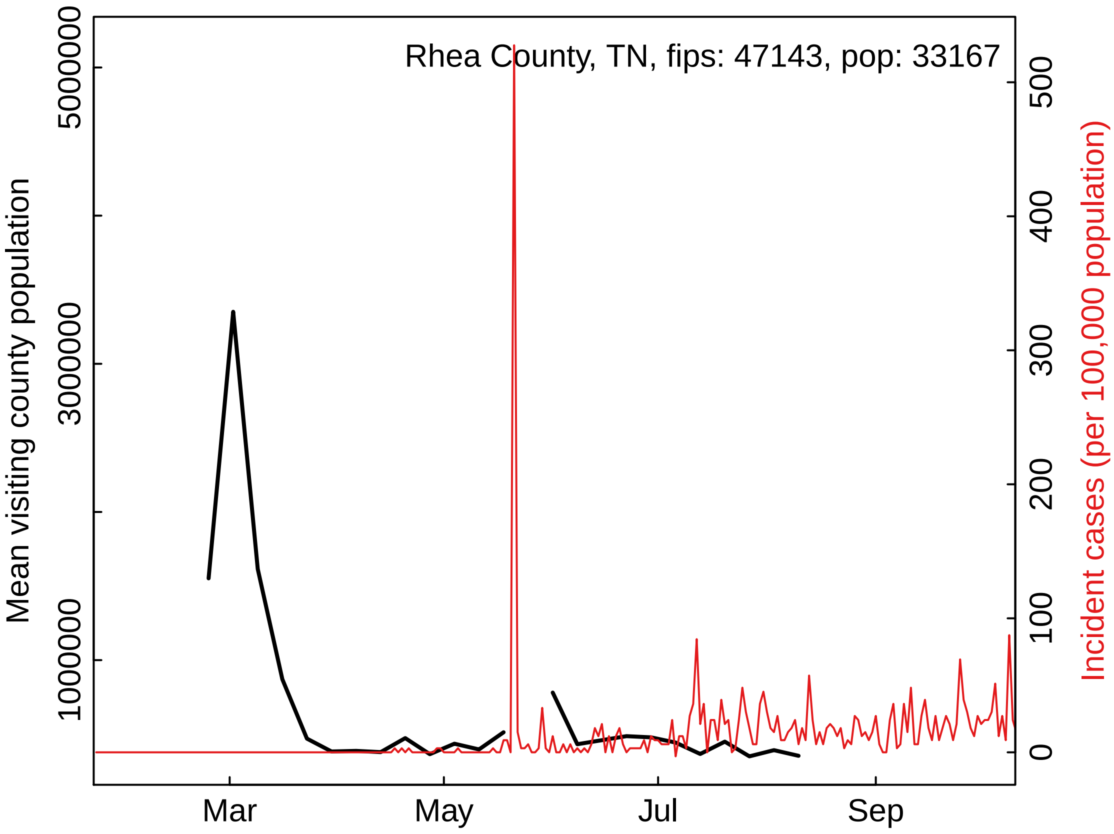

Rhea County, TN, fips: 47143, pop: 33167
Mean visiting county population
1000000
3000000
5000000
Incident cases (per 100,000 population)
0
100
200
300
400
500
Mar
May
Jul
Sep

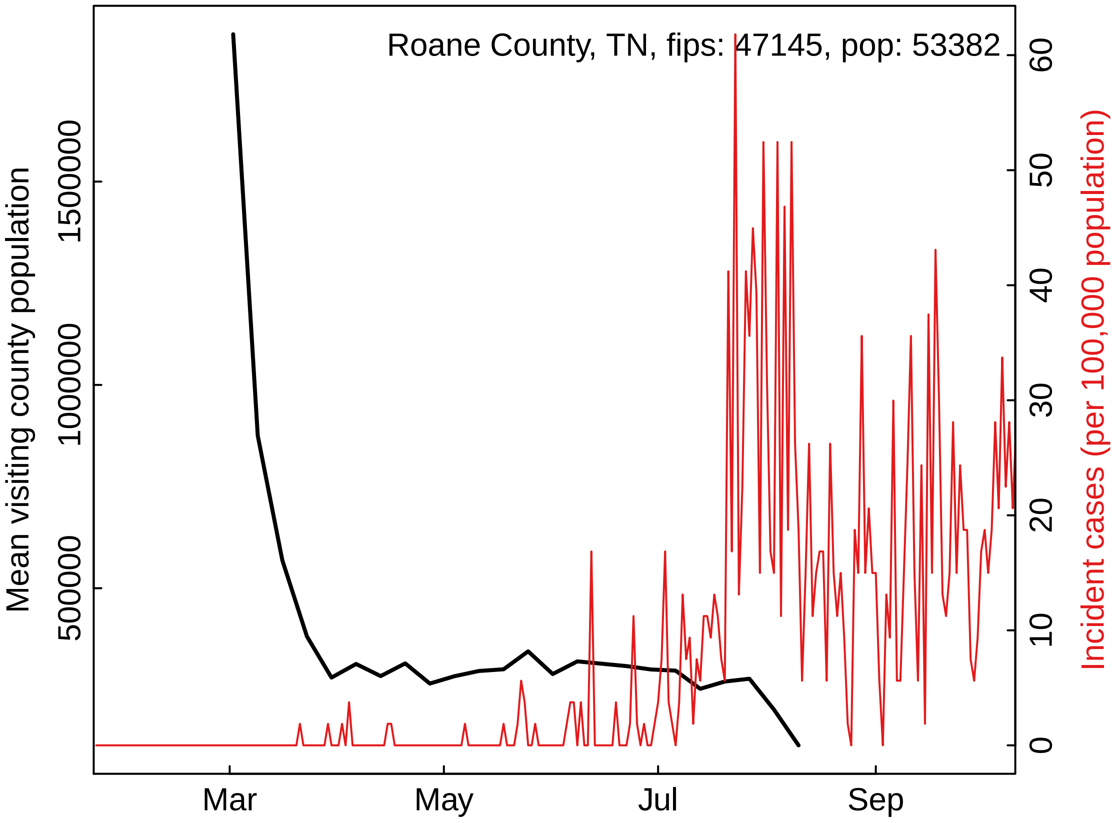

Roane County, TN, fips: 47145, pop: 53382
Mean visiting county population
500000
1000000
1500000
Incident cases (per 100,000 population)
0
10
20
30
40
50
60
Mar
May
Jul
Sep

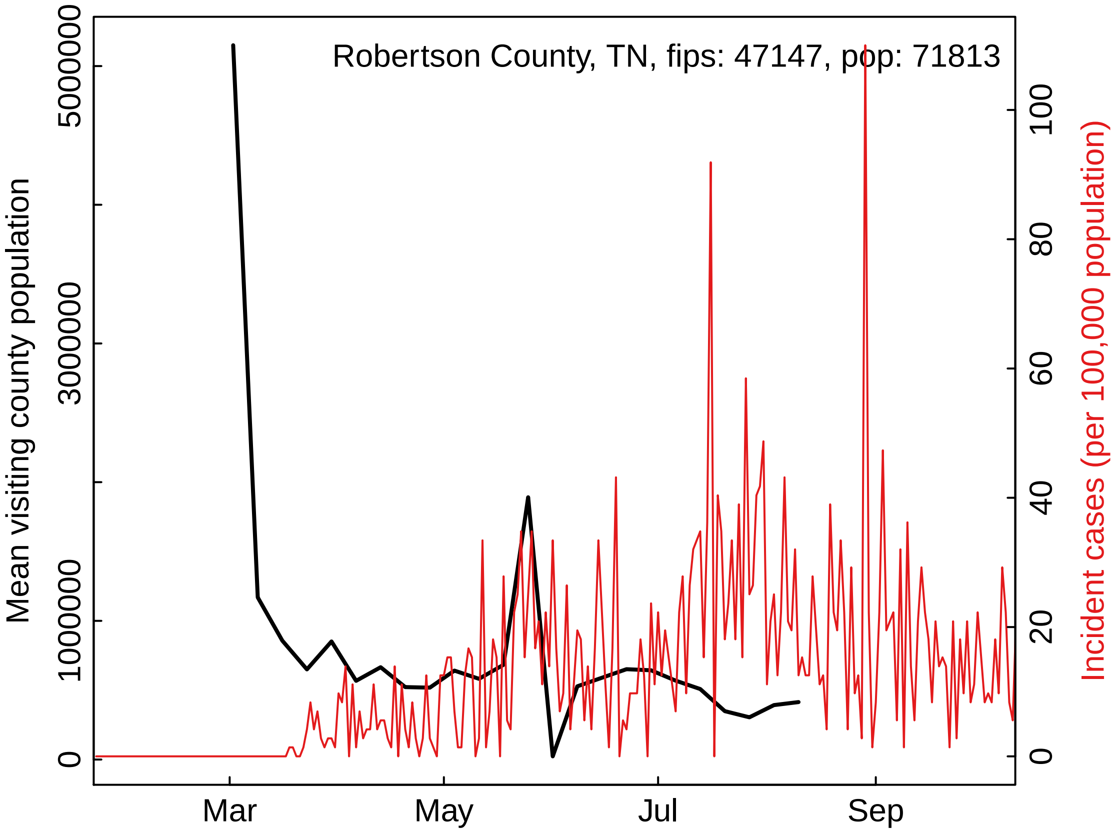

Robertson County, TN, fips: 47147, pop: 71813
Mean visiting county population
0
1000000
3000000
5000000
Incident cases (per 100,000 population)
0
20
40
60
80
100
Mar
May
Jul
Sep

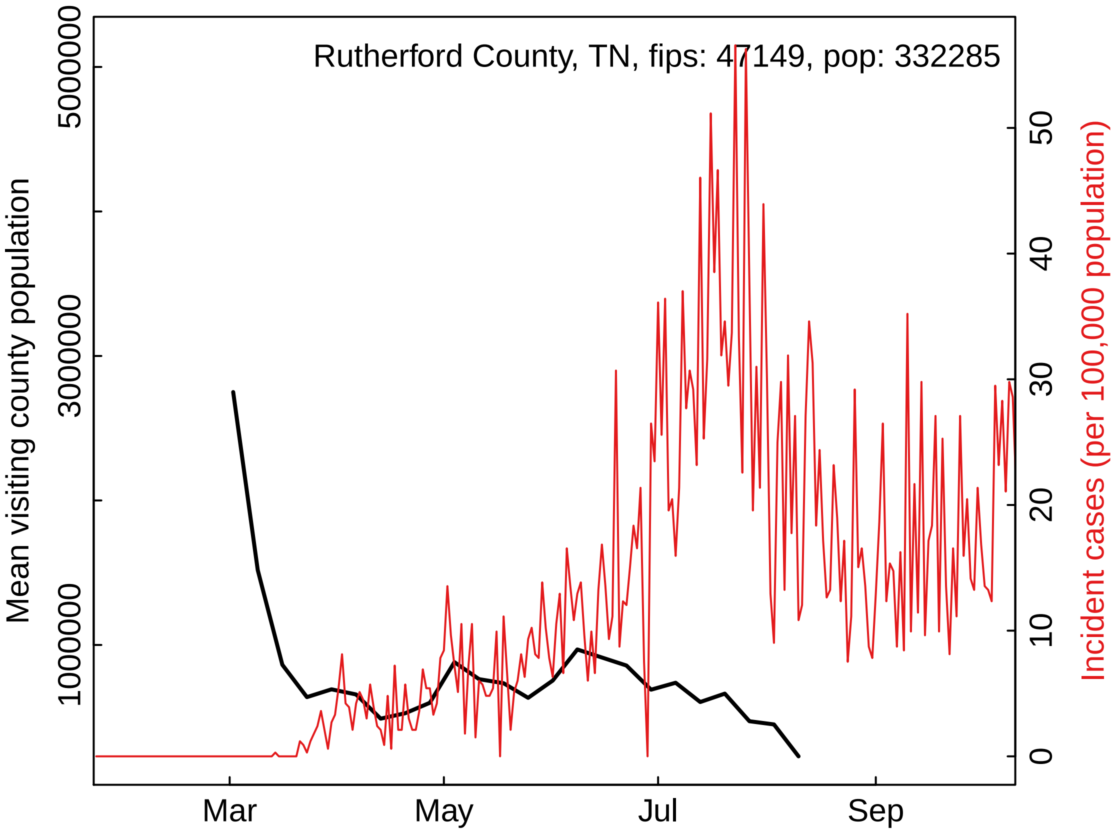

Rutherford County, TN, fips: 47149, pop: 332285
Mean visiting county population
1000000
3000000
5000000
Mar
May
Jul
Sep
0
10
20
30
40
50
Incident cases (per 100,000 population)

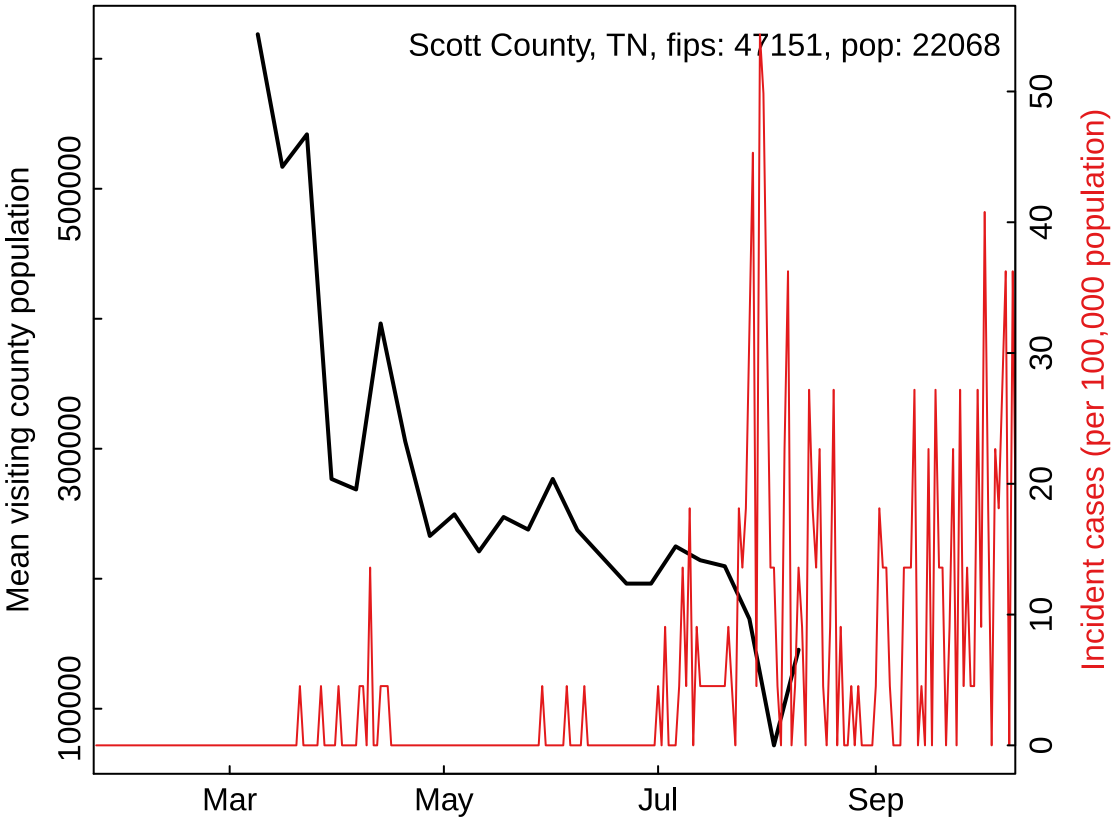

Scott County, TN, fips: 47151, pop: 22068
Mean visiting county population
100000
300000
500000
Incident cases (per 100,000 population)
0
10
20
30
40
50
Mar
May
Jul
Sep

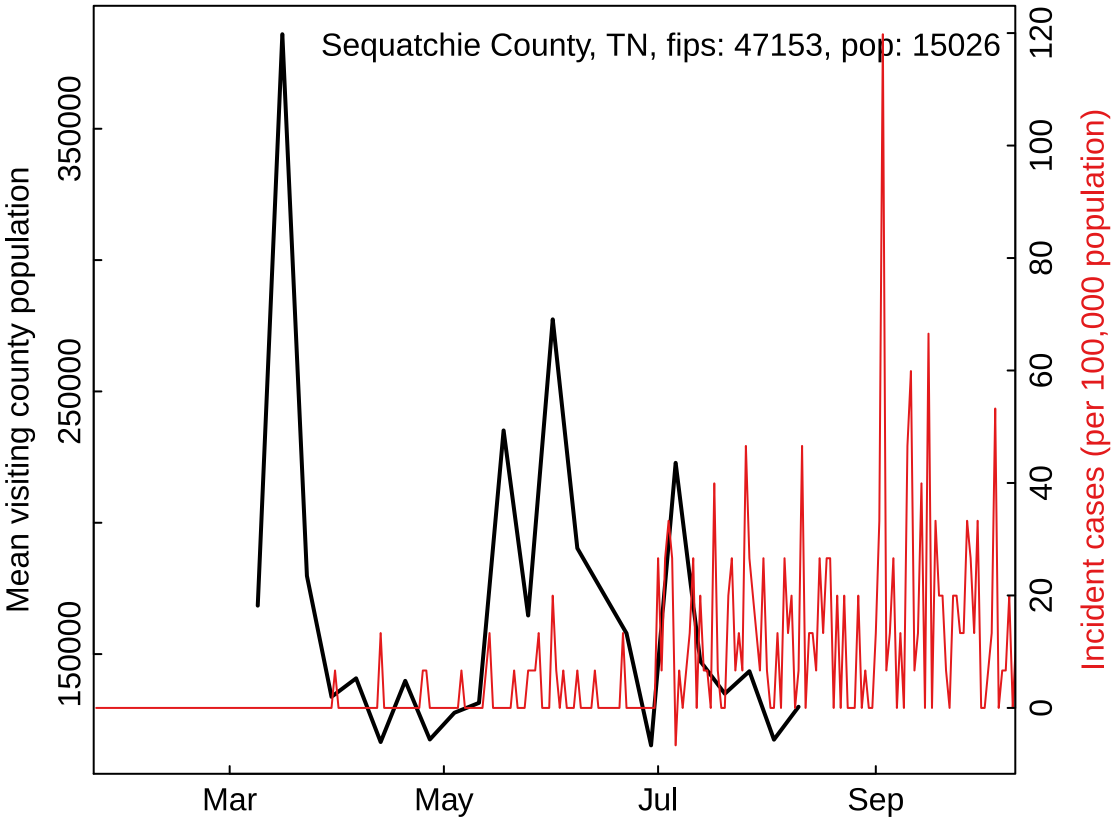

Sequatchie County, TN, fips: 47153, pop: 15026
Mean visiting county population
150000
250000
350000
Incident cases (per 100,000 population)
0
20
40
60
80
100
120
Mar
May
Jul
Sep

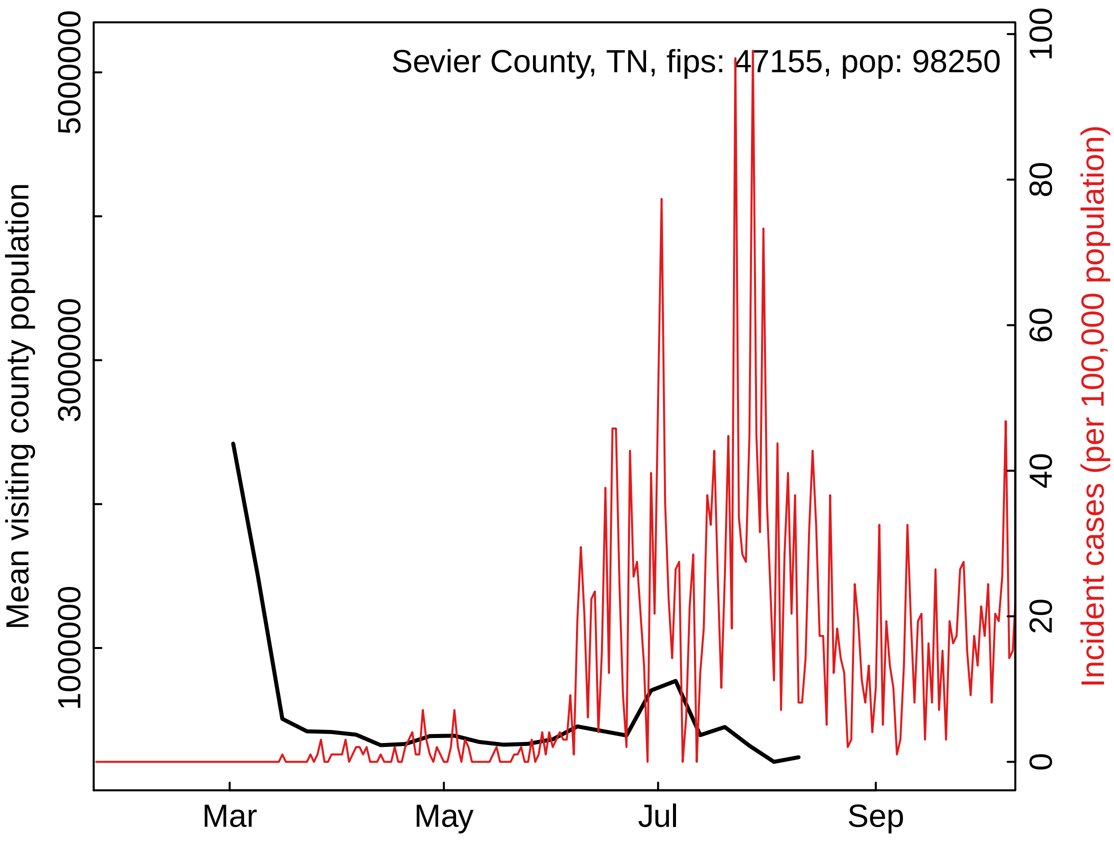
Sevier County, TN, fips: 47155, pop: 98250
Mean visiting county population
1000000
3000000
5000000
Incident cases (per 100,000 population)
0
20
40
60
80
100
Mar
May
Jul
Sep

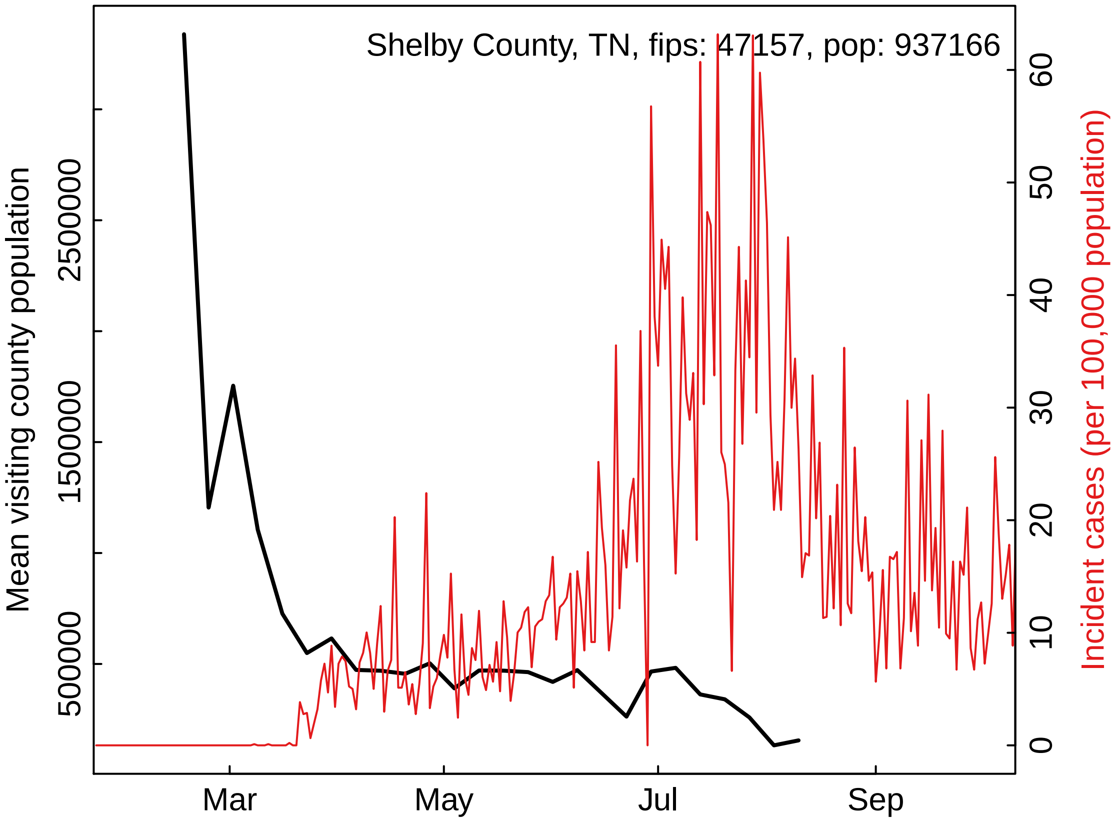

Shelby County, TN, fips: 47157, pop: 937166
Mean visiting county population
500000
1500000
2500000
Incident cases (per 100,000 population)
0
10
20
30
40
50
60
Mar
May
Jul
Sep

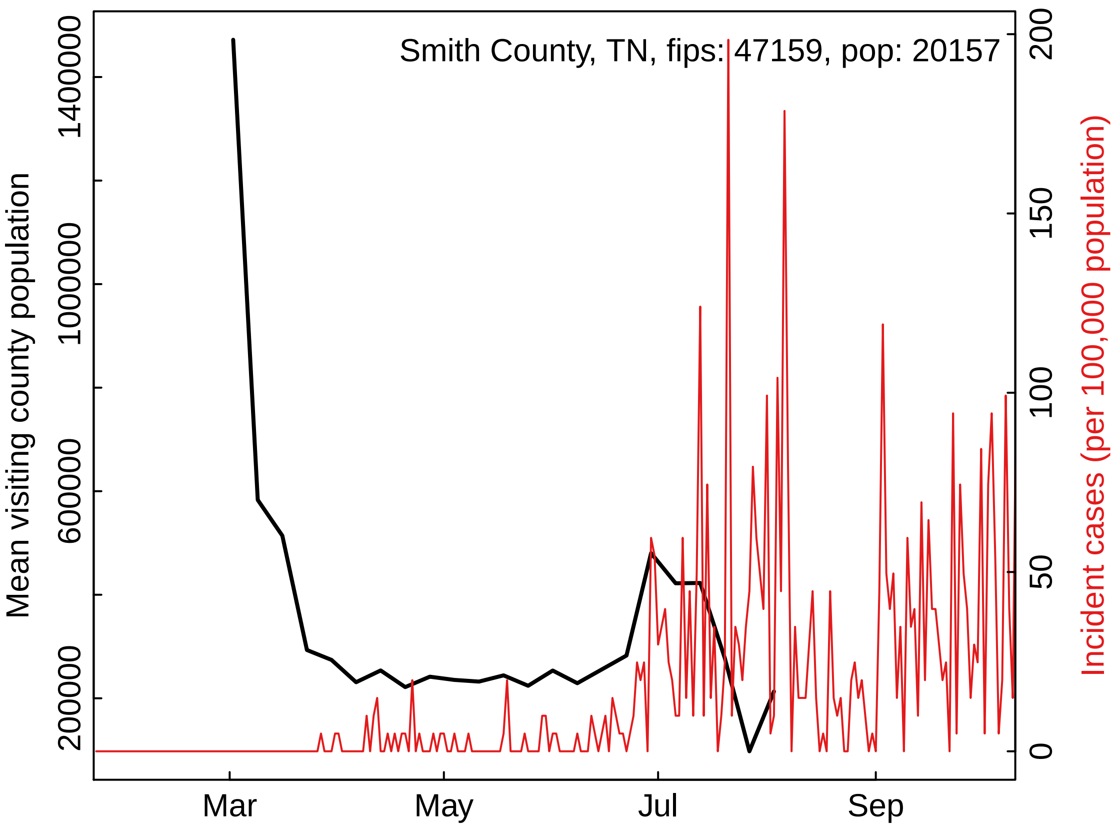
Smith County, TN, fips: 47159, pop: 20157
Mean visiting county population
200000
600000
1000000
1400000
Incident cases (per 100,000 population)
0
50
100
150
200
Mar
May
Jul
Sep

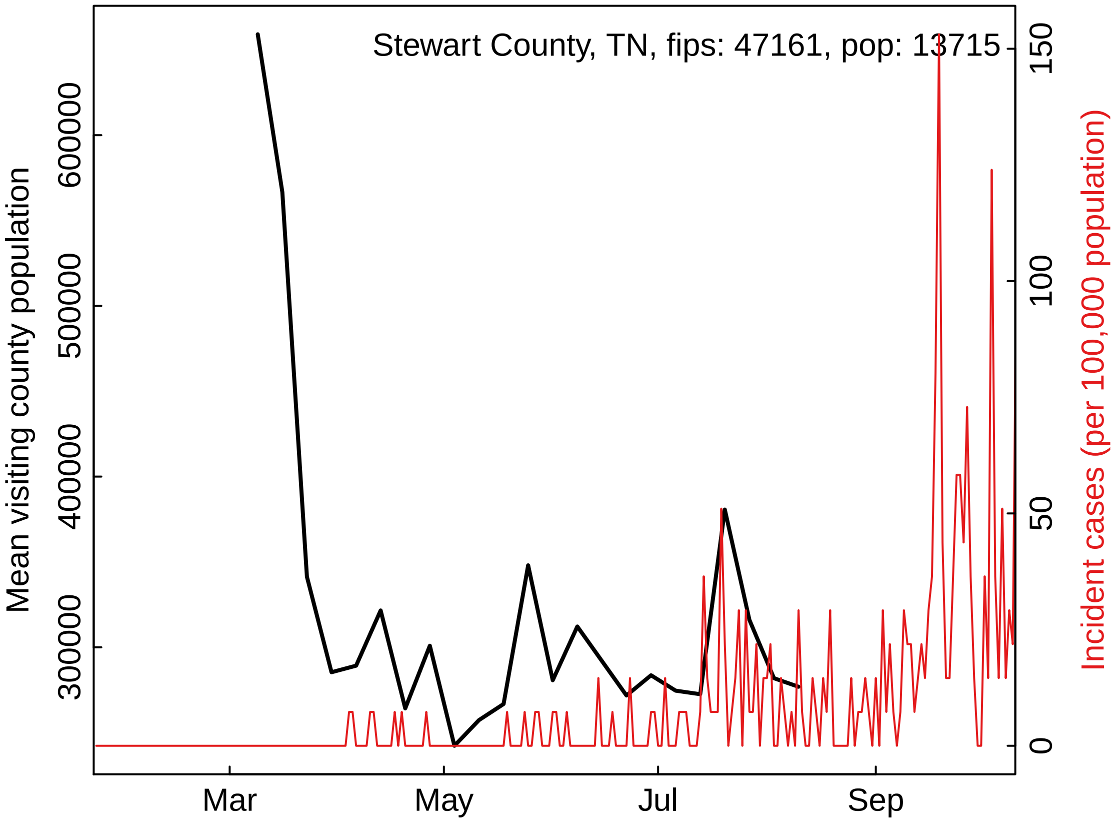

Stewart County, TN, fips: 47161, pop: 13715
Mean visiting county population
300000
400000
500000
600000
Incident cases (per 100,000 population)
0
50
100
150
Mar
May
Jul
Sep

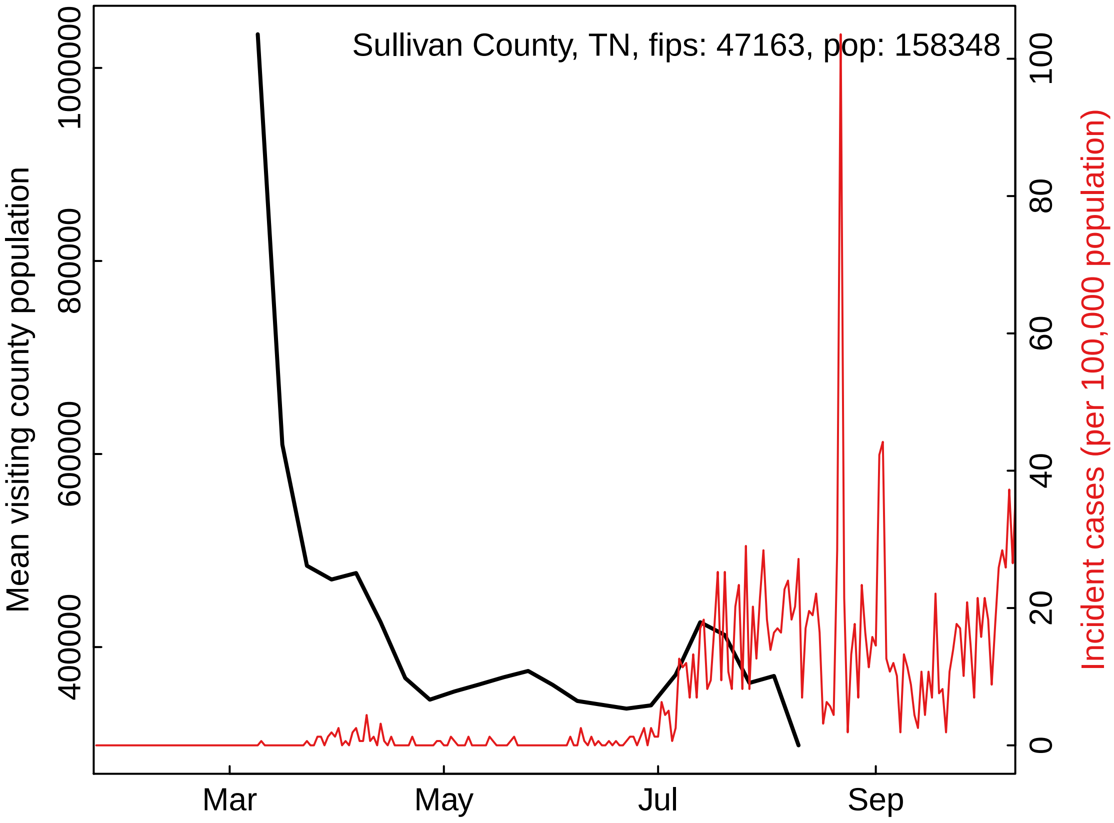

Sullivan County, TN, fips: 47163, pop: 158348
Mean visiting county population
400000
600000
800000
1000000
Incident cases (per 100,000 population)
0
20
40
60
80
100
Mar
May
Jul
Sep

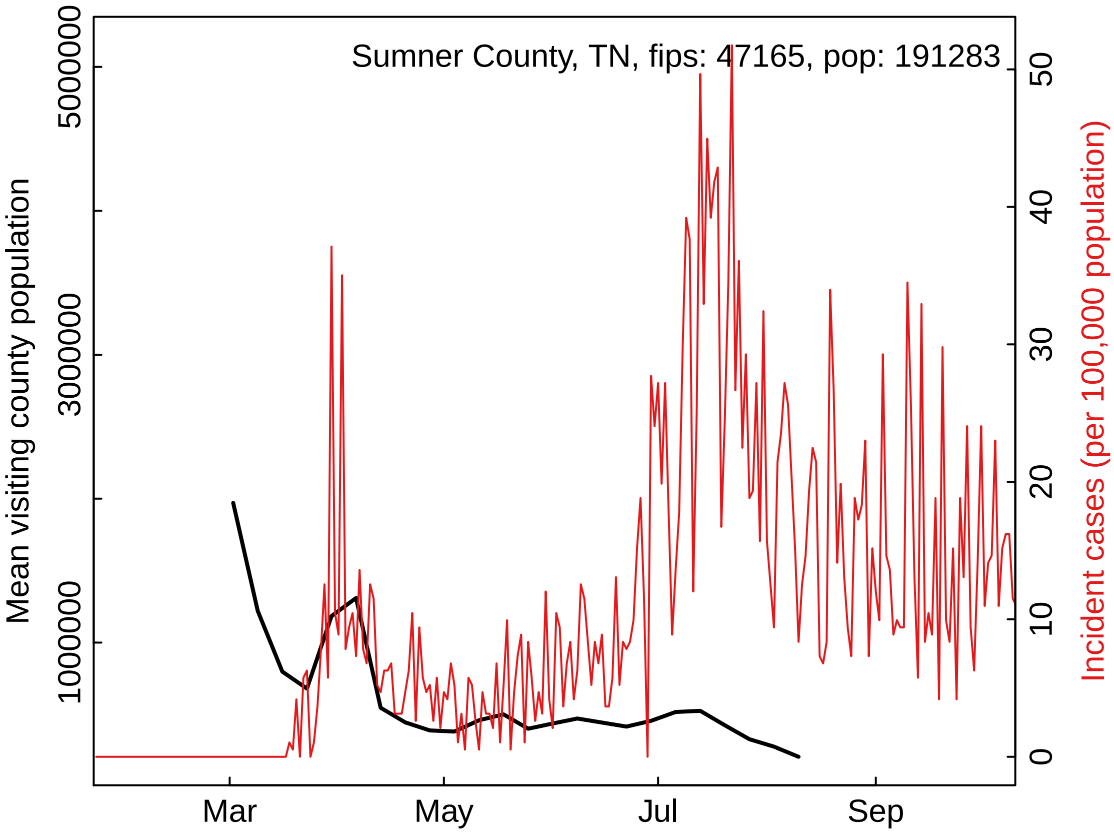

Sumner County, TN, fips: 47165, pop: 191283
Mean visiting county population
1000000
3000000
5000000
Incident cases (per 100,000 population)
0
10
20
30
40
50
Mar
May
Jul
Sep

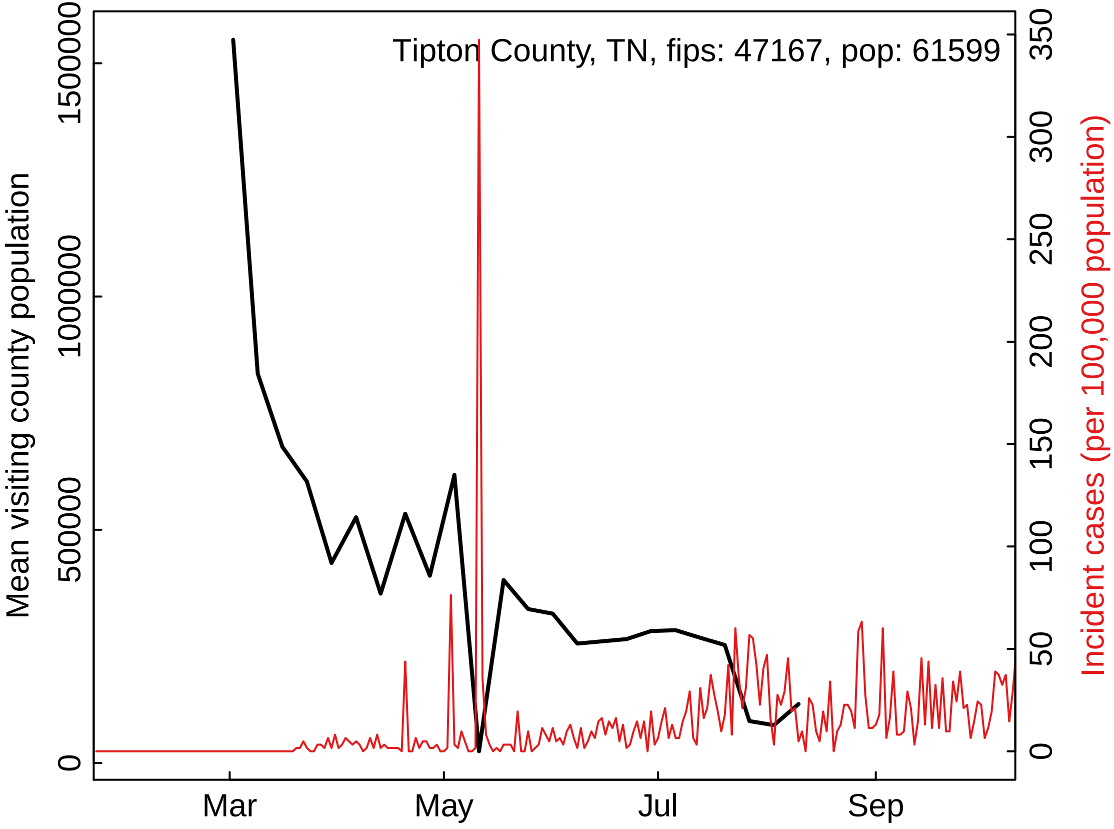

Tipton County, TN, fips: 47167, pop: 61599
Mean visiting county population
0
500000
1000000
1500000
Incident cases (per 100,000 population)
0
50
100
150
200
250
300
350
Mar
May
Jul
Sep

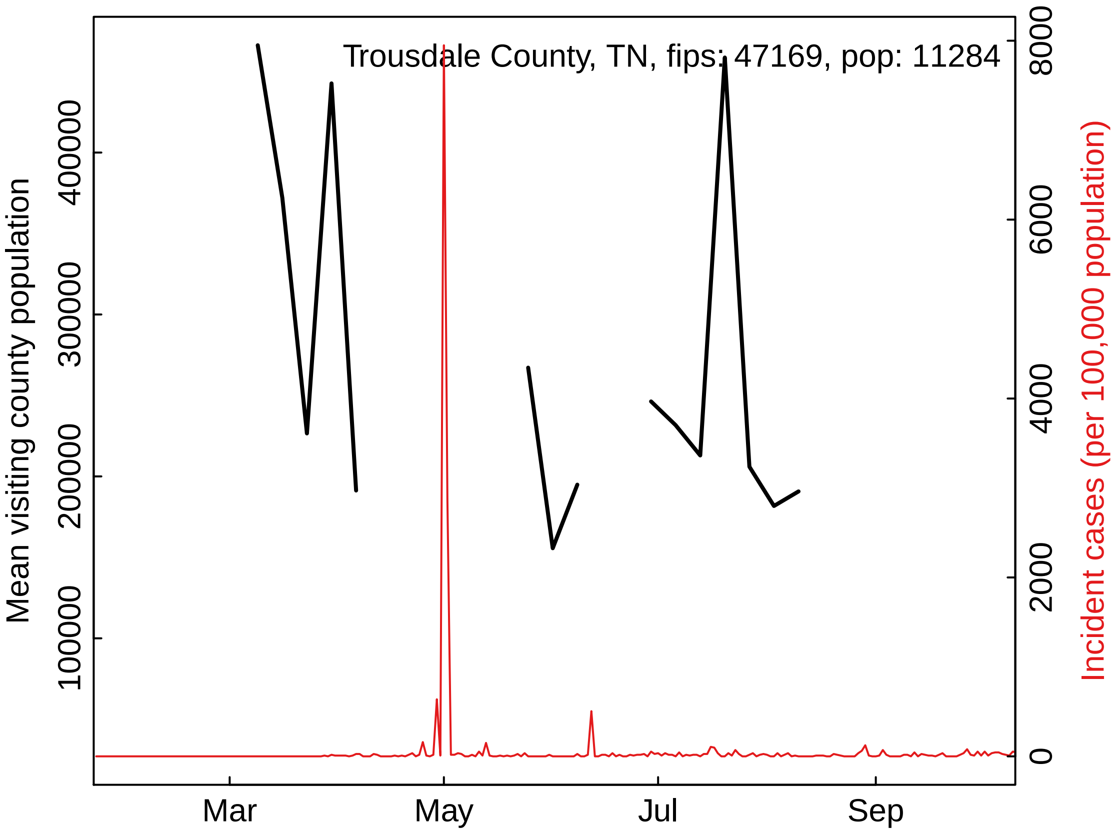
Trousdale County, TN, fips: 47169, pop: 11284
Mean visiting county population
100000
200000
300000
400000
Incident cases (per 100,000 population)
0
2000
4000
6000
8000
Mar
May
Jul
Sep

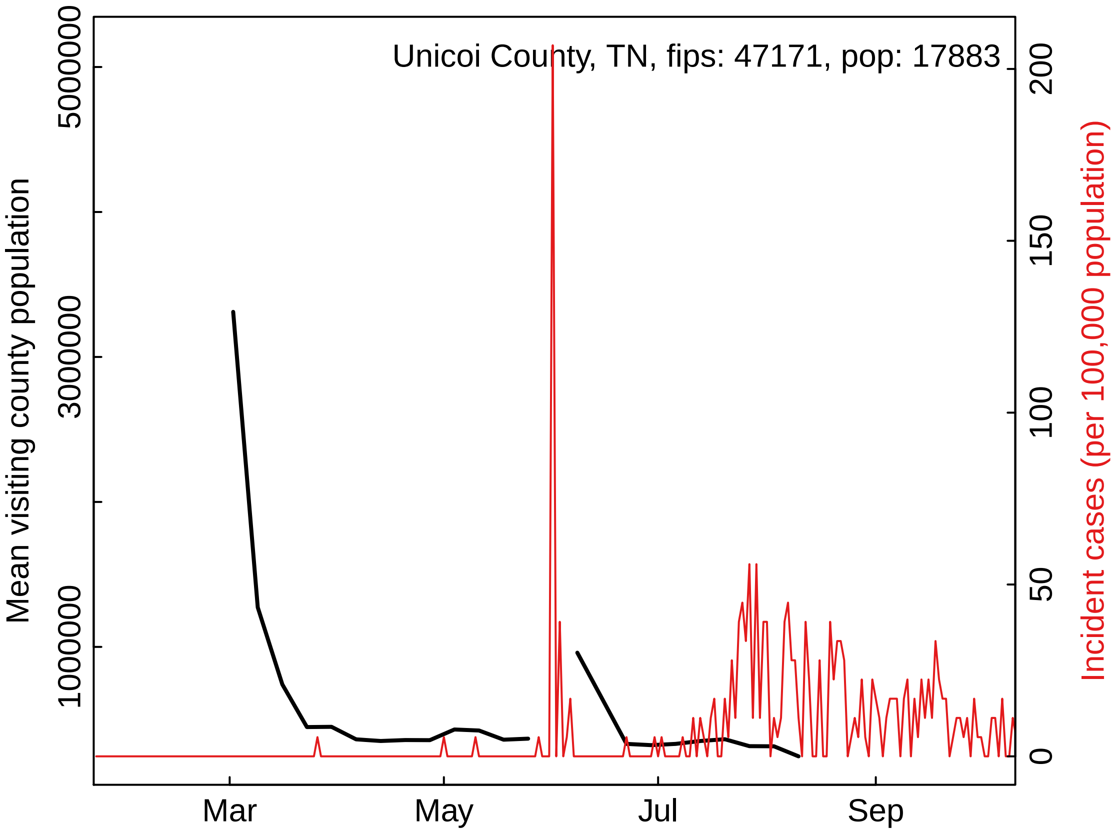

Unicoi County, TN, fips: 47171, pop: 17883
Mean visiting county population
1000000
3000000
5000000
Incident cases (per 100,000 population)
0
50
100
150
200
Mar
May
Jul
Sep

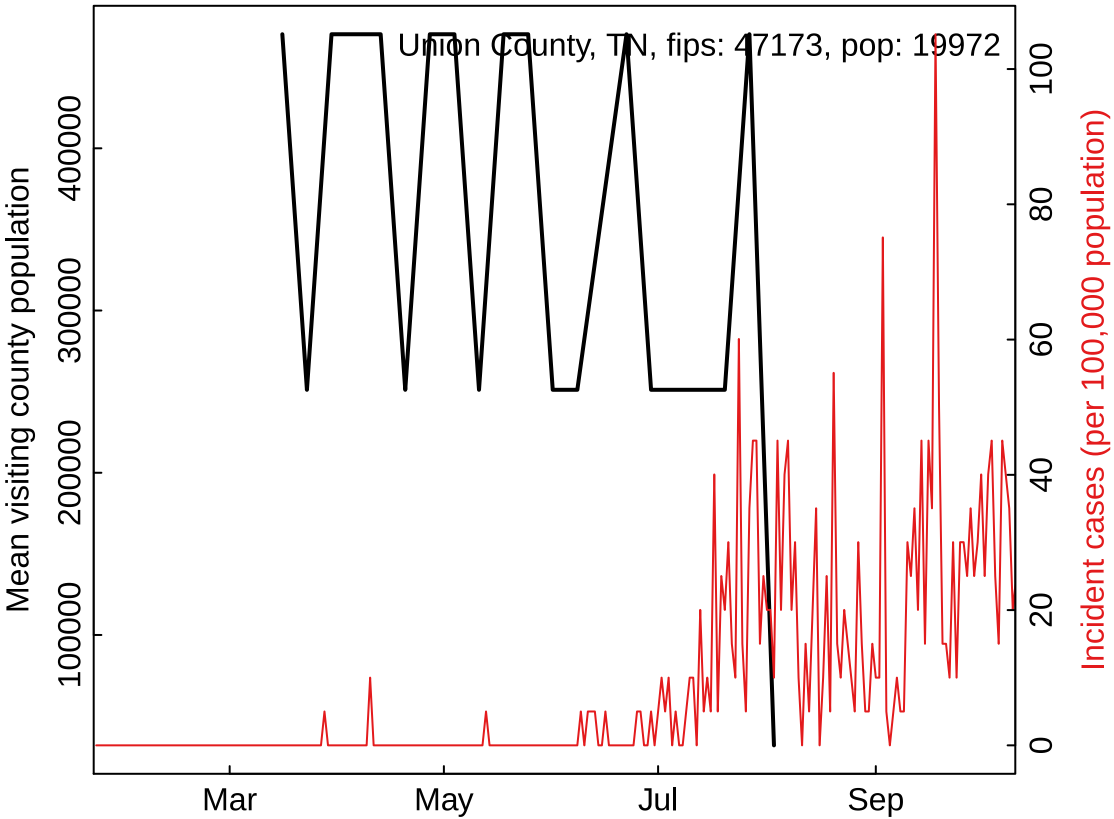

Union County, TN, fips: 47173, pop: 19972
Mean visiting county population
100000
200000
300000
400000
Incident cases (per 100,000 population)
0
20
40
60
80
100
Mar
May
Jul
Sep

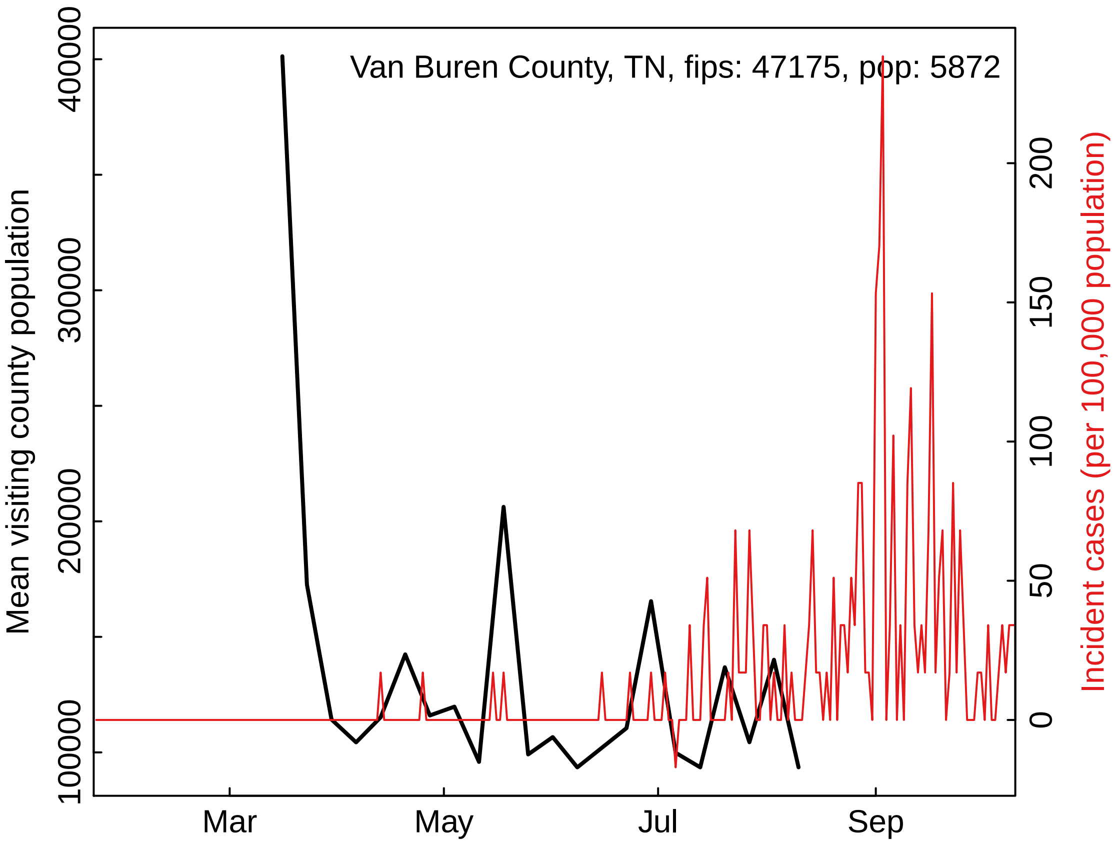
Van Buren County, TN, fips: 47175, pop: 5872
Mean visiting county population
100000
200000
300000
400000
Incident cases (per 100,000 population)
0
50
100
150
200
Mar
May
Jul
Sep

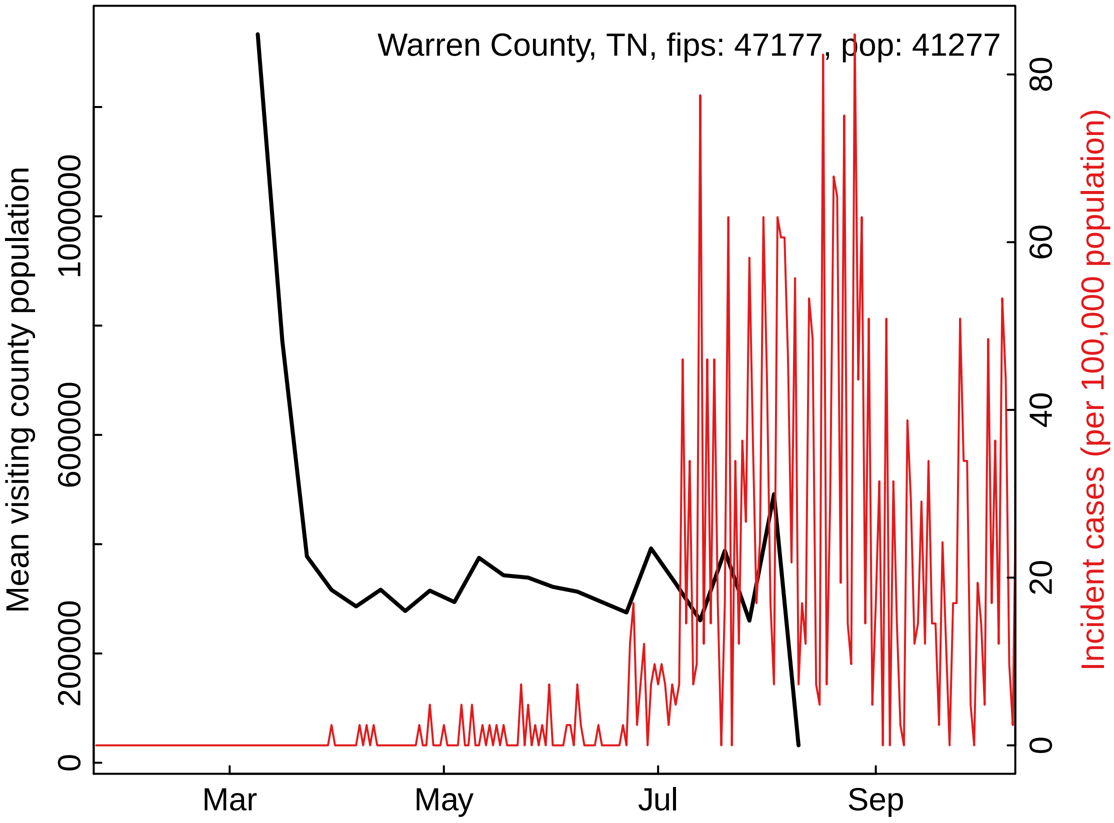

Warren County, TN, fips: 47177, pop: 41277
Mean visiting county population
Incident cases (per 100,000 population)
0
200000
600000
1000000
0
20
40
60
80
Mar
May
Jul
Sep

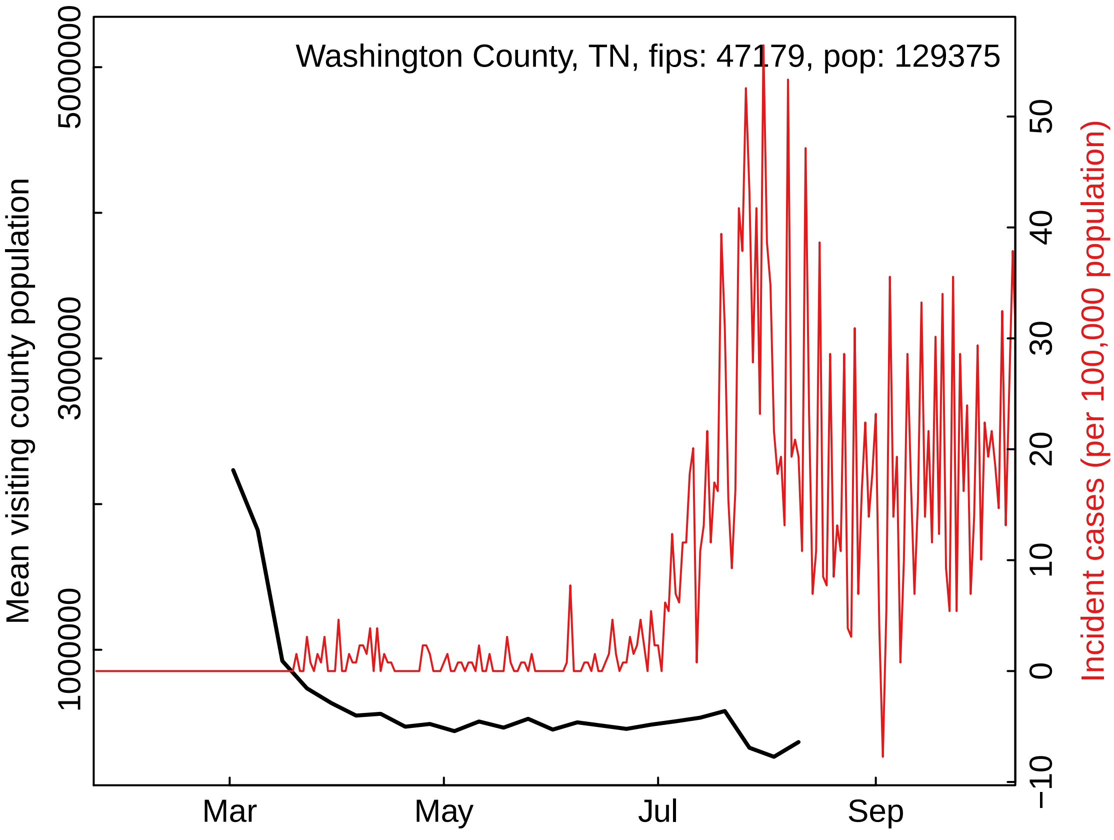

Washington County, TN, fips: 47179, pop: 129375
Mean visiting county population
1000000
3000000
5000000
Incident cases (per 100,000 population)
−10
0
10
20
30
40
50
Mar
May
Jul
Sep

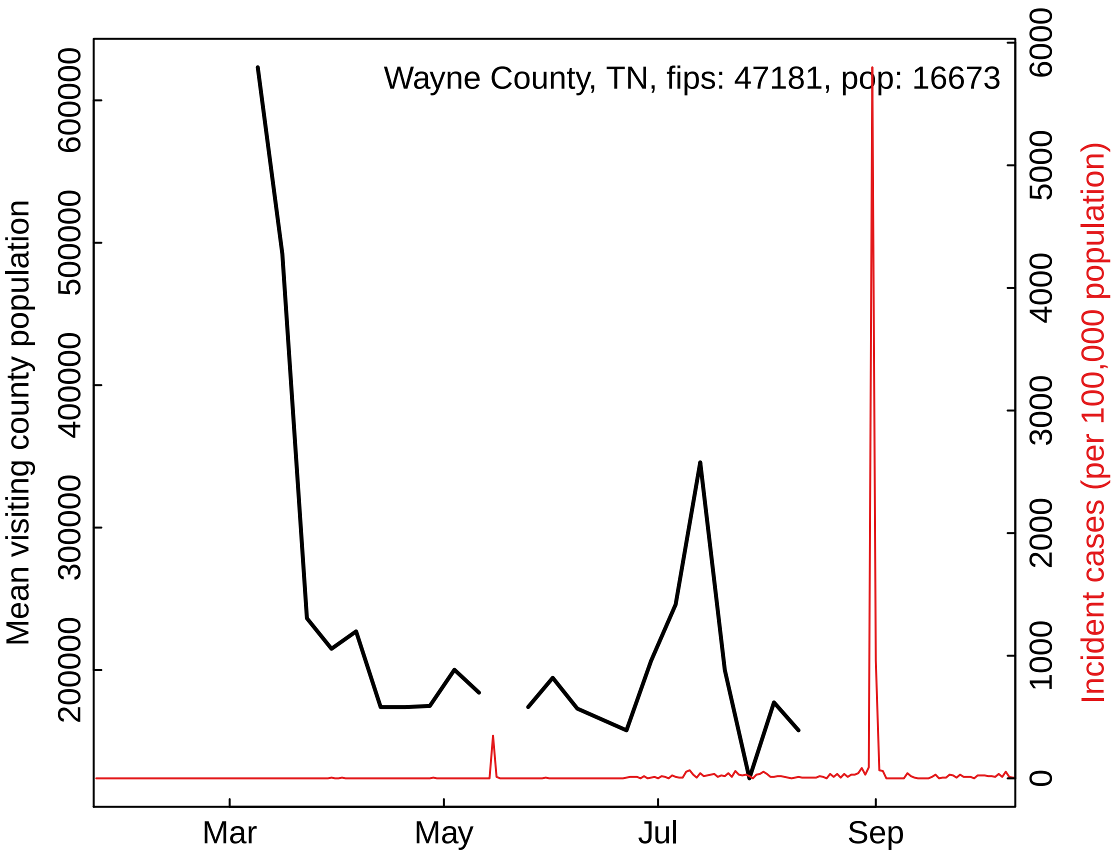
Wayne County, TN, fips: 47181, pop: 16673
Mean visiting county population
200000
300000
400000
500000
600000
Incident cases (per 100,000 population)
0
1000
2000
3000
4000
5000
6000
Mar
May
Jul
Sep

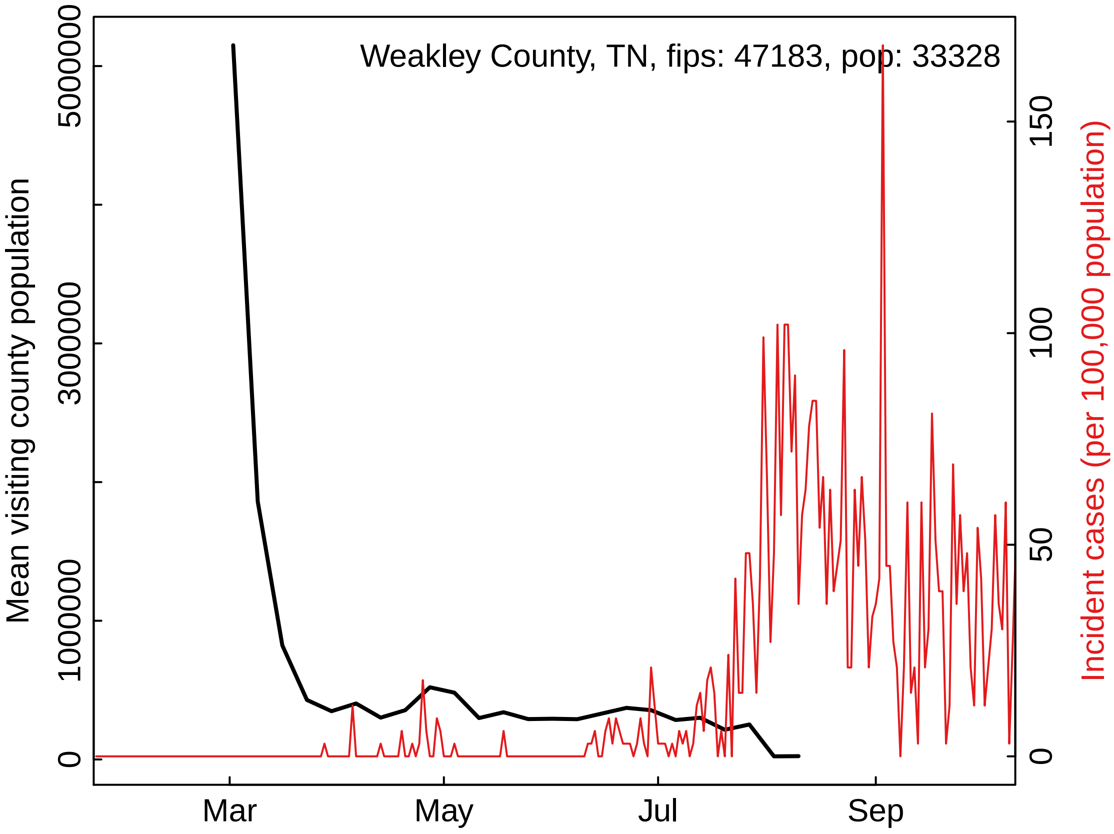

Weakley County, TN, fips: 47183, pop: 33328
Mean visiting county population
0
1000000
3000000
5000000
Incident cases (per 100,000 population)
0
50
100
150
Mar
May
Jul
Sep

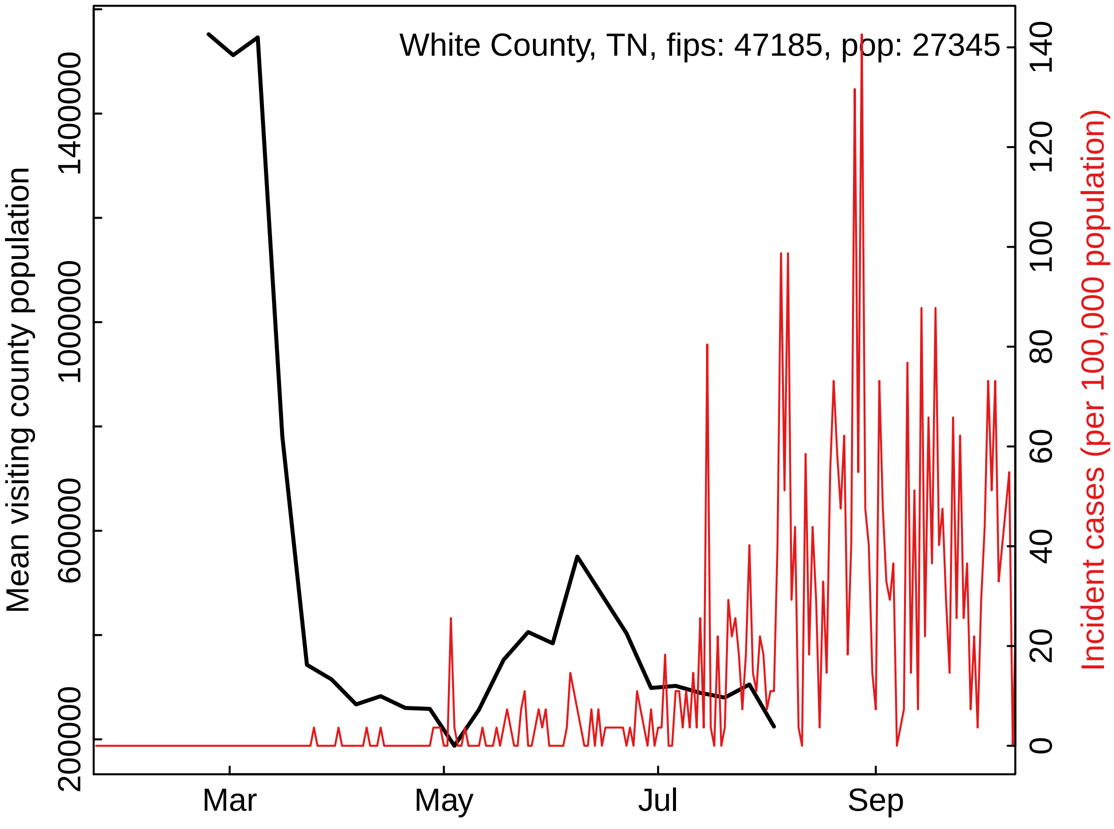

White County, TN, fips: 47185, pop: 27345
Mean visiting county population
200000
600000
1000000
1400000
Mar
May
Jul
Sep
0
20
40
60
80
100
120
140
Incident cases (per 100,000 population)

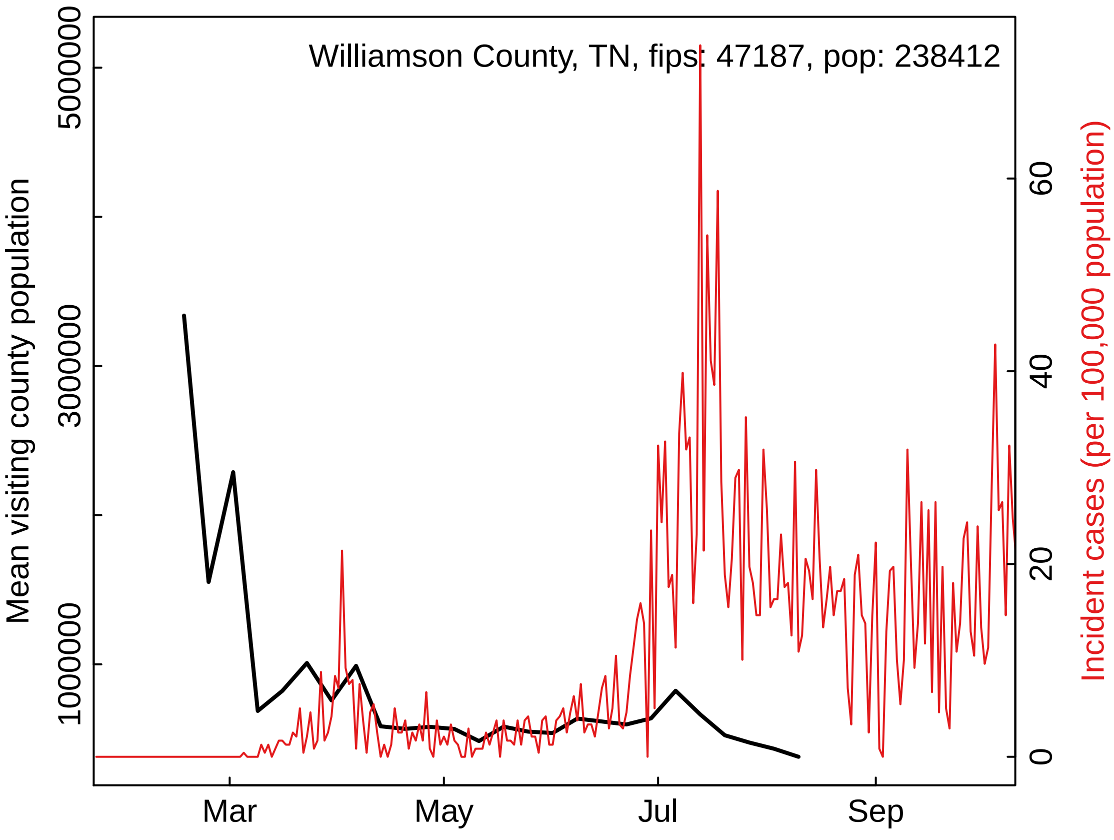

Williamson County, TN, fips: 47187, pop: 238412
Mean visiting county population
1000000
3000000
5000000
Incident cases (per 100,000 population)
0
20
40
60
Mar
May
Jul
Sep

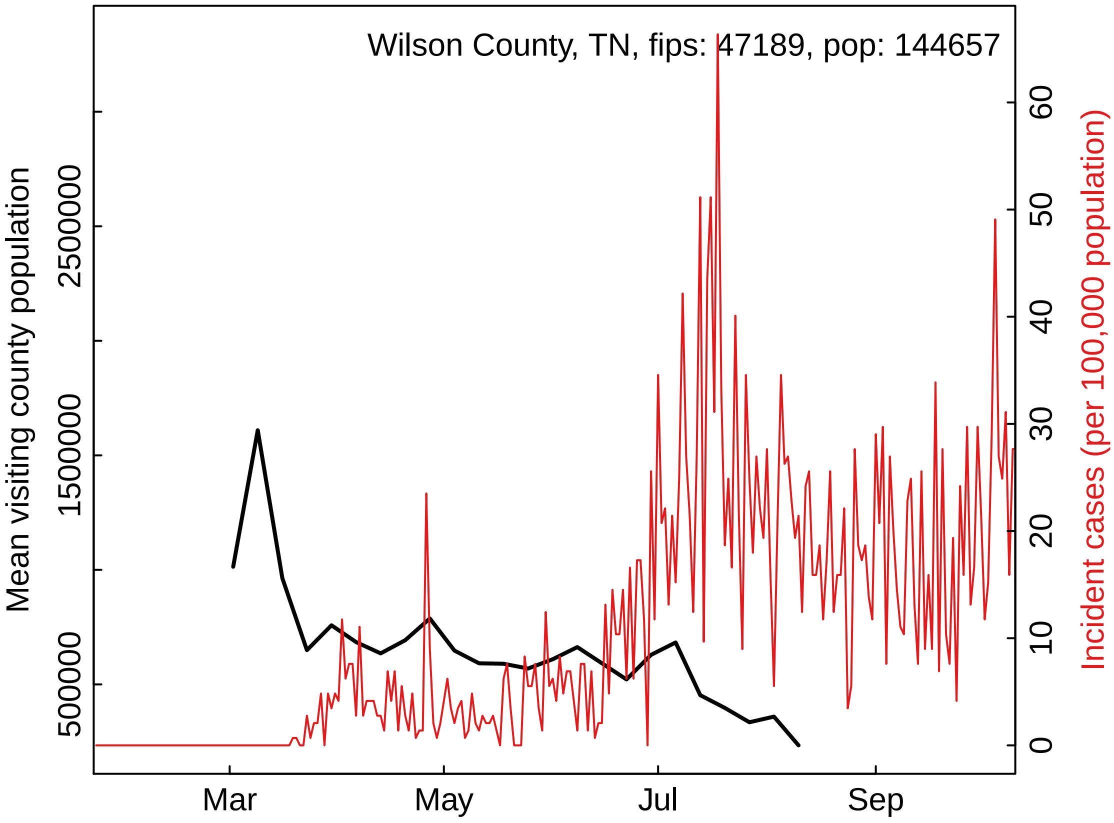

Wilson County, TN, fips: 47189, pop: 144657
Mean visiting county population
500000
1500000
2500000
Incident cases (per 100,000 population)
0
10
20
30
40
50
60
Mar
May
Jul
Sep

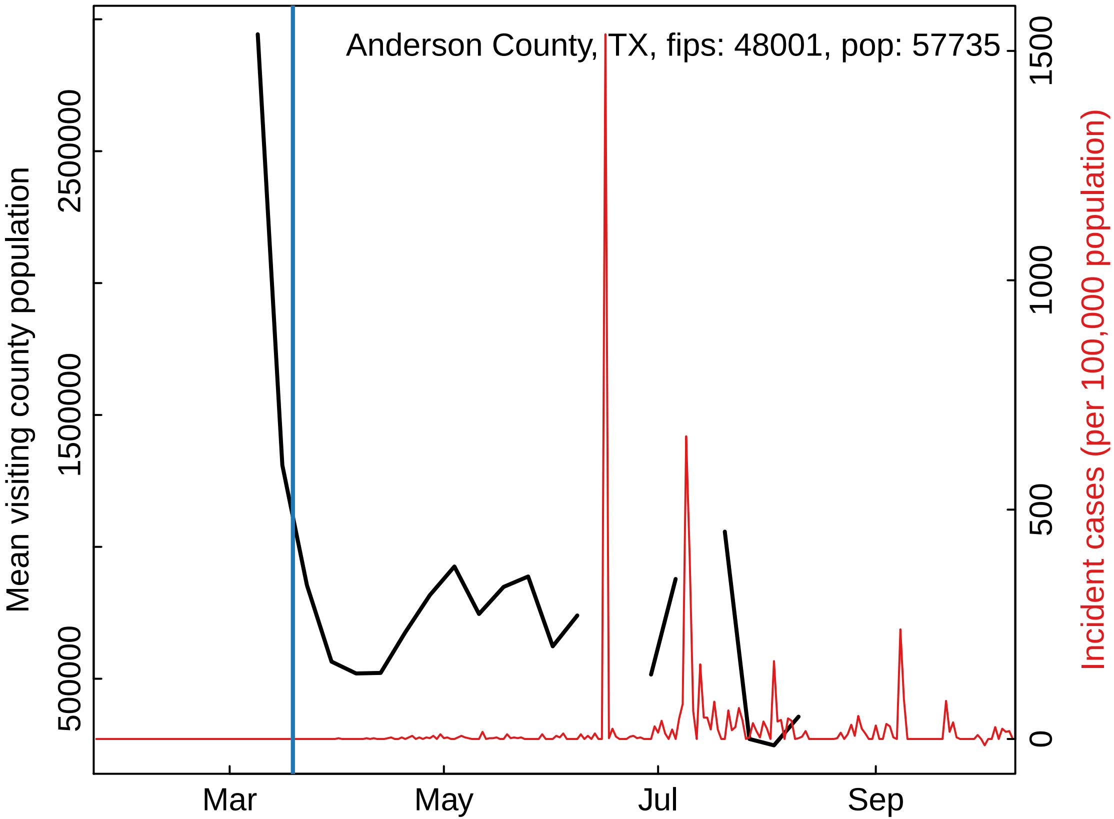
Anderson County, TX, fips: 48001, pop: 57735
Mean visiting county population
500000
1500000
2500000
Incident cases (per 100,000 population)
0
500
1000
1500
Mar
May
Jul
Sep

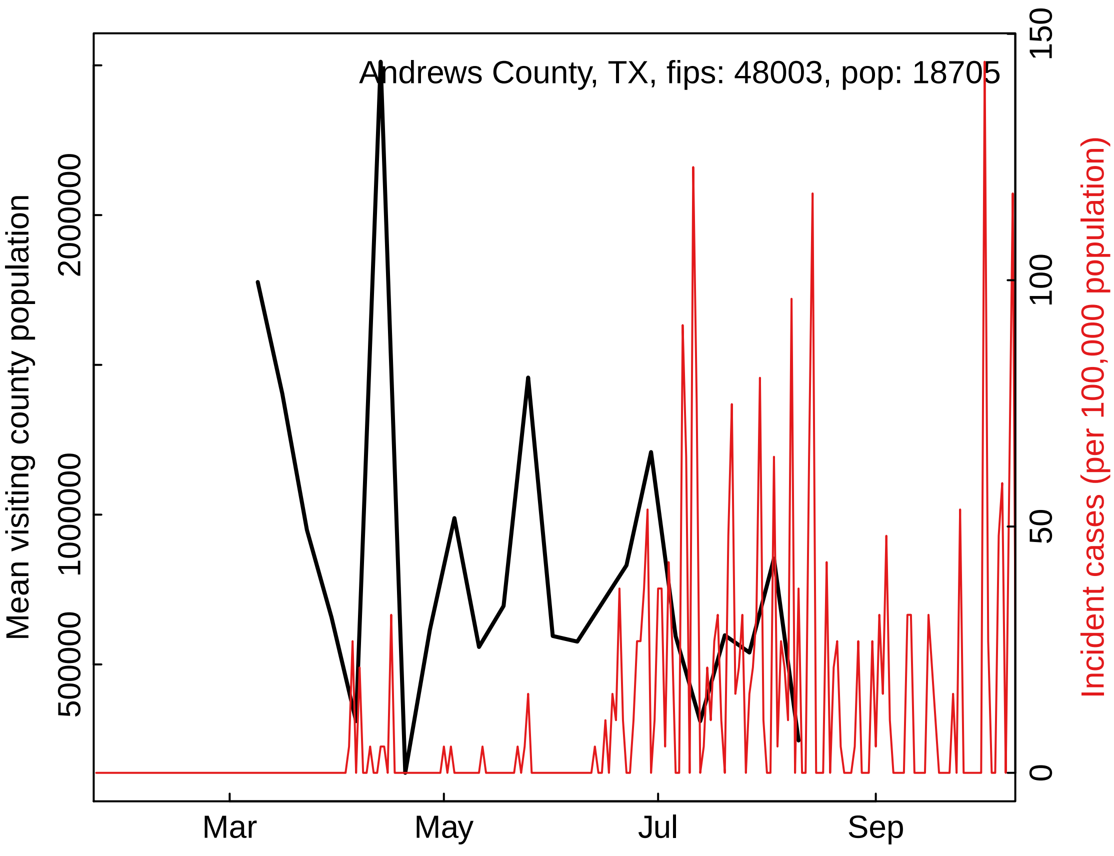
Andrews County, TX, fips: 48003, pop: 18705
Mean visiting county population
500000
1000000
2000000
Mar
May
Jul
Sep
0
50
100
150
Incident cases (per 100,000 population)

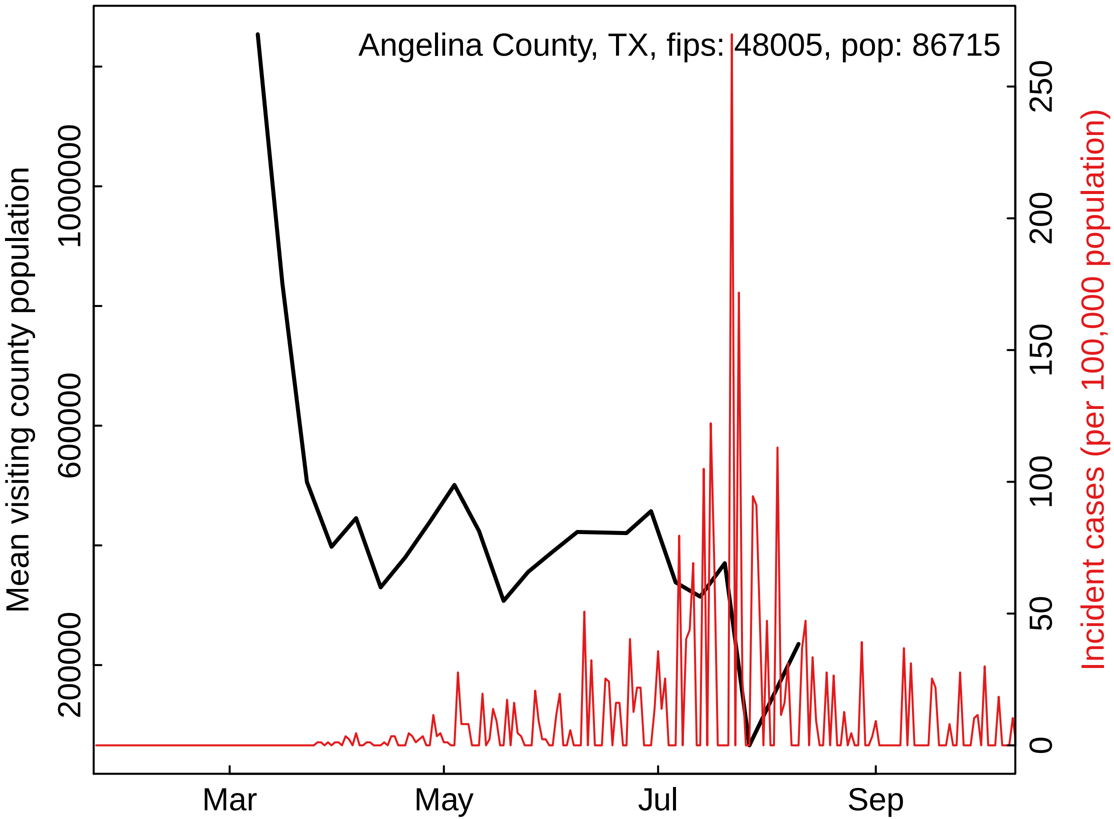

Angelina County, TX, fips: 48005, pop: 86715
Mean visiting county population
200000
600000
1000000
Incident cases (per 100,000 population)
0
50
100
150
200
250
Mar
May
Jul
Sep

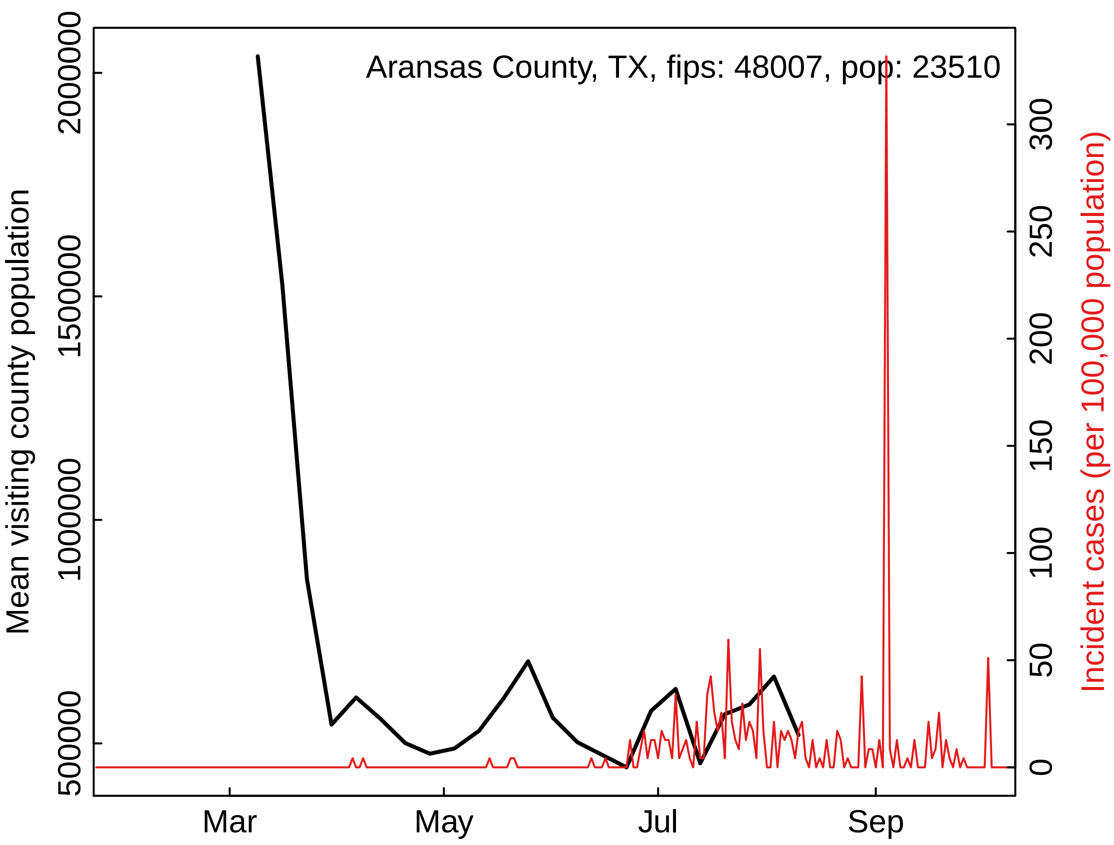

Aransas County, TX, fips: 48007, pop: 23510
Mean visiting county population
500000
1000000
1500000
2000000
Incident cases (per 100,000 population)
0
50
100
150
200
250
300
Mar
May
Jul
Sep

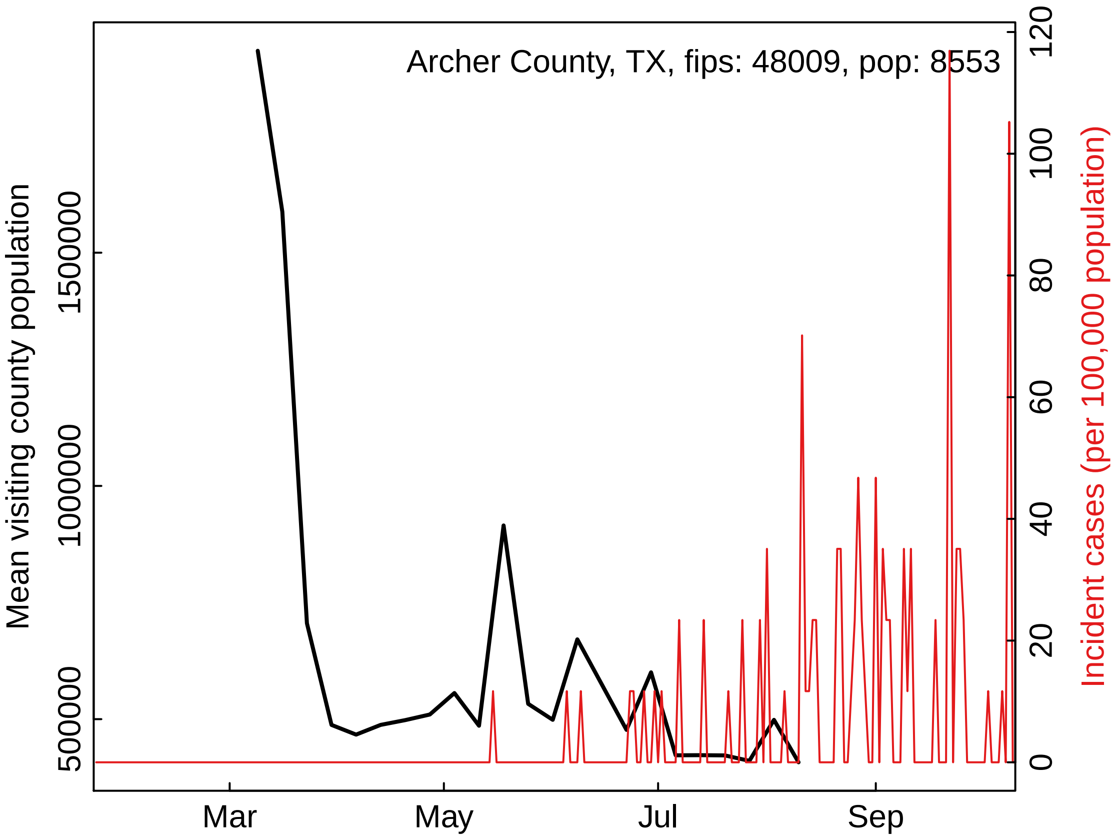

Archer County, TX, fips: 48009, pop: 8553
Mean visiting county population
500000
1000000
1500000
Mar
May
Jul
Sep
0
20
40
60
80
100
120
Incident cases (per 100,000 population)

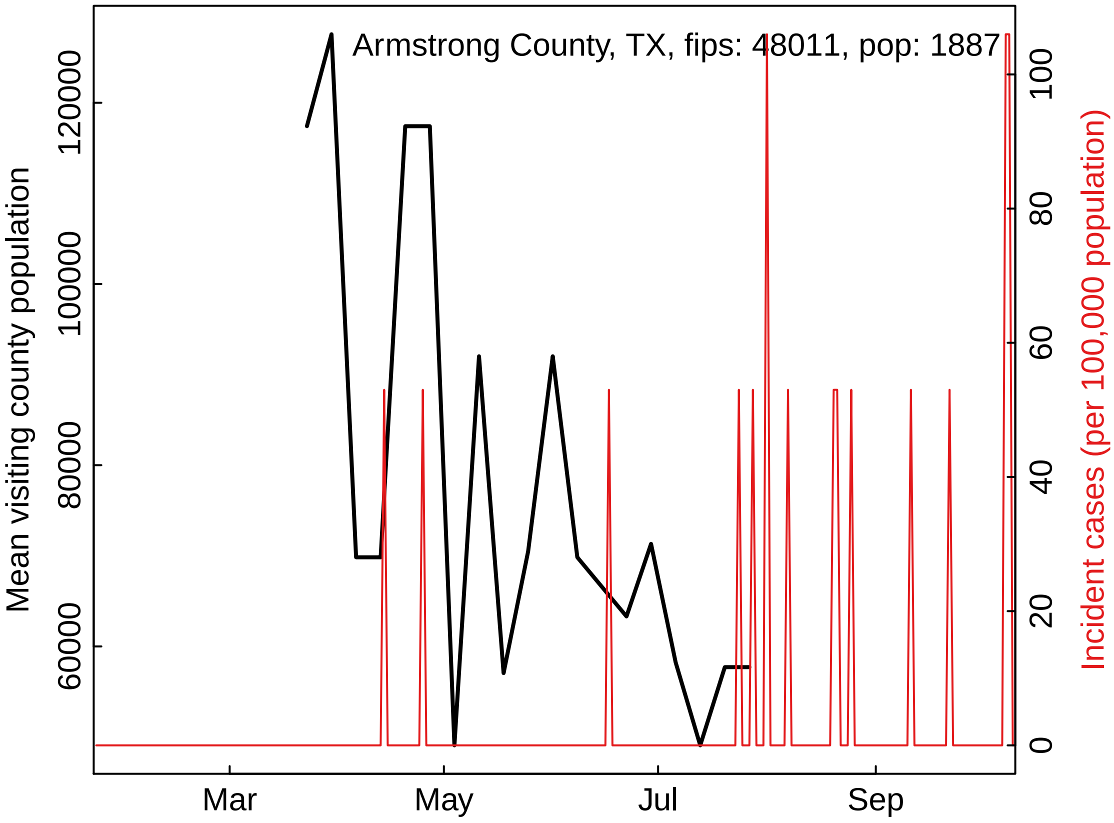

Armstrong County, TX, fips: 48011, pop: 1887
Mean visiting county population
60000
80000
100000
120000
Incident cases (per 100,000 population)
0
20
40
60
80
100
Mar
May
Jul
Sep

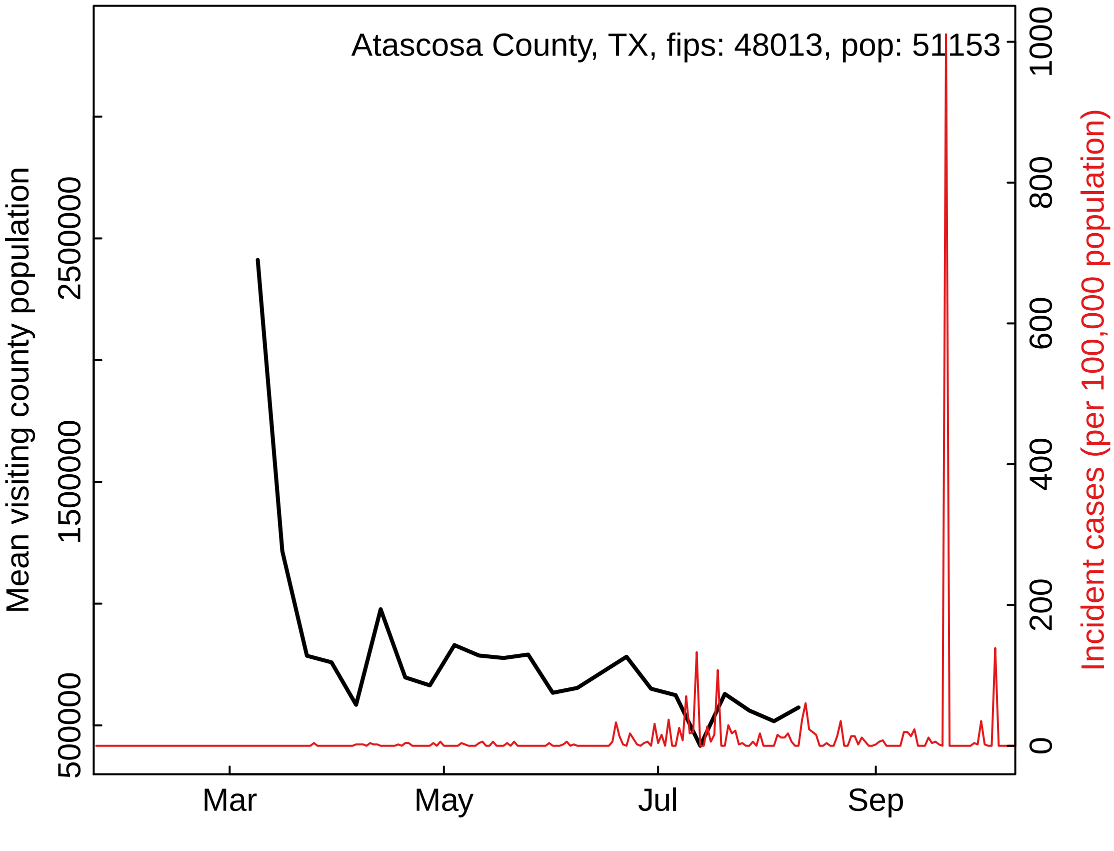

Atascosa County, TX, fips: 48013, pop: 51153
Mean visiting county population
500000
1500000
2500000
Incident cases (per 100,000 population)
0
200
400
600
800
1000
Mar
May
Jul
Sep

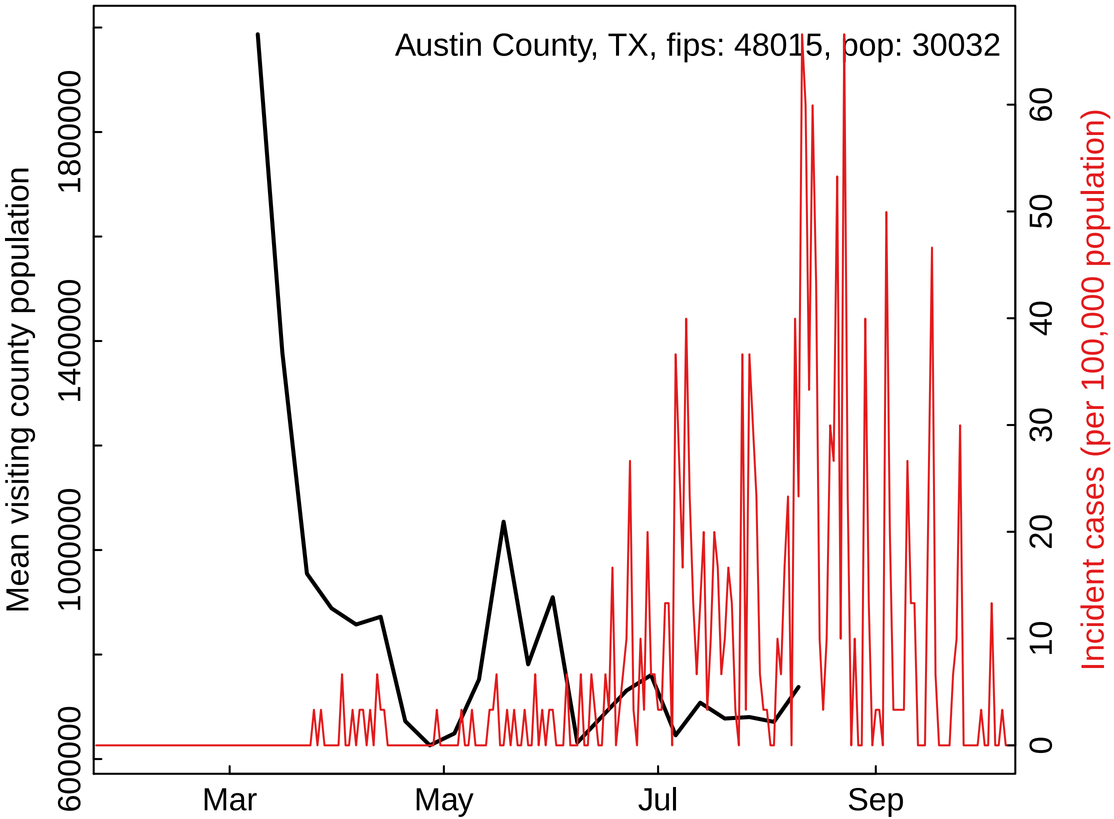

Austin County, TX, fips: 48015, pop: 30032
Mean visiting county population
6000000
10000000
14000000
18000000
Incident cases (per 100,000 population)
0
10
20
30
40
50
60
Mar
May
Jul
Sep

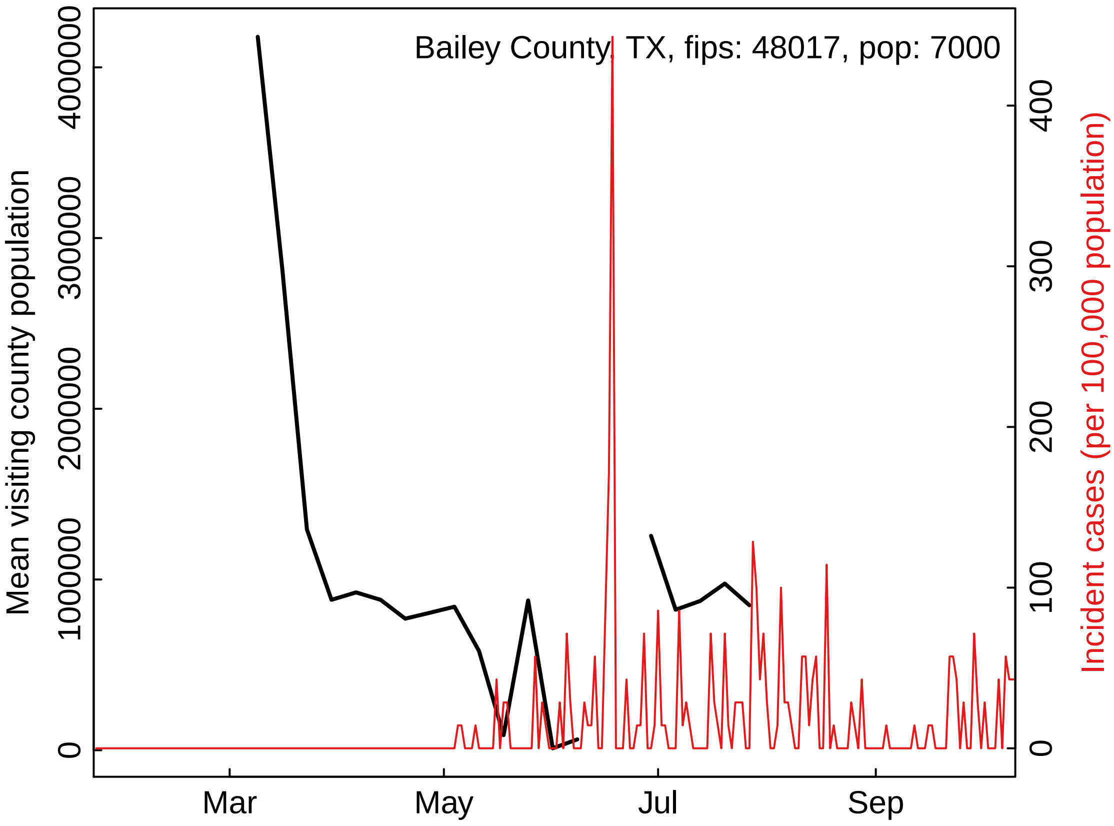

Bailey County, TX, fips: 48017, pop: 7000
Mean visiting county population
0
1000000
2000000
3000000
4000000
Incident cases (per 100,000 population)
0
100
200
300
400
Mar
May
Jul
Sep

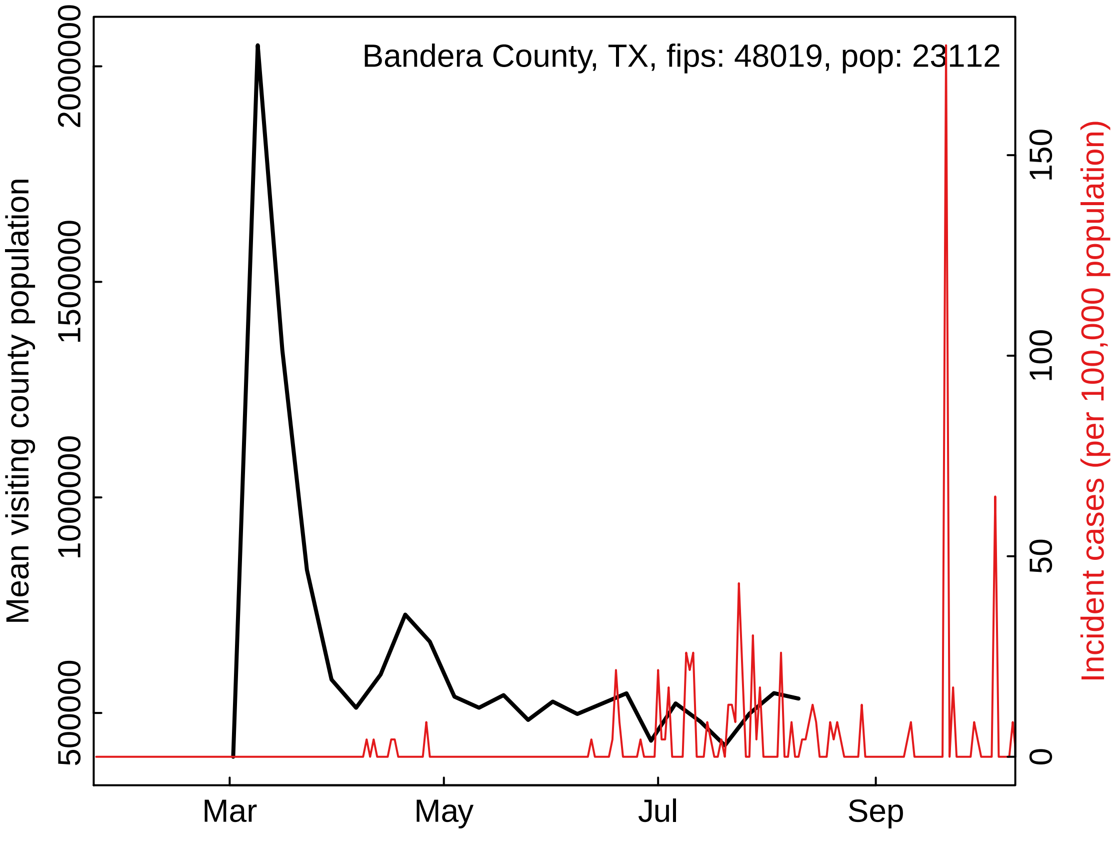

Bandera County, TX, fips: 48019, pop: 23112
Mean visiting county population
500000
1000000
1500000
2000000
Incident cases (per 100,000 population)
0
50
100
150
Mar
May
Jul
Sep

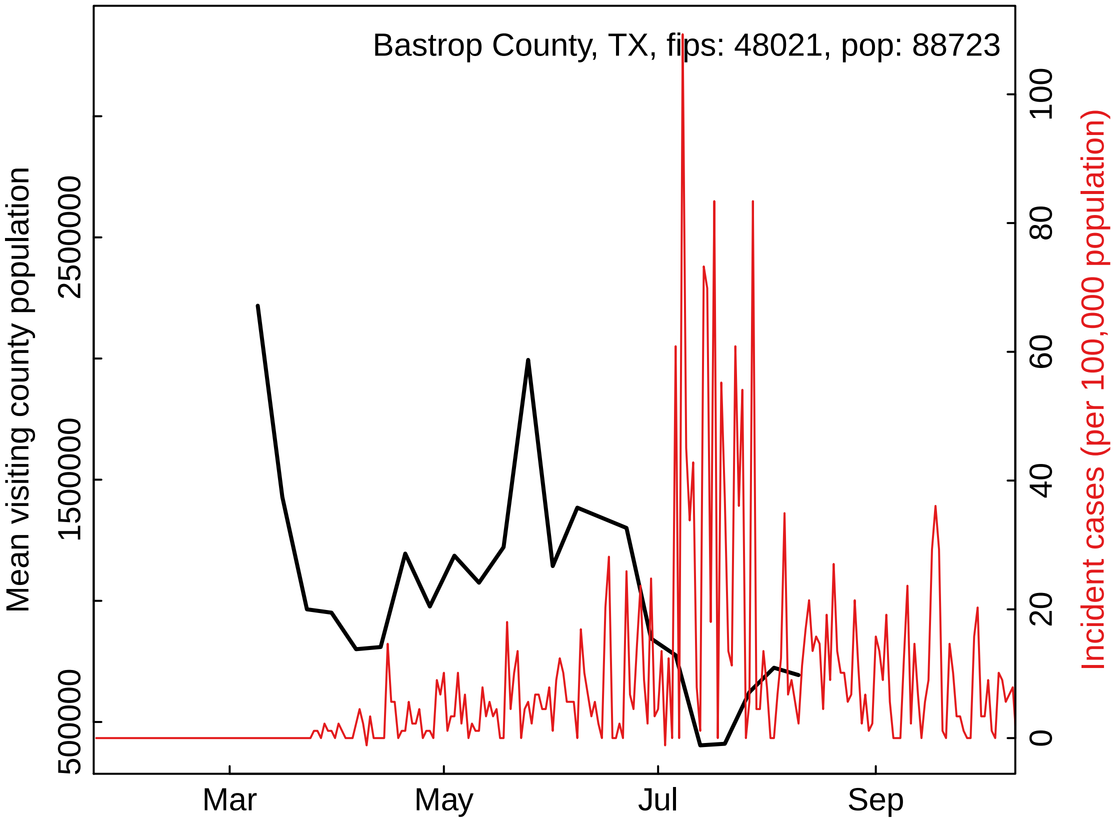

Bastrop County, TX, fips: 48021, pop: 88723
Mean visiting county population
500000
1500000
2500000
Incident cases (per 100,000 population)
0
20
40
60
80
100
Mar
May
Jul
Sep

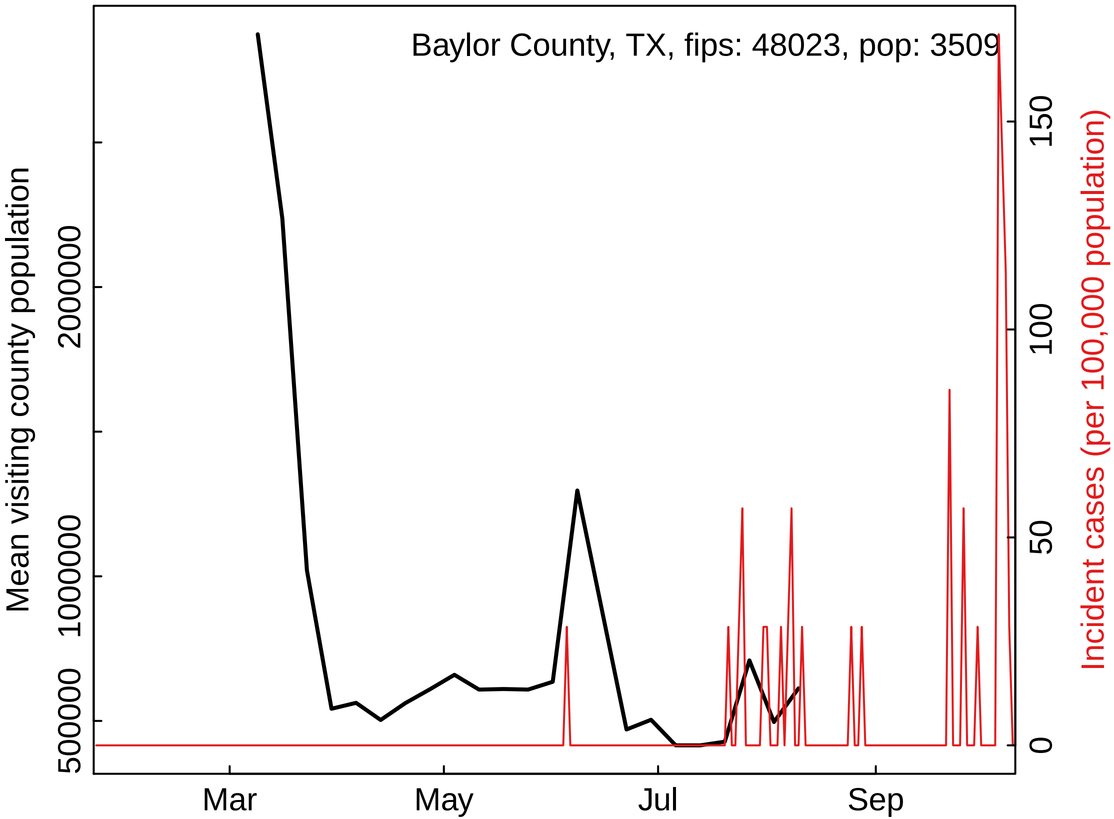

Baylor County, TX, fips: 48023, pop: 3509
Mean visiting county population
500000
1000000
2000000
Incident cases (per 100,000 population)
0
50
100
150
Mar
May
Jul
Sep

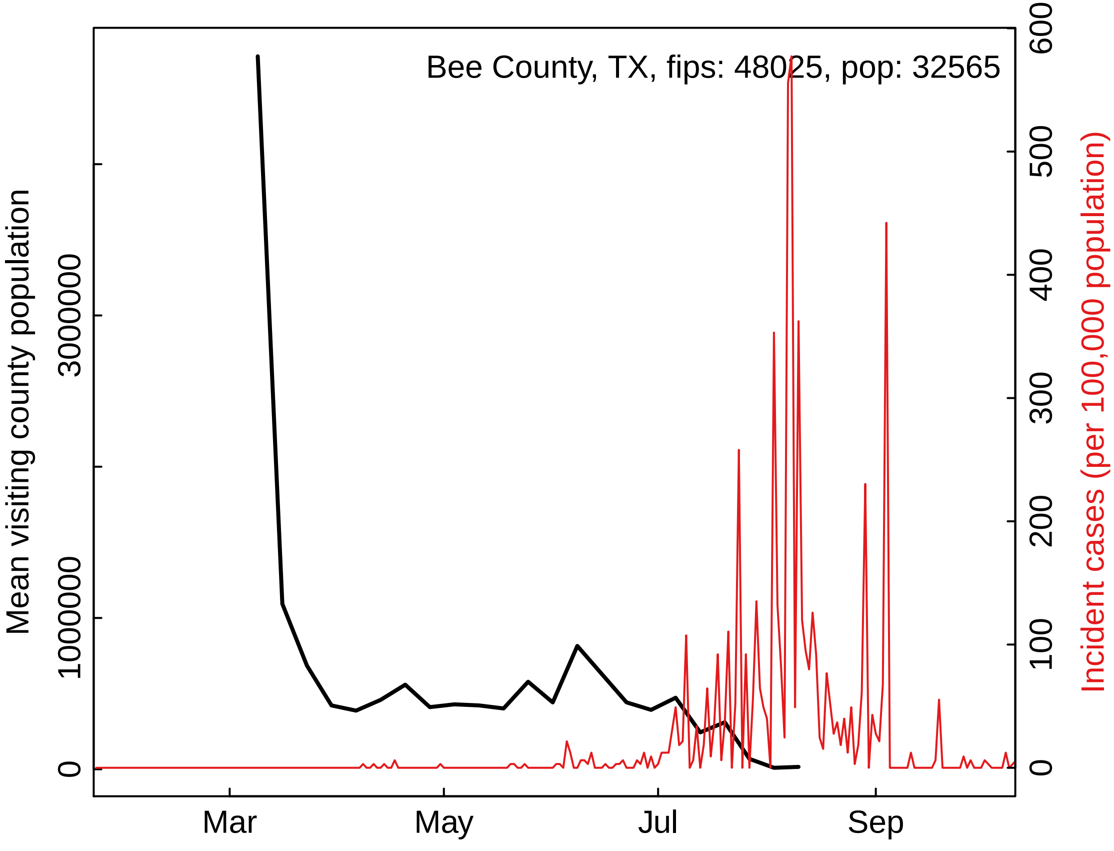

Bee County, TX, fips: 48025, pop: 32565
Mean visiting county population
0
1000000
3000000
Mar
May
Jul
Sep
0
100
200
300
400
500
600
Incident cases (per 100,000 population)

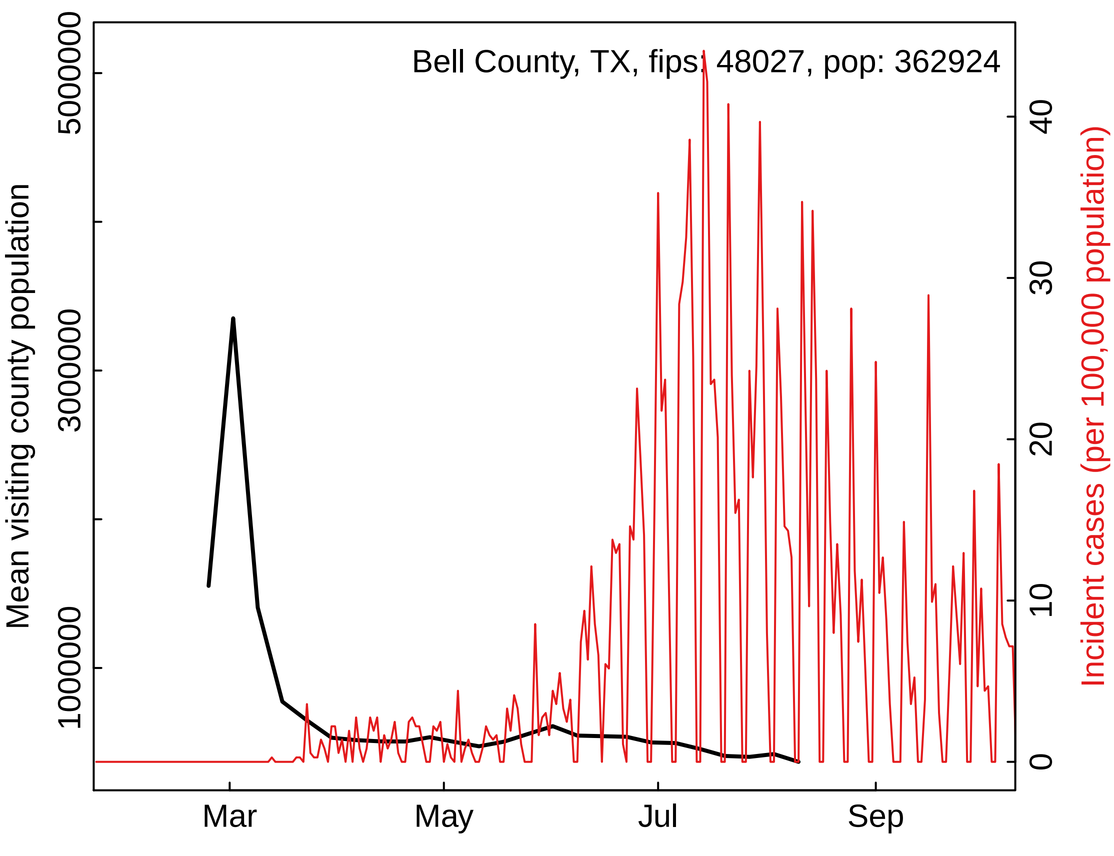

Bell County, TX, fips: 48027, pop: 362924
Mean visiting county population
10000000
30000000
50000000
Incident cases (per 100,000 population)
0
10
20
30
40
Mar
May
Jul
Sep

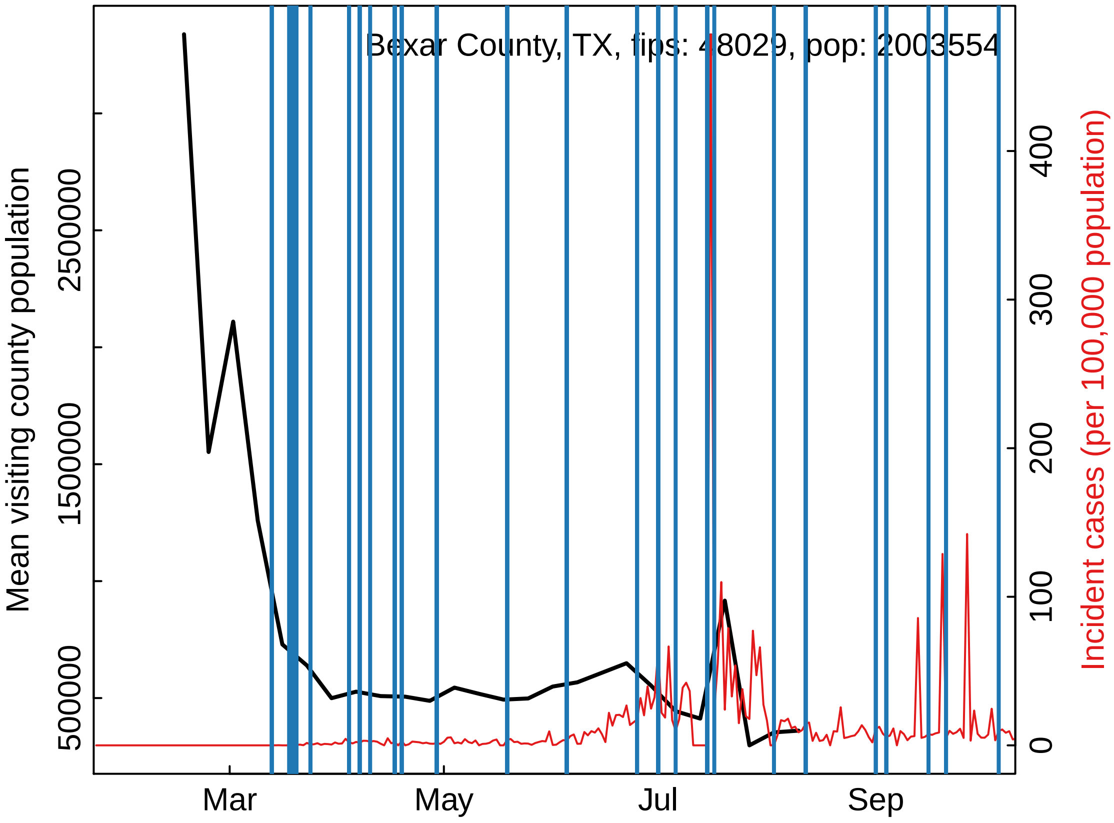

Bexar County, TX, fips: 48029, pop: 2003554
Mean visiting county population
500000
1500000
2500000
Incident cases (per 100,000 population)
0
100
200
300
400
Mar
May
Jul
Sep

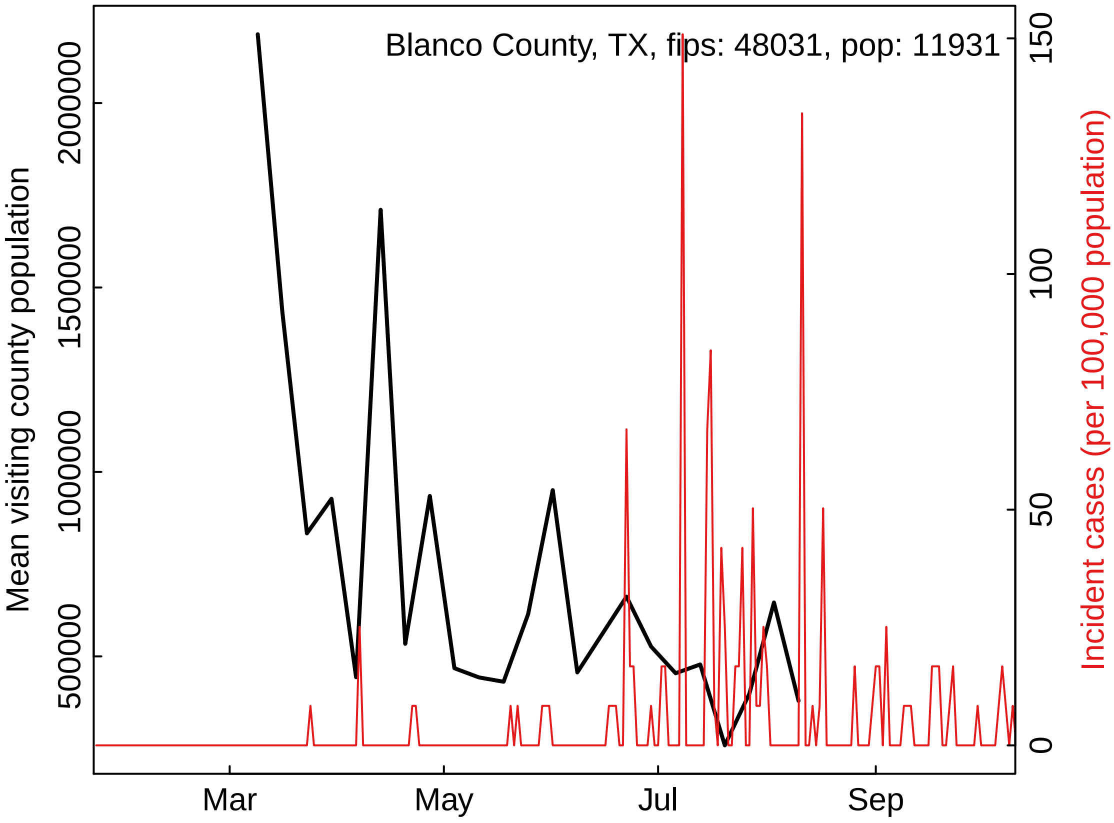
Blanco County, TX, fips: 48031, pop: 11931
Mean visiting county population
500000
1000000
1500000
2000000
Incident cases (per 100,000 population)
0
50
100
150
Mar
May
Jul
Sep

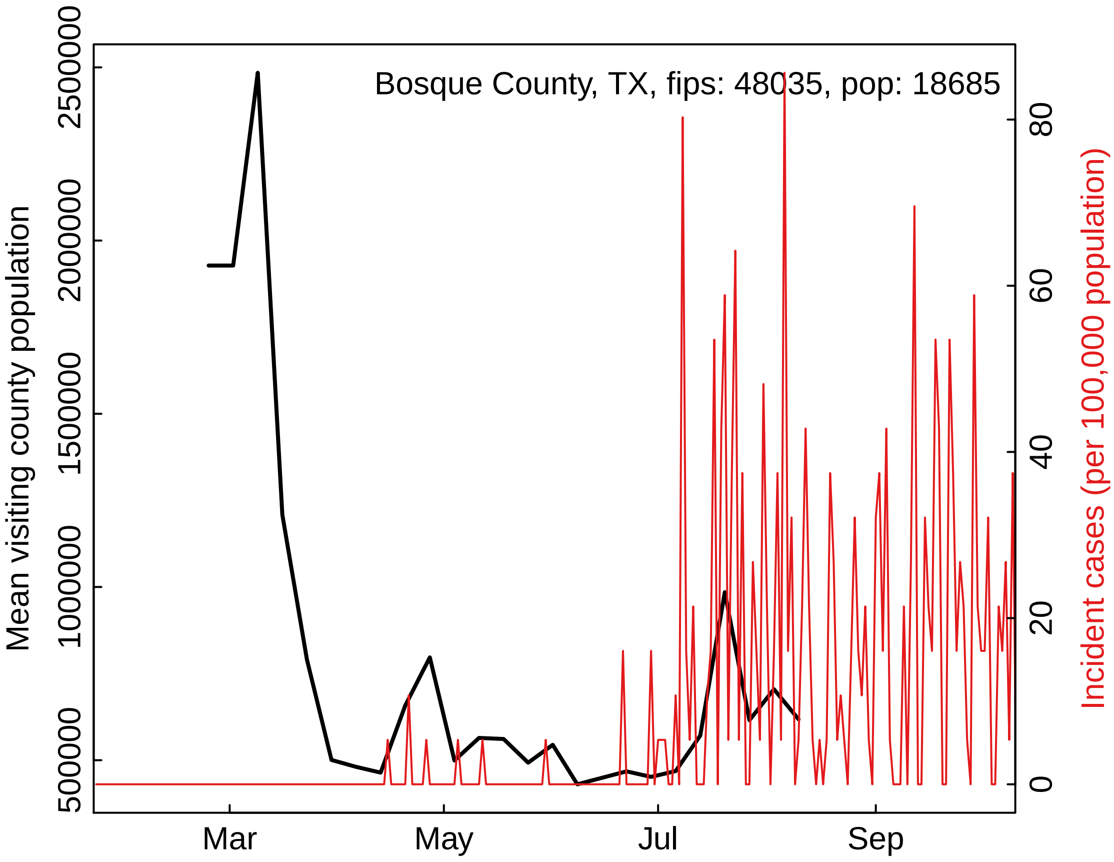

Bosque County, TX, fips: 48035, pop: 18685
Mean visiting county population
500000
1000000
1500000
2000000
2500000
Incident cases (per 100,000 population)
0
20
40
60
80
Mar
May
Jul
Sep

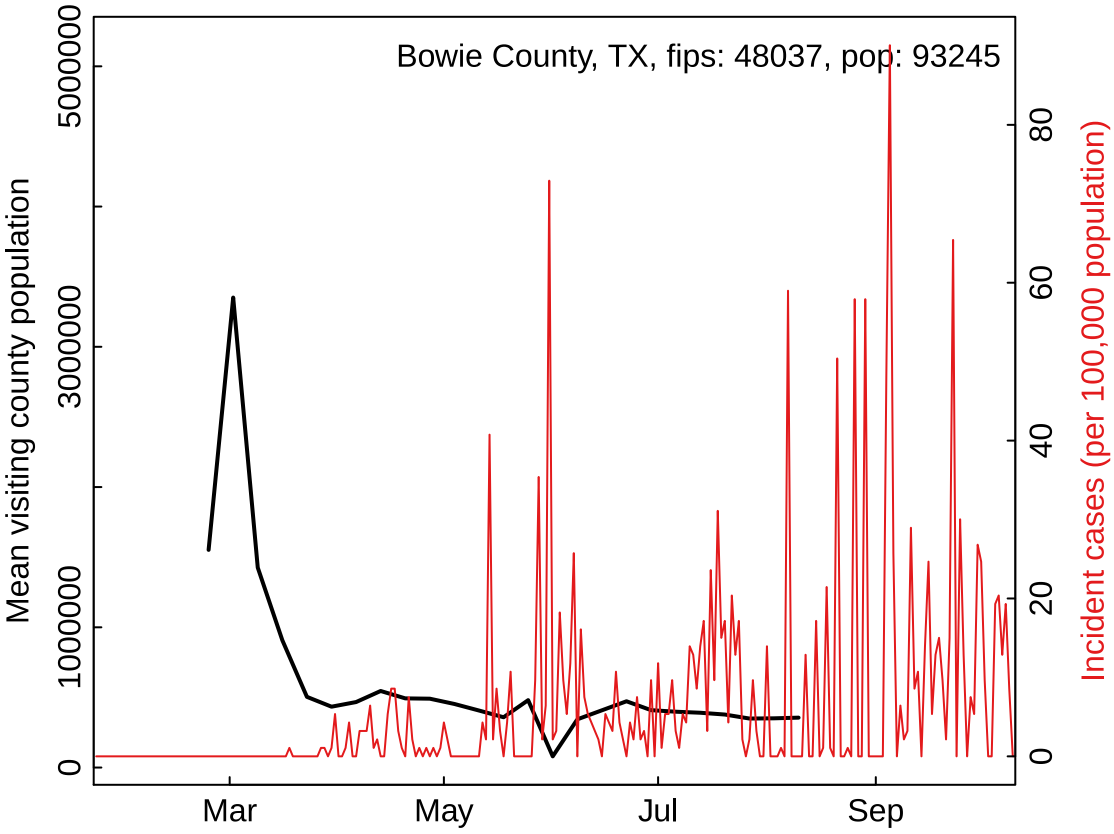

Bowie County, TX, fips: 48037, pop: 93245
Mean visiting county population
0
1000000
3000000
5000000
Incident cases (per 100,000 population)
0
20
40
60
80
Mar
May
Jul
Sep

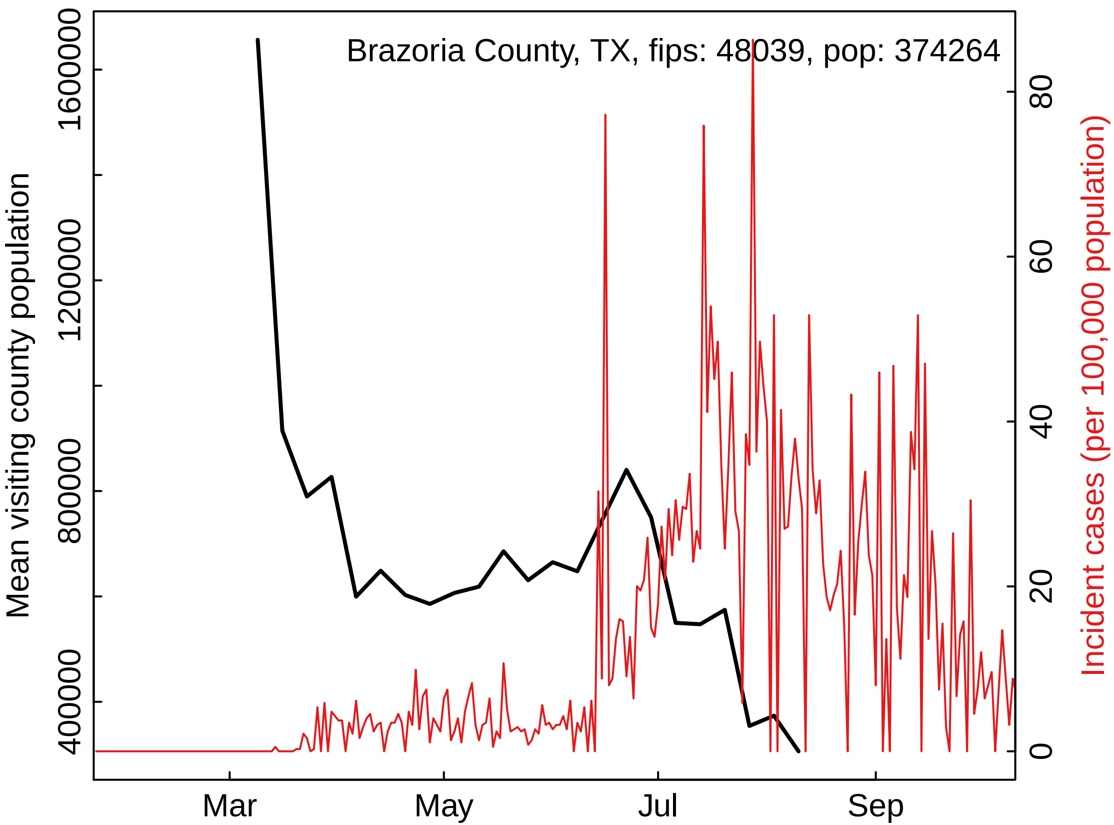

Brazoria County, TX, fips: 48039, pop: 374264
Mean visiting county population
400000
800000
1200000
1600000
Incident cases (per 100,000 population)
0
20
40
60
80
Mar
May
Jul
Sep

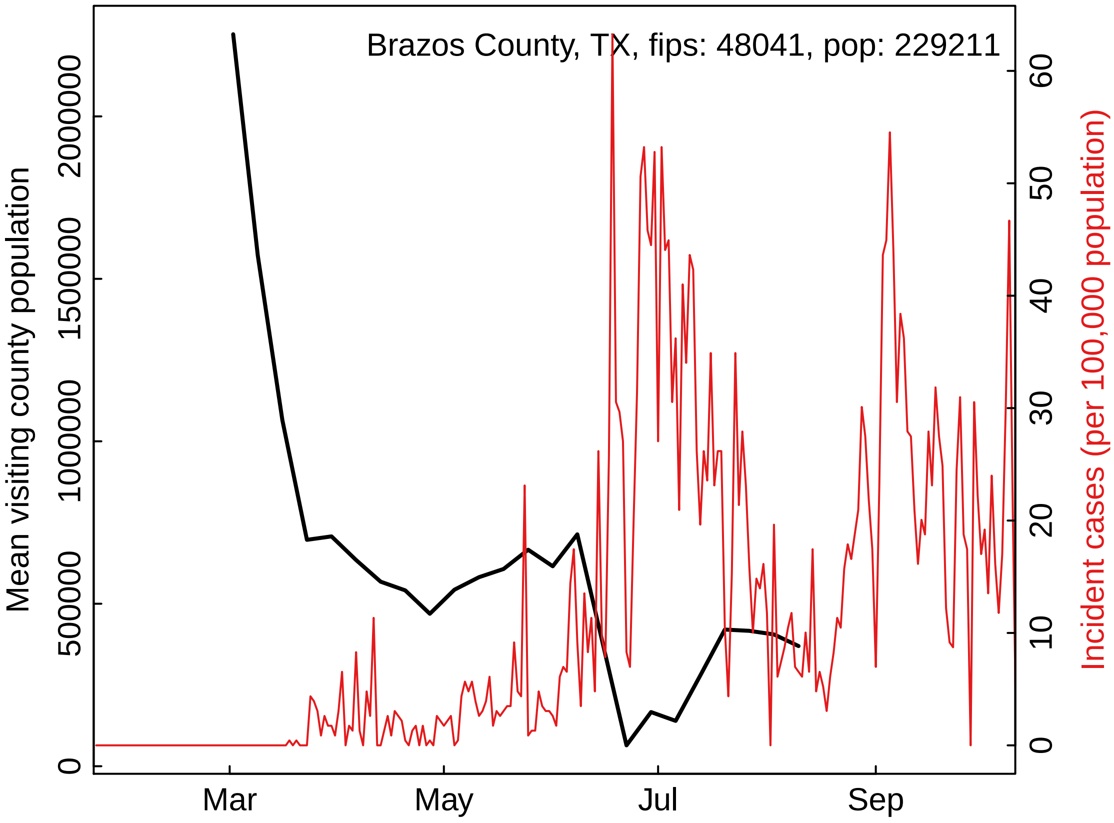
Brazos County, TX, fips: 48041, pop: 229211
Mean visiting county population
0
500000
1000000
1500000
2000000
Incident cases (per 100,000 population)
0
10
20
30
40
50
60
Mar
May
Jul
Sep

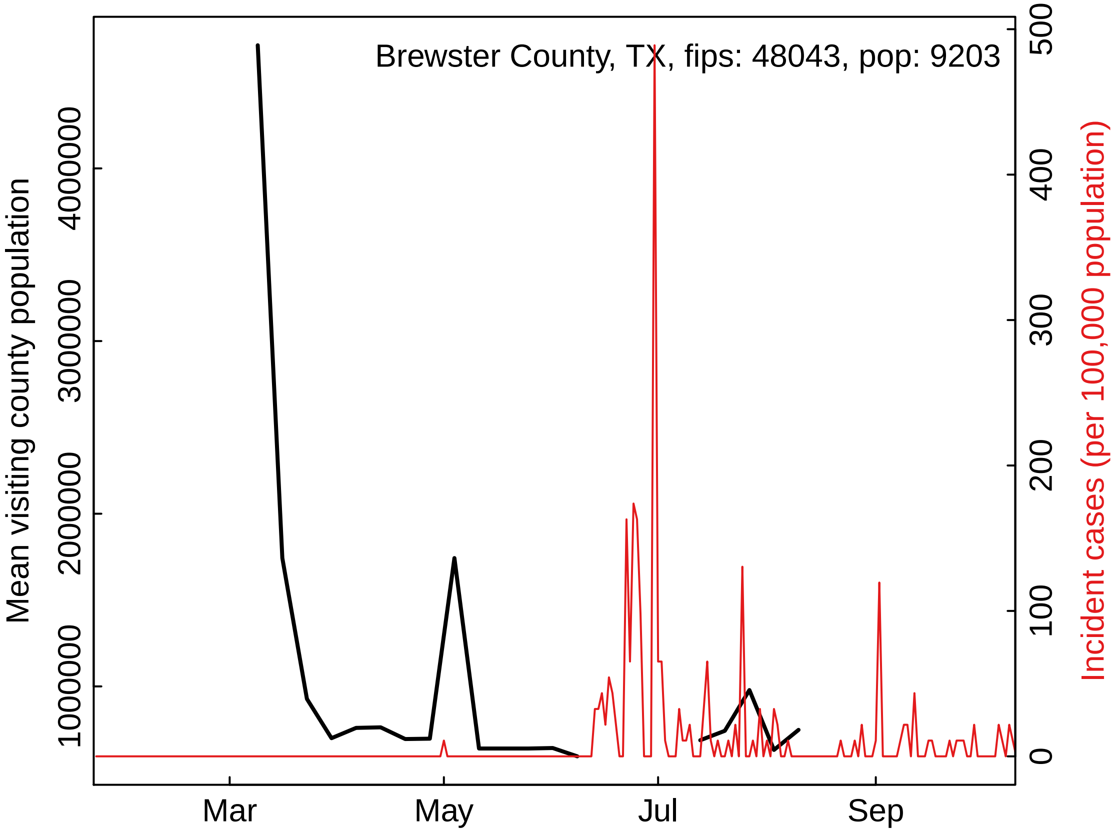
Brewster County, TX, fips: 48043, pop: 9203
Mean visiting county population
1000000
2000000
3000000
4000000
Incident cases (per 100,000 population)
0
100
200
300
400
500
Mar
May
Jul
Sep

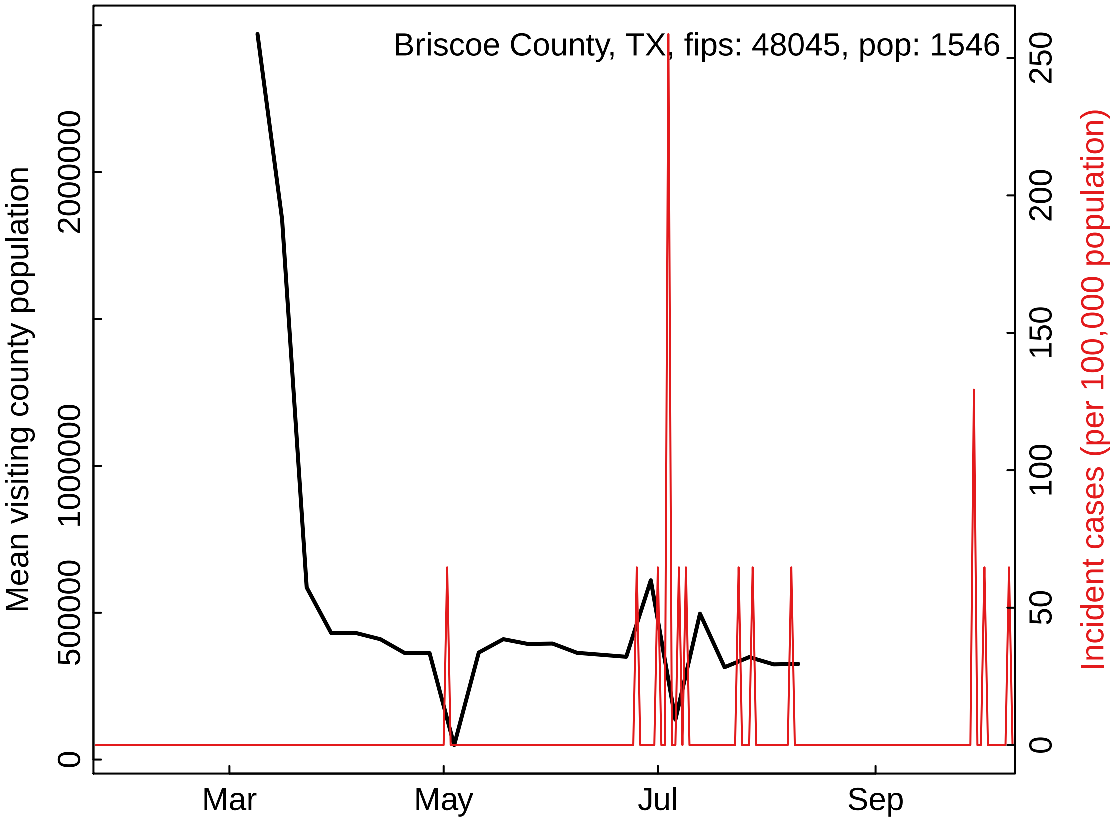

Briscoe County, TX, fips: 48045, pop: 1546
Mean visiting county population
Incident cases (per 100,000 population)
0
500000
1000000
2000000
0
50
100
150
200
250
Mar
May
Jul
Sep

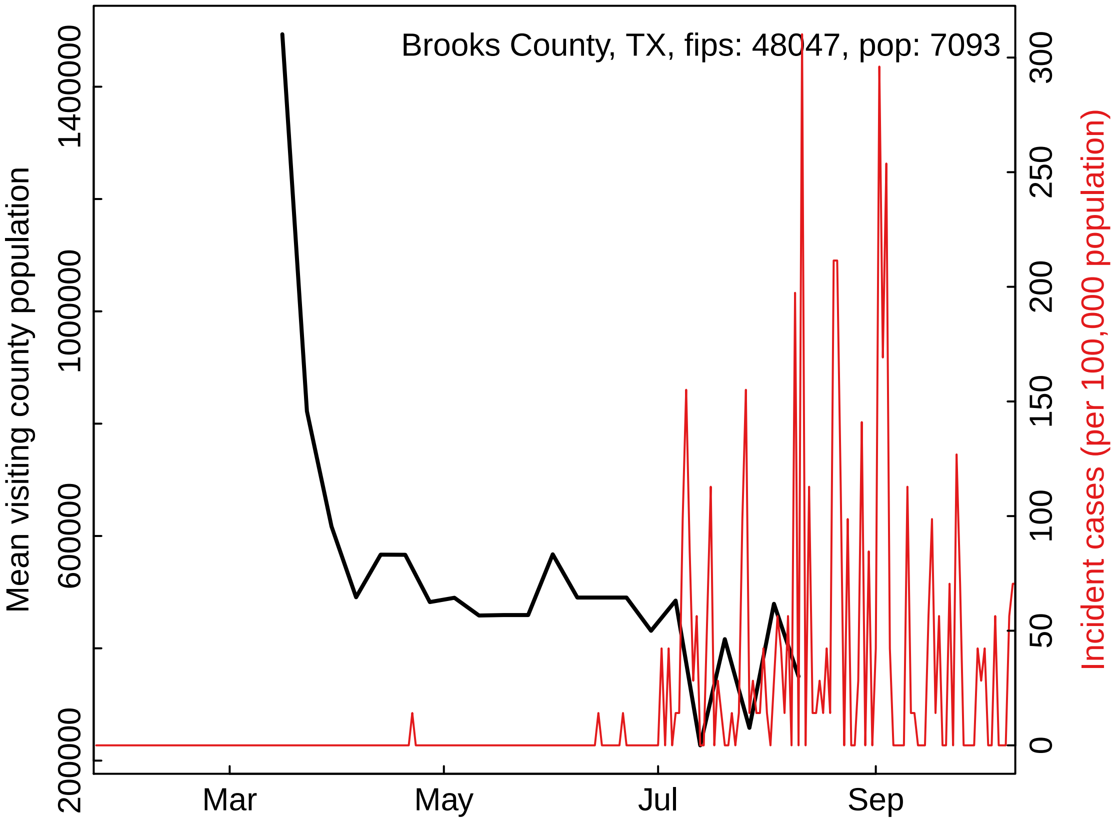

Brooks County, TX, fips: 48047, pop: 7093
Mean visiting county population
200000
600000
1000000
1400000
Incident cases (per 100,000 population)
0
50
100
150
200
250
300
Mar
May
Jul
Sep

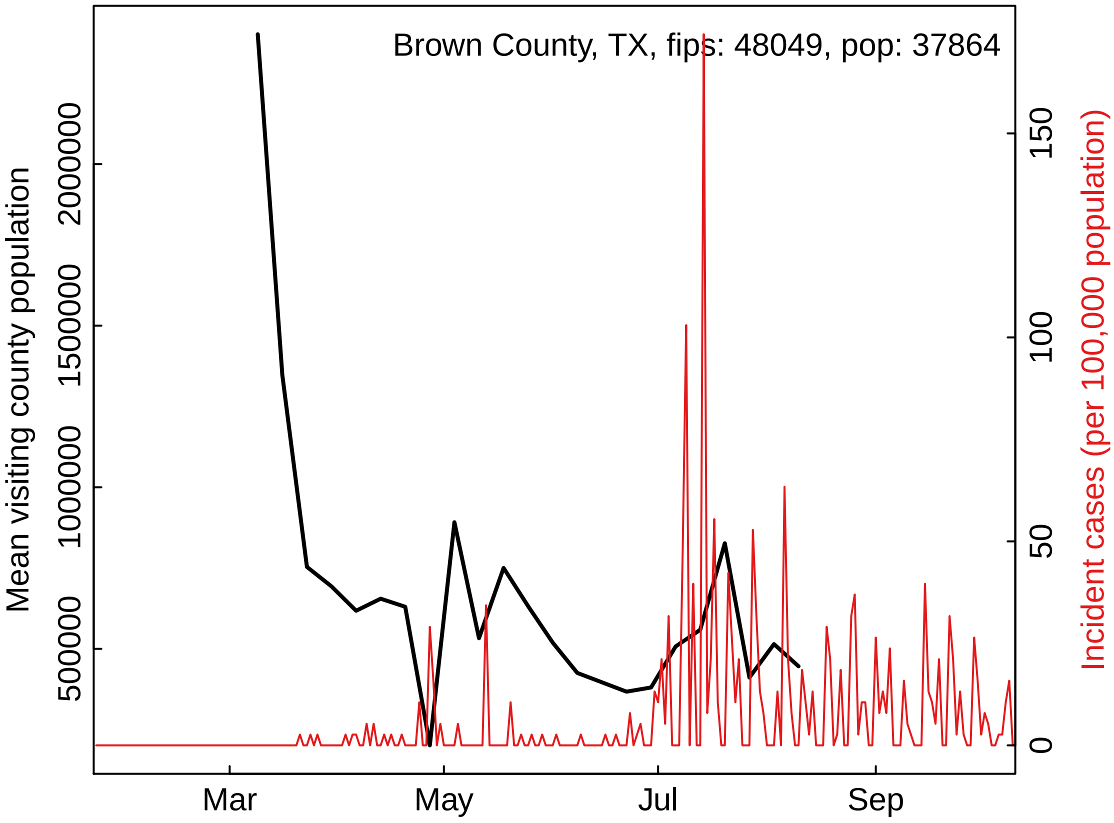
Brown County, TX, fips: 48049, pop: 37864
Mean visiting county population
500000
1000000
1500000
2000000
Incident cases (per 100,000 population)
0
50
100
150
Mar
May
Jul
Sep

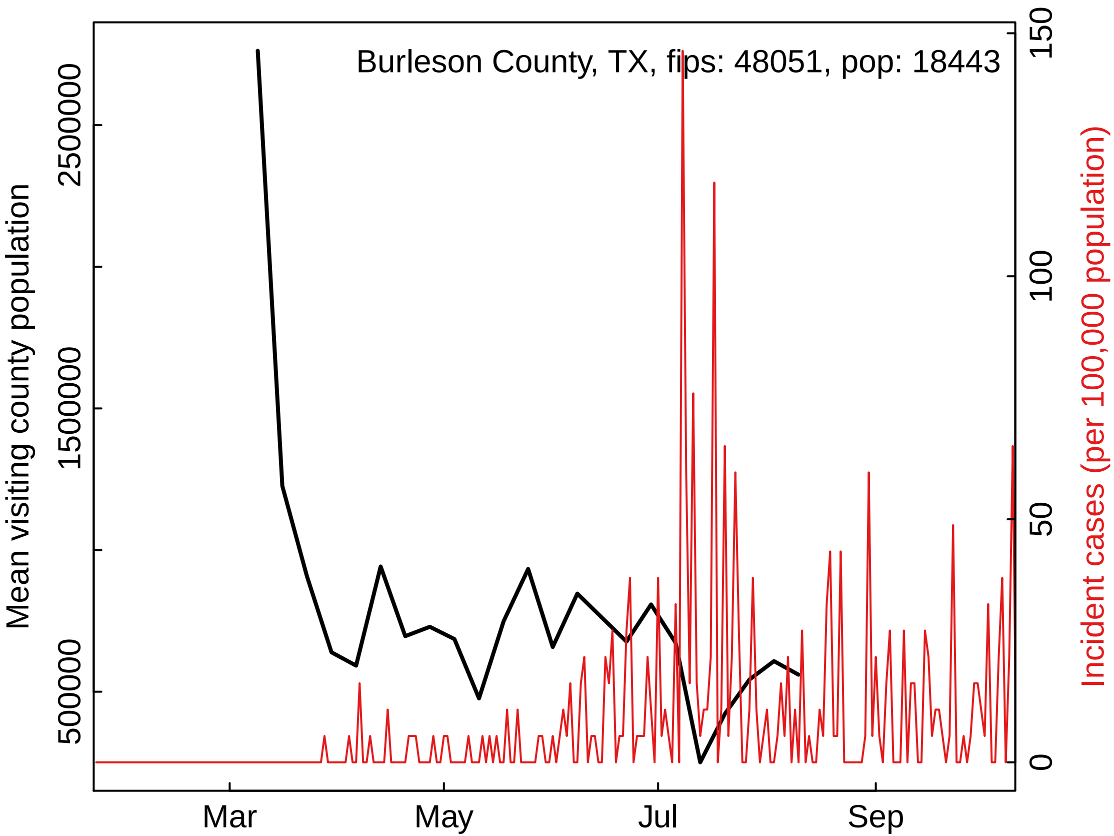

Burleson County, TX, fips: 48051, pop: 18443
Mean visiting county population
500000
1500000
2500000
Incident cases (per 100,000 population)
0
50
100
150
Mar
May
Jul
Sep

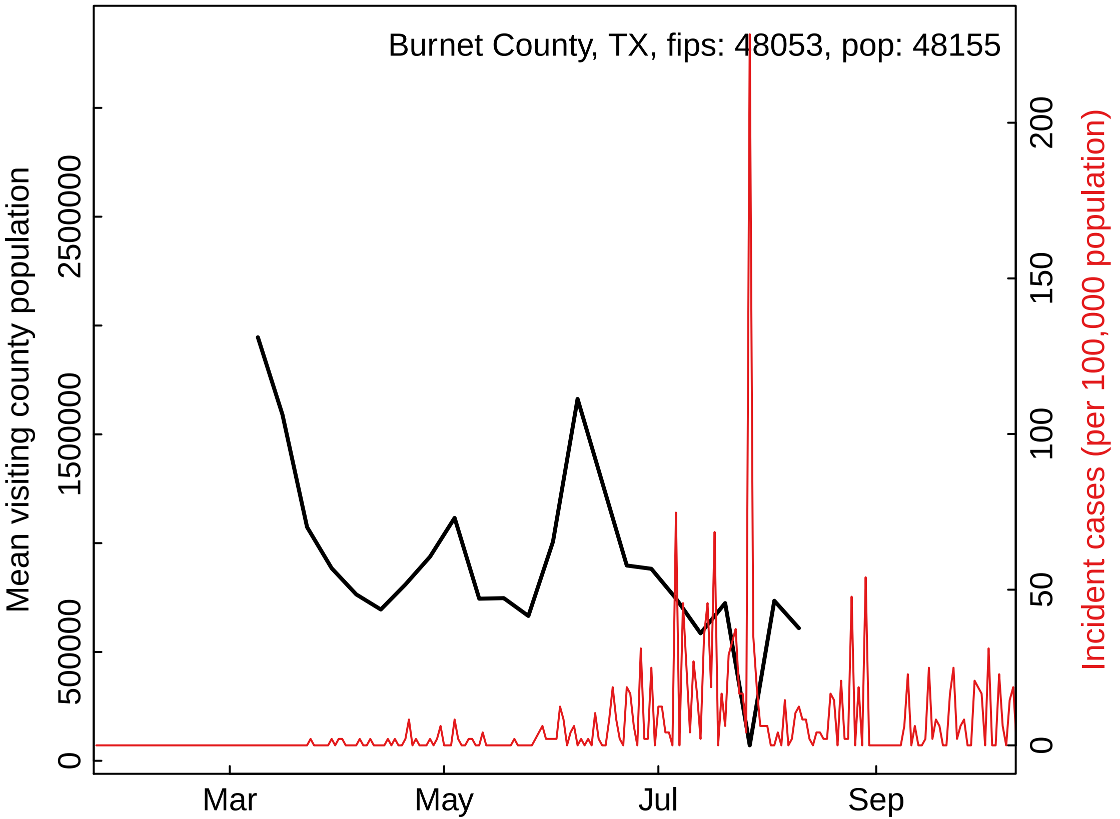

Burnet County, TX, fips: 48053, pop: 48155
Mean visiting county population
0
500000
1500000
2500000
Incident cases (per 100,000 population)
0
50
100
150
200
Mar
May
Jul
Sep

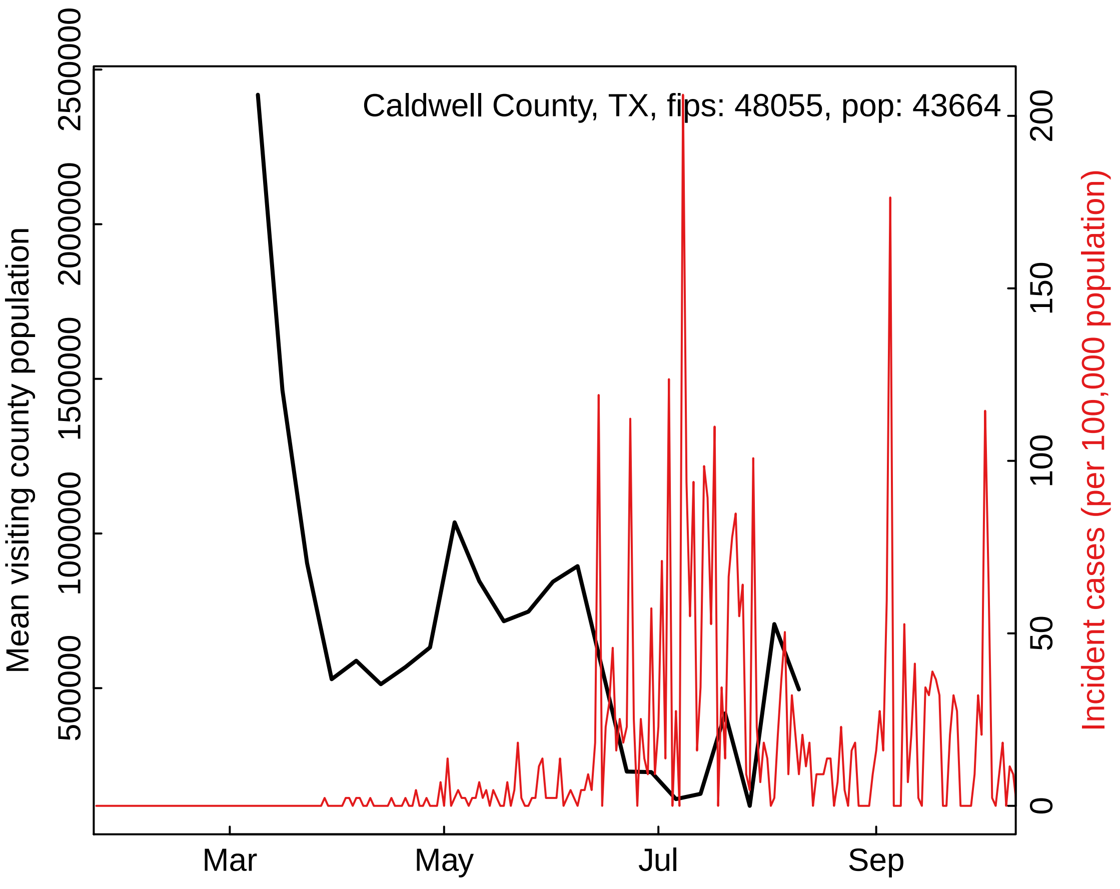

Caldwell County, TX, fips: 48055, pop: 43664
Mean visiting county population
2500000
2000000
1500000
1000000
500000
Incident cases (per 100,000 population)
200
150
100
50
0
Mar
May
Jul
Sep

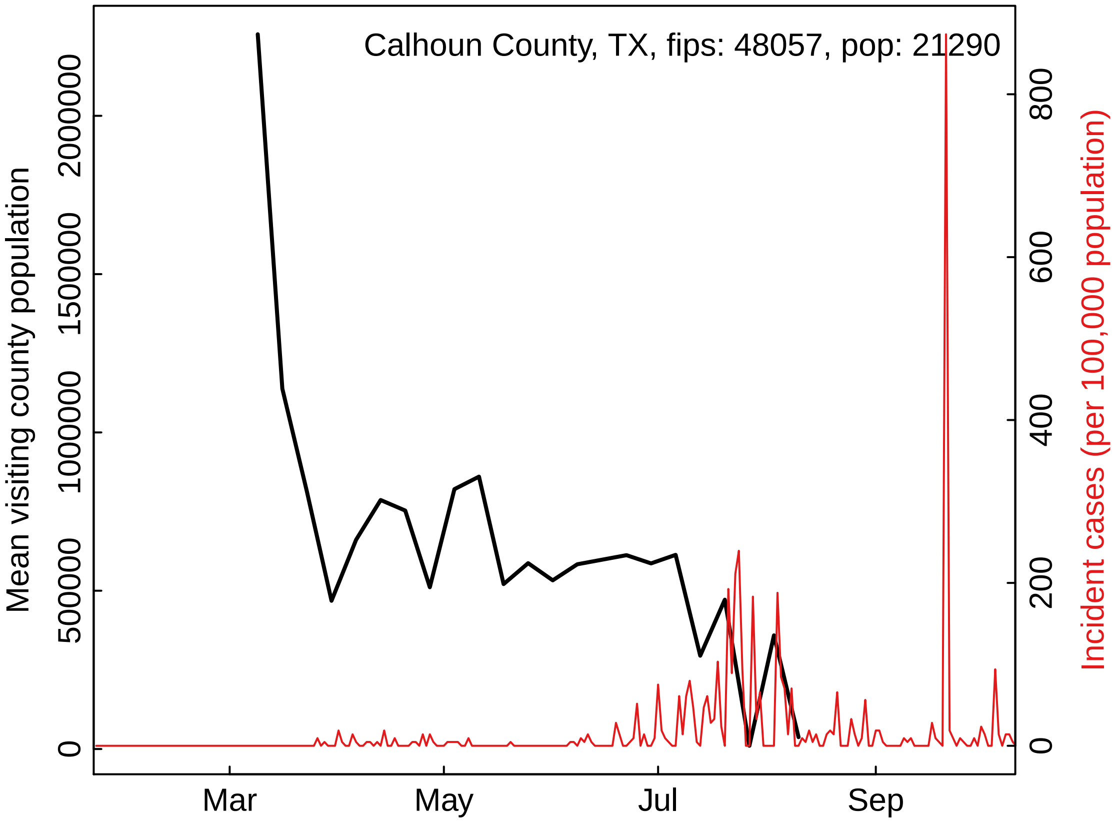

Calhoun County, TX, fips: 48057, pop: 21290
Mean visiting county population
0
500000
1000000
1500000
2000000
Incident cases (per 100,000 population)
0
200
400
600
800
Mar
May
Jul
Sep

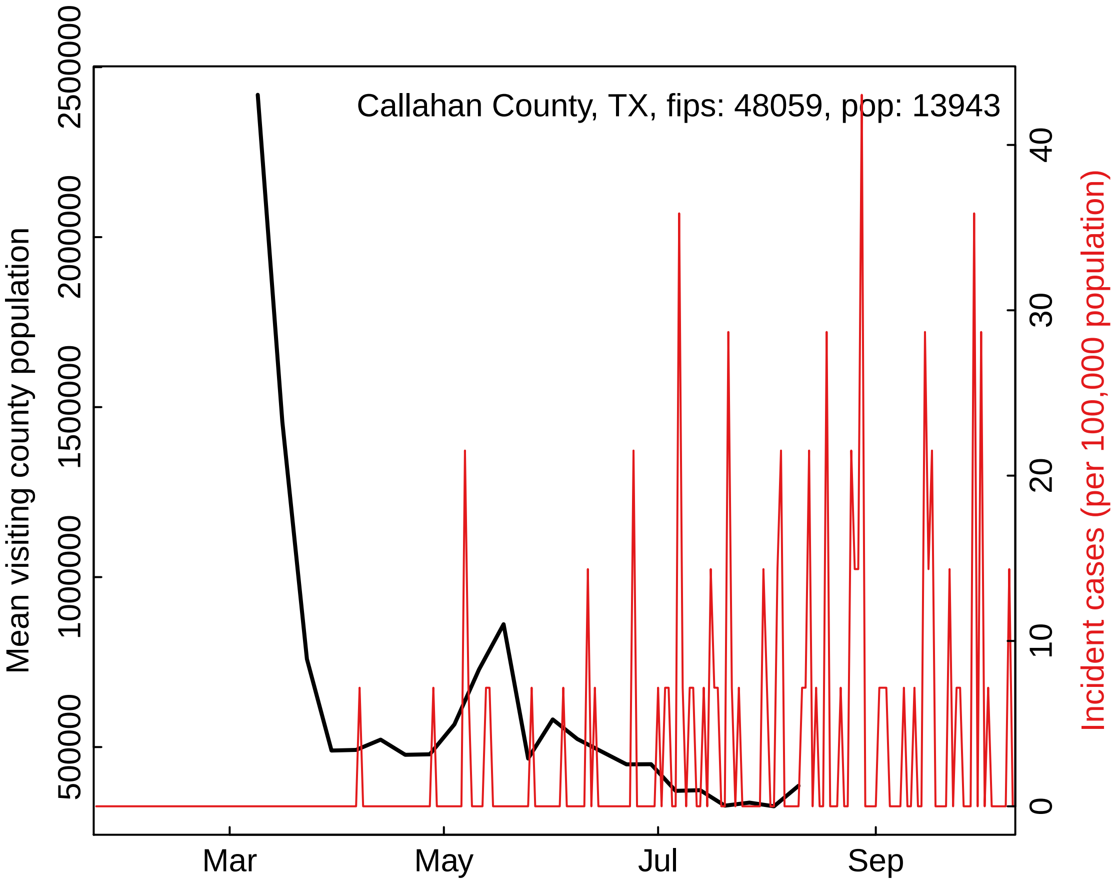
Callahan County, TX, fips: 48059, pop: 13943
Mean visiting county population
2500000
2000000
1500000
1000000
500000
Incident cases (per 100,000 population)
40
30
20
10
0
Mar
May
Jul
Sep

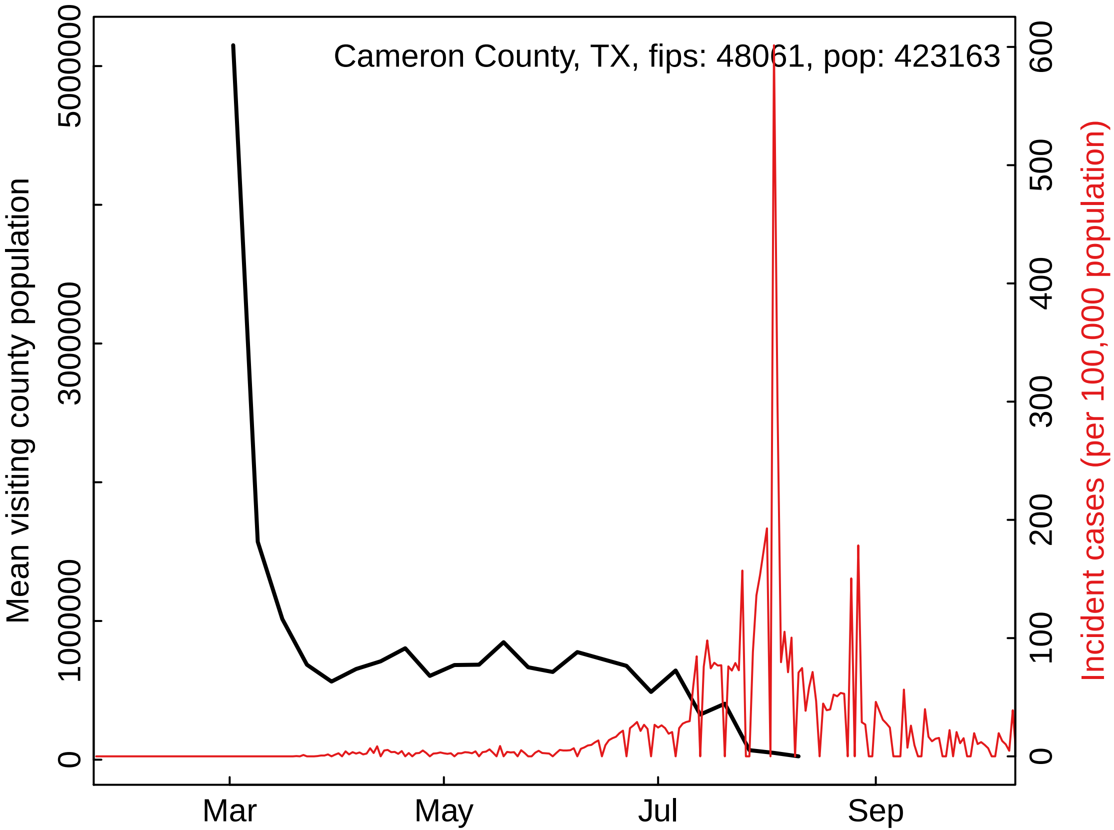
Cameron County, TX, fips: 48061, pop: 423163
Mean visiting county population
0
1000000
3000000
5000000
Incident cases (per 100,000 population)
0
100
200
300
400
500
600
Mar
May
Jul
Sep

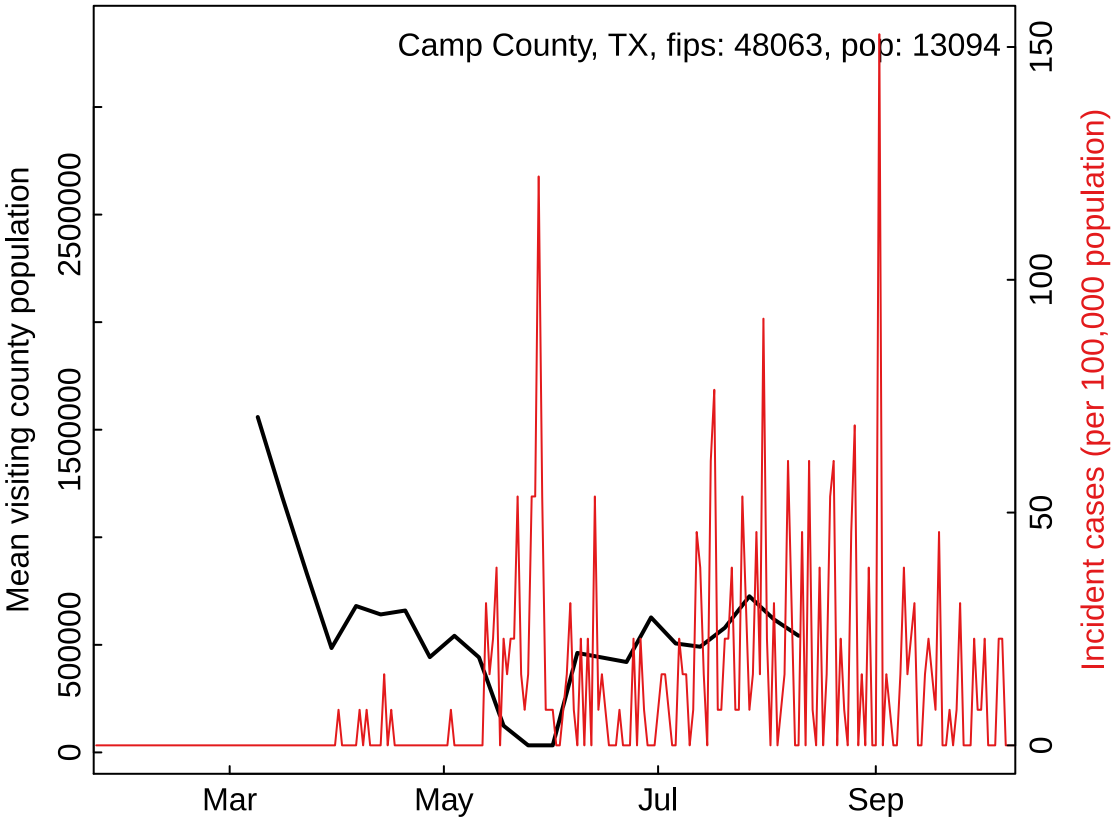
Camp County, TX, fips: 48063, pop: 13094
Mean visiting county population
0
500000
1500000
2500000
Incident cases (per 100,000 population)
0
50
100
150
Mar
May
Jul
Sep

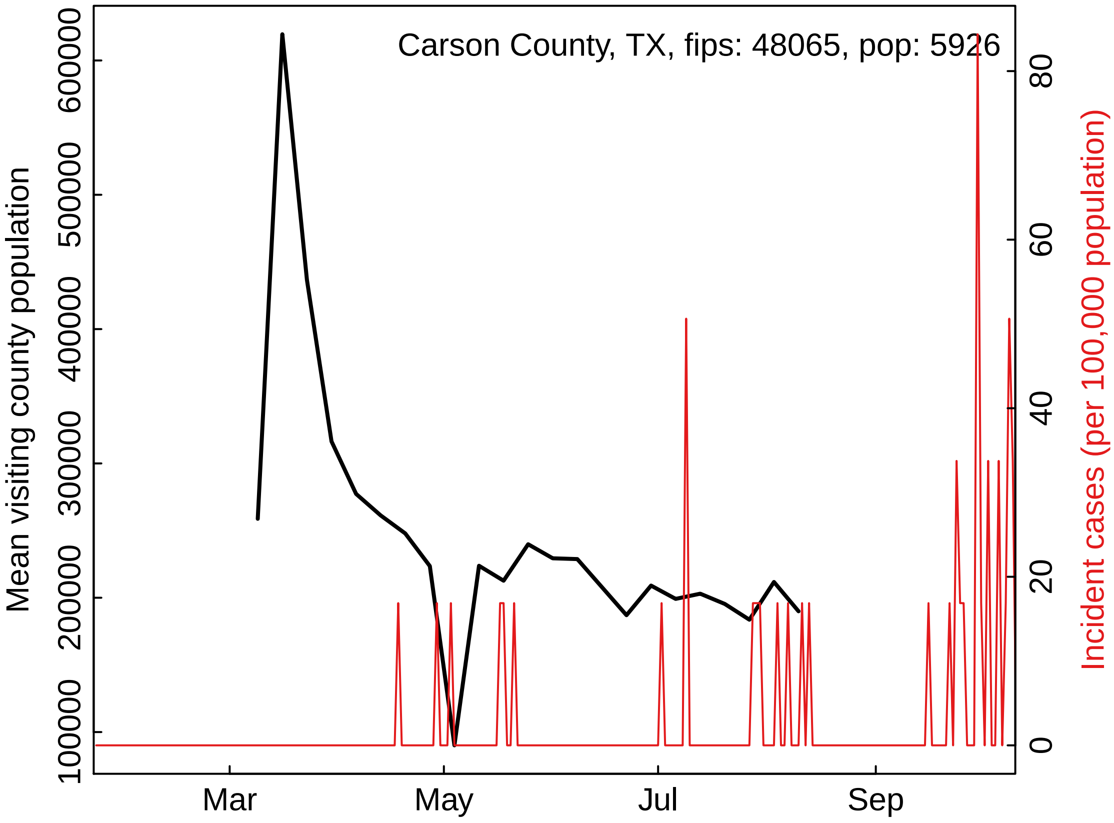

Carson County, TX, fips: 48065, pop: 5926
Mean visiting county population
100000
200000
300000
400000
500000
600000
Incident cases (per 100,000 population)
0
20
40
60
80
Mar
May
Jul
Sep

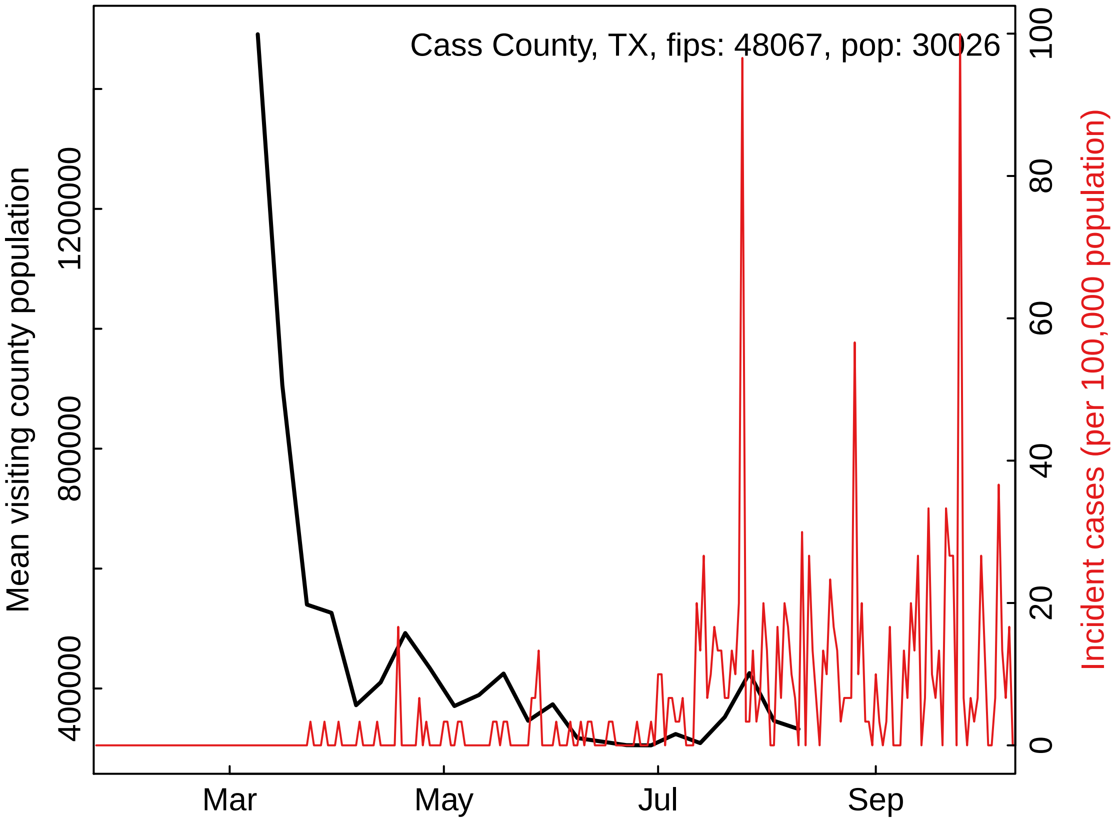

Cass County, TX, fips: 48067, pop: 30026
Mean visiting county population
400000
800000
1200000
Mar
May
Jul
Sep
0
20
40
60
80
100
Incident cases (per 100,000 population)

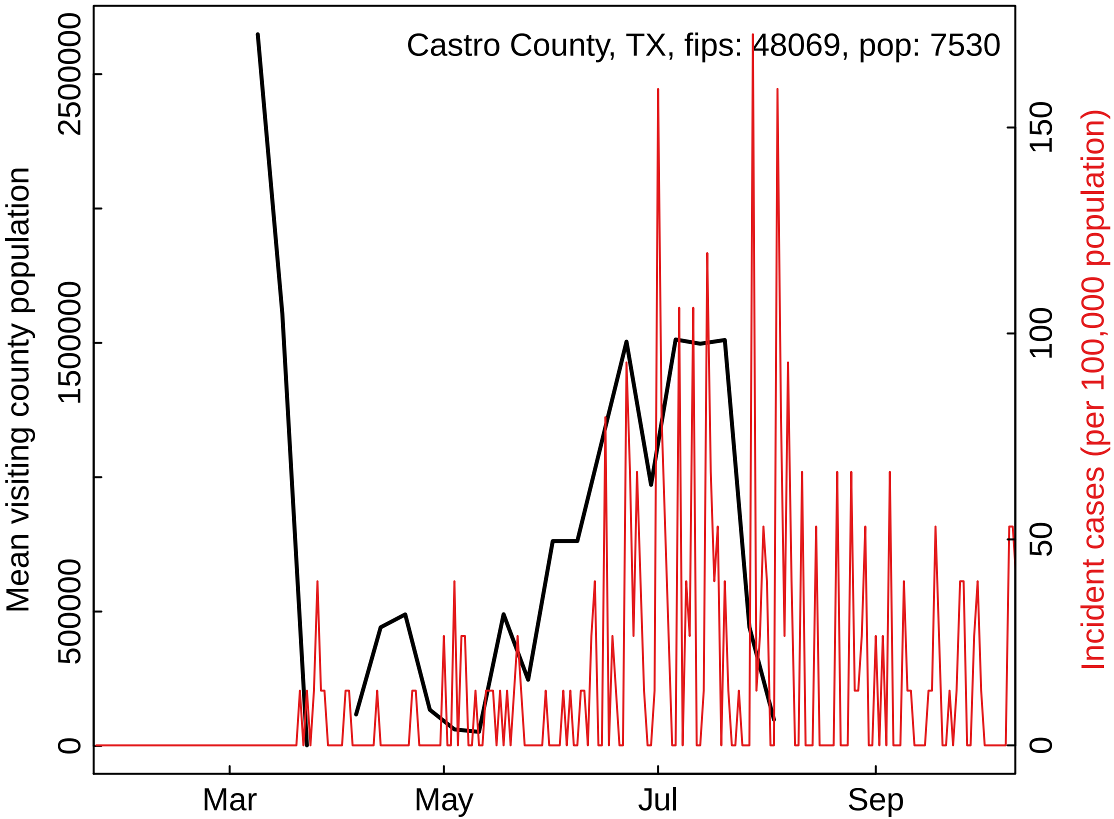

Castro County, TX, fips: 48069, pop: 7530
Mean visiting county population
0
500000
1500000
2500000
Incident cases (per 100,000 population)
0
50
100
150
Mar
May
Jul
Sep

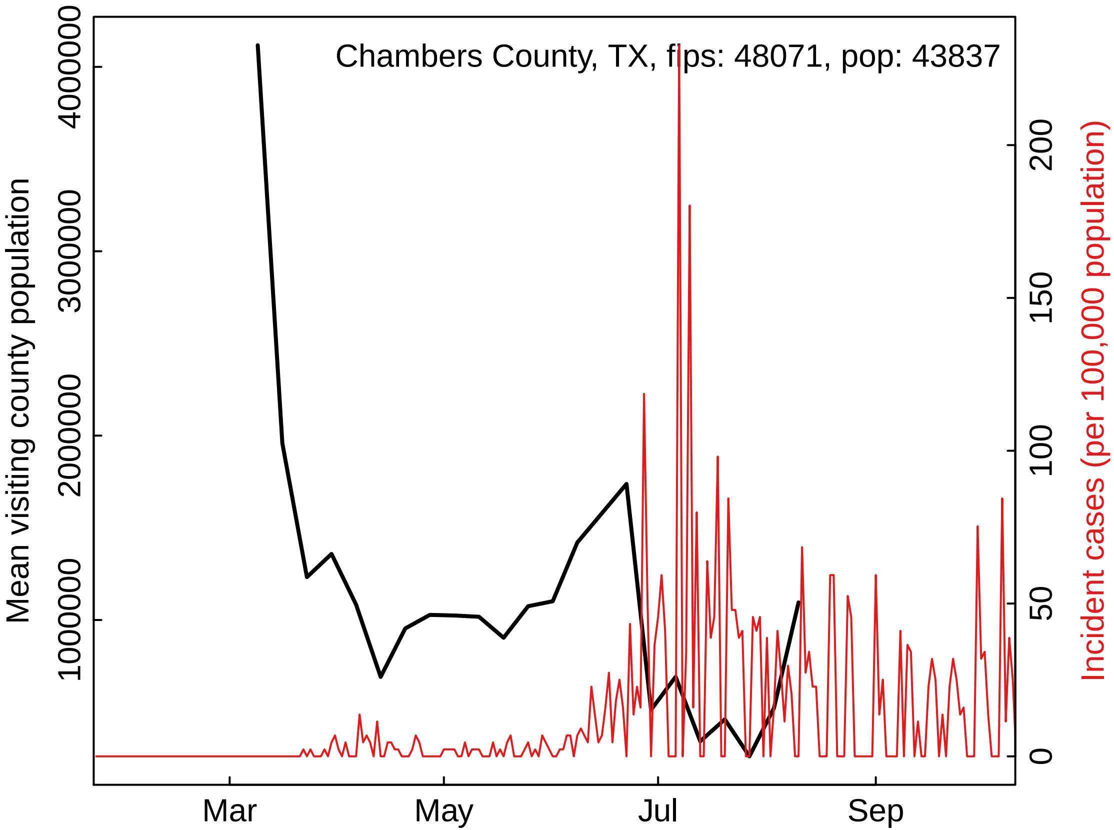

Chambers County, TX, fips: 48071, pop: 43837
Mean visiting county population
1000000
2000000
3000000
4000000
Incident cases (per 100,000 population)
0
50
100
150
200
Mar
May
Jul
Sep

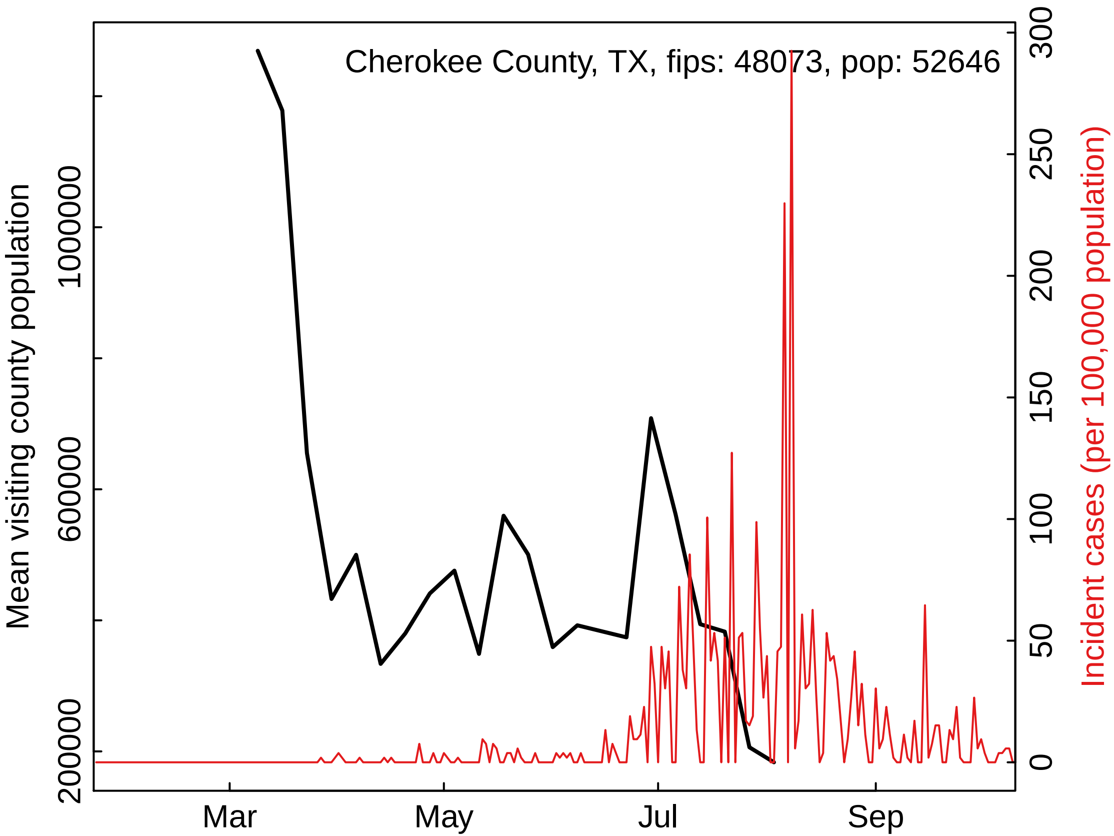
Cherokee County, TX, fips: 48073, pop: 52646
Mean visiting county population
200000
600000
1000000
Incident cases (per 100,000 population)
0
50
100
150
200
250
300
Mar
May
Jul
Sep

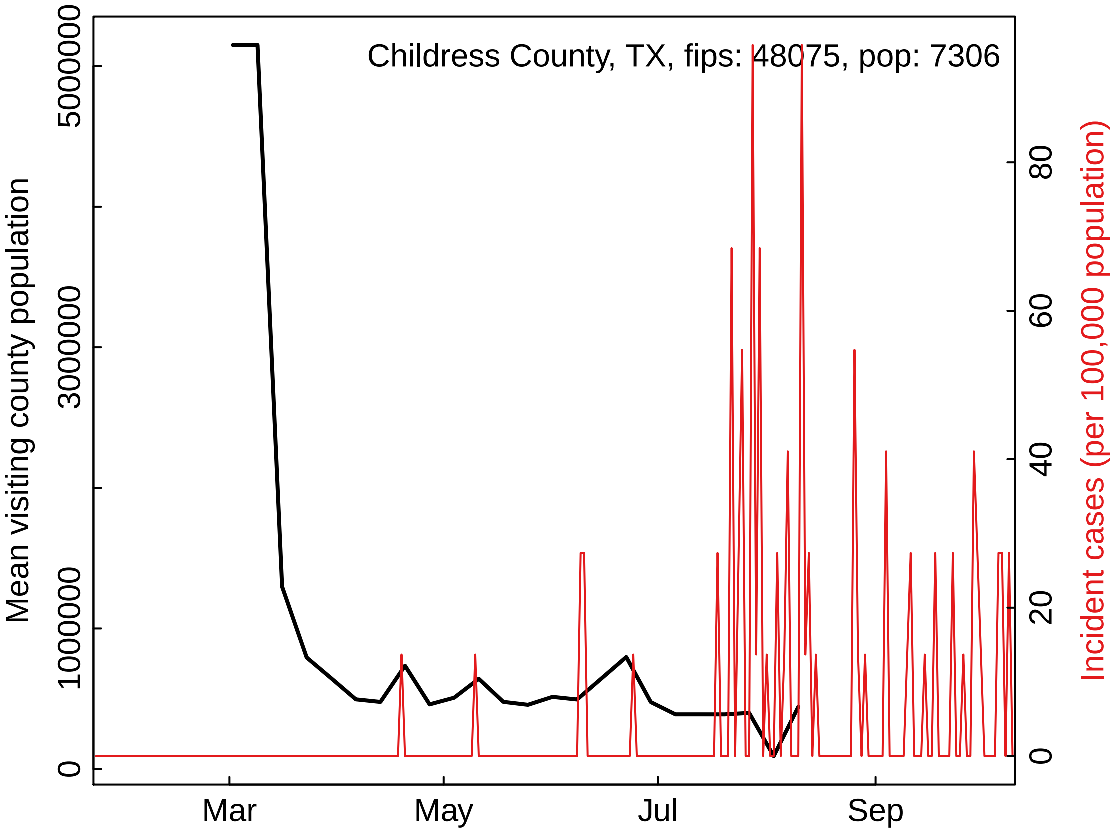

Childress County, TX, fips: 48075, pop: 7306
Mean visiting county population
0
1000000
3000000
5000000
Incident cases (per 100,000 population)
0
20
40
60
80
Mar
May
Jul
Sep

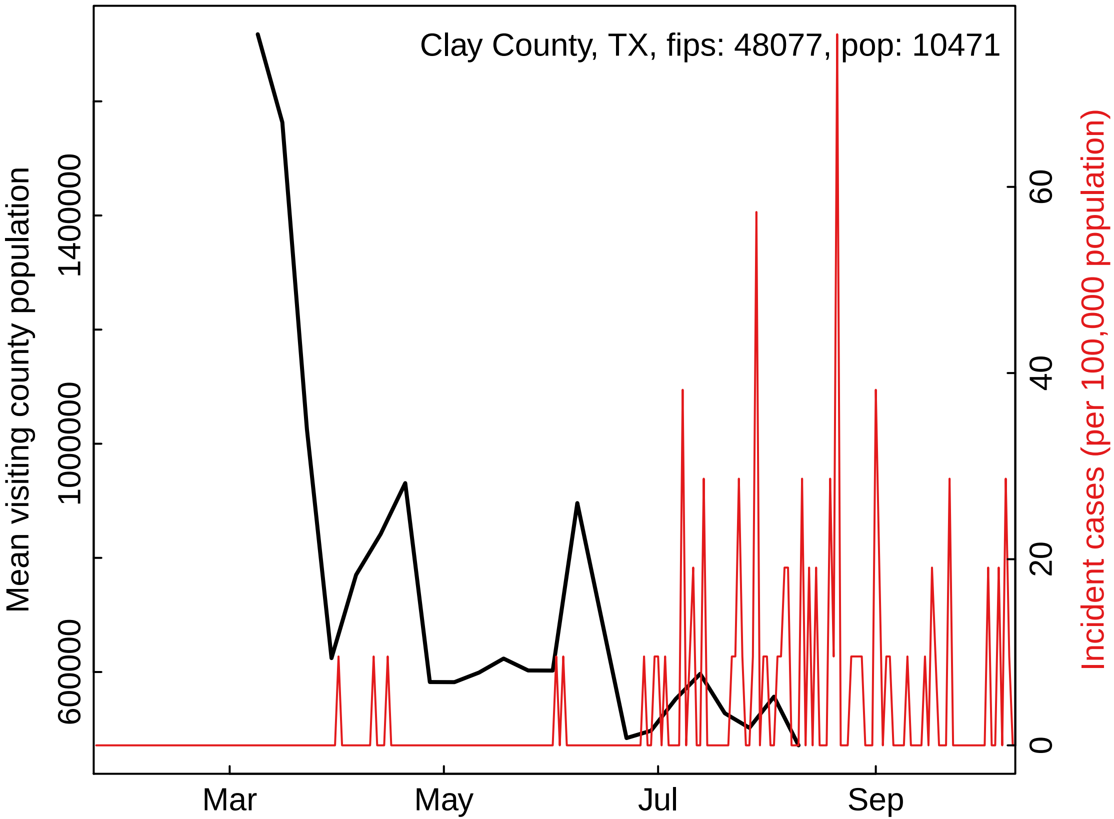

Clay County, TX, fips: 48077, pop: 10471
Mean visiting county population
600000
1000000
1400000
Incident cases (per 100,000 population)
0
20
40
60
Mar
May
Jul
Sep

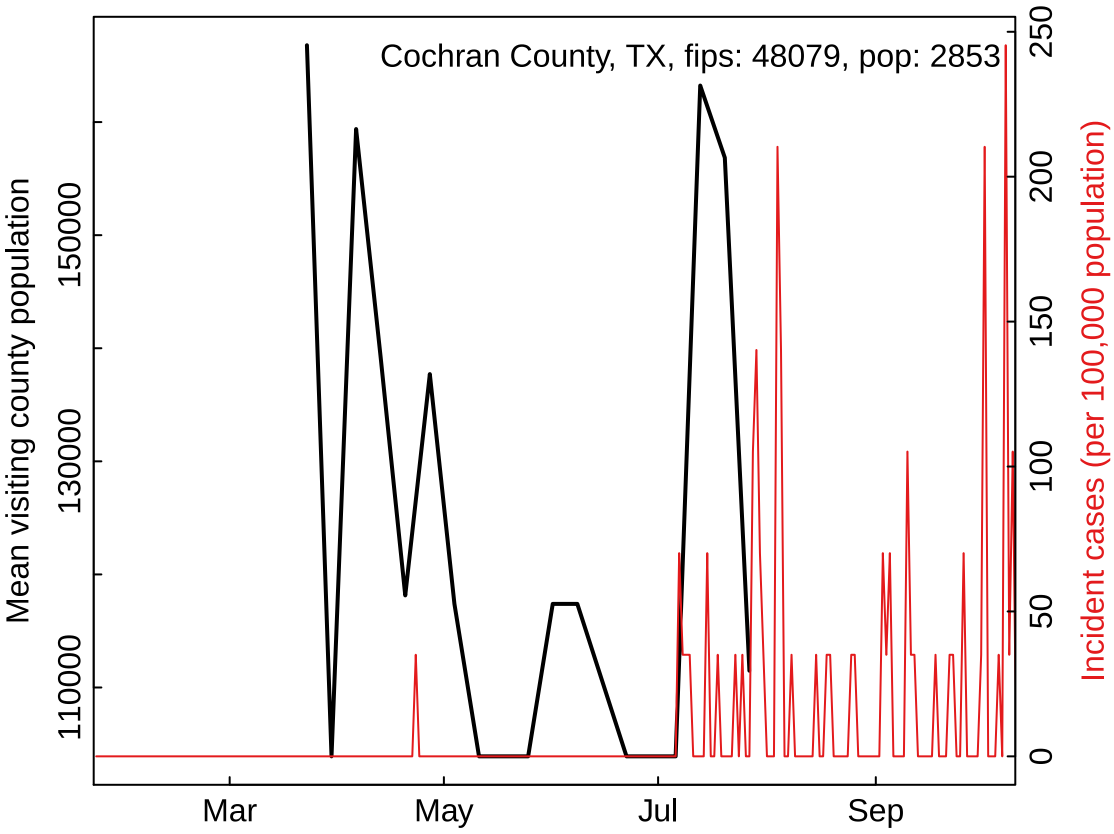
Cochran County, TX, fips: 48079, pop: 2853
Mean visiting county population
110000
130000
150000
Incident cases (per 100,000 population)
0
50
100
150
200
250
Mar
May
Jul
Sep

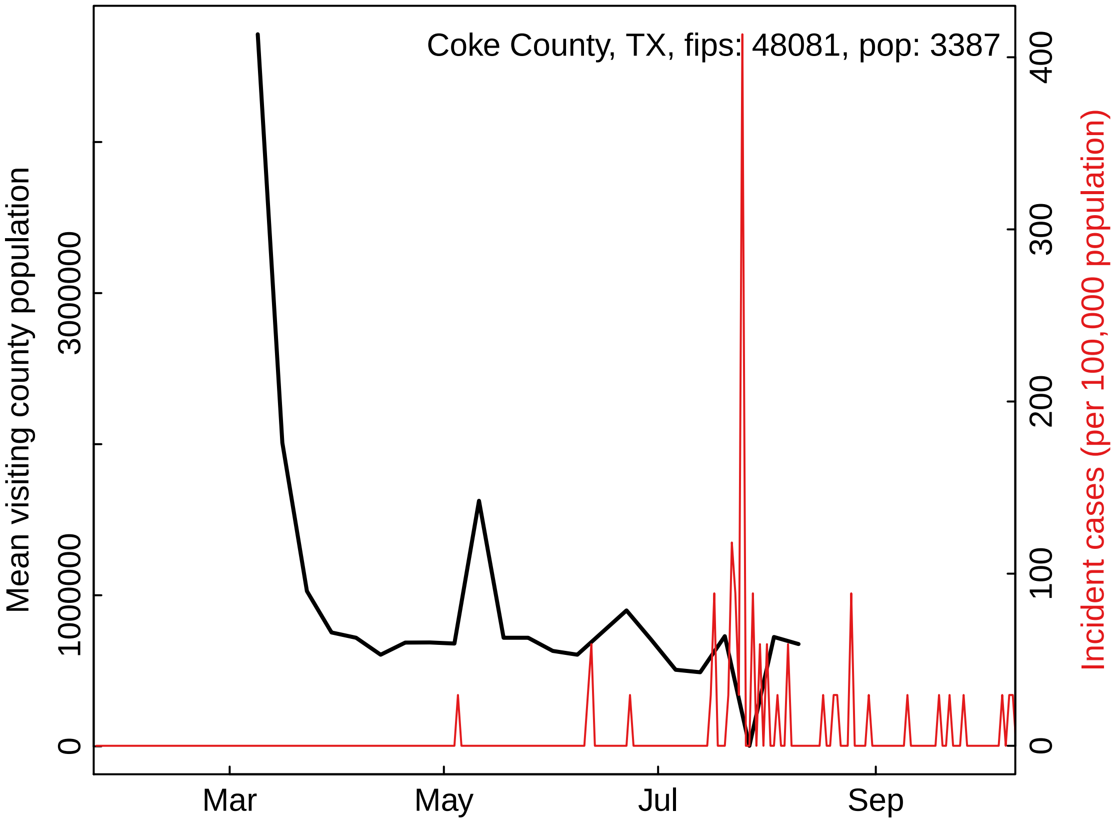

Coke County, TX, fips: 48081, pop: 3387
Mean visiting county population
0
1000000
3000000
Mar
May
Jul
Sep
Incident cases (per 100,000 population)
0
100
200
300
400

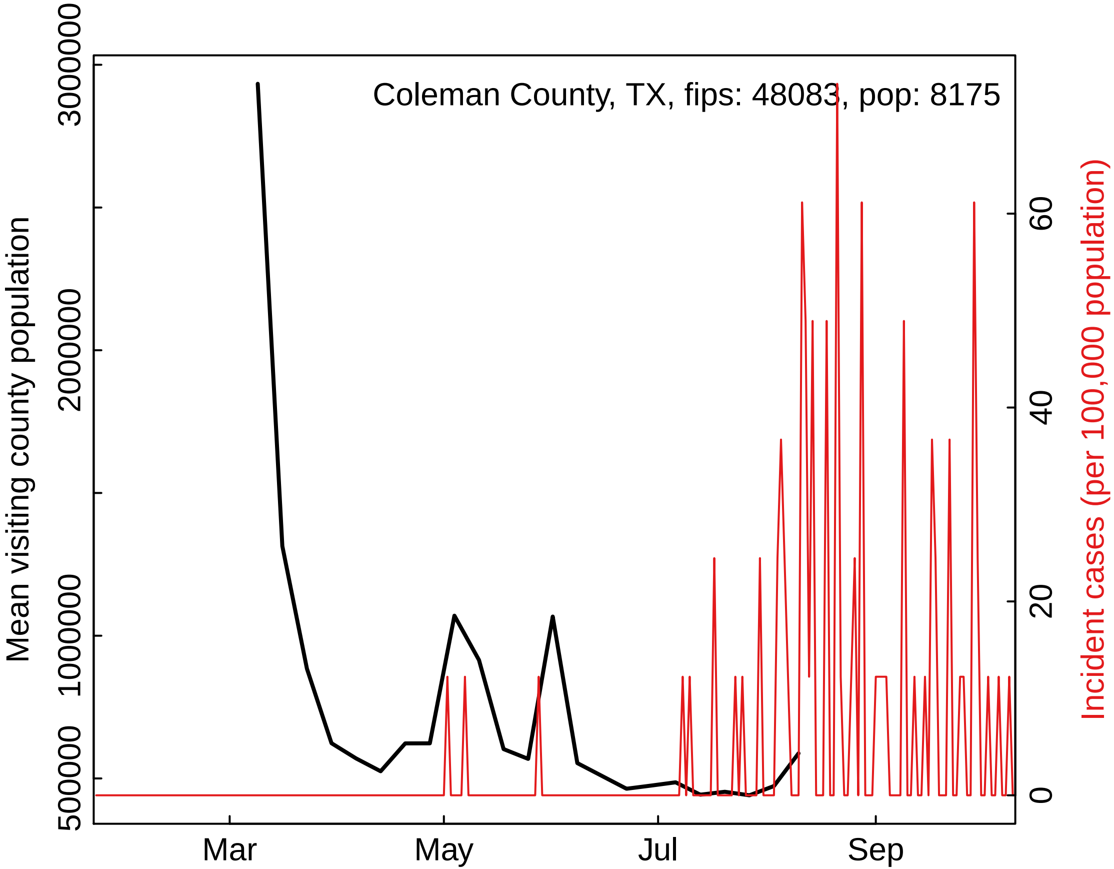

Coleman County, TX, fips: 48083, pop: 8175
Mean visiting county population
500000
1000000
2000000
3000000
Incident cases (per 100,000 population)
0
20
40
60
Mar
May
Jul
Sep

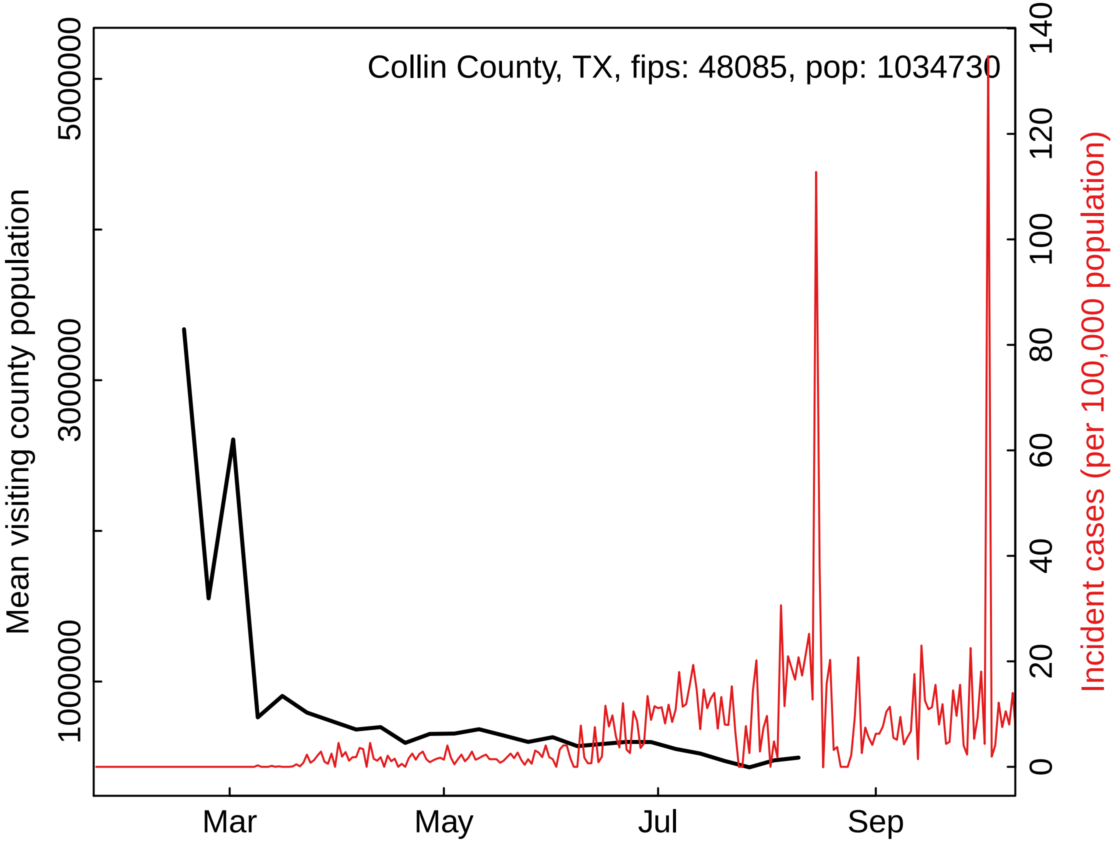
Collin County, TX, fips: 48085, pop: 1034730
Mean visiting county population
10000000
30000000
50000000
Incident cases (per 100,000 population)
0
20
40
60
80
100
120
140
Mar
May
Jul
Sep

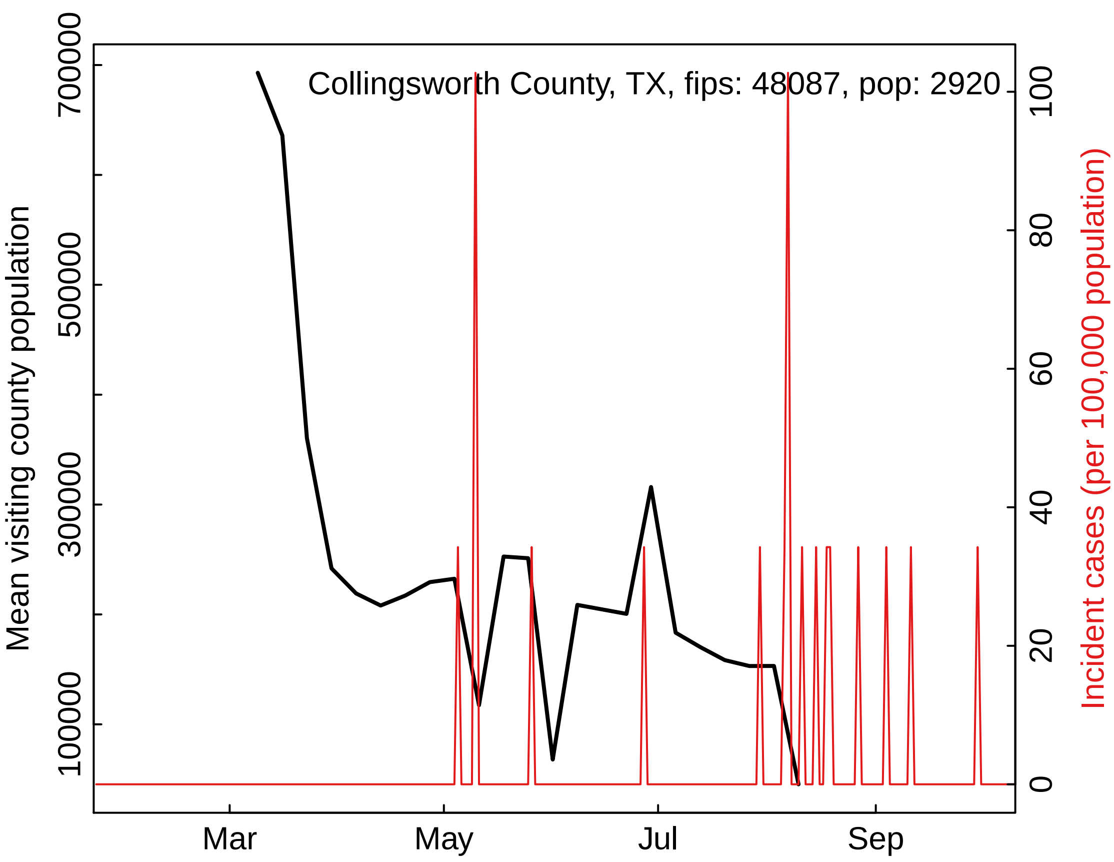

Collingsworth County, TX, fips: 48087, pop: 2920
Mean visiting county population
100000
300000
500000
700000
Incident cases (per 100,000 population)
0
20
40
60
80
100
Mar
May
Jul
Sep

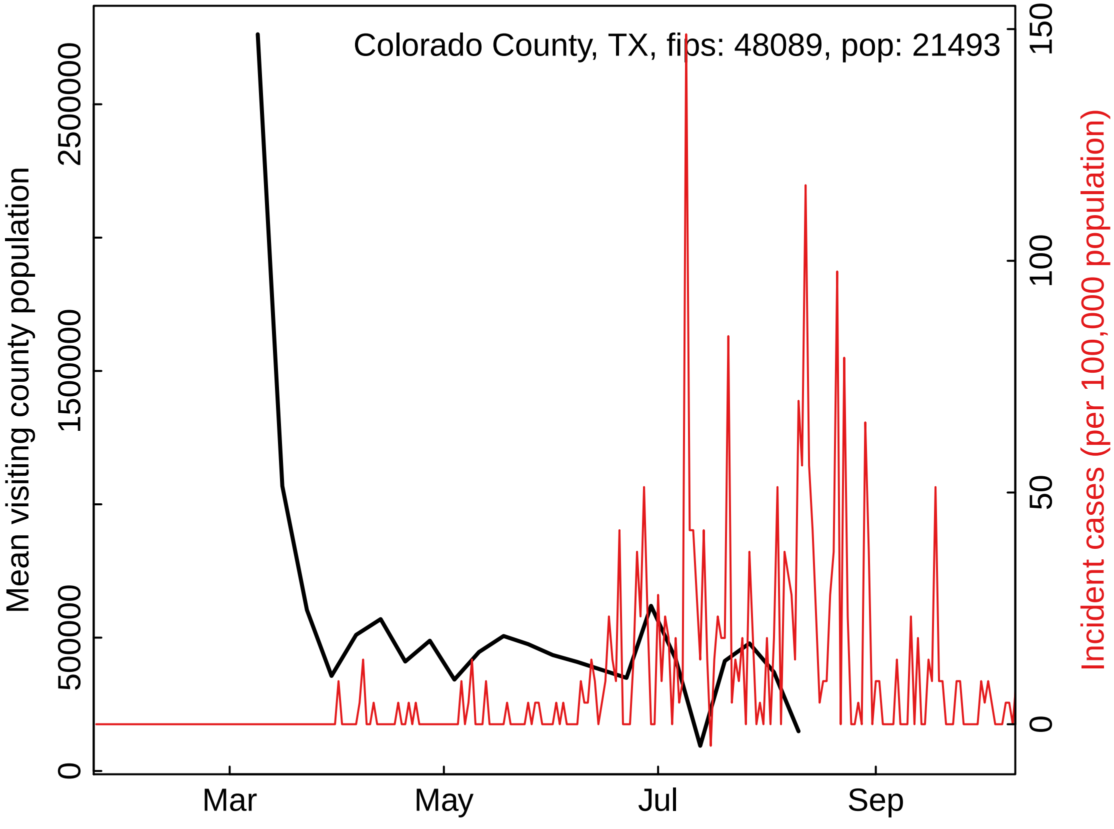

Colorado County, TX, fips: 48089, pop: 21493
Mean visiting county population
0
500000
1500000
2500000
Mar
May
Jul
Sep
Incident cases (per 100,000 population)
0
50
100
150

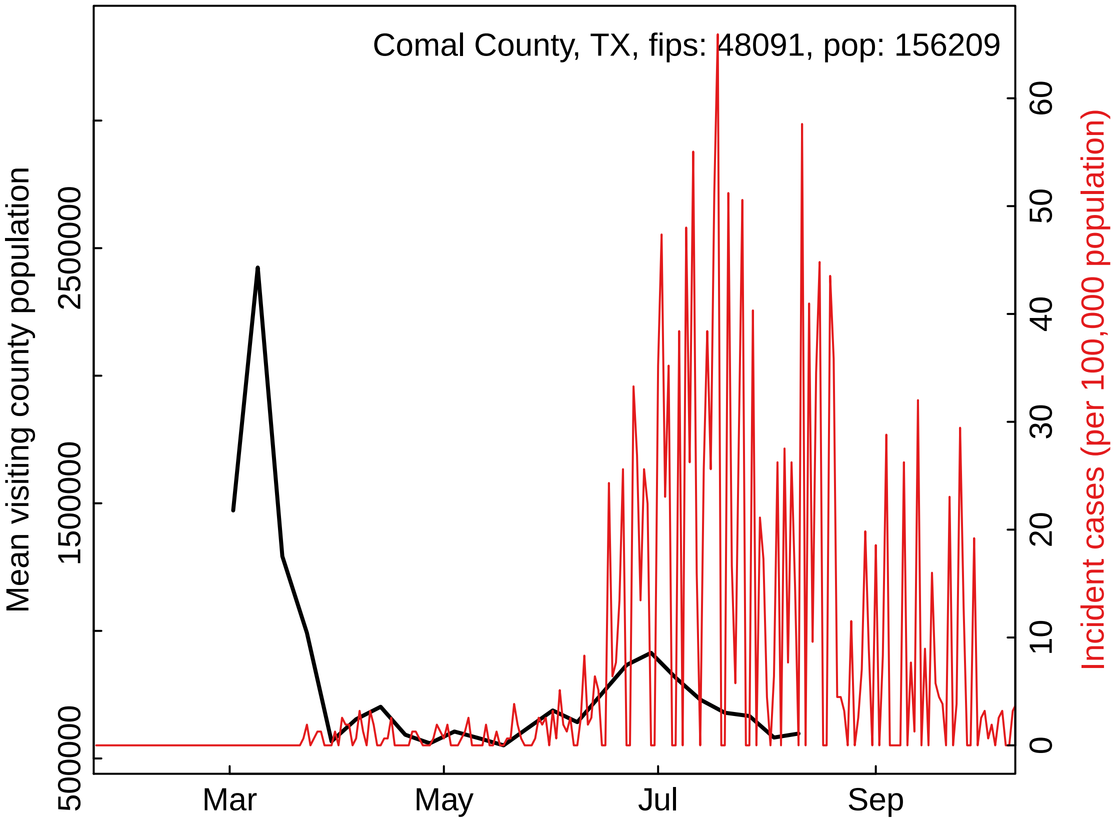

Comal County, TX, fips: 48091, pop: 156209
Mean visiting county population
500000
1500000
2500000
Incident cases (per 100,000 population)
0
10
20
30
40
50
60
Mar
May
Jul
Sep

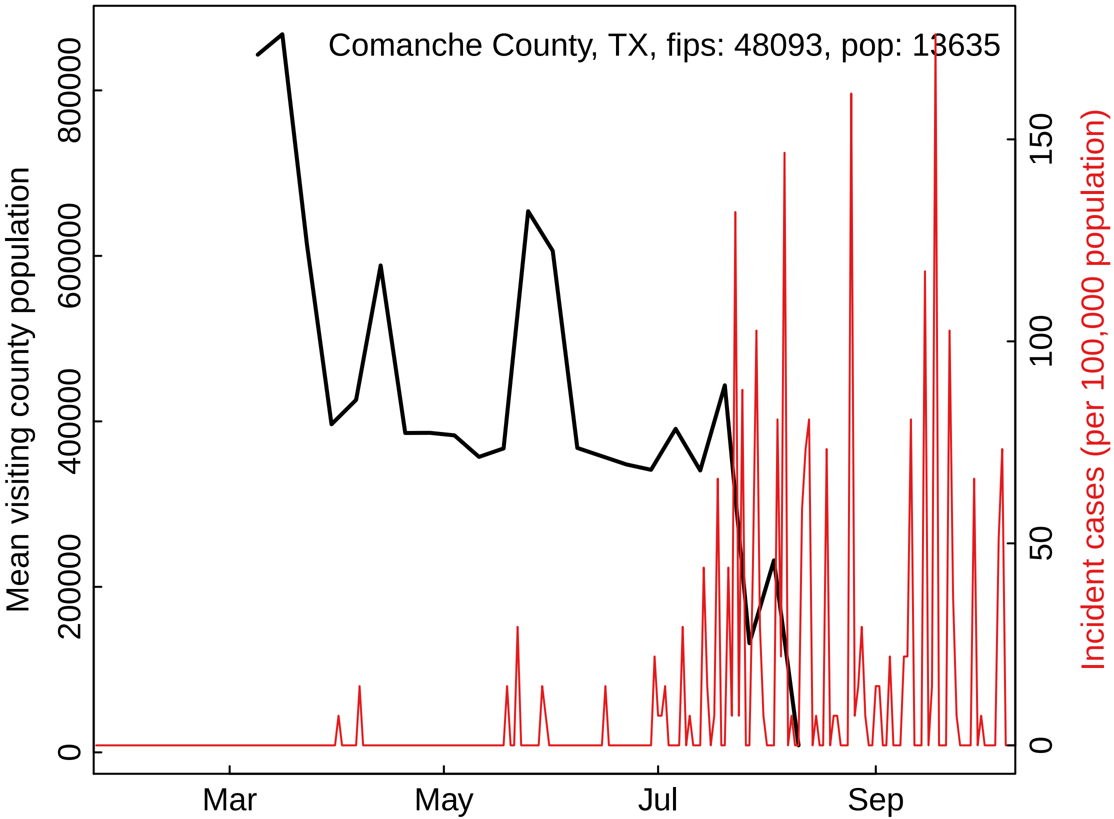

Comanche County, TX, fips: 48093, pop: 13635
Mean visiting county population
0
200000
400000
600000
800000
Incident cases (per 100,000 population)
0
50
100
150
Mar
May
Jul
Sep

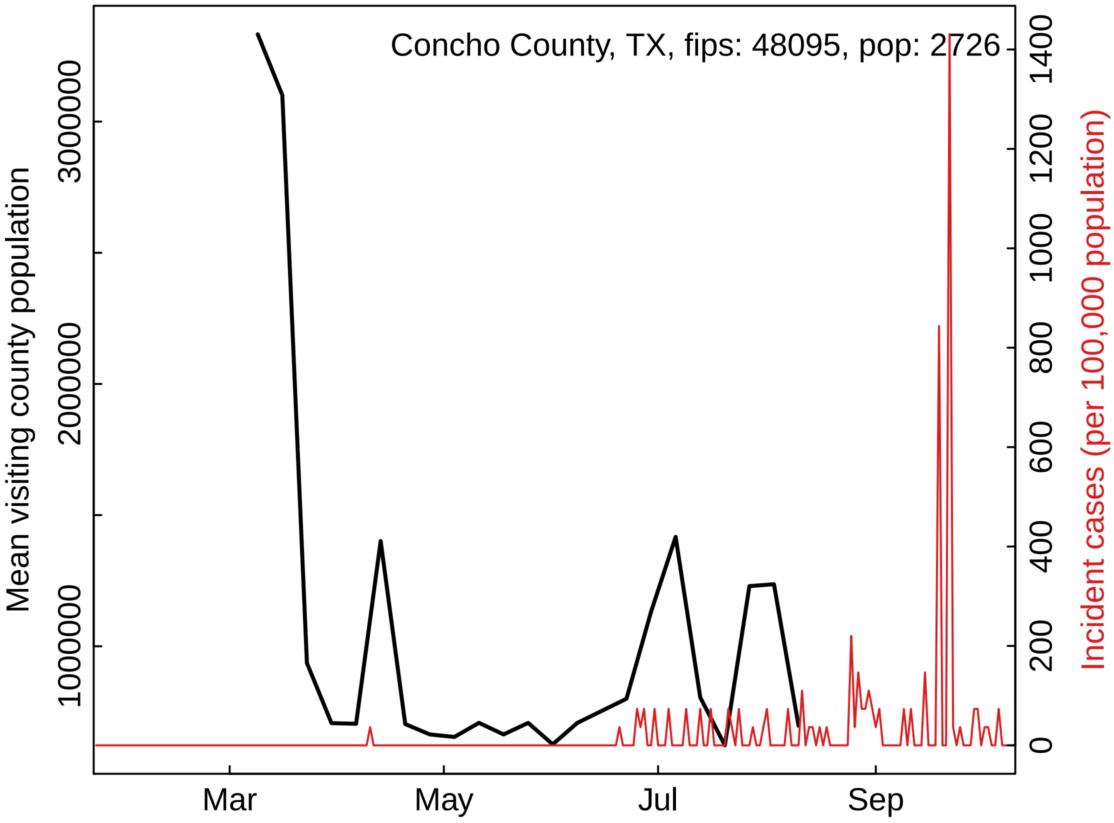

Concho County, TX, fips: 48095, pop: 2726
Mean visiting county population
1000000
2000000
3000000
Incident cases (per 100,000 population)
0
200
400
600
800
1000
1200
1400
Mar
May
Jul
Sep

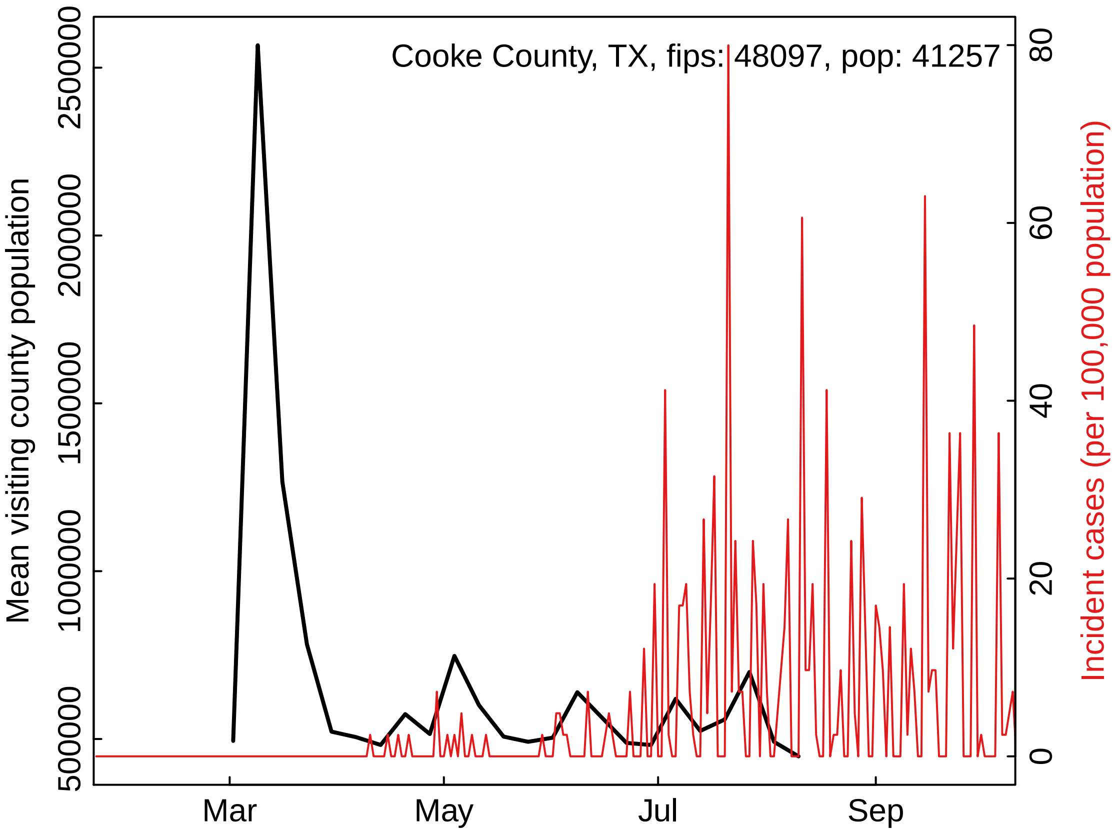
Cooke County, TX, fips: 48097, pop: 41257
Mean visiting county population
500000
1000000
1500000
2000000
2500000
Incident cases (per 100,000 population)
0
20
40
60
80
Mar
May
Jul
Sep

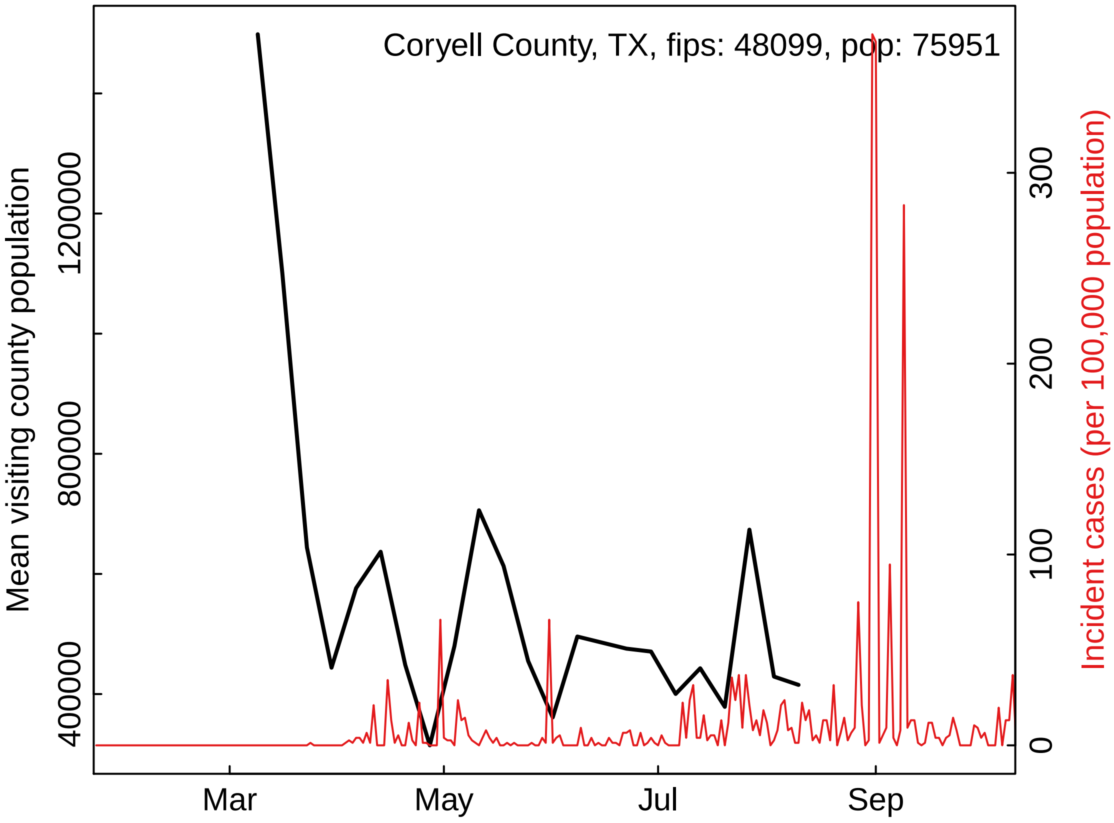

Coryell County, TX, fips: 48099, pop: 75951
Mean visiting county population
400000
800000
1200000
Mar
May
Jul
Sep
0
100
200
300
Incident cases (per 100,000 population)

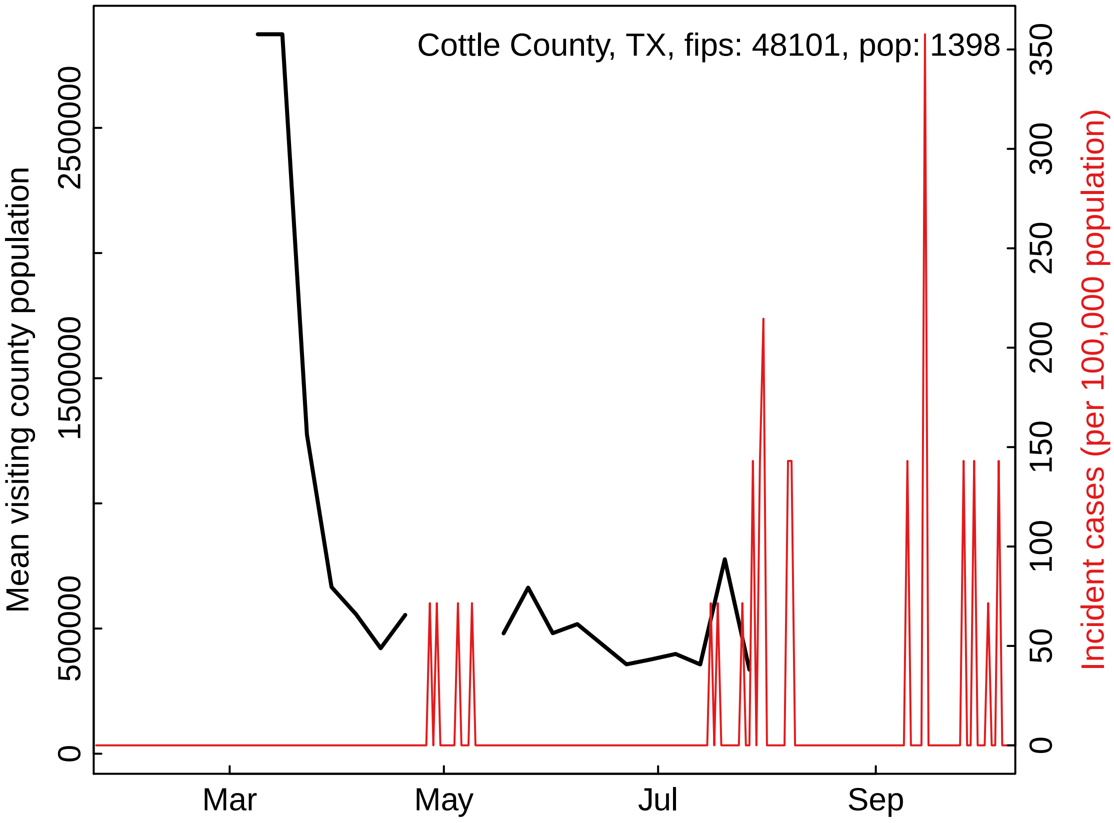

Cottle County, TX, fips: 48101, pop: 1398
Mean visiting county population
0
500000
1500000
2500000
Incident cases (per 100,000 population)
0
50
100
150
200
250
300
350
Mar
May
Jul
Sep

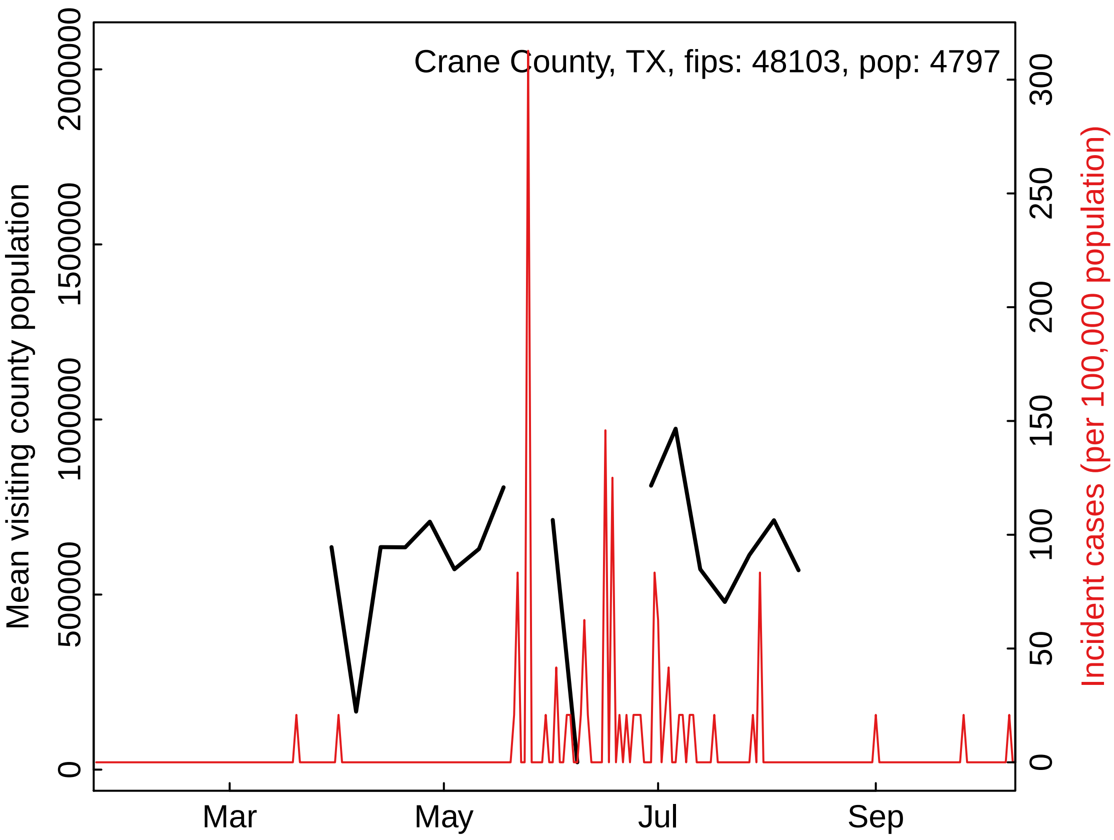

Crane County, TX, fips: 48103, pop: 4797
Mean visiting county population
0
500000
1000000
1500000
2000000
Incident cases (per 100,000 population)
0
50
100
150
200
250
300
Mar
May
Jul
Sep

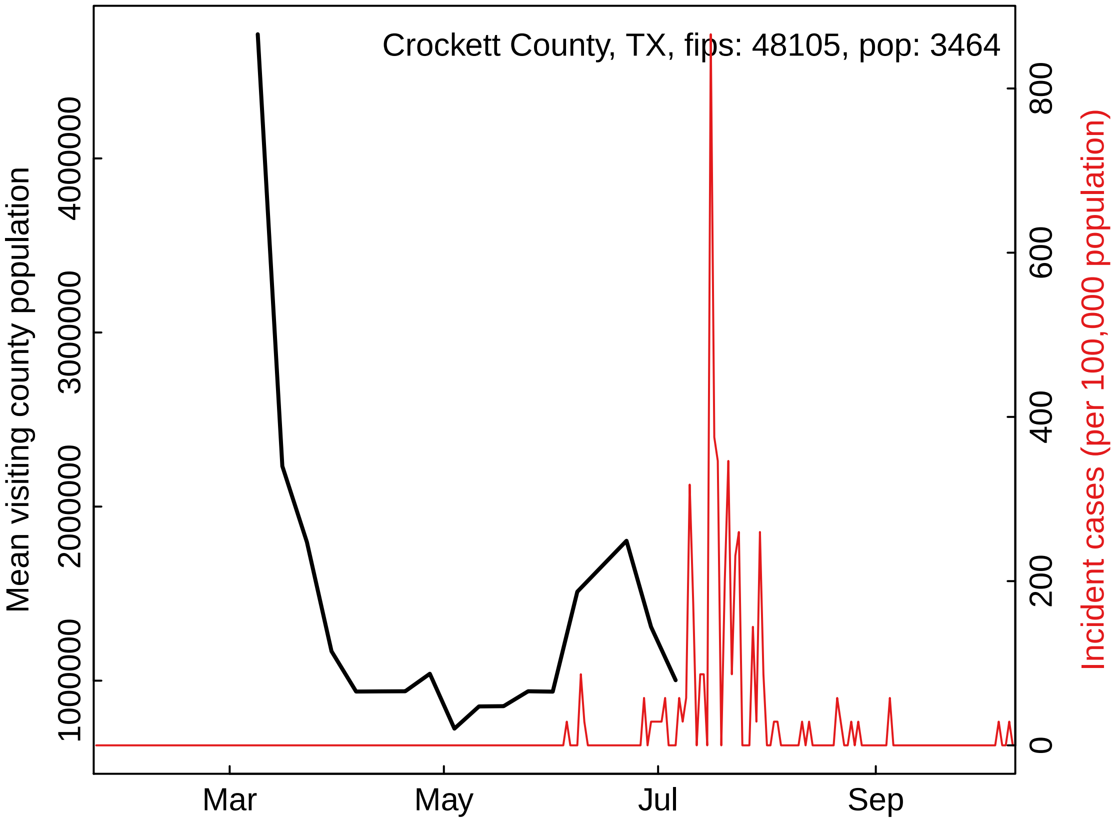
Crockett County, TX, fips: 48105, pop: 3464
Mean visiting county population
10000000
2000000
3000000
4000000
Mar
May
Jul
Sep
0
200
400
600
800
Incident cases (per 100,000 population)

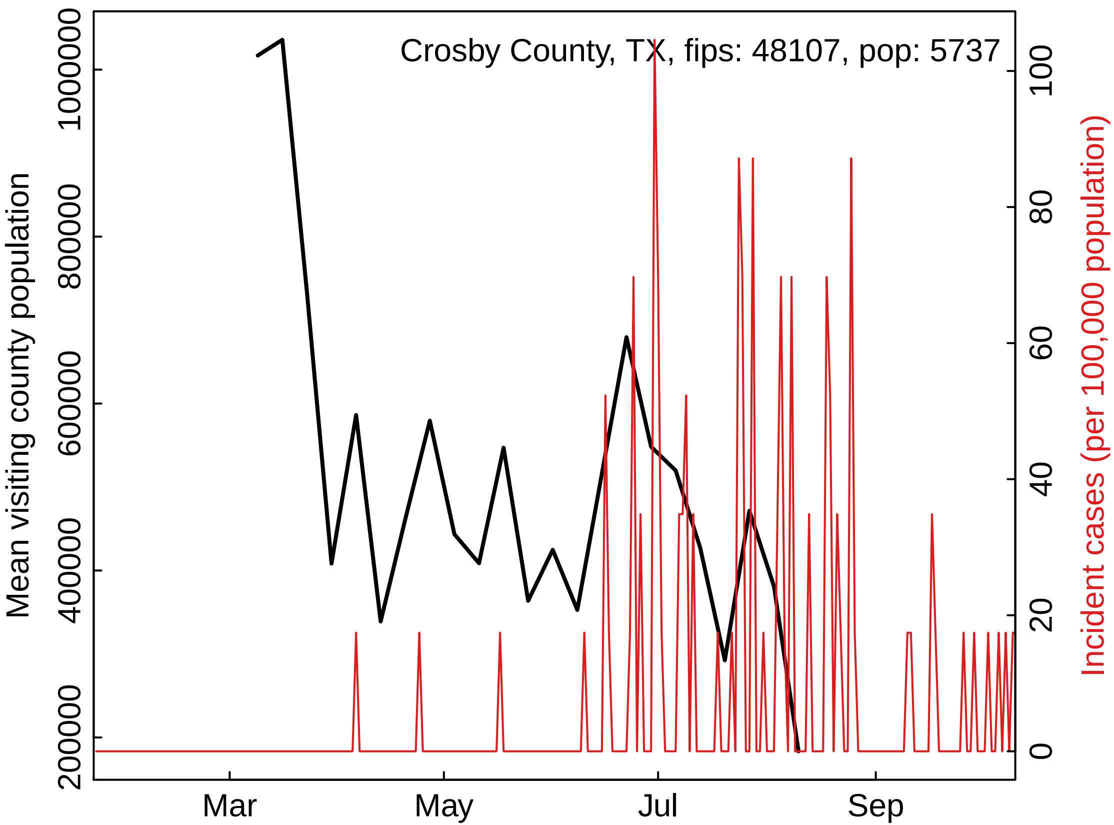
Crosby County, TX, fips: 48107, pop: 5737
Mean visiting county population
Incident cases (per 100,000 population)
200000
400000
600000
800000
1000000
0
20
40
60
80
100
Mar
May
Jul
Sep

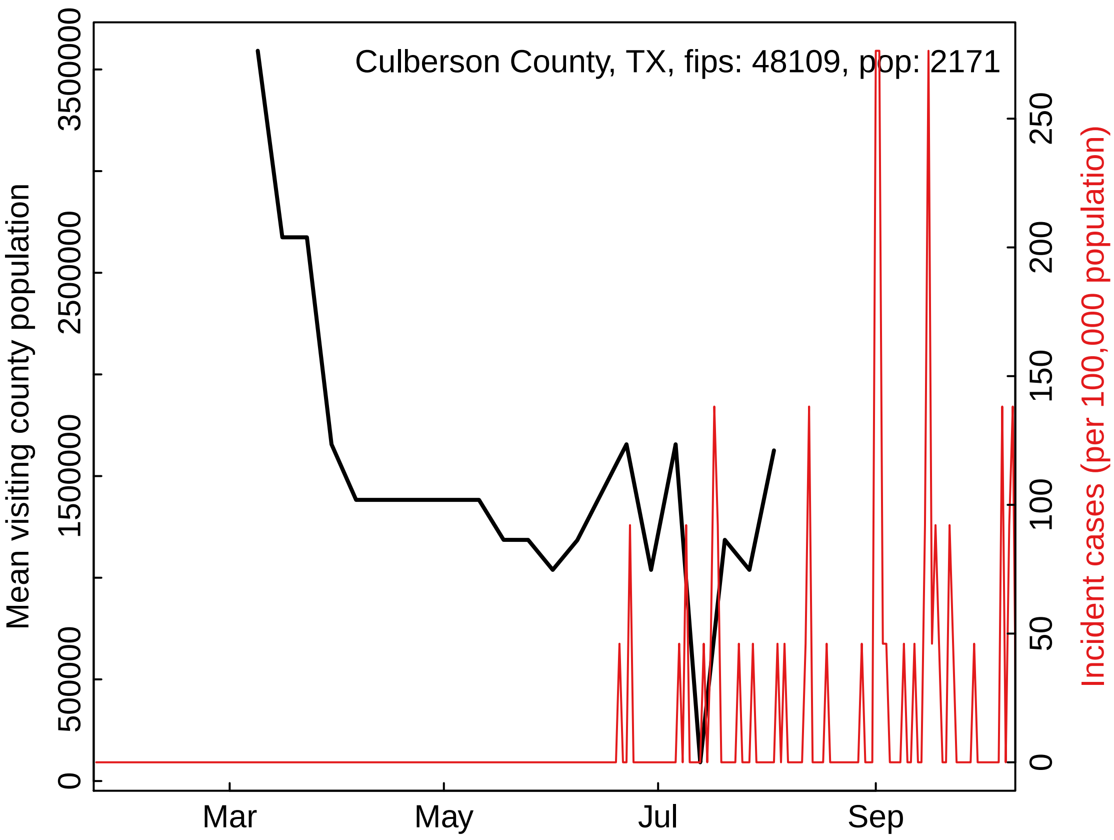

Culberson County, TX, fips: 48109, pop: 2171
Mean visiting county population
0
500000
1500000
2500000
3500000
Incident cases (per 100,000 population)
0
50
100
150
200
250
Mar
May
Jul
Sep

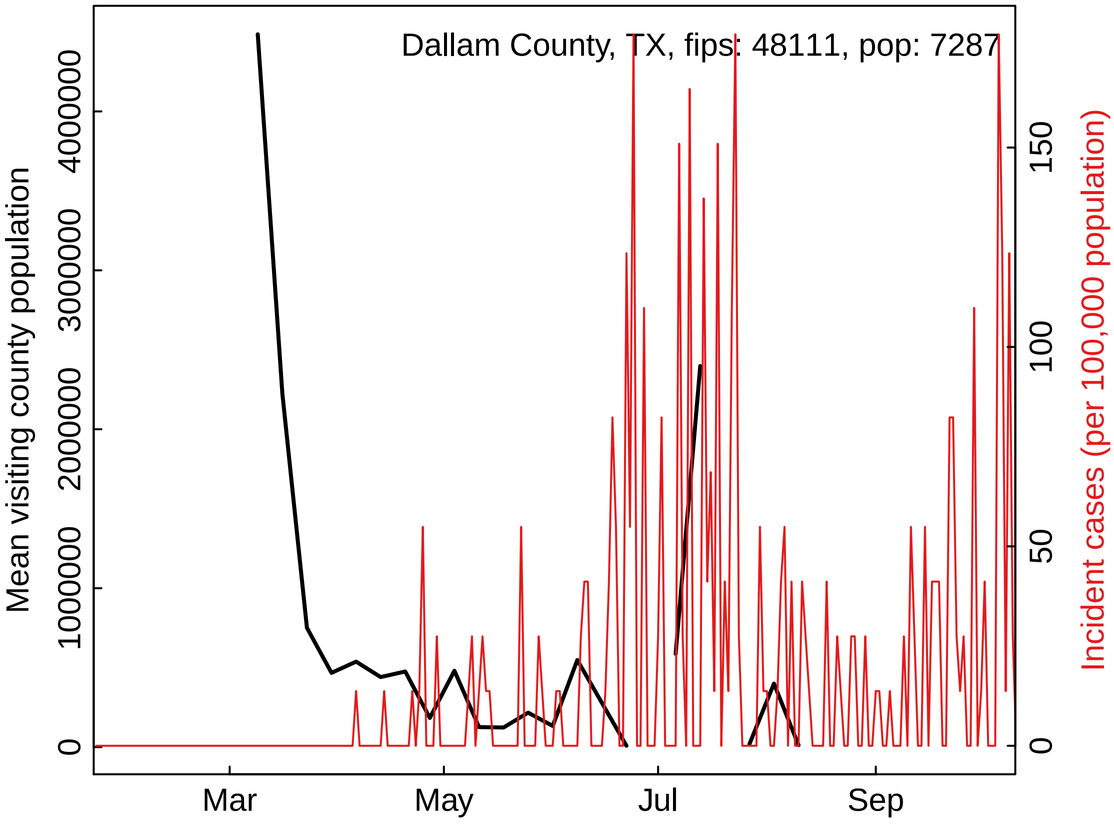

Dallam County, TX, fips: 48111, pop: 7287
Mean visiting county population
0
1000000
2000000
3000000
4000000
Incident cases (per 100,000 population)
0
50
100
150
Mar
May
Jul
Sep

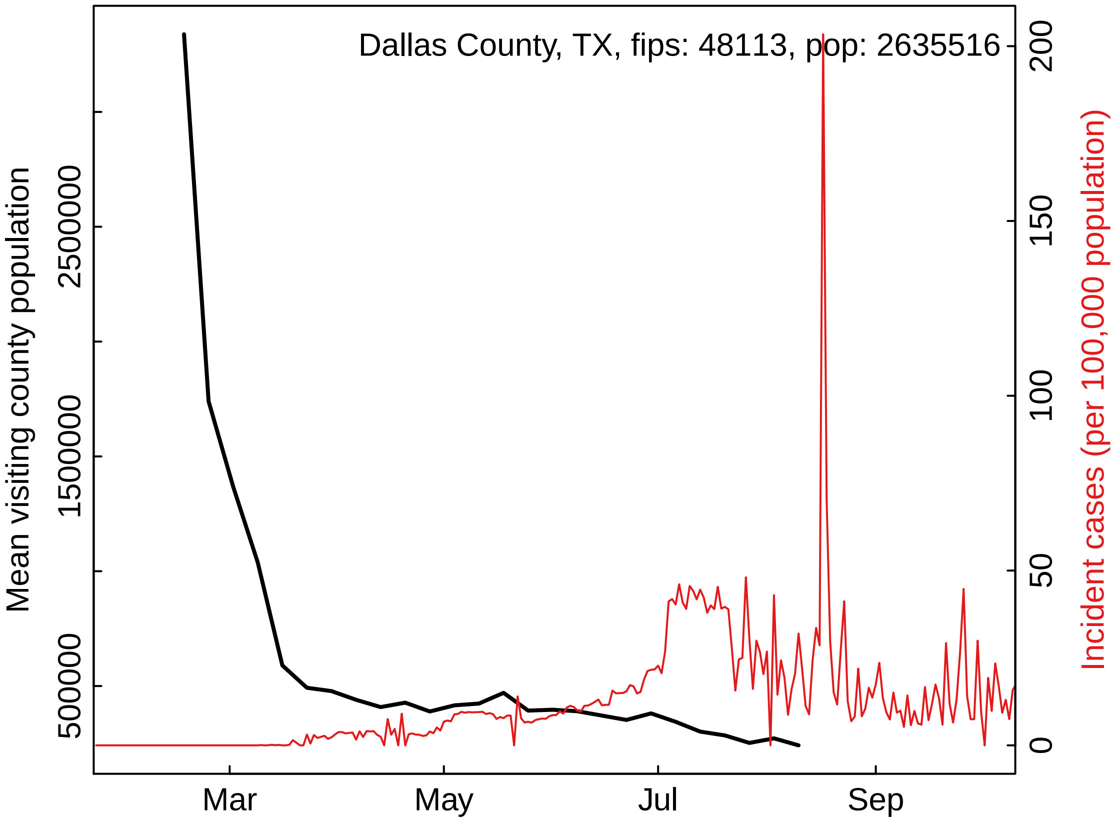

Dallas County, TX, fips: 48113, pop: 2635516
Mean visiting county population
500000
1500000
2500000
Incident cases (per 100,000 population)
0
50
100
150
200
Mar
May
Jul
Sep

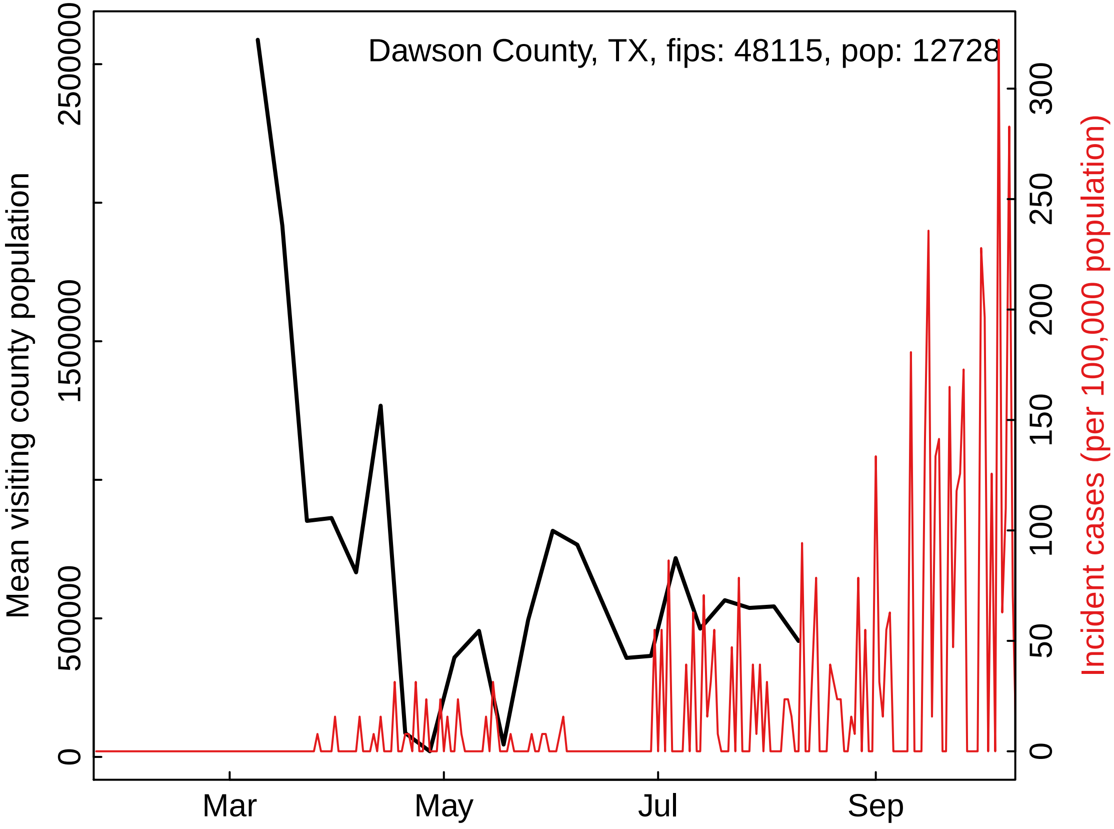
Dawson County, TX, fips: 48115, pop: 12728
Mean visiting county population
0
500000
1500000
2500000
Incident cases (per 100,000 population)
0
50
100
150
200
250
300
Mar
May
Jul
Sep

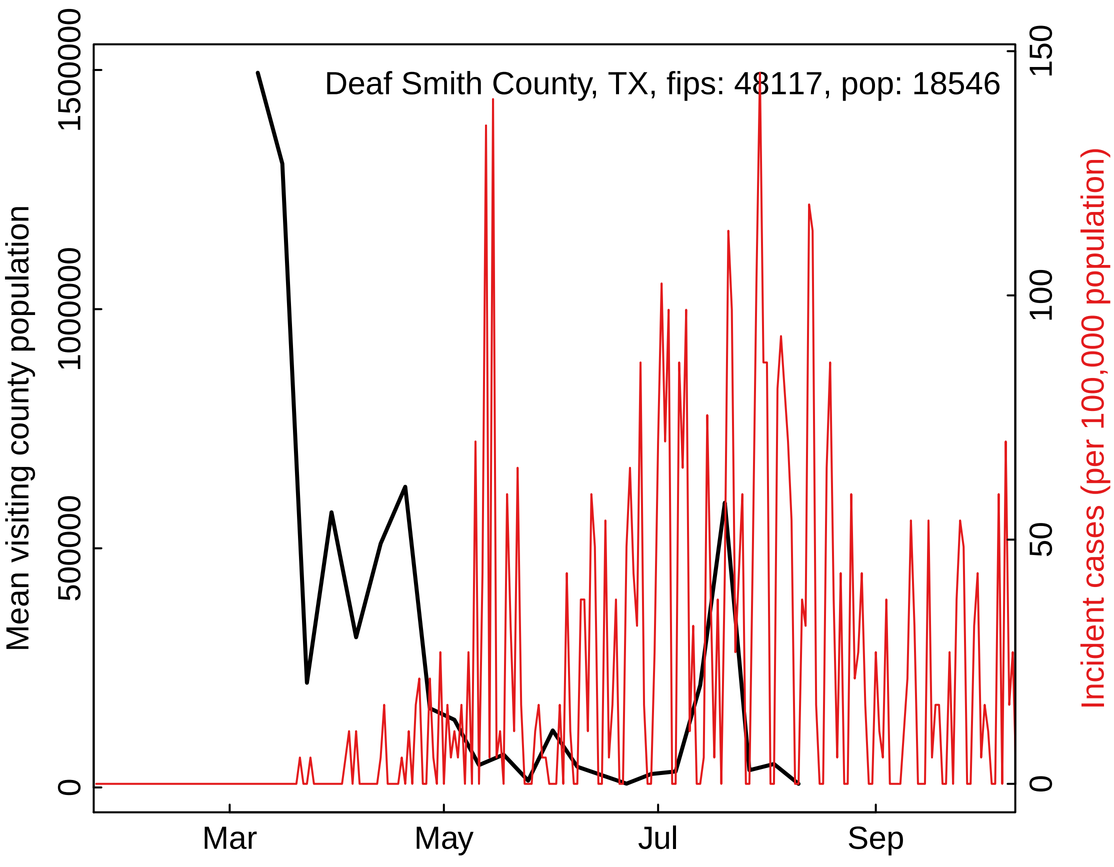
Deaf Smith County, TX, fips: 48117, pop: 18546
Mean visiting county population
0
500000
1000000
1500000
Incident cases (per 100,000 population)
0
50
100
150
Mar
May
Jul
Sep

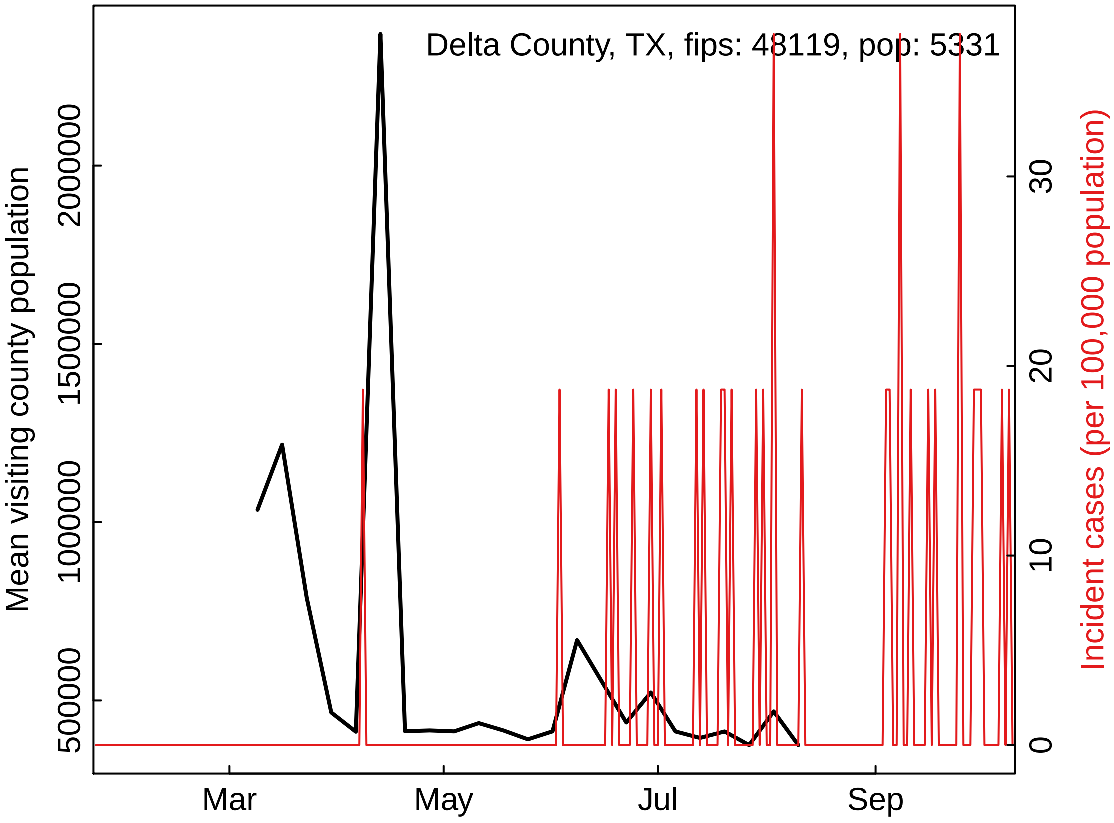
Delta County, TX, fips: 48119, pop: 5331
Mean visiting county population
500000
1000000
1500000
2000000
Incident cases (per 100,000 population)
0
10
20
30
Mar
May
Jul
Sep

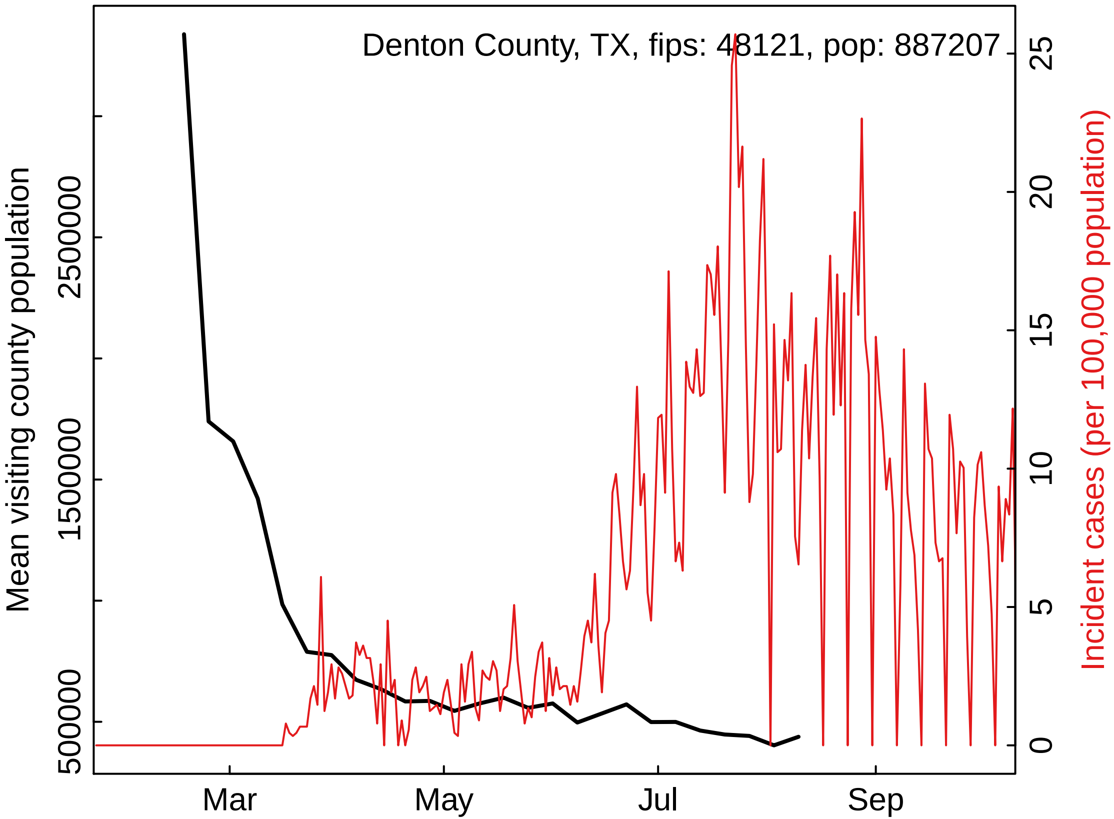

Denton County, TX, fips: 48121, pop: 887207
Mean visiting county population
500000
1500000
2500000
Incident cases (per 100,000 population)
0
5
10
15
20
25
Mar
May
Jul
Sep

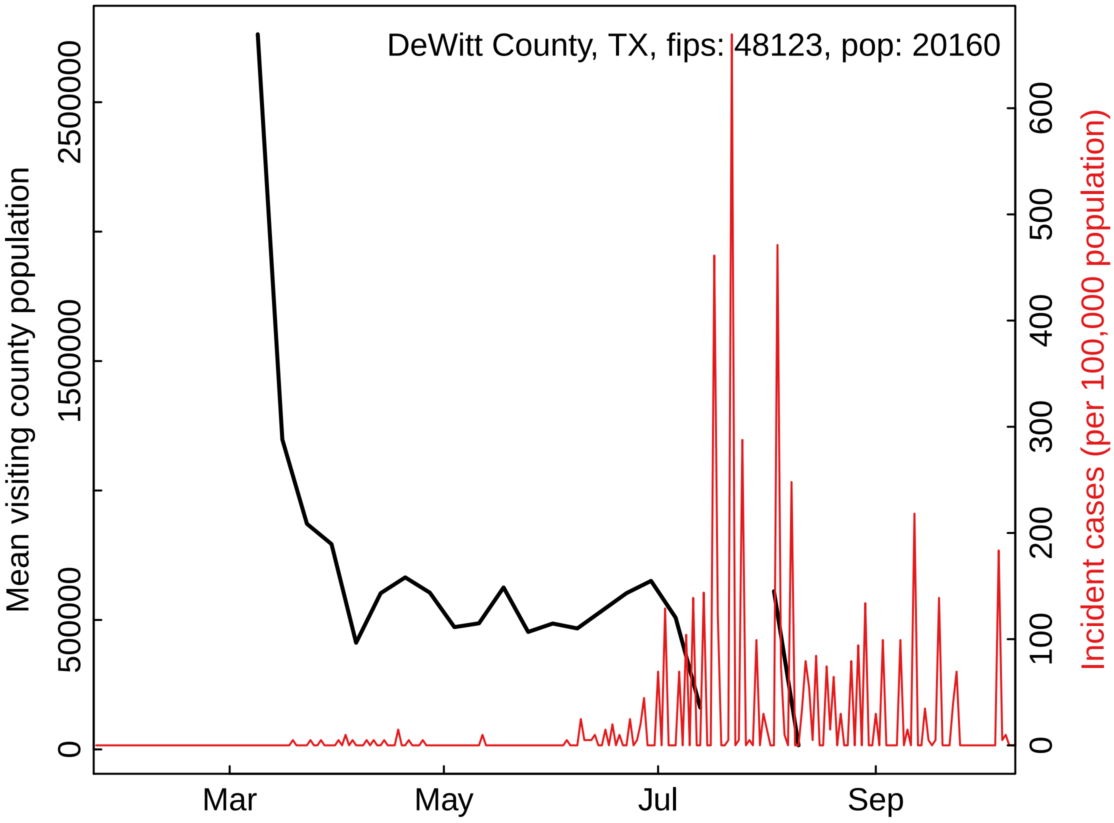

DeWitt County, TX, fips: 48123, pop: 20160
Mean visiting county population
0
500000
1500000
2500000
Incident cases (per 100,000 population)
0
100
200
300
400
500
600
Mar
May
Jul
Sep

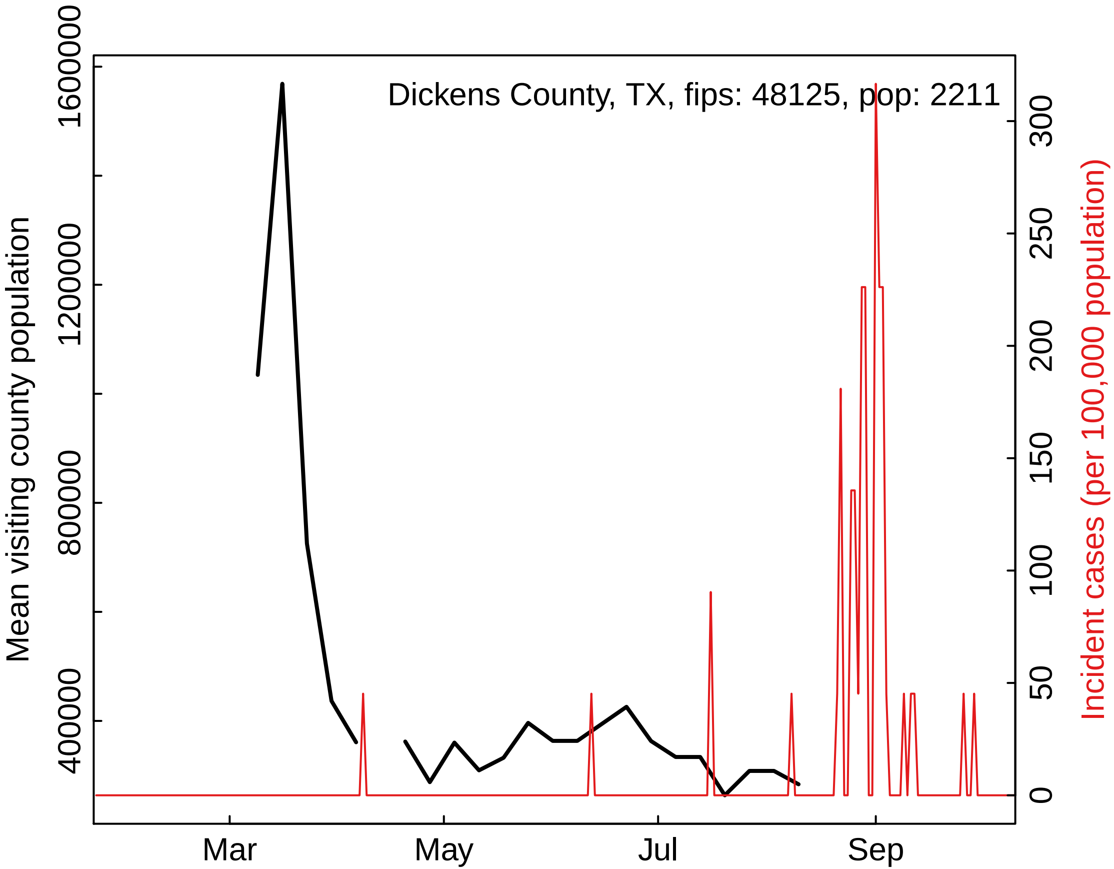

Dickens County, TX, fips: 48125, pop: 2211
Mean visiting county population
400000
800000
1200000
1600000
Incident cases (per 100,000 population)
0
50
100
150
200
250
300
Mar
May
Jul
Sep

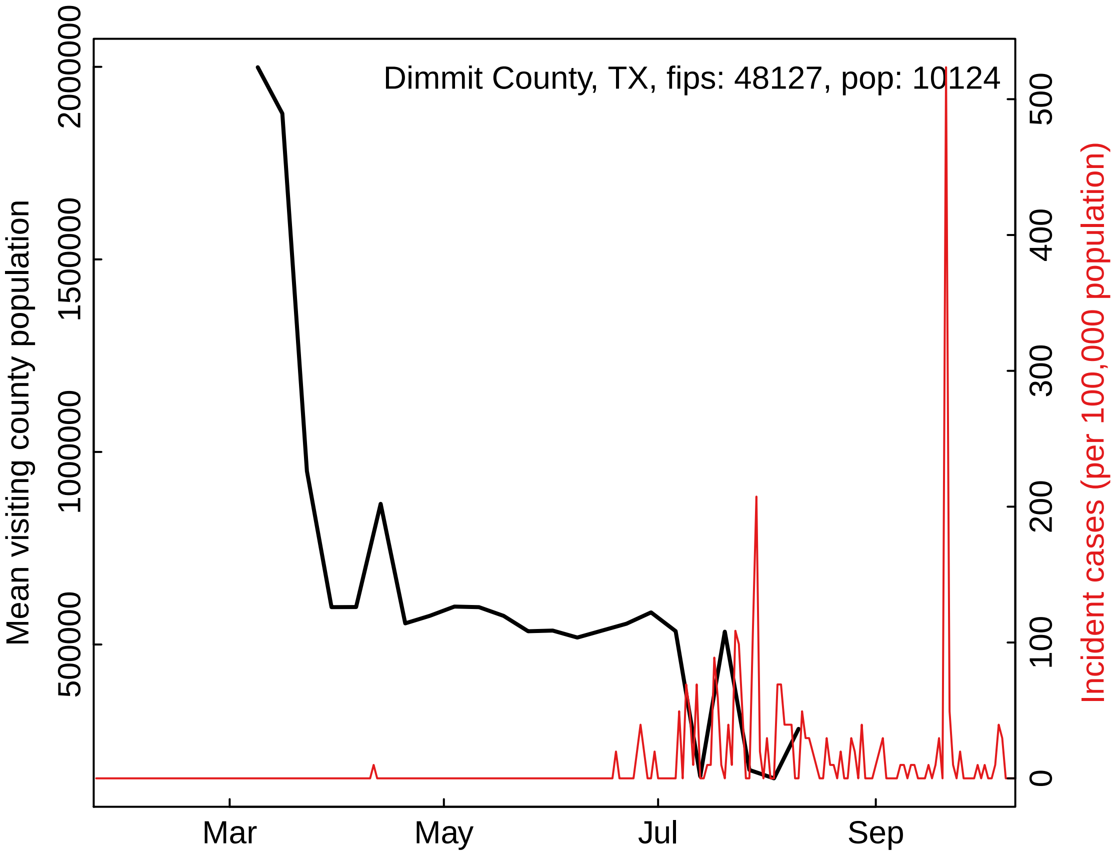

Dimmit County, TX, fips: 48127, pop: 10124
Mean visiting county population
2000000
1500000
1000000
500000
Incident cases (per 100,000 population)
500
400
300
200
100
0
Mar
May
Jul
Sep

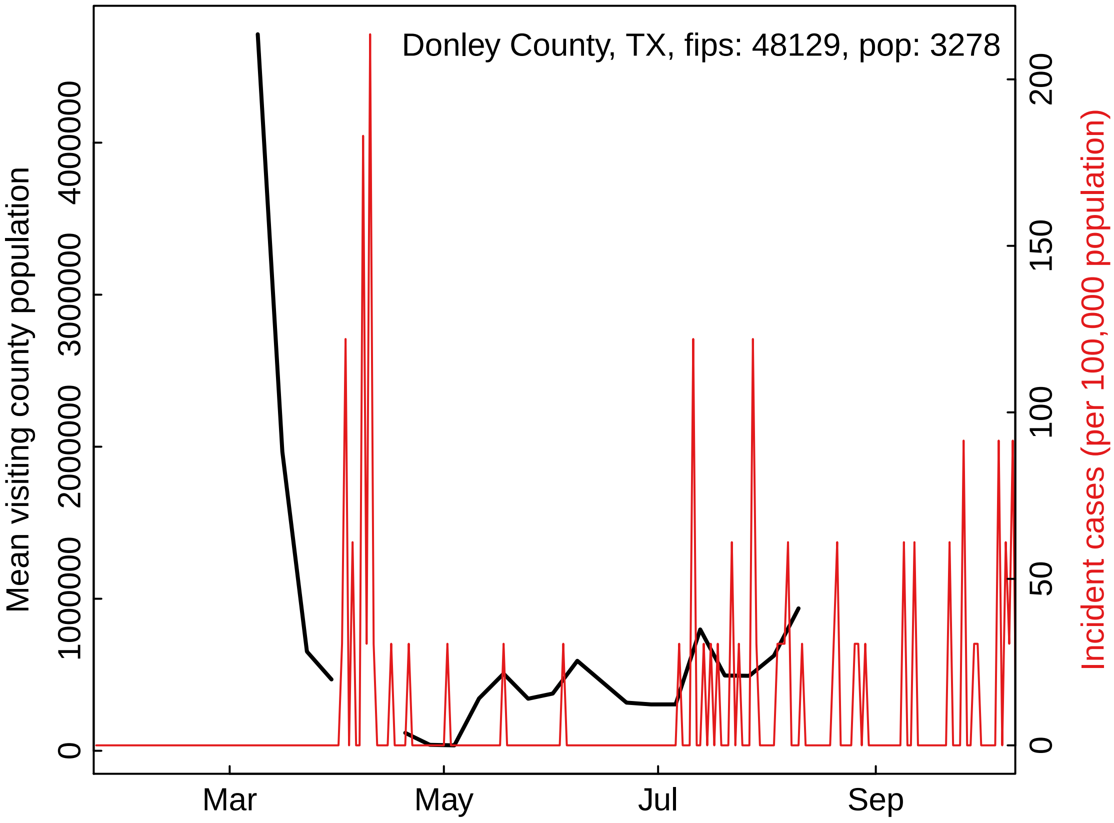

Donley County, TX, fips: 48129, pop: 3278
Mean visiting county population
0
1000000
2000000
3000000
4000000
Incident cases (per 100,000 population)
0
50
100
150
200
Mar
May
Jul
Sep

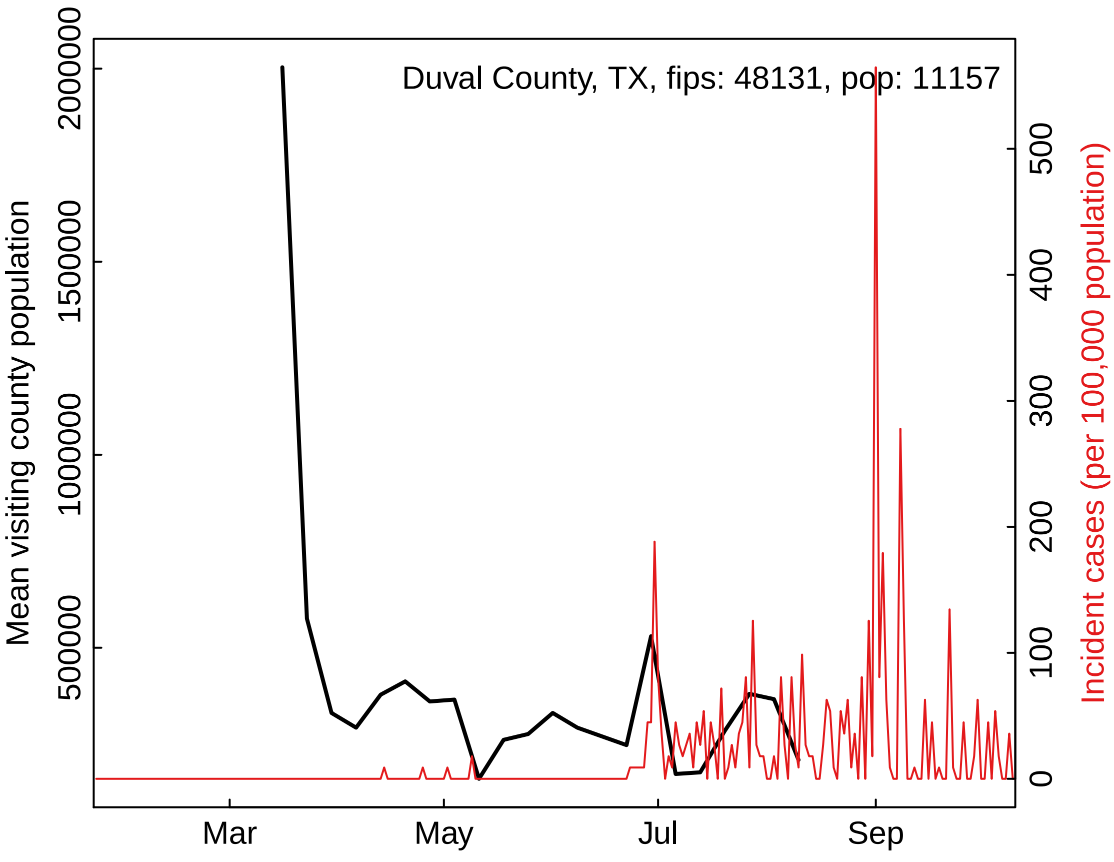
Duval County, TX, fips: 48131, pop: 11157
Mean visiting county population
500000
1000000
1500000
2000000
Incident cases (per 100,000 population)
0
100
200
300
400
500
Mar
May
Jul
Sep

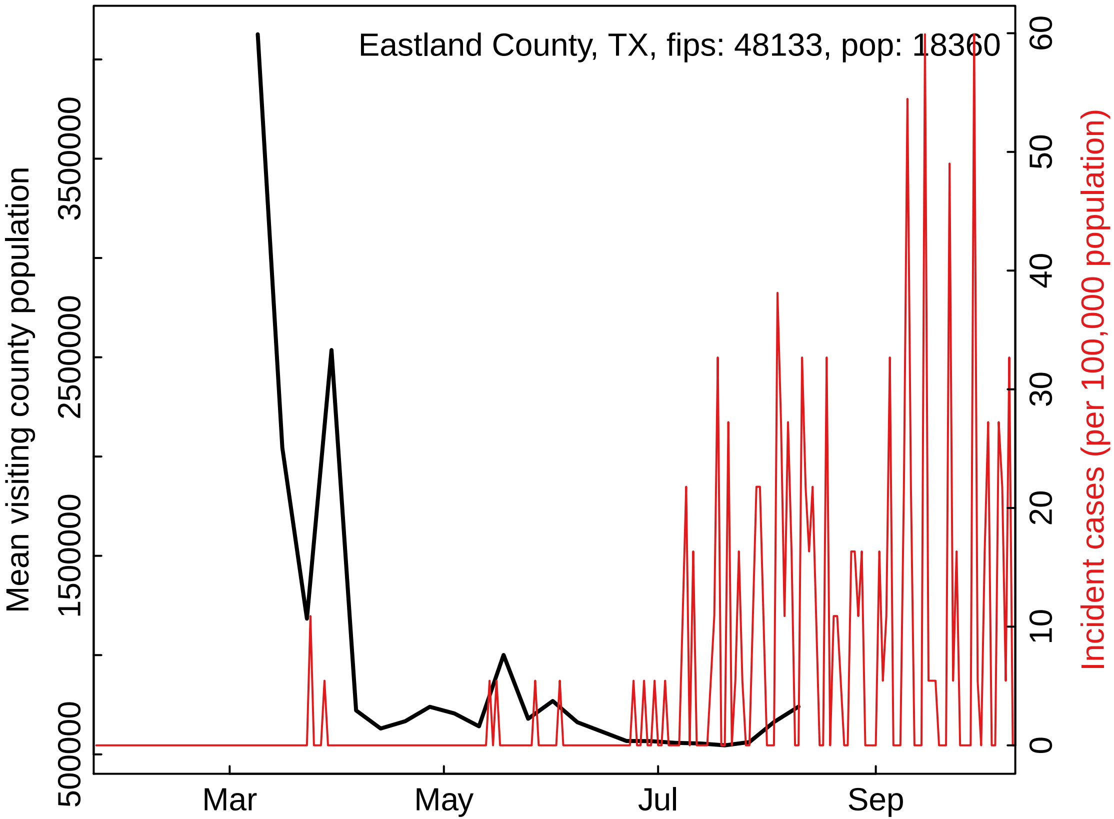
Eastland County, TX, fips: 48133, pop: 18360
Mean visiting county population
500000
1500000
2500000
3500000
Mar
May
Jul
Sep
0
10
20
30
40
50
60
Incident cases (per 100,000 population)

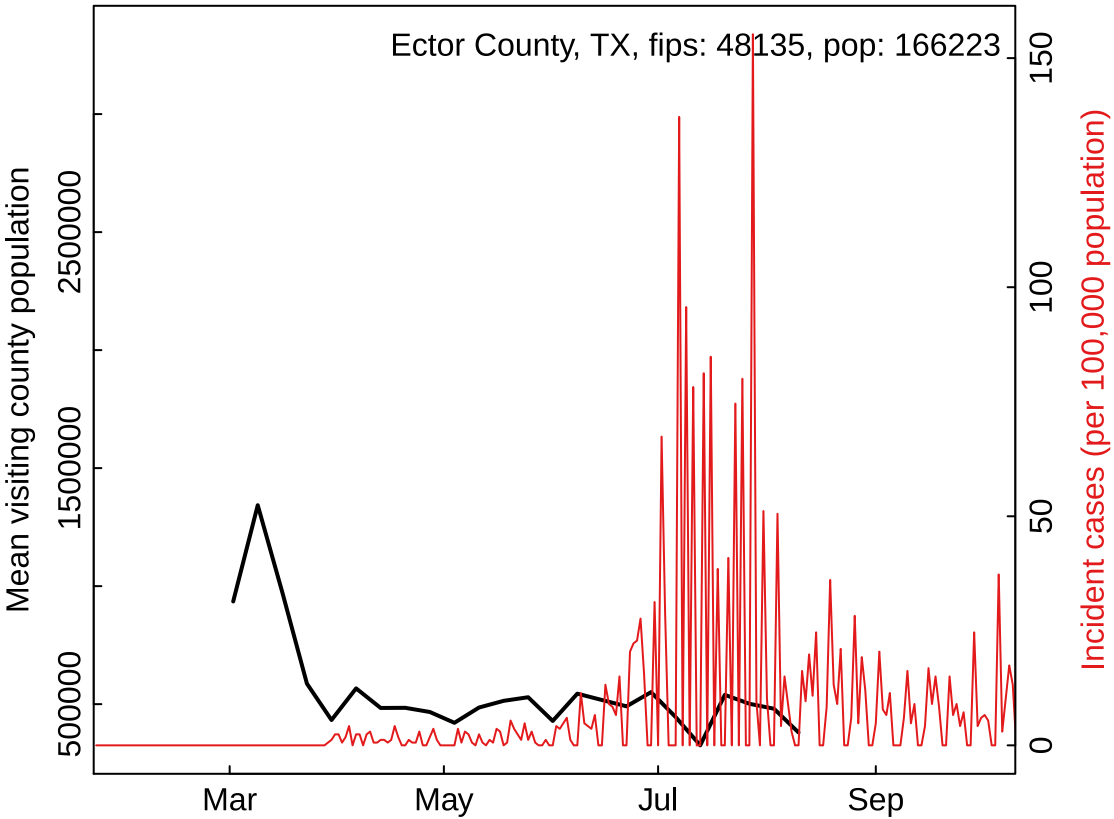

Ector County, TX, fips: 48135, pop: 166223
Mean visiting county population
500000
1500000
2500000
Incident cases (per 100,000 population)
0
50
100
150
Mar
May
Jul
Sep

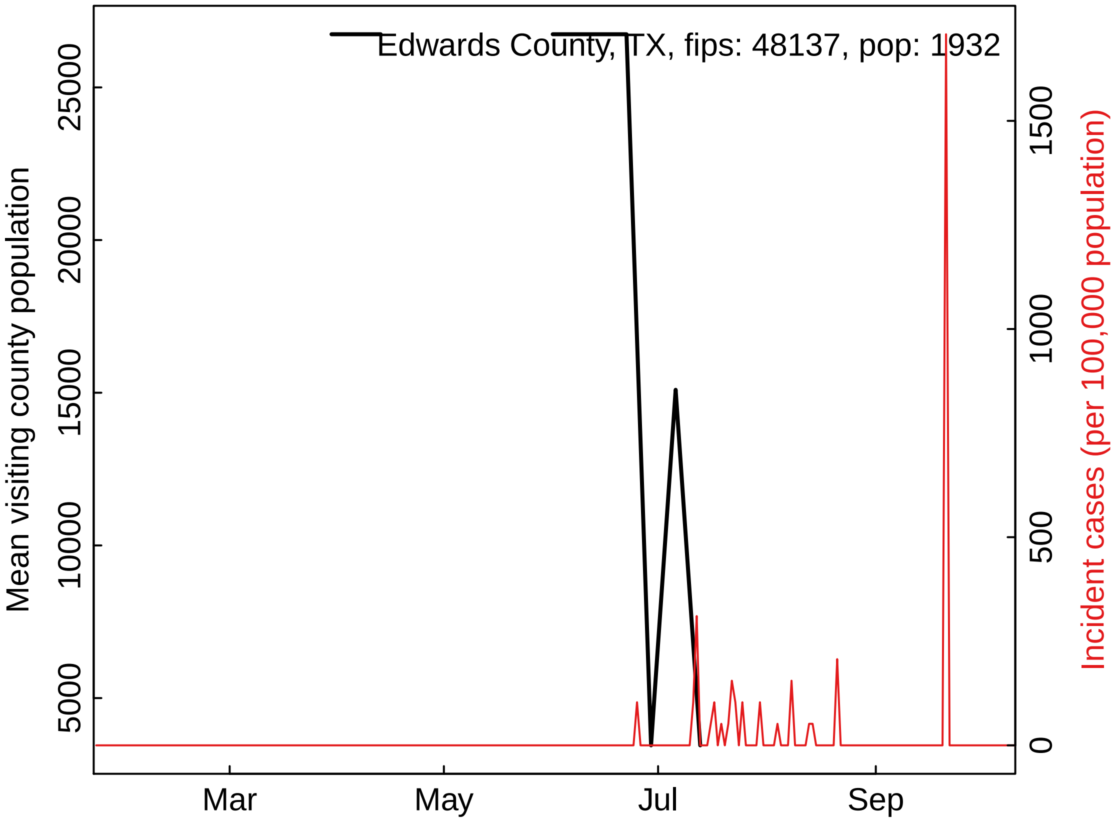

Edwards County, TX, fips: 48137, pop: 1932
Mean visiting county population
5000
10000
15000
20000
25000
Incident cases (per 100,000 population)
0
500
1000
1500
Mar
May
Jul
Sep

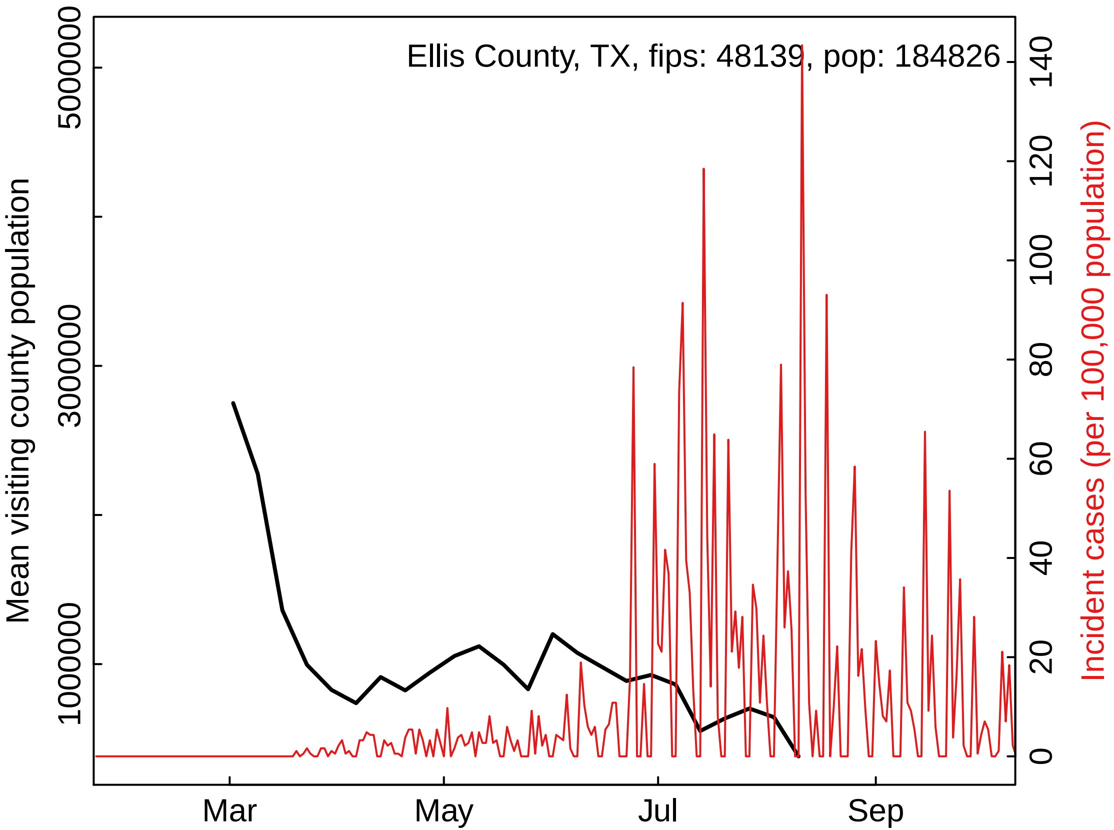
Ellis County, TX, fips: 48139, pop: 184826
Mean visiting county population
1000000
3000000
5000000
Incident cases (per 100,000 population)
0
20
40
60
80
100
120
140
Mar
May
Jul
Sep

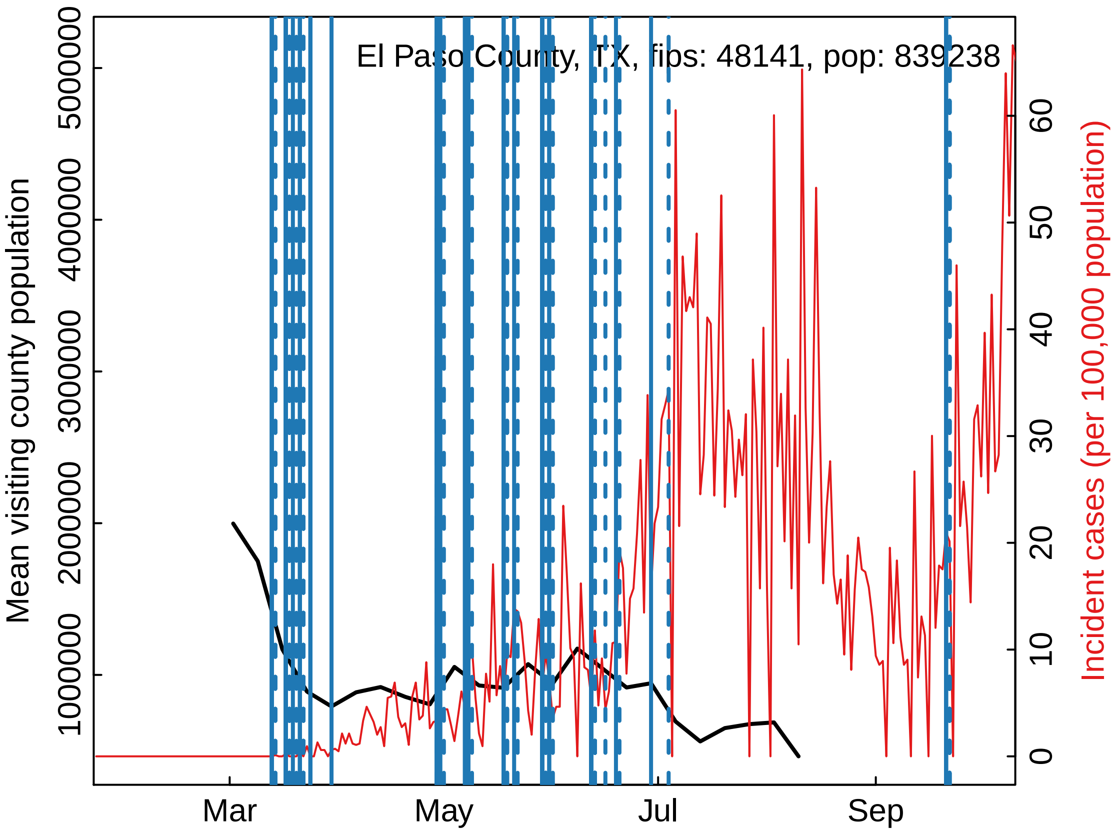

El Paso County, TX, fips: 48141, pop: 839238
Mean visiting county population
1000000
2000000
3000000
4000000
5000000
Incident cases (per 100,000 population)
0
10
20
30
40
50
60
Mar
May
Jul
Sep

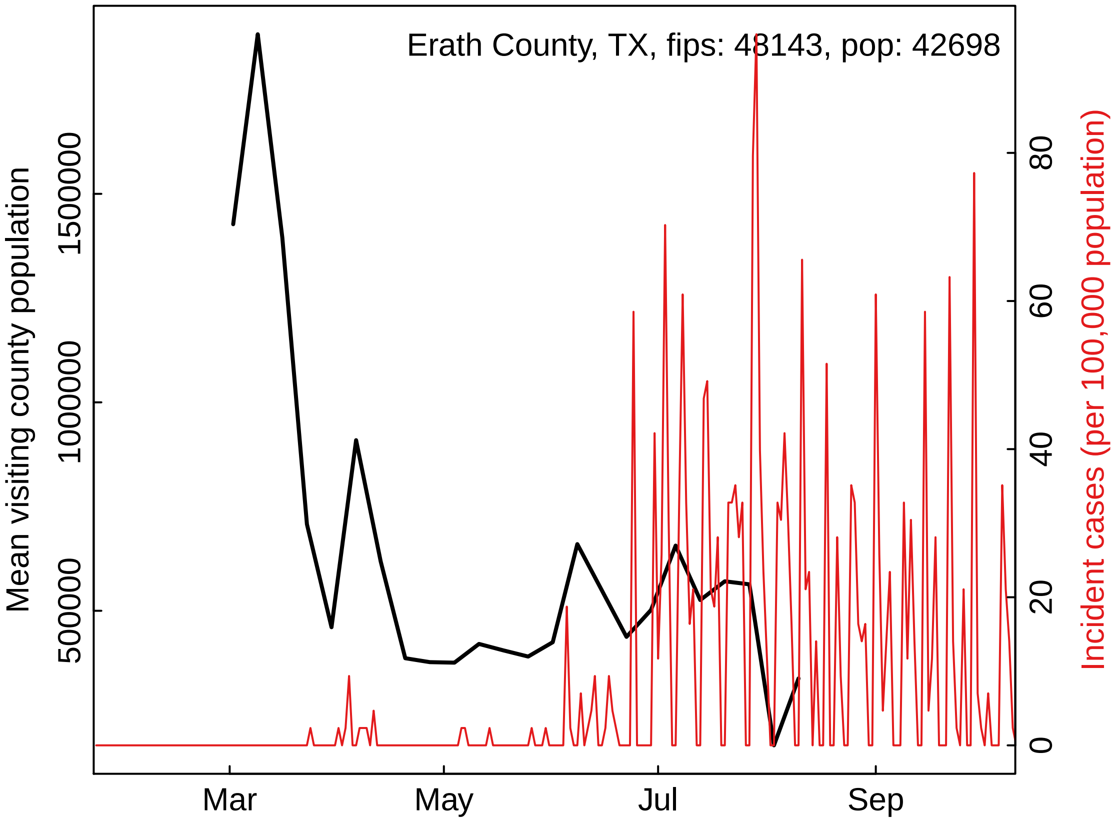

Erath County, TX, fips: 48143, pop: 42698
Mean visiting county population
500000
1000000
1500000
Incident cases (per 100,000 population)
0
20
40
60
80
Mar
May
Jul
Sep

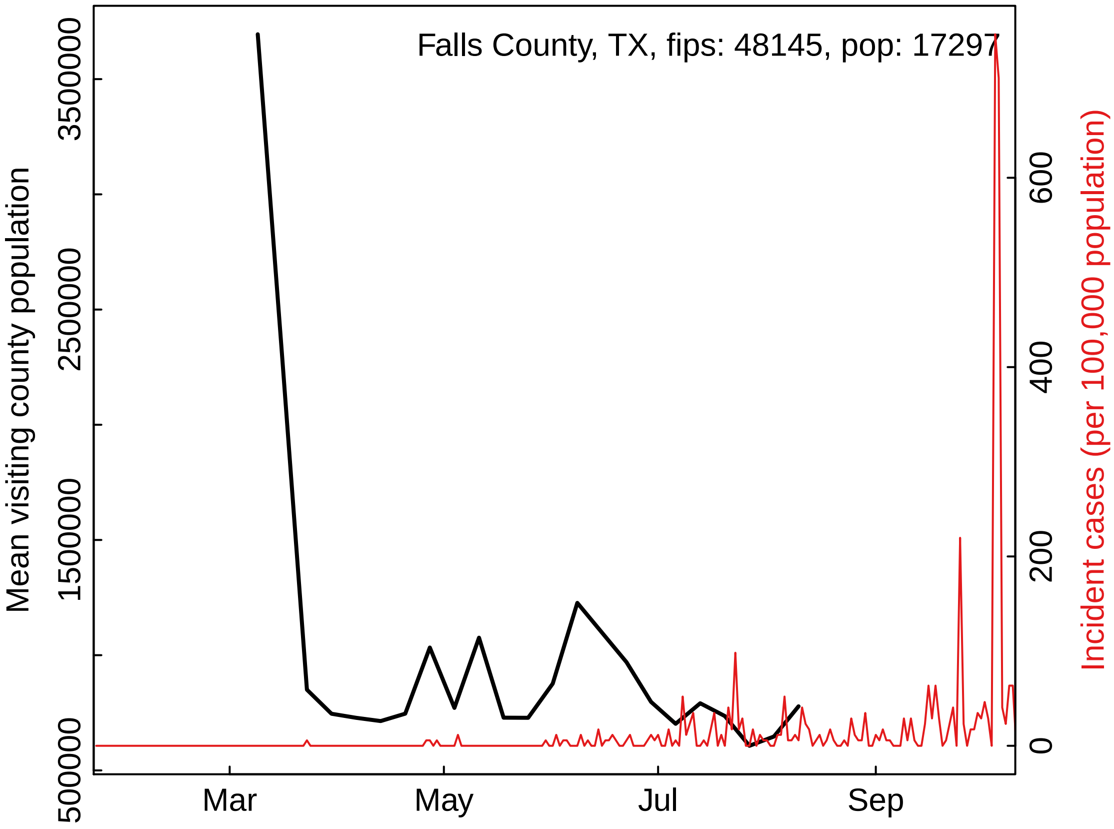
Falls County, TX, fips: 48145, pop: 17297
Mean visiting county population
500000
1500000
2500000
3500000
Incident cases (per 100,000 population)
0
200
400
600
Mar
May
Jul
Sep

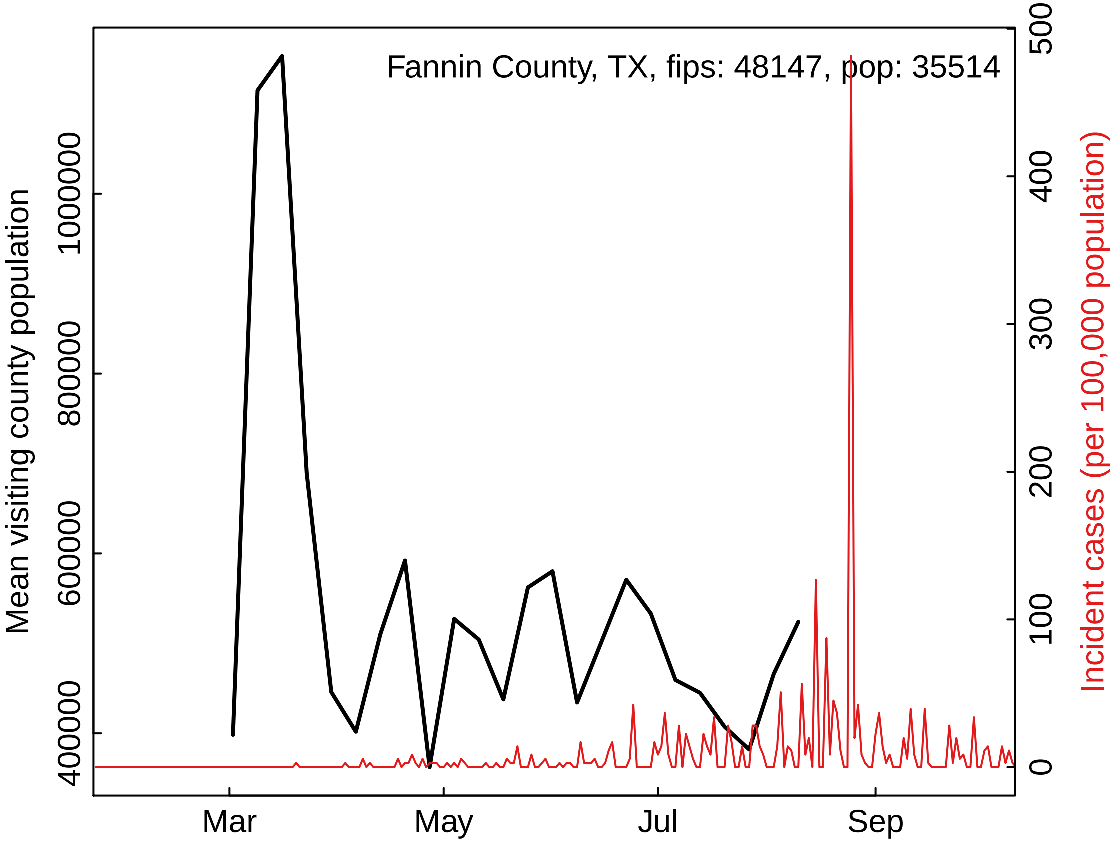
Fannin County, TX, fips: 48147, pop: 35514
Mean visiting county population
400000
600000
800000
1000000
Incident cases (per 100,000 population)
0
100
200
300
400
500
Mar
May
Jul
Sep

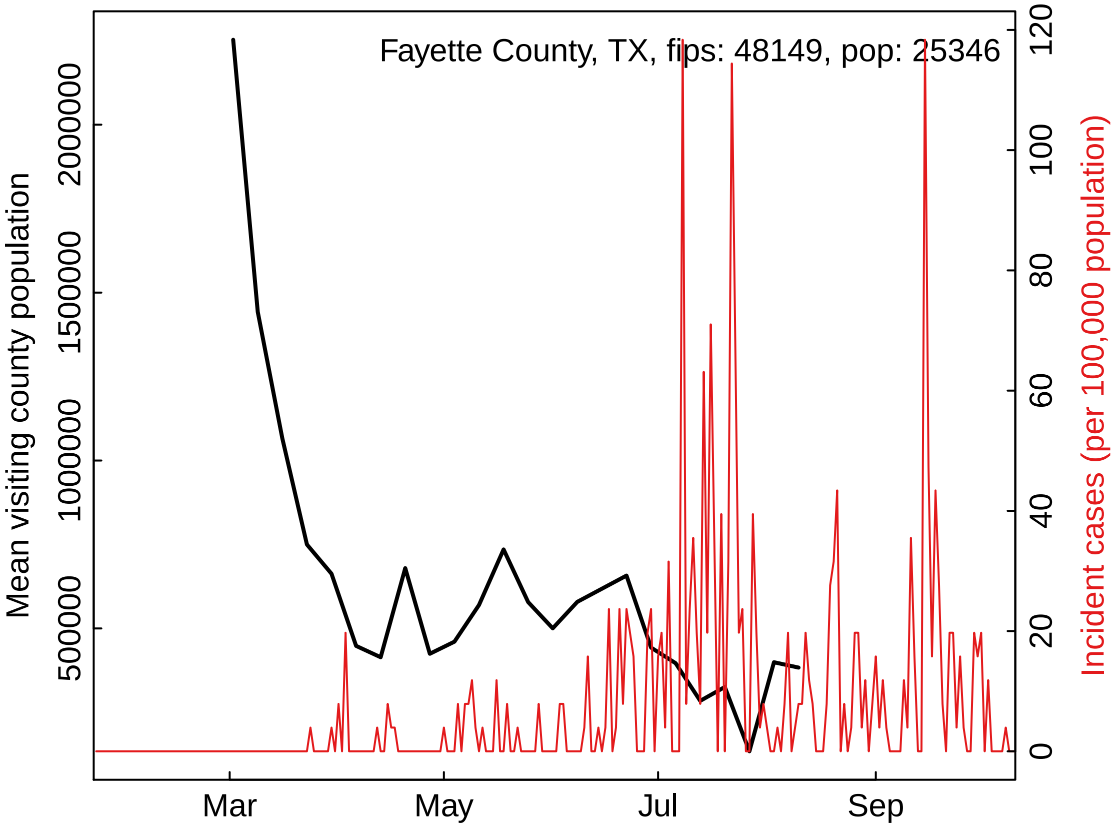
Fayette County, TX, fips: 48149, pop: 25346
Mean visiting county population
500000
1000000
1500000
2000000
Incident cases (per 100,000 population)
0
20
40
60
80
100
120
Mar
May
Jul
Sep

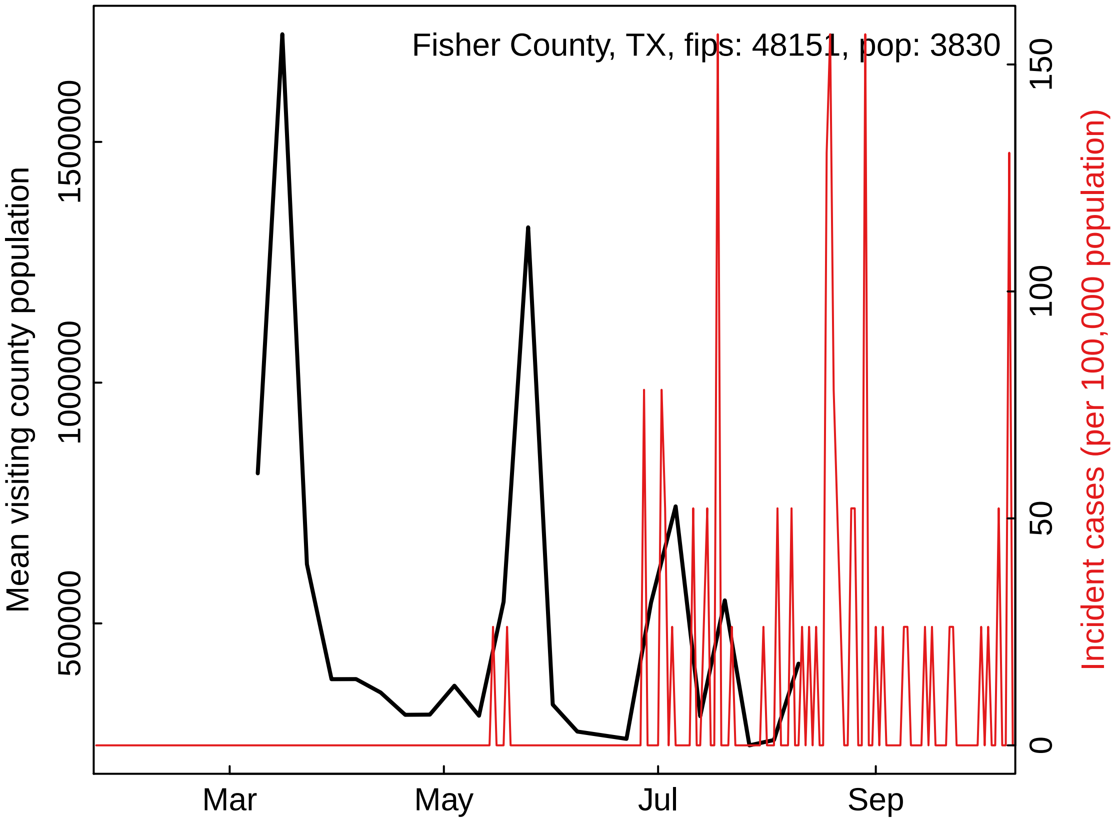
Fisher County, TX, fips: 48151, pop: 3830
Mean visiting county population
500000
1000000
1500000
Incident cases (per 100,000 population)
0
50
100
150
Mar
May
Jul
Sep

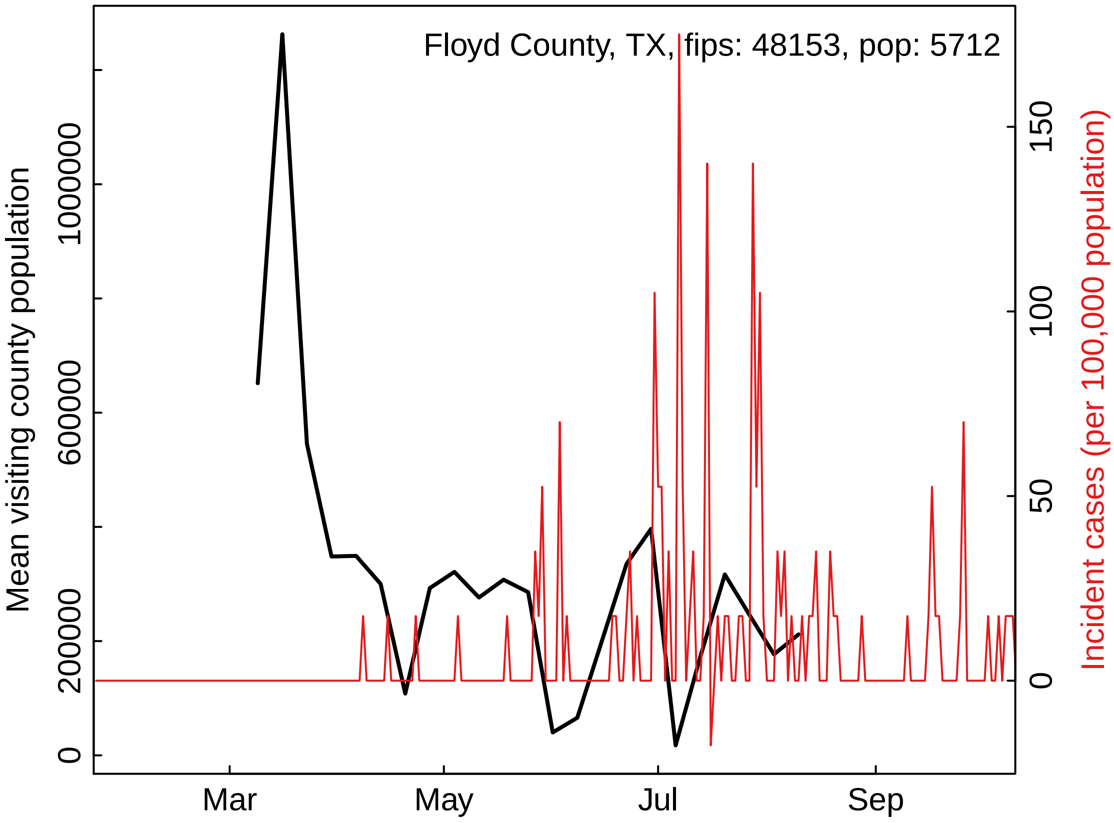

Floyd County, TX, fips: 48153, pop: 5712
Mean visiting county population
0
200000
600000
1000000
Mar
May
Jul
Sep
0
50
100
150
Incident cases (per 100,000 population)

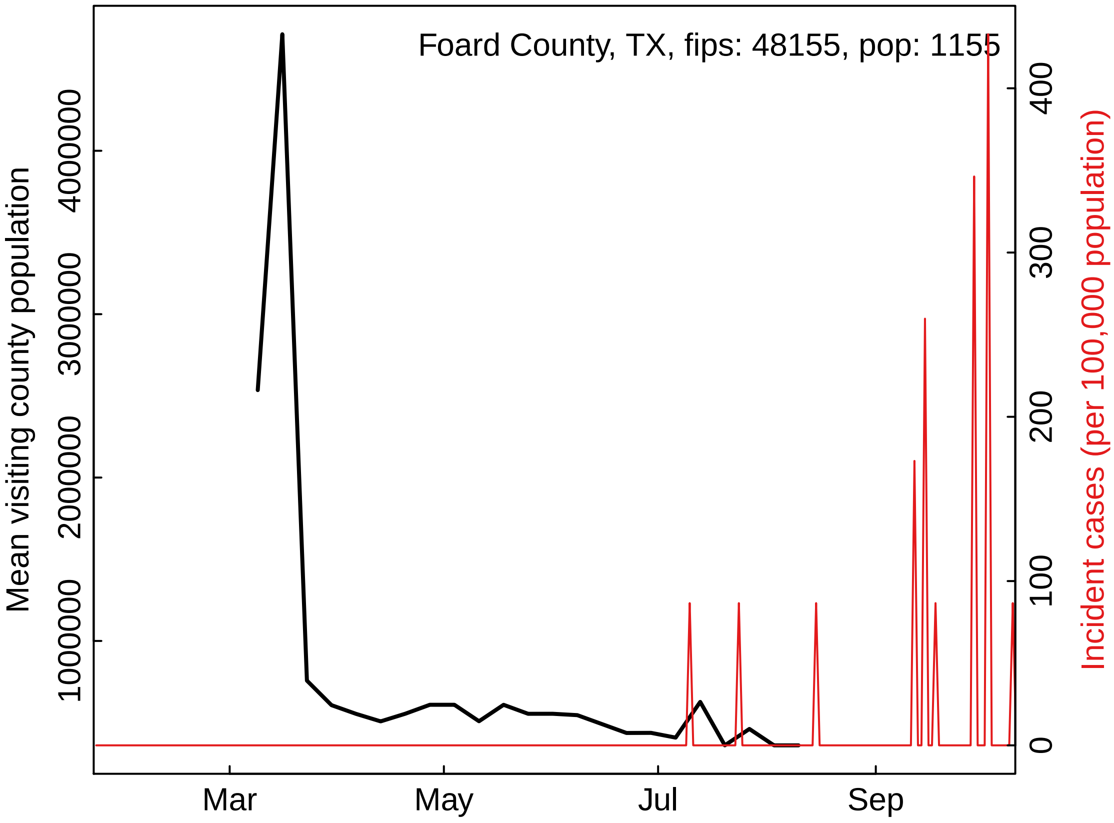

Foard County, TX, fips: 48155, pop: 1155
Mean visiting county population
1000000
2000000
3000000
4000000
Incident cases (per 100,000 population)
0
100
200
300
400
Mar
May
Jul
Sep

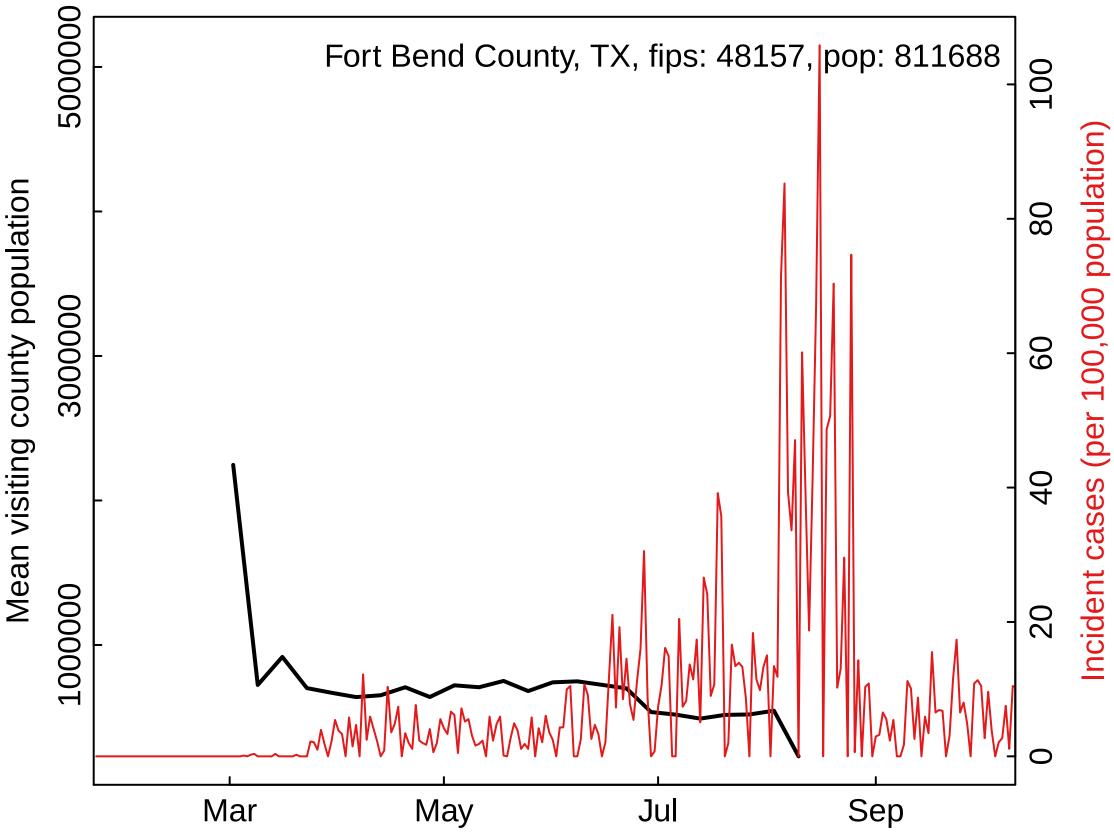

Fort Bend County, TX, fips: 48157, pop: 811688
Mean visiting county population
5000000
3000000
1000000
Mar
May
Jul
Sep
Incident cases (per 100,000 population)
100
80
60
40
20
0

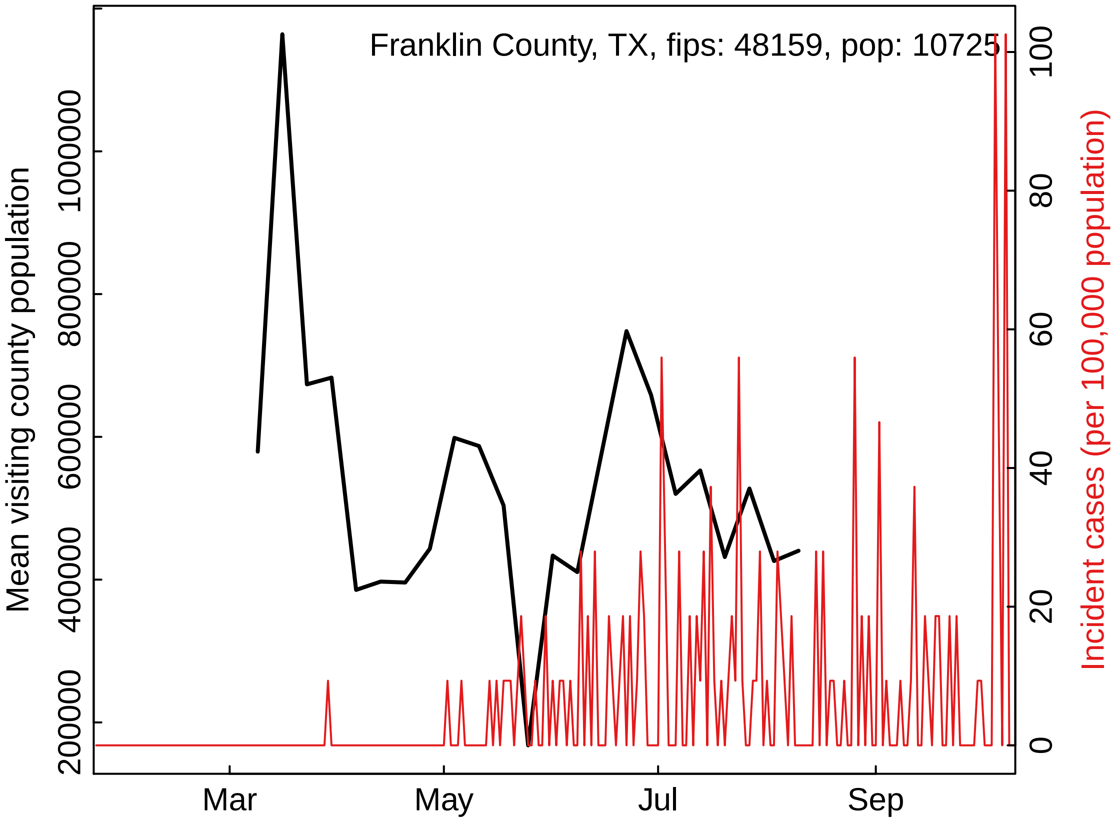

Franklin County, TX, fips: 48159, pop: 10725
Mean visiting county population
200000
400000
600000
800000
1000000
Incident cases (per 100,000 population)
0
20
40
60
80
100
Mar
May
Jul
Sep

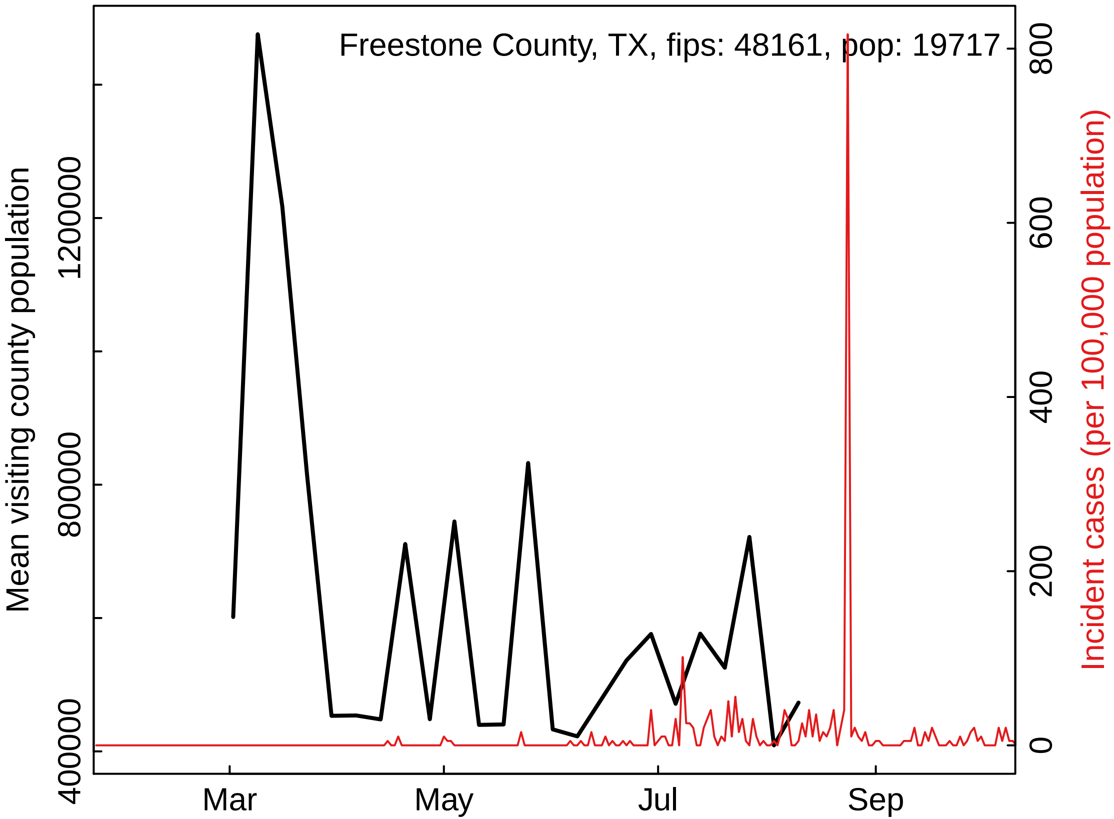

Freestone County, TX, fips: 48161, pop: 19717
Mean visiting county population
400000
800000
1200000
Incident cases (per 100,000 population)
0
200
400
600
800
Mar
May
Jul
Sep

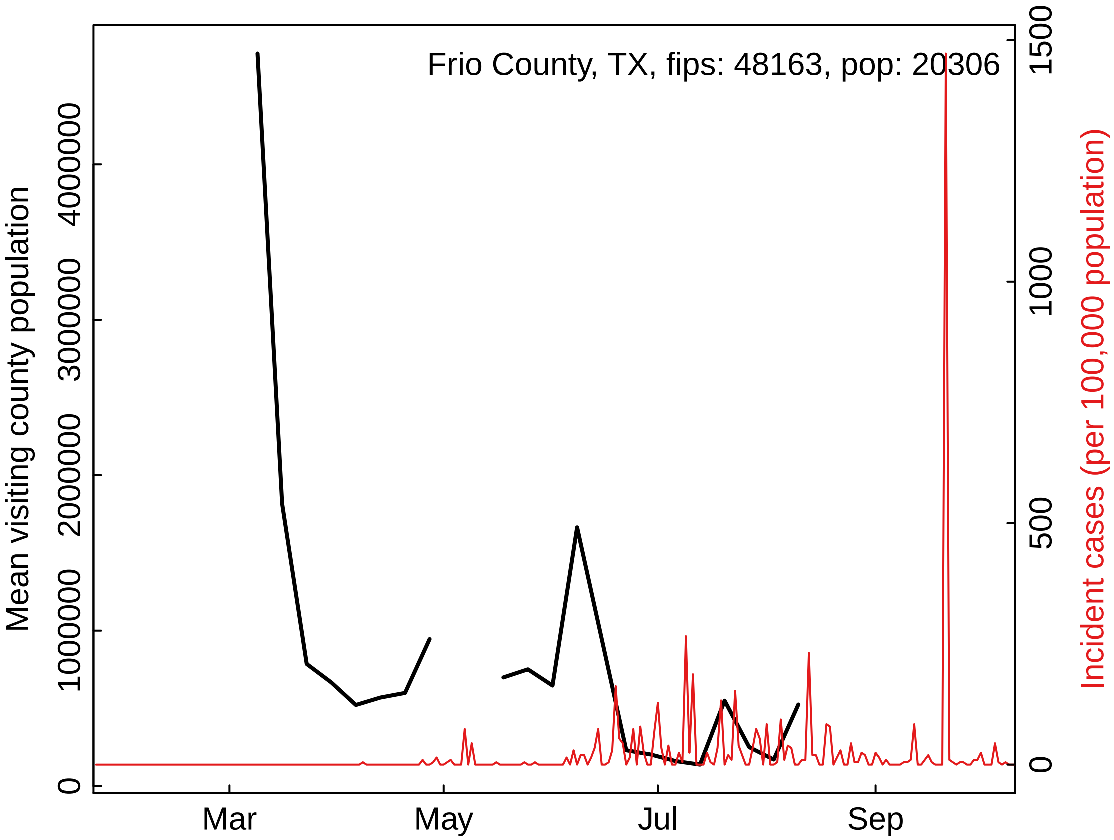
Frio County, TX, fips: 48163, pop: 20306
Mean visiting county population
0
1000000
2000000
3000000
4000000
Incident cases (per 100,000 population)
0
500
1000
1500
Mar
May
Jul
Sep

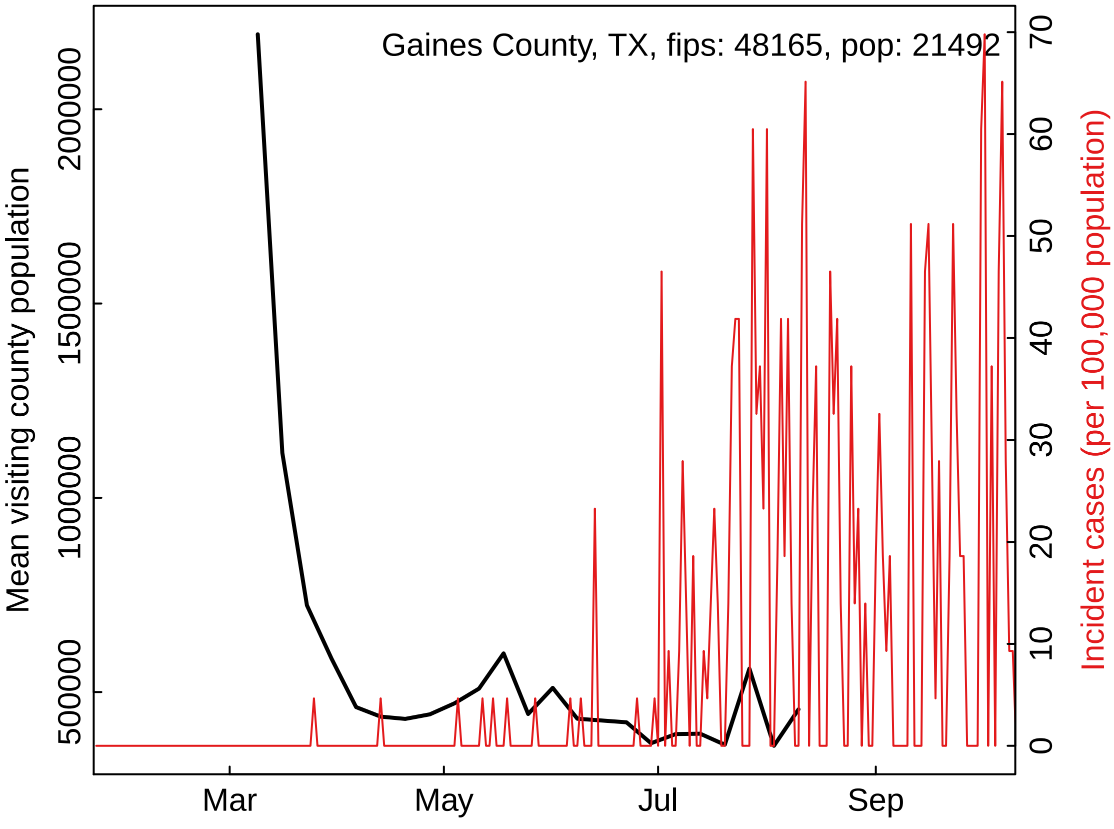

Gaines County, TX, fips: 48165, pop: 21492
Mean visiting county population
500000
1000000
1500000
2000000
Mar
May
Jul
Sep
Incident cases (per 100,000 population)
0
10
20
30
40
50
60
70

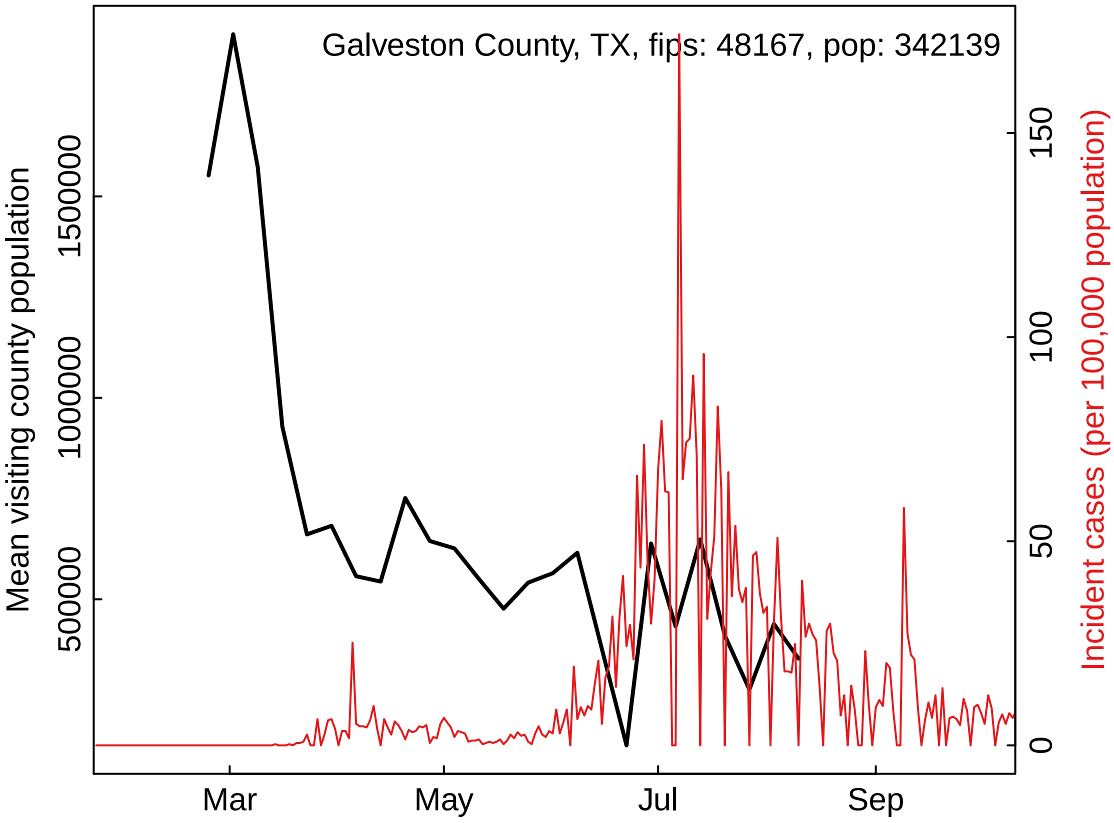

Galveston County, TX, fips: 48167, pop: 342139
Mean visiting county population
500000
1000000
1500000
Incident cases (per 100,000 population)
0
50
100
150
Mar
May
Jul
Sep

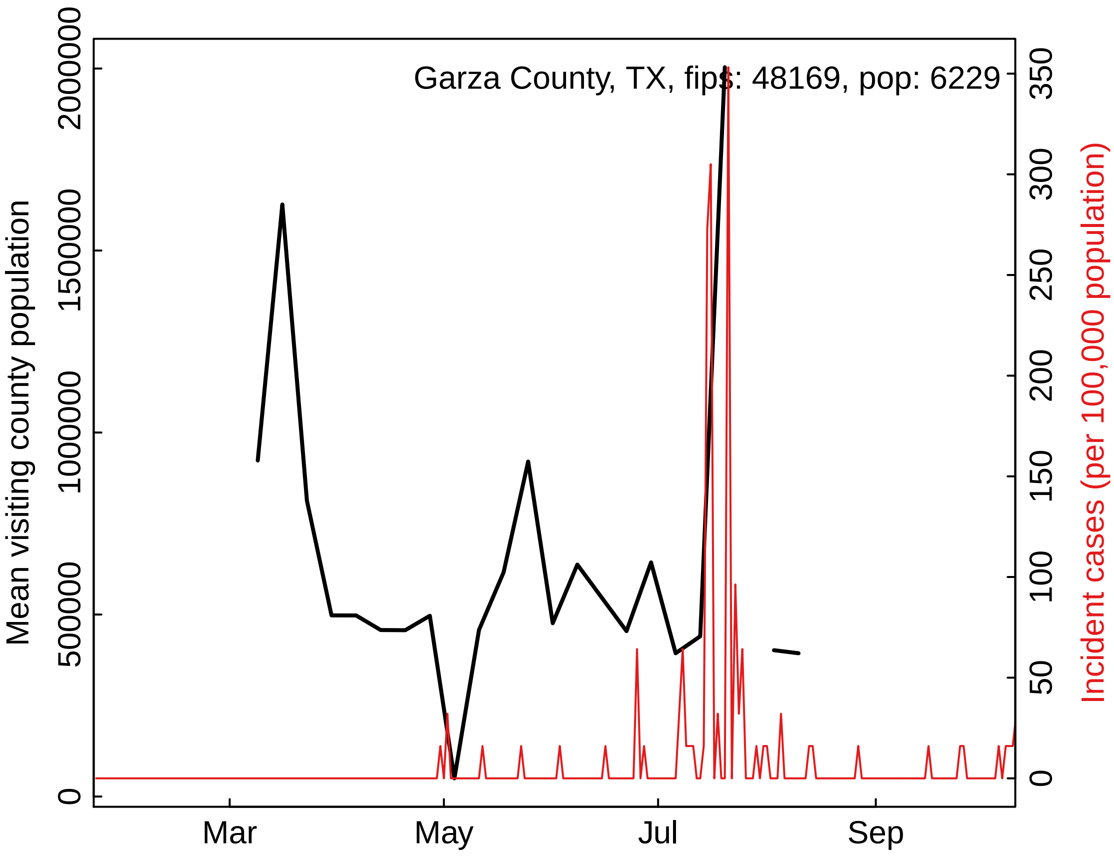

Garza County, TX, fips: 48169, pop: 6229
Mean visiting county population
Incident cases (per 100,000 population)
0
500000
1000000
1500000
2000000
0
50
100
150
200
250
300
350
Mar
May
Jul
Sep

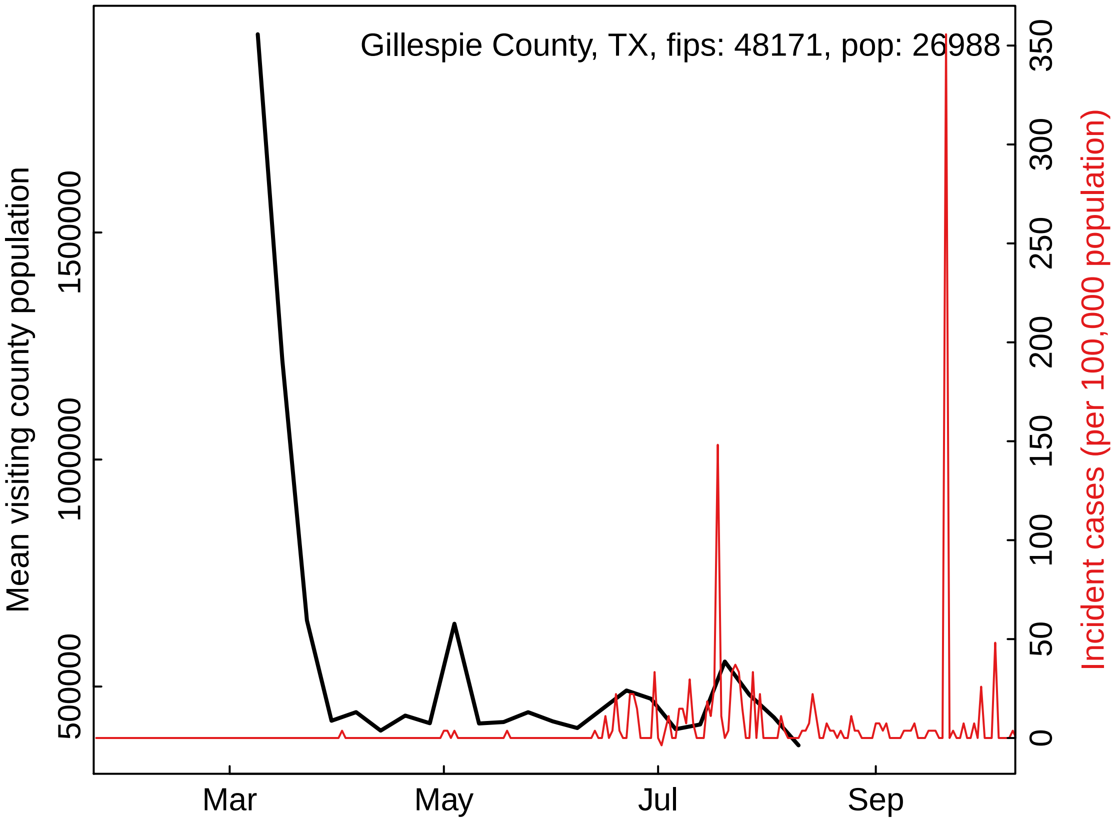

Gillespie County, TX, fips: 48171, pop: 26988
Mean visiting county population
500000
1000000
1500000
Incident cases (per 100,000 population)
0
50
100
150
200
250
300
350
Mar
May
Jul
Sep

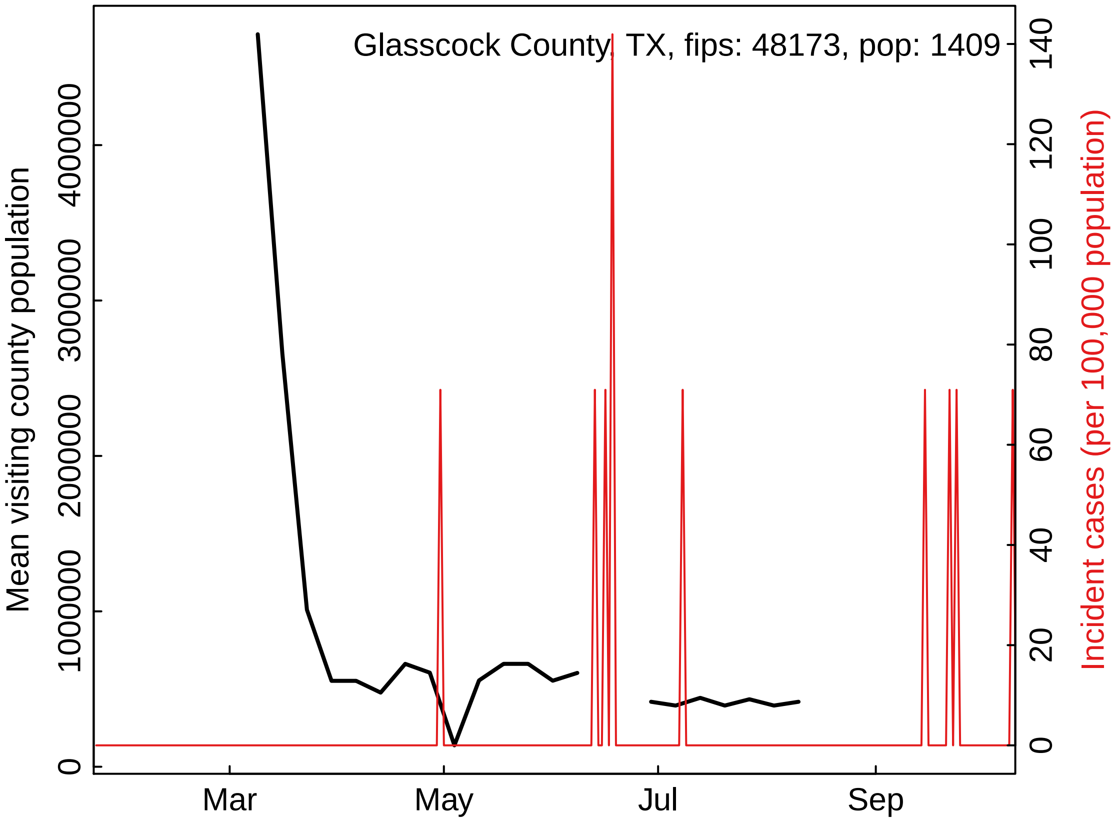

Glasscock County, TX, fips: 48173, pop: 1409
Mean visiting county population
0
1000000
2000000
3000000
4000000
Mar
May
Jul
Sep
0
20
40
60
80
100
120
140
Incident cases (per 100,000 population)

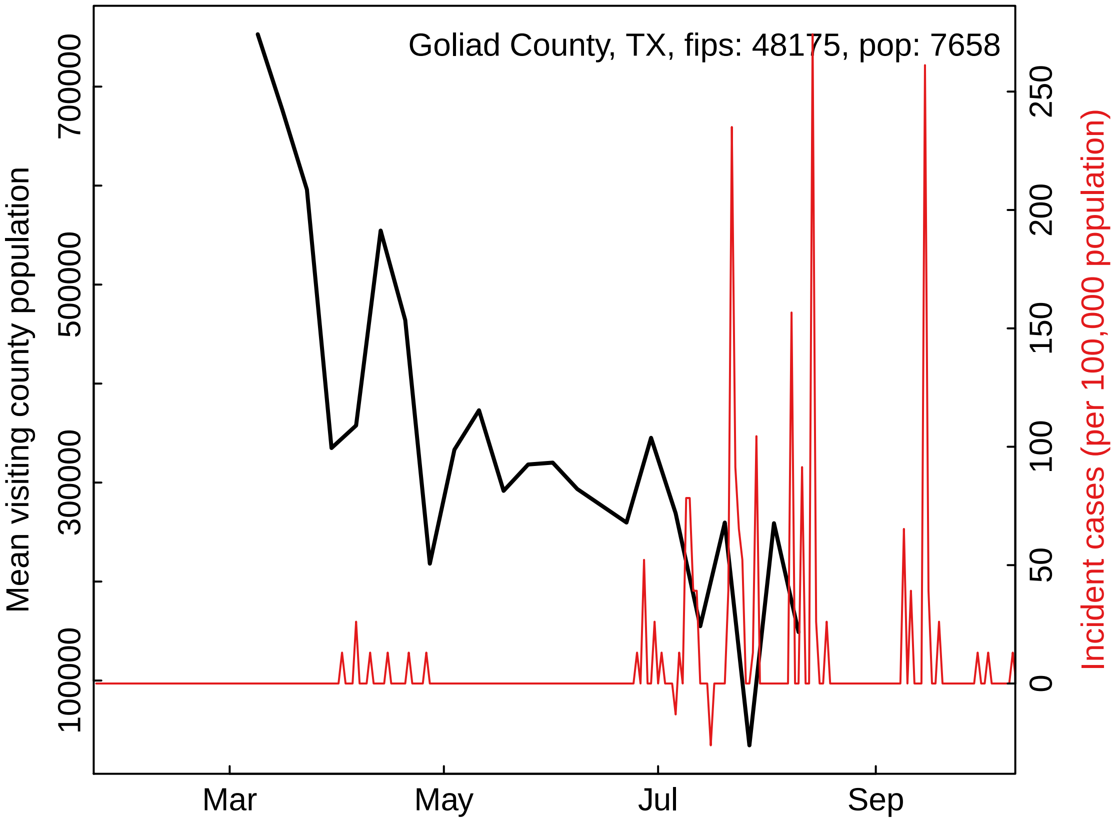

Goliad County, TX, fips: 48175, pop: 7658
Mean visiting county population
100000
300000
500000
700000
Incident cases (per 100,000 population)
0
50
100
150
200
250
Mar
May
Jul
Sep

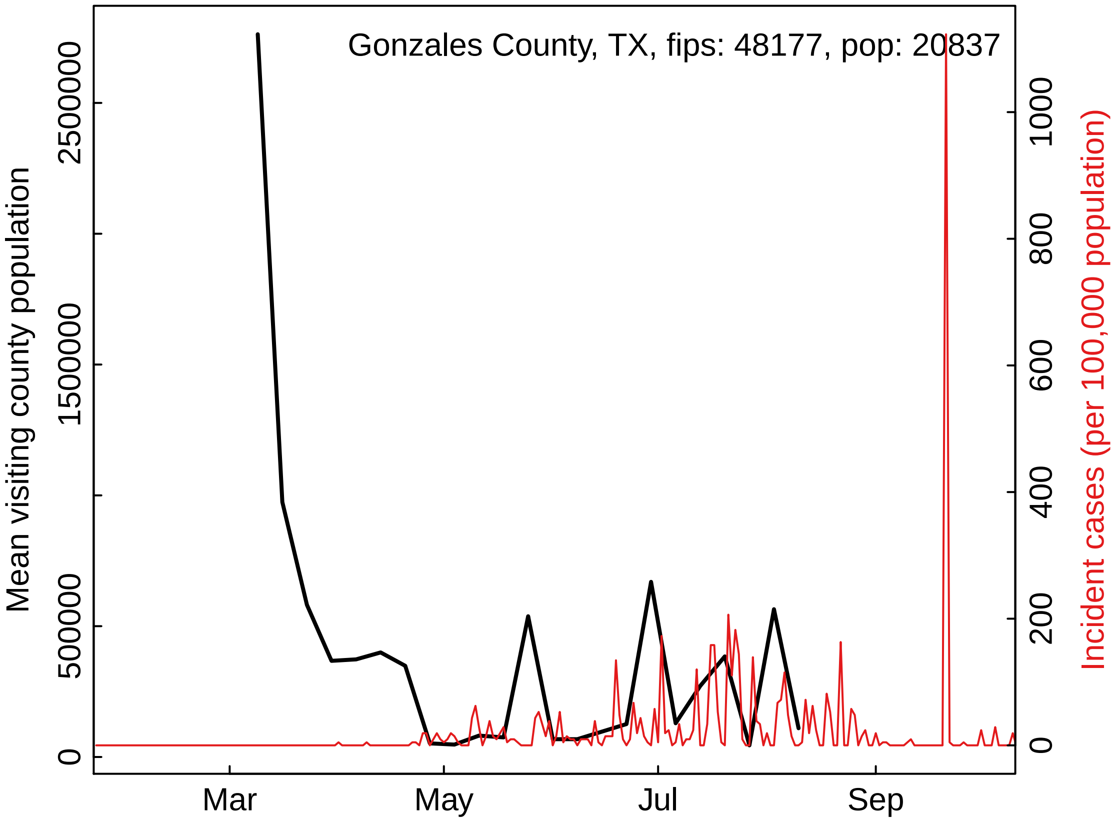

Gonzales County, TX, fips: 48177, pop: 20837
Mean visiting county population
0
500000
1500000
2500000
Incident cases (per 100,000 population)
0
200
400
600
800
1000
Mar
May
Jul
Sep

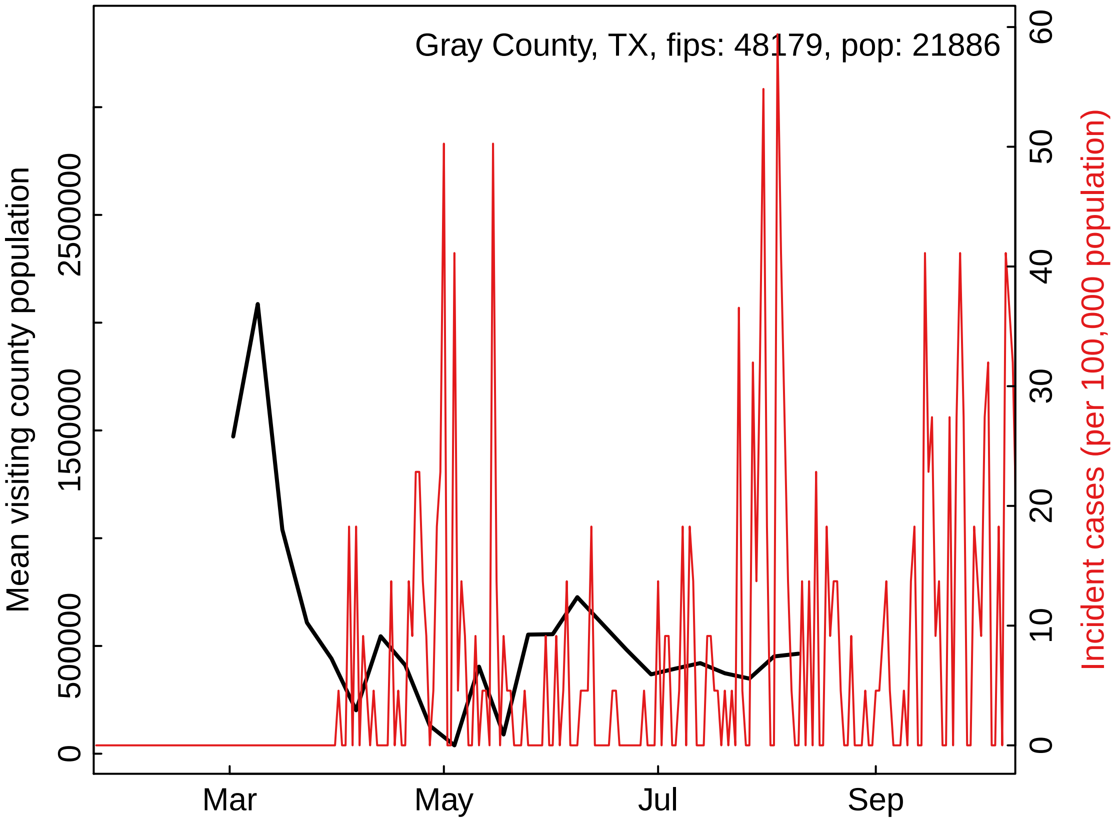

Gray County, TX, fips: 48179, pop: 21886
Mean visiting county population
0
500000
1500000
2500000
Incident cases (per 100,000 population)
0
10
20
30
40
50
60
Mar
May
Jul
Sep

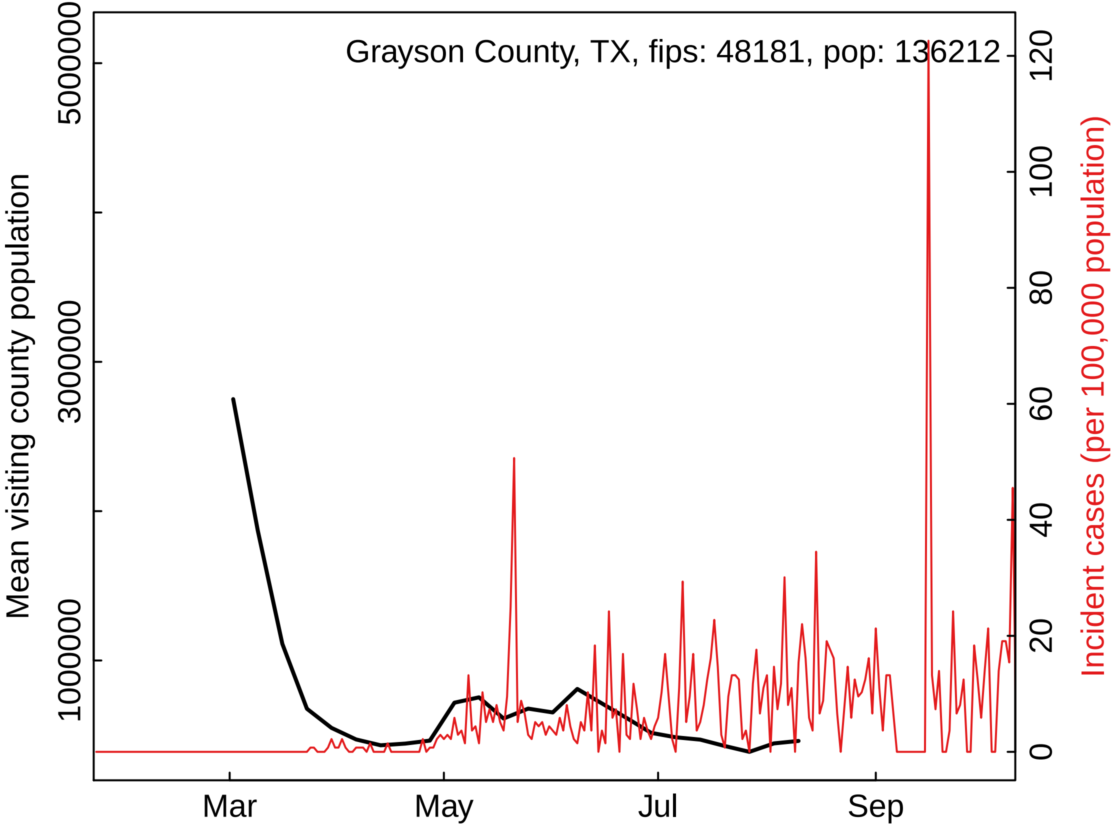

Grayson County, TX, fips: 48181, pop: 136212
Mean visiting county population
1000000
3000000
5000000
Incident cases (per 100,000 population)
0
20
40
60
80
100
120
Mar
May
Jul
Sep

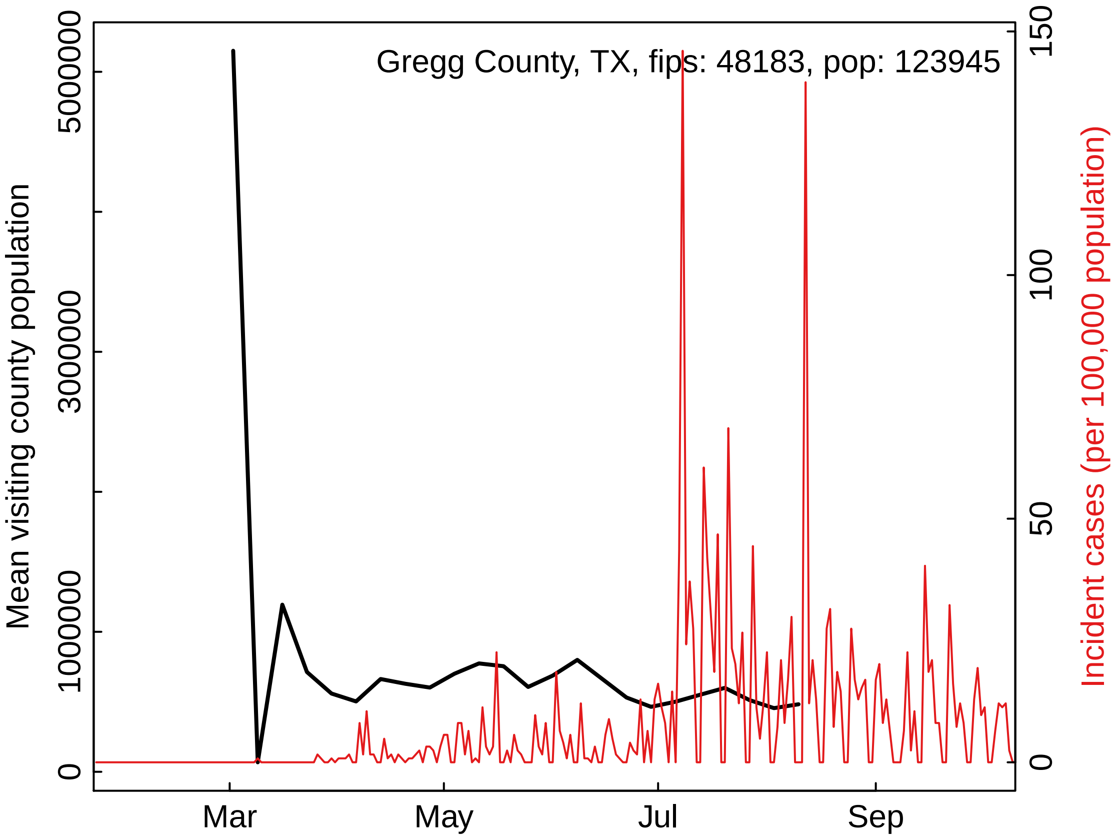

Gregg County, TX, fips: 48183, pop: 123945
Mean visiting county population
0
1000000
3000000
5000000
Incident cases (per 100,000 population)
0
50
100
150
Mar
May
Jul
Sep

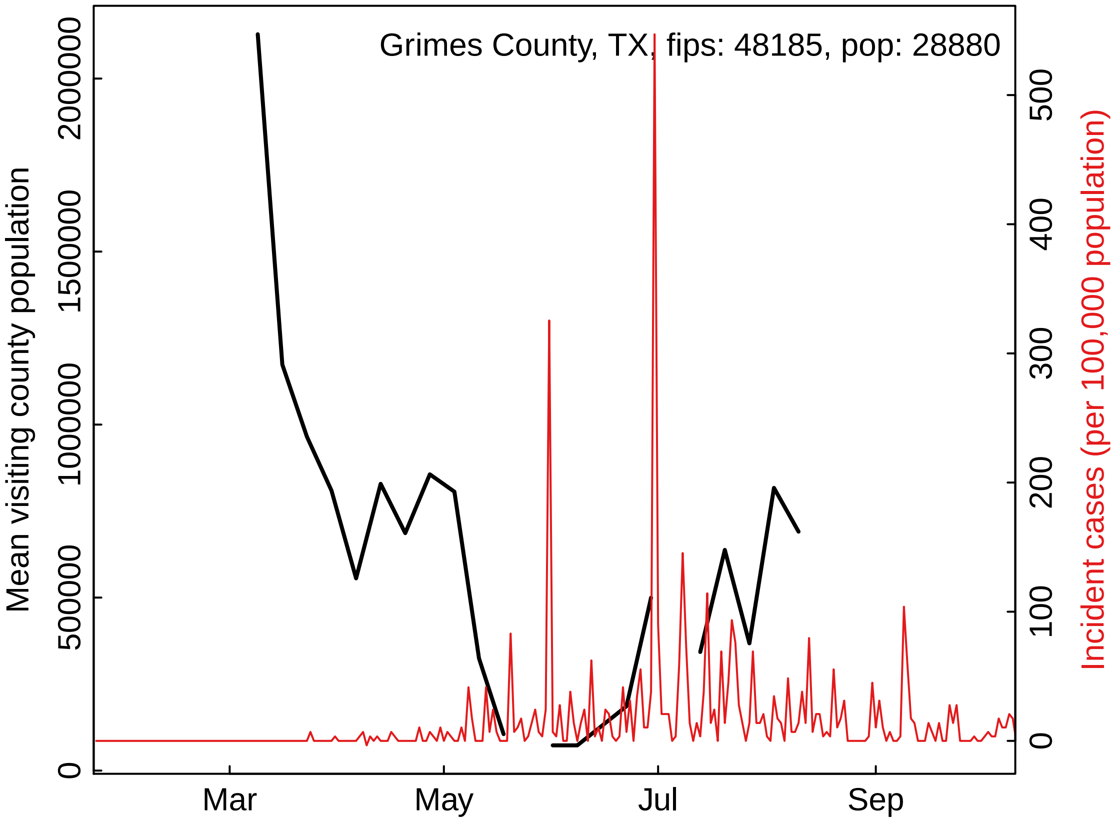

Grimes County, TX, fips: 48185, pop: 28880
Mean visiting county population
0
500000
1000000
1500000
2000000
Incident cases (per 100,000 population)
0
100
200
300
400
500
Mar
May
Jul
Sep

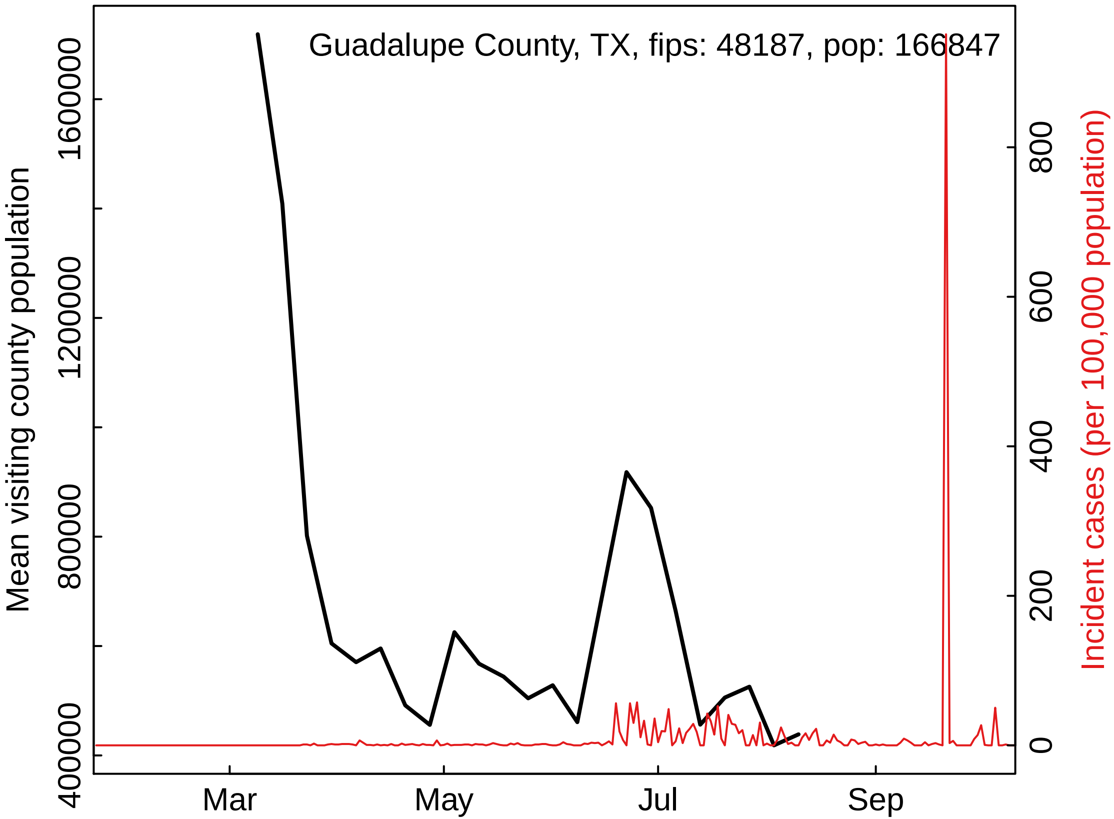
Guadalupe County, TX, fips: 48187, pop: 166847
Mean visiting county population
400000
800000
1200000
1600000
Mar
May
Jul
Sep
0
200
400
600
800
Incident cases (per 100,000 population)

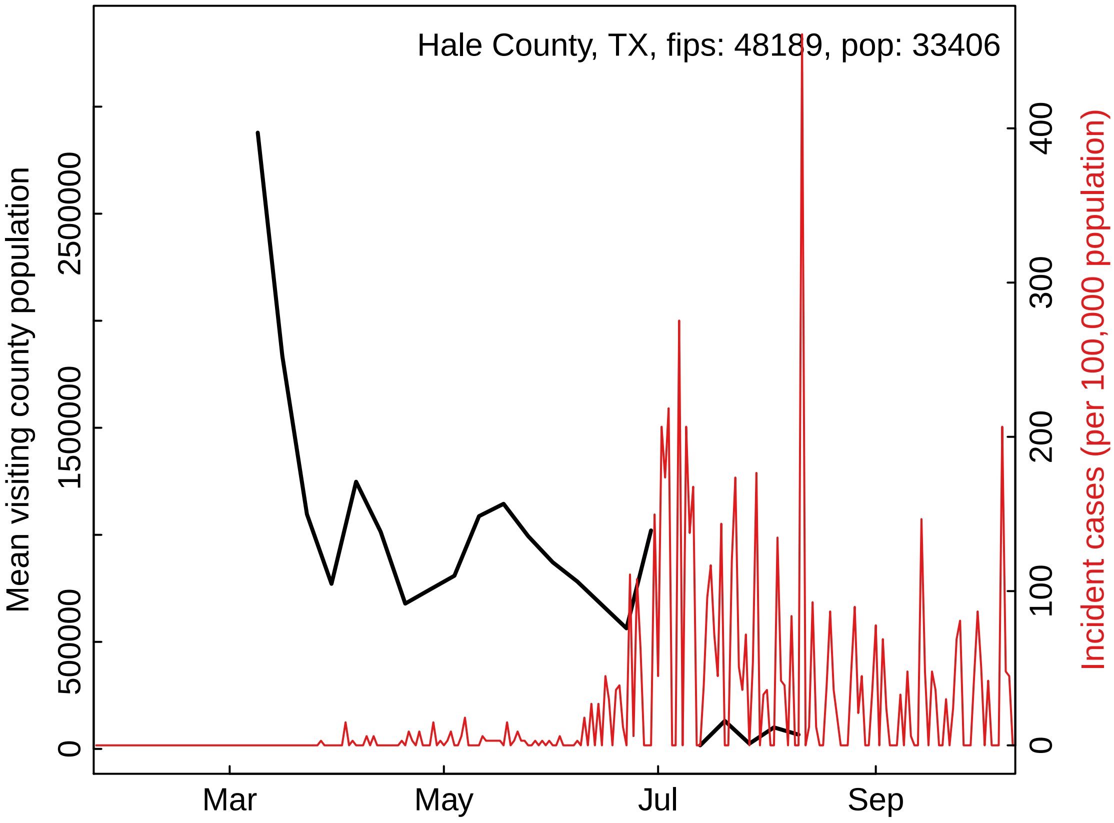
Hale County, TX, fips: 48189, pop: 33406
Mean visiting county population
0
500000
1500000
2500000
Incident cases (per 100,000 population)
0
100
200
300
400
Mar
May
Jul
Sep

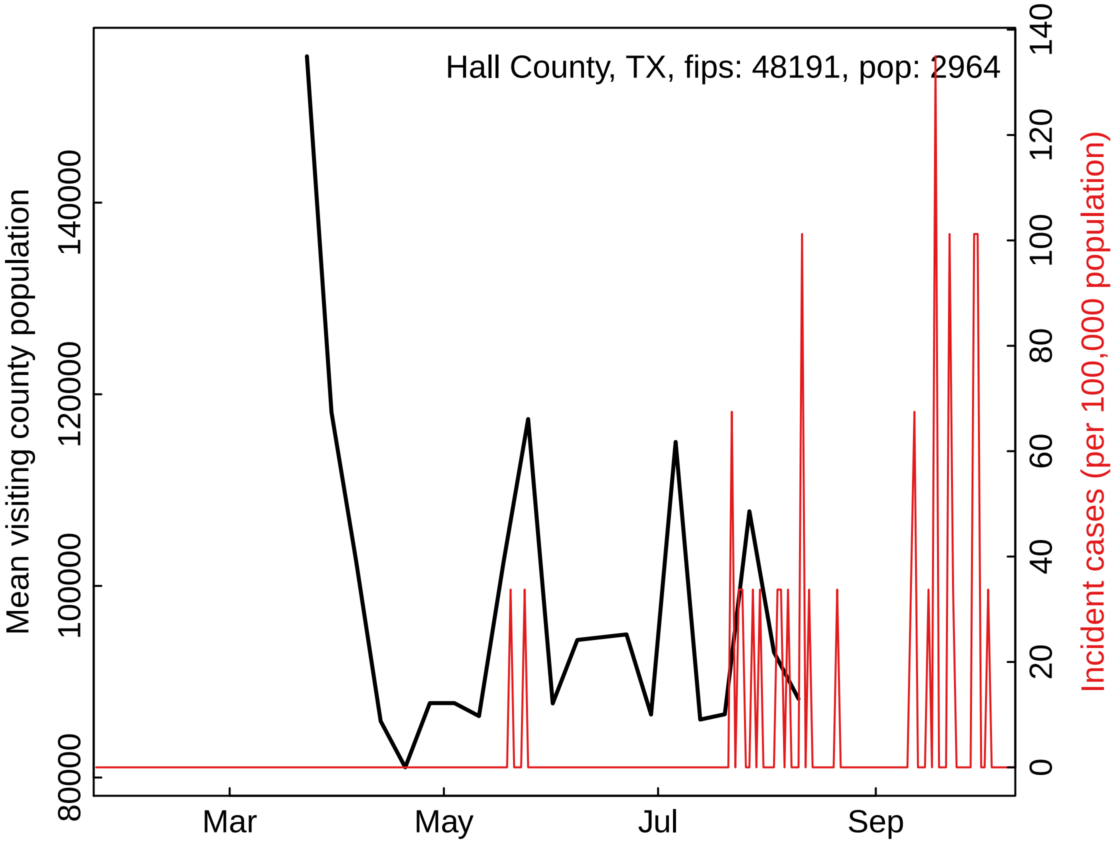

Hall County, TX, fips: 48191, pop: 2964
Mean visiting county population
80000
100000
120000
140000
Mar
May
Jul
Sep
0
20
40
60
80
100
120
140
Incident cases (per 100,000 population)

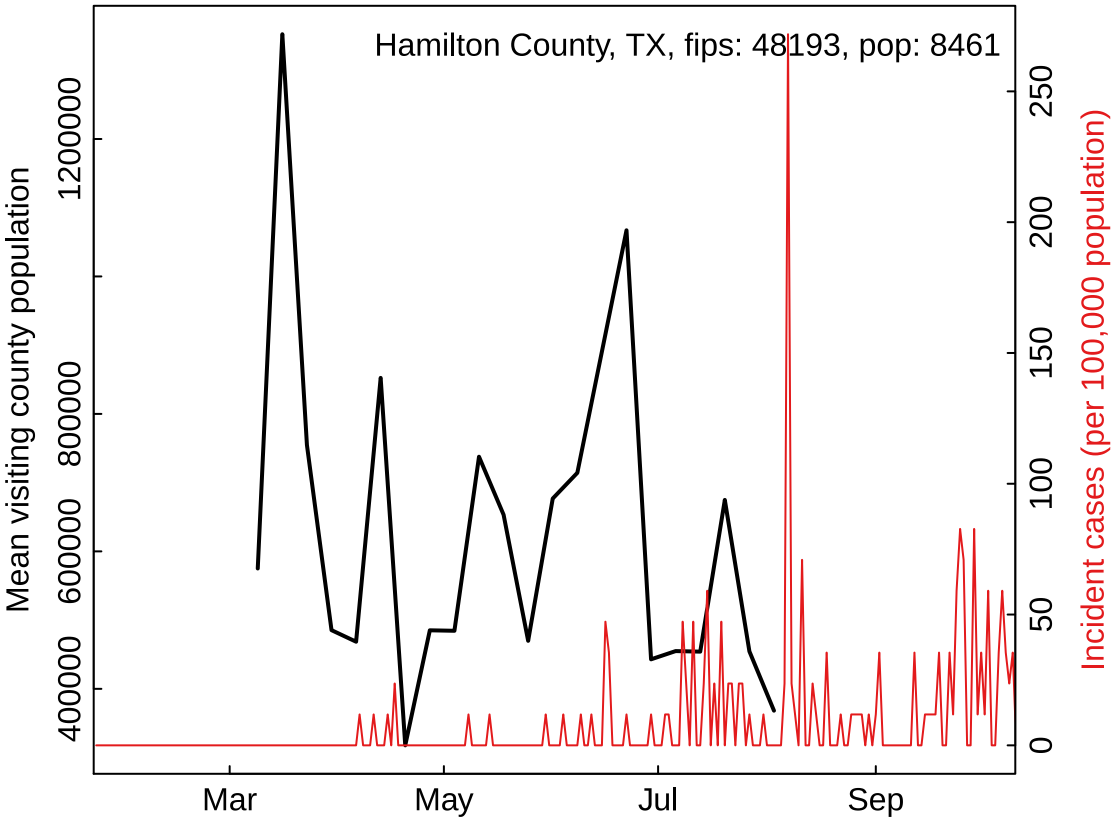

Hamilton County, TX, fips: 48193, pop: 8461
Mean visiting county population
400000
600000
800000
1200000
Incident cases (per 100,000 population)
0
50
100
150
200
250
Mar
May
Jul
Sep

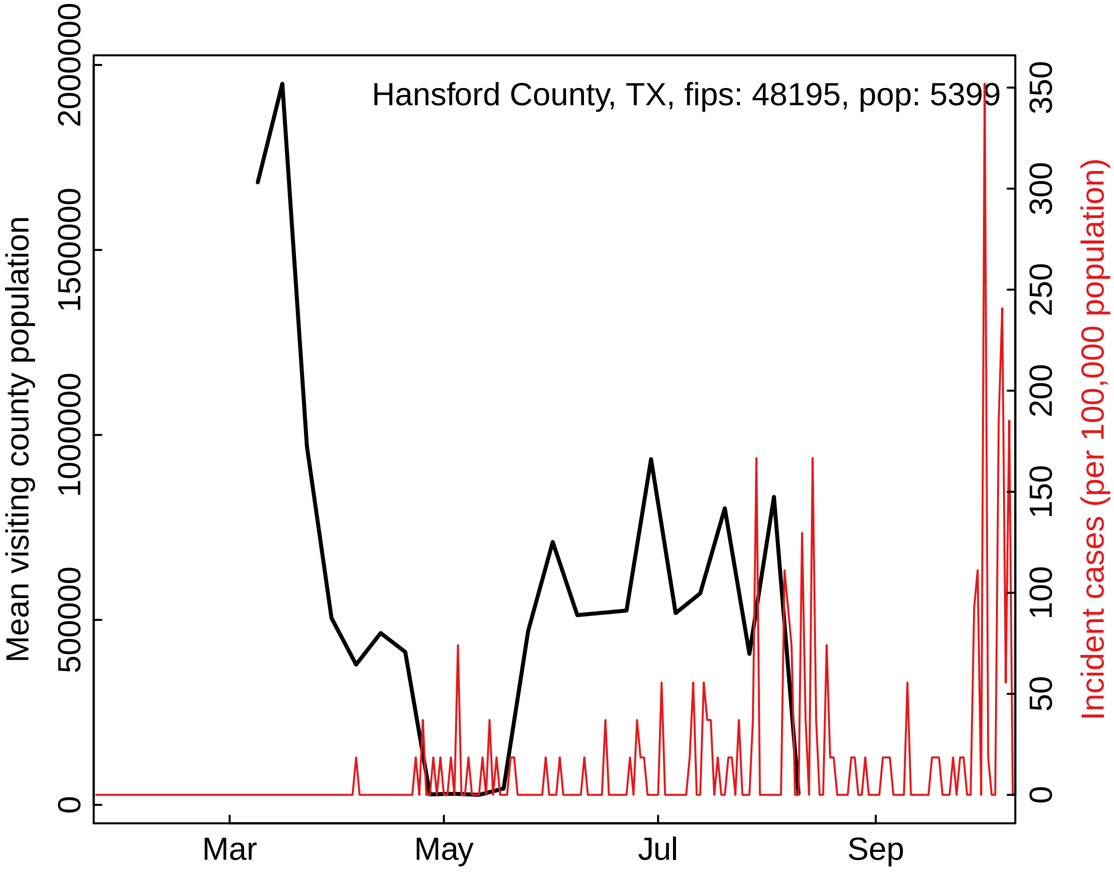

Hansford County, TX, fips: 48195, pop: 5399
Mean visiting county population
0
500000
1000000
1500000
2000000
Incident cases (per 100,000 population)
0
50
100
150
200
250
300
350
Mar
May
Jul
Sep

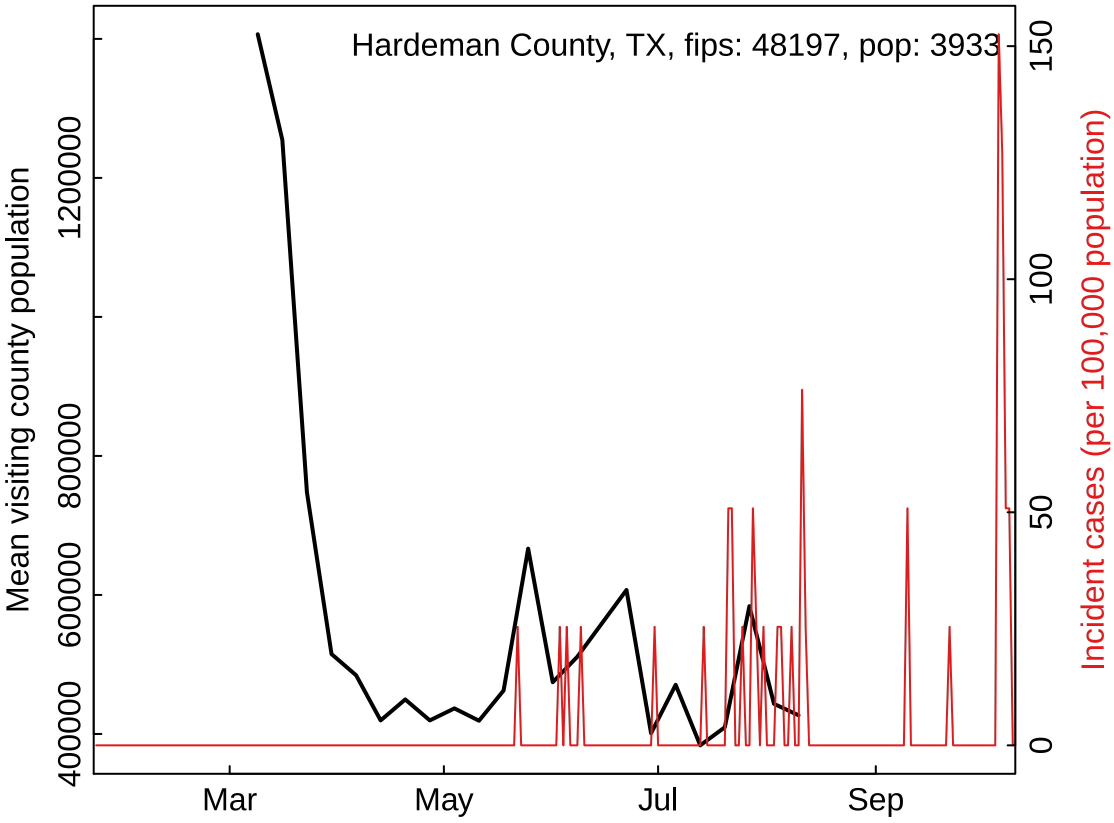

Hardeman County, TX, fips: 48197, pop: 3933
Mean visiting county population
400000
600000
800000
1200000
Incident cases (per 100,000 population)
0
50
100
150
Mar
May
Jul
Sep

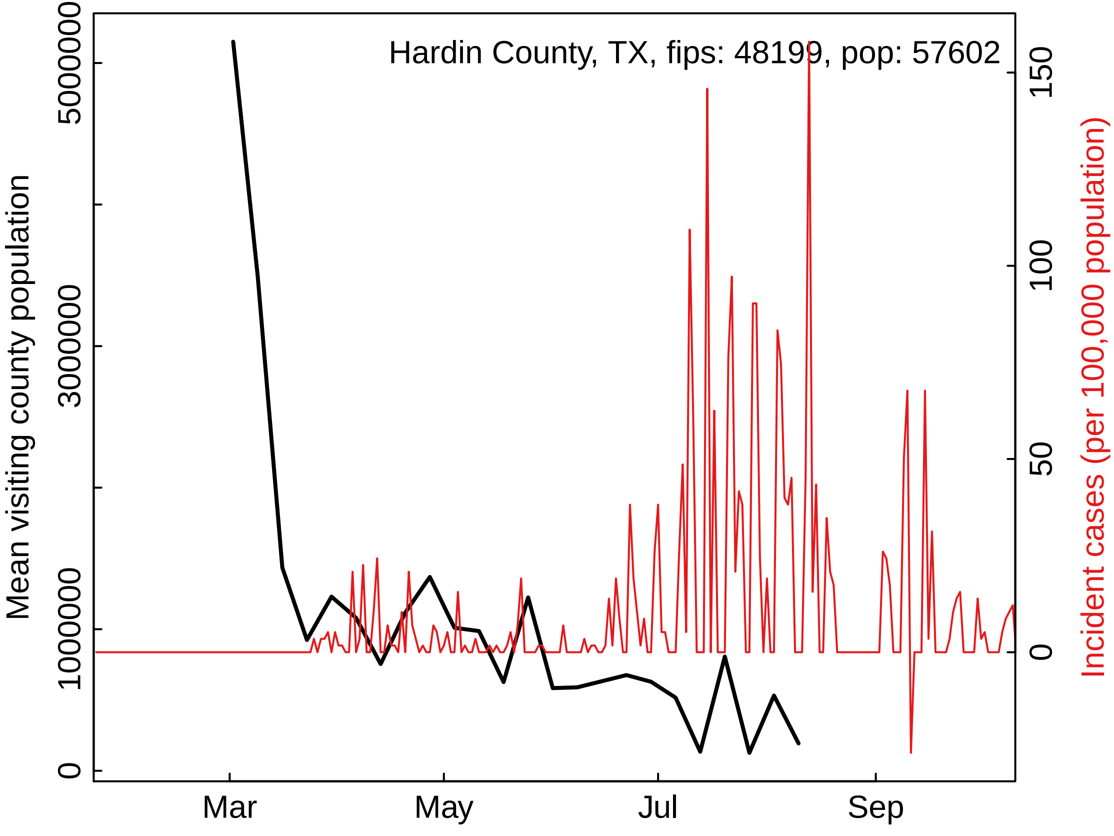

Hardin County, TX, fips: 48199, pop: 57602
Mean visiting county population
0
1000000
3000000
5000000
Incident cases (per 100,000 population)
0
50
100
150
Mar
May
Jul
Sep

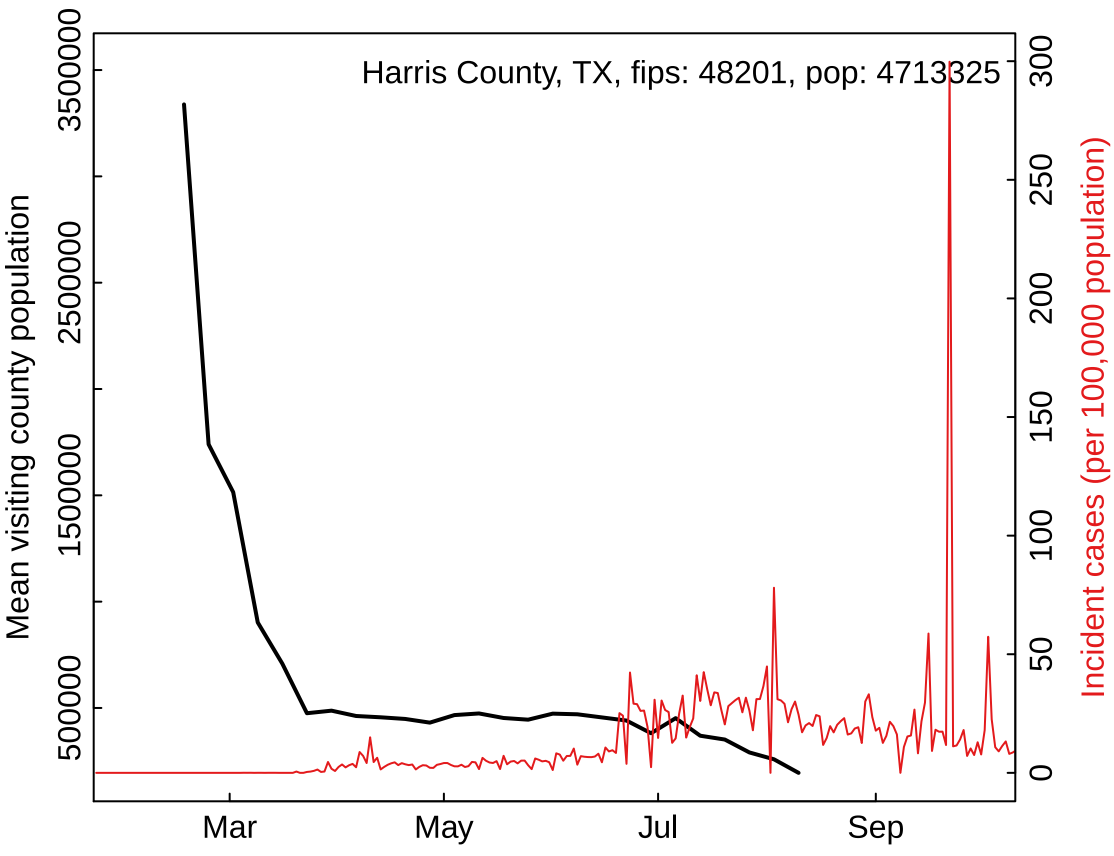

Harris County, TX, fips: 48201, pop: 4713325
Mean visiting county population
500000
1500000
2500000
3500000
Incident cases (per 100,000 population)
0
50
100
150
200
250
300
Mar
May
Jul
Sep

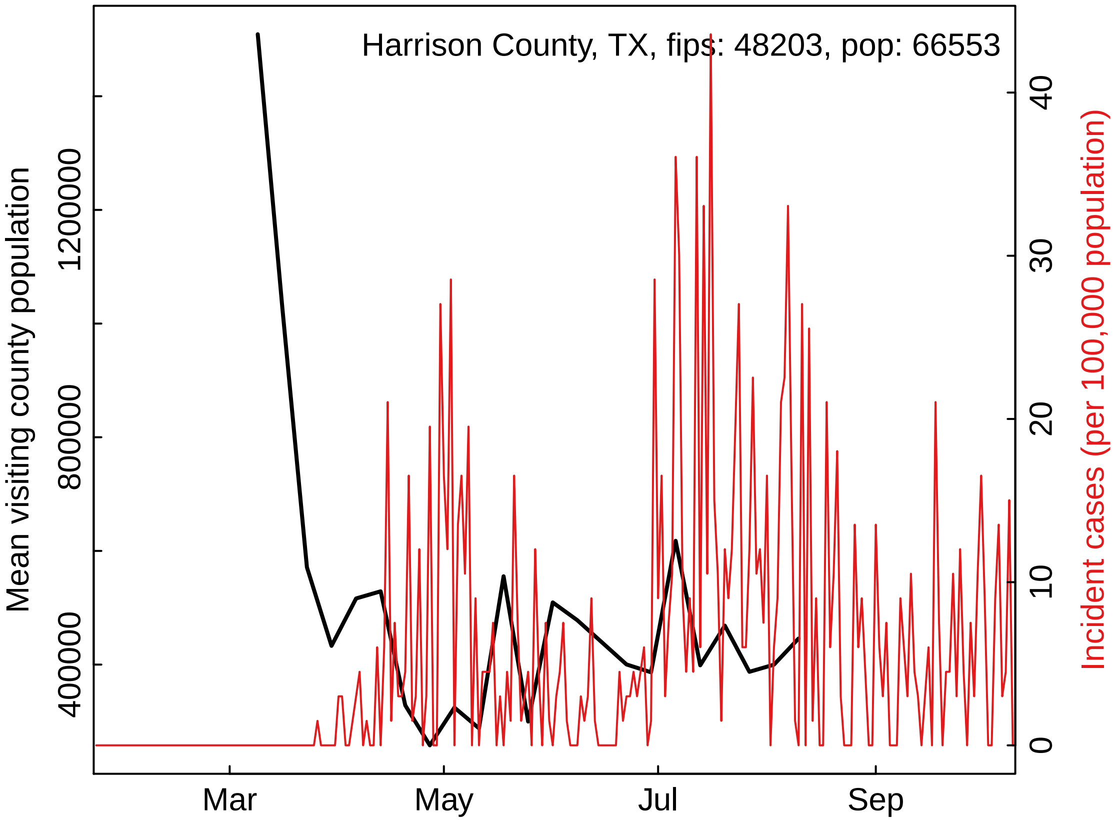

Harrison County, TX, fips: 48203, pop: 66553
Mean visiting county population
400000
800000
1200000
Incident cases (per 100,000 population)
0
10
20
30
40
Mar
May
Jul
Sep

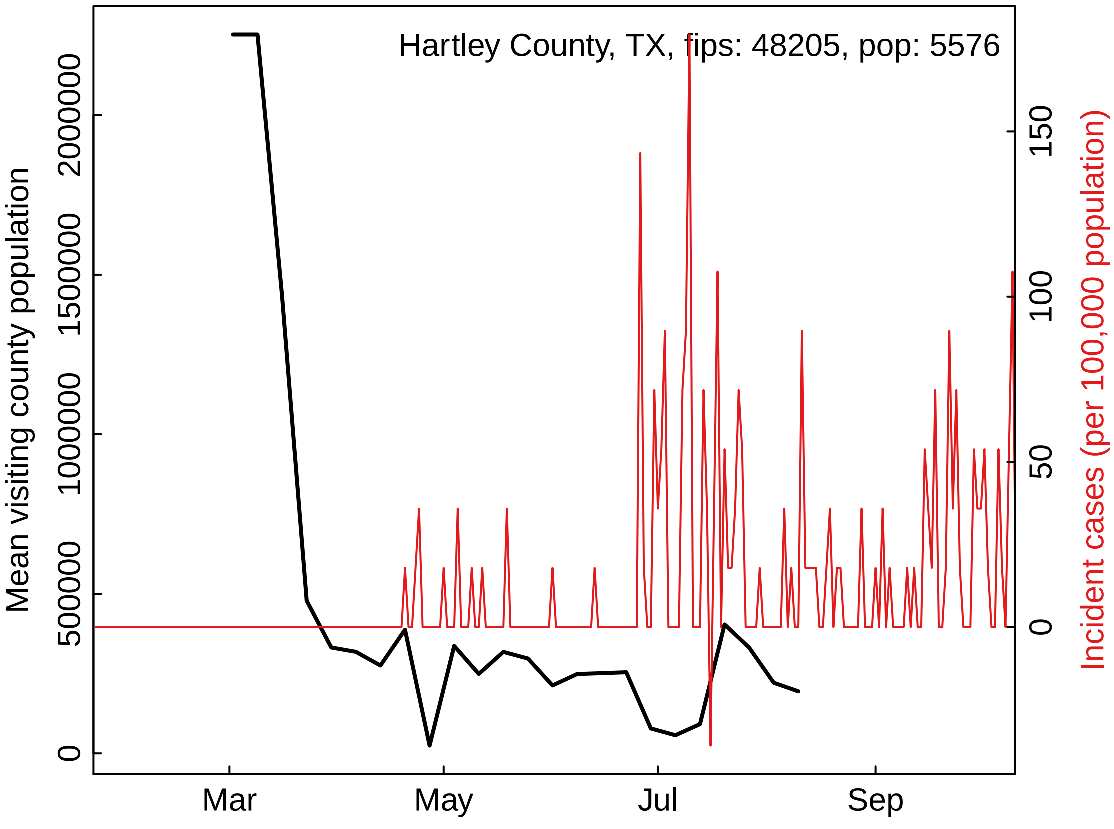

Hartley County, TX, fips: 48205, pop: 5576
Mean visiting county population
0
500000
1000000
1500000
2000000
Incident cases (per 100,000 population)
0
50
100
150
Mar
May
Jul
Sep

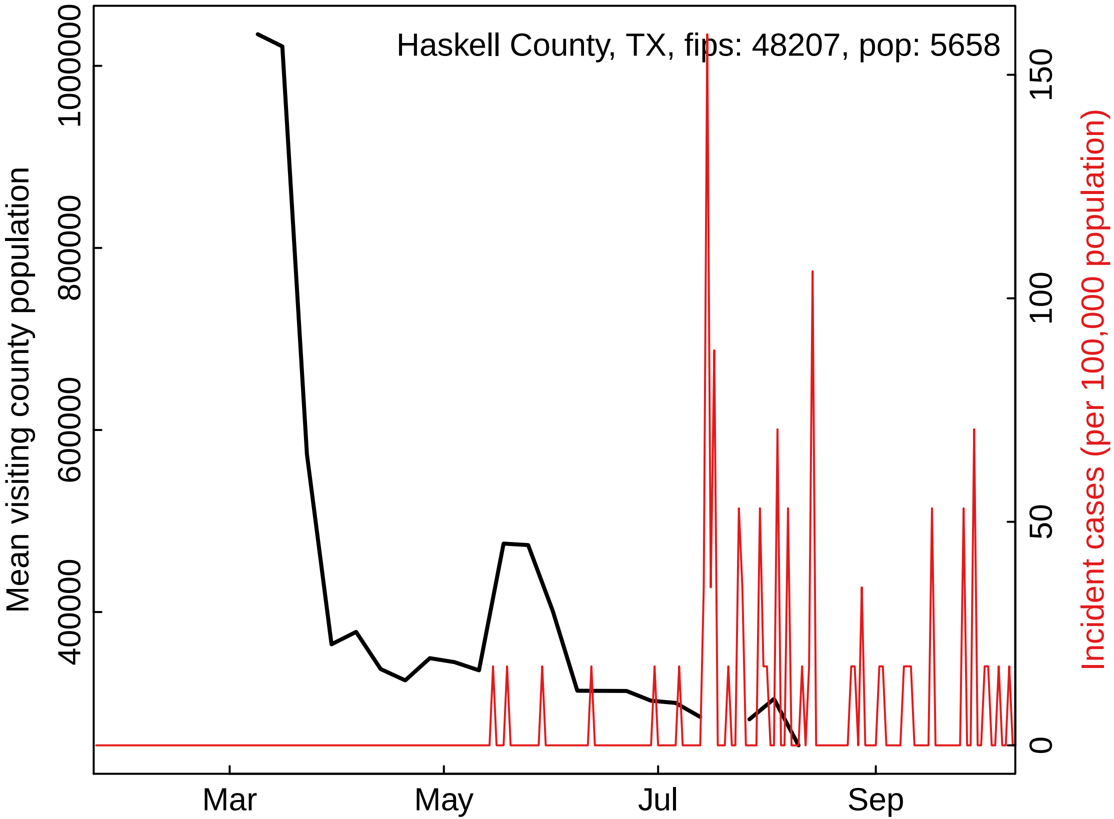
Haskell County, TX, fips: 48207, pop: 5658
Mean visiting county population
400000
600000
800000
1000000
Incident cases (per 100,000 population)
0
50
100
150
Mar
May
Jul
Sep

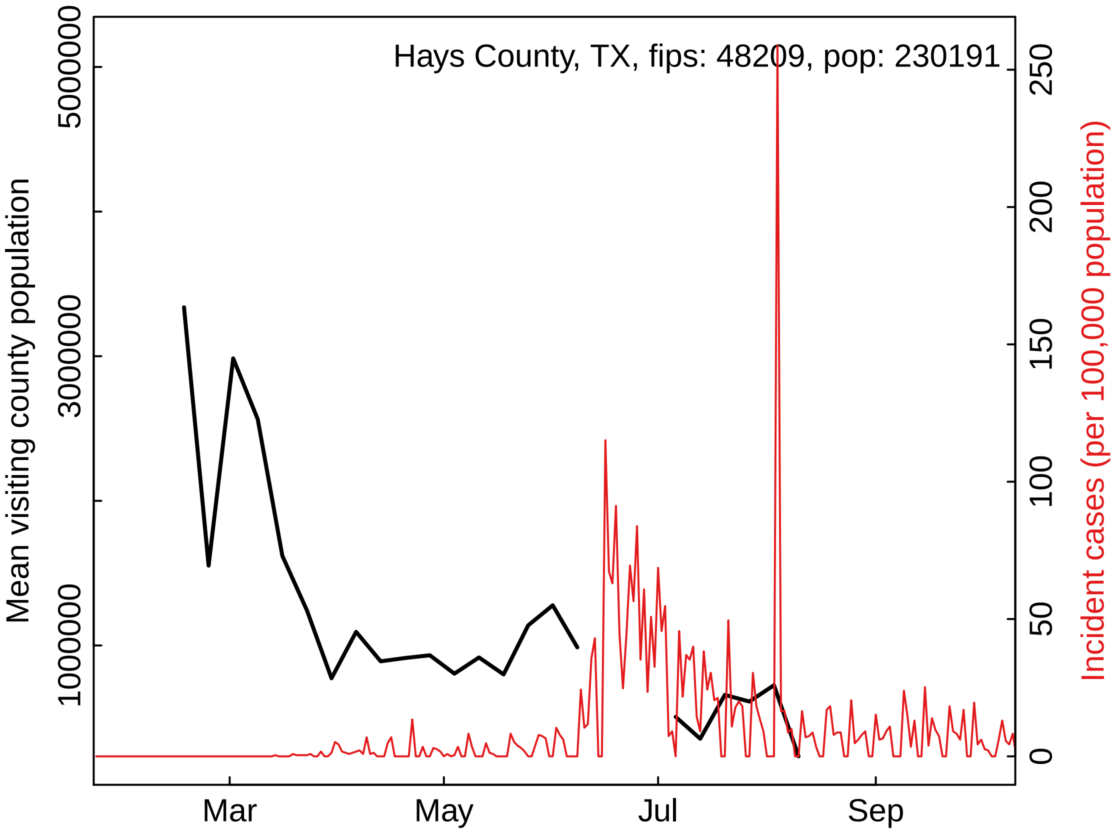

Hays County, TX, fips: 48209, pop: 230191
Mean visiting county population
Incident cases (per 100,000 population)
1000000
3000000
5000000
0
50
100
150
200
250
Mar
May
Jul
Sep

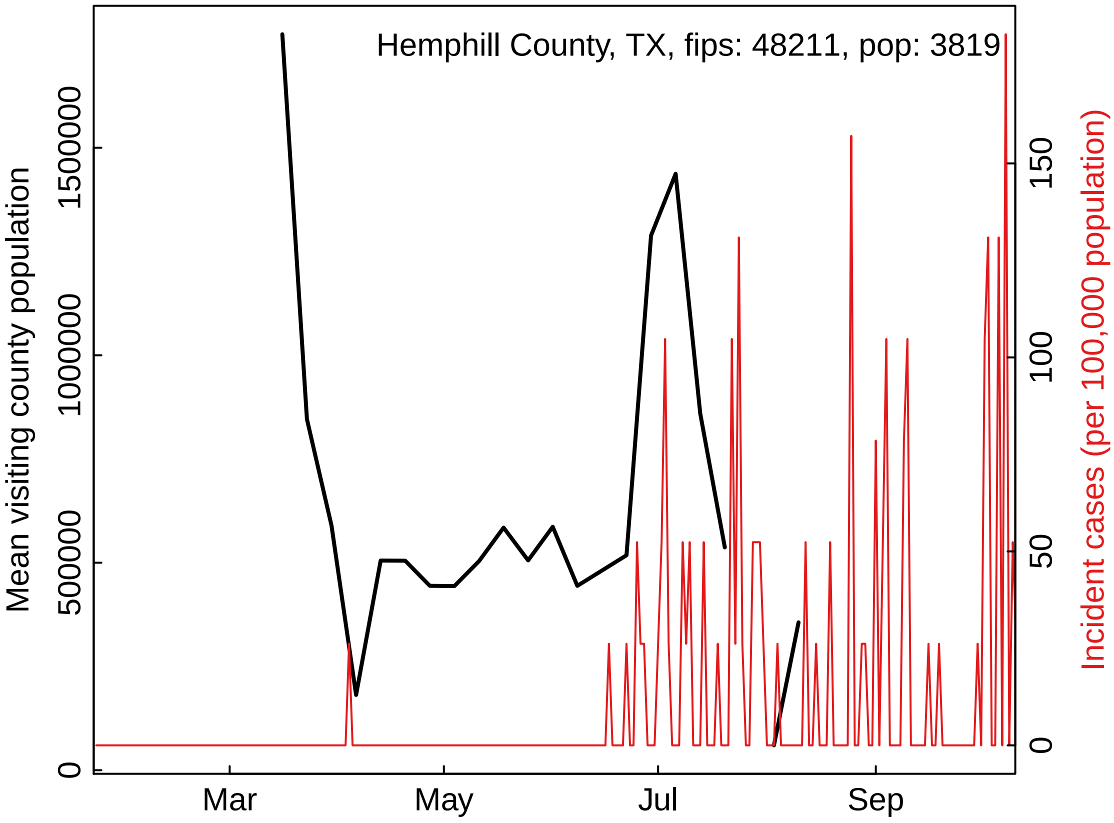

Hemphill County, TX, fips: 48211, pop: 3819
Mean visiting county population
0
500000
1000000
1500000
Incident cases (per 100,000 population)
0
50
100
150
Mar
May
Jul
Sep

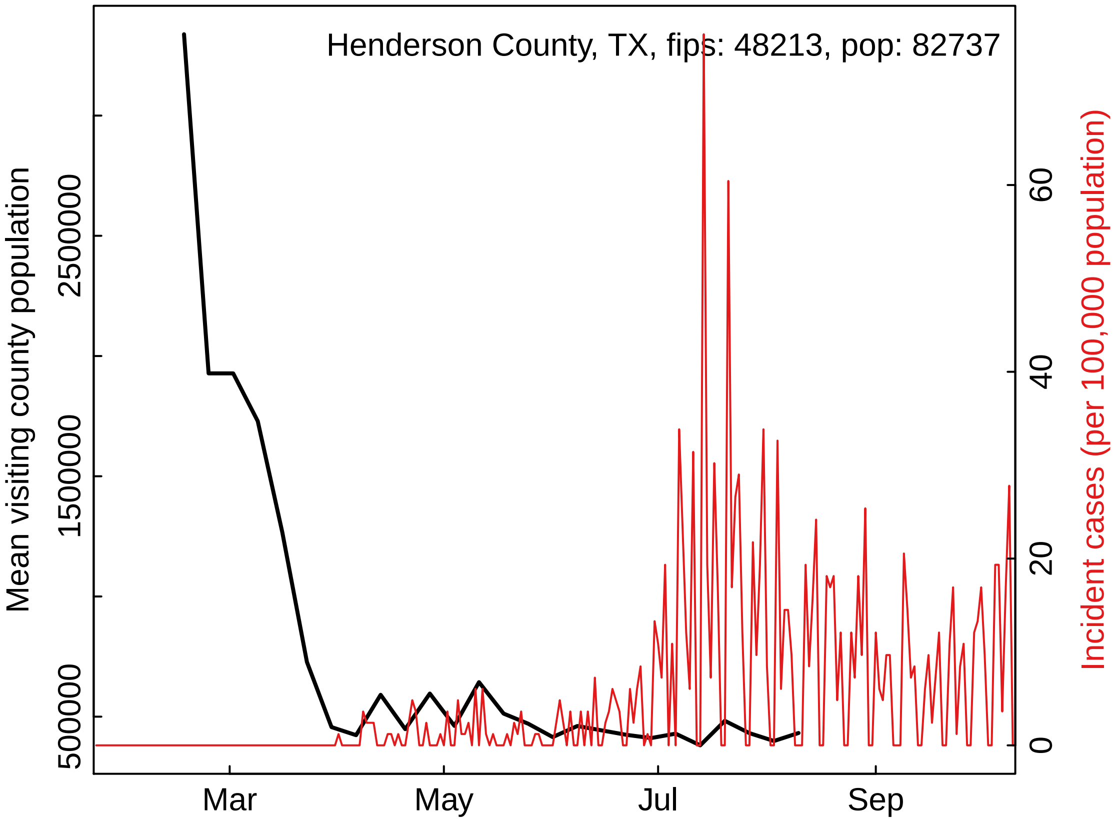
Henderson County, TX, fips: 48213, pop: 82737
Mean visiting county population
500000
1500000
2500000
Incident cases (per 100,000 population)
0
20
40
60
Mar
May
Jul
Sep

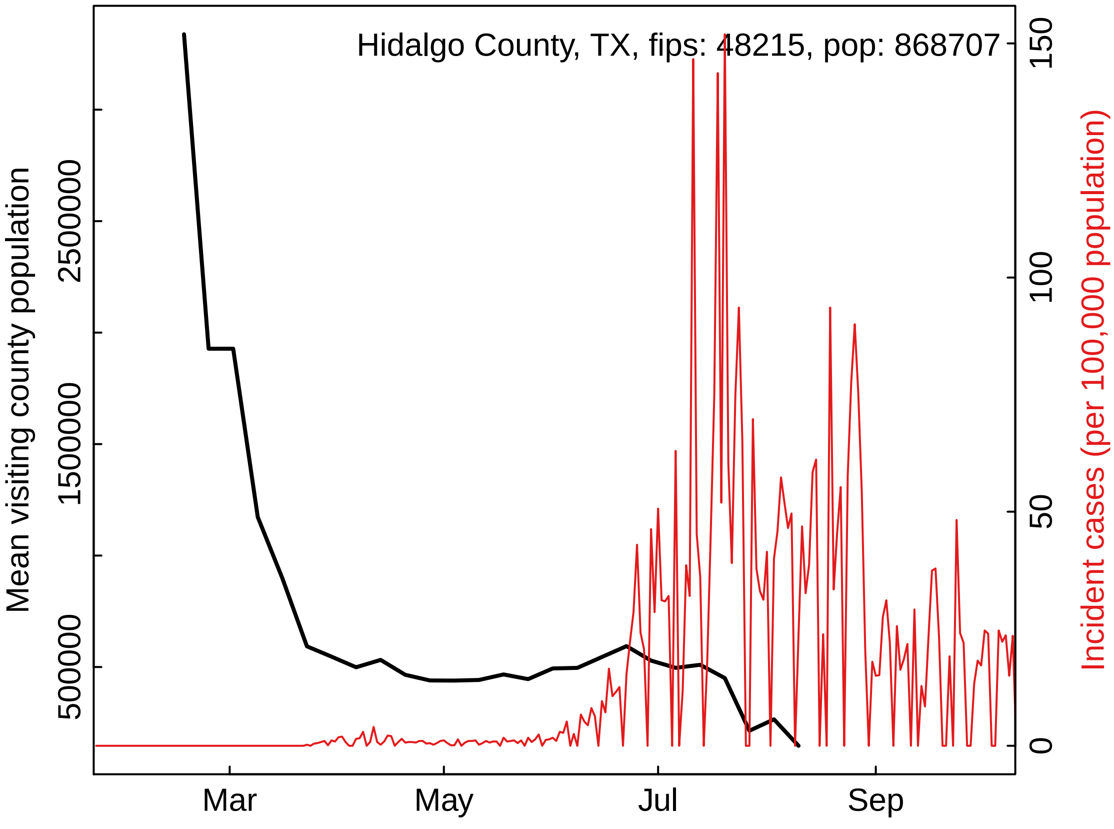
Hidalgo County, TX, fips: 48215, pop: 868707
Mean visiting county population
500000
1500000
2500000
Incident cases (per 100,000 population)
0
50
100
150
Mar
May
Jul
Sep

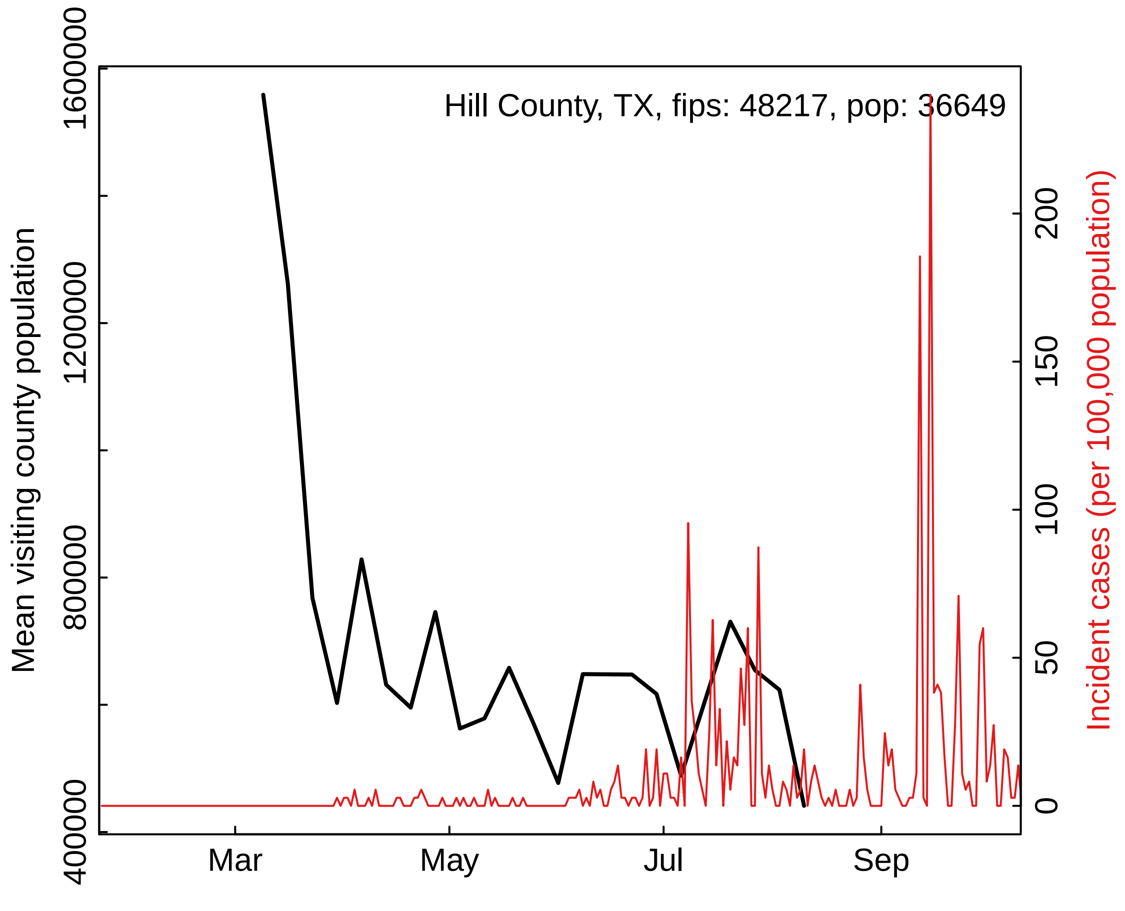
Hill County, TX, fips: 48217, pop: 36649
Mean visiting county population
400000
800000
1200000
1600000
Incident cases (per 100,000 population)
0
50
100
150
200
Mar
May
Jul
Sep

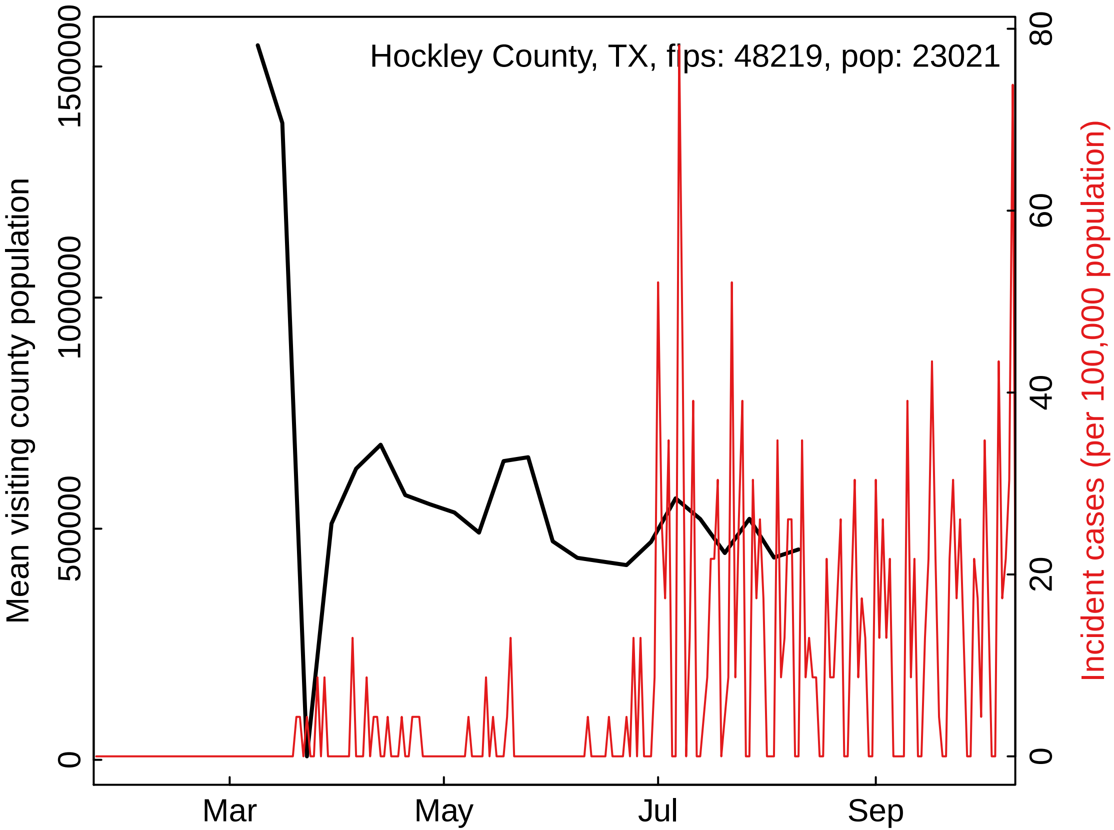

Hockley County, TX, fips: 48219, pop: 23021
Mean visiting county population
0
500000
1000000
1500000
Incident cases (per 100,000 population)
0
20
40
60
80
Mar
May
Jul
Sep

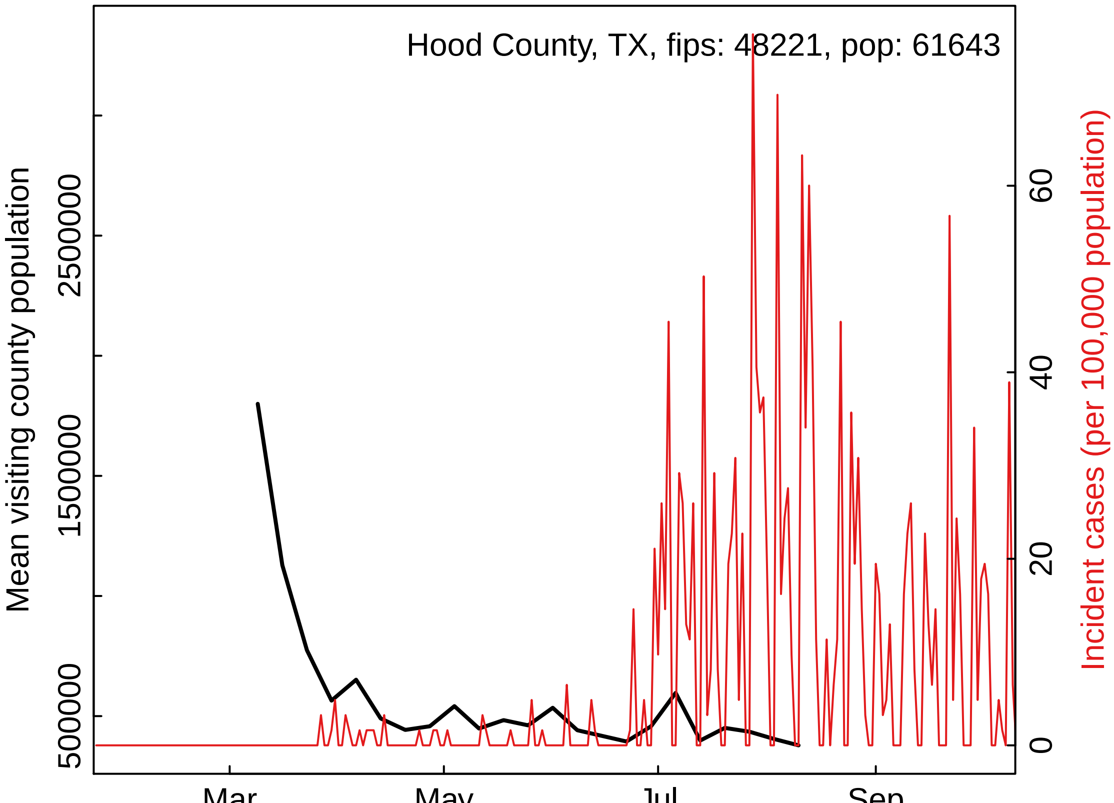

Hood County, TX, fips: 48221, pop: 61643
Mean visiting county population
500000
1500000
2500000
Incident cases (per 100,000 population)
0
20
40
60
Mar
May
Jul
Sep

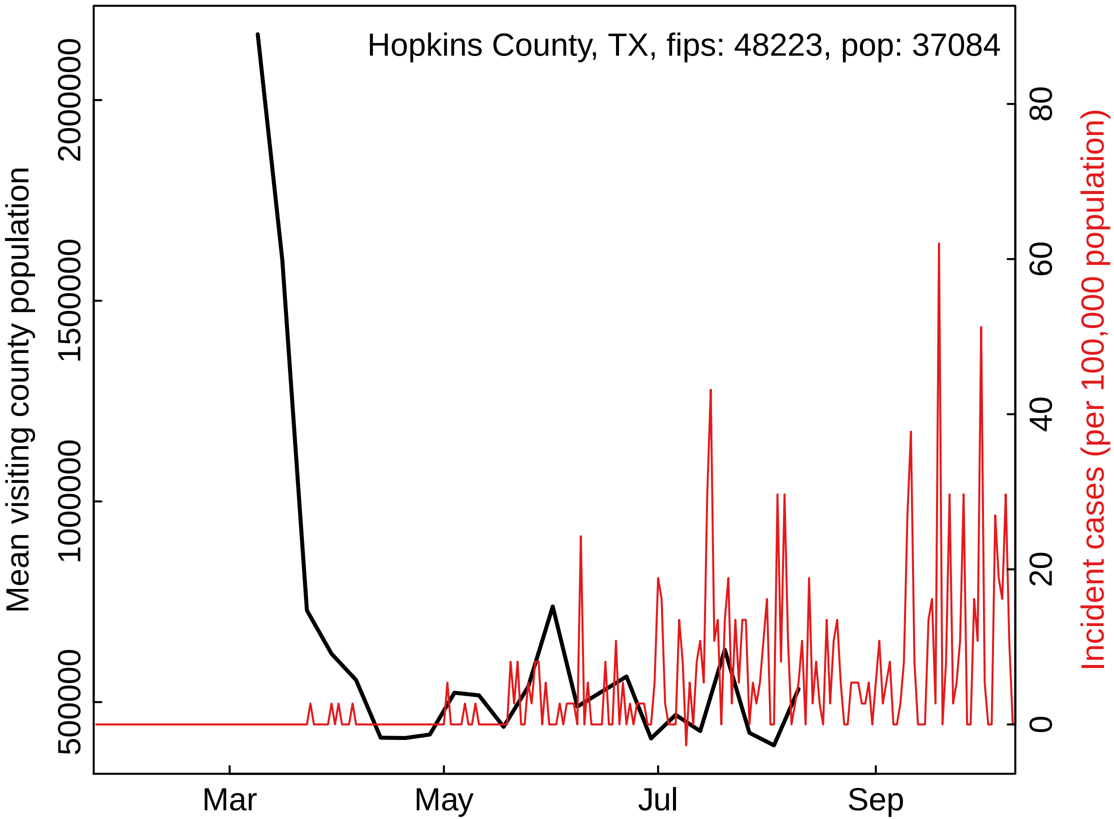

Hopkins County, TX, fips: 48223, pop: 37084
Mean visiting county population
500000
1000000
1500000
2000000
Incident cases (per 100,000 population)
0
20
40
60
80
Mar
May
Jul
Sep

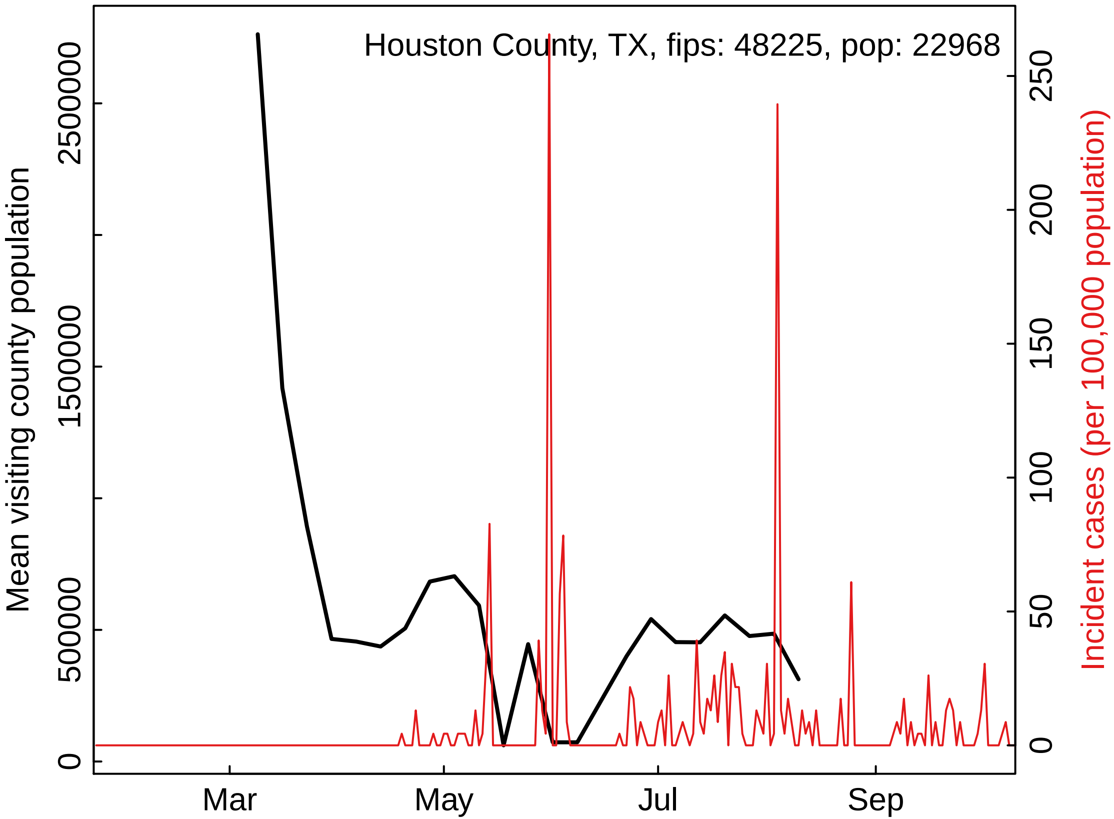
Houston County, TX, fips: 48225, pop: 22968
Mean visiting county population
Incident cases (per 100,000 population)
0
500000
1500000
2500000
0
50
100
150
200
250
Mar
May
Jul
Sep

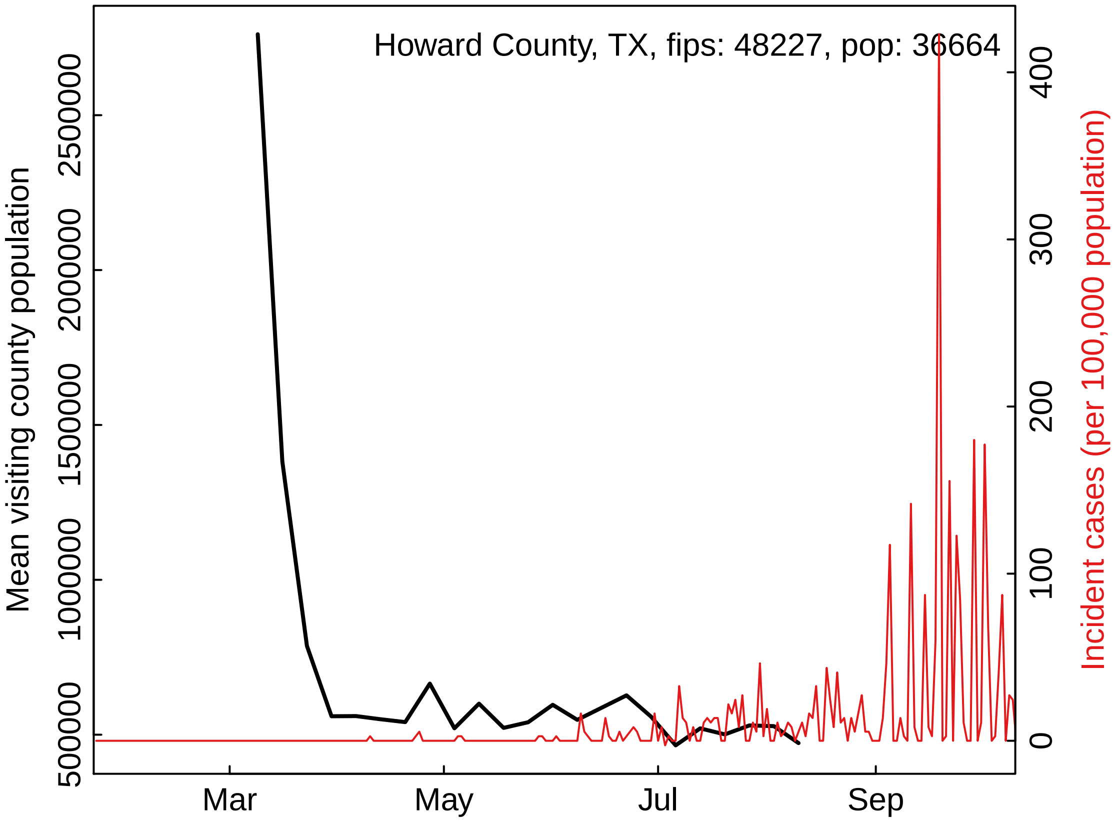

Howard County, TX, fips: 48227, pop: 36664
Mean visiting county population
5000000
1000000
1500000
2000000
2500000
Incident cases (per 100,000 population)
0
100
200
300
400
Mar
May
Jul
Sep

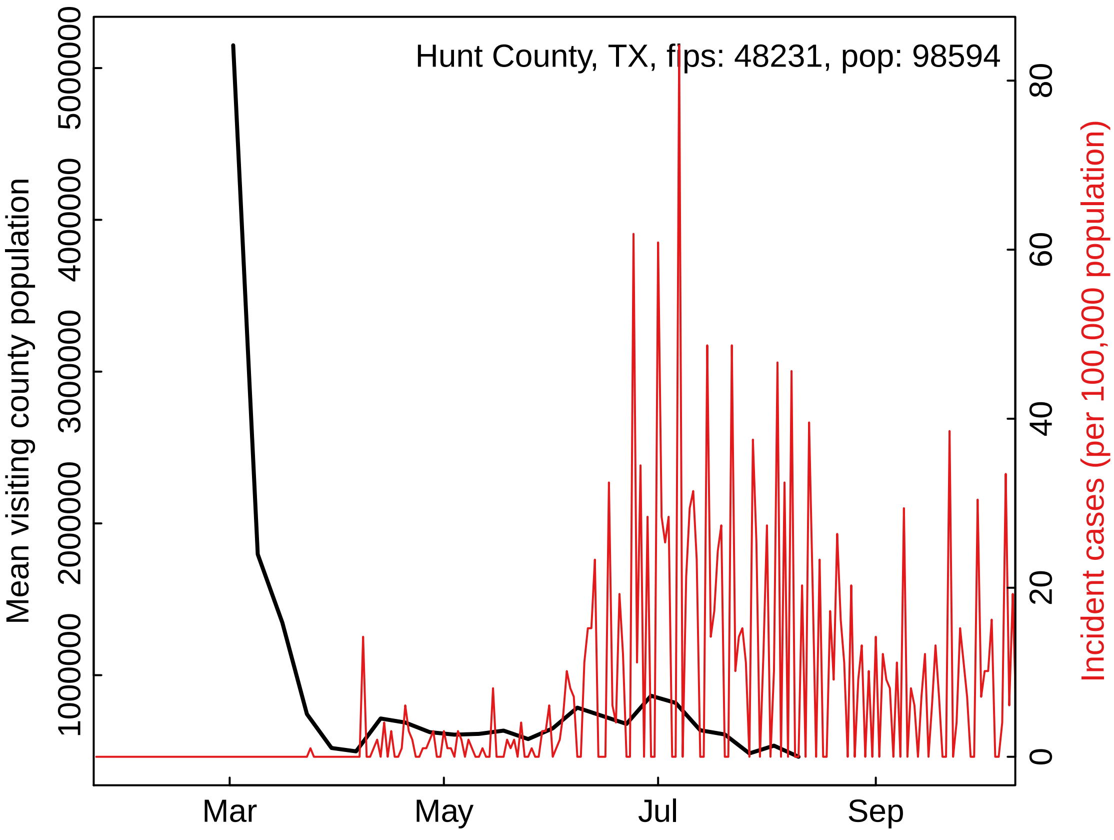

Hunt County, TX, fips: 48231, pop: 98594
Mean visiting county population
Incident cases (per 100,000 population)
1000000
2000000
3000000
4000000
5000000
0
20
40
60
80
Mar
May
Jul
Sep

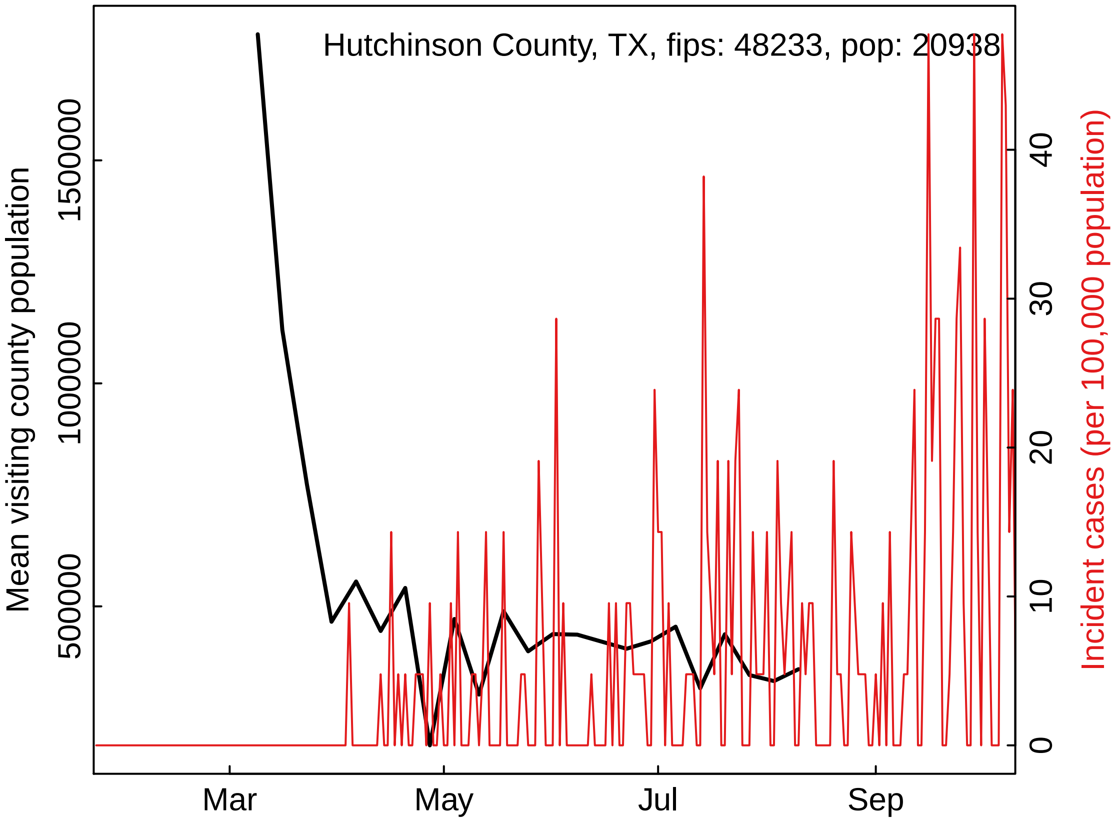

Hutchinson County, TX, fips: 48233, pop: 20938
Mean visiting county population
500000
1000000
1500000
Incident cases (per 100,000 population)
0
10
20
30
40
Mar
May
Jul
Sep

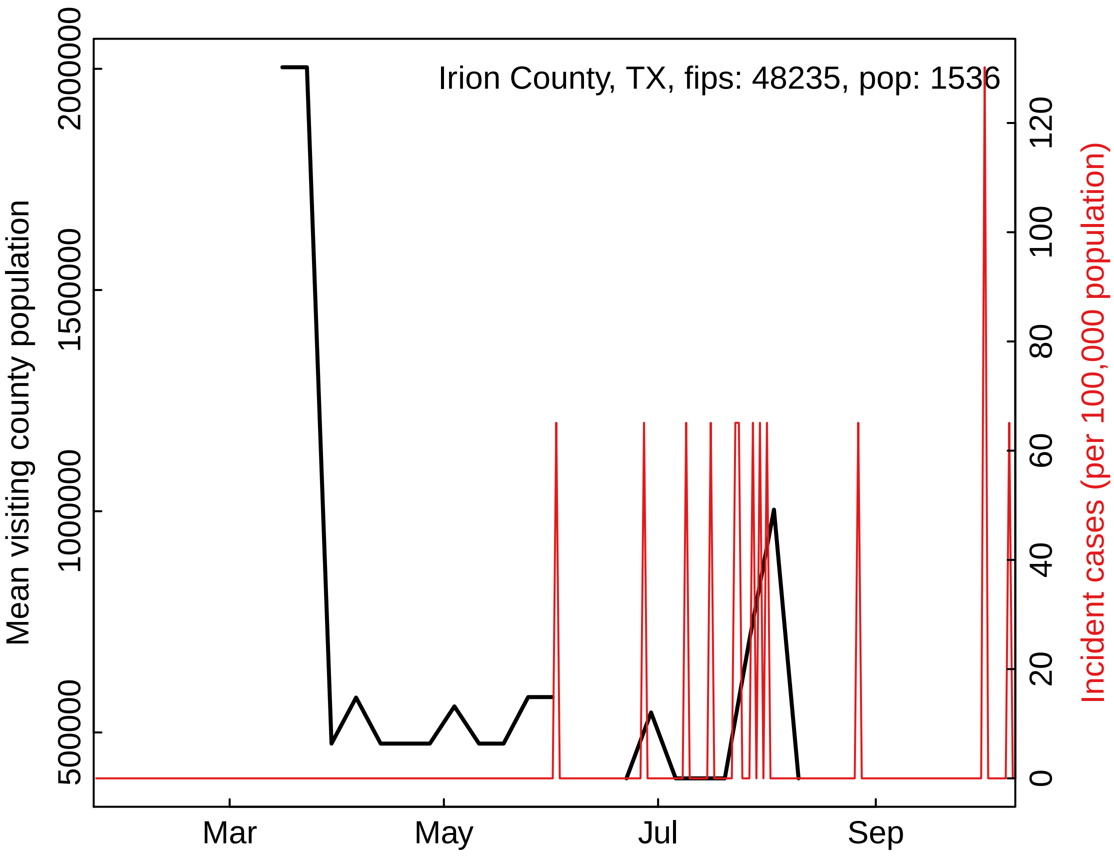

Irion County, TX, fips: 48235, pop: 1536
Mean visiting county population
500000
1000000
1500000
2000000
Incident cases (per 100,000 population)
0
20
40
60
80
100
120
Mar
May
Jul
Sep

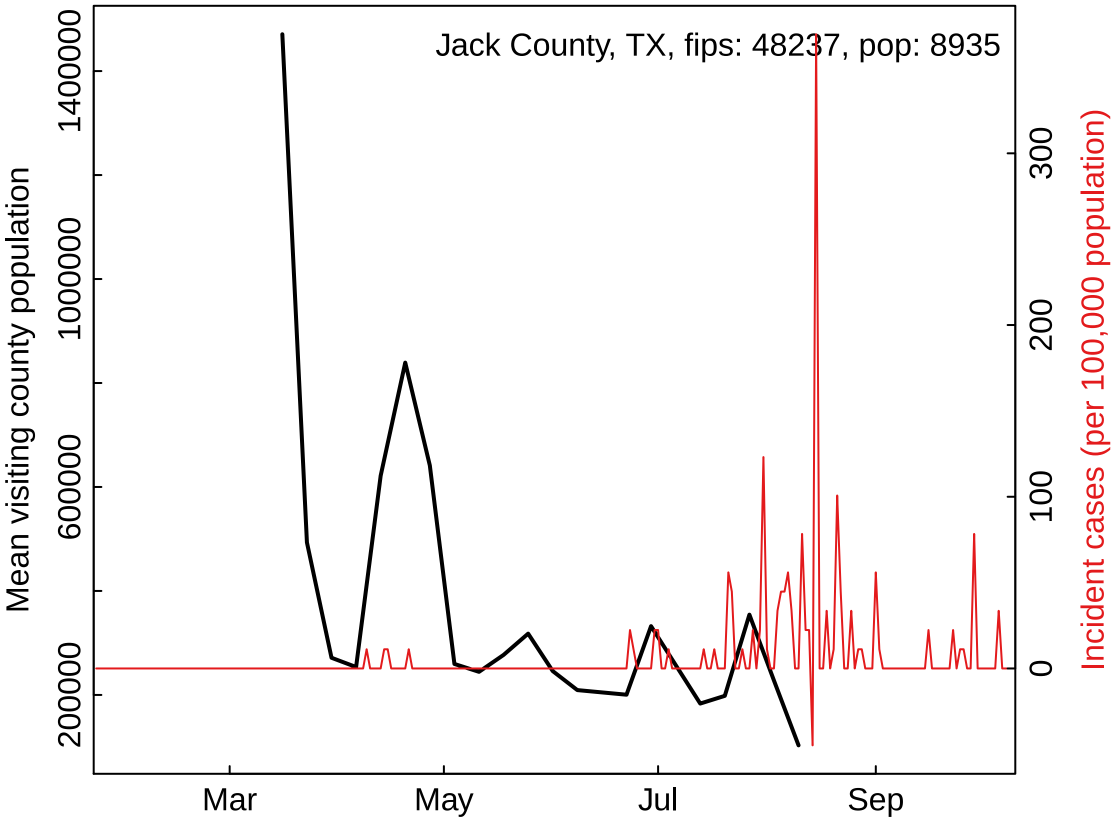

Jack County, TX, fips: 48237, pop: 8935
Mean visiting county population
200000
600000
1000000
1400000
Incident cases (per 100,000 population)
0
100
200
300
Mar
May
Jul
Sep

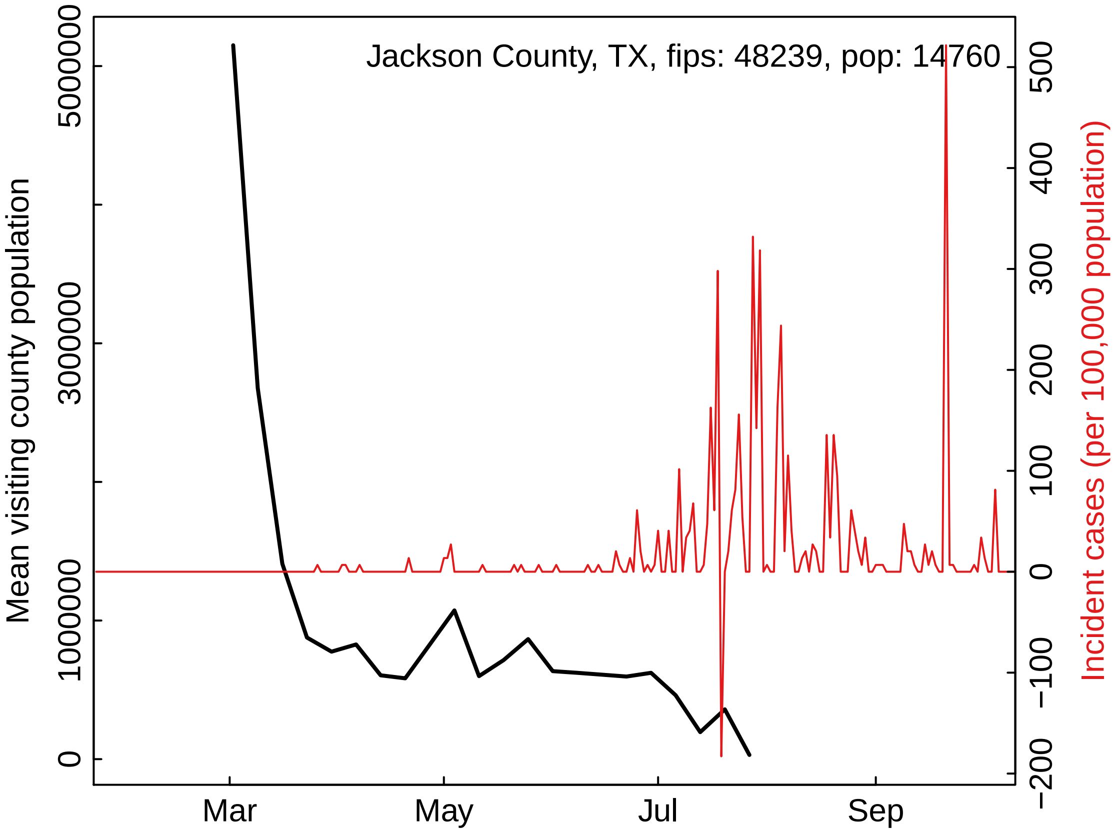

Jackson County, TX, fips: 48239, pop: 14760
Mean visiting county population
0
1000000
3000000
5000000
Incident cases (per 100,000 population)
−200
−100
0
100
200
300
400
500
Mar
May
Jul
Sep

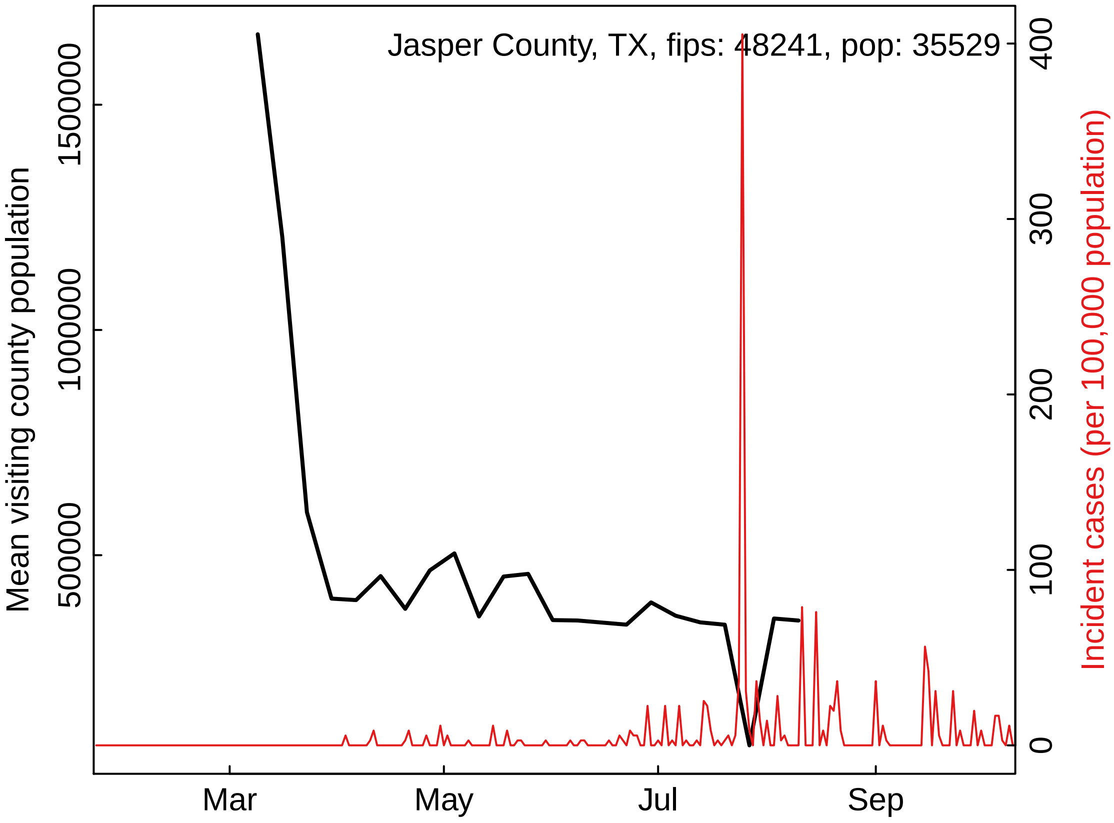

Jasper County, TX, fips: 48241, pop: 35529
Mean visiting county population
500000
1000000
1500000
Incident cases (per 100,000 population)
0
100
200
300
400
Mar
May
Jul
Sep

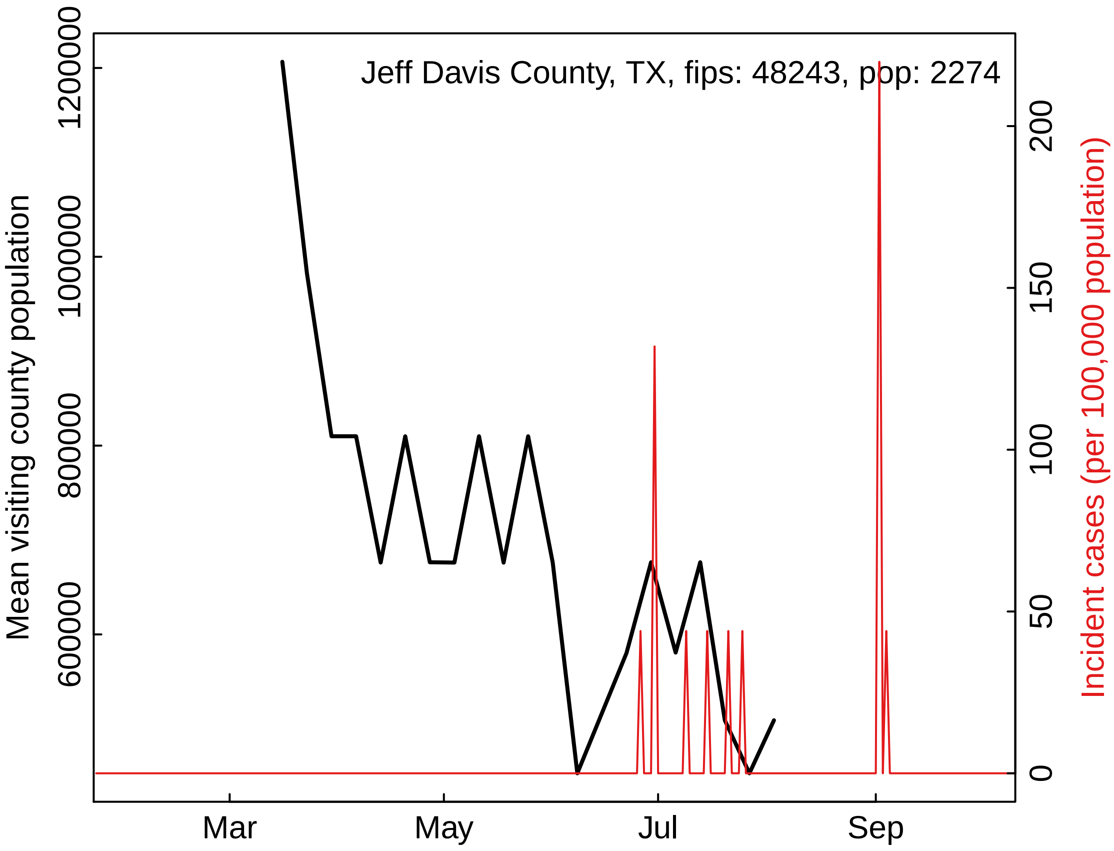

Jeff Davis County, TX, fips: 48243, pop: 2274
Mean visiting county population
600000
800000
1000000
1200000
Incident cases (per 100,000 population)
0
50
100
150
200
Mar
May
Jul
Sep

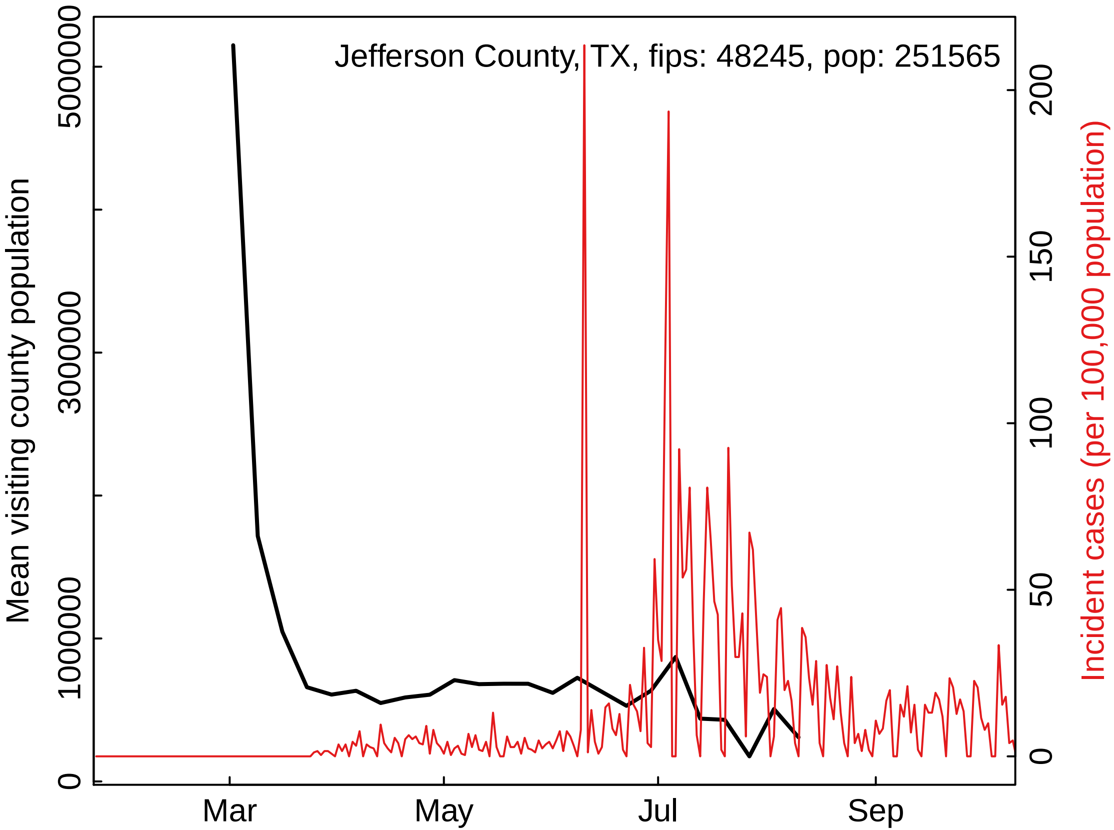

Jefferson County, TX, fips: 48245, pop: 251565
Mean visiting county population
Incident cases (per 100,000 population)
0
1000000
3000000
5000000
0
50
100
150
200
Mar
May
Jul
Sep

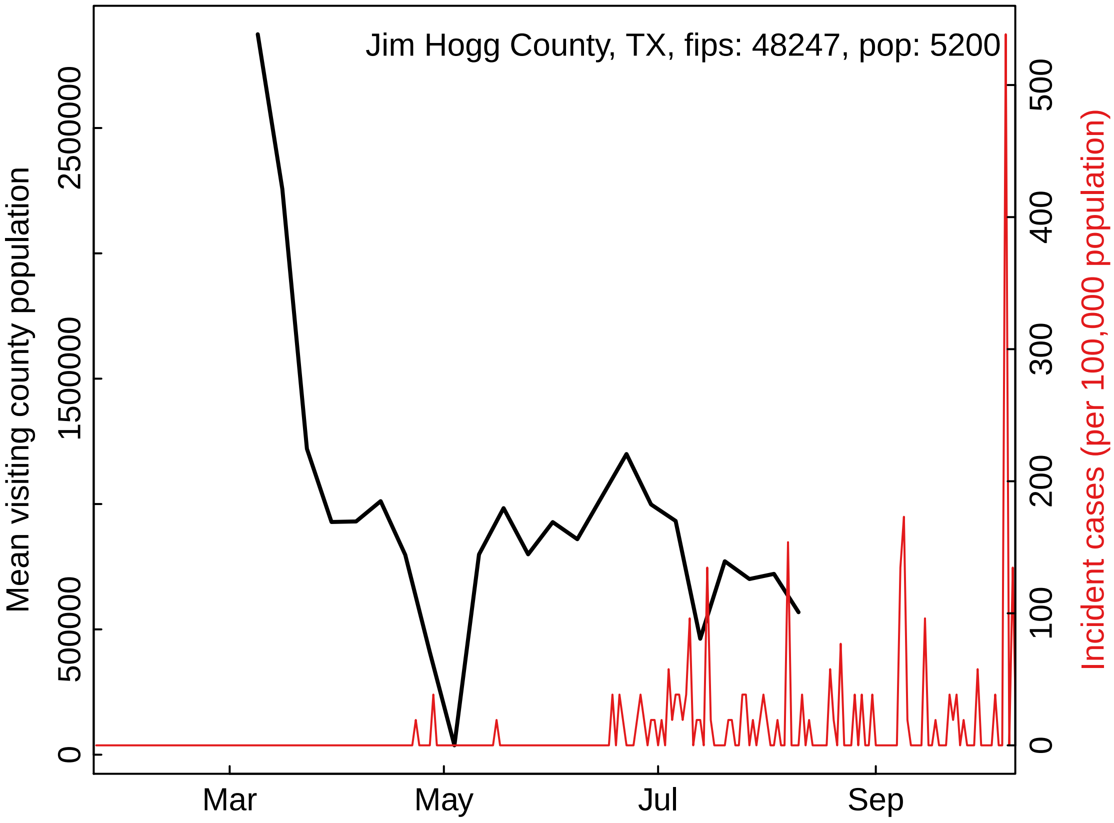
Jim Hogg County, TX, fips: 48247, pop: 5200
Mean visiting county population
0
500000
1500000
2500000
Incident cases (per 100,000 population)
0
100
200
300
400
500
Mar
May
Jul
Sep

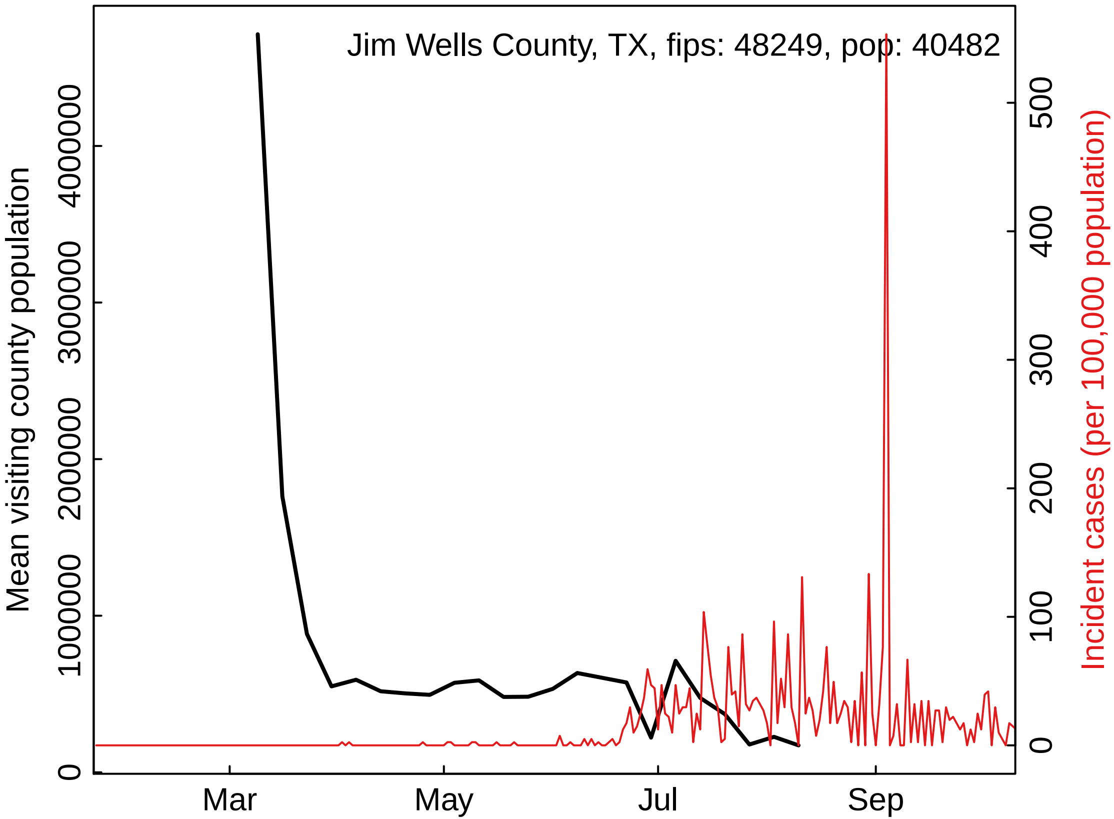

Jim Wells County, TX, fips: 48249, pop: 40482
Mean visiting county population
0
1000000
2000000
3000000
4000000
Mar
May
Jul
Sep
0
100
200
300
400
500
Incident cases (per 100,000 population)

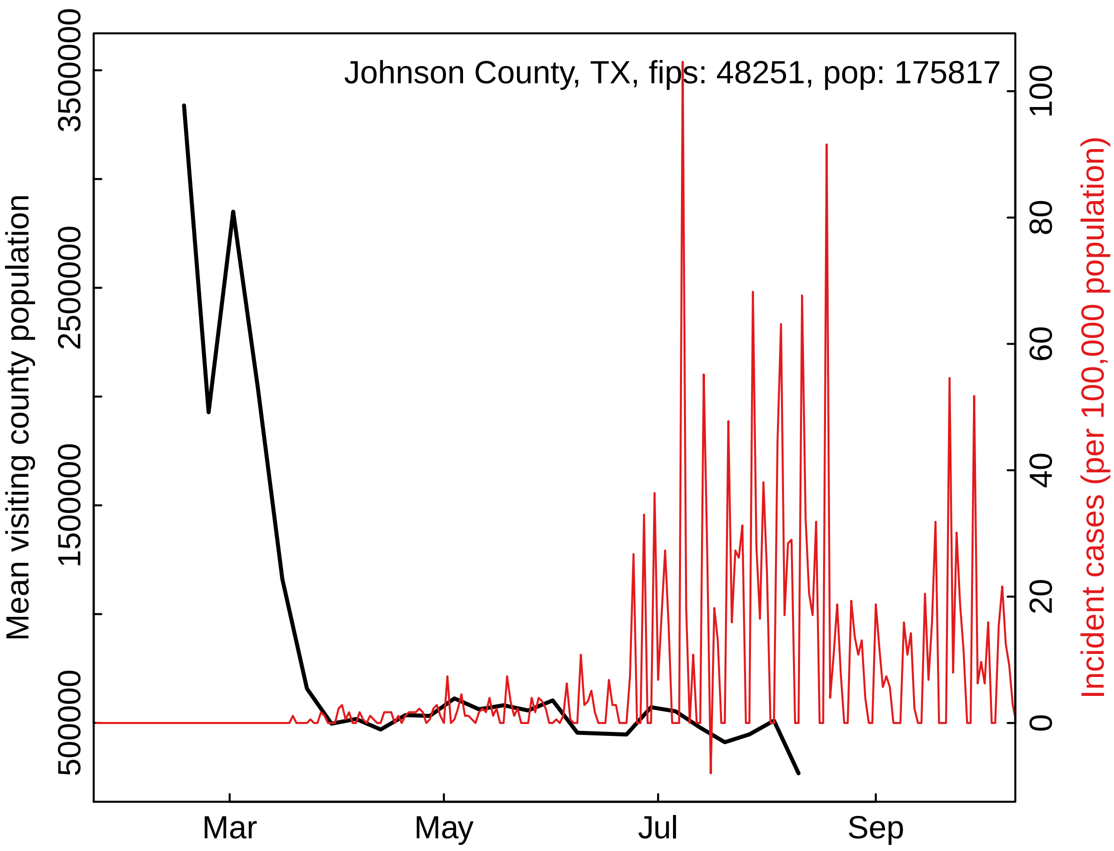

Johnson County, TX, fips: 48251, pop: 175817
Mean visiting county population
500000
1500000
2500000
3500000
Incident cases (per 100,000 population)
0
20
40
60
80
100
Mar
May
Jul
Sep

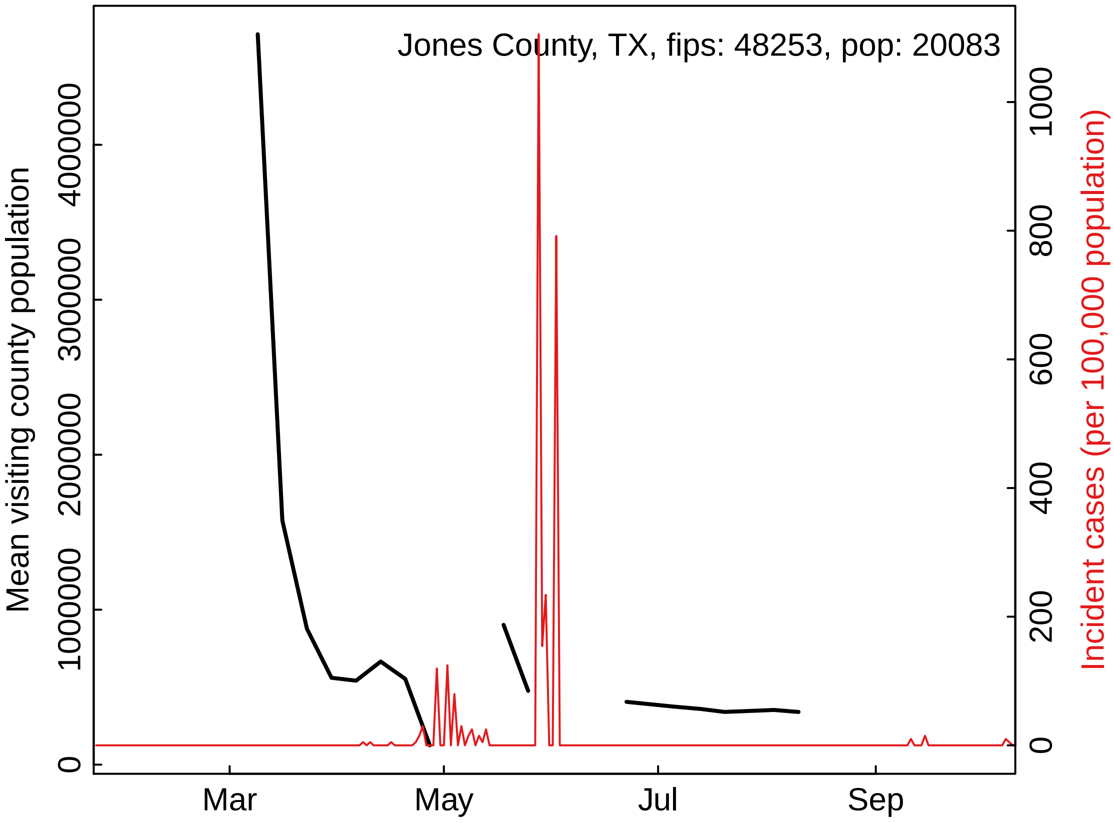

Jones County, TX, fips: 48253, pop: 20083
Mean visiting county population
0
1000000
2000000
3000000
4000000
Incident cases (per 100,000 population)
0
200
400
600
800
1000
Mar
May
Jul
Sep

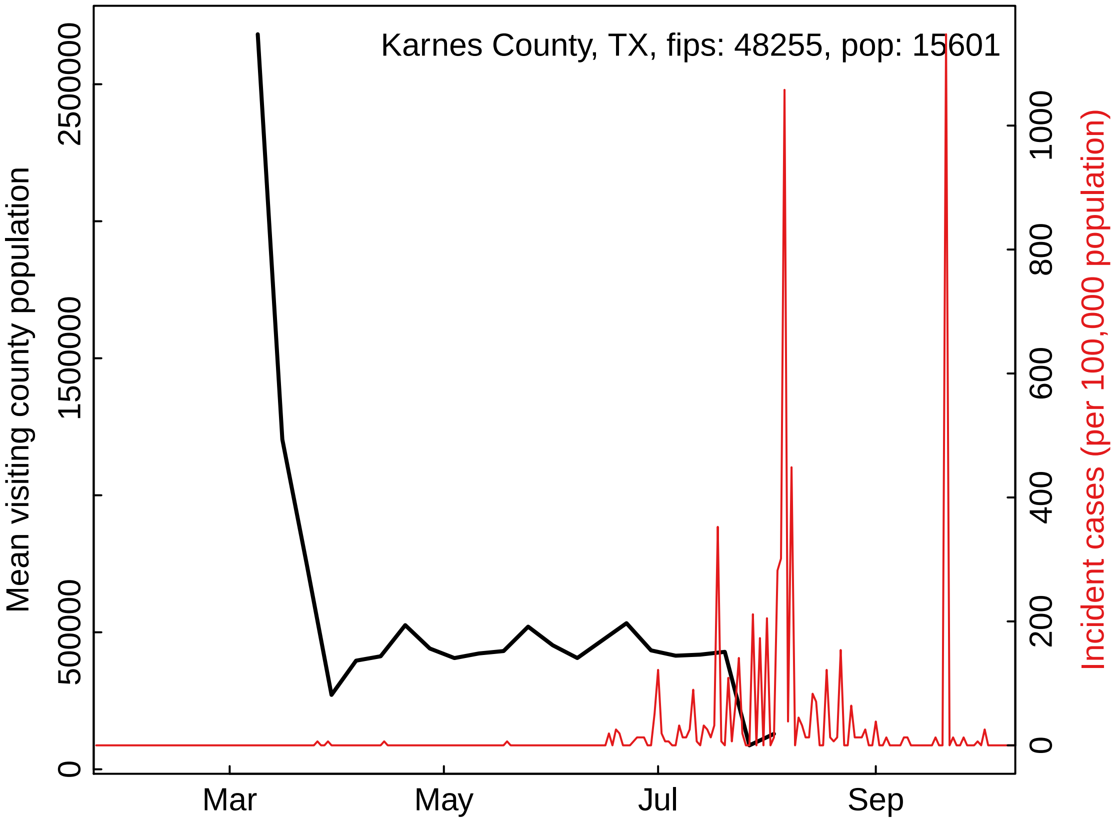

Karnes County, TX, fips: 48255, pop: 15601
Mean visiting county population
0
500000
1500000
2500000
Incident cases (per 100,000 population)
0
200
400
600
800
1000
Mar
May
Jul
Sep

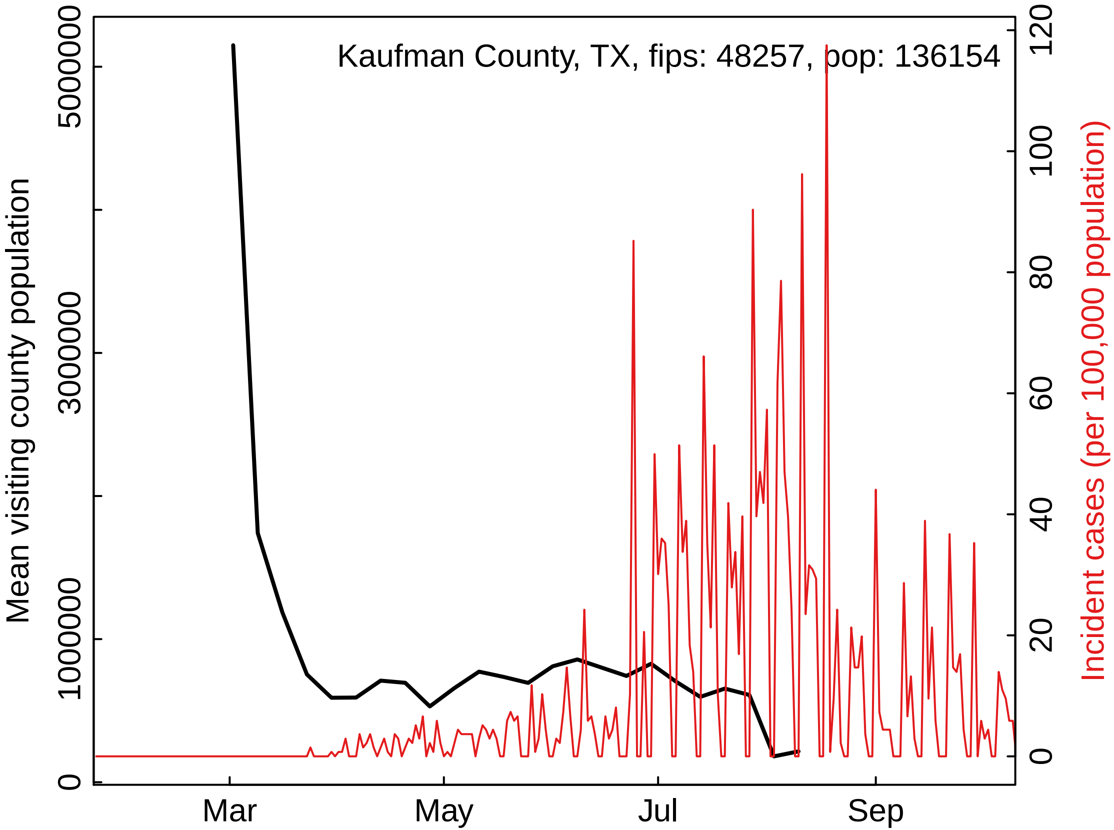
Kaufman County, TX, fips: 48257, pop: 136154
Mean visiting county population
Incident cases (per 100,000 population)
0
1000000
3000000
5000000
0
20
40
60
80
100
120
Mar
May
Jul
Sep

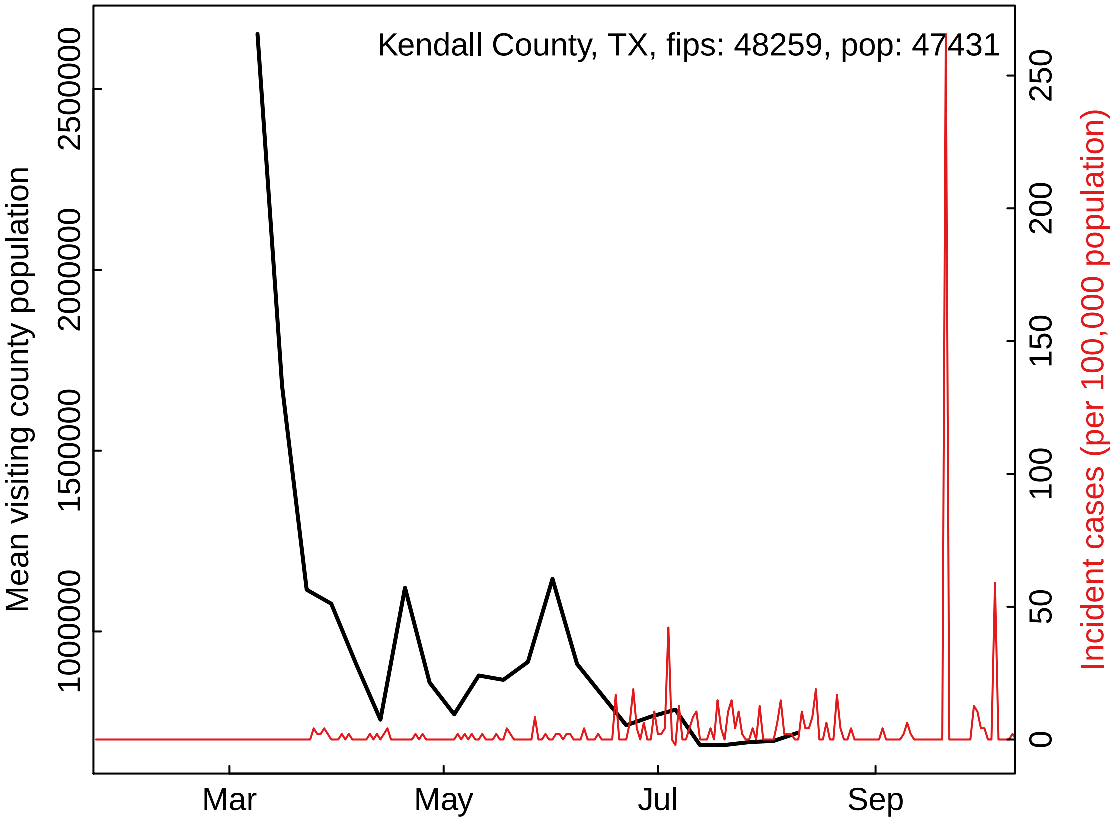

Kendall County, TX, fips: 48259, pop: 47431
Mean visiting county population
1000000
1500000
2000000
2500000
Incident cases (per 100,000 population)
0
50
100
150
200
250
Mar
May
Jul
Sep

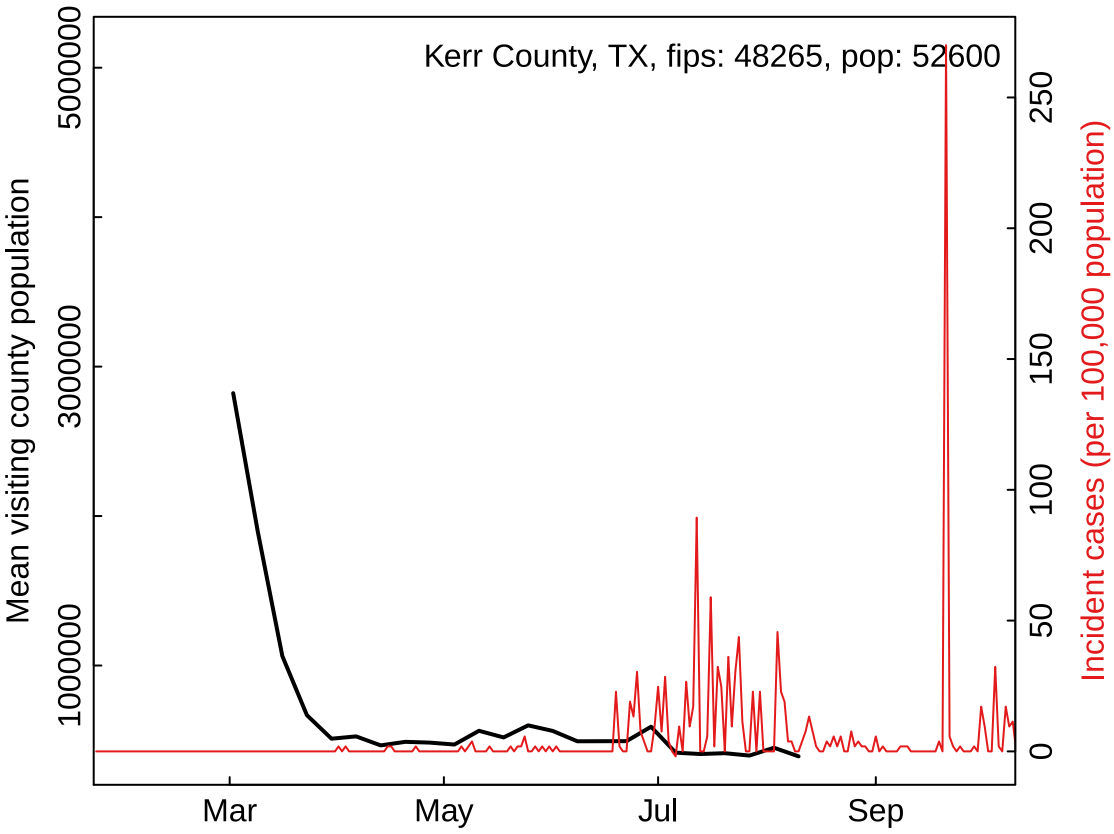
Kerr County, TX, fips: 48265, pop: 52600
Mean visiting county population
5000000
3000000
1000000
Incident cases (per 100,000 population)
250
200
150
100
50
0
Mar
May
Jul
Sep

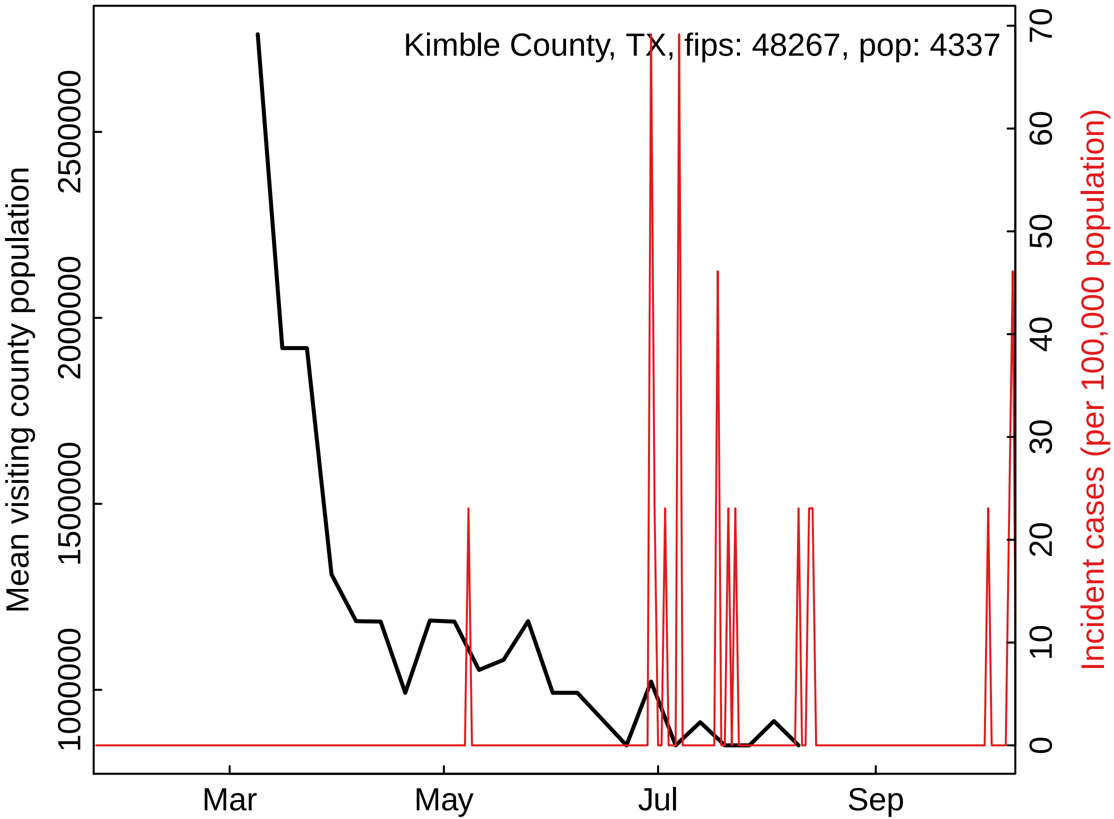
Kimble County, TX, fips: 48267, pop: 4337
Mean visiting county population
1000000
1500000
2000000
2500000
Incident cases (per 100,000 population)
0
10
20
30
40
50
60
70
Mar
May
Jul
Sep

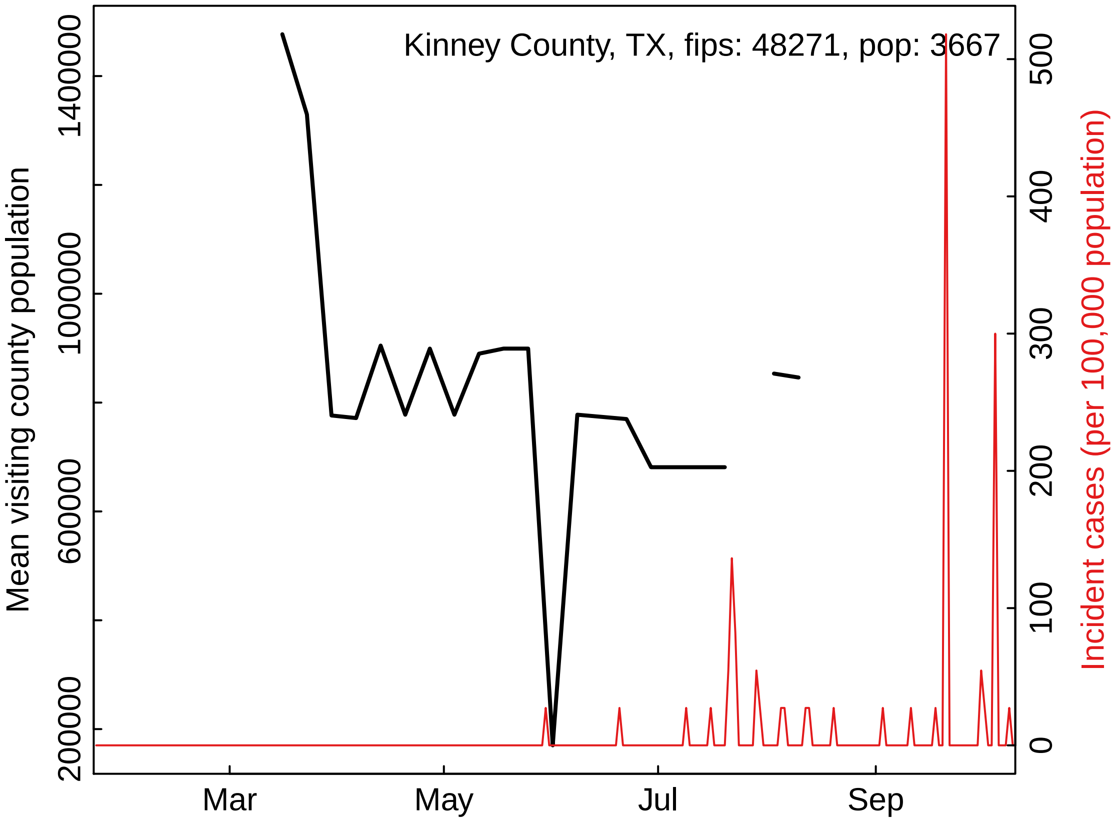
Kinney County, TX, fips: 48271, pop: 3667
Mean visiting county population
200000
600000
1000000
1400000
Incident cases (per 100,000 population)
0
100
200
300
400
500
Mar
May
Jul
Sep

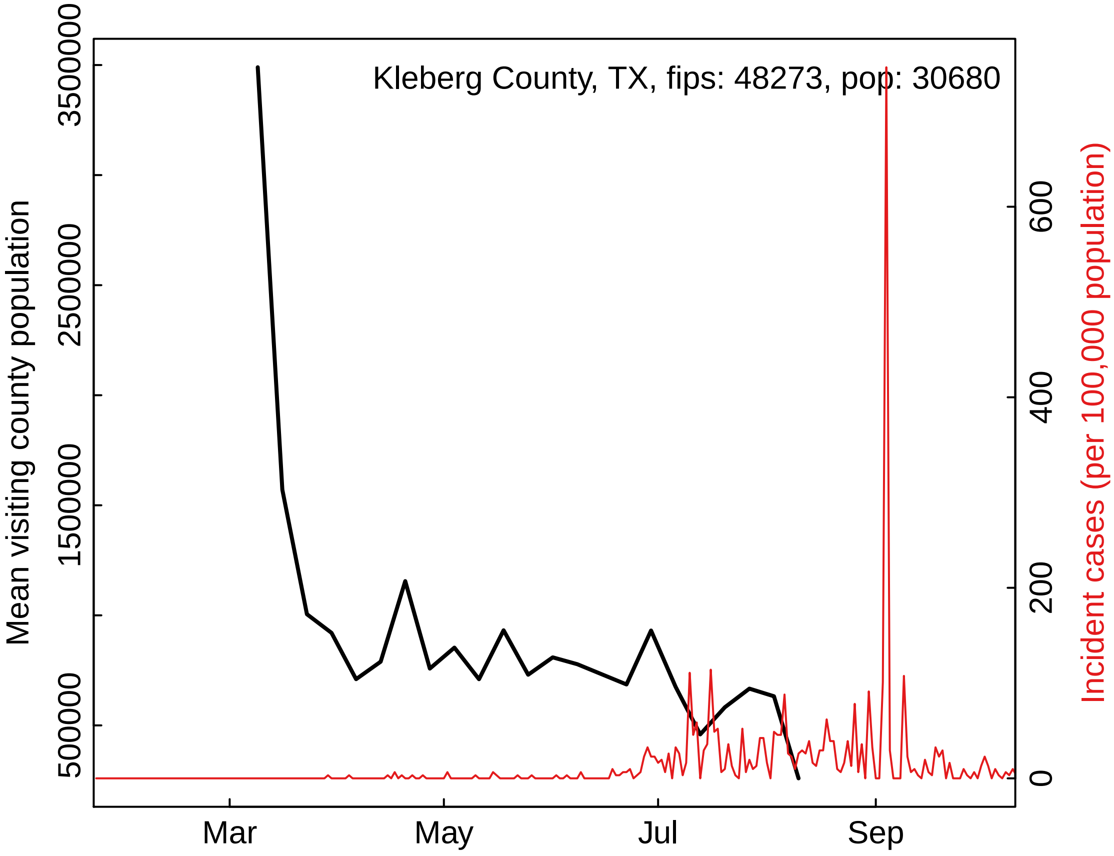
Kleberg County, TX, fips: 48273, pop: 30680
Mean visiting county population
500000
1500000
2500000
3500000
Incident cases (per 100,000 population)
0
200
400
600
Mar
May
Jul
Sep

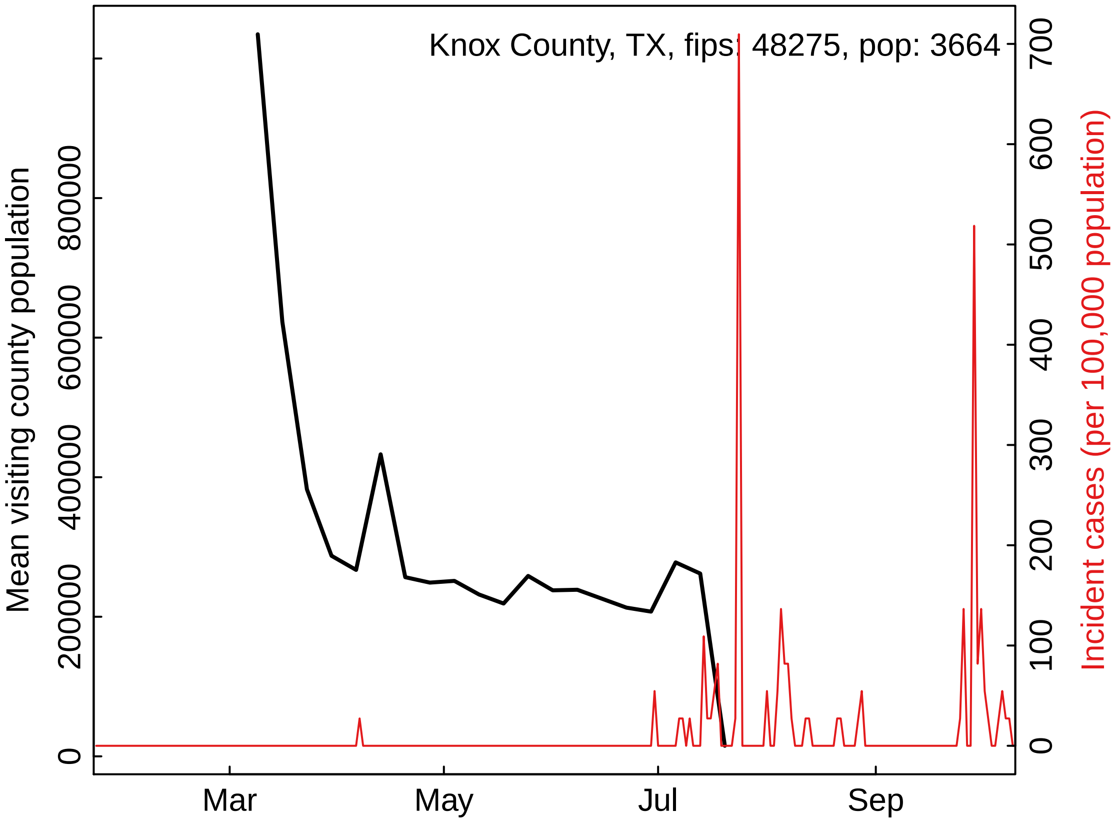
Knox County, TX, fips: 48275, pop: 3664
Mean visiting county population
0
200000
400000
600000
800000
Incident cases (per 100,000 population)
0
100
200
300
400
500
600
700
Mar
May
Jul
Sep

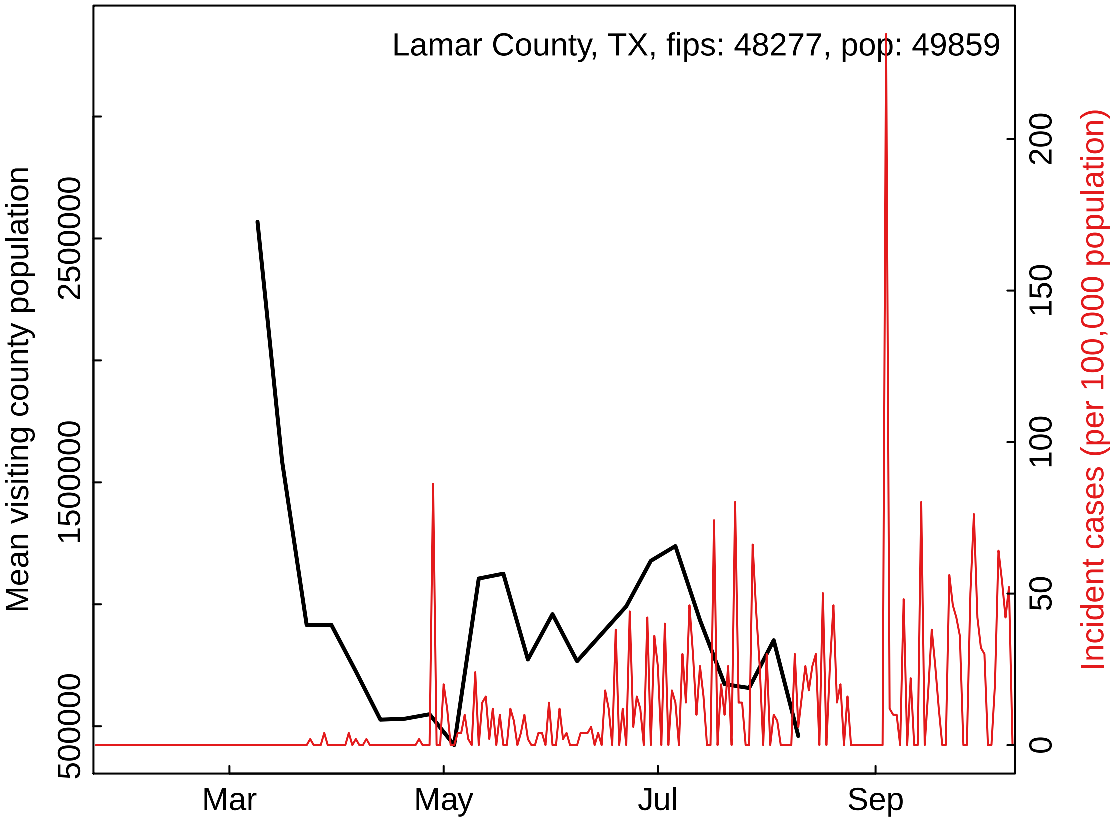

Lamar County, TX, fips: 48277, pop: 49859
Mean visiting county population
500000
1500000
2500000
Incident cases (per 100,000 population)
0
50
100
150
200
Mar
May
Jul
Sep

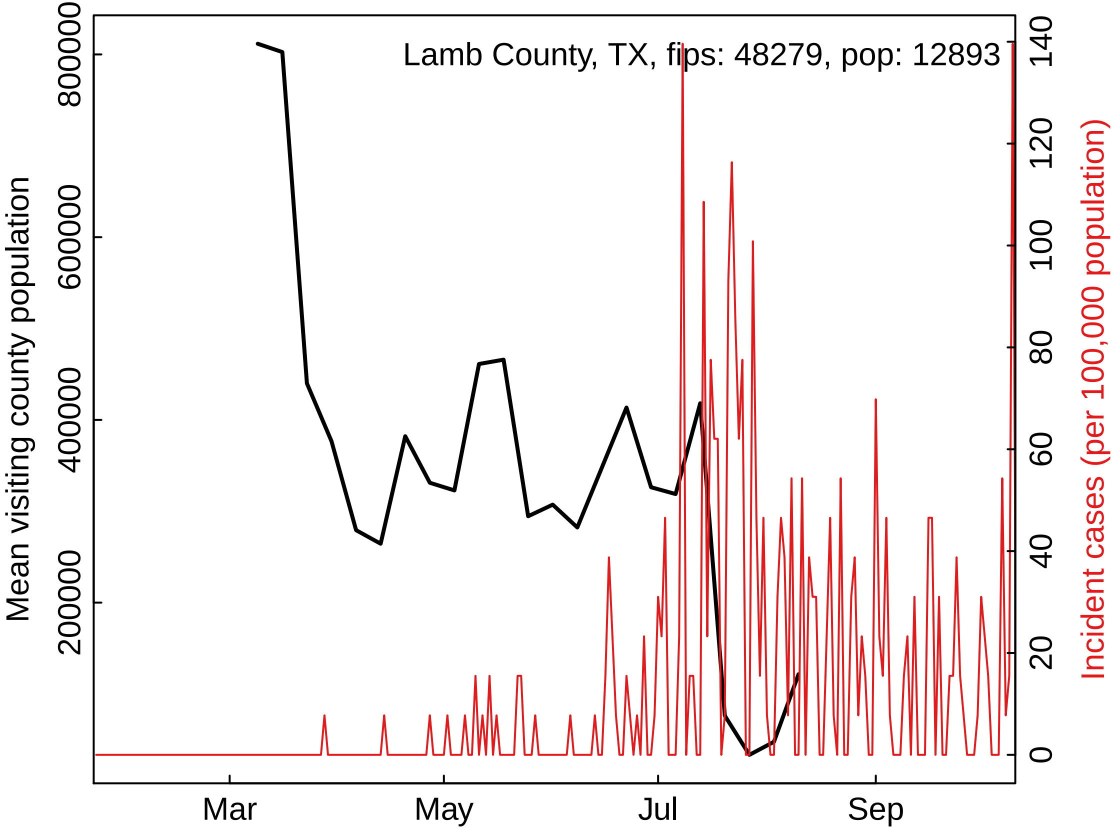

Lamb County, TX, fips: 48279, pop: 12893
Mean visiting county population
200000
400000
600000
800000
Incident cases (per 100,000 population)
0
20
40
60
80
100
120
140
Mar
May
Jul
Sep

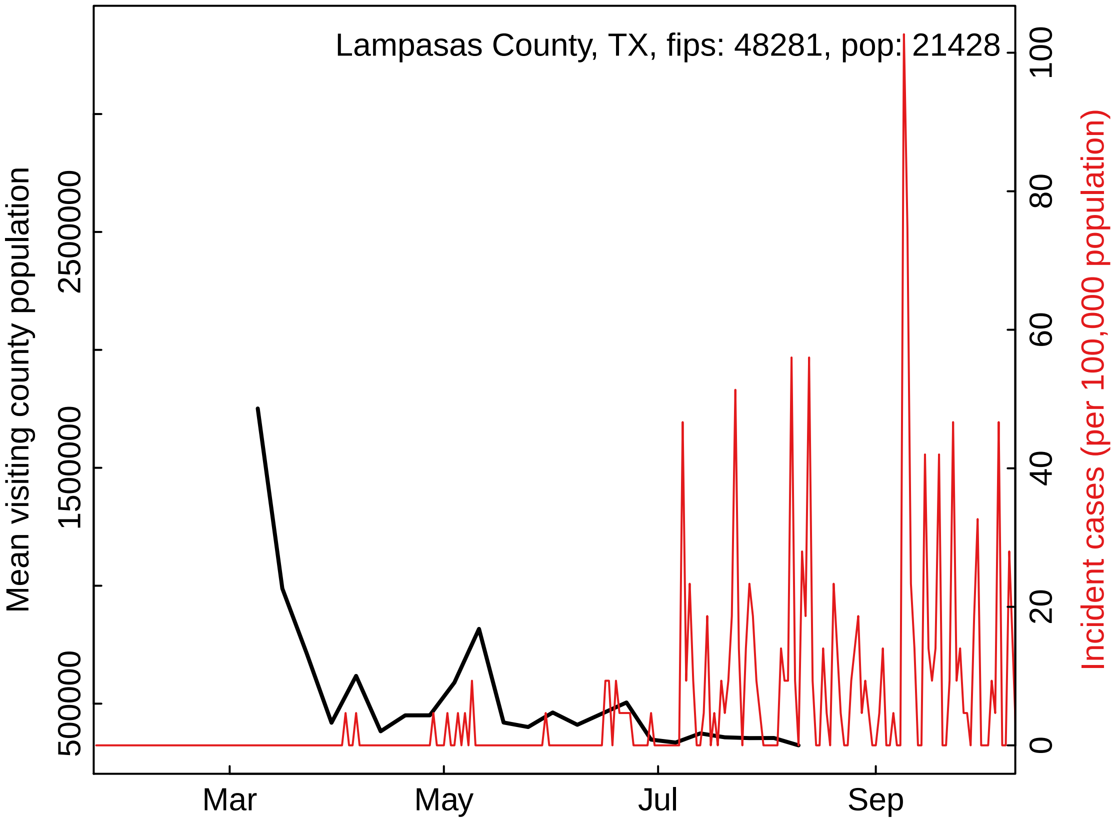

Lampasas County, TX, fips: 48281, pop: 21428
Mean visiting county population
500000
1500000
2500000
Mar
May
Jul
Sep
0
20
40
60
80
100
Incident cases (per 100,000 population)

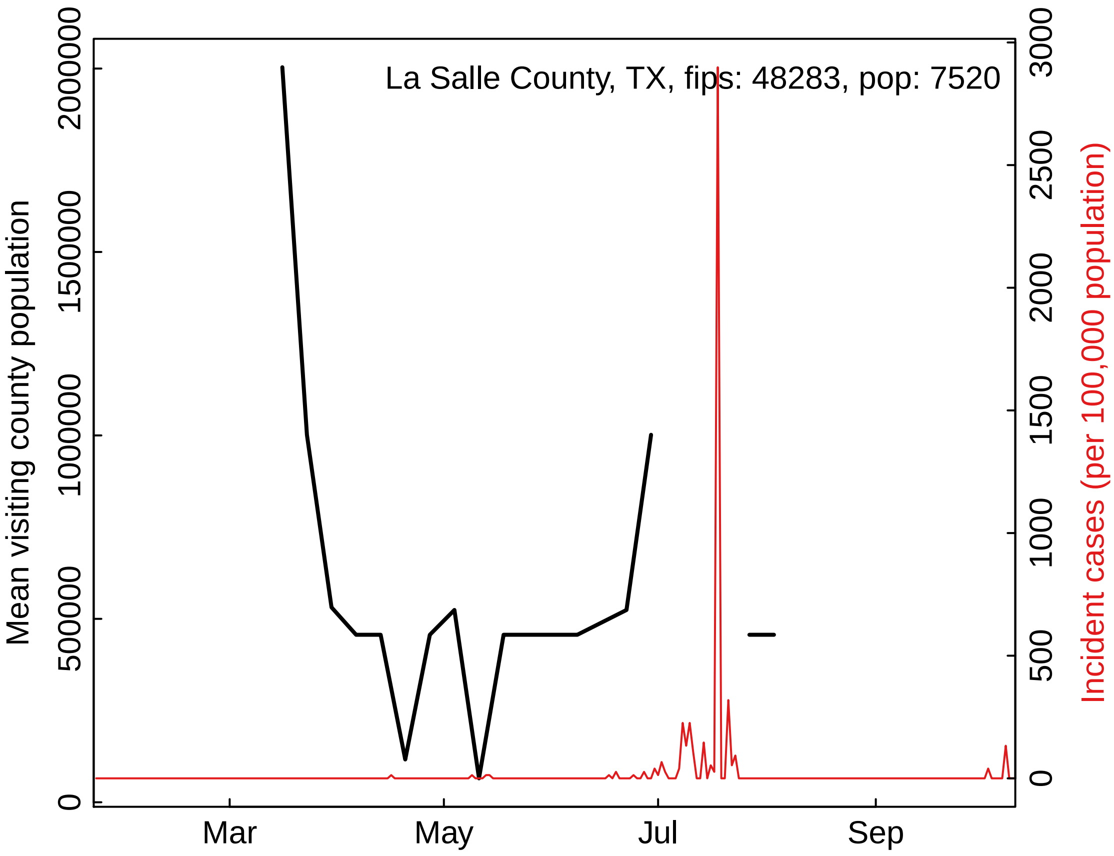

La Salle County, TX, fips: 48283, pop: 7520
Mean visiting county population
0
500000
1000000
1500000
2000000
Incident cases (per 100,000 population)
0
500
1000
1500
2000
2500
3000
Mar
May
Jul
Sep

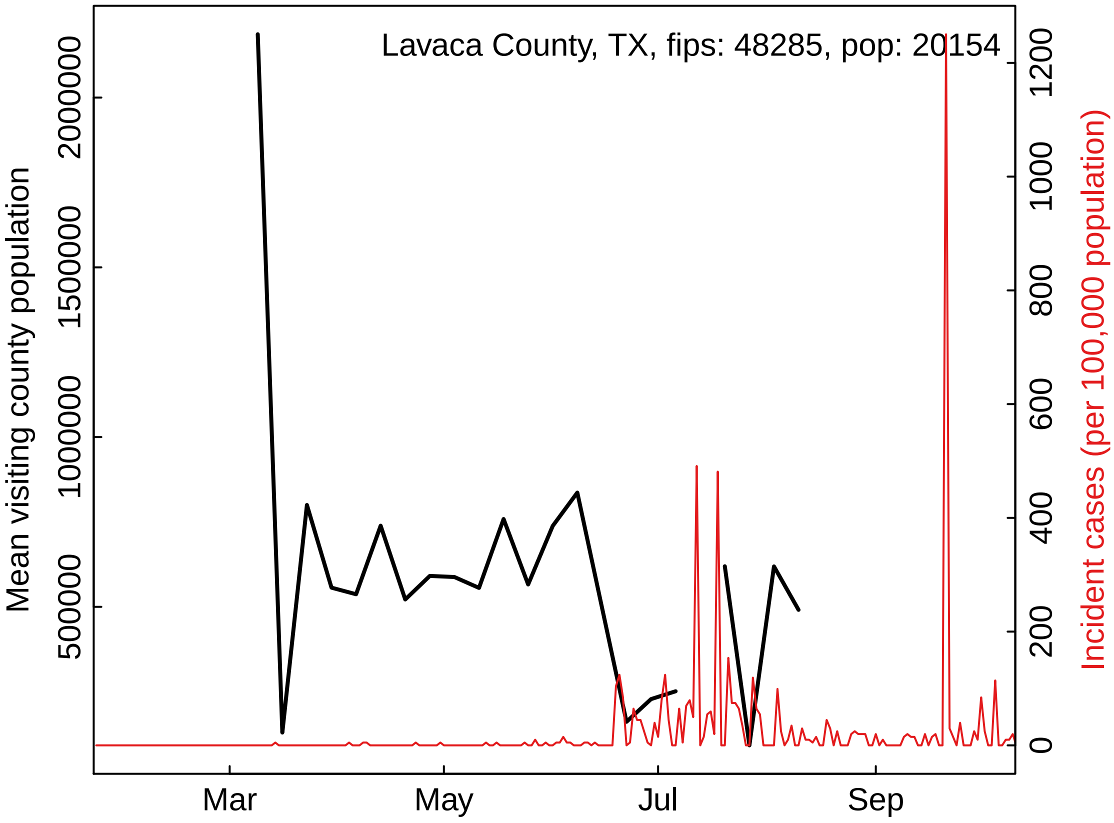

Lavaca County, TX, fips: 48285, pop: 20154
Mean visiting county population
500000
1000000
1500000
2000000
Incident cases (per 100,000 population)
0
200
400
600
800
1000
1200
Mar
May
Jul
Sep

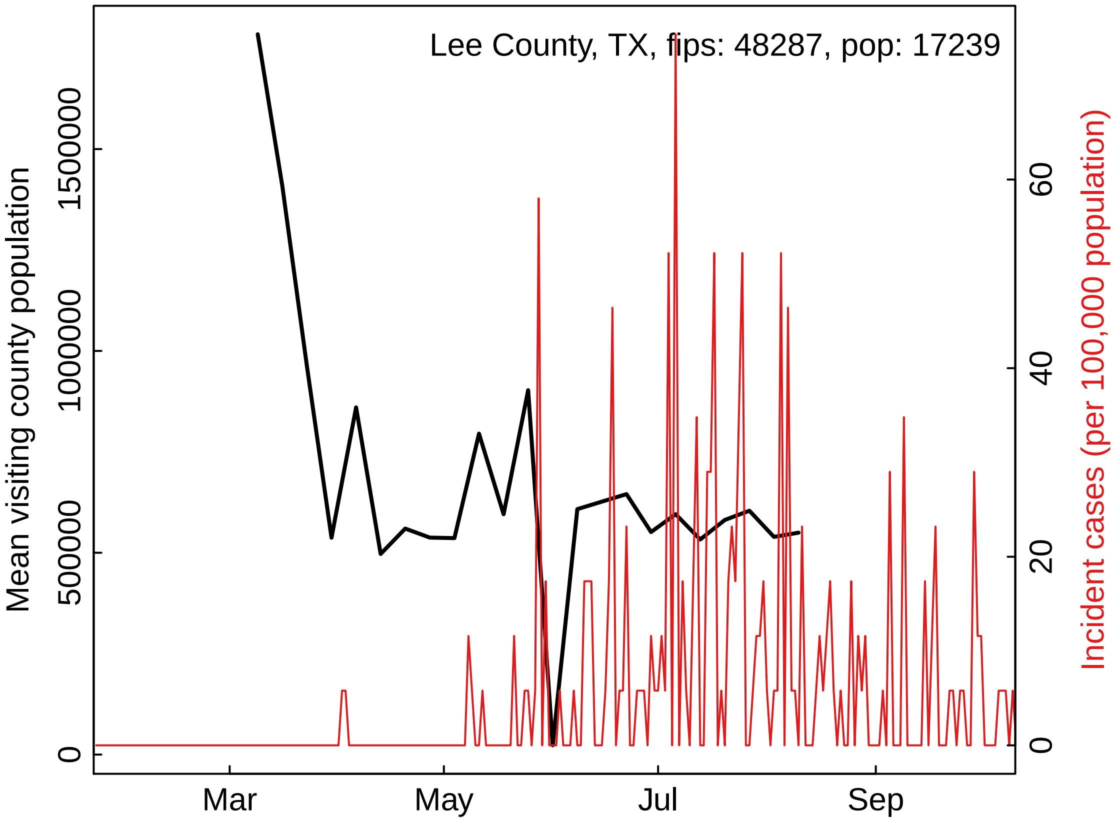

Lee County, TX, fips: 48287, pop: 17239
Mean visiting county population
0
500000
1000000
1500000
Incident cases (per 100,000 population)
0
20
40
60
Mar
May
Jul
Sep

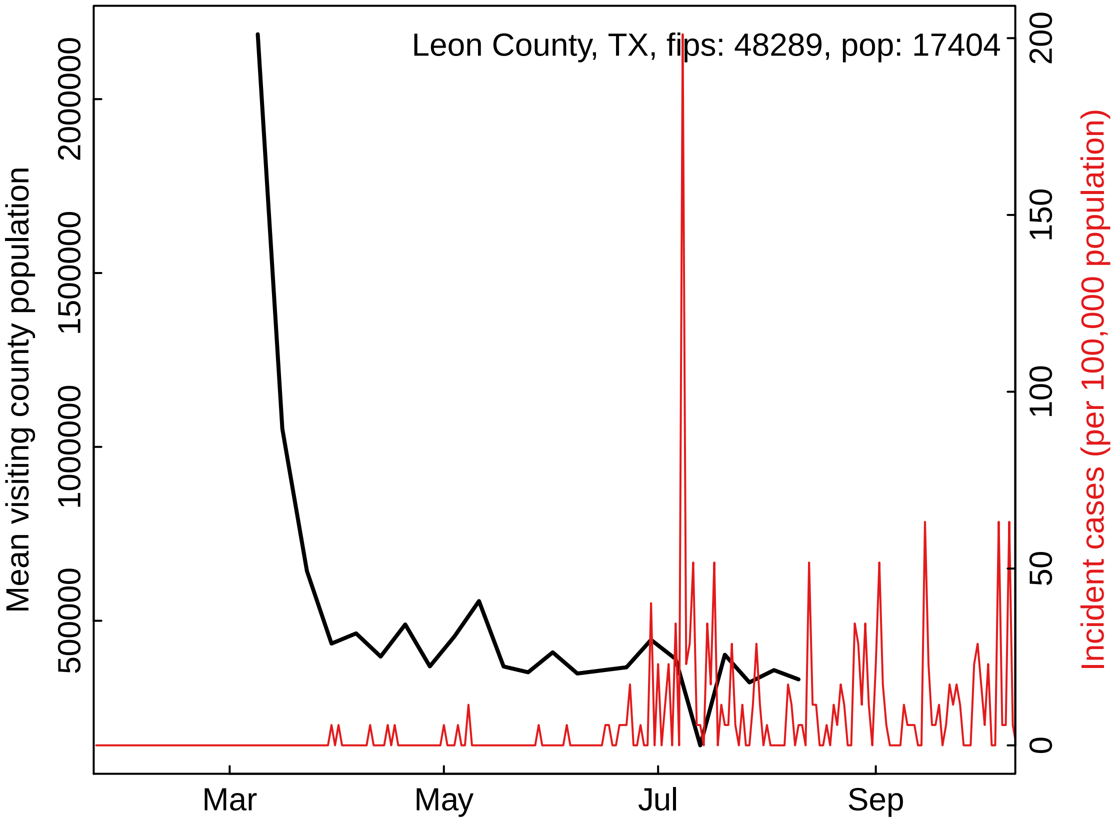

Leon County, TX, fips: 48289, pop: 17404
Mean visiting county population
500000
1000000
1500000
2000000
Incident cases (per 100,000 population)
0
50
100
150
200
Mar
May
Jul
Sep

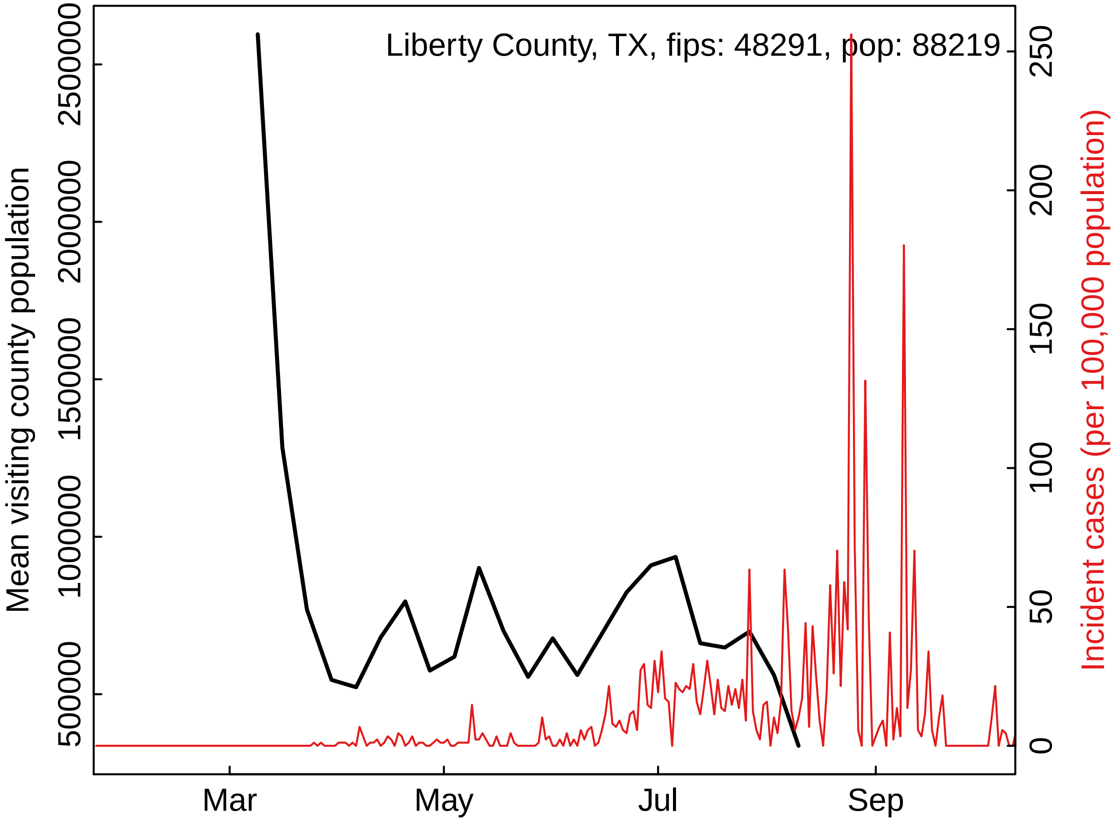

Liberty County, TX, fips: 48291, pop: 88219
Mean visiting county population
Incident cases (per 100,000 population)
500000
1000000
1500000
2000000
2500000
0
50
100
150
200
250
Mar
May
Jul
Sep

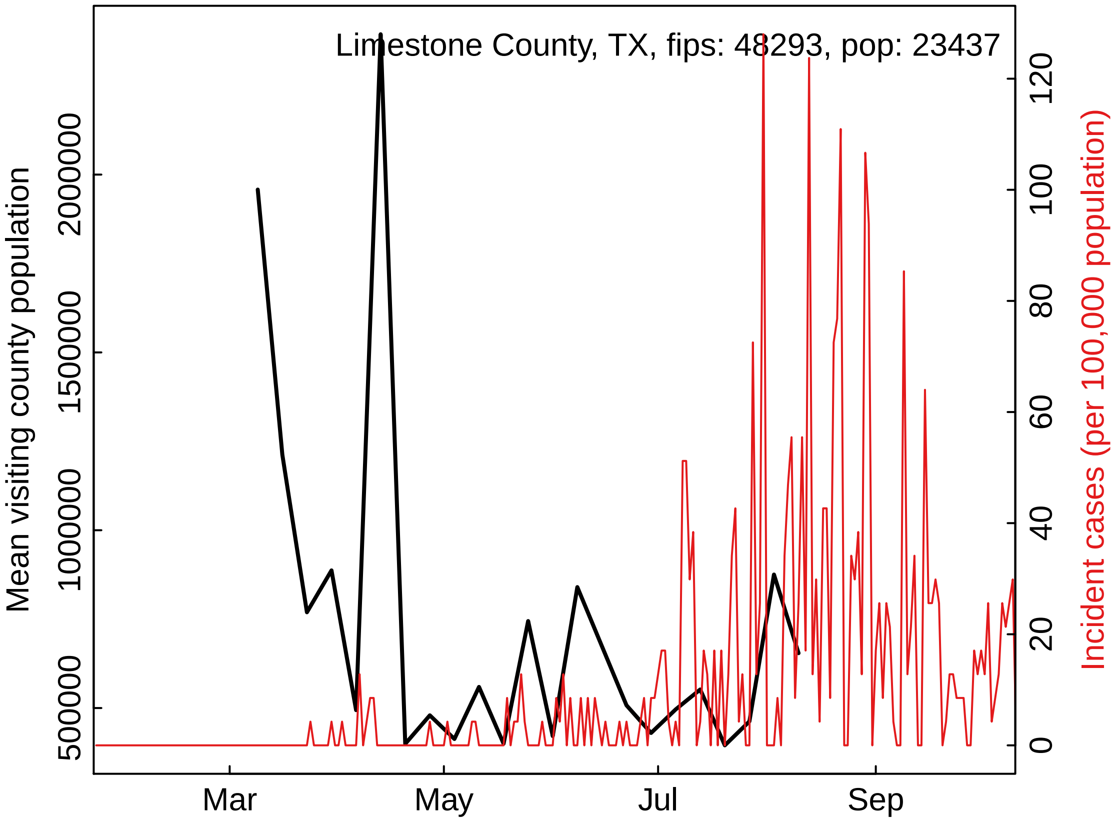

Limestone County, TX, fips: 48293, pop: 23437
Mean visiting county population
Incident cases (per 100,000 population)
500000
1000000
1500000
2000000
0
20
40
60
80
100
120
Mar
May
Jul
Sep

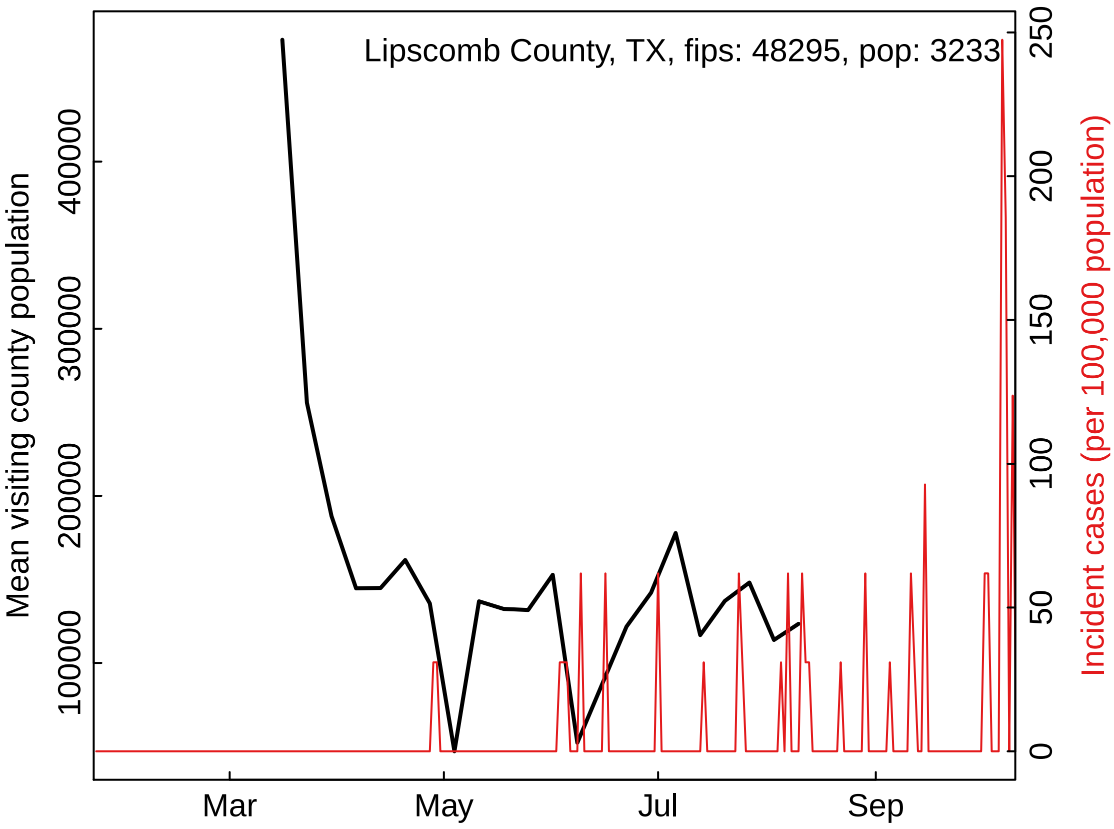
Lipscomb County, TX, fips: 48295, pop: 3233
Mean visiting county population
100000
200000
300000
400000
Incident cases (per 100,000 population)
0
50
100
150
200
250
Mar
May
Jul
Sep

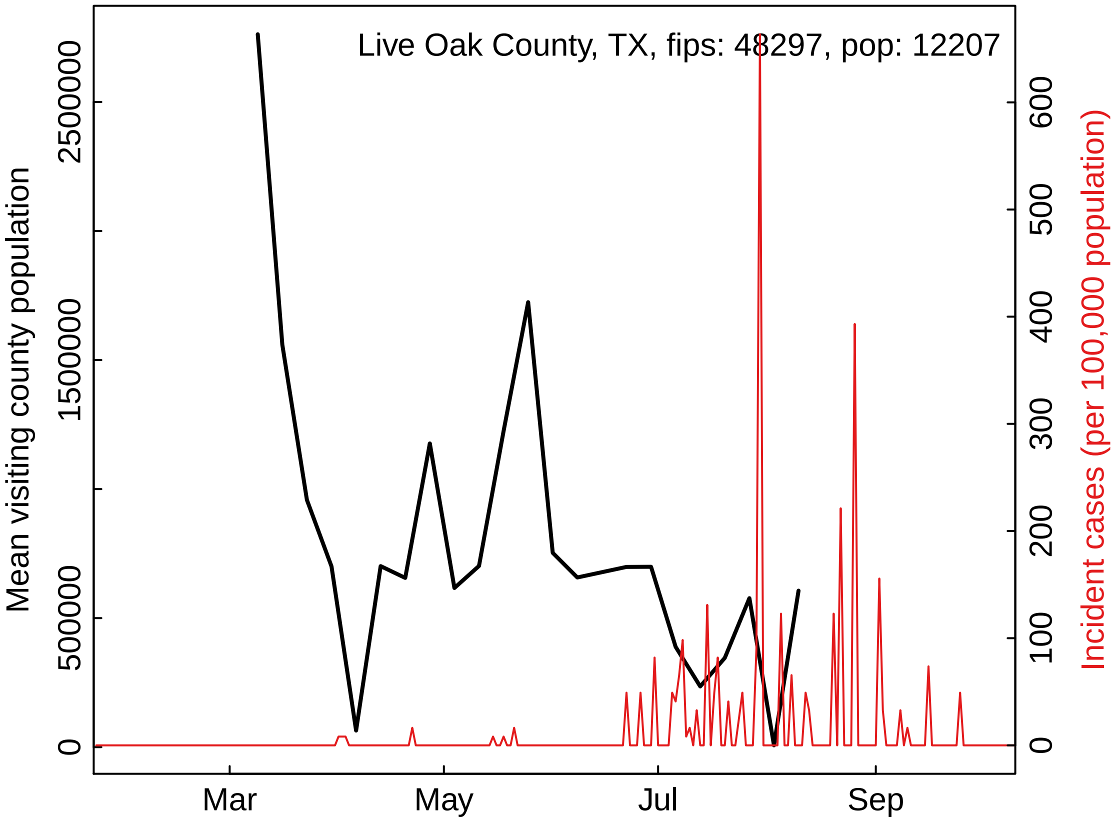

Live Oak County, TX, fips: 48297, pop: 12207
Mean visiting county population
0
500000
1500000
2500000
Incident cases (per 100,000 population)
0
100
200
300
400
500
600
Mar
May
Jul
Sep

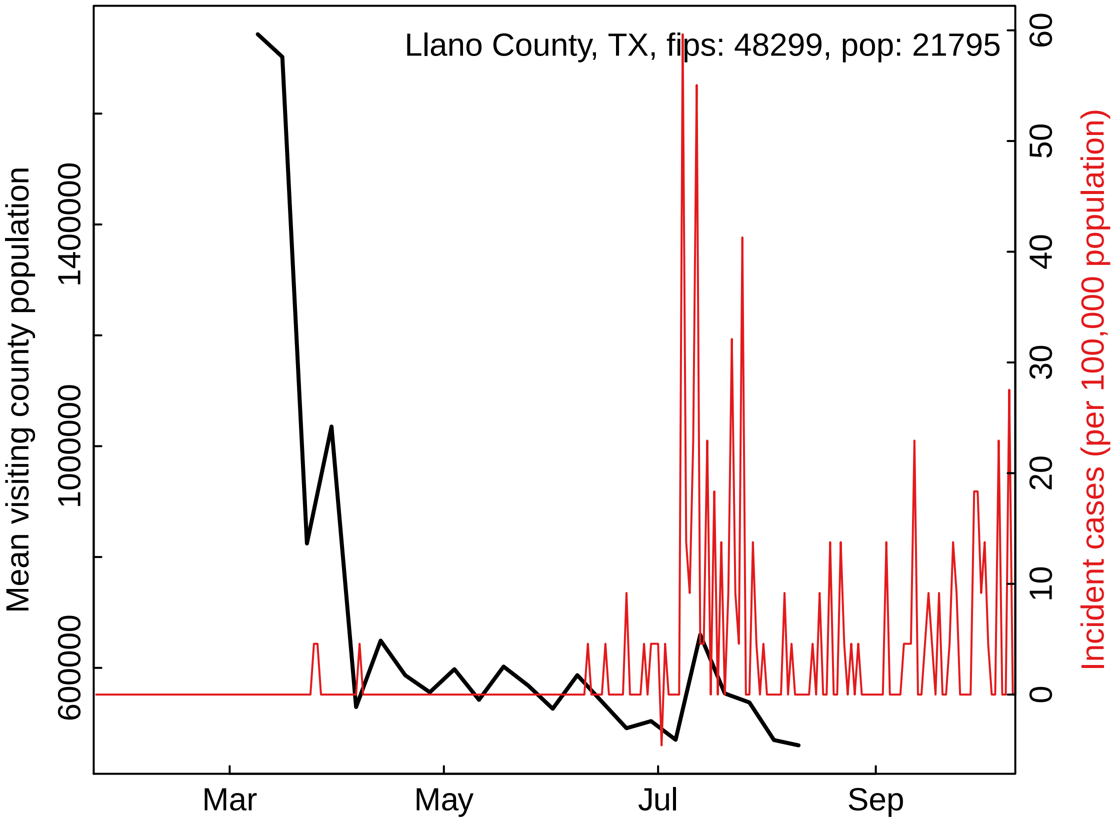

Llano County, TX, fips: 48299, pop: 21795
Mean visiting county population
600000
1000000
1400000
Incident cases (per 100,000 population)
0
10
20
30
40
50
60
Mar
May
Jul
Sep

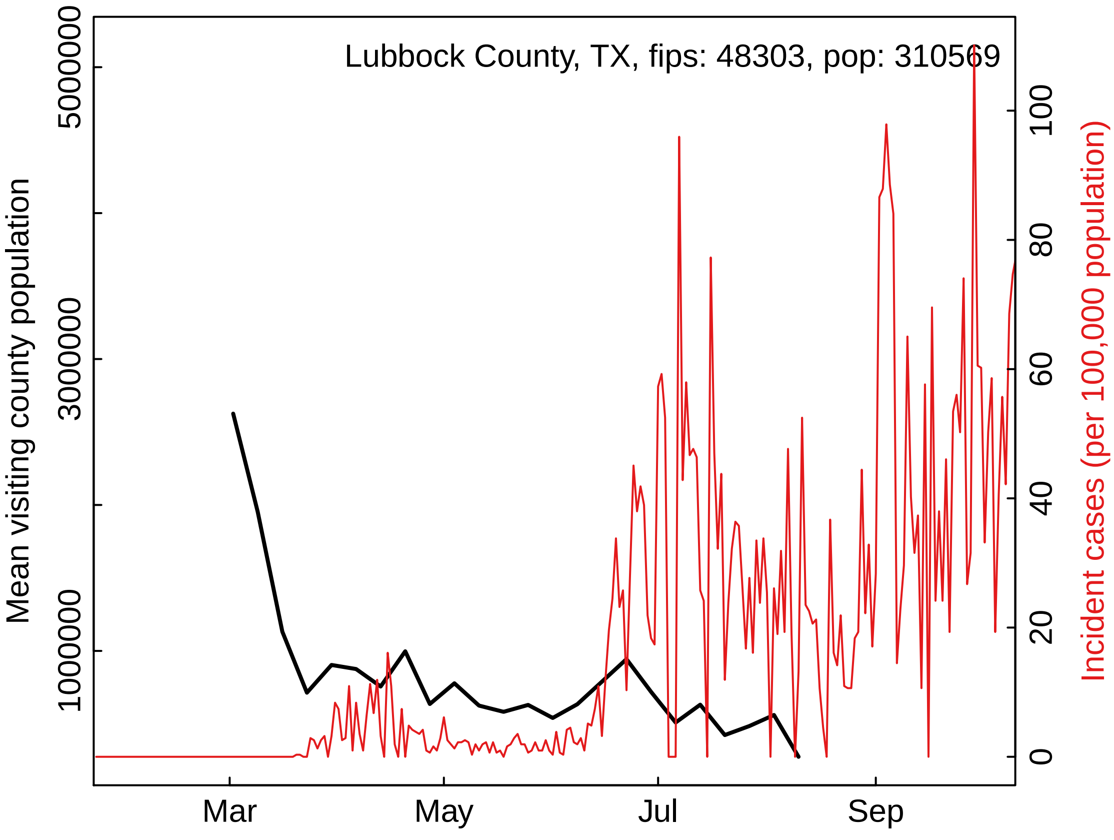

Lubbock County, TX, fips: 48303, pop: 310569
Mean visiting county population
1000000
3000000
5000000
Incident cases (per 100,000 population)
0
20
40
60
80
100
Mar
May
Jul
Sep

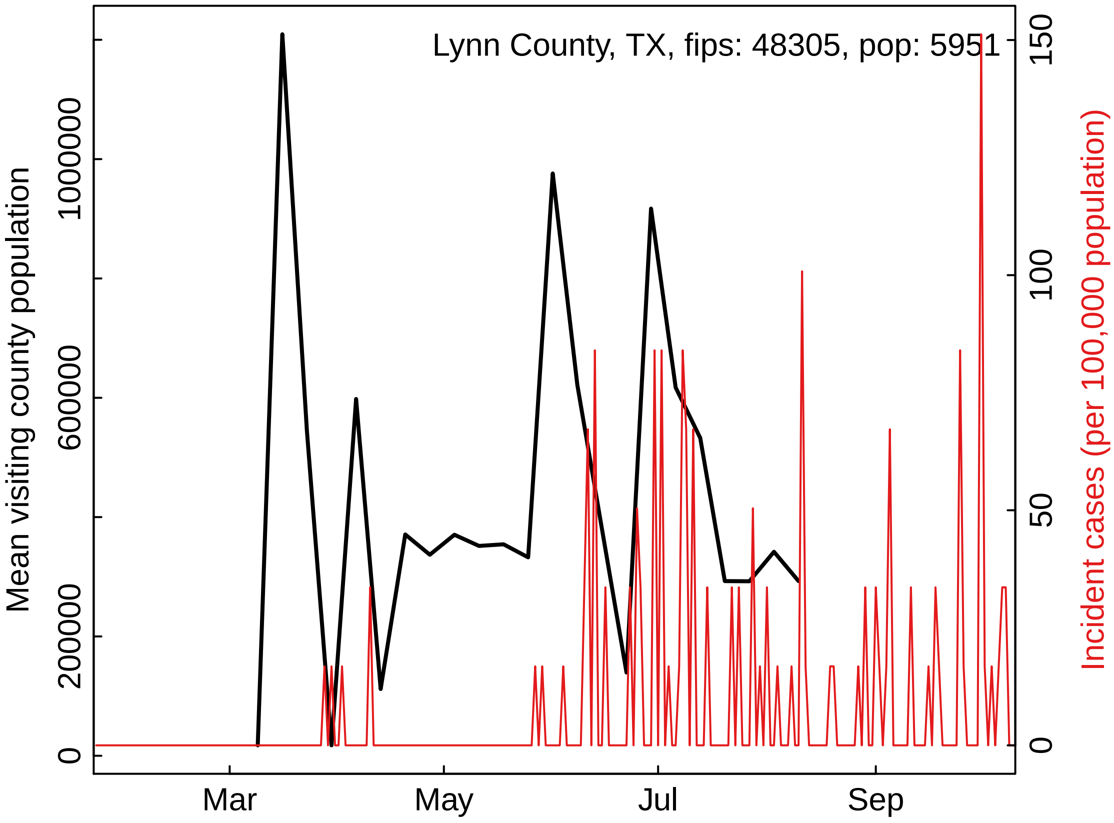

Lynn County, TX, fips: 48305, pop: 5951
Mean visiting county population
0
200000
600000
1000000
Incident cases (per 100,000 population)
0
50
100
150
Mar
May
Jul
Sep

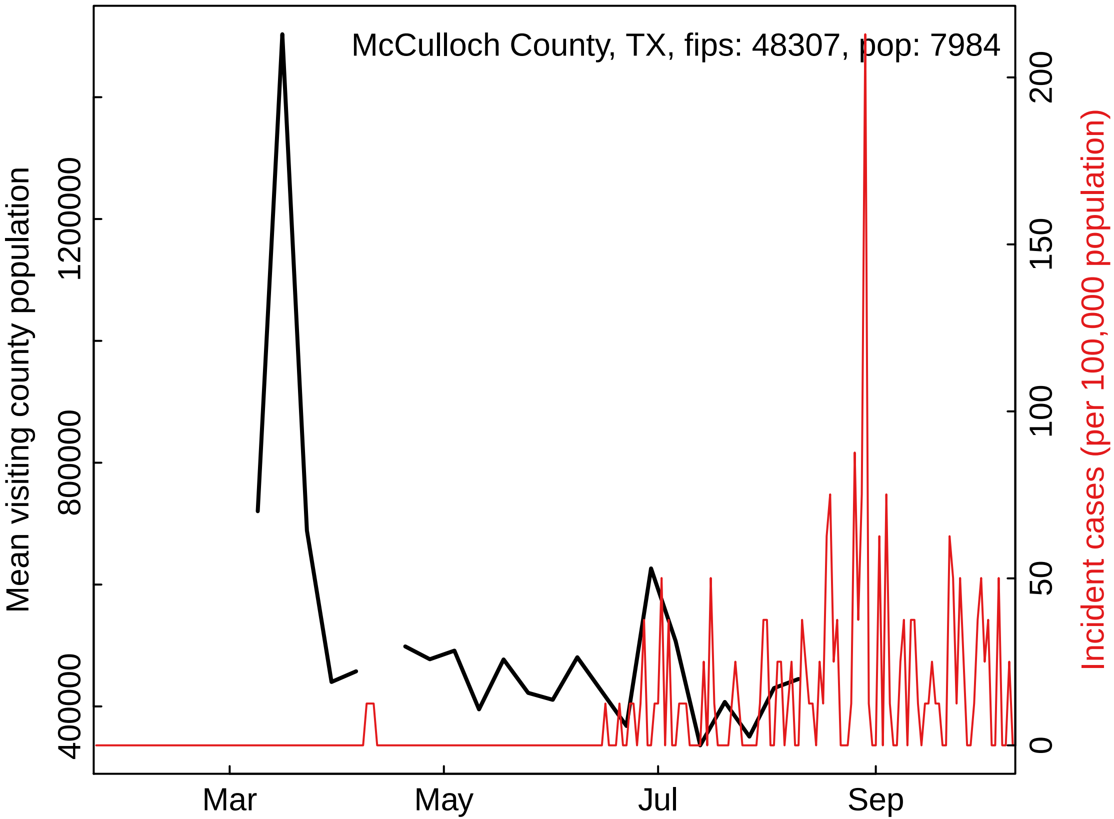
McCulloch County, TX, fips: 48307, pop: 7984
Mean visiting county population
400000
800000
1200000
Incident cases (per 100,000 population)
0
50
100
150
200
Mar
May
Jul
Sep

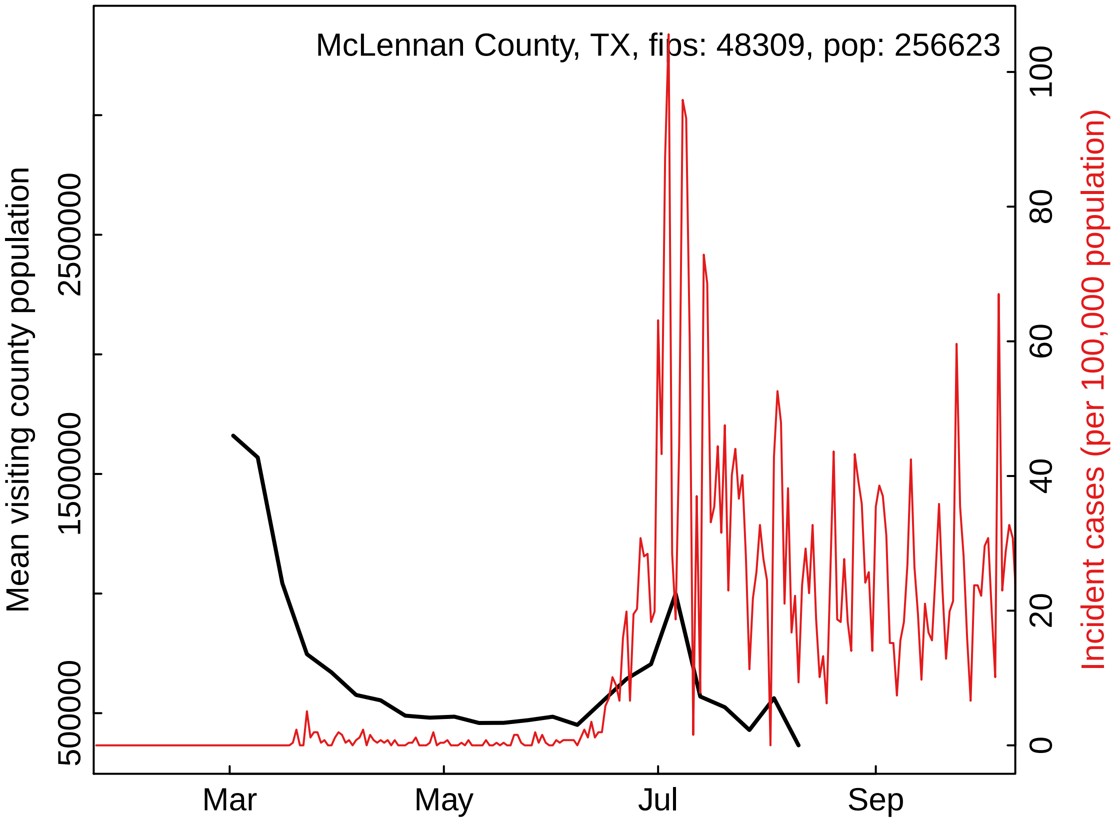
McLennan County, TX, fips: 48309, pop: 256623
Mean visiting county population
500000
1500000
2500000
Mar
May
Jul
Sep
0
20
40
60
80
100
Incident cases (per 100,000 population)

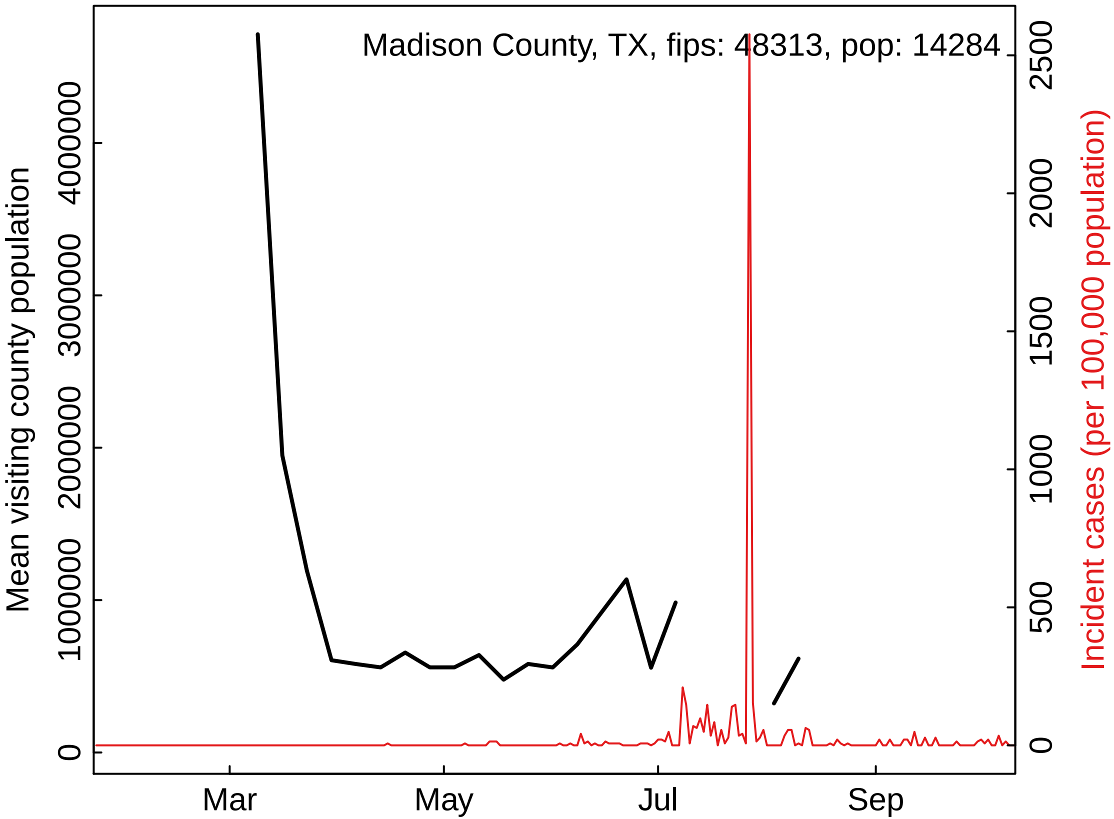
Madison County, TX, fips: 48313, pop: 14284
Mean visiting county population
0
1000000
2000000
3000000
4000000
Incident cases (per 100,000 population)
0
500
1000
1500
2000
2500
Mar
May
Jul
Sep

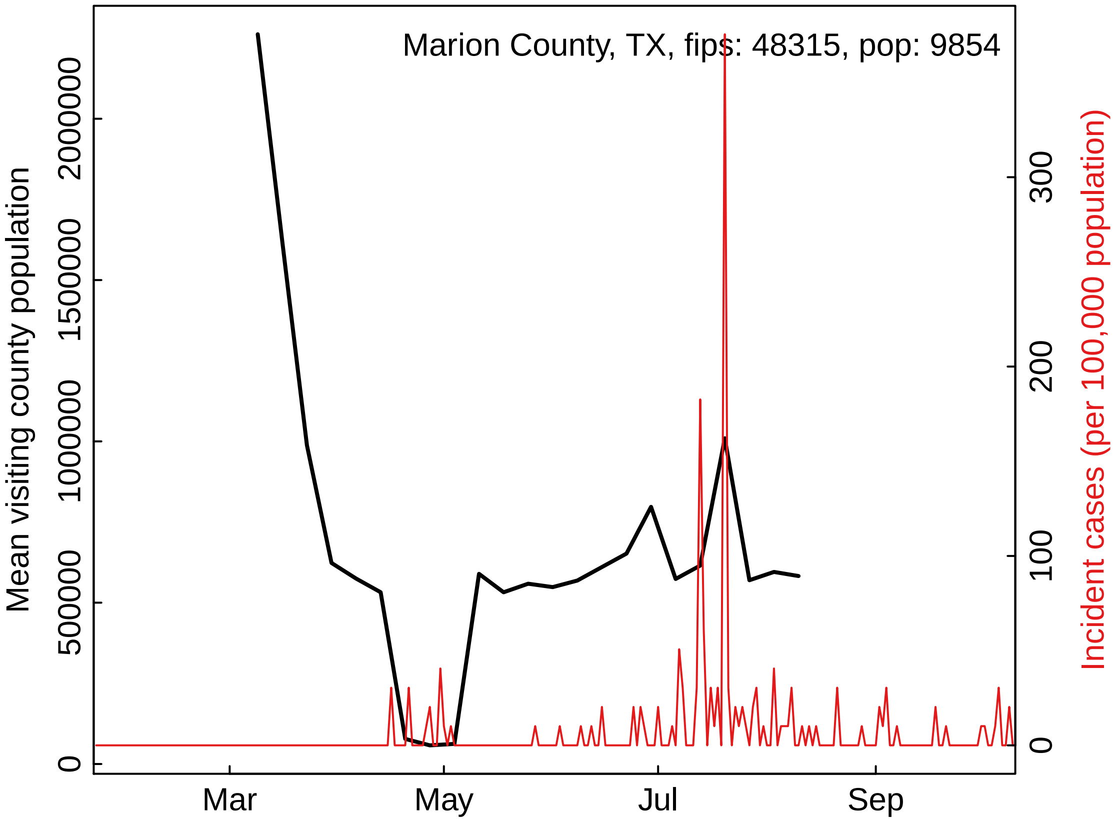

Marion County, TX, fips: 48315, pop: 9854
Mean visiting county population
0
500000
1000000
1500000
2000000
Incident cases (per 100,000 population)
0
100
200
300
Mar
May
Jul
Sep

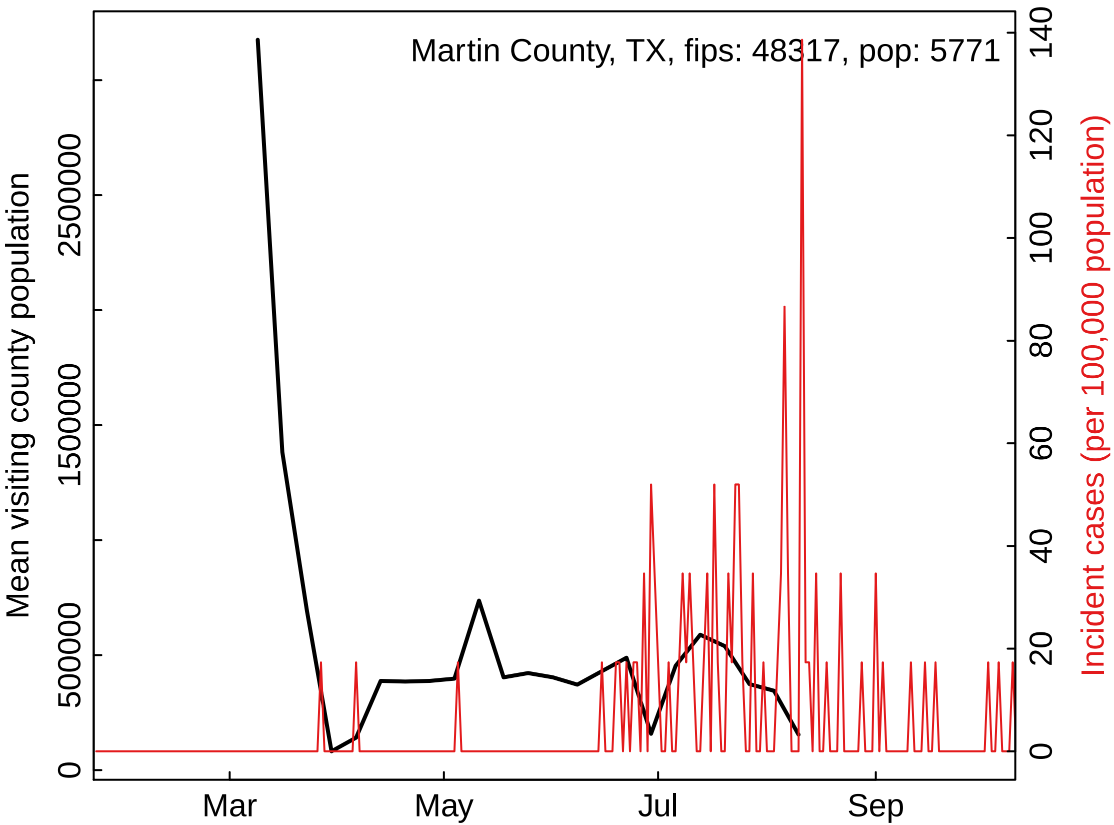
Martin County, TX, fips: 48317, pop: 5771
Mean visiting county population
0
500000
1500000
2500000
Incident cases (per 100,000 population)
0
20
40
60
80
100
120
140
Mar
May
Jul
Sep

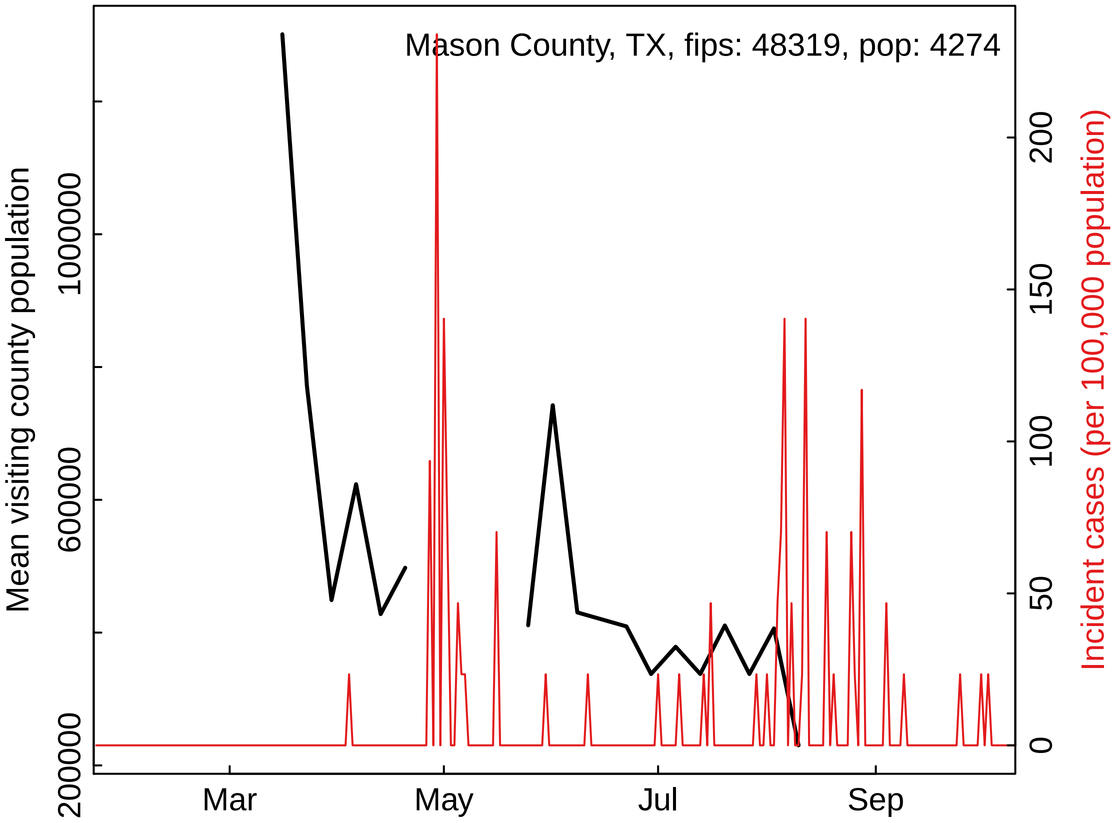

Mason County, TX, fips: 48319, pop: 4274
Mean visiting county population
200000
600000
1000000
Incident cases (per 100,000 population)
0
50
100
150
200
Mar
May
Jul
Sep

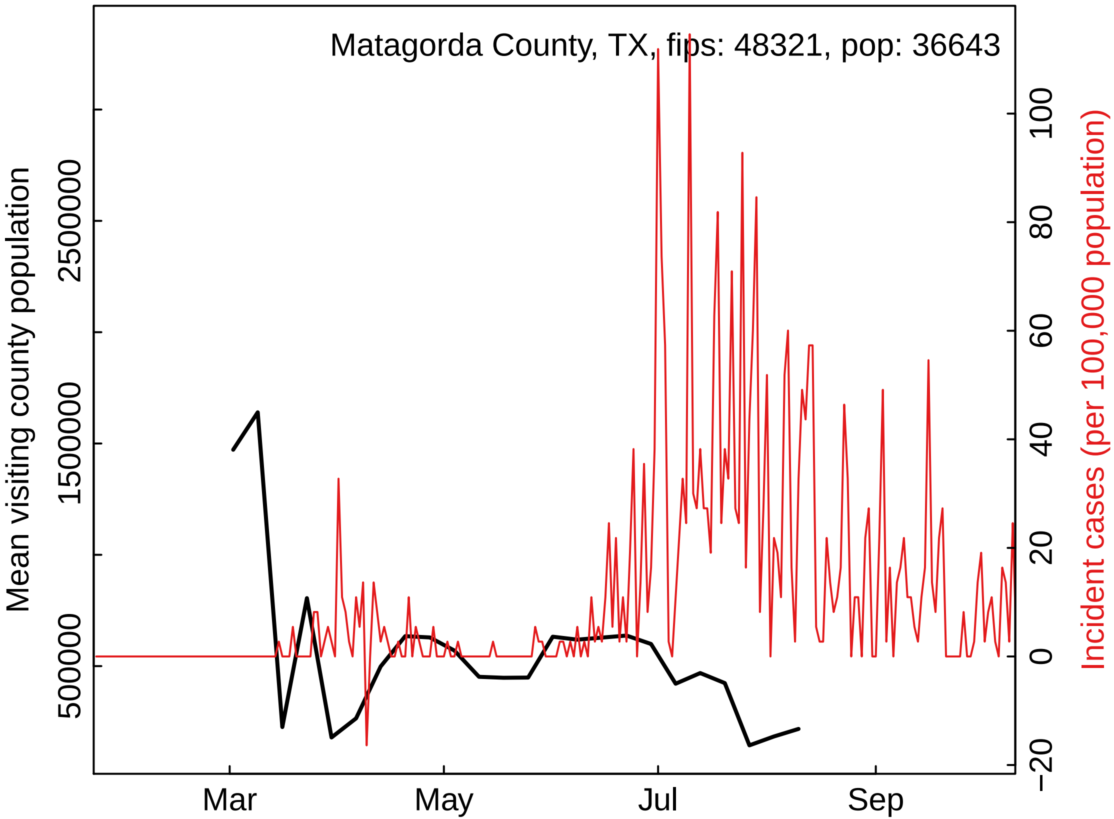

Matagorda County, TX, fips: 48321, pop: 36643
Mean visiting county population
500000
1500000
2500000
Incident cases (per 100,000 population)
−20
0
20
40
60
80
100
Mar
May
Jul
Sep

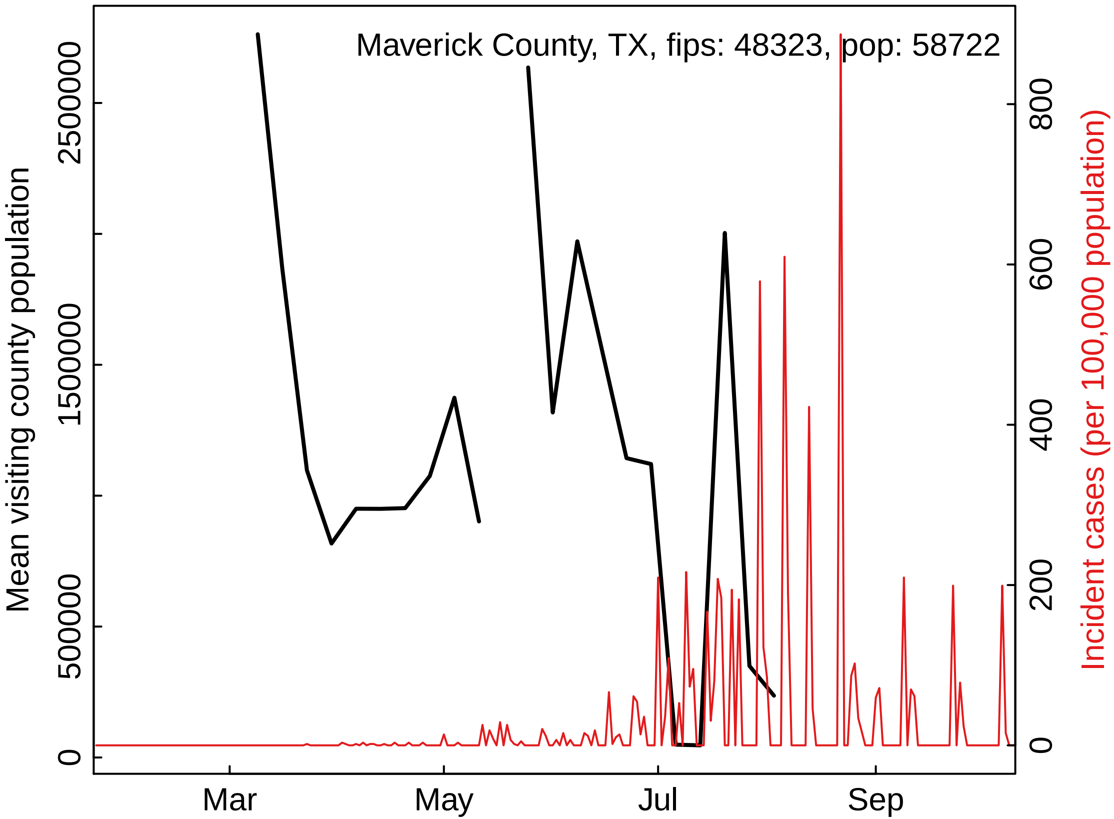
Maverick County, TX, fips: 48323, pop: 58722
Mean visiting county population
Incident cases (per 100,000 population)
0
500000
1500000
2500000
0
200
400
600
800
Mar
May
Jul
Sep

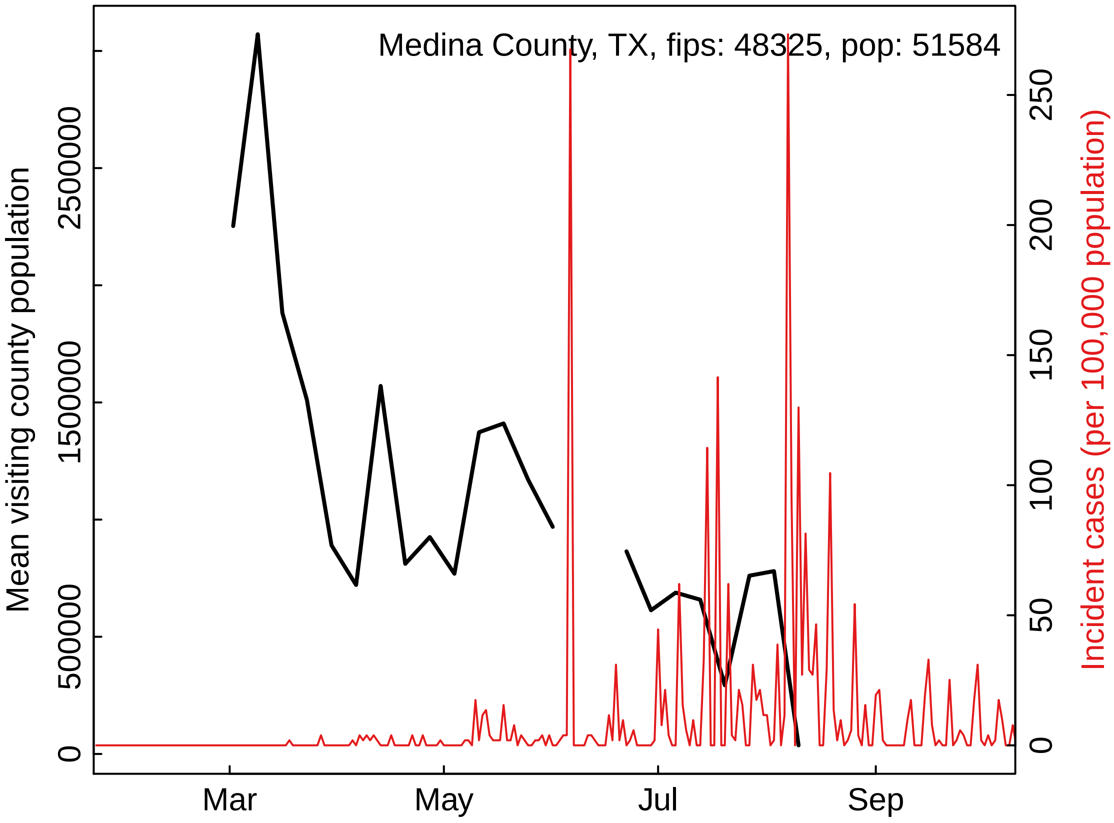
Medina County, TX, fips: 48325, pop: 51584
Mean visiting county population
0
500000
1500000
2500000
Incident cases (per 100,000 population)
0
50
100
150
200
250
Mar
May
Jul
Sep

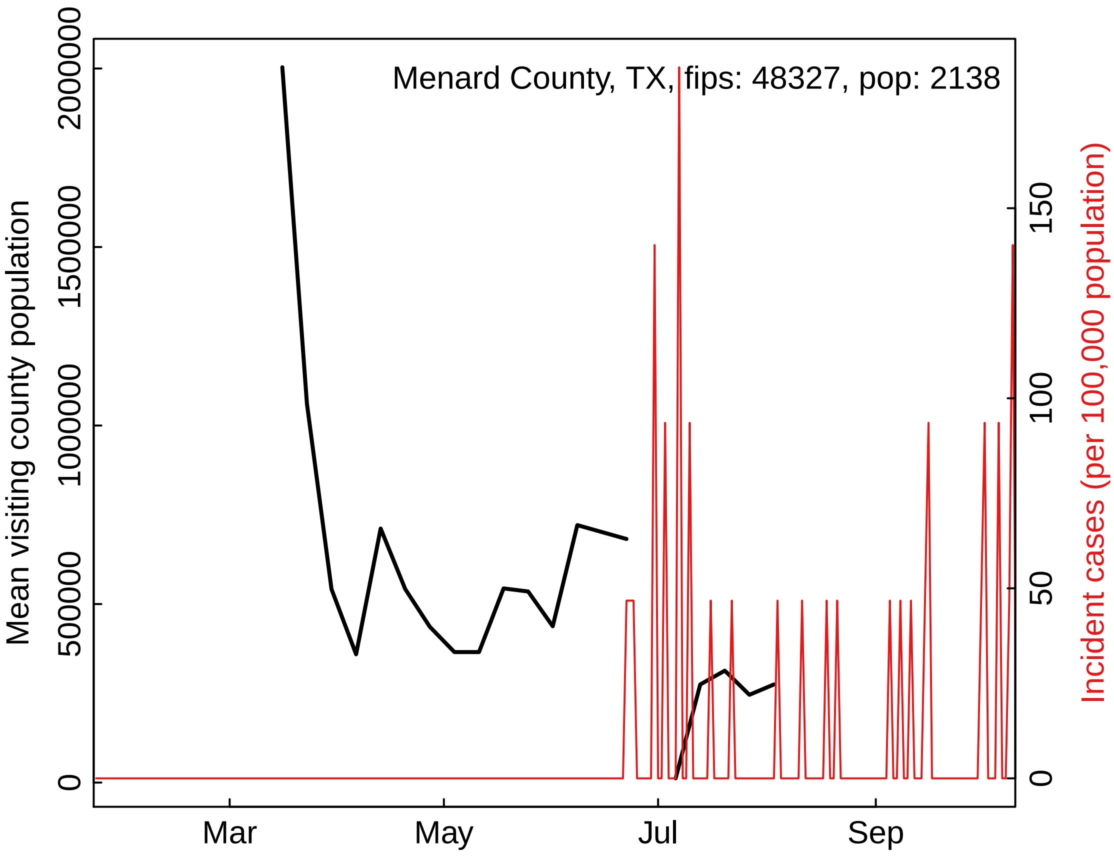

Menard County, TX, fips: 48327, pop: 2138
Mean visiting county population
0
500000
1000000
1500000
2000000
Incident cases (per 100,000 population)
0
50
100
150
Mar
May
Jul
Sep

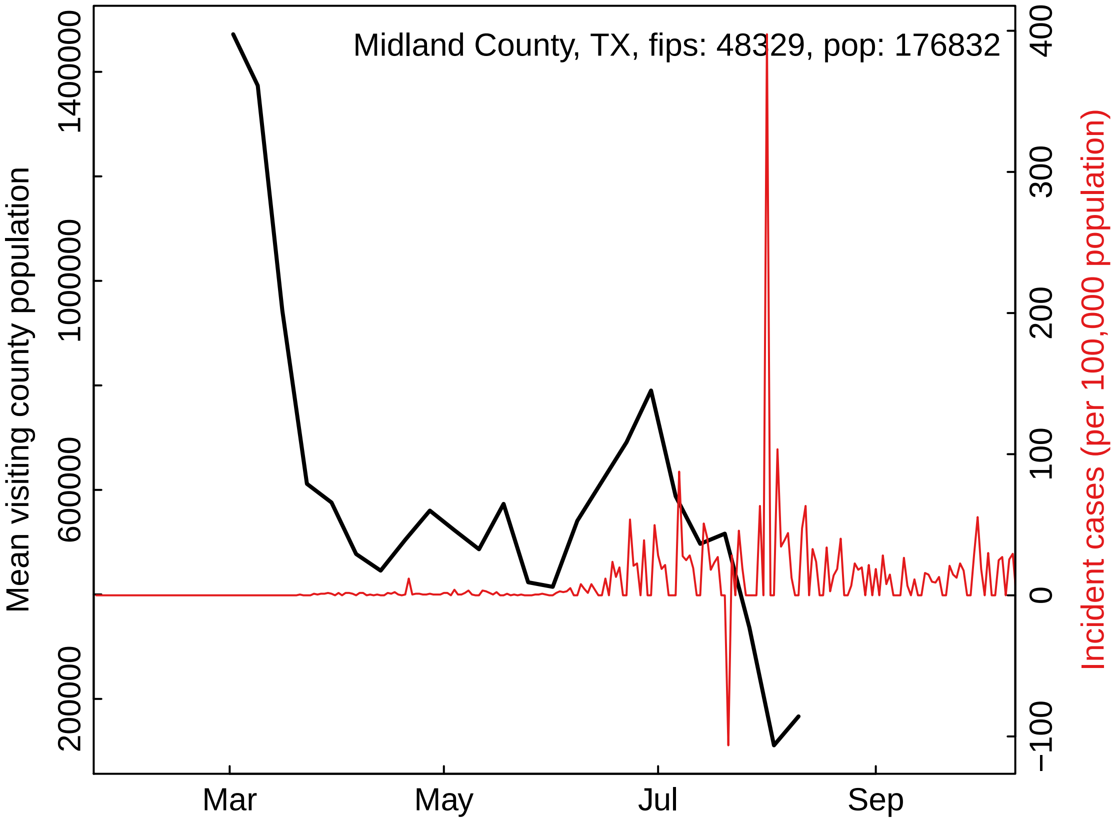
Midland County, TX, fips: 48329, pop: 176832
Mean visiting county population
200000
600000
1000000
1400000
Incident cases (per 100,000 population)
−100
0
100
200
300
400
Mar
May
Jul
Sep

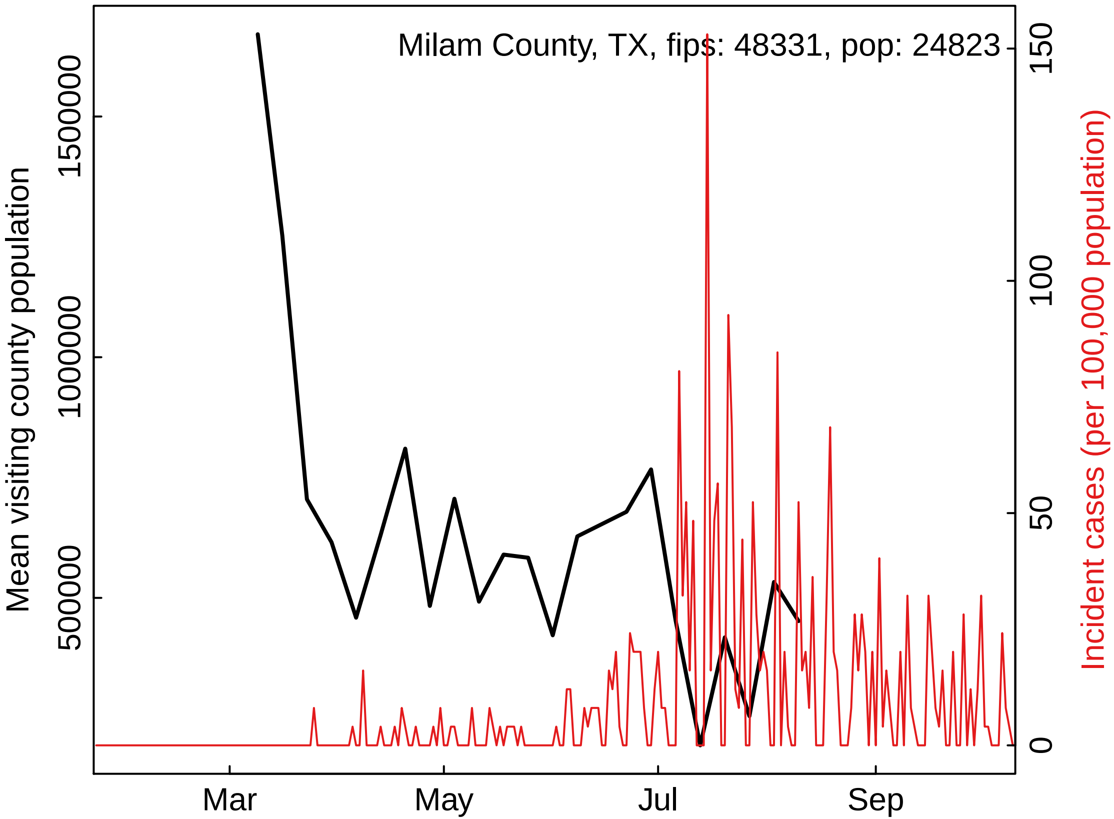

Milam County, TX, fips: 48331, pop: 24823
Mean visiting county population
500000
1000000
1500000
Incident cases (per 100,000 population)
0
50
100
150
Mar
May
Jul
Sep

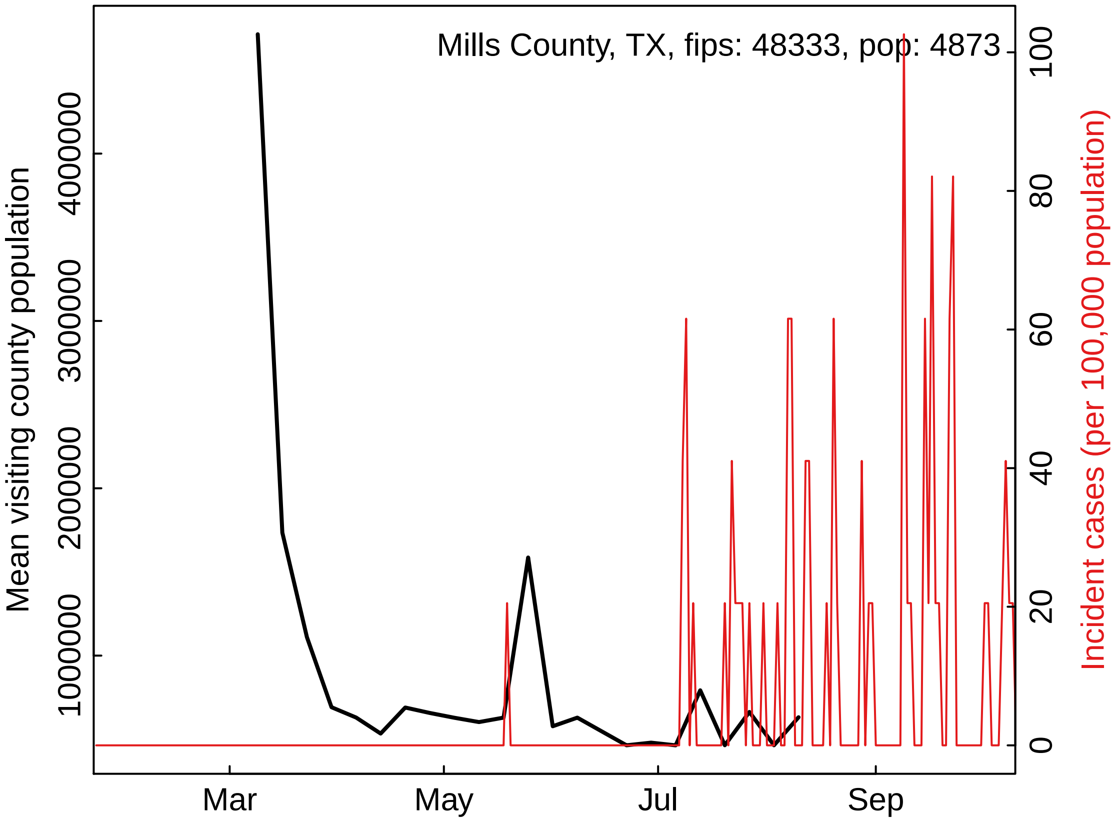

Mills County, TX, fips: 48333, pop: 4873
Mean visiting county population
1000000
2000000
3000000
4000000
Mar
May
Jul
Sep
0
20
40
60
80
100
Incident cases (per 100,000 population)

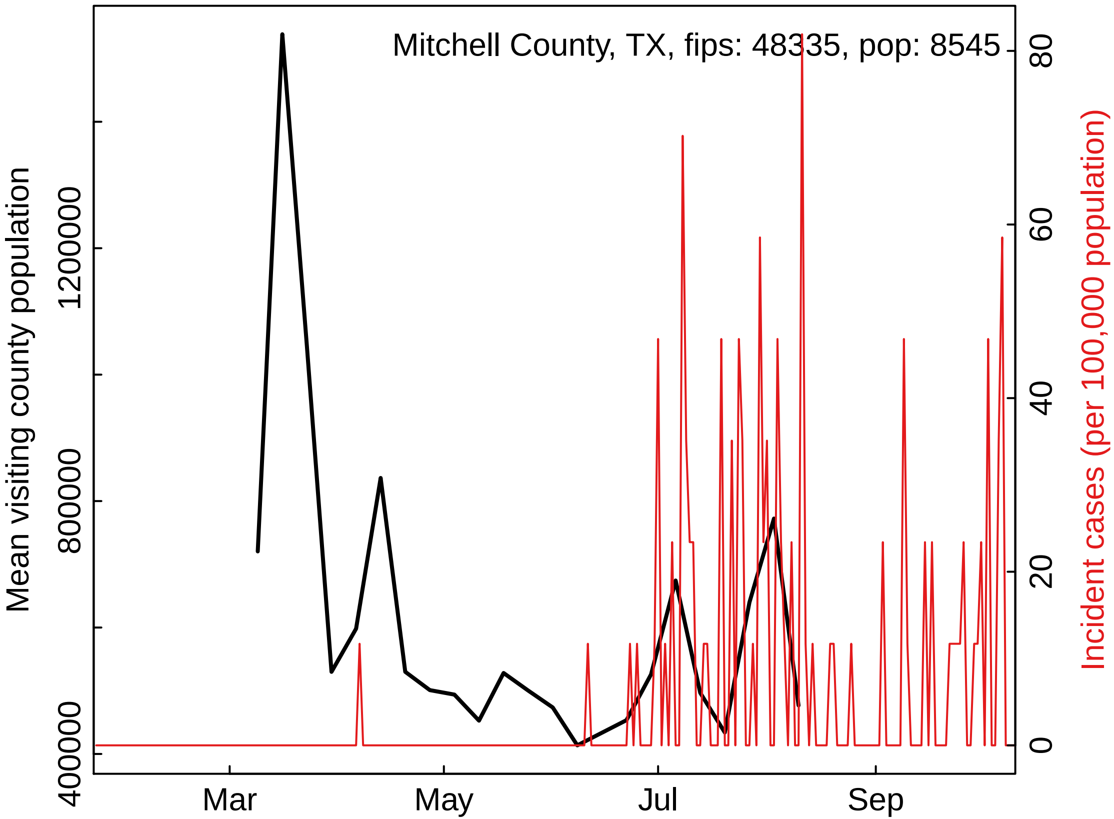

Mitchell County, TX, fips: 48335, pop: 8545
Mean visiting county population
400000
800000
1200000
Incident cases (per 100,000 population)
0
20
40
60
80
Mar
May
Jul
Sep

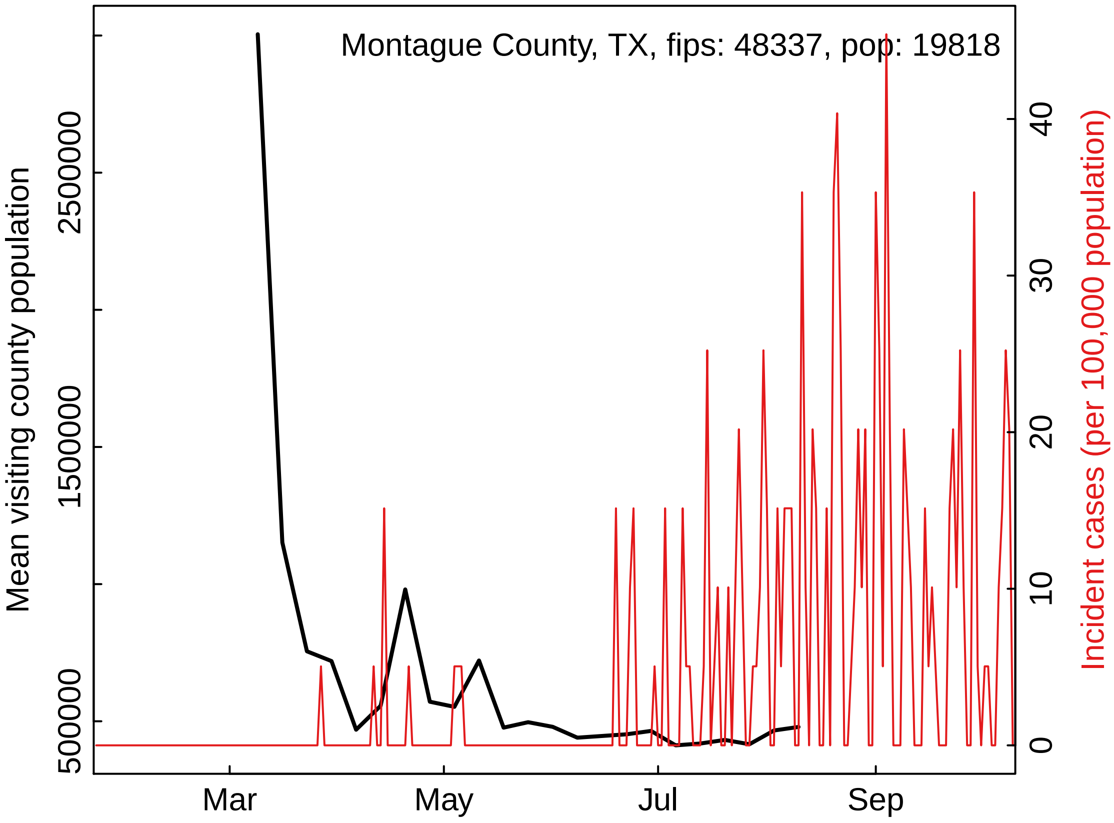

Montague County, TX, fips: 48337, pop: 19818
Mean visiting county population
500000
1500000
2500000
Mar
May
Jul
Sep
0
10
20
30
40
Incident cases (per 100,000 population)

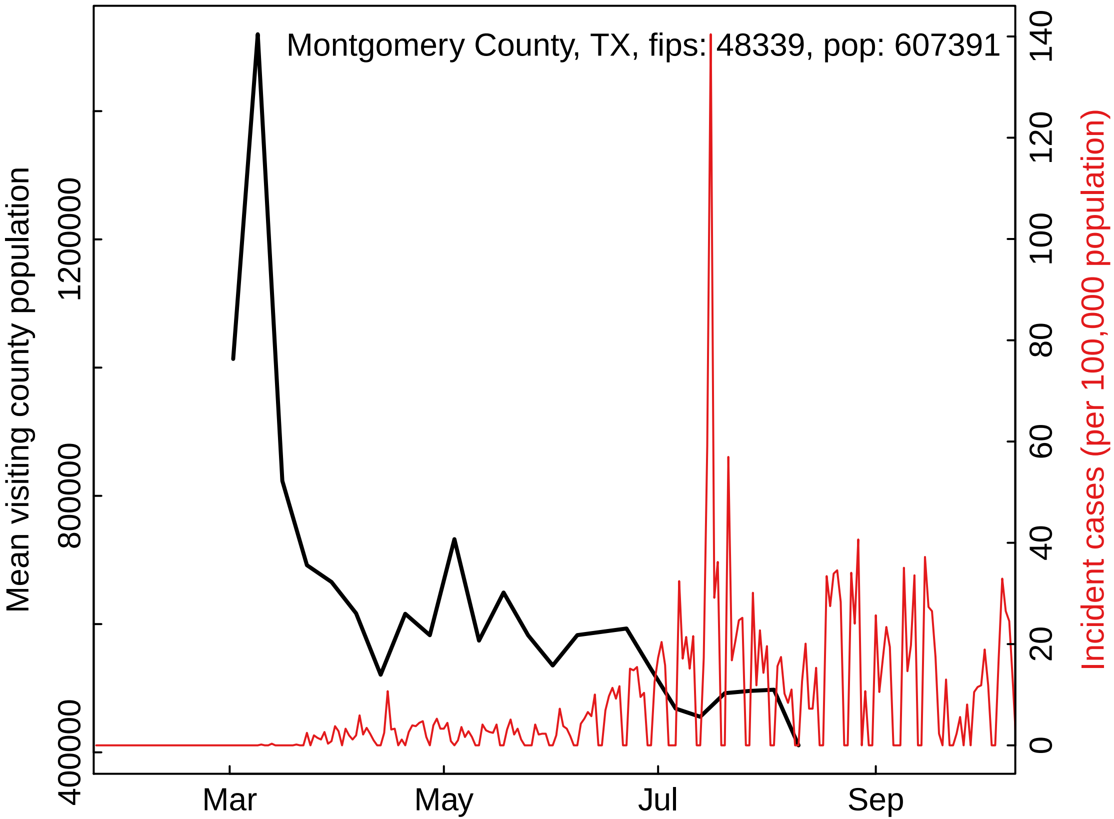
Montgomery County, TX, fips: 48339, pop: 607391
Mean visiting county population
400000
800000
1200000
Mar
May
Jul
Sep
0
20
40
60
80
100
120
140
Incident cases (per 100,000 population)

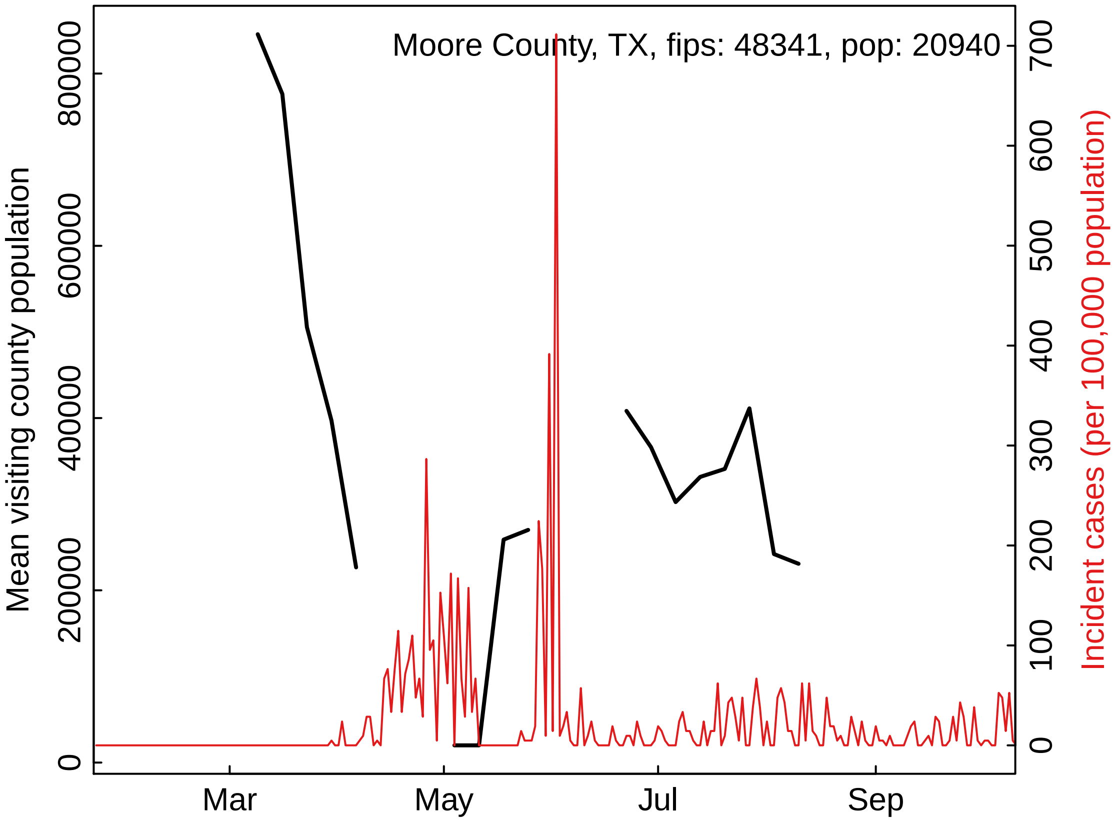
Moore County, TX, fips: 48341, pop: 20940
Mean visiting county population
0
200000
400000
600000
800000
Mar
May
Jul
Sep
0
100
200
300
400
500
600
700
Incident cases (per 100,000 population)

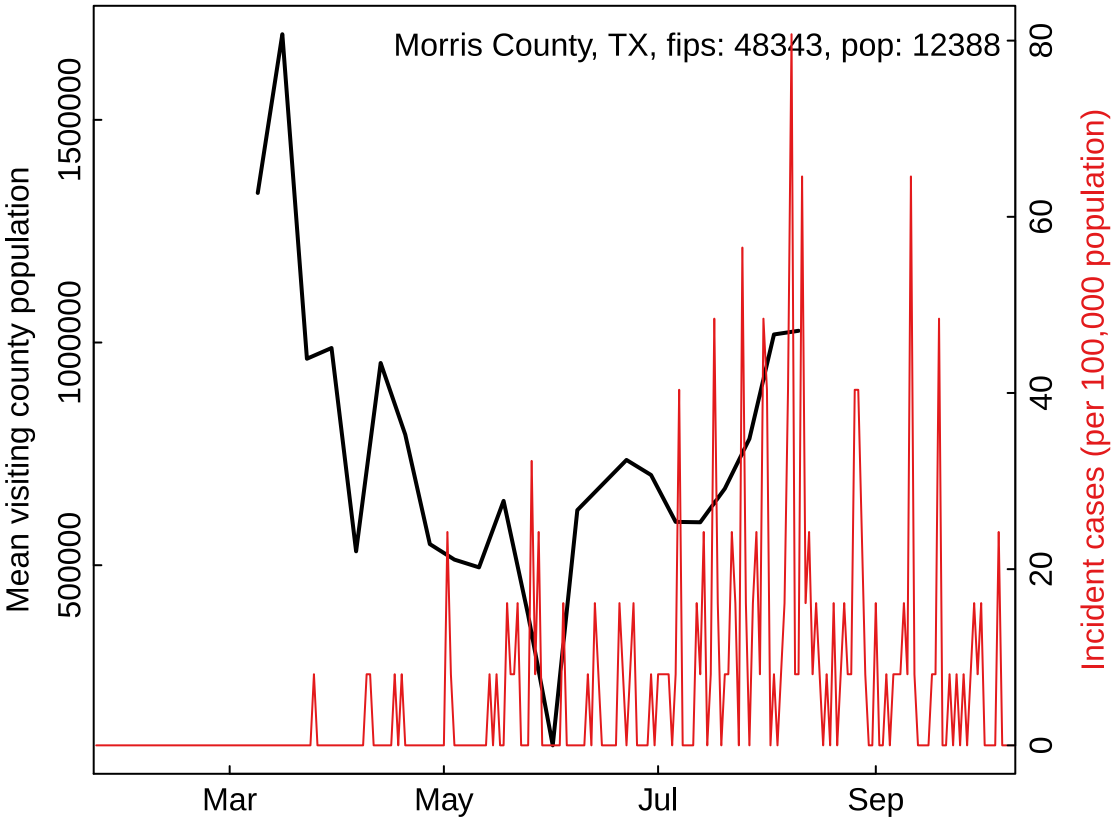

Morris County, TX, fips: 48343, pop: 12388
Mean visiting county population
500000
1000000
1500000
Incident cases (per 100,000 population)
0
20
40
60
80
Mar
May
Jul
Sep

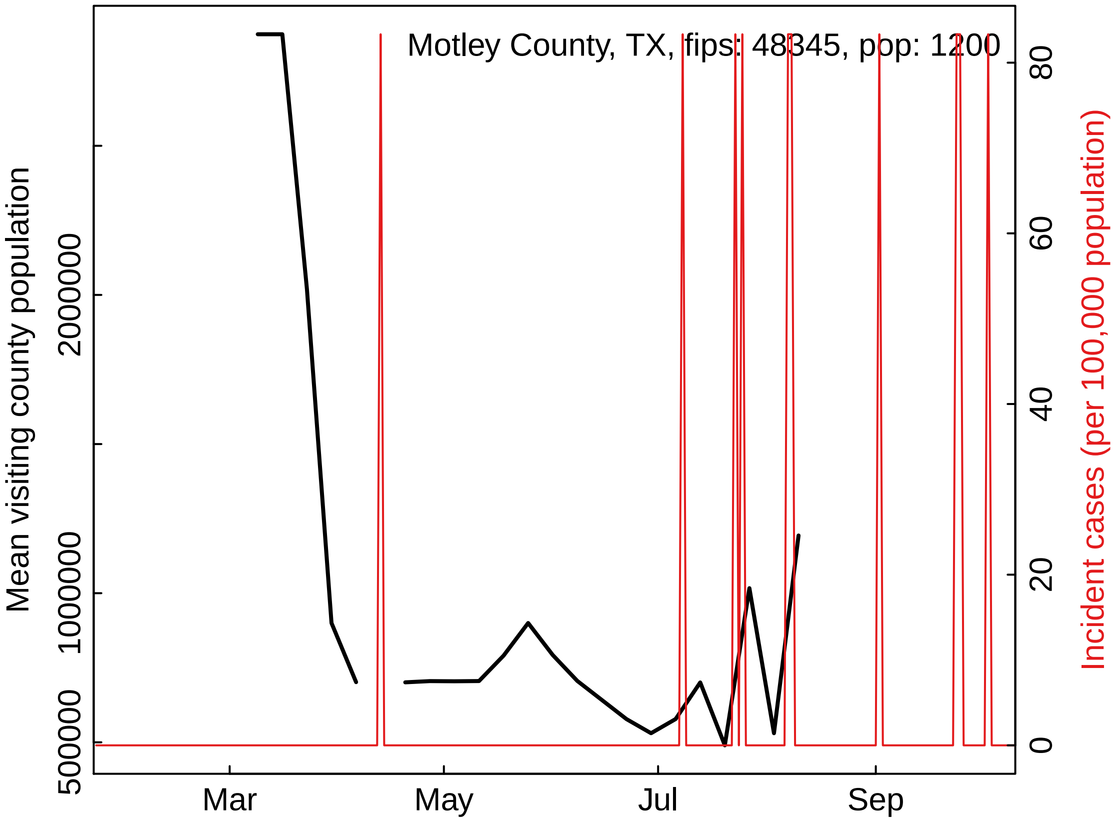

Motley County, TX, fips: 48345, pop: 1200
Mean visiting county population
500000
1000000
2000000
Incident cases (per 100,000 population)
0
20
40
60
80
Mar
May
Jul
Sep

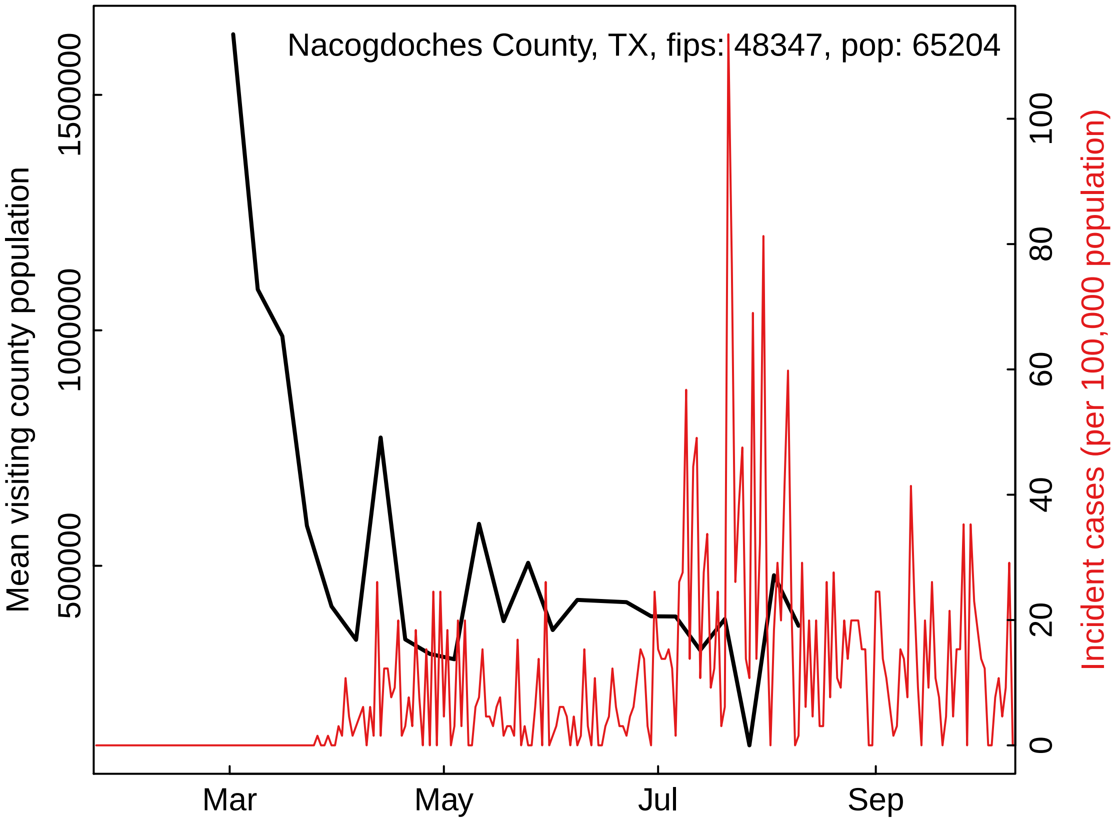
Nacogdoches County, TX, fips: 48347, pop: 65204
Mean visiting county population
500000
1000000
1500000
Incident cases (per 100,000 population)
0
20
40
60
80
100
Mar
May
Jul
Sep

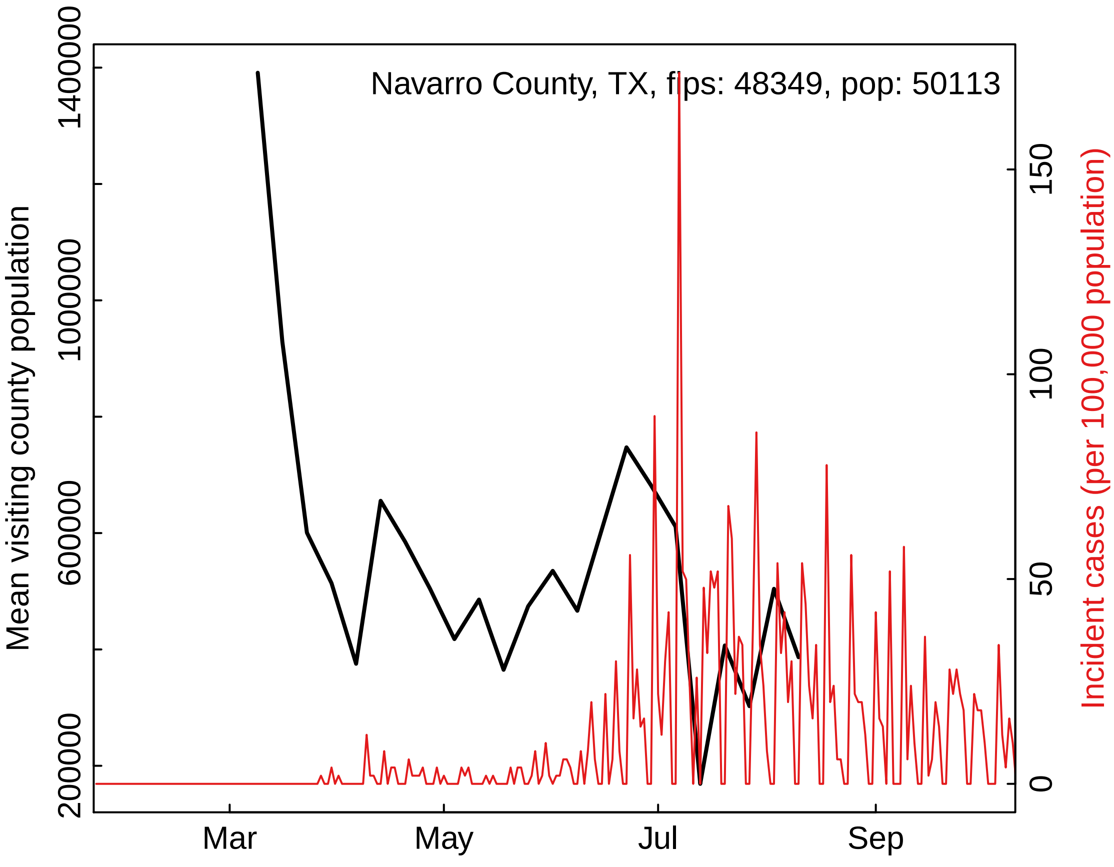

Navarro County, TX, flips: 48349, pop: 50113
Mean visiting county population
Incident cases (per 100,000 population)
200000
600000
1000000
1400000
0
50
100
150
Mar
May
Jul
Sep

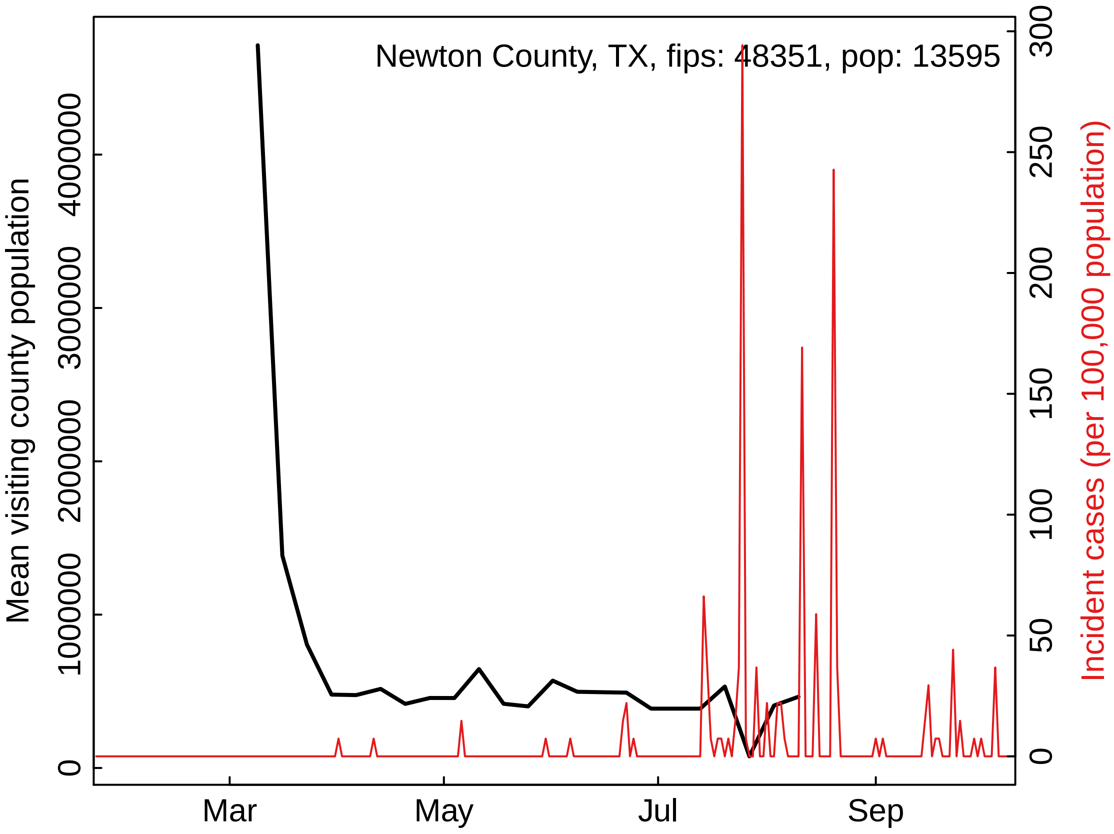

Newton County, TX, fips: 48351, pop: 13595
Mean visiting county population
0
1000000
2000000
3000000
4000000
Incident cases (per 100,000 population)
0
50
100
150
200
250
300
Mar
May
Jul
Sep

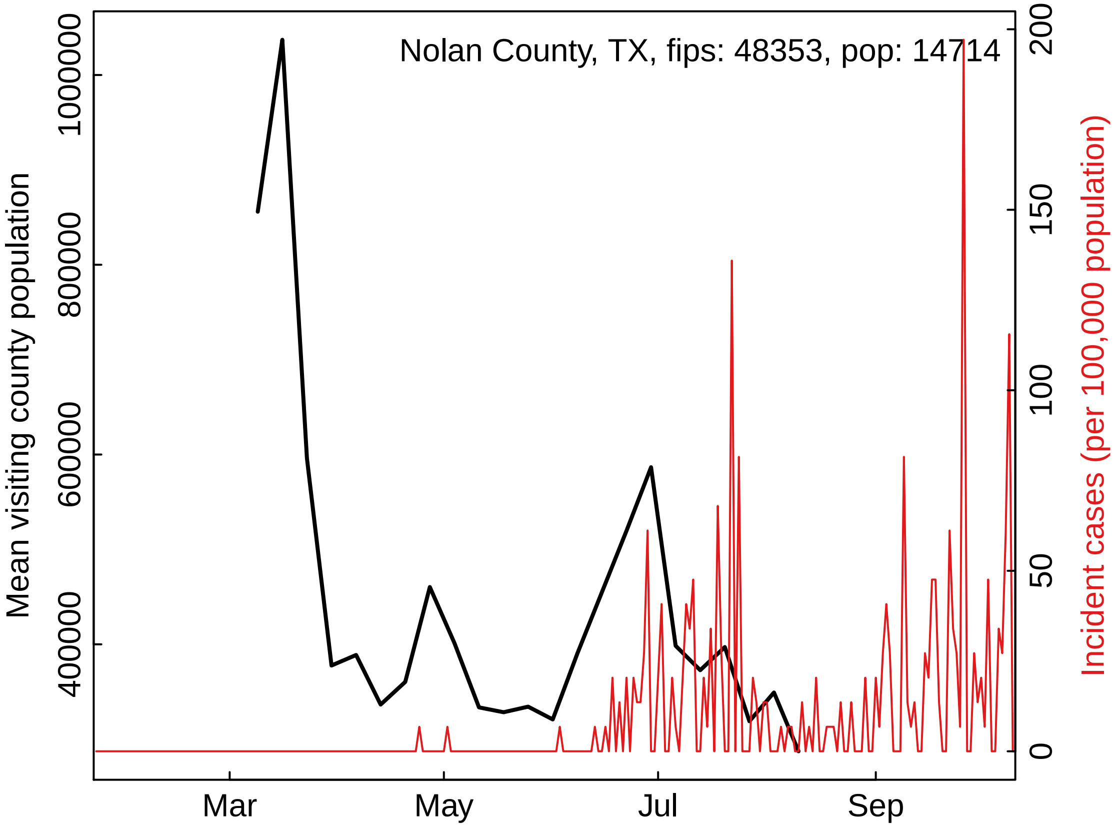
Nolan County, TX, fips: 48353, pop: 14714
Mean visiting county population
400000
600000
800000
1000000
Incident cases (per 100,000 population)
0
50
100
150
200
Mar
May
Jul
Sep

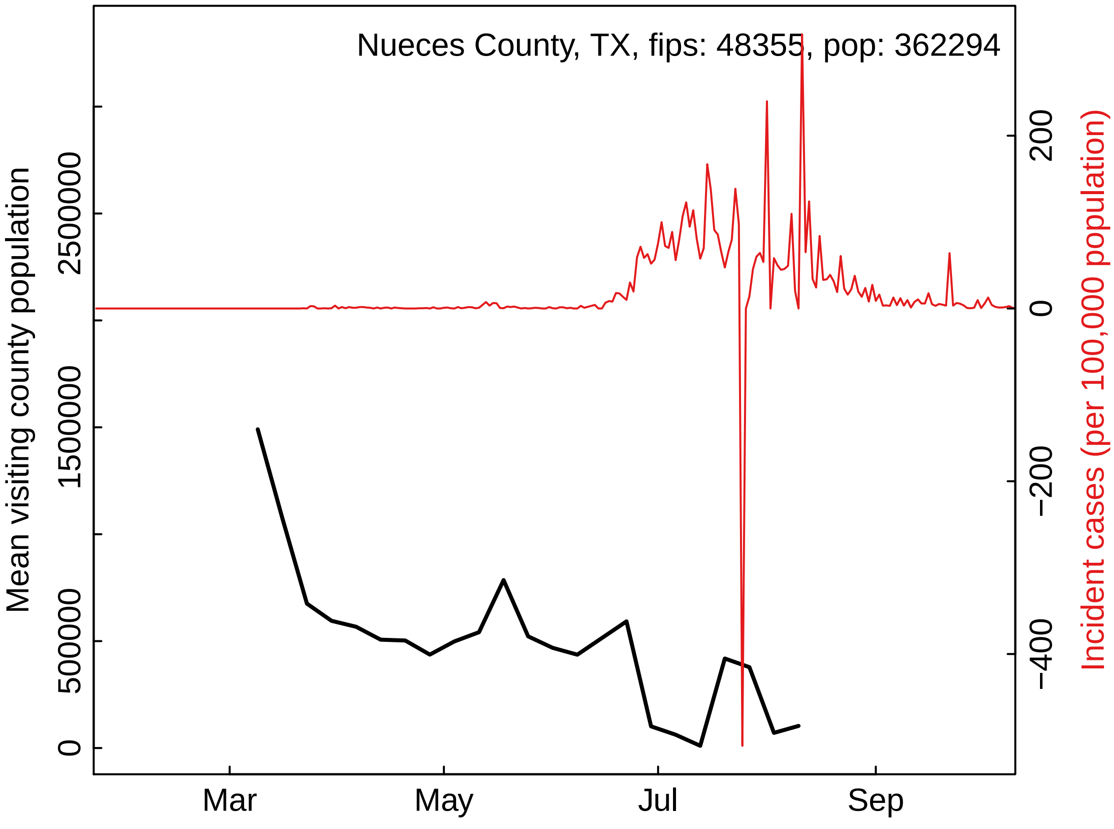

Nueces County, TX, fips: 48355, pop: 362294
Mean visiting county population
0
500000
1500000
2500000
Mar
May
Jul
Sep
Incident cases (per 100,000 population)
−400
−200
0
200

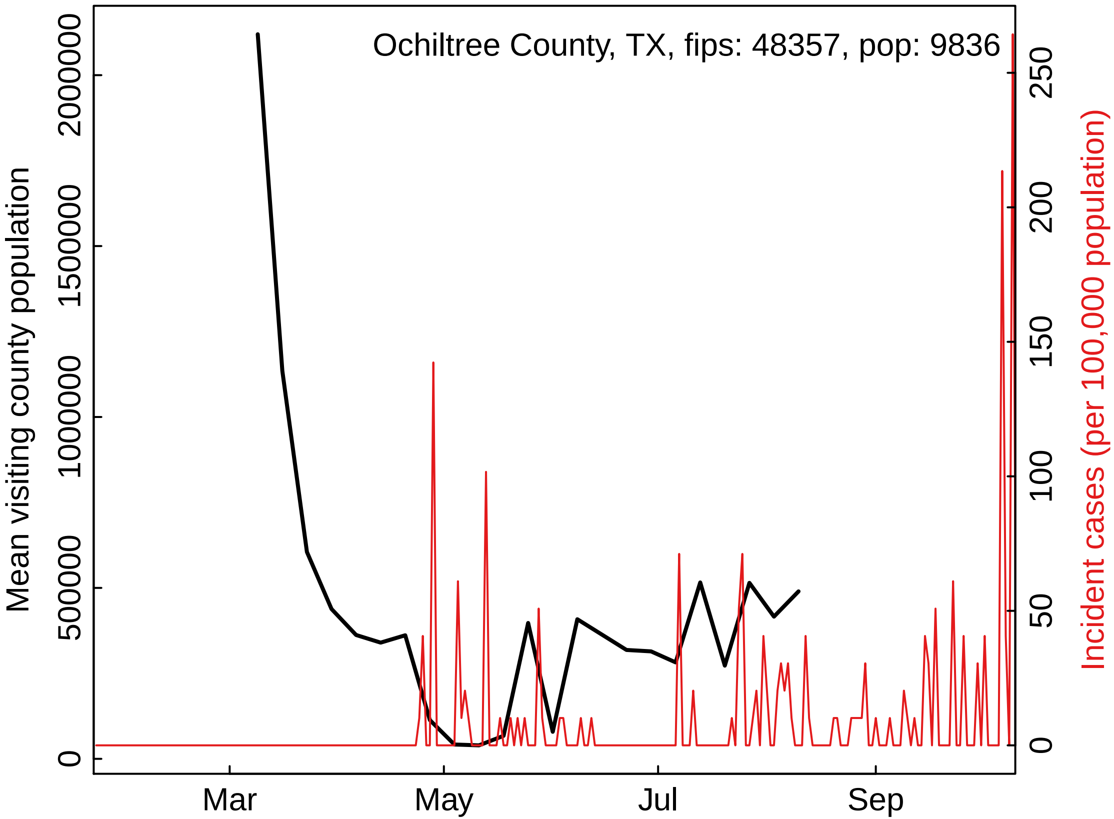
Ochiltree County, TX, fips: 48357, pop: 9836
Mean visiting county population
0
500000
1000000
1500000
2000000
Incident cases (per 100,000 population)
0
50
100
150
200
250
Mar
May
Jul
Sep

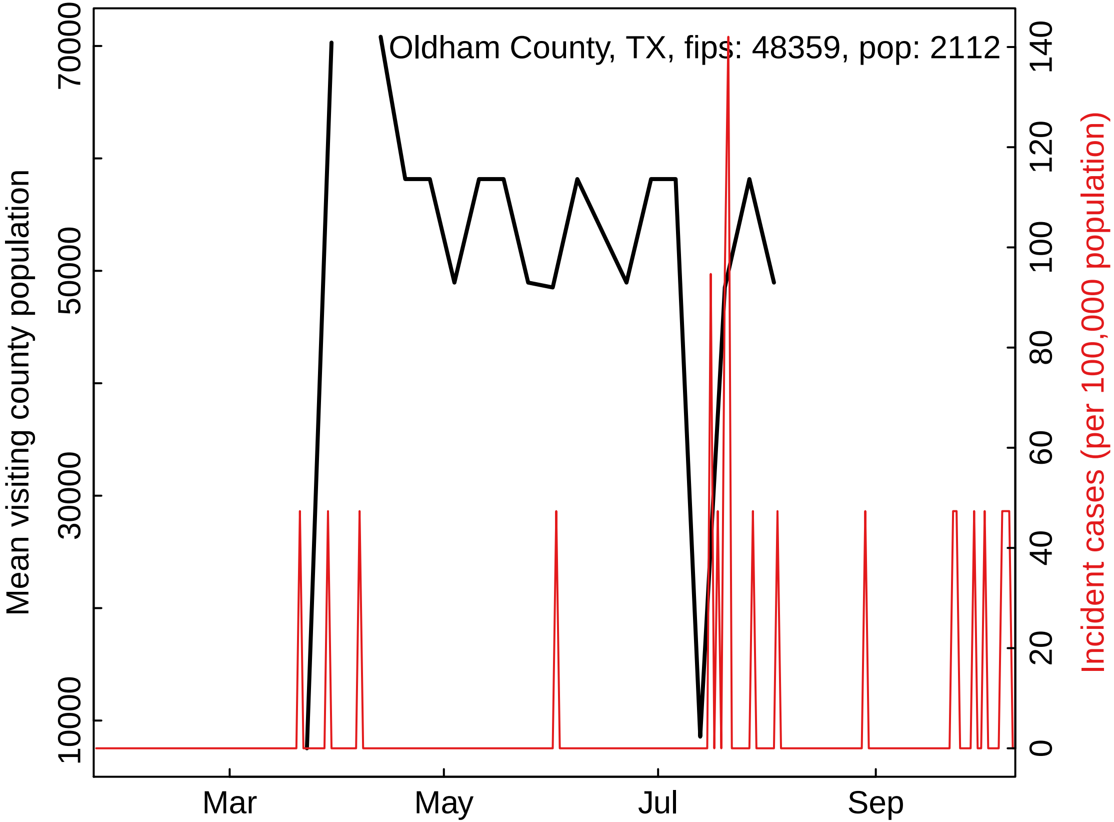
Oldham County, TX, fips: 48359, pop: 2112
Mean visiting county population
10000
30000
50000
70000
Incident cases (per 100,000 population)
0
20
40
60
80
100
120
140
Mar
May
Jul
Sep

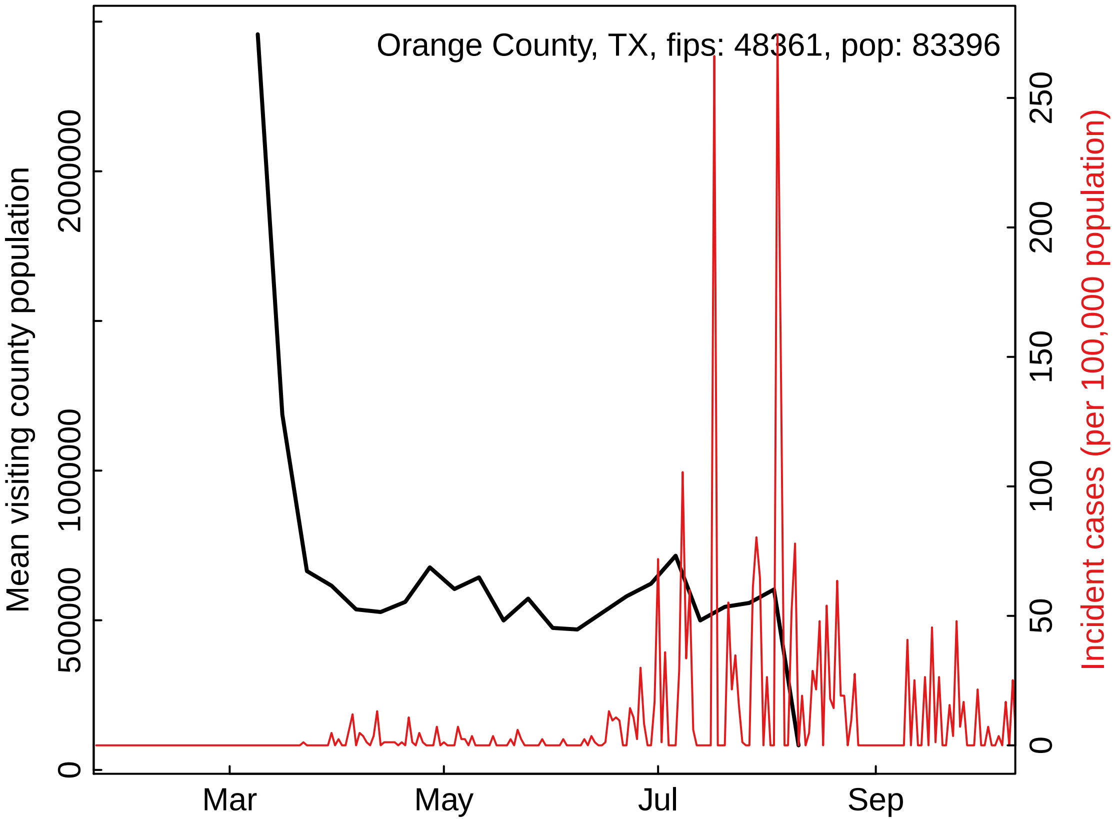

Orange County, TX, fips: 48361, pop: 83396
Mean visiting county population
0
500000
1000000
2000000
Incident cases (per 100,000 population)
0
50
100
150
200
250
Mar
May
Jul
Sep

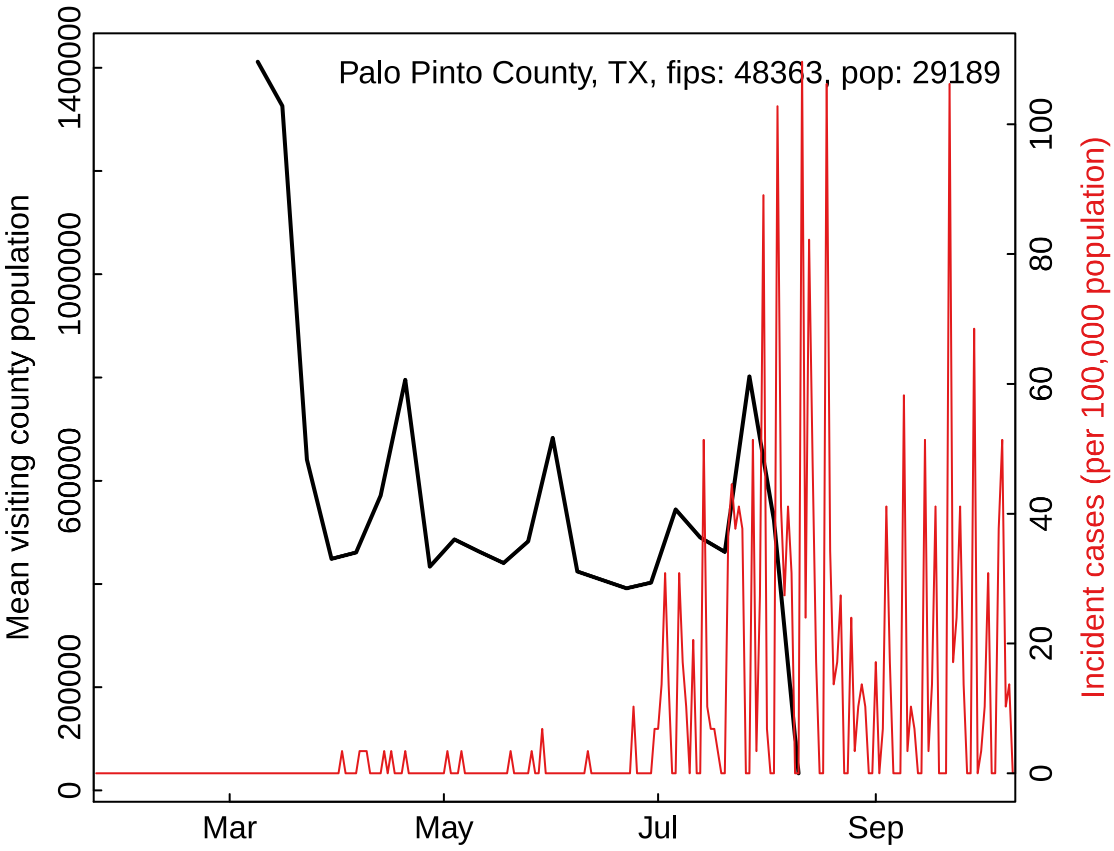
Palo Pinto County, TX, fips: 48363, pop: 29189
Mean visiting county population
0
2000000
6000000
10000000
14000000
Incident cases (per 100,000 population)
0
20
40
60
80
100
Mar
May
Jul
Sep

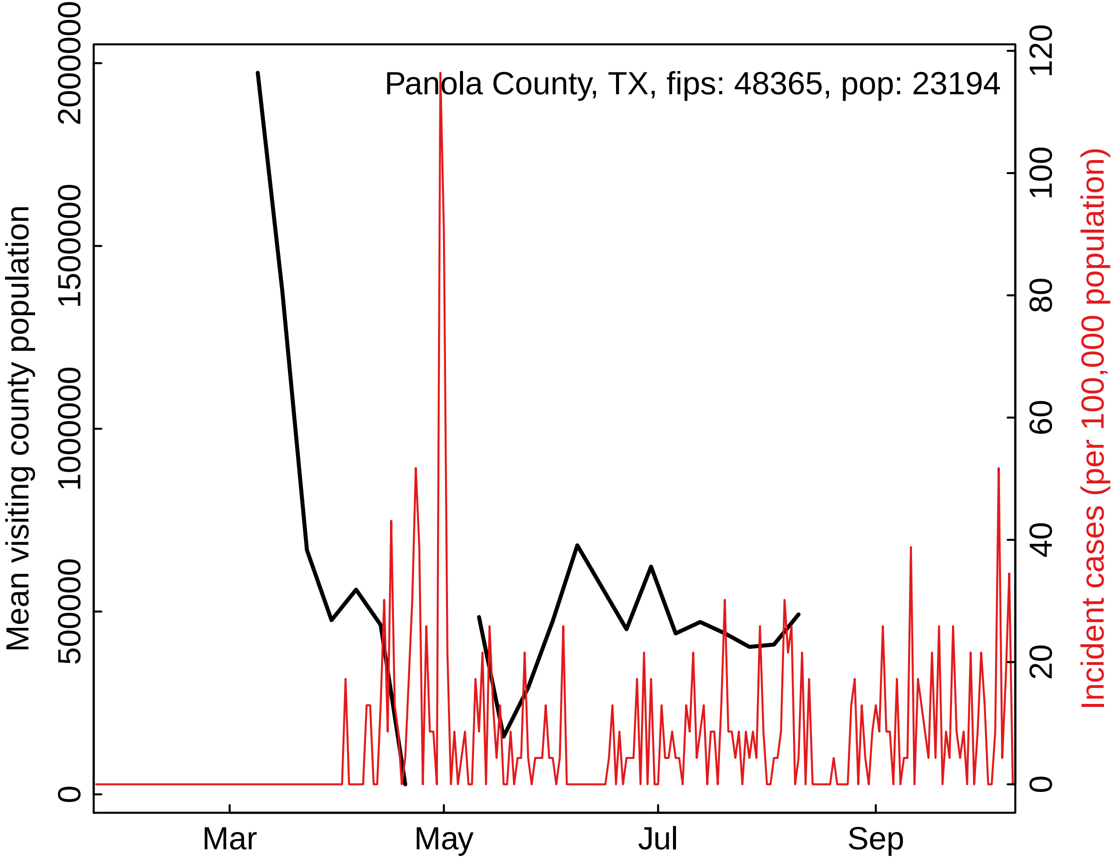

Panola County, TX, fips: 48365, pop: 23194
Mean visiting county population
0
500000
1000000
1500000
2000000
Incident cases (per 100,000 population)
0
20
40
60
80
100
120
Mar
May
Jul
Sep

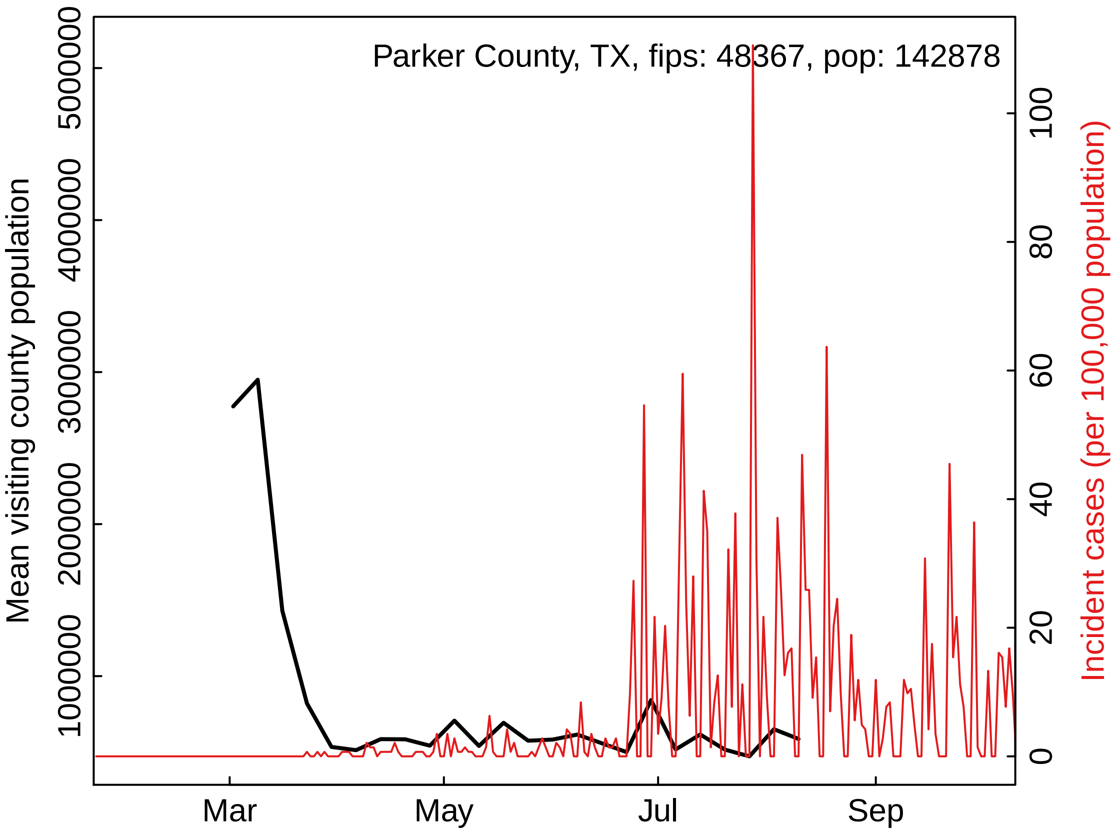

Parker County, TX, fips: 48367, pop: 142878
Mean visiting county population
1000000
2000000
3000000
4000000
5000000
Incident cases (per 100,000 population)
0
20
40
60
80
100
Mar
May
Jul
Sep

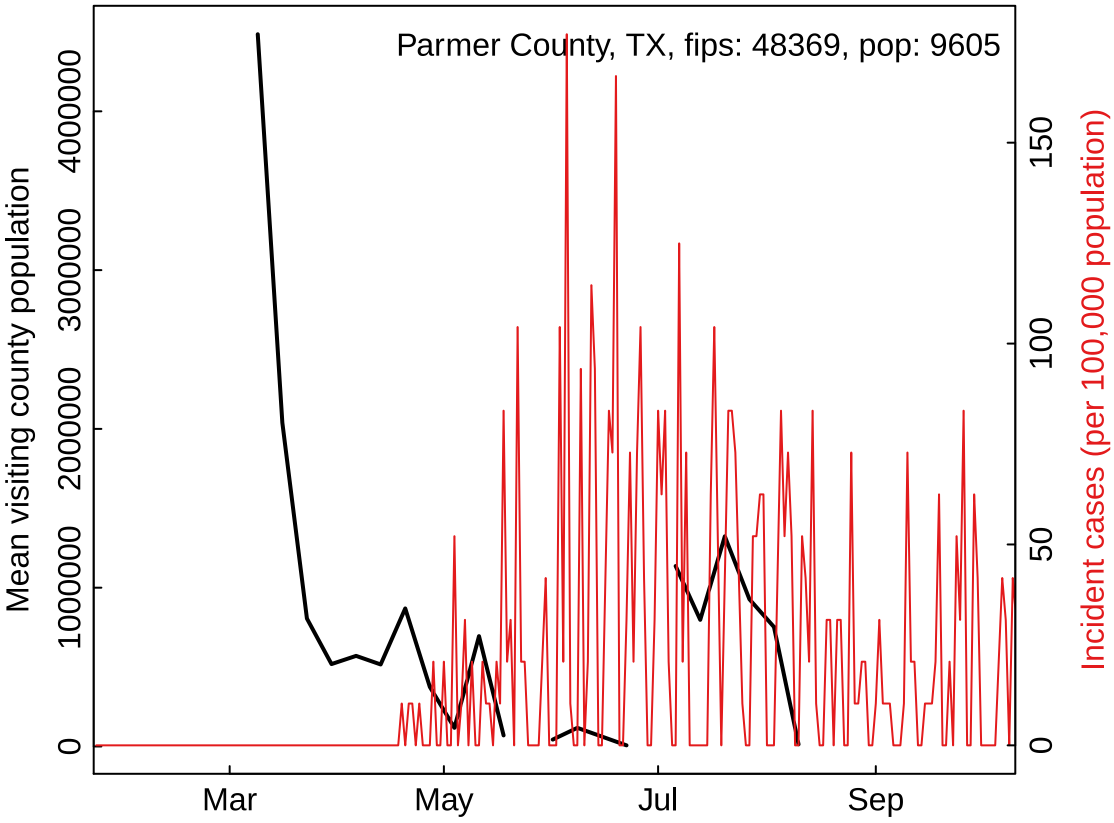

Parmer County, TX, fips: 48369, pop: 9605
Mean visiting county population
0
1000000
2000000
3000000
4000000
Incident cases (per 100,000 population)
0
50
100
150
Mar
May
Jul
Sep

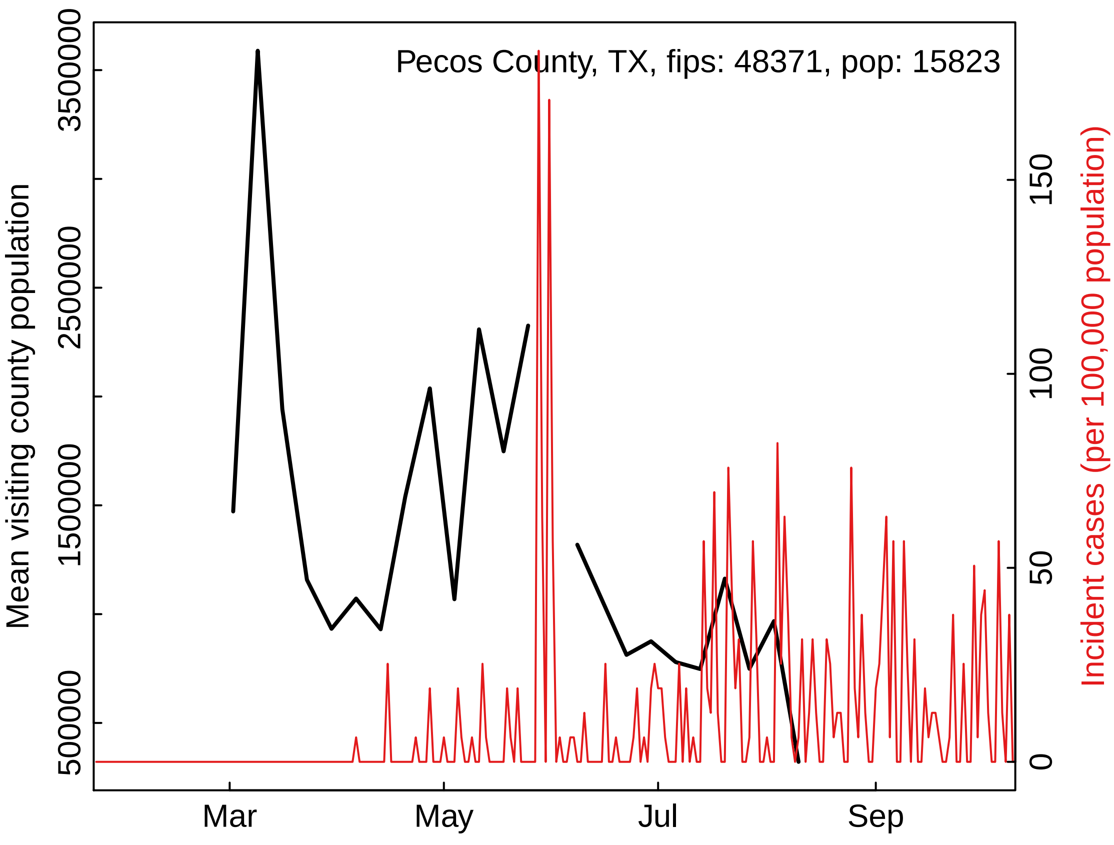
Pecos County, TX, fips: 48371, pop: 15823
Mean visiting county population
500000
1500000
2500000
3500000
Incident cases (per 100,000 population)
0
50
100
150
Mar
May
Jul
Sep

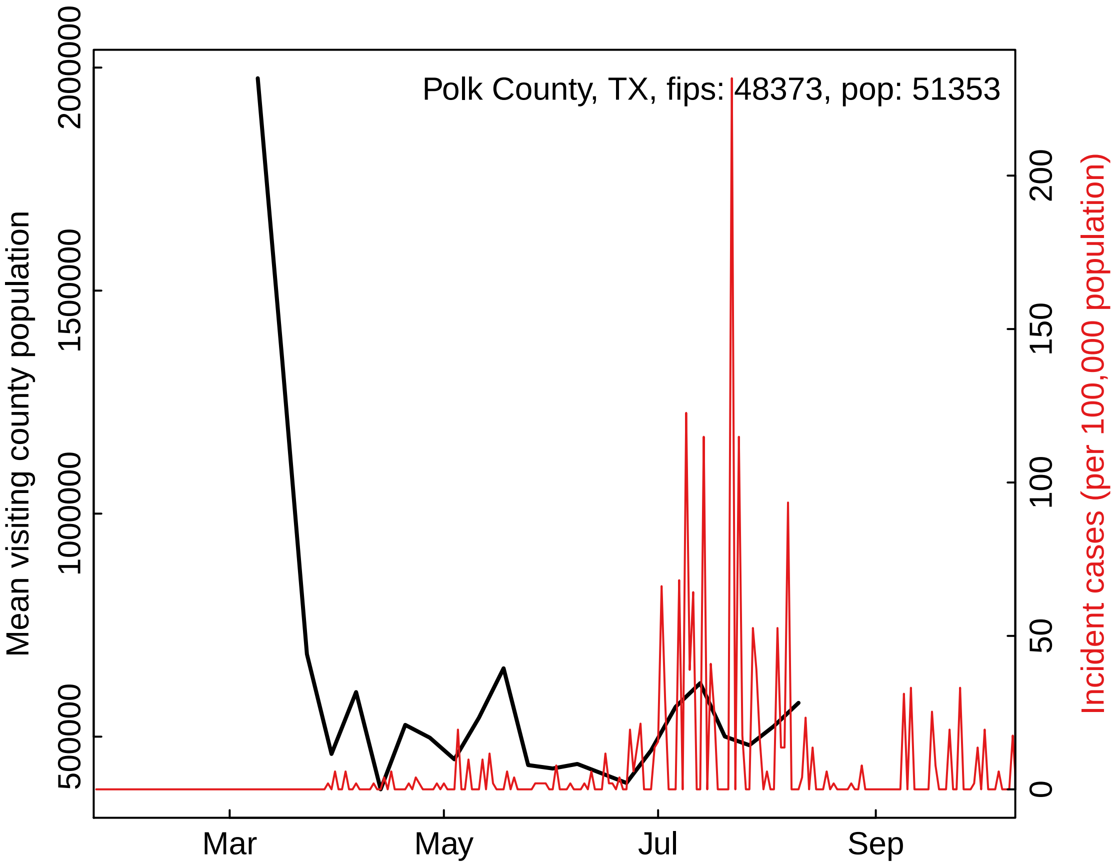
Polk County, TX, fips: 48373, pop: 51353
Mean visiting county population
500000
1000000
1500000
2000000
Incident cases (per 100,000 population)
0
50
100
150
200
Mar
May
Jul
Sep

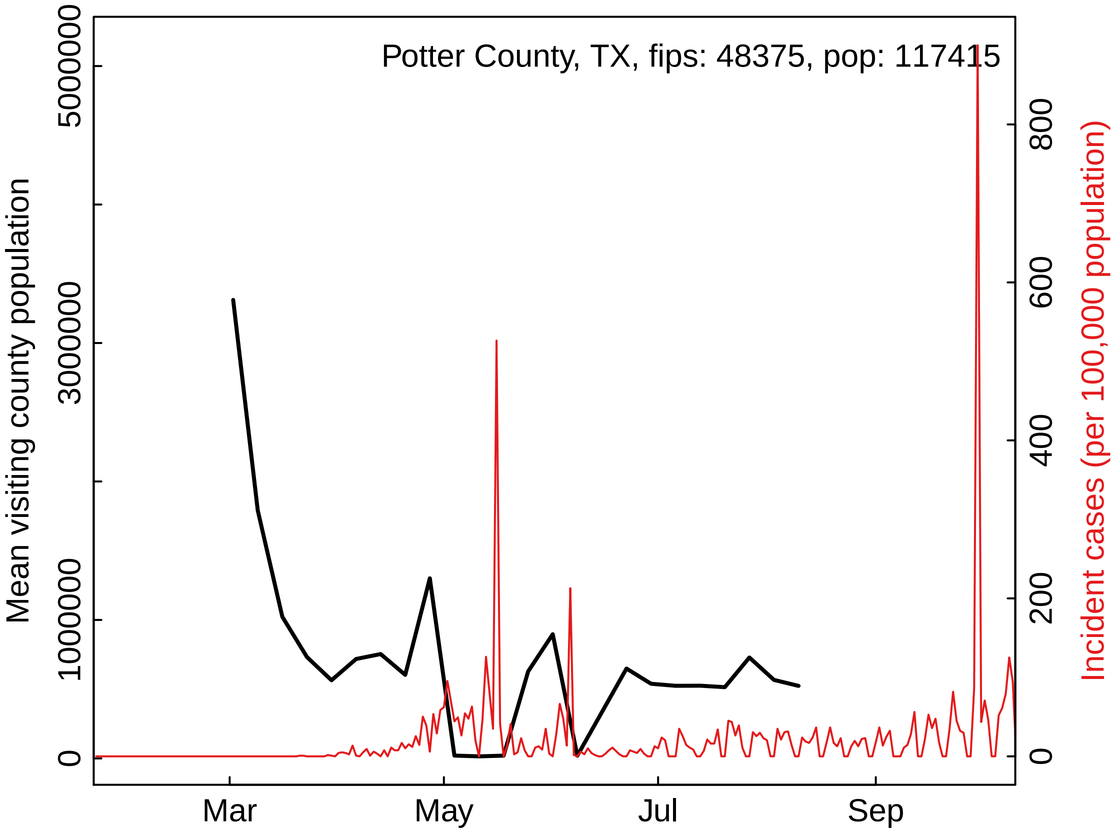

Potter County, TX, fips: 48375, pop: 117415
Mean visiting county population
0
1000000
3000000
5000000
Mar
May
Jul
Sep
0
200
400
600
800
Incident cases (per 100,000 population)

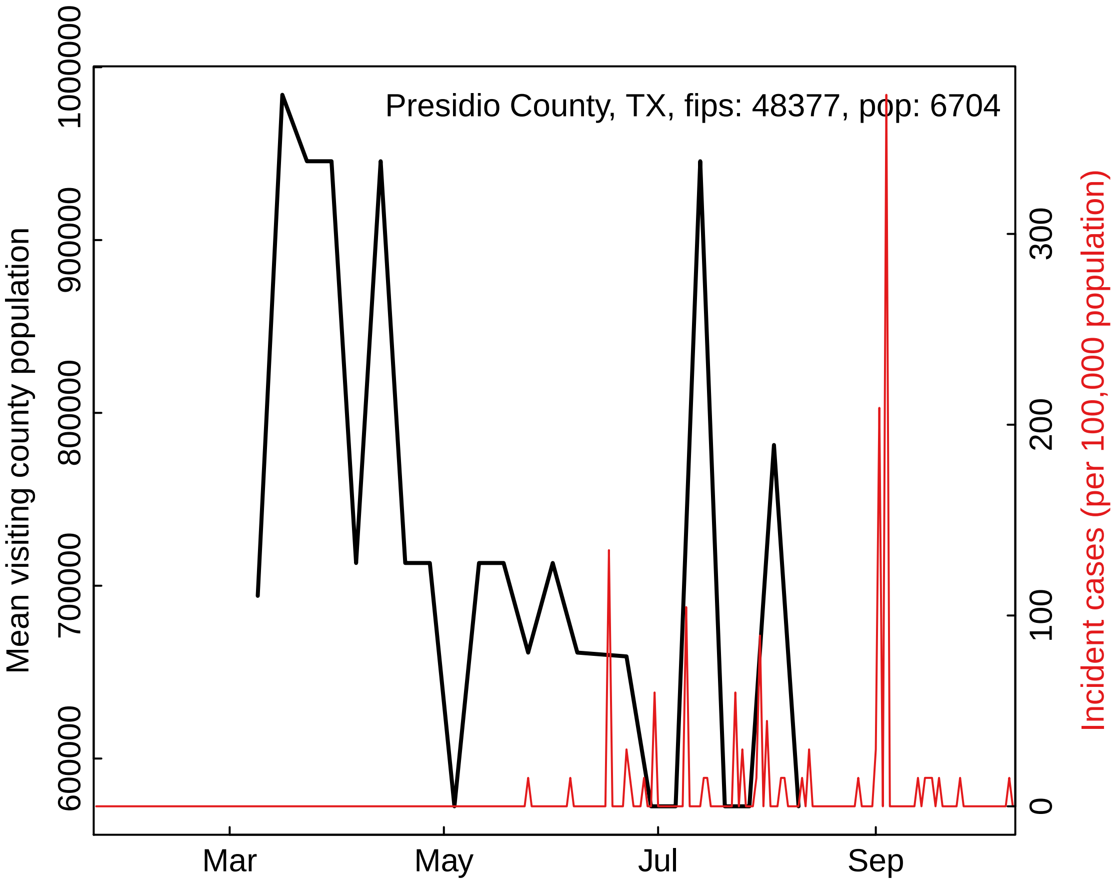

Presidio County, TX, fips: 48377, pop: 6704
Mean visiting county population
600000
700000
800000
900000
1000000
Incident cases (per 100,000 population)
0
100
200
300
Mar
May
Jul
Sep

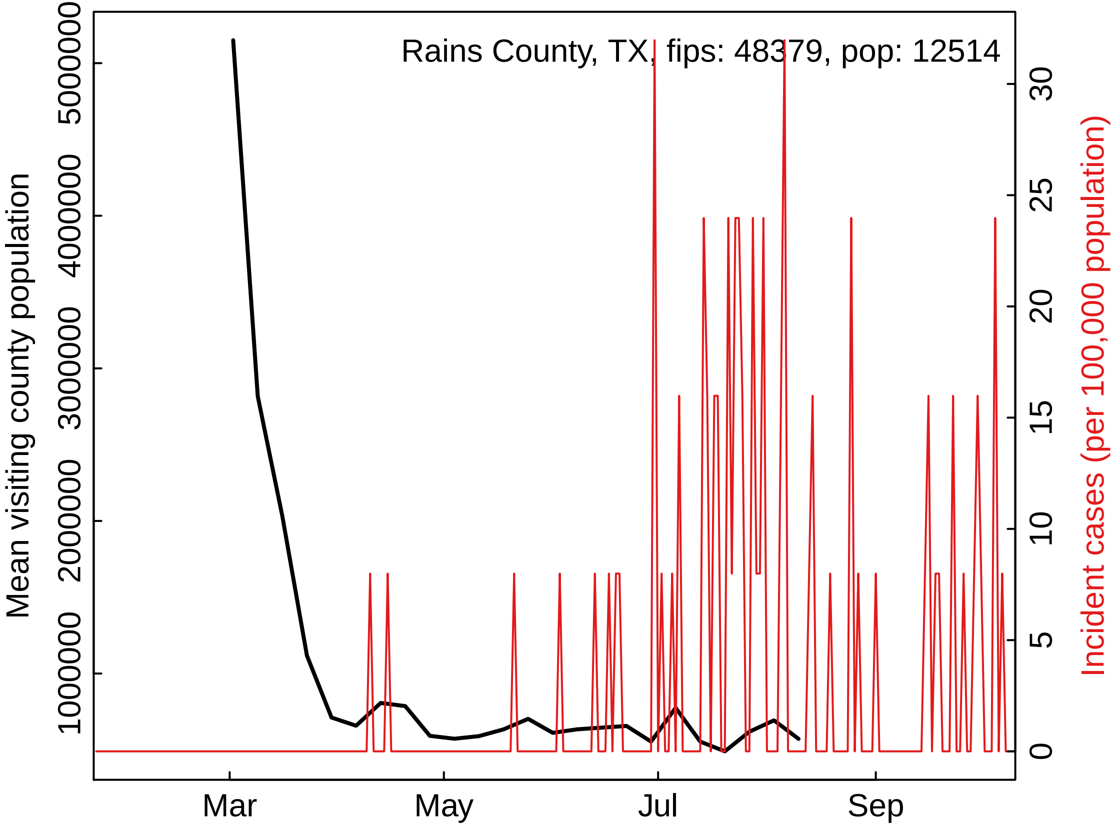

Rains County, TX, fips: 48379, pop: 12514
Mean visiting county population
1000000
2000000
3000000
4000000
5000000
Incident cases (per 100,000 population)
0
5
10
15
20
25
30
Mar
May
Jul
Sep

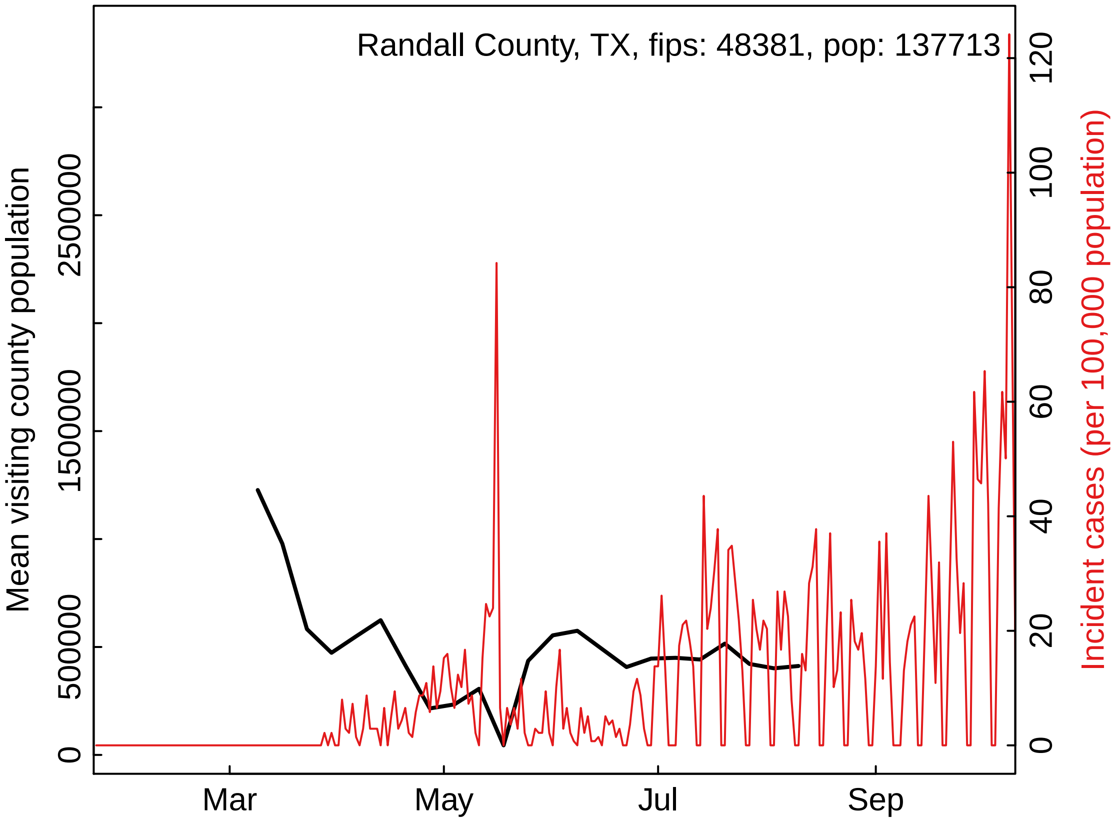

Randall County, TX, fips: 48381, pop: 137713
Mean visiting county population
0
500000
1500000
2500000
Mar
May
Jul
Sep
0
20
40
60
80
100
120
Incident cases (per 100,000 population)

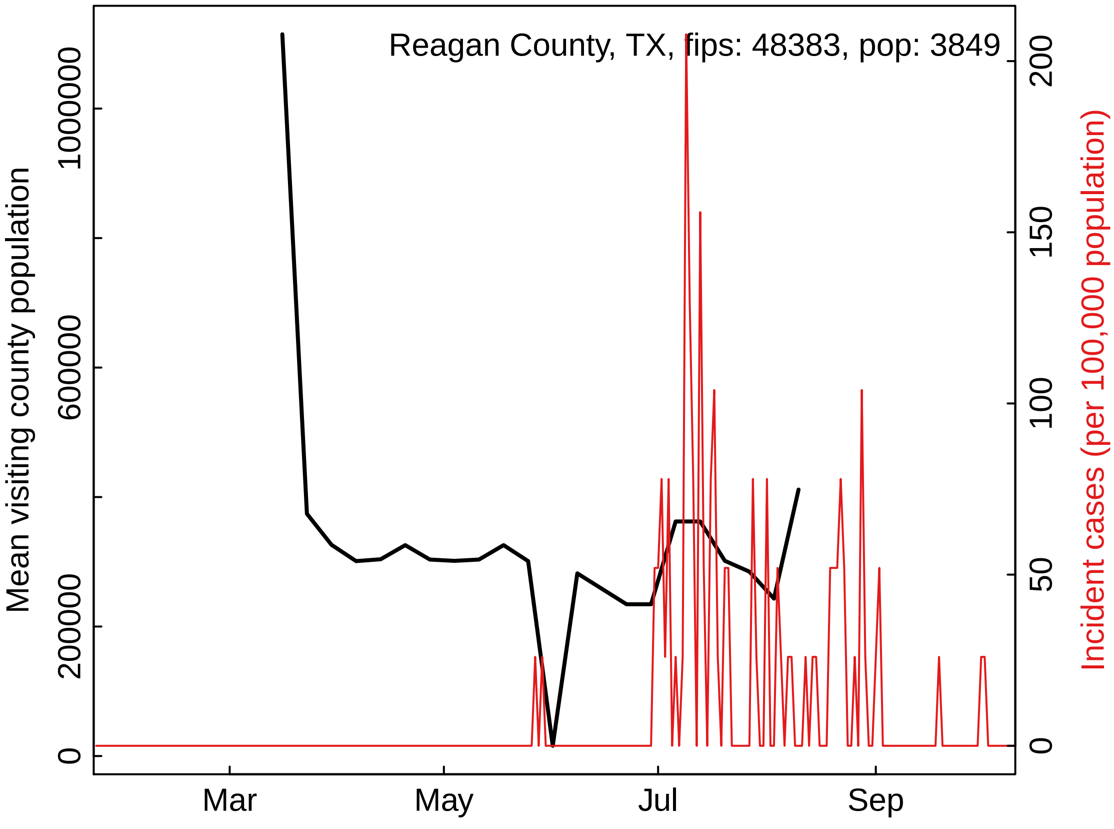
Reagan County, TX, fips: 48383, pop: 3849
Mean visiting county population
0
200000
600000
1000000
Incident cases (per 100,000 population)
0
50
100
150
200
Mar
May
Jul
Sep

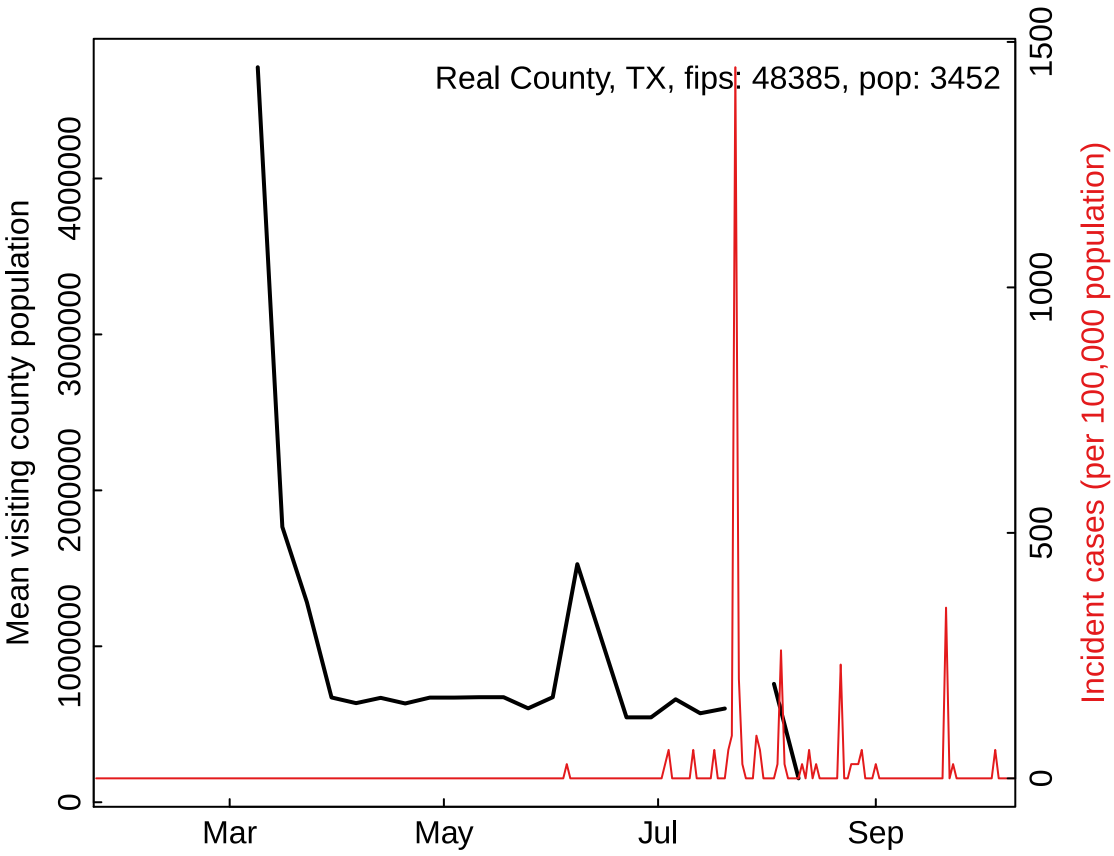

Real County, TX, fips: 48385, pop: 3452
Mean visiting county population
0
1000000
2000000
3000000
4000000
Incident cases (per 100,000 population)
0
500
1000
1500
Mar
May
Jul
Sep

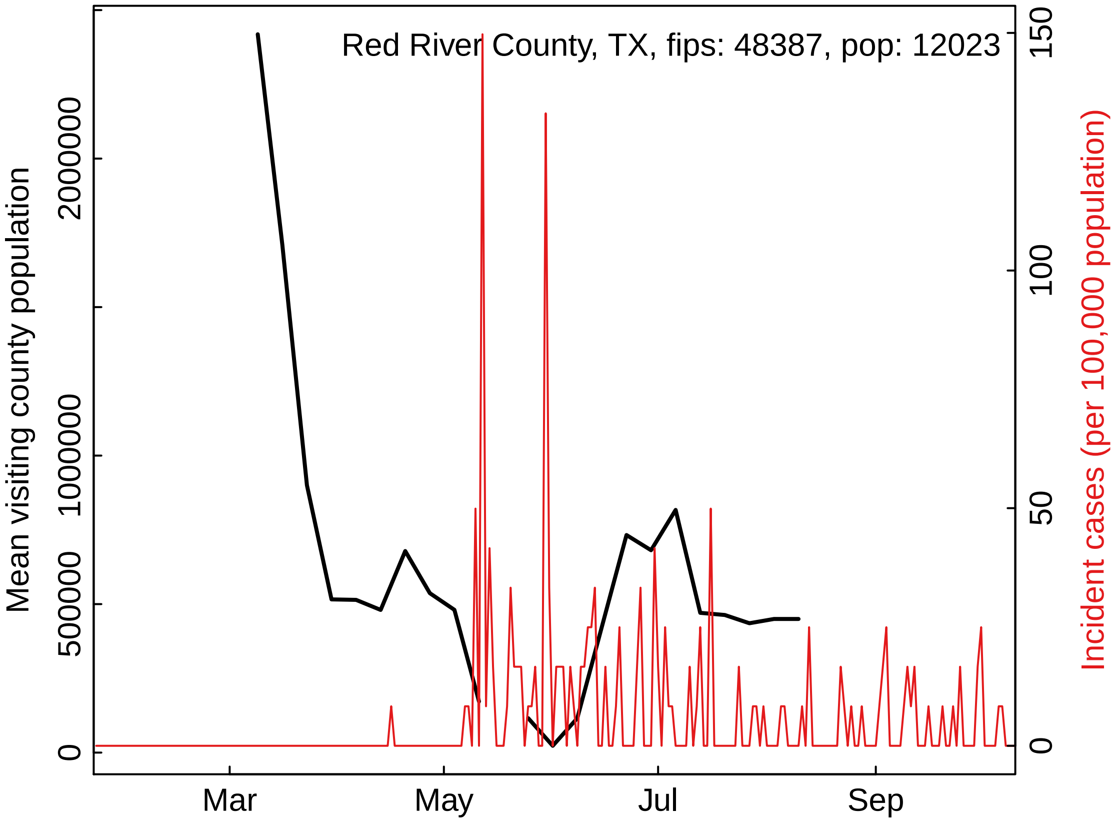

Red River County, TX, fips: 48387, pop: 12023
Mean visiting county population
0
500000
1000000
2000000
Incident cases (per 100,000 population)
0
50
100
150
Mar
May
Jul
Sep

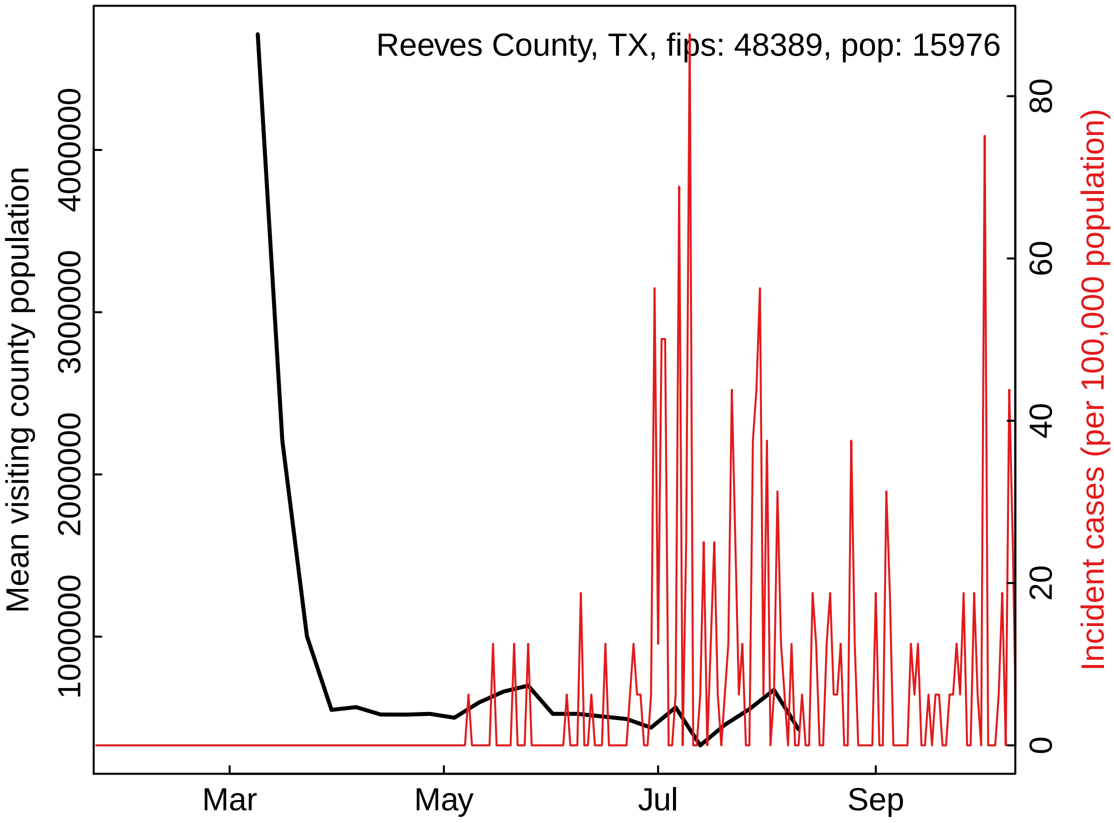
Reeves County, TX, fips: 48389, pop: 15976
Mean visiting county population
1000000
2000000
3000000
4000000
Incident cases (per 100,000 population)
0
20
40
60
80
Mar
May
Jul
Sep

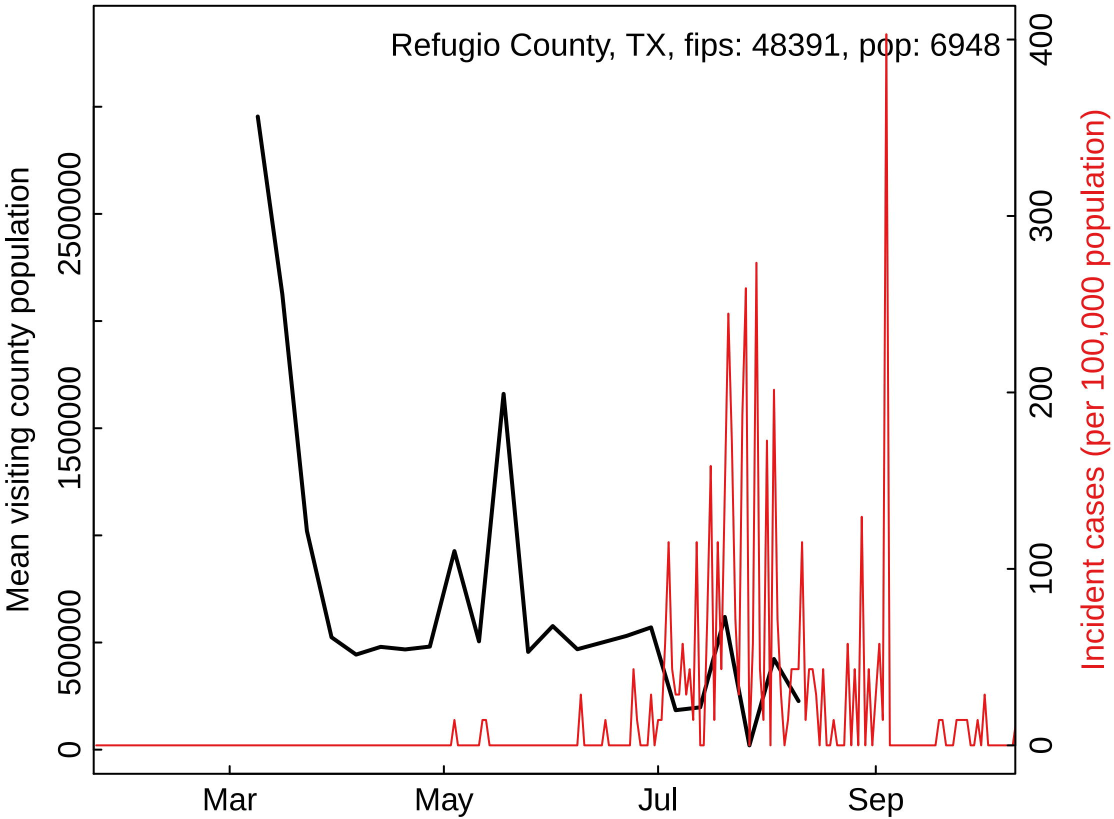

Refugio County, TX, fips: 48391, pop: 6948
Mean visiting county population
0
500000
1500000
2500000
Incident cases (per 100,000 population)
0
100
200
300
400
Mar
May
Jul
Sep

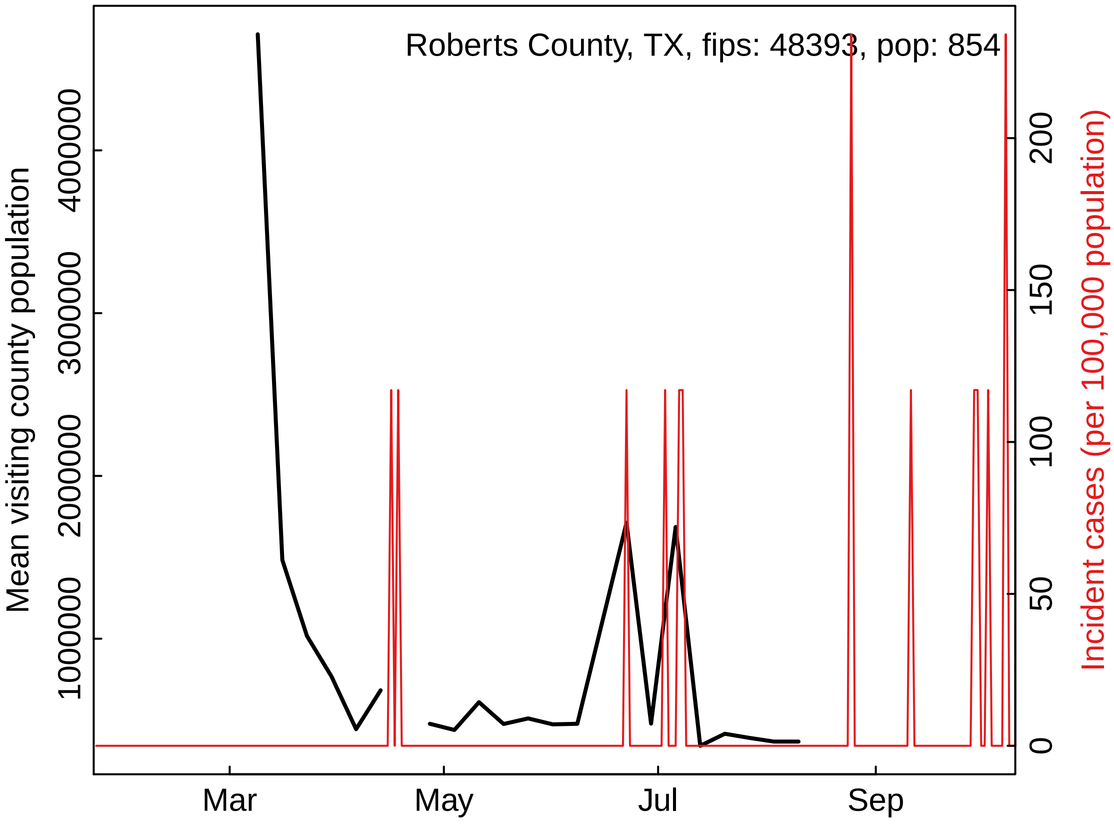

Roberts County, TX, fips: 48393, pop: 854
Mean visiting county population
1000000
2000000
3000000
4000000
Incident cases (per 100,000 population)
0
50
100
150
200
Mar
May
Jul
Sep

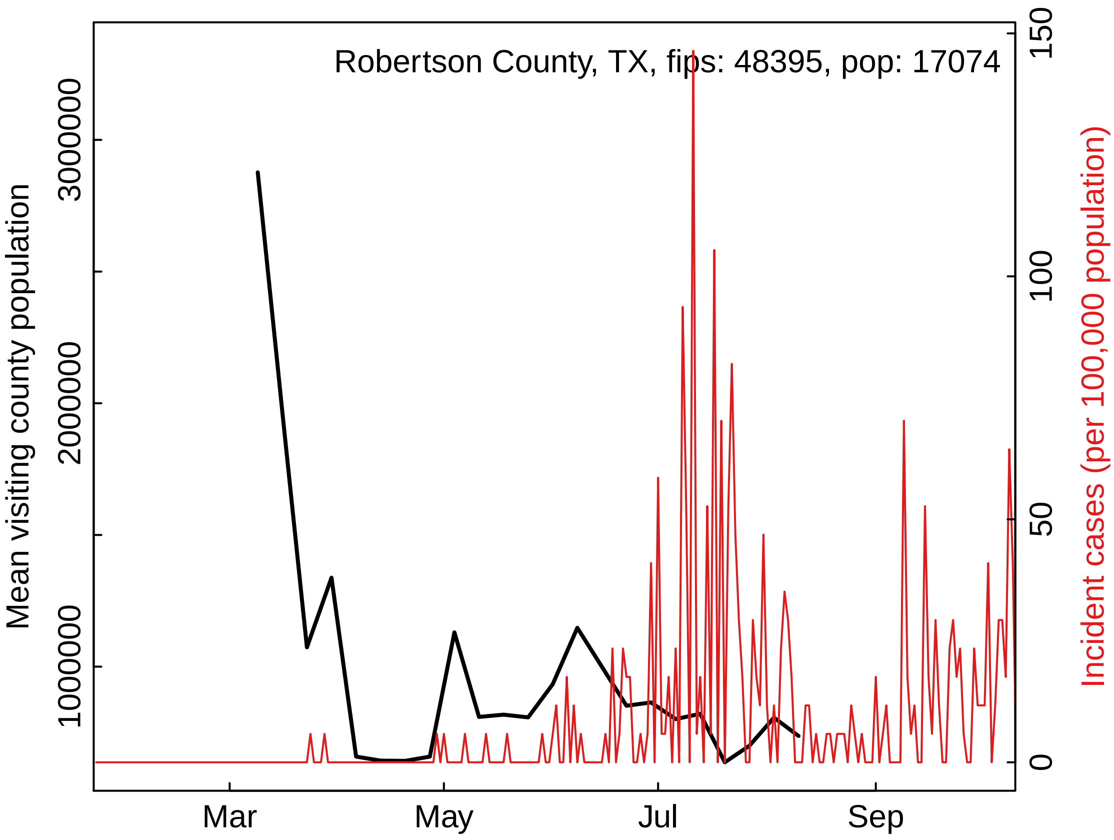

Robertson County, TX, fips: 48395, pop: 17074
Mean visiting county population
1000000
2000000
3000000
Incident cases (per 100,000 population)
0
50
100
150
Mar
May
Jul
Sep

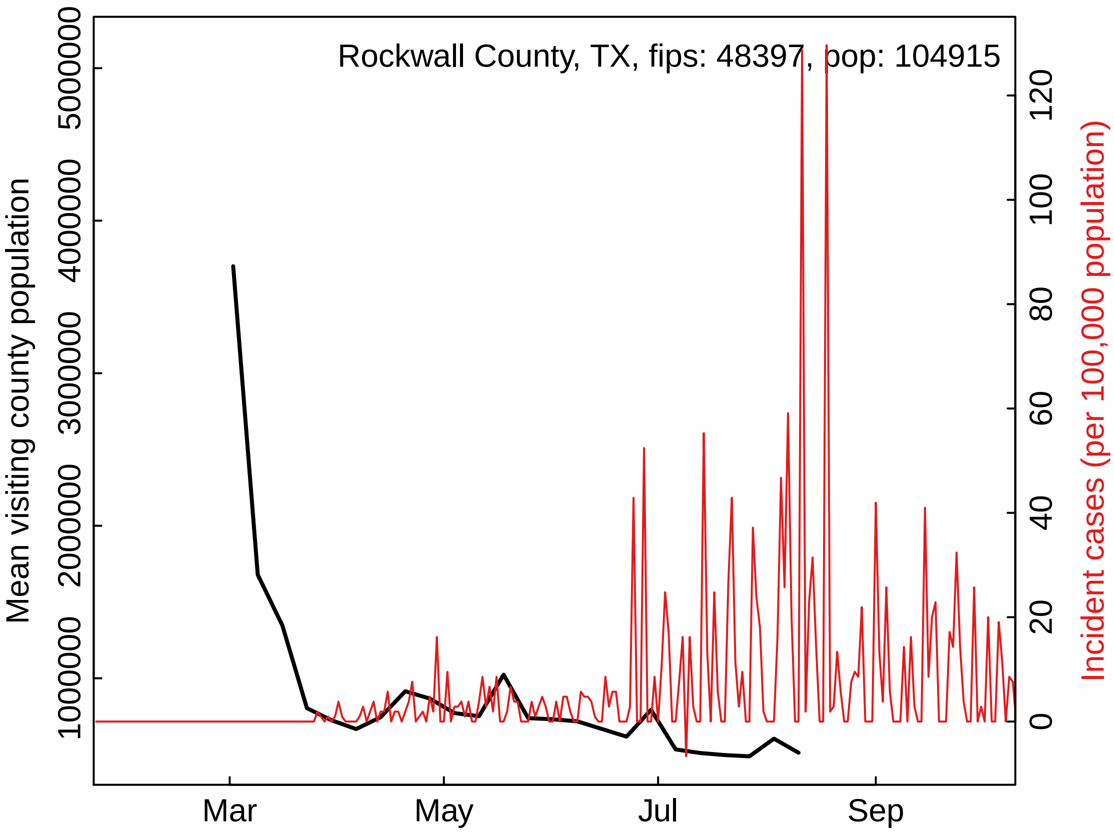
Rockwall County, TX, fips: 48397, pop: 104915
Mean visiting county population
1000000
2000000
3000000
4000000
5000000
Incident cases (per 100,000 population)
0
20
40
60
80
100
120
Mar
May
Jul
Sep

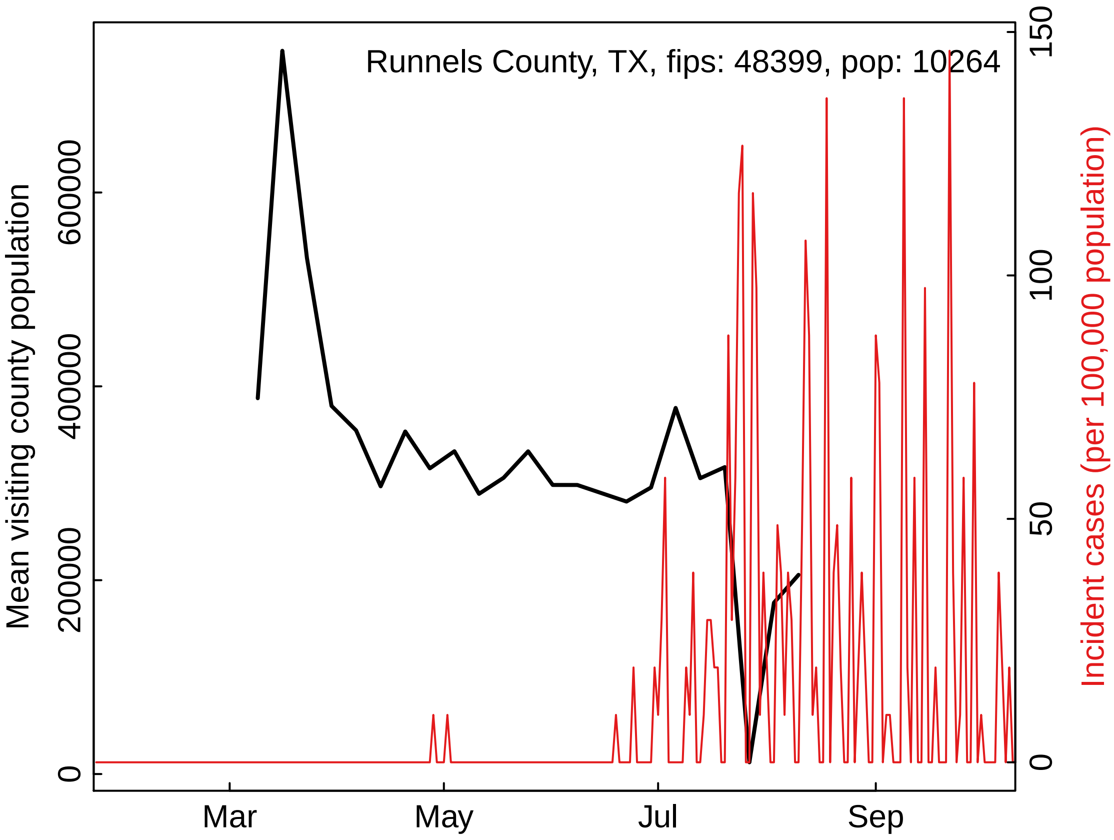
Runnels County, TX, fips: 48399, pop: 10264
Mean visiting county population
0
200000
400000
600000
Incident cases (per 100,000 population)
0
50
100
150
Mar
May
Jul
Sep

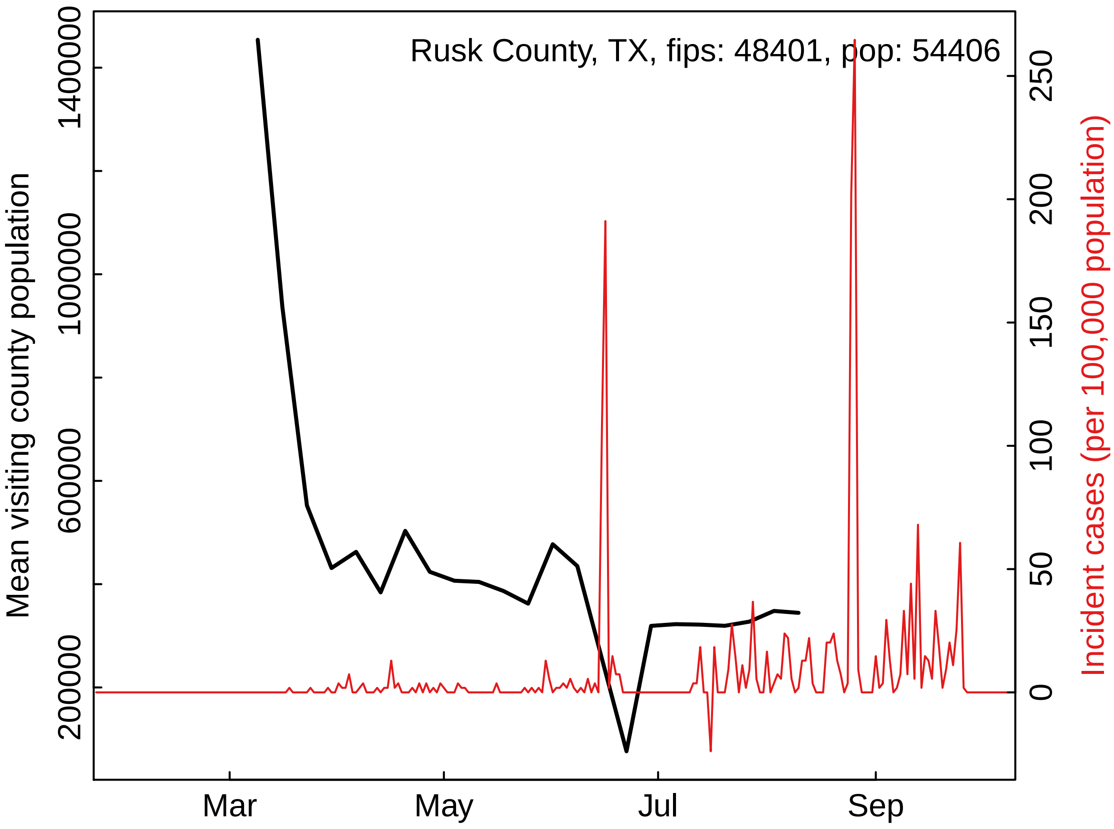

Rusk County, TX, fips: 48401, pop: 54406
Mean visiting county population
200000
600000
1000000
1400000
Incident cases (per 100,000 population)
0
50
100
150
200
250
Mar
May
Jul
Sep

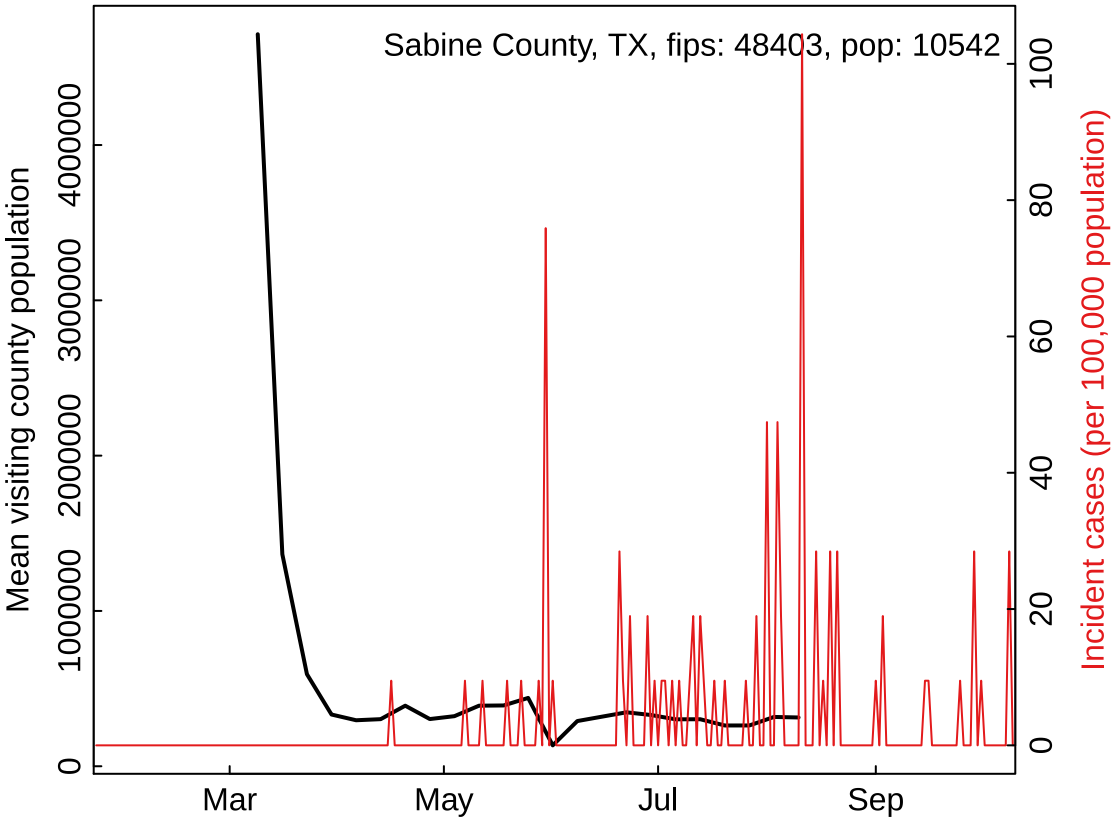
Sabine County, TX, fips: 48403, pop: 10542
Mean visiting county population
0
1000000
2000000
3000000
4000000
Incident cases (per 100,000 population)
0
20
40
60
80
100
Mar
May
Jul
Sep

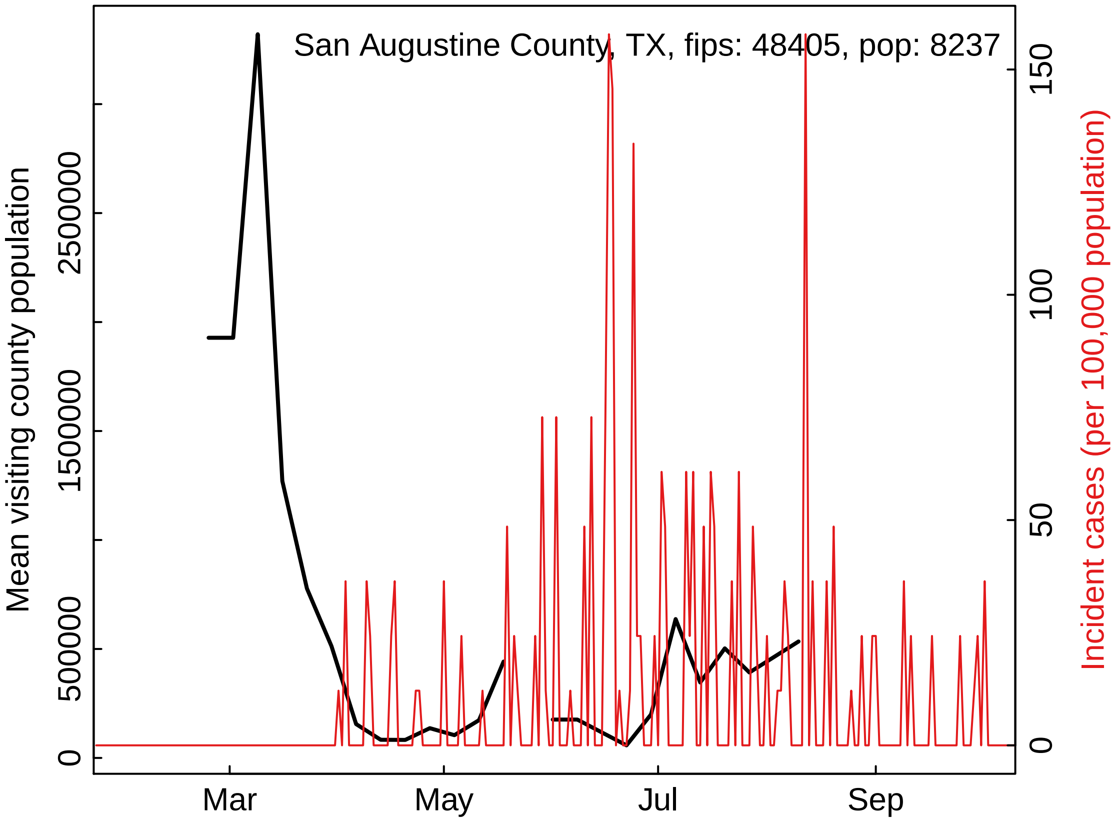

San Augustine County, TX, fips: 48405, pop: 8237
Mean visiting county population
0
500000
1500000
2500000
Incident cases (per 100,000 population)
0
50
100
150
Mar
May
Jul
Sep

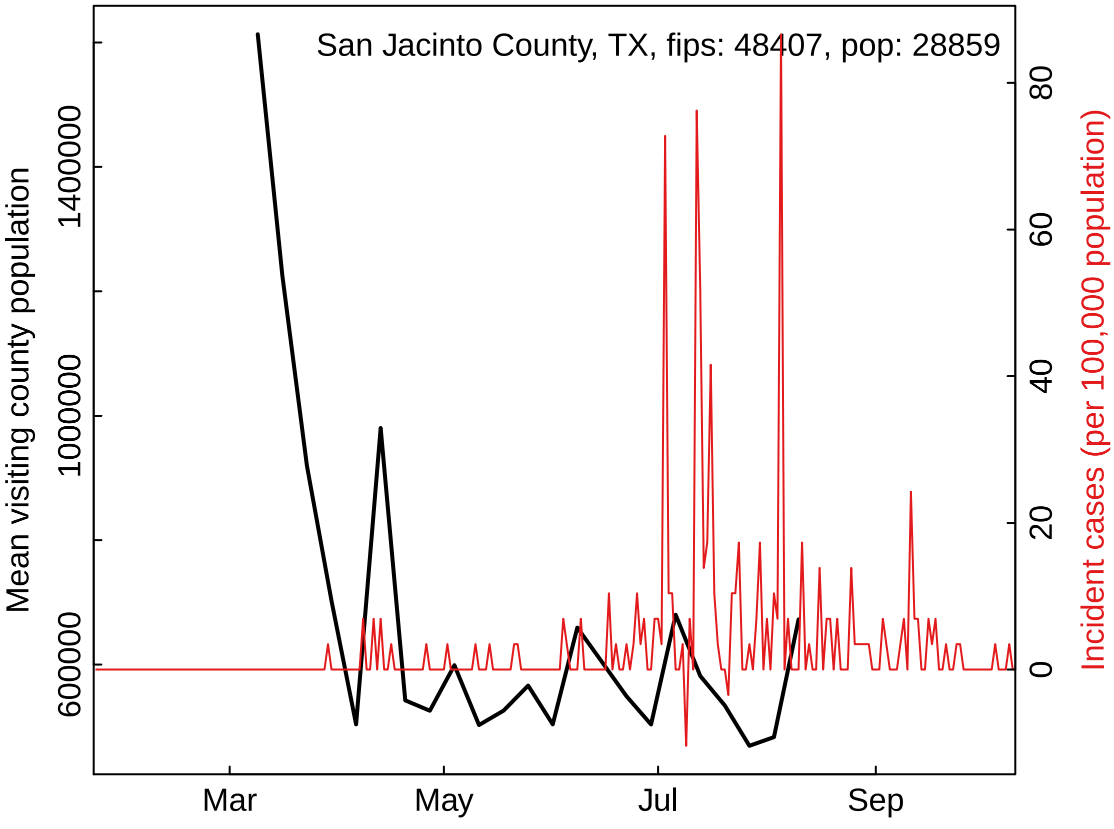
San Jacinto County, TX, fips: 48407, pop: 28859
Mean visiting county population
600000
1000000
1400000
Incident cases (per 100,000 population)
0
20
40
60
80
Mar
May
Jul
Sep

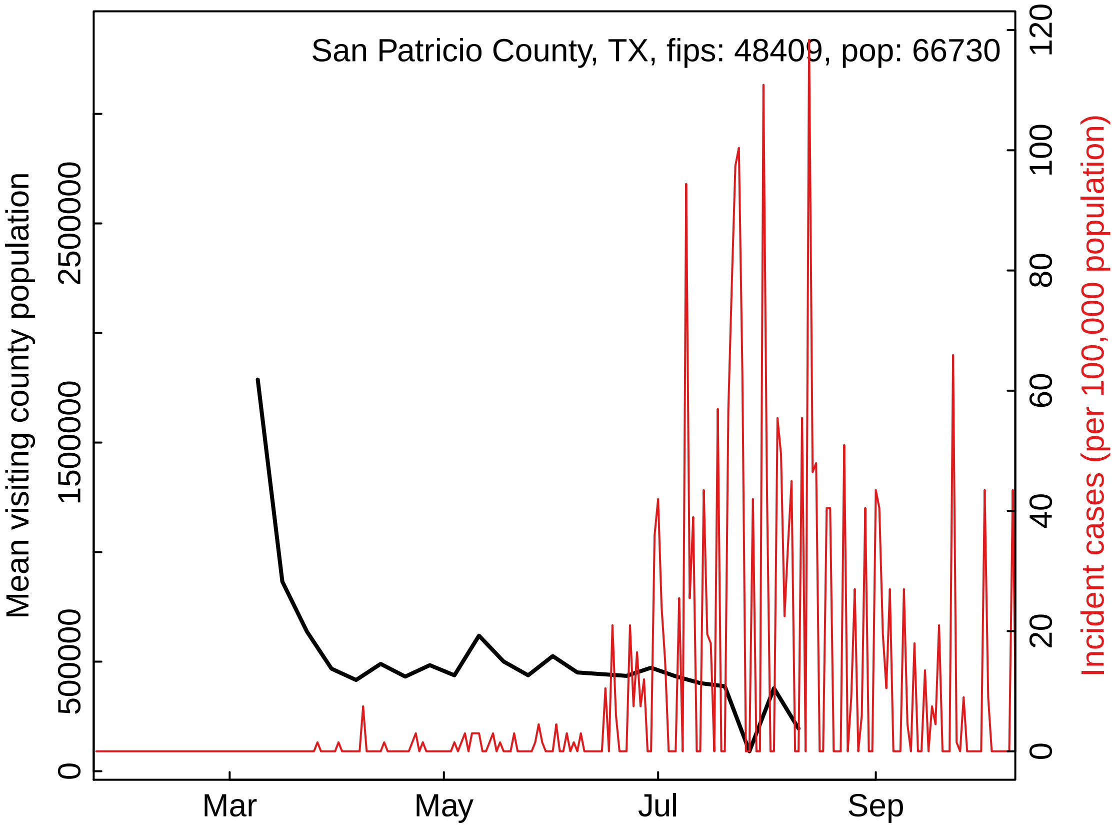

San Patricio County, TX, fips: 48409, pop: 66730
Mean visiting county population
0
500000
1500000
2500000
Mar
May
Jul
Sep
Incident cases (per 100,000 population)
0
20
40
60
80
100
120

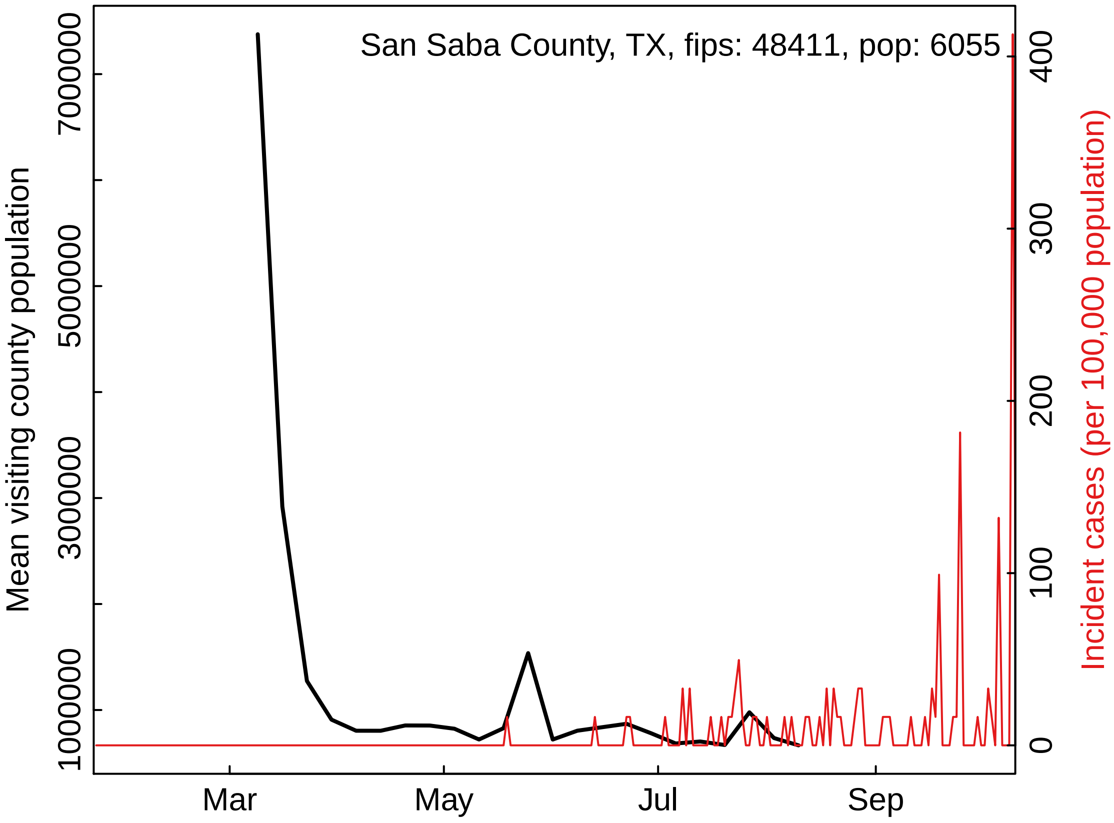

San Saba County, TX, fips: 48411, pop: 6055
Mean visiting county population
1000000
3000000
5000000
7000000
Incident cases (per 100,000 population)
0
100
200
300
400
Mar
May
Jul
Sep

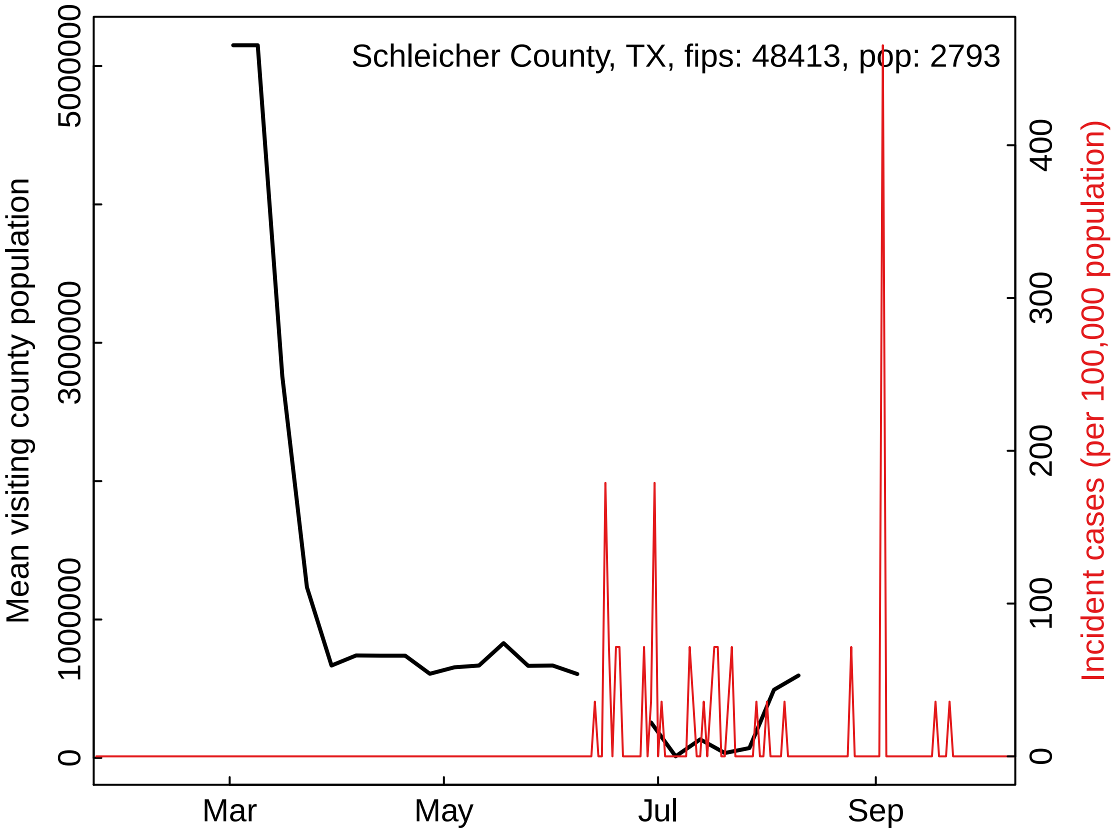

Schleicher County, TX, fips: 48413, pop: 2793
Mean visiting county population
Incident cases (per 100,000 population)
0
1000000
3000000
5000000
0
100
200
300
400
Mar
May
Jul
Sep

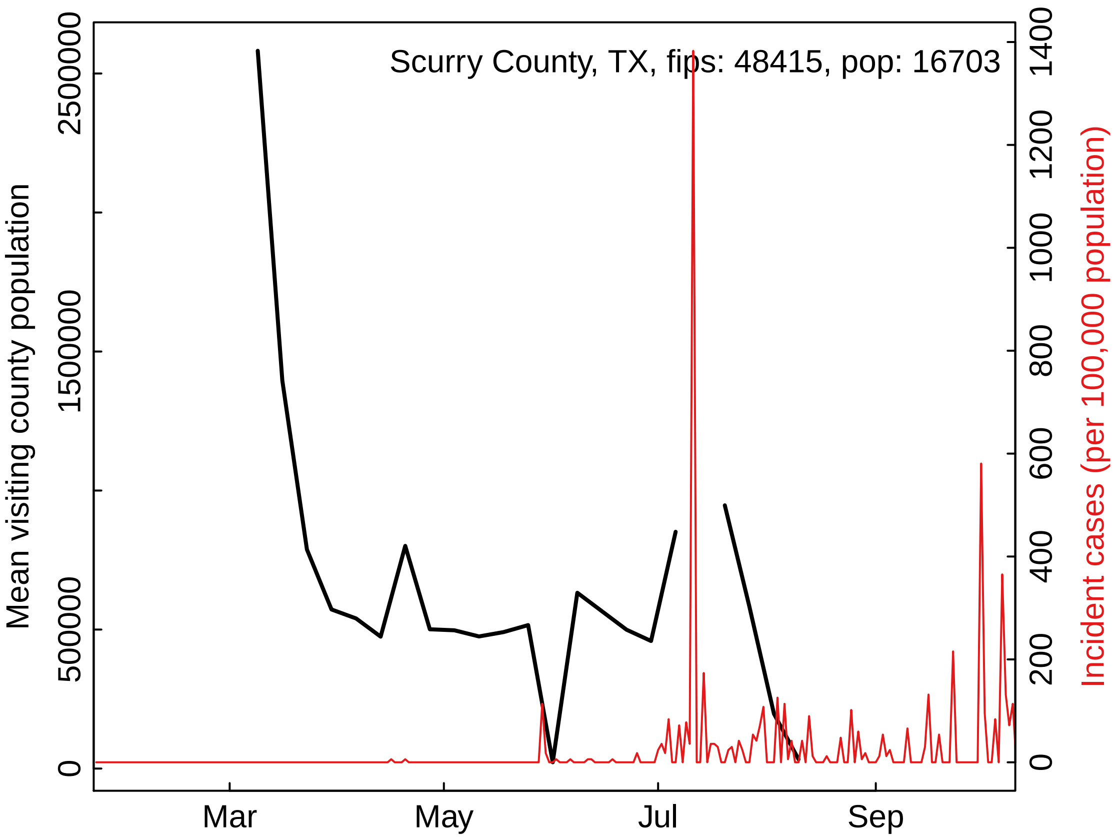
Scurry County, TX, fips: 48415, pop: 16703
Mean visiting county population
0
500000
1500000
2500000
Incident cases (per 100,000 population)
0
200
400
600
800
1000
1200
1400
Mar
May
Jul
Sep

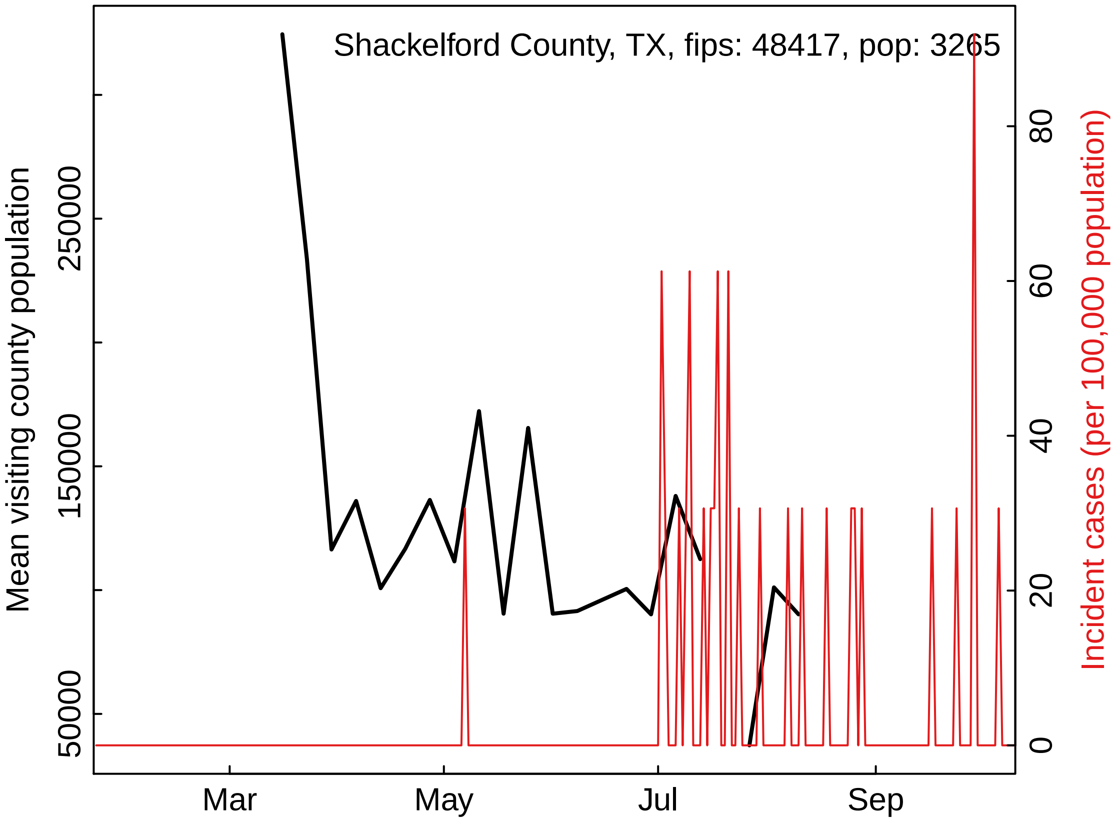
Shackelford County, TX, fips: 48417, pop: 3265
Mean visiting county population
50000
150000
250000
Incident cases (per 100,000 population)
0
20
40
60
80
Mar
May
Jul
Sep

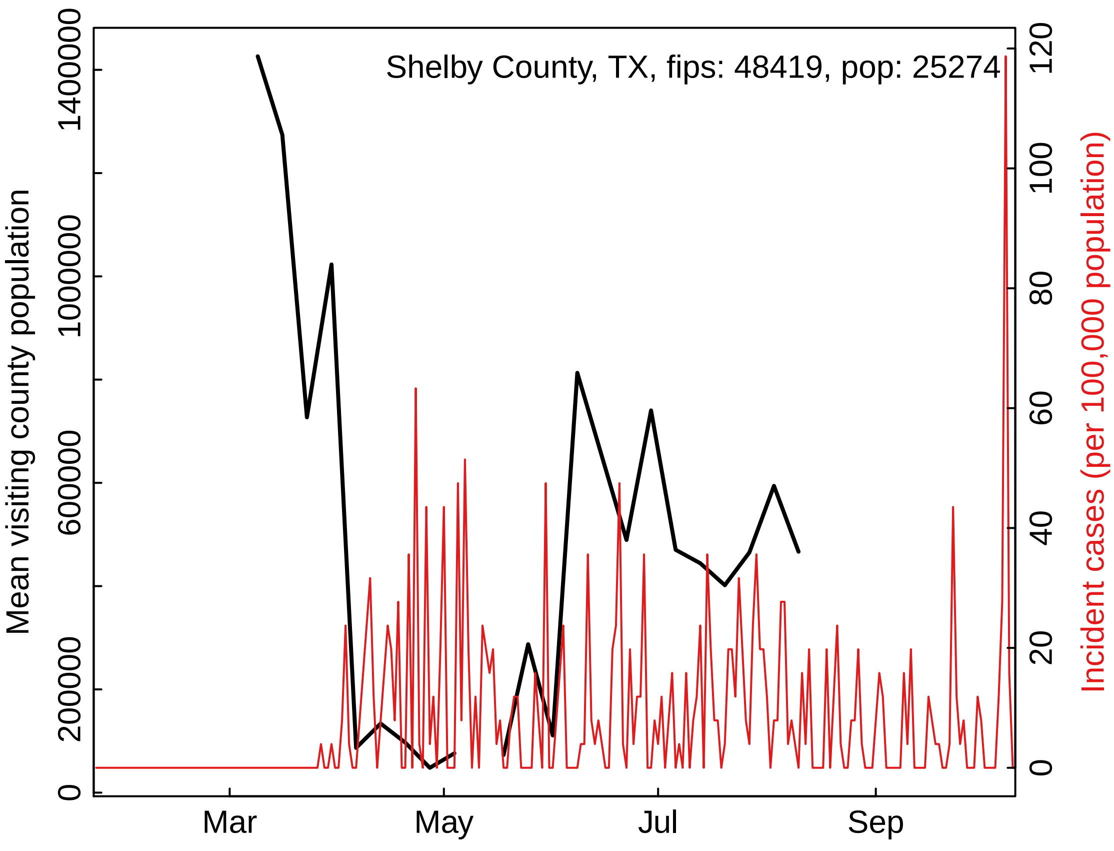

Shelby County, TX, fips: 48419, pop: 25274
Mean visiting county population
0
2000000
6000000
10000000
14000000
Incident cases (per 100,000 population)
0
20
40
60
80
100
120
Mar
May
Jul
Sep

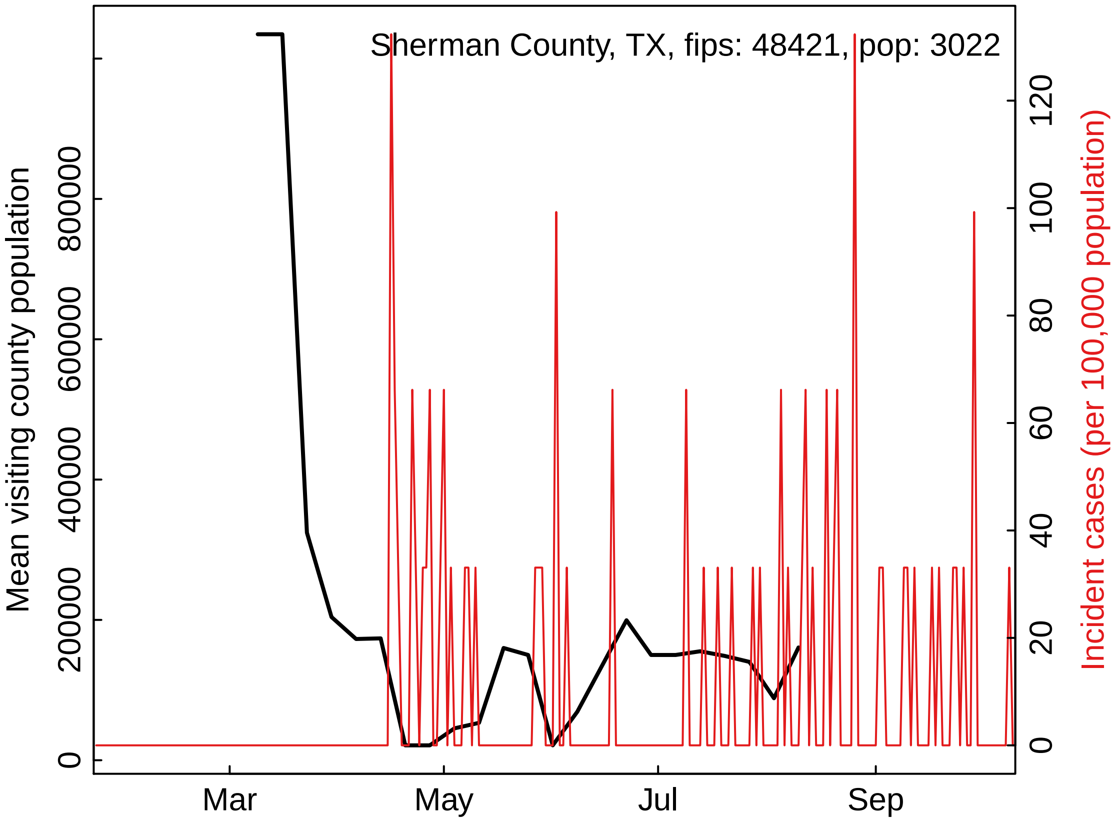
Sherman County, TX, fips: 48421, pop: 3022
Mean visiting county population
0
200000
400000
600000
800000
Incident cases (per 100,000 population)
0
20
40
60
80
100
120
Mar
May
Jul
Sep

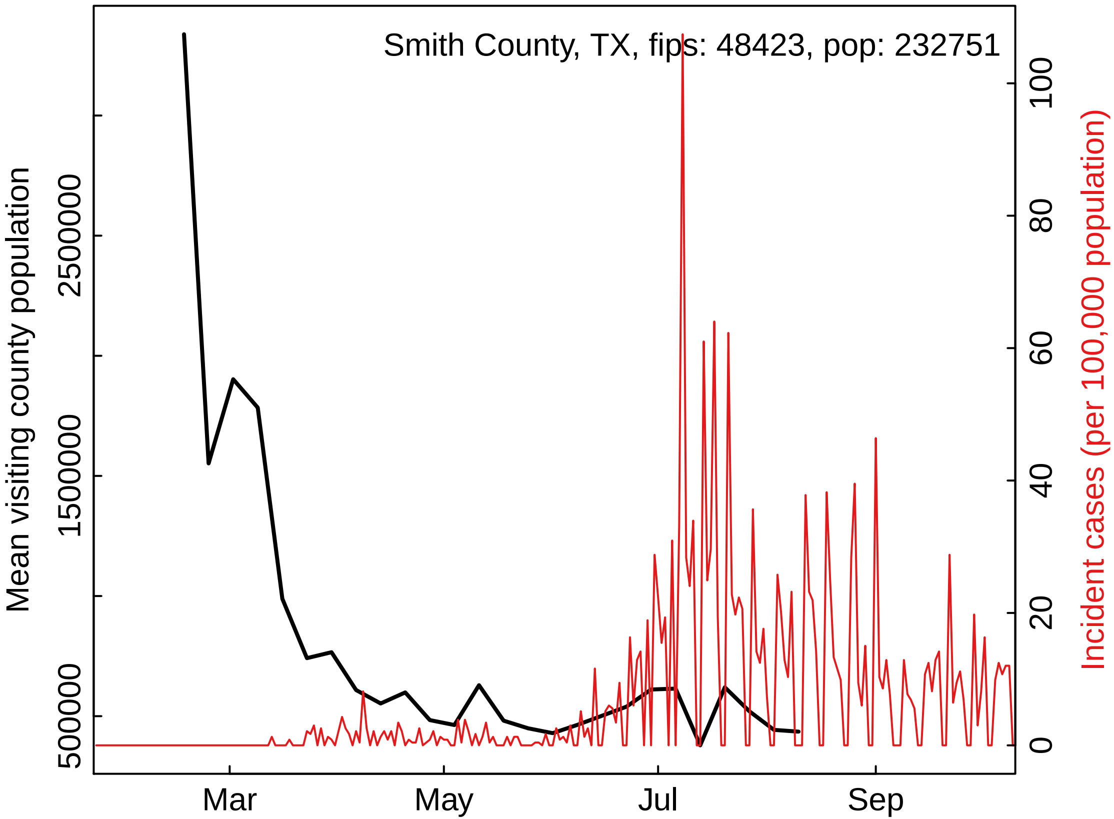

Smith County, TX, fips: 48423, pop: 232751
Mean visiting county population
500000
1500000
2500000
Mar
May
Jul
Sep
Incident cases (per 100,000 population)
0
20
40
60
80
100

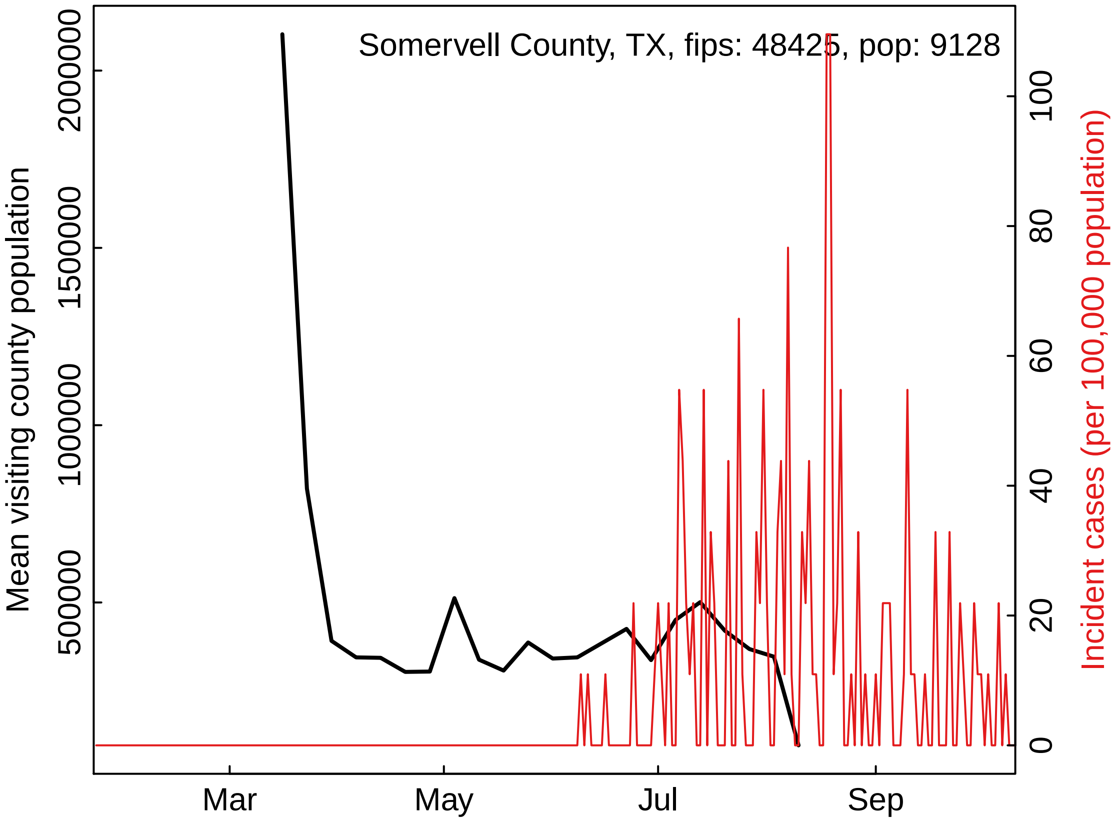

Somervell County, TX, fips: 48425, pop: 9128
Mean visiting county population
500000
1000000
1500000
2000000
Incident cases (per 100,000 population)
0
20
40
60
80
100
Mar
May
Jul
Sep

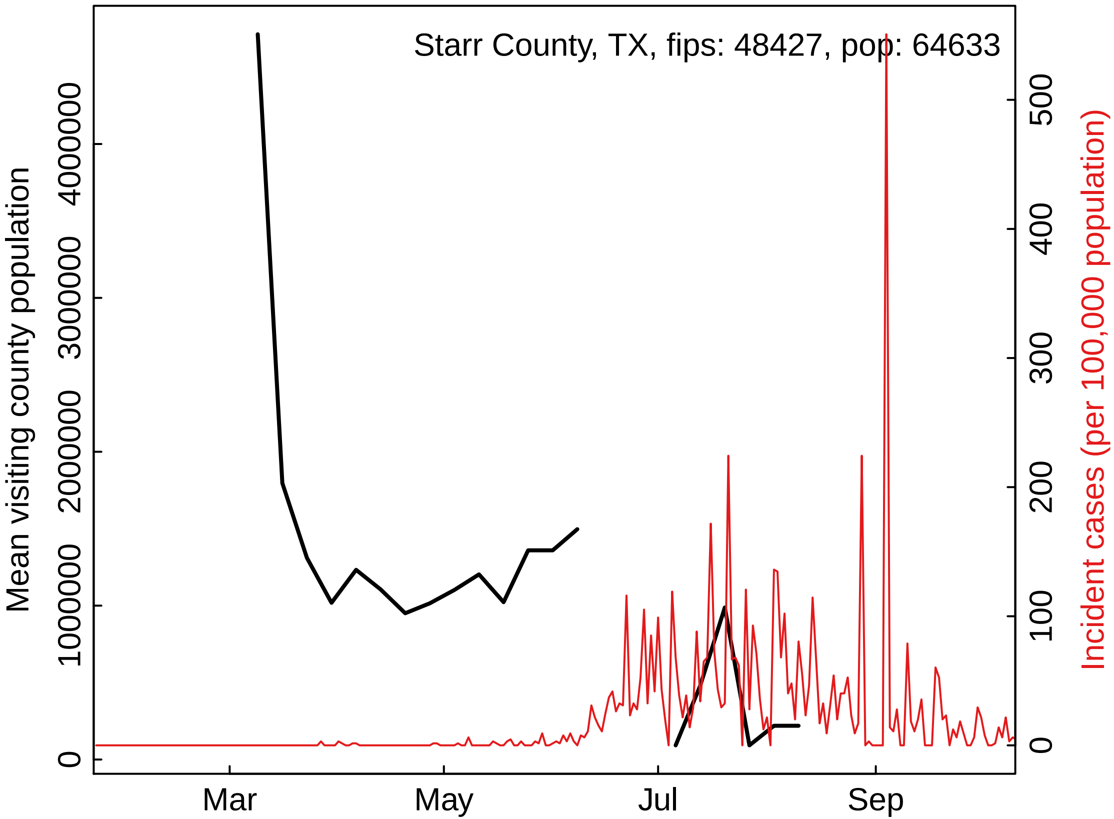

Starr County, TX, fips: 48427, pop: 64633
Mean visiting county population
0
1000000
2000000
3000000
4000000
Mar
May
Jul
Sep
0
100
200
300
400
500
Incident cases (per 100,000 population)

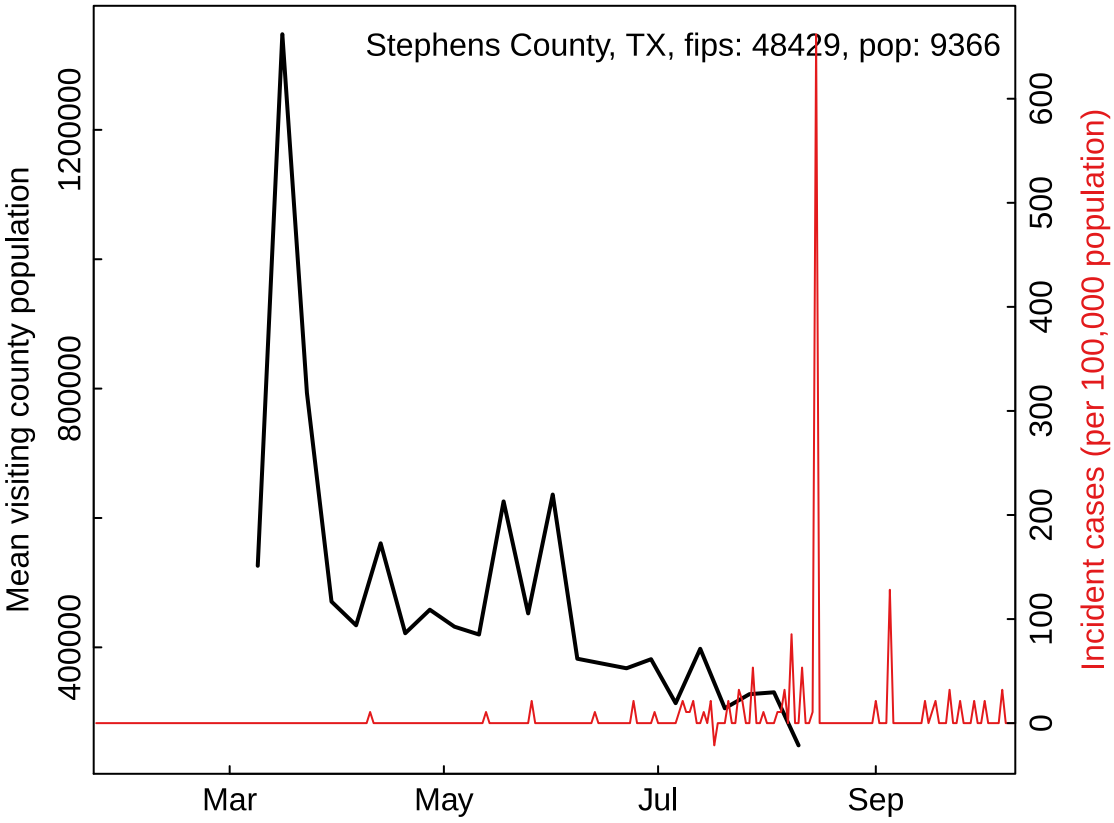

Stephens County, TX, fips: 48429, pop: 9366
Mean visiting county population
400000
800000
1200000
Incident cases (per 100,000 population)
0
100
200
300
400
500
600
Mar
May
Jul
Sep

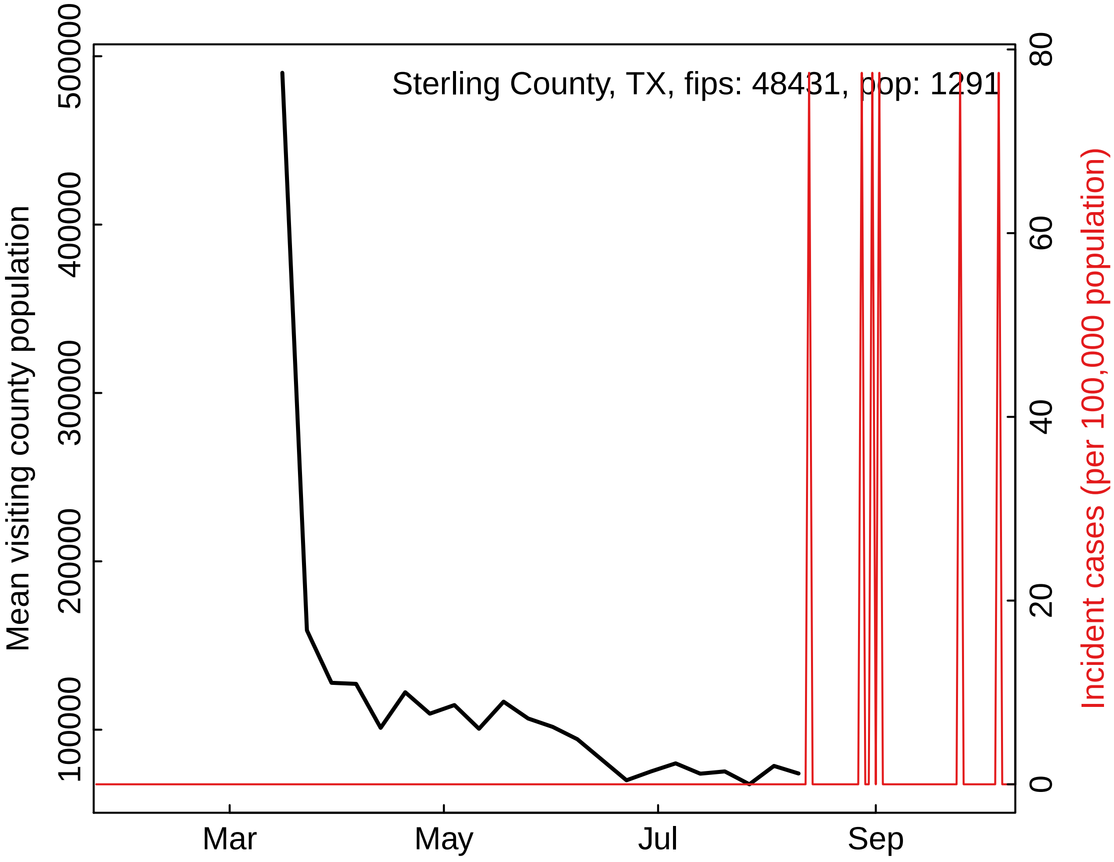
Sterling County, TX, fips: 48431, pop: 1291
Mean visiting county population
100000
200000
300000
400000
500000
Incident cases (per 100,000 population)
0
20
40
60
80
Mar
May
Jul
Sep

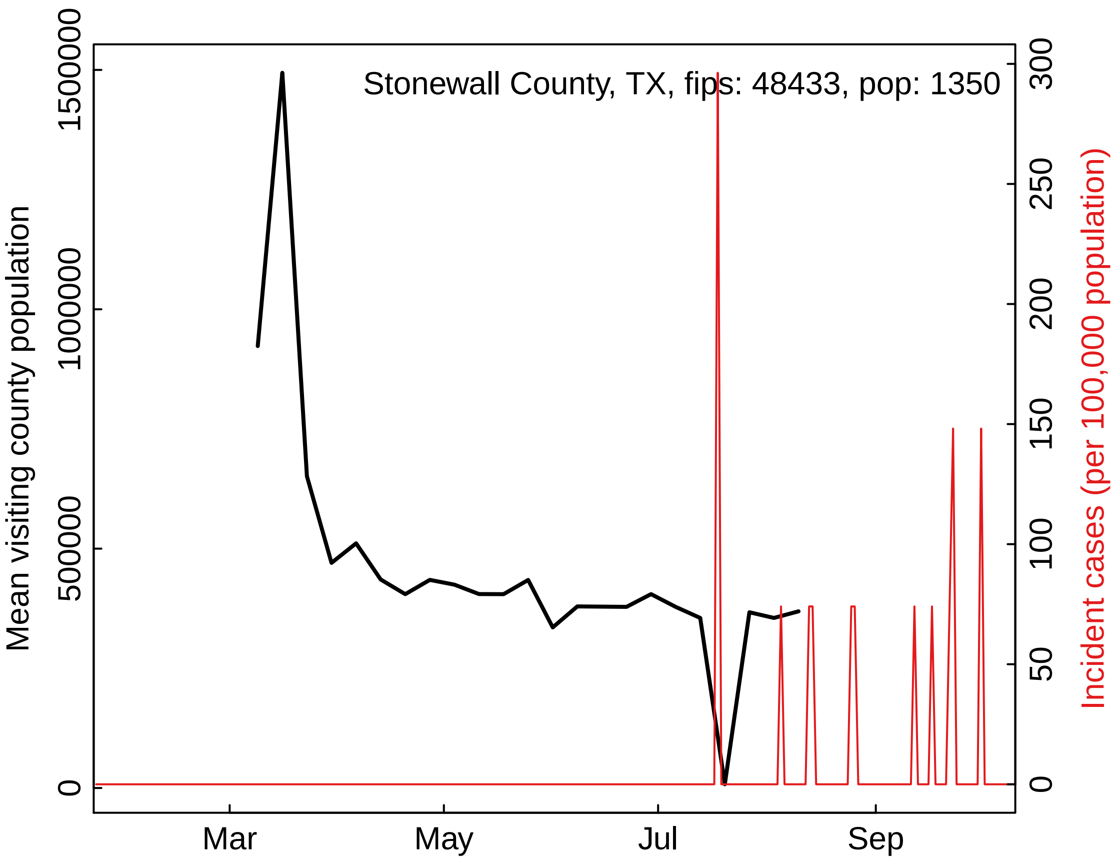

Stonewall County, TX, fips: 48433, pop: 1350
Mean visiting county population
0
500000
1000000
1500000
Incident cases (per 100,000 population)
0
50
100
150
200
250
300
Mar
May
Jul
Sep

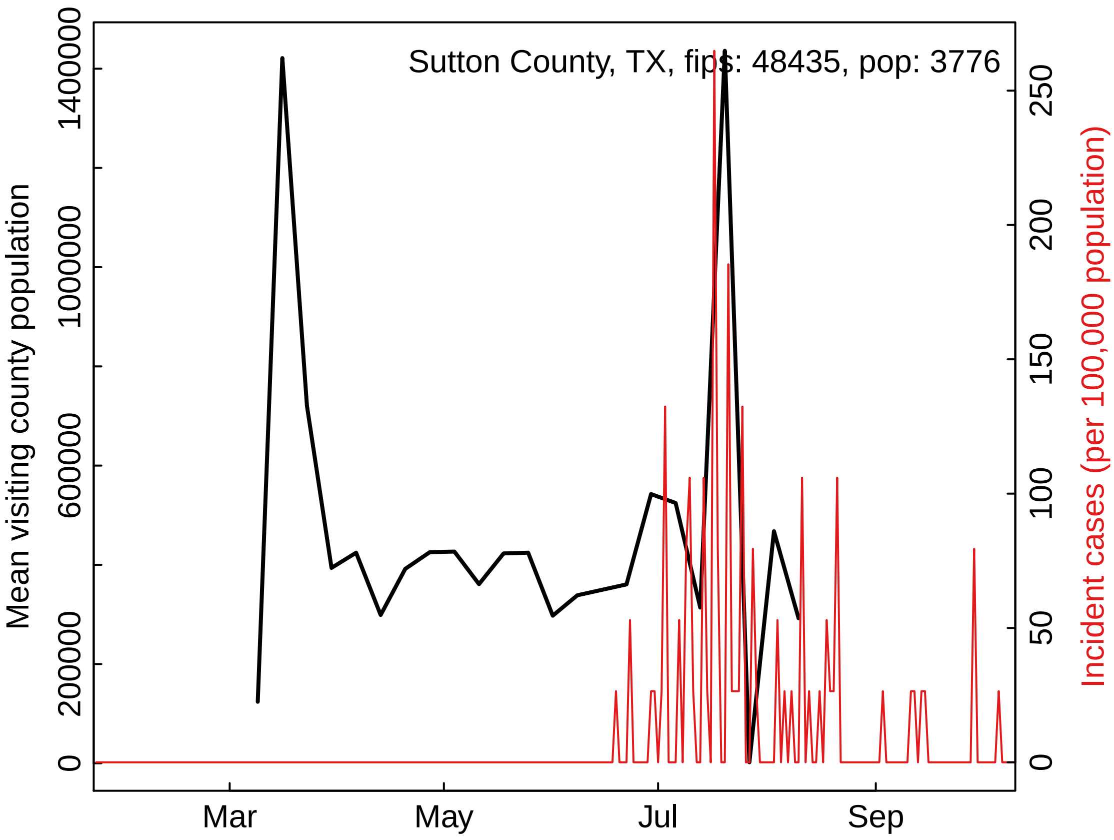

Sutton County, TX, fips: 48435, pop: 3776
Mean visiting county population
0
200000
600000
1000000
1400000
Incident cases (per 100,000 population)
0
50
100
150
200
250
Mar
May
Jul
Sep

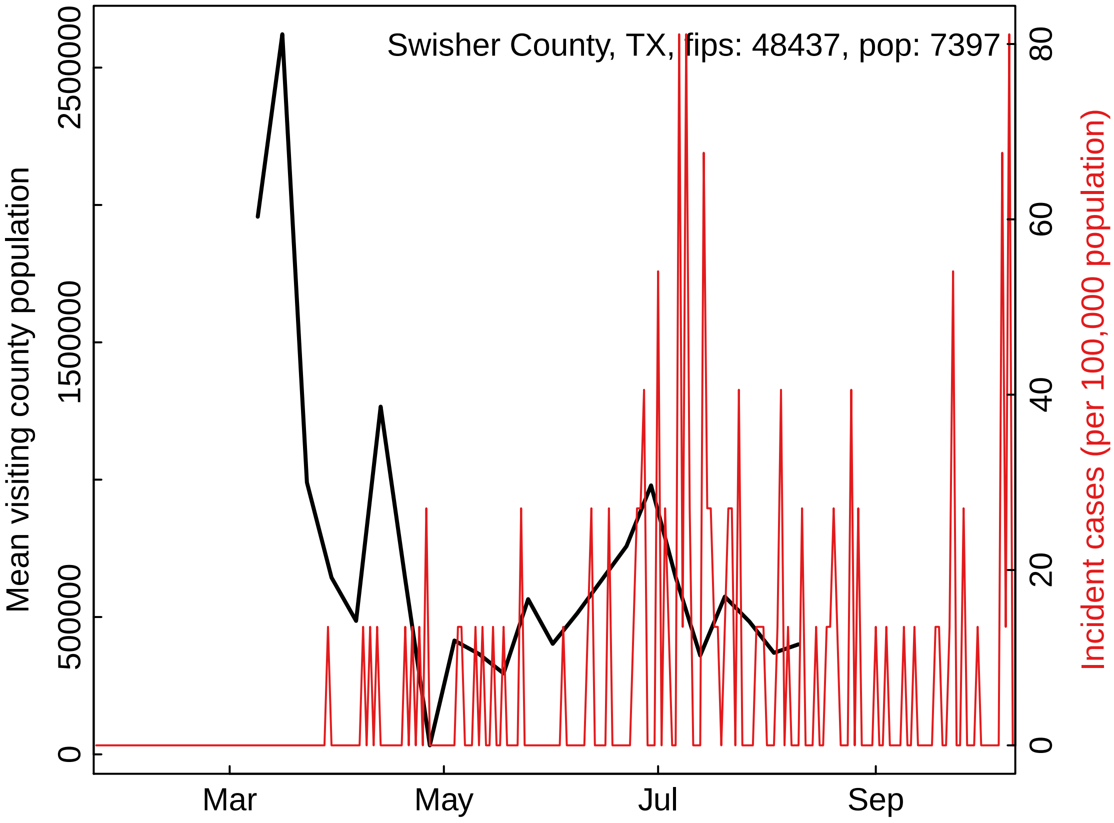
Swisher County, TX, fips: 48437, pop: 7397
Mean visiting county population
0
500000
1500000
2500000
Incident cases (per 100,000 population)
0
20
40
60
80
Mar
May
Jul
Sep

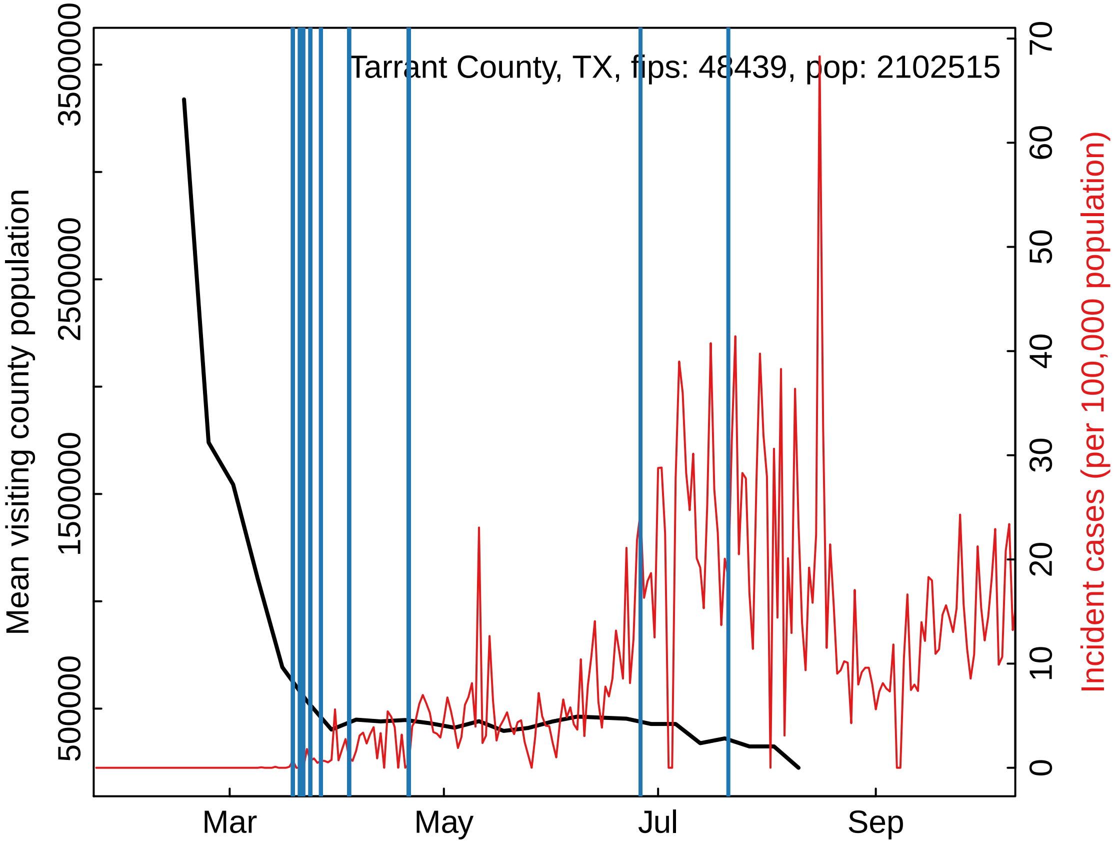
Tarrant County, TX, fips: 48439, pop: 2102515
Mean visiting county population
500000
1500000
2500000
3500000
Incident cases (per 100,000 population)
0
10
20
30
40
50
60
70
Mar
May
Jul
Sep

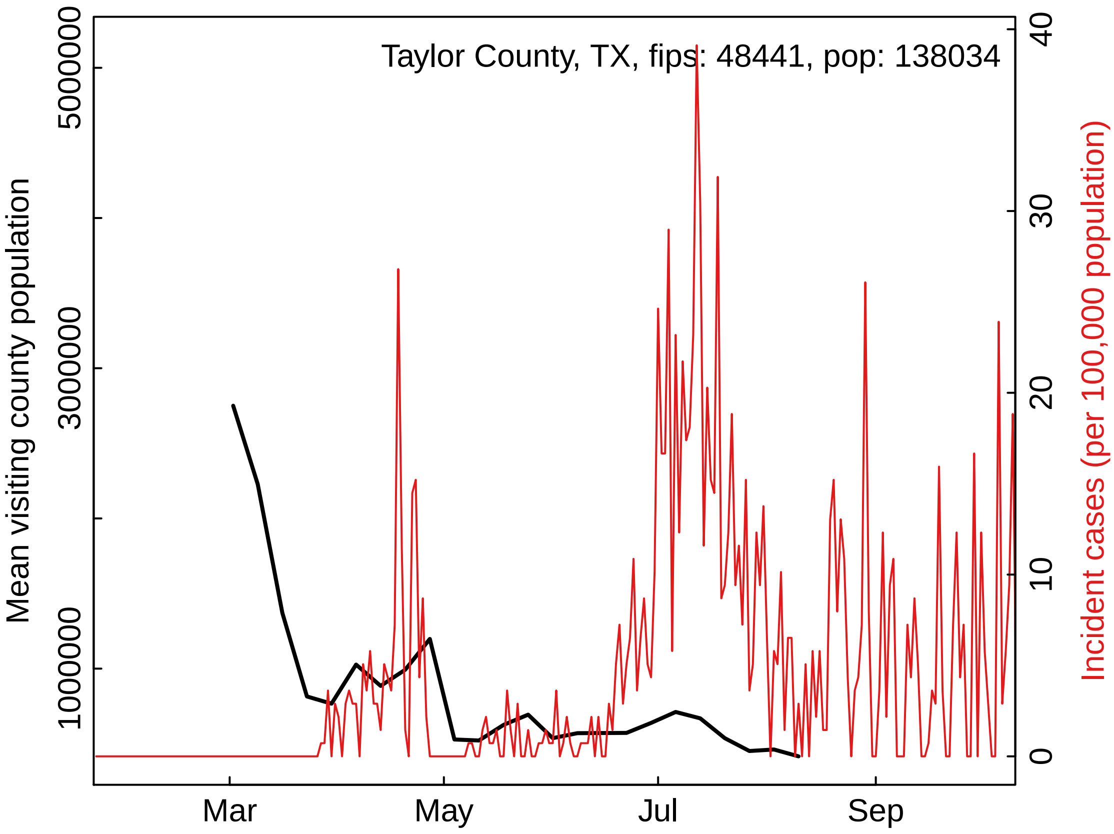

Taylor County, TX, fips: 48441, pop: 138034
Mean visiting county population
Incident cases (per 100,000 population)
1000000
3000000
5000000
0
10
20
30
40
Mar
May
Jul
Sep

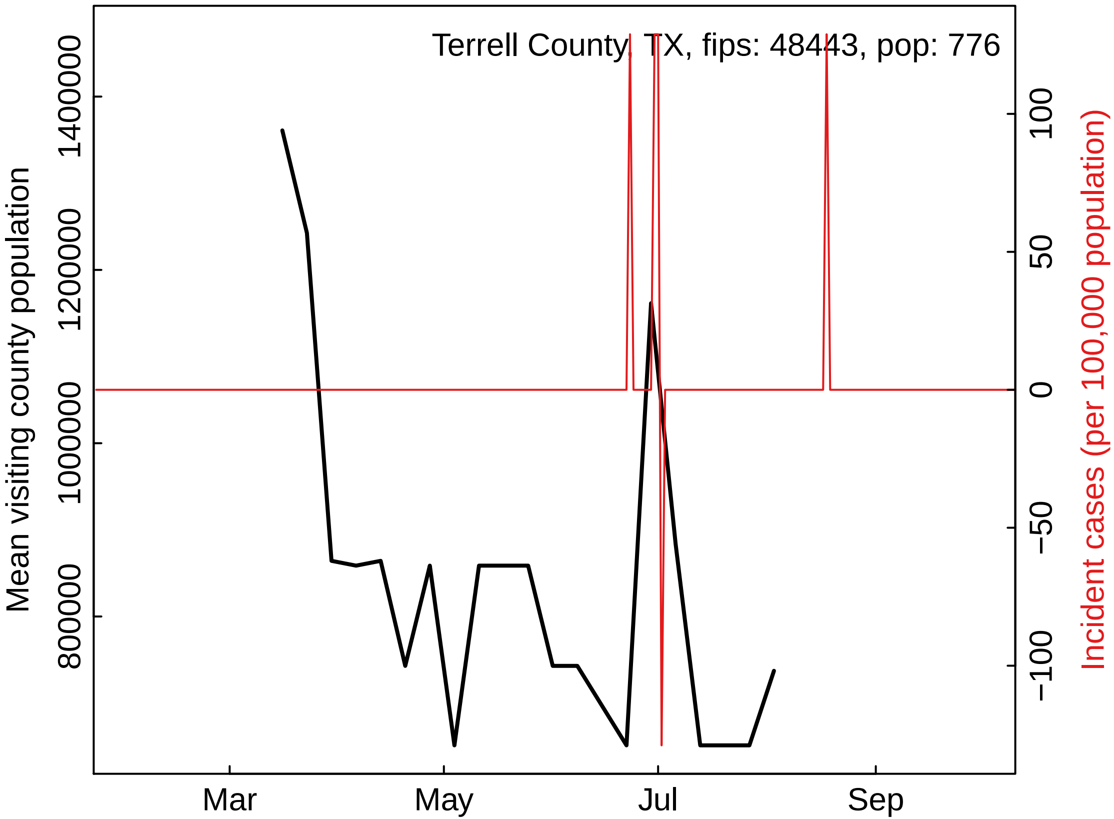
Terrell County, TX, fips: 48443, pop: 776
Mean visiting county population
800000
1000000
1200000
1400000
Incident cases (per 100,000 population)
−100
−50
0
50
100
Mar
May
Jul
Sep

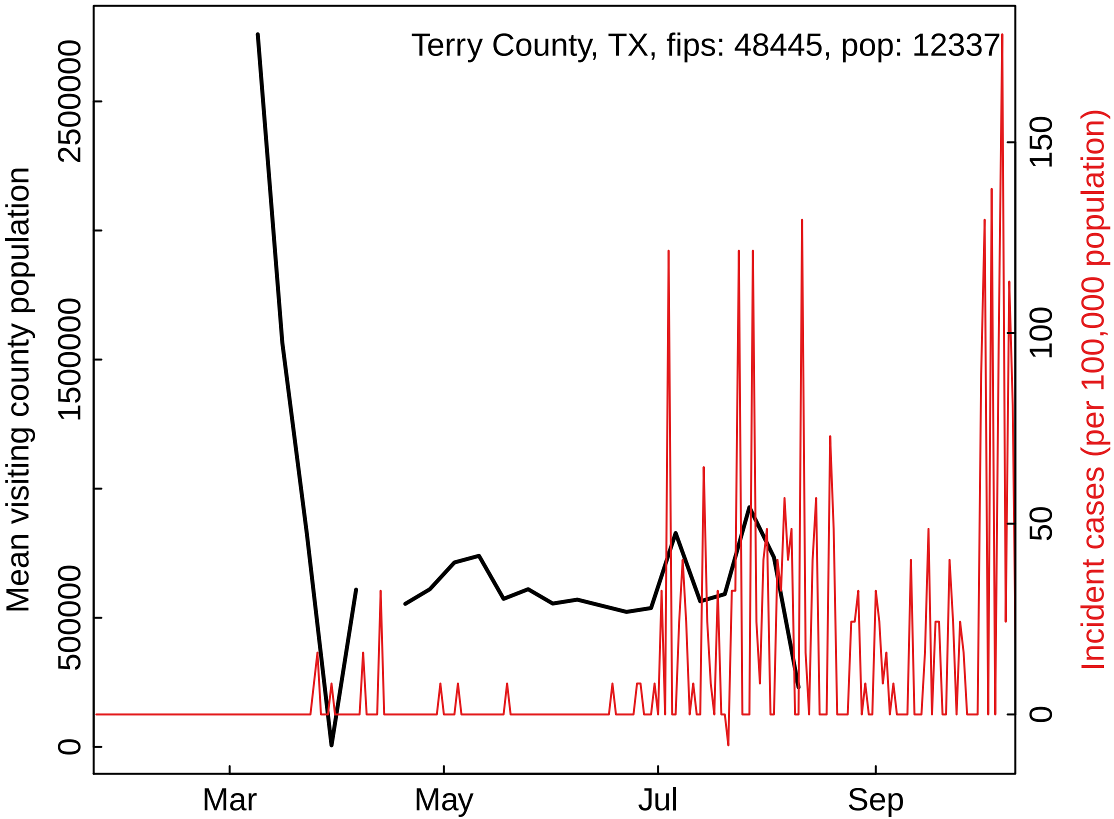

Terry County, TX, fips: 48445, pop: 12337
Mean visiting county population
0
500000
1500000
2500000
Incident cases (per 100,000 population)
0
50
100
150
Mar
May
Jul
Sep

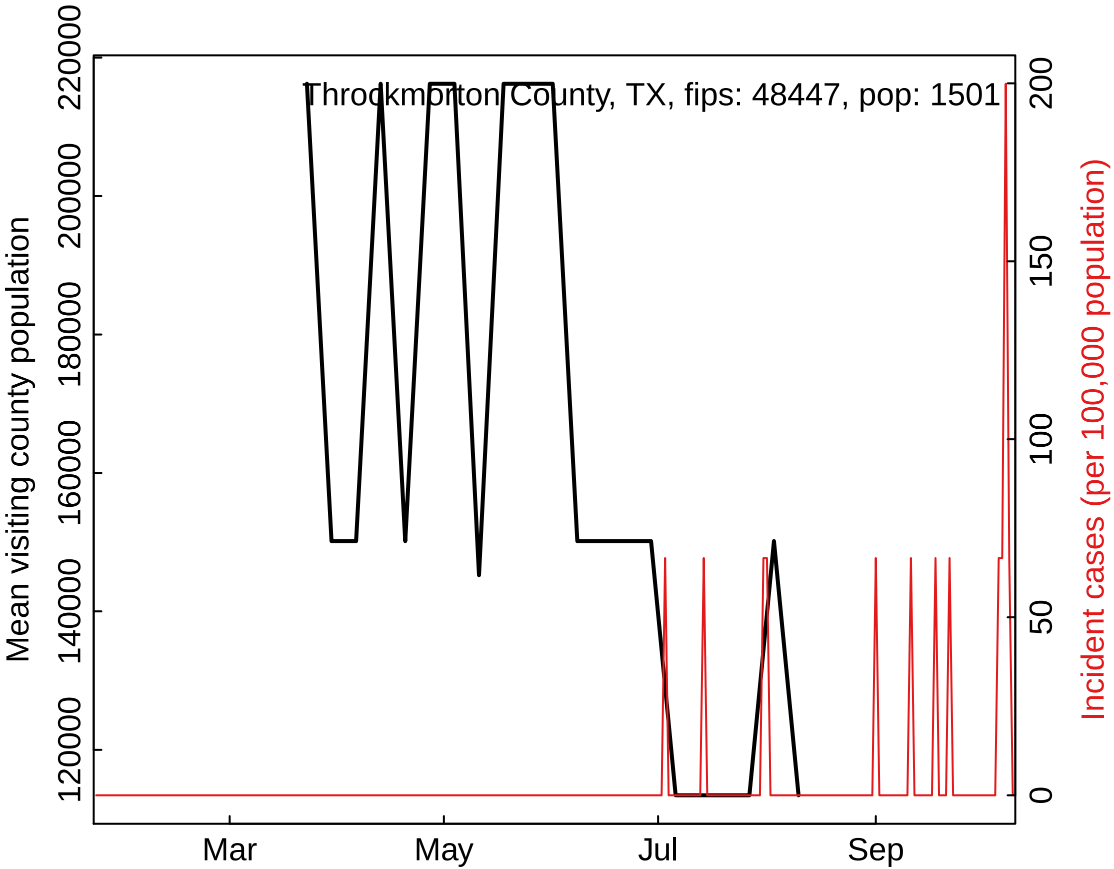

Throckmorton County, TX, fips: 48447, pop: 1501
Mean visiting county population
120000
140000
160000
180000
200000
220000
Incident cases (per 100,000 population)
0
50
100
150
200
Mar
May
Jul
Sep

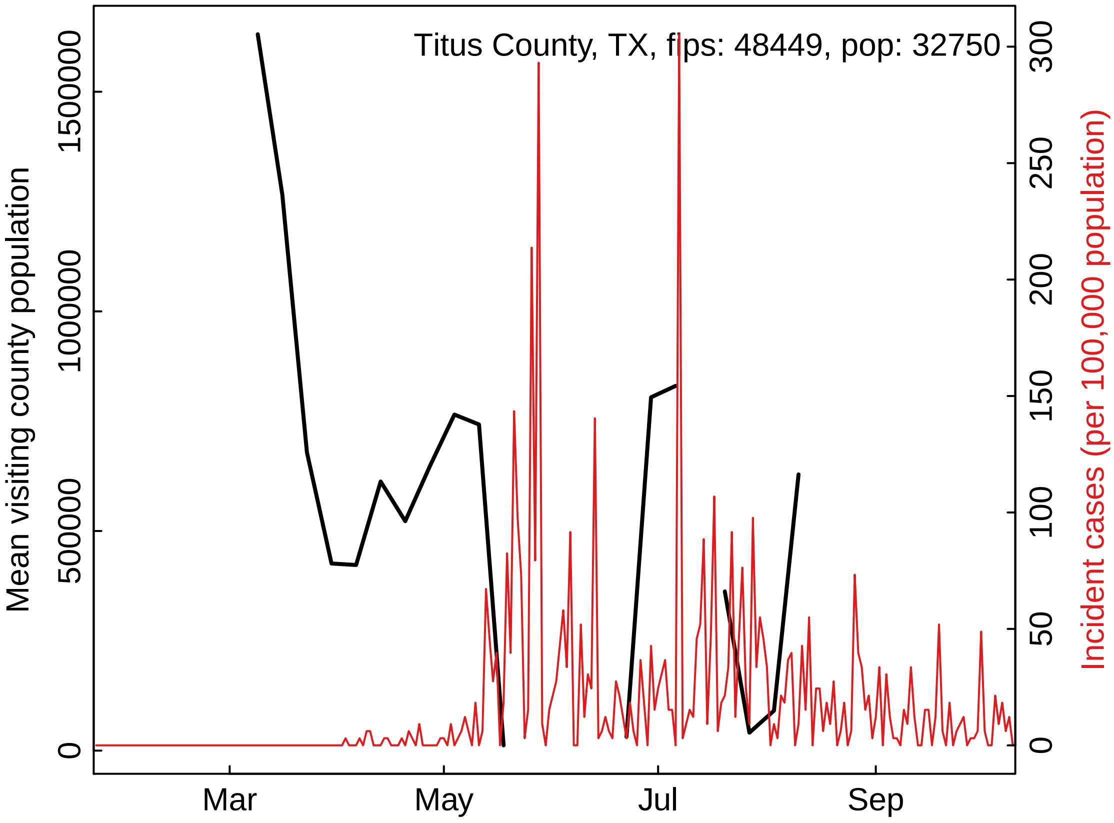

Titus County, TX, fips: 48449, pop: 32750
Mean visiting county population
0
500000
1000000
1500000
Incident cases (per 100,000 population)
0
50
100
150
200
250
300
Mar
May
Jul
Sep

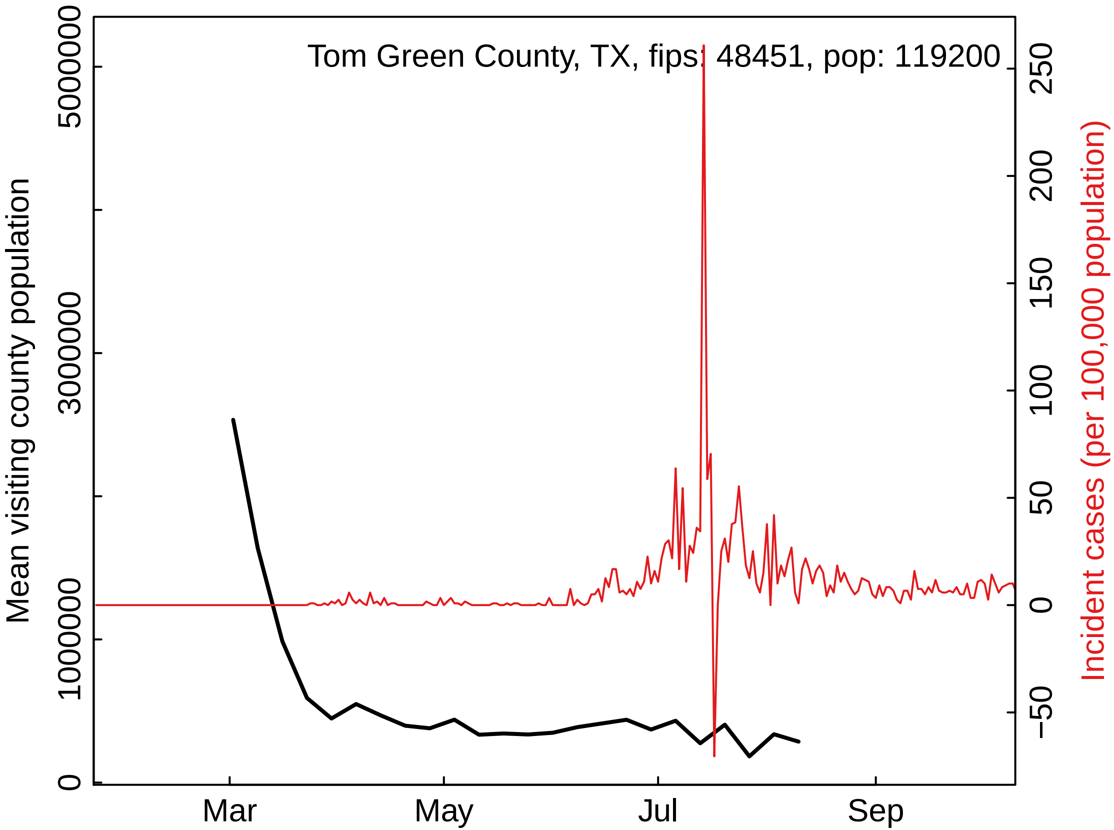
Tom Green County, TX, fips: 48451, pop: 119200
Mean visiting county population
0
1000000
3000000
5000000
Incident cases (per 100,000 population)
−50
0
50
100
150
200
250
Mar
May
Jul
Sep

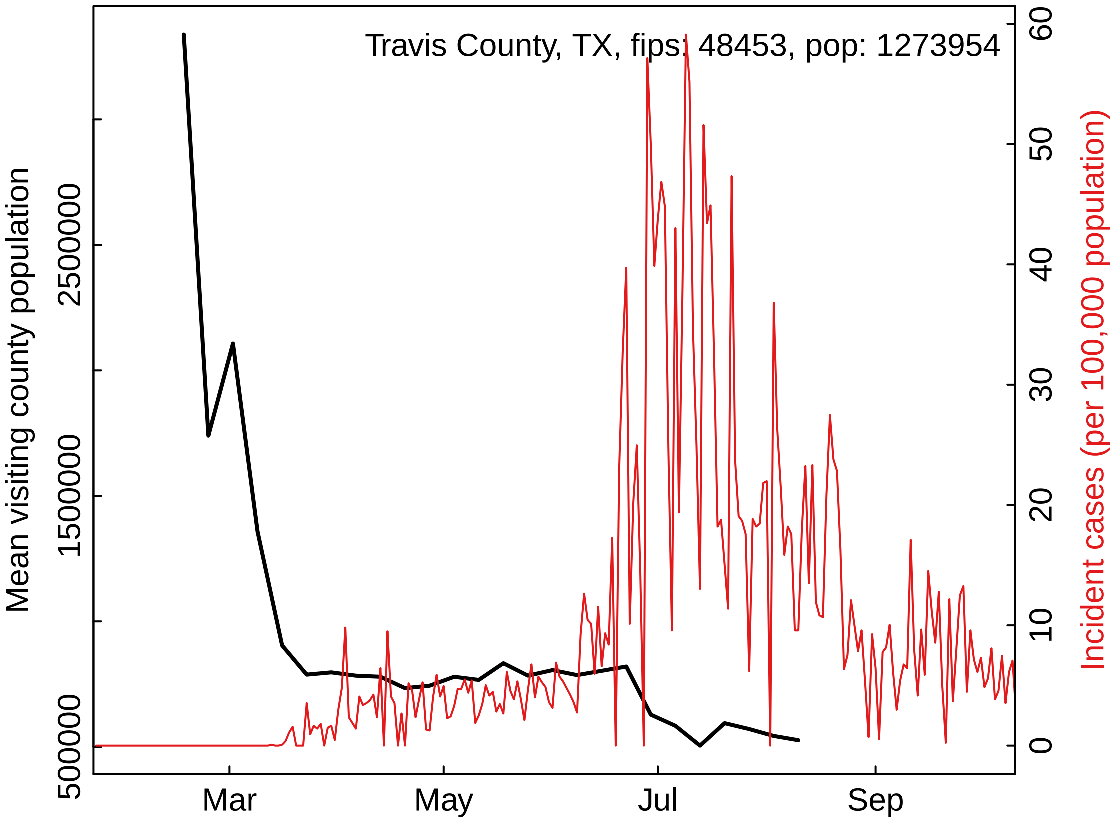
Travis County, TX, fips: 48453, pop: 1273954
Mean visiting county population
500000
1500000
2500000
Mar
May
Jul
Sep
0
10
20
30
40
50
60
Incident cases (per 100,000 population)

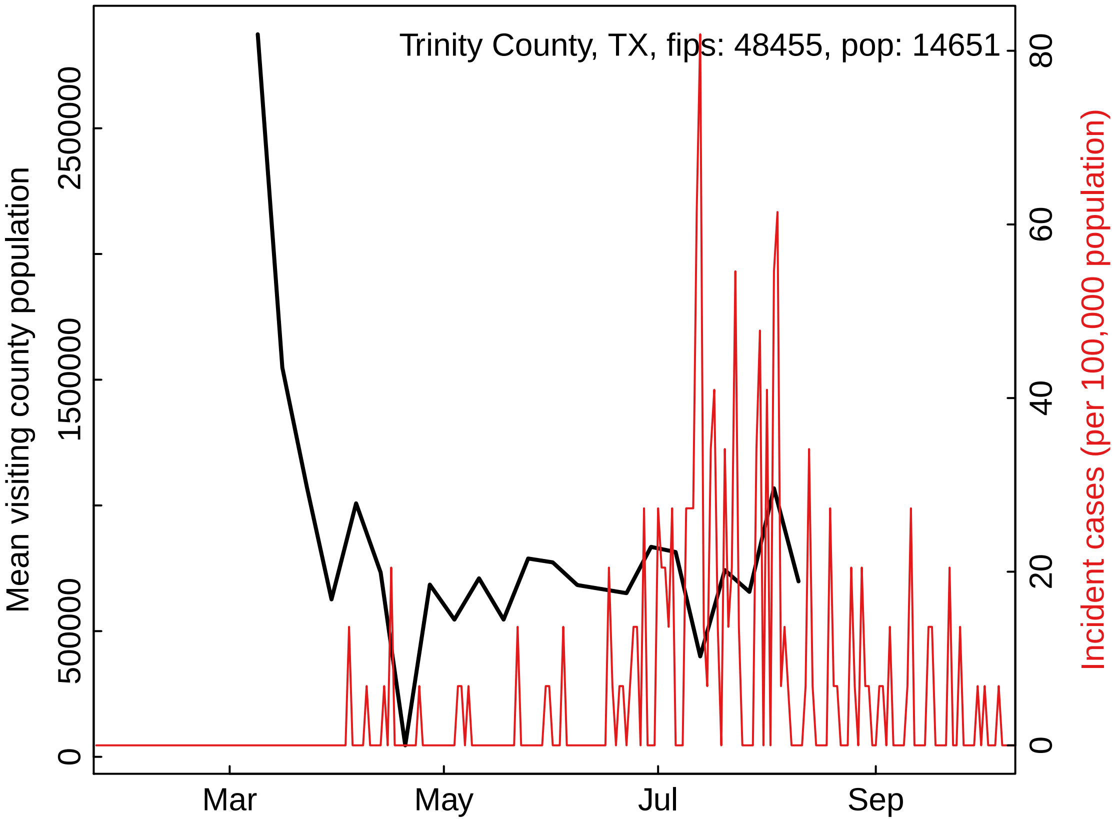

Trinity County, TX, fips: 48455, pop: 14651
Mean visiting county population
0
500000
1500000
2500000
Incident cases (per 100,000 population)
0
20
40
60
80
Mar
May
Jul
Sep

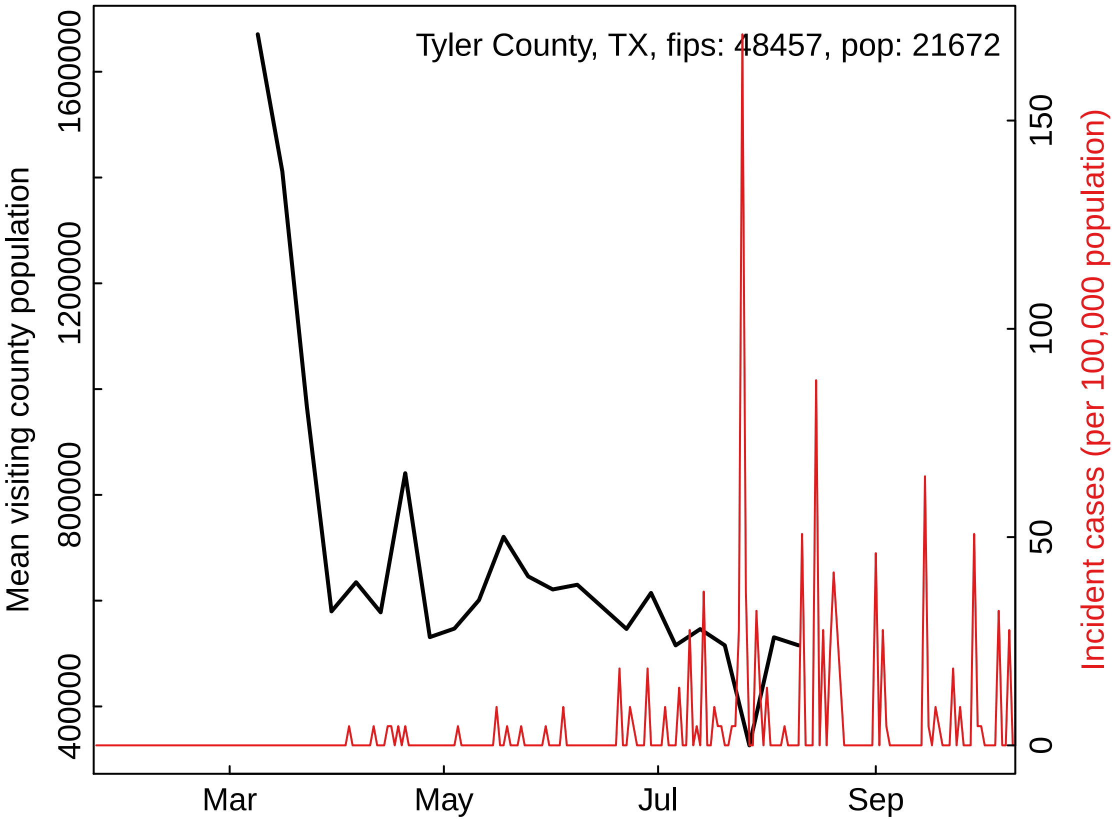

Tyler County, TX, fips: 48457, pop: 21672
Mean visiting county population
400000
800000
1200000
1600000
Incident cases (per 100,000 population)
0
50
100
150
Mar
May
Jul
Sep

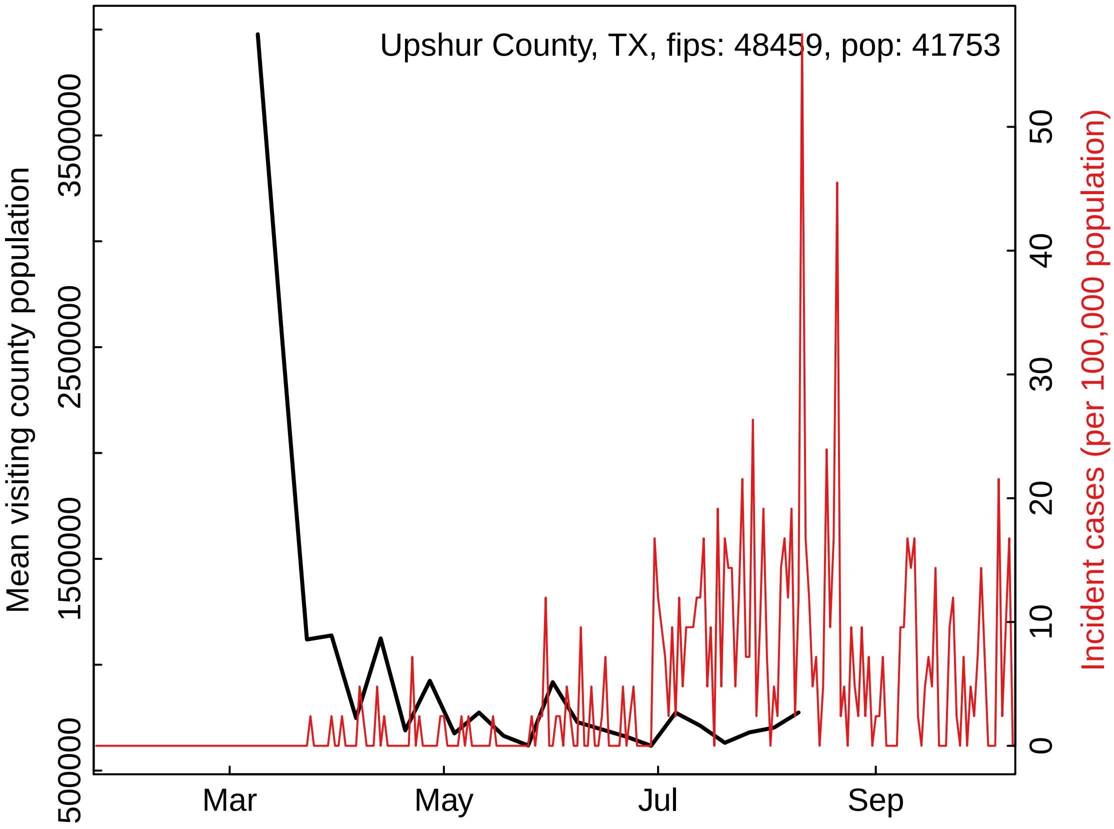

Upshur County, TX, fips: 48459, pop: 41753
Mean visiting county population
5000000
15000000
25000000
35000000
Incident cases (per 100,000 population)
0
10
20
30
40
50
Mar
May
Jul
Sep

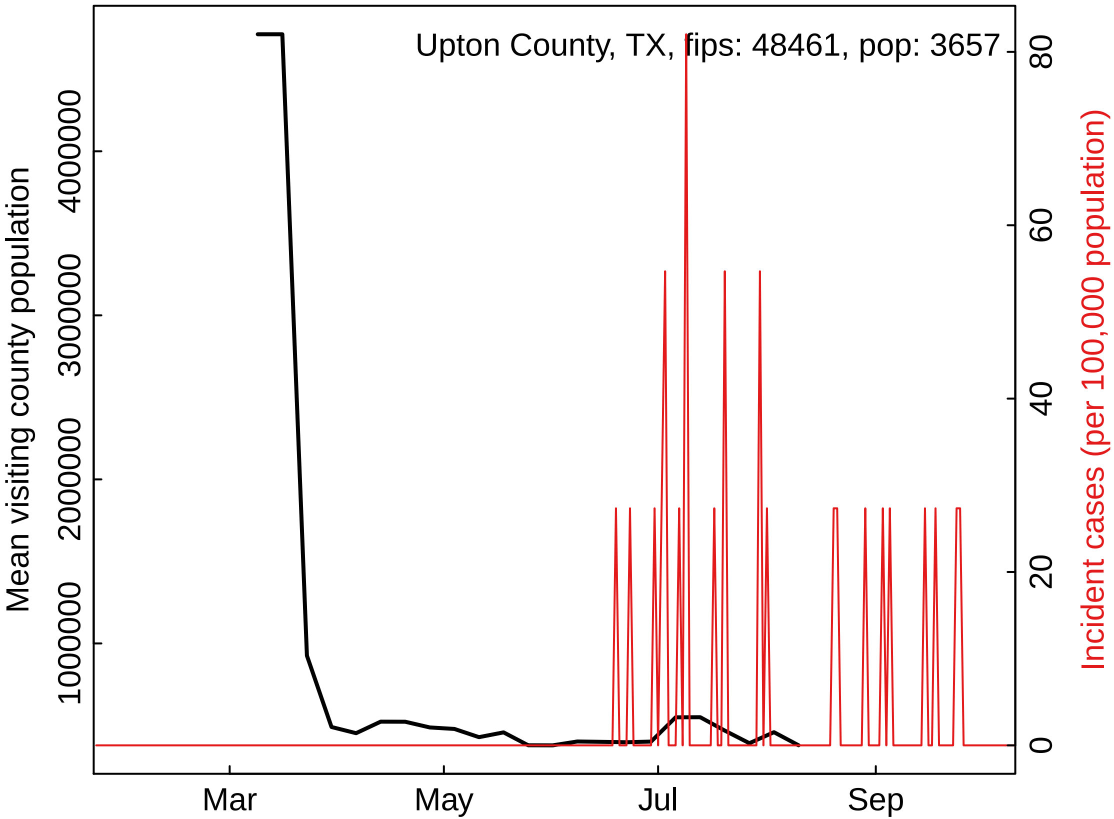

Upton County, TX, fips: 48461, pop: 3657
Mean visiting county population
1000000
2000000
3000000
4000000
Incident cases (per 100,000 population)
0
20
40
60
80
Mar
May
Jul
Sep

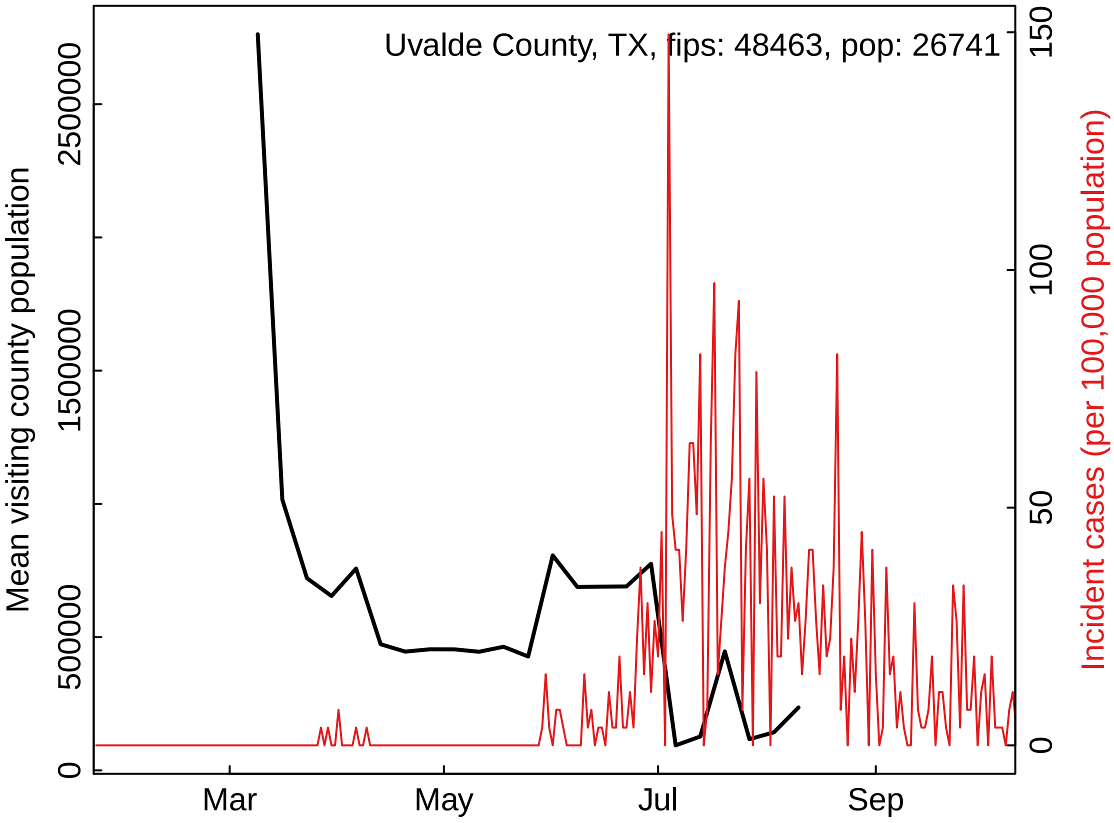

Uvalde County, TX, fips: 48463, pop: 26741
Mean visiting county population
Incident cases (per 100,000 population)
0
500000
1500000
2500000
0
50
100
150
Mar
May
Jul
Sep

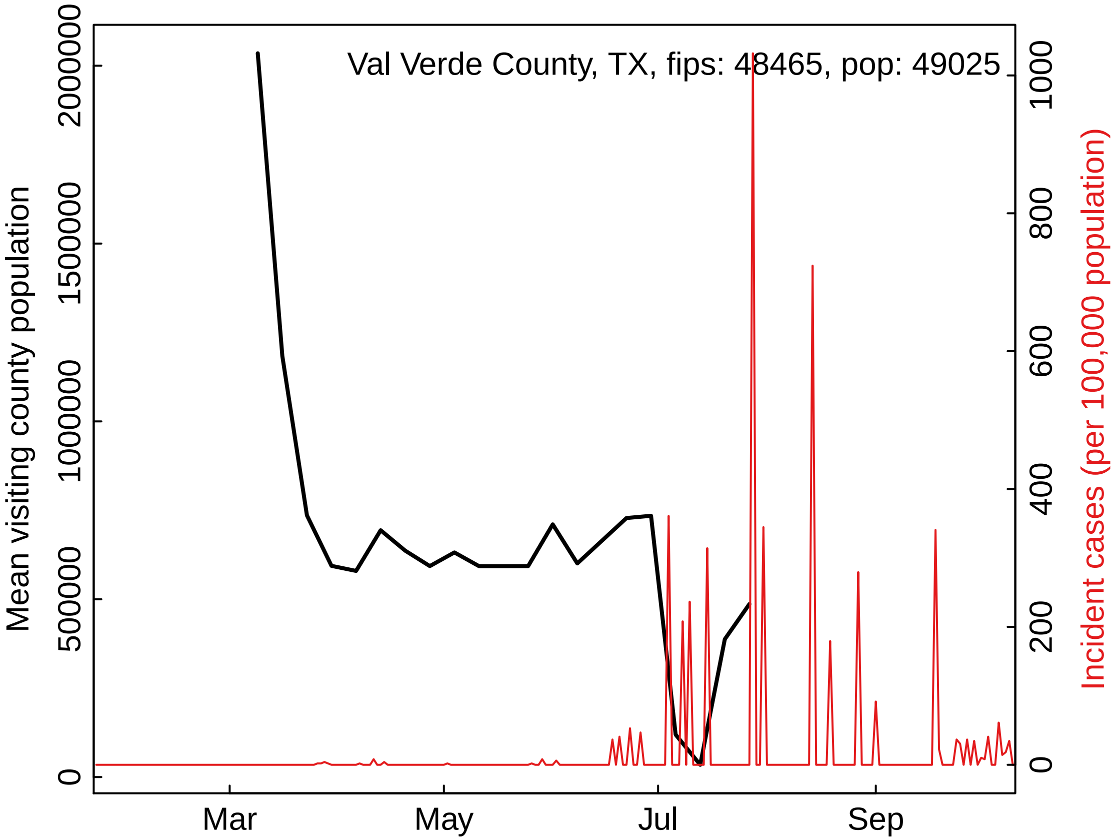

Val Verde County, TX, fips: 48465, pop: 49025
Mean visiting county population
Incident cases (per 100,000 population)
0
500000
1000000
1500000
2000000
0
200
400
600
800
1000
Mar
May
Jul
Sep

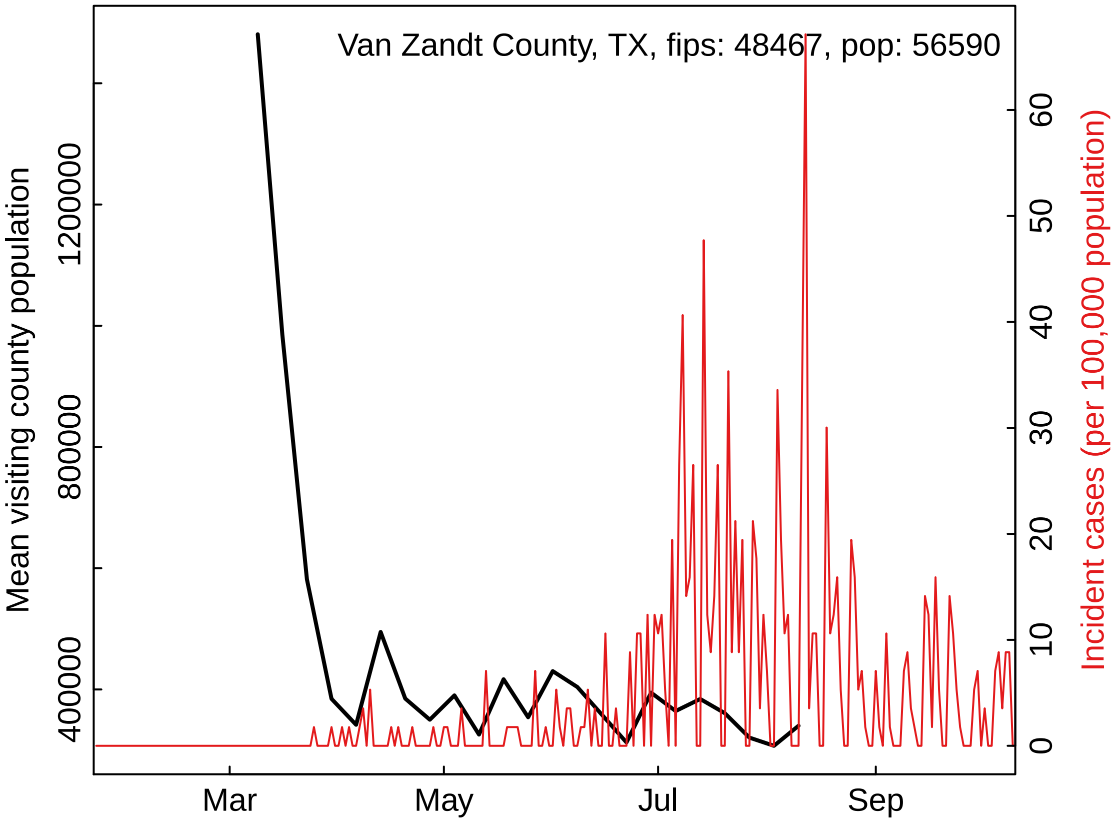

Van Zandt County, TX, fips: 48467, pop: 56590
Mean visiting county population
400000
800000
1200000
0
10
20
30
40
50
60
Incident cases (per 100,000 population)
Mar
May
Jul
Sep

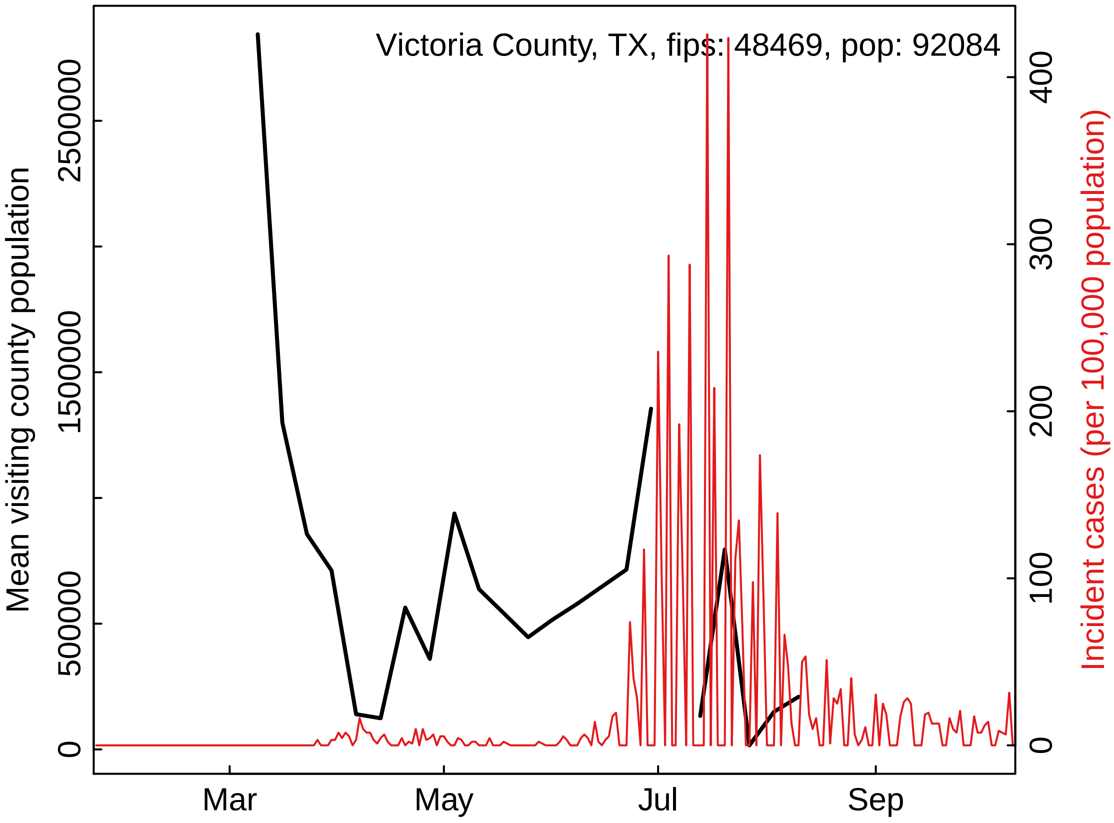

Victoria County, TX, fips: 48469, pop: 92084
Mean visiting county population
0
500000
1500000
2500000
Incident cases (per 100,000 population)
0
100
200
300
400
Mar
May
Jul
Sep

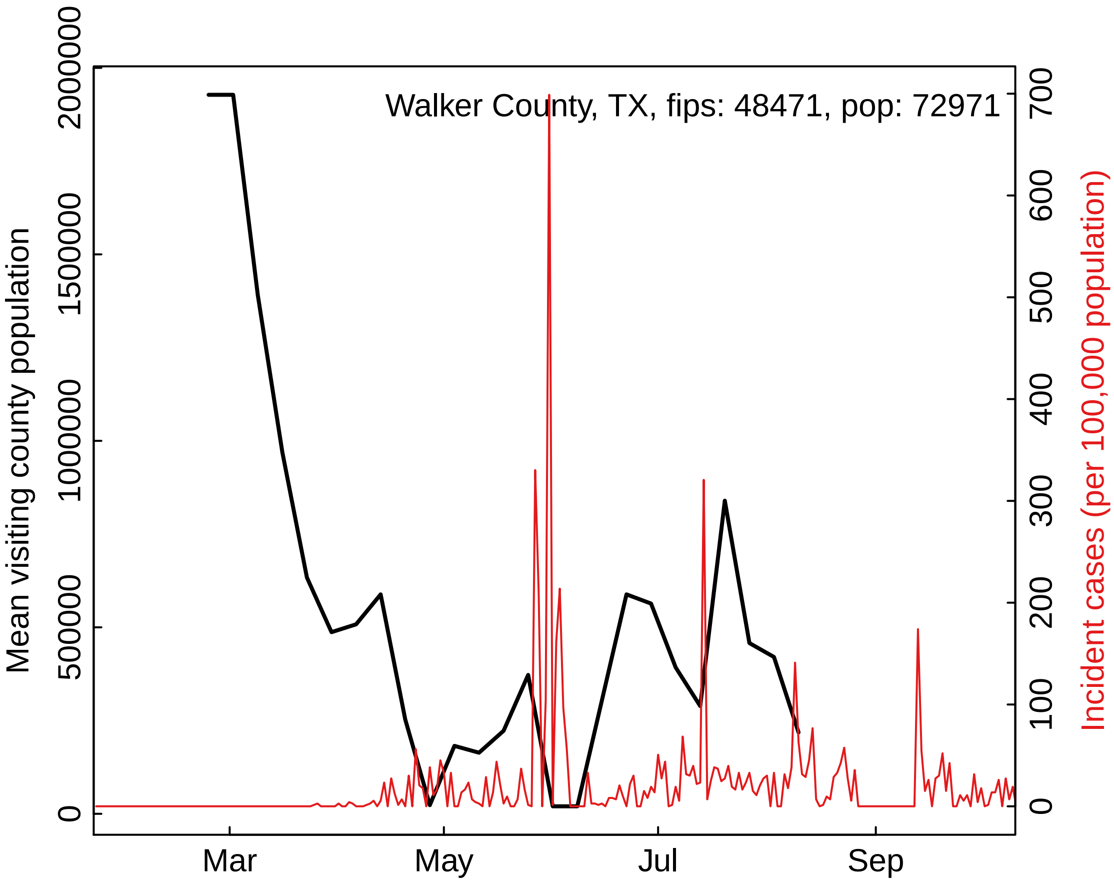

Walker County, TX, fips: 48471, pop: 72971
Mean visiting county population
0
500000
1000000
1500000
2000000
Incident cases (per 100,000 population)
0
100
200
300
400
500
600
700
Mar
May
Jul
Sep

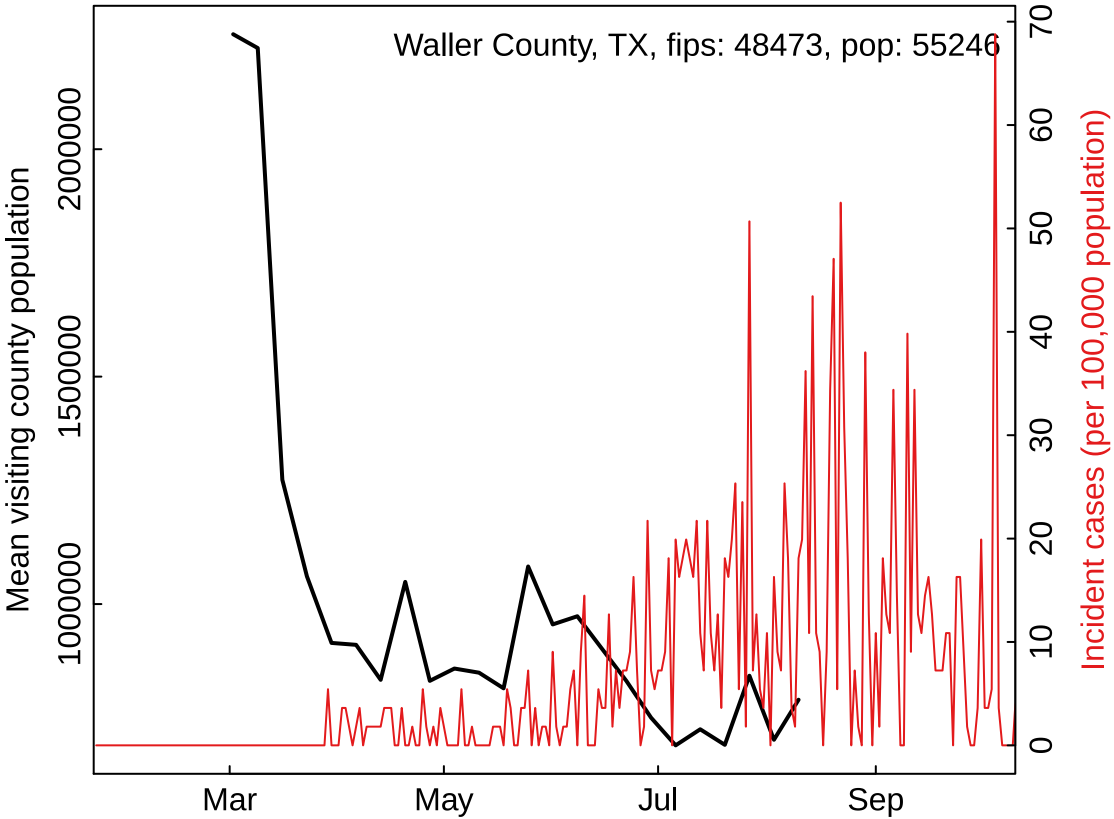

Waller County, TX, fips: 48473, pop: 55246
Mean visiting county population
1000000
1500000
2000000
Incident cases (per 100,000 population)
0
10
20
30
40
50
60
70
Mar
May
Jul
Sep

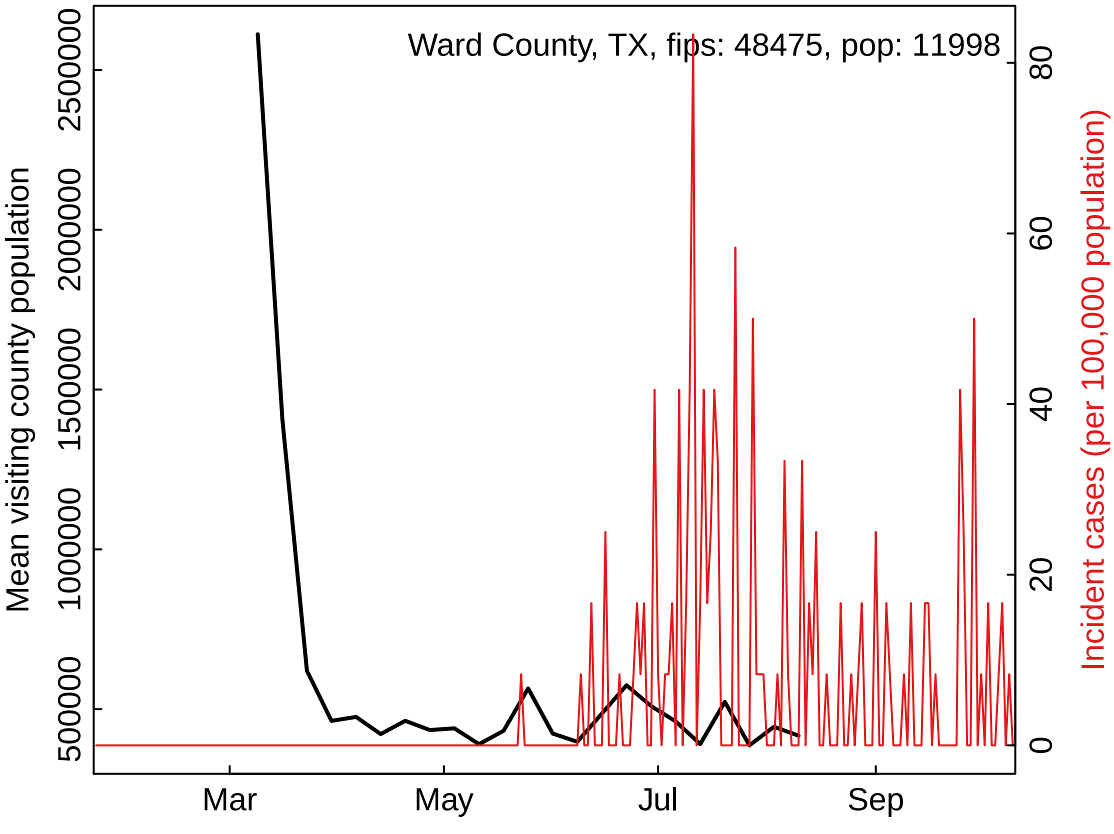

Ward County, TX, fips: 48475, pop: 11998
Mean visiting county population
500000
1000000
1500000
2000000
2500000
Incident cases (per 100,000 population)
0
20
40
60
80
Mar
May
Jul
Sep

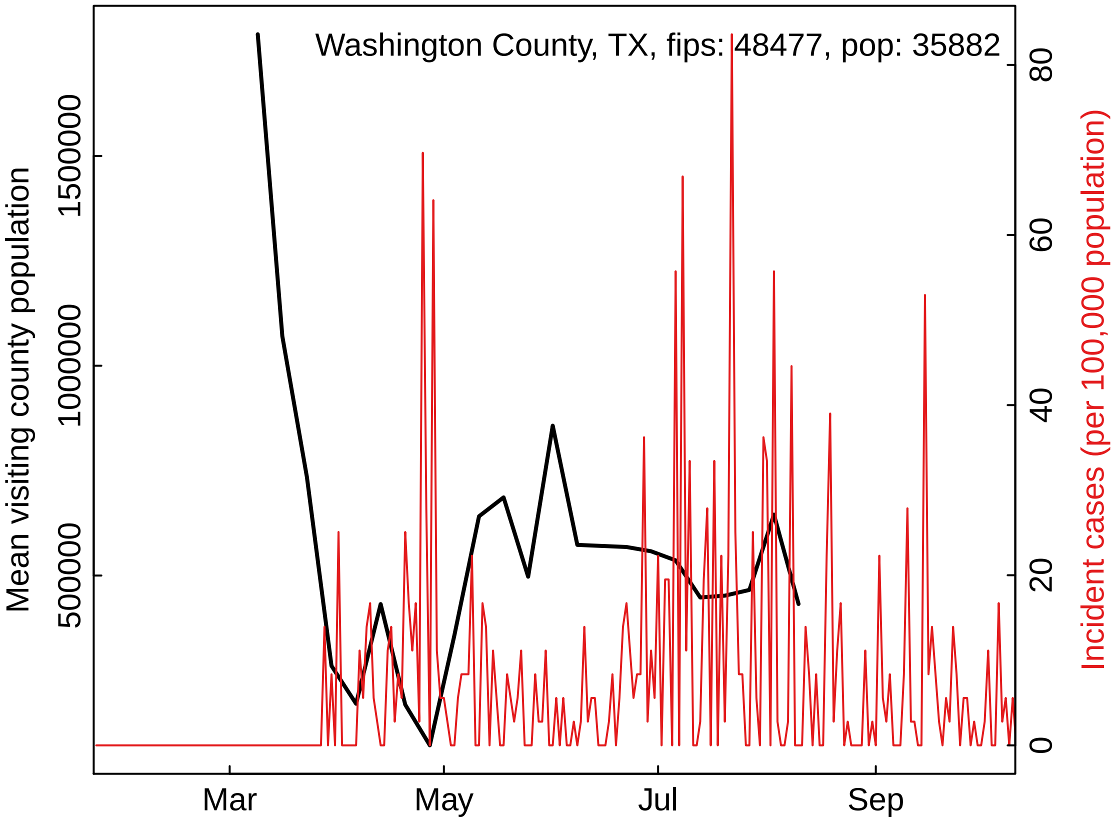
Washington County, TX, fips: 48477, pop: 35882
Mean visiting county population
500000
1000000
1500000
Mar
May
Jul
Sep
0
20
40
60
80
Incident cases (per 100,000 population)

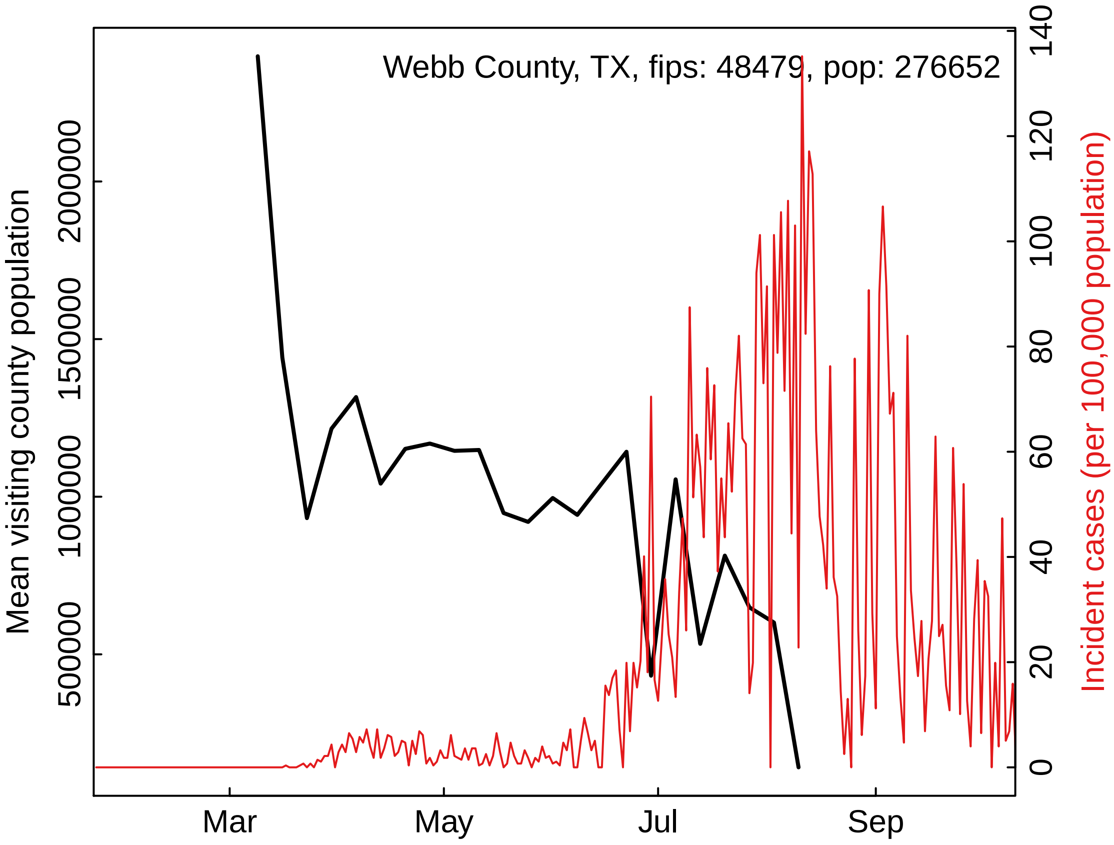
Webb County, TX, fips: 48479, pop: 276652
Mean visiting county population
500000
1000000
1500000
2000000
Incident cases (per 100,000 population)
0
20
40
60
80
100
120
140
Mar
May
Jul
Sep

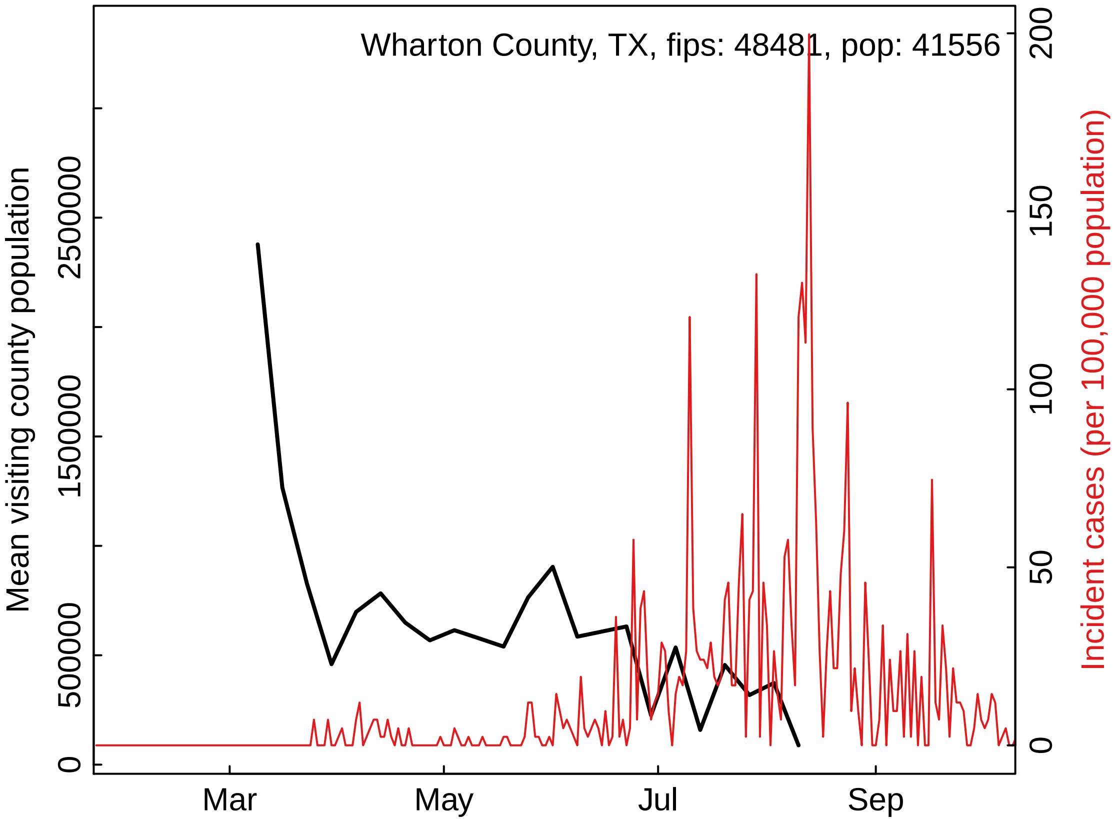

Wharton County, TX, fips: 48481, pop: 41556
Mean visiting county population
0
500000
1500000
2500000
Incident cases (per 100,000 population)
0
50
100
150
200
Mar
May
Jul
Sep

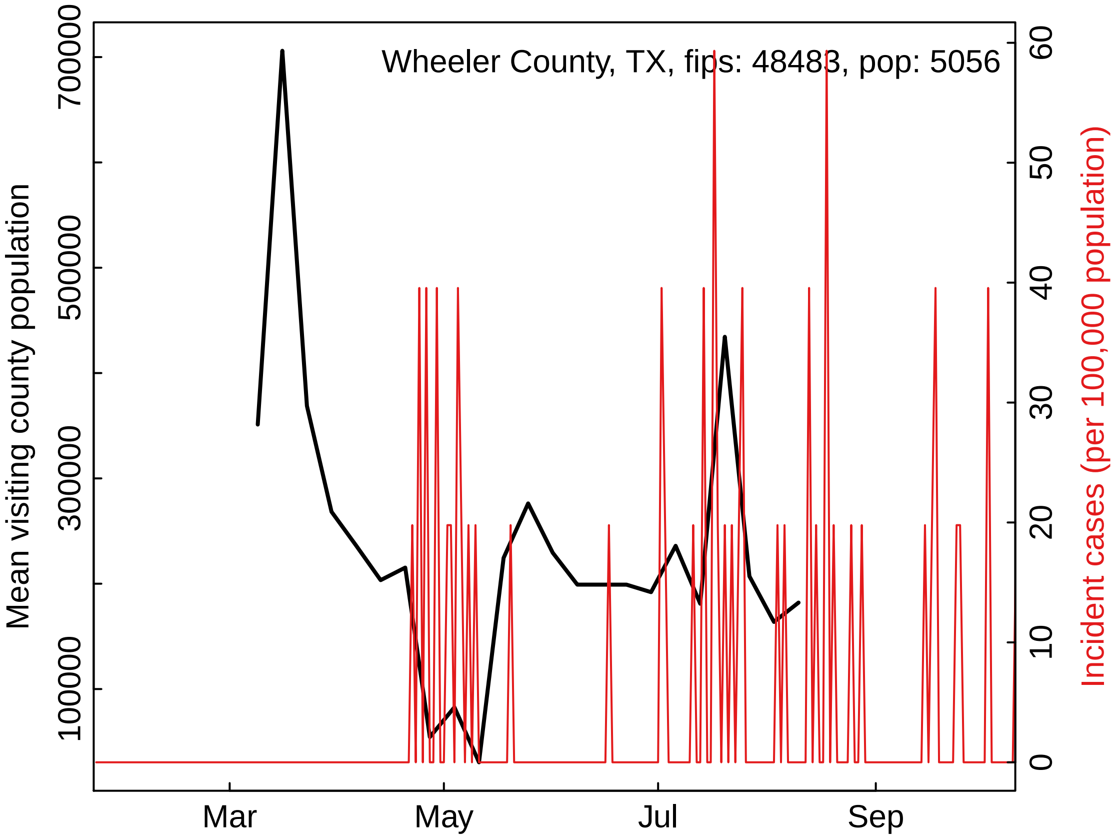
Wheeler County, TX, fips: 48483, pop: 5056
Mean visiting county population
100000
300000
500000
700000
Incident cases (per 100,000 population)
0
10
20
30
40
50
60
Mar
May
Jul
Sep

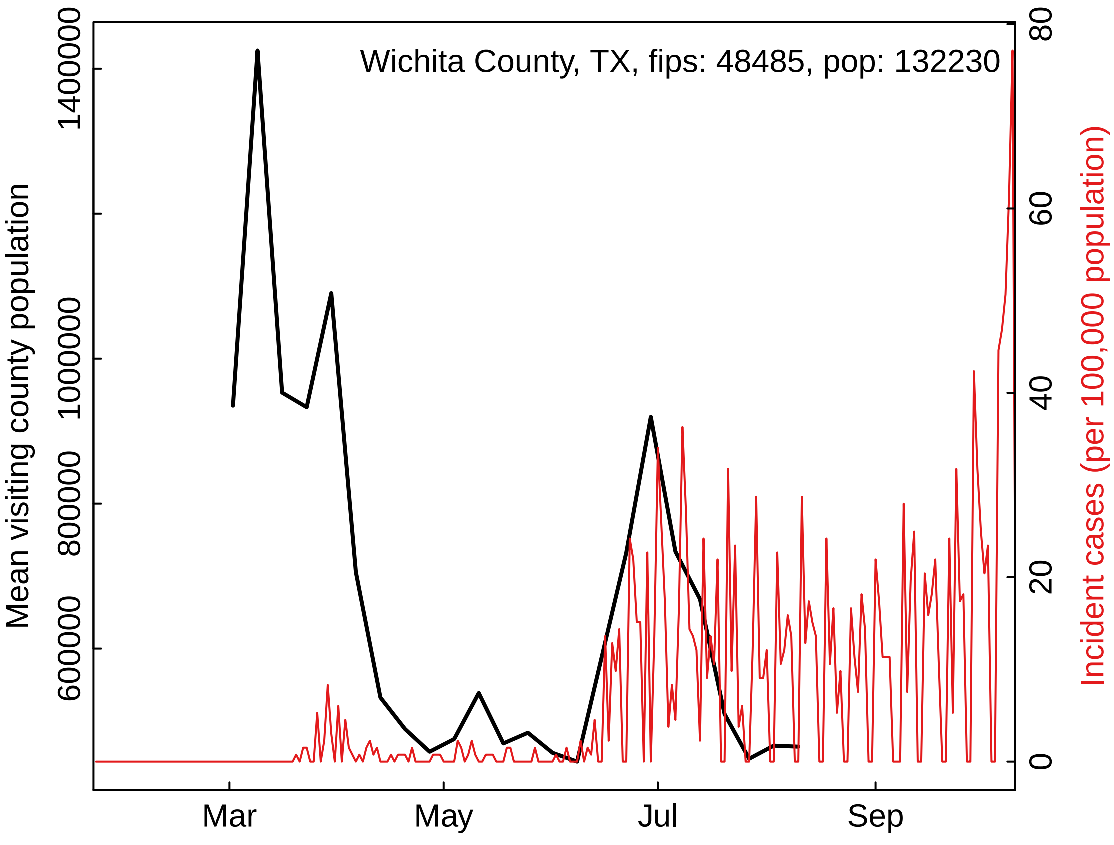

Wichita County, TX, fips: 48485, pop: 132230
Mean visiting county population
600000
800000
1000000
1400000
Incident cases (per 100,000 population)
0
20
40
60
80
Mar
May
Jul
Sep

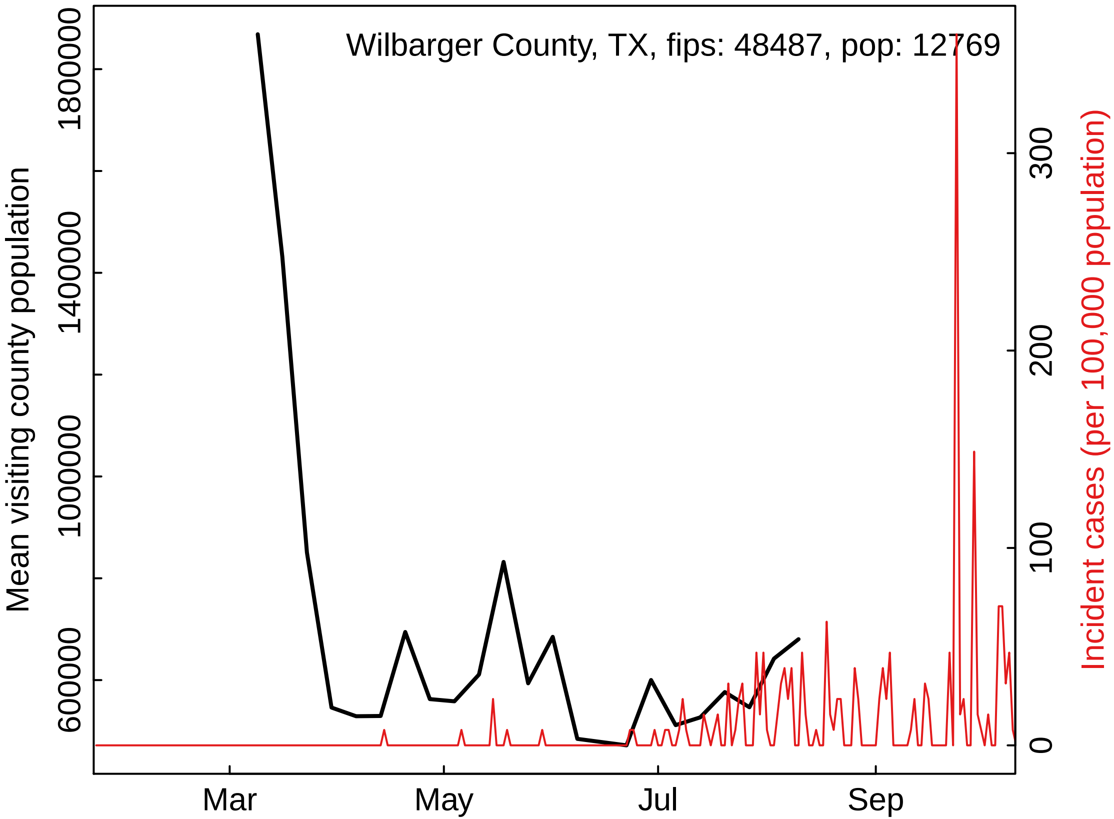

Wilbarger County, TX, fips: 48487, pop: 12769
Mean visiting county population
600000
1000000
1400000
1800000
Incident cases (per 100,000 population)
0
100
200
300
Mar
May
Jul
Sep

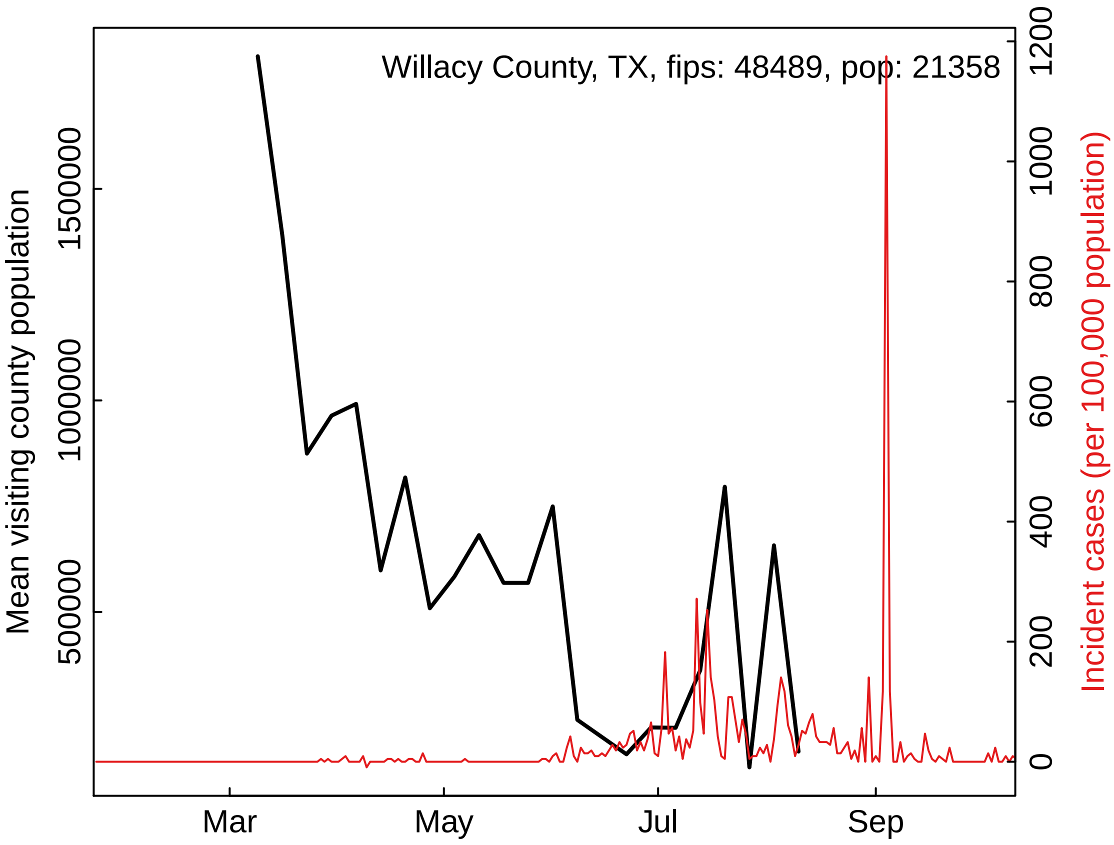

Willacy County, TX, fips: 48489, pop: 21358
Mean visiting county population
500000
1000000
1500000
Incident cases (per 100,000 population)
0
200
400
600
800
1000
1200
Mar
May
Jul
Sep

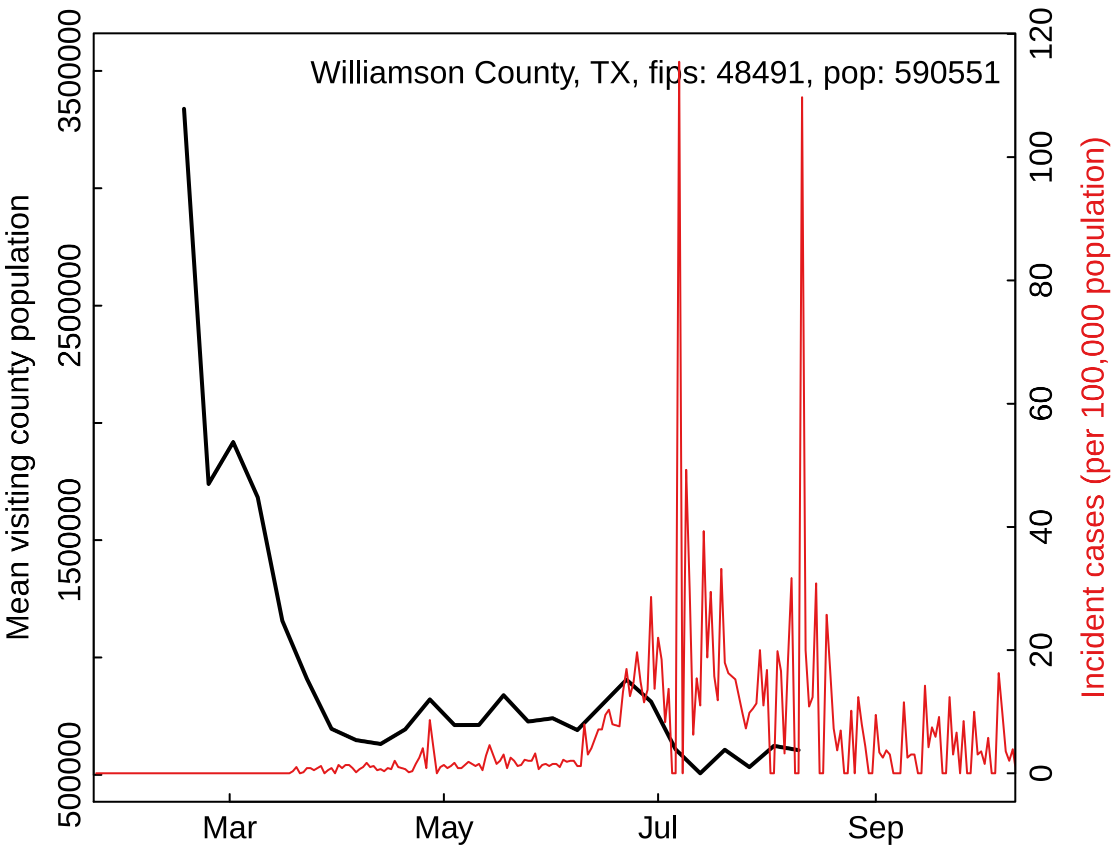
Williamson County, TX, fips: 48491, pop: 590551
Mean visiting county population
5000000
1500000
2500000
3500000
Mar
May
Jul
Sep
0
20
40
60
80
100
120
Incident cases (per 100,000 population)

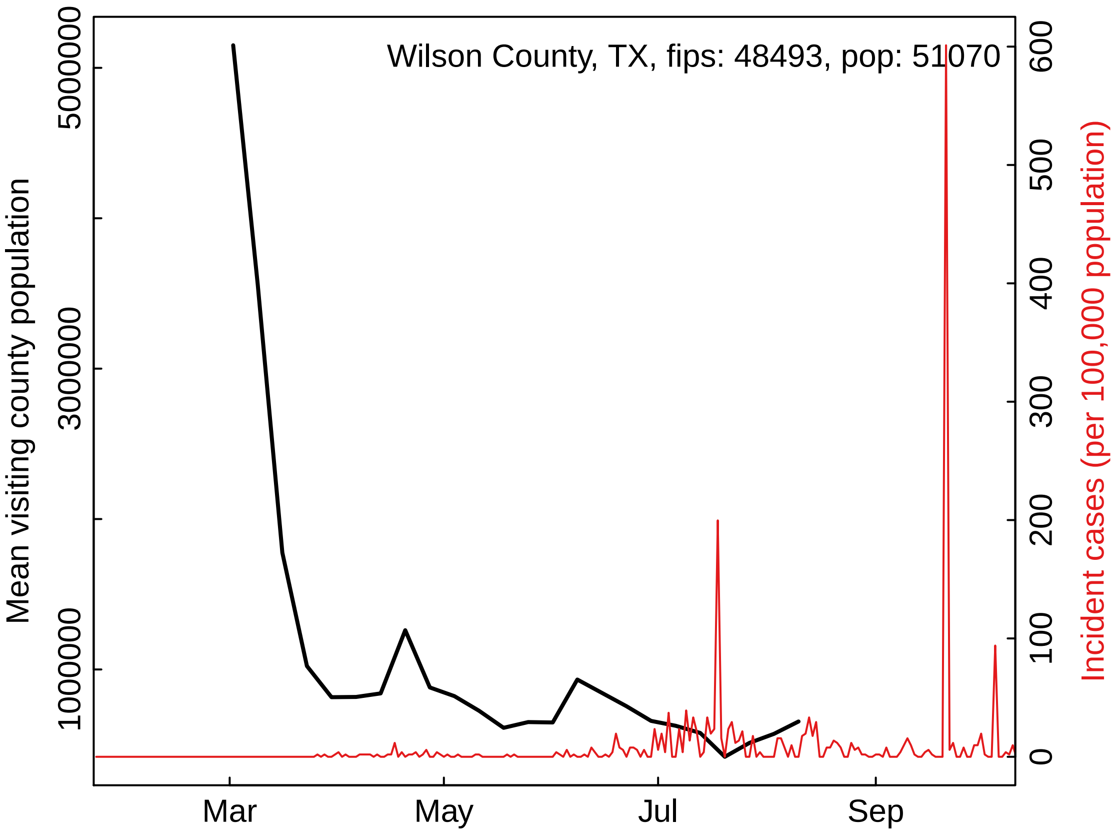
Wilson County, TX, fips: 48493, pop: 51070
Mean visiting county population
1000000
3000000
5000000
Incident cases (per 100,000 population)
0
100
200
300
400
500
600
Mar
May
Jul
Sep

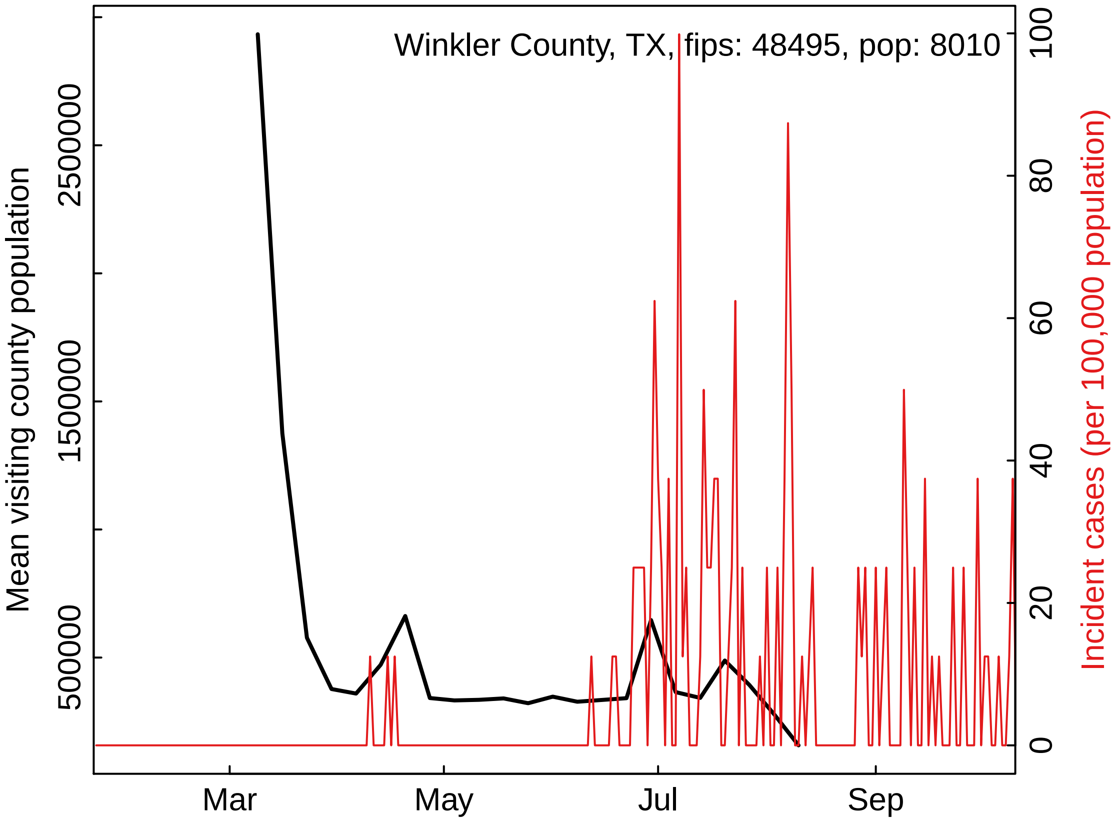

Winkler County, TX, fips: 48495, pop: 8010
Mean visiting county population
500000
1500000
2500000
Mar
May
Jul
Sep
0
20
40
60
80
100
Incident cases (per 100,000 population)

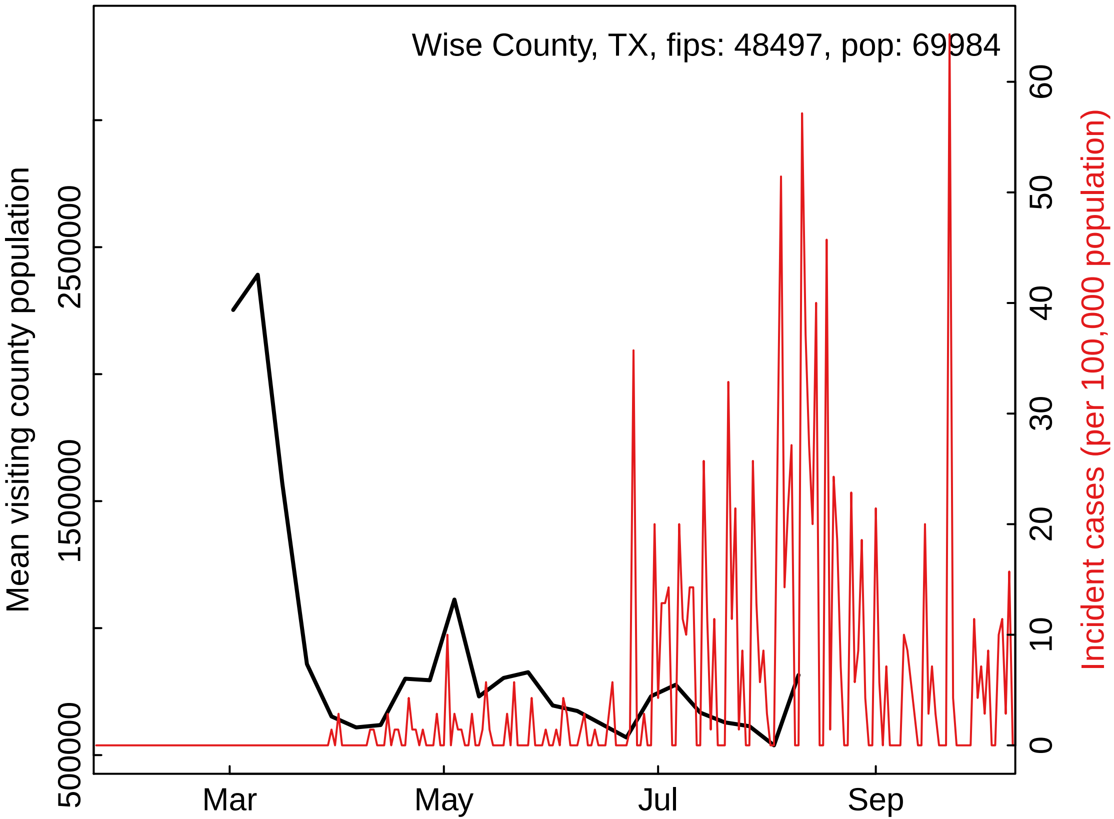

Wise County, TX, fips: 48497, pop: 69984
Mean visiting county population
500000
1500000
2500000
Mar
May
Jul
Sep
0
10
20
30
40
50
60
Incident cases (per 100,000 population)

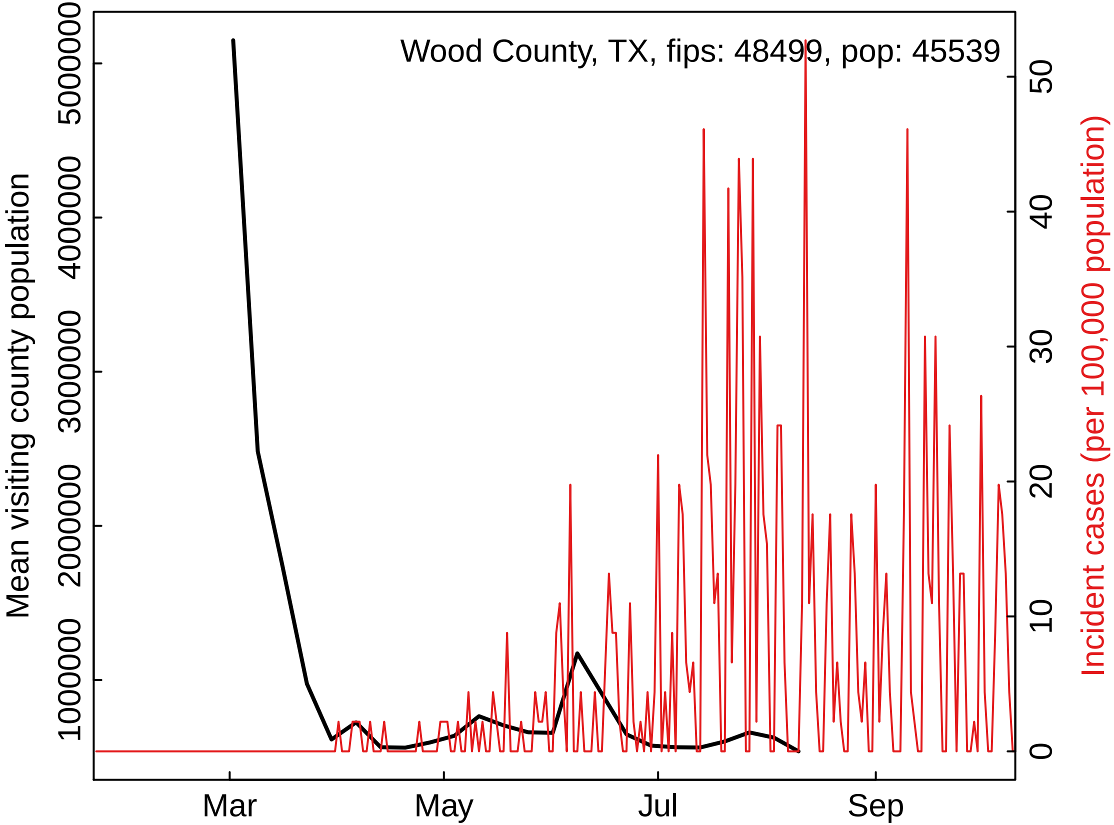

Wood County, TX, fips: 48499, pop: 45539
Mean visiting county population
10000000
20000000
30000000
40000000
50000000
Incident cases (per 100,000 population)
0
10
20
30
40
50
Mar
May
Jul
Sep

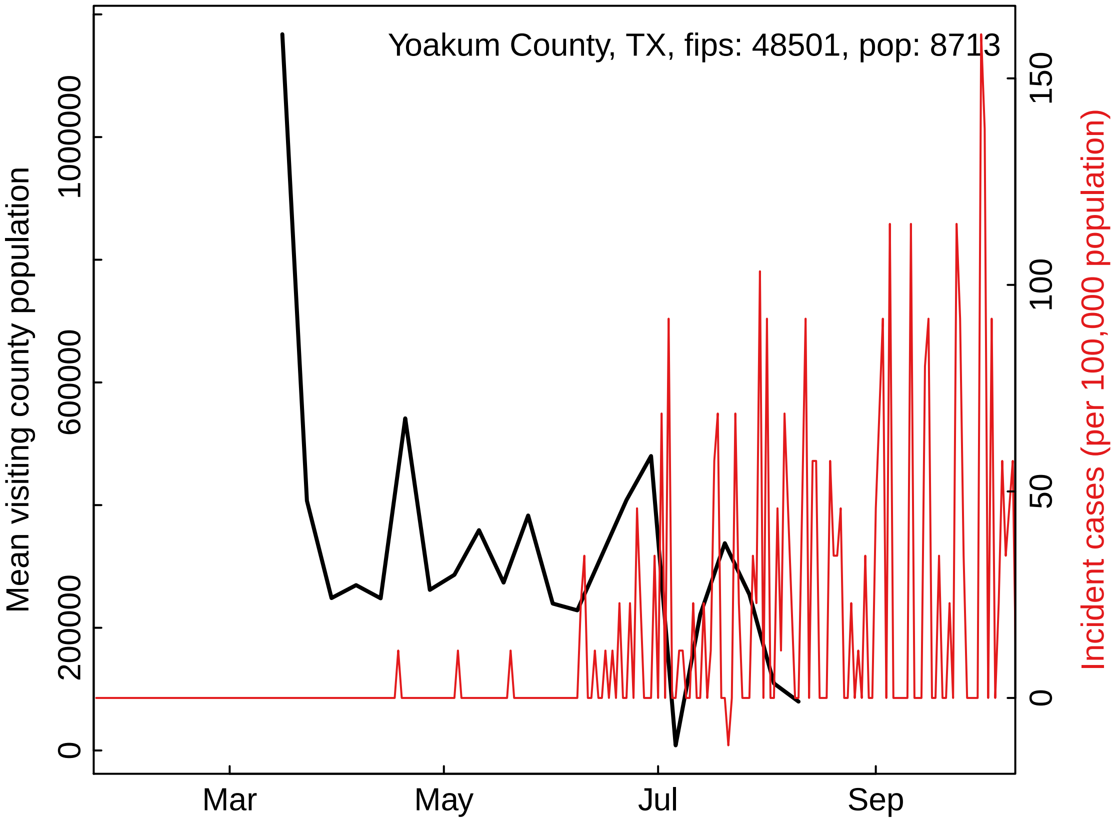

Yoakum County, TX, fips: 48501, pop: 8713
Mean visiting county population
0
200000
600000
1000000
Incident cases (per 100,000 population)
0
50
100
150
Mar
May
Jul
Sep

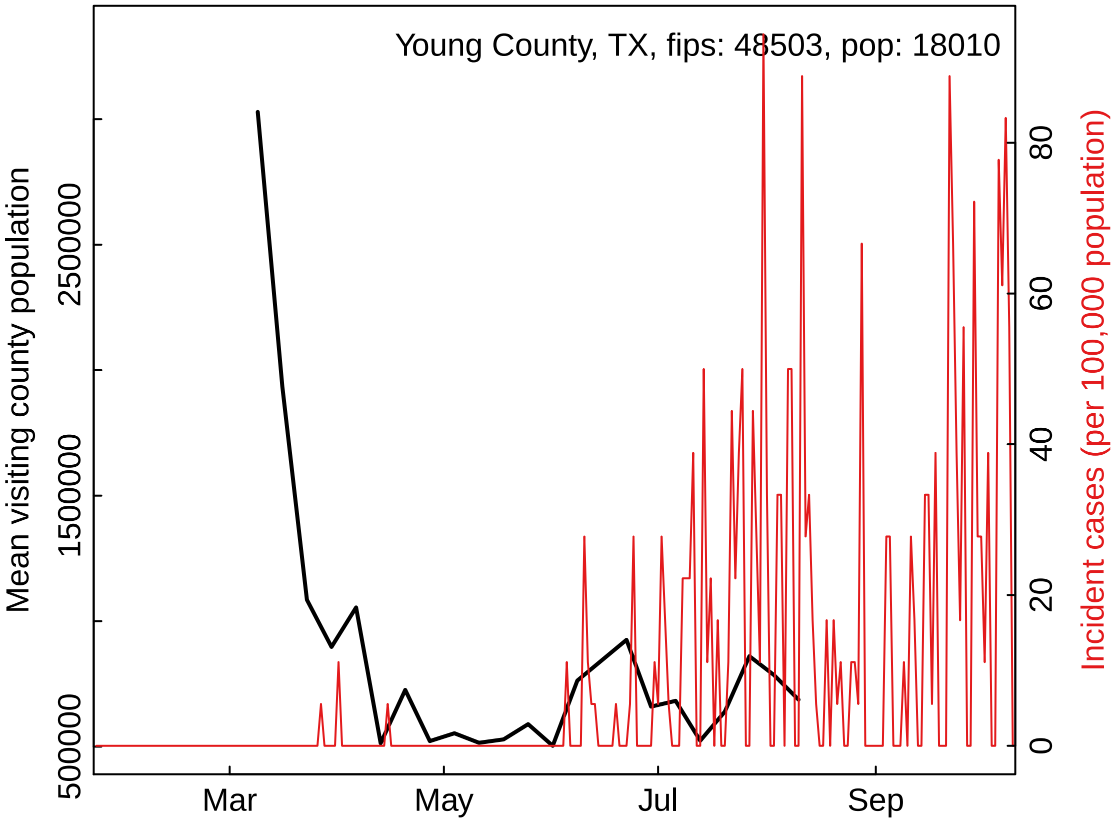

Young County, TX, fips: 48503, pop: 18010
Mean visiting county population
5000000
1500000
2500000
Mar
May
Jul
Sep
0
20
40
60
80
Incident cases (per 100,000 population)

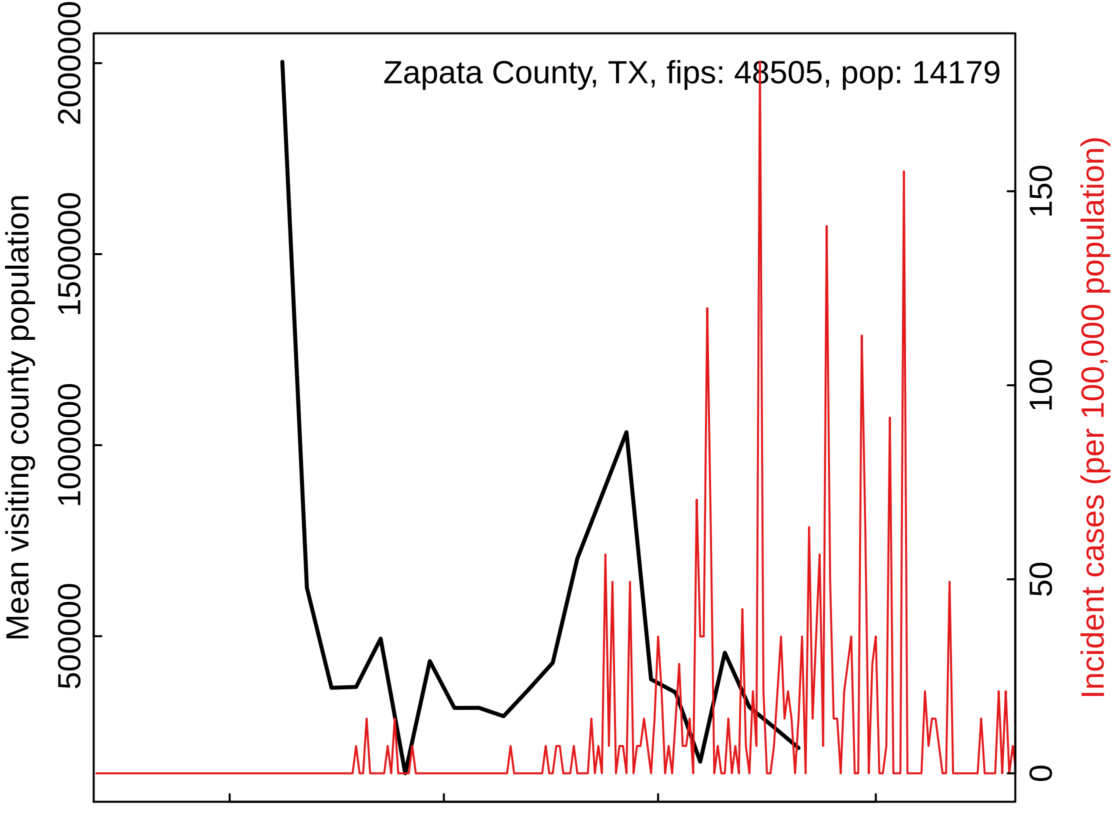

Zapata County, TX, fips: 48505, pop: 14179
Mean visiting county population
Incident cases (per 100,000 population)
2000000
1500000
1000000
500000
150
100
50
0
Mar
May
Jul
Sep

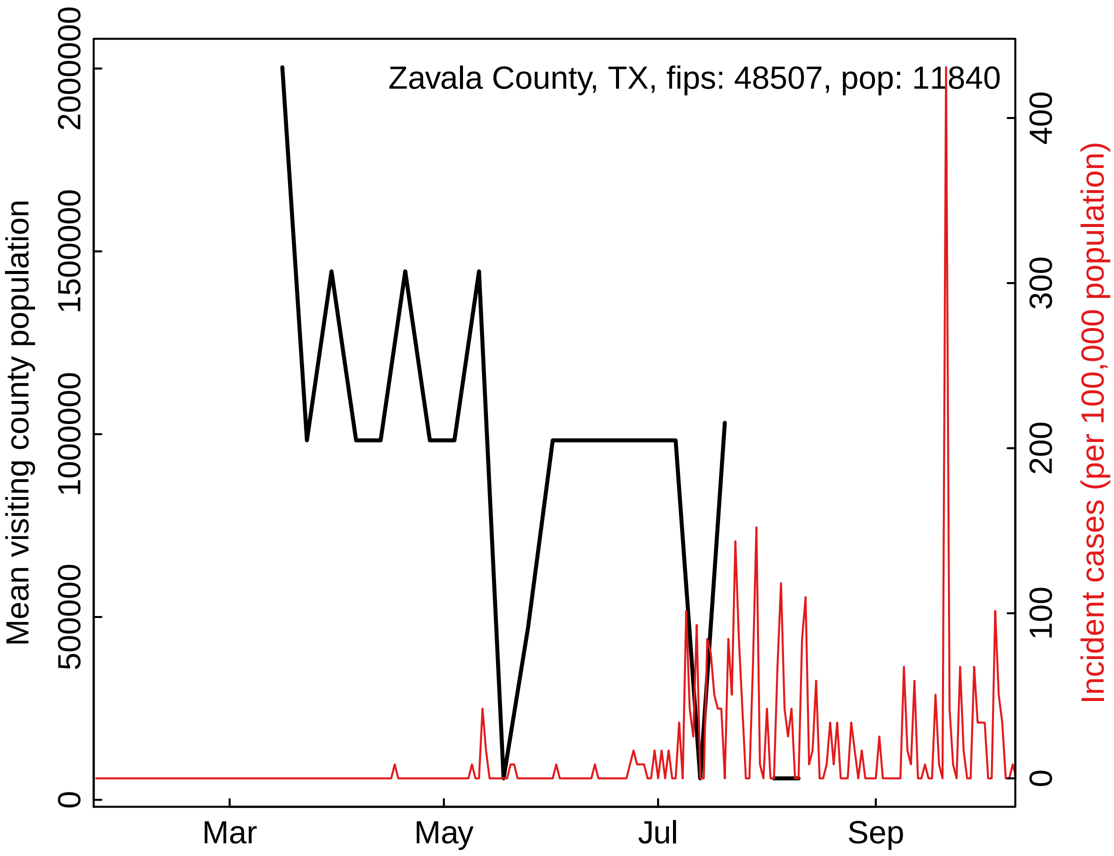

Zavala County, TX, fips: 48507, pop: 11840
Mean visiting county population
Incident cases (per 100,000 population)
0
500000
1000000
1500000
2000000
0
100
200
300
400
Mar
May
Jul
Sep

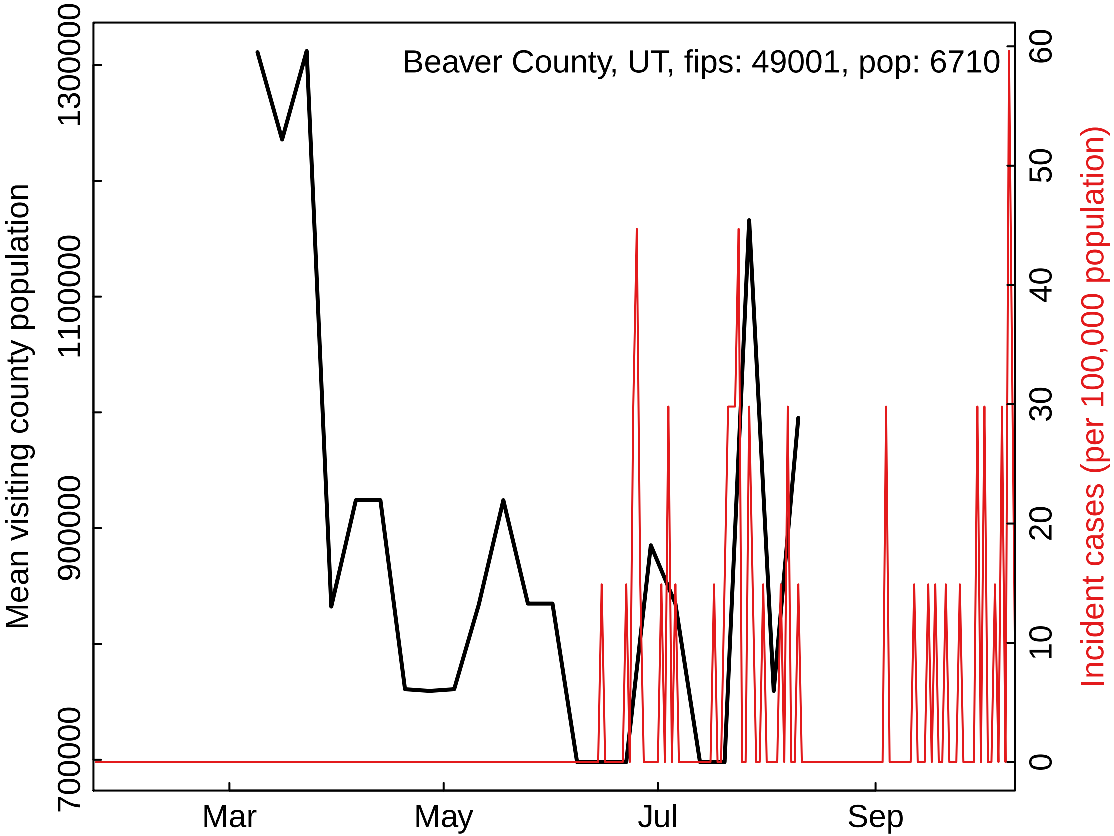

Beaver County, UT, fips: 49001, pop: 6710
Mean visiting county population
Incident cases (per 100,000 population)
700000
900000
1100000
1300000
0
10
20
30
40
50
60
Mar
May
Jul
Sep

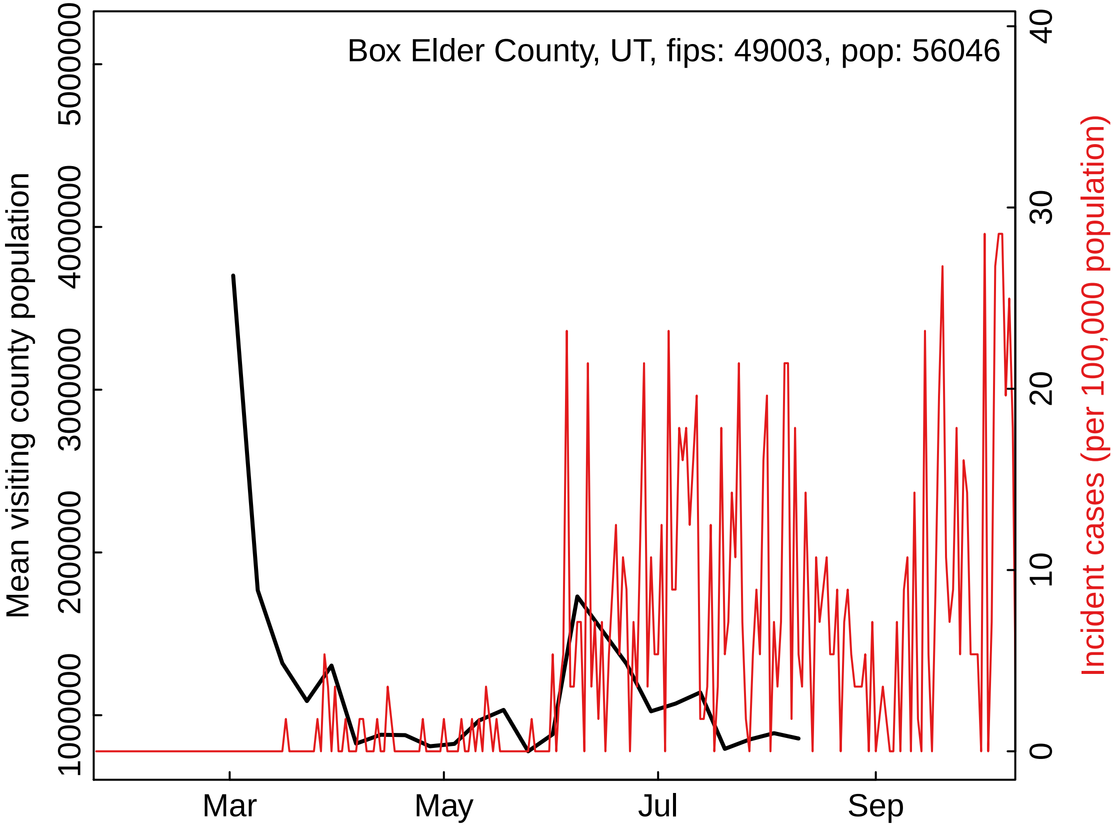

Box Elder County, UT, fips: 49003, pop: 56046
Mean visiting county population
1000000
2000000
3000000
4000000
5000000
Incident cases (per 100,000 population)
0
10
20
30
40
Mar
May
Jul
Sep

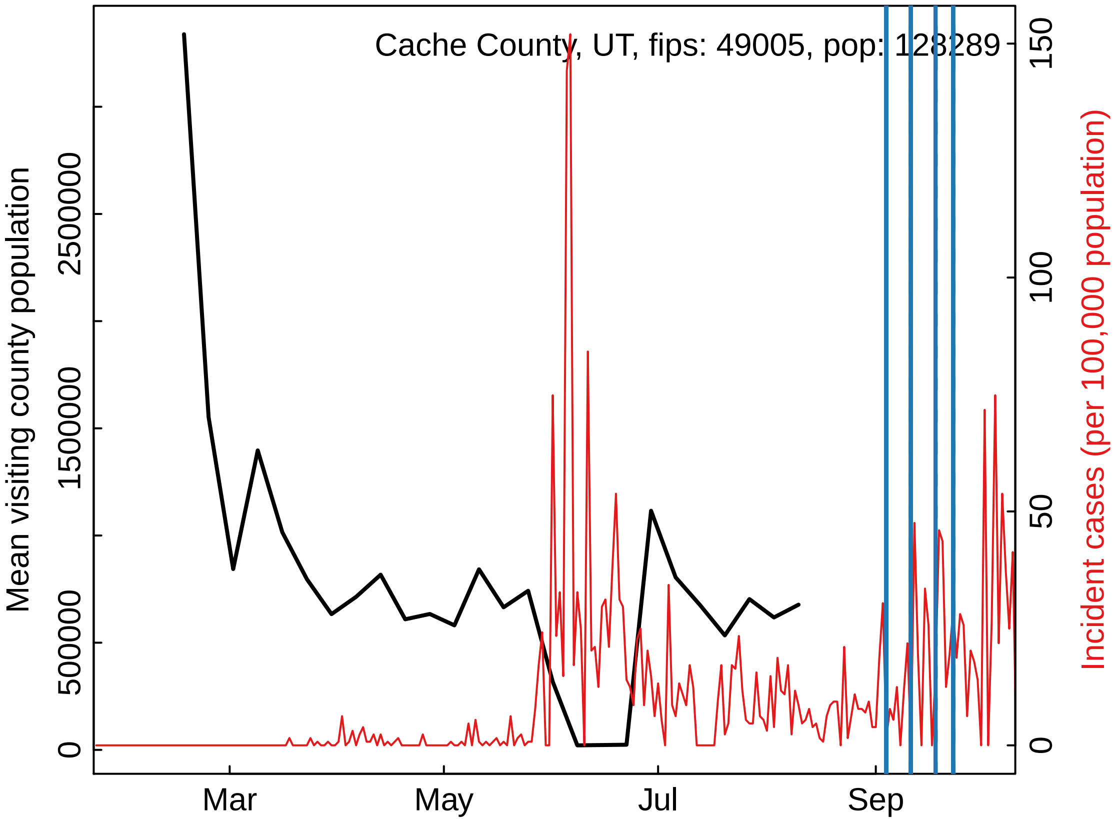

Cache County, UT, fips: 49005, pop: 128289
Mean visiting county population
0
500000
1500000
2500000
Incident cases (per 100,000 population)
0
50
100
150
Mar
May
Jul
Sep

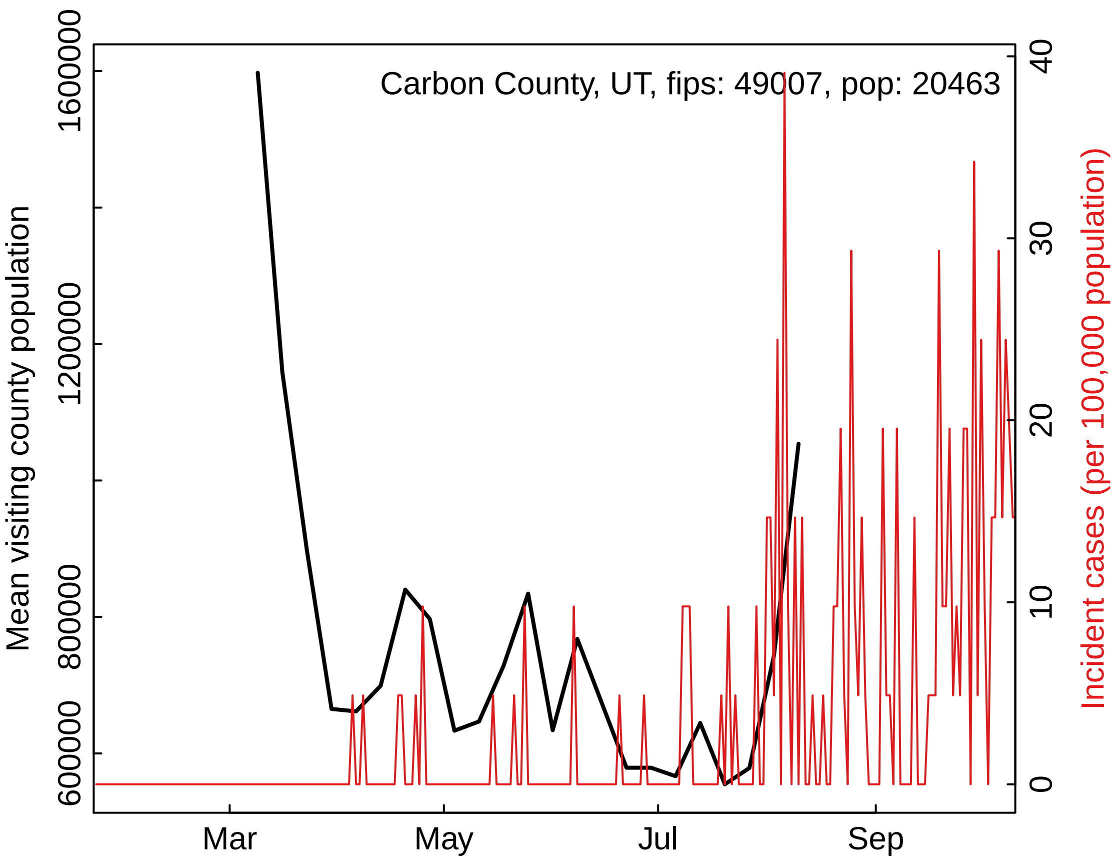

Carbon County, UT, fips: 49007, pop: 20463
Mean visiting county population
600000
800000
1200000
1600000
Incident cases (per 100,000 population)
0
10
20
30
40
Mar
May
Jul
Sep

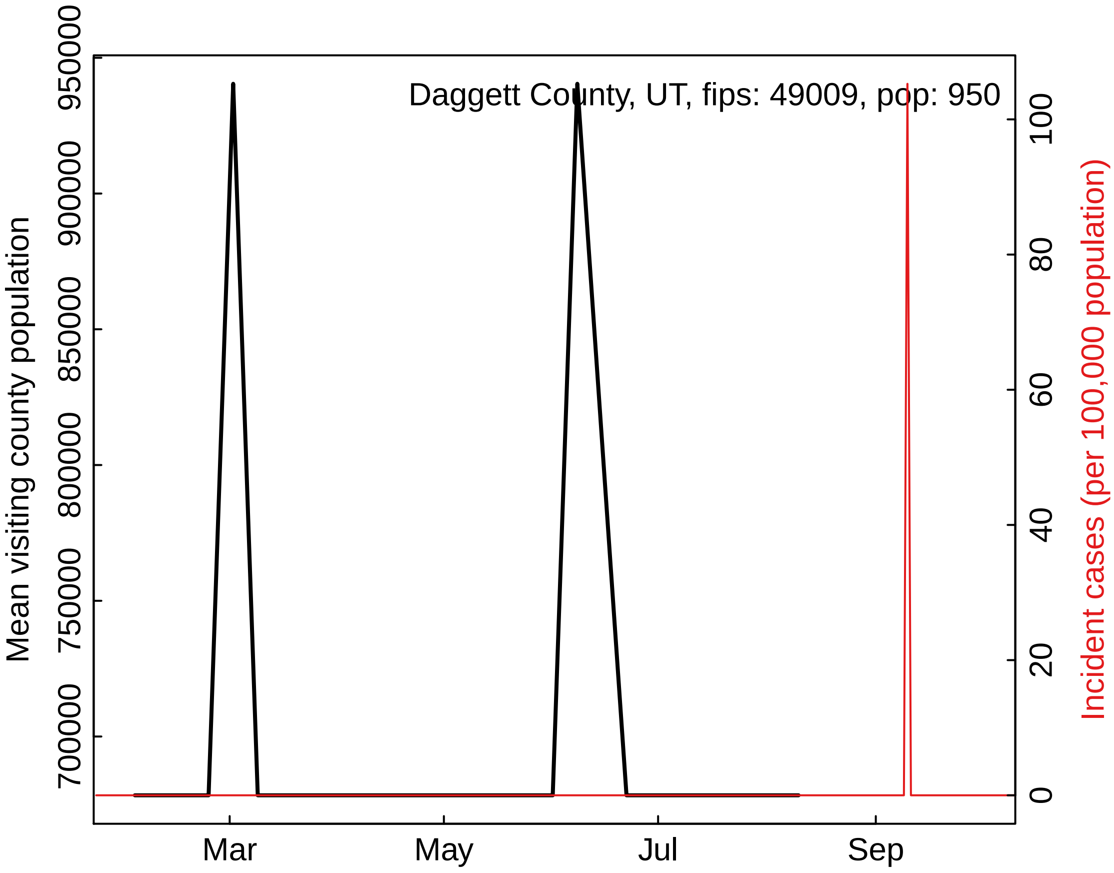

Daggett County, UT, fips: 49009, pop: 950
Mean visiting county population
700000
750000
800000
850000
900000
950000
Mar
May
Jul
Sep
0
20
40
60
80
100
Incident cases (per 100,000 population)

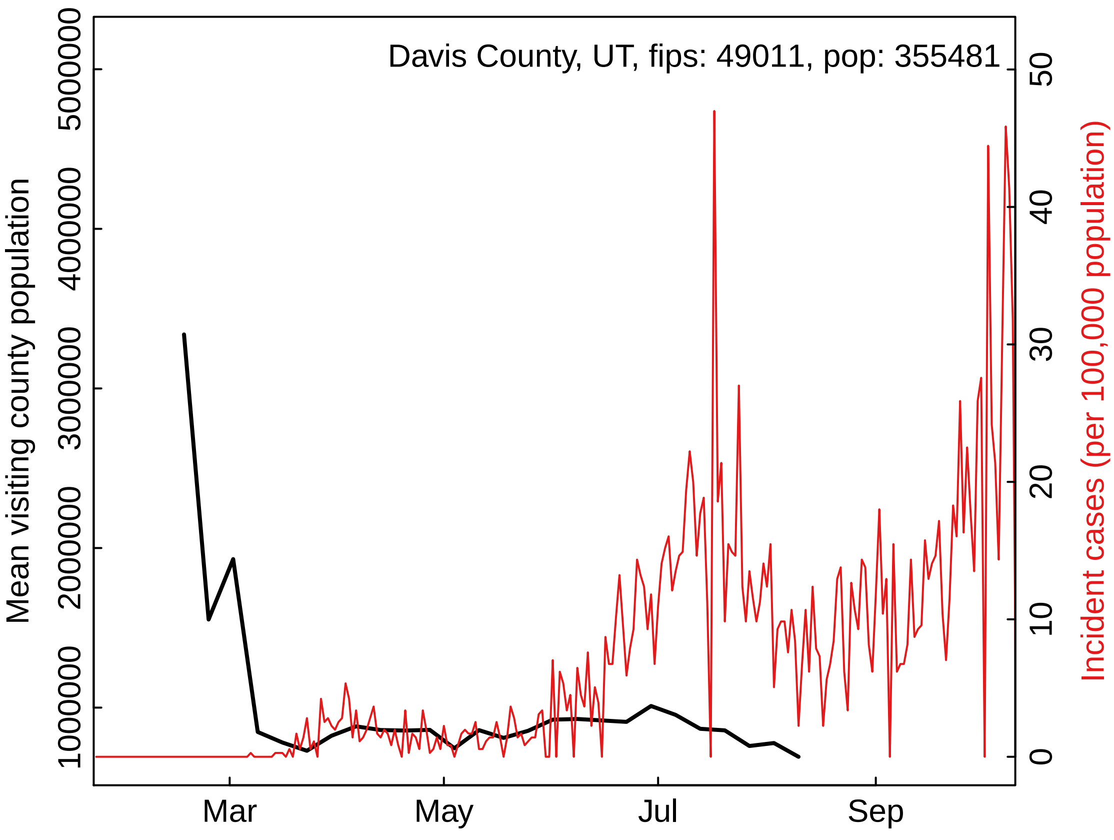

Davis County, UT, fips: 49011, pop: 355481
Mean visiting county population
10000000
20000000
30000000
40000000
50000000
Incident cases (per 100,000 population)
0
10
20
30
40
50
Mar
May
Jul
Sep

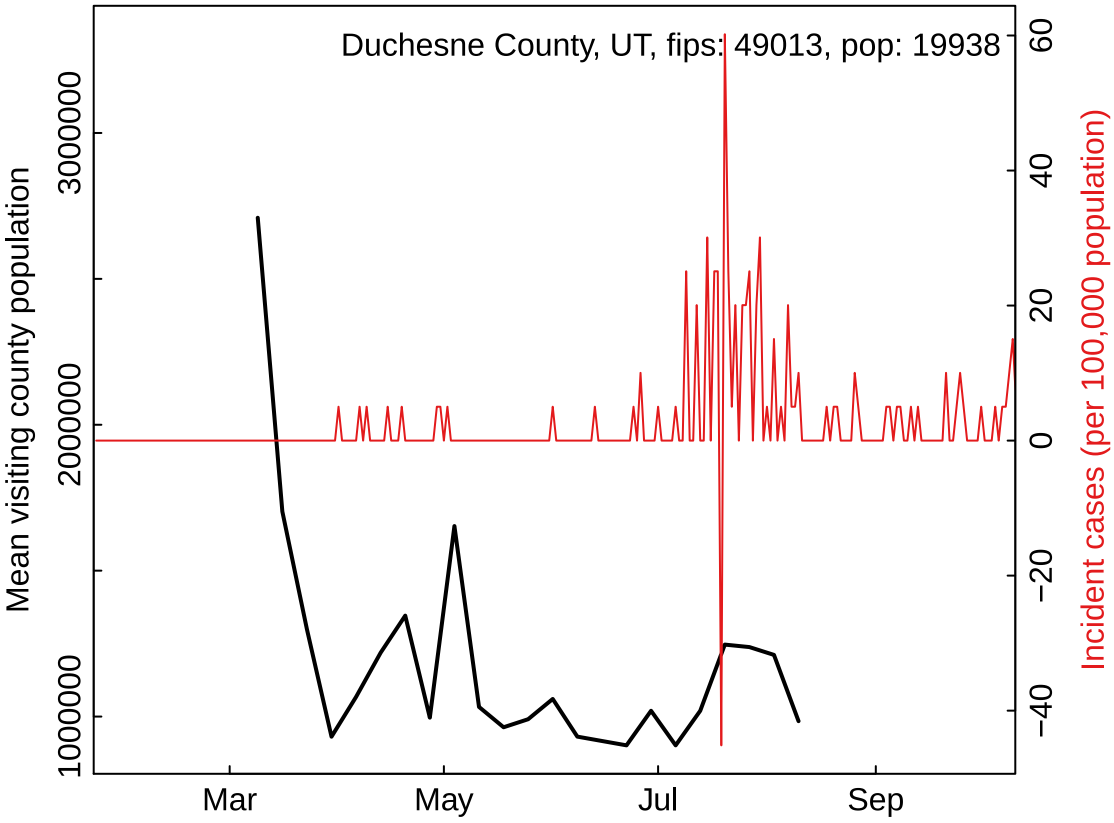

Duchesne County, UT, fips: 49013, pop: 19938
Mean visiting county population
1000000
2000000
3000000
Mar
May
Jul
Sep
Incident cases (per 100,000 population)
−40
−20
0
20
40
60

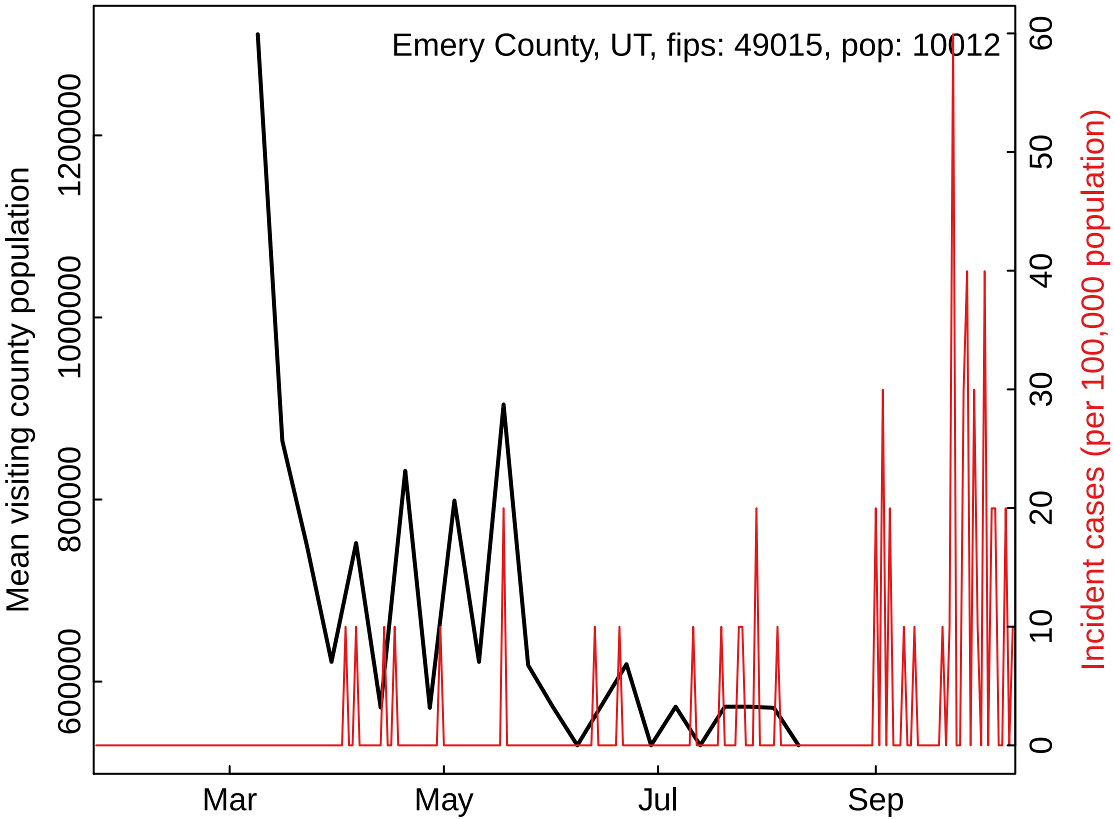
Emery County, UT, fips: 49015, pop: 10012
Mean visiting county population
600000
800000
1000000
1200000
Incident cases (per 100,000 population)
0
10
20
30
40
50
60
Mar
May
Jul
Sep

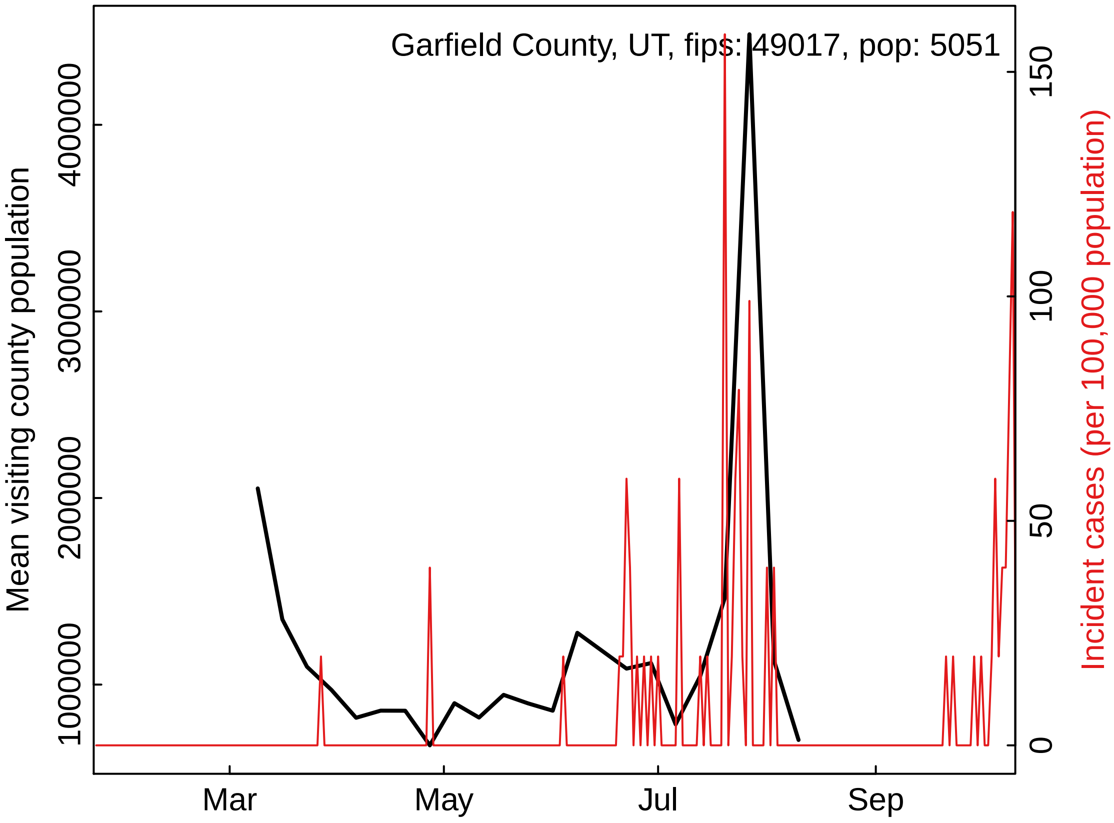

Garfield County, UT, fips: 49017, pop: 5051
Mean visiting county population
1000000
2000000
3000000
4000000
Incident cases (per 100,000 population)
0
50
100
150
Mar
May
Jul
Sep

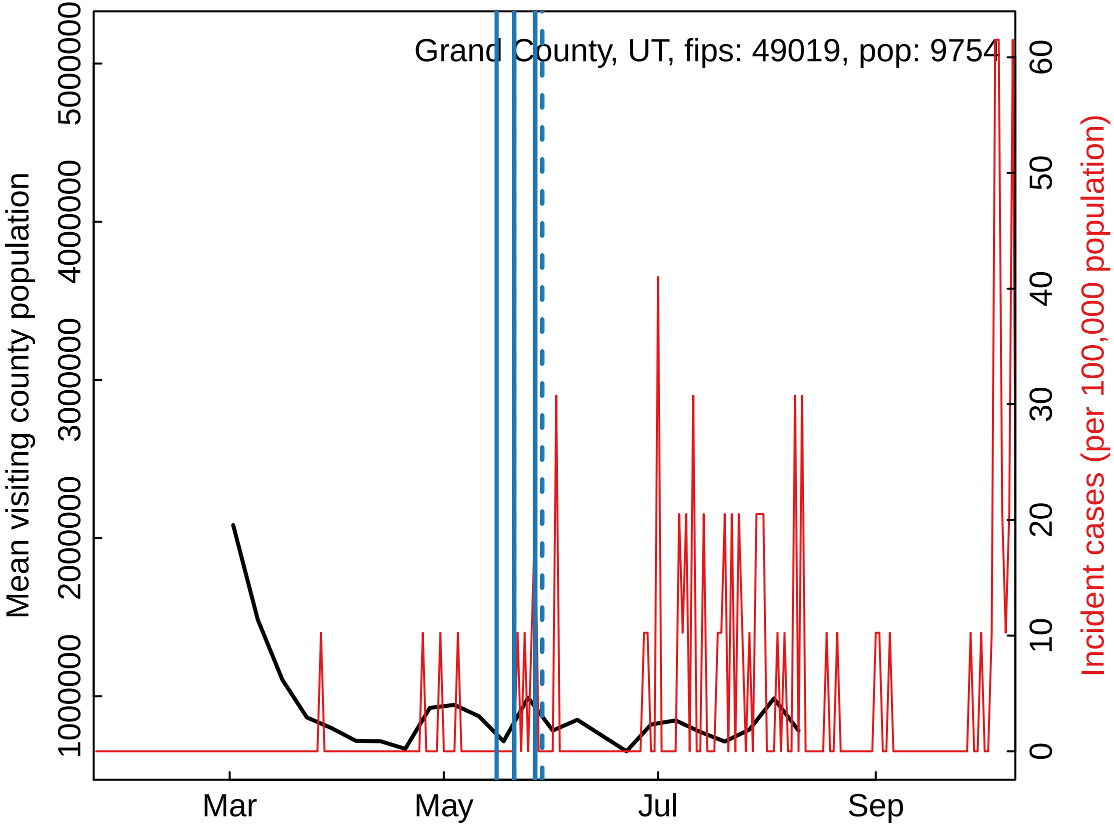
Grand County, UT, fips: 49019, pop: 9754
Mean visiting county population
1000000
2000000
3000000
4000000
5000000
Incident cases (per 100,000 population)
0
10
20
30
40
50
60
Mar
May
Jul
Sep

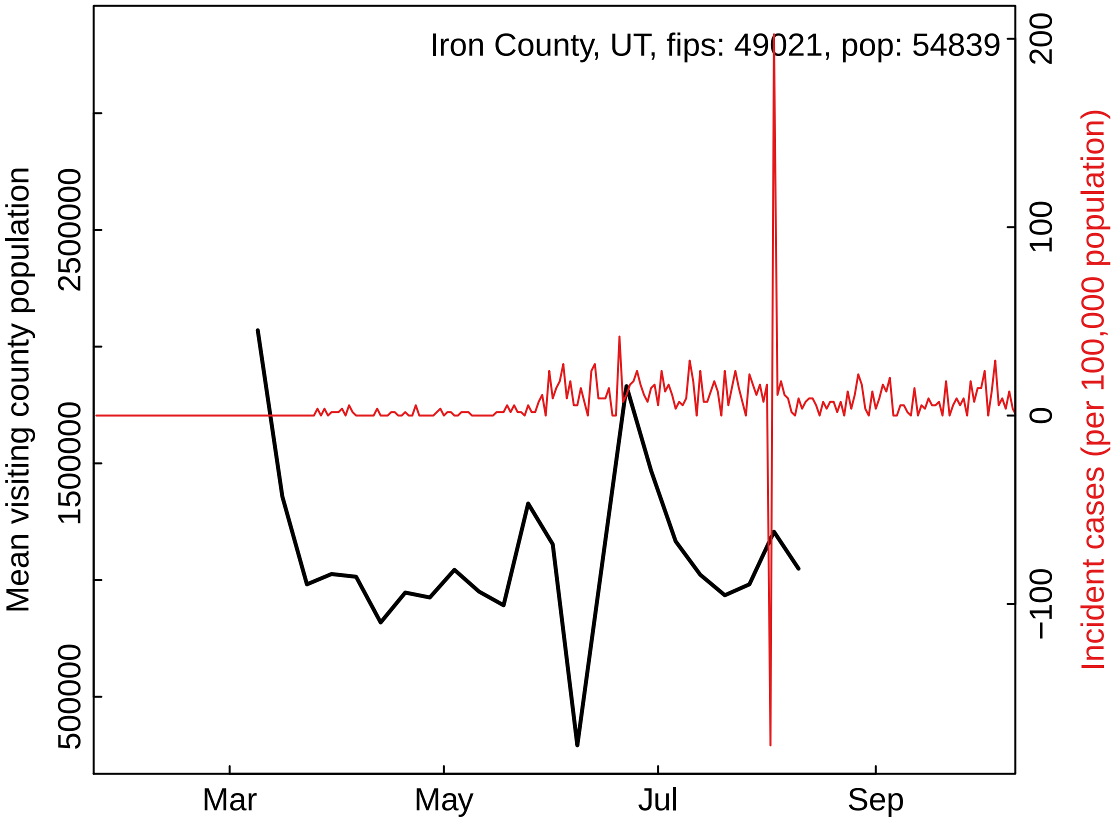

Iron County, UT, fips: 49021, pop: 54839
Mean visiting county population
500000
1500000
2500000
Incident cases (per 100,000 population)
−100
0
100
200
Mar
May
Jul
Sep

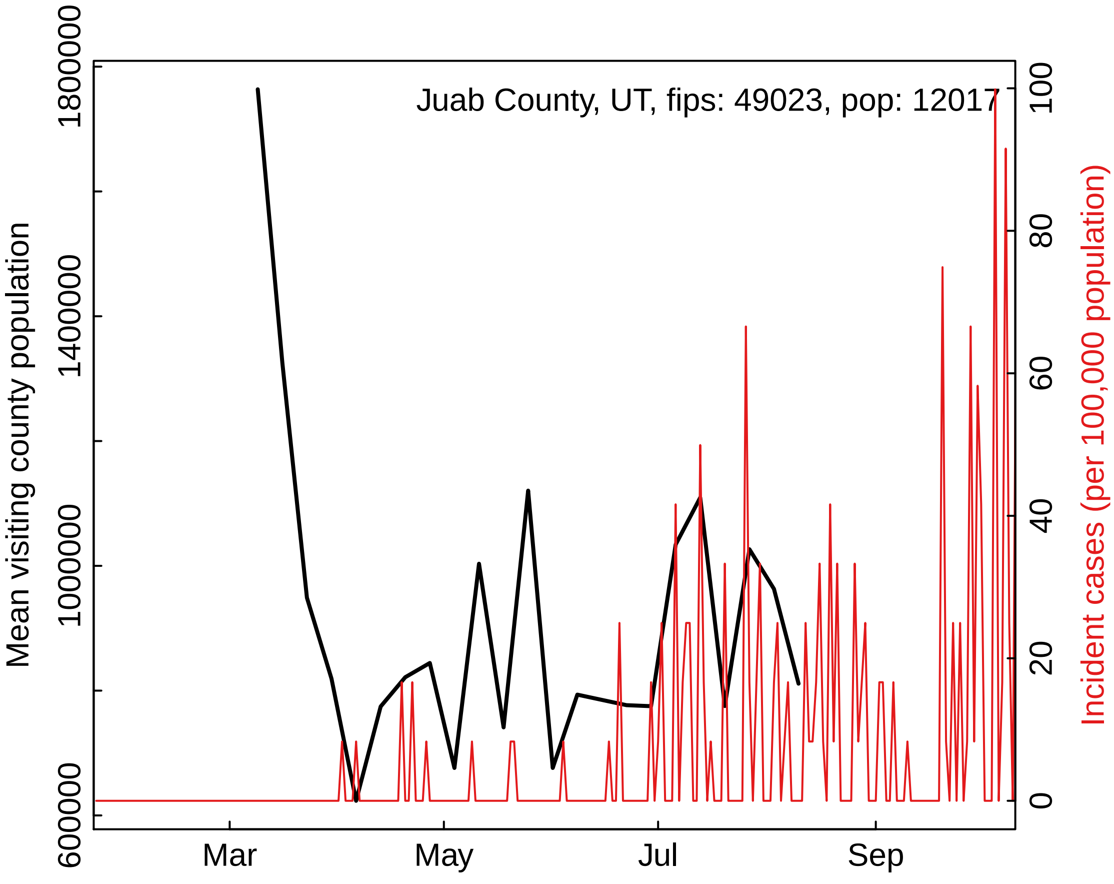

Juab County, UT, fips: 49023, pop: 12017
Mean visiting county population
Incident cases (per 100,000 population)
600000
1000000
1400000
1800000
0
20
40
60
80
100
Mar
May
Jul
Sep

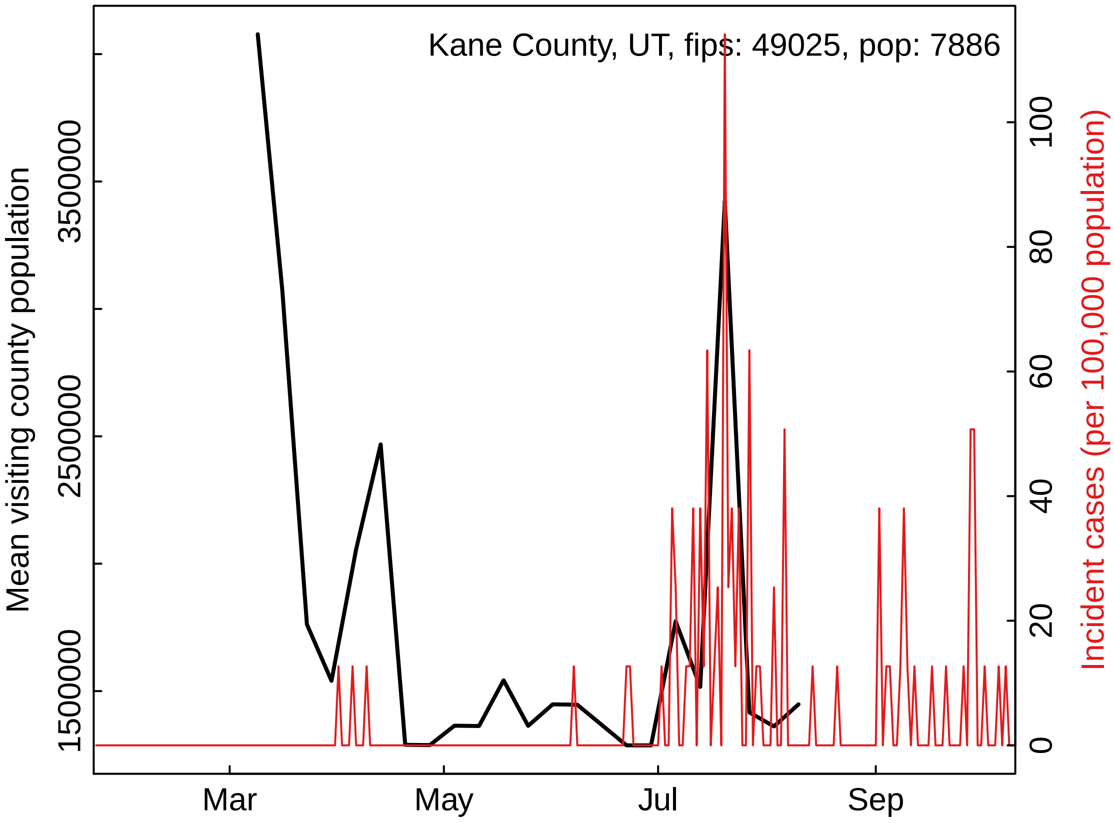

Kane County, UT, fips: 49025, pop: 7886
Mean visiting county population
1500000
2500000
3500000
Incident cases (per 100,000 population)
0
20
40
60
80
100
Mar
May
Jul
Sep

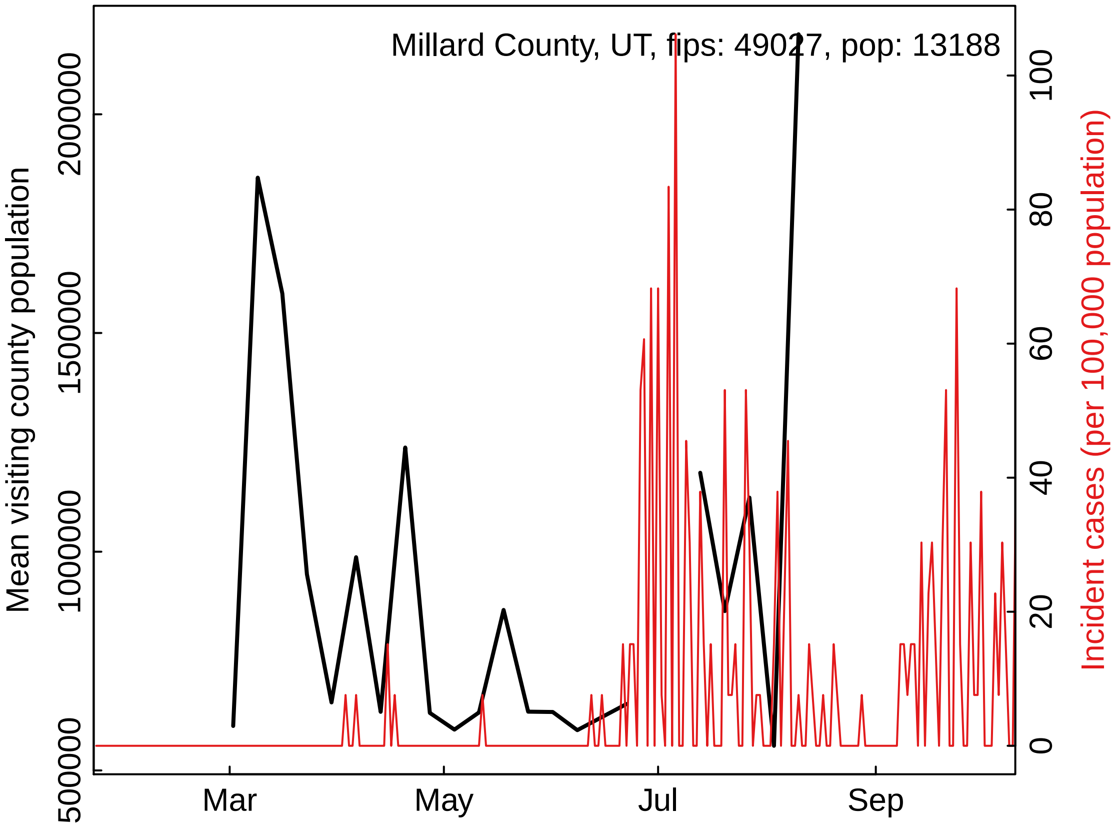

Millard County, UT, fips: 49027, pop: 13188
Mean visiting county population
Incident cases (per 100,000 population)
500000
1000000
1500000
2000000
0
20
40
60
80
100
Mar
May
Jul
Sep

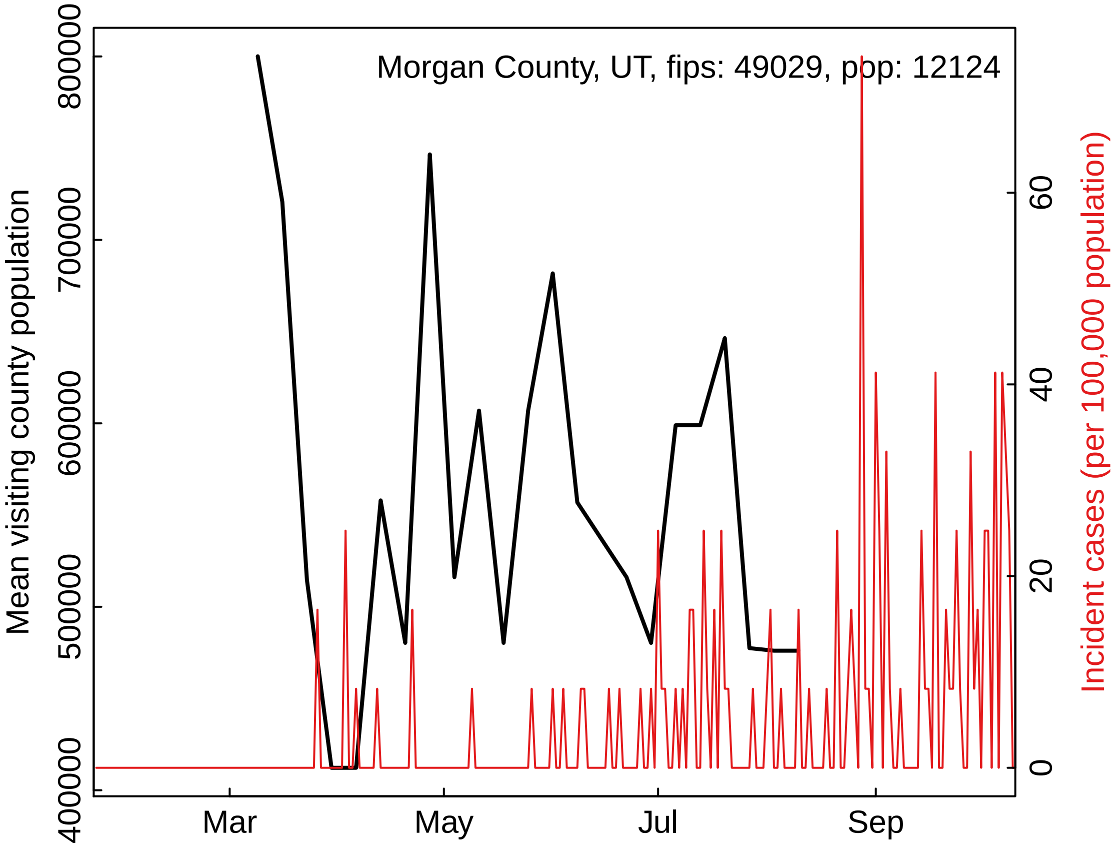

Morgan County, UT, fips: 49029, pop: 12124
Mean visiting county population
400000
500000
600000
700000
800000
Incident cases (per 100,000 population)
0
20
40
60
Mar
May
Jul
Sep

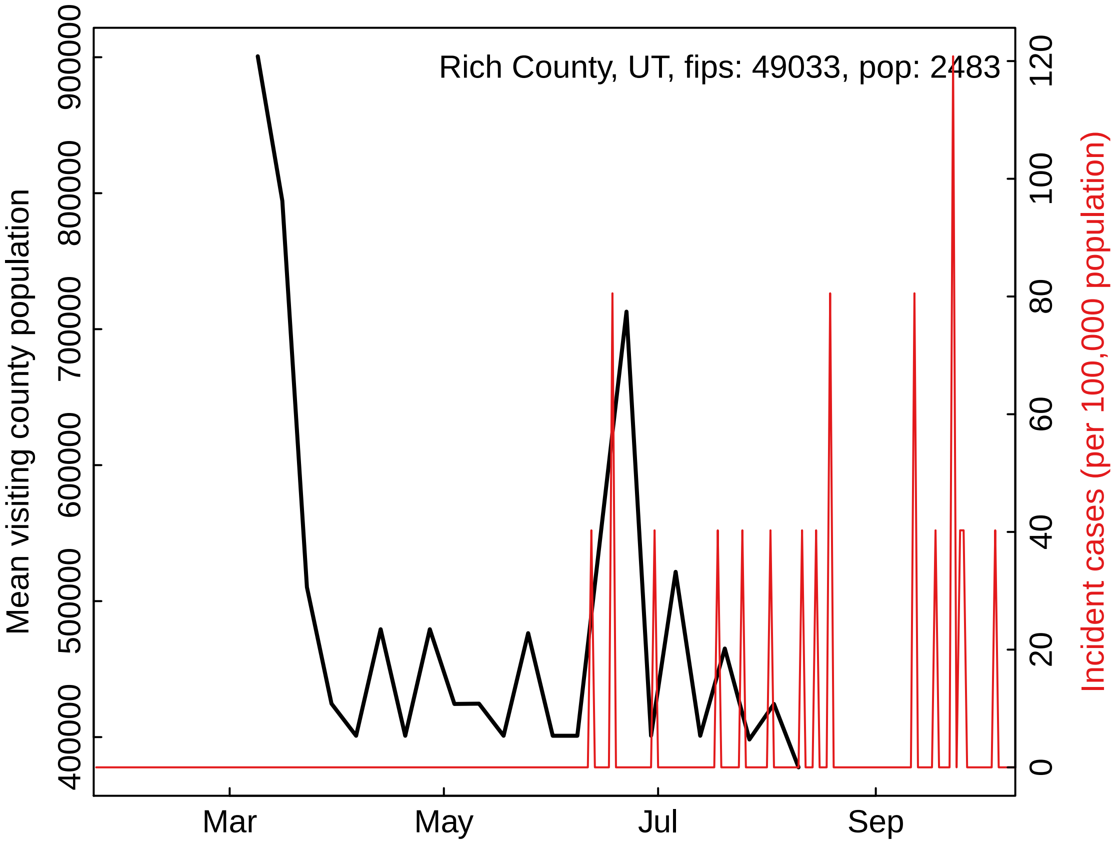

Rich County, UT, fips: 49033, pop: 2483
Mean visiting county population
400000
500000
600000
700000
800000
900000
Incident cases (per 100,000 population)
0
20
40
60
80
100
120
Mar
May
Jul
Sep

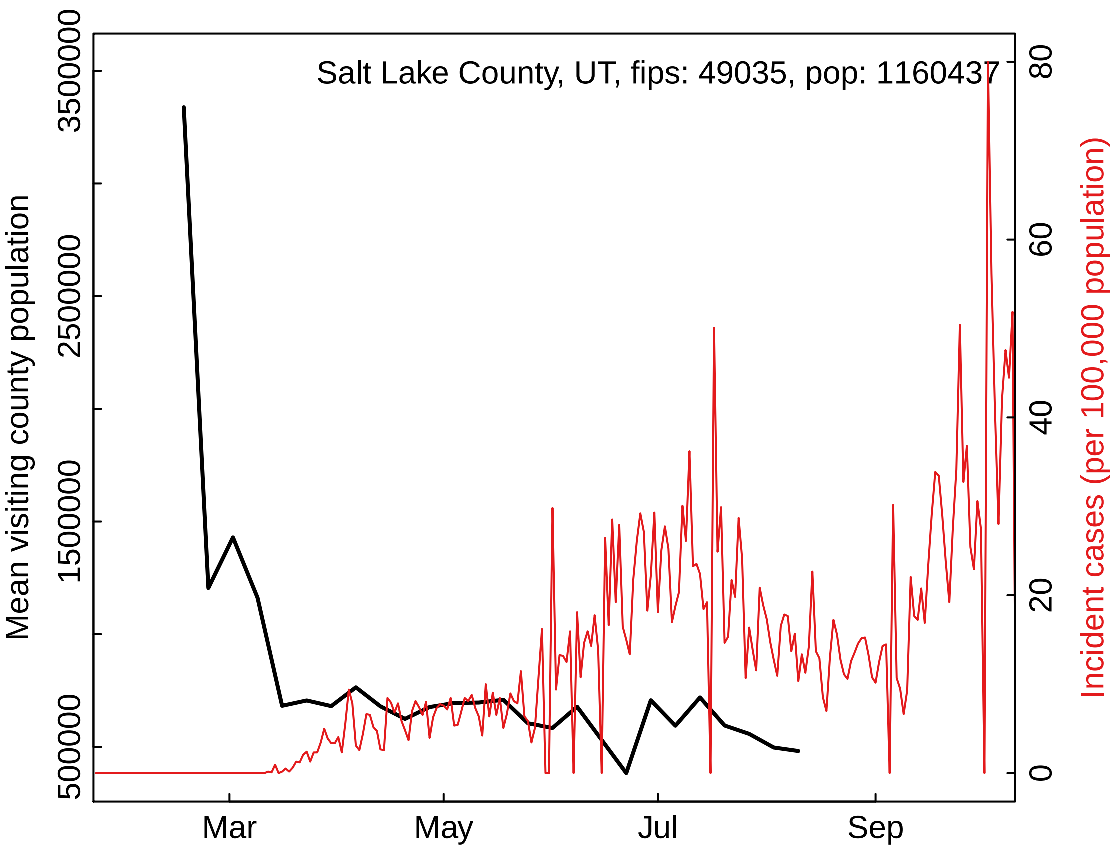

Salt Lake County, UT, fips: 49035, pop: 1160437
Mean visiting county population
500000
1500000
2500000
3500000
Incident cases (per 100,000 population)
0
20
40
60
80
Mar
May
Jul
Sep

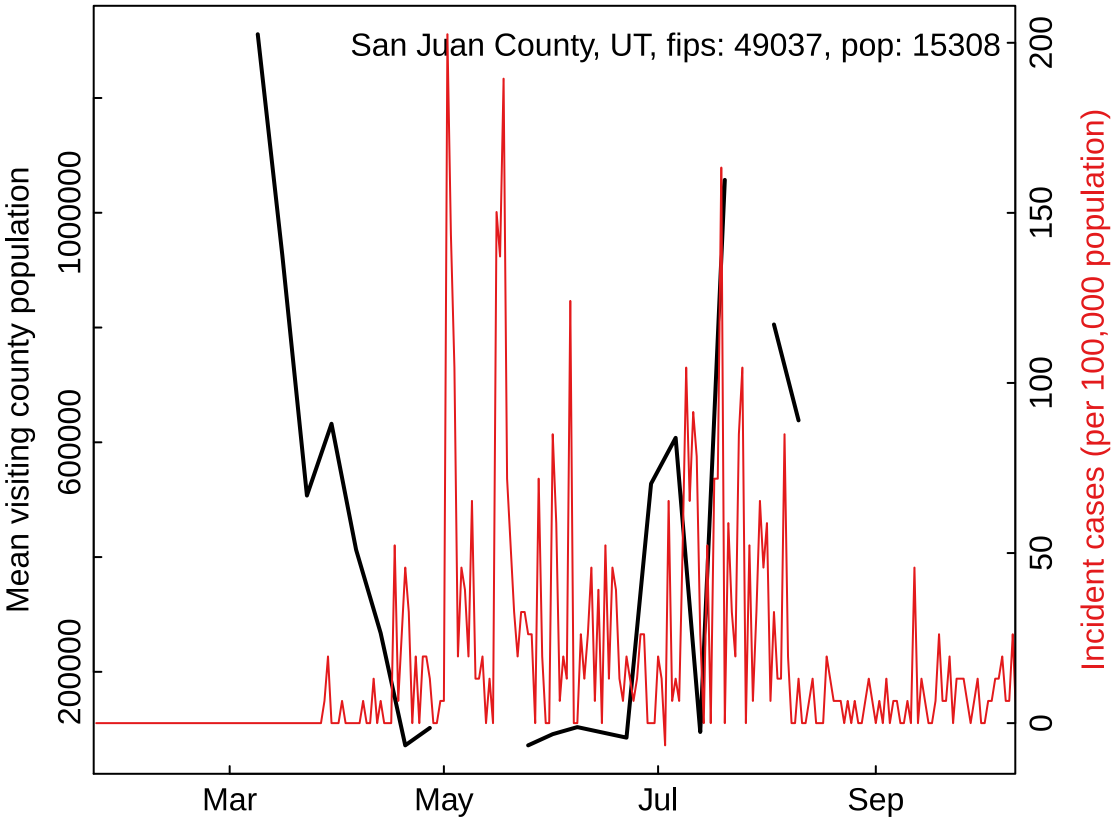
San Juan County, UT, fips: 49037, pop: 15308
Mean visiting county population
200000
600000
1000000
Incident cases (per 100,000 population)
0
50
100
150
200
Mar
May
Jul
Sep

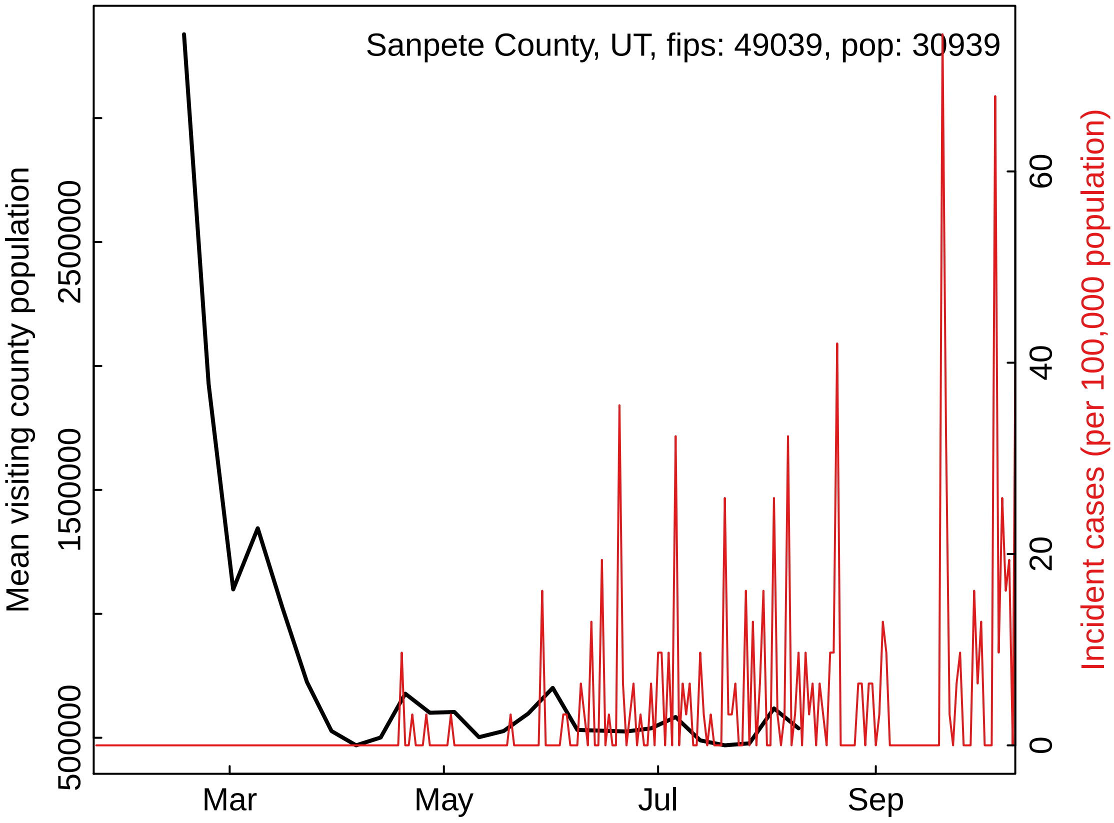
Sanpete County, UT, fips: 49039, pop: 30939
Mean visiting county population
500000
1500000
2500000
Incident cases (per 100,000 population)
0
20
40
60
Mar
May
Jul
Sep

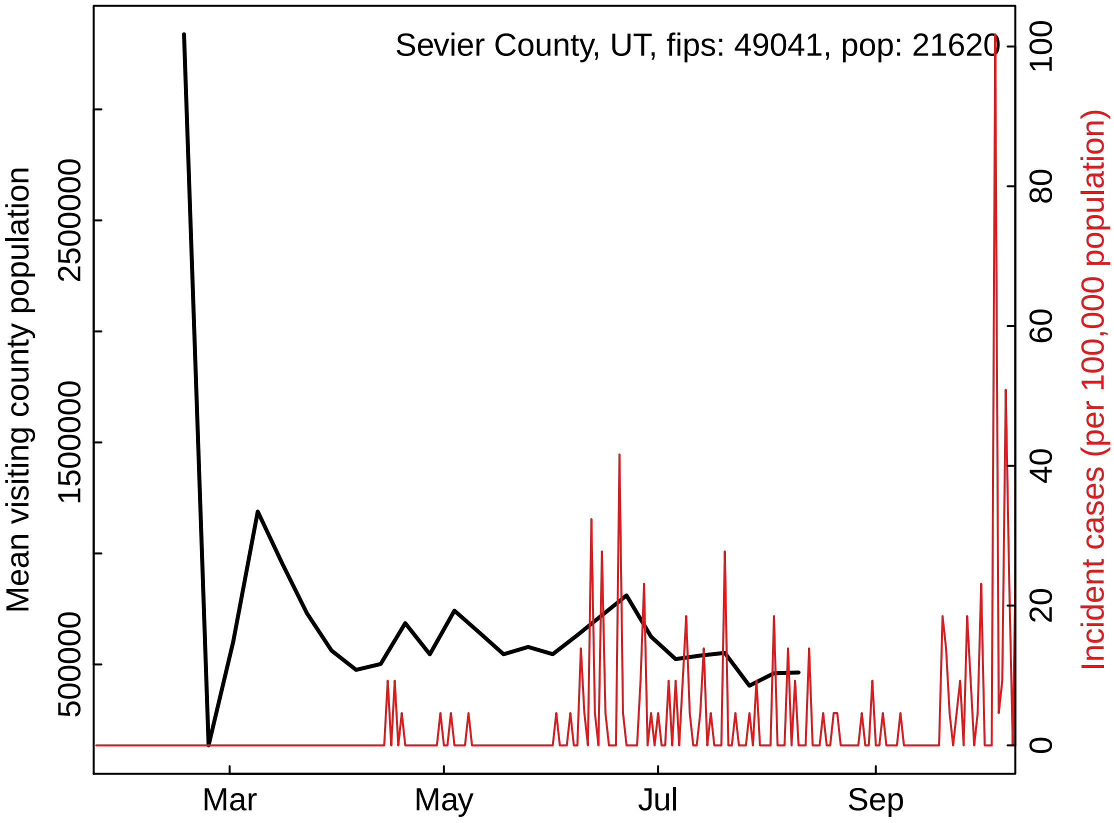

Sevier County, UT, fips: 49041, pop: 21620
Mean visiting county population
500000
1500000
2500000
Mar
May
Jul
Sep
0
20
40
60
80
100
Incident cases (per 100,000 population)

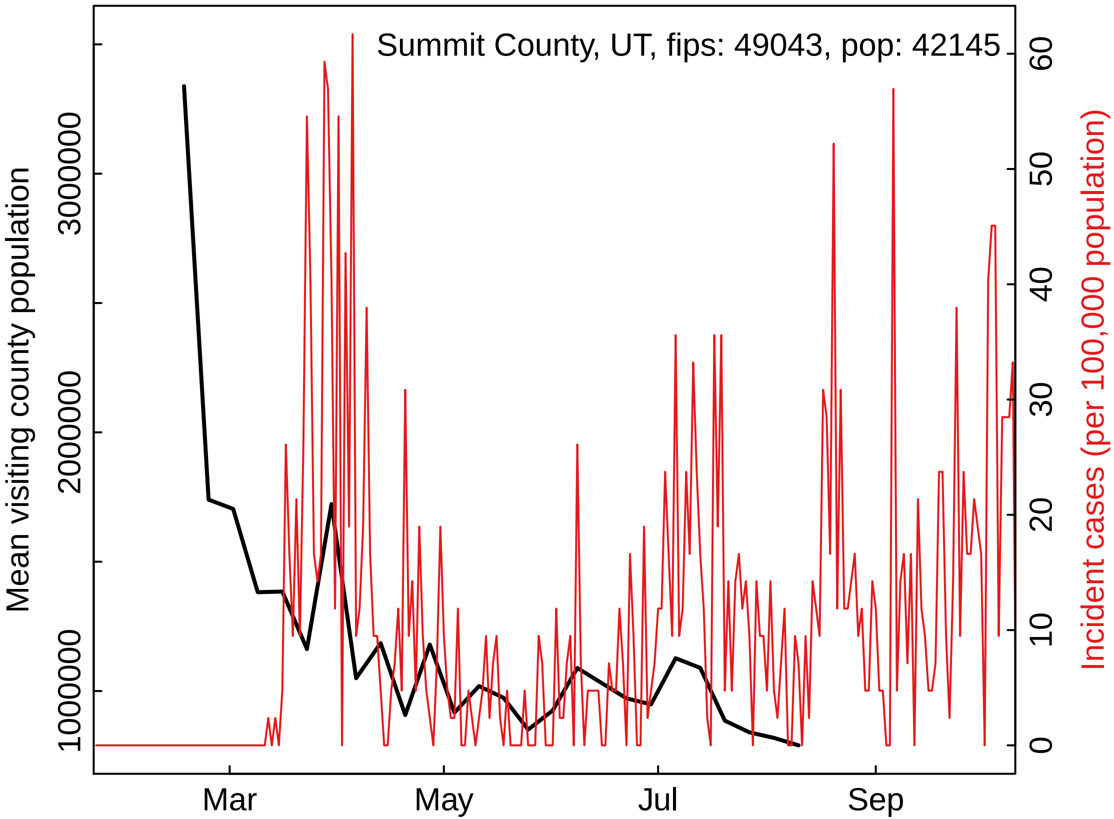

Summit County, UT, fips: 49043, pop: 42145
Mean visiting county population
10000000
20000000
30000000
Incident cases (per 100,000 population)
0
10
20
30
40
50
60
Mar
May
Jul
Sep

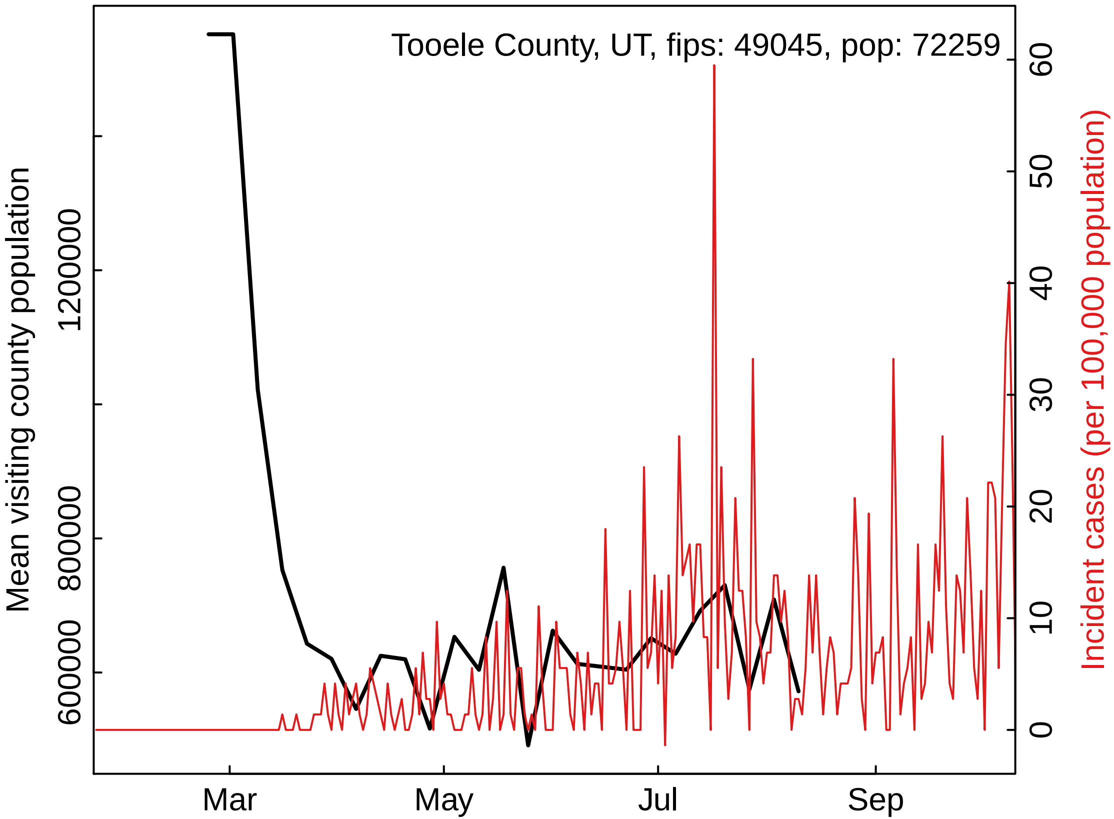
Tooele County, UT, fips: 49045, pop: 72259
Mean visiting county population
600000
800000
1200000
Incident cases (per 100,000 population)
0
10
20
30
40
50
60
Mar
May
Jul
Sep

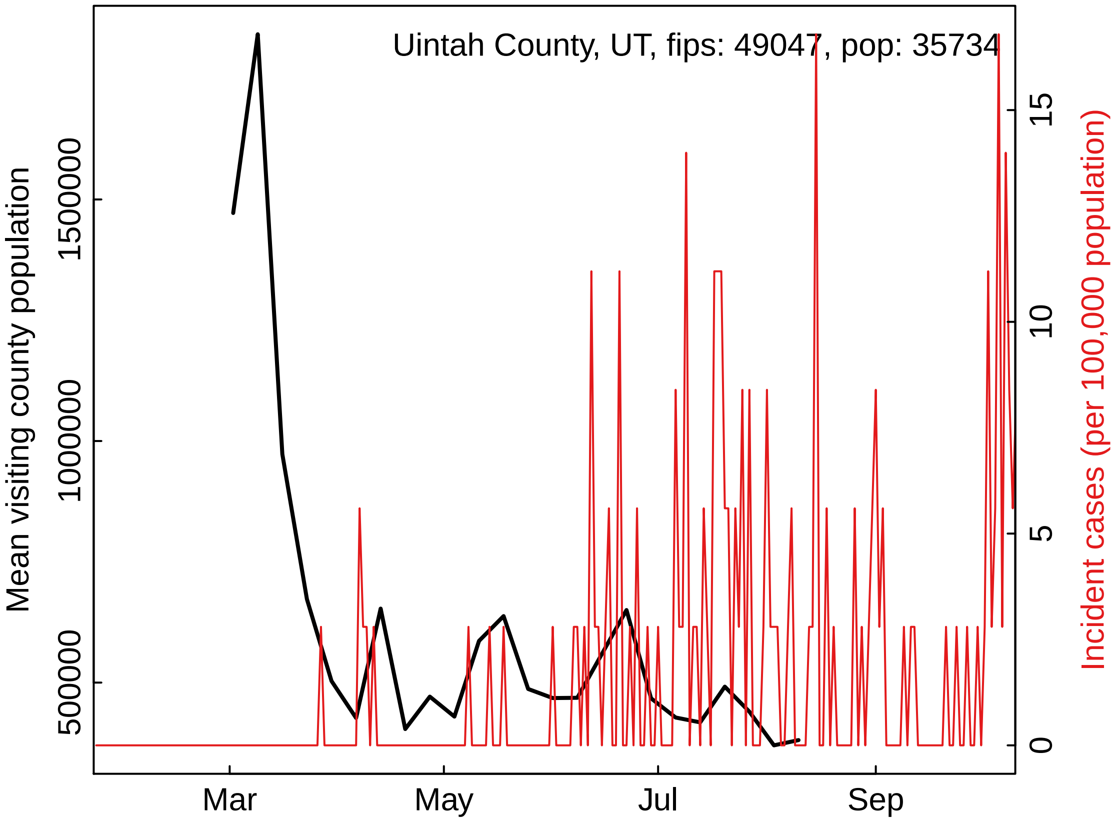

Uintah County, UT, fips: 49047, pop: 35734
Mean visiting county population
500000
1000000
1500000
Incident cases (per 100,000 population)
0
5
10
15
Mar
May
Jul
Sep

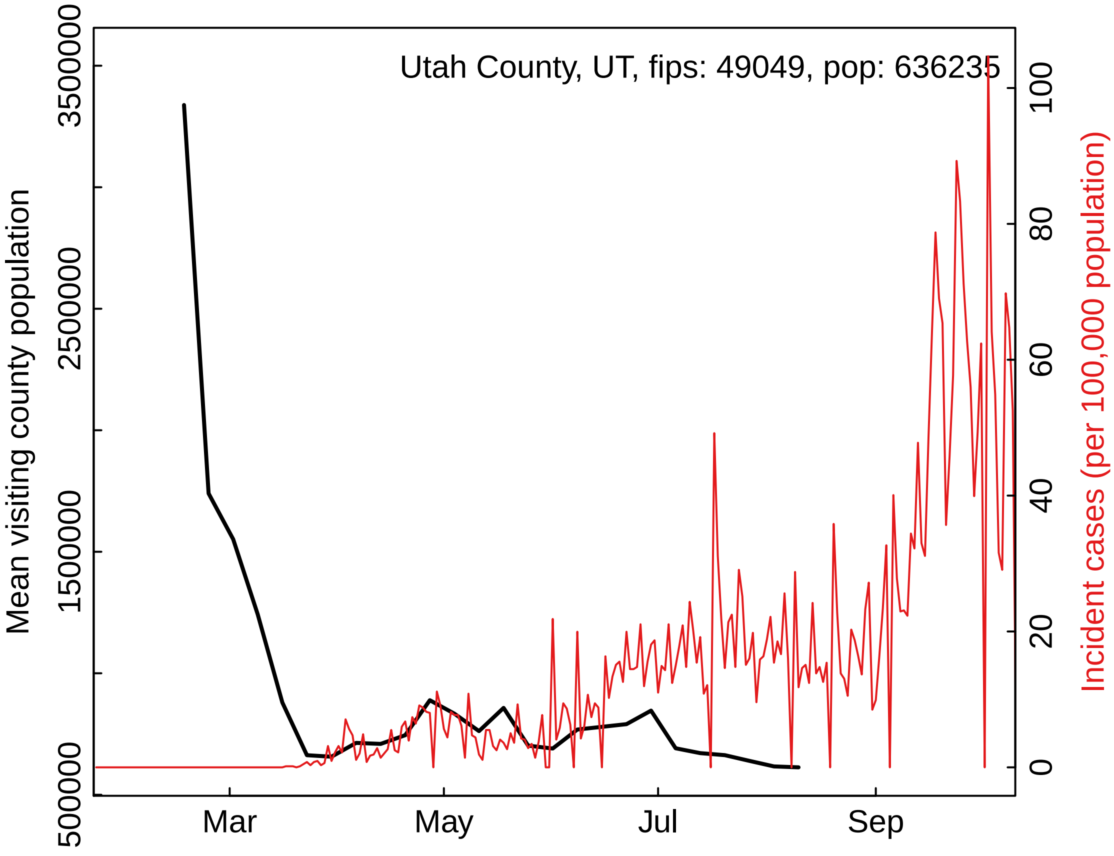

Utah County, UT, fips: 49049, pop: 636235
Mean visiting county population
5000000
15000000
25000000
35000000
Incident cases (per 100,000 population)
0
20
40
60
80
100
Mar
May
Jul
Sep

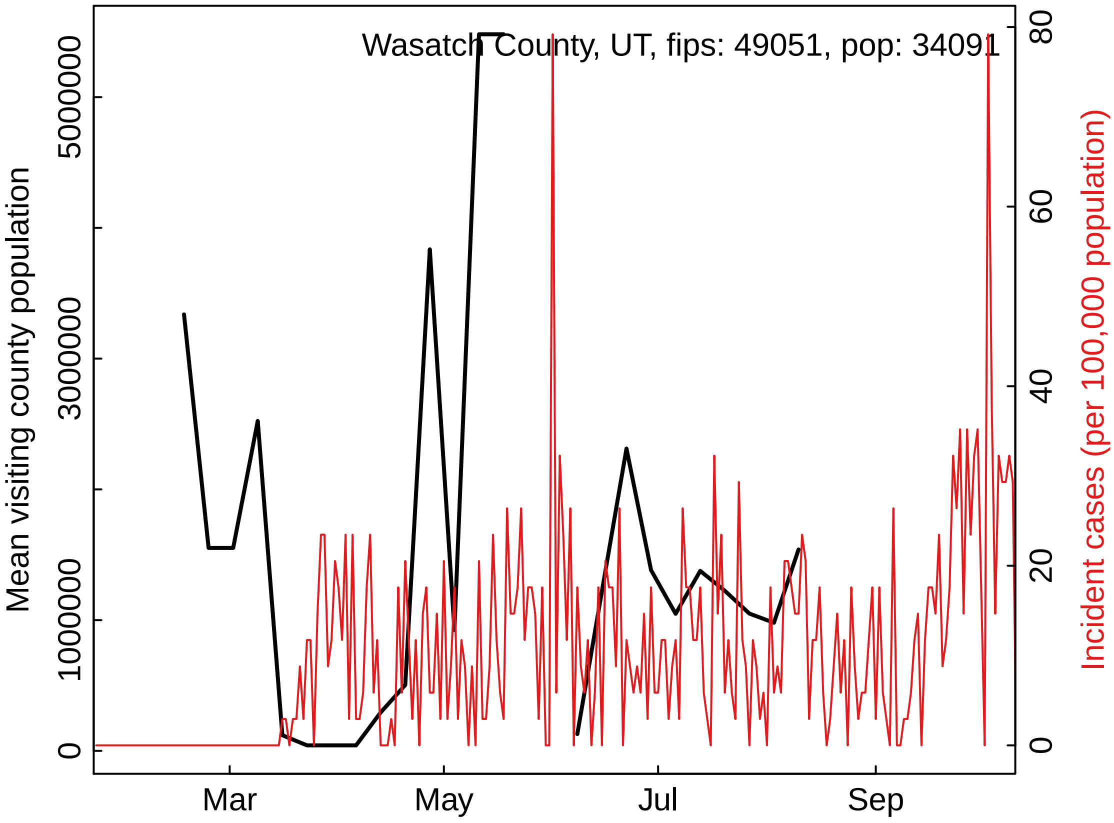

Wasatch County, UT, fips: 49051, pop: 34091
Mean visiting county population
Incident cases (per 100,000 population)
0
10000000
30000000
50000000
0
20
40
60
80
Mar
May
Jul
Sep

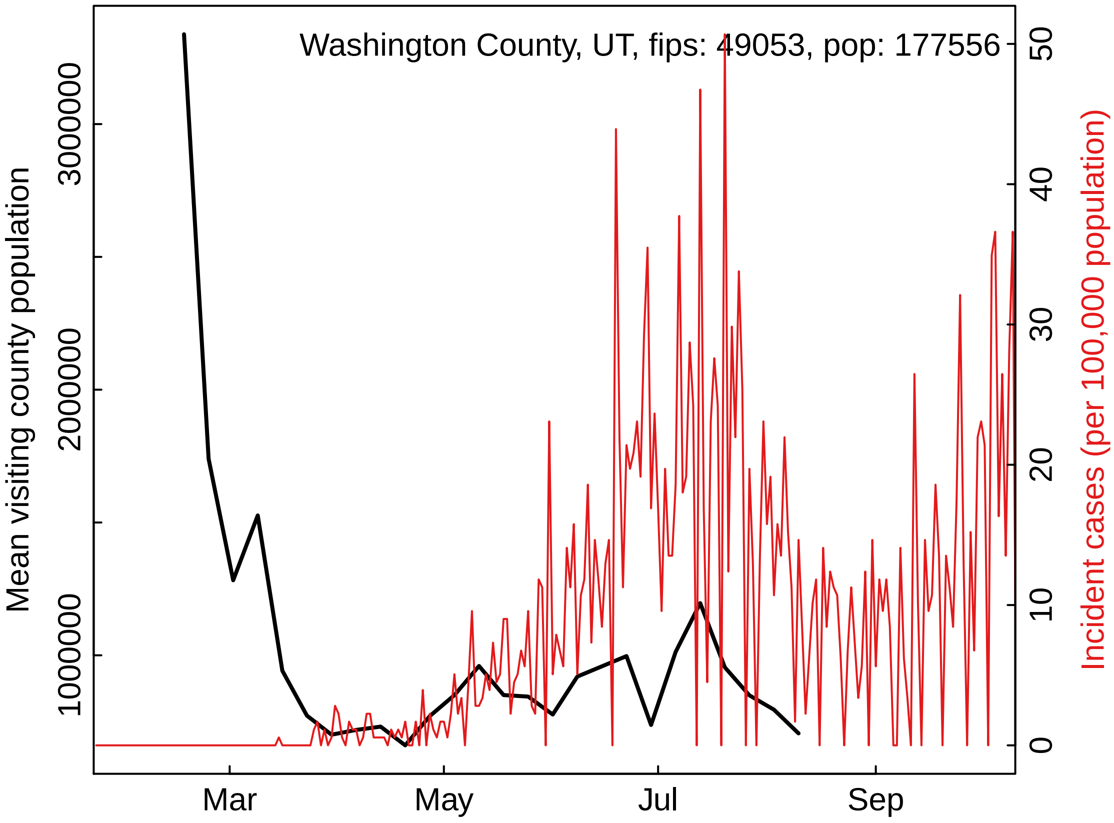
Washington County, UT, fips: 49053, pop: 177556
Mean visiting county population
10000000
20000000
30000000
Incident cases (per 100,000 population)
0
10
20
30
40
50
Mar
May
Jul
Sep

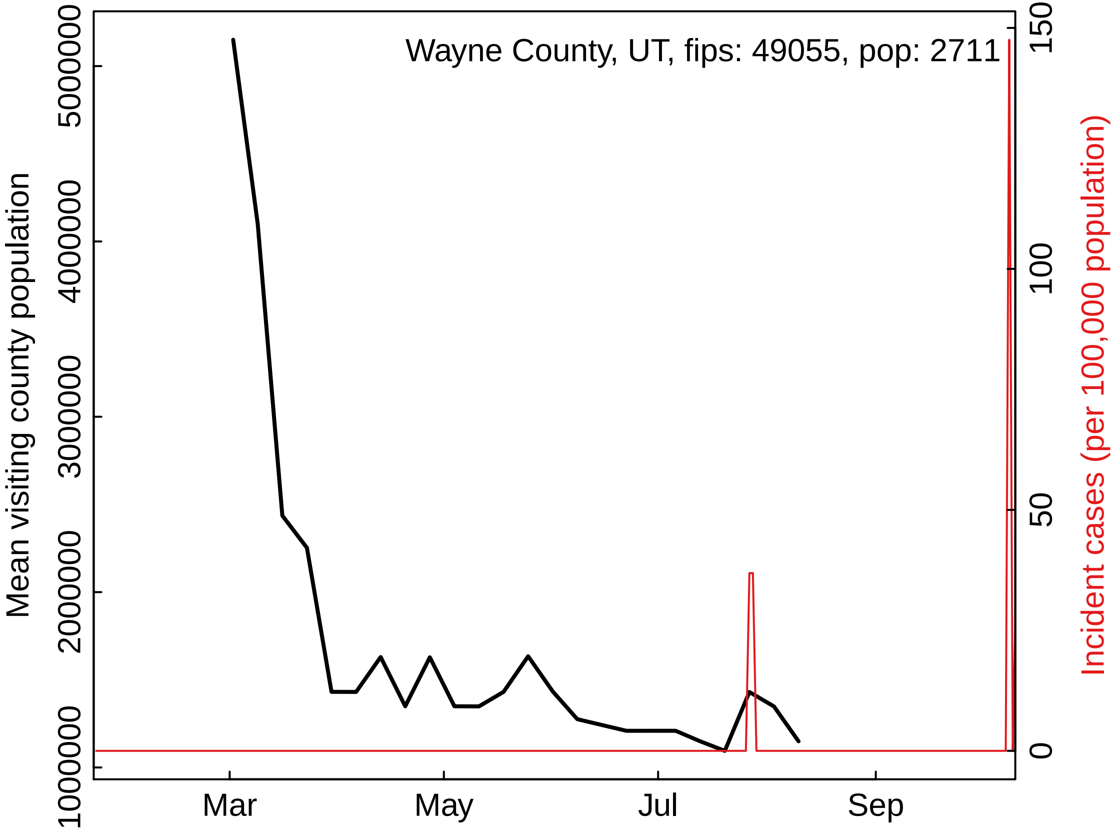

Wayne County, UT, fips: 49055, pop: 2711
Mean visiting county population
1000000
2000000
3000000
4000000
5000000
Incident cases (per 100,000 population)
0
50
100
150
Mar
May
Jul
Sep

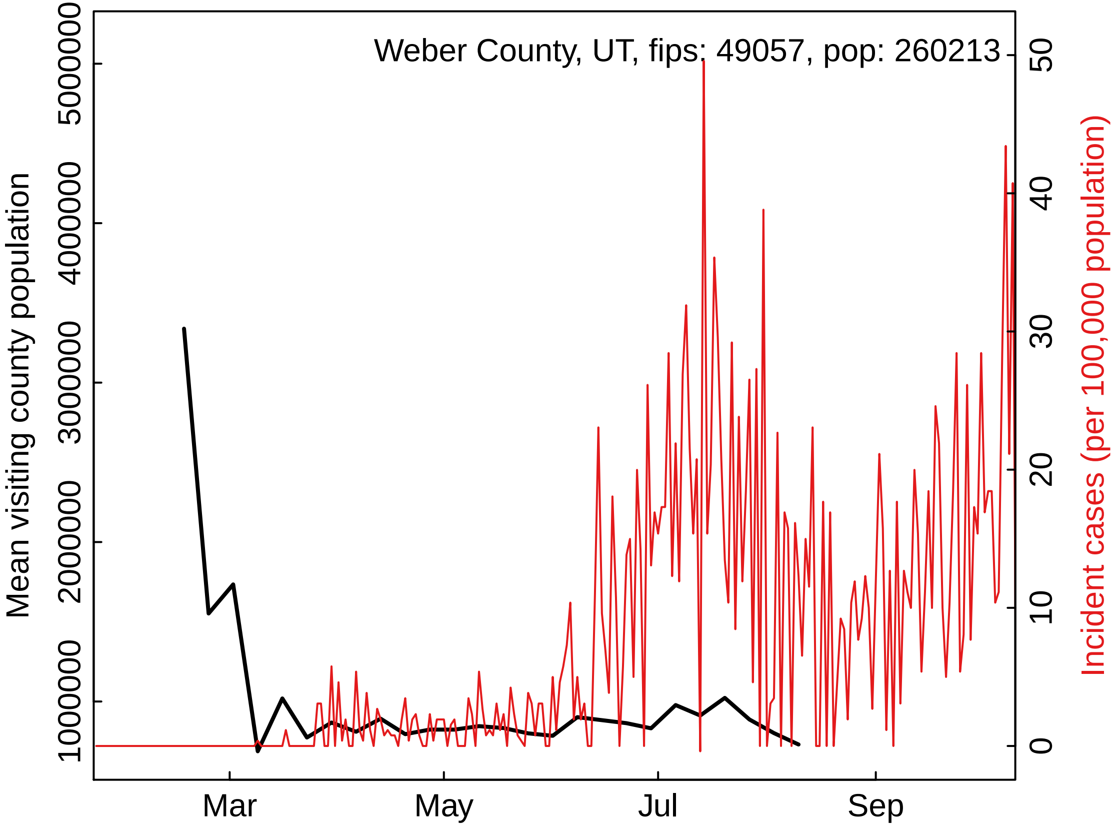

Weber County, UT, fips: 49057, pop: 260213
Mean visiting county population
10000000
20000000
30000000
40000000
50000000
Incident cases (per 100,000 population)
0
10
20
30
40
50
Mar
May
Jul
Sep

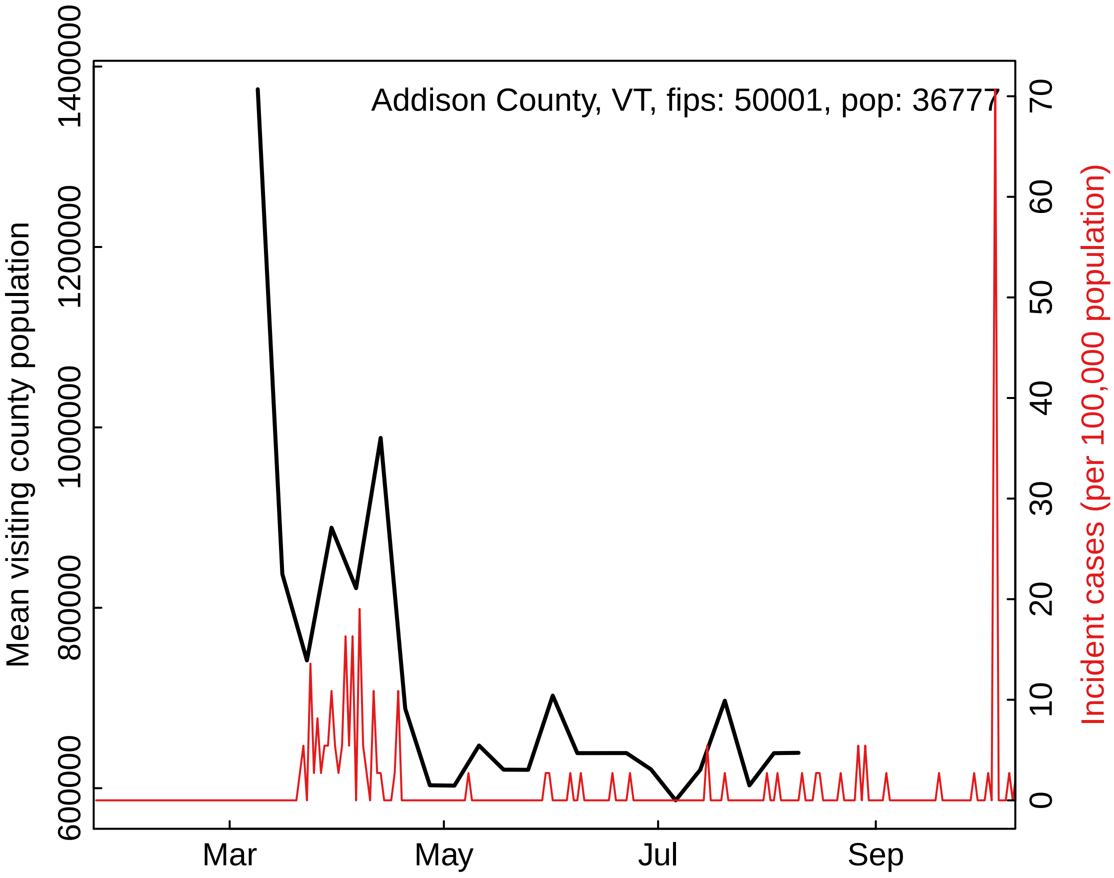
Addison County, VT, fips: 50001, pop: 36777
Mean visiting county population
600000
800000
1000000
1200000
1400000
Mar
May
Jul
Sep
0
10
20
30
40
50
60
70
Incident cases (per 100,000 population)

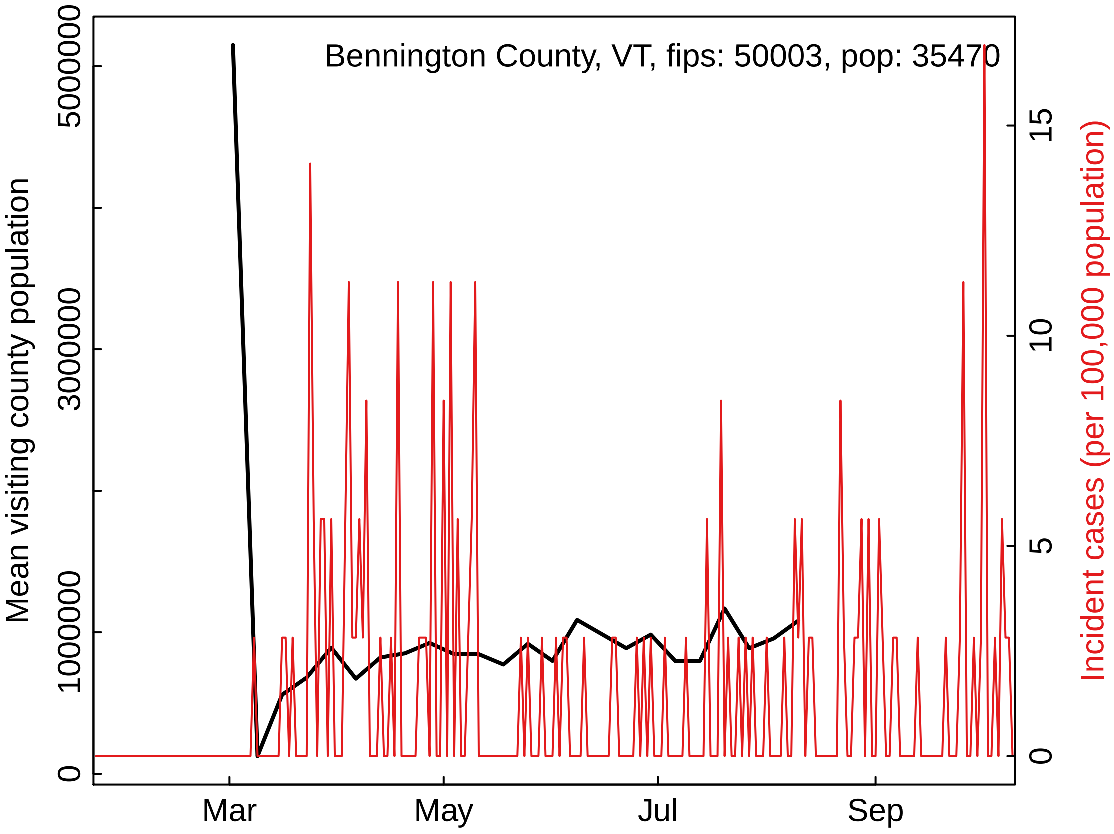

Bennington County, VT, fips: 50003, pop: 35470
Mean visiting county population
0
10000000
30000000
50000000
Incident cases (per 100,000 population)
0
5
10
15
Mar
May
Jul
Sep

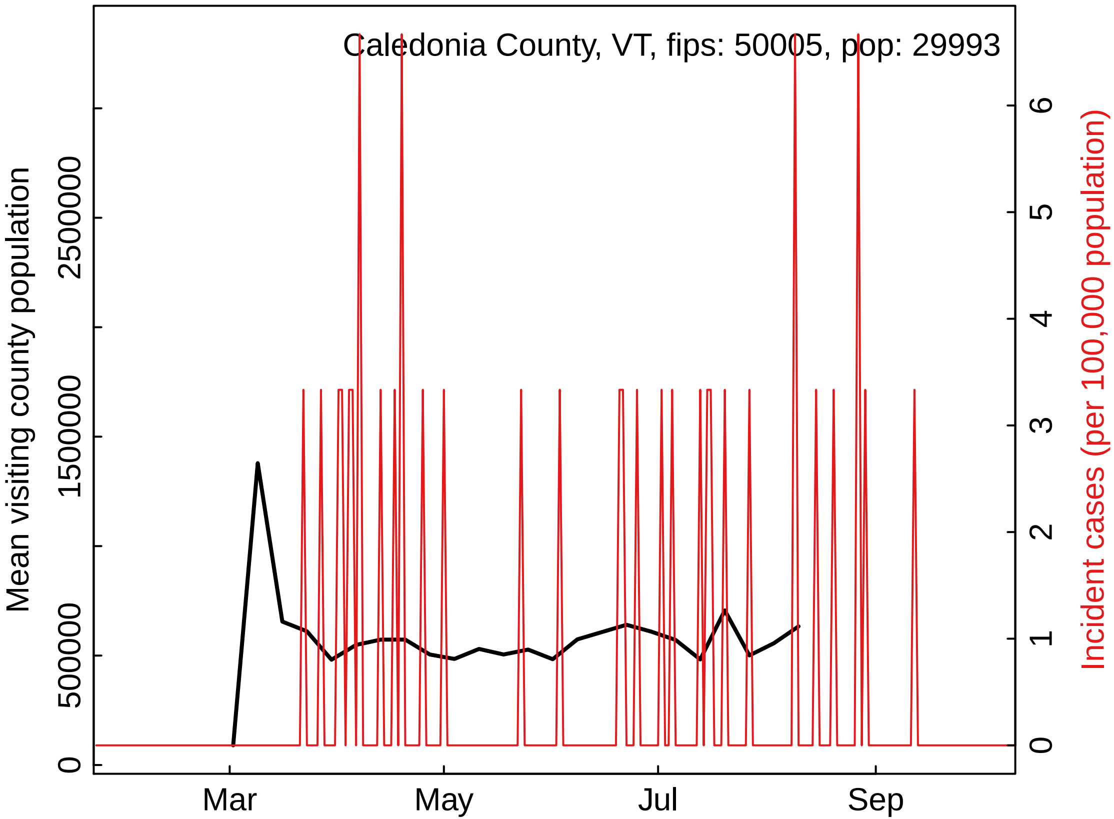

Caledonia County, VT, fips: 50005, pop: 29993
Mean visiting county population
0
500000
1500000
2500000
Incident cases (per 100,000 population)
0
1
2
3
4
5
6
Mar
May
Jul
Sep

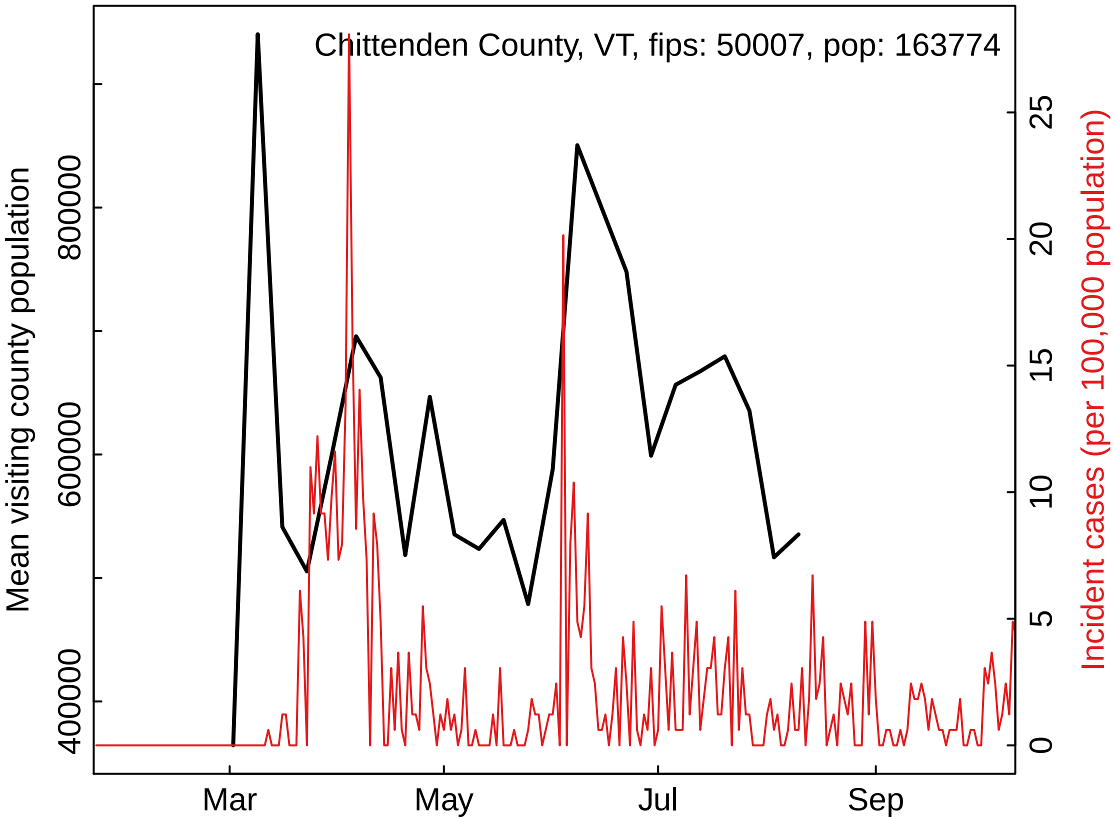
Chittenden County, VT, fips: 50007, pop: 163774
Mean visiting county population
400000
600000
800000
Incident cases (per 100,000 population)
0
5
10
15
20
25
Mar
May
Jul
Sep

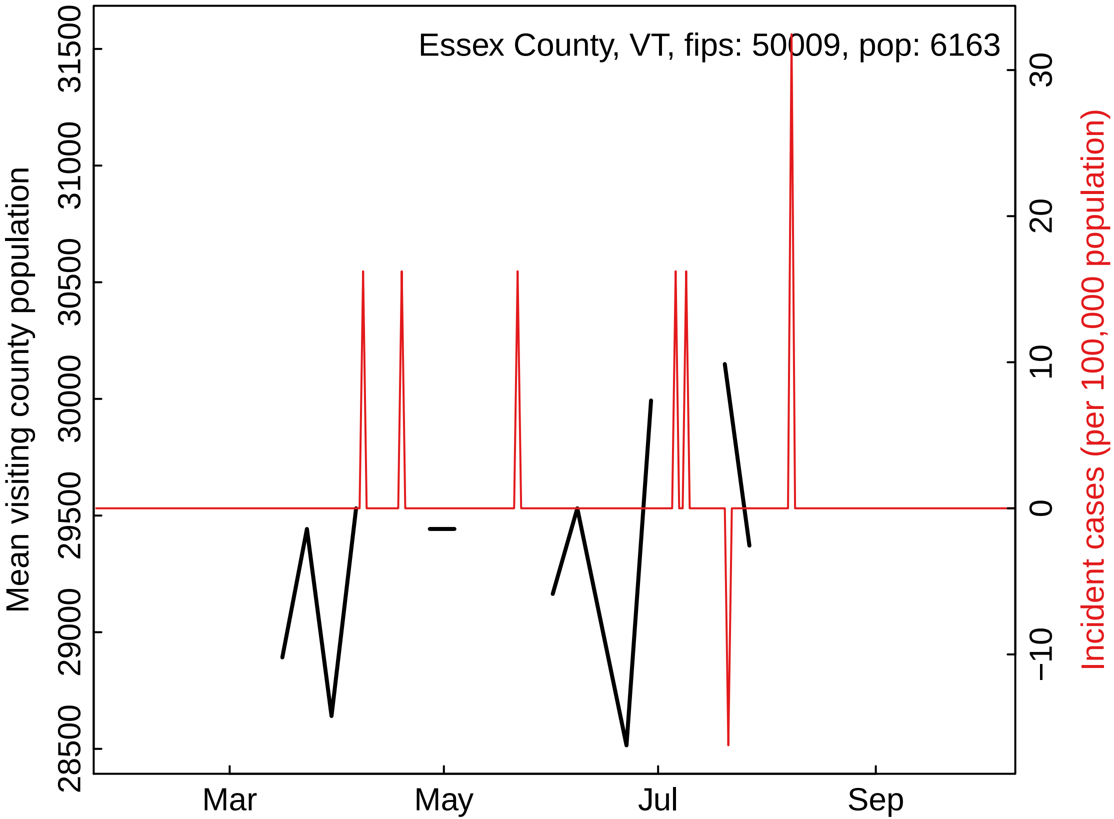

Essex County, VT, fips: 50009, pop: 6163
Mean visiting county population
28500
29000
29500
30000
30500
31000
31500
Incident cases (per 100,000 population)
−10
0
10
20
30
Mar
May
Jul
Sep

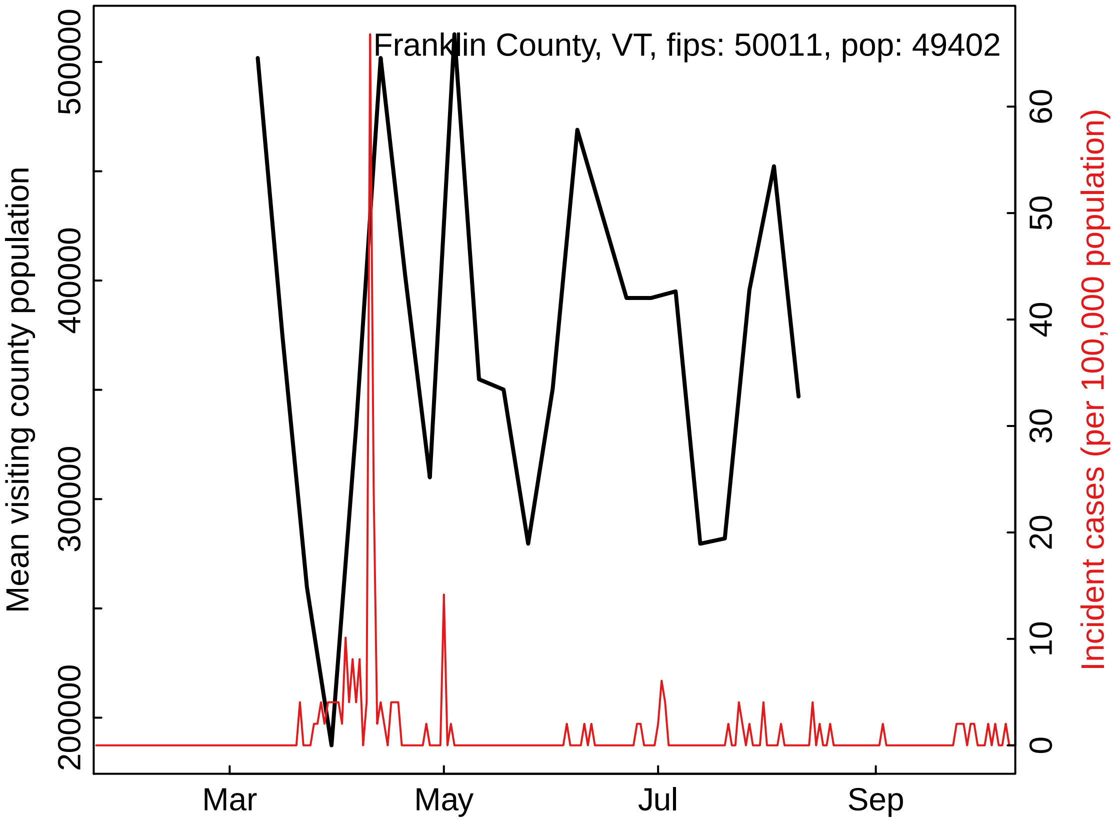

Franklin County, VT, fips: 50011, pop: 49402
Mean visiting county population
200000
300000
400000
500000
Incident cases (per 100,000 population)
0
10
20
30
40
50
60
Mar
May
Jul
Sep

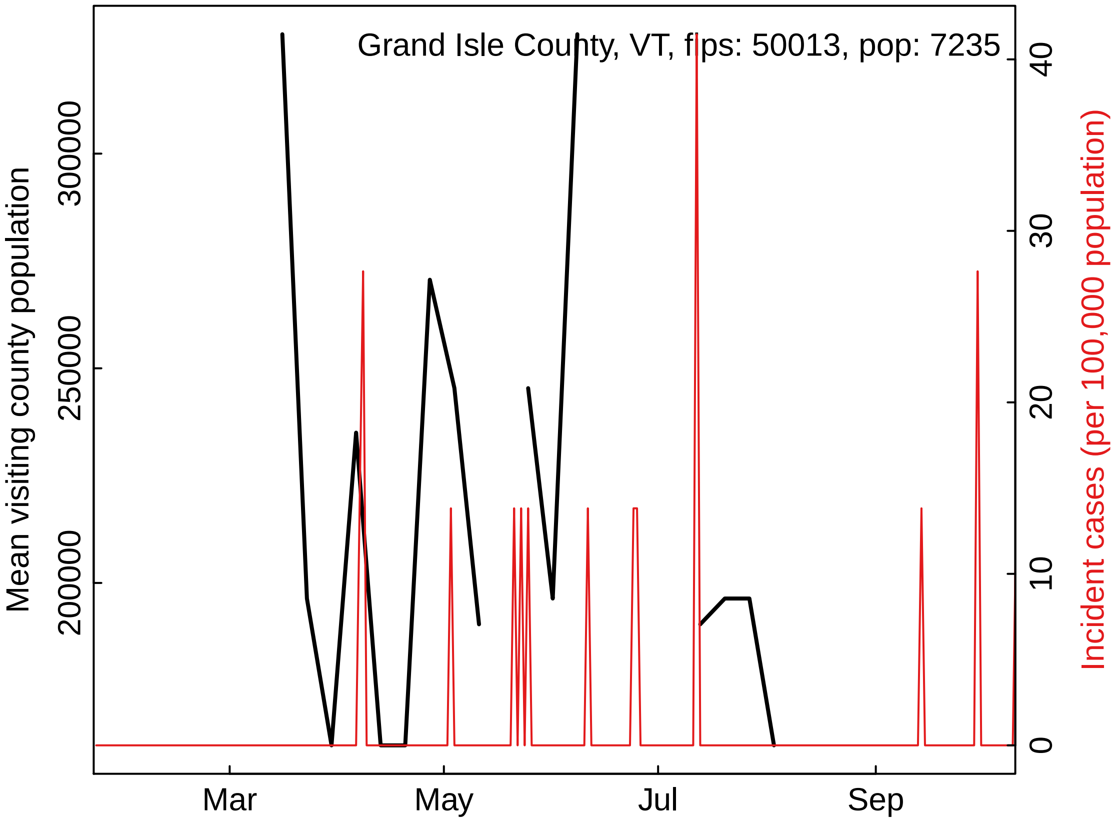
Grand Isle County, VT, flips: 50013, pop: 7235
Mean visiting county population
200000
250000
300000
Incident cases (per 100,000 population)
0
10
20
30
40
Mar
May
Jul
Sep

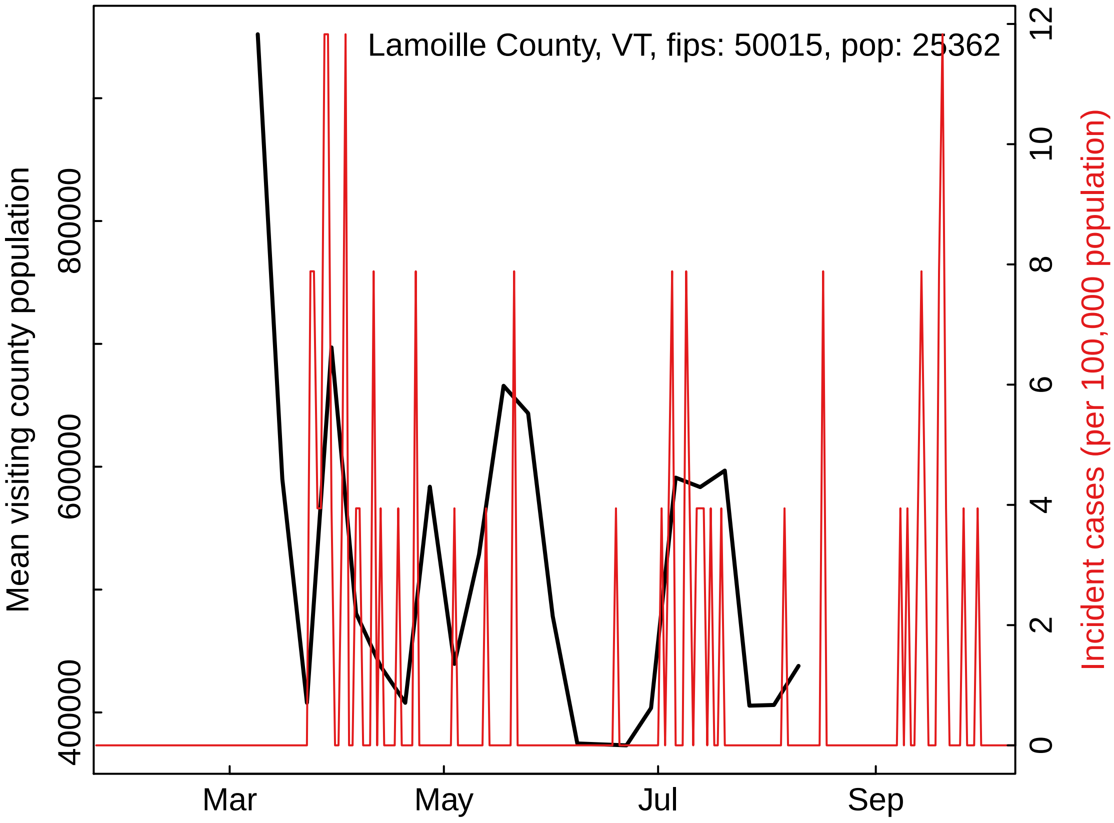

Lamoille County, VT, fips: 50015, pop: 25362
Mean visiting county population
400000
600000
800000
Incident cases (per 100,000 population)
0
2
4
6
8
10
12
Mar
May
Jul
Sep

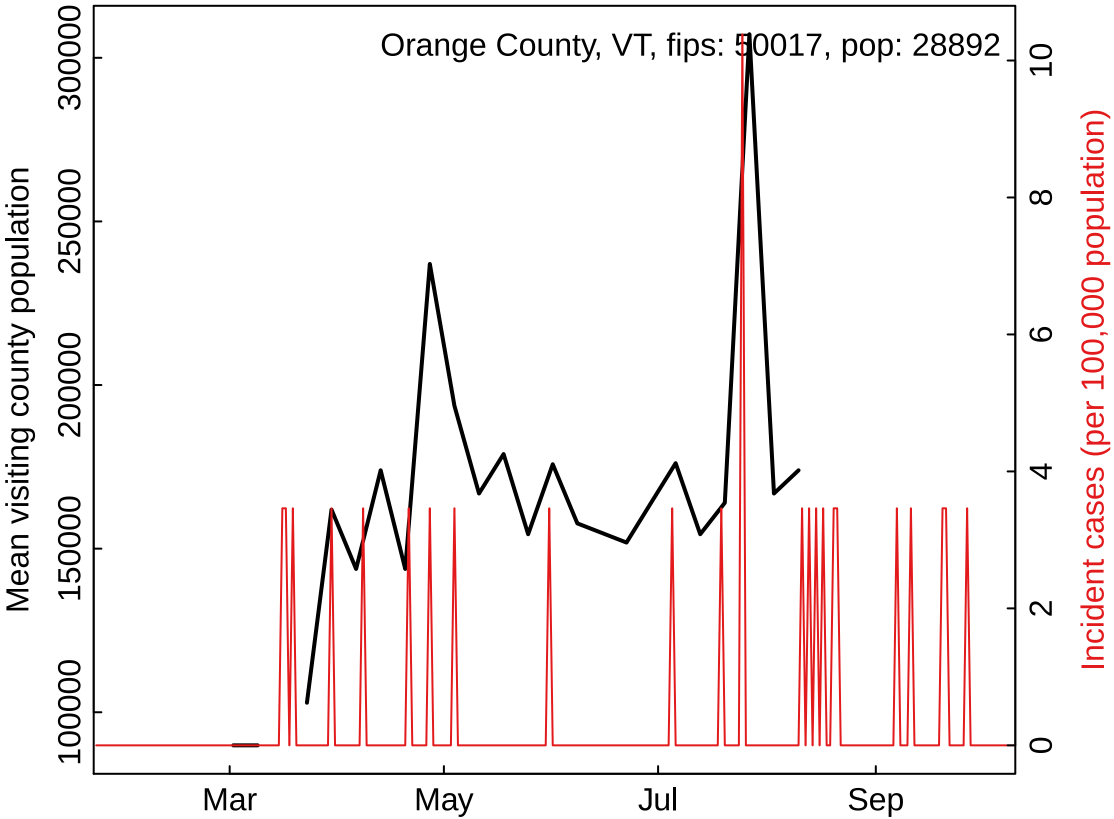
Orange County, VT, fips: 50017, pop: 28892
Mean visiting county population
100000
150000
200000
250000
300000
Incident cases (per 100,000 population)
0
2
4
6
8
10
Mar
May
Jul
Sep

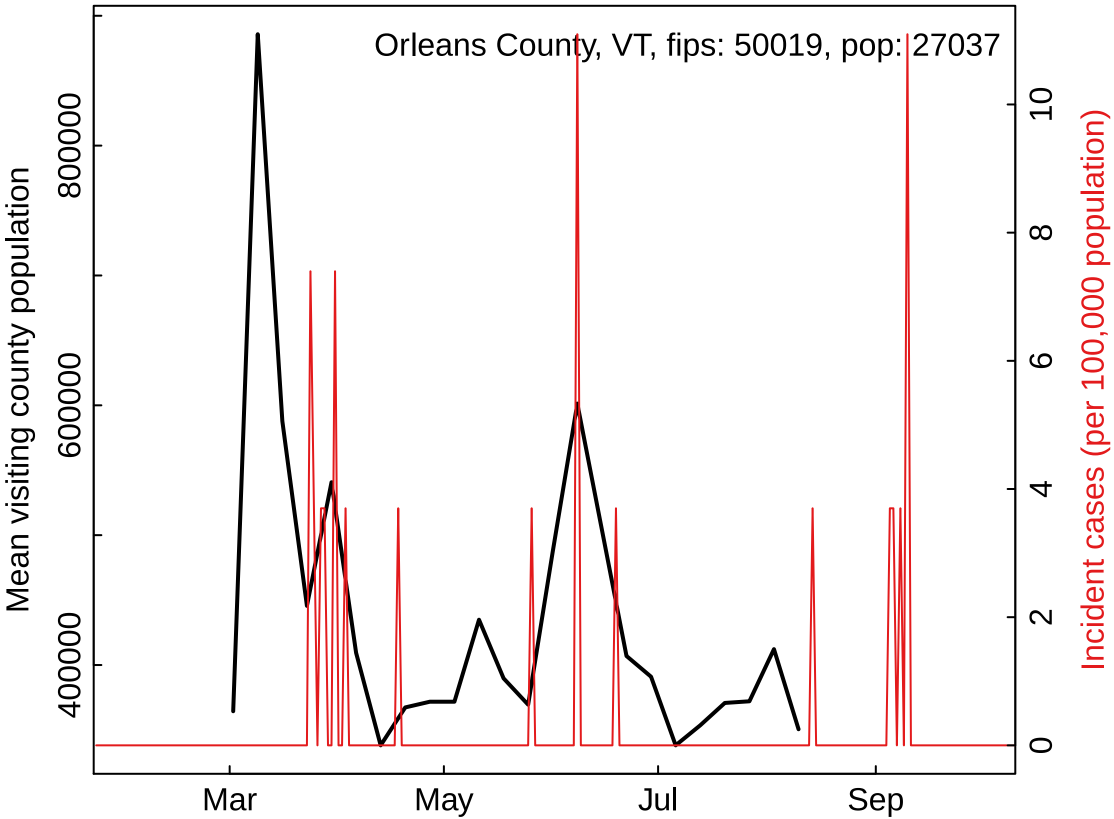

Orleans County, VT, fips: 50019, pop: 27037
Mean visiting county population
400000
600000
800000
Mar
May
Jul
Sep
0
2
4
6
8
10
Incident cases (per 100,000 population)

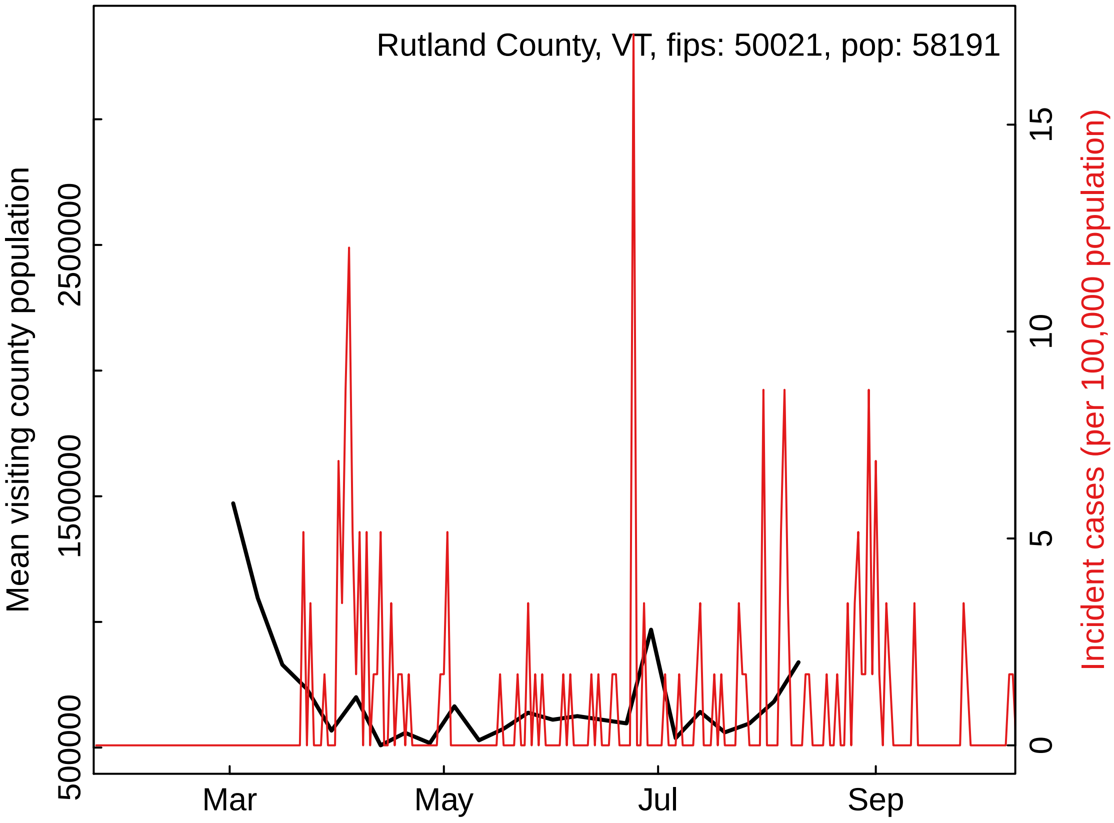
Rutland County, VT, fips: 50021, pop: 58191
Mean visiting county population
500000
1500000
2500000
Incident cases (per 100,000 population)
0
5
10
15
Mar
May
Jul
Sep

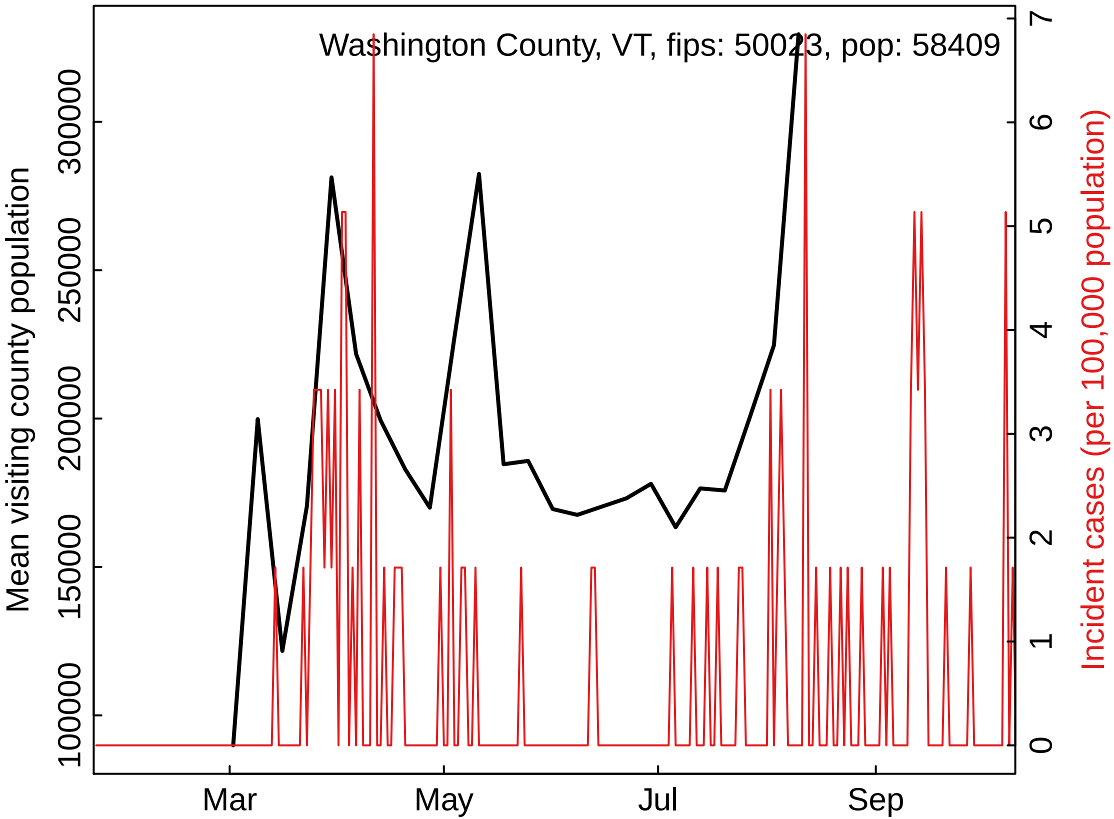

Washington County, VT, fips: 50023, pop: 58409
Mean visiting county population
100000
150000
200000
250000
300000
Incident cases (per 100,000 population)
0
1
2
3
4
5
6
7
Mar
May
Jul
Sep

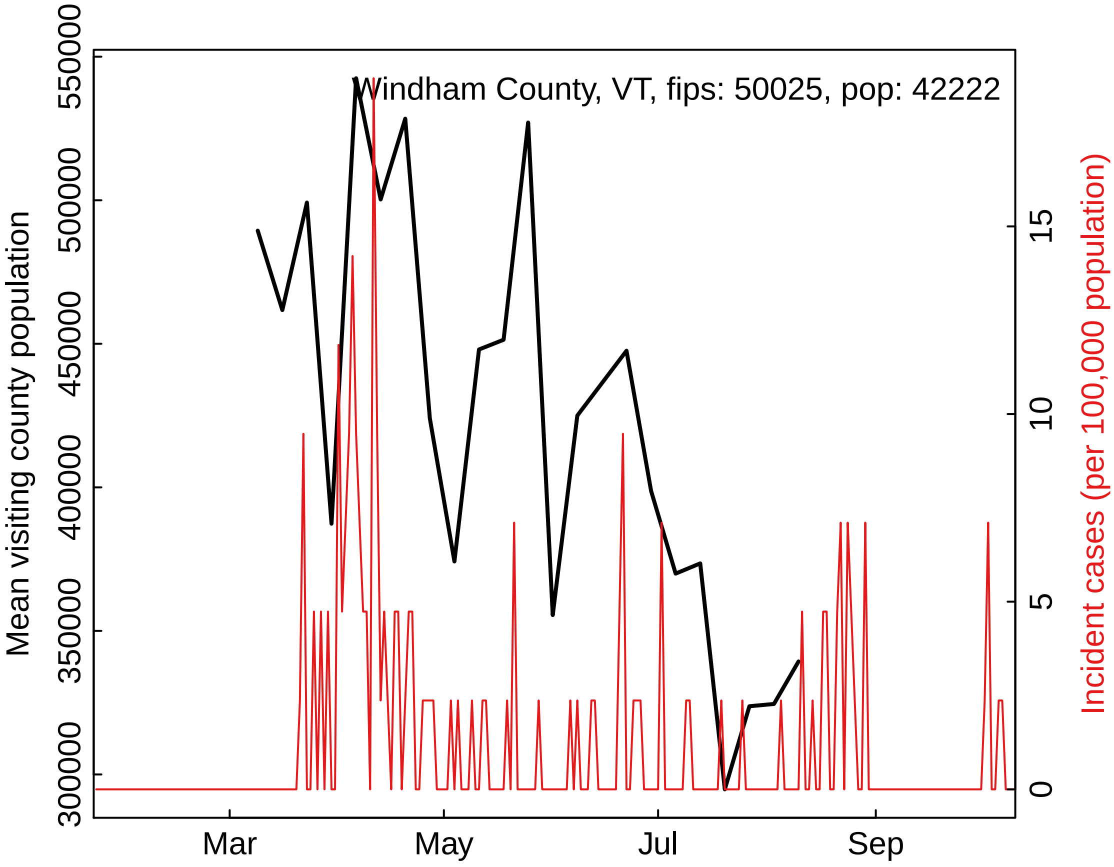

Windham County, VT, fips: 50025, pop: 42222
Mean visiting county population
Incident cases (per 100,000 population)
300000
350000
400000
450000
500000
550000
0
5
10
15
Mar
May
Jul
Sep

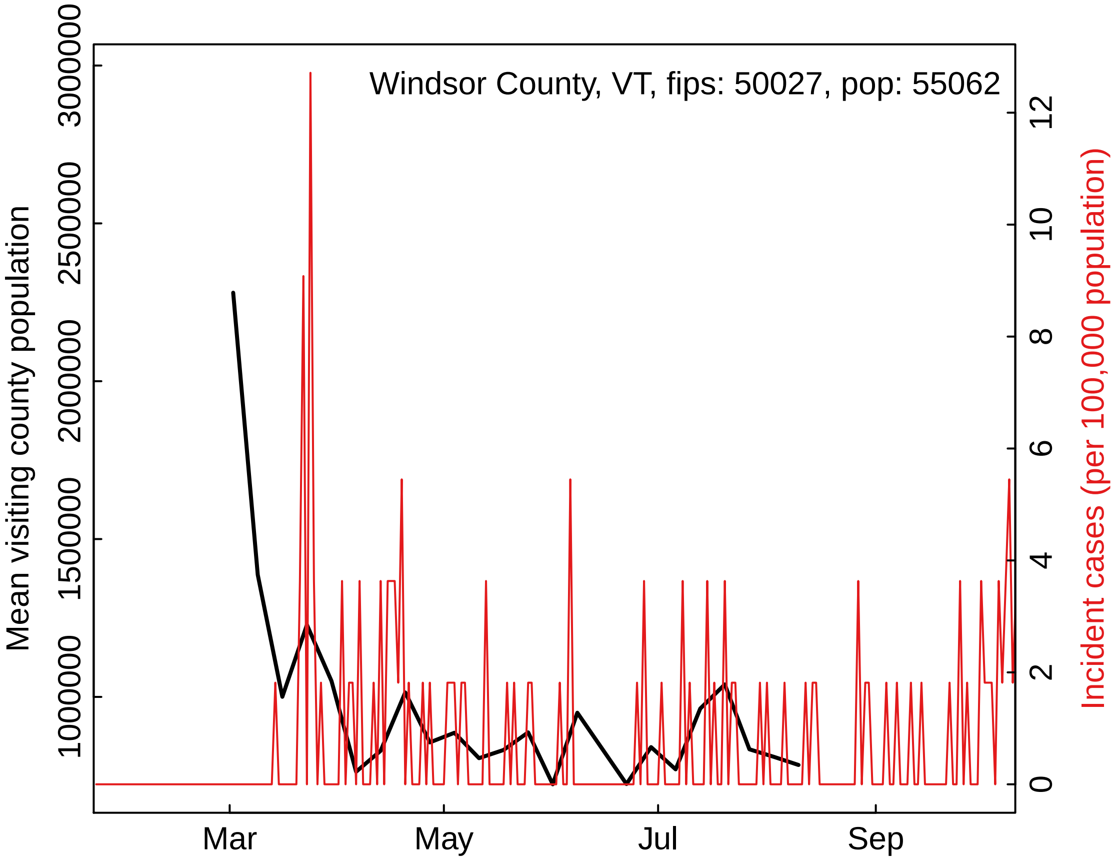

Windsor County, VT, fips: 50027, pop: 55062
Mean visiting county population
Incident cases (per 100,000 population)
10000000
15000000
20000000
25000000
30000000
0
2
4
6
8
10
12
Mar
May
Jul
Sep

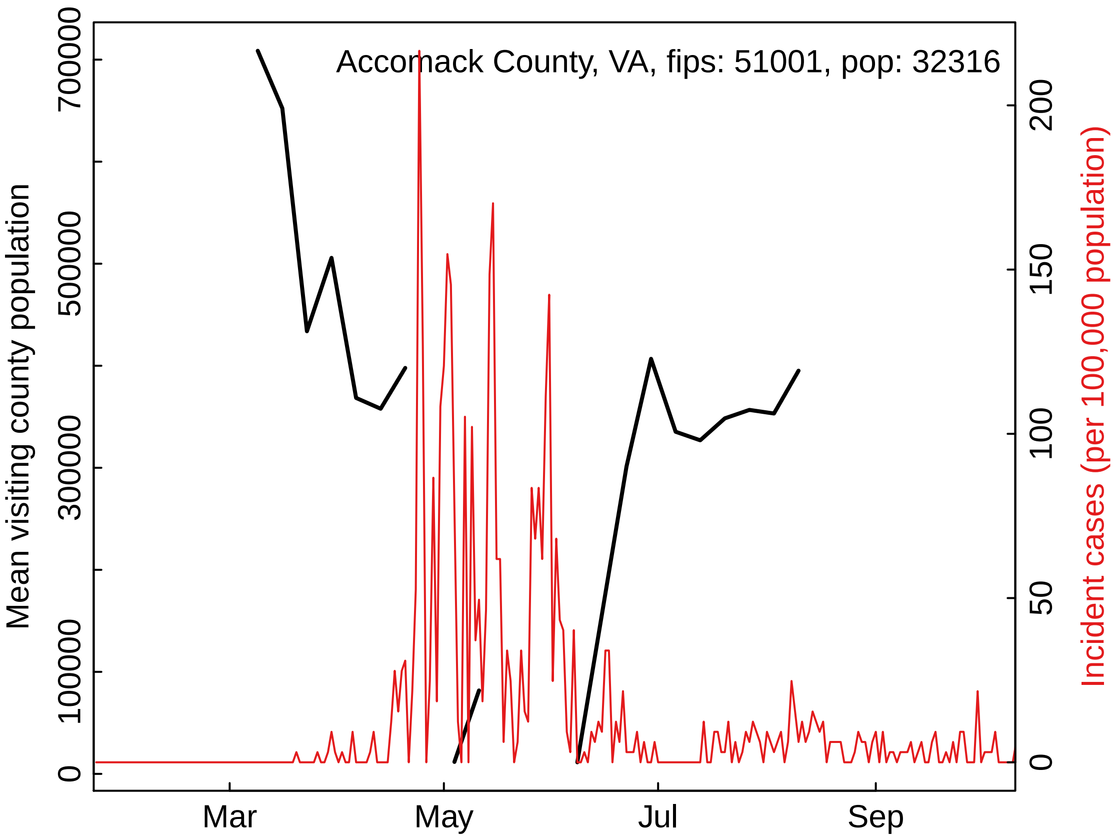
Accomack County, VA, fips: 51001, pop: 32316
Mean visiting county population
Incident cases (per 100,000 population)
0
100000
300000
500000
700000
0
50
100
150
200
Mar
May
Jul
Sep

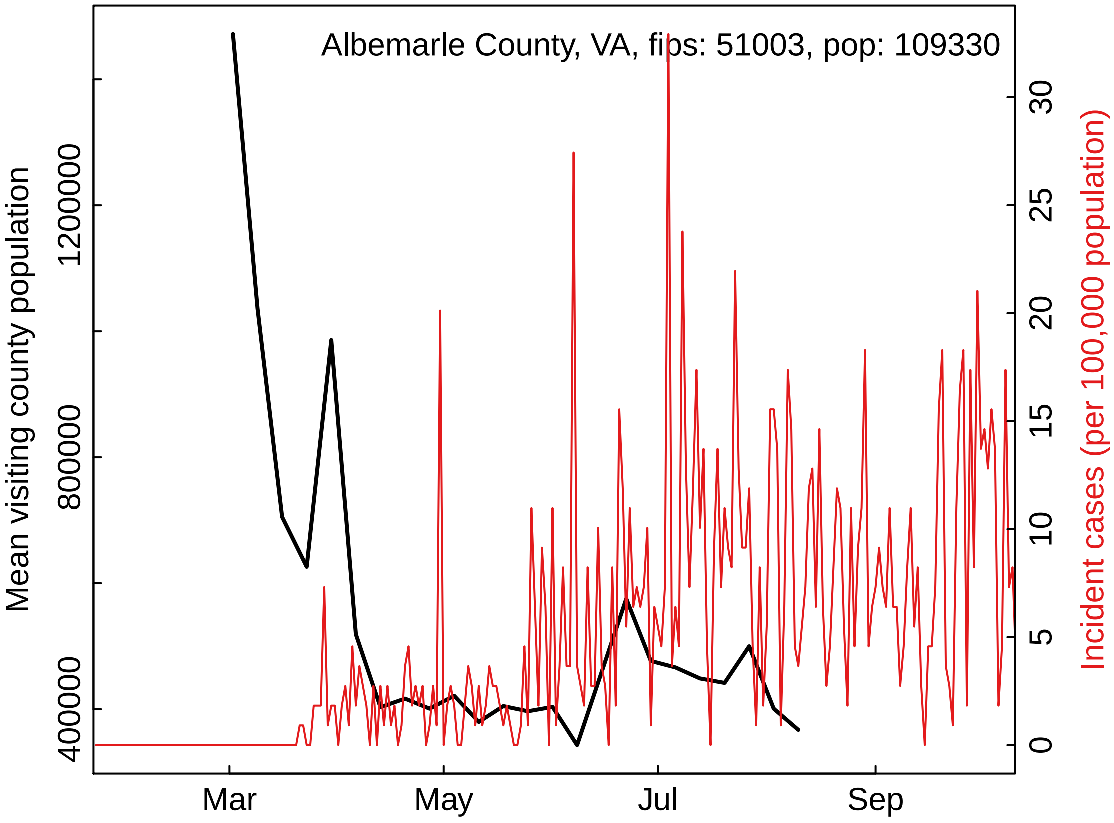
Albemarle County, VA, fips: 51003, pop: 109330
Mean visiting county population
400000
800000
1200000
Mar
May
Jul
Sep
0
5
10
15
20
25
30
Incident cases (per 100,000 population)

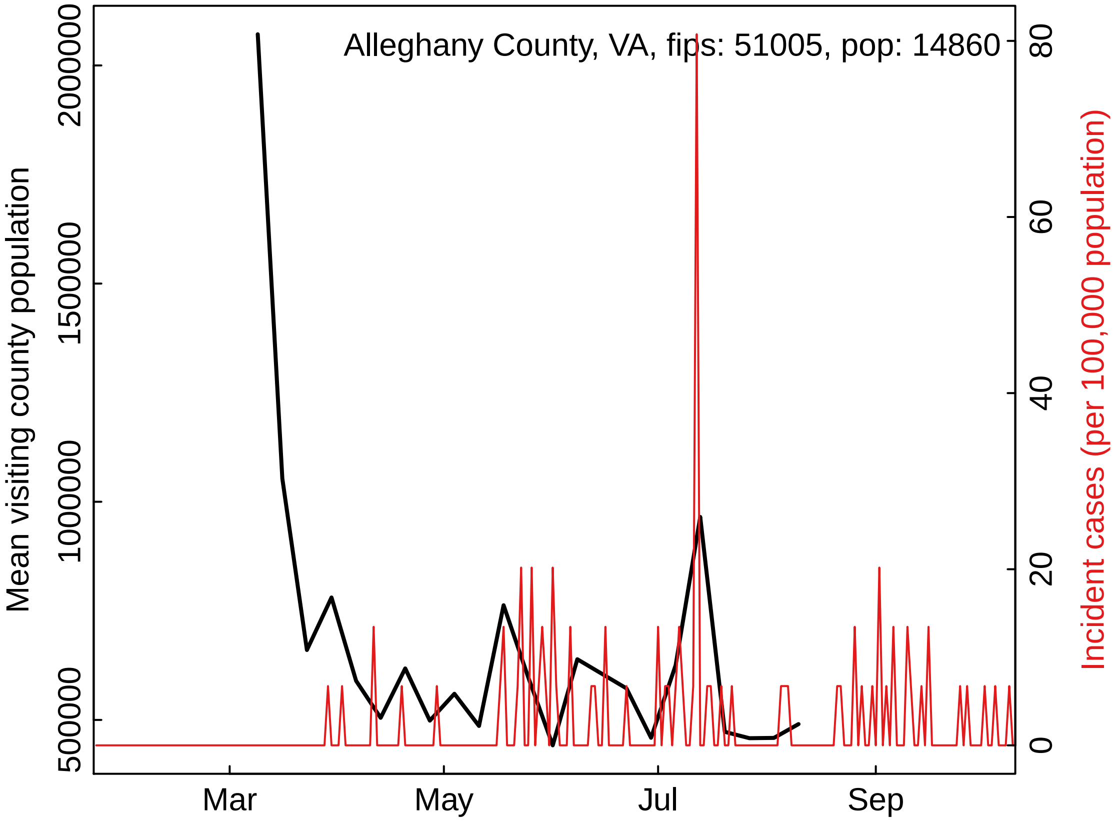

Alleghany County, VA, fips: 51005, pop: 14860
Mean visiting county population
2000000
1500000
1000000
500000
Incident cases (per 100,000 population)
80
60
40
20
0
Mar
May
Jul
Sep

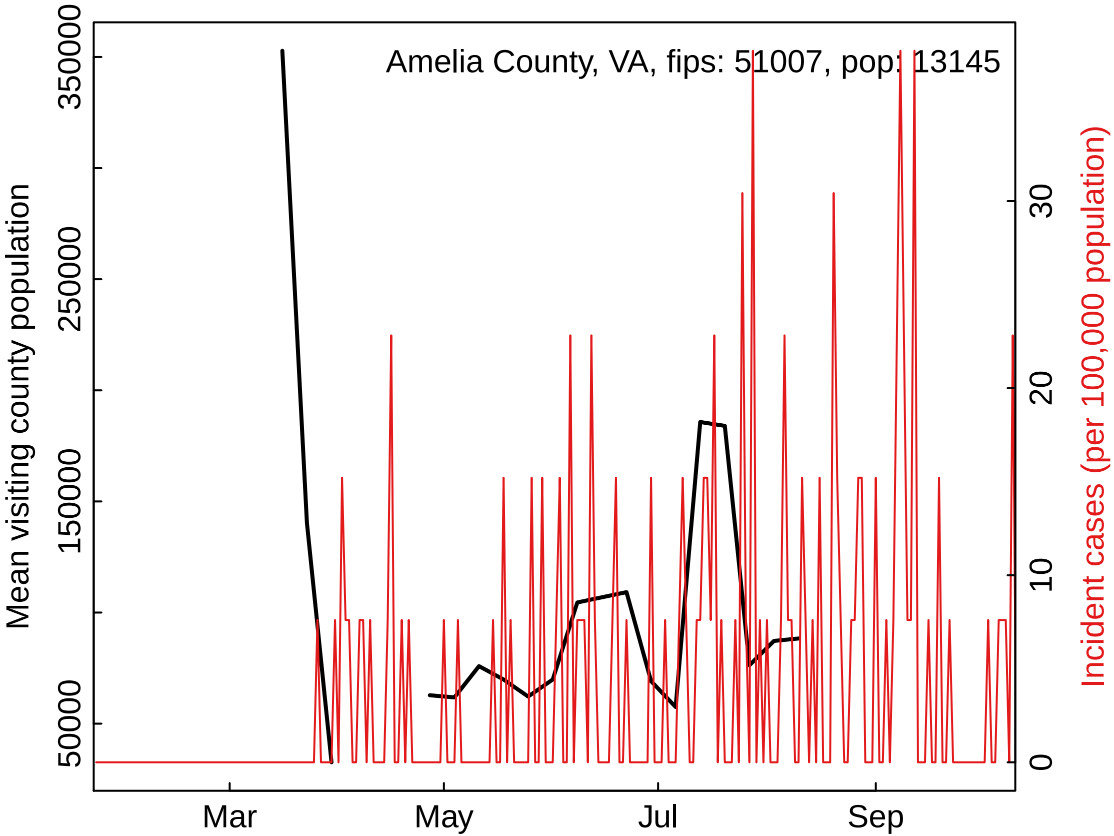
Amelia County, VA, fips: 51007, pop: 13145
Mean visiting county population
50000
150000
250000
350000
Incident cases (per 100,000 population)
0
10
20
30
Mar
May
Jul
Sep

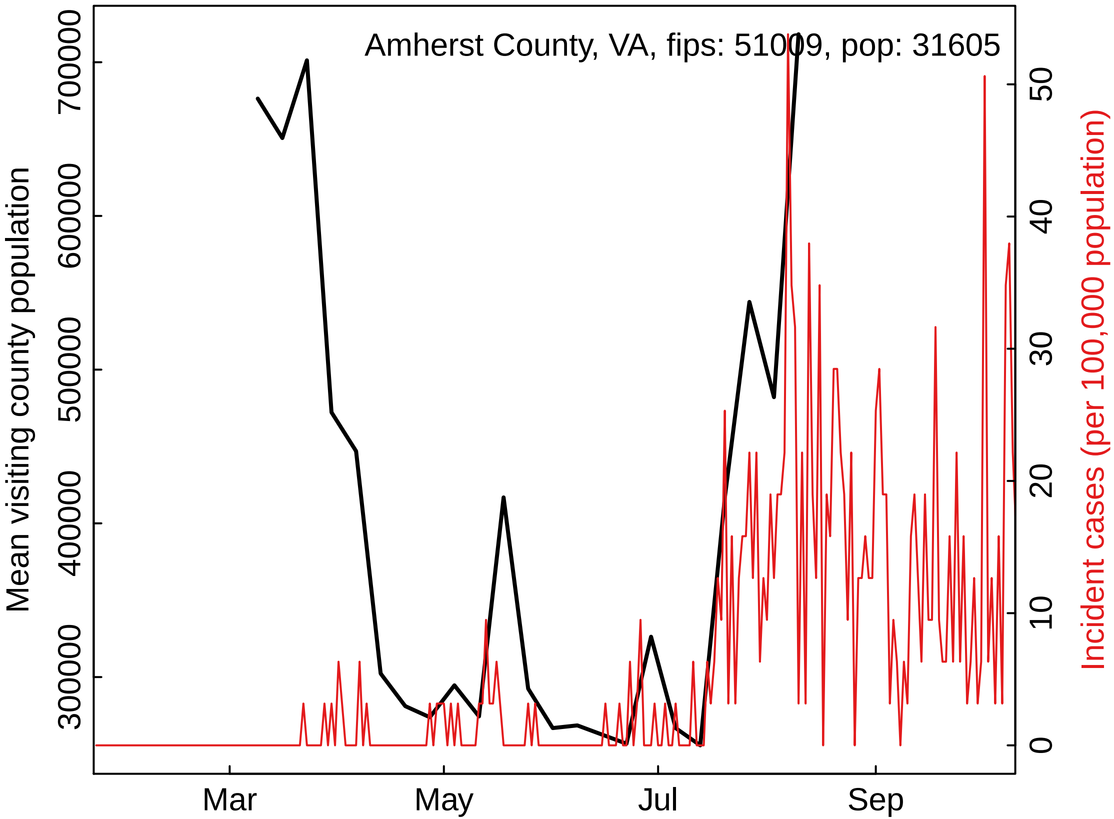

Amherst County, VA, fips: 51009, pop: 31605
Mean visiting county population
Incident cases (per 100,000 population)
300000
400000
500000
600000
700000
0
10
20
30
40
50
Mar
May
Jul
Sep

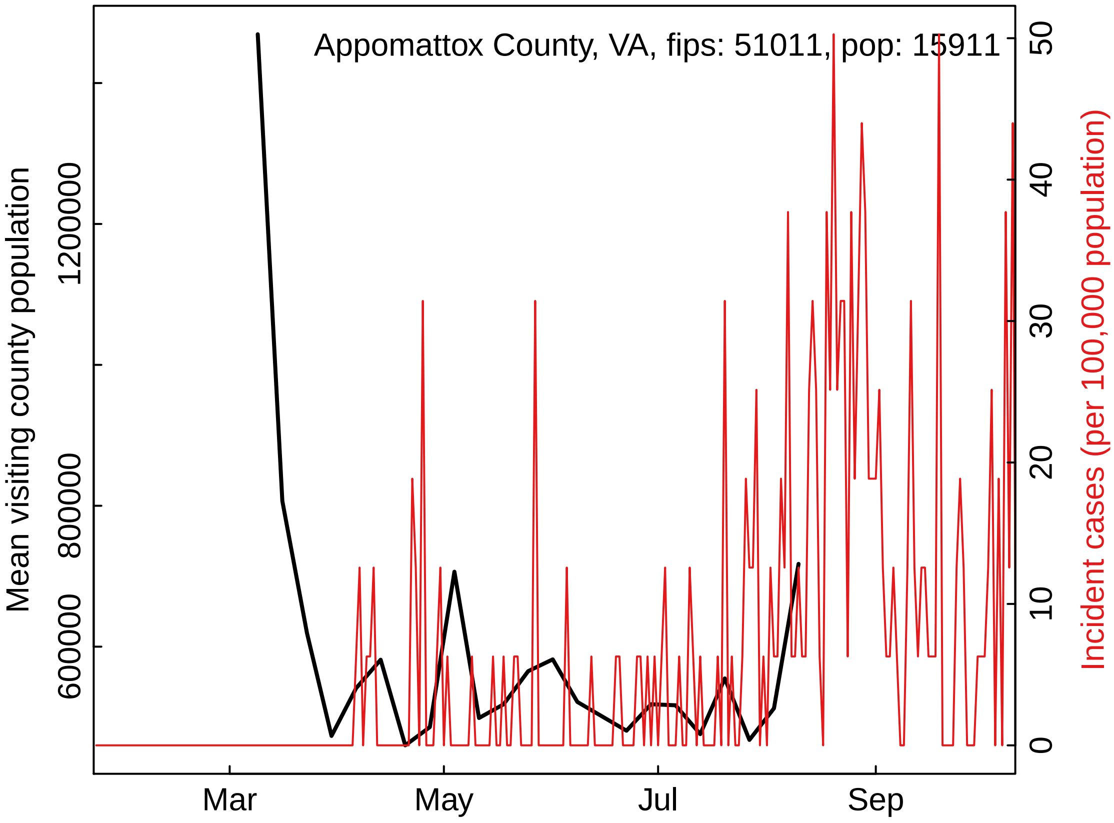

Appomattox County, VA, fips: 51011, pop: 15911
Mean visiting county population
600000
800000
1200000
Incident cases (per 100,000 population)
0
10
20
30
40
50
Mar
May
Jul
Sep

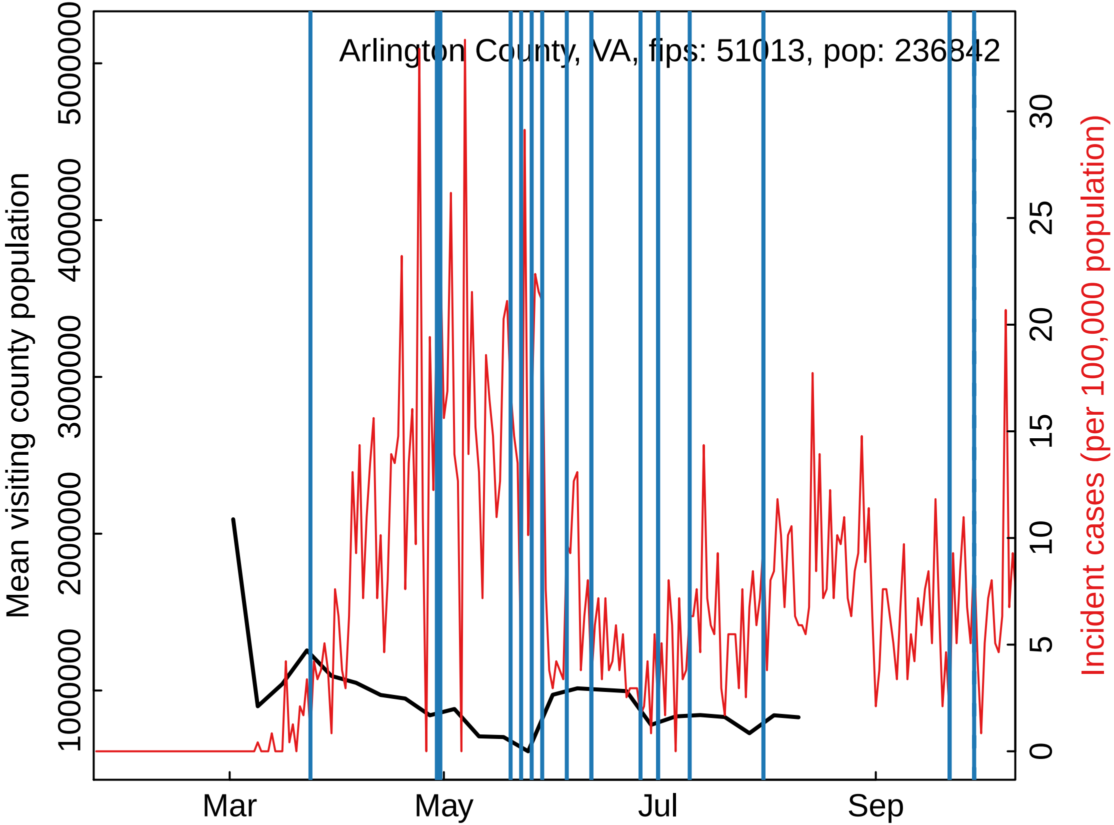

Arlington County, VA, fips: 51013, pop: 236842
Mean visiting county population
10000000
20000000
30000000
40000000
50000000
Incident cases (per 100,000 population)
0
5
10
15
20
25
30
Mar
May
Jul
Sep

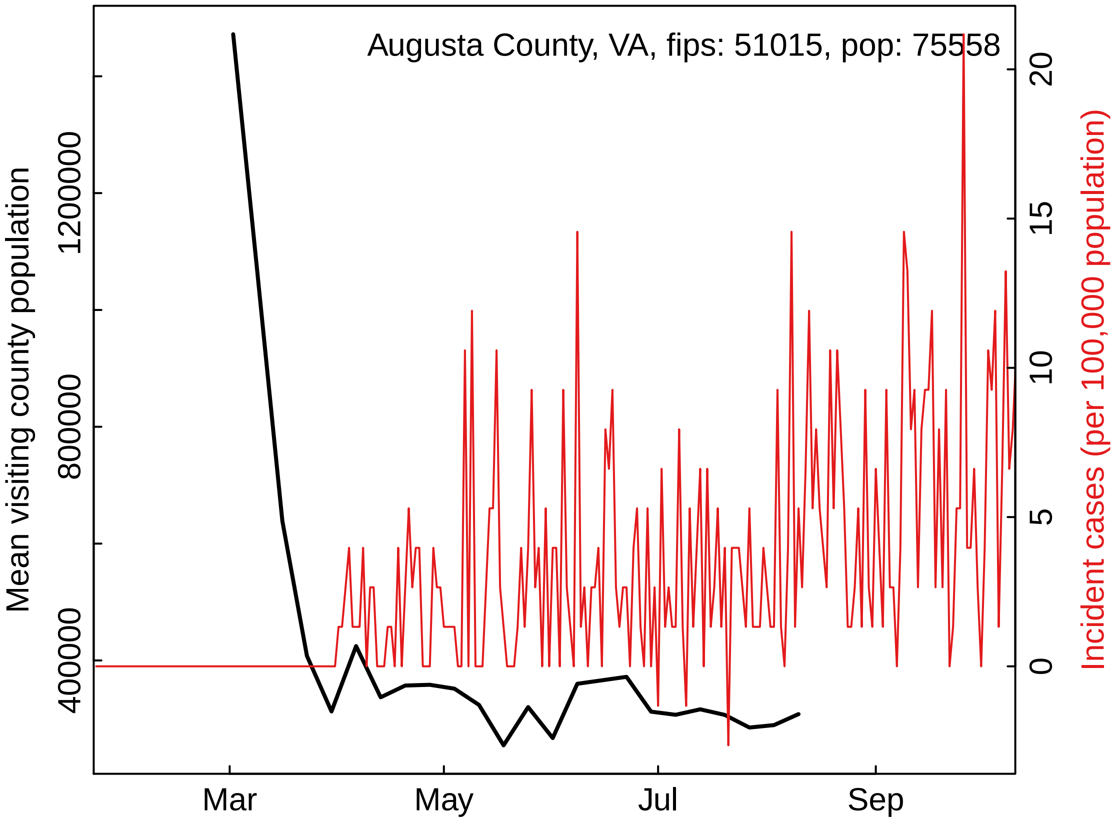

Augusta County, VA, fips: 51015, pop: 75558
Mean visiting county population
400000
800000
1200000
Mar
May
Jul
Sep
0
5
10
15
20
Incident cases (per 100,000 population)

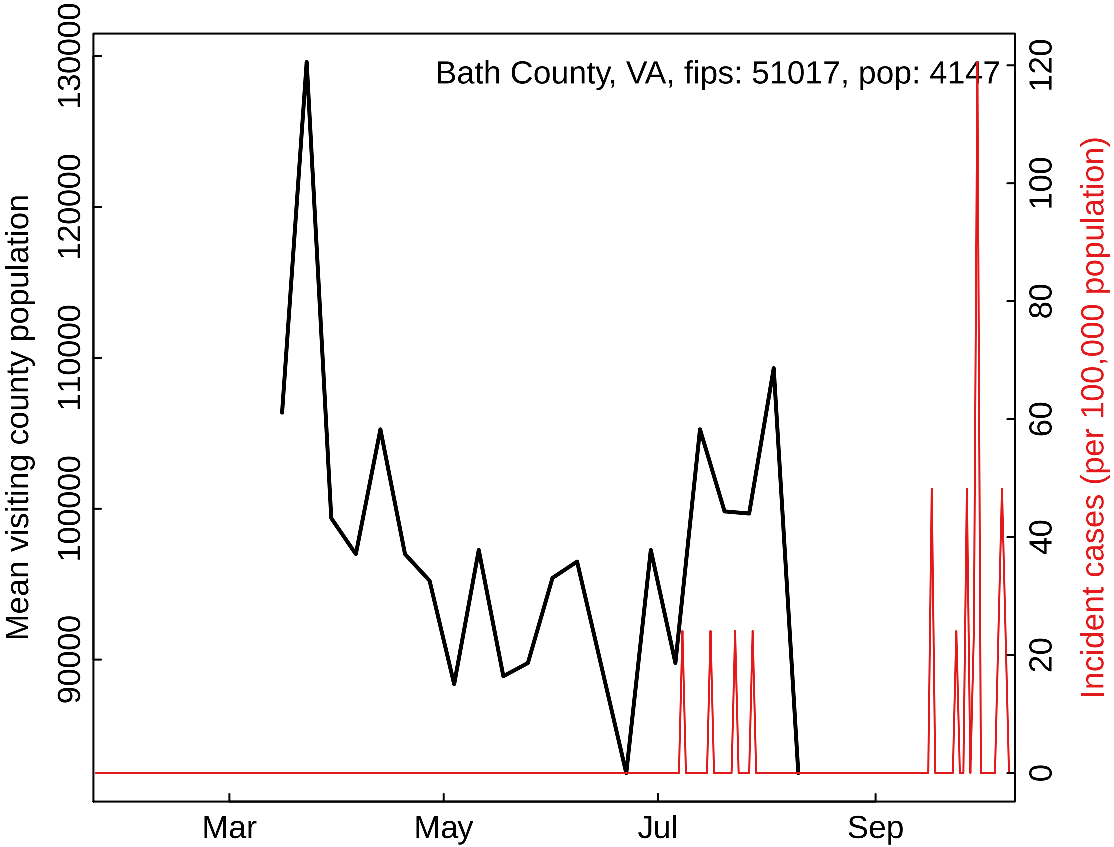

Bath County, VA, fips: 51017, pop: 4147
Mean visiting county population
90000
100000
110000
120000
130000
Incident cases (per 100,000 population)
0
20
40
60
80
100
120
Mar
May
Jul
Sep

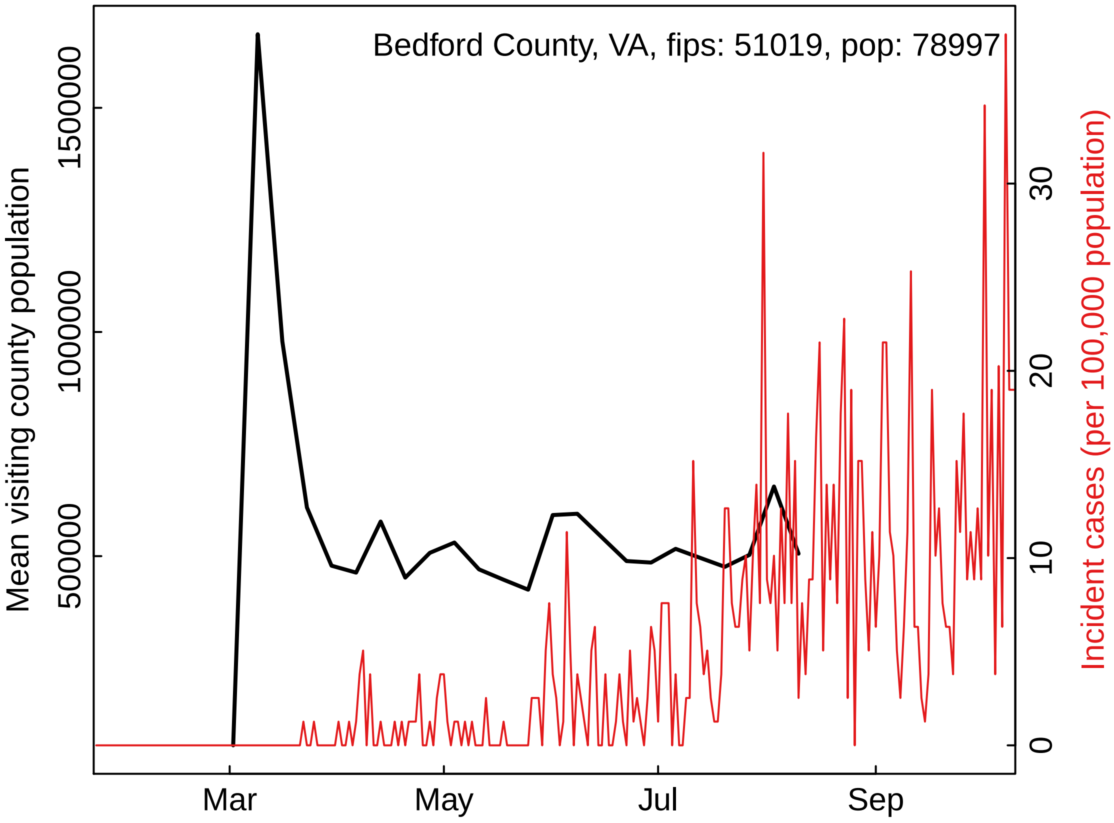

Bedford County, VA, fips: 51019, pop: 78997
Mean visiting county population
Incident cases (per 100,000 population)
500000
1000000
1500000
0
10
20
30
Mar
May
Jul
Sep

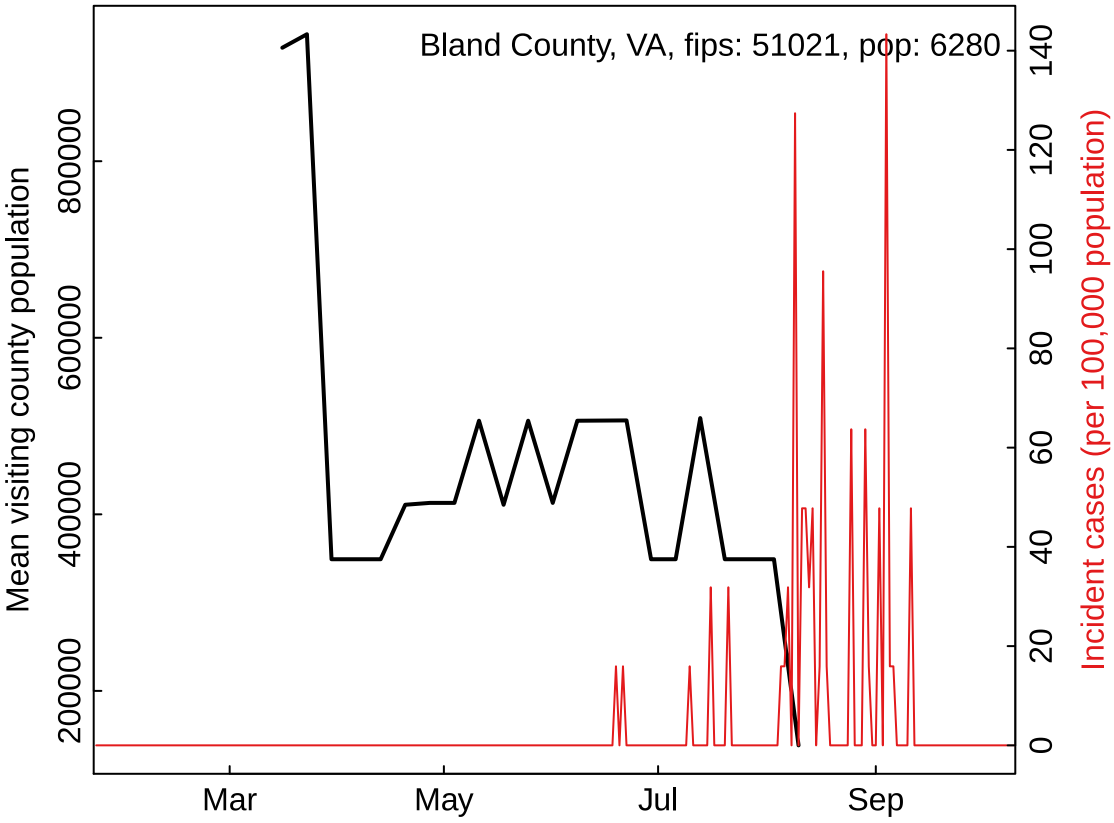

Bland County, VA, fips: 51021, pop: 6280
Mean visiting county population
200000
400000
600000
800000
Incident cases (per 100,000 population)
0
20
40
60
80
100
120
140
Mar
May
Jul
Sep

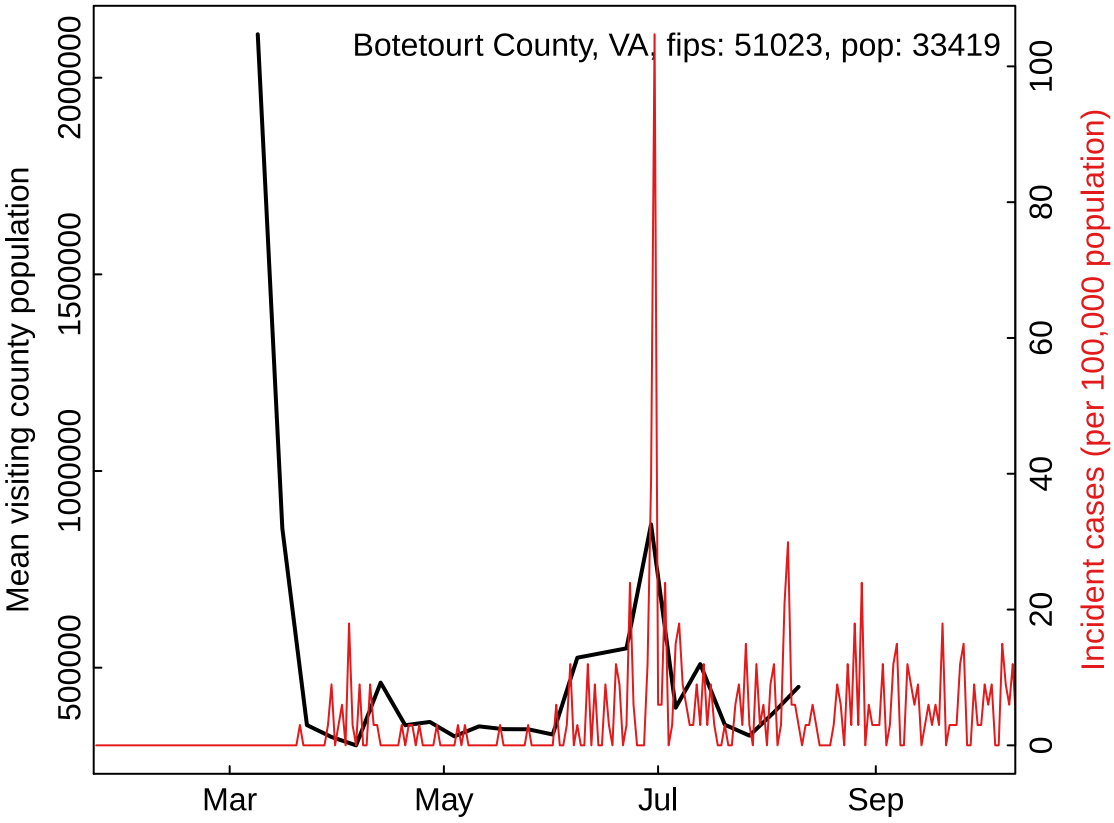

Botetourt County, VA, fips: 51023, pop: 33419
Mean visiting county population
Incident cases (per 100,000 population)
500000
1000000
1500000
2000000
0
20
40
60
80
100
Mar
May
Jul
Sep

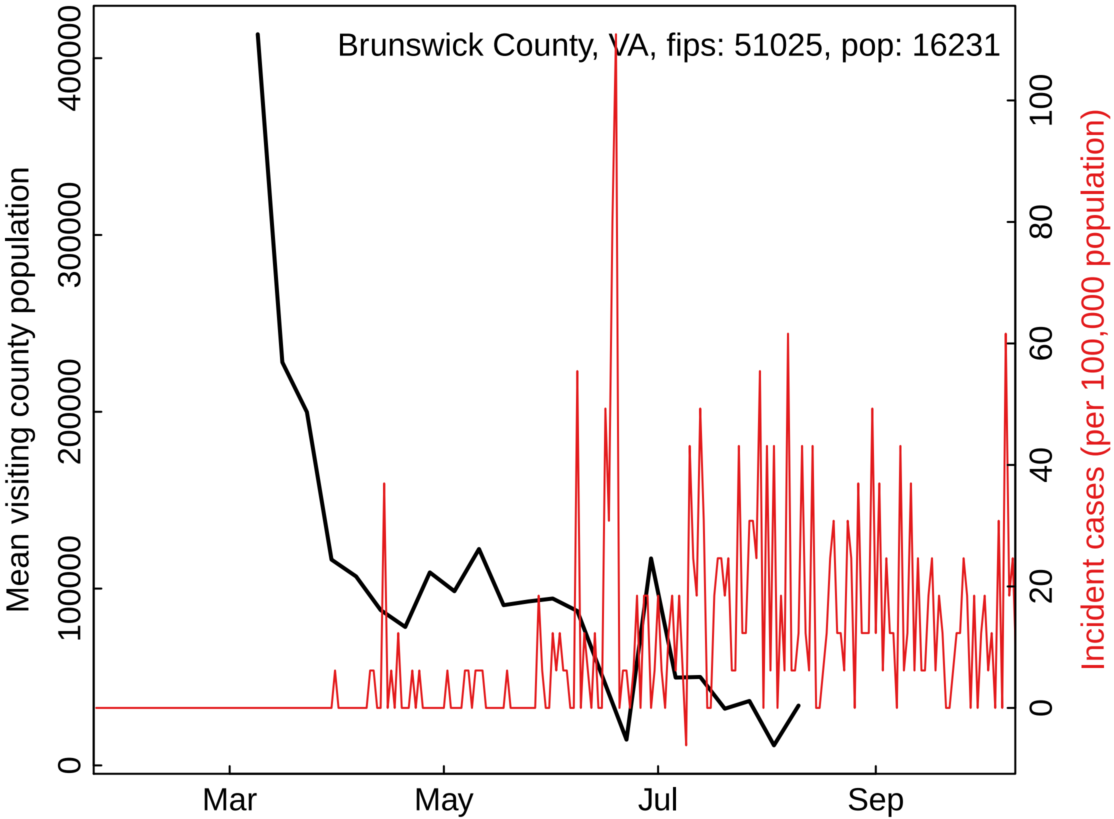

Brunswick County, VA, fips: 51025, pop: 16231
Mean visiting county population
0
100000
200000
300000
400000
Incident cases (per 100,000 population)
0
20
40
60
80
100
Mar
May
Jul
Sep

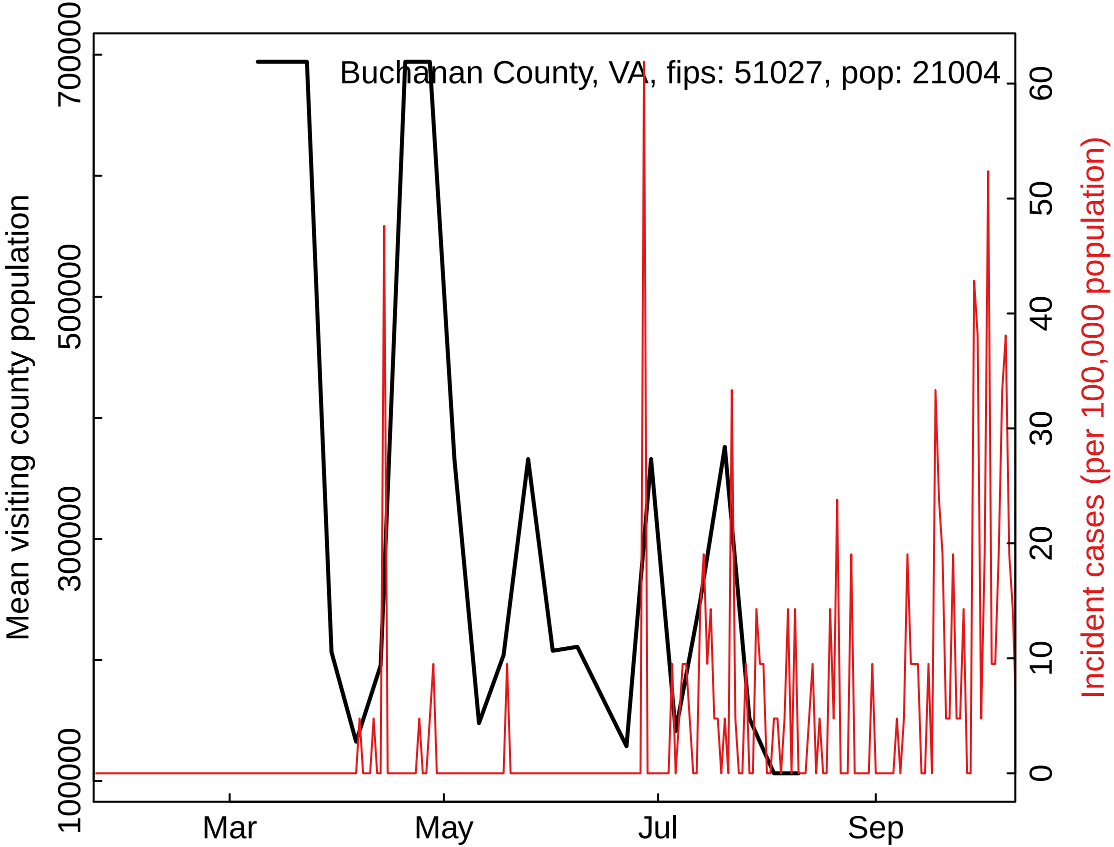

Buchanan County, VA, fips: 51027, pop: 21004
Mean visiting county population
100000
300000
500000
7000000
Incident cases (per 100,000 population)
0
10
20
30
40
50
60
Mar
May
Jul
Sep

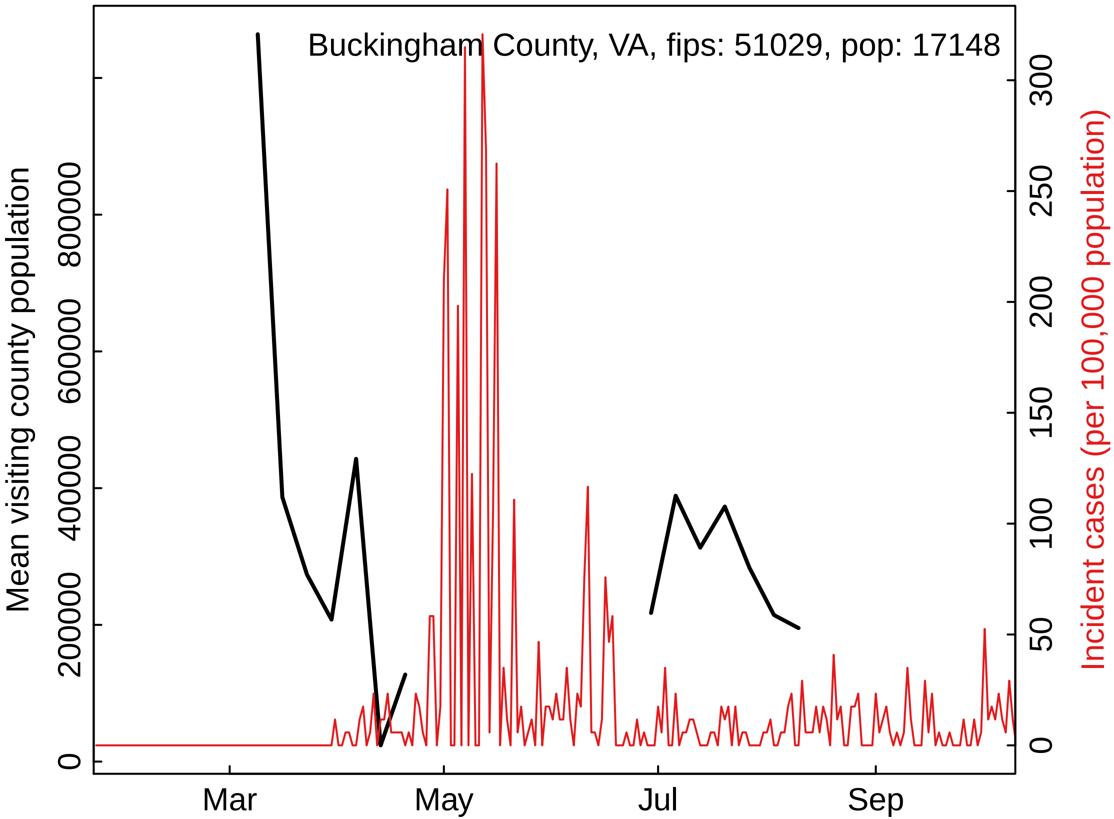

Buckingham County, VA, fips: 51029, pop: 17148
Mean visiting county population
0
200000
400000
600000
800000
Incident cases (per 100,000 population)
0
50
100
150
200
250
300
Mar
May
Jul
Sep

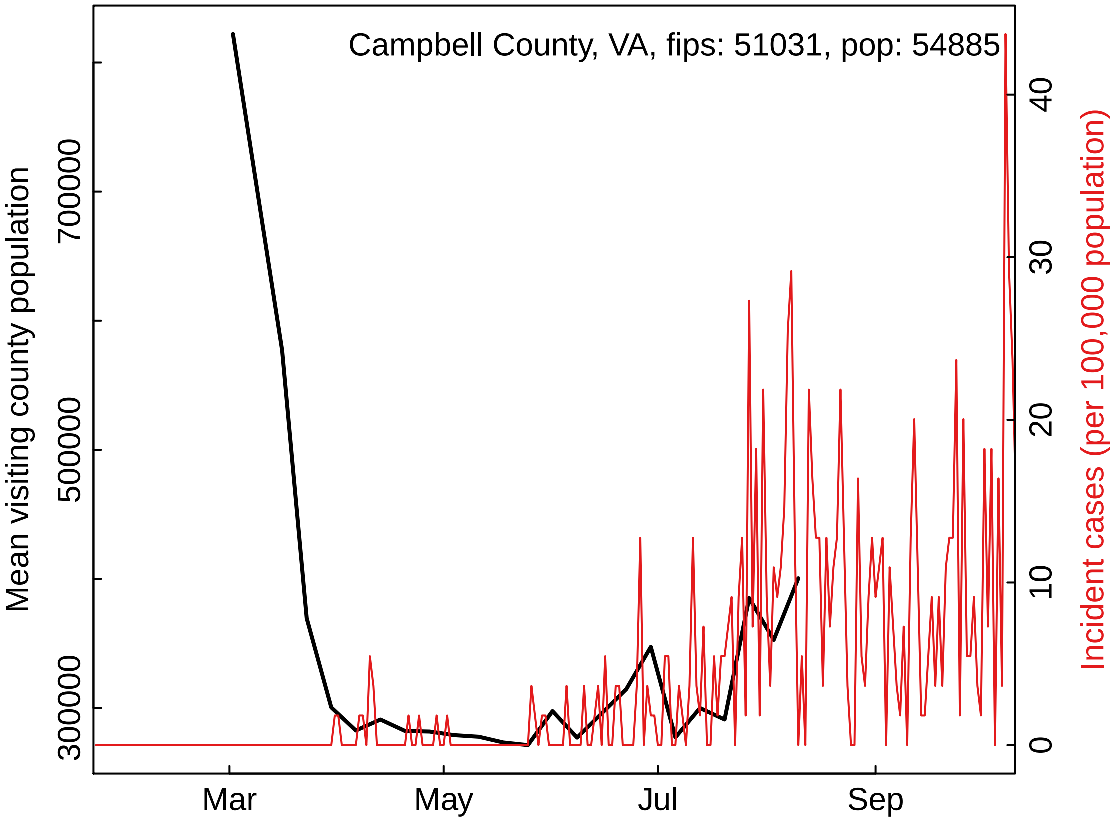

Campbell County, VA, fips: 51031, pop: 54885
Mean visiting county population
300000
500000
700000
Mar
May
Jul
Sep
0
10
20
30
40
Incident cases (per 100,000 population)

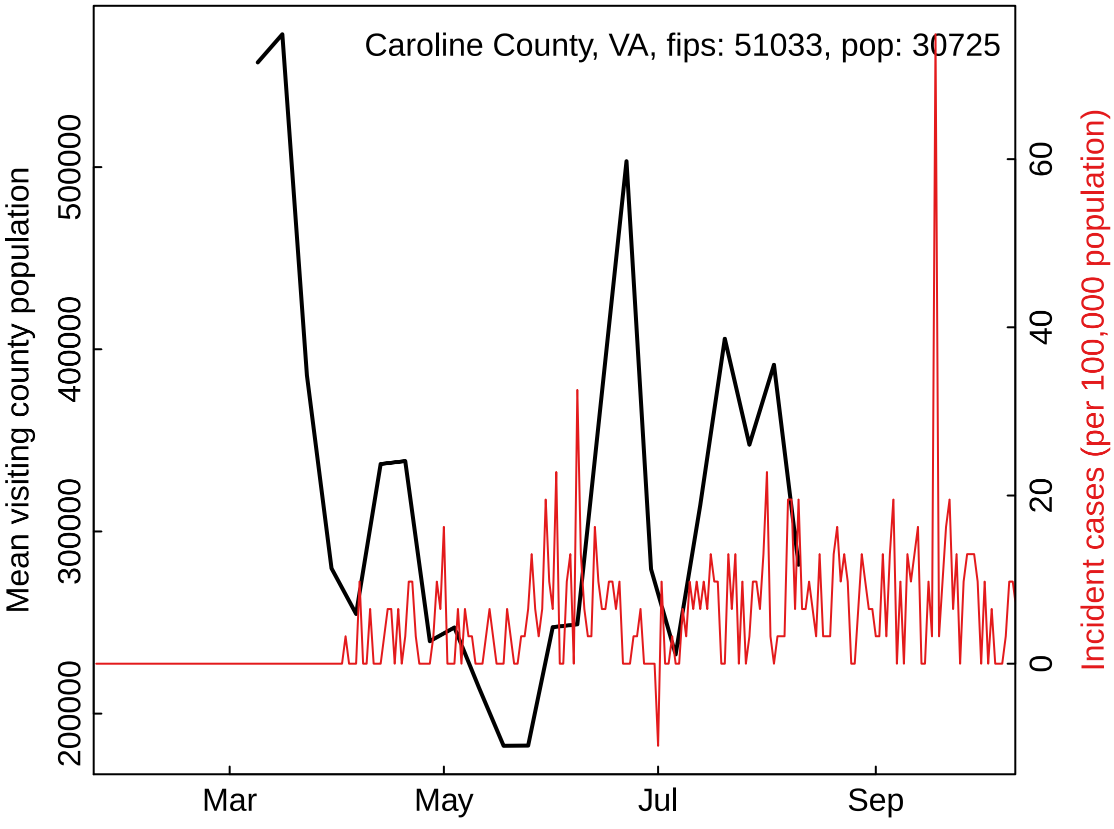

Caroline County, VA, fips: 51033, pop: 30725
Mean visiting county population
200000
300000
400000
500000
Incident cases (per 100,000 population)
0
20
40
60
Mar
May
Jul
Sep

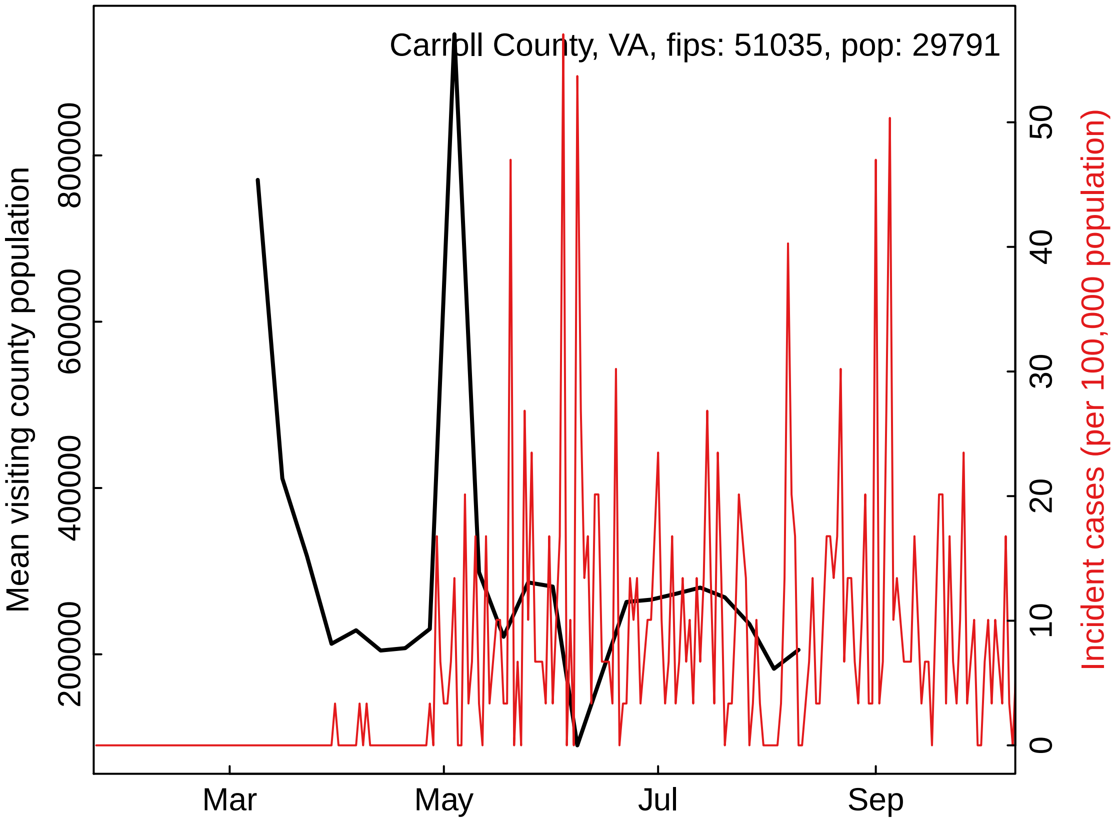

Carroll County, VA, fips: 51035, pop: 29791
Mean visiting county population
200000
400000
600000
800000
Incident cases (per 100,000 population)
0
10
20
30
40
50
Mar
May
Jul
Sep

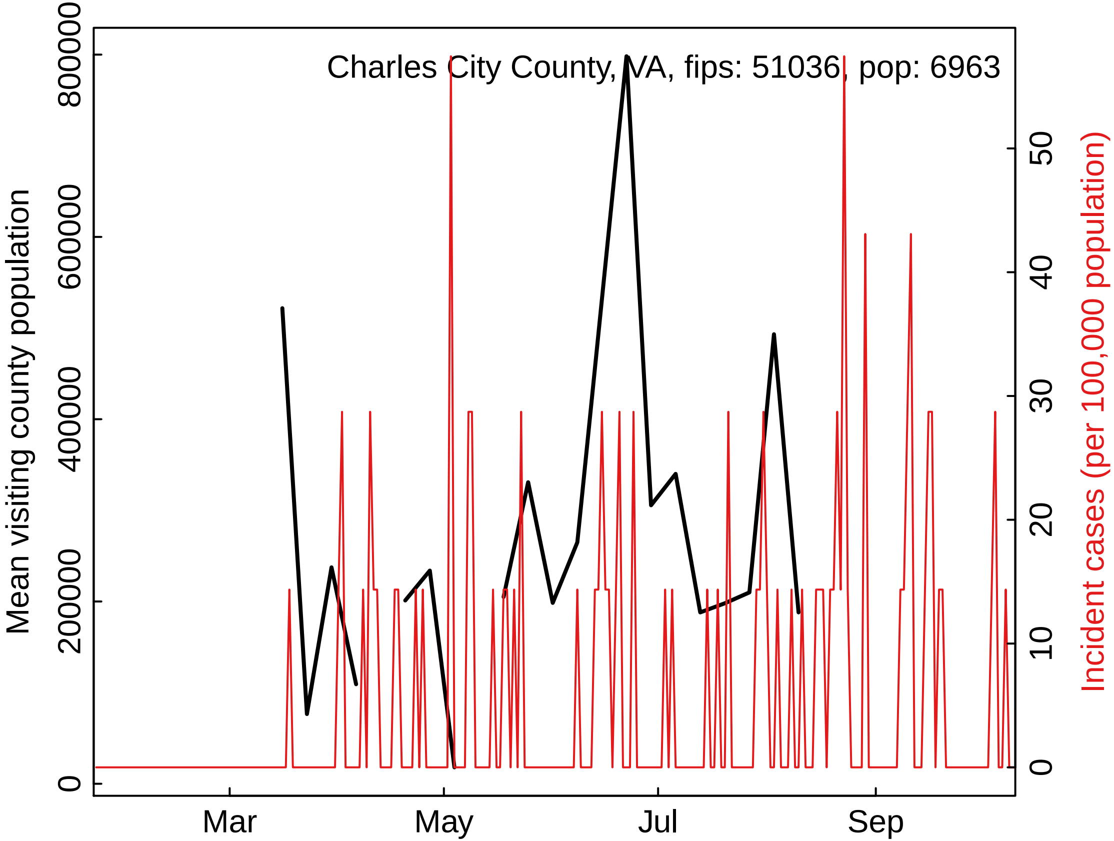
Charles City County, VA, fips: 51036, pop: 6963
Mean visiting county population
0
200000
400000
600000
800000
Incident cases (per 100,000 population)
0
10
20
30
40
50
Mar
May
Jul
Sep

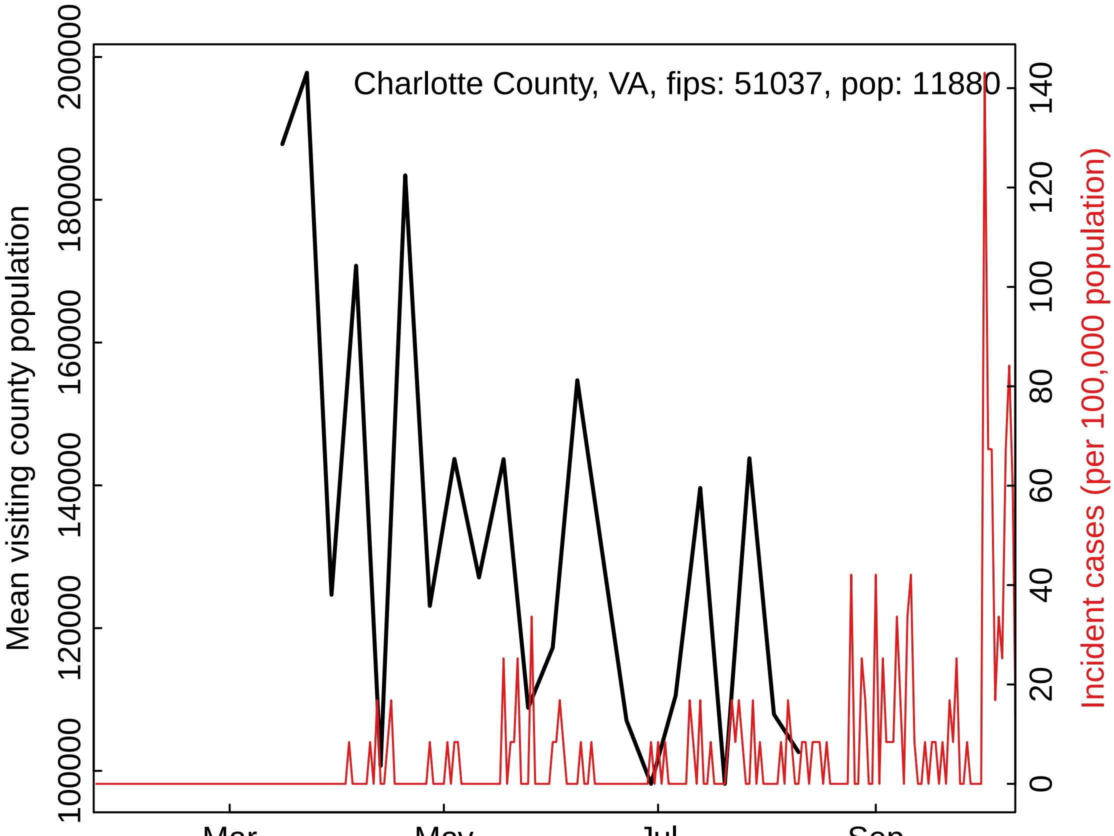

Charlotte County, VA, fips: 51037, pop: 11880
Mean visiting county population
100000
120000
140000
160000
180000
200000
Incident cases (per 100,000 population)
0
20
40
60
80
100
120
140
Mar
May
Jul
Sep

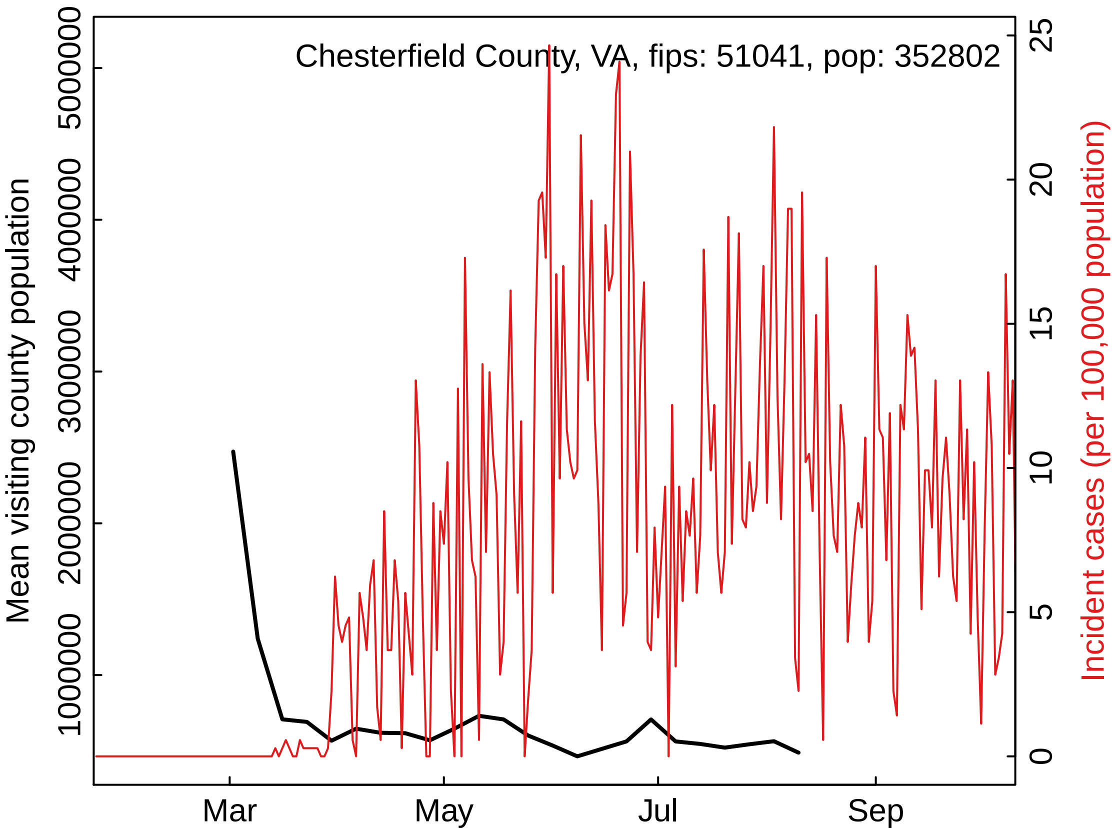
Chesterfield County, VA, fips: 51041, pop: 352802
Mean visiting county population
1000000
2000000
3000000
4000000
5000000
Incident cases (per 100,000 population)
0
5
10
15
20
25
Mar
May
Jul
Sep

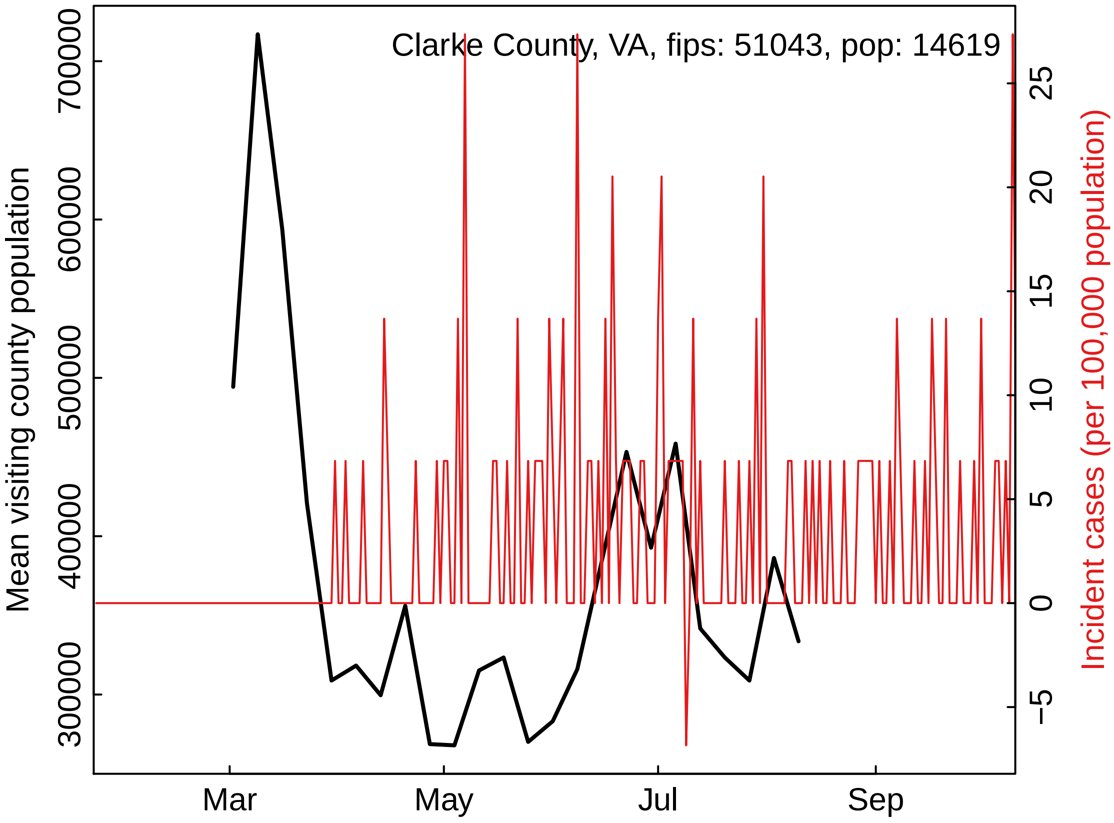

Clarke County, VA, fips: 51043, pop: 14619
Mean visiting county population
300000
400000
500000
600000
700000
Incident cases (per 100,000 population)
−5
0
5
10
15
20
25
Mar
May
Jul
Sep

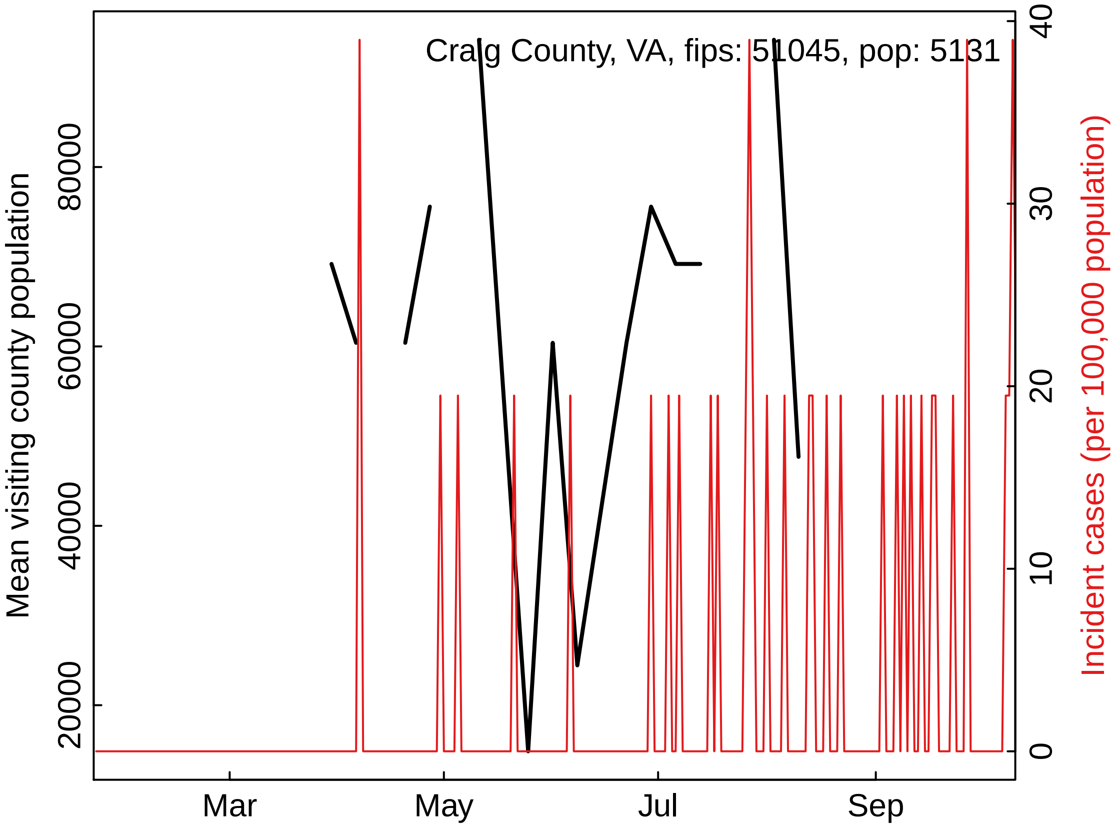

Craig County, VA, fips: 51045, pop: 5131
Mean visiting county population
20000
40000
60000
80000
Incident cases (per 100,000 population)
0
10
20
30
40
Mar
May
Jul
Sep

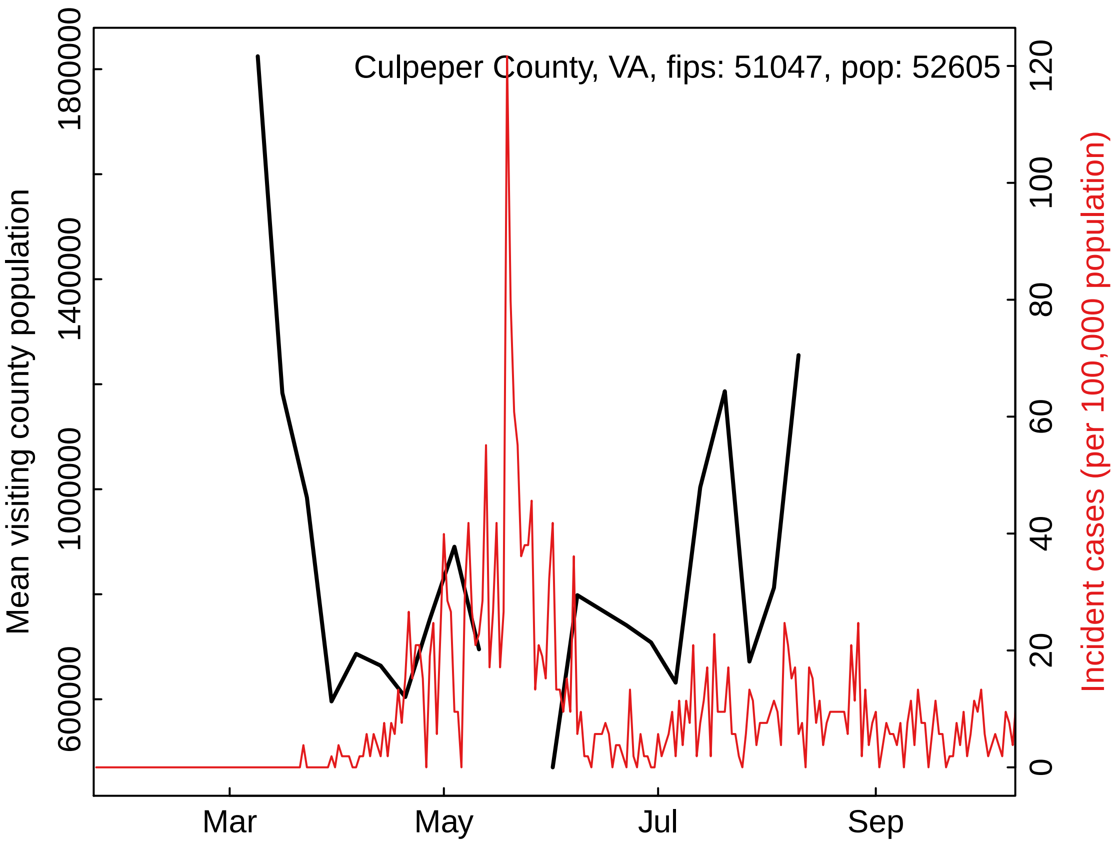

Culpeper County, VA, fips: 51047, pop: 52605
Mean visiting county population
600000
1000000
1400000
1800000
Incident cases (per 100,000 population)
0
20
40
60
80
100
120
Mar
May
Jul
Sep

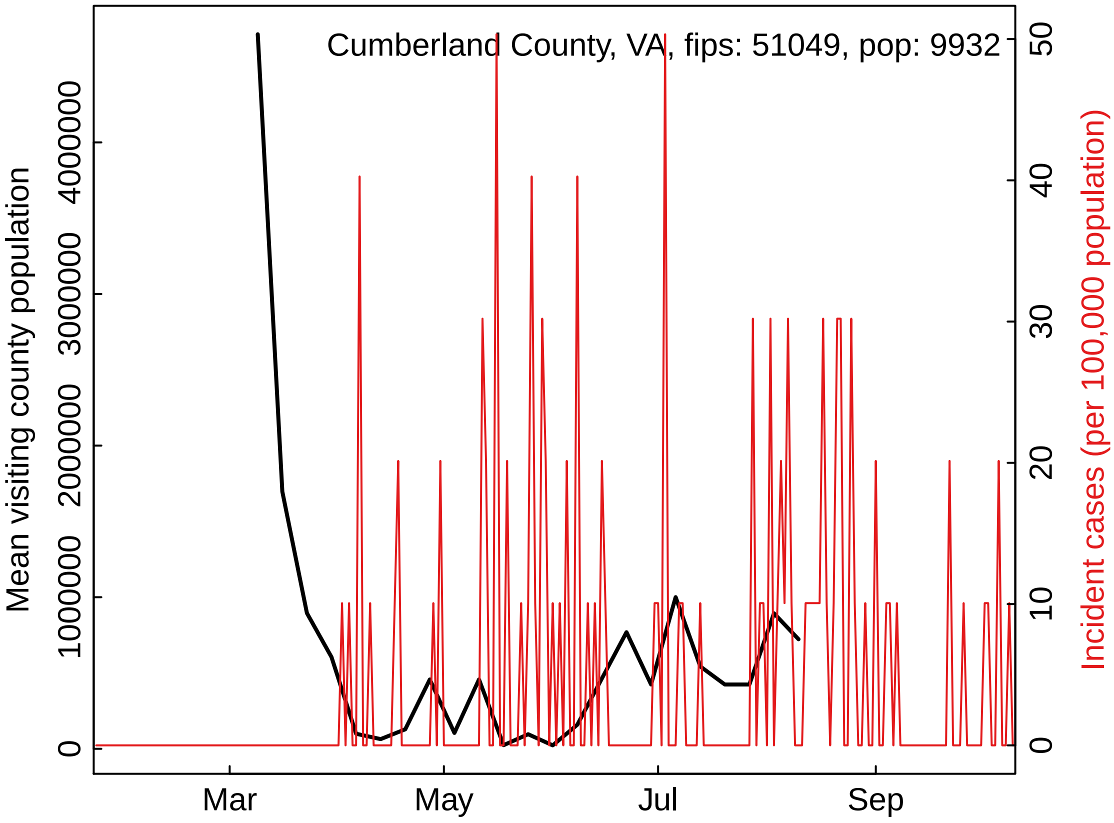

Cumberland County, VA, fips: 51049, pop: 9932
Mean visiting county population
0
1000000
2000000
3000000
4000000
Incident cases (per 100,000 population)
0
10
20
30
40
50
Mar
May
Jul
Sep

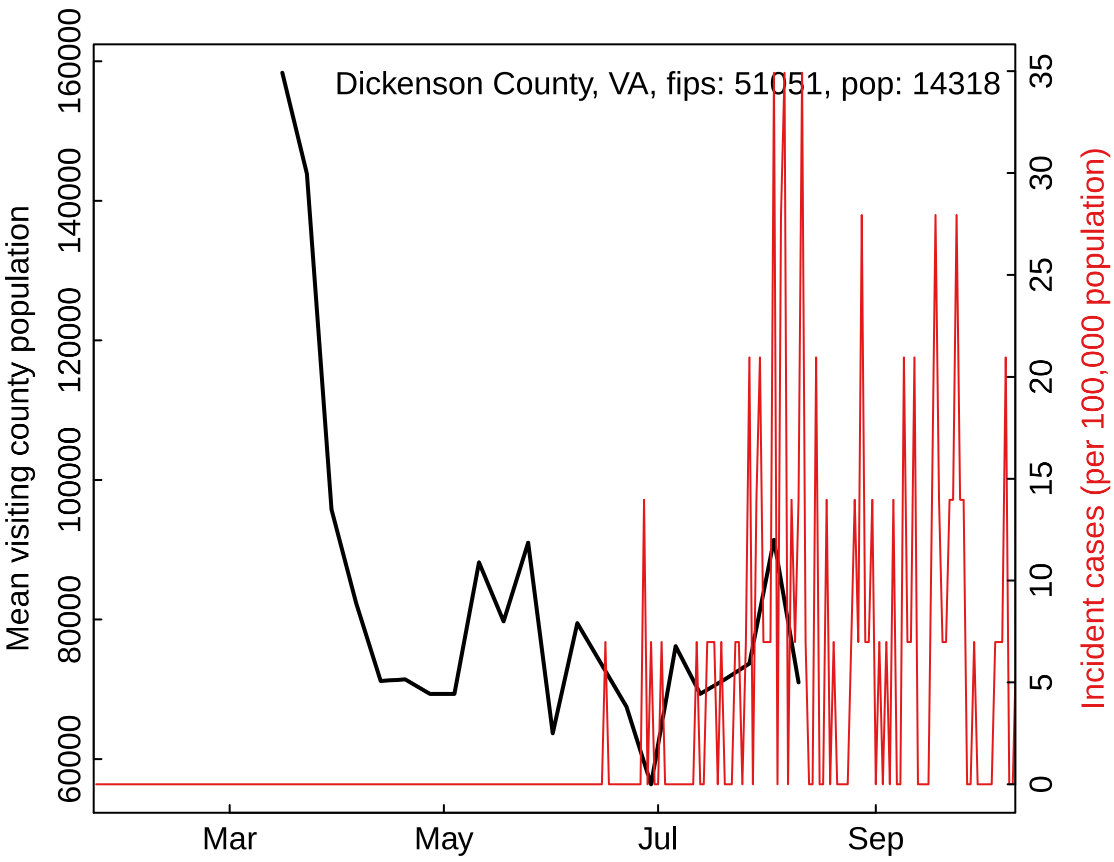

Dickenson County, VA, fips: 51051, pop: 14318
Mean visiting county population
60000
80000
100000
120000
140000
160000
Incident cases (per 100,000 population)
0
5
10
15
20
25
30
35
Mar
May
Jul
Sep

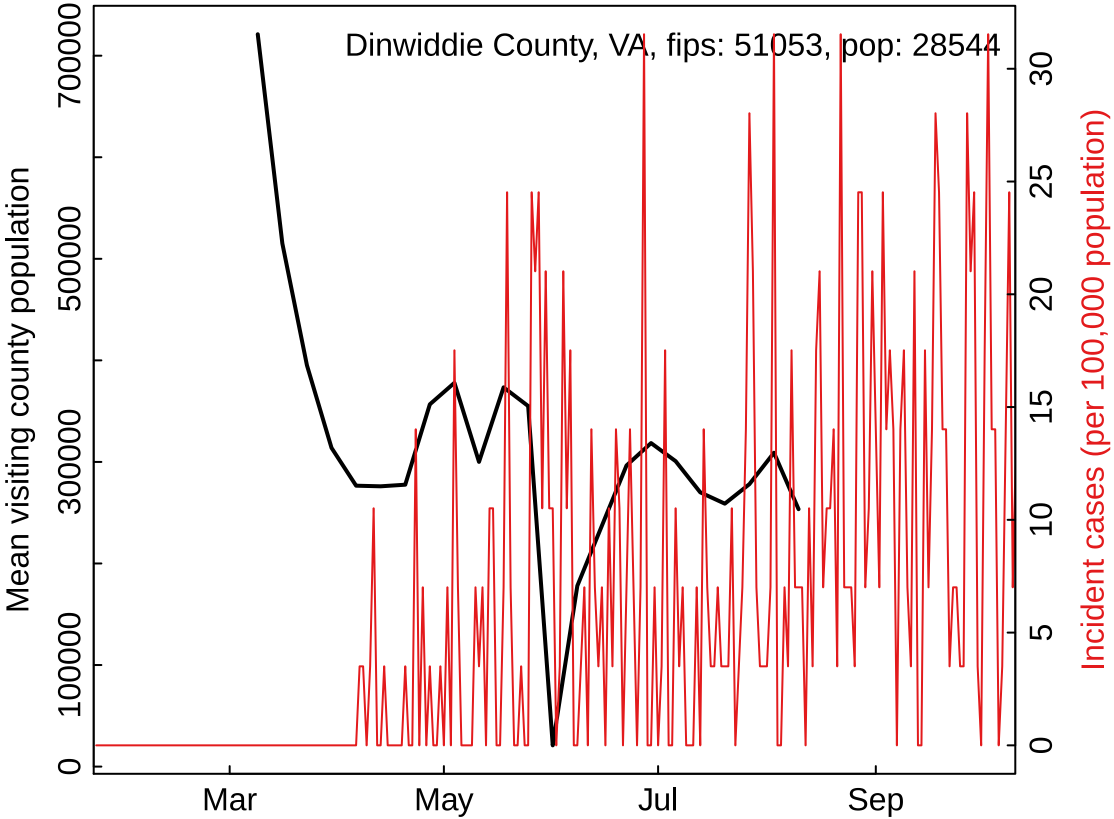

Dinwiddie County, VA, fips: 51053, pop: 28544
Mean visiting county population
0
100000
300000
500000
700000
Incident cases (per 100,000 population)
0
5
10
15
20
25
30
Mar
May
Jul
Sep

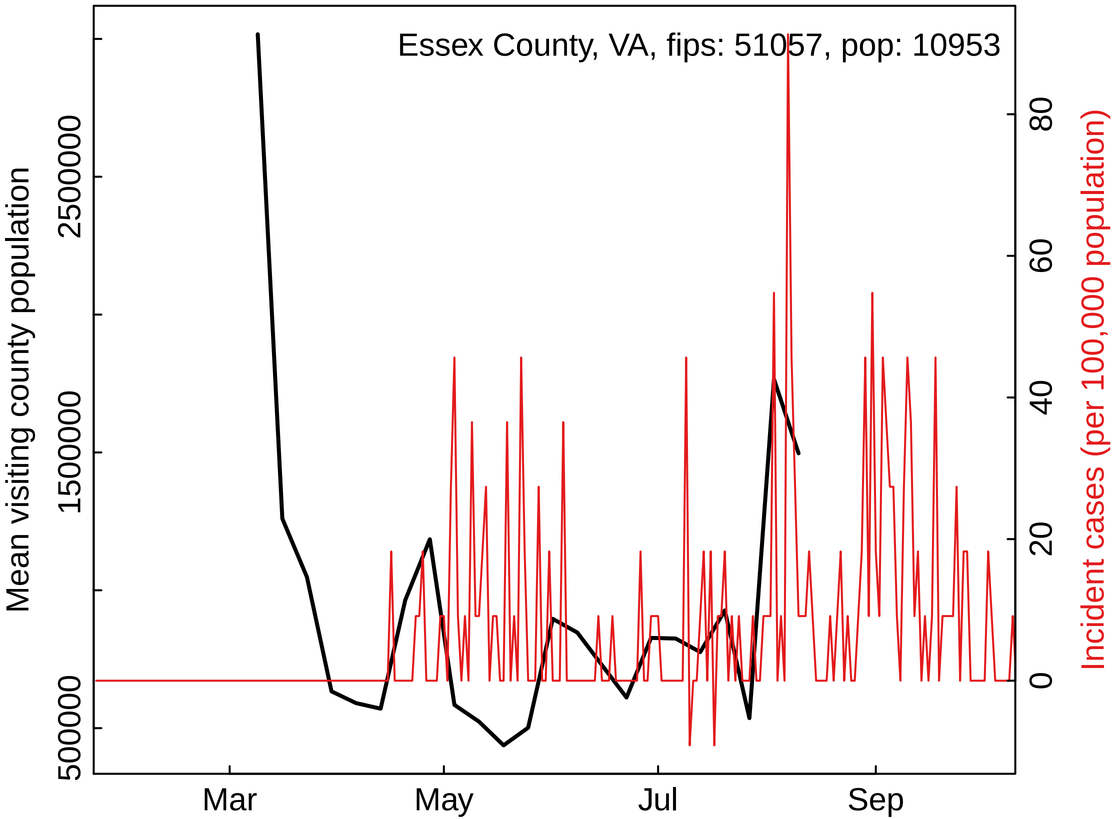
Essex County, VA, fips: 51057, pop: 10953
Mean visiting county population
500000
1500000
2500000
Incident cases (per 100,000 population)
0
20
40
60
80
Mar
May
Jul
Sep

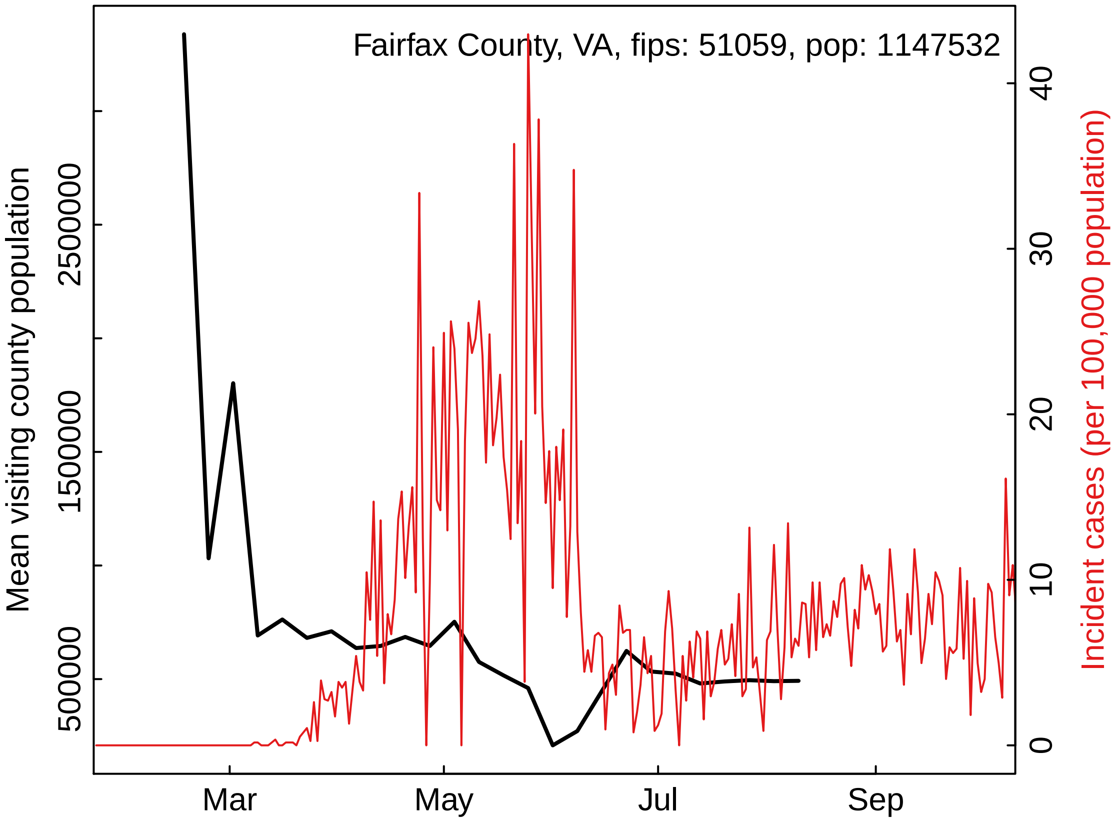

Fairfax County, VA, fips: 51059, pop: 1147532
Mean visiting county population
500000
1500000
2500000
Mar
May
Jul
Sep
0
10
20
30
40
Incident cases (per 100,000 population)

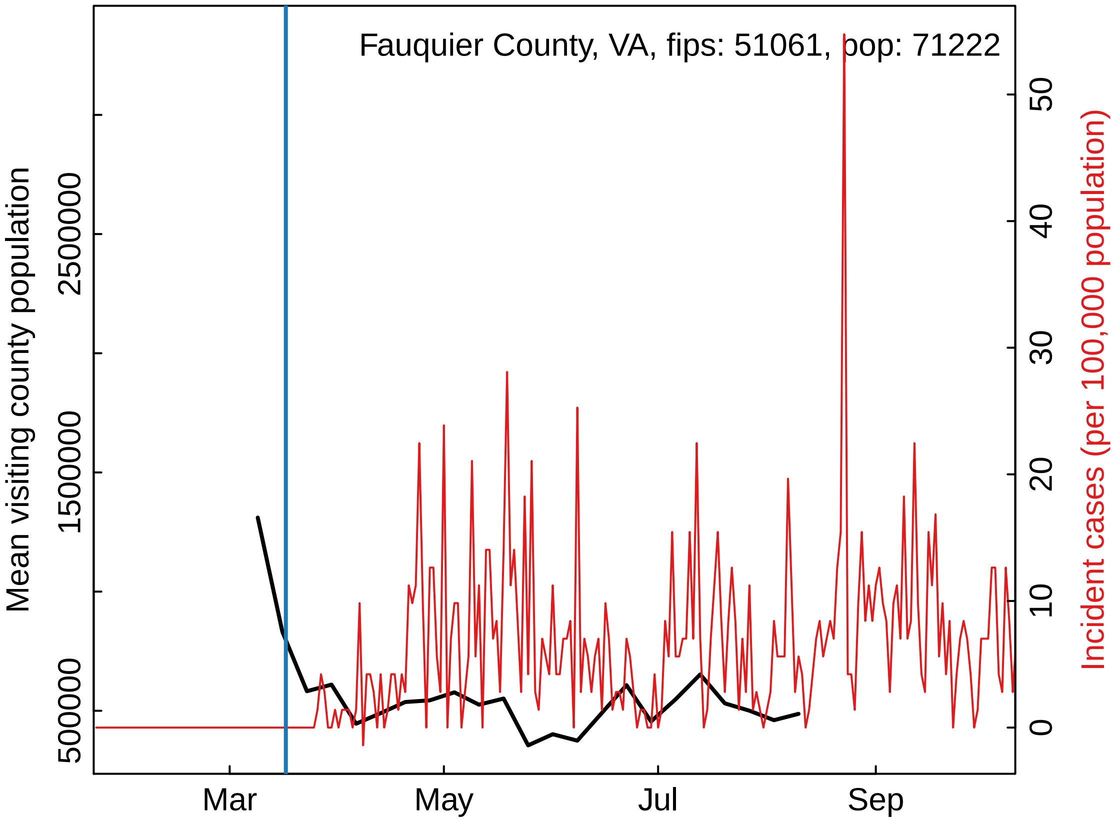

Fauquier County, VA, fips: 51061, pop: 71222
Mean visiting county population
500000
1500000
2500000
Mar
May
Jul
Sep
0
10
20
30
40
50
Incident cases (per 100,000 population)

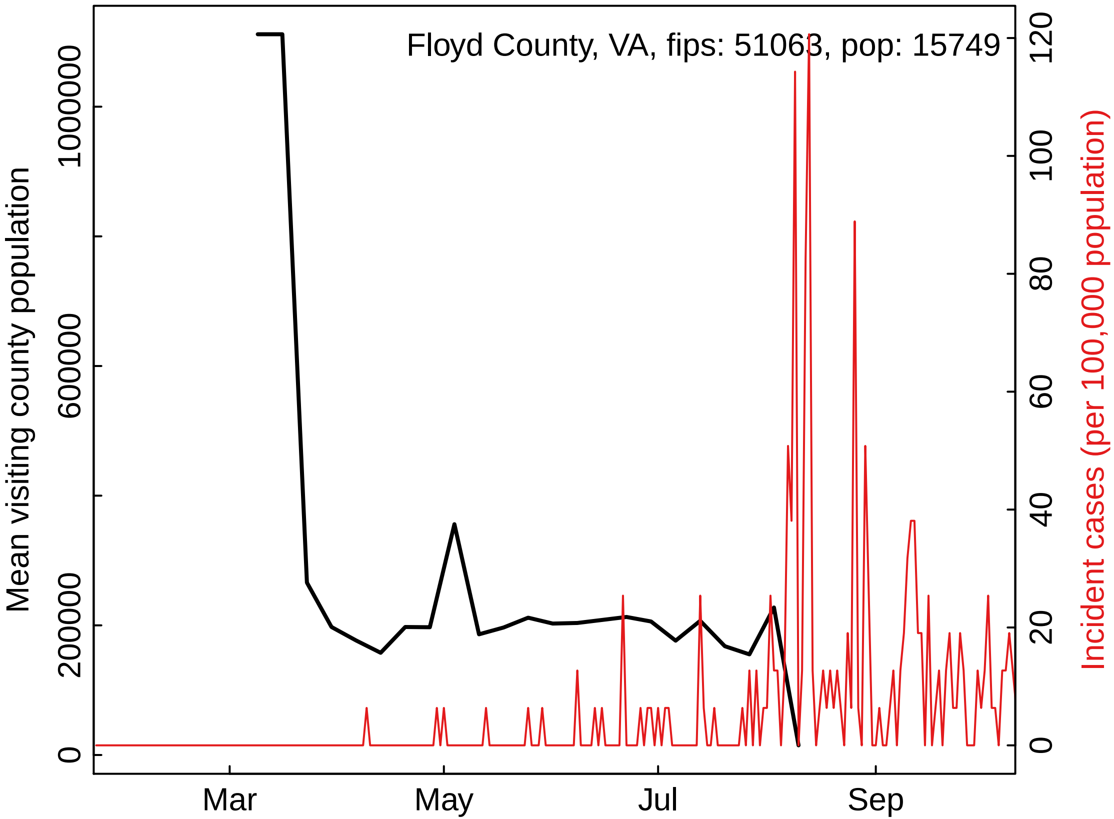

Floyd County, VA, fips: 51063, pop: 15749
Mean visiting county population
0
200000
600000
1000000
Incident cases (per 100,000 population)
0
20
40
60
80
100
120
Mar
May
Jul
Sep

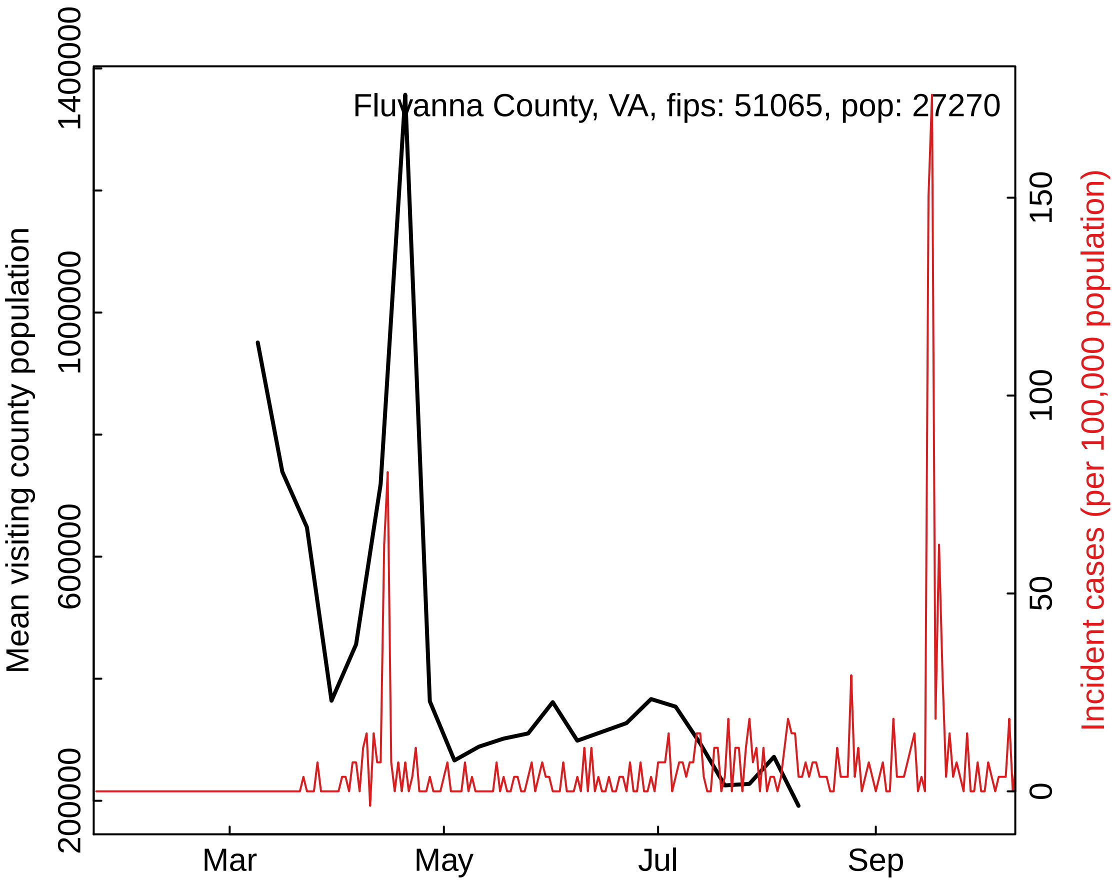

Fluvanna County, VA, fips: 51065, pop: 27270
Mean visiting county population
Incident cases (per 100,000 population)
200000
600000
1000000
1400000
0
50
100
150
Mar
May
Jul
Sep

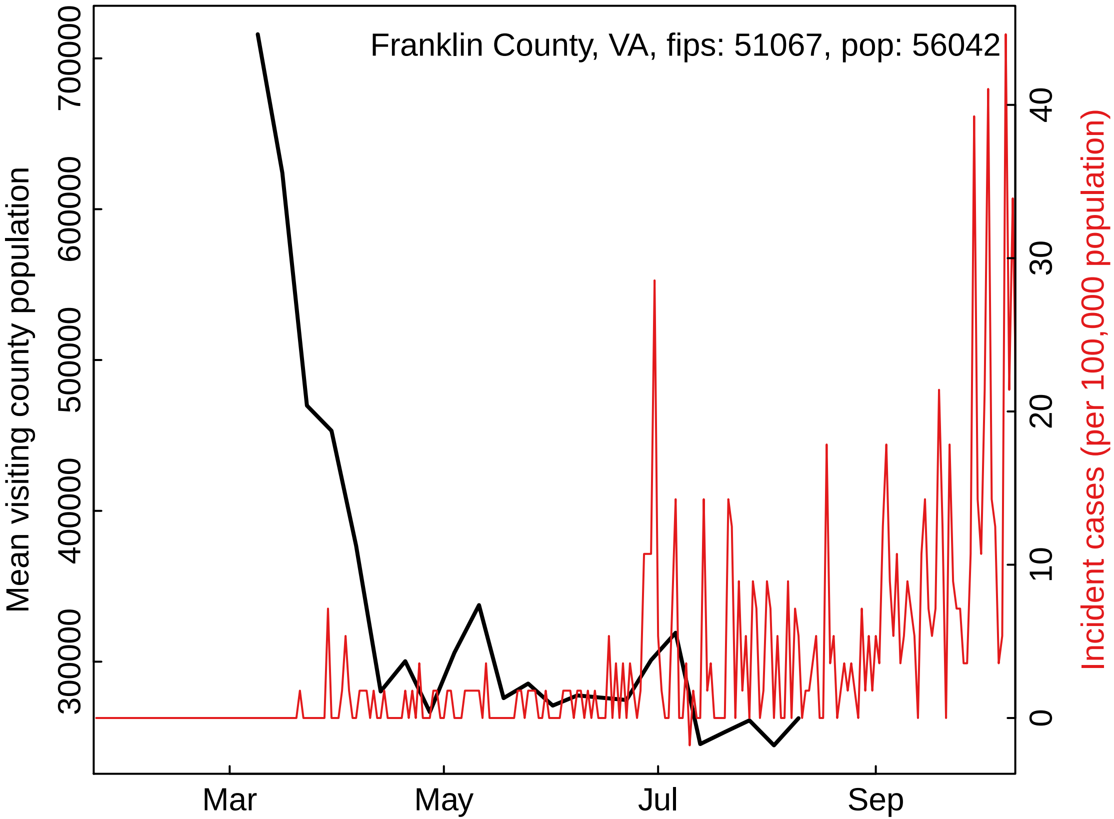
Franklin County, VA, fips: 51067, pop: 56042
Mean visiting county population
300000
400000
500000
600000
700000
Incident cases (per 100,000 population)
0
10
20
30
40
Mar
May
Jul
Sep

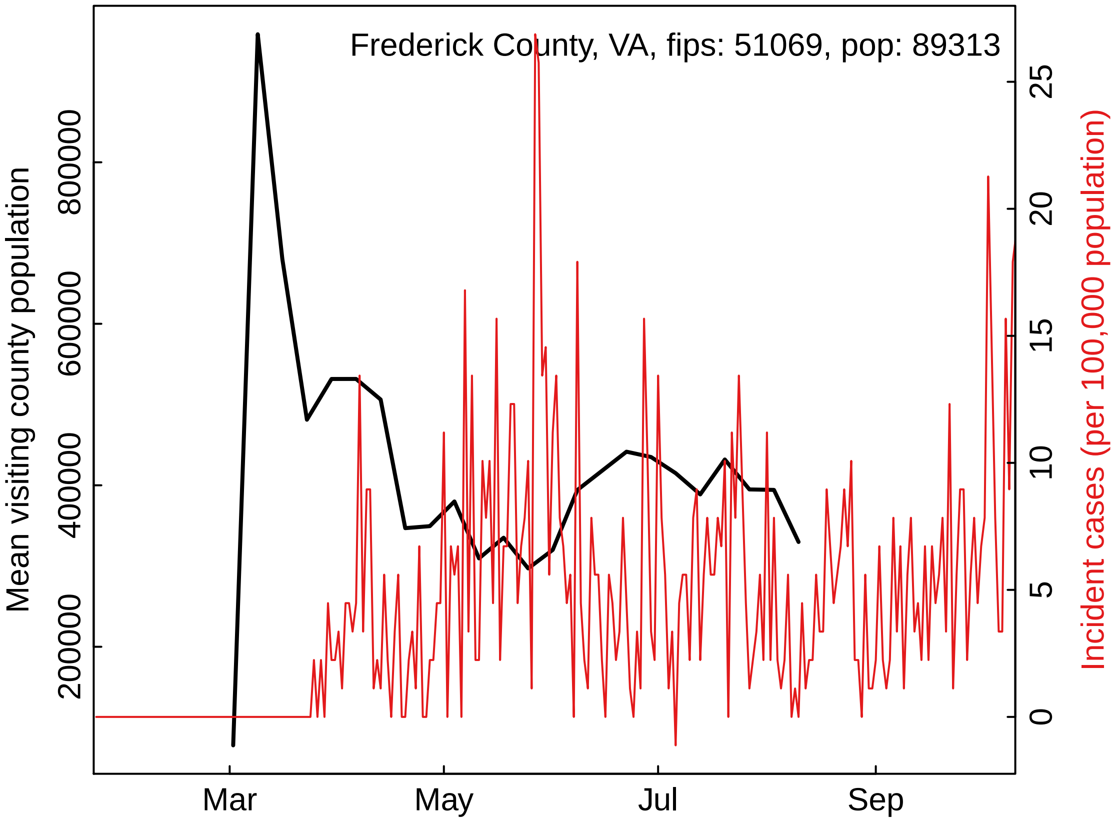

Frederick County, VA, fips: 51069, pop: 89313
Mean visiting county population
200000
400000
600000
800000
Incident cases (per 100,000 population)
0
5
10
15
20
25
Mar
May
Jul
Sep

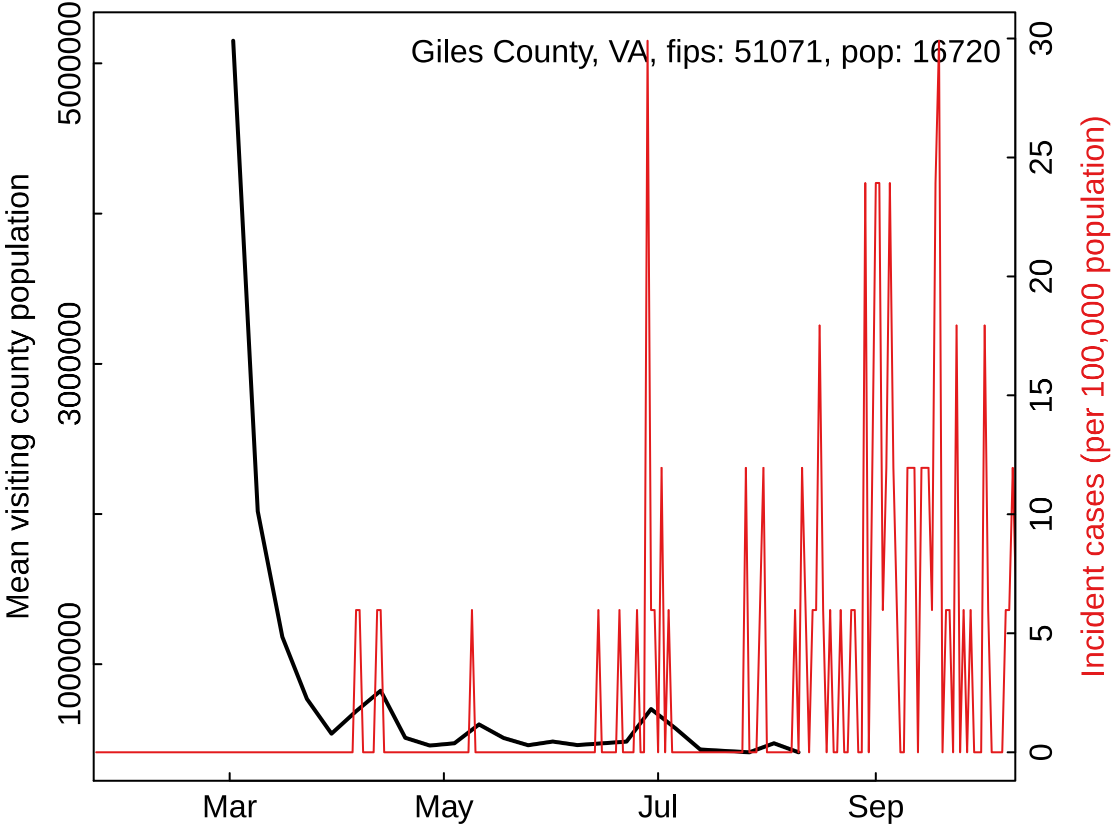
Giles County, VA, fips: 51071, pop: 16720
Mean visiting county population
Incident cases (per 100,000 population)
1000000
3000000
5000000
0
5
10
15
20
25
30
Mar
May
Jul
Sep

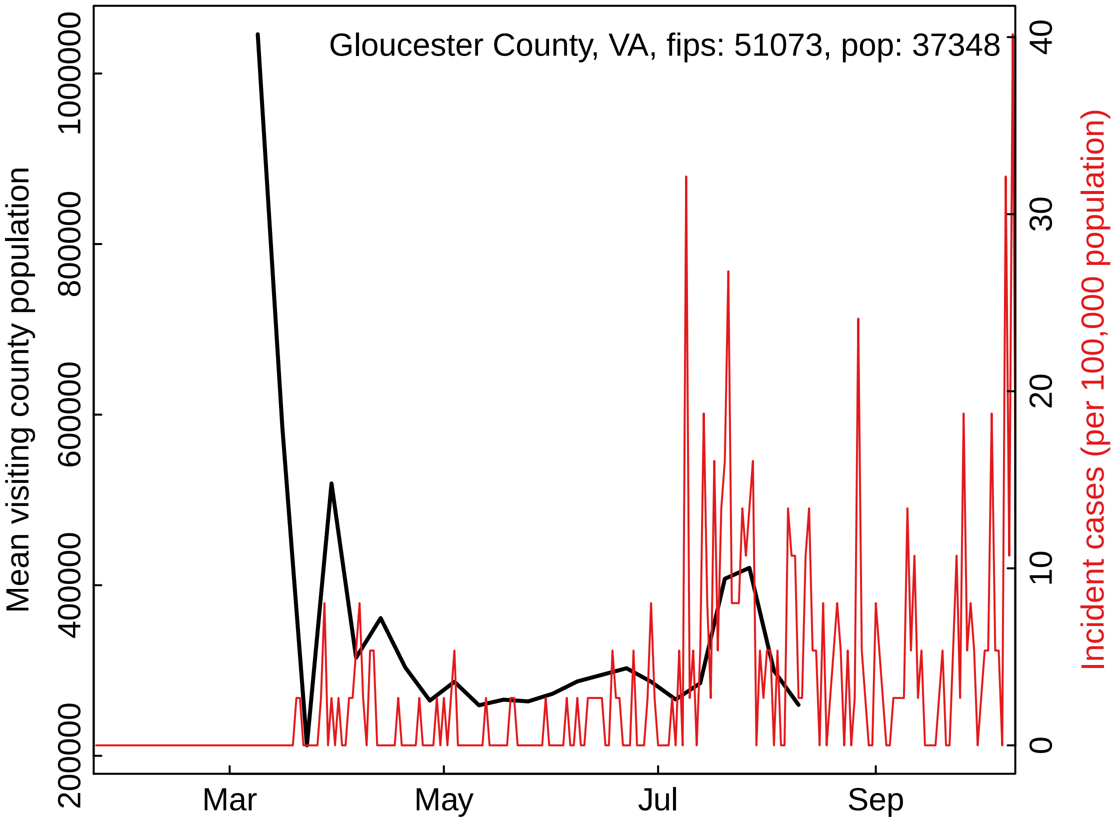
Gloucester County, VA, fips: 51073, pop: 37348
Mean visiting county population
200000
400000
600000
800000
1000000
Incident cases (per 100,000 population)
0
10
20
30
40
Mar
May
Jul
Sep

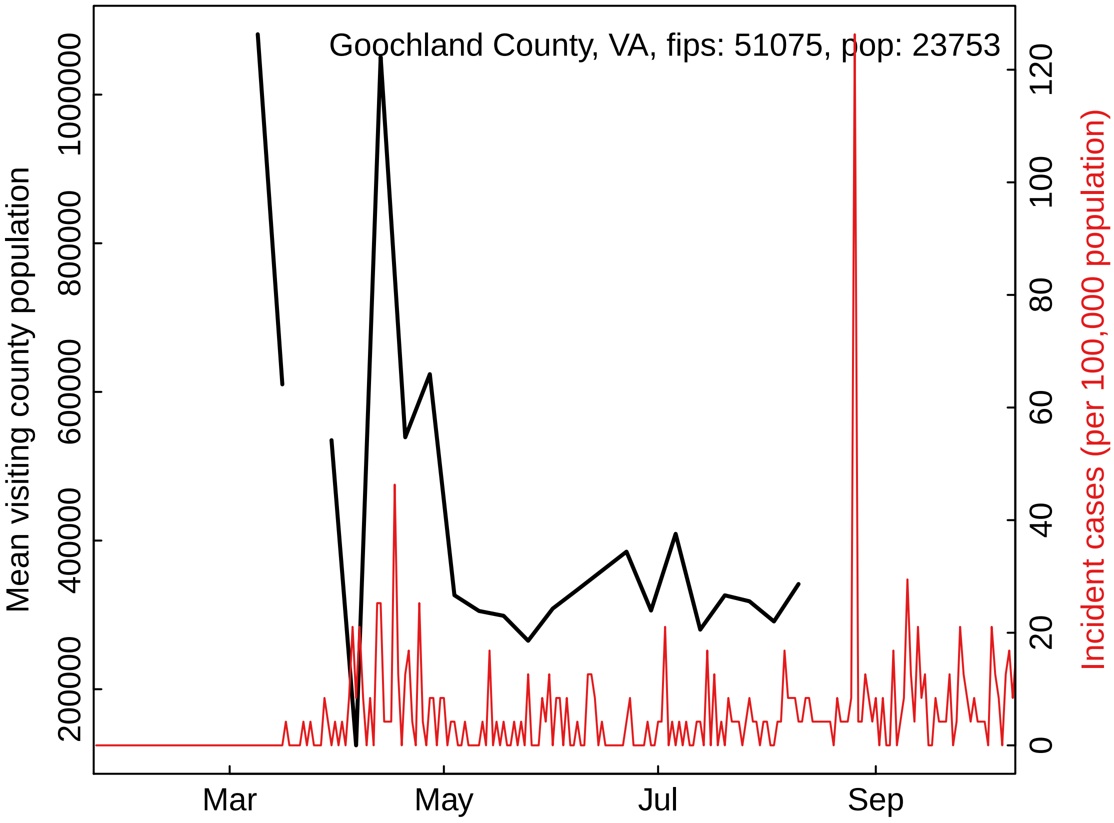

Goochland County, VA, fips: 51075, pop: 23753
Mean visiting county population
200000
400000
600000
800000
1000000
Incident cases (per 100,000 population)
0
20
40
60
80
100
120
Mar
May
Jul
Sep

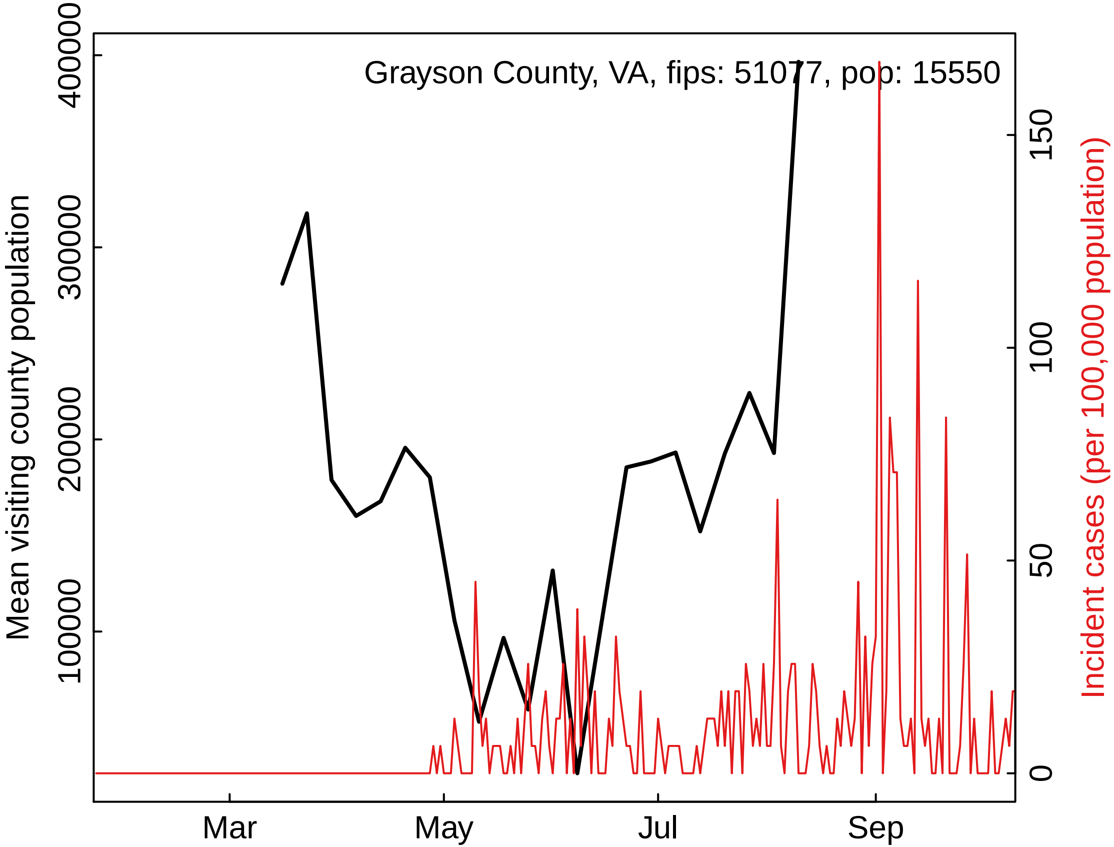
Grayson County, VA, fips: 51077, pop: 15550
Mean visiting county population
Incident cases (per 100,000 population)
100000
200000
300000
400000
0
50
100
150
Mar
May
Jul
Sep

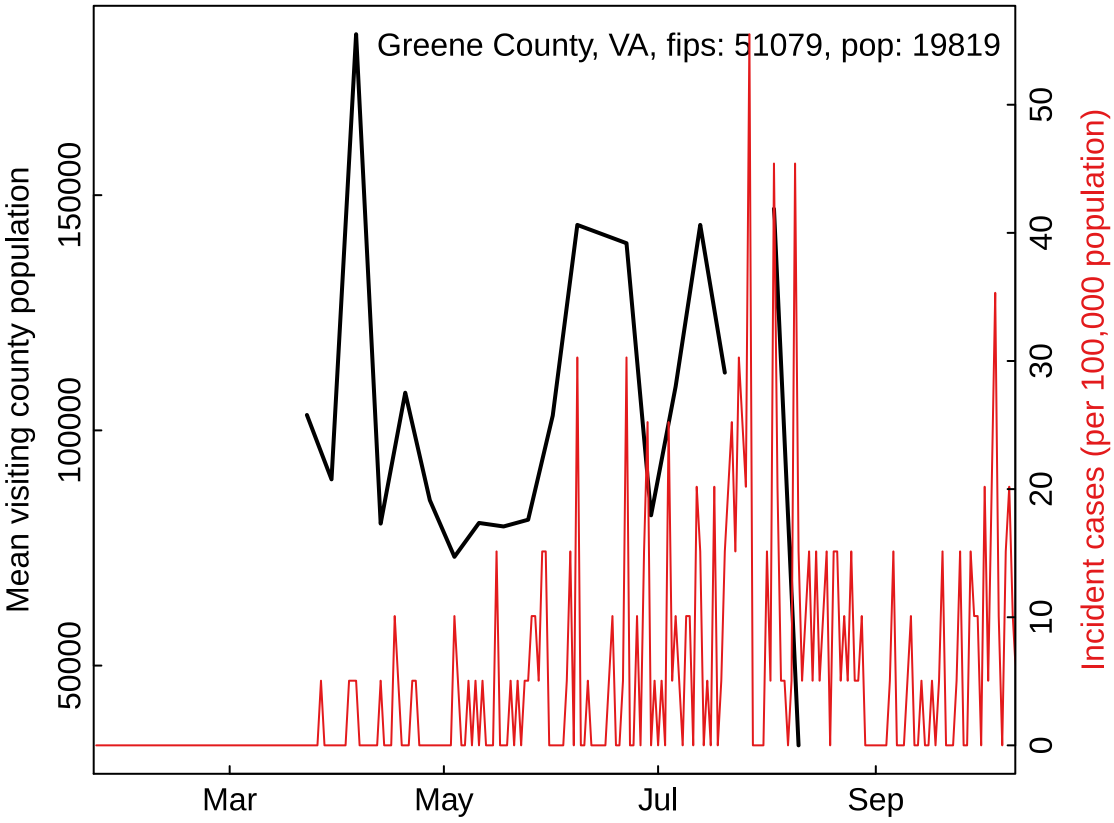

Greene County, VA, fips: 51079, pop: 19819
Mean visiting county population
50000
100000
150000
Incident cases (per 100,000 population)
0
10
20
30
40
50
Mar
May
Jul
Sep

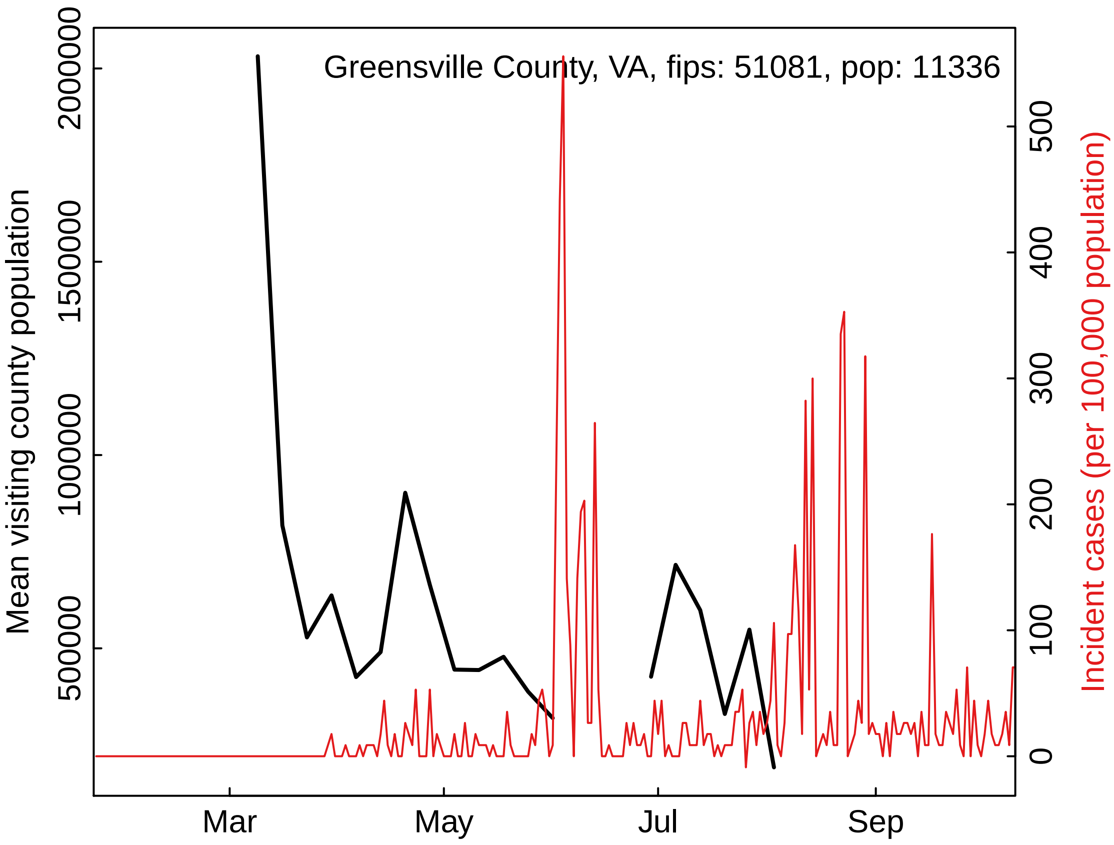

Greensville County, VA, fips: 51081, pop: 11336
Mean visiting county population
500000
1000000
1500000
2000000
Incident cases (per 100,000 population)
0
100
200
300
400
500
Mar
May
Jul
Sep

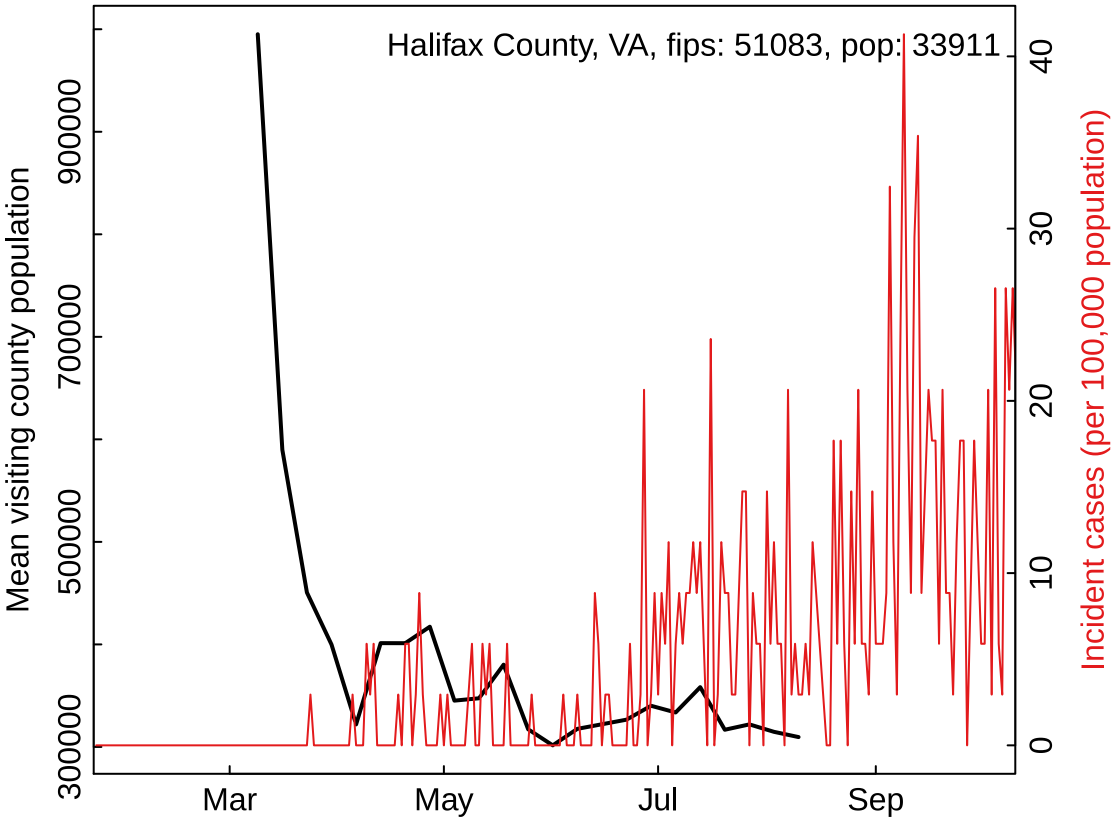
Halifax County, VA, fips: 51083, pop: 33911
Mean visiting county population
300000
500000
7000000
9000000
Mar
May
Jul
Sep
0
10
20
30
40
Incident cases (per 100,000 population)

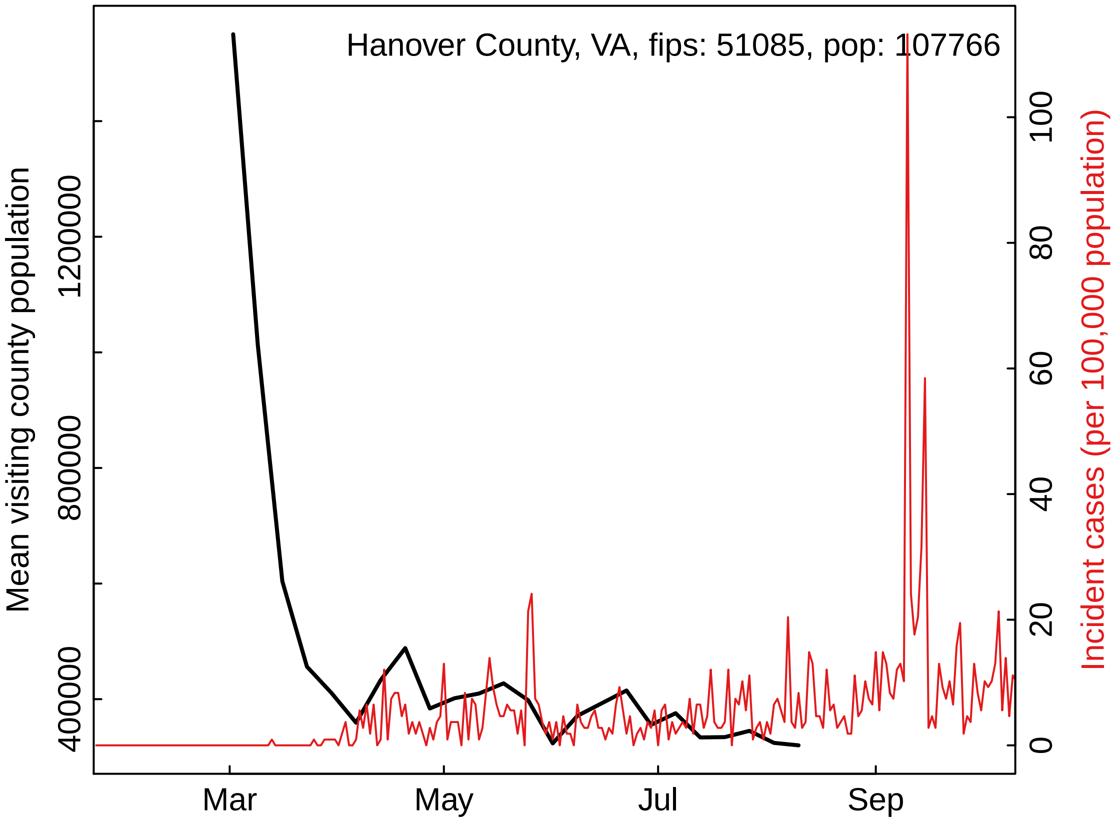

Hanover County, VA, fips: 51085, pop: 107766
Mean visiting county population
400000
800000
1200000
Incident cases (per 100,000 population)
0
20
40
60
80
100
Mar
May
Jul
Sep

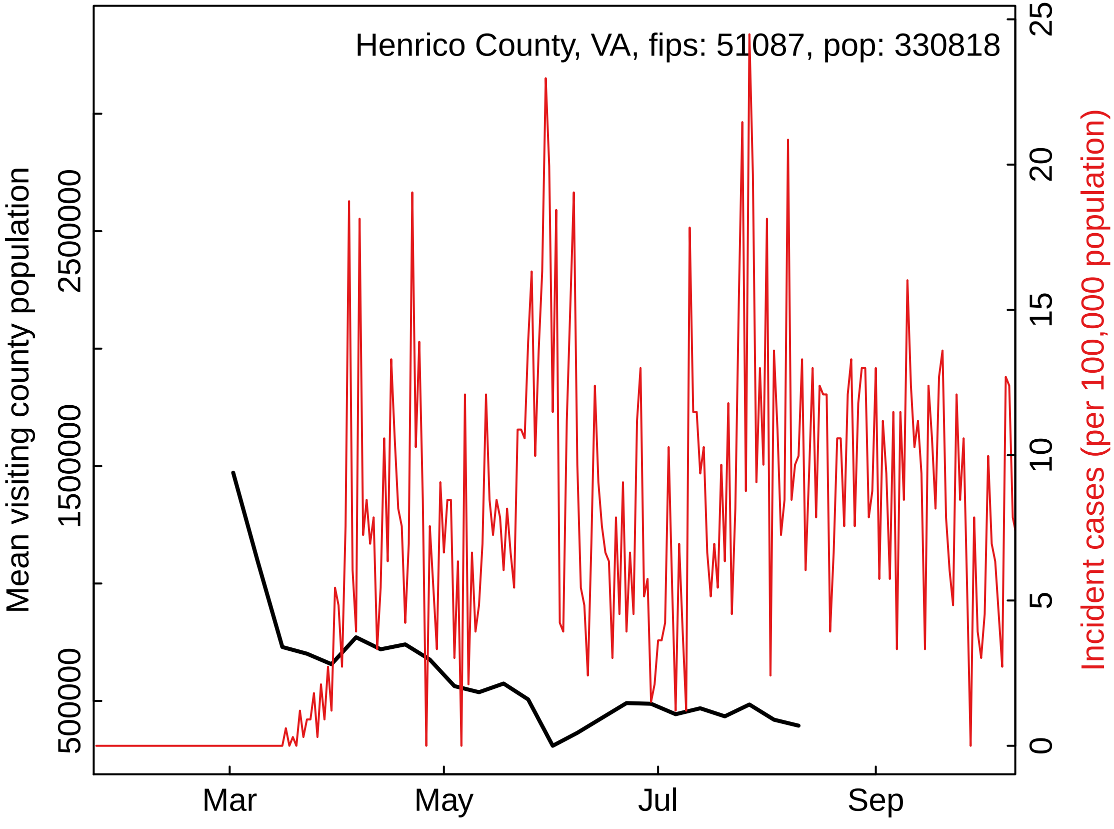

Henrico County, VA, fips: 51087, pop: 330818
Mean visiting county population
500000
1500000
2500000
Incident cases (per 100,000 population)
0
5
10
15
20
25
Mar
May
Jul
Sep

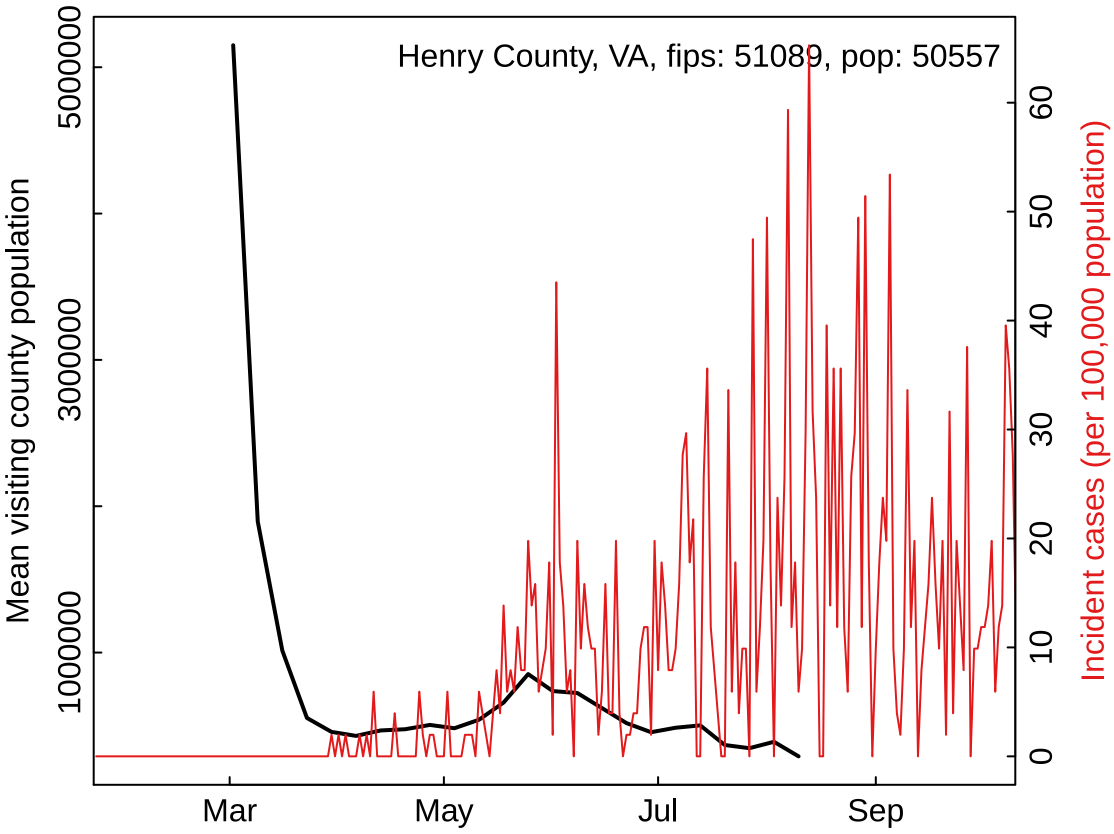

Henry County, VA, fips: 51089, pop: 50557
Mean visiting county population
1000000
3000000
5000000
Incident cases (per 100,000 population)
0
10
20
30
40
50
60
Mar
May
Jul
Sep

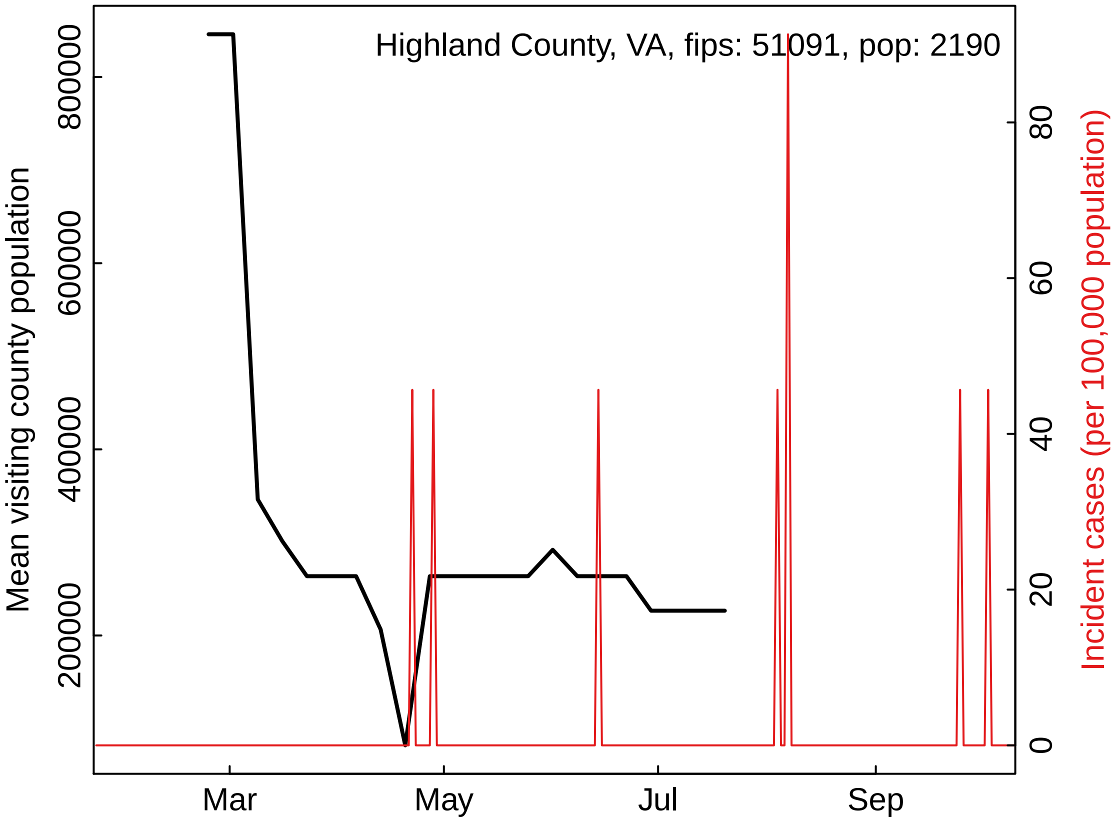
Highland County, VA, fips: 51091, pop: 2190
Mean visiting county population
200000
400000
600000
800000
Incident cases (per 100,000 population)
0
20
40
60
80
Mar
May
Jul
Sep

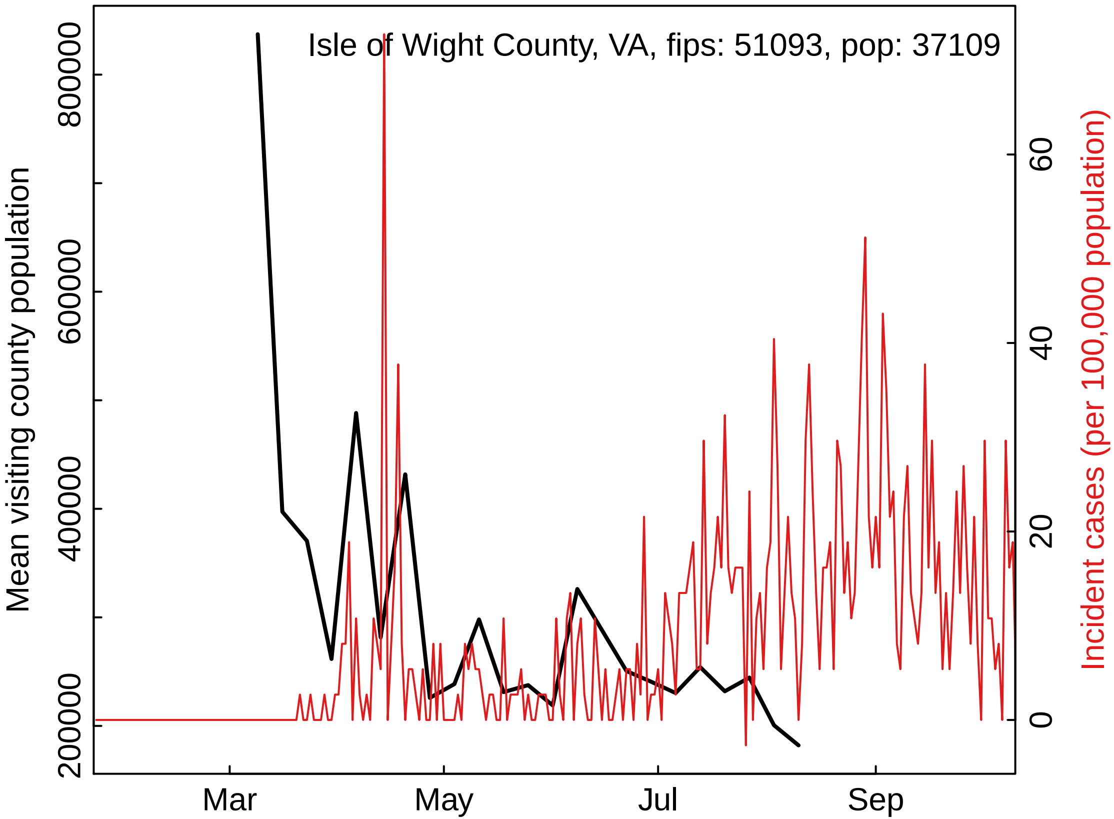

Isle of Wight County, VA, fips: 51093, pop: 37109
Mean visiting county population
200000
400000
600000
800000
Incident cases (per 100,000 population)
0
20
40
60
Mar
May
Jul
Sep

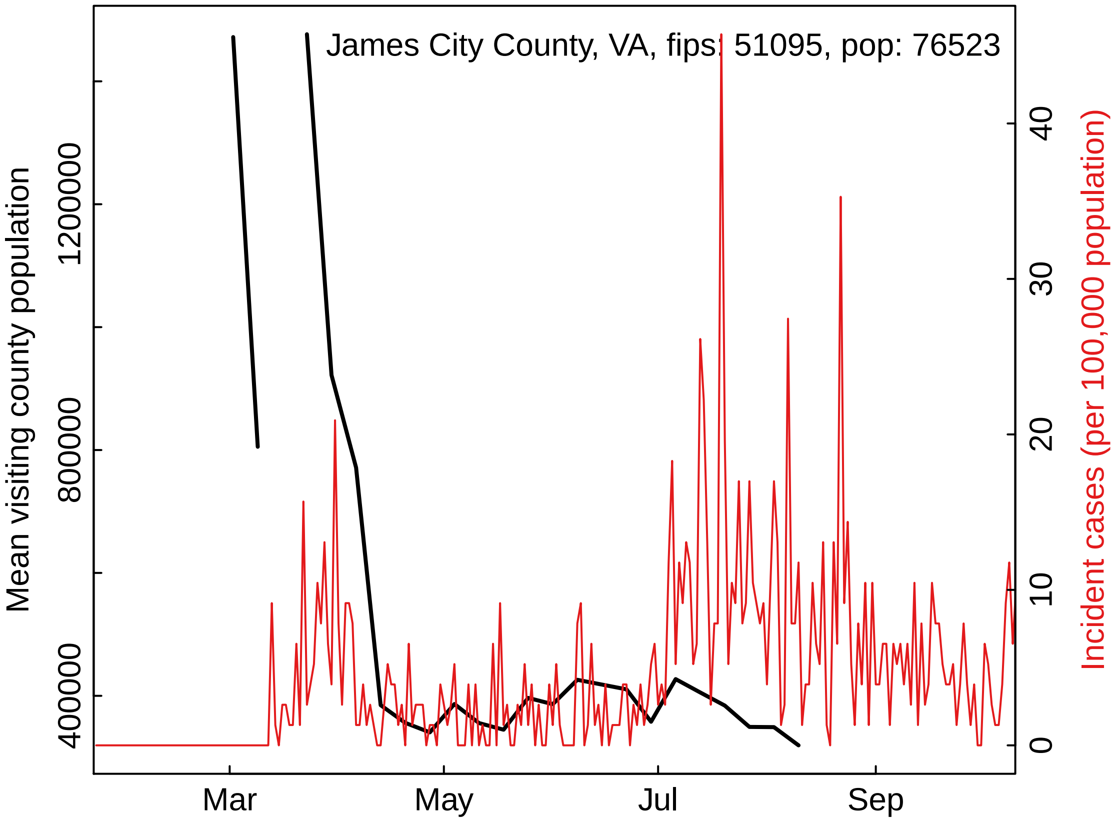

James City County, VA, fips: 51095, pop: 76523
Mean visiting county population
400000
800000
1200000
Incident cases (per 100,000 population)
0
10
20
30
40
Mar
May
Jul
Sep

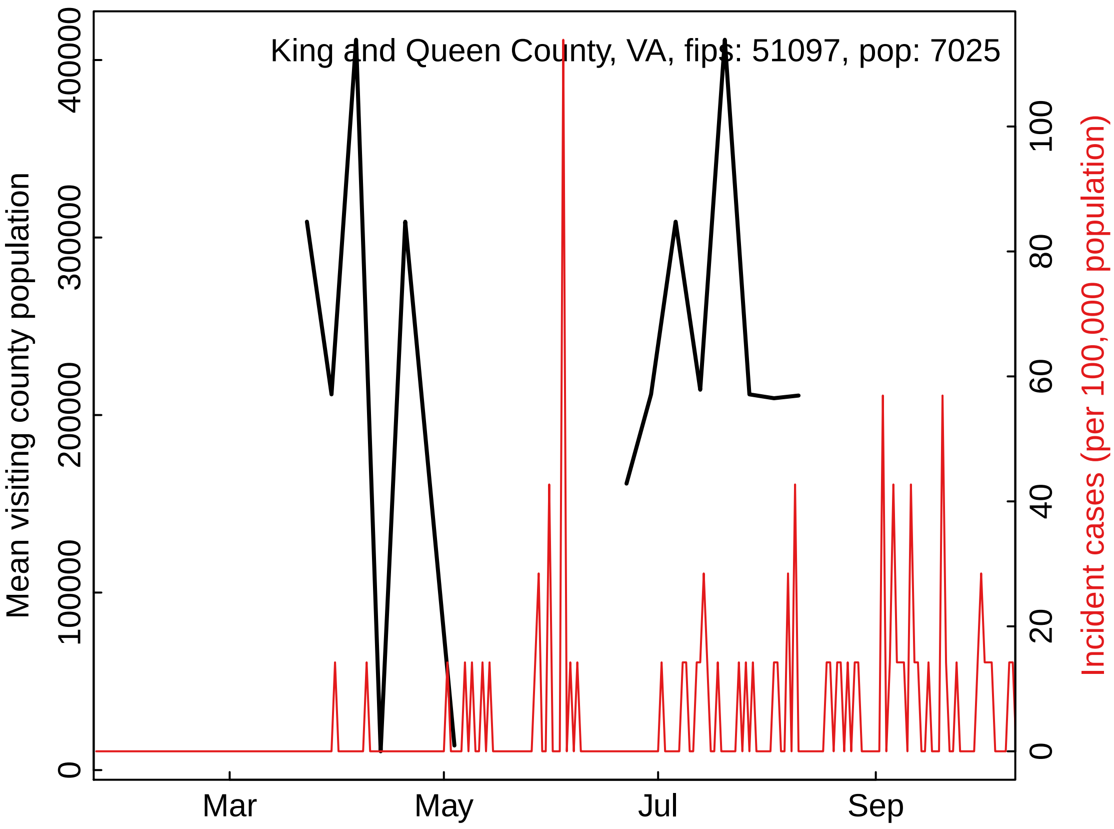

King and Queen County, VA, fips: 51097, pop: 7025
Mean visiting county population
0
100000
200000
300000
400000
Incident cases (per 100,000 population)
0
20
40
60
80
100
Mar
May
Jul
Sep

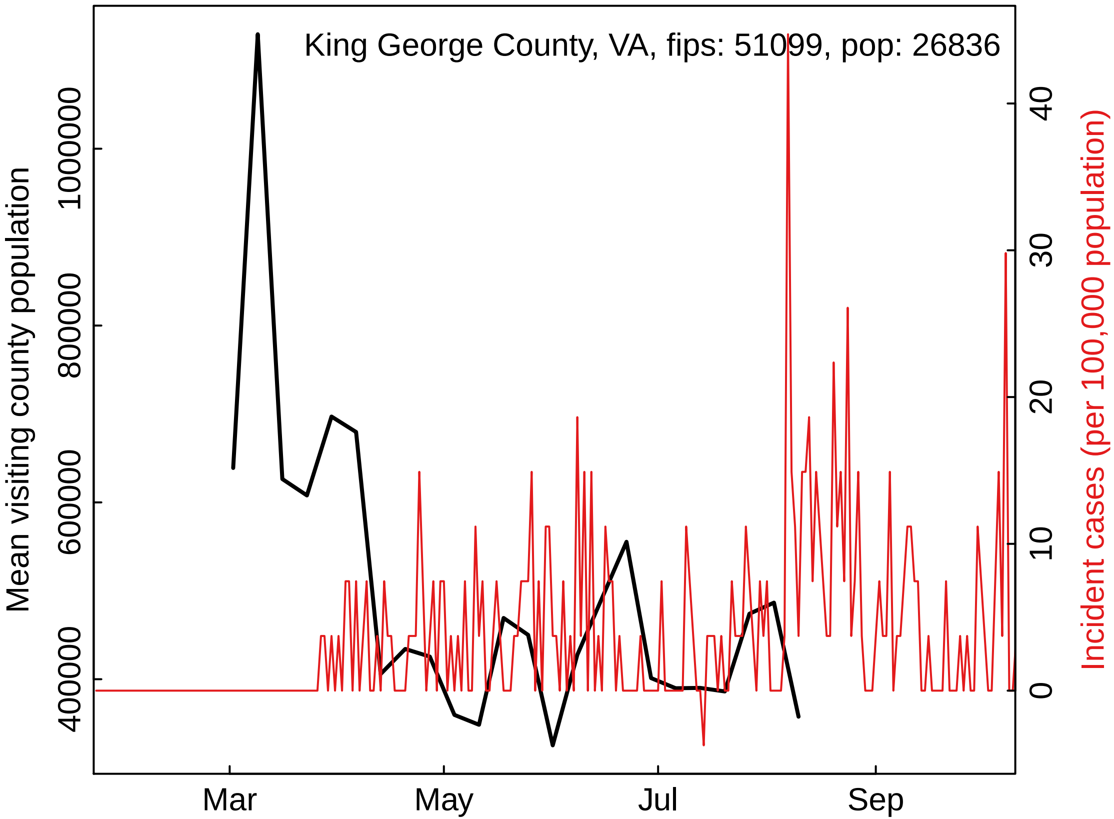
King George County, VA, fips: 51099, pop: 26836
Mean visiting county population
400000
600000
800000
1000000
Incident cases (per 100,000 population)
0
10
20
30
40
Mar
May
Jul
Sep

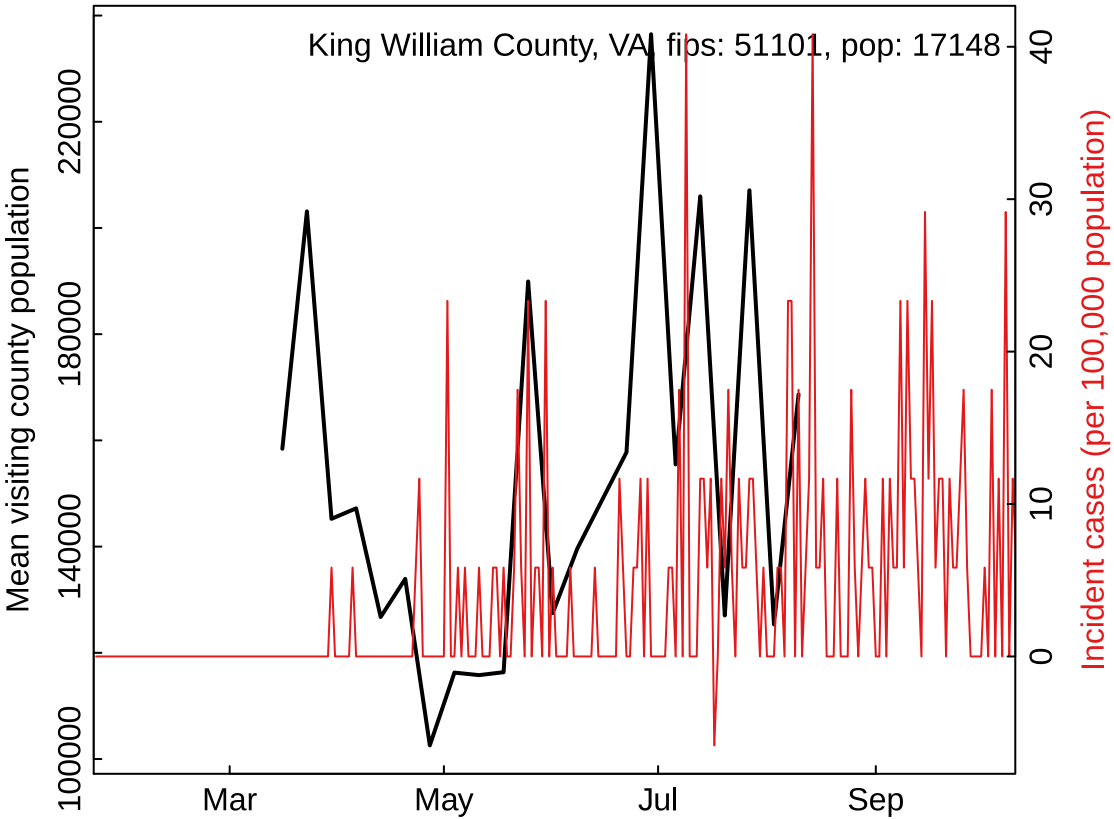
King William County, VA, fips: 51101, pop: 17148
Mean visiting county population
1000000
1400000
1800000
2200000
Mar
May
Jul
Sep
0
10
20
30
40
Incident cases (per 100,000 population)

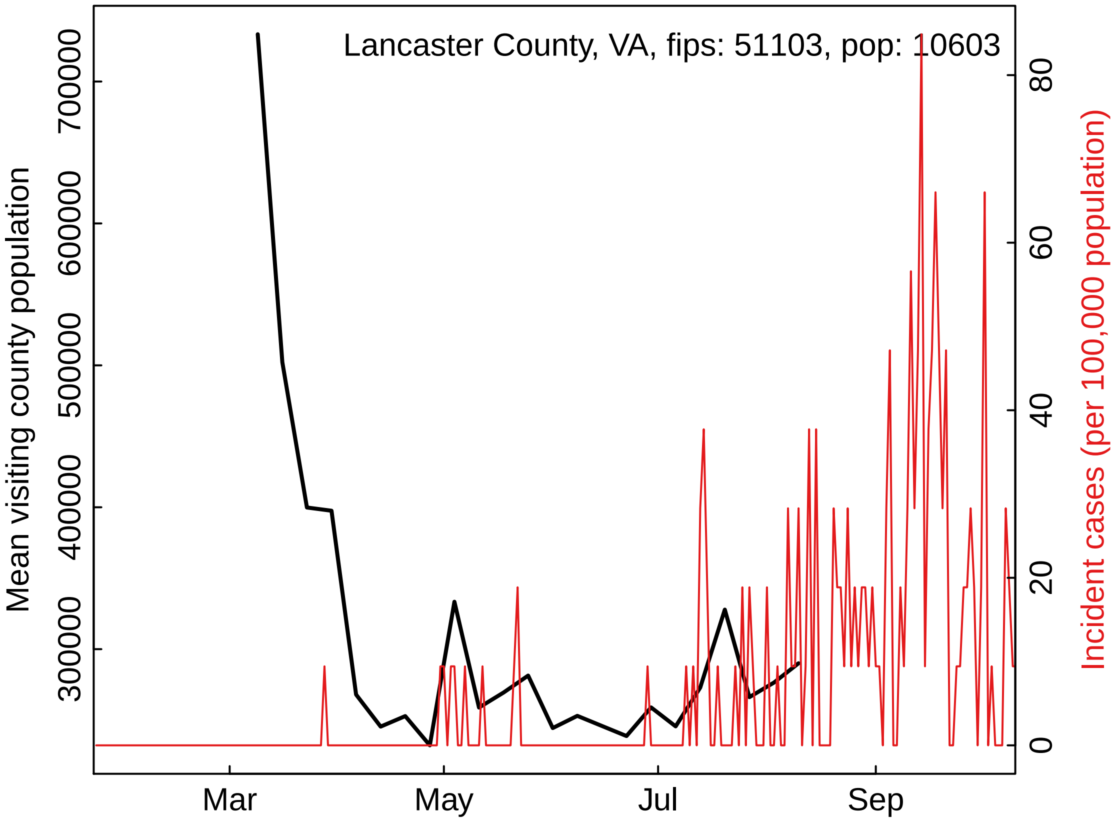

Lancaster County, VA, fips: 51103, pop: 10603
Mean visiting county population
300000
400000
500000
600000
700000
Incident cases (per 100,000 population)
0
20
40
60
80
Mar
May
Jul
Sep

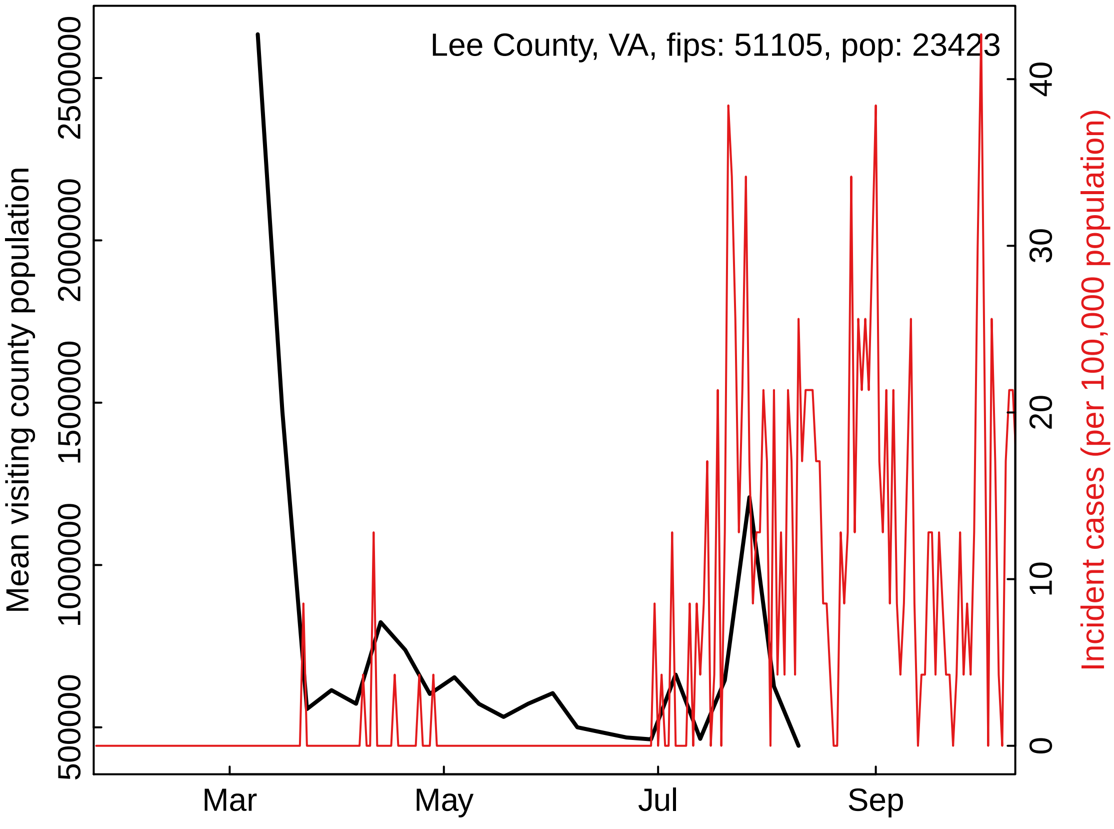

Lee County, VA, fips: 51105, pop: 23423
Mean visiting county population
500000
1000000
1500000
2000000
2500000
Incident cases (per 100,000 population)
0
10
20
30
40
Mar
May
Jul
Sep

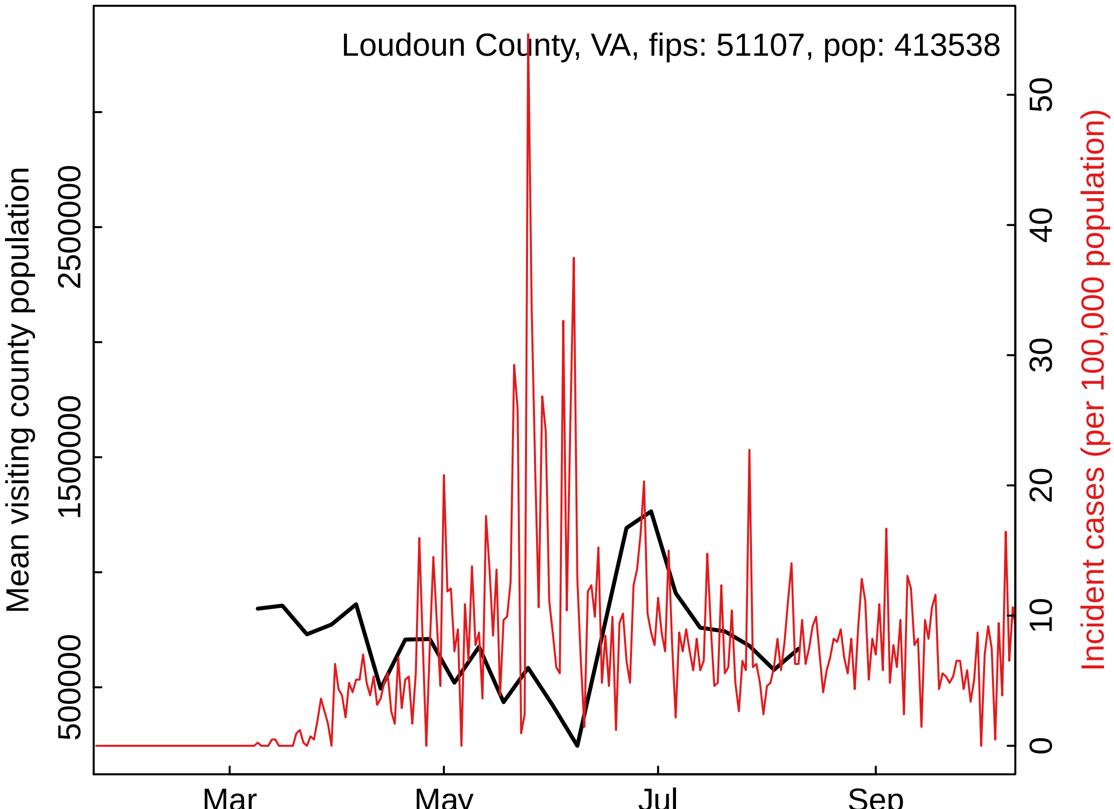
Loudoun County, VA, fips: 51107, pop: 413538
Mean visiting county population
500000
1500000
2500000
Incident cases (per 100,000 population)
0
10
20
30
40
50
Mar
May
Jul
Sep

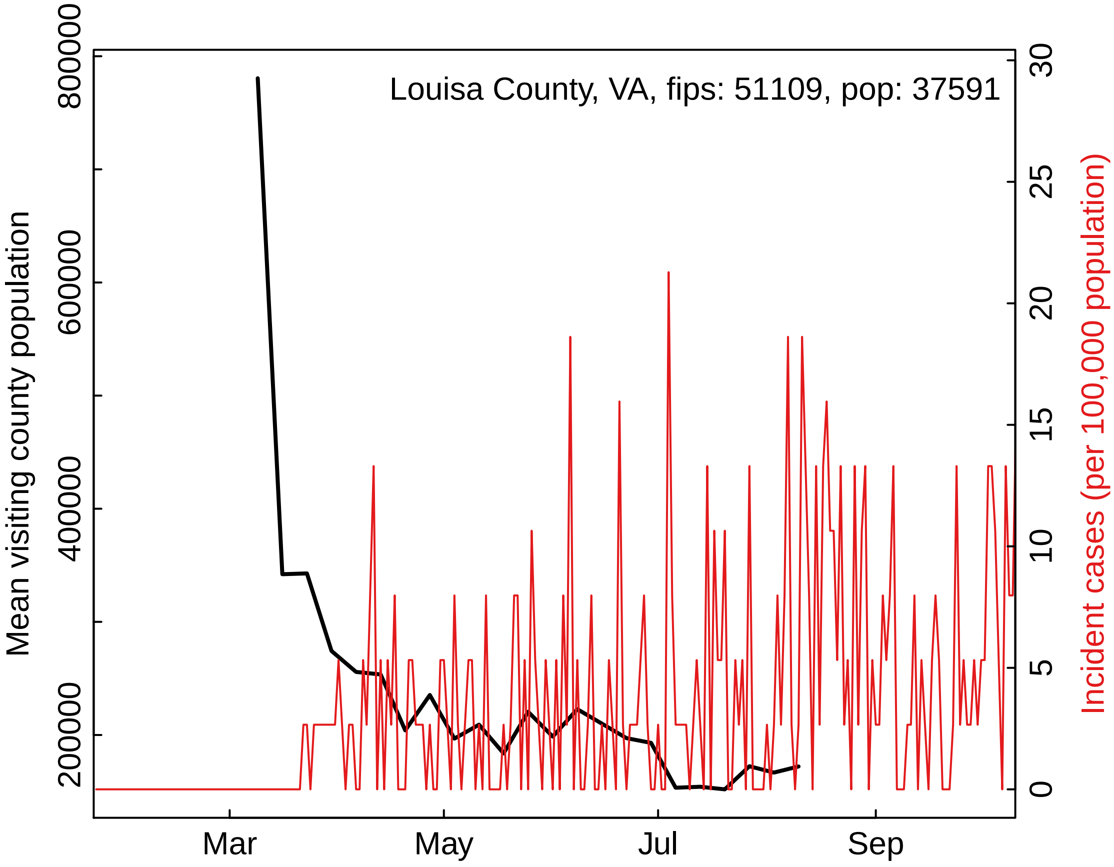

Louisa County, VA, fips: 51109, pop: 37591
Mean visiting county population
200000
400000
600000
800000
Incident cases (per 100,000 population)
0
5
10
15
20
25
30
Mar
May
Jul
Sep

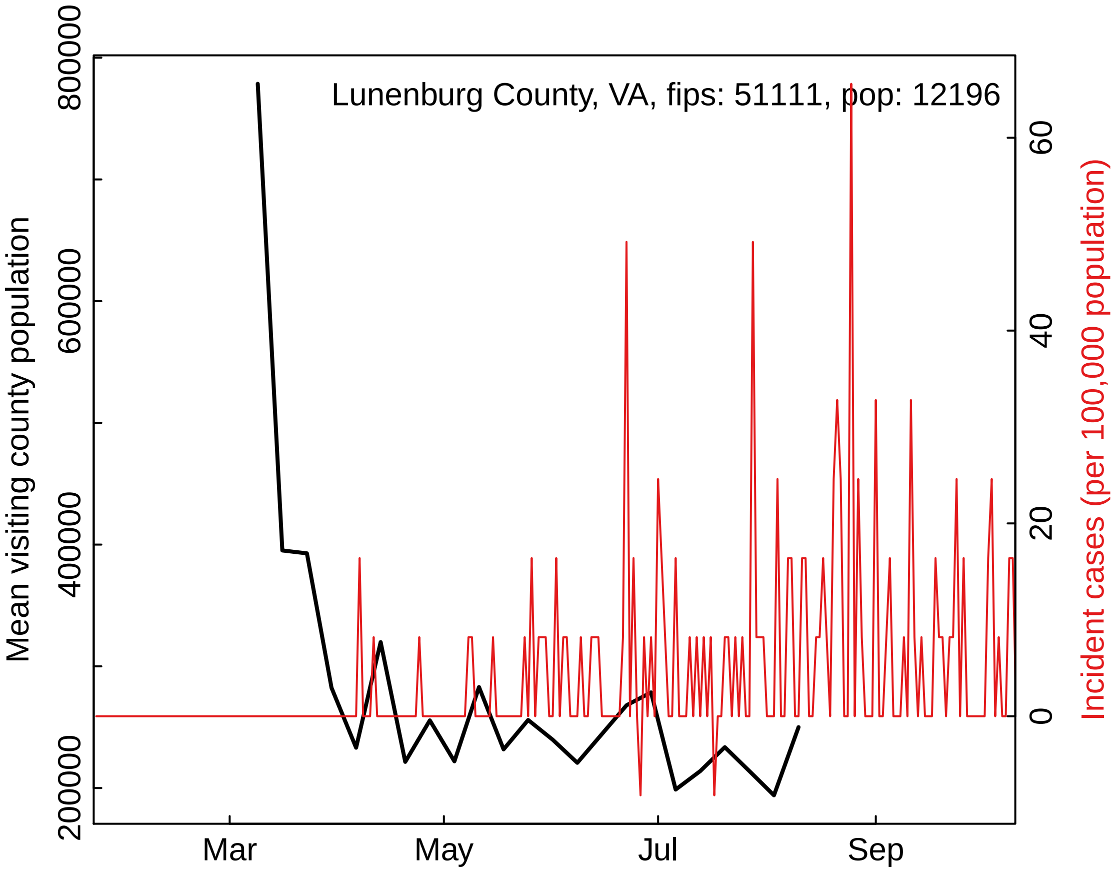

Lunenburg County, VA, fips: 51111, pop: 12196
Mean visiting county population
200000
400000
600000
800000
Incident cases (per 100,000 population)
0
20
40
60
Mar
May
Jul
Sep

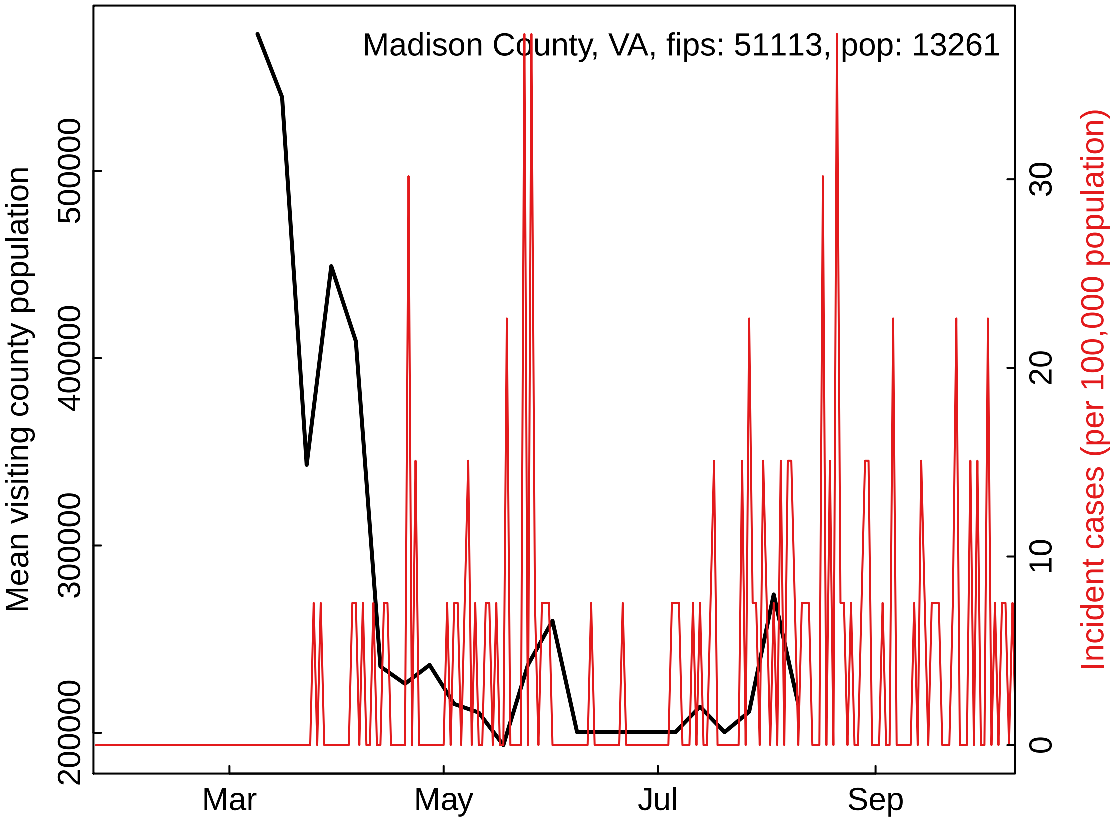

Madison County, VA, fips: 51113, pop: 13261
Mean visiting county population
200000
300000
400000
500000
Incident cases (per 100,000 population)
0
10
20
30
Mar
May
Jul
Sep

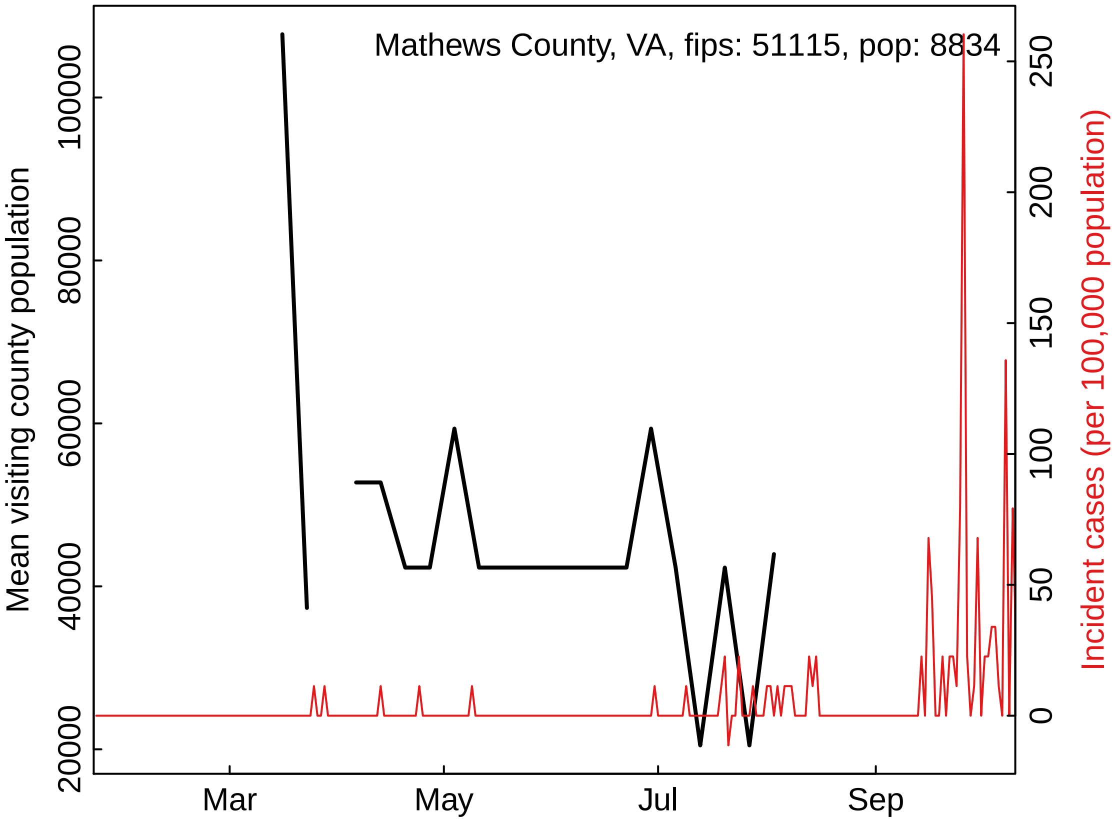
Mathews County, VA, fips: 51115, pop: 8834
Mean visiting county population
20000
40000
60000
80000
100000
Mar
May
Jul
Sep
0
50
100
150
200
250
Incident cases (per 100,000 population)

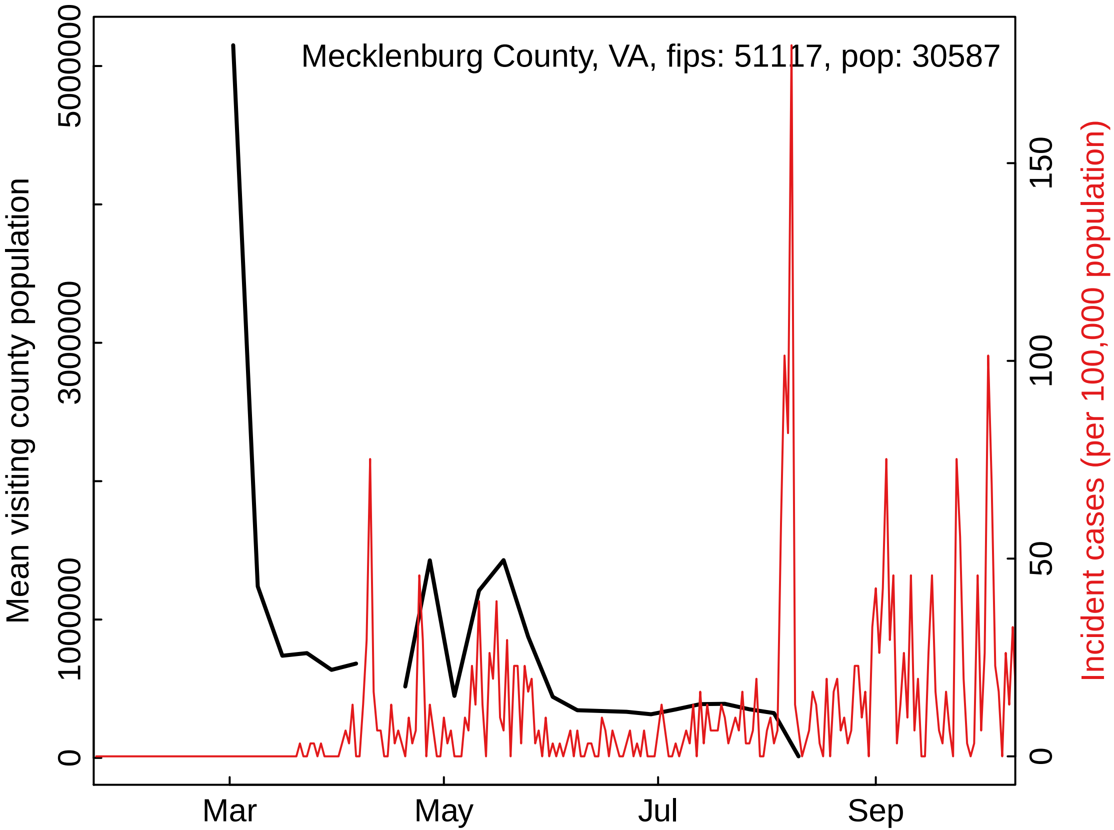
Mecklenburg County, VA, fips: 51117, pop: 30587
Mean visiting county population
0
1000000
3000000
5000000
Incident cases (per 100,000 population)
0
50
100
150
Mar
May
Jul
Sep

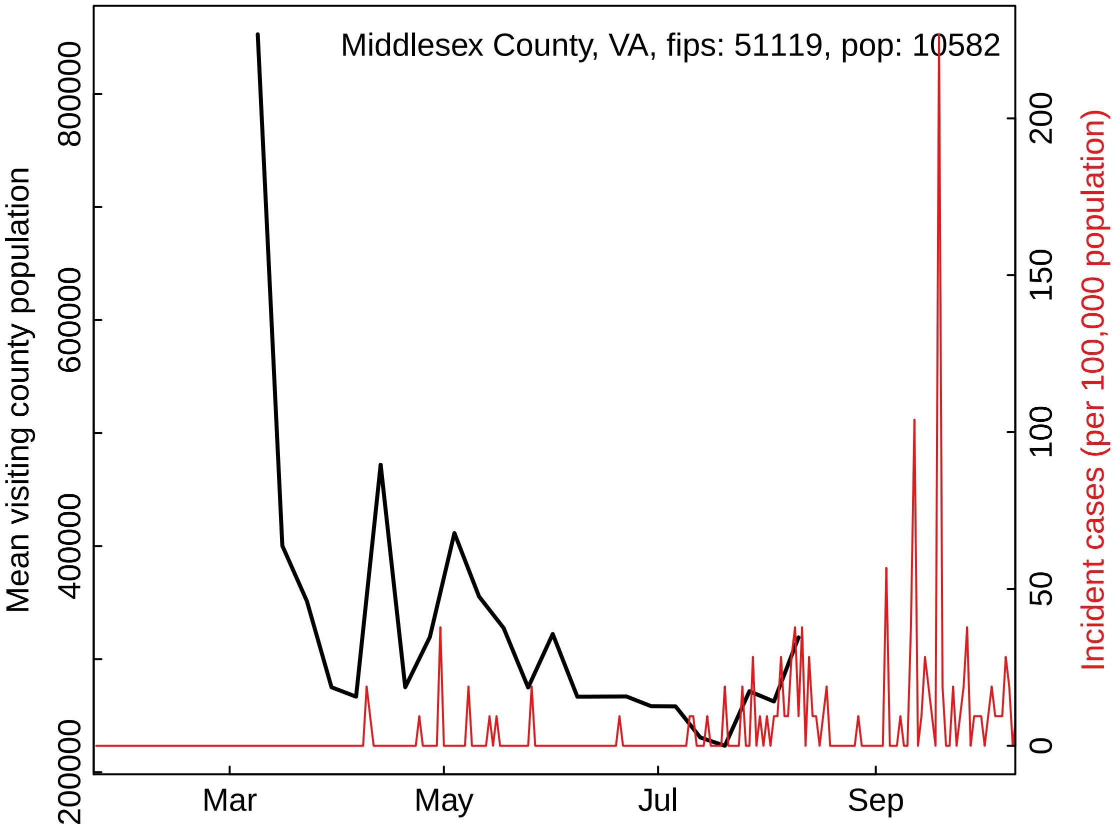
Middlesex County, VA, fips: 51119, pop: 10582
Mean visiting county population
200000
400000
600000
800000
Mar
May
Jul
Sep
0
50
100
150
200
Incident cases (per 100,000 population)

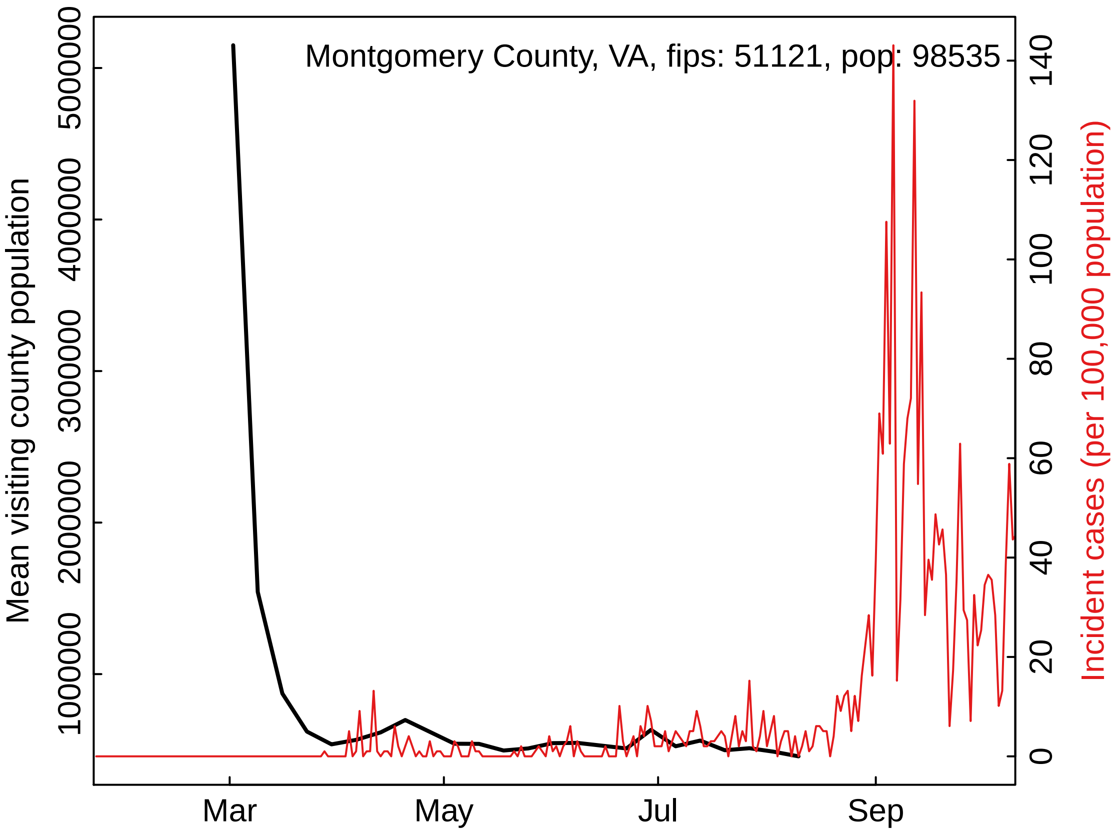
Montgomery County, VA, fips: 51121, pop: 98535
Mean visiting county population
1000000
2000000
3000000
4000000
5000000
Incident cases (per 100,000 population)
0
20
40
60
80
100
120
140
Mar
May
Jul
Sep

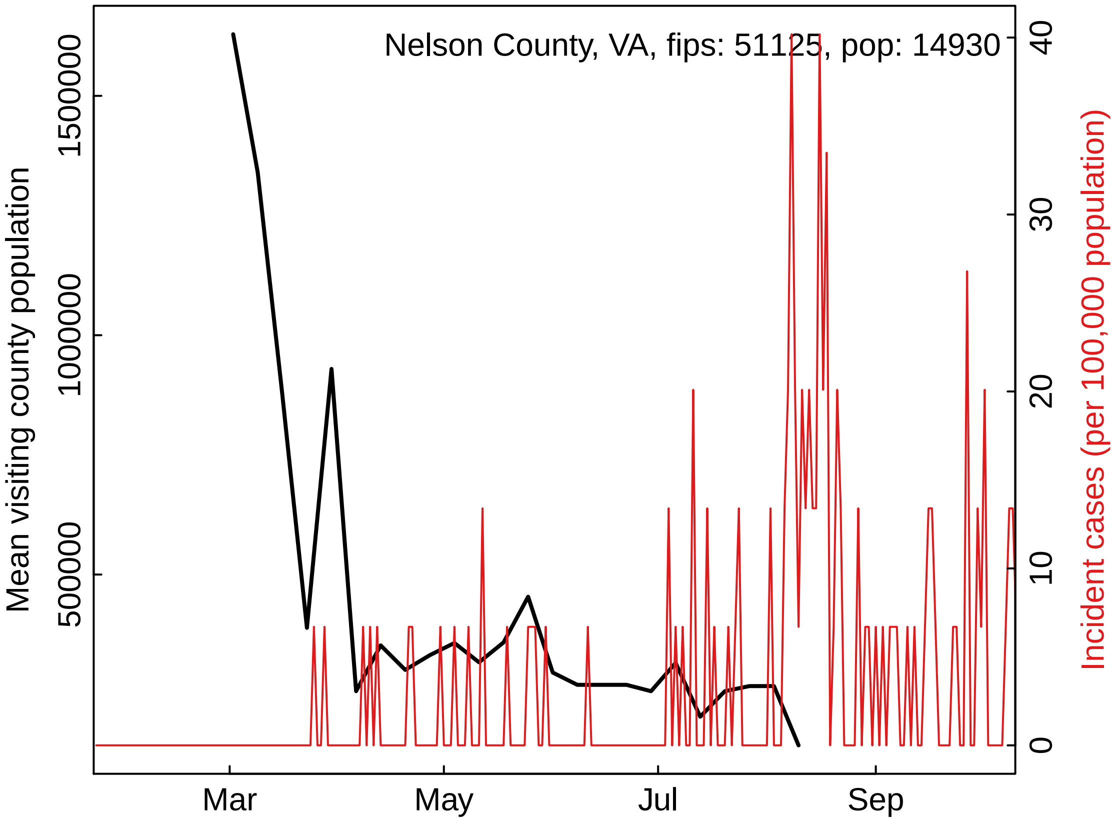

Nelson County, VA, fips: 51125, pop: 14930
Mean visiting county population
Incident cases (per 100,000 population)
500000
1000000
1500000
0
10
20
30
40
Mar
May
Jul
Sep

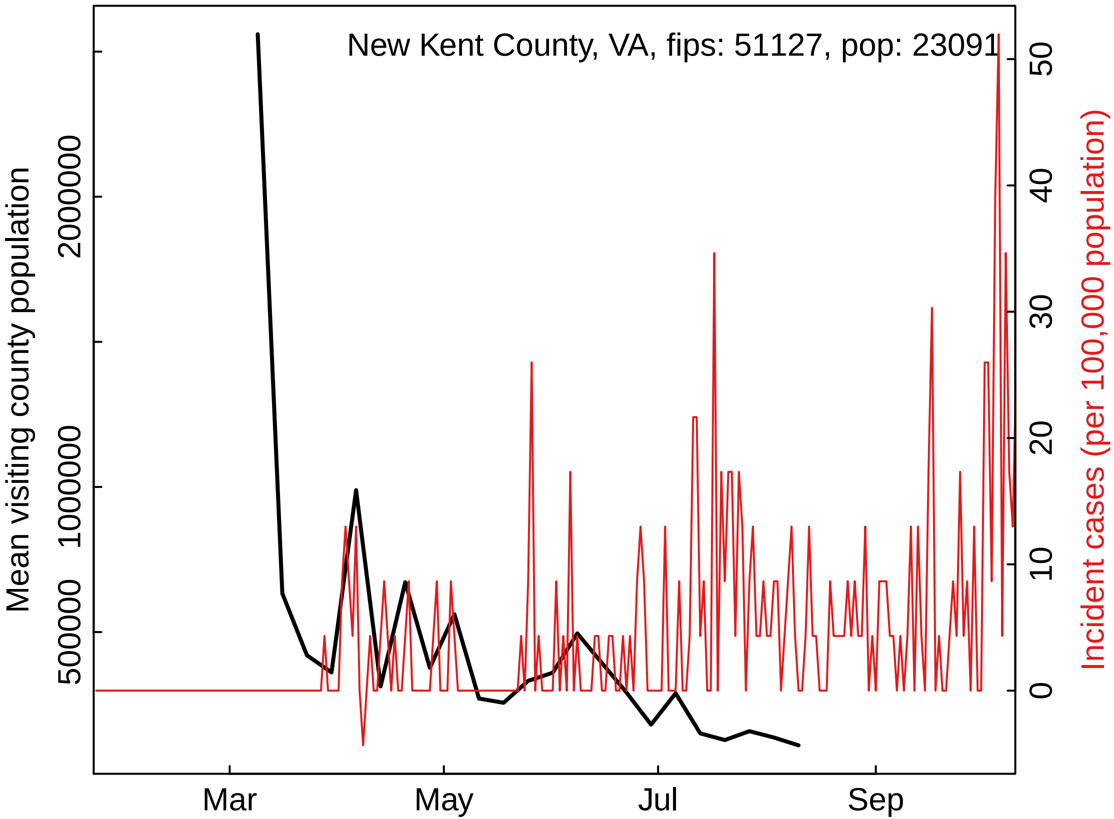

New Kent County, VA, fips: 51127, pop: 23091
Mean visiting county population
500000
1000000
2000000
Incident cases (per 100,000 population)
0
10
20
30
40
50
Mar
May
Jul
Sep

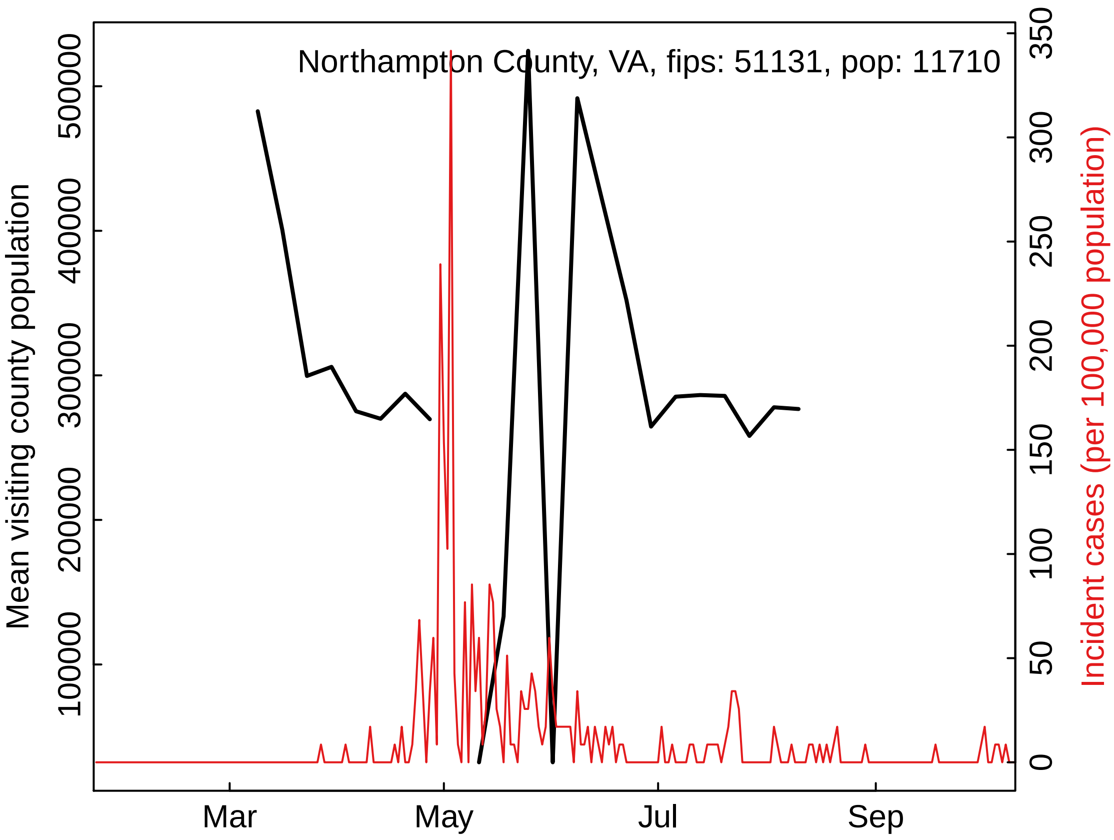

Northampton County, VA, fips: 51131, pop: 11710
Mean visiting county population
100000
200000
300000
400000
500000
Incident cases (per 100,000 population)
0
50
100
150
200
250
300
350
Mar
May
Jul
Sep

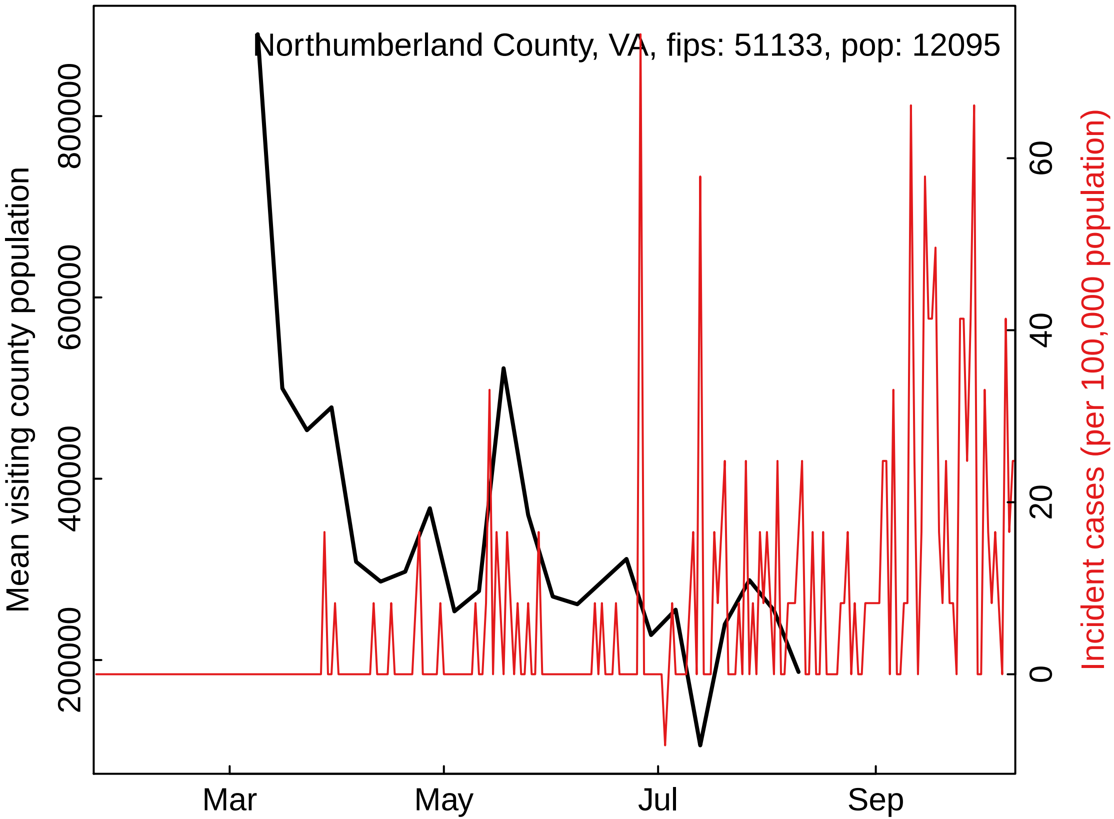

Northumberland County, VA, fips: 51133, pop: 12095
Mean visiting county population
200000
400000
600000
800000
Incident cases (per 100,000 population)
0
20
40
60
Mar
May
Jul
Sep

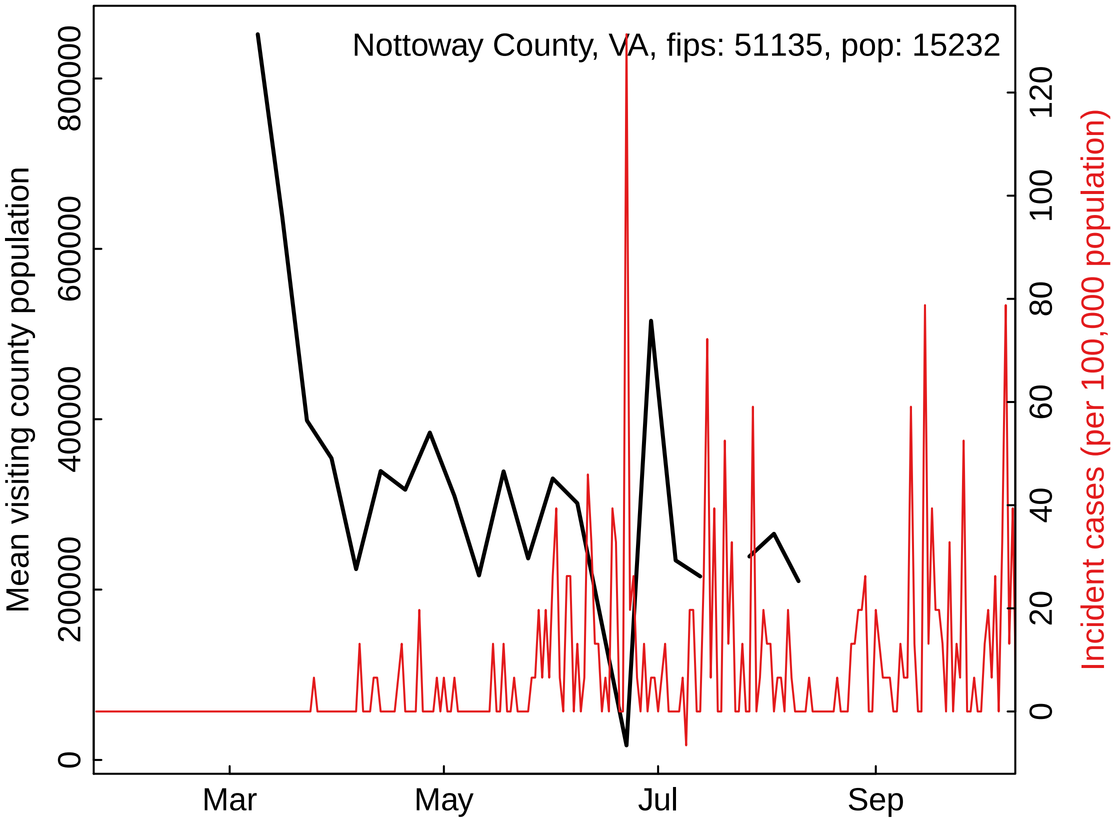

Nottoway County, VA, fips: 51135, pop: 15232
Mean visiting county population
0
200000
400000
600000
800000
Incident cases (per 100,000 population)
0
20
40
60
80
100
120
Mar
May
Jul
Sep

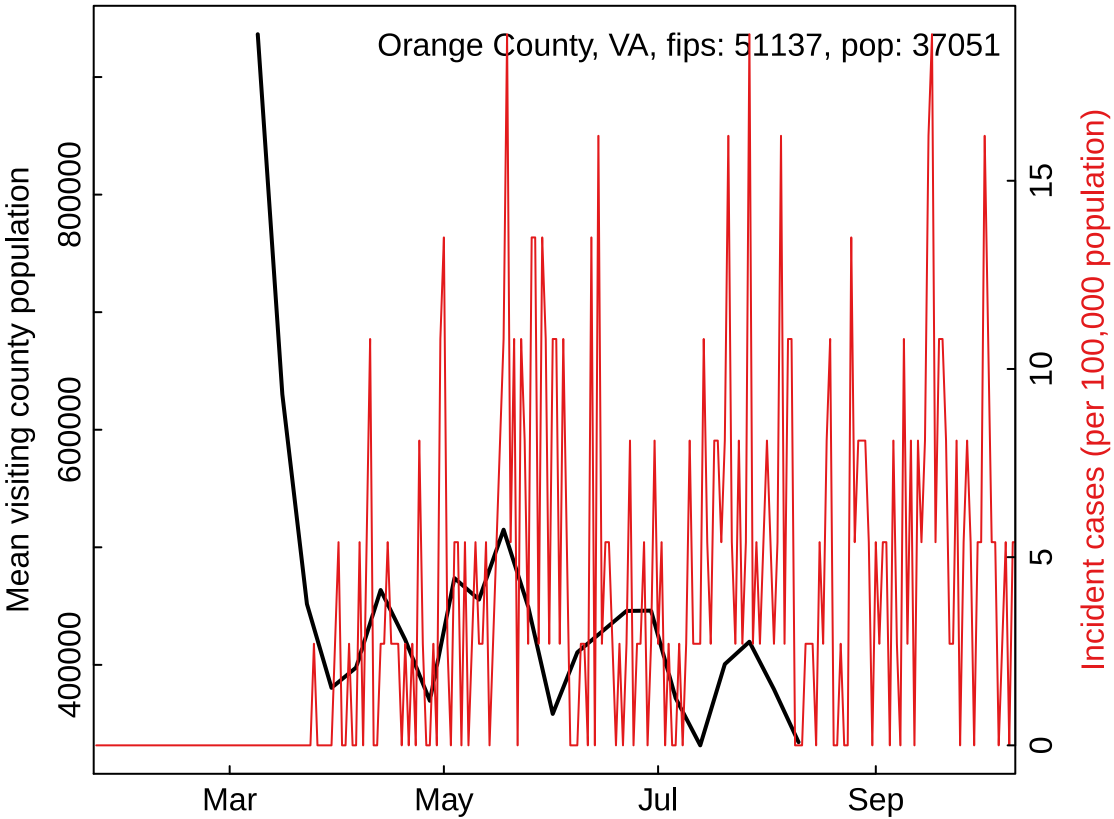

Orange County, VA, fips: 51137, pop: 37051
Mean visiting county population
400000
600000
800000
Incident cases (per 100,000 population)
0
5
10
15
Mar
May
Jul
Sep

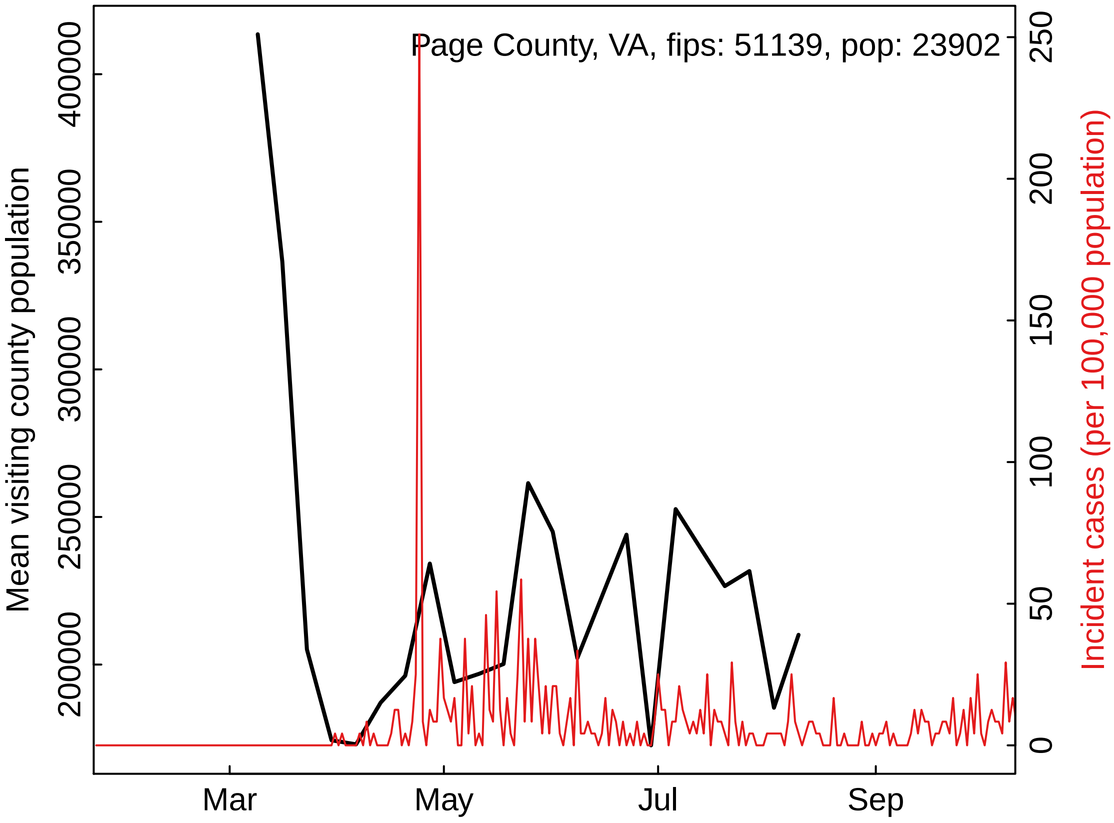

Page County, VA, fips: 51139, pop: 23902
Mean visiting county population
200000
250000
300000
350000
400000
Incident cases (per 100,000 population)
0
50
100
150
200
250
Mar
May
Jul
Sep

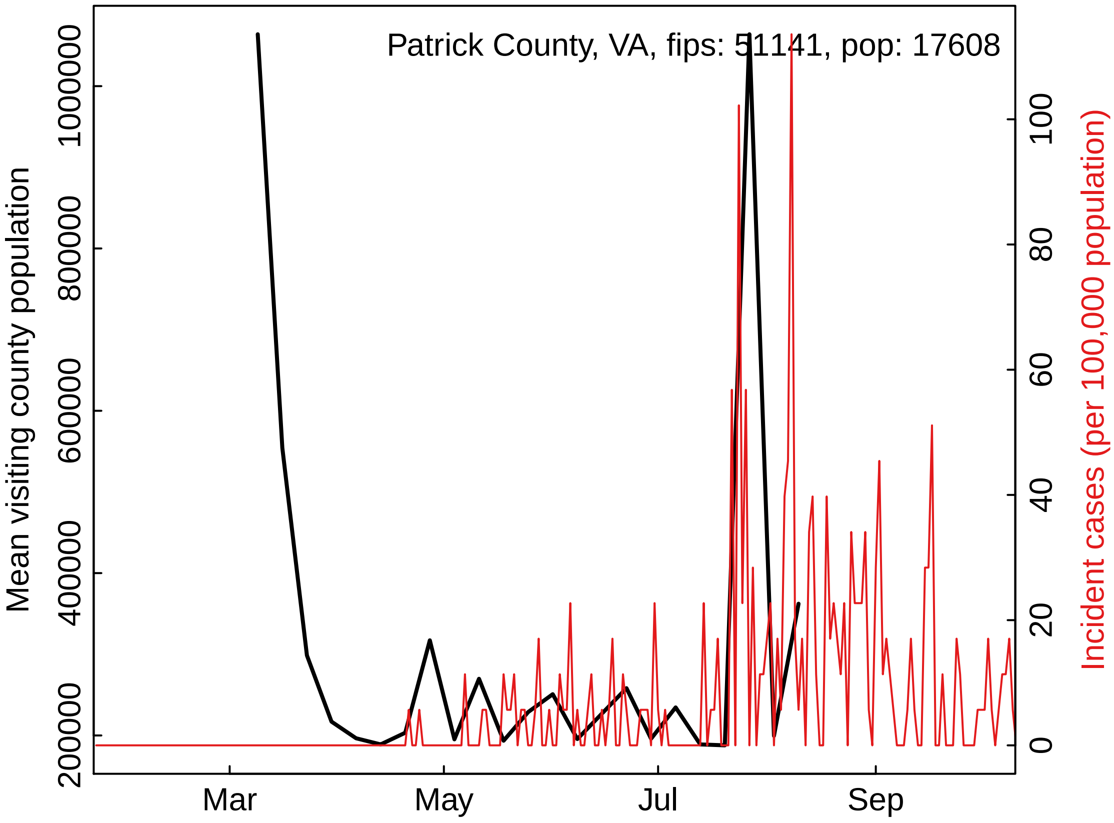

Patrick County, VA, fips: 51141, pop: 17608
Mean visiting county population
200000
400000
600000
800000
1000000
Incident cases (per 100,000 population)
0
20
40
60
80
100
Mar
May
Jul
Sep

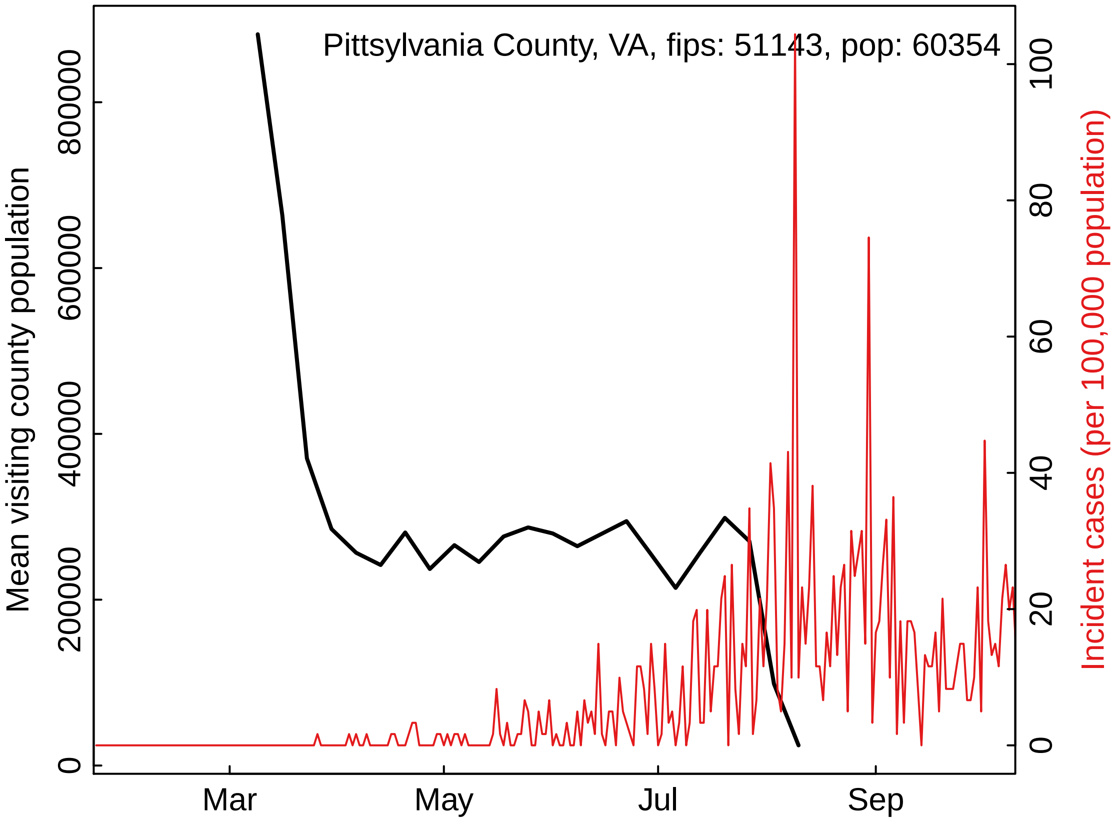
Pittsylvania County, VA, fips: 51143, pop: 60354
Mean visiting county population
0
200000
400000
600000
800000
Mar
May
Jul
Sep
0
20
40
60
80
100
Incident cases (per 100,000 population)

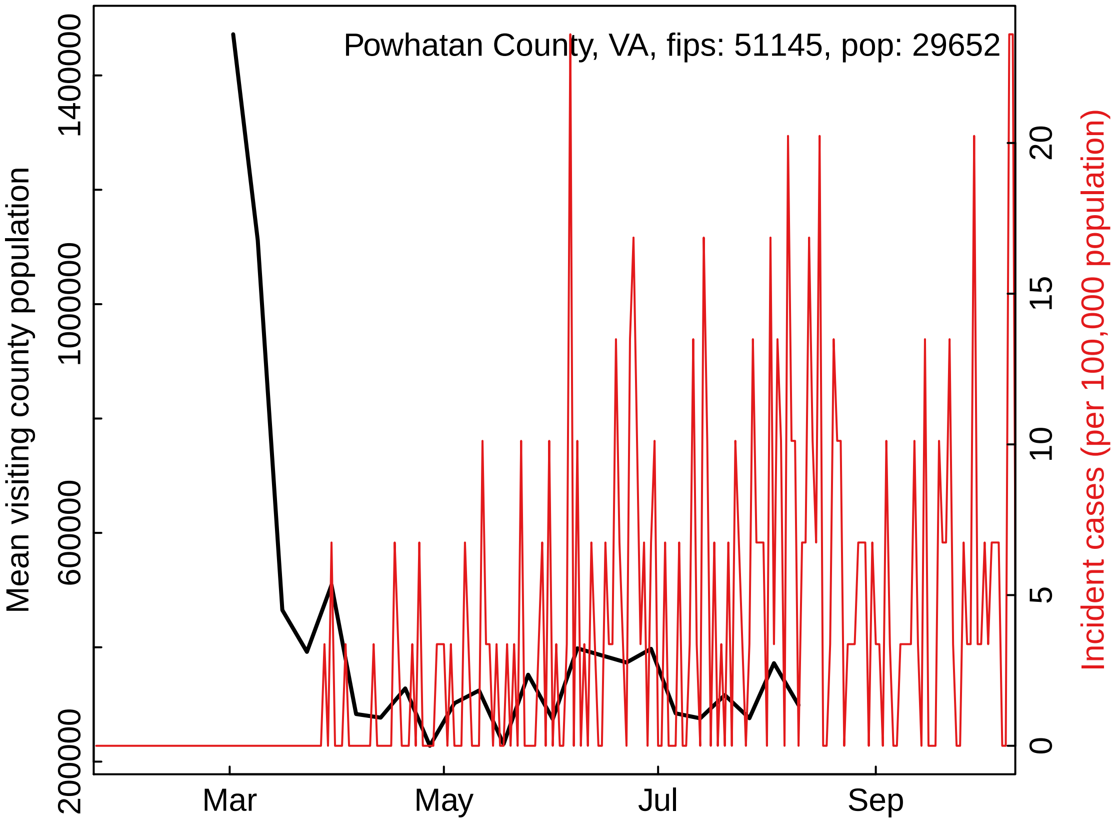

Powhatan County, VA, fips: 51145, pop: 29652
Mean visiting county population
200000
600000
1000000
1400000
Mar
May
Jul
Sep
0
5
10
15
20
Incident cases (per 100,000 population)

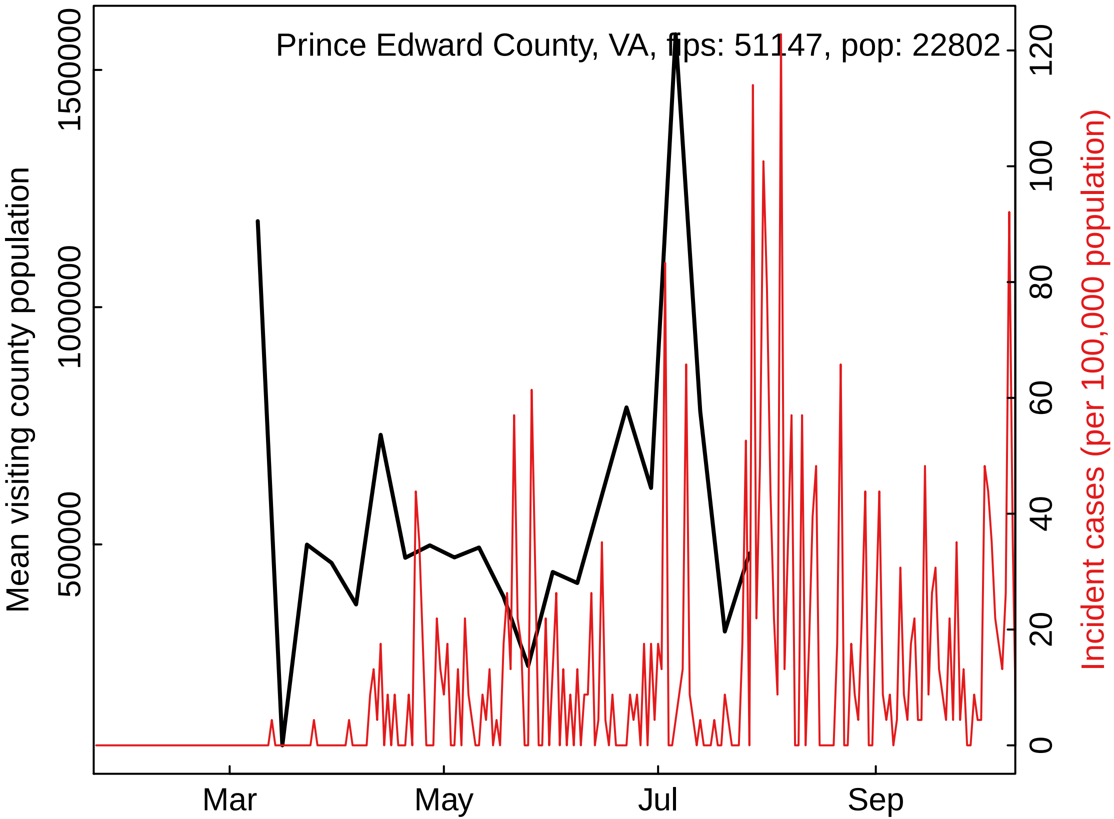

Prince Edward County, VA, fips: 51147, pop: 22802
Mean visiting county population
500000
1000000
1500000
Incident cases (per 100,000 population)
0
20
40
60
80
100
120
Mar
May
Jul
Sep

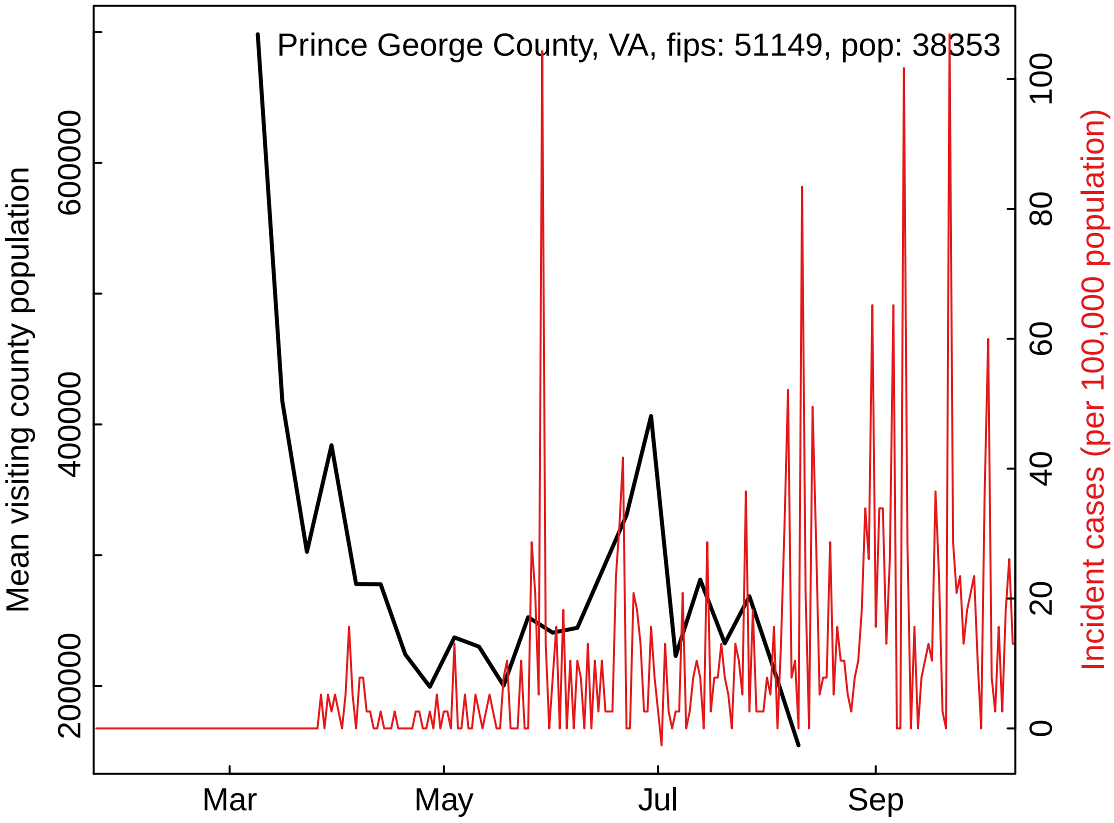
Prince George County, VA, fips: 51149, pop: 38353
Mean visiting county population
200000
400000
600000
Incident cases (per 100,000 population)
0
20
40
60
80
100
Mar
May
Jul
Sep

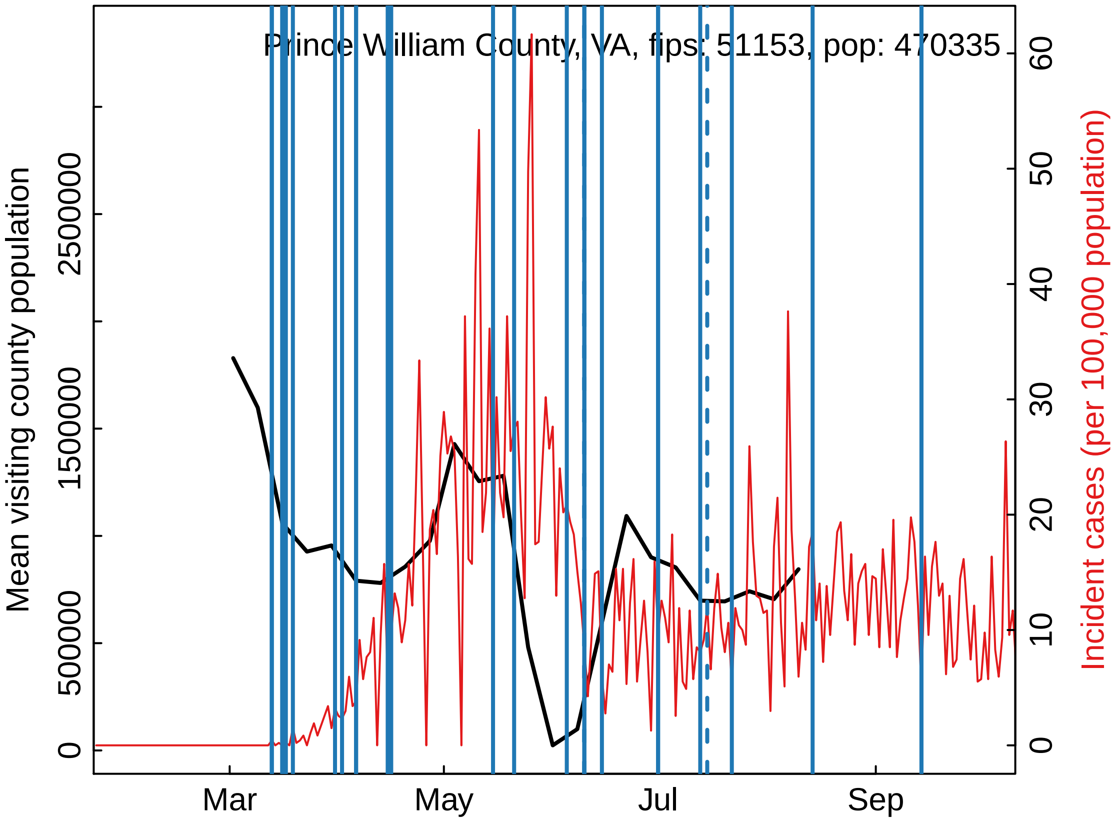

Prince William County, VA, fips: 51153, pop: 470335
Mean visiting county population
Incident cases (per 100,000 population)
0
500000
1500000
2500000
0
10
20
30
40
50
60
Mar
May
Jul
Sep

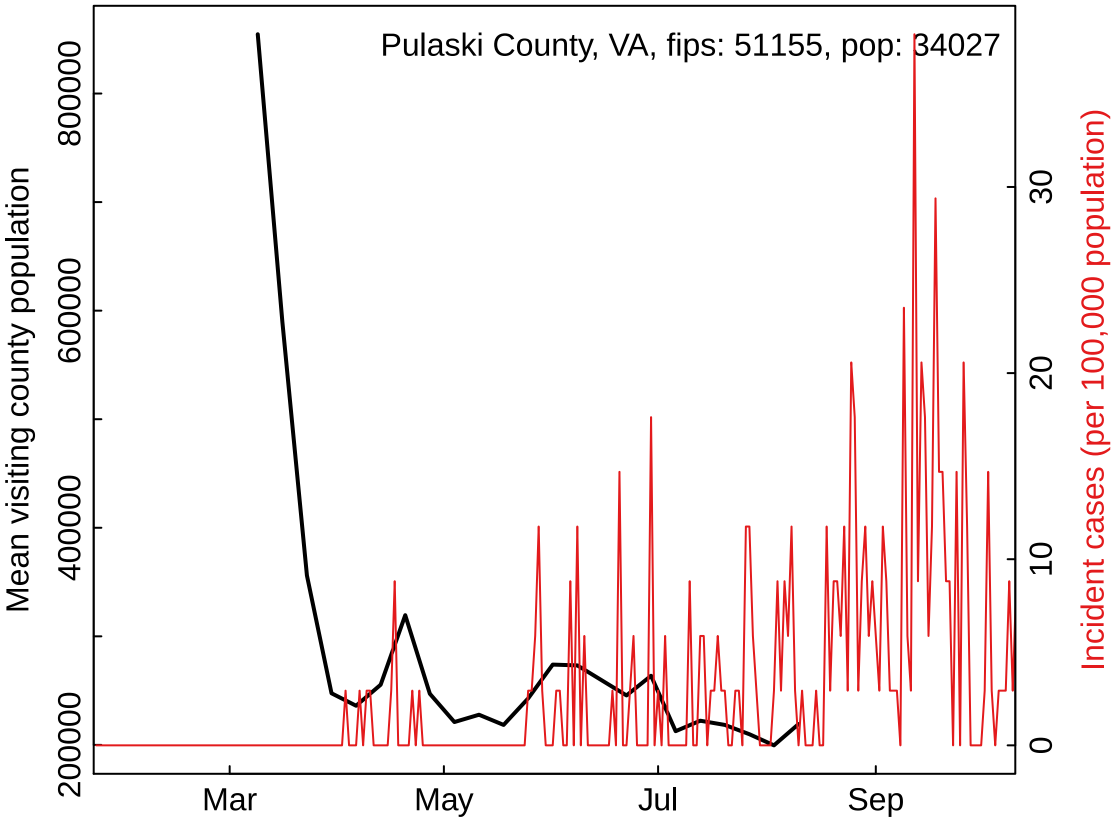

Pulaski County, VA, fips: 51155, pop: 34027
Mean visiting county population
200000
400000
600000
800000
Mar
May
Jul
Sep
0
10
20
30
Incident cases (per 100,000 population)

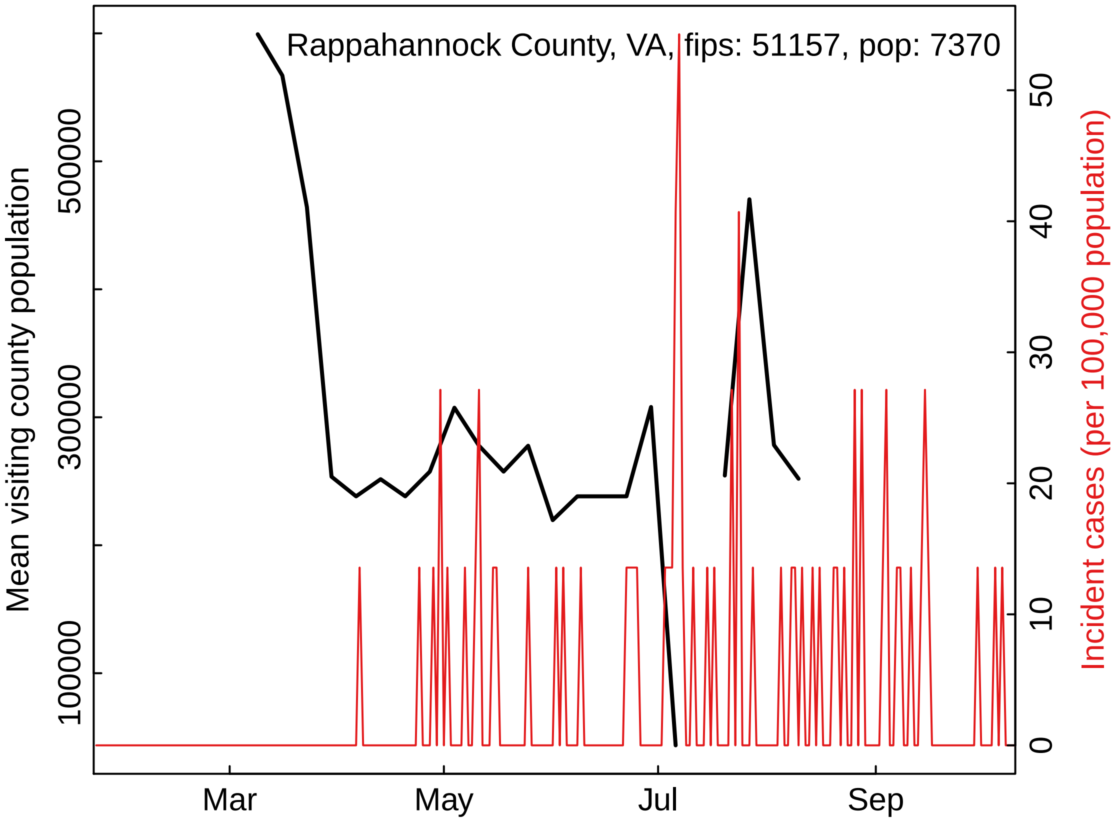

Rappahannock County, VA, fips: 51157, pop: 7370
Mean visiting county population
100000
300000
500000
Incident cases (per 100,000 population)
0
10
20
30
40
50
Mar
May
Jul
Sep

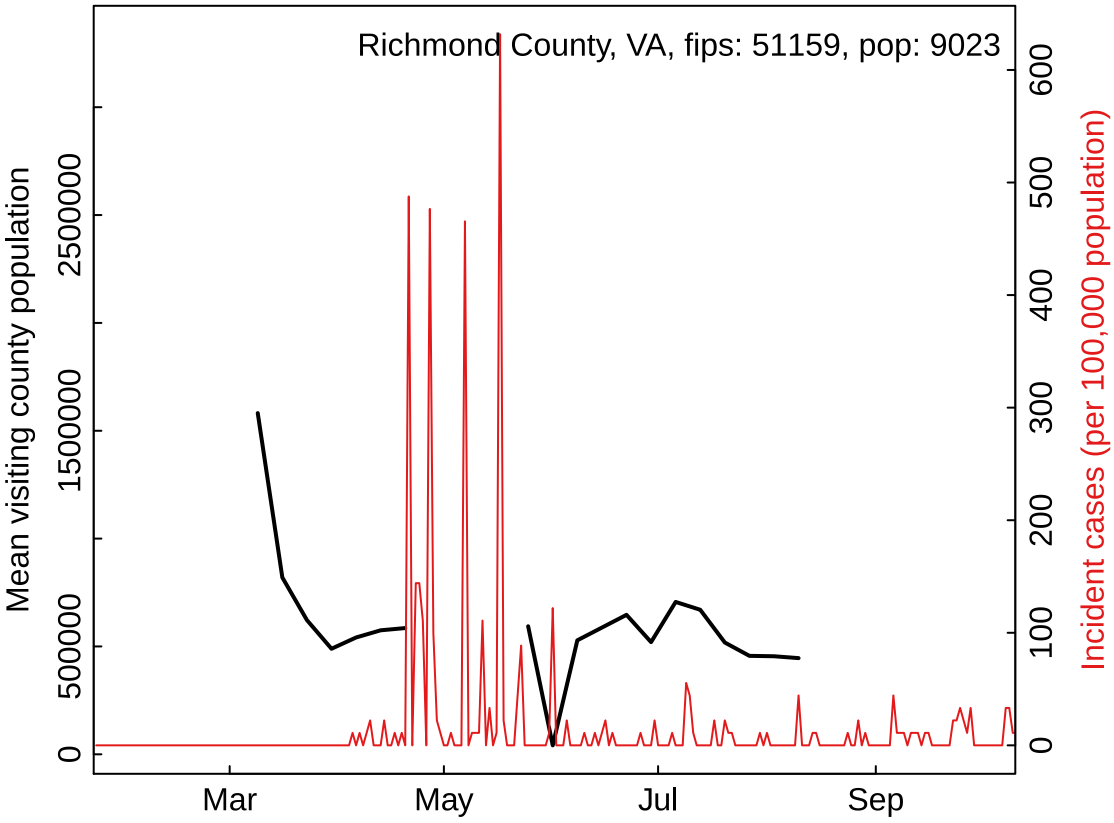

Richmond County, VA, fips: 51159, pop: 9023
Mean visiting county population
0
500000
1500000
2500000
Incident cases (per 100,000 population)
0
100
200
300
400
500
600
Mar
May
Jul
Sep

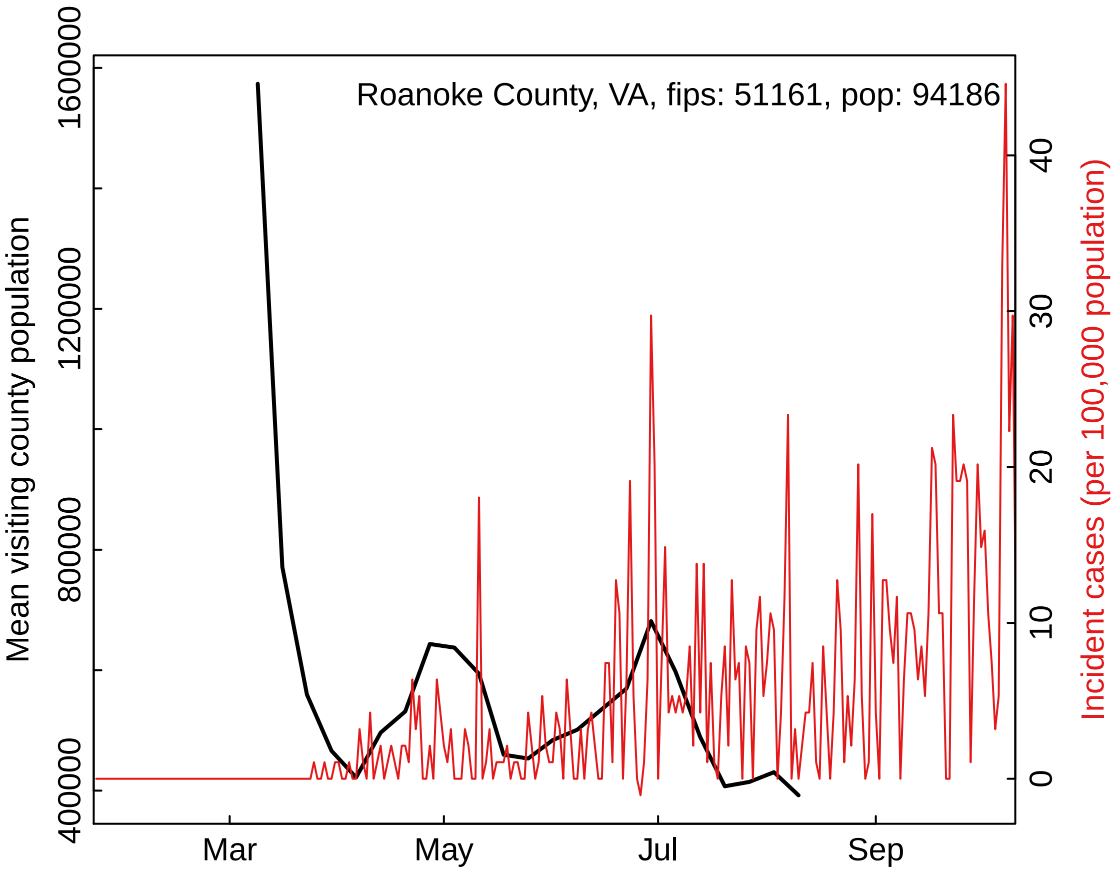
Roanoke County, VA, fips: 51161, pop: 94186
Mean visiting county population
400000
800000
1200000
1600000
Incident cases (per 100,000 population)
0
10
20
30
40
Mar
May
Jul
Sep

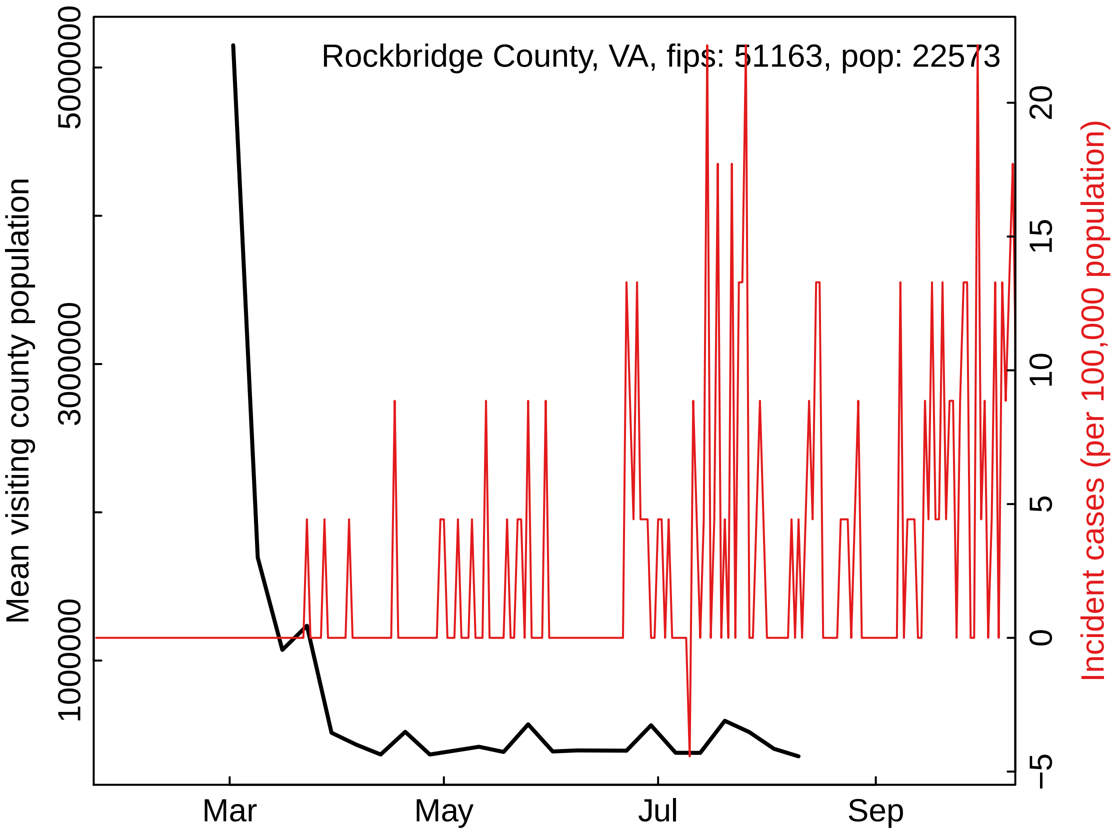
Rockbridge County, VA, fips: 51163, pop: 22573
Mean visiting county population
1000000
3000000
5000000
Incident cases (per 100,000 population)
−5
0
5
10
15
20
Mar
May
Jul
Sep

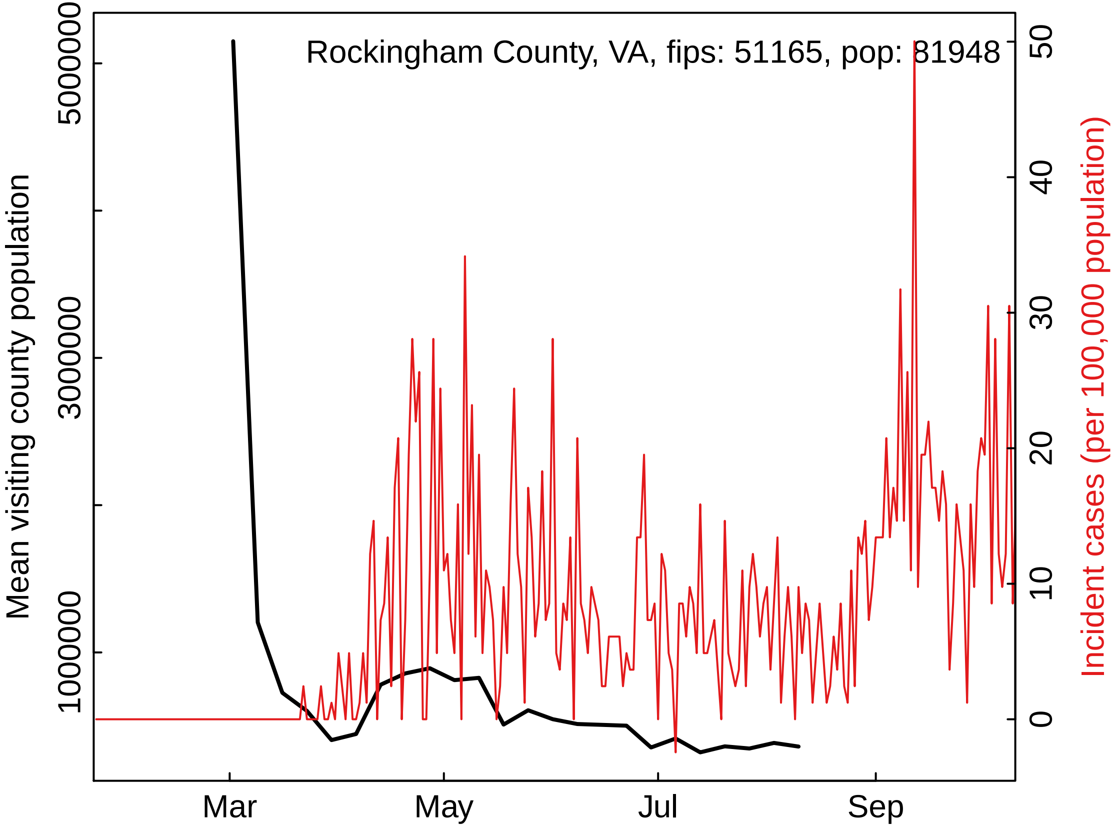

Rockingham County, VA, fips: 51165, pop: 81948
Mean visiting county population
1000000
3000000
5000000
Incident cases (per 100,000 population)
0
10
20
30
40
50
Mar
May
Jul
Sep

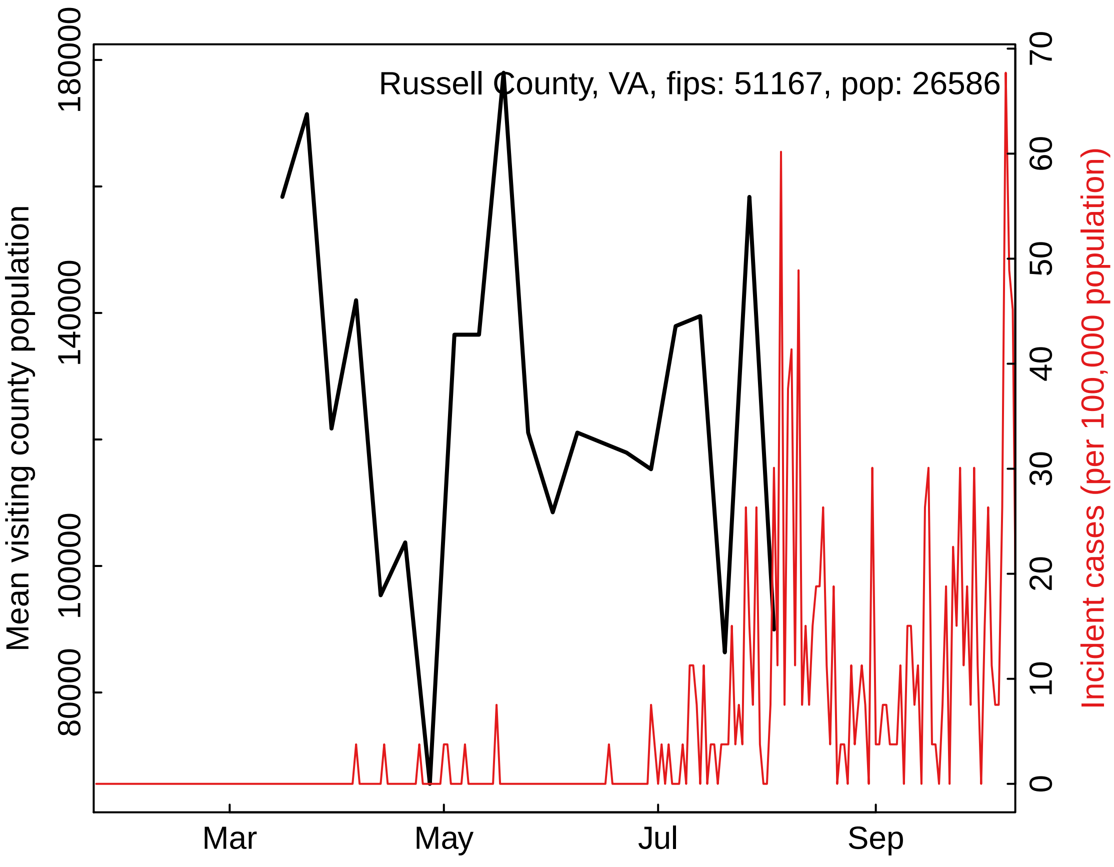

Russell County, VA, fips: 51167, pop: 26586
Mean visiting county population
80000
100000
140000
180000
Incident cases (per 100,000 population)
0
10
20
30
40
50
60
70
Mar
May
Jul
Sep

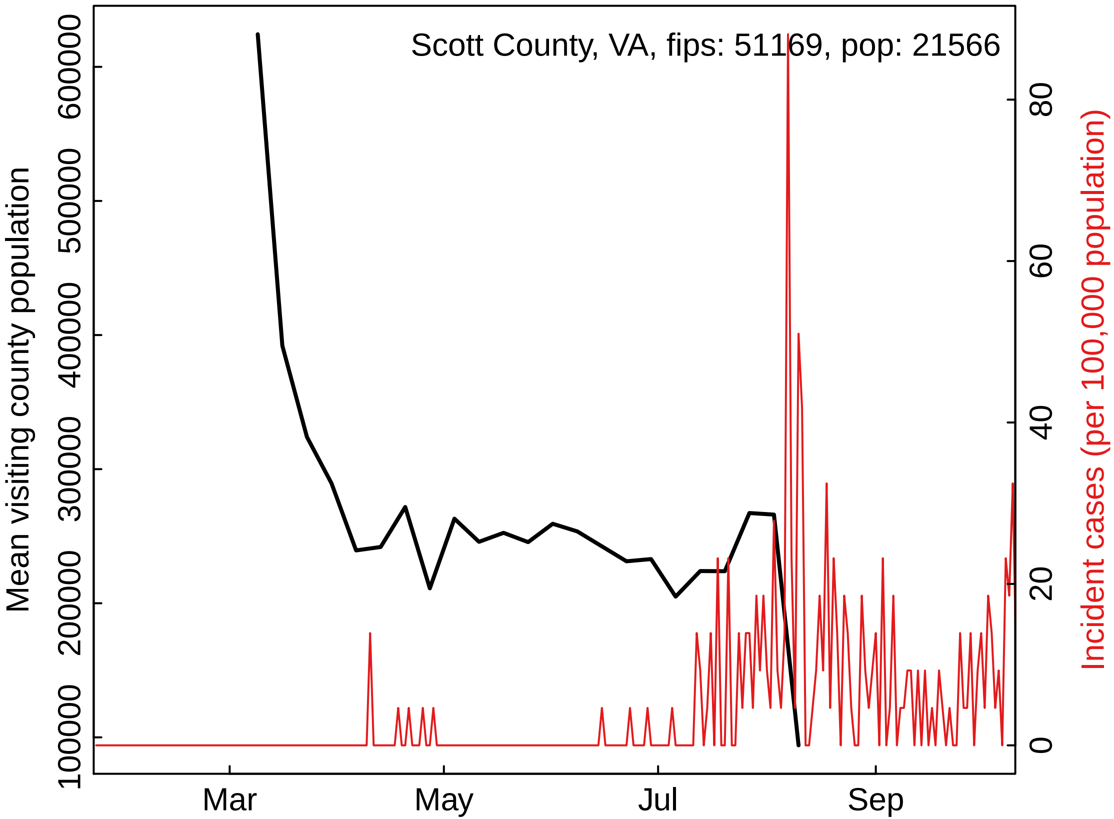
Scott County, VA, fips: 51169, pop: 21566
Mean visiting county population
100000
200000
300000
400000
500000
600000
Mar
May
Jul
Sep
0
20
40
60
80
Incident cases (per 100,000 population)

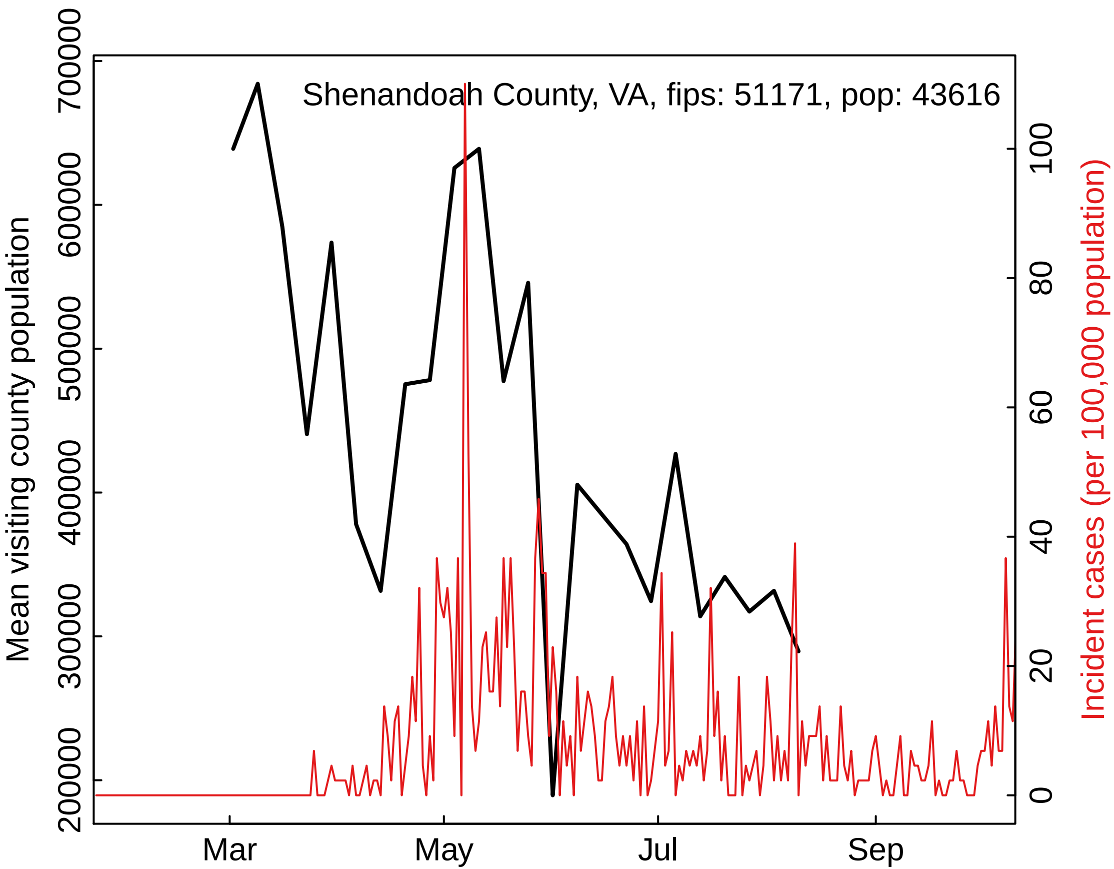

Shenandoah County, VA, fips: 51171, pop: 43616
Mean visiting county population
200000
300000
400000
500000
600000
700000
Incident cases (per 100,000 population)
0
20
40
60
80
100
Mar
May
Jul
Sep

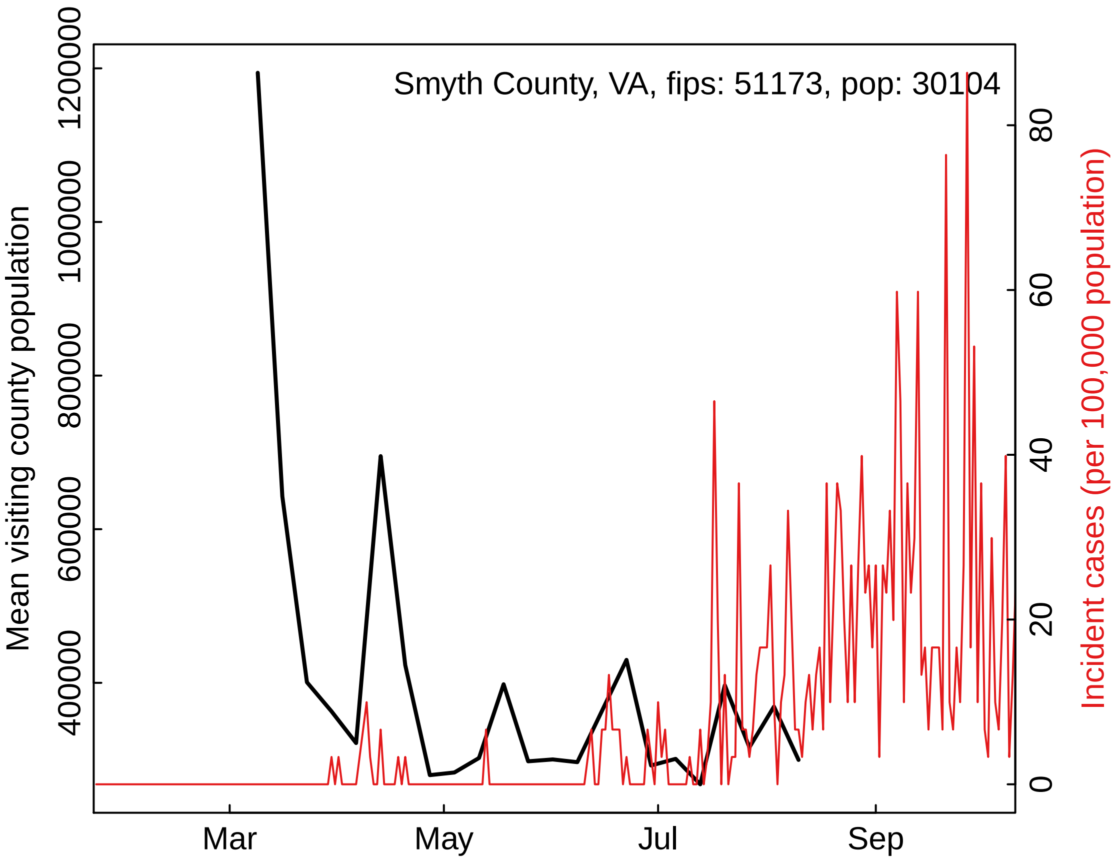

Smyth County, VA, fips: 51173, pop: 30104
Mean visiting county population
400000
600000
800000
1000000
1200000
Incident cases (per 100,000 population)
0
20
40
60
80
Mar
May
Jul
Sep

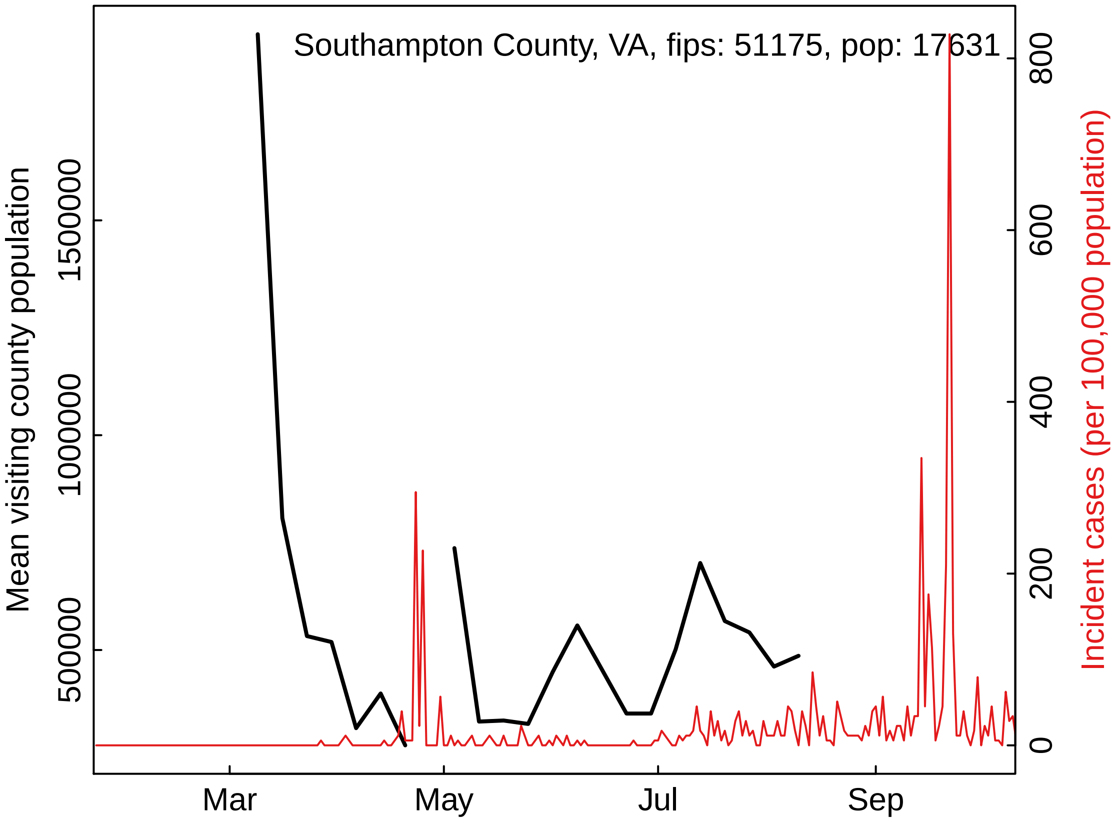
Southampton County, VA, fips: 51175, pop: 17631
Mean visiting county population
500000
1000000
1500000
Incident cases (per 100,000 population)
0
200
400
600
800
Mar
May
Jul
Sep

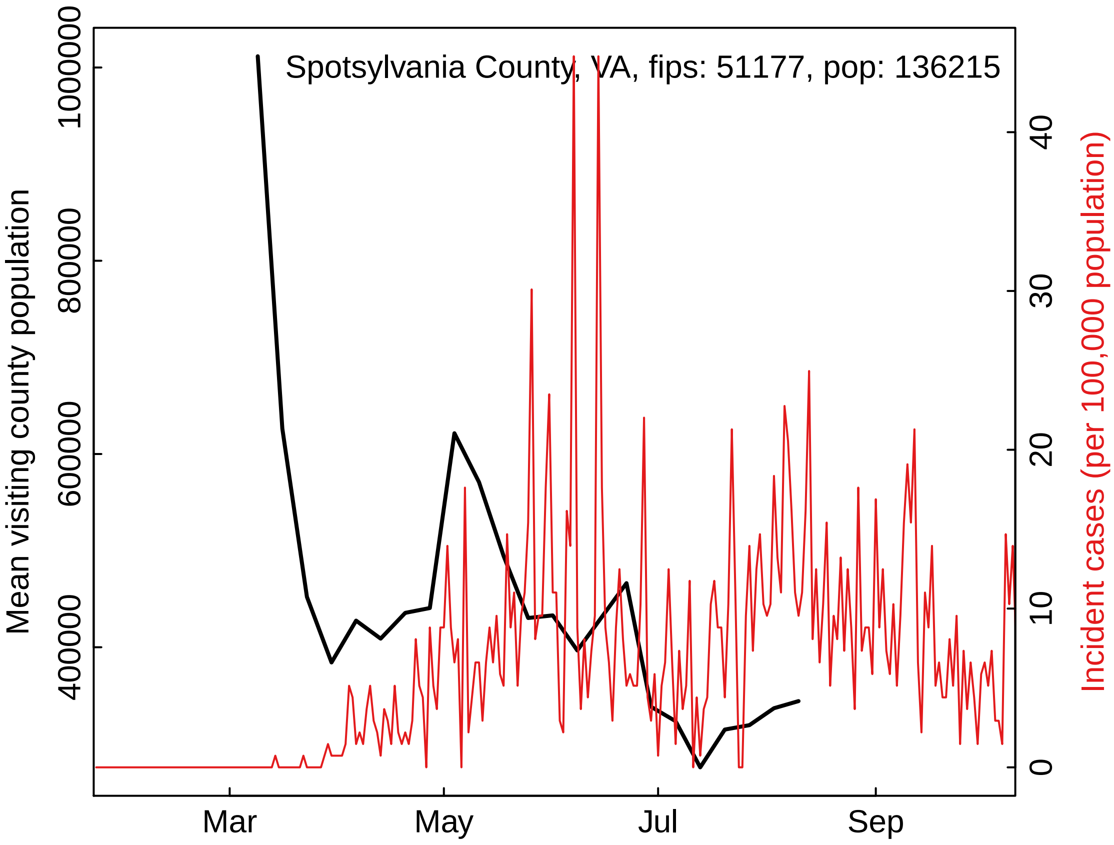

Spotsylvania County, VA, fips: 51177, pop: 136215
Mean visiting county population
1000000
800000
600000
400000
Incident cases (per 100,000 population)
40
30
20
10
0
Mar
May
Jul
Sep

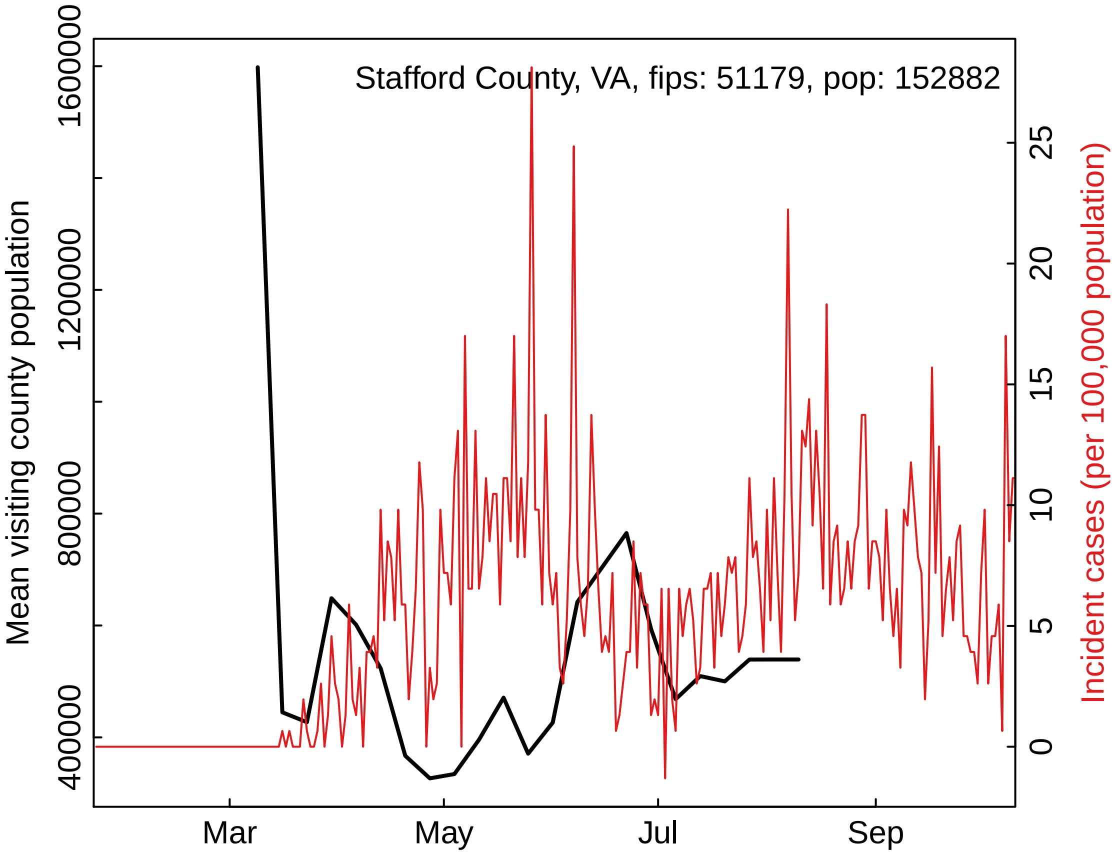

Stafford County, VA, fips: 51179, pop: 152882
Mean visiting county population
400000
800000
1200000
1600000
Incident cases (per 100,000 population)
0
5
10
15
20
25
Mar
May
Jul
Sep

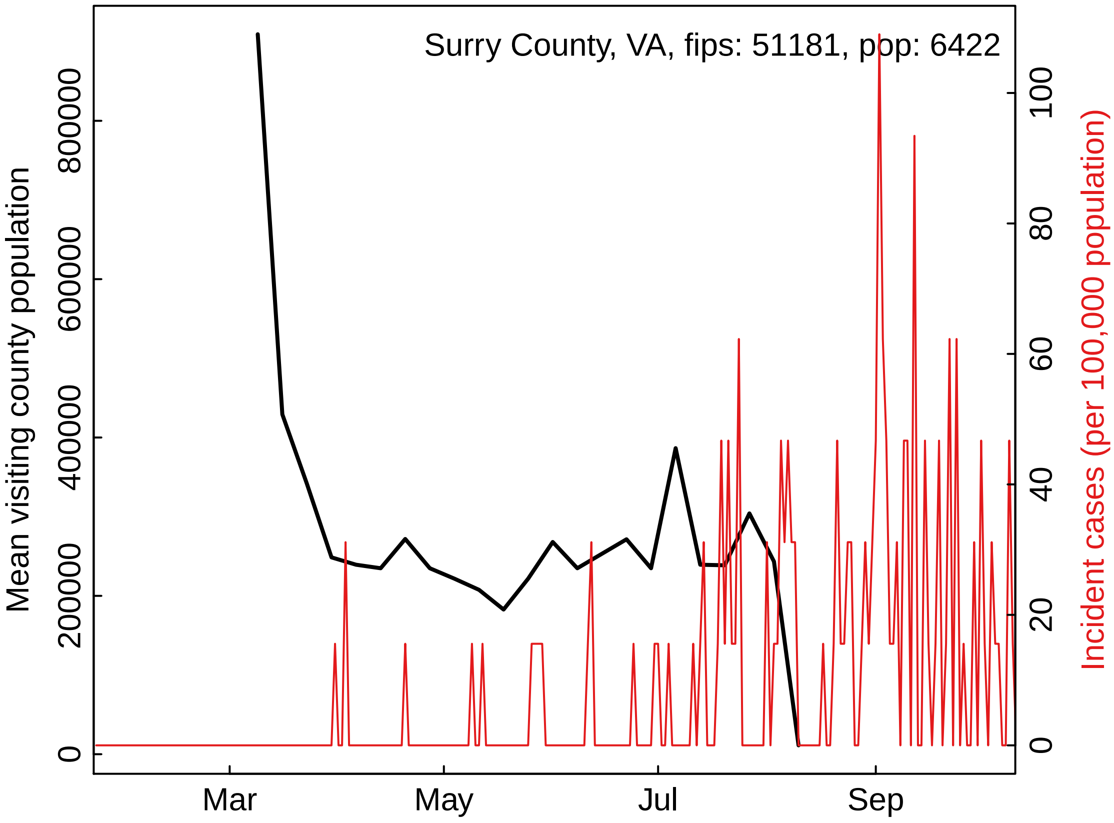

Surry County, VA, fips: 51181, pop: 6422
Mean visiting county population
0
200000
400000
600000
800000
Incident cases (per 100,000 population)
0
20
40
60
80
100
Mar
May
Jul
Sep

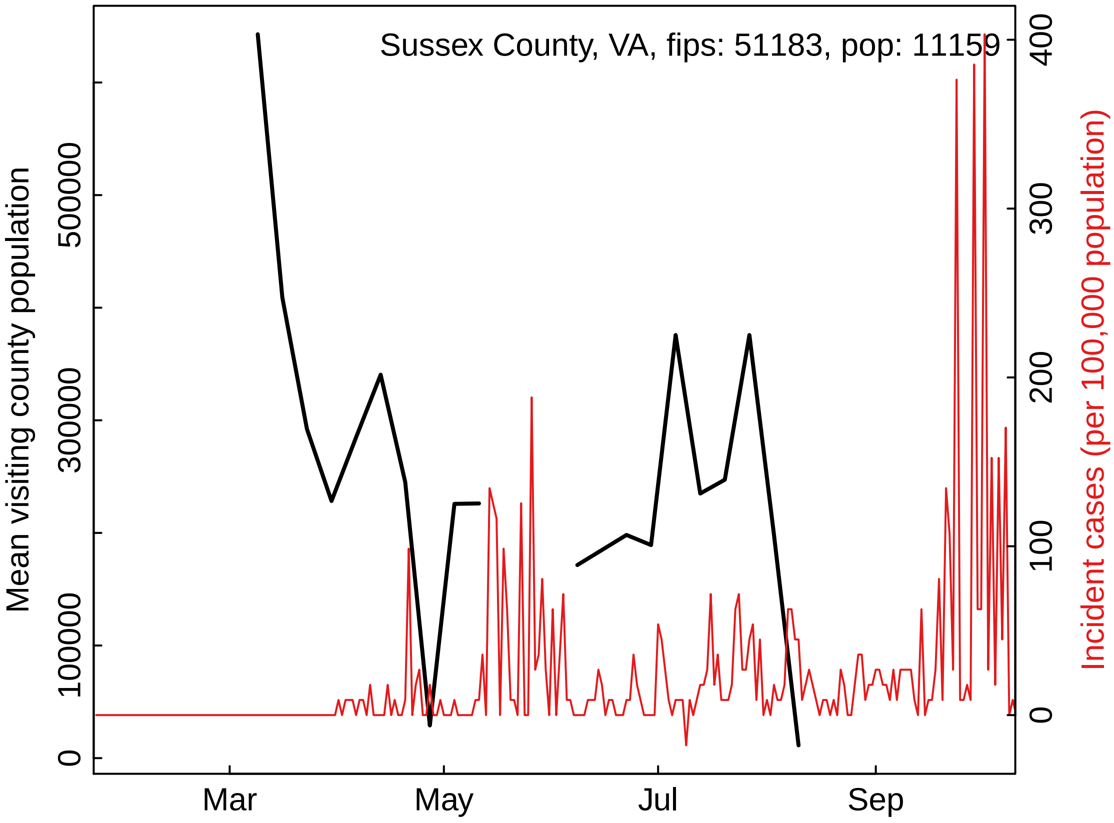
Sussex County, VA, fips: 51183, pop: 11159
Mean visiting county population
0
100000
300000
500000
Incident cases (per 100,000 population)
0
100
200
300
400
Mar
May
Jul
Sep

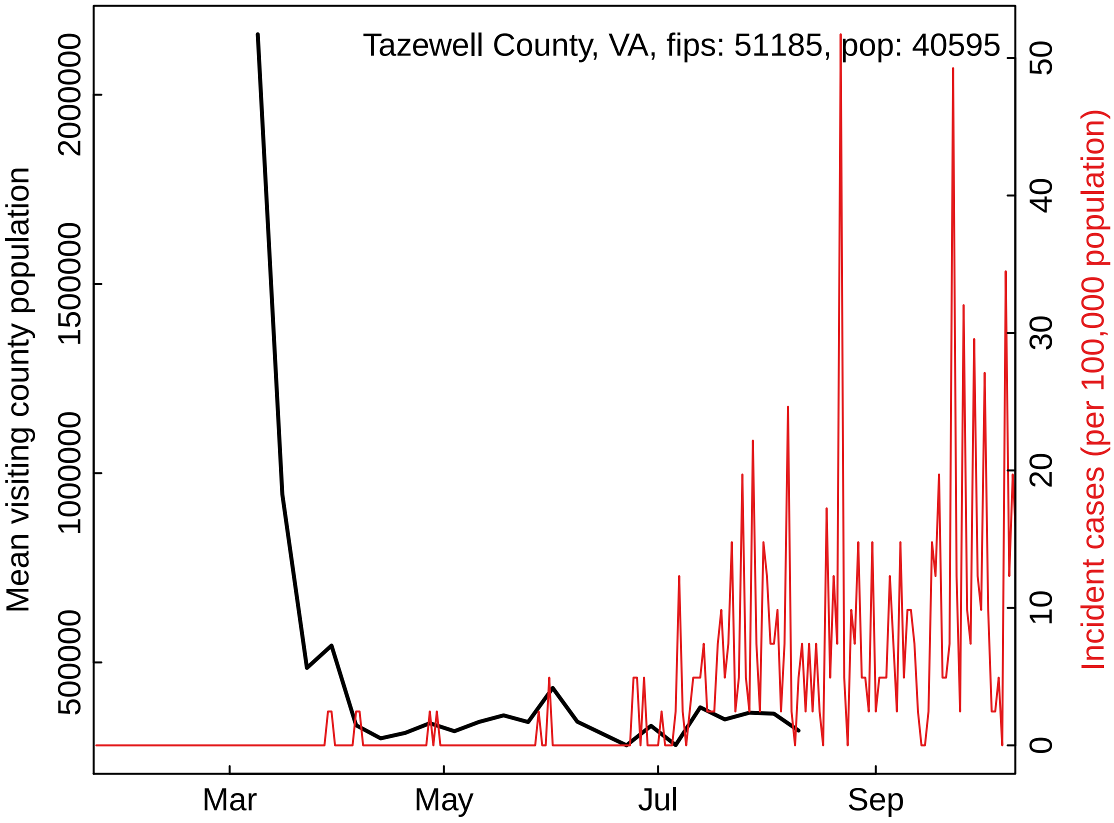
Tazewell County, VA, fips: 51185, pop: 40595
Mean visiting county population
500000
1000000
1500000
2000000
Incident cases (per 100,000 population)
0
10
20
30
40
50
Mar
May
Jul
Sep

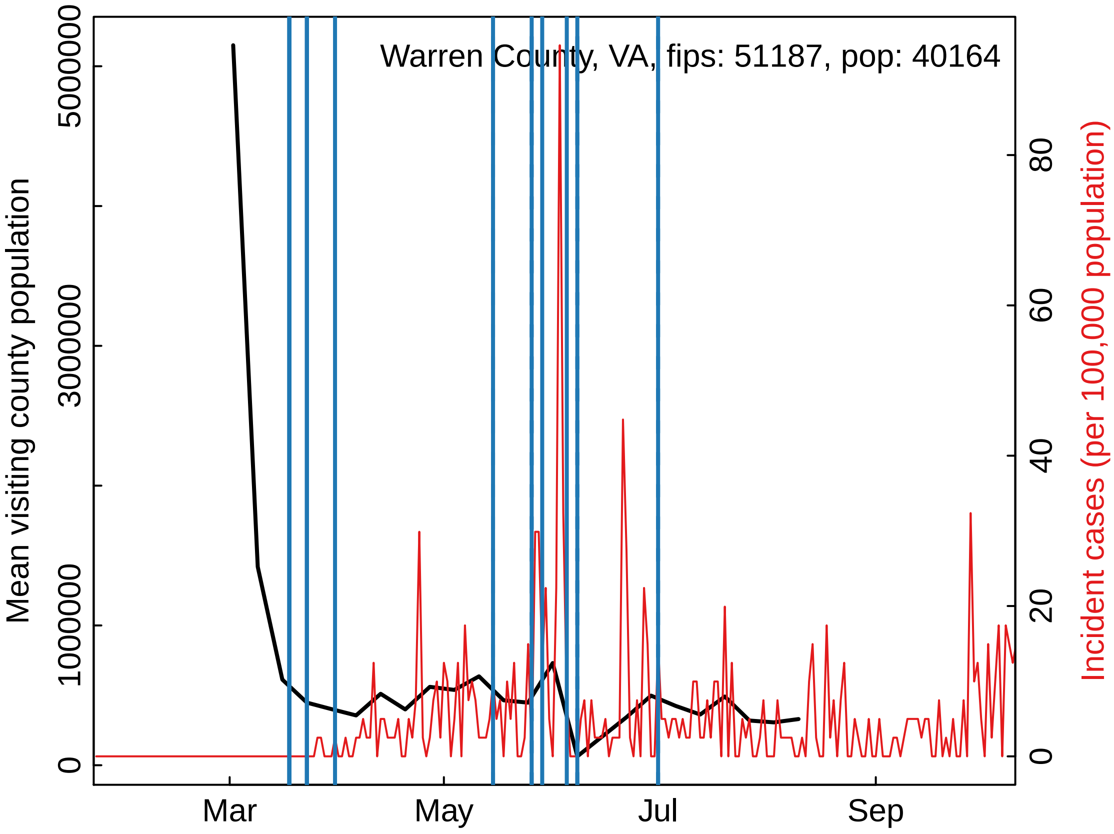
Warren County, VA, fips: 51187, pop: 40164
Mean visiting county population
0
1000000
3000000
5000000
Mar
May
Jul
Sep
Incident cases (per 100,000 population)
0
20
40
60
80

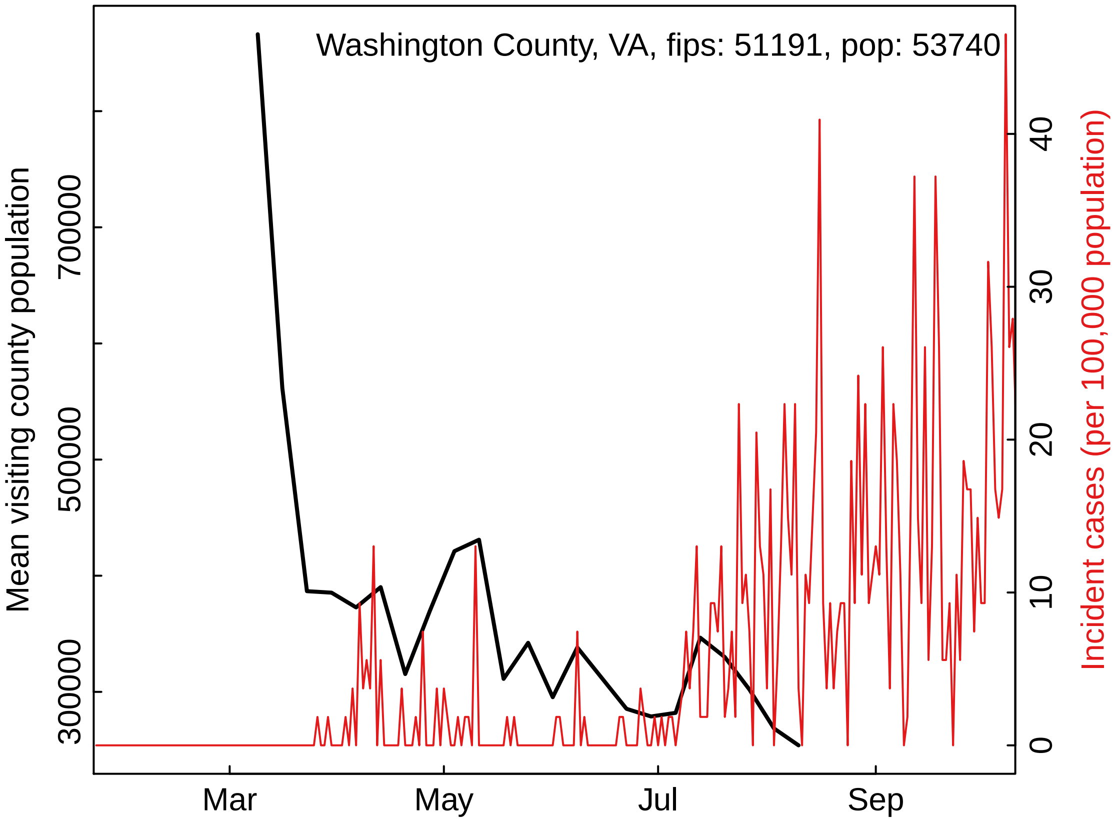

Washington County, VA, fips: 51191, pop: 53740
Mean visiting county population
300000
500000
7000000
Mar
May
Jul
Sep
0
10
20
30
40
Incident cases (per 100,000 population)

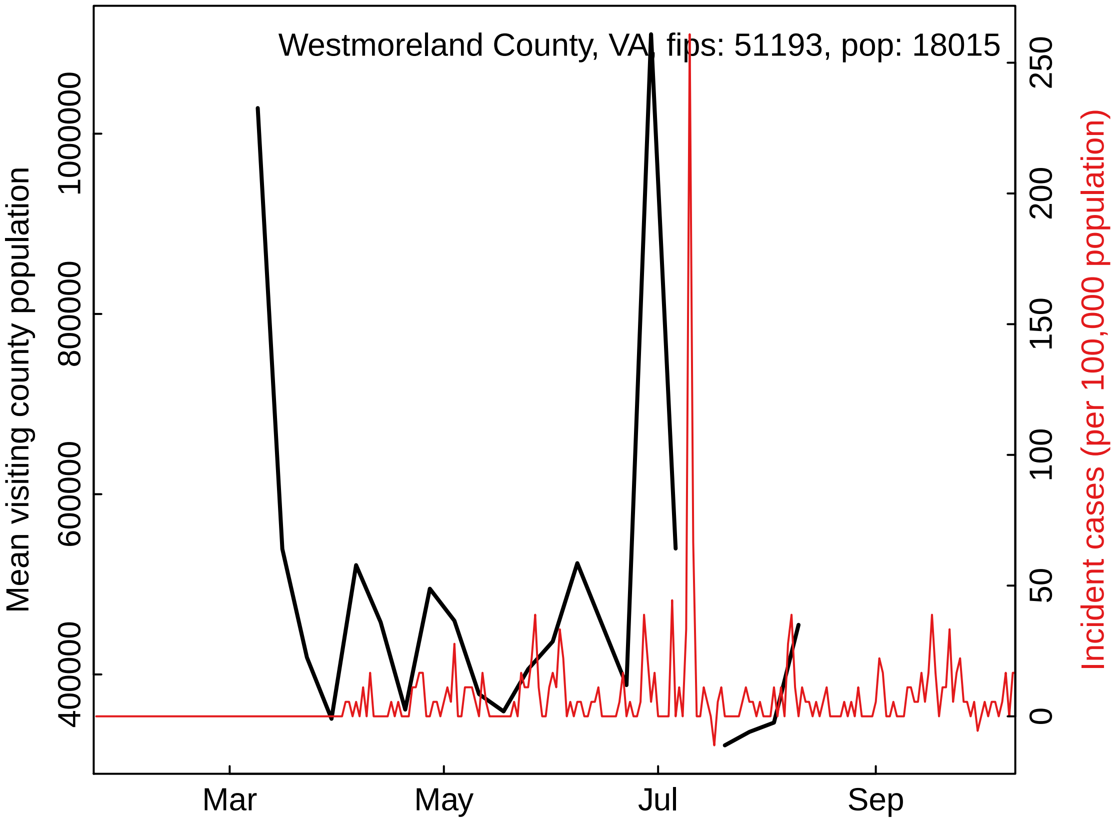

Westmoreland County, VA, fips: 51193, pop: 18015
Mean visiting county population
400000
600000
800000
1000000
Incident cases (per 100,000 population)
0
50
100
150
200
250
Mar
May
Jul
Sep

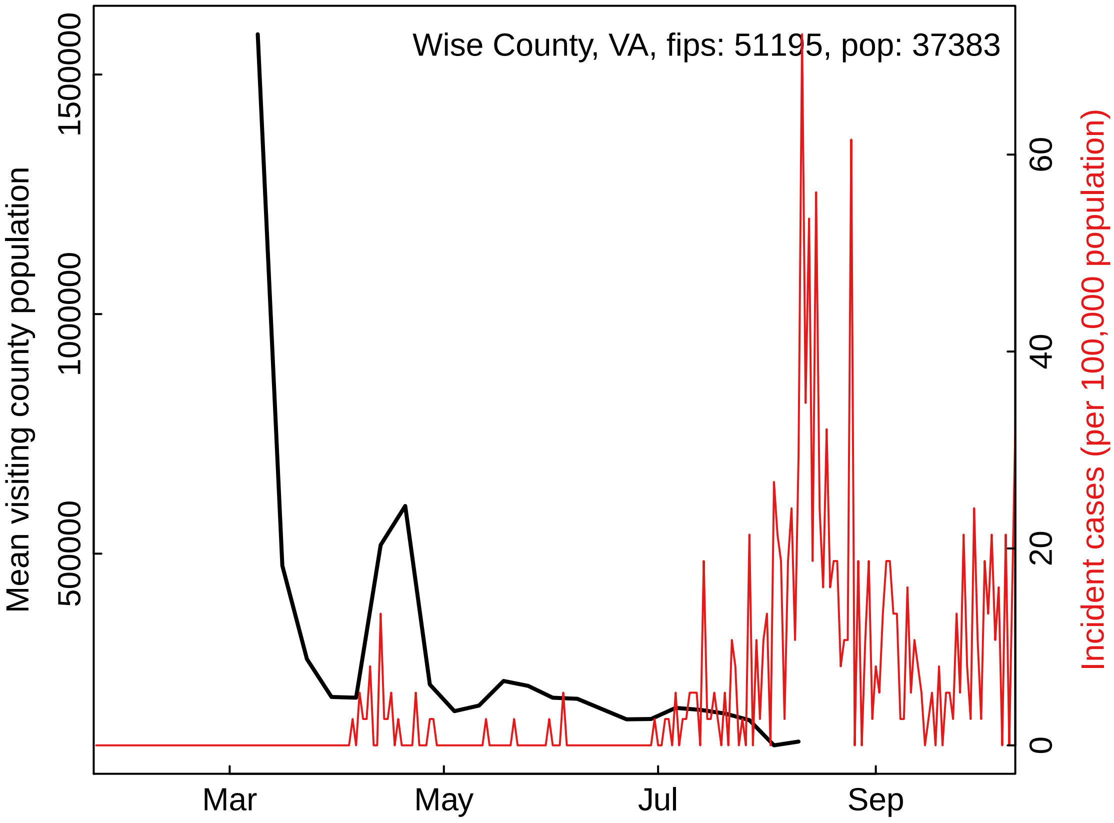

Wise County, VA, fips: 51195, pop: 37383
Mean visiting county population
500000
1000000
1500000
Incident cases (per 100,000 population)
0
20
40
60
Mar
May
Jul
Sep

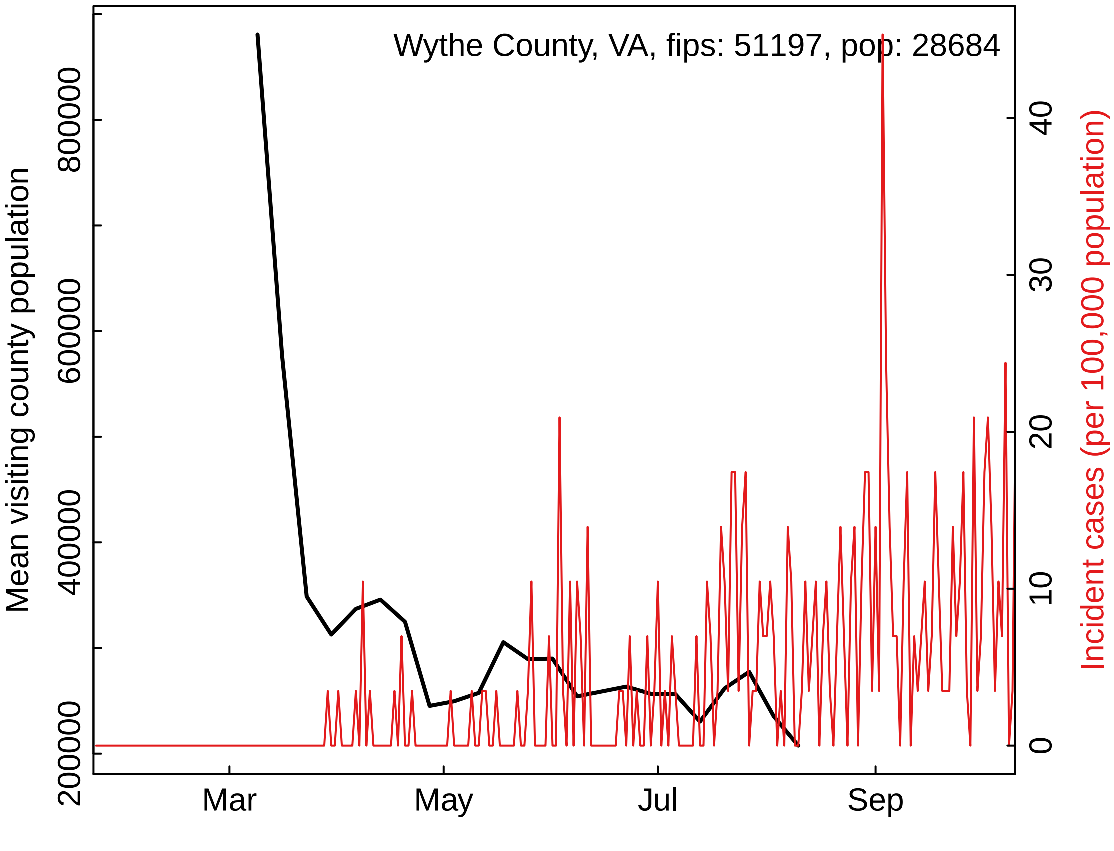

Wythe County, VA, fips: 51197, pop: 28684
Mean visiting county population
200000
400000
600000
800000
Mar
May
Jul
Sep
0
10
20
30
40
Incident cases (per 100,000 population)

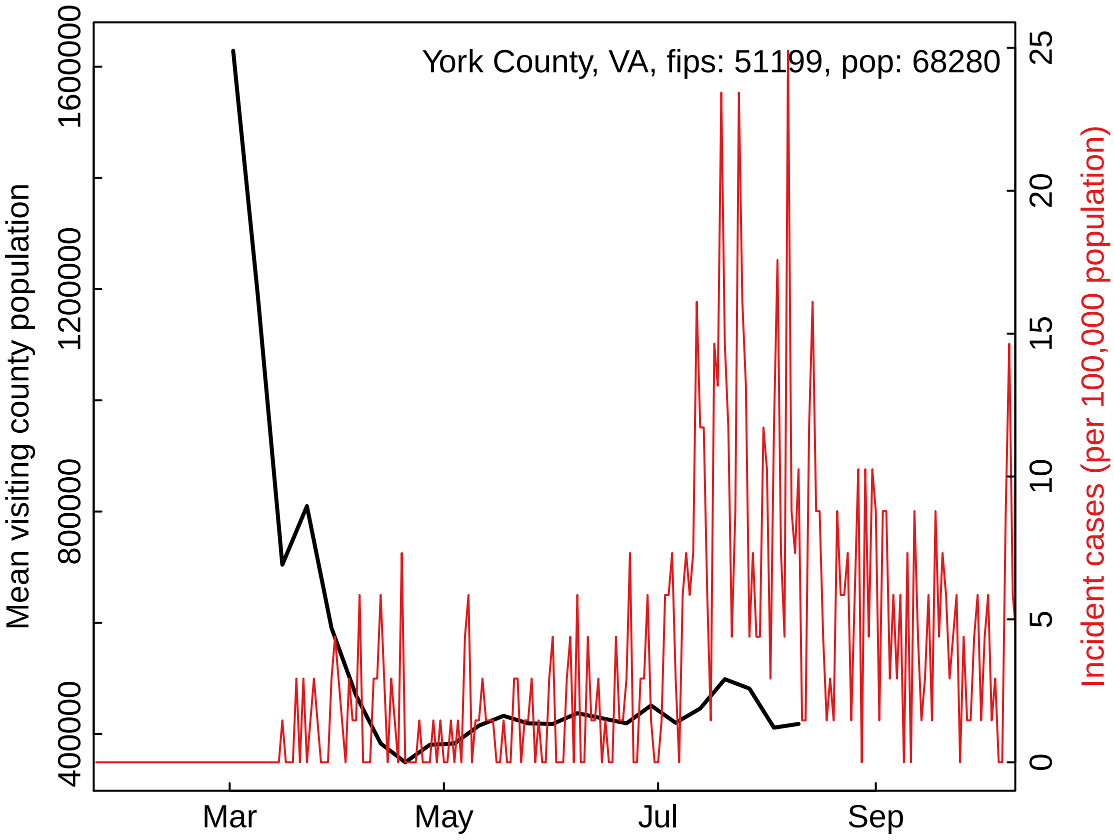
York County, VA, fips: 51199, pop: 68280
Mean visiting county population
400000
800000
1200000
1600000
Incident cases (per 100,000 population)
0
5
10
15
20
25
Mar
May
Jul
Sep

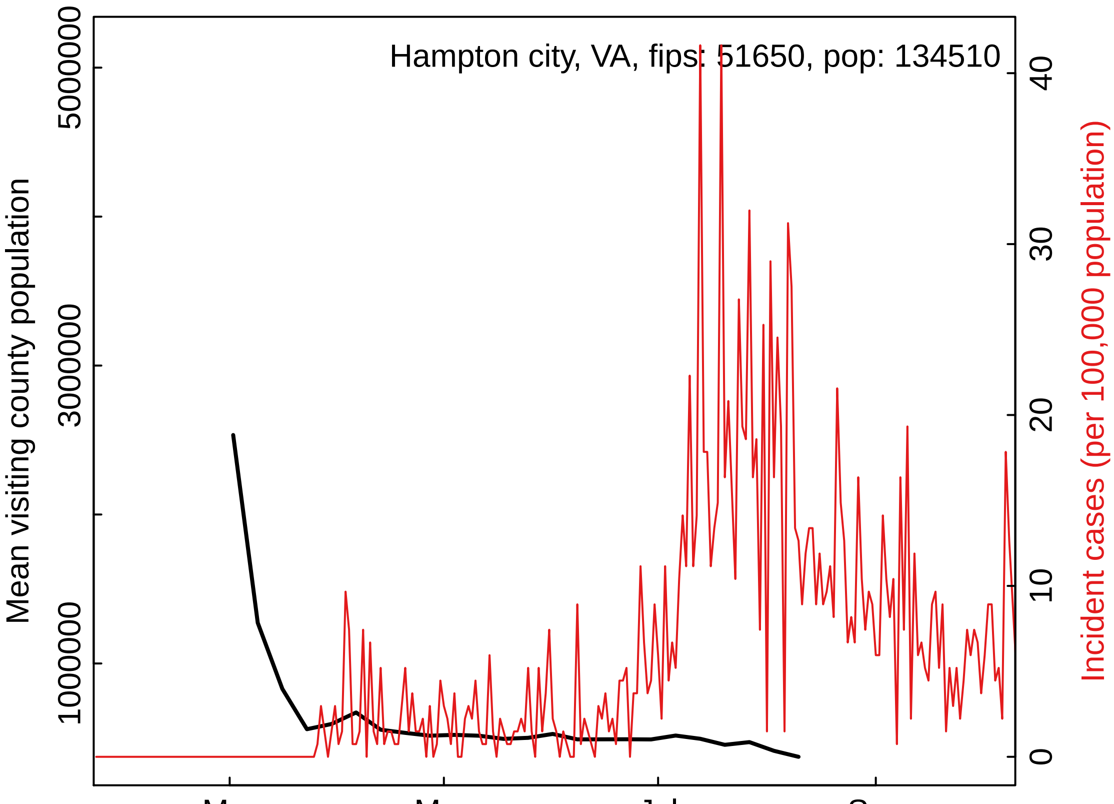

Hampton city, VA, fips: 51650, pop: 134510
Mean visiting county population
1000000
3000000
5000000
Incident cases (per 100,000 population)
0
10
20
30
40
Mar
May
Jul
Sep

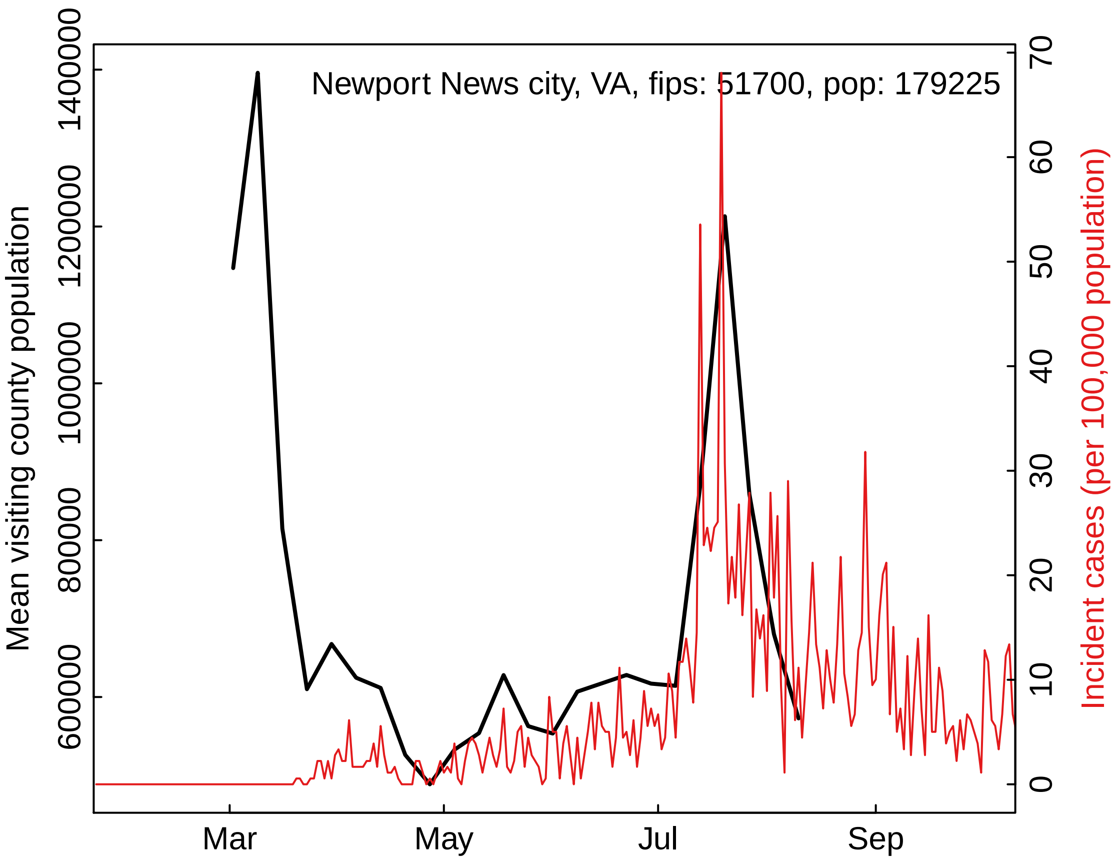

Newport News city, VA, fips: 51700, pop: 179225
Mean visiting county population
600000
800000
1000000
1200000
1400000
Incident cases (per 100,000 population)
0
10
20
30
40
50
60
70
Mar
May
Jul
Sep

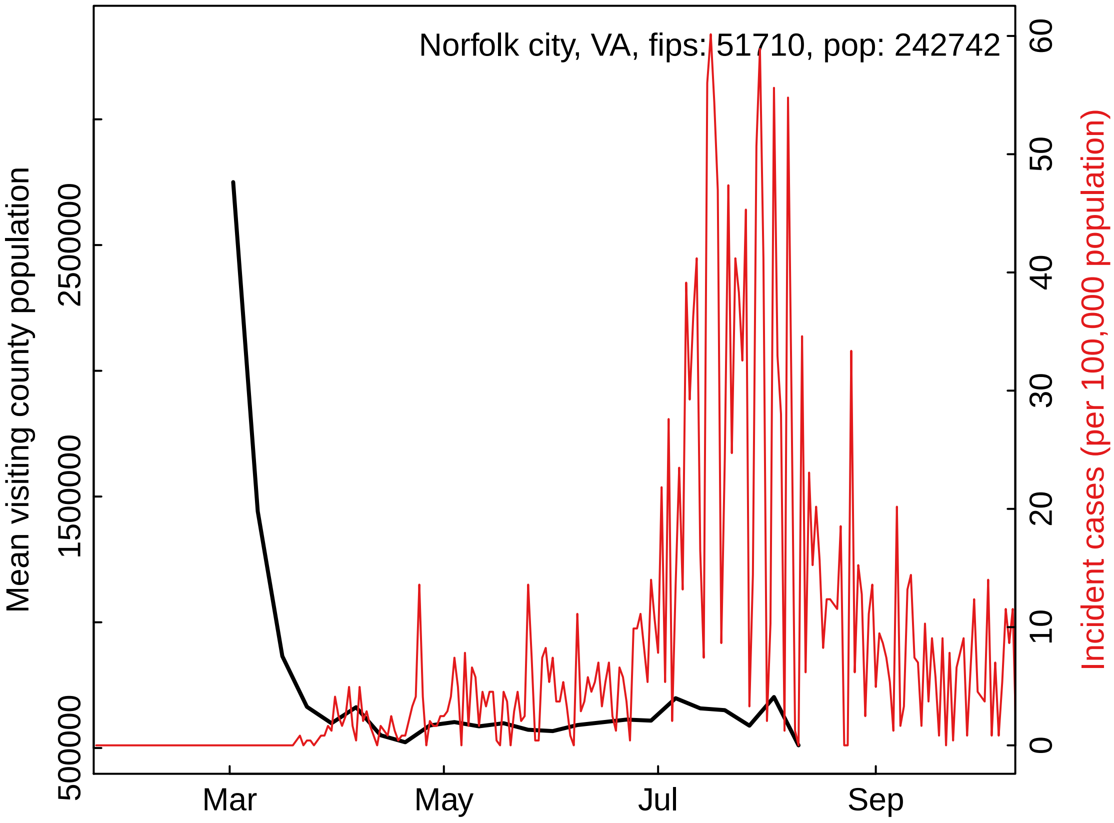
Norfolk city, VA, fips: 51710, pop: 242742
Mean visiting county population
500000
1500000
2500000
Mar
May
Jul
Sep
Incident cases (per 100,000 population)
0
10
20
30
40
50
60

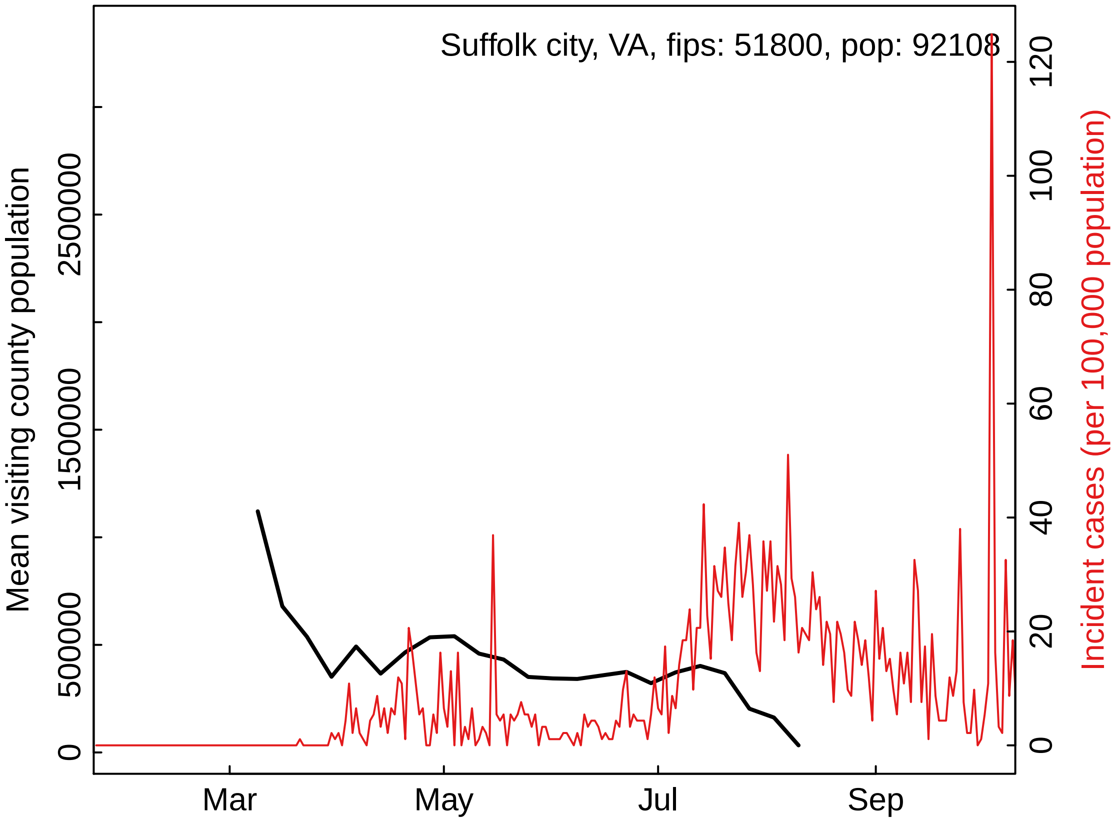

Suffolk city, VA, fips: 51800, pop: 92108
Mean visiting county population
0
500000
1500000
2500000
Mar
May
Jul
Sep
Incident cases (per 100,000 population)
0
20
40
60
80
100
120

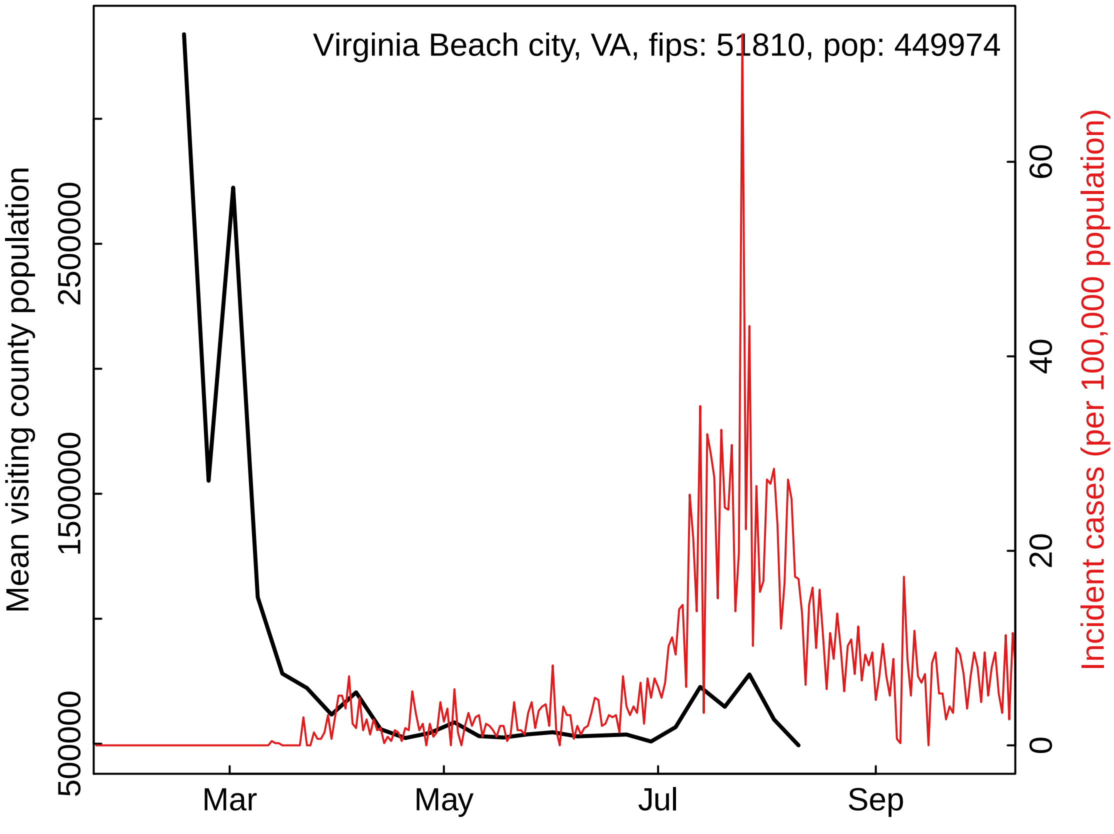

Virginia Beach city, VA, fips: 51810, pop: 449974
Mean visiting county population
500000
1500000
2500000
Incident cases (per 100,000 population)
0
20
40
60
Mar
May
Jul
Sep

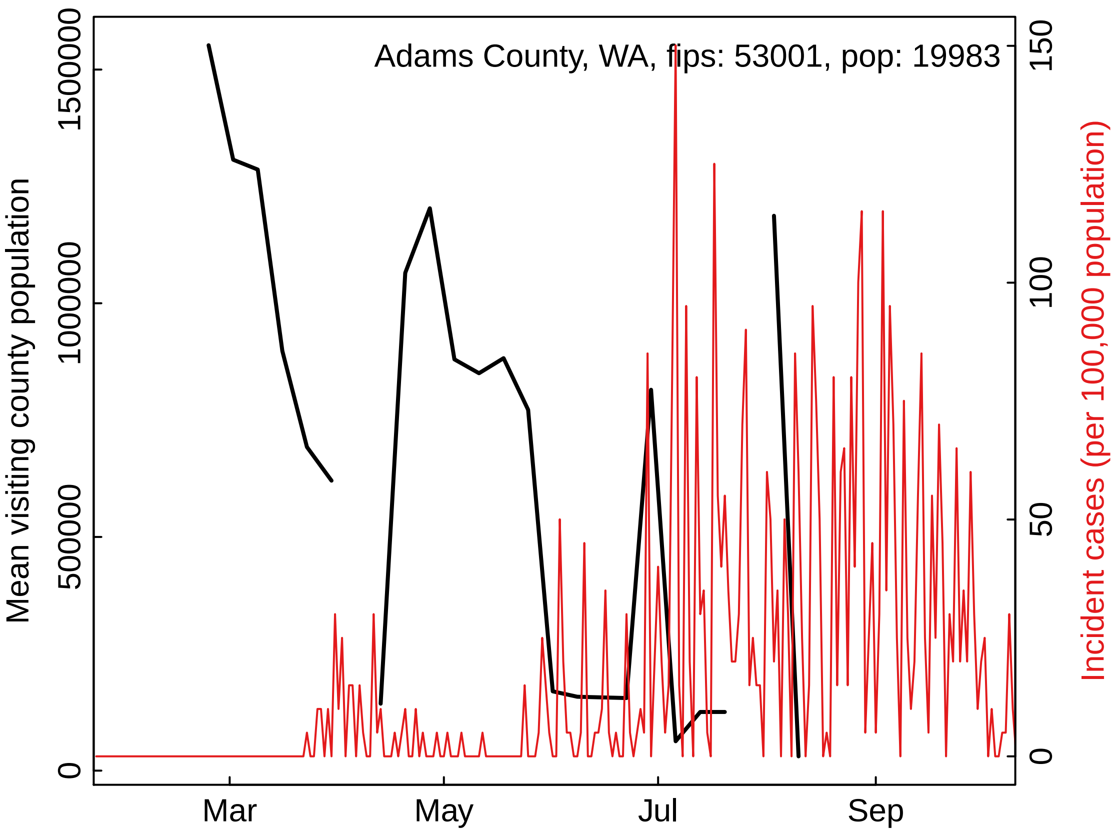

Adams County, WA, fips: 53001, pop: 19983
Mean visiting county population
Incident cases (per 100,000 population)
0
500000
1000000
1500000
0
50
100
150
Mar
May
Jul
Sep

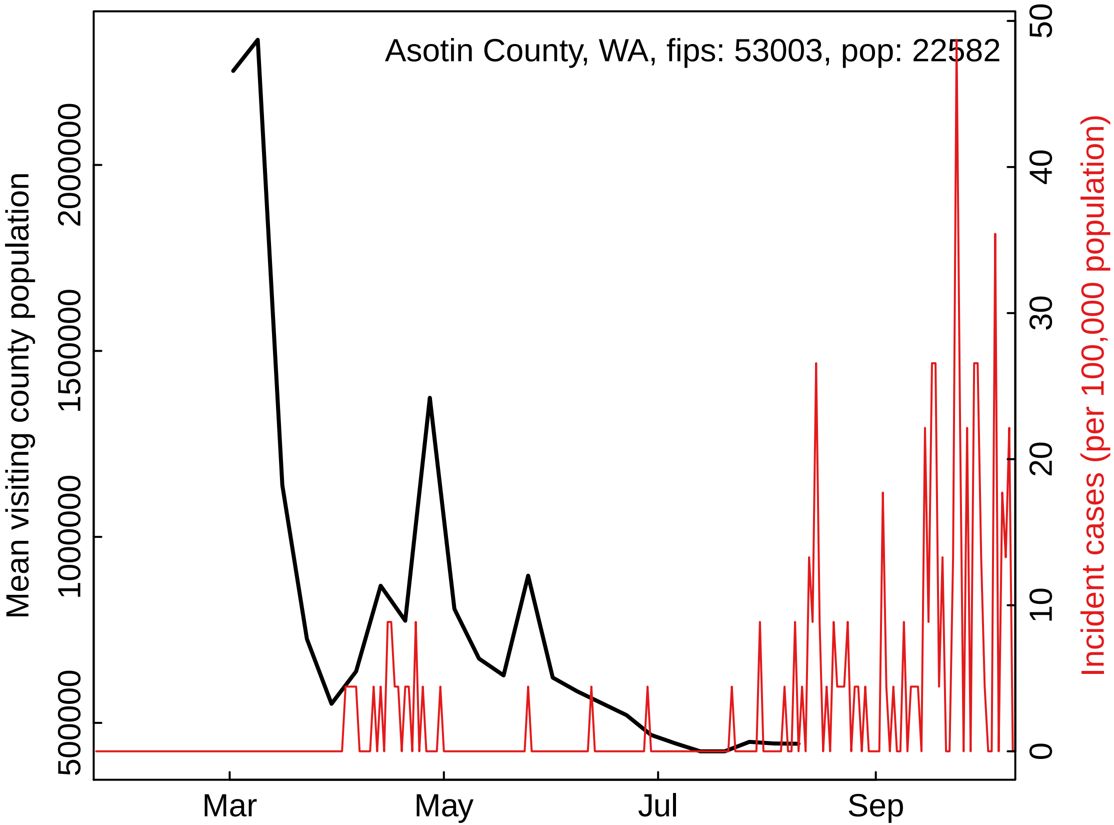

Asotin County, WA, fips: 53003, pop: 22582
Mean visiting county population
500000
1000000
1500000
2000000
Mar
May
Jul
Sep
0
10
20
30
40
50
Incident cases (per 100,000 population)

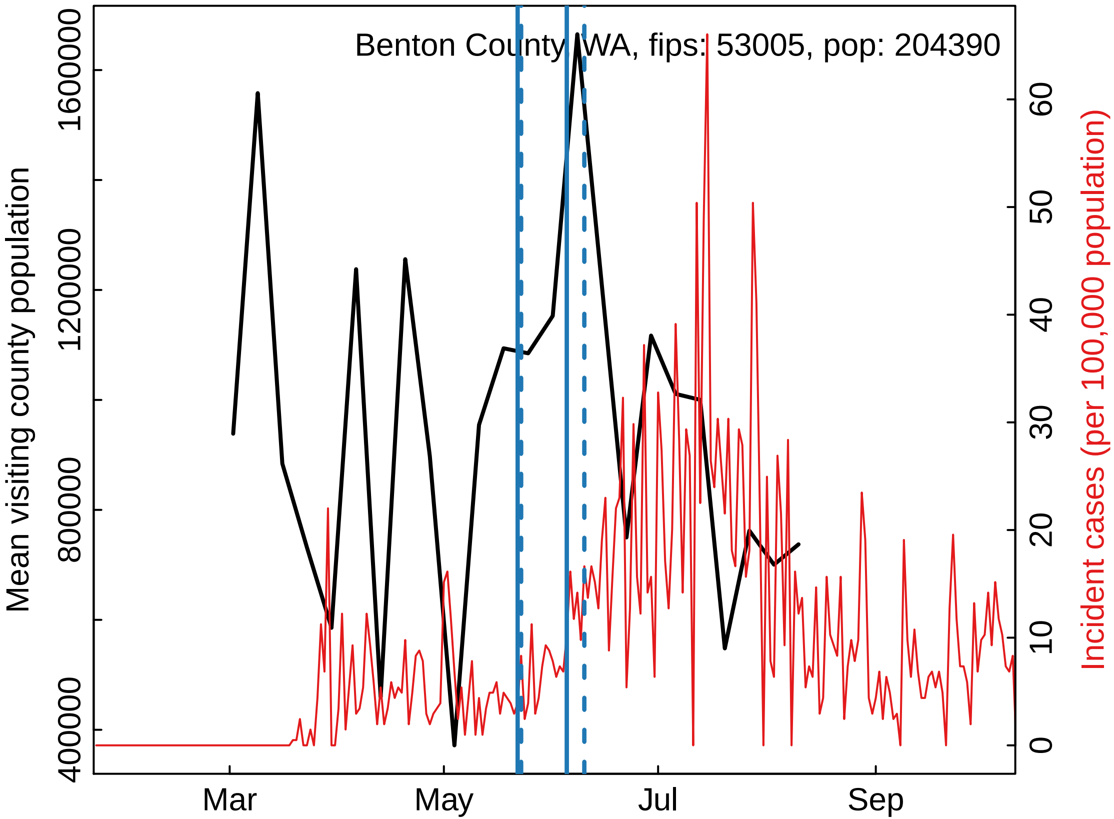

Benton County, WA, fips: 53005, pop: 204390
Mean visiting county population
Incident cases (per 100,000 population)
400000
800000
1200000
1600000
0
10
20
30
40
50
60
Mar
May
Jul
Sep

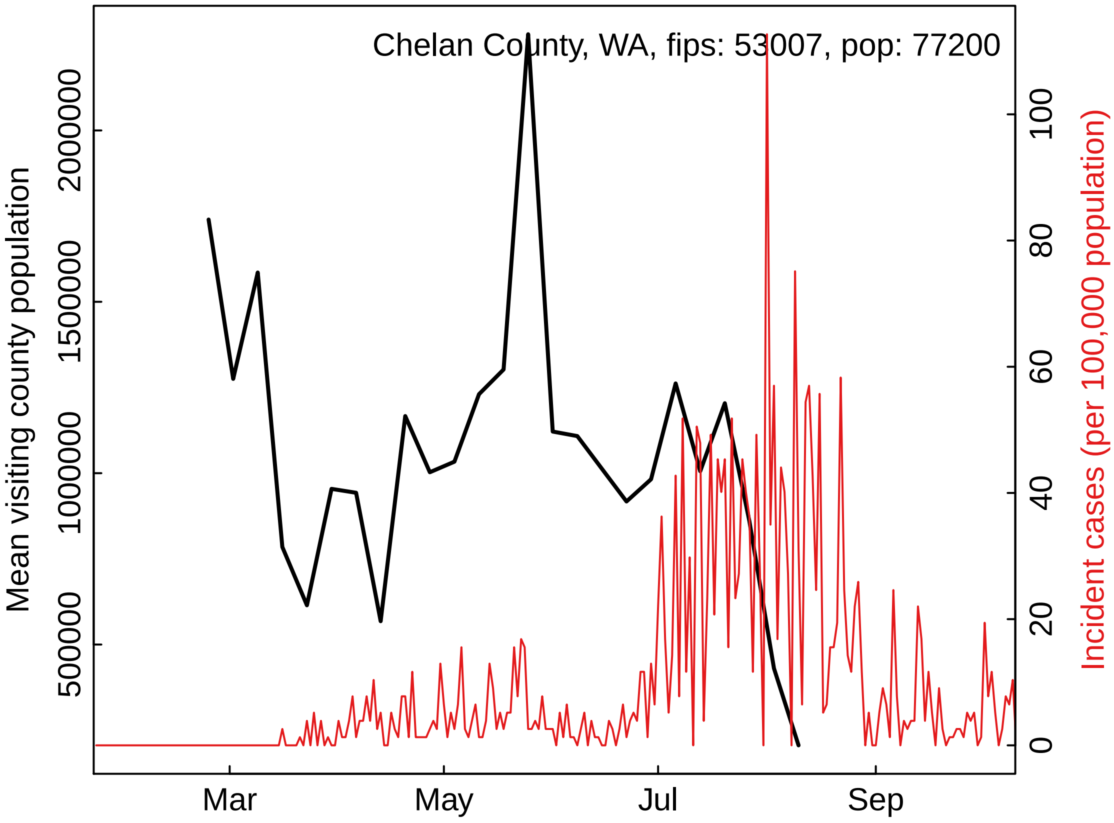

Chelan County, WA, fips: 53007, pop: 77200
Mean visiting county population
500000
1000000
1500000
2000000
Incident cases (per 100,000 population)
0
20
40
60
80
100
Mar
May
Jul
Sep

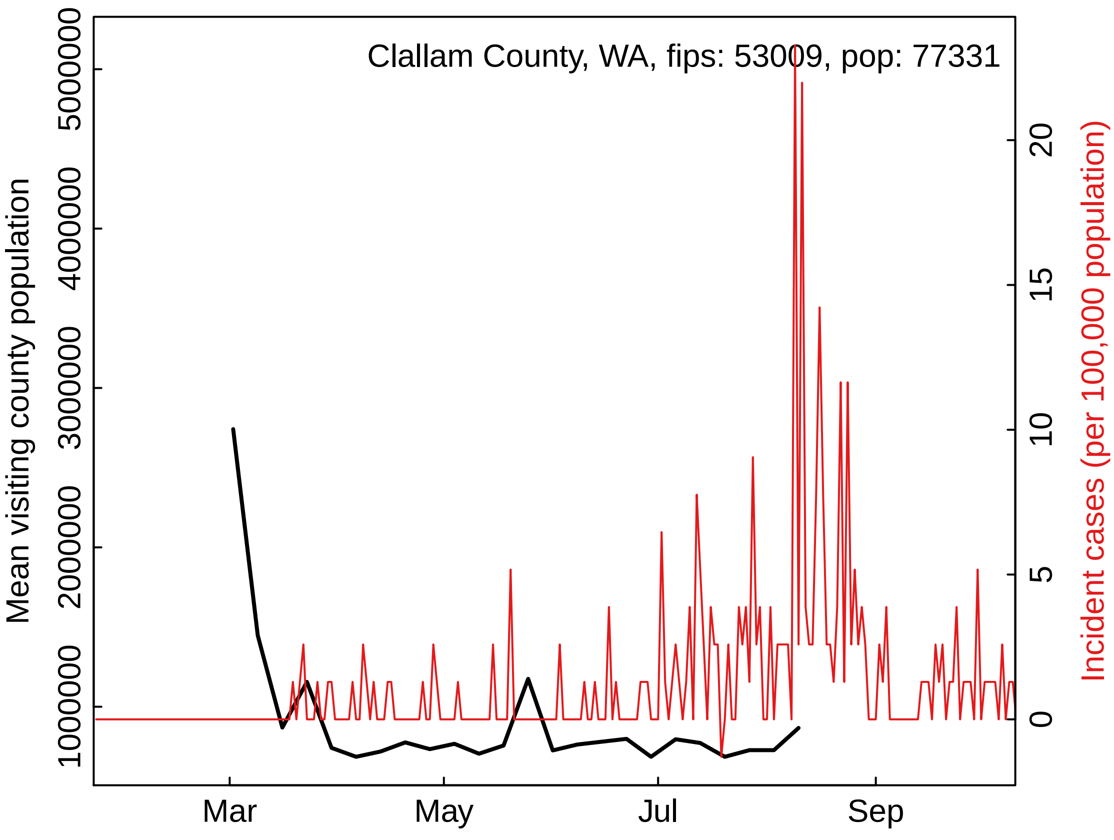

Clallam County, WA, fips: 53009, pop: 77331
Mean visiting county population
10000000
20000000
30000000
40000000
50000000
Incident cases (per 100,000 population)
0
5
10
15
20
Mar
May
Jul
Sep

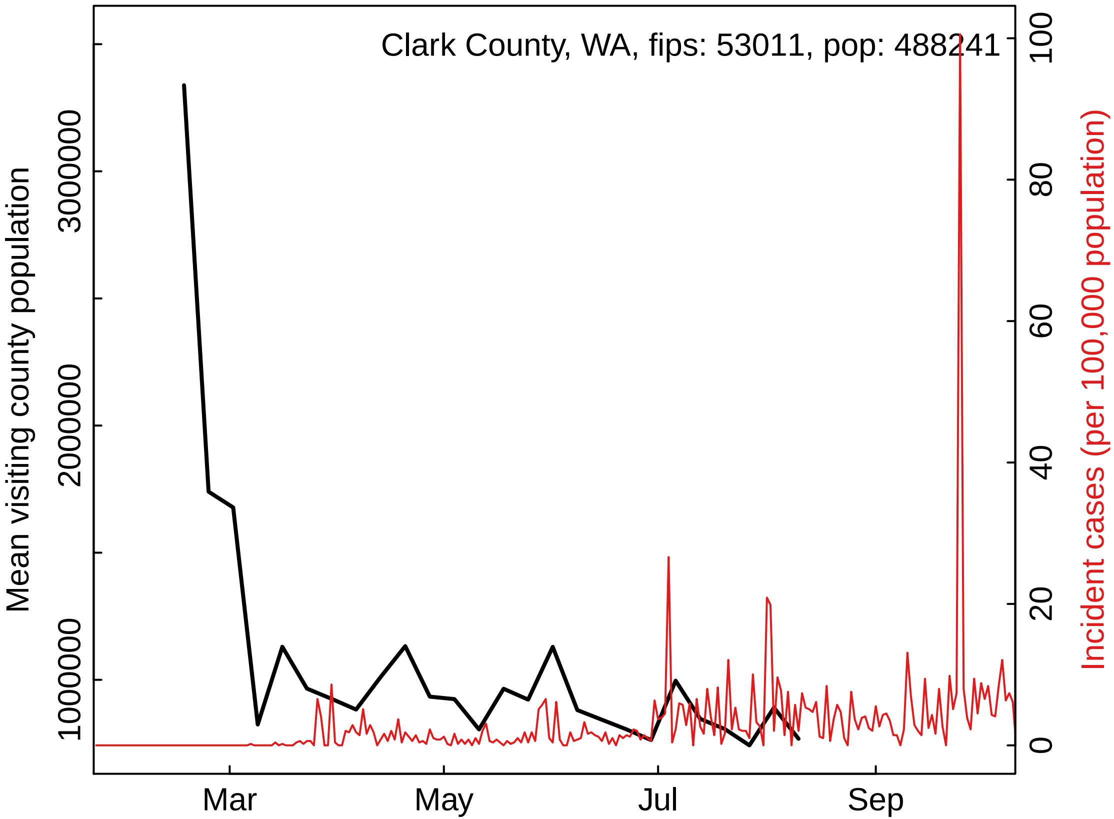

Clark County, WA, fips: 53011, pop: 488241
Mean visiting county population
1000000
2000000
3000000
Incident cases (per 100,000 population)
0
20
40
60
80
100
Mar
May
Jul
Sep

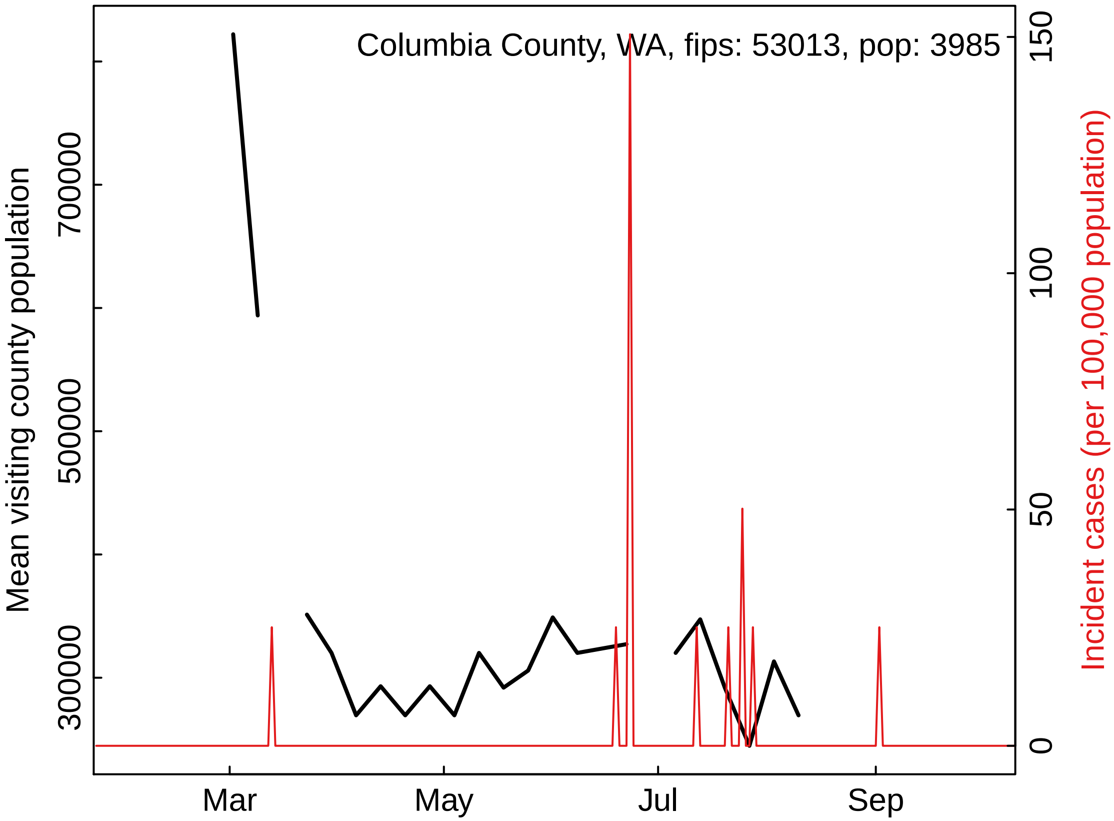

Columbia County, WA, fips: 53013, pop: 3985
Mean visiting county population
300000
500000
700000
Incident cases (per 100,000 population)
0
50
100
150
Mar
May
Jul
Sep

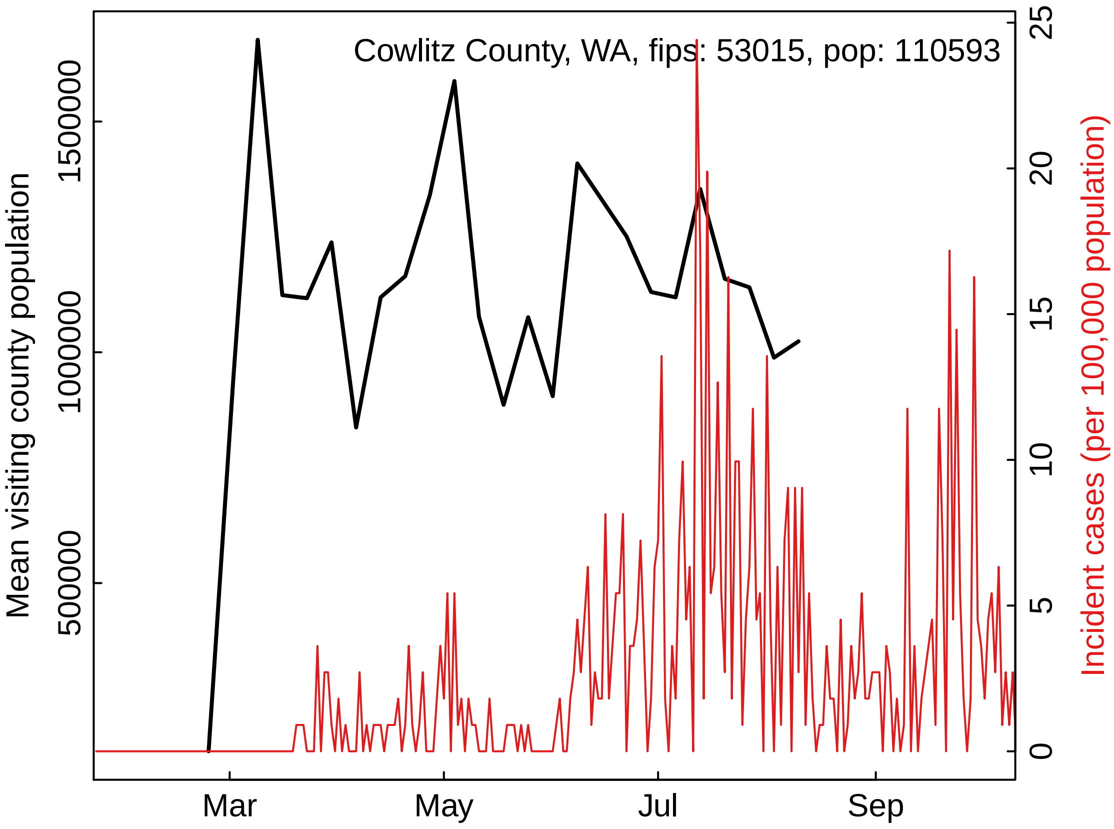

Cowlitz County, WA, fips: 53015, pop: 110593
Mean visiting county population
500000
1000000
1500000
Incident cases (per 100,000 population)
0
5
10
15
20
25
Mar
May
Jul
Sep

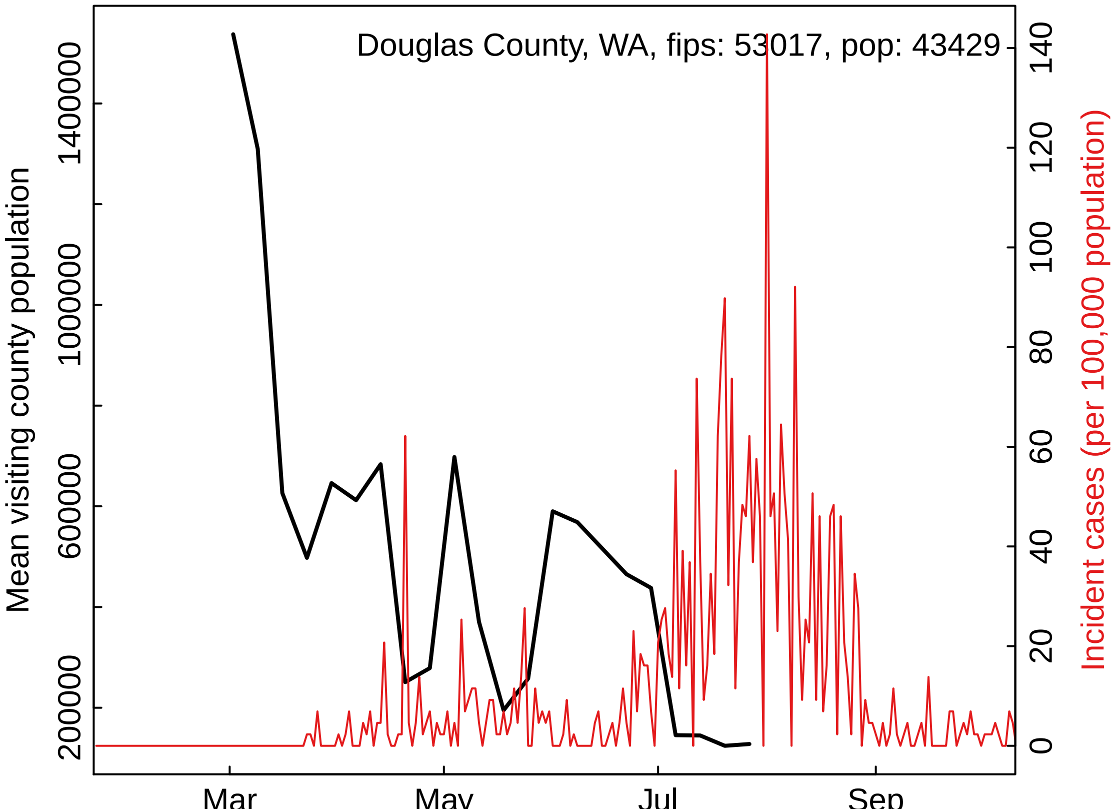

Douglas County, WA, fips: 53017, pop: 43429
Mean visiting county population
200000
600000
1000000
1400000
Incident cases (per 100,000 population)
0
20
40
60
80
100
120
140
Mar
May
Jul
Sep

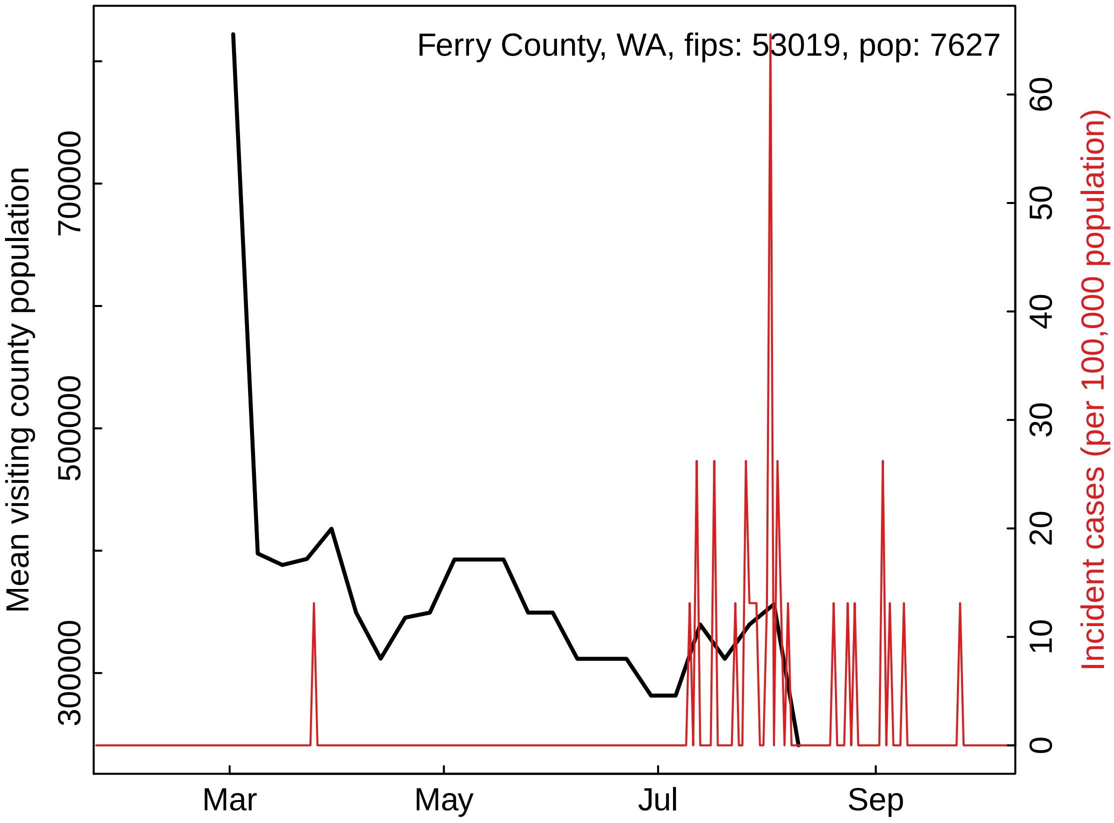

Ferry County, WA, fips: 53019, pop: 7627
Mean visiting county population
300000
500000
700000
Incident cases (per 100,000 population)
0
10
20
30
40
50
60
Mar
May
Jul
Sep

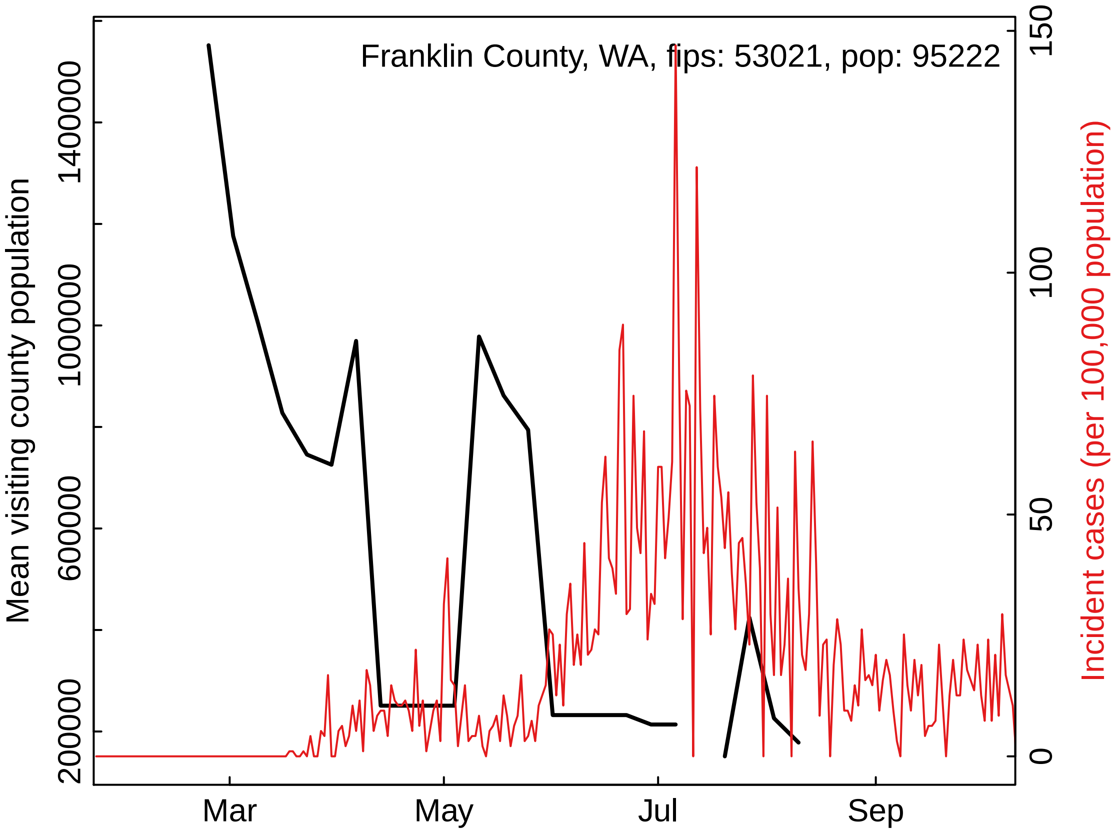

Franklin County, WA, fips: 53021, pop: 95222
Mean visiting county population
200000
600000
1000000
1400000
Mar
May
Jul
Sep
0
50
100
150
Incident cases (per 100,000 population)

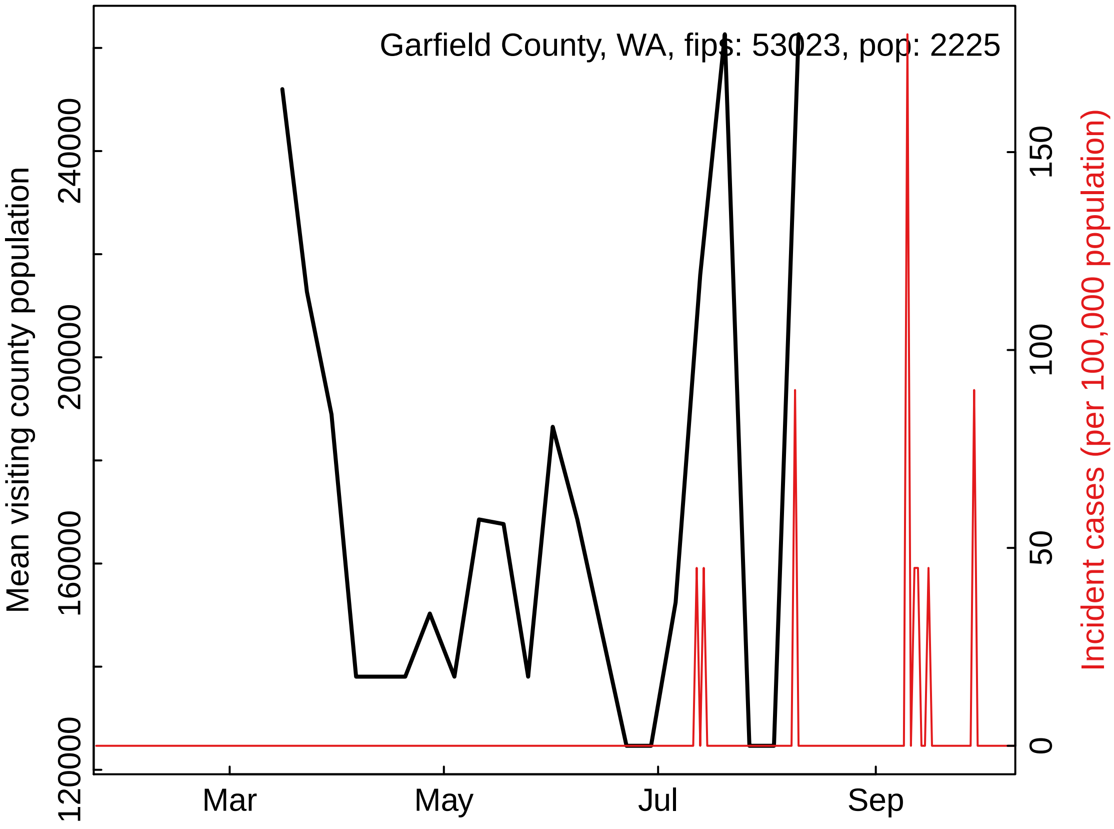
Garfield County, WA, fips: 53023, pop: 2225
Mean visiting county population
120000
160000
200000
240000
Incident cases (per 100,000 population)
0
50
100
150
Mar
May
Jul
Sep

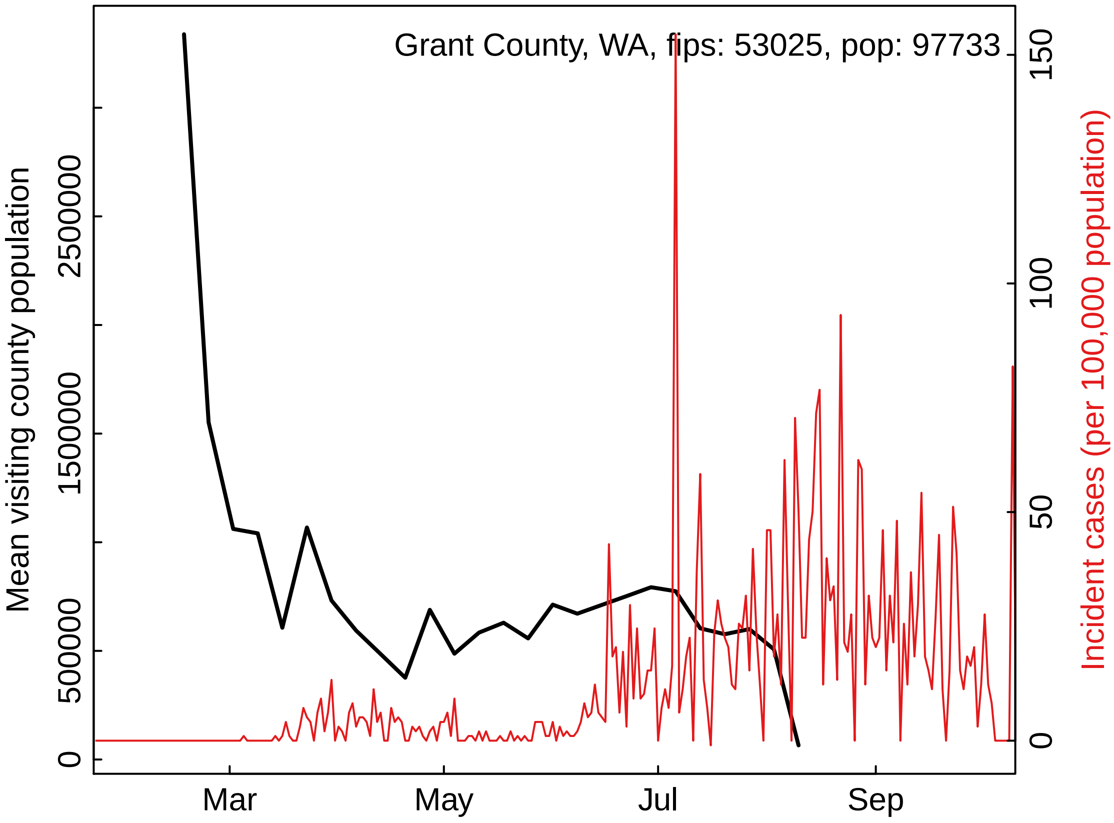

Grant County, WA, fips: 53025, pop: 97733
Mean visiting county population
0
500000
1500000
2500000
Incident cases (per 100,000 population)
0
50
100
150
Mar
May
Jul
Sep

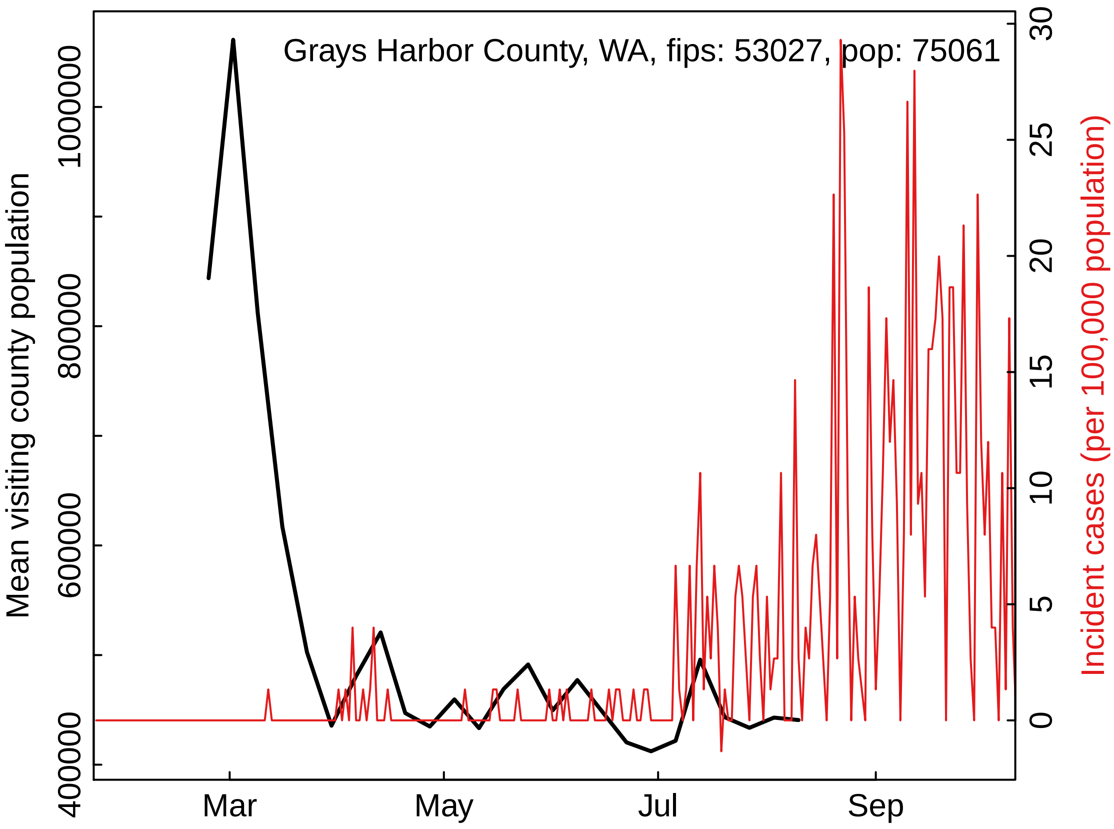

Grays Harbor County, WA, fips: 53027, pop: 75061
Mean visiting county population
400000
600000
800000
1000000
Incident cases (per 100,000 population)
0
5
10
15
20
25
30
Mar
May
Jul
Sep

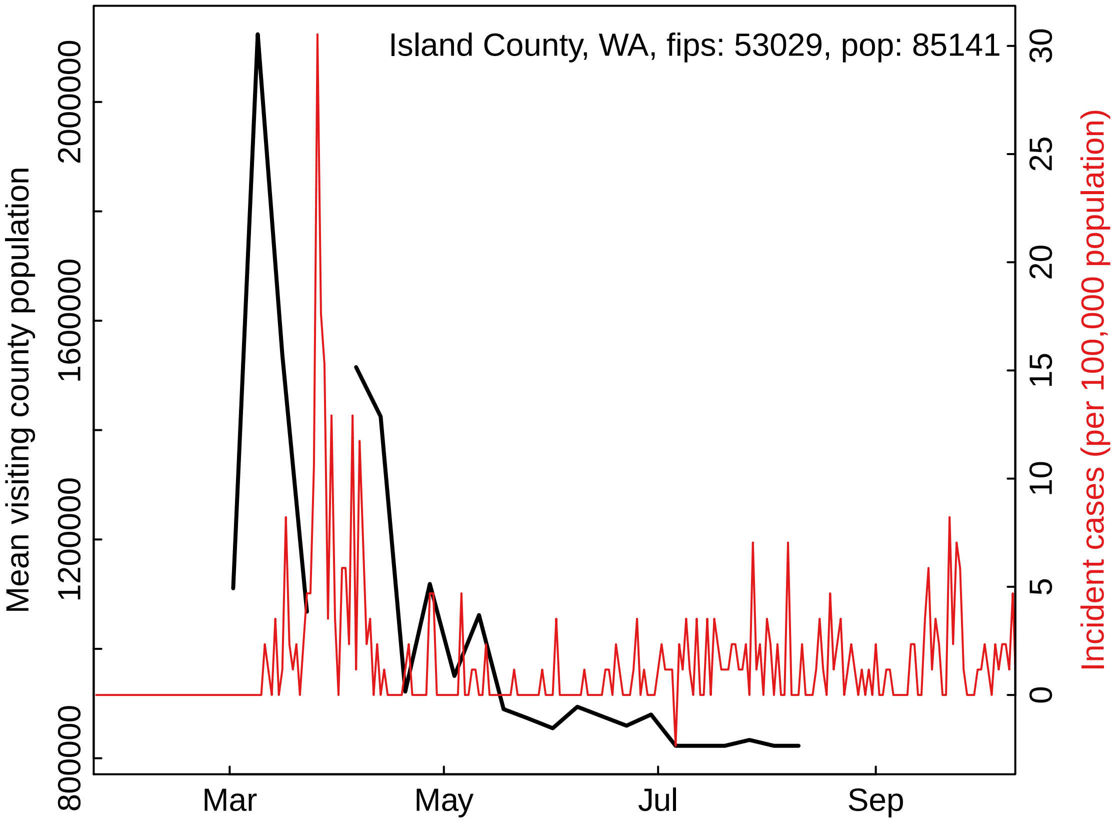

Island County, WA, fips: 53029, pop: 85141
Mean visiting county population
Incident cases (per 100,000 population)
800000
1200000
1600000
2000000
0
5
10
15
20
25
30
Mar
May
Jul
Sep

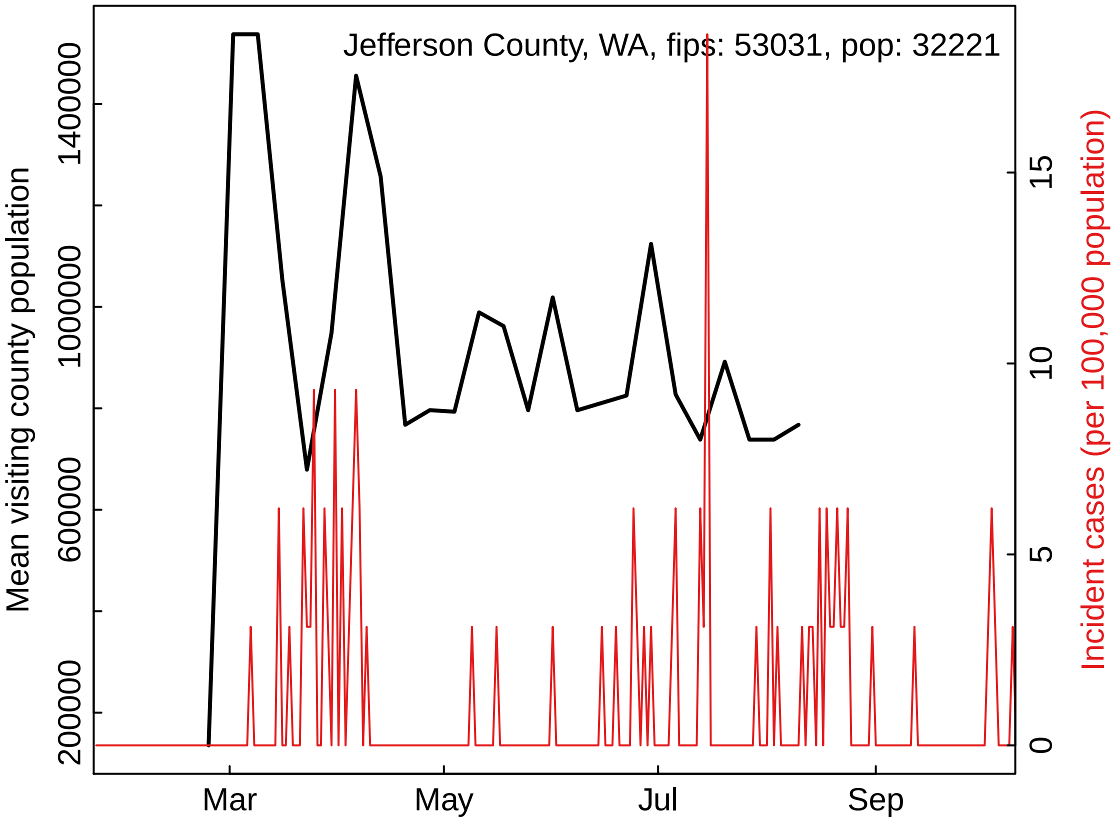
Jefferson County, WA, fips: 53031, pop: 32221
Mean visiting county population
200000
600000
1000000
1400000
Incident cases (per 100,000 population)
0
5
10
15
Mar
May
Jul
Sep

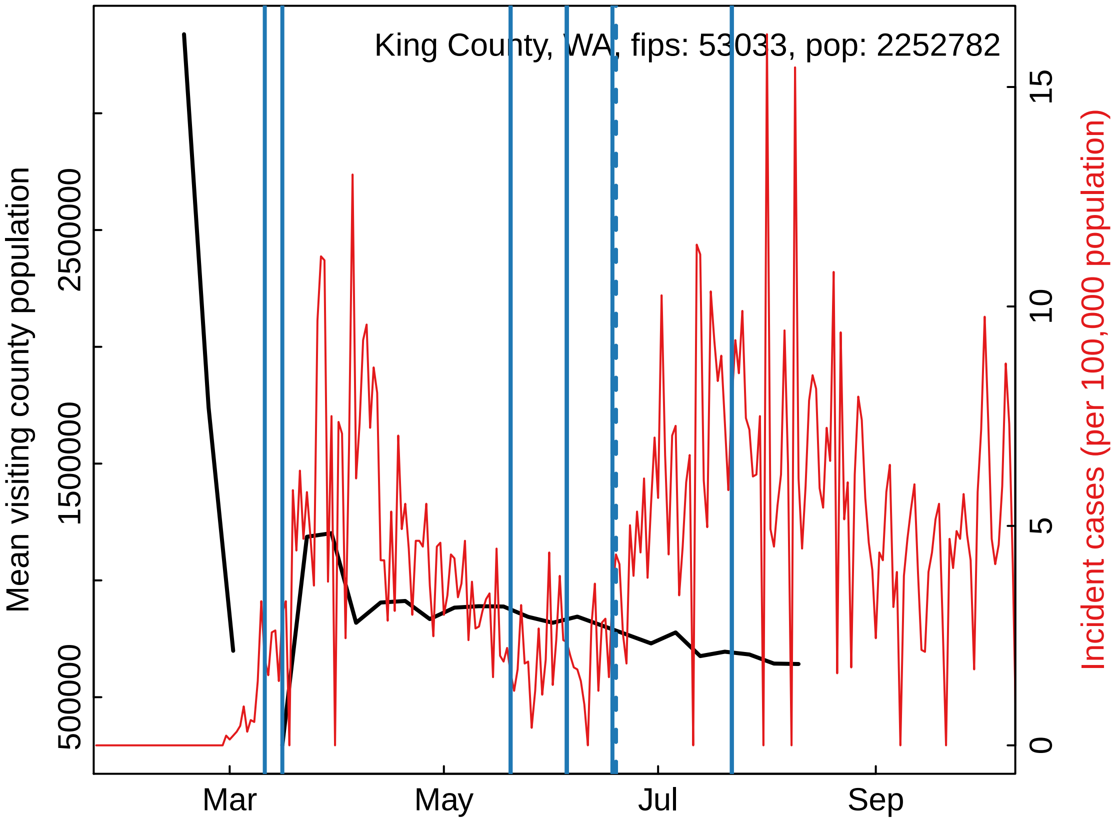
King County, WA, fips: 53033, pop: 2252782
Mean visiting county population
500000
1500000
2500000
Mar
May
Jul
Sep
0
5
10
15
Incident cases (per 100,000 population)

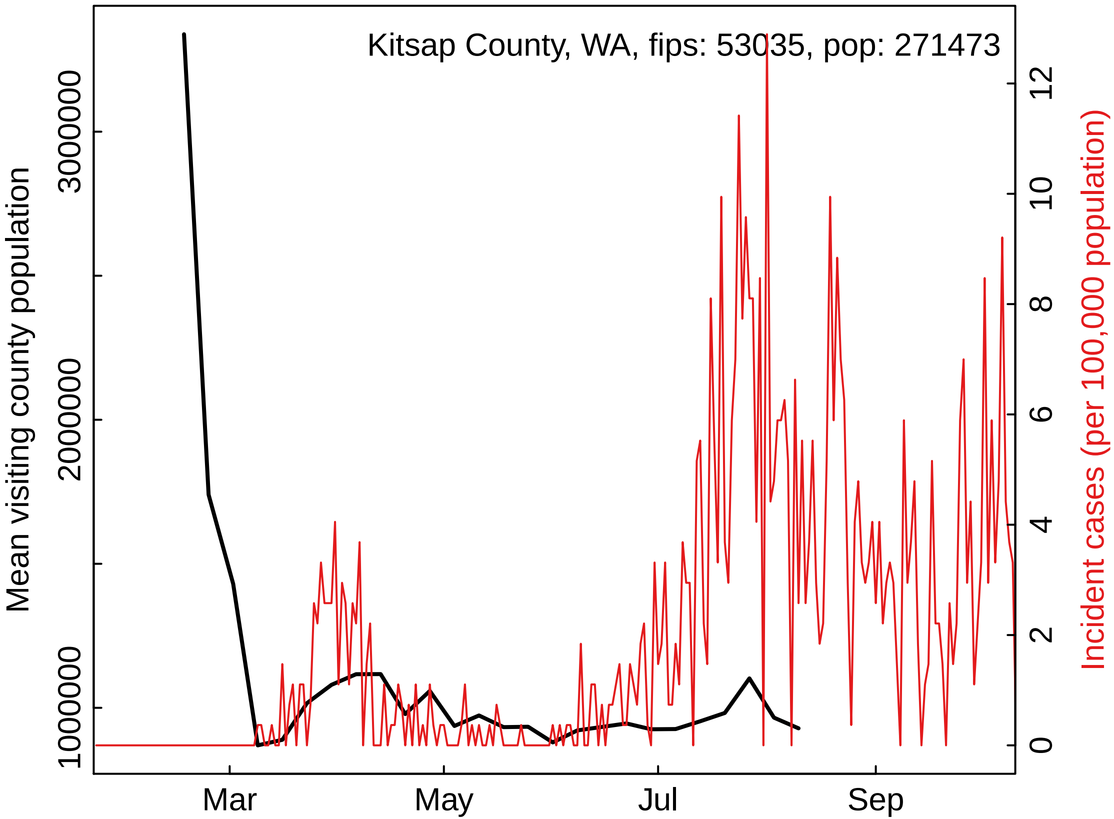

Kitsap County, WA, fips: 53035, pop: 271473
Mean visiting county population
Incident cases (per 100,000 population)
10000000
20000000
30000000
0
2
4
6
8
10
12
Mar
May
Jul
Sep

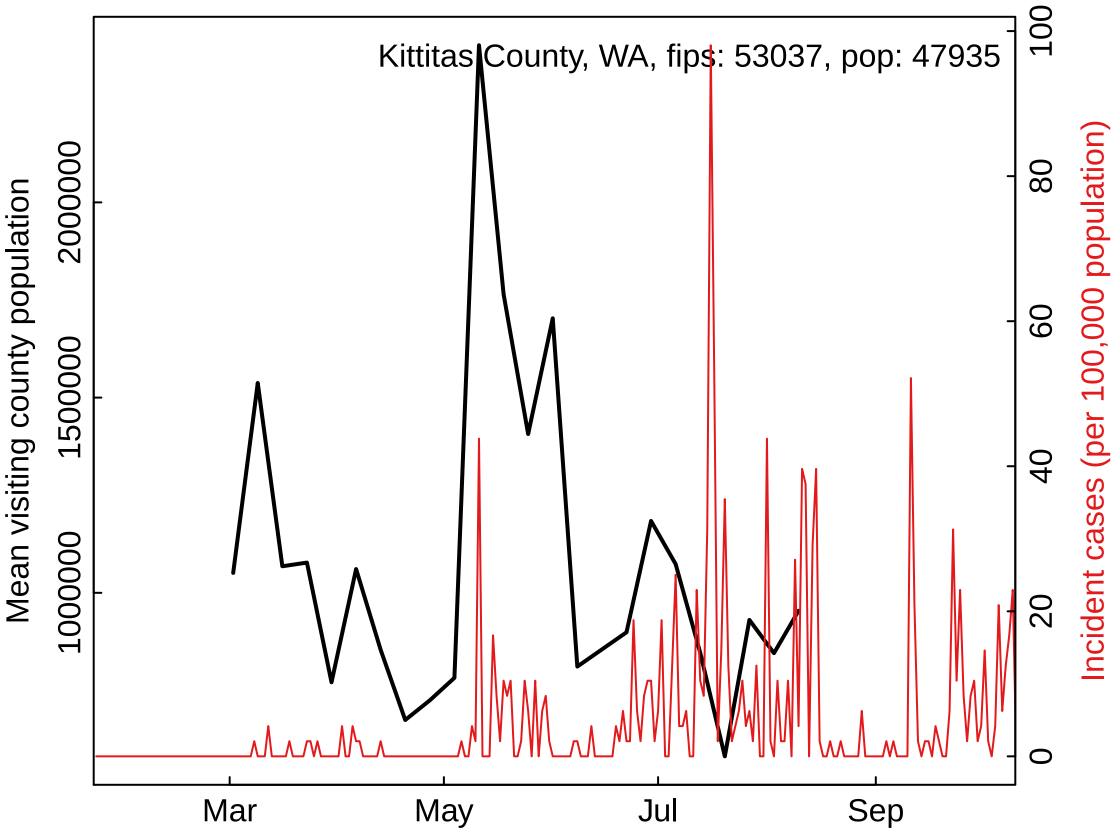
Kittitas County, WA, fips: 53037, pop: 47935
Mean visiting county population
1000000
1500000
2000000
Mar
May
Jul
Sep
0
20
40
60
80
100
Incident cases (per 100,000 population)

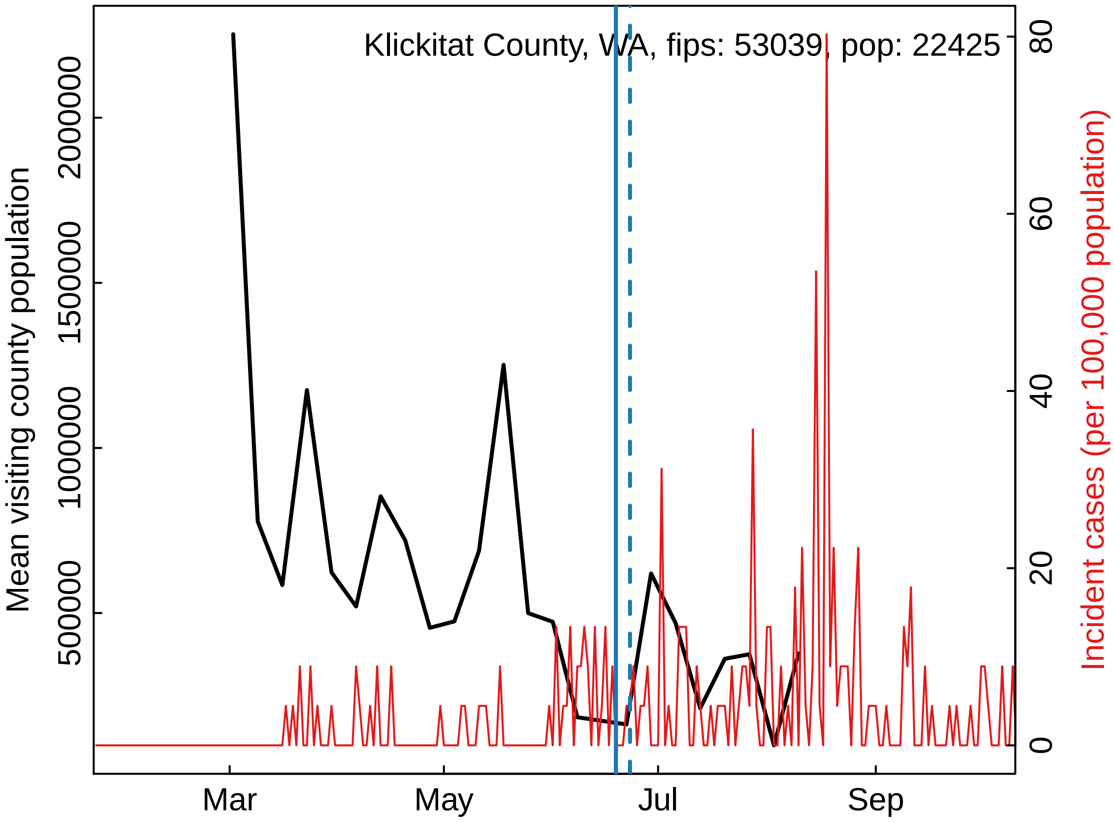

Klickitat County, WA, fips: 53039, pop: 22425
Mean visiting county population
500000
1000000
1500000
2000000
Incident cases (per 100,000 population)
0
20
40
60
80
Mar
May
Jul
Sep

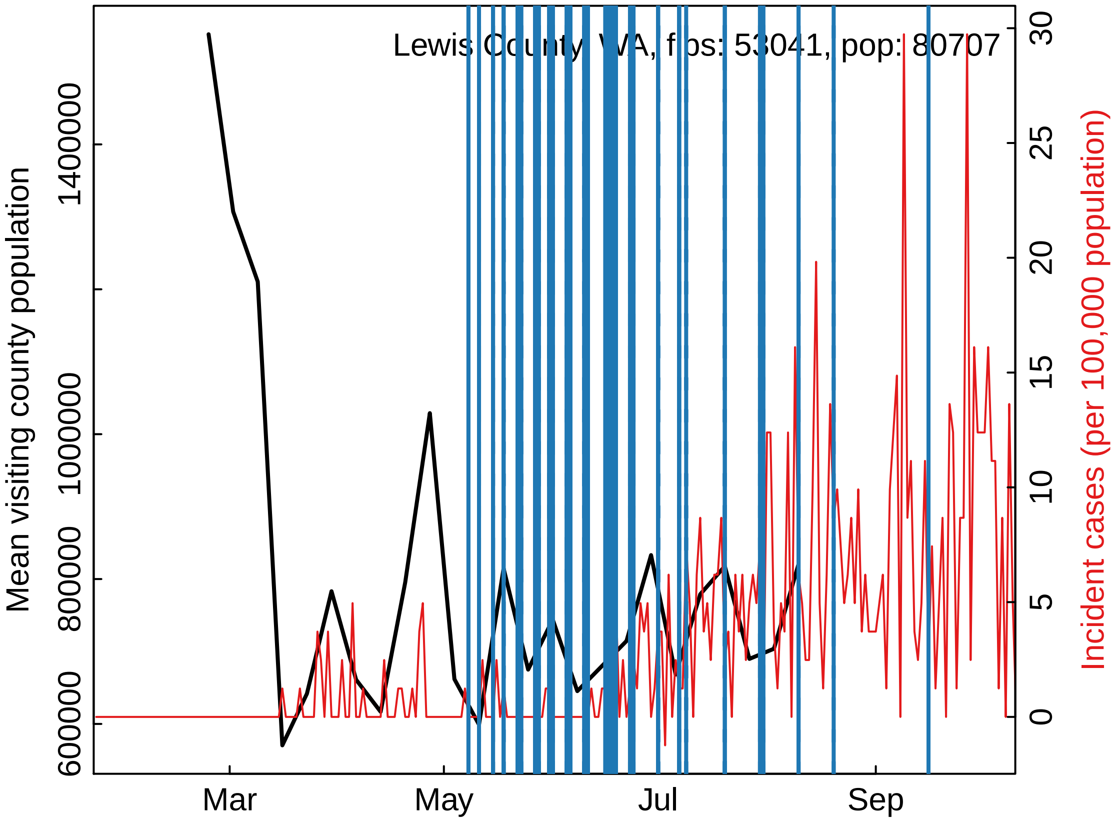

Lewis County, WA, fips: 53041, pop: 80707
Mean visiting county population
600000
800000
1000000
1400000
Mar
May
Jul
Sep
0
5
10
15
20
25
30
Incident cases (per 100,000 population)

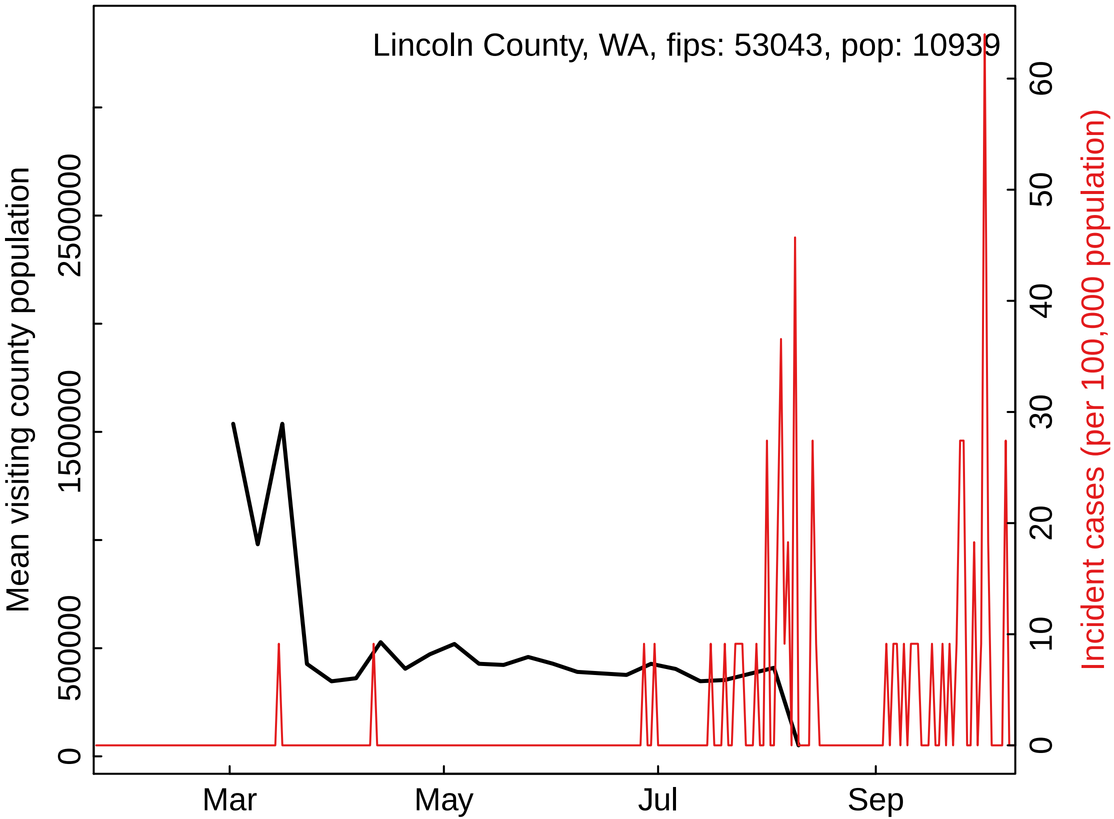

Lincoln County, WA, fips: 53043, pop: 10939
Mean visiting county population
0
500000
1500000
2500000
Incident cases (per 100,000 population)
0
10
20
30
40
50
60
Mar
May
Jul
Sep

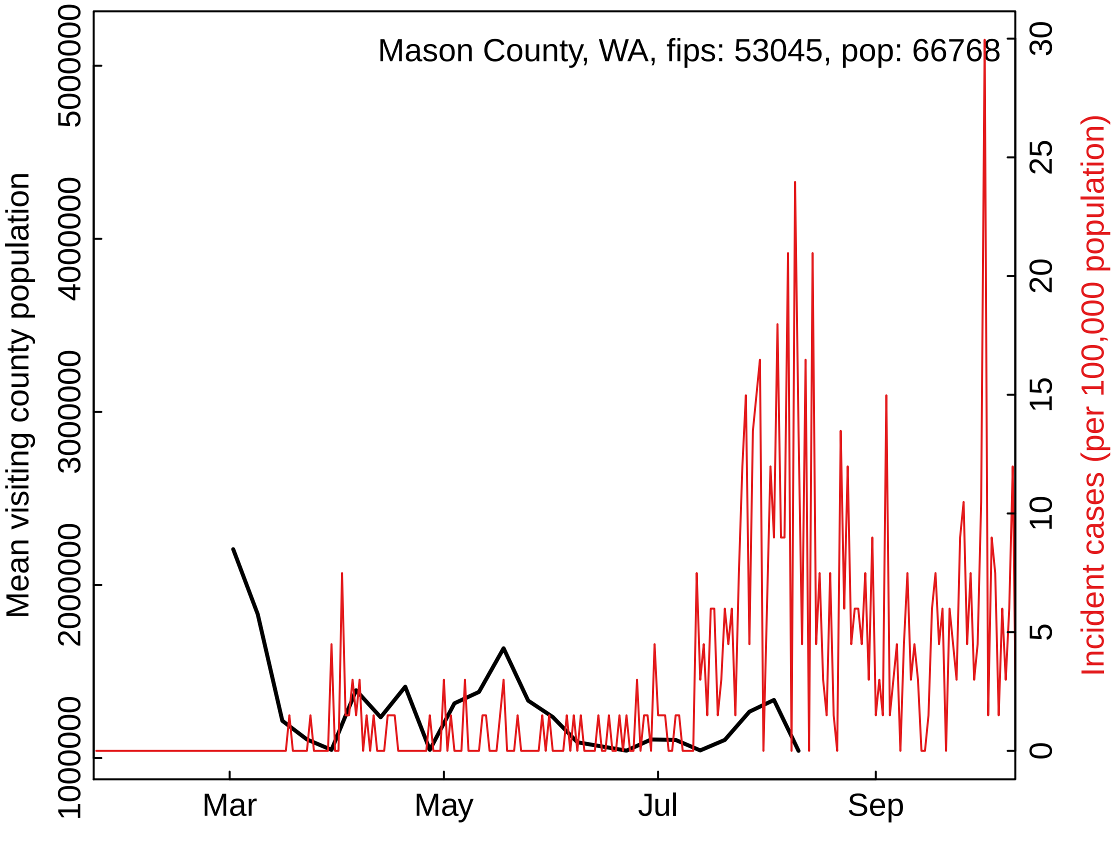
Mason County, WA, fips: 53045, pop: 66768
Mean visiting county population
10000000
20000000
30000000
40000000
50000000
Incident cases (per 100,000 population)
0
5
10
15
20
25
30
Mar
May
Jul
Sep

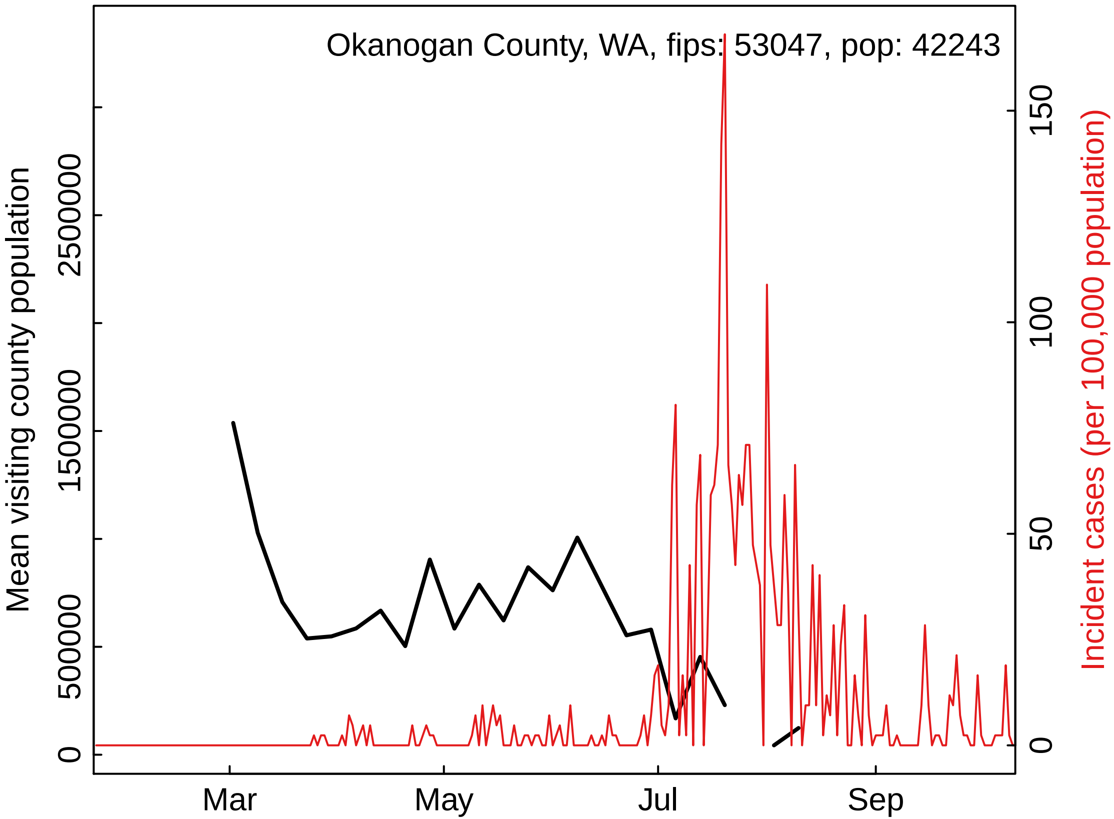

Okanogan County, WA, fips: 53047, pop: 42243
Mean visiting county population
0
500000
1500000
2500000
Incident cases (per 100,000 population)
0
50
100
150
Mar
May
Jul
Sep

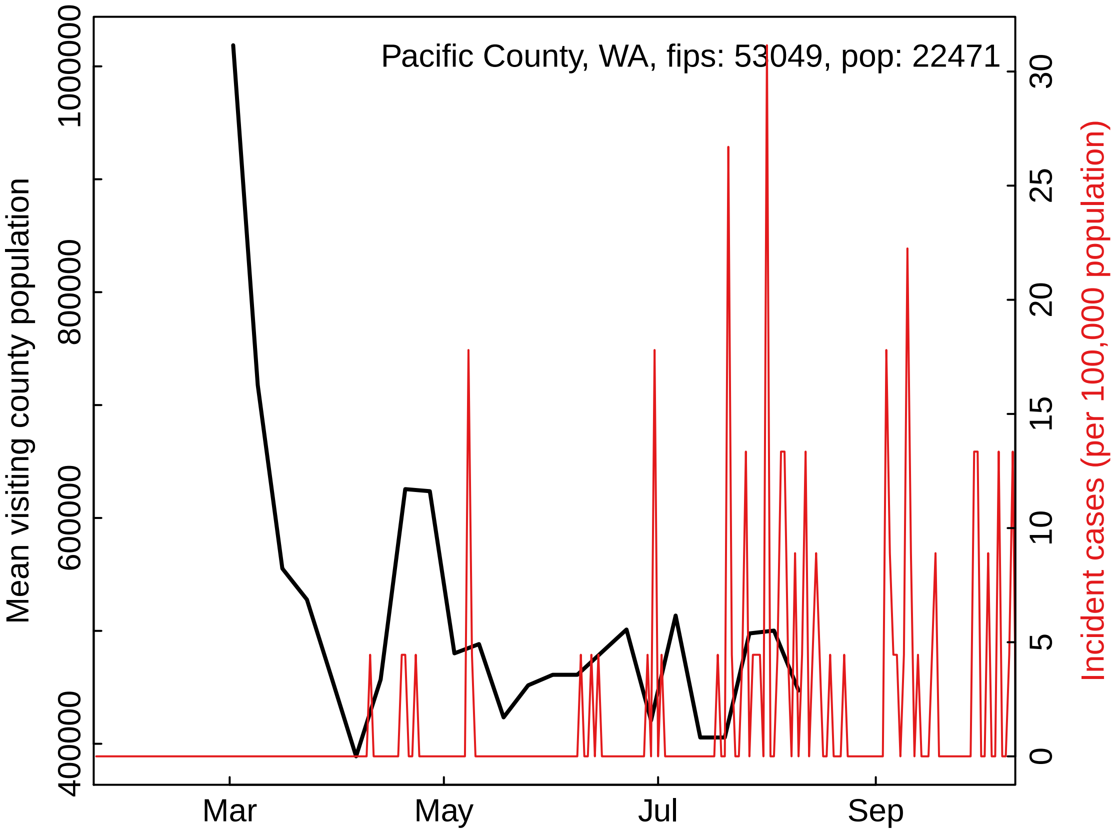
Pacific County, WA, fips: 53049, pop: 22471
Mean visiting county population
400000
600000
800000
1000000
Incident cases (per 100,000 population)
0
5
10
15
20
25
30
Mar
May
Jul
Sep

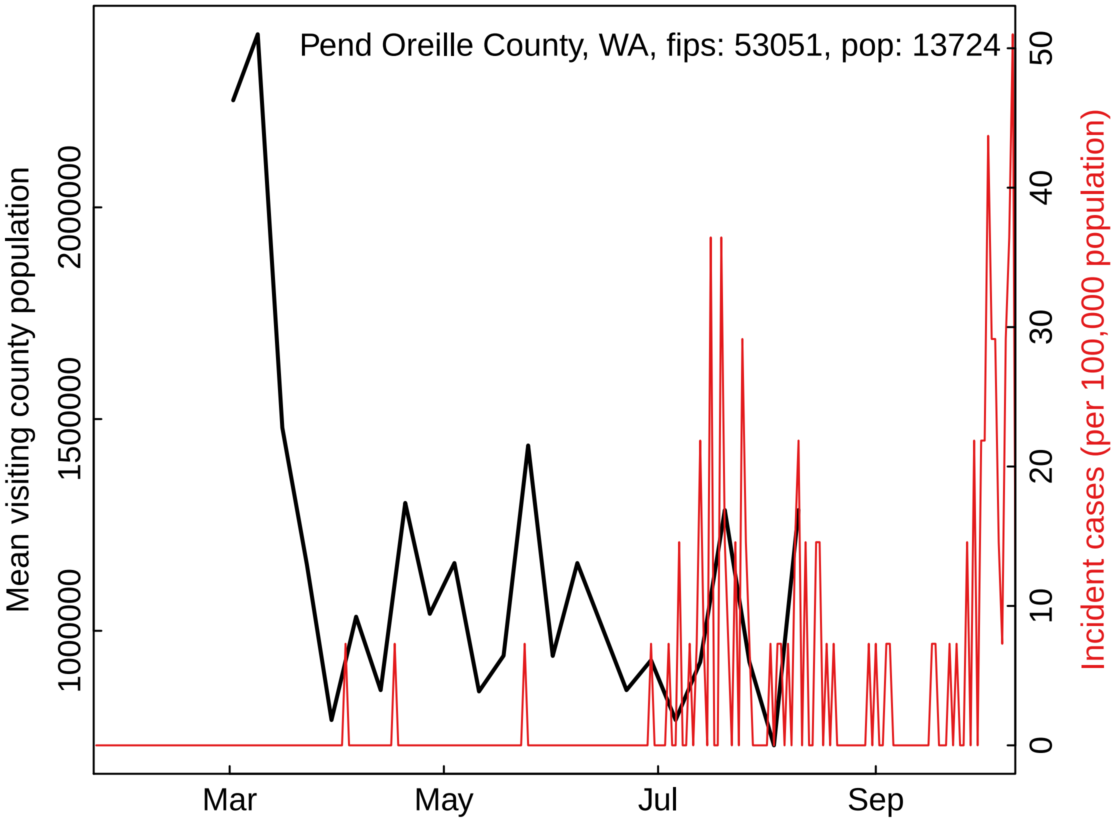
Pend Oreille County, WA, fips: 53051, pop: 13724
Mean visiting county population
1000000
1500000
2000000
Incident cases (per 100,000 population)
0
10
20
30
40
50
Mar
May
Jul
Sep

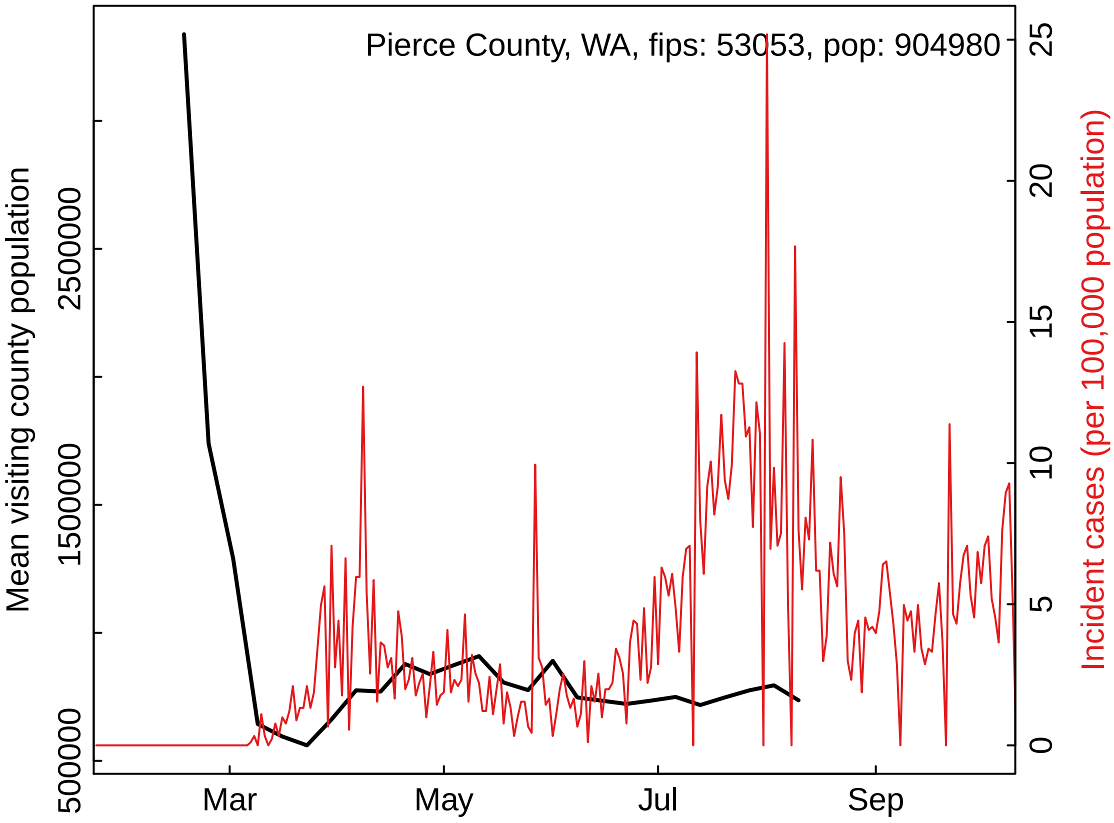

Pierce County, WA, fips: 53053, pop: 904980
Mean visiting county population
Incident cases (per 100,000 population)
500000
1500000
2500000
0
5
10
15
20
25
Mar
May
Jul
Sep

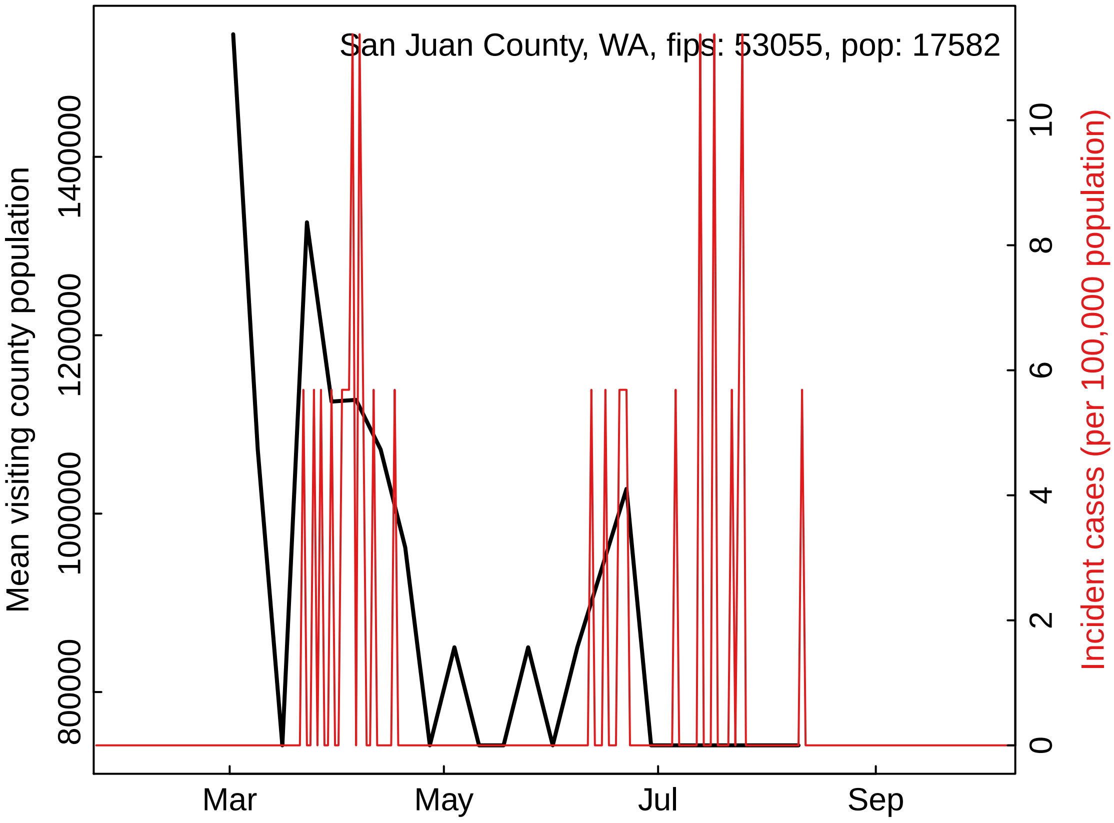

San Juan County, WA, fips: 53055, pop: 17582
Mean visiting county population
800000
1000000
1200000
1400000
Incident cases (per 100,000 population)
0
2
4
6
8
10
Mar
May
Jul
Sep

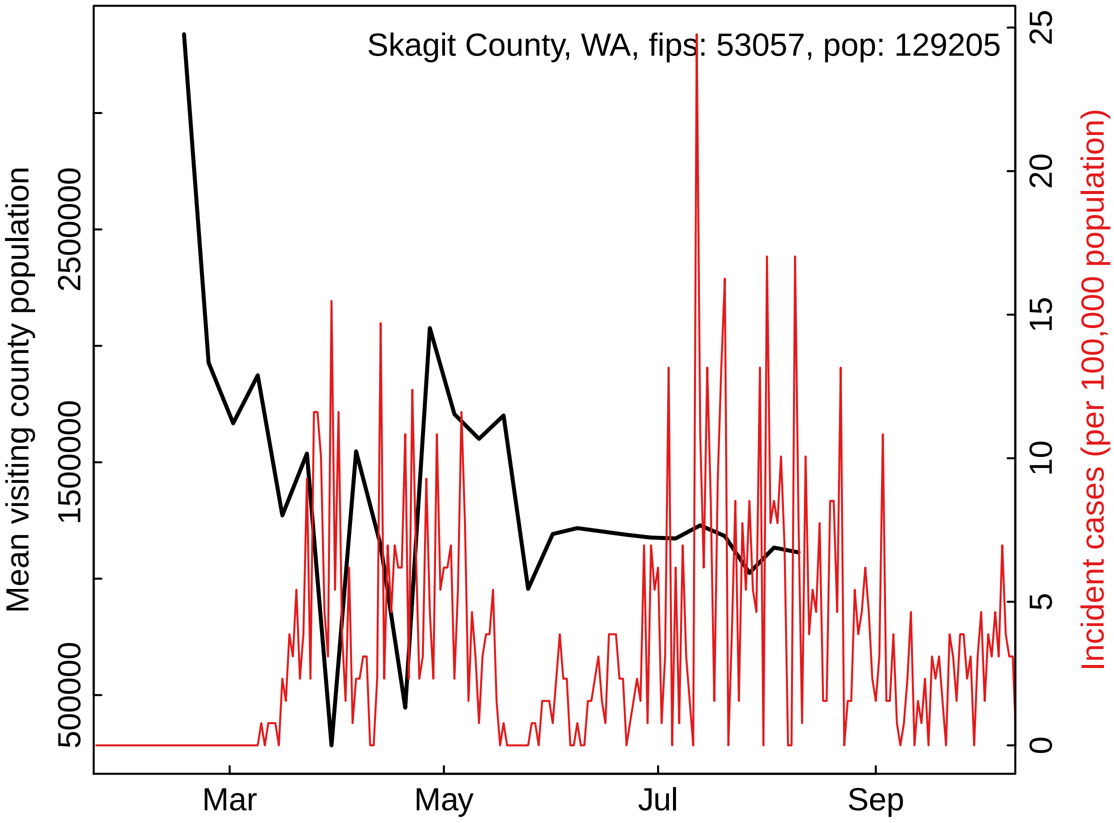

Skagit County, WA, fips: 53057, pop: 129205
Mean visiting county population
500000
1500000
2500000
Incident cases (per 100,000 population)
0
5
10
15
20
25
Mar
May
Jul
Sep

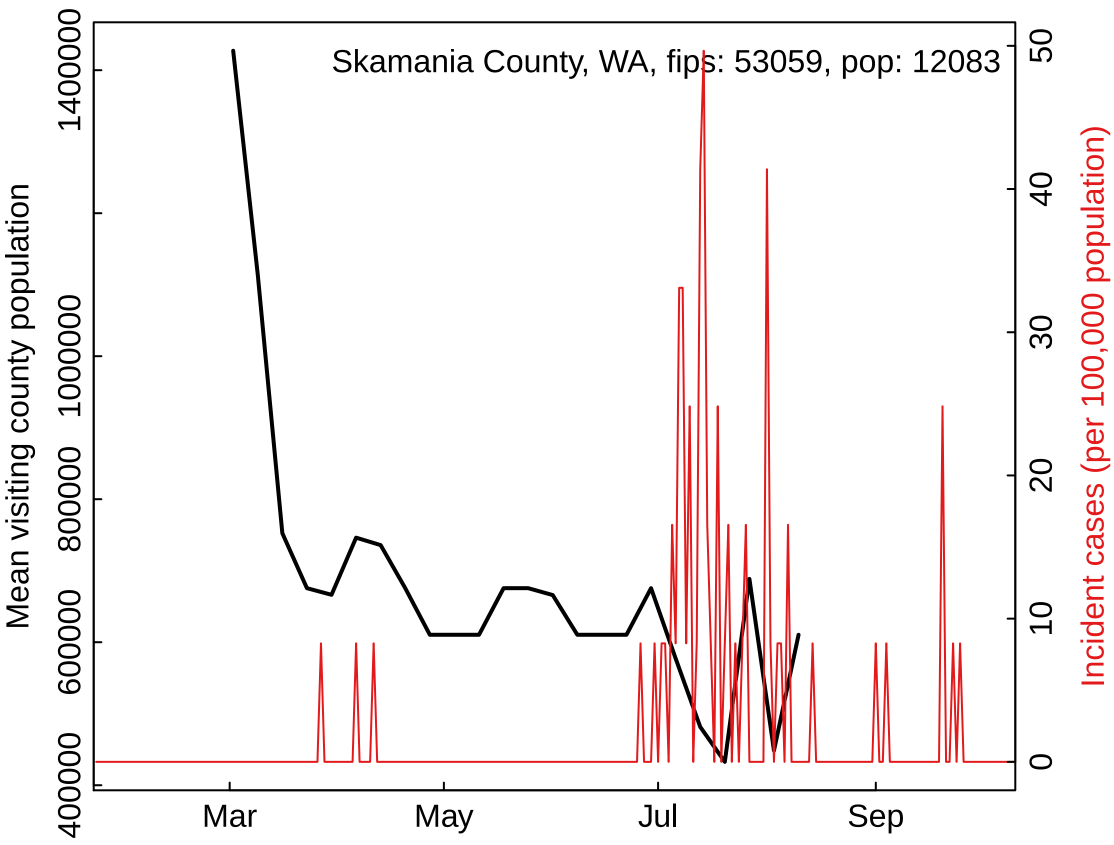
Skamania County, WA, fips: 53059, pop: 12083
Mean visiting county population
400000
600000
800000
1000000
1400000
Incident cases (per 100,000 population)
0
10
20
30
40
50
Mar
May
Jul
Sep

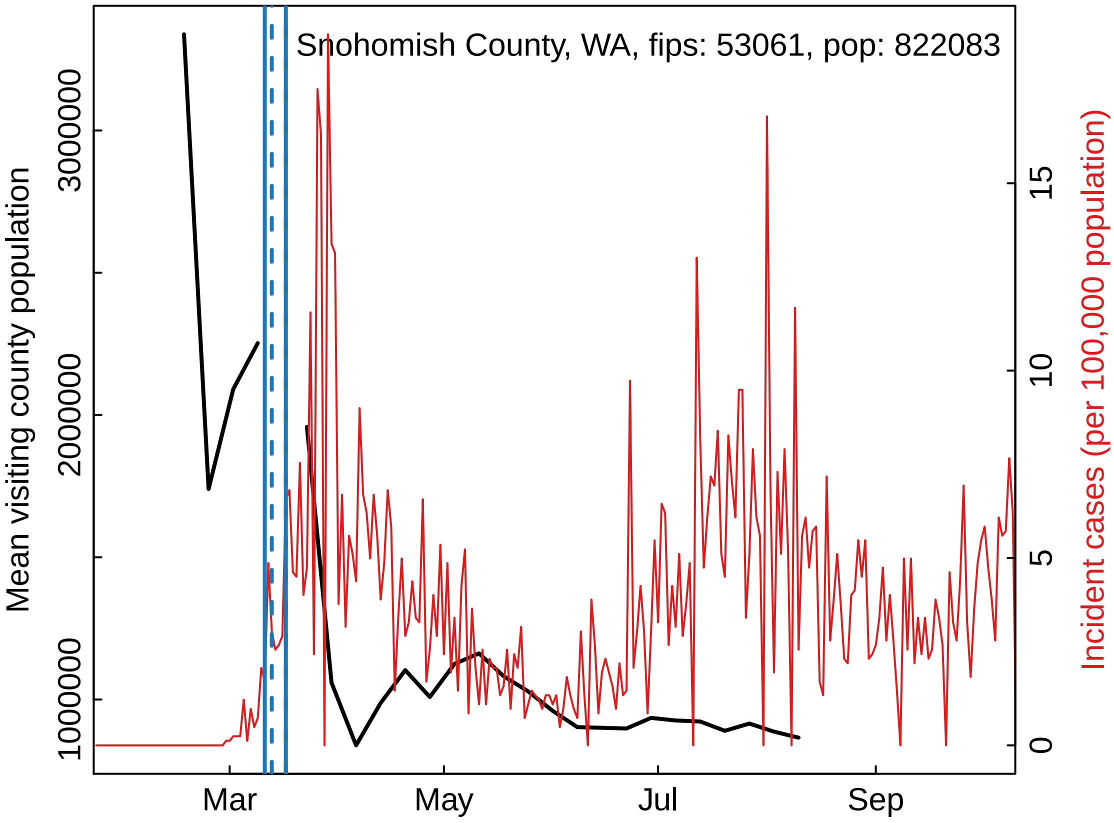

Snohomish County, WA, fips: 53061, pop: 822083
Mean visiting county population
10000000
20000000
30000000
Incident cases (per 100,000 population)
0
5
10
15
Mar
May
Jul
Sep

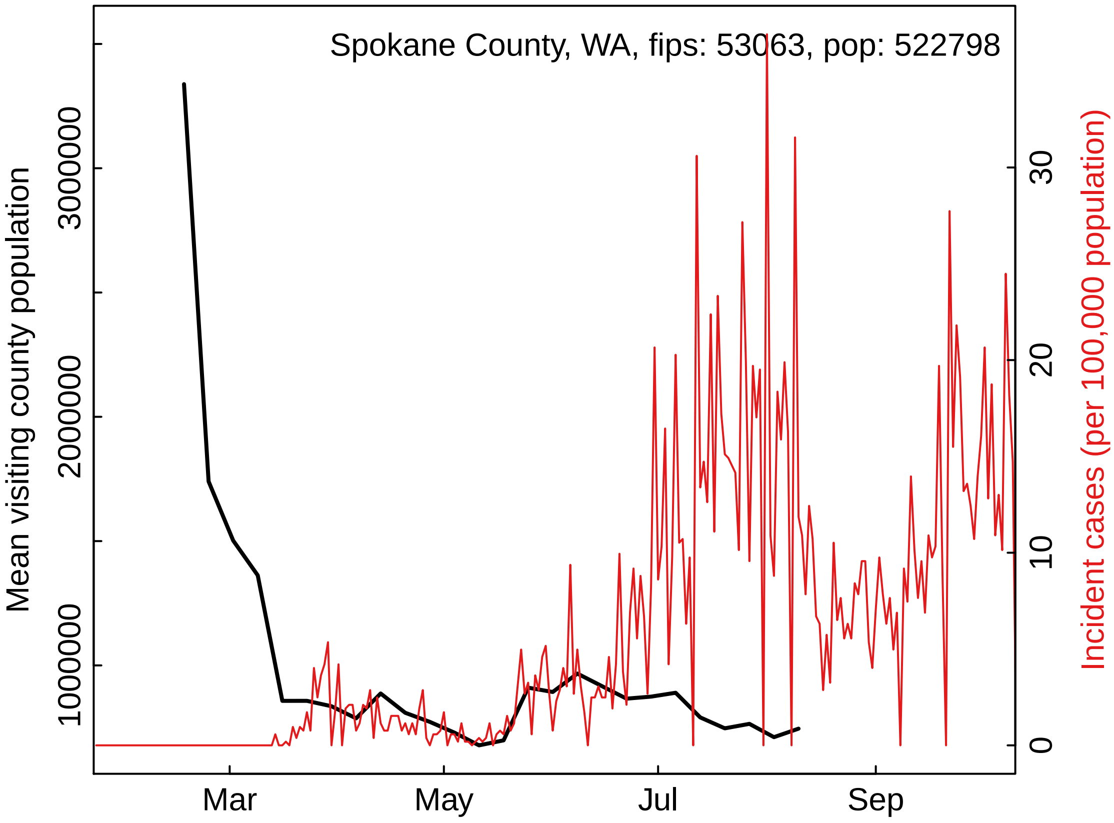

Spokane County, WA, fips: 53063, pop: 522798
Mean visiting county population
10000000
20000000
30000000
Incident cases (per 100,000 population)
0
10
20
30
Mar
May
Jul
Sep

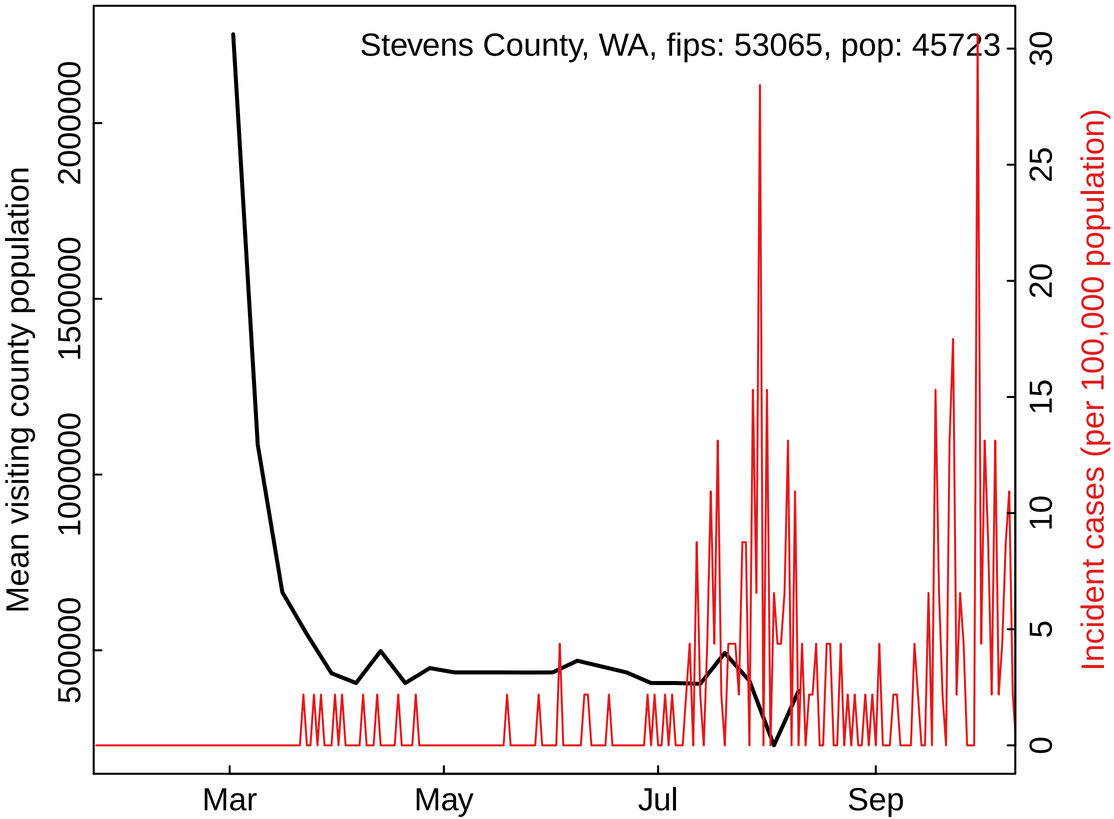

Stevens County, WA, fips: 53065, pop: 45723
Mean visiting county population
500000
1000000
1500000
2000000
Incident cases (per 100,000 population)
0
5
10
15
20
25
30
Mar
May
Jul
Sep

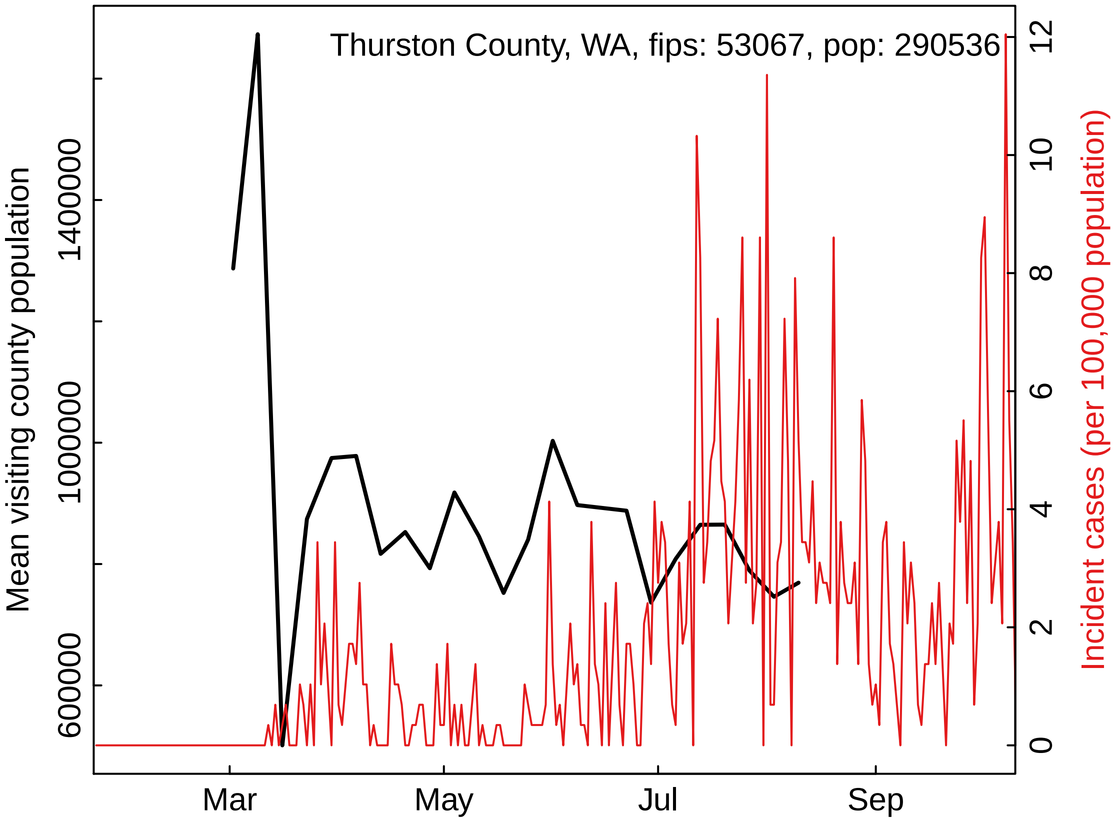

Thurston County, WA, fips: 53067, pop: 290536
Mean visiting county population
600000
1000000
1400000
Incident cases (per 100,000 population)
0
2
4
6
8
10
12
Mar
May
Jul
Sep

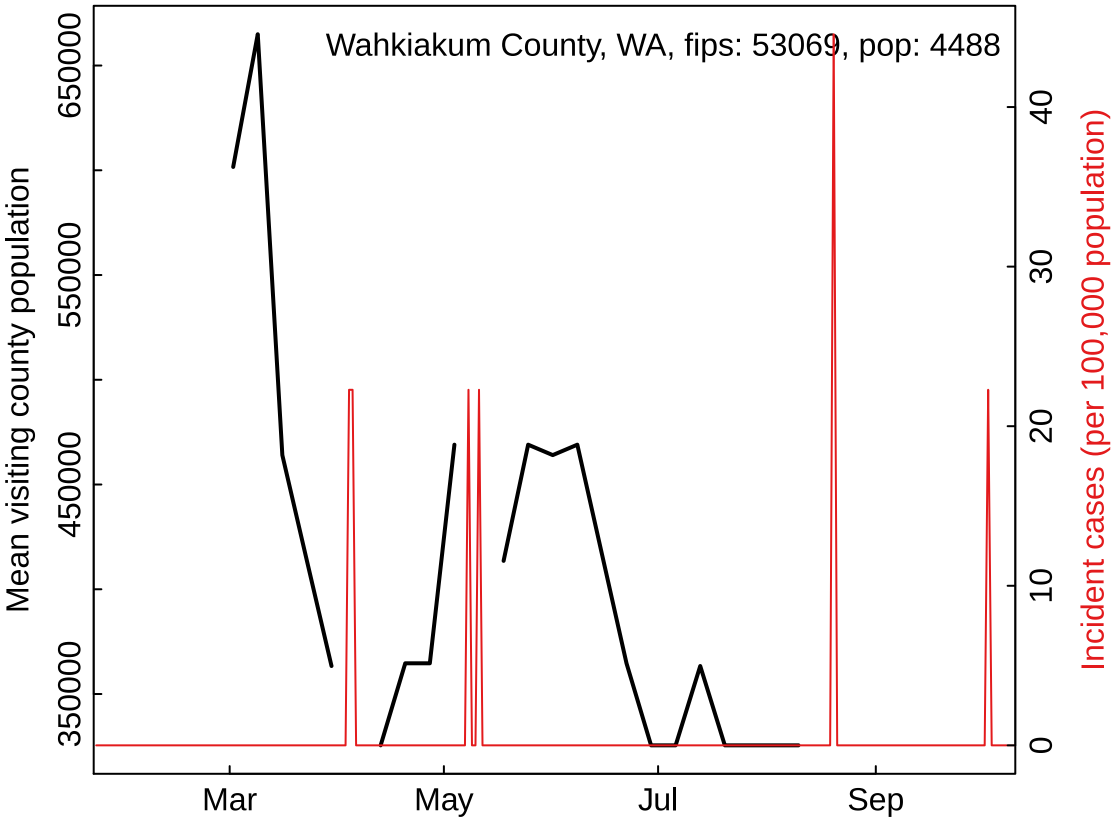

Wahkiakum County, WA, fips: 53069, pop: 4488
Mean visiting county population
650000
550000
450000
350000
Incident cases (per 100,000 population)
40
30
20
10
0
Mar
May
Jul
Sep

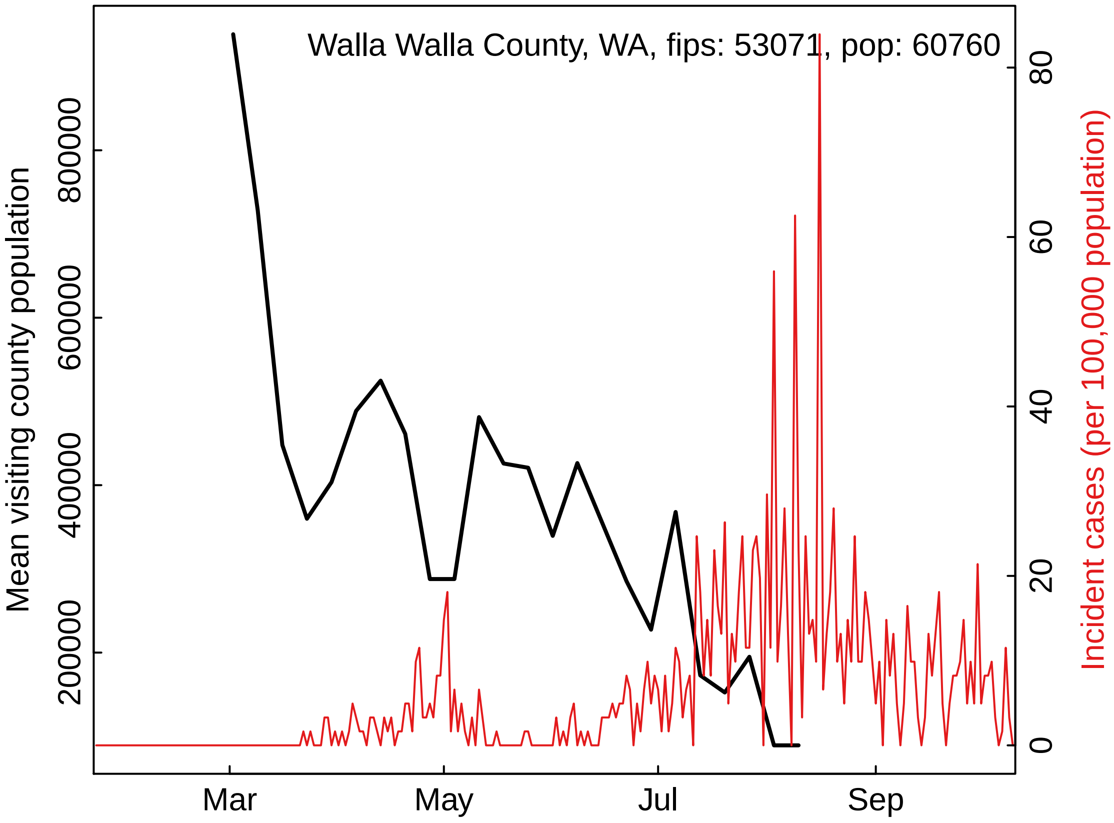

Walla Walla County, WA, fips: 53071, pop: 60760
Mean visiting county population
200000
400000
600000
800000
Incident cases (per 100,000 population)
0
20
40
60
80
Mar
May
Jul
Sep

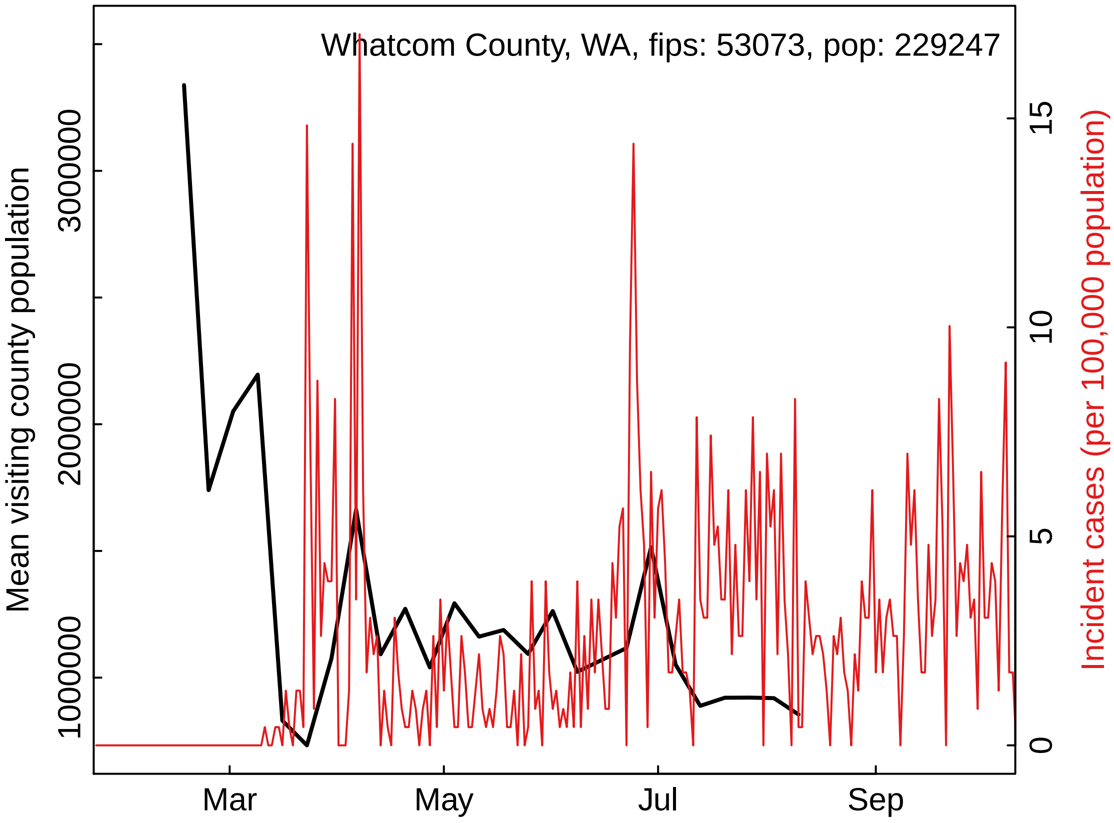

Whatcom County, WA, fips: 53073, pop: 229247
Mean visiting county population
10000000
20000000
30000000
Incident cases (per 100,000 population)
0
5
10
15
Mar
May
Jul
Sep

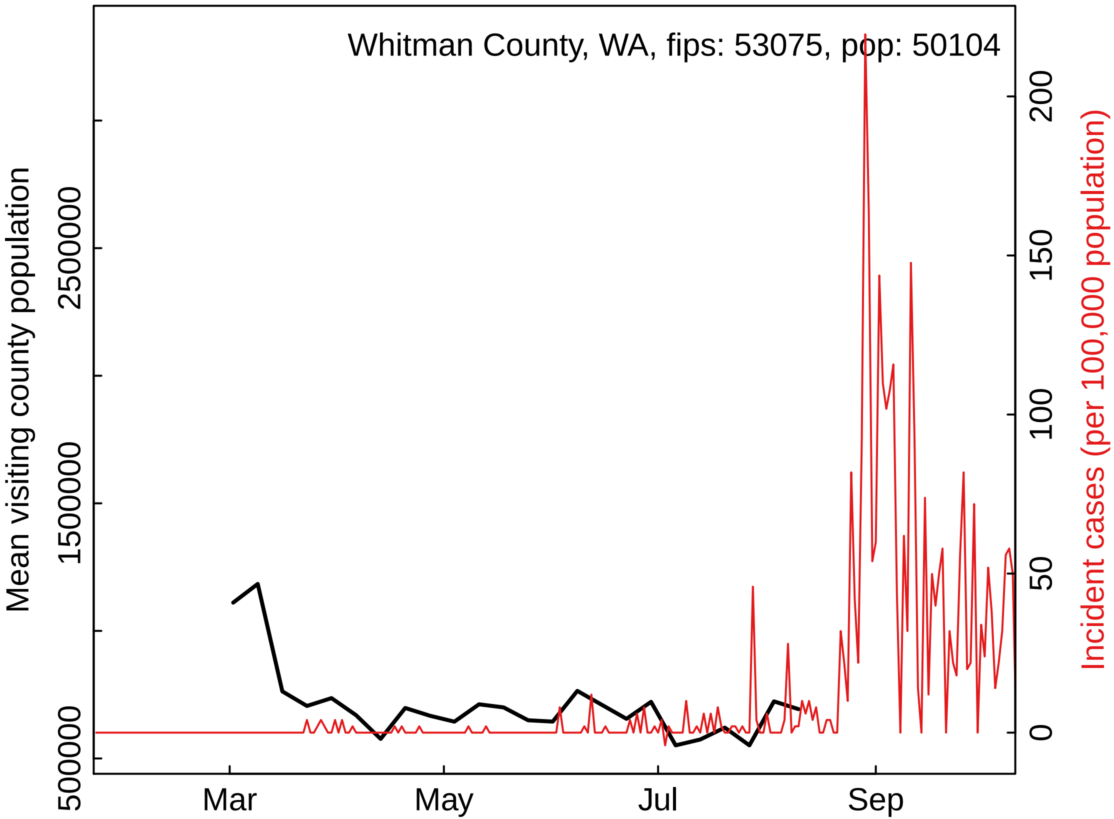

Whitman County, WA, fips: 53075, pop: 50104
Mean visiting county population
500000
1500000
2500000
Mar
May
Jul
Sep
0
50
100
150
200
Incident cases (per 100,000 population)

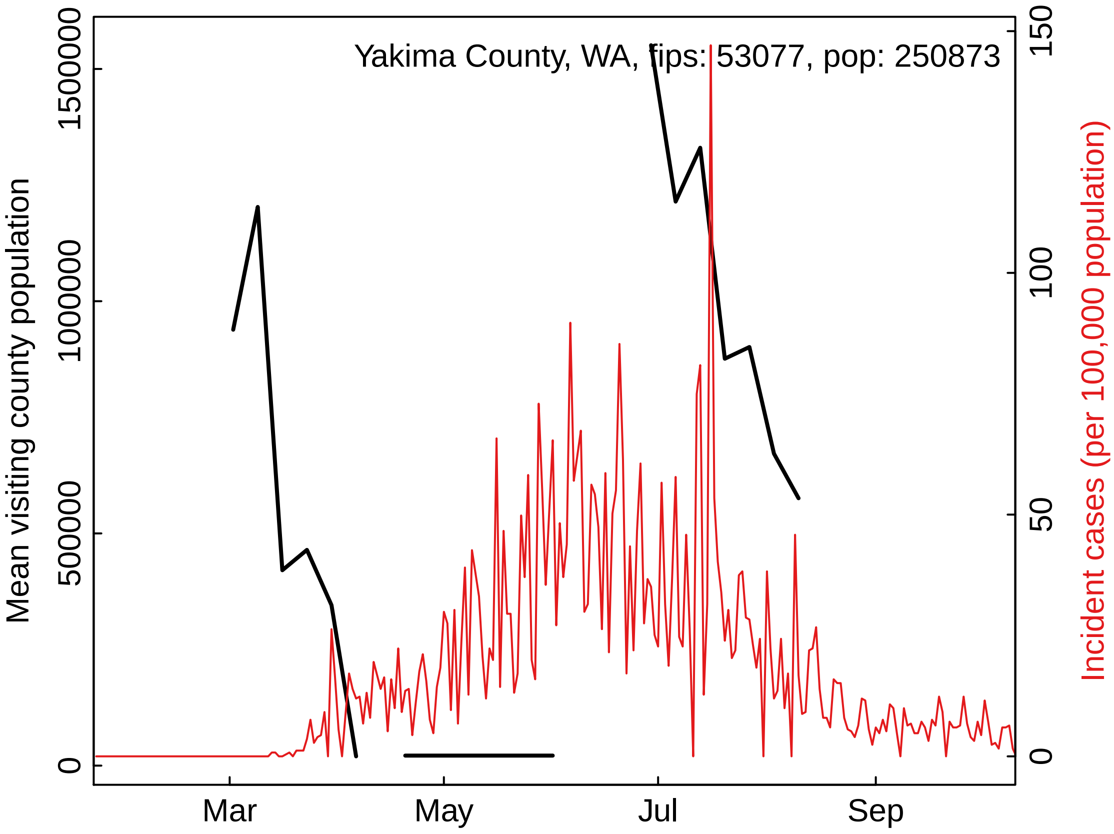
Yakima County, WA, fips: 53077, pop: 250873
Mean visiting county population
0
500000
1000000
1500000
Incident cases (per 100,000 population)
0
50
100
150
Mar
May
Jul
Sep

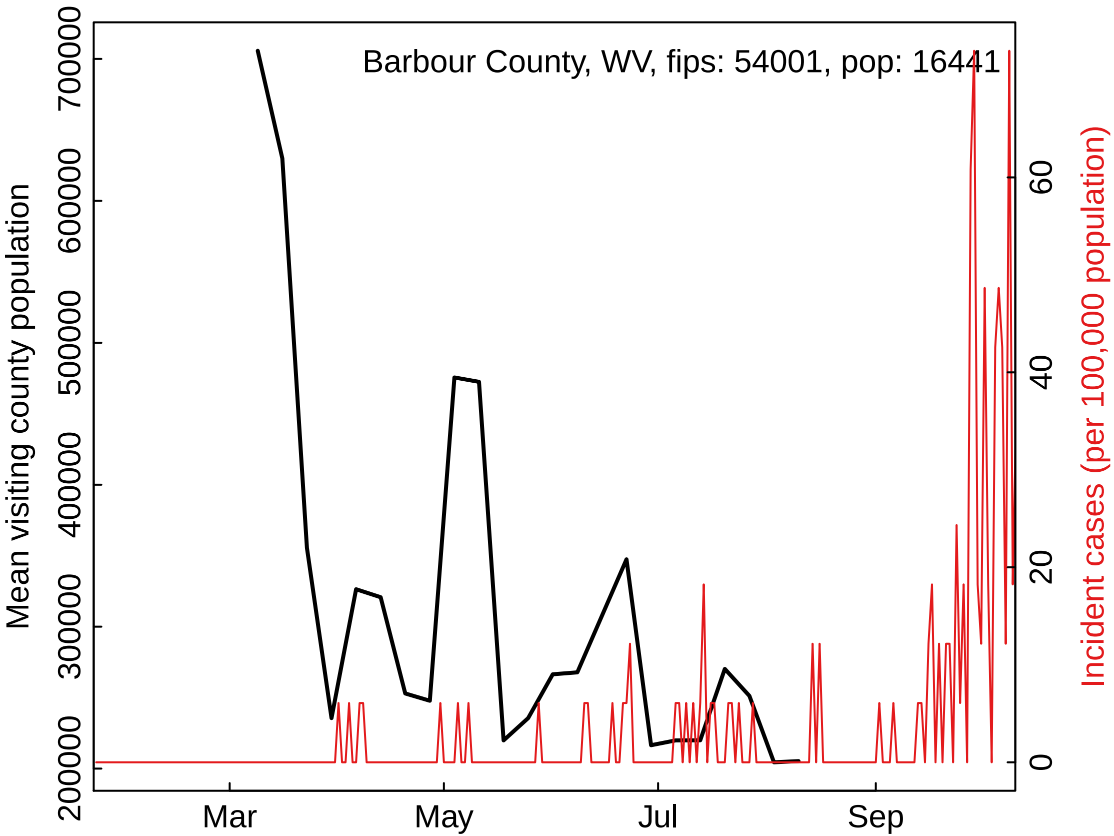

Barbour County, WV, fips: 54001, pop: 16441
Mean visiting county population
200000
300000
400000
500000
600000
700000
Mar
May
Jul
Sep
Incident cases (per 100,000 population)
0
20
40
60

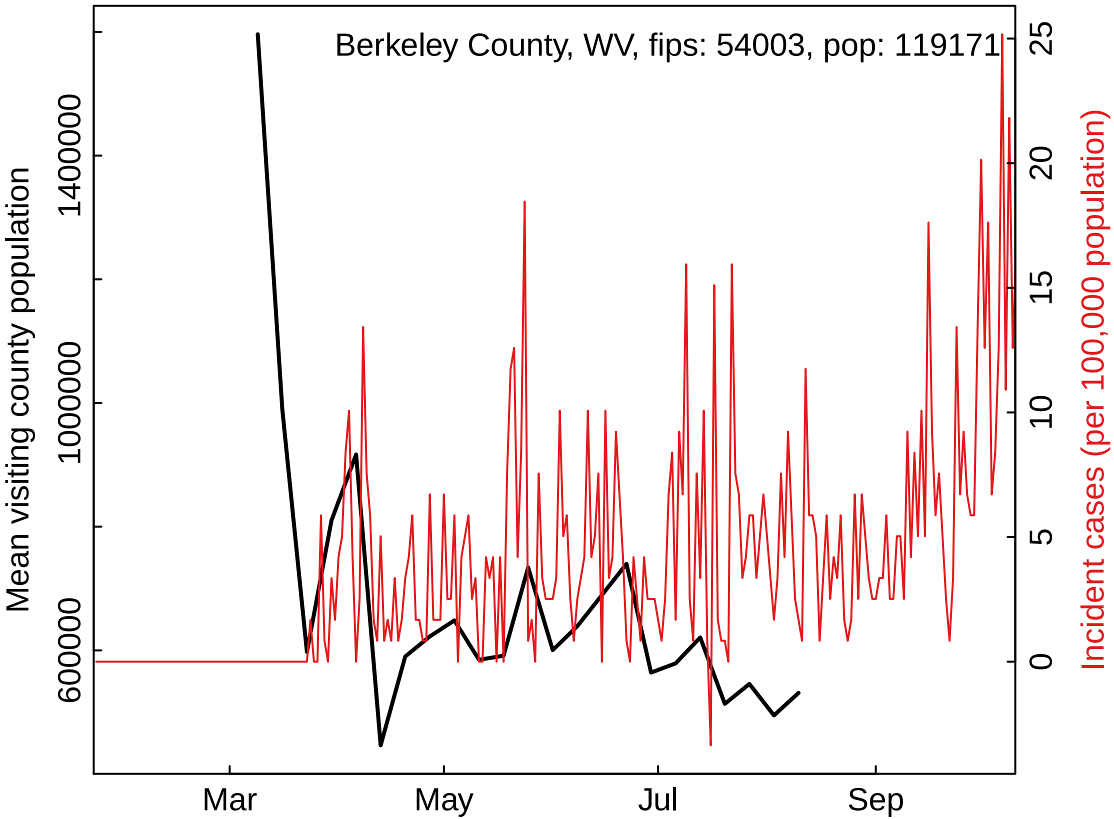
Berkeley County, WV, fips: 54003, pop: 119171
Mean visiting county population
600000
1000000
1400000
Mar
May
Jul
Sep
0
5
10
15
20
25
Incident cases (per 100,000 population)

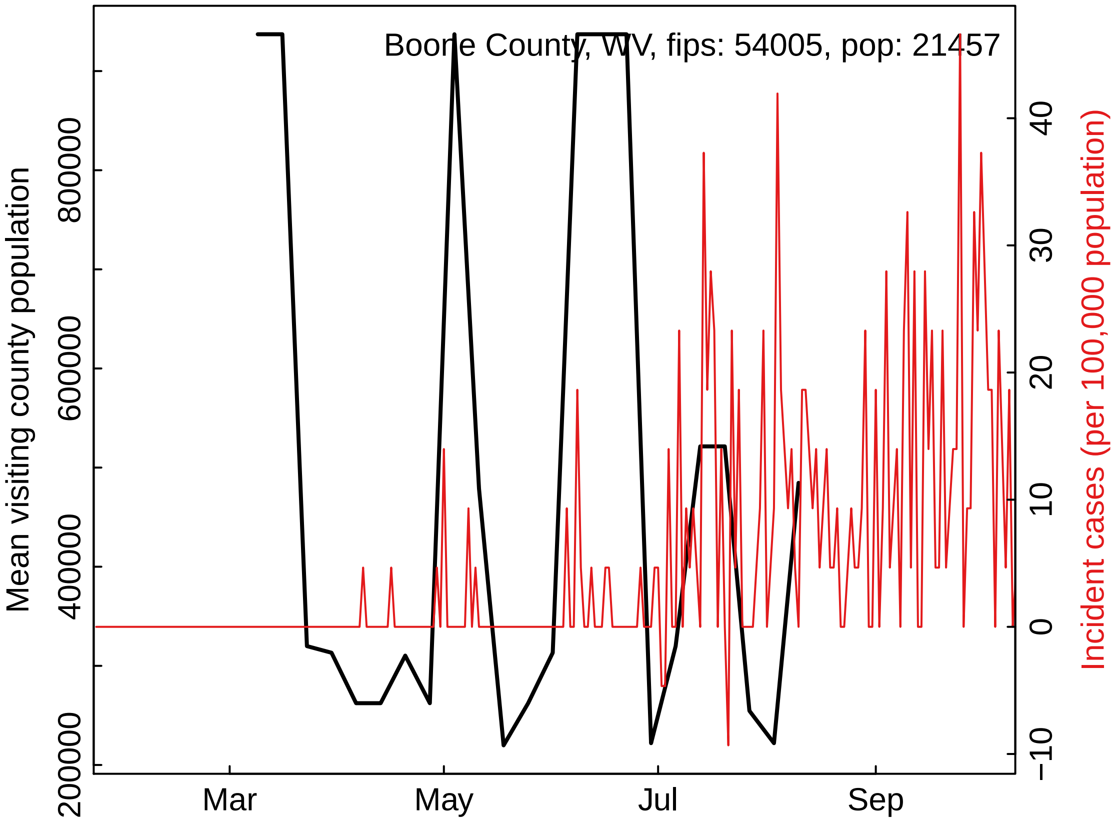

Boone County, WV, fips: 54005, pop: 21457
Mean visiting county population
200000
400000
600000
800000
Mar
May
Jul
Sep
Incident cases (per 100,000 population)
−10
0
10
20
30
40

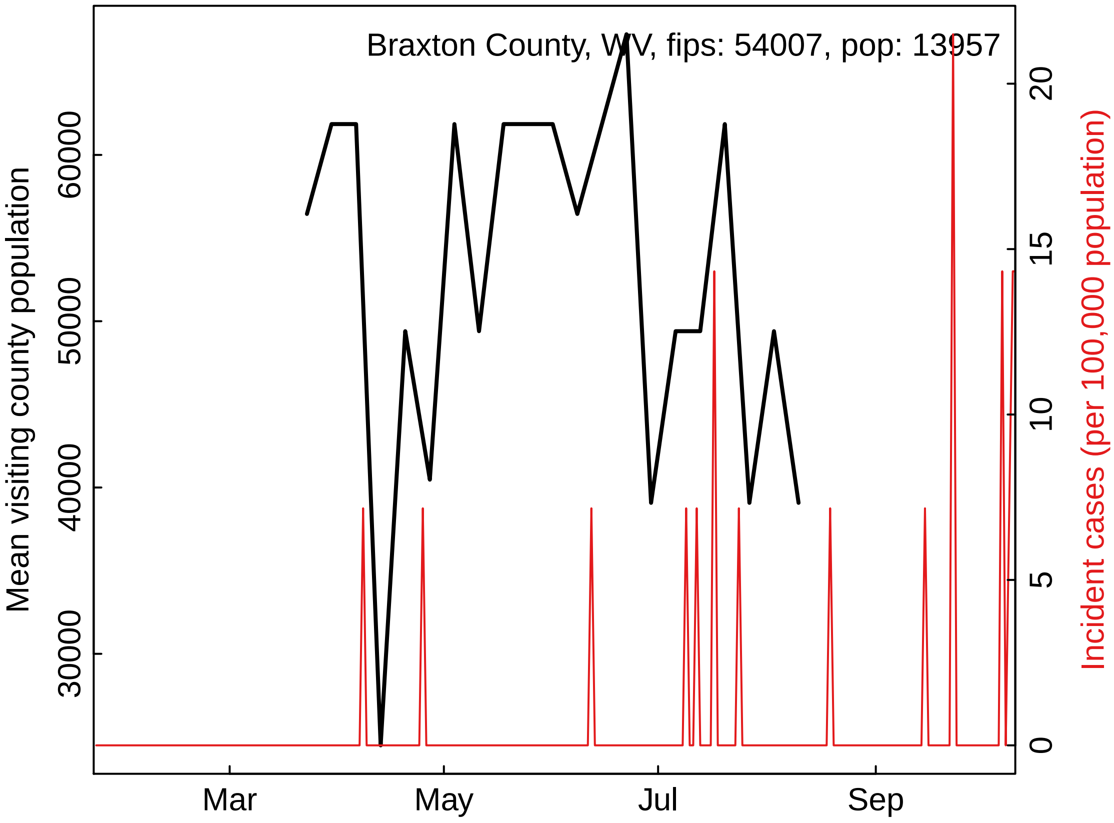

Braxton County, WV, fips: 54007, pop: 13957
Mean visiting county population
30000
40000
50000
60000
Incident cases (per 100,000 population)
0
5
10
15
20
Mar
May
Jul
Sep

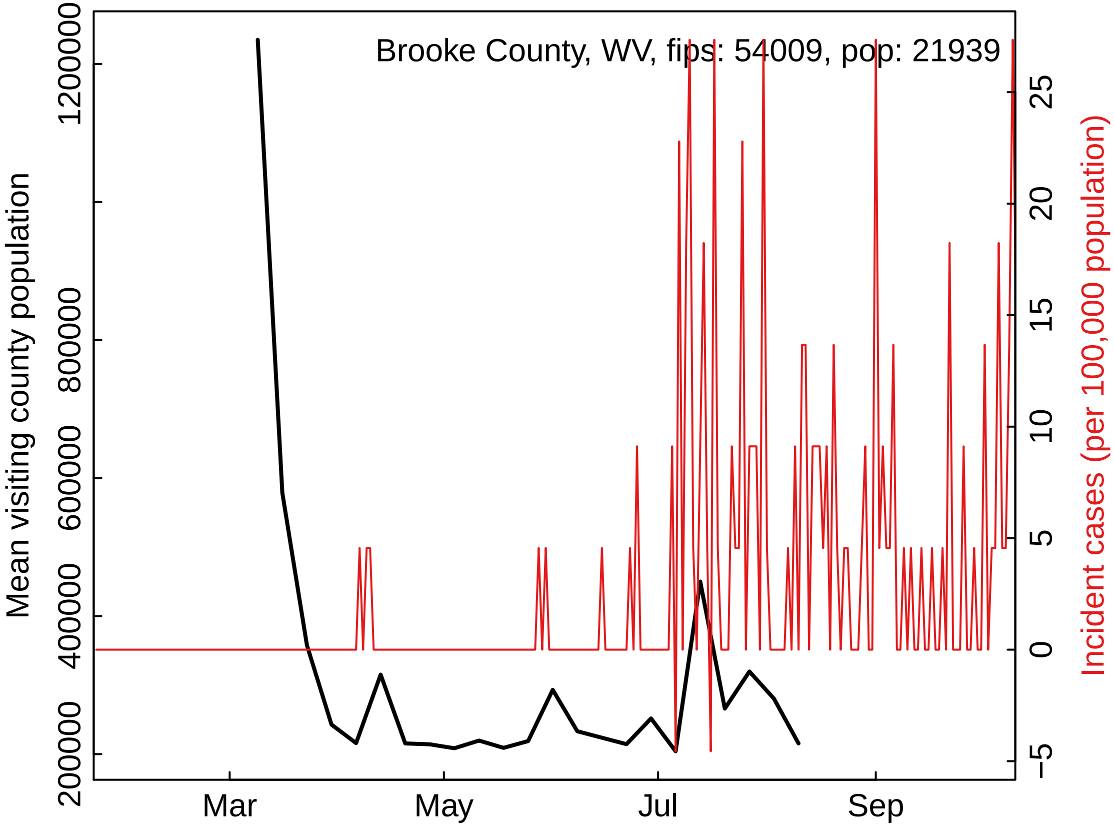
Brooke County, WV, fips: 54009, pop: 21939
Mean visiting county population
200000
400000
600000
800000
1200000
Mar
May
Jul
Sep
−5
0
5
10
15
20
25
Incident cases (per 100,000 population)

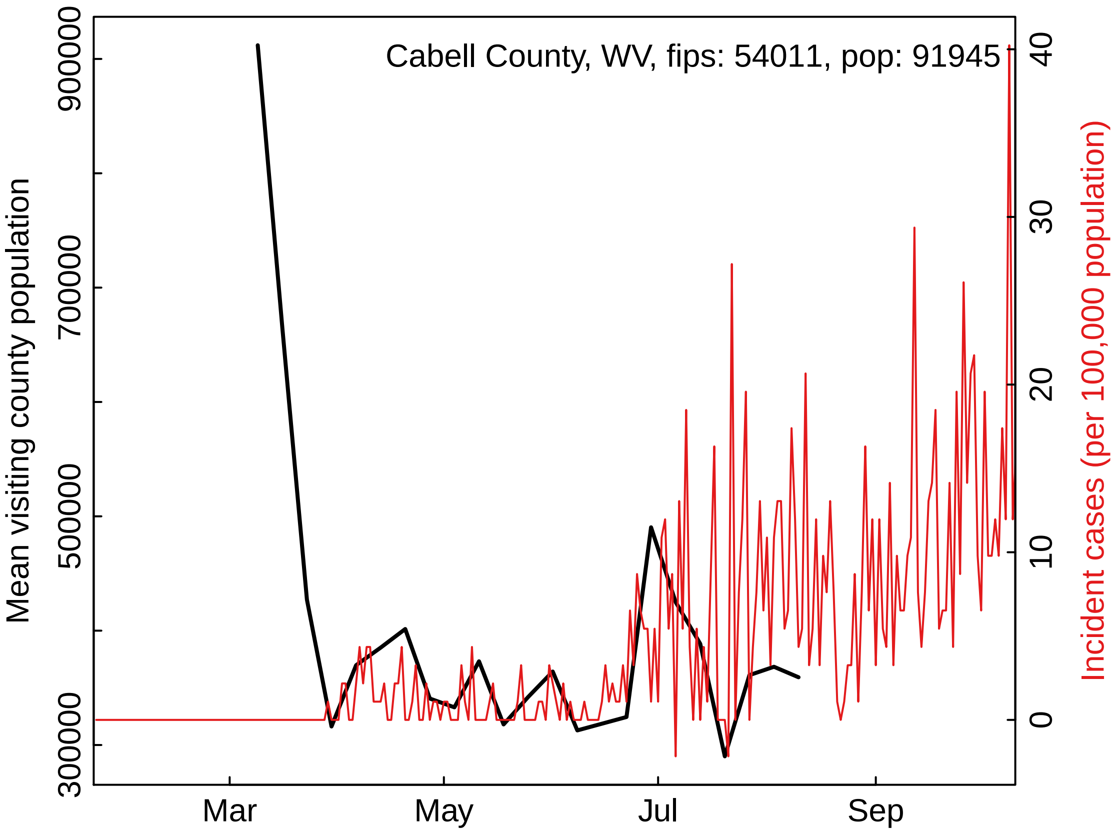

Cabell County, WV, fips: 54011, pop: 91945
Mean visiting county population
300000
500000
700000
900000
Incident cases (per 100,000 population)
0
10
20
30
40
Mar
May
Jul
Sep

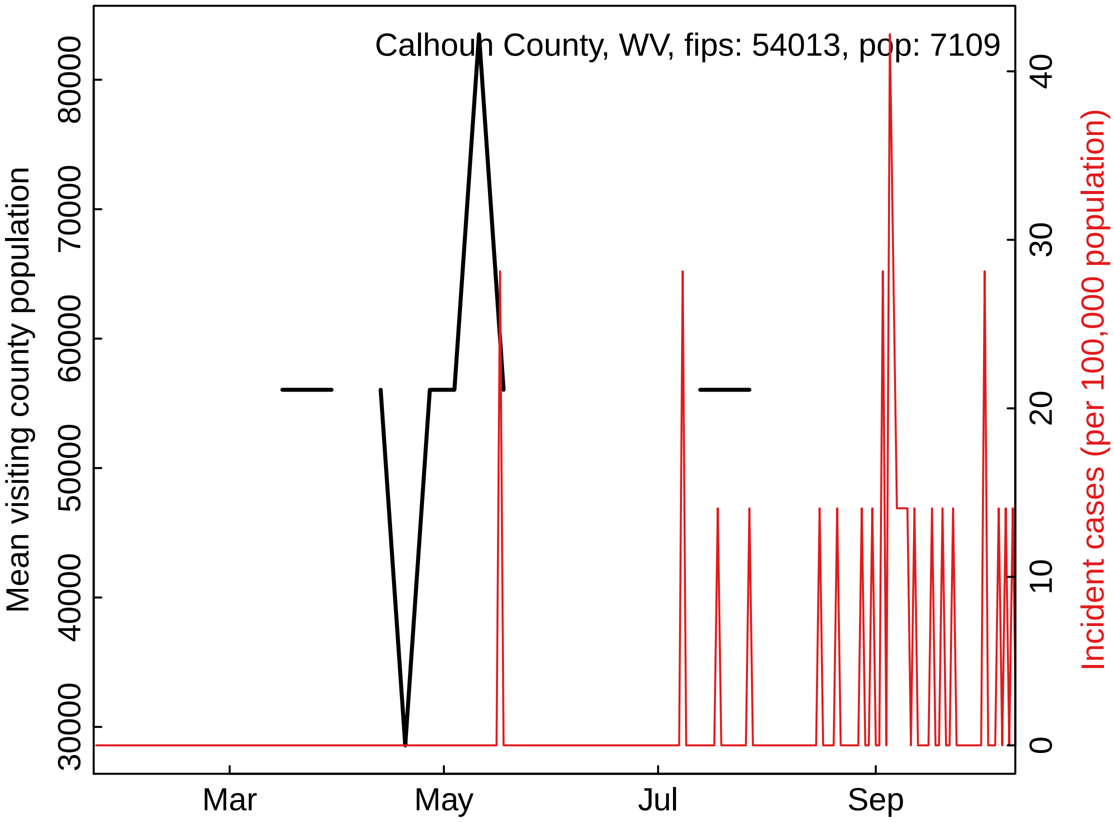

Calhoun County, WV, fips: 54013, pop: 7109
Mean visiting county population
30000
40000
50000
60000
70000
80000
Incident cases (per 100,000 population)
0
10
20
30
40
Mar
May
Jul
Sep

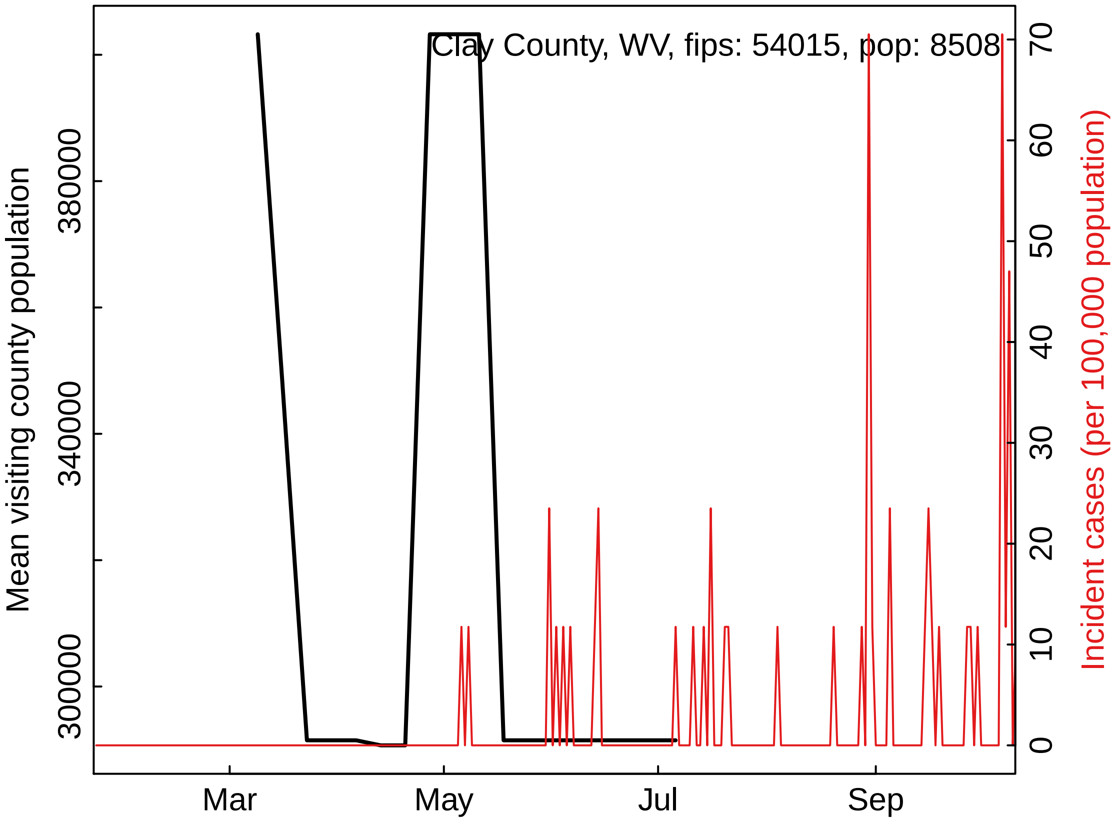

Clay County, WV, fips: 54015, pop: 8508
Mean visiting county population
300000
340000
380000
Incident cases (per 100,000 population)
0
10
20
30
40
50
60
70
Mar
May
Jul
Sep

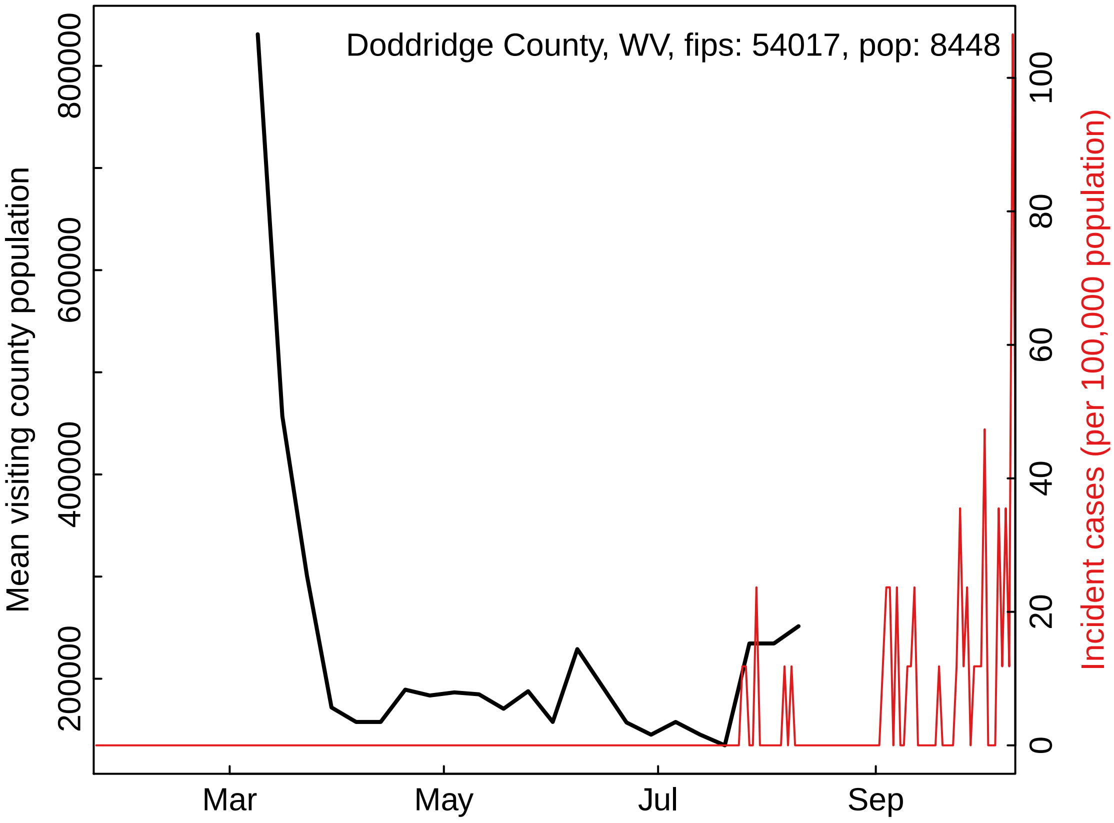

Doddridge County, WV, fips: 54017, pop: 8448
Mean visiting county population
200000
400000
600000
800000
Incident cases (per 100,000 population)
0
20
40
60
80
100
Mar
May
Jul
Sep

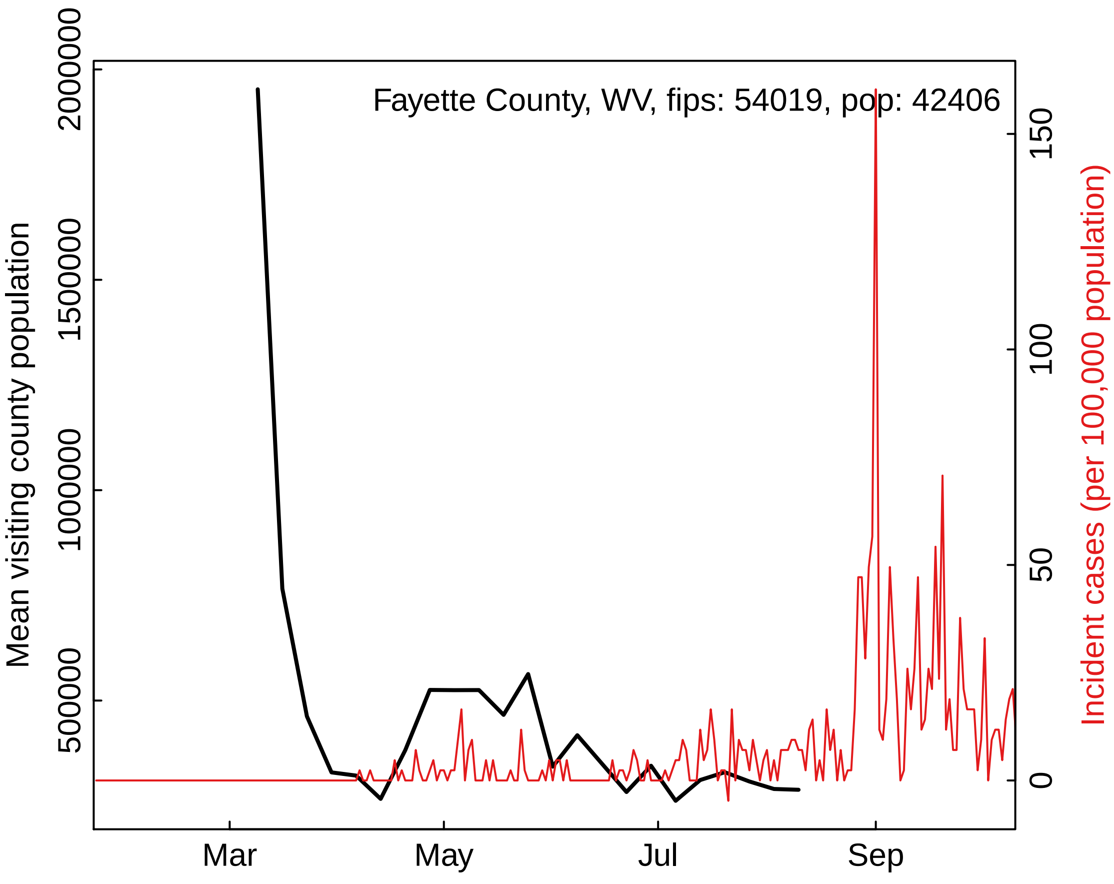

Fayette County, WV, fips: 54019, pop: 42406
Mean visiting county population
500000
1000000
1500000
2000000
Incident cases (per 100,000 population)
0
50
100
150
Mar
May
Jul
Sep

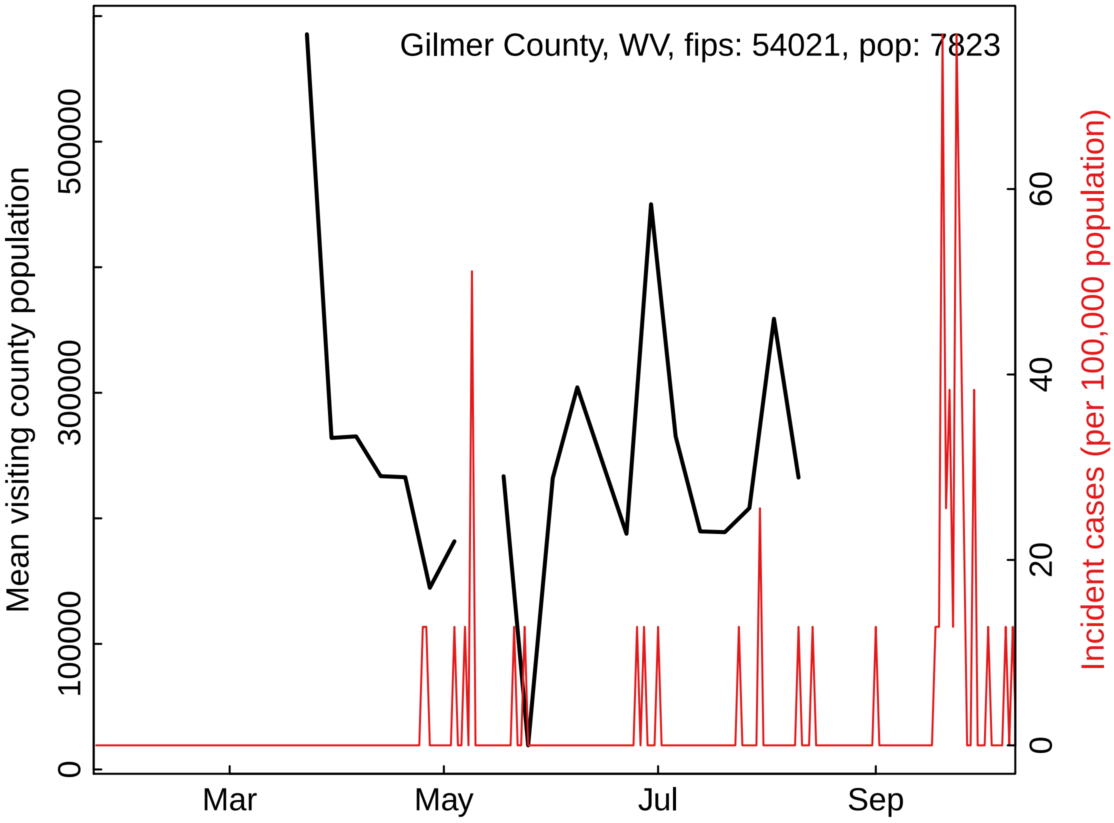

Gilmer County, WV, fips: 54021, pop: 7823
Mean visiting county population
0
100000
300000
500000
Incident cases (per 100,000 population)
0
20
40
60
Mar
May
Jul
Sep

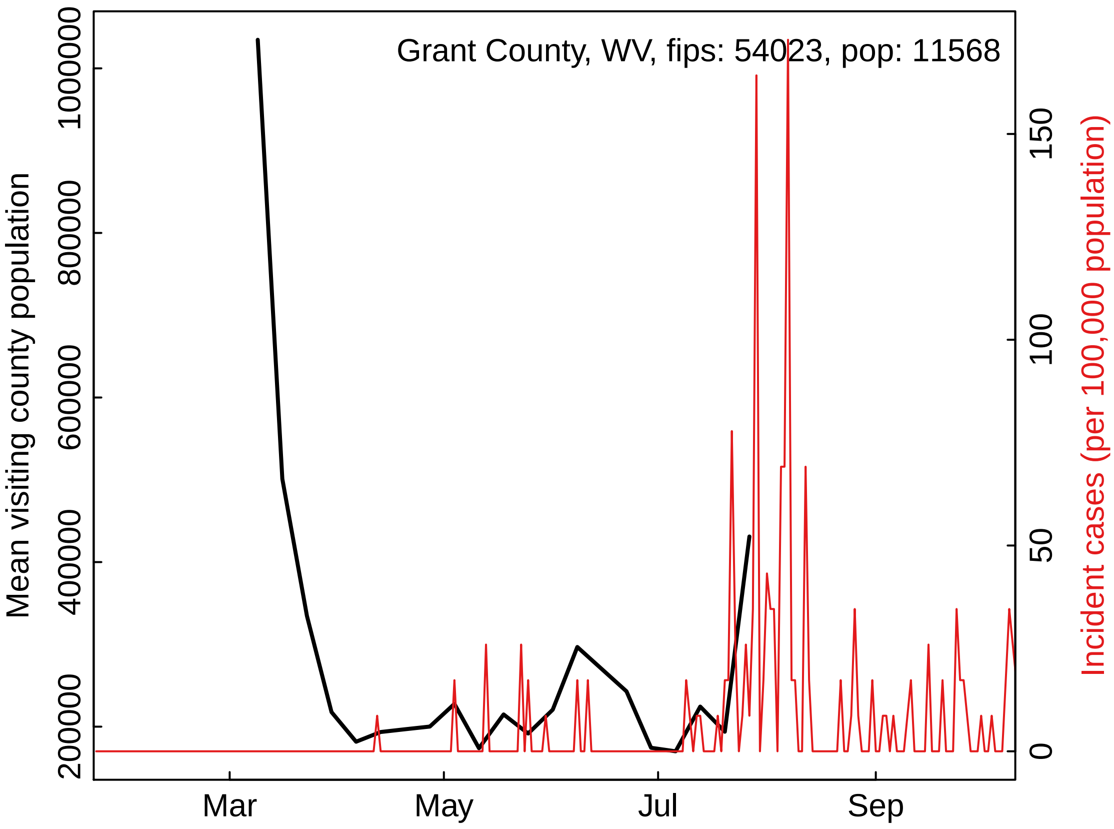

Grant County, WV, fips: 54023, pop: 11568
Mean visiting county population
200000
400000
600000
800000
1000000
Incident cases (per 100,000 population)
0
50
100
150
Mar
May
Jul
Sep

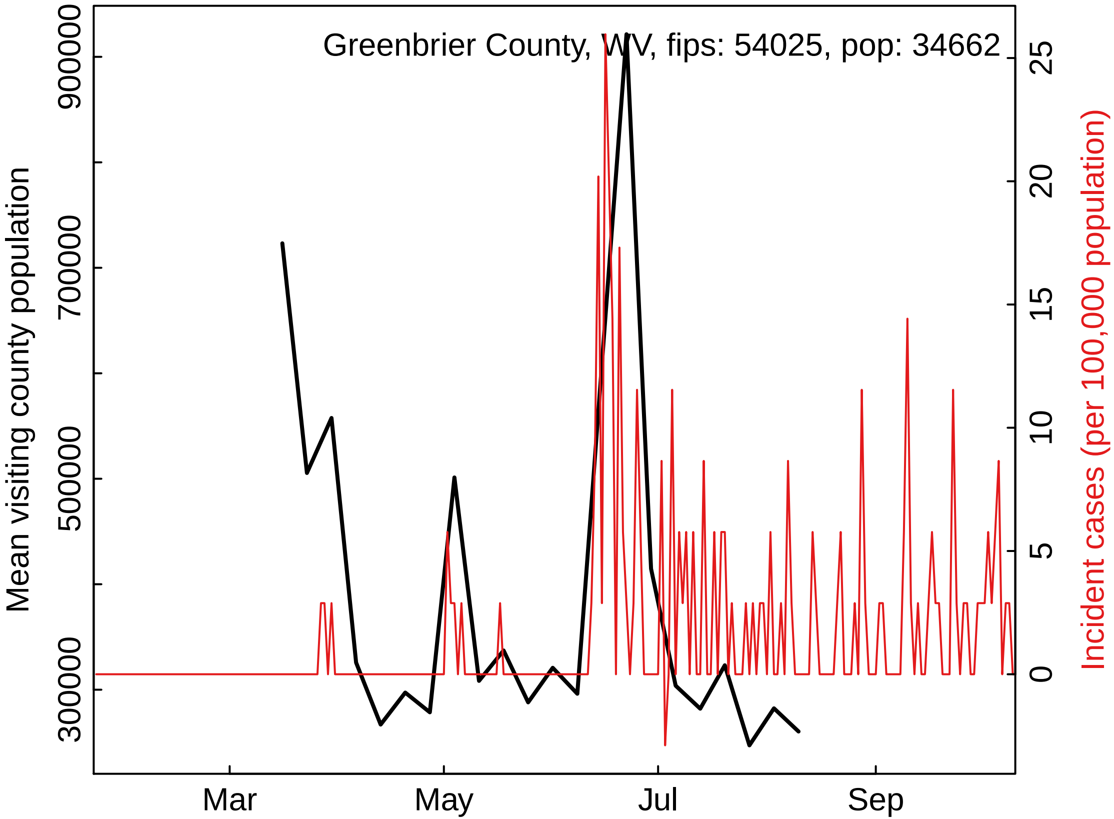

Greenbrier County, WV, fips: 54025, pop: 34662
Mean visiting county population
300000
500000
700000
900000
Incident cases (per 100,000 population)
0
5
10
15
20
25
Mar
May
Jul
Sep

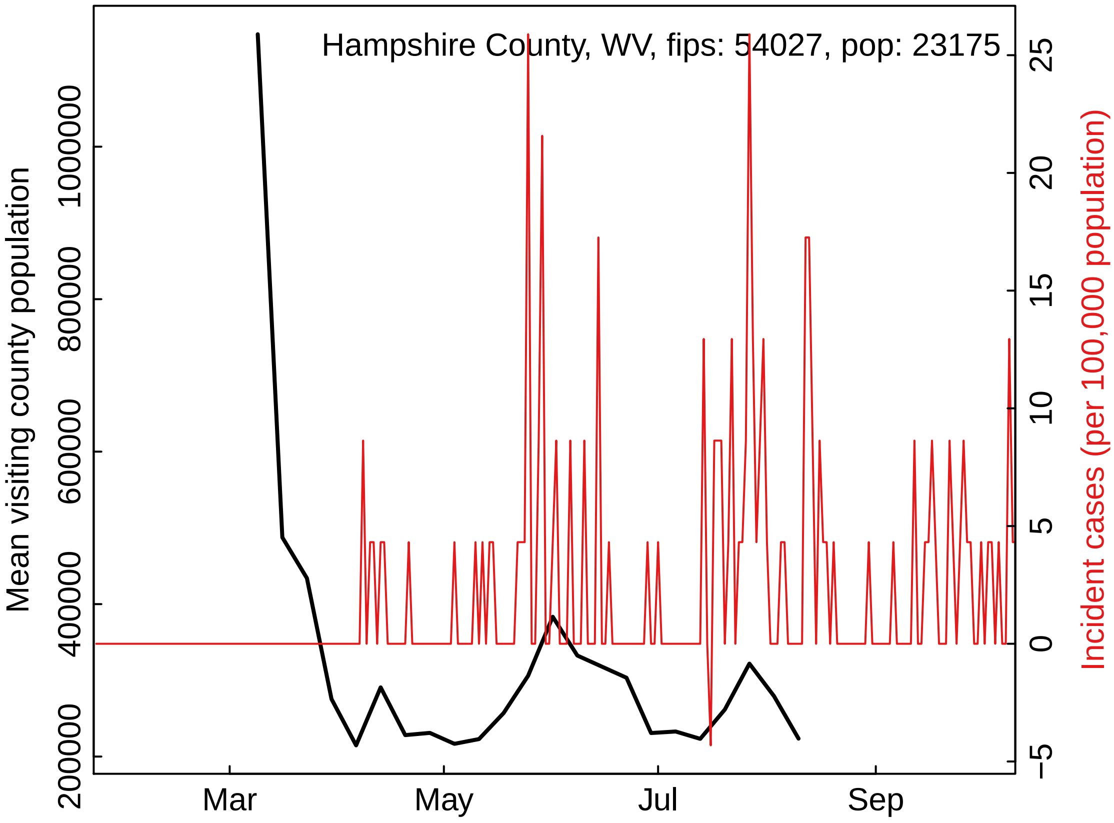

Hampshire County, WV, fips: 54027, pop: 23175
Mean visiting county population
200000
400000
600000
800000
1000000
Incident cases (per 100,000 population)
−5
0
5
10
15
20
25
Mar
May
Jul
Sep

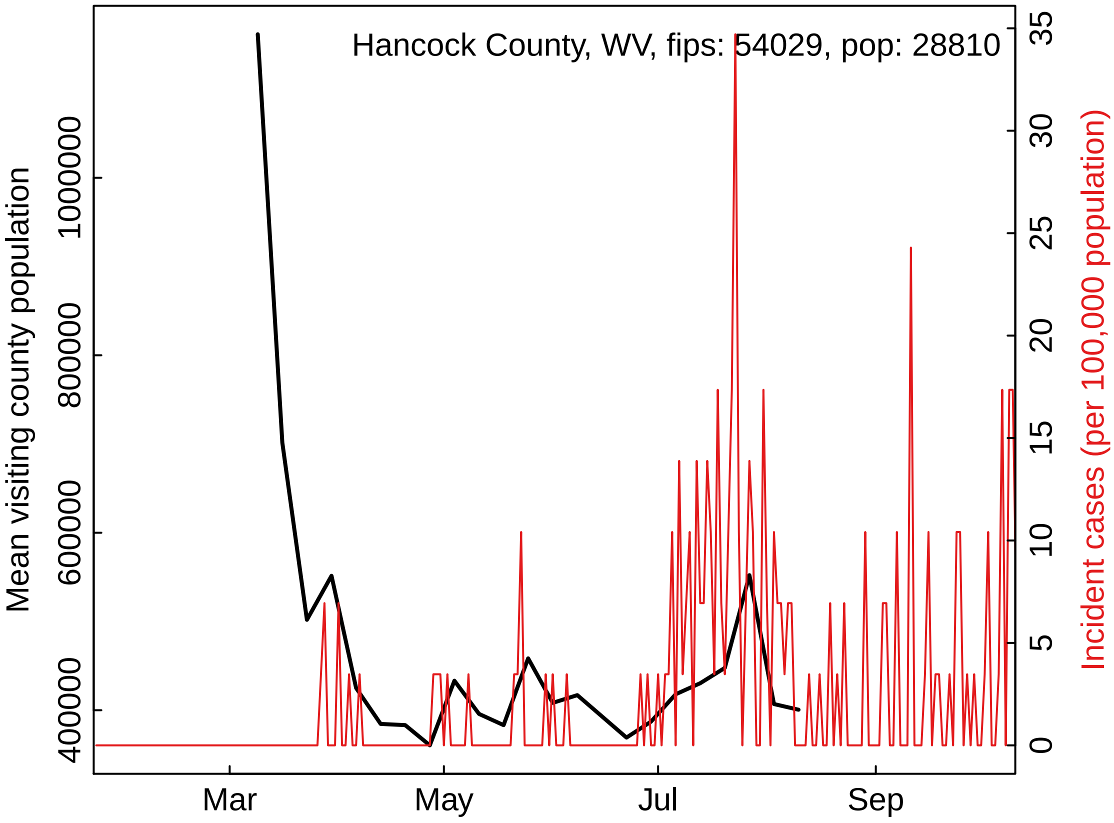
Hancock County, WV, fips: 54029, pop: 28810
Mean visiting county population
400000
600000
800000
1000000
Incident cases (per 100,000 population)
0
5
10
15
20
25
30
35
Mar
May
Jul
Sep

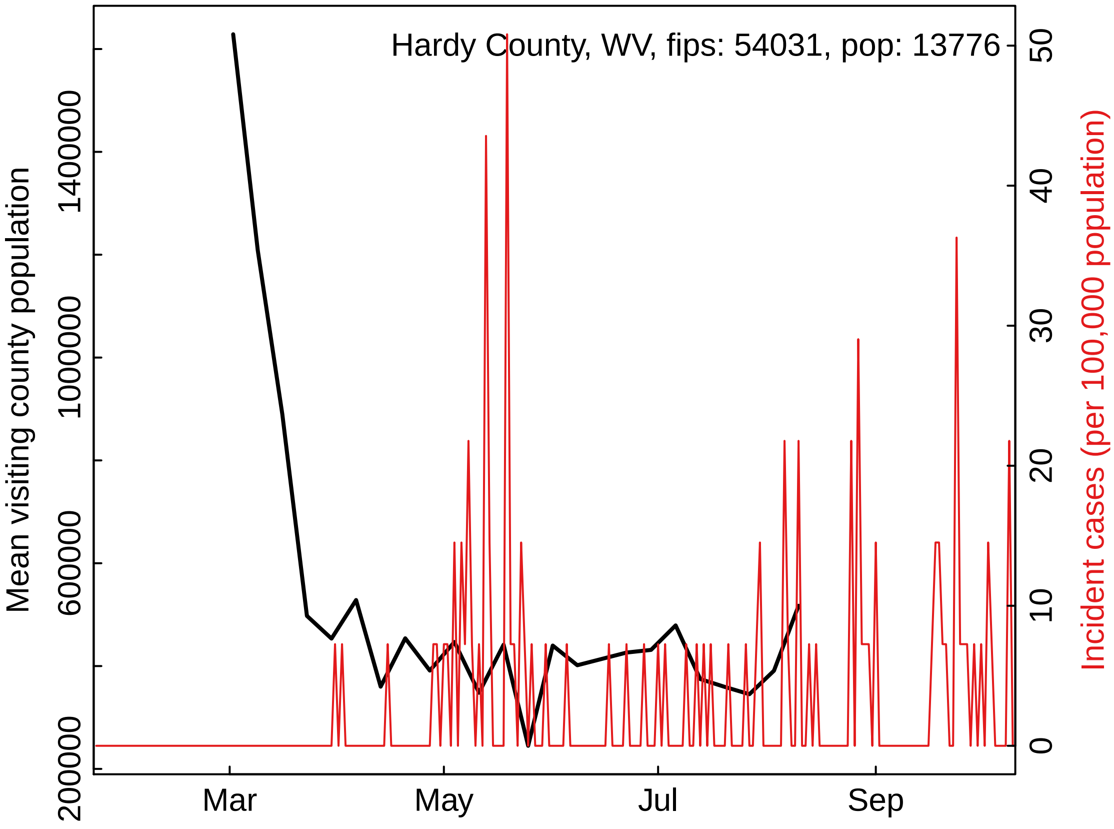

Hardy County, WV, fips: 54031, pop: 13776
Mean visiting county population
200000
600000
1000000
1400000
Incident cases (per 100,000 population)
0
10
20
30
40
50
Mar
May
Jul
Sep

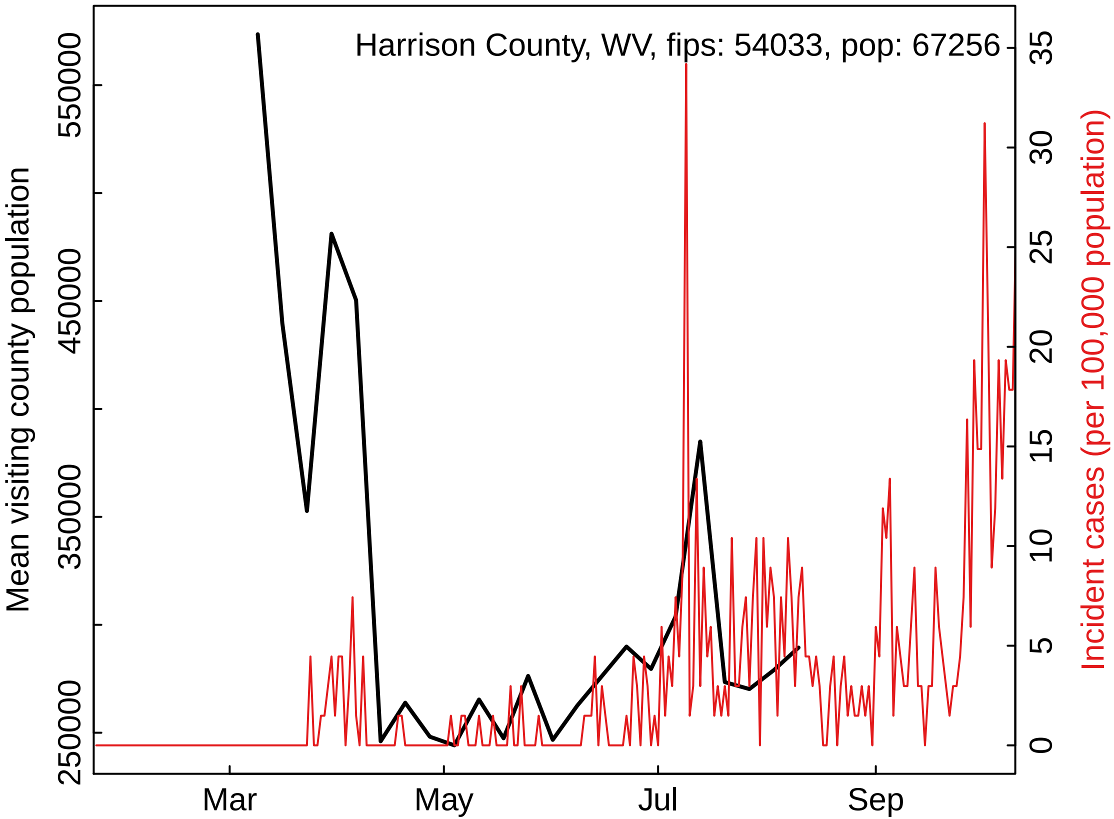

Harrison County, WV, fips: 54033, pop: 67256
Mean visiting county population
250000
350000
450000
550000
Incident cases (per 100,000 population)
0
5
10
15
20
25
30
35
Mar
May
Jul
Sep

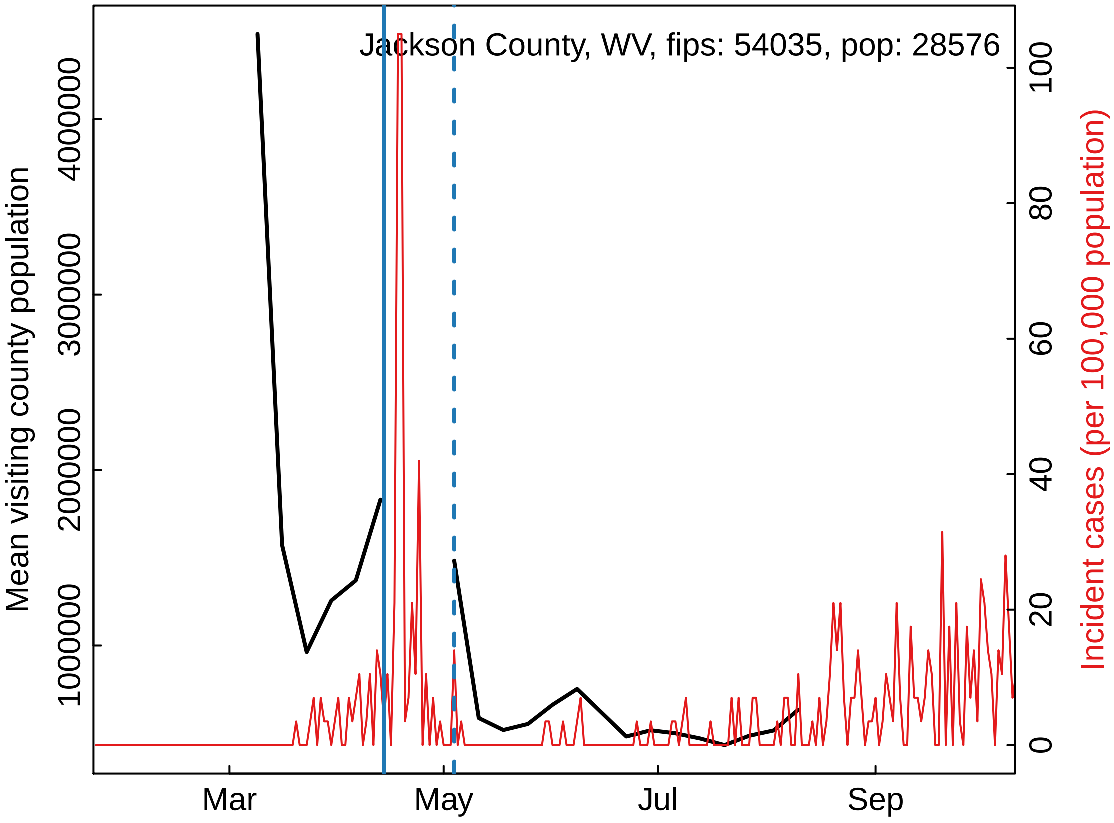

Jackson County, WV, fips: 54035, pop: 28576
Mean visiting county population
1000000
2000000
3000000
4000000
Incident cases (per 100,000 population)
0
20
40
60
80
100
Mar
May
Jul
Sep

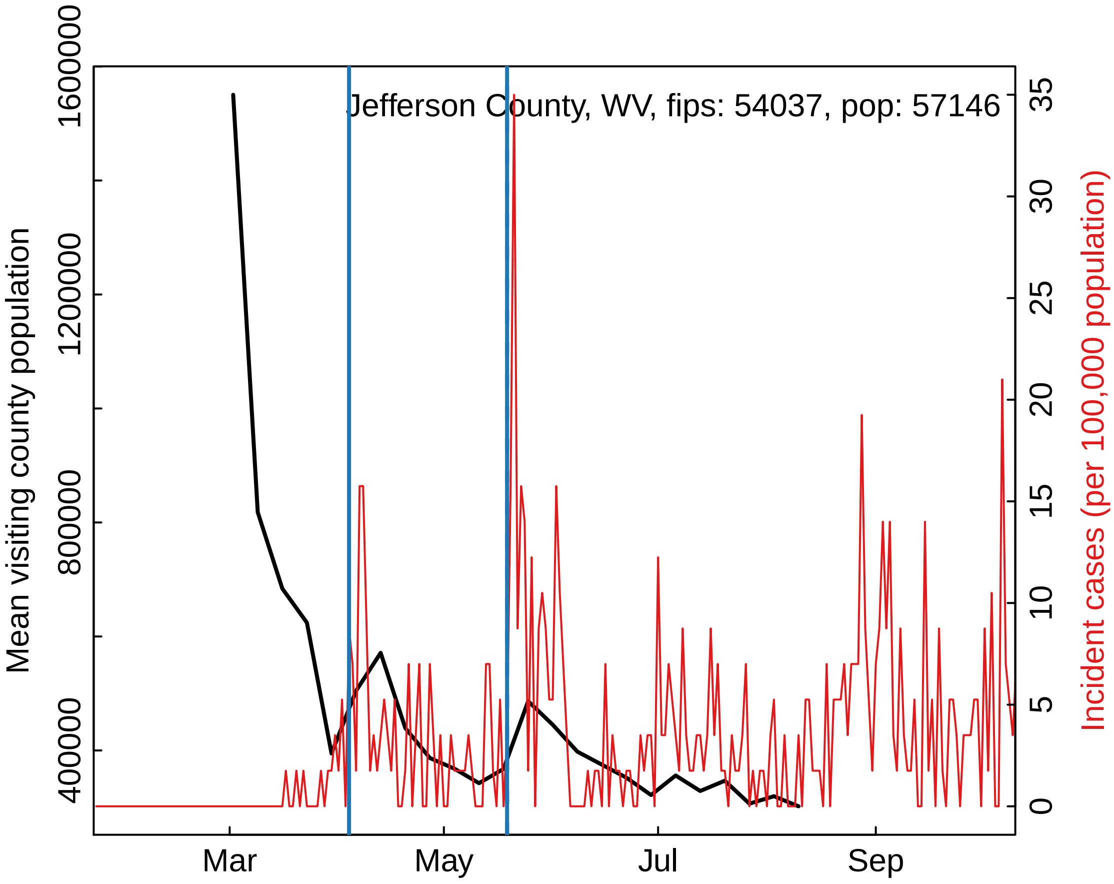
Jefferson County, WV, fips: 54037, pop: 57146
Mean visiting county population
400000
800000
1200000
1600000
Incident cases (per 100,000 population)
0
5
10
15
20
25
30
35
Mar
May
Jul
Sep

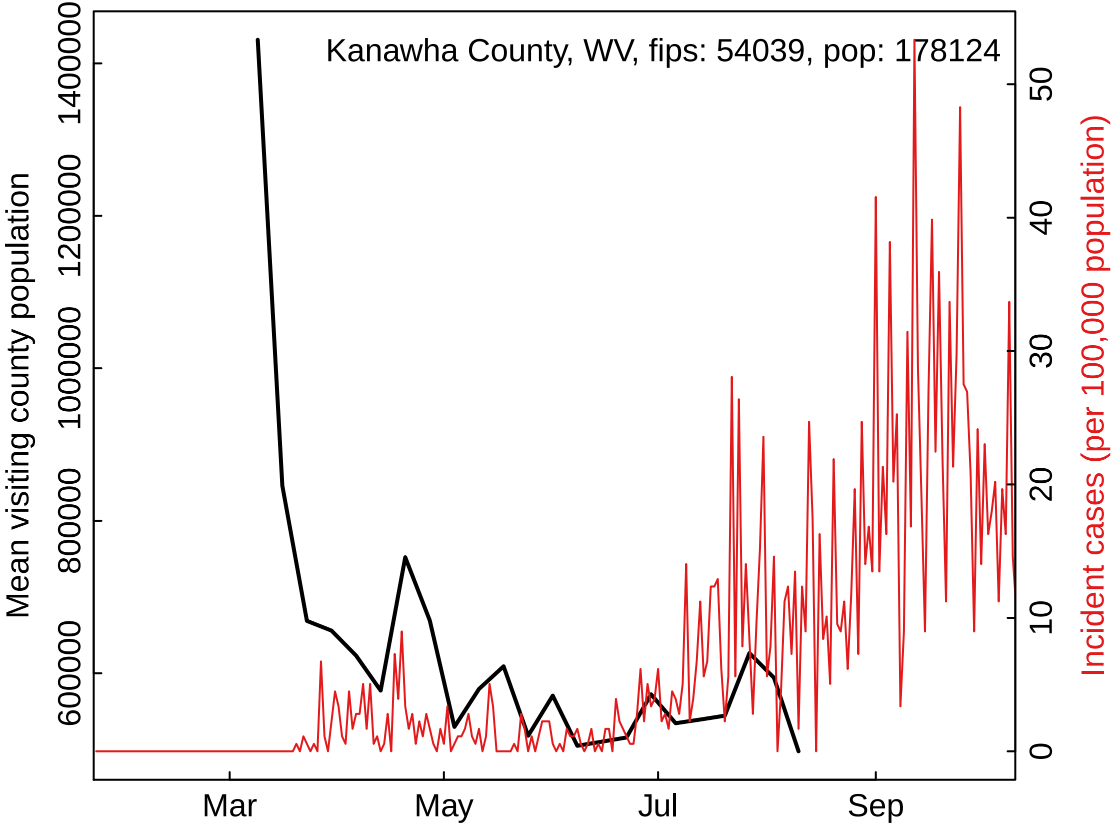

Kanawha County, WV, fips: 54039, pop: 178124
Mean visiting county population
600000
800000
1000000
1200000
1400000
Incident cases (per 100,000 population)
0
10
20
30
40
50
Mar
May
Jul
Sep

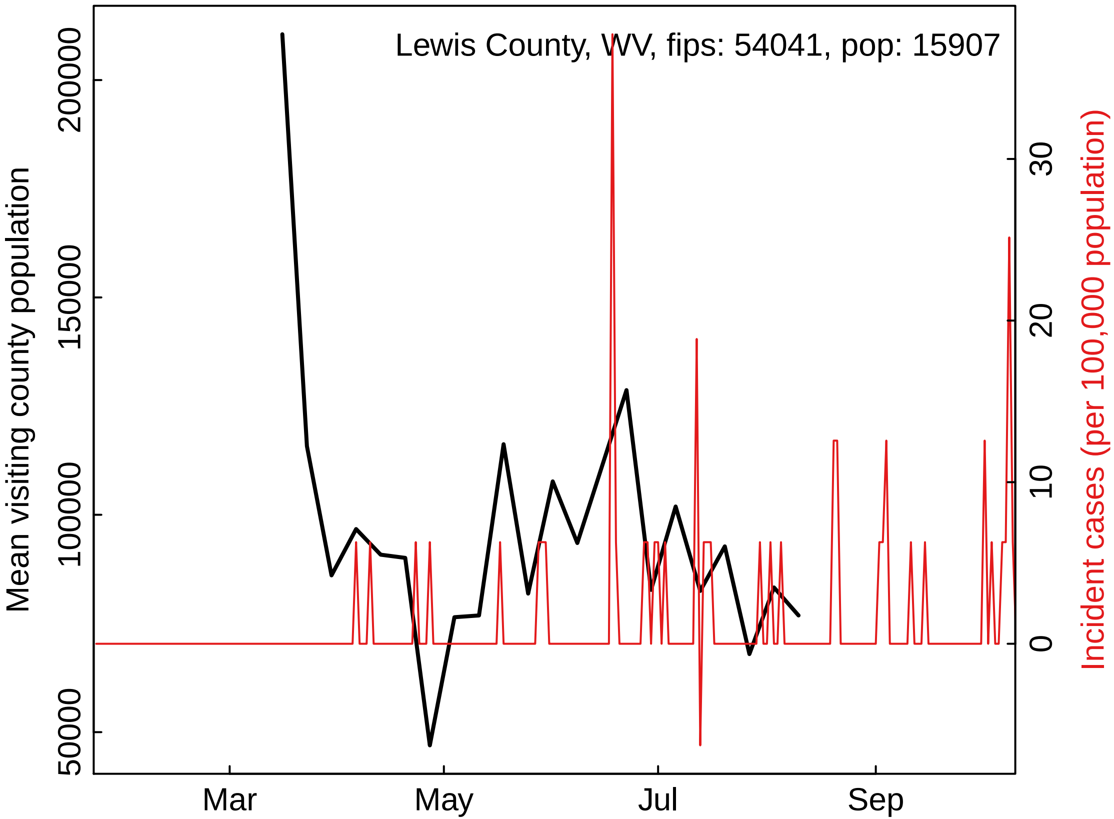
Lewis County, WV, fips: 54041, pop: 15907
Mean visiting county population
50000
100000
150000
200000
Incident cases (per 100,000 population)
0
10
20
30
Mar
May
Jul
Sep

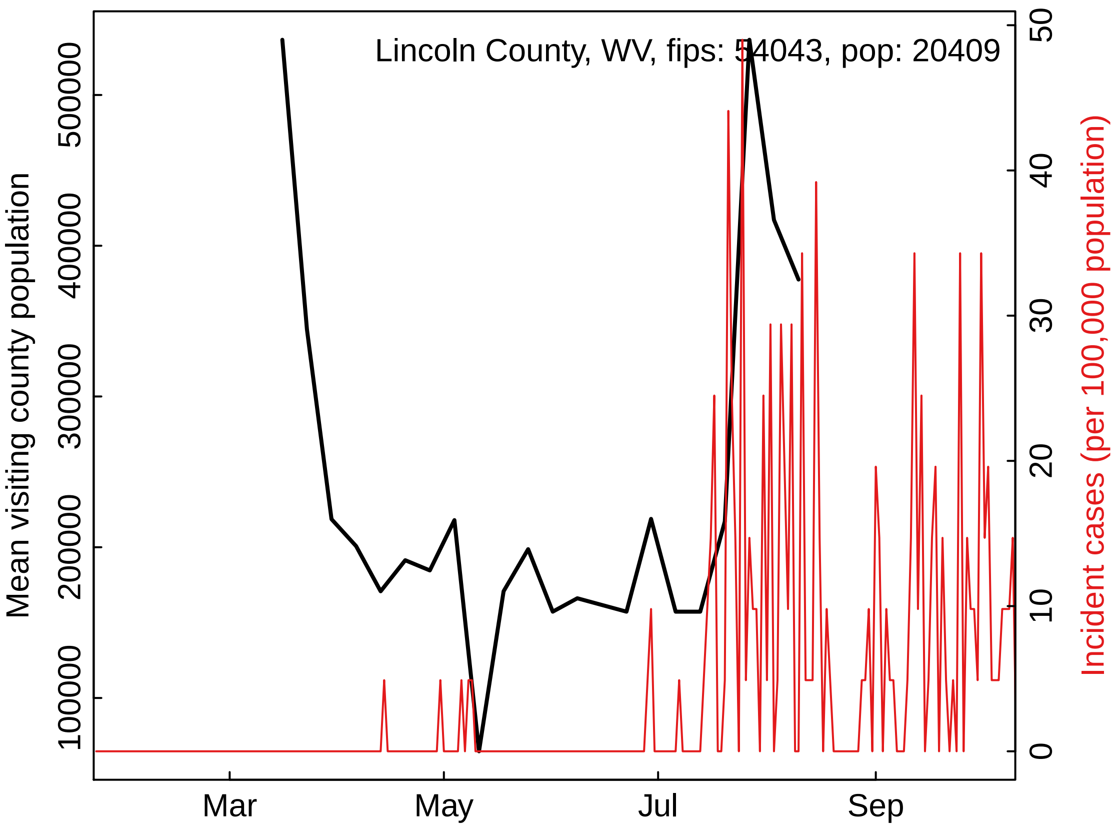

Lincoln County, WV, fips: 54043, pop: 20409
Mean visiting county population
100000
200000
300000
400000
500000
Incident cases (per 100,000 population)
0
10
20
30
40
50
Mar
May
Jul
Sep

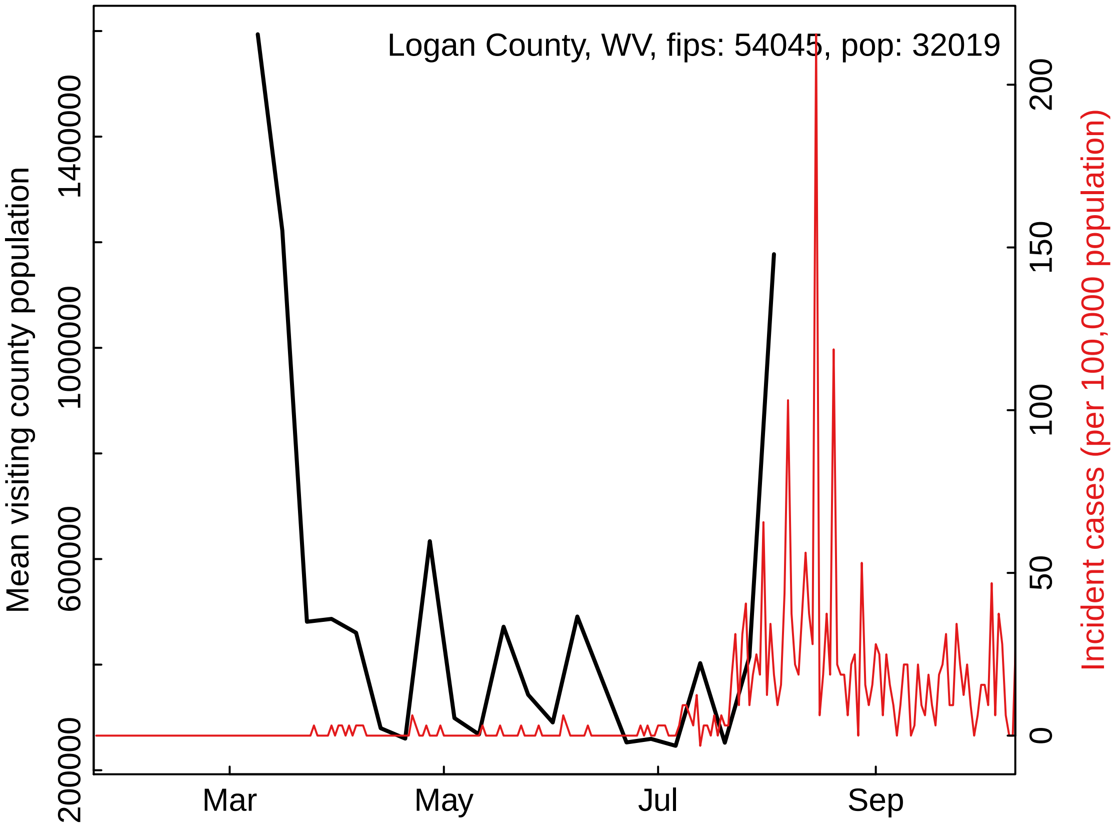

Logan County, WV, fips: 54045, pop: 32019
Mean visiting county population
Incident cases (per 100,000 population)
200000
600000
1000000
1400000
0
50
100
150
200
Mar
May
Jul
Sep

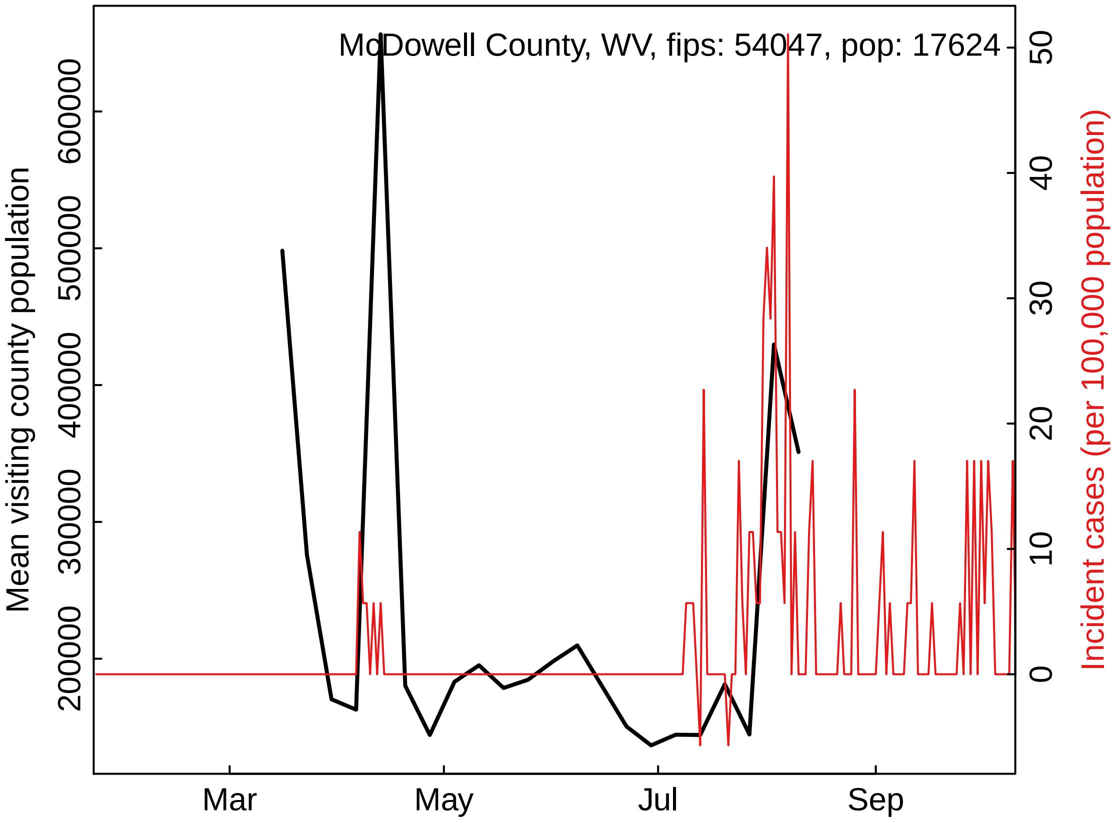
McDowell County, WV, fips: 54047, pop: 17624
Mean visiting county population
200000
300000
400000
500000
600000
Incident cases (per 100,000 population)
0
10
20
30
40
50
Mar
May
Jul
Sep

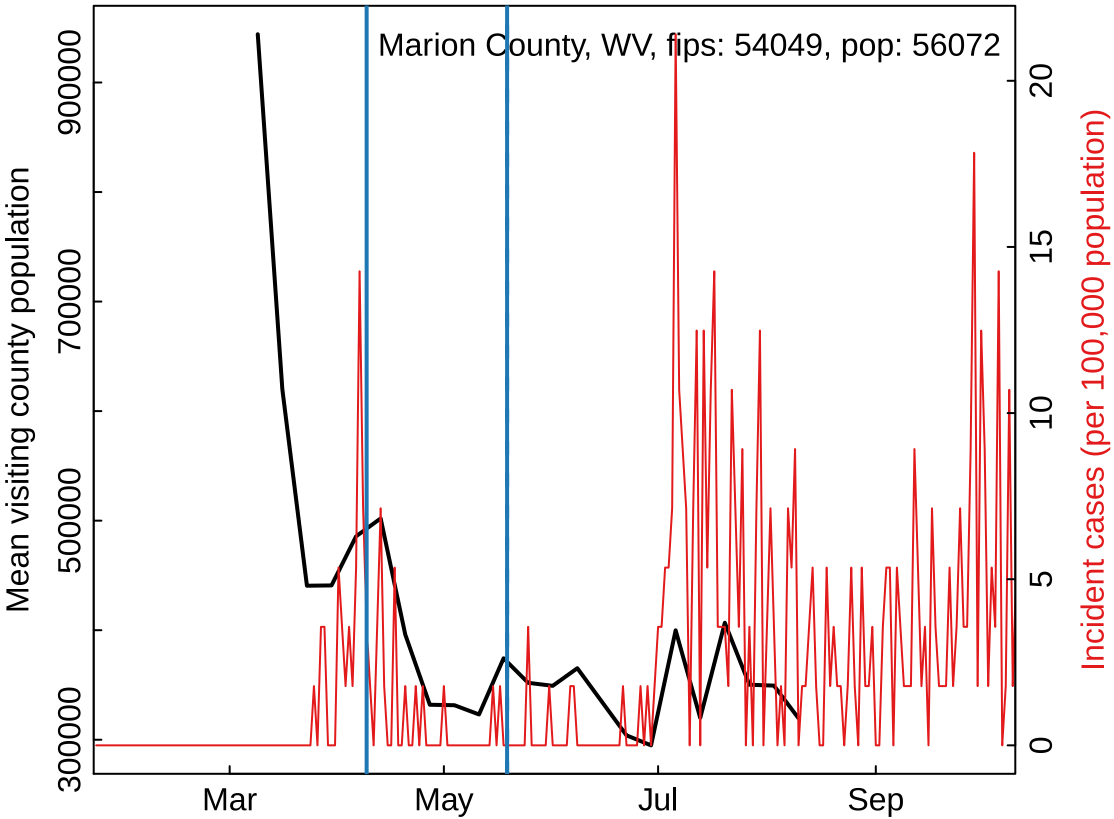

Marion County, WV, fips: 54049, pop: 56072
Mean visiting county population
300000
500000
700000
900000
Mar
May
Jul
Sep
0
5
10
15
20
Incident cases (per 100,000 population)

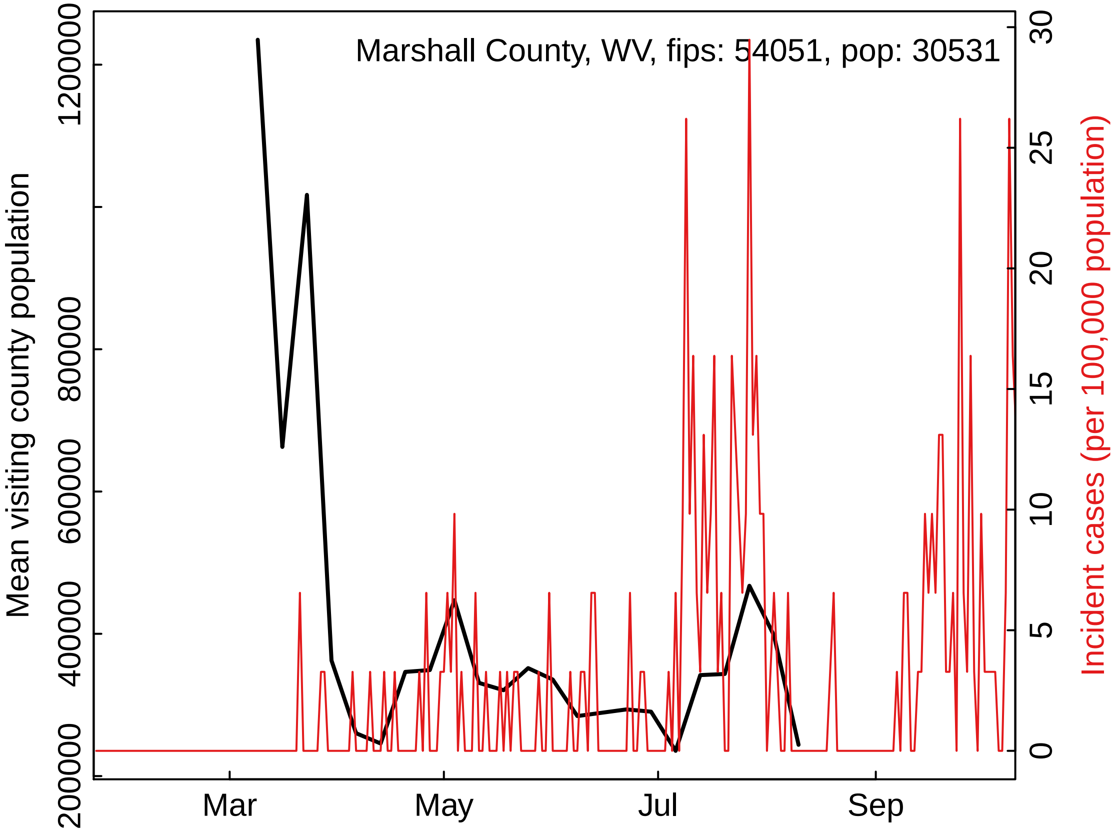
Marshall County, WV, fips: 54051, pop: 30531
Mean visiting county population
200000
400000
600000
800000
1200000
Incident cases (per 100,000 population)
0
5
10
15
20
25
30
Mar
May
Jul
Sep

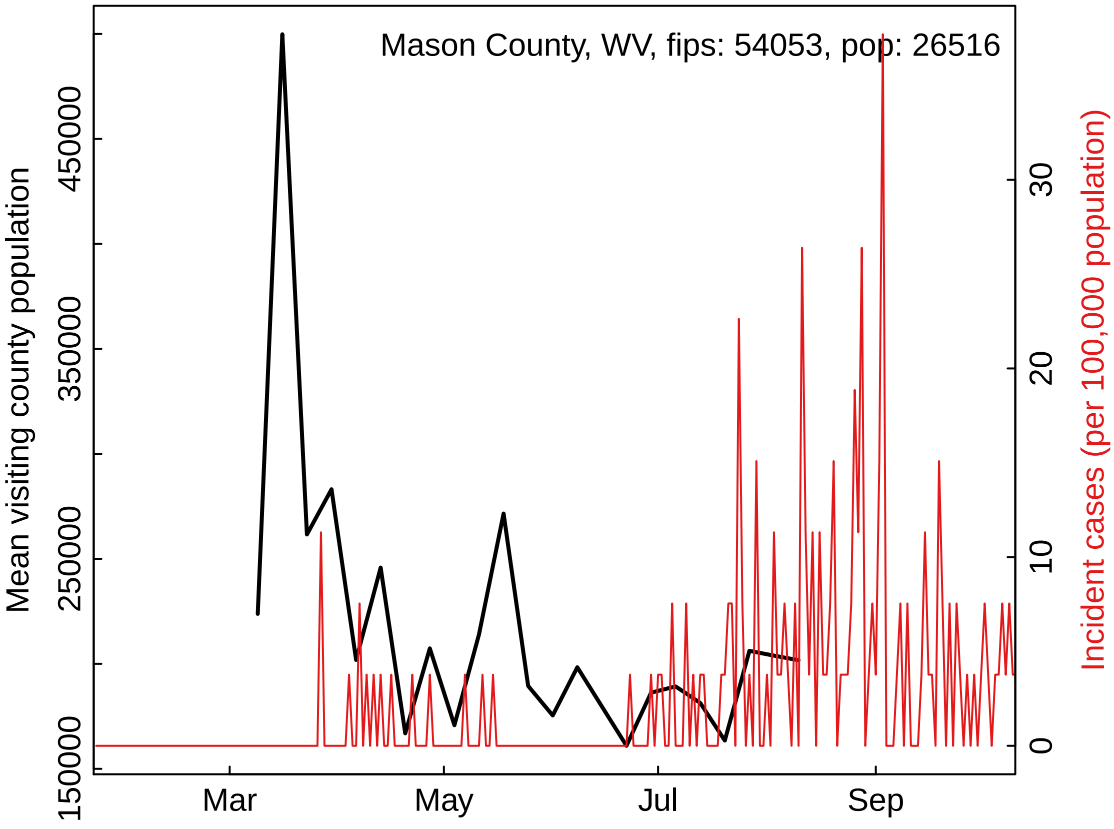
Mason County, WV, fips: 54053, pop: 26516
Mean visiting county population
150000
250000
350000
450000
Incident cases (per 100,000 population)
0
10
20
30
Mar
May
Jul
Sep

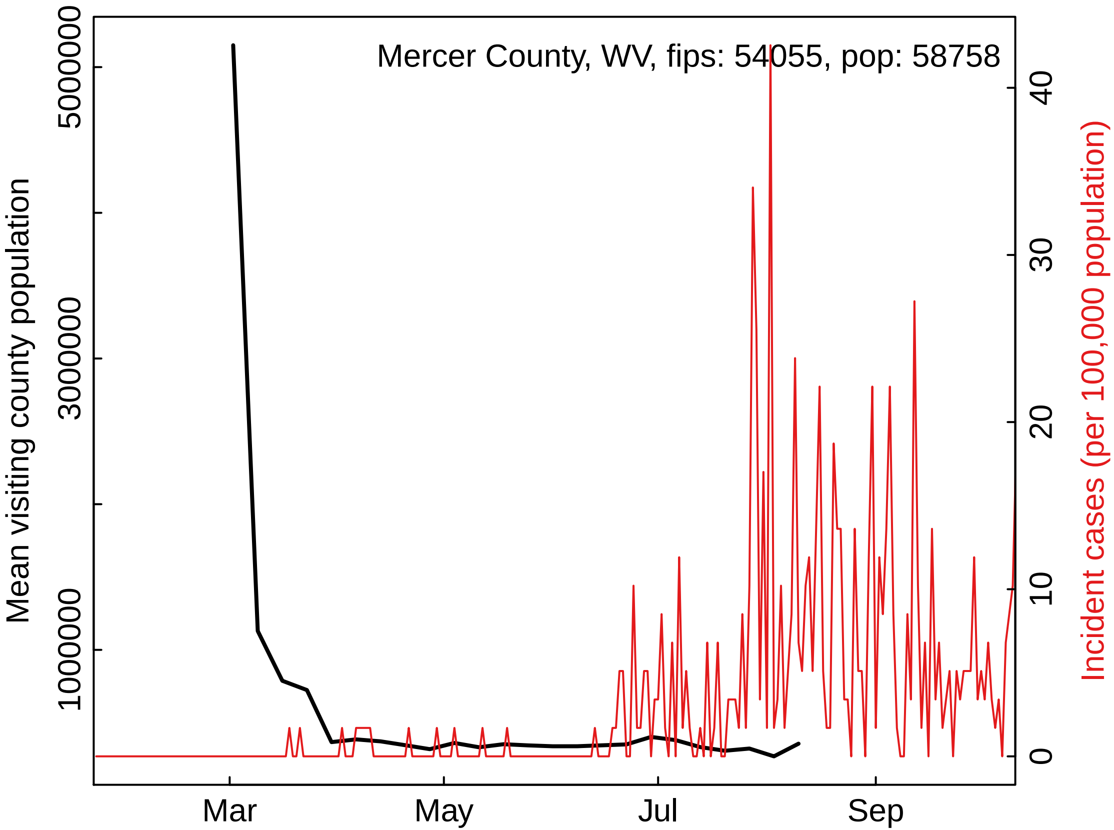

Mercer County, WV, fips: 54055, pop: 58758
Mean visiting county population
1000000
3000000
5000000
Incident cases (per 100,000 population)
0
10
20
30
40
Mar
May
Jul
Sep

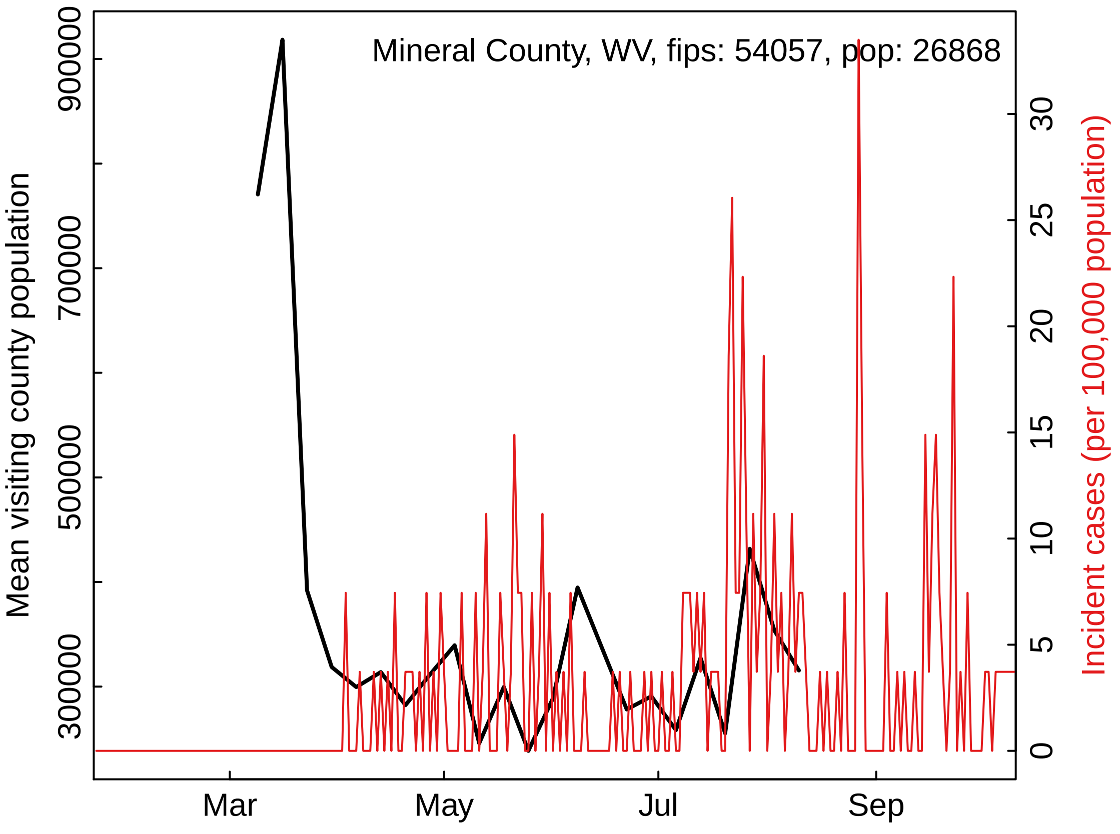

Mineral County, WV, fips: 54057, pop: 26868
Mean visiting county population
300000
500000
700000
900000
Incident cases (per 100,000 population)
0
5
10
15
20
25
30
Mar
May
Jul
Sep

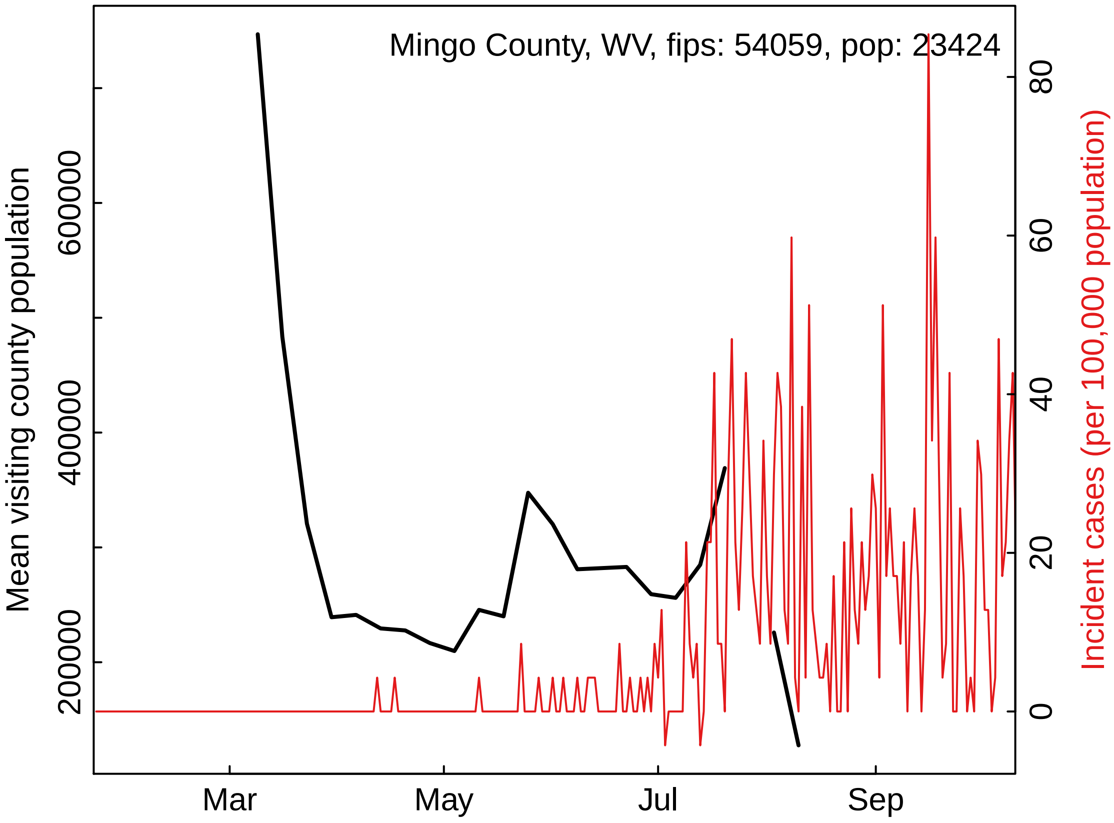
Mingo County, WV, fips: 54059, pop: 23424
Mean visiting county population
200000
400000
600000
Incident cases (per 100,000 population)
0
20
40
60
80
Mar
May
Jul
Sep

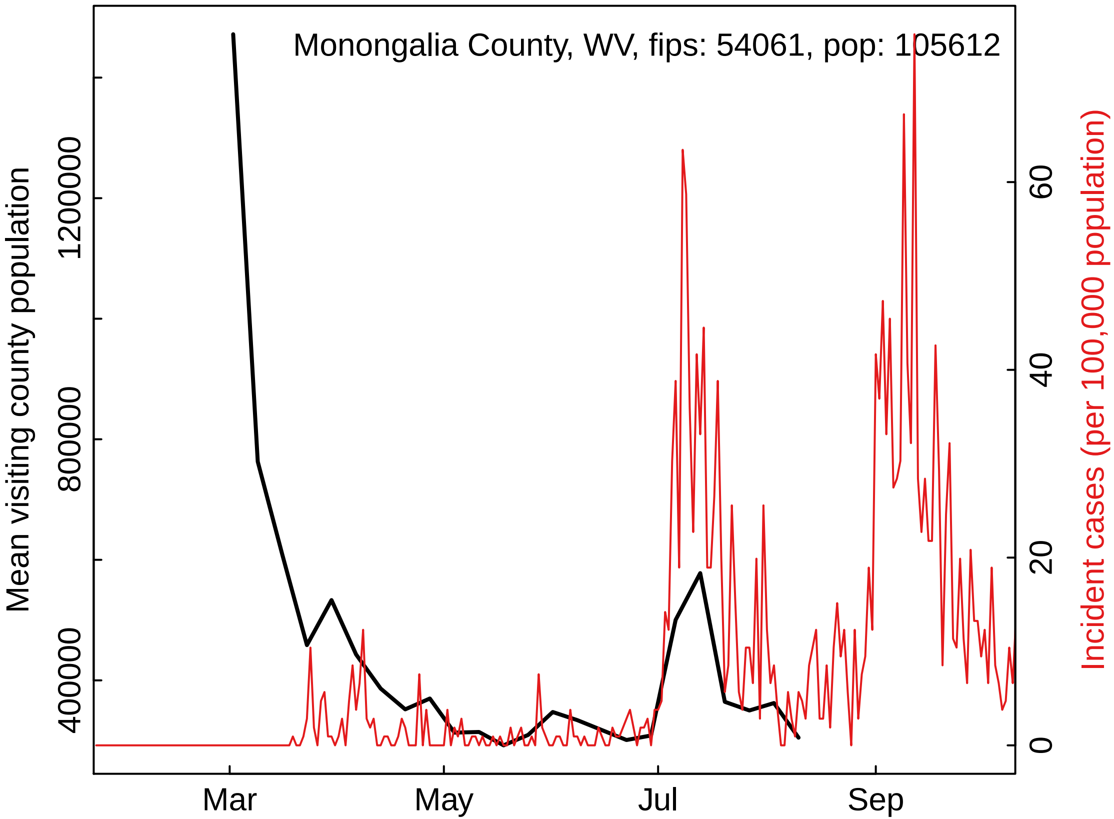

Monongalia County, WV, fips: 54061, pop: 105612
Mean visiting county population
400000
800000
1200000
Incident cases (per 100,000 population)
0
20
40
60
Mar
May
Jul
Sep

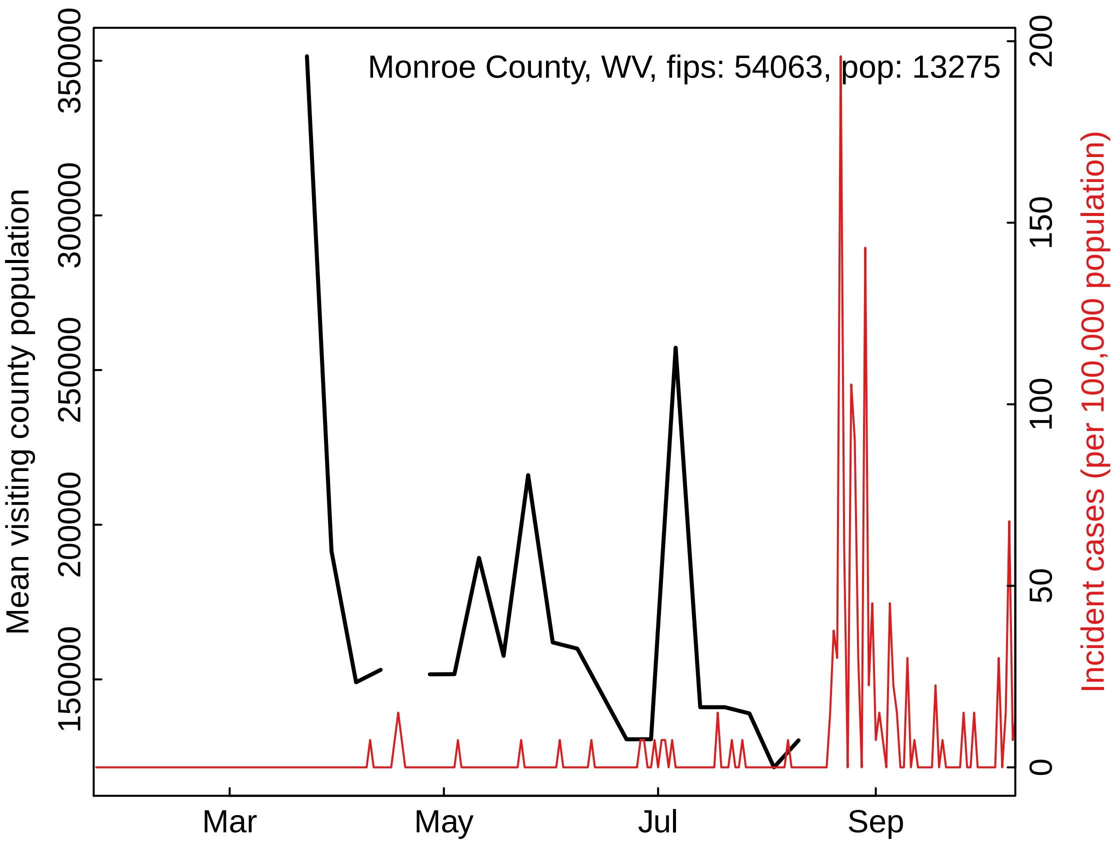

Monroe County, WV, fips: 54063, pop: 13275
Mean visiting county population
150000
200000
250000
300000
350000
Incident cases (per 100,000 population)
0
50
100
150
200
Mar
May
Jul
Sep

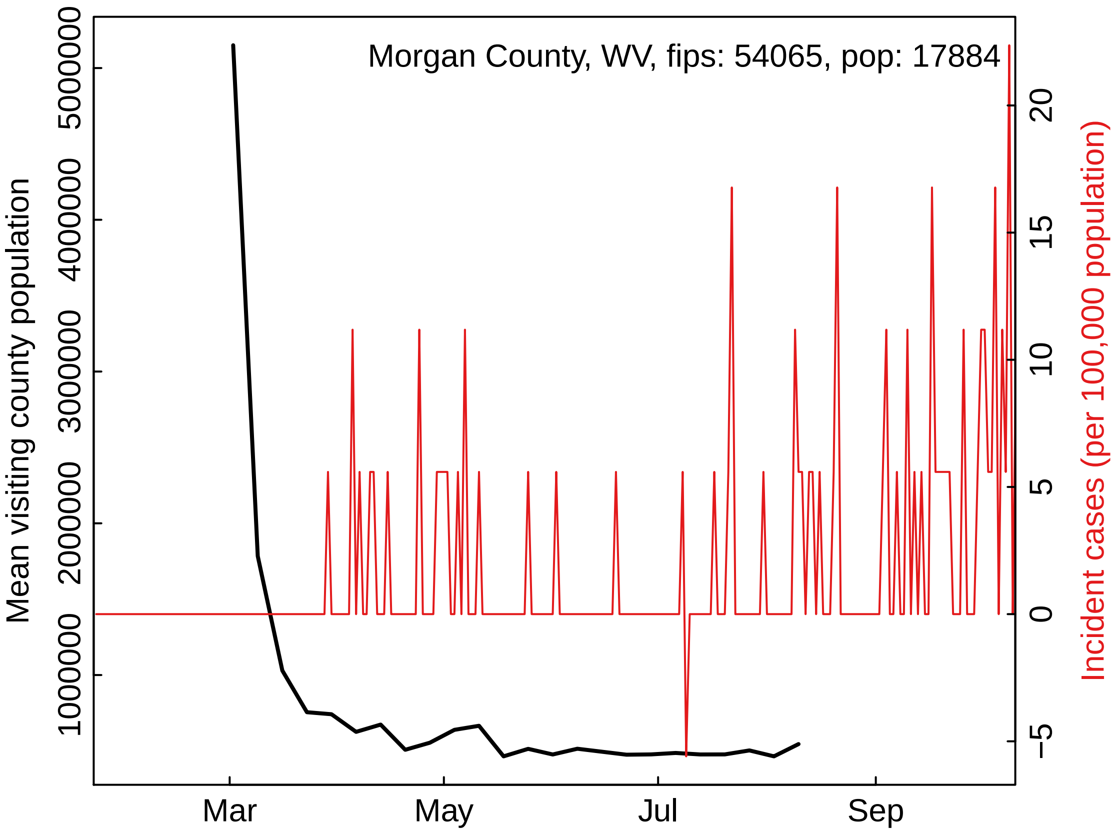

Morgan County, WV, fips: 54065, pop: 17884
Mean visiting county population
1000000
2000000
3000000
4000000
5000000
Incident cases (per 100,000 population)
−5
0
5
10
15
20
Mar
May
Jul
Sep

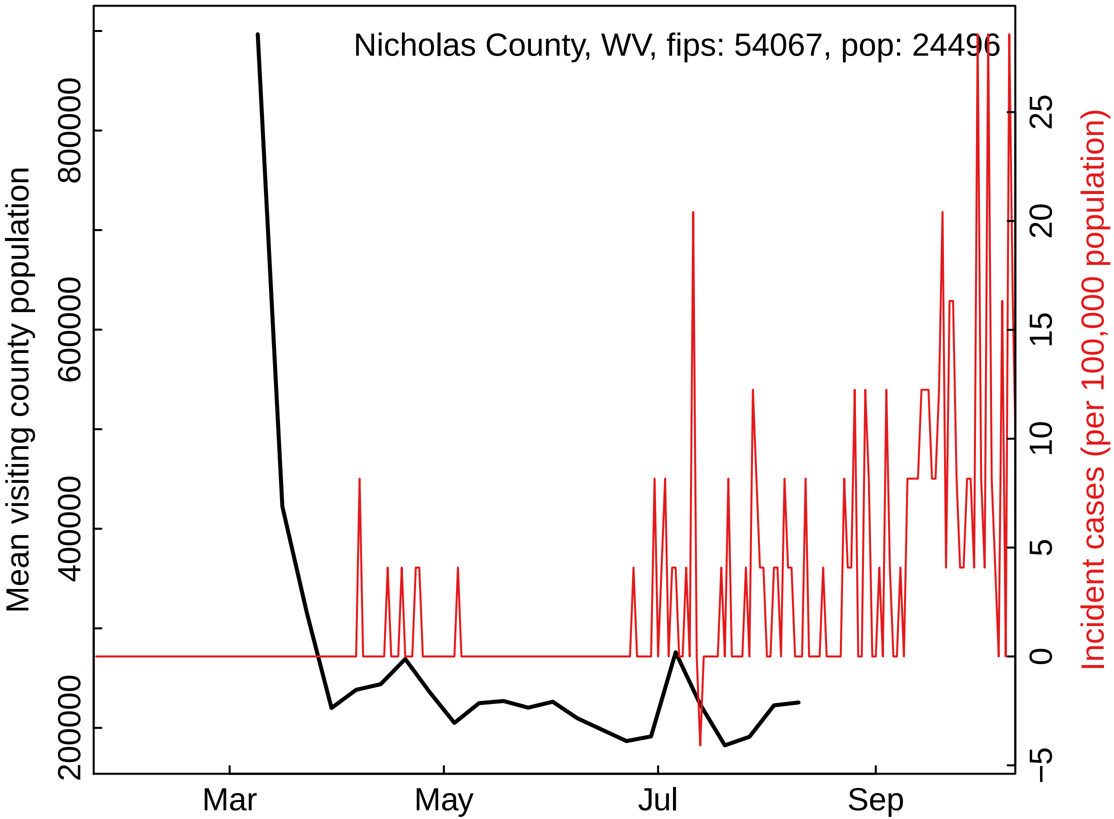

Nicholas County, WV, fips: 54067, pop: 24496
Mean visiting county population
200000
400000
600000
800000
Mar
May
Jul
Sep
Incident cases (per 100,000 population)
−5
0
5
10
15
20
25

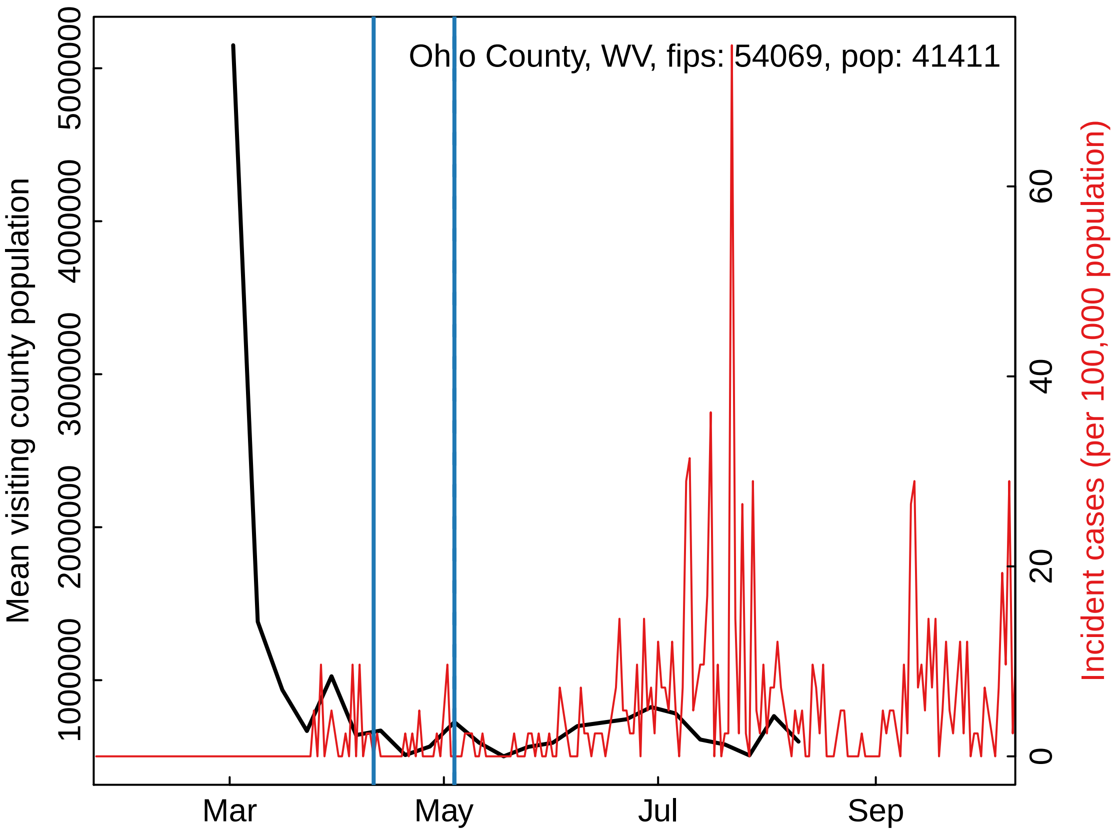
Ohio County, WV, fips: 54069, pop: 41411
Mean visiting county population
1000000
2000000
3000000
4000000
5000000
Incident cases (per 100,000 population)
0
20
40
60
Mar
May
Jul
Sep

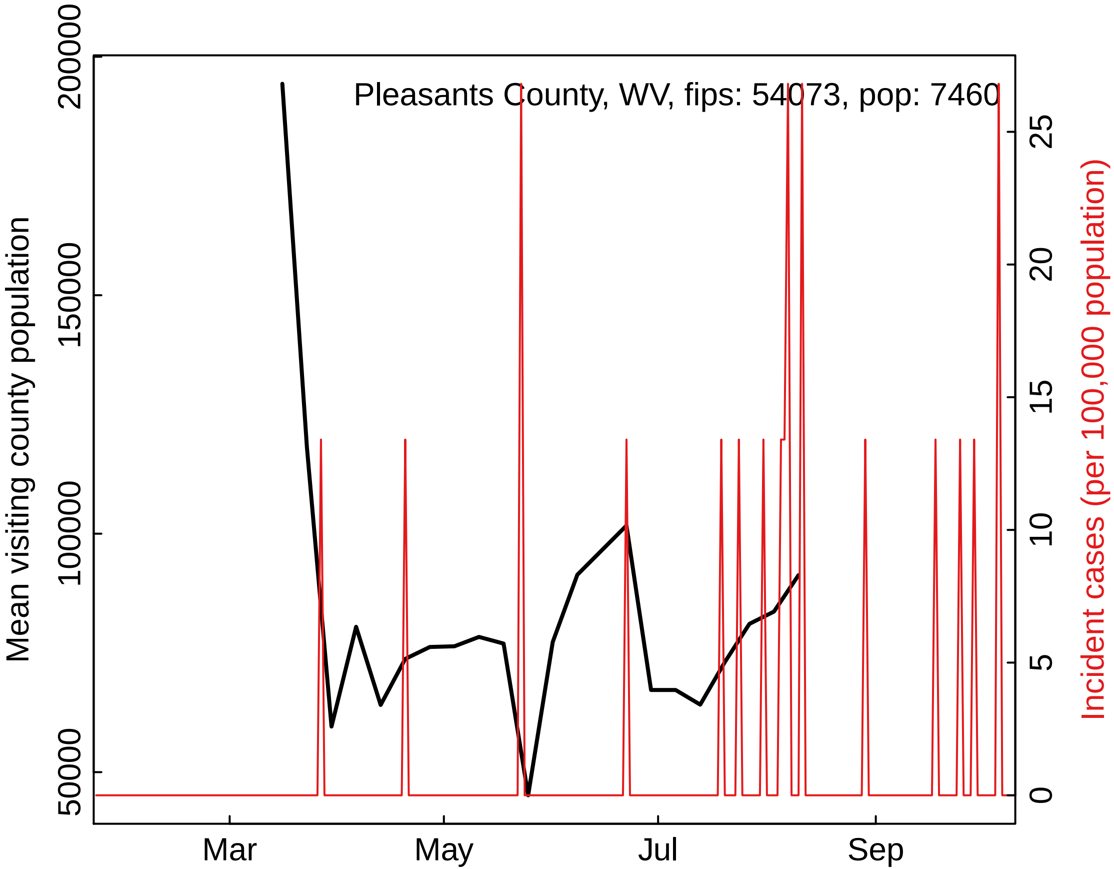
Pleasants County, WV, fips: 54073, pop: 7460
Mean visiting county population
50000
100000
150000
200000
Incident cases (per 100,000 population)
0
5
10
15
20
25
Mar
May
Jul
Sep

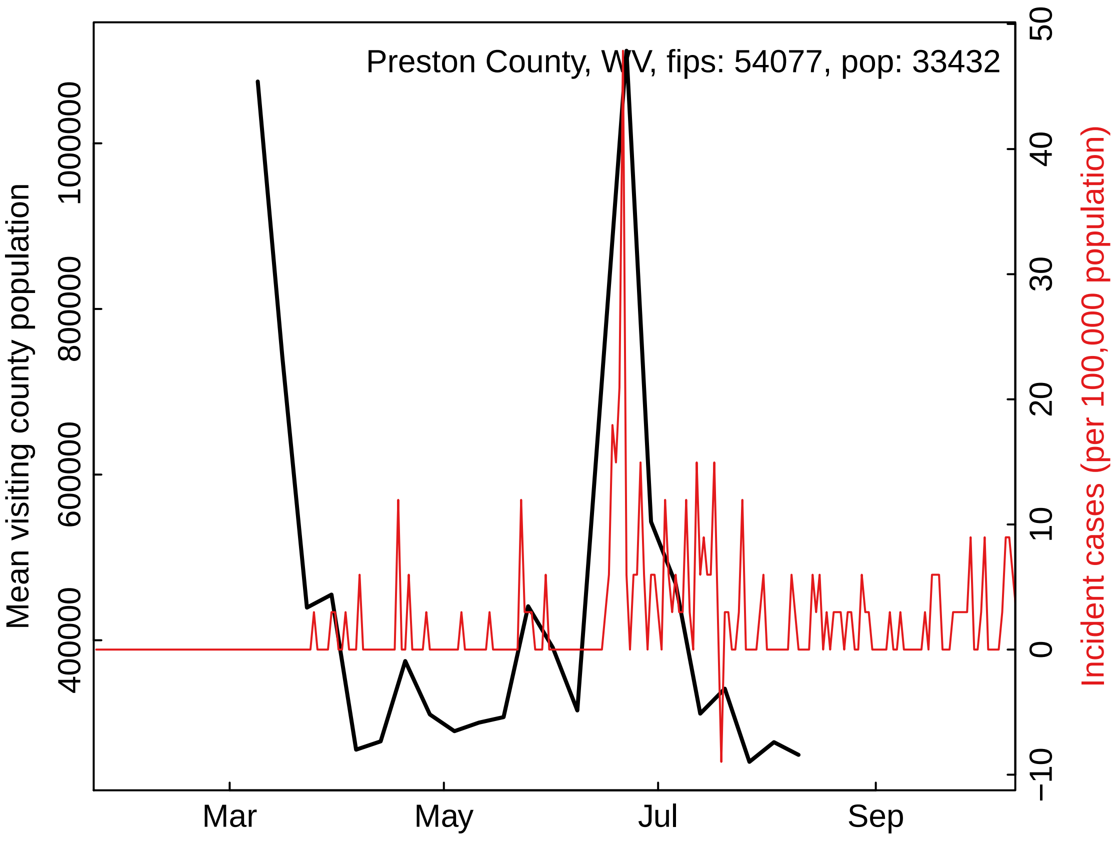

Preston County, WV, fips: 54077, pop: 33432
Mean visiting county population
400000
600000
800000
1000000
Mar
May
Jul
Sep
−10
0
10
20
30
40
50
Incident cases (per 100,000 population)

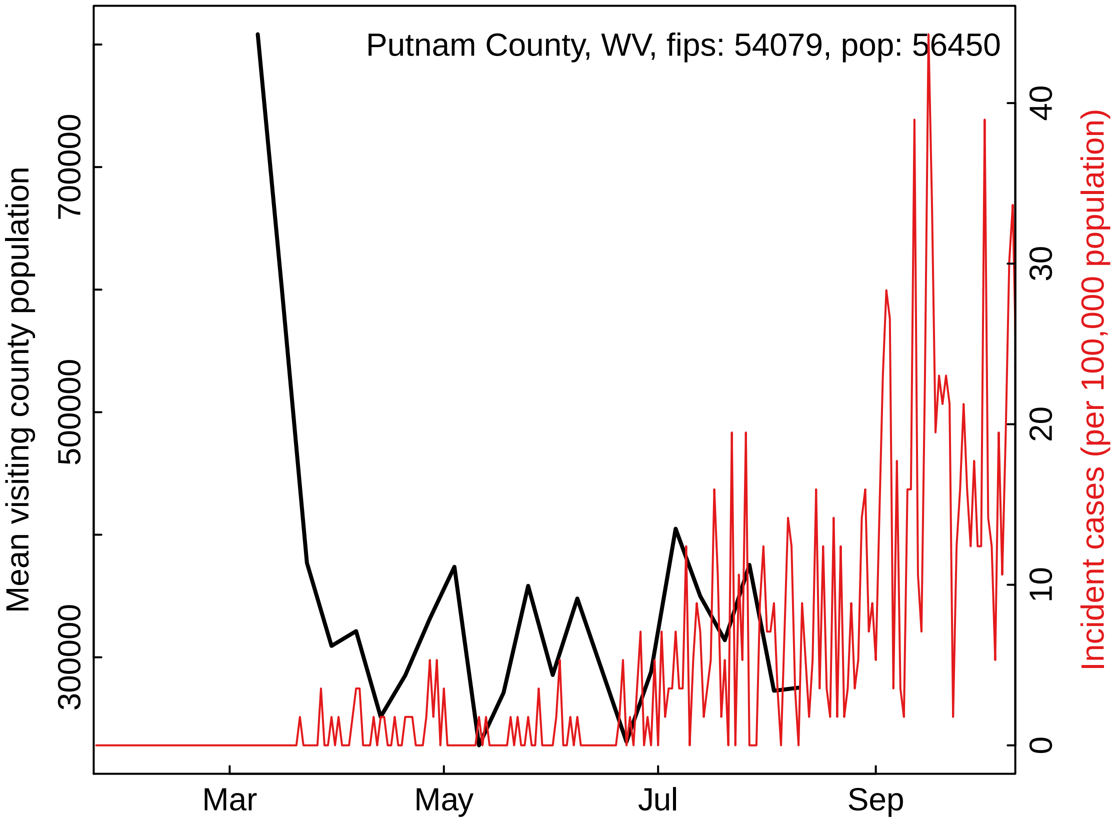
Putnam County, WV, fips: 54079, pop: 56450
Mean visiting county population
300000
500000
700000
Incident cases (per 100,000 population)
0
10
20
30
40
Mar
May
Jul
Sep

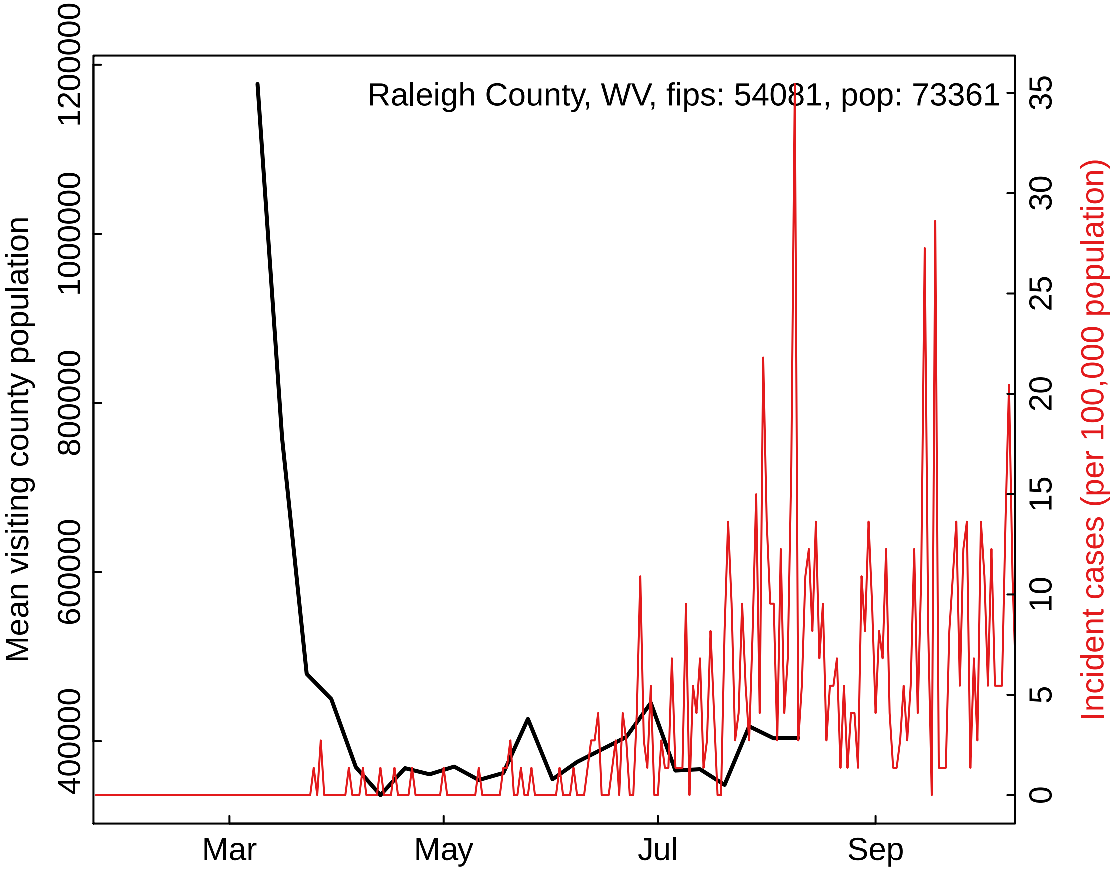
Raleigh County, WV, fips: 54081, pop: 73361
Mean visiting county population
400000
600000
800000
1000000
1200000
Incident cases (per 100,000 population)
0
5
10
15
20
25
30
35
Mar
May
Jul
Sep

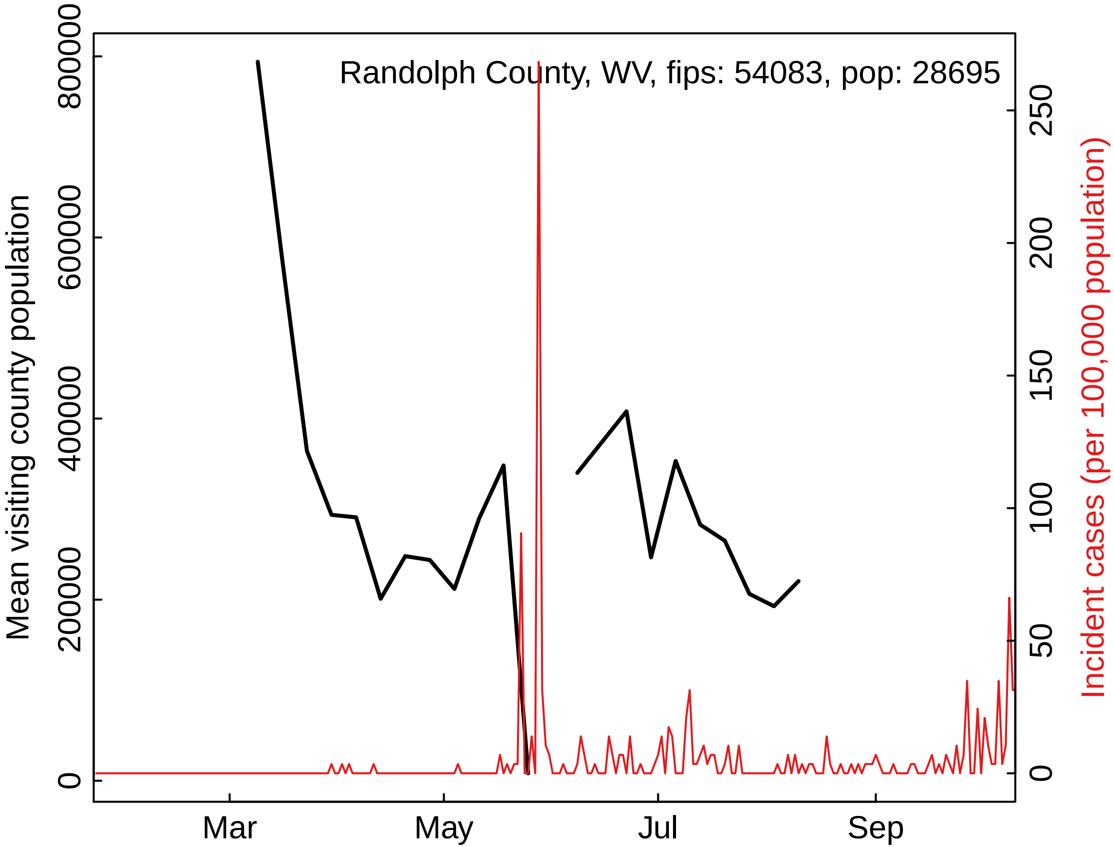

Randolph County, WV, fips: 54083, pop: 28695
Mean visiting county population
0
200000
400000
600000
800000
Incident cases (per 100,000 population)
0
50
100
150
200
250
Mar
May
Jul
Sep

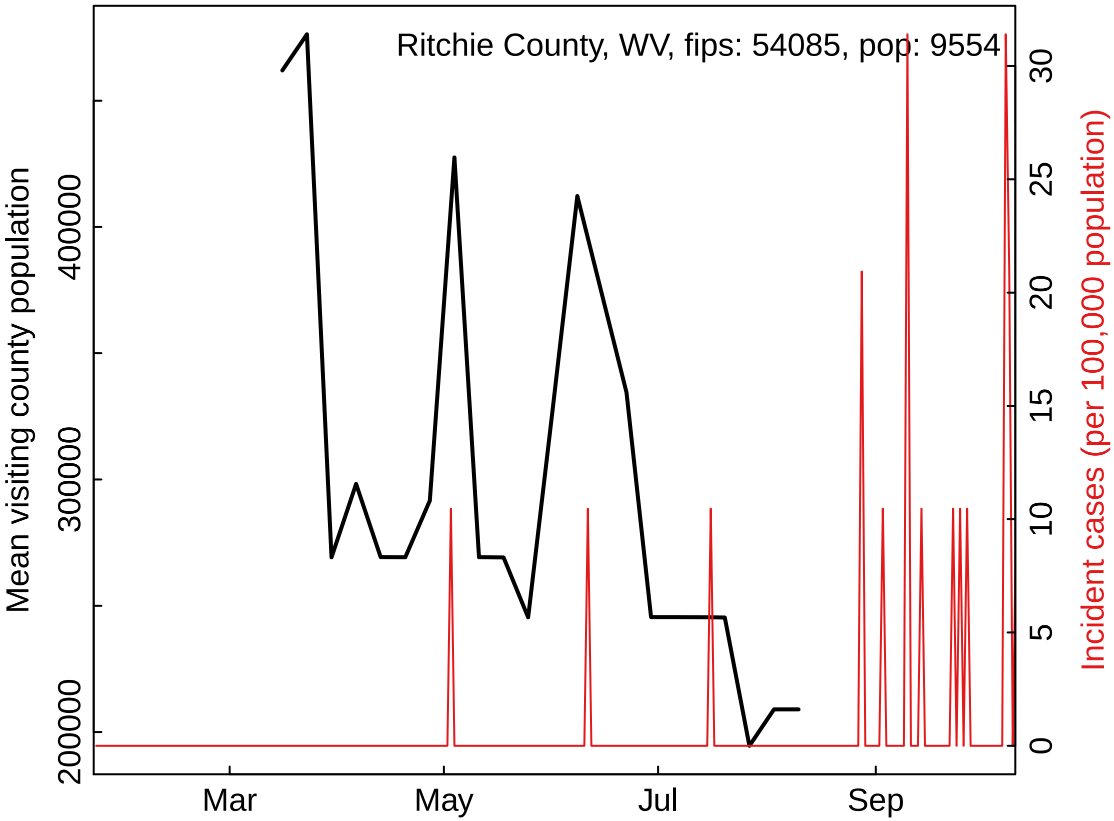
Ritchie County, WV, fips: 54085, pop: 9554
Mean visiting county population
200000
300000
400000
Incident cases (per 100,000 population)
0
5
10
15
20
25
30
Mar
May
Jul
Sep

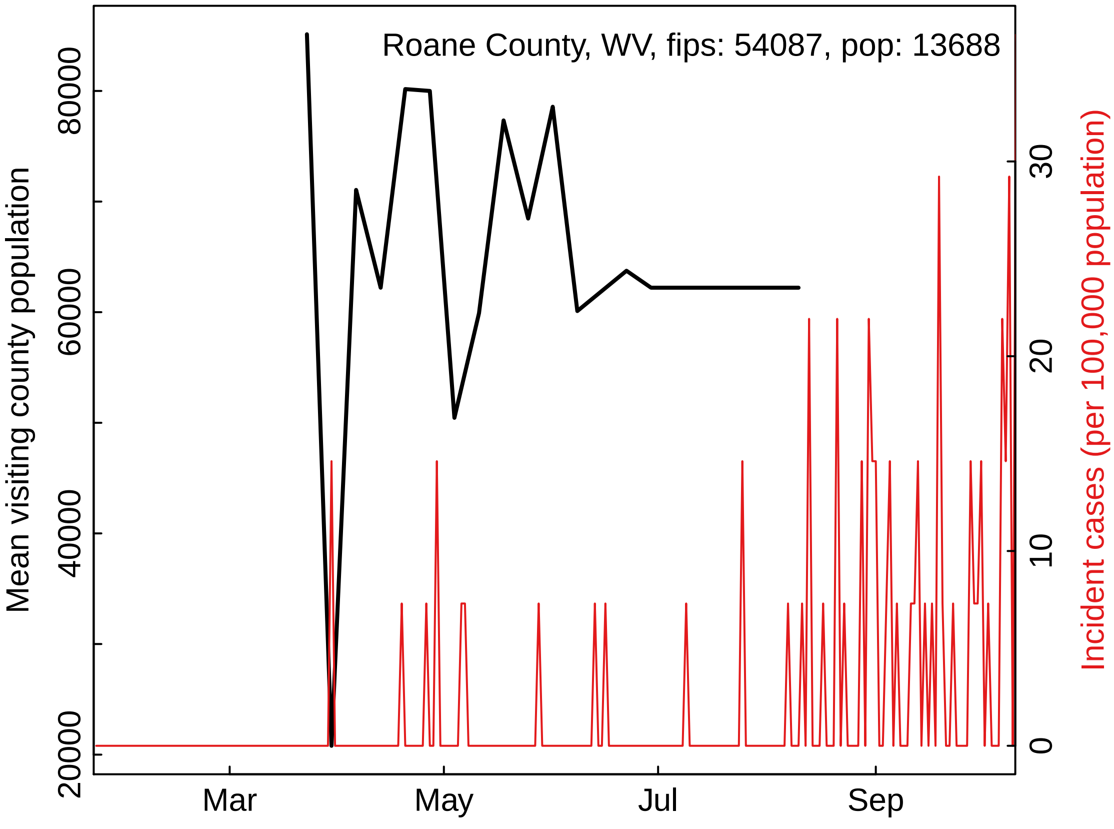
Roane County, WV, fips: 54087, pop: 13688
Mean visiting county population
Incident cases (per 100,000 population)
20000
40000
60000
80000
0
10
20
30
Mar
May
Jul
Sep

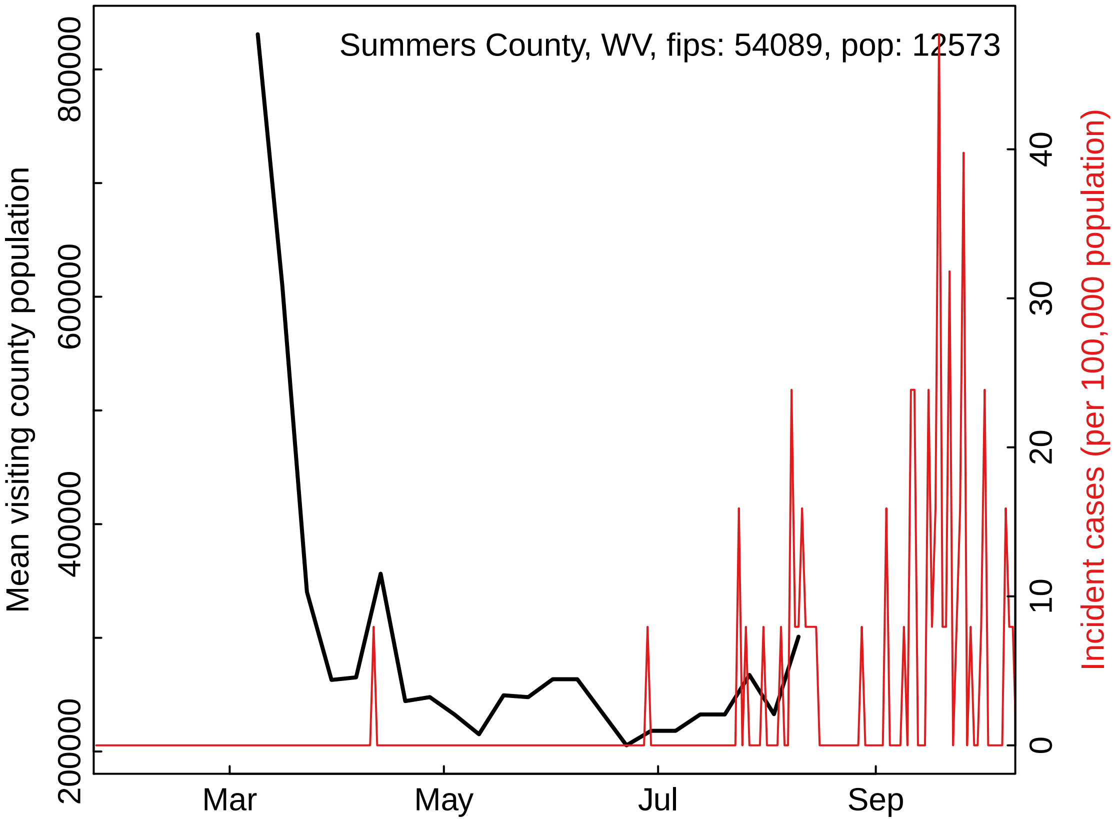

Summers County, WV, fips: 54089, pop: 12573
Mean visiting county population
200000
400000
600000
800000
Incident cases (per 100,000 population)
0
10
20
30
40
Mar
May
Jul
Sep

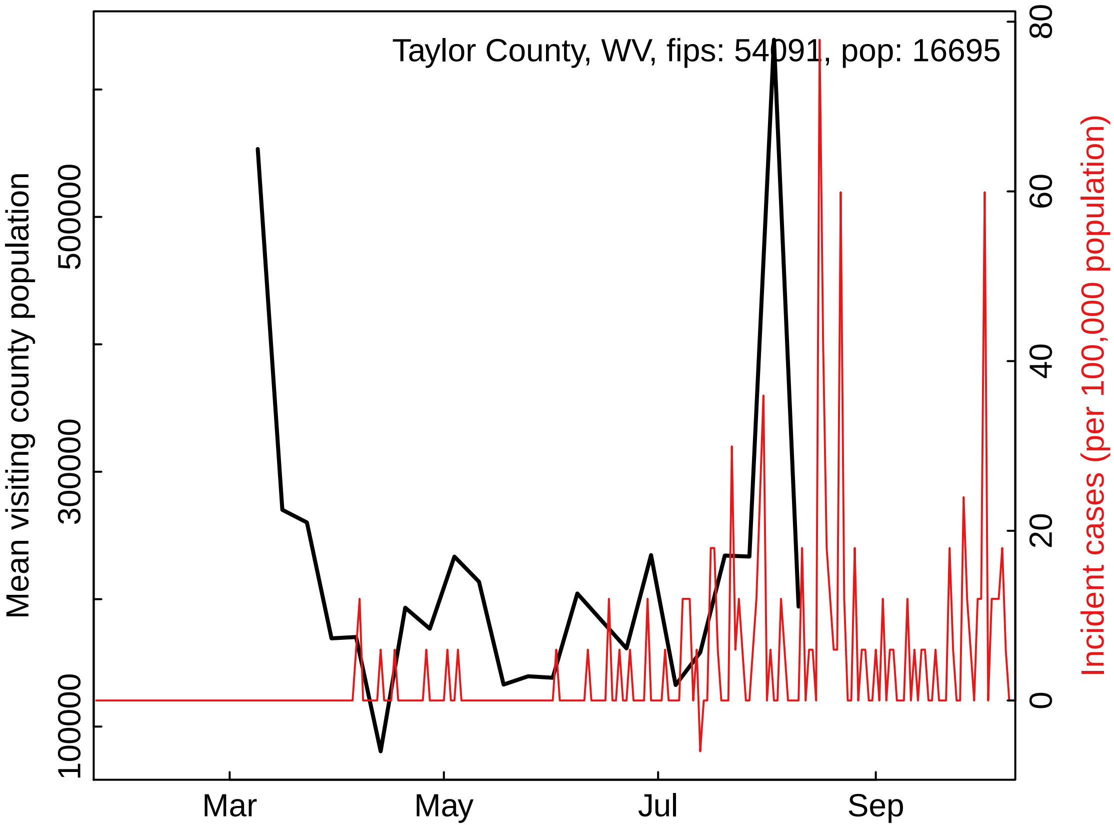

Taylor County, WV, fips: 54091, pop: 16695
Mean visiting county population
100000
300000
500000
Incident cases (per 100,000 population)
0
20
40
60
80
Mar
May
Jul
Sep

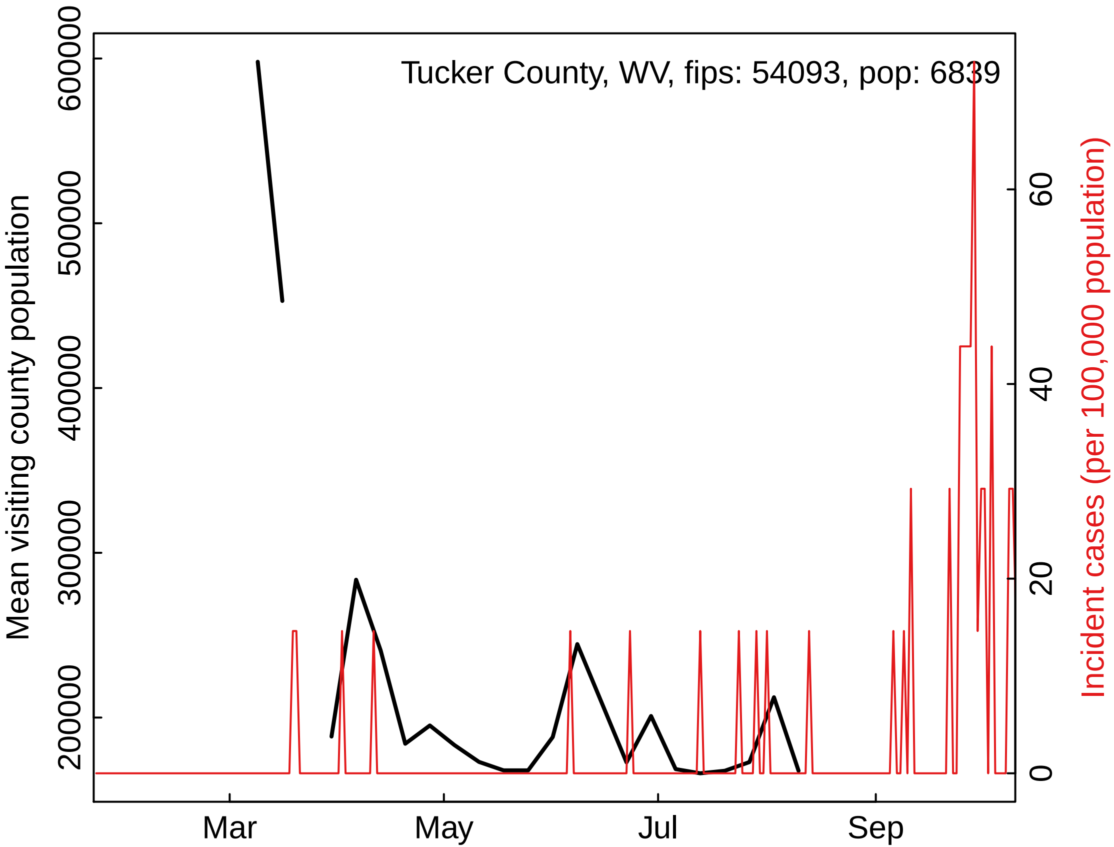

Tucker County, WV, fips: 54093, pop: 6839
Mean visiting county population
200000
300000
400000
500000
600000
Incident cases (per 100,000 population)
0
20
40
60
Mar
May
Jul
Sep

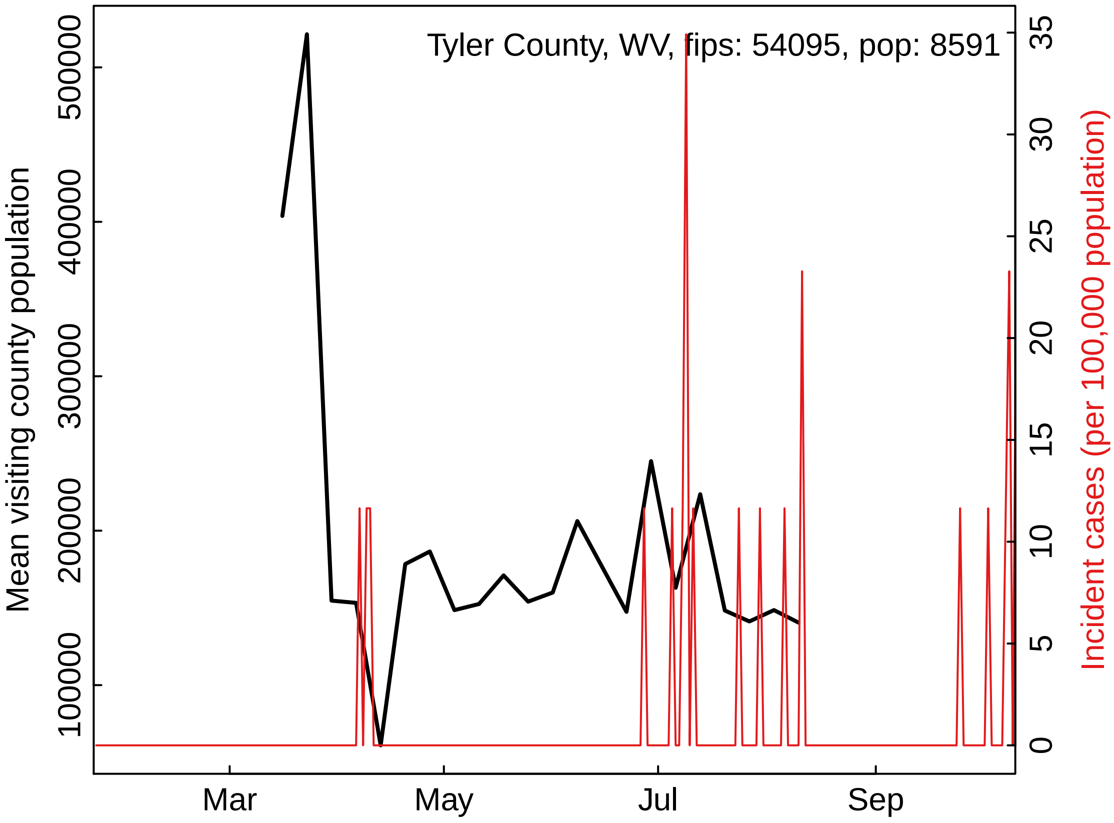

Tyler County, WV, fips: 54095, pop: 8591
Mean visiting county population
100000
200000
300000
400000
500000
Incident cases (per 100,000 population)
0
5
10
15
20
25
30
35
Mar
May
Jul
Sep

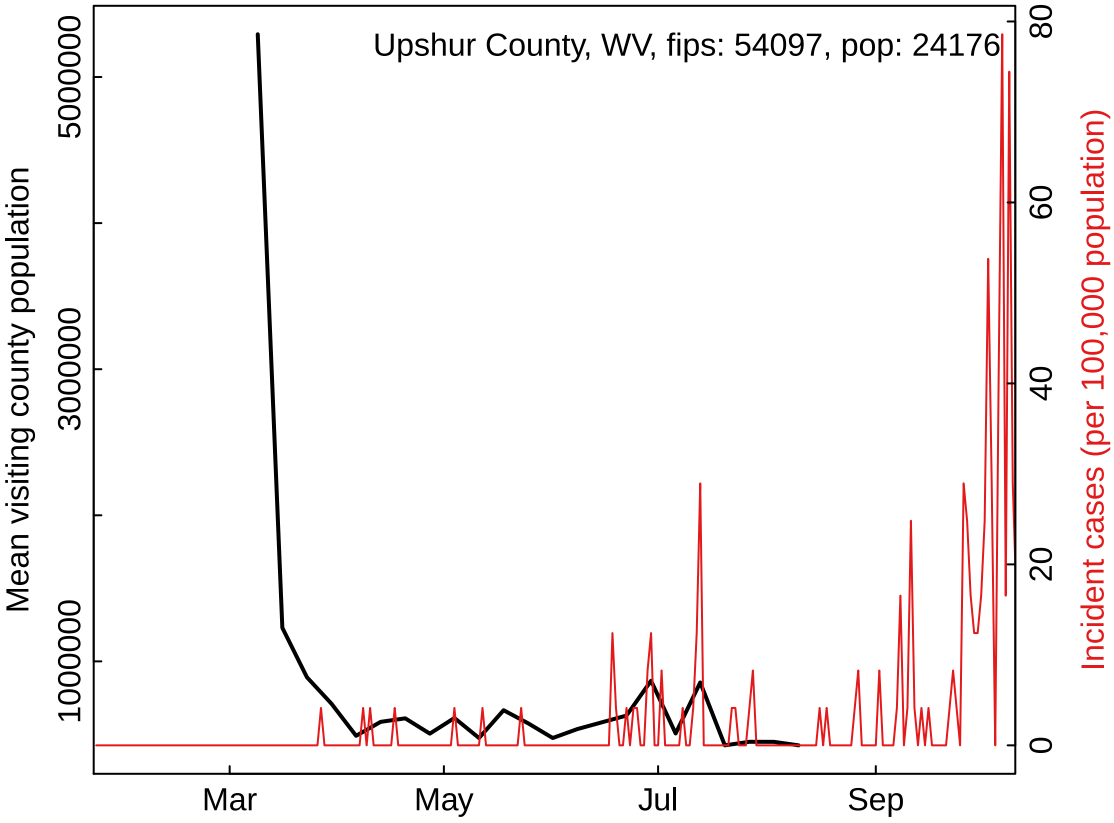

Upshur County, WV, fips: 54097, pop: 24176
Mean visiting county population
1000000
3000000
5000000
Incident cases (per 100,000 population)
0
20
40
60
80
Mar
May
Jul
Sep

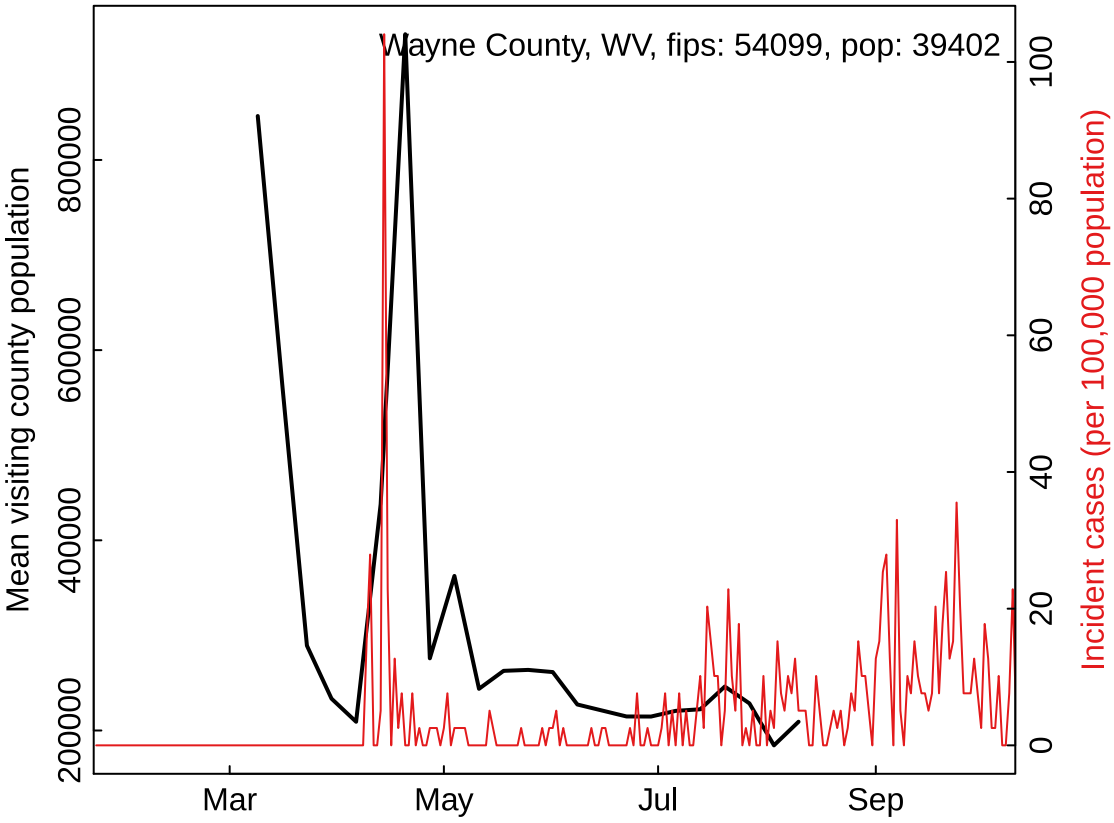
Wayne County, WV, fips: 54099, pop: 39402
Mean visiting county population
200000
400000
600000
800000
Incident cases (per 100,000 population)
0
20
40
60
80
100
Mar
May
Jul
Sep

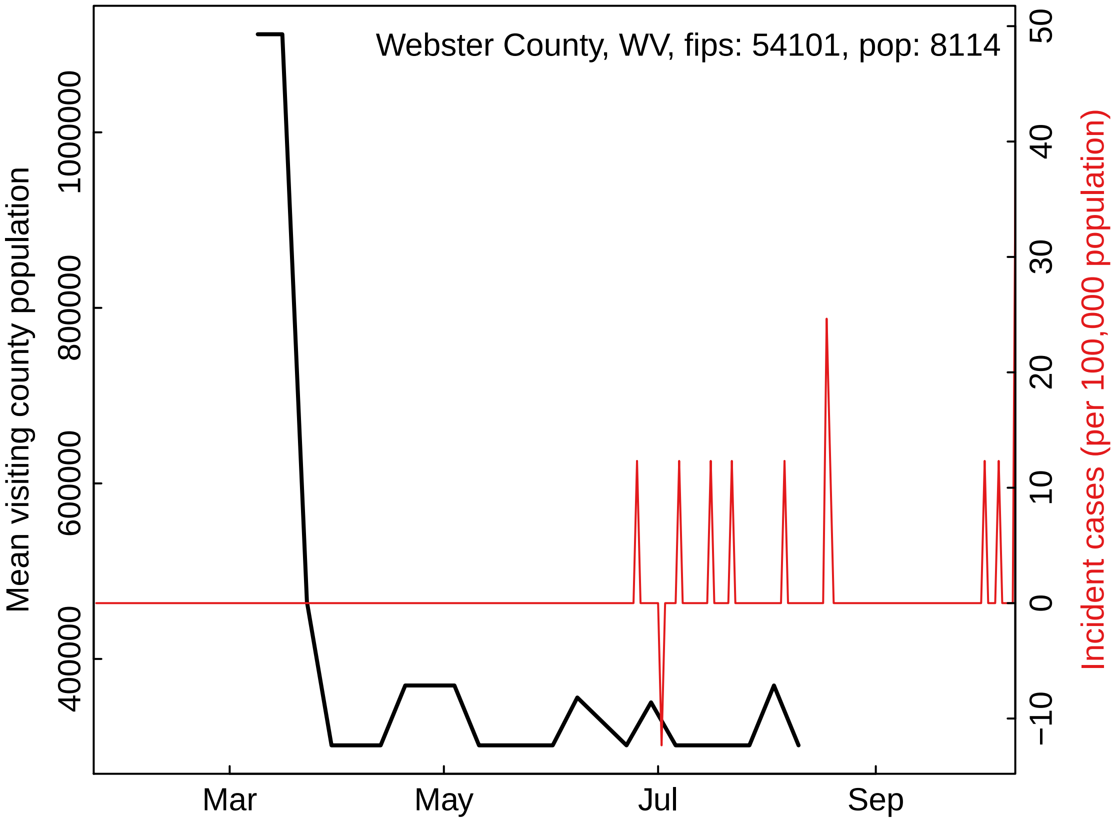

Webster County, WV, fips: 54101, pop: 8114
Mean visiting county population
400000
600000
800000
1000000
Incident cases (per 100,000 population)
−10
0
10
20
30
40
50
Mar
May
Jul
Sep

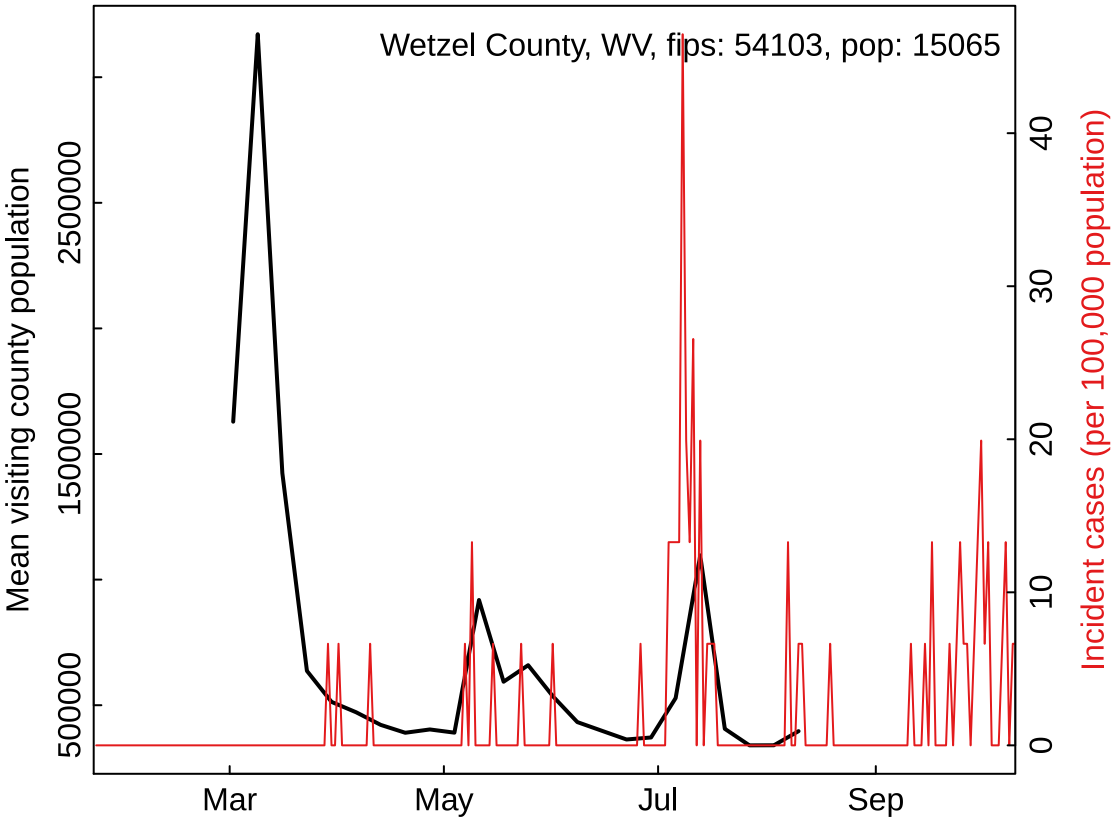

Wetzel County, WV, fips: 54103, pop: 15065
Mean visiting county population
500000
1500000
2500000
Mar
May
Jul
Sep
0
10
20
30
40
Incident cases (per 100,000 population)

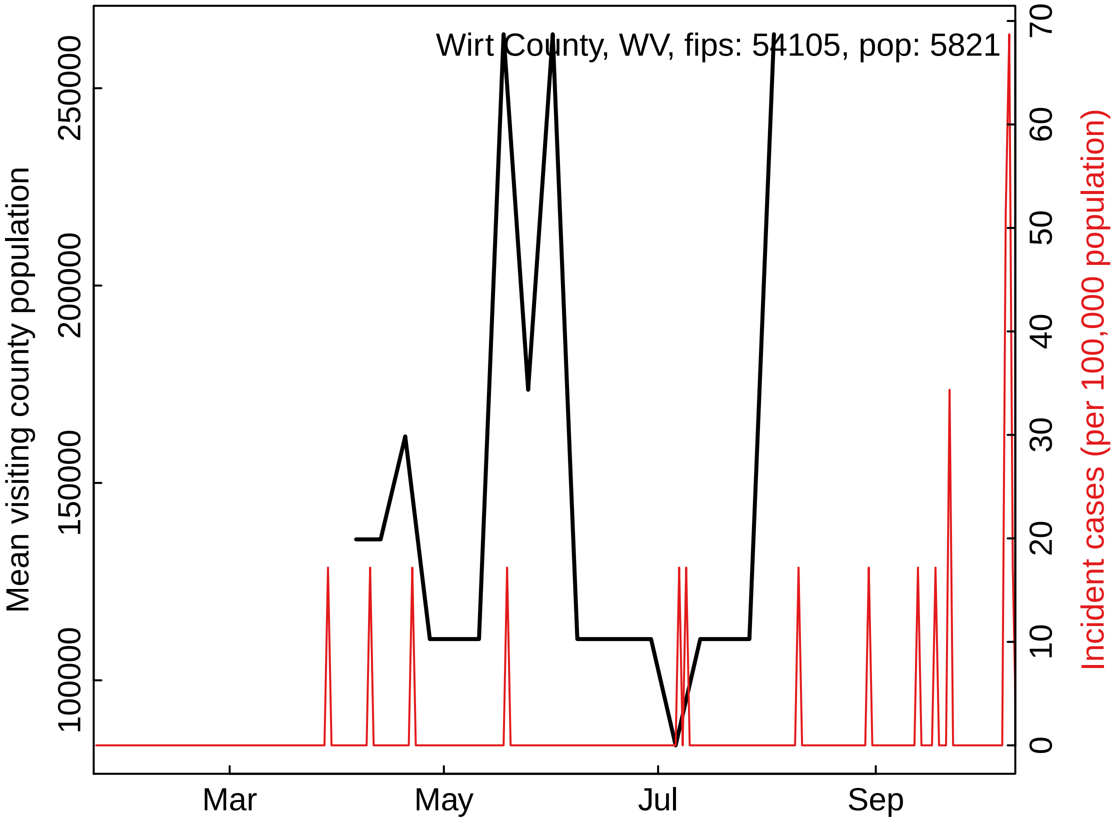
Wirt County, WV, fips: 54105, pop: 5821
Mean visiting county population
100000
150000
200000
250000
Incident cases (per 100,000 population)
0
10
20
30
40
50
60
70
Mar
May
Jul
Sep

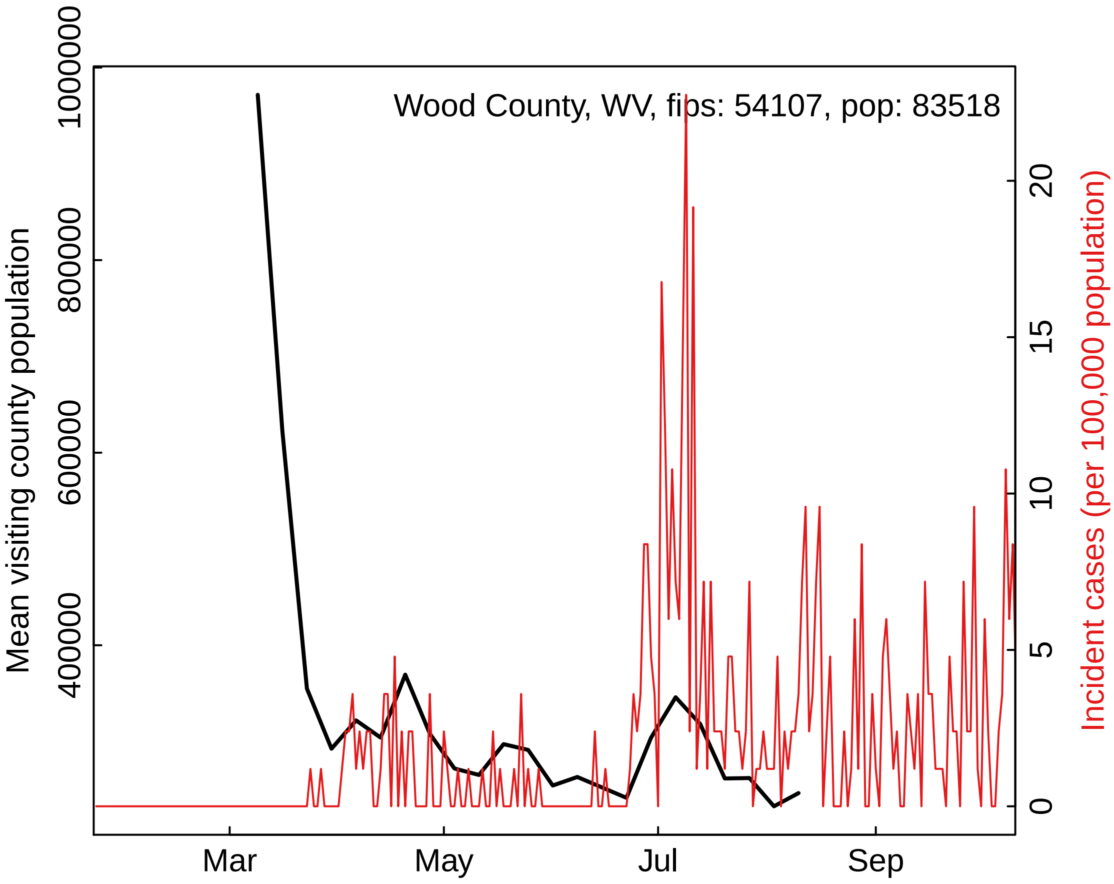
Wood County, WV, fips: 54107, pop: 83518
Mean visiting county population
400000
600000
800000
1000000
Incident cases (per 100,000 population)
0
5
10
15
20
Mar
May
Jul
Sep

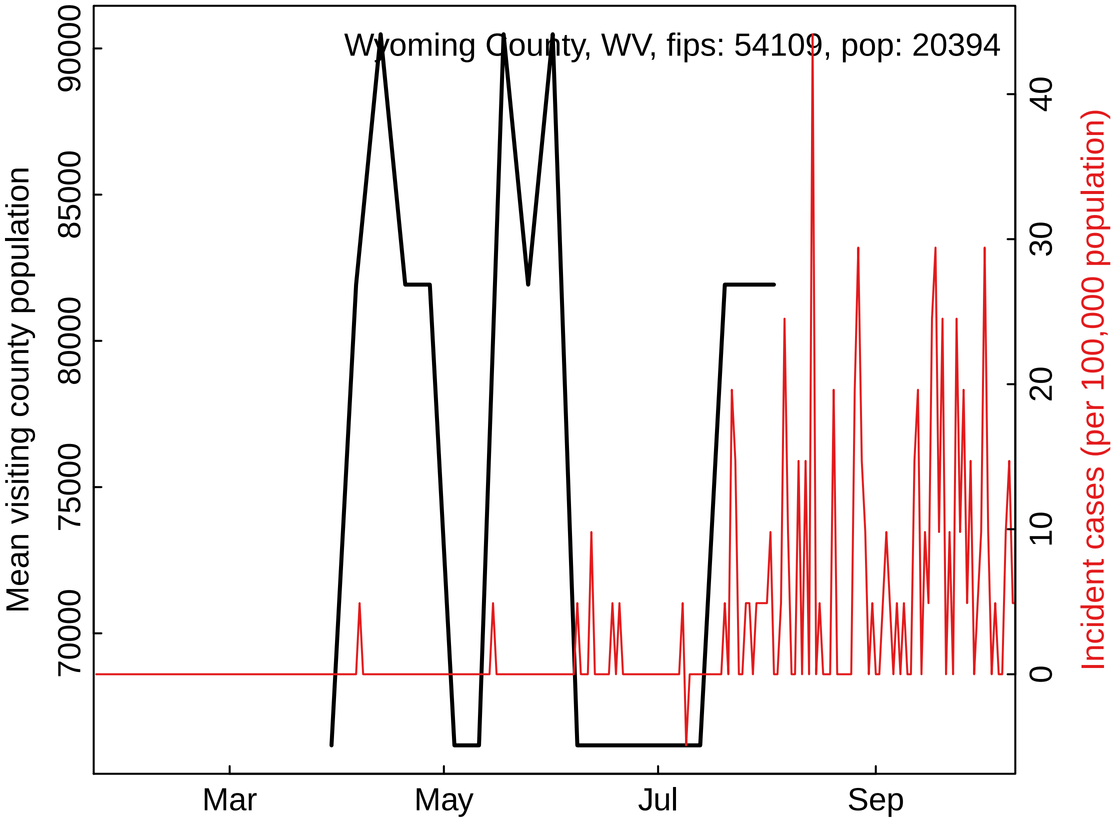
Wyoming County, WV, fips: 54109, pop: 20394
Mean visiting county population
90000
85000
80000
75000
70000
Incident cases (per 100,000 population)
40
30
20
10
0
Mar
May
Jul
Sep

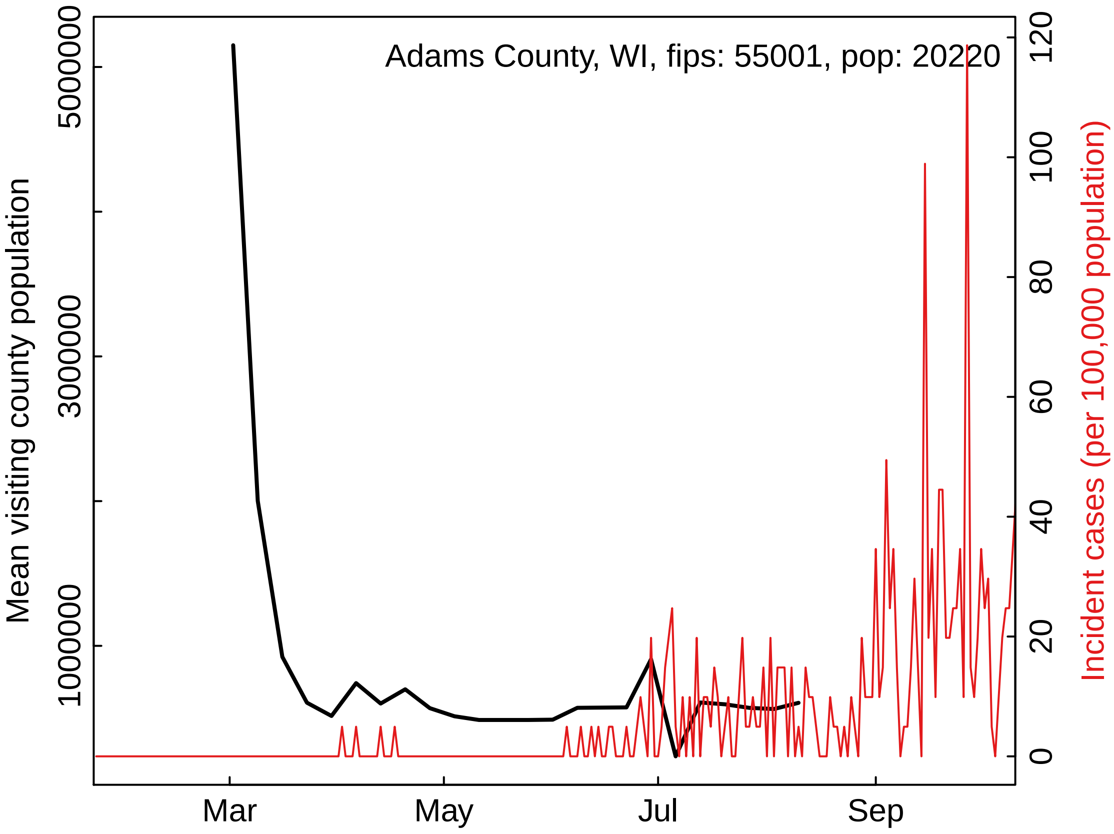
Adams County, WI, fips: 55001, pop: 20220
Mean visiting county population
1000000
3000000
5000000
Incident cases (per 100,000 population)
0
20
40
60
80
100
120
Mar
May
Jul
Sep

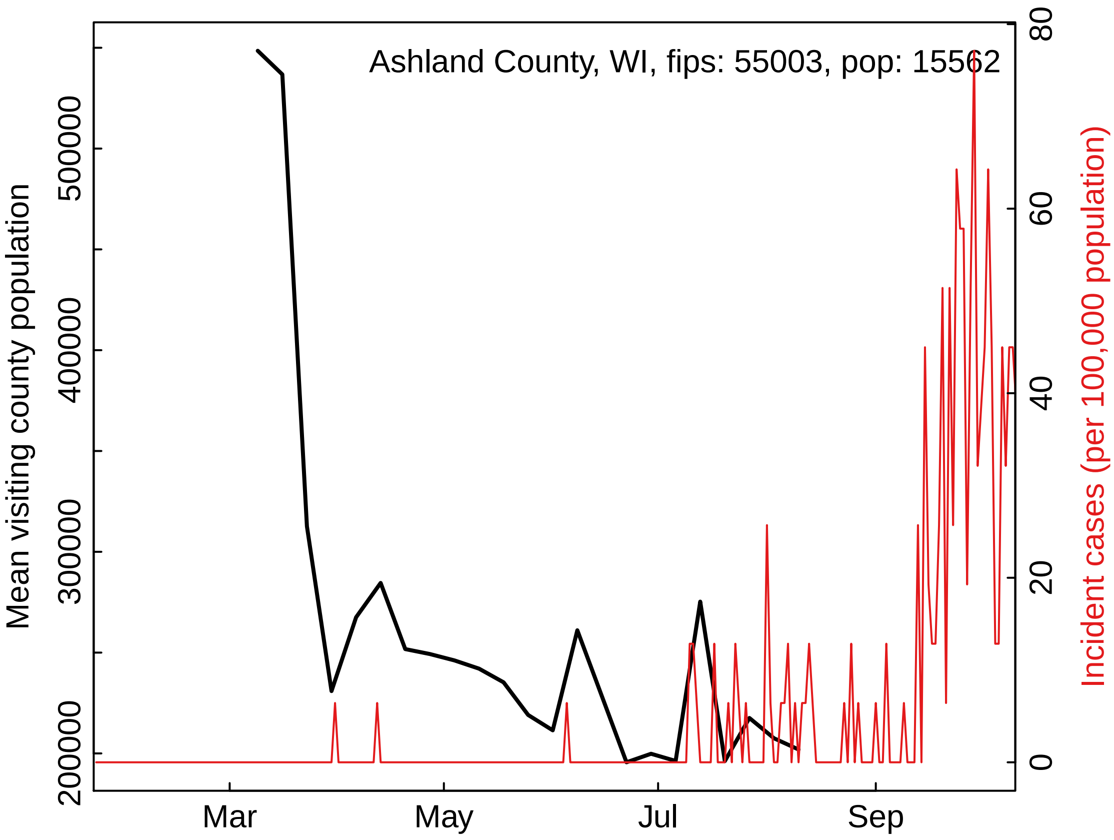

Ashland County, WI, fips: 55003, pop: 15562
Mean visiting county population
200000
300000
400000
500000
Incident cases (per 100,000 population)
0
20
40
60
80
Mar
May
Jul
Sep

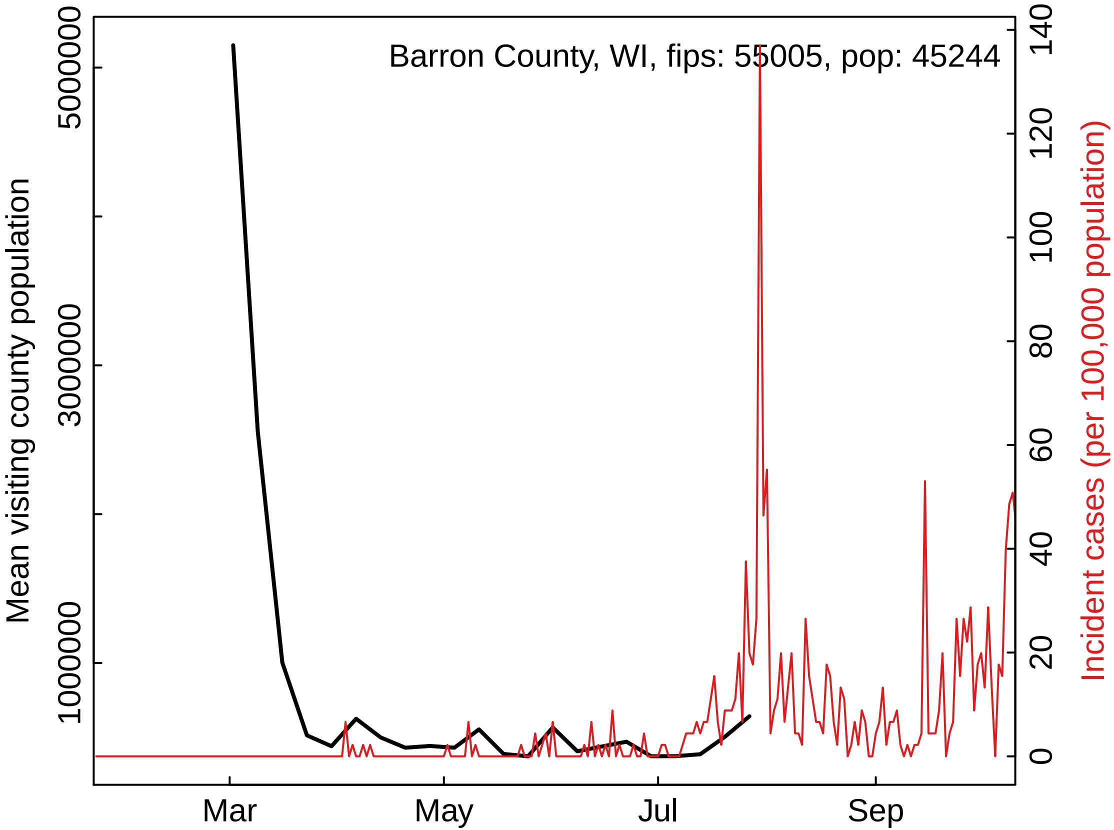

Barron County, WI, fips: 55005, pop: 45244
Mean visiting county population
1000000
3000000
5000000
Mar
May
Jul
Sep
0
20
40
60
80
100
120
140
Incident cases (per 100,000 population)

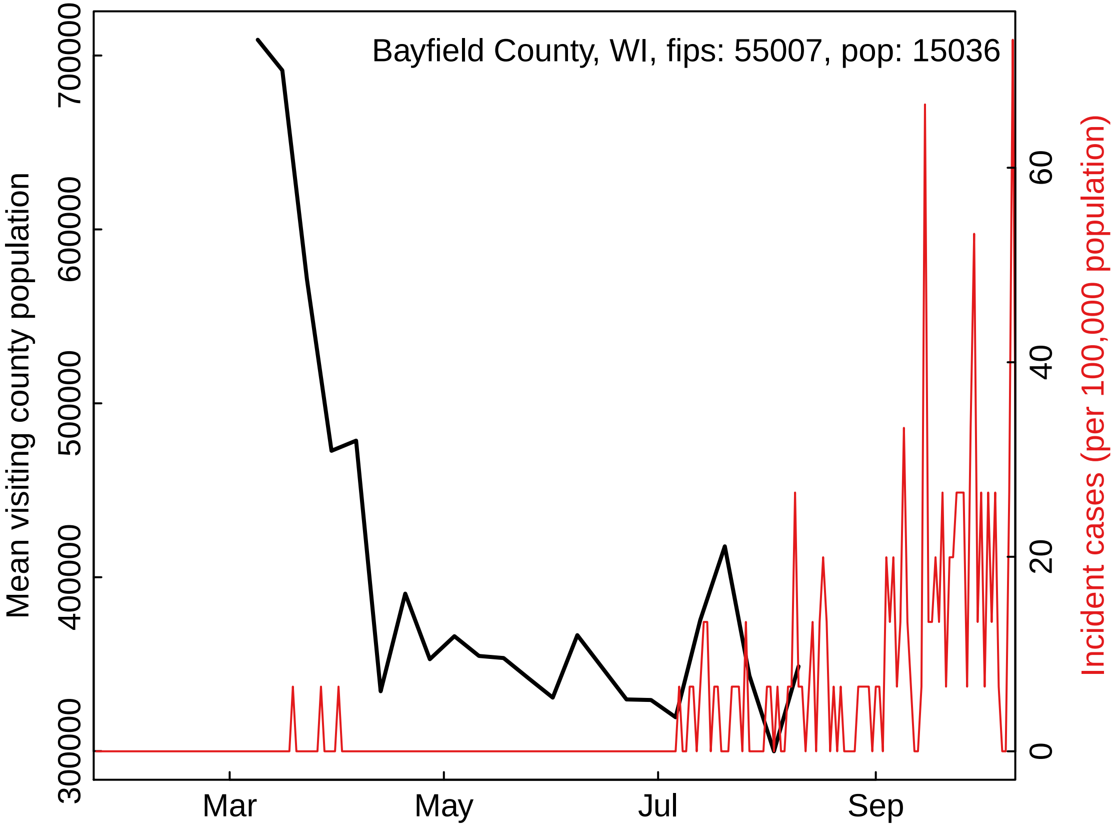

Bayfield County, WI, fips: 55007, pop: 15036
Mean visiting county population
300000
400000
500000
600000
700000
Incident cases (per 100,000 population)
0
20
40
60
Mar
May
Jul
Sep

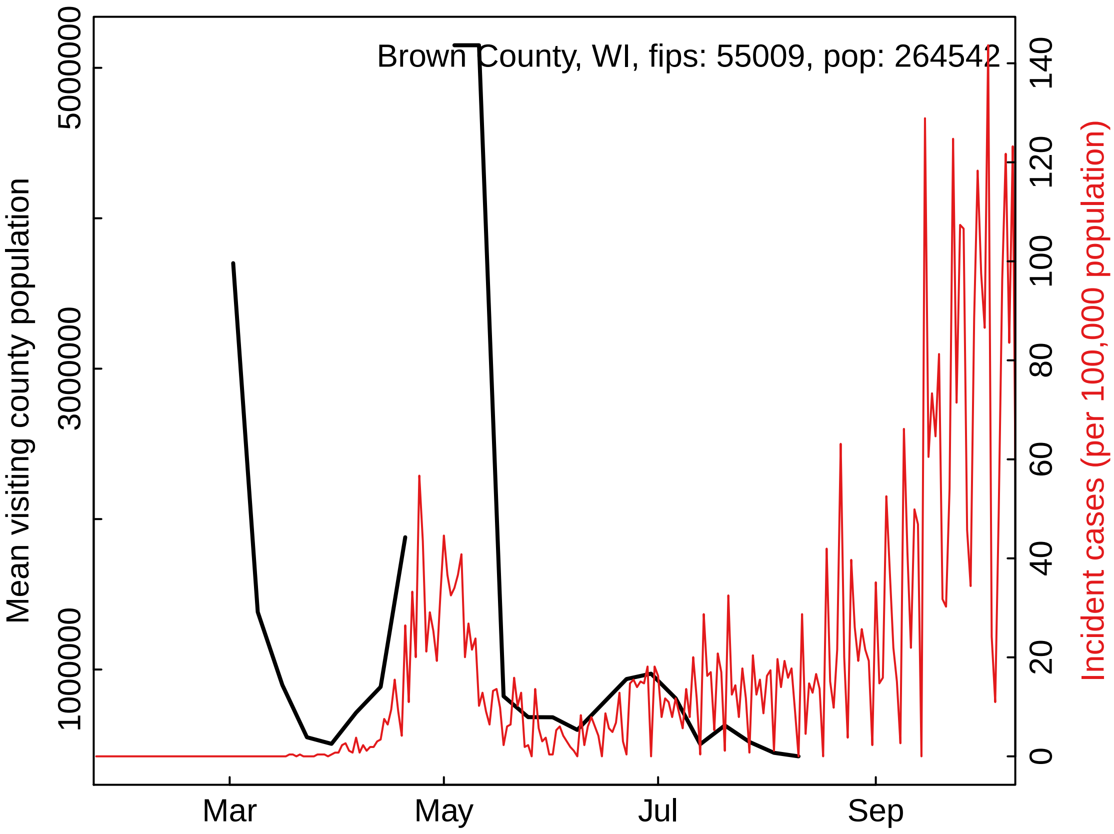
Brown County, WI, fips: 55009, pop: 264542
Mean visiting county population
1000000
3000000
5000000
Incident cases (per 100,000 population)
0
20
40
60
80
100
120
140
Mar
May
Jul
Sep

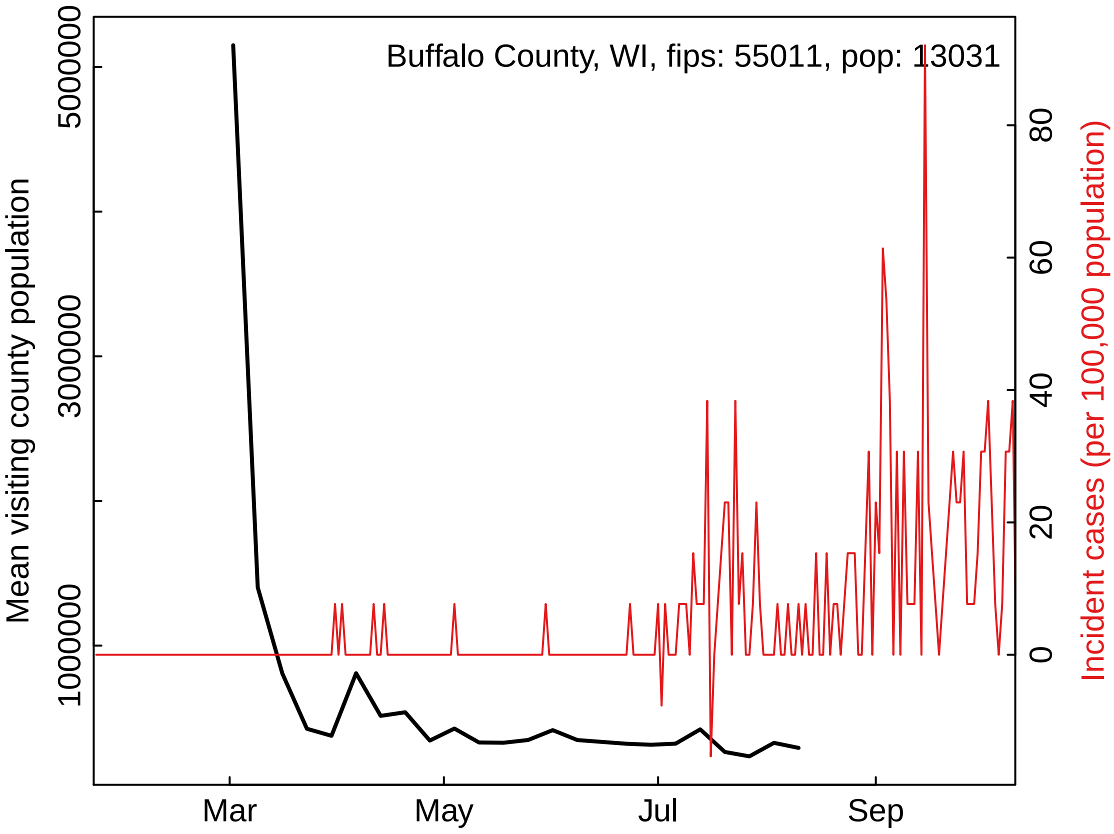

Buffalo County, WI, fips: 55011, pop: 13031
Mean visiting county population
10000000
30000000
50000000
Incident cases (per 100,000 population)
0
20
40
60
80
Mar
May
Jul
Sep

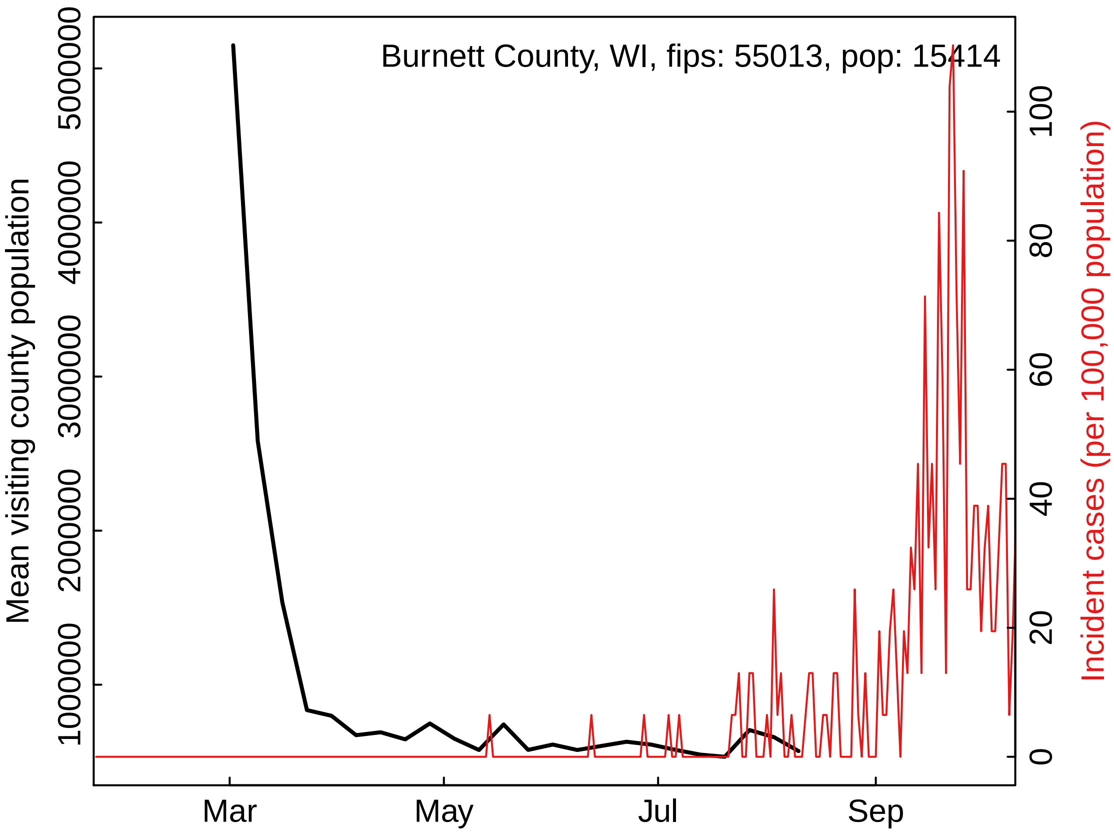

Burnett County, WI, fips: 55013, pop: 15414
Mean visiting county population
1000000
2000000
3000000
4000000
5000000
Incident cases (per 100,000 population)
0
20
40
60
80
100
Mar
May
Jul
Sep

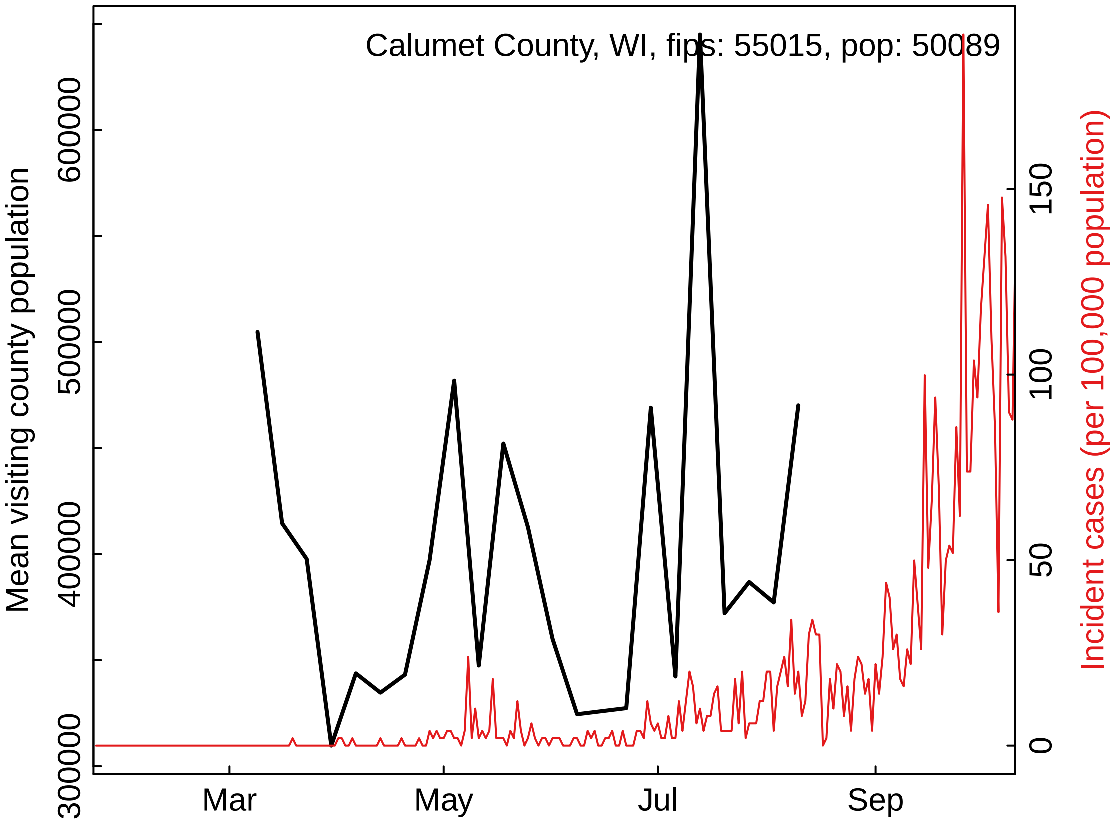

Calumet County, WI, fips: 55015, pop: 50089
Mean visiting county population
Incident cases (per 100,000 population)
3000000
4000000
5000000
6000000
0
50
100
150
Mar
May
Jul
Sep

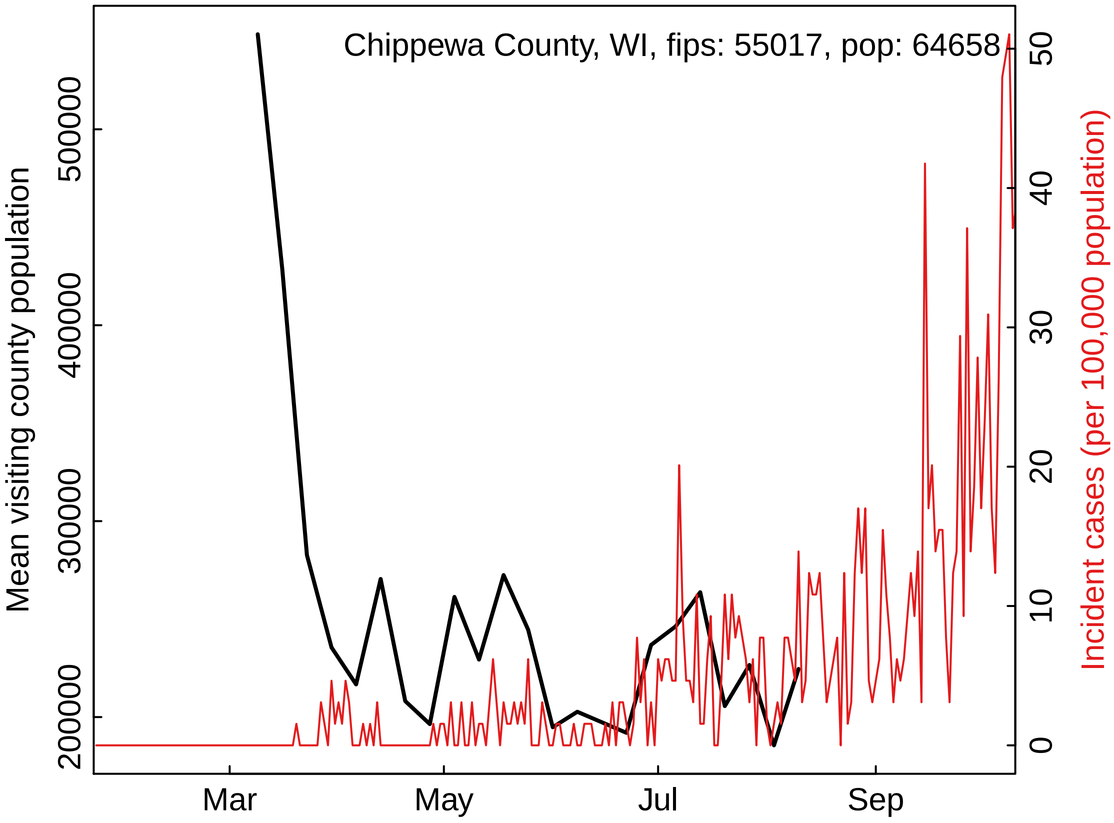

Chippewa County, WI, fips: 55017, pop: 64658
Mean visiting county population
200000
300000
400000
500000
Incident cases (per 100,000 population)
0
10
20
30
40
50
Mar
May
Jul
Sep

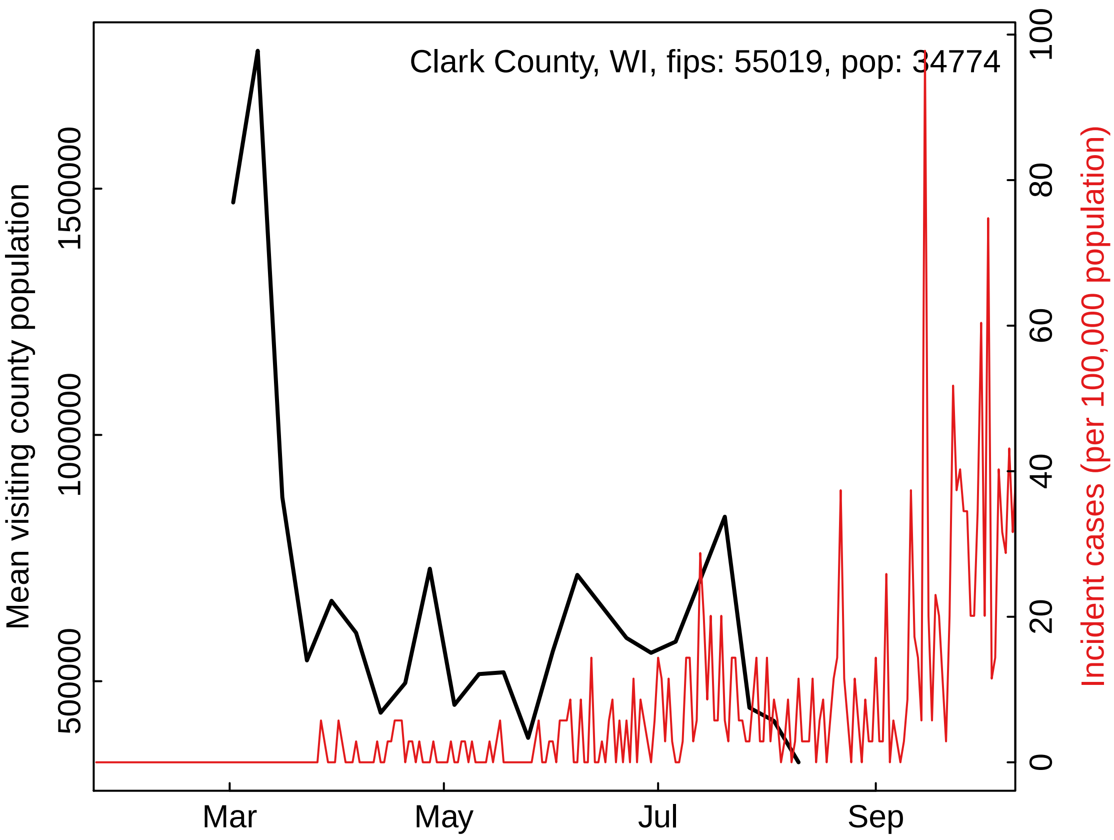

Clark County, WI, fips: 55019, pop: 34774
Mean visiting county population
500000
1000000
1500000
Incident cases (per 100,000 population)
0
20
40
60
80
100
Mar
May
Jul
Sep

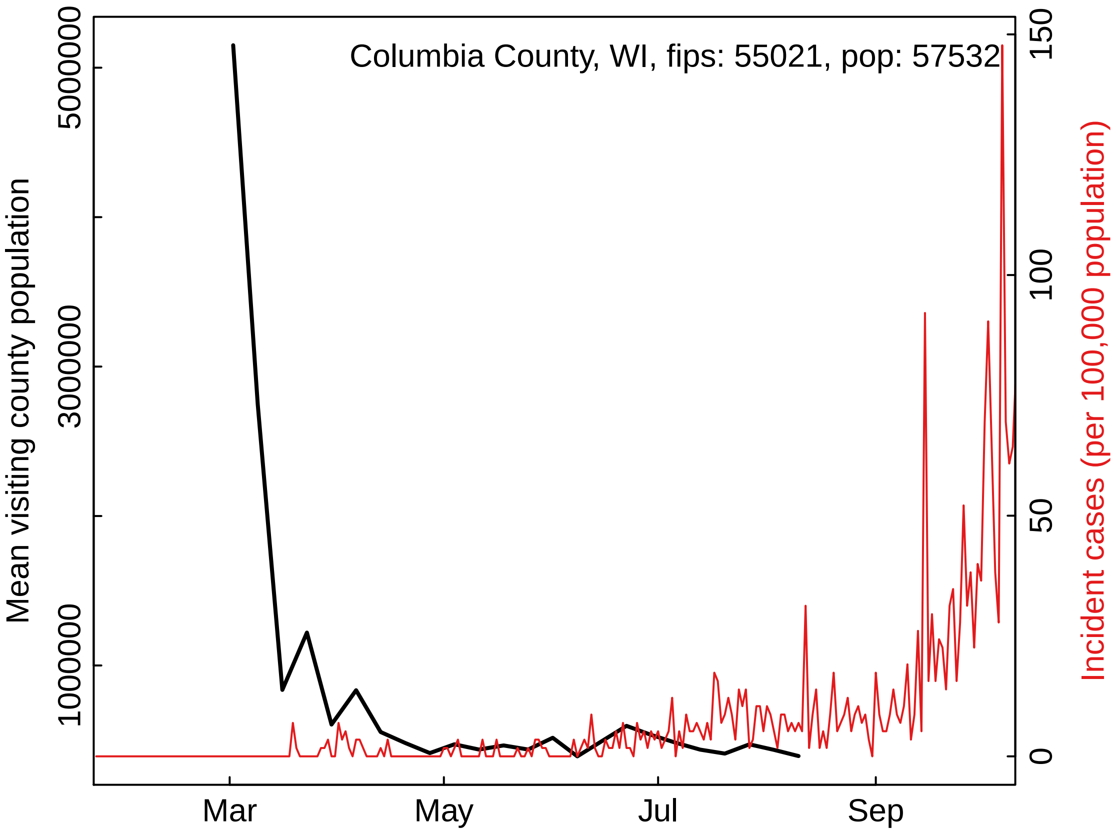

Columbia County, WI, fips: 55021, pop: 57532
Mean visiting county population
1000000
3000000
5000000
Incident cases (per 100,000 population)
0
50
100
150
Mar
May
Jul
Sep

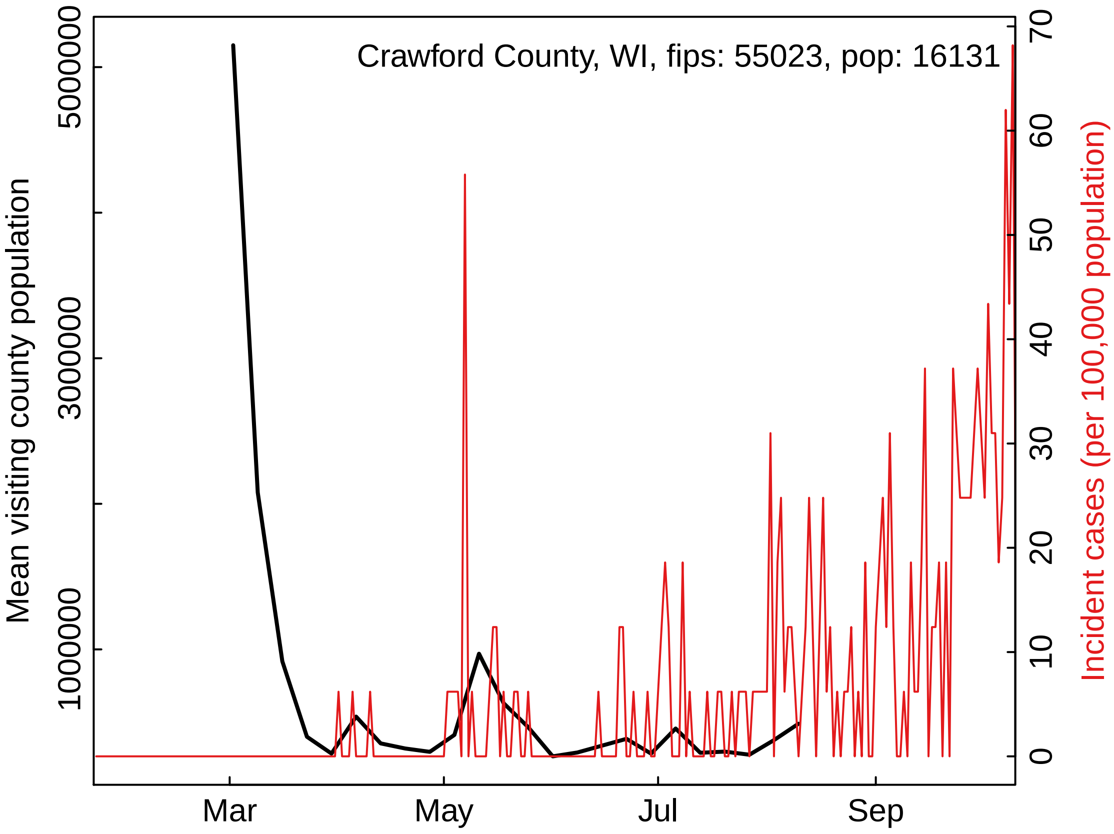

Crawford County, WI, fips: 55023, pop: 16131
Mean visiting county population
1000000
3000000
5000000
Mar
May
Jul
Sep
Incident cases (per 100,000 population)
0
10
20
30
40
50
60
70

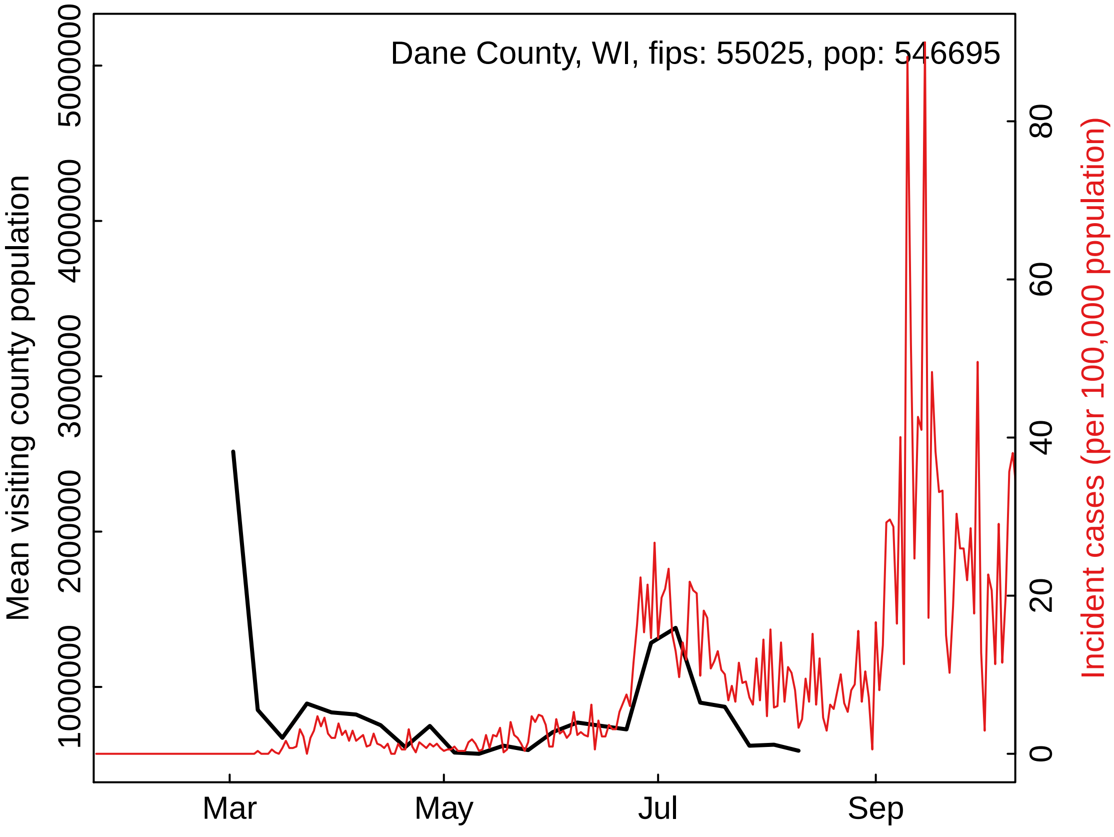

Dane County, WI, fips: 55025, pop: 546695
Mean visiting county population
10000000
20000000
30000000
40000000
50000000
Incident cases (per 100,000 population)
0
20
40
60
80
Mar
May
Jul
Sep

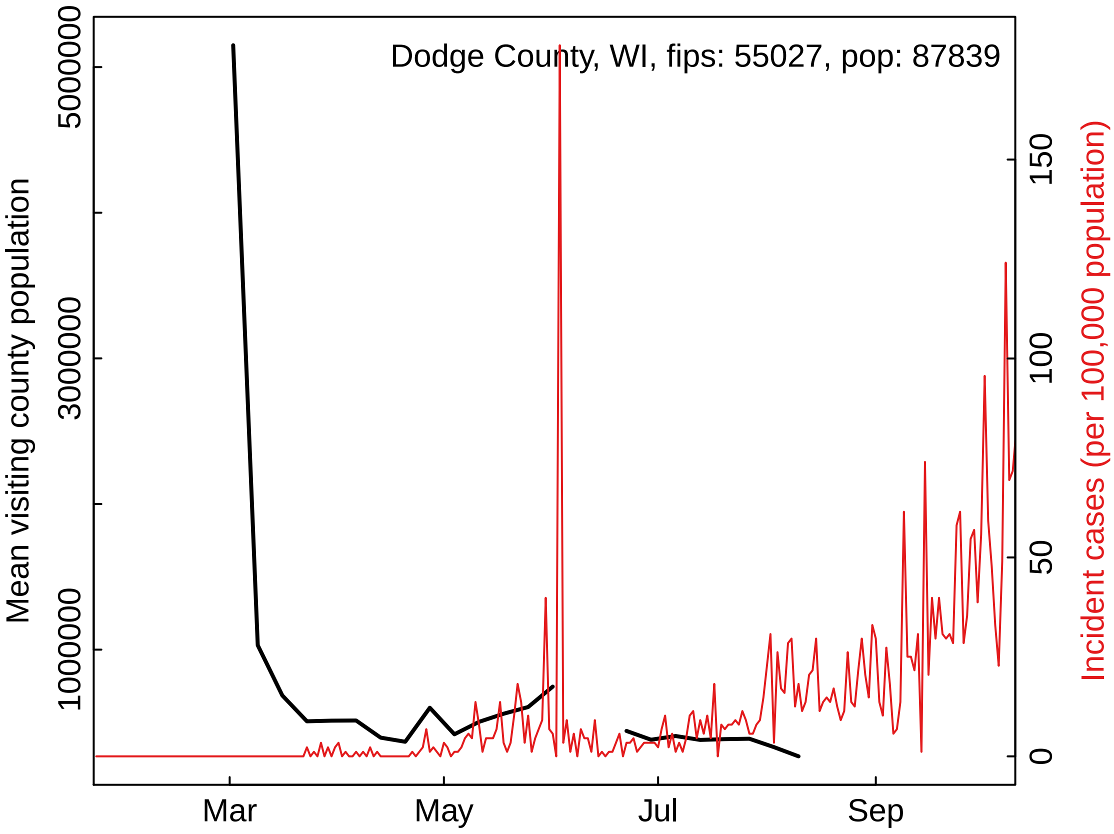
Dodge County, WI, fips: 55027, pop: 87839
Mean visiting county population
1000000
3000000
5000000
Incident cases (per 100,000 population)
0
50
100
150
Mar
May
Jul
Sep

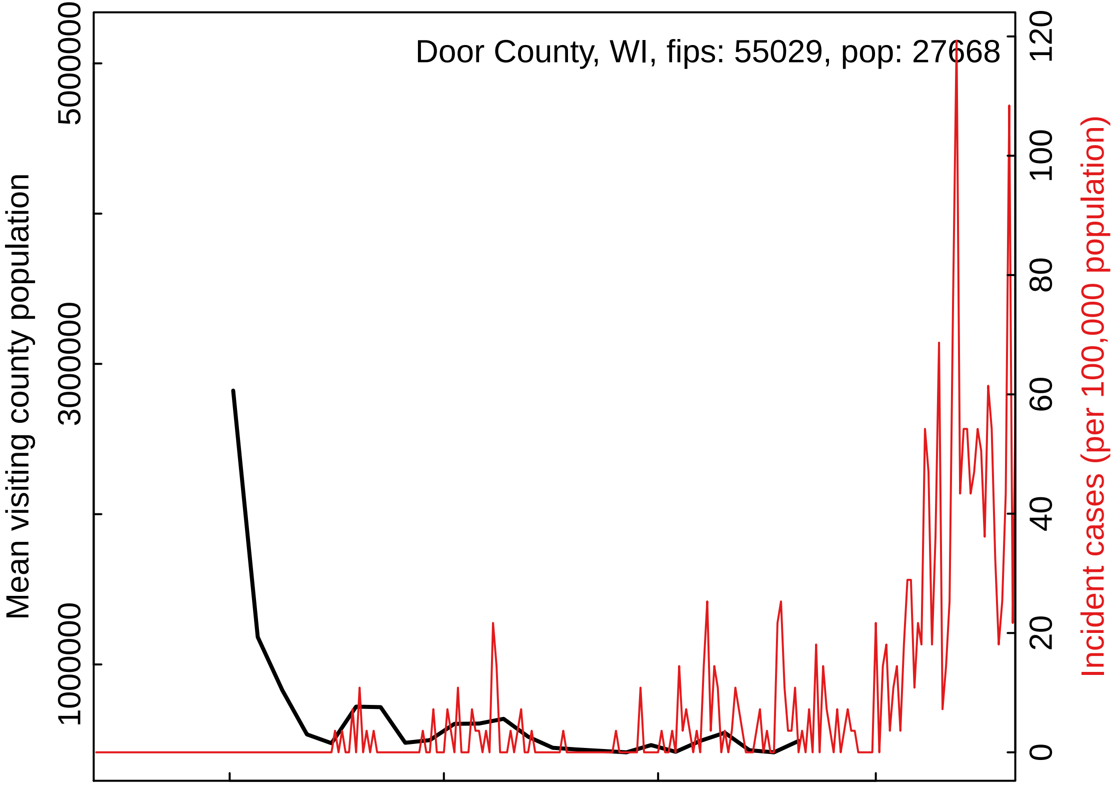
Door County, WI, fips: 55029, pop: 27668
Mean visiting county population
1000000
3000000
5000000
Incident cases (per 100,000 population)
0
20
40
60
80
100
120
Mar
May
Jul
Sep

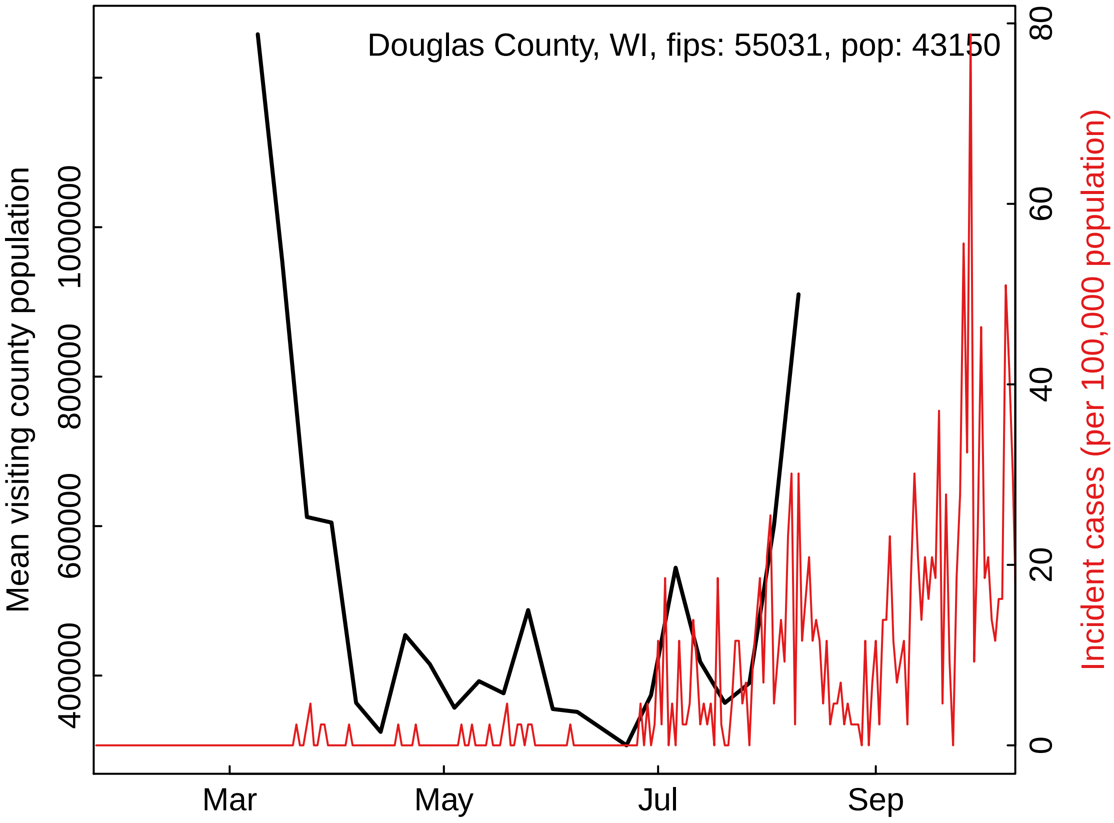

Douglas County, WI, fips: 55031, pop: 43150
Mean visiting county population
400000
600000
800000
1000000
Incident cases (per 100,000 population)
0
20
40
60
80
Mar
May
Jul
Sep

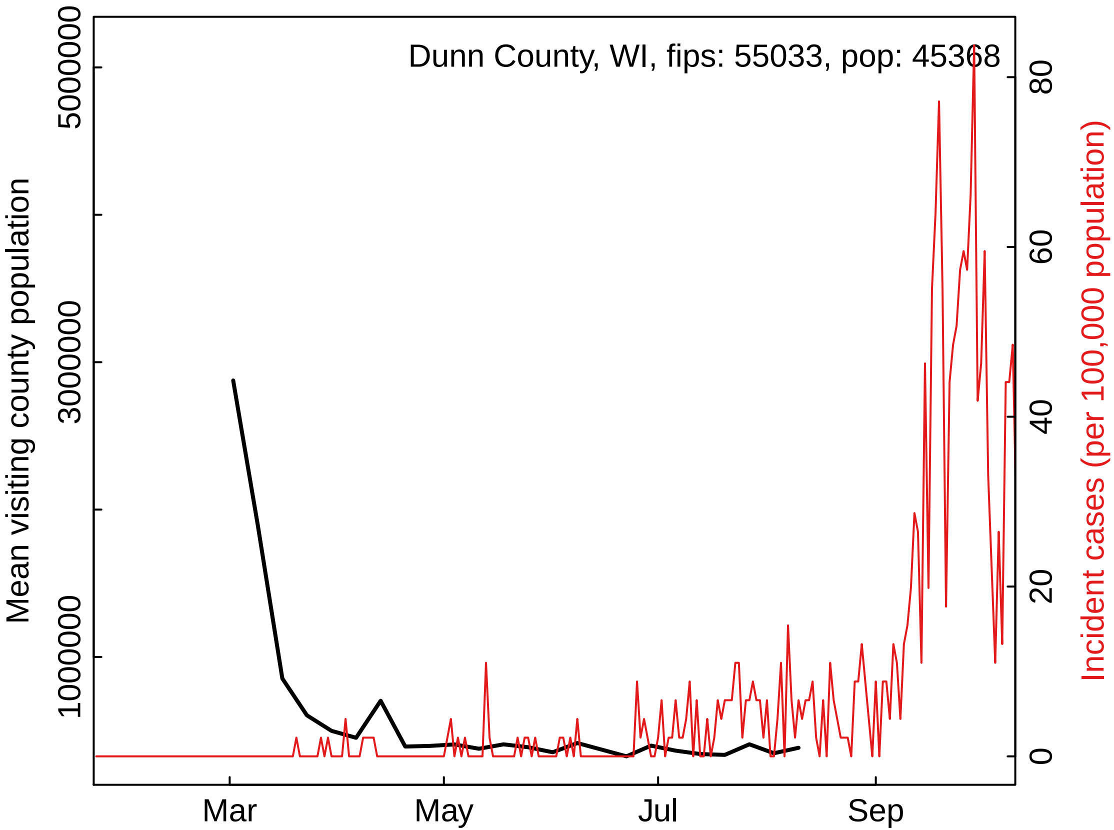

Dunn County, WI, fips: 55033, pop: 45368
Mean visiting county population
1000000
3000000
5000000
Mar
May
Jul
Sep
Incident cases (per 100,000 population)
0
20
40
60
80

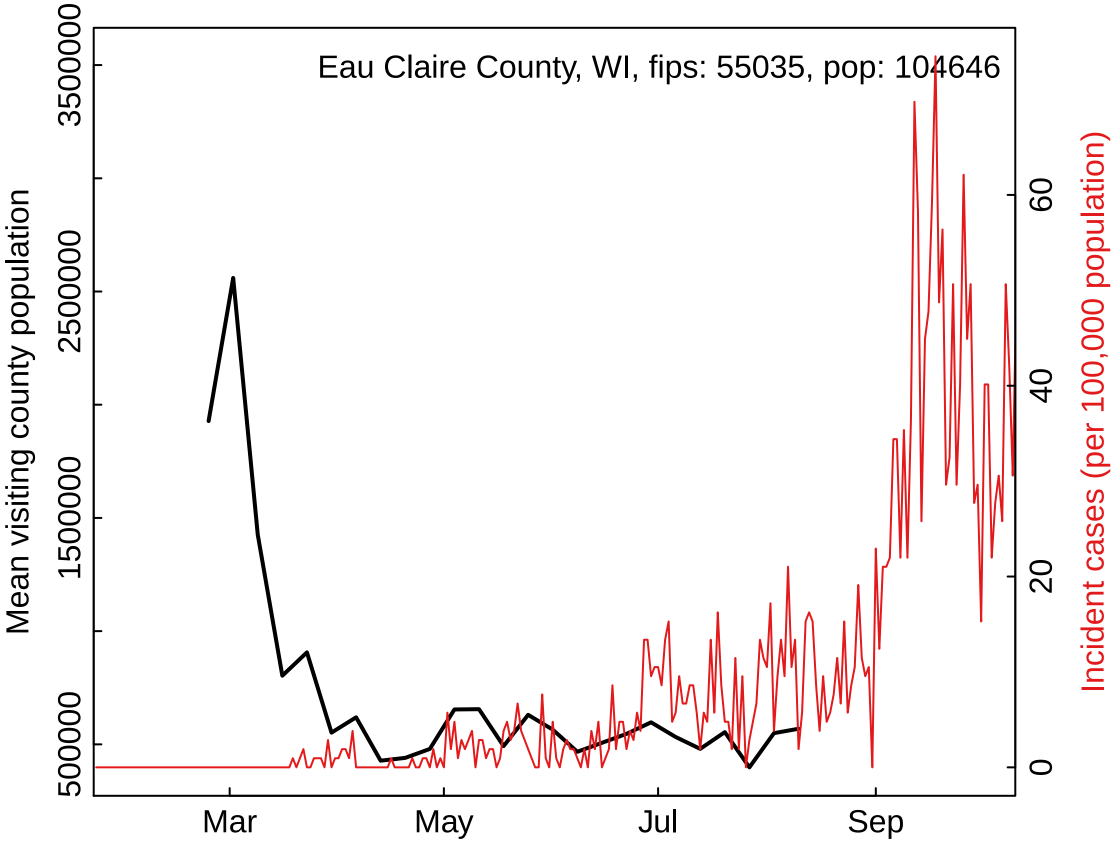

Eau Claire County, WI, fips: 55035, pop: 104646
Mean visiting county population
500000
1500000
2500000
3500000
Incident cases (per 100,000 population)
0
20
40
60
Mar
May
Jul
Sep

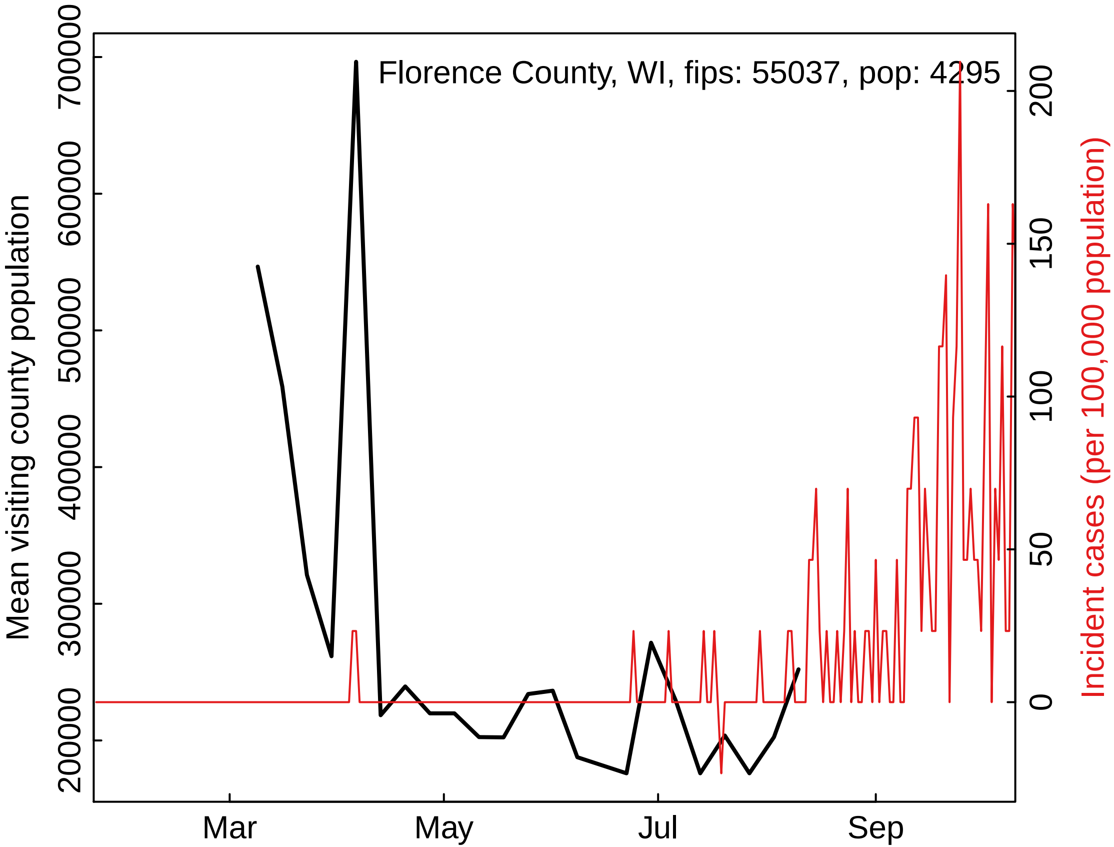

Florence County, WI, fips: 55037, pop: 4295
Mean visiting county population
Incident cases (per 100,000 population)
7000000
6000000
5000000
4000000
3000000
2000000
200
150
100
50
0
Mar
May
Jul
Sep

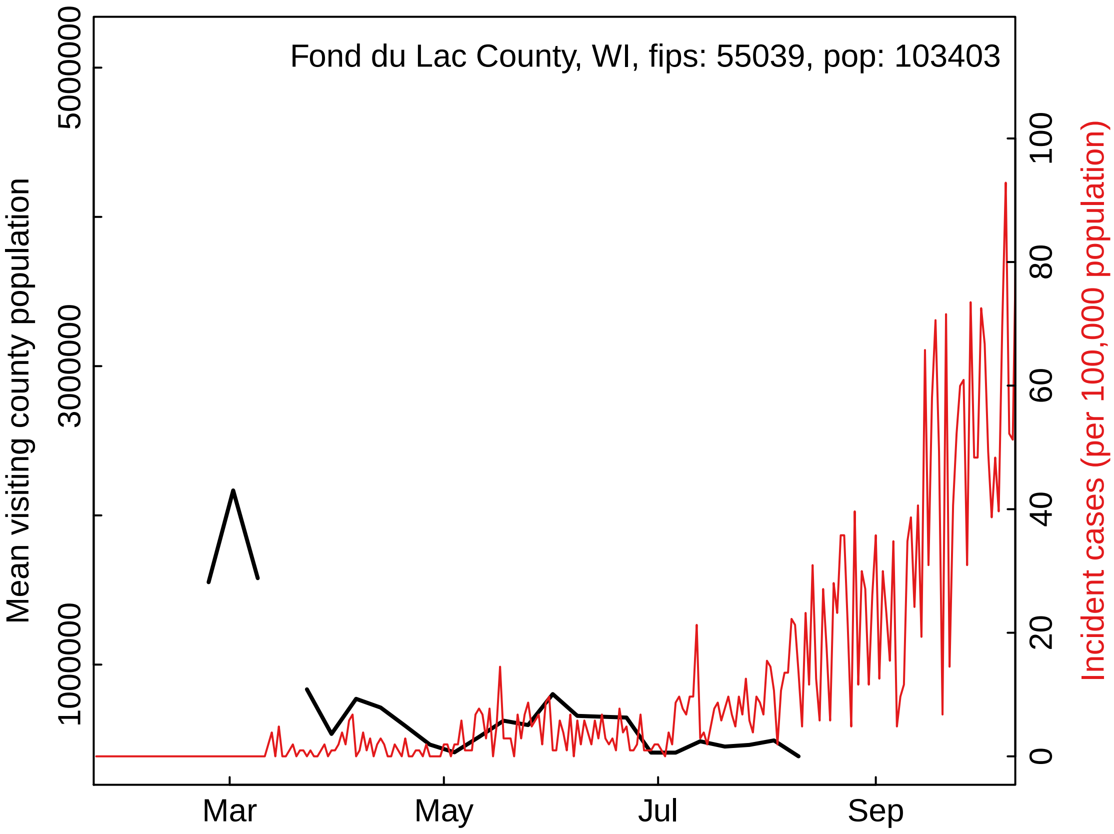

Fond du Lac County, WI, fips: 55039, pop: 103403
Mean visiting county population
1000000
3000000
5000000
Incident cases (per 100,000 population)
0
20
40
60
80
100
Mar
May
Jul
Sep

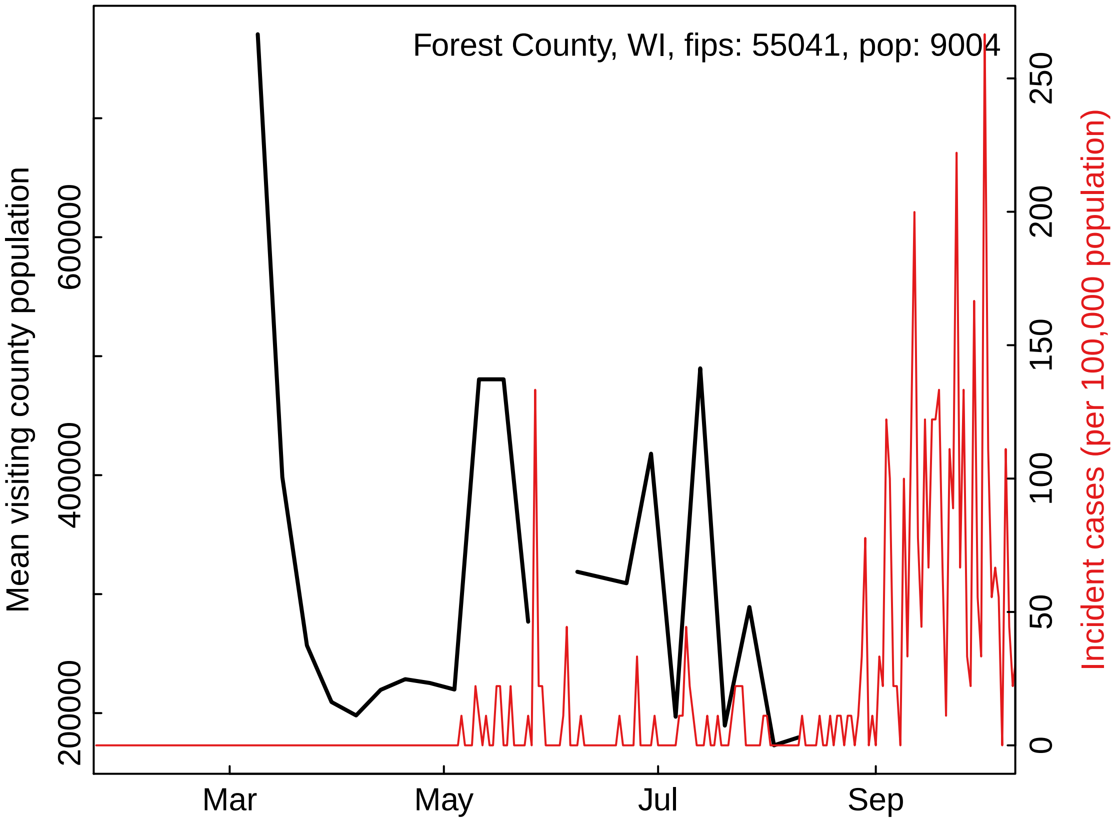

Forest County, WI, fips: 55041, pop: 9004
Mean visiting county population
200000
400000
600000
Mar
May
Jul
Sep
0
50
100
150
200
250
Incident cases (per 100,000 population)

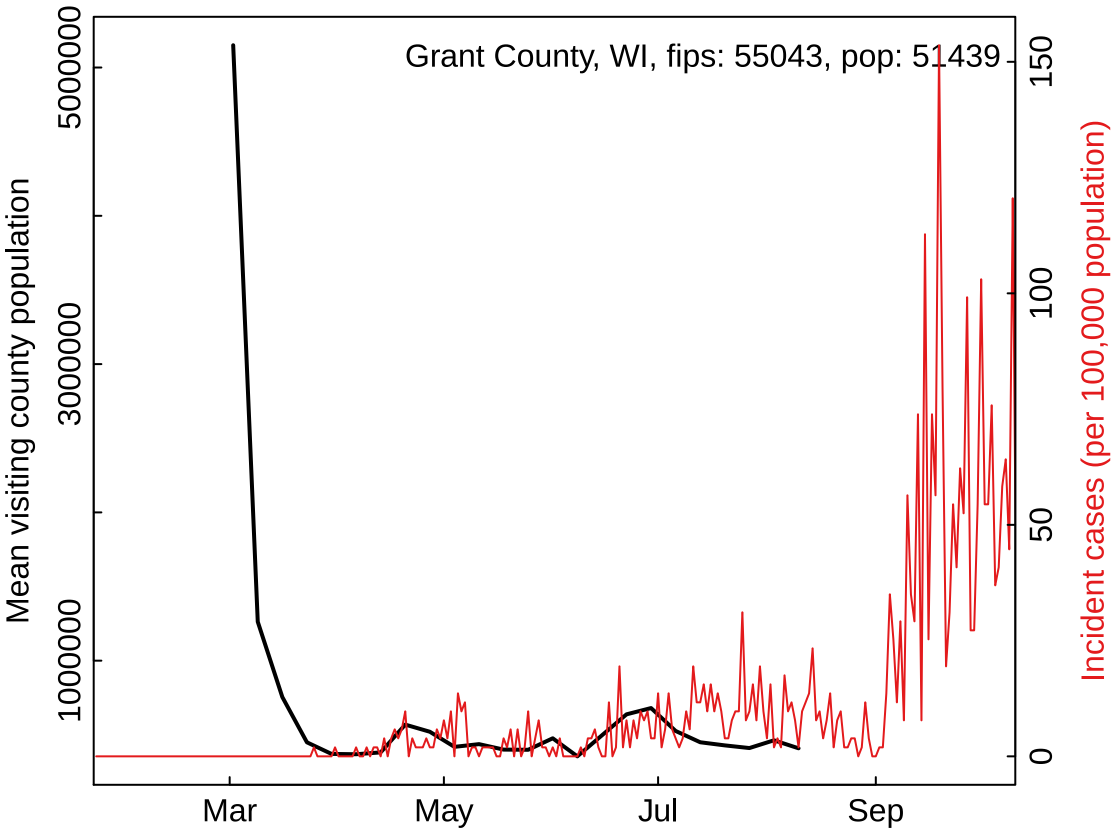

Grant County, WI, fips: 55043, pop: 51439
Mean visiting county population
1000000
3000000
5000000
Incident cases (per 100,000 population)
0
50
100
150
Mar
May
Jul
Sep

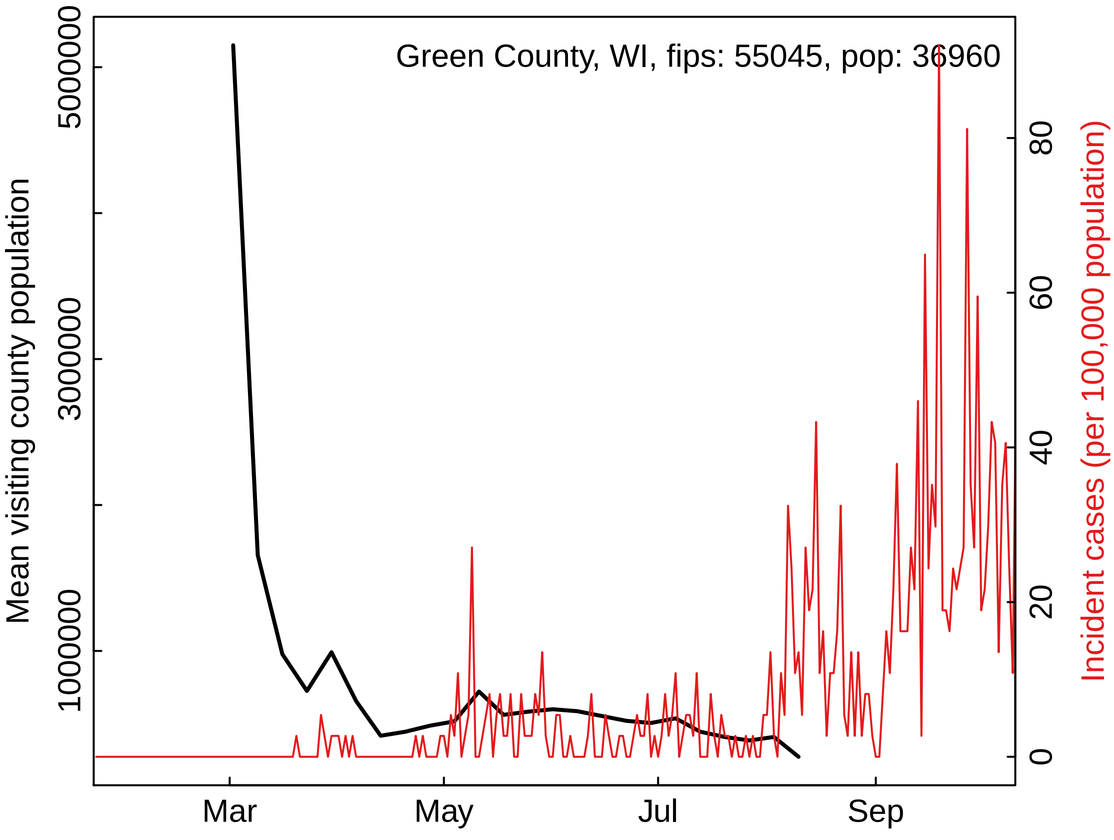

Green County, WI, fips: 55045, pop: 36960
Mean visiting county population
1000000
3000000
5000000
Incident cases (per 100,000 population)
0
20
40
60
80
Mar
May
Jul
Sep

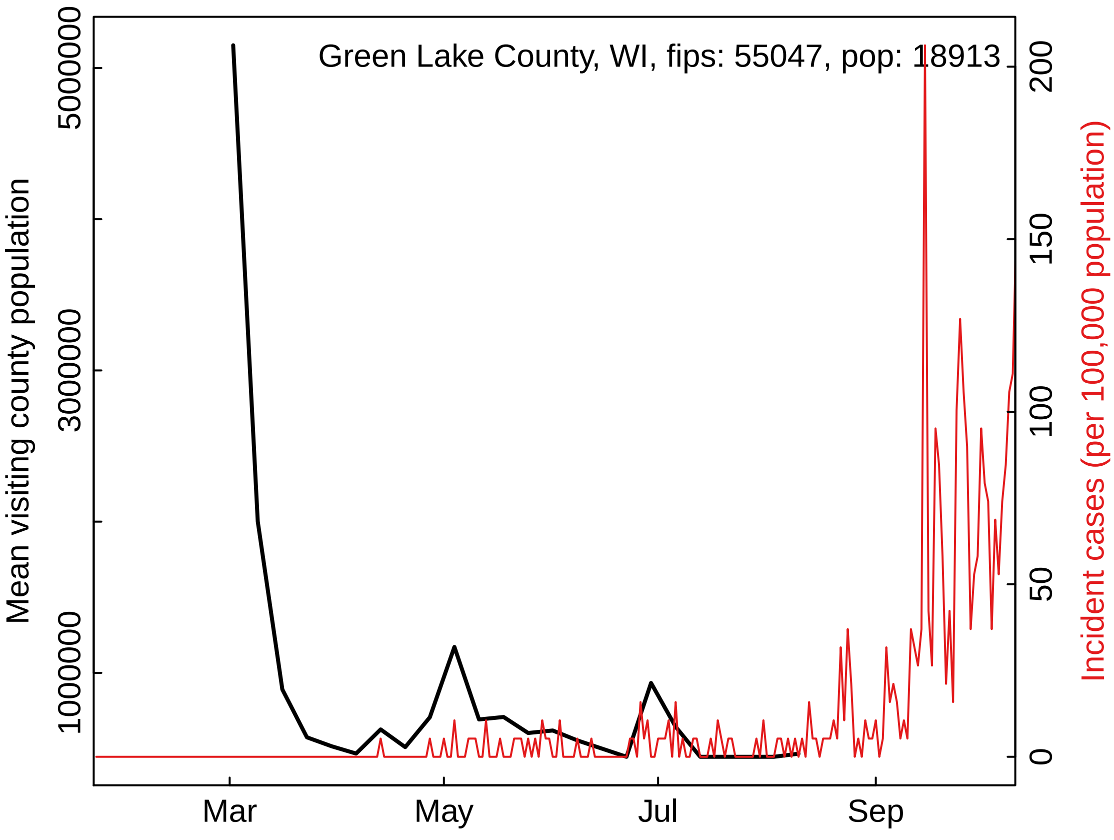
Green Lake County, WI, fips: 55047, pop: 18913
Mean visiting county population
Incident cases (per 100,000 population)
1000000
3000000
5000000
0
50
100
150
200
Mar
May
Jul
Sep

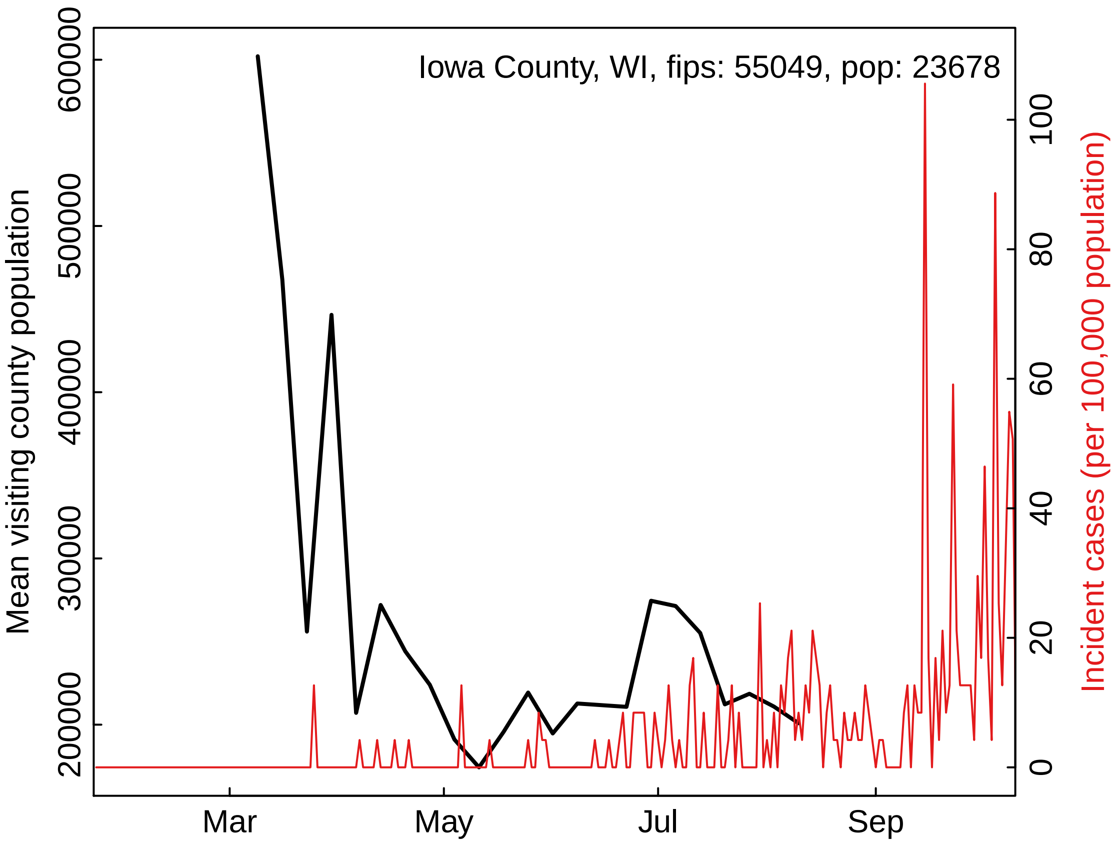

Iowa County, WI, fips: 55049, pop: 23678
Mean visiting county population
200000
300000
400000
500000
600000
Incident cases (per 100,000 population)
0
20
40
60
80
100
Mar
May
Jul
Sep

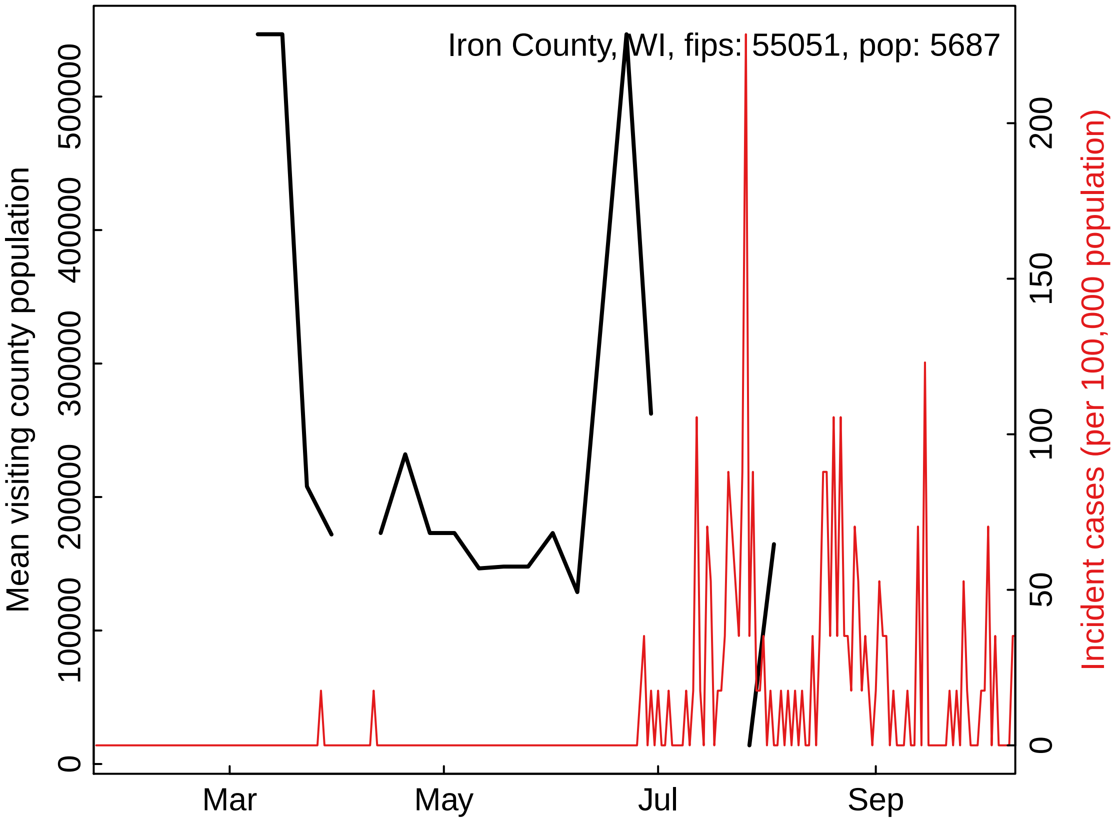
Iron County, WI, fips: 55051, pop: 5687
Mean visiting county population
0
100000
200000
300000
400000
500000
Incident cases (per 100,000 population)
0
50
100
150
200
Mar
May
Jul
Sep

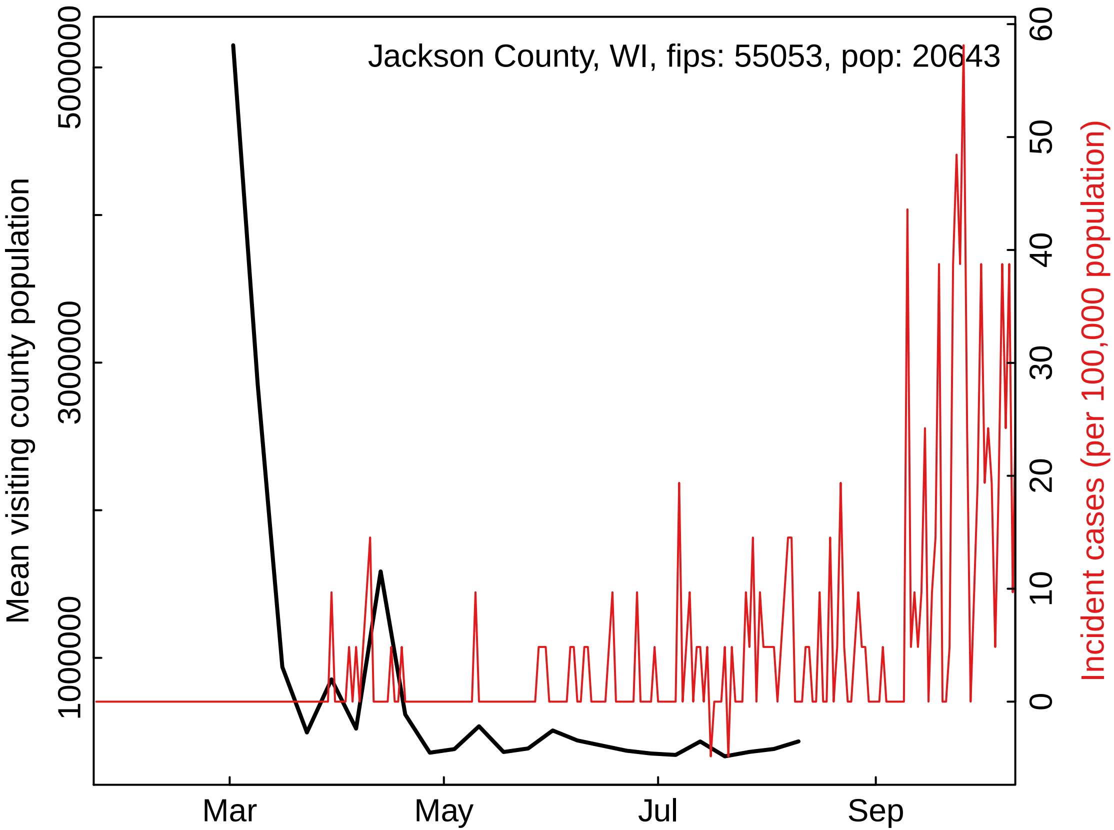

Jackson County, WI, fips: 55053, pop: 20643
Mean visiting county population
1000000
3000000
5000000
Incident cases (per 100,000 population)
0
10
20
30
40
50
60
Mar
May
Jul
Sep

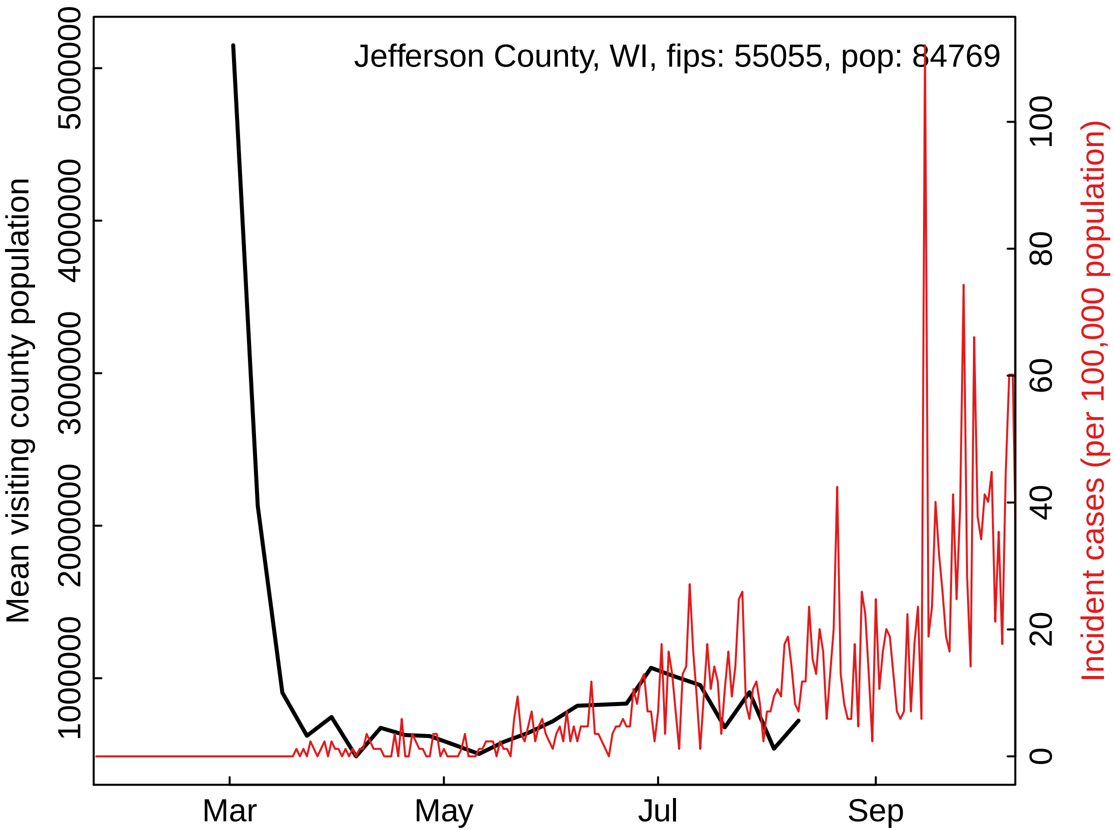

Jefferson County, WI, fips: 55055, pop: 84769
Mean visiting county population
0
1000000
2000000
3000000
4000000
5000000
Incident cases (per 100,000 population)
0
20
40
60
80
100
Mar
May
Jul
Sep

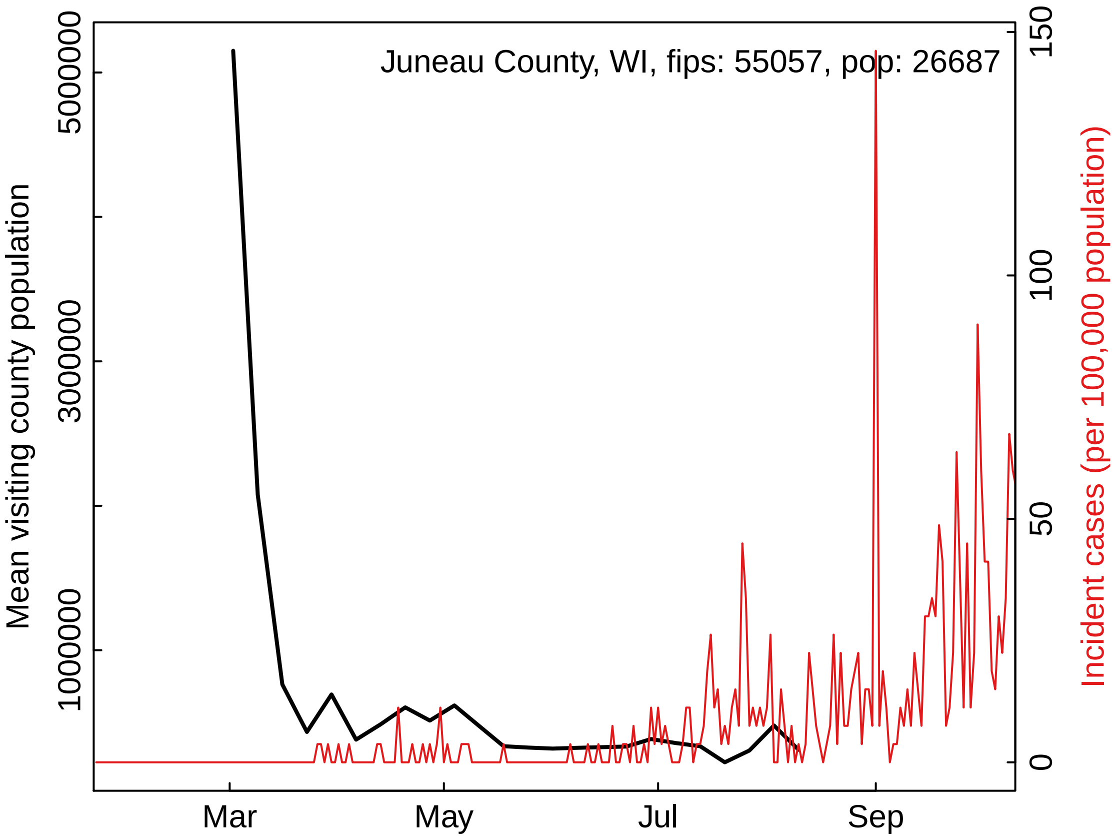

Juneau County, WI, fips: 55057, pop: 26687
Mean visiting county population
1000000
3000000
5000000
Incident cases (per 100,000 population)
0
50
100
150
Mar
May
Jul
Sep

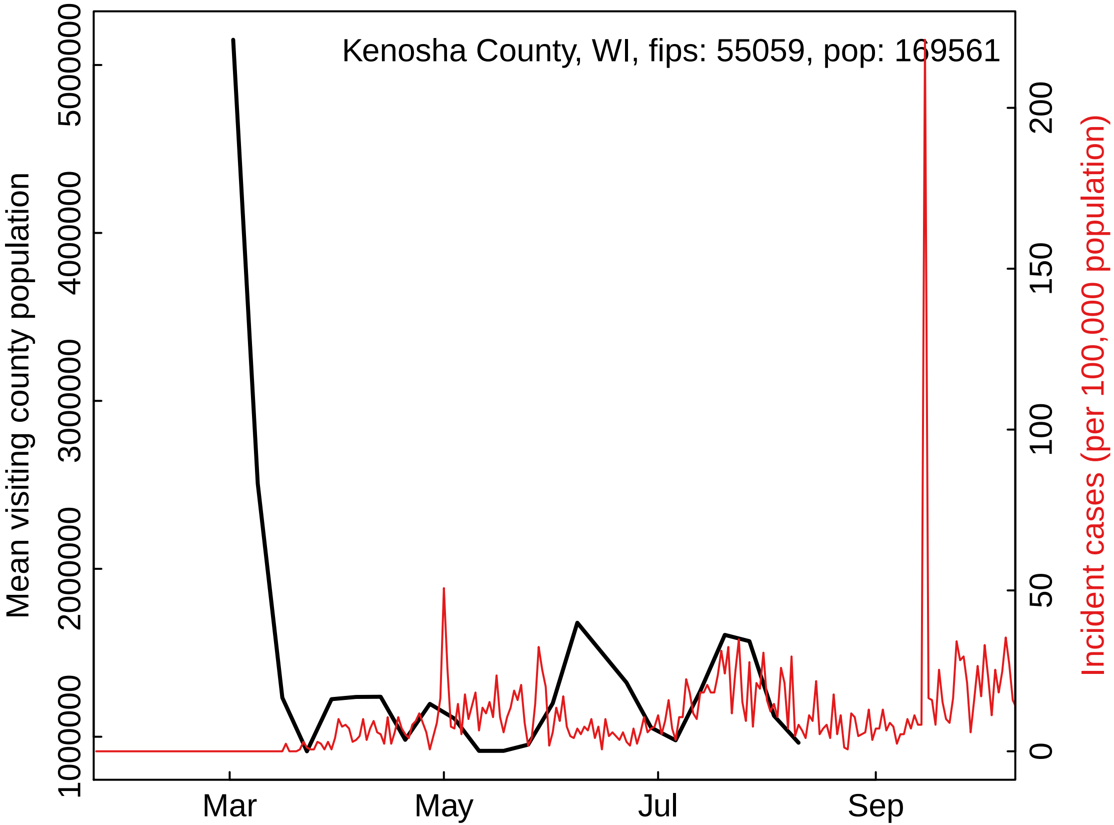

Kenosha County, WI, fips: 55059, pop: 169561
Mean visiting county population
Incident cases (per 100,000 population)
1000000
2000000
3000000
4000000
5000000
0
50
100
150
200
Mar
May
Jul
Sep

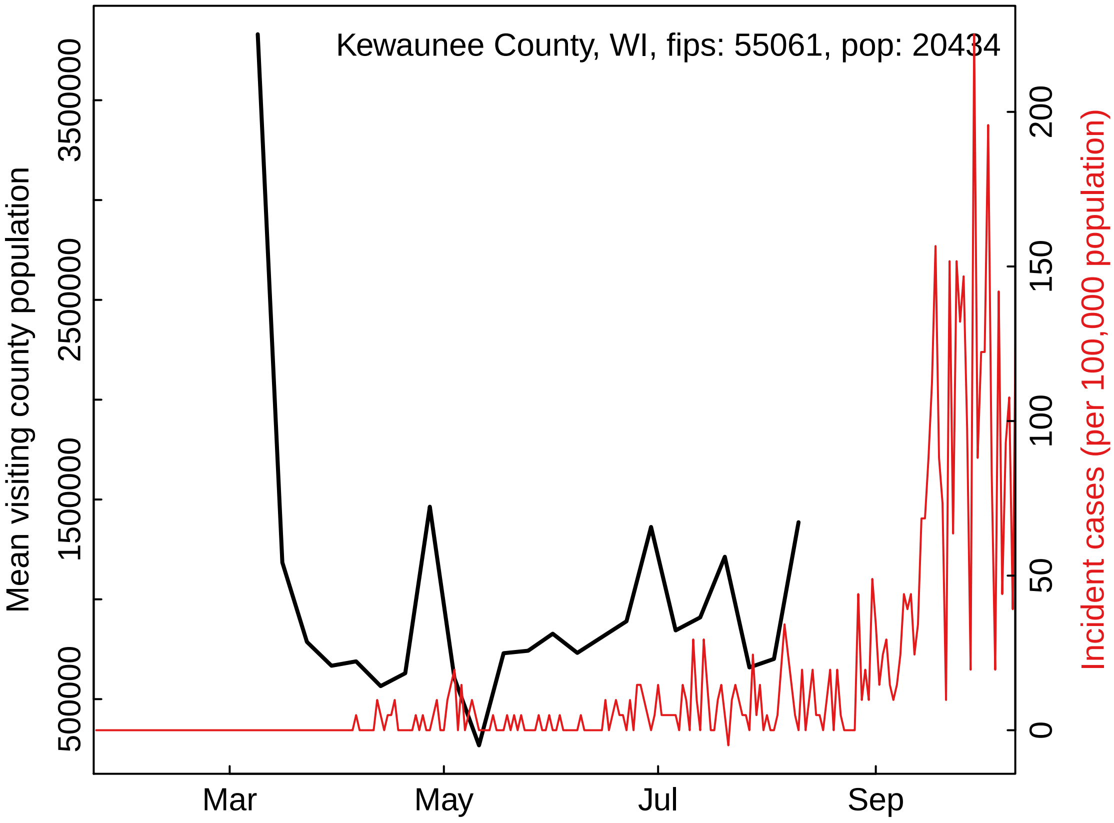

Kewaunee County, WI, fips: 55061, pop: 20434
Mean visiting county population
500000
1500000
2500000
3500000
Incident cases (per 100,000 population)
0
50
100
150
200
Mar
May
Jul
Sep

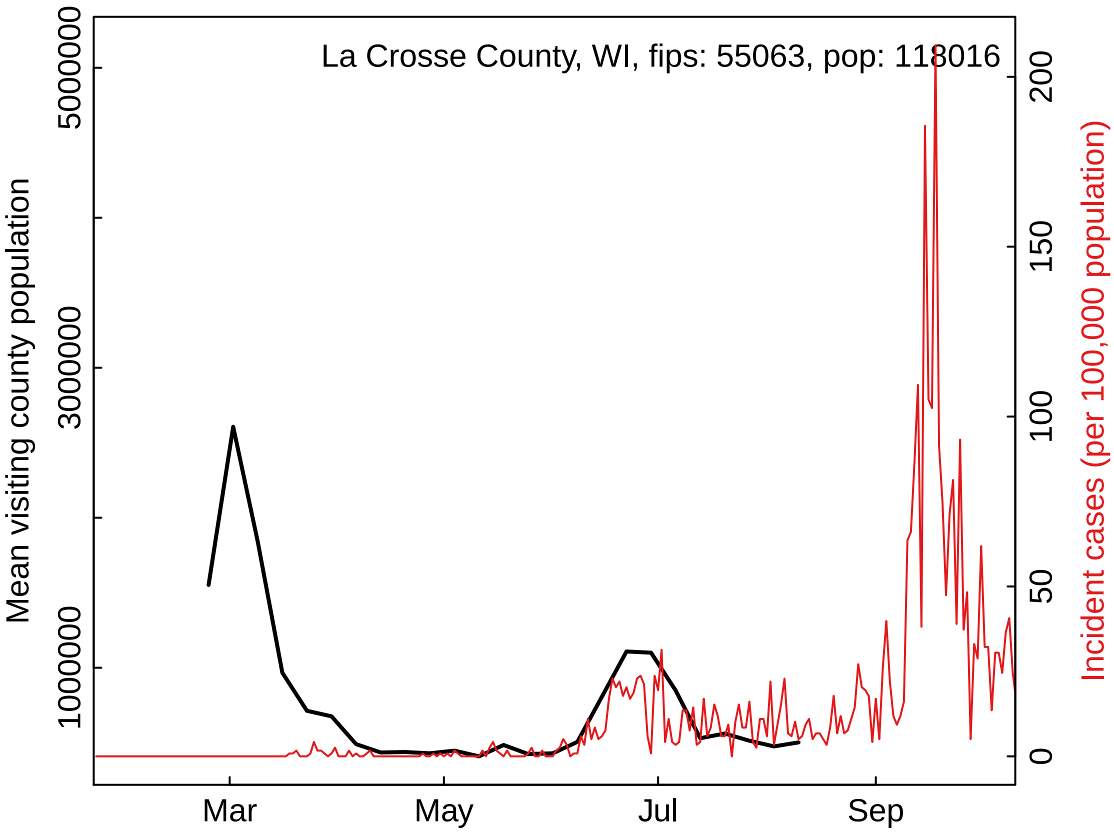

La Crosse County, WI, fips: 55063, pop: 118016
Mean visiting county population
1000000
3000000
5000000
Incident cases (per 100,000 population)
0
50
100
150
200
Mar
May
Jul
Sep

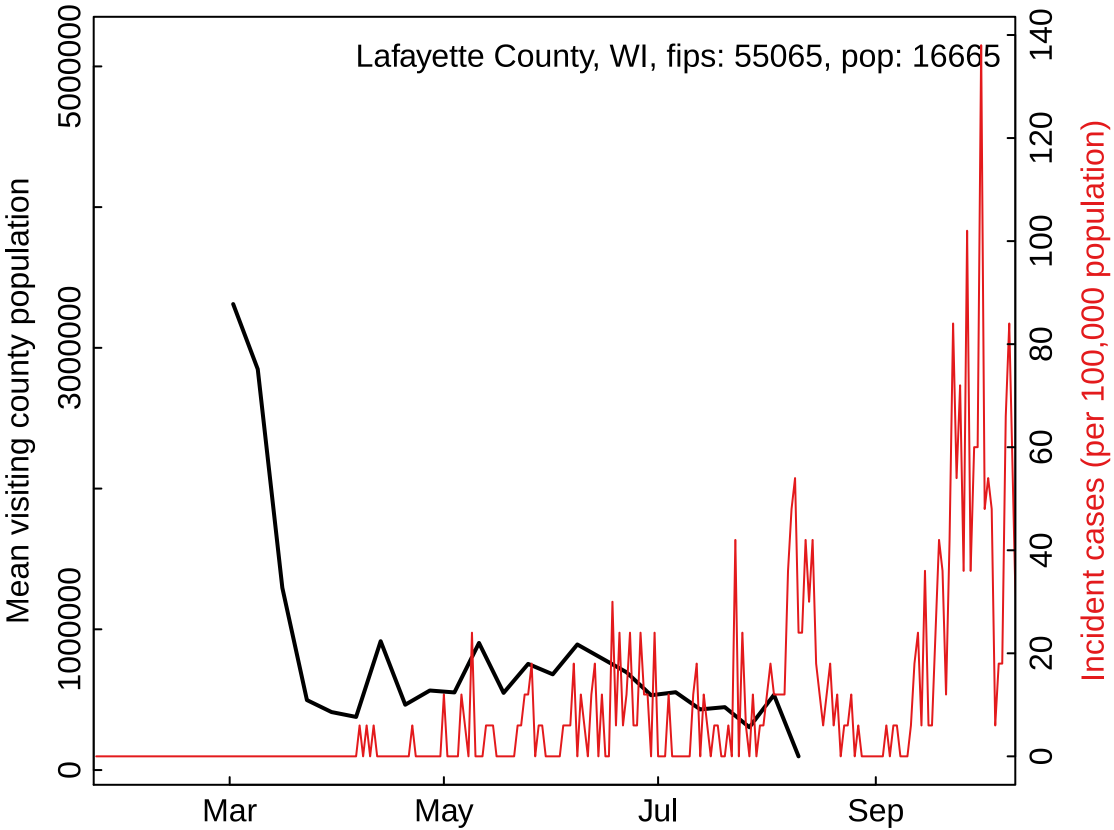

Lafayette County, WI, fips: 55065, pop: 16665
Mean visiting county population
0
1000000
3000000
5000000
Incident cases (per 100,000 population)
0
20
40
60
80
100
120
140
Mar
May
Jul
Sep

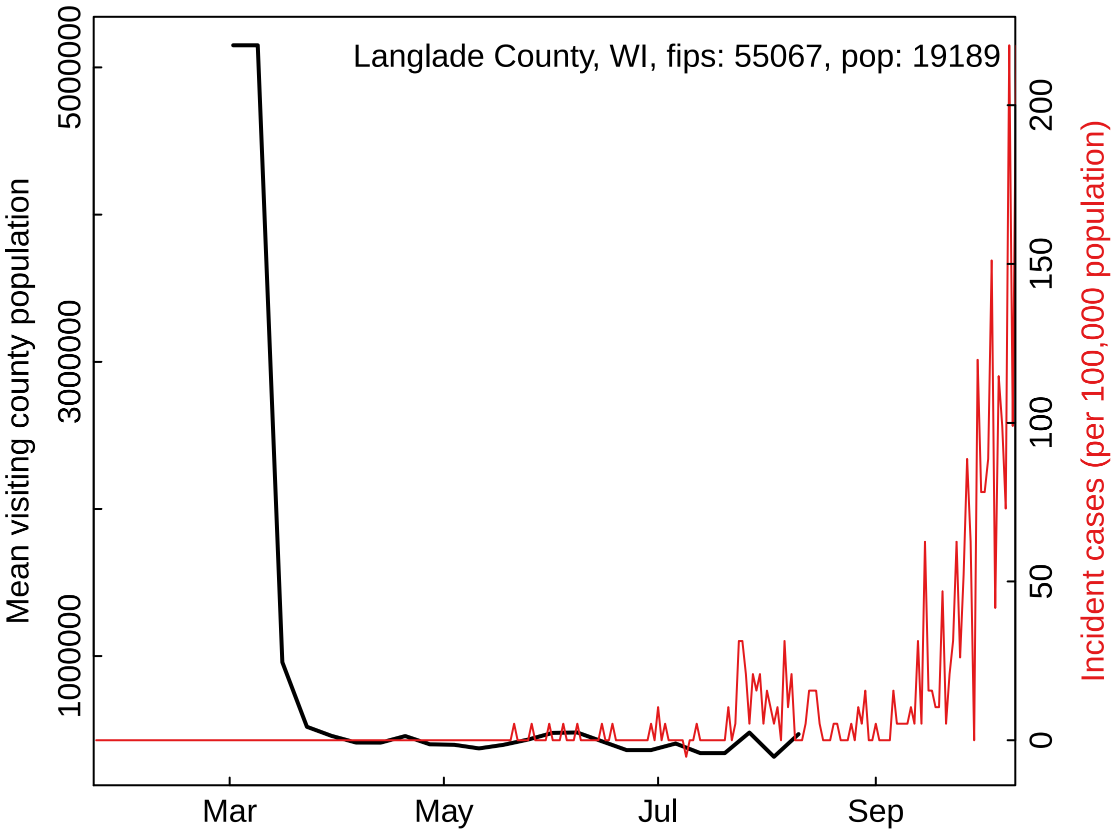

Langlade County, WI, fips: 55067, pop: 19189
Mean visiting county population
1000000
3000000
5000000
Incident cases (per 100,000 population)
0
50
100
150
200
Mar
May
Jul
Sep

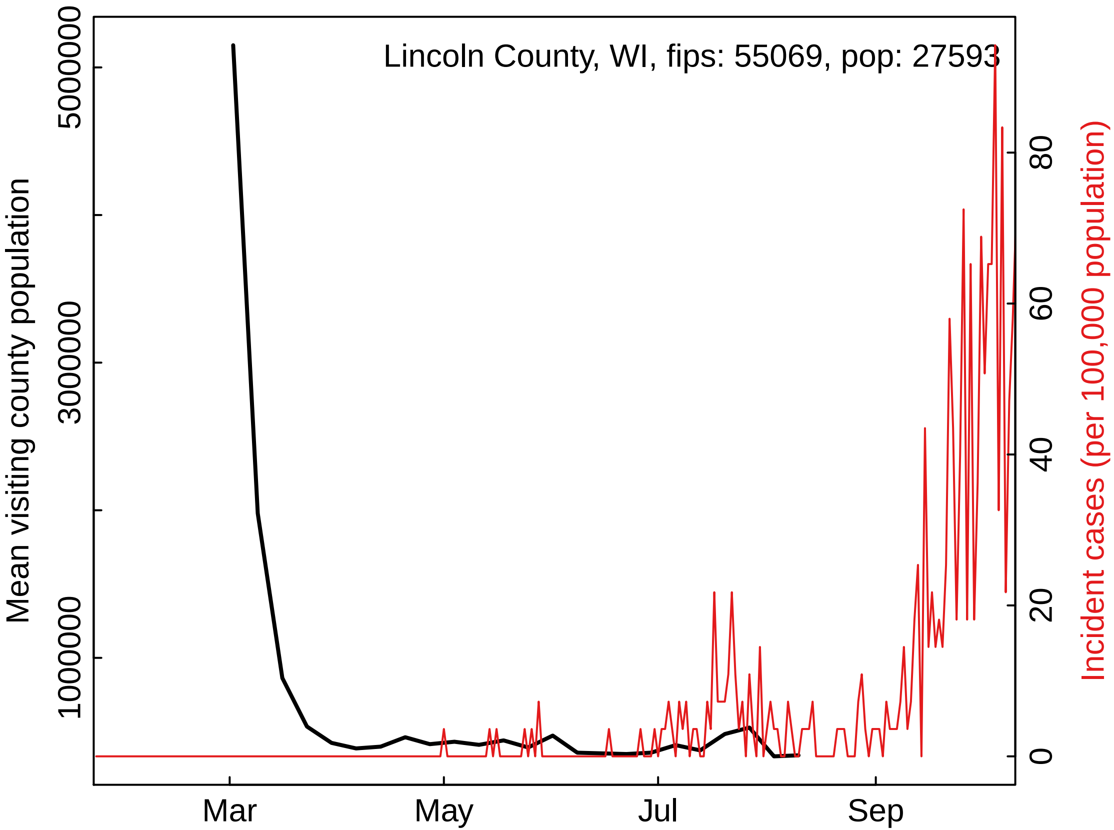
Lincoln County, WI, fips: 55069, pop: 27593
Mean visiting county population
1000000
3000000
5000000
Incident cases (per 100,000 population)
0
20
40
60
80
Mar
May
Jul
Sep

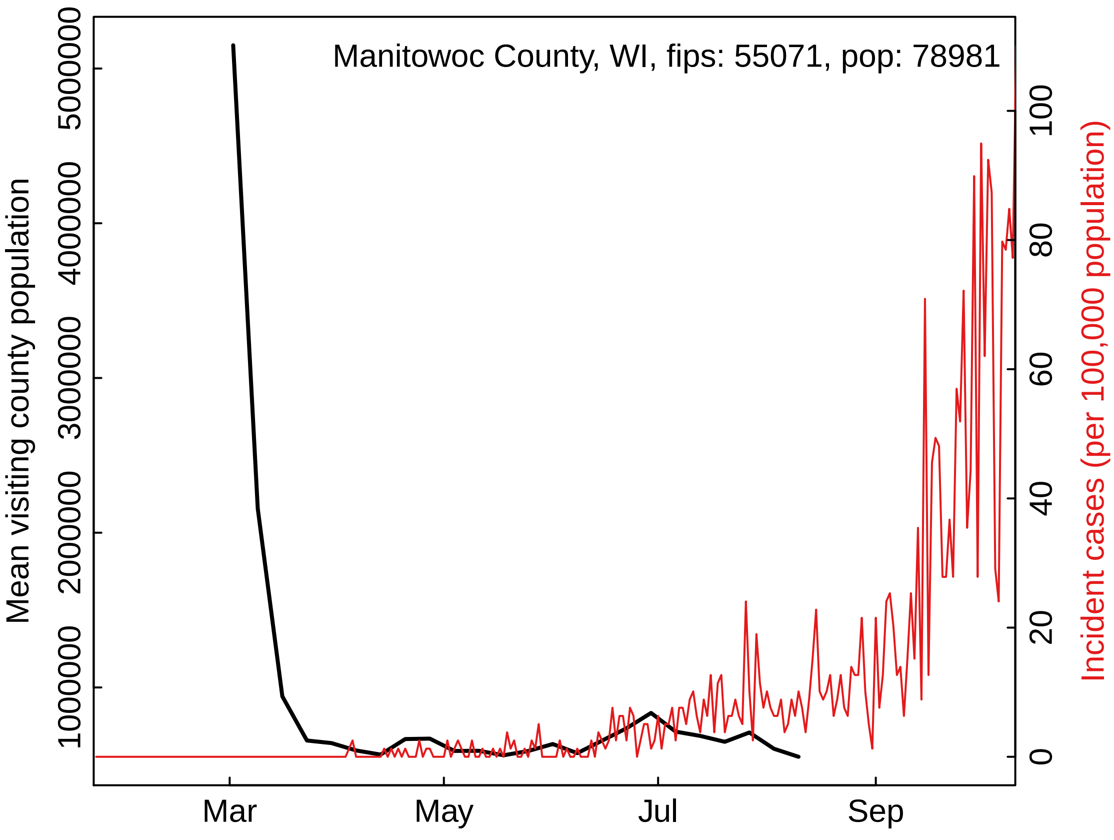

Manitowoc County, WI, fips: 55071, pop: 78981
Mean visiting county population
10000000
20000000
30000000
40000000
50000000
Incident cases (per 100,000 population)
0
20
40
60
80
100
Mar
May
Jul
Sep

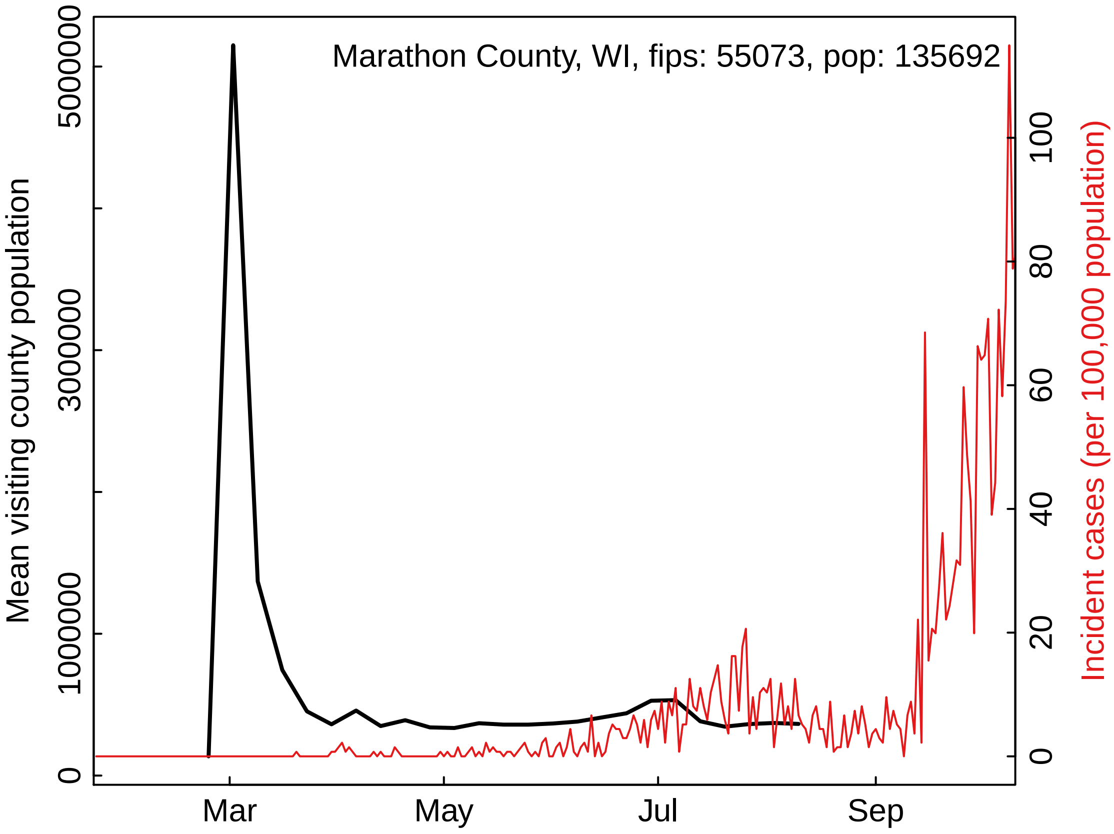

Marathon County, WI, fips: 55073, pop: 135692
Mean visiting county population
0
1000000
3000000
5000000
Incident cases (per 100,000 population)
0
20
40
60
80
100
Mar
May
Jul
Sep

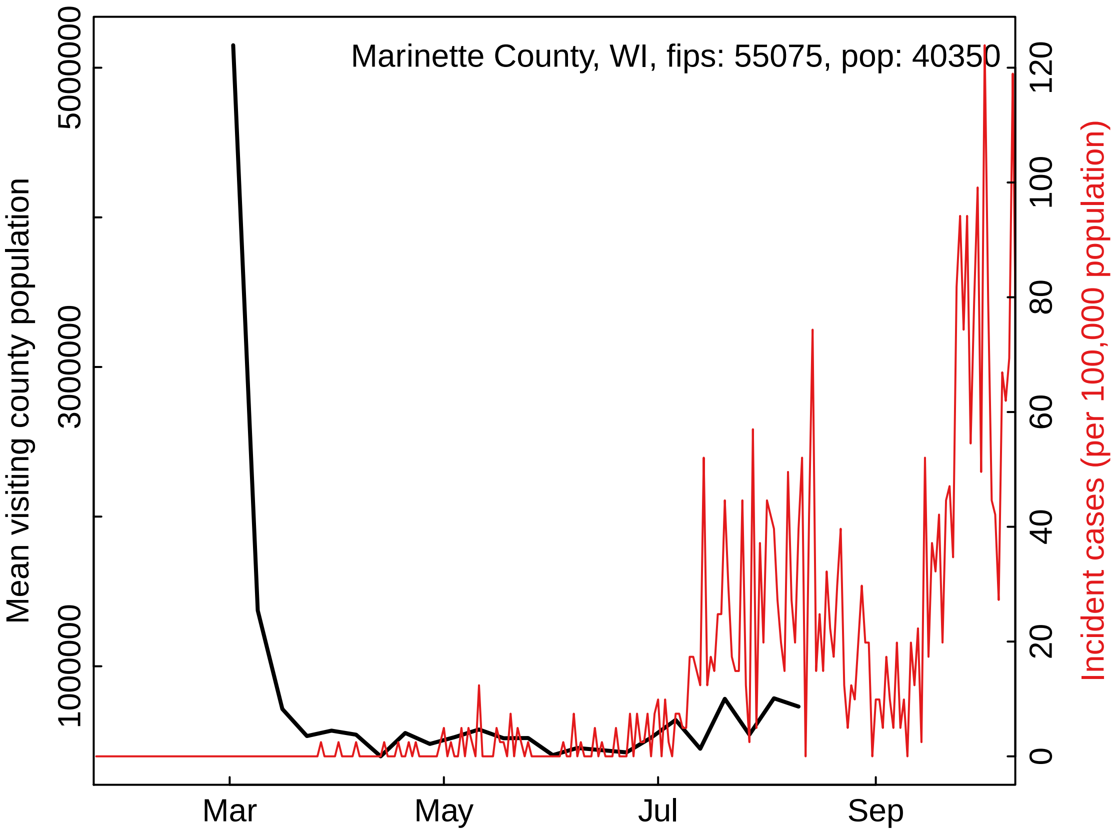

Marinette County, WI, fips: 55075, pop: 40350
Mean visiting county population
1000000
3000000
5000000
Mar
May
Jul
Sep
0
20
40
60
80
100
120
Incident cases (per 100,000 population)

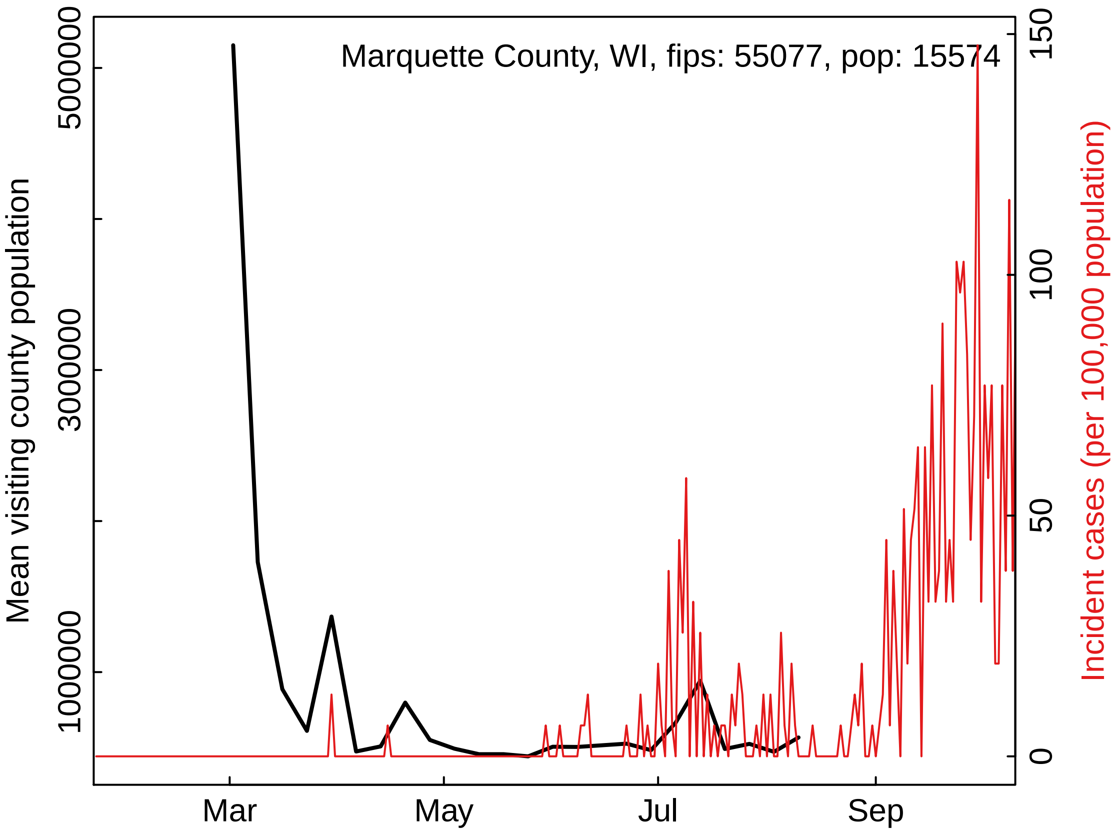

Marquette County, WI, fips: 55077, pop: 15574
Mean visiting county population
1000000
3000000
5000000
Mar
May
Jul
Sep
Incident cases (per 100,000 population)
0
50
100
150

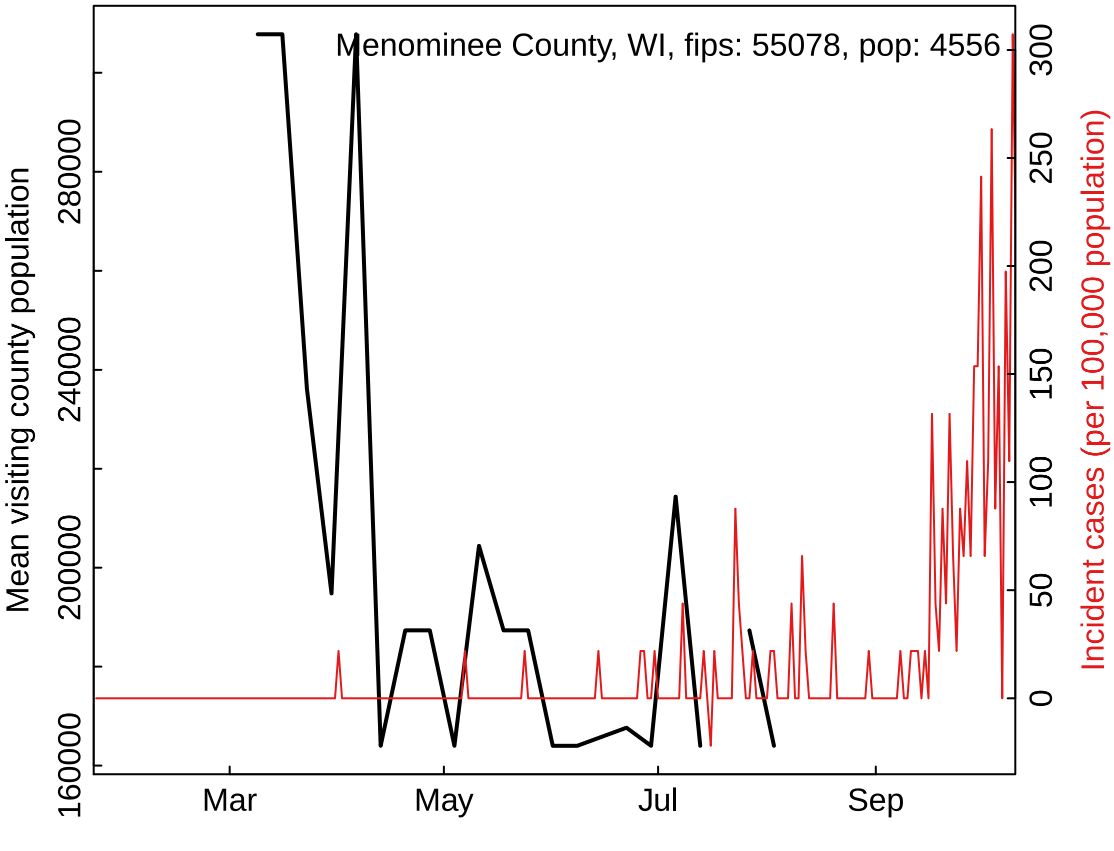

Menominee County, WI, fips: 55078, pop: 4556
Mean visiting county population
160000
200000
240000
280000
Mar
May
Jul
Sep
Incident cases (per 100,000 population)
0
50
100
150
200
250
300

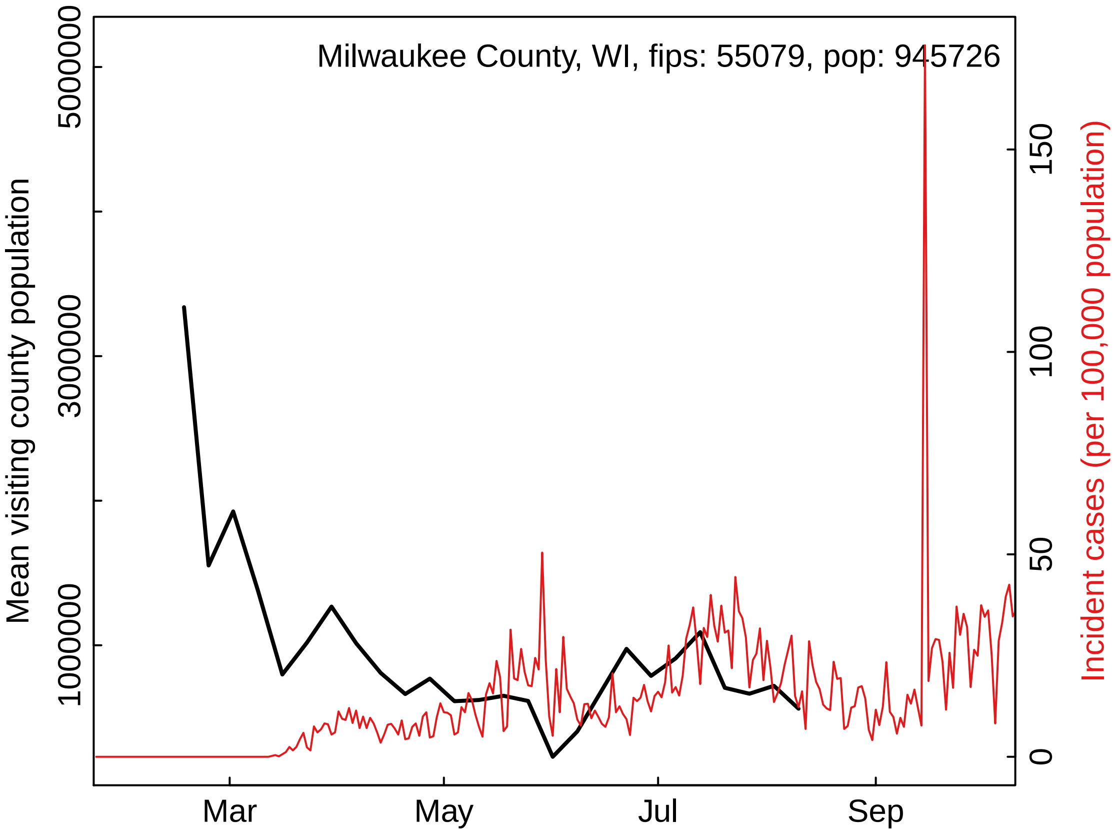

Milwaukee County, WI, fips: 55079, pop: 945726
Mean visiting county population
5000000
3000000
1000000
Incident cases (per 100,000 population)
150
100
50
0
Mar
May
Jul
Sep

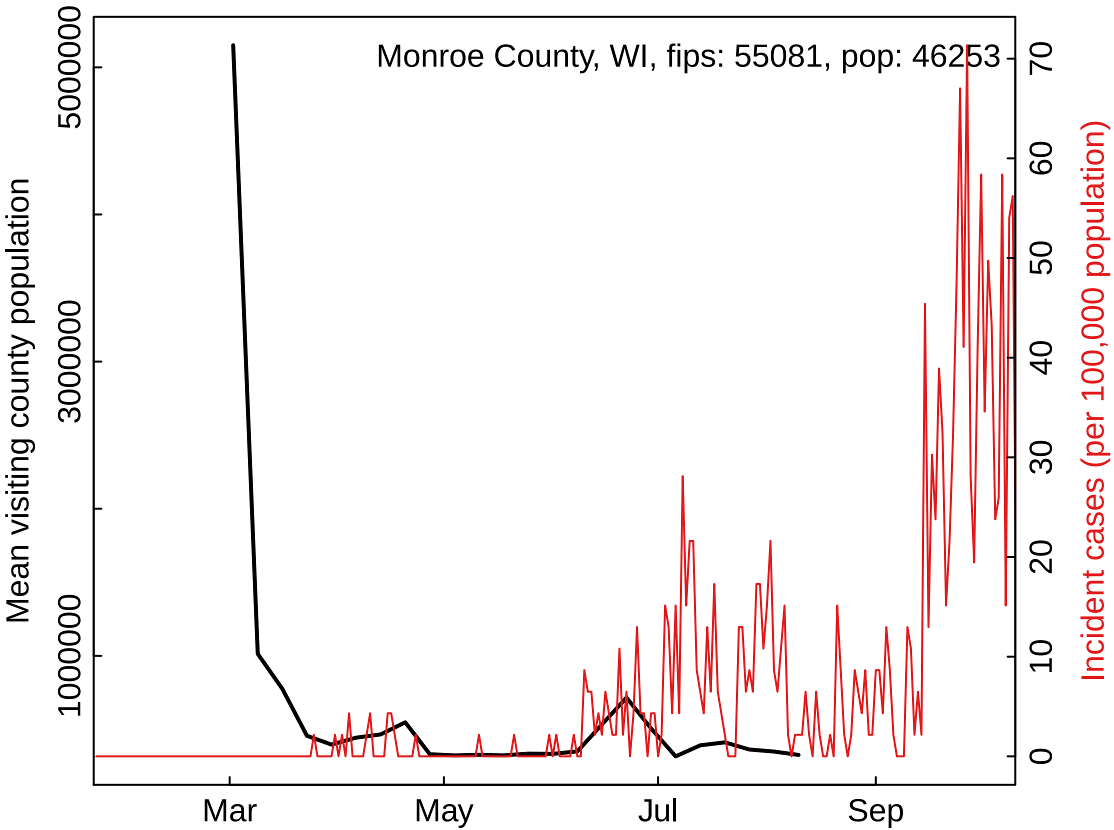

Monroe County, WI, fips: 55081, pop: 46253
Mean visiting county population
1000000
3000000
5000000
Incident cases (per 100,000 population)
0
10
20
30
40
50
60
70
Mar
May
Jul
Sep

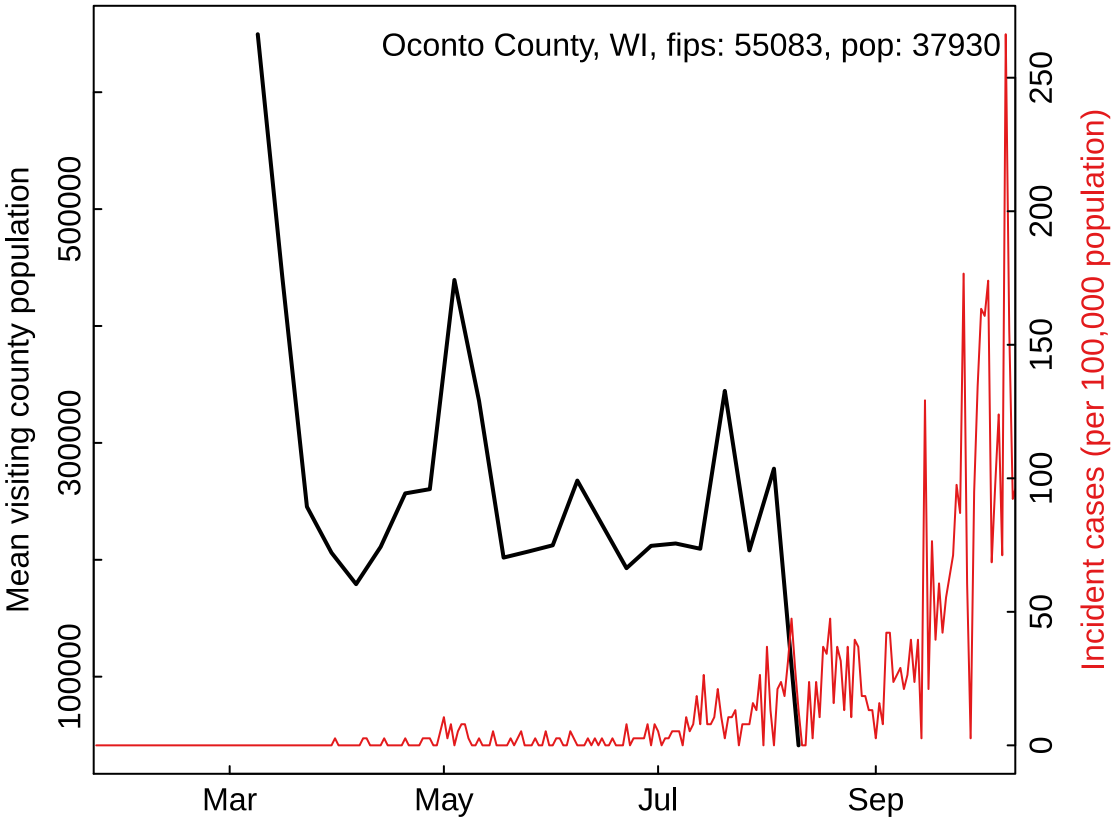

Oconto County, WI, fips: 55083, pop: 37930
Mean visiting county population
100000
300000
500000
Mar
May
Jul
Sep
0
50
100
150
200
250
Incident cases (per 100,000 population)

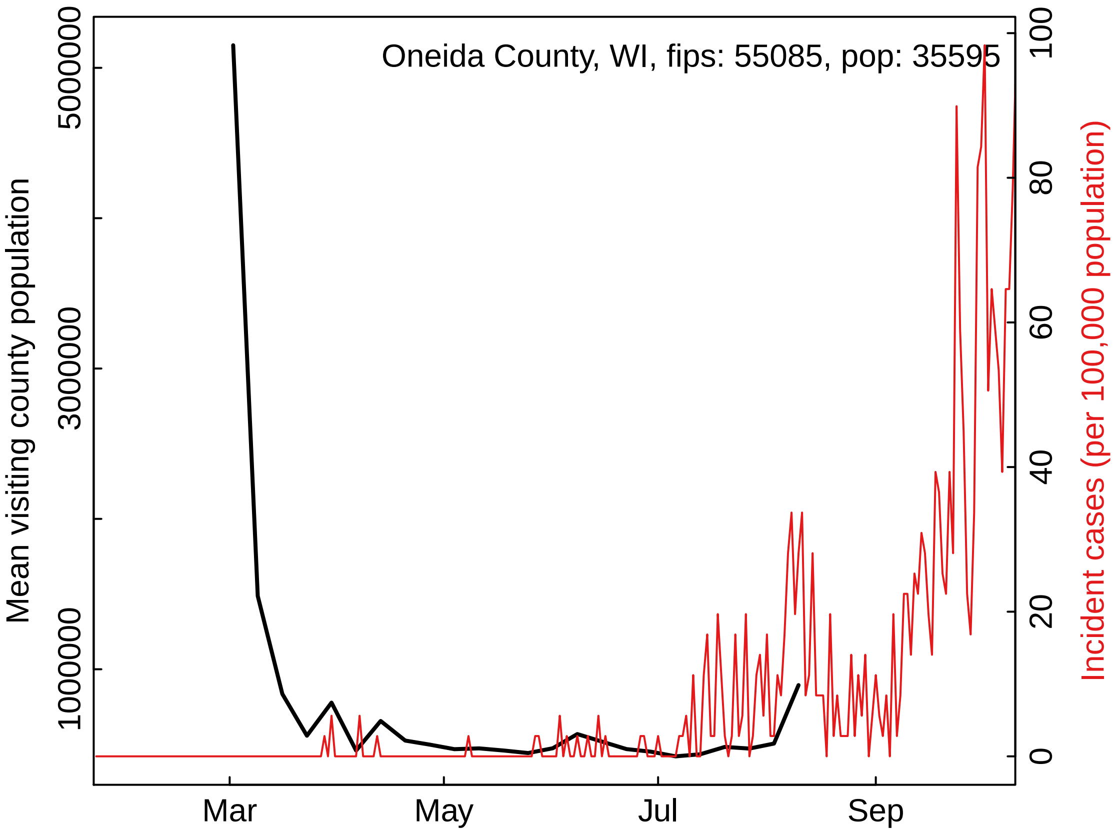

Oneida County, WI, fips: 55085, pop: 35595
Mean visiting county population
1000000
3000000
5000000
Incident cases (per 100,000 population)
0
20
40
60
80
100
Mar
May
Jul
Sep

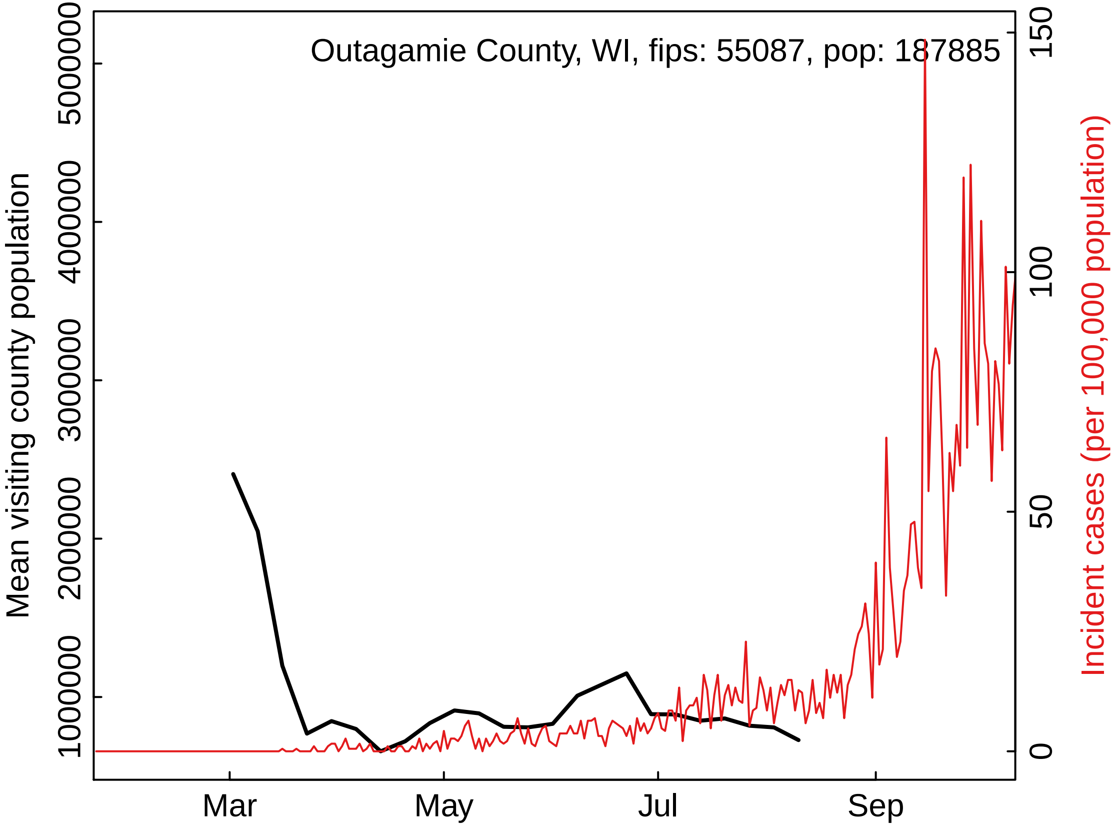
Outagamie County, WI, fips: 55087, pop: 187885
Mean visiting county population
10000000
20000000
30000000
40000000
50000000
Incident cases (per 100,000 population)
0
50
100
150
Mar
May
Jul
Sep

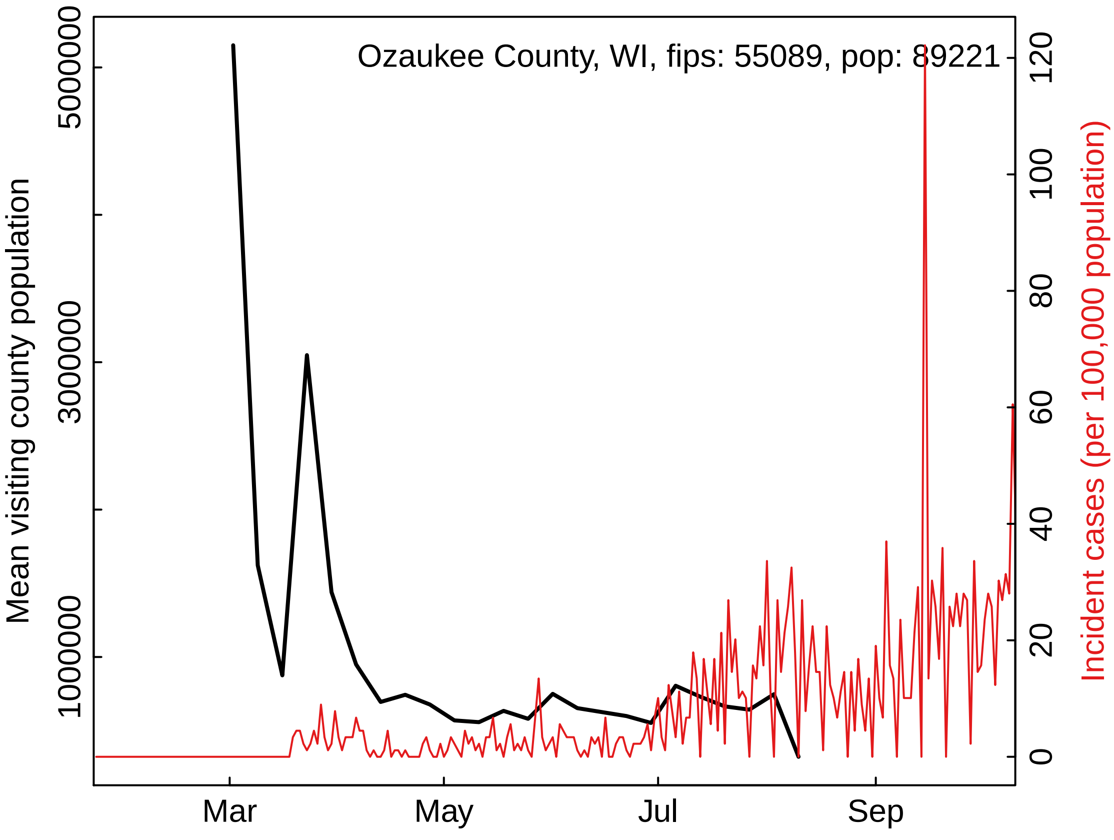

Ozaukee County, WI, fips: 55089, pop: 89221
Mean visiting county population
1000000
3000000
5000000
Mar
May
Jul
Sep
Incident cases (per 100,000 population)
0
20
40
60
80
100
120

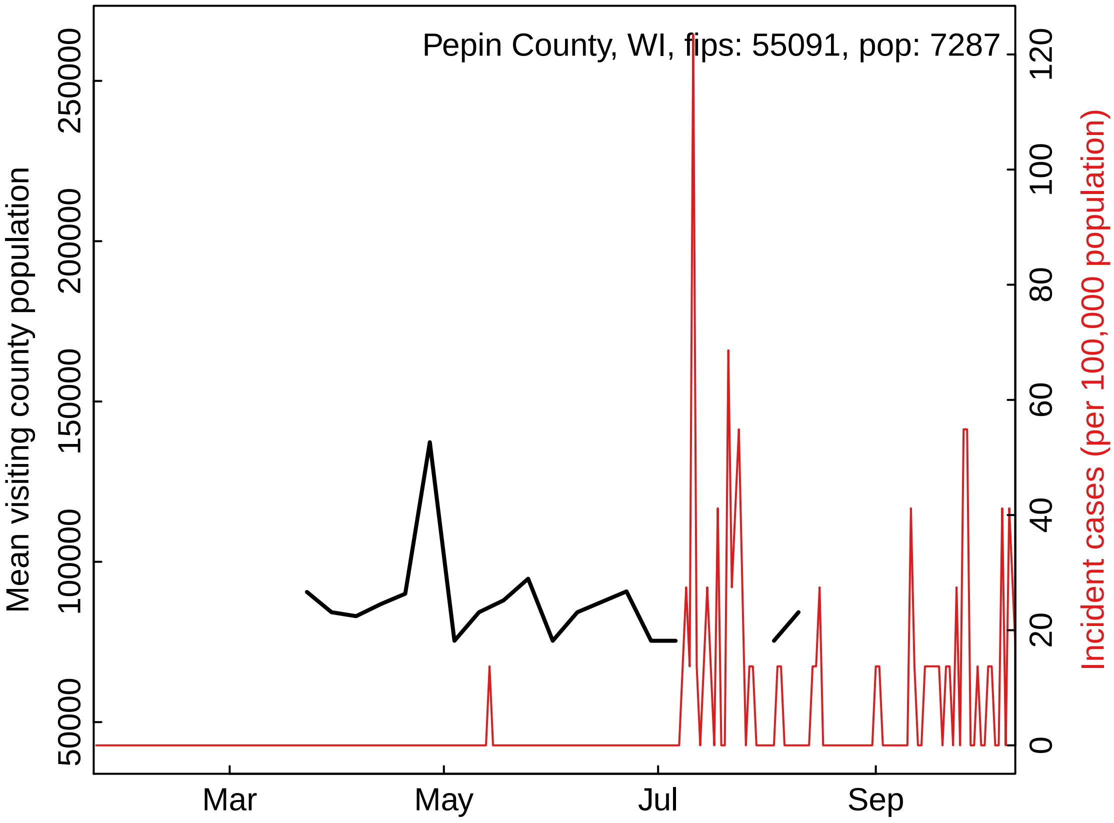

Pepin County, WI, fips: 55091, pop: 7287
Mean visiting county population
50000
100000
150000
200000
250000
Incident cases (per 100,000 population)
0
20
40
60
80
100
120
Mar
May
Jul
Sep

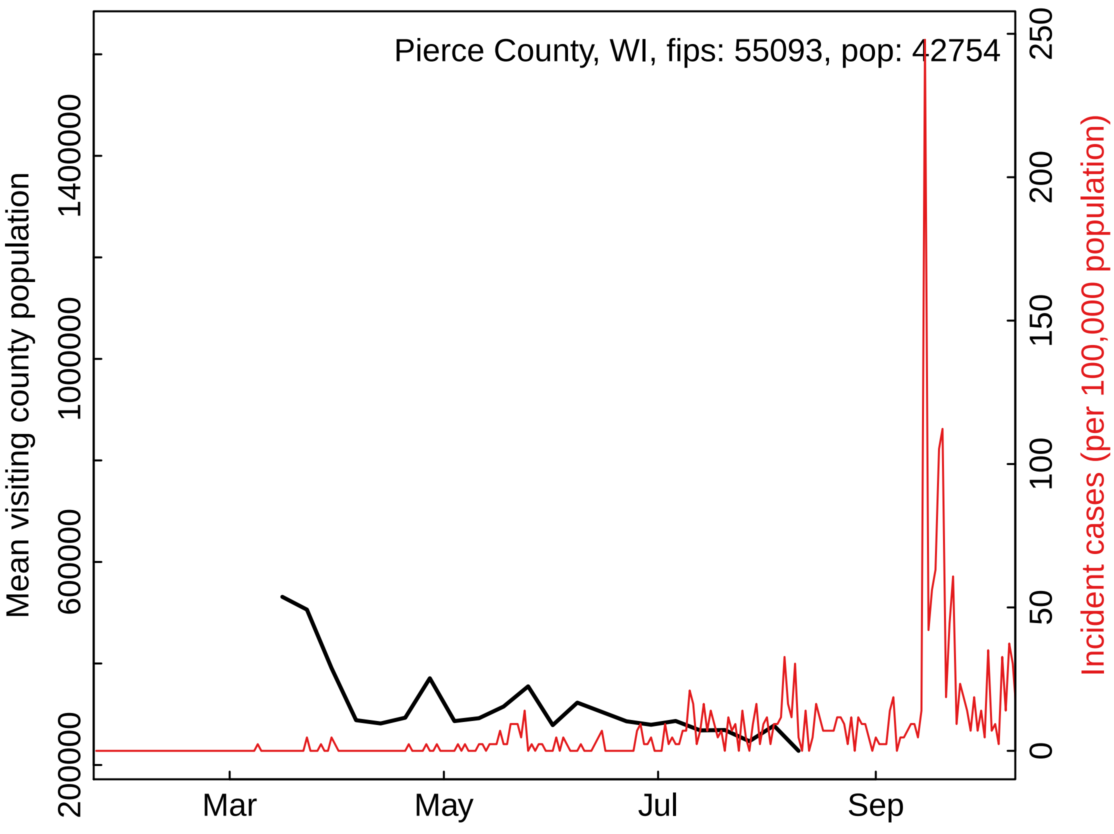

Pierce County, WI, fips: 55093, pop: 42754
Mean visiting county population
200000
600000
1000000
1400000
Incident cases (per 100,000 population)
0
50
100
150
200
250
Mar
May
Jul
Sep

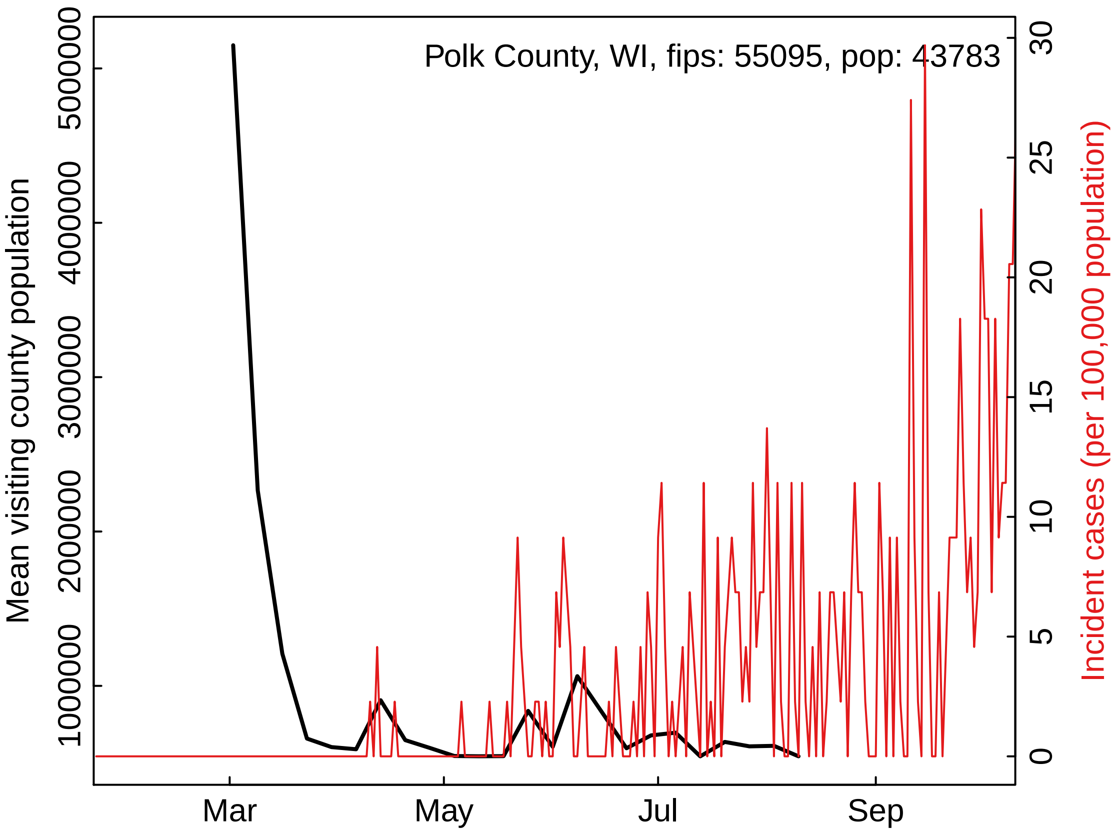

Polk County, WI, fips: 55095, pop: 43783
Mean visiting county population
1000000
2000000
3000000
4000000
5000000
Incident cases (per 100,000 population)
0
5
10
15
20
25
30
Mar
May
Jul
Sep

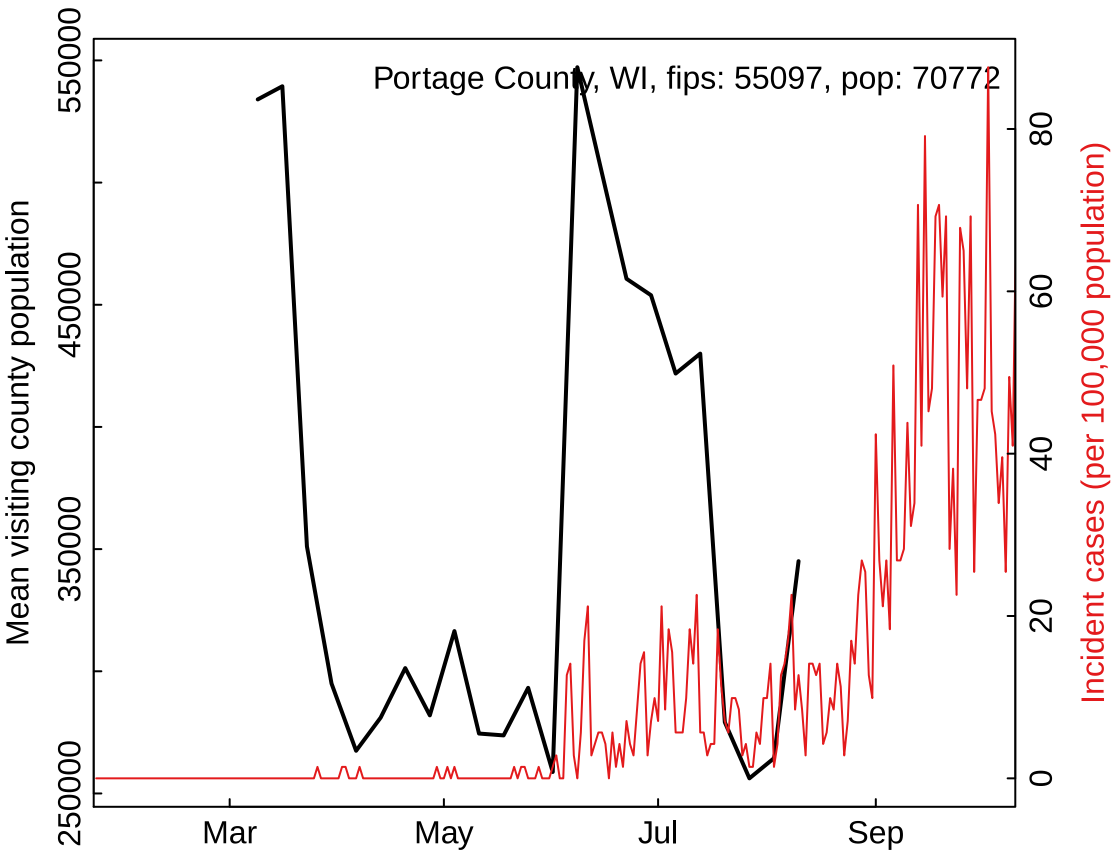

Portage County, WI, fips: 55097, pop: 70772
Mean visiting county population
250000
350000
450000
550000
Incident cases (per 100,000 population)
0
20
40
60
80
Mar
May
Jul
Sep

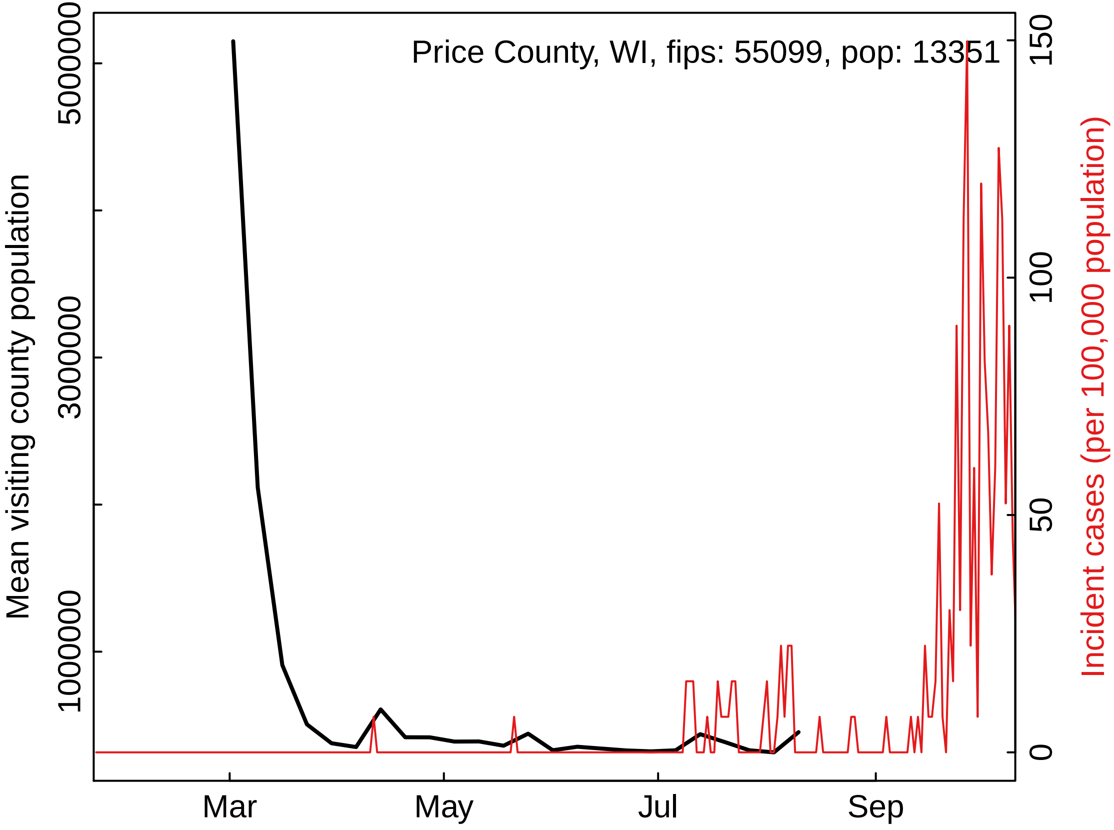

Price County, WI, fips: 55099, pop: 13351
Mean visiting county population
Incident cases (per 100,000 population)
1000000
3000000
5000000
0
50
100
150
Mar
May
Jul
Sep

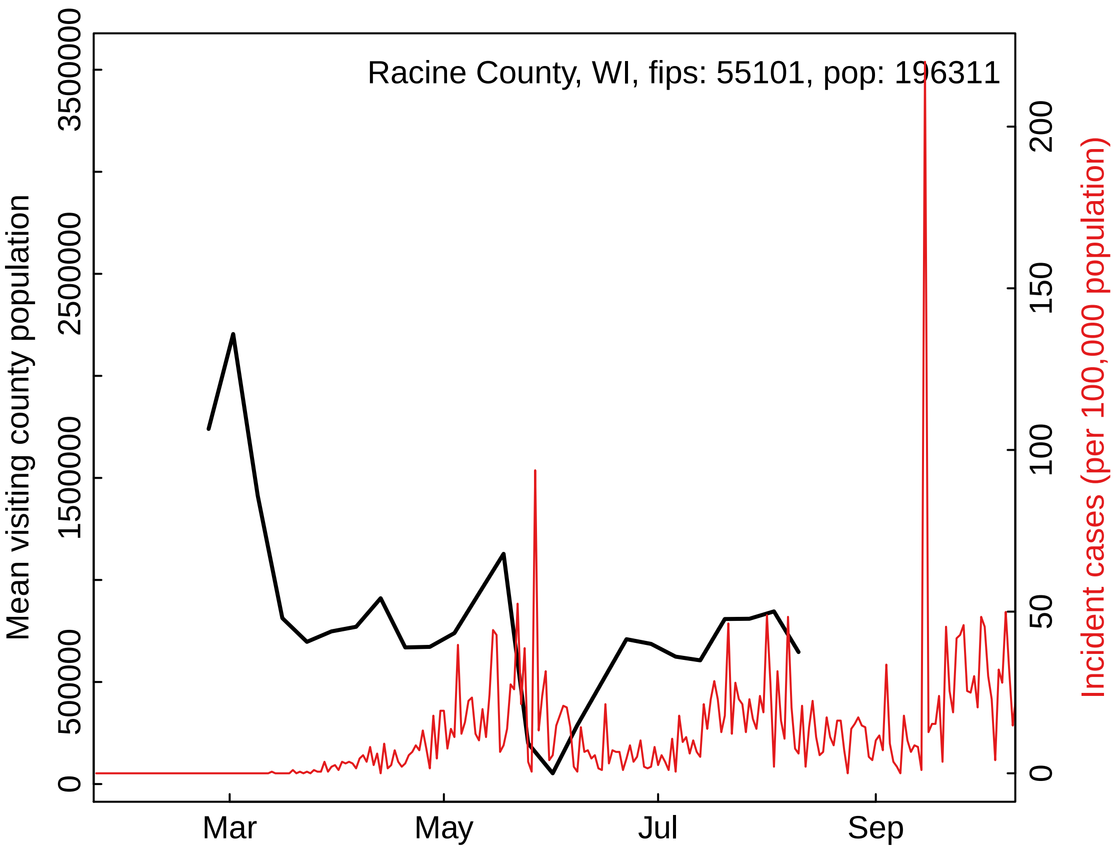

Racine County, WI, fips: 55101, pop: 196311
Mean visiting county population
Incident cases (per 100,000 population)
0
500000
1500000
2500000
3500000
0
50
100
150
200
Mar
May
Jul
Sep

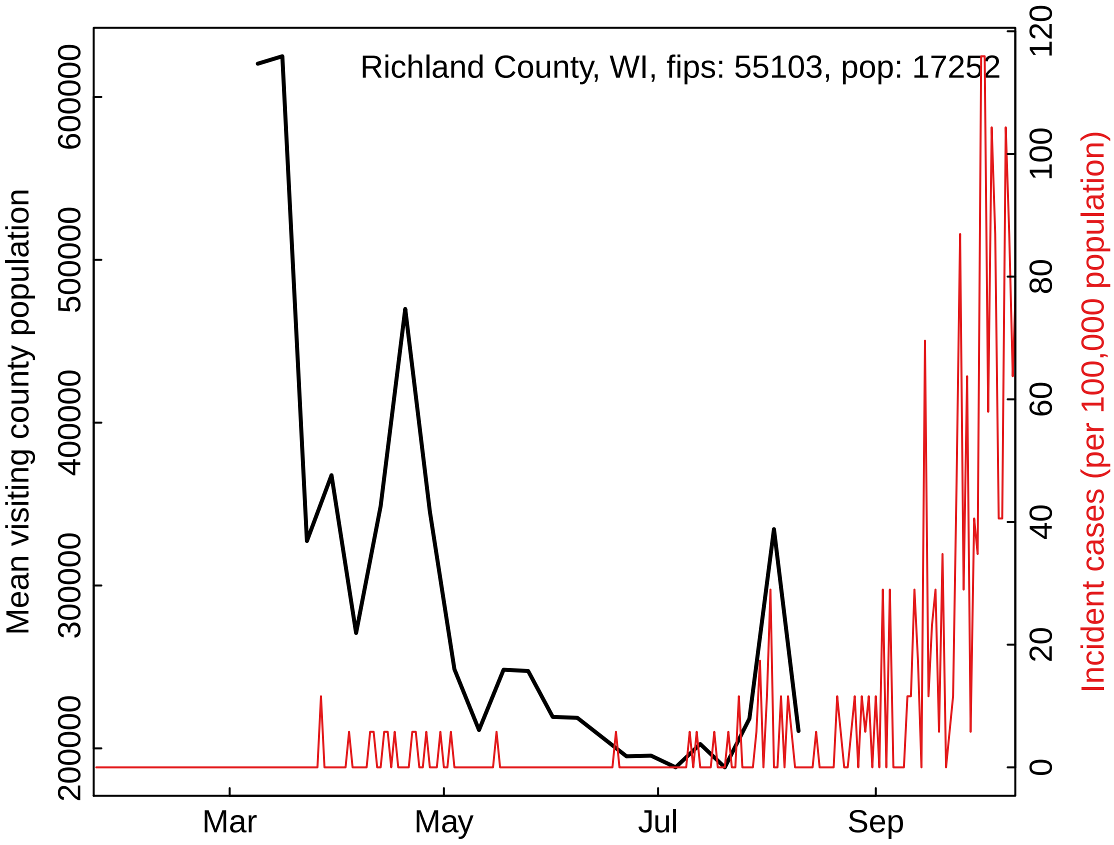

Richland County, WI, fips: 55103, pop: 17252
Mean visiting county population
Incident cases (per 100,000 population)
200000
300000
400000
500000
600000
0
20
40
60
80
100
120
Mar
May
Jul
Sep

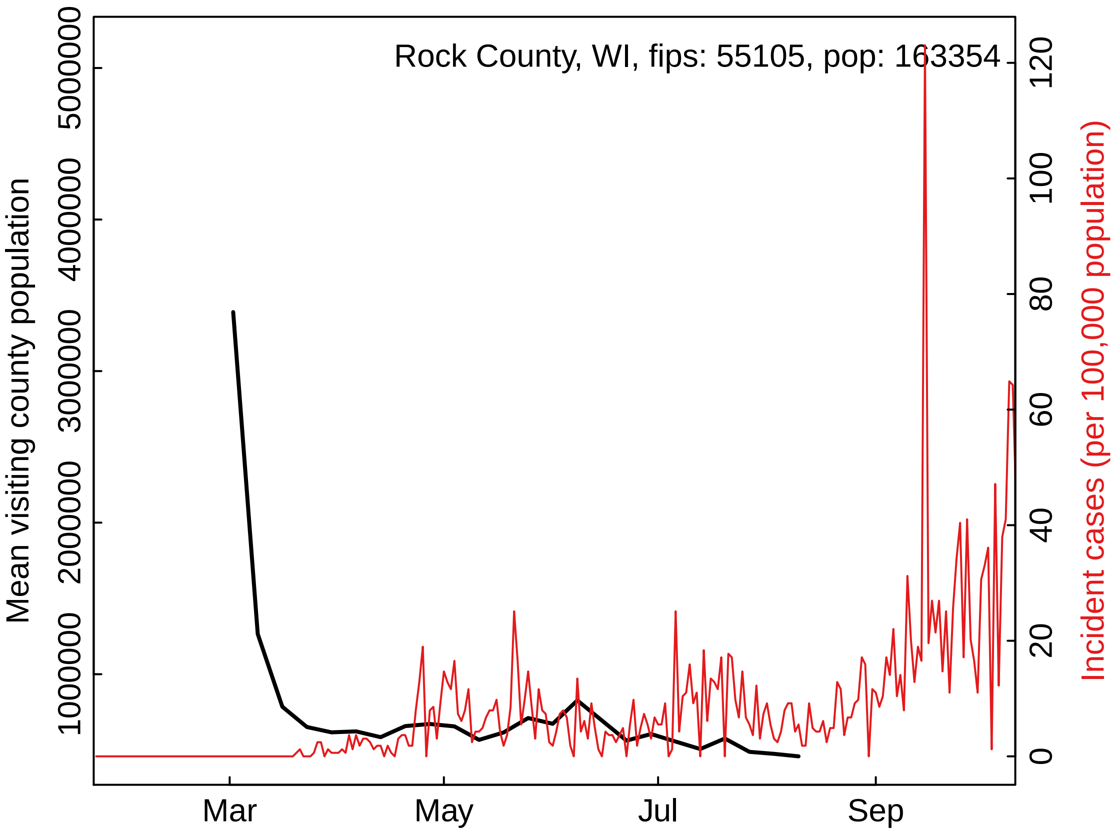

Rock County, WI, fips: 55105, pop: 163354
Mean visiting county population
1000000
2000000
3000000
4000000
5000000
Incident cases (per 100,000 population)
0
20
40
60
80
100
120
Mar
May
Jul
Sep

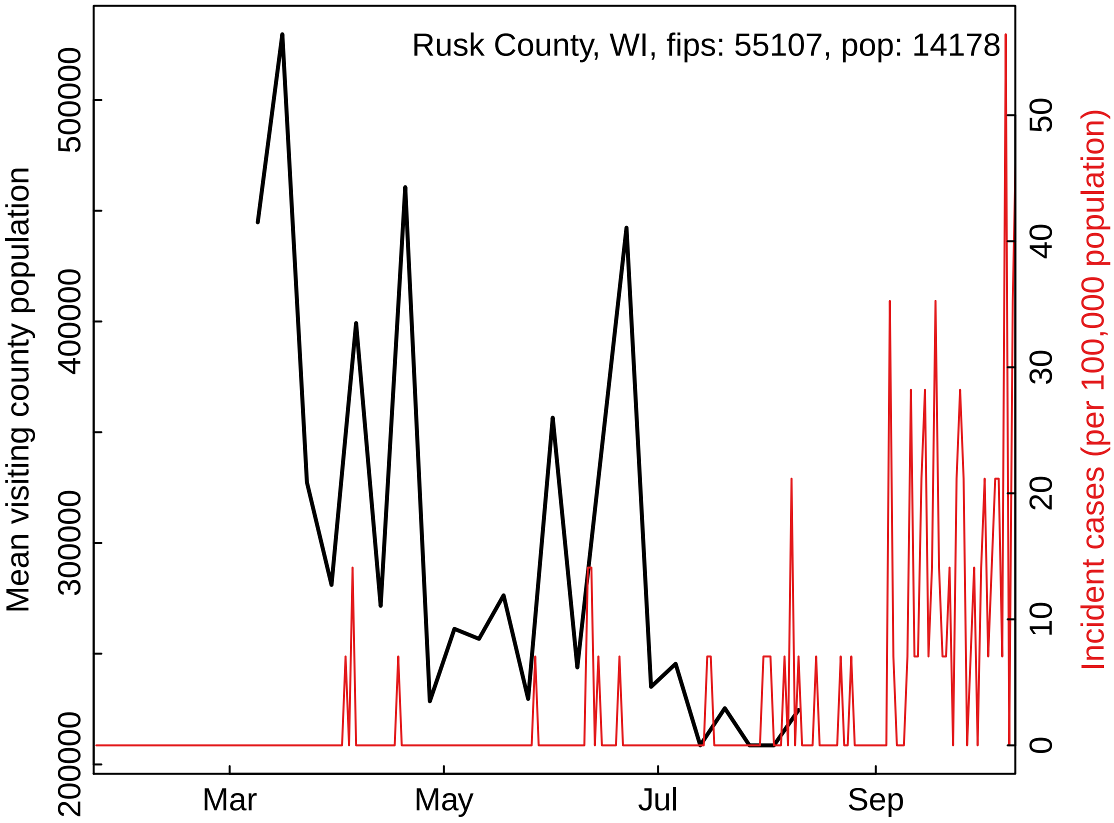

Rusk County, WI, fips: 55107, pop: 14178
Mean visiting county population
200000
300000
400000
500000
Incident cases (per 100,000 population)
0
10
20
30
40
50
Mar
May
Jul
Sep

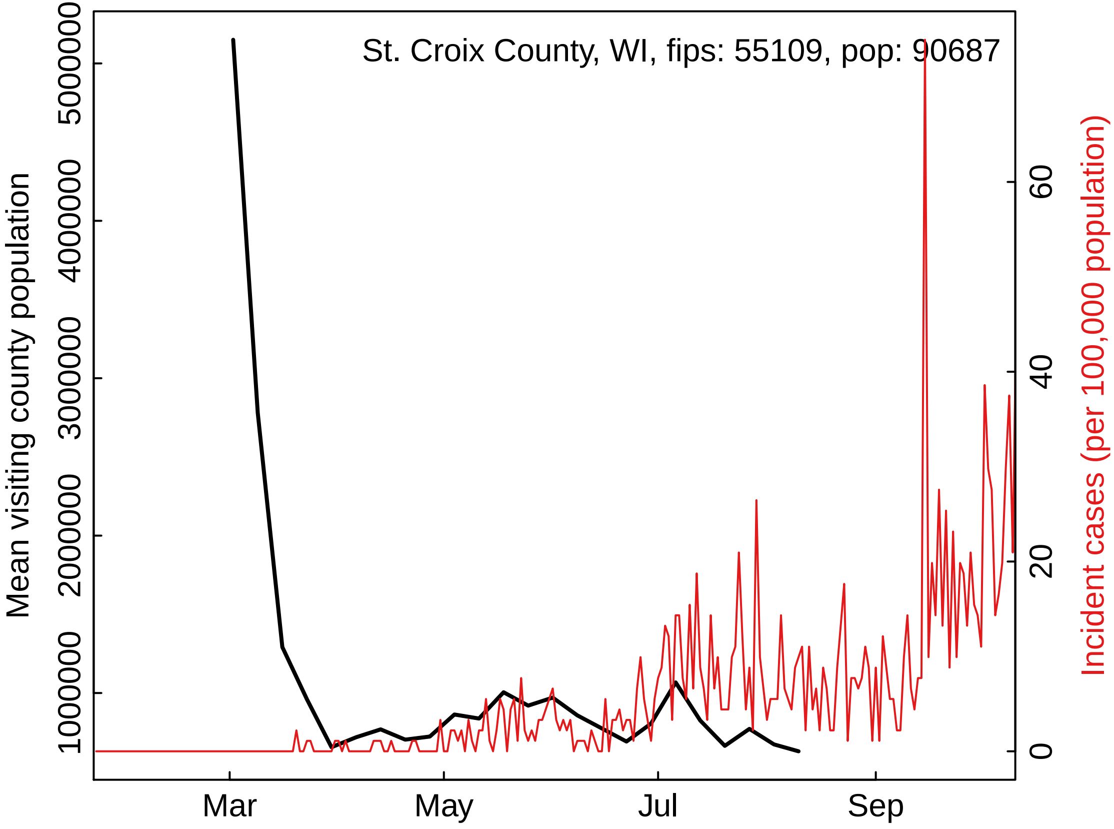
St. Croix County, WI, fips: 55109, pop: 90687
Mean visiting county population
Incident cases (per 100,000 population)
1000000
2000000
3000000
4000000
5000000
0
20
40
60
Mar
May
Jul
Sep

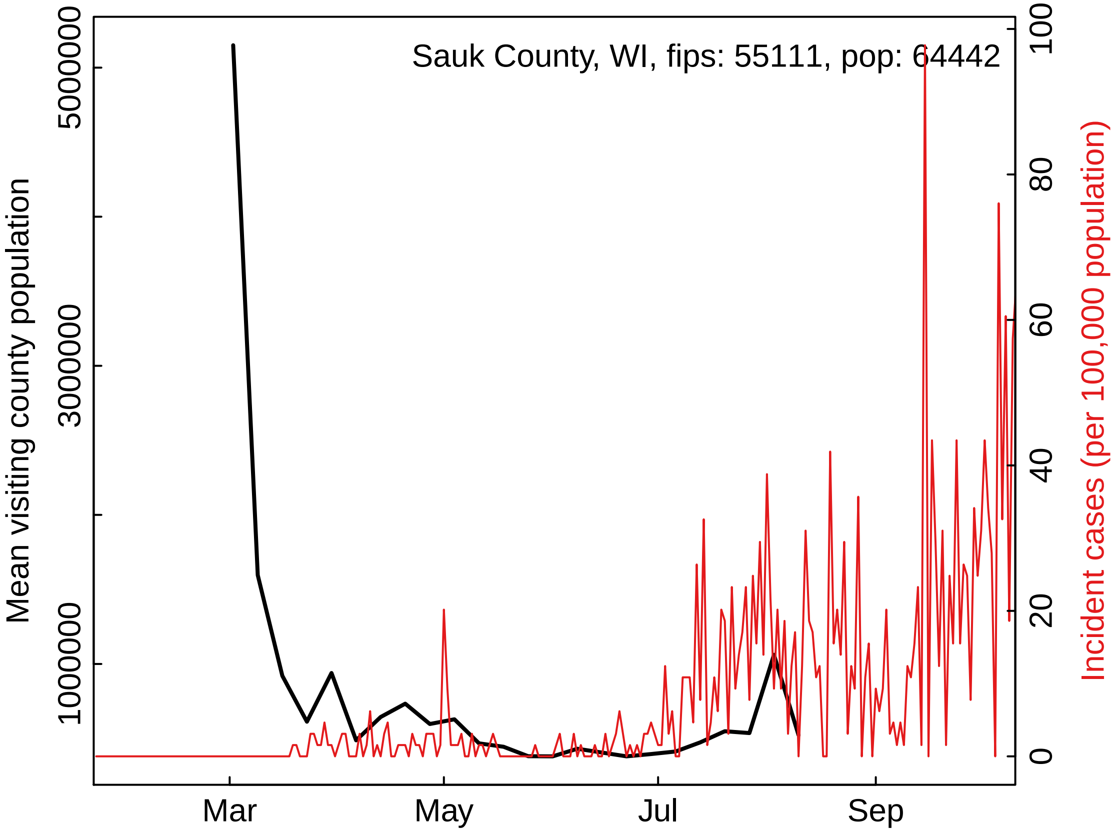
Sauk County, WI, fips: 55111, pop: 64442
Mean visiting county population
Incident cases (per 100,000 population)
1000000
3000000
5000000
0
20
40
60
80
100
Mar
May
Jul
Sep

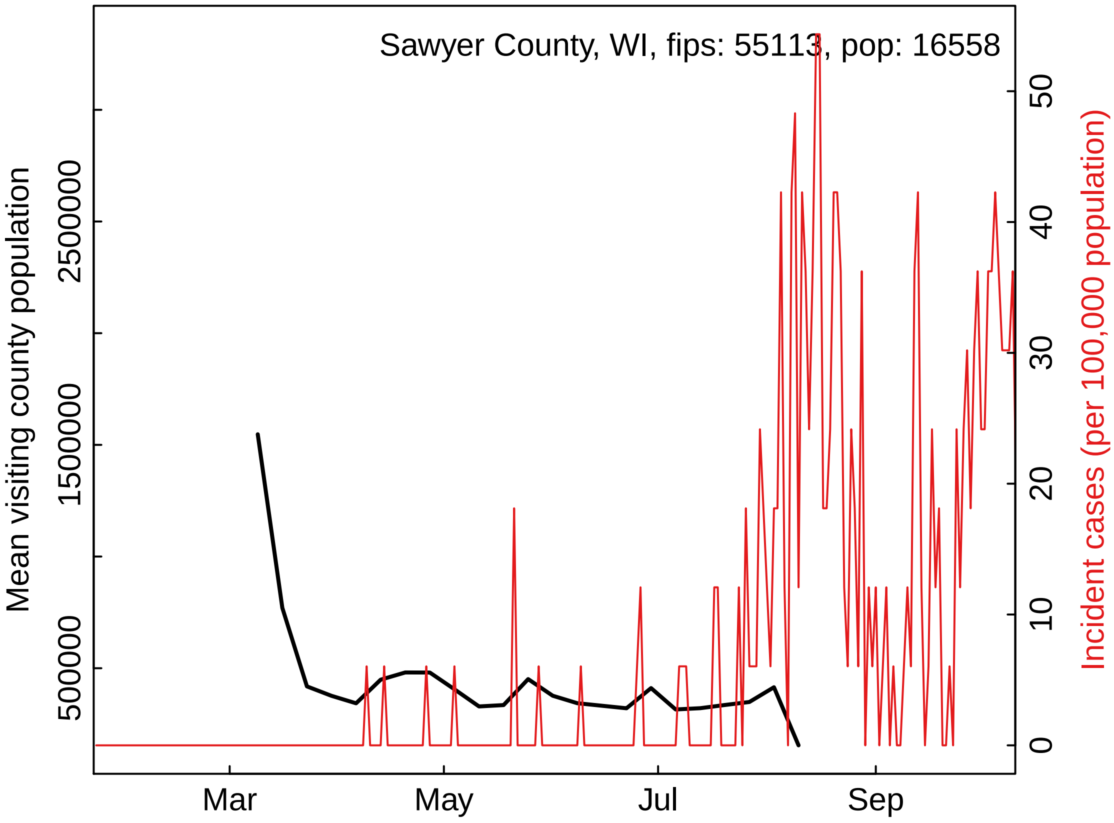

Sawyer County, WI, fips: 55113, pop: 16558
Mean visiting county population
500000
1500000
2500000
Incident cases (per 100,000 population)
0
10
20
30
40
50
Mar
May
Jul
Sep

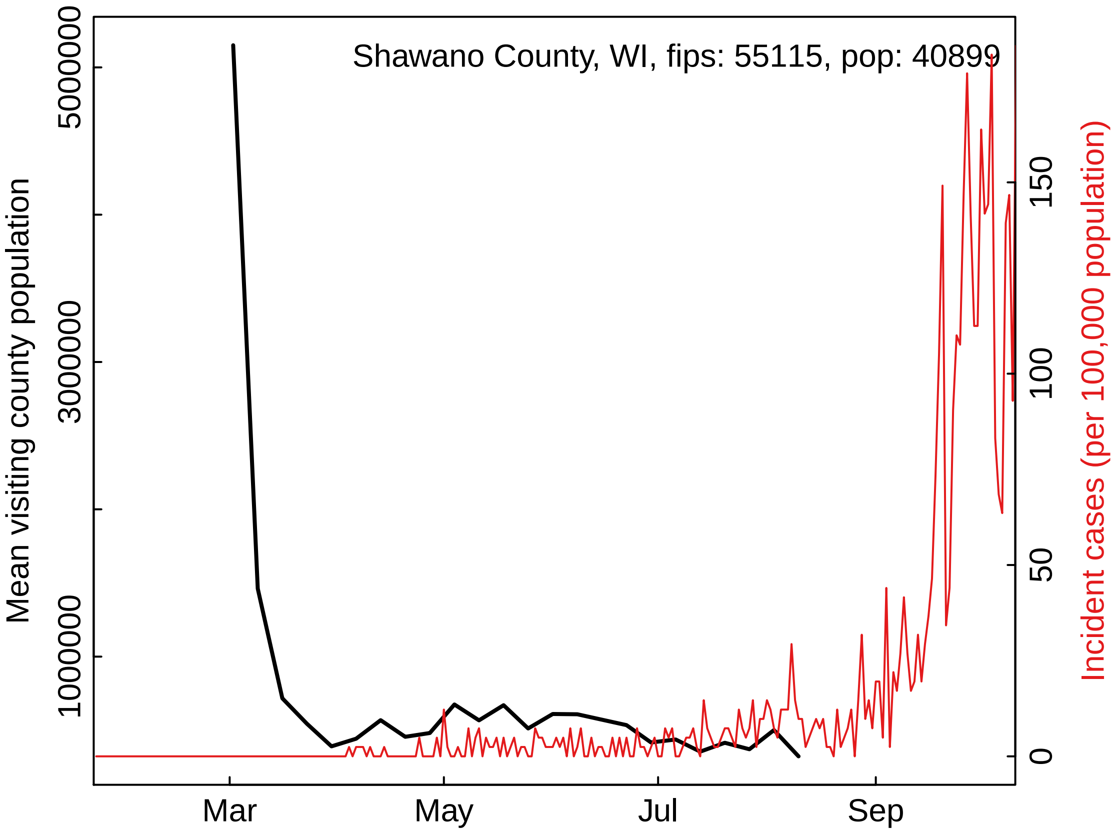

Shawano County, WI, fips: 55115, pop: 40899
Mean visiting county population
1000000
3000000
5000000
Mar
May
Jul
Sep
0
50
100
150
Incident cases (per 100,000 population)

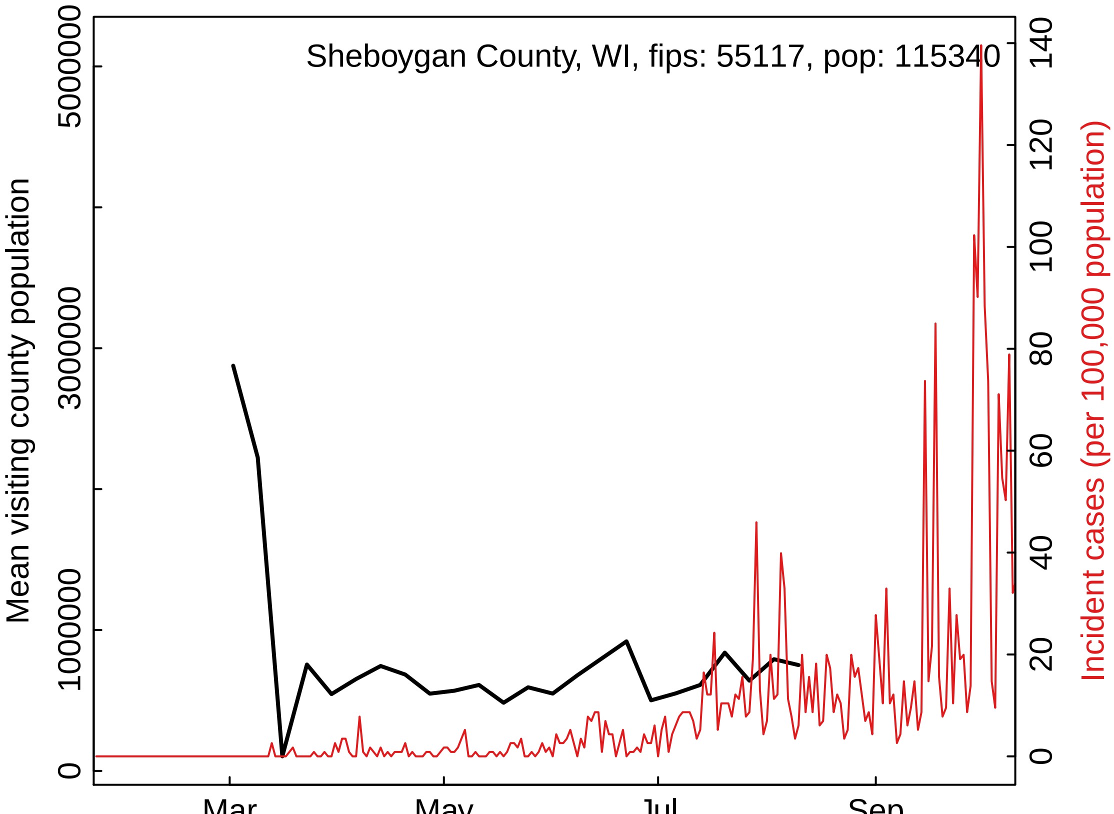
Sheboygan County, WI, fips: 55117, pop: 115340
Mean visiting county population
0
1000000
3000000
5000000
Incident cases (per 100,000 population)
0
20
40
60
80
100
120
140
Mar
May
Jul
Sep

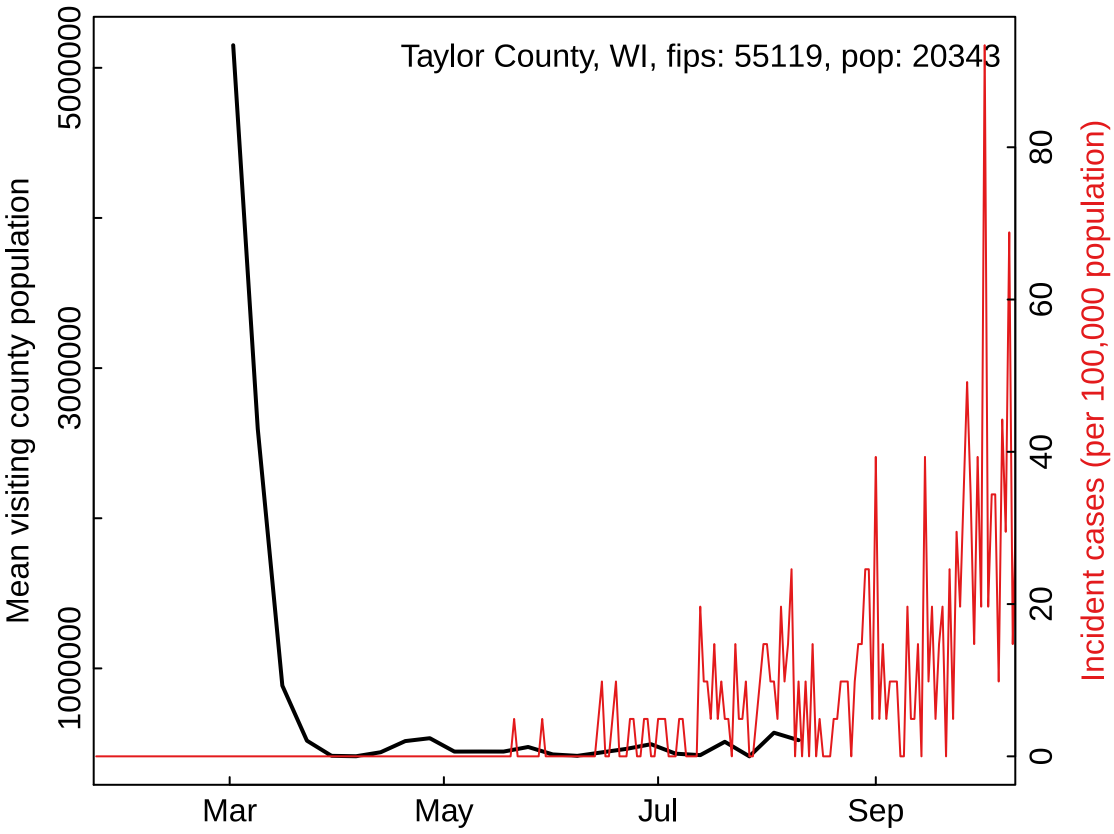

Taylor County, WI, fips: 55119, pop: 20343
Mean visiting county population
1000000
3000000
5000000
Incident cases (per 100,000 population)
0
20
40
60
80
Mar
May
Jul
Sep

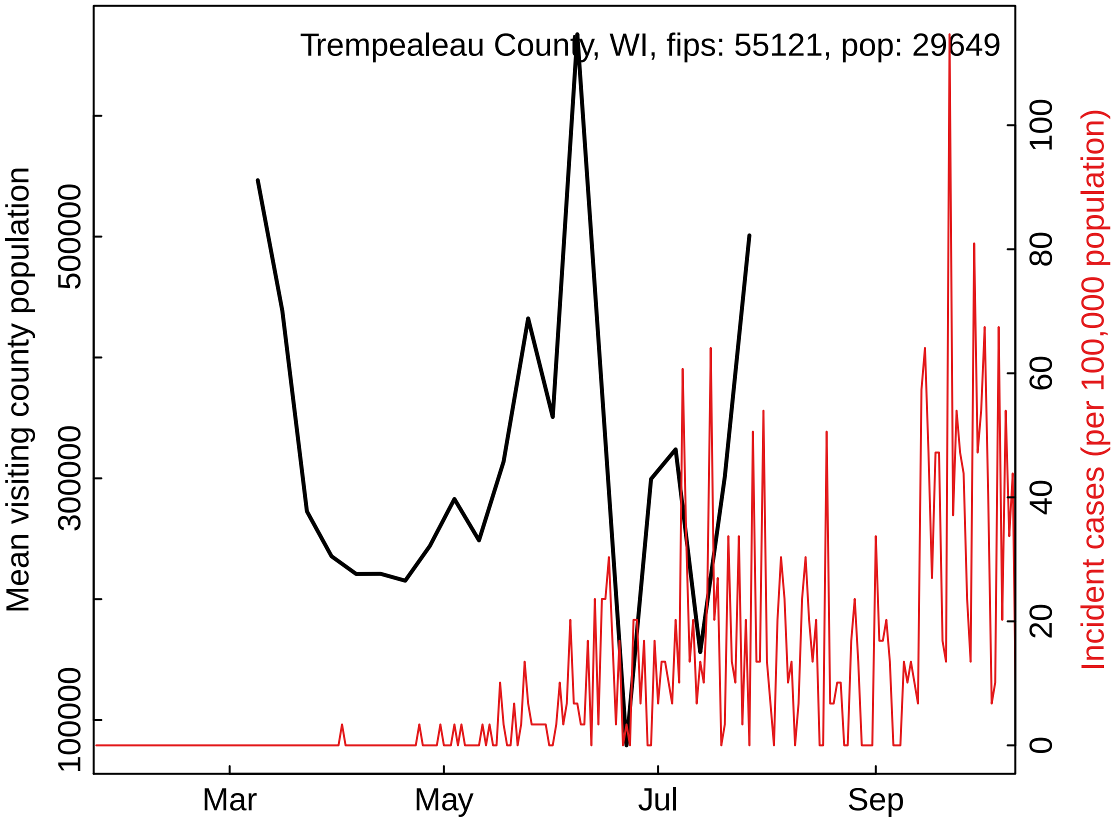

Trempealeau County, WI, fips: 55121, pop: 29649
Mean visiting county population
100000
300000
500000
Incident cases (per 100,000 population)
0
20
40
60
80
100
Mar
May
Jul
Sep

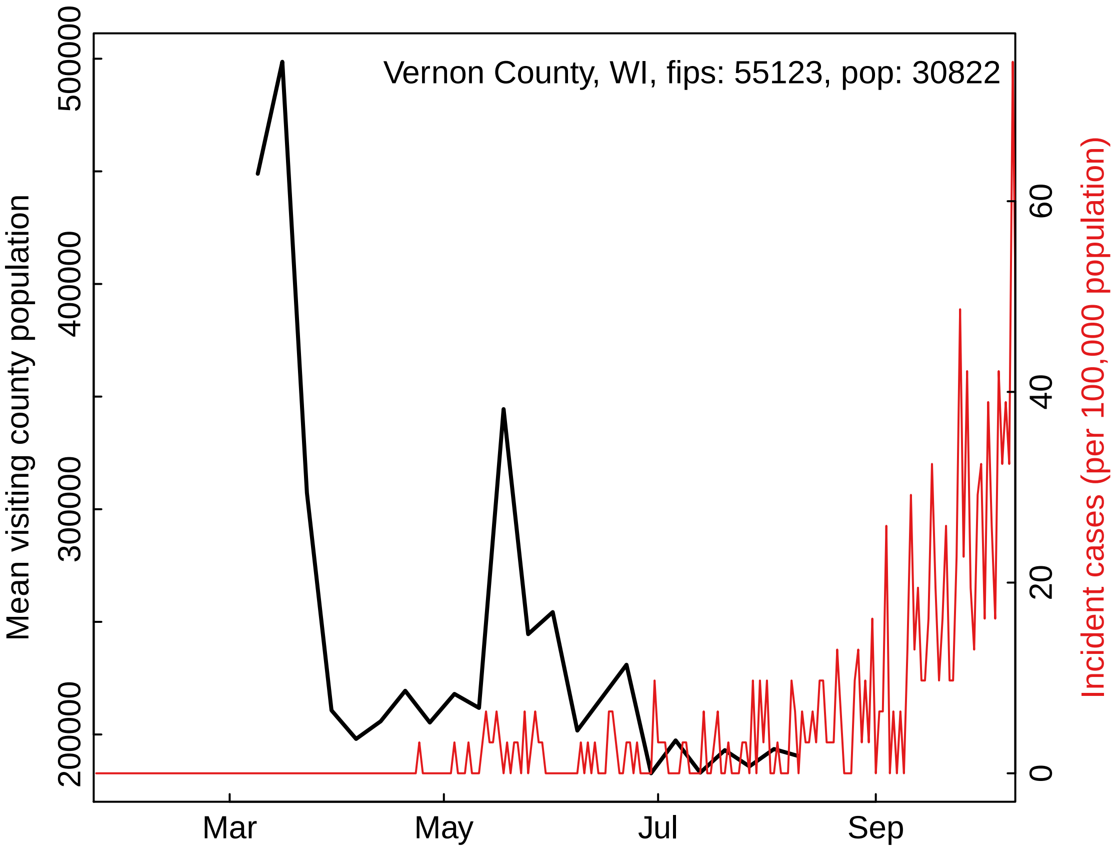
Vernon County, WI, fips: 55123, pop: 30822
Mean visiting county population
200000
300000
400000
500000
Incident cases (per 100,000 population)
0
20
40
60
Mar
May
Jul
Sep

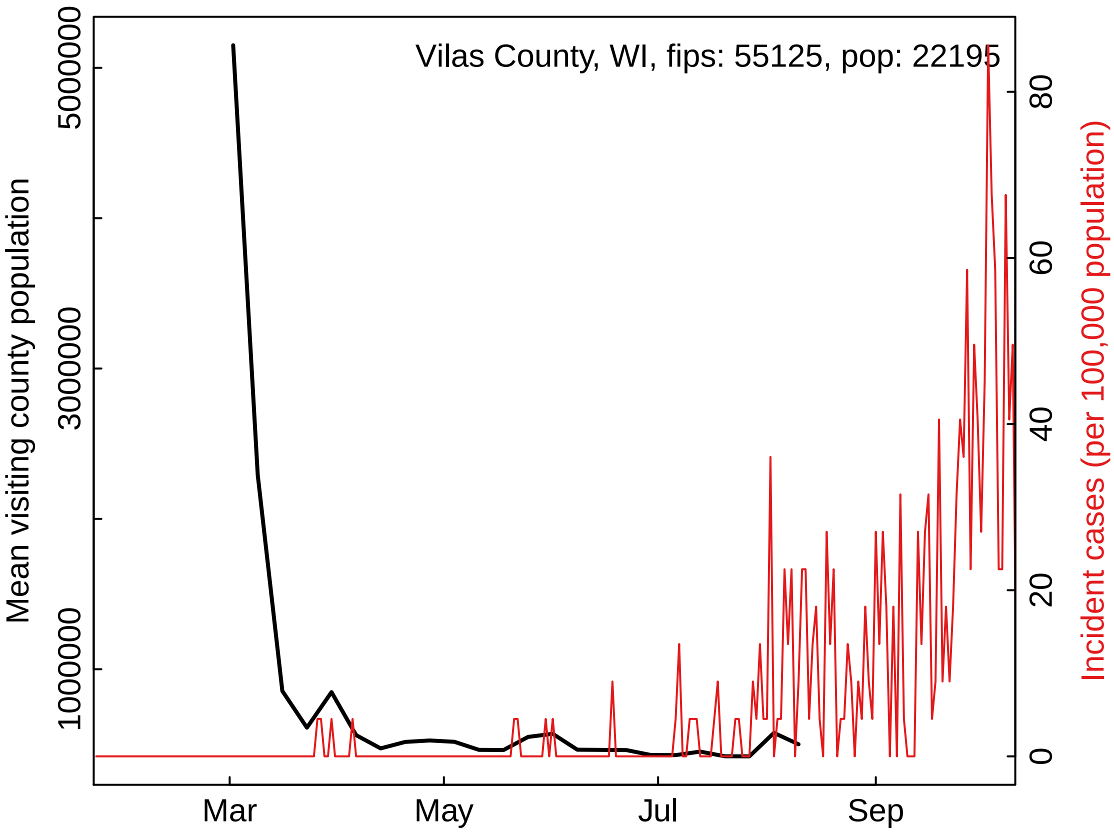

Vilas County, WI, fips: 55125, pop: 22195
Mean visiting county population
1000000
3000000
5000000
Mar
May
Jul
Sep
0
20
40
60
80
Incident cases (per 100,000 population)

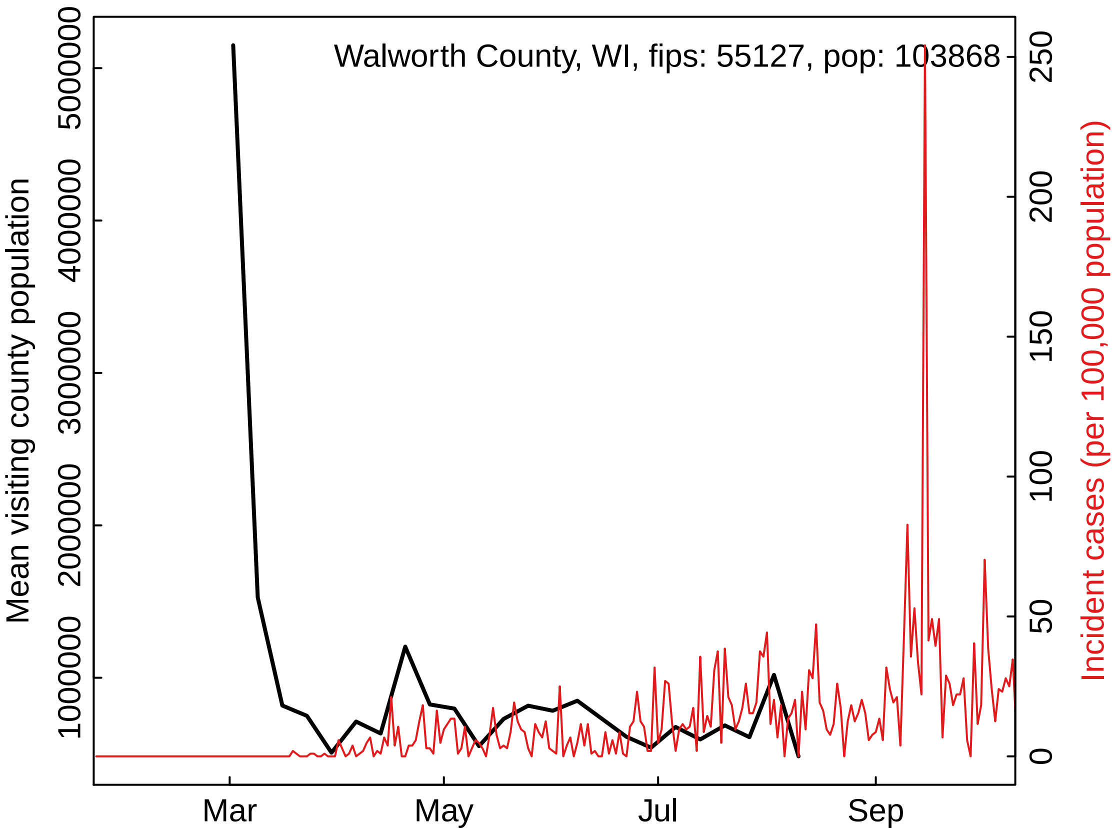

Walworth County, WI, fips: 55127, pop: 103868
Mean visiting county population
Incident cases (per 100,000 population)
1000000
2000000
3000000
4000000
5000000
0
50
100
150
200
250
Mar
May
Jul
Sep

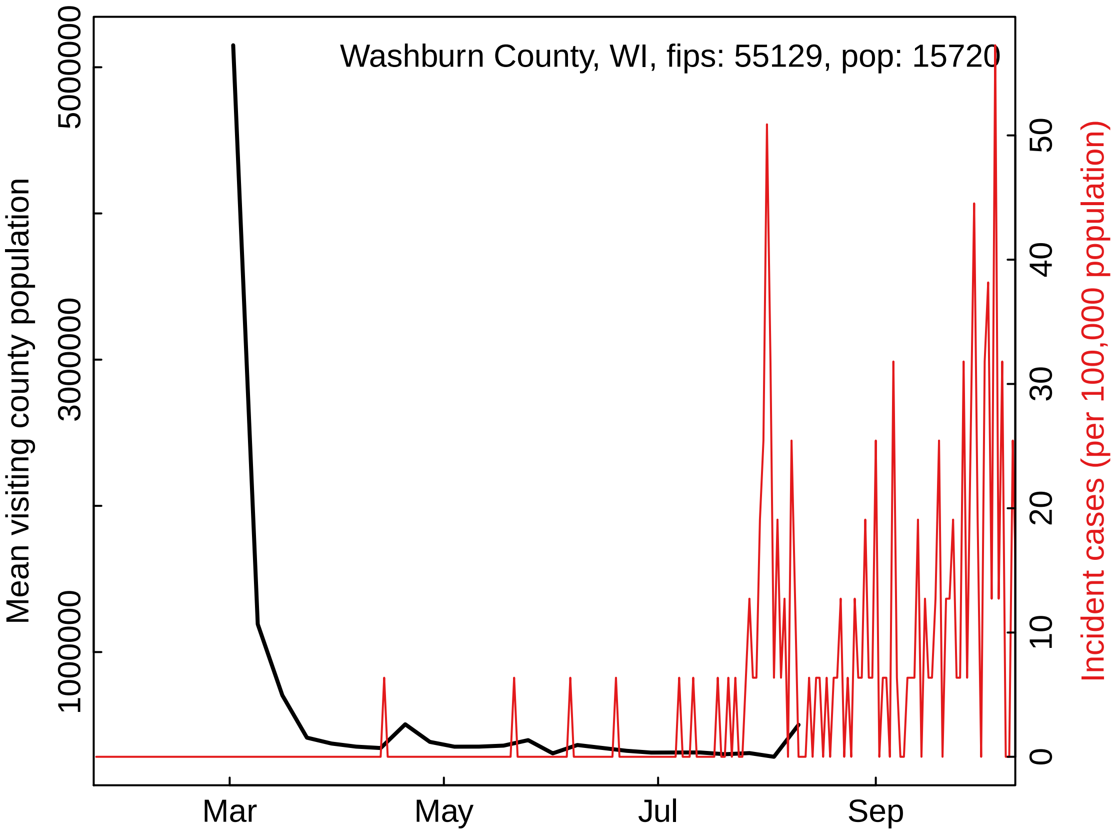

Washburn County, WI, fips: 55129, pop: 15720
Mean visiting county population
1000000
3000000
5000000
Mar
May
Jul
Sep
0
10
20
30
40
50
Incident cases (per 100,000 population)

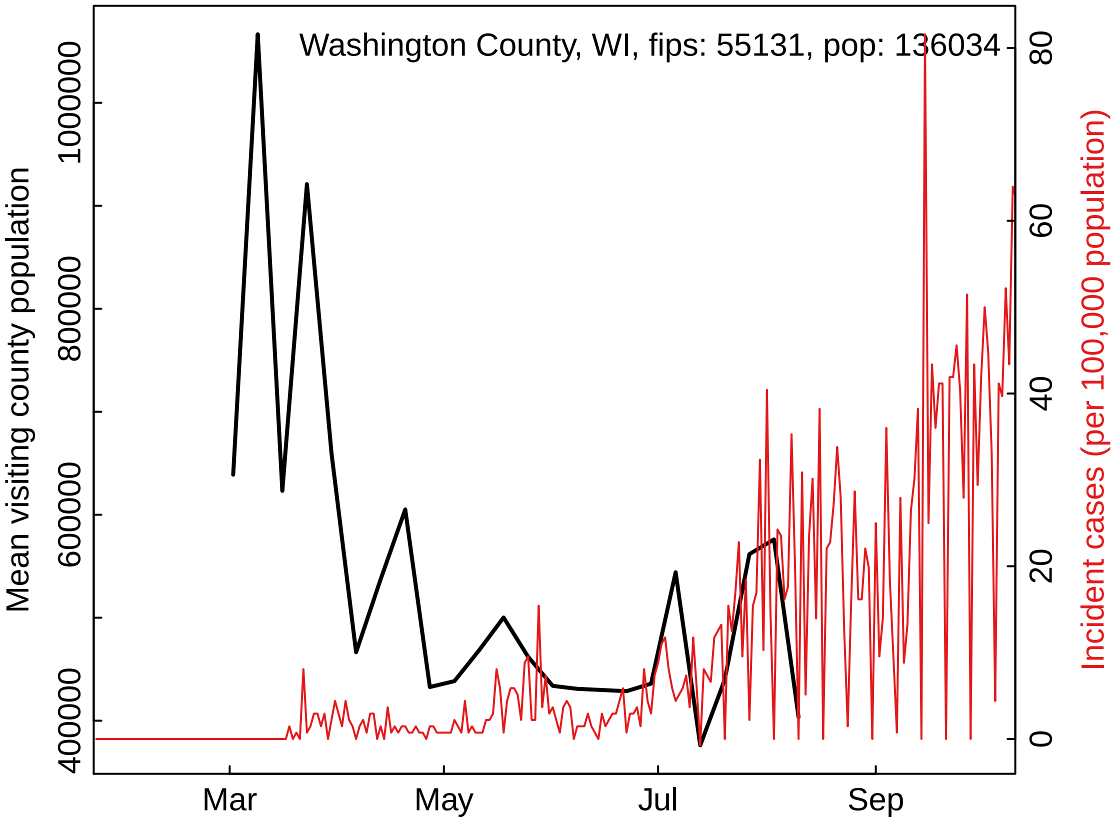

Washington County, WI, fips: 55131, pop: 136034
Mean visiting county population
400000
600000
800000
1000000
Incident cases (per 100,000 population)
0
20
40
60
80
Mar
May
Jul
Sep

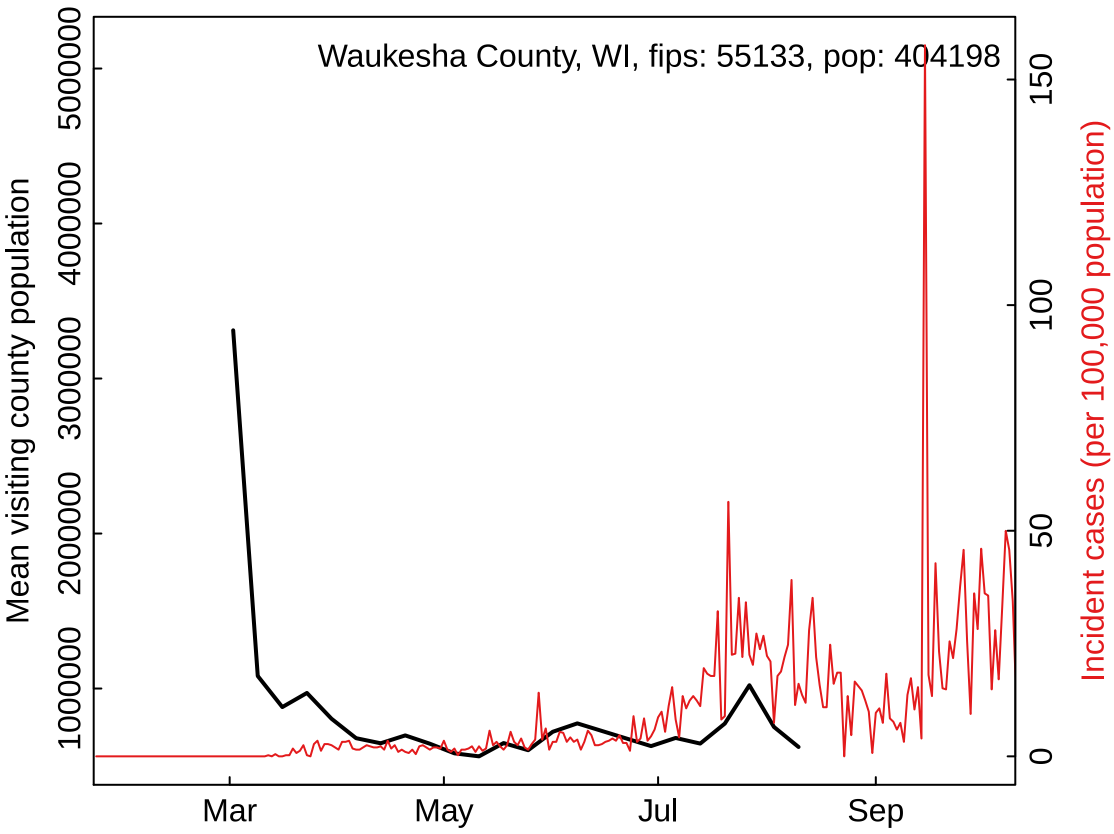

Waukesha County, WI, fips: 55133, pop: 404198
Mean visiting county population
1000000
2000000
3000000
4000000
5000000
Incident cases (per 100,000 population)
0
50
100
150
Mar
May
Jul
Sep

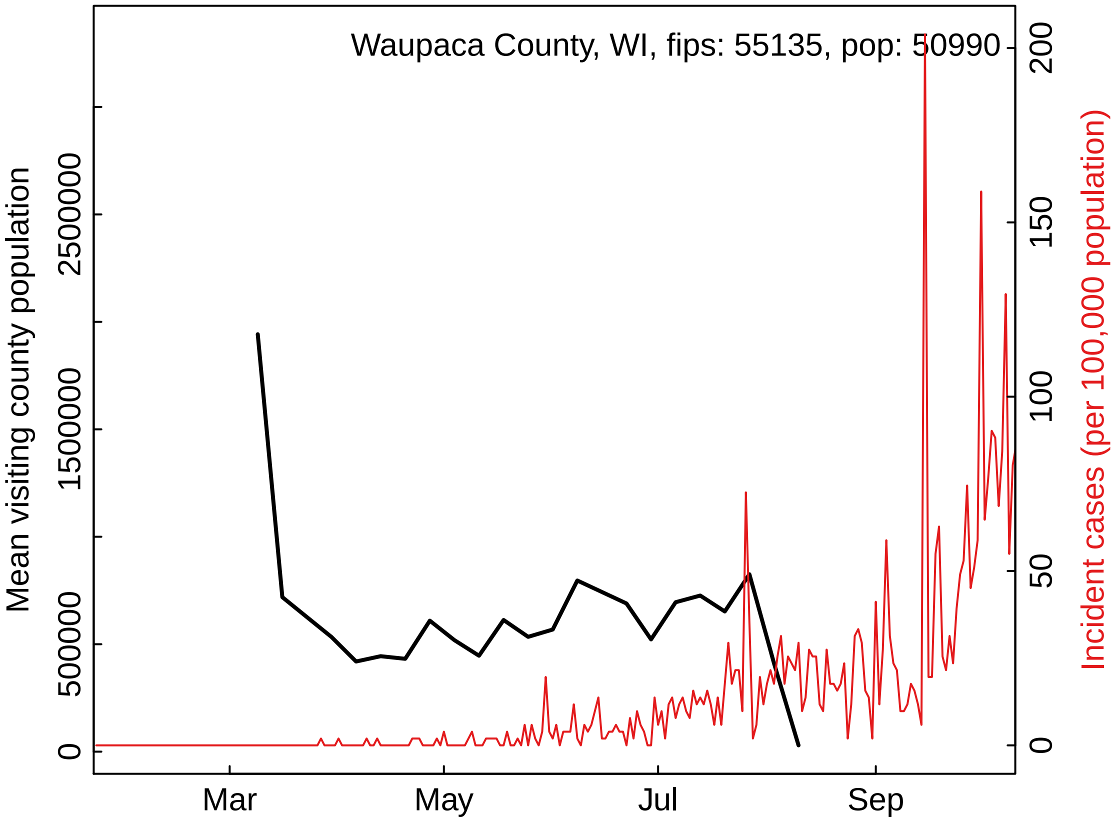
Waupaca County, WI, fips: 55135, pop: 50990
Mean visiting county population
0
500000
1500000
2500000
Incident cases (per 100,000 population)
0
50
100
150
200
Mar
May
Jul
Sep

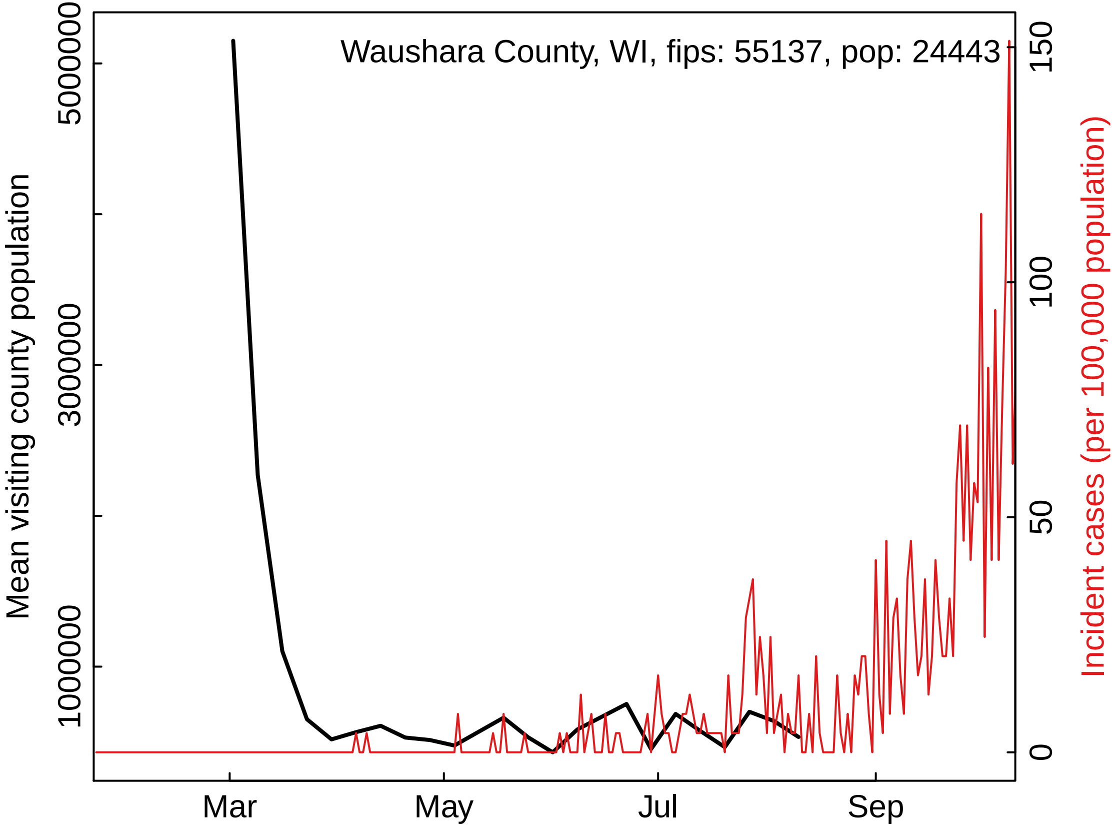

Waushara County, WI, fips: 55137, pop: 24443
Mean visiting county population
1000000
3000000
5000000
Mar
May
Jul
Sep
Incident cases (per 100,000 population)
0
50
100
150

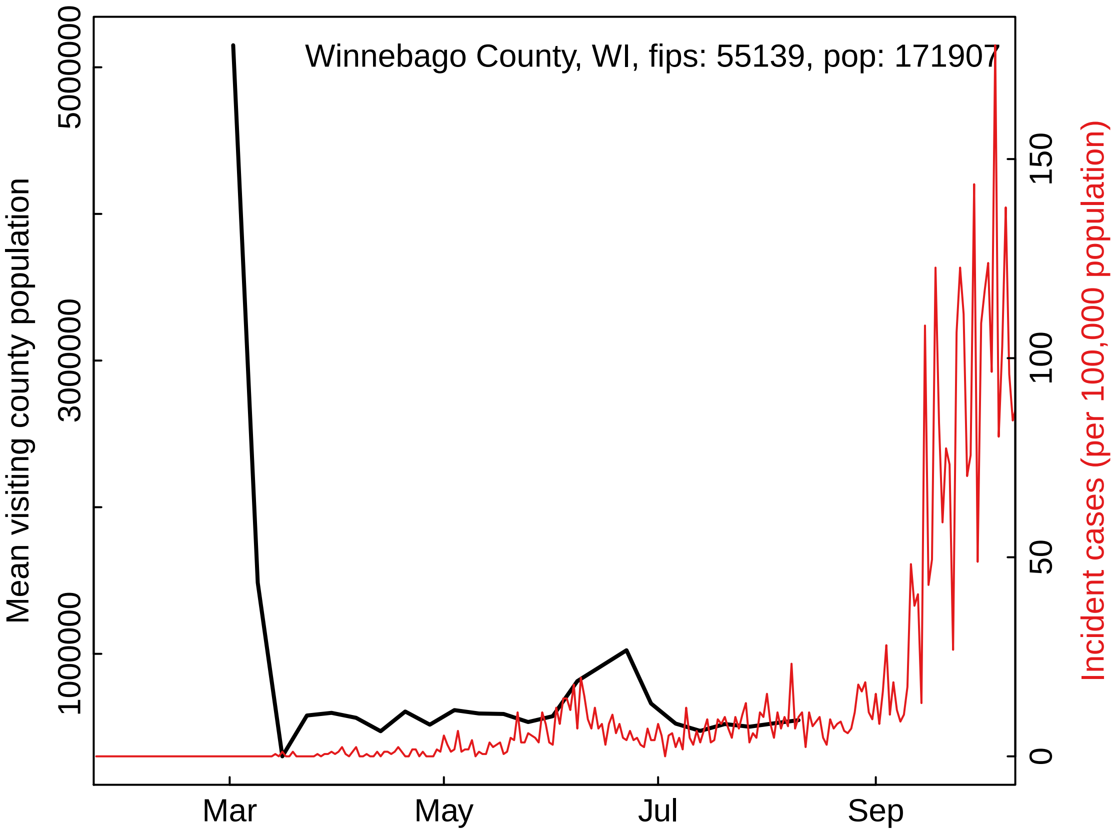

Winnebago County, WI, fips: 55139, pop: 171907
Mean visiting county population
1000000
3000000
5000000
Mar
May
Jul
Sep
Incident cases (per 100,000 population)
0
50
100
150

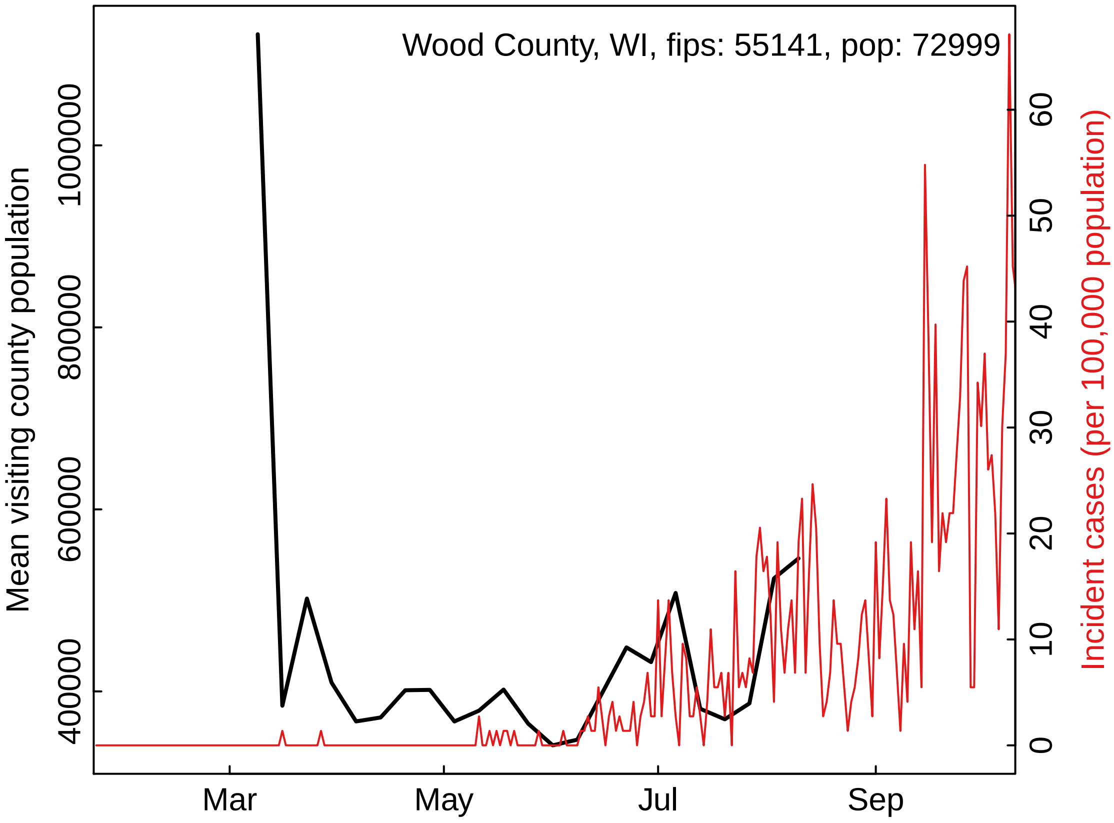

Wood County, WI, fips: 55141, pop: 72999
Mean visiting county population
400000
600000
800000
1000000
Incident cases (per 100,000 population)
0
10
20
30
40
50
60
Mar
May
Jul
Sep

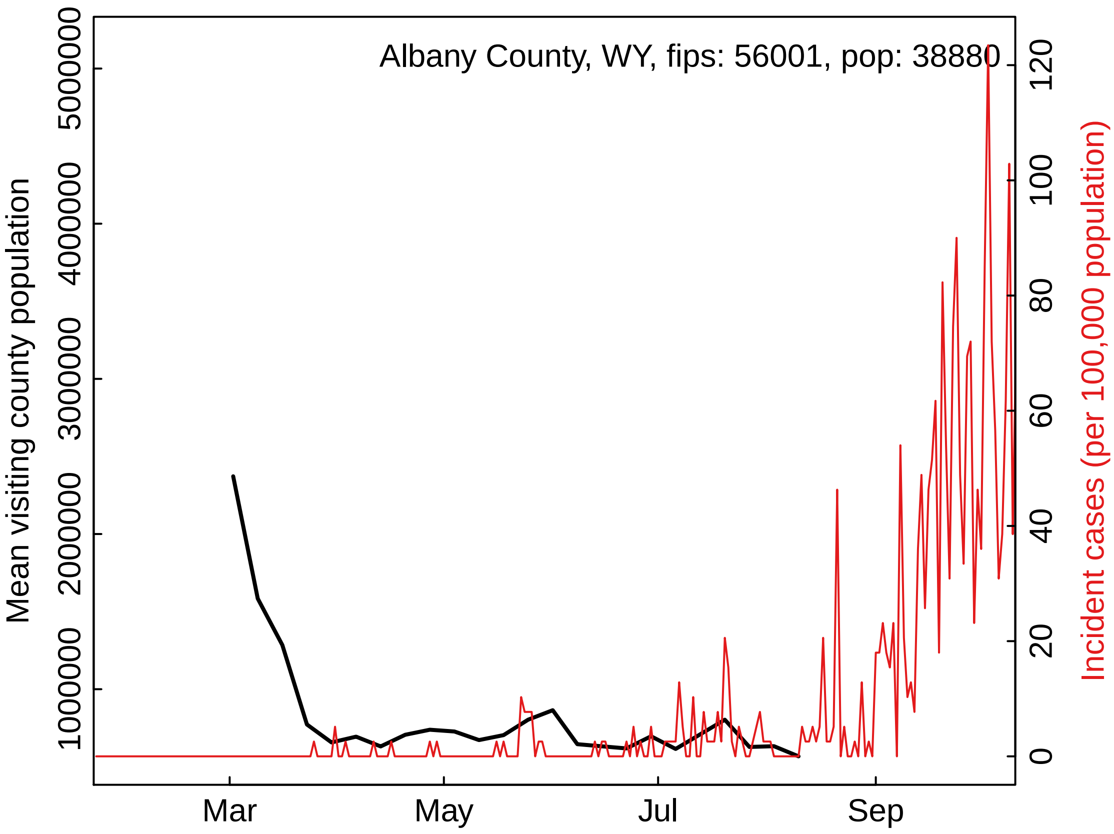

Albany County, WY, fips: 56001, pop: 38880
Mean visiting county population
10000000
20000000
30000000
40000000
50000000
Incident cases (per 100,000 population)
0
20
40
60
80
100
120
Mar
May
Jul
Sep

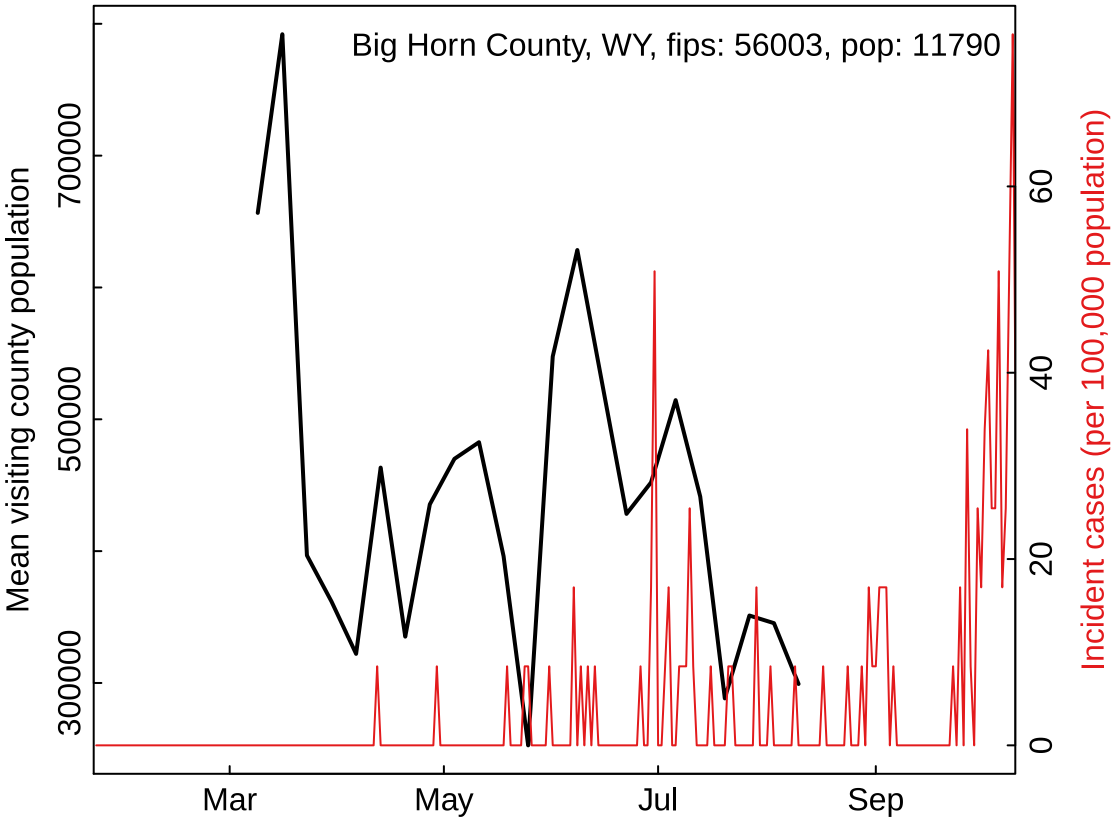
Big Horn County, WY, fips: 56003, pop: 11790
Mean visiting county population
300000
500000
700000
Incident cases (per 100,000 population)
0
20
40
60
Mar
May
Jul
Sep

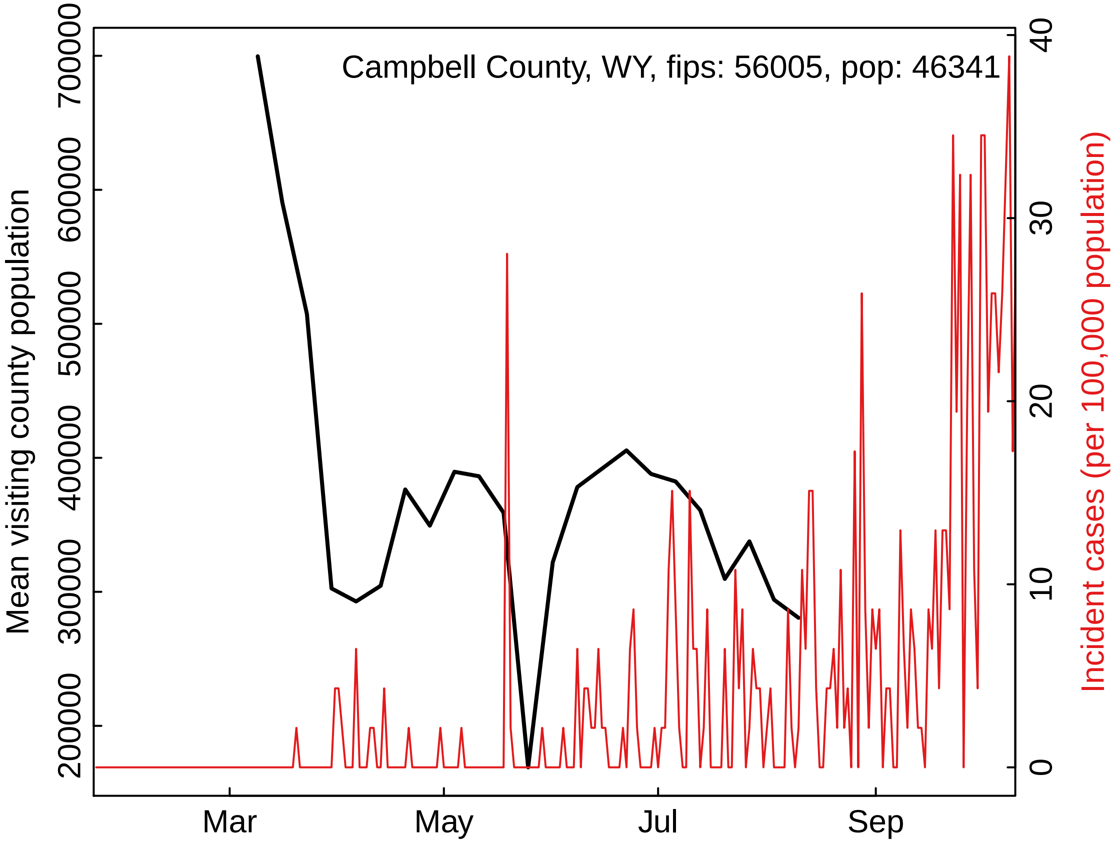
Campbell County, WY, fips: 56005, pop: 46341
Mean visiting county population
200000
300000
400000
500000
600000
700000
Incident cases (per 100,000 population)
0
10
20
30
40
Mar
May
Jul
Sep

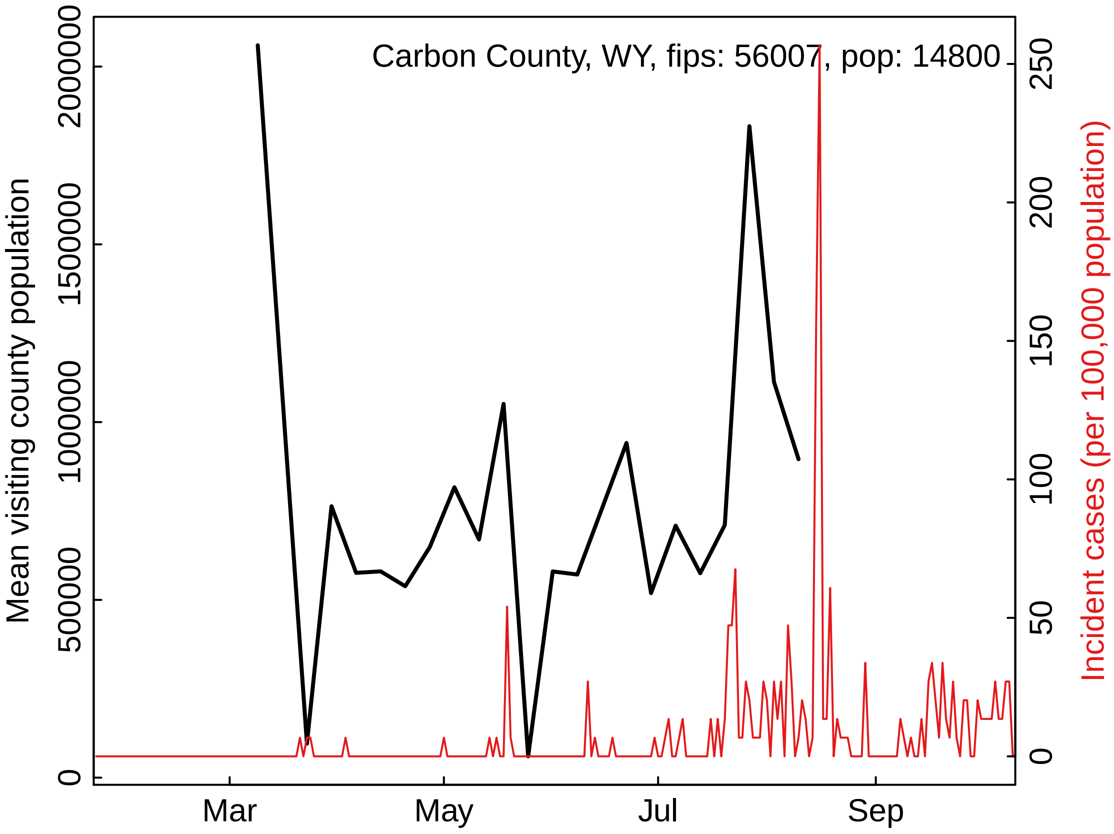

Carbon County, WY, fips: 56007, pop: 14800
Mean visiting county population
Incident cases (per 100,000 population)
0
500000
1000000
1500000
2000000
0
50
100
150
200
250
Mar
May
Jul
Sep

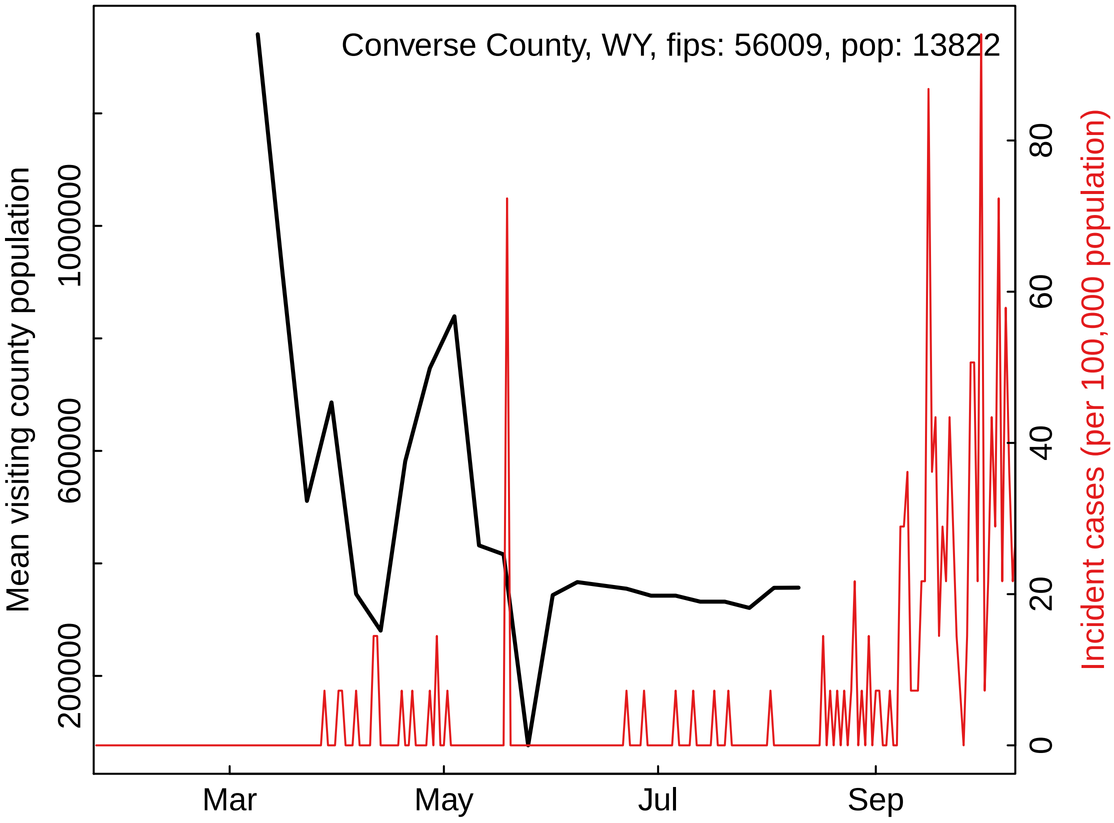
Converse County, WY, fips: 56009, pop: 13822
Mean visiting county population
200000
600000
1000000
Incident cases (per 100,000 population)
0
20
40
60
80
Mar
May
Jul
Sep

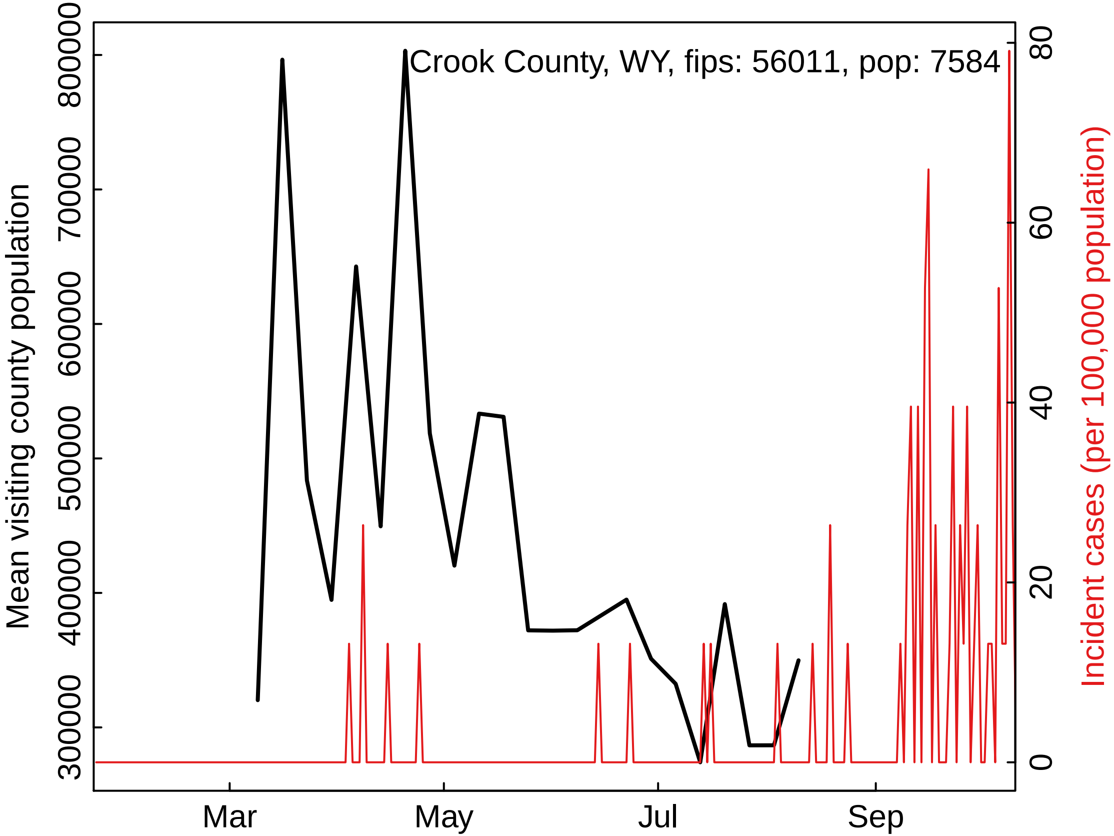
Crook County, WY, fips: 56011, pop: 7584
Mean visiting county population
300000
400000
500000
600000
700000
800000
Incident cases (per 100,000 population)
0
20
40
60
80
Mar
May
Jul
Sep

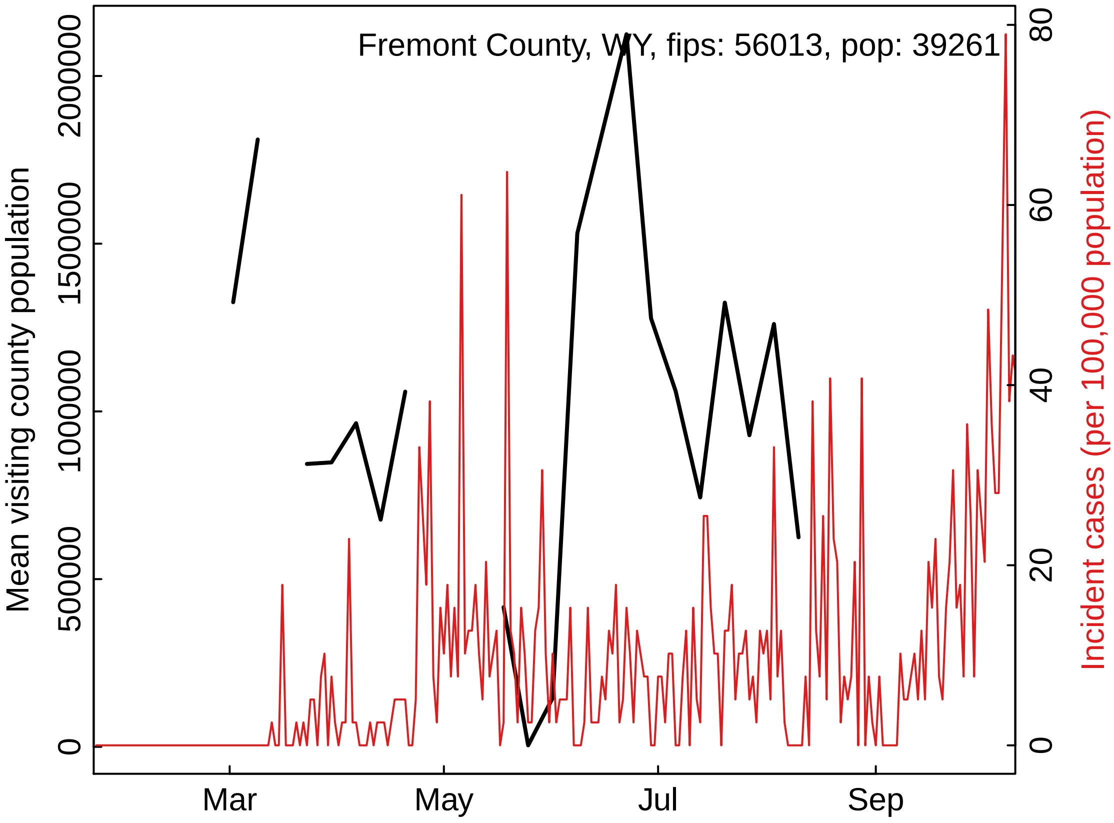

Fremont County, WY, fips: 56013, pop: 39261
Mean visiting county population
0
500000
1000000
1500000
2000000
Mar
May
Jul
Sep
0
20
40
60
80
Incident cases (per 100,000 population)

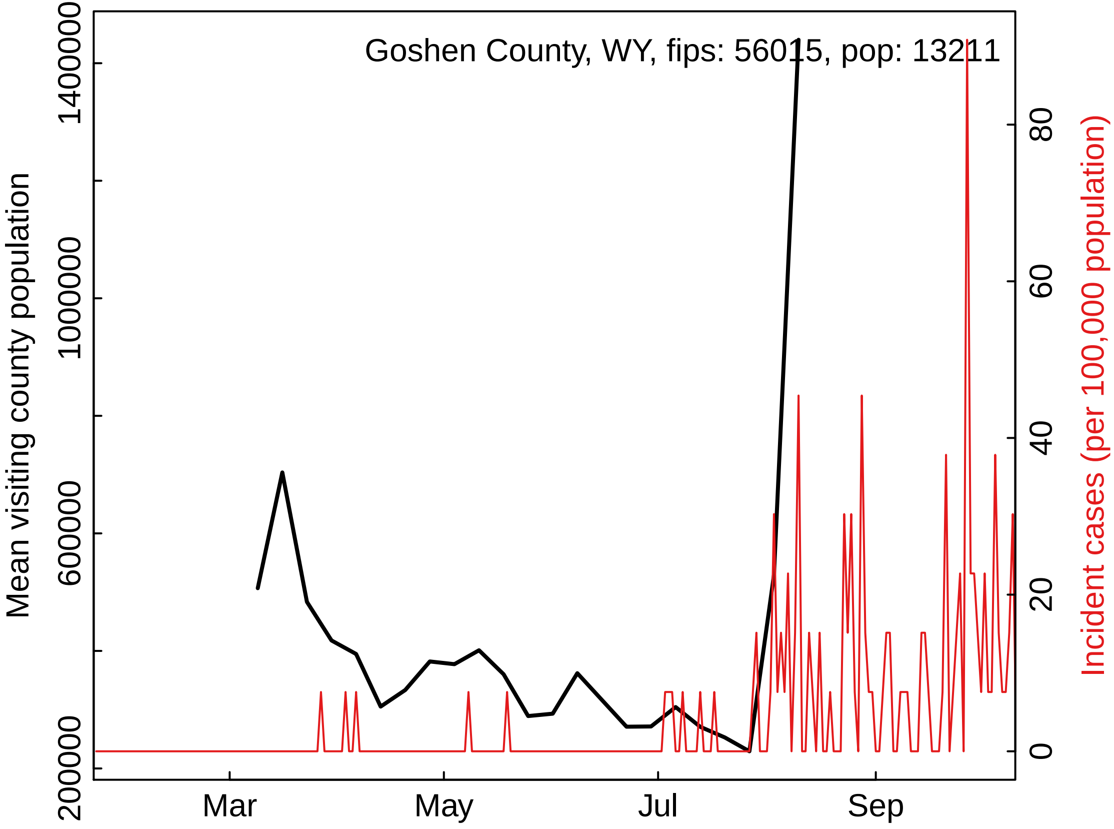

Goshen County, WY, fips: 56015, pop: 13211
Mean visiting county population
Incident cases (per 100,000 population)
200000
600000
1000000
1400000
0
20
40
60
80
Mar
May
Jul
Sep

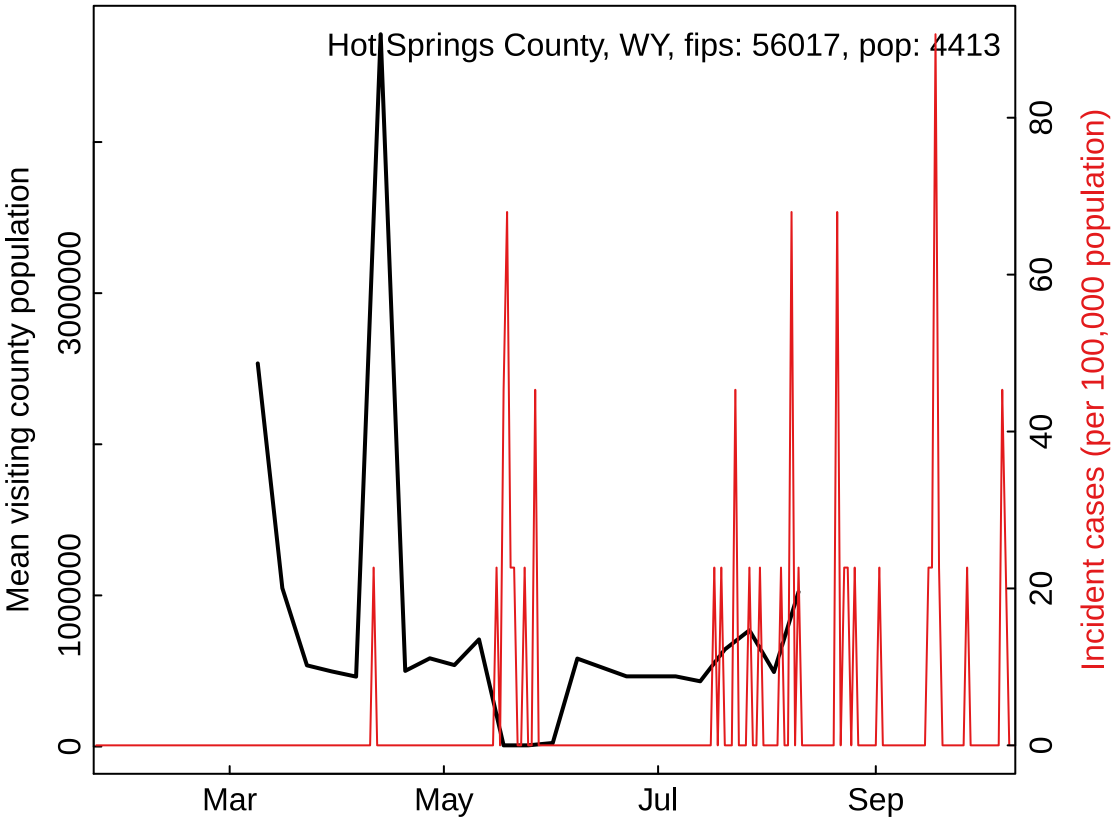

Hot Springs County, WY, fips: 56017, pop: 4413
Mean visiting county population
0
1000000
3000000
Incident cases (per 100,000 population)
0
20
40
60
80
Mar
May
Jul
Sep

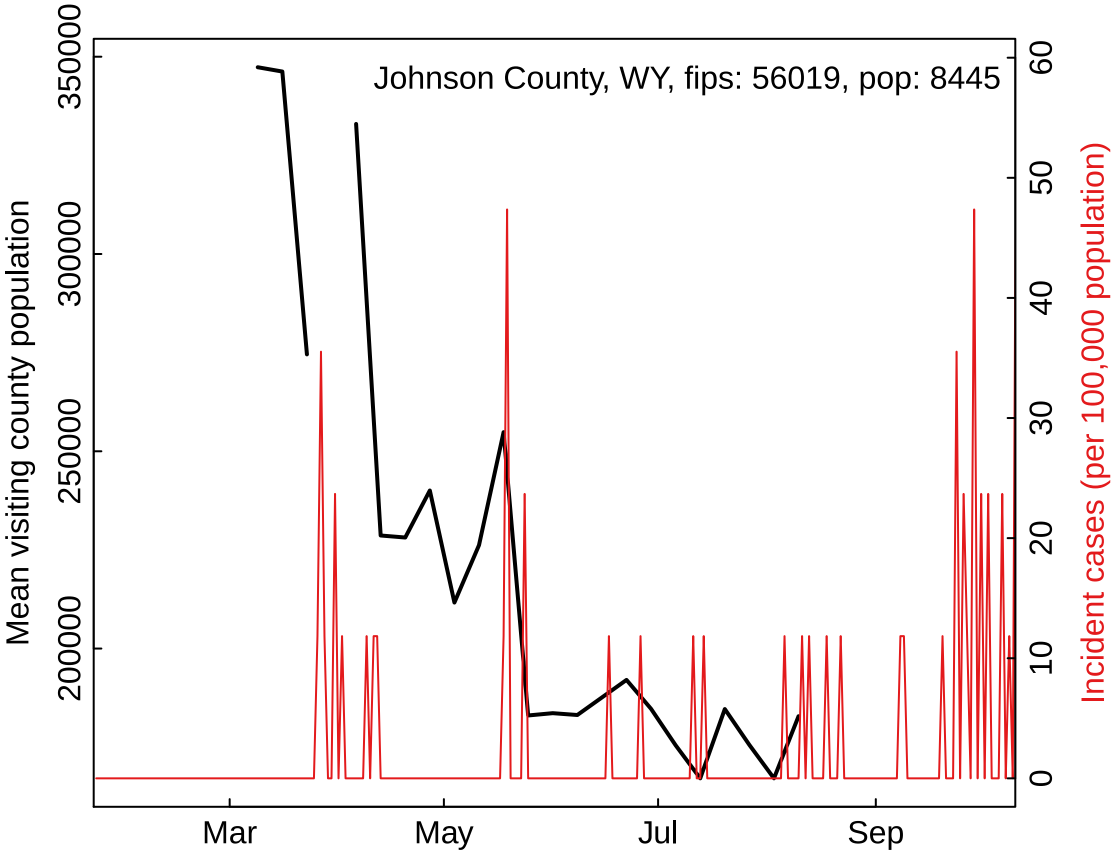
Johnson County, WY, fips: 56019, pop: 8445
Mean visiting county population
Incident cases (per 100,000 population)
200000
250000
300000
350000
0
10
20
30
40
50
60
Mar
May
Jul
Sep

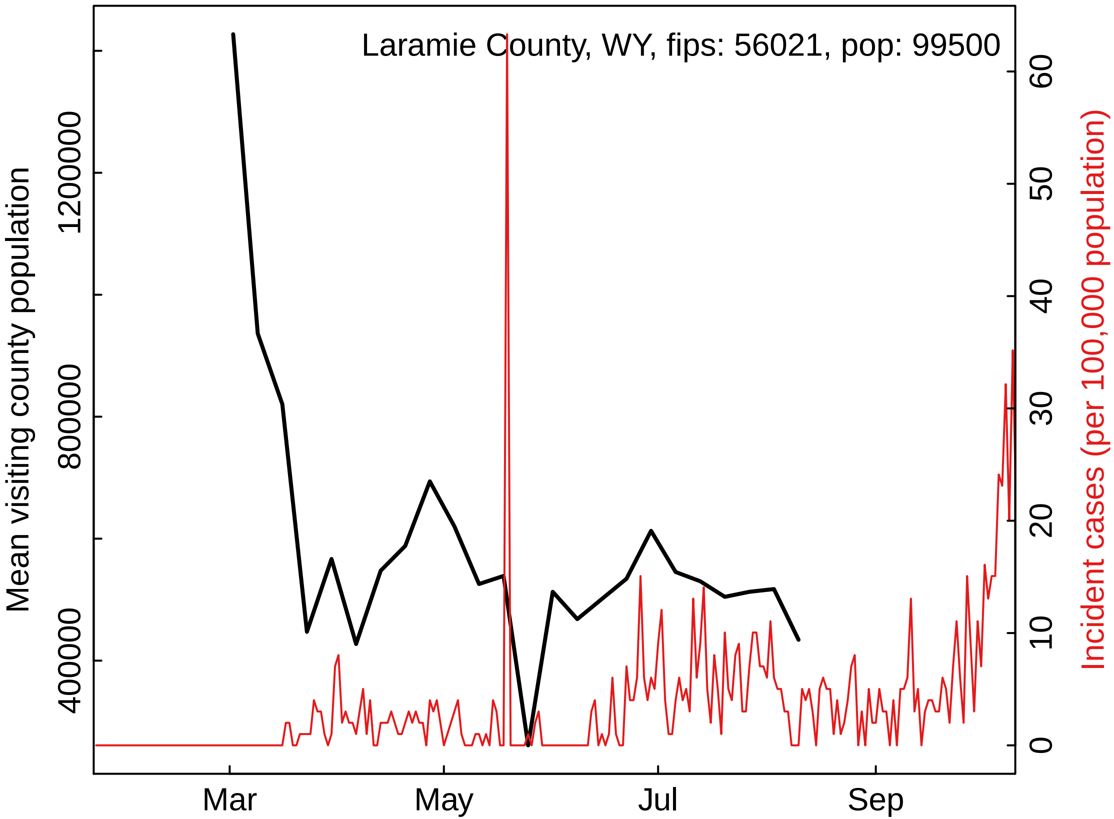

Laramie County, WY, fips: 56021, pop: 99500
Mean visiting county population
400000
800000
1200000
Incident cases (per 100,000 population)
0
10
20
30
40
50
60
Mar
May
Jul
Sep

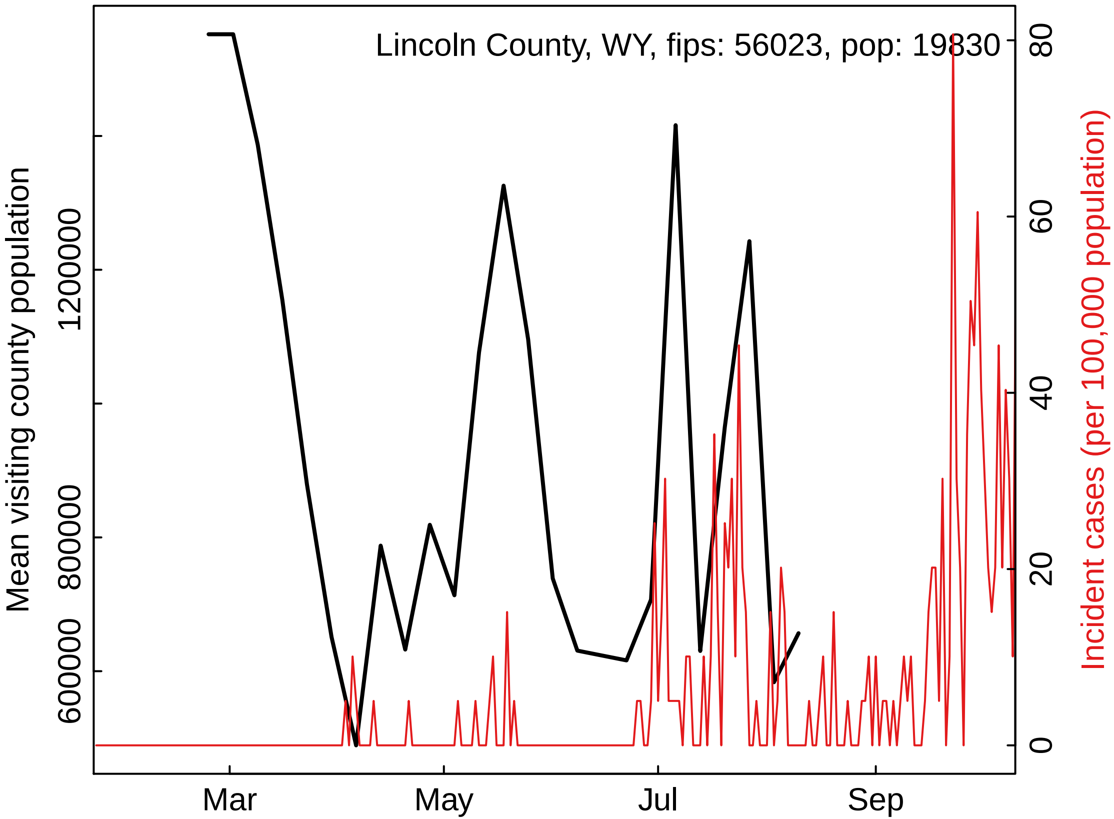

Lincoln County, WY, fips: 56023, pop: 19830
Mean visiting county population
600000
800000
1200000
Incident cases (per 100,000 population)
0
20
40
60
80
Mar
May
Jul
Sep

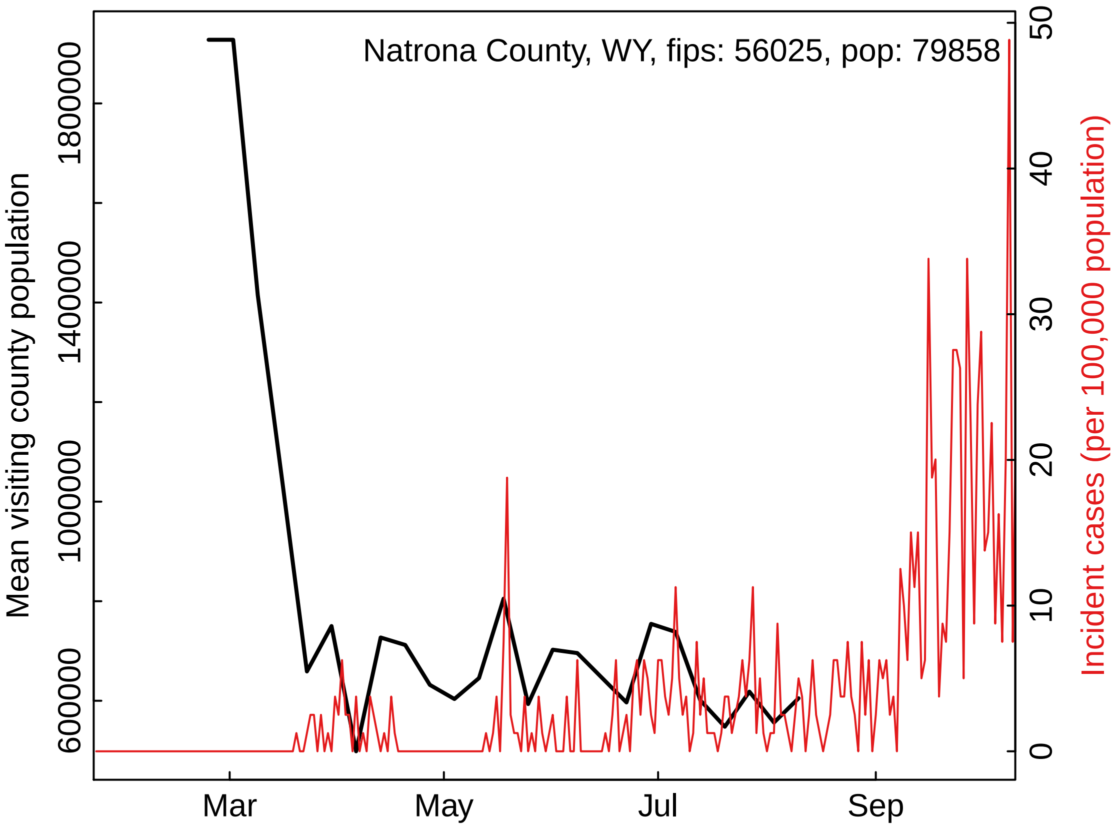

Natrona County, WY, fips: 56025, pop: 79858
Mean visiting county population
600000
1000000
1400000
1800000
Incident cases (per 100,000 population)
0
10
20
30
40
50
Mar
May
Jul
Sep

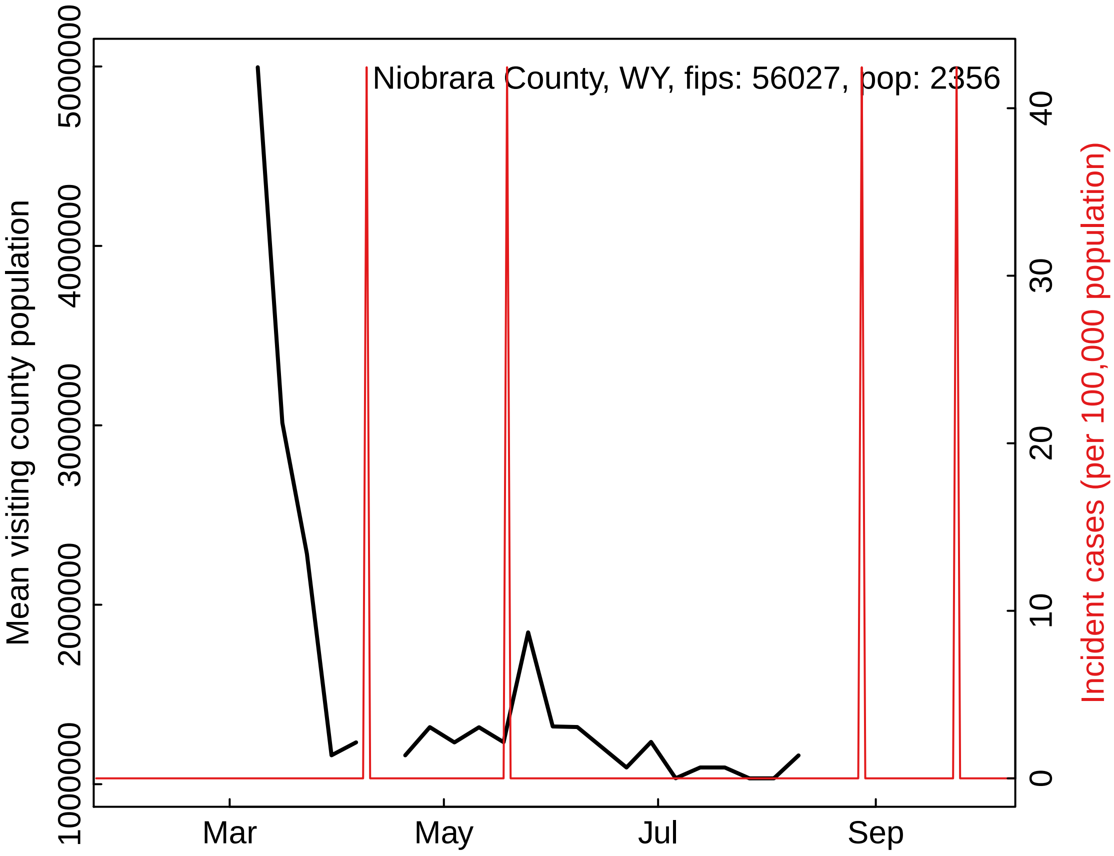
Niobrara County, WY, fips: 56027, pop: 2356
Mean visiting county population
10000000
20000000
30000000
40000000
50000000
Incident cases (per 100,000 population)
0
10
20
30
40
Mar
May
Jul
Sep

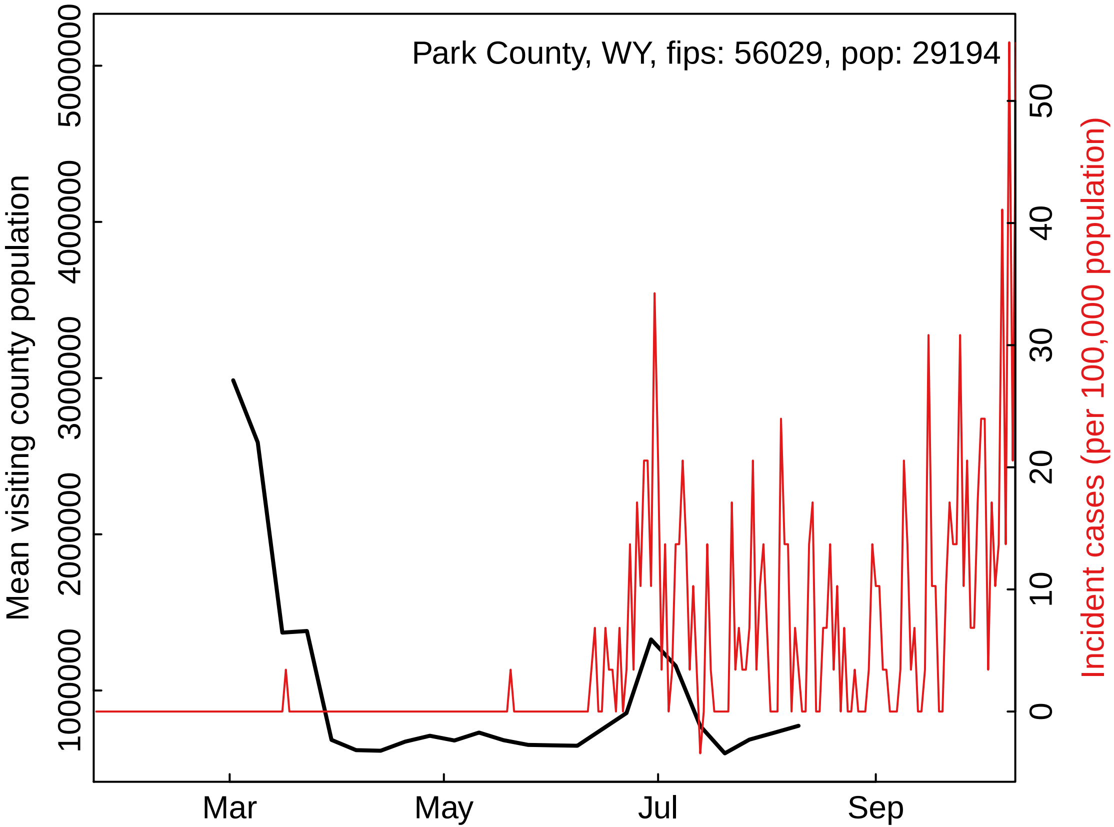

Park County, WY, fips: 56029, pop: 29194
Mean visiting county population
1000000
2000000
3000000
4000000
5000000
Incident cases (per 100,000 population)
0
10
20
30
40
50
Mar
May
Jul
Sep

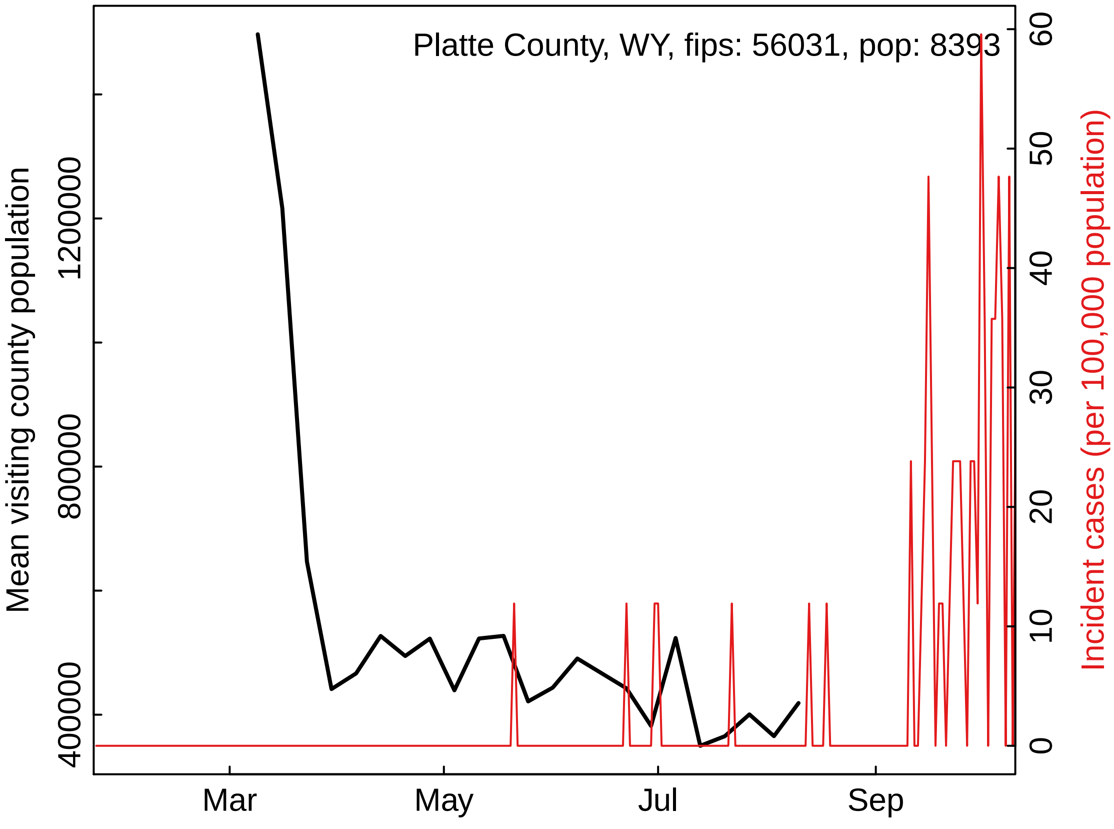
Platte County, WY, fips: 56031, pop: 8393
Mean visiting county population
400000
800000
1200000
Mar
May
Jul
Sep
0
10
20
30
40
50
60
Incident cases (per 100,000 population)

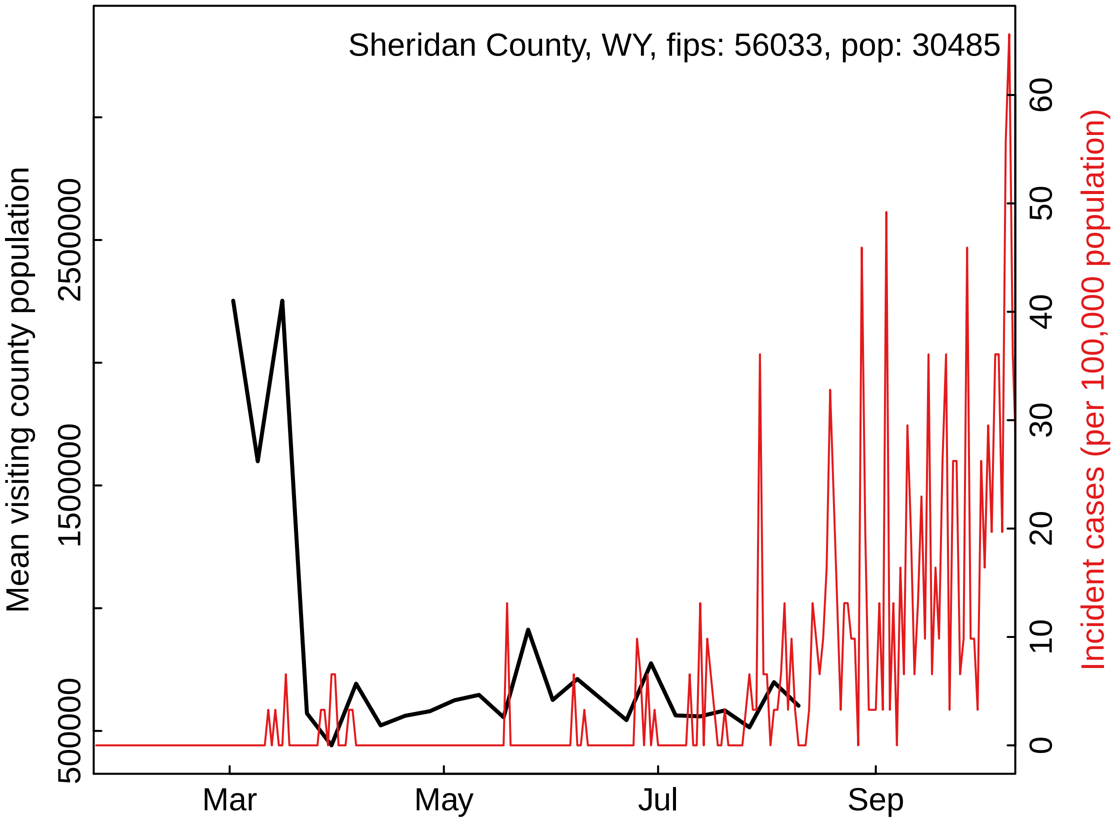

Sheridan County, WY, fips: 56033, pop: 30485
Mean visiting county population
500000
1500000
2500000
Incident cases (per 100,000 population)
0
10
20
30
40
50
60
Mar
May
Jul
Sep

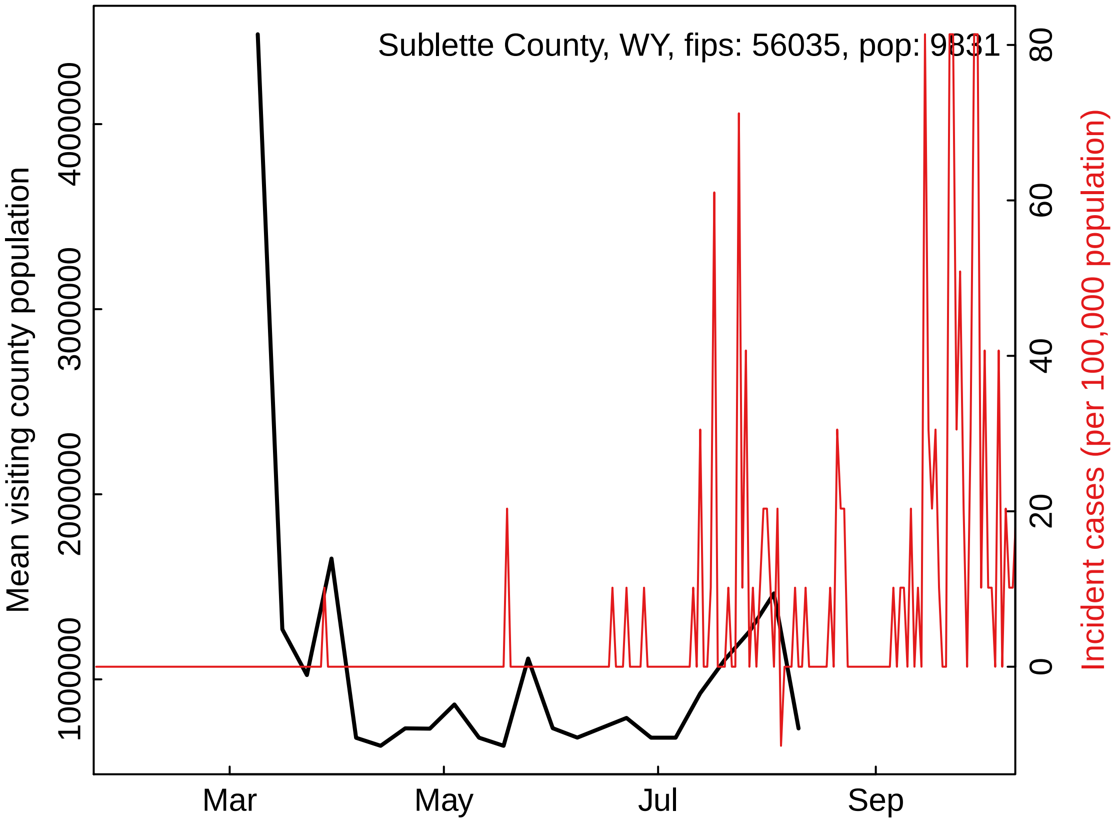

Sublette County, WY, fips: 56035, pop: 9831
Mean visiting county population
1000000
2000000
3000000
4000000
Incident cases (per 100,000 population)
0
20
40
60
80
Mar
May
Jul
Sep

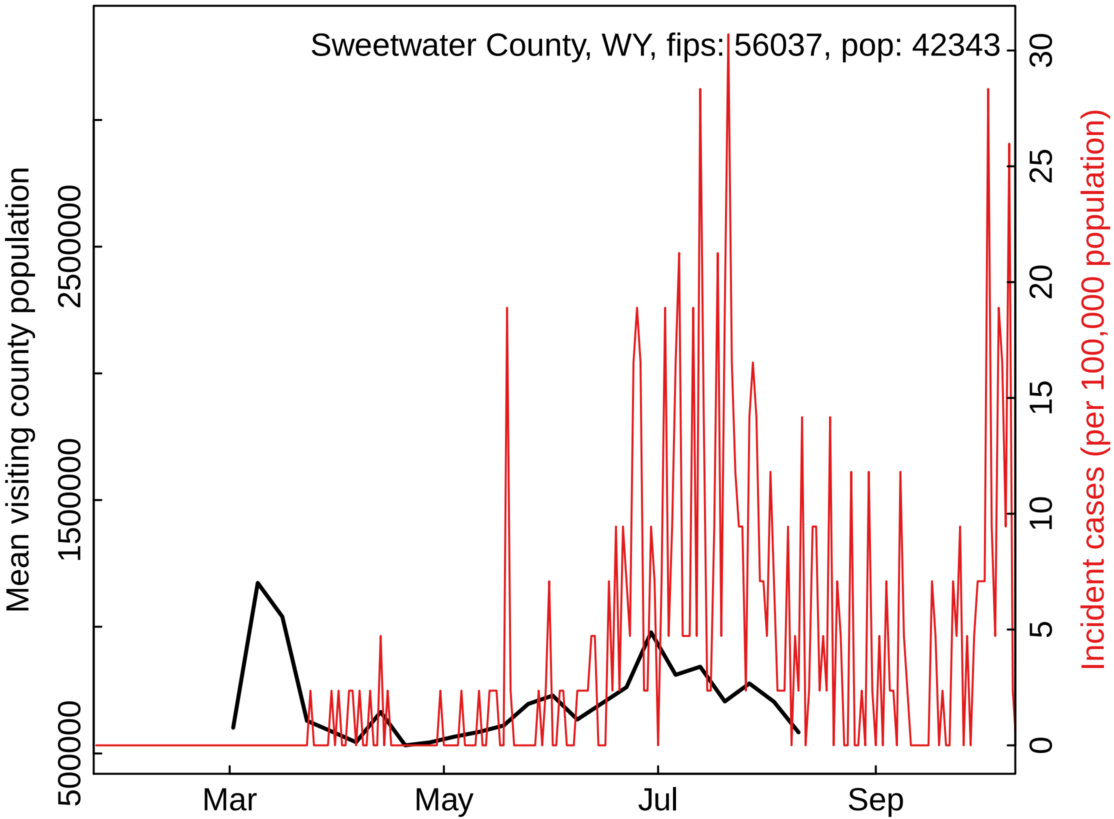

Sweetwater County, WY, fips: 56037, pop: 42343
Mean visiting county population
500000
1500000
2500000
Incident cases (per 100,000 population)
0
5
10
15
20
25
30
Mar
May
Jul
Sep

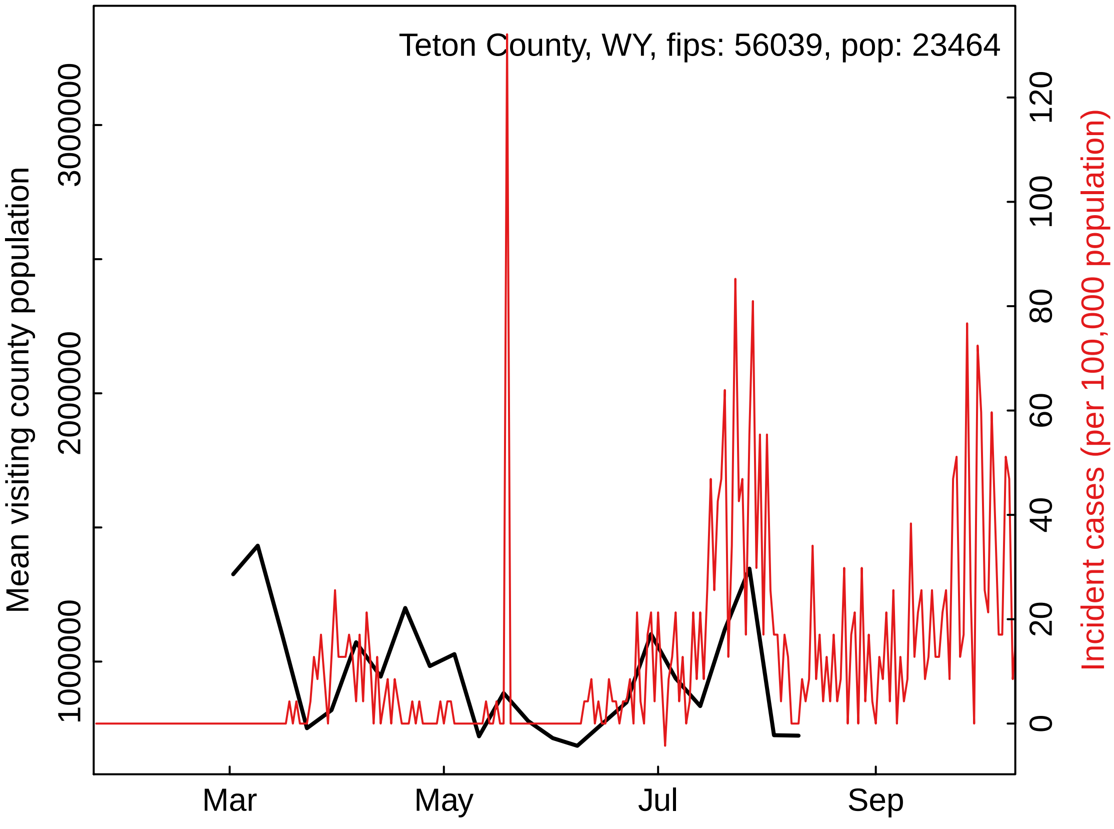
Teton County, WY, fips: 56039, pop: 23464
Mean visiting county population
1000000
2000000
3000000
Incident cases (per 100,000 population)
0
20
40
60
80
100
120
Mar
May
Jul
Sep

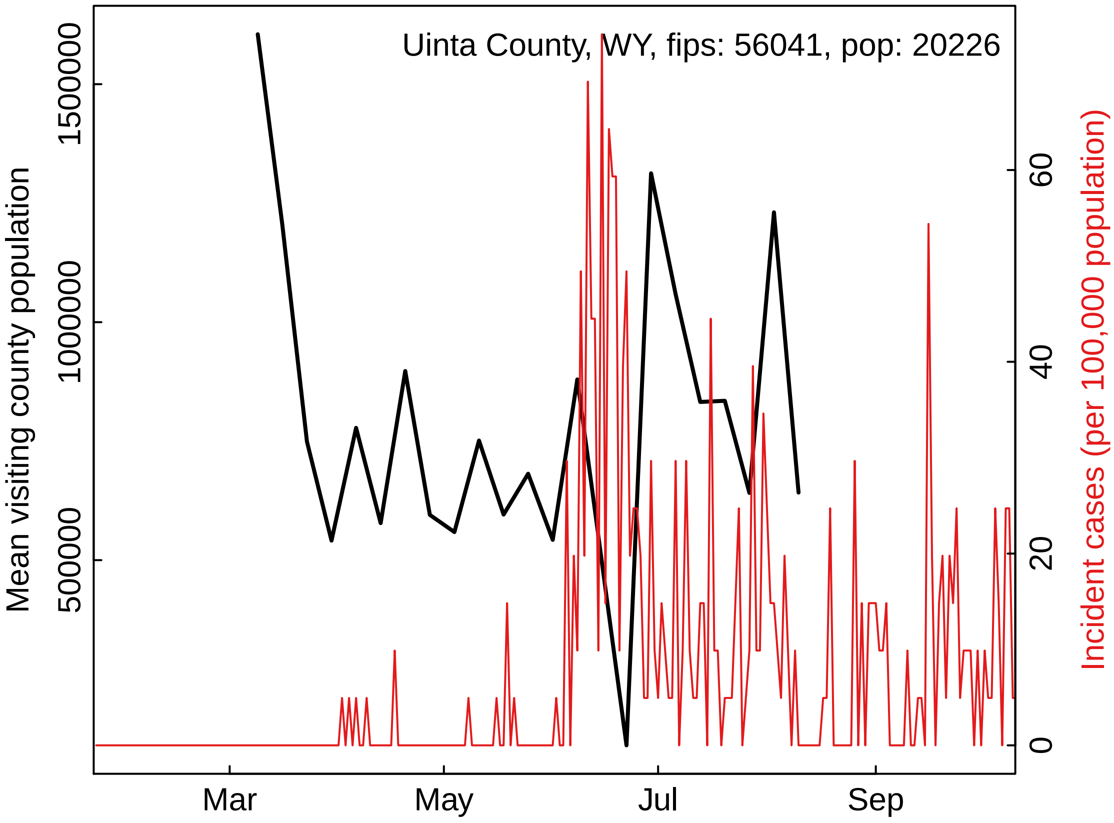

Uinta County, WY, fips: 56041, pop: 20226
Mean visiting county population
500000
1000000
1500000
Incident cases (per 100,000 population)
0
20
40
60
Mar
May
Jul
Sep

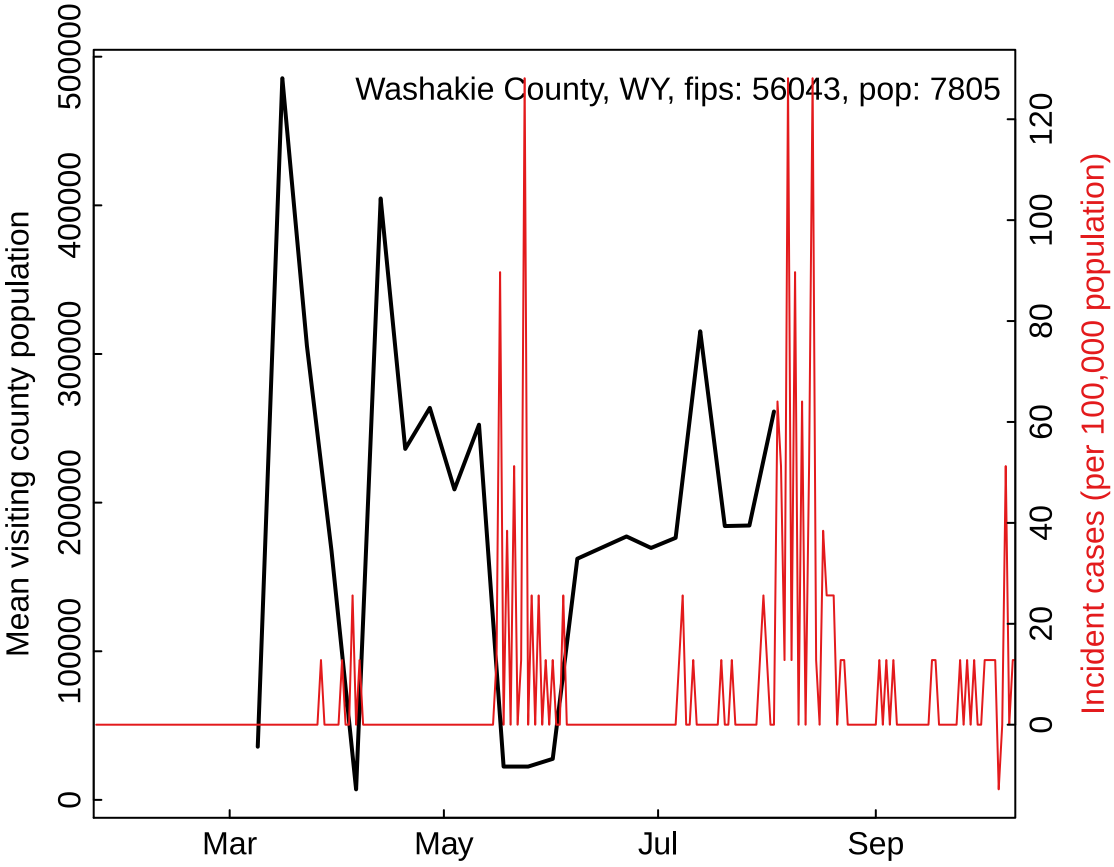

Washakie County, WY, fips: 56043, pop: 7805
Mean visiting county population
0
100000
200000
300000
400000
500000
Incident cases (per 100,000 population)
0
20
40
60
80
100
120
Mar
May
Jul
Sep

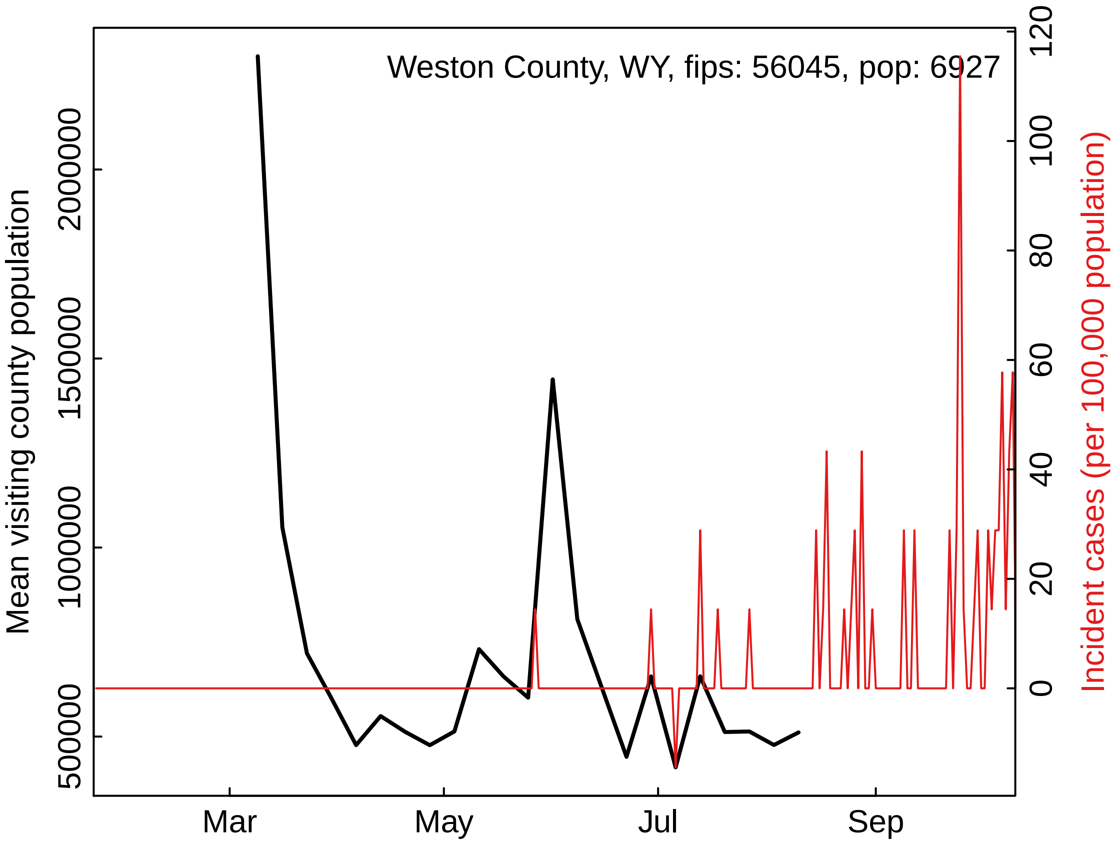
Weston County, WY, fips: 56045, pop: 6927
Mean visiting county population
500000
1000000
1500000
2000000
Incident cases (per 100,000 population)
0
20
40
60
80
100
120
Mar
May
Jul
Sep
